# Probing planetary biodiversity with DNA barcodes: The Noctuoidea of North America

**DOI:** 10.1371/journal.pone.0178548

**Published:** 2017-06-01

**Authors:** Reza Zahiri, J. Donald Lafontaine, B. Christian Schmidt, Jeremy R. deWaard, Evgeny V. Zakharov, Paul D. N. Hebert

**Affiliations:** 1 Canadian Food Inspection Agency, Ottawa Plant Laboratory, Entomology Unit, Ottawa, Ontario, Canada; 2 Centre for Biodiversity Genomics, Biodiversity Institute of Ontario, University of Guelph, Guelph, Ontario, Canada; 3 Agriculture and Agri-Food Canada, Biodiversity Program, Canadian National Collection of Insects, Arachnids, and Nematodes, Ottawa, Ontario, Canada; National Center for Biotechnology Information, UNITED STATES

## Abstract

This study reports the assembly of a DNA barcode reference library for species in the lepidopteran superfamily Noctuoidea from Canada and the USA. Based on the analysis of 69,378 specimens, the library provides coverage for 97.3% of the noctuoid fauna (3565 of 3664 species). In addition to verifying the strong performance of DNA barcodes in the discrimination of these species, the results indicate close congruence between the number of species analyzed (3565) and the number of sequence clusters (3816) recognized by the Barcode Index Number (BIN) system. Distributional patterns across 12 North American ecoregions are examined for the 3251 species that have GPS data while BIN analysis is used to quantify overlap between the noctuoid faunas of North America and other zoogeographic regions. This analysis reveals that 90% of North American noctuoids are endemic and that just 7.5% and 1.8% of BINs are shared with the Neotropics and with the Palearctic, respectively. One third (29) of the latter species are recent introductions and, as expected, they possess low intraspecific divergences.

## Introduction

**Fig 1:**
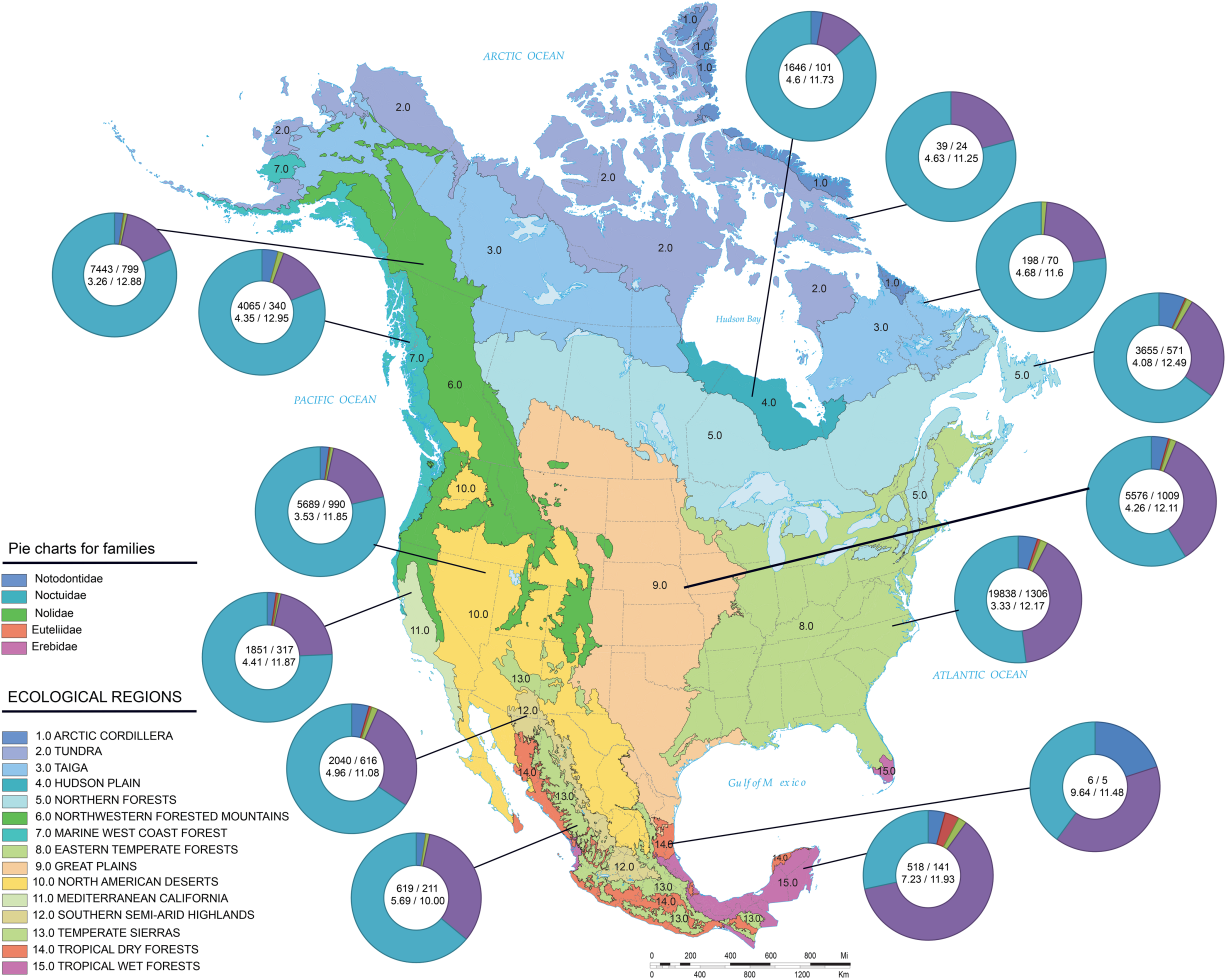
Ecoregions of North America. Maps of North America showing the boundaries of 15 ecoregions [10] which are numbered as follows: 1 Arctic Cordillera; 2 Tundra; 3 Taiga; 4 Hudson plain; 5 Northern forests; 6 Northwestern forested mountains; 7 Marine west coast forests; 8 Eastern temperate forests; 9 Great plains; 10 North American deserts; 11 Mediterranean California; 12 Southern semi-arid highlands; 13 Temperate Sierras; 14 Tropical dry forests; 15 Tropical wet forests. In subsequent analyses, the Arctic Cordillera and Tundra are combined into one region (Arctic) and the Taiga, Hudson plain, and Northern forests are merged to create a Boreal ecoregion. Numbers in each pie chart indicate the number of DNA barcodes from each ecoregion followed by the number of BINs (above) and mean/maximum Nearest-Neighbor distance (below). Modified from [10].

Occupying 14% of the planet’s land surface, Canada and the continental United States (Fig 1) span environments from the high arctic to the subtropics [1, 2]. Past estimates suggest these nations host about 144,000 insect species although approximately a third are still undescribed [2]. With 11,500 described species and perhaps another 2700 species-in-waiting [3], the order Lepidoptera (moths and butterflies) is a substantial component of the fauna. With 3664 named species in 742 genera, the Noctuoidea is the largest superfamily [4, 5], comprising 32% of all Nearctic Lepidoptera [6–9].

Since its inception in 2003 [11], DNA barcoding has gained diverse applications in biodiversity science: detecting new species and accelerating their description [12–14]; revealing cryptic species [15, 16]; linking immature stages with adults [17]; clarifying sexual dimorphisms [18]; and establishing trophic associations [19]. In animals, it employs analysis of the DNA sequence of a standard fragment of the mitochondrial cytochrome c oxidase subunit I gene (COI) as a basis for specimen identification and species discovery [11]. This approach owes its effectiveness to the fact that this gene region is generally characterized by low intraspecific variation and much higher divergence between species. As a consequence, by assembling sequence data for known species (i.e., a DNA barcode reference library), newly encountered specimens can be assigned to a species by comparing their COI barcodes to those in the library. This approach has now gained global acceptance, motivating the assembly of DNA barcode reference libraries for varied groups [20–29], information that is curated and publically available on BOLD, the Barcode of Life Data Systems [30]. Although DNA barcoding is known to deliver high species resolution in Lepidoptera [21–25], most prior studies have examined relatively small geographic areas or only a fraction of the species in a target assemblage [29].

The present study examines the impact on barcode resolution of increasing both taxon coverage and geographic scale. It examines the performance of a reference library that includes records for 97.5% of the noctuoid species known from Canada and the USA. Aside from comprehensive taxonomic coverage, the present library provides a good sense of geographic variation in many of these taxa as it is based on the analysis of nearly 70,000 specimens. Because of the comprehensive taxon coverage and large sample sizes, the present data also provide a good opportunity to test the performance of the Barcode Index Number (BIN) System [31]—an interim taxonomic system that aggregates specimens and their COI sequences into persistent sequence clusters called BINs. By testing the concordance between BIN membership and species boundaries in noctuoids at a continental scale, the constraints of the BIN system for species delineation can be evaluated, aiding its application in lesser known groups. We also examine the frequency of species with deep intraspecific COI divergences with a view towards determining if such cases are often linked to physiographic barriers. Finally, we examine shifts in the composition of noctuoids among the terrestrial ecoregions of North America and use the BIN system to ascertain the level of endemism in the North American noctuoid fauna by examining its overlap with other zoogeographical regions.

## Materials and methods

### Taxonomic coverage

This study recovered DNA barcode records from 69,378 specimens; 43.1% (29,885) derived from Canada, 46.2% (32,032) from the United States, and 10.7% (7,461) from Mexico, and the Neotropical and Palearctic regions (Table 1). The Canadian National Collection of Insects, Arachnids, and Nematodes (CNC) contributed ~18,000 museum specimens (representing 3168 species), while the Biodiversity Institute of Ontario (BIO) provided ~36,500 freshly collected specimens. The remainder (~14,500) derived from both institutional (e.g., National Museum of Natural History, Smithsonian Institution; Canadian Forest Service, Pacific Forestry Centre; University of Pennsylvania; Royal British Columbia Museum; Texas Lepidoptera Survey Research Collection, Houston) and private collections (e.g. D Handfield; JB Sullivan; H Kons, Jr.; R Borth; J Troubridge; T Mustelin; EH Metzler; LG Crabo). All specimens were examined, identified and validated by JDL and BCS; genitalia dissections were made when necessary. Taxonomy (S1 Table) follows the most recent checklist of the Noctuoidea of North America north of Mexico published in 2010 [7] and its three updates [6, 8, 9].

**Table 1:** Noctuoidea of North America summary table.

Family	North American species	Origin of specimens (Nearctics/non-Nearctics)	Barcode coverage (species#/percentage)	Species with no barcodes	# DNA sequences/BINs	Mean NND/intra-specific divergence	% ID success	Species sharing barcodes
**Doidae**	2	2/0	2/100%	0	3/2	8.93/0.00	100.0	0
**Notodontidae**	134	123/1	124/93%	10	2932/147	3.90/0.59	96.8	4
**Euteliidae**	17	15/2	17/100%	0	377/19	3.10/0.18	100.0	0
**Nolidae**	39	38/0	38/97%	1	1140/57	4.24/0.59	94.9	2
**Noctuidae**	2520	2437/16	2453/97%	67	42478/2484	2.47/0.42	92.9	173
**Erebidae**	952	884/47	931/98%	21	22448/1107	2.90/0.53	91.8	76
**Total**	3664	3499/66	3565/97.3%	99	69378/3816	2.67/0.45	92.8	255

Whenever possible, specimens of each species were analyzed from across its range in North America (S1 Dataset). However, coverage for some species could only be obtained by analyzing specimens from outside North America (S2 Table). These ‘extra-territorials’ involved 57 species and were split into three categories: 1) species barcoded from neighbouring countries (e.g., Mexico, Cuba) that likely possess barcodes matching specimens from Canada/USA (S2 Table); 2) species barcoded from a more distant location (e.g., Costa Rica, Panama, or South America) where the barcodes may not match those from Canada/USA (S2 Table); 3) non-indigenous species from Eurasia that are either rare migrants to North America or introduced/invasive species whose populations failed to persist (S3 Table). Species in groups I and II are rare migrants to North America from the Neotropics, most represented by just a single or few specimens collected from the southern United States that are too old for barcode analysis. For species collected from Texas and Arizona, specimens from Mexico were selected as the best representatives with Guatemala as the second choice. For species collected in Florida, specimens from Cuba were selected when possible with the Dominican Republic and Puerto Rico as secondary options. The likely validity of these extra-territorial records as surrogates for barcode data from specimens collected in Canada/USA was legitimized by comparing barcode records from 202 species with data from Canada/United States as well as from nations farther south (S1 Table). As this comparison did not reveal any case of deep intraspecific sequence divergence between specimens from Canada/USA and the other nations, it supports the conclusion that ‘extra-territorials’ will generally provide records valid for inclusion in the North American reference library.

### Sampling strategy across the ecological regions of North America

North America is often partitioned into 15 terrestrial ecoregions (Fig 1) [2]: Arctic Cordillera, Tundra, Taiga, Hudson Plains, Northern Forests, Northwestern Forested Mountains, Marine West Coast Forests, Eastern Temperate Forests, Great Plains, North American Deserts, Mediterranean California, Southern Semi-Arid Highlands, Temperate Sierras, Tropical Dry Forests and Tropical Wet Forests. To better reflect insect phylogeography, our analysis collapsed several of these ecoregions. We merged the Arctic Cordillera and Tundra into an Arctic ecoregion, and merged the Taiga, Hudson Plains and Northern Forests to create a Boreal ecoregion. The analysis of ecoregions employed a dataset with ~53,180 records representing 3252 named species (including species with interim names) with accurate geographic coordinates (S4 Table). To extract points from each of the 12 ecoregions, we employed ArcGIS 10.2.2 [32] to generate a presence/absence data matrix for all North American noctuoid species with GPS information (S5 Table). To perform BIN analysis, we selected those sequences from inside and outside of North America associated with both BIN data and collection data (country name) to generate a dataset with 68,985 records. This data set included 3804 BINs whose occurrence was then assessed in six zoogeographical regions (S6 Table).

### Data acquisition and analysis

DNA extraction, PCR amplification, and sequencing of the COI barcode region were performed at the Canadian Centre for DNA Barcoding (CCDB) and followed standard protocols [33–37]. PCR and sequencing generally used a single pair of primers: LepF1 (ATTCAACCAATCATAAAGATATTGG) and LepR1 (TAAACTTCTGGATGTCCAAAAAATCA) which recovers a 658bp region near the 5′ end of COI including the 648bp barcode region for the animal kingdom [11]. For museum specimens older than ten years, primer pairs designed to amplify smaller overlapping fragments (307bp, 407bp) were employed [37].

Details on all barcoded specimens (e.g., voucher codes, higher taxonomy, repository institutions, voucher images, sequence length, collection dates, and collection data) are provided in S1 Dataset. Residual DNA extracts are stored in the DNA Archive at the Centre for Biodiversity Genomics. GenBank accession numbers for all new sequences are also available in S2 Dataset. Specimen data including images, details on the voucher repositories, GPS coordinates for collection sites, sequence records, trace files, and GenBank accession numbers are available in the Barcode of Life Data Systems (BOLD, www.boldsystems.org) in eight public datasets: DS-NAMNOC1 (dx.doi.org/10.5883/DS-NAMNOC1), DS-NAMNOC2 (dx.doi.org/10.5883/DS-NAMNOC2), DS-NAMNOC3 (dx.doi.org/10.5883/DS-NAMNOC3), DS-NAMNOC4 (dx.doi.org/10.5883/DS-NAMNOC4), DS-NAMNOC5 (dx.doi.org/10.5883/DS-NAMNOC5), DS-NAMNOC6 (dx.doi.org/10.5883/DS-NAMNOC6), DS-NAMNOC7 (dx.doi.org/10.5883/DS-NAMNOC7) and DS-NAMNOC8 (dx.doi.org/10.5883/DS-NAMNOC8). The number of barcode sequences per species varies from 1 to 614 (average = 19.46) (S1 Table). Only sequence records greater than 500bp (range 500bp–658bp) and those that meet length and quality requirements for the BARCODE data standard [38] are included excepting a few short but diagnostic sequences [(*Ectypia mexicana* (307bp); *Hypotrix ocularis* (447bp); *Cydosia nobilitella* (307bp and 370bp); *Cryphia flavipuncta* (307bp); *Sympistis ra* (316bp and 379bp); *Sympistis knudsoni* (407bp); *Grotella margueritaria* (407bp); *Sympistis fortis* (407bp); *Grotella olivacea* (486bp)]. Of the 3671 species known from North America, 102 very rare species lack barcode coverage (S7 Table). They include 23 Erebidae, 67 Noctuidae, 1 Nolidae, and 11 Notodontidae. Fifteen of the species lacking a barcode record are only known from their holotype.

Tests of barcode performance were firstly made at a continental level based on the North American checklist [6–9] and subsequently for each of the 12 ecoregions. Patterns of intra- and interspecific nucleotide sequence variation were examined at various taxonomic levels using the Kimura-2-Parameter (K2P) distance model and the Neighbor-Joining (NJ) algorithm calculated using the analytical tools on BOLD at a continental scale and for each ecoregion. To accommodate for unequal variances and sample sizes in the Nearest-Neighbor (NN) distances and intraspecific data, an unequal variance t-test with random sampling of cases were employed. Finally, a nonparametric correlation test (Spearman) implemented in SPSS v18 (IBM) was used to assess the relationship between the number of species in a genus and the incidence of barcode sharing.

## Results

### Barcode performance

DNA barcodes were obtained for 3565 of the 3664 valid noctuoid species known from North America. No indels, frameshift mutations or stop codons were detected among the 69,378 sequences recovered from these taxa suggesting that they derive from COI rather than a pseudogene. Considered from a continent-wide perspective, 93% of all noctuoid species possess a diagnostic array of barcode sequences (including those species with deep intraspecific divergence) (S1–S8 Trees; Table 1). Barcode performance was slightly higher (96%) when analysis considered the species assemblage within each of the 12 ecoregions (Table 2). The cases of compromised resolution reflected the fact that 255 species (7%) shared their barcode with at least one other species when considered at a continental scale (S8 Table), while the mean incidence of sharing dropped to 4% for the species assemblage in each of the 12 ecoregions (Table 2).

**Table 2:** Mean sequence divergence and mean intraspecific variation at COI among noctuoid species from each of 12 North American ecoregions.

Ecoregions of North America level I	DNA sequences#/ BIN#	Species with high sequence divergence (>2%)/Species with low distance to another species (<2%)	species#/Genus#	Mean/Max NND	Mean/Max intra-specific divergence	% ID success	Species sharing barcodes
**Arctic Cordillera + Tundra**	39/24	18/6	24/14	4.63/11.25	0.39/2.34	100	0
**Boreal**	5499/608	491/104	637/235	3.98/12.49	0.31/4.12	96	26
**Northwestern forested mountains**	7443/747	93/238	788/226	3.40/12.88	0.29/4.53	96	31
**Marine west coast forest**	4065/340	296/24	341/161	4.50/12.95	0.19/2.39	98	7
**Eastern temperate forests**	19838/1247	800/106	1274/391	3.8/12.17	0.33/12.64	93	92
**Great plains**	5576/968	744/111	991/365	4.43/11.85	0.29/3.62	98	23
**North American deserts**	5689/957	731/217	986/304	3.70/11.85	0.39/9.79	97	31
**Mediterranean California**	1851/317	223/27	318/143	4.82/11.87	0.38/3.81	99	2
**Southern semi-arid highlands**	2040/617	444/10	605/255	5.3/11.08	0.33/8.39	99	4
**Temperate Sierras**	619/207	166/1	209/123	5.88/10.00	0.26/4.92	98	5
**Tropical dry forests**	6/5	5/0	5/5	9.64/11.48	0.31/0.31	100	0
**Tropical wet forests**	518/142	134/6	141/98	7.34/11.93	0.23/2.35	100	0
**Total**	53183/6179		2935/658	4.90/11.81	0.38/8.4	96	119

**Fig 2:**
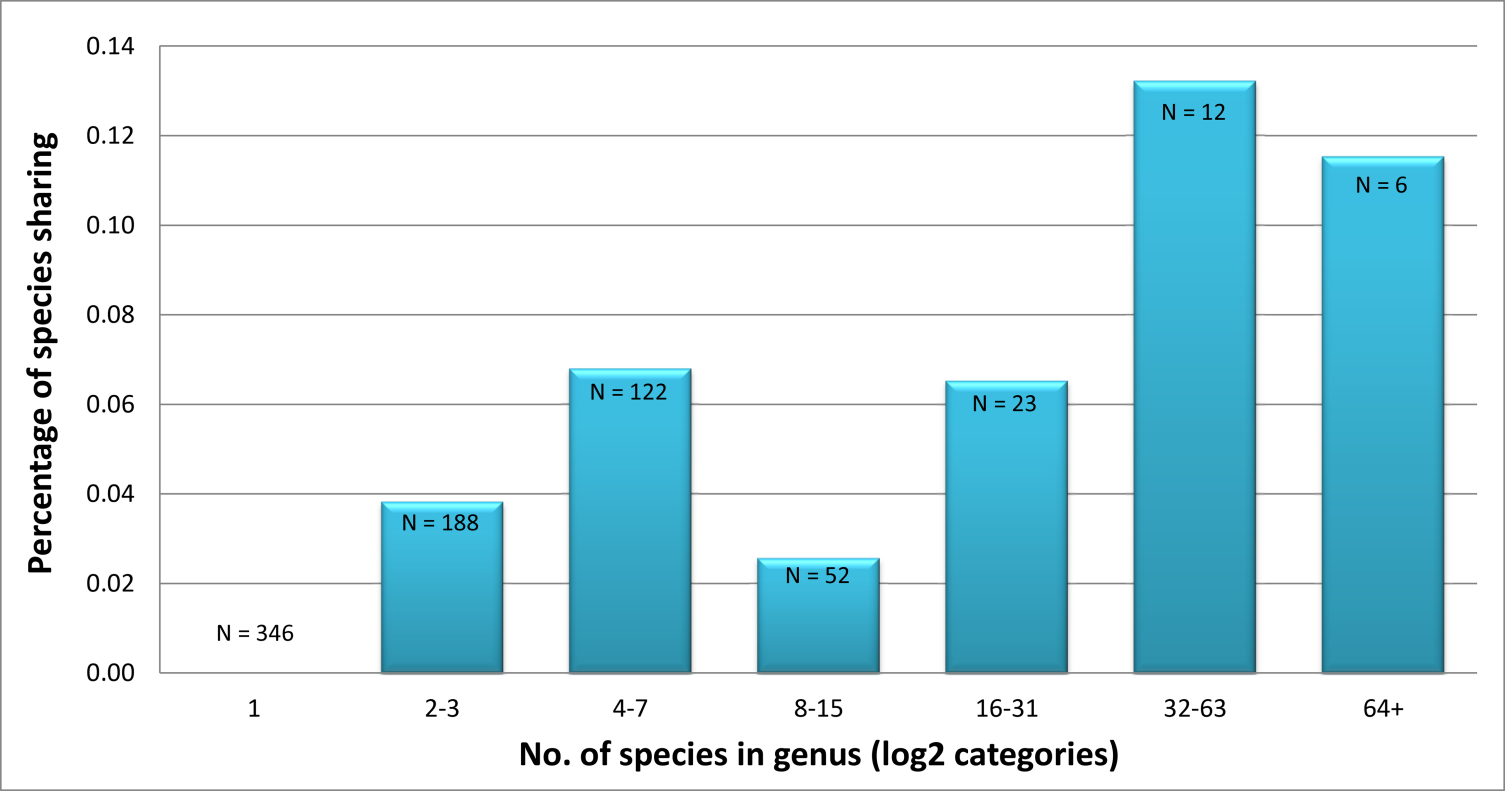
The relationship between the number of species in a genus (plotted on a log_2_ scale categories) and the percentage of barcode sharing. Values at the top of the bars indicate the number of genera in each log_2_ category. Categories are: 1) genera with 1 species, 2) genera with 2–3 species, 3) genera with 4–7 species, 4) genera with 8–15 species, 5) genera with 16–31 species, 6) genera with 32–63 species, and 7) genera with 64 or more species.

Mean NN distances showed limited variation among families, ranging from a low of 2.47% in the Noctuidae to a high of 3.90% in the Notodontidae after excluding the high NN distance (~9%) for the Doidae because it was only represented by two species (Table 1). There was, however, significant variation in barcode performance among families (X^2^ = 38.3, p<0.0001). All species of Doidae (2 species) and Euteliidae (17 species) were unambiguously discriminated by barcode sequences, but barcode sharing in the other families ranged from 3.2% (4/125 species) in the Notodontidae to 5.1% (2/39 species) in the Nolidae, 7.1% (173/2452 species) in the Noctuidae, and 8.2% (76/931 species) in the Erebidae (Table 1 and S8 Table). Barcode sharing was most frequent in genera with many species (Spearman’s rho = 0.432; p<<0.0001); it involved 10.6% (182/1717 species) of the species in the most diverse genera (42 genera with 16–182 species) versus 4.5% (73/1608 species) of the species in genera with fewer taxa (360 genera with 2–15 species). As expected, none of the 346 species in monotypic genera shared their barcodes with any other taxon (Fig 2).

### Cases of barcode sharing

In total, 255 of 3565 species of noctuoids (~7.1%) shared their barcode with at least one other species (S8 Table provides a list). These cases of barcode sharing involved just 55 of the 747 noctuoid genera (7.4%). The 76 cases of barcode sharing among the 931 species of Erebidae involved 14 of its 268 genera (S8 Table). Four genera (*Catocala*– 26, *Grammia–* 18, *Cisthene–* 5, *Haploa*– 5) were responsible for 71% (54 of 76 species) of these cases; the other 22 involved 10 genera with two or three species sharing the same barcode. The most striking cases of barcode sharing in this family involved *Grammia* (50%, 18 of 36 species) and *Catocala* (25%, 26 of 103 species). The 173 cases of barcode sharing among the 2452 species of Noctuidae involved 38 of its 420 genera (S8 Table). Among them, ten genera (*Euxoa*– 24, *Xestia*– 13, *Schinia*– 13, *Abagrotis* –12, *Acronicta*– 11, *Lasionycta*– 10, *Copablepharon*– 9, *Sympistis*– 8, *Lithophane*– 7, *Bellura*– 5) represented 64% (110 out of 173 species) of these cases; the other 63 cases involved 28 genera with two to four species sharing the same barcode. Nine noctuid genera showed a particularly high incidence of barcode sharing with 83% of species in *Bellura* (5 out of 6), 39% of *Copablepharon* (9 of 23), 31% of *Abagrotis* (12 of 39), 26% of *Xestia* (13 of 50), 23% of *Lasionycta* (10 of 43) 16% of *Acronicta*, 14% of *Lithophane*, 13% of *Euxoa*, and 10% of *Schinia*.

### Cases of low barcode divergence

Cases of low sequence divergence were defined as those involving two or more species with less than 1% sequence divergence, but with no evidence of sequence sharing. In total, 14.7% (525 species) of North American noctuoid species showed from 0.15% to 0.99% divergence from their NN (S9 Table). These species belong to 109 genera in four families (S9 Table). Twenty-three genera (*Abagrotis*, *Acronicta*, *Anarta*, *Annaphila*, *Apamea*, *Catocala*, *Copablepharon*, *Dasychira*, *Datana*, *Euxoa*, *Feltia*, *Grammia*, *Hadena*, *Lacinipolia*, *Lasionycta*, *Lithophane*, *Papaipema*, *Schinia*, *Sympistis*, *Virbia*, *Zale*, *Zanclognatha*, *Xestia*) included six or more species with low divergence, but with no evidence of shared sequences (except where noted above). They included seven noctuid genera with many cases of low divergence (*Euxoa–* 56, *Sympistis–* 38, *Lasionycta–* 22, *Lithophane*– 21, *Papaipema–* 19, *Schinia–* 15, *Acronicta*– 11) and two erebid genera (*Catocala–* 21, *Grammia–* 13).

### Cases of deep intraspecific sequence divergence

Deep barcode divergence (>2%) was detected in 135 (3.8%) species, and another 22 species showed sufficient divergence (0.7%–1.99%) for their component specimens to be assigned to two or three BINs (S10 Table). These 157 cases (4.4% of the fauna) involved 12.5% of the noctuoid genera (93 of 747 genera). Most cases involved a species that was partitioned into either two (102 species in 70 genera) or three (31 species in 25 genera) BINs. However, 24 species in 17 genera were placed in four or more BINs (S10 Table). Nine genera included several cases of deep splits including *Abagrotis* (four species in 11 BINs), *Euxoa* (ten species in 25 BINs), *Grammia* (six species in 18 BINs), *Idia* (three species in 13 BINs), *Lacinipolia* (eight species in 30 BINs), *Sympistis* (eight species in 17 BINs), *Virbia* (two species in 22 BINs), and *Xestia* (eight species in 20 BINs). One species showed exceptional diversity; specimens of *Virbia ferruginosa* were assigned to 18 BINs.

### Factors influencing nearest-neighbor distances

#### a) Ecoregions

When the North American fauna was partitioned into the species assemblages from each of 12 ecoregions (Fig 1), barcode resolution improved from 92.8% continent-wide to 96.0% (119 out of 2935 valid species represented sequence sharing and identical haplotypes) (Table 2). When considered at a continental scale, 255 species (7.1%) shared their barcode with one or more species, but barcode sharing dropped to just 119 cases (4.0%) when ecoregions of North America were considered individually. NN distances showed a gradual increase from the north (e.g., Tundra = 4.63%) to the south (e.g., Tropical Wet Forests = 7.34%) (Fig 1, Table 2). While a noticeable decline in NN distance was observed with increasing latitude (Table 3), there was no similar trend with longitude although the average value for the Rocky Mountains ecoregion was slightly lower (~ 0.3%) than for the other regions (Table 4). There were also shifts in the relative diversity of the two major noctuoid families—the Noctuidae dominate the northern half of the continent whereas the Erebidae dominate in the south; this shift in faunal composition likely contributes to the NN pattern with latitude.

**Table 3:** Sequence divergence at COI among North American noctuoid species along a north-south axis.

Zones	#Sequences	#BINs	Mean NND	Mean Intra-specific
**>55°**	3637	276	3.79	0.34
**50° & 55°**	9051	657	3.51	0.24
**45° & 50°**	12739	1090	3.32	0.31
**40° & 45°**	6800	957	3.43	0.36
**35° & 40°**	8214	1283	3.43	0.41
**30° & 35°**	10650	1686	3.73	0.45
**25° & 30°**	3355	698	5.06	0.38
**<25°**	388	75	7.61	0.29
**Total**	54835	3202	4.2	0.3

**Table 4:** Sequence divergence at COI among North American noctuoid species across an east-west axis.

Zones	#Sequences	#BINs	Mean NND	Mean Intra-specific
**>135°**	169	84	4.50	0.73
**125° & 135°**	1504	135	5.25	0.19
**115° & 125°**	14882	1077	3.07	0.32
**105° & 115°**	8200	1354	3.86	0.38
**95° & 105°**	5271	1009	4.43	0.37
**85° & 95°**	4650	753	4.10	0.43
**75° & 85°**	13885	1157	3.63	0.40
**<75°**	6273	636	3.93	0.28
**Total**	54835	3202	4.1	0.4

#### b) Phylogeny and non-indigenous species

It is thought that 35 noctuoid species now found in North America are either migrants or introduced alien species were introduced from Eurasia by human-mediated transport (S3 Table). These species have a significantly (p<0.0001) higher NN distance (x = 5.90%) than native taxa (x = 3.60%), reflecting the fact that many have left their sister taxa behind (S3 Table). As might also be expected, these species have a significantly lower mean intraspecific divergence (x = 0.20%) than native species (x = 0.44%) (S3 Table), reflecting the loss of diversity due to population bottlenecking during their establishment (two-sample t-Test, t = 7.1, p < 0.0001).

### Species boundaries and BIN concordance

Although the BIN count (3816) was just 7% higher than the number of species (3565), this congruence partially reflected the counterbalancing effects of BIN splits and mergers (low-divergence and barcode sharing). In actuality, perfect correspondence between the assignment of specimens to a particular species and their placement in a unique BIN was only evident for 2711 species (76.0%). Another 157 species (including all 135 species with >2% intraspecific sequence divergence) were involved in splits with their members assigned to two (102 species), three (31 species), or more (24 species) BINs (S10 Table and for intra-specific divergences see S11 Table). Another 741 species shared their BIN assignment with at least one other species. Some of these mergers reflected species (255) that shared haplotypes (S8 Table), but most (525) involved species with diagnostic but low barcode divergence (S9 Table). A few cases (40) involved species with mixed barcode sharing and low divergence. Finally, there were 43 species whose members were involved in both BIN splits and mergers. S11 Table reports mean intraspecific divergences, BIN counts and number of specimens analyzed for each species.

Records for 3803 BINs were examined for overlap among zoogeographical regions, an analysis which revealed that 284 (7.5%) are shared with the Neotropics while 70 (1.8%) representing 88 species are shared with the Holarctic. Two-thirds of the latter species (59 species) appear to have natural Holarctic distributions while 29 species are believed to have been introduced as a consequence of human activity.

### Cohesion at higher taxonomic levels

Although barcode sequences generally do not provide robust phylogenetic information beyond the species level [39], most genera formed cohesive clusters. Such genus-level cohesion, or its lack, may provide a useful preliminary assessement of monophyletic assemblages. For example, Acronicta insularis and A. ursini are imbedded within the remainder of Acronicta, but were previously placed in seperate genera, Simyra and Merolonche. Independent molecular markers and morphology has since shown that Simyra and Merolonche fall within the concept of Acronicta [40]. Similarly, barcode results for representative Eriopygini (Noctuidae) flagged the close similarity of species in four putative genera, leading to the recognition of a single unified genus, Hypotrix [41]. Conversely, genera that are split or widely separated in NJ trees may flag non-monophyletic groups in need of revision. For example, morphological study confirms that North American Orthosia represent several genera (JDL, unpubl. data), as suggested by the high COI divergence among its component taxa. Finally, extensive taxon sampling can hint at tribal and subfamily systematics; in the case of the genera currently comprising Eustrotiinae, two separate clusters of genera led to independent confirmation that this subfamily includes two distinct groups that are not closely related (BCS, unpubl. data). In situations, such as the current one, where nearly complete taxon sampling maximizes phylogenetic signal, DNA barcode data have considerable potential to reveal phylogenetic affinities [42].

## Discussion

The present study provides an average of 20× barcode coverage for 97.3% (3565/3664) of the currently recognized noctuoid species in North America. These results indicate that 3310 of these species (92.8%) possess a diagnostic array of DNA barcodes when considered at a continental scale, while barcode resolution rises to 96.0% when examined by ecoregion. About three quarters (76.0%) of these species perfectly coincide with BIN assignments. As reported in other studies [29], many of the cases of discordance involve species with either low sequence divergence from another species or with deep intraspecific divergence. Most species (3412 of 3565) showed a maximum intraspecific distance of less than 2%, but deeper divergence was detected in 157 species (4.4% of the total), and barcode sharing was detected in 255 species (7.1% of the total). Despite these complexities, the resultant DNA barcode library allows the unambiguous identification of 93.0% of currently recognized noctuoid species when considered at a continental level and identification success is 96.0% when analysis examines the species from a particular ecoregion.

Our results reinforce earlier indications that increased geographic sampling does not seriously diminish the performance of DNA barcodes in specimen identification [29, 43, 44]. In fact, the resolution for North American noctuoids is slightly higher than that for the northern half of the continent as Zahiri et al. [29] observed 90.0% resolution in their study of 1541 species of noctuoids from sites across Canada and 95.6% resolution when considered at a provincial scale. Similarly, deWaard et al. [25] found 93% resolution for 400 geometrid species from British Columbia, whereas Hebert et al. [45] observed 99.0% resolution in a study on 1200 species in diverse families of Lepidoptera from southern Ontario. DNA barcodes also distinguished 97% of more than 1000 species from northwestern Costa Rica [46]. Results from the Palearctic indicate similar performance with 93% of 219 species from selected subfamilies of European Geometridae [47], 90% for 185 species of Romanian butterflies [22], 98.5% for 400 species of Bavarian geometrids [23] and 99% for 957 species of butterflies and larger moths from southern Germany [24].

High intraspecific divergences (>2%) were present in 135 species (3.8%) of North American noctuoids, a slightly lower incidence than the 5–8% reported in other Lepidoptera faunas with well-studied taxonomy [16, 22–24, 29, 36]. These deep intraspecific divergences (SI11) may indicate unrecognized sibling species, but may also reflect phylogeographic variation in a single species, divergence linked to bacterial endosymbionts or the recovery of a pseudogene. Cases of deep divergence can arise as a result of introgression following hybridization, paralogous pseudogenes, retained ancestral polymorphisms, and vertically transmitted symbionts [48, 49]. Because mitochondrial genes are inherited maternally, are exposed to little recombination, and have an effective population size (Ne) that is ¼ that of nuclear genes, they are also particularly susceptible to selective sweeps [50, 51]. Because CO1 is a protein-coding gene, pseudogenes can be recognized through translation of the nucleotide sequence to ensure the absence of stop codons or frameshift mutations. The endosymbiont Wolbachia can foster CO1 divergence among infected lineages [52]. Virbia ferruginosa showed an exceptionally high level of COI variation as indicated by its assignment to 18 BINs, but the cause of this diversity remains uncertain. Because morphological studies (BCS) suggest that this variation is not due to cryptic species, there is a need for further work to ascertain if Wolbachia or another agent have provoked recurrent selective sweeps that have created the unusual sequence diversity in this species [44, 53]. Because both Wolbachia and mtDNA are maternally inherited, linkage disequilibrium is inevitable between them [51, 54]. Moreover, Wolbachia has been linked to both a selective sweep of the mtDNA genome and introgression in the butterflies [54, 55]. In such situations, patterns of sequence divergence in the mitochondrial genome inevitably fail to coincide with species boundaries [56–59]. However, discordances between gene trees and morphological traits can also indicate overlooked cryptic species [59]. Factors such as geographic barriers and the fragmentation of the lineages comprising a species during glacial periods can also be an important force in creating unusual patterns of haplotype diversity. All individuals in a monophyletic species have a common ancestor that is shared by individuals of no other species (otherwise it is paraphyletic) [59, 60]. The existence of multiple barcode haplotypes in a single species can reflect high diversity in the original gene pool that created different subpopulations through time. Subpopulations that are phenotypically the same but genotypically slightly diverged have undergone numerous expansion-contraction (isolation and rejoining) events that eventually adapt themselves to various habitats but still look alike morphologically. Lastly, multiple haplotypes may reflect limitations in current molecular technologies. This may explain why a single introduction of Noctua pronuba into Nova Scotia in 1979 has produced a North American population that includes 12 haplotypes with up to 0.9% divergence. Five of these 12 haplotypes are shared with Europe, and several more singletons that might reflect mutational divergence or sequencing error, one widely distributed haplotype (18 specimens from New Brunswick to British Columbia and south to Kansas and North Carolina) is unknown in Europe. Also, the present study revealed that North American populations of Trichoplusia ni show 2.3% barcode divergence from Eurasian specimens, suggesting a taxonomic split may be warranted. A recent study that examined 41,583 barcode sequences from nearly 5000 species of European Lepidoptera revealed that many cases of apparent non-monophyly actually reflect methodological problems including misidentifications, taxonomic oversplitting, overlooked species, and the inherent subjectivity of species delimitations, especially in situations of allopatry [59].

The incidence of barcode sharing in North America uncovered in our study varied among the 747 noctuoid genera, as just 55 genera (7.4%) were involved. Moreover, barcode sharing was highest in Erebidae (8.2%), followed by Noctuidae (7.1%), Nolidae (5.1%) and Notodontidae (3.2%). Our study revealed that 7.15% of North American noctuoid species (255/3565) share their barcode sequence with at least one other species, a pattern that can be explained at least in three ways. First, the lack of divergence may reflect such a recent split that sister taxa lack diagnostic CO1 sequences. Second, barcode sharing can reflect introgression following hybridization between species. Finally, species sharing barcode haplotypes may actually represent only a single polymorphic species as a result of over-splitting, especially in species-rich genera, commonly referred to as “imperfect taxonomy” [49]. Distinct species with shared barcodes can also involve ancestral polymorphisms, often reflecting secondary contact between phylogeographic lineages. Other cases can arise through taxonomic and diagnostics problems such as misidentified specimens or overlooked cryptic taxa. While some instances of barcode sharing may indeed reflect invalid taxonomy, many cases of barcode sharing involve species which show differences in larval or genitalia morphology larval and host plant use. Generally speaking, all cases of barcode sharing and deep intraspecific divergence require detailed investigation to better understand the responsible factors. For example, many of the 157 cases of deep sequence divergences require further investigation to determine if biological attributes covary with barcode clusters, a pattern which would indicate that they are overlooked species.

Our study indicated that just 284 of 3803 BINs (7.5%) of the noctuoid BINs encountered in Canada and the USA also occur in the Neotropics. Overlap with other zoogeographic regions was even lower with 1.8% are shared with the Palearctic Region (88 species), 0.13% with the Ethiopian Region (5 species), 0.13% with the Oriental Region (5 species), and 0.02% with the Australian Region (1 species) (Table 5). While these values may be underestimates, since barcode coverage is not comprehensive in other regions, there is no reason to expect that barcode coverage has been biased against taxa that are shared among regions. Moreover, many Neotropical species known from North America are migrants or accidental/temporary introduced alien species (S3 Table) which act to inflate the overlap. A similar pattern emerges for the 70 BINs (88 species) that are shared between the Nearctic and Palearctic Regions because just 59 are truly Holarctic species while 29 were introduced by humans.

**Table 5:** Noctuoidea BIN distribution among six zoogeographical regions.

Family	BINs#	Nearctic	Neotropical	Palaearctic	Ethiopian	Oriental	Australian
**Doidae**	2	2/100	0/0	0/0	0/0	0/0	0/0
**Notodontidae**	146	131/89.73	21/14.38	2/1.37	0/0	0/0	0/0
**Euteliidae**	18	17/94.44	4/22.22	0/0	0/0	0/0	0/0
**Nolidae**	57	56/98.25	1/1.75	2/3.51	0/0	0/0	0/0
**Erebidae**	1065	997/93.62	101/9.48	12/1.13	3/0.28	1/0.09	0/0
**Noctuidae**	2375	2342/98.61	86/3.62	39/1.64	3/0.13	3/0.13	2/0.08
**NOCTUOIDEA**	3663	3545/96.78%	213/5.81%	55/1.50%	6/0.16%	4/0.11%	2/0.05%

Finally, we consider the correspondence between morphospecies and sequence clusters delineated by the BIN system. The present analysis indicates the strong capacity of the BIN system to estimate species diversity (3816 BINs versus 3565 species with barcode coverage). Our analyses of deep splits suggest that more than 400 undescribed species of Noctuoidea were barcoded in this study. This result suggests the power of BIN analysis to provide rapid estimates of species diversity in poorly studied areas and little known groups, supporting the conclusion of earlier investigations [29, 31]. This result also suggests due to potential discordance between phylogenetic signal in a gene tree and species evolutionary history, biodiversity assessments may be complicated by inaccurate assignment of such cases to a morphospecies [56, 57]. As a result, DNA barcoding could be a key estimator to resolve a long-standing question—how many animal species are there on the planet [61]? However, this capacity will require more large-scale reference libraries such as the one assembled in this study. Overall our continental-scale study supports the conclusion of a recent study [59] that, when used with care and in conjunction with other techniques, DNA barcodes provide powerful addition to the tools available for taxonomic work on animals.

## Supporting information

S1 Dataset Specimen data. Specimen data (vouchers, taxonomy, specimen details, collection data) for North American species in the families Notodontidae + Doidae, Euteliidae, Nolidae, Erebidae, and Noctuidae.
(XLS)

S2 Dataset GenBank accession numbers. (XLS)

S1 Table Checklist for North American noctuoids. The table represents barcode coverage in terms of the number of specimens analyzed and geographic coverage. Species lacking barcode data are in red.
(XLS)

S2 Table Species with extraterritorial barcode records. Forty-eight species barcoded from other nations likely share the same barcode as specimens collected in the USA. For species collected in Texas and Arizona—barcodes from Mexican specimens are the best proxy, followed by Guatemala. For species collected in Florida—barcodes from Cuban specimens are best, followed by those from the Dominican Republic or Puerto Rico. Nine species with barcode records from specimens collected from more distant locations (Costa Rica, Panama, South America) pose a greater risk that their barcode records may not match specimens from the USA.
(XLS)

S3 Table Introduced species into North America. List of introduced species of noctuoids in North America with the approximate date of their arrival.
(XLS)

S4 Table Barcode performance among ecoregions of North America. Data set for the analysis of barcode performance in the discrimination of noctuoid species from the 12 ecoregions of North America.
(XLS)

S5 Table Two-way presence-absence data for 3252 North American noctuoid species in 12 ecoregions of North America. Each region designated by a numeric code: 1 Arctic (Arctic Cordillera + Tundra); 2 Boreal (Taiga + Hudson plain + Northern forests); 3 Northwestern forested mountains; 4 Marine west coast forests; 5 Eastern temperate forests; 6 Great plains; 7 North American deserts; 8 Mediterranean California; 9 Southern semi-arid highlands; 10 Temperate Sierras; 11 Tropical dry forests; 12 Tropical wet forests.
(XLS)

S6 Table Data set for the analysis of BIN overlap among zoogeographical regions. (XLS)

S7 Table Barcode coverage. List of North American noctuoid species without barcode coverage.
(XLS)

S8 Table Barcode sharing. List of North American noctuoid species sharing a barcode haplotype.
(XLS)

S9 Table Low sequence divergence. List of North American noctuoid species with low sequence divergence from another taxon.
(XLS)

S10 Table Deep split. List of North American noctuoid species with deep intraspecific sequence divergence.
(XLS)

S11 Table List of all noctuoid species known from North America with number of BINs, mean intra-specific divergence, and number of specimens per species. (XLS)

S1 Tree Notodontidae and Doidae. NJ tree based on sequence variation in the barcode region of the cytochrome *c* oxidase I gene for North American species in the families Notodontidae and Doidae.
(PDF)

S2 Tree Euteliidae. NJ tree based on sequence variation in the barcode region of the cytochrome *c* oxidase I gene for North American species in the family Euteliidae.
(PDF)

S3 Tree Nolidae. NJ tree based on sequence variation in the barcode region of the cytochrome *c* oxidase I gene for North American species in the family Nolidae.
(PDF)

S4 Tree Erebidae (Arctiinae). NJ tree based on sequence variation in the barcode region of the cytochrome *c* oxidase I gene for North American species in the family Erebidae (Arctiinae).
(PDF)

S5 Tree Erebidae (rest). NJ tree based on sequence variation in the barcode region of the cytochrome *c* oxidase I gene for North American species in the family Erebidae (rest).
(PDF)

S6 Tree Noctuidae-1. NJ tree based on sequence variation in the barcode region of the cytochrome *c* oxidase I gene for North American species in the family Noctuidae-1.
(PDF)

S7 Tree Noctuidae-2. NJ tree based on sequence variation in the barcode region of the cytochrome *c* oxidase I gene for North American species in the family Noctuidae-2.
(PDF)

S8 Tree Noctuidae-3. NJ tree based on sequence variation in the barcode region of the cytochrome *c* oxidase I gene for North American species in the family Noctuidae-3.
(PDF)
