# Supplementary material for: Probing planetary biodiversity with DNA barcodes: The Noctuoidea of North America
Source: PLoS One. 2017 Jun 1;12(6):e0178548. doi: 10.1371/journal.pone.0178548 (PMC5453547; doi:10.1371/journal.pone.0178548)
Supplement: S1 Tree — NJ tree based on sequence variation in the barcode region of the cytochrome c oxidase I gene for North American species in the families Notodontidae and Doidae. (PDF) [file pone.0178548.s014.pdf]

BOLD TaxonID Tree

Title	:	Tree Result - Search (2935 records)
Date	:	19-Dec-2016
Data Type	:	Nucleotide
Distance Model	:	Kimura 2 Parameter
Marker	:	COI-5P
Colourization	:	[blue]=Stop Codons [red]=Contamination or misidentification

Label	:	Sample ID
Label	:	Process ID
Label	:	Taxon
Label	:	Country
Label	:	Province/State
Label	:	Sequence Length
Label	:	Barcode Cluster (BIN)

Sequence Count	:	2935
Species count	:	144
Genus count	:	44
Family count	:	2
Unidentified	:	1

BIN Count	:	149

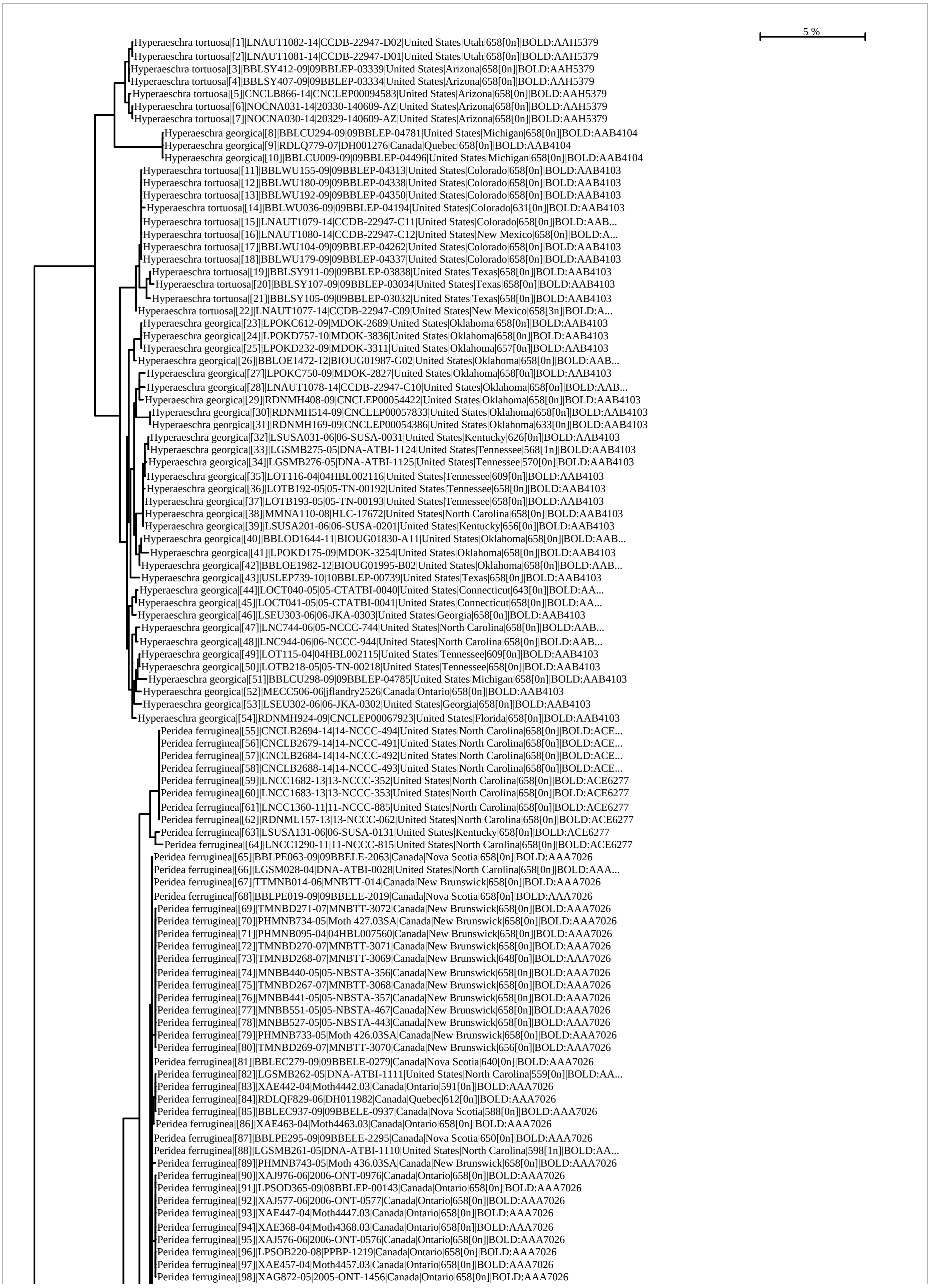
5 %
Hyperaeschra tortuosa|[1]|LNAUT1082-14|CCDB-22947-D02|United States|Utah|658[0n]|BOLD:AAH5379
Hyperaeschra tortuosa|[2]|LNAUT1081-14|CCDB-22947-D01|United States|Utah|658[0n]|BOLD:AAH5379
Hyperaeschra tortuosa|[3]|BBLSY412-09|09BBLEP-03339|United States|Arizona|658[0n]|BOLD:AAH5379
Hyperaeschra tortuosa|[4]|BBLSY407-09|09BBLEP-03334|United States|Arizona|658[0n]|BOLD:AAH5379
Hyperaeschra tortuosa|[5]|CNCLB866-14|CNCLEP00094583|United States|Arizona|658[0n]|BOLD:AAH5379
Hyperaeschra tortuosa|[6]|NOCNA031-14|20330-140609-AZ|United States|Arizona|658[0n]|BOLD:AAH5379
Hyperaeschra tortuosa|[7]|NOCNA030-14|20329-140609-AZ|United States|Arizona|658[0n]|BOLD:AAH5379
Hyperaeschra georgica|[8]|BBLCU294-09|09BBLEP-04781|United States|Michigan|658[0n]|BOLD:AAB4104
Hyperaeschra georgica|[9]|RDLQ779-07|DH001276|Canada|Quebec|658[0n]|BOLD:AAB4104
Hyperaeschra georgica|[10]|BBLCU009-09|09BBLEP-04496|United States|Michigan|658[0n]|BOLD:AAB4104
Hyperaeschra tortuosa|[11]|BBLWU155-09|09BBLEP-04313|United States|Colorado|658[0n]|BOLD:AAB4103
Hyperaeschra tortuosa|[12]|BBLWU180-09|09BBLEP-04338|United States|Colorado|658[0n]|BOLD:AAB4103
Hyperaeschra tortuosa|[13]|BBLWU192-09|09BBLEP-04350|United States|Colorado|658[0n]|BOLD:AAB4103
Hyperaeschra tortuosa|[14]|BBLWU036-09|09BBLEP-04194|United States|Colorado|631[0n]|BOLD:AAB4103
Hyperaeschra tortuosa|[15]|LNAUT1079-14|CCDB-22947-C11|United States|Colorado|658[0n]|BOLD:AAB...
Hyperaeschra tortuosa|[16]|LNAUT1080-14|CCDB-22947-C12|United States|New Mexico|658[0n]|BOLD:A...
Hyperaeschra tortuosa|[17]|BBLWU104-09|09BBLEP-04262|United States|Colorado|658[0n]|BOLD:AAB4103
Hyperaeschra tortuosa|[18]|BBLWU179-09|09BBLEP-04337|United States|Colorado|658[0n]|BOLD:AAB4103
Hyperaeschra tortuosa|[19]|BBLSY911-09|09BBLEP-03838|United States|Texas|658[0n]|BOLD:AAB4103
Hyperaeschra tortuosa|[20]|BBLSY107-09|09BBLEP-03034|United States|Texas|658[0n]|BOLD:AAB4103
Hyperaeschra tortuosa|[21]|BBLSY105-09|09BBLEP-03032|United States|Texas|658[0n]|BOLD:AAB4103
Hyperaeschra tortuosa|[22]|LNAUT1077-14|CCDB-22947-C09|United States|New Mexico|658[3n]|BOLD:A...
Hyperaeschra georgica|[23]|LPOKC612-09|MDOK-2689|United States|Oklahoma|658[0n]|BOLD:AAB4103
Hyperaeschra georgica|[24]|LPOKD757-10|MDOK-3836|United States|Oklahoma|658[0n]|BOLD:AAB4103
Hyperaeschra georgica|[25]|LPOKD232-09|MDOK-3311|United States|Oklahoma|657[0n]|BOLD:AAB4103
Hyperaeschra georgica|[26]|BBLOE1472-12|BIOUG01987-G02|United States|Oklahoma|658[0n]|BOLD:AAB...
Hyperaeschra georgica|[27]|LPOKC750-09|MDOK-2827|United States|Oklahoma|658[0n]|BOLD:AAB4103
Hyperaeschra georgica|[28]|LNAUT1078-14|CCDB-22947-C10|United States|Oklahoma|658[0n]|BOLD:AAB...
Hyperaeschra georgica|[29]|RDNMH408-09|CNCLEP00054422|United States|Oklahoma|658[0n]|BOLD:AAB4103
Hyperaeschra georgica|[30]|RDNMH514-09|CNCLEP00057833|United States|Oklahoma|658[0n]|BOLD:AAB4103
Hyperaeschra georgica|[31]|RDNMH169-09|CNCLEP00054386|United States|Oklahoma|633[0n]|BOLD:AAB4103
Hyperaeschra georgica|[32]|LSUSA031-06|06-SUSA-0031|United States|Kentucky|626[0n]|BOLD:AAB4103
Hyperaeschra georgica|[33]|LGSMB275-05|DNA-ATBI-1124|United States|Tennessee|568[1n]|BOLD:AAB4103
Hyperaeschra georgica|[34]|LGSMB276-05|DNA-ATBI-1125|United States|Tennessee|570[0n]|BOLD:AAB4103
Hyperaeschra georgica|[35]|LOT116-04|04HBL002116|United States|Tennessee|609[0n]|BOLD:AAB4103
Hyperaeschra georgica|[36]|LOTB192-05|05-TN-00192|United States|Tennessee|658[0n]|BOLD:AAB4103
Hyperaeschra georgica|[37]|LOTB193-05|05-TN-00193|United States|Tennessee|658[0n]|BOLD:AAB4103
Hyperaeschra georgica|[38]|MMNA110-08|HLC-17672|United States|North Carolina|658[0n]|BOLD:AAB4103
Hyperaeschra georgica|[39]|LSUSA201-06|06-SUSA-0201|United States|Kentucky|656[0n]|BOLD:AAB4103
Hyperaeschra georgica|[40]|BBLOD1644-11|BIOUG01830-A11|United States|Oklahoma|658[0n]|BOLD:AAB...
Hyperaeschra georgica|[41]|LPOKD175-09|MDOK-3254|United States|Oklahoma|658[0n]|BOLD:AAB4103
Hyperaeschra georgica|[42]|BBLOE1982-12|BIOUG01995-B02|United States|Oklahoma|658[0n]|BOLD:AAB...
Hyperaeschra georgica|[43]|USLEP739-10|10BBLEP-00739|United States|Texas|658[0n]|BOLD:AAB4103
Hyperaeschra georgica|[44]|LOCT040-05|05-CTATBI-0040|United States|Connecticut|643[0n]|BOLD:AA...
Hyperaeschra georgica|[45]|LOCT041-05|05-CTATBI-0041|United States|Connecticut|658[0n]|BOLD:AA...
Hyperaeschra georgica|[46]|LSEU303-06|06-JKA-0303|United States|Georgia|658[0n]|BOLD:AAB4103
Hyperaeschra georgica|[47]|LNC744-06|05-NCCC-744|United States|North Carolina|658[0n]|BOLD:AAB...
Hyperaeschra georgica|[48]|LNC944-06|06-NCCC-944|United States|North Carolina|658[0n]|BOLD:AAB...
Hyperaeschra georgica|[49]|LOT115-04|04HBL002115|United States|Tennessee|609[0n]|BOLD:AAB4103
Hyperaeschra georgica|[50]|LOTB218-05|05-TN-00218|United States|Tennessee|658[0n]|BOLD:AAB4103
Hyperaeschra georgica|[51]|BBLCU298-09|09BBLEP-04785|United States|Michigan|658[0n]|BOLD:AAB4103
Hyperaeschra georgica|[52]|MECC506-06|jflandry2526|Canada|Ontario|658[0n]|BOLD:AAB4103
Hyperaeschra georgica|[53]|LSEU302-06|06-JKA-0302|United States|Georgia|658[0n]|BOLD:AAB4103
Hyperaeschra georgica|[54]|RDNMH924-09|CNCLEP00067923|United States|Florida|658[0n]|BOLD:AAB4103
Peridea ferruginea|[55]|CNCLB2694-14|14-NCCC-494|United States|North Carolina|658[0n]|BOLD:ACE...
Peridea ferruginea|[56]|CNCLB2679-14|14-NCCC-491|United States|North Carolina|658[0n]|BOLD:ACE...
Peridea ferruginea|[57]|CNCLB2684-14|14-NCCC-492|United States|North Carolina|658[0n]|BOLD:ACE...
Peridea ferruginea|[58]|CNCLB2688-14|14-NCCC-493|United States|North Carolina|658[0n]|BOLD:ACE...
Peridea ferruginea|[59]|LNCC1682-13|13-NCCC-352|United States|North Carolina|658[0n]|BOLD:ACE6277
Peridea ferruginea|[60]|LNCC1683-13|13-NCCC-353|United States|North Carolina|658[0n]|BOLD:ACE6277
Peridea ferruginea|[61]|LNCC1360-11|11-NCCC-885|United States|North Carolina|658[0n]|BOLD:ACE6277
Peridea ferruginea|[62]|RDNML157-13|13-NCCC-062|United States|North Carolina|658[0n]|BOLD:ACE6277
Peridea ferruginea|[63]|LSUSA131-06|06-SUSA-0131|United States|Kentucky|658[0n]|BOLD:ACE6277
Peridea ferruginea|[64]|LNCC1290-11|11-NCCC-815|United States|North Carolina|658[0n]|BOLD:ACE6277
Peridea ferruginea|[65]|BBLPE063-09|09BBELE-2063|Canada|Nova Scotia|658[0n]|BOLD:AAA7026
Peridea ferruginea|[66]|LGSM028-04|DNA-ATBI-0028|United States|North Carolina|658[0n]|BOLD:AAA...
Peridea ferruginea|[67]|TTMNB014-06|MNBTT-014|Canada|New Brunswick|658[0n]|BOLD:AAA7026
Peridea ferruginea|[68]|BBLPE019-09|09BBELE-2019|Canada|Nova Scotia|658[0n]|BOLD:AAA7026
Peridea ferruginea|[69]|TMNBD271-07|MNBTT-3072|Canada|New Brunswick|658[0n]|BOLD:AAA7026
Peridea ferruginea|[70]|PHMNB734-05|Moth 427.03SA|Canada|New Brunswick|658[0n]|BOLD:AAA7026
Peridea ferruginea|[71]|PHMNB095-04|04HBL007560|Canada|New Brunswick|658[0n]|BOLD:AAA7026
Peridea ferruginea|[72]|TMNBD270-07|MNBTT-3071|Canada|New Brunswick|658[0n]|BOLD:AAA7026
Peridea ferruginea|[73]|TMNBD268-07|MNBTT-3069|Canada|New Brunswick|648[0n]|BOLD:AAA7026
Peridea ferruginea|[74]|MNBB440-05|05-NBSTA-356|Canada|New Brunswick|658[0n]|BOLD:AAA7026
Peridea ferruginea|[75]|TMNBD267-07|MNBTT-3068|Canada|New Brunswick|658[0n]|BOLD:AAA7026
Peridea ferruginea|[76]|MNBB441-05|05-NBSTA-357|Canada|New Brunswick|658[0n]|BOLD:AAA7026
Peridea ferruginea|[77]|MNBB551-05|05-NBSTA-467|Canada|New Brunswick|658[0n]|BOLD:AAA7026
Peridea ferruginea|[78]|MNBB527-05|05-NBSTA-443|Canada|New Brunswick|658[0n]|BOLD:AAA7026
Peridea ferruginea|[79]|PHMNB733-05|Moth 426.03SA|Canada|New Brunswick|658[0n]|BOLD:AAA7026
Peridea ferruginea|[80]|TMNBD269-07|MNBTT-3070|Canada|New Brunswick|656[0n]|BOLD:AAA7026
Peridea ferruginea|[81]|BBLEC279-09|09BBELE-0279|Canada|Nova Scotia|640[0n]|BOLD:AAA7026
Peridea ferruginea|[82]|LGSMB262-05|DNA-ATBI-1111|United States|North Carolina|559[0n]|BOLD:AA...
Peridea ferruginea|[83]|XAE442-04|Moth4442.03|Canada|Ontario|591[0n]|BOLD:AAA7026
Peridea ferruginea|[84]|RDLQF829-06|DH011982|Canada|Quebec|612[0n]|BOLD:AAA7026
Peridea ferruginea|[85]|BBLEC937-09|09BBELE-0937|Canada|Nova Scotia|588[0n]|BOLD:AAA7026
Peridea ferruginea|[86]|XAE463-04|Moth4463.03|Canada|Ontario|658[0n]|BOLD:AAA7026
Peridea ferruginea|[87]|BBLPE295-09|09BBELE-2295|Canada|Nova Scotia|650[0n]|BOLD:AAA7026
Peridea ferruginea|[88]|LGSMB261-05|DNA-ATBI-1110|United States|North Carolina|598[1n]|BOLD:AA...
Peridea ferruginea|[89]|PHMNB743-05|Moth 436.03SA|Canada|New Brunswick|658[0n]|BOLD:AAA7026
Peridea ferruginea|[90]|XAJ976-06|2006-ONT-0976|Canada|Ontario|658[0n]|BOLD:AAA7026
Peridea ferruginea|[91]|LPSOD365-09|08BBLEP-00143|Canada|Ontario|658[0n]|BOLD:AAA7026
Peridea ferruginea|[92]|XAJ577-06|2006-ONT-0577|Canada|Ontario|658[0n]|BOLD:AAA7026
Peridea ferruginea|[93]|XAE447-04|Moth4447.03|Canada|Ontario|658[0n]|BOLD:AAA7026
Peridea ferruginea|[94]|XAE368-04|Moth4368.03|Canada|Ontario|658[0n]|BOLD:AAA7026
Peridea ferruginea|[95]|XAJ576-06|2006-ONT-0576|Canada|Ontario|658[0n]|BOLD:AAA7026
Peridea ferruginea|[96]|LPSOB220-08|PPBP-1219|Canada|Ontario|658[0n]|BOLD:AAA7026
Peridea ferruginea|[97]|XAE457-04|Moth4457.03|Canada|Ontario|658[0n]|BOLD:AAA7026
Peridea ferruginea|[98]|XAG872-05|2005-ONT-1456|Canada|Ontario|658[0n]|BOLD:AAA7026

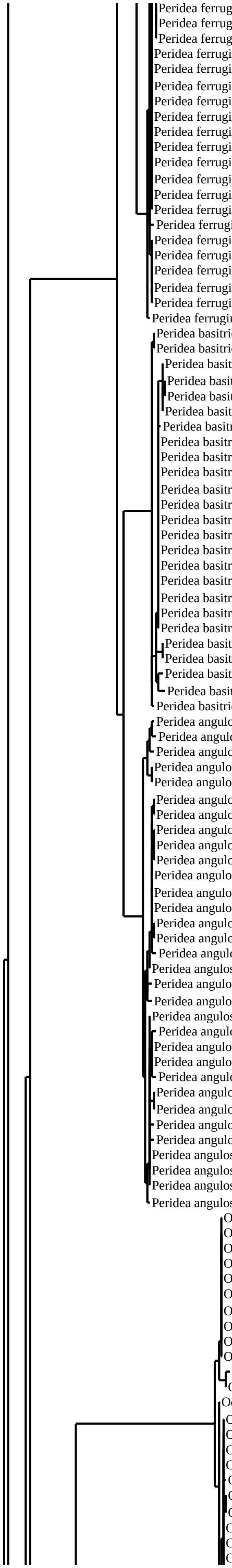

Peridea ferruginea|[96]|LPSOB220-08|PPBP-1219|Canada|Ontario|658[0n]|BOLD:AAA7026
Peridea ferruginea|[97]|XAE457-04|Moth4457.03|Canada|Ontario|658[0n]|BOLD:AAA7026
Peridea ferruginea|[98]|XAG872-05|2005-ONT-1456|Canada|Ontario|658[0n]|BOLD:AAA7026
Peridea ferruginea|[99]|TTMNB017-06|MNBTT-017|Canada|New Brunswick|658[0n]|BOLD:AAA7026
Peridea ferruginea|[100]|TTMNB018-06|MNBTT-018|Canada|New Brunswick|658[0n]|BOLD:AAA7026
Peridea ferruginea|[101]|BBLPE297-09|09BBELE-2297|Canada|Nova Scotia|658[0n]|BOLD:AAA7026
Peridea ferruginea|[102]|BBLEC098-09|09BBELE-0098|Canada|Nova Scotia|658[0n]|BOLD:AAA7026
Peridea ferruginea|[103]|TTMNB244-06|MNBTT-244|Canada|New Brunswick|658[0n]|BOLD:AAA7026
Peridea ferruginea|[104]|MMNA109-08|HLC-17671|United States|North Carolina|658[0n]|BOLD:AAA7026
Peridea ferruginea|[105]|MNBB286-05|05-NBSTA-202|Canada|New Brunswick|658[0n]|BOLD:AAA7026
Peridea ferruginea|[106]|BBLPE011-09|09BBELE-2011|Canada|Nova Scotia|658[0n]|BOLD:AAA7026
Peridea ferruginea|[107]|MNBB238-05|05-NBSTA-154|Canada|New Brunswick|658[0n]|BOLD:AAA7026
Peridea ferruginea|[108]|MNBB237-05|05-NBSTA-153|Canada|New Brunswick|658[0n]|BOLD:AAA7026
Peridea ferruginea|[109]|MNBB284-05|05-NBSTA-200|Canada|New Brunswick|658[0n]|BOLD:AAA7026
Peridea ferruginea|[110]|BBLEC900-09|09BBELE-0900|Canada|Nova Scotia|658[0n]|BOLD:AAA7026
Peridea ferruginea|[111]|BBLPE049-09|09BBELE-2049|Canada|Nova Scotia|658[0n]|BOLD:AAA7026
Peridea ferruginea|[112]|BBLPE012-09|09BBELE-2012|Canada|Nova Scotia|658[0n]|BOLD:AAA7026
Peridea ferruginea|[113]|BBLPE180-09|09BBELE-2180|Canada|Nova Scotia|658[0n]|BOLD:AAA7026
Peridea ferruginea|[114]|BBLEC562-09|09BBELE-0562|Canada|Nova Scotia|639[0n]|BOLD:AAA7026
Peridea ferruginea|[115]|BBLPC993-09|09BBELE-1993|Canada|Nova Scotia|636[0n]|
Peridea ferruginea|[116]|BBLPE152-09|09BBELE-2152|Canada|Nova Scotia|636[0n]|BOLD:AAA7026
Peridea basitriens|[117]|LNCC1359-11|11-NCCC-884|United States|North Carolina|658[0n]|BOLD:ABZ...
Peridea basitriens|[118]|XAE617-04|Moth4617.03|Canada|Ontario|616[0n]|BOLD:ABZ5648
Peridea basitriens|[119]|TMNBD206-07|MNBTT-3007|Canada|New Brunswick|595[0n]|BOLD:ABZ5648
Peridea basitriens|[120]|XAJ493-06|2006-ONT-0493|Canada|Ontario|647[0n]|BOLD:ABZ5648
Peridea basitriens|[121]|LGSMB257-05|DNA-ATBI-1106|United States|Tennessee|561[0n]|BOLD:ABZ5648
Peridea basitriens|[122]|PMG186-03|moth1110.01|Canada|Ontario|617[0n]|BOLD:ABZ5648
Peridea basitriens|[123]|TMG60-03|PERI1.00|Canada|Ontario|639[0n]|BOLD:ABZ5648
Peridea basitriens|[124]|TMNBD205-07|MNBTT-3006|Canada|New Brunswick|641[0n]|BOLD:ABZ5648
Peridea basitriens|[125]|LGSM030-04|DNA-ATBI-0030|United States|North Carolina|656[0n]|BOLD:AB...
Peridea basitriens|[126]|LPSOC319-08|PPBP-2318|Canada|Ontario|658[0n]|BOLD:ABZ5648
Peridea basitriens|[127]|LGSM029-04|DNA-ATBI-0029|United States|North Carolina|658[0n]|BOLD:AB...
Peridea basitriens|[128]|LGSMC745-05|DNA-ATBI-2745|United States|Tennessee|658[0n]|BOLD:ABZ5648
Peridea basitriens|[129]|XAK525-07|HLC-16078|Canada|Ontario|658[0n]|BOLD:ABZ5648
Peridea basitriens|[130]|RDLQG442-06|DH012721|Canada|Quebec|657[0n]|BOLD:ABZ5648
Peridea basitriens|[131]|MMNA107-08|HLC-17669|United States|North Carolina|658[0n]|BOLD:ABZ5648
Peridea basitriens|[132]|LGSMC747-05|DNA-ATBI-2747|United States|Tennessee|658[0n]|BOLD:ABZ5648
Peridea basitriens|[133]|LGSMG735-07|BGS03398|United States|North Carolina|658[0n]|BOLD:ABZ5648
Peridea basitriens|[134]|LGSMC744-05|DNA-ATBI-2744|United States|Tennessee|658[0n]|BOLD:ABZ5648
Peridea basitriens|[135]|TMNBD204-07|MNBTT-3005|Canada|New Brunswick|658[0n]|BOLD:ABZ5648
Peridea basitriens|[136]|XAG102-05|2005-ONT-686|Canada|Ontario|658[0n]|BOLD:ABZ5648
Peridea basitriens|[137]|BBLEC612-09|09BBELE-0612|Canada|Nova Scotia|658[0n]|BOLD:ABZ5648
Peridea basitriens|[138]|XAE415-04|Moth4415.03|Canada|Ontario|658[0n]|BOLD:ABZ5648
Peridea basitriens|[139]|LSUSA193-06|06-SUSA-0193|United States|Kentucky|658[0n]|BOLD:ABZ5648
Peridea basitriens|[140]|LSEU606-06|06-JKA-0606|United States|Georgia|658[0n]|BOLD:ABZ5648
Peridea basitriens|[141]|LGSMC746-05|DNA-ATBI-2746|United States|Tennessee|658[0n]|BOLD:ABZ5648
Peridea angulosa|[142]|XAK524-07|HLC-16077|Canada|Ontario|658[0n]|BOLD:AAB2020
Peridea angulosa|[143]|RDLQB678-05|DH010781|Canada|Quebec|544[1n]|BOLD:AAB2020
Peridea angulosa|[144]|LOCT033-05|05-CTATBI-0033|United States|Connecticut|658[0n]|BOLD:AAB2020
Peridea angulosa|[145]|MNBB552-05|05-NBSTA-468|Canada|New Brunswick|658[0n]|BOLD:AAB2020
Peridea angulosa|[146]|MNBB379-05|05-NBSTA-295|Canada|New Brunswick|658[0n]|BOLD:AAB2020
Peridea angulosa|[147]|LSUSA208-06|06-SUSA-0208|United States|Kentucky|658[0n]|BOLD:AAB2020
Peridea angulosa|[148]|LGSMG736-07|BGS03399|United States|North Carolina|658[0n]|BOLD:AAB2020
Peridea angulosa|[149]|LGSMG738-07|BGS03401|United States|North Carolina|637[0n]|BOLD:AAB2020
Peridea angulosa|[150]|LGSMB258-05|DNA-ATBI-1107|United States|Tennessee|575[0n]|BOLD:AAB2020
Peridea angulosa|[151]|LGSMB260-05|DNA-ATBI-1109|United States|Tennessee|597[0n]|BOLD:AAB2020
Peridea angulosa|[152]|LSUSA260-06|06-SUSA-0260|United States|Kentucky|658[0n]|BOLD:AAB2020
Peridea angulosa|[153]|JRLAA013-09|JRLAA-013|United States|Alabama|658[0n]|BOLD:AAB2020
Peridea angulosa|[154]|BBLSW686-09|09BBLEP-01614|United States|Oklahoma|658[0n]|BOLD:AAB2020
Peridea angulosa|[155]|LOT067-04|04HBL002067|United States|Tennessee|609[0n]|BOLD:AAB2020
Peridea angulosa|[156]|LGSMC742-05|DNA-ATBI-2742|United States|Tennessee|620[0n]|BOLD:AAB2020
Peridea angulosa|[157]|LOFLA531-06|06-FLOR-0531|United States|Florida|658[0n]|BOLD:AAB2020
Peridea angulosa|[158]|USLEP740-10|10BBLEP-00740|United States|Texas|658[0n]|BOLD:AAB2020
Peridea angulosa|[159]|LGSMB259-05|DNA-ATBI-1108|United States|Tennessee|588[0n]|BOLD:AAB2020
Peridea angulosa|[160]|LOT068-04|04HBL002068|United States|Tennessee|609[0n]|BOLD:AAB2020
Peridea angulosa|[161]|LPSO934-08|PPBP-0934|Canada|Ontario|658[0n]|BOLD:AAB2020
Peridea angulosa|[162]|LNC423-05|05-NCCC-423|United States|North Carolina|658[0n]|BOLD:AAB2020
Peridea angulosa|[163]|LOT069-04|04HBL002069|United States|Tennessee|609[0n]|BOLD:AAB2020
Peridea angulosa|[164]|LOT070-04|04HBL002070|United States|Tennessee|609[0n]|BOLD:AAB2020
Peridea angulosa|[165]|LOT066-04|04HBL002066|United States|Tennessee|609[0n]|BOLD:AAB2020
Peridea angulosa|[166]|LOT122-04|04HBL002122|United States|Tennessee|609[0n]|BOLD:AAB2020
Peridea angulosa|[167]|LNCC1684-13|13-NCCC-354|United States|North Carolina|658[0n]|BOLD:AAB2020
Peridea angulosa|[168]|LOT071-04|04HBL002071|United States|Tennessee|609[0n]|BOLD:AAB2020
Peridea angulosa|[169]|LOCT032-05|05-CTATBI-0032|United States|Connecticut|658[0n]|BOLD:AAB2020
Peridea angulosa|[170]|LGSMG737-07|BGS03400|United States|North Carolina|658[0n]|BOLD:AAB2020
Peridea angulosa|[171]|XAJ975-06|2006-ONT-0975|Canada|Ontario|658[0n]|BOLD:AAB2020
Peridea angulosa|[172]|LPSOB096-08|PPBP-1095|Canada|Ontario|658[0n]|BOLD:AAB2020
Peridea angulosa|[173]|LNCB106-06|06-NCC-1062|United States|North Carolina|656[0n]|BOLD:AAB2020
Odontosia elegans|[174]|TMNBD279-07|MNBTT-3080|Canada|New Brunswick|639[0n]|BOLD:AAB3986
Odontosia elegans|[175]|TMNBD281-07|MNBTT-3082|Canada|New Brunswick|617[0n]|BOLD:AAB3986
Odontosia elegans|[176]|MNBB180-05|05-NBSTA-096|Canada|New Brunswick|550[1n]|BOLD:AAB3986
Odontosia elegans|[177]|PHMNB014-03|moth137.02SA|Canada|New Brunswick|639[0n]|BOLD:AAB3986
Odontosia elegans|[178]|LPSOB301-08|PPBP-1300|Canada|Ontario|658[0n]|BOLD:AAB3986
Odontosia elegans|[179]|RDLQF807-06|DH011957|Canada|Quebec|658[0n]|BOLD:AAB3986
Odontosia elegans|[180]|TMNBD277-07|MNBTT-3078|Canada|New Brunswick|656[0n]|BOLD:AAB3986
Odontosia elegans|[181]|LPSOB431-08|PPBP-1430|Canada|Ontario|658[0n]|BOLD:AAB3986
Odontosia elegans|[182]|LPSOB292-08|PPBP-1291|Canada|Ontario|658[0n]|BOLD:AAB3986
Odontosia elegans|[183]|TMNBD280-07|MNBTT-3081|Canada|New Brunswick|644[0n]|BOLD:AAB3986
Odontosia elegans|[184]|PMG185-03|moth749.01|Canada|Ontario|617[0n]|BOLD:AAB3986
Odontosia elegans|[185]|TMG62-03|moth670.01|Canada|Ontario|639[0n]|BOLD:AAB3986
Odontosia elegans|[186]|LOWCB124-05|CGWC-1064|Canada|British Columbia|658[0n]|BOLD:AAB3986
Odontosia elegans|[187]|AWCL046-09|JLB-0244|United States|Idaho|615[0n]|BOLD:AAB3986
Odontosia elegans|[188]|LOWCB126-05|CGWC-1066|Canada|British Columbia|658[0n]|BOLD:AAB3986
Odontosia elegans|[189]|LPMN558-08|08BBLEP-01359|Canada|Manitoba|658[0n]|BOLD:AAB3986
Odontosia elegans|[190]|LBCD263-05|HLC-23083|Canada|British Columbia|658[0n]|BOLD:AAB3986
Odontosia elegans|[191]|LBCB877-05|HLC-21817|Canada|British Columbia|658[0n]|BOLD:AAB3986
Odontosia elegans|[192]|LOWCB127-05|CGWC-1067|Canada|British Columbia|658[0n]|BOLD:AAB3986
Odontosia elegans|[193]|LPMN289-08|08BBLEP-01088|Canada|Manitoba|643[0n]|BOLD:AAB3986
Odontosia elegans|[194]|AWCL047-09|JLB-0245|United States|Idaho|658[0n]|BOLD:AAB3986
Odontosia elegans|[195]|LOWCB128-05|CGWC-1068|Canada|British Columbia|658[0n]|BOLD:AAB3986
Odontosia elegans|[196]|LBCD264-05|HLC-23084|Canada|British Columbia|658[0n]|BOLD:AAB3986

Odontosia elegans|[194]|AWCL047-09|JLB-0245|United States|Idaho|658[0n]|BOLD:AAB3986
Odontosia elegans|[195]|LOWCB128-05|CGWC-1068|Canada|British Columbia|658[0n]|BOLD:AAB3986
Odontosia elegans|[196]|LBCD264-05|HLC-23084|Canada|British Columbia|658[0n]|BOLD:AAB3986
Odontosia elegans|[197]|LOWCB125-05|CGWC-1065|Canada|British Columbia|658[0n]|BOLD:AAB3986
Odontosia elegans|[198]|RDNMH460-09|CNCLEP00054474|United States|Wyoming|658[0n]|BOLD:AAB3986
Odontosia elegans|[199]|XAE513-04|Moth4513.03|Canada|Ontario|658[2n]|BOLD:AAB3986
Pheosia rimosa|[200]|LOCT026-05|05-CTATBI-0026|United States|Connecticut|658[0n]|BOLD:AAA3484
Pheosia rimosa|[201]|LPSOD639-09|08BBLEP-00420|Canada|Ontario|658[0n]|BOLD:AAA3484
Pheosia rimosa|[202]|MHCOL481-09|CHU-APH-007.1|Canada|Manitoba|658[0n]|BOLD:AAA3482
Pheosia rimosa|[203]|LCH207-04|04HBL003207|Canada|Manitoba|658[0n]|BOLD:AAA3482
Pheosia rimosa|[204]|MHCOL478-09|CHU-APH-004.1|Canada|Manitoba|638[0n]|BOLD:AAA3482
Pheosia rimosa|[205]|DSSAW066-06|CHU-APH-005|Canada|Manitoba|650[0n]|
Pheosia rimosa|[206]|DSSAW068-06|CHU-APH-007|Canada|Manitoba|650[0n]|
Pheosia rimosa|[207]|DSSAW065-06|CHU-APH-004|Canada|Manitoba|650[0n]|
Pheosia rimosa|[208]|DSSAW067-06|CHU-APH-006|Canada|Manitoba|650[0n]|
Pheosia rimosa|[209]|MHCOL479-09|CHU-APH-005.1|Canada|Manitoba|658[0n]|BOLD:AAA3482
Pheosia rimosa|[210]|LCH212-04|04HBL003212|Canada|Manitoba|658[0n]|BOLD:AAA3482
Pheosia rimosa|[211]|LCH214-04|04HBL003214|Canada|Manitoba|658[0n]|BOLD:AAA3482
Pheosia rimosa|[212]|MHCOL480-09|CHU-APH-006.1|Canada|Manitoba|658[0n]|BOLD:AAA3482
Pheosia rimosa|[213]|PHLCH736-10|10PROBE-26546|Canada|Manitoba|658[0n]|BOLD:AAA3482
Pheosia rimosa|[214]|LCHQ114-07|07PROBE-10883|Canada|Manitoba|658[0n]|BOLD:AAA3482
Pheosia rimosa|[215]|DSSAW063-06|CHU-APH-002|Canada|Manitoba|655[0n]|
Pheosia rimosa|[216]|CHLEP293-09|09PROBE-09588|Canada|Manitoba|658[0n]|BOLD:AAA3482
Pheosia rimosa|[217]|MHCOL476-09|CHU-APH-002.1|Canada|Manitoba|638[0n]|BOLD:AAA3482
Pheosia rimosa|[218]|LCH208-04|04HBL003208|Canada|Manitoba|658[0n]|BOLD:AAA3482
Pheosia rimosa|[219]|UAMIC1197-13|UAM:Ento:228334|United States|Alaska|658[0n]|BOLD:AAA3482
Pheosia rimosa|[220]|LPMN243-08|08BBLEP-01042|Canada|Manitoba|658[0n]|BOLD:AAA3482
Pheosia rimosa|[221]|MNBB581-05|05-NBSTA-497|Canada|New Brunswick|658[0n]|BOLD:AAA3482
Pheosia rimosa|[222]|BBLCU300-09|09BBLEP-04787|United States|Michigan|658[0n]|BOLD:AAA3482
Pheosia rimosa|[223]|QUNO092-07|2092-100807-WI|United States|Wisconsin|658[0n]|BOLD:AAA3482
Pheosia rimosa|[224]|LPSO345-08|PPBP-0345|Canada|Ontario|658[0n]|BOLD:AAA3482
Pheosia rimosa|[225]|MNBB584-05|05-NBSTA-500|Canada|New Brunswick|658[0n]|BOLD:AAA3482
Pheosia rimosa|[226]|MNBB432-05|05-NBSTA-348|Canada|New Brunswick|658[0n]|BOLD:AAA3482
Pheosia rimosa|[227]|XAD686-05|2005-ONT-101|Canada|Ontario|622[1n]|BOLD:AAA3482
Pheosia rimosa|[228]|PMG187-03|moth979.01|Canada|Ontario|617[0n]|BOLD:AAA3482
Pheosia rimosa|[229]|XAD687-05|2005-ONT-102|Canada|Ontario|634[0n]|BOLD:AAA3482
Pheosia rimosa|[230]|XAE629-04|Moth4629.03|Canada|Ontario|612[0n]|BOLD:AAA3482
Pheosia rimosa|[231]|PHMNB183-04|04HBL007648|Canada|New Brunswick|541[0n]|BOLD:AAA3482
Pheosia rimosa|[232]|MNBB548-05|05-NBSTA-464|Canada|New Brunswick|632[1n]|BOLD:AAA3482
Pheosia rimosa|[233]|MNBB549-05|05-NBSTA-465|Canada|New Brunswick|658[0n]|BOLD:AAA3482
Pheosia rimosa|[234]|XAB015-04|04HBL005015|Canada|Ontario|658[0n]|BOLD:AAA3482
Pheosia rimosa|[235]|TMNBD274-07|MNBTT-3075|Canada|New Brunswick|658[0n]|BOLD:AAA3482
Pheosia rimosa|[236]|MNBB656-05|05-NBSTA-572|Canada|New Brunswick|658[0n]|BOLD:AAA3482
Pheosia rimosa|[237]|IMA013-07|HLC-16980|United States|Massachusetts|658[0n]|BOLD:AAA3482
Pheosia rimosa|[238]|MNBB586-05|05-NBSTA-502|Canada|New Brunswick|658[0n]|BOLD:AAA3482
Pheosia rimosa|[239]|MNBB289-05|05-NBSTA-205|Canada|New Brunswick|658[0n]|BOLD:AAA3482
Pheosia rimosa|[240]|TMNBD276-07|MNBTT-3077|Canada|New Brunswick|655[0n]|BOLD:AAA3482
Pheosia rimosa|[241]|LOCT086-05|05-CTATBI-0086|United States|Connecticut|658[0n]|BOLD:AAA3482
Pheosia rimosa|[242]|XAB017-04|04HBL005017|Canada|Ontario|658[0n]|BOLD:AAA3482
Pheosia rimosa|[243]|MNBB479-05|05-NBSTA-395|Canada|New Brunswick|658[0n]|BOLD:AAA3482
Pheosia rimosa|[244]|BBLPC028-09|09BBELE-1028|Canada|New Brunswick|658[0n]|BOLD:AAA3482
Pheosia rimosa|[245]|PHAUG1786-11|BIOUG01497-D08|Canada|Ontario|658[0n]|BOLD:AAA3482
Pheosia rimosa|[246]|MNBB318-05|05-NBSTA-234|Canada|New Brunswick|658[0n]|BOLD:AAA3482
Pheosia rimosa|[247]|TMNBD273-07|MNBTT-3074|Canada|New Brunswick|657[0n]|BOLD:AAA3482
Pheosia rimosa|[248]|TTMNB245-06|MNBTT-245|Canada|New Brunswick|658[0n]|BOLD:AAA3482
Pheosia rimosa|[249]|MNBB580-05|05-NBSTA-496|Canada|New Brunswick|658[0n]|BOLD:AAA3482
Pheosia rimosa|[250]|MNBB519-05|05-NBSTA-435|Canada|New Brunswick|658[0n]|BOLD:AAA3482
Pheosia rimosa|[251]|MNBB653-05|05-NBSTA-569|Canada|New Brunswick|658[0n]|BOLD:AAA3482
Pheosia rimosa|[252]|BBLEC041-09|09BBELE-0041|Canada|New Brunswick|658[0n]|BOLD:AAA3482
Pheosia rimosa|[253]|XAB273-04|04HBL005273|Canada|Ontario|658[0n]|BOLD:AAA3482
Pheosia rimosa|[254]|MNBB229-05|05-NBSTA-145|Canada|New Brunswick|658[0n]|BOLD:AAA3482
Pheosia rimosa|[255]|MNBB659-05|05-NBSTA-575|Canada|New Brunswick|658[0n]|BOLD:AAA3482
Pheosia rimosa|[256]|MNBB583-05|05-NBSTA-499|Canada|New Brunswick|658[0n]|BOLD:AAA3482
Pheosia rimosa|[257]|PHLCH825-11|10PROBE-26573|Canada|Manitoba|658[0n]|BOLD:AAA3482
Pheosia rimosa|[258]|LCH211-04|04HBL003211|Canada|Manitoba|658[0n]|BOLD:AAA3482
Pheosia rimosa|[259]|LCH209-04|04HBL003209|Canada|Manitoba|658[0n]|BOLD:AAA3482
Pheosia rimosa|[260]|LCH580-04|04HBL003580|Canada|Manitoba|658[0n]|BOLD:AAA3482
Pheosia rimosa|[261]|LCH213-04|04HBL003213|Canada|Manitoba|658[0n]|BOLD:AAA3482
Pheosia rimosa|[262]|CHLEP237-09|09PROBE-09532|Canada|Manitoba|658[0n]|BOLD:AAA3482
Pheosia rimosa|[263]|MHCOL475-09|CHU-APH-001.1|Canada|Manitoba|632[0n]|BOLD:AAA3482
Pheosia rimosa|[264]|DSSAW064-06|CHU-APH-003|Canada|Manitoba|650[0n]|
Pheosia rimosa|[265]|DSSAW062-06|CHU-APH-001|Canada|Manitoba|650[0n]|
Pheosia rimosa|[266]|LCH206-04|04HBL003206|Canada|Manitoba|658[0n]|BOLD:AAA3482
Pheosia rimosa|[267]|MHCOL477-09|CHU-APH-003.1|Canada|Manitoba|658[0n]|BOLD:AAA3482
Pheosia rimosa|[268]|LCH210-04|04HBL003210|Canada|Manitoba|658[0n]|BOLD:AAA3482
Pheosia rimosa|[269]|JGLL033-10|10PROBE-18809|Canada|Manitoba|658[0n]|BOLD:AAA3482
Pheosia rimosa|[270]|MNBB520-05|05-NBSTA-436|Canada|New Brunswick|658[0n]|BOLD:ABZ4474
Pheosia rimosa|[271]|MNBB319-05|05-NBSTA-235|Canada|New Brunswick|658[0n]|BOLD:ABZ4474
Pheosia rimosa|[272]|MNBB657-05|05-NBSTA-573|Canada|New Brunswick|658[0n]|BOLD:ABZ4474
Pheosia rimosa|[273]|MNBB582-05|05-NBSTA-498|Canada|New Brunswick|658[0n]|BOLD:ABZ4474
Pheosia rimosa|[274]|MNBB372-05|05-NBSTA-288|Canada|New Brunswick|658[0n]|BOLD:ABZ4474
Pheosia rimosa|[275]|MNBB161-05|05-NBSTA-077|Canada|New Brunswick|658[0n]|BOLD:ABZ4474
Pheosia rimosa|[276]|MNBB370-05|05-NBSTA-286|Canada|New Brunswick|658[0n]|BOLD:ABZ4474
Pheosia rimosa|[277]|MNBB232-05|05-NBSTA-148|Canada|New Brunswick|607[0n]|BOLD:ABZ4474
Pheosia rimosa|[278]|MNBB658-05|05-NBSTA-574|Canada|New Brunswick|658[0n]|BOLD:ABZ4474
Pheosia rimosa|[279]|MNBB654-05|05-NBSTA-570|Canada|New Brunswick|658[0n]|BOLD:ABZ4474
Pheosia rimosa|[280]|TMNBD275-07|MNBTT-3076|Canada|New Brunswick|658[0n]|BOLD:ABZ4474
Pheosia rimosa|[281]|MNBB545-05|05-NBSTA-461|Canada|New Brunswick|658[0n]|BOLD:ABZ4474
Pheosia rimosa|[282]|MNBB547-05|05-NBSTA-463|Canada|New Brunswick|658[0n]|BOLD:ABZ4474
Pheosia rimosa|[283]|MNBB655-05|05-NBSTA-571|Canada|New Brunswick|658[0n]|BOLD:ABZ5199
Pheosia rimosa|[284]|XAB039-04|04HBL005039|Canada|Ontario|574[0n]|BOLD:ABZ5199
Pheosia rimosa|[285]|TMNBD272-07|MNBTT-3073|Canada|New Brunswick|631[0n]|BOLD:ABZ5199
Pheosia rimosa|[286]|MNBB373-05|05-NBSTA-289|Canada|New Brunswick|658[0n]|BOLD:ABZ5199
Pheosia rimosa|[287]|MNBB546-05|05-NBSTA-462|Canada|New Brunswick|658[0n]|BOLD:ABZ5199
Pheosia rimosa|[288]|MNBB317-05|05-NBSTA-233|Canada|New Brunswick|658[0n]|BOLD:ABZ5199
Pheosia rimosa|[289]|MNBB585-05|05-NBSTA-501|Canada|New Brunswick|658[0n]|BOLD:ABZ5199
Pheosia rimosa|[290]|MNBB230-05|05-NBSTA-146|Canada|New Brunswick|658[0n]|BOLD:ABZ5199
Pheosia rimosa|[291]|XAD642-05|2005-ONT-57|Canada|Ontario|658[0n]|BOLD:ABZ5199
Pheosia rimosa|[292]|XAB549-04|04HBL005549|Canada|Ontario|658[0n]|BOLD:ABZ5199
Pheosia rimosa|[293]|TMG61-03|moth222.01|Canada|Ontario|639[0n]|BOLD:ABZ5199
Pheosia rimosa|[294]|RDLQ777-07|DH007410|Canada|Quebec|646[0n]|BOLD:ABZ5199

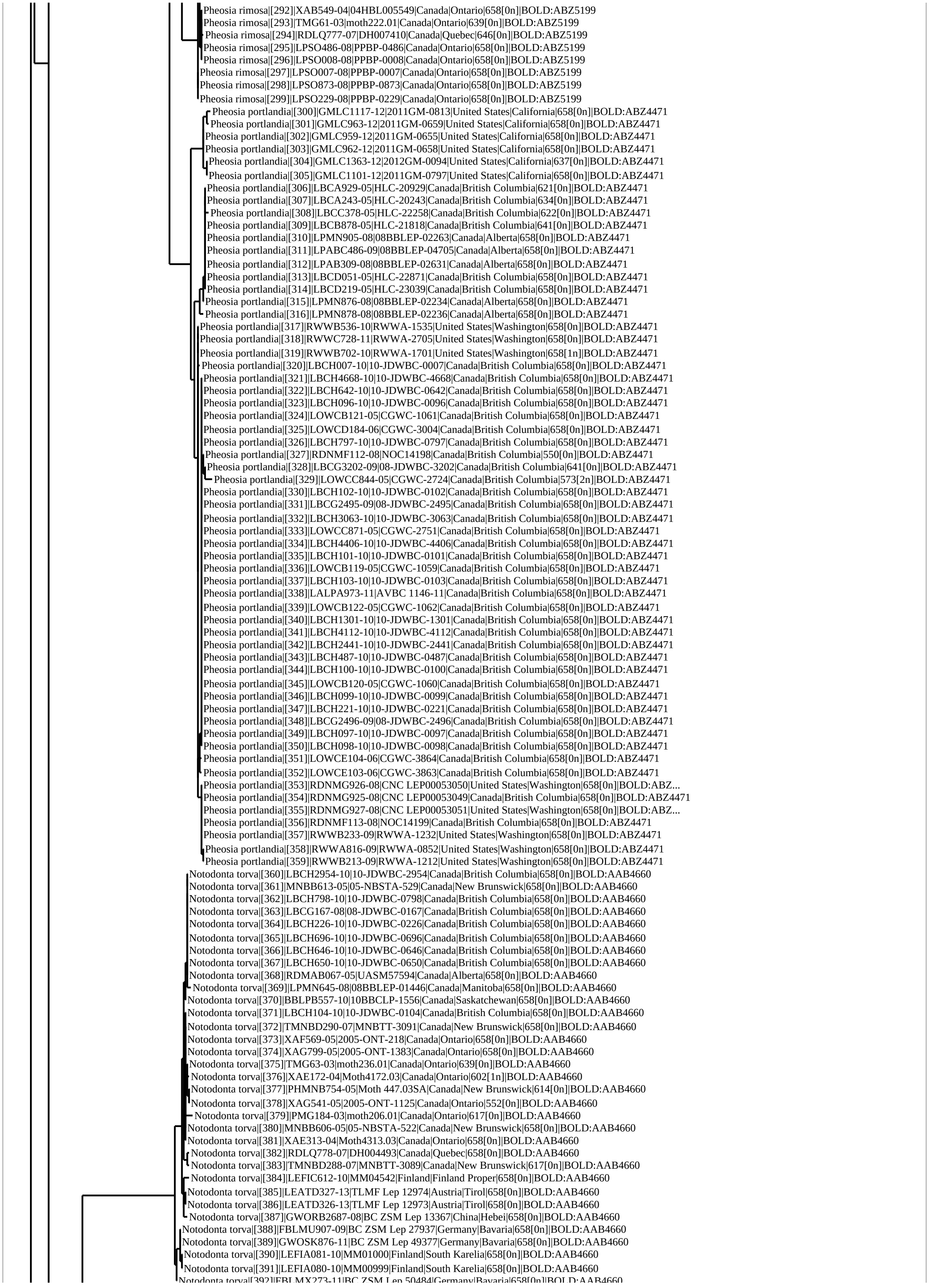
Pheosia rimosa|[292]|XAB549-04|04HBL005549|Canada|Ontario|658[0n]|BOLD:ABZ5199
Pheosia rimosa|[293]|TMG61-03|moth222.01|Canada|Ontario|639[0n]|BOLD:ABZ5199
Pheosia rimosa|[294]|RDLQ777-07|DH007410|Canada|Quebec|646[0n]|BOLD:ABZ5199
Pheosia rimosa|[295]|LPSO486-08|PPBP-0486|Canada|Ontario|658[0n]|BOLD:ABZ5199
Pheosia rimosa|[296]|LPSO008-08|PPBP-0008|Canada|Ontario|658[0n]|BOLD:ABZ5199
Pheosia rimosa|[297]|LPSO007-08|PPBP-0007|Canada|Ontario|658[0n]|BOLD:ABZ5199
Pheosia rimosa|[298]|LPSO873-08|PPBP-0873|Canada|Ontario|658[0n]|BOLD:ABZ5199
Pheosia rimosa|[299]|LPSO229-08|PPBP-0229|Canada|Ontario|658[0n]|BOLD:ABZ5199
Pheosia portlandia|[300]|GMLC1117-12|2011GM-0813|United States|California|658[0n]|BOLD:ABZ4471
Pheosia portlandia|[301]|GMLC963-12|2011GM-0659|United States|California|658[0n]|BOLD:ABZ4471
Pheosia portlandia|[302]|GMLC959-12|2011GM-0655|United States|California|658[0n]|BOLD:ABZ4471
Pheosia portlandia|[303]|GMLC962-12|2011GM-0658|United States|California|658[0n]|BOLD:ABZ4471
Pheosia portlandia|[304]|GMLC1363-12|2012GM-0094|United States|California|637[0n]|BOLD:ABZ4471
Pheosia portlandia|[305]|GMLC1101-12|2011GM-0797|United States|California|658[0n]|BOLD:ABZ4471
Pheosia portlandia|[306]|LBCA929-05|HLC-20929|Canada|British Columbia|621[0n]|BOLD:ABZ4471
Pheosia portlandia|[307]|LBCA243-05|HLC-20243|Canada|British Columbia|634[0n]|BOLD:ABZ4471
Pheosia portlandia|[308]|LBCC378-05|HLC-22258|Canada|British Columbia|622[0n]|BOLD:ABZ4471
Pheosia portlandia|[309]|LBCB878-05|HLC-21818|Canada|British Columbia|641[0n]|BOLD:ABZ4471
Pheosia portlandia|[310]|LPMN905-08|08BBLEP-02263|Canada|Alberta|658[0n]|BOLD:ABZ4471
Pheosia portlandia|[311]|LPABC486-09|08BBLEP-04705|Canada|Alberta|658[0n]|BOLD:ABZ4471
Pheosia portlandia|[312]|LPAB309-08|08BBLEP-02631|Canada|Alberta|658[0n]|BOLD:ABZ4471
Pheosia portlandia|[313]|LBCD051-05|HLC-22871|Canada|British Columbia|658[0n]|BOLD:ABZ4471
Pheosia portlandia|[314]|LBCD219-05|HLC-23039|Canada|British Columbia|658[0n]|BOLD:ABZ4471
Pheosia portlandia|[315]|LPMN876-08|08BBLEP-02234|Canada|Alberta|658[0n]|BOLD:ABZ4471
Pheosia portlandia|[316]|LPMN878-08|08BBLEP-02236|Canada|Alberta|658[0n]|BOLD:ABZ4471
Pheosia portlandia|[317]|RWWB536-10|RWWA-1535|United States|Washington|658[0n]|BOLD:ABZ4471
Pheosia portlandia|[318]|RWWC728-11|RWWA-2705|United States|Washington|658[0n]|BOLD:ABZ4471
Pheosia portlandia|[319]|RWWB702-10|RWWA-1701|United States|Washington|658[1n]|BOLD:ABZ4471
Pheosia portlandia|[320]|LBCH007-10|10-JDWBC-0007|Canada|British Columbia|658[0n]|BOLD:ABZ4471
Pheosia portlandia|[321]|LBCH4668-10|10-JDWBC-4668|Canada|British Columbia|658[0n]|BOLD:ABZ4471
Pheosia portlandia|[322]|LBCH642-10|10-JDWBC-0642|Canada|British Columbia|658[0n]|BOLD:ABZ4471
Pheosia portlandia|[323]|LBCH096-10|10-JDWBC-0096|Canada|British Columbia|658[0n]|BOLD:ABZ4471
Pheosia portlandia|[324]|LOWCB121-05|CGWC-1061|Canada|British Columbia|658[0n]|BOLD:ABZ4471
Pheosia portlandia|[325]|LOWCD184-06|CGWC-3004|Canada|British Columbia|658[0n]|BOLD:ABZ4471
Pheosia portlandia|[326]|LBCH797-10|10-JDWBC-0797|Canada|British Columbia|658[0n]|BOLD:ABZ4471
Pheosia portlandia|[327]|RDNMF112-08|NOC14198|Canada|British Columbia|550[0n]|BOLD:ABZ4471
Pheosia portlandia|[328]|LBCG3202-09|08-JDWBC-3202|Canada|British Columbia|641[0n]|BOLD:ABZ4471
Pheosia portlandia|[329]|LOWCC844-05|CGWC-2724|Canada|British Columbia|573[2n]|BOLD:ABZ4471
Pheosia portlandia|[330]|LBCH102-10|10-JDWBC-0102|Canada|British Columbia|658[0n]|BOLD:ABZ4471
Pheosia portlandia|[331]|LBCG2495-09|08-JDWBC-2495|Canada|British Columbia|658[0n]|BOLD:ABZ4471
Pheosia portlandia|[332]|LBCH3063-10|10-JDWBC-3063|Canada|British Columbia|658[0n]|BOLD:ABZ4471
Pheosia portlandia|[333]|LOWCC871-05|CGWC-2751|Canada|British Columbia|658[0n]|BOLD:ABZ4471
Pheosia portlandia|[334]|LBCH4406-10|10-JDWBC-4406|Canada|British Columbia|658[0n]|BOLD:ABZ4471
Pheosia portlandia|[335]|LBCH101-10|10-JDWBC-0101|Canada|British Columbia|658[0n]|BOLD:ABZ4471
Pheosia portlandia|[336]|LOWCB119-05|CGWC-1059|Canada|British Columbia|658[0n]|BOLD:ABZ4471
Pheosia portlandia|[337]|LBCH103-10|10-JDWBC-0103|Canada|British Columbia|658[0n]|BOLD:ABZ4471
Pheosia portlandia|[338]|LALPA973-11|AVBC 1146-11|Canada|British Columbia|658[0n]|BOLD:ABZ4471
Pheosia portlandia|[339]|LOWCB122-05|CGWC-1062|Canada|British Columbia|658[0n]|BOLD:ABZ4471
Pheosia portlandia|[340]|LBCH1301-10|10-JDWBC-1301|Canada|British Columbia|658[0n]|BOLD:ABZ4471
Pheosia portlandia|[341]|LBCH4112-10|10-JDWBC-4112|Canada|British Columbia|658[0n]|BOLD:ABZ4471
Pheosia portlandia|[342]|LBCH2441-10|10-JDWBC-2441|Canada|British Columbia|658[0n]|BOLD:ABZ4471
Pheosia portlandia|[343]|LBCH487-10|10-JDWBC-0487|Canada|British Columbia|658[0n]|BOLD:ABZ4471
Pheosia portlandia|[344]|LBCH100-10|10-JDWBC-0100|Canada|British Columbia|658[0n]|BOLD:ABZ4471
Pheosia portlandia|[345]|LOWCB120-05|CGWC-1060|Canada|British Columbia|658[0n]|BOLD:ABZ4471
Pheosia portlandia|[346]|LBCH099-10|10-JDWBC-0099|Canada|British Columbia|658[0n]|BOLD:ABZ4471
Pheosia portlandia|[347]|LBCH221-10|10-JDWBC-0221|Canada|British Columbia|658[0n]|BOLD:ABZ4471
Pheosia portlandia|[348]|LBCG2496-09|08-JDWBC-2496|Canada|British Columbia|658[0n]|BOLD:ABZ4471
Pheosia portlandia|[349]|LBCH097-10|10-JDWBC-0097|Canada|British Columbia|658[0n]|BOLD:ABZ4471
Pheosia portlandia|[350]|LBCH098-10|10-JDWBC-0098|Canada|British Columbia|658[0n]|BOLD:ABZ4471
Pheosia portlandia|[351]|LOWCE104-06|CGWC-3864|Canada|British Columbia|658[0n]|BOLD:ABZ4471
Pheosia portlandia|[352]|LOWCE103-06|CGWC-3863|Canada|British Columbia|658[0n]|BOLD:ABZ4471
Pheosia portlandia|[353]|RDNMG926-08|CNC LEP00053050|United States|Washington|658[0n]|BOLD:ABZ...
Pheosia portlandia|[354]|RDNMG925-08|CNC LEP00053049|Canada|British Columbia|658[0n]|BOLD:ABZ4471
Pheosia portlandia|[355]|RDNMG927-08|CNC LEP00053051|United States|Washington|658[0n]|BOLD:ABZ...
Pheosia portlandia|[356]|RDNMF113-08|NOC14199|Canada|British Columbia|658[0n]|BOLD:ABZ4471
Pheosia portlandia|[357]|RWWB233-09|RWWA-1232|United States|Washington|658[0n]|BOLD:ABZ4471
Pheosia portlandia|[358]|RWWA816-09|RWWA-0852|United States|Washington|658[0n]|BOLD:ABZ4471
Pheosia portlandia|[359]|RWWB213-09|RWWA-1212|United States|Washington|658[0n]|BOLD:ABZ4471
Notodonta torva|[360]|LBCH2954-10|10-JDWBC-2954|Canada|British Columbia|658[0n]|BOLD:AAB4660
Notodonta torva|[361]|MNBB613-05|05-NBSTA-529|Canada|New Brunswick|658[0n]|BOLD:AAB4660
Notodonta torva|[362]|LBCH798-10|10-JDWBC-0798|Canada|British Columbia|658[0n]|BOLD:AAB4660
Notodonta torva|[363]|LBCG167-08|08-JDWBC-0167|Canada|British Columbia|658[0n]|BOLD:AAB4660
Notodonta torva|[364]|LBCH226-10|10-JDWBC-0226|Canada|British Columbia|658[0n]|BOLD:AAB4660
Notodonta torva|[365]|LBCH696-10|10-JDWBC-0696|Canada|British Columbia|658[0n]|BOLD:AAB4660
Notodonta torva|[366]|LBCH646-10|10-JDWBC-0646|Canada|British Columbia|658[0n]|BOLD:AAB4660
Notodonta torva|[367]|LBCH650-10|10-JDWBC-0650|Canada|British Columbia|658[0n]|BOLD:AAB4660
Notodonta torva|[368]|RDMAB067-05|UASM57594|Canada|Alberta|658[0n]|BOLD:AAB4660
Notodonta torva|[369]|LPMN645-08|08BBLEP-01446|Canada|Manitoba|658[0n]|BOLD:AAB4660
Notodonta torva|[370]|BBLPB557-10|10BBCLP-1556|Canada|Saskatchewan|658[0n]|BOLD:AAB4660
Notodonta torva|[371]|LBCH104-10|10-JDWBC-0104|Canada|British Columbia|658[0n]|BOLD:AAB4660
Notodonta torva|[372]|TMNBD290-07|MNBTT-3091|Canada|New Brunswick|658[0n]|BOLD:AAB4660
Notodonta torva|[373]|XAF569-05|2005-ONT-218|Canada|Ontario|658[0n]|BOLD:AAB4660
Notodonta torva|[374]|XAG799-05|2005-ONT-1383|Canada|Ontario|658[0n]|BOLD:AAB4660
Notodonta torva|[375]|TMG63-03|moth236.01|Canada|Ontario|639[0n]|BOLD:AAB4660
Notodonta torva|[376]|XAE172-04|Moth4172.03|Canada|Ontario|602[1n]|BOLD:AAB4660
Notodonta torva|[377]|PHMNB754-05|Moth 447.03SA|Canada|New Brunswick|614[0n]|BOLD:AAB4660
Notodonta torva|[378]|XAG541-05|2005-ONT-1125|Canada|Ontario|552[0n]|BOLD:AAB4660
Notodonta torva|[379]|PMG184-03|moth206.01|Canada|Ontario|617[0n]|BOLD:AAB4660
Notodonta torva|[380]|MNBB606-05|05-NBSTA-522|Canada|New Brunswick|658[0n]|BOLD:AAB4660
Notodonta torva|[381]|XAE313-04|Moth4313.03|Canada|Ontario|658[0n]|BOLD:AAB4660
Notodonta torva|[382]|RDLQ778-07|DH004493|Canada|Quebec|658[0n]|BOLD:AAB4660
Notodonta torva|[383]|TMNBD288-07|MNBTT-3089|Canada|New Brunswick|617[0n]|BOLD:AAB4660
Notodonta torva|[384]|LEFIC612-10|MM04542|Finland|Finland Proper|658[0n]|BOLD:AAB4660
Notodonta torva|[385]|LEATD327-13|TLMF Lep 12974|Austria|Tirol|658[0n]|BOLD:AAB4660
Notodonta torva|[386]|LEATD326-13|TLMF Lep 12973|Austria|Tirol|658[0n]|BOLD:AAB4660
Notodonta torva|[387]|GWORB2687-08|BC ZSM Lep 13367|China|Hebei|658[0n]|BOLD:AAB4660
Notodonta torva|[388]|FBLMU907-09|BC ZSM Lep 27937|Germany|Bavaria|658[0n]|BOLD:AAB4660
Notodonta torva|[389]|GWOSK876-11|BC ZSM Lep 49377|Germany|Bavaria|658[0n]|BOLD:AAB4660
Notodonta torva|[390]|LEFIA081-10|MM01000|Finland|South Karelia|658[0n]|BOLD:AAB4660
Notodonta torva|[391]|LEFIA080-10|MM00999|Finland|South Karelia|658[0n]|BOLD:AAB4660
Notodonta torva|[392]|FBLMX273-11|BC ZSM Lep 50484|Germany|Bavaria|658[0n]|BOLD:AAB4660

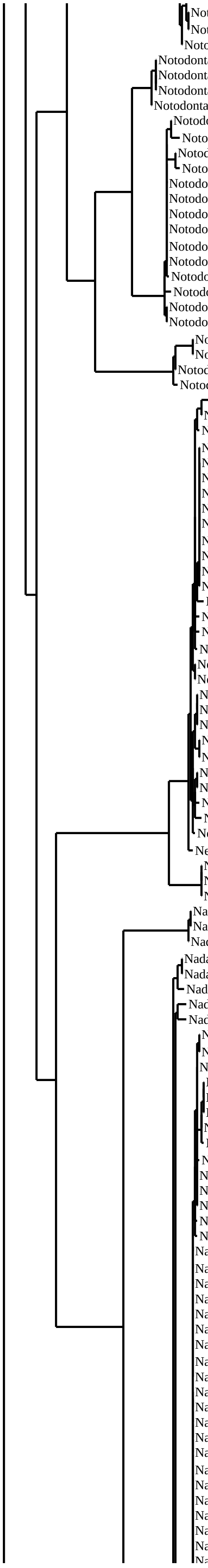

Notodonta torva|[390]|LEFIA081-10|MM01000|Finland|South Karelia|658[0n]|BOLD:AAB4660
Notodonta torva|[391]|LEFIA080-10|MM00999|Finland|South Karelia|658[0n]|BOLD:AAB4660
Notodonta torva|[392]|FBLMX273-11|BC ZSM Lep 50484|Germany|Bavaria|658[0n]|BOLD:AAB4660
Notodonta scitipennis|[393]|BBLPB309-10|10BBCLP-1308|Canada|Ontario|658[0n]|BOLD:ABZ2129
Notodonta scitipennis|[394]|RDLQB187-05|DH010273|Canada|Quebec|580[0n]|BOLD:ABZ2129
Notodonta scitipennis|[395]|RDLQB188-05|DH010274|Canada|Quebec|605[0n]|BOLD:ABZ2129
Notodonta scitipennis|[396]|TMNBD285-07|MNBTT-3086|Canada|New Brunswick|613[0n]|BOLD:ABZ2129
Notodonta scitipennis|[397]|PHMNB039-03|moth211.02SA|Canada|New Brunswick|639[0n]|BOLD:AAB8177
Notodonta scitipennis|[398]|XAJ351-06|2006-ONT-0351|Canada|Ontario|614[0n]|BOLD:AAB8177
Notodonta scitipennis|[399]|XAJ492-06|2006-ONT-0492|Canada|Ontario|656[0n]|BOLD:AAB8177
Notodonta scitipennis|[400]|XAB046-04|04HBL005046|Canada|Ontario|587[0n]|BOLD:AAB8177
Notodonta scitipennis|[401]|TMNBD282-07|MNBTT-3083|Canada|New Brunswick|646[0n]|BOLD:AAB8177
Notodonta scitipennis|[402]|TMNBD283-07|MNBTT-3084|Canada|New Brunswick|647[0n]|BOLD:AAB8177
Notodonta scitipennis|[403]|TMNBD286-07|MNBTT-3087|Canada|New Brunswick|646[0n]|BOLD:AAB8177
Notodonta scitipennis|[404]|TTMNB080-06|MNBTT-080|Canada|New Brunswick|657[0n]|BOLD:AAB8177
Notodonta scitipennis|[405]|XAG645-05|2005-ONT-1229|Canada|Ontario|658[0n]|BOLD:AAB8177
Notodonta scitipennis|[406]|MECD404-06|jflandry2976|Canada|Quebec|658[0n]|BOLD:AAB8177
Notodonta scitipennis|[407]|XAG669-05|2005-ONT-1253|Canada|Ontario|658[0n]|BOLD:AAB8177
Notodonta scitipennis|[408]|TTMNB933-06|MNBTT-933|Canada|New Brunswick|658[0n]|BOLD:AAB8177
Notodonta scitipennis|[409]|TTMNB934-06|MNBTT-934|Canada|New Brunswick|658[0n]|BOLD:AAB8177
Notodonta scitipennis|[410]|TMNBD284-07|MNBTT-3085|Canada|New Brunswick|647[0n]|BOLD:AAB8177
Notodonta pacifica|[411]|RDNMF111-08|NOC14197|Canada|British Columbia|658[0n]|BOLD:AAF2709
Notodonta pacifica|[412]|RDNMF110-08|NOC14196|Canada|British Columbia|609[0n]|BOLD:AAF2709
Notodonta pacifica|[413]|RDNMH156-09|CNCLEP00054373|United States|California|658[0n]|BOLD:AAF2709
Notodonta pacifica|[414]|AWCL049-09|JLB-0247|United States|Idaho|658[0n]|BOLD:AAF2709
Nerice bidentata|[415]|XAK349-06|2006-ONT-1344|Canada|Ontario|615[0n]|BOLD:AAB3168
Nerice bidentata|[416]|RDLQF808-06|DH011958|Canada|Quebec|658[1n]|BOLD:AAB3168
Nerice bidentata|[417]|LOCT224-05|05-CTATBI-0224|United States|Connecticut|619[0n]|BOLD:AAB3168
Nerice bidentata|[418]|XAK443-06|2006-ONT-1438|Canada|Ontario|658[0n]|BOLD:AAB3168
Nerice bidentata|[419]|XAB589-04|04HBL005589|Canada|Ontario|658[0n]|BOLD:AAB3168
Nerice bidentata|[420]|XAK120-06|2006-ONT-1115|Canada|Ontario|658[0n]|BOLD:AAB3168
Nerice bidentata|[421]|LPSOC351-08|PPBP-2350|Canada|Ontario|656[0n]|BOLD:AAB3168
Nerice bidentata|[422]|LPSOB666-08|PPBP-1665|Canada|Ontario|644[0n]|BOLD:AAB3168
Nerice bidentata|[423]|XAK438-06|2006-ONT-1433|Canada|Ontario|658[0n]|BOLD:AAB3168
Nerice bidentata|[424]|XAK607-07|HLC-16160|Canada|Ontario|573[0n]|BOLD:AAB3168
Nerice bidentata|[425]|XAK113-06|2006-ONT-1108|Canada|Ontario|658[0n]|BOLD:AAB3168
Nerice bidentata|[426]|XAJ587-06|2006-ONT-0587|Canada|Ontario|658[0n]|BOLD:AAB3168
Nerice bidentata|[427]|XAJ981-06|2006-ONT-0981|Canada|Ontario|658[0n]|BOLD:AAB3168
Nerice bidentata|[428]|LOCT027-05|05-CTATBI-0027|United States|Connecticut|658[0n]|BOLD:AAB3168
Nerice bidentata|[429]|XAG582-05|2005-ONT-1166|Canada|Ontario|658[0n]|BOLD:AAB3168
Nerice bidentata|[430]|LPSOD320-09|08BBLEP-00098|Canada|Ontario|658[0n]|BOLD:AAB3168
Nerice bidentata|[431]|XAB025-04|04HBL005025|Canada|Ontario|658[0n]|BOLD:AAB3168
Nerice bidentata|[432]|XAJ688-06|2006-ONT-0688|Canada|Ontario|658[0n]|BOLD:AAB3168
Nerice bidentata|[433]|XAE082-04|Moth4082.03|Canada|Ontario|658[0n]|BOLD:AAB3168
Nerice bidentata|[434]|BBLSX575-09|09BBLEP-02503|United States|Oklahoma|658[0n]|BOLD:AAB3168
Nerice bidentata|[435]|BBLSX589-09|09BBLEP-02517|United States|Oklahoma|658[0n]|BOLD:AAB3168
Nerice bidentata|[436]|BBLSU045-09|09BBLEP-04414|United States|Arkansas|658[0n]|BOLD:AAB3168
Nerice bidentata|[437]|BBLSY087-09|09BBLEP-03014|United States|Oklahoma|658[0n]|BOLD:AAB3168
Nerice bidentata|[438]|BBLSX591-09|09BBLEP-02519|United States|Oklahoma|658[0n]|BOLD:AAB3168
Nerice bidentata|[439]|LPSO026-08|PPBP-0026|Canada|Ontario|658[0n]|BOLD:AAB3168
Nerice bidentata|[440]|LSUSA143-06|06-SUSA-0143|United States|Kentucky|658[0n]|BOLD:AAB3168
Nerice bidentata|[441]|LGSMC940-05|DNA-ATBI-4020|United States|Tennessee|658[0n]|BOLD:AAB3168
Nerice bidentata|[442]|LPOKA206-08|MDOK-0206|United States|Oklahoma|658[0n]|BOLD:AAB3168
Nerice bidentata|[443]|LILLA063-11|SNS10IL-00083|United States|Illinois|658[0n]|BOLD:AAB3168
Nerice bidentata|[444]|BBLSX595-09|09BBLEP-02523|United States|Oklahoma|658[0n]|BOLD:AAB3168
Nerice bidentata|[445]|LNCC741-11|11-NCCC-266|United States|North Carolina|658[0n]|BOLD:AAB3168
Nerice bidentata|[446]|LNCB555-09|09-NCCC-025|United States|North Carolina|658[0n]|BOLD:AAB3168
Nerice bidentata|[447]|LNC829-06|06-NCCC-829|United States|North Carolina|658[0n]|BOLD:AAB3168
Nadata oregonensis|[448]|RDNMH155-09|CNCLEP00054372|United States|California|632[0n]|BOLD:AAF7418
Nadata oregonensis|[449]|LOPN180-06|JCM-OSU-0180|United States|Oregon|574[0n]|BOLD:AAF7418
Nadata oregonensis|[450]|RDNMH422-09|CNCLEP00054436|United States|California|658[0n]|BOLD:AAF7418
Nadata gibbosa|[451]|LOFLA883-06|06-FLOR-0883|United States|Florida|658[0n]|BOLD:AAA2279
Nadata gibbosa|[452]|LOFLA882-06|06-FLOR-0882|United States|Florida|658[0n]|BOLD:AAA2279
Nadata gibbosa|[453]|LOFLB009-06|06-FLOR-0949|United States|Florida|658[0n]|BOLD:AAA2279
Nadata gibbosa|[454]|LPOKA168-08|MDOK-0168|United States|Oklahoma|658[0n]|BOLD:AAA2279
Nadata gibbosa|[455]|LOCT035-05|05-CTATBI-0035|United States|Connecticut|658[0n]|BOLD:AAA2279
Nadata gibbosa|[456]|TMNBD264-07|MNBTT-3065|Canada|New Brunswick|646[0n]|BOLD:AAA2279
Nadata gibbosa|[457]|XAE518-04|Moth4518.03|Canada|Ontario|589[0n]|BOLD:AAA2279
Nadata gibbosa|[458]|LBCA233-05|HLC-20233|Canada|British Columbia|621[0n]|BOLD:AAA2279
Nadata gibbosa|[459]|LOPN181-06|JCM-OSU-0181|United States|Oregon|577[2n]|BOLD:AAA2279
Nadata gibbosa|[460]|LOPN222-06|JCM-OSU-0222|United States|Oregon|594[0n]|BOLD:AAA2279
Nadata gibbosa|[461]|RDLQ098-05|DH001310|Canada|Quebec|588[0n]|BOLD:AAA2279
Nadata gibbosa|[462]|LBCB018-05|HLC-20958|Canada|British Columbia|631[0n]|BOLD:AAA2279
Nadata gibbosa|[463]|PMG183-03|moth665.01|Canada|Ontario|617[0n]|BOLD:AAA2279
Nadata gibbosa|[464]|BBLPC621-09|09BBELE-1621|Canada|Nova Scotia|614[0n]|BOLD:AAA2279
Nadata gibbosa|[465]|TMG59-03|moth908.01|Canada|Ontario|639[0n]|BOLD:AAA2279
Nadata gibbosa|[466]|TMNBD266-07|MNBTT-3067|Canada|New Brunswick|648[0n]|BOLD:AAA2279
Nadata gibbosa|[467]|RDMAB064-05|UASM57591|Canada|Alberta|649[0n]|BOLD:AAA2279
Nadata gibbosa|[468]|LBCA778-05|HLC-20778|Canada|British Columbia|658[0n]|BOLD:AAA2279
Nadata gibbosa|[469]|LBCA232-05|HLC-20232|Canada|British Columbia|633[0n]|BOLD:AAA2279
Nadata gibbosa|[470]|LPSOD825-09|08BBLEP-00607|Canada|Ontario|658[0n]|BOLD:AAA2279
Nadata gibbosa|[471]|LPSOB428-08|PPBP-1427|Canada|Ontario|658[0n]|BOLD:AAA2279
Nadata gibbosa|[472]|LPMN561-08|08BBLEP-01362|Canada|Manitoba|658[0n]|BOLD:AAA2279
Nadata gibbosa|[473]|LOPN182-06|JCM-OSU-0182|United States|Oregon|658[0n]|BOLD:AAA2279
Nadata gibbosa|[474]|TMNBD263-07|MNBTT-3064|Canada|New Brunswick|658[0n]|BOLD:AAA2279
Nadata gibbosa|[475]|RDLQ097-05|DH001306|Canada|Quebec|658[0n]|BOLD:AAA2279
Nadata gibbosa|[476]|XAJ415-06|2006-ONT-0415|Canada|Ontario|658[0n]|BOLD:AAA2279
Nadata gibbosa|[477]|RDLQB197-05|DH010283|Canada|Quebec|658[0n]|BOLD:AAA2279
Nadata gibbosa|[478]|LPSOD339-09|08BBLEP-00117|Canada|Ontario|658[0n]|BOLD:AAA2279
Nadata gibbosa|[479]|RWWB966-10|RWWA-1965|United States|Washington|658[0n]|BOLD:AAA2279
Nadata gibbosa|[480]|LOCT034-05|05-CTATBI-0034|United States|Connecticut|658[0n]|BOLD:AAA2279
Nadata gibbosa|[481]|LBCS656-07|UBC-2007-0359|Canada|British Columbia|658[0n]|BOLD:AAA2279
Nadata gibbosa|[482]|XAB084-04|04HBL005084|Canada|Ontario|658[0n]|BOLD:AAA2279
Nadata gibbosa|[483]|PHMNB729-05|Moth 422.03SA|Canada|New Brunswick|658[0n]|BOLD:AAA2279
Nadata gibbosa|[484]|LPSOD826-09|08BBLEP-00608|Canada|Ontario|658[0n]|BOLD:AAA2279
Nadata gibbosa|[485]|RWWB873-10|RWWA-1872|United States|Washington|658[0n]|BOLD:AAA2279
Nadata gibbosa|[486]|LBCS114-07|UBC-2007-0104|Canada|British Columbia|658[0n]|BOLD:AAA2279
Nadata gibbosa|[487]|XAE257-04|Moth4257.03|Canada|Ontario|658[0n]|BOLD:AAA2279
Nadata gibbosa|[488]|LPMN560-08|08BBLEP-01361|Canada|Manitoba|658[0n]|BOLD:AAA2279
Nadata gibbosa|[489]|LBCS115-07|UBC-2007-0105|Canada|British Columbia|658[0n]|BOLD:AAA2279
Nadata gibbosa|[490]|LPMN307-08|08BBLEP-01106|Canada|Manitoba|658[0n]|BOLD:AAA2279

[illegible]
Nadata gibbosa|[488]|LPMN560-08|08BBLEP-01361|Canada|Manitoba|658[0n]|BOLD:AAA2279
Nadata gibbosa|[489]|LBCS115-07|UBC-2007-0105|Canada|British Columbia|658[0n]|BOLD:AAA2279
Nadata gibbosa|[490]|LPMN307-08|08BBLEP-01106|Canada|Manitoba|658[0n]|BOLD:AAA2279
Nadata gibbosa|[491]|LBCB019-05|HLC-20959|Canada|British Columbia|658[0n]|BOLD:AAA2279
Nadata gibbosa|[492]|LPSOC220-08|PPBP-2219|Canada|Ontario|658[0n]|BOLD:AAA2279
Nadata gibbosa|[493]|TMNBD265-07|MNBTT-3066|Canada|New Brunswick|658[0n]|BOLD:AAA2279
Nadata gibbosa|[494]|LALPA367-10|AVBC 369-10|Canada|British Columbia|658[0n]|BOLD:AAA2279
Nadata gibbosa|[495]|LBCS657-07|UBC-2007-0360|Canada|British Columbia|658[0n]|BOLD:AAA2279
Nadata gibbosa|[496]|LBCS655-07|UBC-2007-0358|Canada|British Columbia|658[0n]|BOLD:AAA2279
Nadata gibbosa|[497]|LPSOD431-09|08BBLEP-00210|Canada|Ontario|658[0n]|BOLD:AAA2279
Nadata gibbosa|[498]|LBCB033-05|HLC-20973|Canada|British Columbia|658[0n]|BOLD:AAA2279
Nadata gibbosa|[499]|LPMN305-08|08BBLEP-01104|Canada|Manitoba|658[0n]|BOLD:AAA2279
Nadata gibbosa|[500]|BBLWU040-09|09BBLEP-04198|United States|Colorado|658[0n]|BOLD:AAA2279
Nadata gibbosa|[501]|BBLWU042-09|09BBLEP-04200|United States|Colorado|622[0n]|BOLD:AAA2279
Nadata gibbosa|[502]|BBLWU041-09|09BBLEP-04199|United States|Colorado|658[0n]|BOLD:AAA2279
Nadata gibbosa|[503]|BBLWU039-09|09BBLEP-04197|United States|Colorado|658[0n]|BOLD:AAA2279
Nadata gibbosa|[504]|XAE321-04|Moth4321.03|Canada|Ontario|658[0n]|BOLD:AAA2279
Nadata gibbosa|[505]|LBCA765-05|HLC-20765|Canada|British Columbia|658[0n]|BOLD:AAA2279
Nadata gibbosa|[506]|LBCA776-05|HLC-20776|Canada|British Columbia|658[0n]|BOLD:AAA2279
Nadata gibbosa|[507]|LGSMB264-05|DNA-ATBI-1113|United States|Tennessee|537[0n]|BOLD:AAA2279
Nadata gibbosa|[508]|LGSMB263-05|DNA-ATBI-1112|United States|Tennessee|586[1n]|BOLD:AAA2279
Nadata gibbosa|[509]|LBCH5986-10|10-JDWBC-5986|Canada|British Columbia|623[0n]|BOLD:AAA2279
Nadata gibbosa|[510]|LBCA239-05|HLC-20239|Canada|British Columbia|622[0n]|BOLD:AAA2279
Nadata gibbosa|[511]|LBCA575-05|HLC-20575|Canada|British Columbia|622[0n]|BOLD:AAA2279
Nadata gibbosa|[512]|LBCA236-05|HLC-20236|Canada|British Columbia|636[0n]|BOLD:AAA2279
Nadata gibbosa|[513]|LBCA234-05|HLC-20234|Canada|British Columbia|636[0n]|BOLD:AAA2279
Nadata gibbosa|[514]|LBCA080-05|HLC-20080|Canada|British Columbia|651[0n]|BOLD:AAA2279
Nadata gibbosa|[515]|LBCB028-05|HLC-20968|Canada|British Columbia|650[0n]|BOLD:AAA2279
Nadata gibbosa|[516]|LBCG044-08|08-JDWBC-0044|Canada|British Columbia|658[0n]|BOLD:AAA2279
Nadata gibbosa|[517]|LBCA491-05|HLC-20491|Canada|British Columbia|658[1n]|BOLD:AAA2279
Nadata gibbosa|[518]|LBCA238-05|HLC-20238|Canada|British Columbia|658[1n]|BOLD:AAA2279
Nadata gibbosa|[519]|LBCB136-05|HLC-21076|Canada|British Columbia|658[0n]|BOLD:AAA2279
Nadata gibbosa|[520]|BBLOE1981-12|BIOUG01995-B01|United States|Oklahoma|658[0n]|BOLD:AAA2279
Nadata gibbosa|[521]|LBCA766-05|HLC-20766|Canada|British Columbia|658[0n]|BOLD:AAA2279
Nadata gibbosa|[522]|LBCB130-05|HLC-21070|Canada|British Columbia|658[0n]|BOLD:AAA2279
Nadata gibbosa|[523]|LBCB027-05|HLC-20967|Canada|British Columbia|658[0n]|BOLD:AAA2279
Nadata gibbosa|[524]|LBCH5852-10|10-JDWBC-5852|Canada|British Columbia|658[0n]|BOLD:AAA2279
Nadata gibbosa|[525]|LBCH5929-10|10-JDWBC-5929|Canada|British Columbia|658[0n]|BOLD:AAA2279
Nadata gibbosa|[526]|LBCC375-05|HLC-22255|Canada|British Columbia|658[0n]|BOLD:AAA2279
Nadata gibbosa|[527]|LBCB029-05|HLC-20969|Canada|British Columbia|658[0n]|BOLD:AAA2279
Nadata gibbosa|[528]|LBCB022-05|HLC-20962|Canada|British Columbia|658[0n]|BOLD:AAA2279
Nadata gibbosa|[529]|LPAB265-08|08BBLEP-02587|Canada|Alberta|658[0n]|BOLD:AAA2279
Nadata gibbosa|[530]|LBCH2237-10|10-JDWBC-2237|Canada|British Columbia|658[0n]|BOLD:AAA2279
Nadata gibbosa|[531]|LBCH008-10|10-JDWBC-0008|Canada|British Columbia|658[0n]|BOLD:AAA2279
Nadata gibbosa|[532]|LBCC746-05|HLC-22626|Canada|British Columbia|658[0n]|BOLD:AAA2279
Nadata gibbosa|[533]|LBCB026-05|HLC-20966|Canada|British Columbia|658[0n]|BOLD:AAA2279
Nadata gibbosa|[534]|LBCH799-10|10-JDWBC-0799|Canada|British Columbia|658[0n]|BOLD:AAA2279
Nadata gibbosa|[535]|LBCA043-05|HLC-20043|Canada|British Columbia|658[0n]|BOLD:AAA2279
Nadata gibbosa|[536]|LMH027-06|PFC-2006-0159|Canada|British Columbia|658[0n]|BOLD:AAA2279
Nadata gibbosa|[537]|LBCA764-05|HLC-20764|Canada|British Columbia|658[0n]|BOLD:AAA2279
Nadata gibbosa|[538]|LBCB126-05|HLC-21066|Canada|British Columbia|658[0n]|BOLD:AAA2279
Nadata gibbosa|[539]|LBCA772-05|HLC-20772|Canada|British Columbia|658[0n]|BOLD:AAA2279
Nadata gibbosa|[540]|LBCB127-05|HLC-21067|Canada|British Columbia|658[0n]|BOLD:AAA2279
Nadata gibbosa|[541]|LALPA149-10|AVBC 149-10|Canada|British Columbia|658[0n]|BOLD:AAA2279
Nadata gibbosa|[542]|LBCA770-05|HLC-20770|Canada|British Columbia|658[0n]|BOLD:AAA2279
Nadata gibbosa|[543]|LBCB030-05|HLC-20970|Canada|British Columbia|658[0n]|BOLD:AAA2279
Nadata gibbosa|[544]|LBCG097-08|08-JDWBC-0097|Canada|British Columbia|658[0n]|BOLD:AAA2279
Nadata gibbosa|[545]|LBCA769-05|HLC-20769|Canada|British Columbia|658[0n]|BOLD:AAA2279
Nadata gibbosa|[546]|LBCB128-05|HLC-21068|Canada|British Columbia|658[0n]|BOLD:AAA2279
Nadata gibbosa|[547]|LBCB125-05|HLC-21065|Canada|British Columbia|658[0n]|BOLD:AAA2279
Nadata gibbosa|[548]|LBCB023-05|HLC-20963|Canada|British Columbia|658[0n]|BOLD:AAA2279
Nadata gibbosa|[549]|LBCA774-05|HLC-20774|Canada|British Columbia|658[0n]|BOLD:AAA2279
Nadata gibbosa|[550]|LBCB020-05|HLC-20960|Canada|British Columbia|658[0n]|BOLD:AAA2279
Nadata gibbosa|[551]|LBCC339-05|HLC-22219|Canada|British Columbia|658[0n]|BOLD:AAA2279
Nadata gibbosa|[552]|LBCC373-05|HLC-22253|Canada|British Columbia|658[0n]|BOLD:AAA2279
Nadata gibbosa|[553]|LBCC374-05|HLC-22254|Canada|British Columbia|658[0n]|BOLD:AAA2279
Nadata gibbosa|[554]|LALPA300-10|AVBC 302-10|Canada|British Columbia|658[0n]|BOLD:AAA2279
Nadata gibbosa|[555]|LBCB129-05|HLC-21069|Canada|British Columbia|658[0n]|BOLD:AAA2279
Nadata gibbosa|[556]|LBCH216-10|10-JDWBC-0216|Canada|British Columbia|658[0n]|BOLD:AAA2279
Nadata gibbosa|[557]|LBCA767-05|HLC-20767|Canada|British Columbia|658[0n]|BOLD:AAA2279
Nadata gibbosa|[558]|LBCB017-05|HLC-20957|Canada|British Columbia|658[0n]|BOLD:AAA2279
Nadata gibbosa|[559]|LBCB024-05|HLC-20964|Canada|British Columbia|658[0n]|BOLD:AAA2279
Nadata gibbosa|[560]|LBCB134-05|HLC-21074|Canada|British Columbia|658[0n]|BOLD:AAA2279
Nadata gibbosa|[561]|LBCA773-05|HLC-20773|Canada|British Columbia|658[0n]|BOLD:AAA2279
Nadata gibbosa|[562]|LBCB035-05|HLC-20975|Canada|British Columbia|658[0n]|BOLD:AAA2279
Nadata gibbosa|[563]|LBCA235-05|HLC-20235|Canada|British Columbia|658[0n]|BOLD:AAA2279
Nadata gibbosa|[564]|LBCA775-05|HLC-20775|Canada|British Columbia|658[0n]|BOLD:AAA2279
Nadata gibbosa|[565]|LBCC376-05|HLC-22256|Canada|British Columbia|658[0n]|BOLD:AAA2279
Nadata gibbosa|[566]|LBCB124-05|HLC-21064|Canada|British Columbia|658[0n]|BOLD:AAA2279
Nadata gibbosa|[567]|LBCB133-05|HLC-21073|Canada|British Columbia|658[0n]|BOLD:AAA2279
Nadata gibbosa|[568]|LBCB132-05|HLC-21072|Canada|British Columbia|658[0n]|BOLD:AAA2279
Nadata gibbosa|[569]|LOWCE821-06|CGWC-4581|Canada|British Columbia|658[0n]|BOLD:AAA2279
Nadata gibbosa|[570]|LBCH217-10|10-JDWBC-0217|Canada|British Columbia|658[0n]|BOLD:AAA2279
Nadata gibbosa|[571]|LOWCB118-05|CGWC-1058|Canada|British Columbia|658[0n]|BOLD:AAA2279
Nadata gibbosa|[572]|LOWCB117-05|CGWC-1057|Canada|British Columbia|658[0n]|BOLD:AAA2279
Nadata gibbosa|[573]|LOWCE805-06|CGWC-4565|Canada|British Columbia|658[0n]|BOLD:AAA2279
Nadata gibbosa|[574]|LPSO013-08|PPBP-0013|Canada|Ontario|658[0n]|BOLD:AAA2279
Nadata gibbosa|[575]|LBCB032-05|HLC-20972|Canada|British Columbia|658[0n]|BOLD:AAA2279
Nadata gibbosa|[576]|LBCB021-05|HLC-20961|Canada|British Columbia|658[0n]|BOLD:AAA2279
Nadata gibbosa|[577]|LBCB131-05|HLC-21071|Canada|British Columbia|658[0n]|BOLD:AAA2279
Nadata gibbosa|[578]|LOT119-04|04HBL002119|United States|Tennessee|609[0n]|BOLD:AAA2279
Nadata gibbosa|[579]|LGSMG734-07|BGS03397|United States|Tennessee|658[0n]|BOLD:AAA2279
Nadata gibbosa|[580]|LBCH5082-10|10-JDWBC-5082|Canada|British Columbia|658[0n]|BOLD:AAA2279
Nadata gibbosa|[581]|LBCH5144-10|10-JDWBC-5144|Canada|British Columbia|658[0n]|BOLD:AAA2279
Nadata gibbosa|[582]|XAF804-05|2005-ONT-453|Canada|Ontario|658[0n]|BOLD:AAA2279
Nadata gibbosa|[583]|LBCB135-05|HLC-21075|Canada|British Columbia|658[0n]|BOLD:AAA2279
Nadata gibbosa|[584]|LBCB025-05|HLC-20965|Canada|British Columbia|658[0n]|BOLD:AAA2279
Nadata gibbosa|[585]|LBCC377-05|HLC-22257|Canada|British Columbia|658[0n]|BOLD:AAA2279
Nadata gibbosa|[586]|RDNMH418-09|CNCLEP00054432|United States|Oklahoma|658[0n]|BOLD:AAA2279
Nadata gibbosa|[587]|LBCB034-05|HLC-20974|Canada|British Columbia|658[0n]|BOLD:AAA2279
Nadata gibbosa|[588]|LPSOB061-08|PPBP-1060|Canada|Ontario|658[0n]|BOLD:AAA2279

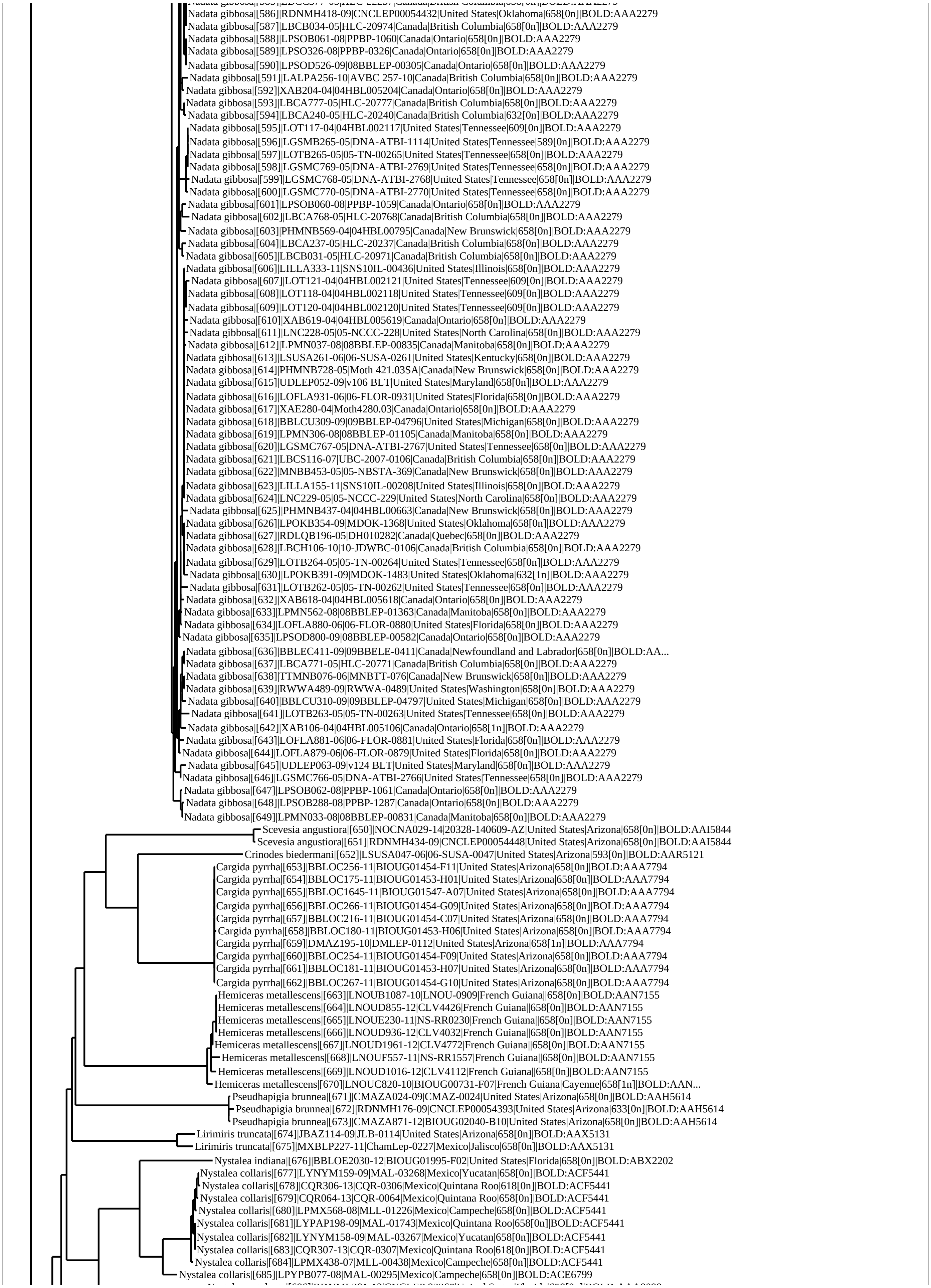
Nadata gibbosa|[586]|RDNMH418-09|CNCLEP00054432|United States|Oklahoma|658[0n]|BOLD:AAA2279
Nadata gibbosa|[587]|LBCB034-05|HLC-20974|Canada|British Columbia|658[0n]|BOLD:AAA2279
Nadata gibbosa|[588]|LPSOB061-08|PPBP-1060|Canada|Ontario|658[0n]|BOLD:AAA2279
Nadata gibbosa|[589]|LPSO326-08|PPBP-0326|Canada|Ontario|658[0n]|BOLD:AAA2279
Nadata gibbosa|[590]|LPSOD526-09|08BBLEP-00305|Canada|Ontario|658[0n]|BOLD:AAA2279
Nadata gibbosa|[591]|LALPA256-10|AVBC 257-10|Canada|British Columbia|658[0n]|BOLD:AAA2279
Nadata gibbosa|[592]|XAB204-04|04HBL005204|Canada|Ontario|658[0n]|BOLD:AAA2279
Nadata gibbosa|[593]|LBCA777-05|HLC-20777|Canada|British Columbia|658[0n]|BOLD:AAA2279
Nadata gibbosa|[594]|LBCA240-05|HLC-20240|Canada|British Columbia|632[0n]|BOLD:AAA2279
Nadata gibbosa|[595]|LOT117-04|04HBL002117|United States|Tennessee|609[0n]|BOLD:AAA2279
Nadata gibbosa|[596]|LGSMB265-05|DNA-ATBI-1114|United States|Tennessee|589[0n]|BOLD:AAA2279
Nadata gibbosa|[597]|LOTB265-05|05-TN-00265|United States|Tennessee|658[0n]|BOLD:AAA2279
Nadata gibbosa|[598]|LGSMC769-05|DNA-ATBI-2769|United States|Tennessee|658[0n]|BOLD:AAA2279
Nadata gibbosa|[599]|LGSMC768-05|DNA-ATBI-2768|United States|Tennessee|658[0n]|BOLD:AAA2279
Nadata gibbosa|[600]|LGSMC770-05|DNA-ATBI-2770|United States|Tennessee|658[0n]|BOLD:AAA2279
Nadata gibbosa|[601]|LPSOB060-08|PPBP-1059|Canada|Ontario|658[0n]|BOLD:AAA2279
Nadata gibbosa|[602]|LBCA768-05|HLC-20768|Canada|British Columbia|658[0n]|BOLD:AAA2279
Nadata gibbosa|[603]|PHMNB569-04|04HBL00795|Canada|New Brunswick|658[0n]|BOLD:AAA2279
Nadata gibbosa|[604]|LBCA237-05|HLC-20237|Canada|British Columbia|658[0n]|BOLD:AAA2279
Nadata gibbosa|[605]|LBCB031-05|HLC-20971|Canada|British Columbia|658[0n]|BOLD:AAA2279
Nadata gibbosa|[606]|LILLA333-11|SNS10IL-00436|United States|Illinois|658[0n]|BOLD:AAA2279
Nadata gibbosa|[607]|LOT121-04|04HBL002121|United States|Tennessee|609[0n]|BOLD:AAA2279
Nadata gibbosa|[608]|LOT118-04|04HBL002118|United States|Tennessee|609[0n]|BOLD:AAA2279
Nadata gibbosa|[609]|LOT120-04|04HBL002120|United States|Tennessee|609[0n]|BOLD:AAA2279
Nadata gibbosa|[610]|XAB619-04|04HBL005619|Canada|Ontario|658[0n]|BOLD:AAA2279
Nadata gibbosa|[611]|LNC228-05|05-NCCC-228|United States|North Carolina|658[0n]|BOLD:AAA2279
Nadata gibbosa|[612]|LPMN037-08|08BBLEP-00835|Canada|Manitoba|658[0n]|BOLD:AAA2279
Nadata gibbosa|[613]|LSUSA261-06|06-SUSA-0261|United States|Kentucky|658[0n]|BOLD:AAA2279
Nadata gibbosa|[614]|PHMNB728-05|Moth 421.03SA|Canada|New Brunswick|658[0n]|BOLD:AAA2279
Nadata gibbosa|[615]|UDLEP052-09|v106 BLT|United States|Maryland|658[0n]|BOLD:AAA2279
Nadata gibbosa|[616]|LOFLA931-06|06-FLOR-0931|United States|Florida|658[0n]|BOLD:AAA2279
Nadata gibbosa|[617]|XAE280-04|Moth4280.03|Canada|Ontario|658[0n]|BOLD:AAA2279
Nadata gibbosa|[618]|BBLCU309-09|09BBLEP-04796|United States|Michigan|658[0n]|BOLD:AAA2279
Nadata gibbosa|[619]|LPMN306-08|08BBLEP-01105|Canada|Manitoba|658[0n]|BOLD:AAA2279
Nadata gibbosa|[620]|LGSMC767-05|DNA-ATBI-2767|United States|Tennessee|658[0n]|BOLD:AAA2279
Nadata gibbosa|[621]|LBCS116-07|UBC-2007-0106|Canada|British Columbia|658[0n]|BOLD:AAA2279
Nadata gibbosa|[622]|MNBB453-05|05-NBSTA-369|Canada|New Brunswick|658[0n]|BOLD:AAA2279
Nadata gibbosa|[623]|LILLA155-11|SNS10IL-00208|United States|Illinois|658[0n]|BOLD:AAA2279
Nadata gibbosa|[624]|LNC229-05|05-NCCC-229|United States|North Carolina|658[0n]|BOLD:AAA2279
Nadata gibbosa|[625]|PHMNB437-04|04HBL00663|Canada|New Brunswick|658[0n]|BOLD:AAA2279
Nadata gibbosa|[626]|LPOKB354-09|MDOK-1368|United States|Oklahoma|658[0n]|BOLD:AAA2279
Nadata gibbosa|[627]|RDLQB196-05|DH010282|Canada|Quebec|658[0n]|BOLD:AAA2279
Nadata gibbosa|[628]|LBCH106-10|10-JDWBC-0106|Canada|British Columbia|658[0n]|BOLD:AAA2279
Nadata gibbosa|[629]|LOTB264-05|05-TN-00264|United States|Tennessee|658[0n]|BOLD:AAA2279
Nadata gibbosa|[630]|LPOKB391-09|MDOK-1483|United States|Oklahoma|632[1n]|BOLD:AAA2279
Nadata gibbosa|[631]|LOTB262-05|05-TN-00262|United States|Tennessee|658[0n]|BOLD:AAA2279
Nadata gibbosa|[632]|XAB618-04|04HBL005618|Canada|Ontario|658[0n]|BOLD:AAA2279
Nadata gibbosa|[633]|LPMN562-08|08BBLEP-01363|Canada|Manitoba|658[0n]|BOLD:AAA2279
Nadata gibbosa|[634]|LOFLA880-06|06-FLOR-0880|United States|Florida|658[0n]|BOLD:AAA2279
Nadata gibbosa|[635]|LPSOD800-09|08BBLEP-00582|Canada|Ontario|658[0n]|BOLD:AAA2279
Nadata gibbosa|[636]|BBLEC411-09|09BBELE-0411|Canada|Newfoundland and Labrador|658[0n]|BOLD:AA...
Nadata gibbosa|[637]|LBCA771-05|HLC-20771|Canada|British Columbia|658[0n]|BOLD:AAA2279
Nadata gibbosa|[638]|TTMNB076-06|MNBTT-076|Canada|New Brunswick|658[0n]|BOLD:AAA2279
Nadata gibbosa|[639]|RWWA489-09|RWWA-0489|United States|Washington|658[0n]|BOLD:AAA2279
Nadata gibbosa|[640]|BBLCU310-09|09BBLEP-04797|United States|Michigan|658[0n]|BOLD:AAA2279
Nadata gibbosa|[641]|LOTB263-05|05-TN-00263|United States|Tennessee|658[0n]|BOLD:AAA2279
Nadata gibbosa|[642]|XAB106-04|04HBL005106|Canada|Ontario|658[1n]|BOLD:AAA2279
Nadata gibbosa|[643]|LOFLA881-06|06-FLOR-0881|United States|Florida|658[0n]|BOLD:AAA2279
Nadata gibbosa|[644]|LOFLA879-06|06-FLOR-0879|United States|Florida|658[0n]|BOLD:AAA2279
Nadata gibbosa|[645]|UDLEP063-09|v124 BLT|United States|Maryland|658[0n]|BOLD:AAA2279
Nadata gibbosa|[646]|LGSMC766-05|DNA-ATBI-2766|United States|Tennessee|658[0n]|BOLD:AAA2279
Nadata gibbosa|[647]|LPSOB062-08|PPBP-1061|Canada|Ontario|658[0n]|BOLD:AAA2279
Nadata gibbosa|[648]|LPSOB288-08|PPBP-1287|Canada|Ontario|658[0n]|BOLD:AAA2279
Nadata gibbosa|[649]|LPMN033-08|08BBLEP-00831|Canada|Manitoba|658[0n]|BOLD:AAA2279
Scevesia angustiora|[650]|NOCNA029-14|20328-140609-AZ|United States|Arizona|658[0n]|BOLD:AAI5844
Scevesia angustiora|[651]|RDNMH434-09|CNCLEP00054448|United States|Arizona|658[0n]|BOLD:AAI5844
Crinodes biedermani|[652]|LSUSA047-06|06-SUSA-0047|United States|Arizona|593[0n]|BOLD:AAR5121
Cargida pyrrha|[653]|BBLOC256-11|BIOUG01454-F11|United States|Arizona|658[0n]|BOLD:AAA7794
Cargida pyrrha|[654]|BBLOC175-11|BIOUG01453-H01|United States|Arizona|658[0n]|BOLD:AAA7794
Cargida pyrrha|[655]|BBLOC1645-11|BIOUG01547-A07|United States|Arizona|658[0n]|BOLD:AAA7794
Cargida pyrrha|[656]|BBLOC266-11|BIOUG01454-G09|United States|Arizona|658[0n]|BOLD:AAA7794
Cargida pyrrha|[657]|BBLOC216-11|BIOUG01454-C07|United States|Arizona|658[0n]|BOLD:AAA7794
Cargida pyrrha|[658]|BBLOC180-11|BIOUG01453-H06|United States|Arizona|658[0n]|BOLD:AAA7794
Cargida pyrrha|[659]|DMAZ195-10|DMLEP-0112|United States|Arizona|658[1n]|BOLD:AAA7794
Cargida pyrrha|[660]|BBLOC254-11|BIOUG01454-F09|United States|Arizona|658[0n]|BOLD:AAA7794
Cargida pyrrha|[661]|BBLOC181-11|BIOUG01453-H07|United States|Arizona|658[0n]|BOLD:AAA7794
Cargida pyrrha|[662]|BBLOC267-11|BIOUG01454-G10|United States|Arizona|658[0n]|BOLD:AAA7794
Hemiceras metallescens|[663]|LNOUB1087-10|LNOU-0909|French Guiana||658[0n]|BOLD:AAN7155
Hemiceras metallescens|[664]|LNOUD855-12|CLV4426|French Guiana||658[0n]|BOLD:AAN7155
Hemiceras metallescens|[665]|LNOUE230-11|NS-RR0230|French Guiana||658[0n]|BOLD:AAN7155
Hemiceras metallescens|[666]|LNOUD936-12|CLV4032|French Guiana||658[0n]|BOLD:AAN7155
Hemiceras metallescens|[667]|LNOUD1961-12|CLV4772|French Guiana||658[0n]|BOLD:AAN7155
Hemiceras metallescens|[668]|LNOUF557-11|NS-RR1557|French Guiana||658[0n]|BOLD:AAN7155
Hemiceras metallescens|[669]|LNOUD1016-12|CLV4112|French Guiana||658[0n]|BOLD:AAN7155
Hemiceras metallescens|[670]|LNOUC820-10|BIOUG00731-F07|French Guiana|Cayenne|658[1n]|BOLD:AAN...
Pseudhapigia brunnea|[671]|CMAZA024-09|CMAZ-0024|United States|Arizona|658[0n]|BOLD:AAH5614
Pseudhapigia brunnea|[672]|RDNMH176-09|CNCLEP00054393|United States|Arizona|633[0n]|BOLD:AAH5614
Pseudhapigia brunnea|[673]|CMAZA871-12|BIOUG02040-B10|United States|Arizona|658[0n]|BOLD:AAH5614
Lirimiris truncata|[674]|JBAZ114-09|JLB-0114|United States|Arizona|658[0n]|BOLD:AAX5131
Lirimiris truncata|[675]|MXBLP227-11|ChamLep-0227|Mexico|Jalisco|658[0n]|BOLD:AAX5131
Nystalea indiana|[676]|BBLOE2030-12|BIOUG01995-F02|United States|Florida|658[0n]|BOLD:ABX2202
Nystalea collaris|[677]|LYNYM159-09|MAL-03268|Mexico|Yucatan|658[0n]|BOLD:ACF5441
Nystalea collaris|[678]|CQR306-13|CQR-0306|Mexico|Quintana Roo|618[0n]|BOLD:ACF5441
Nystalea collaris|[679]|CQR064-13|CQR-0064|Mexico|Quintana Roo|658[0n]|BOLD:ACF5441
Nystalea collaris|[680]|LPMX568-08|MLL-01226|Mexico|Campeche|658[0n]|BOLD:ACF5441
Nystalea collaris|[681]|LYPAP198-09|MAL-01743|Mexico|Quintana Roo|658[0n]|BOLD:ACF5441
Nystalea collaris|[682]|LYNYM158-09|MAL-03267|Mexico|Yucatan|658[0n]|BOLD:ACF5441
Nystalea collaris|[683]|CQR307-13|CQR-0307|Mexico|Quintana Roo|618[0n]|BOLD:ACF5441
Nystalea collaris|[684]|LPMX438-07|MLL-00438|Mexico|Campeche|658[0n]|BOLD:ACF5441
Nystalea collaris|[685]|LPYPB077-08|MAL-00295|Mexico|Campeche|658[0n]|BOLD:ACE6799

Nystalea collaris|[683]|CQR507-13|CQR-0507|Mexico|Quintana Roo|618[0n]|BOLD:ACF5441
Nystalea collaris|[684]|LPMX438-07|MLL-00438|Mexico|Campeche|658[0n]|BOLD:ACF5441
Nystalea collaris|[685]|LPYPB077-08|MAL-00295|Mexico|Campeche|658[0n]|BOLD:ACE6799
Nystalea eutalanta|[686]|RDNML291-13|CNCLEP 92267|United States|Florida|658[0n]|BOLD:AAA8098
Nystalea eutalanta|[687]|CNCLB1615-14|CNCLEP 00113845|United States|Florida|658[0n]|BOLD:AAA8098
Nystalea eutalanta|[688]|CNCLB1683-14|CNCLEP 00113917|Puerto Rico|Mayaguez|658[0n]|BOLD:AAA8098
Nystalea eutalanta|[689]|RDNML292-13|CNCLEP 92268|United States|Florida|658[0n]|BOLD:ACD9122
Rifargia bichorda|[690]|RDNML293-13|CNCLEP 92269|United States|Florida|658[0n]|BOLD:ACD9035
Notela jaliscana|[691]|RDNMH423-09|CNCLEP00054437|United States|Arizona|658[0n]|BOLD:AAF3512
Notela jaliscana|[692]|RDNMH162-09|CNCLEP00054379|United States|Arizona|658[0n]|BOLD:AAF3512
Notela jaliscana|[693]|RDNMH463-09|CNCLEP00054477|United States|Arizona|658[0n]|BOLD:AAF3512
Notela jaliscana|[694]|CMAZA930-12|BIOUG02040-G09|United States|Arizona|658[0n]|BOLD:AAF3512
Dasylophia thyatiroides|[695]|TMNBD346-07|MNBTT-3147|Canada|New Brunswick|658[0n]|BOLD:AAC2023
Dasylophia thyatiroides|[696]|TMNBD347-07|MNBTT-3148|Canada|New Brunswick|658[0n]|BOLD:AAC2023
Dasylophia thyatiroides|[697]|TTMNB932-06|MNBTT-932|Canada|New Brunswick|658[0n]|BOLD:AAC2023
Dasylophia thyatiroides|[698]|TMNBD349-07|MNBTT-3150|Canada|New Brunswick|643[0n]|BOLD:AAC2023
Dasylophia thyatiroides|[699]|LGSMB234-05|DNA-ATBI-1083|United States|Tennessee|545[0n]|BOLD:A...
Dasylophia thyatiroides|[700]|TMNBD350-07|MNBTT-3151|Canada|New Brunswick|646[0n]|BOLD:AAC2023
Dasylophia thyatiroides|[701]|RDLQF819-06|DH011972|Canada|Quebec|658[0n]|BOLD:AAC2023
Dasylophia thyatiroides|[702]|HKONS082-07|1646-COI-07|United States|Florida|655[0n]|BOLD:AAC2023
Dasylophia thyatiroides|[703]|LSUSA034-06|06-SUSA-0034|United States|Kentucky|658[0n]|BOLD:AAC...
Dasylophia thyatiroides|[704]|LGSMC750-05|DNA-ATBI-2750|United States|Tennessee|658[0n]|BOLD:A...
Dasylophia thyatiroides|[705]|TMNBD348-07|MNBTT-3149|Canada|New Brunswick|658[0n]|BOLD:AAC2023
Dasylophia thyatiroides|[706]|RDLQF231-06|DH011311|Canada|Quebec|658[0n]|BOLD:AAC2023
Dasylophia thyatiroides|[707]|LGSMG089-07|BGS03410|United States|North Carolina|658[0n]|BOLD:A...
Dasylophia seriata|[708]|RDNMH409-09|CNCLEP00054423|United States|New Mexico|658[0n]|BOLD:AAI9618
Dasylophia seriata|[709]|RDNMH410-09|CNCLEP00054424|United States|New Mexico|658[0n]|BOLD:AAI9618
Dasylophia anguina|[710]|LGSMB235-05|DNA-ATBI-1084|United States|North Carolina|569[0n]|BOLD:A...
Dasylophia anguina|[711]|LSEU791-06|06-JKA-0791|United States|Missouri|658[0n]|BOLD:AAB7663
Dasylophia anguina|[712]|LSUSA145-06|06-SUSA-0145|United States|Kentucky|658[0n]|BOLD:AAB7663
Dasylophia anguina|[713]|LGSMG746-07|BGS03409|United States|Tennessee|658[0n]|BOLD:AAB7663
Dasylophia anguina|[714]|LGSMC749-05|DNA-ATBI-2749|United States|Tennessee|658[0n]|BOLD:AAB7663
Dasylophia anguina|[715]|LSUSA262-06|06-SUSA-0262|United States|Kentucky|658[0n]|BOLD:AAB7663
Dasylophia anguina|[716]|LPOKC767-09|MDOK-2844|United States|Oklahoma|658[0n]|BOLD:AAB7663
Dasylophia anguina|[717]|LGSMG745-07|BGS03408|United States|Tennessee|658[0n]|BOLD:AAB7663
Dasylophia anguina|[718]|RDNMH433-09|CNCLEP00054447|United States|North Carolina|658[0n]|BOLD:...
Dasylophia anguina|[719]|LPOKD014-09|MDOK-3093|United States|Oklahoma|658[0n]|BOLD:AAB7663
Dasylophia anguina|[720]|LPOKE112-10|MDOK-4190|United States|Oklahoma|658[0n]|BOLD:AAB7663
Dasylophia anguina|[721]|LOFLB442-06|06-FLOR-1382|United States|Florida|658[0n]|BOLD:AAB7663
Dasylophia anguina|[722]|LNCC1348-11|11-NCCC-873|United States|North Carolina|658[0n]|BOLD:AAB...
Dasylophia anguina|[723]|LGSMG744-07|BGS03407|United States|North Carolina|658[0n]|BOLD:AAB7663
Dasylophia anguina|[724]|RDNMH435-09|CNCLEP00054449|United States|Colorado|658[0n]|BOLD:AAB7663
Dasylophia anguina|[725]|LOFLB398-06|06-FLOR-1338|United States|Florida|658[0n]|BOLD:AAB7663
Dasylophia anguina|[726]|LOFLB716-06|06-FLOR-1656|United States|Florida|658[0n]|BOLD:AAB7663
Dasylophia anguina|[727]|LOFLC328-06|06-FLOR-2208|United States|Florida|658[0n]|BOLD:AAB7663
Dasylophia anguina|[728]|LOFLB399-06|06-FLOR-1339|United States|Florida|658[0n]|BOLD:AAB7663
Dasylophia anguina|[729]|LOFLB490-06|06-FLOR-1430|United States|Florida|658[0n]|BOLD:AAB7663
Dasylophia anguina|[730]|LOFLB444-06|06-FLOR-1384|United States|Florida|648[0n]|BOLD:AAB7663
Leuculodes lacteolaria|[731]|LNAUS3263-13|CCDB-20803-F11|United States|Arizona|658[0n]|BOLD:AC...
Doa ampla|[732]|LNAUS3256-13|CCDB-20803-F04|United States|Arizona|658[0n]|BOLD:ACH5042
Doa ampla|[733]|LNAUS3255-13|CCDB-20803-F03|United States|Arizona|658[0n]|BOLD:ACH5042
Doa ampla|[734]|WAGL116-15|DLW-000116|United States|Arizona|658[0n]|BOLD:ACH5042
Heterocampa cubana|[735]|LNAUS4408-13|CCDB-20814-G04|United States|Florida|658[0n]|BOLD:AAA7792
Heterocampa cubana|[736]|LNAUS4412-13|CCDB-20814-G08|United States|Florida|658[0n]|BOLD:AAA7792
Heterocampa cubana|[737]|LNAUS4410-13|CCDB-20814-G06|United States|Florida|658[0n]|BOLD:AAA7792
Ellida caniplaga|[738]|LSEU312-06|06-JKA-0312|United States|Georgia|658[0n]|BOLD:AAA9083
Ellida caniplaga|[739]|LSEU739-06|06-JKA-0739|United States|Georgia|658[0n]|BOLD:AAA9083
Ellida caniplaga|[740]|LGSMG712-07|BGS03375|United States|Tennessee|619[0n]|BOLD:AAA9083
Ellida caniplaga|[741]|LGSMC754-05|DNA-ATBI-2754|United States|Tennessee|618[0n]|BOLD:AAA9083
Ellida caniplaga|[742]|LNCC971-11|11-NCCC-496|United States|North Carolina|658[0n]|BOLD:AAA9083
Ellida caniplaga|[743]|LGSMB236-05|DNA-ATBI-1085|United States|North Carolina|581[1n]|BOLD:AAA...
Ellida caniplaga|[744]|LGSMG739-07|BGS03402|United States|North Carolina|658[0n]|BOLD:AAA9083
Ellida caniplaga|[745]|LOT114-04|04HBL002114|United States|Tennessee|658[0n]|BOLD:AAA9083
Ellida caniplaga|[746]|LOT112-04|04HBL002112|United States|Tennessee|658[0n]|BOLD:AAA9083
Ellida caniplaga|[747]|LOT113-04|04HBL002113|United States|Tennessee|658[0n]|BOLD:AAA9083
Ellida caniplaga|[748]|LOT525-04|04HBL002525|United States|Tennessee|658[0n]|BOLD:AAA9083
Ellida caniplaga|[749]|LPSO153-08|PPBP-0153|Canada|Ontario|658[0n]|BOLD:AAA9083
Ellida caniplaga|[750]|LPSO868-08|PPBP-0868|Canada|Ontario|658[0n]|BOLD:AAA9083
Ellida caniplaga|[751]|LGSMC755-05|DNA-ATBI-2755|United States|Tennessee|658[0n]|BOLD:AAA9083
Ellida caniplaga|[752]|XAB112-04|04HBL005112|Canada|Ontario|658[1n]|BOLD:AAA9083
Ellida caniplaga|[753]|MNBB073-05|HBL008683|Canada|New Brunswick|658[0n]|BOLD:AAA9083
Ellida caniplaga|[754]|XAF426-05|HLC-10467|Canada|Ontario|658[0n]|BOLD:AAA9083
Ellida caniplaga|[755]|XAE307-04|Moth4307.03|Canada|Ontario|658[0n]|BOLD:AAA9083
Ellida caniplaga|[756]|XAB252-04|04HBL005252|Canada|Ontario|658[0n]|BOLD:AAA9083
Ellida caniplaga|[757]|XAB323-04|04HBL005323|Canada|Ontario|658[0n]|BOLD:AAA9083
Ellida caniplaga|[758]|XAF385-05|HLC-10426|Canada|Ontario|658[0n]|BOLD:AAA9083
Ellida caniplaga|[759]|TMG64-03|moth358.01|Canada|Ontario|639[0n]|BOLD:AAA9083
Ellida caniplaga|[760]|PMG174-03|moth326.01|Canada|Ontario|617[0n]|BOLD:AAA9083
Ellida caniplaga|[761]|XAF427-05|HLC-10468|Canada|Ontario|658[0n]|BOLD:AAA9083
Ellida caniplaga|[762]|XAJ837-06|2006-ONT-0837|Canada|Ontario|658[0n]|BOLD:AAA9083
Ellida caniplaga|[763]|XAE349-04|Moth4349.03|Canada|Ontario|658[0n]|BOLD:AAA9083
Ellida caniplaga|[764]|BBLCU013-09|09BBLEP-04500|United States|Michigan|658[0n]|BOLD:AAA9083
Ellida caniplaga|[765]|XAB133-04|04HBL005133|Canada|Ontario|658[0n]|BOLD:AAA9083
Ellida caniplaga|[766]|XAB043-04|04HBL005043|Canada|Ontario|658[0n]|BOLD:AAA9083
Ellida caniplaga|[767]|LPSOB980-08|PPBP-1979|Canada|Ontario|658[0n]|BOLD:AAA9083
Ellida caniplaga|[768]|LPSOC214-08|PPBP-2213|Canada|Ontario|658[0n]|BOLD:AAA9083
Ellida caniplaga|[769]|KPOEC172-08|08OEC-213|Canada|Ontario|658[0n]|BOLD:AAA9083
Ellida caniplaga|[770]|XAJ491-06|2006-ONT-0491|Canada|Ontario|658[0n]|BOLD:AAA9083
Ellida caniplaga|[771]|LPSOC089-08|PPBP-2088|Canada|Ontario|658[0n]|BOLD:AAA9083
Ellida caniplaga|[772]|LPSOC083-08|PPBP-2082|Canada|Ontario|658[0n]|BOLD:AAA9083
Ellida caniplaga|[773]|LPSOC212-08|PPBP-2211|Canada|Ontario|658[0n]|BOLD:AAA9083
Ellida caniplaga|[774]|LPSO256-08|PPBP-0256|Canada|Ontario|658[0n]|BOLD:AAA9083
Ellida caniplaga|[775]|LPSO133-08|PPBP-0133|Canada|Ontario|658[0n]|BOLD:AAA9083
Ellida caniplaga|[776]|LMIS012-05|05-ONMIS-0012|Canada|Ontario|612[1n]|BOLD:AAA9083
Ellida caniplaga|[777]|LPSO545-08|PPBP-0545|Canada|Ontario|658[0n]|BOLD:AAA9083
Misogada unicolor|[778]|LGSMB254-05|DNA-ATBI-1103|United States|Tennessee|535[1n]|BOLD:AAF7371
Misogada unicolor|[779]|LGSMC762-05|DNA-ATBI-2762|United States|Tennessee|658[0n]|BOLD:AAF7371
Misogada unicolor|[780]|LGSMB255-05|DNA-ATBI-1104|United States|Tennessee|571[0n]|BOLD:AAF7371
Litodonta wymola|[781]|JBAZ123-09|JLB-0123|United States|Arizona|658[0n]|BOLD:AAG5373
Litodonta wymola|[782]|BBLOC892-11|BIOUG01467-B07|United States|Arizona|658[0n]|BOLD:AAG5373
Litodonta aonides|[783]|CNCLB1682-14|CNCLEP 00113916|United States|New Mexico|658[0n]|BOLD:ACP...

Litodonta wymola|[781]|JBAZ123-09|JLB-0123|United States|Arizona|658[0n]|BOLD:AAG5373
Litodonta wymola|[782]|BBLOC892-11|BIOUG01467-B07|United States|Arizona|658[0n]|BOLD:AAG5373
Litodonta aonides|[783]|CNCLB1682-14|CNCLEP 00113916|United States|New Mexico|658[0n]|BOLD:ACP...
Litodonta aonides|[784]|CNCLB1798-14|CNCLEP 00114091|United States|Texas|658[0n]|BOLD:ACP4424
Macrurocampa marthesia|[785]|LGSMB288-05|DNA-ATBI-1137|United States|Tennessee|589[1n]|BOLD:AA...
Macrurocampa marthesia|[786]|LOT084-04|04HBL002084|United States|Tennessee|609[0n]|BOLD:AAA9644
Macrurocampa marthesia|[787]|LOT076-04|04HBL002076|United States|Tennessee|609[0n]|BOLD:AAA9644
Macrurocampa marthesia|[788]|LGSMG090-07|BGS03411|United States|Tennessee|658[0n]|BOLD:AAA9644
Macrurocampa marthesia|[789]|LOT083-04|04HBL002083|United States|Tennessee|609[0n]|BOLD:AAA9644
Macrurocampa marthesia|[790]|LOT082-04|04HBL002082|United States|Tennessee|609[0n]|BOLD:AAA9644
Macrurocampa marthesia|[791]|LOT072-04|04HBL002072|United States|Tennessee|609[0n]|BOLD:AAA9644
Macrurocampa marthesia|[792]|RDNMH438-09|CNCLEP00054452|United States|Tennessee|658[0n]|BOLD:A...
Macrurocampa marthesia|[793]|LGSMC751-05|DNA-ATBI-2751|United States|Tennessee|658[0n]|BOLD:AA...
Macrurocampa marthesia|[794]|TMNBD352-07|MNBTT-3153|Canada|New Brunswick|630[0n]|BOLD:AAA9644
Macrurocampa marthesia|[795]|RDLQ771-07|DH007822|Canada|Quebec|653[0n]|BOLD:AAA9644
Macrurocampa marthesia|[796]|NAMUM344-08|AM-93-0030|United States|Maryland|658[0n]|BOLD:AAA9644
Macrurocampa marthesia|[797]|LOT078-04|04HBL002078|United States|Tennessee|609[0n]|BOLD:AAA9644
Macrurocampa marthesia|[798]|LOT080-04|04HBL002080|United States|Tennessee|609[0n]|BOLD:AAA9644
Macrurocampa marthesia|[799]|LOT075-04|04HBL002075|United States|Tennessee|609[1n]|BOLD:AAA9644
Macrurocampa marthesia|[800]|XAE441-04|Moth4441.03|Canada|Ontario|658[0n]|BOLD:AAA9644
Macrurocampa marthesia|[801]|XAE425-04|Moth4425.03|Canada|Ontario|658[0n]|BOLD:AAA9644
Macrurocampa marthesia|[802]|XAF570-05|2005-ONT-219|Canada|Ontario|658[0n]|BOLD:AAA9644
Macrurocampa marthesia|[803]|LOT079-04|04HBL002079|United States|Tennessee|609[0n]|BOLD:AAA9644
Macrurocampa marthesia|[804]|LOT077-04|04HBL002077|United States|Tennessee|609[0n]|BOLD:AAA9644
Macrurocampa marthesia|[805]|LOT074-04|04HBL002074|United States|Tennessee|609[0n]|BOLD:AAA9644
Macrurocampa marthesia|[806]|LGSMB290-05|DNA-ATBI-1139|United States|Tennessee|541[1n]|BOLD:AA...
Macrurocampa marthesia|[807]|LGSMB287-05|DNA-ATBI-1136|United States|Tennessee|597[11n]|
Macrurocampa marthesia|[808]|XAE426-04|Moth4426.03|Canada|Ontario|591[0n]|BOLD:AAA9644
Macrurocampa marthesia|[809]|LPOKB380-09|MDOK-1440|United States|Oklahoma|656[4n]|BOLD:AAA9644
Macrurocampa marthesia|[810]|LPOKB988-09|MDOK-2030|United States|Oklahoma|658[0n]|BOLD:AAA9644
Macrurocampa marthesia|[811]|LPOKB990-09|MDOK-2032|United States|Oklahoma|658[0n]|BOLD:AAA9644
Macrurocampa marthesia|[812]|LOFLA743-06|06-FLOR-0743|United States|Florida|658[0n]|BOLD:AAA9644
Macrurocampa marthesia|[813]|LNCB051-06|06-NCC-1007|United States|North Carolina|658[0n]|BOLD:...
Macrurocampa marthesia|[814]|LOT081-04|04HBL002081|United States|Tennessee|609[0n]|BOLD:AAA9644
Macrurocampa marthesia|[815]|LOT073-04|04HBL002073|United States|Tennessee|658[0n]|BOLD:AAA9644
Macrurocampa marthesia|[816]|LOFLA550-06|06-FLOR-0550|United States|Florida|658[0n]|BOLD:AAA9644
Macrurocampa marthesia|[817]|LOFLB007-06|06-FLOR-0947|United States|Florida|658[0n]|BOLD:AAA9644
Macrurocampa marthesia|[818]|LPOKB984-09|MDOK-2026|United States|Oklahoma|658[0n]|BOLD:AAA9644
Macrurocampa marthesia|[819]|LGSMB289-05|DNA-ATBI-1138|United States|North Carolina|583[1n]|BOLD...
Macrurocampa marthesia|[820]|LPOKB355-09|MDOK-1369|United States|Oklahoma|658[0n]|BOLD:AAA9644
Macrurocampa dorothea|[821]|RDNMK348-11|CNCLEP 84257|United States|New Mexico|658[0n]|BOLD:AAZ...
Macrurocampa dorothea|[822]|RDNMK350-11|CNCLEP 84259|United States|New Mexico|658[0n]|BOLD:AAZ...
Litodonta gigantea|[823]|LNAUT1047-14|CCDB-22947-A03|United States|Arizona|618[0n]|BOLD:ABW6326
Litodonta gigantea|[824]|CMAZA866-12|BIOUG02040-B05|United States|Arizona|658[0n]|BOLD:ABW6326
Litodonta alpina|[825]|LNAUT1050-14|CCDB-22947-A06|United States|Texas|658[0n]|BOLD:ACN8310
Litodonta alpina|[826]|LNAUT1048-14|CCDB-22947-A04|United States|Texas|658[0n]|BOLD:ACN8310
Litodonta alpina|[827]|CNCLB2412-14|CNCLEP 00119554|United States|Texas|658[0n]|BOLD:ACN8310
Heterocampa ruficornis|[828]|AWCLB557-11|AWC-00322|United States|Arizona|658[0n]|BOLD:AAU7970
Heterocampa ruficornis|[829]|CMAZA917-12|BIOUG02040-F08|United States|Arizona|658[0n]|BOLD:AAU...
Lochmaeus manteo|[830]|XAK507-07|HLC-16060|Canada|Ontario|658[0n]|BOLD:AAB1511
Lochmaeus manteo|[831]|LPOKB977-09|MDOK-2019|United States|Oklahoma|658[0n]|BOLD:AAB1511
Lochmaeus manteo|[832]|LPOKA209-08|MDOK-0209|United States|Oklahoma|658[0n]|BOLD:AAB1511
Lochmaeus manteo|[833]|RDLQB677-05|DH010780|Canada|Quebec|658[0n]|BOLD:AAB1511
Lochmaeus manteo|[834]|PMG182-03|HETEROC2.00|Canada|Ontario|617[0n]|BOLD:AAB1511
Lochmaeus manteo|[835]|XAG249-05|2005-ONT-833|Canada|Ontario|505[2n]|BOLD:AAB1511
Lochmaeus manteo|[836]|LGSMB252-05|DNA-ATBI-1101|United States|Tennessee|573[0n]|BOLD:AAB1511
Lochmaeus manteo|[837]|LOT111-04|04HBL002111|United States|Tennessee|609[0n]|BOLD:AAB1511
Lochmaeus manteo|[838]|BBLOE1479-12|BIOUG01987-G09|United States|Oklahoma|658[0n]|BOLD:AAB1511
Lochmaeus manteo|[839]|TMNBD359-07|MNBTT-3160|Canada|New Brunswick|658[0n]|BOLD:AAB1511
Lochmaeus manteo|[840]|LSEU741-06|06-JKA-0741|United States|Georgia|658[0n]|BOLD:AAB1511
Lochmaeus manteo|[841]|TMNBD358-07|MNBTT-3159|Canada|New Brunswick|656[0n]|BOLD:AAB1511
Lochmaeus manteo|[842]|BBLEC904-09|09BBELE-0904|Canada|Nova Scotia|658[0n]|BOLD:AAB1511
Lochmaeus manteo|[843]|LOT501-04|04HBL002501|United States|Tennessee|658[0n]|BOLD:AAB1511
Lochmaeus manteo|[844]|XAG847-05|2005-ONT-1431|Canada|Ontario|658[0n]|BOLD:AAB1511
Lochmaeus manteo|[845]|TMNBD353-07|MNBTT-3154|Canada|New Brunswick|657[0n]|BOLD:AAB1511
Lochmaeus manteo|[846]|JSBIC013-08|HLC-16966|United States|Tennessee|658[0n]|BOLD:AAB1511
Lochmaeus manteo|[847]|PHMNB040-03|moth212.02SA|Canada|New Brunswick|639[0n]|BOLD:AAB1511
Lochmaeus manteo|[848]|MNBB234-05|05-NBSTA-150|Canada|New Brunswick|658[0n]|BOLD:AAB1511
Lochmaeus manteo|[849]|LGSMB253-05|DNA-ATBI-1102|United States|Tennessee|591[0n]|BOLD:AAB1511
Lochmaeus manteo|[850]|BBLOE1474-12|BIOUG01987-G04|United States|Oklahoma|658[0n]|BOLD:AAB1511
Lochmaeus manteo|[851]|LNCC1233-11|11-NCCC-758|United States|North Carolina|658[0n]|BOLD:AAB1511
Lochmaeus manteo|[852]|LNCB073-06|06-NCC-1029|United States|North Carolina|658[0n]|BOLD:AAB1511
Lochmaeus manteo|[853]|RDNMH437-09|CNCLEP00054451|United States|Tennessee|658[0n]|BOLD:AAB1511
Lochmaeus manteo|[854]|LGSMG097-07|BGS03418|United States|Tennessee|658[0n]|BOLD:AAB1511
Lochmaeus manteo|[855]|LNC629-06|06-NCCC-629|United States|North Carolina|658[0n]|BOLD:AAB1511
Lochmaeus manteo|[856]|LPOKB1004-09|MDOK-2046|United States|Oklahoma|658[0n]|BOLD:AAB1511
Lochmaeus manteo|[857]|LNCB074-06|06-NCC-1030|United States|North Carolina|658[0n]|BOLD:AAB1511
Lochmaeus manteo|[858]|BBLOD1646-11|BIOUG01830-B01|United States|Oklahoma|658[0n]|BOLD:AAB1511
Lochmaeus manteo|[859]|LNC628-06|06-NCCC-628|United States|North Carolina|658[0n]|BOLD:AAB1511
Lochmaeus manteo|[860]|RDNMH440-09|CNCLEP00054454|United States|Colorado|658[0n]|BOLD:AAB1511
Lochmaeus manteo|[861]|XAE623-04|Moth4623.03|Canada|Ontario|658[0n]|BOLD:AAB1511
Lochmaeus manteo|[862]|BBLOD1643-11|BIOUG01830-A10|United States|Oklahoma|658[0n]|BOLD:AAB1511
Lochmaeus manteo|[863]|BBLOD1645-11|BIOUG01830-A12|United States|Oklahoma|632[0n]|BOLD:AAB1511
Lochmaeus manteo|[864]|LGSMB251-05|DNA-ATBI-1100|United States|Tennessee|601[0n]|BOLD:AAB1511
Lochmaeus manteo|[865]|LPOKE569-12|BIOUG02922-C12|United States|Oklahoma|632[0n]|BOLD:AAB1511
Lochmaeus manteo|[866]|BBLPE589-09|09BBELE-2589|Canada|Nova Scotia|658[0n]|BOLD:AAB1511
Lochmaeus bilineata|[867]|XAK115-06|2006-ONT-1110|Canada|Ontario|658[0n]|BOLD:AAB3762
Lochmaeus bilineata|[868]|XAK263-06|2006-ONT-1258|Canada|Ontario|658[0n]|BOLD:AAB3762
Lochmaeus bilineata|[869]|XAF820-05|2005-ONT-469|Canada|Ontario|658[0n]|BOLD:AAB3762
Lochmaeus bilineata|[870]|XAE394-04|Moth4394.03|Canada|Ontario|658[0n]|BOLD:AAB3762
Lochmaeus bilineata|[871]|BBLCU206-09|09BBLEP-04693|United States|Michigan|658[0n]|BOLD:AAB3762
Lochmaeus bilineata|[872]|BBLCU222-09|09BBLEP-04709|United States|Michigan|658[0n]|BOLD:AAB3762
Lochmaeus bilineata|[873]|XAK602-07|HLC-16155|Canada|Ontario|594[0n]|BOLD:AAB3762
Lochmaeus bilineata|[874]|XAG110-05|2005-ONT-694|Canada|Ontario|658[0n]|BOLD:AAB3762
Lochmaeus bilineata|[875]|XAE584-04|Moth4584.03|Canada|Ontario|569[0n]|BOLD:AAB3762
Lochmaeus bilineata|[876]|XAE627-04|Moth4627.03|Canada|Ontario|658[0n]|BOLD:AAB3762
Lochmaeus bilineata|[877]|PHMO157-03|moth861.02|Canada|Ontario|639[0n]|BOLD:AAB3762
Lochmaeus bilineata|[878]|XAK603-07|HLC-16156|Canada|Ontario|658[0n]|BOLD:AAB3762
Lochmaeus bilineata|[879]|XAG760-05|2005-ONT-1344|Canada|Ontario|658[0n]|BOLD:AAB3762
Lochmaeus bilineata|[880]|XAG766-05|2005-ONT-1350|Canada|Ontario|658[0n]|BOLD:AAB3762
Lochmaeus bilineata|[881]|XAE354-04|Moth4354.03|Canada|Ontario|658[0n]|BOLD:AAB3762

Lochmaeus bilineata|[879]|XAG760-05|2005-ONT-1344|Canada|Ontario|658[0n]|BOLD:AAB3762
Lochmaeus bilineata|[880]|XAG766-05|2005-ONT-1350|Canada|Ontario|658[0n]|BOLD:AAB3762
Lochmaeus bilineata|[881]|XAE354-04|Moth4354.03|Canada|Ontario|658[0n]|BOLD:AAB3762
Lochmaeus bilineata|[882]|XAB282-04|04HBL005282|Canada|Ontario|658[0n]|BOLD:AAB3762
Lochmaeus bilineata|[883]|XAE308-04|Moth4308.03|Canada|Ontario|658[0n]|BOLD:AAB3762
Lochmaeus bilineata|[884]|XAG879-05|2005-ONT-1463|Canada|Ontario|658[0n]|BOLD:AAB3762
Lochmaeus bilineata|[885]|XAG104-05|2005-ONT-688|Canada|Ontario|658[0n]|BOLD:AAB3762
Lochmaeus bilineata|[886]|LNCB468-07|07-NCNW-0152|United States|North Carolina|658[0n]|BOLD:AA...
Lochmaeus bilineata|[887]|RDNMJ038-10|CNCLEP 73818|United States|Florida|658[0n]|BOLD:AAB3762
Lochmaeus bilineata|[888]|LPOKB393-09|MDOK-1486|United States|Oklahoma|658[0n]|BOLD:ACF1461
Lochmaeus bilineata|[889]|LILLA698-11|SNS10IL-00884|United States|Illinois|658[0n]|BOLD:ACF1461
Lochmaeus bilineata|[890]|LILLA077-11|SNS10IL-00102|United States|Illinois|658[0n]|BOLD:ACF1461
Lochmaeus bilineata|[891]|RDNMH490-09|CNCLEP00057809|United States|Louisiana|631[0n]|BOLD:AAB3762
Lochmaeus bilineata|[892]|LGSMB242-05|DNA-ATBI-1091|United States|Tennessee|569[0n]|BOLD:AAB3762
Lochmaeus bilineata|[893]|BBLOC883-11|BIOUG01467-A10|United States|Arkansas|658[0n]|BOLD:AAB3762
Lochmaeus bilineata|[894]|LPOKA123-08|MDOK-0123|United States|Oklahoma|658[0n]|BOLD:AAB3762
Lochmaeus bilineata|[895]|LGSMC761-05|DNA-ATBI-2761|United States|Tennessee|567[0n]|BOLD:AAB3762
Lochmaeus bilineata|[896]|LGSMC758-05|DNA-ATBI-2758|United States|Tennessee|658[0n]|BOLD:AAB3762
Lochmaeus bilineata|[897]|BBLOC1044-11|BIOUG01468-G04|United States|Arkansas|658[0n]|BOLD:AAB3762
Lochmaeus bilineata|[898]|BBLSX045-09|09BBLEP-01973|United States|Texas|658[0n]|BOLD:ACF3472
Lochmaeus bilineata|[899]|BBLOE1475-12|BIOUG01987-G05|United States|Oklahoma|658[0n]|BOLD:ACF3472
Lochmaeus bilineata|[900]|LPOKE275-11|MDOK-4353|United States|Oklahoma|658[0n]|BOLD:ACF3472
Lochmaeus bilineata|[901]|LPOKE277-11|MDOK-4355|United States|Oklahoma|658[0n]|BOLD:ACF3472
Lochmaeus bilineata|[902]|BBLSW842-09|09BBLEP-01770|United States|Texas|658[0n]|BOLD:ACF3472
Heterocampa zayasi|[903]|RDNML295-13|CNCLEP 92271|United States|Florida|658[0n]|BOLD:ABX8050
Heterocampa zayasi|[904]|RDNML294-13|CNCLEP 92270|United States|Florida|658[0n]|BOLD:ABX8050
Schizura ipomoeae|[905]|LOT491-04|04HBL002491|United States|Tennessee|658[0n]|BOLD:ACE9227
Schizura ipomoeae|[906]|LGSMB245-05|DNA-ATBI-1094|United States|Tennessee|591[2n]|BOLD:ACE9227
Schizura ipomoeae|[907]|LGSMB246-05|DNA-ATBI-1095|United States|Tennessee|615[0n]|BOLD:ACE9227
Schizura ipomoeae|[908]|LSEU560-06|06-JKA-0560|United States|Georgia|658[0n]|BOLD:ACE9227
Schizura ipomoeae|[909]|MMNA108-08|HLC-17670|United States|North Carolina|658[0n]|BOLD:ACE9227
Schizura ipomoeae|[910]|LSUSA186-06|06-SUSA-0186|United States|Kentucky|658[0n]|BOLD:ACE9227
Schizura ipomoeae|[911]|LSEU602-06|06-JKA-0602|United States|Georgia|658[0n]|BOLD:ACE9227
Schizura ipomoeae|[912]|LOT099-04|04HBL002099|United States|Tennessee|609[0n]|BOLD:ACE9227
Schizura ipomoeae|[913]|LGSMG099-07|BGS03420|United States|North Carolina|658[0n]|BOLD:ACE9227
Schizura ipomoeae|[914]|LGSMB244-05|DNA-ATBI-1093|United States|Tennessee|595[0n]|BOLD:ACE9227
Schizura ipomoeae|[915]|LOFLB772-06|06-FLOR-1712|United States|Florida|658[0n]|BOLD:ACE9227
Schizura ipomoeae|[916]|LOFLA903-06|06-FLOR-0903|United States|Florida|600[0n]|BOLD:ACE9227
Schizura ipomoeae|[917]|LNCB132-06|06-NCCC-1088|United States|North Carolina|599[0n]|BOLD:ACE9227
Schizura ipomoeae|[918]|LOT538-04|04HBL002538|United States|Tennessee|596[0n]|BOLD:ACE9227
Schizura ipomoeae|[919]|LGSMB247-05|DNA-ATBI-1096|United States|Tennessee|585[0n]|BOLD:ACE9227
Schizura ipomoeae|[920]|UDLEP102-09|v750 UPM|United States|Maryland|612[0n]|BOLD:ACE9227
Schizura ipomoeae|[921]|LOFLB749-06|06-FLOR-1689|United States|Florida|658[0n]|BOLD:ACE9227
Schizura ipomoeae|[922]|USLEP298-10|10BBLEP-00298|United States|Florida|658[0n]|BOLD:ACE9227
Schizura ipomoeae|[923]|LOT532-04|04HBL002532|United States|Tennessee|515[0n]|BOLD:ACE9227
Schizura ipomoeae|[924]|LGSMG098-07|BGS03419|United States|Tennessee|658[0n]|BOLD:ACE9227
Schizura ipomoeae|[925]|UDLEP211-09|v150 MD|United States|Delaware|658[0n]|BOLD:ACE9227
Schizura ipomoeae|[926]|LNC284-05|05-NCCC-284|United States|North Carolina|658[0n]|BOLD:ACE9227
Schizura ipomoeae|[927]|LOFLB433-06|06-FLOR-1373|United States|Florida|658[0n]|BOLD:ACE9227
Schizura ipomoeae|[928]|LOT123-04|04HBL002123|United States|Tennessee|609[0n]|BOLD:ACE9227
Schizura ipomoeae|[929]|XAK529-07|HLC-16082|Canada|Ontario|658[0n]|BOLD:AAA7094
Schizura ipomoeae|[930]|AHLEP1008-12|2011.08.17.1B.AB.08.C1|United States|Pennsylvania|658[0n]|BO...
Schizura ipomoeae|[931]|RDLQB189-05|DH010275|Canada|Quebec|658[3n]|BOLD:AAA7094
Schizura ipomoeae|[932]|RDLQB192-05|DH010278|Canada|Quebec|571[0n]|BOLD:AAA7094
Schizura ipomoeae|[933]|ALLEP433-13|BIOUG07481-E05|Canada|Ontario|658[0n]|BOLD:AAA7094
Schizura ipomoeae|[934]|PHMO125-03|moth738.02|Canada|Ontario|639[0n]|BOLD:AAA7094
Schizura ipomoeae|[935]|RWWC271-11|RWWA-2248|United States|Washington|658[0n]|BOLD:AAA7094
Schizura ipomoeae|[936]|RWWC987-12|RWWA-3602|United States|Washington|658[0n]|BOLD:AAA7094
Schizura ipomoeae|[937]|RWWC605-11|RWWA-2582|United States|Washington|658[0n]|BOLD:AAA7094
Schizura ipomoeae|[938]|RWWA235-09|RWWA-0235|United States|Washington|658[0n]|BOLD:AAA7094
Schizura ipomoeae|[939]|RWWC366-11|RWWA-2343|United States|Washington|658[0n]|BOLD:AAA7094
Schizura ipomoeae|[940]|RWWC408-11|RWWA-2385|United States|Washington|658[0n]|BOLD:AAA7094
Schizura ipomoeae|[941]|RWWC939-12|RWWA-3490|United States|Washington|658[0n]|BOLD:AAA7094
Schizura ipomoeae|[942]|LHLEP067-06|UBC-2006-0271|Canada|British Columbia|658[0n]|BOLD:AAA7094
Schizura ipomoeae|[943]|LMH030-06|PFC-2006-0135|Canada|British Columbia|658[0n]|BOLD:AAA7094
Schizura ipomoeae|[944]|LHLEP154-06|UBC-2006-1513|Canada|British Columbia|658[0n]|BOLD:AAA7094
Schizura ipomoeae|[945]|LBCB179-05|HLC-21119|Canada|British Columbia|658[0n]|BOLD:AAA7094
Schizura ipomoeae|[946]|LBCC344-05|HLC-22224|Canada|British Columbia|658[0n]|BOLD:AAA7094
Schizura ipomoeae|[947]|LBCA793-05|HLC-20793|Canada|British Columbia|658[0n]|BOLD:AAA7094
Schizura ipomoeae|[948]|LBCA795-05|HLC-20795|Canada|British Columbia|658[0n]|BOLD:AAA7094
Schizura ipomoeae|[949]|LALPA336-10|AVBC 338-10|Canada|British Columbia|658[0n]|BOLD:AAA7094
Schizura ipomoeae|[950]|LALPA475-10|AVBC 477-10|Canada|British Columbia|658[0n]|BOLD:AAA7094
Schizura ipomoeae|[951]|LBCC422-05|HLC-22302|Canada|British Columbia|658[0n]|BOLD:AAA7094
Schizura ipomoeae|[952]|LBCC420-05|HLC-22300|Canada|British Columbia|658[0n]|BOLD:AAA7094
Schizura ipomoeae|[953]|LBCS260-07|UBC-2007-0768|Canada|British Columbia|658[0n]|BOLD:AAA7094
Schizura ipomoeae|[954]|LBCA791-05|HLC-20791|Canada|British Columbia|658[0n]|BOLD:AAA7094
Schizura ipomoeae|[955]|LHLEP153-06|UBC-2006-0741|Canada|British Columbia|658[0n]|BOLD:AAA7094
Schizura ipomoeae|[956]|LBCC419-05|HLC-22299|Canada|British Columbia|658[0n]|BOLD:AAA7094
Schizura ipomoeae|[957]|LALPA366-10|AVBC 368-10|Canada|British Columbia|658[0n]|BOLD:AAA7094
Schizura ipomoeae|[958]|TMNBD213-07|MNBTT-3014|Canada|New Brunswick|658[0n]|BOLD:AAA7094
Schizura ipomoeae|[959]|LBCA792-05|HLC-20792|Canada|British Columbia|600[0n]|BOLD:AAA7094
Schizura ipomoeae|[960]|RDLQB191-05|DH010277|Canada|Quebec|593[0n]|BOLD:AAA7094
Schizura ipomoeae|[961]|TMNBD216-07|MNBTT-3017|Canada|New Brunswick|658[0n]|BOLD:AAA7094
Schizura ipomoeae|[962]|TMNBD217-07|MNBTT-3018|Canada|New Brunswick|658[0n]|BOLD:AAA7094
Schizura ipomoeae|[963]|LOCT030-05|05-CTATBI-0030|United States|Connecticut|658[0n]|BOLD:AAA7094
Schizura ipomoeae|[964]|TMNBD214-07|MNBTT-3015|Canada|New Brunswick|658[0n]|BOLD:AAA7094
Schizura ipomoeae|[965]|LOCT031-05|05-CTATBI-0031|United States|Connecticut|658[0n]|BOLD:AAA7094
Schizura ipomoeae|[966]|TTMNB938-06|MNBTT-938|Canada|New Brunswick|658[0n]|BOLD:AAA7094
Schizura ipomoeae|[967]|RDLQB190-05|DH010276|Canada|Quebec|584[2n]|BOLD:AAA7094
Schizura ipomoeae|[968]|TMNBD215-07|MNBTT-3016|Canada|New Brunswick|630[0n]|BOLD:AAA7094
Schizura ipomoeae|[969]|RWWC1159-13|BIOUG07834-D05|United States|Washington|601[0n]|BOLD:AAA7094
Schizura ipomoeae|[970]|LOFLC129-06|06-FLOR-2009|United States|Florida|658[0n]|BOLD:ACE9227
Schizura biedermani|[971]|CNCLB1446-14|CNCLEP 00113654|United States|Arizona|658[0n]|BOLD:ACN4632
Schizura biedermani|[972]|NOCNA021-14|20320-100609-TX|United States|Texas|658[0n]|BOLD:ACN4632
Schizura biedermani|[973]|NOCNA022-14|20321-100609-TX|United States|Texas|658[0n]|BOLD:ACN4632
Oligocentria pallida|[974]|RWWA772-09|RWWA-0808|United States|Washington|658[0n]|BOLD:AAB7833
Oligocentria pallida|[975]|LBCD314-05|HLC-23134|Canada|British Columbia|648[0n]|BOLD:AAB7833
Oligocentria pallida|[976]|LMH034-06|PFC-2006-0439|Canada|British Columbia|632[0n]|BOLD:AAB7833
Oligocentria pallida|[977]|RDNMH154-09|CNCLEP00054371|United States|Wyoming|644[0n]|BOLD:AAB7833
Oligocentria pallida|[978]|RWWA795-09|RWWA-0831|United States|Washington|658[0n]|BOLD:AAB7833
Oligocentria pallida|[979]|LBCH004-10|10-JDWBC-0004|Canada|British Columbia|658[0n]|BOLD:AAB7833

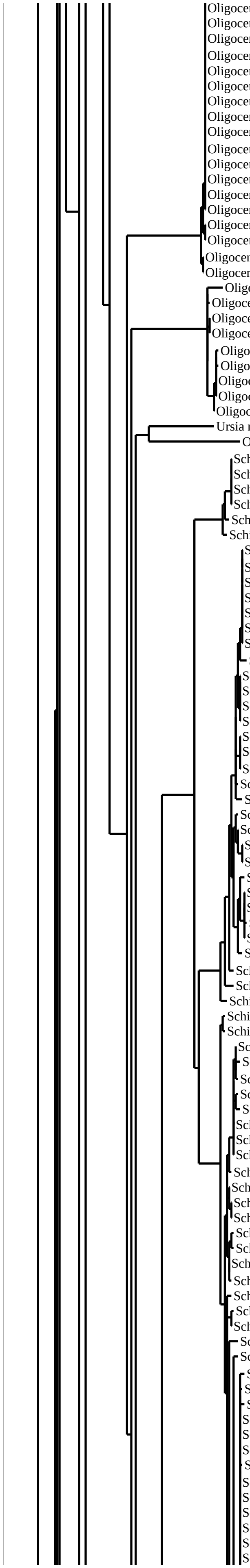

Oligocentria pallida|[977]|RDNMH154-09|CNCLEP00054371|United States|Wyoming|644[0n]|BOLD:AAB7833
Oligocentria pallida|[978]|RWWA795-09|RWWA-0831|United States|Washington|658[0n]|BOLD:AAB7833
Oligocentria pallida|[979]|LBCH004-10|10-JDWBC-0004|Canada|British Columbia|658[0n]|BOLD:AAB7833
Oligocentria pallida|[980]|RWWB182-09|RWWA-1181|United States|Washington|658[0n]|BOLD:AAB7833
Oligocentria pallida|[981]|CNCLB1680-14|CNCLEP 00113914|United States|Wyoming|658[0n]|BOLD:AAB...
Oligocentria pallida|[982]|RWWC405-11|RWWA-2382|United States|Washington|658[0n]|BOLD:AAB7833
Oligocentria pallida|[983]|RWWC025-10|RWWA-2002|United States|Washington|658[0n]|BOLD:AAB7833
Oligocentria pallida|[984]|LPABC961-09|08BBLEP-05372|Canada|Alberta|658[0n]|BOLD:AAB7833
Oligocentria pallida|[985]|LALPA337-10|AVBC 339-10|Canada|British Columbia|658[0n]|BOLD:AAB7833
Oligocentria pallida|[986]|RWWA752-09|RWWA-0788|United States|Washington|658[0n]|BOLD:AAB7833
Oligocentria pallida|[987]|LBCH645-10|10-JDWBC-0645|Canada|British Columbia|658[0n]|BOLD:AAB7833
Oligocentria pallida|[988]|LBCD315-05|HLC-23135|Canada|British Columbia|658[0n]|BOLD:AAB7833
Oligocentria pallida|[989]|RDNMH427-09|CNCLEP00054441|United States|Wyoming|658[0n]|BOLD:AAB7833
Oligocentria pallida|[990]|RWWA729-09|RWWA-0765|United States|Washington|658[0n]|BOLD:AAB7833
Oligocentria pallida|[991]|RDNMH412-09|CNCLEP00054426|United States|Colorado|658[0n]|BOLD:AAB7833
Oligocentria pallida|[992]|RWWA930-09|RWWA-0930|United States|Washington|658[0n]|BOLD:AAB7833
Oligocentria pallida|[993]|LBCA794-05|HLC-20794|Canada|British Columbia|658[0n]|BOLD:AAB7833
Oligocentria pallida|[994]|LBCC023-05|HLC-21903|Canada|British Columbia|658[0n]|BOLD:AAB7833
Oligocentria alpicoides|[995]|CNCLB1571-14|CNCLEP 00113801|United States|Arizona|658[0n]|BOLD:...
Oligocentria alpica|[996]|CNCLB1797-14|CNCLEP 00114090|United States|New Mexico|658[0n]|BOLD:A...
Oligocentria alpica|[997]|NOCNA027-14|20326-100609-TX|United States|Texas|658[0n]|BOLD:AAH5257
Oligocentria alpica|[998]|BBLSW737-09|09BBLEP-01665|United States|Texas|658[0n]|BOLD:AAH5257
Oligocentria alpica|[999]|CNCLB1681-14|CNCLEP 00113915|United States|Arkansas|658[0n]|BOLD:AAH...
Oligocentria alpica|[1000]|BBLSW026-09|09BBLEP-00954|United States|Texas|636[0n]|BOLD:AAH5257
Oligocentria alpica|[1001]|BBLSY127-09|09BBLEP-03054|United States|Texas|658[0n]|BOLD:AAH5257
Oligocentria alpica|[1002]|BBLSW027-09|09BBLEP-00955|United States|Texas|658[0n]|BOLD:AAH5257
Oligocentria alpica|[1003]|NOCNA028-14|20327-100609-TX|United States|Texas|658[0n]|BOLD:AAH5257
Ursia noctuiformis|[1004]|RDNMD453-06|CNCNoctuoidea12785|United States|California|635[0n]|BOLD ...
Oligocentria delicata|[1005]|RDNMH516-09|CNCLEP00057835|United States|Arizona|658[0n]|BOLD:AAK...
Schizura sp.|[1006]|RDNMH430-09|CNCLEP00054444|United States|California|658[0n]|BOLD:ABY7736
Schizura sp.|[1007]|LTOLB021-08|RR-98-1149|United States|California|646[0n]|BOLD:ABY7736
Schizura sp.|[1008]|RDNMH167-09|CNCLEP00054384|United States|California|637[0n]|BOLD:ABY7736
Schizura sp.|[1009]|RDNMB448-05|CNCNoctuoidea10214|Canada|British Columbia|579[0n]|BOLD:ABY7736
Schizura sp.|[1010]|RDNMH161-09|CNCLEP00054378|United States|Nevada|658[0n]|BOLD:ABY7736
Schizura sp.|[1011]|LBCG1105-09|08-JDWBC-1105|Canada|British Columbia|658[0n]|BOLD:ABY7736
Schizura errucata|[1012]|BBLSW037-09|09BBLEP-00965|United States|Texas|658[0n]|BOLD:ABY7735
Schizura errucata|[1013]|BBLSW135-09|09BBLEP-01063|United States|Texas|658[0n]|BOLD:ABY7735
Schizura errucata|[1014]|BBLSW035-09|09BBLEP-00963|United States|Texas|658[0n]|BOLD:ABY7735
Schizura errucata|[1015]|BBLSW735-09|09BBLEP-01663|United States|Texas|658[0n]|BOLD:ABY7735
Schizura errucata|[1016]|USLEP082-10|10BBLEP-00082|United States|Texas|658[0n]|BOLD:ABY7735
Schizura errucata|[1017]|BBLSW729-09|09BBLEP-01657|United States|Texas|658[0n]|BOLD:ABY7735
Schizura errucata|[1018]|BBLSX804-09|09BBLEP-02732|United States|Texas|632[0n]|BOLD:ABY7735
Schizura errucata|[1019]|BBLSZ196-09|09BBLEP-04122|United States|Texas|658[0n]|BOLD:ABY7735
Schizura errucata|[1020]|BBLSY920-09|09BBLEP-03847|United States|Texas|658[0n]|BOLD:ABY7735
Schizura errucata|[1021]|BBLSY126-09|09BBLEP-03053|United States|Texas|658[0n]|BOLD:ABY7735
Schizura errucata|[1022]|BBLSZ187-09|09BBLEP-04113|United States|Texas|658[0n]|BOLD:ABY7735
Schizura errucata|[1023]|CNCLB2094-14|CNCLEP00117936|United States|Texas|658[0n]|BOLD:ABY7735
Schizura errucata|[1024]|CNCLB2095-14|CNCLEP00117937|United States|Texas|658[0n]|BOLD:ABY7735
Schizura errucata|[1025]|CNCLB2093-14|CNCLEP00117935|United States|Texas|658[0n]|BOLD:ABY7735
Schizura errucata|[1026]|CNCLB2096-14|CNCLEP00117938|United States|Texas|658[0n]|BOLD:ABY7735
Schizura errucata|[1027]|CNCLB2414-14|CNCLEP 00119556|United States|Texas|658[0n]|BOLD:ABY7735
Schizura errucata|[1028]|BBLSX764-09|09BBLEP-02692|United States|Texas|658[0n]|BOLD:ABY7735
Schizura errucata|[1029]|BBLSX178-09|09BBLEP-02106|United States|Texas|658[0n]|BOLD:ABY7735
Schizura errucata|[1030]|BBLSZ175-09|09BBLEP-04101|United States|Texas|658[0n]|BOLD:ABY7735
Schizura errucata|[1031]|USLEP081-10|10BBLEP-00081|United States|Texas|658[0n]|BOLD:ABY7735
Schizura errucata|[1032]|USLEP977-10|10BBLEP-00977|United States|Texas|658[0n]|BOLD:ABY7735
Schizura errucata|[1033]|BBLSZ198-09|09BBLEP-04124|United States|Texas|658[0n]|BOLD:ABY7735
Schizura errucata|[1034]|BBLSW720-09|09BBLEP-01648|United States|Texas|658[0n]|BOLD:ABY7735
Schizura errucata|[1035]|BBLSZ139-09|09BBLEP-04065|United States|Texas|658[0n]|BOLD:ABY7735
Schizura errucata|[1036]|BBLSX787-09|09BBLEP-02715|United States|Texas|658[0n]|BOLD:ABY7735
Schizura errucata|[1037]|BBLSY226-09|09BBLEP-03153|United States|Texas|658[0n]|BOLD:ABY7735
Schizura errucata|[1038]|BBLSW716-09|09BBLEP-01644|United States|Texas|658[0n]|BOLD:ABY7735
Schizura errucata|[1039]|BBLSX805-09|09BBLEP-02733|United States|Texas|658[0n]|BOLD:ABY7735
Schizura errucata|[1040]|BBLSX220-09|09BBLEP-02148|United States|Texas|658[0n]|BOLD:ABY7735
Schizura errucata|[1041]|BBLSY231-09|09BBLEP-03158|United States|Texas|658[0n]|BOLD:ABY7735
Schizura unicornis|[1042]|LPOKB427-09|MDOK-1417|United States|Oklahoma|658[1n]|BOLD:AAA3873
Schizura unicornis|[1043]|LMDH133-11|BIOUG01047-B11|United States|Minnesota|658[0n]|BOLD:AAA3873
Schizura unicornis|[1044]|LTOLB019-08|AM-93-0432|United States|Maryland|655[0n]|BOLD:AAA3873
Schizura unicornis|[1045]|PMG189-03|moth609.01|Canada|Ontario|617[0n]|BOLD:AAA3873
Schizura unicornis|[1046]|TMG70-03|moth323.01|Canada|Ontario|617[0n]|BOLD:AAA3873
Schizura unicornis|[1047]|LOWCB160-05|CGWC-1100|Canada|British Columbia|658[0n]|BOLD:AAA3873
Schizura unicornis|[1048]|LPMN265-08|08BBLEP-01064|Canada|Manitoba|658[0n]|BOLD:AAA3873
Schizura unicornis|[1049]|XAJ413-06|2006-ONT-0413|Canada|Ontario|658[0n]|BOLD:AAA3873
Schizura unicornis|[1050]|TMNBD187-07|MNBTT-2988|Canada|New Brunswick|658[0n]|BOLD:AAA3873
Schizura unicornis|[1051]|UDLEP189-09|v650 CV|United States|Delaware|641[0n]|BOLD:AAA3873
Schizura unicornis|[1052]|XAE337-04|Moth4337.03|Canada|Ontario|658[0n]|BOLD:AAA3873
Schizura unicornis|[1053]|BBLOC961-11|BIOUG01467-H04|United States|Texas|658[0n]|BOLD:AAA3873
Schizura unicornis|[1054]|LPOKC771-09|MDOK-2848|United States|Oklahoma|658[0n]|BOLD:AAA3873
Schizura unicornis|[1055]|BBLSU053-09|09BBLEP-04422|United States|Arkansas|658[0n]|BOLD:AAA3873
Schizura unicornis|[1056]|BBLPB681-10|10BBCLP-1680|Canada|Ontario|658[0n]|BOLD:AAA3873
Schizura unicornis|[1057]|TMNBD185-07|MNBTT-2986|Canada|New Brunswick|658[0n]|BOLD:AAA3873
Schizura unicornis|[1058]|XAG183-05|2005-ONT-767|Canada|Ontario|611[0n]|BOLD:AAA3873
Schizura unicornis|[1059]|XAJ501-06|2006-ONT-0501|Canada|Ontario|658[0n]|BOLD:AAA3873
Schizura unicornis|[1060]|LOCT326-05|05-CTATBI-0326|United States|Connecticut|619[0n]|BOLD:AAA...
Schizura unicornis|[1061]|LPSK238-08|08BBLEP-01806|Canada|Saskatchewan|658[0n]|BOLD:AAA3873
Schizura unicornis|[1062]|CNRME4988-12|BIOUG03805-B02|Canada|Manitoba|635[0n]|BOLD:AAA3873
Schizura unicornis|[1063]|UDLEP191-09|v652 CV|United States|Delaware|658[0n]|BOLD:AAA3873
Schizura unicornis|[1064]|MNBB008-05|HBL008618|Canada|New Brunswick|658[0n]|BOLD:AAA3873
Schizura unicornis|[1065]|XAG018-05|2005-ONT-602|Canada|Ontario|629[0n]|BOLD:AAA3873
Schizura unicornis|[1066]|LSUSA171-06|06-SUSA-0171|United States|Kentucky|658[0n]|BOLD:AAA3873
Schizura unicornis|[1067]|USLEP643-10|10BBLEP-00643|United States|Florida|658[0n]|BOLD:AAA3873
Schizura unicornis|[1068]|BBLOB1297-11|BIOUG01417-F02|United States|Florida|658[0n]|BOLD:AAA3873
Schizura unicornis|[1069]|LILLA146-11|SNS10IL-00192|United States|Illinois|658[0n]|BOLD:AAA3873
Schizura unicornis|[1070]|BBLSX527-09|09BBLEP-02455|United States|Oklahoma|658[0n]|BOLD:AAA3873
Schizura unicornis|[1071]|LNC235-05|05-NCCC-235|United States|North Carolina|658[0n]|BOLD:AAA3873
Schizura unicornis|[1072]|LPSO928-08|PPBP-0928|Canada|Ontario|658[0n]|BOLD:AAA3873
Schizura unicornis|[1073]|XAB118-04|04HBL005118|Canada|Ontario|658[0n]|BOLD:AAA3873
Schizura unicornis|[1074]|XAB284-04|04HBL005284|Canada|Ontario|658[0n]|BOLD:AAA3873
Schizura unicornis|[1075]|TTMNB543-06|MNBTT-543|Canada|New Brunswick|658[0n]|BOLD:AAA3873
Schizura unicornis|[1076]|UDLEP264-09|v236 UD|United States|Delaware|658[0n]|BOLD:AAA3873
Schizura unicornis|[1077]|LPSO265-08|PPBP-0265|Canada|Ontario|658[0n]|BOLD:AAA3873

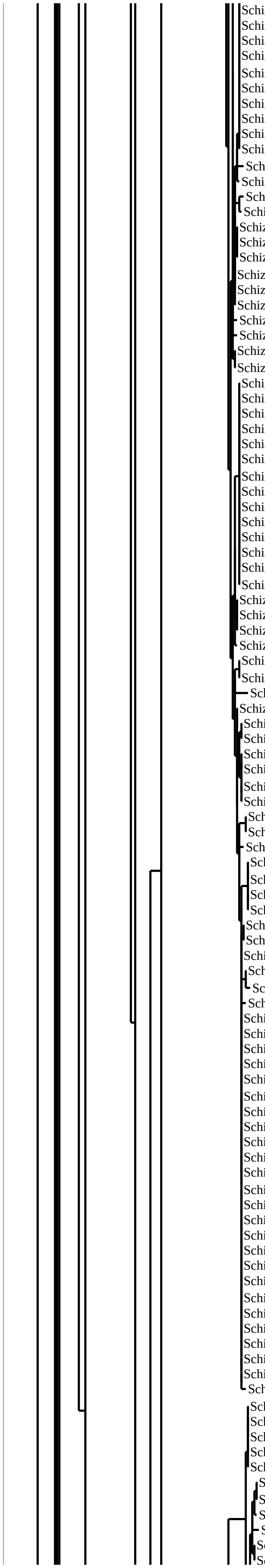

Schizura unicornis|[1075]|TTMNB543-06|MNBTT-543|Canada|New Brunswick|658[0n]|BOLD:AAA3873
Schizura unicornis|[1076]|UDLEP264-09|v236 UD|United States|Delaware|658[0n]|BOLD:AAA3873
Schizura unicornis|[1077]|LPSO265-08|PPBP-0265|Canada|Ontario|658[0n]|BOLD:AAA3873
Schizura unicornis|[1078]|XAG263-05|2005-ONT-847|Canada|Ontario|658[0n]|BOLD:AAA3873
Schizura unicornis|[1079]|USLEP622-10|10BBLEP-00622|United States|Florida|658[0n]|BOLD:AAA3873
Schizura unicornis|[1080]|LOCT039-05|05-CTATBI-0039|United States|Connecticut|658[0n]|BOLD:AAA...
Schizura unicornis|[1081]|BLTIB791-08|BL1208|Canada|Ontario|658[0n]|BOLD:AAA3873
Schizura unicornis|[1082]|MNBB248-05|05-NBSTA-164|Canada|New Brunswick|658[0n]|BOLD:AAA3873
Schizura unicornis|[1083]|XAK009-06|2006-ONT-1004|Canada|Ontario|658[0n]|BOLD:AAA3873
Schizura unicornis|[1084]|TTMNB249-06|MNBTT-249|Canada|New Brunswick|658[0n]|BOLD:AAA3873
Schizura unicornis|[1085]|LPSO089-08|PPBP-0089|Canada|Ontario|648[0n]|BOLD:AAA3873
Schizura unicornis|[1086]|LPSOC368-08|PPBP-2367|Canada|Ontario|655[0n]|BOLD:AAA3873
Schizura unicornis|[1087]|XAE474-04|Moth4474.03|Canada|Ontario|658[0n]|BOLD:AAA3873
Schizura unicornis|[1088]|LPSOB097-08|PPBP-1096|Canada|Ontario|658[0n]|BOLD:AAA3873
Schizura unicornis|[1089]|BBLEC205-09|09BBELE-0205|Canada|Nova Scotia|658[0n]|BOLD:AAA3873
Schizura unicornis|[1090]|MNBB126-05|05-NBSTA-042|Canada|New Brunswick|658[0n]|BOLD:AAA3873
Schizura unicornis|[1091]|LOCT038-05|05-CTATBI-0038|United States|Connecticut|658[0n]|BOLD:AAA...
Schizura unicornis|[1092]|CNROM095-13|BIOUG08978-D04|Canada|Ontario|603[0n]|BOLD:AAA3873
Schizura unicornis|[1093]|LOT088-04|04HBL002088|United States|Tennessee|609[0n]|BOLD:AAA3873
Schizura unicornis|[1094]|LPSO958-08|PPBP-0958|Canada|Ontario|609[0n]|BOLD:AAA3873
Schizura unicornis|[1095]|BBLSU085-09|09BBLEP-04454|United States|Mississippi|658[0n]|BOLD:AAA...
Schizura unicornis|[1096]|XAE568-04|Moth4568.03|Canada|Ontario|597[0n]|BOLD:AAA3873
Schizura unicornis|[1097]|RDNML362-13|CNCLEP 92338|United States|Florida|658[0n]|BOLD:AAA3873
Schizura unicornis|[1098]|BBLPC003-09|09BBELE-1003|Canada|New Brunswick|658[0n]|BOLD:AAA3873
Schizura unicornis|[1099]|LBCC765-05|HLC-22645|Canada|British Columbia|608[0n]|BOLD:AAA3873
Schizura unicornis|[1100]|PHMNB574-04|04HBL00800|Canada|New Brunswick|658[0n]|BOLD:AAA3873
Schizura unicornis|[1101]|TMNBD186-07|MNBTT-2987|Canada|New Brunswick|655[0n]|BOLD:AAA3873
Schizura unicornis|[1102]|TTMNB940-06|MNBTT-940|Canada|New Brunswick|658[0n]|BOLD:AAA3873
Schizura unicornis|[1103]|LALPA274-10|AVBC 275-10|Canada|British Columbia|658[0n]|BOLD:AAA3873
Schizura unicornis|[1104]|MNBB487-05|05-NBSTA-403|Canada|New Brunswick|658[0n]|BOLD:AAA3873
Schizura unicornis|[1105]|TMNBD184-07|MNBTT-2985|Canada|New Brunswick|657[0n]|BOLD:AAA3873
Schizura unicornis|[1106]|LPVIB979-08|PFC-2006-2529|Canada|British Columbia|658[0n]|BOLD:AAA3873
Schizura unicornis|[1107]|LPVIA093-08|PFC-2006-0133|Canada|British Columbia|658[0n]|BOLD:AAA3873
Schizura unicornis|[1108]|TMNBB001-06|MNBTT-941|Canada|New Brunswick|658[0n]|BOLD:AAA3873
Schizura unicornis|[1109]|LALPA500-10|AVBC 502-10|Canada|British Columbia|658[0n]|BOLD:AAA3873
Schizura unicornis|[1110]|MNBB302-05|05-NBSTA-218|Canada|New Brunswick|658[0n]|BOLD:AAA3873
Schizura unicornis|[1111]|LPVIA796-08|PFC-2006-1086|Canada|British Columbia|658[0n]|BOLD:AAA3873
Schizura unicornis|[1112]|LPVIA797-08|PFC-2006-1087|Canada|British Columbia|658[0n]|BOLD:AAA3873
Schizura unicornis|[1113]|RDLQF520-06|DH011669|Canada|Quebec|658[0n]|BOLD:AAA3873
Schizura unicornis|[1114]|TMNBD188-07|MNBTT-2989|Canada|New Brunswick|658[0n]|BOLD:AAA3873
Schizura unicornis|[1115]|XAJ357-06|2006-ONT-0357|Canada|Ontario|614[0n]|BOLD:AAA3873
Schizura unicornis|[1116]|PHMNB575-04|04HBL00801|Canada|New Brunswick|658[0n]|BOLD:AAA3873
Schizura unicornis|[1117]|RWWB895-10|RWWA-1894|United States|Washington|640[0n]|BOLD:AAA3873
Schizura unicornis|[1118]|RWWC1161-13|BIOUG07834-D07|United States|Washington|592[0n]|BOLD:AAA...
Schizura unicornis|[1119]|RDMAB069-05|UASM57596|Canada|Alberta|591[1n]|BOLD:AAA3873
Schizura unicornis|[1120]|BLGSM093-09|BL1646|Canada|Ontario|630[0n]|BOLD:AAA3873
Schizura unicornis|[1121]|XAF559-05|2005-ONT-208|Canada|Ontario|658[0n]|BOLD:AAA3873
Schizura unicornis|[1122]|XAG668-05|2005-ONT-1252|Canada|Ontario|603[2n]|BOLD:AAA3873
Schizura unicornis|[1123]|XAB559-04|04HBL005559|Canada|Ontario|658[0n]|BOLD:AAA3873
Schizura unicornis|[1124]|XAJ670-06|2006-ONT-0670|Canada|Ontario|658[0n]|BOLD:AAA3873
Schizura unicornis|[1125]|LPSOC358-08|PPBP-2357|Canada|Ontario|658[0n]|BOLD:AAA3873
Schizura unicornis|[1126]|BLGSM015-09|BL324|Canada|Ontario|658[0n]|BOLD:AAA3873
Schizura unicornis|[1127]|TTMNB250-06|MNBTT-250|Canada|New Brunswick|658[0n]|BOLD:AAA3873
Schizura unicornis|[1128]|MNBB352-05|05-NBSTA-268|Canada|New Brunswick|658[0n]|BOLD:AAA3873
Schizura unicornis|[1129]|MNBB247-05|05-NBSTA-163|Canada|New Brunswick|570[0n]|BOLD:AAA3873
Schizura unicornis|[1130]|LOWCB567-05|CGWC-1507|Canada|British Columbia|658[0n]|BOLD:AAA3873
Schizura unicornis|[1131]|LOWCB159-05|CGWC-1099|Canada|British Columbia|658[0n]|BOLD:AAA3873
Schizura unicornis|[1132]|LOWCB157-05|CGWC-1097|Canada|British Columbia|658[0n]|BOLD:AAA3873
Schizura unicornis|[1133]|LPSK143-08|08BBLEP-01711|Canada|Saskatchewan|658[0n]|BOLD:AAA3873
Schizura unicornis|[1134]|RWWA933-09|RWWA-0933|United States|Washington|658[0n]|BOLD:AAA3873
Schizura unicornis|[1135]|RWWC345-11|RWWA-2322|United States|Washington|658[0n]|BOLD:AAA3873
Schizura unicornis|[1136]|LBCA606-05|HLC-20606|Canada|British Columbia|633[0n]|BOLD:AAA3873
Schizura unicornis|[1137]|XAE291-04|Moth4291.03|Canada|Ontario|658[0n]|BOLD:AAA3873
Schizura unicornis|[1138]|LPVIA630-08|PFC-2006-0863|Canada|British Columbia|624[0n]|BOLD:AAA3873
Schizura unicornis|[1139]|LOWCB154-05|CGWC-1094|Canada|British Columbia|658[0n]|BOLD:AAA3873
Schizura unicornis|[1140]|LOWCB158-05|CGWC-1098|Canada|British Columbia|648[0n]|BOLD:AAA3873
Schizura unicornis|[1141]|BBLPC248-09|09BBELE-1248|Canada|Nova Scotia|658[0n]|BOLD:AAA3873
Schizura unicornis|[1142]|BBLPB683-10|10BBCLP-1682|Canada|British Columbia|658[0n]|BOLD:AAA3873
Schizura unicornis|[1143]|LBCD022-05|HLC-22842|Canada|British Columbia|658[0n]|BOLD:AAA3873
Schizura unicornis|[1144]|LBCA804-05|HLC-20804|Canada|British Columbia|658[0n]|BOLD:AAA3873
Schizura unicornis|[1145]|LPSOB458-08|PPBP-1457|Canada|Ontario|658[0n]|BOLD:AAA3873
Schizura unicornis|[1146]|LOWCB161-05|CGWC-1101|Canada|British Columbia|658[0n]|BOLD:AAA3873
Schizura unicornis|[1147]|LALPA478-10|AVBC 480-10|Canada|British Columbia|658[0n]|BOLD:AAA3873
Schizura unicornis|[1148]|DUNLP192-08|Dun-08-192|Canada|British Columbia|658[0n]|BOLD:AAA3873
Schizura unicornis|[1149]|LOWCB156-05|CGWC-1096|Canada|British Columbia|658[0n]|BOLD:AAA3873
Schizura unicornis|[1150]|LBCC004-05|HLC-21884|Canada|British Columbia|658[0n]|BOLD:AAA3873
Schizura unicornis|[1151]|LBCA810-05|HLC-20810|Canada|British Columbia|658[0n]|BOLD:AAA3873
Schizura unicornis|[1152]|BBLPC183-09|09BBELE-1183|Canada|Nova Scotia|658[0n]|BOLD:AAA3873
Schizura unicornis|[1153]|BBLPB682-10|10BBCLP-1681|Canada|British Columbia|658[0n]|BOLD:AAA3873
Schizura unicornis|[1154]|LOWCB155-05|CGWC-1095|Canada|British Columbia|658[0n]|BOLD:AAA3873
Schizura unicornis|[1155]|LBCC767-05|HLC-22647|Canada|British Columbia|658[0n]|BOLD:AAA3873
Schizura unicornis|[1156]|LOWCB152-05|CGWC-1092|Canada|British Columbia|658[0n]|BOLD:AAA3873
Schizura unicornis|[1157]|LBCD023-05|HLC-22843|Canada|British Columbia|658[0n]|BOLD:AAA3873
Schizura unicornis|[1158]|LBCB207-05|HLC-21147|Canada|British Columbia|658[0n]|BOLD:AAA3873
Schizura unicornis|[1159]|LBCB141-05|HLC-21081|Canada|British Columbia|658[0n]|BOLD:AAA3873
Schizura unicornis|[1160]|LBCA809-05|HLC-20809|Canada|British Columbia|658[0n]|BOLD:AAA3873
Schizura unicornis|[1161]|LPABC702-09|08BBLEP-04921|Canada|Alberta|658[0n]|BOLD:AAA3873
Schizura unicornis|[1162]|BBLPB680-10|10BBCLP-1679|Canada|British Columbia|658[0n]|BOLD:AAA3873
Schizura unicornis|[1163]|LOWCB153-05|CGWC-1093|Canada|British Columbia|658[0n]|BOLD:AAA3873
Schizura unicornis|[1164]|RWWA647-09|RWWA-0665|United States|Washington|658[0n]|BOLD:AAA3873
Schizura leptinoides|[1165]|TMNBD198-07|MNBTT-2999|Canada|New Brunswick|658[0n]|BOLD:AAB0904
Schizura leptinoides|[1166]|TMNBD195-07|MNBTT-2996|Canada|New Brunswick|648[0n]|BOLD:AAB0904
Schizura leptinoides|[1167]|TMNBD194-07|MNBTT-2995|Canada|New Brunswick|646[0n]|BOLD:AAB0904
Schizura leptinoides|[1168]|TMNBD197-07|MNBTT-2998|Canada|New Brunswick|658[0n]|BOLD:AAB0904
Schizura leptinoides|[1169]|TMNBD196-07|MNBTT-2997|Canada|New Brunswick|658[0n]|BOLD:AAB0904
Schizura leptinoides|[1170]|RDLQB195-05|DH010281|Canada|Quebec|658[0n]|BOLD:AAB0904
Schizura leptinoides|[1171]|RDLQB193-05|DH010279|Canada|Quebec|658[1n]|BOLD:AAB0904
Schizura leptinoides|[1172]|RDLQB194-05|DH010280|Canada|Quebec|658[0n]|BOLD:AAB0904
Schizura leptinoides|[1173]|TMNBB002-06|MNBTT-942|Canada|New Brunswick|656[0n]|BOLD:AAB0904
Schizura leptinoides|[1174]|MNBB669-05|05-NBSTA-585|Canada|New Brunswick|658[0n]|BOLD:AAB0904
Schizura leptinoides|[1175]|RDLQF939-06|DH012119|Canada|Quebec|658[0n]|BOLD:AAB0904

Schizura leptinoides|[1173]|TMNBB002-06|MNBTT-942|Canada|New Brunswick|656[0n]|BOLD:AAB0904
Schizura leptinoides|[1174]|MNBB669-05|05-NBSTA-585|Canada|New Brunswick|658[0n]|BOLD:AAB0904
Schizura leptinoides|[1175]|RDLQF939-06|DH012119|Canada|Quebec|658[0n]|BOLD:AAB0904
Schizura leptinoides|[1176]|PHMO197-03|moth1011.02|Canada|Ontario|639[0n]|BOLD:AAB0904
Schizura leptinoides|[1177]|XAB170-04|04HBL005170|Canada|Ontario|538[0n]|BOLD:AAB0904
Schizura leptinoides|[1178]|PHMO198-03|moth1015.02|Canada|Ontario|639[0n]|BOLD:AAB0904
Schizura leptinoides|[1179]|XAK481-07|HLC-16034|Canada|Ontario|581[1n]|BOLD:AAB0904
Schizura leptinoides|[1180]|XAG703-05|2005-ONT-1287|Canada|Ontario|654[0n]|BOLD:AAB0904
Schizura leptinoides|[1181]|XAG542-05|2005-ONT-1126|Canada|Ontario|564[1n]|BOLD:AAB0904
Schizura leptinoides|[1182]|LGSMB237-05|DNA-ATBI-1086|United States|Tennessee|597[0n]|BOLD:AAB...
Schizura leptinoides|[1183]|LGSMG100-07|BGS03421|United States|Tennessee|658[0n]|BOLD:AAB0904
Schizura leptinoides|[1184]|UDLEP312-09|v334 CS|United States|Pennsylvania|658[0n]|BOLD:AAB0904
Schizura leptinoides|[1185]|LGSMB238-05|DNA-ATBI-1087|United States|Tennessee|658[0n]|BOLD:AAB...
Schizura leptinoides|[1186]|LGSMB243-05|DNA-ATBI-1092|United States|North Carolina|586[0n]|BOLD ...
Schizura leptinoides|[1187]|LPOKE274-11|MDOK-4352|United States|Oklahoma|658[0n]|BOLD:AAB0904
Schizura leptinoides|[1188]|LPSO344-08|PPBP-0344|Canada|Ontario|658[0n]|BOLD:AAB0904
Schizura leptinoides|[1189]|LNCNW113-06|06-NCNW-0113|United States|North Carolina|658[0n]|BOLD ...
Schizura leptinoides|[1190]|LOT497-04|04HBL002497|United States|Tennessee|658[0n]|BOLD:AAB0904
Schizura leptinoides|[1191]|LPOKB1031-09|MDOK-2073|United States|Oklahoma|658[0n]|BOLD:AAB0904
Schizura leptinoides|[1192]|LOTB195-05|05-TN-00195|United States|Tennessee|658[0n]|BOLD:AAB0904
Schizura leptinoides|[1193]|LOFLB523-06|06-FLOR-1463|United States|Florida|658[0n]|BOLD:AAB0904
Schizura leptinoides|[1194]|CNRME1820-12|BIOUG03547-C06|Canada|Manitoba|633[0n]|BOLD:AAB0904
Schizura leptinoides|[1195]|LPMN344-08|08BBLEP-01143|Canada|Manitoba|658[0n]|BOLD:AAB0904
Schizura leptinoides|[1196]|LPMN176-08|08BBLEP-00975|Canada|Manitoba|658[0n]|BOLD:AAB0904
Schizura leptinoides|[1197]|LPMN725-08|08BBLEP-01528|Canada|Manitoba|658[0n]|BOLD:AAB0904
Schizura leptinoides|[1198]|CNRME1843-12|BIOUG03547-E05|Canada|Manitoba|631[0n]|BOLD:AAB0904
Schizura leptinoides|[1199]|RDMAB068-05|UASM57595|Canada|Alberta|636[0n]|BOLD:AAB0904
Schizura concinna|[1200]|LGSMB240-05|DNA-ATBI-1089|United States|Tennessee|578[0n]|BOLD:AAE3774
Schizura concinna|[1201]|LGSMB241-05|DNA-ATBI-1090|United States|Tennessee|584[0n]|BOLD:AAE3774
Schizura concinna|[1202]|LSEU605-06|06-JKA-0605|United States|Georgia|658[0n]|BOLD:AAE3774
Schizura concinna|[1203]|LPOKA065-08|MDOK-0065|United States|Oklahoma|658[0n]|BOLD:AAE3774
Schizura concinna|[1204]|LPOKC770-09|MDOK-2847|United States|Oklahoma|658[0n]|BOLD:AAE3774
Schizura apicalis|[1205]|RDNMF401-08|NOC14487|Canada|Ontario|658[0n]|BOLD:AAF2054
Schizura apicalis|[1206]|RDNMF402-08|NOC14488|Canada|Ontario|658[0n]|BOLD:AAF2054
Schizura apicalis|[1207]|LNAUT2626-14|CCDB-23282-F03|United States|Massachusetts|658[0n]|BOLD:...
Schizura apicalis|[1208]|HKONS529-08|3058-COI-08|United States|Florida|658[0n]|BOLD:AAF2054
Oligocentria semirufescens|[1209]|LOT098-04|04HBL002098|United States|Tennessee|609[0n]|BOLD:A...
Oligocentria semirufescens|[1210]|PHSEP272-11|BIOUG01146-H01|Canada|Ontario|658[0n]|BOLD:AAA8308
Oligocentria semirufescens|[1211]|LGSMG101-07|BGS03422|United States|North Carolina|609[4n]|BOLD...
Oligocentria semirufescens|[1212]|TMNBD192-07|MNBTT-2993|Canada|New Brunswick|646[0n]|BOLD:AAA...
Oligocentria semirufescens|[1213]|TMNBD189-07|MNBTT-2990|Canada|New Brunswick|651[0n]|BOLD:AAA...
Oligocentria semirufescens|[1214]|TMNBD193-07|MNBTT-2994|Canada|New Brunswick|649[0n]|BOLD:AAA...
Oligocentria semirufescens|[1215]|XAB169-04|04HBL005169|Canada|Ontario|555[1n]|BOLD:AAA8308
Oligocentria semirufescens|[1216]|XAB467-04|04HBL005467|Canada|Ontario|578[0n]|BOLD:AAA8308
Oligocentria semirufescens|[1217]|XAK419-06|2006-ONT-1414|Canada|Ontario|658[0n]|BOLD:AAA8308
Oligocentria semirufescens|[1218]|XAG238-05|2005-ONT-822|Canada|Ontario|658[0n]|BOLD:AAA8308
Oligocentria semirufescens|[1219]|RDLQB530-05|DH010616|Canada|Quebec|658[0n]|BOLD:AAA8308
Oligocentria semirufescens|[1220]|LPMN339-08|08BBLEP-01138|Canada|Manitoba|658[0n]|BOLD:AAA8308
Oligocentria semirufescens|[1221]|LMDH181-11|BIOUG01047-F11|United States|Minnesota|658[0n]|BOLD...
Oligocentria semirufescens|[1222]|MNBB297-05|05-NBSTA-213|Canada|New Brunswick|658[0n]|BOLD:AA...
Oligocentria semirufescens|[1223]|TMNBD190-07|MNBTT-2991|Canada|New Brunswick|658[0n]|BOLD:AAA...
Oligocentria semirufescens|[1224]|XAG176-05|2005-ONT-760|Canada|Ontario|658[0n]|BOLD:AAA8308
Oligocentria semirufescens|[1225]|LPMN031-08|08BBLEP-00829|Canada|Manitoba|658[0n]|BOLD:AAA8308
Oligocentria semirufescens|[1226]|PHMNB774-05|Moth 467.03SA|Canada|New Brunswick|658[0n]|BOLD:...
Oligocentria semirufescens|[1227]|TMNBD191-07|MNBTT-2992|Canada|New Brunswick|643[0n]|BOLD:AAA...
Oligocentria semirufescens|[1228]|PHMNB060-03|moth38.02SA|Canada|New Brunswick|639[0n]|BOLD:AA...
Oligocentria semirufescens|[1229]|RWWB962-10|RWWA-1961|United States|Washington|658[0n]|BOLD:A...
Oligocentria semirufescens|[1230]|LBCC421-05|HLC-22301|Canada|British Columbia|658[0n]|BOLD:AA...
Oligocentria semirufescens|[1231]|RWWB954-10|RWWA-1953|United States|Washington|658[0n]|BOLD:A...
Oligocentria semirufescens|[1232]|LHLEP152-06|UBC-2006-0740|Canada|British Columbia|658[0n]|BOLD...
Oligocentria semirufescens|[1233]|LBCA587-05|HLC-20587|Canada|British Columbia|597[0n]|BOLD:AA...
Oligocentria semirufescens|[1234]|LBCC016-05|HLC-21896|Canada|British Columbia|587[0n]|BOLD:AA...
Oligocentria semirufescens|[1235]|LHLEP151-06|UBC-2006-0739|Canada|British Columbia|624[0n]|BOLD...
Oligocentria semirufescens|[1236]|LBCA586-05|HLC-20586|Canada|British Columbia|649[0n]|BOLD:AA...
Oligocentria semirufescens|[1237]|RDNMB447-05|CNCNoctuoidea10213|Canada|British Columbia|581[1n]|...
Oligocentria semirufescens|[1238]|LBCA579-05|HLC-20579|Canada|British Columbia|645[0n]|BOLD:AA...
Oligocentria semirufescens|[1239]|LBCA588-05|HLC-20588|Canada|British Columbia|658[0n]|BOLD:AA...
Oligocentria semirufescens|[1240]|LHLEP149-06|UBC-2006-0737|Canada|British Columbia|658[0n]|BOLD...
Oligocentria semirufescens|[1241]|RWWC455-11|RWWA-2432|United States|Washington|658[0n]|BOLD:A...
Oligocentria semirufescens|[1242]|LBCC020-05|HLC-21900|Canada|British Columbia|658[0n]|BOLD:AA...
Oligocentria semirufescens|[1243]|LBCB180-05|HLC-21120|Canada|British Columbia|658[0n]|BOLD:AA...
Oligocentria semirufescens|[1244]|LBCC423-05|HLC-22303|Canada|British Columbia|658[0n]|BOLD:AA...
Oligocentria semirufescens|[1245]|LBCC022-05|HLC-21902|Canada|British Columbia|658[0n]|BOLD:AA...
Oligocentria semirufescens|[1246]|RWWA821-09|RWWA-0857|United States|Washington|658[0n]|BOLD:A...
Oligocentria semirufescens|[1247]|RWWC433-11|RWWA-2410|United States|Washington|658[0n]|BOLD:A...
Oligocentria semirufescens|[1248]|LBCD024-05|HLC-22844|Canada|British Columbia|658[0n]|BOLD:AA...
Oligocentria semirufescens|[1249]|RWWA457-09|RWWA-0457|United States|Washington|658[0n]|BOLD:A...
Oligocentria semirufescens|[1250]|LBCA589-05|HLC-20589|Canada|British Columbia|658[0n]|BOLD:AA...
Oligocentria semirufescens|[1251]|LBCC424-05|HLC-22304|Canada|British Columbia|658[0n]|BOLD:AA...
Oligocentria semirufescens|[1252]|RWWA692-09|RWWA-0710|United States|Washington|630[0n]|BOLD:A...
Oligocentria semirufescens|[1253]|LBCA797-05|HLC-20797|Canada|British Columbia|658[0n]|BOLD:AA...
Oligocentria semirufescens|[1254]|RWWC1158-13|BIOUG07834-D04|United States|Washington|590[0n]|BOL...
Oligocentria semirufescens|[1255]|LHLEP150-06|UBC-2006-0738|Canada|British Columbia|628[0n]|BOLD...
Oligocentria semirufescens|[1256]|LGSMB239-05|DNA-ATBI-1088|United States|Tennessee|596[0n]|BOLD...
Oligocentria semirufescens|[1257]|LBCD233-05|HLC-23053|Canada|British Columbia|616[0n]|BOLD:AA...
Oligocentria semirufescens|[1258]|LBCC427-05|HLC-22307|Canada|British Columbia|658[0n]|BOLD:AA...
Oligocentria paradisus|[1259]|CMAZA912-12|BIOUG02040-F03|United States|Arizona|658[0n]|BOLD:AA...
Oligocentria paradisus|[1260]|CMAZA894-12|BIOUG02040-D09|United States|Arizona|658[0n]|BOLD:AA...
Oligocentria paradisus|[1261]|CMAZA045-09|CMAZ-0045|United States|Arizona|658[0n]|BOLD:AAG9286
Oligocentria paradisus|[1262]|RDNMH428-09|CNCLEP00054442|United States|Arizona|658[0n]|BOLD:AA...
Oligocentria paradisus|[1263]|RDNMH175-09|CNCLEP00054392|United States|Arizona|636[0n]|BOLD:AA...
Oligocentria paradisus|[1264]|CMAZA227-09|CMAZ-0227|United States|Arizona|658[0n]|BOLD:AAG9286
Euhyparpax rosea|[1265]|CNCLB1637-14|CNCLEP 00113871|United States|New Mexico|658[0n]|BOLD:ACP...
Euhyparpax rosea|[1266]|CNCLB1636-14|CNCLEP 00113870|United States|New Mexico|658[0n]|BOLD:ACP...
Oligocentria lignicolor|[1267]|LOT093-04|04HBL002093|United States|Tennessee|609[0n]|BOLD:AAA8725
Oligocentria lignicolor|[1268]|TTMNB251-06|MNBTT-251|Canada|New Brunswick|658[0n]|BOLD:AAA8725
Oligocentria lignicolor|[1269]|TTMNB252-06|MNBTT-252|Canada|New Brunswick|658[0n]|BOLD:AAA8725
Oligocentria lignicolor|[1270]|BBLEC970-09|09BBELE-0970|Canada|Nova Scotia|658[0n]|BOLD:AAA8725
Oligocentria lignicolor|[1271]|LOT091-04|04HBL002091|United States|Tennessee|658[0n]|BOLD:AAA8725
Oligocentria lignicolor|[1272]|RDLQB203-05|DH010289|Canada|Quebec|658[0n]|BOLD:AAA8725
Oligocentria lignicolor|[1273]|LOT092-04|04HBL002092|United States|Tennessee|658[0n]|BOLD:AAA8725

[illegible]
Oligocentria lignicolor|[1271]|LOT091-04|04HBL002091|United States|Tennessee|658[0n]|BOLD:AAA8725
Oligocentria lignicolor|[1272]|RDLQB203-05|DH010289|Canada|Quebec|658[0n]|BOLD:AAA8725
Oligocentria lignicolor|[1273]|LOT092-04|04HBL002092|United States|Tennessee|658[0n]|BOLD:AAA8725
Oligocentria lignicolor|[1274]|XAE357-04|Moth4357.03|Canada|Ontario|658[0n]|BOLD:AAA8725
Oligocentria lignicolor|[1275]|TMNBD202-07|MNBTT-3003|Canada|New Brunswick|658[0n]|BOLD:AAA8725
Oligocentria lignicolor|[1276]|LSEU740-06|06-JKA-0740|United States|Georgia|658[0n]|BOLD:AAA8725
Oligocentria lignicolor|[1277]|RDLQB202-05|DH010288|Canada|Quebec|658[0n]|BOLD:AAA8725
Oligocentria lignicolor|[1278]|TMNBB003-06|MNBTT-943|Canada|New Brunswick|657[0n]|BOLD:AAA8725
Oligocentria lignicolor|[1279]|PHMO219-03|moth1120.01|Canada|Ontario|639[0n]|BOLD:AAA8725
Oligocentria lignicolor|[1280]|LOT555-04|04HBL002555|United States|Tennessee|603[0n]|BOLD:AAA8725
Oligocentria lignicolor|[1281]|LSEU603-06|06-JKA-0603|United States|Georgia|608[0n]|BOLD:AAA8725
Oligocentria lignicolor|[1282]|TMNBD203-07|MNBTT-3004|Canada|New Brunswick|658[0n]|BOLD:AAA8725
Oligocentria lignicolor|[1283]|PHMNB773-05|Moth 466.03SA|Canada|New Brunswick|658[0n]|BOLD:AAA...
Oligocentria lignicolor|[1284]|MNBB361-05|05-NBSTA-277|Canada|New Brunswick|655[0n]|BOLD:AAA8725
Oligocentria lignicolor|[1285]|TMNBD200-07|MNBTT-3001|Canada|New Brunswick|658[0n]|BOLD:AAA8725
Oligocentria lignicolor|[1286]|MNBB431-05|05-NBSTA-347|Canada|New Brunswick|658[0n]|BOLD:AAA8725
Oligocentria lignicolor|[1287]|RDLQF347-06|DH011414|Canada|Quebec|658[0n]|BOLD:AAA8725
Oligocentria lignicolor|[1288]|LNCC695-11|11-NCCC-220|United States|North Carolina|658[0n]|BOLD ...
Oligocentria lignicolor|[1289]|MNBB614-05|05-NBSTA-530|Canada|New Brunswick|658[0n]|BOLD:AAA8725
Oligocentria lignicolor|[1290]|RDLQB204-05|DH010290|Canada|Quebec|658[0n]|BOLD:AAA8725
Oligocentria lignicolor|[1291]|TTMNB041-06|MNBTT-041|Canada|New Brunswick|658[0n]|BOLD:AAA8725
Oligocentria lignicolor|[1292]|LNCB324-06|06-NCCC-1280|United States|North Carolina|658[0n]|BOLD...
Oligocentria lignicolor|[1293]|RDLQB200-05|DH010286|Canada|Quebec|658[0n]|BOLD:AAA8725
Oligocentria lignicolor|[1294]|RDLQB201-05|DH010287|Canada|Quebec|658[0n]|BOLD:AAA8725
Oligocentria lignicolor|[1295]|TMNBD199-07|MNBTT-3000|Canada|New Brunswick|658[0n]|BOLD:AAA8725
Oligocentria lignicolor|[1296]|LOT090-04|04HBL002090|United States|Tennessee|609[1n]|BOLD:AAA8725
Oligocentria lignicolor|[1297]|LPOKE507-12|BIOUG02920-F08|United States|Oklahoma|632[0n]|BOLD:...
Oligocentria lignicolor|[1298]|TMNBD201-07|MNBTT-3002|Canada|New Brunswick|659[0n]|BOLD:AAA8725
Oligocentria lignicolor|[1299]|LOT097-04|04HBL002097|United States|Tennessee|609[0n]|BOLD:AAA8725
Oligocentria lignicolor|[1300]|LOT096-04|04HBL002096|United States|Tennessee|609[0n]|BOLD:AAA8725
Oligocentria lignicolor|[1301]|LOT095-04|04HBL002095|United States|Tennessee|609[0n]|BOLD:AAA8725
Oligocentria lignicolor|[1302]|LOT089-04|04HBL002089|United States|Tennessee|609[0n]|BOLD:AAA8725
Oligocentria lignicolor|[1303]|LOT094-04|04HBL002094|United States|Tennessee|609[0n]|BOLD:AAA8725
Oligocentria lignicolor|[1304]|TMNBB004-06|MNBTT-944|Canada|New Brunswick|656[0n]|BOLD:AAA8725
Oligocentria lignicolor|[1305]|MNBB177-05|05-NBSTA-093|Canada|New Brunswick|658[0n]|BOLD:AAA8725
Oligocentria lignicolor|[1306]|MNBB251-05|05-NBSTA-167|Canada|New Brunswick|658[0n]|BOLD:AAA8725
Oligocentria lignicolor|[1307]|XAB468-04|04HBL005468|Canada|Ontario|558[1n]|BOLD:AAA8725
Oligocentria lignicolor|[1308]|LGSMB233-05|DNA-ATBI-1082|United States|North Carolina|658[0n]|BOL...
Oligocentria coloradensis|[1309]|RDNMJ300-11|CNCLEP 80208|United States|Arizona|658[0n]|BOLD:A...
Oligocentria coloradensis|[1310]|RDNMJ680-11|CNCLEP 80114|United States|Arizona|658[0n]|BOLD:A...
Oligocentria coloradensis|[1311]|CMAZA935-12|BIOUG02040-H02|United States|Arizona|658[0n]|BOLD ...
Oligocentria coloradensis|[1312]|CMAZA905-12|BIOUG02040-E08|United States|Arizona|658[0n]|BOLD ...
Oligocentria coloradensis|[1313]|CMAZA913-12|BIOUG02040-F04|United States|Arizona|658[0n]|BOLD ...
Oligocentria cerriben|[1314]|RDNMK349-11|CNCLEP 84258|United States|New Mexico|658[0n]|BOLD:AA...
Oligocentria cerriben|[1315]|CNCLB1572-14|CNCLEP 00113802|United States|Arizona|658[0n]|BOLD:A...
Oligocentria coloradensis|[1316]|CMAZA908-12|BIOUG02040-E11|United States|Arizona|658[0n]|BOLD ...
Schizura badia|[1317]|PMG188-03|moth603.01|Canada|Ontario|617[0n]|BOLD:AAC4502
Schizura badia|[1318]|XAB597-04|04HBL005597|Canada|Ontario|658[0n]|BOLD:AAC4502
Schizura badia|[1319]|LOCT044-05|05-CTATBI-0044|United States|Connecticut|658[0n]|BOLD:AAC4502
Schizura badia|[1320]|LOCT043-05|05-CTATBI-0043|United States|Connecticut|658[0n]|BOLD:AAC4502
Schizura badia|[1321]|TTMNB939-06|MNBTT-939|Canada|New Brunswick|658[0n]|BOLD:AAC4502
Schizura badia|[1322]|BBLPE021-09|09BBELE-2021|Canada|Nova Scotia|658[0n]|BOLD:AAC4502
Schizura badia|[1323]|RDLQF697-06|DH011847|Canada|Quebec|637[1n]|BOLD:AAC4502
Schizura badia|[1324]|UDLEP113-09|v771 LPC|United States|Maryland|658[0n]|BOLD:AAC4502
Schizura badia|[1325]|RDLQF508-06|DH011657|Canada|Quebec|658[0n]|BOLD:AAC4502
Schizura badia|[1326]|RDLQF365-06|DH011432|Canada|Quebec|658[0n]|BOLD:AAC4502
Schizura badia|[1327]|RDLQF698-06|DH011848|Canada|Quebec|639[0n]|BOLD:AAC4502
Schizura badia|[1328]|LILLA783-11|SNS10IL-00982|United States|Illinois|658[0n]|BOLD:ACE6695
Schizura badia|[1329]|UDLEP123-09|v790 WCC|United States|Delaware|658[0n]|BOLD:ACF2612
Schizura badia|[1330]|HKONS528-08|3057-COI-08|United States|Florida|658[0n]|BOLD:ACF2612
Hyparpax perophoroides|[1331]|LOFLB440-06|06-FLOR-1380|United States|Florida|658[0n]|BOLD:AAI2220
Hyparpax perophoroides|[1332]|LOFLB518-06|06-FLOR-1458|United States|Florida|654[0n]|BOLD:AAI2220
Hyparpax perophoroides|[1333]|USLEP705-10|10BBLEP-00705|United States|Florida|658[0n]|BOLD:AAI...
Hyparpax perophoroides|[1334]|HKONS531-08|3060-COI-08|United States|Florida|658[0n]|BOLD:AAD3007
Hyparpax perophoroides|[1335]|HKONS532-08|3061-COI-08|United States|Florida|658[0n]|BOLD:AAD3007
Hyparpax aurora|[1336]|LSEU239-06|06-JKA-0239|United States|Georgia|658[0n]|BOLD:AAD3007
Hyparpax aurora|[1337]|HKONS530-08|3059-COI-08|United States|Florida|658[0n]|BOLD:AAD3007
Hyparpax aurora|[1338]|MMNA068-08|HLC-17630|United States|Georgia|658[0n]|BOLD:AAD3007
Hyparpax aurora|[1339]|LNCB053-06|06-NCC-1009|United States|North Carolina|658[0n]|BOLD:AAD3007
Hyparpax aurora|[1340]|LNCB054-06|06-NCC-1010|United States|North Carolina|658[0n]|BOLD:AAD3007
Hyparpax aurora|[1341]|MMNA069-08|HLC-17631|United States|Georgia|658[0n]|BOLD:AAD3007
Hyparpax aurora|[1342]|LNCB052-06|06-NCC-1008|United States|North Carolina|658[0n]|BOLD:AAD3007
Hyparpax aurostriata|[1343]|LTOL809-07|AYK-06-7113|United States|Arizona|658[0n]|BOLD:ABY5577
Hyparpax aurostriata|[1344]|CNCLB1444-14|CNCLEP 00113652|United States|Utah|658[0n]|BOLD:ABY5577
Hyparpax aurostriata|[1345]|LTOL810-07|AYK-06-7151|United States|Arizona|658[0n]|BOLD:ABY5577
Hyparpax aurostriata|[1346]|HKONB515-09|2628-COI-08|United States|Texas|648[0n]|BOLD:ABY5577
Hyparpax aurostriata|[1347]|BBLSW747-09|09BBLEP-01675|United States|Texas|658[0n]|BOLD:ABY5577
Hyparpax aurostriata|[1348]|NOCNA033-14|20332-160609-TX|United States|Texas|658[0n]|BOLD:ABY5577
Hyparpax aurostriata|[1349]|BBLSX794-09|09BBLEP-02722|United States|Texas|658[0n]|BOLD:ABY5577
Hyparpax aurostriata|[1350]|NOCNA034-14|20333-40609-OK|United States|Texas|658[0n]|BOLD:ABY5577
Hyparpax aurostriata|[1351]|RDNMH173-09|CNCLEP00054390|United States|Oklahoma|637[0n]|BOLD:ABY...
Heterocampa incongrua|[1352]|RDNML060-13|CNCLEP 94316|United States|Arizona|658[0n]|BOLD:ACD3748
Heterocampa geronimo|[1353]|AWCLB556-11|AWC-01386|United States|Arizona|613[0n]|BOLD:AAU7969
Heterocampa belfragei|[1354]|CNCLB811-14|CNCLEP00083823|United States|Texas|658[0n]|BOLD:ACM4040
Praeschausia zapata|[1355]|BBLOC081-11|BIOUG01452-H02|United States|Arizona|658[0n]|BOLD:AAY8942
Praeschausia zapata|[1356]|BBLOC803-11|BIOUG01466-C01|United States|Arizona|658[0n]|BOLD:AAY8942
Praeschausia zapata|[1357]|BBLOC322-11|BIOUG01455-D06|United States|Arizona|658[0n]|BOLD:AAY8942
Praeschausia zapata|[1358]|BBLOC543-11|BIOUG01457-G01|United States|Arizona|658[0n]|BOLD:AAY8942
Praeschausia zapata|[1359]|RDNMK019-11|CNCLEP 81142|United States|Arizona|658[0n]|BOLD:AAY8942
Praeschausia zapata|[1360]|RDNMK022-11|CNCLEP 81145|United States|Arizona|658[0n]|BOLD:AAY8942
Baltodonta broui|[1361]|CNCLB1250-14|CNCLEP 00113543|United States|Louisiana|658[0n]|BOLD:ABA2466
Baltodonta broui|[1362]|CNCLB1249-14|CNCLEP 00113542|United States|Louisiana|658[0n]|BOLD:ABA2466
Baltodonta broui|[1363]|LNCC876-11|11-NCCC-401|United States|North Carolina|658[0n]|BOLD:ABA2466
Baltodonta broui|[1364]|LNCC879-11|11-NCCC-404|United States|North Carolina|658[0n]|BOLD:ABA2466
Baltodonta broui|[1365]|LNCC874-11|11-NCCC-399|United States|North Carolina|658[0n]|BOLD:ABA2466
Baltodonta broui|[1366]|LNCC875-11|11-NCCC-400|United States|North Carolina|658[0n]|BOLD:ABA2466
Baltodonta broui|[1367]|LNCC877-11|11-NCCC-402|United States|North Carolina|658[0n]|BOLD:ABA2466
Baltodonta broui|[1368]|LNCC873-11|11-NCCC-398|United States|North Carolina|658[0n]|BOLD:ABA2466
Baltodonta broui|[1369]|LNCC878-11|11-NCCC-403|United States|North Carolina|658[0n]|BOLD:ABA2466
Heterocampa biundata|[1370]|BBLPE421-09|09BBELE-2421|Canada|Newfoundland and Labrador|658[0n]|BOL...
Heterocampa biundata|[1371]|BBLPC198-09|09BBELE-1198|Canada|Nova Scotia|658[0n]|BOLD:AAB0601

[illegible]
Baltodonta broui|[1369]|LNCC878-11|11-NCCC-403|United States|North Carolina|658[0n]|BOLD:ABA2466
Heterocampa biundata|[1370]|BBLPE421-09|09BBELE-2421|Canada|Newfoundland and Labrador|658[0n]|BOL...
Heterocampa biundata|[1371]|BBLPC198-09|09BBELE-1198|Canada|Nova Scotia|658[0n]|BOLD:AAB0601
Heterocampa biundata|[1372]|BBLEC802-09|09BBELE-0802|Canada|Newfoundland and Labrador|658[0n]|BOL...
Heterocampa biundata|[1373]|BBLEC404-09|09BBELE-0404|Canada|Newfoundland and Labrador|658[0n]|BOL...
Heterocampa biundata|[1374]|BBLEC862-09|09BBELE-0862|Canada|Newfoundland and Labrador|658[0n]|BOL...
Heterocampa biundata|[1375]|BBLEC803-09|09BBELE-0803|Canada|Newfoundland and Labrador|658[0n]|BOL...
Heterocampa biundata|[1376]|BBLEC876-09|09BBELE-0876|Canada|Newfoundland and Labrador|658[0n]|BOL...
Heterocampa biundata|[1377]|BBLEC841-09|09BBELE-0841|Canada|Newfoundland and Labrador|633[0n]|BOL...
Heterocampa biundata|[1378]|BBLEC847-09|09BBELE-0847|Canada|Newfoundland and Labrador|633[0n]|BOL...
Heterocampa biundata|[1379]|BBLPC242-09|09BBELE-1242|Canada|Nova Scotia|658[0n]|BOLD:AAB0601
Heterocampa biundata|[1380]|PHMNB037-03|moth207.02SA|Canada|New Brunswick|639[0n]|BOLD:AAB0601
Heterocampa biundata|[1381]|TTMNB937-06|MNBTT-937|Canada|New Brunswick|658[0n]|BOLD:AAB0601
Heterocampa biundata|[1382]|LOCT024-05|05-CTATBI-0024|United States|Connecticut|655[0n]|BOLD:A...
Heterocampa biundata|[1383]|LGSMG095-07|BGS03416|United States|North Carolina|658[0n]|BOLD:AAB...
Heterocampa biundata|[1384]|LGSMC736-05|DNA-ATBI-2736|United States|Tennessee|658[0n]|BOLD:AAB...
Heterocampa biundata|[1385]|LGSMG096-07|BGS03417|United States|Tennessee|656[0n]|BOLD:AAB0601
Heterocampa biundata|[1386]|MNBB022-05|HBL008632|Canada|New Brunswick|596[0n]|BOLD:AAB0601
Heterocampa biundata|[1387]|PHMO376-03|moth1322.02|Canada|Ontario|639[0n]|BOLD:AAB0601
Heterocampa biundata|[1388]|RDLQ772-07|DH011572|Canada|Quebec|633[0n]|BOLD:AAB0601
Heterocampa biundata|[1389]|XAB508-04|04HBL005508|Canada|Ontario|658[0n]|BOLD:AAB0601
Heterocampa biundata|[1390]|XAE413-04|Moth4413.03|Canada|Ontario|658[0n]|BOLD:AAB0601
Heterocampa biundata|[1391]|MNBB067-05|HBL008677|Canada|New Brunswick|658[0n]|BOLD:AAB0601
Heterocampa biundata|[1392]|XAE599-04|Moth4599.03|Canada|Ontario|658[0n]|BOLD:AAB0601
Heterocampa biundata|[1393]|XAB143-04|04HBL005143|Canada|Ontario|658[0n]|BOLD:AAB0601
Heterocampa biundata|[1394]|PHMO372-03|moth1058.02|Canada|Ontario|639[1n]|BOLD:AAB0601
Heterocampa biundata|[1395]|LGSMC739-05|DNA-ATBI-2739|United States|Tennessee|658[0n]|BOLD:AAB...
Heterocampa biundata|[1396]|LGSMC738-05|DNA-ATBI-2738|United States|Tennessee|658[0n]|BOLD:AAB...
Heterocampa biundata|[1397]|LOTB333-05|05-TN-00333|United States|Tennessee|658[0n]|BOLD:AAB0601
Heterocampa biundata|[1398]|LNC126-05|05-NCCC-126|United States|North Carolina|658[0n]|BOLD:AA...
Heterocampa biundata|[1399]|UDLEP117-09|v778 LPC|United States|Maryland|635[0n]|BOLD:AAB0601
Heterocampa biundata|[1400]|LNC125-05|05-NCCC-125|United States|North Carolina|658[0n]|BOLD:AA...
Heterocampa biundata|[1401]|LSEU458-06|06-JKA-0458|United States|Georgia|658[0n]|BOLD:AAB0601
Heterocampa biundata|[1402]|LGSMC737-05|DNA-ATBI-2737|United States|Tennessee|658[0n]|BOLD:AAB...
Heterocampa varia|[1403]|LNAUT2627-14|CCDB-23282-F04|United States|Massachusetts|658[0n]|BOLD:...
Heterocampa varia|[1404]|LNAUT2628-14|CCDB-23282-F05|United States|Massachusetts|658[0n]|BOLD:...
Heterocampa varia|[1405]|HKONS719-08|3435-COI-08|United States|Florida|658[0n]|BOLD:AAC2340
Heterocampa varia|[1406]|HKONS720-08|3436-COI-08|United States|Florida|658[0n]|BOLD:AAC2340
Heterocampa astartoides|[1407]|BBLSY927-09|09BBLEP-03854|United States|Texas|658[0n]|BOLD:AAC2340
Heterocampa astartoides|[1408]|USLEP675-10|10BBLEP-00675|United States|Texas|658[0n]|BOLD:AAC2340
Heterocampa astartoides|[1409]|USLEP677-10|10BBLEP-00677|United States|Texas|658[0n]|BOLD:AAC2340
Heterocampa astartoides|[1410]|USLEP676-10|10BBLEP-00676|United States|Texas|658[0n]|BOLD:AAC2340
Heterocampa astartoides|[1411]|CNCLB2413-14|CNCLEP 00119555|United States|Texas|658[0n]|BOLD:A...
Heterocampa astartoides|[1412]|BBLSZ173-09|09BBLEP-04099|United States|Texas|658[0n]|BOLD:AAC2340
Heterocampa astartoides|[1413]|RDNMH413-09|CNCLEP00054427|United States|Texas|658[0n]|BOLD:AAC...
Heterocampa astartoides|[1414]|BBLSZ172-09|09BBLEP-04098|United States|Texas|636[0n]|BOLD:AAC2340
Heterocampa astarte|[1415]|LOFLA663-06|06-FLOR-0663|United States|Florida|658[0n]|BOLD:AAC2339
Heterocampa astarte|[1416]|LOFLB377-06|06-FLOR-1317|United States|Florida|658[0n]|BOLD:AAC2339
Heterocampa astarte|[1417]|LOFLA378-06|06-FLOR-0378|United States|Florida|658[0n]|BOLD:AAC2339
Heterocampa astarte|[1418]|LOFLA370-06|06-FLOR-0370|United States|Florida|658[0n]|BOLD:AAC2339
Heterocampa astarte|[1419]|LOFLA377-06|06-FLOR-0377|United States|Florida|658[0n]|BOLD:AAC2339
Heterocampa astarte|[1420]|LOFLA666-06|06-FLOR-0666|United States|Florida|601[0n]|BOLD:AAC2339
Heterocampa astarte|[1421]|CNCLB2088-14|CNCLEP00117932|United States|Florida|658[0n]|BOLD:AAC2339
Heterocampa astarte|[1422]|USLEP296-10|10BBLEP-00296|United States|Florida|658[0n]|BOLD:AAC2339
Heterocampa astarte|[1423]|LOFLC096-06|06-FLOR-1976|United States|Florida|658[0n]|BOLD:AAC2339
Heterocampa astarte|[1424]|MILEQ191-11|11-MISC-666|United States|Georgia|633[0n]|BOLD:AAC2339
Heterocampa astarte|[1425]|CNCLB2086-14|CNCLEP00117930|United States|Florida|658[0n]|BOLD:AAC2339
Heterocampa astarte|[1426]|CNCLB2085-14|CNCLEP00117929|United States|Florida|658[0n]|BOLD:AAC2339
Heterocampa astarte|[1427]|LOFLA734-06|06-FLOR-0734|United States|Florida|658[0n]|BOLD:AAC2339
Heterocampa astarte|[1428]|LOFLA233-06|06-FLOR-0233|United States|Florida|658[0n]|BOLD:AAC2339
Heterocampa astarte|[1429]|LOFLA894-06|06-FLOR-0894|United States|Florida|658[0n]|BOLD:AAC2339
Heterocampa astarte|[1430]|USLEP680-10|10BBLEP-00680|United States|Florida|632[0n]|BOLD:AAC2339
Heterocampa astarte|[1431]|CNCLB2084-14|CNCLEP00117928|United States|Florida|658[0n]|BOLD:AAC2339
Heterocampa astarte|[1432]|LOFLB497-06|06-FLOR-1437|United States|Florida|658[0n]|BOLD:AAC2339
Heterocampa astarte|[1433]|LOFLA379-06|06-FLOR-0379|United States|Florida|658[0n]|BOLD:AAC2339
Heterocampa astarte|[1434]|CNCLB2087-14|CNCLEP00117931|United States|Florida|658[0n]|BOLD:AAC2339
Heterocampa astarte|[1435]|USLEP599-10|10BBLEP-00599|United States|Florida|658[0n]|BOLD:AAC2339
Heterocampa secessionis|[1436]|BBLSY907-09|09BBLEP-03834|United States|Texas|658[0n]|BOLD:AAH5419
Heterocampa obliqua|[1437]|LPOKC753-09|MDOK-2830|United States|Oklahoma|658[0n]|BOLD:AAB0487
Heterocampa obliqua|[1438]|LPOKC607-09|MDOK-2684|United States|Oklahoma|658[0n]|BOLD:AAB0486
Heterocampa obliqua|[1439]|LPOKB966-09|MDOK-2008|United States|Oklahoma|658[0n]|BOLD:AAB0486
Heterocampa obliqua|[1440]|LPOKB1012-09|MDOK-2054|United States|Oklahoma|658[0n]|BOLD:AAB0486
Heterocampa obliqua|[1441]|LOFLA383-06|06-FLOR-0383|United States|Florida|658[0n]|BOLD:AAB0486
Heterocampa obliqua|[1442]|LOFLA896-06|06-FLOR-0896|United States|Florida|658[0n]|BOLD:AAB0486
Heterocampa obliqua|[1443]|LOFLA897-06|06-FLOR-0897|United States|Florida|658[0n]|BOLD:AAB0486
Heterocampa obliqua|[1444]|LOFLA917-06|06-FLOR-0917|United States|Florida|658[0n]|BOLD:AAB0486
Heterocampa obliqua|[1445]|LOFLA849-06|06-FLOR-0849|United States|Florida|658[0n]|BOLD:AAB0486
Heterocampa obliqua|[1446]|LGSMB284-05|DNA-ATBI-1133|United States|Tennessee|586[2n]|BOLD:AAB0486
Heterocampa obliqua|[1447]|LNC243-05|05-NCCC-243|United States|North Carolina|658[0n]|BOLD:AAB...
Heterocampa obliqua|[1448]|LOFLB006-06|06-FLOR-0946|United States|Florida|658[0n]|BOLD:AAB0486
Heterocampa obliqua|[1449]|LNCB127-06|06-NCCC-1083|United States|North Carolina|658[0n]|BOLD:A...
Heterocampa obliqua|[1450]|LNCB128-06|06-NCCC-1084|United States|North Carolina|658[0n]|BOLD:A...
Heterocampa obliqua|[1451]|BBLEC138-09|09BBELE-0138|Canada|Nova Scotia|658[0n]|BOLD:AAB0486
Heterocampa obliqua|[1452]|LOFLA893-06|06-FLOR-0893|United States|Florida|658[0n]|BOLD:AAB0486
Heterocampa obliqua|[1453]|RDNMH431-09|CNCLEP00054445|United States|Tennessee|658[0n]|BOLD:AAB...
Heterocampa obliqua|[1454]|LTOLB013-08|CWM-94-0317|United States|Maryland|658[0n]|BOLD:AAB0486
Heterocampa obliqua|[1455]|LOFLA892-06|06-FLOR-0892|United States|Florida|658[0n]|BOLD:AAB0486
Heterocampa obliqua|[1456]|LOFLA898-06|06-FLOR-0898|United States|Florida|658[0n]|BOLD:AAB0486
Heterocampa obliqua|[1457]|LOFLB443-06|06-FLOR-1383|United States|Florida|658[0n]|BOLD:AAB0486
Heterocampa obliqua|[1458]|LOFLA389-06|06-FLOR-0389|United States|Florida|658[0n]|BOLD:AAB0486
Heterocampa obliqua|[1459]|LOFLA895-06|06-FLOR-0895|United States|Florida|658[0n]|BOLD:AAB0486
Heterocampa obliqua|[1460]|LOT086-04|04HBL002086|United States|Tennessee|609[0n]|BOLD:AAB0486
Heterocampa obliqua|[1461]|LOFLA371-06|06-FLOR-0371|United States|Florida|658[0n]|BOLD:AAB0486
Heterocampa obliqua|[1462]|LILLA835-11|SNS10IL-01051|United States|Illinois|658[0n]|BOLD:AAB0486
Heterocampa obliqua|[1463]|LGSMB286-05|DNA-ATBI-1135|United States|Tennessee|658[0n]|BOLD:AAB0486
Heterocampa obliqua|[1464]|LGSMB285-05|DNA-ATBI-1134|United States|Tennessee|568[0n]|BOLD:AAB0486
Heterocampa obliqua|[1465]|LOT087-04|04HBL002087|United States|Tennessee|609[0n]|BOLD:AAB0486
Heterocampa obliqua|[1466]|LOT085-04|04HBL002085|United States|Tennessee|609[0n]|BOLD:AAB0486
Heterocampa obliqua|[1467]|LOT528-04|04HBL002528|United States|Tennessee|658[0n]|BOLD:AAB0486
Heterocampa obliqua|[1468]|RDNMH468-09|CNCLEP00054482|United States|Tennessee|658[0n]|BOLD:AAB...
[illegible]

[illegible]
Heterocampa obliqua|[1467]|LOT528-04|04HBL002528|United States|Tennessee|658[0n]|BOLD:AAB0486
Heterocampa obliqua|[1468]|RDNMH468-09|CNCLEP00054482|United States|Tennessee|658[0n]|BOLD:AAB...
Heterocampa obliqua|[1469]|LOTB302-05|05-TN-00302|United States|Tennessee|658[0n]|BOLD:AAB0486
Heterocampa obliqua|[1470]|LGSMG091-07|BGS03412|United States|Tennessee|658[0n]|BOLD:AAB0486
Heterocampa obliqua|[1471]|LOT527-04|04HBL002527|United States|Tennessee|658[0n]|BOLD:AAB0486
Heterocampa obliqua|[1472]|LILLA752-11|SNS10IL-00949|United States|Illinois|658[0n]|BOLD:AAB0486
Heterocampa obliqua|[1473]|MILEQ250-11|11-MISC-725|United States|Alabama|658[0n]|BOLD:AAB0486
Heterocampa obliqua|[1474]|MILEQ249-11|11-MISC-724|United States|Alabama|658[0n]|BOLD:AAB0486
Heterocampa obliqua|[1475]|MILEQ251-11|11-MISC-726|United States|Alabama|649[0n]|BOLD:AAB0486
Heterocampa obliqua|[1476]|LPOKB398-09|MDOK-1500|United States|Oklahoma|658[0n]|BOLD:AAB0486
Heterocampa subrotata|[1477]|LPSO535-08|PPBP-0535|Canada|Ontario|658[0n]|BOLD:AAA6576
Heterocampa subrotata|[1478]|LPSOB085-08|PPBP-1084|Canada|Ontario|658[0n]|BOLD:AAA6576
Heterocampa subrotata|[1479]|LPSO410-08|PPBP-0410|Canada|Ontario|658[0n]|BOLD:AAA6576
Heterocampa subrotata|[1480]|LPSOB088-08|PPBP-1087|Canada|Ontario|658[0n]|BOLD:AAA6576
Heterocampa subrotata|[1481]|LPSO412-08|PPBP-0412|Canada|Ontario|658[0n]|BOLD:AAA6576
Heterocampa subrotata|[1482]|LPSO531-08|PPBP-0531|Canada|Ontario|658[0n]|BOLD:AAA6576
Heterocampa subrotata|[1483]|LPSO488-08|PPBP-0488|Canada|Ontario|658[0n]|BOLD:AAA6576
Heterocampa subrotata|[1484]|LPSOB084-08|PPBP-1083|Canada|Ontario|658[0n]|BOLD:AAA6576
Heterocampa subrotata|[1485]|LPSOB091-08|PPBP-1090|Canada|Ontario|658[0n]|BOLD:AAA6576
Heterocampa subrotata|[1486]|LPSO490-08|PPBP-0490|Canada|Ontario|658[0n]|BOLD:AAA6576
Heterocampa subrotata|[1487]|LPSO530-08|PPBP-0530|Canada|Ontario|658[0n]|BOLD:AAA6576
Heterocampa subrotata|[1488]|LPSOB086-08|PPBP-1085|Canada|Ontario|658[0n]|BOLD:AAA6576
Heterocampa subrotata|[1489]|LPSO540-08|PPBP-0540|Canada|Ontario|658[0n]|BOLD:AAA6576
Heterocampa subrotata|[1490]|LPSO469-08|PPBP-0469|Canada|Ontario|658[0n]|BOLD:AAA6576
Heterocampa subrotata|[1491]|LPSO532-08|PPBP-0532|Canada|Ontario|658[0n]|BOLD:AAA6576
Heterocampa subrotata|[1492]|LPSO884-08|PPBP-0884|Canada|Ontario|658[0n]|BOLD:AAA6576
Heterocampa subrotata|[1493]|LPSO373-08|PPBP-0373|Canada|Ontario|658[0n]|BOLD:AAA6576
Heterocampa subrotata|[1494]|LPSO548-08|PPBP-0548|Canada|Ontario|658[0n]|BOLD:AAA6576
Heterocampa subrotata|[1495]|LPSO957-08|PPBP-0957|Canada|Ontario|658[0n]|BOLD:AAA6576
Heterocampa subrotata|[1496]|LPSO539-08|PPBP-0539|Canada|Ontario|658[0n]|BOLD:AAA6576
Heterocampa subrotata|[1497]|LPSO501-08|PPBP-0501|Canada|Ontario|658[0n]|BOLD:AAA6576
Heterocampa subrotata|[1498]|LPSO536-08|PPBP-0536|Canada|Ontario|658[0n]|BOLD:AAA6576
Heterocampa subrotata|[1499]|LPSOB073-08|PPBP-1072|Canada|Ontario|658[0n]|BOLD:AAA6576
Heterocampa subrotata|[1500]|LPSO415-08|PPBP-0415|Canada|Ontario|658[0n]|BOLD:AAA6576
Heterocampa subrotata|[1501]|LPSO538-08|PPBP-0538|Canada|Ontario|658[0n]|BOLD:AAA6576
Heterocampa subrotata|[1502]|LPSO956-08|PPBP-0956|Canada|Ontario|658[0n]|BOLD:AAA6576
Heterocampa subrotata|[1503]|LPSO418-08|PPBP-0418|Canada|Ontario|658[0n]|BOLD:AAA6576
Heterocampa subrotata|[1504]|LPSO176-08|PPBP-0176|Canada|Ontario|658[0n]|BOLD:AAA6576
Heterocampa subrotata|[1505]|LPSO164-08|PPBP-0164|Canada|Ontario|658[0n]|BOLD:AAA6576
Heterocampa subrotata|[1506]|LPSOB089-08|PPBP-1088|Canada|Ontario|658[0n]|BOLD:AAA6576
Heterocampa subrotata|[1507]|LPSO356-08|PPBP-0356|Canada|Ontario|658[0n]|BOLD:AAA6576
Heterocampa subrotata|[1508]|LPSO163-08|PPBP-0163|Canada|Ontario|658[0n]|BOLD:AAA6576
Heterocampa subrotata|[1509]|LPSO413-08|PPBP-0413|Canada|Ontario|656[0n]|BOLD:AAA6576
Heterocampa subrotata|[1510]|LPSO420-08|PPBP-0420|Canada|Ontario|631[1n]|BOLD:AAA6576
Heterocampa subrotata|[1511]|LPSO358-08|PPBP-0358|Canada|Ontario|658[0n]|BOLD:AAA6576
Heterocampa subrotata|[1512]|LPSO529-08|PPBP-0529|Canada|Ontario|658[0n]|BOLD:AAA6576
Heterocampa subrotata|[1513]|LPSOB098-08|PPBP-1097|Canada|Ontario|658[0n]|BOLD:AAA6576
Heterocampa subrotata|[1514]|LPSO559-08|PPBP-0559|Canada|Ontario|658[0n]|BOLD:AAA6576
Heterocampa subrotata|[1515]|LPSOB090-08|PPBP-1089|Canada|Ontario|658[0n]|BOLD:AAA6576
Heterocampa subrotata|[1516]|LGSMB283-05|DNA-ATBI-1132|United States|Tennessee|578[0n]|BOLD:AA...
Heterocampa subrotata|[1517]|LPSO416-08|PPBP-0416|Canada|Ontario|658[0n]|BOLD:AAA6576
Heterocampa subrotata|[1518]|LPSOB083-08|PPBP-1082|Canada|Ontario|658[0n]|BOLD:AAA6576
Heterocampa subrotata|[1519]|LPSO419-08|PPBP-0419|Canada|Ontario|658[0n]|BOLD:AAA6576
Heterocampa subrotata|[1520]|LPSO411-08|PPBP-0411|Canada|Ontario|658[0n]|BOLD:AAA6576
Heterocampa subrotata|[1521]|LPSO414-08|PPBP-0414|Canada|Ontario|658[0n]|BOLD:AAA6576
Heterocampa subrotata|[1522]|LPSO541-08|PPBP-0541|Canada|Ontario|658[0n]|BOLD:AAA6576
Heterocampa subrotata|[1523]|LPSO534-08|PPBP-0534|Canada|Ontario|658[0n]|BOLD:AAA6576
Heterocampa subrotata|[1524]|LPSO375-08|PPBP-0375|Canada|Ontario|658[0n]|BOLD:AAA6576
Heterocampa subrotata|[1525]|LPSO533-08|PPBP-0533|Canada|Ontario|658[0n]|BOLD:AAA6576
Heterocampa subrotata|[1526]|LPSO528-08|PPBP-0528|Canada|Ontario|658[0n]|BOLD:AAA6576
Heterocampa subrotata|[1527]|LPSO537-08|PPBP-0537|Canada|Ontario|658[0n]|BOLD:AAA6576
Heterocampa subrotata|[1528]|LPSO426-08|PPBP-0426|Canada|Ontario|658[0n]|BOLD:AAA6576
Heterocampa subrotata|[1529]|LPSOB087-08|PPBP-1086|Canada|Ontario|653[0n]|BOLD:AAA6576
Heterocampa subrotata|[1530]|LSUSA236-06|06-SUSA-0236|United States|Kentucky|658[0n]|BOLD:AAA6576
Heterocampa subrotata|[1531]|LSUSA259-06|06-SUSA-0259|United States|Kentucky|658[0n]|BOLD:AAA6576
Heterocampa subrotata|[1532]|LPOKB786-09|MDOK-1828|United States|Oklahoma|658[0n]|BOLD:AAA6576
Heterocampa subrotata|[1533]|BBLOD1840-11|BIOUG01841-B05|United States|Arizona|658[1n]|BOLD:AA...
Heterocampa subrotata|[1534]|BBLSX172-09|09BBLEP-02100|United States|Oklahoma|658[0n]|BOLD:AAA...
Heterocampa ditta|[1535]|CNCLB1442-14|CNCLEP 00113650|United States|Arizona|658[0n]|BOLD:ACO8223
Heterocampa benitensis|[1536]|HKONB243-09|3740-COI-08|United States|Texas|658[0n]|BOLD:AAF2046
Heterocampa benitensis|[1537]|HKONB244-09|3741-COI-08|United States|Texas|658[0n]|BOLD:AAF2046
Heterocampa benitensis|[1538]|HKONB242-09|3739-COI-08|United States|Texas|640[0n]|BOLD:AAF2046
Heterocampa lunata|[1539]|RDNMJ295-11|CNCLEP 80203|United States|Arizona|658[0n]|BOLD:AAK5892
Heterocampa lunata|[1540]|RDNMH174-09|CNCLEP00054391|United States|Colorado|640[0n]|BOLD:AAK5892
Heterocampa umbrata|[1541]|LPSOD363-09|08BBLEP-00141|Canada|Ontario|658[0n]|BOLD:AAB3178
Heterocampa umbrata|[1542]|TMNBD209-07|MNBTT-3010|Canada|New Brunswick|658[0n]|BOLD:AAB3178
Heterocampa umbrata|[1543]|RDLQB213-05|DH010299|Canada|Quebec|597[0n]|BOLD:AAB3178
Heterocampa umbrata|[1544]|RDLQB211-05|DH010297|Canada|Quebec|558[0n]|BOLD:AAB3178
Heterocampa umbrata|[1545]|PMG181-03|HETEROC1.00|Canada|Ontario|617[0n]|BOLD:AAB3178
Heterocampa umbrata|[1546]|MNBB243-05|05-NBSTA-159|Canada|New Brunswick|589[4n]|BOLD:AAB3178
Heterocampa umbrata|[1547]|PHMNB018-03|moth148.02SA|Canada|New Brunswick|639[0n]|BOLD:AAB3178
Heterocampa umbrata|[1548]|PHNXN767-14|BIOUG10777-A07|Canada|Ontario|592[0n]|BOLD:AAB3178
Heterocampa umbrata|[1549]|PHMNB668-04|04HBL00894|Canada|New Brunswick|658[0n]|BOLD:AAB3178
Heterocampa umbrata|[1550]|TTMNB935-06|MNBTT-935|Canada|New Brunswick|658[0n]|BOLD:AAB3178
Heterocampa umbrata|[1551]|TMNBD208-07|MNBTT-3009|Canada|New Brunswick|658[0n]|BOLD:AAB3178
Heterocampa umbrata|[1552]|TTMNB936-06|MNBTT-936|Canada|New Brunswick|658[0n]|BOLD:AAB3178
Heterocampa umbrata|[1553]|TMNBD210-07|MNBTT-3011|Canada|New Brunswick|658[0n]|BOLD:AAB3178
Heterocampa umbrata|[1554]|LPSOD505-09|08BBLEP-00284|Canada|Ontario|658[0n]|BOLD:AAB3178
Heterocampa umbrata|[1555]|TMNBD211-07|MNBTT-3012|Canada|New Brunswick|658[0n]|BOLD:AAB3178
Heterocampa umbrata|[1556]|TMNBD212-07|MNBTT-3013|Canada|New Brunswick|658[0n]|BOLD:AAB3178
Heterocampa umbrata|[1557]|XAK153-06|2006-ONT-1148|Canada|Ontario|658[0n]|BOLD:AAB3178
Heterocampa umbrata|[1558]|LGSMC765-05|DNA-ATBI-2765|United States|Tennessee|658[0n]|BOLD:AAB3178
Heterocampa umbrata|[1559]|LGSMG092-07|BGS03413|United States|North Carolina|658[0n]|BOLD:AAB3178
Heterocampa umbrata|[1560]|LOT549-04|04HBL002549|United States|Tennessee|658[0n]|BOLD:AAB3178
Heterocampa umbrata|[1561]|LOCT028-05|05-CTATBI-0028|United States|Connecticut|658[0n]|BOLD:AA...
Heterocampa umbrata|[1562]|LNC123-05|05-NCCC-123|United States|North Carolina|658[0n]|BOLD:AAB...
Heterocampa umbrata|[1563]|LGSMC764-05|DNA-ATBI-2764|United States|Tennessee|658[0n]|BOLD:AAB3178
Heterocampa umbrata|[1564]|LOCT029-05|05-CTATBI-0029|United States|Connecticut|658[1n]|BOLD:AA...
Heterocampa umbrata|[1565]|LGSMB277-05|DNA-ATBI-1126|United States|Tennessee|658[0n]|BOLD:AAB3178
Heterocampa umbrata|[1566]|USLEP679-10|10BBLEP-00679|United States|Florida|658[0n]|BOLD:AAB3178
[illegible]

Heterocampa umbrata|[1564]|LOC1029-03|03-CTATBI-0029|United States|Connecticut|658[1n]|BOLD:AA...
Heterocampa umbrata|[1565]|LGSMB277-05|DNA-ATBI-1126|United States|Tennessee|658[0n]|BOLD:AAB3178
Heterocampa umbrata|[1566]|USLEP679-10|10BBLEP-00679|United States|Florida|658[0n]|BOLD:AAB3178
Heterocampa umbrata|[1567]|RDNMJ037-10|CNCLEP 73817|United States|Florida|658[0n]|BOLD:AAB3178
Heterocampa umbrata|[1568]|LOFLA232-06|06-FLOR-0232|United States|Florida|658[0n]|BOLD:AAB3178
Heterocampa umbrata|[1569]|USLEP678-10|10BBLEP-00678|United States|Florida|658[0n]|BOLD:AAB3178
Heterocampa umbrata|[1570]|JRLAA027-09|JRLAA-027|United States|Alabama|658[0n]|BOLD:AAB3178
Heterocampa umbrata|[1571]|LGSMG093-07|BGS03414|United States|Tennessee|658[0n]|BOLD:AAB3178
Heterocampa umbrata|[1572]|USLEP681-10|10BBLEP-00681|United States|Florida|658[0n]|BOLD:AAB3178
Heterocampa umbrata|[1573]|LNC124-05|05-NCCC-124|United States|North Carolina|658[0n]|BOLD:AAB...
Heterocampa umbrata|[1574]|LSEU252-06|06-JKA-0252|United States|Georgia|658[0n]|BOLD:AAB3178
Heterocampa umbrata|[1575]|BBLSZ231-09|09BBLEP-04157|United States|Oklahoma|658[0n]|BOLD:AAB3178
Heterocampa umbrata|[1576]|BBLSZ100-09|09BBLEP-04026|United States|Oklahoma|658[0n]|BOLD:AAB3178
Heterocampa umbrata|[1577]|BBLSZ224-09|09BBLEP-04150|United States|Oklahoma|658[0n]|BOLD:AAB3178
Heterocampa umbrata|[1578]|RDNMH415-09|CNCLEP00054429|United States|Texas|658[0n]|BOLD:AAB3178
Heterocampa averna|[1579]|CMAZA040-09|CMAZ-0040|United States|Arizona|658[0n]|BOLD:AAH5620
Heterocampa averna|[1580]|RDNMH177-09|CNCLEP00054394|United States|Arizona|658[0n]|BOLD:AAH5620
Heterocampa averna|[1581]|RDNMH160-09|CNCLEP00054377|United States|Arizona|658[0n]|BOLD:AAH5620
Heterocampa averna|[1582]|RDNMH466-09|CNCLEP00054480|United States|Arizona|658[0n]|BOLD:AAH5620
Heterocampa amanda|[1583]|CNCLB1573-14|CNCLEP 00113803|United States|Arizona|658[0n]|BOLD:ACM4546
Heterocampa amanda|[1584]|CNCLB806-14|CNCLEP00083818|United States|Arizona|658[0n]|BOLD:ACM4546
Heterocampa amanda|[1585]|CNCLB805-14|CNCLEP00083817|United States|Arizona|658[0n]|BOLD:ACM4546
Heterocampa amanda|[1586]|CNCLB809-14|CNCLEP00083821|United States|Arizona|658[0n]|BOLD:ACM4546
Heterocampa amanda|[1587]|CNCLB808-14|CNCLEP00083820|United States|Arizona|658[0n]|BOLD:ACM4546
Heterocampa amanda|[1588]|CNCLB807-14|CNCLEP00083819|United States|Arizona|599[0n]|BOLD:ACM4546
Heterocampa guttivitta|[1589]|XAF765-05|2005-ONT-414|Canada|Ontario|535[0n]|BOLD:AAA3773
Heterocampa guttivitta|[1590]|LGSMC741-05|DNA-ATBI-2741|United States|Tennessee|658[0n]|BOLD:A...
Heterocampa guttivitta|[1591]|LGSMC740-05|DNA-ATBI-2740|United States|Tennessee|658[0n]|BOLD:A...
Heterocampa guttivitta|[1592]|LPSOB788-08|PPBP-1787|Canada|Ontario|658[0n]|BOLD:AAA3773
Heterocampa guttivitta|[1593]|LPSO009-08|PPBP-0009|Canada|Ontario|658[0n]|BOLD:AAA3773
Heterocampa guttivitta|[1594]|XAF472-05|2005-ONT-121|Canada|Ontario|658[0n]|BOLD:AAA3773
Heterocampa guttivitta|[1595]|XAB394-04|04HBL005394|Canada|Ontario|658[0n]|BOLD:AAA3773
Heterocampa guttivitta|[1596]|XAB395-04|04HBL005395|Canada|Ontario|658[0n]|BOLD:AAA3773
Heterocampa guttivitta|[1597]|XAF328-05|HLC-10369|Canada|Ontario|658[0n]|BOLD:AAA3773
Heterocampa guttivitta|[1598]|LPSOC079-08|PPBP-2078|Canada|Ontario|658[0n]|BOLD:AAA3773
Heterocampa guttivitta|[1599]|XAF736-05|2005-ONT-385|Canada|Ontario|596[1n]|BOLD:AAA3773
Heterocampa guttivitta|[1600]|TMG68-03|moth140.01|Canada|Ontario|639[0n]|BOLD:AAA3773
Heterocampa guttivitta|[1601]|XAE397-04|Moth4397.03|Canada|Ontario|567[0n]|BOLD:AAA3773
Heterocampa guttivitta|[1602]|LPSO526-08|PPBP-0526|Canada|Ontario|622[0n]|BOLD:AAA3773
Heterocampa guttivitta|[1603]|LPSO361-08|PPBP-0361|Canada|Ontario|658[0n]|BOLD:AAA3773
Heterocampa guttivitta|[1604]|LPSO527-08|PPBP-0527|Canada|Ontario|658[0n]|BOLD:AAA3773
Heterocampa guttivitta|[1605]|XAE241-04|Moth4241.03|Canada|Ontario|658[0n]|BOLD:AAA3773
Heterocampa guttivitta|[1606]|XAB361-04|04HBL005361|Canada|Ontario|658[0n]|BOLD:AAA3773
Heterocampa guttivitta|[1607]|LPSOB967-08|PPBP-1966|Canada|Ontario|658[0n]|BOLD:AAA3773
Heterocampa guttivitta|[1608]|XAE221-04|Moth4221.03|Canada|Ontario|658[0n]|BOLD:AAA3773
Heterocampa guttivitta|[1609]|XAB509-04|04HBL005509|Canada|Ontario|658[0n]|BOLD:AAA3773
Heterocampa guttivitta|[1610]|LPSOD352-09|08BBLEP-00130|Canada|Ontario|658[0n]|BOLD:AAA3773
Heterocampa guttivitta|[1611]|LPSOD318-09|08BBLEP-00096|Canada|Ontario|658[0n]|BOLD:AAA3773
Heterocampa guttivitta|[1612]|LPSOD272-09|08BBLEP-00050|Canada|Ontario|608[0n]|BOLD:AAA3773
Heterocampa guttivitta|[1613]|LPSOD251-09|08BBLEP-00029|Canada|Ontario|658[0n]|BOLD:AAA3773
Heterocampa guttivitta|[1614]|LPSO232-08|PPBP-0232|Canada|Ontario|658[0n]|BOLD:AAA3773
Heterocampa guttivitta|[1615]|LPSO239-08|PPBP-0239|Canada|Ontario|658[0n]|BOLD:AAA3773
Heterocampa guttivitta|[1616]|CNCLB2635-14|14-NCCC-381|United States|North Carolina|658[0n]|BOLD...
Heterocampa guttivitta|[1617]|LNCC393-10|10-NCCC-488|United States|North Carolina|658[0n]|BOLD ...
Heterocampa guttivitta|[1618]|CNCLB2636-14|14-NCCC-382|United States|North Carolina|658[0n]|BOLD...
Heterocampa guttivitta|[1619]|LPOKB151-09|MDOK-1338|United States|Oklahoma|658[0n]|BOLD:AAA3773
Heterocampa guttivitta|[1620]|LPSO030-08|PPBP-0030|Canada|Ontario|658[0n]|BOLD:AAA3773
Heterocampa guttivitta|[1621]|XAB547-04|04HBL005547|Canada|Ontario|658[0n]|BOLD:AAA3773
Heterocampa guttivitta|[1622]|XAE240-04|Moth4240.03|Canada|Ontario|658[0n]|BOLD:AAA3773
Heterocampa guttivitta|[1623]|LOT109-04|04HBL002109|United States|Tennessee|609[1n]|BOLD:AAA3773
Heterocampa guttivitta|[1624]|LOTB194-05|05-TN-00194|United States|Tennessee|658[0n]|BOLD:AAA3773
Heterocampa guttivitta|[1625]|LOT107-04|04HBL002107|United States|Tennessee|609[0n]|BOLD:AAA3773
Heterocampa guttivitta|[1626]|LPOKB971-09|MDOK-2013|United States|Oklahoma|658[0n]|BOLD:AAA3773
Heterocampa guttivitta|[1627]|LOT103-04|04HBL002103|United States|Tennessee|609[0n]|BOLD:AAA3773
Heterocampa guttivitta|[1628]|LOT100-04|04HBL002100|United States|Tennessee|609[0n]|BOLD:AAA3773
Heterocampa guttivitta|[1629]|LSEU604-06|06-JKA-0604|United States|Georgia|658[0n]|BOLD:AAA3773
Heterocampa guttivitta|[1630]|LSEU304-06|06-JKA-0304|United States|Georgia|658[0n]|BOLD:AAA3773
Heterocampa guttivitta|[1631]|LOT106-04|04HBL002106|United States|Tennessee|609[0n]|BOLD:AAA3773
Heterocampa guttivitta|[1632]|LPOKB1003-09|MDOK-2045|United States|Oklahoma|658[0n]|BOLD:AAA3773
Heterocampa guttivitta|[1633]|LPOKC759-09|MDOK-2836|United States|Oklahoma|658[0n]|BOLD:AAA3773
Heterocampa guttivitta|[1634]|LILLA153-11|SNS10IL-00201|United States|Illinois|658[0n]|BOLD:AA...
Heterocampa guttivitta|[1635]|LOT105-04|04HBL002105|United States|Tennessee|609[0n]|BOLD:AAA3773
Heterocampa guttivitta|[1636]|LOT102-04|04HBL002102|United States|Tennessee|609[0n]|BOLD:AAA3773
Heterocampa guttivitta|[1637]|LOT108-04|04HBL002108|United States|Tennessee|609[0n]|BOLD:AAA3773
Heterocampa guttivitta|[1638]|LGSMC743-05|DNA-ATBI-2743|United States|Tennessee|515[0n]|BOLD:A...
Heterocampa guttivitta|[1639]|LGSMB279-05|DNA-ATBI-1128|United States|Tennessee|589[10n]|BOLD:...
Heterocampa guttivitta|[1640]|LGSMB281-05|DNA-ATBI-1130|United States|Tennessee|598[13n]|
Heterocampa guttivitta|[1641]|LGSMB280-05|DNA-ATBI-1129|United States|Tennessee|596[6n]|BOLD:A...
Heterocampa guttivitta|[1642]|LPOKC627-09|MDOK-2704|United States|Oklahoma|658[0n]|BOLD:AAA3773
Heterocampa guttivitta|[1643]|LPOKD074-09|MDOK-3153|United States|Oklahoma|658[0n]|BOLD:AAA3773
Heterocampa guttivitta|[1644]|LPOKC615-09|MDOK-2692|United States|Oklahoma|658[0n]|BOLD:AAA3773
Heterocampa guttivitta|[1645]|LPOKA235-08|MDOK-0235|United States|Oklahoma|658[0n]|BOLD:AAA3773
Heterocampa guttivitta|[1646]|LOTB354-05|05-TN-00354|United States|Tennessee|658[0n]|BOLD:AAA3773
Heterocampa guttivitta|[1647]|LILLA149-11|SNS10IL-00197|United States|Illinois|658[0n]|BOLD:AA...
Heterocampa guttivitta|[1648]|LILLA025-11|SNS10IL-00034|United States|Illinois|658[0n]|BOLD:AA...
Heterocampa guttivitta|[1649]|LPOKB348-09|MDOK-1257|United States|Oklahoma|658[0n]|BOLD:AAA3773
Heterocampa guttivitta|[1650]|CNFDF743-14|BIOUG10838-G06|Canada|New Brunswick|562[1n]|BOLD:AAA...
Heterocampa guttivitta|[1651]|RDNMH436-09|CNCLEP00054450|United States|North Carolina|658[0n]|BOL...
Heterocampa guttivitta|[1652]|KPOEC038-08|08OEC-141|Canada|Ontario|658[0n]|BOLD:AAA3773
Heterocampa guttivitta|[1653]|CNSLD555-12|BIOUG02743-A01|Canada|Ontario|632[0n]|BOLD:AAA3773
Heterocampa guttivitta|[1654]|TMNBD355-07|MNBTT-3156|Canada|New Brunswick|631[0n]|BOLD:AAA3773
Heterocampa guttivitta|[1655]|TMG69-03|moth468.01|Canada|Ontario|639[0n]|BOLD:AAA3773
Heterocampa guttivitta|[1656]|CNBRN536-14|BIOUG10845-C03|Canada|Nova Scotia|585[1n]|BOLD:AAA3773
Heterocampa guttivitta|[1657]|TMNBD354-07|MNBTT-3155|Canada|New Brunswick|630[0n]|BOLD:AAA3773
Heterocampa guttivitta|[1658]|TMNBD356-07|MNBTT-3157|Canada|New Brunswick|590[0n]|BOLD:AAA3773
Heterocampa guttivitta|[1659]|KPOEC152-08|08OEC-193|Canada|Ontario|658[0n]|BOLD:AAA3773
Heterocampa guttivitta|[1660]|PHMNB440-04|04HBL00666|Canada|New Brunswick|658[0n]|BOLD:AAA3773
Heterocampa guttivitta|[1661]|XAE239-04|Moth4239.03|Canada|Ontario|658[0n]|BOLD:AAA3773
Heterocampa guttivitta|[1662]|XAG897-05|2005-ONT-1481|Canada|Ontario|658[0n]|BOLD:AAA3773
Heterocampa guttivitta|[1663]|TTMNB059-06|MNBTT-059|Canada|New Brunswick|658[0n]|BOLD:AAA3773
Heterocampa guttivitta|[1664]|PHMNB456-04|04HBL00682|Canada|New Brunswick|658[0n]|BOLD:AAA3773
[illegible]

Heterocampa guttivitta|[1662]|XAG897-05|2005-ONT-1481|Canada|Ontario|658[0n]|BOLD:AAA3773
Heterocampa guttivitta|[1663]|TTMNB059-06|MNBTT-059|Canada|New Brunswick|658[0n]|BOLD:AAA3773
Heterocampa guttivitta|[1664]|PHMNB456-04|04HBL00682|Canada|New Brunswick|658[0n]|BOLD:AAA3773
Heterocampa guttivitta|[1665]|XAB367-04|04HBL005367|Canada|Ontario|658[0n]|BOLD:AAA3773
Heterocampa guttivitta|[1666]|XAJ423-06|2006-ONT-0423|Canada|Ontario|658[0n]|BOLD:AAA3773
Heterocampa guttivitta|[1667]|PHMNB526-04|04HBL00752|Canada|New Brunswick|658[0n]|BOLD:AAA3773
Heterocampa guttivitta|[1668]|XAK285-06|2006-ONT-1280|Canada|Ontario|658[0n]|BOLD:AAA3773
Heterocampa guttivitta|[1669]|KPOEC122-08|08OEC-068|Canada|Ontario|658[0n]|BOLD:AAA3773
Heterocampa guttivitta|[1670]|XAJ459-06|2006-ONT-0459|Canada|Ontario|658[0n]|BOLD:AAA3773
Heterocampa guttivitta|[1671]|PHMNB572-04|04HBL00798|Canada|New Brunswick|658[0n]|BOLD:AAA3773
Heterocampa guttivitta|[1672]|PHMNB577-04|04HBL00803|Canada|New Brunswick|658[0n]|BOLD:AAA3773
Heterocampa guttivitta|[1673]|XAE222-04|Moth4222.03|Canada|Ontario|658[0n]|BOLD:AAA3773
Heterocampa guttivitta|[1674]|PHMNB442-04|04HBL00668|Canada|New Brunswick|658[0n]|BOLD:AAA3773
Heterocampa guttivitta|[1675]|XAF405-05|HLC-10446|Canada|Ontario|658[0n]|BOLD:AAA3773
Heterocampa guttivitta|[1676]|LOCT025-05|05-CTATBI-0025|United States|Connecticut|658[0n]|BOLD ...
Heterocampa guttivitta|[1677]|LGSMB282-05|DNA-ATBI-1131|United States|Tennessee|577[0n]|BOLD:A...
Heterocampa guttivitta|[1678]|PMG179-03|moth286.01|Canada|Ontario|617[0n]|BOLD:AAA3773
Heterocampa guttivitta|[1679]|XAE522-04|Moth4522.03|Canada|Ontario|658[4n]|BOLD:AAA3773
Heterocampa guttivitta|[1680]|PHMNB627-04|04HBL00853|Canada|New Brunswick|658[0n]|BOLD:AAA3773
Heterocampa guttivitta|[1681]|PHMNB439-04|04HBL00665|Canada|New Brunswick|658[0n]|BOLD:AAA3773
Heterocampa guttivitta|[1682]|PHMNB570-04|04HBL00796|Canada|New Brunswick|658[0n]|BOLD:AAA3773
Heterocampa guttivitta|[1683]|TTMNB542-06|MNBTT-542|Canada|New Brunswick|658[0n]|BOLD:AAA3773
Heterocampa guttivitta|[1684]|PHMNB426-04|04HBL00652|Canada|New Brunswick|658[0n]|BOLD:AAA3773
Heterocampa guttivitta|[1685]|PHMNB323-04|04HBL00549|Canada|New Brunswick|658[0n]|BOLD:AAA3773
Heterocampa guttivitta|[1686]|KPOEC128-08|08OEC-107|Canada|Ontario|658[0n]|BOLD:AAA3773
Heterocampa guttivitta|[1687]|XAD607-05|2005-ONT-22|Canada|Ontario|651[0n]|BOLD:AAA3773
Heterocampa guttivitta|[1688]|KPOEC083-08|08OEC-242|Canada|Ontario|658[0n]|BOLD:AAA3773
Heterocampa guttivitta|[1689]|XAB393-04|04HBL005393|Canada|Ontario|658[0n]|BOLD:AAA3773
Heterocampa guttivitta|[1690]|PMG180-03|moth782.01|Canada|Ontario|617[1n]|BOLD:AAA3773
Heterocampa guttivitta|[1691]|PHMNB359-04|04HBL00585|Canada|New Brunswick|658[0n]|BOLD:AAA3773
Heterocampa guttivitta|[1692]|XAB556-04|04HBL005556|Canada|Ontario|658[0n]|BOLD:AAA3773
Heterocampa guttivitta|[1693]|XAB396-04|04HBL005396|Canada|Ontario|658[0n]|BOLD:AAA3773
Heterocampa guttivitta|[1694]|BLTIB162-08|BL237|Canada|Ontario|658[0n]|BOLD:AAA3773
Heterocampa guttivitta|[1695]|XAB555-04|04HBL005555|Canada|Ontario|658[0n]|BOLD:AAA3773
Heterocampa guttivitta|[1696]|XAE163-04|Moth4163.03|Canada|Ontario|658[0n]|BOLD:AAA3773
Heterocampa guttivitta|[1697]|LGSMG094-07|BGS03415|United States|North Carolina|658[0n]|BOLD:A...
Heterocampa guttivitta|[1698]|TTMNB248-06|MNBTT-248|Canada|New Brunswick|658[0n]|BOLD:AAA3773
Heterocampa guttivitta|[1699]|LOT101-04|04HBL002101|United States|Tennessee|609[0n]|BOLD:AAA3773
Heterocampa guttivitta|[1700]|LOT104-04|04HBL002104|United States|Tennessee|609[0n]|BOLD:AAA3773
Heterocampa guttivitta|[1701]|LOT110-04|04HBL002110|United States|Tennessee|609[0n]|BOLD:AAA3773
Didugua argentilinea|[1702]|QUNOD021-10|7020-COI-09|United States|Texas|658[0n]|BOLD:AAE0865
Didugua argentilinea|[1703]|HKONB252-09|3749-COI-08|United States|Texas|640[0n]|BOLD:AAE0865
Elasmia packardii|[1704]|RDNMK083-11|CNCLEP 81206|United States|Arizona|658[0n]|BOLD:AAI1926
Elasmia packardii|[1705]|CMAZA1059-12|BIOUG02042-C06|United States|Arizona|658[0n]|BOLD:AAI1926
Elasmia packardii|[1706]|RDNMK085-11|CNCLEP 81208|United States|New Mexico|658[0n]|BOLD:AAI1926
Elasmia packardii|[1707]|RDNMK086-11|CNCLEP 81209|United States|New Mexico|658[0n]|BOLD:AAI1926
Elasmia packardii|[1708]|RDNMK081-11|CNCLEP 81204|United States|New Mexico|658[0n]|BOLD:AAI1926
Elasmia packardii|[1709]|RDNMK084-11|CNCLEP 81207|United States|New Mexico|658[0n]|BOLD:AAI1926
Elasmia packardii|[1710]|CMAZA971-12|BIOUG02041-D01|United States|Arizona|658[1n]|BOLD:AAI1926
Elasmia packardii|[1711]|RDNMK080-11|CNCLEP 81203|United States|Arizona|658[0n]|BOLD:AAI1926
Elasmia packardii|[1712]|RDNMH432-09|CNCLEP00054446|United States|Oklahoma|658[0n]|BOLD:AAI1926
Elasmia packardii|[1713]|RDNMK082-11|CNCLEP 81205|United States|New Mexico|658[0n]|BOLD:AAI1926
Elasmia cave|[1714]|LPOKD097-09|MDOK-3176|United States|Oklahoma|658[0n]|BOLD:AAA6562
Elasmia cave|[1715]|LPOKB1016-09|MDOK-2058|United States|Oklahoma|658[0n]|BOLD:AAA6562
Elasmia cave|[1716]|RDNMH407-09|CNCLEP00054421|United States|Oklahoma|658[0n]|BOLD:AAA6562
Elasmia cave|[1717]|LPOKA462-09|MDOK-0462|United States|Oklahoma|658[0n]|BOLD:AAA6562
Elasmia cave|[1718]|LPOKE257-10|MDOK-4335|United States|Oklahoma|658[0n]|BOLD:AAA6562
Elasmia cave|[1719]|RDNMK078-11|CNCLEP 81201|United States|New Mexico|658[0n]|BOLD:AAA6562
Elasmia cave|[1720]|RDNMK077-11|CNCLEP 81200|United States|New Mexico|658[0n]|BOLD:AAA6562
Elasmia cave|[1721]|RDNMK079-11|CNCLEP 81202|United States|Texas|658[0n]|BOLD:AAA6562
Elasmia mandela|[1722]|HKONB146-08|3643-COI-08|United States|Texas|658[0n]|BOLD:AAA6562
Symmerista zacualpana|[1723]|RDNMJ422-11|CNCLEP 79950|United States|Arizona|631[0n]|BOLD:AAG5414
Symmerista zacualpana|[1724]|RDNMH881-09|CNCLEP00067189|United States|Arizona|658[1n]|BOLD:AAG...
Symmerista zacualpana|[1725]|RDNMK244-11|CNCLEP 84153|United States|Arizona|658[0n]|BOLD:ABY8276
Symmerista sp.|[1726]|AWCLB552-11|AWC-01229|United States||658[1n]|BOLD:AAG5416
Symmerista sp.|[1727]|RDNMH883-09|CNCLEP00067191|United States|Arizona|658[5n]|BOLD:AAG5416
Symmerista zacualpana|[1728]|RDNMJ421-11|CNCLEP 79949|United States|New Mexico|658[0n]|BOLD:AA...
Symmerista sp.|[1729]|RDNMH419-09|CNCLEP00054433|United States|New Mexico|658[0n]|BOLD:AAG5413
Symmerista sp.|[1730]|RDNMH880-09|CNCLEP00067188|United States|Arizona|658[0n]|BOLD:AAG5413
Symmerista sp.|[1731]|RDNMJ671-11|CNCLEP 80105|United States|Arizona|658[0n]|BOLD:AAG5415
Symmerista sp.|[1732]|RDNMH882-09|CNCLEP00067190|United States|Arizona|658[0n]|BOLD:AAG5415
Symmerista sp.|[1733]|HKONB541-09|2654-COI-08|United States|Texas|658[0n]|BOLD:AAG5412
Symmerista suavis|[1734]|RDNMH172-09|CNCLEP00054389|United States|Colorado|639[0n]|BOLD:AAK0998
Symmerista canicosta|[1735]|TMNBD219-07|MNBTT-3020|Canada|New Brunswick|658[0n]|BOLD:AAB3596
Symmerista canicosta|[1736]|RDNMG366-08|NOC15213|Canada|Ontario|658[0n]|BOLD:AAB3596
Symmerista canicosta|[1737]|RDNMG367-08|NOC15214|Canada|Ontario|658[0n]|BOLD:AAB3596
Symmerista canicosta|[1738]|RDNMG363-08|NOC15210|Canada|Ontario|658[0n]|BOLD:AAB3596
Symmerista canicosta|[1739]|MNBB488-05|05-NBSTA-404|Canada|New Brunswick|658[0n]|BOLD:AAB3596
Symmerista canicosta|[1740]|TMNBD218-07|MNBTT-3019|Canada|New Brunswick|594[0n]|BOLD:AAB3596
Symmerista canicosta|[1741]|MNBB436-05|05-NBSTA-352|Canada|New Brunswick|615[0n]|BOLD:AAB3596
Symmerista canicosta|[1742]|TMNBD313-07|MNBTT-3114|Canada|New Brunswick|631[0n]|BOLD:AAB3596
Symmerista canicosta|[1743]|LNC382-05|05-NCCC-382|United States|North Carolina|588[2n]|BOLD:AA...
Symmerista canicosta|[1744]|LNC383-05|05-NCCC-383|United States|North Carolina|586[4n]|BOLD:AA...
Symmerista canicosta|[1745]|TMNBD223-07|MNBTT-3024|Canada|New Brunswick|647[0n]|BOLD:AAB3596
Symmerista canicosta|[1746]|TMNBD335-07|MNBTT-3136|Canada|New Brunswick|638[0n]|BOLD:AAB3596
Symmerista canicosta|[1747]|TMNBD317-07|MNBTT-3118|Canada|New Brunswick|658[0n]|BOLD:AAB3596
Symmerista canicosta|[1748]|TMNBD319-07|MNBTT-3120|Canada|New Brunswick|658[0n]|BOLD:AAB3596
Symmerista canicosta|[1749]|TMNBD220-07|MNBTT-3021|Canada|New Brunswick|658[0n]|BOLD:AAB3596
Symmerista canicosta|[1750]|RDNMG365-08|NOC15212|Canada|New Brunswick|658[0n]|BOLD:AAB3596
Symmerista canicosta|[1751]|LNCC1395-11|11-NCCC-920|United States|North Carolina|658[0n]|BOLD:...
Symmerista canicosta|[1752]|TMNBD330-07|MNBTT-3131|Canada|New Brunswick|610[0n]|BOLD:AAB3596
Symmerista canicosta|[1753]|TMNBD316-07|MNBTT-3117|Canada|New Brunswick|658[0n]|BOLD:AAB3596
Symmerista canicosta|[1754]|LNC381-05|05-NCCC-381|United States|North Carolina|553[0n]|BOLD:AA...
Symmerista canicosta|[1755]|TMNBD309-07|MNBTT-3110|Canada|New Brunswick|656[0n]|BOLD:AAB3596
Symmerista canicosta|[1756]|LSEU737-06|06-JKA-0737|United States|Georgia|658[0n]|BOLD:AAB3596
Symmerista canicosta|[1757]|LNC380-05|05-NCCC-380|United States|North Carolina|556[0n]|BOLD:AA...
Symmerista albifrons|[1758]|LTOLB143-08|CWM-94-0347|United States|Maryland|658[0n]|BOLD:AAB3596
Symmerista albifrons|[1759]|LPOKD707-10|MDOK-3786|United States|Oklahoma|658[0n]|BOLD:AAB3596
Symmerista albifrons|[1760]|TMNBD318-07|MNBTT-3119|Canada|New Brunswick|658[0n]|BOLD:AAB3596
Symmerista albifrons|[1761]|MILEQ252-11|11-MISC-727|United States|Alabama|658[0n]|BOLD:AAB3596
Symmerista albifrons|[1762]|RDNMG361-08|NOC15208|United States|Georgia|658[0n]|BOLD:AAB3596

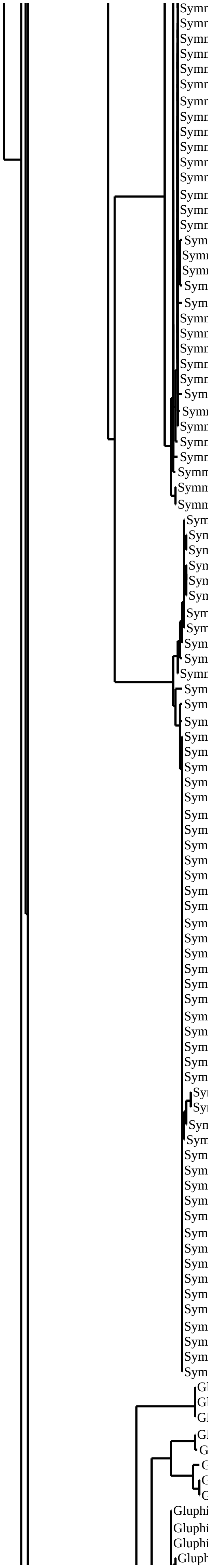

Symmerista albifrons|[1760]|TMNBD318-07|MNBTT-3119|Canada|New Brunswick|658[0n]|BOLD:AAB3596
Symmerista albifrons|[1761]|MILEQ252-11|11-MISC-727|United States|Alabama|658[0n]|BOLD:AAB3596
Symmerista albifrons|[1762]|RDNMG361-08|NOC15208|United States|Georgia|658[0n]|BOLD:AAB3596
Symmerista albifrons|[1763]|LPOKD766-10|MDOK-3845|United States|Oklahoma|658[0n]|BOLD:AAB3596
Symmerista albifrons|[1764]|RDLQF458-06|DH011565|Canada|Quebec|658[0n]|BOLD:AAB3596
Symmerista albifrons|[1765]|LNC092-05|05-NCCC-092|United States|North Carolina|658[0n]|BOLD:AA...
Symmerista albifrons|[1766]|LGSMG743-07|BGS03406|United States|Tennessee|658[0n]|BOLD:AAB3596
Symmerista albifrons|[1767]|RDLQF233-06|DH011313|Canada|Quebec|658[0n]|BOLD:AAB3596
Symmerista albifrons|[1768]|TMNBD321-07|MNBTT-3122|Canada|New Brunswick|658[0n]|BOLD:AAB3596
Symmerista albifrons|[1769]|RDNML163-13|13-NCCC-068|United States|North Carolina|658[0n]|BOLD:...
Symmerista albifrons|[1770]|LTOL800-07|CWM-94-0181|United States|Maryland|658[0n]|BOLD:AAB3596
Symmerista albifrons|[1771]|LPOKD706-10|MDOK-3785|United States|Oklahoma|658[0n]|BOLD:AAB3596
Symmerista albifrons|[1772]|RDLQ104-05|DH003038|Canada|Quebec|658[0n]|BOLD:AAB3596
Symmerista albifrons|[1773]|LNCB558-09|09-NCCC-028|United States|North Carolina|658[0n]|BOLD:A...
Symmerista albifrons|[1774]|LPSOC216-08|PPBP-2215|Canada|Ontario|658[0n]|BOLD:AAB3596
Symmerista albifrons|[1775]|MILEQ253-11|11-MISC-728|United States|Alabama|636[0n]|BOLD:AAB3596
Symmerista albifrons|[1776]|TMNBD327-07|MNBTT-3128|Canada|New Brunswick|635[0n]|BOLD:AAB3596
Symmerista albifrons|[1777]|LNC377-05|05-NCCC-377|United States|North Carolina|568[0n]|BOLD:AA...
Symmerista albifrons|[1778]|LPOKD735-10|MDOK-3814|United States|Oklahoma|658[0n]|BOLD:AAB3596
Symmerista abita|[1779]|CNCLB1248-14|CNCLEP 00113541|United States|Louisiana|658[0n]|BOLD:AAB3596
Symmerista abita|[1780]|CNCLB1247-14|CNCLEP 00113540|United States|Louisiana|658[0n]|BOLD:AAB3596
Symmerista albifrons|[1781]|RDNMG360-08|NOC15207|United States|Georgia|658[0n]|BOLD:AAB3596
Symmerista albifrons|[1782]|RDNMG362-08|NOC15209|United States|Georgia|658[0n]|BOLD:AAB3596
Symmerista albifrons|[1783]|RDNMH518-09|CNCLEP00057837|United States|North Carolina|658[0n]|BOLD...
Symmerista albifrons|[1784]|LNCB576-09|09-NCCC-046|United States|North Carolina|658[0n]|BOLD:A...
Symmerista albifrons|[1785]|LGSMG713-07|BGS03376|United States|Tennessee|658[0n]|BOLD:AAB3596
Symmerista albifrons|[1786]|LGSMG742-07|BGS03405|United States|Tennessee|658[0n]|BOLD:AAB3596
Symmerista albifrons|[1787]|TMNBD325-07|MNBTT-3126|Canada|New Brunswick|655[0n]|BOLD:AAB3596
Symmerista albifrons|[1788]|LPSO744-08|PPBP-0744|Canada|Ontario|658[0n]|BOLD:AAB3596
Symmerista albifrons|[1789]|LPOKE071-10|MDOK-4149|United States|Oklahoma|658[0n]|BOLD:AAB3596
Symmerista albifrons|[1790]|MILEQ254-11|11-MISC-729|United States|Alabama|658[0n]|BOLD:AAB3596
Symmerista albifrons|[1791]|LNC376-05|05-NCCC-376|United States|North Carolina|555[0n]|BOLD:AA...
Symmerista albifrons|[1792]|LNC093-05|05-NCCC-093|United States|North Carolina|658[0n]|BOLD:AA...
Symmerista leucitys|[1793]|LNCC1006-11|11-NCCC-531|United States|North Carolina|658[0n]|BOLD:A...
Symmerista leucitys|[1794]|LGSMB249-05|DNA-ATBI-1098|United States|Tennessee|572[0n]|BOLD:AAA7013
Symmerista leucitys|[1795]|LSEU736-06|06-JKA-0736|United States|Georgia|658[0n]|BOLD:AAA7013
Symmerista leucitys|[1796]|LGSMB248-05|DNA-ATBI-1097|United States|North Carolina|537[0n]|BOLD ...
Symmerista leucitys|[1797]|LNC378-05|05-NCCC-378|United States|North Carolina|576[0n]|BOLD:AAA...
Symmerista leucitys|[1798]|LGSMB250-05|DNA-ATBI-1099|United States|Tennessee|566[0n]|BOLD:AAA7013
Symmerista leucitys|[1799]|LSEU738-06|06-JKA-0738|United States|Georgia|658[0n]|BOLD:AAA7013
Symmerista leucitys|[1800]|LNCC1168-11|11-NCCC-693|United States|North Carolina|658[0n]|BOLD:A...
Symmerista leucitys|[1801]|LNCC1007-11|11-NCCC-532|United States|North Carolina|658[0n]|BOLD:A...
Symmerista leucitys|[1802]|MMNA086-08|HLC-17648|United States|North Carolina|658[0n]|BOLD:AAA7013
Symmerista leucitys|[1803]|LNC379-05|05-NCCC-379|United States|North Carolina|511[0n]|BOLD:AAA...
Symmerista leucitys|[1804]|RDLQ099-05|DH001503|Canada|Quebec|579[1n]|BOLD:AAA7013
Symmerista leucitys|[1805]|TMNBD341-07|MNBTT-3142|Canada|New Brunswick|658[0n]|BOLD:AAA7013
Symmerista leucitys|[1806]|LPSOC287-08|PPBP-2286|Canada|Ontario|658[0n]|BOLD:AAA7013
Symmerista leucitys|[1807]|TMNBD345-07|MNBTT-3146|Canada|New Brunswick|658[0n]|BOLD:AAA7013
Symmerista leucitys|[1808]|TMNBD222-07|MNBTT-3023|Canada|New Brunswick|655[0n]|BOLD:AAA7013
Symmerista leucitys|[1809]|TMNBD333-07|MNBTT-3134|Canada|New Brunswick|655[0n]|BOLD:AAA7013
Symmerista leucitys|[1810]|PMG190-03|moth1151.01|Canada|Ontario|617[0n]|BOLD:AAA7013
Symmerista leucitys|[1811]|TMNBD339-07|MNBTT-3140|Canada|New Brunswick|659[0n]|BOLD:AAA7013
Symmerista leucitys|[1812]|LNCC1200-11|11-NCCC-725|United States|North Carolina|658[0n]|BOLD:A...
Symmerista leucitys|[1813]|TMNBD331-07|MNBTT-3132|Canada|New Brunswick|658[0n]|BOLD:AAA7013
Symmerista leucitys|[1814]|LPSOC309-08|PPBP-2308|Canada|Ontario|658[0n]|BOLD:AAA7013
Symmerista leucitys|[1815]|TMNBD326-07|MNBTT-3127|Canada|New Brunswick|657[0n]|BOLD:AAA7013
Symmerista leucitys|[1816]|LPSOC308-08|PPBP-2307|Canada|Ontario|658[0n]|BOLD:AAA7013
Symmerista leucitys|[1817]|TMNBD338-07|MNBTT-3139|Canada|New Brunswick|658[0n]|BOLD:AAA7013
Symmerista leucitys|[1818]|TMNBD343-07|MNBTT-3144|Canada|New Brunswick|658[0n]|BOLD:AAA7013
Symmerista leucitys|[1819]|TMNBD328-07|MNBTT-3129|Canada|New Brunswick|658[0n]|BOLD:AAA7013
Symmerista leucitys|[1820]|TMNBD332-07|MNBTT-3133|Canada|New Brunswick|658[0n]|BOLD:AAA7013
Symmerista leucitys|[1821]|LPSOC310-08|PPBP-2309|Canada|Ontario|658[0n]|BOLD:AAA7013
Symmerista leucitys|[1822]|LPSOB969-08|PPBP-1968|Canada|Ontario|658[0n]|BOLD:AAA7013
Symmerista leucitys|[1823]|RDNMG364-08|NOC15211|Canada|New Brunswick|658[0n]|BOLD:AAA7013
Symmerista leucitys|[1824]|TMNBD312-07|MNBTT-3113|Canada|New Brunswick|618[0n]|BOLD:AAA7013
Symmerista leucitys|[1825]|TMNBD310-07|MNBTT-3111|Canada|New Brunswick|618[0n]|BOLD:AAA7013
Symmerista leucitys|[1826]|PHMO371-03|moth874.02|Canada|Ontario|639[0n]|BOLD:AAA7013
Symmerista leucitys|[1827]|PHMO217-03|moth1100.02|Canada|Ontario|639[0n]|BOLD:AAA7013
Symmerista leucitys|[1828]|TMG67-03|moth1183.01|Canada|Ontario|639[0n]|BOLD:AAA7013
Symmerista leucitys|[1829]|RDLQF940-06|DH012120|Canada|Quebec|620[0n]|BOLD:AAA7013
Symmerista leucitys|[1830]|TMNBD340-07|MNBTT-3141|Canada|New Brunswick|621[0n]|BOLD:AAA7013
Symmerista leucitys|[1831]|TMNBD311-07|MNBTT-3112|Canada|New Brunswick|578[0n]|BOLD:AAA7013
Symmerista leucitys|[1832]|TMNBD336-07|MNBTT-3137|Canada|New Brunswick|588[0n]|BOLD:AAA7013
Symmerista leucitys|[1833]|TMNBD315-07|MNBTT-3116|Canada|New Brunswick|632[0n]|BOLD:AAA7013
Symmerista leucitys|[1834]|TMNBD324-07|MNBTT-3125|Canada|New Brunswick|621[0n]|BOLD:AAA7013
Symmerista leucitys|[1835]|TMNBD344-07|MNBTT-3145|Canada|New Brunswick|645[0n]|BOLD:AAA7013
Symmerista leucitys|[1836]|XAE414-04|Moth4414.03|Canada|Ontario|658[0n]|BOLD:AAA7013
Symmerista leucitys|[1837]|TMNBD221-07|MNBTT-3022|Canada|New Brunswick|658[0n]|BOLD:AAA7013
Symmerista leucitys|[1838]|TMNBD307-07|MNBTT-3108|Canada|New Brunswick|658[0n]|BOLD:AAA7013
Symmerista leucitys|[1839]|TMNBD334-07|MNBTT-3135|Canada|New Brunswick|658[0n]|BOLD:AAA7013
Symmerista leucitys|[1840]|XAF801-05|2005-ONT-450|Canada|Ontario|658[0n]|BOLD:AAA7013
Symmerista leucitys|[1841]|TMNBD323-07|MNBTT-3124|Canada|New Brunswick|658[0n]|BOLD:AAA7013
Symmerista leucitys|[1842]|RDLQ103-05|DH005439|Canada|Quebec|658[0n]|BOLD:AAA7013
Symmerista leucitys|[1843]|XAI067-05|0102-ONT-0067|Canada|Ontario|658[0n]|BOLD:AAA7013
Symmerista leucitys|[1844]|RDLQ194-05|05HBLT0194|Canada|Quebec|658[0n]|BOLD:AAA7013
Symmerista leucitys|[1845]|TMNBD322-07|MNBTT-3123|Canada|New Brunswick|657[0n]|BOLD:AAA7013
Symmerista leucitys|[1846]|TMNBD337-07|MNBTT-3138|Canada|New Brunswick|658[0n]|BOLD:AAA7013
Symmerista leucitys|[1847]|RDLQF853-06|DH012014|Canada|Quebec|658[0n]|BOLD:AAA7013
Symmerista leucitys|[1848]|XAE466-04|Moth4466.03|Canada|Ontario|658[0n]|BOLD:AAA7013
Gluphisia sp.|[1849]|RDNMH515-09|CNCLEP00057834|United States|Wyoming|658[0n]|BOLD:AAC5384
Gluphisia sp.|[1850]|RDNMH464-09|CNCLEP00054478|United States|Colorado|658[0n]|BOLD:AAC5384
Gluphisia sp.|[1851]|RDNMH414-09|CNCLEP00054428|United States|Wyoming|658[0n]|BOLD:AAC5384
Gluphisia severa|[1852]|RDMAB694-06|UASM2598|Canada|Alberta|658[0n]|BOLD:ABZ7451
Gluphisia severa|[1853]|JMMMB540-13|BIOUG06722-A02|United States|California|658[0n]|BOLD:ABZ7451
Gluphisia avimacula|[1854]|TTMNB920-06|MNBTT-920|Canada|New Brunswick|658[0n]|BOLD:ABZ7450
Gluphisia avimacula|[1855]|TMNBD297-07|MNBTT-3098|Canada|New Brunswick|658[0n]|BOLD:ABZ7450
Gluphisia avimacula|[1856]|TMNBD298-07|MNBTT-3099|Canada|New Brunswick|658[0n]|BOLD:ABZ7450
Gluphisia lintneri|[1857]|TTMNB929-06|MNBTT-929|Canada|New Brunswick|658[0n]|BOLD:AAB5830
Gluphisia lintneri|[1858]|TMNBD299-07|MNBTT-3100|Canada|New Brunswick|658[0n]|BOLD:AAB5830
Gluphisia lintneri|[1859]|TTMNB928-06|MNBTT-928|Canada|New Brunswick|658[0n]|BOLD:AAB5830
Gluphisia lintneri|[1860]|PHMO023-03|moth72.02|Canada|Ontario|639[0n]|BOLD:AAB5830

Gluphisia lintneri|[1858]|TMNBD299-07|MNBTT-3100|Canada|New Brunswick|658[0n]|BOLD:AAB5830
Gluphisia lintneri|[1859]|TTMNB928-06|MNBTT-928|Canada|New Brunswick|658[0n]|BOLD:AAB5830
Gluphisia lintneri|[1860]|PHMO023-03|moth72.02|Canada|Ontario|639[0n]|BOLD:AAB5830
Gluphisia lintneri|[1861]|PMG178-03|moth156.01|Canada|Ontario|617[0n]|BOLD:AAB5830
Gluphisia lintneri|[1862]|TMG65-03|moth111.02|Canada|Ontario|639[0n]|BOLD:AAB5830
Gluphisia lintneri|[1863]|XAD683-05|2005-ONT-98|Canada|Ontario|658[0n]|BOLD:AAB5830
Gluphisia lintneri|[1864]|XAJ165-06|2006-ONT-0165|Canada|Ontario|658[0n]|BOLD:AAB5830
Gluphisia lintneri|[1865]|XAJ127-06|2006-ONT-0127|Canada|Ontario|658[0n]|BOLD:AAB5830
Gluphisia lintneri|[1866]|LMDH022-11|BIOUG01046-A07|United States|Minnesota|658[0n]|BOLD:AAB5830
Gluphisia lintneri|[1867]|XAJ137-06|2006-ONT-0137|Canada|Ontario|633[0n]|BOLD:AAB5830
Gluphisia lintneri|[1868]|XAJ092-06|2006-ONT-0092|Canada|Ontario|658[0n]|BOLD:AAB5830
Gluphisia lintneri|[1869]|XAE131-04|Moth4131.03|Canada|Ontario|658[0n]|BOLD:AAB5830
Gluphisia lintneri|[1870]|XAJ055-06|2006-ONT-0055|Canada|Ontario|658[0n]|BOLD:AAB5830
Gluphisia lintneri|[1871]|XAF133-05|HBL008949|Canada|Ontario|658[0n]|BOLD:AAB5830
Gluphisia lintneri|[1872]|XAJ113-06|2006-ONT-0113|Canada|Ontario|658[0n]|BOLD:AAB5830
Gluphisia lintneri|[1873]|XAJ170-06|2006-ONT-0170|Canada|Ontario|658[0n]|BOLD:AAB5830
Gluphisia lintneri|[1874]|XAJ067-06|2006-ONT-0067|Canada|Ontario|658[0n]|BOLD:AAB5830
Gluphisia lintneri|[1875]|XAF193-05|HLC-10234|Canada|Ontario|658[0n]|BOLD:AAB5830
Gluphisia lintneri|[1876]|TTMNB926-06|MNBTT-926|Canada|New Brunswick|658[0n]|BOLD:AAB5830
Gluphisia lintneri|[1877]|XAE130-04|Moth4130.03|Canada|Ontario|658[0n]|BOLD:AAB5830
Gluphisia avimacula|[1878]|TTMNB924-06|MNBTT-924|Canada|New Brunswick|658[0n]|BOLD:ABZ0941
Gluphisia avimacula|[1879]|TTMNB923-06|MNBTT-923|Canada|New Brunswick|658[0n]|BOLD:ABZ0941
Gluphisia avimacula|[1880]|TTMNB922-06|MNBTT-922|Canada|New Brunswick|658[0n]|BOLD:ABZ0941
Gluphisia avimacula|[1881]|TTMNB921-06|MNBTT-921|Canada|New Brunswick|658[0n]|BOLD:ABZ0941
Gluphisia avimacula|[1882]|TTMNB925-06|MNBTT-925|Canada|New Brunswick|658[0n]|BOLD:ABZ0941
Gluphisia avimacula|[1883]|LPSOB388-08|PPBP-1387|Canada|Ontario|658[0n]|BOLD:ABZ0941
Gluphisia avimacula|[1884]|BBLPB639-10|10BBCLP-1638|Canada|Saskatchewan|658[0n]|BOLD:ABZ0941
Gluphisia avimacula|[1885]|RDLQ773-07|DH009248|Canada|Quebec|632[0n]|BOLD:ABZ0941
Gluphisia avimacula|[1886]|TTMNB927-06|MNBTT-927|Canada|New Brunswick|658[0n]|BOLD:ABZ0941
Gluphisia avimacula|[1887]|LPSOB456-08|PPBP-1455|Canada|Ontario|658[0n]|BOLD:ABZ0941
Gluphisia avimacula|[1888]|LPSOB343-08|PPBP-1342|Canada|Ontario|658[0n]|BOLD:ABZ0941
Gluphisia severa|[1889]|LBCA019-05|HLC-20019|Canada|British Columbia|658[0n]|BOLD:AAC5383
Gluphisia severa|[1890]|LBCA020-05|HLC-20020|Canada|British Columbia|658[0n]|BOLD:AAC5383
Gluphisia severa|[1891]|LBCA279-05|HLC-20279|Canada|British Columbia|658[0n]|BOLD:AAC5383
Gluphisia severa|[1892]|LBCA144-05|HLC-20144|Canada|British Columbia|658[0n]|BOLD:AAC5383
Gluphisia severa|[1893]|RDMAB695-06|UASM2638|Canada|Alberta|621[0n]|BOLD:AAC5383
Gluphisia severa|[1894]|LBCH5281-10|10-JDWBC-5281|Canada|British Columbia|658[0n]|BOLD:AAC5383
Gluphisia severa|[1895]|DUNLP191-08|Dun-08-191|Canada|British Columbia|658[0n]|BOLD:AAC5383
Gluphisia severa|[1896]|RDNMH467-09|CNCLEP00054481|United States|California|658[0n]|BOLD:AAC5383
Gluphisia severa|[1897]|JMMMB183-11|BIOUG00848-H04|United States|California|658[0n]|BOLD:AAC5383
Cerura scitiscripta|[1898]|LPSOB723-08|PPBP-1722|Canada|Ontario|609[0n]|BOLD:AAE9718
Cerura scitiscripta|[1899]|LPOKD733-10|MDOK-3812|United States|Oklahoma|658[0n]|BOLD:AAE9718
Cerura candida|[1900]|RDNMF622-08|NOC14708|United States|Missouri|640[0n]|BOLD:AAE9718
Cerura candida|[1901]|CNCLB801-14|CNCLEP00083813|United States|Texas|407[0n]|BOLD:AAE9718
Cerura candida|[1902]|CNCLB2387-14|CNCLEP 00119529|United States|Texas|658[0n]|BOLD:AAE9718
Cerura candida|[1903]|LPOKC732-09|MDOK-2809|United States|Oklahoma|658[0n]|BOLD:AAE9718
Cerura scitiscripta|[1904]|RDNMF551-08|NOC14637|Canada|Ontario|640[0n]|BOLD:AAE9718
Cerura scitiscripta|[1905]|XAJ421-06|2006-ONT-0421|Canada|Ontario|658[0n]|BOLD:AAE9718
Cerura scitiscripta|[1906]|RDLQ775-07|DH001233|Canada|Quebec|656[0n]|BOLD:AAE9718
Cerura rarata|[1907]|LOCRC001-08|08-CRBS-565|Costa Rica|Alajuela|621[0n]|BOLD:AAA6718
Cerura rarata|[1908]|CNCLB862-14|CNCLEP00093345|Costa Rica|Puntarenas|658[0n]|BOLD:AAA6718
Cerura rarata|[1909]|LOCRB940-08|08-CRBS-564|Costa Rica|Alajuela|658[0n]|BOLD:AAA6718
Cerura rarata|[1910]|LOCRB939-08|08-CRBS-563|Costa Rica|Alajuela|658[0n]|BOLD:AAA6718
Cerura rarata|[1911]|MILEP502-10|10-MISC-407|Ecuador|Orellana|658[0n]|BOLD:AAU1593
Cerura rarata|[1912]|MILEP419-10|10-MISC-324|Ecuador|Orellana|658[0n]|BOLD:AAU1593
Cerura rarata|[1913]|PTNOT246-10|BC-PTh0152|Paraguay|Presidente Hayes|658[0n]|BOLD:AAM3909
Furcula occidentalis|[1914]|CHIP595-12|BIOUG01237-B02|Canada|Manitoba|658[0n]|BOLD:AAA8926
Furcula occidentalis|[1915]|CHLEP219-09|09PROBE-09514|Canada|Manitoba|658[0n]|BOLD:AAA8926
Furcula occidentalis|[1916]|LCH217-04|04HBL003217|Canada|Manitoba|658[0n]|BOLD:AAA8926
Furcula occidentalis|[1917]|JGLL012-10|10PROBE-18734|Canada|Manitoba|658[0n]|BOLD:AAA8926
Furcula occidentalis|[1918]|CHLEP242-09|09PROBE-09537|Canada|Manitoba|658[0n]|BOLD:AAA8926
Furcula occidentalis|[1919]|CHLEP066-09|09PROBE-09361|Canada|Manitoba|658[0n]|BOLD:AAA8926
Furcula occidentalis|[1920]|PHLCH737-10|10PROBE-26547|Canada|Manitoba|658[0n]|BOLD:AAA8926
Furcula occidentalis|[1921]|CHLEP069-09|09PROBE-09364|Canada|Manitoba|658[0n]|BOLD:AAA8926
Furcula occidentalis|[1922]|JGLL011-10|10PROBE-18733|Canada|Manitoba|658[0n]|BOLD:AAA8926
Furcula occidentalis|[1923]|CHLEP084-09|09PROBE-09379|Canada|Manitoba|658[0n]|BOLD:AAA8926
Furcula occidentalis|[1924]|CHLEP085-09|09PROBE-09380|Canada|Manitoba|658[0n]|BOLD:AAA8926
Furcula occidentalis|[1925]|CHLEP290-09|09PROBE-09585|Canada|Manitoba|658[0n]|BOLD:AAA8926
Furcula occidentalis|[1926]|JGLL035-10|10PROBE-18811|Canada|Manitoba|658[0n]|BOLD:AAA8926
Furcula occidentalis|[1927]|JGLL028-10|10PROBE-18772|Canada|Manitoba|658[0n]|BOLD:AAA8926
Furcula occidentalis|[1928]|CHLEP086-09|09PROBE-09381|Canada|Manitoba|658[0n]|BOLD:AAA8926
Furcula occidentalis|[1929]|CHLEP081-09|09PROBE-09376|Canada|Manitoba|658[0n]|BOLD:AAA8926
Furcula occidentalis|[1930]|JGLL031-10|10PROBE-18807|Canada|Manitoba|658[0n]|BOLD:AAA8926
Furcula occidentalis|[1931]|CHLEP249-09|09PROBE-09544|Canada|Manitoba|644[0n]|BOLD:AAA8926
Furcula occidentalis|[1932]|CHLEP067-09|09PROBE-09362|Canada|Manitoba|658[0n]|BOLD:AAA8926
Furcula occidentalis|[1933]|LCHP167-07|07PROBE-00569|Canada|Manitoba|656[0n]|BOLD:AAA8926
Furcula occidentalis|[1934]|BBLPB296-10|10BBCLP-1295|Canada|Saskatchewan|658[0n]|BOLD:AAA8926
Furcula occidentalis|[1935]|BBLPB299-10|10BBCLP-1298|Canada|Saskatchewan|658[0n]|BOLD:AAA8926
Furcula occidentalis|[1936]|LPMN585-08|08BBLEP-01386|Canada|Manitoba|658[0n]|BOLD:AAA8926
Furcula occidentalis|[1937]|LPMN715-08|08BBLEP-01518|Canada|Manitoba|658[0n]|BOLD:AAA8926
Furcula occidentalis|[1938]|XAG583-05|2005-ONT-1167|Canada|Ontario|658[0n]|BOLD:AAA8926
Furcula occidentalis|[1939]|XAK536-07|HLC-16089|Canada|Ontario|658[0n]|BOLD:AAA8926
Furcula occidentalis|[1940]|PMG176-03|FURC2.00|Canada|Ontario|617[0n]|BOLD:AAA8926
Furcula occidentalis|[1941]|XAJ503-06|2006-ONT-0503|Canada|Ontario|634[0n]|BOLD:AAA8926
Furcula occidentalis|[1942]|BLTIB933-08|BL1353|Canada|Ontario|658[0n]|BOLD:AAA8926
Furcula occidentalis|[1943]|XAK244-06|2006-ONT-1239|Canada|Ontario|658[0n]|BOLD:AAA8926
Furcula occidentalis|[1944]|XAD630-05|2005-ONT-45|Canada|Ontario|658[0n]|BOLD:AAA8926
Furcula occidentalis|[1945]|XAK534-07|HLC-16087|Canada|Ontario|658[0n]|BOLD:AAA8926
Furcula occidentalis|[1946]|XAJ598-06|2006-ONT-0598|Canada|Ontario|658[0n]|BOLD:AAA8926
Furcula occidentalis|[1947]|XAK533-07|HLC-16086|Canada|Ontario|657[0n]|BOLD:AAA8926
Furcula occidentalis|[1948]|XAK505-07|HLC-16058|Canada|Ontario|655[0n]|BOLD:AAA8926
Furcula occidentalis|[1949]|PHMNB050-03|moth236.02SA|Canada|New Brunswick|639[0n]|BOLD:AAA8926
Furcula occidentalis|[1950]|TMNBD225-07|MNBTT-3026|Canada|New Brunswick|658[0n]|BOLD:AAA8926
Furcula occidentalis|[1951]|RDLQH087-06|DH013325|Canada|Quebec|658[0n]|BOLD:AAA8926
Furcula occidentalis|[1952]|LPSO422-08|PPBP-0422|Canada|Ontario|657[0n]|BOLD:AAA8926
Furcula occidentalis|[1953]|LPSOD458-09|08BBLEP-00237|Canada|Ontario|658[0n]|BOLD:AAA8926
Furcula occidentalis|[1954]|PHMNB731-05|Moth 424.03SA|Canada|New Brunswick|658[0n]|BOLD:AAA8926
Furcula occidentalis|[1955]|LPSO653-08|PPBP-0653|Canada|Ontario|657[0n]|BOLD:AAA8926
Furcula occidentalis|[1956]|LPSO421-08|PPBP-0421|Canada|Ontario|658[0n]|BOLD:AAA8926
Furcula occidentalis|[1957]|XAK535-07|HLC-16088|Canada|Ontario|658[0n]|BOLD:AAA8926
Furcula occidentalis|[1958]|LPSO423-08|PPBP-0423|Canada|Ontario|657[0n]|BOLD:AAA8926

Furcula occidentalis|[1956]|LPSO421-08|PPBP-0421|Canada|Ontario|658[0n]|BOLD:AAA8926
Furcula occidentalis|[1957]|XAK535-07|HLC-16088|Canada|Ontario|658[0n]|BOLD:AAA8926
Furcula occidentalis|[1958]|LPSO423-08|PPBP-0423|Canada|Ontario|657[0n]|BOLD:AAA8926
Furcula occidentalis|[1959]|LPSO425-08|PPBP-0425|Canada|Ontario|658[0n]|BOLD:AAA8926
Furcula occidentalis|[1960]|BBLPB295-10|10BBCLP-1294|Canada|Ontario|658[0n]|BOLD:AAA8926
Furcula occidentalis|[1961]|LPSO424-08|PPBP-0424|Canada|Ontario|658[0n]|BOLD:AAA8926
Furcula occidentalis|[1962]|LBCW050-08|08-JDWWI-0050|Canada|British Columbia|658[0n]|BOLD:AAA8926
Furcula occidentalis|[1963]|BBLSY542-09|09BBLEP-03469|United States|New Mexico|658[0n]|BOLD:AA...
Furcula furcula|[1964]|RDMAB265-05|UASM41462|Canada|Alberta|658[0n]|BOLD:ACF1064
Furcula furcula|[1965]|RDMAB152-05|UASM22722|Canada|Alberta|658[0n]|BOLD:ACF1064
Furcula furcula|[1966]|RDMAB145-05|UASM58242|Canada|Alberta|658[0n]|BOLD:ACF1064
Furcula furcula|[1967]|LBCB548-05|HLC-21488|Canada|British Columbia|658[0n]|BOLD:ACF1064
Furcula furcula|[1968]|LBCA304-05|HLC-20304|Canada|British Columbia|658[0n]|BOLD:ACF1064
Furcula furcula|[1969]|RDMAB150-05|UASM2749|Canada|Alberta|658[0n]|BOLD:ACF1064
Furcula furcula|[1970]|RDMAB149-05|UASM2739|Canada|Alberta|658[0n]|BOLD:ACF1064
Furcula furcula|[1971]|BBLPB294-10|10BBCLP-1293|Canada|Alberta|658[0n]|BOLD:ACF1064
Furcula furcula|[1972]|RDMAB147-05|UASM2744|Canada|Alberta|582[0n]|BOLD:ACF1064
Furcula furcula|[1973]|RDMAB153-05|UASM56875|Canada|Alberta|658[0n]|BOLD:ACF1064
Furcula furcula|[1974]|LBCC916-05|HLC-22796|Canada|British Columbia|658[0n]|BOLD:ACF1064
Furcula furcula|[1975]|RDMAB151-05|UASM56898|Canada|Alberta|658[0n]|BOLD:ACF1064
Furcula furcula|[1976]|LBCD492-05|HLC-23312|Canada|British Columbia|658[0n]|BOLD:ACF1064
Furcula furcula|[1977]|RDMAB138-05|UASM56892|Canada|Alberta|658[0n]|BOLD:ACF1064
Furcula furcula|[1978]|RDMAB146-05|UASM2716|Canada|Alberta|658[0n]|BOLD:ACF1064
Furcula furcula|[1979]|RDMAB144-05|UASM2769|Canada|Alberta|658[0n]|BOLD:ACF1064
Furcula furcula|[1980]|LOWCE202-06|CGWC-3962|Canada|British Columbia|658[0n]|BOLD:ACF1064
Furcula meridionalis|[1981]|CNCLB2373-14|CNCLEP 00119517|United States|Texas|658[0n]|BOLD:AAB0477
Furcula cinerea|[1982]|RDNMH713-09|CNCLEP00063146|Canada|Ontario|658[0n]|BOLD:AAB0477
Furcula cinerea|[1983]|XAE253-04|Moth4253.03|Canada|Ontario|658[0n]|BOLD:AAB0477
Furcula cinerea|[1984]|TMNBD300-07|MNBTT-3101|Canada|New Brunswick|658[0n]|BOLD:AAB0477
Furcula cinerea|[1985]|TTMNB931-06|MNBTT-931|Canada|New Brunswick|658[0n]|BOLD:AAB0477
Furcula cinerea|[1986]|TTMNB930-06|MNBTT-930|Canada|New Brunswick|658[0n]|BOLD:AAB0477
Furcula cinerea|[1987]|TMNBD301-07|MNBTT-3102|Canada|New Brunswick|646[0n]|BOLD:AAB0477
Furcula cinerea|[1988]|LOCT042-05|05-CTATBI-0042|United States|Connecticut|658[0n]|BOLD:AAB0477
Furcula cinerea|[1989]|MNBB447-05|05-NBSTA-363|Canada|New Brunswick|658[0n]|BOLD:AAB0477
Furcula cinerea|[1990]|MNBB060-05|HBL008670|Canada|New Brunswick|658[0n]|BOLD:AAB0477
Furcula cinerea|[1991]|RDLQG037-06|DH012168|Canada|Quebec|658[0n]|BOLD:AAB0477
Furcula cinerea|[1992]|LSUSA091-06|06-SUSA-0091|United States|Kentucky|657[0n]|BOLD:AAB0477
Furcula cinerea|[1993]|LTOL567-07|AM-93-0430|United States|Maryland|658[0n]|BOLD:AAB0477
Furcula cinerea|[1994]|LPSOD241-09|08BBLEP-00019|Canada|Ontario|658[0n]|BOLD:AAB0477
Furcula cinerea|[1995]|MNBB016-05|HBL008626|Canada|New Brunswick|658[0n]|BOLD:AAB0477
Furcula cinerea|[1996]|BBLSW671-09|09BBLEP-01599|United States|Oklahoma|658[0n]|BOLD:AAB0477
Furcula cinerea|[1997]|LPOKC755-09|MDOK-2832|United States|Oklahoma|658[0n]|BOLD:AAB0477
Furcula nivea|[1998]|CNCLB1912-14|CNCLEP00093279|United States|Arizona|658[0n]|BOLD:ACR1205
Furcula cinerea|[1999]|LPOKD221-09|MDOK-3300|United States|Oklahoma|657[0n]|BOLD:ABZ0858
Furcula sp.|[2000]|RDNME425-08|LEP037849|Canada|Alberta|658[0n]|BOLD:ABZ0858
Furcula sp.|[2001]|BBLOC880-11|BIOUG01467-A07|United States|Arkansas|658[0n]|BOLD:ABZ0858
Furcula sp.|[2002]|RDNME424-08|LEP037848|Canada|Alberta|658[0n]|BOLD:ABZ0858
Furcula sp.|[2003]|BBLOC879-11|BIOUG01467-A06|United States|Arkansas|658[0n]|BOLD:ABZ0858
Furcula sp.|[2004]|BBLOC915-11|BIOUG01467-D06|United States|Arkansas|658[0n]|BOLD:ABZ0858
Furcula sp.|[2005]|RDMAB334-05|UASM77809|Canada|Alberta|591[0n]|BOLD:ABZ0858
Furcula sp.|[2006]|RDNME428-08|LEP037852|Canada|Alberta|658[0n]|BOLD:ABZ0858
Furcula sp.|[2007]|BBLOC914-11|BIOUG01467-D05|United States|Arkansas|658[0n]|BOLD:ABZ0858
Furcula sp.|[2008]|BBLOC882-11|BIOUG01467-A09|United States|Arkansas|658[0n]|BOLD:ABZ0858
Furcula sp.|[2009]|BBLPA611-10|10BBCLP-0611|Canada|Ontario|658[0n]|BOLD:ABZ0858
Furcula cinerea|[2010]|LOCBC386-06|06-BLLOC-2266|United States|California|613[0n]|BOLD:ACF3625
Furcula cinereoides|[2011]|LOCBC431-06|06-BLLOC-2311|United States|California|658[0n]|BOLD:ACF...
Furcula cinereoides|[2012]|LOCBE246-06|06-BLLOC-4065|United States|California|658[0n]|BOLD:ACF...
Furcula cinereoides|[2013]|LOCBD244-06|06-BLLOC-3064|United States|California|656[0n]|BOLD:ACF...
Furcula cinereoides|[2014]|LOCBD744-06|06-BLLOC-3564|United States|California|662[0n]|BOLD:ACF...
Furcula cinerea|[2015]|LOCBC395-06|06-BLLOC-2275|United States|California|657[0n]|BOLD:ACF3625
Furcula cinereoides|[2016]|LOCBE315-06|06-BLLOC-4134|United States|California|658[0n]|BOLD:ACF...
Furcula cinereoides|[2017]|LOCBE245-06|06-BLLOC-4064|United States|California|658[0n]|BOLD:ACF...
Furcula cinereoides|[2018]|LOCBE392-06|06-BLLOC-4211|United States|California|595[0n]|BOLD:ACF...
Furcula placida|[2019]|RDNME422-08|LEP037846|United States|Nevada|658[0n]|BOLD:ACF3625
Furcula cinereoides|[2020]|RDNME427-08|LEP037851|United States|Oregon|658[0n]|BOLD:ACF3625
Furcula placida|[2021]|RDNMH425-09|CNCLEP00054439|United States|California|658[0n]|BOLD:ACF3625
Furcula cinereoides|[2022]|RDNME426-08|LEP037850|Canada|British Columbia|658[0n]|BOLD:ACF3625
Furcula cinereoides|[2023]|RDNME423-08|LEP037847|United States|Washington|658[0n]|BOLD:ACF3625
Furcula placida|[2024]|RDNMH166-09|CNCLEP00054383|United States|California|658[1n]|BOLD:ACF3625
Furcula modesta|[2025]|RDLQG468-06|DH012761|Canada|Quebec|656[0n]|BOLD:ACF2674
Furcula modesta|[2026]|XAJ317-06|2006-ONT-0317|Canada|Ontario|658[0n]|BOLD:ACF2674
Furcula modesta|[2027]|XAD611-05|2005-ONT-26|Canada|Ontario|658[0n]|BOLD:ACF2674
Furcula modesta|[2028]|XAJ406-06|2006-ONT-0406|Canada|Ontario|658[0n]|BOLD:ACF2674
Furcula modesta|[2029]|XAE471-04|Moth4471.03|Canada|Ontario|574[0n]|BOLD:ACF2674
Furcula modesta|[2030]|PMG177-03|FURC1.00|Canada|Ontario|617[0n]|BOLD:ACF2674
Furcula modesta|[2031]|XAB036-04|04HBL005036|Canada|Ontario|617[0n]|BOLD:ACF2674
Furcula modesta|[2032]|XAG644-05|2005-ONT-1228|Canada|Ontario|658[0n]|BOLD:ACF2674
Furcula modesta|[2033]|XAG346-05|2005-ONT-930|Canada|Ontario|658[0n]|BOLD:ACF2674
Furcula modesta|[2034]|RDLQH086-06|DH013324|Canada|Quebec|658[0n]|BOLD:ACF2674
Furcula modesta|[2035]|RDLQB186-05|DH010272|Canada|Quebec|658[0n]|BOLD:ACF2674
Furcula modesta|[2036]|XAD615-05|2005-ONT-30|Canada|Ontario|658[0n]|BOLD:ACF2674
Furcula modesta|[2037]|TMNBD304-07|MNBTT-3105|Canada|New Brunswick|658[0n]|BOLD:ACF2674
Furcula modesta|[2038]|LMIS007-05|05-ONMIS-0007|Canada|Ontario|658[0n]|BOLD:ACF2674
Furcula modesta|[2039]|XAF420-05|HLC-10461|Canada|Ontario|658[0n]|BOLD:ACF2674
Furcula modesta|[2040]|XAK286-06|2006-ONT-1281|Canada|Ontario|658[0n]|BOLD:ACF2674
Furcula modesta|[2041]|XAD661-05|2005-ONT-76|Canada|Ontario|658[0n]|BOLD:ACF2674
Furcula modesta|[2042]|XAK506-07|HLC-16059|Canada|Ontario|658[0n]|BOLD:ACF2674
Furcula modesta|[2043]|XAF421-05|HLC-10462|Canada|Ontario|658[0n]|BOLD:ACF2674
Furcula modesta|[2044]|XAK508-07|HLC-16061|Canada|Ontario|658[0n]|BOLD:ACF2674
Furcula modesta|[2045]|XAE252-04|Moth4252.03|Canada|Ontario|658[0n]|BOLD:ACF2674
Furcula modesta|[2046]|XAE087-04|Moth4087.03|Canada|Ontario|658[0n]|BOLD:ACF2674
Furcula modesta|[2047]|XAE088-04|Moth4088.03|Canada|Ontario|658[0n]|BOLD:ACF2674
Furcula modesta|[2048]|BBLEC136-09|09BBELE-0136|Canada|Nova Scotia|658[0n]|BOLD:ACF2674
Furcula modesta|[2049]|TMNBD305-07|MNBTT-3106|Canada|New Brunswick|658[0n]|BOLD:ACF2674
Furcula modesta|[2050]|TMNBD302-07|MNBTT-3103|Canada|New Brunswick|658[0n]|BOLD:ACF2674
Furcula modesta|[2051]|TMNBD306-07|MNBTT-3107|Canada|New Brunswick|658[0n]|BOLD:ACF2674
Furcula modesta|[2052]|TMNBD303-07|MNBTT-3104|Canada|New Brunswick|659[0n]|BOLD:ACF2674
Furcula modesta|[2053]|LBCA602-05|HLC-20602|Canada|British Columbia|658[0n]|BOLD:ACF2674
Furcula modesta|[2054]|LPMN311-08|08BBLEP-01110|Canada|Manitoba|658[0n]|BOLD:ACF2674
Furcula scolopendrina|[2055]|LOWCB151-05|CGWC-1091|Canada|British Columbia|658[0n]|BOLD:AAA6237
Furcula scolopendrina|[2056]|LOWCB145-05|CGWC-1085|Canada|British Columbia|658[0n]|BOLD:AAA6237

Furcula modesta|[2054]|LPMN311-08|08BBLEP-01110|Canada|Manitoba|658[0n]|BOLD:ACF2674
Furcula scolopendrina|[2055]|LOWCB151-05|CGWC-1091|Canada|British Columbia|658[0n]|BOLD:AAA6237
Furcula scolopendrina|[2056]|LOWCB145-05|CGWC-1085|Canada|British Columbia|658[0n]|BOLD:AAA6237
Furcula scolopendrina|[2057]|RDMAB066-05|UASM57593|Canada|Alberta|635[0n]|BOLD:AAA6237
Furcula scolopendrina|[2058]|RDMAB119-05|UASM41263|Canada|Alberta|630[0n]|BOLD:AAA6237
Furcula scolopendrina|[2059]|RDMAB140-05|UASM56888|Canada|Alberta|578[1n]|BOLD:AAA6237
Furcula scolopendrina|[2060]|RDMAB065-05|UASM57592|Canada|Alberta|593[0n]|BOLD:AAA6237
Furcula scolopendrina|[2061]|RDMAB143-05|UASM58305|Canada|Alberta|636[0n]|BOLD:AAA6237
Furcula scolopendrina|[2062]|RDMAB141-05|UASM56901|Canada|Alberta|658[0n]|BOLD:AAA6237
Furcula scolopendrina|[2063]|RDMAB259-05|UASM41461|Canada|Alberta|658[0n]|BOLD:AAA6237
Furcula scolopendrina|[2064]|RDMAB142-05|UASM2814|Canada|Alberta|658[0n]|BOLD:AAA6237
Furcula scolopendrina|[2065]|LOWCB146-05|CGWC-1086|Canada|British Columbia|658[0n]|BOLD:AAA6237
Furcula scolopendrina|[2066]|LOWCB150-05|CGWC-1090|Canada|British Columbia|658[0n]|BOLD:AAA6237
Furcula scolopendrina|[2067]|RDMAB496-06|UASM58442|Canada|Alberta|658[0n]|BOLD:AAA6237
Furcula scolopendrina|[2068]|LOWCB148-05|CGWC-1088|Canada|British Columbia|658[0n]|BOLD:AAA6237
Furcula scolopendrina|[2069]|LOWCB147-05|CGWC-1087|Canada|British Columbia|658[0n]|BOLD:AAA6237
Furcula scolopendrina|[2070]|LOWC381-05|CGWC-0381|Canada|British Columbia|658[0n]|BOLD:AAA6237
Furcula scolopendrina|[2071]|LPSK241-08|08BBLEP-01809|Canada|Saskatchewan|658[0n]|BOLD:AAA6237
Furcula scolopendrina|[2072]|RDMAB139-05|UASM58110|Canada|Alberta|658[0n]|BOLD:AAA6237
Furcula scolopendrina|[2073]|LOWCB149-05|CGWC-1089|Canada|British Columbia|658[0n]|BOLD:AAA6237
Furcula scolopendrina|[2074]|RDMAB120-05|UASM41264|Canada|Alberta|658[0n]|BOLD:AAA6237
Furcula scolopendrina|[2075]|LPSK242-08|08BBLEP-01810|Canada|Saskatchewan|658[0n]|BOLD:AAA6237
Furcula scolopendrina|[2076]|LPSK244-08|08BBLEP-01812|Canada|Saskatchewan|658[0n]|BOLD:AAA6237
Furcula scolopendrina|[2077]|LPSK243-08|08BBLEP-01811|Canada|Saskatchewan|658[0n]|BOLD:AAA6237
Furcula scolopendrina|[2078]|LPSOB127-08|PPBP-1126|Canada|Ontario|658[0n]|BOLD:AAA6237
Furcula scolopendrina|[2079]|BBLPB297-10|10BBCLP-1296|Canada|Saskatchewan|658[0n]|BOLD:AAA6237
Furcula scolopendrina|[2080]|BBLPB298-10|10BBCLP-1297|Canada|Saskatchewan|658[0n]|BOLD:AAA6237
Furcula scolopendrina|[2081]|RDMAB328-05|UASM77820|Canada|Alberta|658[0n]|BOLD:AAA6237
Furcula scolopendrina|[2082]|RDNMH178-09|CNCLEP00054395|United States|Colorado|640[0n]|BOLD:AA...
Furcula scolopendrina|[2083]|CMAZA901-12|BIOUG02040-E04|United States|Arizona|658[0n]|BOLD:AAA...
Furcula scolopendrina|[2084]|CMAZA810-10|CMAZ-0810|United States|Arizona|658[0n]|BOLD:AAA6237
Furcula scolopendrina|[2085]|RDNMH420-09|CNCLEP00054434|United States|Arizona|658[0n]|BOLD:AAA...
Furcula scolopendrina|[2086]|LOCBD959-06|06-BLLOC-3780|United States|California|658[0n]|BOLD:A...
Furcula scolopendrina|[2087]|LOCBC138-06|06-BLLOC-2018|United States|California|658[0n]|BOLD:A...
Furcula scolopendrina|[2088]|LOCBD029-06|06-BLLOC-2849|United States|California|658[0n]|BOLD:A...
Furcula scolopendrina|[2089]|LOCBC425-06|06-BLLOC-2305|United States|California|658[0n]|BOLD:A...
Furcula scolopendrina|[2090]|LOCBD172-06|06-BLLOC-2992|United States|California|658[0n]|BOLD:A...
Furcula scolopendrina|[2091]|LBCC026-05|HLC-21906|Canada|British Columbia|658[0n]|BOLD:AAA6237
Furcula scolopendrina|[2092]|LALPA399-10|AVBC 401-10|Canada|British Columbia|658[0n]|BOLD:AAA6237
Furcula scolopendrina|[2093]|LBCB356-05|HLC-21296|Canada|British Columbia|658[0n]|BOLD:AAA6237
Furcula scolopendrina|[2094]|LOCBD242-06|06-BLLOC-3062|United States|California|658[0n]|BOLD:A...
Furcula scolopendrina|[2095]|LALPA331-10|AVBC 333-10|Canada|British Columbia|658[0n]|BOLD:AAA6237
Furcula scolopendrina|[2096]|LBCG3222-09|08-JDWBC-3222|Canada|British Columbia|658[0n]|BOLD:AA...
Furcula scolopendrina|[2097]|LBCG3224-09|08-JDWBC-3224|Canada|British Columbia|658[0n]|BOLD:AA...
Furcula scolopendrina|[2098]|LBCG138-08|08-JDWBC-0138|Canada|British Columbia|658[0n]|BOLD:AAA...
Furcula scolopendrina|[2099]|LBCC025-05|HLC-21905|Canada|British Columbia|658[0n]|BOLD:AAA6237
Furcula scolopendrina|[2100]|LBCA210-05|HLC-20210|Canada|British Columbia|658[0n]|BOLD:AAA6237
Furcula scolopendrina|[2101]|LBCA546-05|HLC-20546|Canada|British Columbia|658[0n]|BOLD:AAA6237
Furcula scolopendrina|[2102]|LBCG3225-09|08-JDWBC-3225|Canada|British Columbia|658[0n]|BOLD:AA...
Furcula scolopendrina|[2103]|LALPA400-10|AVBC 402-10|Canada|British Columbia|658[0n]|BOLD:AAA6237
Furcula scolopendrina|[2104]|LOCBD171-06|06-BLLOC-2991|United States|California|658[0n]|BOLD:A...
Furcula scolopendrina|[2105]|LBCG3226-09|08-JDWBC-3226|Canada|British Columbia|658[0n]|BOLD:AA...
Furcula scolopendrina|[2106]|LBCB077-05|HLC-21017|Canada|British Columbia|658[0n]|BOLD:AAA6237
Furcula scolopendrina|[2107]|LBCC411-05|HLC-22291|Canada|British Columbia|658[0n]|BOLD:AAA6237
Furcula scolopendrina|[2108]|LOCBD553-06|06-BLLOC-3373|United States|California|658[0n]|BOLD:A...
Furcula scolopendrina|[2109]|LBCG3223-09|08-JDWBC-3223|Canada|British Columbia|658[0n]|BOLD:AA...
Furcula scolopendrina|[2110]|RWWB746-10|RWWA-1745|United States|Washington|658[0n]|BOLD:AAA6237
Furcula scolopendrina|[2111]|JMMMB184-11|BIOUG00848-H05|United States|California|649[0n]|BOLD:...
Furcula scolopendrina|[2112]|LBCG2540-09|08-JDWBC-2540|Canada|British Columbia|658[0n]|BOLD:AA...
Furcula scolopendrina|[2113]|LBCG166-08|08-JDWBC-0166|Canada|British Columbia|658[0n]|BOLD:AAA...
Furcula scolopendrina|[2114]|RWWB623-10|RWWA-1622|United States|Washington|658[0n]|BOLD:AAA6237
Furcula scolopendrina|[2115]|RWWA490-09|RWWA-0490|United States|Washington|658[0n]|BOLD:AAA6237
Furcula scolopendrina|[2116]|RWWB862-10|RWWA-1861|United States|Washington|658[0n]|BOLD:AAA6237
Furcula scolopendrina|[2117]|RWWA922-09|RWWA-0722|United States|Washington|658[0n]|BOLD:AAA6237
Furcula scolopendrina|[2118]|RWWA156-09|RWWA-0156|United States|Washington|658[0n]|BOLD:AAA6237
Furcula scolopendrina|[2119]|LOCBD030-06|06-BLLOC-2850|United States|California|658[0n]|BOLD:A...
Furcula scolopendrina|[2120]|RWWA061-09|RWWA-0061|United States|Washington|658[0n]|BOLD:AAA6237
Furcula scolopendrina|[2121]|RWWC237-11|RWWA-2214|United States|Washington|658[0n]|BOLD:AAA6237
Furcula scolopendrina|[2122]|RWWA396-09|RWWA-0396|United States|Washington|658[0n]|BOLD:AAA6237
Furcula scolopendrina|[2123]|GMLC961-12|2011GM-0657|United States|California|658[0n]|BOLD:AAA6237
Furcula scolopendrina|[2124]|GMLC1141-12|2011GM-0837|United States|California|658[0n]|BOLD:AAA...
Furcula scolopendrina|[2125]|GMLC1355-12|2012GM-0086|United States|California|612[0n]|BOLD:AAA...
Furcula scolopendrina|[2126]|GMLC1132-12|2011GM-0828|United States|California|658[0n]|BOLD:AAA...
Furcula scolopendrina|[2127]|GMLC1220-12|2011GM-0916|United States|California|658[0n]|BOLD:AAA...
Furcula scolopendrina|[2128]|LBCG165-08|08-JDWBC-0165|Canada|British Columbia|658[0n]|BOLD:AAA...
Furcula scolopendrina|[2129]|RDNMH159-09|CNCLEP00054376|United States|California|530[0n]|
Furcula scolopendrina|[2130]|LBCG3227-09|08-JDWBC-3227|Canada|British Columbia|570[11n]|
Furcula borealis|[2131]|XAG667-05|2005-ONT-1251|Canada|Ontario|611[2n]|BOLD:ACF2678
Furcula borealis|[2132]|RDLQF551-06|DH011700|Canada|Quebec|658[0n]|BOLD:ACF2678
Furcula borealis|[2133]|RDLQF232-06|DH011312|Canada|Quebec|658[0n]|BOLD:ACF2678
Furcula borealis|[2134]|LSEU406-06|06-JKA-0406|United States|Georgia|658[0n]|BOLD:ACF2678
Furcula borealis|[2135]|LGSMG741-07|BGS03404|United States|North Carolina|658[0n]|BOLD:ACF2678
Furcula borealis|[2136]|LSUSA009-06|06-SUSA-0009|United States|Kentucky|658[0n]|BOLD:ACF2678
Furcula borealis|[2137]|CNCLB2909-14|14-NCCC-588|United States|North Carolina|658[0n]|BOLD:ACF...
Furcula borealis|[2138]|LGSMG740-07|BGS03403|United States|North Carolina|658[0n]|BOLD:ACF2678
Furcula borealis|[2139]|XAK293-06|2006-ONT-1288|Canada|Ontario|658[0n]|BOLD:ACF2678
Furcula borealis|[2140]|XAJ590-06|2006-ONT-0590|Canada|Ontario|658[0n]|BOLD:ACF2678
Furcula borealis|[2141]|XAJ591-06|2006-ONT-0591|Canada|Ontario|658[0n]|BOLD:ACF2678
Furcula borealis|[2142]|XAK007-06|2006-ONT-1002|Canada|Ontario|658[0n]|BOLD:ACF2678
Furcula borealis|[2143]|XAE624-04|Moth4624.03|Canada|Ontario|658[0n]|BOLD:ACF2678
Furcula borealis|[2144]|XAJ588-06|2006-ONT-0588|Canada|Ontario|658[0n]|BOLD:ACF2678
Furcula borealis|[2145]|XAF653-05|2005-ONT-302|Canada|Ontario|658[0n]|BOLD:ACF2678
Furcula borealis|[2146]|XAJ589-06|2006-ONT-0589|Canada|Ontario|658[0n]|BOLD:ACF2678
Furcula borealis|[2147]|XAJ592-06|2006-ONT-0592|Canada|Ontario|658[0n]|BOLD:ACF2678
Furcula borealis|[2148]|XAG702-05|2005-ONT-1286|Canada|Ontario|658[0n]|BOLD:ACF2678
Furcula borealis|[2149]|XAK114-06|2006-ONT-1109|Canada|Ontario|658[0n]|BOLD:ACF2678
Furcula borealis|[2150]|XAE109-04|Moth4109.03|Canada|Ontario|658[0n]|BOLD:ACF2678
Furcula borealis|[2151]|PHMO225-03|moth1149.01|Canada|Ontario|639[0n]|BOLD:ACF2678
Furcula borealis|[2152]|TMG66-03|FURC3.00|Canada|Ontario|639[0n]|BOLD:ACF2678
Furcula borealis|[2153]|XAE586-04|Moth4586.03|Canada|Ontario|576[0n]|BOLD:ACF2678
Furcula borealis|[2154]|XAJ593-06|2006-ONT-0593|Canada|Ontario|658[0n]|BOLD:ACF2678

[illegible]
Furcula borealis|[2152]|TMG66-03|FURC3.00|Canada|Ontario|639[0n]|BOLD:ACF2678
Furcula borealis|[2153]|XAE586-04|Moth4586.03|Canada|Ontario|576[0n]|BOLD:ACF2678
Furcula borealis|[2154]|XAJ593-06|2006-ONT-0593|Canada|Ontario|658[0n]|BOLD:ACF2678
Furcula borealis|[2155]|PMG175-03|moth576.01|Canada|Ontario|617[0n]|BOLD:ACF2678
Furcula borealis|[2156]|XAB364-04|04HBL005364|Canada|Ontario|658[0n]|BOLD:ACF2678
Datana integerrima|[2157]|LOFLC197-06|06-FLOR-2077|United States|Florida|658[0n]|BOLD:AAA7653
Datana integerrima|[2158]|LOFLB428-06|06-FLOR-1368|United States|Florida|658[0n]|BOLD:AAA7653
Datana integerrima|[2159]|LOFLA727-06|06-FLOR-0727|United States|Florida|658[0n]|BOLD:AAA7653
Datana integerrima|[2160]|LOFLC364-06|06-FLOR-2244|United States|Florida|658[0n]|BOLD:AAA7653
Datana integerrima|[2161]|LOFLB805-06|06-FLOR-1745|United States|Florida|658[0n]|BOLD:AAA7653
Datana integerrima|[2162]|LOFLA730-06|06-FLOR-0730|United States|Florida|658[0n]|BOLD:AAA7653
Datana integerrima|[2163]|LOFLA726-06|06-FLOR-0726|United States|Florida|658[0n]|BOLD:AAA7653
Datana integerrima|[2164]|LOFLB519-06|06-FLOR-1459|United States|Florida|658[0n]|BOLD:AAA7653
Datana integerrima|[2165]|LOFLA733-06|06-FLOR-0733|United States|Florida|658[0n]|BOLD:AAA7653
Datana integerrima|[2166]|LOFLA884-06|06-FLOR-0884|United States|Florida|658[0n]|BOLD:AAA7653
Datana integerrima|[2167]|LOFLA284-06|06-FLOR-0284|United States|Florida|658[0n]|BOLD:AAA7653
Datana integerrima|[2168]|LOFLA724-06|06-FLOR-0724|United States|Florida|658[0n]|BOLD:AAA7653
Datana integerrima|[2169]|LOFLA749-06|06-FLOR-0749|United States|Florida|658[0n]|BOLD:AAA7653
Datana integerrima|[2170]|LOFLB524-06|06-FLOR-1464|United States|Florida|658[0n]|BOLD:AAA7653
Datana integerrima|[2171]|LOFLA753-06|06-FLOR-0753|United States|Florida|658[0n]|BOLD:AAA7653
Datana integerrima|[2172]|LOFLA725-06|06-FLOR-0725|United States|Florida|658[0n]|BOLD:AAA7653
Datana integerrima|[2173]|LOFLA516-06|06-FLOR-0516|United States|Florida|658[0n]|BOLD:AAA7653
Datana integerrima|[2174]|LOFLA231-06|06-FLOR-0231|United States|Florida|658[0n]|BOLD:AAA7653
Datana integerrima|[2175]|LOFLA752-06|06-FLOR-0752|United States|Florida|658[0n]|BOLD:AAA7653
Datana integerrima|[2176]|LOFLB492-06|06-FLOR-1432|United States|Florida|658[0n]|BOLD:AAA7653
Datana integerrima|[2177]|LOFLB445-06|06-FLOR-1385|United States|Florida|658[0n]|BOLD:AAA7653
Datana integerrima|[2178]|LOFLC297-06|06-FLOR-2177|United States|Florida|632[0n]|BOLD:AAA7653
Datana integerrima|[2179]|LOFLC296-06|06-FLOR-2176|United States|Florida|632[0n]|BOLD:AAA7653
Datana integerrima|[2180]|RDNMJ039-10|CNCLEP 73819|United States|Florida|658[1n]|BOLD:AAA7653
Datana integerrima|[2181]|LOFLA723-06|06-FLOR-0723|United States|Florida|658[0n]|BOLD:AAA7653
Datana integerrima|[2182]|LOFLA731-06|06-FLOR-0731|United States|Florida|658[0n]|BOLD:AAA7653
Datana integerrima|[2183]|LOFLA728-06|06-FLOR-0728|United States|Florida|658[0n]|BOLD:AAA7653
Datana integerrima|[2184]|LOFLA751-06|06-FLOR-0751|United States|Florida|634[0n]|BOLD:AAA7653
Datana integerrima|[2185]|LNCC949-11|11-NCCC-474|United States|North Carolina|658[0n]|BOLD:AAA...
Datana integerrima|[2186]|LOFLA750-06|06-FLOR-0750|United States|Florida|658[0n]|BOLD:AAA7653
Datana integerrima|[2187]|LOFLA754-06|06-FLOR-0754|United States|Florida|658[0n]|BOLD:AAA7653
Datana integerrima|[2188]|LOFLA732-06|06-FLOR-0732|United States|Florida|658[0n]|BOLD:AAA7653
Datana integerrima|[2189]|LNCC045-10|10-NCCC-140|United States|North Carolina|658[0n]|BOLD:AAA...
Datana integerrima|[2190]|LNCC963-11|11-NCCC-488|United States|North Carolina|658[0n]|BOLD:AAA...
Datana integerrima|[2191]|LNCC950-11|11-NCCC-475|United States|North Carolina|658[0n]|BOLD:AAA...
Datana integerrima|[2192]|LNCC1401-11|11-NCCC-926|United States|North Carolina|658[0n]|BOLD:AA...
Datana integerrima|[2193]|LNCC1357-11|11-NCCC-882|United States|North Carolina|658[0n]|BOLD:AA...
Datana integerrima|[2194]|LNCC1355-11|11-NCCC-880|United States|North Carolina|658[0n]|BOLD:AA...
Datana integerrima|[2195]|LNCB208-06|06-NCCC-1164|United States|North Carolina|658[0n]|BOLD:AA...
Datana integerrima|[2196]|LNCC1358-11|11-NCCC-883|United States|North Carolina|658[0n]|BOLD:AA...
Datana integerrima|[2197]|LNCB249-06|06-NCCC-1205|United States|North Carolina|658[0n]|BOLD:AA...
Datana integerrima|[2198]|LNCC962-11|11-NCCC-487|United States|North Carolina|658[0n]|BOLD:AAA...
Datana integerrima|[2199]|LOFLA929-06|06-FLOR-0929|United States|Florida|658[0n]|BOLD:AAA7653
Datana integerrima|[2200]|LOFLA729-06|06-FLOR-0729|United States|Florida|658[0n]|BOLD:AAA7653
Datana integerrima|[2201]|CMAZA502-10|CMAZ-0502|United States|Arizona|658[0n]|BOLD:AAA7653
Datana integerrima|[2202]|BBLPA577-10|10BBCLP-0577|Canada|Ontario|658[0n]|BOLD:AAA7653
Datana integerrima|[2203]|XAD750-05|2005-ONT-549|Canada|Ontario|658[0n]|BOLD:AAA7653
Datana integerrima|[2204]|LPOKB385-09|MDOK-1469|United States|Oklahoma|648[0n]|BOLD:AAA7653
Datana integerrima|[2205]|LGSMB269-05|DNA-ATBI-1118|United States|Tennessee|580[0n]|BOLD:AAA7653
Datana integerrima|[2206]|LILLA277-11|SNS10IL-00368|United States|Illinois|658[0n]|BOLD:AAA7653
Datana integerrima|[2207]|LSUSA237-06|06-SUSA-0237|United States|Kentucky|658[0n]|BOLD:AAA7653
Datana integerrima|[2208]|LPSOD1049-09|08MZPP-115|Canada|Ontario|658[0n]|BOLD:AAA7653
Datana integerrima|[2209]|BBLPA580-10|10BBCLP-0580|Canada|Ontario|658[0n]|BOLD:AAA7653
Datana integerrima|[2210]|LPOKB397-09|MDOK-1498|United States|Oklahoma|658[0n]|BOLD:AAA7653
Datana integerrima|[2211]|LPSOB059-08|PPBP-1058|Canada|Ontario|658[0n]|BOLD:AAA7653
Datana integerrima|[2212]|XAK253-06|2006-ONT-1248|Canada|Ontario|658[0n]|BOLD:AAA7653
Datana integerrima|[2213]|LPOKB1014-09|MDOK-2056|United States|Oklahoma|658[0n]|BOLD:AAA7653
Datana integerrima|[2214]|LSUSA255-06|06-SUSA-0255|United States|Kentucky|658[0n]|BOLD:AAA7653
Datana integerrima|[2215]|LPOKB1013-09|MDOK-2055|United States|Oklahoma|658[0n]|BOLD:AAA7653
Datana integerrima|[2216]|RDNMH461-09|CNCLEP00054475|United States|Oklahoma|658[0n]|BOLD:AAA7653
Datana integerrima|[2217]|MILEQ244-11|11-MISC-719|United States|Alabama|658[0n]|BOLD:AAA7653
Datana integerrima|[2218]|BBLPA579-10|10BBCLP-0579|Canada|Ontario|658[0n]|BOLD:AAA7653
Datana integerrima|[2219]|LPOKA237-08|MDOK-0237|United States|Oklahoma|658[0n]|BOLD:AAA7653
Datana integerrima|[2220]|BBLPA581-10|10BBCLP-0581|Canada|Ontario|658[0n]|BOLD:AAA7653
Datana integerrima|[2221]|BBLPA578-10|10BBCLP-0578|Canada|Ontario|658[0n]|BOLD:AAA7653
Datana integerrima|[2222]|RDNMI035-10|CNCLEP 69825|Canada|Ontario|658[0n]|BOLD:AAA7653
Datana integerrima|[2223]|LGSMB270-05|DNA-ATBI-1119|United States|Tennessee|557[1n]|BOLD:AAA7653
Datana contracta|[2224]|MILEQ238-11|11-MISC-713|United States|Alabama|658[0n]|BOLD:AAA7653
Datana contracta|[2225]|MILEQ241-11|11-MISC-716|United States|Alabama|634[0n]|BOLD:AAA7653
Datana contracta|[2226]|MILEQ240-11|11-MISC-715|United States|Alabama|658[0n]|BOLD:AAA7653
Datana contracta|[2227]|CNCLB1913-14|CNCLEP00117780|United States|Florida|658[0n]|BOLD:AAA7653
Datana contracta|[2228]|LNCC1354-11|11-NCCC-879|United States|North Carolina|658[0n]|BOLD:AAA7653
Datana contracta|[2229]|LNCC1356-11|11-NCCC-881|United States|North Carolina|658[0n]|BOLD:AAA7653
Datana contracta|[2230]|LNCC1400-11|11-NCCC-925|United States|North Carolina|658[0n]|BOLD:AAA7653
Datana contracta|[2231]|MILEQ245-11|11-MISC-720|United States|Alabama|658[0n]|BOLD:AAA7653
Datana contracta|[2232]|LNCC1353-11|11-NCCC-878|United States|North Carolina|658[0n]|BOLD:AAA7653
Datana contracta|[2233]|LGSMB268-05|DNA-ATBI-1117|United States|Tennessee|543[2n]|BOLD:AAA7653
Datana contracta|[2234]|MILEQ243-11|11-MISC-718|United States|Alabama|658[0n]|BOLD:AAA7653
Datana contracta|[2235]|LOT064-04|04HBL002064|United States|Tennessee|609[0n]|BOLD:AAA7653
Datana contracta|[2236]|MILEQ239-11|11-MISC-714|United States|Alabama|658[0n]|BOLD:AAA7653
Datana contracta|[2237]|MILEQ242-11|11-MISC-717|United States|Alabama|658[0n]|BOLD:AAA7653
Datana contracta|[2238]|LOT060-04|04HBL002060|United States|Tennessee|609[0n]|BOLD:AAA7653
Datana contracta|[2239]|CNCLA2574-13|CNCLEP00102109|Canada|Ontario|658[0n]|BOLD:AAA7653
Datana contracta|[2240]|CNCLA2578-13|CNCLEP00102113|Canada|Ontario|658[0n]|BOLD:AAA7653
Datana contracta|[2241]|BBLCU254-09|09BBLEP-04741|United States|Michigan|658[0n]|BOLD:AAA7653
Datana contracta|[2242]|CNCLA2576-13|CNCLEP00102111|Canada|Ontario|658[0n]|BOLD:AAA7653
Datana contracta|[2243]|CNCLA2575-13|CNCLEP00102110|Canada|Ontario|658[0n]|BOLD:AAA7653
Datana contracta|[2244]|CNCLA2577-13|CNCLEP00102112|Canada|Ontario|658[0n]|BOLD:AAA7653
Datana contracta|[2245]|RDNMI040-10|CNCLEP 69830|Canada|Ontario|658[0n]|BOLD:AAA7653
Datana contracta|[2246]|MILEQ246-11|11-MISC-721|United States|Alabama|658[0n]|BOLD:AAA7653
Datana contracta|[2247]|LOT059-04|04HBL002059|United States|Tennessee|609[0n]|BOLD:AAA7653
Datana drexelii|[2248]|MILEQ237-11|11-MISC-712|United States|Alabama|658[0n]|BOLD:AAA7653
Datana modesta|[2249]|LOFLA318-06|06-FLOR-0318|United States|Florida|658[0n]|BOLD:AAA7653
Datana modesta|[2250]|MILEQ212-11|11-MISC-687|United States|Georgia|658[0n]|BOLD:AAA7653
Datana modesta|[2251]|LOFLA381-06|06-FLOR-0381|United States|Florida|658[0n]|BOLD:AAA7653
Datana modesta|[2252]|LOFLA330-06|06-FLOR-0330|United States|Florida|658[0n]|BOLD:AAA7653

Datana modesta|[2249]|LOFLA318-06|06-FLOR-0318|United States|Florida|658[0n]|BOLD:AAA7653
Datana modesta|[2250]|MILEQ212-11|11-MISC-687|United States|Georgia|658[0n]|BOLD:AAA7653
Datana modesta|[2251]|LOFLA381-06|06-FLOR-0381|United States|Florida|658[0n]|BOLD:AAA7653
Datana modesta|[2252]|LOFLA330-06|06-FLOR-0330|United States|Florida|658[0n]|BOLD:AAA7653
Datana modesta|[2253]|CNCLB1914-14|CNCLEP00117781|United States|Florida|658[0n]|BOLD:AAA7653
Datana modesta|[2254]|LNCB851-09|09-MISC-036|United States|Georgia|658[0n]|BOLD:AAA7653
Datana modesta|[2255]|CNCLB1915-14|CNCLEP00117782|United States|Florida|658[0n]|BOLD:AAA7653
Datana modesta|[2256]|CNCLB1917-14|CNCLEP00117784|United States|Florida|658[0n]|BOLD:AAA7653
Datana drexelii|[2257]|LGSMG732-07|BGS03395|United States|Tennessee|658[0n]|BOLD:AAA7653
Datana drexelii|[2258]|CNCLA2573-13|CNCLEP00102108|Canada|Ontario|658[0n]|BOLD:AAA7653
Datana drexelii|[2259]|LOT508-04|04HBL002508|United States|Tennessee|658[0n]|BOLD:AAA7653
Datana drexelii|[2260]|MILEQ235-11|11-MISC-710|United States|Alabama|658[0n]|BOLD:AAA7653
Datana drexelii|[2261]|CNCLA2572-13|CNCLEP00102107|Canada|Ontario|658[0n]|BOLD:AAA7653
Datana drexelii|[2262]|CNCLA2571-13|CNCLEP00102106|Canada|Ontario|658[0n]|BOLD:AAA7653
Datana drexelii|[2263]|LOT493-04|04HBL002493|United States|Tennessee|658[2n]|BOLD:AAA7653
Datana drexelii|[2264]|LGSMB271-05|DNA-ATBI-1120|United States|Tennessee|569[0n]|BOLD:AAA7653
Datana drexelii|[2265]|LOT061-04|04HBL002061|United States|Tennessee|609[0n]|BOLD:AAA7653
Datana drexelii|[2266]|RDLQG124-06|DH012288|Canada|Quebec|632[0n]|BOLD:AAA7653
Datana drexelii|[2267]|LTOL609-07|AM-93-0431|United States|Maryland|658[0n]|BOLD:AAA7653
Datana drexelii|[2268]|LNCC105-10|10-NCCC-200|United States|North Carolina|658[0n]|BOLD:AAA7653
Datana drexelii|[2269]|CNCLB1916-14|CNCLEP00117783|United States|Florida|658[0n]|BOLD:AAA7653
Datana drexelii|[2270]|MILEQ228-11|11-MISC-703|United States|Alabama|658[0n]|BOLD:AAA7653
Datana drexelii|[2271]|RDLQG125-06|DH012289|Canada|Quebec|658[0n]|BOLD:AAA7653
Datana drexelii|[2272]|LGSMG731-07|BGS03394|United States|Tennessee|658[0n]|BOLD:AAA7653
Datana drexelii|[2273]|LGSMB272-05|DNA-ATBI-1121|United States|Tennessee|536[0n]|BOLD:AAA7653
Datana drexelii|[2274]|LNCC1399-11|11-NCCC-924|United States|North Carolina|658[0n]|BOLD:AAA7653
Datana drexelii|[2275]|LNCC106-10|10-NCCC-201|United States|North Carolina|658[0n]|BOLD:AAA7653
Datana drexelii|[2276]|MILEQ236-11|11-MISC-711|United States|Alabama|658[0n]|BOLD:AAA7653
Datana angusii|[2277]|LNCB333-06|06-NCCC-1289|United States|North Carolina|658[0n]|BOLD:ACH9674
Datana angusii|[2278]|LGSMG733-07|BGS03396|United States|Tennessee|658[0n]|BOLD:ACH9674
Datana angusii|[2279]|LNCC1351-11|11-NCCC-876|United States|North Carolina|658[0n]|BOLD:ACH9674
Datana angusii|[2280]|LNCC1352-11|11-NCCC-877|United States|North Carolina|658[0n]|BOLD:ACH9674
Datana angusii|[2281]|MILEQ209-11|11-MISC-684|United States|Georgia|658[0n]|BOLD:ACH9674
Datana angusii|[2282]|LSUSA147-06|06-SUSA-0147|United States|Kentucky|658[0n]|BOLD:ACH9674
Datana angusii|[2283]|RDNMI029-10|CNCLEP 69819|Canada|Ontario|658[0n]|BOLD:ACH9674
Datana angusii|[2284]|RDNMI034-10|CNCLEP 69824|Canada|Ontario|658[0n]|BOLD:ACH9674
Datana angusii|[2285]|CNCLA2570-13|CNCLEP00102105|Canada|Ontario|658[0n]|BOLD:ACH9674
Datana angusii|[2286]|CNCLA2568-13|CNCLEP00102103|Canada|Ontario|658[0n]|BOLD:ACH9674
Datana angusii|[2287]|MILEQ248-11|11-MISC-723|United States|Alabama|658[0n]|BOLD:ACH9674
Datana angusii|[2288]|MILEQ247-11|11-MISC-722|United States|Alabama|658[0n]|BOLD:ACH9674
Datana angusii|[2289]|CNCLA2566-13|CNCLEP00102101|Canada|Ontario|658[0n]|BOLD:ACH9674
Datana angusii|[2290]|CNCLA567-13|CNCLEP00098999|Canada|Ontario|658[0n]|BOLD:ACH9674
Datana angusii|[2291]|CNCLA2569-13|CNCLEP00102104|Canada|Ontario|658[0n]|BOLD:ACH9674
Datana angusii|[2292]|RDNMI030-10|CNCLEP 69820|Canada|Ontario|658[0n]|BOLD:ACH9674
Datana ministra|[2293]|XAJ825-06|2006-ONT-0825|Canada|Ontario|658[0n]|BOLD:ACH9674
Datana ministra|[2294]|RDNMI032-10|CNCLEP 69822|Canada|Ontario|658[0n]|BOLD:ACH9674
Datana ministra|[2295]|LPSO866-08|PPBP-0866|Canada|Ontario|658[0n]|BOLD:ACH9674
Datana ministra|[2296]|RDNMI031-10|CNCLEP 69821|Canada|Ontario|658[0n]|BOLD:ACH9674
Datana ministra|[2297]|LPMN063-08|08BBLEP-00861|Canada|Manitoba|658[0n]|BOLD:ACH9674
Datana ministra|[2298]|RDNMI033-10|CNCLEP 69823|Canada|Ontario|658[0n]|BOLD:ACH9674
Datana ministra|[2299]|PMG173-03|DATA1.00|Canada|Ontario|617[0n]|BOLD:ACH9674
Datana ministra|[2300]|RDNMI039-10|CNCLEP 69829|Canada|Ontario|658[0n]|BOLD:ACH9674
Datana ministra|[2301]|RDLQB198-05|DH010284|Canada|Quebec|658[0n]|BOLD:ACH9674
Datana ministra|[2302]|CNCLA566-13|CNCLEP00098998|Canada|Ontario|658[0n]|BOLD:ACH9674
Datana ministra|[2303]|LPSOD319-09|08BBLEP-00097|Canada|Ontario|658[0n]|BOLD:ACH9674
Datana ministra|[2304]|XAE411-04|Moth4411.03|Canada|Ontario|658[0n]|BOLD:ACH9674
Datana ministra|[2305]|PHMO129-03|moth758.02|Canada|Ontario|639[0n]|BOLD:ACH9674
Datana ministra|[2306]|PHMO172-03|moth907.01|Canada|Ontario|639[0n]|BOLD:ACH9674
Datana ministra|[2307]|TMG58-03|moth972.01|Canada|Ontario|639[0n]|BOLD:ACH9674
Datana ministra|[2308]|PHMNB010-03|moth130.02SA|Canada|New Brunswick|639[0n]|BOLD:ACH9674
Datana ministra|[2309]|PHMNB012-03|moth135.02SA|Canada|New Brunswick|639[0n]|BOLD:ACH9674
Datana ministra|[2310]|TMNBD224-07|MNBTT-3025|Canada|New Brunswick|658[0n]|BOLD:ACH9674
Datana ministra|[2311]|PHMNB067-03|moth55.02SA|Canada|New Brunswick|639[0n]|BOLD:ACH9674
Datana ministra|[2312]|PHMNB063-03|moth44.02SA|Canada|New Brunswick|639[0n]|BOLD:ACH9674
Datana ministra|[2313]|LPSOD442-09|08BBLEP-00221|Canada|Ontario|658[0n]|BOLD:ACH9674
Datana ministra|[2314]|LOT533-04|04HBL002533|United States|Tennessee|616[2n]|BOLD:ACH9674
Datana ministra|[2315]|LTOL611-07|UNK-93-4036|United States|Maryland|646[0n]|BOLD:ACH9674
Datana ministra|[2316]|LPSOB426-08|PPBP-1425|Canada|Ontario|658[0n]|BOLD:ACH9674
Datana ministra|[2317]|MILEQ210-11|11-MISC-685|United States|Georgia|658[0n]|BOLD:ACH9674
Datana ministra|[2318]|LTOL612-07|SWC-93-4021|United States|West Virginia|658[0n]|BOLD:ACH9674
Datana ministra|[2319]|MILEP339-10|10-MISC-244|United States|Alabama|658[0n]|BOLD:ACH9674
Datana ministra|[2320]|XAJ824-06|2006-ONT-0824|Canada|Ontario|658[0n]|BOLD:ACH9674
Datana ministra|[2321]|LTOL613-07|UNK-93-4025|United States|Maryland|594[0n]|BOLD:ACH9674
Datana ministra|[2322]|LGSMB274-05|DNA-ATBI-1123|United States|Tennessee|561[1n]|BOLD:ACH9674
Datana ministra|[2323]|LGSMB273-05|DNA-ATBI-1122|United States|Tennessee|584[0n]|BOLD:ACH9674
Datana ministra|[2324]|LOT063-04|04HBL002063|United States|Tennessee|609[0n]|BOLD:ACH9674
Datana ministra|[2325]|LOT062-04|04HBL002062|United States|Tennessee|609[0n]|BOLD:ACH9674
Datana ministra|[2326]|LOT065-04|04HBL002065|United States|Tennessee|609[0n]|BOLD:ACH9674
Datana angusii|[2327]|MILEQ233-11|11-MISC-708|United States|Alabama|658[0n]|BOLD:ACH9674
Datana angusii|[2328]|MILEQ234-11|11-MISC-709|United States|Alabama|658[0n]|BOLD:ACH9674
Datana angusii|[2329]|MILEQ230-11|11-MISC-705|United States|Alabama|658[0n]|BOLD:ACH9674
Datana angusii|[2330]|MILEQ231-11|11-MISC-706|United States|Alabama|658[0n]|BOLD:ACH9674
Datana angusii|[2331]|MILEQ232-11|11-MISC-707|United States|Alabama|658[0n]|BOLD:ACH9674
Datana angusii|[2332]|MILEQ229-11|11-MISC-704|United States|Alabama|658[0n]|BOLD:ACH9674
Datana angusii|[2333]|LNCC1397-11|11-NCCC-922|United States|North Carolina|658[0n]|BOLD:ACH9674
Datana angusii|[2334]|LNCC1350-11|11-NCCC-875|United States|North Carolina|658[0n]|BOLD:ACH9674
Datana angusii|[2335]|LGSMB266-05|DNA-ATBI-1115|United States|Tennessee|536[0n]|BOLD:ACH9674
Datana angusii|[2336]|LNCC044-10|10-NCCC-139|United States|North Carolina|658[0n]|BOLD:ACH9674
Datana angusii|[2337]|LNCC1349-11|11-NCCC-874|United States|North Carolina|658[0n]|BOLD:ACH9674
Datana angusii|[2338]|LNCC694-11|11-NCCC-219|United States|North Carolina|658[0n]|BOLD:ACH9674
Datana angusii|[2339]|LNCC1398-11|11-NCCC-923|United States|North Carolina|658[0n]|BOLD:ACH9674
Datana angusii|[2340]|LNCB334-06|06-NCCC-1290|United States|North Carolina|658[0n]|BOLD:ACH9674
Datana angusii|[2341]|LNCC964-11|11-NCCC-489|United States|North Carolina|658[0n]|BOLD:ACH9674
Datana angusii|[2342]|LNCC1396-11|11-NCCC-921|United States|North Carolina|658[0n]|BOLD:ACH9674
Datana rotunda|[2343]|CMAZA824-10|CMAZ-0824|United States|Arizona|658[0n]|BOLD:ACH9674
Datana rotunda|[2344]|CMAZA379-10|CMAZ-0379|United States|Arizona|658[0n]|BOLD:ACH9674
Datana rotunda|[2345]|RDNMK275-11|CNCLEP 84184|United States|Arizona|658[0n]|BOLD:ACH9674
Datana neomexicana|[2346]|RDNML289-13|CNCLEP 92265|United States|New Mexico|658[0n]|BOLD:ACH9674
Datana neomexicana|[2347]|RDNML290-13|CNCLEP 92266|United States|New Mexico|547[0n]|BOLD:ACH9674
Datana perspicua|[2348]|BBLOE1417-12|BIOUG01987-B07|United States|Texas|658[0n]|BOLD:ACH9674
Datana perspicua|[2349]|RDNMH462-09|CNCLEP00054476|Mexico|Nuevo Leon|658[0n]|BOLD:ACH9674
Datana [illegible]|[2350]|CNCLB2488-14|CNCLEP [illegible]|United States|New Mexico|658[0n]|BOLD:ACH[illegible]

Datana neomexicana|[2347]|RDNML290-13|CNCLEP 92266|United States|New Mexico|547[0n]|BOLD:ACH9674
Datana perspicua|[2348]|BBLOE1417-12|BIOUG01987-B07|United States|Texas|658[0n]|BOLD:ACH9674
Datana perspicua|[2349]|RDNMH462-09|CNCLEP00054476|Mexico|Nuevo Leon|658[0n]|BOLD:ACH9674
Datana perspicua|[2350]|CNCLB2488-14|CNCLEP 00119612|United States|New Mexico|658[0n]|BOLD:ACH...
Datana perspicua|[2351]|CNCLB2489-14|CNCLEP 00119613|United States|New Mexico|658[0n]|BOLD:ACH...
Datana perspicua|[2352]|LOCBC872-06|06-BLLOC-2752|United States|California|658[0n]|BOLD:ACH9674
Datana perspicua|[2353]|CMAZA822-10|CMAZ-0822|United States|Arizona|658[0n]|BOLD:ACH9674
Datana perspicua|[2354]|RDNMJ266-11|CNCLEP 70289|United States|Arizona|658[0n]|BOLD:ACH9674
Datana perspicua|[2355]|RDNMJ265-11|CNCLEP 70288|United States|Arizona|658[0n]|BOLD:ACH9674
Datana perspicua|[2356]|CMAZA830-10|CMAZ-0830|United States|Arizona|658[0n]|BOLD:ACH9674
Datana perspicua|[2357]|CMAZA823-10|CMAZ-0823|United States|Arizona|641[0n]|BOLD:ACH9674
Datana robusta|[2358]|LNAUV261-16|CCDB-29062-F11|United States|South Carolina|611[0n]|BOLD:ACH...
Datana robusta|[2359]|CNCLB3812-16|CNCLEP 00119901|United States|Florida|658[0n]|BOLD:ACH9674
Datana robusta|[2360]|CNCLB3811-16|CNCLEP 00119900|United States|Florida|658[0n]|BOLD:ACH9674
Datana diffidens|[2361]|LNAUT2570-14|CCDB-23282-A07|United States|Texas|550[0n]|BOLD:ACH9674
Datana diffidens|[2362]|CNCLB2453-14|CNCLEP 00119577|United States|Texas|658[0n]|BOLD:ACH9674
Datana diffidens|[2363]|LNAUT2571-14|CCDB-23282-A08|United States|Texas|658[1n]|BOLD:ACH9674
Datana major|[2364]|LOFLA306-06|06-FLOR-0306|United States|Florida|658[0n]|BOLD:ACH9674
Datana major|[2365]|MILEQ211-11|11-MISC-686|United States|Georgia|658[0n]|BOLD:ACH9674
Datana major|[2366]|LNCB247-06|06-NCCC-1203|United States|North Carolina|604[0n]|BOLD:ACH9674
Datana major|[2367]|RDNMH923-09|CNCLEP00067922|United States|Florida|658[0n]|BOLD:ACH9674
Datana major|[2368]|LNC262-05|05-NCCC-262|United States|North Carolina|658[0n]|BOLD:ACH9674
Datana major|[2369]|LNCB248-06|06-NCCC-1204|United States|North Carolina|658[0n]|BOLD:ACH9674
Datana major|[2370]|MILEQ213-11|11-MISC-688|United States|Georgia|658[0n]|BOLD:ACH9674
Datana major|[2371]|LOFLA322-06|06-FLOR-0322|United States|Florida|658[0n]|BOLD:ACH9674
Datana ranaeceps|[2372]|LNC912-06|06-NCCC-912|United States|North Carolina|658[0n]|BOLD:ACH4491
Datana ranaeceps|[2373]|LNC914-06|06-NCCC-914|United States|North Carolina|658[0n]|BOLD:ACH4491
Datana ranaeceps|[2374]|LNC913-06|06-NCCC-913|United States|North Carolina|658[0n]|BOLD:ACH4491
Datana perspicua|[2375]|BBLOE1974-12|BIOUG01995-A06|United States|Texas|658[0n]|BOLD:ACH9676
Datana perspicua|[2376]|MILEQ207-11|11-MISC-682|United States|Georgia|658[0n]|BOLD:ACH9676
Datana perspicua|[2377]|LILLA848-11|SNS10IL-01069|United States|Illinois|658[0n]|BOLD:ACH9676
Datana perspicua|[2378]|LNCC1890-13|13-NCCC-560|United States|North Carolina|658[0n]|BOLD:ACH9676
Datana perspicua|[2379]|MILEQ208-11|11-MISC-683|United States|Georgia|658[0n]|BOLD:ACH9676
Datana perspicua|[2380]|BBLOE1444-12|BIOUG01987-D10|United States|Texas|658[0n]|BOLD:ACH9676
Datana perspicua|[2381]|LILLA432-11|SNS10IL-00558|United States|Illinois|658[0n]|BOLD:ACH9676
Datana perspicua|[2382]|RDNMF511-08|NOC14597|Canada|Ontario|658[0n]|BOLD:ACH9676
Datana perspicua|[2383]|MILEQ206-11|11-MISC-681|United States|Georgia|658[0n]|BOLD:ACH9676
Datana perspicua|[2384]|RDNMH416-09|CNCLEP00054430|United States|Oklahoma|658[0n]|BOLD:ACH9676
Datana perspicua|[2385]|XAJ922-06|2006-ONT-0922|Canada|Ontario|630[0n]|BOLD:ACH9676
Datana perspicua|[2386]|RDNMF509-08|NOC14595|Canada|Ontario|640[0n]|BOLD:ACH9676
Datana perspicua|[2387]|RDNMF510-08|NOC14596|Canada|Ontario|658[0n]|BOLD:ACH9676
Datana perspicua|[2388]|MILEQ205-11|11-MISC-680|United States|Georgia|630[0n]|BOLD:ACH9676
Datana perspicua|[2389]|BBLCU261-09|09BBLEP-04748|United States|Illinois|658[0n]|BOLD:ACH9676
Datana ranaeceps|[2390]|CNCLB1640-14|CNCLEP 00113874|United States|Florida|658[0n]|BOLD:ACH4491
Theroa zethus|[2391]|CMAZA517-10|CMAZ-0517|United States|Arizona|658[0n]|BOLD:AAP6001
Theroa zethus|[2392]|RDNMJ569-11|CNCLEP 80097|United States|Arizona|658[0n]|BOLD:AAP6001
Theroa zethus|[2393]|CMAZA970-12|BIOUG02041-C12|United States|Arizona|658[0n]|BOLD:AAP6001
Theroa zethus|[2394]|CMAZA518-10|CMAZ-0518|United States|Arizona|658[0n]|BOLD:AAP6001
Litodonta nr. hydromeli|[2395]|RDNML059-13|CNCLEP 92207|United States|Florida|658[0n]|BOLD:ACD...
Litodonta hydromeli|[2396]|BBLSW829-09|09BBLEP-01757|United States|Texas|658[0n]|BOLD:ACF0193
Litodonta hydromeli|[2397]|USLEP1153-10|10BBLEP-01153|United States|Texas|658[0n]|BOLD:ACF0193
Litodonta hydromeli|[2398]|LPOKD215-09|MDOK-3294|United States|Oklahoma|657[0n]|BOLD:ACF0193
Litodonta hydromeli|[2399]|BBLSX250-09|09BBLEP-02178|United States|Texas|658[0n]|BOLD:ACF0193
Litodonta hydromeli|[2400]|BBLSW531-09|09BBLEP-01459|United States|Oklahoma|658[0n]|BOLD:ACF0193
Litodonta contrasta|[2401]|NOCNA026-14|20325-300509-TX|United States|Texas|658[0n]|BOLD:ACF0193
Litodonta hydromeli|[2402]|BBLSW060-09|09BBLEP-00988|United States|Texas|656[0n]|BOLD:ACF0193
Litodonta hydromeli|[2403]|RDNMH465-09|CNCLEP00054479|United States|Oklahoma|658[0n]|BOLD:ACF0193
Litodonta hydromeli|[2404]|BBLOE1697-12|BIOUG01990-B02|United States|Oklahoma|658[0n]|BOLD:ACF...
Litodonta hydromeli|[2405]|USLEP1151-10|10BBLEP-01151|United States|Texas|658[0n]|BOLD:ACF0193
Litodonta contrasta|[2406]|NOCNA023-14|20322-210509-TX|United States|Texas|658[0n]|BOLD:ACF0193
Litodonta hydromeli|[2407]|BBLSX111-09|09BBLEP-02039|United States|Oklahoma|636[0n]|BOLD:ACF0193
Litodonta hydromeli|[2408]|BBLOD532-11|BIOUG01565-D03|United States|Texas|658[0n]|BOLD:AAF6854
Litodonta hydromeli|[2409]|HKONB098-08|3595-COI-08|United States|Texas|658[0n]|BOLD:AAF6854
Litodonta hydromeli|[2410]|HKONB097-08|3594-COI-08|United States|Texas|658[0n]|BOLD:AAF6854
Litodonta hydromeli|[2411]|BBLOD529-11|BIOUG01565-C12|United States|Texas|640[0n]|BOLD:AAF6854
Litodonta hydromeli|[2412]|HKONB096-08|3593-COI-08|United States|Texas|658[0n]|BOLD:AAF6854
Litodonta contrasta|[2413]|LNAUT1060-14|CCDB-22947-B04|United States|Arizona|658[0n]|BOLD:ACN8403
Litodonta contrasta|[2414]|LNAUT1061-14|CCDB-22947-B05|United States|Arizona|658[0n]|BOLD:ACN8403
Litodonta contrasta|[2415]|LNAUT1057-14|CCDB-22947-B01|United States|Arizona|658[0n]|BOLD:ACN8403
Afilia oslari|[2416]|CMAZA1004-12|BIOUG02041-F10|United States|Arizona|658[0n]|BOLD:ABW3024
Afilia oslari|[2417]|CMAZA933-12|BIOUG02040-G12|United States|Arizona|658[0n]|BOLD:ABW3024
Afilia oslari|[2418]|CMAZA1219-12|BIOUG02043-H11|United States|Arizona|658[0n]|BOLD:ABW3024
Afilia oslari|[2419]|CMAZA997-12|BIOUG02041-F03|United States|Arizona|658[0n]|BOLD:ABW3024
Elymiotis notodontoides|[2420]|CNCLB864-14|CNCLEP00094030|United States|Texas|658[0n]|BOLD:AAC...
Elymiotis notodontoides|[2421]|CNCLB863-14|CNCLEP00094029|United States|Texas|658[0n]|BOLD:AAC...
Notodontidae_unassigned|[2422]|TML120-14|CCDB-17964-A12|United States|California|658[0n]|BOLD:...
Gluphisia wrightii|[2423]|CMAZA806-10|CMAZ-0806|United States|Arizona|658[0n]|BOLD:AAA2247
Gluphisia wrightii|[2424]|RDNMJ373-11|CNCLEP 80281|United States|Arizona|658[0n]|BOLD:AAA2247
Gluphisia wrightii|[2425]|IAWLB644-11|IAWAZ-1077|United States|Arizona|658[0n]|BOLD:AAA2247
Gluphisia wrightii|[2426]|RDNMJ374-11|CNCLEP 80282|United States|Arizona|658[0n]|BOLD:AAA2247
Gluphisia wrightii|[2427]|CNCLB765-14|CNCLEP00030194|United States|Arizona|658[0n]|BOLD:AAA2247
Gluphisia wrightii|[2428]|IAWLB643-11|IAWAZ-1076|United States|Arizona|632[0n]|BOLD:AAA2247
Gluphisia septentrionis|[2429]|BBLSX376-09|09BBLEP-02304|United States|Oklahoma|658[0n]|BOLD:A...
Gluphisia septentrionis|[2430]|LOWCB143-05|CGWC-1083|Canada|British Columbia|658[0n]|BOLD:AAA2247
Gluphisia septentrionis|[2431]|LPMN182-08|08BBLEP-00981|Canada|Manitoba|658[0n]|BOLD:AAA2247
Gluphisia septentrionis|[2432]|LOWCE855-06|CGWC-4615|Canada|British Columbia|658[0n]|BOLD:AAA2247
Gluphisia septentrionis|[2433]|LBCH4428-10|10-JDWBC-4428|Canada|British Columbia|658[0n]|BOLD:...
Gluphisia septentrionis|[2434]|LOWCB142-05|CGWC-1082|Canada|British Columbia|603[4n]|BOLD:AAA2247
Gluphisia septentrionis|[2435]|LPABC917-09|08BBLEP-05328|Canada|Alberta|658[0n]|BOLD:AAA2247
Gluphisia septentrionis|[2436]|AWCL048-09|JLB-0246|United States|Idaho|658[0n]|BOLD:AAA2247
Gluphisia septentrionis|[2437]|LBCA372-05|HLC-20372|Canada|British Columbia|658[0n]|BOLD:AAA2247
Gluphisia septentrionis|[2438]|LPMNB564-09|08BBLEP-05642|Canada|Manitoba|634[0n]|BOLD:AAA2247
Gluphisia septentrionis|[2439]|LPVIB846-08|PFC-2006-2353|Canada|British Columbia|658[0n]|BOLD:...
Gluphisia septentrionis|[2440]|LPMN191-08|08BBLEP-00990|Canada|Manitoba|658[0n]|BOLD:AAA2247
Gluphisia septentrionis|[2441]|LPMN529-08|08BBLEP-01328|Canada|Manitoba|658[0n]|BOLD:AAA2247
Gluphisia septentrionis|[2442]|LBCH5288-10|10-JDWBC-5288|Canada|British Columbia|658[0n]|BOLD:...
Gluphisia septentrionis|[2443]|LPMN314-08|08BBLEP-01113|Canada|Manitoba|658[0n]|BOLD:AAA2247
Gluphisia septentrionis|[2444]|LBCB201-05|HLC-21141|Canada|British Columbia|658[0n]|BOLD:AAA2247
Gluphisia septentrionis|[2445]|RDMAB028-05|UASM57573|Canada|Alberta|658[0n]|BOLD:AAA2247
Gluphisia septentrionis|[2446]|LBCA609-05|HLC-20609|Canada|British Columbia|649[0n]|BOLD:AAA2247
Gluphisia septentrionis|[2447]|LBCB072-05|HLC-21012|Canada|British Columbia|658[0n]|BOLD:AAA2247
[illegible]

Gluphisia septentrionis|[2445]|RDMAB028-05|UASM57573|Canada|Alberta|658[0n]|BOLD:AAA2247
Gluphisia septentrionis|[2446]|LBCA609-05|HLC-20609|Canada|British Columbia|649[0n]|BOLD:AAA2247
Gluphisia septentrionis|[2447]|LBCB072-05|HLC-21012|Canada|British Columbia|658[0n]|BOLD:AAA2247
Gluphisia septentrionis|[2448]|LBCA212-05|HLC-20212|Canada|British Columbia|658[0n]|BOLD:AAA2247
Gluphisia septentrionis|[2449]|LPABB451-08|08BBLEP-03716|Canada|Alberta|658[0n]|BOLD:AAA2247
Gluphisia septentrionis|[2450]|LPMN189-08|08BBLEP-00988|Canada|Manitoba|658[0n]|BOLD:AAA2247
Gluphisia septentrionis|[2451]|LPSOB382-08|PPBP-1381|Canada|Ontario|658[0n]|BOLD:AAA2247
Gluphisia septentrionis|[2452]|LPMN199-08|08BBLEP-00998|Canada|Manitoba|658[0n]|BOLD:AAA2247
Gluphisia septentrionis|[2453]|LPMN196-08|08BBLEP-00995|Canada|Manitoba|658[0n]|BOLD:AAA2247
Gluphisia septentrionis|[2454]|LPMN318-08|08BBLEP-01117|Canada|Manitoba|658[0n]|BOLD:AAA2247
Gluphisia septentrionis|[2455]|BLGSM019-09|BL328|Canada|Ontario|658[0n]|BOLD:AAA2247
Gluphisia septentrionis|[2456]|BBLPE616-09|09BBELE-2616|Canada|Nova Scotia|655[0n]|BOLD:AAA2247
Gluphisia septentrionis|[2457]|TMNBD296-07|MNBTT-3097|Canada|New Brunswick|658[0n]|BOLD:AAA2247
Gluphisia septentrionis|[2458]|LPMN799-08|08BBLEP-01602|Canada|Manitoba|658[0n]|BOLD:AAA2247
Gluphisia septentrionis|[2459]|BBLEC224-09|09BBELE-0224|Canada|Nova Scotia|658[0n]|BOLD:AAA2247
Gluphisia septentrionis|[2460]|BBLSY563-09|09BBLEP-03490|United States|New Mexico|658[0n]|BOLD ...
Gluphisia septentrionis|[2461]|CMAZA394-10|CMAZ-0394|United States|Arizona|658[0n]|BOLD:AAA2247
Gluphisia septentrionis|[2462]|LPMN252-08|08BBLEP-01051|Canada|Manitoba|658[0n]|BOLD:AAA2247
Gluphisia septentrionis|[2463]|LPMN185-08|08BBLEP-00984|Canada|Manitoba|658[0n]|BOLD:AAA2247
Gluphisia septentrionis|[2464]|TMNBD294-07|MNBTT-3095|Canada|New Brunswick|631[0n]|BOLD:AAA2247
Gluphisia septentrionis|[2465]|XAB255-04|04HBL005255|Canada|Ontario|658[0n]|BOLD:AAA2247
Gluphisia septentrionis|[2466]|XAE434-04|Moth4434.03|Canada|Ontario|613[1n]|BOLD:AAA2247
Gluphisia septentrionis|[2467]|PHMO074-03|moth374.01|Canada|Ontario|639[2n]|BOLD:AAA2247
Gluphisia septentrionis|[2468]|BBLPE512-09|09BBELE-2512|Canada|Newfoundland and Labrador|626[0n]|...
Gluphisia septentrionis|[2469]|XAG011-05|2005-ONT-595|Canada|Ontario|612[0n]|BOLD:AAA2247
Gluphisia septentrionis|[2470]|XAJ254-06|2006-ONT-0254|Canada|Ontario|658[0n]|BOLD:AAA2247
Gluphisia septentrionis|[2471]|XAF671-05|2005-ONT-320|Canada|Ontario|658[0n]|BOLD:AAA2247
Gluphisia septentrionis|[2472]|RDLQB563-05|DH010649|Canada|Quebec|658[0n]|BOLD:AAA2247
Gluphisia septentrionis|[2473]|LPSOB403-08|PPBP-1402|Canada|Ontario|657[0n]|BOLD:AAA2247
Gluphisia septentrionis|[2474]|BLTIB846-08|BL1265|Canada|Ontario|658[1n]|BOLD:AAA2247
Gluphisia septentrionis|[2475]|BBLCU046-09|09BBLEP-04533|United States|Michigan|658[0n]|BOLD:A...
Gluphisia septentrionis|[2476]|LPMN342-08|08BBLEP-01141|Canada|Manitoba|658[0n]|BOLD:AAA2247
Gluphisia septentrionis|[2477]|BBLCU174-09|09BBLEP-04661|United States|Michigan|658[0n]|BOLD:A...
Gluphisia septentrionis|[2478]|XAB330-04|04HBL005330|Canada|Ontario|658[0n]|BOLD:AAA2247
Gluphisia septentrionis|[2479]|LPMN539-08|08BBLEP-01338|Canada|Manitoba|658[0n]|BOLD:AAA2247
Gluphisia septentrionis|[2480]|XAK329-06|2006-ONT-1324|Canada|Ontario|658[0n]|BOLD:AAA2247
Gluphisia septentrionis|[2481]|BLTIB718-08|BL1004|Canada|Ontario|658[0n]|BOLD:AAA2247
Gluphisia septentrionis|[2482]|LPSOB116-08|PPBP-1115|Canada|Ontario|658[0n]|BOLD:AAA2247
Gluphisia septentrionis|[2483]|BLTIB916-08|BL1336|Canada|Ontario|658[0n]|BOLD:AAA2247
Gluphisia septentrionis|[2484]|LPMN328-08|08BBLEP-01127|Canada|Manitoba|655[0n]|BOLD:AAA2247
Gluphisia septentrionis|[2485]|TTMNB246-06|MNBTT-246|Canada|New Brunswick|658[0n]|BOLD:AAA2247
Gluphisia septentrionis|[2486]|XAB605-04|04HBL005605|Canada|Ontario|658[0n]|BOLD:AAA2247
Gluphisia septentrionis|[2487]|XAD667-05|2005-ONT-82|Canada|Ontario|658[0n]|BOLD:AAA2247
Gluphisia septentrionis|[2488]|LPMN608-08|08BBLEP-01409|Canada|Manitoba|658[0n]|BOLD:AAA2247
Gluphisia septentrionis|[2489]|BLTIB1055-08|BL1063|Canada|Ontario|658[0n]|BOLD:AAA2247
Gluphisia septentrionis|[2490]|LPSOB381-08|PPBP-1380|Canada|Ontario|658[0n]|BOLD:AAA2247
Gluphisia septentrionis|[2491]|XAF532-05|2005-ONT-181|Canada|Ontario|658[0n]|BOLD:AAA2247
Gluphisia septentrionis|[2492]|LPMN315-08|08BBLEP-01114|Canada|Manitoba|658[0n]|BOLD:AAA2247
Gluphisia septentrionis|[2493]|LPSOD418-09|08BBLEP-00197|Canada|Ontario|658[0n]|BOLD:AAA2247
Gluphisia septentrionis|[2494]|LPMN170-08|08BBLEP-00969|Canada|Manitoba|658[0n]|BOLD:AAA2247
Gluphisia septentrionis|[2495]|XAG086-05|2005-ONT-670|Canada|Ontario|658[0n]|BOLD:AAA2247
Gluphisia septentrionis|[2496]|LPMN570-08|08BBLEP-01371|Canada|Manitoba|658[0n]|BOLD:AAA2247
Gluphisia septentrionis|[2497]|XAG087-05|2005-ONT-671|Canada|Ontario|658[0n]|BOLD:AAA2247
Gluphisia septentrionis|[2498]|KPOEC078-08|08OEC-237|Canada|Ontario|652[0n]|BOLD:AAA2247
Gluphisia septentrionis|[2499]|BBLPC204-09|09BBELE-1204|Canada|Nova Scotia|658[0n]|BOLD:AAA2247
Gluphisia septentrionis|[2500]|BBLPC239-09|09BBELE-1239|Canada|Nova Scotia|658[0n]|BOLD:AAA2247
Gluphisia septentrionis|[2501]|BBLPC178-09|09BBELE-1178|Canada|Nova Scotia|658[0n]|BOLD:AAA2247
Gluphisia septentrionis|[2502]|RDMAB029-05|UASM57574|Canada|Alberta|634[0n]|BOLD:AAA2247
Gluphisia septentrionis|[2503]|BBLPC216-09|09BBELE-1216|Canada|Nova Scotia|655[0n]|BOLD:AAA2247
Gluphisia septentrionis|[2504]|BBLPC235-09|09BBELE-1235|Canada|Nova Scotia|631[0n]|BOLD:AAA2247
Gluphisia septentrionis|[2505]|BBLEC629-09|09BBELE-0629|Canada|Nova Scotia|658[0n]|BOLD:AAA2247
Gluphisia septentrionis|[2506]|BBLEC631-09|09BBELE-0631|Canada|Nova Scotia|658[0n]|BOLD:AAA2247
Gluphisia septentrionis|[2507]|BBLPC240-09|09BBELE-1240|Canada|Nova Scotia|658[0n]|BOLD:AAA2247
Gluphisia septentrionis|[2508]|BBLPC218-09|09BBELE-1218|Canada|Nova Scotia|658[0n]|BOLD:AAA2247
Gluphisia septentrionis|[2509]|BBLPC209-09|09BBELE-1209|Canada|Nova Scotia|658[0n]|BOLD:AAA2247
Gluphisia septentrionis|[2510]|BBLPC181-09|09BBELE-1181|Canada|Nova Scotia|614[0n]|BOLD:AAA2247
Gluphisia septentrionis|[2511]|LPSOB399-08|PPBP-1398|Canada|Ontario|658[0n]|BOLD:AAA2247
Gluphisia septentrionis|[2512]|XAE440-04|Moth4440.03|Canada|Ontario|658[0n]|BOLD:AAA2247
Gluphisia septentrionis|[2513]|BBLCU325-09|09BBLEP-04812|United States|Michigan|658[0n]|BOLD:A...
Gluphisia septentrionis|[2514]|LMIS023-05|05-ONMIS-0023|Canada|Ontario|658[0n]|BOLD:AAA2247
Gluphisia septentrionis|[2515]|BBLWU005-09|09BBLEP-04163|United States|Colorado|658[0n]|BOLD:A...
Gluphisia septentrionis|[2516]|BBLCU270-09|09BBLEP-04757|United States|Illinois|658[0n]|BOLD:A...
Gluphisia septentrionis|[2517]|BBLCU269-09|09BBLEP-04756|United States|Illinois|658[0n]|BOLD:A...
Gluphisia septentrionis|[2518]|LPOKB392-09|MDOK-1484|United States|Oklahoma|658[0n]|BOLD:AAA2247
Gluphisia septentrionis|[2519]|LPOKA537-09|MDOK-0537|United States|Oklahoma|658[0n]|BOLD:AAA2247
Gluphisia septentrionis|[2520]|LPOKB341-09|MDOK-1360|United States|Oklahoma|658[0n]|BOLD:AAA2247
Gluphisia septentrionis|[2521]|LPOKE290-11|MDOK-4368|United States|Oklahoma|658[0n]|BOLD:AAA2247
Gluphisia septentrionis|[2522]|LPOKC762-09|MDOK-2839|United States|Oklahoma|658[0n]|BOLD:AAA2247
Gluphisia septentrionis|[2523]|BBLSW546-09|09BBLEP-01474|United States|Oklahoma|645[0n]|BOLD:A...
Gluphisia septentrionis|[2524]|USLEP072-10|10BBLEP-00072|United States|Colorado|630[0n]|BOLD:A...
Gluphisia septentrionis|[2525]|LPOKB480-09|MDOK-1487|United States|Oklahoma|629[0n]|BOLD:AAA2247
Gluphisia septentrionis|[2526]|BBLCU268-09|09BBLEP-04755|United States|Illinois|636[0n]|BOLD:A...
Gluphisia septentrionis|[2527]|BBLSX278-09|09BBLEP-02206|United States|Oklahoma|658[0n]|BOLD:A...
Gluphisia septentrionis|[2528]|BBLSW562-09|09BBLEP-01490|United States|Oklahoma|611[0n]|BOLD:A...
Gluphisia septentrionis|[2529]|LPSOB115-08|PPBP-1114|Canada|Ontario|658[0n]|BOLD:AAA2247
Gluphisia septentrionis|[2530]|LILLA658-11|SNS10IL-00836|United States|Illinois|658[0n]|BOLD:A...
Gluphisia septentrionis|[2531]|BBLSX703-09|09BBLEP-02631|United States|Texas|658[0n]|BOLD:AAA2247
Gluphisia septentrionis|[2532]|CNCLB2877-14|14-NCCC-574|United States|North Carolina|658[0n]|BOLD...
Gluphisia septentrionis|[2533]|RDMAB365-05|UASM77830|Canada|Alberta|657[0n]|BOLD:AAA2247
Gluphisia septentrionis|[2534]|LPMN567-08|08BBLEP-01368|Canada|Manitoba|657[0n]|BOLD:AAA2247
Gluphisia septentrionis|[2535]|RDMAB353-05|UASM7383|Canada|Alberta|658[0n]|BOLD:AAA2247
Gluphisia septentrionis|[2536]|LPMN200-08|08BBLEP-00999|Canada|Manitoba|658[0n]|BOLD:AAA2247
Gluphisia septentrionis|[2537]|LPMN251-08|08BBLEP-01050|Canada|Manitoba|658[0n]|BOLD:AAA2247
Gluphisia septentrionis|[2538]|LPMN574-08|08BBLEP-01375|Canada|Manitoba|658[0n]|BOLD:AAA2247
Gluphisia septentrionis|[2539]|BBLPB281-10|10BBCLP-1280|Canada|Saskatchewan|658[0n]|BOLD:AAA2247
Gluphisia septentrionis|[2540]|LPMN091-08|08BBLEP-00889|Canada|Manitoba|658[0n]|BOLD:AAA2247
Gluphisia septentrionis|[2541]|LPMN197-08|08BBLEP-00996|Canada|Manitoba|658[0n]|BOLD:AAA2247
Gluphisia septentrionis|[2542]|BBLEC897-09|09BBELE-0897|Canada|Nova Scotia|658[0n]|BOLD:AAA2247
Gluphisia septentrionis|[2543]|LPMN537-08|08BBLEP-01336|Canada|Manitoba|658[0n]|BOLD:AAA2247
Gluphisia septentrionis|[2544]|LOCT037-05|05-CTATBI-0037|United States|Connecticut|658[0n]|BOLD ...
Gluphisia septentrionis|[2545]|CNCLB2879-14|14-NCCC-575|United States|North Carolina|658[0n]|BOLD...

Gluphisia septentrionis|[2543]|LPMN537-08|08BBLEP-01336|Canada|Manitoba|658[0n]|BOLD:AAA2247
Gluphisia septentrionis|[2544]|LOCT037-05|05-CTATBI-0037|United States|Connecticut|658[0n]|BOLD ...
Gluphisia septentrionis|[2545]|CNCLB2879-14|14-NCCC-575|United States|North Carolina|658[0n]|BOLD...
Gluphisia septentrionis|[2546]|LOCT036-05|05-CTATBI-0036|United States|Connecticut|658[0n]|BOLD ...
Gluphisia septentrionis|[2547]|LTOL043-06|CWM-94-0297|United States|Maryland|658[0n]|BOLD:AAA2247
Gluphisia septentrionis|[2548]|LMDH196-11|BIOUG01047-H02|United States|Minnesota|635[0n]|BOLD:...
Gluphisia septentrionis|[2549]|BBLCU340-09|09BBLEP-04827|United States|Michigan|634[0n]|BOLD:A...
Gluphisia septentrionis|[2550]|MNBB398-05|05-NBSTA-314|Canada|New Brunswick|580[0n]|BOLD:AAA2247
Gluphisia septentrionis|[2551]|PHMO055-03|moth360.01|Canada|Ontario|617[0n]|BOLD:AAA2247
Gluphisia septentrionis|[2552]|LPMN568-08|08BBLEP-01369|Canada|Manitoba|621[0n]|BOLD:AAA2247
Gluphisia septentrionis|[2553]|BBLEC887-09|09BBELE-0887|Canada|Nova Scotia|658[0n]|BOLD:AAA2247
Gluphisia septentrionis|[2554]|PHMNB049-03|moth235.02SA|Canada|New Brunswick|639[0n]|BOLD:AAA2247
Gluphisia septentrionis|[2555]|PHMNB022-03|moth159.02SA|Canada|New Brunswick|639[0n]|BOLD:AAA2247
Gluphisia septentrionis|[2556]|LOWCB134-05|CGWC-1074|Canada|British Columbia|566[0n]|BOLD:AAA2247
Gluphisia septentrionis|[2557]|PHMNB242-04|04HBL007707|Canada|New Brunswick|563[0n]|BOLD:AAA2247
Gluphisia septentrionis|[2558]|LPSOB398-08|PPBP-1397|Canada|Ontario|658[0n]|BOLD:AAA2247
Gluphisia septentrionis|[2559]|LOWCB137-05|CGWC-1077|Canada|British Columbia|565[0n]|BOLD:AAA2247
Gluphisia septentrionis|[2560]|LBCH805-10|10-JDWBC-0805|Canada|British Columbia|658[0n]|BOLD:A...
Gluphisia septentrionis|[2561]|LBCA214-05|HLC-20214|Canada|British Columbia|658[0n]|BOLD:AAA2247
Gluphisia septentrionis|[2562]|LMDH134-11|BIOUG01047-B12|United States|Minnesota|658[0n]|BOLD:...
Gluphisia septentrionis|[2563]|LOWCB132-05|CGWC-1072|Canada|British Columbia|658[0n]|BOLD:AAA2247
Gluphisia septentrionis|[2564]|LPMN190-08|08BBLEP-00989|Canada|Manitoba|658[0n]|BOLD:AAA2247
Gluphisia septentrionis|[2565]|BBLEC933-09|09BBELE-0933|Canada|Nova Scotia|658[0n]|BOLD:AAA2247
Gluphisia septentrionis|[2566]|LPSOB401-08|PPBP-1400|Canada|Ontario|658[0n]|BOLD:AAA2247
Gluphisia septentrionis|[2567]|LPMN576-08|08BBLEP-01377|Canada|Manitoba|658[0n]|BOLD:AAA2247
Gluphisia septentrionis|[2568]|LPMN249-08|08BBLEP-01048|Canada|Manitoba|658[0n]|BOLD:AAA2247
Gluphisia septentrionis|[2569]|LPSOB221-08|PPBP-1220|Canada|Ontario|658[0n]|BOLD:AAA2247
Gluphisia septentrionis|[2570]|PHMNB573-04|04HBL00799|Canada|New Brunswick|658[0n]|BOLD:AAA2247
Gluphisia septentrionis|[2571]|LPSO925-08|PPBP-0925|Canada|Ontario|658[0n]|BOLD:AAA2247
Gluphisia septentrionis|[2572]|LPMN534-08|08BBLEP-01333|Canada|Manitoba|658[0n]|BOLD:AAA2247
Gluphisia septentrionis|[2573]|LPMN346-08|08BBLEP-01145|Canada|Manitoba|658[0n]|BOLD:AAA2247
Gluphisia septentrionis|[2574]|BBLEC704-09|09BBELE-0704|Canada|Nova Scotia|658[0n]|BOLD:AAA2247
Gluphisia septentrionis|[2575]|LPMN180-08|08BBLEP-00979|Canada|Manitoba|658[0n]|BOLD:AAA2247
Gluphisia septentrionis|[2576]|LPMN531-08|08BBLEP-01330|Canada|Manitoba|658[0n]|BOLD:AAA2247
Gluphisia septentrionis|[2577]|MNBB559-05|05-NBSTA-475|Canada|New Brunswick|658[0n]|BOLD:AAA2247
Gluphisia septentrionis|[2578]|BBLEC701-09|09BBELE-0701|Canada|Nova Scotia|655[0n]|BOLD:AAA2247
Gluphisia septentrionis|[2579]|BBLEC060-09|09BBELE-0060|Canada|New Brunswick|658[0n]|BOLD:AAA2247
Gluphisia septentrionis|[2580]|LPSOD842-09|08BBLEP-00624|Canada|Ontario|658[0n]|BOLD:AAA2247
Gluphisia septentrionis|[2581]|LPMN533-08|08BBLEP-01332|Canada|Manitoba|658[0n]|BOLD:AAA2247
Gluphisia septentrionis|[2582]|LPMN575-08|08BBLEP-01376|Canada|Manitoba|658[0n]|BOLD:AAA2247
Gluphisia septentrionis|[2583]|LBCH117-10|10-JDWBC-0117|Canada|British Columbia|658[0n]|BOLD:A...
Gluphisia septentrionis|[2584]|LPMN178-08|08BBLEP-00977|Canada|Manitoba|658[0n]|BOLD:AAA2247
Gluphisia septentrionis|[2585]|LBCH2942-10|10-JDWBC-2942|Canada|British Columbia|658[0n]|BOLD:...
Gluphisia septentrionis|[2586]|LOWCB140-05|CGWC-1080|Canada|British Columbia|658[0n]|BOLD:AAA2247
Gluphisia septentrionis|[2587]|LBCA213-05|HLC-20213|Canada|British Columbia|658[0n]|BOLD:AAA2247
Gluphisia septentrionis|[2588]|LPMN183-08|08BBLEP-00982|Canada|Manitoba|658[0n]|BOLD:AAA2247
Gluphisia septentrionis|[2589]|LBCC009-05|HLC-21889|Canada|British Columbia|658[0n]|BOLD:AAA2247
Gluphisia septentrionis|[2590]|LOWCC897-05|CGWC-2777|Canada|British Columbia|658[0n]|BOLD:AAA2247
Gluphisia septentrionis|[2591]|LPSO062-08|PPBP-0062|Canada|Ontario|658[0n]|BOLD:AAA2247
Gluphisia septentrionis|[2592]|LBCH3032-10|10-JDWBC-3032|Canada|British Columbia|658[0n]|BOLD:...
Gluphisia septentrionis|[2593]|BBLEC735-09|09BBELE-0735|Canada|Nova Scotia|658[0n]|BOLD:AAA2247
Gluphisia septentrionis|[2594]|LPSOB584-08|PPBP-1583|Canada|Ontario|658[0n]|BOLD:AAA2247
Gluphisia septentrionis|[2595]|LPMN566-08|08BBLEP-01367|Canada|Manitoba|656[0n]|BOLD:AAA2247
Gluphisia septentrionis|[2596]|LBCC002-05|HLC-21882|Canada|British Columbia|658[0n]|BOLD:AAA2247
Gluphisia septentrionis|[2597]|LPMN171-08|08BBLEP-00970|Canada|Manitoba|658[0n]|BOLD:AAA2247
Gluphisia septentrionis|[2598]|LPMN535-08|08BBLEP-01334|Canada|Manitoba|658[0n]|BOLD:AAA2247
Gluphisia septentrionis|[2599]|BBLEC693-09|09BBELE-0693|Canada|Nova Scotia|658[0n]|BOLD:AAA2247
Gluphisia septentrionis|[2600]|LOWCB138-05|CGWC-1078|Canada|British Columbia|658[0n]|BOLD:AAA2247
Gluphisia septentrionis|[2601]|BBLPB640-10|10BBCLP-1639|Canada|British Columbia|658[0n]|BOLD:A...
Gluphisia septentrionis|[2602]|BBLEC725-09|09BBELE-0725|Canada|Nova Scotia|655[0n]|BOLD:AAA2247
Gluphisia septentrionis|[2603]|LPSOD578-09|08BBLEP-00359|Canada|Ontario|658[0n]|BOLD:AAA2247
Gluphisia septentrionis|[2604]|LOWCB133-05|CGWC-1073|Canada|British Columbia|658[0n]|BOLD:AAA2247
Gluphisia septentrionis|[2605]|BBLPB283-10|10BBCLP-1282|Canada|Saskatchewan|658[0n]|BOLD:AAA2247
Gluphisia septentrionis|[2606]|LOWCD141-06|CGWC-2961|Canada|British Columbia|658[0n]|BOLD:AAA2247
Gluphisia septentrionis|[2607]|LOWCB135-05|CGWC-1075|Canada|British Columbia|658[0n]|BOLD:AAA2247
Gluphisia septentrionis|[2608]|BBLEC223-09|09BBELE-0223|Canada|Nova Scotia|643[0n]|BOLD:AAA2247
Gluphisia septentrionis|[2609]|BBLPC188-09|09BBELE-1188|Canada|Nova Scotia|654[0n]|BOLD:AAA2247
Gluphisia septentrionis|[2610]|LOWCB136-05|CGWC-1076|Canada|British Columbia|658[0n]|BOLD:AAA2247
Gluphisia septentrionis|[2611]|LPMN536-08|08BBLEP-01335|Canada|Manitoba|658[0n]|BOLD:AAA2247
Gluphisia septentrionis|[2612]|BBLPB284-10|10BBCLP-1283|Canada|Saskatchewan|658[0n]|BOLD:AAA2247
Gluphisia septentrionis|[2613]|BBLEC929-09|09BBELE-0929|Canada|Nova Scotia|658[0n]|BOLD:AAA2247
Gluphisia septentrionis|[2614]|MNAF481-08|CNCLEP00040466|Canada|Manitoba|658[0n]|BOLD:AAA2247
Gluphisia septentrionis|[2615]|LPSOD717-09|08BBLEP-00499|Canada|Ontario|658[0n]|BOLD:AAA2247
Gluphisia septentrionis|[2616]|LPMN530-08|08BBLEP-01329|Canada|Manitoba|658[0n]|BOLD:AAA2247
Gluphisia septentrionis|[2617]|LPMN586-08|08BBLEP-01387|Canada|Manitoba|658[0n]|BOLD:AAA2247
Gluphisia septentrionis|[2618]|BLTIB1067-08|BL1076|Canada|Ontario|658[0n]|BOLD:AAA2247
Gluphisia septentrionis|[2619]|LPMN319-08|08BBLEP-01118|Canada|Manitoba|658[0n]|BOLD:AAA2247
Gluphisia septentrionis|[2620]|LPMN173-08|08BBLEP-00972|Canada|Manitoba|658[0n]|BOLD:AAA2247
Gluphisia septentrionis|[2621]|LPMN214-08|08BBLEP-01013|Canada|Manitoba|658[0n]|BOLD:AAA2247
Gluphisia septentrionis|[2622]|LPMN571-08|08BBLEP-01372|Canada|Manitoba|609[0n]|BOLD:AAA2247
Gluphisia septentrionis|[2623]|LPMN068-08|08BBLEP-00866|Canada|Manitoba|658[0n]|BOLD:AAA2247
Gluphisia septentrionis|[2624]|LMDH164-11|BIOUG01047-E06|United States|Minnesota|658[0n]|BOLD:...
Gluphisia septentrionis|[2625]|LPSOB327-08|PPBP-1326|Canada|Ontario|658[0n]|BOLD:AAA2247
Gluphisia septentrionis|[2626]|LPSOB400-08|PPBP-1399|Canada|Ontario|658[0n]|BOLD:AAA2247
Gluphisia septentrionis|[2627]|PHMNB571-04|04HBL00797|Canada|New Brunswick|658[0n]|BOLD:AAA2247
Gluphisia septentrionis|[2628]|LPMN349-08|08BBLEP-01148|Canada|Manitoba|658[0n]|BOLD:AAA2247
Gluphisia septentrionis|[2629]|LPSOB402-08|PPBP-1401|Canada|Ontario|658[0n]|BOLD:AAA2247
Gluphisia septentrionis|[2630]|MNBB326-05|05-NBSTA-242|Canada|New Brunswick|562[1n]|BOLD:AAA2247
Gluphisia septentrionis|[2631]|TTMNB247-06|MNBTT-247|Canada|New Brunswick|657[0n]|BOLD:AAA2247
Gluphisia septentrionis|[2632]|BBLPB282-10|10BBCLP-1281|Canada|Saskatchewan|658[0n]|BOLD:AAA2247
Gluphisia septentrionis|[2633]|TMNBD292-07|MNBTT-3093|Canada|New Brunswick|626[0n]|BOLD:AAA2247
Gluphisia septentrionis|[2634]|LMDH179-11|BIOUG01047-F09|United States|Minnesota|658[0n]|BOLD:...
Gluphisia septentrionis|[2635]|BBLWU172-09|09BBLEP-04330|United States|Colorado|658[0n]|BOLD:A...
Gluphisia septentrionis|[2636]|RDNMH163-09|CNCLEP00054380|United States|Colorado|604[0n]|BOLD:...
Gluphisia septentrionis|[2637]|BBLEC222-09|09BBELE-0222|Canada|Nova Scotia|658[0n]|BOLD:AAA2247
Gluphisia septentrionis|[2638]|BBLPB280-10|10BBCLP-1279|Canada|Saskatchewan|658[0n]|BOLD:AAA2247
Gluphisia septentrionis|[2639]|LPMN316-08|08BBLEP-01115|Canada|Manitoba|658[0n]|BOLD:AAA2247
Gluphisia septentrionis|[2640]|BBLPB279-10|10BBCLP-1278|Canada|Saskatchewan|658[0n]|BOLD:AAA2247
Gluphisia septentrionis|[2641]|LPMN532-08|08BBLEP-01331|Canada|Manitoba|658[0n]|BOLD:AAA2247
Gluphisia septentrionis|[2642]|LPMN198-08|08BBLEP-00997|Canada|Manitoba|658[0n]|BOLD:AAA2247
Gluphisia septentrionis|[2643]|LPMN538-08|08BBLEP-01337|Canada|Manitoba|658[0n]|BOLD:AAA2247

Gluphisia septentrionis|[2641]|LPMN532-08|08BBLEP-01331|Canada|Manitoba|658[0n]|BOLD:AAA2247
Gluphisia septentrionis|[2642]|LPMN198-08|08BBLEP-00997|Canada|Manitoba|658[0n]|BOLD:AAA2247
Gluphisia septentrionis|[2643]|LPMN538-08|08BBLEP-01337|Canada|Manitoba|658[0n]|BOLD:AAA2247
Gluphisia septentrionis|[2644]|LPMN184-08|08BBLEP-00983|Canada|Manitoba|658[0n]|BOLD:AAA2247
Gluphisia septentrionis|[2645]|LPMN179-08|08BBLEP-00978|Canada|Manitoba|658[0n]|BOLD:AAA2247
Gluphisia septentrionis|[2646]|LPMN186-08|08BBLEP-00985|Canada|Manitoba|658[0n]|BOLD:AAA2247
Phryganidia californica|[2647]|GMLC1239-12|2011GM-0935|United States|California|658[0n]|BOLD:A...
Phryganidia californica|[2648]|GMLC101-09|2008GM-0110|United States|California|658[0n]|BOLD:AA...
Clostera brucei|[2649]|CHLEP289-09|09PROBE-09584|Canada|Manitoba|658[0n]|BOLD:AAB0492
Clostera brucei|[2650]|CHLEP080-09|09PROBE-09375|Canada|Manitoba|658[0n]|BOLD:AAB0492
Clostera brucei|[2651]|CHLEP073-09|09PROBE-09368|Canada|Manitoba|658[0n]|BOLD:AAB0492
Clostera brucei|[2652]|CHLEP070-09|09PROBE-09365|Canada|Manitoba|658[0n]|BOLD:AAB0492
Clostera brucei|[2653]|CHLEP239-09|09PROBE-09534|Canada|Manitoba|658[0n]|BOLD:AAB0492
Clostera brucei|[2654]|CHLEP251-09|09PROBE-09546|Canada|Manitoba|658[0n]|BOLD:AAB0492
Clostera brucei|[2655]|CHLEP079-09|09PROBE-09374|Canada|Manitoba|658[0n]|BOLD:AAB0492
Clostera brucei|[2656]|LCH215-04|04HBL003215|Canada|Manitoba|658[0n]|BOLD:AAB0492
Clostera brucei|[2657]|CHLEP104-09|09PROBE-09399|Canada|Manitoba|658[0n]|BOLD:AAB0492
Clostera brucei|[2658]|CHLEP243-09|09PROBE-09538|Canada|Manitoba|658[0n]|BOLD:AAB0492
Clostera brucei|[2659]|CHLEP250-09|09PROBE-09545|Canada|Manitoba|643[0n]|BOLD:AAB0492
Clostera brucei|[2660]|CHLEP072-09|09PROBE-09367|Canada|Manitoba|658[0n]|BOLD:AAB0492
Clostera brucei|[2661]|CHLEP240-09|09PROBE-09535|Canada|Manitoba|658[0n]|BOLD:AAB0492
Clostera brucei|[2662]|CHLEP088-09|09PROBE-09383|Canada|Manitoba|658[0n]|BOLD:AAB0492
Clostera brucei|[2663]|CHLEP241-09|09PROBE-09536|Canada|Manitoba|658[0n]|BOLD:AAB0492
Clostera brucei|[2664]|CHLEP089-09|09PROBE-09384|Canada|Manitoba|658[0n]|BOLD:AAB0492
Clostera brucei|[2665]|CHLEP252-09|09PROBE-09547|Canada|Manitoba|658[0n]|BOLD:AAB0492
Clostera brucei|[2666]|LCH581-04|04HBL003581|Canada|Manitoba|658[0n]|BOLD:AAB0492
Clostera brucei|[2667]|CHLEP238-09|09PROBE-09533|Canada|Manitoba|658[0n]|BOLD:AAB0492
Clostera brucei|[2668]|CHLEP244-09|09PROBE-09539|Canada|Manitoba|658[0n]|BOLD:AAB0492
Clostera brucei|[2669]|LCHP980-07|07PROBE-10742|Canada|Manitoba|658[0n]|BOLD:AAB0492
Clostera brucei|[2670]|CHLEP071-09|09PROBE-09366|Canada|Manitoba|658[0n]|BOLD:AAB0492
Clostera brucei|[2671]|CHLEP288-09|09PROBE-09583|Canada|Manitoba|658[0n]|BOLD:AAB0492
Clostera brucei|[2672]|RDLQ782-07|DH008582|Canada|Newfoundland and Labrador|655[0n]|BOLD:AAB0492
Clostera brucei|[2673]|RDNMH164-09|CNCLEP00054381|United States|Colorado|636[0n]|BOLD:AAB0492
Clostera brucei|[2674]|LCH216-04|04HBL003216|Canada|Manitoba|658[0n]|BOLD:AAB0492
Clostera brucei|[2675]|RDNMH165-09|CNCLEP00054382|United States|California|641[0n]|BOLD:AAB0492
Clostera brucei|[2676]|RDNMH429-09|CNCLEP00054443|United States|California|658[0n]|BOLD:AAB0492
Clostera brucei|[2677]|RDNMH426-09|CNCLEP00054440|United States|California|658[0n]|BOLD:AAB0492
Clostera brucei|[2678]|LBCA026-05|HLC-20026|Canada|British Columbia|658[0n]|BOLD:AAB0492
Clostera brucei|[2679]|RDNMH421-09|CNCLEP00054435|United States|Wyoming|658[0n]|BOLD:AAB0492
Clostera brucei|[2680]|LBCH2959-10|10-JDWBC-2959|Canada|British Columbia|641[0n]|BOLD:AAB0492
Clostera brucei|[2681]|LOWCE258-06|CGWC-4018|Canada|British Columbia|658[0n]|BOLD:AAB0492
Clostera brucei|[2682]|LOWCE263-06|CGWC-4023|Canada|British Columbia|658[0n]|BOLD:AAB0492
Clostera albosigma|[2683]|TTMNB915-06|MNBTT-915|Canada|New Brunswick|658[0n]|BOLD:AAA5417
Clostera albosigma|[2684]|LILLA001-11|SNS10IL-00001|United States|Illinois|658[0n]|BOLD:AAA5417
Clostera albosigma|[2685]|BBLPB269-10|10BBCLP-1268|Canada|British Columbia|658[0n]|BOLD:AAA5417
Clostera albosigma|[2686]|XAK330-06|2006-ONT-1325|Canada|Ontario|658[0n]|BOLD:AAA5417
Clostera albosigma|[2687]|LBCA027-05|HLC-20027|Canada|British Columbia|658[0n]|BOLD:AAA5417
Clostera albosigma|[2688]|XAF409-05|HLC-10450|Canada|Ontario|658[0n]|BOLD:AAA5417
Clostera albosigma|[2689]|LBCH2956-10|10-JDWBC-2956|Canada|British Columbia|658[0n]|BOLD:AAA5417
Clostera albosigma|[2690]|RDLQB512-05|DH010598|Canada|Quebec|658[0n]|BOLD:AAA5417
Clostera albosigma|[2691]|XAJ139-06|2006-ONT-0139|Canada|Ontario|658[0n]|BOLD:AAA5417
Clostera albosigma|[2692]|LBCH011-10|10-JDWBC-0011|Canada|British Columbia|658[0n]|BOLD:AAA5417
Clostera albosigma|[2693]|LPABB207-08|08BBLEP-03472|Canada|Alberta|658[0n]|BOLD:AAA5417
Clostera albosigma|[2694]|BBLEC698-09|09BBELE-0698|Canada|Nova Scotia|658[0n]|BOLD:AAA5417
Clostera albosigma|[2695]|BBLPB265-10|10BBCLP-1264|Canada|Alberta|658[0n]|BOLD:AAA5417
Clostera albosigma|[2696]|LBCG181-08|08-JDWBC-0181|Canada|British Columbia|658[0n]|BOLD:AAA5417
Clostera albosigma|[2697]|TMNBD255-07|MNBTT-3056|Canada|New Brunswick|658[0n]|BOLD:AAA5417
Clostera albosigma|[2698]|MNBB439-05|05-NBSTA-355|Canada|New Brunswick|658[0n]|BOLD:AAA5417
Clostera albosigma|[2699]|LBCH119-10|10-JDWBC-0119|Canada|British Columbia|658[0n]|BOLD:AAA5417
Clostera albosigma|[2700]|CNCLB2793-14|14-NCCC-554|United States|North Carolina|658[0n]|BOLD:A...
Clostera albosigma|[2701]|CNCLB2791-14|14-NCCC-552|United States|North Carolina|658[0n]|BOLD:A...
Clostera albosigma|[2702]|TMNBD252-07|MNBTT-3053|Canada|New Brunswick|658[0n]|BOLD:AAA5417
Clostera albosigma|[2703]|XAB283-04|04HBL005283|Canada|Ontario|658[0n]|BOLD:AAA5417
Clostera albosigma|[2704]|LPABC916-09|08BBLEP-05327|Canada|Alberta|658[0n]|BOLD:AAA5417
Clostera albosigma|[2705]|LBCH3028-10|10-JDWBC-3028|Canada|British Columbia|658[0n]|BOLD:AAA5417
Clostera albosigma|[2706]|XAG090-05|2005-ONT-674|Canada|Ontario|658[0n]|BOLD:AAA5417
Clostera albosigma|[2707]|LBCH2919-10|10-JDWBC-2919|Canada|British Columbia|658[0n]|BOLD:AAA5417
Clostera albosigma|[2708]|BBLPC111-09|09BBELE-1111|Canada|New Brunswick|658[0n]|BOLD:AAA5417
Clostera albosigma|[2709]|XAE065-04|Moth4065.03|Canada|Ontario|658[0n]|BOLD:AAA5417
Clostera albosigma|[2710]|TMNBD256-07|MNBTT-3057|Canada|New Brunswick|658[0n]|BOLD:AAA5417
Clostera albosigma|[2711]|MNBB665-05|05-NBSTA-581|Canada|New Brunswick|658[0n]|BOLD:AAA5417
Clostera albosigma|[2712]|TMNBD253-07|MNBTT-3054|Canada|New Brunswick|658[0n]|BOLD:AAA5417
Clostera albosigma|[2713]|LBCH653-10|10-JDWBC-0653|Canada|British Columbia|658[0n]|BOLD:AAA5417
Clostera albosigma|[2714]|XAB634-04|04HBL005634|Canada|Ontario|658[0n]|BOLD:AAA5417
Clostera albosigma|[2715]|LPMN921-08|08BBLEP-02279|Canada|Alberta|658[0n]|BOLD:AAA5417
Clostera albosigma|[2716]|LPSOB513-08|PPBP-1512|Canada|Ontario|658[0n]|BOLD:AAA5417
Clostera albosigma|[2717]|BLTIB1018-08|BL1458|Canada|Ontario|658[0n]|BOLD:AAA5417
Clostera albosigma|[2718]|MNBB172-05|05-NBSTA-088|Canada|New Brunswick|658[0n]|BOLD:AAA5417
Clostera albosigma|[2719]|XAF432-05|HLC-10473|Canada|Ontario|658[0n]|BOLD:AAA5417
Clostera albosigma|[2720]|TMNBD254-07|MNBTT-3055|Canada|New Brunswick|658[0n]|BOLD:AAA5417
Clostera albosigma|[2721]|BBLEC050-09|09BBELE-0050|Canada|New Brunswick|658[0n]|BOLD:AAA5417
Clostera albosigma|[2722]|XAF346-05|HLC-10387|Canada|Ontario|658[0n]|BOLD:AAA5417
Clostera albosigma|[2723]|LBCH806-10|10-JDWBC-0806|Canada|British Columbia|658[0n]|BOLD:AAA5417
Clostera albosigma|[2724]|BBLPB264-10|10BBCLP-1263|Canada|Alberta|658[0n]|BOLD:AAA5417
Clostera albosigma|[2725]|PMG171-03|moth232.01|Canada|Ontario|617[0n]|BOLD:AAA5417
Clostera albosigma|[2726]|MNBB607-05|05-NBSTA-523|Canada|New Brunswick|556[4n]|BOLD:AAA5417
Clostera albosigma|[2727]|LOWCB112-05|CGWC-1052|Canada|British Columbia|556[0n]|BOLD:AAA5417
Clostera albosigma|[2728]|LBCA365-05|HLC-20365|Canada|British Columbia|600[2n]|BOLD:AAA5417
Clostera albosigma|[2729]|XAE600-04|Moth4600.03|Canada|Ontario|562[0n]|BOLD:AAA5417
Clostera albosigma|[2730]|LPSOB580-08|PPBP-1579|Canada|Ontario|658[0n]|BOLD:AAA5417
Clostera albosigma|[2731]|XAH082-05|2005-ONT-1665|Canada|Ontario|658[0n]|BOLD:AAA5417
Clostera albosigma|[2732]|PHMNB576-04|04HBL00802|Canada|New Brunswick|658[0n]|BOLD:AAA5417
Clostera albosigma|[2733]|LOWCB111-05|CGWC-1051|Canada|British Columbia|615[0n]|BOLD:AAA5417
Clostera albosigma|[2734]|LBCG3219-09|08-JDWBC-3219|Canada|British Columbia|632[0n]|BOLD:AAA5417
Clostera albosigma|[2735]|RDNMH411-09|CNCLEP00054425|United States|Colorado|658[0n]|BOLD:AAA5417
Clostera albosigma|[2736]|TMNBB042-06|MNBTT-982|Canada|New Brunswick|658[0n]|BOLD:AAA5417
Clostera albosigma|[2737]|BBLPC041-09|09BBELE-1041|Canada|New Brunswick|658[0n]|BOLD:AAA5417
Clostera albosigma|[2738]|BBLPE528-09|09BBELE-2528|Canada|Newfoundland and Labrador|658[0n]|BOLD...
Clostera albosigma|[2739]|BBLPC747-09|09BBELE-1747|Canada|Newfoundland and Labrador|658[0n]|BOLD...
Clostera albosigma|[2740]|XAJ500-06|2006-ONT-0500|Canada|Ontario|658[0n]|BOLD:AAA5417
Clostera albosigma|[2741]|LPSOB514-08|PPBP-1513|Canada|Ontario|658[0n]|BOLD:AAA5417

Clostera albosigma|[2739]|BBLPC747-09|09BBELE-1747|Canada|Newfoundland and Labrador|658[0n]|BOLD...
Clostera albosigma|[2740]|XAJ500-06|2006-ONT-0500|Canada|Ontario|658[0n]|BOLD:AAA5417
Clostera albosigma|[2741]|LPSOB514-08|PPBP-1513|Canada|Ontario|658[0n]|BOLD:AAA5417
Clostera albosigma|[2742]|LPABC701-09|08BBLEP-04920|Canada|Alberta|658[0n]|BOLD:AAA5417
Clostera albosigma|[2743]|LPMN896-08|08BBLEP-02254|Canada|Alberta|658[0n]|BOLD:AAA5417
Clostera albosigma|[2744]|LPMN906-08|08BBLEP-02264|Canada|Alberta|658[0n]|BOLD:AAA5417
Clostera albosigma|[2745]|LPABC665-09|08BBLEP-04884|Canada|Alberta|632[0n]|BOLD:AAA5417
Clostera albosigma|[2746]|XAF408-05|HLC-10449|Canada|Ontario|658[0n]|BOLD:AAA5417
Clostera albosigma|[2747]|BBLPB263-10|10BBCLP-1262|Canada|Saskatchewan|668[0n]|BOLD:AAA5417
Clostera albosigma|[2748]|BBLPB260-10|10BBCLP-1259|Canada|Saskatchewan|658[0n]|BOLD:AAA5417
Clostera albosigma|[2749]|TMG55-03|moth246.01|Canada|Ontario|639[0n]|BOLD:AAA5417
Clostera albosigma|[2750]|TMG54-03|CLOS2.00|Canada|Ontario|639[0n]|BOLD:AAA5417
Clostera albosigma|[2751]|XAG089-05|2005-ONT-673|Canada|Ontario|658[0n]|BOLD:AAA5417
Clostera albosigma|[2752]|XAF320-05|HLC-10361|Canada|Ontario|658[0n]|BOLD:AAA5417
Clostera albosigma|[2753]|LMIS021-05|05-ONMIS-0021|Canada|Ontario|658[0n]|BOLD:AAA5417
Clostera albosigma|[2754]|BBLPB267-10|10BBCLP-1266|Canada|Ontario|658[0n]|BOLD:AAA5417
Clostera albosigma|[2755]|LPSOB695-08|PPBP-1694|Canada|Ontario|656[0n]|BOLD:AAA5417
Clostera albosigma|[2756]|LPMN326-08|08BBLEP-01125|Canada|Manitoba|658[0n]|BOLD:AAA5417
Clostera albosigma|[2757]|BBLPB257-10|10BBCLP-1256|Canada|Saskatchewan|658[0n]|BOLD:AAA5417
Clostera albosigma|[2758]|BBLPB262-10|10BBCLP-1261|Canada|Saskatchewan|658[0n]|BOLD:AAA5417
Clostera albosigma|[2759]|LPSOB593-08|PPBP-1592|Canada|Ontario|658[0n]|BOLD:AAA5417
Clostera albosigma|[2760]|BBLPB261-10|10BBCLP-1260|Canada|Saskatchewan|658[0n]|BOLD:AAA5417
Clostera albosigma|[2761]|LMDH176-11|BIOUG01047-F06|United States|Minnesota|658[0n]|BOLD:AAA5417
Clostera albosigma|[2762]|LPSOD798-09|08BBLEP-00580|Canada|Ontario|658[0n]|BOLD:AAA5417
Clostera albosigma|[2763]|XAG022-05|2005-ONT-606|Canada|Ontario|658[0n]|BOLD:AAA5417
Clostera albosigma|[2764]|LPMNB234-09|08BBLEP-05078|Canada|Manitoba|658[0n]|BOLD:AAA5417
Clostera albosigma|[2765]|BBLPB266-10|10BBCLP-1265|Canada|Ontario|572[0n]|BOLD:AAA5417
Clostera albosigma|[2766]|XAB221-04|04HBL005221|Canada|Ontario|658[0n]|BOLD:AAA5417
Clostera albosigma|[2767]|XAB594-04|04HBL005594|Canada|Ontario|658[0n]|BOLD:AAA5417
Clostera albosigma|[2768]|RDNMH171-09|CNCLEP00054388|United States|Colorado|636[0n]|BOLD:AAA5417
Clostera paraphora|[2769]|CNCLB857-14|CNCLEP00092182|United States|Colorado|658[0n]|BOLD:ACM4004
Clostera paraphora|[2770]|CNCLB858-14|CNCLEP00092183|United States|Colorado|658[1n]|BOLD:ACM4004
Clostera paraphora|[2771]|CNCLB797-14|CNCLEP00083809|United States|Colorado|658[0n]|BOLD:ACM4004
Clostera paraphora|[2772]|CNCLB798-14|CNCLEP00083810|United States|Colorado|658[0n]|BOLD:ACM4004
Clostera paraphora|[2773]|CNCLB799-14|CNCLEP00083811|United States|Colorado|658[0n]|BOLD:ACM4004
Clostera paraphora|[2774]|TML121-14|CCDB-17964-B01|United States|California|658[0n]|BOLD:ACM4004
Clostera inornata|[2775]|IAWLB640-11|IAWAZ-1073|United States|Arizona|560[0n]|BOLD:AAZ0998
Clostera inornata|[2776]|IAWLB639-11|IAWAZ-1072|United States|Arizona|658[0n]|BOLD:AAZ0998
Clostera inornata|[2777]|CMAZA929-12|BIOUG02040-G08|United States|Arizona|658[0n]|BOLD:AAZ0998
Clostera inclusa|[2778]|LPSO350-08|PPBP-0350|Canada|Ontario|658[0n]|BOLD:AAC4151
Clostera inclusa|[2779]|LPSO365-08|PPBP-0365|Canada|Ontario|658[0n]|BOLD:AAC4151
Clostera inclusa|[2780]|LPOKB396-09|MDOK-1497|United States|Oklahoma|658[0n]|BOLD:AAC4151
Clostera inclusa|[2781]|LPSOB095-08|PPBP-1094|Canada|Ontario|658[0n]|BOLD:AAC4151
Clostera inclusa|[2782]|LPSO837-08|PPBP-0837|Canada|Ontario|658[0n]|BOLD:AAC4151
Clostera inclusa|[2783]|LPSO737-08|PPBP-0737|Canada|Ontario|648[0n]|BOLD:AAC4151
Clostera inclusa|[2784]|LPSO366-08|PPBP-0366|Canada|Ontario|658[0n]|BOLD:AAC4151
Clostera inclusa|[2785]|LPSOB092-08|PPBP-1091|Canada|Ontario|658[0n]|BOLD:AAC4151
Clostera inclusa|[2786]|LPOKA965-09|MDOK-1060|United States|Oklahoma|658[0n]|BOLD:AAC4151
Clostera inclusa|[2787]|LPSO867-08|PPBP-0867|Canada|Ontario|658[0n]|BOLD:AAC4151
Clostera inclusa|[2788]|LPSO270-08|PPBP-0270|Canada|Ontario|658[0n]|BOLD:AAC4151
Clostera inclusa|[2789]|LPSO156-08|PPBP-0156|Canada|Ontario|658[0n]|BOLD:AAC4151
Clostera inclusa|[2790]|LSUSA232-06|06-SUSA-0232|United States|Kentucky|656[0n]|BOLD:AAC4151
Clostera inclusa|[2791]|LILLA076-11|SNS10IL-00101|United States|Illinois|658[0n]|BOLD:AAC4151
Clostera inclusa|[2792]|BBLOB1118-11|BIOUG01415-G01|United States|West Virginia|658[0n]|BOLD:A...
Clostera inclusa|[2793]|BBLCU329-09|09BBLEP-04816|United States|Michigan|658[0n]|BOLD:AAC4151
Clostera inclusa|[2794]|BBLSX301-09|09BBLEP-02229|United States|Oklahoma|658[0n]|BOLD:AAC4151
Clostera strigosa|[2795]|LPSOB137-08|PPBP-1136|Canada|Ontario|655[0n]|BOLD:ABY6578
Clostera strigosa|[2796]|LPSOB348-08|PPBP-1347|Canada|Ontario|657[0n]|BOLD:ABY6578
Clostera strigosa|[2797]|XAB380-04|04HBL005380|Canada|Ontario|658[0n]|BOLD:ABY6578
Clostera strigosa|[2798]|XAJ507-06|2006-ONT-0507|Canada|Ontario|626[0n]|BOLD:ABY6578
Clostera strigosa|[2799]|XAK616-07|HLC-16169|Canada|Ontario|593[0n]|BOLD:ABY6578
Clostera strigosa|[2800]|RDLQ781-07|DH005160|Canada|Quebec|643[0n]|BOLD:ABY6578
Clostera strigosa|[2801]|TTMNB918-06|MNBTT-918|Canada|New Brunswick|658[0n]|BOLD:ABY6578
Clostera strigosa|[2802]|TTMNB919-06|MNBTT-919|Canada|New Brunswick|658[0n]|BOLD:ABY6578
Clostera strigosa|[2803]|TMNBD257-07|MNBTT-3058|Canada|New Brunswick|658[0n]|BOLD:ABY6578
Clostera strigosa|[2804]|TMNBD258-07|MNBTT-3059|Canada|New Brunswick|658[0n]|BOLD:ABY6578
Clostera strigosa|[2805]|TTMNB917-06|MNBTT-917|Canada|New Brunswick|658[0n]|BOLD:ABY6578
Clostera strigosa|[2806]|TTMNB916-06|MNBTT-916|Canada|New Brunswick|658[0n]|BOLD:ABY6578
Clostera n. sp. nr. apicalis|[2807]|BBLOE1407-12|BIOUG01987-A09|United States|California|658[0n]|...
Clostera n. sp. nr. apicalis|[2808]|BBLOE1230-12|BIOUG01985-B10|United States|California|642[0n]|...
Clostera n. sp. nr. apicalis|[2809]|GMLC605-11|2011GM-0301|United States|California|658[0n]|BOLD...
Clostera n. sp. nr. apicalis|[2810]|GMLC423-11|2011GM-0119|United States|California|658[0n]|BOLD...
Clostera n. sp. nr. apicalis|[2811]|GMLC698-11|2011GM-0394|United States|California|658[0n]|BOLD...
Clostera n. sp. nr. apicalis|[2812]|GMLC458-11|2011GM-0154|United States|California|658[0n]|BOLD...
Clostera n. sp. nr. apicalis|[2813]|BBLOE1408-12|BIOUG01987-A10|United States|California|658[0n]|...
Clostera n. sp. nr. apicalis|[2814]|GMLC916-12|2011GM-0612|United States|California|658[0n]|BOLD...
Clostera n. sp. nr. apicalis|[2815]|GMLC1199-12|2011GM-0895|United States|California|658[0n]|BOLD...
Clostera n. sp. nr. apicalis|[2816]|GMLC382-11|2011GM-0078|United States|California|658[0n]|BOLD...
Clostera n. sp. nr. apicalis|[2817]|GMLC1121-12|2011GM-0817|United States|California|658[0n]|BOLD...
Clostera n. sp. nr. apicalis|[2818]|LOCBD298-06|06-BLLOC-3118|United States|California|658[0n]|BO...
Clostera n. sp. nr. apicalis|[2819]|LOCB632-06|06-BLLOC-632|United States|California|658[0n]|BOLD...
Clostera n. sp. nr. apicalis|[2820]|LOCB671-06|06-BLLOC-671|United States|California|658[0n]|BOLD...
Clostera n. sp. nr. apicalis|[2821]|LOCBD550-06|06-BLLOC-3370|United States|California|658[0n]|BO...
Clostera n. sp. nr. apicalis|[2822]|LOCB637-06|06-BLLOC-637|United States|California|658[0n]|BOLD...
Clostera n. sp. nr. apicalis|[2823]|LOCB444-06|06-BLLOC-444|United States|California|658[0n]|BOLD...
Clostera n. sp. nr. apicalis|[2824]|LOCBB264-06|06-BLLOC-1204|United States|California|658[0n]|BO...
Clostera n. sp. nr. apicalis|[2825]|LOCBB575-06|06-BLLOC-1515|United States|California|658[0n]|BO...
Clostera n. sp. nr. apicalis|[2826]|LOCBB577-06|06-BLLOC-1517|United States|California|658[0n]|BO...
Clostera n. sp. nr. apicalis|[2827]|LOCBD299-06|06-BLLOC-3119|United States|California|658[0n]|BO...
Clostera n. sp. nr. apicalis|[2828]|LOCBB652-06|06-BLLOC-1592|United States|California|658[0n]|BO...
Clostera n. sp. nr. apicalis|[2829]|LOCBD602-06|06-BLLOC-3422|United States|California|658[0n]|BO...
Clostera n. sp. nr. apicalis|[2830]|LOCBB265-06|06-BLLOC-1205|United States|California|658[0n]|BO...
Clostera n. sp. nr. apicalis|[2831]|LOCBB266-06|06-BLLOC-1206|United States|California|658[0n]|BO...
Clostera n. sp. nr. apicalis|[2832]|LOCBB576-06|06-BLLOC-1516|United States|California|658[0n]|BO...
Clostera n. sp. nr. apicalis|[2833]|LOCBD897-06|06-BLLOC-3718|United States|California|658[0n]|BO...
Clostera n. sp. nr. apicalis|[2834]|LOCBE253-06|06-BLLOC-4072|United States|California|655[0n]|BO...
Clostera n. sp. nr. apicalis|[2835]|LOCBD213-06|06-BLLOC-3033|United States|California|658[0n]|BO...
Clostera n. sp. nr. apicalis|[2836]|LOCBD290-06|06-BLLOC-3110|United States|California|658[0n]|BO...
Clostera n. sp. nr. apicalis|[2837]|LOCBD567-06|06-BLLOC-3387|United States|California|658[0n]|BO...
Clostera n. sp. nr. apicalis|[2838]|LOCBD718-06|06-BLLOC-3538|United States|California|663[0n]|BO...
Clostera n. sp. nr. apicalis|[2839]|LOCBD209-06|06-BLLOC-3029|United States|California|636[0n]|BO...

Clostera n. sp. nr. apicalis|[2837]|LOCBD567-06|06-BLLOC-3387|United States|California|658[0n]|BO...
Clostera n. sp. nr. apicalis|[2838]|LOCBD718-06|06-BLLOC-3538|United States|California|663[0n]|BO...
Clostera n. sp. nr. apicalis|[2839]|LOCBD209-06|06-BLLOC-3029|United States|California|636[0n]|BO...
Clostera n. sp. nr. apicalis|[2840]|LOCB665-06|06-BLLOC-665|United States|California|658[0n]|BOLD...
Clostera n. sp. nr. apicalis|[2841]|LOCBB267-06|06-BLLOC-1207|United States|California|658[0n]|BO...
Clostera n. sp. nr. apicalis|[2842]|LOCBC456-06|06-BLLOC-2336|United States|California|658[0n]|BO...
Clostera n. sp. nr. apicalis|[2843]|LOCBE425-06|06-BLLOC-4244|United States|California|587[0n]|BO...
Clostera n. sp. nr. apicalis|[2844]|LOCBD552-06|06-BLLOC-3372|United States|California|658[0n]|BO...
Clostera n. sp. nr. apicalis|[2845]|LOCB666-06|06-BLLOC-666|United States|California|658[0n]|BOLD...
Clostera n. sp. nr. apicalis|[2846]|LOCBD546-06|06-BLLOC-3366|United States|California|658[0n]|BO...
Clostera n. sp. nr. apicalis|[2847]|LOCBC140-06|06-BLLOC-2020|United States|California|658[0n]|BO...
Clostera n. sp. nr. apicalis|[2848]|LOCBC454-06|06-BLLOC-2334|United States|California|658[0n]|BO...
Clostera n. sp. nr. apicalis|[2849]|LOCBC139-06|06-BLLOC-2019|United States|California|658[0n]|BO...
Clostera n. sp. nr. apicalis|[2850]|LTOL813-07|NB-06-0013D|United States|California|658[0n]|BOLD...
Clostera n. sp. nr. apicalis|[2851]|LOCBD658-06|06-BLLOC-3478|United States|California|658[0n]|BO...
Clostera n. sp. nr. apicalis|[2852]|LOCBE140-06|06-BLLOC-3959|United States|California|658[0n]|BO...
Clostera n. sp. nr. apicalis|[2853]|LOCBD288-06|06-BLLOC-3108|United States|California|658[0n]|BO...
Clostera n. sp. nr. apicalis|[2854]|LOCBD715-06|06-BLLOC-3535|United States|California|663[0n]|BO...
Clostera n. sp. nr. apicalis|[2855]|LOCBF018-13|BIOUG06016-B06|United States|California|593[0n]|B...
Clostera n. sp. nr. apicalis|[2856]|LTOL812-07|NB-06-0013|United States|California|658[0n]|BOLD ...
Clostera n. sp. nr. apicalis|[2857]|LOCBD468-06|06-BLLOC-3288|United States|California|657[0n]|BO...
Clostera n. sp. nr. apicalis|[2858]|LOCBF013-13|BIOUG06016-B01|United States|California|614[0n]|B...
Clostera n. sp. nr. apicalis|[2859]|LOCBF330-13|BIOUG06176-D09|United States|California|531[0n]|B...
Clostera n. sp. nr. apicalis|[2860]|LOCBF331-13|BIOUG06176-D10|United States|California|531[0n]|B...
Clostera n. sp. nr. apicalis|[2861]|LOCBF005-13|BIOUG06016-A05|United States|California|570[0n]|B...
Clostera apicalis|[2862]|LNCC1569-13|13-NCCC-239|United States|North Carolina|658[0n]|BOLD:AAA...
Clostera apicalis|[2863]|CNCLB2795-14|14-NCCC-553|United States|North Carolina|658[0n]|BOLD:AA...
Clostera apicalis|[2864]|RDNMH517-09|CNCLEP00057836|United States|California|658[0n]|BOLD:AAA4924
Clostera apicalis|[2865]|LOWCE374-06|CGWC-4134|Canada|British Columbia|658[0n]|BOLD:AAA4924
Clostera apicalis|[2866]|RDNMH170-09|CNCLEP00054387|United States|California|658[0n]|BOLD:AAA4924
Clostera apicalis|[2867]|LTOL811-07|RR-98-1201|United States|California|658[0n]|BOLD:AAA4924
Clostera apicalis|[2868]|RWWA293-09|RWWA-0293|United States|Washington|658[0n]|BOLD:AAA4924
Clostera apicalis|[2869]|JMMMB169-11|BIOUG00848-G02|United States|California|658[0n]|BOLD:AAA4924
Clostera apicalis|[2870]|RWWB688-10|RWWA-1687|United States|Washington|658[0n]|BOLD:AAA4924
Clostera apicalis|[2871]|RDNMH157-09|CNCLEP00054374|United States|California|658[0n]|BOLD:AAA4924
Clostera apicalis|[2872]|RWWA445-09|RWWA-0445|United States|Washington|658[0n]|BOLD:AAA4924
Clostera apicalis|[2873]|BBLPB253-10|10BBCLP-1252|Canada|Alberta|658[0n]|BOLD:AAA4924
Clostera apicalis|[2874]|BBLPB256-10|10BBCLP-1255|Canada|Alberta|658[0n]|BOLD:AAA4924
Clostera apicalis|[2875]|BBLPB254-10|10BBCLP-1253|Canada|Alberta|658[0n]|BOLD:AAA4924
Clostera apicalis|[2876]|BBLPB255-10|10BBCLP-1254|Canada|Alberta|658[0n]|BOLD:AAA4924
Clostera apicalis|[2877]|BBLPB252-10|10BBCLP-1251|Canada|Alberta|658[0n]|BOLD:AAA4924
Clostera apicalis|[2878]|LBCA308-05|HLC-20308|Canada|British Columbia|658[0n]|BOLD:AAA4924
Clostera apicalis|[2879]|LBCA548-05|HLC-20548|Canada|British Columbia|658[0n]|BOLD:AAA4924
Clostera apicalis|[2880]|LOWCE386-06|CGWC-4146|Canada|British Columbia|609[0n]|BOLD:AAA4924
Clostera apicalis|[2881]|LOWCB113-05|CGWC-1053|Canada|British Columbia|578[0n]|BOLD:AAA4924
Clostera apicalis|[2882]|LOWCE257-06|CGWC-4017|Canada|British Columbia|658[0n]|BOLD:AAA4924
Clostera apicalis|[2883]|RWWA272-09|RWWA-0272|United States|Washington|622[1n]|BOLD:AAA4924
Clostera apicalis|[2884]|RWWB869-10|RWWA-1868|United States|Washington|635[0n]|BOLD:AAA4924
Clostera apicalis|[2885]|CNCLA7371-13|CNCLEP00092617|Canada|Alberta|658[0n]|BOLD:AAA4924
Clostera apicalis|[2886]|RWWA379-09|RWWA-0379|United States|Washington|658[0n]|BOLD:AAA4924
Clostera apicalis|[2887]|RWWA249-09|RWWA-0249|United States|Washington|658[0n]|BOLD:AAA4924
Clostera apicalis|[2888]|LALPA306-10|AVBC 308-10|Canada|British Columbia|658[0n]|BOLD:AAA4924
Clostera apicalis|[2889]|XAG675-05|2005-ONT-1259|Canada|Ontario|658[0n]|BOLD:AAA4924
Clostera apicalis|[2890]|LALPA847-11|AVBC 1020-11|Canada|British Columbia|658[0n]|BOLD:AAA4924
Clostera apicalis|[2891]|LALPA248-10|AVBC 249-10|Canada|British Columbia|658[0n]|BOLD:AAA4924
Clostera apicalis|[2892]|LOWCB114-05|CGWC-1054|Canada|British Columbia|658[0n]|BOLD:AAA4924
Clostera apicalis|[2893]|LBCH5289-10|10-JDWBC-5289|Canada|British Columbia|658[0n]|BOLD:AAA4924
Clostera apicalis|[2894]|LALPA231-10|AVBC 232-10|Canada|British Columbia|658[0n]|BOLD:AAA4924
Clostera apicalis|[2895]|LOWCE343-06|CGWC-4103|Canada|British Columbia|658[0n]|BOLD:AAA4924
Clostera apicalis|[2896]|LALPA132-10|AVBC 132-10|Canada|British Columbia|658[0n]|BOLD:AAA4924
Clostera apicalis|[2897]|LPVIB845-08|PFC-2006-2352|Canada|British Columbia|658[0n]|BOLD:AAA4924
Clostera apicalis|[2898]|XAE173-04|Moth4173.03|Canada|Ontario|658[0n]|BOLD:AAA4924
Clostera apicalis|[2899]|LPSOB451-08|PPBP-1450|Canada|Ontario|658[0n]|BOLD:AAA4924
Clostera apicalis|[2900]|LPSOB239-08|PPBP-1238|Canada|Ontario|658[0n]|BOLD:AAA4924
Clostera apicalis|[2901]|XAB007-04|04HBL005007|Canada|Ontario|606[0n]|BOLD:AAA4924
Clostera apicalis|[2902]|TTMNB914-06|MNBTT-914|Canada|New Brunswick|658[0n]|BOLD:AAA4924
Clostera apicalis|[2903]|TMNBD259-07|MNBTT-3060|Canada|New Brunswick|658[0n]|BOLD:AAA4924
Clostera apicalis|[2904]|PHMNB496-04|04HBL00722|Canada|New Brunswick|658[0n]|BOLD:AAA4924
Clostera apicalis|[2905]|TMNBD260-07|MNBTT-3061|Canada|New Brunswick|658[0n]|BOLD:AAA4924
Clostera apicalis|[2906]|BLTIB825-08|BL1243|Canada|Ontario|658[0n]|BOLD:AAA4924
Clostera apicalis|[2907]|PMG172-03|moth284.01|Canada|Ontario|617[0n]|BOLD:AAA4924
Clostera apicalis|[2908]|XAF805-05|2005-ONT-454|Canada|Ontario|588[0n]|BOLD:AAA4924
Clostera apicalis|[2909]|TMG56-03|moth297.01|Canada|Ontario|639[0n]|BOLD:AAA4924
Clostera apicalis|[2910]|LBCG3204-09|08-JDWBC-3204|Canada|British Columbia|639[0n]|BOLD:AAA4924
Clostera apicalis|[2911]|TMG57-03|CLOS1.00|Canada|Ontario|639[0n]|BOLD:AAA4924
Clostera apicalis|[2912]|LBCG3220-09|08-JDWBC-3220|Canada|British Columbia|641[0n]|BOLD:AAA4924
Clostera apicalis|[2913]|XAG257-05|2005-ONT-841|Canada|Ontario|658[0n]|BOLD:AAA4924
Clostera apicalis|[2914]|RDLQ780-07|DH009490|Canada|Quebec|658[0n]|BOLD:AAA4924
Clostera apicalis|[2915]|XAG088-05|2005-ONT-672|Canada|Ontario|658[0n]|BOLD:AAA4924
Clostera apicalis|[2916]|XAG349-05|2005-ONT-933|Canada|Ontario|658[0n]|BOLD:AAA4924
Clostera apicalis|[2917]|BLTIB045-08|BL0075|Canada|Ontario|658[0n]|BOLD:AAA4924
Clostera apicalis|[2918]|LPSOB178-08|PPBP-1177|Canada|Ontario|658[0n]|BOLD:AAA4924
Clostera apicalis|[2919]|XAJ877-06|2006-ONT-0877|Canada|Ontario|658[0n]|BOLD:AAA4924
Clostera apicalis|[2920]|LPSOB349-08|PPBP-1348|Canada|Ontario|658[0n]|BOLD:AAA4924
Clostera apicalis|[2921]|XAG606-05|2005-ONT-1190|Canada|Ontario|658[0n]|BOLD:AAA4924
Clostera apicalis|[2922]|XAE296-04|Moth4296.03|Canada|Ontario|658[0n]|BOLD:AAA4924
Clostera apicalis|[2923]|BBLPB268-10|10BBCLP-1267|Canada|Ontario|658[0n]|BOLD:AAA4924
Clostera apicalis|[2924]|BBLPB259-10|10BBCLP-1258|Canada|Saskatchewan|658[0n]|BOLD:AAA4924
Clostera apicalis|[2925]|LPSOD380-09|08BBLEP-00159|Canada|Ontario|658[0n]|BOLD:AAA4924
Clostera apicalis|[2926]|LPSOB340-08|PPBP-1339|Canada|Ontario|658[0n]|BOLD:AAA4924
Clostera apicalis|[2927]|TMNBD261-07|MNBTT-3062|Canada|New Brunswick|658[0n]|BOLD:AAA4924
Clostera apicalis|[2928]|TMNBD262-07|MNBTT-3063|Canada|New Brunswick|658[0n]|BOLD:AAA4924
Clostera apicalis|[2929]|LPSOB453-08|PPBP-1452|Canada|Ontario|658[0n]|BOLD:AAA4924
Clostera apicalis|[2930]|LPMN732-08|08BBLEP-01535|Canada|Manitoba|658[0n]|BOLD:AAA4924
Clostera apicalis|[2931]|BLTIB841-08|BL1260|Canada|Ontario|658[0n]|BOLD:AAA4924
Clostera apicalis|[2932]|XAE116-04|Moth4116.03|Canada|Ontario|658[0n]|BOLD:AAA4924
Clostera apicalis|[2933]|BBLPB258-10|10BBCLP-1257|Canada|Saskatchewan|658[0n]|BOLD:AAA4924
Clostera apicalis|[2934]|BLTIB917-08|BL1337|Canada|Ontario|658[0n]|BOLD:AAA4924
Clostera apicalis|[2935]|LPSOB114-08|PPBP-1113|Canada|Ontario|655[0n]|BOLD:AAA4924
