# Supplementary material for: Probing planetary biodiversity with DNA barcodes: The Noctuoidea of North America
Source: PLoS One. 2017 Jun 1;12(6):e0178548. doi: 10.1371/journal.pone.0178548 (PMC5453547; doi:10.1371/journal.pone.0178548)
Supplement: S2 Tree — NJ tree based on sequence variation in the barcode region of the cytochrome c oxidase I gene for North American species in the family Euteliidae. (PDF) [file pone.0178548.s015.pdf]

BOLD TaxonID Tree

Title	:	Tree Result - Search (377 records)
Date	:	21-April-2016
Data Type	:	Nucleotide
Distance Model	:	Kimura 2 Parameter
Marker	:	COI-5P

Label	:	Process ID
Label	:	Taxon
Label	:	Country
Label	:	Province/State
Label	:	Sequence Length
Label	:	Barcode Cluster (BIN)

Sequence Count	:	377
Species count	:	19
Genus count	:	3
Family count	:	1
Unidentified	:	0

BIN Count	:	19

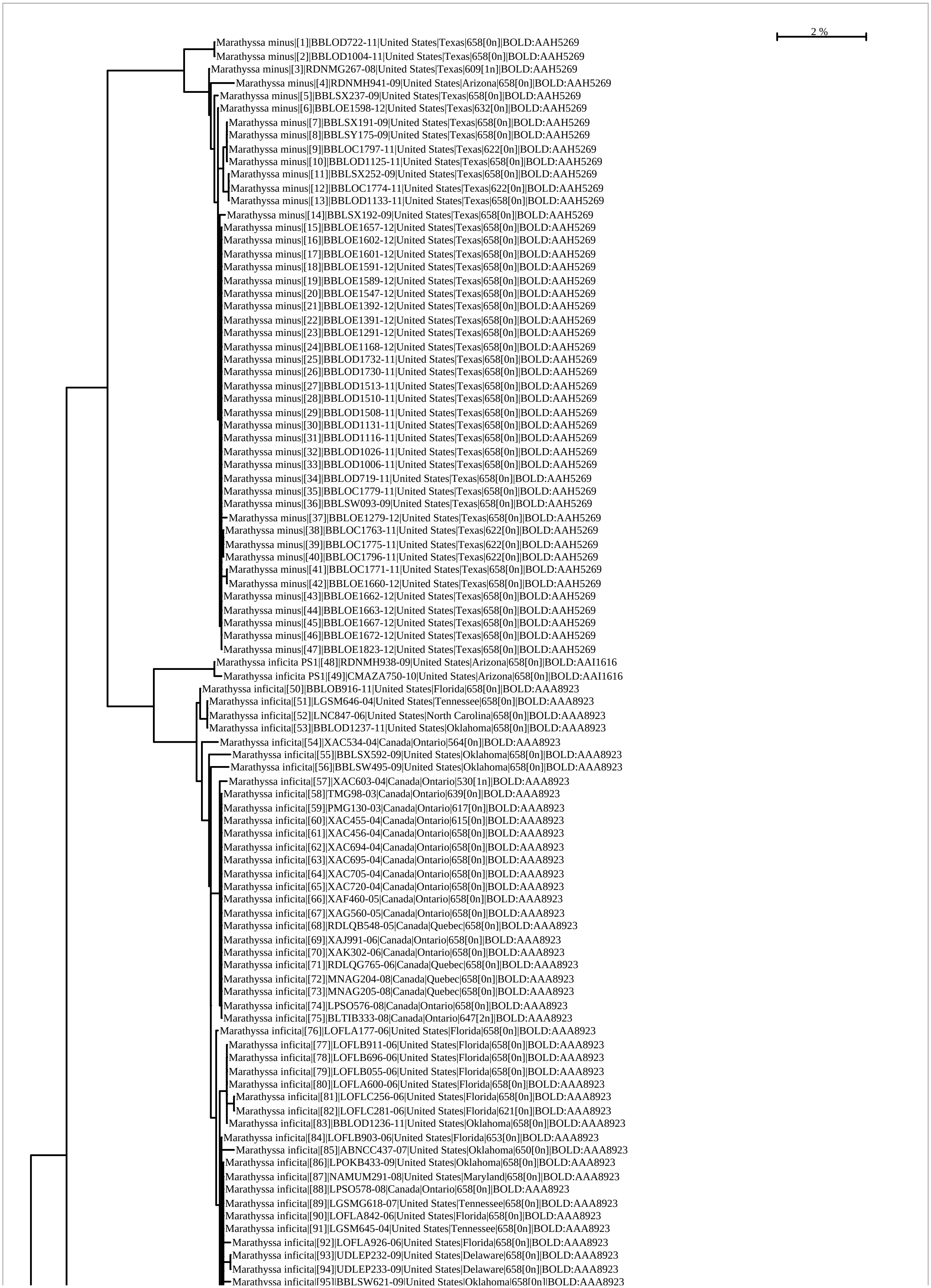
2 %
Marathyssa minus|[1]|BBLOD722-11|United States|Texas|658[0n]|BOLD:AAH5269
Marathyssa minus|[2]|BBLOD1004-11|United States|Texas|658[0n]|BOLD:AAH5269
Marathyssa minus|[3]|RDNMG267-08|United States|Texas|609[1n]|BOLD:AAH5269
Marathyssa minus|[4]|RDNMH941-09|United States|Arizona|658[0n]|BOLD:AAH5269
Marathyssa minus|[5]|BBLSX237-09|United States|Texas|658[0n]|BOLD:AAH5269
Marathyssa minus|[6]|BBLOE1598-12|United States|Texas|632[0n]|BOLD:AAH5269
Marathyssa minus|[7]|BBLSX191-09|United States|Texas|658[0n]|BOLD:AAH5269
Marathyssa minus|[8]|BBLSY175-09|United States|Texas|658[0n]|BOLD:AAH5269
Marathyssa minus|[9]|BBLOC1797-11|United States|Texas|622[0n]|BOLD:AAH5269
Marathyssa minus|[10]|BBLOD1125-11|United States|Texas|658[0n]|BOLD:AAH5269
Marathyssa minus|[11]|BBLSX252-09|United States|Texas|658[0n]|BOLD:AAH5269
Marathyssa minus|[12]|BBLOC1774-11|United States|Texas|622[0n]|BOLD:AAH5269
Marathyssa minus|[13]|BBLOD1133-11|United States|Texas|658[0n]|BOLD:AAH5269
Marathyssa minus|[14]|BBLSX192-09|United States|Texas|658[0n]|BOLD:AAH5269
Marathyssa minus|[15]|BBLOE1657-12|United States|Texas|658[0n]|BOLD:AAH5269
Marathyssa minus|[16]|BBLOE1602-12|United States|Texas|658[0n]|BOLD:AAH5269
Marathyssa minus|[17]|BBLOE1601-12|United States|Texas|658[0n]|BOLD:AAH5269
Marathyssa minus|[18]|BBLOE1591-12|United States|Texas|658[0n]|BOLD:AAH5269
Marathyssa minus|[19]|BBLOE1589-12|United States|Texas|658[0n]|BOLD:AAH5269
Marathyssa minus|[20]|BBLOE1547-12|United States|Texas|658[0n]|BOLD:AAH5269
Marathyssa minus|[21]|BBLOE1392-12|United States|Texas|658[0n]|BOLD:AAH5269
Marathyssa minus|[22]|BBLOE1391-12|United States|Texas|658[0n]|BOLD:AAH5269
Marathyssa minus|[23]|BBLOE1291-12|United States|Texas|658[0n]|BOLD:AAH5269
Marathyssa minus|[24]|BBLOE1168-12|United States|Texas|658[0n]|BOLD:AAH5269
Marathyssa minus|[25]|BBLOD1732-11|United States|Texas|658[0n]|BOLD:AAH5269
Marathyssa minus|[26]|BBLOD1730-11|United States|Texas|658[0n]|BOLD:AAH5269
Marathyssa minus|[27]|BBLOD1513-11|United States|Texas|658[0n]|BOLD:AAH5269
Marathyssa minus|[28]|BBLOD1510-11|United States|Texas|658[0n]|BOLD:AAH5269
Marathyssa minus|[29]|BBLOD1508-11|United States|Texas|658[0n]|BOLD:AAH5269
Marathyssa minus|[30]|BBLOD1131-11|United States|Texas|658[0n]|BOLD:AAH5269
Marathyssa minus|[31]|BBLOD1116-11|United States|Texas|658[0n]|BOLD:AAH5269
Marathyssa minus|[32]|BBLOD1026-11|United States|Texas|658[0n]|BOLD:AAH5269
Marathyssa minus|[33]|BBLOD1006-11|United States|Texas|658[0n]|BOLD:AAH5269
Marathyssa minus|[34]|BBLOD719-11|United States|Texas|658[0n]|BOLD:AAH5269
Marathyssa minus|[35]|BBLOC1779-11|United States|Texas|658[0n]|BOLD:AAH5269
Marathyssa minus|[36]|BBLSW093-09|United States|Texas|658[0n]|BOLD:AAH5269
Marathyssa minus|[37]|BBLOE1279-12|United States|Texas|658[0n]|BOLD:AAH5269
Marathyssa minus|[38]|BBLOC1763-11|United States|Texas|622[0n]|BOLD:AAH5269
Marathyssa minus|[39]|BBLOC1775-11|United States|Texas|622[0n]|BOLD:AAH5269
Marathyssa minus|[40]|BBLOC1796-11|United States|Texas|622[0n]|BOLD:AAH5269
Marathyssa minus|[41]|BBLOC1771-11|United States|Texas|658[0n]|BOLD:AAH5269
Marathyssa minus|[42]|BBLOE1660-12|United States|Texas|658[0n]|BOLD:AAH5269
Marathyssa minus|[43]|BBLOE1662-12|United States|Texas|658[0n]|BOLD:AAH5269
Marathyssa minus|[44]|BBLOE1663-12|United States|Texas|658[0n]|BOLD:AAH5269
Marathyssa minus|[45]|BBLOE1667-12|United States|Texas|658[0n]|BOLD:AAH5269
Marathyssa minus|[46]|BBLOE1672-12|United States|Texas|658[0n]|BOLD:AAH5269
Marathyssa minus|[47]|BBLOE1823-12|United States|Texas|658[0n]|BOLD:AAH5269
Marathyssa inficita PS1|[48]|RDNMH938-09|United States|Arizona|658[0n]|BOLD:AAI1616
Marathyssa inficita PS1|[49]|CMAZA750-10|United States|Arizona|658[0n]|BOLD:AAI1616
Marathyssa inficita|[50]|BBLOB916-11|United States|Florida|658[0n]|BOLD:AAA8923
Marathyssa inficita|[51]|LGSM646-04|United States|Tennessee|658[0n]|BOLD:AAA8923
Marathyssa inficita|[52]|LNC847-06|United States|North Carolina|658[0n]|BOLD:AAA8923
Marathyssa inficita|[53]|BBLOD1237-11|United States|Oklahoma|658[0n]|BOLD:AAA8923
Marathyssa inficita|[54]|XAC534-04|Canada|Ontario|564[0n]|BOLD:AAA8923
Marathyssa inficita|[55]|BBLSX592-09|United States|Oklahoma|658[0n]|BOLD:AAA8923
Marathyssa inficita|[56]|BBLSW495-09|United States|Oklahoma|658[0n]|BOLD:AAA8923
Marathyssa inficita|[57]|XAC603-04|Canada|Ontario|530[1n]|BOLD:AAA8923
Marathyssa inficita|[58]|TMG98-03|Canada|Ontario|639[0n]|BOLD:AAA8923
Marathyssa inficita|[59]|PMG130-03|Canada|Ontario|617[0n]|BOLD:AAA8923
Marathyssa inficita|[60]|XAC455-04|Canada|Ontario|615[0n]|BOLD:AAA8923
Marathyssa inficita|[61]|XAC456-04|Canada|Ontario|658[0n]|BOLD:AAA8923
Marathyssa inficita|[62]|XAC694-04|Canada|Ontario|658[0n]|BOLD:AAA8923
Marathyssa inficita|[63]|XAC695-04|Canada|Ontario|658[0n]|BOLD:AAA8923
Marathyssa inficita|[64]|XAC705-04|Canada|Ontario|658[0n]|BOLD:AAA8923
Marathyssa inficita|[65]|XAC720-04|Canada|Ontario|658[0n]|BOLD:AAA8923
Marathyssa inficita|[66]|XAF460-05|Canada|Ontario|658[0n]|BOLD:AAA8923
Marathyssa inficita|[67]|XAG560-05|Canada|Ontario|658[0n]|BOLD:AAA8923
Marathyssa inficita|[68]|RDLQB548-05|Canada|Quebec|658[0n]|BOLD:AAA8923
Marathyssa inficita|[69]|XAJ991-06|Canada|Ontario|658[0n]|BOLD:AAA8923
Marathyssa inficita|[70]|XAK302-06|Canada|Ontario|658[0n]|BOLD:AAA8923
Marathyssa inficita|[71]|RDLQG765-06|Canada|Quebec|658[0n]|BOLD:AAA8923
Marathyssa inficita|[72]|MNAG204-08|Canada|Quebec|658[0n]|BOLD:AAA8923
Marathyssa inficita|[73]|MNAG205-08|Canada|Quebec|658[0n]|BOLD:AAA8923
Marathyssa inficita|[74]|LPSO576-08|Canada|Ontario|658[0n]|BOLD:AAA8923
Marathyssa inficita|[75]|BLTIB333-08|Canada|Ontario|647[2n]|BOLD:AAA8923
Marathyssa inficita|[76]|LOFLA177-06|United States|Florida|658[0n]|BOLD:AAA8923
Marathyssa inficita|[77]|LOFLB911-06|United States|Florida|658[0n]|BOLD:AAA8923
Marathyssa inficita|[78]|LOFLB696-06|United States|Florida|658[0n]|BOLD:AAA8923
Marathyssa inficita|[79]|LOFLB055-06|United States|Florida|658[0n]|BOLD:AAA8923
Marathyssa inficita|[80]|LOFLA600-06|United States|Florida|658[0n]|BOLD:AAA8923
Marathyssa inficita|[81]|LOFLC256-06|United States|Florida|658[0n]|BOLD:AAA8923
Marathyssa inficita|[82]|LOFLC281-06|United States|Florida|621[0n]|BOLD:AAA8923
Marathyssa inficita|[83]|BBLOD1236-11|United States|Oklahoma|658[0n]|BOLD:AAA8923
Marathyssa inficita|[84]|LOFLB903-06|United States|Florida|653[0n]|BOLD:AAA8923
Marathyssa inficita|[85]|ABNCC437-07|United States|Oklahoma|650[0n]|BOLD:AAA8923
Marathyssa inficita|[86]|LPOKB433-09|United States|Oklahoma|658[0n]|BOLD:AAA8923
Marathyssa inficita|[87]|NAMUM291-08|United States|Maryland|658[0n]|BOLD:AAA8923
Marathyssa inficita|[88]|LPSO578-08|Canada|Ontario|658[0n]|BOLD:AAA8923
Marathyssa inficita|[89]|LGSMG618-07|United States|Tennessee|658[0n]|BOLD:AAA8923
Marathyssa inficita|[90]|LOFLA842-06|United States|Florida|658[0n]|BOLD:AAA8923
Marathyssa inficita|[91]|LGSM645-04|United States|Tennessee|658[0n]|BOLD:AAA8923
Marathyssa inficita|[92]|LOFLA926-06|United States|Florida|658[0n]|BOLD:AAA8923
Marathyssa inficita|[93]|UDLEP232-09|United States|Delaware|658[0n]|BOLD:AAA8923
Marathyssa inficita|[94]|UDLEP233-09|United States|Delaware|658[0n]|BOLD:AAA8923
Marathyssa inficita|[95]|BBLSW621-09|United States|Oklahoma|658[0n]|BOLD:AAA8923

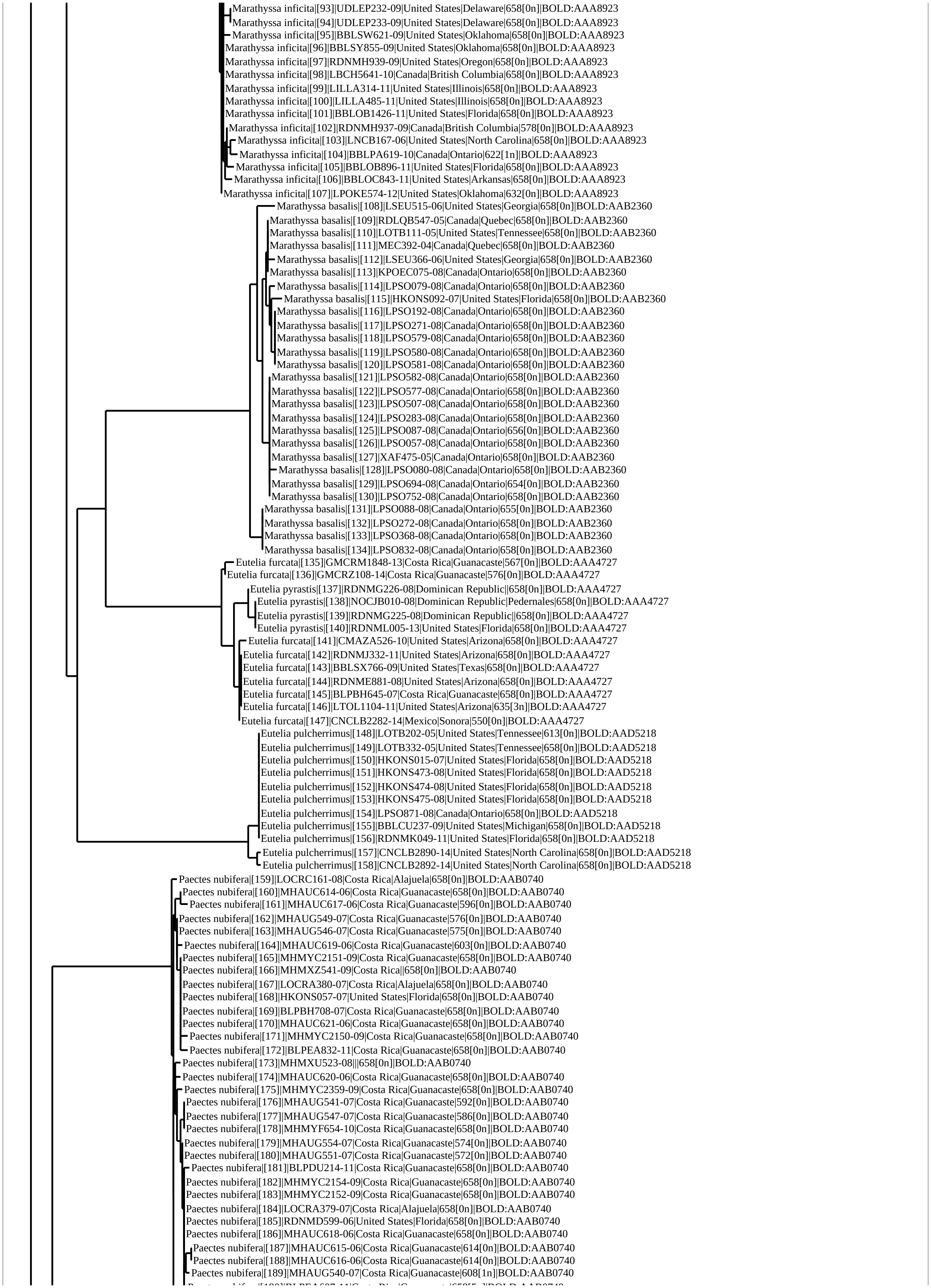
Marathyssa inficita|[93]|UDLEP232-09|United States|Delaware|658[0n]|BOLD:AAA8923
Marathyssa inficita|[94]|UDLEP233-09|United States|Delaware|658[0n]|BOLD:AAA8923
Marathyssa inficita|[95]|BBLSW621-09|United States|Oklahoma|658[0n]|BOLD:AAA8923
Marathyssa inficita|[96]|BBLSY855-09|United States|Oklahoma|658[0n]|BOLD:AAA8923
Marathyssa inficita|[97]|RDNMH939-09|United States|Oregon|658[0n]|BOLD:AAA8923
Marathyssa inficita|[98]|LBCH5641-10|Canada|British Columbia|658[0n]|BOLD:AAA8923
Marathyssa inficita|[99]|LILLA314-11|United States|Illinois|658[0n]|BOLD:AAA8923
Marathyssa inficita|[100]|LILLA485-11|United States|Illinois|658[0n]|BOLD:AAA8923
Marathyssa inficita|[101]|BBLOB1426-11|United States|Florida|658[0n]|BOLD:AAA8923
Marathyssa inficita|[102]|RDNMH937-09|Canada|British Columbia|578[0n]|BOLD:AAA8923
Marathyssa inficita|[103]|LNCB167-06|United States|North Carolina|658[0n]|BOLD:AAA8923
Marathyssa inficita|[104]|BBLPA619-10|Canada|Ontario|622[1n]|BOLD:AAA8923
Marathyssa inficita|[105]|BBLOB896-11|United States|Florida|658[0n]|BOLD:AAA8923
Marathyssa inficita|[106]|BBLOC843-11|United States|Arkansas|658[0n]|BOLD:AAA8923
Marathyssa inficita|[107]|LPOKE574-12|United States|Oklahoma|632[0n]|BOLD:AAA8923
Marathyssa basalis|[108]|LSEU515-06|United States|Georgia|658[0n]|BOLD:AAB2360
Marathyssa basalis|[109]|RDLQB547-05|Canada|Quebec|658[0n]|BOLD:AAB2360
Marathyssa basalis|[110]|LOTB111-05|United States|Tennessee|658[0n]|BOLD:AAB2360
Marathyssa basalis|[111]|MEC392-04|Canada|Quebec|658[0n]|BOLD:AAB2360
Marathyssa basalis|[112]|LSEU366-06|United States|Georgia|658[0n]|BOLD:AAB2360
Marathyssa basalis|[113]|KPOEC075-08|Canada|Ontario|658[0n]|BOLD:AAB2360
Marathyssa basalis|[114]|LPSO079-08|Canada|Ontario|658[0n]|BOLD:AAB2360
Marathyssa basalis|[115]|HKONS092-07|United States|Florida|658[0n]|BOLD:AAB2360
Marathyssa basalis|[116]|LPSO192-08|Canada|Ontario|658[0n]|BOLD:AAB2360
Marathyssa basalis|[117]|LPSO271-08|Canada|Ontario|658[0n]|BOLD:AAB2360
Marathyssa basalis|[118]|LPSO579-08|Canada|Ontario|658[0n]|BOLD:AAB2360
Marathyssa basalis|[119]|LPSO580-08|Canada|Ontario|658[0n]|BOLD:AAB2360
Marathyssa basalis|[120]|LPSO581-08|Canada|Ontario|658[0n]|BOLD:AAB2360
Marathyssa basalis|[121]|LPSO582-08|Canada|Ontario|658[0n]|BOLD:AAB2360
Marathyssa basalis|[122]|LPSO577-08|Canada|Ontario|658[0n]|BOLD:AAB2360
Marathyssa basalis|[123]|LPSO507-08|Canada|Ontario|658[0n]|BOLD:AAB2360
Marathyssa basalis|[124]|LPSO283-08|Canada|Ontario|658[0n]|BOLD:AAB2360
Marathyssa basalis|[125]|LPSO087-08|Canada|Ontario|656[0n]|BOLD:AAB2360
Marathyssa basalis|[126]|LPSO057-08|Canada|Ontario|658[0n]|BOLD:AAB2360
Marathyssa basalis|[127]|XAF475-05|Canada|Ontario|658[0n]|BOLD:AAB2360
Marathyssa basalis|[128]|LPSO080-08|Canada|Ontario|658[0n]|BOLD:AAB2360
Marathyssa basalis|[129]|LPSO694-08|Canada|Ontario|654[0n]|BOLD:AAB2360
Marathyssa basalis|[130]|LPSO752-08|Canada|Ontario|658[0n]|BOLD:AAB2360
Marathyssa basalis|[131]|LPSO088-08|Canada|Ontario|655[0n]|BOLD:AAB2360
Marathyssa basalis|[132]|LPSO272-08|Canada|Ontario|658[0n]|BOLD:AAB2360
Marathyssa basalis|[133]|LPSO368-08|Canada|Ontario|658[0n]|BOLD:AAB2360
Marathyssa basalis|[134]|LPSO832-08|Canada|Ontario|658[0n]|BOLD:AAB2360
Eutelia furcata|[135]|GMCRM1848-13|Costa Rica|Guanacaste|567[0n]|BOLD:AAA4727
Eutelia furcata|[136]|GMCRZ108-14|Costa Rica|Guanacaste|576[0n]|BOLD:AAA4727
Eutelia pyrastis|[137]|RDNMG226-08|Dominican Republic||658[0n]|BOLD:AAA4727
Eutelia pyrastis|[138]|NOCJB010-08|Dominican Republic|Pedernales|658[0n]|BOLD:AAA4727
Eutelia pyrastis|[139]|RDNMG225-08|Dominican Republic||658[0n]|BOLD:AAA4727
Eutelia pyrastis|[140]|RDNML005-13|United States|Florida|658[0n]|BOLD:AAA4727
Eutelia furcata|[141]|CMAZA526-10|United States|Arizona|658[0n]|BOLD:AAA4727
Eutelia furcata|[142]|RDNMJ332-11|United States|Arizona|658[0n]|BOLD:AAA4727
Eutelia furcata|[143]|BBLSX766-09|United States|Texas|658[0n]|BOLD:AAA4727
Eutelia furcata|[144]|RDNME881-08|United States|Arizona|658[0n]|BOLD:AAA4727
Eutelia furcata|[145]|BLPBH645-07|Costa Rica|Guanacaste|658[0n]|BOLD:AAA4727
Eutelia furcata|[146]|LTOL1104-11|United States|Arizona|635[3n]|BOLD:AAA4727
Eutelia furcata|[147]|CNCLB2282-14|Mexico|Sonora|550[0n]|BOLD:AAA4727
Eutelia pulcherrimus|[148]|LOTB202-05|United States|Tennessee|613[0n]|BOLD:AAD5218
Eutelia pulcherrimus|[149]|LOTB332-05|United States|Tennessee|658[0n]|BOLD:AAD5218
Eutelia pulcherrimus|[150]|HKONS015-07|United States|Florida|658[0n]|BOLD:AAD5218
Eutelia pulcherrimus|[151]|HKONS473-08|United States|Florida|658[0n]|BOLD:AAD5218
Eutelia pulcherrimus|[152]|HKONS474-08|United States|Florida|658[0n]|BOLD:AAD5218
Eutelia pulcherrimus|[153]|HKONS475-08|United States|Florida|658[0n]|BOLD:AAD5218
Eutelia pulcherrimus|[154]|LPSO871-08|Canada|Ontario|658[0n]|BOLD:AAD5218
Eutelia pulcherrimus|[155]|BBLCU237-09|United States|Michigan|658[0n]|BOLD:AAD5218
Eutelia pulcherrimus|[156]|RDNMK049-11|United States|Florida|658[0n]|BOLD:AAD5218
Eutelia pulcherrimus|[157]|CNCLB2890-14|United States|North Carolina|658[0n]|BOLD:AAD5218
Eutelia pulcherrimus|[158]|CNCLB2892-14|United States|North Carolina|658[0n]|BOLD:AAD5218
Paectes nubifera|[159]|LOCRC161-08|Costa Rica|Alajuela|658[0n]|BOLD:AAB0740
Paectes nubifera|[160]|MHAUC614-06|Costa Rica|Guanacaste|658[0n]|BOLD:AAB0740
Paectes nubifera|[161]|MHAUC617-06|Costa Rica|Guanacaste|596[0n]|BOLD:AAB0740
Paectes nubifera|[162]|MHAUG549-07|Costa Rica|Guanacaste|576[0n]|BOLD:AAB0740
Paectes nubifera|[163]|MHAUG546-07|Costa Rica|Guanacaste|575[0n]|BOLD:AAB0740
Paectes nubifera|[164]|MHAUC619-06|Costa Rica|Guanacaste|603[0n]|BOLD:AAB0740
Paectes nubifera|[165]|MHMYC2151-09|Costa Rica|Guanacaste|658[0n]|BOLD:AAB0740
Paectes nubifera|[166]|MHMXZ541-09|Costa Rica||658[0n]|BOLD:AAB0740
Paectes nubifera|[167]|LOCRA380-07|Costa Rica|Alajuela|658[0n]|BOLD:AAB0740
Paectes nubifera|[168]|HKONS057-07|United States|Florida|658[0n]|BOLD:AAB0740
Paectes nubifera|[169]|BLPBH708-07|Costa Rica|Guanacaste|658[0n]|BOLD:AAB0740
Paectes nubifera|[170]|MHAUC621-06|Costa Rica|Guanacaste|658[0n]|BOLD:AAB0740
Paectes nubifera|[171]|MHMYC2150-09|Costa Rica|Guanacaste|658[0n]|BOLD:AAB0740
Paectes nubifera|[172]|BLPEA832-11|Costa Rica|Guanacaste|658[0n]|BOLD:AAB0740
Paectes nubifera|[173]|MHMXU523-08|||658[0n]|BOLD:AAB0740
Paectes nubifera|[174]|MHAUC620-06|Costa Rica|Guanacaste|658[0n]|BOLD:AAB0740
Paectes nubifera|[175]|MHMYC2359-09|Costa Rica|Guanacaste|658[0n]|BOLD:AAB0740
Paectes nubifera|[176]|MHAUG541-07|Costa Rica|Guanacaste|592[0n]|BOLD:AAB0740
Paectes nubifera|[177]|MHAUG547-07|Costa Rica|Guanacaste|586[0n]|BOLD:AAB0740
Paectes nubifera|[178]|MHMYF654-10|Costa Rica|Guanacaste|658[0n]|BOLD:AAB0740
Paectes nubifera|[179]|MHAUG554-07|Costa Rica|Guanacaste|574[0n]|BOLD:AAB0740
Paectes nubifera|[180]|MHAUG551-07|Costa Rica|Guanacaste|572[0n]|BOLD:AAB0740
Paectes nubifera|[181]|BLPDU214-11|Costa Rica|Guanacaste|658[0n]|BOLD:AAB0740
Paectes nubifera|[182]|MHMYC2154-09|Costa Rica|Guanacaste|658[0n]|BOLD:AAB0740
Paectes nubifera|[183]|MHMYC2152-09|Costa Rica|Guanacaste|658[0n]|BOLD:AAB0740
Paectes nubifera|[184]|LOCRA379-07|Costa Rica|Alajuela|658[0n]|BOLD:AAB0740
Paectes nubifera|[185]|RDNMD599-06|United States|Florida|658[0n]|BOLD:AAB0740
Paectes nubifera|[186]|MHAUC618-06|Costa Rica|Guanacaste|658[0n]|BOLD:AAB0740
Paectes nubifera|[187]|MHAUC615-06|Costa Rica|Guanacaste|614[0n]|BOLD:AAB0740
Paectes nubifera|[188]|MHAUC616-06|Costa Rica|Guanacaste|614[0n]|BOLD:AAB0740
Paectes nubifera|[189]|MHAUG540-07|Costa Rica|Guanacaste|608[1n]|BOLD:AAB0740

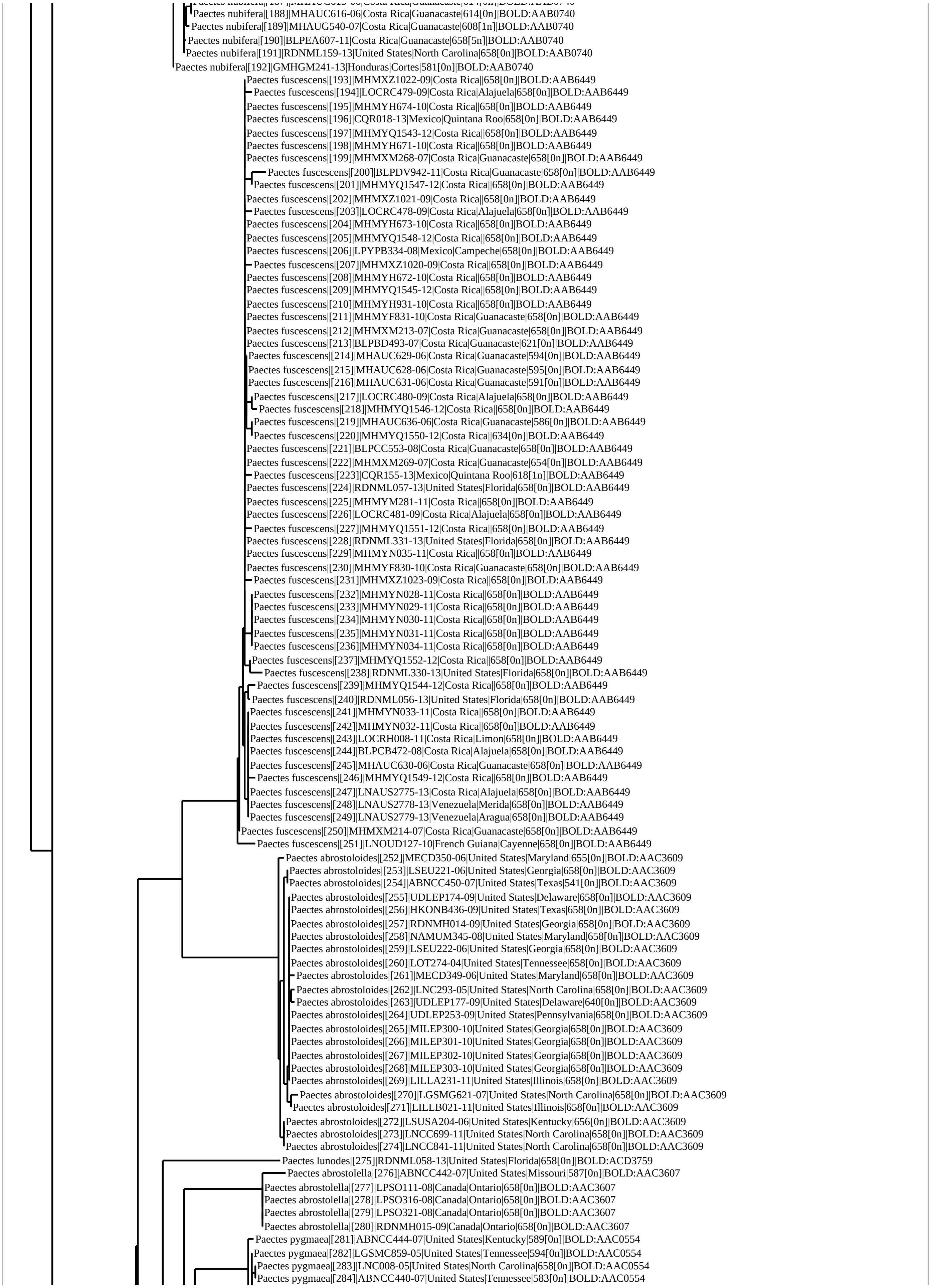
Paectes nubifera|[188]|MHAUC616-06|Costa Rica|Guanacaste|614[0n]|BOLD:AAB0740
Paectes nubifera|[189]|MHAUG540-07|Costa Rica|Guanacaste|608[1n]|BOLD:AAB0740
Paectes nubifera|[190]|BLPEA607-11|Costa Rica|Guanacaste|658[5n]|BOLD:AAB0740
Paectes nubifera|[191]|RDNML159-13|United States|North Carolina|658[0n]|BOLD:AAB0740
Paectes nubifera|[192]|GMHGM241-13|Honduras|Cortes|581[0n]|BOLD:AAB0740
Paectes fuscescens|[193]|MHMXZ1022-09|Costa Rica||658[0n]|BOLD:AAB6449
Paectes fuscescens|[194]|LOCRC479-09|Costa Rica|Alajuela|658[0n]|BOLD:AAB6449
Paectes fuscescens|[195]|MHMYH674-10|Costa Rica||658[0n]|BOLD:AAB6449
Paectes fuscescens|[196]|CQR018-13|Mexico|Quintana Roo|658[0n]|BOLD:AAB6449
Paectes fuscescens|[197]|MHMYQ1543-12|Costa Rica||658[0n]|BOLD:AAB6449
Paectes fuscescens|[198]|MHMYH671-10|Costa Rica||658[0n]|BOLD:AAB6449
Paectes fuscescens|[199]|MHMXM268-07|Costa Rica|Guanacaste|658[0n]|BOLD:AAB6449
Paectes fuscescens|[200]|BLPDV942-11|Costa Rica|Guanacaste|658[0n]|BOLD:AAB6449
Paectes fuscescens|[201]|MHMYQ1547-12|Costa Rica||658[0n]|BOLD:AAB6449
Paectes fuscescens|[202]|MHMXZ1021-09|Costa Rica||658[0n]|BOLD:AAB6449
Paectes fuscescens|[203]|LOCRC478-09|Costa Rica|Alajuela|658[0n]|BOLD:AAB6449
Paectes fuscescens|[204]|MHMYH673-10|Costa Rica||658[0n]|BOLD:AAB6449
Paectes fuscescens|[205]|MHMYQ1548-12|Costa Rica||658[0n]|BOLD:AAB6449
Paectes fuscescens|[206]|LPYPB334-08|Mexico|Campeche|658[0n]|BOLD:AAB6449
Paectes fuscescens|[207]|MHMXZ1020-09|Costa Rica||658[0n]|BOLD:AAB6449
Paectes fuscescens|[208]|MHMYH672-10|Costa Rica||658[0n]|BOLD:AAB6449
Paectes fuscescens|[209]|MHMYQ1545-12|Costa Rica||658[0n]|BOLD:AAB6449
Paectes fuscescens|[210]|MHMYH931-10|Costa Rica||658[0n]|BOLD:AAB6449
Paectes fuscescens|[211]|MHMYF831-10|Costa Rica|Guanacaste|658[0n]|BOLD:AAB6449
Paectes fuscescens|[212]|MHMXM213-07|Costa Rica|Guanacaste|658[0n]|BOLD:AAB6449
Paectes fuscescens|[213]|BLPBD493-07|Costa Rica|Guanacaste|621[0n]|BOLD:AAB6449
Paectes fuscescens|[214]|MHAUC629-06|Costa Rica|Guanacaste|594[0n]|BOLD:AAB6449
Paectes fuscescens|[215]|MHAUC628-06|Costa Rica|Guanacaste|595[0n]|BOLD:AAB6449
Paectes fuscescens|[216]|MHAUC631-06|Costa Rica|Guanacaste|591[0n]|BOLD:AAB6449
Paectes fuscescens|[217]|LOCRC480-09|Costa Rica|Alajuela|658[0n]|BOLD:AAB6449
Paectes fuscescens|[218]|MHMYQ1546-12|Costa Rica||658[0n]|BOLD:AAB6449
Paectes fuscescens|[219]|MHAUC636-06|Costa Rica|Guanacaste|586[0n]|BOLD:AAB6449
Paectes fuscescens|[220]|MHMYQ1550-12|Costa Rica||634[0n]|BOLD:AAB6449
Paectes fuscescens|[221]|BLPCC553-08|Costa Rica|Guanacaste|658[0n]|BOLD:AAB6449
Paectes fuscescens|[222]|MHMXM269-07|Costa Rica|Guanacaste|654[0n]|BOLD:AAB6449
Paectes fuscescens|[223]|CQR155-13|Mexico|Quintana Roo|618[1n]|BOLD:AAB6449
Paectes fuscescens|[224]|RDNML057-13|United States|Florida|658[0n]|BOLD:AAB6449
Paectes fuscescens|[225]|MHMYM281-11|Costa Rica||658[0n]|BOLD:AAB6449
Paectes fuscescens|[226]|LOCRC481-09|Costa Rica|Alajuela|658[0n]|BOLD:AAB6449
Paectes fuscescens|[227]|MHMYQ1551-12|Costa Rica||658[0n]|BOLD:AAB6449
Paectes fuscescens|[228]|RDNML331-13|United States|Florida|658[0n]|BOLD:AAB6449
Paectes fuscescens|[229]|MHMYN035-11|Costa Rica||658[0n]|BOLD:AAB6449
Paectes fuscescens|[230]|MHMYF830-10|Costa Rica|Guanacaste|658[0n]|BOLD:AAB6449
Paectes fuscescens|[231]|MHMXZ1023-09|Costa Rica||658[0n]|BOLD:AAB6449
Paectes fuscescens|[232]|MHMYN028-11|Costa Rica||658[0n]|BOLD:AAB6449
Paectes fuscescens|[233]|MHMYN029-11|Costa Rica||658[0n]|BOLD:AAB6449
Paectes fuscescens|[234]|MHMYN030-11|Costa Rica||658[0n]|BOLD:AAB6449
Paectes fuscescens|[235]|MHMYN031-11|Costa Rica||658[0n]|BOLD:AAB6449
Paectes fuscescens|[236]|MHMYN034-11|Costa Rica||658[0n]|BOLD:AAB6449
Paectes fuscescens|[237]|MHMYQ1552-12|Costa Rica||658[0n]|BOLD:AAB6449
Paectes fuscescens|[238]|RDNML330-13|United States|Florida|658[0n]|BOLD:AAB6449
Paectes fuscescens|[239]|MHMYQ1544-12|Costa Rica||658[0n]|BOLD:AAB6449
Paectes fuscescens|[240]|RDNML056-13|United States|Florida|658[0n]|BOLD:AAB6449
Paectes fuscescens|[241]|MHMYN033-11|Costa Rica||658[0n]|BOLD:AAB6449
Paectes fuscescens|[242]|MHMYN032-11|Costa Rica||658[0n]|BOLD:AAB6449
Paectes fuscescens|[243]|LOCRH008-11|Costa Rica|Limon|658[0n]|BOLD:AAB6449
Paectes fuscescens|[244]|BLPCB472-08|Costa Rica|Alajuela|658[0n]|BOLD:AAB6449
Paectes fuscescens|[245]|MHAUC630-06|Costa Rica|Guanacaste|658[0n]|BOLD:AAB6449
Paectes fuscescens|[246]|MHMYQ1549-12|Costa Rica||658[0n]|BOLD:AAB6449
Paectes fuscescens|[247]|LNAUS2775-13|Costa Rica|Alajuela|658[0n]|BOLD:AAB6449
Paectes fuscescens|[248]|LNAUS2778-13|Venezuela|Merida|658[0n]|BOLD:AAB6449
Paectes fuscescens|[249]|LNAUS2779-13|Venezuela|Aragua|658[0n]|BOLD:AAB6449
Paectes fuscescens|[250]|MHMXM214-07|Costa Rica|Guanacaste|658[0n]|BOLD:AAB6449
Paectes fuscescens|[251]|LNOUD127-10|French Guiana|Cayenne|658[0n]|BOLD:AAB6449
Paectes abrostoloides|[252]|MECD350-06|United States|Maryland|655[0n]|BOLD:AAC3609
Paectes abrostoloides|[253]|LSEU221-06|United States|Georgia|658[0n]|BOLD:AAC3609
Paectes abrostoloides|[254]|ABNCC450-07|United States|Texas|541[0n]|BOLD:AAC3609
Paectes abrostoloides|[255]|UDLEP174-09|United States|Delaware|658[0n]|BOLD:AAC3609
Paectes abrostoloides|[256]|HKONB436-09|United States|Texas|658[0n]|BOLD:AAC3609
Paectes abrostoloides|[257]|RDNMH014-09|United States|Georgia|658[0n]|BOLD:AAC3609
Paectes abrostoloides|[258]|NAMUM345-08|United States|Maryland|658[0n]|BOLD:AAC3609
Paectes abrostoloides|[259]|LSEU222-06|United States|Georgia|658[0n]|BOLD:AAC3609
Paectes abrostoloides|[260]|LOT274-04|United States|Tennessee|658[0n]|BOLD:AAC3609
Paectes abrostoloides|[261]|MECD349-06|United States|Maryland|658[0n]|BOLD:AAC3609
Paectes abrostoloides|[262]|LNC293-05|United States|North Carolina|658[0n]|BOLD:AAC3609
Paectes abrostoloides|[263]|UDLEP177-09|United States|Delaware|640[0n]|BOLD:AAC3609
Paectes abrostoloides|[264]|UDLEP253-09|United States|Pennsylvania|658[0n]|BOLD:AAC3609
Paectes abrostoloides|[265]|MILEP300-10|United States|Georgia|658[0n]|BOLD:AAC3609
Paectes abrostoloides|[266]|MILEP301-10|United States|Georgia|658[0n]|BOLD:AAC3609
Paectes abrostoloides|[267]|MILEP302-10|United States|Georgia|658[0n]|BOLD:AAC3609
Paectes abrostoloides|[268]|MILEP303-10|United States|Georgia|658[0n]|BOLD:AAC3609
Paectes abrostoloides|[269]|LILLA231-11|United States|Illinois|658[0n]|BOLD:AAC3609
Paectes abrostoloides|[270]|LGSMG621-07|United States|North Carolina|658[0n]|BOLD:AAC3609
Paectes abrostoloides|[271]|LILLB021-11|United States|Illinois|658[0n]|BOLD:AAC3609
Paectes abrostoloides|[272]|LSUSA204-06|United States|Kentucky|656[0n]|BOLD:AAC3609
Paectes abrostoloides|[273]|LNCC699-11|United States|North Carolina|658[0n]|BOLD:AAC3609
Paectes abrostoloides|[274]|LNCC841-11|United States|North Carolina|658[0n]|BOLD:AAC3609
Paectes lunodes|[275]|RDNML058-13|United States|Florida|658[0n]|BOLD:ACD3759
Paectes abrostolella|[276]|ABNCC442-07|United States|Missouri|587[0n]|BOLD:AAC3607
Paectes abrostolella|[277]|LPSO111-08|Canada|Ontario|658[0n]|BOLD:AAC3607
Paectes abrostolella|[278]|LPSO316-08|Canada|Ontario|658[0n]|BOLD:AAC3607
Paectes abrostolella|[279]|LPSO321-08|Canada|Ontario|658[0n]|BOLD:AAC3607
Paectes abrostolella|[280]|RDNMH015-09|Canada|Ontario|658[0n]|BOLD:AAC3607
Paectes pygmaea|[281]|ABNCC444-07|United States|Kentucky|589[0n]|BOLD:AAC0554
Paectes pygmaea|[282]|LGSMC859-05|United States|Tennessee|594[0n]|BOLD:AAC0554
Paectes pygmaea|[283]|LNC008-05|United States|North Carolina|658[0n]|BOLD:AAC0554
Paectes pygmaea|[284]|ABNCC440-07|United States|Tennessee|583[0n]|BOLD:AAC0554

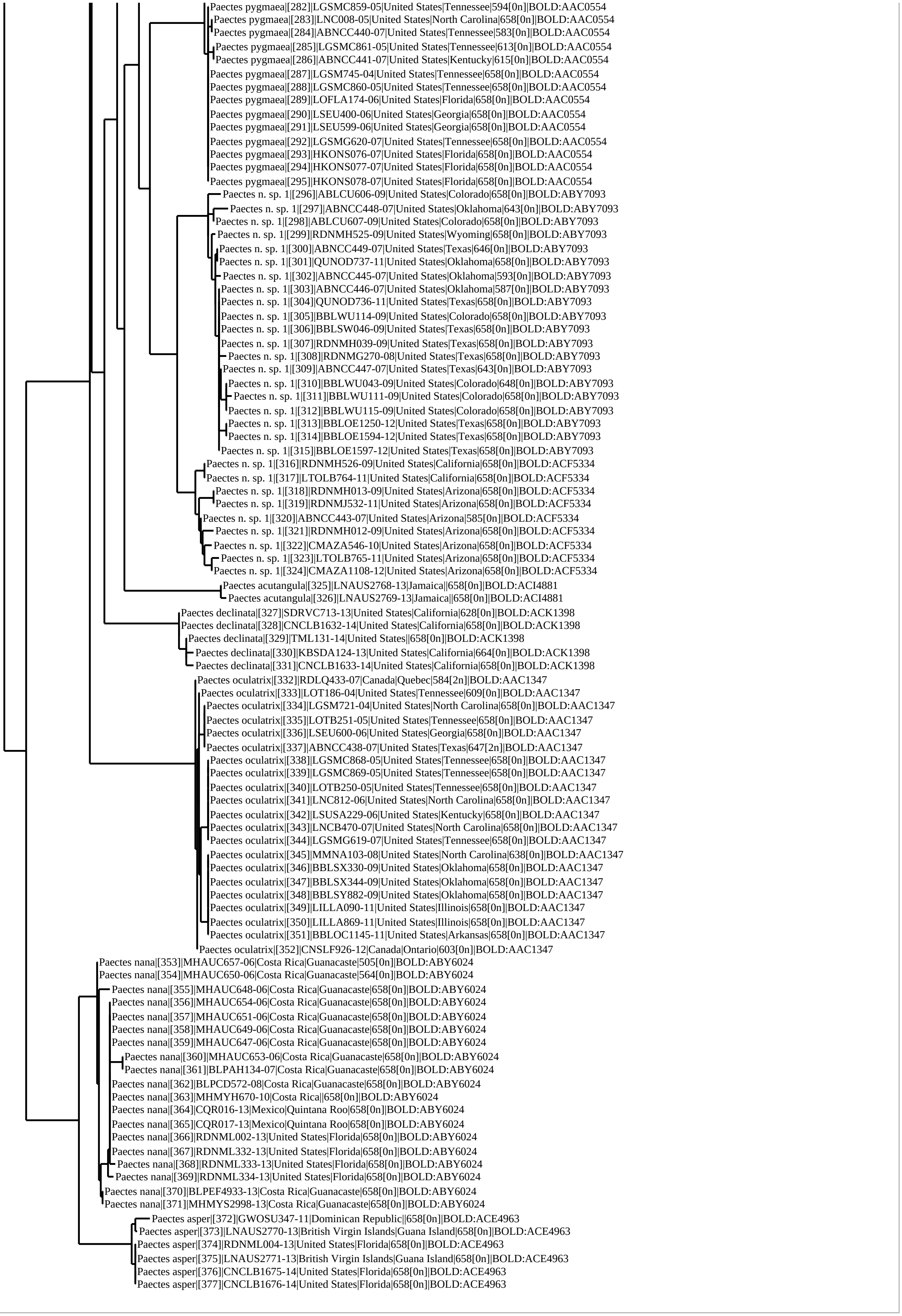
Paectes pygmaea|[282]|LGSMC859-05|United States|Tennessee|594[0n]|BOLD:AAC0554
Paectes pygmaea|[283]|LNC008-05|United States|North Carolina|658[0n]|BOLD:AAC0554
Paectes pygmaea|[284]|ABNCC440-07|United States|Tennessee|583[0n]|BOLD:AAC0554
Paectes pygmaea|[285]|LGSMC861-05|United States|Tennessee|613[0n]|BOLD:AAC0554
Paectes pygmaea|[286]|ABNCC441-07|United States|Kentucky|615[0n]|BOLD:AAC0554
Paectes pygmaea|[287]|LGSM745-04|United States|Tennessee|658[0n]|BOLD:AAC0554
Paectes pygmaea|[288]|LGSMC860-05|United States|Tennessee|658[0n]|BOLD:AAC0554
Paectes pygmaea|[289]|LOFLA174-06|United States|Florida|658[0n]|BOLD:AAC0554
Paectes pygmaea|[290]|LSEU400-06|United States|Georgia|658[0n]|BOLD:AAC0554
Paectes pygmaea|[291]|LSEU599-06|United States|Georgia|658[0n]|BOLD:AAC0554
Paectes pygmaea|[292]|LGSMG620-07|United States|Tennessee|658[0n]|BOLD:AAC0554
Paectes pygmaea|[293]|HKONS076-07|United States|Florida|658[0n]|BOLD:AAC0554
Paectes pygmaea|[294]|HKONS077-07|United States|Florida|658[0n]|BOLD:AAC0554
Paectes pygmaea|[295]|HKONS078-07|United States|Florida|658[0n]|BOLD:AAC0554
Paectes n. sp. 1|[296]|ABLCU606-09|United States|Colorado|658[0n]|BOLD:ABY7093
Paectes n. sp. 1|[297]|ABNCC448-07|United States|Oklahoma|643[0n]|BOLD:ABY7093
Paectes n. sp. 1|[298]|ABLCU607-09|United States|Colorado|658[0n]|BOLD:ABY7093
Paectes n. sp. 1|[299]|RDNMH525-09|United States|Wyoming|658[0n]|BOLD:ABY7093
Paectes n. sp. 1|[300]|ABNCC449-07|United States|Texas|646[0n]|BOLD:ABY7093
Paectes n. sp. 1|[301]|QUNOD737-11|United States|Oklahoma|658[0n]|BOLD:ABY7093
Paectes n. sp. 1|[302]|ABNCC445-07|United States|Oklahoma|593[0n]|BOLD:ABY7093
Paectes n. sp. 1|[303]|ABNCC446-07|United States|Oklahoma|587[0n]|BOLD:ABY7093
Paectes n. sp. 1|[304]|QUNOD736-11|United States|Texas|658[0n]|BOLD:ABY7093
Paectes n. sp. 1|[305]|BBLWU114-09|United States|Colorado|658[0n]|BOLD:ABY7093
Paectes n. sp. 1|[306]|BBLSW046-09|United States|Texas|658[0n]|BOLD:ABY7093
Paectes n. sp. 1|[307]|RDNMH039-09|United States|Texas|658[0n]|BOLD:ABY7093
Paectes n. sp. 1|[308]|RDNMG270-08|United States|Texas|658[0n]|BOLD:ABY7093
Paectes n. sp. 1|[309]|ABNCC447-07|United States|Texas|643[0n]|BOLD:ABY7093
Paectes n. sp. 1|[310]|BBLWU043-09|United States|Colorado|648[0n]|BOLD:ABY7093
Paectes n. sp. 1|[311]|BBLWU111-09|United States|Colorado|658[0n]|BOLD:ABY7093
Paectes n. sp. 1|[312]|BBLWU115-09|United States|Colorado|658[0n]|BOLD:ABY7093
Paectes n. sp. 1|[313]|BBLOE1250-12|United States|Texas|658[0n]|BOLD:ABY7093
Paectes n. sp. 1|[314]|BBLOE1594-12|United States|Texas|658[0n]|BOLD:ABY7093
Paectes n. sp. 1|[315]|BBLOE1597-12|United States|Texas|658[0n]|BOLD:ABY7093
Paectes n. sp. 1|[316]|RDNMH526-09|United States|California|658[0n]|BOLD:ACF5334
Paectes n. sp. 1|[317]|LTOLB764-11|United States|California|658[0n]|BOLD:ACF5334
Paectes n. sp. 1|[318]|RDNMH013-09|United States|Arizona|658[0n]|BOLD:ACF5334
Paectes n. sp. 1|[319]|RDNMJ532-11|United States|Arizona|658[0n]|BOLD:ACF5334
Paectes n. sp. 1|[320]|ABNCC443-07|United States|Arizona|585[0n]|BOLD:ACF5334
Paectes n. sp. 1|[321]|RDNMH012-09|United States|Arizona|658[0n]|BOLD:ACF5334
Paectes n. sp. 1|[322]|CMAZA546-10|United States|Arizona|658[0n]|BOLD:ACF5334
Paectes n. sp. 1|[323]|LTOLB765-11|United States|Arizona|658[0n]|BOLD:ACF5334
Paectes n. sp. 1|[324]|CMAZA1108-12|United States|Arizona|658[0n]|BOLD:ACF5334
Paectes acutangula|[325]|LNAUS2768-13|Jamaica||658[0n]|BOLD:ACI4881
Paectes acutangula|[326]|LNAUS2769-13|Jamaica||658[0n]|BOLD:ACI4881
Paectes declinata|[327]|SDRVC713-13|United States|California|628[0n]|BOLD:ACK1398
Paectes declinata|[328]|CNCLB1632-14|United States|California|658[0n]|BOLD:ACK1398
Paectes declinata|[329]|TML131-14|United States||658[0n]|BOLD:ACK1398
Paectes declinata|[330]|KBSDA124-13|United States|California|664[0n]|BOLD:ACK1398
Paectes declinata|[331]|CNCLB1633-14|United States|California|658[0n]|BOLD:ACK1398
Paectes oculatrix|[332]|RDLQ433-07|Canada|Quebec|584[2n]|BOLD:AAC1347
Paectes oculatrix|[333]|LOT186-04|United States|Tennessee|609[0n]|BOLD:AAC1347
Paectes oculatrix|[334]|LGSM721-04|United States|North Carolina|658[0n]|BOLD:AAC1347
Paectes oculatrix|[335]|LOTB251-05|United States|Tennessee|658[0n]|BOLD:AAC1347
Paectes oculatrix|[336]|LSEU600-06|United States|Georgia|658[0n]|BOLD:AAC1347
Paectes oculatrix|[337]|ABNCC438-07|United States|Texas|647[2n]|BOLD:AAC1347
Paectes oculatrix|[338]|LGSMC868-05|United States|Tennessee|658[0n]|BOLD:AAC1347
Paectes oculatrix|[339]|LGSMC869-05|United States|Tennessee|658[0n]|BOLD:AAC1347
Paectes oculatrix|[340]|LOTB250-05|United States|Tennessee|658[0n]|BOLD:AAC1347
Paectes oculatrix|[341]|LNC812-06|United States|North Carolina|658[0n]|BOLD:AAC1347
Paectes oculatrix|[342]|LSUSA229-06|United States|Kentucky|658[0n]|BOLD:AAC1347
Paectes oculatrix|[343]|LNCB470-07|United States|North Carolina|658[0n]|BOLD:AAC1347
Paectes oculatrix|[344]|LGSMG619-07|United States|Tennessee|658[0n]|BOLD:AAC1347
Paectes oculatrix|[345]|MMNA103-08|United States|North Carolina|638[0n]|BOLD:AAC1347
Paectes oculatrix|[346]|BBLSX330-09|United States|Oklahoma|658[0n]|BOLD:AAC1347
Paectes oculatrix|[347]|BBLSX344-09|United States|Oklahoma|658[0n]|BOLD:AAC1347
Paectes oculatrix|[348]|BBLSY882-09|United States|Oklahoma|658[0n]|BOLD:AAC1347
Paectes oculatrix|[349]|LILLA090-11|United States|Illinois|658[0n]|BOLD:AAC1347
Paectes oculatrix|[350]|LILLA869-11|United States|Illinois|658[0n]|BOLD:AAC1347
Paectes oculatrix|[351]|BBLOC1145-11|United States|Arkansas|658[0n]|BOLD:AAC1347
Paectes oculatrix|[352]|CNSLF926-12|Canada|Ontario|603[0n]|BOLD:AAC1347
Paectes nana|[353]|MHAUC657-06|Costa Rica|Guanacaste|505[0n]|BOLD:ABY6024
Paectes nana|[354]|MHAUC650-06|Costa Rica|Guanacaste|564[0n]|BOLD:ABY6024
Paectes nana|[355]|MHAUC648-06|Costa Rica|Guanacaste|658[0n]|BOLD:ABY6024
Paectes nana|[356]|MHAUC654-06|Costa Rica|Guanacaste|658[0n]|BOLD:ABY6024
Paectes nana|[357]|MHAUC651-06|Costa Rica|Guanacaste|658[0n]|BOLD:ABY6024
Paectes nana|[358]|MHAUC649-06|Costa Rica|Guanacaste|658[0n]|BOLD:ABY6024
Paectes nana|[359]|MHAUC647-06|Costa Rica|Guanacaste|658[0n]|BOLD:ABY6024
Paectes nana|[360]|MHAUC653-06|Costa Rica|Guanacaste|658[0n]|BOLD:ABY6024
Paectes nana|[361]|BLPAH134-07|Costa Rica|Guanacaste|658[0n]|BOLD:ABY6024
Paectes nana|[362]|BLPCD572-08|Costa Rica|Guanacaste|658[0n]|BOLD:ABY6024
Paectes nana|[363]|MHMYH670-10|Costa Rica||658[0n]|BOLD:ABY6024
Paectes nana|[364]|CQR016-13|Mexico|Quintana Roo|658[0n]|BOLD:ABY6024
Paectes nana|[365]|CQR017-13|Mexico|Quintana Roo|658[0n]|BOLD:ABY6024
Paectes nana|[366]|RDNML002-13|United States|Florida|658[0n]|BOLD:ABY6024
Paectes nana|[367]|RDNML332-13|United States|Florida|658[0n]|BOLD:ABY6024
Paectes nana|[368]|RDNML333-13|United States|Florida|658[0n]|BOLD:ABY6024
Paectes nana|[369]|RDNML334-13|United States|Florida|658[0n]|BOLD:ABY6024
Paectes nana|[370]|BLPEF4933-13|Costa Rica|Guanacaste|658[0n]|BOLD:ABY6024
Paectes nana|[371]|MHMYS2998-13|Costa Rica|Guanacaste|658[0n]|BOLD:ABY6024
Paectes asper|[372]|GWOSU347-11|Dominican Republic||658[0n]|BOLD:ACE4963
Paectes asper|[373]|LNAUS2770-13|British Virgin Islands|Guana Island|658[0n]|BOLD:ACE4963
Paectes asper|[374]|RDNML004-13|United States|Florida|658[0n]|BOLD:ACE4963
Paectes asper|[375]|LNAUS2771-13|British Virgin Islands|Guana Island|658[0n]|BOLD:ACE4963
Paectes asper|[376]|CNCLB1675-14|United States|Florida|658[0n]|BOLD:ACE4963
Paectes asper|[377]|CNCLB1676-14|United States|Florida|658[0n]|BOLD:ACE4963
