# Supplementary material for: Probing planetary biodiversity with DNA barcodes: The Noctuoidea of North America
Source: PLoS One. 2017 Jun 1;12(6):e0178548. doi: 10.1371/journal.pone.0178548 (PMC5453547; doi:10.1371/journal.pone.0178548)
Supplement: S3 Tree — NJ tree based on sequence variation in the barcode region of the cytochrome c oxidase I gene for North American species in the family Nolidae. (PDF) [file pone.0178548.s016.pdf]

# BOLD TaxonID Tree

| | | |
|---|---|---|
| Title | : | Tree Result - Search (1140 records) |
| Date | : | 21-April-2016 |
| Data Type | : | Nucleotide |
| Distance Model | : | Kimura 2 Parameter |
| Marker | : | COI-5P |

| | | |
|---|---|---|
| Label | : | Process ID |
| Label | : | Taxon |
| Label | : | Country |
| Label | : | Province/State |
| Label | : | Sequence Length |
| Label | : | Barcode Cluster (BIN) |

| | | |
|---|---|---|
| Sequence Count | : | 1140 |
| Species count | : | 43 |
| Genus count | : | 11 |
| Family count | : | 1 |
| Unidentified | : | 0 |

| | | |
|---|---|---|
| BIN Count | : | 57 |

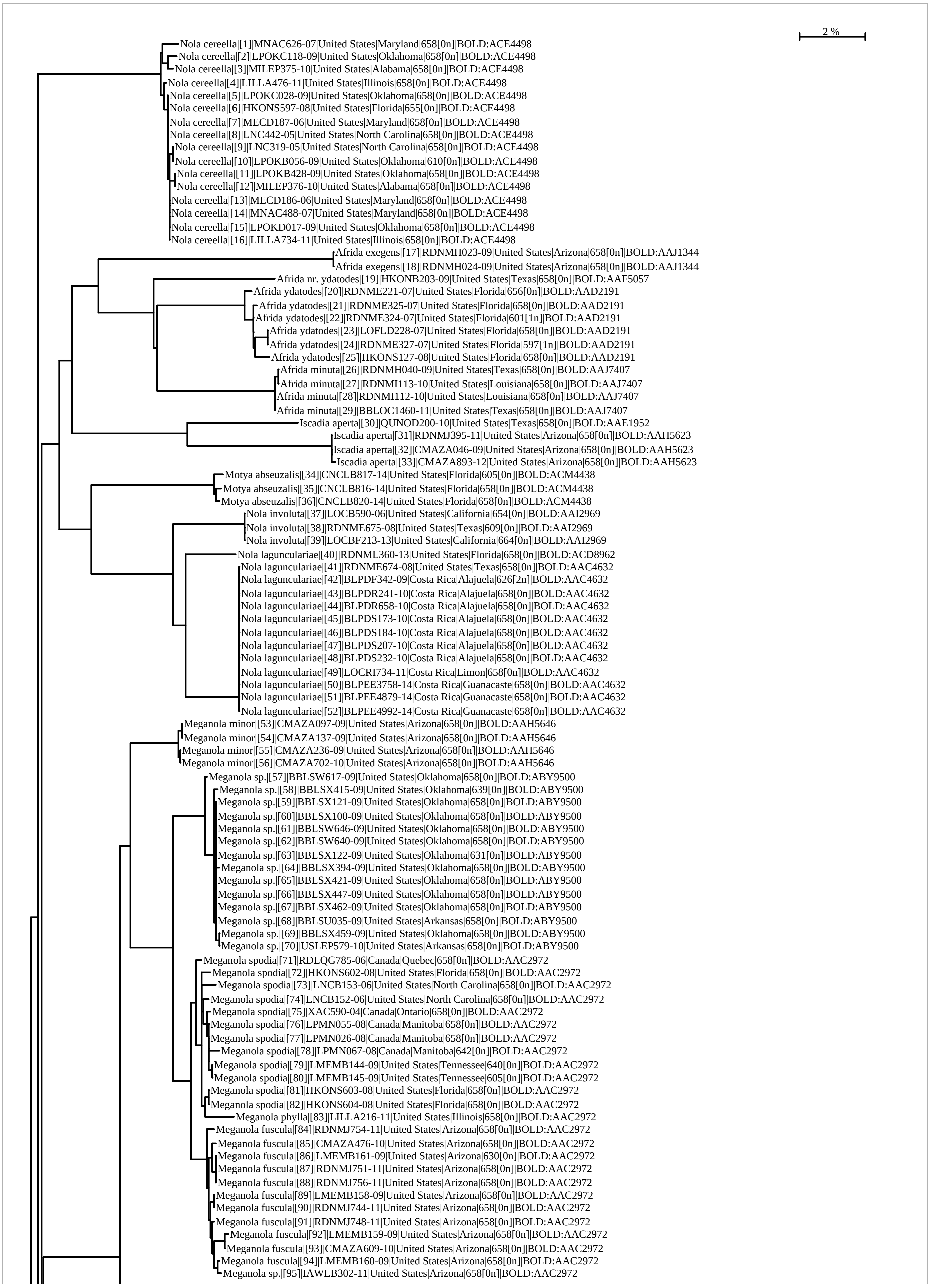
2 %
Nola cereella|[1]|MNAC626-07|United States|Maryland|658[0n]|BOLD:ACE4498
Nola cereella|[2]|LPOKC118-09|United States|Oklahoma|658[0n]|BOLD:ACE4498
Nola cereella|[3]|MILEP375-10|United States|Alabama|658[0n]|BOLD:ACE4498
Nola cereella|[4]|LILLA476-11|United States|Illinois|658[0n]|BOLD:ACE4498
Nola cereella|[5]|LPOKC028-09|United States|Oklahoma|658[0n]|BOLD:ACE4498
Nola cereella|[6]|HKONS597-08|United States|Florida|655[0n]|BOLD:ACE4498
Nola cereella|[7]|MECD187-06|United States|Maryland|658[0n]|BOLD:ACE4498
Nola cereella|[8]|LNC442-05|United States|North Carolina|658[0n]|BOLD:ACE4498
Nola cereella|[9]|LNC319-05|United States|North Carolina|658[0n]|BOLD:ACE4498
Nola cereella|[10]|LPOKB056-09|United States|Oklahoma|610[0n]|BOLD:ACE4498
Nola cereella|[11]|LPOKB428-09|United States|Oklahoma|658[0n]|BOLD:ACE4498
Nola cereella|[12]|MILEP376-10|United States|Alabama|658[0n]|BOLD:ACE4498
Nola cereella|[13]|MECD186-06|United States|Maryland|658[0n]|BOLD:ACE4498
Nola cereella|[14]|MNAC488-07|United States|Maryland|658[0n]|BOLD:ACE4498
Nola cereella|[15]|LPOKD017-09|United States|Oklahoma|658[0n]|BOLD:ACE4498
Nola cereella|[16]|LILLA734-11|United States|Illinois|658[0n]|BOLD:ACE4498
Afrida exegens|[17]|RDNMH023-09|United States|Arizona|658[0n]|BOLD:AAJ1344
Afrida exegens|[18]|RDNMH024-09|United States|Arizona|658[0n]|BOLD:AAJ1344
Afrida nr. ydatodes|[19]|HKONB203-09|United States|Texas|658[0n]|BOLD:AAF5057
Afrida ydatodes|[20]|RDNME221-07|United States|Florida|656[0n]|BOLD:AAD2191
Afrida ydatodes|[21]|RDNME325-07|United States|Florida|658[0n]|BOLD:AAD2191
Afrida ydatodes|[22]|RDNME324-07|United States|Florida|601[1n]|BOLD:AAD2191
Afrida ydatodes|[23]|LOFLD228-07|United States|Florida|658[0n]|BOLD:AAD2191
Afrida ydatodes|[24]|RDNME327-07|United States|Florida|597[1n]|BOLD:AAD2191
Afrida ydatodes|[25]|HKONS127-08|United States|Florida|658[0n]|BOLD:AAD2191
Afrida minuta|[26]|RDNMH040-09|United States|Texas|658[0n]|BOLD:AAJ7407
Afrida minuta|[27]|RDNMI113-10|United States|Louisiana|658[0n]|BOLD:AAJ7407
Afrida minuta|[28]|RDNMI112-10|United States|Louisiana|658[0n]|BOLD:AAJ7407
Afrida minuta|[29]|BBLOC1460-11|United States|Texas|658[0n]|BOLD:AAJ7407
Iscadia aperta|[30]|QUNOD200-10|United States|Texas|658[0n]|BOLD:AAE1952
Iscadia aperta|[31]|RDNMJ395-11|United States|Arizona|658[0n]|BOLD:AAH5623
Iscadia aperta|[32]|CMAZA046-09|United States|Arizona|658[0n]|BOLD:AAH5623
Iscadia aperta|[33]|CMAZA893-12|United States|Arizona|658[0n]|BOLD:AAH5623
Motya abseuzalis|[34]|CNCLB817-14|United States|Florida|605[0n]|BOLD:ACM4438
Motya abseuzalis|[35]|CNCLB816-14|United States|Florida|658[0n]|BOLD:ACM4438
Motya abseuzalis|[36]|CNCLB820-14|United States|Florida|658[0n]|BOLD:ACM4438
Nola involuta|[37]|LOCB590-06|United States|California|654[0n]|BOLD:AAI2969
Nola involuta|[38]|RDNME675-08|United States|Texas|609[0n]|BOLD:AAI2969
Nola involuta|[39]|LOCBF213-13|United States|California|664[0n]|BOLD:AAI2969
Nola laguncularriae|[40]|RDNML360-13|United States|Florida|658[0n]|BOLD:ACD8962
Nola laguncularriae|[41]|RDNME674-08|United States|Texas|658[0n]|BOLD:AAC4632
Nola laguncularriae|[42]|BLPDF342-09|Costa Rica|Alajuela|626[2n]|BOLD:AAC4632
Nola laguncularriae|[43]|BLPDR241-10|Costa Rica|Alajuela|658[0n]|BOLD:AAC4632
Nola laguncularriae|[44]|BLPDR658-10|Costa Rica|Alajuela|658[0n]|BOLD:AAC4632
Nola laguncularriae|[45]|BLPDS173-10|Costa Rica|Alajuela|658[0n]|BOLD:AAC4632
Nola laguncularriae|[46]|BLPDS184-10|Costa Rica|Alajuela|658[0n]|BOLD:AAC4632
Nola laguncularriae|[47]|BLPDS207-10|Costa Rica|Alajuela|658[0n]|BOLD:AAC4632
Nola laguncularriae|[48]|BLPDS232-10|Costa Rica|Alajuela|658[0n]|BOLD:AAC4632
Nola laguncularriae|[49]|LOCRI734-11|Costa Rica|Limon|658[0n]|BOLD:AAC4632
Nola laguncularriae|[50]|BLPEE3758-14|Costa Rica|Guanacaste|658[0n]|BOLD:AAC4632
Nola laguncularriae|[51]|BLPEE4879-14|Costa Rica|Guanacaste|658[0n]|BOLD:AAC4632
Nola laguncularriae|[52]|BLPEE4992-14|Costa Rica|Guanacaste|658[0n]|BOLD:AAC4632
Meganola minor|[53]|CMAZA097-09|United States|Arizona|658[0n]|BOLD:AAH5646
Meganola minor|[54]|CMAZA137-09|United States|Arizona|658[0n]|BOLD:AAH5646
Meganola minor|[55]|CMAZA236-09|United States|Arizona|658[0n]|BOLD:AAH5646
Meganola minor|[56]|CMAZA702-10|United States|Arizona|658[0n]|BOLD:AAH5646
Meganola sp.|[57]|BBLSW617-09|United States|Oklahoma|658[0n]|BOLD:ABY9500
Meganola sp.|[58]|BBLSX415-09|United States|Oklahoma|639[0n]|BOLD:ABY9500
Meganola sp.|[59]|BBLSX121-09|United States|Oklahoma|658[0n]|BOLD:ABY9500
Meganola sp.|[60]|BBLSX100-09|United States|Oklahoma|658[0n]|BOLD:ABY9500
Meganola sp.|[61]|BBLSW646-09|United States|Oklahoma|658[0n]|BOLD:ABY9500
Meganola sp.|[62]|BBLSW640-09|United States|Oklahoma|658[0n]|BOLD:ABY9500
Meganola sp.|[63]|BBLSX122-09|United States|Oklahoma|631[0n]|BOLD:ABY9500
Meganola sp.|[64]|BBLSX394-09|United States|Oklahoma|658[0n]|BOLD:ABY9500
Meganola sp.|[65]|BBLSX421-09|United States|Oklahoma|658[0n]|BOLD:ABY9500
Meganola sp.|[66]|BBLSX447-09|United States|Oklahoma|658[0n]|BOLD:ABY9500
Meganola sp.|[67]|BBLSX462-09|United States|Oklahoma|658[0n]|BOLD:ABY9500
Meganola sp.|[68]|BBLSU035-09|United States|Arkansas|658[0n]|BOLD:ABY9500
Meganola sp.|[69]|BBLSX459-09|United States|Oklahoma|658[0n]|BOLD:ABY9500
Meganola sp.|[70]|USLEP579-10|United States|Arkansas|658[0n]|BOLD:ABY9500
Meganola spodia|[71]|RDLQG785-06|Canada|Quebec|658[0n]|BOLD:AAC2972
Meganola spodia|[72]|HKONS602-08|United States|Florida|658[0n]|BOLD:AAC2972
Meganola spodia|[73]|LNCB153-06|United States|North Carolina|658[0n]|BOLD:AAC2972
Meganola spodia|[74]|LNCB152-06|United States|North Carolina|658[0n]|BOLD:AAC2972
Meganola spodia|[75]|XAC590-04|Canada|Ontario|658[0n]|BOLD:AAC2972
Meganola spodia|[76]|LPMN055-08|Canada|Manitoba|658[0n]|BOLD:AAC2972
Meganola spodia|[77]|LPMN026-08|Canada|Manitoba|658[0n]|BOLD:AAC2972
Meganola spodia|[78]|LPMN067-08|Canada|Manitoba|642[0n]|BOLD:AAC2972
Meganola spodia|[79]|LMEMB144-09|United States|Tennessee|640[0n]|BOLD:AAC2972
Meganola spodia|[80]|LMEMB145-09|United States|Tennessee|605[0n]|BOLD:AAC2972
Meganola spodia|[81]|HKONS603-08|United States|Florida|658[0n]|BOLD:AAC2972
Meganola spodia|[82]|HKONS604-08|United States|Florida|658[0n]|BOLD:AAC2972
Meganola phylla|[83]|LILLA216-11|United States|Illinois|658[0n]|BOLD:AAC2972
Meganola fuscula|[84]|RDNMJ754-11|United States|Arizona|658[0n]|BOLD:AAC2972
Meganola fuscula|[85]|CMAZA476-10|United States|Arizona|658[0n]|BOLD:AAC2972
Meganola fuscula|[86]|LMEMB161-09|United States|Arizona|630[0n]|BOLD:AAC2972
Meganola fuscula|[87]|RDNMJ751-11|United States|Arizona|658[0n]|BOLD:AAC2972
Meganola fuscula|[88]|RDNMJ756-11|United States|Arizona|658[0n]|BOLD:AAC2972
Meganola fuscula|[89]|LMEMB158-09|United States|Arizona|658[0n]|BOLD:AAC2972
Meganola fuscula|[90]|RDNMJ744-11|United States|Arizona|658[0n]|BOLD:AAC2972
Meganola fuscula|[91]|RDNMJ748-11|United States|Arizona|658[0n]|BOLD:AAC2972
Meganola fuscula|[92]|LMEMB159-09|United States|Arizona|658[0n]|BOLD:AAC2972
Meganola fuscula|[93]|CMAZA609-10|United States|Arizona|658[0n]|BOLD:AAC2972
Meganola fuscula|[94]|LMEMB160-09|United States|Arizona|658[0n]|BOLD:AAC2972
Meganola sp.|[95]|IAWLB302-11|United States|Arizona|658[0n]|BOLD:AAC2972

Meganola fuscula|[93]|CMAZA609-10|United States|Arizona|658[0n]|BOLD:AAC2972
Meganola fuscula|[94]|LMEMB160-09|United States|Arizona|658[0n]|BOLD:AAC2972
Meganola sp.|[95]|IAWLB302-11|United States|Arizona|658[0n]|BOLD:AAC2972
Meganola dentata|[96]|IAWL360-09|United States|Arizona|658[0n]|BOLD:AAH4765
Meganola dentata|[97]|LMEMB163-09|United States|Arizona|658[0n]|BOLD:AAH4765
Meganola dentata|[98]|CMAZA1169-12|United States|Arizona|658[0n]|BOLD:AAH4765
Meganola dentata|[99]|RDNMK639-11|United States|Arizona|658[0n]|BOLD:AAH4765
Meganola dentata|[100]|IAWLB303-11|United States|Arizona|658[0n]|BOLD:AAH4765
Meganola dentata|[101]|RDNMJ758-11|United States|Arizona|658[0n]|BOLD:AAH4765
Meganola dentata|[102]|RDNMJ757-11|United States|Arizona|658[0n]|BOLD:AAH4765
Meganola dentata|[103]|RDNMJ755-11|United States|Arizona|658[0n]|BOLD:AAH4765
Meganola dentata|[104]|RDNMJ752-11|United States|Arizona|658[0n]|BOLD:AAH4765
Meganola dentata|[105]|RDNMJ750-11|United States|Arizona|658[0n]|BOLD:AAH4765
Meganola dentata|[106]|RDNMI119-10|United States|Arizona|658[0n]|BOLD:AAH4765
Meganola dentata|[107]|RDNMI054-10|United States|Arizona|658[0n]|BOLD:AAH4765
Meganola dentata|[108]|RDNMI053-10|United States|Arizona|658[0n]|BOLD:AAH4765
Meganola dentata|[109]|RDNMI052-10|United States|Arizona|658[0n]|BOLD:AAH4765
Meganola dentata|[110]|RDNMK642-11|United States|Arizona|628[0n]|BOLD:AAH4765
Meganola dentata|[111]|RDNMK643-11|United States|Arizona|632[0n]|BOLD:AAH4765
Meganola dentata|[112]|RDNMK644-11|United States|Arizona|658[0n]|BOLD:AAH4765
Meganola dentata|[113]|RDNMK646-11|United States|Arizona|658[0n]|BOLD:AAH4765
Meganola dentata|[114]|RDNMK648-11|United States|Arizona|658[0n]|BOLD:AAH4765
Meganola dentata|[115]|LMEMB162-09|United States|Arizona|658[0n]|BOLD:AAH4765
Meganola dentata|[116]|RDNMK848-12|Mexico|Sonora|658[0n]|BOLD:AAH4765
Meganola conspicua|[117]|RDNMJ259-11|United States|Arizona|658[1n]|BOLD:AAT9076
Meganola conspicua|[118]|RDNMJ372-11|United States|Arizona|658[0n]|BOLD:AAT9076
Meganola conspicua|[119]|RDNMJ745-11|United States|Arizona|658[0n]|BOLD:AAT9076
Meganola sp.|[120]|LTOL789-07|United States|California|658[0n]|BOLD:ABZ6759
Meganola sp.|[121]|LTOL791-07|United States|California|658[0n]|BOLD:AAC5041
Meganola sp.|[122]|PSAT145-10|United States|California|658[0n]|BOLD:AAC5041
Meganola sp.|[123]|LOCBB093-06|United States|California|658[0n]|BOLD:AAC5041
Meganola sp.|[124]|LOCBB004-06|United States|California|658[0n]|BOLD:AAC5041
Meganola sp.|[125]|LOCB881-06|United States|California|658[0n]|BOLD:AAC5041
Meganola sp.|[126]|LOCB420-06|United States|California|658[0n]|BOLD:AAC5041
Meganola sp.|[127]|LOCBB094-06|United States|California|632[0n]|BOLD:AAC5041
Meganola sp.|[128]|LOCBB095-06|United States|California|658[0n]|BOLD:AAC5041
Meganola sp.|[129]|PSAT146-10|United States|California|658[0n]|BOLD:AAC5041
Meganola minuscula|[130]|PSAT144-10|United States|Washington|658[0n]|BOLD:ACE7504
Meganola minuscula|[131]|RDNMJ110-10|United States|Washington|658[0n]|BOLD:ACE7504
Meganola minuscula|[132]|RDNMJ111-10|United States|Washington|658[0n]|BOLD:ACE7504
Meganola sp.|[133]|RDNMJ753-11|United States|Arizona|658[0n]|BOLD:ABZ6763
Meganola sp.|[134]|RDNMG243-08|United States|Texas|658[0n]|BOLD:AAC5042
Meganola sp.|[135]|RDNMG242-08|United States|Texas|658[0n]|BOLD:AAC5042
Meganola sp.|[136]|HKONB549-09|United States|Texas|658[0n]|BOLD:AAC5042
Meganola sp.|[137]|HKONB550-09|United States|Texas|658[0n]|BOLD:AAC5042
Meganola sp.|[138]|BBLSY198-09|United States|Texas|658[0n]|BOLD:AAC5042
Meganola sp.|[139]|BBLSY171-09|United States|Texas|658[0n]|BOLD:AAC5042
Meganola sp.|[140]|BBLSY172-09|United States|Texas|658[0n]|BOLD:AAC5042
Meganola sp.|[141]|BBLSY199-09|United States|Texas|658[0n]|BOLD:AAC5042
Meganola sp.|[142]|BBLSY266-09|United States|Texas|658[0n]|BOLD:AAC5042
Meganola sp.|[143]|BBLSX783-09|United States|Texas|658[0n]|BOLD:AAC5042
Meganola sp.|[144]|BBLSX833-09|United States|Texas|658[0n]|BOLD:AAC5042
Meganola sp.|[145]|QUNOD108-10|United States|Texas|658[0n]|BOLD:AAC5042
Meganola sp.|[146]|QUNOD752-11|United States|Texas|658[0n]|BOLD:AAC5042
Meganola sp.|[147]|RDNMI055-10|United States|Arizona|658[0n]|BOLD:AAC5042
Meganola sp.|[148]|RDNMJ749-11|United States|Arizona|658[0n]|BOLD:AAC5042
Meganola sp.|[149]|RDNMJ393-11|United States|Arizona|658[0n]|BOLD:AAC5042
Meganola sp.|[150]|IAWLB634-11|United States|Arizona|591[0n]|BOLD:AAC5042
Meganola sp.|[151]|CMAZA496-10|United States|Arizona|658[0n]|BOLD:AAC5042
Meganola sp.|[152]|RDNMJ746-11|United States|Arizona|658[0n]|BOLD:AAC5042
Meganola sp.|[153]|QUNOD754-11|United States|Arizona|658[0n]|BOLD:AAC5042
Meganola sp.|[154]|RDNMK640-11|United States|Arizona|658[0n]|BOLD:AAC5042
Meganola sp.|[155]|RDNMK645-11|United States|Arizona|658[0n]|BOLD:AAC5042
Meganola sp.|[156]|QUNOD753-11|United States|Arizona|658[0n]|BOLD:AAC5042
Meganola sp.|[157]|RDNMK641-11|United States|Arizona|658[0n]|BOLD:AAC5042
Meganola sp.|[158]|RDNMK647-11|United States|Arizona|658[0n]|BOLD:AAC5042
Meganola phylla|[159]|HKONS633-08|United States|Florida|658[0n]|BOLD:ABZ5427
Meganola phylla|[160]|LOFLA631-06|United States|Florida|658[0n]|BOLD:ABZ5427
Meganola phylla|[161]|HKONS632-08|United States|Florida|658[0n]|BOLD:ABZ5427
Meganola phylla|[162]|HKONS634-08|United States|Florida|658[0n]|BOLD:ABZ5427
Meganola phylla|[163]|HKONS631-08|United States|Florida|658[0n]|BOLD:ABZ5427
Meganola phylla|[164]|LOT211-04|United States|Tennessee|609[0n]|BOLD:ABZ5427
Meganola phylla|[165]|LOT210-04|United States|Tennessee|609[0n]|BOLD:ABZ5427
Meganola phylla|[166]|ABCNA594-07|United States|Florida|577[0n]|BOLD:ABZ5427
Meganola phylla|[167]|LTOL806-07|United States|Maryland|658[0n]|BOLD:ABZ5427
Meganola phylla|[168]|LTOL807-07|United States|Maryland|658[0n]|BOLD:ABZ5427
Meganola phylla|[169]|LGSMD240-05|United States|Tennessee|658[0n]|BOLD:ABZ5427
Meganola phylla|[170]|LGSMD238-05|United States|Tennessee|658[0n]|BOLD:ABZ5427
Meganola phylla|[171]|LSEU561-06|United States|Georgia|658[0n]|BOLD:ABZ5427
Meganola phylla|[172]|HKONS608-08|United States|Florida|658[0n]|BOLD:ABZ5427
Meganola phylla|[173]|HKONS609-08|United States|Florida|658[0n]|BOLD:ABZ5427
Meganola phylla|[174]|LPSO313-08|Canada|Ontario|658[0n]|BOLD:ABZ5427
Meganola phylla|[175]|LNCB592-09|United States|North Carolina|658[0n]|BOLD:ABZ5427
Meganola phylla|[176]|LPOKC574-09|United States|Oklahoma|658[0n]|BOLD:ABZ5427
Meganola phylla|[177]|LILLA017-11|United States|Illinois|658[0n]|BOLD:ABZ5427
Meganola phylla|[178]|LILLA124-11|United States|Illinois|658[0n]|BOLD:ABZ5427
Meganola phylla|[179]|LSEU369-06|United States|Georgia|658[0n]|BOLD:ABZ5427
Meganola phylla|[180]|LPSO466-08|Canada|Ontario|658[0n]|BOLD:ABZ5427
Meganola phylla|[181]|LGSMC678-05|United States|Tennessee|658[0n]|BOLD:ABZ5427
Meganola phylla|[182]|LSEU370-06|United States|Georgia|658[0n]|BOLD:ABZ5427
Meganola phylla|[183]|LTOL039-06|United States|Maryland|658[0n]|BOLD:ABZ5427
Meganola phylla|[184]|LOTB533-05|United States|Tennessee|658[0n]|BOLD:ABZ5427
Meganola phylla|[185]|LOTB532-05|United States|Tennessee|658[0n]|BOLD:ABZ5427
Meganola phylla|[186]|LOTB531-05|United States|Tennessee|658[0n]|BOLD:ABZ5427
Meganola phylla|[187]|LOTB529-05|United States|Tennessee|658[0n]|BOLD:ABZ5427
Meganola phylla|[188]|LOTB528-05|United States|Tennessee|658[0n]|BOLD:ABZ5427
Meganola phylla|[189]|LGSMC679-05|United States|Tennessee|658[0n]|BOLD:ABZ5427
Meganola phylla|[190]|LGSMC677-05|United States|Tennessee|658[0n]|BOLD:ABZ5427
Meganola phylla|[191]|XAF316-04|Canada|Ontario|658[0n]|BOLD:ABZ5427

Meganola phylla|[188]|LOTB528-05|United States|Tennessee|658[0n]|BOLD:ABZ5427
Meganola phylla|[189]|LGSMC679-05|United States|Tennessee|658[0n]|BOLD:ABZ5427
Meganola phylla|[190]|LGSMC677-05|United States|Tennessee|658[0n]|BOLD:ABZ5427
Meganola phylla|[191]|XAE316-04|Canada|Ontario|658[0n]|BOLD:ABZ5427
Meganola phylla|[192]|LGSM416-04|United States|North Carolina|658[0n]|BOLD:ABZ5427
Meganola phylla|[193]|LGSMD237-05|United States|Tennessee|627[0n]|BOLD:ABZ5427
Meganola phylla|[194]|LGSMD239-05|United States|Tennessee|658[0n]|BOLD:ABZ5427
Meganola phylla|[195]|LTOL804-07|United States|Maryland|658[0n]|BOLD:ABZ5427
Meganola phylla|[196]|LTOL808-07|United States|Maryland|658[0n]|BOLD:ABZ5427
Meganola phylla|[197]|LPSO718-08|Canada|Ontario|658[0n]|BOLD:ABZ5427
Meganola phylla|[198]|RDNMG117-08|United States|Georgia|658[0n]|BOLD:ABZ5427
Meganola phylla|[199]|LPOKD759-10|United States|Oklahoma|658[0n]|BOLD:ABZ5427
Meganola phylla|[200]|LGSM417-04|United States|North Carolina|658[0n]|BOLD:ABZ5427
Meganola phylla|[201]|LNCC663-11|United States|North Carolina|658[0n]|BOLD:ABZ5427
Meganola phylla|[202]|LNCC680-11|United States|North Carolina|658[0n]|BOLD:ABZ5427
Meganola sp.|[203]|BBLOB1144-11|United States|Florida|658[0n]|BOLD:AAA8651
Meganola sp.|[204]|LOFLB923-06|United States|Florida|658[0n]|BOLD:AAA8651
Meganola sp.|[205]|LOFLB915-06|United States|Florida|658[0n]|BOLD:AAA8651
Meganola sp.|[206]|LOFLB645-06|United States|Florida|658[0n]|BOLD:AAA8651
Meganola sp.|[207]|LOFLB641-06|United States|Florida|658[0n]|BOLD:AAA8651
Meganola sp.|[208]|LOFLB631-06|United States|Florida|658[0n]|BOLD:AAA8651
Meganola sp.|[209]|LOFLB568-06|United States|Florida|658[0n]|BOLD:AAA8651
Meganola sp.|[210]|LOFLB421-06|United States|Florida|658[0n]|BOLD:AAA8651
Meganola sp.|[211]|LOFLB331-06|United States|Florida|658[0n]|BOLD:AAA8651
Meganola sp.|[212]|LOFLB150-06|United States|Florida|658[0n]|BOLD:AAA8651
Meganola sp.|[213]|LOFLB088-06|United States|Florida|658[0n]|BOLD:AAA8651
Meganola sp.|[214]|LOFLB082-06|United States|Florida|658[0n]|BOLD:AAA8651
Meganola sp.|[215]|LOFLB931-06|United States|Florida|656[0n]|BOLD:AAA8651
Meganola sp.|[216]|LOFLC061-06|United States|Florida|658[0n]|BOLD:AAA8651
Meganola sp.|[217]|LOFLC306-06|United States|Florida|658[0n]|BOLD:AAA8651
Meganola sp.|[218]|USLEP177-10|United States|Florida|658[0n]|BOLD:AAA8651
Meganola sp.|[219]|USLEP178-10|United States|Florida|658[0n]|BOLD:AAA8651
Meganola sp.|[220]|USLEP317-10|United States|Florida|658[0n]|BOLD:AAA8651
Meganola sp.|[221]|HKONS656-08|United States|Florida|609[0n]|BOLD:AAA8651
Meganola sp.|[222]|LSEU493-06|United States|Georgia|658[0n]|BOLD:AAA8651
Meganola sp.|[223]|RDNMG118-08|United States|Georgia|658[0n]|BOLD:AAA8651
Meganola sp.|[224]|RDNMG122-08|United States|Georgia|658[0n]|BOLD:AAA8651
Meganola sp.|[225]|RDNMG123-08|United States|Georgia|658[0n]|BOLD:AAA8651
Meganola sp.|[226]|HKONS644-08|United States|Florida|658[0n]|BOLD:AAA8651
Meganola sp.|[227]|RDNMG119-08|United States|Georgia|658[0n]|BOLD:AAA8651
Meganola sp.|[228]|HKONS606-08|United States|Florida|658[0n]|BOLD:AAA8651
Meganola sp.|[229]|MNAC302-07|United States|Florida|658[0n]|BOLD:AAA8651
Meganola sp.|[230]|LOFLB314-06|United States|Florida|658[0n]|BOLD:AAA8651
Meganola sp.|[231]|LOFLB078-06|United States|Florida|658[0n]|BOLD:AAA8651
Meganola sp.|[232]|LOFLB047-06|United States|Florida|658[0n]|BOLD:AAA8651
Meganola sp.|[233]|LOFLA939-06|United States|Florida|658[0n]|BOLD:AAA8651
Meganola sp.|[234]|LOFLA821-06|United States|Florida|658[0n]|BOLD:AAA8651
Meganola sp.|[235]|LOFLA786-06|United States|Florida|658[0n]|BOLD:AAA8651
Meganola sp.|[236]|LOFLA782-06|United States|Florida|658[0n]|BOLD:AAA8651
Meganola sp.|[237]|LOFLA176-06|United States|Florida|658[0n]|BOLD:AAA8651
Meganola sp.|[238]|LOFLA141-06|United States|Florida|658[0n]|BOLD:AAA8651
Meganola sp.|[239]|LOFLA779-06|United States|Florida|658[0n]|BOLD:AAA8651
Meganola sp.|[240]|HKONS655-08|United States|Florida|587[0n]|BOLD:AAA8651
Meganola sp.|[241]|LOFLB073-06|United States|Florida|658[0n]|BOLD:AAA8651
Meganola sp.|[242]|MNAB145-07|United States|Florida|627[0n]|BOLD:AAA8651
Meganola sp.|[243]|USLEP984-10|United States|Florida|649[0n]|BOLD:AAA8651
Meganola sp.|[244]|HKONS643-08|United States|Florida|658[0n]|BOLD:AAA8651
Meganola sp.|[245]|HKONS635-08|United States|Florida|658[0n]|BOLD:AAA8651
Meganola sp.|[246]|LOFLB353-06|United States|Florida|658[0n]|BOLD:AAA8651
Meganola sp.|[247]|LOFLB908-06|United States|Florida|658[0n]|BOLD:AAA8651
Meganola sp.|[248]|MNAB146-07|United States|Florida|637[0n]|BOLD:AAA8651
Meganola sp.|[249]|LMEMB157-09|United States|Mississippi|539[0n]|BOLD:AAA8651
Meganola sp.|[250]|USLEP578-10|United States|Florida|658[0n]|BOLD:AAA8651
Meganola sp.|[251]|USLEP580-10|United States|Florida|658[0n]|BOLD:AAA8651
Meganola sp.|[252]|USLEP581-10|United States|Florida|658[0n]|BOLD:AAA8651
Meganola sp.|[253]|USLEP926-10|United States|Florida|658[0n]|BOLD:AAA8651
Meganola sp.|[254]|BBLOB1807-11|United States|Florida|658[0n]|BOLD:AAA8651
Meganola minuscula|[255]|BBLCU352-09|United States|Michigan|658[0n]|BOLD:ABX5445
Meganola minuscula|[256]|BLTIB973-08|Canada|Ontario|658[0n]|BOLD:ABX5445
Meganola minuscula|[257]|BBLCU161-09|United States|Michigan|658[0n]|BOLD:ABX5445
Meganola minuscula|[258]|LGSMG623-07|United States|North Carolina|658[0n]|BOLD:ABX5445
Meganola minuscula|[259]|XAD734-05|Canada|Ontario|658[0n]|BOLD:ABX5445
Meganola minuscula|[260]|LGSMD241-05|United States|Tennessee|658[0n]|BOLD:ABX5445
Meganola minuscula|[261]|LOTB530-05|United States|Tennessee|658[0n]|BOLD:ABX5445
Meganola minuscula|[262]|LOTB527-05|United States|Tennessee|658[0n]|BOLD:ABX5445
Meganola minuscula|[263]|XAC711-04|Canada|Ontario|658[0n]|BOLD:ABX5445
Meganola minuscula|[264]|LGSMG624-07|United States|Tennessee|658[0n]|BOLD:ABX5445
Meganola minuscula|[265]|PMG132-03|Canada|Ontario|617[0n]|BOLD:ABX5445
Meganola minuscula|[266]|LGSMD236-05|United States|Tennessee|611[0n]|BOLD:ABX5445
Meganola minuscula|[267]|BBLCU355-09|United States|Michigan|658[0n]|BOLD:ABX5445
Meganola minuscula|[268]|BBLCU354-09|United States|Michigan|658[0n]|BOLD:ABX5445
Meganola minuscula|[269]|BBLCU347-09|United States|Michigan|658[0n]|BOLD:ABX5445
Meganola minuscula|[270]|BBLCU344-09|United States|Michigan|643[0n]|BOLD:ABX5445
Meganola minuscula|[271]|BBLCU356-09|United States|Michigan|632[0n]|BOLD:ABX5445
Meganola minuscula|[272]|LOTB534-05|United States|Tennessee|658[0n]|BOLD:ABX5445
Meganola minuscula|[273]|BBLCU359-09|United States|Michigan|658[0n]|BOLD:ABX5445
Meganola minuscula|[274]|HKONS641-08|United States|Florida|658[0n]|BOLD:ABX5445
Meganola minuscula|[275]|HKONS642-08|United States|Florida|658[0n]|BOLD:ABX5445
Meganola minuscula|[276]|HKONS640-08|United States|Florida|658[0n]|BOLD:ABX5445
Meganola minuscula|[277]|LNC197-05|United States|North Carolina|658[0n]|BOLD:ABX5445
Meganola minuscula|[278]|LNC198-05|United States|North Carolina|658[0n]|BOLD:ABX5445
Meganola minuscula|[279]|ABCNA593-07|United States|Florida|577[0n]|BOLD:ABX5445
Meganola minuscula|[280]|LPOKA940-09|United States|Oklahoma|658[0n]|BOLD:ABX5445
Meganola minuscula|[281]|LPOKA953-09|United States|Oklahoma|658[0n]|BOLD:ABX5445
Meganola minuscula|[282]|LPOKA954-09|United States|Oklahoma|658[0n]|BOLD:ABX5445
Meganola minuscula|[283]|LPOKA1000-09|United States|Oklahoma|658[0n]|BOLD:ABX5445
Meganola minuscula|[284]|LMEMB151-09|United States|Mississippi|658[0n]|BOLD:ABX5445
Meganola minuscula|[285]|LNCC972-11|United States|North Carolina|658[0n]|BOLD:ABX5445
Meganola minuscula|[286]|LNCC1120-11|United States|North Carolina|658[0n]|BOLD:ABX5445

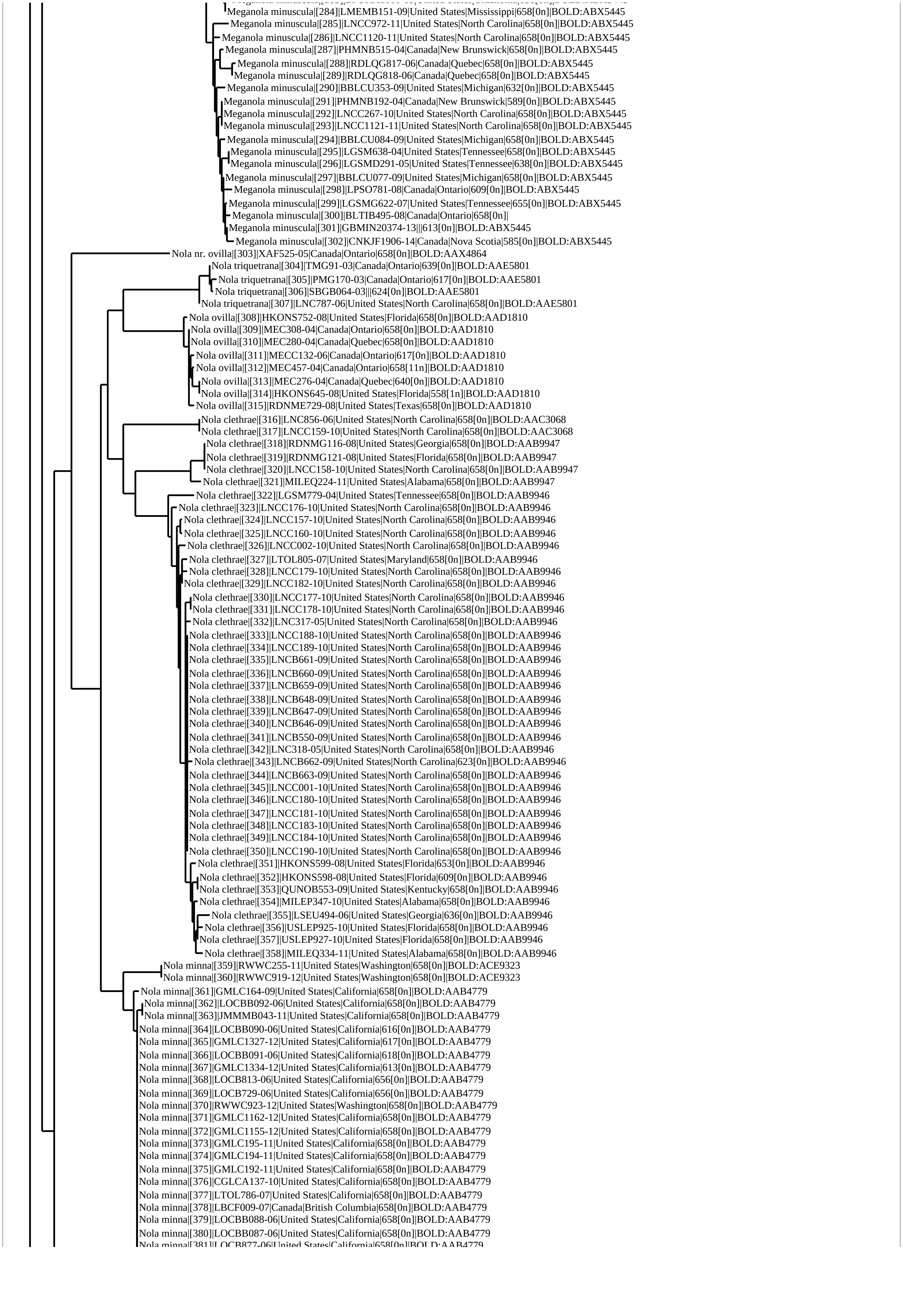
Meganola minuscula|[284]|LMEMB151-09|United States|Mississippi|658[0n]|BOLD:ABX5445
Meganola minuscula|[285]|LNCC972-11|United States|North Carolina|658[0n]|BOLD:ABX5445
Meganola minuscula|[286]|LNCC1120-11|United States|North Carolina|658[0n]|BOLD:ABX5445
Meganola minuscula|[287]|PHMNB515-04|Canada|New Brunswick|658[0n]|BOLD:ABX5445
Meganola minuscula|[288]|RDLQG817-06|Canada|Quebec|658[0n]|BOLD:ABX5445
Meganola minuscula|[289]|RDLQG818-06|Canada|Quebec|658[0n]|BOLD:ABX5445
Meganola minuscula|[290]|BBLCU353-09|United States|Michigan|632[0n]|BOLD:ABX5445
Meganola minuscula|[291]|PHMNB192-04|Canada|New Brunswick|589[0n]|BOLD:ABX5445
Meganola minuscula|[292]|LNCC267-10|United States|North Carolina|658[0n]|BOLD:ABX5445
Meganola minuscula|[293]|LNCC1121-11|United States|North Carolina|658[0n]|BOLD:ABX5445
Meganola minuscula|[294]|BBLCU084-09|United States|Michigan|658[0n]|BOLD:ABX5445
Meganola minuscula|[295]|LGSM638-04|United States|Tennessee|658[0n]|BOLD:ABX5445
Meganola minuscula|[296]|LGSMD291-05|United States|Tennessee|638[0n]|BOLD:ABX5445
Meganola minuscula|[297]|BBLCU077-09|United States|Michigan|658[0n]|BOLD:ABX5445
Meganola minuscula|[298]|LPSO781-08|Canada|Ontario|609[0n]|BOLD:ABX5445
Meganola minuscula|[299]|LGSMG622-07|United States|Tennessee|655[0n]|BOLD:ABX5445
Meganola minuscula|[300]|BLTIB495-08|Canada|Ontario|658[0n]|
Meganola minuscula|[301]|GBMIN20374-13|||613[0n]|BOLD:ABX5445
Meganola minuscula|[302]|CNKJF1906-14|Canada|Nova Scotia|585[0n]|BOLD:ABX5445
Nola nr. ovilla|[303]|XAF525-05|Canada|Ontario|658[0n]|BOLD:AAX4864
Nola triquetrana|[304]|TMG91-03|Canada|Ontario|639[0n]|BOLD:AAE5801
Nola triquetrana|[305]|PMG170-03|Canada|Ontario|617[0n]|BOLD:AAE5801
Nola triquetrana|[306]|SBGB064-03|||624[0n]|BOLD:AAE5801
Nola triquetrana|[307]|LNC787-06|United States|North Carolina|658[0n]|BOLD:AAE5801
Nola ovilla|[308]|HKONS752-08|United States|Florida|658[0n]|BOLD:AAD1810
Nola ovilla|[309]|MEC308-04|Canada|Ontario|658[0n]|BOLD:AAD1810
Nola ovilla|[310]|MEC280-04|Canada|Quebec|658[0n]|BOLD:AAD1810
Nola ovilla|[311]|MECC132-06|Canada|Ontario|617[0n]|BOLD:AAD1810
Nola ovilla|[312]|MEC457-04|Canada|Ontario|658[11n]|BOLD:AAD1810
Nola ovilla|[313]|MEC276-04|Canada|Quebec|640[0n]|BOLD:AAD1810
Nola ovilla|[314]|HKONS645-08|United States|Florida|558[1n]|BOLD:AAD1810
Nola ovilla|[315]|RDNME729-08|United States|Texas|658[0n]|BOLD:AAD1810
Nola clethrae|[316]|LNC856-06|United States|North Carolina|658[0n]|BOLD:AAC3068
Nola clethrae|[317]|LNCC159-10|United States|North Carolina|658[0n]|BOLD:AAC3068
Nola clethrae|[318]|RDNMG116-08|United States|Georgia|658[0n]|BOLD:AAB9947
Nola clethrae|[319]|RDNMG121-08|United States|Florida|658[0n]|BOLD:AAB9947
Nola clethrae|[320]|LNCC158-10|United States|North Carolina|658[0n]|BOLD:AAB9947
Nola clethrae|[321]|MILEQ224-11|United States|Alabama|658[0n]|BOLD:AAB9947
Nola clethrae|[322]|LGSM779-04|United States|Tennessee|658[0n]|BOLD:AAB9946
Nola clethrae|[323]|LNCC176-10|United States|North Carolina|658[0n]|BOLD:AAB9946
Nola clethrae|[324]|LNCC157-10|United States|North Carolina|658[0n]|BOLD:AAB9946
Nola clethrae|[325]|LNCC160-10|United States|North Carolina|658[0n]|BOLD:AAB9946
Nola clethrae|[326]|LNCC002-10|United States|North Carolina|658[0n]|BOLD:AAB9946
Nola clethrae|[327]|LTOL805-07|United States|Maryland|658[0n]|BOLD:AAB9946
Nola clethrae|[328]|LNCC179-10|United States|North Carolina|658[0n]|BOLD:AAB9946
Nola clethrae|[329]|LNCC182-10|United States|North Carolina|658[0n]|BOLD:AAB9946
Nola clethrae|[330]|LNCC177-10|United States|North Carolina|658[0n]|BOLD:AAB9946
Nola clethrae|[331]|LNCC178-10|United States|North Carolina|658[0n]|BOLD:AAB9946
Nola clethrae|[332]|LNC317-05|United States|North Carolina|658[0n]|BOLD:AAB9946
Nola clethrae|[333]|LNCC188-10|United States|North Carolina|658[0n]|BOLD:AAB9946
Nola clethrae|[334]|LNCC189-10|United States|North Carolina|658[0n]|BOLD:AAB9946
Nola clethrae|[335]|LNCB661-09|United States|North Carolina|658[0n]|BOLD:AAB9946
Nola clethrae|[336]|LNCB660-09|United States|North Carolina|658[0n]|BOLD:AAB9946
Nola clethrae|[337]|LNCB659-09|United States|North Carolina|658[0n]|BOLD:AAB9946
Nola clethrae|[338]|LNCB648-09|United States|North Carolina|658[0n]|BOLD:AAB9946
Nola clethrae|[339]|LNCB647-09|United States|North Carolina|658[0n]|BOLD:AAB9946
Nola clethrae|[340]|LNCB646-09|United States|North Carolina|658[0n]|BOLD:AAB9946
Nola clethrae|[341]|LNCB550-09|United States|North Carolina|658[0n]|BOLD:AAB9946
Nola clethrae|[342]|LNC318-05|United States|North Carolina|658[0n]|BOLD:AAB9946
Nola clethrae|[343]|LNCB662-09|United States|North Carolina|623[0n]|BOLD:AAB9946
Nola clethrae|[344]|LNCB663-09|United States|North Carolina|658[0n]|BOLD:AAB9946
Nola clethrae|[345]|LNCC001-10|United States|North Carolina|658[0n]|BOLD:AAB9946
Nola clethrae|[346]|LNCC180-10|United States|North Carolina|658[0n]|BOLD:AAB9946
Nola clethrae|[347]|LNCC181-10|United States|North Carolina|658[0n]|BOLD:AAB9946
Nola clethrae|[348]|LNCC183-10|United States|North Carolina|658[0n]|BOLD:AAB9946
Nola clethrae|[349]|LNCC184-10|United States|North Carolina|658[0n]|BOLD:AAB9946
Nola clethrae|[350]|LNCC190-10|United States|North Carolina|658[0n]|BOLD:AAB9946
Nola clethrae|[351]|HKONS599-08|United States|Florida|653[0n]|BOLD:AAB9946
Nola clethrae|[352]|HKONS598-08|United States|Florida|609[0n]|BOLD:AAB9946
Nola clethrae|[353]|QUNOB553-09|United States|Kentucky|658[0n]|BOLD:AAB9946
Nola clethrae|[354]|MILEP347-10|United States|Alabama|658[0n]|BOLD:AAB9946
Nola clethrae|[355]|LSEU494-06|United States|Georgia|636[0n]|BOLD:AAB9946
Nola clethrae|[356]|USLEP925-10|United States|Florida|658[0n]|BOLD:AAB9946
Nola clethrae|[357]|USLEP927-10|United States|Florida|658[0n]|BOLD:AAB9946
Nola clethrae|[358]|MILEQ334-11|United States|Alabama|658[0n]|BOLD:AAB9946
Nola minna|[359]|RWWC255-11|United States|Washington|658[0n]|BOLD:ACE9323
Nola minna|[360]|RWWC919-12|United States|Washington|658[0n]|BOLD:ACE9323
Nola minna|[361]|GMLC164-09|United States|California|658[0n]|BOLD:AAB4779
Nola minna|[362]|LOCBB092-06|United States|California|658[0n]|BOLD:AAB4779
Nola minna|[363]|JMMMB043-11|United States|California|658[0n]|BOLD:AAB4779
Nola minna|[364]|LOCBB090-06|United States|California|616[0n]|BOLD:AAB4779
Nola minna|[365]|GMLC1327-12|United States|California|617[0n]|BOLD:AAB4779
Nola minna|[366]|LOCBB091-06|United States|California|618[0n]|BOLD:AAB4779
Nola minna|[367]|GMLC1334-12|United States|California|613[0n]|BOLD:AAB4779
Nola minna|[368]|LOCB813-06|United States|California|656[0n]|BOLD:AAB4779
Nola minna|[369]|LOCB729-06|United States|California|656[0n]|BOLD:AAB4779
Nola minna|[370]|RWWC923-12|United States|Washington|658[0n]|BOLD:AAB4779
Nola minna|[371]|GMLC1162-12|United States|California|658[0n]|BOLD:AAB4779
Nola minna|[372]|GMLC1155-12|United States|California|658[0n]|BOLD:AAB4779
Nola minna|[373]|GMLC195-11|United States|California|658[0n]|BOLD:AAB4779
Nola minna|[374]|GMLC194-11|United States|California|658[0n]|BOLD:AAB4779
Nola minna|[375]|GMLC192-11|United States|California|658[0n]|BOLD:AAB4779
Nola minna|[376]|CGLCA137-10|United States|California|658[0n]|BOLD:AAB4779
Nola minna|[377]|LTOL786-07|United States|California|658[0n]|BOLD:AAB4779
Nola minna|[378]|LBCF009-07|Canada|British Columbia|658[0n]|BOLD:AAB4779
Nola minna|[379]|LOCBB088-06|United States|California|658[0n]|BOLD:AAB4779
Nola minna|[380]|LOCBB087-06|United States|California|658[0n]|BOLD:AAB4779
Nola minna|[381]|LOCB877-06|United States|California|658[0n]|BOLD:AAB4779

Nola minna|[379]|LOCBB088-06|United States|California|658[0n]|BOLD:AAB4779
Nola minna|[380]|LOCBB087-06|United States|California|658[0n]|BOLD:AAB4779
Nola minna|[381]|LOCB877-06|United States|California|658[0n]|BOLD:AAB4779
Nola minna|[382]|LOCB876-06|United States|California|658[0n]|BOLD:AAB4779
Nola minna|[383]|LOCB817-06|United States|California|658[0n]|BOLD:AAB4779
Nola minna|[384]|LOCB807-06|United States|California|658[0n]|BOLD:AAB4779
Nola minna|[385]|LOCB805-06|United States|California|658[0n]|BOLD:AAB4779
Nola minna|[386]|LOCB804-06|United States|California|658[0n]|BOLD:AAB4779
Nola minna|[387]|LOCB803-06|United States|California|658[0n]|BOLD:AAB4779
Nola minna|[388]|LOCB725-06|United States|California|658[0n]|BOLD:AAB4779
Nola minna|[389]|LOCBB007-06|United States|California|658[0n]|BOLD:AAB4779
Nola minna|[390]|LTOL790-07|United States|California|658[0n]|BOLD:AAB4779
Nola minna|[391]|LTOL785-07|United States|California|658[0n]|BOLD:AAB4779
Nola minna|[392]|LOCB806-06|United States|California|658[0n]|BOLD:AAB4779
Nola minna|[393]|GBMIN20433-13|||620[0n]|BOLD:AAB4779
Nola minna|[394]|LOCBF229-13|United States|California|617[0n]|BOLD:AAB4779
Nola minna|[395]|LOCBF274-13|United States|California|616[0n]|BOLD:AAB4779
Nola minna|[396]|LOCBF2170-13|United States|California|609[0n]|BOLD:AAB4779
Nola cucullatella|[397]|CGUKB131-09|United Kingdom|England|646[0n]|BOLD:AAC2943
Nola cucullatella|[398]|CGUKD503-09|United Kingdom|England|658[0n]|BOLD:AAC2943
Nola cucullatella|[399]|CGUKA296-09|United Kingdom|England|632[0n]|BOLD:AAC2943
Nola cucullatella|[400]|CGUKB135-09|United Kingdom|England|658[0n]|BOLD:AAC2943
Nola cucullatella|[401]|RDNMD746-06|Denmark||658[0n]|BOLD:AAC2943
Nola cucullatella|[402]|LENOA1192-11|France|Haute Normandie|658[0n]|BOLD:AAC2943
Nola cucullatella|[403]|CGUKC005-09|United Kingdom|Wales|658[0n]|BOLD:AAC2943
Nola cucullatella|[404]|LEFIC788-10|Finland|Finland Proper|658[0n]|BOLD:AAC2943
Nola cucullatella|[405]|LEFIE701-10|Finland|Aland Islands|658[0n]|BOLD:AAC2943
Nola cucullatella|[406]|CGUKB555-09|United Kingdom|England|658[0n]|BOLD:AAC2943
Nola cucullatella|[407]|LON452-08|Norway|Akershus|657[0n]|BOLD:AAC2943
Nola cucullatella|[408]|RDNMD724-06|Denmark||658[0n]|BOLD:AAC2943
Nola cucullatella|[409]|CGUKC424-09|United Kingdom|England|612[0n]|BOLD:AAC2943
Nola cucullatella|[410]|LEFIC237-10|Finland|Uusimaa|658[0n]|BOLD:AAC2943
Nola cucullatella|[411]|CGUKD373-09|United Kingdom|England|658[0n]|BOLD:AAC2943
Nola cucullatella|[412]|GWOTD196-12|Germany|Mecklenburg-Vorpommern|658[0n]|BOLD:AAC2943
Nola cucullatella|[413]|IBLAO574-12|Spain|Castilla-La Mancha|658[0n]|BOLD:AAC2943
Nola cucullatella|[414]|IBLAO739-12|Spain|Andalusia|658[0n]|BOLD:AAC2943
Nola cucullatella|[415]|GWOTL131-13|Germany|Saarland|658[0n]|BOLD:AAC2943
Nola cucullatella|[416]|GWOTL132-13|Germany|Saarland|658[0n]|BOLD:AAC2943
Nola cucullatella|[417]|NLLEA585-12|Netherlands|South Holland|606[0n]|BOLD:AAC2943
Nola cucullatella|[418]|GWOR4216-09|Germany|Bavaria|658[0n]|BOLD:AAC2943
Nola cucullatella|[419]|FBLMU514-09|Germany|Bavaria|658[0n]|BOLD:AAC2943
Nola cucullatella|[420]|FBLMU515-09|Germany|Bavaria|658[0n]|BOLD:AAC2943
Nola cucullatella|[421]|PHLAE252-11|Austria|Tirol|658[0n]|BOLD:AAC2943
Nola cucullatella|[422]|GBLAB621-13|Germany|Brandenburg|658[0n]|BOLD:AAC2943
Nola cucullatella|[423]|LEATB524-13|Austria|Tirol|658[0n]|BOLD:AAC2943
Nola cucullatella|[424]|GBLAD420-14|Germany|Bavaria|658[0n]|BOLD:AAC2943
Nola cilicoides|[425]|RDNMC568-06|United States|Wyoming|592[1n]|BOLD:AAB4095
Nola cilicoides|[426]|RDNMC569-06|Canada|Alberta|658[0n]|BOLD:AAB4095
Nola cilicoides|[427]|XAJ736-06|Canada|Ontario|632[0n]|BOLD:AAB4095
Nola cilicoides|[428]|XAJ784-06|Canada|Ontario|658[0n]|BOLD:AAB4095
Nola cilicoides|[429]|RDLQG891-06|Canada|Quebec|658[0n]|BOLD:AAB4095
Nola cilicoides|[430]|LMEM073-09|United States|Alabama|573[1n]|BOLD:AAB4095
Nola cilicoides|[431]|LGSMC983-05|United States|Tennessee|606[0n]|BOLD:AAB4095
Nola cilicoides|[432]|LMEM071-09|United States|Alabama|658[0n]|BOLD:AAB4095
Nola cilicoides|[433]|LMEM072-09|United States|Alabama|658[0n]|BOLD:AAB4095
Nola cilicoides|[434]|LMEM081-09|United States|Alabama|658[0n]|BOLD:AAB4095
Nola cilicoides|[435]|BLTIB666-08|Canada|Ontario|658[0n]|BOLD:AAB4095
Nola cilicoides|[436]|LPAB531-08|Canada|Alberta|658[0n]|BOLD:AAB4095
Nola cilicoides|[437]|MNAF857-08|Canada|Manitoba|658[0n]|BOLD:AAB4095
Nola cilicoides|[438]|MNAF856-08|Canada|Manitoba|658[0n]|BOLD:AAB4095
Nola cilicoides|[439]|MNAF855-08|Canada|Manitoba|658[0n]|BOLD:AAB4095
Nola cilicoides|[440]|MNAF517-08|Canada|Manitoba|658[0n]|BOLD:AAB4095
Nola cilicoides|[441]|MNAF516-08|Canada|Manitoba|658[0n]|BOLD:AAB4095
Nola cilicoides|[442]|MNAF056-08|Canada|Manitoba|658[0n]|BOLD:AAB4095
Nola cilicoides|[443]|MNAF055-08|Canada|Manitoba|658[0n]|BOLD:AAB4095
Nola cilicoides|[444]|XAJ746-06|Canada|Ontario|657[0n]|BOLD:AAB4095
Nola cilicoides|[445]|XAJ738-06|Canada|Ontario|658[0n]|BOLD:AAB4095
Nola cilicoides|[446]|RDNMC566-06|Canada|Ontario|658[0n]|BOLD:AAB4095
Nola cilicoides|[447]|XAB488-04|Canada|Ontario|658[0n]|BOLD:AAB4095
Nola cilicoides|[448]|RDNMC567-06|Canada|Ontario|658[0n]|BOLD:AAB4095
Nola cilicoides|[449]|XAE593-04|Canada|Ontario|598[0n]|BOLD:AAB4095
Nola cilicoides|[450]|BLTIB618-08|Canada|Ontario|631[0n]|BOLD:AAB4095
Nola cilicoides|[451]|XAE592-04|Canada|Ontario|546[0n]|BOLD:AAB4095
Nola cilicoides|[452]|JSJUL1701-11|Canada|Ontario|620[0n]|BOLD:AAB4095
Nola pustulata|[453]|LTOL799-07|United States|Maryland|658[0n]|BOLD:ABZ7898
Nola pustulata|[454]|LSEU082-06|United States|Georgia|574[0n]|BOLD:ABZ7898
Nola pustulata|[455]|LTOL803-07|United States|Maryland|658[0n]|BOLD:ABZ7898
Nola pustulata|[456]|LTOL802-07|United States|Maryland|658[0n]|BOLD:ABZ7898
Nola pustulata|[457]|LMEM074-09|United States|Alabama|658[1n]|BOLD:ABZ7898
Nola pustulata|[458]|CNCLB1459-14|United States|Louisiana|658[0n]|BOLD:ABZ7898
Nola nr. pustulata|[459]|HKONS601-08|United States|Florida|658[0n]|BOLD:AAD8210
Nola nr. pustulata|[460]|CNCLB1461-14|United States|Louisiana|658[0n]|BOLD:AAD8210
Nola nr. pustulata|[461]|HKONS639-08|United States|Florida|658[0n]|BOLD:AAD8210
Nola nr. pustulata|[462]|MILEP374-10|United States|Alabama|658[0n]|BOLD:AAD8210
Nola nr. pustulata|[463]|LNCB599-09|United States|North Carolina|658[0n]|BOLD:AAD8210
Nola nr. pustulata|[464]|HKONS638-08|United States|Florida|658[0n]|BOLD:AAD8210
Nola nr. pustulata|[465]|HKONS637-08|United States|Florida|658[0n]|BOLD:AAD8210
Nola nr. pustulata|[466]|HKONS600-08|United States|Florida|658[0n]|BOLD:AAD8210
Nola nr. pustulata|[467]|LNC005-05|United States|North Carolina|658[0n]|BOLD:AAD8210
Nola nr. pustulata|[468]|CNCLB1460-14|United States|Louisiana|585[2n]|BOLD:AAD8210
Nola nr. pustulata|[469]|CNCLB1464-14|United States|Louisiana|658[0n]|BOLD:AAD8210
Cephalospargeta elongata|[470]|HKONB192-09|United States|Texas|658[0n]|BOLD:AAE9740
Cephalospargeta elongata|[471]|MHAYA063-06|Costa Rica|Guanacaste|622[0n]|BOLD:AAE9740
Cephalospargeta elongata|[472]|BLPBG148-07|Costa Rica|Guanacaste|658[0n]|BOLD:AAE9740
Cephalospargeta elongata|[473]|BLPBH667-07|Costa Rica|Guanacaste|658[1n]|BOLD:AAE9740
Cephalospargeta elongata|[474]|HKONB177-08|United States|Texas|658[0n]|BOLD:AAE9740
Cephalospargeta elongata|[475]|HKONB178-08|United States|Texas|658[0n]|BOLD:AAE9740
Cephalospargeta elongata|[476]|HKONB193-09|United States|Texas|658[0n]|BOLD:AAE9740

Cephalospargeta elongata|[474]|HKONB177-08|United States|Texas|658[0n]|BOLD:AAE9740
Cephalospargeta elongata|[475]|HKONB178-08|United States|Texas|658[0n]|BOLD:AAE9740
Cephalospargeta elongata|[476]|HKONB193-09|United States|Texas|658[0n]|BOLD:AAE9740
Garella nilotica|[477]|BLPDW494-11|Costa Rica|Guanacaste|658[0n]|BOLD:AAA0951
Garella nilotica|[478]|BLPDU1013-11|Costa Rica|Guanacaste|658[0n]|BOLD:AAA0951
Garella nilotica|[479]|BLPDL1909-10|Costa Rica|Guanacaste|658[0n]|BOLD:AAA0951
Garella nilotica|[480]|RDLQC239-06|Canada|Quebec|658[0n]|BOLD:AAA0951
Garella nilotica|[481]|BLPDW538-11|Costa Rica|Guanacaste|658[0n]|BOLD:AAA0951
Garella nilotica|[482]|MHMYS2843-13|Costa Rica|Guanacaste|658[0n]|BOLD:AAA0951
Garella nilotica|[483]|MHMYS2844-13|Costa Rica|Guanacaste|658[0n]|BOLD:AAA0951
Garella nilotica|[484]|HKONS071-07|United States|Florida|658[1n]|BOLD:AAA0951
Garella nilotica|[485]|MHMYS2846-13|Costa Rica|Guanacaste|658[0n]|BOLD:AAA0951
Garella nilotica|[486]|MHMYS2849-13|Costa Rica|Guanacaste|658[0n]|BOLD:AAA0951
Garella nilotica|[487]|BLPDV527-11|Costa Rica|Guanacaste|658[0n]|BOLD:AAA0951
Garella nilotica|[488]|BLPDM182-10|Costa Rica|Guanacaste|658[0n]|BOLD:AAA0951
Garella nilotica|[489]|BLPDK944-09|Costa Rica|Guanacaste|658[0n]|BOLD:AAA0951
Garella nilotica|[490]|MHMYS2847-13|Costa Rica|Guanacaste|658[0n]|BOLD:AAA0951
Garella nilotica|[491]|MHMYS3389-13|Costa Rica|Guanacaste|658[0n]|BOLD:AAA0951
Garella nilotica|[492]|BLPDM552-10|Costa Rica|Guanacaste|658[0n]|BOLD:AAA0951
Garella nilotica|[493]|MHMYE396-09|Costa Rica|Guanacaste|658[0n]|BOLD:AAA0951
Garella nilotica|[494]|BLPDU653-11|Costa Rica|Guanacaste|658[0n]|BOLD:AAA0951
Garella nilotica|[495]|BLPDV536-11|Costa Rica|Guanacaste|658[0n]|BOLD:AAA0951
Garella nilotica|[496]|BLPDW496-11|Costa Rica|Guanacaste|658[0n]|BOLD:AAA0951
Garella nilotica|[497]|BLPDW500-11|Costa Rica|Guanacaste|658[0n]|BOLD:AAA0951
Garella nilotica|[498]|MHMYS2853-13|Costa Rica|Guanacaste|658[0n]|BOLD:AAA0951
Garella nilotica|[499]|MHMYS2855-13|Costa Rica|Guanacaste|658[0n]|BOLD:AAA0951
Garella nilotica|[500]|BLPDL1868-10|Costa Rica|Guanacaste|658[0n]|BOLD:AAA0951
Garella nilotica|[501]|RDLQD449-06|Canada|Quebec|658[0n]|BOLD:AAA0951
Garella nilotica|[502]|BLPDT1825-10|Costa Rica|Guanacaste|658[0n]|BOLD:AAA0951
Garella nilotica|[503]|BLPDU989-11|Costa Rica|Guanacaste|658[0n]|BOLD:AAA0951
Garella nilotica|[504]|BLPDW490-11|Costa Rica|Guanacaste|658[0n]|BOLD:AAA0951
Garella nilotica|[505]|MHMYS2841-13|Costa Rica|Guanacaste|658[0n]|BOLD:AAA0951
Garella nilotica|[506]|MHMYS2842-13|Costa Rica|Guanacaste|658[0n]|BOLD:AAA0951
Garella nilotica|[507]|BLPDW463-11|Costa Rica|Guanacaste|658[0n]|BOLD:AAA0951
Garella nilotica|[508]|BLPDU009-11|Costa Rica|Guanacaste|658[0n]|BOLD:AAA0951
Garella nilotica|[509]|BLPDK1630-09|Costa Rica|Guanacaste|658[0n]|BOLD:AAA0951
Garella nilotica|[510]|LPOKB883-09|United States|Oklahoma|658[0n]|BOLD:AAA0951
Garella nilotica|[511]|LILLA970-11|United States|Illinois|658[0n]|BOLD:AAA0951
Garella nilotica|[512]|BLPEF2726-13|Costa Rica|Guanacaste|658[0n]|BOLD:AAA0951
Garella nilotica|[513]|BLPEF2847-13|Costa Rica|Guanacaste|658[0n]|BOLD:AAA0951
Garella nilotica|[514]|MHMYS3081-13|Costa Rica|Guanacaste|658[0n]|BOLD:AAA0951
Garella nilotica|[515]|MHMYS3082-13|Costa Rica|Guanacaste|658[0n]|BOLD:AAA0951
Garella nilotica|[516]|BLPDW919-11|Costa Rica|Guanacaste|658[0n]|BOLD:AAA0951
Garella nilotica|[517]|BLPDW926-11|Costa Rica|Guanacaste|658[0n]|BOLD:AAA0951
Garella nilotica|[518]|BLPDW015-11|Costa Rica|Guanacaste|658[0n]|BOLD:AAA0951
Garella nilotica|[519]|BLPDT1774-10|Costa Rica|Guanacaste|658[0n]|BOLD:AAA0951
Garella nilotica|[520]|BLPDK977-09|Costa Rica|Guanacaste|658[0n]|BOLD:AAA0951
Garella nilotica|[521]|BLPDW955-11|Costa Rica|Guanacaste|658[0n]|BOLD:AAA0951
Garella nilotica|[522]|BLPDK1631-09|Costa Rica|Guanacaste|658[0n]|BOLD:AAA0951
Garella nilotica|[523]|BLPDW497-11|Costa Rica|Guanacaste|658[0n]|BOLD:AAA0951
Garella nilotica|[524]|BBLSX433-09|United States|Oklahoma|658[0n]|BOLD:AAA0951
Garella nilotica|[525]|LPOKC268-09|United States|Oklahoma|658[0n]|BOLD:AAA0951
Garella nilotica|[526]|LPOKE510-12|United States|Oklahoma|611[0n]|BOLD:AAA0951
Garella nilotica|[527]|MHMYS2946-13|Costa Rica|Guanacaste|658[0n]|BOLD:AAA0951
Garella nilotica|[528]|MHMYS2952-13|Costa Rica|Guanacaste|658[0n]|BOLD:AAA0951
Garella nilotica|[529]|MHMYS3385-13|Costa Rica|Guanacaste|658[0n]|BOLD:AAA0951
Garella nilotica|[530]|BLPDU079-11|Costa Rica|Guanacaste|658[0n]|BOLD:AAA0951
Garella nilotica|[531]|LPOKC873-09|United States|Oklahoma|658[0n]|BOLD:AAA0951
Garella nilotica|[532]|BLPCK233-08|Costa Rica|Alajuela|658[0n]|BOLD:AAA0951
Garella nilotica|[533]|BLPDV1215-11|Costa Rica|Guanacaste|658[0n]|BOLD:AAA0951
Garella nilotica|[534]|BLPDW469-11|Costa Rica|Guanacaste|658[0n]|BOLD:AAA0951
Garella nilotica|[535]|BLPEF4857-13|Costa Rica|Guanacaste|658[0n]|BOLD:AAA0951
Garella nilotica|[536]|BLPEF5692-13|Costa Rica||658[0n]|BOLD:AAA0951
Garella nilotica|[537]|AWCLB147-10|United States|Arizona|658[0n]|BOLD:AAA0951
Garella nilotica|[538]|BLPDK1079-09|Costa Rica|Guanacaste|658[0n]|BOLD:AAA0951
Garella nilotica|[539]|LPOKA806-09|United States|Oklahoma|658[0n]|BOLD:AAA0951
Garella nilotica|[540]|BLPDU037-11|Costa Rica|Guanacaste|658[0n]|BOLD:AAA0951
Garella nilotica|[541]|BLPDW455-11|Costa Rica|Guanacaste|658[0n]|BOLD:AAA0951
Garella nilotica|[542]|BLPDW934-11|Costa Rica|Guanacaste|658[0n]|BOLD:AAA0951
Garella nilotica|[543]|BLPDW954-11|Costa Rica|Guanacaste|658[0n]|BOLD:AAA0951
Garella nilotica|[544]|MHMYS3149-13|Costa Rica|Guanacaste|658[0n]|BOLD:AAA0951
Garella nilotica|[545]|MHMYS3150-13|Costa Rica|Guanacaste|658[0n]|BOLD:AAA0951
Garella nilotica|[546]|BLPDW470-11|Costa Rica|Guanacaste|658[0n]|BOLD:AAA0951
Garella nilotica|[547]|BLPDU085-11|Costa Rica|Guanacaste|658[0n]|BOLD:AAA0951
Garella nilotica|[548]|CMAZA070-09|United States|Arizona|658[0n]|BOLD:AAA0951
Garella nilotica|[549]|BLPDG862-09|Costa Rica|Guanacaste|658[0n]|BOLD:AAA0951
Garella nilotica|[550]|BLPDW560-11|Costa Rica|Guanacaste|658[0n]|BOLD:AAA0951
Garella nilotica|[551]|BLPEF5693-13|Costa Rica||658[0n]|BOLD:AAA0951
Garella nilotica|[552]|BLPEF5699-13|Costa Rica||658[0n]|BOLD:AAA0951
Garella nilotica|[553]|BLPDW957-11|Costa Rica|Guanacaste|658[0n]|BOLD:AAA0951
Garella nilotica|[554]|MHMYK2683-11|Costa Rica||658[0n]|BOLD:AAA0951
Garella nilotica|[555]|BLPDW456-11|Costa Rica|Guanacaste|658[0n]|BOLD:AAA0951
Garella nilotica|[556]|AWCLB266-10|United States|Arizona|658[0n]|BOLD:AAA0951
Garella nilotica|[557]|BLPDK1092-09|Costa Rica|Guanacaste|658[0n]|BOLD:AAA0951
Garella nilotica|[558]|RDLQE283-06|Canada|Quebec|658[0n]|BOLD:AAA0951
Garella nilotica|[559]|BBLSX667-09|United States|Oklahoma|658[0n]|BOLD:AAA0951
Garella nilotica|[560]|MHMYS2851-13|Costa Rica|Guanacaste|658[0n]|BOLD:AAA0951
Garella nilotica|[561]|MHMYS3079-13|Costa Rica|Guanacaste|658[0n]|BOLD:AAA0951
Garella nilotica|[562]|MHMYS3151-13|Costa Rica|Guanacaste|658[0n]|BOLD:AAA0951
Garella nilotica|[563]|MHMYS3152-13|Costa Rica|Guanacaste|658[0n]|BOLD:AAA0951
Garella nilotica|[564]|BLPEF5875-13|Costa Rica||658[0n]|BOLD:AAA0951
Garella nilotica|[565]|MHMYS2758-13|Costa Rica|Guanacaste|658[0n]|BOLD:AAA0951
Garella nilotica|[566]|BLPDW475-11|Costa Rica|Guanacaste|658[0n]|BOLD:AAA0951
Garella nilotica|[567]|BLPDU107-11|Costa Rica|Guanacaste|658[0n]|BOLD:AAA0951
Garella nilotica|[568]|BLPDL1823-10|Costa Rica|Guanacaste|658[0n]|BOLD:AAA0951
Garella nilotica|[569]|HKONB196-09|United States|Texas|658[0n]|BOLD:AAA0951
Garella nilotica|[570]|MHMYS2953-13|Costa Rica|Guanacaste|658[0n]|BOLD:AAA0951
Garella nilotica|[571]|BLPDK1247-09|Costa Rica|Guanacaste|658[0n]|BOLD:AAA0951

Garella nilotica|[569]|HKONB196-09|United States|Texas|658[0n]|BOLD:AAA0951
Garella nilotica|[570]|MHMYS2953-13|Costa Rica|Guanacaste|658[0n]|BOLD:AAA0951
Garella nilotica|[571]|BLPDK1247-09|Costa Rica|Guanacaste|658[0n]|BOLD:AAA0951
Garella nilotica|[572]|BLPDH296-09|Costa Rica|Guanacaste|658[0n]|BOLD:AAA0951
Garella nilotica|[573]|BLPDK1603-09|Costa Rica|Guanacaste|658[0n]|BOLD:AAA0951
Garella nilotica|[574]|BBLPA618-10|Canada|Ontario|658[0n]|BOLD:AAA0951
Garella nilotica|[575]|BLPDW457-11|Costa Rica|Guanacaste|658[0n]|BOLD:AAA0951
Garella nilotica|[576]|LILLA942-11|United States|Illinois|658[0n]|BOLD:AAA0951
Garella nilotica|[577]|CMAZA1192-12|United States|Arizona|658[0n]|BOLD:AAA0951
Garella nilotica|[578]|MHMYS3294-13|Costa Rica|Guanacaste|658[0n]|BOLD:AAA0951
Garella nilotica|[579]|MHMYS3295-13|Costa Rica|Guanacaste|658[0n]|BOLD:AAA0951
Garella nilotica|[580]|BLPDW477-11|Costa Rica|Guanacaste|658[0n]|BOLD:AAA0951
Garella nilotica|[581]|BLPDU325-11|Costa Rica|Guanacaste|658[0n]|BOLD:AAA0951
Garella nilotica|[582]|BLPDL1824-10|Costa Rica|Guanacaste|658[0n]|BOLD:AAA0951
Garella nilotica|[583]|LOCRC432-08|Costa Rica|Guanacaste|658[0n]|BOLD:AAA0951
Garella nilotica|[584]|BLPDW531-11|Costa Rica|Guanacaste|658[0n]|BOLD:AAA0951
Garella nilotica|[585]|BLPDW939-11|Costa Rica|Guanacaste|658[0n]|BOLD:AAA0951
Garella nilotica|[586]|MHMYS2759-13|Costa Rica|Guanacaste|658[0n]|BOLD:AAA0951
Garella nilotica|[587]|MHMYS2838-13|Costa Rica|Guanacaste|658[0n]|BOLD:AAA0951
Garella nilotica|[588]|BLPEE216-12|Costa Rica|Guanacaste|658[0n]|BOLD:AAA0951
Garella nilotica|[589]|BLPEE1636-12|Costa Rica|Guanacaste|658[0n]|BOLD:AAA0951
Garella nilotica|[590]|BLPDW458-11|Costa Rica|Guanacaste|658[0n]|BOLD:AAA0951
Garella nilotica|[591]|BBLPA700-10|Canada|Ontario|658[0n]|BOLD:AAA0951
Garella nilotica|[592]|BLPDK1249-09|Costa Rica|Guanacaste|658[0n]|BOLD:AAA0951
Garella nilotica|[593]|BLPBH230-07|Costa Rica|Guanacaste|658[0n]|BOLD:AAA0951
Garella nilotica|[594]|BLPEE024-12|Costa Rica|Guanacaste|658[0n]|BOLD:AAA0951
Garella nilotica|[595]|MHMYS2947-13|Costa Rica|Guanacaste|658[0n]|BOLD:AAA0951
Garella nilotica|[596]|MHMYS3296-13|Costa Rica|Guanacaste|658[0n]|BOLD:AAA0951
Garella nilotica|[597]|MHMYS3382-13|Costa Rica|Guanacaste|658[0n]|BOLD:AAA0951
Garella nilotica|[598]|BLPDL1864-10|Costa Rica|Guanacaste|658[0n]|BOLD:AAA0951
Garella nilotica|[599]|BBUSA791-09|United States|Arizona|658[0n]|BOLD:AAA0951
Garella nilotica|[600]|BLPDL1875-10|Costa Rica|Guanacaste|658[0n]|BOLD:AAA0951
Garella nilotica|[601]|BLPDU951-11|Costa Rica|Guanacaste|658[0n]|BOLD:AAA0951
Garella nilotica|[602]|BLPDW487-11|Costa Rica|Guanacaste|658[0n]|BOLD:AAA0951
Garella nilotica|[603]|MHMYS2839-13|Costa Rica|Guanacaste|658[0n]|BOLD:AAA0951
Garella nilotica|[604]|MHMYS2840-13|Costa Rica|Guanacaste|658[0n]|BOLD:AAA0951
Garella nilotica|[605]|LPOKC490-09|United States|Oklahoma|658[0n]|BOLD:AAA0951
Garella nilotica|[606]|BLPDK1235-09|Costa Rica|Guanacaste|658[0n]|BOLD:AAA0951
Garella nilotica|[607]|BLPDK1340-09|Costa Rica|Guanacaste|658[0n]|BOLD:AAA0951
Garella nilotica|[608]|BLPDU001-11|Costa Rica|Guanacaste|658[0n]|BOLD:AAA0951
Garella nilotica|[609]|BLPDW461-11|Costa Rica|Guanacaste|658[0n]|BOLD:AAA0951
Garella nilotica|[610]|BLPEF123-12|Costa Rica|Alajuela|658[0n]|BOLD:AAA0951
Garella nilotica|[611]|BLPEF1498-12|Costa Rica|Guanacaste|658[0n]|BOLD:AAA0951
Garella nilotica|[612]|MHMYS3383-13|Costa Rica|Guanacaste|658[0n]|BOLD:AAA0951
Garella nilotica|[613]|MHMYS3384-13|Costa Rica|Guanacaste|658[0n]|BOLD:AAA0951
Garella nilotica|[614]|MHMYS2956-13|Costa Rica|Guanacaste|658[0n]|BOLD:AAA0951
Garella nilotica|[615]|MHMYS2993-13|Costa Rica|Guanacaste|658[0n]|BOLD:AAA0951
Garella nilotica|[616]|BLPDW549-11|Costa Rica|Guanacaste|658[0n]|BOLD:AAA0951
Garella nilotica|[617]|BLPDW552-11|Costa Rica|Guanacaste|658[0n]|BOLD:AAA0951
Garella nilotica|[618]|BLPDW454-11|Costa Rica|Guanacaste|658[0n]|BOLD:AAA0951
Garella nilotica|[619]|BLPDT1831-10|Costa Rica|Guanacaste|658[0n]|BOLD:AAA0951
Garella nilotica|[620]|BLPDK1067-09|Costa Rica|Guanacaste|658[0n]|BOLD:AAA0951
Garella nilotica|[621]|RDLQC238-06|Canada|Quebec|658[0n]|BOLD:AAA0951
Garella nilotica|[622]|BLPEF2417-13|Costa Rica|Guanacaste|616[0n]|BOLD:AAA0951
Garella nilotica|[623]|LOCBB247-06|United States|California|658[0n]|BOLD:AAA0951
Garella nilotica|[624]|GBMIN20357-13|||616[0n]|BOLD:AAA0951
Garella nilotica|[625]|MHMYS2848-13|Costa Rica|Guanacaste|621[0n]|BOLD:AAA0951
Garella nilotica|[626]|BLPDK425-09|Costa Rica|Guanacaste|622[0n]|BOLD:AAA0951
Garella nilotica|[627]|LOCRC433-08|Costa Rica|Guanacaste|658[0n]|BOLD:AAA0951
Garella nilotica|[628]|BLPDV1232-11|Costa Rica|Guanacaste|658[0n]|BOLD:AAA0951
Garella nilotica|[629]|BLPED124-11|Costa Rica|Guanacaste|658[0n]|BOLD:AAA0951
Garella nilotica|[630]|MHMYS2850-13|Costa Rica|Guanacaste|623[0n]|BOLD:AAA0951
Garella nilotica|[631]|BLPDW486-11|Costa Rica|Guanacaste|658[0n]|BOLD:AAA0951
Garella nilotica|[632]|BLPDU598-11|Costa Rica|Guanacaste|658[0n]|BOLD:AAA0951
Garella nilotica|[633]|BLPDL1854-10|Costa Rica|Guanacaste|658[0n]|BOLD:AAA0951
Garella nilotica|[634]|HKONS625-08|United States|Florida|658[0n]|BOLD:AAA0951
Garella nilotica|[635]|BLPDJ176-09|Costa Rica|Guanacaste|636[0n]|BOLD:AAA0951
Garella nilotica|[636]|BLPDL1840-10|Costa Rica|Guanacaste|630[0n]|BOLD:AAA0951
Garella nilotica|[637]|BLPDU101-11|Costa Rica|Guanacaste|658[0n]|BOLD:AAA0951
Garella nilotica|[638]|BLPDW546-11|Costa Rica|Guanacaste|658[0n]|BOLD:AAA0951
Garella nilotica|[639]|LOCRC431-08|Costa Rica|Guanacaste|658[0n]|BOLD:AAA0951
Garella nilotica|[640]|BLPDM153-10|Costa Rica|Guanacaste|658[0n]|BOLD:AAA0951
Garella nilotica|[641]|BLPEF1501-12|Costa Rica|Guanacaste|658[0n]|BOLD:AAA0951
Garella nilotica|[642]|MHMYS3297-13|Costa Rica|Guanacaste|633[0n]|BOLD:AAA0951
Garella nilotica|[643]|BLPEF2849-13|Costa Rica|Guanacaste|658[0n]|BOLD:AAA0951
Garella nilotica|[644]|BLPEF4856-13|Costa Rica|Guanacaste|658[0n]|BOLD:AAA0951
Garella nilotica|[645]|BLPDW468-11|Costa Rica|Guanacaste|658[0n]|BOLD:AAA0951
Garella nilotica|[646]|BLPDU070-11|Costa Rica|Guanacaste|658[0n]|BOLD:AAA0951
Garella nilotica|[647]|LPOKD094-09|United States|Oklahoma|658[0n]|BOLD:AAA0951
Garella nilotica|[648]|HKONS070-07|United States|Florida|655[0n]|BOLD:AAA0951
Garella nilotica|[649]|BLPDW526-11|Costa Rica|Guanacaste|658[0n]|BOLD:AAA0951
Garella nilotica|[650]|MHMYS3298-13|Costa Rica|Guanacaste|658[0n]|BOLD:AAA0951
Garella nilotica|[651]|HKONS626-08|United States|Florida|658[0n]|BOLD:AAA0951
Garella nilotica|[652]|BLPDK886-09|Costa Rica|Guanacaste|658[0n]|BOLD:AAA0951
Garella nilotica|[653]|BLPDM623-10|Costa Rica|Guanacaste|658[0n]|BOLD:AAA0951
Garella nilotica|[654]|BLPDV656-11|Costa Rica|Guanacaste|658[0n]|BOLD:AAA0951
Garella nilotica|[655]|BLPDW509-11|Costa Rica|Guanacaste|658[0n]|BOLD:AAA0951
Garella nilotica|[656]|BLPDW520-11|Costa Rica|Guanacaste|658[0n]|BOLD:AAA0951
Garella nilotica|[657]|MHMYS2948-13|Costa Rica|Guanacaste|658[0n]|BOLD:AAA0951
Garella nilotica|[658]|MHMYS2949-13|Costa Rica|Guanacaste|658[0n]|BOLD:AAA0951
Garella nilotica|[659]|LPOKA719-09|United States|Oklahoma|658[0n]|BOLD:AAA0951
Garella nilotica|[660]|BBLSX440-09|United States|Oklahoma|658[0n]|BOLD:AAA0951
Garella nilotica|[661]|BLPDR724-10|Costa Rica|Alajuela|658[0n]|BOLD:AAA0951
Garella nilotica|[662]|BLPDV727-11|Costa Rica|Guanacaste|658[0n]|BOLD:AAA0951
Garella nilotica|[663]|BLPDW522-11|Costa Rica|Guanacaste|658[0n]|BOLD:AAA0951
Garella nilotica|[664]|BLPDW530-11|Costa Rica|Guanacaste|658[0n]|BOLD:AAA0951
Garella nilotica|[665]|MHMYS2950-13|Costa Rica|Guanacaste|658[0n]|BOLD:AAA0951
Garella nilotica|[666]|MHMYS2951-13|Costa Rica|Guanacaste|658[0n]|BOLD:AAA0951

Garella nilotica|[664]|BLPDW530-11|Costa Rica|Guanacaste|658[0n]|BOLD:AAA0951
Garella nilotica|[665]|MHMYS2950-13|Costa Rica|Guanacaste|658[0n]|BOLD:AAA0951
Garella nilotica|[666]|MHMYS2951-13|Costa Rica|Guanacaste|658[0n]|BOLD:AAA0951
Garella nilotica|[667]|MHMYS2994-13|Costa Rica|Guanacaste|658[0n]|BOLD:AAA0951
Garella nilotica|[668]|MHMYS3080-13|Costa Rica|Guanacaste|658[0n]|BOLD:AAA0951
Garella nilotica|[669]|BLPDV1202-11|Costa Rica|Guanacaste|658[0n]|BOLD:AAA0951
Garella nilotica|[670]|LPOKD791-10|United States|Oklahoma|658[0n]|BOLD:AAA0951
Garella nilotica|[671]|BBLSX683-09|United States|Arizona|658[0n]|BOLD:AAA0951
Garella nilotica|[672]|BLPDW480-11|Costa Rica|Guanacaste|658[0n]|BOLD:AAA0951
Garella nilotica|[673]|BLPDW543-11|Costa Rica|Guanacaste|658[0n]|BOLD:AAA0951
Garella nilotica|[674]|BLPDW547-11|Costa Rica|Guanacaste|658[0n]|BOLD:AAA0951
Garella nilotica|[675]|MHMYS2954-13|Costa Rica|Guanacaste|658[0n]|BOLD:AAA0951
Garella nilotica|[676]|MHMYS2955-13|Costa Rica|Guanacaste|658[0n]|BOLD:AAA0951
Garella nilotica|[677]|BLPDW557-11|Costa Rica|Guanacaste|658[0n]|BOLD:AAA0951
Garella nilotica|[678]|BLPDW917-11|Costa Rica|Guanacaste|658[0n]|BOLD:AAA0951
Garella nilotica|[679]|BLPDW501-11|Costa Rica|Guanacaste|658[0n]|BOLD:AAA0951
Garella nilotica|[680]|BLPDW508-11|Costa Rica|Guanacaste|658[0n]|BOLD:AAA0951
Garella nilotica|[681]|LPOKC542-09|United States|Oklahoma|658[0n]|BOLD:AAA0951
Garella nilotica|[682]|BLPDK1088-09|Costa Rica|Guanacaste|658[0n]|BOLD:AAA0951
Garella nilotica|[683]|BLPDT1517-10|Costa Rica|Guanacaste|658[0n]|BOLD:AAA0951
Garella nilotica|[684]|BLPDX958-11|Costa Rica|Guanacaste|658[0n]|BOLD:AAA0951
Garella nilotica|[685]|BLPDX104-11|Costa Rica|Guanacaste|658[0n]|BOLD:AAA0951
Garella nilotica|[686]|BLPDT1724-10|Costa Rica|Guanacaste|658[0n]|BOLD:AAA0951
Garella nilotica|[687]|BLPDV666-11|Costa Rica|Guanacaste|658[4n]|BOLD:AAA0951
Garella nilotica|[688]|LOCRC434-08|Costa Rica|Guanacaste|658[0n]|BOLD:AAA0951
Garella nilotica|[689]|MHMYS2845-13|Costa Rica|Guanacaste|658[0n]|BOLD:AAA0951
Garella nilotica|[690]|MHMYS3386-13|Costa Rica|Guanacaste|658[0n]|BOLD:AAA0951
Garella nilotica|[691]|MHMYS2852-13|Costa Rica|Guanacaste|615[0n]|BOLD:AAA0951
Garella nilotica|[692]|LPOKC903-09|United States|Oklahoma|614[0n]|BOLD:AAA0951
Garella nilotica|[693]|MHMYS2992-13|Costa Rica|Guanacaste|658[0n]|BOLD:AAA0951
Garella nilotica|[694]|IAWL413-09|United States|Arizona|658[4n]|BOLD:AAA0951
Garella nilotica|[695]|BLPDV1203-11|Costa Rica|Guanacaste|658[0n]|BOLD:AAA0951
Garella nilotica|[696]|BLPDW545-11|Costa Rica|Guanacaste|658[0n]|BOLD:AAA0951
Garella nilotica|[697]|BCMI188-11|Israel||658[0n]|BOLD:AAA0951
Garella nilotica|[698]|BCMI328-11|Israel||658[0n]|BOLD:AAA0951
Baileya doubledayi|[699]|TMNBB085-06|Canada|New Brunswick|658[0n]|BOLD:ABX6740
Baileya doubledayi|[700]|LPSOD757-09|Canada|Ontario|658[0n]|BOLD:ABX6740
Baileya doubledayi|[701]|LPSOD765-09|Canada|Ontario|658[0n]|BOLD:ABX6740
Baileya doubledayi|[702]|LNCB469-07|United States|North Carolina|658[0n]|BOLD:ABX6740
Baileya doubledayi|[703]|LNCB158-06|United States|North Carolina|658[0n]|BOLD:ABX6740
Baileya doubledayi|[704]|LOCT268-05|United States|Connecticut|658[0n]|BOLD:ABX6740
Baileya doubledayi|[705]|HKONS075-07|United States|Florida|658[0n]|BOLD:ABX6740
Baileya doubledayi|[706]|HKONS610-08|United States|Florida|658[0n]|BOLD:ABX6740
Baileya doubledayi|[707]|RDNMK217-11|Canada|New Brunswick|658[0n]|BOLD:ABX6740
Baileya doubledayi|[708]|RDNMK216-11|Canada|Ontario|658[0n]|BOLD:AAC3802
Baileya doubledayi|[709]|XAG531-05|Canada|Ontario|539[0n]|BOLD:AAC3802
Baileya doubledayi|[710]|MECB136-04|Canada|Quebec|658[0n]|BOLD:AAC3802
Baileya doubledayi|[711]|MECB137-04|Canada|Quebec|658[0n]|BOLD:AAC3802
Baileya doubledayi|[712]|RDLQG318-06|Canada|Quebec|658[0n]|BOLD:AAC3802
Baileya doubledayi|[713]|RDNMK214-11|Canada|Ontario|658[0n]|BOLD:AAC3802
Baileya doubledayi|[714]|RDNMK215-11|Canada|Ontario|658[0n]|BOLD:AAC3802
Baileya doubledayi|[715]|RDNMK218-11|Canada|Ontario|658[0n]|BOLD:AAC3802
Baileya dormitans|[716]|XAB377-04|Canada|Ontario|658[0n]|BOLD:AAB0524
Baileya dormitans|[717]|XAK191-06|Canada|Ontario|658[0n]|BOLD:AAB0524
Baileya dormitans|[718]|KPOEC159-08|Canada|Ontario|658[1n]|BOLD:AAB0524
Baileya dormitans|[719]|XAJ445-06|Canada|Ontario|658[0n]|BOLD:AAB0524
Baileya dormitans|[720]|PHMO104-03|Canada|Ontario|639[2n]|BOLD:AAB0524
Baileya dormitans|[721]|XAB633-04|Canada|Ontario|658[0n]|BOLD:AAB0524
Baileya dormitans|[722]|XAE480-04|Canada|Ontario|658[0n]|BOLD:AAB0524
Baileya dormitans|[723]|MNBB047-05|Canada|New Brunswick|658[0n]|BOLD:AAB0524
Baileya dormitans|[724]|RDNMK210-11|Canada|Ontario|658[0n]|BOLD:AAB0524
Baileya dormitans|[725]|PHMO094-03|Canada|Ontario|639[0n]|BOLD:AAB0524
Baileya dormitans|[726]|XAB208-04|Canada|Ontario|658[0n]|BOLD:AAB0524
Baileya dormitans|[727]|XAB319-04|Canada|Ontario|658[0n]|BOLD:AAB0524
Baileya dormitans|[728]|XAB504-04|Canada|Ontario|658[0n]|BOLD:AAB0524
Baileya dormitans|[729]|XAC466-04|Canada|Ontario|658[0n]|BOLD:AAB0524
Baileya dormitans|[730]|XAF553-05|Canada|Ontario|658[0n]|BOLD:AAB0524
Baileya dormitans|[731]|XAD726-05|Canada|Ontario|658[0n]|BOLD:AAB0524
Baileya dormitans|[732]|XAJ396-06|Canada|Ontario|658[0n]|BOLD:AAB0524
Baileya dormitans|[733]|XAJ504-06|Canada|Ontario|658[0n]|BOLD:AAB0524
Baileya dormitans|[734]|XAK188-06|Canada|Ontario|658[0n]|BOLD:AAB0524
Baileya dormitans|[735]|RDLQF228-06|Canada|Quebec|658[0n]|BOLD:AAB0524
Baileya dormitans|[736]|RDLQG257-06|Canada|Quebec|658[0n]|BOLD:AAB0524
Baileya dormitans|[737]|RDNMK212-11|Canada|Ontario|658[0n]|BOLD:AAB0524
Baileya dormitans|[738]|RDNMK213-11|Canada|Ontario|658[0n]|BOLD:AAB0524
Baileya acadiana|[739]|CNCLA5259-13|United States|Mississippi|658[1n]|BOLD:AAB0524
Baileya dormitans|[740]|LNCC1628-13|United States|North Carolina|658[5n]|BOLD:AAB0524
Baileya dormitans|[741]|HKONS612-08|United States|Florida|609[0n]|BOLD:AAB0524
Baileya dormitans|[742]|BBLOC1244-11|United States|Arkansas|658[0n]|BOLD:AAB0524
Baileya dormitans|[743]|HKONS611-08|United States|Florida|658[0n]|BOLD:AAB0524
Baileya dormitans|[744]|LPOKB796-09|United States|Oklahoma|658[2n]|BOLD:AAB0524
Baileya dormitans|[745]|LNCC1405-11|United States|North Carolina|658[0n]|BOLD:AAB0524
Baileya dormitans|[746]|LNCC1367-11|United States|North Carolina|658[0n]|BOLD:AAB0524
Baileya dormitans|[747]|LNCC1342-11|United States|North Carolina|658[0n]|BOLD:AAB0524
Baileya dormitans|[748]|LPOKE144-10|United States|Oklahoma|658[0n]|BOLD:AAB0524
Baileya dormitans|[749]|LPOKE135-10|United States|Oklahoma|658[0n]|BOLD:AAB0524
Baileya dormitans|[750]|LPOKE115-10|United States|Oklahoma|658[0n]|BOLD:AAB0524
Baileya dormitans|[751]|LPOKE110-10|United States|Oklahoma|658[0n]|BOLD:AAB0524
Baileya dormitans|[752]|LPOKE108-10|United States|Oklahoma|658[0n]|BOLD:AAB0524
Baileya dormitans|[753]|LPOKA210-08|United States|Oklahoma|658[0n]|BOLD:AAB0524
Baileya dormitans|[754]|LPOKA089-08|United States|Oklahoma|658[0n]|BOLD:AAB0524
Baileya dormitans|[755]|LNC288-05|United States|North Carolina|658[0n]|BOLD:AAB0524
Baileya dormitans|[756]|LOTB323-05|United States|Tennessee|658[0n]|BOLD:AAB0524
Baileya dormitans|[757]|LOTB152-05|United States|Tennessee|658[0n]|BOLD:AAB0524
Baileya dormitans|[758]|LPOKA489-09|United States|Oklahoma|588[0n]|BOLD:AAB0524
Baileya dormitans|[759]|LGSM752-04|United States|Tennessee|658[0n]|BOLD:AAB0524
Baileya dormitans|[760]|LGSM670-04|United States|Tennessee|658[0n]|BOLD:AAB0524
Baileya dormitans|[761]|LSUSA240-06|United States|Kentucky|658[0n]|BOLD:AAB0524

Baileya dormitans|[759]|LGSM752-04|United States|Tennessee|658[0n]|BOLD:AAB0524
Baileya dormitans|[760]|LGSM670-04|United States|Tennessee|658[0n]|BOLD:AAB0524
Baileya dormitans|[761]|LSUSA240-06|United States|Kentucky|658[0n]|BOLD:AAB0524
Baileya acadiana|[762]|CNCLA5258-13|United States|Mississippi|658[0n]|BOLD:AAB0524
Baileya dormitans|[763]|LNCC1627-13|United States|North Carolina|658[0n]|BOLD:AAB0524
Baileya dormitans|[764]|LNCC1629-13|United States|North Carolina|658[5n]|BOLD:AAB0524
Baileya levitans|[765]|LGSM652-04|United States|Tennessee|658[0n]|BOLD:ACE9706
Baileya levitans|[766]|LILLA489-11|United States|Illinois|658[0n]|BOLD:ACE9706
Baileya levitans|[767]|LNCC659-11|United States|North Carolina|658[0n]|BOLD:ACE9706
Baileya levitans|[768]|LGSMC379-05|United States|Tennessee|658[0n]|BOLD:ACE9706
Baileya levitans|[769]|LOTB142-05|United States|Tennessee|613[0n]|BOLD:ACE9706
Baileya levitans|[770]|HKONB411-09|United States|Indiana|658[1n]|BOLD:ACE9706
Baileya levitans|[771]|RDNMK208-11|Canada|Ontario|658[0n]|BOLD:ACE9706
Baileya levitans|[772]|RDNMK209-11|Canada|Ontario|658[0n]|BOLD:ACE9706
Baileya levitans|[773]|RDNMK211-11|Canada|Ontario|658[0n]|BOLD:ACE9706
Baileya levitans|[774]|LOT333-04|United States|Tennessee|658[0n]|BOLD:ACE9706
Baileya levitans|[775]|LSEU765-06|United States|Georgia|658[0n]|BOLD:ACE9706
Baileya levitans|[776]|GBMIN20382-13|||671[0n]|BOLD:ACE9706
Baileya levitans|[777]|LNCC1831-13|United States|North Carolina|658[0n]|BOLD:ACE9706
Baileya ellesyoo|[778]|LSEU344-06|United States|Georgia|658[0n]|BOLD:ABZ8053
Baileya ellesyoo|[779]|LSEU345-06|United States|Georgia|658[2n]|BOLD:ABZ8053
Baileya ellesyoo|[780]|CNCLA5261-13|United States|Mississippi|658[0n]|BOLD:ABZ8053
Baileya ellesyoo|[781]|CNCLA5263-13|United States|Kentucky|658[0n]|BOLD:ABZ8053
Baileya ellesyoo|[782]|LNCC1832-13|United States|North Carolina|658[0n]|BOLD:ABZ8053
Baileya ellesyoo|[783]|LNCC1833-13|United States|North Carolina|658[0n]|BOLD:ABZ8053
Baileya ellesyoo|[784]|LNCC1834-13|United States|North Carolina|658[0n]|BOLD:ABZ8053
Baileya ophthalmica|[785]|TMNBB090-06|Canada|New Brunswick|658[1n]|BOLD:AAA6592
Baileya ophthalmica|[786]|UDLEP296-09|United States|Pennsylvania|658[0n]|BOLD:AAA6592
Baileya ophthalmica|[787]|PHMO108-03|Canada|Ontario|639[0n]|BOLD:AAA6592
Baileya ophthalmica|[788]|LNCC1341-11|United States|North Carolina|658[0n]|BOLD:AAA6592
Baileya ophthalmica|[789]|CNRMC1484-12|Canada|Manitoba|615[0n]|BOLD:AAA6592
Baileya ophthalmica|[790]|RDNMK197-11|Canada|Alberta|658[1n]|BOLD:AAA6592
Baileya ophthalmica|[791]|XAF796-05|Canada|Ontario|658[0n]|BOLD:AAA6592
Baileya ophthalmica|[792]|CNRMD2052-12|Canada|Manitoba|633[0n]|BOLD:AAA6592
Baileya ophthalmica|[793]|RDLQG316-06|Canada|Quebec|658[2n]|BOLD:AAA6592
Baileya ophthalmica|[794]|HKONS132-08|United States|Florida|658[0n]|BOLD:AAA6592
Baileya ophthalmica|[795]|XAB291-04|Canada|Ontario|658[0n]|BOLD:AAA6592
Baileya ophthalmica|[796]|LPSOC102-08|Canada|Ontario|658[0n]|BOLD:AAA6592
Baileya ophthalmica|[797]|LMIS025-05|Canada|Ontario|658[1n]|BOLD:AAA6592
Baileya ophthalmica|[798]|RDNMK205-11|Canada|Ontario|658[0n]|BOLD:AAA6592
Baileya ophthalmica|[799]|RDNMK206-11|Canada|Ontario|658[0n]|BOLD:AAA6592
Baileya ophthalmica|[800]|MEC296-04|Canada|Quebec|658[0n]|BOLD:AAA6592
Baileya ophthalmica|[801]|MEC394-04|Canada|Quebec|658[0n]|BOLD:AAA6592
Baileya ophthalmica|[802]|RDLQF211-06|Canada|Quebec|658[0n]|BOLD:AAA6592
Baileya ophthalmica|[803]|RDLQG256-06|Canada|Quebec|658[0n]|BOLD:AAA6592
Baileya ophthalmica|[804]|UDLEP295-09|United States|Pennsylvania|658[0n]|BOLD:AAA6592
Baileya ophthalmica|[805]|RDNMK191-11|Canada|Ontario|658[0n]|BOLD:AAA6592
Baileya ophthalmica|[806]|RDNMK192-11|Canada|Ontario|658[0n]|BOLD:AAA6592
Baileya ophthalmica|[807]|RDNMK193-11|Canada|Ontario|658[0n]|BOLD:AAA6592
Baileya ophthalmica|[808]|RDNMK194-11|Canada|Ontario|658[0n]|BOLD:AAA6592
Baileya ophthalmica|[809]|RDNMK199-11|Canada|Ontario|658[0n]|BOLD:AAA6592
Baileya ophthalmica|[810]|RDNMK200-11|Canada|Ontario|658[0n]|BOLD:AAA6592
Baileya ophthalmica|[811]|RDNMK201-11|Canada|Ontario|658[0n]|BOLD:AAA6592
Baileya ophthalmica|[812]|RDNMK202-11|Canada|Ontario|658[0n]|BOLD:AAA6592
Baileya ophthalmica|[813]|RDNMK203-11|Canada|Ontario|658[0n]|BOLD:AAA6592
Baileya ophthalmica|[814]|RDNMK207-11|Canada|Ontario|658[0n]|BOLD:AAA6592
Baileya ophthalmica|[815]|RDNMK198-11|Canada|Ontario|658[1n]|BOLD:AAA6592
Baileya ophthalmica|[816]|TMNBB098-06|Canada|New Brunswick|658[2n]|BOLD:AAA6592
Baileya ophthalmica|[817]|LSUSA254-06|United States|Kentucky|658[0n]|BOLD:AAA6592
Baileya ophthalmica|[818]|RDLQF446-06|Canada|Quebec|658[0n]|BOLD:AAA6592
Baileya ophthalmica|[819]|TMNBB100-06|Canada|New Brunswick|658[0n]|BOLD:AAA6592
Baileya ophthalmica|[820]|TMNBB102-06|Canada|New Brunswick|658[0n]|BOLD:AAA6592
Baileya ophthalmica|[821]|TMNBB103-06|Canada|New Brunswick|658[0n]|BOLD:AAA6592
Baileya ophthalmica|[822]|TMNBB104-06|Canada|New Brunswick|658[0n]|BOLD:AAA6592
Baileya ophthalmica|[823]|RDLQF928-06|Canada|Quebec|658[0n]|BOLD:AAA6592
Baileya ophthalmica|[824]|RDLQF930-06|Canada|Quebec|658[0n]|BOLD:AAA6592
Baileya ophthalmica|[825]|RDLQF931-06|Canada|Quebec|658[0n]|BOLD:AAA6592
Baileya ophthalmica|[826]|RDLQG438-06|Canada|Quebec|658[0n]|BOLD:AAA6592
Baileya ophthalmica|[827]|HKONS131-08|United States|Florida|658[0n]|BOLD:AAA6592
Baileya ophthalmica|[828]|LPSO100-08|Canada|Ontario|658[0n]|BOLD:AAA6592
Baileya ophthalmica|[829]|LPSOC048-08|Canada|Ontario|658[0n]|BOLD:AAA6592
Baileya ophthalmica|[830]|LPMN259-08|Canada|Manitoba|658[0n]|BOLD:AAA6592
Baileya ophthalmica|[831]|LPMN275-08|Canada|Manitoba|658[0n]|BOLD:AAA6592
Baileya ophthalmica|[832]|RDNMK195-11|United States|Louisiana|658[0n]|BOLD:AAA6592
Baileya ophthalmica|[833]|TMNBB097-06|Canada|New Brunswick|658[0n]|BOLD:AAA6592
Baileya ophthalmica|[834]|TMNBB099-06|Canada|New Brunswick|658[0n]|BOLD:AAA6592
Baileya ophthalmica|[835]|TMNBB095-06|Canada|New Brunswick|658[0n]|BOLD:AAA6592
Baileya ophthalmica|[836]|TMNBB096-06|Canada|New Brunswick|658[0n]|BOLD:AAA6592
Baileya ophthalmica|[837]|TMNBB092-06|Canada|New Brunswick|658[0n]|BOLD:AAA6592
Baileya ophthalmica|[838]|TMNBB094-06|Canada|New Brunswick|658[0n]|BOLD:AAA6592
Baileya ophthalmica|[839]|TMNBB089-06|Canada|New Brunswick|658[0n]|BOLD:AAA6592
Baileya ophthalmica|[840]|TMNBB091-06|Canada|New Brunswick|658[0n]|BOLD:AAA6592
Baileya ophthalmica|[841]|TMNBB088-06|Canada|New Brunswick|658[0n]|BOLD:AAA6592
Baileya ophthalmica|[842]|TMNBB087-06|Canada|New Brunswick|658[0n]|BOLD:AAA6592
Baileya ophthalmica|[843]|LNC287-05|United States|North Carolina|658[0n]|BOLD:AAA6592
Baileya ophthalmica|[844]|XAD699-05|Canada|Ontario|658[0n]|BOLD:AAA6592
Baileya ophthalmica|[845]|XAF481-05|Canada|Ontario|658[0n]|BOLD:AAA6592
Baileya ophthalmica|[846]|LOCT267-05|United States|Connecticut|658[0n]|BOLD:AAA6592
Baileya ophthalmica|[847]|LGSMC671-05|United States|Tennessee|658[0n]|BOLD:AAA6592
Baileya ophthalmica|[848]|LGSMC670-05|United States|Tennessee|658[0n]|BOLD:AAA6592
Baileya ophthalmica|[849]|LGSMC378-05|United States|Tennessee|658[0n]|BOLD:AAA6592
Baileya ophthalmica|[850]|XAC659-04|Canada|Ontario|658[0n]|BOLD:AAA6592
Baileya ophthalmica|[851]|XAB290-04|Canada|Ontario|658[0n]|BOLD:AAA6592
Baileya ophthalmica|[852]|LOTB296-05|United States|Tennessee|658[0n]|BOLD:AAA6592
Baileya ophthalmica|[853]|LNC286-05|United States|North Carolina|658[0n]|BOLD:AAA6592
Baileya ophthalmica|[854]|TMNBB086-06|Canada|New Brunswick|658[0n]|BOLD:AAA6592
Baileya ophthalmica|[855]|XAB378-04|Canada|Ontario|658[0n]|BOLD:AAA6592
Baileya ophthalmica|[856]|TMNBB093-06|Canada|New Brunswick|617[0n]|BOLD:AAA6592
[illegible]

Baileya ophthalmica|[854]|TMNBB086-06|Canada|New Brunswick|658[0n]|BOLD:AAA6592
Baileya ophthalmica|[855]|XAB378-04|Canada|Ontario|658[0n]|BOLD:AAA6592
Baileya ophthalmica|[856]|TMNBB093-06|Canada|New Brunswick|617[0n]|BOLD:AAA6592
Baileya ophthalmica|[857]|TMNBB101-06|Canada|New Brunswick|622[0n]|BOLD:AAA6592
Baileya ophthalmica|[858]|RDNMK204-11|Canada|Alberta|645[0n]|BOLD:AAA6592
Baileya ophthalmica|[859]|PMG093-03|Canada|Ontario|617[0n]|BOLD:AAA6592
Baileya ophthalmica|[860]|CNRMD2067-12|Canada|Manitoba|633[0n]|BOLD:AAA6592
Baileya ophthalmica|[861]|LNCC1340-11|United States|North Carolina|658[0n]|BOLD:AAA6592
Baileya ophthalmica|[862]|ALLEP295-13|Canada|Ontario|658[0n]|BOLD:AAA6592
Baileya ophthalmica|[863]|RDNMK196-11|United States|Louisiana|645[1n]|BOLD:AAA6592
Baileya ophthalmica|[864]|LGSM684-04|United States|Tennessee|658[0n]|BOLD:AAA6592
Baileya ophthalmica|[865]|RDLQ434-07|Canada|Quebec|615[0n]|BOLD:AAA6592
Baileya ophthalmica|[866]|SSPAC13701-13|Canada|Saskatchewan|585[0n]|BOLD:AAA6592
Baileya australis|[867]|RDNMH035-09|United States|Texas|658[0n]|BOLD:ACE7236
Baileya australis|[868]|RDNMH633-09|United States|Texas|658[0n]|BOLD:ACE7236
Baileya australis|[869]|HKONB435-09|United States|Texas|658[0n]|BOLD:AAA9559
Baileya australis|[870]|BBLSX011-09|United States|Texas|658[0n]|BOLD:AAA9559
Baileya australis|[871]|BBLSW907-09|United States|Texas|658[0n]|BOLD:AAA9559
Baileya australis|[872]|BBLSW897-09|United States|Texas|658[0n]|BOLD:AAA9559
Baileya australis|[873]|HKONB434-09|United States|Texas|658[0n]|BOLD:AAA9559
Baileya australis|[874]|RDNMH034-09|United States|Texas|658[0n]|BOLD:AAA9559
Baileya australis|[875]|BBLSX001-09|United States|Texas|636[0n]|BOLD:AAA9559
Baileya australis|[876]|BBLSX020-09|United States|Texas|658[0n]|BOLD:AAA9559
Baileya australis|[877]|BBLSX071-09|United States|Texas|658[0n]|BOLD:AAA9559
Baileya australis|[878]|BBLSY276-09|United States|Texas|658[0n]|BOLD:AAA9559
Baileya australis|[879]|BBLSY279-09|United States|Texas|658[0n]|BOLD:AAA9559
Baileya australis|[880]|BBLSX789-09|United States|Texas|658[0n]|BOLD:AAA9559
Baileya australis|[881]|BBLSZ141-09|United States|Texas|658[0n]|BOLD:AAA9559
Baileya australis|[882]|USLEP305-10|United States|Texas|658[0n]|BOLD:AAA9559
Baileya australis|[883]|LPSO757-08|Canada|Ontario|658[0n]|BOLD:AAA9559
Baileya australis|[884]|LPOKB189-09|United States|Oklahoma|658[0n]|BOLD:AAA9559
Baileya australis|[885]|HKONB432-09|United States|Indiana|658[0n]|BOLD:AAA9559
Baileya australis|[886]|HKONB431-09|United States|Indiana|658[0n]|BOLD:AAA9559
Baileya australis|[887]|LPSO750-08|Canada|Ontario|658[0n]|BOLD:AAA9559
Baileya australis|[888]|LPSO630-08|Canada|Ontario|658[0n]|BOLD:AAA9559
Baileya australis|[889]|LPSO586-08|Canada|Ontario|658[0n]|BOLD:AAA9559
Baileya australis|[890]|LPSO585-08|Canada|Ontario|658[0n]|BOLD:AAA9559
Baileya australis|[891]|LPSO506-08|Canada|Ontario|658[0n]|BOLD:AAA9559
Baileya australis|[892]|LPSO305-08|Canada|Ontario|658[0n]|BOLD:AAA9559
Baileya australis|[893]|LPSO304-08|Canada|Ontario|658[0n]|BOLD:AAA9559
Baileya australis|[894]|LPSO297-08|Canada|Ontario|658[0n]|BOLD:AAA9559
Baileya australis|[895]|LPSO279-08|Canada|Ontario|658[0n]|BOLD:AAA9559
Baileya australis|[896]|LPSO107-08|Canada|Ontario|658[0n]|BOLD:AAA9559
Baileya australis|[897]|XAK058-06|Canada|Ontario|658[0n]|BOLD:AAA9559
Baileya australis|[898]|XAJ603-06|Canada|Ontario|658[0n]|BOLD:AAA9559
Baileya australis|[899]|XAJ513-06|Canada|Ontario|658[0n]|BOLD:AAA9559
Baileya australis|[900]|XAH283-05|Canada|Ontario|658[0n]|BOLD:AAA9559
Baileya australis|[901]|XAG239-05|Canada|Ontario|658[0n]|BOLD:AAA9559
Baileya australis|[902]|XAE325-04|Canada|Ontario|658[0n]|BOLD:AAA9559
Baileya australis|[903]|XAE288-04|Canada|Ontario|658[0n]|BOLD:AAA9559
Baileya australis|[904]|XAB529-04|Canada|Ontario|658[0n]|BOLD:AAA9559
Baileya australis|[905]|XAC855-04|Canada|Ontario|658[0n]|BOLD:AAA9559
Baileya australis|[906]|XAJ439-06|Canada|Ontario|658[0n]|BOLD:AAA9559
Baileya australis|[907]|LPSO194-08|Canada|Ontario|658[0n]|BOLD:AAA9559
Baileya australis|[908]|PHMO345-03|Canada|Ontario|639[0n]|BOLD:AAA9559
Baileya australis|[909]|LPOKB252-09|United States|Oklahoma|621[0n]|BOLD:AAA9559
Baileya australis|[910]|LILLA344-11|United States|Illinois|658[0n]|BOLD:AAA9559
Baileya australis|[911]|LILLA403-11|United States|Illinois|658[0n]|BOLD:AAA9559
Baileya australis|[912]|LILLA839-11|United States|Illinois|658[0n]|BOLD:AAA9559
Baileya australis|[913]|BBLOC726-11|United States|Arkansas|658[0n]|BOLD:AAA9559
Baileya australis|[914]|LGSM565-04|United States|North Carolina|575[0n]|BOLD:ABY6214
Baileya australis|[915]|LGSM564-04|United States|Tennessee|658[0n]|BOLD:ABY6214
Baileya australis|[916]|LOT539-04|United States|Tennessee|565[0n]|BOLD:ABY6214
Baileya australis|[917]|LOT565-04|United States|Tennessee|655[0n]|BOLD:ABY6214
Baileya australis|[918]|LSEU644-06|United States|Georgia|658[0n]|BOLD:ABY6214
Baileya australis|[919]|QUNOC087-09|United States|Kentucky|658[0n]|BOLD:ABY6214
Baileya australis|[920]|CNCLB1432-14|United States|North Carolina|658[0n]|BOLD:ABY6214
Baileya australis|[921]|CNCLB2707-14|United States|North Carolina|658[0n]|BOLD:ABY6214
Baileya australis|[922]|CNCLB2708-14|United States|North Carolina|658[0n]|BOLD:ABY6214
Nycteola fletcheri|[923]|QUNOD201-10|United States|Texas|548[0n]|BOLD:AAZ1939
Nycteola fletcheri|[924]|BBLOB1509-11|United States|Arizona|658[0n]|BOLD:AAZ1939
Nycteola fletcheri|[925]|BBLOB1518-11|United States|Arizona|658[0n]|BOLD:AAZ1939
Nycteola fletcheri|[926]|BBLOB1519-11|United States|Arizona|658[0n]|BOLD:AAZ1939
Nycteola fletcheri|[927]|BBLOD1150-11|United States|Arizona|658[0n]|BOLD:AAZ1939
Nycteola fletcheri|[928]|CMAZA1152-12|United States|Arizona|658[0n]|BOLD:AAZ1939
Nycteola n. sp.|[929]|LALPA1109-11|Canada|British Columbia|658[0n]|BOLD:AAI3437
Nycteola n. sp.|[930]|LBCS024-07|Canada|British Columbia|658[0n]|BOLD:AAI3437
Nycteola n. sp.|[931]|RDNME283-07|United States|Colorado|591[1n]|BOLD:AAI3437
Nycteola n. sp.|[932]|LALPA091-10|Canada|British Columbia|658[0n]|BOLD:AAI3437
Nycteola n. sp.|[933]|LALPA1100-11|Canada|British Columbia|658[0n]|BOLD:AAI3437
Nycteola n. sp.|[934]|EHL938-12|Canada|British Columbia|658[0n]|BOLD:AAI3437
Nycteola n. sp.|[935]|JMMMB509-13|United States|California|570[0n]|BOLD:AAI3437
Nycteola cinereana|[936]|JMMMB128-11|United States|California|658[0n]|BOLD:AAB6411
Nycteola cinereana|[937]|RDLQ438-07|Canada|Quebec|586[7n]|
Nycteola cinereana|[938]|RDMAB717-06|Canada|Alberta|581[0n]|BOLD:AAB6411
Nycteola cinereana|[939]|LBCG1984-09|Canada|British Columbia|658[0n]|BOLD:AAB6411
Nycteola cinereana|[940]|RDLQ436-07|Canada|Quebec|615[0n]|BOLD:AAB6411
Nycteola cinereana|[941]|LBCA917-05|Canada|British Columbia|658[2n]|BOLD:AAB6411
Nycteola cinereana|[942]|MNBB695-06|Canada|New Brunswick|626[0n]|BOLD:AAB6411
Nycteola cinereana|[943]|LBCS045-07|Canada|British Columbia|658[0n]|BOLD:AAB6411
Nycteola cinereana|[944]|MNBB694-06|Canada|New Brunswick|658[0n]|BOLD:AAB6411
Nycteola cinereana|[945]|RDNMC572-06|Canada|British Columbia|658[0n]|BOLD:AAB6411
Nycteola cinereana|[946]|LBCB401-05|Canada|British Columbia|658[0n]|BOLD:AAB6411
Nycteola cinereana|[947]|JSBIC005-08|Canada|New Brunswick|650[0n]|BOLD:AAB6411
Nycteola cinereana|[948]|MNAA014-07|Canada|British Columbia|637[0n]|BOLD:AAB6411
Nycteola cinereana|[949]|JSBIC006-08|Canada|New Brunswick|645[0n]|BOLD:AAB6411
Nycteola cinereana|[950]|LPABB293-08|Canada|Alberta|658[0n]|BOLD:AAB6411
Nycteola cinereana|[951]|LALPA271-10|Canada|British Columbia|658[0n]|BOLD:AAB6411
Nycteola cinereana|[952]|BBLPD857-10|Canada|British Columbia|658[0n]|BOLD:AAB6411

Nycteola cinereana|[949]|JSBIC006-08|Canada|New Brunswick|643[0n]|BOLD:AAB6411
Nycteola cinereana|[950]|LPABB293-08|Canada|Alberta|658[0n]|BOLD:AAB6411
Nycteola cinereana|[951]|LALPA271-10|Canada|British Columbia|658[0n]|BOLD:AAB6411
Nycteola cinereana|[952]|BBLPD857-10|Canada|British Columbia|658[0n]|BOLD:AAB6411
Nycteola cinereana|[953]|JMMMB144-11|United States|California|658[0n]|BOLD:AAB6411
Nycteola cinereana|[954]|LBCS180-07|Canada|British Columbia|658[0n]|BOLD:AAB6411
Nycteola cinereana|[955]|RDNMH337-09|United States|California|658[0n]|BOLD:AAB6411
Nycteola cinereana|[956]|RDNMD494-06|United States|Colorado|658[0n]|BOLD:AAB6411
Nycteola cinereana|[957]|RDNME730-08|United States|Colorado|658[0n]|BOLD:AAB6411
Nycteola cinereana|[958]|LBCA108-05|Canada|British Columbia|635[0n]|BOLD:AAB6411
Nycteola cinereana|[959]|RDNMC286-05|United States|Colorado|580[1n]|BOLD:AAB6411
Nycteola cinereana|[960]|JMMMB153-11|United States|California|637[0n]|BOLD:AAB6411
Nycteola metaspilella|[961]|LGSMC647-05|United States|Tennessee|573[0n]|BOLD:AAD4611
Nycteola metaspilella|[962]|HKONB425-09|United States|Florida|658[0n]|BOLD:AAD4611
Nycteola metaspilella|[963]|HKONB423-09|United States|Indiana|658[0n]|BOLD:AAD4611
Nycteola metaspilella|[964]|HKONS624-08|United States|Florida|658[0n]|BOLD:AAD4611
Nycteola metaspilella|[965]|HKONS623-08|United States|Florida|658[0n]|BOLD:AAD4611
Nycteola metaspilella|[966]|RDNMC285-05|United States|Florida|577[2n]|BOLD:AAD4611
Nycteola metaspilella|[967]|RDNME284-07|United States|Florida|634[0n]|BOLD:AAD4611
Nycteola metaspilella|[968]|HKONB424-09|United States|Texas|658[0n]|BOLD:AAD4611
Nycteola metaspilella|[969]|LPOKC374-09|United States|Oklahoma|658[0n]|BOLD:AAD4611
Nycteola metaspilella|[970]|LPOKC814-09|United States|Oklahoma|658[0n]|BOLD:AAD4611
Nycteola metaspilella|[971]|LNCC277-10|United States|North Carolina|658[0n]|BOLD:AAD4611
Nycteola metaspilella|[972]|LILLA465-11|United States|Illinois|658[0n]|BOLD:AAD4611
Nycteola metaspilella|[973]|LILLA587-11|United States|Illinois|658[0n]|BOLD:AAD4611
Nycteola metaspilella|[974]|LILLA944-11|United States|Illinois|658[0n]|BOLD:AAD4611
Nycteola fletcheri|[975]|RDNMJ538-11|United States|Arizona|658[0n]|BOLD:AAU3711
Nycteola fletcheri|[976]|RDNMJ826-11|United States|Arizona|658[0n]|BOLD:AAU3711
Nycteola n. sp.|[977]|RDNME281-07|United States|Colorado|615[0n]|BOLD:AAC7851
Nycteola n. sp.|[978]|RDNME1024-08|United States|Colorado|643[0n]|BOLD:AAC7851
Nycteola columbiana|[979]|LALPA1106-11|Canada|British Columbia|658[0n]|BOLD:ABU9715
Nycteola frigidana|[980]|MEC094-04|Canada|Quebec|658[0n]|BOLD:AAC7850
Nycteola frigidana|[981]|RDNMC571-06|Canada|Ontario|658[0n]|BOLD:AAC7850
Nycteola frigidana|[982]|CNWBG3126-13|Canada|Alberta|573[0n]|BOLD:AAC7850
Nycteola frigidana|[983]|LALPA242-10|Canada|British Columbia|658[0n]|BOLD:AAC7850
Nycteola frigidana|[984]|SSPAA9981-13|Canada|Saskatchewan|582[0n]|BOLD:AAC7850
Nycteola frigidana|[985]|CNWBG3106-13|Canada|Alberta|598[0n]|BOLD:AAC7850
Nycteola frigidana|[986]|CNWBG3133-13|Canada|Alberta|576[0n]|BOLD:AAC7850
Nycteola frigidana|[987]|LOWCE172-06|Canada|British Columbia|658[0n]|BOLD:AAC7850
Nycteola frigidana|[988]|BBLPD086-10|Canada|Saskatchewan|658[0n]|BOLD:AAC7850
Nycteola frigidana|[989]|LALPA124-10|Canada|British Columbia|658[0n]|BOLD:AAC7850
Nycteola frigidana|[990]|MNAF432-08|Canada|Manitoba|658[0n]|BOLD:AAC7850
Nycteola frigidana|[991]|MNAF431-08|Canada|Manitoba|658[0n]|BOLD:AAC7850
Nycteola frigidana|[992]|RDNMC570-06|United States|Oregon|658[0n]|BOLD:AAC7850
Nycteola frigidana|[993]|CNWBC137-13|Canada|Alberta|566[0n]|BOLD:AAC7850
Nycteola frigidana|[994]|RDMAB716-06|Canada|Alberta|582[0n]|BOLD:AAC7850
Nycteola frigidana|[995]|LOWCE388-06|Canada|British Columbia|618[0n]|BOLD:AAC7850
Nycteola frigidana|[996]|LOWCE389-06|Canada|British Columbia|612[0n]|BOLD:AAC7850
Nycteola frigidana|[997]|CNPKD971-13|Canada|Ontario|607[0n]|BOLD:AAC7850
Collomena filifera|[998]|BBLOB1270-11|United States|Florida|658[0n]|BOLD:ABA1156
Collomena filifera|[999]|BBLOB1269-11|United States|Florida|658[0n]|BOLD:ABA1156
Collomena filifera|[1000]|BBLOB930-11|United States|Florida|658[0n]|BOLD:ABA1156
Collomena filifera|[1001]|BBLOB1271-11|United States|Florida|658[0n]|BOLD:ABA1156
Collomena filifera|[1002]|CNCLB814-14|United States|Florida|658[0n]|BOLD:ABA1156
Collomena filifera|[1003]|CNCLB815-14|United States|Florida|658[0n]|BOLD:ABA1156
Diphthera festiva|[1004]|MHMYM025-11|Costa Rica||521[0n]|BOLD:AAA3373
Diphthera festiva|[1005]|IAWL056-09|United States|Arizona|658[1n]|BOLD:AAA3373
Diphthera festiva|[1006]|MHAUB232-05|Costa Rica|Guanacaste|495[0n]|BOLD:AAA3373
Diphthera festiva|[1007]|MHAUB233-05|Costa Rica|Guanacaste|519[1n]|BOLD:AAA3373
Diphthera festiva|[1008]|BLPCH667-08|Costa Rica|Guanacaste|658[0n]|BOLD:AAA3373
Diphthera festiva|[1009]|BLPDX1003-11|Costa Rica|Guanacaste|658[0n]|BOLD:AAA3373
Diphthera festiva|[1010]|MHAUB231-05|Costa Rica|Guanacaste|533[0n]|BOLD:AAA3373
Diphthera festiva|[1011]|BLPCJ203-08|Costa Rica|Guanacaste|658[0n]|BOLD:AAA3373
Diphthera festiva|[1012]|BLPDA696-09|Costa Rica|Guanacaste|658[0n]|BOLD:AAA3373
Diphthera festiva|[1013]|BLPDA329-09|Costa Rica|Guanacaste|658[0n]|BOLD:AAA3373
Diphthera festiva|[1014]|BLPCJ204-08|Costa Rica|Guanacaste|658[0n]|BOLD:AAA3373
Diphthera festiva|[1015]|BLPCH671-08|Costa Rica|Guanacaste|658[0n]|BOLD:AAA3373
Diphthera festiva|[1016]|BLPCH665-08|Costa Rica|Guanacaste|658[0n]|BOLD:AAA3373
Diphthera festiva|[1017]|BLPCE823-08|Costa Rica|Alajuela|658[0n]|BOLD:AAA3373
Diphthera festiva|[1018]|BLPCE570-08|Costa Rica|Guanacaste|658[0n]|BOLD:AAA3373
Diphthera festiva|[1019]|BLPCA168-08|Costa Rica|Guanacaste|658[0n]|BOLD:AAA3373
Diphthera festiva|[1020]|BLPCA163-08|Costa Rica|Guanacaste|658[0n]|BOLD:AAA3373
Diphthera festiva|[1021]|BLPBH486-07|Costa Rica|Guanacaste|658[0n]|BOLD:AAA3373
Diphthera festiva|[1022]|BLPBA732-07|Costa Rica|Guanacaste|658[0n]|BOLD:AAA3373
Diphthera festiva|[1023]|BLPAH269-07|Costa Rica|Guanacaste|658[0n]|BOLD:AAA3373
Diphthera festiva|[1024]|BLPAE443-06|Costa Rica|Guanacaste|658[0n]|BOLD:AAA3373
Diphthera festiva|[1025]|BLPAE248-06|Costa Rica|Guanacaste|658[0n]|BOLD:AAA3373
Diphthera festiva|[1026]|BLPDA698-09|Costa Rica|Guanacaste|632[0n]|BOLD:AAA3373
Diphthera festiva|[1027]|BLPDA700-09|Costa Rica|Guanacaste|658[0n]|BOLD:AAA3373
Diphthera festiva|[1028]|BLPDC270-09|Costa Rica|Guanacaste|658[0n]|BOLD:AAA3373
Diphthera festiva|[1029]|BLPDT1644-10|Costa Rica|Guanacaste|658[0n]|BOLD:AAA3373
Diphthera festiva|[1030]|BLPDU861-11|Costa Rica|Guanacaste|658[0n]|BOLD:AAA3373
Diphthera festiva|[1031]|LYHES406-09|Mexico|Yucatan|621[0n]|BOLD:AAA3373
Diphthera festiva|[1032]|LYHES195-09|Mexico|Yucatan|506[0n]|BOLD:AAA3373
Diphthera festiva|[1033]|GWOSX645-11|Cuba|Holguin|658[0n]|BOLD:AAA3373
Diphthera festiva|[1034]|LYHES473-09|Mexico|Yucatan|635[0n]|BOLD:AAA3373
Diphthera festiva|[1035]|BLPEE1987-12|Costa Rica|Guanacaste|658[0n]|BOLD:AAA3373
Diphthera festiva|[1036]|GWOSX647-11|Cuba|Holguin|658[0n]|BOLD:AAA3373
Diphthera festiva|[1037]|GWOSS522-11|Honduras|Atlantida|658[0n]|BOLD:AAA3373
Diphthera festiva|[1038]|GWOSS003-11|Costa Rica|San Jose|658[0n]|BOLD:AAA3373
Diphthera festiva|[1039]|GWOSS002-11|Costa Rica|San Jose|658[0n]|BOLD:AAA3373
Diphthera festiva|[1040]|GWOSS001-11|Dominican Republic||658[0n]|BOLD:AAA3373
Diphthera festiva|[1041]|MHMYM218-11|Costa Rica||658[0n]|BOLD:AAA3373
Diphthera festiva|[1042]|MHMYM028-11|Costa Rica||658[0n]|BOLD:AAA3373
Diphthera festiva|[1043]|MHMYM027-11|Costa Rica||658[0n]|BOLD:AAA3373
Diphthera festiva|[1044]|MHMYM026-11|Costa Rica||658[0n]|BOLD:AAA3373
Diphthera festiva|[1045]|MHMYM024-11|Costa Rica||658[0n]|BOLD:AAA3373
Diphthera festiva|[1046]|MHMYM023-11|Costa Rica||658[0n]|BOLD:AAA3373
Diphthera festiva|[1047]|MHMYM022-11|Costa Rica||658[0n]|BOLD:AAA3373

[illegible]
Diphthera festiva|[1045]|MHMYM024-11|Costa Rica||658[0n]|BOLD:AAA3373
Diphthera festiva|[1046]|MHMYM023-11|Costa Rica||658[0n]|BOLD:AAA3373
Diphthera festiva|[1047]|MHMYM022-11|Costa Rica||658[0n]|BOLD:AAA3373
Diphthera festiva|[1048]|MHMYM021-11|Costa Rica||658[0n]|BOLD:AAA3373
Diphthera festiva|[1049]|MHMYM020-11|Costa Rica||658[0n]|BOLD:AAA3373
Diphthera festiva|[1050]|MHMYM019-11|Costa Rica||658[0n]|BOLD:AAA3373
Diphthera festiva|[1051]|MHMYM018-11|Costa Rica||658[0n]|BOLD:AAA3373
Diphthera festiva|[1052]|MHMYL3690-11|Costa Rica||658[0n]|BOLD:AAA3373
Diphthera festiva|[1053]|BLPDW1180-11|Costa Rica|Guanacaste|658[0n]|BOLD:AAA3373
Diphthera festiva|[1054]|BLPDX593-11|Costa Rica|Guanacaste|658[0n]|BOLD:AAA3373
Diphthera festiva|[1055]|BLPDU862-11|Costa Rica|Guanacaste|658[0n]|BOLD:AAA3373
Diphthera festiva|[1056]|LYHES405-09|Mexico|Yucatan|658[0n]|BOLD:AAA3373
Diphthera festiva|[1057]|LYHES094-09|Mexico|Yucatan|658[0n]|BOLD:AAA3373
Diphthera festiva|[1058]|LMEMB118-09|United States|Texas|658[0n]|BOLD:AAA3373
Diphthera festiva|[1059]|BLPDC271-09|Costa Rica|Guanacaste|658[0n]|BOLD:AAA3373
Diphthera festiva|[1060]|BLPDA432-09|Costa Rica|Alajuela|658[0n]|BOLD:AAA3373
Diphthera festiva|[1061]|BLPCO298-08|Costa Rica|Guanacaste|658[0n]|BOLD:AAA3373
Diphthera festiva|[1062]|BLPCO297-08|Costa Rica|Guanacaste|658[0n]|BOLD:AAA3373
Diphthera festiva|[1063]|MHMXU608-08|||658[0n]|BOLD:AAA3373
Diphthera festiva|[1064]|LPYPB096-08|Mexico|Campeche|658[0n]|BOLD:AAA3373
Diphthera festiva|[1065]|LPYPB094-08|Mexico|Campeche|658[0n]|BOLD:AAA3373
Diphthera festiva|[1066]|LPYPA347-08|Mexico|Yucatan|658[0n]|BOLD:AAA3373
Diphthera festiva|[1067]|LPYPA299-08|Mexico|Yucatan|658[0n]|BOLD:AAA3373
Diphthera festiva|[1068]|BLPCJ525-08|Costa Rica|Guanacaste|658[0n]|BOLD:AAA3373
Diphthera festiva|[1069]|BLPCJ524-08|Costa Rica|Guanacaste|658[0n]|BOLD:AAA3373
Diphthera festiva|[1070]|BLPCI163-08|Costa Rica|Guanacaste|658[0n]|BOLD:AAA3373
Diphthera festiva|[1071]|BLPCI149-08|Costa Rica|Guanacaste|658[0n]|BOLD:AAA3373
Diphthera festiva|[1072]|BLPCI147-08|Costa Rica|Guanacaste|658[0n]|BOLD:AAA3373
Diphthera festiva|[1073]|BLPCI146-08|Costa Rica|Guanacaste|658[0n]|BOLD:AAA3373
Diphthera festiva|[1074]|BLPCI145-08|Costa Rica|Guanacaste|658[0n]|BOLD:AAA3373
Diphthera festiva|[1075]|BLPCH670-08|Costa Rica|Guanacaste|658[0n]|BOLD:AAA3373
Diphthera festiva|[1076]|BLPCH669-08|Costa Rica|Guanacaste|658[0n]|BOLD:AAA3373
Diphthera festiva|[1077]|BLPCH668-08|Costa Rica|Guanacaste|658[0n]|BOLD:AAA3373
Diphthera festiva|[1078]|BLPCH666-08|Costa Rica|Guanacaste|658[0n]|BOLD:AAA3373
Diphthera festiva|[1079]|BLPCE571-08|Costa Rica|Guanacaste|658[0n]|BOLD:AAA3373
Diphthera festiva|[1080]|BLPCE569-08|Costa Rica|Guanacaste|658[0n]|BOLD:AAA3373
Diphthera festiva|[1081]|BLPCE568-08|Costa Rica|Guanacaste|658[0n]|BOLD:AAA3373
Diphthera festiva|[1082]|MHMXQ661-08|Costa Rica|Guanacaste|658[0n]|BOLD:AAA3373
Diphthera festiva|[1083]|BLPCC517-08|Costa Rica|Guanacaste|658[0n]|BOLD:AAA3373
Diphthera festiva|[1084]|BLPCC504-08|Costa Rica|Guanacaste|658[0n]|BOLD:AAA3373
Diphthera festiva|[1085]|BLPCB936-08|Costa Rica|Alajuela|658[0n]|BOLD:AAA3373
Diphthera festiva|[1086]|BLPCB933-08|Costa Rica|Alajuela|658[0n]|BOLD:AAA3373
Diphthera festiva|[1087]|BLPCA922-08|Costa Rica|Guanacaste|658[0n]|BOLD:AAA3373
Diphthera festiva|[1088]|BLPCA169-08|Costa Rica|Guanacaste|658[0n]|BOLD:AAA3373
Diphthera festiva|[1089]|BLPCA167-08|Costa Rica|Guanacaste|658[0n]|BOLD:AAA3373
Diphthera festiva|[1090]|BLPCA166-08|Costa Rica|Guanacaste|658[0n]|BOLD:AAA3373
Diphthera festiva|[1091]|BLPCA165-08|Costa Rica|Guanacaste|658[0n]|BOLD:AAA3373
Diphthera festiva|[1092]|BLPCA164-08|Costa Rica|Guanacaste|658[0n]|BOLD:AAA3373
Diphthera festiva|[1093]|BLPCA162-08|Costa Rica|Guanacaste|658[0n]|BOLD:AAA3373
Diphthera festiva|[1094]|HKONS186-08|United States|Florida|658[0n]|BOLD:AAA3373
Diphthera festiva|[1095]|BLPBH490-07|Costa Rica|Guanacaste|658[0n]|BOLD:AAA3373
Diphthera festiva|[1096]|BLPBH489-07|Costa Rica|Guanacaste|658[0n]|BOLD:AAA3373
Diphthera festiva|[1097]|BLPBH488-07|Costa Rica|Guanacaste|658[0n]|BOLD:AAA3373
Diphthera festiva|[1098]|BLPBH487-07|Costa Rica|Guanacaste|658[0n]|BOLD:AAA3373
Diphthera festiva|[1099]|BLPBH485-07|Costa Rica|Guanacaste|658[0n]|BOLD:AAA3373
Diphthera festiva|[1100]|BLPBH484-07|Costa Rica|Guanacaste|658[0n]|BOLD:AAA3373
Diphthera festiva|[1101]|BLPBH483-07|Costa Rica|Guanacaste|658[0n]|BOLD:AAA3373
Diphthera festiva|[1102]|BLPBB639-07|Costa Rica|Guanacaste|658[0n]|BOLD:AAA3373
Diphthera festiva|[1103]|BLPAH268-07|Costa Rica|Guanacaste|658[0n]|BOLD:AAA3373
Diphthera festiva|[1104]|BLPAH266-07|Costa Rica|Guanacaste|658[0n]|BOLD:AAA3373
Diphthera festiva|[1105]|BLPAF447-07|Costa Rica|Guanacaste|658[0n]|BOLD:AAA3373
Diphthera festiva|[1106]|BLPAF446-07|Costa Rica|Guanacaste|658[0n]|BOLD:AAA3373
Diphthera festiva|[1107]|BLPAF445-07|Costa Rica|Guanacaste|658[0n]|BOLD:AAA3373
Diphthera festiva|[1108]|BLPAE926-07|Costa Rica|Guanacaste|658[0n]|BOLD:AAA3373
Diphthera festiva|[1109]|BLPAE925-07|Costa Rica|Guanacaste|658[0n]|BOLD:AAA3373
Diphthera festiva|[1110]|BLPAE924-07|Costa Rica|Guanacaste|658[0n]|BOLD:AAA3373
Diphthera festiva|[1111]|BLPAE249-06|Costa Rica|Guanacaste|658[0n]|BOLD:AAA3373
Diphthera festiva|[1112]|BLPAE247-06|Costa Rica|Guanacaste|658[0n]|BOLD:AAA3373
Diphthera festiva|[1113]|BLPAE246-06|Costa Rica|Guanacaste|658[0n]|BOLD:AAA3373
Diphthera festiva|[1114]|LOFLA108-06|United States|Florida|658[0n]|BOLD:AAA3373
Diphthera festiva|[1115]|BLPAA713-06|Costa Rica|Guanacaste|658[0n]|BOLD:AAA3373
Diphthera festiva|[1116]|MHAUC218-06|Costa Rica|Guanacaste|658[0n]|BOLD:AAA3373
Diphthera festiva|[1117]|MHAUC217-06|Costa Rica|Guanacaste|658[0n]|BOLD:AAA3373
Diphthera festiva|[1118]|MHAUC216-06|Costa Rica|Guanacaste|658[0n]|BOLD:AAA3373
Diphthera festiva|[1119]|MHAUC215-06|Costa Rica|Guanacaste|658[0n]|BOLD:AAA3373
Diphthera festiva|[1120]|MHAUB235-05|Costa Rica|Guanacaste|658[0n]|BOLD:AAA3373
Diphthera festiva|[1121]|MHAUB234-05|Costa Rica|Guanacaste|658[0n]|BOLD:AAA3373
Diphthera festiva|[1122]|MHAUB230-05|Costa Rica|Guanacaste|658[0n]|BOLD:AAA3373
Diphthera festiva|[1123]|BLPCB934-08|Costa Rica|Alajuela|658[0n]|BOLD:AAA3373
Diphthera festiva|[1124]|IAWL055-09|United States|Arizona|658[0n]|BOLD:AAA3373
Diphthera festiva|[1125]|IAWL057-09|United States|Arizona|658[0n]|BOLD:AAA3373
Diphthera festiva|[1126]|BLPAH267-07|Costa Rica|Guanacaste|658[0n]|BOLD:AAA3373
Diphthera festiva|[1127]|BLPCA923-08|Costa Rica|Guanacaste|658[0n]|BOLD:AAA3373
Diphthera festiva|[1128]|MHAUC219-06|Costa Rica|Guanacaste|658[0n]|BOLD:AAA3373
Diphthera festiva|[1129]|BLPCB935-08|Costa Rica|Alajuela|658[0n]|BOLD:AAA3373
Diphthera festiva|[1130]|BLPCE572-08|Costa Rica|Guanacaste|658[0n]|BOLD:AAA3373
Diphthera festiva|[1131]|BLPCI148-08|Costa Rica|Guanacaste|658[0n]|BOLD:AAA3373
Diphthera festiva|[1132]|BLPDA702-09|Costa Rica|Guanacaste|633[0n]|BOLD:AAA3373
Diphthera festiva|[1133]|BLPDA699-09|Costa Rica|Guanacaste|632[0n]|BOLD:AAA3373
Diphthera festiva|[1134]|BLPDA697-09|Costa Rica|Guanacaste|658[0n]|BOLD:AAA3373
Diphthera festiva|[1135]|MHMXM120-07|Costa Rica|Guanacaste|630[0n]|BOLD:AAA3373
Diphthera festiva|[1136]|LPYPB095-08|Mexico|Quintana Roo|648[0n]|BOLD:AAA3373
Diphthera festiva|[1137]|MHAUB229-05|Costa Rica|Guanacaste|595[0n]|BOLD:AAA3373
Diphthera festiva|[1138]|GBMIN20383-13|||613[0n]|BOLD:AAA3373
Diphthera festiva|[1139]|GBMIN20425-13|||620[0n]|BOLD:AAA3373
Diphthera festiva|[1140]|GBMIN20438-13|||619[1n]|BOLD:AAA3373
