# Supplementary material for: Probing planetary biodiversity with DNA barcodes: The Noctuoidea of North America
Source: PLoS One. 2017 Jun 1;12(6):e0178548. doi: 10.1371/journal.pone.0178548 (PMC5453547; doi:10.1371/journal.pone.0178548)
Supplement: S4 Tree — NJ tree based on sequence variation in the barcode region of the cytochrome c oxidase I gene for North American species in the family Erebidae (Arctiinae). (PDF) [file pone.0178548.s017.pdf]

BOLD TaxonID Tree

Title	:	Tree Result - Search (5774 records)
Date	:	20-Dec-2016
Data Type	:	Nucleotide
Distance Model	:	Kimura 2 Parameter
Marker	:	COI-5P
Colourization	:	[blue]=Stop Codons [red]=Contamination or misidentification

Label	:	Sample ID
Label	:	Process ID
Label	:	Taxon
Label	:	Country
Label	:	Province/State
Label	:	Sequence Length
Label	:	Barcode Cluster (BIN)

Sequence Count	:	5774
Species count	:	316
Genus count	:	100
Family count	:	1
Unidentified	:	30

BIN Count	:	324

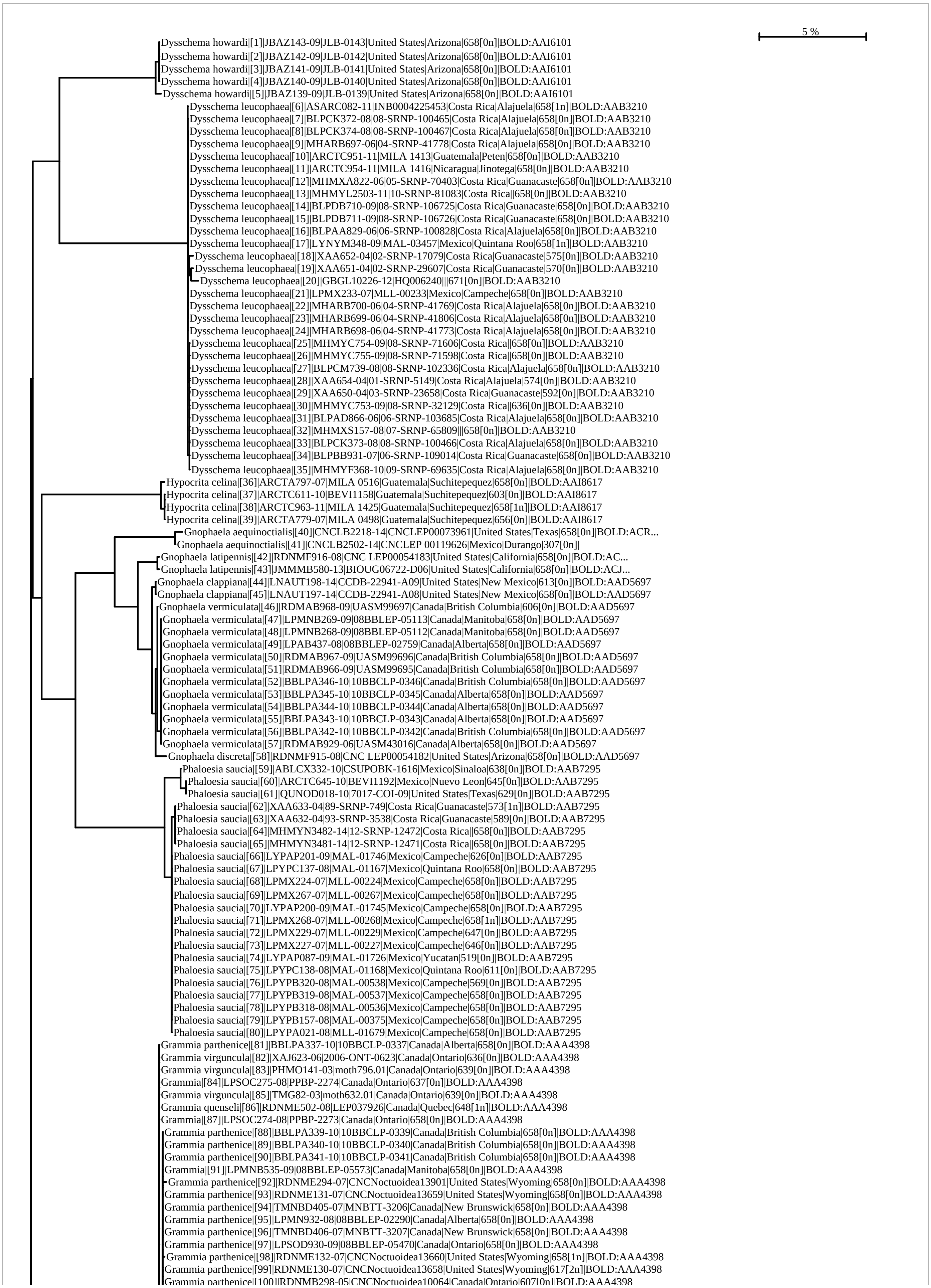
5 %
Dysschema howardi|[1]|JBAZ143-09|JLB-0143|United States|Arizona|658[0n]|BOLD:AAI6101
Dysschema howardi|[2]|JBAZ142-09|JLB-0142|United States|Arizona|658[0n]|BOLD:AAI6101
Dysschema howardi|[3]|JBAZ141-09|JLB-0141|United States|Arizona|658[0n]|BOLD:AAI6101
Dysschema howardi|[4]|JBAZ140-09|JLB-0140|United States|Arizona|658[0n]|BOLD:AAI6101
Dysschema howardi|[5]|JBAZ139-09|JLB-0139|United States|Arizona|658[0n]|BOLD:AAI6101
Dysschema leucophaea|[6]|ASARC082-11|INB0004225453|Costa Rica|Alajuela|658[1n]|BOLD:AAB3210
Dysschema leucophaea|[7]|BLPCK372-08|08-SRNP-100465|Costa Rica|Alajuela|658[0n]|BOLD:AAB3210
Dysschema leucophaea|[8]|BLPCK374-08|08-SRNP-100467|Costa Rica|Alajuela|658[0n]|BOLD:AAB3210
Dysschema leucophaea|[9]|MHARB697-06|04-SRNP-41778|Costa Rica|Alajuela|658[0n]|BOLD:AAB3210
Dysschema leucophaea|[10]|ARCTC951-11|MILA 1413|Guatemala|Peten|658[0n]|BOLD:AAB3210
Dysschema leucophaea|[11]|ARCTC954-11|MILA 1416|Nicaragua|Jinotega|658[0n]|BOLD:AAB3210
Dysschema leucophaea|[12]|MHMXA822-06|05-SRNP-70403|Costa Rica|Guanacaste|658[0n]|BOLD:AAB3210
Dysschema leucophaea|[13]|MHMYL2503-11|10-SRNP-81083|Costa Rica||658[0n]|BOLD:AAB3210
Dysschema leucophaea|[14]|BLPDB710-09|08-SRNP-106725|Costa Rica|Guanacaste|658[0n]|BOLD:AAB3210
Dysschema leucophaea|[15]|BLPDB711-09|08-SRNP-106726|Costa Rica|Guanacaste|658[0n]|BOLD:AAB3210
Dysschema leucophaea|[16]|BLPAA829-06|06-SRNP-100828|Costa Rica|Alajuela|658[0n]|BOLD:AAB3210
Dysschema leucophaea|[17]|LYNYM348-09|MAL-03457|Mexico|Quintana Roo|658[1n]|BOLD:AAB3210
Dysschema leucophaea|[18]|XAA652-04|02-SRNP-17079|Costa Rica|Guanacaste|575[0n]|BOLD:AAB3210
Dysschema leucophaea|[19]|XAA651-04|02-SRNP-29607|Costa Rica|Guanacaste|570[0n]|BOLD:AAB3210
Dysschema leucophaea|[20]|GBGL10226-12|HQ006240|||671[0n]|BOLD:AAB3210
Dysschema leucophaea|[21]|LPMX233-07|MLL-00233|Mexico|Campeche|658[0n]|BOLD:AAB3210
Dysschema leucophaea|[22]|MHARB700-06|04-SRNP-41769|Costa Rica|Alajuela|658[0n]|BOLD:AAB3210
Dysschema leucophaea|[23]|MHARB699-06|04-SRNP-41806|Costa Rica|Alajuela|658[0n]|BOLD:AAB3210
Dysschema leucophaea|[24]|MHARB698-06|04-SRNP-41773|Costa Rica|Alajuela|658[0n]|BOLD:AAB3210
Dysschema leucophaea|[25]|MHMYC754-09|08-SRNP-71606|Costa Rica||658[0n]|BOLD:AAB3210
Dysschema leucophaea|[26]|MHMYC755-09|08-SRNP-71598|Costa Rica||658[0n]|BOLD:AAB3210
Dysschema leucophaea|[27]|BLPCM739-08|08-SRNP-102336|Costa Rica|Alajuela|658[0n]|BOLD:AAB3210
Dysschema leucophaea|[28]|XAA654-04|01-SRNP-5149|Costa Rica|Alajuela|574[0n]|BOLD:AAB3210
Dysschema leucophaea|[29]|XAA650-04|03-SRNP-23658|Costa Rica|Guanacaste|592[0n]|BOLD:AAB3210
Dysschema leucophaea|[30]|MHMYC753-09|08-SRNP-32129|Costa Rica||636[0n]|BOLD:AAB3210
Dysschema leucophaea|[31]|BLPAD866-06|06-SRNP-103685|Costa Rica|Alajuela|658[0n]|BOLD:AAB3210
Dysschema leucophaea|[32]|MHMXS157-08|07-SRNP-65809|||658[0n]|BOLD:AAB3210
Dysschema leucophaea|[33]|BLPCK373-08|08-SRNP-100466|Costa Rica|Alajuela|658[0n]|BOLD:AAB3210
Dysschema leucophaea|[34]|BLPBB931-07|06-SRNP-109014|Costa Rica|Guanacaste|658[0n]|BOLD:AAB3210
Dysschema leucophaea|[35]|MHMYF368-10|09-SRNP-69635|Costa Rica|Alajuela|658[0n]|BOLD:AAB3210
Hypocrita celina|[36]|ARCTA797-07|MILA 0516|Guatemala|Suchitepequez|658[0n]|BOLD:AAI8617
Hypocrita celina|[37]|ARCTC611-10|BEVI1158|Guatemala|Suchitepequez|603[0n]|BOLD:AAI8617
Hypocrita celina|[38]|ARCTC963-11|MILA 1425|Guatemala|Suchitepequez|658[1n]|BOLD:AAI8617
Hypocrita celina|[39]|ARCTA779-07|MILA 0498|Guatemala|Suchitepequez|656[0n]|BOLD:AAI8617
Gnophaela aequinoctialis|[40]|CNCLB2218-14|CNCLEP00073961|United States|Texas|658[0n]|BOLD:ACR...
Gnophaela aequinoctialis|[41]|CNCLB2502-14|CNCLEP 00119626|Mexico|Durango|307[0n]|
Gnophaela latipennis|[42]|RDNMF916-08|CNC LEP00054183|United States|California|658[0n]|BOLD:AC...
Gnophaela latipennis|[43]|JMMMB580-13|BIOUG06722-D06|United States|California|658[0n]|BOLD:ACJ...
Gnophaela clappiana|[44]|LNAUT198-14|CCDB-22941-A09|United States|New Mexico|613[0n]|BOLD:AAD5697
Gnophaela clappiana|[45]|LNAUT197-14|CCDB-22941-A08|United States|New Mexico|658[0n]|BOLD:AAD5697
Gnophaela vermiculata|[46]|RDMAB968-09|UASM99697|Canada|British Columbia|606[0n]|BOLD:AAD5697
Gnophaela vermiculata|[47]|LPMNB269-09|08BBLEP-05113|Canada|Manitoba|658[0n]|BOLD:AAD5697
Gnophaela vermiculata|[48]|LPMNB268-09|08BBLEP-05112|Canada|Manitoba|658[0n]|BOLD:AAD5697
Gnophaela vermiculata|[49]|LPAB437-08|08BBLEP-02759|Canada|Alberta|658[0n]|BOLD:AAD5697
Gnophaela vermiculata|[50]|RDMAB967-09|UASM99696|Canada|British Columbia|658[0n]|BOLD:AAD5697
Gnophaela vermiculata|[51]|RDMAB966-09|UASM99695|Canada|British Columbia|658[0n]|BOLD:AAD5697
Gnophaela vermiculata|[52]|BBLPA346-10|10BBCLP-0346|Canada|British Columbia|658[0n]|BOLD:AAD5697
Gnophaela vermiculata|[53]|BBLPA345-10|10BBCLP-0345|Canada|Alberta|658[0n]|BOLD:AAD5697
Gnophaela vermiculata|[54]|BBLPA344-10|10BBCLP-0344|Canada|Alberta|658[0n]|BOLD:AAD5697
Gnophaela vermiculata|[55]|BBLPA343-10|10BBCLP-0343|Canada|Alberta|658[0n]|BOLD:AAD5697
Gnophaela vermiculata|[56]|BBLPA342-10|10BBCLP-0342|Canada|British Columbia|658[0n]|BOLD:AAD5697
Gnophaela vermiculata|[57]|RDMAB929-06|UASM43016|Canada|Alberta|658[0n]|BOLD:AAD5697
Gnophaela discreta|[58]|RDNMF915-08|CNC LEP00054182|United States|Arizona|658[0n]|BOLD:AAD5697
Phaloesia saucia|[59]|ABLCX332-10|CSUPOBK-1616|Mexico|Sinaloa|638[0n]|BOLD:AAB7295
Phaloesia saucia|[60]|ARCTC645-10|BEVI1192|Mexico|Nuevo Leon|645[0n]|BOLD:AAB7295
Phaloesia saucia|[61]|QUNOD018-10|7017-COI-09|United States|Texas|629[0n]|BOLD:AAB7295
Phaloesia saucia|[62]|XAA633-04|89-SRNP-749|Costa Rica|Guanacaste|573[1n]|BOLD:AAB7295
Phaloesia saucia|[63]|XAA632-04|93-SRNP-3538|Costa Rica|Guanacaste|589[0n]|BOLD:AAB7295
Phaloesia saucia|[64]|MHMYN3482-14|12-SRNP-12472|Costa Rica||658[0n]|BOLD:AAB7295
Phaloesia saucia|[65]|MHMYN3481-14|12-SRNP-12471|Costa Rica||658[0n]|BOLD:AAB7295
Phaloesia saucia|[66]|LYPAP201-09|MAL-01746|Mexico|Campeche|626[0n]|BOLD:AAB7295
Phaloesia saucia|[67]|LPYPC137-08|MAL-01167|Mexico|Quintana Roo|658[0n]|BOLD:AAB7295
Phaloesia saucia|[68]|LPMX224-07|MLL-00224|Mexico|Campeche|658[0n]|BOLD:AAB7295
Phaloesia saucia|[69]|LPMX267-07|MLL-00267|Mexico|Campeche|658[0n]|BOLD:AAB7295
Phaloesia saucia|[70]|LYPAP200-09|MAL-01745|Mexico|Campeche|658[0n]|BOLD:AAB7295
Phaloesia saucia|[71]|LPMX268-07|MLL-00268|Mexico|Campeche|658[1n]|BOLD:AAB7295
Phaloesia saucia|[72]|LPMX229-07|MLL-00229|Mexico|Campeche|647[0n]|BOLD:AAB7295
Phaloesia saucia|[73]|LPMX227-07|MLL-00227|Mexico|Campeche|646[0n]|BOLD:AAB7295
Phaloesia saucia|[74]|LYPAP087-09|MAL-01726|Mexico|Yucatan|519[0n]|BOLD:AAB7295
Phaloesia saucia|[75]|LPYPC138-08|MAL-01168|Mexico|Quintana Roo|611[0n]|BOLD:AAB7295
Phaloesia saucia|[76]|LPYPB320-08|MAL-00538|Mexico|Campeche|569[0n]|BOLD:AAB7295
Phaloesia saucia|[77]|LPYPB319-08|MAL-00537|Mexico|Campeche|658[0n]|BOLD:AAB7295
Phaloesia saucia|[78]|LPYPB318-08|MAL-00536|Mexico|Campeche|658[0n]|BOLD:AAB7295
Phaloesia saucia|[79]|LPYPB157-08|MAL-00375|Mexico|Campeche|658[0n]|BOLD:AAB7295
Phaloesia saucia|[80]|LPYPA021-08|MLL-01679|Mexico|Campeche|658[0n]|BOLD:AAB7295
Grammia parthenice|[81]|BBLPA337-10|10BBCLP-0337|Canada|Alberta|658[0n]|BOLD:AAA4398
Grammia virguncula|[82]|XAJ623-06|2006-ONT-0623|Canada|Ontario|636[0n]|BOLD:AAA4398
Grammia virguncula|[83]|PHMO141-03|moth796.01|Canada|Ontario|639[0n]|BOLD:AAA4398
Grammia|[84]|LPSOC275-08|PPBP-2274|Canada|Ontario|637[0n]|BOLD:AAA4398
Grammia virguncula|[85]|TMG82-03|moth632.01|Canada|Ontario|639[0n]|BOLD:AAA4398
Grammia quenseli|[86]|RDNME502-08|LEP037926|Canada|Quebec|648[1n]|BOLD:AAA4398
Grammia|[87]|LPSOC274-08|PPBP-2273|Canada|Ontario|658[0n]|BOLD:AAA4398
Grammia parthenice|[88]|BBLPA339-10|10BBCLP-0339|Canada|British Columbia|658[0n]|BOLD:AAA4398
Grammia parthenice|[89]|BBLPA340-10|10BBCLP-0340|Canada|British Columbia|658[0n]|BOLD:AAA4398
Grammia parthenice|[90]|BBLPA341-10|10BBCLP-0341|Canada|British Columbia|658[0n]|BOLD:AAA4398
Grammia|[91]|LPMNB535-09|08BBLEP-05573|Canada|Manitoba|658[0n]|BOLD:AAA4398
Grammia parthenice|[92]|RDNME294-07|CNCNoctuoidea13901|United States|Wyoming|658[0n]|BOLD:AAA4398
Grammia parthenice|[93]|RDNME131-07|CNCNoctuoidea13659|United States|Wyoming|658[0n]|BOLD:AAA4398
Grammia parthenice|[94]|TMNBD405-07|MNBTT-3206|Canada|New Brunswick|658[0n]|BOLD:AAA4398
Grammia parthenice|[95]|LPMN932-08|08BBLEP-02290|Canada|Alberta|658[0n]|BOLD:AAA4398
Grammia parthenice|[96]|TMNBD406-07|MNBTT-3207|Canada|New Brunswick|658[0n]|BOLD:AAA4398
Grammia parthenice|[97]|LPSOD930-09|08BBLEP-05470|Canada|Ontario|658[0n]|BOLD:AAA4398
Grammia parthenice|[98]|RDNME132-07|CNCNoctuoidea13660|United States|Wyoming|658[1n]|BOLD:AAA4398
Grammia parthenice|[99]|RDNME130-07|CNCNoctuoidea13658|United States|Wyoming|617[2n]|BOLD:AAA4398
Grammia parthenice|[100]|RDNMB298-05|CNCNoctuoidea10064|Canada|Ontario|607[0n]|BOLD:AAA4398

Grammia parthenice|[98]|RDNME132-07|CNCNoctuoidea13660|United States|Wyoming|658[1n]|BOLD:AAA4398
Grammia parthenice|[99]|RDNME130-07|CNCNoctuoidea13658|United States|Wyoming|617[2n]|BOLD:AAA4398
Grammia parthenice|[100]|RDNMB298-05|CNCNoctuoidea10064|Canada|Ontario|607[0n]|BOLD:AAA4398
Grammia parthenice|[101]|RDNMB300-05|CNCNoctuoidea10066|Canada|Alberta|566[1n]|BOLD:AAA4398
Grammia|[102]|CNWBG3146-13|BIOUG06594-B09|Canada|Alberta|567[0n]|BOLD:AAA4398
Grammia|[103]|BBLPA338-10|10BBCLP-0338|Canada|Alberta|658[0n]|BOLD:AAA4398
Grammia parthenice|[104]|RDMAB872-06|BCSC349|Canada|Ontario|658[0n]|BOLD:AAA4398
Grammia virguncula|[105]|RDMAB844-06|BCSC321|Canada|Saskatchewan|658[0n]|BOLD:AAA4398
Grammia parthenice|[106]|RDLQB599-05|DH010702|Canada|Quebec|658[0n]|BOLD:AAA4398
Grammia parthenice|[107]|LPSK212-08|08BBLEP-01780|Canada|Saskatchewan|658[0n]|BOLD:AAA4398
Grammia parthenice|[108]|LPMN168-08|08BBLEP-00967|Canada|Manitoba|658[0n]|BOLD:AAA4398
Grammia virguncula|[109]|XAF584-05|2005-ONT-233|Canada|Ontario|658[0n]|BOLD:AAA4398
Grammia virguncula|[110]|XAE346-04|Moth4346.03|Canada|Ontario|658[0n]|BOLD:AAA4398
Grammia|[111]|LPSOC276-08|PPBP-2275|Canada|Ontario|658[0n]|BOLD:AAA4398
Grammia|[112]|LPSOC277-08|PPBP-2276|Canada|Ontario|658[0n]|BOLD:AAA4398
Grammia|[113]|LPSOC033-08|PPBP-2032|Canada|Ontario|658[0n]|BOLD:AAA4398
Grammia parthenice|[114]|RDMAB816-06|BCSC293|Canada|Alberta|658[0n]|BOLD:AAA4398
Grammia margo|[115]|RDMAB792-06|BCSC269|Canada|Alberta|658[0n]|BOLD:AAA4398
Grammia|[116]|LPSOD1044-09|08BBLEP-05971|Canada|Ontario|658[0n]|BOLD:AAA4398
Grammia virguncula|[117]|XAF519-05|2005-ONT-168|Canada|Ontario|658[0n]|BOLD:AAA4398
Grammia parthenice|[118]|RDNME463-08|LEP037887|United States|Colorado|658[0n]|BOLD:AAA4398
Grammia parthenice|[119]|RDNME462-08|LEP037886|United States|Colorado|658[0n]|BOLD:AAA4398
Grammia virguncula|[120]|RDNMB396-05|CNCNoctuoidea10162|Canada|Ontario|658[0n]|BOLD:AAA4398
Grammia virguncula|[121]|XAB585-04|04HBL005585|Canada|Ontario|658[0n]|BOLD:AAA4398
Grammia parthenice|[122]|RDNME461-08|LEP037885|United States|Colorado|658[0n]|BOLD:AAA4398
Grammia virguncula|[123]|RDMAB846-06|BCSC323|United States|Pennsylvania|658[0n]|BOLD:AAA4398
Grammia parthenice|[124]|XAI126-05|0102-ONT-0126|Canada|Ontario|658[0n]|BOLD:AAA4398
Grammia parthenice|[125]|LPSK018-08|08BBLEP-00721|Canada|Saskatchewan|658[0n]|BOLD:AAA4398
Grammia virguncula|[126]|RDNMB397-05|CNCNoctuoidea10163|Canada|Ontario|658[0n]|BOLD:AAA4398
Grammia virguncula|[127]|XAB553-04|04HBL005553|Canada|Ontario|658[0n]|BOLD:AAA4398
Grammia virguncula|[128]|XAF471-05|2005-ONT-120|Canada|Ontario|658[0n]|BOLD:AAA4398
Grammia|[129]|LPSOC131-08|PPBP-2130|Canada|Ontario|631[0n]|BOLD:AAA4398
Grammia virguncula|[130]|XAE476-04|Moth4476.03|Canada|Ontario|574[0n]|BOLD:AAA4398
Grammia virguncula|[131]|RDNMC565-06|CNCNoctuoidea12105|Canada|Ontario|658[0n]|BOLD:AAA4398
Grammia virguncula|[132]|PMG008-03|moth691.01|Canada|Ontario|617[0n]|BOLD:AAA4398
Grammia virguncula|[133]|RDMAB852-06|BCSC329|United States|Vermont|563[0n]|BOLD:AAA4398
Grammia parthenice|[134]|RDNME558-08|LEP037982|United States|Colorado|658[0n]|BOLD:AAA4398
Grammia virguncula|[135]|RDMAB845-06|BCSC322|Canada|Nova Scotia|596[0n]|BOLD:AAA4398
Grammia virguncula|[136]|RDMAB851-06|BCSC328|Canada|Nova Scotia|658[0n]|BOLD:AAA4398
Grammia virguncula|[137]|RDMAB818-06|BCSC295|Canada|Nova Scotia|658[0n]|BOLD:AAA4398
Grammia virguncula|[138]|RDMAB817-06|BCSC294|Canada|Nova Scotia|658[0n]|BOLD:AAA4398
Grammia figurata|[139]|LOCT021-05|05-CTATBI-0021|United States|Connecticut|618[0n]|BOLD:AAA4398
Grammia figurata|[140]|RDMAB805-06|BCSC282|United States|Pennsylvania|604[0n]|BOLD:AAA4398
Grammia f-pallida|[141]|RDNMG082-08|NOC15023|United States|Colorado|658[0n]|BOLD:AAA4398
Grammia figurata|[142]|QUNOB533-09|5795-060708-KY|United States|Kentucky|658[0n]|BOLD:AAA4398
Grammia f-pallida|[143]|RDMAB803-06|BCSC280|United States|Oklahoma|658[0n]|BOLD:AAA4398
Grammia f-pallida|[144]|RDMAB802-06|BCSC279|United States|Colorado|658[0n]|BOLD:AAA4398
Grammia f-pallida|[145]|RDMAB801-06|BCSC278|United States|Texas|572[1n]|BOLD:AAA4398
Grammia figurata|[146]|RDMAB917-06|BCSC391|United States|Kansas|658[0n]|BOLD:AAA4398
Grammia figurata|[147]|RDNMB378-05|CNCNoctuoidea10144|Canada|Ontario|658[0n]|BOLD:AAA4398
Grammia virguncula|[148]|LPSOC145-08|PPBP-2144|Canada|Ontario|658[0n]|BOLD:AAA4398
Grammia virguncula|[149]|XAK158-06|2006-ONT-1153|Canada|Ontario|658[0n]|BOLD:AAA4398
Grammia figurata|[150]|RDNM100-05|CNCNoctuoidea6650|Canada|Ontario|658[0n]|BOLD:AAA4398
Grammia figurata|[151]|RDMAB791-06|BCSC268|Canada|Ontario|658[0n]|BOLD:AAA4398
Grammia virguncula|[152]|XAB274-04|04HBL005274|Canada|Ontario|658[0n]|BOLD:AAA4398
Grammia virguncula|[153]|XAJ686-06|2006-ONT-0686|Canada|Ontario|658[0n]|BOLD:AAA4398
Grammia virguncula|[154]|XAJ721-06|2006-ONT-0721|Canada|Ontario|658[0n]|BOLD:AAA4398
Grammia virguncula|[155]|XAB552-04|04HBL005552|Canada|Ontario|658[0n]|BOLD:AAA4398
Grammia virguncula|[156]|XAB586-04|04HBL005586|Canada|Ontario|606[0n]|BOLD:AAA4398
Grammia virguncula|[157]|XAJ685-06|2006-ONT-0685|Canada|Ontario|658[0n]|BOLD:AAA4398
Grammia virguncula|[158]|LPSOB786-08|PPBP-1785|Canada|Ontario|658[0n]|BOLD:AAA4398
Grammia figurata|[159]|RDNMB307-05|CNCNoctuoidea10073|Canada|Ontario|613[0n]|BOLD:AAA4398
Grammia figurata|[160]|RDNM099-05|CNCNoctuoidea6649|Canada|Ontario|658[0n]|BOLD:AAA4398
Grammia virguncula|[161]|RDMAB835-06|BCSC312|United States|Wisconsin|522[1n]|BOLD:AAA4398
Grammia figurata|[162]|RDNME377-08|LEP037801|United States|Georgia|658[0n]|BOLD:AAA4398
Grammia figurata|[163]|RDMAB820-06|BCSC297|United States|Illinois|574[1n]|BOLD:AAA4398
Grammia figurata|[164]|RDMAB784-06|BCSC261|United States|Pennsylvania|658[0n]|BOLD:AAA4398
Grammia figurata|[165]|RDNME380-08|LEP037804|United States|Georgia|658[0n]|BOLD:AAA4398
Grammia figurata|[166]|RDNME379-08|LEP037803|United States|Georgia|658[0n]|BOLD:AAA4398
Grammia figurata|[167]|RDNME378-08|LEP037802|United States|Georgia|658[0n]|BOLD:AAA4398
Grammia figurata|[168]|LNCC290-10|10-NCCC-290|United States|North Carolina|658[0n]|BOLD:AAA4398
Grammia|[169]|BBLSY215-09|09BBLEP-03142|United States|Texas|658[0n]|BOLD:AAA4398
Grammia figurata|[170]|USLEP658-10|10BBLEP-00658|United States|Texas|658[0n]|BOLD:AAA4398
Grammia|[171]|BBLSY214-09|09BBLEP-03141|United States|Texas|642[0n]|BOLD:AAA4398
Grammia|[172]|BBLSY213-09|09BBLEP-03140|United States|Texas|658[0n]|BOLD:AAA4398
Grammia|[173]|BBLSX762-09|09BBLEP-02690|United States|Texas|658[0n]|BOLD:AAA4398
Grammia figurata|[174]|USLEP660-10|10BBLEP-00660|United States|Colorado|658[0n]|BOLD:AAA4398
Grammia figurata|[175]|USLEP659-10|10BBLEP-00659|United States|Texas|658[0n]|BOLD:AAA4398
Grammia|[176]|BBLSY022-09|09BBLEP-02949|United States|Oklahoma|658[0n]|BOLD:AAA4398
Grammia f-pallida|[177]|HKONB409-09|3906-COI-08|United States|Texas|658[0n]|BOLD:AAA4398
Grammia figurata|[178]|RDMAB804-06|BCSC281|United States|Oklahoma|585[0n]|BOLD:AAA4398
Grammia figurata|[179]|USLEP657-10|10BBLEP-00657|United States|Colorado|658[0n]|BOLD:AAA4398
Grammia parthenice|[180]|LOWCB187-05|CGWC-1127|Canada|British Columbia|658[0n]|BOLD:AAA4398
Grammia parthenice|[181]|LOWCB185-05|CGWC-1125|Canada|British Columbia|658[0n]|BOLD:AAA4398
Grammia parthenice|[182]|LOWCB189-05|CGWC-1129|Canada|British Columbia|658[0n]|BOLD:AAA4398
Grammia parthenice|[183]|LOWCB188-05|CGWC-1128|Canada|British Columbia|658[0n]|BOLD:AAA4398
Grammia parthenice|[184]|LOWCB184-05|CGWC-1124|Canada|British Columbia|658[0n]|BOLD:AAA4398
Grammia parthenice|[185]|RDMAB871-06|BCSC348|Canada|Alberta|658[0n]|BOLD:AAA4398
Grammia virguncula|[186]|RDMAB843-06|BCSC320|Canada|Ontario|658[0n]|BOLD:AAA4398
Grammia parthenice|[187]|LOWCB186-05|CGWC-1126|Canada|British Columbia|658[0n]|BOLD:AAA4398
Grammia virguncula|[188]|RDLQG144-06|DH012315|Canada|Quebec|614[0n]|BOLD:AAA4398
Grammia virguncula|[189]|RDMAB853-06|BCSC330|United States|Vermont|658[0n]|BOLD:AAA4398
Grammia virguncula|[190]|RDLQB700-05|DH010803|Canada|Quebec|658[0n]|BOLD:AAA4398
Grammia virguncula|[191]|XAF726-05|2005-ONT-375|Canada|Ontario|658[0n]|BOLD:AAA4398
Grammia virguncula|[192]|RDLQF825-06|DH011978|Canada|Quebec|658[0n]|BOLD:AAA4398
Grammia virguncula|[193]|RDLQF826-06|DH011979|Canada|Quebec|658[0n]|BOLD:AAA4398
Grammia virguncula|[194]|RDLQB818-05|DH010905|Canada|Quebec|617[0n]|BOLD:AAA4398
Grammia quenseli|[195]|RDNME653-08|LEP038077|Canada|Newfoundland and Labrador|658[0n]|BOLD:AAA...
Grammia quenseli|[196]|MHLEP033-07|CHU06-LEP-033|Canada|Manitoba|658[0n]|BOLD:AAA4398
Grammia quenseli|[197]|LCHP920-07|07PROBE-10682|Canada|Manitoba|658[0n]|BOLD:AAA4398
Grammia virguncula|[198]|RDLQF827-06|DH011980|Canada|Quebec|658[0n]|BOLD:AAA4398
Grammia virguncula|[199]|TTMNB027-06|MNBTT-027|Canada|New Brunswick|658[0n]|BOLD:AAA4398
[illegible]

Grammia quenseli|[197]|LCHP920-07|07PROBE-10682|Canada|Manitoba|658[0n]|BOLD:AAA4398
Grammia virguncula|[198]|RDLQF827-06|DH011980|Canada|Quebec|658[0n]|BOLD:AAA4398
Grammia virguncula|[199]|TTMNB027-06|MNBTT-027|Canada|New Brunswick|658[0n]|BOLD:AAA4398
Grammia virguncula|[200]|BLGSM056-09|BL375|Canada|Ontario|632[0n]|BOLD:AAA4398
Grammia virguncula|[201]|TMNBB029-06|MNBTT-969|Canada|New Brunswick|658[0n]|BOLD:AAA4398
Grammia virguncula|[202]|RDLQB699-05|DH010802|Canada|Quebec|581[0n]|BOLD:AAA4398
Grammia|[203]|BBLPA336-10|10BBCLP-0336|Canada|Alberta|658[0n]|BOLD:AAA4398
Grammia|[204]|BBLPA335-10|10BBCLP-0335|Canada|Alberta|658[0n]|BOLD:AAA4398
Grammia williamsii|[205]|RDMAB893-06|BCSC370|Canada|Alberta|658[0n]|BOLD:AAA4398
Grammia williamsii|[206]|RDMAB892-06|BCSC369|Canada|Alberta|658[0n]|BOLD:AAA4398
Grammia williamsii|[207]|RDMAB891-06|BCSC368|Canada|Alberta|658[0n]|BOLD:AAA4398
Grammia williamsii|[208]|RDMAB890-06|BCSC367|Canada|Alberta|658[0n]|BOLD:AAA4398
Grammia favorita|[209]|RDNME296-07|CNCNoctuoidea13903|United States|Colorado|658[0n]|BOLD:AAA4398
Grammia favorita|[210]|RDNME297-07|CNCNoctuoidea13904|United States|Colorado|658[0n]|BOLD:AAA4398
Grammia favorita|[211]|RDNME295-07|CNCNoctuoidea13902|United States|Colorado|593[0n]|BOLD:AAA4398
Grammia favorita|[212]|RDMAB785-06|BCSC262|United States|Colorado|588[1n]|BOLD:AAA4398
Grammia virguncula|[213]|RDMAB842-06|BCSC319|Canada|Alberta|658[0n]|BOLD:AAA4398
Grammia virguncula|[214]|RDNMB395-05|CNCNoctuoidea10161|Canada|Alberta|658[0n]|BOLD:AAA4398
Grammia virguncula|[215]|LPMN021-08|08BBLEP-00819|Canada|Manitoba|658[0n]|BOLD:AAA4398
Grammia virguncula|[216]|LPMN018-08|08BBLEP-00816|Canada|Manitoba|658[0n]|BOLD:AAA4398
Grammia virguncula|[217]|RDMAB841-06|BCSC318|United States|Wyoming|658[0n]|BOLD:AAA4398
Grammia virguncula|[218]|RDMAB834-06|BCSC311|United States|Wisconsin|515[0n]|BOLD:AAA4398
Grammia virguncula|[219]|XAF794-05|2005-ONT-443|Canada|Ontario|658[0n]|BOLD:AAA4398
Grammia virguncula|[220]|RDNMG079-08|NOC15020|United States|South Dakota|648[0n]|BOLD:AAA4398
Grammia virguncula|[221]|RDNMG078-08|NOC15019|United States|South Dakota|648[1n]|BOLD:AAA4398
Grammia virguncula|[222]|RDLQB208-05|DH010294|Canada|Quebec|658[0n]|BOLD:AAA4398
Grammia virguncula|[223]|GWORT799-10|BC AB Lep 00039|United States||658[0n]|BOLD:AAA4398
Grammia virguncula|[224]|GWORT798-10|BC AB Lep 00038|United States||658[0n]|BOLD:AAA4398
Grammia virguncula|[225]|RDMAB819-06|BCSC296|Canada|Nova Scotia|658[0n]|BOLD:AAA4398
Grammia virguncula|[226]|LPMN167-08|08BBLEP-00966|Canada|Manitoba|658[0n]|BOLD:AAA4398
Grammia virguncula|[227]|LPMN024-08|08BBLEP-00822|Canada|Manitoba|658[0n]|BOLD:AAA4398
Grammia virguncula|[228]|LPMN023-08|08BBLEP-00821|Canada|Manitoba|658[0n]|BOLD:AAA4398
Grammia virguncula|[229]|LPMN022-08|08BBLEP-00820|Canada|Manitoba|658[0n]|BOLD:AAA4398
Grammia virguncula|[230]|LPMN020-08|08BBLEP-00818|Canada|Manitoba|658[0n]|BOLD:AAA4398
Grammia virguncula|[231]|LPMN019-08|08BBLEP-00817|Canada|Manitoba|658[0n]|BOLD:AAA4398
Grammia virguncula|[232]|LPMN017-08|08BBLEP-00815|Canada|Manitoba|658[0n]|BOLD:AAA4398
Grammia virguncula|[233]|LPMN016-08|08BBLEP-00814|Canada|Manitoba|658[0n]|BOLD:AAA4398
Grammia virguncula|[234]|LPMN015-08|08BBLEP-00813|Canada|Manitoba|658[0n]|BOLD:AAA4398
Grammia virguncula|[235]|LPMN014-08|08BBLEP-00812|Canada|Manitoba|658[0n]|BOLD:AAA4398
Grammia virguncula|[236]|XAF737-05|2005-ONT-386|Canada|Ontario|506[1n]|BOLD:AAA4398
Grammia virguncula|[237]|TMNBB030-06|MNBTT-970|Canada|New Brunswick|658[0n]|BOLD:AAA4398
Grammia virguncula|[238]|RDMAB848-06|BCSC325|Canada|Alberta|658[0n]|BOLD:AAA4398
Grammia virguncula|[239]|RDMAB850-06|BCSC327|Canada|Alberta|658[0n]|BOLD:AAA4398
Grammia williamsii|[240]|RDMAB879-06|BCSC356|Canada|Alberta|658[0n]|BOLD:AAA4398
Grammia williamsii|[241]|RDMAB909-06|BCSC383|Canada|Alberta|658[0n]|BOLD:AAA4398
Grammia elongata|[242]|RDMAB918-06|BCSC392|Canada|Alberta|658[0n]|BOLD:AAA4398
Grammia margo|[243]|RDMAB773-06|BCSC250|Canada|British Columbia|590[0n]|BOLD:AAA4398
Grammia virguncula|[244]|RDMAB854-06|BCSC331|Canada|Alberta|572[0n]|BOLD:AAA4398
Grammia williamsii|[245]|RDNMB370-05|CNCNoctuoidea10136|Canada|Alberta|572[0n]|BOLD:AAA4398
Grammia virguncula|[246]|RDMAB847-06|BCSC324|Canada|Yukon Territory|573[2n]|BOLD:AAA4398
Grammia margo|[247]|RDMAB833-06|BCSC310|Canada|British Columbia|658[0n]|BOLD:AAA4398
Grammia margo|[248]|RDMAB793-06|BCSC270|Canada|Alberta|658[0n]|BOLD:AAA4398
Grammia f-pallida|[249]|RDNMG081-08|NOC15022|United States|Colorado|658[0n]|BOLD:AAA4398
Grammia virguncula|[250]|RDLQB796-05|DH010883|Canada|Quebec|617[0n]|BOLD:AAA4398
Grammia quenseli|[251]|RDNME652-08|LEP038076|Canada|Newfoundland and Labrador|648[3n]|BOLD:AAA...
Grammia franconia|[252]|RDLQB209-05|DH010295|Canada|Quebec|658[0n]|BOLD:AAA4398
Grammia williamsii|[253]|RDLQB206-05|DH010292|Canada|Quebec|658[0n]|BOLD:AAA4398
Grammia williamsii|[254]|RDLQB205-05|DH010291|Canada|Quebec|658[0n]|BOLD:AAA4398
Grammia speciosa|[255]|LCHP190-07|07PROBE-00117|Canada|Manitoba|658[0n]|BOLD:AAA4398
Grammia speciosa|[256]|LCHP189-07|07PROBE-00116|Canada|Manitoba|658[0n]|BOLD:AAA4398
Grammia speciosa|[257]|LCHP111-07|07PROBE-00552|Canada|Manitoba|658[0n]|BOLD:AAA4398
Grammia quenseli|[258]|RDNME840-08|LEP041305|Canada|Quebec|639[2n]|BOLD:AAA4398
Grammia quenseli|[259]|RDMAB866-06|BCSC343|Canada|Manitoba|658[0n]|BOLD:AAA4398
Grammia quenseli|[260]|RDMAB867-06|BCSC344|Canada|Manitoba|658[0n]|BOLD:AAA4398
Grammia williamsii|[261]|RDMAB878-06|BCSC355|Canada|Alberta|658[0n]|BOLD:AAA4398
Grammia williamsii|[262]|RDMAB907-06|BCSC381|Canada|Alberta|658[0n]|BOLD:AAA4398
Grammia quenseli|[263]|RDMAB862-06|BCSC339|Canada|British Columbia|658[0n]|BOLD:AAA4398
Grammia williamsii|[264]|RDMAB880-06|BCSC357|Canada|Alberta|658[0n]|BOLD:AAA4398
Grammia williamsii|[265]|RDLQB210-05|DH010296|Canada|Quebec|658[0n]|BOLD:AAA4398
Grammia quenseli|[266]|MHLEP081-07|CHU06-LEP-081|Canada|Manitoba|658[0n]|BOLD:AAA4398
Grammia quenseli|[267]|RDMAB865-06|BCSC342|Canada|Manitoba|658[0n]|BOLD:AAA4398
Grammia quenseli|[268]|MHLEP083-07|CHU06-LEP-083|Canada|Manitoba|658[0n]|BOLD:AAA4398
Grammia franconia|[269]|RDMAB911-06|BCSC385|Canada|Ontario|658[0n]|BOLD:AAA4398
Grammia franconia|[270]|RDNMB368-05|CNCNoctuoidea10134|Canada|Ontario|658[0n]|BOLD:AAA4398
Grammia f-pallida|[271]|RDNMG080-08|NOC15021|United States|Colorado|658[0n]|BOLD:AAA4398
Grammia virguncula|[272]|RDMAB857-06|BCSC334|Canada|Alberta|658[0n]|BOLD:AAA4398
Grammia quenseli|[273]|LCHQ893-08|07WNP-10785|Canada|Manitoba|658[0n]|BOLD:AAA4398
Grammia williamsii|[274]|RDMAB908-06|BCSC382|Canada|Alberta|658[0n]|BOLD:AAA4398
Grammia virguncula|[275]|RDMAB856-06|BCSC333|Canada|Alberta|658[0n]|BOLD:AAA4398
Grammia franconia|[276]|RDNMB309-05|CNCNoctuoidea10075|Canada|Ontario|615[0n]|BOLD:AAA4398
Grammia quenseli|[277]|RDNME842-08|LEP031953|United States|New Hampshire|591[0n]|BOLD:AAA4398
Grammia franconia|[278]|RDNMB308-05|CNCNoctuoidea10074|Canada|Ontario|583[0n]|BOLD:AAA4398
Grammia f-pallida|[279]|RDNME129-07|CNCNoctuoidea13657|United States|Colorado|658[1n]|BOLD:AAA...
Grammia figurata|[280]|RDNMB379-05|CNCNoctuoidea10145|United States|Colorado|503[0n]|
Grammia quenseli|[281]|RDMAB863-06|BCSC340|Canada|British Columbia|585[0n]|BOLD:AAA4398
Grammia quenseli|[282]|RDNME841-08|LEP041306|United States|New Hampshire|587[0n]|BOLD:AAA4398
Grammia quenseli|[283]|RDNME839-08|LEP041304|Canada|Quebec|587[0n]|BOLD:AAA4398
Grammia williamsii|[284]|BBLCU308-09|09BBLEP-04795|United States|Michigan|658[0n]|BOLD:AAA4398
Grammia williamsii|[285]|RDLQB207-05|DH010293|Canada|Quebec|658[0n]|BOLD:AAA4398
Grammia virguncula|[286]|RDMAB858-06|BCSC335|Canada|Quebec|658[0n]|BOLD:AAA4398
Grammia quenseli|[287]|JGLL045-10|10PROBE-19309|Canada|Manitoba|658[0n]|BOLD:AAA4398
Grammia parthenice|[288]|RDMAB873-06|BCSC350|United States|Georgia|571[1n]|BOLD:AAA8807
Grammia parthenice|[289]|RDMAB737-06|BCSC406|United States|Georgia|563[0n]|BOLD:AAA8807
Grammia parthenice|[290]|LNCNW014-06|06-NCNW-0014|United States|North Carolina|658[0n]|BOLD:AA...
Grammia parthenice|[291]|XAI125-05|0102-ONT-0125|Canada|Ontario|545[1n]|BOLD:AAA8807
Grammia parthenice|[292]|XAI124-05|0102-ONT-0124|Canada|Ontario|556[0n]|BOLD:AAA8807
Grammia parthenice|[293]|LNCNW032-06|06-NCNW-0032|United States|North Carolina|658[0n]|BOLD:AA...
Grammia parthenice|[294]|XAK296-06|2006-ONT-1291|Canada|Ontario|658[0n]|BOLD:AAA8807
Grammia parthenice|[295]|LNC429-05|05-NCCC-429|United States|North Carolina|617[0n]|BOLD:AAA8807
Grammia parthenice|[296]|LSUSA022-06|06-SUSA-0022|United States|Kentucky|658[0n]|BOLD:AAA8807
Grammia parthenice|[297]|RDLQB600-05|DH010703|Canada|Quebec|658[0n]|BOLD:AAA8807
Grammia parthenice|[298]|LNC428-05|05-NCCC-428|United States|North Carolina|658[0n]|BOLD:AAA8807
Grammia parthenice|[299]|XAG850-05|2005-ONT-1434|Canada|Ontario|658[0n]|BOLD:AAA8807

Grammia parthenice|[297]|RDLQB600-05|DH010703|Canada|Quebec|658[0n]|BOLD:AAA8807
Grammia parthenice|[298]|LNC428-05|05-NCCC-428|United States|North Carolina|658[0n]|BOLD:AAA8807
Grammia parthenice|[299]|XAG850-05|2005-ONT-1434|Canada|Ontario|658[0n]|BOLD:AAA8807
Grammia nevadensis|[300]|LOWCD480-06|CGWC-3300|Canada|British Columbia|512[0n]|BOLD:ABZ6253
Grammia nevadensis|[301]|LOWCD481-06|CGWC-3301|Canada|British Columbia|513[0n]|BOLD:ABZ6253
Grammia nevadensis|[302]|LOWCD483-06|CGWC-3303|Canada|British Columbia|514[0n]|BOLD:ABZ6253
Grammia nevadensis|[303]|RDNMB402-05|CNCNoctuoidea10168|Canada|British Columbia|509[0n]|BOLD:A...
Grammia yavapai|[304]|CNCLB2211-14|CNCLEP00118013|United States|Arizona|658[0n]|BOLD:ABZ6253
Grammia yavapai|[305]|CNCLB2210-14|CNCLEP00118012|United States|Arizona|658[0n]|BOLD:ABZ6253
Grammia yavapai|[306]|RDNME128-07|CNCNoctuoidea13656|United States|Arizona|283[0n]|
Grammia williamsii tooele|[307]|RDMAB828-06|BCSC305|United States|Utah|520[1n]|BOLD:ABZ5637
Grammia williamsii tooele|[308]|RDMAB829-06|BCSC306|United States|Utah|658[0n]|BOLD:ABZ5637
Grammia williamsii tooele|[309]|RDMAB827-06|BCSC304|United States|Utah|658[0n]|BOLD:ABZ5637
Grammia williamsii tooele|[310]|RDMAB826-06|BCSC303|United States|Utah|658[0n]|BOLD:ABZ5637
Grammia williamsii tooele|[311]|RDMAB825-06|BCSC302|United States|Utah|658[0n]|BOLD:ABZ5637
Grammia williamsii tooele|[312]|RDMAB824-06|BCSC301|United States|Utah|658[0n]|BOLD:ABZ5637
Grammia fergusoni|[313]|CNCLB1000-14|CNCLEP00113408|United States|California|658[0n]|BOLD:ABZ6253
Grammia fergusoni|[314]|CNCLB999-14|CNCLEP00113407|United States|California|658[0n]|BOLD:ABZ6253
Grammia fergusoni|[315]|CNCLB998-14|CNCLEP00113406|United States|California|658[0n]|BOLD:ABZ6253
Grammia nevadensis|[316]|RDNMB399-05|CNCNoctuoidea10165|Canada|British Columbia|571[2n]|BOLD:A...
Grammia nevadensis|[317]|LOWCD462-06|CGWC-3282|Canada|British Columbia|579[0n]|BOLD:ABZ6253
Grammia nevadensis|[318]|LOWCD475-06|CGWC-3295|Canada|British Columbia|604[0n]|BOLD:ABZ6253
Grammia nevadensis|[319]|LOWCD471-06|CGWC-3291|Canada|British Columbia|561[0n]|BOLD:ABZ6253
Grammia nevadensis|[320]|LBCH6337-10|10-JDWBC-6337|Canada|British Columbia|658[1n]|BOLD:ABZ6253
Grammia nevadensis|[321]|LBCH6960-10|10-JDWBC-6960|Canada|British Columbia|658[0n]|BOLD:ABZ6253
Grammia nevadensis|[322]|LBCH6958-10|10-JDWBC-6958|Canada|British Columbia|658[0n]|BOLD:ABZ6253
Grammia nevadensis|[323]|LBCH6957-10|10-JDWBC-6957|Canada|British Columbia|658[0n]|BOLD:ABZ6253
Grammia nevadensis|[324]|LBCH6954-10|10-JDWBC-6954|Canada|British Columbia|658[0n]|BOLD:ABZ6253
Grammia nevadensis|[325]|LBCH6863-10|10-JDWBC-6863|Canada|British Columbia|658[0n]|BOLD:ABZ6253
Grammia nevadensis|[326]|LBCH6858-10|10-JDWBC-6858|Canada|British Columbia|658[0n]|BOLD:ABZ6253
Grammia nevadensis|[327]|LBCH6594-10|10-JDWBC-6594|Canada|British Columbia|658[0n]|BOLD:ABZ6253
Grammia nevadensis|[328]|LBCH6473-10|10-JDWBC-6473|Canada|British Columbia|658[0n]|BOLD:ABZ6253
Grammia nevadensis|[329]|LBCH6415-10|10-JDWBC-6415|Canada|British Columbia|658[0n]|BOLD:ABZ6253
Grammia nevadensis|[330]|LBCH6334-10|10-JDWBC-6334|Canada|British Columbia|658[0n]|BOLD:ABZ6253
Grammia nevadensis|[331]|LBCH7572-10|10-JDWBC-7572|Canada|British Columbia|658[0n]|BOLD:ABZ6253
Grammia nevadensis|[332]|LBCH7567-10|10-JDWBC-7567|Canada|British Columbia|658[0n]|BOLD:ABZ6253
Grammia nevadensis|[333]|LBCH7714-10|10-JDWBC-7714|Canada|British Columbia|658[0n]|BOLD:ABZ6253
Grammia nevadensis|[334]|LBCH6039-10|10-JDWBC-6039|Canada|British Columbia|658[0n]|BOLD:ABZ6253
Grammia nevadensis|[335]|LBCH7868-10|10-JDWBC-7868|Canada|British Columbia|658[0n]|BOLD:ABZ6253
Grammia nevadensis|[336]|LBCH7573-10|10-JDWBC-7573|Canada|British Columbia|658[0n]|BOLD:ABZ6253
Grammia nevadensis|[337]|LBCG2898-09|08-JDWBC-2898|Canada|British Columbia|658[0n]|BOLD:ABZ6253
Grammia nevadensis|[338]|LBCG2896-09|08-JDWBC-2896|Canada|British Columbia|658[0n]|BOLD:ABZ6253
Grammia nevadensis|[339]|LBCH7568-10|10-JDWBC-7568|Canada|British Columbia|658[0n]|BOLD:ABZ6253
Grammia nevadensis|[340]|LOWCD469-06|CGWC-3289|Canada|British Columbia|590[0n]|BOLD:ABZ6253
Grammia nevadensis|[341]|LOWCD464-06|CGWC-3284|Canada|British Columbia|598[0n]|BOLD:ABZ6253
Grammia nevadensis|[342]|LBCH6593-10|10-JDWBC-6593|Canada|British Columbia|658[0n]|BOLD:ABZ6253
Grammia nevadensis|[343]|LBCH6591-10|10-JDWBC-6591|Canada|British Columbia|658[0n]|BOLD:ABZ6253
Grammia nevadensis|[344]|LOWCD478-06|CGWC-3298|Canada|British Columbia|608[0n]|BOLD:ABZ6253
Grammia behrii|[345]|RDMAB768-06|BCSC245|United States|Oregon|658[0n]|BOLD:ABZ6253
Grammia nevadensis|[346]|LOWCB191-05|CGWC-1131|Canada|British Columbia|658[0n]|BOLD:ABZ6253
Grammia nevadensis|[347]|LBCG486-08|08-JDWBC-0486|Canada|British Columbia|658[0n]|BOLD:ABZ6253
Grammia nevadensis|[348]|LBCG484-08|08-JDWBC-0484|Canada|British Columbia|658[0n]|BOLD:ABZ6253
Grammia nevadensis|[349]|LBCH7298-10|10-JDWBC-7298|Canada|British Columbia|635[0n]|BOLD:ABZ6253
Grammia nevadensis|[350]|LBCH7517-10|10-JDWBC-7517|Canada|British Columbia|632[0n]|BOLD:ABZ6253
Grammia nevadensis|[351]|LBCH7390-10|10-JDWBC-7390|Canada|British Columbia|630[0n]|BOLD:ABZ6253
Grammia nevadensis|[352]|LBCH7296-10|10-JDWBC-7296|Canada|British Columbia|627[0n]|BOLD:ABZ6253
Grammia nevadensis|[353]|LOWCD468-06|CGWC-3288|Canada|British Columbia|613[0n]|BOLD:ABZ6253
Grammia nevadensis|[354]|LOWCD474-06|CGWC-3294|Canada|British Columbia|610[0n]|BOLD:ABZ6253
Grammia nevadensis|[355]|LOWCD460-06|CGWC-3280|Canada|British Columbia|612[0n]|BOLD:ABZ6253
Grammia nevadensis|[356]|LBCH7514-10|10-JDWBC-7514|Canada|British Columbia|626[0n]|BOLD:ABZ6253
Grammia nevadensis|[357]|RDNMB398-05|CNCNoctuoidea10164|Canada|Alberta|611[0n]|BOLD:ABZ6253
Grammia nevadensis|[358]|LBCH6331-10|10-JDWBC-6331|Canada|British Columbia|619[28n]|BOLD:ABZ6253
Grammia nevadensis|[359]|LBCH7118-10|10-JDWBC-7118|Canada|British Columbia|658[0n]|BOLD:ABZ6253
Grammia nevadensis|[360]|RDNMB418-05|CNCNoctuoidea10184|United States|Oregon|658[0n]|BOLD:ABZ6253
Grammia williamsii|[361]|RDMAB772-06|BCSC249|United States|Nevada|658[0n]|BOLD:ABZ6253
Grammia williamsii|[362]|RDMAB883-06|BCSC360|United States|Nevada|658[0n]|BOLD:ABZ6253
Grammia williamsii|[363]|RDMAB882-06|BCSC359|United States|Nevada|658[0n]|BOLD:ABZ6253
Grammia nevadensis|[364]|RDMAB807-06|BCSC284|United States|Utah|658[0n]|BOLD:ABZ6253
Grammia nevadensis|[365]|RDNMG075-08|NOC15016|United States|Colorado|658[0n]|BOLD:ABZ6253
Grammia nevadensis|[366]|LBCH6471-10|10-JDWBC-6471|Canada|British Columbia|658[0n]|BOLD:ABZ6253
Grammia nevadensis|[367]|LBCH6470-10|10-JDWBC-6470|Canada|British Columbia|635[0n]|BOLD:ABZ6253
Grammia nevadensis|[368]|LBCH6862-10|10-JDWBC-6862|Canada|British Columbia|658[0n]|BOLD:ABZ6253
Grammia nevadensis|[369]|LBCH6861-10|10-JDWBC-6861|Canada|British Columbia|658[0n]|BOLD:ABZ6253
Grammia nevadensis|[370]|LBCH6857-10|10-JDWBC-6857|Canada|British Columbia|658[0n]|BOLD:ABZ6253
Grammia nevadensis|[371]|LBCH6590-10|10-JDWBC-6590|Canada|British Columbia|658[0n]|BOLD:ABZ6253
Grammia nevadensis|[372]|LBCH6092-10|10-JDWBC-6092|Canada|British Columbia|658[0n]|BOLD:ABZ6253
Grammia nevadensis|[373]|LBCH5991-10|10-JDWBC-5991|Canada|British Columbia|658[0n]|BOLD:ABZ6253
Grammia nevadensis|[374]|LBCH7297-10|10-JDWBC-7297|Canada|British Columbia|658[0n]|BOLD:ABZ6253
Grammia nevadensis|[375]|LBCH7223-10|10-JDWBC-7223|Canada|British Columbia|658[0n]|BOLD:ABZ6253
Grammia nevadensis|[376]|LBCH7867-10|10-JDWBC-7867|Canada|British Columbia|658[0n]|BOLD:ABZ6253
Grammia nevadensis|[377]|LBCH7866-10|10-JDWBC-7866|Canada|British Columbia|658[0n]|BOLD:ABZ6253
Grammia nevadensis|[378]|LBCH7097-10|10-JDWBC-7097|Canada|British Columbia|658[0n]|BOLD:ABZ6253
Grammia nevadensis|[379]|LBCH6959-10|10-JDWBC-6959|Canada|British Columbia|658[0n]|BOLD:ABZ6253
Grammia nevadensis|[380]|LBCH7864-10|10-JDWBC-7864|Canada|British Columbia|658[0n]|BOLD:ABZ6253
Grammia nevadensis|[381]|LBCH7863-10|10-JDWBC-7863|Canada|British Columbia|658[0n]|BOLD:ABZ6253
Grammia nevadensis|[382]|LBCH6475-10|10-JDWBC-6475|Canada|British Columbia|658[0n]|BOLD:ABZ6253
Grammia nevadensis|[383]|LBCH6474-10|10-JDWBC-6474|Canada|British Columbia|658[0n]|BOLD:ABZ6253
Grammia nevadensis|[384]|LBCH6472-10|10-JDWBC-6472|Canada|British Columbia|658[0n]|BOLD:ABZ6253
Grammia nevadensis|[385]|LBCH6411-10|10-JDWBC-6411|Canada|British Columbia|658[0n]|BOLD:ABZ6253
Grammia nevadensis|[386]|LBCH6409-10|10-JDWBC-6409|Canada|British Columbia|658[0n]|BOLD:ABZ6253
Grammia nevadensis|[387]|LBCH6336-10|10-JDWBC-6336|Canada|British Columbia|658[0n]|BOLD:ABZ6253
Grammia nevadensis|[388]|LBCH6333-10|10-JDWBC-6333|Canada|British Columbia|658[0n]|BOLD:ABZ6253
Grammia nevadensis|[389]|LBCH7566-10|10-JDWBC-7566|Canada|British Columbia|658[0n]|BOLD:ABZ6253
Grammia nevadensis|[390]|LBCH7518-10|10-JDWBC-7518|Canada|British Columbia|658[0n]|BOLD:ABZ6253
Grammia nevadensis|[391]|LBCH7718-10|10-JDWBC-7718|Canada|British Columbia|658[0n]|BOLD:ABZ6253
Grammia nevadensis|[392]|LBCH7713-10|10-JDWBC-7713|Canada|British Columbia|658[0n]|BOLD:ABZ6253
Grammia nevadensis|[393]|LBCH7712-10|10-JDWBC-7712|Canada|British Columbia|658[0n]|BOLD:ABZ6253
Grammia nevadensis|[394]|LBCH7711-10|10-JDWBC-7711|Canada|British Columbia|658[0n]|BOLD:ABZ6253
Grammia nevadensis|[395]|LBCH6330-10|10-JDWBC-6330|Canada|British Columbia|658[0n]|BOLD:ABZ6253
Grammia nevadensis|[396]|LBCH7716-10|10-JDWBC-7716|Canada|British Columbia|658[0n]|BOLD:ABZ6253
Grammia nevadensis|[397]|LBCH7715-10|10-JDWBC-7715|Canada|British Columbia|658[0n]|BOLD:ABZ6253
Grammia nevadensis|[398]|LBCH7870-10|10-JDWBC-7870|Canada|British Columbia|658[0n]|BOLD:ABZ6253
Grammia nevadensis|[399]|LBCH7869-10|10-JDWBC-7869|Canada|British Columbia|658[0n]|BOLD:ABZ6253

Grammia nevadensis|[396]|LBCH7716-10|10-JDWBC-7716|Canada|British Columbia|658[0n]|BOLD:ABZ6253
Grammia nevadensis|[397]|LBCH7715-10|10-JDWBC-7715|Canada|British Columbia|658[0n]|BOLD:ABZ6253
Grammia nevadensis|[398]|LBCH7870-10|10-JDWBC-7870|Canada|British Columbia|658[0n]|BOLD:ABZ6253
Grammia nevadensis|[399]|LBCH7869-10|10-JDWBC-7869|Canada|British Columbia|658[0n]|BOLD:ABZ6253
Grammia nevadensis|[400]|LBCH6860-10|10-JDWBC-6860|Canada|British Columbia|658[0n]|BOLD:ABZ6253
Grammia nevadensis|[401]|LBCH6859-10|10-JDWBC-6859|Canada|British Columbia|658[0n]|BOLD:ABZ6253
Grammia nevadensis|[402]|LOWCB192-05|CGWC-1132|Canada|British Columbia|658[0n]|BOLD:ABZ6253
Grammia nevadensis|[403]|LOWCB190-05|CGWC-1130|Canada|British Columbia|658[0n]|BOLD:ABZ6253
Grammia nevadensis|[404]|LBCH6856-10|10-JDWBC-6856|Canada|British Columbia|658[0n]|BOLD:ABZ6253
Grammia nevadensis|[405]|LBCH6592-10|10-JDWBC-6592|Canada|British Columbia|658[0n]|BOLD:ABZ6253
Grammia nevadensis|[406]|LBCH7521-10|10-JDWBC-7521|Canada|British Columbia|658[0n]|BOLD:ABZ6253
Grammia nevadensis|[407]|LBCH7519-10|10-JDWBC-7519|Canada|British Columbia|658[0n]|BOLD:ABZ6253
Grammia nevadensis|[408]|LBCH7300-10|10-JDWBC-7300|Canada|British Columbia|658[0n]|BOLD:ABZ6253
Grammia nevadensis|[409]|LBCH7299-10|10-JDWBC-7299|Canada|British Columbia|658[0n]|BOLD:ABZ6253
Grammia nevadensis|[410]|LBCH7302-10|10-JDWBC-7302|Canada|British Columbia|658[0n]|BOLD:ABZ6253
Grammia nevadensis|[411]|LBCH7301-10|10-JDWBC-7301|Canada|British Columbia|658[0n]|BOLD:ABZ6253
Grammia nevadensis|[412]|LBCH7464-10|10-JDWBC-7464|Canada|British Columbia|658[0n]|BOLD:ABZ6253
Grammia nevadensis|[413]|LBCH7303-10|10-JDWBC-7303|Canada|British Columbia|658[0n]|BOLD:ABZ6253
Grammia nevadensis|[414]|LOWCD459-06|CGWC-3279|Canada|British Columbia|658[0n]|BOLD:ABZ6253
Grammia nevadensis|[415]|LOWCD458-06|CGWC-3278|Canada|British Columbia|658[0n]|BOLD:ABZ6253
Grammia nevadensis|[416]|LOWCD465-06|CGWC-3285|Canada|British Columbia|658[0n]|BOLD:ABZ6253
Grammia nevadensis|[417]|LOWCD463-06|CGWC-3283|Canada|British Columbia|658[0n]|BOLD:ABZ6253
Grammia nevadensis|[418]|LOWCD467-06|CGWC-3287|Canada|British Columbia|658[0n]|BOLD:ABZ6253
Grammia nevadensis|[419]|LOWCD470-06|CGWC-3290|Canada|British Columbia|658[0n]|BOLD:ABZ6253
Grammia nevadensis|[420]|LOWCD472-06|CGWC-3292|Canada|British Columbia|658[0n]|BOLD:ABZ6253
Grammia nevadensis|[421]|LOWCD473-06|CGWC-3293|Canada|British Columbia|658[0n]|BOLD:ABZ6253
Grammia nevadensis|[422]|LOWCD476-06|CGWC-3296|Canada|British Columbia|658[0n]|BOLD:ABZ6253
Grammia nevadensis|[423]|LOWCD477-06|CGWC-3297|Canada|British Columbia|658[0n]|BOLD:ABZ6253
Grammia nevadensis|[424]|LOWCD479-06|CGWC-3299|Canada|British Columbia|658[0n]|BOLD:ABZ6253
Grammia nevadensis|[425]|LOWCD482-06|CGWC-3302|Canada|British Columbia|658[0n]|BOLD:ABZ6253
Grammia nevadensis|[426]|LBCG2105-09|08-JDWBC-2105|Canada|British Columbia|658[0n]|BOLD:ABZ6253
Grammia nevadensis|[427]|LBCH6468-10|10-JDWBC-6468|Canada|British Columbia|658[0n]|BOLD:ABZ6253
Grammia nevadensis|[428]|LBCH7717-10|10-JDWBC-7717|Canada|British Columbia|658[0n]|BOLD:ABZ6253
Grammia nevadensis|[429]|LOWCD461-06|CGWC-3281|Canada|British Columbia|656[0n]|BOLD:ABZ6253
Grammia nevadensis|[430]|LBCH6469-10|10-JDWBC-6469|Canada|British Columbia|658[0n]|BOLD:ABZ6253
Grammia nevadensis|[431]|LBCH7865-10|10-JDWBC-7865|Canada|British Columbia|658[0n]|BOLD:ABZ6253
Grammia nevadensis|[432]|RDMAB763-06|BCSC240|Canada|British Columbia|658[0n]|BOLD:ABZ6253
Grammia nevadensis|[433]|LBCH6758-10|10-JDWBC-6758|Canada|British Columbia|658[0n]|BOLD:ABZ6253
Grammia nevadensis|[434]|LBCH6588-10|10-JDWBC-6588|Canada|British Columbia|658[0n]|BOLD:ABZ6253
Grammia nevadensis|[435]|LBCH6589-10|10-JDWBC-6589|Canada|British Columbia|658[0n]|BOLD:ABZ6253
Grammia nevadensis|[436]|LBCH6587-10|10-JDWBC-6587|Canada|British Columbia|658[0n]|BOLD:ABZ6253
Grammia nevadensis|[437]|RDMAB765-06|BCSC242|Canada|Alberta|658[0n]|BOLD:ABZ6253
Grammia nevadensis|[438]|LOWCB193-05|CGWC-1133|Canada|British Columbia|658[0n]|BOLD:ABZ6253
Grammia nevadensis|[439]|LBCG485-08|08-JDWBC-0485|Canada|British Columbia|658[0n]|BOLD:ABZ6253
Grammia nevadensis|[440]|LBCG237-08|08-JDWBC-0237|Canada|British Columbia|658[0n]|BOLD:ABZ6253
Grammia ornata|[441]|RDNMB374-05|CNCNoctuoidea10140|United States|Oregon|553[0n]|BOLD:ABZ6253
Grammia ornata|[442]|RDNMB377-05|CNCNoctuoidea10143|United States|Oregon|580[0n]|BOLD:ABZ6253
Grammia ornata|[443]|RDNMB372-05|CNCNoctuoidea10138|United States|Oregon|602[0n]|BOLD:ABZ6253
Grammia nevadensis|[444]|RDNMB401-05|CNCNoctuoidea10167|Canada|British Columbia|577[4n]|BOLD:A...
Grammia nevadensis|[445]|LBCH7516-10|10-JDWBC-7516|Canada|British Columbia|642[0n]|BOLD:ABZ6253
Grammia nevadensis|[446]|LBCH7520-10|10-JDWBC-7520|Canada|British Columbia|658[0n]|BOLD:ABZ6253
Grammia nevadensis|[447]|LBCH6332-10|10-JDWBC-6332|Canada|British Columbia|658[0n]|BOLD:ABZ6253
Grammia nevadensis|[448]|LBCH7569-10|10-JDWBC-7569|Canada|British Columbia|658[0n]|BOLD:ABZ6253
Grammia nevadensis|[449]|LBCH7570-10|10-JDWBC-7570|Canada|British Columbia|658[0n]|BOLD:ABZ6253
Grammia nevadensis|[450]|LBCH6410-10|10-JDWBC-6410|Canada|British Columbia|658[0n]|BOLD:ABZ6253
Grammia nevadensis|[451]|LBCH6412-10|10-JDWBC-6412|Canada|British Columbia|658[0n]|BOLD:ABZ6253
Grammia nevadensis|[452]|LBCH6416-10|10-JDWBC-6416|Canada|British Columbia|658[0n]|BOLD:ABZ6253
Grammia nevadensis|[453]|LBCH6955-10|10-JDWBC-6955|Canada|British Columbia|658[0n]|BOLD:ABZ6253
Grammia nevadensis|[454]|LBCH6956-10|10-JDWBC-6956|Canada|British Columbia|658[0n]|BOLD:ABZ6253
Grammia nevadensis|[455]|LOWCD466-06|CGWC-3286|Canada|British Columbia|658[0n]|BOLD:ABZ6253
Grammia nevadensis|[456]|LBCH6961-10|10-JDWBC-6961|Canada|British Columbia|658[0n]|BOLD:ABZ6253
Grammia nevadensis|[457]|LBCG2897-09|08-JDWBC-2897|Canada|British Columbia|658[0n]|BOLD:ABZ6253
Grammia nevadensis|[458]|RDNMB420-05|CNCNoctuoidea10186|Canada|British Columbia|571[1n]|BOLD:A...
Grammia nevadensis|[459]|LBCH6836-10|10-JDWBC-6836|Canada|British Columbia|658[0n]|BOLD:ABZ6253
Grammia nevadensis|[460]|LBCH7571-10|10-JDWBC-7571|Canada|British Columbia|658[0n]|BOLD:ABZ6253
Grammia nevadensis|[461]|RDMAB764-06|BCSC241|Canada|British Columbia|569[1n]|BOLD:ABZ6253
Grammia nevadensis|[462]|RDNMB417-05|CNCNoctuoidea10183|United States|Washington|658[0n]|BOLD:...
Grammia behrii|[463]|RDNMB416-05|CNCNoctuoidea10182|United States|Oregon|658[0n]|BOLD:ABZ6253
Grammia behrii|[464]|RDNMB415-05|CNCNoctuoidea10181|United States|Oregon|658[0n]|BOLD:ABZ6253
Grammia nevadensis|[465]|RDMAB810-06|BCSC287|United States|Colorado|602[0n]|BOLD:ABZ6253
Grammia nevadensis|[466]|RDMAB809-06|BCSC286|United States|Colorado|595[0n]|BOLD:ABZ6253
Grammia nevadensis|[467]|RDMAB808-06|BCSC285|United States|Colorado|601[0n]|BOLD:ABZ6253
Grammia nevadensis|[468]|RDNMB422-05|CNCNoctuoidea10188|Canada|Saskatchewan|658[0n]|BOLD:ABZ6253
Grammia nevadensis|[469]|RDNMB421-05|CNCNoctuoidea10187|Canada|Saskatchewan|658[0n]|BOLD:ABZ6253
Grammia nevadensis|[470]|RDNMB400-05|CNCNoctuoidea10166|Canada|Alberta|533[1n]|BOLD:ABZ6253
Grammia nevadensis|[471]|RDMAB766-06|BCSC243|Canada|Alberta|658[0n]|BOLD:ABZ6253
Grammia incorrupta|[472]|RDNMG073-08|NOC15014|United States|Colorado|658[0n]|BOLD:ABZ6253
Grammia quenseli|[473]|RDMAB749-06|BCSC418|Canada|Yukon Territory|585[0n]|BOLD:ABZ6253
Grammia quenseli|[474]|RDMAB859-06|BCSC336|Canada|Yukon Territory|599[0n]|BOLD:ABZ6253
Grammia yukona|[475]|RDMAB783-06|BCSC260|Canada|Yukon Territory|658[0n]|BOLD:ABZ6253
Grammia yukona|[476]|RDMAB782-06|BCSC259|Canada|Yukon Territory|658[0n]|BOLD:ABZ6253
Grammia quenseli|[477]|RDNME385-08|LEP037809|Canada|Yukon Territory|658[0n]|BOLD:ABZ6253
Grammia ornata|[478]|RDMAB757-06|BCSC234|Canada|British Columbia|658[0n]|BOLD:ABZ6253
Grammia ornata|[479]|RDMAB758-06|BCSC235|Canada|British Columbia|658[0n]|BOLD:ABZ6253
Grammia ornata|[480]|JMMMB435-11|BIOUG00851-E07|United States|California|658[0n]|BOLD:ABZ6253
Grammia ornata|[481]|JBAZ059-09|JLB-0059|United States|California|658[0n]|BOLD:ABZ6253
Grammia hewletti|[482]|RDNME123-07|CNCNoctuoidea13651|United States|California|658[0n]|BOLD:AB...
Grammia hewletti|[483]|RDNME125-07|CNCNoctuoidea13653|United States|California|602[0n]|BOLD:AB...
Grammia hewletti|[484]|JBAZ016-09|JLB-0016|United States|California|658[0n]|BOLD:ABZ6253
Grammia hewletti|[485]|RDNME126-07|CNCNoctuoidea13654|United States|California|658[0n]|BOLD:AB...
Grammia hewletti|[486]|JBAZ019-09|JLB-0019|United States|California|658[0n]|BOLD:ABZ6253
Grammia hewletti|[487]|JBAZ018-09|JLB-0018|United States|California|658[0n]|BOLD:ABZ6253
Grammia hewletti|[488]|JBAZ017-09|JLB-0017|United States|California|658[0n]|BOLD:ABZ6253
Grammia hewletti|[489]|JBAZ015-09|JLB-0015|United States|California|658[0n]|BOLD:ABZ6253
Grammia hewletti|[490]|JBAZ014-09|JLB-0014|United States|California|658[0n]|BOLD:ABZ6253
Grammia ornata|[491]|GMLC293-11|2009GM-0162|United States|California|658[0n]|BOLD:ABZ6253
Grammia ornata|[492]|GMLC257-11|2009GM-0126|United States|California|615[0n]|BOLD:ABZ6253
Grammia ornata|[493]|GMLC542-11|2011GM-0238|United States|California|658[0n]|BOLD:ABZ6253
Grammia ornata|[494]|GMLC299-11|2009GM-0168|United States|California|658[0n]|BOLD:ABZ6253
Grammia ornata|[495]|GMLC533-11|2011GM-0229|United States|California|658[0n]|BOLD:ABZ6253
Grammia ornata|[496]|GMLC489-11|2011GM-0185|United States|California|658[0n]|BOLD:ABZ6253
Grammia ornata|[497]|GMLC358-11|2011GM-0054|United States|California|658[0n]|BOLD:ABZ6253
Grammia ornata|[498]|RDNMB373-05|CNCNoctuoidea10139|United States|Oregon|597[0n]|BOLD:ABZ6253

Grammia ornata|[496]|GMLC489-11|2011GM-0185|United States|California|658[0n]|BOLD:ABZ6253
Grammia ornata|[497]|GMLC358-11|2011GM-0054|United States|California|658[0n]|BOLD:ABZ6253
Grammia ornata|[498]|RDNMB373-05|CNCNoctuoidea10139|United States|Oregon|597[0n]|BOLD:ABZ6253
Grammia ornata|[499]|RDNMB375-05|CNCNoctuoidea10141|United States|California|591[0n]|BOLD:ABZ6253
Grammia ornata|[500]|CGLCA003-10|CCGBOLD00003|United States|California|658[0n]|BOLD:ABZ6253
Grammia ornata|[501]|CGLCA005-10|CCGBOLD00005|United States|California|658[0n]|BOLD:ABZ6253
Grammia ornata|[502]|CGLCA004-10|CCGBOLD00004|United States|California|658[0n]|BOLD:ABZ6253
Grammia ornata|[503]|CGLCA002-10|CCGBOLD00002|United States|California|658[0n]|BOLD:ABZ6253
Grammia ursina|[504]|CGLCA194-10|CCGBOLD00194|United States|California|658[0n]|BOLD:ABZ6253
Grammia ursina|[505]|CGLCA193-10|CCGBOLD00193|United States|California|658[0n]|BOLD:ABZ6253
Grammia ursina|[506]|CGLCA192-10|CCGBOLD00192|United States|California|658[0n]|BOLD:ABZ6253
Grammia williamsii|[507]|RDMAB887-06|BCSC364|United States|Wyoming|658[0n]|BOLD:ABZ6253
Grammia williamsii|[508]|RDMAB806-06|BCSC283|United States|Colorado|609[0n]|BOLD:ABZ6253
Grammia williamsii|[509]|RDMAB888-06|BCSC365|United States|Wyoming|658[0n]|BOLD:ABZ6253
Grammia williamsii|[510]|RDMAB889-06|BCSC366|United States|Arizona|658[0n]|BOLD:ABZ6253
Grammia cervinoides|[511]|RDNME347-07|CNCNoctuoidea13954|United States|Colorado|657[0n]|BOLD:A...
Grammia williamsii|[512]|RDMAB914-06|BCSC388|Canada|Alberta|658[0n]|BOLD:ABZ6253
Grammia williamsii|[513]|RDNMB369-05|CNCNoctuoidea10135|Canada|Alberta|589[0n]|BOLD:ABZ6253
Grammia blakei|[514]|RDMAB753-06|BCSC230|Canada|Alberta|658[0n]|BOLD:ABZ6253
Grammia blakei|[515]|RDMAB813-06|BCSC290|Canada|Alberta|658[0n]|BOLD:ABZ6253
Grammia blakei|[516]|RDNMG529-08|CNC LEP00052353|Canada|Alberta|658[0n]|BOLD:ABZ6253
Grammia blakei|[517]|RDNMG528-08|CNC LEP00052352|Canada|Alberta|658[0n]|BOLD:ABZ6253
Grammia williamsii|[518]|LPABB500-08|08BBLEP-03765|Canada|Alberta|631[0n]|BOLD:ABZ6253
Grammia williamsii|[519]|RDMAB915-06|BCSC389|Canada|Alberta|658[0n]|BOLD:ABZ6253
Grammia williamsii|[520]|RDMAB913-06|BCSC387|Canada|Alberta|658[0n]|BOLD:ABZ6253
Grammia williamsii|[521]|RDMAB912-06|BCSC386|Canada|Alberta|658[0n]|BOLD:ABZ6253
Grammia williamsii|[522]|RDMAB905-06|BCSC27|Canada|Alberta|658[0n]|BOLD:ABZ6253
Grammia williamsii|[523]|RDMAB904-06|BCSC26|Canada|Alberta|658[0n]|BOLD:ABZ6253
Grammia williamsii|[524]|RDMAB903-06|BCSC25|Canada|Alberta|658[0n]|BOLD:ABZ6253
Grammia williamsii|[525]|RDMAB896-06|BCSC373|Canada|Alberta|658[0n]|BOLD:ABZ6253
Grammia williamsii|[526]|RDMAB894-06|BCSC371|Canada|Alberta|658[0n]|BOLD:ABZ6253
Grammia bowmani|[527]|RDMAB771-06|BCSC248|United States|Colorado|658[0n]|BOLD:ABZ6253
Grammia bowmani|[528]|RDMAB770-06|BCSC247|United States|Colorado|657[0n]|BOLD:ABZ6253
Grammia williamsii|[529]|RDMAB886-06|BCSC363|United States|Wyoming|658[0n]|BOLD:ABZ6253
Grammia blakei|[530]|RDNMG527-08|CNC LEP00052351|Canada|Alberta|658[0n]|BOLD:ABZ6253
Grammia elongata|[531]|RDMAB774-06|BCSC251|Canada|British Columbia|519[2n]|BOLD:ABZ6253
Grammia complicata|[532]|RDNMB460-05|CNCNoctuoidea10226|Canada|British Columbia|658[0n]|BOLD:A...
Grammia complicata|[533]|RDMAB755-06|BCSC232|Canada|British Columbia|658[0n]|BOLD:ABZ6253
Grammia complicata|[534]|RDMAB756-06|BCSC233|United States|Washington|597[0n]|BOLD:ABZ6253
Grammia complicata|[535]|RDMAB754-06|BCSC231|Canada|British Columbia|658[0n]|BOLD:ABZ6253
Grammia ornata|[536]|LBCH5154-10|10-JDWBC-5154|Canada|British Columbia|658[0n]|BOLD:ABZ6253
Grammia ornata|[537]|LBCH5078-10|10-JDWBC-5078|Canada|British Columbia|658[0n]|BOLD:ABZ6253
Grammia ornata|[538]|LBCH5075-10|10-JDWBC-5075|Canada|British Columbia|658[0n]|BOLD:ABZ6253
Grammia elongata|[539]|RDMAB775-06|BCSC252|Canada|Alberta|658[0n]|BOLD:ABZ6253
Grammia ornata|[540]|RDNMB376-05|CNCNoctuoidea10142|Canada|British Columbia|579[0n]|BOLD:ABZ6253
Grammia ornata|[541]|LBCH5073-10|10-JDWBC-5073|Canada|British Columbia|658[0n]|BOLD:ABZ6253
Grammia ornata|[542]|LBCH5270-10|10-JDWBC-5270|Canada|British Columbia|658[0n]|BOLD:ABZ6253
Grammia ornata|[543]|LBCH5269-10|10-JDWBC-5269|Canada|British Columbia|658[0n]|BOLD:ABZ6253
Grammia ornata|[544]|LBCH5267-10|10-JDWBC-5267|Canada|British Columbia|658[0n]|BOLD:ABZ6253
Grammia ornata|[545]|LBCH5274-10|10-JDWBC-5274|Canada|British Columbia|658[0n]|BOLD:ABZ6253
Grammia ornata|[546]|LBCH5275-10|10-JDWBC-5275|Canada|British Columbia|658[0n]|BOLD:ABZ6253
Grammia ornata|[547]|LBCH5079-10|10-JDWBC-5079|Canada|British Columbia|658[0n]|BOLD:ABZ6253
Grammia ornata|[548]|LBCH5273-10|10-JDWBC-5273|Canada|British Columbia|640[0n]|BOLD:ABZ6253
Grammia ornata|[549]|LBCH5272-10|10-JDWBC-5272|Canada|British Columbia|658[0n]|BOLD:ABZ6253
Grammia ornata|[550]|LBCH5271-10|10-JDWBC-5271|Canada|British Columbia|658[0n]|BOLD:ABZ6253
Grammia ornata|[551]|LBCH5268-10|10-JDWBC-5268|Canada|British Columbia|658[0n]|BOLD:ABZ6253
Grammia ornata|[552]|LBCH5081-10|10-JDWBC-5081|Canada|British Columbia|658[0n]|BOLD:ABZ6253
Grammia ornata|[553]|LBCH5080-10|10-JDWBC-5080|Canada|British Columbia|658[0n]|BOLD:ABZ6253
Grammia ornata|[554]|LBCH5077-10|10-JDWBC-5077|Canada|British Columbia|658[0n]|BOLD:ABZ6253
Grammia ornata|[555]|LBCH5076-10|10-JDWBC-5076|Canada|British Columbia|658[0n]|BOLD:ABZ6253
Grammia ornata|[556]|LBCH5074-10|10-JDWBC-5074|Canada|British Columbia|658[0n]|BOLD:ABZ6253
Grammia ornata|[557]|LBCG018-08|08-JDWBC-0018|Canada|British Columbia|658[0n]|BOLD:ABZ6253
Grammia ornata|[558]|LBCG017-08|08-JDWBC-0017|Canada|British Columbia|658[0n]|BOLD:ABZ6253
Grammia ornata|[559]|LPVIC108-08|PFC-2006-2684|Canada|British Columbia|658[0n]|BOLD:ABZ6253
Grammia elongata|[560]|RDMAB910-06|BCSC384|Canada|British Columbia|584[0n]|BOLD:ABZ6253
Grammia williamsii|[561]|RDMAB897-06|BCSC374|Canada|Alberta|658[0n]|BOLD:ABZ6253
Grammia williamsii|[562]|RDMAB898-06|BCSC375|Canada|Alberta|658[0n]|BOLD:ABZ6253
Grammia williamsii|[563]|RDMAB916-06|BCSC390|Canada|Alberta|658[0n]|BOLD:ABZ6253
Grammia williamsii|[564]|RDMAB895-06|BCSC372|Canada|Alberta|658[0n]|BOLD:ABZ6253
Grammia williamsii|[565]|RDMAB901-06|BCSC378|Canada|British Columbia|511[1n]|BOLD:ABZ6253
Grammia williamsii|[566]|RDMAB900-06|BCSC377|Canada|British Columbia|634[0n]|BOLD:ABZ6253
Grammia williamsii|[567]|RDMAB902-06|BCSC379|Canada|British Columbia|658[0n]|BOLD:ABZ6253
Grammia williamsii|[568]|RDNMB899-06|BCSC376|Canada|British Columbia|658[0n]|BOLD:ABZ6253
Grammia williamsii|[569]|RDMAB884-06|BCSC361|United States|Wyoming|658[0n]|BOLD:ABZ6253
Grammia brillians|[570]|RDMAB767-06|BCSC244|United States|Utah|658[0n]|BOLD:ABZ6253
Grammia williamsii|[571]|RDMAB885-06|BCSC362|United States|Wyoming|658[0n]|BOLD:ABZ6253
Grammia virguncula|[572]|RDNMB394-05|CNCNoctuoidea10160|Canada|Quebec|522[0n]|BOLD:ABZ6253
Grammia virgo|[573]|LSEU003-06|06-JKA-0003|United States|Georgia|658[0n]|BOLD:ABZ6253
Grammia quenseli|[574]|MHLEP084-07|CHU06-LEP-084|Canada|Manitoba|658[0n]|BOLD:ABZ6253
Grammia incorrupta|[575]|BBLSW356-09|09BBLEP-01284|United States|Arizona|658[0n]|BOLD:ABZ6253
Grammia anna|[576]|RDMAB855-06|BCSC332|Canada|Ontario|658[0n]|BOLD:AAD6928
Grammia anna|[577]|LNCC064-10|10-NCCC-159|United States|North Carolina|658[0n]|BOLD:AAD6928
Grammia anna|[578]|RDLQB557-05|DH010643|Canada|Quebec|658[0n]|BOLD:AAD6928
Grammia anna|[579]|LNCC063-10|10-NCCC-158|United States|North Carolina|658[0n]|BOLD:AAD6928
Grammia anna|[580]|LSEU004-06|06-JKA-0004|United States|North Carolina|581[1n]|BOLD:AAD6928
Grammia anna|[581]|LSEU396-06|06-JKA-0396|United States|Georgia|622[0n]|BOLD:AAD6928
Grammia anna|[582]|BBLCU202-09|09BBLEP-04689|United States|Michigan|658[0n]|BOLD:AAD6928
Grammia anna|[583]|LSEU005-06|06-JKA-0005|United States|North Carolina|658[0n]|BOLD:AAD6928
Grammia anna|[584]|LNCC062-10|10-NCCC-157|United States|North Carolina|658[0n]|BOLD:AAD6928
Grammia anna|[585]|LSEU395-06|06-JKA-0395|United States|Georgia|658[0n]|BOLD:AAD6928
Grammia anna|[586]|LNCC061-10|10-NCCC-156|United States|North Carolina|658[0n]|BOLD:AAD6928
Grammia virgo|[587]|GWORT809-10|BC AB Lep 00049|Canada|Ontario|658[0n]|BOLD:AAA7209
Grammia virgo|[588]|XAJ937-06|2006-ONT-0937|Canada|Ontario|658[0n]|BOLD:AAA7209
Grammia virgo|[589]|LNC427-05|05-NCCC-427|United States|North Carolina|658[0n]|BOLD:AAA7209
Grammia williamsii|[590]|RDMAB881-06|BCSC358|Canada|Alberta|658[0n]|BOLD:AAA7209
Grammia incorrupta|[591]|CMAZA054-09|CMAZ-0054|United States|Arizona|658[0n]|BOLD:ACF3388
Grammia incorrupta|[592]|CMAZA828-10|CMAZ-0828|United States|Arizona|658[0n]|BOLD:ACF3388
Grammia quenseli|[593]|RDMAB868-06|BCSC345|Canada|Manitoba|658[0n]|BOLD:ACF3388
Grammia incorrupta|[594]|BBLSW763-09|09BBLEP-01691|United States|Texas|658[0n]|BOLD:ABZ4332
Grammia phyllira|[595]|RDNMB371-05|CNCNoctuoidea10137|Canada|Ontario|658[0n]|BOLD:AAC5868
Grammia phyllira|[596]|RDMAB787-06|BCSC264|Canada|Ontario|658[0n]|BOLD:AAC5868
Grammia phyllira|[597]|RDMAB786-06|BCSC263|United States|Colorado|658[0n]|BOLD:ACF3893
Grammia phyllira|[598]|RDMAB788-06|BCSC265|Canada|Ontario|658[0n]|BOLD:ABX5768

[illegible]
Grammia phyllira|[596]|RDMAB787-06|BCSC264|Canada|Ontario|658[0n]|BOLD:AAC5868
Grammia phyllira|[597]|RDMAB786-06|BCSC263|United States|Colorado|658[0n]|BOLD:ACF3893
Grammia phyllira|[598]|RDMAB788-06|BCSC265|Canada|Ontario|658[0n]|BOLD:ABX5768
Grammia phyllira|[599]|RDNME133-07|CNCNoctuoidea13661|United States|Florida|658[0n]|BOLD:ABZ5913
Grammia phyllira|[600]|RDMAB830-06|BCSC307|United States|Kentucky|601[0n]|BOLD:ABZ5913
Grammia phyllira|[601]|LPOKC727-09|MDOK-2804|United States|Oklahoma|658[0n]|BOLD:ABZ5913
Grammia phyllira|[602]|LPOKB365-09|MDOK-1380|United States|Oklahoma|658[0n]|BOLD:ABZ5913
Grammia phyllira|[603]|LSUSA123-06|06-SUSA-0123|United States|Kentucky|656[0n]|BOLD:ABZ5913
Grammia incorrupta|[604]|RDMAB778-06|BCSC255|United States|Colorado|658[0n]|BOLD:ABZ5913
Grammia phyllira|[605]|RDMAB790-06|BCSC267|Canada|Alberta|658[0n]|BOLD:ABZ5913
Grammia phyllira|[606]|RDMAB789-06|BCSC266|Canada|Alberta|658[0n]|BOLD:ABZ5913
Grammia phyllira|[607]|BBLSU023-09|09BBLEP-04392|United States|Arkansas|658[0n]|BOLD:ABZ5913
Grammia placentia|[608]|RDMAB800-06|BCSC277|United States|Georgia|658[0n]|BOLD:AAD1597
Grammia placentia|[609]|LSEU018-06|06-JKA-0018|United States|Georgia|603[0n]|BOLD:AAD1597
Grammia placentia|[610]|MMNA121-08|HLC-17683|United States|Georgia|658[0n]|BOLD:AAD1597
Grammia placentia|[611]|RDMAB799-06|BCSC276|United States|Georgia|658[0n]|BOLD:AAD1597
Grammia placentia|[612]|RDMAB821-06|BCSC298|United States|Florida|658[0n]|BOLD:AAD1597
Grammia placentia|[613]|LSEU019-06|06-JKA-0019|United States|Georgia|596[0n]|BOLD:AAD1597
Grammia placentia|[614]|LSEU020-06|06-JKA-0020|United States|Georgia|593[0n]|BOLD:AAD1597
Grammia placentia|[615]|QUNO090-07|2090-120507-FL|United States|Florida|658[0n]|BOLD:AAD1597
Grammia virgo|[616]|LGSM624-04|DNA-ATBI-0624|United States|North Carolina|609[0n]|BOLD:AAA7208
Grammia virgo|[617]|LOT148-04|04HBL002148|United States|Tennessee|609[0n]|BOLD:AAA7208
Grammia virgo|[618]|LGSMB293-05|DNA-ATBI-1142|United States|North Carolina|549[1n]|BOLD:AAA7208
Grammia virgo|[619]|LGSMG118-07|BGS03439|United States|North Carolina|658[0n]|BOLD:AAA7208
Grammia virgo|[620]|LNC426-05|05-NCCC-426|United States|North Carolina|658[0n]|BOLD:AAA7208
Grammia virgo|[621]|LGSMB294-05|DNA-ATBI-1143|United States|North Carolina|653[0n]|BOLD:AAA7208
Grammia virgo|[622]|RDMAB877-06|BCSC354|United States||658[0n]|BOLD:AAA7208
Grammia virgo|[623]|LPMN707-08|08BBLEP-01510|Canada|Manitoba|658[0n]|BOLD:AAA7208
Grammia virgo|[624]|RDNMB299-05|CNCNoctuoidea10065|Canada|Alberta|599[0n]|BOLD:AAA7208
Grammia virgo|[625]|LPMN556-08|08BBLEP-01357|Canada|Manitoba|658[0n]|BOLD:AAA7208
Grammia virgo|[626]|TMNBD408-07|MNBTT-3209|Canada|New Brunswick|658[0n]|BOLD:AAA7208
Grammia virgo|[627]|TMNBB031-06|MNBTT-971|Canada|New Brunswick|656[0n]|BOLD:AAA7208
Grammia virgo|[628]|PHMNB139-04|04HBL007604|Canada|New Brunswick|658[0n]|BOLD:AAA7208
Grammia virgo|[629]|MNBB134-05|05-NBSTA-050|Canada|New Brunswick|658[0n]|BOLD:AAA7208
Grammia virgo|[630]|TMNBD407-07|MNBTT-3208|Canada|New Brunswick|658[0n]|BOLD:AAA7208
Grammia virgo|[631]|TMNBD409-07|MNBTT-3210|Canada|New Brunswick|657[0n]|BOLD:AAA7208
Grammia virgo|[632]|TMNBD411-07|MNBTT-3212|Canada|New Brunswick|658[0n]|BOLD:AAA7208
Grammia virgo|[633]|TMNBB032-06|MNBTT-972|Canada|New Brunswick|658[0n]|BOLD:AAA7208
Grammia virgo|[634]|TMNBD410-07|MNBTT-3211|Canada|New Brunswick|646[0n]|BOLD:AAA7208
Grammia virgo|[635]|PHMNB042-03|moth217.02SA|Canada|New Brunswick|639[0n]|BOLD:AAA7208
Grammia virgo|[636]|LPMN288-08|08BBLEP-01087|Canada|Manitoba|658[0n]|BOLD:AAA7208
Grammia virgo|[637]|XAJ938-06|2006-ONT-0938|Canada|Ontario|656[0n]|BOLD:AAA7208
Grammia virgo|[638]|BBLEC563-09|09BBELE-0563|Canada|Nova Scotia|658[0n]|BOLD:AAA7208
Grammia virgo|[639]|RDMAB875-06|BCSC352|Canada|Ontario|658[0n]|BOLD:AAA7208
Grammia virgo|[640]|BBLEC208-09|09BBELE-0208|Canada|Nova Scotia|658[0n]|BOLD:AAA7208
Grammia virgo|[641]|RDMAB814-06|BCSC291|Canada|Alberta|658[0n]|BOLD:AAA7208
Grammia virgo|[642]|LPMNB560-09|08BBLEP-05595|Canada|Manitoba|658[0n]|BOLD:AAA7208
Grammia virgo|[643]|PHMNB714-05|Moth 407.03SA|Canada|New Brunswick|658[0n]|BOLD:AAA7208
Grammia virgo|[644]|MNBB644-05|05-NBSTA-560|Canada|New Brunswick|658[0n]|BOLD:AAA7208
Grammia virgo|[645]|XAI129-05|0102-ONT-0129|Canada|Ontario|603[0n]|BOLD:AAA7208
Grammia virgo|[646]|XAI128-05|0102-ONT-0128|Canada|Ontario|616[0n]|BOLD:AAA7208
Grammia virgo|[647]|XAB080-04|04HBL005080|Canada|Ontario|658[0n]|BOLD:AAA7208
Grammia virgo|[648]|RDMAB876-06|BCSC353|United States|Pennsylvania|658[0n]|BOLD:AAA7208
Grammia virgo|[649]|XAI127-05|0102-ONT-0127|Canada|Ontario|658[0n]|BOLD:AAA7208
Grammia virgo|[650]|RDLQB523-05|DH010609|Canada|Quebec|658[0n]|BOLD:AAA7208
Grammia virgo|[651]|XAK037-06|2006-ONT-1032|Canada|Ontario|658[0n]|BOLD:AAA7208
Grammia virgo|[652]|XAK038-06|2006-ONT-1033|Canada|Ontario|658[0n]|BOLD:AAA7208
Grammia virgo|[653]|XAJ939-06|2006-ONT-0939|Canada|Ontario|658[0n]|BOLD:AAA7208
Grammia virgo|[654]|XAJ993-06|2006-ONT-0993|Canada|Ontario|658[0n]|BOLD:AAA7208
Grammia virgo|[655]|XAB105-04|04HBL005105|Canada|Ontario|658[0n]|BOLD:AAA7208
Grammia virgo|[656]|XAB184-04|04HBL005184|Canada|Ontario|658[0n]|BOLD:AAA7208
Grammia virgo|[657]|XAJ936-06|2006-ONT-0936|Canada|Ontario|658[0n]|BOLD:AAA7208
Grammia virgo|[658]|QUNOC088-09|5919-030708-KY|United States|Kentucky|658[0n]|BOLD:AAA7208
Grammia virgo|[659]|XAG896-05|2005-ONT-1480|Canada|Ontario|658[0n]|BOLD:AAA7208
Grammia virgo|[660]|PMG007-03|APAN2.01|Canada|Ontario|617[0n]|BOLD:AAA7208
Grammia allectans|[661]|RDMAB769-06|BCSC246|United States|Arizona|658[0n]|BOLD:AAY2209
Grammia ursina|[662]|RDMAB781-06|BCSC258|United States|California|658[0n]|BOLD:AAB9250
Grammia ursina|[663]|RDMAB780-06|BCSC257|United States|California|658[0n]|BOLD:AAB9250
Grammia incorrupta|[664]|BBLSX760-09|09BBLEP-02688|United States|Texas|658[0n]|BOLD:AAA7787
Grammia incorrupta|[665]|RDNMG076-08|NOC15017|United States|Colorado|658[0n]|BOLD:AAA7787
Grammia nevadensis|[666]|RDNMG072-08|NOC15013|United States|Colorado|658[0n]|BOLD:AAA7787
Grammia nevadensis|[667]|RDNMG071-08|NOC15012|United States|Colorado|658[0n]|BOLD:AAA7787
Grammia incorrupta|[668]|RDMAB779-06|BCSC256|United States|Colorado|658[0n]|BOLD:AAA7787
Grammia incorrupta|[669]|LSUSA039-06|06-SUSA-0039|United States|Texas|658[0n]|BOLD:AAA7787
Grammia incorrupta|[670]|BBLSW024-09|09BBLEP-00952|United States|Texas|658[0n]|BOLD:AAA7787
Grammia incorrupta|[671]|RDMAB777-06|BCSC254|United States|New Mexico|658[0n]|BOLD:ABY4300
Grammia incorrupta|[672]|RDNME484-08|LEP037908|United States|Colorado|658[0n]|BOLD:ABY4300
Grammia incorrupta|[673]|CNCLB1524-14|CNCLEP 00113754|United States|Texas|585[0n]|BOLD:ABY4300
Grammia incorrupta|[674]|CMAZA356-10|CMAZ-0356|United States|Arizona|658[0n]|BOLD:ABY4300
Grammia incorrupta|[675]|RDNMG074-08|NOC15015|United States|Colorado|658[0n]|BOLD:ABY4300
Grammia incorrupta|[676]|CMAZA829-10|CMAZ-0829|United States|Arizona|658[0n]|BOLD:ABY4300
Grammia incorrupta|[677]|CNCLB1525-14|CNCLEP 00113755|United States|Texas|658[0n]|BOLD:ABY4300
Grammia incorrupta|[678]|RDMAB776-06|BCSC253|United States|Arizona|658[0n]|BOLD:ABY4300
Grammia incorrupta|[679]|CMAZA096-09|CMAZ-0096|United States|Arizona|658[0n]|BOLD:ABY4300
Grammia incorrupta|[680]|JBAZ103-09|JLB-0103|United States|Arizona|658[0n]|BOLD:ABY4300
Grammia incorrupta|[681]|BBLSW023-09|09BBLEP-00951|United States|Texas|658[0n]|BOLD:ABY4300
Grammia incorrupta|[682]|BBLSW022-09|09BBLEP-00950|United States|Texas|658[0n]|BOLD:ABY4300
Grammia philipiana|[683]|CNCLB080-14|CFIA-01080|Canada|Yukon Territory|658[0n]|
Grammia philipiana|[684]|CNCLB079-14|CFIA-01079|Canada|Yukon Territory|658[0n]|
Holarctia obliterata|[685]|RDMAB838-06|BCSC315|Canada|Alberta|658[0n]|BOLD:AAE3362
Holarctia obliterata|[686]|LPABC813-09|08BBLEP-05032|Canada|Alberta|658[0n]|BOLD:AAE3362
Holarctia obliterata|[687]|RDMAB837-06|BCSC314|Canada|Alberta|658[0n]|BOLD:AAE3362
Holarctia obliterata|[688]|RDMAB836-06|BCSC313|Canada|Alberta|540[0n]|BOLD:AAE3362
Apantesis phalerata|[689]|BBLOC115-11|BIOUG01453-C01|United States|Florida|658[0n]|BOLD:ABY9320
Apantesis phalerata|[690]|LOFLB334-06|06-FLOR-1274|United States|Florida|658[0n]|BOLD:ABY9320
Apantesis phalerata|[691]|CNCLB2673-14|14-NCCC-724|United States|Florida|658[0n]|BOLD:ABY9320
Apantesis phalerata|[692]|CNCLB2675-14|14-NCCC-725|United States|Florida|658[0n]|BOLD:ABY9320
Apantesis phalerata|[693]|LOFLB122-06|06-FLOR-1062|United States|Florida|658[0n]|BOLD:ABY9320
Apantesis phalerata|[694]|BBLOC119-11|BIOUG01453-C05|United States|Florida|658[0n]|BOLD:ABY9320
Apantesis phalerata|[695]|BBLOB1660-11|BIOUG01421-D09|United States|Florida|658[0n]|BOLD:ABY9320
Apantesis phalerata|[696]|BBLOB1329-11|BIOUG01417-H10|United States|Florida|658[0n]|BOLD:ABY9320
Apantesis phalerata|[697]|CNCLB2667-14|14-NCCC-668|United States|North Carolina|658[0n]|BOLD:A...
[illegible]

Apantesis phalerata|[695]|BBLOB1660-11|BIOUG01421-D09|United States|Florida|658[0n]|BOLD:ABY9320
Apantesis phalerata|[696]|BBLOB1329-11|BIOUG01417-H10|United States|Florida|658[0n]|BOLD:ABY9320
Apantesis phalerata|[697]|CNCLB2667-14|14-NCCC-668|United States|North Carolina|658[0n]|BOLD:A...
Apantesis phalerata|[698]|CNCLB2670-14|14-NCCC-722|United States|Florida|658[0n]|BOLD:ABY9320
Apantesis phalerata|[699]|LOFLC194-06|06-FLOR-2074|United States|Florida|658[0n]|BOLD:ABY9320
Apantesis phalerata|[700]|LOFLB513-06|06-FLOR-1453|United States|Florida|658[0n]|BOLD:ABY9320
Apantesis phalerata|[701]|BBLOB255-11|BIOUG01369-F05|United States|Florida|658[0n]|BOLD:ABY9320
Apantesis phalerata|[702]|LOFLA928-06|06-FLOR-0928|United States|Florida|652[0n]|BOLD:ABY9320
Apantesis phalerata|[703]|CNCLB2676-14|14-NCCC-726|United States|Florida|658[0n]|BOLD:ABY9320
Apantesis phalerata|[704]|CNCLB2671-14|14-NCCC-723|United States|Florida|658[0n]|BOLD:ABY9320
Apantesis phalerata|[705]|CNCLB2665-14|14-NCCC-727|United States|Florida|658[0n]|BOLD:ABY9320
Apantesis phalerata|[706]|LGSMB322-05|DNA-ATBI-1171|United States|North Carolina|597[1n]|BOLD:...
Apantesis phalerata|[707]|LOCT022-05|05-CTATBI-0022|United States|Connecticut|658[0n]|BOLD:ACF...
Apantesis phalerata|[708]|LGSMB325-05|DNA-ATBI-1174|United States|Tennessee|565[0n]|BOLD:ABY9321
Apantesis phalerata|[709]|LGSMB323-05|DNA-ATBI-1172|United States|Tennessee|578[0n]|BOLD:ABY9321
Apantesis phalerata|[710]|LGSMB324-05|DNA-ATBI-1173|United States|Tennessee|578[0n]|BOLD:ABY9321
Apantesis phalerata|[711]|LNCB320-06|06-NCCC-1276|United States|North Carolina|658[0n]|BOLD:AB...
Apantesis phalerata|[712]|LNCC1241-11|11-NCCC-766|United States|North Carolina|658[0n]|BOLD:AB...
Apantesis phalerata|[713]|XAJ424-06|2006-ONT-0424|Canada|Ontario|658[0n]|BOLD:ABY9321
Apantesis phalerata|[714]|XAJ340-06|2006-ONT-0340|Canada|Ontario|658[0n]|BOLD:ABY9321
Apantesis phalerata|[715]|XAK254-06|2006-ONT-1249|Canada|Ontario|658[0n]|BOLD:ABY9321
Apantesis phalerata|[716]|XAG251-05|2005-ONT-835|Canada|Ontario|658[0n]|BOLD:ABY9321
Apantesis phalerata|[717]|XAJ496-06|2006-ONT-0496|Canada|Ontario|658[0n]|BOLD:ABY9321
Apantesis phalerata|[718]|XAG010-05|2005-ONT-594|Canada|Ontario|619[0n]|BOLD:ABY9321
Apantesis phalerata|[719]|XAG515-05|2005-ONT-1099|Canada|Ontario|658[0n]|BOLD:ABY9321
Apantesis phalerata|[720]|CNCLB2669-14|14-NCCC-600|United States|North Carolina|658[0n]|BOLD:A...
Apantesis phalerata|[721]|XAK255-06|2006-ONT-1250|Canada|Ontario|658[0n]|BOLD:ABY9321
Apantesis phalerata|[722]|CNCLB2666-14|14-NCCC-478|United States|North Carolina|658[0n]|BOLD:A...
Apantesis phalerata|[723]|LNCC1240-11|11-NCCC-765|United States|North Carolina|658[0n]|BOLD:AB...
Apantesis phalerata|[724]|LNC218-05|05-NCCC-218|United States|North Carolina|658[0n]|BOLD:ABY9321
Apantesis phalerata|[725]|BBLSU012-09|09BBLEP-04381|United States|Arkansas|658[0n]|BOLD:ABY9321
Apantesis phalerata|[726]|XAF687-05|2005-ONT-336|Canada|Ontario|658[0n]|BOLD:ABY9321
Apantesis phalerata|[727]|LNCB094-06|06-NCC-1050|United States|North Carolina|658[0n]|BOLD:ABY...
Apantesis phalerata|[728]|LNCC1236-11|11-NCCC-761|United States|North Carolina|658[0n]|BOLD:AB...
Apantesis phalerata|[729]|LNCB130-06|06-NCCC-1086|United States|North Carolina|658[0n]|BOLD:AB...
Apantesis phalerata|[730]|RDMAB741-06|BCSC410|United States|Kentucky|571[0n]|BOLD:ABY9321
Apantesis phalerata|[731]|LNCB131-06|06-NCCC-1087|United States|North Carolina|658[0n]|BOLD:AB...
Apantesis phalerata|[732]|LPOKD453-09|MDOK-3532|United States|Oklahoma|658[0n]|BOLD:ABY9321
Apantesis phalerata|[733]|LPOKB357-09|MDOK-1371|United States|Oklahoma|658[0n]|BOLD:ABY9321
Apantesis phalerata|[734]|LILLA402-11|SNS10IL-00527|United States|Illinois|658[0n]|BOLD:ABY9321
Apantesis phalerata|[735]|BBLSZ113-09|09BBLEP-04039|United States|Oklahoma|658[0n]|BOLD:ABY9321
Apantesis phalerata|[736]|BBLSZ115-09|09BBLEP-04041|United States|Oklahoma|658[0n]|BOLD:ABY9321
Apantesis phalerata|[737]|BBLSZ116-09|09BBLEP-04042|United States|Oklahoma|658[0n]|BOLD:ABY9321
Apantesis phalerata|[738]|BBLSZ117-09|09BBLEP-04043|United States|Oklahoma|658[0n]|BOLD:ABY9321
Apantesis phalerata|[739]|LPOKB140-09|MDOK-1282|United States|Oklahoma|658[0n]|BOLD:ABY9321
Apantesis phalerata|[740]|RDNMH020-09|CNCLEP00032071|United States|Mississippi|658[0n]|BOLD:AB...
Apantesis phalerata|[741]|LPOKD405-09|MDOK-3484|United States|Oklahoma|658[0n]|BOLD:ABY9321
Apantesis phalerata|[742]|RDNMH021-09|CNCLEP00032072|United States|Louisiana|658[0n]|BOLD:ABY9321
Apantesis phalerata|[743]|BBLSZ122-09|09BBLEP-04048|United States|Oklahoma|658[0n]|BOLD:ABY9321
Apantesis phalerata|[744]|BBLSZ110-09|09BBLEP-04036|United States|Oklahoma|658[0n]|BOLD:ABY9321
Apantesis phalerata|[745]|LPOKB311-09|MDOK-1325|United States|Oklahoma|658[0n]|BOLD:ABY9321
Apantesis phalerata|[746]|LPOKC725-09|MDOK-2802|United States|Oklahoma|658[0n]|BOLD:ABY9321
Apantesis phalerata|[747]|RDNMB381-05|CNCNoctuoidea10147|Canada|Ontario|658[0n]|BOLD:ABY9321
Apantesis phalerata|[748]|LPOKB133-09|MDOK-1256|United States|Oklahoma|658[0n]|BOLD:ABY9321
Apantesis phalerata|[749]|RDMAB832-06|BCSC309|United States|Kentucky|658[0n]|BOLD:ABY9321
Apantesis phalerata|[750]|RDMAB831-06|BCSC308|United States|Kentucky|658[0n]|BOLD:ABY9321
Apantesis phalerata|[751]|RDNMF990-08|CNC LEP00054257|United States|Mississippi|658[0n]|BOLD:A...
Apantesis phalerata|[752]|RDNMH128-09|CNCLEP00054352|United States|Texas|658[1n]|BOLD:ABY9321
Apantesis phalerata|[753]|RDNMH126-09|CNCLEP00054354|United States|Texas|658[0n]|BOLD:ABY9321
Apantesis phalerata|[754]|LNC737-06|05-NCCC-737|United States|North Carolina|656[0n]|BOLD:ABY9321
Apantesis phalerata|[755]|LPOKB138-09|MDOK-1272|United States|Oklahoma|658[0n]|BOLD:ABY9321
Apantesis phalerata|[756]|RDNMF985-08|CNC LEP00054252|United States|Missouri|658[0n]|BOLD:ABY9321
Apantesis phalerata|[757]|RDNMH127-09|CNCLEP00054353|United States|Texas|658[0n]|BOLD:ABY9321
Apantesis phalerata|[758]|RDNMH286-09|CNCLEP00054595|United States|Alabama|658[0n]|BOLD:ABY9321
Apantesis phalerata|[759]|RDNMH285-09|CNCLEP00054594|United States|Missouri|658[0n]|BOLD:ABY9321
Apantesis carlotta|[760]|RDMAB811-06|BCSC288|United States|Pennsylvania|658[0n]|BOLD:AAC8380
Apantesis carlotta|[761]|USLEP655-10|10BBLEP-00655|United States|Colorado|658[0n]|BOLD:AAC8380
Apantesis carlotta|[762]|BBLSX956-09|09BBLEP-02884|United States|Oklahoma|638[0n]|BOLD:AAC8380
Apantesis carlotta|[763]|RDNMF945-08|CNC LEP00054212|United States|Kansas|658[0n]|BOLD:AAC8380
Apantesis carlotta|[764]|USLEP654-10|10BBLEP-00654|United States|Colorado|658[0n]|BOLD:AAC8380
Apantesis carlotta|[765]|LNCC1238-11|11-NCCC-763|United States|North Carolina|658[0n]|BOLD:AAC...
Apantesis carlotta|[766]|LNCC386-10|10-NCCC-481|United States|North Carolina|658[0n]|BOLD:AAC8380
Apantesis carlotta|[767]|LNCC1237-11|11-NCCC-762|United States|North Carolina|658[0n]|BOLD:AAC...
Apantesis carlotta|[768]|LNCNW010-06|06-NCNW-0010|United States|North Carolina|658[0n]|BOLD:AA...
Apantesis carlotta|[769]|RDMAB822-06|BCSC299|United States|Pennsylvania|594[1n]|BOLD:AAC8380
Apantesis carlotta|[770]|LNCNW011-06|06-NCNW-0011|United States|North Carolina|658[0n]|BOLD:AA...
Apantesis carlotta|[771]|LNCNW009-06|06-NCNW-0009|United States|North Carolina|658[0n]|BOLD:AA...
Apantesis carlotta|[772]|USLEP652-10|10BBLEP-00652|United States|Colorado|658[0n]|BOLD:AAC8380
Apantesis carlotta|[773]|RDNME556-08|LEP037980|United States|Colorado|658[0n]|BOLD:AAC8380
Apantesis carlotta|[774]|RDMAB823-06|BCSC300|United States|Pennsylvania|602[0n]|BOLD:AAC8380
Apantesis carlotta|[775]|BBLSY021-09|09BBLEP-02948|United States|Oklahoma|658[0n]|BOLD:AAC8380
Apantesis carlotta|[776]|USLEP653-10|10BBLEP-00653|United States|Colorado|658[0n]|BOLD:AAC8380
Apantesis carlotta|[777]|RDNMF987-08|CNC LEP00054254|United States|Kansas|658[0n]|BOLD:AAC8380
Apantesis nais|[778]|RDNMH645-09|CNCLEP00062969|United States|Louisiana|658[0n]|BOLD:AAA8555
Apantesis nais|[779]|RDNMH644-09|CNCLEP00062968|United States|Louisiana|658[0n]|BOLD:AAA8555
Apantesis nais|[780]|HKONB393-09|3890-COI-08|United States|Louisiana|658[0n]|BOLD:AAA8555
Apantesis nais|[781]|HKONB392-09|3889-COI-08|United States|Texas|658[0n]|BOLD:AAA8555
Apantesis vittata|[782]|LSUSA021-06|06-SUSA-0021|United States|Kentucky|658[0n]|BOLD:AAA8555
Apantesis vittata|[783]|LSUSA176-06|06-SUSA-0176|United States|Kentucky|658[0n]|BOLD:AAA8555
Apantesis vittata|[784]|LOFLA335-06|06-FLOR-0335|United States|Florida|658[0n]|BOLD:AAA8555
Apantesis vittata|[785]|LOFLA099-06|06-FLOR-0099|United States|Florida|658[0n]|BOLD:AAA8555
Apantesis nr. nais|[786]|HKONB402-09|3899-COI-08|United States|Texas|658[0n]|BOLD:AAA8555
Apantesis nr. nais|[787]|HKONB403-09|3900-COI-08|United States|Louisiana|658[0n]|BOLD:AAA8555
Apantesis vittata|[788]|LOFLA524-06|06-FLOR-0524|United States|Florida|658[0n]|BOLD:AAA8555
Apantesis vittata|[789]|LOFLA289-06|06-FLOR-0289|United States|Florida|658[0n]|BOLD:AAA8555
Apantesis vittata|[790]|LOFLA295-06|06-FLOR-0295|United States|Florida|658[0n]|BOLD:AAA8555
Apantesis vittata|[791]|LOFLA288-06|06-FLOR-0288|United States|Florida|658[0n]|BOLD:AAA8555
Apantesis vittata|[792]|LOFLA287-06|06-FLOR-0287|United States|Florida|658[0n]|BOLD:AAA8555
Apantesis vittata|[793]|CNCLB2681-14|14-NCCC-734|United States|Florida|658[0n]|BOLD:AAA8555
Apantesis vittata|[794]|LOFLA299-06|06-FLOR-0299|United States|Florida|658[0n]|BOLD:AAA8555
Apantesis vittata|[795]|LOFLA296-06|06-FLOR-0296|United States|Florida|658[0n]|BOLD:AAA8555
Apantesis vittata|[796]|LOFLA323-06|06-FLOR-0323|United States|Florida|658[0n]|BOLD:AAA8555
Apantesis vittata|[797]|LOFLA683-06|06-FLOR-0683|United States|Florida|658[0n]|BOLD:AAA8555

Apantesis vittata|[795]|LOFLA296-06|06-FLOR-0296|United States|Florida|658[0n]|BOLD:AAA8555
Apantesis vittata|[796]|LOFLA323-06|06-FLOR-0323|United States|Florida|658[0n]|BOLD:AAA8555
Apantesis vittata|[797]|LOFLA683-06|06-FLOR-0683|United States|Florida|658[0n]|BOLD:AAA8555
Apantesis vittata|[798]|BBLOC113-11|BIOUG01453-B11|United States|Florida|658[0n]|BOLD:AAA8555
Apantesis vittata|[799]|LNCC697-11|11-NCCC-222|United States|North Carolina|658[0n]|BOLD:AAA8555
Apantesis vittata|[800]|LNCB240-06|06-NCCC-1196|United States|North Carolina|658[0n]|BOLD:AAA8555
Apantesis vittata|[801]|LNC219-05|05-NCCC-219|United States|North Carolina|658[0n]|BOLD:AAA8555
Apantesis vittata|[802]|LNC220-05|05-NCCC-220|United States|North Carolina|658[0n]|BOLD:AAA8555
Apantesis vittata|[803]|LOFLA235-06|06-FLOR-0235|United States|Florida|658[0n]|BOLD:AAA8555
Apantesis sp.|[804]|RDNMH884-09|CNCLEP00067192|United States|Georgia|658[0n]|BOLD:AAA8555
Apantesis vittata|[805]|LOFLA298-06|06-FLOR-0298|United States|Florida|658[0n]|BOLD:AAA8555
Apantesis vittata|[806]|LNCC918-11|11-NCCC-443|United States|North Carolina|658[0n]|BOLD:AAA8555
Apantesis vittata|[807]|RDNMH642-09|CNCLEP00062966|United States|Louisiana|658[0n]|BOLD:AAA8555
Apantesis vittata|[808]|RDNMH643-09|CNCLEP00062967|United States|Louisiana|658[0n]|BOLD:AAA8555
Apantesis vittata|[809]|LOFLA919-06|06-FLOR-0919|United States|Florida|627[0n]|BOLD:AAA8555
Apantesis vittata|[810]|CNCLB2680-14|14-NCCC-733|United States|Florida|658[0n]|BOLD:AAA8555
Apantesis vittata|[811]|CNCLB2678-14|14-NCCC-732|United States|Florida|658[0n]|BOLD:AAA8555
Apantesis vittata|[812]|BBLOB1303-11|BIOUG01417-F08|United States|Florida|658[0n]|BOLD:AAA8555
Apantesis vittata|[813]|LOFLC195-06|06-FLOR-2075|United States|Florida|658[0n]|BOLD:AAA8555
Apantesis vittata|[814]|LOFLA384-06|06-FLOR-0384|United States|Florida|658[0n]|BOLD:AAA8555
Apantesis vittata|[815]|LOFLA385-06|06-FLOR-0385|United States|Florida|658[0n]|BOLD:AAA8555
Apantesis vittata|[816]|LOFLA684-06|06-FLOR-0684|United States|Florida|658[0n]|BOLD:AAA8555
Apantesis vittata|[817]|RDNME328-07|CNCNoctuoidea13935|United States|Florida|658[0n]|BOLD:AAA8555
Apantesis vittata|[818]|LOFLA374-06|06-FLOR-0374|United States|Florida|656[0n]|BOLD:AAA8555
Apantesis vittata|[819]|LOFLA373-06|06-FLOR-0373|United States|Florida|658[0n]|BOLD:AAA8555
Apantesis vittata|[820]|LOFLA331-06|06-FLOR-0331|United States|Florida|658[0n]|BOLD:AAA8555
Apantesis vittata|[821]|LOFLA294-06|06-FLOR-0294|United States|Florida|658[0n]|BOLD:AAA8555
Apantesis vittata|[822]|CNCLB2683-14|14-NCCC-735|United States|Florida|658[0n]|BOLD:AAA8555
Apantesis vittata|[823]|LOFLB388-06|06-FLOR-1328|United States|Florida|608[0n]|BOLD:AAA8555
Apantesis vittata|[824]|LOFLC128-06|06-FLOR-2008|United States|Florida|658[0n]|BOLD:AAA8555
Apantesis vittata|[825]|USLEP656-10|10BBLEP-00656|United States|Florida|658[0n]|BOLD:AAA8555
Apantesis vittata|[826]|LOFLC130-06|06-FLOR-2010|United States|Florida|658[0n]|BOLD:AAA8555
Apantesis vittata|[827]|LOFLB208-06|06-FLOR-1148|United States|Florida|658[0n]|BOLD:AAA8555
Apantesis vittata|[828]|LSEU492-06|06-JKA-0492|United States|Georgia|658[0n]|BOLD:AAA8555
Apantesis vittata|[829]|LOFLB817-06|06-FLOR-1757|United States|Florida|658[0n]|BOLD:AAA8555
Apantesis vittata|[830]|BBLOB807-11|BIOUG01412-D11|United States|Florida|658[0n]|BOLD:AAA8555
Apantesis vittata|[831]|BBLOB808-11|BIOUG01412-D12|United States|Florida|658[0n]|BOLD:AAA8555
Apantesis vittata|[832]|BBLOB1302-11|BIOUG01417-F07|United States|Florida|658[0n]|BOLD:AAA8555
Apantesis vittata|[833]|BBLOB1305-11|BIOUG01417-F10|United States|Florida|658[0n]|BOLD:AAA8555
Apantesis vittata|[834]|BBLOE1722-12|BIOUG01990-D03|United States|Florida|658[0n]|BOLD:AAA8555
Apantesis vittata|[835]|LOFLA297-06|06-FLOR-0297|United States|Florida|658[0n]|BOLD:AAA8555
Apantesis vittata|[836]|LOFLB268-06|06-FLOR-1208|United States|Florida|658[0n]|BOLD:AAA8555
Apantesis vittata|[837]|BBLOB238-11|BIOUG01369-D12|United States|Florida|658[0n]|BOLD:AAA8555
Apantesis vittata|[838]|LOFLB389-06|06-FLOR-1329|United States|Florida|632[0n]|BOLD:AAA8555
Apantesis vittata|[839]|LOFLB833-06|06-FLOR-1773|United States|Florida|674[0n]|BOLD:AAA8555
Apantesis vittata|[840]|CNCLB2663-14|14-NCCC-731|United States|Florida|658[0n]|BOLD:AAA8555
Apantesis vittata|[841]|CNCLB2662-14|14-NCCC-730|United States|Florida|658[0n]|BOLD:AAA8555
Apantesis vittata|[842]|CNCLB2661-14|14-NCCC-729|United States|Florida|658[0n]|BOLD:AAA8555
Apantesis vittata|[843]|CNCLB2659-14|14-NCCC-728|United States|Florida|658[0n]|BOLD:AAA8555
Apantesis vittata|[844]|LOFLC196-06|06-FLOR-2076|United States|Florida|658[0n]|BOLD:AAA8555
Apantesis nais|[845]|RDNME340-07|CNCNoctuoidea13947|Canada|Ontario|658[1n]|BOLD:AAA8555
Apantesis nais|[846]|RDNMB382-05|CNCNoctuoidea10148|Canada|Ontario|658[0n]|BOLD:AAA8555
Apantesis nais|[847]|LOCT023-05|05-CTATBI-0023|United States|Connecticut|658[0n]|BOLD:AAA8555
Apantesis nais|[848]|RDNME458-08|LEP037882|United States|Maryland|658[0n]|BOLD:AAA8555
Apantesis nais|[849]|LNCC1239-11|11-NCCC-764|United States|North Carolina|658[0n]|BOLD:AAA8555
Apantesis nais|[850]|RDNMH885-09|CNCLEP00067193|United States|Georgia|658[0n]|BOLD:AAA8555
Apantesis nais|[851]|LGSMB319-05|DNA-ATBI-1168|United States|North Carolina|579[0n]|BOLD:AAA8555
Apantesis nais|[852]|RDNMH886-09|CNCLEP00067194|United States|Georgia|658[0n]|BOLD:AAA8555
Apantesis nais|[853]|LGSMB321-05|DNA-ATBI-1170|United States|Tennessee|585[0n]|BOLD:AAA8555
Apantesis nais|[854]|LSEU342-06|06-JKA-0342|United States|Georgia|658[0n]|BOLD:AAA8555
Apantesis nais|[855]|RDMAB812-06|BCSC289|United States|New Jersey|658[0n]|BOLD:AAA8555
Apantesis nais|[856]|CNCLB2655-14|14-NCCC-565|United States|North Carolina|658[0n]|BOLD:AAA8555
Apantesis nais|[857]|LGSMB326-05|DNA-ATBI-1175|United States|Tennessee|658[4n]|BOLD:AAA8555
Apantesis nais|[858]|LOT125-04|04HBL002125|United States|Tennessee|609[0n]|BOLD:AAA8555
Apantesis nais|[859]|LILLA327-11|SNS10IL-00429|United States|Illinois|658[0n]|BOLD:AAA8555
Apantesis nais|[860]|LGSMG117-07|BGS03438|United States|North Carolina|658[0n]|BOLD:AAA8555
Apantesis nais|[861]|CNCLB2658-14|14-NCCC-567|United States|North Carolina|658[0n]|BOLD:AAA8555
Apantesis nais|[862]|CNCLB2657-14|14-NCCC-566|United States|North Carolina|658[0n]|BOLD:AAA8555
Apantesis nais|[863]|CNCLB2654-14|14-NCCC-564|United States|North Carolina|658[0n]|BOLD:AAA8555
Apantesis nais|[864]|LPOKB378-09|MDOK-1436|United States|Oklahoma|658[0n]|BOLD:AAA8555
Apantesis nais|[865]|LPOKB363-09|MDOK-1377|United States|Oklahoma|658[0n]|BOLD:AAA8555
Apantesis nais|[866]|LPOKD364-09|MDOK-3443|United States|Oklahoma|658[0n]|BOLD:AAA8555
Apantesis nais|[867]|LPOKD177-09|MDOK-3256|United States|Oklahoma|658[0n]|BOLD:AAA8555
Apantesis nais|[868]|LPOKC726-09|MDOK-2803|United States|Oklahoma|658[0n]|BOLD:AAA8555
Apantesis nais|[869]|LPOKB132-09|MDOK-1254|United States|Oklahoma|658[0n]|BOLD:AAA8555
Apantesis nais|[870]|LGSMB320-05|DNA-ATBI-1169|United States|Tennessee|599[0n]|BOLD:AAA8555
Apantesis nais|[871]|PHMO314-03|moth2374.02|Canada|Ontario|639[0n]|BOLD:AAA8555
Grammia doris|[872]|GWNC607-07|CNCLEP00034169|Canada|New Brunswick|658[0n]|BOLD:AAE3399
Grammia doris|[873]|RDMAB760-06|BCSC237|Canada|Alberta|658[0n]|BOLD:AAE3399
Grammia doris|[874]|RDMAB759-06|BCSC236|Canada|Alberta|658[0n]|BOLD:AAE3399
Grammia doris|[875]|HKONB382-09|3879-COI-08|United States|Texas|658[0n]|BOLD:ACF2433
Grammia doris|[876]|RDMAB815-06|BCSC292|United States|Florida|658[0n]|BOLD:ACF2433
Grammia arge|[877]|LPOKB970-09|MDOK-2012|United States|Oklahoma|658[0n]|BOLD:AAB7789
Grammia arge|[878]|RDMAB761-06|BCSC238|United States|Pennsylvania|658[0n]|BOLD:AAB7789
Grammia arge|[879]|XAG252-05|2005-ONT-836|Canada|Ontario|658[0n]|BOLD:AAB7789
Grammia arge|[880]|XAG101-05|2005-ONT-685|Canada|Ontario|658[0n]|BOLD:AAB7789
Grammia arge|[881]|LNCNW016-06|06-NCNW-0016|United States|North Carolina|658[1n]|BOLD:AAB7789
Grammia arge|[882]|LNCNW015-06|06-NCNW-0015|United States|North Carolina|656[0n]|BOLD:AAB7789
Grammia arge|[883]|RDMAB762-06|BCSC239|Canada|Ontario|658[0n]|BOLD:AAB7789
Grammia arge|[884]|XAH311-05|2005-ONT-1894|Canada|Ontario|658[0n]|BOLD:AAB7789
Grammia arge|[885]|RDLQH040-06|DH013277|Canada|Quebec|656[0n]|BOLD:AAB7789
Grammia arge|[886]|XAK266-06|2006-ONT-1261|Canada|Ontario|658[0n]|BOLD:AAB7789
Grammia arge|[887]|XAK292-06|2006-ONT-1287|Canada|Ontario|658[0n]|BOLD:AAB7789
Grammia arge|[888]|XAJ402-06|2006-ONT-0402|Canada|Ontario|658[0n]|BOLD:AAB7789
Grammia arge|[889]|XAG083-05|2005-ONT-667|Canada|Ontario|658[0n]|BOLD:AAB7789
Grammia arge|[890]|XAG178-05|2005-ONT-762|Canada|Ontario|658[0n]|BOLD:AAB7789
Grammia arge|[891]|XAG232-05|2005-ONT-816|Canada|Ontario|658[0n]|BOLD:AAB7789
Grammia arge|[892]|XAE625-04|Moth4625.03|Canada|Ontario|572[0n]|BOLD:AAB7789
Grammia arge|[893]|XAG233-05|2005-ONT-817|Canada|Ontario|629[1n]|BOLD:AAB7789
Grammia arge|[894]|PMG006-03|APAN1.01|Canada|Ontario|617[0n]|BOLD:AAB7789
Notarctia proxima|[895]|LOCBD961-06|06-BLLOC-3782|United States|California|612[0n]|BOLD:AAA1371
Notarctia proxima|[896]|LOCBC165-06|06-BLLOC-2045|United States|California|658[0n]|BOLD:AAA1371
[illegible]

Grammia arge|[894]|PMG006-05|APANT.01|Canada|Ontario|617[0n]|BOLD:AAB7789
Notarctia proxima|[895]|LOCBD961-06|06-BLLOC-3782|United States|California|612[0n]|BOLD:AAA1371
Notarctia proxima|[896]|LOCBC165-06|06-BLLOC-2045|United States|California|658[0n]|BOLD:AAA1371
Notarctia proxima|[897]|LOCBC152-06|06-BLLOC-2032|United States|California|658[0n]|BOLD:AAA1371
Notarctia proxima|[898]|RDNME119-07|CNCNoctuoidea13647|United States|California|610[0n]|BOLD:A...
Notarctia proxima|[899]|LOCBC149-06|06-BLLOC-2029|United States|California|606[0n]|BOLD:AAA1371
Notarctia proxima|[900]|GMLC896-12|2011GM-0592|United States|California|631[0n]|BOLD:AAA1371
Notarctia proxima|[901]|LOCBC150-06|06-BLLOC-2030|United States|California|603[0n]|BOLD:AAA1371
Notarctia proxima|[902]|LOCB753-06|06-BLLOC-753|United States|California|658[0n]|BOLD:AAA1371
Notarctia proxima|[903]|LOCB766-06|06-BLLOC-766|United States|California|658[0n]|BOLD:AAA1371
Notarctia proxima|[904]|LOCBC151-06|06-BLLOC-2031|United States|California|626[0n]|BOLD:AAA1371
Notarctia proxima|[905]|LOCBC153-06|06-BLLOC-2033|United States|California|626[0n]|BOLD:AAA1371
Notarctia proxima|[906]|LOCB534-06|06-BLLOC-534|United States|California|606[0n]|BOLD:AAA1371
Notarctia proxima|[907]|LOCB913-06|06-BLLOC-913|United States|California|614[0n]|BOLD:AAA1371
Notarctia proxima|[908]|LOCBC154-06|06-BLLOC-2034|United States|California|610[0n]|BOLD:AAA1371
Notarctia proxima|[909]|LOCBC148-06|06-BLLOC-2028|United States|California|614[0n]|BOLD:AAA1371
Notarctia proxima|[910]|RDNME118-07|CNCNoctuoidea13646|United States|California|658[0n]|BOLD:A...
Notarctia proxima|[911]|RDMAB798-06|BCSC275|United States|California|581[0n]|BOLD:AAA1371
Notarctia proxima|[912]|LOCBC155-06|06-BLLOC-2035|United States|California|658[0n]|BOLD:AAA1371
Notarctia proxima|[913]|RDMAB797-06|BCSC274|United States|Utah|584[1n]|BOLD:AAA1371
Notarctia proxima|[914]|RDMAB796-06|BCSC273|United States|California|571[1n]|BOLD:AAA1371
Notarctia proxima|[915]|LOCBC465-06|06-BLLOC-2345|United States|California|658[0n]|BOLD:AAA1371
Notarctia proxima|[916]|LOCBB378-06|06-BLLOC-1318|United States|California|658[0n]|BOLD:AAA1371
Notarctia proxima|[917]|LOCBB377-06|06-BLLOC-1317|United States|California|658[0n]|BOLD:AAA1371
Notarctia proxima|[918]|LOCBB375-06|06-BLLOC-1315|United States|California|658[0n]|BOLD:AAA1371
Notarctia proxima|[919]|LOCBD297-06|06-BLLOC-3117|United States|California|658[0n]|BOLD:AAA1371
Notarctia proxima|[920]|GMLC048-09|2008GM-0048|United States|California|658[0n]|BOLD:AAA1371
Notarctia proxima|[921]|LOCB533-06|06-BLLOC-533|United States|California|658[0n]|BOLD:AAA1371
Notarctia proxima|[922]|LOCB823-06|06-BLLOC-823|United States|California|658[0n]|BOLD:AAA1371
Notarctia proxima|[923]|LOCB767-06|06-BLLOC-767|United States|California|658[0n]|BOLD:AAA1371
Notarctia proxima|[924]|LOCBC778-06|06-BLLOC-2658|United States|California|658[0n]|BOLD:AAA1371
Notarctia proxima|[925]|GMLC575-11|2011GM-0271|United States|California|658[0n]|BOLD:AAA1371
Notarctia proxima|[926]|GMLC537-11|2011GM-0233|United States|California|658[0n]|BOLD:AAA1371
Notarctia proxima|[927]|LOCBC939-06|06-BLLOC-2819|United States|California|658[0n]|BOLD:AAA1371
Notarctia proxima|[928]|LOCBC936-06|06-BLLOC-2816|United States|California|658[0n]|BOLD:AAA1371
Notarctia proxima|[929]|GMLC367-11|2011GM-0063|United States|California|658[0n]|BOLD:AAA1371
Notarctia proxima|[930]|JBAZ030-09|JLB-0030|United States|California|658[0n]|BOLD:AAA1371
Notarctia proxima|[931]|GMLC949-12|2011GM-0645|United States|California|658[0n]|BOLD:AAA1371
Notarctia proxima|[932]|GMLC892-12|2011GM-0588|United States|California|658[0n]|BOLD:AAA1371
Notarctia proxima|[933]|LOCBF2369-13|BIOUG06952-F06|United States|California|603[0n]|BOLD:AAA1371
Notarctia arizoniensis|[934]|RDNME397-08|LEP037821|United States|California|658[0n]|BOLD:AAA1371
Notarctia arizoniensis|[935]|RDNMF917-08|CNC LEP00054184|United States|Colorado|658[0n]|BOLD:A...
Cycnia collaris|[936]|LNAUT2889-14|CCDB-23279-E08|United States|Nebraska|447[0n]|BOLD:AAF7685
Cycnia collaris|[937]|RDNMH964-09|CNCLEP00067972|United States|Kansas|658[0n]|BOLD:AAF7685
Cycnia collaris|[938]|HKONS235-08|3140-COI-07|United States|Florida|632[3n]|BOLD:AAF7685
Cycnia collaris|[939]|BBLOE1455-12|BIOUG01987-E09|United States|Texas|658[0n]|BOLD:AAF7685
Cycnia collaris|[940]|LNAUT2887-14|CCDB-23279-E06|United States|Nebraska|658[0n]|BOLD:AAF7685
Cycnia collaris|[941]|LNAUT2888-14|CCDB-23279-E07|United States|Nebraska|658[0n]|BOLD:AAF7685
Cycnia collaris|[942]|LPOKD725-10|MDOK-3804|United States|Oklahoma|658[0n]|BOLD:AAF7685
Cycnia collaris|[943]|LNAUT2885-14|CCDB-23279-E04|United States|Nebraska|658[0n]|BOLD:AAF7685
Cycnia collaris|[944]|LNAUT2886-14|CCDB-23279-E05|United States|Nebraska|658[0n]|BOLD:AAF7685
Cycnia collaris|[945]|QUNOD390-10|9104-250610-FL|United States|Florida|658[0n]|BOLD:AAF7685
Cycnia collaris|[946]|HKONS081-07|1645-COI-07|United States|Florida|648[0n]|BOLD:AAF7685
Cycnia collaris|[947]|HKONS237-08|3142-COI-07|United States|Florida|658[0n]|BOLD:AAF7685
Cycnia collaris|[948]|RDNMH365-09|CNCLEP00057774|United States|Florida|658[0n]|BOLD:AAF7685
Hyperborea czekanowskii|[949]|RDNME099-07|CNCNoctuoidea13627|Russia||626[0n]|BOLD:AAD2643
Hyperborea czekanowskii|[950]|RDNME098-07|CNCNoctuoidea13626|Russia||626[0n]|BOLD:AAD2643
Hyperborea czekanowskii|[951]|GWORT830-10|BC AB Lep 00070|Russia|Magadanskaya Oblast|658[0n]|BOLD...
Hyperborea czekanowskii|[952]|GWORT829-10|BC AB Lep 00069|Russia|Magadanskaya Oblast|658[0n]|BOLD...
Hyperborea czekanowskii|[953]|GWORT828-10|BC AB Lep 00068|Russia|Magadanskaya Oblast|658[0n]|BOLD...
Hyperborea czekanowskii|[954]|GWORT827-10|BC AB Lep 00067|Russia|Magadanskaya Oblast|658[0n]|BOLD...
Hyperborea czekanowskii|[955]|GWORT826-10|BC AB Lep 00066|Russia||658[0n]|BOLD:AAD2643
Holoarctia puengeleri|[956]|CNCLB2219-14|CNCLEP00118020|United States|Alaska|658[0n]|BOLD:AAE5387
Holoarctia puengeleri|[957]|RDNMK242-11|CNCLEP 84151|Russia|Kamchatskiy Kray|658[0n]|BOLD:AAE5387
Holoarctia puengeleri|[958]|RDNME097-07|CNCNoctuoidea13625|Sweden||591[5n]|BOLD:AAE5387
Holoarctia puengeleri|[959]|LEFIL247-10|MM19247|Sweden||658[0n]|BOLD:AAE5387
Holoarctia puengeleri|[960]|LEFIL246-10|MM19246|Sweden||658[0n]|BOLD:AAE5387
Holoarctia puengeleri|[961]|LON180-08|NHMO-06181|Norway|Finnmark|657[0n]|BOLD:AAE5387
Holoarctia puengeleri|[962]|LON1180-12|NHMO-2012.004|Norway|Finnmark|658[0n]|BOLD:AAE5387
Holoarctia n. sp.|[963]|RDNME381-08|LEP037805|Canada|Yukon Territory|658[0n]|BOLD:ABY8300
Holoarctia n. sp.|[964]|RDNMK701-11|CNCLEP 81847|Canada|Yukon Territory|658[0n]|BOLD:ABY8300
Holoarctia sordida|[965]|RDMAB933-06|UASM57344|Canada|Alberta|658[0n]|BOLD:ABY8300
Holoarctia sordida|[966]|RDNMK125-11|CNCLEP 80412|Canada|Alberta|658[0n]|BOLD:ABY8300
Holoarctia sordida|[967]|RDMAB932-06|BCSC399|Canada|Alberta|658[0n]|BOLD:ABY8300
Neoarctia beanii|[968]|LPABC095-09|08BBLEP-04314|Canada|Alberta|658[0n]|BOLD:AAE4175
Neoarctia beanii|[969]|RDNMG070-08|NOC15011|Canada|Alberta|658[0n]|BOLD:AAE4175
Neoarctia beanii|[970]|RDNMG069-08|NOC15010|Canada|Alberta|658[0n]|BOLD:AAE4175
Neoarctia beanii|[971]|LBCH1561-10|10-JDWBC-1561|Canada|British Columbia|658[0n]|BOLD:AAE4175
Neoarctia beanii|[972]|RDMAB934-06|BCSC400|Canada|Alberta|658[0n]|BOLD:AAE4175
Neoarctia brucei|[973]|RDNMB304-05|CNCNoctuoidea10070|United States|Washington|582[0n]|BOLD:AA...
Neoarctia brucei|[974]|RDMAB919-06|BCSC393|Canada|British Columbia|658[0n]|BOLD:AAE4175
Neoarctia lafontainei|[975]|RDNMF381-08|NOC14467|Canada|Yukon Territory|582[0n]|BOLD:ABY7044
Neoarctia brucei|[976]|RDNMB303-05|CNCNoctuoidea10069|United States|Colorado|580[0n]|BOLD:ABY7044
Neoarctia brucei|[977]|QUNOD684-11|9398-CO|United States|Colorado|658[0n]|BOLD:ABY7044
Pararctia subnebulosa|[978]|RDNMF382-08|NOC14468|Canada|Yukon Territory|609[0n]|BOLD:AAF7314
Pararctia subnebulosa|[979]|GWORT775-10|BC AB Lep 00015|Russia||658[0n]|BOLD:ACF2202
Pararctia subnebulosa|[980]|GWORT776-10|BC AB Lep 00016|Russia||658[0n]|BOLD:ACF2202
Pararctia subnebulosa|[981]|RDNMI100-10|CNCLEP 68110|Russia|Chukot Autonomous Okrug|658[0n]|BOLD...
Pararctia yarrowii|[982]|RDNMK702-11|CNCLEP 81848|Canada|Yukon Territory|658[0n]|BOLD:ACF2201
Pararctia yarrowii|[983]|RDNMB301-05|CNCNoctuoidea10067|Canada|Alberta|527[0n]|BOLD:AAD4311
Pararctia yarrowii|[984]|RDMAB393-05|BCSC66|Canada|Alberta|658[0n]|BOLD:AAD4311
Pararctia yarrowii|[985]|GWORT781-10|BC AB Lep 00021|Canada|Alberta|658[0n]|BOLD:AAD4311
Pararctia yarrowii|[986]|BBLPA389-10|10BBCLP-0389|Canada|Alberta|658[0n]|BOLD:AAD4311
Pararctia yarrowii|[987]|BBLPA388-10|10BBCLP-0388|Canada|Alberta|658[0n]|BOLD:AAD4311
Pararctia lapponica|[988]|RDNME836-08|LEP041301|Canada|Newfoundland and Labrador|573[0n]|BOLD:...
Pararctia lapponica|[989]|RDMAB392-05|BCSC65|Canada|Alberta|658[0n]|BOLD:AAD4311
Pararctia yarrowii|[990]|RDMAB385-05|BCSC59|Canada|Alberta|658[0n]|BOLD:AAD4311
Pararctia lapponica|[991]|RDNME835-08|LEP041300|Canada|Newfoundland and Labrador|603[0n]|BOLD:...
Pararctia lapponica|[992]|RDNME834-08|LEP041299|Canada|Newfoundland and Labrador|584[0n]|BOLD:...
Pararctia lapponica|[993]|QUNOD685-11|9399-WY|United States|Wyoming|658[0n]|BOLD:AAD4311
Platyprepia virginalis|[994]|RDNMF827-08|CNC LEP00053188|United States|Oregon|609[0n]|BOLD:AAE...
Platyprepia virginalis|[995]|RDNMF826-08|CNC LEP00053187|United States|Oregon|658[0n]|BOLD:AAE...
Platyprepia virginalis|[996]|RDMAB961-09|UASM99714|United States|Oregon|658[0n]|BOLD:AAE4732

Platyprepia virginalis|[994]|RDNMF827-08|CNC LEP00053188|United States|Oregon|609[0n]|BOLD:AAE...
Platyprepia virginalis|[995]|RDNMF826-08|CNC LEP00053187|United States|Oregon|658[0n]|BOLD:AAE...
Platyprepia virginalis|[996]|RDMAB961-09|UASM99714|United States|Oregon|658[0n]|BOLD:AAE4732
Platyprepia virginalis|[997]|RDMAB935-06|BCSC401|Canada|British Columbia|658[0n]|BOLD:AAE4732
Platarctia parthenos|[998]|LGSMB307-05|DNA-ATBI-1156|United States|North Carolina|595[0n]|BOLD ...
Platarctia parthenos|[999]|LOWCE072-06|CGWC-3832|Canada|British Columbia|658[1n]|BOLD:AAB2132
Acerbia alpina|[1000]|RDNMC461-05|CNCNoctuoidea12094|Canada|Northwest Territories|658[0n]|BOLD ...
Acerbia alpina|[1001]|LEFIC312-10|MM03814|Finland|Lapland|658[0n]|BOLD:AAD7179
Acerbia alpina|[1002]|LEFIC311-10|MM03813|Finland|Lapland|658[0n]|BOLD:AAD7179
Acerbia alpina|[1003]|LEFIF141-10|MM10600|Finland||658[0n]|BOLD:AAD7179
Platarctia parthenos|[1004]|LPABC843-09|08BBLEP-05062|Canada|Alberta|658[0n]|BOLD:AAB2131
Platarctia parthenos|[1005]|LBCG1934-09|08-JDWBC-1934|Canada|British Columbia|658[0n]|BOLD:AAB...
Platarctia parthenos|[1006]|LOWCD148-06|CGWC-2968|Canada|British Columbia|648[0n]|BOLD:AAB2131
Platarctia parthenos|[1007]|BBLPA316-10|10BBCLP-0316|Canada|British Columbia|658[0n]|BOLD:AAB2131
Platarctia parthenos|[1008]|BBLPA315-10|10BBCLP-0315|Canada|British Columbia|658[0n]|BOLD:AAB2131
Platarctia parthenos|[1009]|BBLPA313-10|10BBCLP-0313|Canada|British Columbia|658[0n]|BOLD:AAB2131
Platarctia parthenos|[1010]|LBCG639-09|08-JDWBC-0639|Canada|British Columbia|658[0n]|BOLD:AAB2131
Platarctia parthenos|[1011]|RDNME358-07|CNCNoctuoidea13965|Canada|Yukon Territory|658[0n]|BOLD ...
Platarctia parthenos|[1012]|LCHP026-07|07PROBE-00086|Canada|Manitoba|658[0n]|BOLD:AAB2131
Platarctia parthenos|[1013]|LCHP700-07|07PROBE-10382|Canada|Manitoba|658[0n]|BOLD:AAB2131
Platarctia parthenos|[1014]|LCHP897-07|07PROBE-10659|Canada|Manitoba|658[0n]|BOLD:AAB2131
Platarctia parthenos|[1015]|RDLQB183-05|DH010269|Canada|Quebec|632[1n]|BOLD:AAB2131
Platarctia parthenos|[1016]|LPMN520-08|08BBLEP-01319|Canada|Manitoba|658[0n]|BOLD:AAB2131
Platarctia parthenos|[1017]|DIMC088-09|DS-Test-088|Canada|Ontario|666[0n]|
Platarctia parthenos|[1018]|LPAB308-08|08BBLEP-02630|Canada|Alberta|658[0n]|BOLD:AAB2131
Platarctia parthenos|[1019]|LPABC922-09|08BBLEP-05333|Canada|Alberta|658[0n]|BOLD:AAB2131
Platarctia parthenos|[1020]|RDMAB923-06|BCSC396|Canada|Alberta|657[0n]|BOLD:AAB2131
Platarctia parthenos|[1021]|LPSOD396-09|08BBLEP-00175|Canada|Ontario|658[0n]|BOLD:AAB2131
Platarctia parthenos|[1022]|MNAC787-07|CNCLEP00027528|Canada|Quebec|658[0n]|BOLD:AAB2131
Platarctia parthenos|[1023]|LPMN555-08|08BBLEP-01356|Canada|Manitoba|658[0n]|BOLD:AAB2131
Platarctia parthenos|[1024]|RDLQF472-06|DH011621|Canada|Quebec|658[0n]|BOLD:AAB2131
Platarctia parthenos|[1025]|RDLQB182-05|DH010268|Canada|Quebec|658[0n]|BOLD:AAB2131
Platarctia parthenos|[1026]|BBLPA318-10|10BBCLP-0318|Canada|Ontario|658[0n]|BOLD:AAB2131
Platarctia parthenos|[1027]|BBLPA317-10|10BBCLP-0317|Canada|Alberta|658[0n]|BOLD:AAB2131
Platarctia parthenos|[1028]|LPMN554-08|08BBLEP-01355|Canada|Manitoba|658[0n]|BOLD:AAB2131
Platarctia parthenos|[1029]|LPMN451-08|08BBLEP-01250|Canada|Manitoba|658[0n]|BOLD:AAB2131
Platarctia parthenos|[1030]|BBLPA314-10|10BBCLP-0314|Canada|Ontario|658[0n]|BOLD:AAB2131
Platarctia parthenos|[1031]|BBLPA312-10|10BBCLP-0312|Canada|Saskatchewan|658[0n]|BOLD:AAB2131
Platarctia parthenos|[1032]|LPSOD828-09|08BBLEP-00610|Canada|Ontario|658[0n]|BOLD:AAB2131
Platarctia parthenos|[1033]|BBLPC976-09|09BBELE-1976|Canada|Newfoundland and Labrador|658[0n]|BOL...
Platarctia parthenos|[1034]|RDLQF857-06|DH012018|Canada|Quebec|658[0n]|BOLD:AAB2131
Platarctia parthenos|[1035]|RDLQF473-06|DH011622|Canada|Quebec|658[0n]|BOLD:AAB2131
Platarctia parthenos|[1036]|LPMN553-08|08BBLEP-01354|Canada|Manitoba|658[0n]|BOLD:AAB2131
Parasemia plantaginis|[1037]|RDNME164-07|CNCNoctuoidea13692|Canada|Alberta|658[3n]|BOLD:AAB6883
Parasemia plantaginis|[1038]|RDMAB736-06|BCS_DNA405|Canada|Alberta|632[0n]|BOLD:AAB6883
Parasemia plantaginis|[1039]|ABKWR046-07|KENWR 6557|United States|Alaska|658[0n]|BOLD:AAB6883
Parasemia plantaginis|[1040]|LOWCE082-06|CGWC-3842|Canada|British Columbia|658[0n]|BOLD:AAB6883
Parasemia plantaginis|[1041]|LPSK106-08|08BBLEP-01674|Canada|Saskatchewan|658[0n]|BOLD:AAB6883
Parasemia plantaginis|[1042]|RDNME158-07|CNCNoctuoidea13686|Canada|Alberta|658[0n]|BOLD:AAB6883
Parasemia plantaginis|[1043]|RDNME163-07|CNCNoctuoidea13691|Canada|Alberta|658[0n]|BOLD:AAB6883
Parasemia plantaginis|[1044]|LOWCE081-06|CGWC-3841|Canada|British Columbia|658[0n]|BOLD:AAB6883
Parasemia plantaginis|[1045]|LOWCE083-06|CGWC-3843|Canada|British Columbia|658[0n]|BOLD:AAB6883
Parasemia plantaginis|[1046]|RDNME165-07|CNCNoctuoidea13693|Canada|Alberta|617[0n]|BOLD:AAB6883
Parasemia plantaginis|[1047]|BBLPA387-10|10BBCLP-0387|Canada|British Columbia|634[0n]|BOLD:AAB...
Parasemia plantaginis|[1048]|BBLPA386-10|10BBCLP-0386|Canada|British Columbia|658[0n]|BOLD:AAB...
Parasemia plantaginis|[1049]|BBLPA385-10|10BBCLP-0385|Canada|British Columbia|658[0n]|BOLD:AAB...
Parasemia plantaginis|[1050]|LPSK105-08|08BBLEP-01673|Canada|Saskatchewan|658[0n]|BOLD:AAB6883
Parasemia plantaginis|[1051]|LPSK104-08|08BBLEP-01672|Canada|Saskatchewan|658[0n]|BOLD:AAB6883
Arctia caja|[1052]|RDNMB388-05|CNCNoctuoidea10154|Canada|Ontario|616[0n]|BOLD:AAA8530
Arctia caja|[1053]|LNCC229-10|10-NCCC-419|United States|North Carolina|658[0n]|BOLD:AAA8530
Arctia caja|[1054]|XAK250-06|2006-ONT-1245|Canada|Ontario|658[0n]|BOLD:AAA8530
Arctia caja americana|[1055]|RDLQB836-05|DH010923|Canada|Quebec|621[0n]|BOLD:AAA8530
Arctia caja americana|[1056]|RDLQB571-05|DH010674|Canada|Quebec|658[0n]|BOLD:AAA8530
Arctia caja|[1057]|TMNBD404-07|MNBTT-3205|Canada|New Brunswick|658[0n]|BOLD:AAA8530
Arctia caja|[1058]|XAG617-05|2005-ONT-1201|Canada|Ontario|658[0n]|BOLD:AAA8530
Arctia caja|[1059]|XAG615-05|2005-ONT-1199|Canada|Ontario|658[0n]|BOLD:AAA8530
Arctia caja|[1060]|XAG579-05|2005-ONT-1163|Canada|Ontario|658[0n]|BOLD:AAA8530
Arctia caja|[1061]|XAG404-05|2005-ONT-988|Canada|Ontario|658[0n]|BOLD:AAA8530
Arctia brachyptera|[1062]|RDNMB383-05|CNCNoctuoidea10149|Canada|Yukon Territory|569[1n]|BOLD:A...
Arctia caja|[1063]|RDNMB386-05|CNCNoctuoidea10152|Canada|British Columbia|516[1n]|BOLD:AAA8530
Arctia caja|[1064]|RDNME157-07|CNCNoctuoidea13685|United States|Utah|617[1n]|BOLD:AAA8530
Arctia opulenta|[1065]|RDNMB384-05|CNCNoctuoidea10150|Canada|Yukon Territory|585[0n]|BOLD:AAA8530
Arctia sp.|[1066]|RDNMG140-08|NOC14893|United States|Colorado|633[0n]|BOLD:AAA8530
Arctia caja|[1067]|RDNMB387-05|CNCNoctuoidea10153|United States|Oregon|592[1n]|BOLD:AAA8530
Arctia caja|[1068]|RDMAB388-05|BCSC62|Canada|Alberta|658[0n]|BOLD:AAA8530
Arctia caja|[1069]|RDNMB385-05|CNCNoctuoidea10151|United States|Nevada|612[0n]|BOLD:AAA8530
Arctia brachyptera|[1070]|RDNMK540-11|CNCLEP 81969|Canada|Northwest Territories|658[0n]|BOLD:A...
Arctia opulenta|[1071]|CNCLB082-14|CFIA-01082|Canada|Yukon Territory|658[0n]|
Arctia opulenta|[1072]|CNCLB081-14|CFIA-01081|Canada|Yukon Territory|658[0n]|
Arctia caja|[1073]|RDMAB386-05|BCSC60|Canada|British Columbia|658[1n]|BOLD:AAA8530
Arctia caja|[1074]|RDMAB387-05|BCSC61|Canada|Alberta|658[0n]|BOLD:AAA8530
Arctia caja|[1075]|RWWB952-10|RWWA-1951|United States|Washington|634[0n]|BOLD:AAA8530
Arctia caja|[1076]|RWWA776-09|RWWA-0812|United States|Washington|658[0n]|BOLD:AAA8530
Arctia caja|[1077]|RWWA832-09|RWWA-0868|United States|Washington|658[0n]|BOLD:AAA8530
Arctia caja|[1078]|RWWA728-09|RWWA-0764|United States|Washington|658[0n]|BOLD:AAA8530
Arctia caja|[1079]|RWWC467-11|RWWA-2444|United States|Washington|658[0n]|BOLD:AAA8530
Sonorarctia fervida|[1080]|CNCLB2209-14|CNCLEP00114014|United States|Arizona|658[0n]|BOLD:ACR3453
Sonorarctia fervida|[1081]|CNCLB2208-14|CNCLEP00114013|United States|Arizona|550[0n]|BOLD:ACR3453
Spilosoma latipennis|[1082]|RDNME651-08|LEP038075|Canada|Ontario|658[0n]|BOLD:AAB6210
Spilosoma latipennis|[1083]|RDNME650-08|LEP038074|Canada|Ontario|658[0n]|BOLD:AAB6210
Spilosoma latipennis|[1084]|LSUSA219-06|06-SUSA-0219|United States|Kentucky|658[0n]|BOLD:AAB6210
Spilosoma latipennis|[1085]|XAK169-06|2006-ONT-1164|Canada|Ontario|658[0n]|BOLD:AAB6210
Spilosoma latipennis|[1086]|XAK170-06|2006-ONT-1165|Canada|Ontario|658[0n]|BOLD:AAB6210
Spilosoma latipennis|[1087]|LPSOB730-08|PPBP-1729|Canada|Ontario|658[0n]|BOLD:AAB6210
Spilosoma latipennis|[1088]|XAF672-05|2005-ONT-321|Canada|Ontario|658[0n]|BOLD:AAB6210
Spilosoma latipennis|[1089]|XAB304-04|04HBL005304|Canada|Ontario|658[0n]|BOLD:AAB6210
Spilosoma latipennis|[1090]|PHMO123-03|moth735.02|Canada|Ontario|639[1n]|BOLD:AAB6210
Spilosoma latipennis|[1091]|LPSO952-08|PPBP-0952|Canada|Ontario|646[0n]|BOLD:AAB6210
Spilosoma latipennis|[1092]|PHMO090-03|moth569.02|Canada|Ontario|639[0n]|BOLD:AAB6210
Spilosoma latipennis|[1093]|LPSO136-08|PPBP-0136|Canada|Ontario|658[0n]|BOLD:AAB6210
Spilosoma latipennis|[1094]|XAI003-05|0102-ONT-0003|Canada|Ontario|658[0n]|BOLD:AAB6210
Spilosoma latipennis|[1095]|LGSMC702-05|DNA-ATBI-2702|United States|Tennessee|658[0n]|BOLD:AAB...
Spilosoma latipennis|[1096]|MMNA122-08|HLC-17684|United States|North Carolina|636[0n]|BOLD:AAB...

Spilosoma latipennis|[1093]|LPSO150-08|PPBP-0150|Canada|Ontario|658[0n]|BOLD:AAB6210
Spilosoma latipennis|[1094]|XAI003-05|0102-ONT-0003|Canada|Ontario|658[0n]|BOLD:AAB6210
Spilosoma latipennis|[1095]|LGSMC702-05|DNA-ATBI-2702|United States|Tennessee|658[0n]|BOLD:AAB...
Spilosoma latipennis|[1096]|MMNA122-08|HLC-17684|United States|North Carolina|636[0n]|BOLD:AAB...
Spilosoma latipennis|[1097]|LSEU405-06|06-JKA-0405|United States|Georgia|658[0n]|BOLD:AAB6210
Spilosoma latipennis|[1098]|LPSO353-08|PPBP-0353|Canada|Ontario|658[0n]|BOLD:AAB6210
Spilosoma latipennis|[1099]|XAI002-05|0102-ONT-0002|Canada|Ontario|658[0n]|BOLD:AAB6210
Spilosoma latipennis|[1100]|XAK168-06|2006-ONT-1163|Canada|Ontario|658[0n]|BOLD:AAB6210
Spilosoma latipennis|[1101]|PHMO086-03|moth548.02|Canada|Ontario|639[1n]|BOLD:AAB6210
Spilosoma vestalis|[1102]|LOCBF1299-13|BIOUG06805-D05|United States|California|588[0n]|BOLD:AA...
Spilosoma vestalis|[1103]|LOCBF1298-13|BIOUG06805-D04|United States|California|593[0n]|BOLD:AA...
Spilosoma vestalis|[1104]|LOCBF1295-13|BIOUG06805-D01|United States|California|593[0n]|BOLD:AA...
Spilosoma vestalis|[1105]|LOPN224-06|JCM-OSU-0224|United States|Oregon|593[0n]|BOLD:AAD0201
Spilosoma vestalis|[1106]|GMLC544-11|2011GM-0240|United States|California|658[0n]|BOLD:AAD0201
Spilosoma vestalis|[1107]|GMLC491-11|2011GM-0187|United States|California|658[0n]|BOLD:AAD0201
Spilosoma vestalis|[1108]|GMLC435-11|2011GM-0131|United States|California|658[0n]|BOLD:AAD0201
Spilosoma vestalis|[1109]|GMLC426-11|2011GM-0122|United States|California|658[0n]|BOLD:AAD0201
Spilosoma vestalis|[1110]|LOCBB385-06|06-BLLOC-1325|United States|California|658[0n]|BOLD:AAD0201
Spilosoma vestalis|[1111]|LOCBB384-06|06-BLLOC-1324|United States|California|658[0n]|BOLD:AAD0201
Spilosoma vestalis|[1112]|LOCBB383-06|06-BLLOC-1323|United States|California|658[0n]|BOLD:AAD0201
Spilosoma vestalis|[1113]|GMLC437-11|2011GM-0133|United States|California|658[0n]|BOLD:AAD0201
Spilosoma vestalis|[1114]|GMLC541-11|2011GM-0237|United States|California|658[0n]|BOLD:AAD0201
Spilosoma vestalis|[1115]|GMLC391-11|2011GM-0087|United States|California|658[0n]|BOLD:AAD0201
Spilosoma vestalis|[1116]|JBAZ038-09|JLB-0038|United States|California|658[0n]|BOLD:AAD0201
Spilosoma vestalis|[1117]|JBAZ037-09|JLB-0037|United States|California|623[0n]|BOLD:AAD0201
Spilosoma vestalis|[1118]|GMLC436-11|2011GM-0132|United States|California|658[0n]|BOLD:AAD0201
Spilosoma vestalis|[1119]|RDNME184-07|CNCNoctuoidea13712|United States|California|642[1n]|BOLD ...
Spilosoma vestalis|[1120]|RDNME392-08|LEP037816|United States|California|658[0n]|BOLD:AAD0201
Spilosoma vestalis|[1121]|JMMMB181-11|BIOUG00848-H02|United States|California|658[0n]|BOLD:AAD...
Alexicles aspersa|[1122]|RDNMG145-08|NOC14898|United States|New Mexico|658[0n]|BOLD:AAW1860
Spilosoma vagans|[1123]|RDNMB392-05|CNCNoctuoidea10158|United States|California|570[1n]|BOLD:A...
Spilosoma vagans|[1124]|RDNME155-07|CNCNoctuoidea13683|United States|California|658[0n]|BOLD:A...
Spilosoma vagans|[1125]|RDNME154-07|CNCNoctuoidea13682|United States|California|658[0n]|BOLD:A...
Spilosoma vagans|[1126]|RDNMB389-05|CNCNoctuoidea10155|United States|California|591[0n]|BOLD:A...
Spilosoma vagans|[1127]|RDNME391-08|LEP037815|United States|California|658[0n]|BOLD:AAB4577
Spilosoma vagans|[1128]|RDNME390-08|LEP037814|United States|California|658[0n]|BOLD:AAB4577
Spilosoma vagans|[1129]|RDNME389-08|LEP037813|United States|California|658[0n]|BOLD:AAB4577
Spilosoma vagans|[1130]|JMMMB178-11|BIOUG00848-G11|United States|California|658[0n]|BOLD:AAB4577
Spilosoma pteridis|[1131]|RWWC1146-13|BIOUG07834-C04|United States|Washington|593[0n]|BOLD:AAB...
Spilosoma pteridis|[1132]|RWWC1132-13|BIOUG07834-B02|United States|Washington|567[0n]|BOLD:AAB...
Spilosoma vagans|[1133]|JBAZ053-09|JLB-0053|United States|California|658[0n]|BOLD:AAB4578
Spilosoma pteridis|[1134]|RDMAB939-06|BCSC405|United States|Oregon|535[0n]|BOLD:AAB4578
Spilosoma vagans|[1135]|GMLC1313-12|2012GM-0044|United States|California|614[0n]|BOLD:AAB4578
Spilosoma vagans|[1136]|RDMAB410-05|BCSC83|United States|Oregon|658[0n]|BOLD:AAB4578
Spilosoma vagans|[1137]|RDNMB390-05|CNCNoctuoidea10156|United States|California|581[0n]|BOLD:A...
Spilosoma pteridis|[1138]|RDMAB391-05|BCSC64|Canada|British Columbia|561[0n]|BOLD:AAB4578
Spilosoma vagans|[1139]|RDNME162-07|CNCNoctuoidea13690|United States|California|658[0n]|BOLD:A...
Spilosoma vagans|[1140]|RDNME161-07|CNCNoctuoidea13689|United States|California|658[0n]|BOLD:A...
Spilosoma vagans|[1141]|GMLC214-11|2009GM-0083|United States|California|625[0n]|BOLD:AAB4578
Spilosoma vagans|[1142]|RDMAB409-05|BCSC82|United States|California|658[0n]|BOLD:AAB4578
Spilosoma vagans|[1143]|GMLC314-11|2011GM-0010|United States|California|658[0n]|BOLD:AAB4578
Spilosoma vagans|[1144]|GMLC490-11|2011GM-0186|United States|California|658[0n]|BOLD:AAB4578
Spilosoma vagans|[1145]|GMLC337-11|2011GM-0033|United States|California|658[0n]|BOLD:AAB4578
Spilosoma vagans|[1146]|GMLC336-11|2011GM-0032|United States|California|658[0n]|BOLD:AAB4578
Spilosoma vagans|[1147]|GMLC328-11|2011GM-0024|United States|California|658[0n]|BOLD:AAB4578
Spilosoma vagans|[1148]|GMLC315-11|2011GM-0011|United States|California|658[0n]|BOLD:AAB4578
Spilosoma vagans|[1149]|GMLC313-11|2011GM-0009|United States|California|658[0n]|BOLD:AAB4578
Spilosoma vagans|[1150]|RDNME156-07|CNCNoctuoidea13684|United States|California|617[2n]|BOLD:A...
Spilosoma vagans|[1151]|RDNME167-07|CNCNoctuoidea13695|Canada|British Columbia|658[0n]|BOLD:AA...
Spilosoma vagans|[1152]|RDNME168-07|CNCNoctuoidea13696|Canada|British Columbia|658[0n]|BOLD:AA...
Spilosoma pteridis|[1153]|RDNME146-07|CNCNoctuoidea13674|Canada|British Columbia|611[0n]|BOLD:...
Spilosoma vagans|[1154]|RDMAB411-05|BCSC84|Canada|British Columbia|658[0n]|BOLD:AAB4578
Spilosoma vagans|[1155]|LPAB261-08|08BBLEP-02583|Canada|Alberta|658[0n]|BOLD:AAB4578
Spilosoma vagans|[1156]|LPABB567-08|08BBLEP-03832|Canada|Alberta|658[0n]|BOLD:AAB4578
Spilosoma vagans|[1157]|LPABB428-08|08BBLEP-03693|Canada|Alberta|658[0n]|BOLD:AAB4578
Spilosoma vagans|[1158]|RDNMB391-05|CNCNoctuoidea10157|Canada|British Columbia|658[0n]|BOLD:AA...
Spilosoma vagans|[1159]|LBCG027-08|08-JDWBC-0027|Canada|British Columbia|658[0n]|BOLD:AAB4578
Spilosoma vagans|[1160]|RDNME151-07|CNCNoctuoidea13679|United States|Oregon|615[0n]|BOLD:AAB4578
Spilosoma vagans|[1161]|RDNME152-07|CNCNoctuoidea13680|United States|Oregon|657[0n]|BOLD:AAB4578
Spilosoma vagans|[1162]|RDNME166-07|CNCNoctuoidea13694|Canada|British Columbia|658[0n]|BOLD:AA...
Spilosoma pteridis|[1163]|RDNME147-07|CNCNoctuoidea13675|Canada|British Columbia|381[0n]|BOLD:...
Spilosoma pteridis|[1164]|RDNMB393-05|CNCNoctuoidea10159|United States|Oregon|586[0n]|BOLD:AAB...
Spilosoma pteridis|[1165]|RDNME144-07|CNCNoctuoidea13672|Canada|Alberta|616[0n]|BOLD:AAB4578
Spilosoma vagans|[1166]|RDNME153-07|CNCNoctuoidea13681|United States|Oregon|618[0n]|BOLD:AAB4578
Spilosoma pteridis|[1167]|LPVIC113-08|PFC-2006-2690|Canada|British Columbia|621[0n]|BOLD:AAB4578
Spilosoma pteridis|[1168]|LPVIC112-08|PFC-2006-2689|Canada|British Columbia|616[0n]|BOLD:AAB4578
Spilosoma pteridis|[1169]|RDNMH319-09|CNCLEP00057728|Canada|British Columbia|658[0n]|BOLD:AAB4578
Seirarctia echo|[1170]|RDNME547-08|LEP037971|United States|Florida|658[0n]|BOLD:AAD3649
Seirarctia echo|[1171]|LOFLB796-06|06-FLOR-1736|United States|Florida|658[0n]|BOLD:AAD3649
Seirarctia echo|[1172]|LOFLB335-06|06-FLOR-1275|United States|Florida|658[0n]|BOLD:AAD3649
Seirarctia echo|[1173]|LOFLB227-06|06-FLOR-1167|United States|Florida|658[0n]|BOLD:AAD3649
Seirarctia echo|[1174]|LOFLB226-06|06-FLOR-1166|United States|Florida|658[0n]|BOLD:AAD3649
Seirarctia echo|[1175]|LOFLC093-06|06-FLOR-1973|United States|Florida|658[0n]|BOLD:AAD3649
Seirarctia echo|[1176]|LOFLC097-06|06-FLOR-1977|United States|Florida|658[0n]|BOLD:AAD3649
Spilosoma dubia|[1177]|LNAUT2646-14|CCDB-23282-G11|United States|Massachusetts|658[0n]|BOLD:AA...
Spilosoma dubia|[1178]|KPOEC113-08|08OEC-058|Canada|Ontario|658[0n]|BOLD:AAC8394
Spilosoma dubia|[1179]|RDNME341-07|CNCNoctuoidea13948|Canada|Ontario|658[0n]|BOLD:AAC8394
Spilosoma dubia|[1180]|RDNME173-07|CNCNoctuoidea13701|Canada|Alberta|638[0n]|BOLD:AAC8394
Spilosoma dubia|[1181]|RDNME174-07|CNCNoctuoidea13702|Canada|Alberta|638[2n]|BOLD:AAC8394
Spilosoma dubia|[1182]|RDNME172-07|CNCNoctuoidea13700|Canada|Alberta|641[0n]|BOLD:AAC8394
Spilosoma sp.|[1183]|QUNOD391-10|9105-120310-FL|United States|Florida|658[0n]|BOLD:AAC8394
Spilosoma dubia|[1184]|LNC733-06|05-NCCC-733|United States|North Carolina|658[0n]|BOLD:AAC8394
Spilosoma dubia|[1185]|LNC732-06|05-NCCC-732|United States|North Carolina|658[0n]|BOLD:AAC8394
Spilosoma dubia|[1186]|LNC731-06|05-NCCC-731|United States|North Carolina|658[0n]|BOLD:AAC8394
Spilosoma congrua|[1187]|LOT134-04|04HBL002134|United States|Tennessee|609[0n]|BOLD:AAA4294
Spilosoma congrua|[1188]|LOT132-04|04HBL002132|United States|Tennessee|609[0n]|BOLD:AAA4294
Spilosoma congrua|[1189]|LOT131-04|04HBL002131|United States|Tennessee|609[0n]|BOLD:AAA4294
Spilosoma congrua|[1190]|LOT130-04|04HBL002130|United States|Tennessee|609[0n]|BOLD:AAA4294
Spilosoma congrua|[1191]|XAK117-06|2006-ONT-1112|Canada|Ontario|658[0n]|BOLD:AAA4294
Spilosoma congrua|[1192]|LMDH132-11|BIOUG01047-B10|United States|Minnesota|658[0n]|BOLD:AAA4294
Spilosoma congrua|[1193]|BLTIB025-08|BL0052|Canada|Ontario|658[0n]|BOLD:AAA4294
Spilosoma congrua|[1194]|LPSO241-08|PPBP-0241|Canada|Ontario|658[0n]|BOLD:AAA4294
Spilosoma congrua|[1195]|BBLCU296-09|09BBLEP-04783|United States|Michigan|658[0n]|BOLD:AAA4294

Spilosoma congrua|[1193]|BLTIB025-08|BL0052|Canada|Ontario|658[0n]|BOLD:AAA4294
Spilosoma congrua|[1194]|LPSO241-08|PPBP-0241|Canada|Ontario|658[0n]|BOLD:AAA4294
Spilosoma congrua|[1195]|BBLCU296-09|09BBLEP-04783|United States|Michigan|658[0n]|BOLD:AAA4294
Spilosoma congrua|[1196]|TMG77-03|moth289.01|Canada|Ontario|639[0n]|BOLD:AAA4294
Spilosoma congrua|[1197]|LGSMB295-05|DNA-ATBI-1144|United States|Tennessee|564[1n]|BOLD:AAA4294
Spilosoma congrua|[1198]|LOFLB522-06|06-FLOR-1462|United States|Florida|658[0n]|BOLD:AAA4294
Spilosoma congrua|[1199]|LOFLB252-06|06-FLOR-1192|United States|Florida|658[0n]|BOLD:AAA4294
Spilosoma congrua|[1200]|LOFLC345-06|06-FLOR-2225|United States|Florida|658[0n]|BOLD:AAA4294
Spilosoma congrua|[1201]|LOFLC317-06|06-FLOR-2197|United States|Florida|658[0n]|BOLD:AAA4294
Spilosoma congrua|[1202]|LOFLC314-06|06-FLOR-2194|United States|Florida|658[0n]|BOLD:AAA4294
Spilosoma congrua|[1203]|LNC104-05|05-NCCC-104|United States|North Carolina|636[0n]|BOLD:AAA4294
Spilosoma congrua|[1204]|LNC736-06|05-NCCC-736|United States|North Carolina|658[0n]|BOLD:AAA4294
Spilosoma congrua|[1205]|LNC734-06|05-NCCC-734|United States|North Carolina|658[0n]|BOLD:AAA4294
Spilosoma congrua|[1206]|LGSMG115-07|BGS03436|United States|North Carolina|658[0n]|BOLD:AAA4294
Spilosoma congrua|[1207]|LOTB341-05|05-TN-00341|United States|Tennessee|658[0n]|BOLD:AAA4294
Spilosoma congrua|[1208]|CNCLB1379-14|14-NCCC-228|United States|North Carolina|658[0n]|BOLD:AA...
Spilosoma congrua|[1209]|LOTB504-05|05-TN-00504|United States|Tennessee|658[0n]|BOLD:AAA4294
Spilosoma congrua|[1210]|LOTB502-05|05-TN-00502|United States|Tennessee|658[0n]|BOLD:AAA4294
Spilosoma congrua|[1211]|LOTB506-05|05-TN-00506|United States|Tennessee|658[0n]|BOLD:AAA4294
Spilosoma congrua|[1212]|LOTB505-05|05-TN-00505|United States|Tennessee|658[0n]|BOLD:AAA4294
Spilosoma congrua|[1213]|LOTB508-05|05-TN-00508|United States|Tennessee|658[0n]|BOLD:AAA4294
Spilosoma congrua|[1214]|LOTB507-05|05-TN-00507|United States|Tennessee|658[0n]|BOLD:AAA4294
Spilosoma congrua|[1215]|LOTB510-05|05-TN-00510|United States|Tennessee|658[0n]|BOLD:AAA4294
Spilosoma congrua|[1216]|LOTB509-05|05-TN-00509|United States|Tennessee|658[0n]|BOLD:AAA4294
Spilosoma congrua|[1217]|LOTB512-05|05-TN-00512|United States|Tennessee|658[0n]|BOLD:AAA4294
Spilosoma congrua|[1218]|LOTB511-05|05-TN-00511|United States|Tennessee|658[0n]|BOLD:AAA4294
Spilosoma congrua|[1219]|LOTB313-05|05-TN-00313|United States|Tennessee|658[0n]|BOLD:AAA4294
Spilosoma congrua|[1220]|LGSMC714-05|DNA-ATBI-2714|United States|Tennessee|658[0n]|BOLD:AAA4294
Spilosoma congrua|[1221]|LOTB513-05|05-TN-00513|United States|Tennessee|658[0n]|BOLD:AAA4294
Spilosoma congrua|[1222]|LOTB514-05|05-TN-00514|United States|Tennessee|658[0n]|BOLD:AAA4294
Spilosoma congrua|[1223]|LOTB515-05|05-TN-00515|United States|Tennessee|658[0n]|BOLD:AAA4294
Spilosoma congrua|[1224]|LOTB516-05|05-TN-00516|United States|Tennessee|658[0n]|BOLD:AAA4294
Spilosoma congrua|[1225]|LOCT016-05|05-CTATBI-0016|United States|Connecticut|658[0n]|BOLD:AAA4294
Spilosoma congrua|[1226]|LOCT017-05|05-CTATBI-0017|United States|Connecticut|658[0n]|BOLD:AAA4294
Spilosoma congrua|[1227]|LGSMC713-05|DNA-ATBI-2713|United States|Tennessee|658[0n]|BOLD:AAA4294
Spilosoma congrua|[1228]|LOCT019-05|05-CTATBI-0019|United States|Connecticut|658[0n]|BOLD:AAA4294
Spilosoma congrua|[1229]|LOT133-04|04HBL002133|United States|Tennessee|658[0n]|BOLD:AAA4294
Spilosoma congrua|[1230]|LGSMC711-05|DNA-ATBI-2711|United States|Tennessee|658[0n]|BOLD:AAA4294
Spilosoma congrua|[1231]|LGSMB296-05|DNA-ATBI-1145|United States|Tennessee|658[0n]|BOLD:AAA4294
Spilosoma congrua|[1232]|LOT135-04|04HBL002135|United States|Tennessee|658[0n]|BOLD:AAA4294
Spilosoma congrua|[1233]|LOTB503-05|05-TN-00503|United States|Tennessee|658[0n]|BOLD:AAA4294
Spilosoma congrua|[1234]|LOTB501-05|05-TN-00501|United States|Tennessee|658[0n]|BOLD:AAA4294
Spilosoma congrua|[1235]|LGSMC712-05|DNA-ATBI-2712|United States|Tennessee|658[0n]|BOLD:AAA4294
Spilosoma congrua|[1236]|LGSMG116-07|BGS03437|United States|Tennessee|658[0n]|BOLD:AAA4294
Spilosoma congrua|[1237]|LNAUT2647-14|CCDB-23282-G12|United States|Massachusetts|658[0n]|BOLD:...
Spilosoma congrua|[1238]|TTMNB545-06|MNBTT-545|Canada|New Brunswick|658[0n]|BOLD:AAA4294
Spilosoma congrua|[1239]|LPMN269-08|08BBLEP-01068|Canada|Manitoba|658[0n]|BOLD:AAA4294
Spilosoma congrua|[1240]|PHMNB580-04|04HBL00806|Canada|New Brunswick|658[0n]|BOLD:AAA4294
Spilosoma congrua|[1241]|PHMNB579-04|04HBL00805|Canada|New Brunswick|658[0n]|BOLD:AAA4294
Spilosoma congrua|[1242]|LPSOB135-08|PPBP-1134|Canada|Ontario|658[0n]|BOLD:AAA4294
Spilosoma congrua|[1243]|LPSOB134-08|PPBP-1133|Canada|Ontario|658[0n]|BOLD:AAA4294
Spilosoma congrua|[1244]|LPSOD871-09|08BBLEP-00653|Canada|Ontario|658[0n]|BOLD:AAA4294
Spilosoma congrua|[1245]|LPOKB144-09|MDOK-1305|United States|Oklahoma|658[0n]|BOLD:AAA4294
Spilosoma congrua|[1246]|BBLPA376-10|10BBCLP-0376|Canada|Saskatchewan|658[0n]|BOLD:AAA4294
Spilosoma congrua|[1247]|BBLPA375-10|10BBCLP-0375|Canada|Saskatchewan|658[0n]|BOLD:AAA4294
Spilosoma congrua|[1248]|BBLOC878-11|BIOUG01467-A05|United States|Arkansas|658[0n]|BOLD:AAA4294
Spilosoma congrua|[1249]|LPSOD872-09|08BBLEP-00654|Canada|Ontario|658[0n]|BOLD:AAA4294
Spilosoma congrua|[1250]|TMNBD388-07|MNBTT-3189|Canada|New Brunswick|658[0n]|BOLD:AAA4294
Spilosoma congrua|[1251]|TMNBD389-07|MNBTT-3190|Canada|New Brunswick|658[0n]|BOLD:AAA4294
Spilosoma congrua|[1252]|TMNBD390-07|MNBTT-3191|Canada|New Brunswick|658[0n]|BOLD:AAA4294
Spilosoma congrua|[1253]|TMNBD392-07|MNBTT-3193|Canada|New Brunswick|658[0n]|BOLD:AAA4294
Spilosoma congrua|[1254]|TMNBD393-07|MNBTT-3194|Canada|New Brunswick|658[0n]|BOLD:AAA4294
Spilosoma congrua|[1255]|RDNME342-07|CNCNoctuoidea13949|Canada|Ontario|658[0n]|BOLD:AAA4294
Spilosoma congrua|[1256]|ALLEP109-13|BIOUG06755-B02|Canada|Ontario|658[0n]|BOLD:AAA4294
Spilosoma congrua|[1257]|ALLEP110-13|BIOUG06755-B03|Canada|Ontario|658[0n]|BOLD:AAA4294
Spilosoma congrua|[1258]|TMNBB012-06|MNBTT-952|Canada|New Brunswick|658[0n]|BOLD:AAA4294
Spilosoma congrua|[1259]|TMNBB013-06|MNBTT-953|Canada|New Brunswick|658[0n]|BOLD:AAA4294
Spilosoma congrua|[1260]|LPSOB335-08|PPBP-1334|Canada|Ontario|658[0n]|BOLD:AAA4294
Spilosoma congrua|[1261]|XAF495-05|2005-ONT-144|Canada|Ontario|658[0n]|BOLD:AAA4294
Spilosoma congrua|[1262]|LPSOD848-09|08BBLEP-00630|Canada|Ontario|658[0n]|BOLD:AAA4294
Spilosoma congrua|[1263]|LSEU792-06|06-JKA-0792|United States|Missouri|658[0n]|BOLD:AAA4294
Spilosoma congrua|[1264]|LPSOD870-09|08BBLEP-00652|Canada|Ontario|658[0n]|BOLD:AAA4294
Spilosoma congrua|[1265]|LPOKB145-09|MDOK-1306|United States|Oklahoma|658[0n]|BOLD:AAA4294
Spilosoma congrua|[1266]|LPSOB136-08|PPBP-1135|Canada|Ontario|652[0n]|BOLD:AAA4294
Spilosoma congrua|[1267]|LPSOB696-08|PPBP-1695|Canada|Ontario|646[0n]|BOLD:AAA4294
Spilosoma congrua|[1268]|TMNBD395-07|MNBTT-3196|Canada|New Brunswick|646[0n]|BOLD:AAA4294
Spilosoma congrua|[1269]|TMNBD387-07|MNBTT-3188|Canada|New Brunswick|646[0n]|BOLD:AAA4294
Spilosoma congrua|[1270]|RDLQ762-07|DH006622|Canada|Quebec|652[0n]|BOLD:AAA4294
Spilosoma congrua|[1271]|LSEU341-06|06-JKA-0341|United States|Georgia|658[0n]|BOLD:AAA4294
Spilosoma congrua|[1272]|LILLA005-11|SNS10IL-00008|United States|Illinois|658[0n]|BOLD:AAA4294
Spilosoma congrua|[1273]|LNCC868-11|11-NCCC-393|United States|North Carolina|658[0n]|BOLD:AAA4294
Spilosoma congrua|[1274]|LNCC867-11|11-NCCC-392|United States|North Carolina|658[0n]|BOLD:AAA4294
Spilosoma congrua|[1275]|LOFLB381-06|06-FLOR-1321|United States|Florida|647[0n]|BOLD:AAA4294
Spilosoma congrua|[1276]|LOFLB379-06|06-FLOR-1319|United States|Florida|658[0n]|BOLD:AAA4294
Spilosoma congrua|[1277]|LOFLB380-06|06-FLOR-1320|United States|Florida|658[0n]|BOLD:AAA4294
Spilosoma congrua|[1278]|LOFLB382-06|06-FLOR-1322|United States|Florida|658[0n]|BOLD:AAA4294
Spilosoma congrua|[1279]|LOFLC318-06|06-FLOR-2198|United States|Florida|658[1n]|BOLD:AAA4294
Spilosoma congrua|[1280]|LSEU558-06|06-JKA-0558|United States|Georgia|658[0n]|BOLD:AAA4294
Spilosoma congrua|[1281]|LNC735-06|05-NCCC-735|United States|North Carolina|635[0n]|BOLD:AAA4294
Spilosoma congrua|[1282]|BBLSW476-09|09BBLEP-01404|United States|Oklahoma|631[0n]|BOLD:AAA4294
Spilosoma congrua|[1283]|RDNME192-07|CNCNoctuoidea13799|Canada|Alberta|617[1n]|BOLD:AAA4294
Spilosoma congrua|[1284]|RDNME191-07|CNCNoctuoidea13798|Canada|Alberta|620[0n]|BOLD:AAA4294
Spilosoma congrua|[1285]|PMG017-03|moth306.01|Canada|Ontario|617[0n]|BOLD:AAA4294
Leptarctia californiae|[1286]|RDNMF391-08|NOC14477|Canada|British Columbia|658[0n]|BOLD:AAE5275
Leptarctia californiae|[1287]|RDNMF390-08|NOC14476|Canada|British Columbia|658[0n]|BOLD:AAE5275
Leptarctia californiae|[1288]|RDNMF389-08|NOC14475|Canada|British Columbia|658[0n]|BOLD:AAE5275
Leptarctia californiae|[1289]|RDNMF388-08|NOC14474|Canada|British Columbia|658[0n]|BOLD:AAE5275
Leptarctia californiae|[1290]|RDNML070-13|CNCLEP 94294|United States|Arizona|658[0n]|BOLD:AAM8613
Leptarctia californiae|[1291]|CNCLB2198-14|CNCLEP00118002|United States|Arizona|658[0n]|BOLD:A...
Leptarctia californiae|[1292]|CNCLB2157-14|CNCLEP00117745|United States|Arizona|658[0n]|BOLD:A...
Leptarctia californiae|[1293]|CNCLB1835-14|CNCLEP00117962|United States|Arizona|658[1n]|BOLD:A...
Leptarctia californiae|[1294]|USLEP903-10|10BBLEP-00903|United States|Colorado|658[0n]|BOLD:AA...
Hypercompe scribonia|[1295]|LPSOD925-09|08BBLEP-00961|Canada|Ontario|658[0n]|BOLD:ABZ5715

[illegible]
Leptarctia californiae|[1293]|CNCLB1835-14|CNCLEP00117962|United States|Arizona|658[1n]|BOLD:A...
Leptarctia californiae|[1294]|USLEP903-10|10BBLEP-00903|United States|Colorado|658[0n]|BOLD:AA...
Hypercompe scribonia|[1295]|LPSOD925-09|08BBLEP-00961|Canada|Ontario|658[0n]|BOLD:ABZ5715
Hypercompe scribonia|[1296]|BBLPA602-10|10BBCLP-0602|Canada|Ontario|658[0n]|BOLD:ABZ5715
Hypercompe scribonia|[1297]|LPSOD1054-09|08MZPP-122|Canada|Ontario|658[0n]|BOLD:ABZ5715
Hypercompe scribonia|[1298]|LOT127-04|04HBL002127|United States|Tennessee|609[0n]|BOLD:ABZ5715
Hypercompe scribonia|[1299]|LOT126-04|04HBL002126|United States|Tennessee|609[0n]|BOLD:ABZ5715
Hypercompe scribonia|[1300]|LOT550-04|04HBL002550|United States|Tennessee|616[0n]|BOLD:ABZ5715
Hypercompe scribonia|[1301]|LGSMB328-05|DNA-ATBI-1177|United States|North Carolina|595[0n]|BOLD ...
Hypercompe scribonia|[1302]|JRLAA001-09|JRLAA-001|United States|Georgia|658[0n]|BOLD:ABZ5715
Hypercompe scribonia|[1303]|RDMAB750-06|BCSC419|United States|Kentucky|658[0n]|BOLD:ABZ5715
Hypercompe scribonia|[1304]|BBLOB630-11|BIOUG01398-E12|United States|Florida|658[0n]|BOLD:ABZ5715
Hypercompe scribonia|[1305]|LSEU632-06|06-JKA-0632|United States|Georgia|658[0n]|BOLD:ABZ5715
Hypercompe scribonia|[1306]|LNC905-06|06-NCCC-905|United States|North Carolina|658[0n]|BOLD:AB...
Hypercompe scribonia|[1307]|LOFLB834-06|06-FLOR-1774|United States|Florida|658[0n]|BOLD:ABZ5715
Hypercompe caudata|[1308]|RDNME137-07|CNCNoctuoidea13665|United States|Texas|658[0n]|BOLD:AAA1342
Hypercompe caudata|[1309]|ARCTA766-07|MILA 0485|Guatemala|Baja Verapaz|655[0n]|BOLD:AAA1342
Hypercompe caudata|[1310]|RDNMH185-09|CNCLEP00054402|Mexico|Veracruz|633[0n]|BOLD:AAA1342
Hypercompe caudata|[1311]|ARCTD605-12|MILA 1730|Costa Rica|Guanacaste|658[0n]|BOLD:AAA1342
Hypercompe caudata|[1312]|ARCTB336-08|MILA 0995|Guatemala|Suchitepequez|656[0n]|BOLD:AAA1342
Hypercompe caudata|[1313]|ARCTA622-07|MILA 0341|Guatemala|Suchitepequez|658[0n]|BOLD:AAA1342
Hypercompe caudata|[1314]|ARCTA620-07|MILA 0339|Guatemala|Quezaltenango|658[0n]|BOLD:AAA1342
Hypercompe caudata|[1315]|ARCTA024-07|MILA 0024|Nicaragua|Jinotega|621[0n]|BOLD:AAA1342
Hypercompe caudata|[1316]|ARCTB320-08|MILA 0979|Guatemala|Izabal|658[0n]|BOLD:AAA1342
Hypercompe caudata|[1317]|ARCTA042-07|MILA 0042|Nicaragua|Granada|658[0n]|BOLD:AAA1342
Hypercompe caudata|[1318]|ARCTA205-07|MILA 0205|Venezuela|Merida|658[0n]|BOLD:AAA1342
Hypercompe caudata|[1319]|ARCTA040-07|MILA 0040|Nicaragua|Nueva Segovia|658[0n]|BOLD:AAA1342
Hypercompe caudata|[1320]|ARCTA011-07|MILA 0011|Nicaragua|Jinotega|658[0n]|BOLD:AAA1342
Hypercompe caudata|[1321]|ARCTA851-07|MILA 0570|Nicaragua|Madriz|658[0n]|BOLD:AAA1342
Hypercompe extrema|[1322]|RDNMK158-11|CNCLEP 80445|United States|Arizona|658[0n]|BOLD:AAY8846
Hypercompe permaculata|[1323]|BBLOC1364-11|BIOUG01542-A11|United States|California|658[0n]|BOLD ...
Hypercompe permaculata|[1324]|BBLOC1378-11|BIOUG01542-C01|United States|California|658[0n]|BOLD ...
Hypercompe permaculata|[1325]|BBLOC1363-11|BIOUG01542-A10|United States|California|629[0n]|BOLD ...
Hypercompe permaculata|[1326]|BBLOC1362-11|BIOUG01542-A09|United States|California|636[0n]|BOLD ...
Hypercompe permaculata|[1327]|JBAZ060-09|JLB-0060|United States|California|658[0n]|BOLD:AAD0396
Hypercompe permaculata|[1328]|RDNME143-07|CNCNoctuoidea13671|Canada|British Columbia|626[0n]|BOLD...
Hypercompe permaculata|[1329]|RDNME139-07|CNCNoctuoidea13667|United States|Colorado|630[0n]|BOLD...
Hypercompe permaculata|[1330]|RDNME138-07|CNCNoctuoidea13666|Canada|Alberta|658[0n]|BOLD:AAD0396
Hypercompe permaculata|[1331]|RDMAB519-06|UASM58480|Canada|Alberta|658[0n]|BOLD:AAD0396
Hypercompe permaculata|[1332]|RDMAB926-06|UASM58669|Canada|Alberta|658[0n]|BOLD:AAD0396
Hypercompe permaculata|[1333]|RDNME141-07|CNCNoctuoidea13669|United States|Arizona|615[0n]|BOLD ...
Hypercompe permaculata|[1334]|ARCTA021-07|MILA 0021|United States|Arizona|613[0n]|BOLD:AAD0396
Hypercompe permaculata|[1335]|AWCLB091-10|AWC-00227|United States|Colorado|658[0n]|BOLD:AAD0396
Arachnis zuni|[1336]|RDNMG137-08|NOC14890|United States|New Mexico|658[0n]|BOLD:AAJ8879
Arachnis picta|[1337]|GMLC269-11|2009GM-0138|United States|California|658[0n]|BOLD:AAB5413
Arachnis picta|[1338]|GMLC266-11|2009GM-0135|United States|California|634[0n]|BOLD:AAB5413
Arachnis picta|[1339]|LOCBB381-06|06-BLLOC-1321|United States|California|658[0n]|BOLD:AAB5413
Arachnis picta|[1340]|LOCBB382-06|06-BLLOC-1322|United States|California|658[0n]|BOLD:AAB5413
Arachnis picta|[1341]|LOCBB379-06|06-BLLOC-1319|United States|California|658[0n]|BOLD:AAB5413
Arachnis picta|[1342]|RDNMG147-08|NOC14900|United States|California|658[0n]|BOLD:AAB5413
Arachnis picta|[1343]|LOCBB380-06|06-BLLOC-1320|United States|California|658[0n]|BOLD:AAB5413
Arachnis picta|[1344]|RDNMG146-08|NOC14899|United States|California|658[0n]|BOLD:AAB5413
Arachnis picta|[1345]|JBAZ035-09|JLB-0035|United States|California|658[0n]|BOLD:AAB5413
Arachnis citra|[1346]|RDNME140-07|CNCNoctuoidea13668|United States|Colorado|647[0n]|BOLD:AAD2256
Arachnis citra|[1347]|RDNME135-07|CNCNoctuoidea13663|United States|Colorado|658[0n]|BOLD:AAD2256
Arachnis citra|[1348]|RDNME134-07|CNCNoctuoidea13662|United States|Colorado|658[0n]|BOLD:AAD2256
Arachnis citra|[1349]|RDNME790-08|LEP041255|United States|Utah|658[0n]|BOLD:AAD2256
Arachnis picta|[1350]|RDNME786-08|LEP041251|United States|New Mexico|658[0n]|BOLD:ACF2921
Arachnis picta|[1351]|RDNMG151-08|NOC14904|United States|Colorado|658[0n]|BOLD:ACF2921
Arachnis picta|[1352]|RDNMG150-08|NOC14903|United States|Colorado|658[0n]|BOLD:ACF2921
Arachnis picta|[1353]|RDNMH392-09|CNCLEP00054406|United States|Colorado|658[0n]|BOLD:ACF2921
Arachnis picta|[1354]|RDNMF979-08|CNC LEP00054246|United States|Colorado|658[0n]|BOLD:ACF2921
Arachnis picta|[1355]|RDNMF980-08|CNC LEP00054247|United States|Colorado|658[0n]|BOLD:ACF2921
Arachnis picta|[1356]|RDNMI169-10|CNCLEP 69769|United States|Utah|658[0n]|BOLD:ABY6889
Arachnis picta|[1357]|RDNMG153-08|NOC14906|United States|Colorado|658[0n]|BOLD:ABZ4863
Arachnis picta|[1358]|RDNMG149-08|NOC14902|United States|Colorado|658[0n]|BOLD:ABZ4863
Arachnis picta|[1359]|RDNMG152-08|NOC14905|United States|Colorado|658[0n]|BOLD:ABZ4863
Arachnis picta|[1360]|RDNMG148-08|NOC14901|United States|Colorado|658[0n]|BOLD:ABZ4863
Arachnis picta|[1361]|RDNMG141-08|NOC14894|United States|Colorado|658[0n]|BOLD:ABZ4863
Arachnis picta|[1362]|RDNMH393-09|CNCLEP00054407|United States|Colorado|658[0n]|BOLD:ABZ4863
Arachnis picta|[1363]|RDNME464-08|LEP037888|United States|Colorado|658[0n]|BOLD:ABZ4863
Arachnis picta|[1364]|DMAZ030-09|DMTRN-0124|United States|Arizona|658[0n]|BOLD:AAB5410
Arachnis picta|[1365]|RDMAB936-06|BCSC402|United States|Arizona|658[0n]|BOLD:AAB5410
Arachnis picta|[1366]|RDNMH372-09|CNCLEP00057781|United States|Arizona|658[0n]|BOLD:AAB5410
Arachnis picta|[1367]|AWCLB078-10|AWC-00579|United States|Arizona|658[0n]|BOLD:AAB5410
Arachnis nedyma|[1368]|CNCLB662-14|CNCLEP00078768|United States|Arizona|550[0n]|BOLD:ACQ3669
Arachnis aulaea|[1369]|CMAZA563-10|CMAZ-0563|United States|Arizona|658[0n]|BOLD:ABY6795
Arachnis aulaea|[1370]|DMAZ253-10|BIOUG00716-D09|United States|Arizona|658[0n]|BOLD:ABY6795
Arachnis aulaea|[1371]|AWCL061-09|JLB-0259|United States|Arizona|658[0n]|BOLD:ABY6795
Arachnis aulaea|[1372]|DMAZ194-10|DMLEP-0111|United States|Arizona|658[0n]|BOLD:ABY6795
Arachnis aulaea|[1373]|AWCLB077-10|AWC-00692|United States|Arizona|658[0n]|BOLD:ABY6795
Arachnis aulaea|[1374]|AWCLB075-10|AWC-01408|United States|Arizona|658[0n]|BOLD:ABY6795
Arachnis aulaea|[1375]|AWCLB073-10|AWC-01134|United States|Arizona|658[0n]|BOLD:ABY6795
Arachnis aulaea|[1376]|AWCLB068-10|AWC-01513|United States|Arizona|658[0n]|BOLD:ABY6795
Arachnis aulaea|[1377]|RDNME472-08|LEP037896|United States|Arizona|658[0n]|BOLD:ABY6795
Arachnis aulaea|[1378]|AWCL060-09|JLB-0258|United States|Arizona|658[0n]|BOLD:ABY6795
Arachnis aulaea|[1379]|AWCL007-09|JLB-0205|United States|Arizona|658[0n]|BOLD:ABY6795
Arachnis aulaea|[1380]|AWCLB076-10|AWC-01409|United States|Arizona|658[0n]|BOLD:ABY6795
Arachnis aulaea|[1381]|AWCLB074-10|AWC-01407|United States|Arizona|658[0n]|BOLD:ABY6795
Arachnis aulaea|[1382]|AWCLB069-10|AWC-01512|United States|Arizona|658[0n]|BOLD:ABY6795
Arachnis aulaea|[1383]|AWCLB067-10|AWC-01514|United States|Arizona|658[0n]|BOLD:ABY6795
Arachnis aulaea|[1384]|AWCLB065-10|AWC-01516|United States|Arizona|658[0n]|BOLD:ABY6795
Arachnis aulaea|[1385]|AWCLB064-10|AWC-01517|United States|Arizona|658[0n]|BOLD:ABY6795
Arachnis aulaea|[1386]|RDNME473-08|LEP037897|United States|Arizona|658[0n]|BOLD:ABY6795
Arachnis aulaea|[1387]|RDNME471-08|LEP037895|United States|Arizona|658[0n]|BOLD:ABY6795
Arachnis aulaea|[1388]|DMAZ170-10|DMLEP-0069|United States|Arizona|658[0n]|BOLD:ABY6795
Arachnis aulaea|[1389]|AWCLB071-10|AWC-01510|United States|Arizona|658[0n]|BOLD:ABY6795
Arachnis aulaea|[1390]|JBAZ106-09|JLB-0106|United States|Arizona|658[0n]|BOLD:ABY6795
Arachnis aulaea|[1391]|AWCLB070-10|AWC-01511|United States|Arizona|658[0n]|BOLD:ABY6795
Arachnis aulaea|[1392]|AWCLB063-10|AWC-01518|United States|Arizona|632[0n]|BOLD:ABY6795
Arachnis aulaea|[1393]|AWCLB066-10|AWC-01515|United States|Arizona|658[0n]|BOLD:ABY6795
Arachnis aulaea|[1394]|AWCLB072-10|AWC-00956|United States|Arizona|658[0n]|BOLD:ABY6795
[illegible]

Arachnis aulaea|[1392]|AWCLB063-10|AWC-01518|United States|Arizona|632[0n]|BOLD:ABY6795
Arachnis aulaea|[1393]|AWCLB066-10|AWC-01515|United States|Arizona|658[0n]|BOLD:ABY6795
Arachnis aulaea|[1394]|AWCLB072-10|AWC-00956|United States|Arizona|658[0n]|BOLD:ABY6795
Arachnis aulaea|[1395]|AWCLB062-10|AWC-01519|United States|Arizona|658[0n]|BOLD:ABY6795
Hypercompe oslari|[1396]|RDNME449-08|LEP037873|United States|Texas|566[0n]|BOLD:AAA5815
Hypercompe oslari|[1397]|HKONB132-08|3629-COI-08|United States|Texas|658[0n]|BOLD:AAA5815
Hypercompe sp.|[1398]|AWCLB083-10|AWC-01505|United States|Arizona|658[0n]|BOLD:AAL7185
Hypercompe suffusa|[1399]|CMAZA528-10|CMAZ-0528|United States|Arizona|658[0n]|BOLD:AAC2092
Hypercompe suffusa|[1400]|RDNME475-08|LEP037899|United States|Arizona|658[0n]|BOLD:AAC2092
Hypercompe suffusa|[1401]|AWCLB082-10|AWC-01506|United States|Arizona|658[0n]|BOLD:AAC2092
Hypercompe suffusa|[1402]|AWCLB088-10|AWC-00200|United States|Arizona|658[0n]|BOLD:AAC2092
Hypercompe suffusa|[1403]|RDNME136-07|CNCNoctuoidea13664|United States|Arizona|658[0n]|BOLD:AA...
Hypercompe suffusa|[1404]|AWCLB090-10|AWC-00651|United States|Arizona|658[0n]|BOLD:AAC2092
Hypercompe suffusa|[1405]|CMAZA388-10|CMAZ-0388|United States|Arizona|658[0n]|BOLD:AAC2092
Hypercompe suffusa|[1406]|AWCLB089-10|AWC-00193|United States|Arizona|658[0n]|BOLD:AAC2092
Hypercompe suffusa|[1407]|AWCLB085-10|AWC-01013|United States|Arizona|658[0n]|BOLD:AAC2092
Hypercompe suffusa|[1408]|AWCLB086-10|AWC-00652|United States|Arizona|658[0n]|BOLD:AAC2092
Hypercompe suffusa|[1409]|AWCL080-09|JLB-0279|United States|Arizona|658[0n]|BOLD:AAC2092
Hypercompe suffusa|[1410]|RDNME476-08|LEP037900|United States|Arizona|658[0n]|BOLD:AAC2092
Hypercompe suffusa|[1411]|AWCLB084-10|AWC-01504|United States|Arizona|658[0n]|BOLD:AAC2092
Hypercompe suffusa|[1412]|AWCLB081-10|AWC-01507|United States|Arizona|658[0n]|BOLD:AAC2092
Hypercompe suffusa|[1413]|AWCLB080-10|AWC-01508|United States|Arizona|658[0n]|BOLD:AAC2092
Pyrrharctia isabella|[1414]|LHLEP291-06|UBC-2006-0724|Canada|British Columbia|657[0n]|BOLD:ACF...
Pyrrharctia isabella|[1415]|LHLEP058-06|UBC-2006-0259|Canada|British Columbia|577[0n]|BOLD:ACF...
Pyrrharctia isabella|[1416]|LILLA041-11|SNS10IL-00056|United States|Illinois|658[0n]|BOLD:ACF1163
Pyrrharctia isabella|[1417]|RDLQG212-06|DH012388|Canada|Quebec|538[1n]|BOLD:ACF1163
Pyrrharctia isabella|[1418]|LPMN708-08|08BBLEP-01511|Canada|Manitoba|658[0n]|BOLD:ACF1163
Pyrrharctia isabella|[1419]|LOTB517-05|05-TN-00517|United States|Tennessee|606[0n]|BOLD:ACF1163
Pyrrharctia isabella|[1420]|PHMNB712-05|Moth 405.03SA|Canada|New Brunswick|616[0n]|BOLD:ACF1163
Pyrrharctia isabella|[1421]|QUNOC089-09|5920-070708-KY|United States|Kentucky|658[0n]|BOLD:ACF...
Pyrrharctia isabella|[1422]|LOT128-04|04HBL002128|United States|Tennessee|609[0n]|BOLD:ACF1163
Pyrrharctia isabella|[1423]|TMNBB011-06|MNBTT-951|Canada|New Brunswick|658[0n]|BOLD:ACF1163
Pyrrharctia isabella|[1424]|LHLEP295-06|UBC-2006-0930|Canada|British Columbia|629[0n]|BOLD:ACF...
Pyrrharctia isabella|[1425]|LOWCE046-06|CGWC-3806|Canada|British Columbia|658[0n]|BOLD:ACF1163
Pyrrharctia isabella|[1426]|RWWB781-10|RWWA-1780|United States|Washington|658[0n]|BOLD:ACF1163
Pyrrharctia isabella|[1427]|RWWA534-09|RWWA-0552|United States|Washington|658[0n]|BOLD:ACF1163
Pyrrharctia isabella|[1428]|BBLEC567-09|09BBELE-0567|Canada|Nova Scotia|641[0n]|BOLD:ACF1163
Pyrrharctia isabella|[1429]|RDNME201-07|CNCNoctuoidea13808|United States|California|617[0n]|BOLD...
Pyrrharctia isabella|[1430]|TMNBD385-07|MNBTT-3186|Canada|New Brunswick|658[0n]|BOLD:ACF1163
Pyrrharctia isabella|[1431]|LGSMC748-05|DNA-ATBI-2748|United States|Tennessee|658[0n]|BOLD:ACF...
Pyrrharctia isabella|[1432]|XAK121-06|2006-ONT-1116|Canada|Ontario|658[0n]|BOLD:ACF1163
Pyrrharctia isabella|[1433]|LPOKB351-09|MDOK-1364|United States|Oklahoma|658[0n]|BOLD:ACF1163
Pyrrharctia isabella|[1434]|RDLQG002-06|DH012122|Canada|Quebec|658[0n]|BOLD:ACF1163
Pyrrharctia isabella|[1435]|BBLEC568-09|09BBELE-0568|Canada|Nova Scotia|658[0n]|BOLD:ACF1163
Pyrrharctia isabella|[1436]|TMNBD383-07|MNBTT-3184|Canada|New Brunswick|654[0n]|BOLD:ACF1163
Pyrrharctia isabella|[1437]|LPSO927-08|PPBP-0927|Canada|Ontario|658[0n]|BOLD:ACF1163
Pyrrharctia isabella|[1438]|BBLPC231-09|09BBELE-1231|Canada|Nova Scotia|658[0n]|BOLD:ACF1163
Pyrrharctia isabella|[1439]|BBLPC217-09|09BBELE-1217|Canada|Nova Scotia|658[0n]|BOLD:ACF1163
Pyrrharctia isabella|[1440]|BBLEC569-09|09BBELE-0569|Canada|Nova Scotia|658[0n]|BOLD:ACF1163
Pyrrharctia isabella|[1441]|LALPA378-10|AVBC 380-10|Canada|British Columbia|658[0n]|BOLD:ACF1163
Pyrrharctia isabella|[1442]|RWWC326-11|RWWA-2303|United States|Washington|658[0n]|BOLD:ACF1163
Pyrrharctia isabella|[1443]|LOWCE039-06|CGWC-3799|Canada|British Columbia|658[0n]|BOLD:ACF1163
Pyrrharctia isabella|[1444]|RWWA424-09|RWWA-0424|United States|Washington|658[0n]|BOLD:ACF1163
Pyrrharctia isabella|[1445]|RWWA462-09|RWWA-0462|United States|Washington|658[0n]|BOLD:ACF1163
Pyrrharctia isabella|[1446]|RWWA468-09|RWWA-0468|United States|Washington|658[0n]|BOLD:ACF1163
Pyrrharctia isabella|[1447]|LHLEP057-06|UBC-2006-0258|Canada|British Columbia|658[0n]|BOLD:ACF...
Pyrrharctia isabella|[1448]|LHLEP059-06|UBC-2006-0260|Canada|British Columbia|658[0n]|BOLD:ACF...
Pyrrharctia isabella|[1449]|LHLEP060-06|UBC-2006-0261|Canada|British Columbia|658[0n]|BOLD:ACF...
Pyrrharctia isabella|[1450]|LHLEP292-06|UBC-2006-0725|Canada|British Columbia|657[0n]|BOLD:ACF...
Pyrrharctia isabella|[1451]|LHLEP294-06|UBC-2006-0727|Canada|British Columbia|657[0n]|BOLD:ACF...
Pyrrharctia isabella|[1452]|LHLEP296-06|UBC-2006-1166|Canada|British Columbia|657[0n]|BOLD:ACF...
Pyrrharctia isabella|[1453]|LHLEP293-06|UBC-2006-0726|Canada|British Columbia|653[0n]|BOLD:ACF...
Pyrrharctia isabella|[1454]|TMNBD386-07|MNBTT-3187|Canada|New Brunswick|656[0n]|BOLD:ACF1163
Pyrrharctia isabella|[1455]|LALPA207-10|AVBC 208-10|Canada|British Columbia|658[0n]|BOLD:ACF1163
Pyrrharctia isabella|[1456]|LPSO230-08|PPBP-0230|Canada|Ontario|658[0n]|BOLD:ACF1163
Pyrrharctia isabella|[1457]|RWWC382-11|RWWA-2359|United States|Washington|658[0n]|BOLD:ACF1163
Pyrrharctia isabella|[1458]|LPSOB064-08|PPBP-1063|Canada|Ontario|658[0n]|BOLD:AAA4533
Pyrrharctia isabella|[1459]|LPSO515-08|PPBP-0515|Canada|Ontario|658[0n]|BOLD:AAA4533
Pyrrharctia isabella|[1460]|LGSM621-04|DNA-ATBI-0621|United States|North Carolina|609[0n]|BOLD ...
Pyrrharctia isabella|[1461]|LOT129-04|04HBL002129|United States|Tennessee|609[0n]|BOLD:AAA4533
Pyrrharctia isabella|[1462]|LOFLB209-06|06-FLOR-1149|United States|Florida|658[0n]|BOLD:AAA4533
Pyrrharctia isabella|[1463]|USLEP586-10|10BBLEP-00586|United States|Florida|658[0n]|BOLD:AAA4533
Pyrrharctia isabella|[1464]|RDMAB740-06|BCSC409|United States|Kentucky|658[0n]|BOLD:AAA4533
Pyrrharctia isabella|[1465]|XAB104-04|04HBL005104|Canada|Ontario|606[2n]|BOLD:AAA4533
Pyrrharctia isabella|[1466]|LGSMB306-05|DNA-ATBI-1155|United States|North Carolina|584[1n]|BOLD ...
Pyrrharctia isabella|[1467]|BBLPC990-09|09BBELE-1990|Canada|Nova Scotia|633[0n]|
Pyrrharctia isabella|[1468]|LGSM622-04|DNA-ATBI-0622|United States|North Carolina|576[0n]|BOLD ...
Pyrrharctia isabella|[1469]|LGSMB305-05|DNA-ATBI-1154|United States|North Carolina|564[0n]|BOLD ...
Pyrrharctia isabella|[1470]|XAB622-04|04HBL005622|Canada|Ontario|658[0n]|BOLD:AAA4533
Pyrrharctia isabella|[1471]|LNCNW013-06|06-NCNW-0013|United States|North Carolina|658[0n]|BOLD ...
Pyrrharctia isabella|[1472]|LOTB518-05|05-TN-00518|United States|Tennessee|658[1n]|BOLD:AAA4533
Pyrrharctia isabella|[1473]|RDMAB927-06|UASM57202|Canada|Alberta|658[0n]|BOLD:AAA4533
Pyrrharctia isabella|[1474]|LNCNW034-06|06-NCNW-0034|United States|North Carolina|658[0n]|BOLD ...
Pyrrharctia isabella|[1475]|XAE359-04|Moth4359.03|Canada|Ontario|658[0n]|BOLD:AAA4533
Pyrrharctia isabella|[1476]|XAE372-04|Moth4372.03|Canada|Ontario|658[0n]|BOLD:AAA4533
Pyrrharctia isabella|[1477]|BBLPC251-09|09BBELE-1251|Canada|Nova Scotia|658[0n]|BOLD:AAA4533
Pyrrharctia isabella|[1478]|LPSO514-08|PPBP-0514|Canada|Ontario|658[0n]|BOLD:AAA4533
Pyrrharctia isabella|[1479]|LPMN762-08|08BBLEP-01565|Canada|Manitoba|658[0n]|BOLD:AAA4533
Pyrrharctia isabella|[1480]|LPSO872-08|PPBP-0872|Canada|Ontario|658[0n]|BOLD:AAA4533
Pyrrharctia isabella|[1481]|LPSOB065-08|PPBP-1064|Canada|Ontario|658[0n]|BOLD:AAA4533
Pyrrharctia isabella|[1482]|LGSMC728-05|DNA-ATBI-2728|United States|Tennessee|658[0n]|BOLD:AAA...
Pyrrharctia isabella|[1483]|LGSMG114-07|BGS03435|United States|Tennessee|658[0n]|BOLD:AAA4533
Pyrrharctia isabella|[1484]|TMNBD382-07|MNBTT-3183|Canada|New Brunswick|658[0n]|BOLD:AAA4533
Pyrrharctia isabella|[1485]|TMNBD384-07|MNBTT-3185|Canada|New Brunswick|658[0n]|BOLD:AAA4533
Pyrrharctia isabella|[1486]|LGSMC729-05|DNA-ATBI-2729|United States|Tennessee|658[0n]|BOLD:AAA...
Pyrrharctia isabella|[1487]|BLTIB225-08|BL406|Canada|Ontario|658[0n]|BOLD:AAA4533
Pyrrharctia isabella|[1488]|LPSOC128-08|PPBP-2127|Canada|Ontario|658[0n]|BOLD:AAA4533
Pyrrharctia isabella|[1489]|LPSOC140-08|PPBP-2139|Canada|Ontario|658[0n]|BOLD:AAA4533
Pyrrharctia isabella|[1490]|TMNBB010-06|MNBTT-950|Canada|New Brunswick|658[0n]|BOLD:AAA4533
Pyrrharctia isabella|[1491]|LPSO325-08|PPBP-0325|Canada|Ontario|658[0n]|BOLD:AAA4533
Pyrrharctia isabella|[1492]|XAK157-06|2006-ONT-1152|Canada|Ontario|658[0n]|BOLD:AAA4533
Pyrrharctia isabella|[1493]|XAB082-04|04HBL005082|Canada|Ontario|658[0n]|BOLD:AAA4533
Pyrrharctia isabella|[1494]|LOCT020-05|05-CTATBI-0020|United States|Connecticut|658[0n]|BOLD:A...

Pyrrharctia isabella|[1492]|XAK157-06|2006-ONT-1152|Canada|Ontario|658[0n]|BOLD:AAA4533
Pyrrharctia isabella|[1493]|XAB082-04|04HBL005082|Canada|Ontario|658[0n]|BOLD:AAA4533
Pyrrharctia isabella|[1494]|LOCT020-05|05-CTATBI-0020|United States|Connecticut|658[0n]|BOLD:A...
Pyrrharctia isabella|[1495]|BLTIB428-08|BL675|Canada|Ontario|658[0n]|BOLD:AAA4533
Pyrrharctia isabella|[1496]|XAF693-05|2005-ONT-342|Canada|Ontario|658[0n]|BOLD:AAA4533
Pyrrharctia isabella|[1497]|XAJ935-06|2006-ONT-0935|Canada|Ontario|658[0n]|BOLD:AAA4533
Pyrrharctia isabella|[1498]|BBLOD1681-11|BIOUG01830-D12|United States|Texas|658[0n]|BOLD:AAA4533
Pyrrharctia isabella|[1499]|LILLA612-11|SNS10IL-00785|United States|Illinois|658[0n]|BOLD:AAA4533
Pyrrharctia isabella|[1500]|XAB183-04|04HBL005183|Canada|Ontario|658[0n]|BOLD:AAA4533
Pyrrharctia isabella|[1501]|LGSMC730-05|DNA-ATBI-2730|United States|Tennessee|575[0n]|BOLD:AAA...
Pyrrharctia isabella|[1502]|BLTIB226-08|BL407|Canada|Ontario|658[0n]|BOLD:AAA4533
Pyrrharctia isabella|[1503]|TMG76-03|moth540.01|Canada|Ontario|639[0n]|BOLD:AAA4533
Pyrrharctia isabella|[1504]|PMG016-03|moth305.01|Canada|Ontario|617[0n]|BOLD:AAA4533
Phragmatobia fuliginosa|[1505]|ABKWR039-07|KENWR 5144|United States|Alaska|658[0n]|BOLD:AAA6178
Phragmatobia assimilans|[1506]|LMIS016-05|05-ONMIS-0016|Canada|Ontario|658[0n]|BOLD:ACF3777
Phragmatobia assimilans|[1507]|TTMNB067-06|MNBTT-067|Canada|New Brunswick|658[0n]|BOLD:ACF3777
Phragmatobia assimilans|[1508]|TTMNB066-06|MNBTT-066|Canada|New Brunswick|658[0n]|BOLD:ACF3777
Phragmatobia assimilans|[1509]|TTMNB065-06|MNBTT-065|Canada|New Brunswick|658[0n]|BOLD:ACF3777
Phragmatobia assimilans|[1510]|TTMNB064-06|MNBTT-064|Canada|New Brunswick|658[0n]|BOLD:ACF3777
Phragmatobia assimilans|[1511]|TTMNB063-06|MNBTT-063|Canada|New Brunswick|658[0n]|BOLD:ACF3777
Phragmatobia assimilans|[1512]|TTMNB062-06|MNBTT-062|Canada|New Brunswick|658[0n]|BOLD:ACF3777
Phragmatobia assimilans|[1513]|TMNBB024-06|MNBTT-964|Canada|New Brunswick|658[0n]|BOLD:ACF3777
Phragmatobia assimilans|[1514]|LPSOB594-08|PPBP-1593|Canada|Ontario|658[0n]|BOLD:ACF3777
Phragmatobia assimilans|[1515]|LPSOB395-08|PPBP-1394|Canada|Ontario|658[0n]|BOLD:ACF3777
Phragmatobia assimilans|[1516]|LPSOB325-08|PPBP-1324|Canada|Ontario|658[0n]|BOLD:ACF3777
Phragmatobia assimilans|[1517]|RDLQ768-07|DH009494|Canada|Quebec|658[0n]|BOLD:ACF3777
Phragmatobia assimilans|[1518]|TMNBB027-06|MNBTT-967|Canada|New Brunswick|658[0n]|BOLD:ACF3777
Phragmatobia assimilans|[1519]|TMNBB026-06|MNBTT-966|Canada|New Brunswick|658[0n]|BOLD:ACF3777
Phragmatobia assimilans|[1520]|TMNBB025-06|MNBTT-965|Canada|New Brunswick|658[0n]|BOLD:ACF3777
Phragmatobia assimilans|[1521]|LMDH098-11|BIOUG01046-G11|United States|Minnesota|658[0n]|BOLD:...
Phragmatobia assimilans|[1522]|TMNBB023-06|MNBTT-963|Canada|New Brunswick|658[0n]|BOLD:ACF3777
Phragmatobia assimilans|[1523]|TMNBB022-06|MNBTT-962|Canada|New Brunswick|658[0n]|BOLD:ACF3777
Phragmatobia assimilans|[1524]|RDLQ767-07|DH009273|Canada|Quebec|658[0n]|BOLD:ACF3777
Phragmatobia assimilans|[1525]|RDMAB920-06|UASM24734|Canada|Alberta|658[0n]|BOLD:ACF3777
Phragmatobia assimilans|[1526]|LPSOB138-08|PPBP-1137|Canada|Ontario|658[0n]|BOLD:ACF3777
Phragmatobia assimilans|[1527]|LPSOB133-08|PPBP-1132|Canada|Ontario|658[0n]|BOLD:ACF3777
Phragmatobia assimilans|[1528]|LPSOB131-08|PPBP-1130|Canada|Ontario|658[0n]|BOLD:ACF3777
Phragmatobia assimilans|[1529]|LPSOB118-08|PPBP-1117|Canada|Ontario|658[0n]|BOLD:ACF3777
Phragmatobia assimilans|[1530]|BBLPB275-10|10BBCLP-1274|Canada|Saskatchewan|658[0n]|BOLD:ACF3777
Phragmatobia assimilans|[1531]|TMNBB028-06|MNBTT-968|Canada|New Brunswick|658[0n]|BOLD:ACF3777
Phragmatobia lineata|[1532]|RDNME441-08|LEP037865|United States|Ohio|646[0n]|BOLD:AAA6177
Phragmatobia lineata|[1533]|RDNMH377-09|BIRD22041|Canada|Alberta|658[0n]|BOLD:AAA6177
Phragmatobia lineata|[1534]|RDNME442-08|LEP037866|United States|Ohio|646[1n]|BOLD:AAA6177
Phragmatobia fuliginosa|[1535]|RDNMG136-08|NOC14889|United States|California|658[0n]|BOLD:AAA6177
Phragmatobia fuliginosa|[1536]|XAG012-05|2005-ONT-596|Canada|Ontario|658[0n]|BOLD:AAA6177
Phragmatobia fuliginosa|[1537]|RWWA953-09|RWWA-0953|United States|Washington|658[0n]|BOLD:AAA6177
Phragmatobia fuliginosa|[1538]|XAG231-05|2005-ONT-815|Canada|Ontario|658[0n]|BOLD:AAA6177
Phragmatobia fuliginosa|[1539]|BBLPE566-09|09BBELE-2566|Canada|Nova Scotia|658[0n]|BOLD:AAA6177
Phragmatobia fuliginosa|[1540]|BBLPE585-09|09BBELE-2585|Canada|Nova Scotia|658[0n]|BOLD:AAA6177
Phragmatobia fuliginosa|[1541]|BBLPE598-09|09BBELE-2598|Canada|Nova Scotia|658[0n]|BOLD:AAA6177
Phragmatobia fuliginosa|[1542]|BBLPE563-09|09BBELE-2563|Canada|Nova Scotia|638[0n]|BOLD:AAA6177
Phragmatobia fuliginosa|[1543]|RWWC514-11|RWWA-2491|United States|Washington|658[0n]|BOLD:AAA6177
Phragmatobia fuliginosa|[1544]|MNBB399-05|05-NBSTA-315|Canada|New Brunswick|658[0n]|BOLD:AAA6177
Phragmatobia fuliginosa|[1545]|MNBB618-05|05-NBSTA-534|Canada|New Brunswick|658[0n]|BOLD:AAA6177
Phragmatobia fuliginosa|[1546]|XAG427-05|2005-ONT-1011|Canada|Ontario|658[0n]|BOLD:AAA6177
Phragmatobia fuliginosa|[1547]|BBLPE584-09|09BBELE-2584|Canada|Nova Scotia|658[0n]|BOLD:AAA6177
Phragmatobia fuliginosa|[1548]|RDNME827-08|LEP041292|Canada|Alberta|658[0n]|BOLD:AAA6177
Phragmatobia fuliginosa|[1549]|RWWC1019-12|RWWA-3679|United States|Washington|658[0n]|BOLD:AAA...
Phragmatobia fuliginosa rubricosa|[1550]|RDLQ106-05|DH007139|Canada|Quebec|658[0n]|BOLD:AAA6177
Phragmatobia fuliginosa rubricosa|[1551]|RDLQB740-05|DH010655|Canada|Quebec|658[0n]|BOLD:AAA6177
Phragmatobia fuliginosa|[1552]|XAJ854-06|2006-ONT-0854|Canada|Ontario|658[0n]|BOLD:AAA6177
Phragmatobia fuliginosa|[1553]|TMNBB015-06|MNBTT-955|Canada|New Brunswick|658[0n]|BOLD:AAA6177
Phragmatobia fuliginosa|[1554]|TMNBB016-06|MNBTT-956|Canada|New Brunswick|658[0n]|BOLD:AAA6177
Phragmatobia fuliginosa|[1555]|TMNBB017-06|MNBTT-957|Canada|New Brunswick|658[0n]|BOLD:AAA6177
Phragmatobia fuliginosa|[1556]|TMNBB018-06|MNBTT-958|Canada|New Brunswick|658[0n]|BOLD:AAA6177
Phragmatobia fuliginosa|[1557]|TMNBB021-06|MNBTT-961|Canada|New Brunswick|658[0n]|BOLD:AAA6177
Phragmatobia fuliginosa|[1558]|XAK064-06|2006-ONT-1059|Canada|Ontario|658[0n]|BOLD:AAA6177
Phragmatobia fuliginosa|[1559]|RDLQG269-06|DH012477|Canada|Quebec|658[0n]|BOLD:AAA6177
Phragmatobia fuliginosa|[1560]|XAB035-04|04HBL005035|Canada|Ontario|658[0n]|BOLD:AAA6177
Phragmatobia fuliginosa rubricosa|[1561]|RDLQB511-05|DH010597|Canada|Quebec|658[0n]|BOLD:AAA6177
Phragmatobia fuliginosa|[1562]|XAJ924-06|2006-ONT-0924|Canada|Ontario|658[0n]|BOLD:AAA6177
Phragmatobia fuliginosa|[1563]|XAJ931-06|2006-ONT-0931|Canada|Ontario|658[0n]|BOLD:AAA6177
Phragmatobia fuliginosa|[1564]|XAD765-05|2005-ONT-564|Canada|Ontario|658[0n]|BOLD:AAA6177
Phragmatobia fuliginosa|[1565]|RDMAB390-05|UASM41320|Canada|Alberta|658[0n]|BOLD:AAA6177
Phragmatobia fuliginosa|[1566]|RDMAB389-05|BCSC63|Canada|Alberta|658[0n]|BOLD:AAA6177
Phragmatobia fuliginosa|[1567]|BLTIB957-08|BL1386|Canada|Ontario|658[0n]|BOLD:AAA6177
Phragmatobia fuliginosa|[1568]|XAK014-06|2006-ONT-1009|Canada|Ontario|658[0n]|BOLD:AAA6177
Phragmatobia fuliginosa|[1569]|XAB480-04|04HBL005480|Canada|Ontario|658[0n]|BOLD:AAA6177
Phragmatobia fuliginosa|[1570]|RDNMB305-05|CNCNoctuoidea10071|United States|Washington|658[0n]|BO...
Phragmatobia fuliginosa|[1571]|RDMAB319-05|UASM77802|United States|Illinois|553[1n]|BOLD:AAA6177
Phragmatobia fuliginosa|[1572]|RDLQ107-05|DH004615|Canada|Quebec|609[0n]|BOLD:AAA6177
Phragmatobia fuliginosa|[1573]|XAG444-05|2005-ONT-1028|Canada|Ontario|658[0n]|BOLD:AAA6177
Phragmatobia fuliginosa|[1574]|TMNBB020-06|MNBTT-960|Canada|New Brunswick|612[0n]|BOLD:AAA6177
Phragmatobia fuliginosa rubricosa|[1575]|RDLQ105-05|DH006364|Canada|Quebec|609[0n]|BOLD:AAA6177
Phragmatobia fuliginosa|[1576]|RDNM109-05|CNCNoctuoidea6659|Canada|Ontario|658[0n]|BOLD:AAA6177
Phragmatobia fuliginosa|[1577]|XAB481-04|04HBL005481|Canada|Ontario|658[0n]|BOLD:AAA6177
Phragmatobia fuliginosa|[1578]|CNEII643-13|BIOUG04567-A09|Canada|Alberta|595[0n]|BOLD:AAA6177
Phragmatobia fuliginosa rubricosa|[1579]|RDLQF686-06|DH011836|Canada|Quebec|601[0n]|BOLD:AAA6177
Phragmatobia fuliginosa|[1580]|TMNBB019-06|MNBTT-959|Canada|New Brunswick|596[0n]|BOLD:AAA6177
Phragmatobia fuliginosa|[1581]|PMG015-03|PHRA1.01|Canada|Ontario|617[0n]|BOLD:AAA6177
Estigmene albida|[1582]|DMAZ252-10|BIOUG00716-D08|United States|Arizona|658[0n]|BOLD:AAB1407
Estigmene albida|[1583]|JBAZ109-09|JLB-0109|United States|Arizona|658[0n]|BOLD:AAB1407
Estigmene albida|[1584]|RDMAB931-06|BCSC398|United States|Arizona|658[0n]|BOLD:AAB1407
Estigmene albida|[1585]|CMAZA549-10|CMAZ-0549|United States|Arizona|658[0n]|BOLD:AAB1407
Estigmene albida|[1586]|QUNOD017-10|7016-COI-09|United States|Texas|658[0n]|BOLD:AAB1407
Estigmene albida|[1587]|CMAZA550-10|CMAZ-0550|United States|Arizona|658[0n]|BOLD:AAB1407
Estigmene albida|[1588]|RDNMF981-08|CNC LEP00054248|United States|Colorado|658[0n]|BOLD:AAB1407
Estigmene acrea|[1589]|XAB241-04|04HBL005241|Canada|Ontario|573[0n]|BOLD:AAB1406
Estigmene acrea|[1590]|XAG234-05|2005-ONT-818|Canada|Ontario|658[0n]|BOLD:AAB1406
Estigmene acrea|[1591]|RDLQ757-07|DH011386|Canada|Quebec|658[0n]|BOLD:AAB1406
Estigmene acrea|[1592]|UDLEP094-09|v732 UPM|United States|Maryland|610[0n]|BOLD:AAB1406
Estigmene acrea|[1593]|XAB069-04|04HBL005069|Canada|Ontario|624[1n]|BOLD:AAB1406
[illegible]

Estigmene acrea|[1591]|RDLQ757-07|DH011386|Canada|Quebec|658[0n]|BOLD:AAB1406
Estigmene acrea|[1592]|UDLEP094-09|v732 UPM|United States|Maryland|610[0n]|BOLD:AAB1406
Estigmene acrea|[1593]|XAB069-04|04HBL005069|Canada|Ontario|624[1n]|BOLD:AAB1406
Estigmene acrea|[1594]|XAB359-04|04HBL005359|Canada|Ontario|658[0n]|BOLD:AAB1406
Estigmene acrea|[1595]|LPSOC129-08|PPBP-2128|Canada|Ontario|658[0n]|BOLD:AAB1406
Estigmene acrea|[1596]|RDNME307-07|CNCNoctuoidea13914|United States|Oregon|658[0n]|BOLD:AAB1406
Estigmene acrea|[1597]|LPMN169-08|08BBLEP-00968|Canada|Manitoba|658[0n]|BOLD:AAB1406
Estigmene acrea|[1598]|XAK174-06|2006-ONT-1169|Canada|Ontario|658[0n]|BOLD:AAB1406
Estigmene acrea|[1599]|XAK147-06|2006-ONT-1142|Canada|Ontario|658[0n]|BOLD:AAB1406
Estigmene acrea|[1600]|XAJ463-06|2006-ONT-0463|Canada|Ontario|658[0n]|BOLD:AAB1406
Estigmene acrea|[1601]|LPSOC027-08|PPBP-2026|Canada|Ontario|658[0n]|BOLD:AAB1406
Estigmene acrea|[1602]|LPSOC130-08|PPBP-2129|Canada|Ontario|658[0n]|BOLD:AAB1406
Estigmene acrea|[1603]|XAF697-05|2005-ONT-346|Canada|Ontario|658[0n]|BOLD:AAB1406
Estigmene acrea|[1604]|XAJ314-06|2006-ONT-0314|Canada|Ontario|658[0n]|BOLD:AAB1406
Estigmene acrea|[1605]|XAG248-05|2005-ONT-832|Canada|Ontario|658[0n]|BOLD:AAB1406
Estigmene acrea|[1606]|TZBCA172-06|OMAFRA06-107|Canada|Ontario|658[0n]|BOLD:AAB1406
Estigmene acrea|[1607]|XAB001-04|04HBL005001|Canada|Ontario|658[0n]|BOLD:AAB1406
Estigmene acrea|[1608]|XAJ531-06|2006-ONT-0531|Canada|Ontario|658[0n]|BOLD:AAB1406
Estigmene acrea|[1609]|RDLQB842-05|DH010929|Canada|Quebec|616[0n]|BOLD:AAB1406
Estigmene acrea|[1610]|TMNBD430-07|MNBTT-3231|Canada|New Brunswick|658[0n]|BOLD:AAB1406
Estigmene acrea|[1611]|DUNLP002-08|Dun-08-002|Canada|British Columbia|658[0n]|BOLD:AAB1406
Estigmene acrea|[1612]|LPVIC102-08|PFC-2006-2677|Canada|British Columbia|658[0n]|BOLD:AAB1406
Estigmene acrea|[1613]|LPVIC100-08|PFC-2006-2675|Canada|British Columbia|658[0n]|BOLD:AAB1406
Estigmene acrea|[1614]|RDMAB928-06|UASM57426|Canada|Alberta|658[0n]|BOLD:AAB1406
Estigmene acrea|[1615]|DMAZ187-10|DMLEP-0104|United States|Arizona|658[0n]|BOLD:AAB1406
Estigmene acrea|[1616]|LILLA989-11|SNS10IL-01210|United States|Illinois|658[0n]|BOLD:AAB1406
Estigmene acrea|[1617]|XAG664-05|2005-ONT-1248|Canada|Ontario|658[1n]|BOLD:AAB1406
Estigmene acrea|[1618]|XAJ532-06|2006-ONT-0532|Canada|Ontario|658[0n]|BOLD:AAB1406
Estigmene acrea|[1619]|LPMN246-08|08BBLEP-01045|Canada|Manitoba|658[0n]|BOLD:AAB1406
Estigmene acrea|[1620]|BBLSY924-09|09BBLEP-03851|United States|Texas|658[0n]|BOLD:AAB1406
Estigmene acrea|[1621]|JBAZ108-09|JLB-0108|United States|Arizona|658[0n]|BOLD:AAB1406
Estigmene acrea|[1622]|LPOKB134-09|MDOK-1258|United States|Oklahoma|658[0n]|BOLD:AAB1406
Estigmene acrea|[1623]|USLEP1190-10|10BBLEP-01190|United States|Texas|658[0n]|BOLD:AAB1406
Estigmene acrea|[1624]|BBLSW028-09|09BBLEP-00956|United States|Texas|658[0n]|BOLD:AAB1406
Estigmene acrea|[1625]|BBLSW710-09|09BBLEP-01638|United States|Texas|658[0n]|BOLD:AAB1406
Estigmene acrea|[1626]|BBLSW709-09|09BBLEP-01637|United States|Texas|658[0n]|BOLD:AAB1406
Spilosoma virginica|[1627]|MNBB486-05|05-NBSTA-402|Canada|New Brunswick|658[0n]|BOLD:AAA3348
Spilosoma virginica|[1628]|USLEP761-10|10BBLEP-00761|United States|Florida|658[0n]|BOLD:ACE7664
Spilosoma virginica|[1629]|LOFLB520-06|06-FLOR-1460|United States|Florida|655[0n]|BOLD:ACE7664
Spilosoma virginica|[1630]|LOFLC192-06|06-FLOR-2072|United States|Florida|658[0n]|BOLD:ACE7664
Spilosoma virginica|[1631]|LGSMB297-05|DNA-ATBI-1146|United States|Tennessee|590[0n]|BOLD:ACE7664
Spilosoma virginica|[1632]|PHJUN4075-12|BIOUG02430-G06|Canada|Ontario|610[0n]|BOLD:ACE7664
Spilosoma virginica|[1633]|LPSO243-08|PPBP-0243|Canada|Ontario|658[0n]|BOLD:ACE7664
Spilosoma virginica|[1634]|MECD371-06|jflandry2943|United States|Maryland|656[0n]|BOLD:ACE7664
Spilosoma virginica|[1635]|LMEM945-09|RBMIS-1040|United States|Mississippi|658[0n]|BOLD:ACE7664
Spilosoma virginica|[1636]|LSUSA011-06|06-SUSA-0011|United States|Kentucky|657[0n]|BOLD:ACE7664
Spilosoma virginica|[1637]|UDLEP118-09|v781 LPC|United States|Maryland|658[0n]|BOLD:ACE7664
Spilosoma virginica|[1638]|LSEU633-06|06-JKA-0633|United States|Georgia|658[0n]|BOLD:ACE7664
Spilosoma virginica|[1639]|LNCB516-07|07-NCNW-0200|United States|North Carolina|658[0n]|BOLD:A...
Spilosoma virginica|[1640]|LNC268-05|05-NCCC-268|United States|North Carolina|658[0n]|BOLD:ACE...
Spilosoma virginica|[1641]|LPSO012-08|PPBP-0012|Canada|Ontario|658[0n]|BOLD:ACE7664
Spilosoma virginica|[1642]|LPSO011-08|PPBP-0011|Canada|Ontario|658[0n]|BOLD:ACE7664
Spilosoma virginica|[1643]|UDLEP217-09|v161 MD|United States|Delaware|658[0n]|BOLD:ACE7664
Spilosoma virginica|[1644]|UDLEP143-09|v831 Mill|United States|Pennsylvania|658[0n]|BOLD:ACE7664
Spilosoma virginica|[1645]|PHJUN4074-12|BIOUG02430-G05|Canada|Ontario|632[0n]|BOLD:ACE7664
Spilosoma virginica|[1646]|UDLEP142-09|v829 Mill|United States|Pennsylvania|658[0n]|BOLD:ACE7664
Spilosoma virginica|[1647]|MECD370-06|jflandry2942|United States|Maryland|658[0n]|BOLD:ACE7664
Spilosoma virginica|[1648]|LNC267-05|05-NCCC-267|United States|North Carolina|658[0n]|BOLD:ACE...
Spilosoma virginica|[1649]|LILLA794-11|SNS10IL-00998|United States|Illinois|658[0n]|BOLD:ACE7664
Spilosoma virginica|[1650]|LPOKB147-09|MDOK-1313|United States|Oklahoma|658[0n]|BOLD:ACE7664
Spilosoma virginica|[1651]|USLEP663-10|10BBLEP-00663|United States|Arkansas|658[0n]|BOLD:ACE7664
Spilosoma virginica|[1652]|LPVIA201-08|PFC-2006-0282|Canada|British Columbia|513[0n]|BOLD:AAA3347
Spilosoma virginica|[1653]|BLTIB176-08|BL255|Canada|Ontario|658[0n]|BOLD:AAA3347
Spilosoma|[1654]|CNCLB2365-14|CNCLEP 00119509|Mexico|Chiapas|658[0n]|BOLD:AAA3347
Spilosoma|[1655]|CNCLB2363-14|CNCLEP 00119507|Mexico|Chiapas|658[0n]|BOLD:AAA3347
Spilosoma virginica|[1656]|LPMN459-08|08BBLEP-01258|Canada|Manitoba|658[0n]|BOLD:AAA3347
Spilosoma virginica|[1657]|RDNME170-07|CNCNoctuoidea13698|Canada|Alberta|638[0n]|BOLD:AAA3347
Spilosoma virginica|[1658]|TTMNB544-06|MNBTT-544|Canada|New Brunswick|658[0n]|BOLD:AAA3347
Spilosoma virginica|[1659]|RDLQ763-07|DH004867|Canada|Quebec|658[0n]|BOLD:AAA3347
Spilosoma virginica|[1660]|XAE468-04|Moth4468.03|Canada|Ontario|658[0n]|BOLD:AAA3347
Spilosoma virginica|[1661]|XAK450-06|2006-ONT-1445|Canada|Ontario|626[0n]|BOLD:AAA3347
Spilosoma virginica|[1662]|XAD608-05|2005-ONT-23|Canada|Ontario|658[0n]|BOLD:AAA3347
Spilosoma virginica|[1663]|MNBB329-05|05-NBSTA-245|Canada|New Brunswick|658[0n]|BOLD:AAA3347
Spilosoma virginica|[1664]|PHMNB233-04|04HBL007698|Canada|New Brunswick|658[0n]|BOLD:AAA3347
Spilosoma virginica|[1665]|BBLPC250-09|09BBELE-1250|Canada|Nova Scotia|658[0n]|BOLD:AAA3347
Spilosoma virginica|[1666]|LPSK109-08|08BBLEP-01677|Canada|Saskatchewan|658[0n]|BOLD:AAA3347
Spilosoma virginica|[1667]|LPMN820-08|08BBLEP-01623|Canada|Manitoba|658[0n]|BOLD:AAA3347
Spilosoma virginica|[1668]|LMDH123-11|BIOUG01047-B01|United States|Minnesota|658[0n]|BOLD:AAA3347
Spilosoma virginica|[1669]|RDNME171-07|CNCNoctuoidea13699|Canada|Alberta|638[1n]|BOLD:AAA3347
Spilosoma virginica|[1670]|TMG78-03|moth355.01|Canada|Ontario|639[0n]|BOLD:AAA3347
Spilosoma virginica|[1671]|LHLEP054-06|UBC-2006-0255|Canada|British Columbia|589[0n]|BOLD:AAA3347
Spilosoma virginica|[1672]|RWWA420-09|RWWA-0420|United States|Washington|658[0n]|BOLD:AAA3347
Spilosoma virginica|[1673]|LHLEP052-06|UBC-2006-0253|Canada|British Columbia|658[0n]|BOLD:AAA3347
Spilosoma virginica|[1674]|LPVIB910-08|PFC-2006-2439|Canada|British Columbia|645[0n]|BOLD:AAA3347
Spilosoma virginica|[1675]|LHLEP056-06|UBC-2006-0257|Canada|British Columbia|658[0n]|BOLD:AAA3347
Spilosoma virginica|[1676]|LMH003-06|PFC-2006-0005|Canada|British Columbia|635[0n]|BOLD:AAA3347
Spilosoma virginica|[1677]|LPMN962-08|08BBLEP-02320|Canada|Alberta|658[0n]|BOLD:AAA3347
Spilosoma virginica|[1678]|LBCA241-05|HLC-20241|Canada|British Columbia|658[0n]|BOLD:AAA3347
Spilosoma virginica|[1679]|LPMN925-08|08BBLEP-02283|Canada|Alberta|654[0n]|BOLD:AAA3347
Spilosoma virginica|[1680]|BBLPA373-10|10BBCLP-0373|Canada|British Columbia|658[0n]|BOLD:AAA3347
Spilosoma virginica|[1681]|LBCB145-05|HLC-21085|Canada|British Columbia|658[0n]|BOLD:AAA3347
Spilosoma virginica|[1682]|LBCB143-05|HLC-21083|Canada|British Columbia|658[0n]|BOLD:AAA3347
Spilosoma virginica|[1683]|LBCB142-05|HLC-21082|Canada|British Columbia|658[0n]|BOLD:AAA3347
Spilosoma virginica|[1684]|BBLPA372-10|10BBCLP-0372|Canada|British Columbia|658[0n]|BOLD:AAA3347
Spilosoma virginica|[1685]|RDNME183-07|CNCNoctuoidea13711|Canada|British Columbia|642[0n]|BOLD ...
Spilosoma virginica|[1686]|LOWCB547-05|CGWC-1487|Canada|British Columbia|582[0n]|BOLD:AAA3347
Spilosoma virginica|[1687]|LOWCE191-06|CGWC-3951|Canada|British Columbia|658[0n]|BOLD:AAA3347
Spilosoma virginica|[1688]|LBCB630-05|HLC-21570|Canada|British Columbia|658[0n]|BOLD:AAA3347
Spilosoma virginica|[1689]|LBCB144-05|HLC-21084|Canada|British Columbia|658[0n]|BOLD:AAA3347
Spilosoma virginica|[1690]|LBCA545-05|HLC-20545|Canada|British Columbia|658[0n]|BOLD:AAA3347
Spilosoma virginica|[1691]|LBCA494-05|HLC-20494|Canada|British Columbia|658[0n]|BOLD:AAA3347
Spilosoma virginica|[1692]|LPVIB911-08|PFC-2006-2441|Canada|British Columbia|647[0n]|BOLD:AAA3347
Spilosoma virginica|[1693]|LPVIB912-08|PFC-2006-2442|Canada|British Columbia|646[0n]|BOLD:AAA3347

Spilosoma virginica|[1691]|LBCA494-05|HLC-20494|Canada|British Columbia|658[0n]|BOLD:AAA3347
Spilosoma virginica|[1692]|LPVIB911-08|PFC-2006-2441|Canada|British Columbia|647[0n]|BOLD:AAA3347
Spilosoma virginica|[1693]|LPVIB912-08|PFC-2006-2442|Canada|British Columbia|646[0n]|BOLD:AAA3347
Spilosoma virginica|[1694]|LPVIA200-08|PFC-2006-0274|Canada|British Columbia|658[0n]|BOLD:AAA3347
Spilosoma virginica|[1695]|LPVIA202-08|PFC-2006-0283|Canada|British Columbia|658[0n]|BOLD:AAA3347
Spilosoma virginica|[1696]|LPVIA264-08|PFC-2006-0350|Canada|British Columbia|658[0n]|BOLD:AAA3347
Spilosoma virginica|[1697]|LPSK481-08|08BBLEP-02049|Canada|Saskatchewan|658[0n]|BOLD:AAA3347
Spilosoma virginica|[1698]|LBCS030-07|UBC-2007-0053|Canada|British Columbia|658[0n]|BOLD:AAA3347
Spilosoma virginica|[1699]|RWWB265-09|RWWA-1264|United States|Washington|658[0n]|BOLD:AAA3347
Spilosoma virginica|[1700]|LBCS443-07|UBC-2007-0199|Canada|British Columbia|658[0n]|BOLD:AAA3347
Spilosoma virginica|[1701]|LALPA109-10|AVBC 109-10|Canada|British Columbia|658[0n]|BOLD:AAA3347
Spilosoma virginica|[1702]|LALPA110-10|AVBC 110-10|Canada|British Columbia|658[0n]|BOLD:AAA3347
Spilosoma virginica|[1703]|RWWA155-09|RWWA-0155|United States|Washington|658[0n]|BOLD:AAA3347
Spilosoma virginica|[1704]|LPVIA265-08|PFC-2006-0351|Canada|British Columbia|658[0n]|BOLD:AAA3347
Spilosoma virginica|[1705]|LHLEP053-06|UBC-2006-0254|Canada|British Columbia|658[0n]|BOLD:AAA3347
Spilosoma virginica|[1706]|LHLEP055-06|UBC-2006-0256|Canada|British Columbia|658[0n]|BOLD:AAA3347
Spilosoma virginica|[1707]|RWWA021-09|RWWA-0021|United States|Washington|658[0n]|BOLD:AAA3347
Spilosoma virginica|[1708]|LBCS444-07|UBC-2007-0200|Canada|British Columbia|658[0n]|BOLD:AAA3347
Spilosoma virginica|[1709]|LPGVA733-08|UBC-2006-2076|Canada|British Columbia|658[0n]|BOLD:AAA3347
Spilosoma virginica|[1710]|LPMN309-08|08BBLEP-01108|Canada|Manitoba|658[0n]|BOLD:AAA3347
Spilosoma virginica|[1711]|RWWC217-11|RWWA-2194|United States|Washington|658[0n]|BOLD:AAA3347
Spilosoma virginica|[1712]|LMH028-06|PFC-2006-0149|Canada|British Columbia|658[0n]|BOLD:AAA3347
Spilosoma virginica|[1713]|LBCA544-05|HLC-20544|Canada|British Columbia|658[0n]|BOLD:AAA3347
Spilosoma virginica|[1714]|LBCA493-05|HLC-20493|Canada|British Columbia|658[0n]|BOLD:AAA3347
Spilosoma virginica|[1715]|UDLEP327-09|v365 Amb|United States|Pennsylvania|658[0n]|BOLD:AAA3347
Spilosoma virginica|[1716]|XAE444-04|Moth4444.03|Canada|Ontario|658[0n]|BOLD:AAA3347
Spilosoma virginica|[1717]|PHMNB540-04|04HBL00766|Canada|New Brunswick|658[1n]|BOLD:AAA3347
Spilosoma virginica|[1718]|TTMNB254-06|MNBTT-254|Canada|New Brunswick|658[0n]|BOLD:AAA3347
Spilosoma virginica|[1719]|TMNBD396-07|MNBTT-3197|Canada|New Brunswick|649[0n]|BOLD:AAA3347
Spilosoma virginica|[1720]|MNBB502-05|05-NBSTA-418|Canada|New Brunswick|658[0n]|BOLD:AAA3347
Spilosoma virginica|[1721]|MNBB281-05|05-NBSTA-197|Canada|New Brunswick|658[0n]|BOLD:AAA3347
Spilosoma virginica|[1722]|XAE467-04|Moth4467.03|Canada|Ontario|658[0n]|BOLD:AAA3347
Spilosoma virginica|[1723]|PHMNB436-04|04HBL00662|Canada|New Brunswick|658[0n]|BOLD:AAA3347
Spilosoma virginica|[1724]|PHMNB529-04|04HBL00755|Canada|New Brunswick|658[0n]|BOLD:AAA3347
Spilosoma virginica|[1725]|XAF648-05|2005-ONT-297|Canada|Ontario|658[0n]|BOLD:AAA3347
Spilosoma virginica|[1726]|XAG710-05|2005-ONT-1294|Canada|Ontario|658[0n]|BOLD:AAA3347
Spilosoma virginica|[1727]|LPMN598-08|08BBLEP-01399|Canada|Manitoba|658[0n]|BOLD:AAA3347
Spilosoma virginica|[1728]|UDLEP245-09|v200 CV|United States|Delaware|658[0n]|BOLD:AAA3347
Spilosoma virginica|[1729]|LPSO240-08|PPBP-0240|Canada|Ontario|658[0n]|BOLD:AAA3347
Spilosoma virginica|[1730]|LPSOD641-09|08BBLEP-00422|Canada|Ontario|658[0n]|BOLD:AAA3347
Spilosoma virginica|[1731]|BLTIB093-08|BL0150|Canada|Ontario|658[0n]|BOLD:AAA3347
Spilosoma virginica|[1732]|BBLPA374-10|10BBCLP-0374|Canada|Saskatchewan|658[0n]|BOLD:AAA3347
Spilosoma virginica|[1733]|PHMNB726-05|Moth 419.03SA|Canada|New Brunswick|658[0n]|BOLD:AAA3347
Spilosoma virginica|[1734]|TMNBD391-07|MNBTT-3192|Canada|New Brunswick|658[0n]|BOLD:AAA3347
Spilosoma virginica|[1735]|TMNBD397-07|MNBTT-3198|Canada|New Brunswick|658[0n]|BOLD:AAA3347
Spilosoma virginica|[1736]|TMNBD398-07|MNBTT-3199|Canada|New Brunswick|658[0n]|BOLD:AAA3347
Spilosoma virginica|[1737]|LOCT223-05|05-CTATBI-0223|United States|Connecticut|658[0n]|BOLD:AA...
Spilosoma virginica|[1738]|XAK172-06|2006-ONT-1167|Canada|Ontario|658[0n]|BOLD:AAA3347
Spilosoma virginica|[1739]|LPSOB713-08|PPBP-1712|Canada|Ontario|658[0n]|BOLD:AAA3347
Spilosoma virginica|[1740]|XAK346-06|2006-ONT-1341|Canada|Ontario|658[0n]|BOLD:AAA3347
Spilosoma virginica|[1741]|XAD701-05|2005-ONT-500|Canada|Ontario|658[0n]|BOLD:AAA3347
Spilosoma virginica|[1742]|XAD702-05|2005-ONT-501|Canada|Ontario|658[0n]|BOLD:AAA3347
Spilosoma virginica|[1743]|XAD703-05|2005-ONT-502|Canada|Ontario|658[0n]|BOLD:AAA3347
Spilosoma virginica|[1744]|XAG230-05|2005-ONT-814|Canada|Ontario|658[0n]|BOLD:AAA3347
Spilosoma virginica|[1745]|XAB125-04|04HBL005125|Canada|Ontario|658[0n]|BOLD:AAA3347
Spilosoma virginica|[1746]|XAB141-04|04HBL005141|Canada|Ontario|658[0n]|BOLD:AAA3347
Spilosoma virginica|[1747]|XAB154-04|04HBL005154|Canada|Ontario|658[0n]|BOLD:AAA3347
Spilosoma virginica|[1748]|LPSO475-08|PPBP-0475|Canada|Ontario|656[0n]|BOLD:AAA3347
Spilosoma virginica|[1749]|TZBCA314-07|234-211|Canada|Ontario|656[0n]|BOLD:AAA3347
Spilosoma virginica|[1750]|XAK171-06|2006-ONT-1166|Canada|Ontario|658[0n]|BOLD:AAA3347
Spilosoma virginica|[1751]|XAB140-04|04HBL005140|Canada|Ontario|658[0n]|BOLD:AAA3347
Spilosoma virginica|[1752]|LPSO354-08|PPBP-0354|Canada|Ontario|658[0n]|BOLD:AAA3347
Spilosoma virginica|[1753]|TZBCA171-06|OMAFRA06-106|Canada|Ontario|622[0n]|BOLD:AAA3347
Spilosoma virginica|[1754]|BBLCU267-09|09BBLEP-04754|United States|Illinois|623[0n]|BOLD:AAA3347
Spilosoma virginica|[1755]|PHMNB057-03|moth34.02SA|Canada|New Brunswick|639[0n]|BOLD:AAA3347
Spilosoma virginica|[1756]|PHMNB043-03|moth219.02SA|Canada|New Brunswick|639[0n]|BOLD:AAA3347
Spilosoma virginica|[1757]|TMG80-03|moth357.01|Canada|Ontario|639[0n]|BOLD:AAA3347
Spilosoma virginica|[1758]|TMG79-03|moth547.01|Canada|Ontario|639[0n]|BOLD:AAA3347
Spilosoma virginica|[1759]|TMNBD394-07|MNBTT-3195|Canada|New Brunswick|646[0n]|BOLD:AAA3347
Spilosoma virginica|[1760]|PMG018-03|moth373.01|Canada|Ontario|617[0n]|BOLD:AAA3347
Hyphantria cunea|[1761]|CNROJ039-13|BIOUG08614-G03|Canada|Ontario|537[1n]|BOLD:AAA2436
Hyphantria cunea|[1762]|CNROT015-13|BIOUG09600-C12|Canada|Ontario|606[0n]|BOLD:AAA2436
Hyphantria cunea|[1763]|CNROJ005-13|BIOUG06036-H11|Canada|Ontario|550[0n]|BOLD:AAA2436
Hyphantria cunea|[1764]|GMGSU038-13|BIOUG04567-B03|United States|Tennessee|561[0n]|BOLD:AAA2436
Hyphantria cunea|[1765]|CNGBH029-14|BIOUG10208-G10|Canada|Ontario|576[1n]|BOLD:AAA2436
Hyphantria cunea|[1766]|CNPPI1867-12|BIOUG03567-C12|Canada|Ontario|612[0n]|BOLD:AAA2436
Hyphantria cunea|[1767]|CNROJ038-13|BIOUG08614-G02|Canada|Ontario|603[0n]|BOLD:AAA2436
Hyphantria cunea|[1768]|CNPPI1863-12|BIOUG03567-C08|Canada|Ontario|618[0n]|BOLD:AAA2436
Hyphantria cunea|[1769]|CNPPI1887-12|BIOUG03567-E08|Canada|Ontario|618[0n]|BOLD:AAA2436
Hyphantria cunea|[1770]|CNROT012-13|BIOUG09600-C09|Canada|Ontario|606[0n]|BOLD:AAA2436
Hyphantria cunea|[1771]|CNROT013-13|BIOUG09600-C10|Canada|Ontario|606[0n]|BOLD:AAA2436
Hyphantria cunea|[1772]|CNROT014-13|BIOUG09600-C11|Canada|Ontario|606[0n]|BOLD:AAA2436
Hyphantria cunea|[1773]|CNROT016-13|BIOUG09600-D01|Canada|Ontario|606[0n]|BOLD:AAA2436
Hyphantria cunea|[1774]|CNGBH032-14|BIOUG10208-H01|Canada|Ontario|591[0n]|BOLD:AAA2436
Hyphantria cunea|[1775]|CNPPI1871-12|BIOUG03567-D04|Canada|Ontario|605[0n]|BOLD:AAA2436
Hyphantria cunea|[1776]|UDLEP141-09|v828 Mill|United States|Pennsylvania|658[0n]|BOLD:AAA2436
Hyphantria cunea|[1777]|CNPPG919-12|BIOUG03567-B08|Canada|Ontario|620[0n]|BOLD:AAA2436
Hyphantria cunea|[1778]|CNPPI1862-12|BIOUG03567-C07|Canada|Ontario|620[0n]|BOLD:AAA2436
Hyphantria cunea|[1779]|CNPPI1883-12|BIOUG03567-E04|Canada|Ontario|620[0n]|BOLD:AAA2436
Hyphantria cunea|[1780]|CNPPH1202-12|BIOUG03567-B12|Canada|Ontario|621[0n]|BOLD:AAA2436
Hyphantria cunea|[1781]|CNPPI1870-12|BIOUG03567-D03|Canada|Ontario|624[0n]|BOLD:AAA2436
Hyphantria cunea|[1782]|CNPPI1882-12|BIOUG03567-E03|Canada|Ontario|624[0n]|BOLD:AAA2436
Hyphantria cunea|[1783]|CNPPI1895-12|BIOUG03567-F04|Canada|Ontario|624[0n]|BOLD:AAA2436
Hyphantria cunea|[1784]|CNPPI1886-12|BIOUG03567-E07|Canada|Ontario|620[0n]|BOLD:AAA2436
Hyphantria cunea|[1785]|CNPPH1199-12|BIOUG03567-B09|Canada|Ontario|630[0n]|BOLD:AAA2436
Hyphantria cunea|[1786]|CNPPI1872-12|BIOUG03567-D05|Canada|Ontario|627[0n]|BOLD:AAA2436
Hyphantria cunea|[1787]|CNPPI1894-12|BIOUG03567-F03|Canada|Ontario|626[0n]|BOLD:AAA2436
Hyphantria cunea|[1788]|CNPPH1203-12|BIOUG03567-C01|Canada|Ontario|627[0n]|BOLD:AAA2436
Hyphantria cunea|[1789]|CNPPH1201-12|BIOUG03567-B11|Canada|Ontario|627[0n]|BOLD:AAA2436
Hyphantria cunea|[1790]|CNPPH1200-12|BIOUG03567-B10|Canada|Ontario|627[0n]|BOLD:AAA2436
Hyphantria cunea|[1791]|CNPPG918-12|BIOUG03567-B07|Canada|Ontario|630[0n]|BOLD:AAA2436
Hyphantria cunea|[1792]|CNPPI1880-12|BIOUG03567-E01|Canada|Ontario|630[0n]|BOLD:AAA2436
Hyphantria cunea|[1793]|CNPPI1866-12|BIOUG03567-C11|Canada|Ontario|630[0n]|BOLD:AAA2436

[illegible]
Hyphantria cunea|[1791]|CNPPG918-12|BIOUG03567-B07|Canada|Ontario|630[0n]|BOLD:AAA2436
Hyphantria cunea|[1792]|CNPPI1880-12|BIOUG03567-E01|Canada|Ontario|630[0n]|BOLD:AAA2436
Hyphantria cunea|[1793]|CNPPI1866-12|BIOUG03567-C11|Canada|Ontario|630[0n]|BOLD:AAA2436
Hyphantria cunea|[1794]|CNPPI1869-12|BIOUG03567-D02|Canada|Ontario|630[0n]|BOLD:AAA2436
Hyphantria cunea|[1795]|CNPPI1868-12|BIOUG03567-D01|Canada|Ontario|630[0n]|BOLD:AAA2436
Hyphantria cunea|[1796]|CNPPI1865-12|BIOUG03567-C10|Canada|Ontario|630[0n]|BOLD:AAA2436
Hyphantria cunea|[1797]|CNPPI1864-12|BIOUG03567-C09|Canada|Ontario|630[0n]|BOLD:AAA2436
Hyphantria cunea|[1798]|CNPPH1207-12|BIOUG03567-C05|Canada|Ontario|625[0n]|BOLD:AAA2436
Hyphantria cunea|[1799]|CNPPH1204-12|BIOUG03567-C02|Canada|Ontario|630[0n]|BOLD:AAA2436
Hyphantria cunea|[1800]|CNPPI1897-12|BIOUG03567-F06|Canada|Ontario|630[0n]|BOLD:AAA2436
Hyphantria cunea|[1801]|CNPPI1890-12|BIOUG03567-E11|Canada|Ontario|630[0n]|BOLD:AAA2436
Hyphantria cunea|[1802]|CNPPI1874-12|BIOUG03567-D07|Canada|Ontario|630[0n]|BOLD:AAA2436
Hyphantria cunea|[1803]|CNPPI1873-12|BIOUG03567-D06|Canada|Ontario|630[0n]|BOLD:AAA2436
Hyphantria cunea|[1804]|CNPPI1876-12|BIOUG03567-D09|Canada|Ontario|630[0n]|BOLD:AAA2436
Hyphantria cunea|[1805]|CNPPI1875-12|BIOUG03567-D08|Canada|Ontario|627[0n]|BOLD:AAA2436
Hyphantria cunea|[1806]|CNPPI1877-12|BIOUG03567-D10|Canada|Ontario|630[0n]|BOLD:AAA2436
Hyphantria cunea|[1807]|CNPPI1878-12|BIOUG03567-D11|Canada|Ontario|630[0n]|BOLD:AAA2436
Hyphantria cunea|[1808]|CNPPI1879-12|BIOUG03567-D12|Canada|Ontario|630[0n]|BOLD:AAA2436
Hyphantria cunea|[1809]|CNPPI1881-12|BIOUG03567-E02|Canada|Ontario|630[0n]|BOLD:AAA2436
Hyphantria cunea|[1810]|CNPPI1884-12|BIOUG03567-E05|Canada|Ontario|630[0n]|BOLD:AAA2436
Hyphantria cunea|[1811]|CNPPI1885-12|BIOUG03567-E06|Canada|Ontario|629[0n]|BOLD:AAA2436
Hyphantria cunea|[1812]|CNPPI1888-12|BIOUG03567-E09|Canada|Ontario|627[0n]|BOLD:AAA2436
Hyphantria cunea|[1813]|CNPPI1889-12|BIOUG03567-E10|Canada|Ontario|627[0n]|BOLD:AAA2436
Hyphantria cunea|[1814]|CNPPI1891-12|BIOUG03567-E12|Canada|Ontario|630[0n]|BOLD:AAA2436
Hyphantria cunea|[1815]|CNPPI1892-12|BIOUG03567-F01|Canada|Ontario|630[0n]|BOLD:AAA2436
Hyphantria cunea|[1816]|CNPPI1893-12|BIOUG03567-F02|Canada|Ontario|630[0n]|BOLD:AAA2436
Hyphantria cunea|[1817]|CNPPI1896-12|BIOUG03567-F05|Canada|Ontario|630[0n]|BOLD:AAA2436
Hyphantria cunea|[1818]|CNPPI1898-12|BIOUG03567-F07|Canada|Ontario|630[0n]|BOLD:AAA2436
Hyphantria cunea|[1819]|CNPPI1899-12|BIOUG03567-F08|Canada|Ontario|630[0n]|BOLD:AAA2436
Hyphantria cunea|[1820]|BBLPA630-10|10BBCLP-0630|Canada|Ontario|658[0n]|BOLD:AAA2436
Hyphantria cunea|[1821]|XAB628-04|04HBL005628|Canada|Ontario|658[0n]|BOLD:AAA2436
Hyphantria cunea|[1822]|TMNBD399-07|MNBTT-3200|Canada|New Brunswick|658[0n]|BOLD:AAA2436
Hyphantria cunea|[1823]|XAB167-04|04HBL005167|Canada|Ontario|658[0n]|BOLD:AAA2436
Hyphantria cunea|[1824]|CNPPH1205-12|BIOUG03567-C03|Canada|Ontario|625[0n]|BOLD:AAA2436
Hyphantria cunea|[1825]|PHMNB019-03|moth153.02SA|Canada|New Brunswick|639[0n]|BOLD:AAA2436
Hyphantria cunea|[1826]|PHMNB085-03|moth180.02SA|Canada|New Brunswick|639[4n]|BOLD:AAA2436
Hyphantria cunea|[1827]|RDNMG143-08|NOC14896|United States|New Mexico|658[0n]|BOLD:AAA2436
Hyphantria cunea|[1828]|BBLWU062-09|09BBLEP-04220|United States|Colorado|658[0n]|BOLD:AAA2436
Hyphantria cunea|[1829]|USLEP704-10|10BBLEP-00704|United States|Colorado|658[0n]|BOLD:AAA2436
Hyphantria cunea|[1830]|USLEP703-10|10BBLEP-00703|United States|Colorado|658[0n]|BOLD:AAA2436
Hyphantria cunea|[1831]|BBLSX716-09|09BBLEP-02644|United States|Arizona|658[0n]|BOLD:ACE3204
Hyphantria cunea|[1832]|RDNMG026-08|NOC14967|United States|Arizona|658[0n]|BOLD:ACE3204
Hyphantria cunea|[1833]|BBLSY421-09|09BBLEP-03348|United States|Arizona|658[0n]|BOLD:ACE3204
Hyphantria cunea|[1834]|BBLSX715-09|09BBLEP-02643|United States|Arizona|658[0n]|BOLD:ACE3204
Hyphantria cunea|[1835]|LOFLA192-06|06-FLOR-0192|United States|Florida|658[0n]|BOLD:ACE3204
Hyphantria cunea|[1836]|LOFLA756-06|06-FLOR-0756|United States|Florida|658[0n]|BOLD:ACE3204
Hyphantria cunea|[1837]|RDNMH933-09|CNCLEP00067932|United States|Florida|658[0n]|BOLD:ACE3204
Hyphantria cunea|[1838]|RDNMH934-09|CNCLEP00067933|United States|Florida|658[0n]|BOLD:ACE3204
Hyphantria cunea|[1839]|LPOKB425-09|MDOK-1413|United States|Oklahoma|658[0n]|BOLD:ACE3204
Hyphantria cunea|[1840]|BBLOD1191-11|BIOUG01824-C09|United States|Texas|658[0n]|BOLD:ACE3204
Hyphantria cunea|[1841]|RDNMG029-08|NOC14970|United States|Oklahoma|658[0n]|BOLD:ACE3204
Hyphantria cunea|[1842]|RDNMH936-09|CNCLEP00067935|Canada|Ontario|658[0n]|BOLD:ACE3204
Hyphantria cunea|[1843]|LMEM947-09|RBMIS-1042|United States|Alabama|658[0n]|BOLD:ACE3204
Hyphantria cunea|[1844]|LOFLA105-06|06-FLOR-0105|United States|Florida|658[0n]|BOLD:ACE3204
Hyphantria cunea|[1845]|RDNMH579-09|CNCLEP00062906|United States|Arizona|658[0n]|BOLD:ACE3204
Hyphantria cunea|[1846]|RDNMG025-08|NOC14966|United States|Arizona|658[0n]|BOLD:ACE3204
Hyphantria cunea|[1847]|CMAZA1069-12|BIOUG02042-D04|United States|Arizona|658[0n]|BOLD:ACE3204
Hyphantria cunea|[1848]|RDNMF923-08|CNC LEP00054190|United States|Arizona|658[0n]|BOLD:ACE3204
Hyphantria cunea|[1849]|RDNMF992-08|CNC LEP00054259|United States|Georgia|652[0n]|BOLD:ACE3204
Hyphantria cunea|[1850]|RDMAB372-05|UASM77838|Canada|Alberta|588[0n]|BOLD:ACE3204
Hyphantria cunea|[1851]|RDNMB426-05|CNCNoctuoidea10192|Canada|Saskatchewan|587[2n]|BOLD:ACE3204
Hyphantria cunea|[1852]|LHLEP289-06|UBC-2006-0163|Canada|British Columbia|649[0n]|BOLD:ACE3204
Hyphantria cunea|[1853]|RDNMB427-05|CNCNoctuoidea10193|Canada|British Columbia|658[0n]|BOLD:AC...
Hyphantria cunea|[1854]|LOPN145-06|JCM-OSU-0145|United States|Washington|658[0n]|BOLD:ACE3204
Hyphantria cunea|[1855]|LHLEP287-06|UBC-2006-0161|Canada|British Columbia|657[0n]|BOLD:ACE3204
Hyphantria cunea|[1856]|LHLEP288-06|UBC-2006-0162|Canada|British Columbia|657[0n]|BOLD:ACE3204
Hyphantria cunea|[1857]|RDMAB100-05|UASM41980|Canada|Alberta|658[0n]|BOLD:ACE3204
Hyphantria cunea|[1858]|RDMAB101-05|UASM41981|Canada|Alberta|658[0n]|BOLD:ACE3204
Hyphantria cunea|[1859]|RDMAB428-05|BCSC101|Canada|Alberta|658[0n]|BOLD:ACE3204
Hyphantria cunea|[1860]|LHLEP290-06|UBC-2006-0803|Canada|British Columbia|649[0n]|BOLD:ACE3204
Hyphantria cunea|[1861]|RDNMG027-08|NOC14968|United States|Nevada|658[0n]|BOLD:ACE3204
Hyphantria cunea|[1862]|RDNMB425-05|CNCNoctuoidea10191|United States|California|658[0n]|BOLD:A...
Hyphantria cunea|[1863]|LALPA258-10|AVBC 259-10|Canada|British Columbia|658[0n]|BOLD:ACE3204
Hyphantria cunea|[1864]|LALPA246-10|AVBC 247-10|Canada|British Columbia|658[0n]|BOLD:ACE3204
Hyphantria cunea|[1865]|LALPA163-10|AVBC 163-10|Canada|British Columbia|658[0n]|BOLD:ACE3204
Hyphantria cunea|[1866]|RWWC361-11|RWWA-2338|United States|Washington|658[0n]|BOLD:ACE3204
Hyphantria cunea|[1867]|CNBPQ467-13|BIOUG05875-E04|Canada|Ontario|552[0n]|BOLD:AAA2435
Hyphantria cunea|[1868]|RDNME753-08|LEP041218|United States|Georgia|658[0n]|BOLD:AAA2435
Hyphantria cunea|[1869]|CNPPF1471-12|BIOUG03567-A08|Canada|Ontario|606[0n]|BOLD:AAA2435
Hyphantria cunea|[1870]|CNPPF1481-12|BIOUG03567-B06|Canada|Ontario|614[0n]|BOLD:AAA2435
Hyphantria cunea|[1871]|CNPPF1476-12|BIOUG03567-B01|Canada|Ontario|618[0n]|BOLD:AAA2435
Hyphantria cunea|[1872]|CNPPF1480-12|BIOUG03567-B05|Canada|Ontario|624[0n]|BOLD:AAA2435
Hyphantria cunea|[1873]|CNPPJ1375-12|BIOUG03567-F10|Canada|Ontario|630[0n]|BOLD:AAA2435
Hyphantria cunea|[1874]|LPSOC390-08|PPBP-2389|Canada|Ontario|658[0n]|BOLD:AAA2435
Hyphantria cunea|[1875]|LPSOC389-08|PPBP-2388|Canada|Ontario|658[0n]|BOLD:AAA2435
Hyphantria cunea|[1876]|CNPPF1474-12|BIOUG03567-A11|Canada|Ontario|624[0n]|BOLD:AAA2435
Hyphantria cunea|[1877]|CNPPF1470-12|BIOUG03567-A07|Canada|Ontario|630[0n]|BOLD:AAA2435
Hyphantria cunea|[1878]|CNPPF1472-12|BIOUG03567-A09|Canada|Ontario|630[0n]|BOLD:AAA2435
Hyphantria cunea|[1879]|CNPPF1475-12|BIOUG03567-A12|Canada|Ontario|624[0n]|BOLD:AAA2435
Hyphantria cunea|[1880]|BLTIB175-08|BL254|Canada|Ontario|658[0n]|BOLD:AAA2435
Hyphantria cunea|[1881]|LPOKA1004-09|MDOK-1099|United States|Oklahoma|658[0n]|BOLD:AAA2435
Hyphantria cunea|[1882]|LPSO052-08|PPBP-0052|Canada|Ontario|658[0n]|BOLD:AAA2435
Hyphantria cunea|[1883]|RDNMH935-09|CNCLEP00067934|Canada|Ontario|658[0n]|BOLD:AAA2435
Hyphantria cunea|[1884]|XAF465-05|2005-ONT-114|Canada|Ontario|658[0n]|BOLD:AAA2435
Hyphantria cunea|[1885]|XAJ419-06|2006-ONT-0419|Canada|Ontario|658[0n]|BOLD:AAA2435
Hyphantria cunea|[1886]|XAB579-04|04HBL005579|Canada|Ontario|658[0n]|BOLD:AAA2435
Hyphantria cunea|[1887]|LMEM952-09|RBMIS-1047|United States|Mississippi|658[0n]|BOLD:AAA2435
Hyphantria cunea|[1888]|BBLSX951-09|09BBLEP-02879|United States|Oklahoma|658[0n]|BOLD:AAA2435
Hyphantria cunea|[1889]|RDNME754-08|LEP041219|United States|Georgia|658[0n]|BOLD:AAA2435
Hyphantria cunea|[1890]|LPOKA990-09|MDOK-1085|United States|Oklahoma|658[0n]|BOLD:AAA2435
Hyphantria cunea|[1891]|LPOKA968-09|MDOK-1063|United States|Oklahoma|658[0n]|BOLD:AAA2435
Hyphantria cunea|[1892]|CNPPJ1374-12|BIOUG03567-F09|Canada|Ontario|636[0n]|BOLD:AAA2435

Hyphantria cunea|[1890]|LPOKA990-09|MDOK-1085|United States|Oklahoma|658[0n]|BOLD:AAA2435
Hyphantria cunea|[1891]|LPOKA968-09|MDOK-1063|United States|Oklahoma|658[0n]|BOLD:AAA2435
Hyphantria cunea|[1892]|CNPPJ1374-12|BIOUG03567-F09|Canada|Ontario|636[0n]|BOLD:AAA2435
Hyphantria cunea|[1893]|PHMO369-03|moth432.02|Canada|Ontario|639[0n]|BOLD:AAA2435
Hyphantria cunea|[1894]|XAF466-05|2005-ONT-115|Canada|Ontario|658[0n]|BOLD:AAA2435
Hyphantria cunea|[1895]|CNPPF1469-12|BIOUG03567-A06|Canada|Ontario|630[0n]|BOLD:AAA2435
Hyphantria cunea|[1896]|CNPPF1473-12|BIOUG03567-A10|Canada|Ontario|630[0n]|BOLD:AAA2435
Hyphantria cunea|[1897]|RDNME747-08|LEP042294|Canada|Quebec|658[0n]|BOLD:AAA2435
Hyphantria cunea|[1898]|CNPPF1477-12|BIOUG03567-B02|Canada|Ontario|630[0n]|BOLD:AAA2435
Hyphantria cunea|[1899]|XAB570-04|04HBL005570|Canada|Ontario|658[0n]|BOLD:AAA2435
Hyphantria cunea|[1900]|XAB152-04|04HBL005152|Canada|Ontario|658[0n]|BOLD:AAA2435
Hyphantria cunea|[1901]|XAF561-05|2005-ONT-210|Canada|Ontario|658[0n]|BOLD:AAA2435
Hyphantria cunea|[1902]|XAF467-05|2005-ONT-116|Canada|Ontario|658[0n]|BOLD:AAA2435
Hyphantria cunea|[1903]|XAK112-06|2006-ONT-1107|Canada|Ontario|658[0n]|BOLD:AAA2435
Hyphantria cunea|[1904]|LPSOC049-08|PPBP-2048|Canada|Ontario|658[0n]|BOLD:AAA2435
Hyphantria cunea|[1905]|RDNMB423-05|CNCNoctuoidea10189|Canada|Ontario|658[0n]|BOLD:AAA2435
Hyphantria cunea|[1906]|RDMAB415-05|BCSC88|United States|Texas|600[0n]|BOLD:AAA2435
Hyphantria cunea|[1907]|MECD369-06|jflandry2941|United States|Maryland|658[0n]|BOLD:AAA2435
Hyphantria cunea|[1908]|RDNMG028-08|NOC14969|United States|Oklahoma|658[0n]|BOLD:AAA2435
Hyphantria cunea|[1909]|LPOKB293-09|MDOK-1302|United States|Oklahoma|658[0n]|BOLD:AAA2435
Hyphantria cunea|[1910]|LMEM954-09|RBMIS-1049|United States|Mississippi|658[0n]|BOLD:AAA2435
Hyphantria cunea|[1911]|LMEM951-09|RBMIS-1046|United States|Mississippi|658[0n]|BOLD:AAA2435
Hyphantria cunea|[1912]|LMEM950-09|RBMIS-1045|United States|Louisiana|658[0n]|BOLD:AAA2435
Hyphantria cunea|[1913]|LMEM949-09|RBMIS-1044|United States|Mississippi|658[0n]|BOLD:AAA2435
Hyphantria cunea|[1914]|LMEM948-09|RBMIS-1043|United States|Mississippi|658[0n]|BOLD:AAA2435
Hyphantria cunea|[1915]|LPOKA059-08|MDOK-0059|United States|Oklahoma|657[0n]|BOLD:AAA2435
Hyphantria cunea|[1916]|BBLSX915-09|09BBLEP-02843|United States|Arizona|658[0n]|BOLD:AAA2435
Hyphantria cunea|[1917]|LPOKA995-09|MDOK-1090|United States|Oklahoma|658[0n]|BOLD:AAA2435
Hyphantria cunea|[1918]|LPOKA966-09|MDOK-1061|United States|Oklahoma|658[0n]|BOLD:AAA2435
Hyphantria cunea|[1919]|RDNMJ003-10|CNCLEP 73783|United States|Florida|658[0n]|BOLD:AAA2435
Hyphantria cunea|[1920]|RDNMJ002-10|CNCLEP 73782|United States|Florida|658[0n]|BOLD:AAA2435
Hyphantria cunea|[1921]|LOFLA341-06|06-FLOR-0341|United States|Florida|658[0n]|BOLD:AAA2435
Hyphantria cunea|[1922]|RDLQF858-06|DH012019|Canada|Quebec|658[0n]|BOLD:AAA2435
Hyphantria cunea|[1923]|RDNMH123-09|CNCLEP00054342|United States|North Carolina|658[0n]|BOLD:A...
Hyphantria cunea|[1924]|XAE225-04|Moth4225.03|Canada|Ontario|658[0n]|BOLD:AAA2435
Hyphantria cunea|[1925]|PHMNB372-04|04HBL00598|Canada|New Brunswick|658[0n]|BOLD:AAA2435
Hyphantria cunea|[1926]|PHMNB474-04|04HBL00700|Canada|New Brunswick|658[0n]|BOLD:AAA2435
Hyphantria cunea|[1927]|UDLEP127-09|v805 UDE|United States|Delaware|655[0n]|BOLD:AAA2435
Hyphantria cunea|[1928]|LPSOD270-09|08BBLEP-00048|Canada|Ontario|658[0n]|BOLD:AAA2435
Hyphantria cunea|[1929]|LPSOD328-09|08BBLEP-00106|Canada|Ontario|658[0n]|BOLD:AAA2435
Hyphantria cunea|[1930]|XAE356-04|Moth4356.03|Canada|Ontario|658[0n]|BOLD:AAA2435
Hyphantria cunea|[1931]|RDNME745-08|LEP042292|Canada|New Brunswick|658[0n]|BOLD:AAA2435
Hyphantria cunea|[1932]|RDNME748-08|LEP042295|Canada|Quebec|658[0n]|BOLD:AAA2435
Hyphantria cunea|[1933]|RDNMJ800-11|CNCLEP 80328|Canada|Quebec|658[0n]|BOLD:AAA2435
Hyphantria cunea|[1934]|RDNMB424-05|CNCNoctuoidea10190|United States|Maryland|658[0n]|BOLD:AAA...
Hyphantria cunea|[1935]|LPSO400-08|PPBP-0400|Canada|Ontario|658[0n]|BOLD:AAA2435
Hyphantria cunea|[1936]|XAB265-04|04HBL005265|Canada|Ontario|658[0n]|BOLD:AAA2435
Hyphantria cunea|[1937]|BLTIB151-08|BL225|Canada|Ontario|658[0n]|BOLD:AAA2435
Hyphantria cunea|[1938]|BLTIB152-08|BL226|Canada|Ontario|658[0n]|BOLD:AAA2435
Hyphantria cunea|[1939]|LPSOC132-08|PPBP-2131|Canada|Ontario|658[0n]|BOLD:AAA2435
Hyphantria cunea|[1940]|RDLQG447-06|DH012731|Canada|Quebec|658[0n]|BOLD:AAA2435
Hyphantria cunea|[1941]|LOCT018-05|05-CTATBI-0018|United States|Connecticut|658[0n]|BOLD:AAA2435
Hyphantria cunea|[1942]|XAF562-05|2005-ONT-211|Canada|Ontario|658[0n]|BOLD:AAA2435
Hyphantria cunea|[1943]|XAF563-05|2005-ONT-212|Canada|Ontario|658[0n]|BOLD:AAA2435
Hyphantria cunea|[1944]|XAF659-05|2005-ONT-308|Canada|Ontario|658[0n]|BOLD:AAA2435
Hyphantria cunea|[1945]|XAF660-05|2005-ONT-309|Canada|Ontario|658[0n]|BOLD:AAA2435
Hyphantria cunea|[1946]|XAJ535-06|2006-ONT-0535|Canada|Ontario|657[0n]|BOLD:AAA2435
Hyphantria cunea|[1947]|XAK453-06|2006-ONT-1448|Canada|Ontario|658[0n]|BOLD:AAA2435
Hyphantria cunea|[1948]|XAB087-04|04HBL005087|Canada|Ontario|658[0n]|BOLD:AAA2435
Hyphantria cunea|[1949]|XAB091-04|04HBL005091|Canada|Ontario|658[0n]|BOLD:AAA2435
Hyphantria cunea|[1950]|XAB155-04|04HBL005155|Canada|Ontario|658[0n]|BOLD:AAA2435
Hyphantria cunea|[1951]|XAB334-04|04HBL005334|Canada|Ontario|658[0n]|BOLD:AAA2435
Hyphantria cunea|[1952]|XAB076-04|04HBL005076|Canada|Ontario|658[0n]|BOLD:AAA2435
Hyphantria cunea|[1953]|PHMNB375-04|04HBL00601|Canada|New Brunswick|658[0n]|BOLD:AAA2435
Hyphantria cunea|[1954]|PHMNB377-04|04HBL00603|Canada|New Brunswick|658[0n]|BOLD:AAA2435
Hyphantria cunea|[1955]|PHMNB606-04|04HBL00832|Canada|New Brunswick|658[0n]|BOLD:AAA2435
Hyphantria cunea|[1956]|PHMNB007-03|moth120.02SA|Canada|New Brunswick|639[0n]|BOLD:AAA2435
Hyphantria cunea|[1957]|PHMNB086-03|moth231.02SA|Canada|New Brunswick|639[2n]|BOLD:AAA2435
Hyphantria cunea|[1958]|XAB349-04|04HBL005349|Canada|Ontario|658[0n]|BOLD:AAA2435
Hyphantria cunea|[1959]|XAJ691-06|2006-ONT-0691|Canada|Ontario|658[0n]|BOLD:AAA2435
Hyphantria cunea|[1960]|XAF824-05|2005-ONT-473|Canada|Ontario|658[0n]|BOLD:AAA2435
Hyphantria cunea|[1961]|XAF713-05|2005-ONT-362|Canada|Ontario|658[0n]|BOLD:AAA2435
Hyphantria cunea|[1962]|XAF701-05|2005-ONT-350|Canada|Ontario|658[0n]|BOLD:AAA2435
Hyphantria cunea|[1963]|XAK173-06|2006-ONT-1168|Canada|Ontario|658[0n]|BOLD:AAA2435
Hyphantria cunea|[1964]|RDNME211-07|CNCNoctuoidea13818|United States|Virginia|619[1n]|BOLD:AAA...
Hyphantria cunea|[1965]|LOTB318-05|05-TN-00318|United States|Tennessee|658[0n]|BOLD:AAA2435
Hyphantria cunea|[1966]|XAB386-04|04HBL005386|Canada|Ontario|658[0n]|BOLD:AAA2435
Hyphantria cunea|[1967]|XAB101-04|04HBL005101|Canada|Ontario|658[0n]|BOLD:AAA2435
Hyphantria cunea|[1968]|LPSOB387-08|PPBP-1386|Canada|Ontario|658[0n]|BOLD:AAA2435
Hyphantria cunea|[1969]|LPSOB386-08|PPBP-1385|Canada|Ontario|658[0n]|BOLD:AAA2435
Hyphantria cunea|[1970]|LPSO489-08|PPBP-0489|Canada|Ontario|658[0n]|BOLD:AAA2435
Hyphantria cunea|[1971]|BLGSM064-09|BL385|Canada|Ontario|658[0n]|BOLD:AAA2435
Hyphantria cunea|[1972]|PHMNB374-04|04HBL00600|Canada|New Brunswick|658[0n]|BOLD:AAA2435
Hyphantria cunea|[1973]|PHMNB376-04|04HBL00602|Canada|New Brunswick|658[0n]|BOLD:AAA2435
Hyphantria cunea|[1974]|BLGSM057-09|BL376|Canada|Ontario|658[0n]|BOLD:AAA2435
Hyphantria cunea|[1975]|RDNME739-08|LEP042286|Canada|New Brunswick|658[0n]|BOLD:AAA2435
Hyphantria cunea|[1976]|RDNME740-08|LEP042287|Canada|New Brunswick|658[0n]|BOLD:AAA2435
Hyphantria cunea|[1977]|RDNME744-08|LEP042291|Canada|New Brunswick|658[0n]|BOLD:AAA2435
Hyphantria cunea|[1978]|TMNBD400-07|MNBTT-3201|Canada|New Brunswick|658[0n]|BOLD:AAA2435
Hyphantria cunea|[1979]|TMNBD401-07|MNBTT-3202|Canada|New Brunswick|658[0n]|BOLD:AAA2435
Hyphantria cunea|[1980]|TMNBD402-07|MNBTT-3203|Canada|New Brunswick|658[0n]|BOLD:AAA2435
Hyphantria cunea|[1981]|TMNBD403-07|MNBTT-3204|Canada|New Brunswick|658[0n]|BOLD:AAA2435
Hyphantria cunea|[1982]|BLTIB216-08|BL394|Canada|Ontario|658[0n]|BOLD:AAA2435
Hyphantria cunea|[1983]|BLTIB224-08|BL405|Canada|Ontario|658[0n]|BOLD:AAA2435
Hyphantria cunea|[1984]|BLTIB329-08|BL520|Canada|Ontario|658[0n]|BOLD:AAA2435
Hyphantria cunea|[1985]|BLTIB330-08|BL521|Canada|Ontario|658[0n]|BOLD:AAA2435
Hyphantria cunea|[1986]|TMNBB014-06|MNBTT-954|Canada|New Brunswick|658[0n]|BOLD:AAA2435
Hyphantria cunea|[1987]|XAK167-06|2006-ONT-1162|Canada|Ontario|658[0n]|BOLD:AAA2435
Hyphantria cunea|[1988]|XAB090-04|04HBL005090|Canada|Ontario|658[0n]|BOLD:AAA2435
Hyphantria cunea|[1989]|XAF464-05|2005-ONT-113|Canada|Ontario|658[0n]|BOLD:AAA2435
Hyphantria cunea|[1990]|XAF531-05|2005-ONT-180|Canada|Ontario|658[0n]|BOLD:AAA2435
Hyphantria cunea|[1991]|XAF560-05|2005-ONT-209|Canada|Ontario|658[0n]|BOLD:AAA2435
Hyphantria cunea|[1992]|XAJ534-06|2006-ONT-0534|Canada|Ontario|658[0n]|BOLD:AAA2435

Hyphantria cunea|[1990]|XAF531-05|2005-ONT-180|Canada|Ontario|658[0n]|BOLD:AAA2435
Hyphantria cunea|[1991]|XAF560-05|2005-ONT-209|Canada|Ontario|658[0n]|BOLD:AAA2435
Hyphantria cunea|[1992]|XAJ534-06|2006-ONT-0534|Canada|Ontario|658[0n]|BOLD:AAA2435
Hyphantria cunea|[1993]|XAK435-06|2006-ONT-1430|Canada|Ontario|658[0n]|BOLD:AAA2435
Hyphantria cunea|[1994]|XAF823-05|2005-ONT-472|Canada|Ontario|658[0n]|BOLD:AAA2435
Hyphantria cunea|[1995]|XAB237-04|04HBL005237|Canada|Ontario|658[0n]|BOLD:AAA2435
Hyphantria cunea|[1996]|XAB264-04|04HBL005264|Canada|Ontario|658[0n]|BOLD:AAA2435
Hyphantria cunea|[1997]|XAB263-04|04HBL005263|Canada|Ontario|658[0n]|BOLD:AAA2435
Hyphantria cunea|[1998]|XAB163-04|04HBL005163|Canada|Ontario|658[0n]|BOLD:AAA2435
Hyphantria cunea|[1999]|XAB340-04|04HBL005340|Canada|Ontario|658[0n]|BOLD:AAA2435
Hyphantria cunea|[2000]|XAB569-04|04HBL005569|Canada|Ontario|658[0n]|BOLD:AAA2435
Hyphantria cunea|[2001]|PHMNB764-05|Moth 457.03SA|Canada|New Brunswick|658[0n]|BOLD:AAA2435
Hyphantria cunea|[2002]|BLTIB255-08|BL437|Canada|Ontario|656[0n]|BOLD:AAA2435
Hyphantria cunea|[2003]|PHMO458-03|moth560.01|Canada|Ontario|639[0n]|BOLD:AAA2435
Hyphantria cunea|[2004]|TMG81-03|moth661.01|Canada|Ontario|639[0n]|BOLD:AAA2435
Hyphantria cunea|[2005]|BLTIB235-08|BL416|Canada|Ontario|608[2n]|BOLD:AAA2435
Hyphantria cunea|[2006]|LPSOD301-09|08BBLEP-00079|Canada|Ontario|634[0n]|BOLD:AAA2435
Hyphantria cunea|[2007]|RDNME212-07|CNCNoctuoidea13819|United States|Florida|617[0n]|BOLD:AAA2435
Hyphantria cunea|[2008]|PMG011-03|moth589.01|Canada|Ontario|617[0n]|BOLD:AAA2435
Xenosoma flaviceps|[2009]|ARCTA919-07|MILA 0638|Guatemala|Suchitepequez|658[0n]|BOLD:ABA4318
Purius superpulverea|[2010]|QUNOD003-10|7002-COI-09|United States|Texas|658[0n]|BOLD:AAH8013
Purius superpulverea|[2011]|QUNOD002-10|7001-COI-09|United States|Texas|658[0n]|BOLD:AAH8013
Virbia ostenta|[2012]|CMAZA782-10|CMAZ-0782|United States|Arizona|658[0n]|BOLD:AAE9998
Virbia ostenta|[2013]|CMAZA964-12|BIOUG02041-C06|United States|Arizona|658[0n]|BOLD:AAE9998
Virbia ostenta|[2014]|AWCL075-09|JLB-0274|United States|Arizona|658[0n]|BOLD:AAE9998
Virbia ostenta|[2015]|RDNMJ515-11|CNCLEP 80043|United States|Arizona|658[0n]|BOLD:AAE9998
Virbia ostenta|[2016]|RDNME542-08|LEP037966|United States|Arizona|658[0n]|BOLD:AAE9998
Virbia ostenta|[2017]|RDNMJ292-11|CNCLEP 80200|United States|Arizona|658[0n]|BOLD:AAE9998
Virbia ostenta|[2018]|RDNME543-08|LEP037967|United States|Arizona|658[0n]|BOLD:AAE9998
Virbia ostenta|[2019]|RDNME537-08|LEP037961|United States|Arizona|658[0n]|BOLD:AAE9998
Virbia ostenta|[2020]|RDNME536-08|LEP037960|United States|New Mexico|658[0n]|BOLD:AAE9998
Virbia opella|[2021]|RDNME335-07|CNCNoctuoidea13942|United States|Massachusetts|503[0n]|BOLD:A...
Virbia opella|[2022]|RDNME337-07|CNCNoctuoidea13944|United States|New Jersey|591[0n]|BOLD:ACF4451
Virbia opella|[2023]|RDNME339-07|CNCNoctuoidea13946|United States|Florida|592[0n]|BOLD:ACF4451
Virbia opella|[2024]|RDNME334-07|CNCNoctuoidea13941|United States|Massachusetts|583[0n]|BOLD:A...
Virbia opella|[2025]|BBLSX155-09|09BBLEP-02083|United States|Oklahoma|658[0n]|BOLD:ACF4451
Virbia opella|[2026]|RDNMH545-09|CNCLEP00057864|United States|Louisiana|658[0n]|BOLD:ACF4451
Virbia opella|[2027]|BBLOB1848-11|BIOUG01425-D07|United States|Florida|658[0n]|BOLD:ACF4451
Virbia sp.|[2028]|LTOLB292-09|CWM-94-0195|United States|Maryland|658[0n]|BOLD:ACF4453
Virbia sp.|[2029]|LTOLB1376-11|CWM-94-0186|United States|Maryland|658[0n]|BOLD:ACF4453
Virbia fergusoni|[2030]|RDNML297-13|CNCLEP 92273|United States|Florida|658[0n]|BOLD:AAA4930
Virbia fergusoni|[2031]|BBLOB1472-11|BIOUG01419-D11|United States|Florida|658[0n]|BOLD:AAA4930
Virbia fergusoni|[2032]|BBLOB430-11|BIOUG01396-E02|United States|Florida|658[0n]|BOLD:AAA4930
Virbia fergusoni|[2033]|RDNME207-07|CNCNoctuoidea13814|United States|Florida|614[0n]|BOLD:AAA4930
Virbia fergusoni|[2034]|RDNML296-13|CNCLEP 92272|United States|Florida|658[0n]|BOLD:AAA4930
Virbia fergusoni|[2035]|BBLOB1237-11|BIOUG01417-A02|United States|Florida|658[1n]|BOLD:AAA4930
Virbia fergusoni|[2036]|USLEP1006-10|10BBLEP-01006|United States|Florida|658[0n]|BOLD:AAA4930
Virbia fergusoni|[2037]|HKONS542-08|3071-COI-08|United States|Florida|658[1n]|BOLD:AAA4930
Virbia fergusoni|[2038]|RDNME343-07|CNCNoctuoidea13950|United States|Florida|593[0n]|BOLD:AAA4930
Virbia fergusoni|[2039]|HKONS718-08|3434-COI-08|United States|Florida|658[0n]|BOLD:AAA4930
Virbia fergusoni|[2040]|HKONS717-08|3433-COI-08|United States|Florida|658[0n]|BOLD:AAA4930
Virbia fergusoni|[2041]|HKONS716-08|3432-COI-08|United States|Florida|658[0n]|BOLD:AAA4930
Virbia fergusoni|[2042]|HKONS715-08|3431-COI-08|United States|Florida|658[0n]|BOLD:AAA4930
Virbia fergusoni|[2043]|HKONS714-08|3430-COI-08|United States|Florida|658[0n]|BOLD:AAA4930
Virbia fergusoni|[2044]|HKONS713-08|3429-COI-08|United States|Florida|658[0n]|BOLD:AAA4930
Virbia fergusoni|[2045]|HKONS712-08|3428-COI-08|United States|Florida|658[0n]|BOLD:AAA4930
Virbia fergusoni|[2046]|JMZCA074-08|JMZG4-004|United States|Florida|658[0n]|BOLD:AAA4930
Virbia opella|[2047]|CNSLE002-12|BIOUG02738-G07|Canada|Ontario|615[0n]|BOLD:ACF4452
Virbia opella|[2048]|CNSLP1125-13|BIOUG07571-F08|Canada|Ontario|613[0n]|BOLD:ACF4452
Virbia opella|[2049]|CNGIL690-13|BIOUG07571-E05|Canada|British Columbia|613[0n]|BOLD:ACF4452
Virbia opella|[2050]|RDNMH546-09|CNCLEP00057865|United States|Arkansas|658[0n]|BOLD:ACF4452
Virbia opella|[2051]|RDNME332-07|CNCNoctuoidea13939|Canada|Ontario|658[0n]|BOLD:ACF4452
Virbia opella|[2052]|RDNME331-07|CNCNoctuoidea13938|Canada|Ontario|658[0n]|BOLD:ACF4452
Virbia opella|[2053]|RDNME345-07|CNCNoctuoidea13952|Canada|Ontario|658[1n]|BOLD:ACF4452
Virbia opella|[2054]|RDNME333-07|CNCNoctuoidea13940|Canada|Ontario|658[0n]|BOLD:ACF4452
Virbia opella|[2055]|RDNME346-07|CNCNoctuoidea13953|Canada|Ontario|658[0n]|BOLD:ACF4452
Virbia opella|[2056]|RDNME330-07|CNCNoctuoidea13937|Canada|Ontario|658[0n]|BOLD:ACF4452
Virbia opella|[2057]|RDNMB414-05|CNCNoctuoidea10180|Canada|Ontario|658[0n]|BOLD:ACF4452
Virbia sp. 2|[2058]|HKONS552-08|3081-COI-08|United States|Florida|650[0n]|BOLD:ABY7337
Virbia sp. 2|[2059]|HKONS551-08|3080-COI-08|United States|Florida|658[0n]|BOLD:ABY7337
Virbia sp. 2|[2060]|HKONS550-08|3079-COI-08|United States|Florida|658[1n]|BOLD:ABY7337
Virbia opella|[2061]|LGSMG108-07|BGS03429|United States|North Carolina|658[0n]|BOLD:ABY7337
Virbia opella|[2062]|GMGSQ085-13|BIOUG04561-C05|United States|Tennessee|593[0n]|BOLD:ABY7337
Virbia opella|[2063]|HKONB448-09|3945-COI-08|United States|Kentucky|658[0n]|BOLD:ABY7337
Virbia opella|[2064]|LNCB002-06|06-NCC-958|United States|North Carolina|658[0n]|BOLD:ABY7337
Virbia opella|[2065]|LNCB001-06|06-NCCC-957|United States|North Carolina|658[0n]|BOLD:ABY7337
Virbia opella|[2066]|LSEU611-06|06-JKA-0611|United States|Georgia|609[0n]|BOLD:ABY7337
Virbia opella|[2067]|LSEU610-06|06-JKA-0610|United States|Georgia|658[0n]|BOLD:ABY7337
Virbia opella|[2068]|LNC184-05|05-NCCC-184|United States|North Carolina|658[0n]|BOLD:ABY7337
Virbia opella|[2069]|LNC183-05|05-NCCC-183|United States|North Carolina|658[0n]|BOLD:ABY7337
Virbia opella|[2070]|LNCB157-06|06-NCCC-1113|United States|North Carolina|658[0n]|BOLD:ABY7337
Virbia opella|[2071]|LNCB156-06|06-NCCC-1112|United States|North Carolina|658[0n]|BOLD:ABY7337
Virbia opella|[2072]|LNCB155-06|06-NCCC-1111|United States|North Carolina|658[0n]|BOLD:ABY7337
Virbia opella|[2073]|IAWLB388-11|IAWAZ-1296|United States|Virginia|658[0n]|BOLD:ABY7337
Virbia opella|[2074]|LNCNW031-06|06-NCNW-0031|United States|North Carolina|658[0n]|BOLD:ABY7337
Virbia opella|[2075]|LNCNW076-06|06-NCNW-0076|United States|North Carolina|658[0n]|BOLD:ABY7337
Virbia opella|[2076]|LNCB760-09|09-NCCC-230|United States|North Carolina|658[0n]|BOLD:ABY7337
Virbia opella|[2077]|LNCB761-09|09-NCCC-231|United States|North Carolina|658[0n]|BOLD:ABY7337
Virbia opella|[2078]|LGSMB311-05|DNA-ATBI-1160|United States|Tennessee|558[1n]|BOLD:ABY7337
Virbia opella|[2079]|RDNME771-08|LEP041236|United States|Georgia|570[0n]|BOLD:ABY7337
Virbia opella|[2080]|HKONS549-08|3078-COI-08|United States|Florida|658[0n]|BOLD:ABY7337
Virbia opella|[2081]|HKONS232-08|3137-COI-07|United States|Florida|658[0n]|BOLD:ABY7337
Virbia opella|[2082]|HKONS231-08|3136-COI-07|United States|Florida|658[0n]|BOLD:ABY7337
Virbia laeta|[2083]|BBLOD1500-11|BIOUG01828-E09|United States|Texas|658[0n]|BOLD:AAA7235
Virbia laeta|[2084]|RDMAB414-05|BCSC87|United States|Texas|603[0n]|BOLD:AAA7235
Virbia laeta|[2085]|BBLOC384-11|BIOUG01456-A09|United States|Texas|658[0n]|BOLD:AAA7235
Virbia laeta|[2086]|LOFLA812-06|06-FLOR-0812|United States|Florida|658[0n]|BOLD:AAA7235
Virbia laeta|[2087]|MNAB089-07|CNCLEP00025680|United States|Florida|582[0n]|BOLD:AAA7235
Virbia laeta|[2088]|RDNME544-08|LEP037968|United States|Alabama|658[0n]|BOLD:AAA7235
Virbia laeta|[2089]|LSEU006-06|06-JKA-0006|United States|Florida|520[2n]|BOLD:AAA7235
Virbia laeta|[2090]|LOFLD482-07|HLC-17098|United States|Florida|658[0n]|BOLD:AAA7235
Virbia laeta|[2091]|LOFLA819-06|06-FLOR-0819|United States|Florida|657[0n]|BOLD:AAA7235
[illegible]

Virbia laeta|[2089]|LSEU006-06|06-JKA-0006|United States|Florida|520[2n]|BOLD:AAA7235
Virbia laeta|[2090]|LOFLD482-07|HLC-17098|United States|Florida|658[0n]|BOLD:AAA7235
Virbia laeta|[2091]|LOFLA819-06|06-FLOR-0819|United States|Florida|657[0n]|BOLD:AAA7235
Virbia laeta|[2092]|LOFLA353-06|06-FLOR-0353|United States|Florida|658[0n]|BOLD:AAA7235
Virbia laeta|[2093]|RDNMH549-09|CNCLEP00057868|United States|Louisiana|564[0n]|BOLD:AAA7235
Virbia laeta|[2094]|MNAB223-07|CNCLEP00025814|United States|Florida|632[0n]|BOLD:AAA7235
Virbia laeta|[2095]|RDNMH550-09|CNCLEP00057869|United States|Louisiana|658[0n]|BOLD:AAA7235
Virbia laeta|[2096]|RDNMH547-09|CNCLEP00057866|United States|Louisiana|658[0n]|BOLD:AAA7235
Virbia laeta|[2097]|JMZCA073-08|JMZG3-003|United States|Florida|658[0n]|BOLD:AAA7235
Virbia laeta|[2098]|LOFLA338-06|06-FLOR-0338|United States|Florida|658[0n]|BOLD:AAA7235
Virbia laeta|[2099]|LOFLA245-06|06-FLOR-0245|United States|Florida|658[0n]|BOLD:AAA7235
Virbia laeta|[2100]|LOFLA073-06|06-FLOR-0073|United States|Florida|656[0n]|BOLD:AAA7235
Virbia laeta|[2101]|HKONS226-08|3131-COI-07|United States|Florida|658[0n]|BOLD:AAA7235
Virbia laeta|[2102]|LSEU009-06|06-JKA-0009|United States|Florida|525[3n]|BOLD:AAA7235
Virbia laeta|[2103]|LNC247-05|05-NCCC-247|United States|North Carolina|658[0n]|BOLD:AAA7235
Virbia laeta|[2104]|LNC248-05|05-NCCC-248|United States|North Carolina|580[0n]|BOLD:AAA7235
Virbia laeta|[2105]|JMZCA075-08|JMZG5-005|United States|Florida|658[0n]|BOLD:AAA7235
Virbia laeta|[2106]|BBLOB1845-11|BIOUG01425-D04|United States|Florida|658[0n]|BOLD:AAA7235
Virbia laeta|[2107]|LSEU008-06|06-JKA-0008|United States|Florida|544[0n]|BOLD:AAA7235
Virbia laeta|[2108]|LOFLD001-07|HLC-16575|United States|Florida|658[0n]|BOLD:AAA7235
Virbia laeta|[2109]|BBLOB1142-11|BIOUG01416-A02|United States|Florida|658[0n]|BOLD:AAA7235
Virbia laeta|[2110]|BBLOB1828-11|BIOUG01425-B11|United States|Florida|658[0n]|BOLD:AAA7235
Virbia laeta|[2111]|LOFLB868-06|06-FLOR-1808|United States|Florida|658[0n]|BOLD:AAA7235
Virbia laeta|[2112]|BBLOB422-11|BIOUG01396-D06|United States|Florida|658[0n]|BOLD:AAA7235
Virbia laeta|[2113]|LOFLB551-06|06-FLOR-1491|United States|Florida|658[0n]|BOLD:AAA7235
Virbia laeta|[2114]|LOFLB363-06|06-FLOR-1303|United States|Florida|658[0n]|BOLD:AAA7235
Virbia laeta|[2115]|LOFLC303-06|06-FLOR-2183|United States|Florida|658[0n]|BOLD:AAA7235
Virbia laeta|[2116]|LOFLC007-06|06-FLOR-1887|United States|Florida|658[0n]|BOLD:AAA7235
Virbia laeta|[2117]|LOFLB925-06|06-FLOR-1865|United States|Florida|658[0n]|BOLD:AAA7235
Virbia laeta|[2118]|LOFLD022-07|HLC-16596|United States|Florida|658[0n]|BOLD:AAA7235
Virbia laeta|[2119]|LOFLB373-06|06-FLOR-1313|United States|Florida|658[0n]|BOLD:AAA7235
Virbia laeta|[2120]|LOFLD006-07|HLC-16580|United States|Florida|658[0n]|BOLD:AAA7235
Virbia laeta|[2121]|BBLOB1607-11|BIOUG01420-H03|United States|Florida|658[0n]|BOLD:AAA7235
Virbia laeta|[2122]|RDNME206-07|CNCNoctuoidea13813|United States|Florida|658[0n]|BOLD:AAA7235
Virbia laeta|[2123]|LOFLD027-07|HLC-16601|United States|Florida|658[0n]|BOLD:AAA7235
Virbia laeta|[2124]|BBLOB1778-11|BIOUG01424-F08|United States|Florida|658[0n]|BOLD:AAA7235
Virbia laeta|[2125]|BBLOB1775-11|BIOUG01424-F05|United States|Florida|658[0n]|BOLD:AAA7235
Virbia laeta|[2126]|ABLCW012-10|CSUPOBK-0012|United States|Alabama|658[0n]|BOLD:AAA7235
Virbia laeta|[2127]|LOFLA630-06|06-FLOR-0630|United States|Florida|658[0n]|BOLD:AAA7235
Virbia laeta|[2128]|BBLOB1751-11|BIOUG01424-D05|United States|Florida|658[0n]|BOLD:AAA7235
Virbia laeta|[2129]|LOFLB872-06|06-FLOR-1812|United States|Florida|585[0n]|BOLD:AAA7235
Virbia costata|[2130]|RDNMK622-11|CNCLEP 79886|United States|New Mexico|658[0n]|BOLD:ABA2376
Virbia costata|[2131]|CMAZA553-10|CMAZ-0553|United States|Arizona|658[0n]|BOLD:AAH4921
Virbia costata|[2132]|CMAZA555-10|CMAZ-0555|United States|Arizona|658[0n]|BOLD:AAH4921
Virbia costata|[2133]|CMAZA554-10|CMAZ-0554|United States|Arizona|658[0n]|BOLD:AAH4921
Virbia costata|[2134]|DMAZ050-09|DMTRN-0144|United States|Arizona|658[0n]|BOLD:AAH4921
Virbia costata|[2135]|AWCL076-09|JLB-0275|United States|Arizona|658[0n]|BOLD:AAH4921
Virbia costata|[2136]|BBLSX823-09|09BBLEP-02751|United States|Texas|658[0n]|BOLD:ABZ7418
Virbia costata|[2137]|BBLSY929-09|09BBLEP-03856|United States|Texas|658[0n]|BOLD:ABZ7418
Virbia costata|[2138]|BBLSY922-09|09BBLEP-03849|United States|Texas|658[0n]|BOLD:ABZ7418
Virbia costata|[2139]|BBLSY152-09|09BBLEP-03079|United States|Texas|658[0n]|BOLD:ABZ7418
Virbia costata|[2140]|BBLSY151-09|09BBLEP-03078|United States|Texas|658[0n]|BOLD:ABZ7418
Virbia costata|[2141]|BBLSY150-09|09BBLEP-03077|United States|Texas|658[0n]|BOLD:ABZ7418
Virbia costata|[2142]|BBLSX825-09|09BBLEP-02753|United States|Texas|658[0n]|BOLD:ABZ7418
Virbia costata|[2143]|BBLSY921-09|09BBLEP-03848|United States|Texas|658[0n]|BOLD:ABZ7418
Virbia costata|[2144]|USLEP1007-10|10BBLEP-01007|United States|Texas|658[0n]|BOLD:ABZ7418
Virbia costata|[2145]|HKONB314-09|3811-COI-08|United States|Texas|658[0n]|BOLD:AAF1345
Virbia costata|[2146]|RDNME539-08|LEP037963|United States|Texas|658[0n]|BOLD:AAF1345
Virbia costata|[2147]|RDNME538-08|LEP037962|United States|Texas|658[0n]|BOLD:AAF1345
Virbia laeta|[2148]|TMNBD381-07|MNBTT-3182|Canada|New Brunswick|658[0n]|BOLD:AAA7234
Virbia laeta|[2149]|TTMNB253-06|MNBTT-253|Canada|New Brunswick|658[0n]|BOLD:AAA7234
Virbia laeta treatii|[2150]|RDLQG443-06|DH012727|Canada|Quebec|658[0n]|BOLD:AAA7234
Virbia laeta|[2151]|RDNME818-08|LEP041283|Canada|Ontario|658[0n]|BOLD:AAA7234
Virbia laeta|[2152]|MNAF437-08|CNCLEP00040422|Canada|Manitoba|658[0n]|BOLD:AAA7234
Virbia laeta|[2153]|TMNBD380-07|MNBTT-3181|Canada|New Brunswick|658[0n]|BOLD:AAA7234
Virbia laeta|[2154]|RDNME819-08|LEP041284|Canada|Ontario|658[0n]|BOLD:AAA7234
Virbia laeta|[2155]|MNAF477-08|CNCLEP00040462|Canada|Manitoba|658[0n]|BOLD:AAA7234
Virbia laeta|[2156]|TMNBD451-07|MNBTT-3252|Canada|New Brunswick|646[0n]|BOLD:AAA7234
Virbia laeta|[2157]|PHMO229-03|moth1162.02|Canada|Ontario|639[0n]|BOLD:AAA7234
Virbia laeta|[2158]|LPMNB546-09|08BBLEP-05584|Canada|Manitoba|658[0n]|BOLD:AAA7234
Virbia laeta|[2159]|MNAF479-08|CNCLEP00040464|Canada|Manitoba|658[0n]|BOLD:AAA7234
Virbia laeta|[2160]|MNAF478-08|CNCLEP00040463|Canada|Manitoba|658[0n]|BOLD:AAA7234
Virbia laeta|[2161]|MNAF480-08|CNCLEP00040465|Canada|Manitoba|658[0n]|BOLD:AAA7234
Virbia laeta|[2162]|LPMN046-08|08BBLEP-00844|Canada|Manitoba|658[0n]|BOLD:AAA7234
Virbia ferruginosa|[2163]|RDLQB386-05|DH010472|Canada|Quebec|658[0n]|BOLD:AAA4487
Virbia nigricans|[2164]|LNAUT190-14|CCDB-22941-A01|United States|New Jersey|611[0n]|BOLD:ACN8965
Virbia immaculata|[2165]|RDNMH552-09|CNCLEP00057871|United States|Louisiana|658[0n]|BOLD:AAC0912
Virbia immaculata|[2166]|RDNMB413-05|CNCNoctuoidea10179|Canada|Ontario|658[0n]|BOLD:AAC0912
Virbia aurantiaca|[2167]|LOFLD451-07|HLC-17067|United States|Florida|608[0n]|BOLD:AAA4929
Virbia aurantiaca|[2168]|LOFLB574-06|06-FLOR-1514|United States|Florida|658[0n]|BOLD:AAA4929
Virbia ferruginosa|[2169]|RDNME500-08|LEP037924|Canada|Quebec|658[0n]|BOLD:AAA4492
Virbia ferruginosa|[2170]|RDNMB412-05|CNCNoctuoidea10178|Canada|Ontario|658[0n]|BOLD:AAA4486
Virbia ferruginosa|[2171]|RDNME796-08|LEP041261|Canada|Ontario|658[0n]|BOLD:AAA4493
Virbia|[2172]|USLEP1008-10|10BBLEP-01008|United States|Arkansas|658[0n]|BOLD:AAM8626
Virbia rubicundaria|[2173]|LILLA598-11|SNS10IL-00765|United States|Illinois|658[0n]|BOLD:AAA4491
Virbia ferruginosa|[2174]|RDNME499-08|LEP037923|Canada|Quebec|658[0n]|BOLD:AAA4491
Virbia ferruginosa|[2175]|RDLQG147-06|DH012318|Canada|Quebec|658[3n]|BOLD:AAA4489
Virbia ferruginosa|[2176]|RDLQG146-06|DH012317|Canada|Quebec|594[0n]|BOLD:AAA4489
Virbia ferruginosa|[2177]|RDLQG149-06|DH012320|Canada|Quebec|592[0n]|BOLD:ABY6325
Virbia ferruginosa|[2178]|CNCLA560-13|CNCLEP00098992|Canada|Ontario|658[0n]|BOLD:ACG1066
Virbia ferruginosa|[2179]|CNSLP1113-13|BIOUG07571-E08|Canada|Ontario|661[0n]|BOLD:ACI8955
Virbia ferruginosa|[2180]|RDNMB411-05|CNCNoctuoidea10177|Canada|Ontario|658[0n]|BOLD:ABZ2730
Virbia ferruginosa|[2181]|CNSLP1119-13|BIOUG07571-F02|Canada|Ontario|661[0n]|BOLD:ACI9057
Virbia ferruginosa|[2182]|RDNME486-08|LEP037910|Canada|Quebec|658[0n]|BOLD:AAA4490
Virbia lamae|[2183]|QUNOD683-11|9397-MN|United States|Minnesota|658[0n]|BOLD:ACE4283
Virbia immaculata|[2184]|RDNMH554-09|CNCLEP00057873|United States|Louisiana|658[0n]|BOLD:ABZ1841
Virbia immaculata|[2185]|RDNMH553-09|CNCLEP00057872|United States|Louisiana|658[0n]|BOLD:ABZ1841
Virbia ferruginosa|[2186]|XAJ882-06|2006-ONT-0882|Canada|Ontario|658[0n]|BOLD:ABZ1841
Virbia ferruginosa|[2187]|XAJ889-06|2006-ONT-0889|Canada|Ontario|658[0n]|BOLD:ABZ1841
Virbia ferruginosa|[2188]|CNSLP1116-13|BIOUG07571-E11|Canada|Ontario|661[0n]|BOLD:ACP3165
Virbia ferruginosa|[2189]|CNSLP1114-13|BIOUG07571-E09|Canada|Ontario|661[0n]|BOLD:ACP3165
Virbia immaculata|[2190]|RDNMH551-09|CNCLEP00057870|United States|Louisiana|658[0n]|BOLD:ABZ1843
Virbia immaculata|[2191]|RDNME487-08|LEP037911|Canada|Ontario|658[0n]|BOLD:ABZ1843

Virbia ferruginosa|[2189]|CNSLP1114-13|BIOUG07571-E09|Canada|Ontario|661[0n]|BOLD:ACP3165
Virbia immaculata|[2190]|RDNMH551-09|CNCLEP00057870|United States|Louisiana|658[0n]|BOLD:ABZ1843
Virbia immaculata|[2191]|RDNME487-08|LEP037911|Canada|Ontario|658[0n]|BOLD:ABZ1843
Virbia ferruginosa|[2192]|BBLOC1628-11|BIOUG01546-H01|United States|Texas|658[0n]|BOLD:ACE5534
Virbia ferruginosa|[2193]|CNSLP1118-13|BIOUG07571-F01|Canada|Ontario|661[0n]|BOLD:ACE5534
Virbia ferruginosa|[2194]|RDNME797-08|LEP041262|Canada|Ontario|658[0n]|BOLD:ACE5534
Virbia ferruginosa sp. 2|[2195]|HKONB321-09|3818-COI-08|United States|Louisiana|658[0n]|BOLD:A...
Virbia ferruginosa|[2196]|CNSLP1117-13|BIOUG07571-E12|Canada|Ontario|661[0n]|BOLD:ACI9202
Virbia ferruginosa|[2197]|CNSLP1115-13|BIOUG07571-E10|Canada|Ontario|661[0n]|BOLD:ACI9202
Virbia ferruginosa|[2198]|RDLQG150-06|DH012321|Canada|Quebec|611[3n]|BOLD:ACF1306
Virbia ferruginosa|[2199]|RDNME495-08|LEP037919|Canada|Ontario|658[0n]|BOLD:ACE4285
Virbia ferruginosa|[2200]|RDNME209-07|CNCNoctuoidea13816|Canada|Ontario|658[2n]|BOLD:ABZ2731
Virbia ferruginosa|[2201]|RDLQB534-05|DH010620|Canada|Quebec|658[0n]|BOLD:ABZ2731
Virbia ferruginosa|[2202]|RDNME501-08|LEP037925|Canada|Quebec|658[0n]|BOLD:ABZ2731
Virbia ferruginosa|[2203]|RDNME498-08|LEP037922|Canada|Quebec|658[0n]|BOLD:ABZ2731
Virbia ferruginosa|[2204]|RDNME497-08|LEP037921|Canada|Quebec|658[0n]|BOLD:ABZ2731
Virbia ferruginosa|[2205]|RDLQG151-06|DH012322|Canada|Quebec|627[0n]|BOLD:ABY5771
Virbia ferruginosa|[2206]|CNRME1795-12|BIOUG03547-A05|Canada|Manitoba|661[0n]|BOLD:ABY5771
Virbia ferruginosa|[2207]|RDLQG148-06|DH012319|Canada|Quebec|640[1n]|BOLD:AAA4485
Virbia ferruginosa|[2208]|XAJ883-06|2006-ONT-0883|Canada|Ontario|658[0n]|BOLD:AAA4485
Virbia ferruginosa|[2209]|CNRME2806-12|BIOUG03588-F06|Canada|Manitoba|627[0n]|BOLD:AAA4485
Virbia ferruginosa|[2210]|RDMAB377-05|BCSC51|Canada|Alberta|658[0n]|BOLD:AAA4485
Virbia ferruginosa|[2211]|RDMAB362-05|UASM78177|Canada|Alberta|658[0n]|BOLD:AAA4485
Virbia ferruginosa|[2212]|RDMAB361-05|UASM78179|Canada|Alberta|601[0n]|BOLD:AAA4485
Virbia ferruginosa|[2213]|CNRME716-12|BIOUG03023-E11|Canada|Manitoba|635[0n]|BOLD:AAA4485
Virbia ferruginosa|[2214]|CNRME713-12|BIOUG03023-E08|Canada|Manitoba|636[0n]|BOLD:AAA4485
Virbia ferruginosa|[2215]|CNRME720-12|BIOUG03023-F03|Canada|Manitoba|636[0n]|BOLD:AAA4485
Virbia ferruginosa|[2216]|CNRME737-12|BIOUG03023-G08|Canada|Manitoba|636[0n]|BOLD:AAA4485
Virbia ferruginosa|[2217]|CNRME751-12|BIOUG03023-H10|Canada|Manitoba|636[0n]|BOLD:AAA4485
Virbia ferruginosa|[2218]|CNRME744-12|BIOUG03023-H03|Canada|Manitoba|634[0n]|BOLD:AAA4485
Virbia ferruginosa|[2219]|CNRME2764-12|BIOUG03588-B12|Canada|Manitoba|637[0n]|BOLD:AAA4485
Virbia ferruginosa|[2220]|CNRME2747-12|BIOUG03588-A07|Canada|Manitoba|633[0n]|BOLD:AAA4485
Virbia ferruginosa|[2221]|CNRME2748-12|BIOUG03588-A08|Canada|Manitoba|633[0n]|BOLD:AAA4485
Virbia ferruginosa|[2222]|CNRME2759-12|BIOUG03588-B07|Canada|Manitoba|633[0n]|BOLD:AAA4485
Virbia ferruginosa|[2223]|CNRME2766-12|BIOUG03588-C02|Canada|Manitoba|633[0n]|BOLD:AAA4485
Virbia ferruginosa|[2224]|CNRME2795-12|BIOUG03588-E07|Canada|Manitoba|644[0n]|BOLD:AAA4485
Virbia ferruginosa|[2225]|CNRME2810-12|BIOUG03588-F10|Canada|Manitoba|633[0n]|BOLD:AAA4485
Virbia ferruginosa|[2226]|CNRME736-12|BIOUG03023-G07|Canada|Manitoba|632[0n]|BOLD:AAA4485
Virbia ferruginosa|[2227]|CNRME2761-12|BIOUG03588-B09|Canada|Manitoba|630[0n]|BOLD:AAA4485
Virbia ferruginosa|[2228]|LPMN618-08|08BBLEP-01419|Canada|Manitoba|658[0n]|BOLD:AAA4485
Virbia ferruginosa|[2229]|RDLQ398-05|DH010081|Canada|Quebec|572[1n]|BOLD:AAA4485
Virbia ferruginosa|[2230]|RDLQG152-06|DH012323|Canada|Quebec|621[8n]|
Virbia ferruginosa|[2231]|TMNBB006-06|MNBTT-946|Canada|New Brunswick|658[0n]|BOLD:AAA4485
Virbia ferruginosa|[2232]|TMNBB007-06|MNBTT-947|Canada|New Brunswick|658[0n]|BOLD:AAA4485
Virbia ferruginosa|[2233]|TMNBB008-06|MNBTT-948|Canada|New Brunswick|658[0n]|BOLD:AAA4485
Virbia ferruginosa|[2234]|RDLQ766-07|DH006360|Canada|Quebec|641[0n]|BOLD:AAA4485
Virbia ferruginosa|[2235]|TMNBB009-06|MNBTT-949|Canada|New Brunswick|658[0n]|BOLD:AAA4485
Virbia ferruginosa|[2236]|RDNMB409-05|CNCNoctuoidea10175|Canada|Alberta|616[0n]|BOLD:AAA4485
Virbia nigricans|[2237]|CNCLB1028-14|CNCLEP00113436|United States|Ohio|540[0n]|BOLD:AAA4485
Virbia ferruginosa|[2238]|RDLQ396-05|DH010079|Canada|Quebec|548[0n]|BOLD:AAA4485
Virbia ferruginosa|[2239]|XAJ894-06|2006-ONT-0894|Canada|Ontario|658[0n]|BOLD:AAA4485
Virbia ferruginosa|[2240]|RDNME496-08|LEP037920|Canada|Ontario|658[0n]|BOLD:AAA4485
Virbia sp.|[2241]|RDNME540-08|LEP037964|United States|Wisconsin|658[0n]|BOLD:AAA4485
Virbia ferruginosa|[2242]|RDNME798-08|LEP041263|Canada|Ontario|658[0n]|BOLD:AAA4485
Virbia ferruginosa|[2243]|RDNME801-08|LEP041266|Canada|Ontario|658[0n]|BOLD:AAA4485
Virbia ferruginosa|[2244]|RDNME799-08|LEP041264|Canada|Ontario|658[0n]|BOLD:AAA4485
Virbia ferruginosa|[2245]|RDNME795-08|LEP041260|Canada|Ontario|658[0n]|BOLD:AAA4485
Virbia ferruginosa|[2246]|RDNME507-08|LEP037931|Canada|Ontario|658[0n]|BOLD:AAA4485
Virbia ferruginosa|[2247]|RDNME505-08|LEP037929|Canada|Ontario|658[0n]|BOLD:AAA4485
Virbia ferruginosa|[2248]|RDNME493-08|LEP037917|Canada|Ontario|658[0n]|BOLD:AAA4485
Virbia ferruginosa|[2249]|RDNME492-08|LEP037916|Canada|Ontario|658[0n]|BOLD:AAA4485
Virbia ferruginosa|[2250]|LPMN236-08|08BBLEP-01035|Canada|Manitoba|658[0n]|BOLD:AAA4485
Virbia ferruginosa|[2251]|LPMN235-08|08BBLEP-01034|Canada|Manitoba|658[0n]|BOLD:AAA4485
Virbia ferruginosa|[2252]|LPMN234-08|08BBLEP-01033|Canada|Manitoba|658[0n]|BOLD:AAA4485
Virbia ferruginosa|[2253]|CNCLA559-13|CNCLEP00098991|Canada|Ontario|658[0n]|BOLD:AAA4485
Virbia ferruginosa|[2254]|RDNMB410-05|CNCNoctuoidea10176|Canada|Ontario|658[0n]|BOLD:AAA4485
Virbia ferruginosa|[2255]|LPMN233-08|08BBLEP-01032|Canada|Manitoba|658[0n]|BOLD:AAA4485
Virbia nigricans|[2256]|CNCLB1027-14|CNCLEP00113435|United States|Ohio|658[0n]|BOLD:AAA4485
Virbia rubicundaria|[2257]|LILLA860-11|SNS10IL-01081|United States|Illinois|658[0n]|BOLD:AAA4485
Virbia immaculata|[2258]|HKONB400-09|3897-COI-08|United States|Indiana|658[0n]|BOLD:AAA4485
Virbia immaculata|[2259]|RDMAB746-06|BCSC415|United States|Kentucky|658[0n]|BOLD:AAA4485
Virbia rubicundaria|[2260]|LILLA113-11|SNS10IL-00154|United States|Illinois|658[0n]|BOLD:AAA4485
Virbia immaculata|[2261]|RDNME768-08|LEP041233|United States|South Dakota|658[0n]|BOLD:AAA4485
Virbia immaculata|[2262]|XAK314-06|2006-ONT-1309|Canada|Ontario|651[0n]|BOLD:AAA4485
Virbia immaculata|[2263]|RDNME354-07|CNCNoctuoidea13961|Canada|Ontario|658[0n]|BOLD:AAA4485
Virbia immaculata|[2264]|XAK313-06|2006-ONT-1308|Canada|Ontario|658[0n]|BOLD:AAA4485
Virbia immaculata|[2265]|RDNME344-07|CNCNoctuoidea13951|Canada|Ontario|643[2n]|BOLD:AAA4485
Virbia immaculata|[2266]|CNCLA562-13|CNCLEP00098994|Canada|Ontario|658[0n]|BOLD:AAA4485
Virbia immaculata|[2267]|CNCLA561-13|CNCLEP00098993|Canada|Ontario|658[0n]|BOLD:AAA4485
Virbia immaculata|[2268]|RDNME820-08|LEP041285|Canada|Ontario|658[0n]|BOLD:AAA4485
Virbia immaculata|[2269]|RDNME494-08|LEP037918|Canada|Ontario|658[0n]|BOLD:AAA4485
Virbia immaculata|[2270]|MEC419-04|jflandry0419|Canada|Quebec|658[0n]|BOLD:AAA4485
Virbia marginata|[2271]|RDNMF936-08|CNC LEP00054203|United States|Arizona|622[0n]|BOLD:AAA4485
Virbia marginata|[2272]|RDNMF940-08|CNC LEP00054207|United States|Arizona|658[0n]|BOLD:AAA4485
Virbia lamae|[2273]|RDMAB376-05|BCSC50|Canada|Nova Scotia|610[0n]|BOLD:AAA4485
Virbia lamae|[2274]|CNCLA556-13|CNCLEP00098988|Canada|New Brunswick|658[0n]|BOLD:AAA4485
Virbia ferruginosa|[2275]|RDNMC251-05|CNCNoctuoidea11885|Canada|New Brunswick|543[0n]|BOLD:AAA...
Virbia lamae|[2276]|CNCLA558-13|CNCLEP00098990|Canada|New Brunswick|658[0n]|BOLD:AAA4485
Virbia lamae|[2277]|CNCLA557-13|CNCLEP00098989|Canada|New Brunswick|658[0n]|BOLD:AAA4485
Virbia lamae|[2278]|CNCLA554-13|CNCLEP00098986|Canada|New Brunswick|658[0n]|BOLD:AAA4485
Virbia lamae|[2279]|CNCLA555-13|CNCLEP00098987|Canada|New Brunswick|658[0n]|BOLD:AAA4485
Virbia virbia sp. 4|[2280]|QUNOD676-11|9390-MN|United States|Minnesota|658[0n]|BOLD:AAA4485
Virbia ferruginosa|[2281]|QUNOD682-11|9396-MN|United States|Minnesota|658[0n]|BOLD:AAA4485
Virbia ferruginosa|[2282]|QUNOD673-11|9387-MN|United States|Minnesota|658[0n]|BOLD:AAA4485
Virbia virbia sp. 4|[2283]|QUNOD677-11|9391-MN|United States|Minnesota|658[0n]|BOLD:AAA4485
Virbia ferruginosa sp. 2|[2284]|HKONB320-09|3817-COI-08|United States|Louisiana|622[0n]|BOLD:A...
Virbia ferruginosa|[2285]|RDLQG145-06|DH012316|Canada|Quebec|630[0n]|BOLD:AAA4485
Virbia ferruginosa|[2286]|CNRME2798-12|BIOUG03588-E10|Canada|Manitoba|632[0n]|BOLD:AAA4485
Virbia ferruginosa|[2287]|MNAF854-08|CNCLEP00040839|Canada|Manitoba|658[0n]|BOLD:AAA4485
Virbia ferruginosa|[2288]|SSPAC3403-13|BIOUG07628-F12|Canada|Saskatchewan|588[0n]|BOLD:AAA4485
Virbia ferruginosa|[2289]|RDMAB425-05|BCSC98|Canada|Alberta|658[0n]|BOLD:AAA4485
Virbia ferruginosa|[2290]|CNRME2746-12|BIOUG03588-A06|Canada|Manitoba|615[0n]|BOLD:AAA4485
Virbia ferruginosa|[2291]|RDNME490-08|LEP037914|Canada|British Columbia|658[0n]|BOLD:AAA4485

Virbia ferruginosa|[2288]|SSPAC3403-13|BIOUG07628-F12|Canada|Saskatchewan|[illegible][0n]|BOLD:AAA4485
Virbia ferruginosa|[2289]|RDMAB425-05|BCSC98|Canada|Alberta|658[0n]|BOLD:AAA4485
Virbia ferruginosa|[2290]|CNRME2746-12|BIOUG03588-A06|Canada|Manitoba|615[0n]|BOLD:AAA4485
Virbia ferruginosa|[2291]|RDNME490-08|LEP037914|Canada|British Columbia|658[0n]|BOLD:AAA4485
Virbia ferruginosa|[2292]|RDNME489-08|LEP037913|Canada|British Columbia|658[0n]|BOLD:AAA4485
Virbia ferruginosa|[2293]|CNRME735-12|BIOUG03023-G06|Canada|Manitoba|635[0n]|BOLD:AAA4485
Virbia ferruginosa|[2294]|CNRME4980-12|BIOUG03805-A06|Canada|Manitoba|635[0n]|BOLD:AAA4485
Virbia ferruginosa|[2295]|CNPAD1001-13|BIOUG04568-C05|Canada|Saskatchewan|632[0n]|BOLD:AAA4485
Virbia ferruginosa|[2296]|CNRME2817-12|BIOUG03588-G05|Canada|Manitoba|632[0n]|BOLD:AAA4485
Virbia ferruginosa|[2297]|BBLPA480-10|10BBCLP-0480|Canada|British Columbia|632[0n]|BOLD:AAA4485
Virbia ferruginosa|[2298]|SSPAC080-13|BIOUG06892-C03|Canada|Saskatchewan|583[0n]|BOLD:AAA4485
Virbia ferruginosa|[2299]|SSPAB918-13|BIOUG06035-H09|Canada|Saskatchewan|607[1n]|BOLD:AAA4485
Virbia sp.|[2300]|RDMAB359-05|UASM78173|Canada|Alberta|541[0n]|BOLD:AAA4485
Virbia ferruginosa|[2301]|SSPAC3404-13|BIOUG07628-G01|Canada|Saskatchewan|602[0n]|BOLD:AAA4485
Virbia ferruginosa|[2302]|SSPAC3406-13|BIOUG07628-G03|Canada|Saskatchewan|593[0n]|BOLD:AAA4485
Virbia ferruginosa|[2303]|SSPAC3405-13|BIOUG07628-G02|Canada|Saskatchewan|600[0n]|BOLD:AAA4485
Virbia ferruginosa|[2304]|SSPAB917-13|BIOUG06035-H08|Canada|Saskatchewan|604[0n]|BOLD:AAA4485
Virbia ferruginosa|[2305]|CNEID3325-12|BIOUG03742-A11|Canada|Alberta|640[0n]|BOLD:AAA4485
Virbia ferruginosa|[2306]|RDLQB381-05|DH010467|Canada|Quebec|541[1n]|BOLD:AAA4485
Virbia ferruginosa|[2307]|RDLQB385-05|DH010471|Canada|Quebec|555[0n]|BOLD:AAA4485
Virbia ferruginosa|[2308]|RDLQB383-05|DH010469|Canada|Quebec|658[0n]|BOLD:AAA4485
Virbia ferruginosa|[2309]|RDLQB382-05|DH010468|Canada|Quebec|551[0n]|BOLD:AAA4485
Virbia ferruginosa|[2310]|RDLQB380-05|DH010466|Canada|Quebec|585[0n]|BOLD:AAA4485
Virbia ferruginosa|[2311]|BBLPA482-10|10BBCLP-0482|Canada|British Columbia|658[0n]|BOLD:AAA4485
Virbia ferruginosa|[2312]|BBLPA481-10|10BBCLP-0481|Canada|British Columbia|632[0n]|BOLD:AAA4485
Virbia n. sp.|[2313]|RDMAB360-05|UASM78169|Canada|Alberta|611[0n]|BOLD:AAA4485
Virbia ferruginosa|[2314]|RDMAB424-05|BCSC97|Canada|Alberta|658[0n]|BOLD:AAA4485
Virbia ferruginosa|[2315]|RDNME488-08|LEP037912|Canada|British Columbia|658[0n]|BOLD:AAA4485
Virbia ferruginosa|[2316]|QUNOD681-11|9395-MN|United States|Minnesota|658[0n]|BOLD:AAA4485
Virbia ferruginosa|[2317]|RDNME491-08|LEP037915|Canada|British Columbia|658[0n]|BOLD:AAA4485
Virbia ferruginosa|[2318]|LPSOD951-09|08BBLEP-05491|Canada|Ontario|658[0n]|BOLD:AAA4485
Virbia ferruginosa|[2319]|LOWCB548-05|CGWC-1488|Canada|British Columbia|658[0n]|BOLD:AAA4485
Virbia ferruginosa|[2320]|LOWCB549-05|CGWC-1489|Canada|British Columbia|658[0n]|BOLD:AAA4485
Virbia ferruginosa|[2321]|BBLPA483-10|10BBCLP-0483|Canada|Ontario|658[0n]|BOLD:AAA4485
Virbia ferruginosa|[2322]|BBLPA484-10|10BBCLP-0484|Canada|British Columbia|658[0n]|BOLD:AAA4485
Virbia ferruginosa|[2323]|LOWCB558-05|CGWC-1498|Canada|British Columbia|658[0n]|BOLD:AAA4485
Virbia ferruginosa|[2324]|LOWCB559-05|CGWC-1499|Canada|British Columbia|658[0n]|BOLD:AAA4485
Virbia ferruginosa|[2325]|LOWCB561-05|CGWC-1501|Canada|British Columbia|658[0n]|BOLD:AAA4485
Virbia ferruginosa|[2326]|LBCH6104-10|10-JDWBC-6104|Canada|British Columbia|658[0n]|BOLD:AAA4485
Virbia rindgei|[2327]|RDNMJ094-10|CNCLEP 73454|United States|Wyoming|658[0n]|BOLD:AAA4485
Virbia ferruginosa|[2328]|LOWCC878-05|CGWC-2758|Canada|British Columbia|658[0n]|BOLD:AAA4485
Virbia ferruginosa|[2329]|RDLQB384-05|DH010470|Canada|Quebec|658[0n]|BOLD:AAA4485
Virbia ferruginosa|[2330]|RDMAB426-05|BCSC99|Canada|Alberta|658[0n]|BOLD:AAA4485
Virbia sp.|[2331]|RDMAB429-05|BCSC102|Canada|Alberta|658[1n]|BOLD:AAA4485
Virbia ferruginosa|[2332]|LOWCB557-05|CGWC-1497|Canada|British Columbia|658[0n]|BOLD:AAA4485
Virbia ferruginosa|[2333]|LOWCB554-05|CGWC-1494|Canada|British Columbia|658[0n]|BOLD:AAA4485
Virbia ferruginosa|[2334]|LOWCB552-05|CGWC-1492|Canada|British Columbia|578[0n]|BOLD:AAA4485
Virbia ferruginosa|[2335]|LOWCB555-05|CGWC-1495|Canada|British Columbia|571[0n]|BOLD:AAA4485
Virbia ferruginosa|[2336]|LOWCB560-05|CGWC-1500|Canada|British Columbia|577[0n]|BOLD:AAA4485
Virbia ferruginosa|[2337]|LOWCB553-05|CGWC-1493|Canada|British Columbia|658[0n]|BOLD:AAA4485
Virbia ferruginosa|[2338]|LOWCB551-05|CGWC-1491|Canada|British Columbia|542[0n]|BOLD:AAA4485
Virbia ferruginosa|[2339]|LOWCB550-05|CGWC-1490|Canada|British Columbia|540[0n]|BOLD:AAA4485
Virbia ferruginosa|[2340]|RDMAB427-05|BCSC100|Canada|Alberta|658[0n]|BOLD:AAA4485
Virbia ferruginosa|[2341]|RDNMB408-05|CNCNoctuoidea10174|Canada|British Columbia|616[0n]|BOLD:...
Virbia virbia sp. 4|[2342]|QUNOD674-11|9388-MN|United States|Minnesota|619[0n]|BOLD:AAA4485
Virbia marginata|[2343]|RDNMF939-08|CNC LEP00054206|United States|Arizona|658[0n]|BOLD:AAA4485
Virbia ferruginosa|[2344]|LOWCB556-05|CGWC-1496|Canada|British Columbia|658[0n]|BOLD:AAA4485
Virbia ferruginosa|[2345]|CNRME1797-12|BIOUG03547-A07|Canada|Manitoba|634[0n]|BOLD:AAA4485
Virbia ferruginosa|[2346]|CNPAD996-13|BIOUG04568-B12|Canada|Saskatchewan|635[0n]|BOLD:AAA4485
Virbia aurantiaca|[2347]|HKONB159-08|3656-COI-08|United States|Texas|658[0n]|BOLD:AAA4931
Virbia aurantiaca|[2348]|HKONB160-08|3657-COI-08|United States|Texas|658[0n]|BOLD:AAA4931
Virbia aurantiaca|[2349]|LOFLB900-06|06-FLOR-1840|United States|Florida|545[2n]|BOLD:AAA4928
Virbia aurantiaca|[2350]|BBLSZ136-09|09BBLEP-04062|United States|Oklahoma|658[0n]|BOLD:AAA4928
Virbia rubicundaria|[2351]|RDNME541-08|LEP037965|United States|Florida|577[1n]|BOLD:AAA4928
Virbia aurantiaca|[2352]|LOFLC104-06|06-FLOR-1984|United States|Florida|657[0n]|BOLD:AAA4928
Virbia aurantiaca|[2353]|LOFLC361-06|06-FLOR-2241|United States|Florida|658[0n]|BOLD:AAA4928
Virbia aurantiaca|[2354]|LOFLC384-06|06-FLOR-2264|United States|Florida|658[0n]|BOLD:AAA4928
Virbia aurantiaca|[2355]|LOFLB159-06|06-FLOR-1099|United States|Florida|658[0n]|BOLD:AAA4928
Virbia aurantiaca|[2356]|LOFLB182-06|06-FLOR-1122|United States|Florida|658[0n]|BOLD:AAA4928
Virbia aurantiaca|[2357]|LOFLB349-06|06-FLOR-1289|United States|Florida|658[0n]|BOLD:AAA4928
Virbia aurantiaca|[2358]|BBLOB1773-11|BIOUG01424-F03|United States|Florida|658[0n]|BOLD:AAA4928
Virbia aurantiaca|[2359]|BBLOB1787-11|BIOUG01424-G05|United States|Florida|658[0n]|BOLD:AAA4928
Virbia aurantiaca|[2360]|BBLOB1251-11|BIOUG01417-B04|United States|Florida|658[0n]|BOLD:AAA4928
Virbia aurantiaca|[2361]|LOFLD026-07|HLC-16600|United States|Florida|658[0n]|BOLD:AAA4928
Virbia aurantiaca|[2362]|LOFLC344-06|06-FLOR-2224|United States|Florida|621[0n]|BOLD:AAA4928
Virbia aurantiaca|[2363]|LOCT015-05|05-CTATBI-0015|United States|Connecticut|658[0n]|BOLD:AAA4928
Virbia aurantiaca|[2364]|MILEQ203-11|11-MISC-678|United States|Georgia|658[0n]|BOLD:AAA4928
Virbia aurantiaca|[2365]|BBLSX331-09|09BBLEP-02259|United States|Oklahoma|658[0n]|BOLD:AAA4928
Virbia aurantiaca|[2366]|BBLSU062-09|09BBLEP-04431|United States|Arkansas|658[0n]|BOLD:AAA4928
Virbia aurantiaca|[2367]|LOFLC220-06|06-FLOR-2100|United States|Florida|631[2n]|BOLD:AAA4928
Virbia aurantiaca|[2368]|LOFLB415-06|06-FLOR-1355|United States|Florida|627[0n]|BOLD:AAA4928
Virbia aurantiaca|[2369]|LOFLD019-07|HLC-16593|United States|Florida|658[0n]|BOLD:AAA4928
Virbia sp.|[2370]|HKONB328-09|3825-COI-08|United States|Texas|658[0n]|BOLD:AAA4928
Virbia aurantiaca|[2371]|LOCT014-05|05-CTATBI-0014|United States|Connecticut|658[0n]|BOLD:AAA4928
Virbia aurantiaca|[2372]|LNCB382-06|06-NCCC-1338|United States|North Carolina|658[0n]|BOLD:AAA...
Virbia aurantiaca|[2373]|LNCB216-06|06-NCCC-1172|United States|North Carolina|658[0n]|BOLD:AAA...
Virbia aurantiaca|[2374]|LOFLC385-06|06-FLOR-2265|United States|Florida|658[1n]|BOLD:AAA4928
Virbia aurantiaca|[2375]|LOFLB091-06|06-FLOR-1031|United States|Florida|658[0n]|BOLD:AAA4928
Virbia aurantiaca|[2376]|LOFLB160-06|06-FLOR-1100|United States|Florida|658[0n]|BOLD:AAA4928
Virbia aurantiaca|[2377]|LOFLC222-06|06-FLOR-2102|United States|Florida|658[0n]|BOLD:AAA4928
Virbia aurantiaca|[2378]|LOFLB414-06|06-FLOR-1354|United States|Florida|658[0n]|BOLD:AAA4928
Virbia aurantiaca|[2379]|LOFLB642-06|06-FLOR-1582|United States|Florida|658[0n]|BOLD:AAA4928
Virbia aurantiaca|[2380]|LOFLB869-06|06-FLOR-1809|United States|Florida|658[0n]|BOLD:AAA4928
Virbia aurantiaca|[2381]|LOFLC444-06|06-FLOR-2324|United States|Florida|658[0n]|BOLD:AAA4928
Virbia aurantiaca|[2382]|LOFLC453-06|06-FLOR-2333|United States|Florida|658[0n]|BOLD:AAA4928
Virbia aurantiaca|[2383]|BBLOB1601-11|BIOUG01420-G09|United States|Florida|658[0n]|BOLD:AAA4928
Virbia aurantiaca|[2384]|LOFLD018-07|HLC-16592|United States|Florida|658[0n]|BOLD:AAA4928
Virbia aurantiaca|[2385]|LOFLB410-06|06-FLOR-1350|United States|Florida|658[0n]|BOLD:AAA4928
Virbia aurantiaca|[2386]|LOFLC049-06|06-FLOR-1929|United States|Florida|658[0n]|BOLD:AAA4928
Virbia aurantiaca|[2387]|LOFLC059-06|06-FLOR-1939|United States|Florida|658[0n]|BOLD:AAA4928
Virbia aurantiaca|[2388]|LOFLB084-06|06-FLOR-1024|United States|Florida|658[0n]|BOLD:AAA4928
Virbia aurantiaca|[2389]|IMA015-07|HLC-16982|United States|Massachusetts|634[0n]|BOLD:AAA4928
Virbia aurantiaca|[2390]|HKONS209-08|3114-COI-07|United States|Florida|658[0n]|BOLD:AAA4928

Virbia aurantiaca|[2388]|LOFLB084-06|06-FLOR-1024|United States|Florida|658[0n]|BOLD:AAA4928
Virbia aurantiaca|[2389]|IMA015-07|HLC-16982|United States|Massachusetts|634[0n]|BOLD:AAA4928
Virbia aurantiaca|[2390]|HKONS209-08|3114-COI-07|United States|Florida|658[0n]|BOLD:AAA4928
Virbia aurantiaca|[2391]|HKONS207-08|3112-COI-07|United States|Florida|658[0n]|BOLD:AAA4928
Virbia aurantiaca|[2392]|RDMAB422-05|BCSC95|Canada|Manitoba|658[0n]|BOLD:AAA4928
Virbia aurantiaca|[2393]|MNAG040-08|CNCLEP00041025|Canada|Manitoba|658[0n]|BOLD:AAA4928
Virbia aurantiaca|[2394]|RDMAB423-05|BCSC96|Canada|Manitoba|658[0n]|BOLD:AAA4928
Virbia aurantiaca|[2395]|MNAG039-08|CNCLEP00041024|Canada|Manitoba|658[0n]|BOLD:AAA4928
Virbia aurantiaca|[2396]|MNAG038-08|CNCLEP00041023|Canada|Manitoba|658[0n]|BOLD:AAA4928
Virbia aurantiaca|[2397]|MNAG037-08|CNCLEP00041022|Canada|Manitoba|658[0n]|BOLD:AAA4928
Virbia aurantiaca|[2398]|MNAG036-08|CNCLEP00041021|Canada|Manitoba|658[0n]|BOLD:AAA4928
Virbia aurantiaca|[2399]|RDNMB405-05|CNCNoctuoidea10171|Canada|Manitoba|658[0n]|BOLD:AAA4928
Virbia sp.|[2400]|HKONB412-09|3909-COI-08|United States||658[0n]|BOLD:AAA4928
Virbia aurantiaca|[2401]|BBLSZ161-09|09BBLEP-04087|United States|Texas|658[0n]|BOLD:AAA4928
Virbia aurantiaca|[2402]|LPOKB410-09|MDOK-1395|United States|Oklahoma|658[0n]|BOLD:AAA4928
Virbia aurantiaca|[2403]|BBLSZ156-09|09BBLEP-04082|United States|Texas|658[0n]|BOLD:AAA4928
Virbia aurantiaca|[2404]|RDNMB407-05|CNCNoctuoidea10173|Canada|Ontario|658[0n]|BOLD:AAA4928
Virbia aurantiaca|[2405]|RDNMB404-05|CNCNoctuoidea10170|Canada|Ontario|658[0n]|BOLD:AAA4928
Virbia aurantiaca|[2406]|MILEQ204-11|11-MISC-679|United States|Georgia|658[0n]|BOLD:AAA4928
Virbia aurantiaca|[2407]|LNCNW077-06|06-NCNW-0077|United States|North Carolina|658[0n]|BOLD:AA...
Virbia aurantiaca|[2408]|BBLSY153-09|09BBLEP-03080|United States|Texas|658[0n]|BOLD:AAA4928
Virbia aurantiaca|[2409]|RDNME769-08|LEP041234|United States|Tennessee|658[0n]|BOLD:AAA4928
Virbia aurantiaca|[2410]|LNCB850-09|09-MISC-035|United States|Georgia|658[0n]|BOLD:AAA4928
Virbia aurantiaca|[2411]|CNPPC1857-12|BIOUG03216-B12|Canada|Ontario|622[2n]|BOLD:AAA4928
Virbia aurantiaca|[2412]|BBLPA593-10|10BBCLP-0593|Canada|Ontario|658[0n]|BOLD:AAA4928
Virbia aurantiaca|[2413]|CNPPC1856-12|BIOUG03216-B11|Canada|Ontario|632[0n]|BOLD:AAA4928
Virbia aurantiaca|[2414]|LPSO819-08|PPBP-0819|Canada|Ontario|658[0n]|BOLD:AAA4928
Virbia aurantiaca|[2415]|LGSMC763-05|DNA-ATBI-2763|United States|Tennessee|612[0n]|BOLD:AAA4928
Virbia aurantiaca|[2416]|HKONS214-08|3119-COI-07|United States|Florida|658[0n]|BOLD:AAA4928
Virbia aurantiaca|[2417]|USLEP545-10|10BBLEP-00545|United States|Arkansas|658[0n]|BOLD:AAA4928
Virbia aurantiaca|[2418]|LOFLA772-06|06-FLOR-0772|United States|Florida|658[0n]|BOLD:AAA4928
Virbia aurantiaca|[2419]|LNCC857-11|11-NCCC-382|United States|North Carolina|658[0n]|BOLD:AAA4928
Virbia aurantiaca|[2420]|LGSMG109-07|BGS03430|United States|North Carolina|658[0n]|BOLD:AAA4928
Virbia aurantiaca|[2421]|LPOKA300-08|MDOK-0300|United States|Oklahoma|658[0n]|BOLD:AAA4928
Virbia aurantiaca|[2422]|BBLSY876-09|09BBLEP-03803|United States|Oklahoma|658[0n]|BOLD:AAA4928
Virbia aurantiaca|[2423]|HKONS212-08|3117-COI-07|United States|Florida|644[0n]|BOLD:AAA4928
Virbia aurantiaca|[2424]|HKONS208-08|3113-COI-07|United States|Florida|658[0n]|BOLD:AAA4928
Virbia aurantiaca|[2425]|LOFLA934-06|06-FLOR-0934|United States|Florida|658[0n]|BOLD:AAA4928
Virbia aurantiaca|[2426]|LPOKB771-09|MDOK-1813|United States|Oklahoma|655[1n]|BOLD:AAA4928
Virbia aurantiaca|[2427]|BBLSX325-09|09BBLEP-02253|United States|Oklahoma|658[0n]|BOLD:AAA4928
Virbia aurantiaca|[2428]|LPOKB437-09|MDOK-1428|United States|Oklahoma|658[0n]|BOLD:AAA4928
Virbia aurantiaca|[2429]|LPOKE128-10|MDOK-4206|United States|Oklahoma|658[0n]|BOLD:AAA4928
Virbia aurantiaca|[2430]|LNCC858-11|11-NCCC-383|United States|North Carolina|658[0n]|BOLD:AAA4928
Virbia aurantiaca|[2431]|BBLOB747-11|BIOUG01399-G10|United States|Oklahoma|658[0n]|BOLD:AAA4928
Virbia aurantiaca|[2432]|USLEP544-10|10BBLEP-00544|United States|Arkansas|658[0n]|BOLD:AAA4928
Virbia aurantiaca|[2433]|HKONS213-08|3118-COI-07|United States|Florida|658[0n]|BOLD:AAA4928
Virbia aurantiaca|[2434]|BBLSW801-09|09BBLEP-01729|United States|Oklahoma|658[0n]|BOLD:AAA4928
Virbia aurantiaca|[2435]|LPOKB797-09|MDOK-1839|United States|Oklahoma|658[0n]|BOLD:AAA4928
Virbia aurantiaca|[2436]|LPOKB728-09|MDOK-1770|United States|Oklahoma|658[0n]|BOLD:AAA4928
Virbia aurantiaca|[2437]|LPOKA391-09|MDOK-0391|United States|Oklahoma|658[0n]|BOLD:AAA4928
Virbia aurantiaca|[2438]|LPOKE314-11|MDOK-4392|United States|Oklahoma|658[0n]|BOLD:AAA4928
Virbia fragilis|[2439]|RDNME510-08|LEP037934|United States|Colorado|658[0n]|BOLD:AAA4928
Virbia fragilis|[2440]|RDNME508-08|LEP037932|United States|Wyoming|658[0n]|BOLD:AAA4928
Virbia fragilis|[2441]|RDNME509-08|LEP037933|United States|Colorado|658[0n]|BOLD:AAA4928
Virbia fragilis|[2442]|RDNME506-08|LEP037930|United States|Utah|658[0n]|BOLD:AAA4928
Virbia aurantiaca|[2443]|BBLEC996-09|09BBELE-0996|Canada|Nova Scotia|658[0n]|BOLD:AAA4928
Virbia aurantiaca|[2444]|BBLOD1035-11|BIOUG01820-F07|United States|Arizona|658[0n]|BOLD:AAA4928
Virbia aurantiaca|[2445]|BBLOD1164-11|BIOUG01824-A06|United States|Arizona|658[0n]|BOLD:AAA4928
Virbia aurantiaca|[2446]|BBLOD1034-11|BIOUG01820-F06|United States|Arizona|658[0n]|BOLD:AAA4928
Virbia aurantiaca|[2447]|BBLOD1030-11|BIOUG01820-F02|United States|Arizona|658[0n]|BOLD:AAA4928
Virbia aurantiaca|[2448]|BBLOD1037-11|BIOUG01820-F09|United States|Arizona|658[0n]|BOLD:AAA4928
Virbia aurantiaca|[2449]|BBLOD1036-11|BIOUG01820-F08|United States|Arizona|658[0n]|BOLD:AAA4928
Virbia aurantiaca|[2450]|BBLOD1031-11|BIOUG01820-F03|United States|Arizona|658[0n]|BOLD:AAA4928
Virbia aurantiaca|[2451]|BBLOD1063-11|BIOUG01820-H11|United States|Arizona|658[0n]|BOLD:AAA4928
Virbia aurantiaca|[2452]|BBLOD1163-11|BIOUG01824-A05|United States|Arizona|658[0n]|BOLD:AAA4928
Virbia aurantiaca|[2453]|BBLOD1148-11|BIOUG01822-H01|United States|Arizona|658[0n]|BOLD:AAA4928
Virbia aurantiaca|[2454]|BBLOD736-11|BIOUG01567-E05|United States|Arizona|658[0n]|BOLD:AAA4928
Virbia aurantiaca|[2455]|LPSK197-08|08BBLEP-01765|Canada|Saskatchewan|658[0n]|BOLD:AAA4928
Virbia aurantiaca|[2456]|LPSK632-08|08BBLEP-02200|Canada|Saskatchewan|658[0n]|BOLD:AAA4928
Virbia aurantiaca|[2457]|LPSK196-08|08BBLEP-01764|Canada|Saskatchewan|658[0n]|BOLD:AAA4928
Virbia aurantiaca|[2458]|BBLPA591-10|10BBCLP-0591|Canada|Ontario|658[0n]|BOLD:AAA4928
Virbia aurantiaca|[2459]|BBLPA590-10|10BBCLP-0590|Canada|Ontario|623[0n]|BOLD:AAA4928
Virbia aurantiaca|[2460]|RDNMB406-05|CNCNoctuoidea10172|Canada|Ontario|658[0n]|BOLD:AAA4928
Virbia aurantiaca|[2461]|LPSK200-08|08BBLEP-01768|Canada|Saskatchewan|658[0n]|BOLD:AAA4928
Virbia aurantiaca|[2462]|LPSK400-08|08BBLEP-01968|Canada|Saskatchewan|658[0n]|BOLD:AAA4928
Pseudosphex leovazquezae|[2463]|CNCLB1876-14|CNCLEP00117600|Mexico|Chiapas|658[0n]|BOLD:AAA1455
Pseudosphex leovazquezae|[2464]|ARCTC839-11|MILA 1301|Guatemala|Izabal|658[0n]|BOLD:AAA1455
Pseudosphex leovazquezae|[2465]|LYRIO397-10|MAL-05408|Mexico|Yucatan|658[0n]|BOLD:AAA1455
Pseudosphex leovazquezae|[2466]|LPYPA393-08|MLL-01863|Mexico|Campeche|658[0n]|BOLD:AAA1455
Empyreuma pugione|[2467]|LNAUT202-14|CCDB-22941-B01|United States|Florida|658[0n]|BOLD:AAX7433
Empyreuma pugione|[2468]|LNAUT205-14|CCDB-22941-B04|United States|Florida|658[0n]|BOLD:AAX7433
Empyreuma pugione|[2469]|LNAUT203-14|CCDB-22941-B02|United States|Florida|658[0n]|BOLD:AAX7433
Empyreuma pugione|[2470]|RDNML363-13|CNCLEP 92339|United States|Florida|658[0n]|BOLD:AAX7433
Empyreuma pugione|[2471]|LNAUT204-14|CCDB-22941-B03|United States|Florida|658[0n]|BOLD:AAX7433
Empyreuma pugione|[2472]|LNAUT206-14|CCDB-22941-B05|United States|Florida|658[0n]|BOLD:AAX7433
Empyreuma pugione|[2473]|LSEU155-06|06-JKA-0155|United States||535[1n]|BOLD:AAX7433
Bertholdia|[2474]|LNAUT2881-14|CCDB-23279-D12|United States|Arizona|423[0n]|
Bertholdia trigona|[2475]|CMAZA552-10|CMAZ-0552|United States|Arizona|658[0n]|BOLD:AAE3757
Bertholdia|[2476]|LNAUT2883-14|CCDB-23279-E02|United States|Arizona|658[0n]|BOLD:AAE3757
Bertholdia|[2477]|LNAUT2884-14|CCDB-23279-E03|United States|Arizona|658[0n]|BOLD:AAE3757
Bertholdia|[2478]|LNAUT2882-14|CCDB-23279-E01|United States|Arizona|658[0n]|BOLD:AAE3757
Bertholdia|[2479]|LNAUT2880-14|CCDB-23279-D11|United States|Arizona|658[0n]|BOLD:AAE3757
Bertholdia trigona|[2480]|AWCL072-09|JLB-0270|United States|Arizona|630[0n]|BOLD:AAE3757
Bertholdia trigona|[2481]|AWCL073-09|JLB-0272|United States|Arizona|658[0n]|BOLD:AAE3757
Bertholdia trigona|[2482]|RDNME468-08|LEP037892|United States|Arizona|658[0n]|BOLD:AAE3757
Bertholdia sp. 1|[2483]|NOCNA003-14|20302-140609-TX|United States|Texas|658[0n]|BOLD:AAE3757
Bertholdia sp. 1|[2484]|NOCNA002-14|20301-140609-TX|United States|Texas|658[0n]|BOLD:AAE3757
Bertholdia trigona|[2485]|AWCL071-09|JLB-0269|United States|Arizona|658[0n]|BOLD:AAE3757
Bertholdia trigona|[2486]|ARCTB342-08|MILA 1001|Guatemala|Izabal|658[0n]|BOLD:ACR9271
Bertholdia trigona|[2487]|ARCTB333-08|MILA 0992|Guatemala|Baja Verapaz|658[0n]|BOLD:ACR9271
Bertholdia flammea|[2488]|CNCLB1840-14|CNCLEP00117967|Mexico|Chiapas|658[2n]|BOLD:ACR9271
Bertholdia flammea|[2489]|CNCLB1839-14|CNCLEP00117966|Mexico|Chiapas|658[0n]|BOLD:ACR9271
Bertholdia trigona|[2490]|RDNMH182-09|CNCLEP00054399|United States|Colorado|624[0n]|BOLD:ACR9271

Bertholdia flammea|[2488]|CNCLB1840-14|CNCLEP00117967|Mexico|Chiapas|658[2n]|BOLD:ACR9271
Bertholdia flammea|[2489]|CNCLB1839-14|CNCLEP00117966|Mexico|Chiapas|658[0n]|BOLD:ACR9271
Bertholdia trigona|[2490]|RDNMH182-09|CNCLEP00054399|United States|Colorado|624[0n]|BOLD:ACR9271
Bertholdia sp.|[2491]|RDNMH181-09|CNCLEP00054398|United States|Arizona|640[0n]|BOLD:ACR9271
Bertholdia trigona|[2492]|RDNMH180-09|CNCLEP00054397|United States|Texas|637[0n]|BOLD:ACR9271
Cisthene subrufa|[2493]|HKONB167-08|3664-COI-08|United States|Texas|658[0n]|BOLD:ABY4997
Cisthene subrufa|[2494]|RDNMF901-08|CNC LEP00054168|United States|Texas|609[0n]|BOLD:ABY4997
Cisthene subrufa|[2495]|HKONB330-09|3827-COI-08|United States|Texas|658[0n]|BOLD:ABY4997
Cisthene subrufa|[2496]|ABLCW138-10|CSUPOBK-0138|United States|Texas|658[0n]|BOLD:ABY4997
Cisthene subrufa|[2497]|ABLCW139-10|CSUPOBK-0139|United States|Texas|658[0n]|BOLD:ABY4997
Cisthene subrufa|[2498]|ABLCW137-10|CSUPOBK-0137|United States|Texas|658[0n]|BOLD:ABY4997
Cisthene subrufa|[2499]|ABLCW141-10|CSUPOBK-0141|United States|Texas|658[0n]|BOLD:ABY4997
Cisthene subrufa|[2500]|ABLCW142-10|CSUPOBK-0142|United States|Texas|658[0n]|BOLD:ABY4997
Cisthene subrufa|[2501]|RDNMF902-08|CNC LEP00054169|United States|Texas|658[0n]|BOLD:ABY4997
Cisthene subrufa|[2502]|HKONB168-08|3665-COI-08|United States|Texas|658[0n]|BOLD:ABY4997
Cisthene subrufa|[2503]|HKONB166-08|3663-COI-08|United States|Texas|658[0n]|BOLD:ABY4997
Cisthene perrosea|[2504]|ABLCW128-10|CSUPOBK-0128|United States|California|646[0n]|BOLD:ABX6342
Cisthene perrosea|[2505]|RDNMH751-09|CNCLEP00063184|United States|California|658[0n]|BOLD:ABX6342
Cisthene perrosea|[2506]|ABLCW129-10|CSUPOBK-0129|United States|California|658[0n]|BOLD:ABX6342
Cisthene perrosea|[2507]|ABLCW127-10|CSUPOBK-0127|United States|California|658[0n]|BOLD:ABX6342
Cisthene angelus|[2508]|BBLSY660-09|09BBLEP-03587|United States|Arizona|589[0n]|BOLD:AAD5301
Cisthene angelus|[2509]|LMEM932-09|RBMIS-1027|United States|Arizona|658[0n]|BOLD:AAD5301
Cisthene angelus|[2510]|ABLCW081-10|CSUPOBK-0081|United States|Utah|658[0n]|BOLD:AAD5301
Cisthene angelus|[2511]|ABLCW080-10|CSUPOBK-0080|United States|Utah|658[0n]|BOLD:AAD5301
Cisthene angelus|[2512]|ABLCW084-10|CSUPOBK-0084|United States|Utah|658[0n]|BOLD:AAD5301
Cisthene angelus|[2513]|ABLCW083-10|CSUPOBK-0083|United States|Utah|658[0n]|BOLD:AAD5301
Cisthene angelus|[2514]|ABLCW079-10|CSUPOBK-0079|United States|Utah|658[0n]|BOLD:AAD5301
Cisthene angelus|[2515]|ABLCW082-10|CSUPOBK-0082|United States|Utah|658[0n]|BOLD:AAD5301
Cisthene angelus|[2516]|RDNMH345-09|CNCLEP00057754|United States|Arizona|658[0n]|BOLD:AAD5301
Cisthene angelus|[2517]|BBLOC299-11|BIOUG01455-B07|United States|Arizona|658[0n]|BOLD:AAD5301
Cisthene angelus|[2518]|BBLOC336-11|BIOUG01455-E08|United States|Arizona|658[0n]|BOLD:AAD5301
Cisthene angelus|[2519]|BBLOC337-11|BIOUG01455-E09|United States|Arizona|658[0n]|BOLD:AAD5301
Cisthene angelus|[2520]|BBLOC341-11|BIOUG01455-F01|United States|Arizona|658[0n]|BOLD:AAD5301
Cisthene angelus|[2521]|BBLOC342-11|BIOUG01455-F02|United States|Arizona|658[0n]|BOLD:AAD5301
Cisthene angelus|[2522]|BBLOC351-11|BIOUG01455-F11|United States|Arizona|658[0n]|BOLD:AAD5301
Cisthene angelus|[2523]|BBLOC404-11|BIOUG01456-C05|United States|Arizona|658[0n]|BOLD:AAD5301
Cisthene angelus|[2524]|BBLOC786-11|BIOUG01466-A08|United States|Arizona|658[0n]|BOLD:AAD5301
Cisthene angelus|[2525]|BBLOC787-11|BIOUG01466-A09|United States|Arizona|658[0n]|BOLD:AAD5301
Cisthene angelus|[2526]|LMEM930-09|RBMIS-1025|United States|Arizona|658[0n]|BOLD:AAD5301
Cisthene angelus|[2527]|LMEM931-09|RBMIS-1026|United States|Arizona|658[0n]|BOLD:AAD5301
Cisthene angelus|[2528]|LMEM933-09|RBMIS-1028|United States|Arizona|658[0n]|BOLD:AAD5301
Cisthene angelus|[2529]|LMEM934-09|RBMIS-1029|United States|Arizona|658[0n]|BOLD:AAD5301
Cisthene angelus|[2530]|BBLSY689-09|09BBLEP-03616|United States|Arizona|658[0n]|BOLD:AAD5301
Cisthene angelus|[2531]|BBLSY741-09|09BBLEP-03668|United States|Arizona|658[0n]|BOLD:AAD5301
Cisthene angelus|[2532]|BBLSY742-09|09BBLEP-03669|United States|Arizona|658[0n]|BOLD:AAD5301
Cisthene angelus|[2533]|BBLSY836-09|09BBLEP-03763|United States|Arizona|658[0n]|BOLD:AAD5301
Cisthene angelus|[2534]|BBLOC1611-11|BIOUG01546-F08|United States|Arizona|658[0n]|BOLD:AAD5301
Cisthene angelus|[2535]|BBLOC1622-11|BIOUG01546-G07|United States|Arizona|658[0n]|BOLD:AAD5301
Cisthene angelus|[2536]|BBLSY987-09|09BBLEP-03914|United States|Arizona|658[0n]|BOLD:AAD5301
Cisthene angelus|[2537]|BBLOB091-11|BIOUG01367-H07|United States|Arizona|658[0n]|BOLD:AAD5301
Cisthene angelus|[2538]|BBLOB156-11|BIOUG01368-F01|United States|Arizona|658[0n]|BOLD:AAD5301
Cisthene angelus|[2539]|BBLOB301-11|BIOUG01370-B04|United States|Arizona|658[0n]|BOLD:AAD5301
Cisthene angelus|[2540]|BBLOE1648-12|BIOUG01989-E12|United States|Arizona|658[0n]|BOLD:AAD5301
Cisthene angelus|[2541]|BBLSY823-09|09BBLEP-03750|United States|Arizona|620[0n]|BOLD:AAD5301
Cisthene angelus|[2542]|BBLSY601-09|09BBLEP-03528|United States|Arizona|589[0n]|BOLD:AAD5301
Cisthene angelus|[2543]|BBLSY661-09|09BBLEP-03588|United States|Arizona|589[0n]|BOLD:AAD5301
Cisthene angelus|[2544]|BBLSY758-09|09BBLEP-03685|United States|Arizona|632[0n]|BOLD:AAD5301
Cisthene angelus|[2545]|BBLSY954-09|09BBLEP-03881|United States|Arizona|635[0n]|BOLD:AAD5301
Cisthene angelus|[2546]|RDNMH346-09|CNCLEP00057755|United States|Arizona|658[0n]|BOLD:AAD5301
Cisthene angelus|[2547]|BBLSW240-09|09BBLEP-01168|United States|Arizona|658[0n]|BOLD:AAD5301
Cisthene angelus|[2548]|BBLSW239-09|09BBLEP-01167|United States|Arizona|658[0n]|BOLD:AAD5301
Cisthene angelus|[2549]|BBLSW200-09|09BBLEP-01128|United States|Arizona|658[0n]|BOLD:AAD5301
Cisthene faustinula|[2550]|LNAUT254-14|CCDB-22941-F05|United States|California|589[0n]|BOLD:AA...
Cisthene sp.|[2551]|LOCBC718-06|06-BLLOC-2598|United States|California|626[0n]|BOLD:AAG6219
Cisthene sp.|[2552]|LOCBC003-06|06-BLLOC-1883|United States|California|658[0n]|BOLD:AAG6219
Cisthene sp.|[2553]|LOCBF3281-14|BIOUG11929-C04|United States|California|561[0n]|BOLD:AAG6219
Cisthene sp.|[2554]|LOCBC720-06|06-BLLOC-2600|United States|California|599[0n]|BOLD:AAG6219
Cisthene sp.|[2555]|LOCBF3282-14|BIOUG11929-C05|United States|California|540[0n]|BOLD:AAG6219
Cisthene sp.|[2556]|LOCBC483-06|06-BLLOC-2363|United States|California|658[0n]|BOLD:AAG6219
Cisthene sp.|[2557]|LOCBC723-06|06-BLLOC-2603|United States|California|658[0n]|BOLD:AAG6219
Cisthene sp.|[2558]|LOCBC717-06|06-BLLOC-2597|United States|California|658[0n]|BOLD:AAG6219
Cisthene sp.|[2559]|LOCBC480-06|06-BLLOC-2360|United States|California|658[0n]|BOLD:AAG6219
Cisthene sp.|[2560]|LOCBB936-06|06-BLLOC-1876|United States|California|658[0n]|BOLD:AAG6219
Cisthene sp.|[2561]|LOCBC008-06|06-BLLOC-1888|United States|California|658[0n]|BOLD:AAG6219
Cisthene sp.|[2562]|LOCBC001-06|06-BLLOC-1881|United States|California|658[0n]|BOLD:AAG6219
Cisthene sp.|[2563]|LOCBC719-06|06-BLLOC-2599|United States|California|658[0n]|BOLD:AAG6219
Cisthene sp.|[2564]|LOCBC721-06|06-BLLOC-2601|United States|California|658[0n]|BOLD:AAG6219
Cisthene sp.|[2565]|LOCBC722-06|06-BLLOC-2602|United States|California|658[0n]|BOLD:AAG6219
Cisthene sp.|[2566]|LOCBF3283-14|BIOUG11929-C06|United States|California|564[0n]|BOLD:AAG6219
Cisthene sp.|[2567]|LOCBB935-06|06-BLLOC-1875|United States|California|656[0n]|BOLD:AAG6219
Cisthene sp.|[2568]|LOCBF3280-14|BIOUG11929-C03|United States|California|555[0n]|BOLD:AAG6219
Cisthene sp.|[2569]|LOCBB937-06|06-BLLOC-1877|United States|California|658[0n]|BOLD:AAG6219
Cisthene sp.|[2570]|LOCBB938-06|06-BLLOC-1878|United States|California|658[0n]|BOLD:AAG6219
Cisthene sp.|[2571]|LOCBB934-06|06-BLLOC-1874|United States|California|658[0n]|BOLD:AAG6219
Cisthene sp.|[2572]|LOCBF3279-14|BIOUG11929-C02|United States|California|561[0n]|BOLD:AAG6219
Cisthene plumbea|[2573]|LMEM051-09|RBMIS-0051|United States|Louisiana|658[0n]|BOLD:AAC4269
Cisthene plumbea|[2574]|LMEM050-09|RBMIS-0050|United States|Louisiana|658[0n]|BOLD:AAC4269
Cisthene plumbea|[2575]|LMEM049-09|RBMIS-0049|United States|Louisiana|658[1n]|BOLD:AAC4269
Cisthene plumbea|[2576]|LSEU612-06|06-JKA-0612|United States|Georgia|658[0n]|BOLD:AAC4269
Cisthene striata|[2577]|LNAUT1270-14|CCDB-22953-C12|United States|Georgia|658[0n]|BOLD:AAC4269
Cisthene striata|[2578]|LNAUT1272-14|CCDB-22953-D02|United States|Georgia|658[0n]|BOLD:AAC4269
Cisthene plumbea|[2579]|LMEM055-09|RBMIS-0055|United States|Alabama|658[1n]|BOLD:AAC4269
Cisthene plumbea|[2580]|LMEM053-09|RBMIS-0053|United States|Alabama|658[0n]|BOLD:AAC4269
Cisthene plumbea|[2581]|LMEM054-09|RBMIS-0054|United States|Alabama|658[0n]|BOLD:AAC4269
Cisthene plumbea|[2582]|LMEM056-09|RBMIS-0056|United States|Georgia|658[0n]|BOLD:AAC4269
Cisthene plumbea|[2583]|LMEM057-09|RBMIS-0057|United States|Georgia|658[0n]|BOLD:AAC4269
Cisthene plumbea|[2584]|LPOKD202-09|MDOK-3281|United States|Oklahoma|657[0n]|BOLD:AAC4269
Cisthene plumbea|[2585]|LPOKC305-09|MDOK-2382|United States|Oklahoma|658[0n]|BOLD:AAC4269
Cisthene striata|[2586]|RDNMH915-09|CNCLEP00067914|United States|Florida|658[0n]|BOLD:AAC4269
Cisthene plumbea|[2587]|LMEM058-09|RBMIS-0058|United States|Georgia|658[1n]|BOLD:AAC4269
Cisthene plumbea|[2588]|LSUSA150-06|06-SUSA-0150|United States|Kentucky|656[0n]|BOLD:AAC4269
Cisthene plumbea|[2589]|LSUSA141-06|06-SUSA-0141|United States|Kentucky|658[0n]|BOLD:AAC4269

Cisthene plumbea|[2587]|LMEM058-09|RBMIS-0058|United States|Georgia|658[1n]|BOLD:AAC4269
Cisthene plumbea|[2588]|LSUSA150-06|06-SUSA-0150|United States|Kentucky|656[0n]|BOLD:AAC4269
Cisthene plumbea|[2589]|LSUSA141-06|06-SUSA-0141|United States|Kentucky|658[0n]|BOLD:AAC4269
Cisthene plumbea|[2590]|LNCNW105-06|06-NCNW-0105|United States|North Carolina|658[0n]|BOLD:AAC...
Cisthene plumbea|[2591]|LNCNW103-06|06-NCNW-0103|United States|North Carolina|658[0n]|BOLD:AAC...
Cisthene plumbea|[2592]|LMEM048-09|RBMIS-0048|United States|Louisiana|658[0n]|BOLD:AAC4269
Cisthene striata|[2593]|RDNMH918-09|CNCLEP00067917|United States|Florida|636[0n]|BOLD:AAC4269
Cisthene plumbea|[2594]|USLEP542-10|10BBLEP-00542|United States|Florida|658[0n]|BOLD:AAC4269
Cisthene plumbea|[2595]|HKONS228-08|3133-COI-07|United States|Florida|658[1n]|BOLD:AAC4269
Cisthene plumbea|[2596]|LPOKA186-08|MDOK-0186|United States|Oklahoma|658[0n]|BOLD:AAC4269
Cisthene plumbea|[2597]|RDNMH350-09|CNCLEP00057759|United States|Arkansas|658[0n]|BOLD:AAC4269
Cisthene striata|[2598]|LNAUT1271-14|CCDB-22953-D01|United States|Georgia|658[0n]|BOLD:AAC4269
Cisthene plumbea|[2599]|BBLSU038-09|09BBLEP-04407|United States|Arkansas|658[0n]|BOLD:AAC4269
Cisthene plumbea|[2600]|BBLSU024-09|09BBLEP-04393|United States|Arkansas|658[0n]|BOLD:AAC4269
Cisthene plumbea|[2601]|RDNMH349-09|CNCLEP00057758|United States|Mississippi|658[0n]|BOLD:AAC4269
Cisthene striata|[2602]|RDNMH919-09|CNCLEP00067918|United States|Florida|658[0n]|BOLD:AAC4269
Cisthene striata|[2603]|RDNMH917-09|CNCLEP00067916|United States|Florida|658[0n]|BOLD:AAC4269
Cisthene plumbea|[2604]|HKONS218-08|3123-COI-07|United States|Florida|658[0n]|BOLD:AAC4269
Cisthene plumbea|[2605]|LMEM052-09|RBMIS-0052|United States|Louisiana|658[0n]|BOLD:AAC4269
Cisthene plumbea|[2606]|LMEM059-09|RBMIS-0059|United States|Louisiana|658[0n]|BOLD:AAC4269
Cisthene plumbea|[2607]|HKONB163-08|3660-COI-08|United States|Texas|658[0n]|BOLD:AAC4269
Cisthene plumbea|[2608]|USLEP543-10|10BBLEP-00543|United States|Florida|658[0n]|BOLD:AAC4269
Cisthene plumbea|[2609]|USLEP505-10|10BBLEP-00505|United States|Florida|658[0n]|BOLD:AAC4269
Cisthene plumbea|[2610]|USLEP233-10|10BBLEP-00233|United States|Florida|658[0n]|BOLD:AAC4269
Cisthene sp. T1|[2611]|NAMUM006-08|AYK-06-7153|United States|Arizona|656[0n]|BOLD:AAC4255
Cisthene sp. T1|[2612]|RDNMJ501-11|CNCLEP 80029|United States|Arizona|658[0n]|BOLD:AAC4255
Cisthene sp. T1|[2613]|RDNMJ494-11|CNCLEP 80022|United States|Arizona|658[0n]|BOLD:AAC4255
Cisthene sp. T1|[2614]|RDNMJ491-11|CNCLEP 80019|United States|Arizona|658[0n]|BOLD:AAC4255
Cisthene sp. T1|[2615]|RDNMJ488-11|CNCLEP 80016|United States|Arizona|658[0n]|BOLD:AAC4255
Cisthene sp. T1|[2616]|RDNMJ486-11|CNCLEP 80014|United States|Arizona|658[0n]|BOLD:AAC4255
Cisthene sp. T1|[2617]|LMEM929-09|RBMIS-1024|United States|New Mexico|658[0n]|BOLD:AAC4255
Cisthene sp. T1|[2618]|LMEM918-09|RBMIS-1013|United States|New Mexico|658[0n]|BOLD:AAC4255
Cisthene sp. T1|[2619]|DMAZ048-09|DMTRN-0142|United States|Arizona|658[0n]|BOLD:AAC4255
Cisthene sp. T1|[2620]|BBLOC343-11|BIOUG01455-F03|United States|Arizona|658[0n]|BOLD:AAC4255
Cisthene sp. T1|[2621]|BBLOC324-11|BIOUG01455-D08|United States|Arizona|658[0n]|BOLD:AAC4255
Cisthene dorsimacula|[2622]|ABLCW114-10|CSUPOBK-0114|United States|California|658[0n]|BOLD:ABZ...
Cisthene dorsimacula|[2623]|ABLCW115-10|CSUPOBK-0115|United States|California|658[0n]|BOLD:ABZ...
Cisthene dorsimacula|[2624]|ABLCW112-10|CSUPOBK-0112|United States|California|658[0n]|BOLD:ABZ...
Cisthene dorsimacula|[2625]|ABLCW111-10|CSUPOBK-0111|United States|California|658[0n]|BOLD:ABZ...
Cisthene dorsimacula|[2626]|RDNMF903-08|CNC LEP00054170|United States|California|658[0n]|BOLD:...
Cisthene dorsimacula|[2627]|ABLCW113-10|CSUPOBK-0113|United States|California|658[0n]|BOLD:ABZ...
Cisthene dorsimacula|[2628]|ABLCW110-10|CSUPOBK-0110|United States|California|658[0n]|BOLD:ABZ...
Cisthene dorsimacula|[2629]|LOCBF3335-14|BIOUG11929-G10|United States|California|546[0n]|BOLD:...
Cisthene sp. T2|[2630]|ABLCW122-10|CSUPOBK-0122|United States|Arizona|658[0n]|BOLD:AAC4271
Cisthene sp. T2|[2631]|ABLCW123-10|CSUPOBK-0123|United States|Arizona|658[0n]|BOLD:AAC4271
Cisthene sp. T2|[2632]|CMAZA081-09|CMAZ-0081|United States|Arizona|658[0n]|BOLD:AAC4271
Cisthene sp. T2|[2633]|CMAZA019-09|CMAZ-0019|United States|Arizona|658[0n]|BOLD:AAC4271
Cisthene sp. T2|[2634]|RDNMJ497-11|CNCLEP 80025|United States|Arizona|658[0n]|BOLD:AAC4271
Cisthene sp. T2|[2635]|RDNMJ492-11|CNCLEP 80020|United States|Arizona|658[0n]|BOLD:AAC4271
Cisthene sp. T2|[2636]|RDNMJ487-11|CNCLEP 80015|United States|Arizona|658[0n]|BOLD:AAC4271
Cisthene sp. T2|[2637]|ABLCW125-10|CSUPOBK-0125|United States|Arizona|658[0n]|BOLD:AAC4271
Cisthene sp. T2|[2638]|RDNMJ496-11|CNCLEP 80024|United States|Arizona|658[0n]|BOLD:AAC4271
Cisthene sp. T2|[2639]|RDNMJ495-11|CNCLEP 80023|United States|Arizona|658[0n]|BOLD:AAC4271
Cisthene sp. T2|[2640]|CMAZA632-10|CMAZ-0632|United States|Arizona|658[0n]|BOLD:AAC4271
Cisthene sp. T2|[2641]|ABLCX139-10|CSUPOBK-1423|Mexico|Sonora|658[0n]|BOLD:AAC4271
Cisthene sp. T2|[2642]|ABLCW124-10|CSUPOBK-0124|United States|Arizona|605[0n]|BOLD:AAC4271
Cisthene sp. T2|[2643]|ABLCW109-10|CSUPOBK-0109|Mexico|Sonora|658[0n]|BOLD:AAC4271
Cisthene sp. T2|[2644]|ABLCW108-10|CSUPOBK-0108|Mexico|Sonora|658[0n]|BOLD:AAC4271
Cisthene sp. T2|[2645]|RDNMJ500-11|CNCLEP 80028|United States|Arizona|658[0n]|BOLD:AAC4271
Cisthene sp. T2|[2646]|RDNMJ498-11|CNCLEP 80026|United States|Arizona|658[0n]|BOLD:AAC4271
Cisthene sp. T2|[2647]|RDNMH348-09|CNCLEP00057757|United States|Arizona|658[0n]|BOLD:AAC4271
Cisthene sp. T2|[2648]|RDNMH347-09|CNCLEP00057756|United States|Arizona|658[0n]|BOLD:AAC4271
Cisthene tenuifascia|[2649]|BBLSW086-09|09BBLEP-01014|United States|Texas|658[0n]|BOLD:AAC4270
Cisthene tenuifascia|[2650]|LPOKD452-09|MDOK-3531|United States|Oklahoma|658[0n]|BOLD:AAC4270
Cisthene tenuifascia|[2651]|LPOKA334-08|MDOK-0334|United States|Oklahoma|658[0n]|BOLD:AAC4270
Cisthene tenuifascia|[2652]|BBLSX810-09|09BBLEP-02738|United States|Texas|658[0n]|BOLD:AAC4270
Cisthene tenuifascia|[2653]|ABLCW102-10|CSUPOBK-0102|United States|Oklahoma|658[0n]|BOLD:AAC4270
Cisthene tenuifascia|[2654]|ABLCW100-10|CSUPOBK-0100|United States|Oklahoma|658[0n]|BOLD:AAC4270
Cisthene tenuifascia|[2655]|ABLCW104-10|CSUPOBK-0104|United States|Oklahoma|658[0n]|BOLD:AAC4270
Cisthene tenuifascia|[2656]|ABLCW103-10|CSUPOBK-0103|United States|Oklahoma|658[0n]|BOLD:AAC4270
Cisthene tenuifascia|[2657]|LPOKD384-09|MDOK-3463|United States|Oklahoma|658[0n]|BOLD:AAC4270
Cisthene tenuifascia|[2658]|LPOKA332-08|MDOK-0332|United States|Oklahoma|658[0n]|BOLD:AAC4270
Cisthene tenuifascia|[2659]|BBLSX819-09|09BBLEP-02747|United States|Texas|658[0n]|BOLD:AAC4270
Cisthene tenuifascia|[2660]|HKONB461-09|3958-COI-08|United States|Texas|658[0n]|BOLD:AAC4270
Cisthene tenuifascia|[2661]|BBLSY288-09|09BBLEP-03215|United States|Texas|634[0n]|BOLD:AAC4270
Cisthene tenuifascia|[2662]|LMEM915-09|RBMIS-1010|United States|Texas|638[0n]|BOLD:AAC4270
Cisthene tenuifascia|[2663]|ABLCW101-10|CSUPOBK-0101|United States|Oklahoma|658[0n]|BOLD:AAC4270
Cisthene tenuifascia|[2664]|BBLSY196-09|09BBLEP-03123|United States|Texas|658[0n]|BOLD:AAC4270
Cisthene tenuifascia|[2665]|BBLSY195-09|09BBLEP-03122|United States|Texas|658[0n]|BOLD:AAC4270
Cisthene tenuifascia|[2666]|BBLSX785-09|09BBLEP-02713|United States|Texas|658[0n]|BOLD:AAC4270
Cisthene tenuifascia|[2667]|BBLSX780-09|09BBLEP-02708|United States|Texas|658[0n]|BOLD:AAC4270
Cisthene tenuifascia|[2668]|BBLSX778-09|09BBLEP-02706|United States|Texas|658[0n]|BOLD:AAC4270
Cisthene tenuifascia|[2669]|ABLCW105-10|CSUPOBK-0105|United States|Oklahoma|658[0n]|BOLD:AAC4270
Cisthene tenuifascia|[2670]|HKONB161-08|3658-COI-08|United States|Texas|658[0n]|BOLD:AAC4270
Cisthene tenuifascia|[2671]|LMEM916-09|RBMIS-1011|United States|Texas|658[0n]|BOLD:AAC4270
Cisthene tenuifascia|[2672]|LPOKB764-09|MDOK-1806|United States|Oklahoma|658[1n]|BOLD:AAC4270
Cisthene deserta|[2673]|RDNMH755-09|CNCLEP00063188|United States|California|658[0n]|BOLD:AAJ1065
Cisthene deserta|[2674]|RDNMH754-09|CNCLEP00063187|United States|California|658[0n]|BOLD:AAJ1065
Cisthene deserta|[2675]|ABLCW126-10|CSUPOBK-0126|United States|California|658[0n]|BOLD:AAJ1065
Cisthene kentuckiensis|[2676]|LSUSA100-06|06-SUSA-0100|United States|Kentucky|658[0n]|BOLD:AAC...
Cisthene kentuckiensis|[2677]|LNCB638-09|09-NCCC-108|United States|North Carolina|658[0n]|BOLD ...
Cisthene kentuckiensis|[2678]|LMEM926-09|RBMIS-1021|United States|Georgia|658[0n]|BOLD:AAC4253
Cisthene kentuckiensis|[2679]|LNCB784-09|09-NCCC-254|United States|North Carolina|658[0n]|BOLD ...
Cisthene kentuckiensis|[2680]|LNCB727-09|09-NCCC-197|United States|North Carolina|658[1n]|BOLD ...
Cisthene kentuckiensis|[2681]|RDNMH351-09|CNCLEP00057760|United States|Alabama|658[0n]|BOLD:AA...
Cisthene kentuckiensis|[2682]|LMEM925-09|RBMIS-1020|United States|Georgia|658[0n]|BOLD:AAC4253
Cisthene kentuckiensis|[2683]|LMEM927-09|RBMIS-1022|United States|Georgia|658[0n]|BOLD:AAC4253
Cisthene kentuckiensis|[2684]|LMEM928-09|RBMIS-1023|United States|Georgia|658[0n]|BOLD:AAC4253
Cisthene kentuckiensis|[2685]|LNCB755-09|09-NCCC-225|United States|North Carolina|658[0n]|BOLD ...
Cisthene kentuckiensis|[2686]|HKONS225-08|3130-COI-07|United States|Florida|658[0n]|BOLD:AAC4253
Cisthene kentuckiensis|[2687]|HKONS224-08|3129-COI-07|United States|Florida|658[0n]|BOLD:AAC4253
Cisthene unifascia|[2688]|ABLCW140-10|CSUPOBK-0140|United States|Texas|658[0n]|BOLD:AAE2719
Cisthene unifascia|[2689]|BBLSX352-09|09BBLEP-02280|United States|Oklahoma|658[0n]|BOLD:AAE2719

Cisthene kentuckiensis|[2687]|HKONS224-08|3129-COI-07|United States|Florida|658[0n]|BOLD:AAC4253
Cisthene unifascia|[2688]|ABLCW140-10|CSUPOBK-0140|United States|Texas|658[0n]|BOLD:AAE2719
Cisthene unifascia|[2689]|BBLSX352-09|09BBLEP-02280|United States|Oklahoma|658[0n]|BOLD:AAE2719
Cisthene unifascia|[2690]|BBLSZ108-09|09BBLEP-04034|United States|Oklahoma|658[0n]|BOLD:AAE2719
Cisthene unifascia|[2691]|HKONB401-09|3898-COI-08|United States|Texas|658[0n]|BOLD:AAE2719
Cisthene unifascia|[2692]|LMEM939-09|RBMIS-1034|United States|Mississippi|658[0n]|BOLD:AAE2719
Cisthene unifascia|[2693]|LMEM936-09|RBMIS-1031|United States|Mississippi|658[0n]|BOLD:AAE2719
Cisthene unifascia|[2694]|LMEM935-09|RBMIS-1030|United States|Mississippi|658[0n]|BOLD:AAE2719
Cisthene unifascia|[2695]|BBLSX503-09|09BBLEP-02431|United States|Oklahoma|658[0n]|BOLD:AAE2719
Cisthene unifascia|[2696]|IAWL697-09|IAWAZ-0542|United States|Texas|658[0n]|BOLD:AAE2719
Cisthene unifascia|[2697]|LPOKB680-09|MDOK-1722|United States|Oklahoma|658[0n]|BOLD:AAE2719
Cisthene picta|[2698]|ABLCW096-10|CSUPOBK-0096|United States|Oklahoma|658[0n]|BOLD:AAC4254
Cisthene picta|[2699]|LNAUT1264-14|CCDB-22953-C06|United States|Texas|658[0n]|BOLD:AAC4254
Cisthene picta|[2700]|LPOKA060-08|MDOK-0060|United States|Oklahoma|658[0n]|BOLD:AAC4254
Cisthene picta|[2701]|ABLCW094-10|CSUPOBK-0094|United States|Oklahoma|658[0n]|BOLD:AAC4254
Cisthene picta|[2702]|LPOKB799-09|MDOK-1841|United States|Oklahoma|658[0n]|BOLD:AAC4254
Cisthene picta|[2703]|RDNMH752-09|CNCLEP00063185|United States|Texas|658[0n]|BOLD:AAC4254
Cisthene picta|[2704]|ABLCW097-10|CSUPOBK-0097|United States|Oklahoma|658[0n]|BOLD:AAC4254
Cisthene picta|[2705]|ABLCW099-10|CSUPOBK-0099|United States|Oklahoma|658[0n]|BOLD:AAC4254
Cisthene picta|[2706]|HKONB399-09|3896-COI-08|United States|Indiana|658[0n]|BOLD:AAC4254
Cisthene picta|[2707]|BBLSX374-09|09BBLEP-02302|United States|Oklahoma|658[0n]|BOLD:AAC4254
Cisthene picta|[2708]|ABLCW098-10|CSUPOBK-0098|United States|Oklahoma|658[0n]|BOLD:AAC4254
Cisthene picta|[2709]|ABLCW095-10|CSUPOBK-0095|United States|Oklahoma|658[0n]|BOLD:AAC4254
Cisthene picta|[2710]|LPOKB798-09|MDOK-1840|United States|Oklahoma|658[0n]|BOLD:AAC4254
Cisthene martini|[2711]|ABLCW090-10|CSUPOBK-0090|United States|Arizona|658[0n]|BOLD:ABZ0372
Cisthene martini|[2712]|ABLCW091-10|CSUPOBK-0091|United States|Arizona|658[0n]|BOLD:ABZ0372
Cisthene juanita|[2713]|IAWL658-09|IAWAZ-0519|United States|Arizona|619[1n]|BOLD:ABZ0372
Cisthene juanita|[2714]|LMEM923-09|RBMIS-1018|United States|Arizona|658[0n]|BOLD:ABZ0372
Cisthene juanita|[2715]|LMEM924-09|RBMIS-1019|United States|Arizona|658[0n]|BOLD:ABZ0372
Cisthene juanita|[2716]|CMAZA112-09|CMAZ-0112|United States|Arizona|658[0n]|BOLD:ABZ0372
Cisthene juanita|[2717]|LMEM922-09|RBMIS-1017|United States|Arizona|568[0n]|BOLD:ABZ0372
Cisthene juanita|[2718]|LMEM921-09|RBMIS-1016|United States|Arizona|647[0n]|BOLD:ABZ0372
Cisthene juanita|[2719]|RDNMH756-09|CNCLEP00063189|United States|Arizona|658[0n]|BOLD:ABZ0372
Cisthene juanita|[2720]|ABLCW092-10|CSUPOBK-0092|United States|Arizona|658[0n]|BOLD:ABZ0372
Cisthene juanita|[2721]|CMAZA1226-12|BIOUG02044-A07|United States|Arizona|658[0n]|BOLD:ABZ0372
Cisthene juanita|[2722]|ABLCW093-10|CSUPOBK-0093|United States|Arizona|658[0n]|BOLD:ABZ0372
Cisthene juanita|[2723]|ABLCW023-10|CSUPOBK-0023|United States|Arizona|658[0n]|BOLD:ABZ0372
Cisthene juanita|[2724]|ABLCW022-10|CSUPOBK-0022|United States|Arizona|658[0n]|BOLD:ABZ0372
Cisthene juanita|[2725]|ABLCW021-10|CSUPOBK-0021|United States|Arizona|658[0n]|BOLD:ABZ0372
Cisthene juanita|[2726]|ABLCW020-10|CSUPOBK-0020|United States|Arizona|658[0n]|BOLD:ABZ0372
Cisthene barnesii|[2727]|RDNMF900-08|CNC LEP00054167|United States|Colorado|658[0n]|BOLD:ACE9970
Cisthene barnesii|[2728]|ABLCW009-10|CSUPOBK-0009|United States|Colorado|658[0n]|BOLD:ACE9970
Cisthene barnesii|[2729]|ABLCW010-10|CSUPOBK-0010|United States|Colorado|658[0n]|BOLD:ACE9970
Cisthene barnesii|[2730]|ABLCW008-10|CSUPOBK-0008|United States|Colorado|658[0n]|BOLD:ACE9970
Cisthene martini|[2731]|LMEM067-09|RBMIS-0067|United States|Arizona|544[2n]|BOLD:AAA2038
Cisthene barnesii|[2732]|LMEM919-09|RBMIS-1014|United States|New Mexico|658[0n]|BOLD:AAA2038
Cisthene barnesii|[2733]|ABLCW029-10|CSUPOBK-0029|United States|Wyoming|658[0n]|BOLD:AAA2038
Cisthene barnesii|[2734]|PSAT131-10|CNCLEP 70015|United States|Colorado|658[1n]|BOLD:AAA2038
Cisthene barnesii|[2735]|ABLCW028-10|CSUPOBK-0028|United States|Wyoming|658[0n]|BOLD:AAA2038
Cisthene barnesii|[2736]|ABLCW027-10|CSUPOBK-0027|United States|Wyoming|658[0n]|BOLD:AAA2038
Cisthene barnesii|[2737]|ABLCW026-10|CSUPOBK-0026|United States|Wyoming|658[0n]|BOLD:AAA2038
Cisthene barnesii|[2738]|ABLCW025-10|CSUPOBK-0025|United States|Wyoming|658[0n]|BOLD:AAA2038
Cisthene barnesii|[2739]|RDNMH368-09|CNCLEP00057777|United States|Colorado|658[0n]|BOLD:AAA2038
Cisthene barnesii|[2740]|PSAT132-10|CNCLEP 70016|United States|Colorado|658[0n]|BOLD:AAA2038
Cisthene martini|[2741]|RDNMH356-09|CNCLEP00057765|United States|Arizona|658[0n]|BOLD:AAA2038
Cisthene martini|[2742]|LMEM066-09|RBMIS-0066|United States|Arizona|658[2n]|BOLD:AAA2038
Cisthene martini|[2743]|CMAZA436-10|CMAZ-0436|United States|Arizona|658[0n]|BOLD:AAA2038
Cisthene martini|[2744]|ABLCW086-10|CSUPOBK-0086|United States|Arizona|658[0n]|BOLD:AAA2038
Cisthene coronado|[2745]|LNAUT248-14|CCDB-22941-E11|United States|Arizona|658[0n]|BOLD:AAA2038
Cisthene martini|[2746]|CMAZA012-09|CMAZ-0012|United States|Arizona|658[0n]|BOLD:AAA2038
Cisthene martini|[2747]|RDNMH343-09|CNCLEP00057752|United States|Arizona|658[0n]|BOLD:AAA2038
Cisthene martini|[2748]|LMEM068-09|RBMIS-0068|United States|Arizona|658[0n]|BOLD:AAA2038
Cisthene martini|[2749]|ABLCW088-10|CSUPOBK-0088|United States|Arizona|658[0n]|BOLD:AAA2038
Cisthene coronado|[2750]|LNAUT250-14|CCDB-22941-F01|United States|Arizona|658[0n]|BOLD:AAA2038
Cisthene martini|[2751]|RDNMJ493-11|CNCLEP 80021|United States|Arizona|658[0n]|BOLD:AAA2038
Cisthene barnesii|[2752]|LMEM917-09|RBMIS-1012|United States|New Mexico|658[0n]|BOLD:AAA2038
Cisthene martini|[2753]|RDNMJ269-11|CNCLEP 70292|United States|Arizona|658[0n]|BOLD:AAA2038
Cisthene martini|[2754]|RDNMJ489-11|CNCLEP 80017|United States|Arizona|658[0n]|BOLD:AAA2038
Cisthene martini|[2755]|RDNMJ490-11|CNCLEP 80018|United States|Arizona|658[0n]|BOLD:AAA2038
Cisthene martini|[2756]|BBLSX725-09|09BBLEP-02653|United States|Arizona|637[0n]|BOLD:AAA2038
Cisthene martini|[2757]|ABLCW087-10|CSUPOBK-0087|United States|Arizona|658[0n]|BOLD:AAA2038
Cisthene martini|[2758]|RDNMH342-09|CNCLEP00057751|United States|Arizona|658[0n]|BOLD:AAA2038
Cisthene martini|[2759]|LMEM065-09|RBMIS-0065|United States|Arizona|658[0n]|BOLD:AAA2038
Cisthene martini|[2760]|ABLCW089-10|CSUPOBK-0089|United States|Arizona|658[0n]|BOLD:AAA2038
Cisthene coronado|[2761]|LNAUT251-14|CCDB-22941-F02|United States|Arizona|658[0n]|BOLD:AAA2038
Cisthene coronado|[2762]|LNAUT249-14|CCDB-22941-E12|United States|Arizona|658[0n]|BOLD:AAA2038
Cisthene martini|[2763]|BBLOB862-11|BIOUG01413-A07|United States|Arizona|658[0n]|BOLD:AAA2038
Cisthene martini|[2764]|BBLSW169-09|09BBLEP-01097|United States|Arizona|658[0n]|BOLD:AAA2038
Cisthene martini|[2765]|BBLSX724-09|09BBLEP-02652|United States|Arizona|658[0n]|BOLD:AAA2038
Cisthene martini|[2766]|RDNMJ499-11|CNCLEP 80027|United States|Arizona|658[0n]|BOLD:AAA2038
Cisthene martini|[2767]|BBLSW168-09|09BBLEP-01096|United States|Arizona|658[0n]|BOLD:AAA2038
Cisthene martini|[2768]|BBLSY361-09|09BBLEP-03288|United States|Arizona|614[0n]|BOLD:AAA2038
Cisthene martini|[2769]|BBLSW167-09|09BBLEP-01095|United States|Arizona|658[0n]|BOLD:AAA2038
Cisthene subjecta|[2770]|LOFLB354-06|06-FLOR-1294|United States|Florida|658[0n]|BOLD:AAB0728
Cisthene subjecta|[2771]|LOFLB352-06|06-FLOR-1292|United States|Florida|658[0n]|BOLD:AAB0728
Cisthene subjecta|[2772]|LOFLD401-07|HLC-17017|United States|Florida|658[0n]|BOLD:AAB0728
Cisthene subjecta|[2773]|MNAB346-07|CNCLEP00025942|United States|Florida|658[0n]|BOLD:AAB0728
Cisthene subjecta|[2774]|BBLOB1804-11|BIOUG01424-H10|United States|Florida|658[0n]|BOLD:AAB0728
Cisthene subjecta|[2775]|BBLOB1753-11|BIOUG01424-D07|United States|Florida|658[0n]|BOLD:AAB0728
Cisthene subjecta|[2776]|LOFLC082-06|06-FLOR-1962|United States|Florida|658[0n]|BOLD:AAB0728
Cisthene subjecta|[2777]|LOFLC103-06|06-FLOR-1983|United States|Florida|658[0n]|BOLD:AAB0728
Cisthene subjecta|[2778]|MNAB039-07|CNCLEP00025630|United States|Florida|613[0n]|BOLD:AAB0728
Cisthene subjecta|[2779]|LOFLB092-06|06-FLOR-1032|United States|Florida|658[0n]|BOLD:AAB0728
Cisthene subjecta|[2780]|LOFLA811-06|06-FLOR-0811|United States|Florida|658[0n]|BOLD:AAB0728
Cisthene subjecta|[2781]|LOFLA798-06|06-FLOR-0798|United States|Florida|658[0n]|BOLD:AAB0728
Cisthene subjecta|[2782]|LOFLD293-07|HLC-16868|United States|Florida|658[0n]|BOLD:AAB0728
Cisthene subjecta|[2783]|LOFLA506-06|06-FLOR-0506|United States|Florida|658[0n]|BOLD:AAB0728
Cisthene subjecta|[2784]|LOFLA479-06|06-FLOR-0479|United States|Florida|658[0n]|BOLD:AAB0728
Cisthene subjecta|[2785]|LOFLD330-07|HLC-16905|United States|Florida|658[0n]|BOLD:AAB0728
Cisthene subjecta|[2786]|LOFLA486-06|06-FLOR-0486|United States|Florida|658[0n]|BOLD:AAB0728
Cisthene subjecta|[2787]|USLEP508-10|10BBLEP-00508|United States|Florida|658[0n]|BOLD:AAB0728
Cisthene subjecta|[2788]|USLEP507-10|10BBLEP-00507|United States|Florida|658[0n]|BOLD:AAB0728
[illegible]

Cisthene subjecta|[2786]|LOFLA486-06|06-FLOR-0486|United States|Florida|658[0n]|BOLD:AAB0728
Cisthene subjecta|[2787]|USLEP508-10|10BBLEP-00508|United States|Florida|658[0n]|BOLD:AAB0728
Cisthene subjecta|[2788]|USLEP507-10|10BBLEP-00507|United States|Florida|658[0n]|BOLD:AAB0728
Cisthene subjecta|[2789]|LOFLD421-07|HLC-17037|United States|Florida|658[0n]|BOLD:AAB0728
Cisthene subjecta|[2790]|LOFLA255-06|06-FLOR-0255|United States|Florida|658[0n]|BOLD:AAB0728
Cisthene subjecta|[2791]|LOFLD032-07|HLC-16606|United States|Florida|658[0n]|BOLD:AAB0728
Cisthene subjecta|[2792]|MNAC280-07|CNCLEP00026246|United States|Florida|658[0n]|BOLD:AAB0728
Cisthene subjecta|[2793]|LOFLA356-06|06-FLOR-0356|United States|Florida|658[0n]|BOLD:AAB0728
Cisthene subjecta|[2794]|LOFLB050-06|06-FLOR-0990|United States|Florida|658[0n]|BOLD:AAB0728
Cisthene subjecta|[2795]|MNAC279-07|CNCLEP00026245|United States|Florida|656[0n]|BOLD:AAB0728
Cisthene subjecta|[2796]|LOFLA508-06|06-FLOR-0508|United States|Florida|658[0n]|BOLD:AAB0728
Cisthene subjecta|[2797]|LOFLB045-06|06-FLOR-0985|United States|Florida|658[0n]|BOLD:AAB0728
Cisthene subjecta|[2798]|LOFLB029-06|06-FLOR-0969|United States|Florida|658[0n]|BOLD:AAB0728
Cisthene subjecta|[2799]|LNC802-06|06-NCCC-802|United States|North Carolina|658[0n]|BOLD:AAB0728
Cisthene subjecta|[2800]|LMEM944-09|RBMIS-1039|United States|Georgia|658[0n]|BOLD:AAB0728
Cisthene subjecta|[2801]|LMEM943-09|RBMIS-1038|United States|Georgia|658[0n]|BOLD:AAB0728
Cisthene subjecta|[2802]|LMEM942-09|RBMIS-1037|United States|Mississippi|658[0n]|BOLD:AAB0728
Cisthene subjecta|[2803]|LSEU491-06|06-JKA-0491|United States|Georgia|658[0n]|BOLD:AAB0728
Cisthene subjecta|[2804]|USLEP506-10|10BBLEP-00506|United States|Florida|658[0n]|BOLD:AAB0728
Cisthene subjecta|[2805]|HKONS229-08|3134-COI-07|United States|Florida|658[0n]|BOLD:AAB0728
Cisthene packardii|[2806]|LILLA820-11|SNS10IL-01028|United States|Illinois|658[1n]|BOLD:AAB7941
Cisthene packardii|[2807]|LSUSA097-06|06-SUSA-0097|United States|Kentucky|658[0n]|BOLD:AAB7941
Cisthene packardii|[2808]|LGSMD422-05|DNA-ATBI-3436|United States|Tennessee|658[0n]|BOLD:AAB7941
Cisthene packardii|[2809]|LGSMD421-05|DNA-ATBI-3435|United States|Tennessee|658[0n]|BOLD:AAB7941
Cisthene packardii|[2810]|LSEU559-06|06-JKA-0559|United States|Georgia|658[0n]|BOLD:AAB7941
Cisthene packardii|[2811]|LGSMC757-05|DNA-ATBI-2757|United States|Tennessee|658[0n]|BOLD:AAB7941
Cisthene packardii|[2812]|LGSMG107-07|BGS03428|United States|Tennessee|658[0n]|BOLD:AAB7941
Cisthene packardii|[2813]|LNC166-05|05-NCCC-166|United States|North Carolina|658[0n]|BOLD:AAB7941
Cisthene packardii|[2814]|LNC165-05|05-NCCC-165|United States|North Carolina|658[0n]|BOLD:AAB7941
Cisthene packardii|[2815]|LOFLA626-06|06-FLOR-0626|United States|Florida|658[0n]|BOLD:AAB7941
Cisthene packardii|[2816]|LOFLA260-06|06-FLOR-0260|United States|Florida|658[0n]|BOLD:AAB7941
Cisthene packardii|[2817]|LOFLA244-06|06-FLOR-0244|United States|Florida|658[0n]|BOLD:AAB7941
Cisthene packardii|[2818]|HKONS219-08|3124-COI-07|United States|Florida|658[0n]|BOLD:AAB7941
Cisthene packardii|[2819]|MILEQ304-11|11-MISC-779|United States|Alabama|658[0n]|BOLD:AAB7941
Cisthene packardii|[2820]|LMEM060-09|RBMIS-0060|United States|Mississippi|604[4n]|BOLD:AAB7941
Cisthene conjuncta|[2821]|ABLCW085-10|CSUPOBK-0085|United States|Texas|634[0n]|BOLD:AAB7941
Cisthene packardii|[2822]|LPOKD404-09|MDOK-3483|United States|Oklahoma|658[0n]|BOLD:AAB7941
Cisthene packardii|[2823]|LPOKD455-09|MDOK-3534|United States|Oklahoma|627[0n]|BOLD:AAB7941
Cisthene packardii|[2824]|LMEM064-09|RBMIS-0064|United States|Mississippi|658[0n]|BOLD:AAB7941
Cisthene packardii|[2825]|LMEM063-09|RBMIS-0063|United States|Mississippi|658[0n]|BOLD:AAB7941
Cisthene packardii|[2826]|LMEM062-09|RBMIS-0062|United States|Mississippi|658[0n]|BOLD:AAB7941
Cisthene packardii|[2827]|LMEM061-09|RBMIS-0061|United States|Alabama|658[0n]|BOLD:AAB7941
Cisthene packardii|[2828]|LPOKD430-09|MDOK-3509|United States|Oklahoma|658[0n]|BOLD:AAB7941
Cisthene packardii|[2829]|LPOKB762-09|MDOK-1804|United States|Oklahoma|658[0n]|BOLD:AAB7941
Cisthene liberomacula|[2830]|LOCBD014-06|06-BLLOC-2834|United States|California|658[0n]|BOLD:A...
Cisthene liberomacula|[2831]|LOCBD964-06|06-BLLOC-3785|United States|California|658[0n]|BOLD:A...
Cisthene liberomacula|[2832]|LOCBD011-06|06-BLLOC-2831|United States|California|658[0n]|BOLD:A...
Cisthene liberomacula|[2833]|LOCBC702-06|06-BLLOC-2582|United States|California|658[0n]|BOLD:A...
Cisthene liberomacula|[2834]|LOCBC700-06|06-BLLOC-2580|United States|California|658[0n]|BOLD:A...
Cisthene liberomacula|[2835]|LOCBC513-06|06-BLLOC-2393|United States|California|658[0n]|BOLD:A...
Cisthene liberomacula|[2836]|LOCBC701-06|06-BLLOC-2581|United States|California|658[0n]|BOLD:A...
Cisthene liberomacula|[2837]|LOCBD016-06|06-BLLOC-2836|United States|California|658[0n]|BOLD:A...
Cisthene liberomacula|[2838]|LOCBC710-06|06-BLLOC-2590|United States|California|658[0n]|BOLD:A...
Cisthene liberomacula|[2839]|LOCBC714-06|06-BLLOC-2594|United States|California|658[0n]|BOLD:A...
Cisthene liberomacula|[2840]|LOCBD017-06|06-BLLOC-2837|United States|California|658[0n]|BOLD:A...
Cisthene liberomacula|[2841]|LOCBD020-06|06-BLLOC-2840|United States|California|658[0n]|BOLD:A...
Cisthene liberomacula|[2842]|LOCBC704-06|06-BLLOC-2584|United States|California|658[0n]|BOLD:A...
Cisthene liberomacula|[2843]|LOCBC004-06|06-BLLOC-1884|United States|California|658[0n]|BOLD:A...
Cisthene liberomacula|[2844]|BBLOE1117-12|BIOUG01984-A04|United States|California|658[0n]|BOLD ...
Cisthene liberomacula|[2845]|ABLCW131-10|CSUPOBK-0131|United States|California|658[0n]|BOLD:AA...
Cisthene liberomacula|[2846]|CGLCA198-10|CCGBOLD00198|United States|California|658[0n]|BOLD:AA...
Cisthene liberomacula|[2847]|CGLCA199-10|CCGBOLD00199|United States|California|658[0n]|BOLD:AA...
Cisthene liberomacula|[2848]|CGLCA197-10|CCGBOLD00197|United States|California|658[0n]|BOLD:AA...
Cisthene liberomacula|[2849]|ABLCW135-10|CSUPOBK-0135|United States|California|658[0n]|BOLD:AA...
Cisthene liberomacula|[2850]|RDNMF905-08|CNC LEP00054172|United States|California|658[0n]|BOLD ...
Cisthene liberomacula|[2851]|LOCBC699-06|06-BLLOC-2579|United States|California|658[0n]|BOLD:A...
Cisthene liberomacula|[2852]|LOCBC706-06|06-BLLOC-2586|United States|California|658[0n]|BOLD:A...
Cisthene liberomacula|[2853]|LOCBD018-06|06-BLLOC-2838|United States|California|658[0n]|BOLD:A...
Cisthene liberomacula|[2854]|LOCBD844-06|06-BLLOC-3664|United States|California|658[0n]|BOLD:A...
Cisthene liberomacula|[2855]|ABLCW136-10|CSUPOBK-0136|United States|California|658[0n]|BOLD:AA...
Cisthene liberomacula|[2856]|ABLCW134-10|CSUPOBK-0134|United States|California|658[0n]|BOLD:AA...
Cisthene liberomacula|[2857]|ABLCW133-10|CSUPOBK-0133|United States|California|658[0n]|BOLD:AA...
Cisthene liberomacula|[2858]|ABLCW132-10|CSUPOBK-0132|United States|California|658[0n]|BOLD:AA...
Cisthene liberomacula|[2859]|CGLCA196-10|CCGBOLD00196|United States|California|658[0n]|BOLD:AA...
Cisthene liberomacula|[2860]|CGLCA195-10|CCGBOLD00195|United States|California|658[0n]|BOLD:AA...
Cisthene liberomacula|[2861]|LOCBC711-06|06-BLLOC-2591|United States|California|658[0n]|BOLD:A...
Cisthene liberomacula|[2862]|LOCBD015-06|06-BLLOC-2835|United States|California|658[0n]|BOLD:A...
Cisthene liberomacula|[2863]|LOCBC697-06|06-BLLOC-2577|United States|California|658[0n]|BOLD:A...
Cisthene liberomacula|[2864]|LOCBC696-06|06-BLLOC-2576|United States|California|658[0n]|BOLD:A...
Cisthene liberomacula|[2865]|LOCBC006-06|06-BLLOC-1886|United States|California|658[0n]|BOLD:A...
Cisthene liberomacula|[2866]|LOCBD013-06|06-BLLOC-2833|United States|California|658[0n]|BOLD:A...
Cisthene liberomacula|[2867]|LOCBC005-06|06-BLLOC-1885|United States|California|658[0n]|BOLD:A...
Cisthene liberomacula|[2868]|LOCBD012-06|06-BLLOC-2832|United States|California|658[0n]|BOLD:A...
Cisthene liberomacula|[2869]|LOCBC709-06|06-BLLOC-2589|United States|California|658[0n]|BOLD:A...
Cisthene liberomacula|[2870]|LOCBC703-06|06-BLLOC-2583|United States|California|658[0n]|BOLD:A...
Cisthene liberomacula|[2871]|LOCBC007-06|06-BLLOC-1887|United States|California|658[0n]|BOLD:A...
Cisthene liberomacula|[2872]|LOCBC002-06|06-BLLOC-1882|United States|California|658[0n]|BOLD:A...
Cisthene liberomacula|[2873]|BBLOD223-11|BIOUG01562-B03|United States|California|658[0n]|BOLD:...
Cisthene liberomacula|[2874]|BBLOD222-11|BIOUG01562-B02|United States|California|658[0n]|BOLD:...
Cisthene liberomacula|[2875]|BBLOD210-11|BIOUG01562-A02|United States|California|658[0n]|BOLD:...
Cisthene liberomacula|[2876]|RDNMF904-08|CNC LEP00054171|United States|California|658[0n]|BOLD ...
Cisthene liberomacula|[2877]|BBLOD323-11|BIOUG01563-B08|United States|California|658[0n]|BOLD:...
Cisthene liberomacula|[2878]|BBLOD230-11|BIOUG01562-B10|United States|California|658[0n]|BOLD:...
Cisthene liberomacula|[2879]|BBLOE1115-12|BIOUG01984-A02|United States|California|658[0n]|BOLD ...
Cisthene liberomacula|[2880]|LOCBC695-06|06-BLLOC-2575|United States|California|658[0n]|BOLD:A...
Cisthene liberomacula|[2881]|LOCBC473-06|06-BLLOC-2353|United States|California|658[0n]|BOLD:A...
Cisthene liberomacula|[2882]|LOCBC705-06|06-BLLOC-2585|United States|California|658[0n]|BOLD:A...
Cisthene liberomacula|[2883]|LOCBC698-06|06-BLLOC-2578|United States|California|658[0n]|BOLD:A...
Cisthene liberomacula|[2884]|LOCBC707-06|06-BLLOC-2587|United States|California|658[0n]|BOLD:A...
Cisthene liberomacula|[2885]|LOCBC712-06|06-BLLOC-2592|United States|California|658[0n]|BOLD:A...
Cisthene liberomacula|[2886]|LOCBD010-06|06-BLLOC-2830|United States|California|658[0n]|BOLD:A...
Cisthene liberomacula|[2887]|LOCBD019-06|06-BLLOC-2839|United States|California|658[0n]|BOLD:A...
Cisthene liberomacula|[2888]|LOCBC713-06|06-BLLOC-2593|United States|California|600[0n]|BOLD:A...

Cisthene liberomacula|[2886]|LOCBD010-06|06-BLLOC-2830|United States|California|658[0n]|BOLD:A...
Cisthene liberomacula|[2887]|LOCBD019-06|06-BLLOC-2839|United States|California|658[0n]|BOLD:A...
Cisthene liberomacula|[2888]|LOCBC713-06|06-BLLOC-2593|United States|California|600[0n]|BOLD:A...
Cisthene liberomacula|[2889]|LOCBC708-06|06-BLLOC-2588|United States|California|643[0n]|BOLD:A...
Cisthene liberomacula|[2890]|BBLOD319-11|BIOUG01563-B04|United States|California|658[0n]|BOLD:...
Cisthene liberomacula|[2891]|BBLOD873-11|BIOUG01568-H11|United States|California|658[0n]|BOLD:...
Haploa contigua|[2892]|RDMAB969-09|UASM99698|Canada|Ontario|658[0n]|BOLD:ABX5655
Haploa contigua|[2893]|LNCC977-11|11-NCCC-502|United States|North Carolina|658[0n]|BOLD:ABX5655
Haploa contigua|[2894]|RDNMF047-08|NOC14133|Canada|Quebec|658[0n]|BOLD:ABX5655
Haploa clymene|[2895]|LILLA349-11|SNS10IL-00459|United States|Illinois|658[0n]|BOLD:ABZ0257
Haploa clymene|[2896]|LGSMB300-05|DNA-ATBI-1149|United States|Tennessee|583[0n]|BOLD:ABZ0257
Haploa clymene|[2897]|LNCC1869-13|13-NCCC-539|United States|North Carolina|658[0n]|BOLD:ABZ0254
Haploa clymene|[2898]|LNCC1347-11|11-NCCC-872|United States|North Carolina|658[0n]|BOLD:ABZ0254
Haploa clymene|[2899]|LNCC1346-11|11-NCCC-871|United States|North Carolina|658[0n]|BOLD:ABZ0254
Haploa clymene|[2900]|LNCC060-10|10-NCCC-155|United States|North Carolina|570[0n]|BOLD:ABZ0254
Haploa lecontei|[2901]|TAMIC832-10|TAMUICEGR-0832|United States|New York|658[0n]|BOLD:ABZ0254
Haploa lecontei|[2902]|PHMNB024-03|moth160.02SA|Canada|New Brunswick|639[0n]|BOLD:ABZ0254
Haploa confusa|[2903]|MNBB564-05|05-NBSTA-480|Canada|New Brunswick|658[0n]|BOLD:ABZ0254
Haploa lecontei|[2904]|MNBB139-05|05-NBSTA-055|Canada|New Brunswick|658[0n]|BOLD:ABZ0254
Haploa clymene|[2905]|LILLA595-11|SNS10IL-00762|United States|Illinois|658[0n]|BOLD:ABZ0254
Haploa clymene|[2906]|LNCC1872-13|13-NCCC-542|United States|North Carolina|658[0n]|BOLD:ABZ0254
Haploa lecontei|[2907]|BLTIB865-08|BL1284|Canada|Ontario|658[0n]|BOLD:ABZ0254
Haploa clymene|[2908]|LPOKB996-09|MDOK-2038|United States|Oklahoma|658[0n]|BOLD:ABZ0254
Haploa clymene|[2909]|LILLA636-11|SNS10IL-00812|United States|Illinois|658[0n]|BOLD:ABZ0254
Haploa clymene|[2910]|LNCC1871-13|13-NCCC-541|United States|North Carolina|658[0n]|BOLD:ABZ0254
Haploa clymene|[2911]|LNCC1393-11|11-NCCC-918|United States|North Carolina|658[0n]|BOLD:ABZ0254
Haploa clymene|[2912]|QUNOC090-09|5921-020708-KY|United States|Kentucky|653[0n]|BOLD:ABZ0254
Haploa clymene|[2913]|LNCC1870-13|13-NCCC-540|United States|North Carolina|658[0n]|BOLD:ABZ0254
Haploa clymene|[2914]|LNC279-05|05-NCCC-279|United States|North Carolina|658[0n]|BOLD:ABZ0254
Haploa clymene|[2915]|LNCC1391-11|11-NCCC-916|United States|North Carolina|658[0n]|BOLD:ABZ0254
Haploa clymene|[2916]|LNCC1390-11|11-NCCC-915|United States|North Carolina|658[0n]|BOLD:ABZ0254
Haploa clymene|[2917]|LNCC1345-11|11-NCCC-870|United States|North Carolina|658[0n]|BOLD:ABZ0254
Haploa clymene|[2918]|LNCC058-10|10-NCCC-153|United States|North Carolina|658[0n]|BOLD:ABZ0254
Haploa lecontei|[2919]|LSEU704-06|06-JKA-0704|United States|Georgia|658[0n]|BOLD:AAA8684
Haploa lecontei|[2920]|JSBIC011-08|HLC-16964|United States|North Carolina|658[0n]|BOLD:AAA8684
Haploa lecontei|[2921]|LOT138-04|04HBL002138|United States|Tennessee|609[0n]|BOLD:AAA8684
Haploa lecontei|[2922]|BBLEC286-09|09BBELE-0286|Canada|Nova Scotia|565[0n]|BOLD:AAA8684
Haploa lecontei|[2923]|PHMNB213-04|04HBL007678|Canada|New Brunswick|609[0n]|BOLD:AAA8684
Haploa lecontei|[2924]|BBLEC571-09|09BBELE-0571|Canada|Nova Scotia|614[0n]|BOLD:AAA8684
Haploa confusa|[2925]|BLTIB770-08|BL1165|Canada|Ontario|658[0n]|BOLD:AAA8684
Haploa confusa|[2926]|BLTIB816-08|BL1234|Canada|Ontario|658[0n]|BOLD:AAA8684
Haploa confusa|[2927]|TMG75-03|moth1129.01|Canada|Ontario|639[0n]|BOLD:AAA8684
Haploa lecontei|[2928]|PHMNB032-03|moth192.02SA|Canada|New Brunswick|639[0n]|BOLD:AAA8684
Haploa lecontei|[2929]|BBLPE296-09|09BBELE-2296|Canada|Nova Scotia|648[0n]|BOLD:AAA8684
Haploa lecontei|[2930]|BBLEC259-09|09BBELE-0259|Canada|Nova Scotia|658[0n]|BOLD:AAA8684
Haploa lecontei|[2931]|RDLQB371-05|DH010457|Canada|Quebec|622[3n]|BOLD:AAA8684
Haploa lecontei|[2932]|BBLEC285-09|09BBELE-0285|Canada|Nova Scotia|658[0n]|BOLD:AAA8684
Haploa lecontei|[2933]|BBLEC271-09|09BBELE-0271|Canada|Nova Scotia|638[0n]|BOLD:AAA8684
Haploa clymene|[2934]|LNCNW033-06|06-NCNW-0033|United States|North Carolina|658[0n]|BOLD:AAA8684
Haploa lecontei|[2935]|MNBB254-05|05-NBSTA-170|Canada|New Brunswick|658[0n]|BOLD:AAA8684
Haploa lecontei|[2936]|PHMNB146-04|04HBL007611|Canada|New Brunswick|658[0n]|BOLD:AAA8684
Haploa lecontei|[2937]|MNBB400-05|05-NBSTA-316|Canada|New Brunswick|658[0n]|BOLD:AAA8684
Haploa lecontei|[2938]|MNBB676-05|05-NBSTA-592|Canada|New Brunswick|658[0n]|BOLD:AAA8684
Haploa lecontei|[2939]|PHMNB232-04|04HBL007697|Canada|New Brunswick|658[0n]|BOLD:AAA8684
Haploa lecontei|[2940]|BBLEC590-09|09BBELE-0590|Canada|Nova Scotia|658[0n]|BOLD:AAA8684
Haploa lecontei|[2941]|QUNOB537-09|5799-020708-KY|United States|Kentucky|658[0n]|BOLD:AAA8684
Haploa lecontei|[2942]|QUNOB539-09|5801-010708-KY|United States|Kentucky|658[0n]|BOLD:AAA8684
Haploa lecontei|[2943]|QUNOB541-09|5803-030708-KY|United States|Kentucky|658[0n]|BOLD:AAA8684
Haploa lecontei|[2944]|QUNOB542-09|5804-020708-KY|United States|Kentucky|658[0n]|BOLD:AAA8684
Haploa lecontei|[2945]|BBLPE254-09|09BBELE-2254|Canada|Nova Scotia|658[0n]|BOLD:AAA8684
Haploa clymene|[2946]|LNCC1392-11|11-NCCC-917|United States|North Carolina|658[0n]|BOLD:AAA8684
Haploa lecontei|[2947]|BBLPE294-09|09BBELE-2294|Canada|Nova Scotia|658[0n]|BOLD:AAA8684
Haploa lecontei|[2948]|QUNOB536-09|5798-020708-KY|United States|Kentucky|658[0n]|BOLD:AAA8684
Haploa lecontei|[2949]|PHMNB218-04|04HBL007683|Canada|New Brunswick|658[0n]|BOLD:AAA8684
Haploa lecontei|[2950]|TMNBD370-07|MNBTT-3171|Canada|New Brunswick|621[0n]|BOLD:AAA8684
Haploa lecontei|[2951]|BBLEC340-09|09BBELE-0340|Canada|Nova Scotia|651[0n]|BOLD:AAA8684
Haploa lecontei|[2952]|PHMNB145-04|04HBL007610|Canada|New Brunswick|658[0n]|BOLD:AAA8684
Haploa lecontei|[2953]|PHMNB144-04|04HBL007609|Canada|New Brunswick|658[0n]|BOLD:AAA8684
Haploa lecontei|[2954]|BBLEC290-09|09BBELE-0290|Canada|Nova Scotia|658[0n]|BOLD:AAA8684
Haploa lecontei|[2955]|BBLEC289-09|09BBELE-0289|Canada|Nova Scotia|658[0n]|BOLD:AAA8684
Haploa lecontei|[2956]|BBLEC258-09|09BBELE-0258|Canada|Nova Scotia|658[0n]|BOLD:AAA8684
Haploa clymene|[2957]|LNCC059-10|10-NCCC-154|United States|North Carolina|658[0n]|BOLD:AAA8684
Haploa lecontei militaris|[2958]|LGSMB299-05|DNA-ATBI-1148|United States|North Carolina|564[0n]|B...
Haploa lecontei militaris|[2959]|LOT137-04|04HBL002137|United States|Tennessee|609[0n]|BOLD:AA...
Haploa lecontei militaris|[2960]|LOT136-04|04HBL002136|United States|Tennessee|609[0n]|BOLD:AA...
Haploa lecontei|[2961]|QUNOB540-09|5802-010708-KY|United States|Kentucky|658[0n]|BOLD:AAA8684
Haploa lecontei|[2962]|QUNOB538-09|5800-010708-KY|United States|Kentucky|658[0n]|BOLD:AAA8684
Haploa lecontei|[2963]|LSEU703-06|06-JKA-0703|United States|Georgia|658[0n]|BOLD:AAA8684
Haploa colona|[2964]|LNCC917-11|11-NCCC-442|United States|North Carolina|658[0n]|BOLD:AAA8684
Haploa lecontei|[2965]|JSBIC012-08|HLC-16965|United States|North Carolina|658[0n]|BOLD:AAA8684
Haploa lecontei|[2966]|JSBIC009-08|HLC-16962|United States|North Carolina|658[0n]|BOLD:AAA8684
Haploa reversa|[2967]|HKONB381-09|3878-COI-08|United States|Louisiana|658[0n]|BOLD:AAA8684
Haploa lecontei|[2968]|BLTIB662-08|BL943|Canada|Ontario|635[0n]|BOLD:AAA8684
Haploa lecontei|[2969]|RDMAB529-06|UASM58496|Canada|Alberta|622[0n]|BOLD:AAA8684
Haploa confusa|[2970]|MNBB626-05|05-NBSTA-542|Canada|New Brunswick|658[0n]|BOLD:AAA8684
Haploa confusa|[2971]|BBLPC253-09|09BBELE-1253|Canada|Nova Scotia|658[0n]|BOLD:AAA8684
Haploa confusa|[2972]|TMNBD379-07|MNBTT-3180|Canada|New Brunswick|658[0n]|BOLD:AAA8684
Haploa lecontei|[2973]|RDLQG446-06|DH012730|Canada|Quebec|658[0n]|BOLD:AAA8684
Haploa confusa|[2974]|XAB119-04|04HBL005119|Canada|Ontario|658[0n]|BOLD:AAA8684
Haploa confusa|[2975]|PHMNB074-03|moth75.02SA|Canada|New Brunswick|639[0n]|BOLD:AAA8684
Haploa confusa|[2976]|MNBB203-05|05-NBSTA-119|Canada|New Brunswick|621[0n]|BOLD:AAA8684
Haploa reversa|[2977]|LPOKB389-09|MDOK-1479|United States|Oklahoma|658[0n]|BOLD:AAA8684
Haploa lecontei|[2978]|TMNBD376-07|MNBTT-3177|Canada|New Brunswick|658[0n]|BOLD:AAA8684
Haploa lecontei|[2979]|TMNBD375-07|MNBTT-3176|Canada|New Brunswick|658[0n]|BOLD:AAA8684
Haploa lecontei|[2980]|MNBB362-05|05-NBSTA-278|Canada|New Brunswick|658[0n]|BOLD:AAA8684
Haploa confusa|[2981]|MNBB253-05|05-NBSTA-169|Canada|New Brunswick|658[0n]|BOLD:AAA8684
Haploa lecontei|[2982]|JSBIC010-08|HLC-16963|United States|North Carolina|658[0n]|BOLD:AAA8684
Haploa lecontei|[2983]|RDNM112-05|CNCNoctuoidea6662|Canada|Ontario|658[0n]|BOLD:AAA8684
Haploa lecontei|[2984]|RDMAB528-06|UASM58495|Canada|Alberta|656[0n]|BOLD:AAA8684
Haploa lecontei|[2985]|RDLQB697-05|DH010800|Canada|Quebec|658[0n]|BOLD:AAA8684
Haploa lecontei|[2986]|TMNBD374-07|MNBTT-3175|Canada|New Brunswick|658[0n]|BOLD:AAA8684
Haploa lecontei|[2987]|TMNBD373-07|MNBTT-3174|Canada|New Brunswick|657[0n]|BOLD:AAA8684
Haploa confusa|[2988]|TMNBD372-07|MNBTT-3173|Canada|New Brunswick|656[0n]|BOLD:AAA8684

Haploa lecontei|[2985]|[illegible]|Canada|Quebec|658[0n]|BOLD:AAA8684
Haploa lecontei|[2986]|TMNBD374-07|MNBTT-3175|Canada|New Brunswick|658[0n]|BOLD:AAA8684
Haploa lecontei|[2987]|TMNBD373-07|MNBTT-3174|Canada|New Brunswick|657[0n]|BOLD:AAA8684
Haploa confusa|[2988]|TMNBD372-07|MNBTT-3173|Canada|New Brunswick|656[0n]|BOLD:AAA8684
Haploa lecontei|[2989]|PHMNB767-05|Moth 460.03SA|Canada|New Brunswick|658[0n]|BOLD:AAA8684
Haploa lecontei|[2990]|PHMNB765-05|Moth 458.03SA|Canada|New Brunswick|658[0n]|BOLD:AAA8684
Haploa confusa|[2991]|BBLPC262-09|09BBELE-1262|Canada|Nova Scotia|658[0n]|BOLD:AAA8684
Haploa lecontei|[2992]|PHMNB441-04|04HBL00667|Canada|New Brunswick|658[0n]|BOLD:AAA8684
Haploa colona|[2993]|LNCB124-06|06-NCCC-1080|United States|North Carolina|658[0n]|BOLD:AAA8684
Haploa colona|[2994]|LNC080-05|05-NCCC-080|United States|North Carolina|658[0n]|BOLD:AAA8684
Haploa lecontei|[2995]|LNCC980-11|11-NCCC-505|United States|North Carolina|658[0n]|BOLD:AAA8684
Haploa lecontei|[2996]|LNCC979-11|11-NCCC-504|United States|North Carolina|658[0n]|BOLD:AAA8684
Haploa lecontei|[2997]|LNCC978-11|11-NCCC-503|United States|North Carolina|658[0n]|BOLD:AAA8684
Haploa colona|[2998]|HKONB380-09|3877-COI-08|United States|Texas|658[0n]|BOLD:AAA8684
Haploa lecontei militaris|[2999]|LSUSA195-06|06-SUSA-0195|United States|Kentucky|658[0n]|BOLD:...
Haploa lecontei|[3000]|RDNM110-05|CNCNoctuoidea6660|Canada|Ontario|658[0n]|BOLD:AAA8684
Haploa confusa|[3001]|XAK591-07|HLC-16144|Canada|Ontario|596[0n]|BOLD:AAA8684
Haploa lecontei|[3002]|LPOKB973-09|MDOK-2015|United States|Oklahoma|658[0n]|BOLD:AAA8684
Haploa lecontei militaris|[3003]|LGSMB298-05|DNA-ATBI-1147|United States|North Carolina|595[0n]|B...
Haploa lecontei|[3004]|RDMAB930-06|UASM7031|Canada|Alberta|658[0n]|BOLD:AAA8684
Haploa confusa|[3005]|BBLEC299-09|09BBELE-0299|Canada|Nova Scotia|658[0n]|BOLD:AAA8684
Haploa lecontei|[3006]|QUNOB535-09|5797-020708-KY|United States|Kentucky|658[0n]|BOLD:AAA8684
Haploa lecontei|[3007]|LNCC981-11|11-NCCC-506|United States|North Carolina|658[0n]|BOLD:AAA8684
Haploa confusa|[3008]|TMNBD377-07|MNBTT-3178|Canada|New Brunswick|658[0n]|BOLD:AAA8684
Haploa confusa|[3009]|TMNBD378-07|MNBTT-3179|Canada|New Brunswick|658[0n]|BOLD:AAA8684
Haploa lecontei|[3010]|LPOKB972-09|MDOK-2014|United States|Oklahoma|658[0n]|BOLD:AAA8684
Haploa confusa|[3011]|XAK008-06|2006-ONT-1003|Canada|Ontario|658[0n]|BOLD:AAA8684
Haploa confusa|[3012]|XAK047-06|2006-ONT-1042|Canada|Ontario|658[0n]|BOLD:AAA8684
Haploa confusa|[3013]|XAK048-06|2006-ONT-1043|Canada|Ontario|658[0n]|BOLD:AAA8684
Haploa confusa|[3014]|BLTIB918-08|BL1338|Canada|Ontario|658[0n]|BOLD:AAA8684
Haploa lecontei|[3015]|JSBIC008-08|HLC-16961|United States|North Carolina|658[0n]|BOLD:AAA8684
Haploa lecontei|[3016]|RDNM111-05|CNCNoctuoidea6661|Canada|Ontario|658[0n]|BOLD:AAA8684
Haploa lecontei|[3017]|RDNM113-05|CNCNoctuoidea6663|Canada|Ontario|658[0n]|BOLD:AAA8684
Haploa lecontei|[3018]|XAB012-04|04HBL005012|Canada|Ontario|658[0n]|BOLD:AAA8684
Haploa lecontei|[3019]|XAB196-04|04HBL005196|Canada|Ontario|658[0n]|BOLD:AAA8684
Haploa reversa|[3020]|RDNMF055-08|NOC14141|United States|New Mexico|658[0n]|BOLD:AAA8684
Haploa lecontei|[3021]|PHMO378-03|moth1377.02|Canada|Ontario|639[0n]|BOLD:AAA8684
Haploa lecontei|[3022]|TMG74-03|moth1075.01|Canada|Ontario|639[0n]|BOLD:AAA8684
Haploa confusa|[3023]|XAK511-07|HLC-16064|Canada|Ontario|658[0n]|BOLD:AAA8684
Haploa confusa|[3024]|MNBB075-05|HBL008685|Canada|New Brunswick|658[0n]|BOLD:AAA8684
Haploa confusa|[3025]|PMG010-03|HAPL1.00|Canada|Ontario|617[0n]|BOLD:AAA8684
Myrmecopsis strigosa|[3026]|CNCLB1874-14|CNCLEP00117598|Mexico|Chiapas|658[0n]|BOLD:AAJ3373
Apelopoda mecrida|[3027]|ARCTB198-08|MILA 0857|Guatemala|Quezaltenango|658[0n]|BOLD:AAR3994
Apeplopoda mecrida|[3028]|CNCLB2156-14|CNCLEP00117744|Mexico|Sonora|658[0n]|BOLD:ACR5051
Apeplopoda mecrida|[3029]|CNCLB2437-14|Knudson 004|United States|Texas|658[0n]|BOLD:ACR5051
Phoenicoprocta hampsonii|[3030]|RDNME549-08|LEP037973|United States|Arizona|658[0n]|BOLD:AAK1344
Phoenicoprocta hampsonii|[3031]|CMAZA1214-12|BIOUG02043-H06|United States|Arizona|658[0n]|BOLD ...
Phoenicoprocta lydia|[3032]|ARCTB331-08|MILA 0990|Guatemala|Zacapa|658[0n]|BOLD:AAA0317
Phoenicoprocta lydia|[3033]|BLPAE389-06|06-SRNP-104148|Costa Rica|Guanacaste|658[0n]|BOLD:AAA0317
Phoenicoprocta lydia|[3034]|ARCTD045-11|MILA 1550|Guatemala|Zacapa|614[0n]|BOLD:AAA0317
Phoenicoprocta lydia|[3035]|ARCTB324-08|MILA 0983|Guatemala|Zacapa|621[0n]|BOLD:AAA0317
Phoenicoprocta lydia|[3036]|BLPAE391-06|06-SRNP-104150|Costa Rica|Guanacaste|658[0n]|BOLD:AAA0317
Phoenicoprocta lydia|[3037]|BLPCA737-08|07-SRNP-105342|Costa Rica|Guanacaste|658[0n]|BOLD:AAA0317
Phoenicoprocta lydia|[3038]|BLPCA735-08|07-SRNP-105340|Costa Rica|Guanacaste|658[0n]|BOLD:AAA0317
Phoenicoprocta lydia|[3039]|BLPCA018-08|07-SRNP-104623|Costa Rica|Guanacaste|658[0n]|BOLD:AAA0317
Phoenicoprocta lydia|[3040]|BLPEE1435-12|12-SRNP-101434|Costa Rica|Guanacaste|658[0n]|BOLD:AAA...
Phoenicoprocta lydia|[3041]|BLPDJ451-09|09-SRNP-104833|Costa Rica|Guanacaste|658[0n]|BOLD:AAA0317
Phoenicoprocta lydia|[3042]|BLPCB004-08|07-SRNP-105549|Costa Rica|Guanacaste|658[0n]|BOLD:AAA0317
Phoenicoprocta lydia|[3043]|BLPAG339-07|06-SRNP-105978|Costa Rica|Guanacaste|658[0n]|BOLD:AAA0317
Phoenicoprocta lydia|[3044]|BLPAG147-07|06-SRNP-105786|Costa Rica|Guanacaste|658[0n]|BOLD:AAA0317
Phoenicoprocta lydia|[3045]|BLPEE1194-12|12-SRNP-101193|Costa Rica|Guanacaste|658[0n]|BOLD:AAA...
Phoenicoprocta lydia|[3046]|BLPEE1193-12|12-SRNP-101192|Costa Rica|Guanacaste|658[0n]|BOLD:AAA...
Phoenicoprocta lydia|[3047]|BLPED2392-12|11-SRNP-105994|Costa Rica|Guanacaste|658[0n]|BOLD:AAA...
Phoenicoprocta lydia|[3048]|BLPDK622-09|09-SRNP-105550|Costa Rica|Guanacaste|658[0n]|BOLD:AAA0317
Phoenicoprocta lydia|[3049]|BLPCB003-08|07-SRNP-105548|Costa Rica|Guanacaste|658[0n]|BOLD:AAA0317
Phoenicoprocta lydia|[3050]|BLPCO742-08|08-SRNP-104219|Costa Rica|Guanacaste|658[0n]|BOLD:AAA0317
Phoenicoprocta lydia|[3051]|BLPCA738-08|07-SRNP-105343|Costa Rica|Guanacaste|658[0n]|BOLD:AAA0317
Phoenicoprocta lydia|[3052]|BLPCA390-08|07-SRNP-104995|Costa Rica|Guanacaste|658[0n]|BOLD:AAA0317
Phoenicoprocta lydia|[3053]|BLPCA025-08|07-SRNP-104630|Costa Rica|Guanacaste|658[0n]|BOLD:AAA0317
Phoenicoprocta lydia|[3054]|BLPCA021-08|07-SRNP-104626|Costa Rica|Guanacaste|658[0n]|BOLD:AAA0317
Phoenicoprocta lydia|[3055]|BLPCA020-08|07-SRNP-104625|Costa Rica|Guanacaste|658[0n]|BOLD:AAA0317
Phoenicoprocta lydia|[3056]|BLPCA019-08|07-SRNP-104624|Costa Rica|Guanacaste|658[0n]|BOLD:AAA0317
Phoenicoprocta lydia|[3057]|BLPBB016-07|06-SRNP-108099|Costa Rica|Guanacaste|658[0n]|BOLD:AAA0317
Phoenicoprocta lydia|[3058]|BLPCA736-08|07-SRNP-105341|Costa Rica|Guanacaste|658[0n]|BOLD:AAA0317
Phoenicoprocta lydia|[3059]|BLPCA732-08|07-SRNP-105337|Costa Rica|Guanacaste|658[0n]|BOLD:AAA0317
Phoenicoprocta lydia|[3060]|BLPAE390-06|06-SRNP-104149|Costa Rica|Guanacaste|658[0n]|BOLD:AAA0317
Phoenicoprocta lydia|[3061]|BLPAE388-06|06-SRNP-104147|Costa Rica|Guanacaste|658[0n]|BOLD:AAA0317
Phoenicoprocta lydia|[3062]|BLPAE392-06|06-SRNP-104151|Costa Rica|Guanacaste|658[0n]|BOLD:AAA0317
Phoenicoprocta lydia|[3063]|BLPAE393-06|06-SRNP-104152|Costa Rica|Guanacaste|658[0n]|BOLD:AAA0317
Phoenicoprocta lydia|[3064]|BLPAE394-06|06-SRNP-104153|Costa Rica|Guanacaste|658[0n]|BOLD:AAA0317
Phoenicoprocta lydia|[3065]|BLPAE395-06|06-SRNP-104154|Costa Rica|Guanacaste|658[0n]|BOLD:AAA0317
Phoenicoprocta lydia|[3066]|BLPAE397-06|06-SRNP-104156|Costa Rica|Guanacaste|658[0n]|BOLD:AAA0317
Phoenicoprocta lydia|[3067]|BLPAE398-06|06-SRNP-104157|Costa Rica|Guanacaste|658[0n]|BOLD:AAA0317
Phoenicoprocta lydia|[3068]|BLPAE399-06|06-SRNP-104158|Costa Rica|Guanacaste|658[0n]|BOLD:AAA0317
Phoenicoprocta lydia|[3069]|BLPAE400-06|06-SRNP-104159|Costa Rica|Guanacaste|658[0n]|BOLD:AAA0317
Phoenicoprocta lydia|[3070]|BLPAE401-06|06-SRNP-104160|Costa Rica|Guanacaste|658[0n]|BOLD:AAA0317
Phoenicoprocta lydia|[3071]|BLPAE402-06|06-SRNP-104161|Costa Rica|Guanacaste|658[0n]|BOLD:AAA0317
Phoenicoprocta lydia|[3072]|BLPDA665-09|08-SRNP-105740|Costa Rica|Guanacaste|658[0n]|BOLD:AAA0317
Phoenicoprocta lydia|[3073]|BLPAE396-06|06-SRNP-104155|Costa Rica|Guanacaste|658[0n]|BOLD:AAA0317
Phoenicoprocta lydia|[3074]|BLPDA666-09|08-SRNP-105741|Costa Rica|Guanacaste|658[1n]|BOLD:AAA0317
Phoenicoprocta lydia|[3075]|BLPBH279-07|07-SRNP-104038|Costa Rica|Guanacaste|645[0n]|BOLD:AAA0317
Phoenicoprocta lydia|[3076]|BLPED2122-12|11-SRNP-105724|Costa Rica|Guanacaste|658[0n]|BOLD:AAA...
Phoenicoprocta lydia|[3077]|BLPDJ256-09|09-SRNP-104638|Costa Rica|Guanacaste|658[0n]|BOLD:AAA0317
Phoenicoprocta lydia|[3078]|BLPDI883-09|09-SRNP-104315|Costa Rica|Guanacaste|658[0n]|BOLD:AAA0317
Phoenicoprocta lydia|[3079]|BLPEE1013-12|12-SRNP-101012|Costa Rica|Guanacaste|658[0n]|BOLD:AAA...
Phoenicoprocta lydia|[3080]|BLPEE1012-12|12-SRNP-101011|Costa Rica|Guanacaste|658[0n]|BOLD:AAA...
Haemanota prophaea|[3081]|LNOUD124-10|BIOUG00736-H01|French Guiana|Cayenne|658[0n]|BOLD:AAU7943
Haemanota prophaea|[3082]|ARCTB068-08|MILA 0727|French Guiana||658[0n]|BOLD:AAI6533
Haemanota prophaea|[3083]|ARCTB009-08|MILA 0668|French Guiana||658[0n]|BOLD:AAI6533
Haemanota prophaea|[3084]|ARCTB078-08|MILA 0737|French Guiana||658[0n]|BOLD:AAI6533
Psilopleura polia|[3085]|CNCLB2438-14|Knudson 005|United States|Texas|658[0n]|BOLD:AAE8066
Psilopleura vittata|[3086]|BLPDK1129-09|09-SRNP-106057|Costa Rica|Guanacaste|658[0n]|BOLD:AAE8066
Psilopleura vittata|[3087]|BLPCA037-08|07-SRNP-104642|Costa Rica|Guanacaste|658[0n]|BOLD:AAE8066

Psilopleura polia|[3085]|CNCLB2438-14|Knudson 005|United States|Texas|658[0n]|BOLD:AAE8066
Psilopleura vittata|[3086]|BLPDK1129-09|09-SRNP-106057|Costa Rica|Guanacaste|658[0n]|BOLD:AAE8066
Psilopleura vittata|[3087]|BLPCA037-08|07-SRNP-104642|Costa Rica|Guanacaste|658[0n]|BOLD:AAE8066
Psilopleura vittata|[3088]|BLPAA419-06|06-SRNP-100418|Costa Rica|Guanacaste|658[0n]|BOLD:AAE8066
Psilopleura vittata|[3089]|BLPDK798-09|09-SRNP-105726|Costa Rica|Guanacaste|658[0n]|BOLD:AAE8066
Psilopleura vittata|[3090]|GWOTK254-12|BC ZSM Lep add 57590|Mexico|Oaxaca|633[0n]|BOLD:AAE8066
Psilopleura vittata|[3091]|MHMYN6282-14|14-SRNP-12282|Costa Rica||623[0n]|BOLD:AAE8066
Psilopleura vittata|[3092]|CNCLB1898-14|CNCLEP00117622|Guatemala||658[0n]|BOLD:AAE8066
Psilopleura vittata|[3093]|CNCLB1892-14|CNCLEP00117616|Guatemala||658[0n]|BOLD:AAE8066
Psilopleura vittata|[3094]|CNCLB1891-14|CNCLEP00117615|Mexico|Chiapas|658[0n]|BOLD:AAE8066
Pareuchaetes insulata|[3095]|HKONB227-09|3724-COI-08|United States|Texas|658[0n]|BOLD:AAA1317
Pareuchaetes insulata|[3096]|LSEU017-06|06-JKA-0017|United States|Florida|515[1n]|BOLD:AAA1317
Pareuchaetes insulata|[3097]|HKONB228-09|3725-COI-08|United States|Texas|640[0n]|BOLD:AAA1317
Pareuchaetes insulata|[3098]|LSEU015-06|06-JKA-0015|United States|Florida|513[1n]|BOLD:AAA1317
Pareuchaetes insulata|[3099]|LSEU016-06|06-JKA-0016|United States|Florida|524[0n]|BOLD:AAA1317
Pareuchaetes insulata|[3100]|LSEU014-06|06-JKA-0014|United States|Florida|546[1n]|BOLD:AAA1317
Pareuchaetes insulata|[3101]|BBLOB452-11|BIOUG01396-F12|United States|Florida|619[0n]|BOLD:AAA...
Pareuchaetes insulata|[3102]|LOFLC203-06|06-FLOR-2083|United States|Florida|655[0n]|BOLD:AAA1317
Pareuchaetes insulata|[3103]|LOFLC329-06|06-FLOR-2209|United States|Florida|658[0n]|BOLD:AAA1317
Crambidia sp.|[3104]|ABLCW162-10|CSUPOBK-0162|United States|Arizona|658[0n]|BOLD:AAH4869
Crambidia sp.|[3105]|ABLCW161-10|CSUPOBK-0161|United States|Arizona|658[0n]|BOLD:AAH4869
Pagara simplex|[3106]|ABLCW284-10|CSUPOBK-0284|United States|Mississippi|658[0n]|BOLD:AAD0349
Pagara simplex|[3107]|LNC443-05|05-NCCC-443|United States|North Carolina|658[0n]|BOLD:AAD0349
Pagara simplex|[3108]|HKONS234-08|3139-COI-07|United States|Florida|658[0n]|BOLD:AAD0349
Pagara simplex|[3109]|LTOLB312-09|CWM-94-0385|United States|Maryland|658[0n]|BOLD:AAD0349
Pagara simplex|[3110]|BBLOB273-11|BIOUG01369-G11|United States|Florida|658[0n]|BOLD:AAD0349
Pagara simplex|[3111]|USLEP531-10|10BBLEP-00531|United States|Arkansas|658[0n]|BOLD:AAD0349
Pagara simplex|[3112]|BBLOB745-11|BIOUG01399-G08|United States|Oklahoma|658[0n]|BOLD:AAD0349
Pagara simplex|[3113]|LNC444-05|05-NCCC-444|United States|North Carolina|658[0n]|BOLD:AAD0349
Pagara simplex|[3114]|LNCC1414-11|11-NCCC-939|United States|North Carolina|658[0n]|BOLD:AAD0349
Pagara simplex|[3115]|LPOKB179-09|MDOK-1155|United States|Oklahoma|658[0n]|BOLD:AAD0349
Pagara simplex|[3116]|LILLA842-11|SNS10IL-01062|United States|Illinois|658[0n]|BOLD:AAD0349
Pagara simplex|[3117]|LPOKA178-08|MDOK-0178|United States|Oklahoma|658[0n]|BOLD:AAD0349
Pagara simplex|[3118]|LILLA919-11|SNS10IL-01140|United States|Illinois|658[0n]|BOLD:AAD0349
Pagara simplex|[3119]|ABLCW285-10|CSUPOBK-0285|United States|Mississippi|603[0n]|BOLD:AAD0349
Pagara simplex|[3120]|HKONS233-08|3138-COI-07|United States|Florida|658[0n]|BOLD:AAD0349
Pagara simplex|[3121]|RDNMH352-09|CNCLEP00057761|United States|Mississippi|658[0n]|BOLD:AAD0349
Utetheisa ornatrix|[3122]|LOFLB826-06|06-FLOR-1766|United States|Florida|658[0n]|BOLD:AAA1412
Utetheisa ornatrix|[3123]|LOFLB830-06|06-FLOR-1770|United States|Florida|656[0n]|BOLD:AAA1412
Utetheisa ornatrix|[3124]|USLEP744-10|10BBLEP-00744|United States|Florida|634[0n]|BOLD:AAA1412
Utetheisa ornatrix|[3125]|USLEP743-10|10BBLEP-00743|United States|Florida|658[0n]|BOLD:AAA1412
Utetheisa ornatrix|[3126]|USLEP742-10|10BBLEP-00742|United States|Florida|658[0n]|BOLD:AAA1412
Utetheisa ornatrix|[3127]|LOFLB825-06|06-FLOR-1765|United States|Florida|656[0n]|BOLD:AAA1412
Utetheisa ornatrix|[3128]|LOFLB392-06|06-FLOR-1332|United States|Florida|658[0n]|BOLD:AAA1412
Utetheisa ornatrix|[3129]|BBLOB841-11|BIOUG01412-G09|United States|Florida|658[0n]|BOLD:AAA1412
Utetheisa ornatrix|[3130]|LPOKE136-10|MDOK-4214|United States|Oklahoma|658[0n]|BOLD:AAA1412
Utetheisa ornatrix|[3131]|MNAC154-07|CNCLEP00026118|United States|Florida|658[0n]|BOLD:AAA1412
Utetheisa ornatrix|[3132]|BBLOB844-11|BIOUG01412-G12|United States|Florida|658[0n]|BOLD:AAA1412
Utetheisa ornatrix|[3133]|LOFLB827-06|06-FLOR-1767|United States|Florida|656[0n]|BOLD:AAA1412
Utetheisa ornatrix|[3134]|USLEP741-10|10BBLEP-00741|United States|Florida|658[0n]|BOLD:AAA1412
Utetheisa ornatrix|[3135]|LOFLB206-06|06-FLOR-1146|United States|Florida|674[0n]|BOLD:AAA1412
Utetheisa ornatrix|[3136]|HKONB275-09|3772-COI-08|United States|Texas|638[0n]|BOLD:AAA1412
Utetheisa ornatrix|[3137]|BBLOB1126-11|BIOUG01415-G09|United States|Florida|658[0n]|BOLD:AAA1412
Utetheisa ornatrix|[3138]|BBLOB1818-11|BIOUG01425-B01|United States|Florida|658[0n]|BOLD:AAA1412
Utetheisa ornatrix|[3139]|BBLOB843-11|BIOUG01412-G11|United States|Florida|658[0n]|BOLD:AAA1412
Utetheisa ornatrix|[3140]|BBLOB842-11|BIOUG01412-G10|United States|Florida|658[0n]|BOLD:AAA1412
Utetheisa ornatrix|[3141]|LOFLB829-06|06-FLOR-1769|United States|Florida|658[0n]|BOLD:AAA1412
Utetheisa ornatrix|[3142]|LOFLB824-06|06-FLOR-1764|United States|Florida|658[0n]|BOLD:AAA1412
Utetheisa ornatrix|[3143]|USLEP747-10|10BBLEP-00747|United States|Florida|658[0n]|BOLD:AAA1412
Utetheisa ornatrix|[3144]|USLEP746-10|10BBLEP-00746|United States|Florida|658[0n]|BOLD:AAA1412
Utetheisa ornatrix|[3145]|USLEP745-10|10BBLEP-00745|United States|Florida|658[0n]|BOLD:AAA1412
Utetheisa ornatrix|[3146]|LNC049-05|05-NCCC-049|United States|North Carolina|658[0n]|BOLD:AAA1412
Utetheisa ornatrix|[3147]|LOFLA930-06|06-FLOR-0930|United States|Florida|658[0n]|BOLD:AAA1412
Utetheisa ornatrix|[3148]|LOFLB828-06|06-FLOR-1768|United States|Florida|658[0n]|BOLD:AAA1412
Dodia tarandus|[3149]|RDMAB420-05|BCSC93|Canada|Alberta|658[0n]|BOLD:AAC2146
Dodia tarandus|[3150]|RDNME213-07|CNCNoctuoidea13820|Canada|Alberta|617[0n]|BOLD:AAC2146
Dodia albertae|[3151]|RDMAB416-05|BCSC89|Canada|Yukon Territory|583[0n]|BOLD:AAC2146
Dodia albertae|[3152]|RDMAB417-05|BCSC90|Canada|Alberta|546[0n]|BOLD:AAC2146
Dodia verticalis|[3153]|RDNMG902-08|CNC LEP00019115|Canada|Yukon Territory|658[0n]|BOLD:AAC2146
Dodia|[3154]|CNCLB191-14|CNCLEP00113273|Canada|Yukon Territory|658[0n]|BOLD:AAC2146
Dodia verticalis|[3155]|TIPSY590-12|STG116|Canada|Yukon Territory|658[0n]|BOLD:AAC2146
Dodia verticalis|[3156]|TIPSY589-12|STG115|Canada|Yukon Territory|658[0n]|BOLD:AAC2146
Dodia albertae|[3157]|RDNME838-08|LEP041303|Canada|Newfoundland and Labrador|609[0n]|BOLD:ABZ1341
Dodia albertae|[3158]|RDNME837-08|LEP041302|Canada|Newfoundland and Labrador|598[0n]|BOLD:ABZ1341
Dodia albertae|[3159]|RDMAB421-05|BCSC94|Canada|Yukon Territory|601[0n]|BOLD:ABZ1341
Dodia albertae|[3160]|RDMAB419-05|BCSC92|Canada|Alberta|658[0n]|BOLD:ABZ1341
Dodia albertae|[3161]|RDMAB418-05|BCSC91|Canada|Alberta|658[0n]|BOLD:ABZ1341
Dodia albertae|[3162]|RDNME217-07|CNCNoctuoidea13824|Canada|Alberta|658[0n]|BOLD:ABZ1341
Dodia albertae|[3163]|RDNME216-07|CNCNoctuoidea13823|Canada|Alberta|658[0n]|BOLD:ABZ1341
Dodia albertae|[3164]|RDNME215-07|CNCNoctuoidea13822|Canada|Alberta|658[0n]|BOLD:ABZ1341
Dodia albertae|[3165]|RDNME214-07|CNCNoctuoidea13821|Canada|Alberta|658[0n]|BOLD:ABZ1341
Dodia albertae|[3166]|TIPSY588-12|STG114|Canada|Yukon Territory|658[0n]|BOLD:ABZ1341
Dodia kononenkoi|[3167]|RDNMF520-08|NOC14606|Canada|Yukon Territory|652[0n]|BOLD:AAF5396
Dodia kononenkoi|[3168]|TIPSY578-12|STG104|Canada|Yukon Territory|658[0n]|BOLD:AAF5396
Dodia kononenkoi|[3169]|RDNMF522-08|NOC14608|Canada|Yukon Territory|652[0n]|BOLD:AAF5396
Tyria jacobaeae|[3170]|LEFIL049-10|MM19049|Russia|Volgogradskaya Oblast|658[0n]|BOLD:AAB5189
Tyria jacobaeae|[3171]|RWWC599-11|RWWA-2576|United States|Washington|658[0n]|BOLD:AAB5189
Tyria jacobaeae|[3172]|NARCA456-10|Arcec 32342|Austria|Vorarlberg|658[0n]|BOLD:AAB5189
Tyria jacobaeae|[3173]|GBGL16402-14|KF533506|||654[0n]|BOLD:AAB5189
Tyria jacobaeae|[3174]|LEFIF138-10|MM10597|Finland||658[0n]|BOLD:AAB5189
Tyria jacobaeae|[3175]|NARCA455-10|Arcec 32341|Austria|Vorarlberg|658[0n]|BOLD:AAB5189
Tyria jacobaeae|[3176]|LOWCE193-06|CGWC-3953|Canada|British Columbia|658[0n]|BOLD:AAB5189
Tyria jacobaeae|[3177]|RDMAB921-06|BCSC394|Canada|Nova Scotia|658[0n]|BOLD:AAB5189
Tyria jacobaeae|[3178]|NARCA457-10|Arcec 32343|Austria|Vorarlberg|658[0n]|BOLD:AAB5189
Tyria jacobaeae|[3179]|NARCA186-09|Arcec 32075|Austria|Vorarlberg|658[0n]|BOLD:AAB5189
Tyria jacobaeae|[3180]|NARCA184-09|Arcec 32073|France||658[0n]|BOLD:AAB5189
Tyria jacobaeae|[3181]|CGUKC346-09|UKLB25H06|United Kingdom|England|658[0n]|BOLD:AAB5189
Tyria jacobaeae|[3182]|CGUKB177-09|UKLB13E02|United Kingdom|England|658[0n]|BOLD:AAB5189
Tyria jacobaeae|[3183]|ODOPE714-11|BC ZSM Lep 50355|Germany|Bavaria|658[0n]|BOLD:AAB5189
Tyria jacobaeae|[3184]|GWOSK783-11|BC ZSM Lep 48999|Germany|Bavaria|658[0n]|BOLD:AAB5189
Tyria jacobaeae|[3185]|NARCA188-09|Arcec 32077|Belgium||658[0n]|BOLD:AAB5189
Tyria jacobaeae|[3186]|NARCA187-09|Arcec 32076|United Kingdom||658[0n]|BOLD:AAB5189
Tyria jacobaeae|[3187]|NLLEA235-12|RMNH.INS.538850|Netherlands|South Holland|658[0n]|BOLD:AAB5189

Tyria jacobaeae|[3185]|NARCA188-09|Arcec 32077|Belgium||658[0n]|BOLD:AAB5189
Tyria jacobaeae|[3186]|NARCA187-09|Arcec 32076|United Kingdom||658[0n]|BOLD:AAB5189
Tyria jacobaeae|[3187]|NLLEA235-12|RMNH.INS.538850|Netherlands|South Holland|658[0n]|BOLD:AAB5189
Tyria jacobaeae|[3188]|NARCA189-09|Arcec 32078|Norway|Vestfold|658[0n]|BOLD:AAB5189
Tyria jacobaeae|[3189]|LON1108-12|NHMO-2011.169|Norway|Ostfold|658[0n]|BOLD:AAB5189
Tyria jacobaeae|[3190]|NLLEA325-12|RMNH.INS.538940|Netherlands|South Holland|658[0n]|BOLD:AAB5189
Tyria jacobaeae|[3191]|NARCA185-09|Arcec 32074|Germany|Lower Saxony|658[0n]|BOLD:AAB5189
Tyria jacobaeae|[3192]|RWWB830-10|RWWA-1829|United States|Washington|658[0n]|BOLD:AAB5189
Tyria jacobaeae|[3193]|LEATG176-14|TLMF Lep 13963|Austria|Tirol|658[0n]|BOLD:AAB5189
Tyria jacobaeae|[3194]|RWWA094-09|RWWA-0094|United States|Washington|658[0n]|BOLD:AAB5189
Tyria jacobaeae|[3195]|LON217-08|NHMO-07006|Norway|Vestfold|657[0n]|BOLD:AAB5189
Tyria jacobaeae|[3196]|CGUKD338-09|UKLB36E02|United Kingdom|England|658[0n]|BOLD:AAB5189
Tyria jacobaeae|[3197]|CGUKA053-09|UKLB1E07|United Kingdom|England|658[0n]|BOLD:AAB5189
Tyria jacobaeae|[3198]|CGUKA235-09|UKLB3E01|United Kingdom|England|658[0n]|BOLD:AAB5189
Tyria jacobaeae|[3199]|LON960-12|NHMO Lep2011.020|Norway||658[0n]|BOLD:AAB5189
Tyria jacobaeae|[3200]|NARCA183-09|Arcec 32072|Slovenia||658[0n]|BOLD:AAB5189
Tyria jacobaeae|[3201]|PHLAD763-11|TLMF Lep 04128|Austria|Vorarlberg|658[0n]|BOLD:AAB5189
Tyria jacobaeae|[3202]|PHLAD764-11|TLMF Lep 04129|Austria|Vorarlberg|658[0n]|BOLD:AAB5189
Tyria jacobaeae|[3203]|GBLAB174-13|BC ZSM Lep 75800|Germany|Saarland|658[0n]|BOLD:AAB5189
Tyria jacobaeae|[3204]|NLLEA236-12|RMNH.INS.538851|Netherlands|South Holland|658[0n]|BOLD:AAB5189
Tyria jacobaeae|[3205]|LEFIL050-10|MM19050|Russia|Orenburgskaya Oblast|658[0n]|BOLD:AAB5189
Tyria jacobaeae|[3206]|LEATG175-14|TLMF Lep 13962|Austria|Tirol|658[0n]|BOLD:AAB5189
Tyria jacobaeae|[3207]|RWWA134-09|RWWA-0134|United States|Washington|648[0n]|BOLD:AAB5189
Tyria jacobaeae|[3208]|RDNMG966-08|CNC LEP00053090|Canada|Nova Scotia|642[0n]|BOLD:AAB5189
Tyria jacobaeae|[3209]|CGUKB552-09|UKLB17E01|United Kingdom|England|582[0n]|BOLD:AAB5189
Tyria jacobaeae|[3210]|CGUKB1020-09|UKLB19D12|United Kingdom|England|619[0n]|BOLD:AAB5189
Eupseudosoma involuta|[3211]|CNCLB2201-14|CNCLEP00118005|Dominican Republic|Barahona|658[0n]|BOLD...
Eupseudosoma involuta|[3212]|ARCTD148-11|BEVI1453|Cuba|Matanzas|658[0n]|BOLD:AAG6225
Eupseudosoma involuta|[3213]|BBLOE2033-12|BIOUG01995-F05|United States|Florida|658[0n]|BOLD:AA...
Eupseudosoma involuta|[3214]|CNCLB2199-14|CNCLEP00118003|United States|Florida|658[0n]|BOLD:AA...
Eupseudosoma involuta|[3215]|CNCLB2200-14|CNCLEP00118004|United States|Florida|658[0n]|BOLD:AA...
Eupseudosoma involuta|[3216]|LPYPC373-08|MLL-00573|Mexico|Campeche|614[0n]|BOLD:AAG6225
Eupseudosoma involuta|[3217]|LPMX245-07|MLL-00245|Mexico|Campeche|630[0n]|BOLD:AAG6225
Euerythra trimaculata|[3218]|RDNMF949-08|CNC LEP00054216|United States|Texas|658[0n]|BOLD:AAE2937
Euerythra phasma|[3219]|LPOKB482-09|MDOK-1489|United States|Oklahoma|658[0n]|BOLD:AAE2937
Euerythra phasma|[3220]|LPOKC758-09|MDOK-2835|United States|Oklahoma|658[0n]|BOLD:AAE2937
Euerythra phasma|[3221]|LPOKB1034-09|MDOK-2076|United States|Oklahoma|658[0n]|BOLD:AAE2937
Euerythra phasma|[3222]|LPOKE087-10|MDOK-4165|United States|Oklahoma|658[0n]|BOLD:AAE2937
Euerythra phasma|[3223]|LSUSA130-06|06-SUSA-0130|United States|Kentucky|658[0n]|BOLD:AAE2937
Euerythra phasma|[3224]|RDMAB937-06|BCSC403|United States|Florida|536[1n]|BOLD:AAE2937
Euerythra phasma|[3225]|BBLSW489-09|09BBLEP-01417|United States|Oklahoma|658[0n]|BOLD:AAE2937
Euerythra phasma|[3226]|BBLSX321-09|09BBLEP-02249|United States|Oklahoma|658[0n]|BOLD:AAE2937
Euerythra phasma|[3227]|BBLSW488-09|09BBLEP-01416|United States|Oklahoma|658[0n]|BOLD:AAE2937
Eucereon erythrolepsis|[3228]|CNCLB1884-14|CNCLEP00117608|Mexico|Chiapas|658[0n]|BOLD:AAB8816
Eucereon erythrolepsis|[3229]|ARCTC841-11|MILA 1303|Guatemala|Suchitepequez|658[0n]|BOLD:AAB8816
Eucereon erythrolepsis|[3230]|GWOTK256-12|BC ZSM Lep add 57592|Mexico|Oaxaca|658[0n]|BOLD:AAB8816
Eucereon erythrolepsis|[3231]|GWOTK252-12|BC ZSM Lep add 57588|Mexico|Oaxaca|658[0n]|BOLD:AAB8816
Eucereon erythrolepsis|[3232]|CNCLB1888-14|CNCLEP00117612|Guatemala||658[0n]|BOLD:AAB8816
Eucereon erythrolepsis|[3233]|CNCLB1887-14|CNCLEP00117611|Guatemala|San Marcos|658[0n]|BOLD:AA...
Eucereon erythrolepsis|[3234]|CNCLB1886-14|CNCLEP00117610|Mexico|Veracruz|658[0n]|BOLD:AAC2374
Eucereon erythrolepsis|[3235]|CNCLB1885-14|CNCLEP00117609|Mexico|Chiapas|658[0n]|BOLD:AAC2374
Eucereon erythrolepsis|[3236]|CNCLB2503-14|CNCLEP 00119627|Mexico|Veracruz|658[0n]|BOLD:AAB5395
Episcepsis inornata|[3237]|CNCLB1872-14|CNCLEP00117999|Mexico|Chiapas|658[0n]|BOLD:AAF4526
Episcepsis inornata|[3238]|CNCLB1873-14|CNCLEP00118000|Mexico|Chiapas|658[0n]|BOLD:AAF4526
Episcepsis inornata|[3239]|CNCLB1871-14|CNCLEP00117998|Mexico|Chiapas|658[0n]|BOLD:AAF4526
Didasys belae|[3240]|NOCNA082-14|20381-180713-FL|United States|Florida|658[0n]|BOLD:ACN6763
Macrocneme chrysitis|[3241]|CNCLB2207-14|CNCLEP00118011|Mexico|Chiapas|658[0n]|BOLD:AAA1436
Macrocneme chrysitis|[3242]|CNCLB2204-14|CNCLEP00118008|Mexico|Chiapas|658[0n]|BOLD:AAA1436
Macrocneme chrysitis|[3243]|CNCLB2205-14|CNCLEP00118009|Mexico|Chiapas|407[0n]|BOLD:ACR1030
Macrocneme chrysitis|[3244]|CNCLB2203-14|CNCLEP00118007|Mexico|Chiapas|407[0n]|
Macrocneme chrysitis|[3245]|CNCLB2206-14|CNCLEP00118010|Mexico|Chiapas|658[0n]|BOLD:ABZ8392
Macrocneme chrysitis|[3246]|CNCLB2202-14|CNCLEP00118006|Mexico|Chiapas|658[0n]|BOLD:ABZ8392
Eucereon myrina|[3247]|CNCLB2155-14|CNCLEP00117743|Mexico|Sonora|658[0n]|BOLD:ACR1626
Eucereon myrina|[3248]|CNCLB1883-14|CNCLEP00117607|Mexico|Oaxaca|658[0n]|BOLD:ACR1626
Eucereon myrina|[3249]|CNCLB1882-14|CNCLEP00117606|Mexico|Chiapas|658[0n]|BOLD:ACR1626
Eucereon myrina|[3250]|CNCLB1881-14|CNCLEP00117605|Mexico|Chiapas|658[0n]|BOLD:ACR1626
Eucereon myrina|[3251]|CNCLB1880-14|CNCLEP00117604|Mexico|Chiapas|658[0n]|BOLD:ACR1626
Eucereon myrina|[3252]|CNCLB1879-14|CNCLEP00117603|Mexico|Chiapas|658[0n]|BOLD:ACR1626
Nelphe carolina|[3253]|BBLOB451-11|BIOUG01396-F11|United States|Florida|658[0n]|BOLD:ACE8501
Nelphe carolina|[3254]|BBLOB449-11|BIOUG01396-F09|United States|Florida|658[0n]|BOLD:ACE8501
Nelphe carolina|[3255]|BBLOB447-11|BIOUG01396-F07|United States|Florida|658[0n]|BOLD:ACE8501
Nelphe carolina|[3256]|RDNML302-13|CNCLEP 92278|United States|Florida|658[0n]|BOLD:ACE8501
Nelphe carolina|[3257]|RDNML303-13|CNCLEP 92279|United States|Florida|658[0n]|BOLD:ACE8501
Nelphe carolina|[3258]|BBLOE2034-12|BIOUG01995-F06|United States|Florida|658[0n]|BOLD:ACE8501
Nelphe carolina|[3259]|BBLOB1321-11|BIOUG01417-H02|United States|Florida|658[0n]|BOLD:ACE8501
Nelphe carolina|[3260]|BBLOB503-11|BIOUG01397-C04|United States|Florida|658[0n]|BOLD:ACE8501
Nelphe carolina|[3261]|BBLOB456-11|BIOUG01396-G04|United States|Florida|658[0n]|BOLD:ACE8501
Nelphe carolina|[3262]|BBLOB441-11|BIOUG01396-F01|United States|Florida|658[0n]|BOLD:ACE8501
Nelphe carolina|[3263]|BBLOB438-11|BIOUG01396-E10|United States|Florida|658[0n]|BOLD:ACE8501
Nelphe|[3264]|CNCLB1896-14|CNCLEP00117620|Dominican Republic|Barahona|658[0n]|BOLD:ACR1972
Nelphe relegatum|[3265]|BLPAD858-06|06-SRNP-103677|Costa Rica|Alajuela|632[0n]|BOLD:AAA9439
Nelphe relegatum|[3266]|MHARB893-06|06-SRNP-4616|Costa Rica|Alajuela|658[0n]|BOLD:AAA9439
Nelphe relegatum|[3267]|MHARB895-06|06-SRNP-4618|Costa Rica|Alajuela|658[0n]|BOLD:AAA9439
Nelphe relegatum|[3268]|ARCTC784-11|MILA 1246|Costa Rica|Cartago|658[0n]|BOLD:AAA9439
Nelphe relegatum|[3269]|BLPDD379-09|08-SRNP-108274|Costa Rica|Guanacaste|658[0n]|BOLD:AAA9439
Nelphe relegatum|[3270]|BLPEE3970-14|13-SRNP-103406|Costa Rica|Guanacaste|613[0n]|BOLD:AAA9439
Nelphe relegatum|[3271]|MHMYN6280-14|14-SRNP-70265|Costa Rica||610[0n]|BOLD:AAA9439
Nelphe relegatum|[3272]|MHARB894-06|06-SRNP-4617|Costa Rica|Alajuela|623[0n]|BOLD:AAA9439
Nelphe relegatum|[3273]|BLPAG786-07|06-SRNP-106425|Costa Rica|Guanacaste|658[0n]|BOLD:AAA9439
Nelphe relegatum|[3274]|BLPCG697-08|07-SRNP-110942|Costa Rica|Guanacaste|658[0n]|BOLD:AAA9439
Nelphe relegatum|[3275]|BLPCC568-08|07-SRNP-107053|Costa Rica|Guanacaste|658[0n]|BOLD:AAA9439
Nelphe relegatum|[3276]|BLPCC122-08|07-SRNP-106607|Costa Rica|Guanacaste|658[0n]|BOLD:AAA9439
Nelphe relegatum|[3277]|BLPED1717-12|11-SRNP-105319|Costa Rica|Guanacaste|658[0n]|BOLD:AAA9439
Nelphe relegatum|[3278]|BLPBH161-07|07-SRNP-103920|Costa Rica|Guanacaste|658[0n]|BOLD:AAA9439
Nelphe relegatum|[3279]|MHARB591-06|04-SRNP-47617|Costa Rica|Guanacaste|658[0n]|BOLD:AAA9439
Nelphe relegatum|[3280]|BLPAB230-06|06-SRNP-101169|Costa Rica|Alajuela|658[0n]|BOLD:AAA9439
Nelphe relegatum|[3281]|BLPDC076-09|08-SRNP-107031|Costa Rica|Guanacaste|658[0n]|BOLD:AAA9439
Nelphe relegatum|[3282]|BLPAA804-06|06-SRNP-100803|Costa Rica|Alajuela|658[0n]|BOLD:AAA9439
Nelphe relegatum|[3283]|LYHES397-09|MAL-03694|Mexico|Quintana Roo|658[0n]|BOLD:AAA9439
Nelphe relegatum|[3284]|MHMYL2465-11|10-SRNP-72772|Costa Rica||658[0n]|BOLD:AAA9439
Nelphe relegatum|[3285]|BLPDS404-10|10-SRNP-107028|Costa Rica|Alajuela|658[0n]|BOLD:AAA9439
Nelphe relegatum|[3286]|MHMYC616-09|08-SRNP-32294|Costa Rica|Guanacaste|658[0n]|BOLD:AAA9439

Nelphe relegatum|[3284]|MHMYL2465-11|10-SRNP-72772|Costa Rica||658[0n]|BOLD:AAA9439
Nelphe relegatum|[3285]|BLPDS404-10|10-SRNP-107028|Costa Rica|Alajuela|658[0n]|BOLD:AAA9439
Nelphe relegatum|[3286]|MHMYC616-09|08-SRNP-32294|Costa Rica|Guanacaste|658[0n]|BOLD:AAA9439
Nelphe relegatum|[3287]|BLPAB723-06|06-SRNP-101662|Costa Rica|Guanacaste|658[0n]|BOLD:AAA9439
Nelphe relegatum|[3288]|BLPCG698-08|07-SRNP-110943|Costa Rica|Guanacaste|658[0n]|BOLD:AAA9439
Nelphe relegatum|[3289]|BLPCJ333-08|07-SRNP-113398|Costa Rica|Guanacaste|658[0n]|BOLD:AAA9439
Nelphe relegatum|[3290]|BLPCE349-08|07-SRNP-108714|Costa Rica|Guanacaste|658[0n]|BOLD:AAA9439
Nelphe relegatum|[3291]|BLPCE348-08|07-SRNP-108713|Costa Rica|Guanacaste|658[0n]|BOLD:AAA9439
Nelphe relegatum|[3292]|ARCTC782-11|MILA 1244|Nicaragua|Granada|658[0n]|BOLD:AAA9439
Nelphe relegatum|[3293]|ARCTC781-11|MILA 1243|Nicaragua|Granada|658[0n]|BOLD:AAA9439
Nelphe relegatum|[3294]|ARCTC780-11|MILA 1242|Nicaragua|Granada|658[0n]|BOLD:AAA9439
Nelphe relegatum|[3295]|ARCTC766-11|MILA 1228|Guatemala|Izabal|658[0n]|BOLD:AAA9439
Nelphe relegatum|[3296]|BLPCD203-08|07-SRNP-107628|Costa Rica|Guanacaste|658[0n]|BOLD:AAA9439
Nelphe relegatum|[3297]|MHMYN3327-14|13-SRNP-22731|Costa Rica||658[0n]|BOLD:AAA9439
Nelphe relegatum|[3298]|MHMYN3326-14|13-SRNP-22730|Costa Rica||658[0n]|BOLD:AAA9439
Nelphe relegatum|[3299]|BLPCC569-08|07-SRNP-107054|Costa Rica|Guanacaste|658[0n]|BOLD:AAA9439
Nelphe relegatum|[3300]|BLPCC567-08|07-SRNP-107052|Costa Rica|Guanacaste|658[0n]|BOLD:AAA9439
Nelphe relegatum|[3301]|BLPCC121-08|07-SRNP-106606|Costa Rica|Guanacaste|658[0n]|BOLD:AAA9439
Nelphe relegatum|[3302]|MHMYN6279-14|14-SRNP-70266|Costa Rica||658[0n]|BOLD:AAA9439
Nelphe relegatum|[3303]|BCIGE758-12|YB-BCI48319|Panama|Panama|658[0n]|BOLD:AAA9439
Nelphe relegatum|[3304]|BLPEE2872-14|13-SRNP-102308|Costa Rica|Guanacaste|658[0n]|BOLD:AAA9439
Nelphe relegatum|[3305]|BLPBA323-07|06-SRNP-107466|Costa Rica|Guanacaste|658[0n]|BOLD:AAA9439
Nelphe relegatum|[3306]|MHARB592-06|04-SRNP-49033|Costa Rica|Guanacaste|658[0n]|BOLD:AAA9439
Nelphe relegatum|[3307]|BLPED1718-12|11-SRNP-105320|Costa Rica|Guanacaste|658[0n]|BOLD:AAA9439
Nelphe relegatum|[3308]|LPMX279-07|MLL-00279|Mexico|Campeche|658[0n]|BOLD:AAA9439
Nelphe relegatum|[3309]|BLPBD536-07|07-SRNP-100535|Costa Rica|Guanacaste|658[0n]|BOLD:AAA9439
Nelphe relegatum|[3310]|CNCLB1895-14|CNCLEP00117619|Mexico|Quintana Roo|658[0n]|BOLD:AAA9439
Nelphe relegatum|[3311]|BLPEE2069-12|12-SRNP-102068|Costa Rica|Guanacaste|658[0n]|BOLD:AAA9439
Nelphe relegatum|[3312]|MHARB594-06|05-SRNP-45092|Costa Rica|Guanacaste|658[0n]|BOLD:AAA9439
Nelphe relegatum|[3313]|MHARB593-06|04-SRNP-47618|Costa Rica|Guanacaste|658[0n]|BOLD:AAA9439
Nelphe relegatum|[3314]|MHARB589-06|04-SRNP-47446|Costa Rica|Guanacaste|658[0n]|BOLD:AAA9439
Nelphe relegatum|[3315]|MHARB588-06|04-SRNP-47447|Costa Rica|Guanacaste|658[0n]|BOLD:AAA9439
Nelphe relegatum|[3316]|BLPDD315-09|08-SRNP-108210|Costa Rica|Alajuela|658[0n]|BOLD:AAA9439
Nelphe relegatum|[3317]|BLPDU1229-11|10-SRNP-110651|Costa Rica|Guanacaste|658[0n]|BOLD:AAA9439
Nelphe relegatum|[3318]|BLPDH845-09|09-SRNP-103329|Costa Rica|Alajuela|658[0n]|BOLD:AAA9439
Dahana atripennis|[3319]|MNAB219-07|CNCLEP00025810|United States|Florida|637[0n]|BOLD:AAI9695
Dahana atripennis|[3320]|HKONS390-08|1860-COI-07|United States|Florida|658[0n]|BOLD:AAI9695
Lymire edwardsii|[3321]|RDNML300-13|CNCLEP 92276|United States|Florida|658[0n]|BOLD:AAM8591
Lymire edwardsii|[3322]|BBLOB1266-11|BIOUG01417-C07|United States|Florida|658[0n]|BOLD:AAM8591
Lymire edwardsii|[3323]|RDNML301-13|CNCLEP 92277|United States|Florida|658[0n]|BOLD:AAM8591
Lymire edwardsii|[3324]|BBLOB1265-11|BIOUG01417-C06|United States|Florida|658[0n]|BOLD:AAM8591
Lymire edwardsii|[3325]|USLEP068-10|10BBLEP-00068|United States|Florida|658[0n]|BOLD:AAM8591
Ctenucha venosa|[3326]|CMAZA049-09|CMAZ-0049|United States|Arizona|655[0n]|BOLD:AAH5420
Ctenucha venosa|[3327]|BBLSZ163-09|09BBLEP-04089|United States|Texas|658[0n]|BOLD:AAH5420
Ctenucha venosa|[3328]|CMAZA927-12|BIOUG02040-G06|United States|Arizona|658[0n]|BOLD:AAH5420
Ctenucha venosa|[3329]|BBLSY912-09|09BBLEP-03839|United States|Texas|658[0n]|BOLD:AAH5420
Ctenucha venosa|[3330]|RDNMJ506-11|CNCLEP 80034|United States|Arizona|658[0n]|BOLD:AAH5420
Ctenucha venosa|[3331]|DMAZ254-10|BIOUG00716-D10|United States|Arizona|658[0n]|BOLD:AAH5420
Ctenucha venosa|[3332]|RDNMH375-09|CNCLEP00057784|United States|Arizona|658[0n]|BOLD:AAH5420
Ctenucha brunnea|[3333]|LNAUT229-14|CCDB-22941-D04|United States|California|550[0n]|BOLD:ACQ7295
Ctenucha brunnea|[3334]|CNCLB1007-14|CNCLEP00113415|United States|California|305[0n]|
Ctenucha rubroscapus|[3335]|RDNME444-08|LEP037868|United States|Oregon|648[0n]|BOLD:AAF1986
Ctenucha rubroscapus|[3336]|BBLOC986-11|BIOUG01468-B06|United States|California|658[0n]|BOLD:A...
Ctenucha rubroscapus|[3337]|RDNMF907-08|CNC LEP00054174|United States|Oregon|658[0n]|BOLD:AAF1986
Ctenucha rubroscapus|[3338]|RDNMF906-08|CNC LEP00054173|United States|Oregon|658[0n]|BOLD:AAF1986
Ctenucha cressonana|[3339]|RDNMF908-08|CNC LEP00054175|United States|Colorado|658[0n]|BOLD:AAR...
Ctenucha virginica|[3340]|XAB640-04|04HBL005640|Canada|Ontario|558[0n]|BOLD:AAA6017
Ctenucha virginica|[3341]|XAD697-05|2005-ONT-496|Canada|Ontario|658[0n]|BOLD:AAA6017
Ctenucha virginica|[3342]|RBINA708-13|BIOUG07951-C03|Canada|Ontario|606[0n]|BOLD:AAA6017
Ctenucha virginica|[3343]|BLTIB988-08|BL1425|Canada|Ontario|658[0n]|BOLD:AAA6017
Ctenucha virginica|[3344]|MNBB454-05|05-NBSTA-370|Canada|New Brunswick|616[0n]|BOLD:AAA6017
Ctenucha virginica|[3345]|BLTIB610-08|BL890|Canada|Ontario|634[0n]|BOLD:AAA6017
Ctenucha virginica|[3346]|LPSOB704-08|PPBP-1703|Canada|Ontario|651[0n]|BOLD:AAA6017
Ctenucha virginica|[3347]|TMNBD423-07|MNBTT-3224|Canada|New Brunswick|658[0n]|BOLD:AAA6017
Ctenucha virginica|[3348]|TMNBD422-07|MNBTT-3223|Canada|New Brunswick|658[0n]|BOLD:AAA6017
Ctenucha virginica|[3349]|TMNBD421-07|MNBTT-3222|Canada|New Brunswick|658[0n]|BOLD:AAA6017
Ctenucha virginica|[3350]|TMNBD420-07|MNBTT-3221|Canada|New Brunswick|657[0n]|BOLD:AAA6017
Ctenucha virginica|[3351]|TMNBD419-07|MNBTT-3220|Canada|New Brunswick|658[0n]|BOLD:AAA6017
Ctenucha virginica|[3352]|PHMNB759-05|Moth 452.03SA|Canada|New Brunswick|658[0n]|BOLD:AAA6017
Ctenucha virginica|[3353]|PHMNB724-05|Moth 417.03SA|Canada|New Brunswick|658[0n]|BOLD:AAA6017
Ctenucha virginica|[3354]|TTMNB256-06|MNBTT-256|Canada|New Brunswick|658[0n]|BOLD:AAA6017
Ctenucha virginica|[3355]|LPSOD1047-09|08MZPP-108|Canada|Ontario|658[0n]|BOLD:AAA6017
Ctenucha virginica|[3356]|BBLPE303-09|09BBELE-2303|Canada|Newfoundland and Labrador|658[0n]|BOLD...
Ctenucha virginica|[3357]|BBLPE302-09|09BBELE-2302|Canada|Newfoundland and Labrador|658[0n]|BOLD...
Ctenucha virginica|[3358]|RDMAB924-06|BCSC397|Canada|Alberta|658[0n]|BOLD:AAA6017
Ctenucha virginica|[3359]|BBLPE408-09|09BBELE-2408|Canada|Newfoundland and Labrador|658[0n]|BOLD...
Ctenucha virginica|[3360]|LPMN788-08|08BBLEP-01591|Canada|Manitoba|658[0n]|BOLD:AAA6017
Ctenucha virginica|[3361]|BLGSM034-09|BL343|Canada|Ontario|658[0n]|BOLD:AAA6017
Ctenucha virginica|[3362]|MNBB200-05|05-NBSTA-116|Canada|New Brunswick|658[0n]|BOLD:AAA6017
Ctenucha virginica|[3363]|BBLPC230-09|09BBELE-1230|Canada|Nova Scotia|658[0n]|BOLD:AAA6017
Ctenucha virginica|[3364]|LPMN300-08|08BBLEP-01099|Canada|Manitoba|658[0n]|BOLD:AAA6017
Ctenucha virginica|[3365]|LMDH175-11|BIOUG01047-F05|United States|Minnesota|658[0n]|BOLD:AAA6017
Ctenucha virginica|[3366]|BLTIB284-08|BL469|Canada|Ontario|658[0n]|BOLD:AAA6017
Ctenucha virginica|[3367]|LPMN203-08|08BBLEP-01002|Canada|Manitoba|658[0n]|BOLD:AAA6017
Ctenucha virginica|[3368]|PHMNB458-04|04HBL00684|Canada|New Brunswick|658[0n]|BOLD:AAA6017
Ctenucha virginica|[3369]|LPMN292-08|08BBLEP-01091|Canada|Manitoba|658[0n]|BOLD:AAA6017
Ctenucha virginica|[3370]|BBLEC683-09|09BBELE-0683|Canada|Nova Scotia|658[0n]|BOLD:AAA6017
Ctenucha virginica|[3371]|XAF456-05|2005-ONT-105|Canada|Ontario|658[0n]|BOLD:AAA6017
Ctenucha virginica|[3372]|BBLCU170-09|09BBLEP-04657|United States|Michigan|658[0n]|BOLD:AAA6017
Ctenucha virginica|[3373]|PHMNB353-04|04HBL00579|Canada|New Brunswick|658[0n]|BOLD:AAA6017
Ctenucha virginica|[3374]|MNBB130-05|05-NBSTA-046|Canada|New Brunswick|658[0n]|BOLD:AAA6017
Ctenucha virginica|[3375]|MNBB129-05|05-NBSTA-045|Canada|New Brunswick|658[0n]|BOLD:AAA6017
Ctenucha virginica|[3376]|PHMNB140-04|04HBL007605|Canada|New Brunswick|658[0n]|BOLD:AAA6017
Ctenucha virginica|[3377]|BLTIB231-08|BL412|Canada|Ontario|657[0n]|BOLD:AAA6017
Ctenucha virginica|[3378]|BLTIB221-08|BL400|Canada|Ontario|658[0n]|BOLD:AAA6017
Ctenucha virginica|[3379]|BLTIB220-08|BL399|Canada|Ontario|658[0n]|BOLD:AAA6017
Ctenucha virginica|[3380]|BLTIB215-08|BL318|Canada|Ontario|658[0n]|BOLD:AAA6017
Ctenucha virginica|[3381]|LPMN294-08|08BBLEP-01093|Canada|Manitoba|658[0n]|BOLD:AAA6017
Ctenucha virginica|[3382]|LPMN293-08|08BBLEP-01092|Canada|Manitoba|658[0n]|BOLD:AAA6017
Ctenucha virginica|[3383]|BLTIB196-08|BL287|Canada|Ontario|658[0n]|BOLD:AAA6017
Ctenucha virginica|[3384]|BBLPA603-10|10BBCLP-0603|Canada|Ontario|658[0n]|BOLD:AAA6017
Ctenucha virginica|[3385]|BLTIB283-08|BL468|Canada|Ontario|658[0n]|BOLD:AAA6017
Ctenucha virginica|[3386]|BLTIB232-08|BL413|Canada|Ontario|658[0n]|BOLD:AAA6017

Ctenucha virginica|[3384]|BBLPA603-10|10BBCLP-0603|Canada|Ontario|658[0n]|BOLD:AAA6017
Ctenucha virginica|[3385]|BLTIB283-08|BL468|Canada|Ontario|658[0n]|BOLD:AAA6017
Ctenucha virginica|[3386]|BLTIB232-08|BL413|Canada|Ontario|658[0n]|BOLD:AAA6017
Ctenucha virginica|[3387]|BBLEC090-09|09BBELE-0090|Canada|Nova Scotia|658[0n]|BOLD:AAA6017
Ctenucha virginica|[3388]|RDLQG445-06|DH012729|Canada|Quebec|658[0n]|BOLD:AAA6017
Ctenucha virginica|[3389]|TMNBB035-06|MNBTT-975|Canada|New Brunswick|658[0n]|BOLD:AAA6017
Ctenucha virginica|[3390]|TMNBB036-06|MNBTT-976|Canada|New Brunswick|658[0n]|BOLD:AAA6017
Ctenucha virginica|[3391]|TMNBB037-06|MNBTT-977|Canada|New Brunswick|658[0n]|BOLD:AAA6017
Ctenucha virginica|[3392]|LPSOB771-08|PPBP-1770|Canada|Ontario|658[0n]|BOLD:AAA6017
Ctenucha virginica|[3393]|BLTIB444-08|BL691|Canada|Ontario|658[0n]|BOLD:AAA6017
Ctenucha virginica|[3394]|XAF692-05|2005-ONT-341|Canada|Ontario|658[0n]|BOLD:AAA6017
Ctenucha virginica|[3395]|XAK439-06|2006-ONT-1434|Canada|Ontario|658[0n]|BOLD:AAA6017
Ctenucha virginica|[3396]|BBLPE368-09|09BBELE-2368|Canada|Newfoundland and Labrador|658[0n]|BOLD...
Ctenucha virginica|[3397]|XAD696-05|2005-ONT-495|Canada|Ontario|658[0n]|BOLD:AAA6017
Ctenucha virginica|[3398]|XAD698-05|2005-ONT-497|Canada|Ontario|658[0n]|BOLD:AAA6017
Ctenucha virginica|[3399]|LPSOB796-08|PPBP-1795|Canada|Ontario|658[0n]|BOLD:AAA6017
Ctenucha virginica|[3400]|LPSOB797-08|PPBP-1796|Canada|Ontario|658[0n]|BOLD:AAA6017
Ctenucha virginica|[3401]|XAJ658-06|2006-ONT-0658|Canada|Ontario|658[0n]|BOLD:AAA6017
Ctenucha virginica|[3402]|XAB554-04|04HBL005554|Canada|Ontario|658[0n]|BOLD:AAA6017
Ctenucha virginica|[3403]|PHMNB072-03|moth73.02SA|Canada|New Brunswick|639[0n]|BOLD:AAA6017
Ctenucha virginica|[3404]|PHMO088-03|moth563.01|Canada|Ontario|639[0n]|BOLD:AAA6017
Ctenucha virginica|[3405]|PHMO003-03|CTEN1.01|Canada|Ontario|639[0n]|BOLD:AAA6017
Ctenucha virginica|[3406]|PMG002-03|moth605.01|Canada|Ontario|617[0n]|BOLD:AAA6017
Cisseps fulvicollis|[3407]|BBLOC646-11|BIOUG01458-G09|United States|California|658[0n]|BOLD:AA...
Cisseps fulvicollis|[3408]|SMTPD4587-13|BIOUG08650-G09|Canada|Ontario|540[0n]|BOLD:AAA4200
Cisseps fulvicollis|[3409]|LPSOC388-08|PPBP-2387|Canada|Ontario|658[0n]|BOLD:AAA4200
Cisseps fulvicollis|[3410]|LNC499-06|05-NCCC-499|United States|North Carolina|600[4n]|BOLD:AAA...
Cisseps fulvicollis|[3411]|LPAB074-08|08BBLEP-02396|Canada|Alberta|658[0n]|BOLD:AAA4200
Cisseps fulvicollis|[3412]|LBCW013-08|08-JDWWI-0013|Canada|British Columbia|658[0n]|BOLD:AAA4200
Cisseps fulvicollis|[3413]|LOWCE036-06|CGWC-3796|Canada|British Columbia|618[0n]|BOLD:AAA4200
Cisseps fulvicollis|[3414]|LOWCE074-06|CGWC-3834|Canada|British Columbia|658[0n]|BOLD:AAA4200
Cisseps fulvicollis|[3415]|LOWCE076-06|CGWC-3836|Canada|British Columbia|658[0n]|BOLD:AAA4200
Cisseps fulvicollis|[3416]|LPABB802-09|08BBLEP-04122|Canada|Alberta|658[0n]|BOLD:AAA4200
Cisseps fulvicollis|[3417]|LOWCE067-06|CGWC-3827|Canada|British Columbia|649[0n]|BOLD:AAA4200
Cisseps fulvicollis|[3418]|LOWCE035-06|CGWC-3795|Canada|British Columbia|658[0n]|BOLD:AAA4200
Cisseps fulvicollis|[3419]|LOWCE029-06|CGWC-3789|Canada|British Columbia|658[0n]|BOLD:AAA4200
Cisseps fulvicollis|[3420]|RWWA843-09|RWWA-0879|United States|Washington|658[0n]|BOLD:AAA4200
Cisseps fulvicollis|[3421]|RWWC529-11|RWWA-2506|United States|Washington|658[0n]|BOLD:AAA4200
Cisseps fulvicollis|[3422]|LPMN873-08|08BBLEP-02231|Canada|Alberta|658[0n]|BOLD:AAA4200
Cisseps fulvicollis|[3423]|LPMN872-08|08BBLEP-02230|Canada|Alberta|658[0n]|BOLD:AAA4200
Cisseps fulvicollis|[3424]|LPMN871-08|08BBLEP-02229|Canada|Alberta|658[0n]|BOLD:AAA4200
Cisseps fulvicollis|[3425]|LBCW012-08|08-JDWWI-0012|Canada|British Columbia|658[0n]|BOLD:AAA4200
Cisseps fulvicollis|[3426]|RWWC452-11|RWWA-2429|United States|Washington|658[0n]|BOLD:AAA4200
Cisseps fulvicollis|[3427]|LPABB032-08|08BBLEP-03297|Canada|Alberta|658[0n]|BOLD:AAA4200
Cisseps fulvicollis|[3428]|LEFIA1075-10|AM-94-0396|United States|Maryland|670[0n]|BOLD:AAA4200
Cisseps fulvicollis|[3429]|XAG915-05|2005-ONT-1499|Canada|Ontario|658[0n]|BOLD:AAA4200
Cisseps fulvicollis|[3430]|RWWC423-11|RWWA-2400|United States|Washington|658[0n]|BOLD:AAA4200
Cisseps fulvicollis|[3431]|MNBB455-05|05-NBSTA-371|Canada|New Brunswick|658[0n]|BOLD:AAA4200
Cisseps fulvicollis|[3432]|MNBB633-05|05-NBSTA-549|Canada|New Brunswick|658[0n]|BOLD:AAA4200
Cisseps fulvicollis|[3433]|MNBB630-05|05-NBSTA-546|Canada|New Brunswick|658[0n]|BOLD:AAA4200
Cisseps fulvicollis|[3434]|MNBB629-05|05-NBSTA-545|Canada|New Brunswick|658[0n]|BOLD:AAA4200
Cisseps fulvicollis|[3435]|MNBB673-05|05-NBSTA-589|Canada|New Brunswick|658[0n]|BOLD:AAA4200
Cisseps fulvicollis|[3436]|MNBB634-05|05-NBSTA-550|Canada|New Brunswick|658[0n]|BOLD:AAA4200
Cisseps fulvicollis|[3437]|MNBB674-05|05-NBSTA-590|Canada|New Brunswick|658[0n]|BOLD:AAA4200
Cisseps fulvicollis|[3438]|PHMNB369-04|04HBL00595|Canada|New Brunswick|658[0n]|BOLD:AAA4200
Cisseps fulvicollis|[3439]|PHMNB358-04|04HBL00584|Canada|New Brunswick|658[0n]|BOLD:AAA4200
Cisseps fulvicollis|[3440]|BBLEC469-09|09BBELE-0469|Canada|New Brunswick|658[0n]|BOLD:AAA4200
Cisseps fulvicollis|[3441]|XAG388-05|2005-ONT-972|Canada|Ontario|658[0n]|BOLD:AAA4200
Cisseps fulvicollis|[3442]|PHMNB605-04|04HBL00831|Canada|New Brunswick|658[0n]|BOLD:AAA4200
Cisseps fulvicollis|[3443]|PHMNB473-04|04HBL00699|Canada|New Brunswick|658[0n]|BOLD:AAA4200
Cisseps fulvicollis|[3444]|PHMNB462-04|04HBL00688|Canada|New Brunswick|658[0n]|BOLD:AAA4200
Cisseps fulvicollis|[3445]|BBLEC494-09|09BBELE-0494|Canada|New Brunswick|658[0n]|BOLD:AAA4200
Cisseps fulvicollis|[3446]|BBLPC013-09|09BBELE-1013|Canada|New Brunswick|658[0n]|BOLD:AAA4200
Cisseps fulvicollis|[3447]|BBLPC020-09|09BBELE-1020|Canada|New Brunswick|658[0n]|BOLD:AAA4200
Cisseps fulvicollis|[3448]|LOFLC121-06|06-FLOR-2001|United States|Florida|658[0n]|BOLD:AAA4200
Cisseps fulvicollis|[3449]|BBLPC025-09|09BBELE-1025|Canada|New Brunswick|658[0n]|BOLD:AAA4200
Cisseps fulvicollis|[3450]|BBLPC360-09|09BBELE-1360|Canada|New Brunswick|658[0n]|BOLD:AAA4200
Cisseps fulvicollis|[3451]|BBLPC419-09|09BBELE-1419|Canada|New Brunswick|658[0n]|BOLD:AAA4200
Cisseps fulvicollis|[3452]|BBLPC637-09|09BBELE-1637|Canada|Newfoundland and Labrador|658[0n]|BOLD...
Cisseps fulvicollis|[3453]|BBLPC467-09|09BBELE-1467|Canada|New Brunswick|658[0n]|BOLD:AAA4200
Cisseps fulvicollis|[3454]|BBLPE540-09|09BBELE-2540|Canada|Newfoundland and Labrador|658[0n]|BOLD...
Cisseps fulvicollis|[3455]|BBLPE511-09|09BBELE-2511|Canada|Newfoundland and Labrador|658[0n]|BOLD...
Cisseps fulvicollis|[3456]|BBLPE500-09|09BBELE-2500|Canada|Newfoundland and Labrador|658[0n]|BOLD...
Cisseps fulvicollis|[3457]|BBLPE451-09|09BBELE-2451|Canada|Newfoundland and Labrador|658[0n]|BOLD...
Cisseps fulvicollis|[3458]|BBLOC099-11|BIOUG01453-A09|United States|Florida|658[0n]|BOLD:AAA4200
Cisseps fulvicollis|[3459]|BBLPE557-09|09BBELE-2557|Canada|Newfoundland and Labrador|658[0n]|BOLD...
Cisseps fulvicollis|[3460]|MECC498-06|jflandry2518|Canada|Quebec|658[0n]|BOLD:AAA4200
Cisseps fulvicollis|[3461]|NSBUG180-07|2007-01-663-45|Canada|Nova Scotia|657[0n]|BOLD:AAA4200
Cisseps fulvicollis|[3462]|LNCNW051-06|06-NCNW-0051|United States|North Carolina|658[0n]|BOLD:...
Cisseps fulvicollis|[3463]|NSBUG103-07|2007-01-664-2|Canada|Nova Scotia|657[0n]|BOLD:AAA4200
Cisseps fulvicollis|[3464]|BBLOB1547-11|BIOUG01420-C03|United States|Florida|658[0n]|BOLD:AAA4200
Cisseps fulvicollis|[3465]|LGSMC725-05|DNA-ATBI-2725|United States|Tennessee|658[0n]|BOLD:AAA4200
Cisseps fulvicollis|[3466]|LPSOD166-08|08MZPP-017|Canada|Ontario|656[0n]|BOLD:AAA4200
Cisseps fulvicollis|[3467]|LGSMG123-07|BGS03444|United States|North Carolina|658[0n]|BOLD:AAA4200
Cisseps fulvicollis|[3468]|RWWA611-09|RWWA-0629|United States|Washington|658[0n]|BOLD:AAA4200
Cisseps fulvicollis|[3469]|TMNBD427-07|MNBTT-3228|Canada|New Brunswick|658[0n]|BOLD:AAA4200
Cisseps fulvicollis|[3470]|TMNBD426-07|MNBTT-3227|Canada|New Brunswick|658[0n]|BOLD:AAA4200
Cisseps fulvicollis|[3471]|TMNBD425-07|MNBTT-3226|Canada|New Brunswick|658[0n]|BOLD:AAA4200
Cisseps fulvicollis|[3472]|TMNBD424-07|MNBTT-3225|Canada|New Brunswick|658[0n]|BOLD:AAA4200
Cisseps fulvicollis|[3473]|LPABB023-08|08BBLEP-03288|Canada|Alberta|658[0n]|BOLD:AAA4200
Cisseps fulvicollis|[3474]|LGSMC722-05|DNA-ATBI-2722|United States|Tennessee|658[0n]|BOLD:AAA4200
Cisseps fulvicollis|[3475]|TMNBD428-07|MNBTT-3229|Canada|New Brunswick|658[0n]|BOLD:AAA4200
Cisseps fulvicollis|[3476]|LGSMC724-05|DNA-ATBI-2724|United States|Tennessee|658[0n]|BOLD:AAA4200
Cisseps fulvicollis|[3477]|LGSMC726-05|DNA-ATBI-2726|United States|Tennessee|658[0n]|BOLD:AAA4200
Cisseps fulvicollis|[3478]|NCCH059-11|BIOUG01573-E11|Canada|Ontario|673[0n]|BOLD:AAA4200
Cisseps fulvicollis|[3479]|NCCH061-11|BIOUG01573-F01|Canada|Ontario|673[0n]|BOLD:AAA4200
Cisseps fulvicollis|[3480]|LPABB320-08|08BBLEP-03585|Canada|Alberta|658[0n]|BOLD:AAA4200
Cisseps fulvicollis|[3481]|BBLPA604-10|10BBCLP-0604|Canada|Ontario|658[0n]|BOLD:AAA4200
Cisseps fulvicollis|[3482]|LOWCE073-06|CGWC-3833|Canada|British Columbia|658[0n]|BOLD:AAA4200
Cisseps fulvicollis|[3483]|LOWCE077-06|CGWC-3837|Canada|British Columbia|658[0n]|BOLD:AAA4200
Cisseps fulvicollis|[3484]|RDLQ764-07|DH007392|Canada|Quebec|658[0n]|BOLD:AAA4200
Cisseps fulvicollis|[3485]|XAF630-05|2005-ONT-279|Canada|Ontario|658[0n]|BOLD:AAA4200
[illegible]

Cisseps fulvicollis|[3483]|LOWCE077-06|CGWC-3837|Canada|British Columbia|658[0n]|BOLD:AAA4200
Cisseps fulvicollis|[3484]|RDLQ764-07|DH007392|Canada|Quebec|658[0n]|BOLD:AAA4200
Cisseps fulvicollis|[3485]|XAF630-05|2005-ONT-279|Canada|Ontario|658[0n]|BOLD:AAA4200
Cisseps fulvicollis|[3486]|XAF703-05|2005-ONT-352|Canada|Ontario|658[0n]|BOLD:AAA4200
Cisseps fulvicollis|[3487]|XAJ457-06|2006-ONT-0457|Canada|Ontario|658[0n]|BOLD:AAA4200
Cisseps fulvicollis|[3488]|XAK326-06|2006-ONT-1321|Canada|Ontario|658[0n]|BOLD:AAA4200
Cisseps fulvicollis|[3489]|LILLA486-11|SNS10IL-00623|United States|Illinois|658[0n]|BOLD:AAA4200
Cisseps fulvicollis|[3490]|BLTIB1033-08|BL1475|Canada|Ontario|658[0n]|BOLD:AAA4200
Cisseps fulvicollis|[3491]|XAJ391-06|2006-ONT-0391|Canada|Ontario|658[0n]|BOLD:AAA4200
Cisseps fulvicollis|[3492]|LILLA524-11|SNS10IL-00670|United States|Illinois|658[0n]|BOLD:AAA4200
Cisseps fulvicollis|[3493]|TTMNB257-06|MNBTT-257|Canada|New Brunswick|656[0n]|BOLD:AAA4200
Cisseps fulvicollis|[3494]|LTOL017-06|AM-93-0003|United States|Maryland|658[0n]|BOLD:AAA4200
Cisseps fulvicollis|[3495]|MNBB675-05|05-NBSTA-591|Canada|New Brunswick|658[0n]|BOLD:AAA4200
Cisseps fulvicollis|[3496]|MNBB631-05|05-NBSTA-547|Canada|New Brunswick|658[0n]|BOLD:AAA4200
Cisseps fulvicollis|[3497]|LPABC671-09|08BBLEP-04890|Canada|Alberta|658[0n]|BOLD:AAA4200
Cisseps fulvicollis|[3498]|LPMN874-08|08BBLEP-02232|Canada|Alberta|658[0n]|BOLD:AAA4200
Cisseps fulvicollis|[3499]|LPMN870-08|08BBLEP-02228|Canada|Alberta|652[0n]|BOLD:AAA4200
Cisseps fulvicollis|[3500]|BBLEC501-09|09BBELE-0501|Canada|New Brunswick|636[0n]|BOLD:AAA4200
Cisseps fulvicollis|[3501]|LOFLB430-06|06-FLOR-1370|United States|Florida|634[0n]|BOLD:AAA4200
Cisseps fulvicollis|[3502]|LNC500-06|05-NCCC-500|United States|North Carolina|578[0n]|BOLD:AAA...
Cisseps fulvicollis|[3503]|LGSMC727-05|DNA-ATBI-2727|United States|Tennessee|619[0n]|BOLD:AAA4200
Cisseps fulvicollis|[3504]|LGSMC723-05|DNA-ATBI-2723|United States|Tennessee|617[0n]|BOLD:AAA4200
Cisseps fulvicollis|[3505]|LGSMB308-05|DNA-ATBI-1157|United States|Tennessee|609[0n]|BOLD:AAA4200
Cisseps fulvicollis|[3506]|TMG90-03|moth611.01|Canada|Ontario|639[0n]|BOLD:AAA4200
Cisseps fulvicollis|[3507]|BBLPC715-09|09BBELE-1715|Canada|Newfoundland and Labrador|658[0n]|BOLD...
Cisseps fulvicollis|[3508]|BBLPC785-09|09BBELE-1785|Canada|Newfoundland and Labrador|658[0n]|BOLD...
Cisseps fulvicollis|[3509]|BBLPE489-09|09BBELE-2489|Canada|Newfoundland and Labrador|658[0n]|BOLD...
Cisseps fulvicollis|[3510]|BBLPC981-09|09BBELE-1981|Canada|Newfoundland and Labrador|658[0n]|BOLD...
Cisseps fulvicollis|[3511]|LNCNW050-06|06-NCNW-0050|United States|North Carolina|658[0n]|BOLD:...
Cisseps fulvicollis|[3512]|BBLPA888-10|10BBCLP-0888|Canada|Alberta|658[0n]|BOLD:AAA4200
Cisseps fulvicollis|[3513]|LNAUT235-14|CCDB-22941-D10|Canada|Saskatchewan|658[0n]|BOLD:AAA4200
Cisseps fulvicollis|[3514]|LPOKE472-12|BIOUG02920-C09|United States|Oklahoma|621[0n]|BOLD:AAA4200
Cisseps fulvicollis|[3515]|BBLPE497-09|09BBELE-2497|Canada|Newfoundland and Labrador|635[0n]|BOLD...
Cisseps fulvicollis|[3516]|PMG001-03|moth599.01|Canada|Ontario|617[0n]|BOLD:AAA4200
Syntomeida melanthus|[3517]|QUNOD103-10|7102-COI-09|United States|Texas|658[0n]|BOLD:AAA1416
Syntomeida melanthus|[3518]|QUNOD102-10|7101-COI-09|United States|Texas|658[0n]|BOLD:AAA1416
Syntomeida ipomoeae|[3519]|LOFLB267-06|06-FLOR-1207|United States|Florida|658[0n]|BOLD:AAA1416
Syntomeida ipomoeae|[3520]|LOFLC122-06|06-FLOR-2002|United States|Florida|658[0n]|BOLD:AAA1416
Syntomeida ipomoeae|[3521]|LOFLB265-06|06-FLOR-1205|United States|Florida|658[0n]|BOLD:AAA1416
Syntomeida ipomoeae|[3522]|LOFLB266-06|06-FLOR-1206|United States|Florida|658[0n]|BOLD:AAA1416
Syntomeida ipomoeae|[3523]|LOFLC112-06|06-FLOR-1992|United States|Florida|657[2n]|BOLD:AAA1416
Syntomeida ipomoeae|[3524]|HKONS353-08|3258-COI-08|United States|Florida|658[0n]|BOLD:AAA1416
Syntomeida syntomoides|[3525]|MHARB285-05|01-SRNP-249|Costa Rica|Alajuela|658[0n]|BOLD:AAK1348
Syntomeida syntomoides|[3526]|MHMYN3277-14|13-SRNP-77733|Costa Rica||658[0n]|BOLD:AAK1348
Syntomeida syntomoides|[3527]|MHMYL2551-11|10-SRNP-75938|Costa Rica||658[0n]|BOLD:AAK1348
Syntomeida syntomoides|[3528]|MHMYL2434-11|10-SRNP-67932|Costa Rica||658[0n]|BOLD:AAK1348
Syntomeida syntomoides|[3529]|MHMYN3276-14|13-SRNP-77732|Costa Rica||658[0n]|BOLD:AAK1348
Syntomeida syntomoides|[3530]|CNCLB1635-14|CNCLEP 00113869|United States|Florida|658[0n]|BOLD:...
Syntomeida syntomoides|[3531]|CNCLB1634-14|CNCLEP 00113868|United States|Florida|658[0n]|BOLD:...
Syntomeida syntomoides|[3532]|ARCTB174-08|MILA 0833|Guatemala|Izabal|658[0n]|BOLD:AAK1348
Syntomeida syntomoides|[3533]|MHMYQ2034-12|12-SRNP-75173|Costa Rica||658[0n]|BOLD:AAK1348
Cosmosoma myrodora|[3534]|LSUSA269-06|06-SUSA-0269|United States|Florida|658[0n]|BOLD:AAE4005
Cosmosoma myrodora|[3535]|HKONS086-07|1650-COI-07|United States|Florida|645[0n]|BOLD:AAE4005
Cosmosoma festivum|[3536]|BLPEF1289-12|12-SRNP-103473|Costa Rica|Guanacaste|658[0n]|BOLD:AAA1329
Cosmosoma festivum|[3537]|BLPCD184-08|07-SRNP-107609|Costa Rica|Guanacaste|658[0n]|BOLD:AAA1329
Cosmosoma festivum|[3538]|LYHES422-09|MAL-03719|Mexico|Yucatan|626[0n]|BOLD:AAA1329
Cosmosoma festivum|[3539]|BLPED1713-12|11-SRNP-105315|Costa Rica|Guanacaste|658[0n]|BOLD:AAA1329
Cosmosoma festivum|[3540]|BLPED1712-12|11-SRNP-105314|Costa Rica|Guanacaste|658[0n]|BOLD:AAA1329
Cosmosoma festivum|[3541]|BLPDL1940-10|09-SRNP-109322|Costa Rica|Guanacaste|658[0n]|BOLD:AAA1329
Cosmosoma festivum|[3542]|BLPDY609-11|10-SRNP-115161|Costa Rica|Guanacaste|658[0n]|BOLD:AAA1329
Cosmosoma festivum|[3543]|LYPAP342-09|MAL-01887|Mexico|Quintana Roo|658[0n]|BOLD:AAA1329
Cosmosoma festivum|[3544]|BLPCO167-08|08-SRNP-103644|Costa Rica|Guanacaste|658[0n]|BOLD:AAA1329
Cosmosoma festivum|[3545]|BLPCB002-08|07-SRNP-105547|Costa Rica|Guanacaste|658[0n]|BOLD:AAA1329
Cosmosoma festivum|[3546]|BLPCI252-08|07-SRNP-112377|Costa Rica|Guanacaste|658[0n]|BOLD:AAA1329
Cosmosoma festivum|[3547]|BLPAH505-07|06-SRNP-107084|Costa Rica|Guanacaste|658[0n]|BOLD:AAA1329
Cosmosoma festivum|[3548]|BLPDU301-11|10-SRNP-109723|Costa Rica|Guanacaste|658[0n]|BOLD:AAA1329
Cosmosoma festivum|[3549]|LYHES484-09|MAL-03781|Mexico|Yucatan|658[0n]|BOLD:AAA1329
Cosmosoma festivum|[3550]|BLPDU743-11|10-SRNP-110165|Costa Rica|Guanacaste|658[0n]|BOLD:AAA1329
Cosmosoma festivum|[3551]|LYPIE228-09|MAL-01209|Mexico|Quintana Roo|658[0n]|BOLD:AAA1329
Cosmosoma festivum|[3552]|BLPDK1383-09|09-SRNP-106311|Costa Rica|Guanacaste|658[0n]|BOLD:AAA1329
Cosmosoma festivum|[3553]|LYHES540-09|MAL-03837|Mexico|Quintana Roo|646[0n]|BOLD:AAA1329
Cosmosoma festivum|[3554]|BLPBH284-07|07-SRNP-104043|Costa Rica|Guanacaste|646[0n]|BOLD:AAA1329
Cosmosoma festivum|[3555]|LYNYM276-09|MAL-03385|Mexico|Quintana Roo|658[0n]|BOLD:AAA1329
Cosmosoma festivum|[3556]|LPYPB274-08|MAL-00492|Mexico|Quintana Roo|643[0n]|BOLD:AAA1329
Cosmosoma festivum|[3557]|LPYPB169-08|MAL-00387|Mexico|Campeche|658[0n]|BOLD:AAA1329
Cosmosoma teuthras|[3558]|LEPPA467-12|MACN-Bar-Lep-ct 02973|Argentina|Formosa|658[0n]|BOLD:AAA...
Cosmosoma teuthras|[3559]|ARCTD770-12|MILA 1895|Paraguay|Cordillera|658[0n]|BOLD:AAA1312
Cosmosoma teuthras|[3560]|LEMMZ250-10|MMZ0250|Brazil|Parana|658[0n]|BOLD:AAA1312
Cosmosoma teuthras|[3561]|LEPPA684-13|MACN-Bar-Lep-ct 04069|Argentina|Formosa|609[0n]|BOLD:AAA...
Cosmosoma teuthras|[3562]|LEMMZ476-11|MMZ0476|Brazil|Parana|658[0n]|BOLD:AAA1312
Cosmosoma teuthras|[3563]|LEMMZ829-11|MMZ0829|Brazil|Parana|658[0n]|BOLD:AAA1312
Cosmosoma teuthras|[3564]|ARCTD767-12|MILA 1892|Paraguay|Cordillera|658[0n]|BOLD:AAA1312
Cosmosoma teuthras|[3565]|ARCTC824-11|MILA 1286|Guatemala|Izabal|658[0n]|BOLD:AAA1312
Cosmosoma teuthras|[3566]|ARCTB211-08|MILA 0870|Guatemala|Suchitepequez|658[0n]|BOLD:AAA1312
Cosmosoma teuthras|[3567]|BCIAR153-10|YB-BCI22944|Panama|Panama|658[0n]|BOLD:AAA1312
Cosmosoma teuthras|[3568]|BCIAR758-13|YB-BCI63104|Panama|Panama|658[0n]|BOLD:AAA1312
Cosmosoma teuthras|[3569]|BCIAR750-13|YB-BCI59891|Panama|Panama|658[0n]|BOLD:AAA1312
Cosmosoma teuthras|[3570]|BCIAR518-12|YB-BCI37506|Panama|Panama|630[0n]|BOLD:AAA1312
Cosmosoma teuthras|[3571]|BCIAR526-12|YB-BCI37997|Panama|Panama|658[0n]|BOLD:AAA1312
Cosmosoma teuthras|[3572]|BCIAR184-10|YB-BCI7504|Panama|Panama|658[0n]|BOLD:AAA1312
Cosmosoma teuthras|[3573]|BCIAR759-13|YB-BCI63294|Panama|Panama|658[0n]|BOLD:AAA1312
Cosmosoma teuthras|[3574]|BCIAR704-13|YB-BCI33532|Panama|Panama|658[0n]|BOLD:AAA1312
Cosmosoma teuthras|[3575]|BCIAR182-10|YB-BCI7441|Panama|Panama|658[0n]|BOLD:AAA1312
Cosmosoma teuthras|[3576]|LYPAP064-09|MAL-01703|Mexico|Quintana Roo|658[0n]|BOLD:AAA1312
Cosmosoma teuthras|[3577]|CQR022-13|CQR-0022|Mexico|Quintana Roo|658[0n]|BOLD:AAA1312
Cosmosoma teuthras|[3578]|LPYPC048-08|MLL-02172|Mexico|Yucatan|658[0n]|BOLD:AAA1312
Cosmosoma teuthras|[3579]|LPYPB170-08|MAL-00388|Mexico|Campeche|658[0n]|BOLD:AAA1312
Cosmosoma teuthras|[3580]|LPYPB016-08|MLL-01046|Mexico|Campeche|658[0n]|BOLD:AAA1312
Poliopastea evelina|[3581]|MHARB775-06|05-SRNP-24898|Costa Rica|Guanacaste|658[0n]|BOLD:AAA4849
Poliopastea evelina|[3582]|MHARB778-06|05-SRNP-24901|Costa Rica|Guanacaste|658[0n]|BOLD:AAA4849
Poliopastea evelina|[3583]|MHARB777-06|05-SRNP-24907|Costa Rica|Guanacaste|658[0n]|BOLD:AAA4849
Poliopastea clavipes|[3584]|MHARB669-06|01-SRNP-15668|Costa Rica|Guanacaste|658[5n]|BOLD:AAA1307
Poliopastea clavipes|[3585]|BLPED1476-12|11-SRNP-105078|Costa Rica|Guanacaste|658[0n]|BOLD:AAA...

Poliopastea evelina|[3583]|MHARB777-06|05-SRNP-24907|Costa Rica|Guanacaste|658[0n]|BOLD:AAA4849
Poliopastea clavipes|[3584]|MHARB669-06|01-SRNP-15668|Costa Rica|Guanacaste|658[5n]|BOLD:AAA1307
Poliopastea clavipes|[3585]|BLPED1476-12|11-SRNP-105078|Costa Rica|Guanacaste|658[0n]|BOLD:AAA...
Poliopastea clavipes|[3586]|BLPEF3233-13|12-SRNP-104971|Costa Rica|Guanacaste|658[0n]|BOLD:AAA...
Poliopastea clavipes|[3587]|BLPDA674-09|08-SRNP-105749|Costa Rica|Guanacaste|630[0n]|BOLD:AAA1307
Poliopastea clavipes|[3588]|BLPEE5290-14|14-SRNP-100430|Costa Rica|Guanacaste|658[0n]|BOLD:AAA...
Poliopastea clavipes|[3589]|BLPBH296-07|07-SRNP-104055|Costa Rica|Guanacaste|645[0n]|BOLD:AAA1307
Poliopastea clavipes|[3590]|XAA148-04|95-SRNP-4693.15|Costa Rica|Guanacaste|572[1n]|BOLD:AAA1307
Poliopastea clavipes|[3591]|BLPCA023-08|07-SRNP-104628|Costa Rica|Guanacaste|658[0n]|BOLD:AAA1307
Poliopastea clavipes|[3592]|LYRIO393-10|MAL-05404|Mexico|Yucatan|658[0n]|BOLD:AAA1307
Poliopastea clavipes|[3593]|LYRIO391-10|MAL-05402|Mexico|Yucatan|658[0n]|BOLD:AAA1307
Poliopastea clavipes|[3594]|BLPCA024-08|07-SRNP-104629|Costa Rica|Guanacaste|658[0n]|BOLD:AAA1307
Poliopastea clavipes|[3595]|CNCLB1864-14|CNCLEP00117991|Guatemala||658[0n]|BOLD:AAA1307
Poliopastea clavipes|[3596]|BLPEE3671-14|13-SRNP-103107|Costa Rica|Guanacaste|658[0n]|BOLD:AAA...
Poliopastea clavipes|[3597]|BLPAC643-06|06-SRNP-102522|Costa Rica|Guanacaste|658[0n]|BOLD:AAA1307
Poliopastea clavipes|[3598]|CNCLB2507-14|CNCLEP 00119631|Mexico|Nuevo Leon|658[0n]|BOLD:AAA1307
Poliopastea clavipes|[3599]|BLPDA672-09|08-SRNP-105747|Costa Rica|Guanacaste|631[0n]|BOLD:AAA1307
Poliopastea clavipes|[3600]|CQR579-13|CQR-0579|Mexico|Quintana Roo|658[0n]|BOLD:AAA1307
Poliopastea clavipes|[3601]|BLPCN826-08|08-SRNP-103363|Costa Rica|Guanacaste|632[0n]|BOLD:AAA1307
Poliopastea clavipes|[3602]|MHARB672-06|01-SRNP-15666|Costa Rica|Guanacaste|658[4n]|BOLD:AAA1307
Poliopastea clavipes|[3603]|BLPEE2256-14|13-SRNP-101692|Costa Rica|Guanacaste|658[0n]|BOLD:AAA...
Poliopastea clavipes|[3604]|LYRIO392-10|MAL-05403|Mexico|Yucatan|658[0n]|BOLD:AAA1307
Poliopastea clavipes|[3605]|BLPAG737-07|06-SRNP-106376|Costa Rica|Guanacaste|658[0n]|BOLD:AAA1307
Poliopastea clavipes|[3606]|BLPCD185-08|07-SRNP-107610|Costa Rica|Guanacaste|658[0n]|BOLD:AAA1307
Poliopastea clavipes|[3607]|XAA144-04|03-SRNP-12358|Costa Rica|Guanacaste|662[0n]|BOLD:AAA1307
Poliopastea clavipes|[3608]|BLPAG738-07|06-SRNP-106377|Costa Rica|Guanacaste|658[0n]|BOLD:AAA1307
Poliopastea clavipes|[3609]|LYRIO394-10|MAL-05405|Mexico|Yucatan|658[0n]|BOLD:AAA1307
Poliopastea clavipes|[3610]|CNCLB1865-14|CNCLEP00117992|Mexico|Chiapas|658[0n]|BOLD:AAA1307
Poliopastea clavipes|[3611]|BLPCN824-08|08-SRNP-103361|Costa Rica|Guanacaste|658[0n]|BOLD:AAA1307
Poliopastea clavipes|[3612]|BLPCN825-08|08-SRNP-103362|Costa Rica|Guanacaste|658[0n]|BOLD:AAA1307
Poliopastea clavipes|[3613]|BLPCN827-08|08-SRNP-103364|Costa Rica|Guanacaste|658[0n]|BOLD:AAA1307
Poliopastea clavipes|[3614]|BLPCC109-08|07-SRNP-106594|Costa Rica|Guanacaste|658[0n]|BOLD:AAA1307
Poliopastea clavipes|[3615]|BLPCD186-08|07-SRNP-107611|Costa Rica|Guanacaste|658[0n]|BOLD:AAA1307
Poliopastea clavipes|[3616]|BLPCD189-08|07-SRNP-107614|Costa Rica|Guanacaste|658[0n]|BOLD:AAA1307
Poliopastea clavipes|[3617]|BLPCD190-08|07-SRNP-107615|Costa Rica|Guanacaste|658[0n]|BOLD:AAA1307
Poliopastea clavipes|[3618]|BLPCD191-08|07-SRNP-107616|Costa Rica|Guanacaste|658[0n]|BOLD:AAA1307
Poliopastea clavipes|[3619]|BLPDK456-09|09-SRNP-105384|Costa Rica|Guanacaste|658[0n]|BOLD:AAA1307
Poliopastea clavipes|[3620]|BLPCE555-08|07-SRNP-108920|Costa Rica|Guanacaste|658[0n]|BOLD:AAA1307
Poliopastea clavipes|[3621]|BLPEF5828-13|13-SRNP-10377|Costa Rica||658[0n]|BOLD:AAA1307
Poliopastea clavipes|[3622]|BLPDM067-10|09-SRNP-109444|Costa Rica|Guanacaste|658[0n]|BOLD:AAA1307
Poliopastea clavipes|[3623]|BLPDM607-10|09-SRNP-109984|Costa Rica|Guanacaste|658[0n]|BOLD:AAA1307
Poliopastea clavipes|[3624]|BLPCD188-08|07-SRNP-107613|Costa Rica|Guanacaste|658[0n]|BOLD:AAA1307
Poliopastea clavipes|[3625]|BLPDA673-09|08-SRNP-105748|Costa Rica|Guanacaste|648[0n]|BOLD:AAA1307
Poliopastea clavipes|[3626]|BLPDK1131-09|09-SRNP-106059|Costa Rica|Guanacaste|658[0n]|BOLD:AAA...
Poliopastea clavipes|[3627]|CNCLB1861-14|CNCLEP00117988|Mexico|Chiapas|658[0n]|BOLD:AAA1307
Poliopastea clavipes|[3628]|BLPEF4889-13|13-SRNP-100839|Costa Rica|Guanacaste|605[0n]|BOLD:AAA...
Poliopastea clavipes|[3629]|MHARB673-06|03-SRNP-12360|Costa Rica|Guanacaste|658[0n]|BOLD:AAA1307
Poliopastea clavipes|[3630]|MHARB671-06|03-SRNP-12357|Costa Rica|Guanacaste|658[0n]|BOLD:AAA1307
Poliopastea clavipes|[3631]|MHARB670-06|03-SRNP-12356|Costa Rica|Guanacaste|658[0n]|BOLD:AAA1307
Poliopastea clavipes|[3632]|LPYPA043-08|MLL-01701|Mexico|Campeche|658[0n]|BOLD:AAA1307
Neoplynes eudora|[3633]|HKONS141-08|1705-COI-07|United States|Florida|624[0n]|BOLD:AAX6590
Lerina incarnata|[3634]|RDNMH099-09|CNCLEP00054318|United States|Arizona|557[0n]|BOLD:AAZ2819
Ectypia mexicana|[3635]|CNCLB2374-14|CNCLEP 00119518|United States|Texas|307[0n]|
Ectypia sp.|[3636]|RDNME481-08|LEP037905|United States|Colorado|658[0n]|BOLD:AAI9299
Ectypia sp.|[3637]|RDNME479-08|LEP037903|United States|Colorado|658[0n]|BOLD:AAI9299
Ectypia clio|[3638]|CMAZA351-10|CMAZ-0351|United States|Arizona|658[0n]|BOLD:AAD9681
Ectypia clio|[3639]|RDNMF957-08|CNC LEP00054224|United States|Wyoming|658[0n]|BOLD:AAD9681
Ectypia clio|[3640]|JBAZ069-09|JLB-0069|United States|California|658[0n]|BOLD:AAD9681
Ectypia clio|[3641]|RDNMF948-08|CNC LEP00054215|United States|Texas|658[0n]|BOLD:AAD9681
Ectypia clio|[3642]|CMAZA372-10|CMAZ-0372|United States|Arizona|658[0n]|BOLD:AAD9681
Ectypia clio|[3643]|RDNMF956-08|CNC LEP00054223|United States|Arizona|658[0n]|BOLD:ABY4367
Ectypia clio|[3644]|RDNMF955-08|CNC LEP00054222|United States|California|658[0n]|BOLD:AAD9682
Ectypia bivittata|[3645]|BBLSW708-09|09BBLEP-01636|United States|Texas|658[0n]|BOLD:AAH5313
Euchaetes zella|[3646]|RDNME828-08|LEP041293|United States|Arizona|658[0n]|BOLD:AAI4947
Euchaetes zella|[3647]|RDNMH959-09|CNCLEP00067967|United States|Arizona|658[0n]|BOLD:AAI4947
Euchaetes zella|[3648]|RDNMF988-08|CNC LEP00054255|United States|Arizona|658[0n]|BOLD:AAI4947
Pygarctia neomexicana|[3649]|NOCNA001-14|20300-100609-TX|United States|Texas|658[0n]|BOLD:ACN6475
Euchaetes perlevis|[3650]|BBLOC231-11|BIOUG01454-D10|United States|Arizona|658[0n]|BOLD:AAI4946
Euchaetes perlevis|[3651]|BBLOC408-11|BIOUG01456-C09|United States|Arizona|658[0n]|BOLD:AAI4946
Euchaetes perlevis|[3652]|BBLOC273-11|BIOUG01454-H04|United States|Arizona|658[0n]|BOLD:AAI4946
Euchaetes perlevis|[3653]|RDNMH958-09|CNCLEP00067966|United States|Arizona|658[0n]|BOLD:AAI4946
Euchaetes perlevis|[3654]|CMAZA1088-12|BIOUG02042-E11|United States|Arizona|658[0n]|BOLD:AAI4946
Euchaetes fusca|[3655]|RDNMI131-10|CNCLEP 69731|United States|Arizona|658[0n]|BOLD:AAL8835
Euchaetes fusca|[3656]|RDNMH960-09|CNCLEP00067968|United States|Arizona|658[0n]|BOLD:AAL8835
Euchaetes fusca|[3657]|RDNMI132-10|CNCLEP 69732|United States|Arizona|658[0n]|BOLD:AAL8835
Euchaetes fusca|[3658]|AWCLB007-10|AWC-00648|United States|Arizona|658[0n]|BOLD:AAL8835
Pygarctia murina|[3659]|RDNME447-08|LEP037871|United States|Arizona|609[0n]|BOLD:AAF5095
Pygarctia murina|[3660]|RDNMF953-08|CNC LEP00054220|United States|Arizona|658[0n]|BOLD:AAF5095
Pygarctia murina|[3661]|CMAZA525-10|CMAZ-0525|United States|Arizona|658[0n]|BOLD:AAF5095
Pygarctia murina|[3662]|BBLOC1614-11|BIOUG01546-F11|United States|Arizona|658[0n]|BOLD:AAF5095
Pygarctia murina|[3663]|BBLOE1799-12|BIOUG01991-B09|United States|Arizona|658[0n]|BOLD:AAF5095
Pygarctia murina|[3664]|BBLOE1812-12|BIOUG01991-C10|United States|Arizona|658[0n]|BOLD:AAF5095
Pygarctia murina|[3665]|BBLOC540-11|BIOUG01457-F10|United States|Arizona|633[0n]|BOLD:AAF5095
Pygarctia murina|[3666]|RDNMJ702-11|CNCLEP 80136|United States|Arizona|658[0n]|BOLD:AAF5095
Pygarctia murina|[3667]|BBLOD1893-11|BIOUG01841-F10|United States|Arizona|658[0n]|BOLD:AAF5095
Pygarctia murina|[3668]|RDNME548-08|LEP037972|United States|New Mexico|658[0n]|BOLD:AAF5095
Pygarctia murina|[3669]|CMAZA524-10|CMAZ-0524|United States|Arizona|658[0n]|BOLD:AAF5095
Pygarctia murina|[3670]|BBLOC900-11|BIOUG01467-C03|United States|Arizona|658[0n]|BOLD:AAF5095
Pygarctia murina|[3671]|BBLOC888-11|BIOUG01467-B03|United States|Arizona|658[0n]|BOLD:AAF5095
Pygarctia murina|[3672]|BBLOC887-11|BIOUG01467-B02|United States|Arizona|658[0n]|BOLD:AAF5095
Pygarctia murina|[3673]|BBLOC445-11|BIOUG01456-F10|United States|Arizona|658[0n]|BOLD:AAF5095
Pygarctia murina|[3674]|CMAZA1087-12|BIOUG02042-E10|United States|Arizona|658[0n]|BOLD:AAF5095
Pygarctia murina|[3675]|BBLOC185-11|BIOUG01453-H11|United States|Arizona|658[0n]|BOLD:AAF5095
Pygarctia murina|[3676]|DMAZ175-10|DMLEP-0074|United States|Arizona|658[0n]|BOLD:AAF5095
Pygarctia sp. 2|[3677]|NOCNA016-14|20315-100609-TX|United States|Texas|658[0n]|BOLD:ABZ1939
Pygarctia sp. 2|[3678]|NOCNA017-14|20316-160609-TX|United States|Texas|658[0n]|BOLD:ABZ1939
Pygarctia pterygostigma|[3679]|RDNMK619-11|CNCLEP 79883|United States|New Mexico|658[0n]|BOLD:...
Pygarctia pterygostigma|[3680]|RDNMK611-11|CNCLEP 79875|United States|New Mexico|658[0n]|BOLD:...
Pygarctia pterygostigma|[3681]|RDNMK621-11|CNCLEP 79885|United States|New Mexico|658[0n]|BOLD:...
Pygarctia pterygostigma|[3682]|BBLSW064-09|09BBLEP-00992|United States|Texas|658[0n]|BOLD:ABZ1939
Pygarctia pterygostigma|[3683]|BBLSW065-09|09BBLEP-00993|United States|Texas|658[0n]|BOLD:ABZ1939
Pygarctia pterygostigma|[3684]|RDNMK609-11|CNCLEP 79873|United States|New Mexico|658[0n]|BOLD:...
Pygarctia pterygostigma|[3685]|RDNMK617-11|CNCLEP 79881|United States|New Mexico|658[0n]|BOLD:...

Pygarctia pterygostigma|[3682]|BBLSW064-09|09BBLEP-00992|United States|Texas|658[0n]|BOLD:ABZ1939
Pygarctia pterygostigma|[3683]|BBLSW065-09|09BBLEP-00993|United States|Texas|658[0n]|BOLD:ABZ1939
Pygarctia pterygostigma|[3684]|RDNMK609-11|CNCLEP 79873|United States|New Mexico|658[0n]|BOLD:...
Pygarctia pterygostigma|[3685]|RDNMK617-11|CNCLEP 79881|United States|New Mexico|658[0n]|BOLD:...
Pygarctia pterygostigma|[3686]|RDNMK618-11|CNCLEP 79882|United States|New Mexico|658[0n]|BOLD:...
Pygarctia pterygostigma|[3687]|RDNMK620-11|CNCLEP 79884|United States|New Mexico|658[0n]|BOLD:...
Pygarctia pterygostigma|[3688]|BBLSW740-09|09BBLEP-01668|United States|Texas|658[0n]|BOLD:ABZ1939
Pygarctia flavidorsalis|[3689]|HKONB246-09|3743-COI-08|United States|Texas|636[0n]|BOLD:AAD6507
Pygarctia flavidorsalis|[3690]|HKONB245-09|3742-COI-08|United States|Texas|658[0n]|BOLD:AAD6507
Pygarctia roseicapitis|[3691]|CMAZA548-10|CMAZ-0548|United States|Arizona|658[0n]|BOLD:AAL4668
Pygarctia roseicapitis|[3692]|CMAZA989-12|BIOUG02041-E07|United States|Arizona|658[0n]|BOLD:AA...
Pygarctia roseicapitis|[3693]|DMAZ144-10|DMLEP-0036|United States|Arizona|658[0n]|BOLD:AAL4668
Pygarctia roseicapitis|[3694]|RDNMK277-11|CNCLEP 84186|United States|Arizona|658[0n]|BOLD:AAL4668
Pygarctia roseicapitis|[3695]|CMAZA939-12|BIOUG02040-H06|United States|Arizona|635[0n]|BOLD:AA...
Pygarctia roseicapitis|[3696]|RDNMJ504-11|CNCLEP 80032|United States|Arizona|658[0n]|BOLD:AAL4668
Pygarctia roseicapitis|[3697]|RDNME443-08|LEP037867|United States|Arizona|658[0n]|BOLD:AAL4668
Pygarctia roseicapitis|[3698]|RDNMK276-11|CNCLEP 84185|United States|Arizona|658[0n]|BOLD:AAL4668
Pygarctia roseicapitis|[3699]|CMAZA511-10|CMAZ-0511|United States|Arizona|658[0n]|BOLD:AAL4668
Pygarctia roseicapitis|[3700]|CMAZA966-12|BIOUG02041-C08|United States|Arizona|658[0n]|BOLD:AA...
Pygarctia roseicapitis|[3701]|CMAZA1075-12|BIOUG02042-D10|United States|Arizona|658[0n]|BOLD:A...
Pygarctia abdominalis|[3702]|RDNMH963-09|CNCLEP00067971|United States|Florida|658[0n]|BOLD:AAD...
Pygarctia sp. 1|[3703]|NOCNA015-14|20314-090609-TX|United States|Texas|658[0n]|BOLD:AAD0348
Pygarctia spraguei|[3704]|RDNMF394-08|NOC14480|United States|Colorado|658[0n]|BOLD:AAD0348
Pygarctia spraguei|[3705]|RDNMF392-08|NOC14478|Canada|Manitoba|658[0n]|BOLD:AAD0348
Pygarctia abdominalis|[3706]|RDNMH961-09|CNCLEP00067969|United States|Indiana|658[0n]|BOLD:AAD...
Pygarctia spraguei|[3707]|RDNMH648-09|CNCLEP00062972|United States|Louisiana|658[0n]|BOLD:AAD0348
Pygarctia spraguei|[3708]|RDNMH647-09|CNCLEP00062971|United States|Louisiana|658[0n]|BOLD:AAD0348
Pygarctia spraguei|[3709]|RDNMF395-08|NOC14481|United States|Colorado|658[0n]|BOLD:AAD0348
Pygarctia spraguei|[3710]|RDNMF393-08|NOC14479|Canada|Manitoba|658[0n]|BOLD:AAD0348
Pygarctia spraguei|[3711]|RDNMH962-09|CNCLEP00067970|United States|Colorado|658[0n]|BOLD:AAD0348
Pygarctia abdominalis|[3712]|HKONS677-08|3393-COI-08|United States|Florida|658[0n]|BOLD:AAD0348
Pygarctia abdominalis|[3713]|HKONS676-08|3392-COI-08|United States|Florida|658[0n]|BOLD:AAD0348
Pygarctia spraguei|[3714]|RDNME450-08|LEP037874|Canada|Manitoba|658[0n]|BOLD:AAD0348
Pygarctia eglenensis|[3715]|CNCLB1025-14|CNCLEP00113433|United States|Arizona|658[0n]|BOLD:AAD...
Pygarctia eglenensis|[3716]|CNCLB1024-14|CNCLEP00113432|United States|Texas|658[0n]|BOLD:AAD0348
Pygarctia abdominalis|[3717]|RDNMH649-09|CNCLEP00062973|United States|Louisiana|658[0n]|BOLD:A...
Pygarctia abdominalis|[3718]|LOFLA922-06|06-FLOR-0922|United States|Florida|658[0n]|BOLD:AAD0348
Pygarctia lorula|[3719]|NOCNA007-14|20306-110609-TX|United States|Texas|658[0n]|BOLD:AAH5312
Pygarctia lorula|[3720]|RDNMK616-11|CNCLEP 79880|United States|New Mexico|658[0n]|BOLD:AAH5312
Pygarctia lorula|[3721]|NOCNA006-14|20305-110609-TX|United States|Texas|658[0n]|BOLD:AAH5312
Pygarctia lorula|[3722]|BBLSW764-09|09BBLEP-01692|United States|Texas|658[0n]|BOLD:AAH5312
Pygarctia lorula|[3723]|BBLSW703-09|09BBLEP-01631|United States|Texas|658[0n]|BOLD:AAH5312
Euchaetes helena|[3724]|CNCLB1018-14|CNCLEP00113426|Mexico|Coahuila|658[0n]|BOLD:ACM4587
Euchaetes helena|[3725]|CNCLB1017-14|CNCLEP00113425|Mexico|Coahuila|658[0n]|BOLD:ACM4587
Euchaetes helena|[3726]|CNCLB1016-14|CNCLEP00113424|United States|Texas|658[0n]|BOLD:ACM4587
Euchaetes helena|[3727]|CNCLB1015-14|CNCLEP00113423|United States|Texas|658[0n]|BOLD:ACM4587
Euchaetes gigantea|[3728]|ARCTB363-08|MILA 1022|Guatemala|Suchitepequez|658[0n]|BOLD:AAE4505
Euchaetes gigantea|[3729]|ARCTC001-09|MILA 1031|Guatemala|Suchitepequez|658[0n]|BOLD:AAE4505
Euchaetes gigantea|[3730]|ARCTA695-07|MILA 0414|Guatemala|Suchitepequez|654[0n]|BOLD:AAE4505
Euchaetes gigantea|[3731]|ARCTA693-07|MILA 0412|Guatemala|Suchitepequez|655[0n]|BOLD:AAE4505
Euchaetes expressa|[3732]|HKONB241-09|3738-COI-08|United States|Texas|658[0n]|BOLD:AAF1159
Euchaetes expressa|[3733]|HKONB240-09|3737-COI-08|United States|Texas|658[0n]|BOLD:AAF1159
Euchaetes expressa|[3734]|HKONB239-09|3736-COI-08|United States|Texas|640[0n]|BOLD:AAF1159
Euchaetes gigantea|[3735]|CNCLB2225-14|CNCLEP00083257|Mexico|Sonora|658[0n]|BOLD:ACR2764
Euchaetes gigantea|[3736]|CNCLB2224-14|CNCLEP00083258|Mexico|Sonora|658[0n]|BOLD:ACR2764
Euchaetes gigantea|[3737]|CNCLB2223-14|CNCLEP00083256|Mexico|Sonora|658[0n]|BOLD:ACR2764
Euchaetes albicosta|[3738]|CNCLB1030-14|CNCLEP00113438|Mexico|Queretaro|658[0n]|BOLD:ACM4567
Pygoctenucha pyrrhoura|[3739]|CNCLB2165-14|CNCLEP00108542_rerun|United States|Arizona|515[0n]|BOL...
Pygoctenucha terminalis|[3740]|CNCLB658-14|CNCLEP00108539|United States|Arizona|658[0n]|BOLD:A...
Pygoctenucha terminalis|[3741]|CNCLB656-14|CNCLEP00108537|United States|Arizona|658[0n]|BOLD:A...
Pygoctenucha terminalis|[3742]|CNCLB657-14|CNCLEP00108538|Mexico|Chihuahua|658[0n]|BOLD:ACM3970
Pygoctenucha pyrrhoura|[3743]|TML124-14|CCDB-17965-A03|United States||658[0n]|BOLD:ACM3970
Euchaetes polingi|[3744]|NOCNA014-14|20313-170609-TX|United States|Texas|658[0n]|BOLD:AAI4945
Euchaetes polingi|[3745]|NOCNA013-14|20312-110609-TX|United States|Texas|658[0n]|BOLD:AAI4945
Euchaetes polingi|[3746]|CNCLB785-14|CNCLEP00083261|United States|Texas|658[0n]|BOLD:AAI4945
Euchaetes bolteri|[3747]|HKONB250-09|3747-COI-08|United States|Texas|658[0n]|BOLD:AAI4945
Euchaetes bolteri|[3748]|BBLOC1994-11|BIOUG01550-F11|United States|Texas|658[0n]|BOLD:AAI4945
Euchaetes bolteri|[3749]|RDNME448-08|LEP037872|United States|Texas|642[0n]|BOLD:AAI4945
Euchaetes bolteri|[3750]|QUNOD010-10|7009-COI-09|United States|Texas|639[0n]|BOLD:AAI4945
Euchaetes castalla|[3751]|CNCLB2229-14|CNCLEP00118026|Mexico|Sonora|658[0n]|BOLD:AAD6506
Euchaetes castalla|[3752]|RDNME554-08|LEP037978|United States|Arizona|658[0n]|BOLD:AAD6506
Euchaetes elegans|[3753]|CNCLB1020-14|CNCLEP00113428|United States|California|658[0n]|BOLD:ACM...
Euchaetes elegans|[3754]|CNCLB1019-14|CNCLEP00113427|United States|California|658[0n]|BOLD:ACM...
Euchaetes antica|[3755]|RDNME503-08|LEP037927|United States|Arizona|658[0n]|BOLD:AAJ0969
Euchaetes antica|[3756]|CMAZA914-12|BIOUG02040-F05|United States|Arizona|658[0n]|BOLD:AAJ0969
Euchaetes antica|[3757]|AWCLB002-10|AWC-01502|United States|Arizona|658[0n]|BOLD:AAJ0969
Euchaetes antica|[3758]|CMAZA560-10|CMAZ-0560|United States|Arizona|658[0n]|BOLD:AAJ0969
Euchaetes antica|[3759]|AWCLB004-10|AWC-00199|United States|Arizona|614[0n]|BOLD:AAJ0969
Euchaetes antica|[3760]|AWCLB003-10|AWC-01501|United States|Arizona|658[0n]|BOLD:AAJ0969
Euchaetes antica|[3761]|AWCLB001-10|AWC-01503|United States|Arizona|658[0n]|BOLD:AAJ0969
Euchaetes egle|[3762]|XAI013-05|0102-ONT-0013|Canada|Ontario|611[3n]|BOLD:AAC2978
Euchaetes egle|[3763]|XAK451-06|2006-ONT-1446|Canada|Ontario|658[0n]|BOLD:AAC2978
Euchaetes egle|[3764]|XAD752-05|2005-ONT-551|Canada|Ontario|658[0n]|BOLD:AAC2978
Euchaetes egle|[3765]|BLTIB564-08|BL842|Canada|Ontario|658[0n]|BOLD:AAC2978
Euchaetes egle|[3766]|XAI011-05|0102-ONT-0011|Canada|Ontario|658[0n]|BOLD:AAC2978
Euchaetes egle|[3767]|LNCC1122-11|11-NCCC-647|United States|North Carolina|658[0n]|BOLD:AAC2978
Euchaetes egle|[3768]|XAI014-05|0102-ONT-0014|Canada|Ontario|658[0n]|BOLD:AAC2978
Euchaetes egle|[3769]|LPOKD115-09|MDOK-3194|United States|Oklahoma|658[0n]|BOLD:AAC2978
Euchaetes egle|[3770]|LILLA779-11|SNS10IL-00978|United States|Illinois|658[0n]|BOLD:AAC2978
Euchaetes egle|[3771]|LILLA882-11|SNS10IL-01103|United States|Illinois|658[0n]|BOLD:AAC2978
Euchaetes egle|[3772]|XAI012-05|0102-ONT-0012|Canada|Ontario|516[2n]|BOLD:AAC2978
Euchaetes egle|[3773]|XAE390-04|Moth4390.03|Canada|Ontario|658[0n]|BOLD:AAC2978
Euchaetes egle|[3774]|TMG89-03|EUCHA1.00|Canada|Ontario|639[0n]|BOLD:AAC2978
Euchaetes egle|[3775]|TMG88-03|moth651.01|Canada|Ontario|639[0n]|BOLD:AAC2978
Euchaetes egle|[3776]|PMG005-03|moth706.01|Canada|Ontario|617[0n]|BOLD:AAC2978
Syntomeida epilais|[3777]|LNAUT210-14|CCDB-22941-B09|United States|Florida|658[0n]|BOLD:AAC4951
Syntomeida epilais|[3778]|LNAUT211-14|CCDB-22941-B10|United States|Florida|658[0n]|BOLD:AAC4951
Syntomeida epilais|[3779]|LNAUT212-14|CCDB-22941-B11|United States|Florida|658[0n]|BOLD:AAC4951
Syntomeida epilais|[3780]|LNAUT213-14|CCDB-22941-B12|United States|Florida|658[0n]|BOLD:AAC4951
Syntomeida epilais|[3781]|LSUSA073-06|06-SUSA-0073|United States|Florida|571[0n]|BOLD:AAC4951
Syntomeida epilais|[3782]|GBGL8444-12|GU258439|||1176[150n]|BOLD:AAC4951
Syntomeida epilais|[3783]|LYHES547-09|MAL-03844|Mexico|Quintana Roo|628[0n]|BOLD:AAC4951
Syntomeida epilais|[3784]|ARCTB155-08|MILA 0814|Guatemala|Izabal|658[0n]|BOLD:AAC4951

Syntomeida epilais|[3782]|GBGL8444-12|GU258439|||1176[150n]|BOLD:AAC4951
Syntomeida epilais|[3783]|LYHES547-09|MAL-03844|Mexico|Quintana Roo|628[0n]|BOLD:AAC4951
Syntomeida epilais|[3784]|ARCTB155-08|MILA 0814|Guatemala|Izabal|658[0n]|BOLD:AAC4951
Syntomeida epilais|[3785]|LPYPB165-08|MAL-00383|Mexico|Quintana Roo|658[0n]|BOLD:AAC4951
Syntomeida epilais|[3786]|LYPAP202-09|MAL-01747|Mexico|Quintana Roo|566[0n]|BOLD:AAC4951
Syntomeida epilais|[3787]|LYHES559-09|MAL-03856|Mexico|Quintana Roo|649[0n]|BOLD:AAC4951
Syntomeida epilais|[3788]|ARCTB145-08|MILA 0804|Guatemala|Baja Verapaz|658[0n]|BOLD:AAC4951
Syntomeida epilais|[3789]|LPYPB649-08|MLL-01961|Mexico|Campeche|658[0n]|BOLD:AAC4951
Syntomeida epilais|[3790]|LPYPB166-08|MAL-00384|Mexico|Campeche|636[0n]|BOLD:AAC4951
Syntomeida epilais|[3791]|LPYPB164-08|MAL-00382|Mexico|Campeche|658[0n]|BOLD:AAC4951
Syntomeida epilais|[3792]|ARCTB135-08|MILA 0794|Guatemala|Peten|658[0n]|BOLD:AAC4951
Syntomeida epilais|[3793]|CQR819-13|CQR-0819|Mexico|Quintana Roo|658[0n]|BOLD:AAC4951
Kodiosoma fulvum|[3794]|CNCLB766-14|CNCLEP00030272|United States|Arizona|658[0n]|BOLD:ACM4565
Trocodima fuscipes|[3795]|RDNMH017-09|CNCLEP00032068|United States|Arizona|658[0n]|BOLD:AAH4893
Trocodima fuscipes|[3796]|IAWL889-09|IAWAZ-0686|United States|Arizona|658[0n]|BOLD:AAH4893
Cycnia tenera|[3797]|LBCA088-05|HLC-20888|Canada|British Columbia|658[0n]|BOLD:AAB0274
Cycnia tenera|[3798]|LPABC722-09|08BBLEP-04941|Canada|Alberta|658[0n]|BOLD:AAB0274
Cycnia tenera|[3799]|LBCB208-05|HLC-21148|Canada|British Columbia|658[0n]|BOLD:AAB0274
Cycnia tenera|[3800]|XAE506-04|Moth4506.03|Canada|Ontario|653[7n]|BOLD:AAB0274
Cycnia tenera|[3801]|XAE459-04|Moth4459.03|Canada|Ontario|658[0n]|BOLD:AAB0274
Cycnia tenera|[3802]|LGSMB331-05|DNA-ATBI-1180|United States|Tennessee|567[0n]|BOLD:AAB0274
Cycnia tenera|[3803]|BLTIB084-08|BL0126|Canada|Ontario|561[0n]|BOLD:AAB0274
Cycnia tenera|[3804]|BLTIB072-08|BL0112|Canada|Ontario|610[0n]|BOLD:AAB0274
Cycnia tenera|[3805]|TMG86-03|CYCN1.00|Canada|Ontario|639[0n]|BOLD:AAB0274
Cycnia tenera|[3806]|XAE275-04|Moth4275.03|Canada|Ontario|658[0n]|BOLD:AAB0274
Cycnia tenera|[3807]|LPMN050-08|08BBLEP-00848|Canada|Manitoba|658[0n]|BOLD:AAB0274
Cycnia tenera|[3808]|LNC021-05|05-NCCC-021|United States|North Carolina|658[0n]|BOLD:AAB0274
Cycnia tenera|[3809]|XAE412-04|Moth4412.03|Canada|Ontario|658[0n]|BOLD:AAB0274
Cycnia tenera|[3810]|LSUSA177-06|06-SUSA-0177|United States|Kentucky|658[0n]|BOLD:AAB0274
Cycnia tenera|[3811]|LNCC869-11|11-NCCC-394|United States|North Carolina|658[0n]|BOLD:AAB0274
Cycnia tenera|[3812]|LGSMC756-05|DNA-ATBI-2756|United States|Tennessee|658[0n]|BOLD:AAB0274
Cycnia tenera|[3813]|LSUSA218-06|06-SUSA-0218|United States|Kentucky|658[0n]|BOLD:AAB0274
Cycnia tenera|[3814]|LPOKB693-09|MDOK-1735|United States|Oklahoma|658[0n]|BOLD:AAB0274
Cycnia tenera|[3815]|LPOKA147-08|MDOK-0147|United States|Oklahoma|658[0n]|BOLD:AAB0274
Cycnia tenera|[3816]|LILLA206-11|SNS10IL-00276|United States|Illinois|658[0n]|BOLD:AAB0274
Cycnia tenera|[3817]|LPSOB432-08|PPBP-1431|Canada|Ontario|658[0n]|BOLD:AAB0274
Cycnia tenera|[3818]|LPSOB433-08|PPBP-1432|Canada|Ontario|658[0n]|BOLD:AAB0274
Cycnia tenera|[3819]|LOCT010-05|05-CTATBI-0010|United States|Connecticut|658[0n]|BOLD:AAB0274
Cycnia tenera|[3820]|LOCT011-05|05-CTATBI-0011|United States|Connecticut|658[0n]|BOLD:AAB0274
Cycnia tenera|[3821]|BLTIB461-08|BL708|Canada|Ontario|658[0n]|BOLD:AAB0274
Cycnia tenera|[3822]|XAK259-06|2006-ONT-1254|Canada|Ontario|658[0n]|BOLD:AAB0274
Cycnia tenera|[3823]|XAK449-06|2006-ONT-1444|Canada|Ontario|658[0n]|BOLD:AAB0274
Cycnia tenera|[3824]|LILLA407-11|SNS10IL-00532|United States|Illinois|658[0n]|BOLD:AAB0274
Cycnia tenera|[3825]|XAF827-05|2005-ONT-476|Canada|Ontario|658[0n]|BOLD:AAB0274
Cycnia tenera|[3826]|BLTIB885-08|BL1304|Canada|Ontario|658[0n]|BOLD:AAB0274
Cycnia tenera|[3827]|XAJ915-06|2006-ONT-0915|Canada|Ontario|658[0n]|BOLD:AAB0274
Cycnia tenera|[3828]|XAB244-04|04HBL005244|Canada|Ontario|658[0n]|BOLD:AAB0274
Cycnia tenera|[3829]|XAJ834-06|2006-ONT-0834|Canada|Ontario|658[0n]|BOLD:AAB0274
Cycnia tenera|[3830]|XAB086-04|04HBL005086|Canada|Ontario|658[0n]|BOLD:AAB0274
Cycnia tenera|[3831]|XAJ627-06|2006-ONT-0627|Canada|Ontario|658[0n]|BOLD:AAB0274
Cycnia tenera|[3832]|LNCNW019-06|06-NCNW-0019|United States|North Carolina|601[0n]|BOLD:AAB0274
Cycnia tenera|[3833]|PMG004-03|moth525.01|Canada|Ontario|617[0n]|BOLD:AAB0274
Cycnia oregonensis|[3834]|XAJ296-06|2006-ONT-0296|Canada|Ontario|658[0n]|BOLD:AAB1681
Cycnia oregonensis|[3835]|XAB281-04|04HBL005281|Canada|Ontario|658[0n]|BOLD:AAB1681
Cycnia oregonensis|[3836]|XAJ601-06|2006-ONT-0601|Canada|Ontario|658[0n]|BOLD:AAB1681
Cycnia oregonensis|[3837]|CNCLB1023-14|CNCLEP00113431|Mexico|Coahuila|658[0n]|BOLD:AAB1681
Cycnia oregonensis|[3838]|CNCLB1022-14|CNCLEP00113430|Mexico|Coahuila|614[0n]|BOLD:AAB1681
Cycnia oregonensis|[3839]|BLTIB051-08|BL0082|Canada|Ontario|582[1n]|BOLD:AAB1681
Cycnia oregonensis|[3840]|RDNMC703-06|CNCNoctuoidea12243|United States|Washington|658[0n]|BOLD ...
Cycnia oregonensis|[3841]|BLTIB063-08|BL0102|Canada|Ontario|658[0n]|BOLD:AAB1681
Cycnia oregonensis|[3842]|XAI005-05|0102-ONT-0005|Canada|Ontario|658[0n]|BOLD:AAB1681
Cycnia oregonensis|[3843]|XAJ600-06|2006-ONT-0600|Canada|Ontario|658[0n]|BOLD:AAB1681
Cycnia oregonensis|[3844]|XAK287-06|2006-ONT-1282|Canada|Ontario|658[0n]|BOLD:AAB1681
Cycnia oregonensis|[3845]|LPOKD761-10|MDOK-3840|United States|Oklahoma|658[0n]|BOLD:AAB1681
Cycnia oregonensis|[3846]|LPOKD786-10|MDOK-3865|United States|Oklahoma|658[0n]|BOLD:AAB1681
Cycnia oregonensis|[3847]|XAJ657-06|2006-ONT-0657|Canada|Ontario|658[0n]|BOLD:AAB1681
Cycnia oregonensis|[3848]|XAB063-04|04HBL005063|Canada|Ontario|658[0n]|BOLD:AAB1681
Cycnia oregonensis|[3849]|XAK224-06|2006-ONT-1219|Canada|Ontario|653[0n]|BOLD:AAB1681
Cycnia oregonensis|[3850]|TMG87-03|moth732.01|Canada|Ontario|639[0n]|BOLD:AAB1681
Cycnia oregonensis|[3851]|PHMNB141-04|04HBL007606|Canada|New Brunswick|658[0n]|BOLD:AAB1681
Cycnia oregonensis|[3852]|RDLQG003-06|DH012123|Canada|Quebec|658[0n]|BOLD:AAB1681
Cycnia oregonensis|[3853]|RDMAB925-06|UASM58591|Canada|Alberta|658[0n]|BOLD:AAB1681
Cycnia oregonensis|[3854]|LOWCB663-05|CGWC-1603|Canada|British Columbia|658[0n]|BOLD:AAB1681
Cycnia oregonensis|[3855]|LOWCB664-05|CGWC-1604|Canada|British Columbia|658[0n]|BOLD:AAB1681
Cycnia oregonensis|[3856]|LOWCB665-05|CGWC-1605|Canada|British Columbia|658[0n]|BOLD:AAB1681
Cycnia oregonensis|[3857]|LBCH5615-10|10-JDWBC-5615|Canada|British Columbia|658[0n]|BOLD:AAB1681
Cycnia oregonensis|[3858]|LBCH5693-10|10-JDWBC-5693|Canada|British Columbia|658[0n]|BOLD:AAB1681
Cycnia oregonensis|[3859]|LBCH5439-10|10-JDWBC-5439|Canada|British Columbia|658[0n]|BOLD:AAB1681
Cycnia oregonensis|[3860]|XAB243-04|04HBL005243|Canada|Ontario|658[0n]|BOLD:AAB1681
Cycnia oregonensis|[3861]|XAI004-05|0102-ONT-0004|Canada|Ontario|601[1n]|BOLD:AAB1681
Cycnia oregonensis|[3862]|LOWCB666-05|CGWC-1606|Canada|British Columbia|658[0n]|BOLD:AAB1681
Cycnia oregonensis|[3863]|XAJ354-06|2006-ONT-0354|Canada|Ontario|658[0n]|BOLD:AAB1681
Cycnia oregonensis|[3864]|XAG491-05|2005-ONT-1075|Canada|Ontario|658[0n]|BOLD:AAB1681
Cycnia oregonensis|[3865]|LPMN009-08|08BBLEP-00807|Canada|Manitoba|649[0n]|BOLD:AAB1681
Cycnia oregonensis|[3866]|XAI007-05|0102-ONT-0007|Canada|Ontario|658[0n]|BOLD:AAB1681
Cycnia oregonensis|[3867]|XAI006-05|0102-ONT-0006|Canada|Ontario|658[0n]|BOLD:AAB1681
Cycnia oregonensis|[3868]|LBCH5808-10|10-JDWBC-5808|Canada|British Columbia|658[0n]|BOLD:AAB1681
Cycnia oregonensis|[3869]|XAK243-06|2006-ONT-1238|Canada|Ontario|658[0n]|BOLD:AAB1681
Cycnia oregonensis|[3870]|PMG003-03|moth542.01|Canada|Ontario|617[0n]|BOLD:AAB1681
Biturix venosata|[3871]|RDNMF912-08|CNC LEP00054179|||658[0n]|BOLD:AAC1902
Biturix venosata|[3872]|RDNMH106-09|CNCLEP00054325|United States|Texas|647[0n]|BOLD:AAC1902
Horama panthalon|[3873]|ARMOT074-12|MACN-Bar-Lep-ct 04147|Argentina|Formosa|658[0n]|BOLD:AAD1684
Horama panthalon|[3874]|LNAUT220-14|CCDB-22941-C07|United States|Texas|658[0n]|BOLD:AAD1684
Horama panthalon|[3875]|LNAUT217-14|CCDB-22941-C04|United States|Texas|658[0n]|BOLD:AAD1684
Horama panthalon|[3876]|ARCTC020-09|MILA 1050|Jamaica||658[0n]|BOLD:AAD1684
Horama panthalon|[3877]|ARCTD054-11|MILA 1559|Jamaica|Cornwall|658[0n]|BOLD:AAD1684
Horama panthalon texana|[3878]|BBLOD1728-11|BIOUG01830-H11|United States|Texas|658[0n]|BOLD:AA...
Horama panthalon|[3879]|LNAUT216-14|CCDB-22941-C03|United States|Texas|612[0n]|BOLD:AAD1684
Horama panthalon|[3880]|LNAUT219-14|CCDB-22941-C06|United States|Texas|658[0n]|BOLD:AAD1684
Horama panthalon|[3881]|LNAUT218-14|CCDB-22941-C05|United States|Texas|658[0n]|BOLD:AAD1684
Horama panthalon texana|[3882]|ARCTD055-11|MILA 1560|Guatemala|Peten|658[0n]|BOLD:AAD1684
Pseudocharis minima|[3883]|RDNMF911-08|CNC LEP00054178|United States|Texas|609[0n]|BOLD:AAX7058
Horama plumipes|[3884]|BLPDS392-10|10-SRNP-107016|Costa Rica|Alajuela|658[0n]|BOLD:AAA8734

Horama panthalon texana|[3882]|ARCTD055-11|MILA 1560|Guatemala|Peten|658[0n]|BOLD:AAD1684
Pseudocharis minima|[3883]|RDNMF911-08|CNC LEP00054178|United States|Texas|609[0n]|BOLD:AAX7058
Horama plumipes|[3884]|BLPDS392-10|10-SRNP-107016|Costa Rica|Alajuela|658[0n]|BOLD:AAA8734
Horama plumipes|[3885]|BLPBF934-07|07-SRNP-102813|Costa Rica|Guanacaste|597[0n]|BOLD:AAA8734
Horama plumipes|[3886]|XAA254-04|03-SRNP-4072|Costa Rica|Guanacaste|665[0n]|BOLD:AAA8734
Horama plumipes|[3887]|LPYPC208-08|MLL-00693|Mexico|Campeche|658[0n]|BOLD:AAA8734
Horama plumipes|[3888]|LPMX174-07|MLL-00174|Mexico|Campeche|658[0n]|BOLD:AAA8734
Horama plumipes|[3889]|MHARB274-05|05-SRNP-40916|Costa Rica|Alajuela|658[0n]|BOLD:AAA8734
Horama plumipes|[3890]|ARCTB290-08|MILA 0949|Guatemala|Izabal|658[0n]|BOLD:AAA8734
Horama plumipes|[3891]|ARCTB299-08|MILA 0958|Guatemala|Izabal|658[0n]|BOLD:AAA8734
Horama plumipes|[3892]|ARCTB310-08|MILA 0969|Guatemala|Izabal|658[0n]|BOLD:AAA8734
Horama plumipes|[3893]|MHMYO391-11|11-SRNP-80119|Costa Rica||658[0n]|BOLD:AAA8734
Horama plumipes|[3894]|MHMYC570-09|08-SRNP-21416|Costa Rica|Guanacaste|658[0n]|BOLD:AAA8734
Horama plumipes|[3895]|MHMYC571-09|08-SRNP-35721|Costa Rica|Guanacaste|658[0n]|BOLD:AAA8734
Horama plumipes|[3896]|MHMYC572-09|08-SRNP-21415|Costa Rica|Guanacaste|658[0n]|BOLD:AAA8734
Horama plumipes|[3897]|BLPCM029-08|08-SRNP-101626|Costa Rica|Guanacaste|658[0n]|BOLD:AAA8734
Horama plumipes|[3898]|MHARB275-05|05-SRNP-40917|Costa Rica|Alajuela|658[0n]|BOLD:AAA8734
Horama plumipes|[3899]|MHARB276-05|05-SRNP-40915|Costa Rica|Alajuela|658[0n]|BOLD:AAA8734
Horama plumipes|[3900]|MHARB277-05|05-SRNP-21133|Costa Rica|Guanacaste|658[0n]|BOLD:AAA8734
Horama plumipes|[3901]|MHARB440-05|05-SRNP-23383|Costa Rica|Guanacaste|658[0n]|BOLD:AAA8734
Horama plumipes|[3902]|MHARB441-05|05-SRNP-23384|Costa Rica|Guanacaste|658[0n]|BOLD:AAA8734
Horama plumipes|[3903]|MHARB442-05|05-SRNP-23382|Costa Rica|Guanacaste|658[0n]|BOLD:AAA8734
Horama plumipes|[3904]|MHMYL2405-11|10-SRNP-35156|Costa Rica||658[0n]|BOLD:AAA8734
Horama plumipes|[3905]|MHMYL2549-11|10-SRNP-22033|Costa Rica||658[0n]|BOLD:AAA8734
Horama plumipes|[3906]|LYNYM301-09|MAL-03410|Mexico|Quintana Roo|658[0n]|BOLD:AAA8734
Horama plumipes|[3907]|LPMX781-08|MLL-01439|Mexico|Campeche|658[0n]|BOLD:AAA8734
Horama plumipes|[3908]|LPMX799-08|MLL-01457|Mexico|Campeche|658[0n]|BOLD:AAA8734
Horama plumipes|[3909]|LPMX806-08|MLL-01464|Mexico|Campeche|658[0n]|BOLD:AAA8734
Horama plumipes|[3910]|LPMX827-08|MLL-01485|Mexico|Campeche|658[0n]|BOLD:AAA8734
Horama plumipes|[3911]|LPMX882-08|MLL-01540|Mexico|Campeche|658[0n]|BOLD:AAA8734
Horama plumipes|[3912]|MHARB278-05|05-SRNP-21132|Costa Rica|Guanacaste|658[2n]|BOLD:AAA8734
Horama plumipes|[3913]|LPYPC147-08|MLL-00632|Mexico|Campeche|588[0n]|BOLD:AAA8734
Horama plumipes|[3914]|LPYPB639-08|MLL-01951|Mexico|Campeche|658[0n]|BOLD:AAA8734
Horama plumipes|[3915]|LPYPB406-08|MLL-01154|Mexico|Campeche|658[0n]|BOLD:AAA8734
Horama plumipes|[3916]|LPYPB367-08|MLL-01115|Mexico|Campeche|658[0n]|BOLD:AAA8734
Horama plumipes|[3917]|LPYPB354-08|MLL-01102|Mexico|Campeche|658[0n]|BOLD:AAA8734
Horama plumipes|[3918]|LPYPB184-08|MAL-00402|Mexico|Quintana Roo|658[0n]|BOLD:AAA8734
Horama plumipes|[3919]|LPYPB183-08|MAL-00401|Mexico|Quintana Roo|658[0n]|BOLD:AAA8734
Horama plumipes|[3920]|LPYPA603-08|MLL-00945|Mexico|Campeche|658[0n]|BOLD:AAA8734
Horama plumipes|[3921]|LPYPA467-08|MLL-01937|Mexico|Campeche|658[0n]|BOLD:AAA8734
Horama plumipes|[3922]|LPYPA466-08|MLL-01936|Mexico|Campeche|658[0n]|BOLD:AAA8734
Horama plumipes|[3923]|LPYPA104-08|MLL-01762|Mexico|Campeche|658[0n]|BOLD:AAA8734
Horama plumipes|[3924]|LPYPA095-08|MLL-01753|Mexico|Campeche|658[0n]|BOLD:AAA8734
Horama plumipes|[3925]|LPYPA036-08|MLL-01694|Mexico|Campeche|658[0n]|BOLD:AAA8734
Horama plumipes|[3926]|LPMX981-08|MLL-01639|Mexico|Campeche|658[0n]|BOLD:AAA8734
Horama plumipes|[3927]|LPMX972-08|MLL-01630|Mexico|Campeche|658[0n]|BOLD:AAA8734
Leucanopsis perdentata|[3928]|JBAZ133-09|JLB-0133|United States|Arizona|658[0n]|BOLD:AAE7304
Leucanopsis perdentata|[3929]|RDNME470-08|LEP037894|United States|Arizona|658[0n]|BOLD:AAE7304
Leucanopsis perdentata|[3930]|RDNME474-08|LEP037898|United States|Arizona|658[0n]|BOLD:AAE7304
Leucanopsis perdentata|[3931]|RDNME469-08|LEP037893|United States|Arizona|658[0n]|BOLD:AAE7304
Hypocrisias minima|[3932]|RDNMH369-09|CNCLEP00057778|United States|Arizona|658[0n]|BOLD:AAK3573
Leucanopsis lurida|[3933]|ARCTB867-09|BEVI0762|Mexico|Michoacan|658[0n]|BOLD:ABY5309
Leucanopsis lurida|[3934]|ARCTB866-09|BEVI0761|Mexico|Michoacan|658[0n]|BOLD:ABY5309
Leucanopsis lurida|[3935]|RDNME480-08|LEP037904|United States|Arizona|658[0n]|BOLD:ABY5309
Pseudohemihyalea ambigua|[3936]|RDNMF933-08|CNC LEP00054200|United States|Arizona|609[0n]|BOLD ...
Pseudohemihyalea ambigua|[3937]|RDNME440-08|LEP037864|United States|Colorado|658[0n]|BOLD:AAE6662
Pseudohemihyalea ambigua|[3938]|RDNMF932-08|CNC LEP00054199|United States|Arizona|658[0n]|BOLD ...
Apocrisias thaumasta|[3939]|RDNMH100-09|CNCLEP00054319|United States|Arizona|634[0n]|BOLD:AAJ1353
Apocrisias thaumasta|[3940]|JBAZ136-09|JLB-0136|United States|Arizona|636[0n]|BOLD:AAJ1353
Apocrisias thaumasta|[3941]|CMAZA500-10|CMAZ-0500|United States|Arizona|658[0n]|BOLD:AAJ1353
Apocrisias thaumasta|[3942]|CMAZA865-12|BIOUG02040-B04|United States|Arizona|658[0n]|BOLD:AAJ1353
Apocrisias thaumasta|[3943]|DMAZ190-10|DMLEP-0107|United States|Arizona|658[0n]|BOLD:AAJ1353
Apocrisias thaumasta|[3944]|DMAZ189-10|DMLEP-0106|United States|Arizona|658[0n]|BOLD:AAJ1353
Apocrisias thaumasta|[3945]|AWCLB029-10|AWC-01301|United States|Arizona|658[0n]|BOLD:AAJ1353
Apocrisias thaumasta|[3946]|AWCLB028-10|AWC-01491|United States|Arizona|658[0n]|BOLD:AAJ1353
Apocrisias thaumasta|[3947]|AWCLB027-10|AWC-01492|United States|Arizona|658[0n]|BOLD:AAJ1353
Apocrisias thaumasta|[3948]|AWCLB026-10|AWC-01493|United States|Arizona|658[0n]|BOLD:AAJ1353
Apocrisias thaumasta|[3949]|AWCLB024-10|AWC-01495|United States|Arizona|658[0n]|BOLD:AAJ1353
Apocrisias thaumasta|[3950]|AWCLB023-10|AWC-01496|United States|Arizona|658[0n]|BOLD:AAJ1353
Pseudohemihyalea splendens|[3951]|CNCLB1013-14|CNCLEP00113421|United States|Arizona|658[0n]|BOLD...
Pseudohemihyalea splendens|[3952]|CNCLB1014-14|CNCLEP00113422|United States|Arizona|658[0n]|BOLD...
Pseudohemihyalea splendens|[3953]|CNCLB1012-14|CNCLEP00113420|United States|Arizona|658[0n]|BOLD...
Pseudohemihyalea labecula|[3954]|AWCLB020-10|AWC-01264|United States|Arizona|658[0n]|BOLD:AAD4222
Pseudohemihyalea labecula|[3955]|RDNME482-08|LEP037906|United States|Colorado|658[0n]|BOLD:AAD...
Pseudohemihyalea labecula|[3956]|AWCLB022-10|AWC-00988|United States|New Mexico|614[0n]|BOLD:A...
Pseudohemihyalea labecula|[3957]|RDNMG315-08|NOC15162|United States|Texas|658[0n]|BOLD:AAD4222
Pseudohemihyalea labecula|[3958]|RDNMG318-08|NOC15165|United States|Texas|658[0n]|BOLD:AAD4222
Pseudohemihyalea labecula|[3959]|RDNMG317-08|NOC15164|United States|Texas|658[0n]|BOLD:AAD4222
Pseudohemihyalea labecula|[3960]|RDNME467-08|LEP037891|United States|New Mexico|658[0n]|BOLD:A...
Pseudohemihyalea labecula|[3961]|RDNME465-08|LEP037889|United States|Colorado|658[0n]|BOLD:AAD...
Pseudohemihyalea labecula|[3962]|QUNOD028-10|7027-COI-09|United States|Texas|658[0n]|BOLD:AAD4222
Pseudohemihyalea labecula|[3963]|AWCLB021-10|AWC-00987|United States|New Mexico|658[0n]|BOLD:A...
Pseudohemihyalea labecula|[3964]|AWCLB019-10|AWC-01265|United States|Arizona|658[0n]|BOLD:AAD4222
Pseudohemihyalea edwardsii|[3965]|CMAZA094-09|CMAZ-0094|United States|Arizona|658[0n]|BOLD:AAC...
Pseudohemihyalea edwardsii|[3966]|RDNME415-08|LEP037839|United States|New Mexico|658[0n]|BOLD:...
Pseudohemihyalea edwardsii|[3967]|JBAZ036-09|JLB-0036|United States|California|658[0n]|BOLD:AA...
Pseudohemihyalea edwardsii|[3968]|NAMUM001-08|RR-99-1309|United States|California|656[1n]|BOLD ...
Pseudohemihyalea edwardsii|[3969]|LOCBB388-06|06-BLLOC-1328|United States|California|658[0n]|BOLD...
Pseudohemihyalea edwardsii|[3970]|LOCBB389-06|06-BLLOC-1329|United States|California|658[0n]|BOLD...
Pseudohemihyalea edwardsii|[3971]|LOCBB387-06|06-BLLOC-1327|United States|California|658[0n]|BOLD...
Pseudohemihyalea edwardsii|[3972]|GMLC283-11|2009GM-0152|United States|California|658[0n]|BOLD ...
Pseudohemihyalea edwardsii|[3973]|GMLC270-11|2009GM-0139|United States|California|658[0n]|BOLD ...
Pseudohemihyalea edwardsii|[3974]|RDNME414-08|LEP037838|United States|Oregon|658[0n]|BOLD:AAC3480
Pseudohemihyalea edwardsii|[3975]|RDNME454-08|LEP037878|United States|Oregon|658[0n]|BOLD:AAC3480
Pseudohemihyalea edwardsii|[3976]|GMLC282-11|2009GM-0151|United States|California|658[0n]|BOLD ...
Pseudohemihyalea edwardsii|[3977]|LOCBB391-06|06-BLLOC-1331|United States|California|658[0n]|BOLD...
Pseudohemihyalea edwardsii|[3978]|LOCBB390-06|06-BLLOC-1330|United States|California|658[0n]|BOLD...
Pseudohemihyalea edwardsii|[3979]|AWCLB015-10|AWC-00701|United States|Arizona|658[0n]|BOLD:AAC...
Pseudohemihyalea edwardsii|[3980]|AWCLB014-10|AWC-00699|United States|Arizona|658[0n]|BOLD:AAC...
Pseudohemihyalea edwardsii|[3981]|RDNME466-08|LEP037890|United States|Arizona|658[0n]|BOLD:AAC...
Pseudohemihyalea edwardsii|[3982]|CMAZA583-10|CMAZ-0583|United States|Arizona|658[0n]|BOLD:AAC...
Pseudohemihyalea edwardsii|[3983]|DMAZ046-09|DMTRN-0140|United States|Arizona|658[0n]|BOLD:AAC...
Pseudohemihyalea edwardsii|[3984]|JBAZ111-09|JLB-0111|United States|Arizona|658[0n]|BOLD:AAC3480

Pseudohemihyalea edwardsii|[3981]|RDNME466-08|LEP037890|United States|Arizona|658[0n]|BOLD:AAC...
Pseudohemihyalea edwardsii|[3982]|CMAZA583-10|CMAZ-0583|United States|Arizona|658[0n]|BOLD:AAC...
Pseudohemihyalea edwardsii|[3983]|DMAZ046-09|DMTRN-0140|United States|Arizona|658[0n]|BOLD:AAC...
Pseudohemihyalea edwardsii|[3984]|JBAZ111-09|JLB-0111|United States|Arizona|658[0n]|BOLD:AAC3480
Pseudohemihyalea edwardsii|[3985]|AWCLB018-10|AWC-01271|United States|Arizona|658[0n]|BOLD:AAC...
Pseudohemihyalea edwardsii|[3986]|AWCLB017-10|AWC-00698|United States|Arizona|658[0n]|BOLD:AAC...
Pseudohemihyalea edwardsii|[3987]|AWCLB016-10|AWC-00700|United States|Arizona|658[0n]|BOLD:AAC...
Pseudohemihyalea edwardsii|[3988]|AWCLB013-10|AWC-01497|United States|Arizona|658[0n]|BOLD:AAC...
Pseudohemihyalea edwardsii|[3989]|AWCLB012-10|AWC-01498|United States|Arizona|658[0n]|BOLD:AAC...
Pseudohemihyalea edwardsii|[3990]|AWCLB011-10|AWC-01499|United States|Arizona|658[0n]|BOLD:AAC...
Pseudohemihyalea edwardsii|[3991]|AWCLB010-10|AWC-01500|United States|Arizona|658[0n]|BOLD:AAC...
Halysidota cinctipes|[3992]|RDNML299-13|CNCLEP 92275|United States|Florida|658[0n]|BOLD:ACD9081
Halysidota cinctipes|[3993]|RDNML298-13|CNCLEP 92274|United States|Florida|658[0n]|BOLD:ACD9081
Halysidota cinctipes|[3994]|CNCLB1967-14|CNCLEP00081225|United States|Florida|658[0n]|BOLD:ACD...
Halysidota davisii|[3995]|CMAZA872-12|BIOUG02040-B11|United States|Arizona|658[0n]|BOLD:ABX5604
Halysidota davisii|[3996]|JBAZ110-09|JLB-0110|United States|Arizona|658[0n]|BOLD:ABX5604
Halysidota davisii|[3997]|LSEU030-06|06-JKA-0030|United States|Texas|658[0n]|BOLD:ABX5604
Halysidota davisii|[3998]|LSEU029-06|06-JKA-0029|United States|Texas|658[0n]|BOLD:ABX5604
Halysidota schausi|[3999]|CNCLB1033-14|CNCLEP00113441|United States|Texas|658[0n]|BOLD:AAB0674
Halysidota schausi|[4000]|CNCLB1032-14|CNCLEP00113440|United States|Texas|658[0n]|BOLD:AAB0674
Halysidota schausi|[4001]|CNCLB1969-14|CNCLEP00113440_rerun|United States|Texas|658[0n]|BOLD:A...
Halysidota schausi|[4002]|CNCLB783-14|CNCLEP00081384|United States|Texas|658[0n]|BOLD:AAB0674
Halysidota harrisii|[4003]|LGSMC973-05|DNA-ATBI-4053|United States|Tennessee|587[0n]|BOLD:ABZ7359
Halysidota harrisii|[4004]|TMFL001-06|06-TMFL-00001|United States|Florida|658[0n]|BOLD:ABZ7359
Halysidota harrisii|[4005]|LGSMC934-05|DNA-ATBI-4014|United States|Tennessee|658[0n]|BOLD:ABZ7359
Halysidota harrisii|[4006]|LGSMC935-05|DNA-ATBI-4015|United States|Tennessee|658[3n]|BOLD:ABZ7359
Halysidota harrisii|[4007]|BBLSX102-09|09BBLEP-02030|United States|Oklahoma|658[0n]|BOLD:ABZ7359
Halysidota harrisii|[4008]|LGSMG120-07|BGS03441|United States|Tennessee|658[0n]|BOLD:ABZ7359
Halysidota harrisii|[4009]|TMFL002-06|06-TMFL-00002|United States|Florida|658[0n]|BOLD:ABZ7359
Halysidota harrisii|[4010]|BBLSW528-09|09BBLEP-01456|United States|Oklahoma|658[0n]|BOLD:ABZ7359
Halysidota harrisii|[4011]|UDLEP329-09|v367 Amb|United States|Pennsylvania|658[0n]|BOLD:ABZ7359
Halysidota harrisii|[4012]|LNCNW039-06|06-NCNW-0039|United States|North Carolina|658[0n]|BOLD:...
Halysidota harrisii|[4013]|LPOKB1009-09|MDOK-2051|United States|Oklahoma|658[0n]|BOLD:ABZ7359
Halysidota harrisii|[4014]|LGSMC721-05|DNA-ATBI-2721|United States|Tennessee|574[0n]|BOLD:ABZ7359
Halysidota harrisii|[4015]|BBLSX101-09|09BBLEP-02029|United States|Oklahoma|658[0n]|BOLD:ABZ7359
Halysidota harrisii|[4016]|BBLSW527-09|09BBLEP-01455|United States|Oklahoma|658[0n]|BOLD:ABZ7359
Halysidota tessellaris|[4017]|PHNXG1219-13|BIOUG09289-G07|Canada|Ontario|582[0n]|BOLD:AAA3425
Halysidota tessellaris|[4018]|HPPPE1542-13|BIOUG07371-D08|Canada|Nova Scotia|563[0n]|BOLD:AAA3425
Halysidota tessellaris|[4019]|PHNXG099-13|BIOUG09268-A04|Canada|Ontario|567[0n]|BOLD:AAA3425
Halysidota tessellaris|[4020]|PHNGT815-13|BIOUG08321-E07|Canada|Ontario|547[0n]|BOLD:AAA3425
Halysidota tessellaris|[4021]|PHNGT827-13|BIOUG08321-F07|Canada|Ontario|548[0n]|BOLD:AAA3425
Halysidota tessellaris|[4022]|PHNXG101-13|BIOUG09268-A06|Canada|Ontario|567[1n]|BOLD:AAA3425
Halysidota tessellaris|[4023]|PHNXG1223-13|BIOUG09289-G11|Canada|Ontario|582[0n]|BOLD:AAA3425
Halysidota tessellaris|[4024]|GMGSV008-13|BIOUG04567-B04|United States|Tennessee|581[0n]|BOLD:...
Halysidota tessellaris|[4025]|GMGSJ368-12|BIOUG02884-B08|United States|Tennessee|621[0n]|BOLD:...
Halysidota tessellaris|[4026]|PHNGT820-13|BIOUG08321-E12|Canada|Ontario|554[0n]|BOLD:AAA3425
Halysidota tessellaris|[4027]|PHNGT825-13|BIOUG08321-F05|Canada|Ontario|554[0n]|BOLD:AAA3425
Halysidota tessellaris|[4028]|PHNGT824-13|BIOUG08321-F04|Canada|Ontario|554[0n]|BOLD:AAA3425
Halysidota tessellaris|[4029]|BLTIB447-08|BL694|Canada|Ontario|658[0n]|BOLD:AAA3425
Halysidota tessellaris|[4030]|BBLOB1629-11|BIOUG01421-B02|United States|Florida|658[0n]|BOLD:A...
Halysidota tessellaris|[4031]|LOT139-04|04HBL002139|United States|Tennessee|609[0n]|BOLD:AAA3425
Halysidota tessellaris|[4032]|LOT141-04|04HBL002141|United States|Tennessee|609[0n]|BOLD:AAA3425
Halysidota tessellaris|[4033]|LOT143-04|04HBL002143|United States|Tennessee|609[0n]|BOLD:AAA3425
Halysidota tessellaris|[4034]|PHMNB198-04|04HBL007663|Canada|New Brunswick|609[0n]|BOLD:AAA3425
Halysidota tessellaris|[4035]|PHNXG109-13|BIOUG09268-B02|Canada|Ontario|597[0n]|BOLD:AAA3425
Halysidota tessellaris|[4036]|GMGSX010-13|BIOUG04567-B05|United States|Tennessee|585[0n]|BOLD:...
Halysidota tessellaris|[4037]|LOT140-04|04HBL002140|United States|Tennessee|609[0n]|BOLD:AAA3425
Halysidota tessellaris|[4038]|XAD751-05|2005-ONT-550|Canada|Ontario|658[0n]|BOLD:AAA3425
Halysidota tessellaris|[4039]|PHNGT817-13|BIOUG08321-E09|Canada|Ontario|580[0n]|BOLD:AAA3425
Halysidota tessellaris|[4040]|CNSLR015-13|BIOUG06046-G09|Canada|Ontario|588[0n]|BOLD:AAA3425
Halysidota tessellaris|[4041]|SMTPD5420-13|BIOUG08661-E01|Canada|Ontario|606[0n]|BOLD:AAA3425
Halysidota tessellaris|[4042]|PHNGT818-13|BIOUG08321-E10|Canada|Ontario|583[3n]|BOLD:AAA3425
Halysidota tessellaris|[4043]|GMGSR093-13|BIOUG04722-B02|United States|Tennessee|636[0n]|BOLD:...
Halysidota tessellaris|[4044]|CNSLQ048-13|BIOUG06957-B03|Canada|Ontario|585[0n]|BOLD:AAA3425
Halysidota tessellaris|[4045]|USLEP445-10|10BBLEP-00445|United States|Arkansas|658[0n]|BOLD:AA...
Halysidota tessellaris|[4046]|LOFLA214-06|06-FLOR-0214|United States|Florida|658[0n]|BOLD:AAA3425
Halysidota tessellaris|[4047]|LOFLA525-06|06-FLOR-0525|United States|Florida|658[0n]|BOLD:AAA3425
Halysidota tessellaris|[4048]|BBLOB386-11|BIOUG01396-A06|United States|Florida|658[0n]|BOLD:AA...
Halysidota tessellaris|[4049]|LOFLA853-06|06-FLOR-0853|United States|Florida|658[0n]|BOLD:AAA3425
Halysidota tessellaris|[4050]|USLEP444-10|10BBLEP-00444|United States|Florida|658[0n]|BOLD:AAA...
Halysidota tessellaris|[4051]|LOFLA218-06|06-FLOR-0218|United States|Florida|658[0n]|BOLD:AAA3425
Halysidota tessellaris|[4052]|BBLPA574-10|10BBCLP-0574|Canada|Ontario|632[0n]|BOLD:AAA3425
Halysidota tessellaris|[4053]|MNBB235-05|05-NBSTA-151|Canada|New Brunswick|658[0n]|BOLD:AAA3425
Halysidota tessellaris|[4054]|MNBB197-05|05-NBSTA-113|Canada|New Brunswick|658[0n]|BOLD:AAA3425
Halysidota tessellaris|[4055]|MNBB355-05|05-NBSTA-271|Canada|New Brunswick|658[0n]|BOLD:AAA3425
Halysidota tessellaris|[4056]|MNBB294-05|05-NBSTA-210|Canada|New Brunswick|658[0n]|BOLD:AAA3425
Halysidota tessellaris|[4057]|MNBB433-05|05-NBSTA-349|Canada|New Brunswick|658[0n]|BOLD:AAA3425
Halysidota tessellaris|[4058]|MNBB385-05|05-NBSTA-301|Canada|New Brunswick|658[0n]|BOLD:AAA3425
Halysidota tessellaris|[4059]|LILLA382-11|SNS10IL-00500|United States|Illinois|658[0n]|BOLD:AA...
Halysidota tessellaris|[4060]|BBLPA576-10|10BBCLP-0576|Canada|Ontario|658[0n]|BOLD:AAA3425
Halysidota tessellaris|[4061]|LPSOC284-08|PPBP-2283|Canada|Ontario|658[0n]|BOLD:AAA3425
Halysidota tessellaris|[4062]|BLTIB394-08|BL630|Canada|Ontario|658[0n]|BOLD:AAA3425
Halysidota tessellaris|[4063]|LNCC1702-13|13-NCCC-372|United States|North Carolina|658[0n]|BOLD ...
Halysidota tessellaris|[4064]|BBLSZ098-09|09BBLEP-04024|United States|Oklahoma|658[0n]|BOLD:AA...
Halysidota tessellaris|[4065]|XAB088-04|04HBL005088|Canada|Ontario|658[0n]|BOLD:AAA3425
Halysidota tessellaris|[4066]|LPSOC273-08|PPBP-2272|Canada|Ontario|658[0n]|BOLD:AAA3425
Halysidota tessellaris|[4067]|LSUSA258-06|06-SUSA-0258|United States|Kentucky|658[0n]|BOLD:AAA...
Halysidota tessellaris|[4068]|LGSM620-04|DNA-ATBI-0620|United States|North Carolina|658[0n]|BOLD...
Halysidota tessellaris|[4069]|TTMNB255-06|MNBTT-255|Canada|New Brunswick|658[0n]|BOLD:AAA3425
Halysidota tessellaris|[4070]|UDLEP202-09|v678 UD|United States|Delaware|658[0n]|BOLD:AAA3425
Halysidota tessellaris|[4071]|USLEP448-10|10BBLEP-00448|United States|Florida|658[0n]|BOLD:AAA...
Halysidota tessellaris|[4072]|MNBB086-05|05-NBSTA-002|Canada|New Brunswick|658[0n]|BOLD:AAA3425
Halysidota tessellaris|[4073]|GMGST029-13|BIOUG04567-B02|United States|Tennessee|593[0n]|BOLD:...
Halysidota tessellaris|[4074]|GMGSJ366-12|BIOUG02884-B06|United States|Tennessee|621[0n]|BOLD:...
Halysidota tessellaris|[4075]|LGSMB314-05|DNA-ATBI-1163|United States|Tennessee|596[0n]|BOLD:A...
Halysidota tessellaris|[4076]|LOFLA856-06|06-FLOR-0856|United States|Florida|605[0n]|BOLD:AAA3425
Halysidota tessellaris|[4077]|BBLSZ104-09|09BBLEP-04030|United States|Oklahoma|658[0n]|BOLD:AA...
Halysidota tessellaris|[4078]|RDNME416-08|LEP037840|United States|Maryland|658[0n]|BOLD:AAA3425
Halysidota tessellaris|[4079]|LPOKB125-09|MDOK-1229|United States|Oklahoma|658[0n]|BOLD:AAA3425
Halysidota tessellaris|[4080]|CNSLF698-12|BIOUG03835-C10|Canada|Ontario|631[0n]|BOLD:AAA3425
Halysidota tessellaris|[4081]|LGSMB315-05|DNA-ATBI-1164|United States|Tennessee|601[0n]|BOLD:A...
Halysidota tessellaris|[4082]|CNSLG087-12|BIOUG03817-F08|Canada|Ontario|637[0n]|BOLD:AAA3425
Halysidota tessellaris|[4083]|LNC266-05|05-NCCC-266|United States|North Carolina|619[0n]|BOLD:...

Halysidota tessellaris|[4081]|LGSMB315-05|DNA-ATBI-1164|United States|Tennessee|601[0n]|BOLD:A...
Halysidota tessellaris|[4082]|CNSLG087-12|BIOUG03817-F08|Canada|Ontario|637[0n]|BOLD:AAA3425
Halysidota tessellaris|[4083]|LNC266-05|05-NCCC-266|United States|North Carolina|619[0n]|BOLD:...
Halysidota tessellaris|[4084]|PHMNB033-03|moth197.02SA|Canada|New Brunswick|639[0n]|BOLD:AAA3425
Halysidota tessellaris|[4085]|PHMNB457-04|04HBL00683|Canada|New Brunswick|621[1n]|BOLD:AAA3425
Halysidota tessellaris|[4086]|PHMNB730-05|Moth 423.03SA|Canada|New Brunswick|621[1n]|BOLD:AAA3425
Halysidota tessellaris|[4087]|LPOKD290-09|MDOK-3369|United States|Oklahoma|657[1n]|BOLD:AAA3425
Halysidota tessellaris|[4088]|MNBB087-05|05-NBSTA-003|Canada|New Brunswick|658[0n]|BOLD:AAA3425
Halysidota tessellaris|[4089]|MNBB088-05|05-NBSTA-004|Canada|New Brunswick|658[0n]|BOLD:AAA3425
Halysidota tessellaris|[4090]|MNBB089-05|05-NBSTA-005|Canada|New Brunswick|658[0n]|BOLD:AAA3425
Halysidota tessellaris|[4091]|MNBB090-05|05-NBSTA-006|Canada|New Brunswick|658[0n]|BOLD:AAA3425
Halysidota tessellaris|[4092]|MNBB091-05|05-NBSTA-007|Canada|New Brunswick|658[0n]|BOLD:AAA3425
Halysidota tessellaris|[4093]|MNBB196-05|05-NBSTA-112|Canada|New Brunswick|658[0n]|BOLD:AAA3425
Halysidota tessellaris|[4094]|MNBB236-05|05-NBSTA-152|Canada|New Brunswick|658[0n]|BOLD:AAA3425
Halysidota tessellaris|[4095]|MNBB290-05|05-NBSTA-206|Canada|New Brunswick|658[0n]|BOLD:AAA3425
Halysidota tessellaris|[4096]|MNBB291-05|05-NBSTA-207|Canada|New Brunswick|658[0n]|BOLD:AAA3425
Halysidota tessellaris|[4097]|MNBB292-05|05-NBSTA-208|Canada|New Brunswick|658[0n]|BOLD:AAA3425
Halysidota tessellaris|[4098]|MNBB295-05|05-NBSTA-211|Canada|New Brunswick|658[0n]|BOLD:AAA3425
Halysidota tessellaris|[4099]|MNBB328-05|05-NBSTA-244|Canada|New Brunswick|658[0n]|BOLD:AAA3425
Halysidota tessellaris|[4100]|MNBB357-05|05-NBSTA-273|Canada|New Brunswick|658[0n]|BOLD:AAA3425
Halysidota tessellaris|[4101]|MNBB358-05|05-NBSTA-274|Canada|New Brunswick|658[0n]|BOLD:AAA3425
Halysidota tessellaris|[4102]|MNBB382-05|05-NBSTA-298|Canada|New Brunswick|658[0n]|BOLD:AAA3425
Halysidota tessellaris|[4103]|MNBB384-05|05-NBSTA-300|Canada|New Brunswick|658[0n]|BOLD:AAA3425
Halysidota tessellaris|[4104]|MNBB434-05|05-NBSTA-350|Canada|New Brunswick|658[0n]|BOLD:AAA3425
Halysidota tessellaris|[4105]|MNBB435-05|05-NBSTA-351|Canada|New Brunswick|658[0n]|BOLD:AAA3425
Halysidota tessellaris|[4106]|MNBB623-05|05-NBSTA-539|Canada|New Brunswick|658[0n]|BOLD:AAA3425
Halysidota tessellaris|[4107]|MNBB624-05|05-NBSTA-540|Canada|New Brunswick|658[0n]|BOLD:AAA3425
Halysidota tessellaris|[4108]|LOFLA887-06|06-FLOR-0887|United States|Florida|658[0n]|BOLD:AAA3425
Halysidota tessellaris|[4109]|MNBB557-05|05-NBSTA-473|Canada|New Brunswick|658[0n]|BOLD:AAA3425
Halysidota tessellaris|[4110]|LOFLB008-06|06-FLOR-0948|United States|Florida|658[0n]|BOLD:AAA3425
Halysidota tessellaris|[4111]|USLEP443-10|10BBLEP-00443|United States|Florida|658[0n]|BOLD:AAA...
Halysidota tessellaris|[4112]|USLEP447-10|10BBLEP-00447|United States|Florida|658[0n]|BOLD:AAA...
Halysidota tessellaris|[4113]|LOFLA095-06|06-FLOR-0095|United States|Florida|658[0n]|BOLD:AAA3425
Halysidota tessellaris|[4114]|LOFLA096-06|06-FLOR-0096|United States|Florida|658[0n]|BOLD:AAA3425
Halysidota tessellaris|[4115]|LOFLA097-06|06-FLOR-0097|United States|Florida|658[0n]|BOLD:AAA3425
Halysidota tessellaris|[4116]|LOFLA098-06|06-FLOR-0098|United States|Florida|658[0n]|BOLD:AAA3425
Halysidota tessellaris|[4117]|BBLCU312-09|09BBLEP-04799|United States|Michigan|658[0n]|BOLD:AA...
Halysidota tessellaris|[4118]|LOFLA327-06|06-FLOR-0327|United States|Florida|658[0n]|BOLD:AAA3425
Halysidota tessellaris|[4119]|LOFLA328-06|06-FLOR-0328|United States|Florida|658[0n]|BOLD:AAA3425
Halysidota tessellaris|[4120]|LOFLA380-06|06-FLOR-0380|United States|Florida|658[0n]|BOLD:AAA3425
Halysidota tessellaris|[4121]|LOFLA523-06|06-FLOR-0523|United States|Florida|657[0n]|BOLD:AAA3425
Halysidota tessellaris|[4122]|LOFLA680-06|06-FLOR-0680|United States|Florida|658[0n]|BOLD:AAA3425
Halysidota tessellaris|[4123]|LOFLA854-06|06-FLOR-0854|United States|Florida|658[0n]|BOLD:AAA3425
Halysidota tessellaris|[4124]|LOFLA855-06|06-FLOR-0855|United States|Florida|658[0n]|BOLD:AAA3425
Halysidota tessellaris|[4125]|LOFLA886-06|06-FLOR-0886|United States|Florida|658[0n]|BOLD:AAA3425
Halysidota tessellaris|[4126]|UDLEP112-09|v768 LPC|United States|Maryland|658[0n]|BOLD:AAA3425
Halysidota tessellaris|[4127]|UDLEP130-09|v813 CS|United States|Pennsylvania|658[0n]|BOLD:AAA3425
Halysidota tessellaris|[4128]|UDLEP132-09|v816 CS|United States|Pennsylvania|658[0n]|BOLD:AAA3425
Halysidota tessellaris|[4129]|UDLEP183-09|v641 CV|United States|Delaware|658[0n]|BOLD:AAA3425
Halysidota tessellaris|[4130]|UDLEP196-09|v663 UD|United States|Delaware|658[0n]|BOLD:AAA3425
Halysidota tessellaris|[4131]|XAJ919-06|2006-ONT-0919|Canada|Ontario|658[0n]|BOLD:AAA3425
Halysidota tessellaris|[4132]|LGSM619-04|DNA-ATBI-0619|United States|North Carolina|658[0n]|BOLD...
Halysidota tessellaris|[4133]|LPSOD1067-09|08MZPP-172|Canada|Ontario|658[0n]|BOLD:AAA3425
Halysidota tessellaris|[4134]|LOT142-04|04HBL002142|United States|Tennessee|658[0n]|BOLD:AAA3425
Halysidota tessellaris|[4135]|BBLOB1638-11|BIOUG01421-B11|United States|Florida|658[0n]|BOLD:A...
Halysidota tessellaris|[4136]|BBLOB1645-11|BIOUG01421-C06|United States|Florida|658[0n]|BOLD:A...
Halysidota tessellaris|[4137]|TMNBD412-07|MNBTT-3213|Canada|New Brunswick|658[0n]|BOLD:AAA3425
Halysidota tessellaris|[4138]|TMNBD413-07|MNBTT-3214|Canada|New Brunswick|658[0n]|BOLD:AAA3425
Halysidota tessellaris|[4139]|TMNBD414-07|MNBTT-3215|Canada|New Brunswick|658[0n]|BOLD:AAA3425
Halysidota tessellaris|[4140]|TMNBD415-07|MNBTT-3216|Canada|New Brunswick|658[0n]|BOLD:AAA3425
Halysidota tessellaris|[4141]|TMNBD416-07|MNBTT-3217|Canada|New Brunswick|658[0n]|BOLD:AAA3425
Halysidota tessellaris|[4142]|LPOKD030-09|MDOK-3109|United States|Oklahoma|658[0n]|BOLD:AAA3425
Halysidota tessellaris|[4143]|XAJ832-06|2006-ONT-0832|Canada|Ontario|658[0n]|BOLD:AAA3425
Halysidota tessellaris|[4144]|BBLPA573-10|10BBCLP-0573|Canada|Ontario|658[0n]|BOLD:AAA3425
Halysidota tessellaris|[4145]|LOFLA213-06|06-FLOR-0213|United States|Florida|658[0n]|BOLD:AAA3425
Halysidota tessellaris|[4146]|TMFL003-06|06-TMFL-00003|United States|Florida|658[0n]|BOLD:AAA3425
Halysidota tessellaris|[4147]|LOTB350-05|05-TN-00350|United States|Tennessee|658[0n]|BOLD:AAA3425
Halysidota tessellaris|[4148]|LPOKB350-09|MDOK-1363|United States|Oklahoma|658[0n]|BOLD:AAA3425
Halysidota tessellaris|[4149]|XAF544-05|2005-ONT-193|Canada|Ontario|658[0n]|BOLD:AAA3425
Halysidota tessellaris|[4150]|BLTIB1087-08|BL1096|Canada|Ontario|658[0n]|BOLD:AAA3425
Halysidota tessellaris|[4151]|LILLA398-11|SNS10IL-00520|United States|Illinois|658[0n]|BOLD:AA...
Halysidota tessellaris|[4152]|BBLSZ105-09|09BBLEP-04031|United States|Oklahoma|658[0n]|BOLD:AA...
Halysidota tessellaris|[4153]|LOFLA529-06|06-FLOR-0529|United States|Florida|658[0n]|BOLD:AAA3425
Halysidota tessellaris|[4154]|BBLSZ106-09|09BBLEP-04032|United States|Oklahoma|658[0n]|BOLD:AA...
Halysidota tessellaris|[4155]|USLEP446-10|10BBLEP-00446|United States|Florida|658[0n]|BOLD:AAA...
Halysidota tessellaris|[4156]|MNBB556-05|05-NBSTA-472|Canada|New Brunswick|658[0n]|BOLD:AAA3425
Halysidota tessellaris|[4157]|LGSMG119-07|BGS03440|United States|North Carolina|658[0n]|BOLD:A...
Halysidota tessellaris|[4158]|RDMAB739-06|BCSC408|United States|Kentucky|658[0n]|BOLD:AAA3425
Halysidota tessellaris|[4159]|MNBB383-05|05-NBSTA-299|Canada|New Brunswick|658[0n]|BOLD:AAA3425
Halysidota tessellaris|[4160]|MNBB293-05|05-NBSTA-209|Canada|New Brunswick|658[0n]|BOLD:AAA3425
Halysidota tessellaris|[4161]|LPSOC285-08|PPBP-2284|Canada|Ontario|642[0n]|BOLD:AAA3425
Halysidota tessellaris|[4162]|LPOKB383-09|MDOK-1467|United States|Oklahoma|654[0n]|BOLD:AAA3425
Halysidota tessellaris|[4163]|PHMNB052-03|moth245.02SA|Canada|New Brunswick|639[0n]|BOLD:AAA3425
Halysidota tessellaris|[4164]|TMG83-03|HALY1.00|Canada|Ontario|639[0n]|BOLD:AAA3425
Halysidota tessellaris|[4165]|BBLEC325-09|09BBELE-0325|Canada|Nova Scotia|658[0n]|BOLD:AAA3425
Halysidota tessellaris|[4166]|MNBB510-05|05-NBSTA-426|Canada|New Brunswick|658[0n]|BOLD:AAA3425
Halysidota tessellaris|[4167]|MNBB509-05|05-NBSTA-425|Canada|New Brunswick|658[0n]|BOLD:AAA3425
Halysidota tessellaris|[4168]|MNBB508-05|05-NBSTA-424|Canada|New Brunswick|658[0n]|BOLD:AAA3425
Halysidota tessellaris|[4169]|PHNGT821-13|BIOUG08321-F01|Canada|Ontario|584[0n]|BOLD:AAA3425
Halysidota tessellaris|[4170]|GMGSR100-13|BIOUG04722-B09|United States|Tennessee|632[0n]|BOLD:...
Halysidota tessellaris|[4171]|MNBB625-05|05-NBSTA-541|Canada|New Brunswick|658[0n]|BOLD:AAA3425
Halysidota tessellaris|[4172]|LPOKA102-08|MDOK-0102|United States|Oklahoma|658[0n]|BOLD:AAA3425
Halysidota tessellaris|[4173]|BBLPA575-10|10BBCLP-0575|Canada|Ontario|658[0n]|BOLD:AAA3425
Halysidota tessellaris|[4174]|XAK462-06|2006-ONT-1457|Canada|Ontario|658[0n]|BOLD:AAA3425
Halysidota tessellaris|[4175]|XAB587-04|04HBL005587|Canada|Ontario|658[0n]|BOLD:AAA3425
Halysidota tessellaris|[4176]|MNBB356-05|05-NBSTA-272|Canada|New Brunswick|658[0n]|BOLD:AAA3425
Halysidota tessellaris|[4177]|MNBB085-05|05-NBSTA-001|Canada|New Brunswick|658[0n]|BOLD:AAA3425
Halysidota tessellaris|[4178]|MNBB507-05|05-NBSTA-423|Canada|New Brunswick|658[0n]|BOLD:AAA3425
Halysidota tessellaris|[4179]|PMG009-03|moth898.01|Canada|Ontario|617[0n]|BOLD:AAA3425
Leucanopsis longa|[4180]|LNC425-05|05-NCCC-425|United States|North Carolina|658[0n]|BOLD:ABZ1735
Leucanopsis longa|[4181]|LSEU001-06|06-JKA-0001|United States|Georgia|658[0n]|BOLD:ABZ1735
Leucanopsis longa|[4182]|LNC022-05|05-NCCC-022|United States|North Carolina|658[0n]|BOLD:ABZ1735
Leucanopsis longa|[4183]|LSEU002-06|06-JKA-0002|United States|Georgia|601[0n]|BOLD:ABZ1735

Leucanopsis longa|[4180]|LNC423-05|05-NCCC-423|United States|North Carolina|658[0n]|BOLD:ABZ1735
Leucanopsis longa|[4181]|LSEU001-06|06-JKA-0001|United States|Georgia|658[0n]|BOLD:ABZ1735
Leucanopsis longa|[4182]|LNC022-05|05-NCCC-022|United States|North Carolina|658[0n]|BOLD:ABZ1735
Leucanopsis longa|[4183]|LSEU002-06|06-JKA-0002|United States|Georgia|601[0n]|BOLD:ABZ1735
Leucanopsis longa|[4184]|LOFLA336-06|06-FLOR-0336|United States|Florida|658[0n]|BOLD:ABZ1735
Leucanopsis longa|[4185]|USLEP1053-10|10BBLEP-01053|United States|Florida|658[0n]|BOLD:ABZ1735
Leucanopsis longa|[4186]|USLEP1055-10|10BBLEP-01055|United States|Florida|658[0n]|BOLD:ABZ1735
Leucanopsis longa|[4187]|BBLOB626-11|BIOUG01398-E08|United States|Florida|658[0n]|BOLD:ABZ1735
Leucanopsis longa|[4188]|BBLOC199-11|BIOUG01454-B02|United States|Florida|658[0n]|BOLD:ABZ1735
Leucanopsis longa|[4189]|BBLOB1530-11|BIOUG01420-A10|United States|Florida|658[0n]|BOLD:ABZ1735
Leucanopsis longa|[4190]|BBLOB1533-11|BIOUG01420-B01|United States|Florida|658[0n]|BOLD:ABZ1735
Leucanopsis longa|[4191]|BBLOB1538-11|BIOUG01420-B06|United States|Florida|658[0n]|BOLD:ABZ1735
Leucanopsis longa|[4192]|BBLOB1705-11|BIOUG01421-H06|United States|Florida|658[0n]|BOLD:ABZ1735
Leucanopsis longa|[4193]|BBLOB1276-11|BIOUG01417-D05|United States|Florida|658[0n]|BOLD:ABZ1735
Leucanopsis longa|[4194]|BBLOB233-11|BIOUG01369-D07|United States|Florida|658[0n]|BOLD:ABZ1735
Leucanopsis longa|[4195]|BBLOB1309-11|BIOUG01417-G02|United States|Florida|658[0n]|BOLD:ABZ1735
Leucanopsis longa|[4196]|BBLOB448-11|BIOUG01396-F08|United States|Florida|658[0n]|BOLD:ABZ1735
Leucanopsis longa|[4197]|USLEP1054-10|10BBLEP-01054|United States|Florida|658[0n]|BOLD:ABZ1735
Leucanopsis longa|[4198]|LOFLA333-06|06-FLOR-0333|United States|Florida|658[0n]|BOLD:ABZ1735
Leucanopsis longa|[4199]|LOFLC350-06|06-FLOR-2230|United States|Florida|658[0n]|BOLD:ABZ1735
Leucanopsis longa|[4200]|LOFLC292-06|06-FLOR-2172|United States|Florida|658[0n]|BOLD:ABZ1735
Leucanopsis longa|[4201]|LOFLC127-06|06-FLOR-2007|United States|Florida|655[0n]|BOLD:ABZ1735
Leucanopsis longa|[4202]|LOFLC091-06|06-FLOR-1971|United States|Florida|658[0n]|BOLD:ABZ1735
Leucanopsis longa|[4203]|LOFLC295-06|06-FLOR-2175|United States|Florida|632[0n]|BOLD:ABZ1735
Carales arizonensis|[4204]|CMAZA542-10|CMAZ-0542|United States|Arizona|658[0n]|BOLD:ACF0040
Carales arizonensis|[4205]|DMAZ018-09|DMTRN-0112|United States|Arizona|658[0n]|BOLD:ACF0040
Carales arizonensis|[4206]|RDNME453-08|LEP037877|United States|Arizona|658[0n]|BOLD:ACF0040
Carales arizonensis|[4207]|AWCL087-09|JLB-0286|United States|Arizona|658[0n]|BOLD:ACF0040
Carales arizonensis|[4208]|RDNME555-08|LEP037979|United States|Arizona|658[0n]|BOLD:ACF0040
Carales arizonensis|[4209]|AWCLB009-10|AWC-01401|United States|Arizona|658[0n]|BOLD:ACF0040
Carales arizonensis|[4210]|AWCLB008-10|AWC-01402|United States|Arizona|658[0n]|BOLD:ACF0040
Lophocampa annulosa|[4211]|HKONB249-09|3746-COI-08|United States|Texas|658[0n]|BOLD:ACE9243
Lophocampa roseata|[4212]|RDNMG077-08|NOC15018|United States|Arizona|658[0n]|BOLD:ABZ3222
Lophocampa roseata|[4213]|TML123-14|CCDB-17965-A02|United States||658[0n]|BOLD:ABZ3222
Lophocampa roseata|[4214]|RDNMF921-08|CNC LEP00054188|United States|Arizona|609[0n]|BOLD:ABZ3222
Lophocampa roseata|[4215]|LHLEP305-06|UBC-2006-1475|Canada|British Columbia|658[0n]|BOLD:AAC3308
Lophocampa roseata|[4216]|LHLEP304-06|UBC-2006-1221|Canada|British Columbia|657[0n]|BOLD:AAC3308
Lophocampa roseata|[4217]|LHLEP302-06|UBC-2006-1219|Canada|British Columbia|657[0n]|BOLD:AAC3308
Lophocampa roseata|[4218]|LHLEP300-06|UBC-2006-1217|Canada|British Columbia|657[0n]|BOLD:AAC3308
Lophocampa roseata|[4219]|LHLEP303-06|UBC-2006-1220|Canada|British Columbia|657[0n]|BOLD:AAC3308
Lophocampa roseata|[4220]|LHLEP301-06|UBC-2006-1218|Canada|British Columbia|657[0n]|BOLD:AAC3308
Lophocampa roseata|[4221]|LBCS693-07|UBC-2007-0398|Canada|British Columbia|658[0n]|BOLD:AAC3308
Lophocampa roseata|[4222]|LBCS692-07|UBC-2007-0397|Canada|British Columbia|658[0n]|BOLD:AAC3308
Lophocampa roseata|[4223]|LBCS259-07|UBC-2007-0767|Canada|British Columbia|658[0n]|BOLD:AAC3308
Lophocampa roseata|[4224]|LALPA219-10|AVBC 220-10|Canada|British Columbia|658[0n]|BOLD:AAC3308
Lophocampa roseata|[4225]|BBLCU388-09|09BBLEP-04875|United States|Kansas|658[0n]|BOLD:AAC3308
Lophocampa roseata|[4226]|RDNME648-08|LEP038072|United States|Colorado|658[0n]|BOLD:AAC3308
Lophocampa mixta|[4227]|CMAZA889-12|BIOUG02040-D04|United States|Arizona|658[0n]|BOLD:ABZ4590
Lophocampa mixta|[4228]|RDNME457-08|LEP037881|United States|Arizona|658[0n]|BOLD:ABZ4590
Lophocampa mixta|[4229]|IAWLB610-11|IAWAZ-1043|United States|Arizona|658[0n]|BOLD:ABZ4590
Lophocampa mixta|[4230]|CMAZA510-10|CMAZ-0510|United States|Arizona|658[0n]|BOLD:ABZ4590
Lophocampa mixta|[4231]|JBAZ137-09|JLB-0137|United States|Arizona|658[0n]|BOLD:ABZ4590
Lophocampa mixta|[4232]|IAWLB612-11|IAWAZ-1045|United States|Arizona|658[0n]|BOLD:ABZ4590
Lophocampa mixta|[4233]|IAWLB611-11|IAWAZ-1044|United States|Arizona|658[0n]|BOLD:ABZ4590
Lophocampa mixta|[4234]|CMAZA1057-12|BIOUG02042-C04|United States|Arizona|658[0n]|BOLD:ABZ4590
Lophocampa mixta|[4235]|ARCTA099-07|MILA 0099|United States|Arizona|658[0n]|BOLD:ABZ4590
Lophocampa indistincta|[4236]|CNCLB1029-14|CNCLEP00113437|United States|California|658[0n]|BOLD ...
Lophocampa indistincta|[4237]|CNCLB768-14|CNCLEP00031832|United States|California|658[0n]|BOLD ...
Lophocampa bicolor|[4238]|ARCTA361-07|BEVI0079|Mexico||658[0n]|BOLD:AAB3918
Lophocampa caryae|[4239]|CNSLT006-13|BIOUG06036-C06|Canada|Ontario|516[0n]|BOLD:AAB3918
Lophocampa caryae|[4240]|GMOEF016-15|BIOUG22651-G08|Canada|Ontario|528[1n]|BOLD:AAB3918
Lophocampa caryae|[4241]|GMOEF018-15|BIOUG22651-G10|Canada|Ontario|516[0n]|BOLD:AAB3918
Lophocampa caryae|[4242]|PHNXG036-13|BIOUG09267-C12|Canada|Ontario|540[0n]|BOLD:AAB3918
Lophocampa caryae|[4243]|GMOEF001-15|BIOUG22651-F05|Canada|Ontario|540[0n]|BOLD:AAB3918
Lophocampa caryae|[4244]|GMOEF019-15|BIOUG22651-G11|Canada|Ontario|555[1n]|BOLD:AAB3918
Lophocampa caryae|[4245]|GMOEF003-15|BIOUG22651-F07|Canada|Ontario|564[0n]|BOLD:AAB3918
Lophocampa caryae|[4246]|GMOEF390-15|BIOUG24354-A03|Canada|Ontario|576[0n]|BOLD:AAB3918
Lophocampa caryae|[4247]|GMOEF391-15|BIOUG24354-A04|Canada|Ontario|534[1n]|BOLD:AAB3918
Lophocampa caryae|[4248]|GMOEF006-15|BIOUG22651-F10|Canada|Ontario|552[0n]|BOLD:AAB3918
Lophocampa caryae|[4249]|GMOEF010-15|BIOUG22651-G02|Canada|Ontario|552[0n]|BOLD:AAB3918
Lophocampa caryae|[4250]|GMOEF017-15|BIOUG22651-G09|Canada|Ontario|552[0n]|BOLD:AAB3918
Lophocampa caryae|[4251]|PHNGT816-13|BIOUG08321-E08|Canada|Ontario|547[0n]|BOLD:AAB3918
Lophocampa caryae|[4252]|GMOOG001-15|BIOUG22651-C09|Canada|Ontario|543[0n]|BOLD:AAB3918
Lophocampa caryae|[4253]|GMOEF008-15|BIOUG22651-F12|Canada|Ontario|555[0n]|BOLD:AAB3918
Lophocampa caryae|[4254]|GMOEF009-15|BIOUG22651-G01|Canada|Ontario|564[0n]|BOLD:AAB3918
Lophocampa caryae|[4255]|PHNXG107-13|BIOUG09268-A12|Canada|Ontario|576[0n]|BOLD:AAB3918
Lophocampa caryae|[4256]|SMTPJ1607-14|BIOUG16126-H11|Canada|Ontario|564[0n]|BOLD:AAB3918
Lophocampa caryae|[4257]|PHNGT811-13|BIOUG08321-E03|Canada|Ontario|553[0n]|BOLD:AAB3918
Lophocampa caryae|[4258]|LPSO879-08|PPBP-0879|Canada|Ontario|658[0n]|BOLD:AAB3918
Lophocampa caryae|[4259]|LPSO852-08|PPBP-0852|Canada|Ontario|647[0n]|BOLD:AAB3918
Lophocampa caryae|[4260]|GMOOG003-15|BIOUG22651-C11|Canada|Ontario|609[1n]|BOLD:AAB3918
Lophocampa caryae|[4261]|GMOEF022-15|BIOUG22651-H02|Canada|Ontario|555[0n]|BOLD:AAB3918
Lophocampa caryae|[4262]|GMOEF013-15|BIOUG22651-G05|Canada|Ontario|567[0n]|BOLD:AAB3918
Lophocampa caryae|[4263]|PHNGT812-13|BIOUG08321-E04|Canada|Ontario|568[0n]|BOLD:AAB3918
Lophocampa caryae|[4264]|GMOEF392-15|BIOUG24354-A05|Canada|Ontario|567[0n]|BOLD:AAB3918
Lophocampa caryae|[4265]|GMOEF394-15|BIOUG24354-A07|Canada|Ontario|576[0n]|BOLD:AAB3918
Lophocampa caryae|[4266]|GMOEF004-15|BIOUG22651-F08|Canada|Ontario|576[0n]|BOLD:AAB3918
Lophocampa caryae|[4267]|GMOEF393-15|BIOUG24354-A06|Canada|Ontario|576[0n]|BOLD:AAB3918
Lophocampa caryae|[4268]|SMTPO2574-15|BIOUG25463-A05|Canada|Ontario|591[0n]|BOLD:AAB3918
Lophocampa caryae|[4269]|GMOEF002-15|BIOUG22651-F06|Canada|Ontario|567[0n]|BOLD:AAB3918
Lophocampa caryae|[4270]|GMOEF014-15|BIOUG22651-G06|Canada|Ontario|609[0n]|BOLD:AAB3918
Lophocampa caryae|[4271]|GMOEF020-15|BIOUG22651-G12|Canada|Ontario|609[0n]|BOLD:AAB3918
Lophocampa caryae|[4272]|PHNXG033-13|BIOUG09267-C09|Canada|Ontario|603[0n]|BOLD:AAB3918
Lophocampa caryae|[4273]|PHNXG111-13|BIOUG09268-B04|Canada|Ontario|603[0n]|BOLD:AAB3918
Lophocampa caryae|[4274]|GMOEF007-15|BIOUG22651-F11|Canada|Ontario|588[0n]|BOLD:AAB3918
Lophocampa caryae|[4275]|PHNXG106-13|BIOUG09268-A11|Canada|Ontario|600[1n]|BOLD:AAB3918
Lophocampa caryae|[4276]|LPSO853-08|PPBP-0853|Canada|Ontario|609[0n]|BOLD:AAB3918
Lophocampa caryae|[4277]|LGSME009-06|DNA-ATBI-5009|United States|Tennessee|603[0n]|BOLD:AAB3918
Lophocampa caryae|[4278]|CNSLG082-12|BIOUG03817-F03|Canada|Ontario|634[0n]|BOLD:AAB3918
Lophocampa caryae|[4279]|PHNXN054-14|BIOUG10725-E06|Canada|Ontario|639[0n]|BOLD:AAB3918
Lophocampa caryae|[4280]|PHNXN057-14|BIOUG10725-E09|Canada|Ontario|636[0n]|BOLD:AAB3918
Lophocampa caryae|[4281]|PHNXN058-14|BIOUG10725-E10|Canada|Ontario|636[0n]|BOLD:AAB3918
Lophocampa caryae|[4282]|CNSLH029-12|BIOUG03817-G03|Canada|Ontario|637[0n]|BOLD:AAB3918

Lophocampa caryae|[4280]|PHNXN057-14|BIOUG10725-E09|Canada|Ontario|636[0n]|BOLD:AAB3918
Lophocampa caryae|[4281]|PHNXN058-14|BIOUG10725-E10|Canada|Ontario|636[0n]|BOLD:AAB3918
Lophocampa caryae|[4282]|CNSLH029-12|BIOUG03817-G03|Canada|Ontario|637[0n]|BOLD:AAB3918
Lophocampa caryae|[4283]|PHNXN059-14|BIOUG10725-E11|Canada|Ontario|640[0n]|BOLD:AAB3918
Lophocampa caryae|[4284]|TMNBD164-07|MNBTT-2965|Canada|New Brunswick|658[0n]|BOLD:AAB3918
Lophocampa caryae|[4285]|MMNA120-08|HLC-17682|United States|North Carolina|658[0n]|BOLD:AAB3918
Lophocampa caryae|[4286]|MMNA119-08|HLC-17681|United States|North Carolina|658[0n]|BOLD:AAB3918
Lophocampa caryae|[4287]|LPSO880-08|PPBP-0880|Canada|Ontario|658[0n]|BOLD:AAB3918
Lophocampa caryae|[4288]|LPSO854-08|PPBP-0854|Canada|Ontario|658[0n]|BOLD:AAB3918
Lophocampa caryae|[4289]|RDNME411-08|LEP037835|Canada|Ontario|658[0n]|BOLD:AAB3918
Lophocampa caryae|[4290]|TMNBD166-07|MNBTT-2967|Canada|New Brunswick|658[0n]|BOLD:AAB3918
Lophocampa caryae|[4291]|TMNBD165-07|MNBTT-2966|Canada|New Brunswick|658[0n]|BOLD:AAB3918
Lophocampa caryae|[4292]|LPSO640-08|PPBP-0640|Canada|Ontario|658[0n]|BOLD:AAB3918
Lophocampa caryae|[4293]|GMFRY042-15|BIOUG21136-F08|United States|Virginia|632[0n]|BOLD:AAB3918
Lophocampa caryae|[4294]|GMOAG002-15|BIOUG22651-D02|Canada|Ontario|552[0n]|BOLD:AAB3918
Lophocampa caryae|[4295]|GMOOG002-15|BIOUG22651-C10|Canada|Ontario|540[0n]|BOLD:AAB3918
Lophocampa caryae|[4296]|GMOEF388-15|BIOUG24354-A01|Canada|Ontario|540[1n]|BOLD:AAB3918
Lophocampa caryae|[4297]|GMOEF012-15|BIOUG22651-G04|Canada|Ontario|555[0n]|BOLD:AAB3918
Lophocampa caryae|[4298]|GMOEF389-15|BIOUG24354-A02|Canada|Ontario|564[0n]|BOLD:AAB3918
Lophocampa caryae|[4299]|GMOEF021-15|BIOUG22651-H01|Canada|Ontario|564[0n]|BOLD:AAB3918
Lophocampa caryae|[4300]|GMOEF015-15|BIOUG22651-G07|Canada|Ontario|561[0n]|BOLD:AAB3918
Lophocampa caryae|[4301]|CNKJQ1194-14|BIOUG12435-H08|Canada|Nova Scotia|562[0n]|BOLD:AAB3918
Lophocampa caryae|[4302]|GMOEF005-15|BIOUG22651-F09|Canada|Ontario|585[0n]|BOLD:AAB3918
Lophocampa caryae|[4303]|GMOEF011-15|BIOUG22651-G03|Canada|Ontario|579[0n]|BOLD:AAB3918
Lophocampa caryae|[4304]|LPSO639-08|PPBP-0639|Canada|Ontario|658[0n]|BOLD:AAB3918
Lophocampa caryae|[4305]|ARCTD162-11|BEVI1467|United States|Colorado|648[0n]|BOLD:AAB3918
Lophocampa caryae|[4306]|TMNBD163-07|MNBTT-2964|Canada|New Brunswick|658[0n]|BOLD:AAB3918
Lophocampa caryae|[4307]|LPSOB511-08|PPBP-1510|Canada|Ontario|658[0n]|BOLD:AAB3918
Lophocampa caryae|[4308]|TMNBD167-07|MNBTT-2968|Canada|New Brunswick|658[0n]|BOLD:AAB3918
Lophocampa caryae|[4309]|QUNOC091-09|5922-010708-KY|United States|Kentucky|658[0n]|BOLD:AAB3918
Lophocampa caryae|[4310]|RDNME456-08|LEP037880|Canada|Ontario|658[0n]|BOLD:AAB3918
Lophocampa caryae|[4311]|SMTPI7737-14|BIOUG16072-B09|Canada|Ontario|591[0n]|BOLD:AAB3918
Lophocampa caryae|[4312]|SMTPI7735-14|BIOUG16072-B07|Canada|Ontario|558[0n]|BOLD:AAB3918
Lophocampa ingens|[4313]|ARCTA076-07|MILA 0076|United States|Arizona|658[0n]|BOLD:AAJ2273
Lophocampa ingens|[4314]|ARCTA480-07|BEVI0198|Mexico|Nuevo Leon|658[0n]|BOLD:AAJ2273
Lophocampa argentata|[4315]|RDNME203-07|CNCNoctuoidea13810|United States|Arizona|557[0n]|BOLD:...
Lophocampa argentata|[4316]|RDNME413-08|LEP037837|United States|Colorado|658[0n]|BOLD:ACE6855
Lophocampa argentata|[4317]|ARCTA071-07|MILA 0071|United States|Arizona|658[0n]|BOLD:ACE6855
Lophocampa sobrina|[4318]|RDNME417-08|LEP037841|United States|California|658[0n]|BOLD:AAB4184
Lophocampa sobrina|[4319]|RDNME418-08|LEP037842|United States|California|658[0n]|BOLD:AAB4184
Lophocampa argentata|[4320]|LALPA375-10|AVBC 377-10|Canada|British Columbia|658[0n]|BOLD:AAB4184
Lophocampa argentata|[4321]|LALPA385-10|AVBC 387-10|Canada|British Columbia|658[0n]|BOLD:AAB4184
Lophocampa argentata|[4322]|RDNME408-08|LEP037832|Canada|British Columbia|658[0n]|BOLD:AAB4184
Lophocampa argentata|[4323]|RDNME409-08|LEP037833|Canada|British Columbia|658[0n]|BOLD:AAB4184
Lophocampa argentata|[4324]|RDNME410-08|LEP037834|United States|Oregon|658[0n]|BOLD:AAB4184
Lophocampa argentata|[4325]|RWWC658-11|RWWA-2635|United States|Washington|658[0n]|BOLD:AAB4184
Lophocampa argentata|[4326]|DUNLP005-08|Dun-08-005|Canada|British Columbia|658[0n]|BOLD:AAB4184
Lophocampa argentata|[4327]|DUNLP006-08|Dun-08-006|Canada|British Columbia|658[0n]|BOLD:AAB4184
Lophocampa argentata|[4328]|RWWA715-09|RWWA-0751|United States|Washington|658[0n]|BOLD:AAB4184
Lophocampa argentata|[4329]|RWWA743-09|RWWA-0779|United States|Washington|658[0n]|BOLD:AAB4184
Lophocampa argentata|[4330]|LBCS257-07|UBC-2007-0765|Canada|British Columbia|658[0n]|BOLD:AAB4184
Lophocampa argentata|[4331]|LBCS258-07|UBC-2007-0766|Canada|British Columbia|658[0n]|BOLD:AAB4184
Lophocampa argentata|[4332]|RWWC543-11|RWWA-2520|United States|Washington|658[0n]|BOLD:AAB4184
Lophocampa argentata|[4333]|RWWA945-09|RWWA-0945|United States|Washington|658[0n]|BOLD:AAB4184
Lophocampa argentata|[4334]|RWWA731-09|RWWA-0767|United States|Washington|658[0n]|BOLD:AAB4184
Lophocampa argentata|[4335]|LHLEP297-06|UBC-2006-0732|Canada|British Columbia|657[0n]|BOLD:AAB...
Lophocampa argentata|[4336]|LHLEP298-06|UBC-2006-1055|Canada|British Columbia|657[0n]|BOLD:AAB...
Lophocampa argentata|[4337]|LHLEP299-06|UBC-2006-1651|Canada|British Columbia|657[0n]|BOLD:AAB...
Lophocampa argentata|[4338]|RDNME202-07|CNCNoctuoidea13809|Canada|British Columbia|612[0n]|BOLD ...
Lophocampa argentata|[4339]|LALPA374-10|AVBC 376-10|Canada|British Columbia|632[0n]|BOLD:AAB4184
Lophocampa argentata|[4340]|RDNME787-08|LEP041252|United States|California|658[0n]|BOLD:AAB4184
Lophocampa argentata|[4341]|JMMMB179-11|BIOUG00848-G12|United States|California|658[0n]|BOLD:A...
Lophocampa argentata|[4342]|ARCTA072-07|MILA 0072|Canada|British Columbia|658[0n]|BOLD:AAB4184
Lophocampa argentata|[4343]|LBCW031-08|08-JDWWI-0031|Canada|British Columbia|658[0n]|BOLD:AAB4184
Lophocampa argentata|[4344]|LBCW030-08|08-JDWWI-0030|Canada|British Columbia|658[0n]|BOLD:AAB4184
Lophocampa argentata|[4345]|LMH035-06|PFC-2006-0448|Canada|British Columbia|658[0n]|BOLD:AAB4184
Lophocampa pura|[4346]|RDNME545-08|LEP037969|United States|Arizona|658[0n]|BOLD:ABZ1706
Lophocampa pura|[4347]|CMAZA354-10|CMAZ-0354|United States|Arizona|658[0n]|BOLD:ABZ1706
Lophocampa pura|[4348]|IAWLB609-11|IAWAZ-1042|United States|Arizona|658[0n]|BOLD:ABZ1706
Lophocampa pura|[4349]|IAWLB608-11|IAWAZ-1041|United States|Arizona|658[0n]|BOLD:ABZ1706
Lophocampa pura|[4350]|IAWLB607-11|IAWAZ-1040|United States|Arizona|658[0n]|BOLD:ABZ1706
Lophocampa pura|[4351]|IAWLB606-11|IAWAZ-1039|United States|Arizona|658[0n]|BOLD:ABZ1706
Lophocampa pura|[4352]|RDNME546-08|LEP037970|United States|Arizona|658[0n]|BOLD:ABZ1706
Lophocampa pura|[4353]|RDNME452-08|LEP037876|United States|Arizona|658[0n]|BOLD:ABZ1706
Lophocampa pura|[4354]|CMAZA1016-12|BIOUG02041-G10|United States|Arizona|658[0n]|BOLD:ABZ1706
Lophocampa pura|[4355]|CMAZA869-12|BIOUG02040-B08|United States|Arizona|658[0n]|BOLD:ABZ1706
Lophocampa maculata|[4356]|SSEIA7713-13|BIOUG04535-E06|Canada|Alberta|565[3n]|BOLD:AAA3071
Lophocampa maculata|[4357]|CNGIJ628-13|BIOUG06863-A08|Canada|British Columbia|609[0n]|BOLD:AAA...
Lophocampa maculata|[4358]|CNEIH1581-13|BIOUG04567-A07|Canada|Alberta|581[0n]|BOLD:AAA3071
Lophocampa maculata|[4359]|LPSOB427-08|PPBP-1426|Canada|Ontario|609[0n]|BOLD:AAA3071
Lophocampa maculata|[4360]|LPSOC286-08|PPBP-2285|Canada|Ontario|655[0n]|BOLD:AAA3071
Lophocampa maculata|[4361]|LPSOD420-09|08BBLEP-00199|Canada|Ontario|658[0n]|BOLD:AAA3071
Lophocampa maculata|[4362]|LPSOD417-09|08BBLEP-00196|Canada|Ontario|658[0n]|BOLD:AAA3071
Lophocampa maculata|[4363]|LPSOD857-09|08BBLEP-00639|Canada|Ontario|658[0n]|BOLD:AAA3071
Lophocampa maculata|[4364]|LPSOD658-09|08BBLEP-00439|Canada|Ontario|658[0n]|BOLD:AAA3071
Lophocampa maculata|[4365]|LPSOD637-09|08BBLEP-00418|Canada|Ontario|658[0n]|BOLD:AAA3071
Lophocampa maculata|[4366]|LPSOD574-09|08BBLEP-00355|Canada|Ontario|658[0n]|BOLD:AAA3071
Lophocampa maculata|[4367]|BBLPC894-09|09BBELE-1894|Canada|Newfoundland and Labrador|658[0n]|BOLD...
Lophocampa maculata|[4368]|LPMN709-08|08BBLEP-01512|Canada|Manitoba|658[0n]|BOLD:AAA3071
Lophocampa maculata|[4369]|LPMN295-08|08BBLEP-01094|Canada|Manitoba|658[0n]|BOLD:AAA3071
Lophocampa maculata|[4370]|LPMN212-08|08BBLEP-01011|Canada|Manitoba|658[0n]|BOLD:AAA3071
Lophocampa maculata|[4371]|PHMNB595-04|04HBL00821|Canada|New Brunswick|658[0n]|BOLD:AAA3071
Lophocampa maculata|[4372]|PHMNB455-04|04HBL00681|Canada|New Brunswick|658[0n]|BOLD:AAA3071
Lophocampa maculata|[4373]|LPSOD330-09|08BBLEP-00108|Canada|Ontario|658[0n]|BOLD:AAA3071
Lophocampa maculata|[4374]|BBLPE374-09|09BBELE-2374|Canada|Newfoundland and Labrador|658[0n]|BOLD...
Lophocampa maculata|[4375]|LPMN298-08|08BBLEP-01097|Canada|Manitoba|658[0n]|BOLD:AAA3071
Lophocampa maculata|[4376]|LPMN296-08|08BBLEP-01095|Canada|Manitoba|658[0n]|BOLD:AAA3071
Lophocampa maculata|[4377]|LPMN211-08|08BBLEP-01010|Canada|Manitoba|658[0n]|BOLD:AAA3071
Lophocampa maculata|[4378]|BBLEC371-09|09BBELE-0371|Canada|Newfoundland and Labrador|658[0n]|BOLD...
Lophocampa maculata|[4379]|PHMNB448-04|04HBL00674|Canada|New Brunswick|658[0n]|BOLD:AAA3071
Lophocampa maculata|[4380]|PHMNB447-04|04HBL00673|Canada|New Brunswick|658[0n]|BOLD:AAA3071
Lophocampa maculata|[4381]|PHMNB356-04|04HBL00582|Canada|New Brunswick|658[0n]|BOLD:AAA3071
Lophocampa maculata|[4382]|PHMNB324-04|04HBL00550|Canada|New Brunswick|658[0n]|BOLD:AAA3071

[illegible]
Lophocampa maculata|[4380]|PHMNB447-04|04HBL00673|Canada|New Brunswick|658[0n]|BOLD:AAA3071
Lophocampa maculata|[4381]|PHMNB356-04|04HBL00582|Canada|New Brunswick|658[0n]|BOLD:AAA3071
Lophocampa maculata|[4382]|PHMNB324-04|04HBL00550|Canada|New Brunswick|658[0n]|BOLD:AAA3071
Lophocampa maculata|[4383]|PHMNB450-04|04HBL00676|Canada|New Brunswick|658[0n]|BOLD:AAA3071
Lophocampa maculata|[4384]|PHMNB449-04|04HBL00675|Canada|New Brunswick|658[0n]|BOLD:AAA3071
Lophocampa maculata|[4385]|LPMN301-08|08BBLEP-01100|Canada|Manitoba|658[0n]|BOLD:AAA3071
Lophocampa maculata|[4386]|LPMN299-08|08BBLEP-01098|Canada|Manitoba|658[0n]|BOLD:AAA3071
Lophocampa maculata|[4387]|BBLPC966-09|09BBELE-1966|Canada|Newfoundland and Labrador|658[0n]|BOLD...
Lophocampa maculata|[4388]|LPSOD421-09|08BBLEP-00200|Canada|Ontario|658[0n]|BOLD:AAA3071
Lophocampa maculata|[4389]|BBLPA351-10|10BBCLP-0351|Canada|Ontario|658[0n]|BOLD:AAA3071
Lophocampa maculata|[4390]|TMNBD417-07|MNBTT-3218|Canada|New Brunswick|658[0n]|BOLD:AAA3071
Lophocampa maculata|[4391]|TMNBD429-07|MNBTT-3230|Canada|New Brunswick|658[0n]|BOLD:AAA3071
Lophocampa maculata|[4392]|BBLPA349-10|10BBCLP-0349|Canada|Saskatchewan|658[0n]|BOLD:AAA3071
Lophocampa maculata|[4393]|LPABB319-08|08BBLEP-03584|Canada|Alberta|658[0n]|BOLD:AAA3071
Lophocampa maculata|[4394]|LPABB328-08|08BBLEP-03593|Canada|Alberta|658[0n]|BOLD:AAA3071
Lophocampa maculata|[4395]|LPSOC168-08|PPBP-2167|Canada|Ontario|658[0n]|BOLD:AAA3071
Lophocampa maculata|[4396]|TMNBB033-06|MNBTT-973|Canada|New Brunswick|658[0n]|BOLD:AAA3071
Lophocampa maculata|[4397]|TMNBB034-06|MNBTT-974|Canada|New Brunswick|658[0n]|BOLD:AAA3071
Lophocampa maculata|[4398]|LPSOB314-08|PPBP-1313|Canada|Ontario|658[0n]|BOLD:AAA3071
Lophocampa maculata|[4399]|TTMNB057-06|MNBTT-057|Canada|New Brunswick|657[0n]|BOLD:AAA3071
Lophocampa maculata|[4400]|TTMNB060-06|MNBTT-060|Canada|New Brunswick|658[0n]|BOLD:AAA3071
Lophocampa maculata|[4401]|XAB360-04|04HBL005360|Canada|Ontario|658[0n]|BOLD:AAA3071
Lophocampa maculata|[4402]|LPSOB249-08|PPBP-1248|Canada|Ontario|658[0n]|BOLD:AAA3071
Lophocampa maculata|[4403]|BBLPE361-09|09BBELE-2361|Canada|Newfoundland and Labrador|639[0n]|BOLD...
Lophocampa maculata|[4404]|PHMNB423-04|04HBL00649|Canada|New Brunswick|658[1n]|BOLD:AAA3071
Lophocampa maculata|[4405]|BBLEC275-09|09BBELE-0275|Canada|Nova Scotia|658[0n]|BOLD:AAA3071
Lophocampa maculata|[4406]|LBCS124-07|UBC-2007-0115|Canada|British Columbia|658[0n]|BOLD:AAA3071
Lophocampa maculata|[4407]|MMNA117-08|HLC-17679|United States|North Carolina|655[0n]|BOLD:AAA3071
Lophocampa maculata|[4408]|MMNA116-08|HLC-17678|United States|North Carolina|655[0n]|BOLD:AAA3071
Lophocampa maculata|[4409]|GMLC670-11|2011GM-0366|United States|California|658[0n]|BOLD:AAA3071
Lophocampa maculata|[4410]|LPSOD643-09|08BBLEP-00424|Canada|Ontario|634[0n]|BOLD:AAA3071
Lophocampa maculata|[4411]|RWWA068-09|RWWA-0068|United States|Washington|632[0n]|BOLD:AAA3071
Lophocampa maculata|[4412]|LBCA345-05|HLC-20345|Canada|British Columbia|658[0n]|BOLD:AAA3071
Lophocampa maculata|[4413]|LMH026-06|PFC-2006-0147|Canada|British Columbia|658[0n]|BOLD:AAA3071
Lophocampa maculata|[4414]|AWCLB030-10|AWC-01490|United States|California|658[0n]|BOLD:AAA3071
Lophocampa maculata|[4415]|AWCLB031-10|AWC-01489|United States|California|658[0n]|BOLD:AAA3071
Lophocampa maculata|[4416]|AWCLB032-10|AWC-00859|United States|Idaho|658[0n]|BOLD:AAA3071
Lophocampa maculata|[4417]|LBCA740-05|HLC-20740|Canada|British Columbia|658[0n]|BOLD:AAA3071
Lophocampa maculata|[4418]|LBCA779-05|HLC-20779|Canada|British Columbia|658[0n]|BOLD:AAA3071
Lophocampa maculata|[4419]|LPSOD419-09|08BBLEP-00198|Canada|Ontario|658[0n]|BOLD:AAA3071
Lophocampa maculata|[4420]|RDNME394-08|LEP037818|United States|California|658[0n]|BOLD:AAA3071
Lophocampa maculata|[4421]|RDMAB922-06|BCSC395|Canada|Alberta|658[0n]|BOLD:AAA3071
Lophocampa maculata|[4422]|LALPA146-10|AVBC 146-10|Canada|British Columbia|658[0n]|BOLD:AAA3071
Lophocampa maculata|[4423]|LPSOD422-09|08BBLEP-00201|Canada|Ontario|658[0n]|BOLD:AAA3071
Lophocampa maculata|[4424]|LPSOD423-09|08BBLEP-00202|Canada|Ontario|658[0n]|BOLD:AAA3071
Lophocampa maculata|[4425]|LOPN065-06|JCM-OSU-0065|United States|Oregon|658[0n]|BOLD:AAA3071
Lophocampa maculata|[4426]|LOPN066-06|JCM-OSU-0066|United States|Oregon|658[0n]|BOLD:AAA3071
Lophocampa maculata|[4427]|LPSOD573-09|08BBLEP-00354|Canada|Ontario|658[0n]|BOLD:AAA3071
Lophocampa maculata|[4428]|LPSOD873-09|08BBLEP-00655|Canada|Ontario|658[0n]|BOLD:AAA3071
Lophocampa maculata|[4429]|MMNA118-08|HLC-17680|United States|North Carolina|658[0n]|BOLD:AAA3071
Lophocampa maculata|[4430]|LOWCB667-05|CGWC-1607|Canada|British Columbia|658[0n]|BOLD:AAA3071
Lophocampa maculata|[4431]|LGSMG121-07|BGS03442|United States|North Carolina|658[0n]|BOLD:AAA3071
Lophocampa maculata|[4432]|BBLPA347-10|10BBCLP-0347|Canada|Ontario|658[0n]|BOLD:AAA3071
Lophocampa maculata|[4433]|BBLPA348-10|10BBCLP-0348|Canada|British Columbia|658[0n]|BOLD:AAA3071
Lophocampa maculata|[4434]|LBCS123-07|UBC-2007-0114|Canada|British Columbia|658[0n]|BOLD:AAA3071
Lophocampa maculata|[4435]|LBCS125-07|UBC-2007-0116|Canada|British Columbia|658[0n]|BOLD:AAA3071
Lophocampa maculata|[4436]|RWWA723-09|RWWA-0759|United States|Washington|658[0n]|BOLD:AAA3071
Lophocampa maculata|[4437]|RWWA072-09|RWWA-0072|United States|Washington|658[0n]|BOLD:AAA3071
Lophocampa maculata|[4438]|RWWA080-09|RWWA-0080|United States|Washington|658[0n]|BOLD:AAA3071
Lophocampa maculata|[4439]|LOWCE113-06|CGWC-3873|Canada|British Columbia|658[0n]|BOLD:AAA3071
Lophocampa maculata|[4440]|LOWCE117-06|CGWC-3877|Canada|British Columbia|658[0n]|BOLD:AAA3071
Lophocampa maculata|[4441]|LBCS440-07|UBC-2007-0196|Canada|British Columbia|658[0n]|BOLD:AAA3071
Lophocampa maculata|[4442]|LBCS663-07|UBC-2007-0368|Canada|British Columbia|658[0n]|BOLD:AAA3071
Lophocampa maculata|[4443]|LBCS664-07|UBC-2007-0369|Canada|British Columbia|658[0n]|BOLD:AAA3071
Lophocampa maculata|[4444]|LALPA113-10|AVBC 113-10|Canada|British Columbia|658[0n]|BOLD:AAA3071
Lophocampa maculata|[4445]|RDLQG444-06|DH012728|Canada|Quebec|658[0n]|BOLD:AAA3071
Lophocampa maculata|[4446]|LPSOB248-08|PPBP-1247|Canada|Ontario|658[0n]|BOLD:AAA3071
Lophocampa maculata|[4447]|LPSOD237-09|08BBLEP-00015|Canada|Ontario|658[0n]|BOLD:AAA3071
Lophocampa maculata|[4448]|LPSOB793-08|PPBP-1792|Canada|Ontario|658[0n]|BOLD:AAA3071
Lophocampa maculata|[4449]|XAJ656-06|2006-ONT-0656|Canada|Ontario|658[0n]|BOLD:AAA3071
Lophocampa maculata|[4450]|RWWA783-09|RWWA-0819|United States|Washington|658[0n]|BOLD:AAA3071
Lophocampa maculata|[4451]|BBLOC1357-11|BIOUG01542-A04|United States|California|658[0n]|BOLD:A...
Lophocampa maculata|[4452]|BBLOC203-11|BIOUG01454-B06|United States|Texas|658[0n]|BOLD:AAA3071
Lophocampa maculata|[4453]|LMH004-06|PFC-2006-0006|Canada|British Columbia|658[0n]|BOLD:AAA3071
Lophocampa maculata|[4454]|LOWCE118-06|CGWC-3878|Canada|British Columbia|658[0n]|BOLD:AAA3071
Lophocampa maculata|[4455]|LOWCE115-06|CGWC-3875|Canada|British Columbia|658[0n]|BOLD:AAA3071
Lophocampa maculata|[4456]|LOPN221-06|JCM-OSU-0221|United States|Oregon|606[0n]|BOLD:AAA3071
Lophocampa maculata|[4457]|TMG85-03|moth655.01|Canada|Ontario|639[0n]|BOLD:AAA3071
Lophocampa maculata|[4458]|TMG84-03|moth662.01|Canada|Ontario|639[0n]|BOLD:AAA3071
Lophocampa maculata|[4459]|PMG014-03|moth656.01|Canada|Ontario|617[0n]|BOLD:AAA3071
Lophocampa maculata|[4460]|LBCA346-05|HLC-20346|Canada|British Columbia|658[0n]|BOLD:AAA3071
Lophocampa maculata|[4461]|LOT144-04|04HBL002144|United States|Tennessee|609[0n]|BOLD:AAA3071
Lophocampa maculata|[4462]|XAJ495-06|2006-ONT-0495|Canada|Ontario|656[0n]|BOLD:AAA3071
Lophocampa maculata|[4463]|GMLC503-11|2011GM-0199|United States|California|658[0n]|BOLD:AAA3071
Lophocampa maculata|[4464]|LPABB318-08|08BBLEP-03583|Canada|Alberta|658[0n]|BOLD:AAA3071
Lophocampa maculata|[4465]|LPABB327-08|08BBLEP-03592|Canada|Alberta|658[0n]|BOLD:AAA3071
Lophocampa maculata|[4466]|LBCS121-07|UBC-2007-0112|Canada|British Columbia|658[0n]|BOLD:AAA3071
Lophocampa maculata|[4467]|LBCS122-07|UBC-2007-0113|Canada|British Columbia|658[0n]|BOLD:AAA3071
Lophocampa maculata|[4468]|LOWCE114-06|CGWC-3874|Canada|British Columbia|658[0n]|BOLD:AAA3071
Lophocampa maculata|[4469]|LOWCE116-06|CGWC-3876|Canada|British Columbia|658[0n]|BOLD:AAA3071
Lophocampa maculata|[4470]|LBCS665-07|UBC-2007-0370|Canada|British Columbia|658[0n]|BOLD:AAA3071
Lophocampa maculata|[4471]|LBCS666-07|UBC-2007-0371|Canada|British Columbia|658[0n]|BOLD:AAA3071
Lophocampa maculata|[4472]|LBCS667-07|UBC-2007-0372|Canada|British Columbia|658[0n]|BOLD:AAA3071
Lophocampa maculata|[4473]|XAJ624-06|2006-ONT-0624|Canada|Ontario|658[0n]|BOLD:AAA3071
Lophocampa maculata|[4474]|XAB185-04|04HBL005185|Canada|Ontario|658[0n]|BOLD:AAA3071
Lophocampa maculata|[4475]|LBCA280-05|HLC-20280|Canada|British Columbia|658[0n]|BOLD:AAA3071
Lophocampa maculata|[4476]|GMLC379-11|2011GM-0075|United States|California|644[0n]|BOLD:AAA3071
Lophocampa maculata|[4477]|LPABB020-08|08BBLEP-03285|Canada|Alberta|658[0n]|BOLD:AAA3071
Lophocampa maculata|[4478]|LGSMG122-07|BGS03443|United States|North Carolina|658[0n]|BOLD:AAA3071
Lophocampa maculata|[4479]|LOWCB668-05|CGWC-1608|Canada|British Columbia|658[0n]|BOLD:AAA3071
Lophocampa maculata|[4480]|BBLPA350-10|10BBCLP-0350|Canada|British Columbia|658[0n]|BOLD:AAA3071
Lophocampa maculata|[4481]|JBAZ058-09|JLB-0058|United States|California|658[0n]|BOLD:AAA3071
[illegible]

Lophocampa maculata|[4479]|LOWCB668-05|CGWC-1608|Canada|British Columbia|658[0n]|BOLD:AAA3071
Lophocampa maculata|[4480]|BBLPA350-10|10BBCLP-0350|Canada|British Columbia|658[0n]|BOLD:AAA3071
Lophocampa maculata|[4481]|JBAZ058-09|JLB-0058|United States|California|658[0n]|BOLD:AAA3071
Lophocampa maculata|[4482]|RDNME395-08|LEP037819|United States|California|658[0n]|BOLD:AAA3071
Lophocampa maculata|[4483]|LBCC033-05|HLC-21913|Canada|British Columbia|658[0n]|BOLD:AAA3071
Lophocampa maculata|[4484]|LBCC032-05|HLC-21912|Canada|British Columbia|658[0n]|BOLD:AAA3071
Lophocampa maculata|[4485]|LBCC031-05|HLC-21911|Canada|British Columbia|658[0n]|BOLD:AAA3071
Lophocampa maculata|[4486]|LBCC030-05|HLC-21910|Canada|British Columbia|658[0n]|BOLD:AAA3071
Lophocampa maculata|[4487]|LBCC029-05|HLC-21909|Canada|British Columbia|658[0n]|BOLD:AAA3071
Lophocampa maculata|[4488]|LBCD032-05|HLC-22852|Canada|British Columbia|658[0n]|BOLD:AAA3071
Lophocampa maculata|[4489]|LBCB138-05|HLC-21078|Canada|British Columbia|658[0n]|BOLD:AAA3071
Lophocampa maculata|[4490]|LBCB137-05|HLC-21077|Canada|British Columbia|658[0n]|BOLD:AAA3071
Lophocampa maculata|[4491]|BBLCU311-09|09BBLEP-04798|United States|Michigan|658[0n]|BOLD:AAA3071
Lophocampa maculata|[4492]|JMMMB180-11|BIOUG00848-H01|United States|California|658[0n]|BOLD:AA...
Lophocampa maculata|[4493]|ARCTA066-07|MILA 0066|Canada|British Columbia|658[0n]|BOLD:AAA3071
Lophocampa maculata|[4494]|ARCTA065-07|MILA 0065|United States|Arizona|658[0n]|BOLD:AAA3071
Lophocampa maculata|[4495]|LBCA780-05|HLC-20780|Canada|British Columbia|658[0n]|BOLD:AAA3071
Lophocampa maculata|[4496]|LBCA492-05|HLC-20492|Canada|British Columbia|658[0n]|BOLD:AAA3071
Lophocampa maculata|[4497]|XAE389-04|Moth4389.03|Canada|Ontario|658[0n]|BOLD:AAA3071
Lophocampa maculata|[4498]|XAE185-04|Moth4185.03|Canada|Ontario|658[0n]|BOLD:AAA3071
Lophocampa maculata|[4499]|LBCA344-05|HLC-20344|Canada|British Columbia|658[0n]|BOLD:AAA3071
Lophocampa maculata|[4500]|LBCA343-05|HLC-20343|Canada|British Columbia|658[0n]|BOLD:AAA3071
Aclytia heberDHJ02|[4501]|BLPCI330-08|07-SRNP-112455|Costa Rica|Guanacaste|658[0n]|BOLD:AAA1306
Aclytia heberDHJ02|[4502]|BLPCO422-08|08-SRNP-103899|Costa Rica|Guanacaste|658[0n]|BOLD:AAA1306
Aclytia heberDHJ02|[4503]|MHARB241-05|04-SRNP-15304|Costa Rica|Guanacaste|658[0n]|BOLD:AAA1306
Aclytia heberDHJ02|[4504]|BLPAG145-07|06-SRNP-105784|Costa Rica|Guanacaste|658[0n]|BOLD:AAA1306
Aclytia heberDHJ02|[4505]|MHARB243-05|04-SRNP-15310|Costa Rica|Guanacaste|568[0n]|BOLD:AAA1306
Aclytia heber|[4506]|CNCLB1905-14|CNCLEP00117629|Mexico|Chiapas|658[0n]|BOLD:AAA1306
Aclytia heberDHJ02|[4507]|BLPCD108-08|07-SRNP-107533|Costa Rica|Guanacaste|658[0n]|BOLD:AAA1306
Aclytia heber|[4508]|CNCLB2436-14|Knudson 003|United States|Texas|658[0n]|BOLD:AAA1306
Aclytia heberDHJ02|[4509]|BLPDA670-09|08-SRNP-105745|Costa Rica|Guanacaste|632[0n]|BOLD:AAA1306
Aclytia heberDHJ02|[4510]|BLPDA671-09|08-SRNP-105746|Costa Rica|Guanacaste|632[0n]|BOLD:AAA1306
Aclytia heberDHJ02|[4511]|BLPAF262-07|06-SRNP-104961|Costa Rica|Guanacaste|658[3n]|BOLD:AAA1306
Aclytia heberDHJ02|[4512]|BLPCP520-08|08-SRNP-104937|Costa Rica|Guanacaste|658[0n]|BOLD:AAA1306
Aclytia heberDHJ02|[4513]|BLPBC934-07|06-SRNP-109957|Costa Rica|Alajuela|630[1n]|BOLD:AAA1306
Aclytia heberDHJ02|[4514]|BLPCK345-08|08-SRNP-100438|Costa Rica|Alajuela|658[0n]|BOLD:AAA1306
Aclytia heberDHJ02|[4515]|XAA228-04|01-SRNP-15852|Costa Rica|Guanacaste|665[0n]|BOLD:AAA1306
Aclytia heberDHJ02|[4516]|BLPCH074-08|07-SRNP-111259|Costa Rica|Guanacaste|658[0n]|BOLD:AAA1306
Aclytia heberDHJ02|[4517]|BLPCE320-08|07-SRNP-108685|Costa Rica|Guanacaste|658[0n]|BOLD:AAA1306
Aclytia heberDHJ02|[4518]|BLPCJ325-08|07-SRNP-113390|Costa Rica|Guanacaste|658[0n]|BOLD:AAA1306
Aclytia heberDHJ02|[4519]|BLPCH075-08|07-SRNP-111260|Costa Rica|Guanacaste|658[0n]|BOLD:AAA1306
Aclytia heberDHJ02|[4520]|BLPEE2546-14|13-SRNP-101982|Costa Rica|Guanacaste|658[0n]|BOLD:AAA1306
Aclytia heberDHJ02|[4521]|BLPCG690-08|07-SRNP-110935|Costa Rica|Guanacaste|658[0n]|BOLD:AAA1306
Aclytia heberDHJ02|[4522]|BLPEG1521-14|14-SRNP-101126|Costa Rica|Guanacaste|658[0n]|BOLD:AAA1306
Aclytia heberDHJ02|[4523]|BLPCI327-08|07-SRNP-112452|Costa Rica|Guanacaste|658[0n]|BOLD:AAA1306
Aclytia heberDHJ02|[4524]|BLPCI328-08|07-SRNP-112453|Costa Rica|Guanacaste|658[0n]|BOLD:AAA1306
Aclytia heberDHJ02|[4525]|BLPCI251-08|07-SRNP-112376|Costa Rica|Guanacaste|658[0n]|BOLD:AAA1306
Aclytia heberDHJ02|[4526]|BLPCI331-08|07-SRNP-112456|Costa Rica|Guanacaste|658[0n]|BOLD:AAA1306
Aclytia heberDHJ02|[4527]|BLPCI329-08|07-SRNP-112454|Costa Rica|Guanacaste|658[0n]|BOLD:AAA1306
Aclytia heberDHJ02|[4528]|BLPEE2885-14|13-SRNP-102321|Costa Rica|Guanacaste|658[0n]|BOLD:AAA1306
Aclytia heberDHJ02|[4529]|BLPCI332-08|07-SRNP-112457|Costa Rica|Guanacaste|658[0n]|BOLD:AAA1306
Aclytia heberDHJ02|[4530]|BLPDW710-11|10-SRNP-112602|Costa Rica|Guanacaste|658[0n]|BOLD:AAA1306
Aclytia heberDHJ02|[4531]|BLPDW709-11|10-SRNP-112601|Costa Rica|Guanacaste|658[0n]|BOLD:AAA1306
Aclytia heberDHJ02|[4532]|BLPDM316-10|09-SRNP-109693|Costa Rica|Guanacaste|658[0n]|BOLD:AAA1306
Aclytia heberDHJ02|[4533]|MHMYO394-11|11-SRNP-13014|Costa Rica||658[0n]|BOLD:AAA1306
Aclytia heberDHJ02|[4534]|BLPAG335-07|06-SRNP-105974|Costa Rica|Guanacaste|658[0n]|BOLD:AAA1306
Aclytia heberDHJ02|[4535]|BLPCI864-08|07-SRNP-112989|Costa Rica|Guanacaste|658[0n]|BOLD:AAA1306
Aclytia heber|[4536]|CNCLB1901-14|CNCLEP00117625|Guatemala|San Marcos|658[0n]|BOLD:AAA1306
Aclytia heberDHJ02|[4537]|BLPEF2743-13|12-SRNP-104481|Costa Rica|Guanacaste|658[0n]|BOLD:AAA1306
Aclytia heberDHJ02|[4538]|BLPBB911-07|06-SRNP-108994|Costa Rica|Guanacaste|658[0n]|BOLD:AAA1306
Aclytia heberDHJ02|[4539]|BLPEE2887-14|13-SRNP-102323|Costa Rica|Guanacaste|658[0n]|BOLD:AAA1306
Aclytia heberDHJ02|[4540]|BLPAG149-07|06-SRNP-105788|Costa Rica|Guanacaste|658[0n]|BOLD:AAA1306
Aclytia heberDHJ02|[4541]|BLPED2484-12|11-SRNP-106086|Costa Rica|Guanacaste|658[0n]|BOLD:AAA1306
Aclytia heberDHJ02|[4542]|BLPED2393-12|11-SRNP-105995|Costa Rica|Guanacaste|658[0n]|BOLD:AAA1306
Aclytia|[4543]|CNCLB1902-14|CNCLEP00117626|Guatemala|San Marcos|658[0n]|BOLD:AAA1306
Aclytia heberDHJ02|[4544]|MHMYC1355-09|09-SRNP-14167|Costa Rica||658[0n]|BOLD:AAA1306
Aclytia heberDHJ02|[4545]|BLPAE576-06|06-SRNP-104335|Costa Rica|Guanacaste|658[0n]|BOLD:AAA1306
Aclytia heberDHJ02|[4546]|BLPDU402-11|10-SRNP-109824|Costa Rica|Guanacaste|658[0n]|BOLD:AAA1306
Aclytia heberDHJ02|[4547]|MHARB239-05|04-SRNP-12775|Costa Rica|Guanacaste|658[0n]|BOLD:AAA1306
Aclytia heberDHJ02|[4548]|MHARB240-05|04-SRNP-15303|Costa Rica|Guanacaste|658[0n]|BOLD:AAA1306
Aclytia heberDHJ02|[4549]|MHARB242-05|04-SRNP-15311|Costa Rica|Guanacaste|658[0n]|BOLD:AAA1306
Aclytia heberDHJ02|[4550]|MHARB245-05|04-SRNP-15364|Costa Rica|Guanacaste|658[0n]|BOLD:AAA1306
Aclytia heberDHJ02|[4551]|MHARB247-05|04-SRNP-15306|Costa Rica|Guanacaste|658[0n]|BOLD:AAA1306
Aclytia heberDHJ02|[4552]|BLPAG747-07|06-SRNP-106386|Costa Rica|Guanacaste|658[0n]|BOLD:AAA1306
Aclytia heberDHJ02|[4553]|BLPAG749-07|06-SRNP-106388|Costa Rica|Guanacaste|658[0n]|BOLD:AAA1306
Aclytia heberDHJ02|[4554]|BLPDK1130-09|09-SRNP-106058|Costa Rica|Guanacaste|658[0n]|BOLD:AAA1306
Aclytia heberDHJ02|[4555]|BLPDD529-09|08-SRNP-108424|Costa Rica|Guanacaste|658[0n]|BOLD:AAA1306
Aclytia heberDHJ02|[4556]|BLPDD530-09|08-SRNP-108425|Costa Rica|Guanacaste|658[0n]|BOLD:AAA1306
Aclytia heberDHJ02|[4557]|MHARB244-05|04-SRNP-15307|Costa Rica|Guanacaste|658[1n]|BOLD:AAA1306
Aclytia heberDHJ02|[4558]|BLPDC577-09|08-SRNP-107532|Costa Rica|Alajuela|658[0n]|BOLD:AAA1306
Aclytia heberDHJ02|[4559]|BLPCD112-08|07-SRNP-107537|Costa Rica|Guanacaste|658[0n]|BOLD:AAA1306
Aclytia heberDHJ02|[4560]|MHARB246-05|04-SRNP-15305|Costa Rica|Guanacaste|658[1n]|BOLD:AAA1306
Aclytia heberDHJ02|[4561]|BLPEA1026-11|11-SRNP-101493|Costa Rica|Guanacaste|658[1n]|BOLD:AAA1306
Aclytia heberDHJ02|[4562]|BLPEG2134-14|14-SRNP-101739|Costa Rica|Guanacaste|612[0n]|BOLD:AAA1306
Aclytia heberDHJ02|[4563]|BLPBA883-07|06-SRNP-108026|Costa Rica|Guanacaste|621[0n]|BOLD:AAA1306
Aclytia heberDHJ02|[4564]|BLPBH156-07|07-SRNP-103915|Costa Rica|Guanacaste|658[0n]|BOLD:AAA1306
Aclytia heberDHJ02|[4565]|BLPBB001-07|06-SRNP-108084|Costa Rica|Guanacaste|658[0n]|BOLD:AAA1306
Aclytia heberDHJ02|[4566]|BLPEE2547-14|13-SRNP-101983|Costa Rica|Guanacaste|658[0n]|BOLD:AAA1306
Aclytia heberDHJ02|[4567]|BLPAG150-07|06-SRNP-105789|Costa Rica|Guanacaste|658[0n]|BOLD:AAA1306
Aclytia heberDHJ02|[4568]|BLPAG334-07|06-SRNP-105973|Costa Rica|Guanacaste|658[0n]|BOLD:AAA1306
Aclytia heberDHJ02|[4569]|BLPAG151-07|06-SRNP-105790|Costa Rica|Guanacaste|658[0n]|BOLD:AAA1306
Aclytia heberDHJ02|[4570]|BLPCN536-08|08-SRNP-103073|Costa Rica|Guanacaste|658[0n]|BOLD:AAA1306
Aclytia heberDHJ02|[4571]|BLPAA234-06|06-SRNP-100233|Costa Rica|Guanacaste|658[0n]|BOLD:AAA1306
Aclytia heberDHJ02|[4572]|BLPAG748-07|06-SRNP-106387|Costa Rica|Guanacaste|658[0n]|BOLD:AAA1306
Aclytia heberDHJ02|[4573]|BLPED1835-12|11-SRNP-105437|Costa Rica|Guanacaste|658[0n]|BOLD:AAA1306
Aclytia heberDHJ02|[4574]|BLPED1564-12|11-SRNP-105166|Costa Rica|Guanacaste|658[0n]|BOLD:AAA1306
Aclytia heberDHJ02|[4575]|BLPCE319-08|07-SRNP-108684|Costa Rica|Guanacaste|658[0n]|BOLD:AAA1306
Aclytia heberDHJ02|[4576]|BLPCE317-08|07-SRNP-108682|Costa Rica|Guanacaste|658[0n]|BOLD:AAA1306
Aclytia heberDHJ02|[4577]|BLPCD113-08|07-SRNP-107538|Costa Rica|Guanacaste|658[0n]|BOLD:AAA1306
Aclytia heberDHJ02|[4578]|BLPCD107-08|07-SRNP-107532|Costa Rica|Guanacaste|658[0n]|BOLD:AAA1306
Aclytia heberDHJ02|[4579]|BLPCD111-08|07-SRNP-107536|Costa Rica|Guanacaste|658[0n]|BOLD:AAA1306
Aclytia heberDHJ02|[4580]|BLPCD109-08|07-SRNP-107534|Costa Rica|Guanacaste|658[0n]|BOLD:AAA1306
Aclytia heberDHJ02|[4581]|BLPCD106-08|07-SRNP-107531|Costa Rica|Guanacaste|658[0n]|BOLD:AAA1306

Aclytia heberDHJ02|[4579]|BLPCD111-08|07-SRNP-107536|Costa Rica|Guanacaste|658[0n]|BOLD:AAA1306
Aclytia heberDHJ02|[4580]|BLPCD109-08|07-SRNP-107534|Costa Rica|Guanacaste|658[0n]|BOLD:AAA1306
Aclytia heberDHJ02|[4581]|BLPCD106-08|07-SRNP-107531|Costa Rica|Guanacaste|658[0n]|BOLD:AAA1306
Aclytia heberDHJ02|[4582]|BLPCO886-08|08-SRNP-104363|Costa Rica|Guanacaste|658[0n]|BOLD:AAA1306
Aclytia heberDHJ02|[4583]|BLPEE5298-14|14-SRNP-100438|Costa Rica|Guanacaste|658[0n]|BOLD:AAA1306
Aclytia heberDHJ02|[4584]|MHMYS3090-13|13-SRNP-101291|Costa Rica|Guanacaste|658[0n]|BOLD:AAA1306
Aclytia heberDHJ02|[4585]|BLPCA731-08|07-SRNP-105336|Costa Rica|Guanacaste|658[0n]|BOLD:AAA1306
Aclytia heberDHJ02|[4586]|BLPCC102-08|07-SRNP-106587|Costa Rica|Guanacaste|658[0n]|BOLD:AAA1306
Aclytia heberDHJ02|[4587]|BLPCC101-08|07-SRNP-106586|Costa Rica|Guanacaste|658[0n]|BOLD:AAA1306
Aclytia heberDHJ02|[4588]|BLPEE4721-14|13-SRNP-104157|Costa Rica|Guanacaste|658[0n]|BOLD:AAA1306
Aclytia heberDHJ02|[4589]|BLPEE2884-14|13-SRNP-102320|Costa Rica|Guanacaste|658[0n]|BOLD:AAA1306
Aclytia heberDHJ02|[4590]|BLPEE2882-14|13-SRNP-102318|Costa Rica|Guanacaste|658[0n]|BOLD:AAA1306
Aclytia heberDHJ02|[4591]|BLPEE2881-14|13-SRNP-102317|Costa Rica|Guanacaste|658[0n]|BOLD:AAA1306
Aclytia heberDHJ02|[4592]|BLPEE2879-14|13-SRNP-102315|Costa Rica|Guanacaste|658[0n]|BOLD:AAA1306
Aclytia heberDHJ02|[4593]|BLPBA325-07|06-SRNP-107468|Costa Rica|Guanacaste|658[0n]|BOLD:AAA1306
Aclytia heberDHJ02|[4594]|BLPDW042-11|10-SRNP-111934|Costa Rica|Guanacaste|658[0n]|BOLD:AAA1306
Aclytia|[4595]|CNCLB1903-14|CNCLEP00117627|Mexico||658[0n]|BOLD:AAA1306
Aclytia heberDHJ02|[4596]|BLPBE473-07|07-SRNP-101412|Costa Rica|Guanacaste|658[0n]|BOLD:AAA1306
Aclytia heberDHJ02|[4597]|BLPDX1020-11|10-SRNP-114147|Costa Rica|Guanacaste|658[0n]|BOLD:AAA1306
Aclytia heberDHJ02|[4598]|BLPED377-11|11-SRNP-103979|Costa Rica|Guanacaste|658[0n]|BOLD:AAA1306
Aclytia heberDHJ02|[4599]|BLPDX501-11|10-SRNP-113628|Costa Rica|Guanacaste|658[0n]|BOLD:AAA1306
Aclytia heberDHJ02|[4600]|BLPAF266-07|06-SRNP-104965|Costa Rica|Guanacaste|658[0n]|BOLD:AAA1306
Aclytia heberDHJ02|[4601]|BLPAF265-07|06-SRNP-104964|Costa Rica|Guanacaste|658[0n]|BOLD:AAA1306
Aclytia heberDHJ02|[4602]|BLPDL667-10|09-SRNP-108049|Costa Rica|Guanacaste|658[0n]|BOLD:AAA1306
Aclytia heberDHJ02|[4603]|BLPDL539-10|09-SRNP-107921|Costa Rica|Guanacaste|658[0n]|BOLD:AAA1306
Aclytia heberDHJ02|[4604]|BLPDL538-10|09-SRNP-107920|Costa Rica|Guanacaste|658[0n]|BOLD:AAA1306
Opharus muricolor|[4605]|RDNMH124-09|CNCLEP00054343|United States|Arizona|658[0n]|BOLD:AAI1181
Composia fidelissima|[4606]|LPYPB295-08|MAL-00513|Mexico|Quintana Roo|597[0n]|BOLD:AAC3572
Composia fidelissima|[4607]|LYHES523-09|MAL-03820|Mexico|Quintana Roo|658[0n]|BOLD:AAC3572
Composia fidelissima|[4608]|LYHES524-09|MAL-03821|Mexico|Quintana Roo|656[0n]|BOLD:AAC3572
Composia fidelissima|[4609]|LYHES525-09|MAL-03822|Mexico|Quintana Roo|657[0n]|BOLD:AAC3572
Composia fidelissima|[4610]|LYHES526-09|MAL-03823|Mexico|Quintana Roo|656[0n]|BOLD:AAC3572
Composia fidelissima|[4611]|LYHES527-09|MAL-03824|Mexico|Quintana Roo|658[0n]|BOLD:AAC3572
Composia fidelissima|[4612]|LYHES522-09|MAL-03819|Mexico|Quintana Roo|612[0n]|BOLD:AAC3572
Composia fidelissima|[4613]|LYHES521-09|MAL-03818|Mexico|Quintana Roo|658[0n]|BOLD:AAC3572
Composia fidelissima|[4614]|LYHES520-09|MAL-03817|Mexico|Quintana Roo|658[0n]|BOLD:AAC3572
Composia fidelissima|[4615]|RDNMF910-08|CNC LEP00054177|Mexico||658[0n]|BOLD:AAC3572
Composia fidelissima|[4616]|LPYPB831-08|MAL-01049|Mexico|Quintana Roo|658[0n]|BOLD:AAC3572
Composia fidelissima|[4617]|LPYPB292-08|MAL-00510|Mexico|Quintana Roo|658[0n]|BOLD:AAC3572
Agaraea semivitrea|[4618]|QUNOD005-10|7004-COI-09|United States|Texas|658[0n]|BOLD:AAA1378
Agaraea semivitrea|[4619]|CNCLB2375-14|CNCLEP 00119519|United States|Texas|658[0n]|BOLD:AAA1378
Agaraea semivitrea|[4620]|QUNOD004-10|7003-COI-09|United States|Texas|658[0n]|BOLD:AAA1378
Calidota laqueata|[4621]|CNCLB2197-14|CNCLEP00118001|United States|Florida|658[0n]|BOLD:ACM4177
Calidota laqueata|[4622]|CNCLB1010-14|CNCLEP00113418|United States|Florida|658[0n]|BOLD:ACM4177
Calidota laqueata|[4623]|CNCLB1011-14|CNCLEP00113419|United States|Florida|658[0n]|BOLD:ACM4177
Calidota laqueata|[4624]|CNCLB1009-14|CNCLEP00113417|United States|Florida|658[1n]|BOLD:ACM4177
Clemensia sp.|[4625]|MILEP351-10|10-MISC-256|United States|Alabama|658[0n]|BOLD:AAA4334
Clemensia sp.|[4626]|MILEQ303-11|11-MISC-778|United States|Alabama|658[0n]|BOLD:AAA4334
Clemensia sp.|[4627]|MILEQ301-11|11-MISC-776|United States|Alabama|658[0n]|BOLD:AAA4334
Clemensia sp.|[4628]|LNCB498-07|07-NCNW-0182|United States|North Carolina|658[0n]|BOLD:AAA4334
Clemensia sp.|[4629]|LNCB499-07|07-NCNW-0183|United States|North Carolina|658[0n]|BOLD:AAA4334
Clemensia sp.|[4630]|LNCB686-09|09-NCCC-156|United States|North Carolina|658[0n]|BOLD:AAA4334
Clemensia sp.|[4631]|LNC650-06|06-NCCC-650|United States|North Carolina|658[0n]|BOLD:AAA4334
Clemensia sp.|[4632]|LNC649-06|06-NCCC-649|United States|North Carolina|656[0n]|BOLD:AAA4334
Clemensia sp.|[4633]|LNCB685-09|09-NCCC-155|United States|North Carolina|624[0n]|BOLD:AAA4334
Clemensia sp.|[4634]|LNCB005-06|06-NCC-961|United States|North Carolina|630[0n]|BOLD:AAA4334
Clemensia sp.|[4635]|LNCB282-06|06-NCCC-1238|United States|North Carolina|658[0n]|BOLD:AAA4334
Clemensia sp.|[4636]|LNCB281-06|06-NCCC-1237|United States|North Carolina|658[0n]|BOLD:AAA4334
Clemensia sp.|[4637]|LNCC186-10|10-NCCC-281|United States|North Carolina|658[0n]|BOLD:AAA4334
Clemensia sp.|[4638]|LNCB649-09|09-NCCC-119|United States|North Carolina|658[0n]|BOLD:AAA4334
Clemensia sp.|[4639]|LNCB004-06|06-NCC-960|United States|North Carolina|630[0n]|BOLD:AAA4334
Clemensia sp.|[4640]|LNCB723-09|09-NCCC-193|United States|North Carolina|658[0n]|BOLD:AAA4334
Clemensia sp.|[4641]|MILEQ302-11|11-MISC-777|United States|Alabama|658[0n]|BOLD:AAA4334
Clemensia sp.|[4642]|RDNME303-07|CNCNoctuoidea13910|United States|Florida|658[0n]|BOLD:AAA4334
Clemensia sp.|[4643]|RDNME302-07|CNCNoctuoidea13909|United States|Florida|658[0n]|BOLD:AAA4334
Clemensia sp.|[4644]|RDNME299-07|CNCNoctuoidea13906|United States|Florida|658[0n]|BOLD:AAA4334
Clemensia sp.|[4645]|USLEP831-10|10BBLEP-00831|United States|Florida|658[0n]|BOLD:AAA4334
Clemensia sp.|[4646]|USLEP319-10|10BBLEP-00319|United States|Florida|658[0n]|BOLD:AAA4334
Clemensia sp.|[4647]|USLEP316-10|10BBLEP-00316|United States|Florida|658[0n]|BOLD:AAA4334
Clemensia albata|[4648]|ABLCW283-10|CSUPOBK-0283|United States|North Carolina|658[0n]|BOLD:ACF...
Clemensia albata|[4649]|LNCC644-11|11-NCCC-169|United States|North Carolina|658[0n]|BOLD:ACF3441
Clemensia albata|[4650]|LGSMC735-05|DNA-ATBI-2735|United States|Tennessee|658[0n]|BOLD:ACF3441
Clemensia albata|[4651]|LOTB520-05|05-TN-00520|United States|Tennessee|658[0n]|BOLD:ACF3441
Clemensia albata|[4652]|RDNME300-07|CNCNoctuoidea13907|United States|Georgia|614[1n]|BOLD:ACF3441
Clemensia albata|[4653]|LGSMC731-05|DNA-ATBI-2731|United States|Tennessee|605[0n]|BOLD:ACF3441
Clemensia albata|[4654]|LNCB775-09|09-NCCC-245|United States|North Carolina|655[0n]|BOLD:ACF3441
Clemensia albata|[4655]|LNCB776-09|09-NCCC-246|United States|North Carolina|658[0n]|BOLD:ACF3441
Clemensia albata|[4656]|LNCB372-06|06-NCCC-1328|United States|North Carolina|658[0n]|BOLD:ACF3441
Clemensia albata|[4657]|LNCB581-09|09-NCCC-051|United States|North Carolina|658[0n]|BOLD:ACF3441
Clemensia albata|[4658]|LNCB687-09|09-NCCC-157|United States|North Carolina|658[0n]|BOLD:ACF3441
Clemensia albata|[4659]|LNCB721-09|09-NCCC-191|United States|North Carolina|654[0n]|BOLD:ACF3441
Clemensia albata|[4660]|LNCB582-09|09-NCCC-052|United States|North Carolina|658[0n]|BOLD:ACF3441
Clemensia albata|[4661]|LGSMG110-07|BGS03431|United States|Tennessee|658[0n]|BOLD:ACF3441
Clemensia albata|[4662]|LGSMC734-05|DNA-ATBI-2734|United States|Tennessee|658[0n]|BOLD:ACF3441
Clemensia albata|[4663]|LGSMC732-05|DNA-ATBI-2732|United States|Tennessee|658[0n]|BOLD:ACF3441
Clemensia albata|[4664]|LGSMG111-07|BGS03432|United States|Tennessee|658[0n]|BOLD:ACF3441
Clemensia albata|[4665]|BBLCU149-09|09BBLEP-04636|United States|Michigan|658[0n]|BOLD:ACF3441
Clemensia albata|[4666]|GWNC614-07|CNCLEP00034176|Canada|Ontario|658[0n]|BOLD:ACF3441
Clemensia albata|[4667]|LPOKA395-09|MDOK-0395|United States|Oklahoma|658[0n]|BOLD:ACF3441
Clemensia albata|[4668]|RDNMH799-09|CNCLEP00064127|Canada|Ontario|658[0n]|BOLD:ACF3441
Clemensia albata|[4669]|XAK559-07|HLC-16112|Canada|Ontario|593[0n]|BOLD:ACF3441
Clemensia albata|[4670]|LGSMC733-05|DNA-ATBI-2733|United States|North Carolina|617[0n]|BOLD:AC...
Clemensia albata|[4671]|LNCC032-10|10-NCCC-127|United States|North Carolina|658[0n]|BOLD:ACF3441
Clemensia albata|[4672]|LNCB774-09|09-NCCC-244|United States|North Carolina|658[0n]|BOLD:ACF3441
Clemensia albata|[4673]|LNCC681-11|11-NCCC-206|United States|North Carolina|658[0n]|BOLD:ACF3441
Clemensia albata|[4674]|LNCC854-11|11-NCCC-379|United States|North Carolina|658[0n]|BOLD:ACF3441
Clemensia albata|[4675]|LNCC855-11|11-NCCC-380|United States|North Carolina|658[0n]|BOLD:ACF3441
Clemensia albata|[4676]|LPOKC304-09|MDOK-2381|United States|Oklahoma|658[0n]|BOLD:ACF3441
Clemensia albata|[4677]|LILLA157-11|SNS10IL-00210|United States|Illinois|658[0n]|BOLD:ACF3441
Clemensia albata|[4678]|LILLA176-11|SNS10IL-00237|United States|Illinois|658[0n]|BOLD:ACF3441
Clemensia albata|[4679]|LPOKA541-09|MDOK-0541|United States|Oklahoma|658[0n]|BOLD:ACF3441
Clemensia albata|[4680]|LOTB519-05|05-TN-00519|United States|Tennessee|658[0n]|BOLD:ACF3441
[illegible]

Clemensia albata|[4678]|LILLA176-11|SNS10IL-00237|United States|Illinois|658[0n]|BOLD:ACF3441
Clemensia albata|[4679]|LPOKA541-09|MDOK-0541|United States|Oklahoma|658[0n]|BOLD:ACF3441
Clemensia albata|[4680]|LOTB519-05|05-TN-00519|United States|Tennessee|658[0n]|BOLD:ACF3441
Clemensia albata|[4681]|LNCB767-09|09-NCCC-237|United States|North Carolina|633[0n]|BOLD:ACF3441
Clemensia albata|[4682]|LNCB722-09|09-NCCC-192|United States|North Carolina|658[0n]|BOLD:ACF3441
Clemensia albata|[4683]|LNCC185-10|10-NCCC-280|United States|North Carolina|658[0n]|BOLD:ACF3441
Clemensia albata|[4684]|LNCC034-10|10-NCCC-129|United States|North Carolina|658[0n]|BOLD:ACF3441
Clemensia albata|[4685]|LNCC033-10|10-NCCC-128|United States|North Carolina|658[0n]|BOLD:ACF3441
Clemensia albata|[4686]|LNCC031-10|10-NCCC-126|United States|North Carolina|658[0n]|BOLD:ACF3441
Clemensia albata|[4687]|LNCC030-10|10-NCCC-125|United States|North Carolina|658[0n]|BOLD:ACF3441
Clemensia albata|[4688]|LPOKB765-09|MDOK-1807|United States|Oklahoma|658[0n]|BOLD:ACF3441
Clemensia albata|[4689]|CNPAQ109-13|BIOUG07082-F11|Canada|Saskatchewan|579[0n]|BOLD:AAA4333
Clemensia albata|[4690]|LPVIA957-08|PFC-2006-1272|Canada|British Columbia|581[0n]|BOLD:AAA4333
Clemensia albata|[4691]|SSPAB915-13|BIOUG06035-H06|Canada|Saskatchewan|593[0n]|BOLD:AAA4333
Clemensia albata|[4692]|SSPAB913-13|BIOUG06035-H04|Canada|Saskatchewan|582[0n]|BOLD:AAA4333
Clemensia albata|[4693]|RDMAB082-05|UASM57609|Canada|Alberta|633[0n]|BOLD:AAA4333
Clemensia albata|[4694]|LMDH187-11|BIOUG01047-G05|United States|Minnesota|635[0n]|BOLD:AAA4333
Clemensia albata|[4695]|MNAG089-08|CNCLEP00041074|Canada|Manitoba|658[0n]|BOLD:AAA4333
Clemensia albata|[4696]|LBCH605-10|10-JDWBC-0605|Canada|British Columbia|658[0n]|BOLD:AAA4333
Clemensia albata|[4697]|LBCH203-10|10-JDWBC-0203|Canada|British Columbia|658[0n]|BOLD:AAA4333
Clemensia albata|[4698]|LBCH3661-10|10-JDWBC-3661|Canada|British Columbia|658[0n]|BOLD:AAA4333
Clemensia albata|[4699]|LBCH4852-10|10-JDWBC-4852|Canada|British Columbia|658[0n]|BOLD:AAA4333
Clemensia albata|[4700]|TMNBD367-07|MNBTT-3168|Canada|New Brunswick|656[0n]|BOLD:AAA4333
Clemensia albata|[4701]|LNCC974-11|11-NCCC-499|United States|North Carolina|658[0n]|BOLD:AAA4333
Clemensia albata|[4702]|LPSOD999-09|08BBLEP-05632|Canada|Ontario|658[0n]|BOLD:AAA4333
Clemensia albata|[4703]|LPMN395-08|08BBLEP-01194|Canada|Manitoba|658[0n]|BOLD:AAA4333
Clemensia albata|[4704]|LBCC541-05|HLC-22421|Canada|British Columbia|658[0n]|BOLD:AAA4333
Clemensia albata|[4705]|LPVIA194-08|PFC-2006-0262|Canada|British Columbia|658[0n]|BOLD:AAA4333
Clemensia albata|[4706]|LPMN402-08|08BBLEP-01201|Canada|Manitoba|658[0n]|BOLD:AAA4333
Clemensia albata|[4707]|LPMN398-08|08BBLEP-01197|Canada|Manitoba|658[0n]|BOLD:AAA4333
Clemensia albata|[4708]|LPMN367-08|08BBLEP-01166|Canada|Manitoba|658[0n]|BOLD:AAA4333
Clemensia albata|[4709]|SSPAB036-13|BIOUG06035-F11|Canada|Saskatchewan|604[0n]|BOLD:AAA4333
Clemensia albata|[4710]|LNCC976-11|11-NCCC-501|United States|North Carolina|658[0n]|BOLD:AAA4333
Clemensia albata|[4711]|LNCC975-11|11-NCCC-500|United States|North Carolina|658[0n]|BOLD:AAA4333
Clemensia albata|[4712]|MNBB465-05|05-NBSTA-381|Canada|New Brunswick|658[5n]|BOLD:AAA4333
Clemensia albata|[4713]|LPVIA580-08|PFC-2006-0807|Canada|British Columbia|636[0n]|BOLD:AAA4333
Clemensia albata|[4714]|LGSM031-04|DNA-ATBI-0031|United States|North Carolina|658[0n]|BOLD:AAA...
Clemensia albata|[4715]|ABLCW280-10|CSUPOBK-0280|United States|North Carolina|658[0n]|BOLD:AAA...
Clemensia albata|[4716]|ABLCW282-10|CSUPOBK-0282|United States|North Carolina|658[0n]|BOLD:AAA...
Clemensia albata|[4717]|LPGVA732-08|UBC-2006-2061|Canada|British Columbia|658[0n]|BOLD:AAA4333
Clemensia albata|[4718]|LBCB747-05|HLC-21687|Canada|British Columbia|658[0n]|BOLD:AAA4333
Clemensia albata|[4719]|LPVIA577-08|PFC-2006-0804|Canada|British Columbia|658[0n]|BOLD:AAA4333
Clemensia albata|[4720]|LPVIA578-08|PFC-2006-0805|Canada|British Columbia|658[0n]|BOLD:AAA4333
Clemensia albata|[4721]|LPVIA579-08|PFC-2006-0806|Canada|British Columbia|658[0n]|BOLD:AAA4333
Clemensia albata|[4722]|LPVIA849-08|PFC-2006-1150|Canada|British Columbia|658[0n]|BOLD:AAA4333
Clemensia albata|[4723]|LPVIA851-08|PFC-2006-1152|Canada|British Columbia|658[0n]|BOLD:AAA4333
Clemensia albata|[4724]|LALPA401-10|AVBC 403-10|Canada|British Columbia|658[0n]|BOLD:AAA4333
Clemensia albata|[4725]|LALPA437-10|AVBC 439-10|Canada|British Columbia|658[0n]|BOLD:AAA4333
Clemensia albata|[4726]|LALPA459-10|AVBC 461-10|Canada|British Columbia|658[0n]|BOLD:AAA4333
Clemensia albata|[4727]|LALPA499-10|AVBC 501-10|Canada|British Columbia|658[0n]|BOLD:AAA4333
Clemensia albata|[4728]|BBLPD663-10|10BBCLP-2661|Canada|British Columbia|658[0n]|BOLD:AAA4333
Clemensia albata|[4729]|BBLPD738-10|10BBCLP-2736|Canada|British Columbia|658[0n]|BOLD:AAA4333
Clemensia albata|[4730]|BBLPD739-10|10BBCLP-2737|Canada|British Columbia|658[0n]|BOLD:AAA4333
Clemensia albata|[4731]|BBLPD741-10|10BBCLP-2739|Canada|British Columbia|658[0n]|BOLD:AAA4333
Clemensia albata|[4732]|LBCD575-05|HLC-23395|Canada|British Columbia|658[0n]|BOLD:AAA4333
Clemensia albata|[4733]|LBCD576-05|HLC-23396|Canada|British Columbia|658[0n]|BOLD:AAA4333
Clemensia albata|[4734]|LBCD646-05|HLC-23466|Canada|British Columbia|658[0n]|BOLD:AAA4333
Clemensia albata|[4735]|LBCD647-05|HLC-23467|Canada|British Columbia|658[0n]|BOLD:AAA4333
Clemensia albata|[4736]|LBCD873-05|HLC-23693|Canada|British Columbia|658[0n]|BOLD:AAA4333
Clemensia albata|[4737]|LBCD874-05|HLC-23694|Canada|British Columbia|658[0n]|BOLD:AAA4333
Clemensia albata|[4738]|TMNBD368-07|MNBTT-3169|Canada|New Brunswick|658[0n]|BOLD:AAA4333
Clemensia albata|[4739]|LBCH4082-10|10-JDWBC-4082|Canada|British Columbia|658[0n]|BOLD:AAA4333
Clemensia albata|[4740]|LBCS242-07|UBC-2007-0623|Canada|British Columbia|658[0n]|BOLD:AAA4333
Clemensia albata|[4741]|RDLQG613-06|DH012906|Canada|Quebec|658[0n]|BOLD:AAA4333
Clemensia albata|[4742]|RDLQE456-06|MDH002459|Canada|Quebec|656[0n]|BOLD:AAA4333
Clemensia albata|[4743]|XAK581-07|HLC-16134|Canada|Ontario|594[0n]|BOLD:AAA4333
Clemensia albata|[4744]|LBCG2243-09|08-JDWBC-2243|Canada|British Columbia|658[0n]|BOLD:AAA4333
Clemensia albata|[4745]|LBCH3663-10|10-JDWBC-3663|Canada|British Columbia|658[0n]|BOLD:AAA4333
Clemensia albata|[4746]|LBCH3372-10|10-JDWBC-3372|Canada|British Columbia|658[0n]|BOLD:AAA4333
Clemensia albata|[4747]|LBCH745-10|10-JDWBC-0745|Canada|British Columbia|658[0n]|BOLD:AAA4333
Clemensia albata|[4748]|LBCH322-10|10-JDWBC-0322|Canada|British Columbia|658[0n]|BOLD:AAA4333
Clemensia albata|[4749]|LBCH085-10|10-JDWBC-0085|Canada|British Columbia|658[0n]|BOLD:AAA4333
Clemensia albata|[4750]|LBCH204-10|10-JDWBC-0204|Canada|British Columbia|658[0n]|BOLD:AAA4333
Clemensia albata|[4751]|LBCH4853-10|10-JDWBC-4853|Canada|British Columbia|658[0n]|BOLD:AAA4333
Clemensia albata|[4752]|LBCH4654-10|10-JDWBC-4654|Canada|British Columbia|658[0n]|BOLD:AAA4333
Clemensia albata|[4753]|LBCH4488-10|10-JDWBC-4488|Canada|British Columbia|658[0n]|BOLD:AAA4333
Clemensia albata|[4754]|LBCH3832-10|10-JDWBC-3832|Canada|British Columbia|658[0n]|BOLD:AAA4333
Clemensia albata|[4755]|LBCH3830-10|10-JDWBC-3830|Canada|British Columbia|658[0n]|BOLD:AAA4333
Clemensia albata|[4756]|LGSMB317-05|DNA-ATBI-1166|United States|North Carolina|658[0n]|BOLD:AA...
Clemensia albata|[4757]|LGSMB316-05|DNA-ATBI-1165|United States|North Carolina|594[0n]|BOLD:AA...
Clemensia albata|[4758]|TMNBD366-07|MNBTT-3167|Canada|New Brunswick|631[0n]|BOLD:AAA4333
Clemensia albata|[4759]|LBCH3088-10|10-JDWBC-3088|Canada|British Columbia|642[0n]|BOLD:AAA4333
Clemensia albata|[4760]|TMNBD369-07|MNBTT-3170|Canada|New Brunswick|641[0n]|BOLD:AAA4333
Clemensia albata|[4761]|BBLPD740-10|10BBCLP-2738|Canada|British Columbia|622[0n]|BOLD:AAA4333
Clemensia albata|[4762]|LGSM617-04|DNA-ATBI-0617|United States|Tennessee|658[6n]|BOLD:AAA4333
Clemensia albata|[4763]|ABLCW281-10|CSUPOBK-0281|United States|North Carolina|658[0n]|BOLD:AAA...
Clemensia albata|[4764]|ABLCW279-10|CSUPOBK-0279|United States|North Carolina|658[0n]|BOLD:AAA...
Clemensia albata|[4765]|ABLCW278-10|CSUPOBK-0278|United States|North Carolina|658[0n]|BOLD:AAA...
Clemensia albata|[4766]|LBCB331-05|HLC-21271|Canada|British Columbia|658[0n]|BOLD:AAA4333
Clemensia albata|[4767]|MNBB689-05|05-NBSTA-605|Canada|New Brunswick|658[0n]|BOLD:AAA4333
Clemensia albata|[4768]|MNBB497-05|05-NBSTA-413|Canada|New Brunswick|658[0n]|BOLD:AAA4333
Gardinia anopla|[4769]|RDNMJ249-11|CNCLEP 70272|United States|Arizona|657[0n]|BOLD:AAV6822
Eudesmia arida|[4770]|ABLCW272-10|CSUPOBK-0272|United States|Arizona|658[0n]|BOLD:AAH2607
Eudesmia arida|[4771]|RDNMH969-09|CNCLEP00067977|United States|Arizona|658[0n]|BOLD:AAH2607
Eudesmia arida|[4772]|ABLCW274-10|CSUPOBK-0274|United States|Arizona|658[0n]|BOLD:AAH2607
Eudesmia arida|[4773]|ABLCW273-10|CSUPOBK-0273|United States|Arizona|658[0n]|BOLD:AAH2607
Eudesmia arida|[4774]|ABLCW271-10|CSUPOBK-0271|United States|Arizona|658[0n]|BOLD:AAH2607
Eudesmia arida|[4775]|ABLCW270-10|CSUPOBK-0270|United States|Arizona|658[0n]|BOLD:AAH2607
Eudesmia arida|[4776]|RDNMI161-10|CNCLEP 69761|United States|Texas|658[0n]|BOLD:AAH2607
Eudesmia arida|[4777]|QUNOD117-10|7116-COI-09|United States|Texas|658[0n]|BOLD:AAH2607
Eudesmia menea|[4778]|LPYPB409-08|MLL-01157|Mexico|Campeche|658[0n]|BOLD:ACE8030
Eudesmia menea|[4779]|LPYPC064-08|MLL-02188|Mexico|Yucatan|658[0n]|BOLD:ACE8030
Eudesmia menea|[4780]|LPMX088-07|MLL-00088|Mexico|Campeche|658[0n]|BOLD:ACE8030

Eudesmia menea|[4778]|LPYPB409-08|MLL-01157|Mexico|Campeche|658[0n]|BOLD:ACE8030
Eudesmia menea|[4779]|LPYPC064-08|MLL-02188|Mexico|Yucatan|658[0n]|BOLD:ACE8030
Eudesmia menea|[4780]|LPMX088-07|MLL-00088|Mexico|Campeche|658[0n]|BOLD:ACE8030
Eudesmia menea|[4781]|LPMX089-07|MLL-00089|Mexico|Campeche|658[0n]|BOLD:ACE8030
Eudesmia menea|[4782]|LPMX090-07|MLL-00090|Mexico|Campeche|658[0n]|BOLD:ACE8030
Eudesmia menea|[4783]|LPMX092-07|MLL-00092|Mexico|Campeche|658[0n]|BOLD:ACE8030
Eudesmia menea|[4784]|LPMX180-07|MLL-00180|Mexico|Campeche|658[0n]|BOLD:ACE8030
Eudesmia menea|[4785]|LPMX183-07|MLL-00183|Mexico|Campeche|658[0n]|BOLD:ACE8030
Eudesmia menea|[4786]|LPMX184-07|MLL-00184|Mexico|Campeche|658[0n]|BOLD:ACE8030
Eudesmia menea|[4787]|LPMX185-07|MLL-00185|Mexico|Campeche|658[0n]|BOLD:ACE8030
Eudesmia menea|[4788]|LPYPB205-08|MAL-00423|Mexico|Campeche|658[0n]|BOLD:ACE8030
Eudesmia menea|[4789]|LPMX137-07|MLL-00137|Mexico|Campeche|658[0n]|BOLD:ACE8030
Eudesmia menea|[4790]|LPMX138-07|MLL-00138|Mexico|Campeche|658[0n]|BOLD:ACE8030
Eudesmia menea|[4791]|LPMX153-07|MLL-00153|Mexico|Campeche|658[0n]|BOLD:ACE8030
Eudesmia menea|[4792]|LPMX154-07|MLL-00154|Mexico|Campeche|658[0n]|BOLD:ACE8030
Eudesmia menea|[4793]|LPMX155-07|MLL-00155|Mexico|Campeche|658[0n]|BOLD:ACE8030
Eudesmia menea|[4794]|LPMX181-07|MLL-00181|Mexico|Campeche|658[0n]|BOLD:ACE8030
Eudesmia menea|[4795]|LPMX550-08|MLL-01208|Mexico|Campeche|658[0n]|BOLD:ACE8030
Eudesmia menea|[4796]|LYPIE923-09|MAL-01622|Mexico|Yucatan|573[2n]|BOLD:ACE8030
Eudesmia menea|[4797]|LPYPC330-08|MLL-00616|Mexico|Campeche|601[0n]|BOLD:ACE8030
Eudesmia menea|[4798]|LPMX091-07|MLL-00091|Mexico|Campeche|648[0n]|BOLD:ACE8030
Eudesmia menea|[4799]|LPYPB301-08|MAL-00519|Mexico|Campeche|658[0n]|BOLD:ACE8030
Eudesmia menea|[4800]|LPYPB291-08|MAL-00509|Mexico|Campeche|658[0n]|BOLD:ACE8030
Eudesmia menea|[4801]|LPYPB204-08|MAL-00422|Mexico|Campeche|658[0n]|BOLD:ACE8030
Eudesmia menea|[4802]|LPYPB203-08|MAL-00421|Mexico|Campeche|658[0n]|BOLD:ACE8030
Eudesmia menea|[4803]|LPYPB202-08|MAL-00420|Mexico|Campeche|658[0n]|BOLD:ACE8030
Eudesmia menea|[4804]|LPYPB201-08|MAL-00419|Mexico|Campeche|658[0n]|BOLD:ACE8030
Eudesmia menea|[4805]|LPMX517-07|MLL-00517|Mexico|Campeche|658[0n]|BOLD:ACE8030
Eudesmia menea|[4806]|LPYPB027-08|MLL-01057|Mexico|Campeche|658[0n]|BOLD:ACE8030
Rhabdatomis laudamia|[4807]|BLPEB100-11|11-SRNP-101612|Costa Rica|Guanacaste|658[0n]|BOLD:AAA1327
Rhabdatomis laudamia|[4808]|BLPEF4141-13|13-SRNP-100091|Costa Rica|Guanacaste|658[0n]|BOLD:AAA...
Rhabdatomis laudamia|[4809]|BLPEF4624-13|13-SRNP-100574|Costa Rica|Guanacaste|605[0n]|BOLD:AAA...
Rhabdatomis laudamia|[4810]|CNCLB2506-14|CNCLEP 00119630|Mexico|Veracruz|658[0n]|BOLD:AAA1327
Rhabdatomis laudamia|[4811]|BLPDQ892-10|10-SRNP-105518|Costa Rica|Alajuela|658[0n]|BOLD:AAA1327
Rhabdatomis laudamia|[4812]|BLPBA398-07|06-SRNP-107541|Costa Rica|Guanacaste|658[0n]|BOLD:AAA1327
Rhabdatomis laudamia|[4813]|BLPAH395-07|06-SRNP-106974|Costa Rica|Guanacaste|658[0n]|BOLD:AAA1327
Rhabdatomis laudamia|[4814]|BLPCC134-08|07-SRNP-106619|Costa Rica|Guanacaste|658[0n]|BOLD:AAA1327
Rhabdatomis laudamia|[4815]|BLPDQ908-10|10-SRNP-105534|Costa Rica|Alajuela|658[0n]|BOLD:AAA1327
Rhabdatomis laudamia|[4816]|BLPDN1964-10|10-SRNP-102139|Costa Rica|Alajuela|658[0n]|BOLD:AAA1327
Rhabdatomis laudamia|[4817]|BLPCG822-08|07-SRNP-111067|Costa Rica|Guanacaste|658[0n]|BOLD:AAA1327
Rhabdatomis laudamia|[4818]|BLPCG700-08|07-SRNP-110945|Costa Rica|Guanacaste|658[0n]|BOLD:AAA1327
Rhabdatomis laudamia|[4819]|BLPDO365-10|10-SRNP-102947|Costa Rica|Alajuela|658[0n]|BOLD:AAA1327
Rhabdatomis laudamia|[4820]|BLPDK617-09|09-SRNP-105545|Costa Rica|Guanacaste|658[0n]|BOLD:AAA1327
Rhabdatomis laudamia|[4821]|BLPCD080-08|07-SRNP-107505|Costa Rica|Guanacaste|658[0n]|BOLD:AAA1327
Rhabdatomis laudamia|[4822]|BLPDK1774-09|09-SRNP-106702|Costa Rica|Guanacaste|658[0n]|BOLD:AAA...
Rhabdatomis laudamia|[4823]|BLPCF283-08|07-SRNP-109588|Costa Rica|Alajuela|658[0n]|BOLD:AAA1327
Rhabdatomis laudamia|[4824]|BLPCC135-08|07-SRNP-106620|Costa Rica|Guanacaste|658[0n]|BOLD:AAA1327
Rhabdatomis laudamia|[4825]|BLPBC262-07|06-SRNP-109285|Costa Rica|Alajuela|658[0n]|BOLD:AAA1327
Rhabdatomis laudamia|[4826]|BLPBA400-07|06-SRNP-107543|Costa Rica|Guanacaste|658[0n]|BOLD:AAA1327
Rhabdatomis laudamia|[4827]|BLPAH391-07|06-SRNP-106970|Costa Rica|Guanacaste|658[0n]|BOLD:AAA1327
Rhabdatomis laudamia|[4828]|BLPDX386-11|10-SRNP-113513|Costa Rica|Guanacaste|658[0n]|BOLD:AAA1327
Rhabdatomis laudamia|[4829]|BLPDX1006-11|10-SRNP-114133|Costa Rica|Guanacaste|658[0n]|BOLD:AAA...
Rhabdatomis laudamia|[4830]|BLPCG699-08|07-SRNP-110944|Costa Rica|Guanacaste|658[0n]|BOLD:AAA1327
Rhabdatomis laudamia|[4831]|BLPDK1593-09|09-SRNP-106521|Costa Rica|Guanacaste|658[1n]|BOLD:AAA...
Rhabdatomis laudamia|[4832]|BLPDO366-10|10-SRNP-102948|Costa Rica|Alajuela|658[0n]|BOLD:AAA1327
Rhabdatomis laudamia|[4833]|BLPDX468-11|10-SRNP-113595|Costa Rica|Guanacaste|658[0n]|BOLD:AAA1327
Rhabdatomis laudamia|[4834]|BLPEA1028-11|11-SRNP-101495|Costa Rica|Guanacaste|658[1n]|BOLD:AAA...
Rhabdatomis laudamia|[4835]|BLPBB396-07|06-SRNP-108479|Costa Rica|Guanacaste|658[0n]|BOLD:AAA1327
Rhabdatomis laudamia|[4836]|BLPBB397-07|06-SRNP-108480|Costa Rica|Guanacaste|658[0n]|BOLD:AAA1327
Rhabdatomis laudamia|[4837]|BLPEC284-11|11-SRNP-102841|Costa Rica|Alajuela|658[0n]|BOLD:AAA1327
Rhabdatomis laudamia|[4838]|BLPCF284-08|07-SRNP-109589|Costa Rica|Alajuela|658[0n]|BOLD:AAA1327
Rhabdatomis laudamia|[4839]|BLPDK1381-09|09-SRNP-106309|Costa Rica|Guanacaste|658[0n]|BOLD:AAA...
Rhabdatomis laudamia|[4840]|BLPCF783-08|07-SRNP-110088|Costa Rica|Alajuela|658[0n]|BOLD:AAA1327
Rhabdatomis laudamia|[4841]|BLPCD081-08|07-SRNP-107506|Costa Rica|Guanacaste|658[0n]|BOLD:AAA1327
Rhabdatomis laudamia|[4842]|BLPDN2061-10|10-SRNP-102236|Costa Rica|Alajuela|658[0n]|BOLD:AAA1327
Rhabdatomis laudamia|[4843]|BLPAD112-06|06-SRNP-102931|Costa Rica|Guanacaste|658[0n]|BOLD:AAA1327
Rhabdatomis laudamia|[4844]|BLPDQ031-10|10-SRNP-104657|Costa Rica|Alajuela|658[0n]|BOLD:AAA1327
Rhabdatomis laudamia|[4845]|BLPDN165-10|10-SRNP-100340|Costa Rica|Alajuela|658[0n]|BOLD:AAA1327
Rhabdatomis laudamia|[4846]|BLPDN330-10|10-SRNP-100505|Costa Rica|Alajuela|658[0n]|BOLD:AAA1327
Rhabdatomis laudamia|[4847]|BLPDN2332-10|10-SRNP-102507|Costa Rica|Alajuela|658[0n]|BOLD:AAA1327
Rhabdatomis laudamia|[4848]|BLPDQ909-10|10-SRNP-105535|Costa Rica|Alajuela|658[0n]|BOLD:AAA1327
Rhabdatomis laudamia|[4849]|BLPDM1218-10|09-SRNP-110595|Costa Rica|Guanacaste|658[0n]|BOLD:AAA...
Rhabdatomis laudamia|[4850]|BLPDM2312-10|10-SRNP-100049|Costa Rica|Alajuela|658[0n]|BOLD:AAA1327
Rhabdatomis laudamia|[4851]|BLPBA856-07|06-SRNP-107999|Costa Rica|Guanacaste|650[0n]|BOLD:AAA1327
Rhabdatomis laudamia|[4852]|BLPBG297-07|07-SRNP-103116|Costa Rica|Guanacaste|643[0n]|BOLD:AAA1327
Rhabdatomis laudamia|[4853]|BLPEF4143-13|13-SRNP-100093|Costa Rica|Guanacaste|658[0n]|BOLD:AAA...
Rhabdatomis laudamia|[4854]|BLPEF4142-13|13-SRNP-100092|Costa Rica|Guanacaste|658[0n]|BOLD:AAA...
Rhabdatomis laudamia|[4855]|BLPDW581-11|10-SRNP-112473|Costa Rica|Guanacaste|658[0n]|BOLD:AAA1327
Rhabdatomis laudamia|[4856]|BLPDX1007-11|10-SRNP-114134|Costa Rica|Guanacaste|658[0n]|BOLD:AAA...
Rhabdatomis laudamia|[4857]|BLPDX1004-11|10-SRNP-114131|Costa Rica|Guanacaste|658[0n]|BOLD:AAA...
Rhabdatomis laudamia|[4858]|BLPDH861-09|09-SRNP-103345|Costa Rica|Alajuela|658[0n]|BOLD:AAA1327
Rhabdatomis laudamia|[4859]|BLPDX1227-11|10-SRNP-114354|Costa Rica|Guanacaste|658[0n]|BOLD:AAA...
Rhabdatomis laudamia|[4860]|BLPBE838-07|07-SRNP-101777|Costa Rica|Guanacaste|504[0n]|BOLD:AAA1327
Rhabdatomis laudamia|[4861]|BLPDN065-10|10-SRNP-100240|Costa Rica|Alajuela|658[0n]|BOLD:AAA1327
Rhabdatomis laudamia|[4862]|BLPDN670-10|10-SRNP-100845|Costa Rica|Alajuela|658[2n]|BOLD:AAA1327
Rhabdatomis laudamia|[4863]|BLPDO267-10|10-SRNP-102849|Costa Rica|Alajuela|658[1n]|BOLD:AAA1327
Rhabdatomis laudamia|[4864]|BLPDO164-10|10-SRNP-102746|Costa Rica|Alajuela|658[0n]|BOLD:AAA1327
Rhabdatomis laudamia|[4865]|BLPDM997-10|09-SRNP-110374|Costa Rica|Guanacaste|658[0n]|BOLD:AAA1327
Rhabdatomis laudamia|[4866]|BLPDN1644-10|10-SRNP-101819|Costa Rica|Alajuela|658[0n]|BOLD:AAA1327
Rhabdatomis laudamia|[4867]|BLPDN1734-10|10-SRNP-101909|Costa Rica|Alajuela|658[0n]|BOLD:AAA1327
Rhabdatomis laudamia|[4868]|BLPDQ893-10|10-SRNP-105519|Costa Rica|Alajuela|658[0n]|BOLD:AAA1327
Rhabdatomis laudamia|[4869]|LTOLB426-09|KN-06-1098|Costa Rica|San Jose|658[0n]|BOLD:AAA1327
Rhabdatomis laudamia|[4870]|BLPDX047-11|10-SRNP-113174|Costa Rica|Guanacaste|658[0n]|BOLD:AAA1327
Rhabdatomis laudamia|[4871]|BLPDK452-09|09-SRNP-105380|Costa Rica|Guanacaste|622[0n]|BOLD:AAA1327
Rhabdatomis laudamia|[4872]|BLPCP604-08|08-SRNP-105021|Costa Rica|Guanacaste|658[0n]|BOLD:AAA1327
Rhabdatomis laudamia|[4873]|BLPAH467-07|06-SRNP-107046|Costa Rica|Guanacaste|658[0n]|BOLD:AAA1327
Rhabdatomis laudamia|[4874]|BLPDI223-09|09-SRNP-103655|Costa Rica|Alajuela|658[0n]|BOLD:AAA1327
Rhabdatomis laudamia|[4875]|BLPDX1008-11|10-SRNP-114135|Costa Rica|Guanacaste|658[0n]|BOLD:AAA...
Rhabdatomis laudamia|[4876]|BLPEF2919-13|12-SRNP-104657|Costa Rica|Guanacaste|658[0n]|BOLD:AAA...
Rhabdatomis laudamia|[4877]|BLPDX1226-11|10-SRNP-114353|Costa Rica|Guanacaste|658[0n]|BOLD:AAA...
Rhabdatomis laudamia|[4878]|BLPDZ226-11|10-SRNP-115633|Costa Rica|Guanacaste|658[1n]|BOLD:AAA1327
Rhabdatomis laudamia|[4879]|BLPDN1115-10|10-SRNP-101290|Costa Rica|Alajuela|658[0n]|BOLD:AAA1327
Rhabdatomis laudamia|[4880]|BLPBC261-07|06-SRNP-109284|Costa Rica|Alajuela|631[0n]|BOLD:AAA1327

Rhabdatomis laudamia|[4877]|BLPDX1226-11|10-SRNP-114333|Costa Rica|Guanacaste|658[0n]|BOLD:AAA...
Rhabdatomis laudamia|[4878]|BLPDZ226-11|10-SRNP-115633|Costa Rica|Guanacaste|658[1n]|BOLD:AAA1327
Rhabdatomis laudamia|[4879]|BLPDN1115-10|10-SRNP-101290|Costa Rica|Alajuela|658[0n]|BOLD:AAA1327
Rhabdatomis laudamia|[4880]|BLPBC261-07|06-SRNP-109284|Costa Rica|Alajuela|631[0n]|BOLD:AAA1327
Rhabdatomis laudamia|[4881]|BLPDX1005-11|10-SRNP-114132|Costa Rica|Guanacaste|658[0n]|BOLD:AAA...
Rhabdatomis laudamia|[4882]|BLPDX385-11|10-SRNP-113512|Costa Rica|Guanacaste|658[0n]|BOLD:AAA1327
Rhabdatomis laudamia|[4883]|BLPEF2580-13|12-SRNP-104318|Costa Rica|Guanacaste|658[0n]|BOLD:AAA...
Rhabdatomis laudamia|[4884]|BLPAH393-07|06-SRNP-106972|Costa Rica|Guanacaste|658[0n]|BOLD:AAA1327
Rhabdatomis laudamia|[4885]|BLPAH394-07|06-SRNP-106973|Costa Rica|Guanacaste|658[0n]|BOLD:AAA1327
Rhabdatomis laudamia|[4886]|BLPDZ225-11|10-SRNP-115632|Costa Rica|Guanacaste|658[0n]|BOLD:AAA1327
Rhabdatomis laudamia|[4887]|BLPEB432-11|11-SRNP-101944|Costa Rica|Guanacaste|658[0n]|BOLD:AAA1327
Rhabdatomis laudamia|[4888]|BLPEB433-11|11-SRNP-101945|Costa Rica|Guanacaste|658[0n]|BOLD:AAA1327
Rhabdatomis laudamia|[4889]|BLPEB434-11|11-SRNP-101946|Costa Rica|Guanacaste|658[0n]|BOLD:AAA1327
Rhabdatomis laudamia|[4890]|BLPBA053-07|06-SRNP-107196|Costa Rica|Guanacaste|658[0n]|BOLD:AAA1327
Rhabdatomis laudamia|[4891]|BLPBB395-07|06-SRNP-108478|Costa Rica|Guanacaste|658[0n]|BOLD:AAA1327
Rhabdatomis laudamia|[4892]|BLPCP513-08|08-SRNP-104930|Costa Rica|Guanacaste|658[0n]|BOLD:AAA1327
Rhabdatomis laudamia|[4893]|BLPEB902-11|11-SRNP-102414|Costa Rica|Guanacaste|658[0n]|BOLD:AAA1327
Rhabdatomis laudamia|[4894]|BLPEB956-11|11-SRNP-102468|Costa Rica|Guanacaste|658[0n]|BOLD:AAA1327
Rhabdatomis laudamia|[4895]|BLPCP514-08|08-SRNP-104931|Costa Rica|Guanacaste|658[0n]|BOLD:AAA1327
Rhabdatomis laudamia|[4896]|BLPDK1591-09|09-SRNP-106519|Costa Rica|Guanacaste|658[0n]|BOLD:AAA...
Rhabdatomis laudamia|[4897]|BLPDK1592-09|09-SRNP-106520|Costa Rica|Guanacaste|658[0n]|BOLD:AAA...
Rhabdatomis laudamia|[4898]|BLPDK1773-09|09-SRNP-106701|Costa Rica|Guanacaste|658[0n]|BOLD:AAA...
Rhabdatomis laudamia|[4899]|BLPCD079-08|07-SRNP-107504|Costa Rica|Guanacaste|658[0n]|BOLD:AAA1327
Rhabdatomis laudamia|[4900]|BLPCD082-08|07-SRNP-107507|Costa Rica|Guanacaste|658[0n]|BOLD:AAA1327
Rhabdatomis laudamia|[4901]|BLPDK1858-09|09-SRNP-106786|Costa Rica|Guanacaste|658[0n]|BOLD:AAA...
Rhabdatomis laudamia|[4902]|BLPDO364-10|10-SRNP-102946|Costa Rica|Alajuela|658[0n]|BOLD:AAA1327
Rhabdatomis laudamia|[4903]|BLPCG239-08|07-SRNP-110484|Costa Rica|Alajuela|658[0n]|BOLD:AAA1327
Rhabdatomis laudamia|[4904]|BLPDN2106-10|10-SRNP-102281|Costa Rica|Alajuela|658[0n]|BOLD:AAA1327
Rhabdatomis laudamia|[4905]|BLPEA065-11|11-SRNP-100532|Costa Rica||658[0n]|BOLD:AAA1327
Rhabdatomis laudamia|[4906]|BLPAA738-06|06-SRNP-100737|Costa Rica|Alajuela|658[0n]|BOLD:AAA1327
Rhabdatomis laudamia|[4907]|BLPDO616-10|10-SRNP-103199|Costa Rica|Alajuela|658[0n]|BOLD:AAA1327
Rhabdatomis laudamia|[4908]|BLPDN1423-10|10-SRNP-101598|Costa Rica|Alajuela|658[0n]|BOLD:AAA1327
Rhabdatomis laudamia|[4909]|BLPDN1444-10|10-SRNP-101619|Costa Rica|Alajuela|658[0n]|BOLD:AAA1327
Rhabdatomis laudamia|[4910]|BLPDN1927-10|10-SRNP-102102|Costa Rica|Alajuela|658[0n]|BOLD:AAA1327
Rhabdatomis laudamia|[4911]|BLPAD267-06|06-SRNP-103086|Costa Rica|Guanacaste|658[0n]|BOLD:AAA1327
Rhabdatomis laudamia|[4912]|BLPAD769-06|06-SRNP-103588|Costa Rica|Alajuela|658[0n]|BOLD:AAA1327
Rhabdatomis laudamia|[4913]|BLPDN629-10|10-SRNP-100804|Costa Rica|Alajuela|658[0n]|BOLD:AAA1327
Rhabdatomis laudamia|[4914]|BLPEF188-12|12-SRNP-102372|Costa Rica|Alajuela|658[0n]|BOLD:AAA1327
Rhabdatomis laudamia|[4915]|BLPDN1126-10|10-SRNP-101301|Costa Rica|Alajuela|658[0n]|BOLD:AAA1327
Rhabdatomis laudamia|[4916]|BLPDZ694-11|11-SRNP-100306|Costa Rica|Alajuela|658[0n]|BOLD:AAA1327
Rhabdatomis laudamia|[4917]|LTOLB427-09|KN-06-1096|Costa Rica|San Jose|658[0n]|BOLD:AAA1327
Rhabdatomis laudamia|[4918]|BLPDX046-11|10-SRNP-113173|Costa Rica|Guanacaste|658[0n]|BOLD:AAA1327
Rhabdatomis laudamia|[4919]|BLPBA855-07|06-SRNP-107998|Costa Rica|Guanacaste|649[0n]|BOLD:AAA1327
Rhabdatomis laudamia|[4920]|BLPBC925-07|06-SRNP-109948|Costa Rica|Alajuela|658[1n]|BOLD:AAA1327
Rhabdatomis laudamia|[4921]|BLPBC482-07|06-SRNP-109505|Costa Rica|Alajuela|638[0n]|BOLD:AAA1327
Rhabdatomis laudamia|[4922]|BLPDN957-10|10-SRNP-101132|Costa Rica|Alajuela|633[0n]|BOLD:AAA1327
Rhabdatomis laudamia|[4923]|BLPDN1376-10|10-SRNP-101551|Costa Rica|Alajuela|532[0n]|BOLD:AAA1327
Rhabdatomis laudamia|[4924]|BLPBC481-07|06-SRNP-109504|Costa Rica|Alajuela|615[1n]|BOLD:AAA1327
Rhabdatomis laudamia|[4925]|BLPBC479-07|06-SRNP-109502|Costa Rica|Alajuela|633[0n]|BOLD:AAA1327
Rhabdatomis laudamia|[4926]|BLPBC480-07|06-SRNP-109503|Costa Rica|Alajuela|632[0n]|BOLD:AAA1327
Rhabdatomis laudamia|[4927]|BLPDO837-10|10-SRNP-103198|Costa Rica|Alajuela|658[0n]|BOLD:AAA1327
Rhabdatomis laudamia|[4928]|BLPAH468-07|06-SRNP-107047|Costa Rica|Guanacaste|658[0n]|BOLD:AAA1327
Rhabdatomis laudamia|[4929]|BLPBE029-07|07-SRNP-100968|Costa Rica|Guanacaste|658[0n]|BOLD:AAA1327
Rhabdatomis laudamia|[4930]|BLPBE028-07|07-SRNP-100967|Costa Rica|Guanacaste|658[0n]|BOLD:AAA1327
Rhabdatomis laudamia|[4931]|BLPBE027-07|07-SRNP-100966|Costa Rica|Guanacaste|658[0n]|BOLD:AAA1327
Rhabdatomis laudamia|[4932]|BLPDT1706-10|10-SRNP-109133|Costa Rica|Guanacaste|658[0n]|BOLD:AAA...
Rhabdatomis laudamia|[4933]|BLPDX384-11|10-SRNP-113511|Costa Rica|Guanacaste|658[0n]|BOLD:AAA1327
Haematomis uniformis|[4934]|ABLCW251-10|CSUPOBK-0251|United States|Arizona|658[0n]|BOLD:AAI6523
Haematomis uniformis|[4935]|ABLCW250-10|CSUPOBK-0250|United States|Arizona|658[0n]|BOLD:AAI6523
Haematomis uniformis|[4936]|ABLCW249-10|CSUPOBK-0249|United States|Arizona|658[0n]|BOLD:AAI6523
Haematomis uniformis|[4937]|ABLCW246-10|CSUPOBK-0246|United States|Arizona|658[0n]|BOLD:AAI6523
Haematomis uniformis|[4938]|RDNMH370-09|CNCLEP00057779|United States|Arizona|658[0n]|BOLD:AAI6523
Haematomis uniformis|[4939]|ABLCW024-10|CSUPOBK-0024|United States|Arizona|658[0n]|BOLD:AAI6523
Haematomis uniformis|[4940]|ABLCW248-10|CSUPOBK-0248|United States|Arizona|658[0n]|BOLD:AAI6523
Haematomis uniformis|[4941]|ABLCW247-10|CSUPOBK-0247|United States|Arizona|658[0n]|BOLD:AAI6523
Haematomis uniformis|[4942]|RDNMH366-09|CNCLEP00057775|United States|Arizona|658[0n]|BOLD:AAI6523
Bruceia pulverina|[4943]|RDNMG031-08|NOC14972|United States|Wyoming|658[0n]|BOLD:AAD9797
Bruceia pulverina|[4944]|RDNME227-07|CNCNoctuoidea13834|Canada|British Columbia|615[2n]|BOLD:A...
Bruceia pulverina|[4945]|LBCG2899-09|08-JDWBC-2899|Canada|British Columbia|658[0n]|BOLD:AAD9797
Bruceia pulverina|[4946]|LBCH7697-10|10-JDWBC-7697|Canada|British Columbia|658[0n]|BOLD:AAD9797
Bruceia pulverina|[4947]|LBCH6241-10|10-JDWBC-6241|Canada|British Columbia|658[0n]|BOLD:AAD9797
Bruceia pulverina|[4948]|LBCH6133-10|10-JDWBC-6133|Canada|British Columbia|658[0n]|BOLD:AAD9797
Bruceia pulverina|[4949]|RDNME446-08|LEP037870|Canada|British Columbia|549[0n]|BOLD:AAD9797
Bruceia pulverina|[4950]|RDNMG024-08|NOC14965|United States|Colorado|658[0n]|BOLD:AAD9797
Bruceia pulverina|[4951]|ABLCW262-10|CSUPOBK-0262|United States|Colorado|658[0n]|BOLD:AAD9797
Bruceia pulverina|[4952]|ABLCW261-10|CSUPOBK-0261|United States|Colorado|658[0n]|BOLD:AAD9797
Bruceia pulverina|[4953]|ABLCW260-10|CSUPOBK-0260|United States|Colorado|658[0n]|BOLD:AAD9797
Bruceia pulverina|[4954]|ABLCW258-10|CSUPOBK-0258|United States|Wyoming|658[0n]|BOLD:AAD9797
Bruceia pulverina|[4955]|ABLCW072-10|CSUPOBK-0072|United States|Colorado|658[0n]|BOLD:ACE4641
Bruceia pulverina|[4956]|ABLCW070-10|CSUPOBK-0070|United States|Arizona|658[0n]|BOLD:ACE4641
Bruceia pulverina|[4957]|ABLCW069-10|CSUPOBK-0069|United States|New Mexico|658[0n]|BOLD:ACE6091
Bruceia pulverina|[4958]|ABLCW068-10|CSUPOBK-0068|United States|New Mexico|658[0n]|BOLD:ACE6091
Bruceia hubbardi|[4959]|CMAZA696-10|CMAZ-0696|United States|Arizona|658[0n]|BOLD:AAE8103
Bruceia hubbardi|[4960]|RDNME552-08|LEP037976|United States|Arizona|658[0n]|BOLD:AAE8103
Bruceia hubbardi|[4961]|BBLOC488-11|BIOUG01457-B06|United States|Arizona|658[0n]|BOLD:AAE8103
Bruceia hubbardi|[4962]|BBLOC484-11|BIOUG01457-B02|United States|Arizona|658[0n]|BOLD:AAE8103
Bruceia hubbardi|[4963]|BBLOE1810-12|BIOUG01991-C08|United States|Arizona|658[0n]|BOLD:AAE8103
Bruceia hubbardi|[4964]|RDNMG023-08|NOC14964|United States|Nevada|658[0n]|BOLD:AAE8103
Bruceia hubbardi|[4965]|ABLCW259-10|CSUPOBK-0259|United States|Nevada|658[0n]|BOLD:AAE8103
Bruceia hubbardi|[4966]|ABLCW267-10|CSUPOBK-0267|United States|Wyoming|658[0n]|BOLD:AAE8103
Bruceia hubbardi|[4967]|ABLCW268-10|CSUPOBK-0268|United States|Wyoming|658[0n]|BOLD:AAE8103
Bruceia hubbardi|[4968]|RDNMG022-08|NOC14963|United States|Colorado|658[0n]|BOLD:AAE8103
Bruceia hubbardi|[4969]|RDNMG033-08|NOC14974|United States|Utah|649[0n]|BOLD:AAE8103
Bruceia hubbardi|[4970]|RDNMG032-08|NOC14973|United States|Utah|658[0n]|BOLD:AAE8103
Bruceia hubbardi|[4971]|ABLCW266-10|CSUPOBK-0266|United States|Wyoming|658[0n]|BOLD:AAE8103
Bruceia hubbardi|[4972]|ABLCW265-10|CSUPOBK-0265|United States|Colorado|658[0n]|BOLD:AAE8103
Bruceia hubbardi|[4973]|ABLCW264-10|CSUPOBK-0264|United States|Colorado|658[0n]|BOLD:AAE8103
Bruceia hubbardi|[4974]|ABLCW263-10|CSUPOBK-0263|United States|Colorado|658[0n]|BOLD:AAE8103
Bruceia hubbardi|[4975]|ABLCW257-10|CSUPOBK-0257|United States|Wyoming|658[0n]|BOLD:AAE8103
Bruceia NEW|[4976]|LOCBE439-06|06-BLLOC-4258|United States|California|594[0n]|BOLD:AAI3857
Acsala anomala|[4977]|RDNMF753-08|NOC14839|Canada|Yukon Territory|658[0n]|BOLD:AAD5206
Acsala anomala|[4978]|RDNME383-08|LEP037807|Canada|Yukon Territory|658[0n]|BOLD:AAD5206
Acsala anomala|[4979]|RDNME382-08|LEP037806|Canada|Yukon Territory|658[0n]|BOLD:AAD5206

Acsala anomala|[4977]|RDNMF753-08|NOC14839|Canada|Yukon Territory|658[0n]|BOLD:AAD5206
Acsala anomala|[4978]|RDNME383-08|LEP037807|Canada|Yukon Territory|658[0n]|BOLD:AAD5206
Acsala anomala|[4979]|RDNME382-08|LEP037806|Canada|Yukon Territory|658[0n]|BOLD:AAD5206
Acsala anomala|[4980]|RDNMF752-08|NOC14838|Canada|Yukon Territory|658[0n]|BOLD:AAD5206
Acsala anomala|[4981]|RDNMF751-08|NOC14837|Canada|Yukon Territory|658[0n]|BOLD:AAD5206
Acsala anomala|[4982]|RDNME384-08|LEP037808|Canada|Yukon Territory|658[0n]|BOLD:AAD5206
Acsala anomala|[4983]|TIPSY572-12|STG98|Canada|Yukon Territory|658[0n]|BOLD:AAD5206
Inopsis modulata|[4984]|RDNML064-13|CNCLEP 94288|Mexico|Sonora|658[0n]|BOLD:AAW9025
Inopsis modulata|[4985]|RDNML063-13|CNCLEP 94287|Mexico|Sonora|658[0n]|BOLD:AAW9025
Inopsis modulata|[4986]|RDNMJ439-11|CNCLEP 79967|United States|Arizona|658[0n]|BOLD:AAW9025
Agylla septentrionalis|[4987]|RDNMG045-08|NOC14986|United States|Arizona|658[0n]|BOLD:AAF2522
Agylla septentrionalis|[4988]|RDNMG043-08|NOC14984|United States|Arizona|658[0n]|BOLD:AAF2522
Agylla septentrionalis|[4989]|RDNMG287-08|NOC15040|United States|Arizona|658[0n]|BOLD:AAF2522
Agylla septentrionalis|[4990]|RDNMJ271-11|CNCLEP 70294|United States|Arizona|658[0n]|BOLD:AAF2522
Agylla septentrionalis|[4991]|ABLCW148-10|CSUPOBK-0148|United States|Arizona|658[0n]|BOLD:AAF2522
Agylla septentrionalis|[4992]|ABLCW147-10|CSUPOBK-0147|United States|Arizona|658[0n]|BOLD:AAF2522
Agylla septentrionalis|[4993]|ABLCW146-10|CSUPOBK-0146|United States|Arizona|658[0n]|BOLD:AAF2522
Agylla septentrionalis|[4994]|ABLCW145-10|CSUPOBK-0145|United States|Arizona|658[0n]|BOLD:AAF2522
Agylla septentrionalis|[4995]|ABLCW144-10|CSUPOBK-0144|United States|Arizona|658[0n]|BOLD:AAF2522
Agylla septentrionalis|[4996]|ABLCW143-10|CSUPOBK-0143|United States|Arizona|658[0n]|BOLD:AAF2522
Inopsis funerea|[4997]|LNAUT259-14|CCDB-22941-F10|United States|New Mexico|632[0n]|BOLD:AAL3900
Gnamptonychia ventralis|[4998]|RDNML062-13|CNCLEP 94286|Mexico|Sonora|658[0n]|BOLD:AAL3900
Gnamptonychia ventralis|[4999]|RDNMJ440-11|CNCLEP 79968|United States|Arizona|658[0n]|BOLD:AAL...
Gnamptonychia ventralis|[5000]|ABLCW018-10|CSUPOBK-0018|United States|New Mexico|658[0n]|BOLD:...
Gnamptonychia ventralis|[5001]|ABLCW078-10|CSUPOBK-0078|Mexico|San Luis Potosi|658[0n]|BOLD:AA...
Gnamptonychia ventralis|[5002]|JBAZ135-09|JLB-0135|United States|Arizona|658[0n]|BOLD:AAL3900
Gnamptonychia ventralis|[5003]|ABLCW019-10|CSUPOBK-0019|United States|New Mexico|658[0n]|BOLD:...
Gnamptonychia ventralis|[5004]|ABLCW017-10|CSUPOBK-0017|United States|New Mexico|658[0n]|BOLD:...
Gnamptonychia ventralis|[5005]|DMAZ158-10|DMLEP-0057|United States|Arizona|658[0n]|BOLD:AAL3900
Crambidia n. sp. 2|[5006]|RDNMF926-08|CNC LEP00054193|Mexico|Tamaulipas|658[0n]|BOLD:AAB8012
Crambidia impura|[5007]|LMEM911-09|RBMIS-1006|United States|New Mexico|658[0n]|BOLD:AAE4082
Crambidia impura|[5008]|RDNME305-07|CNCNoctuoidea13912|United States|Colorado|658[0n]|BOLD:AAE...
Crambidia impura|[5009]|RDNME460-08|LEP037884|United States|Colorado|658[0n]|BOLD:AAE4082
Crambidia impura|[5010]|RDNME459-08|LEP037883|United States|Colorado|658[0n]|BOLD:AAE4082
Crambidia impura|[5011]|LMEM912-09|RBMIS-1007|United States|New Mexico|658[0n]|BOLD:AAE4082
Crambidia impura|[5012]|ABLCW063-10|CSUPOBK-0063|United States|New Mexico|658[0n]|BOLD:AAE4082
Crambidia impura|[5013]|RDNMF929-08|CNC LEP00054196|United States|Texas|658[0n]|BOLD:AAE4082
Crambidia lithosioides|[5014]|RDNME315-07|CNCNoctuoidea13922|United States|Florida|658[1n]|BOLD ...
Crambidia lithosioides|[5015]|HKONS563-08|3092-COI-08|United States|Florida|658[0n]|BOLD:AAA5564
Crambidia lithosioides|[5016]|LNCC910-11|11-NCCC-435|United States|North Carolina|658[0n]|BOLD ...
Crambidia lithosioides|[5017]|LNC507-06|05-NCCC-507|United States|North Carolina|658[0n]|BOLD:...
Crambidia lithosioides|[5018]|ABLCW191-10|CSUPOBK-0191|United States|Georgia|658[0n]|BOLD:AAA5564
Crambidia lithosioides|[5019]|LPOKD366-09|MDOK-3445|United States|Oklahoma|658[0n]|BOLD:AAA5564
Crambidia lithosioides|[5020]|LNCB497-07|07-NCNW-0181|United States|North Carolina|658[0n]|BOLD ...
Crambidia lithosioides|[5021]|LPOKA294-08|MDOK-0294|United States|Oklahoma|658[0n]|BOLD:AAA5564
Crambidia lithosioides|[5022]|RDNME314-07|CNCNoctuoidea13921|United States|Florida|647[1n]|BOLD ...
Crambidia lithosioides|[5023]|LNCB804-09|09-NCCC-274|United States|North Carolina|620[1n]|BOLD ...
Crambidia lithosioides|[5024]|HKONS540-08|3069-COI-08|United States|Florida|588[0n]|BOLD:AAA5564
Crambidia lithosioides|[5025]|LNC506-06|05-NCCC-506|United States|North Carolina|658[0n]|BOLD:...
Crambidia lithosioides|[5026]|LOFLD043-07|HLC-16617|United States|Florida|658[0n]|BOLD:AAA5564
Crambidia lithosioides|[5027]|HKONS562-08|3091-COI-08|United States|Florida|658[0n]|BOLD:AAA5564
Crambidia lithosioides|[5028]|LOFLB355-06|06-FLOR-1295|United States|Florida|658[0n]|BOLD:AAA5564
Crambidia casta|[5029]|LPABC166-09|08BBLEP-04385|Canada|Alberta|658[6n]|
Crambidia casta|[5030]|LPSOD942-09|08BBLEP-05482|Canada|Ontario|658[0n]|BOLD:AAB4937
Crambidia casta|[5031]|LPSK555-08|08BBLEP-02123|Canada|Saskatchewan|658[0n]|BOLD:AAB4937
Crambidia casta|[5032]|RDMAB398-05|BCSC71|Canada|Alberta|658[0n]|BOLD:AAB4937
Crambidia casta|[5033]|ABLCW034-10|CSUPOBK-0034|United States|Wyoming|658[0n]|BOLD:AAB4937
Crambidia casta|[5034]|ABLCW016-10|CSUPOBK-0016|United States|Wyoming|658[0n]|BOLD:AAB4937
Crambidia casta|[5035]|RDNME403-08|LEP037827|United States|Oregon|658[0n]|BOLD:AAB4937
Crambidia casta|[5036]|RDMAB592-06|UASM58902|Canada|Alberta|658[0n]|BOLD:AAB4937
Crambidia casta|[5037]|RDMAB593-06|UASM58903|Canada|Alberta|658[0n]|BOLD:AAB4937
Crambidia casta|[5038]|RDMAB600-06|UASM58644|Canada|Alberta|658[0n]|BOLD:AAB4937
Crambidia casta|[5039]|RDMAB604-06|UASM58652|Canada|Alberta|658[0n]|BOLD:AAB4937
Crambidia casta|[5040]|LMEM894-09|RBMIS-0989|United States|Wyoming|658[0n]|BOLD:AAB4937
Crambidia casta|[5041]|LPABC151-09|08BBLEP-04370|Canada|Alberta|658[0n]|BOLD:AAB4937
Crambidia casta|[5042]|LBCG3240-09|08-JDWBC-3240|Canada|British Columbia|658[0n]|BOLD:AAB4937
Crambidia casta|[5043]|LBCG2647-09|08-JDWBC-2647|Canada|British Columbia|658[0n]|BOLD:AAB4937
Crambidia casta|[5044]|LBCG2587-09|08-JDWBC-2587|Canada|British Columbia|658[0n]|BOLD:AAB4937
Crambidia casta|[5045]|LBCG2157-09|08-JDWBC-2157|Canada|British Columbia|658[0n]|BOLD:AAB4937
Crambidia casta|[5046]|RDNME304-07|CNCNoctuoidea13911|United States|Colorado|653[0n]|BOLD:AAB4937
Crambidia casta|[5047]|RDNME402-08|LEP037826|United States|Washington|658[0n]|BOLD:AAB4937
Crambidia casta|[5048]|ABLCW179-10|CSUPOBK-0179|United States|Wyoming|658[0n]|BOLD:AAB4937
Crambidia casta|[5049]|ABLCW067-10|CSUPOBK-0067|United States|Colorado|658[0n]|BOLD:AAB4937
Crambidia casta|[5050]|ABLCW066-10|CSUPOBK-0066|United States|Colorado|658[0n]|BOLD:AAB4937
Crambidia casta|[5051]|ABLCW065-10|CSUPOBK-0065|United States|Colorado|658[0n]|BOLD:AAB4937
Crambidia casta|[5052]|ABLCW033-10|CSUPOBK-0033|United States|Wyoming|658[0n]|BOLD:AAB4937
Crambidia casta|[5053]|RDMAB397-05|BCSC70|Canada|Alberta|658[0n]|BOLD:AAB4937
Crambidia casta|[5054]|LPABC153-09|08BBLEP-04372|Canada|Alberta|658[0n]|BOLD:AAB4937
Crambidia casta|[5055]|LMEM910-09|RBMIS-1005|United States|Wyoming|658[0n]|BOLD:AAB4937
Crambidia casta|[5056]|LMEM896-09|RBMIS-0991|United States|Wyoming|658[0n]|BOLD:AAB4937
Crambidia casta|[5057]|LMEM893-09|RBMIS-0988|United States|Wyoming|658[0n]|BOLD:AAB4937
Crambidia casta|[5058]|ABLCW014-10|CSUPOBK-0014|United States|Wyoming|658[0n]|BOLD:AAB4937
Crambidia casta|[5059]|RDMAB680-06|UASM58811|Canada|Alberta|658[0n]|BOLD:AAB4937
Crambidia casta|[5060]|ABLCW002-10|CSUPOBK-0002|United States|Colorado|658[0n]|BOLD:AAB4937
Crambidia casta|[5061]|RDNME557-08|LEP037981|United States|Wyoming|658[0n]|BOLD:AAB4937
Crambidia casta|[5062]|ABLCW178-10|CSUPOBK-0178|United States|Wyoming|658[0n]|BOLD:AAB4937
Crambidia casta|[5063]|ABLCW168-10|CSUPOBK-0168|United States|Wyoming|658[0n]|BOLD:AAB4937
Crambidia casta|[5064]|ABLCW015-10|CSUPOBK-0015|United States|Wyoming|658[0n]|BOLD:AAB4937
Crambidia casta|[5065]|ABLCW013-10|CSUPOBK-0013|United States|Wyoming|658[0n]|BOLD:AAB4937
Crambidia casta|[5066]|ABLCW001-10|CSUPOBK-0001|United States|Colorado|658[0n]|BOLD:AAB4937
Crambidia casta|[5067]|BBLPA889-10|10BBCLP-0889|Canada|Alberta|658[0n]|BOLD:AAB4937
Crambidia casta|[5068]|BBLPB899-10|10BBCLP-1898|Canada|Alberta|658[0n]|BOLD:AAB4937
Crambidia xanthocorpa|[5069]|LSEU634-06|06-JKA-0634|United States|Georgia|658[0n]|BOLD:AAB7303
Crambidia xanthocorpa|[5070]|LGSMG103-07|BGS03424|United States|Tennessee|658[0n]|BOLD:AAB7303
Crambidia xanthocorpa|[5071]|LNCC1557-13|13-NCCC-227|United States|North Carolina|658[0n]|BOLD ...
Crambidia xanthocorpa|[5072]|LOT530-04|04HBL002530|United States|Tennessee|658[0n]|BOLD:AAB7303
Crambidia xanthocorpa|[5073]|ABLCW155-10|CSUPOBK-0155|United States|North Carolina|658[0n]|BOLD ...
Crambidia xanthocorpa|[5074]|LGSMB531-05|DNA-ATBI-1380|United States|Tennessee|658[0n]|BOLD:AA...
Crambidia xanthocorpa|[5075]|LNCC1556-13|13-NCCC-226|United States|North Carolina|658[0n]|BOLD ...
Crambidia xanthocorpa|[5076]|MNAE861-13|CNCLEP00098226|United States|Indiana|658[0n]|BOLD:AAB7303
Crambidia xanthocorpa|[5077]|JSBIC007-08|HLC-16960|United States|Tennessee|658[0n]|BOLD:AAB7303
Crambidia xanthocorpa|[5078]|LMEM907-09|RBMIS-1002|United States|Tennessee|658[0n]|BOLD:AAB7303
Crambidia xanthocorpa|[5079]|LMEM905-09|RBMIS-1000|United States|Tennessee|658[0n]|BOLD:AAB7303

[illegible]
Crambidia xanthocorpa|[5077]|JSBIC007-08|HLC-16960|United States|Tennessee|658[0n]|BOLD:AAB7303
Crambidia xanthocorpa|[5078]|LMEM907-09|RBMIS-1002|United States|Tennessee|658[0n]|BOLD:AAB7303
Crambidia xanthocorpa|[5079]|LMEM905-09|RBMIS-1000|United States|Tennessee|658[0n]|BOLD:AAB7303
Crambidia xanthocorpa|[5080]|LGSMG105-07|BGS03426|United States|Tennessee|658[0n]|BOLD:AAB7303
Crambidia xanthocorpa|[5081]|MNAE862-13|CNCLEP00098227|United States|Indiana|658[0n]|BOLD:AAB7303
Crambidia xanthocorpa|[5082]|LGSMG104-07|BGS03425|United States|Tennessee|658[0n]|BOLD:AAB7303
Crambidia xanthocorpa|[5083]|LMEM904-09|RBMIS-0999|United States|Tennessee|658[0n]|BOLD:AAB7303
Crambidia xanthocorpa|[5084]|LMEM906-09|RBMIS-1001|United States|Tennessee|658[0n]|BOLD:AAB7303
Crambidia xanthocorpa|[5085]|LMEM908-09|RBMIS-1003|United States|Tennessee|658[0n]|BOLD:AAB7303
Crambidia xanthocorpa|[5086]|LSEU635-06|06-JKA-0635|United States|Georgia|658[0n]|BOLD:AAB7303
Crambidia xanthocorpa|[5087]|MNAA737-07|CNCLEP00027229|United States|Tennessee|634[0n]|BOLD:AA...
Crambidia xanthocorpa|[5088]|LOT553-04|04HBL002553|United States|Tennessee|573[0n]|BOLD:AAB7303
Crambidia xanthocorpa|[5089]|LGSMB330-05|DNA-ATBI-1179|United States|Tennessee|559[1n]|BOLD:AA...
Crambidia xanthocorpa|[5090]|LOT552-04|04HBL002552|United States|Tennessee|572[0n]|BOLD:AAB7303
Crambidia xanthocorpa|[5091]|LGSMB329-05|DNA-ATBI-1178|United States|Tennessee|591[0n]|BOLD:AA...
Crambidia xanthocorpa|[5092]|LGSMB532-05|DNA-ATBI-1381|United States|Tennessee|658[0n]|BOLD:AA...
Crambidia xanthocorpa|[5093]|ABLCW154-10|CSUPOBK-0154|United States|North Carolina|658[0n]|BOLD ...
Crambidia nr. pallida|[5094]|HKONB439-09|3936-COI-08|United States|Texas|658[0n]|BOLD:ABZ1042
Crambidia nr. pallida|[5095]|HKONB440-09|3937-COI-08|United States|Texas|658[0n]|BOLD:ABZ1042
Crambidia nr. pallida|[5096]|MILEQ305-11|11-MISC-780|United States|Alabama|658[0n]|BOLD:ABZ1042
Crambidia nr. pallida|[5097]|LNCB830-09|09-MISC-015|United States|Alabama|658[0n]|BOLD:ABZ1042
Crambidia nr. pallida|[5098]|MILEP350-10|10-MISC-255|United States|Alabama|658[0n]|BOLD:ABZ1042
Crambidia nr. pallida|[5099]|MILEP349-10|10-MISC-254|United States|Alabama|658[0n]|BOLD:ABZ1042
Crambidia nr. pallida|[5100]|MILEP348-10|10-MISC-253|United States|Alabama|658[0n]|BOLD:ABZ1042
Crambidia nr. pallida|[5101]|LMEM884-09|RBMIS-0979|United States|Alabama|658[0n]|BOLD:ABZ1042
Crambidia nr. pallida|[5102]|MNAB149-07|CNCLEP00025740|United States|Florida|637[0n]|BOLD:ABZ1042
Crambidia nr. pallida|[5103]|RDNME316-07|CNCNoctuoidea13923|United States|Florida|597[0n]|BOLD ...
Crambidia nr. pallida|[5104]|HKONS544-08|3073-COI-08|United States|Florida|658[0n]|BOLD:ABZ1042
Crambidia nr. pallida|[5105]|MECD372-06|jflandry2944|United States|Maryland|658[0n]|BOLD:ABZ1042
Crambidia nr. pallida|[5106]|MECD373-06|jflandry2945|United States|Maryland|658[0n]|BOLD:ABZ1042
Crambidia nr. pallida|[5107]|BBLOB274-11|BIOUG01369-G12|United States|Florida|658[0n]|BOLD:ABZ...
Crambidia nr. pallida|[5108]|BBLOB924-11|BIOUG01413-F09|United States|Florida|658[0n]|BOLD:ABZ...
Crambidia nr. pallida|[5109]|HKONS543-08|3072-COI-08|United States|Florida|658[0n]|BOLD:ABZ1042
Crambidia nr. pallida|[5110]|RDNME317-07|CNCNoctuoidea13924|United States|Florida|658[0n]|BOLD ...
Crambidia nr. pallida|[5111]|RDNME318-07|CNCNoctuoidea13925|United States|Florida|658[1n]|BOLD ...
Crambidia nr. pallida|[5112]|MNAB148-07|CNCLEP00025739|United States|Florida|637[0n]|BOLD:ABZ1042
Crambidia nr. pallida|[5113]|RDNME219-07|CNCNoctuoidea13826|United States|Florida|616[1n]|BOLD ...
Crambidia nr. pallida|[5114]|HKONS085-07|1649-COI-07|United States|Florida|647[0n]|BOLD:ABZ1042
Crambidia nr. pallida|[5115]|MNAC442-07|CNCLEP00026409|United States|Florida|658[0n]|BOLD:ABZ1042
Crambidia nr. pallida|[5116]|HKONS548-08|3077-COI-08|United States|Florida|658[0n]|BOLD:ABZ1042
Crambidia nr. pallida|[5117]|HKONS547-08|3076-COI-08|United States|Florida|658[0n]|BOLD:ABZ1042
Crambidia nr. pallida|[5118]|HKONS546-08|3075-COI-08|United States|Florida|658[0n]|BOLD:ABZ1042
Crambidia nr. pallida|[5119]|HKONS545-08|3074-COI-08|United States|Florida|658[0n]|BOLD:ABZ1042
Crambidia nr. pallida|[5120]|LOFLB313-06|06-FLOR-1253|United States|Florida|658[0n]|BOLD:ABZ1042
Crambidia nr. pallida|[5121]|MNAC629-07|CNCLEP00027071|United States|Maryland|658[0n]|BOLD:ABZ...
Crambidia pallida|[5122]|XAG585-05|2005-ONT-1169|Canada|Ontario|606[1n]|BOLD:AAA5563
Crambidia pallida|[5123]|XAG505-05|2005-ONT-1089|Canada|Ontario|597[2n]|BOLD:AAA5563
Crambidia pallida|[5124]|LMEM881-09|RBMIS-0976|United States|Mississippi|658[0n]|BOLD:AAA5563
Crambidia pallida|[5125]|LMEM880-09|RBMIS-0975|United States|Mississippi|658[3n]|BOLD:AAA5563
Crambidia pallida|[5126]|LMEM879-09|RBMIS-0974|United States|Mississippi|658[0n]|BOLD:AAA5563
Crambidia pallida|[5127]|LNCB495-07|07-NCNW-0179|United States|North Carolina|658[0n]|BOLD:AAA...
Crambidia pallida|[5128]|MNAC634-07|CNCLEP00027076|United States|Maryland|658[0n]|BOLD:AAA5563
Crambidia pallida|[5129]|MECD374-06|jflandry2946|United States|Maryland|658[0n]|BOLD:AAA5563
Crambidia pallida|[5130]|LNCC911-11|11-NCCC-436|United States|North Carolina|658[0n]|BOLD:AAA5563
Crambidia pallida|[5131]|LNCB287-06|06-NCCC-1243|United States|North Carolina|658[0n]|BOLD:AAA...
Crambidia pallida|[5132]|LNCC909-11|11-NCCC-434|United States|North Carolina|658[0n]|BOLD:AAA5563
Crambidia pallida|[5133]|TMNBD168-07|MNBTT-2969|Canada|New Brunswick|658[0n]|BOLD:AAA5563
Crambidia pallida|[5134]|RDLQB687-05|DH010790|Canada|Quebec|658[0n]|BOLD:AAA5563
Crambidia pallida|[5135]|TMNBD455-07|MNBTT-3256|Canada|New Brunswick|658[0n]|BOLD:AAA5563
Crambidia pallida|[5136]|TMNBD453-07|MNBTT-3254|Canada|New Brunswick|658[0n]|BOLD:AAA5563
Crambidia pallida|[5137]|LNCC358-10|10-NCCC-358|United States|North Carolina|658[0n]|BOLD:AAA5563
Crambidia pallida|[5138]|MNAF858-08|CNCLEP00040843|Canada|Manitoba|658[0n]|BOLD:AAA5563
Crambidia pallida|[5139]|MNAF860-08|CNCLEP00040845|Canada|Manitoba|658[0n]|BOLD:AAA5563
Crambidia pallida|[5140]|BLTIB809-08|BL1227|Canada|Ontario|625[0n]|BOLD:AAA5563
Crambidia pallida|[5141]|MNAF861-08|CNCLEP00040846|Canada|Manitoba|658[0n]|BOLD:AAA5563
Crambidia pallida|[5142]|MNAF859-08|CNCLEP00040844|Canada|Manitoba|658[0n]|BOLD:AAA5563
Crambidia pallida|[5143]|MNAF440-08|CNCLEP00040425|Canada|Manitoba|658[0n]|BOLD:AAA5563
Crambidia pallida|[5144]|MNAF057-08|CNCLEP00038542|Canada|Manitoba|658[0n]|BOLD:AAA5563
Crambidia pallida|[5145]|MNAF441-08|CNCLEP00040426|Canada|Manitoba|658[0n]|BOLD:AAA5563
Crambidia pallida|[5146]|MNAF439-08|CNCLEP00040424|Canada|Manitoba|658[0n]|BOLD:AAA5563
Crambidia pallida|[5147]|MNAF438-08|CNCLEP00040423|Canada|Manitoba|658[0n]|BOLD:AAA5563
Crambidia pallida|[5148]|LPSOD980-09|08BBLEP-05613|Canada|Ontario|658[0n]|BOLD:AAA5563
Crambidia pallida|[5149]|RDLQB683-05|DH010786|Canada|Quebec|613[0n]|BOLD:AAA5563
Crambidia pallida|[5150]|XAK215-06|2006-ONT-1210|Canada|Ontario|658[0n]|BOLD:AAA5563
Crambidia pallida|[5151]|RDLQB542-05|DH010628|Canada|Quebec|658[0n]|BOLD:AAA5563
Crambidia pallida|[5152]|RDLQB684-05|DH010787|Canada|Quebec|658[0n]|BOLD:AAA5563
Crambidia pallida|[5153]|RDLQB685-05|DH010788|Canada|Quebec|658[0n]|BOLD:AAA5563
Crambidia pallida|[5154]|RDLQB890-05|DH006630|Canada|Quebec|658[0n]|BOLD:AAA5563
Crambidia pallida|[5155]|RDLQG686-06|DH012979|Canada|Quebec|656[0n]|BOLD:AAA5563
Crambidia pallida|[5156]|RDLQG883-06|DH013176|Canada|Quebec|658[0n]|BOLD:AAA5563
Crambidia pallida|[5157]|RDLQB686-05|DH010789|Canada|Quebec|658[0n]|BOLD:AAA5563
Crambidia pallida|[5158]|LPMNB238-09|08BBLEP-05082|Canada|Manitoba|658[0n]|BOLD:AAA5563
Crambidia pallida|[5159]|LILLA812-11|SNS10IL-01019|United States|Illinois|658[0n]|BOLD:AAA5563
Crambidia pallida|[5160]|RDLQG754-06|DH013047|Canada|Quebec|658[0n]|BOLD:AAA5563
Crambidia pallida|[5161]|XAJ879-06|2006-ONT-0879|Canada|Ontario|636[0n]|BOLD:AAA5563
Crambidia pallida|[5162]|TMNBD452-07|MNBTT-3253|Canada|New Brunswick|658[0n]|BOLD:AAA5563
Crambidia pallida|[5163]|LILLA777-11|SNS10IL-00975|United States|Illinois|658[0n]|BOLD:AAA5563
Crambidia pallida|[5164]|TMNBD454-07|MNBTT-3255|Canada|New Brunswick|654[0n]|BOLD:AAA5563
Crambidia pallida|[5165]|MNAF442-08|CNCLEP00040427|Canada|Manitoba|658[0n]|BOLD:AAA5563
Crambidia pallida|[5166]|RDLQB682-05|DH010785|Canada|Quebec|623[0n]|BOLD:AAA5563
Crambidia pallida|[5167]|BBLPE619-09|09BBELE-2619|Canada|Nova Scotia|650[0n]|BOLD:AAA5563
Crambidia pallida|[5168]|RDNME407-08|LEP037831|Canada|Ontario|658[0n]|BOLD:AAA5563
Crambidia pallida|[5169]|RDNME406-08|LEP037830|Canada|Ontario|658[0n]|BOLD:AAA5563
Crambidia pallida|[5170]|RDNME405-08|LEP037829|Canada|Ontario|658[0n]|BOLD:AAA5563
Crambidia pallida|[5171]|BBLEC741-09|09BBELE-0741|Canada|Nova Scotia|658[0n]|BOLD:AAA5563
Crambidia pallida|[5172]|BBLEC712-09|09BBELE-0712|Canada|Nova Scotia|658[0n]|BOLD:AAA5563
Crambidia pallida|[5173]|LMEM878-09|RBMIS-0973|United States|Georgia|595[0n]|BOLD:AAA5563
Crambidia pallida|[5174]|LGSMG102-07|BGS03423|United States|Tennessee|658[0n]|BOLD:AAA5563
Crambidia pallida|[5175]|LNCNW106-06|06-NCNW-0106|United States|North Carolina|658[0n]|BOLD:AA...
Crambidia pallida|[5176]|LNCB496-07|07-NCNW-0180|United States|North Carolina|658[0n]|BOLD:AAA...
Crambidia pallida|[5177]|LNCB010-06|06-NCC-966|United States|North Carolina|658[0n]|BOLD:AAA5563
Crambidia pallida|[5178]|LNCB011-06|06-NCC-967|United States|North Carolina|658[0n]|BOLD:AAA5563
[illegible]

Crambidia pallida|[5176]|LNCB496-07|07-NCNW-0180|United States|North Carolina|658[0n]|BOLD:AAA...
Crambidia pallida|[5177]|LNCB010-06|06-NCC-966|United States|North Carolina|658[0n]|BOLD:AAA5563
Crambidia pallida|[5178]|LNCB011-06|06-NCC-967|United States|North Carolina|658[0n]|BOLD:AAA5563
Crambidia pallida|[5179]|LNCC731-11|11-NCCC-256|United States|North Carolina|658[0n]|BOLD:AAA5563
Crambidia pallida|[5180]|RDNME218-07|CNCNoctuoidea13825|United States|Florida|658[0n]|BOLD:AAA...
Crambidia uniformis|[5181]|LMEM876-09|RBMIS-0971|United States|Alabama|658[0n]|BOLD:AAA5563
Crambidia pallida|[5182]|LGSMC759-05|DNA-ATBI-2759|United States|Tennessee|658[0n]|BOLD:AAA5563
Crambidia uniformis|[5183]|LMEM875-09|RBMIS-0970|United States|Alabama|658[0n]|BOLD:AAA5563
Crambidia uniformis|[5184]|LMEM873-09|RBMIS-0968|United States|Mississippi|658[0n]|BOLD:AAA5563
Crambidia pallida|[5185]|LNCC853-11|11-NCCC-378|United States|North Carolina|658[0n]|BOLD:AAA5563
Crambidia pallida|[5186]|HKONB428-09|3925-COI-08|United States|Kentucky|658[0n]|BOLD:AAA5563
Crambidia uniformis|[5187]|LNCC1469-13|13-NCCC-139|United States|North Carolina|658[0n]|BOLD:A...
Crambidia uniformis|[5188]|LNCC1468-13|13-NCCC-138|United States|North Carolina|658[0n]|BOLD:A...
Crambidia pallida|[5189]|LGSMC760-05|DNA-ATBI-2760|United States|Tennessee|658[0n]|BOLD:AAA5563
Crambidia pallida|[5190]|LNCC852-11|11-NCCC-377|United States|North Carolina|658[0n]|BOLD:AAA5563
Crambidia uniformis|[5191]|ABLCW192-10|CSUPOBK-0192|United States|Georgia|658[0n]|BOLD:AAA5563
Crambidia pallida|[5192]|LNCB009-06|06-NCC-965|United States|North Carolina|658[0n]|BOLD:AAA5563
Crambidia pallida|[5193]|HKONS538-08|3067-COI-08|United States|Florida|658[0n]|BOLD:AAA5563
Crambidia pallida|[5194]|HKONS539-08|3068-COI-08|United States|Florida|658[0n]|BOLD:AAA5563
Crambidia pallida|[5195]|HKONS541-08|3070-COI-08|United States|Florida|658[0n]|BOLD:AAA5563
Crambidia pallida|[5196]|HKONS537-08|3066-COI-08|United States|Florida|647[0n]|BOLD:AAA5563
Crambidia pallida|[5197]|LNCB286-06|06-NCCC-1242|United States|North Carolina|658[0n]|BOLD:AAA...
Crambidia pallida|[5198]|LNCC029-10|10-NCCC-124|United States|North Carolina|658[0n]|BOLD:AAA5563
Crambidia dusca|[5199]|LOCBF3526-14|BIOUG11931-G11|United States|California|549[0n]|BOLD:ABY4243
Crambidia dusca|[5200]|LOCBF3529-14|BIOUG11931-H02|United States|California|552[0n]|BOLD:ABY4243
Crambidia dusca|[5201]|LOCBF3500-14|BIOUG11931-E09|United States|California|549[0n]|BOLD:ABY4243
Crambidia dusca|[5202]|LOCBF3779-14|BIOUG12229-E03|United States|California|576[0n]|BOLD:ABY4243
Crambidia dusca|[5203]|LOCBF3459-14|BIOUG11931-B04|United States|California|576[0n]|BOLD:ABY4243
Crambidia dusca|[5204]|LOCBF3475-14|BIOUG11931-C08|United States|California|591[0n]|BOLD:ABY4243
Crambidia dusca|[5205]|LOCBB904-06|06-BLLOC-1844|United States|California|658[0n]|BOLD:ABY4243
Crambidia dusca|[5206]|LOCBD038-06|06-BLLOC-2858|United States|California|658[0n]|BOLD:ABY4243
Crambidia dusca|[5207]|LOCBB903-06|06-BLLOC-1843|United States|California|658[0n]|BOLD:ABY4243
Crambidia dusca|[5208]|ABLCW183-10|CSUPOBK-0183|United States|California|658[0n]|BOLD:ABY4243
Crambidia dusca|[5209]|ABLCW184-10|CSUPOBK-0184|United States|California|658[0n]|BOLD:ABY4243
Crambidia dusca|[5210]|ABLCW181-10|CSUPOBK-0181|United States|California|658[0n]|BOLD:ABY4243
Crambidia dusca|[5211]|ABLCW185-10|CSUPOBK-0185|United States|California|658[0n]|BOLD:ABY4243
Crambidia dusca|[5212]|LOCBC596-06|06-BLLOC-2476|United States|California|658[0n]|BOLD:ABY4243
Crambidia dusca|[5213]|RDNMG038-08|NOC14979|United States|California|658[0n]|BOLD:ABY4243
Crambidia dusca|[5214]|LOCBF2643-13|BIOUG07173-E07|United States|California|658[0n]|BOLD:ABY4243
Crambidia dusca|[5215]|LOCBF2648-13|BIOUG07173-E12|United States|California|658[0n]|BOLD:ABY4243
Crambidia dusca|[5216]|ABLCW182-10|CSUPOBK-0182|United States|California|633[0n]|BOLD:ABY4243
Crambidia dusca|[5217]|LOCBB902-06|06-BLLOC-1842|United States|California|656[0n]|BOLD:ABY4243
Crambidia dusca|[5218]|ABLCW180-10|CSUPOBK-0180|United States|California|658[0n]|BOLD:ABY4243
Crambidia dusca|[5219]|LOCBF435-13|BIOUG06177-E07|United States|California|616[0n]|BOLD:ABY4243
Crambidia myrlosea|[5220]|ABLCW167-10|CSUPOBK-0167|Mexico|Sonora|658[0n]|BOLD:AAB8011
Crambidia myrlosea|[5221]|ABLCW163-10|CSUPOBK-0163|Mexico|Sonora|658[0n]|BOLD:AAB8011
Crambidia myrlosea|[5222]|RDNMG048-08|NOC14989|Mexico|Sonora|658[0n]|BOLD:AAB8011
Crambidia myrlosea|[5223]|TXLEP001-14|BIOUG13574-A01|United States|Texas|582[0n]|BOLD:AAB8011
Crambidia myrlosea|[5224]|ABLCW165-10|CSUPOBK-0165|Mexico|Sonora|658[0n]|BOLD:AAB8011
Crambidia myrlosea|[5225]|ABLCW166-10|CSUPOBK-0166|Mexico|Sonora|658[0n]|BOLD:AAB8011
Crambidia myrlosea|[5226]|ABLCW164-10|CSUPOBK-0164|Mexico|Sonora|658[0n]|BOLD:AAB8011
Crambidia myrlosea|[5227]|HKONB457-09|3954-COI-08|United States|Texas|658[0n]|BOLD:AAB8011
Crambidia myrlosea|[5228]|HKONB152-08|3649-COI-08|United States|Texas|658[0n]|BOLD:AAB8011
Crambidia myrlosea|[5229]|RDNMH122-09|CNCLEP00054341|United States|Texas|621[0n]|BOLD:AAB8011
Crambidia myrlosea|[5230]|HKONB153-08|3650-COI-08|United States|Texas|658[1n]|BOLD:AAB8011
Crambidia myrlosea|[5231]|HKONB151-08|3648-COI-08|United States|Texas|658[0n]|BOLD:AAB8011
Crambidia myrlosea|[5232]|HKONB149-08|3646-COI-08|United States|Texas|658[0n]|BOLD:AAB8011
Crambidia myrlosea|[5233]|HKONB148-08|3645-COI-08|United States|Texas|658[0n]|BOLD:AAB8011
Crambidia myrlosea|[5234]|HKONB147-08|3644-COI-08|United States|Texas|658[0n]|BOLD:AAB8011
Crambidia myrlosea|[5235]|HKONB458-09|3955-COI-08|United States|Texas|658[0n]|BOLD:AAB8011
Crambidia myrlosea|[5236]|RDNMG044-08|NOC14985|Mexico|Nuevo Leon|658[0n]|BOLD:AAB8011
Crambidia myrlosea|[5237]|ABLCW159-10|CSUPOBK-0159|Mexico|Nuevo Leon|658[0n]|BOLD:AAB8011
Crambidia myrlosea|[5238]|ABLCW157-10|CSUPOBK-0157|Mexico|Nuevo Leon|658[0n]|BOLD:AAB8011
Crambidia myrlosea|[5239]|RDNMF930-08|CNC LEP00054197|United States|Texas|507[0n]|BOLD:AAB8011
Crambidia myrlosea|[5240]|BBLSW133-09|09BBLEP-01061|United States|Texas|658[0n]|BOLD:AAB8011
Crambidia pura|[5241]|RDNMF756-08|NOC14842|United States|Wisconsin|658[0n]|BOLD:AAD3975
Crambidia pura|[5242]|LMEM890-09|RBMIS-0985|United States|Mississippi|658[0n]|BOLD:AAD3975
Crambidia pura|[5243]|RDNMF755-08|NOC14841|United States|Mississippi|658[0n]|BOLD:AAD3975
Crambidia pura|[5244]|LMEM889-09|RBMIS-0984|United States|Florida|658[0n]|BOLD:AAD3975
Crambidia pura|[5245]|LMEM887-09|RBMIS-0982|United States|Mississippi|658[0n]|BOLD:AAD3975
Crambidia pura|[5246]|LMEM888-09|RBMIS-0983|United States|Mississippi|658[0n]|BOLD:AAD3975
Crambidia pura|[5247]|RDNME404-08|LEP037828|Canada|Ontario|649[0n]|BOLD:AAD3975
Crambidia n. sp. 1|[5248]|HKONB158-08|3655-COI-08|United States|Texas|658[0n]|BOLD:AAC3365
Crambidia n. sp. 1|[5249]|HKONB157-08|3654-COI-08|United States|Texas|658[0n]|BOLD:AAC3365
Crambidia n. sp. 1|[5250]|HKONB156-08|3653-COI-08|United States|Texas|658[0n]|BOLD:AAC3365
Crambidia n. sp. 1|[5251]|HKONB155-08|3652-COI-08|United States|Texas|658[0n]|BOLD:AAC3365
Crambidia n. sp. 1|[5252]|HKONB154-08|3651-COI-08|United States|Texas|658[0n]|BOLD:AAC3365
Crambidia n. sp. 1|[5253]|HKONB150-08|3647-COI-08|United States|Texas|658[0n]|BOLD:AAC3365
Crambidia cephalica|[5254]|CMAZA601-10|CMAZ-0601|United States|Arizona|658[0n]|BOLD:AAB7304
Crambidia cephalica|[5255]|ABLCW170-10|CSUPOBK-0170|United States|Arizona|658[0n]|BOLD:AAB7304
Crambidia cephalica|[5256]|CMAZA138-09|CMAZ-0138|United States|Arizona|629[0n]|BOLD:AAB7304
Crambidia cephalica|[5257]|RDMAB395-05|BCSC68|United States|Arizona|658[0n]|BOLD:AAB7304
Crambidia cephalica|[5258]|RDMAB396-05|BCSC69|United States|Arizona|658[0n]|BOLD:AAB7304
Crambidia cephalica|[5259]|ABLCW171-10|CSUPOBK-0171|United States|Arizona|658[0n]|BOLD:AAB7304
Crambidia cephalica|[5260]|ABLCW169-10|CSUPOBK-0169|United States|Arizona|658[0n]|BOLD:AAB7304
Crambidia cephalica|[5261]|ABLCW032-10|CSUPOBK-0032|United States|Arizona|658[0n]|BOLD:AAB7304
Crambidia cephalica|[5262]|RDNMF919-08|CNC LEP00054186|United States|Arizona|658[0n]|BOLD:AAB7304
Crambidia cephalica|[5263]|RDNMG041-08|NOC14982|United States|Colorado|658[0n]|BOLD:AAB7304
Crambidia cephalica|[5264]|RDNMG042-08|NOC14983|United States|Colorado|658[0n]|BOLD:AAB7304
Crambidia cephalica|[5265]|LMEM913-09|RBMIS-1008|United States|New Mexico|658[0n]|BOLD:AAB7304
Crambidia cephalica|[5266]|RDNMF928-08|CNC LEP00054195|United States|Colorado|545[0n]|BOLD:AAB...
Crambidia cephalica|[5267]|ABLCW038-10|CSUPOBK-0038|United States|Wyoming|658[0n]|BOLD:AAB7304
Crambidia cephalica|[5268]|ABLCW172-10|CSUPOBK-0172|United States|Wyoming|658[0n]|BOLD:AAB7304
Crambidia cephalica|[5269]|ABLCW173-10|CSUPOBK-0173|United States|Colorado|658[0n]|BOLD:AAB7304
Crambidia cephalica|[5270]|RDNMG040-08|NOC14981|United States|Wyoming|658[0n]|BOLD:AAB7304
Crambidia cephalica|[5271]|RDNME298-07|CNCNoctuoidea13905|United States|Colorado|656[0n]|BOLD:...
Crambidia cephalica|[5272]|ABLCW031-10|CSUPOBK-0031|United States|Wyoming|658[0n]|BOLD:AAB7304
Crambidia cephalica|[5273]|RDMAB394-05|BCSC67|Canada|Alberta|658[0n]|BOLD:AAB7304
Crambidia cephalica|[5274]|ABLCW030-10|CSUPOBK-0030|United States|Wyoming|658[0n]|BOLD:AAB7304
Crambidia cephalica|[5275]|RDNMG039-08|NOC14980|United States|Colorado|658[0n]|BOLD:ABY7022
Crambidia cephalica|[5276]|RDNMG139-08|NOC14892|United States|New Mexico|658[0n]|BOLD:ACE5957
Crambidia cephalica|[5277]|RDNMK612-11|CNCLEP 79876|United States|New Mexico|658[0n]|BOLD:ACE5957
Crambidia cephalica|[5278]|RDNMF918-08|CNC LEP00054185|United States|Arizona|658[0n]|BOLD:ABY7022

Crambidia cephalica|[5276]|RDNMG139-08|NOC14892|United States|New Mexico|658[0n]|BOLD:ACE5957
Crambidia cephalica|[5277]|RDNMK612-11|CNCLEP 79876|United States|New Mexico|658[0n]|BOLD:ACE5957
Crambidia cephalica|[5278]|RDNMF918-08|CNC LEP00054185|United States|Arizona|658[0n]|BOLD:ABY7022
Crambidia cephalica|[5279]|ABLCW061-10|CSUPOBK-0061|United States|New Mexico|658[0n]|BOLD:ABY7022
Crambidia cephalica|[5280]|RDNMK613-11|CNCLEP 79877|United States|New Mexico|658[0n]|BOLD:ABY7022
Crambidia cephalica|[5281]|LMEM914-09|RBMIS-1009|United States|New Mexico|658[0n]|BOLD:ABY7022
Crambidia cephalica|[5282]|LMEM909-09|RBMIS-1004|United States|New Mexico|658[0n]|BOLD:ABY7022
Crambidia cephalica|[5283]|ABLCW062-10|CSUPOBK-0062|United States|New Mexico|658[0n]|BOLD:ABY7022
Crambidia cephalica|[5284]|RDNMJ283-11|CNCLEP 79906|United States|New Mexico|658[0n]|BOLD:ABY7022
Crambidia cephalica|[5285]|RDNME455-08|LEP037879|United States|New Mexico|658[0n]|BOLD:ABY7022
Crambidia cephalica|[5286]|RDNMH367-09|CNCLEP00057776|United States|Colorado|658[0n]|BOLD:ABY7022
Crambidia nr. cephalica|[5287]|LMEM897-09|RBMIS-0992|United States|Tennessee|593[1n]|BOLD:AAB7306
Crambidia nr. cephalica|[5288]|BBLSY205-09|09BBLEP-03132|United States|Texas|658[0n]|BOLD:AAB7306
Crambidia nr. cephalica|[5289]|RDNMJ284-11|CNCLEP 79907|United States|New Mexico|655[0n]|BOLD:...
Crambidia nr. cephalica|[5290]|BBLSX219-09|09BBLEP-02147|United States|Texas|658[0n]|BOLD:AAB7306
Crambidia nr. cephalica|[5291]|LMEM898-09|RBMIS-0993|United States|Tennessee|658[0n]|BOLD:AAB7306
Crambidia nr. cephalica|[5292]|HKONB315-09|3812-COI-08|United States|Texas|658[0n]|BOLD:AAB7306
Crambidia nr. cephalica|[5293]|BBLSY940-09|09BBLEP-03867|United States|Texas|658[0n]|BOLD:AAB7306
Crambidia nr. cephalica|[5294]|BBLSX223-09|09BBLEP-02151|United States|Texas|658[0n]|BOLD:AAB7306
Crambidia nr. cephalica|[5295]|BBLSX217-09|09BBLEP-02145|United States|Texas|658[0n]|BOLD:AAB7306
Crambidia nr. cephalica|[5296]|BBLSX205-09|09BBLEP-02133|United States|Texas|658[0n]|BOLD:AAB7306
Crambidia nr. cephalica|[5297]|BBLSX188-09|09BBLEP-02116|United States|Texas|658[0n]|BOLD:AAB7306
Crambidia nr. cephalica|[5298]|BBLSY273-09|09BBLEP-03200|United States|Texas|658[0n]|BOLD:AAB7306
Crambidia nr. cephalica|[5299]|BBLSY204-09|09BBLEP-03131|United States|Texas|658[0n]|BOLD:AAB7306
Crambidia nr. cephalica|[5300]|BBLSW105-09|09BBLEP-01033|United States|Texas|658[0n]|BOLD:AAB7306
Crambidia nr. cephalica|[5301]|BBLSW749-09|09BBLEP-01677|United States|Texas|658[0n]|BOLD:AAB7306
Crambidia nr. cephalica|[5302]|BBLSX255-09|09BBLEP-02183|United States|Texas|658[0n]|BOLD:AAB7306
Crambidia nr. cephalica|[5303]|BBLSZ144-09|09BBLEP-04070|United States|Texas|658[0n]|BOLD:AAB7306
Crambidia nr. cephalica|[5304]|BBLSX236-09|09BBLEP-02164|United States|Texas|658[0n]|BOLD:AAB7306
Crambidia nr. cephalica|[5305]|BBLSY252-09|09BBLEP-03179|United States|Texas|658[0n]|BOLD:AAB7306
Crambidia nr. cephalica|[5306]|BBLSW106-09|09BBLEP-01034|United States|Texas|658[0n]|BOLD:AAB7306
Crambidia nr. cephalica|[5307]|BBLSW104-09|09BBLEP-01032|United States|Texas|658[0n]|BOLD:AAB7306
Crambidia nr. cephalica|[5308]|BBLSW103-09|09BBLEP-01031|United States|Texas|658[0n]|BOLD:AAB7306
Crambidia nr. cephalica|[5309]|BBLSW750-09|09BBLEP-01678|United States|Texas|658[0n]|BOLD:AAB7306
Crambidia nr. cephalica|[5310]|BBLSW748-09|09BBLEP-01676|United States|Texas|658[0n]|BOLD:AAB7306
Manulea bicolor|[5311]|LOWCB575-05|CGWC-1515|Canada|British Columbia|563[1n]|BOLD:AAA4503
Manulea bicolor|[5312]|LOWCB582-05|CGWC-1522|Canada|British Columbia|560[0n]|BOLD:AAA4503
Manulea bicolor|[5313]|LOWCB570-05|CGWC-1510|Canada|British Columbia|568[0n]|BOLD:AAA4503
Manulea bicolor|[5314]|SSJAC1662-13|BIOUG06046-C08|Canada|Alberta|589[0n]|BOLD:AAA4503
Manulea bicolor|[5315]|LOWCB574-05|CGWC-1514|Canada|British Columbia|575[0n]|BOLD:AAA4503
Manulea bicolor|[5316]|LOWCB584-05|CGWC-1524|Canada|British Columbia|573[0n]|BOLD:AAA4503
Manulea bicolor|[5317]|LMEM869-09|RBMIS-0964|United States|Wyoming|658[0n]|BOLD:AAA4503
Manulea bicolor|[5318]|ABLCW194-10|CSUPOBK-0194|United States|Wyoming|658[0n]|BOLD:AAA4503
Manulea bicolor|[5319]|LOWCB572-05|CGWC-1512|Canada|British Columbia|573[0n]|BOLD:AAA4503
Manulea bicolor|[5320]|LOWCB583-05|CGWC-1523|Canada|British Columbia|567[6n]|
Manulea bicolor|[5321]|LOWCB580-05|CGWC-1520|Canada|British Columbia|587[2n]|BOLD:AAA4503
Manulea bicolor|[5322]|LBCD382-05|HLC-23202|Canada|British Columbia|658[1n]|BOLD:AAA4503
Manulea bicolor|[5323]|LBCH816-10|10-JDWBC-0816|Canada|British Columbia|658[0n]|BOLD:AAA4503
Manulea bicolor|[5324]|LBCH507-10|10-JDWBC-0507|Canada|British Columbia|658[0n]|BOLD:AAA4503
Manulea bicolor|[5325]|LBCH4054-10|10-JDWBC-4054|Canada|British Columbia|658[0n]|BOLD:AAA4503
Manulea bicolor|[5326]|CNJAF2182-12|BIOUG03976-D01|Canada|Alberta|612[0n]|BOLD:AAA4503
Manulea bicolor|[5327]|LBCD384-05|HLC-23204|Canada|British Columbia|617[0n]|BOLD:AAA4503
Manulea bicolor|[5328]|LBCH002-10|10-JDWBC-0002|Canada|British Columbia|658[0n]|BOLD:AAA4503
Manulea bicolor|[5329]|LBCG3241-09|08-JDWBC-3241|Canada|British Columbia|635[0n]|BOLD:AAA4503
Manulea bicolor|[5330]|LBCH6483-10|10-JDWBC-6483|Canada|British Columbia|658[0n]|BOLD:AAA4503
Manulea bicolor|[5331]|LBCH6482-10|10-JDWBC-6482|Canada|British Columbia|658[0n]|BOLD:AAA4503
Manulea bicolor|[5332]|LBCH7147-10|10-JDWBC-7147|Canada|British Columbia|658[0n]|BOLD:AAA4503
Manulea bicolor|[5333]|LBCH7144-10|10-JDWBC-7144|Canada|British Columbia|658[0n]|BOLD:AAA4503
Manulea bicolor|[5334]|LBCH7382-10|10-JDWBC-7382|Canada|British Columbia|658[0n]|BOLD:AAA4503
Manulea bicolor|[5335]|LBCH7378-10|10-JDWBC-7378|Canada|British Columbia|658[0n]|BOLD:AAA4503
Manulea bicolor|[5336]|LBCH7384-10|10-JDWBC-7384|Canada|British Columbia|658[0n]|BOLD:AAA4503
Manulea bicolor|[5337]|LBCH7383-10|10-JDWBC-7383|Canada|British Columbia|658[0n]|BOLD:AAA4503
Manulea bicolor|[5338]|LBCH122-10|10-JDWBC-0122|Canada|British Columbia|658[0n]|BOLD:AAA4503
Manulea bicolor|[5339]|LBCH7509-10|10-JDWBC-7509|Canada|British Columbia|658[0n]|BOLD:AAA4503
Manulea bicolor|[5340]|LBCH700-10|10-JDWBC-0700|Canada|British Columbia|658[0n]|BOLD:AAA4503
Manulea bicolor|[5341]|LBCH699-10|10-JDWBC-0699|Canada|British Columbia|658[0n]|BOLD:AAA4503
Manulea bicolor|[5342]|LBCH702-10|10-JDWBC-0702|Canada|British Columbia|658[0n]|BOLD:AAA4503
Manulea bicolor|[5343]|LBCH701-10|10-JDWBC-0701|Canada|British Columbia|658[0n]|BOLD:AAA4503
Manulea bicolor|[5344]|LBCH704-10|10-JDWBC-0704|Canada|British Columbia|658[0n]|BOLD:AAA4503
Manulea bicolor|[5345]|LBCH703-10|10-JDWBC-0703|Canada|British Columbia|658[0n]|BOLD:AAA4503
Manulea bicolor|[5346]|LBCH6481-10|10-JDWBC-6481|Canada|British Columbia|658[0n]|BOLD:AAA4503
Manulea bicolor|[5347]|LBCH6480-10|10-JDWBC-6480|Canada|British Columbia|658[0n]|BOLD:AAA4503
Manulea bicolor|[5348]|LBCH6479-10|10-JDWBC-6479|Canada|British Columbia|658[0n]|BOLD:AAA4503
Manulea bicolor|[5349]|LBCH6478-10|10-JDWBC-6478|Canada|British Columbia|658[0n]|BOLD:AAA4503
Manulea bicolor|[5350]|LBCH6477-10|10-JDWBC-6477|Canada|British Columbia|658[0n]|BOLD:AAA4503
Manulea bicolor|[5351]|LBCH6476-10|10-JDWBC-6476|Canada|British Columbia|658[0n]|BOLD:AAA4503
Manulea bicolor|[5352]|LBCH6345-10|10-JDWBC-6345|Canada|British Columbia|658[0n]|BOLD:AAA4503
Manulea bicolor|[5353]|LBCH6342-10|10-JDWBC-6342|Canada|British Columbia|658[0n]|BOLD:AAA4503
Manulea bicolor|[5354]|LBCH6341-10|10-JDWBC-6341|Canada|British Columbia|658[0n]|BOLD:AAA4503
Manulea bicolor|[5355]|LBCH6340-10|10-JDWBC-6340|Canada|British Columbia|658[0n]|BOLD:AAA4503
Manulea bicolor|[5356]|LBCH6338-10|10-JDWBC-6338|Canada|British Columbia|658[0n]|BOLD:AAA4503
Manulea bicolor|[5357]|LMEM868-09|RBMIS-0963|United States|Wyoming|658[0n]|BOLD:AAA4503
Manulea bicolor|[5358]|LBCH7513-10|10-JDWBC-7513|Canada|British Columbia|658[0n]|BOLD:AAA4503
Manulea bicolor|[5359]|LBCH7512-10|10-JDWBC-7512|Canada|British Columbia|658[0n]|BOLD:AAA4503
Manulea bicolor|[5360]|LBCD387-05|HLC-23207|Canada|British Columbia|658[0n]|BOLD:AAA4503
Manulea bicolor|[5361]|LBCD380-05|HLC-23200|Canada|British Columbia|658[0n]|BOLD:AAA4503
Manulea bicolor|[5362]|LBCH7742-10|10-JDWBC-7742|Canada|British Columbia|658[0n]|BOLD:AAA4503
Manulea bicolor|[5363]|LBCH7741-10|10-JDWBC-7741|Canada|British Columbia|658[0n]|BOLD:AAA4503
Manulea bicolor|[5364]|LBCH7740-10|10-JDWBC-7740|Canada|British Columbia|658[0n]|BOLD:AAA4503
Manulea bicolor|[5365]|LBCH7739-10|10-JDWBC-7739|Canada|British Columbia|658[0n]|BOLD:AAA4503
Manulea bicolor|[5366]|LBCH7736-10|10-JDWBC-7736|Canada|British Columbia|658[0n]|BOLD:AAA4503
Manulea bicolor|[5367]|LBCH7696-10|10-JDWBC-7696|Canada|British Columbia|658[0n]|BOLD:AAA4503
Manulea bicolor|[5368]|LBCH6276-10|10-JDWBC-6276|Canada|British Columbia|658[0n]|BOLD:AAA4503
Manulea bicolor|[5369]|DUNLP001-08|Dun-08-001|Canada|British Columbia|658[0n]|BOLD:AAA4503
Manulea bicolor|[5370]|LBCH7381-10|10-JDWBC-7381|Canada|British Columbia|658[0n]|BOLD:AAA4503
Manulea bicolor|[5371]|LOWCB578-05|CGWC-1518|Canada|British Columbia|658[0n]|BOLD:AAA4503
Manulea bicolor|[5372]|LOWCB568-05|CGWC-1508|Canada|British Columbia|658[0n]|BOLD:AAA4503
Manulea bicolor|[5373]|LBCH7925-10|10-JDWBC-7925|Canada|British Columbia|658[0n]|BOLD:AAA4503
Manulea bicolor|[5374]|LBCH7921-10|10-JDWBC-7921|Canada|British Columbia|658[0n]|BOLD:AAA4503
Manulea bicolor|[5375]|LBCH7920-10|10-JDWBC-7920|Canada|British Columbia|658[0n]|BOLD:AAA4503
Manulea bicolor|[5376]|LBCH7919-10|10-JDWBC-7919|Canada|British Columbia|658[0n]|BOLD:AAA4503
Manulea bicolor|[5377]|LBCH7918-10|10-JDWBC-7918|Canada|British Columbia|658[0n]|BOLD:AAA4503
[illegible]

Manulea bicolor|[5375]|LBCH7920-10|10-JDWBC-7920|Canada|British Columbia|658[0n]|BOLD:AAA4503
Manulea bicolor|[5376]|LBCH7919-10|10-JDWBC-7919|Canada|British Columbia|658[0n]|BOLD:AAA4503
Manulea bicolor|[5377]|LBCH7918-10|10-JDWBC-7918|Canada|British Columbia|658[0n]|BOLD:AAA4503
Manulea bicolor|[5378]|LBCH7743-10|10-JDWBC-7743|Canada|British Columbia|658[0n]|BOLD:AAA4503
Manulea bicolor|[5379]|LMEM867-09|RBMIS-0962|United States|Wyoming|658[0n]|BOLD:AAA4503
Manulea bicolor|[5380]|LBCH7923-10|10-JDWBC-7923|Canada|British Columbia|658[0n]|BOLD:AAA4503
Manulea bicolor|[5381]|LBCD388-05|HLC-23208|Canada|British Columbia|658[0n]|BOLD:AAA4503
Manulea bicolor|[5382]|LBCD379-05|HLC-23199|Canada|British Columbia|658[0n]|BOLD:AAA4503
Manulea bicolor|[5383]|LBCD378-05|HLC-23198|Canada|British Columbia|658[0n]|BOLD:AAA4503
Manulea bicolor|[5384]|LBCH227-10|10-JDWBC-0227|Canada|British Columbia|658[0n]|BOLD:AAA4503
Manulea bicolor|[5385]|LBCH6629-10|10-JDWBC-6629|Canada|British Columbia|658[0n]|BOLD:AAA4503
Manulea bicolor|[5386]|LBCH6840-10|10-JDWBC-6840|Canada|British Columbia|658[0n]|BOLD:AAA4503
Manulea bicolor|[5387]|LBCH6839-10|10-JDWBC-6839|Canada|British Columbia|658[0n]|BOLD:AAA4503
Manulea bicolor|[5388]|LBCH7143-10|10-JDWBC-7143|Canada|British Columbia|658[0n]|BOLD:AAA4503
Manulea bicolor|[5389]|LBCH6963-10|10-JDWBC-6963|Canada|British Columbia|658[0n]|BOLD:AAA4503
Manulea bicolor|[5390]|LBCH7508-10|10-JDWBC-7508|Canada|British Columbia|658[0n]|BOLD:AAA4503
Manulea bicolor|[5391]|LBCH7465-10|10-JDWBC-7465|Canada|British Columbia|658[0n]|BOLD:AAA4503
Manulea bicolor|[5392]|LBCH7924-10|10-JDWBC-7924|Canada|British Columbia|658[0n]|BOLD:AAA4503
Manulea bicolor|[5393]|LBCH7922-10|10-JDWBC-7922|Canada|British Columbia|658[0n]|BOLD:AAA4503
Manulea bicolor|[5394]|LBCD386-05|HLC-23206|Canada|British Columbia|658[0n]|BOLD:AAA4503
Manulea bicolor|[5395]|LBCD385-05|HLC-23205|Canada|British Columbia|658[0n]|BOLD:AAA4503
Manulea bicolor|[5396]|LBCD383-05|HLC-23203|Canada|British Columbia|658[0n]|BOLD:AAA4503
Manulea bicolor|[5397]|LBCD381-05|HLC-23201|Canada|British Columbia|658[0n]|BOLD:AAA4503
Manulea bicolor|[5398]|LBCD376-05|HLC-23196|Canada|British Columbia|658[0n]|BOLD:AAA4503
Manulea bicolor|[5399]|ABLCW197-10|CSUPOBK-0197|United States|Wyoming|658[0n]|BOLD:AAA4503
Manulea bicolor|[5400]|ABLCW196-10|CSUPOBK-0196|United States|Wyoming|658[0n]|BOLD:AAA4503
Manulea bicolor|[5401]|LBCG474-08|08-JDWBC-0474|Canada|British Columbia|658[0n]|BOLD:AAA4503
Manulea bicolor|[5402]|LBCH7738-10|10-JDWBC-7738|Canada|British Columbia|658[0n]|BOLD:AAA4503
Manulea bicolor|[5403]|LBCH7737-10|10-JDWBC-7737|Canada|British Columbia|658[0n]|BOLD:AAA4503
Manulea bicolor|[5404]|LBCH706-10|10-JDWBC-0706|Canada|British Columbia|658[0n]|BOLD:AAA4503
Manulea bicolor|[5405]|LBCH705-10|10-JDWBC-0705|Canada|British Columbia|658[0n]|BOLD:AAA4503
Manulea bicolor|[5406]|LBCH818-10|10-JDWBC-0818|Canada|British Columbia|658[0n]|BOLD:AAA4503
Manulea bicolor|[5407]|LBCH817-10|10-JDWBC-0817|Canada|British Columbia|658[0n]|BOLD:AAA4503
Manulea bicolor|[5408]|LBCH820-10|10-JDWBC-0820|Canada|British Columbia|658[0n]|BOLD:AAA4503
Manulea bicolor|[5409]|LBCH819-10|10-JDWBC-0819|Canada|British Columbia|658[0n]|BOLD:AAA4503
Manulea bicolor|[5410]|LBCH7511-10|10-JDWBC-7511|Canada|British Columbia|658[0n]|BOLD:AAA4503
Manulea bicolor|[5411]|LBCH7510-10|10-JDWBC-7510|Canada|British Columbia|658[0n]|BOLD:AAA4503
Manulea bicolor|[5412]|LBCH821-10|10-JDWBC-0821|Canada|British Columbia|658[0n]|BOLD:AAA4503
Manulea bicolor|[5413]|LBCH822-10|10-JDWBC-0822|Canada|British Columbia|658[0n]|BOLD:AAA4503
Manulea bicolor|[5414]|LBCH823-10|10-JDWBC-0823|Canada|British Columbia|658[0n]|BOLD:AAA4503
Manulea bicolor|[5415]|LPABB674-08|08BBLEP-03939|Canada|Alberta|658[0n]|BOLD:AAA4503
Manulea bicolor|[5416]|RDMAB379-05|BCSC53|Canada|British Columbia|658[0n]|BOLD:AAA4503
Manulea bicolor|[5417]|BBLPA890-10|10BBCLP-0890|Canada|British Columbia|658[0n]|BOLD:AAA4503
Manulea bicolor|[5418]|BBLPA893-10|10BBCLP-0893|Canada|Alberta|658[0n]|BOLD:AAA4503
Manulea bicolor|[5419]|BBLPA894-10|10BBCLP-0894|Canada|Alberta|658[0n]|BOLD:AAA4503
Manulea bicolor|[5420]|LMDH171-11|BIOUG01047-F01|United States|Minnesota|658[0n]|BOLD:AAA4503
Manulea bicolor|[5421]|LBCG2106-09|08-JDWBC-2106|Canada|British Columbia|658[0n]|BOLD:AAA4503
Manulea bicolor|[5422]|LBCG2588-09|08-JDWBC-2588|Canada|British Columbia|658[0n]|BOLD:AAA4503
Manulea bicolor|[5423]|CNJAF2149-12|BIOUG03976-A04|Canada|Alberta|612[0n]|BOLD:AAA4503
Manulea bicolor|[5424]|CNJAF2162-12|BIOUG03976-B05|Canada|Alberta|612[2n]|BOLD:AAA4503
Manulea bicolor|[5425]|CNJAF2154-12|BIOUG03976-A09|Canada|Alberta|600[0n]|BOLD:AAA4503
Manulea bicolor|[5426]|BBLPA891-10|10BBCLP-0891|Canada|Alberta|658[0n]|BOLD:AAA4503
Manulea bicolor|[5427]|BBLPA892-10|10BBCLP-0892|Canada|Alberta|658[0n]|BOLD:AAA4503
Manulea bicolor|[5428]|CNJAF2169-12|BIOUG03976-B12|Canada|Alberta|632[0n]|BOLD:AAA4503
Manulea bicolor|[5429]|CNJAF2147-12|BIOUG03976-A02|Canada|Alberta|632[0n]|BOLD:AAA4503
Manulea bicolor|[5430]|CNJAF2170-12|BIOUG03976-C01|Canada|Alberta|632[0n]|BOLD:AAA4503
Manulea bicolor|[5431]|CNJAF2146-12|BIOUG03976-A01|Canada|Alberta|630[0n]|BOLD:AAA4503
Manulea bicolor|[5432]|CNJAF2163-12|BIOUG03976-B06|Canada|Alberta|630[1n]|BOLD:AAA4503
Manulea bicolor|[5433]|LBCH7507-10|10-JDWBC-7507|Canada|British Columbia|658[0n]|BOLD:AAA4503
Manulea bicolor|[5434]|LBCH6343-10|10-JDWBC-6343|Canada|British Columbia|643[0n]|BOLD:AAA4503
Manulea bicolor|[5435]|LBCH7428-10|10-JDWBC-7428|Canada|British Columbia|643[0n]|BOLD:AAA4503
Manulea bicolor|[5436]|LMEM865-09|RBMIS-0960|United States|Wyoming|649[0n]|BOLD:AAA4503
Manulea bicolor|[5437]|LOWCB573-05|CGWC-1513|Canada|British Columbia|590[0n]|BOLD:AAA4503
Manulea bicolor|[5438]|LOWCB579-05|CGWC-1519|Canada|British Columbia|577[0n]|BOLD:AAA4503
Manulea bicolor|[5439]|LBCH7149-10|10-JDWBC-7149|Canada|British Columbia|658[0n]|BOLD:AAA4503
Manulea bicolor|[5440]|LPABB154-08|08BBLEP-03419|Canada|Alberta|643[0n]|BOLD:AAA4503
Manulea bicolor|[5441]|LOWCB587-05|CGWC-1527|Canada|British Columbia|604[3n]|BOLD:AAA4503
Manulea bicolor|[5442]|LOWCB571-05|CGWC-1511|Canada|British Columbia|610[2n]|BOLD:AAA4503
Manulea bicolor|[5443]|LOWCB576-05|CGWC-1516|Canada|British Columbia|516[1n]|BOLD:AAA4503
Manulea bicolor|[5444]|LBCH7506-10|10-JDWBC-7506|Canada|British Columbia|640[0n]|BOLD:AAA4503
Manulea bicolor|[5445]|LOWCB585-05|CGWC-1525|Canada|British Columbia|514[3n]|BOLD:AAA4503
Manulea bicolor|[5446]|TMNBD360-07|MNBTT-3161|Canada|New Brunswick|609[0n]|BOLD:AAA4503
Manulea bicolor|[5447]|LOWCB586-05|CGWC-1526|Canada|British Columbia|514[1n]|BOLD:AAA4503
Manulea bicolor|[5448]|LOWCB581-05|CGWC-1521|Canada|British Columbia|616[0n]|BOLD:AAA4503
Manulea bicolor|[5449]|LOWCB569-05|CGWC-1509|Canada|British Columbia|515[0n]|BOLD:AAA4503
Manulea bicolor|[5450]|LBCG240-08|08-JDWBC-0240|Canada|British Columbia|658[0n]|BOLD:AAA4503
Manulea bicolor|[5451]|LGSMB318-05|DNA-ATBI-1167|United States|Tennessee|580[0n]|BOLD:AAA4503
Manulea bicolor|[5452]|RDLQB546-05|DH010632|Canada|Quebec|528[0n]|BOLD:AAA4503
Manulea bicolor|[5453]|RDLQB532-05|DH010618|Canada|Quebec|565[1n]|BOLD:AAA4503
Manulea bicolor|[5454]|RDLQG814-06|DH013107|Canada|Quebec|658[0n]|BOLD:AAA4503
Manulea bicolor|[5455]|MECD366-06|jflandry2938|Canada|Quebec|658[0n]|BOLD:AAA4503
Manulea bicolor|[5456]|MNBB635-05|05-NBSTA-551|Canada|New Brunswick|658[0n]|BOLD:AAA4503
Manulea bicolor|[5457]|MNAC813-07|CNCLEP00027554|Canada|Quebec|658[0n]|BOLD:AAA4503
Manulea bicolor|[5458]|MNAC812-07|CNCLEP00027553|Canada|Quebec|658[0n]|BOLD:AAA4503
Manulea bicolor|[5459]|MNBB632-05|05-NBSTA-548|Canada|New Brunswick|658[0n]|BOLD:AAA4503
Manulea bicolor|[5460]|MEC809-04|jflandry0809|Canada|Quebec|658[0n]|BOLD:AAA4503
Manulea bicolor|[5461]|LBCH6339-10|10-JDWBC-6339|Canada|British Columbia|658[0n]|BOLD:AAA4503
Manulea bicolor|[5462]|LGSM618-04|DNA-ATBI-0618|United States|North Carolina|609[0n]|BOLD:AAA4503
Manulea bicolor|[5463]|ABLCW193-10|CSUPOBK-0193|United States|Tennessee|658[0n]|BOLD:AAA4503
Manulea bicolor|[5464]|SSPAB10005-13|BIOUG06892-A09|Canada|Saskatchewan|528[0n]|BOLD:AAA4503
Manulea bicolor|[5465]|SSPAA2602-13|BIOUG04561-G02|Canada|Saskatchewan|604[0n]|BOLD:AAA4503
Manulea bicolor|[5466]|LBCH7380-10|10-JDWBC-7380|Canada|British Columbia|658[0n]|BOLD:AAA4503
Manulea bicolor|[5467]|ABLCW195-10|CSUPOBK-0195|United States|Wyoming|658[0n]|BOLD:AAA4503
Manulea bicolor|[5468]|LBCG475-08|08-JDWBC-0475|Canada|British Columbia|658[0n]|BOLD:AAA4503
Manulea bicolor|[5469]|LOWCB577-05|CGWC-1517|Canada|British Columbia|658[0n]|BOLD:AAA4503
Manulea bicolor|[5470]|LBCD377-05|HLC-23197|Canada|British Columbia|658[0n]|BOLD:AAA4503
Manulea bicolor|[5471]|LBCH7142-10|10-JDWBC-7142|Canada|British Columbia|658[0n]|BOLD:AAA4503
Manulea bicolor|[5472]|LBCH7379-10|10-JDWBC-7379|Canada|British Columbia|658[0n]|BOLD:AAA4503
Manulea bicolor|[5473]|LBCH7385-10|10-JDWBC-7385|Canada|British Columbia|658[0n]|BOLD:AAA4503
Manulea bicolor|[5474]|LBCH7397-10|10-JDWBC-7397|Canada|British Columbia|658[0n]|BOLD:AAA4503
Manulea bicolor|[5475]|LPMNB326-09|08BBLEP-05170|Canada|Manitoba|658[0n]|BOLD:AAA4503
Manulea bicolor|[5476]|LPMNB447-09|08BBLEP-05447|Canada|Manitoba|658[0n]|BOLD:AAA4503
Manulea bicolor|[5477]|LBCH7146-10|10-JDWBC-7146|Canada|British Columbia|636[0n]|BOLD:AAA4503

Manulea bicolor|[5475]|LPMNB326-09|08BBLEP-05170|Canada|Manitoba|658[0n]|BOLD:AAA4503
Manulea bicolor|[5476]|LPMNB447-09|08BBLEP-05447|Canada|Manitoba|658[0n]|BOLD:AAA4503
Manulea bicolor|[5477]|LBCH7146-10|10-JDWBC-7146|Canada|British Columbia|636[0n]|BOLD:AAA4503
Manulea bicolor|[5478]|RDMAB404-05|BCSC77|Canada|Yukon Territory|658[0n]|BOLD:AAA4503
Manulea bicolor|[5479]|LBCH7148-10|10-JDWBC-7148|Canada|British Columbia|658[0n]|BOLD:AAA4503
Manulea bicolor|[5480]|CNRME2794-12|BIOUG03588-E06|Canada|Manitoba|633[0n]|BOLD:AAA4503
Manulea bicolor|[5481]|LBCH7145-10|10-JDWBC-7145|Canada|British Columbia|642[0n]|BOLD:AAA4503
Manulea bicolor|[5482]|CNRMF3878-12|BIOUG03881-C03|Canada|Manitoba|630[0n]|BOLD:AAA4503
Lycomorphodes sordida|[5483]|BCIAR993-13|YB-BCI30625|Panama|Panama|514[0n]|BOLD:AAA4769
Lycomorphodes sordida|[5484]|CNCLB2504-14|CNCLEP 00119628|Mexico|Veracruz|658[0n]|BOLD:AAA4769
Lycomorphodes sordida|[5485]|BCIAR1019-13|YB-BCI45687|Panama|Panama|658[0n]|BOLD:AAA4769
Lycomorphodes sordida|[5486]|NARCB075-09|ArcCR 40203|Costa Rica|Heredia|658[0n]|BOLD:AAA4769
Lycomorphodes sordida|[5487]|BCIAR493-12|YB-BCI30381|Panama|Panama|658[0n]|BOLD:AAA4769
Lycomorphodes sordida|[5488]|BCIAR1012-13|YB-BCI40797|Panama|Panama|587[0n]|BOLD:AAA4769
Lycomorphodes sordida|[5489]|BCIAR230-10|YB-BCI18462|Panama|Panama|658[0n]|BOLD:AAA4769
Lycomorphodes sordida|[5490]|BCIAR997-13|YB-BCI32342|Panama|Panama|658[0n]|BOLD:AAA4769
Lycomorphodes sordida|[5491]|BCIAR1018-13|YB-BCI45681|Panama|Panama|658[0n]|BOLD:AAA4769
Lycomorphodes sordida|[5492]|BCIAR224-10|YB-BCI18221|Panama|Panama|633[1n]|BOLD:AAA4769
Lycomorphodes sordida|[5493]|BCIAR210-10|YB-BCI17498|Panama|Panama|658[0n]|BOLD:AAA4769
Lycomorphodes sordida|[5494]|BCIAR193-10|YB-BCI11560|Panama|Panama|658[0n]|BOLD:AAA4769
Lycomorpha splendens|[5495]|CNCLB1852-14|CNCLEP00117979|United States|Texas|658[0n]|BOLD:ACN8475
Lycomorpha splendens|[5496]|CNCLB1851-14|CNCLEP00117978|United States|Texas|658[0n]|BOLD:ACN8475
Lycomorpha splendens|[5497]|CNCLB1850-14|CNCLEP00117977|United States|Texas|658[0n]|BOLD:ACN8475
Lycomorpha splendens|[5498]|CNCLB1849-14|CNCLEP00117976|United States|Texas|614[0n]|BOLD:ACN8475
Lycomorpha nr. splendens|[5499]|RDNMH967-09|CNCLEP00067975|United States|Colorado|658[0n]|BOLD ...
Lycomorpha nr. splendens|[5500]|RDNMH968-09|CNCLEP00067976|United States|Colorado|658[0n]|BOLD ...
Lycomorpha nr. splendens|[5501]|ABLCW200-10|CSUPOBK-0200|United States|Colorado|646[0n]|BOLD:A...
Lycomorpha nr. splendens|[5502]|ABLCW201-10|CSUPOBK-0201|United States|Colorado|658[0n]|BOLD:A...
Lycomorpha nr. splendens|[5503]|ABLCW202-10|CSUPOBK-0202|United States|Colorado|646[0n]|BOLD:A...
Lycomorpha nr. splendens|[5504]|ABLCW203-10|CSUPOBK-0203|United States|Colorado|644[0n]|BOLD:A...
Lycomorpha nr. splendens|[5505]|ABLCW199-10|CSUPOBK-0199|United States|Colorado|646[0n]|BOLD:A...
Lycomorpha nr. splendens|[5506]|ABLCW198-10|CSUPOBK-0198|United States|Colorado|646[0n]|BOLD:A...
Lycomorpha pholus|[5507]|XAJ974-06|2006-ONT-0974|Canada|Ontario|658[0n]|BOLD:AAC3139
Lycomorpha pholus|[5508]|RDNMG036-08|NOC14977|United States|Colorado|658[0n]|BOLD:AAC3139
Lycomorpha pholus|[5509]|MNAF323-08|CNCLEP00040308|Canada|Manitoba|658[0n]|BOLD:AAC3139
Lycomorpha pholus|[5510]|CNCLB1853-14|CNCLEP00117980|United States|Texas|658[0n]|BOLD:AAC3139
Lycomorpha pholus|[5511]|ABLCW036-10|CSUPOBK-0036|United States|Colorado|658[0n]|BOLD:AAC3139
Lycomorpha pholus|[5512]|RDNME784-08|LEP041249|United States|Colorado|658[0n]|BOLD:AAC3139
Lycomorpha pholus|[5513]|RDNMG034-08|NOC14975|United States|Colorado|658[0n]|BOLD:AAC3139
Lycomorpha pholus|[5514]|RDNMG125-08|NOC14878|United States|Colorado|658[0n]|BOLD:AAC3139
Lycomorpha pholus|[5515]|RDNMG126-08|NOC14879|United States|Colorado|658[0n]|BOLD:AAC3139
Lycomorpha pholus|[5516]|ABLCW205-10|CSUPOBK-0205|United States|Colorado|645[0n]|BOLD:AAC3139
Lycomorpha pholus|[5517]|ABLCW204-10|CSUPOBK-0204|United States|Colorado|646[0n]|BOLD:AAC3139
Lycomorpha pholus|[5518]|RDNME512-08|LEP037936|Canada|Ontario|658[0n]|BOLD:AAC3139
Lycomorpha pholus|[5519]|RDLQB533-05|DH010619|Canada|Quebec|658[0n]|BOLD:AAC3139
Lycomorpha pholus|[5520]|LPSOD964-09|08BBLEP-05597|Canada|Ontario|658[0n]|BOLD:AAC3139
Lycomorpha pholus|[5521]|RDNME511-08|LEP037935|Canada|Ontario|658[0n]|BOLD:AAC3139
Lycomorpha pholus|[5522]|ABLCW035-10|CSUPOBK-0035|United States|Colorado|658[0n]|BOLD:AAC3139
Lycomorpha pholus|[5523]|RDNMG035-08|NOC14976|United States|Oklahoma|658[0n]|BOLD:AAC3139
Lycomorpha pholus|[5524]|ABLCW207-10|CSUPOBK-0207|United States|Colorado|646[0n]|BOLD:AAC3139
Lycomorpha pholus|[5525]|ABLCW206-10|CSUPOBK-0206|United States|Colorado|645[0n]|BOLD:AAC3139
Lycomorpha sp.|[5526]|RDNMH453-09|CNCLEP00054467|United States|Arizona|658[0n]|BOLD:AAJ2778
Lycomorpha regulus|[5527]|CNCLB1846-14|CNCLEP00117973|United States|Arizona|658[0n]|BOLD:AAH5648
Lycomorpha regulus|[5528]|CNCLB1845-14|CNCLEP00117972|United States|Arizona|658[0n]|BOLD:AAH5648
Lycomorpha regulus|[5529]|CNCLB1843-14|CNCLEP00117970|United States|Arizona|658[0n]|BOLD:AAH5648
Lycomorpha regulus|[5530]|CNCLB1841-14|CNCLEP00117968|United States|Arizona|658[0n]|BOLD:AAH5648
Lycomorpha fulgens|[5531]|RDNMG030-08|NOC14971|United States|Arizona|658[0n]|BOLD:AAH5648
Lycomorpha fulgens|[5532]|CMAZA107-09|CMAZ-0107|United States|Arizona|658[0n]|BOLD:AAH5648
Lycomorpha fulgens|[5533]|CNCLB1844-14|CNCLEP00117971|United States|Arizona|658[1n]|BOLD:AAH5648
Lycomorpha grotei|[5534]|RDNME445-08|LEP037869|United States|Utah|638[0n]|BOLD:AAH5648
Lycomorpha grotei|[5535]|ABLCW006-10|CSUPOBK-0006|United States|Colorado|658[0n]|BOLD:AAH5648
Lycomorpha grotei|[5536]|ABLCW007-10|CSUPOBK-0007|United States|Colorado|658[0n]|BOLD:AAH5648
Lycomorpha grotei|[5537]|ABLCW005-10|CSUPOBK-0005|United States|Colorado|658[0n]|BOLD:AAH5648
Lycomorpha grotei|[5538]|ABLCW004-10|CSUPOBK-0004|United States|Colorado|658[0n]|BOLD:AAH5648
Lycomorpha grotei|[5539]|ABLCW003-10|CSUPOBK-0003|United States|Colorado|658[0n]|BOLD:AAH5648
Lycomorpha grotei|[5540]|RDNMH283-09|CNCLEP00054592|United States|Colorado|658[0n]|BOLD:AAH5648
Ptychoglene coccinea|[5541]|RDNMH451-09|CNCLEP00054465|United States|Arizona|658[0n]|BOLD:AAH2606
Ptychoglene coccinea|[5542]|ABLCW209-10|CSUPOBK-0209|United States|Arizona|658[0n]|BOLD:AAH2606
Ptychoglene phrada|[5543]|RDNMH452-09|CNCLEP00054466|United States|Arizona|658[0n]|BOLD:AAL2268
Ptychoglene|[5544]|CNCLB1923-14|CNCLEP00117789|Mexico|Queretaro|658[0n]|BOLD:AAM9404
Hypoprepia inculta|[5545]|ABLCW242-10|CSUPOBK-0242|United States|Arizona|658[0n]|BOLD:ABY4706
Hypoprepia inculta|[5546]|ABLCW056-10|CSUPOBK-0056|United States|Utah|658[0n]|BOLD:ABY4706
Hypoprepia inculta|[5547]|ABLCW244-10|CSUPOBK-0244|United States|Colorado|658[0n]|BOLD:ABY4706
Hypoprepia inculta|[5548]|ABLCW071-10|CSUPOBK-0071|United States|South Dakota|658[0n]|BOLD:ABY...
Hypoprepia inculta|[5549]|RDNMG037-08|NOC14978|United States|Arizona|658[0n]|BOLD:ABY4706
Hypoprepia inculta|[5550]|RDNME352-07|CNCNoctuoidea13959|United States|Colorado|642[0n]|BOLD:A...
Hypoprepia inculta|[5551]|ABLCW245-10|CSUPOBK-0245|United States|Colorado|658[0n]|BOLD:ABY4706
Hypoprepia inculta|[5552]|CMAZA783-10|CMAZ-0783|United States|Arizona|658[0n]|BOLD:ABY4706
Hypoprepia inculta|[5553]|ABLCW241-10|CSUPOBK-0241|United States|Colorado|658[0n]|BOLD:ABY4706
Hypoprepia inculta|[5554]|ABLCW240-10|CSUPOBK-0240|United States|Colorado|658[0n]|BOLD:ABY4706
Hypoprepia inculta|[5555]|ABLCW055-10|CSUPOBK-0055|United States|Wyoming|658[0n]|BOLD:ABY4706
Hypoprepia miniata|[5556]|LOFLB682-06|06-FLOR-1622|United States|Florida|632[0n]|BOLD:AAA4714
Hypoprepia miniata|[5557]|BBLOB1474-11|BIOUG01419-E01|United States|Florida|658[0n]|BOLD:AAA4714
Hypoprepia miniata|[5558]|LOFLB420-06|06-FLOR-1360|United States|Florida|658[0n]|BOLD:AAA4714
Hypoprepia cadaverosa|[5559]|RDNME477-08|LEP037901|United States|Colorado|658[2n]|BOLD:AAA4714
Hypoprepia miniata|[5560]|RDNME350-07|CNCNoctuoidea13957|United States|Texas|623[3n]|BOLD:AAA4714
Hypoprepia miniata|[5561]|LOWCB563-05|CGWC-1503|Canada|British Columbia|658[6n]|BOLD:AAA4714
Hypoprepia cadaverosa|[5562]|RDNME351-07|CNCNoctuoidea13958|United States|Texas|599[1n]|BOLD:A...
Hypoprepia miniata|[5563]|LOWCB566-05|CGWC-1506|Canada|British Columbia|600[0n]|BOLD:AAA4714
Hypoprepia miniata|[5564]|LOWCB562-05|CGWC-1502|Canada|British Columbia|586[0n]|BOLD:AAA4714
Hypoprepia miniata|[5565]|LBCC462-05|HLC-22342|Canada|British Columbia|658[0n]|BOLD:AAA4714
Hypoprepia miniata|[5566]|LBCC460-05|HLC-22340|Canada|British Columbia|658[0n]|BOLD:AAA4714
Hypoprepia miniata|[5567]|LBCC769-05|HLC-22649|Canada|British Columbia|658[0n]|BOLD:AAA4714
Hypoprepia miniata|[5568]|LOWCB564-05|CGWC-1504|Canada|British Columbia|658[0n]|BOLD:AAA4714
Hypoprepia miniata|[5569]|LOWCB565-05|CGWC-1505|Canada|British Columbia|658[0n]|BOLD:AAA4714
Hypoprepia miniata|[5570]|LALPA865-11|AVBC 1038-11|Canada|British Columbia|658[0n]|BOLD:AAA4714
Hypoprepia miniata|[5571]|RDMAB381-05|BCSC55|Canada|British Columbia|658[0n]|BOLD:AAA4714
Hypoprepia miniata|[5572]|RDMAB382-05|BCSC56|Canada|British Columbia|658[0n]|BOLD:AAA4714
Hypoprepia miniata|[5573]|RDMAB402-05|BCSC75|Canada|British Columbia|658[0n]|BOLD:AAA4714
Hypoprepia miniata|[5574]|LBCC461-05|HLC-22341|Canada|British Columbia|658[0n]|BOLD:AAA4714
Hypoprepia miniata|[5575]|LBCC459-05|HLC-22339|Canada|British Columbia|658[0n]|BOLD:AAA4714
Hypoprepia miniata|[5576]|LBCB632-05|HLC-21572|Canada|British Columbia|658[1n]|BOLD:AAA4714
Hypoprepia miniata|[5577]|LBCC354-05|HLC-22234|Canada|British Columbia|658[0n]|BOLD:AAA4714

Hypoprepia miniata|[5574]|LBCC461-05|HLC-22341|Canada|British Columbia|658[0n]|BOLD:AAA4714
Hypoprepia miniata|[5575]|LBCC459-05|HLC-22339|Canada|British Columbia|658[0n]|BOLD:AAA4714
Hypoprepia miniata|[5576]|LBCB632-05|HLC-21572|Canada|British Columbia|658[1n]|BOLD:AAA4714
Hypoprepia miniata|[5577]|LBCC354-05|HLC-22234|Canada|British Columbia|658[0n]|BOLD:AAA4714
Hypoprepia miniata|[5578]|LBCB631-05|HLC-21571|Canada|British Columbia|658[0n]|BOLD:AAA4714
Hypoprepia cadaverosa|[5579]|RDNME353-07|CNCNoctuoidea13960|United States|Texas|658[0n]|BOLD:A...
Hypoprepia cadaverosa|[5580]|HKONB512-09|2625-COI-08|United States|Texas|592[0n]|BOLD:AAA4714
Hypoprepia cadaverosa|[5581]|HKONB511-09|2624-COI-08|United States|Texas|658[0n]|BOLD:AAA4714
Hypoprepia cadaverosa|[5582]|RDNME478-08|LEP037902|United States|Colorado|658[0n]|BOLD:AAA4714
Hypoprepia cadaverosa|[5583]|ABLCW237-10|CSUPOBK-0237|United States|Oklahoma|658[0n]|BOLD:AAA4714
Hypoprepia cadaverosa|[5584]|ABLCW236-10|CSUPOBK-0236|United States|Oklahoma|658[0n]|BOLD:AAA4714
Hypoprepia cadaverosa|[5585]|ABLCW239-10|CSUPOBK-0239|United States|Oklahoma|658[0n]|BOLD:AAA4714
Hypoprepia cadaverosa|[5586]|ABLCW238-10|CSUPOBK-0238|United States|Oklahoma|658[0n]|BOLD:AAA4714
Hypoprepia cadaverosa|[5587]|ABLCW235-10|CSUPOBK-0235|United States|Oklahoma|658[0n]|BOLD:AAA4714
Hypoprepia miniata|[5588]|BBLSW930-09|09BBLEP-01858|United States|Texas|658[0n]|BOLD:AAA4714
Hypoprepia miniata|[5589]|ABLCW233-10|CSUPOBK-0233|United States|Oklahoma|658[1n]|BOLD:AAA4714
Hypoprepia miniata|[5590]|ABLCW231-10|CSUPOBK-0231|United States|Oklahoma|658[0n]|BOLD:AAA4714
Hypoprepia miniata|[5591]|BBLSY156-09|09BBLEP-03083|United States|Texas|658[0n]|BOLD:AAA4714
Hypoprepia miniata|[5592]|BBLSY155-09|09BBLEP-03082|United States|Texas|658[0n]|BOLD:AAA4714
Hypoprepia miniata|[5593]|BBLSY154-09|09BBLEP-03081|United States|Texas|658[0n]|BOLD:AAA4714
Hypoprepia miniata|[5594]|ABLCW234-10|CSUPOBK-0234|United States|Oklahoma|658[0n]|BOLD:AAA4714
Hypoprepia miniata|[5595]|ABLCW232-10|CSUPOBK-0232|United States|Oklahoma|658[0n]|BOLD:AAA4714
Hypoprepia miniata|[5596]|ABLCW230-10|CSUPOBK-0230|United States|Oklahoma|658[0n]|BOLD:AAA4714
Hypoprepia miniata|[5597]|ABLCW229-10|CSUPOBK-0229|United States|Oklahoma|658[0n]|BOLD:AAA4714
Hypoprepia miniata|[5598]|BBLSW931-09|09BBLEP-01859|United States|Texas|658[0n]|BOLD:AAA4714
Hypoprepia miniata|[5599]|BBLSW929-09|09BBLEP-01857|United States|Texas|658[0n]|BOLD:AAA4714
Hypoprepia miniata|[5600]|RDMAB380-05|BCSC54|Canada|Alberta|580[1n]|BOLD:AAA4714
Hypoprepia miniata|[5601]|LGSMB301-05|DNA-ATBI-1150|United States|Tennessee|583[0n]|BOLD:AAA4714
Hypoprepia miniata|[5602]|LGSMB302-05|DNA-ATBI-1151|United States|Tennessee|580[0n]|BOLD:AAA4714
Hypoprepia miniata|[5603]|ABLCW220-10|CSUPOBK-0220|United States|Florida|658[0n]|BOLD:AAA4714
Hypoprepia miniata|[5604]|ABLCW219-10|CSUPOBK-0219|United States|Florida|658[0n]|BOLD:AAA4714
Hypoprepia miniata|[5605]|LNC549-06|05-NCCC-549|United States|North Carolina|591[4n]|BOLD:AAA4714
Hypoprepia miniata|[5606]|LOFLB685-06|06-FLOR-1625|United States|Florida|631[0n]|BOLD:AAA4714
Hypoprepia miniata|[5607]|LOFLB684-06|06-FLOR-1624|United States|Florida|632[0n]|BOLD:AAA4714
Hypoprepia miniata|[5608]|LNC548-06|05-NCCC-548|United States|North Carolina|590[1n]|BOLD:AAA4714
Hypoprepia miniata|[5609]|LOFLB823-06|06-FLOR-1763|United States|Florida|643[0n]|BOLD:AAA4714
Hypoprepia miniata|[5610]|LOFLB822-06|06-FLOR-1762|United States|Florida|658[0n]|BOLD:AAA4714
Hypoprepia miniata|[5611]|LOFLB683-06|06-FLOR-1623|United States|Florida|674[0n]|BOLD:AAA4714
Hypoprepia miniata|[5612]|LOFLB758-06|06-FLOR-1698|United States|Florida|658[0n]|BOLD:AAA4714
Hypoprepia miniata|[5613]|LOFLB686-06|06-FLOR-1626|United States|Florida|658[0n]|BOLD:AAA4714
Hypoprepia miniata|[5614]|LNCB344-06|06-NCCC-1300|United States|North Carolina|658[0n]|BOLD:AA...
Hypoprepia miniata|[5615]|LNCB328-06|06-NCCC-1284|United States|North Carolina|658[0n]|BOLD:AA...
Hypoprepia miniata|[5616]|LNC275-05|05-NCCC-275|United States|North Carolina|658[0n]|BOLD:AAA4714
Hypoprepia miniata|[5617]|LNC274-05|05-NCCC-274|United States|North Carolina|658[0n]|BOLD:AAA4714
Hypoprepia miniata|[5618]|LNCC1394-11|11-NCCC-919|United States|North Carolina|658[0n]|BOLD:AA...
Hypoprepia miniata|[5619]|LNCB190-06|06-NCCC-1146|United States|North Carolina|658[0n]|BOLD:AA...
Hypoprepia miniata|[5620]|BBLOB1278-11|BIOUG01417-D07|United States|Florida|658[0n]|BOLD:AAA4714
Hypoprepia miniata|[5621]|BBLOB1608-11|BIOUG01420-H04|United States|Florida|658[0n]|BOLD:AAA4714
Hypoprepia miniata|[5622]|LOFLC469-06|06-FLOR-2349|United States|Florida|658[0n]|BOLD:AAA4714
Hypoprepia miniata|[5623]|LOFLB852-06|06-FLOR-1792|United States|Florida|658[0n]|BOLD:AAA4714
Hypoprepia miniata|[5624]|LOFLB787-06|06-FLOR-1727|United States|Florida|658[0n]|BOLD:AAA4714
Hypoprepia miniata|[5625]|LOFLB764-06|06-FLOR-1704|United States|Florida|658[0n]|BOLD:AAA4714
Hypoprepia miniata|[5626]|ABLCW011-10|CSUPOBK-0011|United States|Mississippi|658[0n]|BOLD:AAA4714
Hypoprepia miniata|[5627]|LOFLB131-06|06-FLOR-1071|United States|Florida|658[0n]|BOLD:AAA4714
Hypoprepia miniata|[5628]|LOFLC313-06|06-FLOR-2193|United States|Florida|658[0n]|BOLD:AAA4714
Hypoprepia miniata|[5629]|LOFLC312-06|06-FLOR-2192|United States|Florida|658[0n]|BOLD:AAA4714
Hypoprepia miniata|[5630]|LOFLC311-06|06-FLOR-2191|United States|Florida|658[0n]|BOLD:AAA4714
Hypoprepia miniata|[5631]|LOFLC248-06|06-FLOR-2128|United States|Florida|658[0n]|BOLD:AAA4714
Hypoprepia miniata|[5632]|LOFLC114-06|06-FLOR-1994|United States|Florida|657[0n]|BOLD:AAA4714
Hypoprepia miniata|[5633]|LOFLC110-06|06-FLOR-1990|United States|Florida|658[0n]|BOLD:AAA4714
Hypoprepia miniata|[5634]|HKONS230-08|3135-COI-07|United States|Florida|658[0n]|BOLD:AAA4714
Hypoprepia miniata|[5635]|HKONS189-08|3094-COI-07|United States|Florida|658[0n]|BOLD:AAA4714
Hypoprepia miniata|[5636]|RDNMD603-06|CNCNoctuoidea12935|United States|Florida|658[1n]|BOLD:AA...
Hypoprepia miniata|[5637]|LOT145-04|04HBL002145|United States|Tennessee|609[0n]|BOLD:AAA4714
Hypoprepia miniata|[5638]|LOT146-04|04HBL002146|United States|Tennessee|609[0n]|BOLD:AAA4714
Hypoprepia miniata|[5639]|LOT147-04|04HBL002147|United States|Tennessee|609[0n]|BOLD:AAA4714
Hypoprepia miniata|[5640]|LGSM623-04|DNA-ATBI-0623|United States|North Carolina|658[0n]|BOLD:A...
Hypoprepia miniata|[5641]|ABLCW218-10|CSUPOBK-0218|United States|Florida|658[0n]|BOLD:AAA4714
Hypoprepia miniata|[5642]|ABLCW044-10|CSUPOBK-0044|United States|Colorado|658[0n]|BOLD:AAA4714
Hypoprepia miniata|[5643]|ABLCW041-10|CSUPOBK-0041|United States|Colorado|658[0n]|BOLD:AAA4714
Hypoprepia miniata|[5644]|ABLCW039-10|CSUPOBK-0039|United States|Colorado|658[0n]|BOLD:AAA4714
Hypoprepia miniata|[5645]|ABLCW228-10|CSUPOBK-0228|United States|Colorado|658[0n]|BOLD:AAA4714
Hypoprepia miniata|[5646]|LPSOD1062-09|08MZPP-159|Canada|Ontario|658[0n]|BOLD:AAA4714
Hypoprepia miniata|[5647]|ABLCW043-10|CSUPOBK-0043|United States|Colorado|658[0n]|BOLD:AAA4714
Hypoprepia miniata|[5648]|ABLCW042-10|CSUPOBK-0042|United States|Colorado|658[0n]|BOLD:AAA4714
Hypoprepia miniata|[5649]|LPSK629-08|08BBLEP-02197|Canada|Saskatchewan|658[0n]|BOLD:AAA4714
Hypoprepia miniata|[5650]|ABLCW227-10|CSUPOBK-0227|United States|Colorado|658[0n]|BOLD:AAA4714
Hypoprepia miniata|[5651]|BBLPA586-10|10BBCLP-0586|Canada|Ontario|658[0n]|BOLD:AAA4714
Hypoprepia miniata|[5652]|BBLPA585-10|10BBCLP-0585|Canada|Ontario|658[0n]|BOLD:AAA4714
Hypoprepia miniata|[5653]|MNAF436-08|CNCLEP00040421|Canada|Manitoba|658[0n]|BOLD:AAA4714
Hypoprepia miniata|[5654]|ABLCW040-10|CSUPOBK-0040|United States|Colorado|658[0n]|BOLD:AAA4714
Hypoprepia miniata|[5655]|RDNME349-07|CNCNoctuoidea13956|United States|Iowa|658[0n]|BOLD:AAA4714
Hypoprepia miniata|[5656]|RDNME226-07|CNCNoctuoidea13833|United States|Montana|658[0n]|BOLD:AA...
Hypoprepia miniata|[5657]|BBLPA587-10|10BBCLP-0587|Canada|Ontario|658[0n]|BOLD:AAA4714
Hypoprepia miniata|[5658]|BBLPA588-10|10BBCLP-0588|Canada|Ontario|658[0n]|BOLD:AAA4714
Hypoprepia miniata|[5659]|BBLPA589-10|10BBCLP-0589|Canada|Ontario|658[0n]|BOLD:AAA4714
Hypoprepia miniata|[5660]|MNAF058-08|CNCLEP00038543|Canada|Manitoba|658[0n]|BOLD:AAA4714
Hypoprepia miniata|[5661]|LPOKA608-09|MDOK-0608|United States|Oklahoma|658[0n]|BOLD:AAA4714
Hypoprepia miniata|[5662]|RDLQ769-07|DH006568|Canada|Quebec|658[0n]|BOLD:AAA4714
Hypoprepia miniata|[5663]|RDMAB378-05|BCSC52|Canada|Alberta|658[0n]|BOLD:AAA4714
Hypoprepia miniata|[5664]|RDMAB383-05|BCSC57|United States|Kentucky|658[0n]|BOLD:AAA4714
Hypoprepia miniata|[5665]|RDMAB401-05|BCSC74|Canada|Alberta|658[0n]|BOLD:AAA4714
Hypoprepia miniata|[5666]|RDMAB400-05|BCSC73|Canada|Alberta|658[0n]|BOLD:AAA4714
Hypoprepia miniata|[5667]|LTOL932-08|RFD-93-0437|United States|West Virginia|656[0n]|BOLD:AAA4714
Hypoprepia miniata|[5668]|RDNMD602-06|CNCNoctuoidea12934|United States|Florida|658[0n]|BOLD:AA...
Hypoprepia miniata|[5669]|PHMO381-03|moth1885.02|Canada|Ontario|639[0n]|BOLD:AAA4714
Hypoprepia miniata|[5670]|LSUSA012-06|06-SUSA-0012|United States|Kentucky|658[0n]|BOLD:AAA4714
Hypoprepia miniata|[5671]|LNEL012-06|CNCLEP00007384|Canada|Alberta|658[0n]|BOLD:AAA4714
Hypoprepia miniata|[5672]|PMG013-03|HYPO2.00|Canada|Ontario|617[0n]|BOLD:AAA4714
Hypoprepia fucosa|[5673]|LOT543-04|04HBL002543|United States|Tennessee|560[0n]|BOLD:AAA4714
Hypoprepia fucosa|[5674]|LNCNW070-06|06-NCNW-0070|United States|North Carolina|658[0n]|BOLD:AA...
Hypoprepia fucosa|[5675]|LGSMB303-05|DNA-ATBI-1152|United States|North Carolina|583[0n]|BOLD:A...
Hypoprepia fucosa|[5676]|LOT546-04|04HBL002546|United States|Tennessee|569[0n]|BOLD:AAA4714

Hypoprepia fucosa|[5674]|LNCNW070-06|06-NCNW-0070|United States|North Carolina|658[0n]|BOLD:AA...
Hypoprepia fucosa|[5675]|LGSMB303-05|DNA-ATBI-1152|United States|North Carolina|583[0n]|BOLD:A...
Hypoprepia fucosa|[5676]|LOT546-04|04HBL002546|United States|Tennessee|569[0n]|BOLD:AAA4714
Hypoprepia fucosa|[5677]|LSEU011-06|06-JKA-0011|United States|Georgia|596[0n]|BOLD:AAA4714
Hypoprepia fucosa|[5678]|LGSMB304-05|DNA-ATBI-1153|United States|North Carolina|590[0n]|BOLD:A...
Hypoprepia fucosa|[5679]|BBLOB1875-11|BIOUG01425-F10|United States|Florida|658[0n]|BOLD:AAA4714
Hypoprepia fucosa|[5680]|LMDH184-11|BIOUG01047-G02|United States|Minnesota|658[0n]|BOLD:AAA4714
Hypoprepia fucosa|[5681]|LOT545-04|04HBL002545|United States|Tennessee|597[0n]|BOLD:AAA4714
Hypoprepia fucosa|[5682]|LOT544-04|04HBL002544|United States|Tennessee|587[0n]|BOLD:AAA4714
Hypoprepia fucosa|[5683]|MECD368-06|jflandry2940|United States|Maryland|658[0n]|BOLD:AAA4714
Hypoprepia fucosa|[5684]|LOT542-04|04HBL002542|United States|Tennessee|656[0n]|BOLD:AAA4714
Hypoprepia fucosa|[5685]|LNCB343-06|06-NCCC-1299|United States|North Carolina|658[0n]|BOLD:AAA...
Hypoprepia fucosa|[5686]|LGSMG112-07|BGS03433|United States|North Carolina|658[0n]|BOLD:AAA4714
Hypoprepia fucosa|[5687]|LNCC1224-11|11-NCCC-749|United States|North Carolina|658[0n]|BOLD:AAA...
Hypoprepia fucosa|[5688]|LNCB103-06|06-NCC-1059|United States|North Carolina|658[0n]|BOLD:AAA4714
Hypoprepia fucosa|[5689]|LNCB342-06|06-NCCC-1298|United States|North Carolina|658[0n]|BOLD:AAA...
Hypoprepia fucosa|[5690]|LNC277-05|05-NCCC-277|United States|North Carolina|658[0n]|BOLD:AAA4714
Hypoprepia fucosa|[5691]|LNCB135-06|06-NCCC-1091|United States|North Carolina|658[0n]|BOLD:AAA...
Hypoprepia fucosa|[5692]|XAG138-05|2005-ONT-722|Canada|Ontario|658[1n]|BOLD:AAA4714
Hypoprepia fucosa|[5693]|XAK452-06|2006-ONT-1447|Canada|Ontario|658[0n]|BOLD:AAA4714
Hypoprepia fucosa|[5694]|TMG71-03|HYPO1.00|Canada|Ontario|639[0n]|BOLD:AAA4714
Hypoprepia fucosa|[5695]|XAE433-04|Moth4433.03|Canada|Ontario|658[0n]|BOLD:AAA4714
Hypoprepia fucosa tricolor|[5696]|RDLQB676-05|DH010779|Canada|Quebec|516[0n]|BOLD:AAA4714
Hypoprepia fucosa|[5697]|LNCB243-06|06-NCCC-1199|United States|North Carolina|658[0n]|BOLD:AAA...
Hypoprepia fucosa|[5698]|LNCB244-06|06-NCCC-1200|United States|North Carolina|658[0n]|BOLD:AAA...
Hypoprepia fucosa|[5699]|LNCB191-06|06-NCCC-1147|United States|North Carolina|658[0n]|BOLD:AAA...
Hypoprepia fucosa|[5700]|LNCB192-06|06-NCCC-1148|United States|North Carolina|658[0n]|BOLD:AAA...
Hypoprepia fucosa|[5701]|LSEU010-06|06-JKA-0010|United States|Georgia|579[0n]|BOLD:AAA4714
Hypoprepia fucosa|[5702]|LNC276-05|05-NCCC-276|United States|North Carolina|658[0n]|BOLD:AAA4714
Hypoprepia fucosa|[5703]|ABLCW217-10|CSUPOBK-0217|United States|Florida|658[0n]|BOLD:AAA4714
Hypoprepia fucosa|[5704]|BBLOC091-11|BIOUG01453-A01|United States|Florida|658[0n]|BOLD:AAA4714
Hypoprepia fucosa|[5705]|ABLCW221-10|CSUPOBK-0221|United States|Florida|658[0n]|BOLD:AAA4714
Hypoprepia fucosa|[5706]|ABLCW216-10|CSUPOBK-0216|United States|Florida|658[0n]|BOLD:AAA4714
Hypoprepia fucosa|[5707]|MILEP299-10|10-MISC-204|United States|Georgia|658[0n]|BOLD:AAA4714
Hypoprepia fucosa|[5708]|MILEP298-10|10-MISC-203|United States|Georgia|658[0n]|BOLD:AAA4714
Hypoprepia fucosa|[5709]|HKONS222-08|3127-COI-07|United States|Florida|658[0n]|BOLD:AAA4714
Hypoprepia fucosa|[5710]|RDMAB403-05|BCSC76|Canada|Saskatchewan|577[0n]|BOLD:AAA4714
Hypoprepia fucosa|[5711]|RDMAB412-05|BCSC85|United States|Texas|578[0n]|BOLD:AAA4714
Hypoprepia fucosa|[5712]|TMNBD361-07|MNBTT-3162|Canada|New Brunswick|597[0n]|BOLD:AAA4714
Hypoprepia fucosa|[5713]|RDMAB384-05|BCSC58|Canada|Nova Scotia|555[0n]|BOLD:AAA4714
Hypoprepia fucosa|[5714]|MNAC816-07|CNCLEP00027557|Canada|Quebec|658[0n]|BOLD:AAA4714
Hypoprepia fucosa|[5715]|MNAC814-07|CNCLEP00027555|Canada|Quebec|658[0n]|BOLD:AAA4714
Hypoprepia fucosa|[5716]|TMNBD363-07|MNBTT-3164|Canada|New Brunswick|649[0n]|BOLD:AAA4714
Hypoprepia fucosa|[5717]|TMNBD362-07|MNBTT-3163|Canada|New Brunswick|636[0n]|BOLD:AAA4714
Hypoprepia fucosa|[5718]|MNAC815-07|CNCLEP00027556|Canada|Quebec|658[0n]|BOLD:AAA4714
Hypoprepia fucosa|[5719]|TMNBD365-07|MNBTT-3166|Canada|New Brunswick|658[0n]|BOLD:AAA4714
Hypoprepia fucosa tricolor|[5720]|RDLQB691-05|DH010794|Canada|Quebec|658[0n]|BOLD:AAA4714
Hypoprepia fucosa|[5721]|XAJ926-06|2006-ONT-0926|Canada|Ontario|658[0n]|BOLD:AAA4714
Hypoprepia fucosa|[5722]|XAB188-04|04HBL005188|Canada|Ontario|658[0n]|BOLD:AAA4714
Hypoprepia fucosa|[5723]|TMG72-03|MOTH2.00|Canada|Ontario|639[0n]|BOLD:AAA4714
Hypoprepia fucosa|[5724]|RDMAB747-06|BCSC416|United States|Kentucky|625[2n]|BOLD:AAA4714
Hypoprepia fucosa|[5725]|MECB142-04|jflandry1086|Canada|Quebec|603[0n]|BOLD:AAA4714
Hypoprepia fucosa|[5726]|LPMNB358-09|08BBLEP-05202|Canada|Manitoba|658[0n]|BOLD:AAA4714
Hypoprepia fucosa|[5727]|LPMNB324-09|08BBLEP-05168|Canada|Manitoba|658[0n]|BOLD:AAA4714
Hypoprepia fucosa|[5728]|LPMNB325-09|08BBLEP-05169|Canada|Manitoba|658[0n]|BOLD:AAA4714
Hypoprepia fucosa|[5729]|LILLA904-11|SNS10IL-01125|United States|Illinois|658[0n]|BOLD:AAA4714
Hypoprepia fucosa|[5730]|HEOCT1021-12|BIOUG02500-E05|Canada|Ontario|614[0n]|BOLD:AAA4714
Hypoprepia fucosa tricolor|[5731]|BLTIB780-08|BL1197|Canada|Ontario|658[0n]|BOLD:AAA4714
Hypoprepia fucosa|[5732]|LPMNB389-09|08BBLEP-05233|Canada|Manitoba|627[0n]|BOLD:AAA4714
Hypoprepia fucosa|[5733]|BLTIB1096-08|BL1106|Canada|Ontario|658[1n]|BOLD:AAA4714
Hypoprepia fucosa|[5734]|BBLSX367-09|09BBLEP-02295|United States|Oklahoma|658[0n]|BOLD:AAA4714
Hypoprepia fucosa|[5735]|LPOKC313-09|MDOK-2390|United States|Oklahoma|658[0n]|BOLD:AAA4714
Hypoprepia fucosa|[5736]|BBLSX369-09|09BBLEP-02297|United States|Oklahoma|658[0n]|BOLD:AAA4714
Hypoprepia fucosa|[5737]|BBLSX368-09|09BBLEP-02296|United States|Oklahoma|658[0n]|BOLD:AAA4714
Hypoprepia fucosa|[5738]|ABLCW222-10|CSUPOBK-0222|United States|Oklahoma|658[0n]|BOLD:AAA4714
Hypoprepia fucosa|[5739]|LPMN079-08|08BBLEP-00877|Canada|Manitoba|658[0n]|BOLD:AAA4714
Hypoprepia fucosa|[5740]|HKONB092-08|3589-COI-08|United States|Texas|658[0n]|BOLD:AAA4714
Hypoprepia fucosa|[5741]|HKONB093-08|3590-COI-08|United States|Texas|658[0n]|BOLD:AAA4714
Hypoprepia fucosa|[5742]|LPSOD1052-09|08MZPP-119|Canada|Ontario|658[0n]|BOLD:AAA4714
Hypoprepia fucosa|[5743]|RDMAB748-06|BCSC417|United States|Kentucky|658[0n]|BOLD:AAA4714
Hypoprepia fucosa|[5744]|LPMN992-09|08BBLEP-04033|Canada|Manitoba|658[0n]|BOLD:AAA4714
Hypoprepia fucosa|[5745]|LPMN1001-09|08BBLEP-04042|Canada|Manitoba|658[0n]|BOLD:AAA4714
Hypoprepia fucosa|[5746]|LPMN1003-09|08BBLEP-04044|Canada|Manitoba|658[0n]|BOLD:AAA4714
Hypoprepia fucosa tricolor|[5747]|RDLQB749-05|DH010664|Canada|Quebec|658[0n]|BOLD:AAA4714
Hypoprepia fucosa|[5748]|MNAF476-08|CNCLEP00040461|Canada|Manitoba|658[0n]|BOLD:AAA4714
Hypoprepia fucosa tricolor|[5749]|XAJ881-06|2006-ONT-0881|Canada|Ontario|658[0n]|BOLD:AAA4714
Hypoprepia fucosa|[5750]|MNAF862-08|CNCLEP00040847|Canada|Manitoba|658[0n]|BOLD:AAA4714
Hypoprepia fucosa|[5751]|MNAF863-08|CNCLEP00040848|Canada|Manitoba|658[0n]|BOLD:AAA4714
Hypoprepia fucosa|[5752]|MNAF864-08|CNCLEP00040849|Canada|Manitoba|658[0n]|BOLD:AAA4714
Hypoprepia fucosa|[5753]|TMNBB005-06|MNBTT-945|Canada|New Brunswick|658[0n]|BOLD:AAA4714
Hypoprepia fucosa|[5754]|HKONB094-08|3591-COI-08|United States|Texas|658[0n]|BOLD:AAA4714
Hypoprepia fucosa tricolor|[5755]|LSUSA250-06|06-SUSA-0250|United States|Kentucky|658[0n]|BOLD ...
Hypoprepia fucosa|[5756]|BLTIB781-08|BL1198|Canada|Ontario|658[0n]|BOLD:AAA4714
Hypoprepia fucosa|[5757]|BLTIB782-08|BL1199|Canada|Ontario|658[0n]|BOLD:AAA4714
Hypoprepia fucosa|[5758]|BLTIB877-08|BL1296|Canada|Ontario|658[0n]|BOLD:AAA4714
Hypoprepia fucosa|[5759]|BLTIB931-08|BL1351|Canada|Ontario|658[0n]|BOLD:AAA4714
Hypoprepia fucosa|[5760]|BLTIB932-08|BL1352|Canada|Ontario|658[0n]|BOLD:AAA4714
Hypoprepia fucosa|[5761]|BLTIB986-08|BL1423|Canada|Ontario|658[0n]|BOLD:AAA4714
Hypoprepia fucosa|[5762]|BLTIB987-08|BL1424|Canada|Ontario|658[0n]|BOLD:AAA4714
Hypoprepia fucosa|[5763]|LPMNB338-09|08BBLEP-05182|Canada|Manitoba|658[0n]|BOLD:AAA4714
Hypoprepia fucosa|[5764]|LPMNB352-09|08BBLEP-05196|Canada|Manitoba|658[0n]|BOLD:AAA4714
Hypoprepia fucosa|[5765]|LPMNB382-09|08BBLEP-05226|Canada|Manitoba|658[0n]|BOLD:AAA4714
Hypoprepia fucosa|[5766]|MNAF435-08|CNCLEP00040420|Canada|Manitoba|648[0n]|BOLD:AAA4714
Hypoprepia fucosa|[5767]|TMNBD364-07|MNBTT-3165|Canada|New Brunswick|658[0n]|BOLD:AAA4714
Hypoprepia fucosa|[5768]|LPMNB404-09|08BBLEP-05248|Canada|Manitoba|658[0n]|BOLD:AAA4714
Hypoprepia fucosa|[5769]|LPMNB479-09|08BBLEP-05517|Canada|Manitoba|658[0n]|BOLD:AAA4714
Hypoprepia fucosa|[5770]|LILLA409-11|SNS10IL-00534|United States|Illinois|658[0n]|BOLD:AAA4714
Hypoprepia fucosa|[5771]|XAB151-04|04HBL005151|Canada|Ontario|658[0n]|BOLD:AAA4714
Hypoprepia fucosa|[5772]|BLTIB649-08|BL929|Canada|Ontario|636[0n]|BOLD:AAA4714
Hypoprepia fucosa|[5773]|TMG73-03|moth1164.01|Canada|Ontario|639[0n]|BOLD:AAA4714
Hypoprepia fucosa|[5774]|PMG012-03|moth958.01|Canada|Ontario|617[0n]|BOLD:AAA4714
