# Supplementary material for: Probing planetary biodiversity with DNA barcodes: The Noctuoidea of North America
Source: PLoS One. 2017 Jun 1;12(6):e0178548. doi: 10.1371/journal.pone.0178548 (PMC5453547; doi:10.1371/journal.pone.0178548)
Supplement: S5 Tree — NJ tree based on sequence variation in the barcode region of the cytochrome c oxidase I gene for North American species in the family Erebidae (rest). (PDF) [file pone.0178548.s018.pdf]

BOLD TaxonID Tree

Title	:	Tree Result - Search (16674 records)
Date	:	27-April-2016
Data Type	:	Nucleotide
Distance Model	:	Kimura 2 Parameter
Marker	:	COI-5P

Label	:	Process ID
Label	:	Taxon
Label	:	Country
Label	:	Province/State
Label	:	Sequence Length
Label	:	Barcode Cluster (BIN)

Sequence Count	:	16673
Species count	:	787
Genus count	:	171
Family count	:	1
Unidentified	:	0

BIN Count	:	773

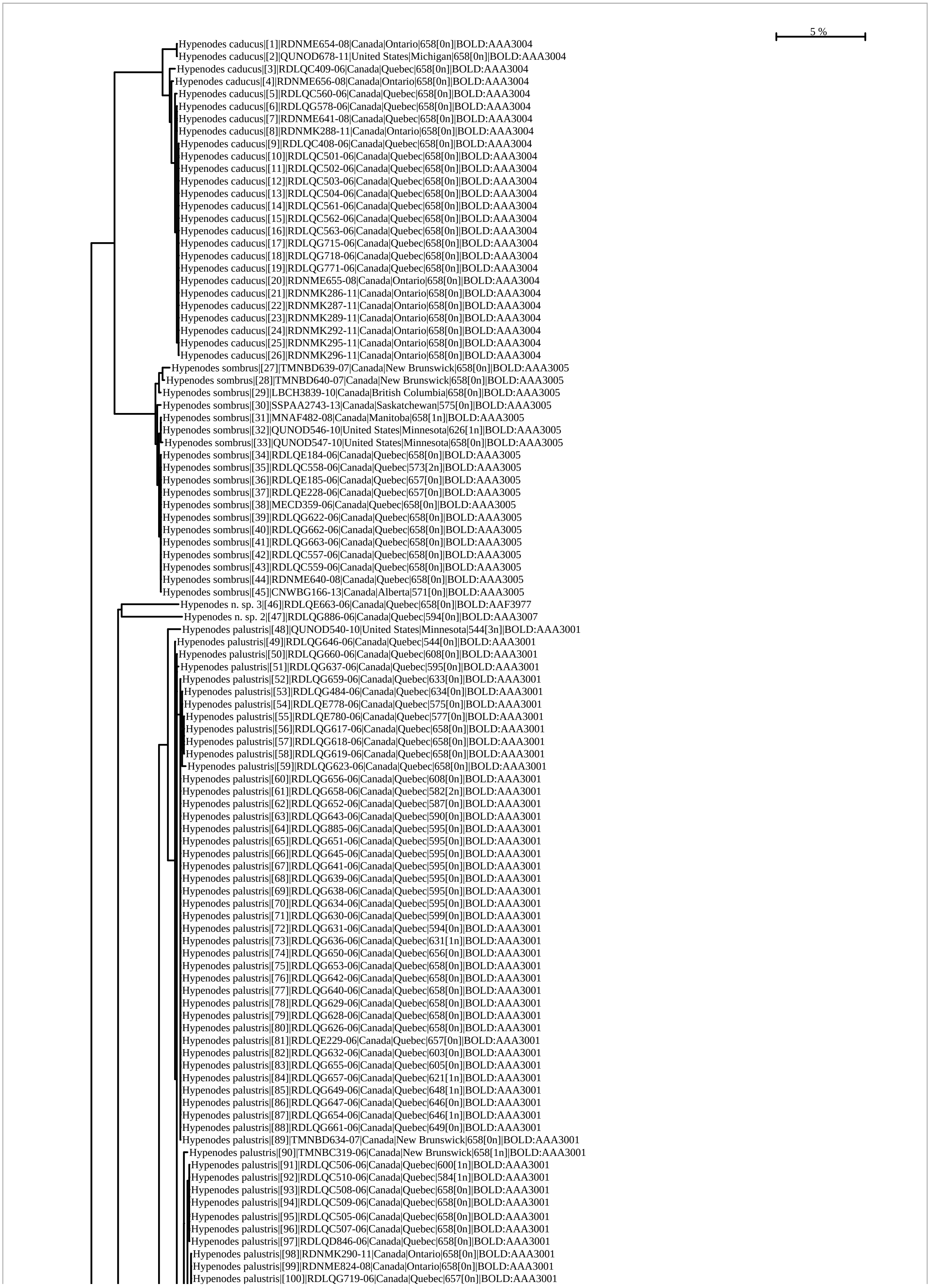
5 %
Hypenodes caducus|[1]|RDNME654-08|Canada|Ontario|658[0n]|BOLD:AAA3004
Hypenodes caducus|[2]|QUNOD678-11|United States|Michigan|658[0n]|BOLD:AAA3004
Hypenodes caducus|[3]|RDLQC409-06|Canada|Quebec|658[0n]|BOLD:AAA3004
Hypenodes caducus|[4]|RDNME656-08|Canada|Ontario|658[0n]|BOLD:AAA3004
Hypenodes caducus|[5]|RDLQC560-06|Canada|Quebec|658[0n]|BOLD:AAA3004
Hypenodes caducus|[6]|RDLQG578-06|Canada|Quebec|658[0n]|BOLD:AAA3004
Hypenodes caducus|[7]|RDNME641-08|Canada|Quebec|658[0n]|BOLD:AAA3004
Hypenodes caducus|[8]|RDNMK288-11|Canada|Ontario|658[0n]|BOLD:AAA3004
Hypenodes caducus|[9]|RDLQC408-06|Canada|Quebec|658[0n]|BOLD:AAA3004
Hypenodes caducus|[10]|RDLQC501-06|Canada|Quebec|658[0n]|BOLD:AAA3004
Hypenodes caducus|[11]|RDLQC502-06|Canada|Quebec|658[0n]|BOLD:AAA3004
Hypenodes caducus|[12]|RDLQC503-06|Canada|Quebec|658[0n]|BOLD:AAA3004
Hypenodes caducus|[13]|RDLQC504-06|Canada|Quebec|658[0n]|BOLD:AAA3004
Hypenodes caducus|[14]|RDLQC561-06|Canada|Quebec|658[0n]|BOLD:AAA3004
Hypenodes caducus|[15]|RDLQC562-06|Canada|Quebec|658[0n]|BOLD:AAA3004
Hypenodes caducus|[16]|RDLQC563-06|Canada|Quebec|658[0n]|BOLD:AAA3004
Hypenodes caducus|[17]|RDLQG715-06|Canada|Quebec|658[0n]|BOLD:AAA3004
Hypenodes caducus|[18]|RDLQG718-06|Canada|Quebec|658[0n]|BOLD:AAA3004
Hypenodes caducus|[19]|RDLQG771-06|Canada|Quebec|658[0n]|BOLD:AAA3004
Hypenodes caducus|[20]|RDNME655-08|Canada|Ontario|658[0n]|BOLD:AAA3004
Hypenodes caducus|[21]|RDNMK286-11|Canada|Ontario|658[0n]|BOLD:AAA3004
Hypenodes caducus|[22]|RDNMK287-11|Canada|Ontario|658[0n]|BOLD:AAA3004
Hypenodes caducus|[23]|RDNMK289-11|Canada|Ontario|658[0n]|BOLD:AAA3004
Hypenodes caducus|[24]|RDNMK292-11|Canada|Ontario|658[0n]|BOLD:AAA3004
Hypenodes caducus|[25]|RDNMK295-11|Canada|Ontario|658[0n]|BOLD:AAA3004
Hypenodes caducus|[26]|RDNMK296-11|Canada|Ontario|658[0n]|BOLD:AAA3004
Hypenodes sombrus|[27]|TMNBD639-07|Canada|New Brunswick|658[0n]|BOLD:AAA3005
Hypenodes sombrus|[28]|TMNBD640-07|Canada|New Brunswick|658[0n]|BOLD:AAA3005
Hypenodes sombrus|[29]|LBCH3839-10|Canada|British Columbia|658[0n]|BOLD:AAA3005
Hypenodes sombrus|[30]|SSPAA2743-13|Canada|Saskatchewan|575[0n]|BOLD:AAA3005
Hypenodes sombrus|[31]|MNAF482-08|Canada|Manitoba|658[1n]|BOLD:AAA3005
Hypenodes sombrus|[32]|QUNOD546-10|United States|Minnesota|626[1n]|BOLD:AAA3005
Hypenodes sombrus|[33]|QUNOD547-10|United States|Minnesota|658[0n]|BOLD:AAA3005
Hypenodes sombrus|[34]|RDLQE184-06|Canada|Quebec|658[0n]|BOLD:AAA3005
Hypenodes sombrus|[35]|RDLQC558-06|Canada|Quebec|573[2n]|BOLD:AAA3005
Hypenodes sombrus|[36]|RDLQE185-06|Canada|Quebec|657[0n]|BOLD:AAA3005
Hypenodes sombrus|[37]|RDLQE228-06|Canada|Quebec|657[0n]|BOLD:AAA3005
Hypenodes sombrus|[38]|MECD359-06|Canada|Quebec|658[0n]|BOLD:AAA3005
Hypenodes sombrus|[39]|RDLQG622-06|Canada|Quebec|658[0n]|BOLD:AAA3005
Hypenodes sombrus|[40]|RDLQG662-06|Canada|Quebec|658[0n]|BOLD:AAA3005
Hypenodes sombrus|[41]|RDLQG663-06|Canada|Quebec|658[0n]|BOLD:AAA3005
Hypenodes sombrus|[42]|RDLQC557-06|Canada|Quebec|658[0n]|BOLD:AAA3005
Hypenodes sombrus|[43]|RDLQC559-06|Canada|Quebec|658[0n]|BOLD:AAA3005
Hypenodes sombrus|[44]|RDNME640-08|Canada|Quebec|658[0n]|BOLD:AAA3005
Hypenodes sombrus|[45]|CNWBG166-13|Canada|Alberta|571[0n]|BOLD:AAA3005
Hypenodes n. sp. 3|[46]|RDLQE663-06|Canada|Quebec|658[0n]|BOLD:AAF3977
Hypenodes n. sp. 2|[47]|RDLQG886-06|Canada|Quebec|594[0n]|BOLD:AAA3007
Hypenodes palustris|[48]|QUNOD540-10|United States|Minnesota|544[3n]|BOLD:AAA3001
Hypenodes palustris|[49]|RDLQG646-06|Canada|Quebec|544[0n]|BOLD:AAA3001
Hypenodes palustris|[50]|RDLQG660-06|Canada|Quebec|608[0n]|BOLD:AAA3001
Hypenodes palustris|[51]|RDLQG637-06|Canada|Quebec|595[0n]|BOLD:AAA3001
Hypenodes palustris|[52]|RDLQG659-06|Canada|Quebec|633[0n]|BOLD:AAA3001
Hypenodes palustris|[53]|RDLQG484-06|Canada|Quebec|634[0n]|BOLD:AAA3001
Hypenodes palustris|[54]|RDLQE778-06|Canada|Quebec|575[0n]|BOLD:AAA3001
Hypenodes palustris|[55]|RDLQE780-06|Canada|Quebec|577[0n]|BOLD:AAA3001
Hypenodes palustris|[56]|RDLQG617-06|Canada|Quebec|658[0n]|BOLD:AAA3001
Hypenodes palustris|[57]|RDLQG618-06|Canada|Quebec|658[0n]|BOLD:AAA3001
Hypenodes palustris|[58]|RDLQG619-06|Canada|Quebec|658[0n]|BOLD:AAA3001
Hypenodes palustris|[59]|RDLQG623-06|Canada|Quebec|658[0n]|BOLD:AAA3001
Hypenodes palustris|[60]|RDLQG656-06|Canada|Quebec|608[0n]|BOLD:AAA3001
Hypenodes palustris|[61]|RDLQG658-06|Canada|Quebec|582[2n]|BOLD:AAA3001
Hypenodes palustris|[62]|RDLQG652-06|Canada|Quebec|587[0n]|BOLD:AAA3001
Hypenodes palustris|[63]|RDLQG643-06|Canada|Quebec|590[0n]|BOLD:AAA3001
Hypenodes palustris|[64]|RDLQG885-06|Canada|Quebec|595[0n]|BOLD:AAA3001
Hypenodes palustris|[65]|RDLQG651-06|Canada|Quebec|595[0n]|BOLD:AAA3001
Hypenodes palustris|[66]|RDLQG645-06|Canada|Quebec|595[0n]|BOLD:AAA3001
Hypenodes palustris|[67]|RDLQG641-06|Canada|Quebec|595[0n]|BOLD:AAA3001
Hypenodes palustris|[68]|RDLQG639-06|Canada|Quebec|595[0n]|BOLD:AAA3001
Hypenodes palustris|[69]|RDLQG638-06|Canada|Quebec|595[0n]|BOLD:AAA3001
Hypenodes palustris|[70]|RDLQG634-06|Canada|Quebec|595[0n]|BOLD:AAA3001
Hypenodes palustris|[71]|RDLQG630-06|Canada|Quebec|599[0n]|BOLD:AAA3001
Hypenodes palustris|[72]|RDLQG631-06|Canada|Quebec|594[0n]|BOLD:AAA3001
Hypenodes palustris|[73]|RDLQG636-06|Canada|Quebec|631[1n]|BOLD:AAA3001
Hypenodes palustris|[74]|RDLQG650-06|Canada|Quebec|656[0n]|BOLD:AAA3001
Hypenodes palustris|[75]|RDLQG653-06|Canada|Quebec|658[0n]|BOLD:AAA3001
Hypenodes palustris|[76]|RDLQG642-06|Canada|Quebec|658[0n]|BOLD:AAA3001
Hypenodes palustris|[77]|RDLQG640-06|Canada|Quebec|658[0n]|BOLD:AAA3001
Hypenodes palustris|[78]|RDLQG629-06|Canada|Quebec|658[0n]|BOLD:AAA3001
Hypenodes palustris|[79]|RDLQG628-06|Canada|Quebec|658[0n]|BOLD:AAA3001
Hypenodes palustris|[80]|RDLQG626-06|Canada|Quebec|658[0n]|BOLD:AAA3001
Hypenodes palustris|[81]|RDLQE229-06|Canada|Quebec|657[0n]|BOLD:AAA3001
Hypenodes palustris|[82]|RDLQG632-06|Canada|Quebec|603[0n]|BOLD:AAA3001
Hypenodes palustris|[83]|RDLQG655-06|Canada|Quebec|605[0n]|BOLD:AAA3001
Hypenodes palustris|[84]|RDLQG657-06|Canada|Quebec|621[1n]|BOLD:AAA3001
Hypenodes palustris|[85]|RDLQG649-06|Canada|Quebec|648[1n]|BOLD:AAA3001
Hypenodes palustris|[86]|RDLQG647-06|Canada|Quebec|646[0n]|BOLD:AAA3001
Hypenodes palustris|[87]|RDLQG654-06|Canada|Quebec|646[1n]|BOLD:AAA3001
Hypenodes palustris|[88]|RDLQG661-06|Canada|Quebec|649[0n]|BOLD:AAA3001
Hypenodes palustris|[89]|TMNBD634-07|Canada|New Brunswick|658[0n]|BOLD:AAA3001
Hypenodes palustris|[90]|TMNBC319-06|Canada|New Brunswick|658[1n]|BOLD:AAA3001
Hypenodes palustris|[91]|RDLQC506-06|Canada|Quebec|600[1n]|BOLD:AAA3001
Hypenodes palustris|[92]|RDLQC510-06|Canada|Quebec|584[1n]|BOLD:AAA3001
Hypenodes palustris|[93]|RDLQC508-06|Canada|Quebec|658[0n]|BOLD:AAA3001
Hypenodes palustris|[94]|RDLQC509-06|Canada|Quebec|658[0n]|BOLD:AAA3001
Hypenodes palustris|[95]|RDLQC505-06|Canada|Quebec|658[0n]|BOLD:AAA3001
Hypenodes palustris|[96]|RDLQC507-06|Canada|Quebec|658[0n]|BOLD:AAA3001
Hypenodes palustris|[97]|RDLQD846-06|Canada|Quebec|658[0n]|BOLD:AAA3001
Hypenodes palustris|[98]|RDNMK290-11|Canada|Ontario|658[0n]|BOLD:AAA3001
Hypenodes palustris|[99]|RDNME824-08|Canada|Ontario|658[0n]|BOLD:AAA3001
Hypenodes palustris|[100]|RDLQG719-06|Canada|Quebec|657[0n]|BOLD:AAA3001

Hypenodes palustris|[98]|RDNMK290-11|Canada|Ontario|658[0n]|BOLD:AAA3001
Hypenodes palustris|[99]|RDNME824-08|Canada|Ontario|658[0n]|BOLD:AAA3001
Hypenodes palustris|[100]|RDLQG719-06|Canada|Quebec|657[0n]|BOLD:AAA3001
Hypenodes palustris|[101]|RDLQG717-06|Canada|Quebec|658[0n]|BOLD:AAA3001
Hypenodes palustris|[102]|RDLQG580-06|Canada|Quebec|658[0n]|BOLD:AAA3001
Hypenodes palustris|[103]|RDLQG470-06|Canada|Quebec|658[0n]|BOLD:AAA3001
Hypenodes palustris|[104]|RDLQE577-06|Canada|Quebec|657[0n]|BOLD:AAA3001
Hypenodes palustris|[105]|MECC493-06|Canada|Quebec|658[0n]|BOLD:AAA3001
Hypenodes palustris|[106]|RDLQC466-06|Canada|Quebec|658[0n]|BOLD:AAA3001
Hypenodes palustris|[107]|RDLQG577-06|Canada|Quebec|658[0n]|BOLD:AAA3001
Hypenodes palustris|[108]|RDLQH208-07|Canada|Quebec|651[0n]|BOLD:AAA3001
Hypenodes palustris|[109]|RDLQG648-06|Canada|Quebec|595[0n]|BOLD:AAA3001
Hypenodes palustris|[110]|MECC494-06|Canada|Quebec|624[0n]|BOLD:AAA3001
Hypenodes palustris|[111]|RDLQH207-07|Canada|Quebec|596[1n]|BOLD:AAA3001
Hypenodes palustris|[112]|RDNMK291-11|Canada|Ontario|618[0n]|BOLD:AAA3001
Hypenodes palustris|[113]|RDNMK294-11|Canada|Ontario|658[0n]|BOLD:AAA3001
Hypenodes palustris|[114]|RDNMK297-11|Canada|Ontario|658[0n]|BOLD:AAA3001
Hypenodes palustris|[115]|RDNMK298-11|Canada|Ontario|658[0n]|BOLD:AAA3001
Hypenodes palustris|[116]|RDLQG867-06|Canada|Quebec|658[0n]|BOLD:AAA3001
Hypenodes palustris|[117]|RDLQG873-06|Canada|Quebec|632[0n]|BOLD:AAA3001
Hypenodes palustris|[118]|RDLQG851-06|Canada|Quebec|595[0n]|BOLD:AAA3001
Hypenodes palustris|[119]|RDLQG869-06|Canada|Quebec|658[0n]|BOLD:AAA3001
Hypenodes palustris|[120]|RDLQG854-06|Canada|Quebec|658[0n]|BOLD:AAA3001
Hypenodes palustris|[121]|RDLQG871-06|Canada|Quebec|658[0n]|BOLD:AAA3001
Hypenodes palustris|[122]|RDLQG878-06|Canada|Quebec|654[0n]|BOLD:AAA3001
Hypenodes palustris|[123]|RDLQG865-06|Canada|Quebec|658[0n]|BOLD:AAA3001
Hypenodes palustris|[124]|RDLQG868-06|Canada|Quebec|658[0n]|BOLD:AAA3001
Hypenodes palustris|[125]|RDLQG872-06|Canada|Quebec|658[0n]|BOLD:AAA3001
Hypenodes palustris|[126]|RDLQG880-06|Canada|Quebec|658[0n]|BOLD:AAA3001
Hypenodes palustris|[127]|RDLQG863-06|Canada|Quebec|658[0n]|BOLD:AAA3001
Hypenodes palustris|[128]|RDLQG860-06|Canada|Quebec|658[0n]|BOLD:AAA3001
Hypenodes palustris|[129]|RDLQG856-06|Canada|Quebec|658[0n]|BOLD:AAA3001
Hypenodes palustris|[130]|RDLQG876-06|Canada|Quebec|658[0n]|BOLD:AAA3001
Hypenodes palustris|[131]|RDLQG881-06|Canada|Quebec|658[0n]|BOLD:AAA3001
Hypenodes palustris|[132]|RDLQG892-06|Canada|Quebec|658[0n]|BOLD:AAA3001
Hypenodes palustris|[133]|RDLQG893-06|Canada|Quebec|658[0n]|BOLD:AAA3001
Hypenodes palustris|[134]|RDLQG603-06|Canada|Quebec|658[0n]|BOLD:AAA3001
Hypenodes palustris|[135]|RDLQG581-06|Canada|Quebec|658[0n]|BOLD:AAA3001
Hypenodes palustris|[136]|RDLQG579-06|Canada|Quebec|632[0n]|BOLD:AAA3001
Hypenodes palustris|[137]|RDLQG716-06|Canada|Quebec|595[0n]|BOLD:AAA3001
Hypenodes palustris|[138]|TMNBD635-07|Canada|New Brunswick|646[0n]|BOLD:AAA3001
Hypenodes palustris|[139]|QUNOD543-10|United States|Minnesota|658[0n]|BOLD:AAA3001
Hypenodes palustris|[140]|RDNMK293-11|Canada|Ontario|658[0n]|BOLD:AAA3001
Hypenodes palustris|[141]|RDLQE183-06|Canada|Quebec|657[0n]|BOLD:AAA3001
Hypenodes palustris|[142]|RDLQG616-06|Canada|Quebec|658[0n]|BOLD:AAA3001
Hypenodes palustris|[143]|RDLQG620-06|Canada|Quebec|658[0n]|BOLD:AAA3001
Hypenodes palustris|[144]|RDLQG621-06|Canada|Quebec|658[0n]|BOLD:AAA3001
Hypenodes palustris|[145]|QUNOD545-10|United States|Minnesota|610[1n]|BOLD:AAA3001
Hypenodes palustris|[146]|MNAO258-11|Canada|Ontario|658[0n]|BOLD:AAA3001
Hypenodes palustris|[147]|SSPAA2736-13|Canada|Saskatchewan|528[0n]|BOLD:AAA3001
Hypenodes palustris|[148]|RDLQH211-07|Canada|Quebec|583[2n]|BOLD:AAA3001
Hypenodes palustris|[149]|RDLQG541-06|Canada|Quebec|658[0n]|BOLD:AAA3001
Hypenodes palustris|[150]|RDLQH209-07|Canada|Quebec|591[0n]|BOLD:AAA3001
Hypenodes palustris|[151]|RDLQH210-07|Canada|Quebec|594[4n]|BOLD:AAA3001
Hypenodes palustris|[152]|RDLQH214-07|Canada|Quebec|586[1n]|BOLD:AAA3001
Hypenodes palustris|[153]|SSPAA2675-13|Canada|Saskatchewan|595[0n]|BOLD:AAA3001
Hypenodes palustris|[154]|MECD356-06|Canada|Quebec|658[0n]|BOLD:AAA3001
Hypenodes palustris|[155]|SSPAA2695-13|Canada|Saskatchewan|566[0n]|BOLD:AAA3001
Hypenodes palustris|[156]|SSPAA2709-13|Canada|Saskatchewan|583[0n]|BOLD:AAA3001
Hypenodes palustris|[157]|SSPAA2679-13|Canada|Saskatchewan|552[0n]|BOLD:AAA3001
Hypenodes palustris|[158]|SSPAA2746-13|Canada|Saskatchewan|580[0n]|BOLD:AAA3001
Hypenodes palustris|[159]|SSPAA2672-13|Canada|Saskatchewan|580[0n]|BOLD:AAA3001
Hypenodes palustris|[160]|SSPAA2692-13|Canada|Saskatchewan|598[0n]|BOLD:AAA3001
Hypenodes palustris|[161]|SSPAA2706-13|Canada|Saskatchewan|554[0n]|BOLD:AAA3001
Hypenodes palustris|[162]|SSPAA2750-13|Canada|Saskatchewan|553[0n]|BOLD:AAA3001
Hypenodes palustris|[163]|SSPAA2751-13|Canada|Saskatchewan|595[0n]|BOLD:AAA3001
Hypenodes palustris|[164]|SSPAA2680-13|Canada|Saskatchewan|554[0n]|BOLD:AAA3001
Hypenodes palustris|[165]|ABKWR018-07|United States|Alaska|658[0n]|BOLD:AAA3001
Hypenodes palustris|[166]|SSPAA2726-13|Canada|Saskatchewan|565[0n]|BOLD:AAA3001
Hypenodes palustris|[167]|SSPAA2669-13|Canada|Saskatchewan|594[0n]|BOLD:AAA3001
Hypenodes palustris|[168]|SSPAA2668-13|Canada|Saskatchewan|593[0n]|BOLD:AAA3001
Hypenodes palustris|[169]|SSPAA2708-13|Canada|Saskatchewan|591[0n]|BOLD:AAA3001
Hypenodes palustris|[170]|SSPAA9951-13|Canada|Saskatchewan|605[0n]|BOLD:AAA3001
Hypenodes palustris|[171]|SSPAA2733-13|Canada|Saskatchewan|586[0n]|BOLD:AAA3001
Hypenodes palustris|[172]|SSPAA2720-13|Canada|Saskatchewan|586[0n]|BOLD:AAA3001
Hypenodes palustris|[173]|SSPAA2712-13|Canada|Saskatchewan|586[0n]|BOLD:AAA3001
Hypenodes palustris|[174]|SSPAA2691-13|Canada|Saskatchewan|588[0n]|BOLD:AAA3001
Hypenodes palustris|[175]|QUNOD541-10|United States|Minnesota|618[0n]|BOLD:AAA3001
Hypenodes palustris|[176]|SSPAA2742-13|Canada|Saskatchewan|603[0n]|BOLD:AAA3001
Hypenodes palustris|[177]|SSPAA9966-13|Canada|Saskatchewan|599[0n]|BOLD:AAA3001
Hypenodes palustris|[178]|SSPAA10028-13|Canada|Saskatchewan|592[0n]|BOLD:AAA3001
Hypenodes n. sp. 5|[179]|RDLQG861-06|Canada|Quebec|612[0n]|BOLD:AAA3006
Hypenodes n. sp. 5|[180]|RDLQG864-06|Canada|Quebec|591[0n]|BOLD:AAA3006
Hypenodes n. sp. 5|[181]|RDLQG875-06|Canada|Quebec|658[0n]|BOLD:AAA3006
Hypenodes n. sp. 5|[182]|RDLQG874-06|Canada|Quebec|658[0n]|BOLD:AAA3006
Hypenodes n. sp. 5|[183]|RDLQG870-06|Canada|Quebec|658[0n]|BOLD:AAA3006
Hypenodes n. sp. 5|[184]|RDLQG866-06|Canada|Quebec|658[0n]|BOLD:AAA3006
Hypenodes n. sp. 5|[185]|RDLQG859-06|Canada|Quebec|658[0n]|BOLD:AAA3006
Hypenodes n. sp. 5|[186]|RDLQG857-06|Canada|Quebec|658[0n]|BOLD:AAA3006
Hypenodes n. sp. 5|[187]|RDLQG853-06|Canada|Quebec|658[0n]|BOLD:AAA3006
Hypenodes n. sp. 5|[188]|RDLQG852-06|Canada|Quebec|658[0n]|BOLD:AAA3006
Hypenodes n. sp. 5|[189]|RDLQG855-06|Canada|Quebec|658[0n]|BOLD:AAA3006
Hypenodes n. sp. 5|[190]|RDLQG858-06|Canada|Quebec|658[0n]|BOLD:AAA3006
Hypenodes n. sp. 5|[191]|RDLQG862-06|Canada|Quebec|658[0n]|BOLD:AAA3006
Hypenodes n. sp. 5|[192]|RDLQG877-06|Canada|Quebec|657[0n]|BOLD:AAA3006
Hypenodes n. sp. 5|[193]|RDLQG879-06|Canada|Quebec|658[0n]|BOLD:AAA3006
Hypenodes n. sp. 4|[194]|LBCH3431-10|Canada|British Columbia|645[0n]|BOLD:AAA3002
Hypenodes n. sp. 4|[195]|CNBPN600-13|Canada|Ontario|537[0n]|BOLD:AAA3002
Hypenodes n. sp. 4|[196]|MNAD421-07|Canada|British Columbia|658[0n]|BOLD:AAA3002
Hypenodes n. sp. 4|[197]|MNAD422-07|Canada|British Columbia|658[0n]|BOLD:AAA3002
Hypenodes n. sp. 4|[198]|LPSOD1011-09|Canada|Ontario|658[0n]|BOLD:AAA3002
Hypenodes n. sp. 4|[199]|LBCH4566-10|Canada|British Columbia|658[0n]|BOLD:AAA3002
Hypenodes n. sp. 4|[200]|LBCH1054-10|Canada|British Columbia|658[0n]|BOLD:AAA3002

Hypenodes n. sp. 4|[198]|LPSOD1011-09|Canada|Ontario|658[0n]|BOLD:AAA3002
Hypenodes n. sp. 4|[199]|LBCH4566-10|Canada|British Columbia|658[0n]|BOLD:AAA3002
Hypenodes n. sp. 4|[200]|LBCH1054-10|Canada|British Columbia|658[0n]|BOLD:AAA3002
Hypenodes n. sp. 4|[201]|LBCH4927-10|Canada|British Columbia|642[0n]|BOLD:AAA3002
Hypenodes n. sp. 4|[202]|CNRME2991-12|Canada|Manitoba|637[0n]|BOLD:AAA3002
Hypenodes n. sp. 4|[203]|CNRME2996-12|Canada|Manitoba|633[0n]|BOLD:AAA3002
Hypenodes n. sp. 4|[204]|CNRME2934-12|Canada|Manitoba|637[0n]|BOLD:AAA3002
Hypenodes n. sp. 4|[205]|CNRME2760-12|Canada|Manitoba|633[0n]|BOLD:AAA3002
Hypenodes n. sp. 4|[206]|CNRME1746-12|Canada|Manitoba|626[0n]|BOLD:AAA3002
Hypenodes n. sp. 4|[207]|CNRMF3935-12|Canada|Manitoba|621[0n]|BOLD:AAA3002
Hypenodes n. sp. 4|[208]|CNRME5146-12|Canada|Manitoba|629[0n]|BOLD:AAA3002
Hypenodes n. sp. 4|[209]|CNRME5148-12|Canada|Manitoba|622[0n]|BOLD:AAA3002
Hypenodes n. sp. 4|[210]|CNRMF3924-12|Canada|Manitoba|621[0n]|BOLD:AAA3002
Hypenodes n. sp. 4|[211]|CNRMF2883-12|Canada|Manitoba|621[0n]|BOLD:AAA3002
Hypenodes n. sp. 4|[212]|CNRMF3941-12|Canada|Manitoba|618[0n]|BOLD:AAA3002
Hypenodes n. sp. 4|[213]|RDLQG627-06|Canada|Quebec|658[0n]|BOLD:AAA3002
Hypenodes n. sp. 4|[214]|CNBPG510-13|Canada|Ontario|612[0n]|BOLD:AAA3002
Hypenodes n. sp. 4|[215]|RDLQC556-06|Canada|Quebec|658[0n]|BOLD:AAA3002
Hypenodes n. sp. 4|[216]|CNRMF3919-12|Canada|Manitoba|621[0n]|BOLD:AAA3002
Hypenodes n. sp. 4|[217]|CNPAE507-13|Canada|Saskatchewan|621[0n]|BOLD:AAA3002
Hypenodes n. sp. 4|[218]|CNRMF3912-12|Canada|Manitoba|621[0n]|BOLD:AAA3002
Hypenodes|[219]|SSPAC788-13|Canada|Saskatchewan|589[0n]|BOLD:AAA3002
Hypenodes|[220]|SSPAC821-13|Canada|Saskatchewan|548[0n]|BOLD:AAA3002
Hypenodes n. sp. 4|[221]|CNPAM634-13|Canada|Saskatchewan|628[3n]|BOLD:AAA3002
Hypenodes n. sp. 4|[222]|RDNMH022-09|Canada|Alberta|658[0n]|BOLD:AAA3002
Hypenodes n. sp. 4|[223]|CNPAM068-13|Canada|Saskatchewan|570[0n]|BOLD:AAA3002
Hypenodes n. sp. 4|[224]|CNPAM064-13|Canada|Saskatchewan|570[0n]|BOLD:AAA3002
Hypenodes n. sp. 4|[225]|CNPAM061-13|Canada|Saskatchewan|570[0n]|BOLD:AAA3002
Hypenodes n. sp. 4|[226]|MNAF117-08|Canada|Manitoba|658[0n]|BOLD:AAA3002
Hypenodes|[227]|SSPAC784-13|Canada|Saskatchewan|559[0n]|BOLD:AAA3002
Hypenodes|[228]|SSPAC829-13|Canada|Saskatchewan|551[0n]|BOLD:AAA3002
Hypenodes n. sp. 4|[229]|CNPAM059-13|Canada|Saskatchewan|583[0n]|BOLD:AAA3002
Hypenodes|[230]|SSPAC823-13|Canada|Saskatchewan|579[0n]|BOLD:AAA3002
Hypenodes|[231]|SSEIB3958-13|Canada|Alberta|594[1n]|BOLD:AAA3002
Hypenodes n. sp. 4|[232]|CNPAM073-13|Canada|Saskatchewan|558[0n]|BOLD:AAA3002
Hypenodes n. sp. 4|[233]|CNPAM067-13|Canada|Saskatchewan|561[0n]|BOLD:AAA3002
Hypenodes|[234]|SSPAC789-13|Canada|Saskatchewan|556[0n]|BOLD:AAA3002
Hypenodes n. sp. 4|[235]|CNPAM633-13|Canada|Saskatchewan|632[1n]|BOLD:AAA3002
Hypenodes n. sp. 4|[236]|CNPAM074-13|Canada|Saskatchewan|594[0n]|BOLD:AAA3002
Hypenodes n. sp. 4|[237]|CNRMF3903-12|Canada|Manitoba|633[0n]|BOLD:AAA3002
Hypenodes n. sp. 4|[238]|CNRME3024-12|Canada|Manitoba|633[0n]|BOLD:AAA3002
Hypenodes n. sp. 4|[239]|CNEID045-12|Canada|Alberta|633[0n]|BOLD:AAA3002
Hypenodes n. sp. 4|[240]|CNPAM628-13|Canada|Saskatchewan|628[1n]|BOLD:AAA3002
Hypenodes n. sp. 4|[241]|CNPAM630-13|Canada|Saskatchewan|630[0n]|BOLD:AAA3002
Hypenodes n. sp. 4|[242]|CNPAE503-13|Canada|Saskatchewan|629[0n]|BOLD:AAA3002
Hypenodes n. sp. 4|[243]|CNPAE501-13|Canada|Saskatchewan|630[0n]|BOLD:AAA3002
Hypenodes n. sp. 4|[244]|CNPAD980-13|Canada|Saskatchewan|630[0n]|BOLD:AAA3002
Hypenodes n. sp. 4|[245]|CNPAM062-13|Canada|Saskatchewan|588[0n]|BOLD:AAA3002
Hypenodes n. sp. 4|[246]|CNPAM058-13|Canada|Saskatchewan|588[0n]|BOLD:AAA3002
Hypenodes|[247]|SSPAC827-13|Canada|Saskatchewan|583[0n]|BOLD:AAA3002
Hypenodes n. sp. 4|[248]|CNPAM627-13|Canada|Saskatchewan|619[4n]|BOLD:AAA3002
Hypenodes|[249]|SSPAC824-13|Canada|Saskatchewan|583[0n]|BOLD:AAA3002
Hypenodes n. sp. 4|[250]|CNPAM069-13|Canada|Saskatchewan|606[0n]|BOLD:AAA3002
Hypenodes|[251]|SSPAC787-13|Canada|Saskatchewan|601[0n]|BOLD:AAA3002
Hypenodes n. sp. 4|[252]|CNPAD957-13|Canada|Saskatchewan|601[0n]|BOLD:AAA3002
Hypenodes n. sp. 4|[253]|CNPAD981-13|Canada|Saskatchewan|627[0n]|BOLD:AAA3002
Hypenodes n. sp. 4|[254]|CNPAM631-13|Canada|Saskatchewan|674[0n]|BOLD:AAA3002
Hypenodes n. sp. 4|[255]|CNRMF3914-12|Canada|Manitoba|613[0n]|BOLD:AAA3002
Hypenodes n. sp. 4|[256]|CNPAM621-13|Canada|Saskatchewan|630[2n]|BOLD:AAA3002
Hypenodes n. sp. 4|[257]|CNPAE510-13|Canada|Saskatchewan|621[0n]|BOLD:AAA3002
Hypenodes n. sp. 4|[258]|CNPAE508-13|Canada|Saskatchewan|621[0n]|BOLD:AAA3002
Hypenodes n. sp. 4|[259]|CNPAE505-13|Canada|Saskatchewan|621[0n]|BOLD:AAA3002
Hypenodes n. sp. 4|[260]|CNPAE500-13|Canada|Saskatchewan|621[0n]|BOLD:AAA3002
Hypenodes n. sp. 4|[261]|CNPAD979-13|Canada|Saskatchewan|621[0n]|BOLD:AAA3002
Hypenodes n. sp. 4|[262]|CNPAD975-13|Canada|Saskatchewan|621[0n]|BOLD:AAA3002
Hypenodes n. sp. 4|[263]|CNPAD974-13|Canada|Saskatchewan|621[0n]|BOLD:AAA3002
Hypenodes n. sp. 4|[264]|CNPAD973-13|Canada|Saskatchewan|621[0n]|BOLD:AAA3002
Hypenodes n. sp. 4|[265]|CNPAD970-13|Canada|Saskatchewan|621[0n]|BOLD:AAA3002
Hypenodes n. sp. 4|[266]|CNPAD953-13|Canada|Saskatchewan|621[0n]|BOLD:AAA3002
Hypenodes n. sp. 4|[267]|CNRMF3934-12|Canada|Manitoba|621[0n]|BOLD:AAA3002
Hypenodes n. sp. 4|[268]|CNRMF3918-12|Canada|Manitoba|621[0n]|BOLD:AAA3002
Hypenodes n. sp. 4|[269]|CNRMF3916-12|Canada|Manitoba|621[0n]|BOLD:AAA3002
Hypenodes n. sp. 4|[270]|CNRMF3909-12|Canada|Manitoba|621[0n]|BOLD:AAA3002
Hypenodes n. sp. 4|[271]|CNRMF3890-12|Canada|Manitoba|621[0n]|BOLD:AAA3002
Hypenodes n. sp. 4|[272]|CNRMF3863-12|Canada|Manitoba|621[0n]|BOLD:AAA3002
Hypenodes n. sp. 4|[273]|CNRME2832-12|Canada|Manitoba|621[0n]|BOLD:AAA3002
Hypenodes n. sp. 4|[274]|CNPAD968-13|Canada|Saskatchewan|617[0n]|BOLD:AAA3002
Hypenodes n. sp. 4|[275]|CNEII011-13|Canada|Alberta|632[0n]|BOLD:AAA3002
Hypenodes n. sp. 4|[276]|CNRMF3867-12|Canada|Manitoba|612[0n]|BOLD:AAA3002
Hypenodes n. sp. 4|[277]|CNRMF3889-12|Canada|Manitoba|621[0n]|BOLD:AAA3002
Hypenodes n. sp. 4|[278]|CNPAD954-13|Canada|Saskatchewan|611[0n]|BOLD:AAA3002
Hypenodes n. sp. 4|[279]|CNPAE504-13|Canada|Saskatchewan|612[0n]|BOLD:AAA3002
Hypenodes n. sp. 4|[280]|CNPAE511-13|Canada|Saskatchewan|611[0n]|BOLD:AAA3002
Hypenodes n. sp. 4|[281]|CNPAM632-13|Canada|Saskatchewan|614[2n]|BOLD:AAA3002
Hypenodes n. sp. 4|[282]|CNPAN046-13|Canada|Saskatchewan|629[0n]|BOLD:AAA3002
Hypenodes|[283]|SSPAC831-13|Canada|Saskatchewan|555[0n]|BOLD:AAA3002
Hypenodes n. sp. 4|[284]|RDLQG624-06|Canada|Quebec|658[0n]|BOLD:AAA3002
Hypenodes n. sp. 4|[285]|TTMNB605-06|Canada|New Brunswick|658[0n]|BOLD:AAA3002
Hypenodes n. sp. 4|[286]|MECD354-06|Canada|Quebec|658[0n]|BOLD:AAA3002
Hypenodes n. sp. 4|[287]|CNPAD967-13|Canada|Saskatchewan|611[0n]|BOLD:AAA3002
Hypenodes n. sp. 4|[288]|CNRME3014-12|Canada|Manitoba|633[0n]|BOLD:AAA3002
Hypenodes n. sp. 4|[289]|CNRMF2881-12|Canada|Manitoba|569[0n]|BOLD:AAA3002
Hypenodes n. sp. 4|[290]|CNPAM066-13|Canada|Saskatchewan|558[0n]|BOLD:AAA3002
Hypenodes n. sp. 4|[291]|RDLQE664-06|Canada|Quebec|656[0n]|BOLD:AAA3002
Hypenodes n. sp. 4|[292]|CNPAM070-13|Canada|Saskatchewan|582[0n]|BOLD:AAA3002
Hypenodes n. sp. 4|[293]|CNRME2995-12|Canada|Manitoba|640[0n]|BOLD:AAA3002
Hypenodes n. sp. 4|[294]|MNAC733-07|Canada|Quebec|636[0n]|BOLD:AAA3002
Hypenodes n. sp. 4|[295]|CNRME3015-12|Canada|Manitoba|637[0n]|BOLD:AAA3002
Hypenodes n. sp. 4|[296]|CNRMF3902-12|Canada|Manitoba|632[0n]|BOLD:AAA3002
Hypenodes n. sp. 4|[297]|CNRMF2867-12|Canada|Manitoba|632[0n]|BOLD:AAA3002
Hypenodes n. sp. 4|[298]|CNRME2983-12|Canada|Manitoba|633[0n]|BOLD:AAA3002
Hypenodes n. sp. 4|[299]|CNRME2932-12|Canada|Manitoba|633[0n]|BOLD:AAA3002
Hypenodes n. sp. 4|[300]|CNRMF706-12|Canada|Manitoba|633[0n]|BOLD:AAA3002

[illegible]
Hypenodes n. sp. 4|[298]|CNRME2983-12|Canada|Manitoba|633[0n]|BOLD:AAA3002
Hypenodes n. sp. 4|[299]|CNRME2932-12|Canada|Manitoba|633[0n]|BOLD:AAA3002
Hypenodes n. sp. 4|[300]|CNRME706-12|Canada|Manitoba|633[0n]|BOLD:AAA3002
Hypenodes n. sp. 4|[301]|CNRME5153-12|Canada|Manitoba|629[0n]|BOLD:AAA3002
Hypenodes n. sp. 4|[302]|CNRMF3940-12|Canada|Manitoba|613[0n]|BOLD:AAA3002
Hypenodes n. sp. 4|[303]|CNRMF3925-12|Canada|Manitoba|621[0n]|BOLD:AAA3002
Hypenodes n. sp. 4|[304]|CNRMF3930-12|Canada|Manitoba|621[0n]|BOLD:AAA3002
Hypenodes n. sp. 4|[305]|CNRMF3921-12|Canada|Manitoba|621[0n]|BOLD:AAA3002
Hypenodes n. sp. 4|[306]|CNRMF3915-12|Canada|Manitoba|621[0n]|BOLD:AAA3002
Hypenodes n. sp. 4|[307]|CNRMF3911-12|Canada|Manitoba|619[0n]|BOLD:AAA3002
Hypenodes n. sp. 4|[308]|CNRMF3908-12|Canada|Manitoba|621[0n]|BOLD:AAA3002
Hypenodes n. sp. 4|[309]|CNRMF3906-12|Canada|Manitoba|621[0n]|BOLD:AAA3002
Hypenodes n. sp. 4|[310]|CNRMF3888-12|Canada|Manitoba|621[0n]|BOLD:AAA3002
Hypenodes n. sp. 4|[311]|CNRMF3882-12|Canada|Manitoba|621[0n]|BOLD:AAA3002
Hypenodes n. sp. 4|[312]|CNRME5143-12|Canada|Manitoba|621[0n]|BOLD:AAA3002
Hypenodes n. sp. 4|[313]|CNRME2984-12|Canada|Manitoba|621[0n]|BOLD:AAA3002
Hypenodes n. sp. 4|[314]|CNRME2833-12|Canada|Manitoba|621[0n]|BOLD:AAA3002
Hypenodes n. sp. 4|[315]|CNPAD987-13|Canada|Saskatchewan|621[0n]|BOLD:AAA3002
Hypenodes n. sp. 4|[316]|CNPAD965-13|Canada|Saskatchewan|621[0n]|BOLD:AAA3002
Hypenodes n. sp. 4|[317]|CNPAE506-13|Canada|Saskatchewan|621[0n]|BOLD:AAA3002
Hypenodes n. sp. 4|[318]|CNPAM057-13|Canada|Saskatchewan|585[0n]|BOLD:AAA3002
Hypenodes n. sp. 4|[319]|CNPAM075-13|Canada|Saskatchewan|594[0n]|BOLD:AAA3002
Hypenodes n. sp. 4|[320]|CNPAM620-13|Canada|Saskatchewan|600[1n]|BOLD:AAA3002
Hypenodes n. sp. 4|[321]|CNBPQ445-13|Canada|Ontario|594[0n]|BOLD:AAA3002
Hypenodes n. sp. 4|[322]|RDLQG491-06|Canada|Quebec|638[0n]|BOLD:AAA3002
Hypenodes n. sp. 4|[323]|MECD357-06|Canada|Quebec|658[0n]|BOLD:AAA3002
Hypenodes n. sp. 4|[324]|CNBPO017-13|Canada|Ontario|597[0n]|BOLD:AAA3002
Hypenodes n. sp. 4|[325]|RDLQH018-06|Canada|Quebec|646[0n]|BOLD:AAA3002
Hypenodes n. sp. 4|[326]|CNRMF3910-12|Canada|Manitoba|632[0n]|BOLD:AAA3002
Hypenodes n. sp. 4|[327]|CNRMF2892-12|Canada|Manitoba|632[0n]|BOLD:AAA3002
Hypenodes n. sp. 4|[328]|CNRME3012-12|Canada|Manitoba|633[0n]|BOLD:AAA3002
Hypenodes n. sp. 4|[329]|CNRMF3920-12|Canada|Manitoba|621[0n]|BOLD:AAA3002
Hypenodes n. sp. 4|[330]|HPPPI099-13|Canada|Nova Scotia|595[0n]|BOLD:AAA3002
Hypenodes|[331]|SSPAA2702-13|Canada|Saskatchewan|536[0n]|BOLD:AAA3002
Hypenodes|[332]|SSPAA2700-13|Canada|Saskatchewan|536[0n]|BOLD:AAA3002
Hypenodes|[333]|SSPAA2721-13|Canada|Saskatchewan|524[0n]|BOLD:AAA3002
Hypenodes|[334]|SSPAA2724-13|Canada|Saskatchewan|536[0n]|BOLD:AAA3002
Hypenodes|[335]|SSPAA2713-13|Canada|Saskatchewan|524[0n]|BOLD:AAA3002
Hypenodes|[336]|SSPAA2662-13|Canada|Saskatchewan|539[0n]|BOLD:AAA3002
Hypenodes n. sp. 4|[337]|MECD353-06|Canada|Quebec|658[0n]|BOLD:AAA3002
Hypenodes n. sp. 4|[338]|MECD358-06|Canada|Quebec|658[0n]|BOLD:AAA3002
Hypenodes|[339]|SSPAA2690-13|Canada|Saskatchewan|598[0n]|BOLD:AAA3002
Hypenodes|[340]|SSPAA2664-13|Canada|Saskatchewan|586[0n]|BOLD:AAA3002
Hypenodes|[341]|SSPAA2698-13|Canada|Saskatchewan|586[0n]|BOLD:AAA3002
Hypenodes|[342]|SSPAA2716-13|Canada|Saskatchewan|552[0n]|BOLD:AAA3002
Hypenodes|[343]|SSPAA2731-13|Canada|Saskatchewan|590[0n]|BOLD:AAA3002
Hypenodes|[344]|SSPAA2688-13|Canada|Saskatchewan|591[0n]|BOLD:AAA3002
Hypenodes|[345]|SSPAA2730-13|Canada|Saskatchewan|598[0n]|BOLD:AAA3002
Hypenodes|[346]|SSPAA2704-13|Canada|Saskatchewan|556[0n]|BOLD:AAA3002
Hypenodes|[347]|SSPAA2671-13|Canada|Saskatchewan|592[0n]|BOLD:AAA3002
Hypenodes|[348]|SSPAA2697-13|Canada|Saskatchewan|574[0n]|BOLD:AAA3002
Hypenodes n. sp. 4|[349]|HPPPJ035-13|Canada|Nova Scotia|591[0n]|BOLD:AAA3002
Hypenodes n. sp. 4|[350]|MECD355-06|Canada|Quebec|658[0n]|BOLD:AAA3002
Hypenodes n. sp. 4|[351]|RDNME702-08|Canada|Quebec|658[0n]|BOLD:AAA3002
Hypenodes|[352]|SSPAA2727-13|Canada|Saskatchewan|587[0n]|BOLD:AAA3002
Hypenodes|[353]|SSPAA2737-13|Canada|Saskatchewan|562[0n]|BOLD:AAA3002
Hypenodes|[354]|SSPAA2681-13|Canada|Saskatchewan|590[0n]|BOLD:AAA3002
Hypenodes|[355]|SSPAA2755-13|Canada|Saskatchewan|581[0n]|BOLD:AAA3002
Hypenodes|[356]|SSPAA2747-13|Canada|Saskatchewan|581[0n]|BOLD:AAA3002
Hypenodes|[357]|SSPAA2661-13|Canada|Saskatchewan|581[0n]|BOLD:AAA3002
Hypenodes|[358]|SSPAA2683-13|Canada|Saskatchewan|600[0n]|BOLD:AAA3002
Hypenodes|[359]|SSPAA2682-13|Canada|Saskatchewan|599[0n]|BOLD:AAA3002
Hypenodes|[360]|SSPAA2741-13|Canada|Saskatchewan|574[0n]|BOLD:AAA3002
Hypenodes|[361]|SSPAA2752-13|Canada|Saskatchewan|587[1n]|BOLD:AAA3002
Hypenodes|[362]|SSPAA2728-13|Canada|Saskatchewan|578[0n]|BOLD:AAA3002
Hypenodes|[363]|SSPAA2674-13|Canada|Saskatchewan|574[0n]|BOLD:AAA3002
Hypenodes|[364]|SSPAA2748-13|Canada|Saskatchewan|596[0n]|BOLD:AAA3002
Hypenodes|[365]|SSPAA2735-13|Canada|Saskatchewan|549[0n]|BOLD:AAA3002
Hypenodes|[366]|SSPAA2701-13|Canada|Saskatchewan|554[0n]|BOLD:AAA3002
Hypenodes|[367]|SSPAA2687-13|Canada|Saskatchewan|553[0n]|BOLD:AAA3002
Hypenodes|[368]|SSPAA2738-13|Canada|Saskatchewan|564[1n]|BOLD:AAA3002
Hypenodes|[369]|SSPAA2693-13|Canada|Saskatchewan|559[0n]|BOLD:AAA3002
Hypenodes|[370]|SSPAA2670-13|Canada|Saskatchewan|559[0n]|BOLD:AAA3002
Hypenodes|[371]|SSPAA2694-13|Canada|Saskatchewan|578[0n]|BOLD:AAA3002
Hypenodes|[372]|SSPAA2676-13|Canada|Saskatchewan|578[0n]|BOLD:AAA3002
Hypenodes|[373]|SSPAA2703-13|Canada|Saskatchewan|590[0n]|BOLD:AAA3002
Hypenodes|[374]|SSPAA2699-13|Canada|Saskatchewan|586[0n]|BOLD:AAA3002
Hypenodes|[375]|SSPAA2707-13|Canada|Saskatchewan|586[0n]|BOLD:AAA3002
Hypenodes|[376]|SSPAA2722-13|Canada|Saskatchewan|586[0n]|BOLD:AAA3002
Hypenodes|[377]|SSPAA2725-13|Canada|Saskatchewan|586[0n]|BOLD:AAA3002
Hypenodes|[378]|SSPAA2673-13|Canada|Saskatchewan|586[0n]|BOLD:AAA3002
Hypenodes|[379]|SSPAA2678-13|Canada|Saskatchewan|586[0n]|BOLD:AAA3002
Hypenodes|[380]|SSPAA2729-13|Canada|Saskatchewan|586[0n]|BOLD:AAA3002
Hypenodes|[381]|SSPAA2740-13|Canada|Saskatchewan|557[0n]|BOLD:AAA3002
Hypenodes|[382]|SSPAA9975-13|Canada|Saskatchewan|564[0n]|BOLD:AAA3002
Hypenodes|[383]|SSPAA10029-13|Canada|Saskatchewan|584[0n]|BOLD:AAA3002
Hypenodes fractilinea|[384]|CNCLB611-14|Canada|Ontario|658[0n]|BOLD:AAB7623
Hypenodes fractilinea|[385]|HKONS154-08|United States|Kentucky|658[0n]|BOLD:AAB7623
Hypenodes fractilinea|[386]|LGSMC690-05|United States|Tennessee|658[0n]|BOLD:AAB7623
Hypenodes fractilinea|[387]|LGSMC689-05|United States|Tennessee|658[0n]|BOLD:AAB7623
Hypenodes fractilinea|[388]|LGSMC676-05|United States|Tennessee|658[0n]|BOLD:AAB7623
Hypenodes fractilinea|[389]|HKONS155-08|United States|Kentucky|632[0n]|BOLD:AAB7623
Hypenodes fractilinea|[390]|BBUSA414-09|United States|Michigan|658[0n]|BOLD:AAB7623
Hypenodes fractilinea|[391]|CNBPF446-13|Canada|Ontario|618[0n]|BOLD:AAB7623
Hypenodes fractilinea|[392]|CNCLB612-14|Canada|Ontario|658[0n]|BOLD:AAB7623
Hypenodes n. sp. 1|[393]|LGSM485-04|United States|Tennessee|543[1n]|BOLD:AAA3003
Hypenodes n. sp. 1|[394]|LPOKC423-09|United States|Oklahoma|628[0n]|BOLD:AAA3003
Hypenodes n. sp. 1|[395]|LPOKB516-09|United States|Oklahoma|658[0n]|BOLD:AAA3003
Hypenodes n. sp. 1|[396]|LGSM484-04|United States|Tennessee|570[0n]|BOLD:AAA3003
Hypenodes n. sp. 1|[397]|RDLQG490-06|Canada|Quebec|598[0n]|BOLD:AAA3003
Hypenodes n. sp. 1|[398]|LPOKB619-09|United States|Oklahoma|658[0n]|BOLD:AAA3003
Hypenodes n. sp. 1|[399]|LPOKD165-09|United States|Oklahoma|658[0n]|BOLD:AAA3003
Hypenodes n. sp. 1|[400]|LPOKD172-09|United States|Oklahoma|658[0n]|BOLD:AAA3003

[illegible]
Hypenodes n. sp. 1|[398]|LPOKB619-09|United States|Oklahoma|658[0n]|BOLD:AAA3003
Hypenodes n. sp. 1|[399]|LPOKD165-09|United States|Oklahoma|658[0n]|BOLD:AAA3003
Hypenodes n. sp. 1|[400]|LPOKD172-09|United States|Oklahoma|658[0n]|BOLD:AAA3003
Hypenodes n. sp. 1|[401]|LPOKD990-10|United States|Oklahoma|658[0n]|BOLD:AAA3003
Hypenodes n. sp. 1|[402]|RDLQE408-06|Canada|Quebec|657[4n]|BOLD:AAA3003
Hypenodes n. sp. 1|[403]|CNCLB997-14|Canada|Ontario|540[0n]|BOLD:AAA3003
Hypenodes franclemonti|[404]|HKONS616-08|United States|Florida|658[0n]|BOLD:AAD1517
Hypenodes franclemonti|[405]|RDNME822-08|Canada|Ontario|658[0n]|BOLD:AAD1517
Hypenodes franclemonti|[406]|QUNOD538-10|United States|Wisconsin|658[0n]|BOLD:AAD1517
Hypenodes franclemonti|[407]|RDLQC410-06|Canada|Quebec|658[0n]|BOLD:AAD1517
Hypenodes franclemonti|[408]|RDLQD847-06|Canada|Quebec|657[0n]|BOLD:AAD1517
Hypenodes franclemonti|[409]|HKONS617-08|United States|Florida|658[0n]|BOLD:AAD1517
Hypenodes franclemonti|[410]|RDNME781-08|United States|Georgia|658[0n]|BOLD:AAD1517
Hypenodes franclemonti|[411]|RDNMI022-10|United States|Florida|658[0n]|BOLD:AAD1517
Hypenodes franclemonti|[412]|RDNME823-08|Canada|Ontario|658[0n]|BOLD:AAD1517
Hypenodes franclemonti|[413]|LNCC1814-13|United States|North Carolina|615[0n]|BOLD:AAD1517
Hypenodes franclemonti|[414]|HKONS618-08|United States|Florida|658[0n]|BOLD:AAD1517
Hypenodes franclemonti|[415]|CNCLB2380-14|United States|Florida|658[0n]|BOLD:AAD1517
Gynaephora rossii|[416]|RDMAB751-06|Canada|British Columbia|643[0n]|BOLD:AAD4553
Gynaephora rossii|[417]|LCHP021-07|Canada|Manitoba|658[0n]|BOLD:AAD4553
Gynaephora rossii|[418]|RDNME643-08|United States|Alaska|649[0n]|BOLD:AAD4553
Gynaephora rossii|[419]|RDNME642-08|Canada|Yukon Territory|658[0n]|BOLD:AAD4553
Gynaephora rossii|[420]|MNAD823-07|Canada|Nunavut|658[0n]|BOLD:AAD4553
Gynaephora rossii|[421]|MNAD824-07|Canada|Nunavut|658[0n]|BOLD:AAD4553
Gynaephora rossii|[422]|RDNME644-08|Canada||658[0n]|BOLD:AAD4553
Gynaephora groenlandica|[423]|RDNMJ571-11|Canada|Yukon Territory|658[0n]|BOLD:AAE6832
Gynaephora groenlandica|[424]|RDNMJ572-11|Canada|Yukon Territory|643[0n]|BOLD:AAE6832
Gynaephora groenlandica|[425]|GRAFW695-12|Greenland||658[0n]|BOLD:AAE6832
Gynaephora groenlandica|[426]|MNAD827-07|Canada|Nunavut|658[0n]|BOLD:AAE6832
Gynaephora groenlandica|[427]|GRAFW903-12|Greenland||658[1n]|BOLD:AAE6832
Gynaephora groenlandica|[428]|GRAFW896-12|Greenland||658[1n]|BOLD:AAE6832
Gynaephora groenlandica|[429]|GRAFW881-12|Greenland||658[1n]|BOLD:AAE6832
Gynaephora groenlandica|[430]|MNAD825-07|Canada|Nunavut|654[0n]|BOLD:AAE6832
Gynaephora groenlandica|[431]|GRAFW900-12|Greenland||658[0n]|BOLD:AAE6832
Gynaephora groenlandica|[432]|GRAFW897-12|Greenland||658[0n]|BOLD:AAE6832
Gynaephora groenlandica|[433]|GRAFW894-12|Greenland||658[0n]|BOLD:AAE6832
Gynaephora groenlandica|[434]|GRAFW892-12|Greenland||658[0n]|BOLD:AAE6832
Gynaephora groenlandica|[435]|GRAFW891-12|Greenland||658[0n]|BOLD:AAE6832
Gynaephora groenlandica|[436]|GRAFW890-12|Greenland||658[0n]|BOLD:AAE6832
Gynaephora groenlandica|[437]|GRAFW888-12|Greenland||658[0n]|BOLD:AAE6832
Gynaephora groenlandica|[438]|GRAFW887-12|Greenland||658[0n]|BOLD:AAE6832
Gynaephora groenlandica|[439]|GRAFW884-12|Greenland||658[0n]|BOLD:AAE6832
Gynaephora groenlandica|[440]|GRAFW878-12|Greenland||658[0n]|BOLD:AAE6832
Gynaephora groenlandica|[441]|GRAFW694-12|Greenland||658[0n]|BOLD:AAE6832
Gynaephora groenlandica|[442]|GRAFW693-12|Greenland||658[0n]|BOLD:AAE6832
Gynaephora groenlandica|[443]|GRAFW692-12|Greenland||658[0n]|BOLD:AAE6832
Gynaephora groenlandica|[444]|GRAFW688-12|Greenland||658[0n]|BOLD:AAE6832
Gynaephora groenlandica|[445]|GRAFW687-12|Greenland||658[0n]|BOLD:AAE6832
Gynaephora groenlandica|[446]|GRAFW684-12|Greenland||658[0n]|BOLD:AAE6832
Gynaephora groenlandica|[447]|GRAFW682-12|Greenland||658[0n]|BOLD:AAE6832
Gynaephora groenlandica|[448]|GRAFW681-12|Greenland||658[0n]|BOLD:AAE6832
Gynaephora groenlandica|[449]|GRAFW680-12|Greenland||658[0n]|BOLD:AAE6832
Gynaephora groenlandica|[450]|GRAFW679-12|Greenland||658[0n]|BOLD:AAE6832
Gynaephora groenlandica|[451]|GRAFW320-11|Greenland||658[0n]|BOLD:AAE6832
Gynaephora groenlandica|[452]|GRAFW319-11|Greenland||658[0n]|BOLD:AAE6832
Gynaephora groenlandica|[453]|GRAFW318-11|Greenland||658[0n]|BOLD:AAE6832
Gynaephora groenlandica|[454]|GRAFW095-11|Greenland||658[0n]|BOLD:AAE6832
Gynaephora groenlandica|[455]|GRAFW071-11|Greenland||658[0n]|BOLD:AAE6832
Gynaephora groenlandica|[456]|GRAFW070-11|Greenland||658[0n]|BOLD:AAE6832
Gynaephora groenlandica|[457]|GRAFW069-11|Greenland||658[0n]|BOLD:AAE6832
Gynaephora groenlandica|[458]|GRAFW068-11|Greenland||658[0n]|BOLD:AAE6832
Gynaephora groenlandica|[459]|GRAFW067-11|Greenland||658[0n]|BOLD:AAE6832
Gynaephora groenlandica|[460]|GRAFW902-12|Greenland||658[1n]|BOLD:AAE6832
Gynaephora groenlandica|[461]|GRAFW901-12|Greenland||658[1n]|BOLD:AAE6832
Gynaephora groenlandica|[462]|GRAFW899-12|Greenland||658[1n]|BOLD:AAE6832
Gynaephora groenlandica|[463]|GRAFW895-12|Greenland||658[1n]|BOLD:AAE6832
Gynaephora groenlandica|[464]|GRAFW893-12|Greenland||658[1n]|BOLD:AAE6832
Gynaephora groenlandica|[465]|GRAFW882-12|Greenland||658[1n]|BOLD:AAE6832
Gynaephora groenlandica|[466]|GRAFW879-12|Greenland||658[1n]|BOLD:AAE6832
Gynaephora groenlandica|[467]|MNAD826-07|Canada|Nunavut|658[0n]|BOLD:AAE6832
Gynaephora groenlandica|[468]|RDNME645-08|Canada||649[1n]|BOLD:AAE6832
Gynaephora groenlandica|[469]|GRAFW317-11|Greenland||642[0n]|BOLD:AAE6832
Gynaephora groenlandica|[470]|GRAFW683-12|Greenland||658[0n]|BOLD:AAE6832
Gynaephora groenlandica|[471]|GRAFW880-12|Greenland||658[1n]|BOLD:AAE6832
Gynaephora groenlandica|[472]|GRAFW883-12|Greenland||658[2n]|BOLD:AAE6832
Gynaephora groenlandica|[473]|GRAFW885-12|Greenland||658[0n]|BOLD:AAE6832
Gynaephora groenlandica|[474]|GRAFW904-12|Greenland||658[0n]|BOLD:AAE6832
Gynaephora groenlandica|[475]|GRAFW905-12|Greenland||658[0n]|BOLD:AAE6832
Gynaephora groenlandica|[476]|GRAFW1019-12|Greenland||658[0n]|BOLD:AAE6832
Gynaephora groenlandica|[477]|GRAFW1020-12|Greenland||658[0n]|BOLD:AAE6832
Gynaephora groenlandica|[478]|GRAFW1021-12|Greenland||658[0n]|BOLD:AAE6832
Catocala residua|[479]|QUNOC016-09|United States|Missouri|658[0n]|BOLD:ACE7470
Catocala residua|[480]|QUNO742-08|United States|Wisconsin|658[0n]|BOLD:ACE7470
Catocala residua|[481]|RDLQB665-05|Canada|Quebec|582[0n]|BOLD:ACE7470
Catocala retecta|[482]|QUNO070-07|United States|Wisconsin|658[0n]|BOLD:ACE7470
Catocala flebilis|[483]|LNCC1278-11|United States|North Carolina|658[0n]|BOLD:ACE7470
Catocala flebilis|[484]|LNCC1279-11|United States|North Carolina|658[0n]|BOLD:ACE7470
Catocala flebilis|[485]|LNCC389-10|United States|North Carolina|658[0n]|BOLD:ACE7470
Catocala residua|[486]|LNCC443-10|United States|North Carolina|658[0n]|BOLD:ACE7470
Catocala retecta|[487]|QUNOD396-10|Canada|Quebec|658[0n]|BOLD:ACE7470
Catocala retecta|[488]|LNCC223-10|United States|North Carolina|658[0n]|BOLD:ACE7470
Catocala retecta|[489]|QUNOD362-10|Canada|Quebec|658[0n]|BOLD:ACE7470
Catocala retecta|[490]|QUNOD395-10|Canada|Quebec|658[0n]|BOLD:ACE7470
Catocala obscura|[491]|QUNOB218-08|United States|Indiana|658[0n]|BOLD:ACE7470
Catocala residua|[492]|QUNOB238-08|United States|Indiana|658[0n]|BOLD:ACE7470
Catocala flebilis|[493]|QUNOB210-08|United States|Indiana|658[0n]|BOLD:ACE7470
Catocala flebilis|[494]|QUNOB211-08|United States|Indiana|658[0n]|BOLD:ACE7470
Catocala retecta|[495]|QUNOB216-08|United States|Indiana|658[0n]|BOLD:ACE7470
Catocala obscura|[496]|QUNOB217-08|United States|Indiana|658[0n]|BOLD:ACE7470
Catocala flebilis|[497]|QUNOB212-08|United States|Indiana|658[0n]|BOLD:ACE7470
Catocala retecta|[498]|QUNOB215-08|United States|Indiana|658[0n]|BOLD:ACE7470
Catocala flebilis|[499]|QUNOB208-08|United States|Indiana|658[0n]|BOLD:ACE7470
[illegible]

Catocala flebilis|[497]|QUNOB212-08|United States|Indiana|658[0n]|BOLD:ACE7470
Catocala retecta|[498]|QUNOB215-08|United States|Indiana|658[0n]|BOLD:ACE7470
Catocala flebilis|[499]|QUNOB208-08|United States|Indiana|658[0n]|BOLD:ACE7470
Catocala flebilis|[500]|QUNOB209-08|United States|Indiana|658[0n]|BOLD:ACE7470
Catocala retecta|[501]|QUNOB193-08|United States|Indiana|658[0n]|BOLD:ACE7470
Catocala flebilis|[502]|QUNOB207-08|United States|Indiana|658[0n]|BOLD:ACE7470
Catocala flebilis|[503]|QUNOB035-08|United States|Indiana|658[0n]|BOLD:ACE7470
Catocala retecta|[504]|QUNOB182-08|United States|Wisconsin|658[0n]|BOLD:ACE7470
Catocala obscura|[505]|QUNO745-08|United States|Wisconsin|658[0n]|BOLD:ACE7470
Catocala obscura|[506]|QUNO747-08|United States|Wisconsin|658[0n]|BOLD:ACE7470
Catocala obscura|[507]|RDLQB753-05|Canada|Quebec|658[0n]|BOLD:ACE7470
Catocala obscura|[508]|RDLQB827-05|Canada|Quebec|658[0n]|BOLD:ACE7470
Catocala retecta|[509]|RDLQ114-05|Canada|Quebec|658[0n]|BOLD:ACE7470
Catocala obscura|[510]|RDLQ115-05|Canada|Quebec|658[0n]|BOLD:ACE7470
Catocala flebilis|[511]|LGSM419-04|United States|Tennessee|658[0n]|BOLD:ACE7470
Catocala retecta|[512]|RDLQB660-05|Canada|Quebec|593[0n]|BOLD:ACE7470
Catocala retecta|[513]|RDLQB662-05|Canada|Quebec|591[0n]|BOLD:ACE7470
Catocala obscura|[514]|RDLQ112-05|Canada|Quebec|590[0n]|BOLD:ACE7470
Catocala retecta|[515]|RDLQB659-05|Canada|Quebec|588[0n]|BOLD:ACE7470
Catocala retecta|[516]|RDLQB661-05|Canada|Quebec|586[0n]|BOLD:ACE7470
Catocala retecta|[517]|RDLQB664-05|Canada|Quebec|591[3n]|BOLD:ACE7470
Catocala retecta|[518]|RDLQ113-05|Canada|Quebec|544[18n]|
Catocala residua|[519]|RDLQB666-05|Canada|Quebec|594[0n]|BOLD:ACE7470
Catocala flebilis|[520]|ABCNA652-07|United States|Tennessee|577[0n]|BOLD:ACE7470
Catocala retecta|[521]|QUNO617-08|United States|Wisconsin|658[0n]|BOLD:ACE7470
Catocala residua|[522]|QUNO741-08|United States|Wisconsin|658[0n]|BOLD:ACE7470
Catocala obscura|[523]|QUNO605-08|United States|Indiana|658[0n]|BOLD:ACE7470
Catocala retecta|[524]|QUNO616-08|United States|Wisconsin|658[0n]|BOLD:ACE7470
Catocala retecta|[525]|LGSMG592-07|United States|Tennessee|658[0n]|BOLD:ACE7470
Catocala obscura|[526]|QUNO069-07|United States|Wisconsin|658[0n]|BOLD:ACE7470
Catocala retecta|[527]|RDLQ111-05|Canada|Quebec|658[1n]|BOLD:ACE7470
Catocala residua|[528]|QUNOB237-08|United States|Indiana|658[0n]|BOLD:ACE7470
Catocala obscura|[529]|QUNO602-08|United States|Indiana|658[0n]|BOLD:ACE7470
Catocala obscura|[530]|QUNO603-08|United States|Indiana|658[0n]|BOLD:ACE7470
Catocala residua|[531]|QUNOB233-08|United States|Indiana|658[0n]|BOLD:ACE7470
Catocala retecta|[532]|QUNOE493-12|United States|Wisconsin|658[0n]|BOLD:ACE7470
Catocala flebilis|[533]|QUNO585-08|United States|Indiana|658[0n]|BOLD:ACE7470
Catocala flebilis|[534]|QUNO586-08|United States|Indiana|658[0n]|BOLD:ACE7470
Catocala flebilis|[535]|LGSM418-04|United States|Tennessee|658[0n]|BOLD:ACE7470
Catocala obscura|[536]|QUNO744-08|United States|Wisconsin|658[0n]|BOLD:ACE7470
Catocala retecta|[537]|RDLQB454-05|Canada|Quebec|658[0n]|BOLD:ACE7470
Catocala obscura|[538]|RDLQB667-05|Canada|Quebec|658[0n]|BOLD:ACE7470
Catocala vidua|[539]|LNCC1325-11|United States|North Carolina|658[0n]|BOLD:ACE7470
Catocala flebilis|[540]|QUNO587-08|United States|Indiana|658[0n]|BOLD:ACE7470
Catocala obscura|[541]|QUNO750-08|United States|Wisconsin|658[0n]|BOLD:ACE7470
Catocala obscura|[542]|QUNO604-08|United States|Indiana|658[0n]|BOLD:ACE7470
Catocala obscura|[543]|QUNOB221-08|United States|Indiana|658[0n]|BOLD:ACE7470
Catocala obscura|[544]|QUNOB224-08|United States|Indiana|658[0n]|BOLD:ACE7470
Catocala judith|[545]|QUNO764-08|United States|Wisconsin|658[0n]|BOLD:ACE7470
Catocala judith|[546]|QUNO765-08|United States|Wisconsin|658[0n]|BOLD:ACE7470
Catocala judith|[547]|QUNO763-08|United States|Wisconsin|658[0n]|BOLD:ACE7470
Catocala judith|[548]|ABCNA284-06|United States|Tennessee|598[1n]|BOLD:ACE7470
Catocala judith|[549]|ABCNA285-06|United States|Tennessee|585[3n]|BOLD:ACE7470
Catocala judith|[550]|ABCNA645-07|United States|Tennessee|564[0n]|BOLD:ACE7470
Catocala judith|[551]|QUNO762-08|United States|Wisconsin|658[0n]|BOLD:ACE7470
Catocala judith|[552]|QUNO766-08|United States|Wisconsin|658[0n]|BOLD:ACE7470
Catocala judith|[553]|QUNO767-08|United States|Wisconsin|658[0n]|BOLD:ACE7470
Catocala judith|[554]|QUNO768-08|United States|Wisconsin|658[0n]|BOLD:ACE7470
Catocala judith|[555]|QUNO769-08|United States|Wisconsin|658[0n]|BOLD:ACE7470
Catocala judith|[556]|QUNO770-08|United States|Wisconsin|658[0n]|BOLD:ACE7470
Catocala judith|[557]|QUNO771-08|United States|Wisconsin|658[0n]|BOLD:ACE7470
Catocala obscura|[558]|QUNO749-08|United States|Wisconsin|658[0n]|BOLD:ACE7470
Catocala obscura|[559]|QUNO607-08|United States|Indiana|658[0n]|BOLD:ACE7470
Catocala obscura|[560]|QUNOB223-08|United States|Indiana|658[0n]|BOLD:ACE7470
Catocala retecta|[561]|QUNO810-08|United States|Wisconsin|658[0n]|BOLD:ACE7470
Catocala obscura|[562]|QUNO746-08|United States|Wisconsin|658[0n]|BOLD:ACE7470
Catocala obscura|[563]|QUNOB225-08|United States|Indiana|658[0n]|BOLD:ACE7470
Catocala nr. residua|[564]|RDLQ116-05|Canada|Quebec|524[0n]|BOLD:ACE7470
Catocala retecta|[565]|RDLQ179-05|Canada|Quebec|525[0n]|BOLD:ACE7470
Catocala luctuosa|[566]|ABCNA135-06|United States|Indiana|594[0n]|BOLD:ACY0830
Catocala lacrymosa|[567]|ABCNA142-06|United States|Indiana|578[0n]|BOLD:AAD6919
Catocala robinsonii|[568]|QUNO598-08|United States|Indiana|658[0n]|BOLD:ACE7470
Catocala robinsonii|[569]|QUNO599-08|United States|Indiana|658[0n]|BOLD:ACE7470
Catocala robinsonii|[570]|QUNO597-08|United States|Indiana|658[0n]|BOLD:ACE7470
Catocala robinsonii|[571]|QUNO596-08|United States|Indiana|658[0n]|BOLD:ACE7470
Catocala retecta|[572]|RDLQ176-05|Canada|Quebec|577[0n]|BOLD:ACE7470
Catocala robinsonii|[573]|ABCNA012-06|United States|Indiana|607[0n]|BOLD:ACE7470
Catocala robinsonii|[574]|ABCNA011-06|United States|South Carolina|579[0n]|BOLD:ACE7470
Catocala robinsonii|[575]|LSUSA112-06|United States|Kentucky|531[0n]|BOLD:ACE7470
Catocala robinsonii|[576]|ABCNA648-07|United States|Indiana|574[0n]|BOLD:ACE7470
Catocala robinsonii|[577]|QUNO574-08|United States|Indiana|658[0n]|BOLD:ACE7470
Catocala robinsonii|[578]|QUNO595-08|United States|Indiana|658[0n]|BOLD:ACE7470
Catocala obscura|[579]|QUNO601-08|United States|Indiana|658[0n]|BOLD:ACE7470
Catocala residua|[580]|QUNO606-08|United States|Indiana|658[0n]|BOLD:ACE7470
Catocala obscura|[581]|QUNO608-08|United States|Indiana|658[0n]|BOLD:ACE7470
Catocala obscura|[582]|QUNO743-08|United States|Wisconsin|658[0n]|BOLD:ACE7470
Catocala robinsonii|[583]|QUNOB125-08|United States|Indiana|658[0n]|BOLD:ACE7470
Catocala robinsonii|[584]|QUNOB239-08|United States|Indiana|658[0n]|BOLD:ACE7470
Catocala robinsonii|[585]|QUNOB240-08|United States|Indiana|658[0n]|BOLD:ACE7470
Catocala robinsonii|[586]|QUNOB241-08|United States|Indiana|658[0n]|BOLD:ACE7470
Catocala robinsonii|[587]|QUNOB242-08|United States|Indiana|658[0n]|BOLD:ACE7470
Catocala robinsonii|[588]|QUNOB243-08|United States|Indiana|658[0n]|BOLD:ACE7470
Catocala robinsonii|[589]|QUNOB244-08|United States|Indiana|658[0n]|BOLD:ACE7470
Catocala robinsonii|[590]|HKONB476-09|United States|Louisiana|658[0n]|BOLD:ACE7470
Catocala robinsonii|[591]|LNCC419-10|United States|North Carolina|658[0n]|BOLD:ACE7470
Catocala robinsonii|[592]|LNCC420-10|United States|North Carolina|658[0n]|BOLD:ACE7470
Catocala robinsonii|[593]|LNCC447-10|United States|North Carolina|658[0n]|BOLD:ACE7470
Catocala obscura|[594]|QUNOB219-08|United States|Indiana|658[0n]|BOLD:ACE7470
Catocala obscura|[595]|QUNOB222-08|United States|Indiana|658[0n]|BOLD:ACE7470
Catocala obscura|[596]|QUNOB226-08|United States|Indiana|658[0n]|BOLD:ACE7470
Catocala obscura|[597]|QUNO618-08|United States|Indiana|658[0n]|BOLD:ACE7470
Catocala residua|[598]|QUNOB235-08|United States|Indiana|658[0n]|BOLD:ACE7470
Catocala residua|[599]|QUNO610-08|United States|Indiana|658[0n]|BOLD:ACE7470
[illegible]

Catocala obscura|[597]|QUNO618-08|United States|Indiana|658[0n]|BOLD:ACE7470
Catocala residua|[598]|QUNOB235-08|United States|Indiana|658[0n]|BOLD:ACE7470
Catocala residua|[599]|QUNO610-08|United States|Indiana|658[0n]|BOLD:ACE7470
Catocala obscura|[600]|QUNOB176-08|United States|Indiana|658[0n]|BOLD:ACE7470
Catocala obscura|[601]|ABCNA654-07|United States|Indiana|577[0n]|BOLD:ACE7470
Catocala obscura|[602]|QUNO611-08|United States|Indiana|658[0n]|BOLD:ACE7470
Catocala residua|[603]|QUNO613-08|United States|Indiana|658[0n]|BOLD:ACE7470
Catocala residua|[604]|QUNO614-08|United States|Indiana|658[0n]|BOLD:ACE7470
Catocala residua|[605]|QUNOB177-08|United States|Indiana|658[0n]|BOLD:ACE7470
Catocala residua|[606]|QUNO612-08|United States|Indiana|658[0n]|BOLD:ACE7470
Catocala obscura|[607]|QUNOB220-08|United States|Indiana|658[0n]|BOLD:ACE7470
Catocala residua|[608]|QUNOB227-08|United States|Indiana|658[0n]|BOLD:ACE7470
Catocala residua|[609]|QUNOB228-08|United States|Indiana|658[0n]|BOLD:ACE7470
Catocala residua|[610]|QUNOB229-08|United States|Indiana|658[0n]|BOLD:ACE7470
Catocala residua|[611]|QUNOB230-08|United States|Indiana|658[0n]|BOLD:ACE7470
Catocala residua|[612]|QUNOB231-08|United States|Indiana|658[0n]|BOLD:ACE7470
Catocala residua|[613]|QUNOB232-08|United States|Indiana|658[0n]|BOLD:ACE7470
Catocala residua|[614]|QUNOB234-08|United States|Indiana|658[0n]|BOLD:ACE7470
Catocala residua|[615]|QUNOB236-08|United States|Indiana|658[0n]|BOLD:ACE7470
Catocala residua|[616]|QUNOD658-10|United States|Mississippi|658[0n]|BOLD:ACE7470
Catocala obscura|[617]|LNCC1292-11|United States|North Carolina|658[0n]|BOLD:ACE7470
Catocala retecta|[618]|QUNOB180-08|United States|Wisconsin|652[0n]|BOLD:ACE7470
Catocala retecta|[619]|QUNOD397-10|Canada|Quebec|658[0n]|BOLD:ACE7470
Catocala residua|[620]|LNCC444-10|United States|North Carolina|658[0n]|BOLD:ACE7470
Catocala retecta|[621]|QUNOB181-08|United States|Wisconsin|658[0n]|BOLD:ACE7470
Catocala retecta|[622]|QUNOB183-08|United States|Wisconsin|658[0n]|BOLD:ACE7470
Catocala retecta|[623]|QUNO809-08|United States|Wisconsin|658[0n]|BOLD:ACE7470
Catocala residua|[624]|QUNOB030-08|United States|Indiana|658[0n]|BOLD:ACE7470
Catocala habilis|[625]|QUNO776-08|United States|Wisconsin|658[0n]|BOLD:ACE7470
Catocala retecta|[626]|QUNO808-08|United States|Wisconsin|658[0n]|BOLD:ACE7470
Catocala habilis|[627]|QUNO772-08|United States|Wisconsin|658[0n]|BOLD:ACE7470
Catocala habilis|[628]|QUNO773-08|United States|Wisconsin|658[0n]|BOLD:ACE7470
Catocala residua|[629]|QUNO609-08|United States|Indiana|658[0n]|BOLD:ACE7470
Catocala retecta|[630]|QUNO615-08|United States|Wisconsin|658[0n]|BOLD:ACE7470
Catocala habilis|[631]|QUNO774-08|United States|Wisconsin|658[0n]|BOLD:ACE7470
Catocala habilis|[632]|QUNO775-08|United States|Wisconsin|658[0n]|BOLD:ACE7470
Catocala retecta|[633]|QUNO071-07|United States|Wisconsin|658[0n]|BOLD:ACE7470
Catocala habilis|[634]|QUNO078-07|United States|Wisconsin|658[0n]|BOLD:ACE7470
Catocala habilis|[635]|RDLQB671-05|Canada|Quebec|658[0n]|BOLD:ACE7470
Catocala habilis|[636]|RDLQB457-05|Canada|Quebec|658[0n]|BOLD:ACE7470
Catocala luctuosa|[637]|QUNOB284-08|United States|Indiana|658[0n]|BOLD:ACE7470
Catocala luctuosa|[638]|QUNOB283-08|United States|Indiana|658[0n]|BOLD:ACE7470
Catocala luctuosa|[639]|QUNOB282-08|United States|Indiana|658[0n]|BOLD:ACE7470
Catocala luctuosa|[640]|QUNOB280-08|United States|Indiana|658[0n]|BOLD:ACE7470
Catocala luctuosa|[641]|QUNOB279-08|United States|Indiana|658[0n]|BOLD:ACE7470
Catocala luctuosa|[642]|QUNOB278-08|United States|Indiana|658[0n]|BOLD:ACE7470
Catocala luctuosa|[643]|QUNOB277-08|United States|Indiana|658[0n]|BOLD:ACE7470
Catocala luctuosa|[644]|QUNOB274-08|United States|Indiana|658[0n]|BOLD:ACE7470
Catocala luctuosa|[645]|QUNOB273-08|United States|Indiana|658[0n]|BOLD:ACE7470
Catocala luctuosa|[646]|QUNOB272-08|United States|Indiana|658[0n]|BOLD:ACE7470
Catocala luctuosa|[647]|QUNOB271-08|United States|Indiana|658[0n]|BOLD:ACE7470
Catocala luctuosa|[648]|QUNOB270-08|United States|Indiana|658[0n]|BOLD:ACE7470
Catocala luctuosa|[649]|QUNOB123-08|United States|Indiana|658[0n]|BOLD:ACE7470
Catocala luctuosa|[650]|QUNOB034-08|United States|Indiana|658[0n]|BOLD:ACE7470
Catocala luctuosa|[651]|QUNOB032-08|United States|Indiana|658[0n]|BOLD:ACE7470
Catocala luctuosa|[652]|QUNOB031-08|United States|Indiana|658[0n]|BOLD:ACE7470
Catocala luctuosa|[653]|QUNO590-08|United States|Indiana|658[0n]|BOLD:ACE7470
Catocala luctuosa|[654]|ABCNA133-06|United States|Indiana|658[0n]|BOLD:ACE7470
Catocala habilis|[655]|ABCNA269-06|United States|Wisconsin|624[0n]|BOLD:ACE7470
Catocala luctuosa|[656]|ABCNA134-06|United States|Indiana|618[0n]|BOLD:ACE7470
Catocala luctuosa|[657]|ABCNA136-06|United States|Indiana|608[0n]|BOLD:ACE7470
Catocala luctuosa|[658]|QUNOB275-08|United States|Indiana|613[0n]|BOLD:ACE7470
Catocala luctuosa|[659]|QUNOD686-11|United States|Missouri|633[0n]|BOLD:ACE7470
Catocala luctuosa|[660]|QUNOD687-11|United States|Missouri|658[0n]|BOLD:ACE7470
Catocala retecta|[661]|RDLQB467-05|Canada|Quebec|658[0n]|BOLD:ACE7470
Catocala angusi|[662]|QUNOB268-08|United States|Indiana|658[0n]|BOLD:ACE7470
Catocala angusi|[663]|QUNOB249-08|United States|Indiana|658[0n]|BOLD:ACE7470
Catocala angusi|[664]|QUNOB261-08|United States|Indiana|658[0n]|BOLD:ACE7470
Catocala angusi|[665]|QUNOB260-08|United States|Indiana|658[0n]|BOLD:ACE7470
Catocala angusi|[666]|QUNOB259-08|United States|Indiana|658[0n]|BOLD:ACE7470
Catocala angusi|[667]|QUNOB257-08|United States|Indiana|658[0n]|BOLD:ACE7470
Catocala angusi|[668]|QUNOB255-08|United States|Indiana|658[0n]|BOLD:ACE7470
Catocala angusi|[669]|QUNOB254-08|United States|Indiana|658[0n]|BOLD:ACE7470
Catocala angusi|[670]|QUNOB253-08|United States|Indiana|658[0n]|BOLD:ACE7470
Catocala angusi|[671]|QUNOB252-08|United States|Indiana|658[0n]|BOLD:ACE7470
Catocala angusi|[672]|QUNOB251-08|United States|Indiana|658[0n]|BOLD:ACE7470
Catocala angusi|[673]|QUNOB250-08|United States|Indiana|658[0n]|BOLD:ACE7470
Catocala angusi|[674]|QUNOB247-08|United States|Indiana|658[0n]|BOLD:ACE7470
Catocala angusi|[675]|QUNOB246-08|United States|Indiana|658[0n]|BOLD:ACE7470
Catocala angusi|[676]|QUNOB245-08|United States|Indiana|658[0n]|BOLD:ACE7470
Catocala angusi|[677]|QUNO582-08|United States|Indiana|658[0n]|BOLD:ACE7470
Catocala angusi|[678]|QUNOB258-08|United States|Indiana|658[0n]|BOLD:ACE7470
Catocala angusi|[679]|QUNO581-08|United States|Indiana|658[0n]|BOLD:ACE7470
Catocala angusi|[680]|QUNO579-08|United States|Indiana|658[0n]|BOLD:ACE7470
Catocala angusi|[681]|ABCNA122-06|United States|Mississippi|620[0n]|BOLD:ACE7470
Catocala angusi|[682]|ABCNA649-07|United States|Indiana|577[0n]|BOLD:ACE7470
Catocala angusi|[683]|ABCNA650-07|United States|Indiana|575[1n]|BOLD:ACE7470
Catocala angusi|[684]|QUNOB248-08|United States|Indiana|658[0n]|BOLD:ACE7470
Catocala angusi|[685]|QUNO575-08|United States|Indiana|658[0n]|BOLD:ACE7470
Catocala angusi|[686]|QUNOB256-08|United States|Indiana|658[0n]|BOLD:ACE7470
Catocala angusi|[687]|QUNOB262-08|United States|Indiana|658[0n]|BOLD:ACE7470
Catocala angusi|[688]|QUNOB263-08|United States|Indiana|658[0n]|BOLD:ACE7470
Catocala angusi|[689]|QUNOB264-08|United States|Indiana|658[0n]|BOLD:ACE7470
Catocala angusi|[690]|QUNOB265-08|United States|Indiana|658[0n]|BOLD:ACE7470
Catocala angusi|[691]|QUNOB266-08|United States|Indiana|658[0n]|BOLD:ACE7470
Catocala angusi|[692]|QUNOB267-08|United States|Indiana|658[0n]|BOLD:ACE7470
Catocala angusi|[693]|QUNOB269-08|United States|Indiana|658[0n]|BOLD:ACE7470
Catocala robinsonii|[694]|CNCLB2768-14|United States|North Carolina|658[0n]|BOLD:ACE7470
Catocala luctuosa|[695]|QUNOD694-11|United States|Massachusetts|658[0n]|BOLD:ACE7470
Catocala habilis|[696]|RDLQB461-05|Canada|Quebec|658[0n]|BOLD:ACE7470
Catocala habilis|[697]|RDLQB456-05|Canada|Quebec|658[0n]|BOLD:ACE7470
Catocala residua|[698]|RDLQB455-05|Canada|Quebec|578[0n]|BOLD:ACE7470
Catocala obscura|[699]|RDLQB668-05|Canada|Quebec|608[0n]|BOLD:ACE7470

Catocala habilis|[697]|RDLQB456-05|Canada|Quebec|658[0n]|BOLD:ACE7470
Catocala residua|[698]|RDLQB455-05|Canada|Quebec|578[0n]|BOLD:ACE7470
Catocala obscura|[699]|RDLQB668-05|Canada|Quebec|608[0n]|BOLD:ACE7470
Catocala residua|[700]|ABCNA129-06|United States|Indiana|612[0n]|BOLD:ACE7470
Catocala habilis|[701]|RDLQB462-05|Canada|Quebec|592[0n]|BOLD:ACE7470
Catocala habilis|[702]|RDLQB672-05|Canada|Quebec|595[0n]|BOLD:ACE7470
Catocala retecta|[703]|RDLQB663-05|Canada|Quebec|585[0n]|BOLD:ACE7470
Catocala habilis|[704]|ABCNA642-07|United States|Kentucky|577[0n]|BOLD:ACE7470
Catocala habilis|[705]|ABCNA641-07|United States|Kentucky|577[0n]|BOLD:ACE7470
Catocala cara|[706]|RDLQB458-05|Canada|Quebec|585[0n]|BOLD:ACE7470
Catocala habilis|[707]|RDLQB669-05|Canada|Quebec|584[0n]|BOLD:ACE7470
Catocala habilis|[708]|RDLQB670-05|Canada|Quebec|586[0n]|BOLD:ACE7470
Catocala habilis|[709]|RDLQB460-05|Canada|Quebec|658[0n]|BOLD:ACE7470
Catocala luctuosa|[710]|ABCNA132-06|United States|Indiana|658[0n]|BOLD:ACE7470
Catocala luctuosa|[711]|QUNOB276-08|United States|Indiana|658[0n]|BOLD:ACE7470
Catocala luctuosa|[712]|QUNOB281-08|United States|Indiana|658[0n]|BOLD:ACE7470
Catocala serena|[713]|QUNO754-08|United States|Wisconsin|658[0n]|BOLD:ACE7468
Catocala serena|[714]|QUNO074-07|United States|Wisconsin|658[0n]|BOLD:ACE7468
Catocala serena|[715]|ABCNA636-07|United States|Kentucky|577[0n]|BOLD:ACE7468
Catocala serena|[716]|QUNO760-08|United States|Wisconsin|658[0n]|BOLD:ACE7468
Catocala serena|[717]|QUNO761-08|United States|Wisconsin|658[0n]|BOLD:ACE7468
Catocala serena|[718]|QUNO758-08|United States|Wisconsin|658[0n]|BOLD:ACE7468
Catocala serena|[719]|QUNO759-08|United States|Wisconsin|658[0n]|BOLD:ACE7468
Catocala serena|[720]|QUNO751-08|United States|Wisconsin|658[0n]|BOLD:ACE7468
Catocala serena|[721]|QUNO073-07|United States|Wisconsin|658[0n]|BOLD:ACE7468
Catocala serena|[722]|QUNO072-07|United States|Wisconsin|658[0n]|BOLD:ACE7468
Catocala serena|[723]|QUNO752-08|United States|Wisconsin|658[0n]|BOLD:ACE7468
Catocala serena|[724]|QUNO753-08|United States|Wisconsin|658[0n]|BOLD:ACE7468
Catocala serena|[725]|QUNO755-08|United States|Wisconsin|658[0n]|BOLD:ACE7468
Catocala serena|[726]|QUNO756-08|United States|Wisconsin|658[0n]|BOLD:ACE7468
Catocala serena|[727]|QUNO757-08|United States|Wisconsin|658[0n]|BOLD:ACE7468
Catocala serena|[728]|QUNOC005-09|United States|Indiana|658[0n]|BOLD:ACE7468
Catocala palaeogama|[729]|QUNO053-07|United States|Wisconsin|504[0n]|
Catocala vidua|[730]|QUNOB124-08|United States|Indiana|658[0n]|BOLD:ACE7470
Catocala vidua|[731]|QUNOB033-08|United States|Indiana|658[0n]|BOLD:ACE7470
Catocala vidua|[732]|QUNO592-08|United States|Indiana|658[0n]|BOLD:ACE7470
Catocala vidua|[733]|ABCNA145-06|United States|Wisconsin|658[0n]|BOLD:ACE7470
Catocala vidua|[734]|ABCNA407-07|United States|Indiana|570[1n]|BOLD:ACE7470
Catocala vidua|[735]|LGSMG593-07|United States|North Carolina|658[0n]|BOLD:ACE7470
Catocala vidua|[736]|QUNO591-08|United States|Indiana|658[0n]|BOLD:ACE7470
Catocala vidua|[737]|LNCC1324-11|United States|North Carolina|658[0n]|BOLD:ACE7470
Catocala palaeogama|[738]|LNCC363-10|United States|North Carolina|658[0n]|BOLD:ACE7470
Catocala palaeogama|[739]|LNCC1174-11|United States|North Carolina|658[0n]|BOLD:ACE7470
Catocala palaeogama|[740]|QUNO806-08|United States|Wisconsin|658[0n]|BOLD:ACE7470
Catocala palaeogama|[741]|QUNO807-08|United States|Wisconsin|658[0n]|BOLD:ACE7470
Catocala palaeogama|[742]|QUNO803-08|United States|Wisconsin|658[0n]|BOLD:ACE7470
Catocala palaeogama|[743]|QUNO804-08|United States|Wisconsin|658[0n]|BOLD:ACE7470
Catocala palaeogama|[744]|QUNO801-08|United States|Wisconsin|658[0n]|BOLD:ACE7470
Catocala palaeogama|[745]|QUNO802-08|United States|Wisconsin|658[0n]|BOLD:ACE7470
Catocala palaeogama|[746]|QUNO719-08|United States|Kentucky|658[0n]|BOLD:ACE7470
Catocala palaeogama|[747]|QUNO722-08|United States|Kentucky|658[0n]|BOLD:ACE7470
Catocala palaeogama|[748]|QUNO055-07|United States|Wisconsin|658[0n]|BOLD:ACE7470
Catocala palaeogama|[749]|QUNO054-07|United States|Wisconsin|658[0n]|BOLD:ACE7470
Catocala palaeogama|[750]|LGSMG594-07|United States|Tennessee|658[0n]|BOLD:ACE7470
Catocala palaeogama|[751]|RDLQB468-05|Canada|Quebec|658[0n]|BOLD:ACE7470
Catocala palaeogama|[752]|LGSM667-04|United States|North Carolina|658[0n]|BOLD:ACE7470
Catocala dejecta|[753]|QUNOD275-10|United States|Georgia|658[0n]|BOLD:ACE7470
Catocala dejecta|[754]|QUNO660-08|United States|Kentucky|658[0n]|BOLD:ACE7470
Catocala dejecta|[755]|ABCNA291-06|United States|Kentucky|609[0n]|BOLD:ACE7470
Catocala dejecta|[756]|ABCNA399-07|United States|Tennessee|595[0n]|BOLD:ACE7470
Catocala dejecta|[757]|LGSM641-04|United States|Tennessee|606[1n]|BOLD:ACE7470
Catocala dejecta|[758]|ABCNA397-07|United States|Kentucky|584[0n]|BOLD:ACE7470
Catocala dejecta|[759]|ABCNA398-07|United States|Tennessee|573[0n]|BOLD:ACE7470
Catocala dejecta|[760]|ABCNA779-07|United States|Virginia|583[0n]|BOLD:ACE7470
Catocala dejecta|[761]|LNCC1293-11|United States|North Carolina|633[0n]|BOLD:ACE7470
Catocala palaeogama|[762]|QUNO578-08|United States|Indiana|658[0n]|BOLD:ACE7470
Catocala palaeogama|[763]|QUNO718-08|United States|Kentucky|658[0n]|BOLD:ACE7470
Catocala palaeogama|[764]|CNSLM015-13|Canada|Ontario|573[0n]|BOLD:ACE7470
Catocala lacrymosa|[765]|MILEQ187-11|United States|Florida|658[0n]|BOLD:ACE7470
Catocala lacrymosa|[766]|MILEQ186-11|United States|Florida|658[0n]|BOLD:ACE7470
Catocala lacrymosa|[767]|MILEQ185-11|United States|Florida|658[0n]|BOLD:ACE7470
Catocala lacrymosa|[768]|MILEQ184-11|United States|Florida|658[0n]|BOLD:ACE7470
Catocala lacrymosa|[769]|MILEQ183-11|United States|Florida|658[0n]|BOLD:ACE7470
Catocala lacrymosa|[770]|MILEQ182-11|United States|Florida|658[0n]|BOLD:ACE7470
Catocala lacrymosa|[771]|LNCC446-10|United States|North Carolina|658[0n]|BOLD:ACE7470
Catocala lacrymosa|[772]|LNCC445-10|United States|North Carolina|658[0n]|BOLD:ACE7470
Catocala lacrymosa|[773]|LPOKD361-09|United States|Oklahoma|658[0n]|BOLD:ACE7470
Catocala lacrymosa|[774]|QUNO638-08|United States|Mississippi|658[0n]|BOLD:ACE7470
Catocala lacrymosa|[775]|QUNO566-08|United States|Indiana|658[0n]|BOLD:ACE7470
Catocala lacrymosa|[776]|QUNO565-08|United States|Indiana|658[0n]|BOLD:ACE7470
Catocala cara|[777]|QUNO560-08|United States|Indiana|658[0n]|BOLD:ACE7470
Catocala lacrymosa|[778]|ABCNA150-06|United States|Indiana|594[0n]|BOLD:ACE7470
Catocala lacrymosa|[779]|LPOKA378-08|United States|Oklahoma|658[0n]|BOLD:ACE7470
Catocala lacrymosa|[780]|ABCNA146-06|United States|Mississippi|617[0n]|BOLD:ACE7470
Catocala lacrymosa|[781]|ABCNA147-06|United States|Indiana|614[0n]|BOLD:ACE7470
Catocala lacrymosa|[782]|ABCNA149-06|United States|Florida|599[0n]|BOLD:ACE7470
Catocala lacrymosa|[783]|ABCNA408-07|United States|Mississippi|595[0n]|BOLD:ACE7470
Catocala lacrymosa|[784]|JRLAA032-09|United States|Alabama|630[0n]|BOLD:ACE7470
Catocala lacrymosa|[785]|MILEQ188-11|United States|North Carolina|658[0n]|BOLD:ACE7470
Catocala lacrymosa|[786]|MILEQ189-11|United States|North Carolina|658[0n]|BOLD:ACE7470
Catocala lacrymosa|[787]|QUNO593-08|United States|Indiana|658[0n]|BOLD:ACE7470
Catocala palaeogama|[788]|CNSLD280-12|Canada|Ontario|632[0n]|BOLD:ACE7470
Catocala palaeogama|[789]|CNSLM016-13|Canada|Ontario|595[0n]|BOLD:ACE7470
Catocala myristica|[790]|QUNO626-08|United States|Mississippi|658[0n]|BOLD:ACE7470
Catocala myristica|[791]|QUNO628-08|United States|Mississippi|658[0n]|BOLD:ACE7470
Catocala agrippina|[792]|QUNO482-08|United States|Texas|658[0n]|BOLD:ACE7470
Catocala agrippina|[793]|QUNO484-08|United States|Texas|658[0n]|BOLD:ACE7470
Catocala agrippina|[794]|QUNO646-08|United States|Louisiana|658[0n]|BOLD:ACE7470
Catocala ulalume|[795]|QUNOD697-11|United States|North Carolina|658[0n]|BOLD:ACE7470
Catocala ulalume|[796]|QUNOB502-09|United States|Alabama|658[0n]|BOLD:ACE7470
Catocala sappho|[797]|QUNO653-08|United States|Mississippi|658[0n]|BOLD:ACE7470
Catocala sappho|[798]|QUNO652-08|United States|Mississippi|658[0n]|BOLD:ACE7470
Catocala ulalume|[799]|QUNO480-08|United States|Texas|658[0n]|BOLD:ACE7470

Catocala sappho|[797]|QUNO653-08|United States|Mississippi|658[0n]|BOLD:ACE7470
Catocala sappho|[798]|QUNO652-08|United States|Mississippi|658[0n]|BOLD:ACE7470
Catocala ulalume|[799]|QUNO480-08|United States|Texas|658[0n]|BOLD:ACE7470
Catocala sappho|[800]|ABCNA280-06|United States|Florida|658[0n]|BOLD:ACE7470
Catocala sappho|[801]|QUNO637-08|United States|Mississippi|658[0n]|BOLD:ACE7470
Catocala sappho|[802]|ABCNA289-06|United States|Kentucky|658[0n]|BOLD:ACE7470
Catocala ulalume|[803]|ABCNA143-06|United States|Mississippi|572[0n]|BOLD:ACE7470
Catocala ulalume|[804]|QUNOB194-08|United States|Indiana|658[0n]|BOLD:ACE7470
Catocala ulalume|[805]|LNCC390-10|United States|North Carolina|658[0n]|BOLD:ACE7470
Catocala sappho|[806]|LNCC961-11|United States|North Carolina|658[0n]|BOLD:ACE7470
Catocala myristica|[807]|QUNO643-08|United States|Mississippi|609[0n]|BOLD:ACE7470
Catocala agrippina|[808]|QUNO570-08|United States|Indiana|658[0n]|BOLD:ACE7470
Catocala agrippina|[809]|ABCNA393-07|United States|Louisiana|593[0n]|BOLD:ACE7470
Catocala agrippina|[810]|QUNO485-08|United States|Texas|658[0n]|BOLD:ACE7470
Catocala agrippina|[811]|QUNO650-08|United States|Mississippi|658[0n]|BOLD:ACE7470
Catocala agrippina|[812]|QUNO644-08|United States|Louisiana|658[0n]|BOLD:ACE7470
Catocala agrippina|[813]|QUNO645-08|United States|Louisiana|658[0n]|BOLD:ACE7470
Catocala agrippina|[814]|QUNO648-08|United States|Louisiana|658[0n]|BOLD:ACE7470
Catocala agrippina|[815]|LPOKC593-09|United States|Oklahoma|658[0n]|BOLD:ACE7470
Catocala agrippina|[816]|QUNO483-08|United States|Texas|658[0n]|BOLD:ACE7470
Catocala agrippina|[817]|QUNO569-08|United States|Indiana|658[0n]|BOLD:ACE7470
Catocala agrippina|[818]|LPOKC597-09|United States|Oklahoma|658[0n]|BOLD:ACE7470
Catocala maestosa|[819]|QUNOB285-08|United States|Indiana|658[0n]|BOLD:ACE7470
Catocala myristica|[820]|QUNOB377-09|United States|Mississippi|658[0n]|BOLD:ACE7470
Catocala myristica|[821]|QUNO630-08|United States|Mississippi|658[0n]|BOLD:ACE7470
Catocala myristica|[822]|QUNO639-08|United States|Mississippi|658[0n]|BOLD:ACE7470
Catocala myristica|[823]|QUNO642-08|United States|Mississippi|658[0n]|BOLD:ACE7470
Catocala myristica|[824]|QUNO641-08|United States|Mississippi|658[0n]|BOLD:ACE7470
Catocala myristica|[825]|QUNO640-08|United States|Mississippi|658[0n]|BOLD:ACE7470
Catocala myristica|[826]|QUNO631-08|United States|Mississippi|658[0n]|BOLD:ACE7470
Catocala myristica|[827]|QUNO629-08|United States|Mississippi|658[0n]|BOLD:ACE7470
Catocala myristica|[828]|ABCNA010-06|United States|Mississippi|658[0n]|BOLD:ACE7470
Catocala myristica|[829]|QUNO627-08|United States|Mississippi|658[0n]|BOLD:ACE7470
Catocala myristica|[830]|QUNO625-08|United States|Mississippi|658[0n]|BOLD:ACE7470
Catocala ulalume|[831]|QUNOB501-09|United States|Alabama|658[0n]|BOLD:ACE7470
Catocala myristica|[832]|QUNOD829-11|United States|South Carolina|658[0n]|BOLD:ACE7470
Catocala maestosa|[833]|BBLOD1852-11|United States|Texas|658[0n]|BOLD:ACE7470
Catocala maestosa|[834]|QUNO481-08|United States|Texas|658[0n]|BOLD:ACE7470
Catocala maestosa|[835]|QUNOB297-09|United States|Wisconsin|658[0n]|BOLD:ACE7470
Catocala maestosa|[836]|BBLOD1854-11|United States|Texas|658[0n]|BOLD:ACE7470
Catocala maestosa|[837]|LPOKA569-09|United States|Oklahoma|633[0n]|BOLD:ACE7470
Catocala maestosa|[838]|ABCNA293-06|United States|Indiana|632[0n]|BOLD:ACE7470
Catocala maestosa|[839]|QUNO588-08|United States|Indiana|658[0n]|BOLD:ACE7470
Catocala maestosa|[840]|QUNO635-08|United States|Mississippi|658[0n]|BOLD:ACE7470
Catocala maestosa|[841]|QUNO633-08|United States|Mississippi|658[0n]|BOLD:ACE7470
Catocala maestosa|[842]|QUNO589-08|United States|Indiana|658[0n]|BOLD:ACE7470
Catocala maestosa|[843]|QUNO556-08|United States|Texas|658[0n]|BOLD:ACE7470
Catocala maestosa|[844]|LPOKD626-09|United States|Oklahoma|658[0n]|BOLD:ACE7470
Catocala maestosa|[845]|QUNO647-08|United States|Louisiana|658[0n]|BOLD:ACE7470
Catocala maestosa|[846]|QUNOB126-08|United States|Indiana|658[0n]|BOLD:ACE7470
Catocala maestosa|[847]|LPOKC599-09|United States|Oklahoma|658[0n]|BOLD:ACE7470
Catocala maestosa|[848]|LPOKD362-09|United States|Oklahoma|658[0n]|BOLD:ACE7470
Catocala maestosa|[849]|LNCC1423-11|United States|North Carolina|658[0n]|BOLD:ACE7470
Catocala maestosa|[850]|BBLOD1836-11|United States|Texas|658[0n]|BOLD:ACE7470
Catocala maestosa|[851]|BBLOD1856-11|United States|Texas|658[0n]|BOLD:ACE7470
Catocala maestosa|[852]|BBLOD1858-11|United States|Texas|658[0n]|BOLD:ACE7470
Catocala neogama euphemia|[853]|ABCNA419-07|United States|Arizona|579[9n]|
Catocala neogama euphemia|[854]|BBLOE1970-12|United States|Arizona|658[0n]|BOLD:ACE7470
Catocala neogama euphemia|[855]|QUNOE152-11|United States|Arizona|658[0n]|BOLD:ACE7470
Catocala neogama euphemia|[856]|QUNOE151-11|United States|Arizona|658[0n]|BOLD:ACE7470
Catocala neogama euphemia|[857]|QUNOE090-11|United States|Arizona|658[0n]|BOLD:ACE7470
Catocala neogama euphemia|[858]|DMAZ234-10|United States|Arizona|658[0n]|BOLD:ACE7470
Catocala neogama euphemia|[859]|DMAZ231-10|United States|Arizona|658[0n]|BOLD:ACE7470
Catocala neogama euphemia|[860]|QUNOD202-10|United States|Texas|658[0n]|BOLD:ACE7470
Catocala neogama euphemia|[861]|ABCNA153-06|United States|Arizona|658[0n]|BOLD:ACE7470
Catocala neogama euphemia|[862]|ABCNA154-06|United States|Arizona|616[0n]|BOLD:ACE7470
Catocala neogama euphemia|[863]|ABCNA155-06|United States|Arizona|620[0n]|BOLD:ACE7470
Catocala neogama euphemia|[864]|CMAZA857-12|United States|Arizona|658[0n]|BOLD:ACE7470
Catocala nebulosa|[865]|ABCNA296-06|United States|Kentucky|658[0n]|BOLD:ACE8372
Catocala nebulosa|[866]|ABCNA295-06|United States|Kentucky|605[1n]|BOLD:ACE8372
Catocala nebulosa|[867]|ABCNA765-07|United States|Kentucky|647[0n]|BOLD:ACE8372
Catocala nebulosa|[868]|QUNO049-07|United States|Wisconsin|658[0n]|BOLD:ACE8372
Catocala nebulosa|[869]|QUNO050-07|United States|Wisconsin|658[0n]|BOLD:ACE8372
Catocala nebulosa|[870]|QUNOB201-08|United States|Indiana|658[0n]|BOLD:ACE8372
Catocala nebulosa|[871]|QUNOB202-08|United States|Indiana|658[0n]|BOLD:ACE8372
Catocala nebulosa|[872]|LNCC1110-11|United States|North Carolina|658[0n]|BOLD:ACE8372
Catocala insolabilis|[873]|QUNO738-08|United States|Wisconsin|658[0n]|BOLD:ACF2562
Catocala insolabilis|[874]|QUNO736-08|United States|Wisconsin|658[0n]|BOLD:ACF2562
Catocala insolabilis|[875]|ABCNA292-06|United States|Indiana|658[0n]|BOLD:ACF2562
Catocala insolabilis|[876]|ABCNA405-07|United States|Tennessee|593[0n]|BOLD:ACF2562
Catocala insolabilis|[877]|QUNO651-08|United States|Mississippi|658[0n]|BOLD:ACF2562
Catocala insolabilis|[878]|QUNO659-08|United States|Kentucky|658[0n]|BOLD:ACF2562
Catocala insolabilis|[879]|QUNO737-08|United States|Wisconsin|658[0n]|BOLD:ACF2562
Catocala insolabilis|[880]|QUNO739-08|United States|Wisconsin|658[0n]|BOLD:ACF2562
Catocala insolabilis|[881]|QUNO740-08|United States|Wisconsin|658[0n]|BOLD:ACF2562
Catocala insolabilis|[882]|QUNOB025-08|United States|Indiana|658[0n]|BOLD:ACF2562
Catocala insolabilis|[883]|QUNOB213-08|United States|Indiana|658[0n]|BOLD:ACF2562
Catocala insolabilis|[884]|QUNOB214-08|United States|Indiana|658[0n]|BOLD:ACF2562
Catocala insolabilis|[885]|LNCC391-10|United States|North Carolina|658[0n]|BOLD:ACF2562
Catocala insolabilis|[886]|CNCLB2736-14|United States|North Carolina|658[0n]|BOLD:ACF2562
Catocala neogama|[887]|QUNOB198-08|United States|Indiana|658[0n]|BOLD:AAA9558
Catocala neogama|[888]|QUNOD715-11|United States|Utah|658[0n]|BOLD:AAA9558
Catocala neogama|[889]|ABCNA151-06|United States|Texas|644[0n]|BOLD:AAA9558
Catocala neogama|[890]|QUNOD132-10|United States|Texas|658[0n]|BOLD:AAA9558
Catocala neogama|[891]|QUNOB197-08|United States|Indiana|658[0n]|BOLD:AAA9558
Catocala neogama|[892]|QUNOB171-08|United States|Wisconsin|658[0n]|BOLD:AAA9558
Catocala neogama|[893]|QUNOB170-08|United States|Wisconsin|658[0n]|BOLD:AAA9558
Catocala neogama|[894]|QUNOB169-08|United States|Wisconsin|658[0n]|BOLD:AAA9558
Catocala neogama|[895]|QUNOB127-08|United States|Wisconsin|658[0n]|BOLD:AAA9558
Catocala neogama|[896]|QUNO576-08|United States|Indiana|658[0n]|BOLD:AAA9558
Catocala neogama|[897]|QUNO552-08|United States|Texas|658[0n]|BOLD:AAA9558
Catocala neogama|[898]|QUNO551-08|United States|Texas|658[0n]|BOLD:AAA9558
Catocala neogama|[899]|QUNO545-08|United States|Texas|658[0n]|BOLD:AAA9558

Catocala neogama|[897]|QUNO552-08|United States|Texas|658[0n]|BOLD:AAA9558
Catocala neogama|[898]|QUNO551-08|United States|Texas|658[0n]|BOLD:AAA9558
Catocala neogama|[899]|QUNO545-08|United States|Texas|658[0n]|BOLD:AAA9558
Catocala neogama|[900]|QUNOD531-10|United States|Texas|658[0n]|BOLD:AAA9558
Catocala neogama|[901]|LNCC1280-11|United States|North Carolina|658[0n]|BOLD:AAA9558
Catocala neogama|[902]|CNCLB2754-14|United States|North Carolina|658[0n]|BOLD:AAA9558
Catocala neogama|[903]|CNCLB2759-14|United States|North Carolina|658[0n]|BOLD:AAA9558
Catocala neogama|[904]|CNCLB2762-14|United States|North Carolina|658[0n]|BOLD:AAA9558
Catocala subnata|[905]|ABCNA298-06|United States|Kentucky|658[0n]|BOLD:AAA9558
Catocala subnata|[906]|LGSM738-04|United States|North Carolina|658[0n]|BOLD:AAA9558
Catocala subnata|[907]|ABCNA416-07|United States|Kentucky|575[0n]|BOLD:AAA9558
Catocala subnata|[908]|LGSMG595-07|United States|Tennessee|658[0n]|BOLD:AAA9558
Catocala subnata|[909]|LGSMG596-07|United States|Tennessee|658[0n]|BOLD:AAA9558
Catocala subnata|[910]|QUNO080-07|United States|Wisconsin|658[0n]|BOLD:AAA9558
Catocala subnata|[911]|QUNO081-07|United States|Wisconsin|658[0n]|BOLD:AAA9558
Catocala subnata|[912]|QUNO784-08|United States|Wisconsin|658[0n]|BOLD:AAA9558
Catocala subnata|[913]|QUNO785-08|United States|Wisconsin|658[0n]|BOLD:AAA9558
Catocala subnata|[914]|QUNO786-08|United States|Wisconsin|658[0n]|BOLD:AAA9558
Catocala subnata|[915]|QUNO787-08|United States|Wisconsin|658[0n]|BOLD:AAA9558
Catocala subnata|[916]|QUNO788-08|United States|Wisconsin|658[0n]|BOLD:AAA9558
Catocala subnata|[917]|QUNO789-08|United States|Wisconsin|658[0n]|BOLD:AAA9558
Catocala subnata|[918]|QUNOB191-08|United States|Indiana|658[0n]|BOLD:AAA9558
Catocala subnata|[919]|QUNOB192-08|United States|Indiana|658[0n]|BOLD:AAA9558
Catocala subnata|[920]|LNCC224-10|United States|North Carolina|658[0n]|BOLD:AAA9558
Catocala neogama|[921]|XAB447-04|Canada|Ontario|658[0n]|BOLD:AAA9558
Catocala neogama|[922]|MECD409-06|Canada|Quebec|658[0n]|BOLD:AAA9558
Catocala neogama|[923]|QUNOB195-08|United States|Indiana|658[0n]|BOLD:AAA9558
Catocala neogama|[924]|CNCLB2747-14|United States|North Carolina|658[0n]|BOLD:AAA9558
Catocala neogama|[925]|CNCLB2748-14|United States|North Carolina|658[0n]|BOLD:AAA9558
Catocala neogama|[926]|QUNOB196-08|United States|Indiana|658[0n]|BOLD:AAA9558
Catocala neogama|[927]|QUNOB168-08|United States|Wisconsin|658[0n]|BOLD:AAA9558
Catocala neogama|[928]|QUNO583-08|United States|Indiana|658[0n]|BOLD:AAA9558
Catocala neogama|[929]|QUNO051-07|United States|Wisconsin|658[0n]|BOLD:AAA9558
Catocala neogama|[930]|MECD407-06|Canada|Quebec|658[0n]|BOLD:AAA9558
Catocala neogama|[931]|RDLQ118-05|Canada|Quebec|658[0n]|BOLD:AAA9558
Catocala neogama|[932]|RDLQ117-05|Canada|Quebec|658[0n]|BOLD:AAA9558
Catocala neogama|[933]|MECD406-06|Canada|Quebec|658[0n]|BOLD:AAA9558
Catocala neogama|[934]|LGSM468-04|United States|Tennessee|658[0n]|BOLD:AAA9558
Catocala neogama|[935]|CNPPB2155-12|Canada|Ontario|586[0n]|BOLD:AAA9558
Catocala neogama|[936]|PHMO359-03|Canada|Ontario|639[0n]|BOLD:AAA9558
Catocala neogama|[937]|CNPPC817-12|Canada|Ontario|569[0n]|BOLD:AAA9558
Catocala neogama|[938]|LGSM469-04|United States|Tennessee|609[0n]|BOLD:AAA9558
Catocala neogama|[939]|RDLQB453-05|Canada|Quebec|591[0n]|BOLD:AAA9558
Catocala neogama|[940]|RDLQB469-05|Canada|Quebec|540[0n]|BOLD:AAA9558
Catocala neogama|[941]|CNPPC1880-12|Canada|Ontario|633[0n]|BOLD:AAA9558
Catocala neogama|[942]|CNCLB2756-14|United States|North Carolina|632[0n]|BOLD:AAA9558
Catocala neogama|[943]|CNCLB2761-14|United States|North Carolina|658[0n]|BOLD:AAA9558
Catocala neogama|[944]|LPOKA039-08|United States|Oklahoma|643[0n]|BOLD:AAA9558
Catocala neogama|[945]|QUNOE562-12|United States|Wisconsin|658[0n]|BOLD:AAA9558
Catocala neogama|[946]|QUNOE081-11|United States|Utah|658[0n]|BOLD:AAA9558
Catocala neogama|[947]|QUNOD716-11|United States|Utah|658[0n]|BOLD:AAA9558
Catocala neogama|[948]|QUNOB199-08|United States|Indiana|658[0n]|BOLD:AAA9558
Catocala neogama|[949]|QUNO636-08|United States|Mississippi|658[0n]|BOLD:AAA9558
Catocala neogama|[950]|QUNO052-07|United States|Wisconsin|658[0n]|BOLD:AAA9558
Catocala neogama|[951]|XAH429-05|Canada|Ontario|658[0n]|BOLD:AAA9558
Catocala neogama|[952]|XAH202-05|Canada|Ontario|658[0n]|BOLD:AAA9558
Catocala neogama|[953]|PHMO354-03|Canada|Ontario|639[0n]|BOLD:AAA9558
Catocala neogama|[954]|CNPPC814-12|Canada|Ontario|556[0n]|BOLD:AAA9558
Catocala neogama|[955]|CNCLB2751-14|United States|North Carolina|658[0n]|BOLD:AAA9558
Catocala neogama|[956]|CNCLB2764-14|United States|North Carolina|658[0n]|BOLD:AAA9558
Catocala atocala|[957]|ABCNA394-07|United States|Louisiana|595[0n]|BOLD:AAE4236
Catocala atocala|[958]|QUNO001-07|United States|Mississippi|658[0n]|BOLD:AAE4236
Catocala atocala|[959]|QUNO002-07|United States|Mississippi|658[0n]|BOLD:AAE4236
Catocala atocala|[960]|QUNO003-07|United States|Mississippi|658[0n]|BOLD:AAE4236
Catocala atocala|[961]|QUNO004-07|United States|Mississippi|658[0n]|BOLD:AAE4236
Catocala piatrix|[962]|QUNOE177-11|United States|California|658[0n]|BOLD:ACF2141
Catocala piatrix|[963]|QUNOE178-11|United States|California|658[0n]|BOLD:ACF2141
Catocala piatrix dionyza|[964]|CMAZA856-12|United States|Arizona|614[0n]|BOLD:ACF2141
Catocala piatrix dionyza|[965]|ABCNA112-06|United States|Texas|609[0n]|BOLD:ACF2141
Catocala piatrix dionyza|[966]|CMAZA095-09|United States|Arizona|658[0n]|BOLD:ACF2141
Catocala piatrix dionyza|[967]|QUNOD133-10|United States|Texas|658[0n]|BOLD:ACF2141
Catocala piatrix dionyza|[968]|QUNOD239-10|United States|Texas|658[0n]|BOLD:ACF2141
Catocala piatrix dionyza|[969]|QUNOC293-10|United States|Arizona|658[0n]|BOLD:ACF2141
Catocala piatrix dionyza|[970]|QUNOD845-11|United States|Texas|658[0n]|BOLD:ACF2141
Catocala piatrix dionyza|[971]|QUNOE150-11|United States|Arizona|658[0n]|BOLD:ACF2141
Catocala piatrix dionyza|[972]|CMAZA945-12|United States|Arizona|658[0n]|BOLD:ACF2141
Catocala piatrix|[973]|LPOKC595-09|United States|Oklahoma|658[0n]|BOLD:AAB7092
Catocala piatrix|[974]|UDLEP223-09|United States|Delaware|658[0n]|BOLD:AAB7092
Catocala piatrix|[975]|LPOKA379-08|United States|Oklahoma|658[0n]|BOLD:AAB7092
Catocala piatrix|[976]|QUNOB200-08|United States|Indiana|658[0n]|BOLD:AAB7092
Catocala piatrix|[977]|QUNOB179-08|United States|Indiana|658[0n]|BOLD:AAB7092
Catocala piatrix|[978]|QUNO573-08|United States|Indiana|658[0n]|BOLD:AAB7092
Catocala piatrix|[979]|QUNOB026-08|United States|Indiana|658[0n]|BOLD:AAB7092
Catocala piatrix|[980]|QUNOB028-08|United States|Indiana|658[0n]|BOLD:AAB7092
Catocala piatrix|[981]|LPOKD093-09|United States|Oklahoma|636[0n]|BOLD:AAB7092
Catocala piatrix|[982]|LPOKA568-09|United States|Oklahoma|636[0n]|BOLD:AAB7092
Catocala piatrix|[983]|QUNOD388-10|United States|Florida|658[0n]|BOLD:AAB7092
Catocala piatrix|[984]|LPOKC594-09|United States|Oklahoma|658[0n]|BOLD:AAB7092
Catocala piatrix|[985]|LPSOD203-09|Canada|Ontario|658[0n]|BOLD:AAB7092
Catocala piatrix|[986]|QUNOB029-08|United States|Indiana|658[0n]|BOLD:AAB7092
Catocala piatrix|[987]|QUNOB027-08|United States|Indiana|658[0n]|BOLD:AAB7092
Catocala piatrix|[988]|QUNO572-08|United States|Indiana|658[0n]|BOLD:AAB7092
Catocala piatrix|[989]|QUNO571-08|United States|Indiana|658[0n]|BOLD:AAB7092
Catocala piatrix|[990]|LPOKC602-09|United States|Oklahoma|658[0n]|BOLD:AAB7092
Catocala piatrix|[991]|MECD393-06|United States|Maryland|658[0n]|BOLD:AAB7092
Catocala piatrix|[992]|QUNO058-07|United States|Wisconsin|648[0n]|BOLD:AAB7092
Catocala piatrix|[993]|QUNOB178-08|United States|Indiana|658[0n]|BOLD:AAB7092
Catocala piatrix|[994]|CNPPD2500-12|Canada|Ontario|639[0n]|BOLD:AAB7092
Catocala antinympha|[995]|QUNOD341-10|United States|Michigan|639[0n]|BOLD:AAB9017
Catocala badia coelebs|[996]|RDLQB569-05|Canada|Quebec|581[4n]|BOLD:AAB9017
Catocala antinympha|[997]|QUNO024-07|United States|Wisconsin|658[0n]|BOLD:AAB9017
Catocala badia|[998]|ABCNA118-06|United States|Wisconsin|658[0n]|BOLD:AAB9017
Catocala badia|[999]|ABCNA117-06|United States|Wisconsin|658[0n]|BOLD:AAB9017

[illegible]
Catocala antinympha|[997]|QUNO024-07|United States|Wisconsin|658[0n]|BOLD:AAB9017
Catocala badia|[998]|ABCNA118-06|United States|Wisconsin|658[0n]|BOLD:AAB9017
Catocala badia|[999]|ABCNA117-06|United States|Wisconsin|658[0n]|BOLD:AAB9017
Catocala badia|[1000]|ABCNA116-06|United States|Wisconsin|658[0n]|BOLD:AAB9017
Catocala badia|[1001]|ABCNA009-06|United States|New Hampshire|657[0n]|BOLD:AAB9017
Catocala badia|[1002]|ABCNA008-06|United States||658[0n]|BOLD:AAB9017
Catocala badia|[1003]|ABCNA007-06|United States|New Hampshire|604[0n]|BOLD:AAB9017
Catocala badia|[1004]|ABCNA006-06|United States|New Hampshire|585[0n]|BOLD:AAB9017
Catocala badia|[1005]|ABCNA119-06|United States|Wisconsin|596[1n]|BOLD:AAB9017
Catocala badia|[1006]|IMA005-07|United States|Massachusetts|645[0n]|BOLD:AAB9017
Catocala badia coelebs|[1007]|QUNOD460-10|United States|Wisconsin|658[0n]|BOLD:AAB9017
Catocala antinympha|[1008]|QUNOE279-12|United States|Minnesota|658[0n]|BOLD:AAB9017
Catocala antinympha|[1009]|BBLPC038-09|Canada|New Brunswick|622[0n]|BOLD:AAB9017
Catocala badia|[1010]|BBLEC533-09|Canada|New Brunswick|658[0n]|BOLD:AAB9017
Catocala antinympha|[1011]|BBLEC532-09|Canada|New Brunswick|658[0n]|BOLD:AAB9017
Catocala badia|[1012]|BBLEC531-09|Canada|New Brunswick|658[0n]|BOLD:AAB9017
Catocala badia|[1013]|BBLEC421-09|Canada|New Brunswick|658[0n]|BOLD:AAB9017
Catocala antinympha|[1014]|BBLEC417-09|Canada|New Brunswick|658[0n]|BOLD:AAB9017
Catocala antinympha|[1015]|BBLEC686-09|Canada|Nova Scotia|642[0n]|BOLD:AAB9017
Catocala antinympha|[1016]|QUNOE407-12|United States|Michigan|658[0n]|BOLD:AAB9017
Catocala muliercula|[1017]|LNC077-05|United States|North Carolina|658[0n]|BOLD:AAB9017
Catocala muliercula|[1018]|ABCNA273-06|United States|Florida|658[0n]|BOLD:AAB9017
Catocala muliercula|[1019]|QUNOB040-08|United States|Texas|658[0n]|BOLD:AAB9017
Catocala muliercula|[1020]|QUNOB039-08|United States|Texas|658[0n]|BOLD:AAB9017
Catocala muliercula|[1021]|ABCNA274-06|United States|Florida|658[0n]|BOLD:AAB9017
Catocala muliercula|[1022]|LNC078-05|United States|North Carolina|658[0n]|BOLD:AAB9017
Catocala muliercula|[1023]|ABCNA639-07|United States|Texas|573[0n]|BOLD:AAB9017
Catocala muliercula|[1024]|ABCNA640-07|United States|Florida|577[0n]|BOLD:AAB9017
Catocala muliercula|[1025]|QUNOB041-08|United States|Texas|658[0n]|BOLD:AAB9017
Catocala muliercula|[1026]|QUNOB042-08|United States|Texas|658[0n]|BOLD:AAB9017
Catocala muliercula|[1027]|BBLOB1634-11|United States|Florida|658[0n]|BOLD:AAB9017
Catocala muliercula|[1028]|NOCNA044-14|United States|Florida|658[0n]|BOLD:AAB9017
Catocala lineella|[1029]|QUNO421-08|United States|Florida|513[0n]|
Catocala lineella|[1030]|BBLOC1035-11|United States|Texas|658[0n]|BOLD:AAA5644
Catocala lineella|[1031]|LOFLA224-06|United States|Florida|658[0n]|BOLD:AAA5644
Catocala lineella|[1032]|ABCNA104-06|United States|Texas|658[0n]|BOLD:AAA5644
Catocala sp.|[1033]|QUNOB586-10|United States|Indiana|658[0n]|BOLD:AAA5644
Catocala lineella|[1034]|LOT240-04|United States|Tennessee|609[0n]|BOLD:AAA5644
Catocala lineella|[1035]|LOT239-04|United States|Tennessee|609[0n]|BOLD:AAA5644
Catocala lineella|[1036]|QUNO404-08|United States|Texas|658[0n]|BOLD:AAA5644
Catocala lineella|[1037]|QUNO554-08|United States|Texas|658[0n]|BOLD:AAA5644
Catocala lineella|[1038]|QUNO414-08|United States|Florida|649[0n]|BOLD:AAA5644
Catocala lineella|[1039]|QUNO185-08|United States|Florida|658[1n]|BOLD:AAA5644
Catocala lineella|[1040]|ABCNA880-08|United States|Texas|621[0n]|BOLD:AAA5644
Catocala lineella|[1041]|QUNO095-08|United States|Florida|649[0n]|BOLD:AAA5644
Catocala lineella|[1042]|QUNO325-08|United States|Florida|658[0n]|BOLD:AAA5644
Catocala lineella|[1043]|QUNO148-08|United States|Florida|658[0n]|BOLD:AAA5644
Catocala lineella|[1044]|LOFLA100-06|United States|Florida|658[0n]|BOLD:AAA5644
Catocala lineella|[1045]|QUNO416-08|United States|Florida|658[1n]|BOLD:AAA5644
Catocala lineella|[1046]|QUNO419-08|United States|Florida|658[0n]|BOLD:AAA5644
Catocala lineella|[1047]|QUNO420-08|United States|Florida|658[0n]|BOLD:AAA5644
Catocala lineella|[1048]|QUNO422-08|United States|Florida|658[0n]|BOLD:AAA5644
Catocala lineella|[1049]|QUNO432-08|United States|Florida|658[0n]|BOLD:AAA5644
Catocala lineella|[1050]|QUNO465-08|United States|Florida|649[1n]|BOLD:AAA5644
Catocala lineella|[1051]|QUNO674-08|United States|Kentucky|658[0n]|BOLD:AAA5644
Catocala lineella|[1052]|LGSM637-04|United States|Tennessee|609[0n]|BOLD:AAA5644
Catocala lineella|[1053]|QUNO423-08|United States|Florida|658[0n]|BOLD:AAA5644
Catocala lineella|[1054]|ABCNA914-08|United States|Wisconsin|658[0n]|BOLD:AAA5644
Catocala lineella|[1055]|QUNO370-08|United States|Florida|658[0n]|BOLD:AAA5644
Catocala lineella|[1056]|ABCNA838-07|United States|Kentucky|594[0n]|BOLD:AAA5644
Catocala lineella|[1057]|QUNO676-08|United States|Kentucky|658[0n]|BOLD:AAA5644
Catocala lineella|[1058]|QUNOB079-08|United States|Texas|658[0n]|BOLD:AAA5644
Catocala lineella|[1059]|QUNOC006-09|United States|Texas|658[0n]|BOLD:AAA5644
Catocala lineella|[1060]|QUNO413-08|United States|Florida|658[0n]|BOLD:AAA5644
Catocala lineella|[1061]|QUNO657-08|United States|Mississippi|658[0n]|BOLD:AAA5644
Catocala lineella|[1062]|ABCNA840-07|United States|Oklahoma|594[1n]|BOLD:AAA5644
Catocala lineella|[1063]|ABCNA865-08|United States|Texas|658[0n]|BOLD:AAA5644
Catocala lineella|[1064]|QUNOB053-08|United States|Texas|658[0n]|BOLD:AAA5644
Catocala sp.|[1065]|QUNOB585-10|United States|Indiana|658[0n]|BOLD:AAA5644
Catocala lineella|[1066]|QUNOB058-08|United States|Texas|658[0n]|BOLD:AAA5644
Catocala lineella|[1067]|LNCC227-10|United States|North Carolina|658[0n]|BOLD:AAA5644
Catocala lineella|[1068]|LNCC228-10|United States|North Carolina|658[0n]|BOLD:AAA5644
Catocala lineella|[1069]|LNCC1343-11|United States|North Carolina|658[0n]|BOLD:AAA5644
Catocala lineella|[1070]|QUNO634-08|United States|Mississippi|658[0n]|BOLD:AAA5644
Catocala lineella|[1071]|QUNOB057-08|United States|Texas|658[0n]|BOLD:AAA5644
Catocala lineella|[1072]|QUNO415-08|United States|Florida|658[0n]|BOLD:AAA5644
Catocala lineella|[1073]|QUNO494-08|United States|Texas|658[0n]|BOLD:AAA5644
Catocala lineella|[1074]|QUNO399-08|United States|Florida|658[0n]|BOLD:AAA5644
Catocala lineella|[1075]|QUNO412-08|United States|Florida|658[0n]|BOLD:AAA5644
Catocala lineella|[1076]|QUNO327-08|United States|Florida|658[0n]|BOLD:AAA5644
Catocala lineella|[1077]|QUNO384-08|United States|Florida|658[0n]|BOLD:AAA5644
Catocala lineella|[1078]|ABCNA915-08|United States|Wisconsin|658[0n]|BOLD:AAA5644
Catocala lineella|[1079]|QUNO321-08|United States|Florida|658[0n]|BOLD:AAA5644
Catocala lineella|[1080]|ABCNA873-08|United States|Florida|658[0n]|BOLD:AAA5644
Catocala lineella|[1081]|ABCNA871-08|United States|Florida|658[0n]|BOLD:AAA5644
Catocala lineella|[1082]|ABCNA867-08|United States|Florida|658[0n]|BOLD:AAA5644
Catocala lineella|[1083]|ABCNA860-08|United States|Texas|658[0n]|BOLD:AAA5644
Catocala lineella|[1084]|QUNO149-08|United States|Florida|658[0n]|BOLD:AAA5644
Catocala lineella|[1085]|ABNCC389-07|United States||658[0n]|BOLD:AAA5644
Catocala lineella|[1086]|XAK427-06|Canada|Ontario|658[0n]|BOLD:AAA5644
Catocala lineella|[1087]|LGSM409-04|United States|North Carolina|658[0n]|BOLD:AAA5644
Catocala lineella|[1088]|LGSM408-04|United States|North Carolina|658[0n]|BOLD:AAA5644
Catocala lineella|[1089]|QUNO410-08|United States|Florida|658[1n]|BOLD:AAA5644
Catocala lineella|[1090]|QUNO398-08|United States|Florida|651[0n]|BOLD:AAA5644
Catocala lineella|[1091]|ABCNA544-07|United States|Kentucky|633[0n]|BOLD:AAA5644
Catocala lineella|[1092]|ABCNA698-07|United States|Oklahoma|594[0n]|BOLD:AAA5644
Catocala lineella|[1093]|ABCNA836-07|United States|Virginia|592[1n]|BOLD:AAA5644
Catocala lineella|[1094]|QUNO183-08|United States|Florida|639[1n]|BOLD:AAA5644
Catocala lineella|[1095]|QUNO411-08|United States|Florida|648[0n]|BOLD:AAA5644
Catocala lineella|[1096]|LNCC1404-11|United States|North Carolina|658[0n]|BOLD:AAA5644
Catocala amica|[1097]|QUNO406-08|United States|Texas|609[0n]|BOLD:AAA5644
Catocala lineella|[1098]|ABCNA539-07|United States|Florida|609[0n]|BOLD:AAA5644
Catocala amica|[1099]|ABCNA861-08|United States|Texas|658[0n]|BOLD:AAA5644

Catocala lineella|[1096]|LNCC1404-11|United States|North Carolina|658[0n]|BOLD:AAA5644
Catocala amica|[1097]|QUNO406-08|United States|Texas|609[0n]|BOLD:AAA5644
Catocala lineella|[1098]|ABCNA539-07|United States|Florida|609[0n]|BOLD:AAA5644
Catocala amica|[1099]|ABCNA861-08|United States|Texas|658[0n]|BOLD:AAA5644
Catocala amica|[1100]|BBLOB1101-11|United States|Florida|658[0n]|BOLD:AAA5644
Catocala amica|[1101]|QUNOB114-08|United States|Texas|658[0n]|BOLD:AAA5644
Catocala amica|[1102]|QUNOB575-10|United States|Texas|658[0n]|BOLD:AAA5644
Catocala amica|[1103]|QUNO555-08|United States|Texas|658[0n]|BOLD:AAA5644
Catocala amica|[1104]|ABCNA875-08|United States|Texas|658[0n]|BOLD:AAA5644
Catocala amica|[1105]|QUNOB113-08|United States|Texas|658[0n]|BOLD:AAA5644
Catocala amica|[1106]|QUNO402-08|United States|Texas|648[0n]|BOLD:AAA5644
Catocala amica|[1107]|ABCNA545-07|United States|Texas|649[0n]|BOLD:AAA5644
Catocala amica|[1108]|QUNOB064-08|United States|Texas|658[0n]|BOLD:AAA5644
Catocala amica|[1109]|QUNO408-08|United States|Texas|644[0n]|BOLD:AAA5644
Catocala amica|[1110]|QUNOB078-08|United States|Texas|658[0n]|BOLD:AAA5644
Catocala amica|[1111]|QUNOB059-08|United States|Texas|658[0n]|BOLD:AAA5644
Catocala amica|[1112]|QUNO533-08|United States|Texas|658[0n]|BOLD:AAA5644
Catocala amica|[1113]|QUNO529-08|United States|Texas|658[0n]|BOLD:AAA5644
Catocala amica|[1114]|QUNO405-08|United States|Texas|658[0n]|BOLD:AAA5644
Catocala amica|[1115]|QUNO394-08|United States|Florida|658[0n]|BOLD:AAA5644
Catocala amica|[1116]|LGSMG605-07|United States|Tennessee|658[0n]|BOLD:AAA5644
Catocala amica|[1117]|ABCNA099-06|United States|Texas|658[0n]|BOLD:AAA5644
Catocala amica|[1118]|LSEU577-06|United States|Georgia|658[0n]|BOLD:AAA5644
Catocala amica|[1119]|LGSM465-04|United States|Tennessee|589[0n]|BOLD:AAA5644
Catocala amica|[1120]|ABCNA702-07|United States|Oklahoma|615[0n]|BOLD:AAA5644
Catocala amica|[1121]|ABCNA835-07|United States|Texas|616[0n]|BOLD:AAA5644
Catocala amica|[1122]|ABCNA100-06|United States|Texas|658[0n]|BOLD:AAA5644
Catocala amica|[1123]|QUNO655-08|United States|Mississippi|658[0n]|BOLD:AAA5644
Catocala amica|[1124]|QUNOB117-08|United States|Texas|658[0n]|BOLD:AAA5644
Catocala amica|[1125]|LNCC1282-11|United States|North Carolina|658[0n]|BOLD:AAA5644
Catocala lineella|[1126]|QUNOB289-09|United States|Indiana|658[0n]|BOLD:AAA5644
Catocala lineella|[1127]|LNCC716-11|United States|North Carolina|658[0n]|BOLD:AAA5644
Catocala amica|[1128]|QUNO396-08|United States|Florida|658[0n]|BOLD:AAA5644
Catocala amica|[1129]|QUNO395-08|United States|Florida|609[1n]|BOLD:AAA5644
Catocala amica|[1130]|QUNO151-08|United States|Florida|658[0n]|BOLD:AAA5644
Catocala amica|[1131]|QUNO184-08|United States|Florida|639[0n]|BOLD:AAA5644
Catocala amica|[1132]|QUNO462-08|United States|Florida|658[0n]|BOLD:AAA5644
Catocala amica|[1133]|LOFLA234-06|United States|Florida|658[0n]|BOLD:AAA5644
Catocala amica|[1134]|QUNO150-08|United States|Florida|658[0n]|BOLD:AAA5644
Catocala amica|[1135]|QUNO152-08|United States|Florida|658[0n]|BOLD:AAA5644
Catocala amica|[1136]|ABCNA922-08|United States|Florida|658[0n]|BOLD:AAA5644
Catocala amica|[1137]|QUNO391-08|United States|Florida|658[0n]|BOLD:AAA5644
Catocala amica|[1138]|QUNO392-08|United States|Florida|658[0n]|BOLD:AAA5644
Catocala amica|[1139]|QUNO397-08|United States|Florida|658[0n]|BOLD:AAA5644
Catocala amica|[1140]|QUNO400-08|United States|Florida|658[0n]|BOLD:AAA5644
Catocala amica|[1141]|QUNO463-08|United States|Florida|658[0n]|BOLD:AAA5644
Catocala amica|[1142]|BBLOB241-11|United States|Florida|658[0n]|BOLD:AAA5644
Catocala amica|[1143]|QUNO403-08|United States|Texas|658[0n]|BOLD:AAA5644
Catocala amica|[1144]|QUNO553-08|United States|Texas|658[0n]|BOLD:AAA5644
Catocala amica|[1145]|LGSM464-04|United States|Tennessee|616[0n]|BOLD:AAA5644
Catocala amica|[1146]|QUNO407-08|United States|Texas|649[0n]|BOLD:AAA5644
Catocala amica|[1147]|ABCNA097-06|United States|Texas|658[0n]|BOLD:AAA5644
Catocala amica|[1148]|LOFLA222-06|United States|Florida|658[0n]|BOLD:AAA5644
Catocala amica|[1149]|ABCNA870-08|United States|Texas|658[0n]|BOLD:AAA5644
Catocala amica|[1150]|QUNO390-08|United States|Florida|658[0n]|BOLD:AAA5644
Catocala amica|[1151]|QUNO409-08|United States|Virginia|658[0n]|BOLD:AAA5644
Catocala amica|[1152]|QUNO534-08|United States|Texas|658[0n]|BOLD:AAA5644
Catocala amica|[1153]|QUNOB020-08|United States|Texas|658[0n]|BOLD:AAA5644
Catocala amica|[1154]|QUNOB115-08|United States|Texas|658[0n]|BOLD:AAA5644
Catocala amica|[1155]|QUNOB577-10|United States|Texas|658[0n]|BOLD:AAA5644
Catocala amica|[1156]|ABCNA874-08|United States|Texas|609[0n]|BOLD:AAA5644
Catocala amica|[1157]|ABCNA098-06|United States|Texas|658[0n]|BOLD:AAA5644
Catocala amica|[1158]|LGSMG604-07|United States|Tennessee|658[0n]|BOLD:AAA5644
Catocala amica|[1159]|QUNO389-08|United States|Florida|658[0n]|BOLD:AAA5644
Catocala amica|[1160]|ABCNA533-07|United States|Florida|649[0n]|BOLD:AAA5644
Catocala amica|[1161]|QUNO528-08|United States|Texas|658[0n]|BOLD:AAA5644
Catocala amica|[1162]|QUNOB116-08|United States|Texas|658[0n]|BOLD:AAA5644
Catocala amica|[1163]|LNCC1163-11|United States|North Carolina|658[0n]|BOLD:AAA5644
Catocala lineella|[1164]|QUNO147-08|United States|Florida|658[0n]|BOLD:AAA5644
Catocala lineella|[1165]|QUNO424-08|United States|Florida|658[0n]|BOLD:AAA5644
Catocala lineella|[1166]|QUNO600-08|United States|Indiana|658[0n]|BOLD:AAA5644
Catocala lineella|[1167]|LNCC712-11|United States|North Carolina|658[0n]|BOLD:AAA5644
Catocala lineella|[1168]|LNCB438-07|United States|North Carolina|658[0n]|BOLD:AAA5644
Catocala lineella|[1169]|QUNO326-08|United States|Florida|658[0n]|BOLD:AAA5644
Catocala lineella|[1170]|QUNO393-08|United States|Florida|658[0n]|BOLD:AAA5644
Catocala lineella|[1171]|QUNO658-08|United States|Mississippi|658[0n]|BOLD:AAA5644
Catocala lineella|[1172]|LNCC553-11|United States|North Carolina|658[0n]|BOLD:AAA5644
Catocala lineella|[1173]|LNCC713-11|United States|North Carolina|658[0n]|BOLD:AAA5644
Catocala lineella|[1174]|ABCNA535-07|United States|Florida|642[0n]|BOLD:AAA5644
Catocala lineella|[1175]|MILEP326-10|United States|Alabama|658[0n]|BOLD:AAA5644
Catocala lineella|[1176]|MILEQ284-11|United States|Alabama|658[0n]|BOLD:AAA5644
Catocala lineella|[1177]|ABCNA856-08|United States|Oklahoma|658[0n]|BOLD:AAA5644
Catocala lineella|[1178]|ABCNA877-08|United States|Texas|609[0n]|BOLD:AAA5644
Catocala lineella|[1179]|ABCNA842-07|United States|Texas|562[0n]|BOLD:AAA5644
Catocala lineella|[1180]|ABCNA847-08|United States|Oklahoma|658[0n]|BOLD:AAA5644
Catocala lineella|[1181]|QUNO675-08|United States|Kentucky|658[0n]|BOLD:AAA5644
Catocala amica|[1182]|QUNOB080-08|United States|Texas|658[0n]|BOLD:AAA5644
Catocala amica|[1183]|QUNOB054-08|United States|Texas|658[0n]|BOLD:AAA5644
Catocala lineella|[1184]|ABCNA101-06|United States|Texas|658[0n]|BOLD:AAA5644
Catocala amica|[1185]|QUNOB576-10|United States|Texas|658[0n]|BOLD:AAA5644
Catocala lineella|[1186]|ABCNA862-08|United States|Texas|609[0n]|BOLD:AAA5644
Catocala lineella|[1187]|ABCNA855-08|United States|Oklahoma|609[0n]|BOLD:AAA5644
Catocala amica|[1188]|QUNOB560-09|United States|Louisiana|658[0n]|BOLD:AAA5644
Catocala lineella|[1189]|QUNO430-08|United States|Florida|658[0n]|BOLD:AAA5644
Catocala lineella|[1190]|MILEP324-10|United States|Alabama|658[0n]|BOLD:AAA5644
Catocala lineella|[1191]|BBLOC1664-11|United States|Texas|658[0n]|BOLD:AAA5644
Catocala jair|[1192]|ABCNA286-06|United States|Florida|658[0n]|BOLD:AAA5644
Catocala jair|[1193]|QUNOE538-12|United States|Florida|658[0n]|BOLD:AAA5644
Catocala n. sp. Schweitzer|[1194]|LNCC714-11|United States|North Carolina|658[0n]|BOLD:ACF0904
Catocala n. sp. Schweitzer|[1195]|LNCC717-11|United States|North Carolina|658[0n]|BOLD:ACF0904
Catocala n. sp. Schweitzer|[1196]|LNCC758-11|United States|North Carolina|658[0n]|BOLD:ACF0904
Catocala n. sp. Schweitzer|[1197]|LNCC760-11|United States|North Carolina|658[0n]|BOLD:ACF0904
Catocala n. sp. Schweitzer|[1198]|QUNO656-08|United States|Mississippi|658[0n]|BOLD:ACF0904
Catocala n. sp. Schweitzer|[1199]|ABCNA873-08|United States|Florida|658[0n]|BOLD:ACF0904

Catocala n. sp. Schweitzer|[1196]|LNCC758-11|United States|North Carolina|658[0n]|BOLD:ACF0904
Catocala n. sp. Schweitzer|[1197]|LNCC760-11|United States|North Carolina|658[0n]|BOLD:ACF0904
Catocala n. sp. Schweitzer|[1198]|QUNO656-08|United States|Mississippi|658[0n]|BOLD:ACF0904
Catocala n. sp. Schweitzer|[1199]|ABCNA872-08|United States|Florida|658[0n]|BOLD:ACF0904
Catocala n. sp. Schweitzer|[1200]|ABCNA536-07|United States|Florida|556[0n]|BOLD:ACF0904
Catocala n. sp. Schweitzer|[1201]|ABCNA879-08|United States|Florida|655[0n]|BOLD:ACF0904
Catocala n. sp. Schweitzer|[1202]|ABCNA876-08|United States|Texas|609[0n]|BOLD:ACF0904
Catocala n. sp. Schweitzer|[1203]|ABCNA534-07|United States|Florida|649[0n]|BOLD:ACF0904
Catocala n. sp. Schweitzer|[1204]|MILEP319-10|United States|Alabama|634[0n]|BOLD:ACF0904
Catocala n. sp. Schweitzer|[1205]|QUNO146-08|United States|Florida|658[0n]|BOLD:ACF0904
Catocala n. sp. Schweitzer|[1206]|QUNO532-08|United States|Texas|658[0n]|BOLD:ACF0904
Catocala n. sp. Schweitzer|[1207]|LNCC759-11|United States|North Carolina|658[0n]|BOLD:ACF0904
Catocala n. sp. Schweitzer|[1208]|LNCC960-11|United States|North Carolina|658[0n]|BOLD:ACF0904
Catocala n. sp. Schweitzer|[1209]|LNCC718-11|United States|North Carolina|658[0n]|BOLD:ACF0904
Catocala n. sp. Schweitzer|[1210]|LNCC757-11|United States|North Carolina|658[0n]|BOLD:ACF0904
Catocala n. sp. Schweitzer|[1211]|MILEP328-10|United States|Alabama|658[0n]|BOLD:ACF0904
Catocala n. sp. Schweitzer|[1212]|LNCC715-11|United States|North Carolina|658[0n]|BOLD:ACF0904
Catocala n. sp. Schweitzer|[1213]|MILEP325-10|United States|Alabama|658[0n]|BOLD:ACF0904
Catocala n. sp. Schweitzer|[1214]|MILEP327-10|United States|Alabama|656[0n]|BOLD:ACF0904
Catocala n. sp. Schweitzer|[1215]|MILEP323-10|United States|Alabama|658[0n]|BOLD:ACF0904
Catocala n. sp. Schweitzer|[1216]|MILEP322-10|United States|Alabama|658[0n]|BOLD:ACF0904
Catocala n. sp. Schweitzer|[1217]|MILEP321-10|United States|Alabama|658[0n]|BOLD:ACF0904
Catocala n. sp. Schweitzer|[1218]|MILEP320-10|United States|Alabama|658[0n]|BOLD:ACF0904
Catocala n. sp. Schweitzer|[1219]|QUNOB089-08|United States|Texas|658[0n]|BOLD:ACF0904
Catocala n. sp. Schweitzer|[1220]|QUNO841-08|United States|Louisiana|658[0n]|BOLD:ACF0904
Catocala n. sp. Schweitzer|[1221]|QUNO540-08|United States|Louisiana|658[0n]|BOLD:ACF0904
Catocala n. sp. Schweitzer|[1222]|QUNO433-08|United States|Florida|658[0n]|BOLD:ACF0904
Catocala n. sp. Schweitzer|[1223]|QUNO431-08|United States|Florida|658[0n]|BOLD:ACF0904
Catocala n. sp. Schweitzer|[1224]|ABCNA878-08|United States|Florida|658[0n]|BOLD:ACF0904
Catocala n. sp. Schweitzer|[1225]|QUNOC326-10|United States|New Jersey|658[0n]|BOLD:ACF0904
Catocala n. sp. Schweitzer|[1226]|LSEU576-06|United States|Georgia|658[0n]|BOLD:ACF0904
Catocala n. sp. Schweitzer|[1227]|ABCNA831-07|United States|Texas|594[2n]|BOLD:ACF0904
Catocala n. sp. Schweitzer|[1228]|ABCNA843-07|United States|Texas|583[1n]|BOLD:ACF0904
Catocala n. sp. Schweitzer|[1229]|QUNO145-08|United States|Florida|658[0n]|BOLD:ACF0904
Catocala n. sp. Schweitzer|[1230]|LNCC552-11|United States|North Carolina|658[0n]|BOLD:ACF0904
Catocala n. sp. Schweitzer|[1231]|MILEQ285-11|United States|Alabama|658[0n]|BOLD:ACF0904
Catocala jair|[1232]|ABCNA863-08|United States|Texas|658[0n]|BOLD:ACF4808
Catocala jair|[1233]|ABCNA834-07|United States|Texas|658[0n]|BOLD:ACF4808
Catocala jair|[1234]|LOFLB814-06|United States|Florida|658[0n]|BOLD:ACF4808
Catocala jair|[1235]|ABCNA841-07|United States|Texas|590[0n]|BOLD:ACF4808
Catocala jair|[1236]|ABNCC390-07|United States||585[0n]|BOLD:ACF4808
Catocala jair|[1237]|QUNO141-08|United States|Florida|658[0n]|BOLD:ACF4808
Catocala jair|[1238]|ABCNA868-08|United States|Texas|658[0n]|BOLD:ACF4808
Catocala jair|[1239]|QUNO526-08|United States|Texas|658[0n]|BOLD:ACF4808
Catocala jair|[1240]|QUNO527-08|United States|Texas|658[0n]|BOLD:ACF4808
Catocala jair|[1241]|QUNOB003-08|United States|Texas|658[0n]|BOLD:ACF4808
Catocala jair|[1242]|QUNOB017-08|United States|Texas|658[0n]|BOLD:ACF4808
Catocala jair|[1243]|QUNOB018-08|United States|Texas|658[0n]|BOLD:ACF4808
Catocala jair|[1244]|QUNOB120-08|United States|Texas|658[0n]|BOLD:ACF4808
Catocala jair|[1245]|QUNOB579-10|United States|Texas|658[0n]|BOLD:ACF4808
Catocala jair|[1246]|QUNOB581-10|United States|Texas|658[0n]|BOLD:ACF4808
Catocala jair|[1247]|QUNO142-08|United States|Florida|658[0n]|BOLD:ACF4808
Catocala sp.|[1248]|QUNOB584-10|United States|Texas|658[0n]|BOLD:ACF4808
Catocala jair|[1249]|QUNOD151-10|United States|Texas|658[0n]|BOLD:ACF4808
Catocala jair|[1250]|ABCNA551-07|United States|Florida|612[3n]|BOLD:ACF4808
Catocala jair|[1251]|QUNOC071-09|United States|Oklahoma|658[1n]|BOLD:ACF4808
Catocala jair|[1252]|QUNOC070-09|United States|Oklahoma|658[0n]|BOLD:ACF4808
Catocala jair|[1253]|QUNOB019-08|United States|Texas|658[0n]|BOLD:ACF4808
Catocala jair|[1254]|QUNOB016-08|United States|Texas|658[0n]|BOLD:ACF4808
Catocala jair|[1255]|QUNO525-08|United States|Texas|658[0n]|BOLD:ACF4808
Catocala jair|[1256]|QUNO524-08|United States|Texas|658[0n]|BOLD:ACF4808
Catocala jair|[1257]|ABCNA844-07|United States|Texas|658[0n]|BOLD:ACF4808
Catocala jair|[1258]|ABCNA103-06|United States|Texas|658[0n]|BOLD:ACF4808
Catocala jair|[1259]|QUNOB582-10|United States|Texas|647[0n]|BOLD:ACF4808
Catocala sp.|[1260]|QUNOB583-10|United States|Texas|658[0n]|BOLD:ACF4808
Catocala jair|[1261]|LOFLB139-06|United States|Florida|658[1n]|BOLD:ACF4808
Catocala jair|[1262]|LOFLB494-06|United States|Florida|658[0n]|BOLD:ACF4808
Catocala jair|[1263]|LOFLC126-06|United States|Florida|658[0n]|BOLD:ACF4808
Catocala jair|[1264]|LOFLB136-06|United States|Florida|658[0n]|BOLD:ACF4808
Catocala jair|[1265]|QUNOB572-10|United States|Florida|658[0n]|BOLD:ACF4808
Catocala jair|[1266]|QUNOB573-10|United States|Florida|658[0n]|BOLD:ACF4808
Catocala jair|[1267]|QUNOC663-13|United States|Florida|658[0n]|BOLD:ACF4808
Catocala jair|[1268]|QUNOB574-10|United States|Florida|658[0n]|BOLD:ACF4808
Catocala jair|[1269]|LNCB620-09|United States|North Carolina|658[0n]|BOLD:ACF4808
Catocala jair|[1270]|LOFLC471-06|United States|Florida|658[0n]|BOLD:ACF4808
Catocala jair|[1271]|LOFLC457-06|United States|Florida|658[0n]|BOLD:ACF4808
Catocala jair|[1272]|LOFLB808-06|United States|Florida|658[0n]|BOLD:ACF4808
Catocala jair|[1273]|LOFLB723-06|United States|Florida|658[0n]|BOLD:ACF4808
Catocala jair|[1274]|LOFLB138-06|United States|Florida|658[0n]|BOLD:ACF4808
Catocala jair|[1275]|LOFLB250-06|United States|Florida|658[0n]|BOLD:ACF4808
Catocala jair|[1276]|LOFLB488-06|United States|Florida|658[0n]|BOLD:ACF4808
Catocala jair|[1277]|LOFLA372-06|United States|Florida|658[0n]|BOLD:ACF4808
Catocala jair|[1278]|LOFLA223-06|United States|Florida|658[0n]|BOLD:ACF4808
Catocala jair|[1279]|LOFLB256-06|United States|Florida|658[0n]|BOLD:ACF4808
Catocala jair|[1280]|LOFLB733-06|United States|Florida|658[0n]|BOLD:ACF4808
Catocala jair|[1281]|QUNOB571-10|United States|Florida|658[0n]|BOLD:ACF4808
Catocala jair|[1282]|QUNOC664-13|United States|Florida|658[0n]|BOLD:ACF4808
Catocala consors|[1283]|QUNOB069-08|United States|Texas|658[0n]|BOLD:AAB8286
Catocala consors|[1284]|QUNOB072-08|United States|Texas|658[0n]|BOLD:AAB8286
Catocala consors|[1285]|QUNO491-08|United States|Texas|658[0n]|BOLD:AAB8286
Catocala consors|[1286]|QUNOB068-08|United States|Texas|658[0n]|BOLD:AAB8286
Catocala consors|[1287]|ABCNA113-06|United States|Florida|658[0n]|BOLD:AAB8286
Catocala consors|[1288]|QUNO550-08|United States|Texas|658[0n]|BOLD:AAB8286
Catocala consors|[1289]|QUNO549-08|United States|Texas|658[0n]|BOLD:AAB8286
Catocala consors|[1290]|QUNO546-08|United States|Texas|658[0n]|BOLD:AAB8286
Catocala consors|[1291]|ABCNA115-06|United States|Florida|658[0n]|BOLD:AAB8286
Catocala consors|[1292]|ABCNA114-06|United States|Florida|658[0n]|BOLD:AAB8286
Catocala consors|[1293]|QUNO547-08|United States|Texas|658[0n]|BOLD:AAB8286
Catocala consors|[1294]|QUNO548-08|United States|Texas|658[0n]|BOLD:AAB8286
Catocala consors|[1295]|ABCNA814-07|United States|Florida|593[1n]|BOLD:AAB8286
Catocala consors|[1296]|ABCNA635-07|United States|Florida|574[0n]|BOLD:AAB8286
Catocala consors|[1297]|QUNOB043-08|United States|Texas|658[0n]|BOLD:AAB8286
Catocala consors|[1298]|QUNOB070-08|United States|Texas|658[0n]|BOLD:AAB8286
[illegible]

Catocala consors|[1296]|ABCNA635-07|United States|Florida|574[0n]|BOLD:AAB8286
Catocala consors|[1297]|QUNOB043-08|United States|Texas|658[0n]|BOLD:AAB8286
Catocala consors|[1298]|QUNOB070-08|United States|Texas|658[0n]|BOLD:AAB8286
Catocala consors|[1299]|QUNOB071-08|United States|Texas|658[0n]|BOLD:AAB8286
Catocala consors|[1300]|HKONB475-09|United States|Louisiana|658[1n]|BOLD:AAB8286
Catocala epione|[1301]|LOT230-04|United States|Tennessee|609[0n]|BOLD:ABZ2889
Catocala epione|[1302]|LOT229-04|United States|Tennessee|609[0n]|BOLD:ABZ2889
Catocala epione|[1303]|QUNO723-08|United States|Kentucky|658[0n]|BOLD:ABZ2889
Catocala epione|[1304]|QUNO796-08|United States|Wisconsin|658[0n]|BOLD:ABZ2889
Catocala epione|[1305]|QUNO797-08|United States|Wisconsin|658[0n]|BOLD:ABZ2889
Catocala epione|[1306]|LSEU594-06|United States|Georgia|658[0n]|BOLD:ABZ2889
Catocala epione|[1307]|ABCNA272-06|United States|Florida|582[0n]|BOLD:ABZ2889
Catocala epione|[1308]|ABCNA271-06|United States|Florida|658[0n]|BOLD:ABZ2889
Catocala epione|[1309]|QUNOB045-08|United States|Texas|658[0n]|BOLD:ABZ2889
Catocala epione|[1310]|LOT233-04|United States|Tennessee|609[0n]|BOLD:ABZ2889
Catocala epione|[1311]|LOT232-04|United States|Tennessee|609[0n]|BOLD:ABZ2889
Catocala epione|[1312]|LOT231-04|United States|Tennessee|609[0n]|BOLD:ABZ2889
Catocala epione|[1313]|JRLAA026-09|United States|Alabama|635[0n]|BOLD:ABZ2889
Catocala epione|[1314]|QUNO724-08|United States|Kentucky|658[0n]|BOLD:ABZ2889
Catocala epione|[1315]|QUNO725-08|United States|Kentucky|658[0n]|BOLD:ABZ2889
Catocala epione|[1316]|QUNO795-08|United States|Wisconsin|658[0n]|BOLD:ABZ2889
Catocala epione|[1317]|QUNO798-08|United States|Wisconsin|658[0n]|BOLD:ABZ2889
Catocala epione|[1318]|LNCC120-10|United States|North Carolina|658[0n]|BOLD:ABZ2889
Catocala epione|[1319]|LNCC1275-11|United States|North Carolina|658[0n]|BOLD:ABZ2889
Catocala epione|[1320]|LNCC1276-11|United States|North Carolina|658[0n]|BOLD:ABZ2889
Catocala texanae|[1321]|ABCNA049-06|United States|Texas|610[0n]|BOLD:ACE8722
Catocala texanae|[1322]|HKONB568-09|United States|Texas|600[0n]|BOLD:ACE8722
Catocala texanae|[1323]|QUNOD024-10|United States|Oklahoma|658[0n]|BOLD:ACE8722
Catocala texanae|[1324]|ABCNA048-06|United States|Texas|593[0n]|BOLD:ACE8722
Catocala texanae|[1325]|QUNOD101-10|United States|Texas|658[0n]|BOLD:ACE8722
Catocala texanae|[1326]|QUNOD125-10|United States|Texas|658[0n]|BOLD:ACE8722
Catocala hermia|[1327]|QUNOE221-12|United States|California|658[0n]|BOLD:AAB0100
Catocala irene|[1328]|QUNOE193-12|United States|California|658[0n]|BOLD:AAB0100
Catocala irene|[1329]|QUNOE194-12|United States|California|658[0n]|BOLD:AAB0100
Catocala irene|[1330]|QUNOE199-12|United States|California|658[0n]|BOLD:AAB0100
Catocala irene|[1331]|QUNOE204-12|United States|California|658[0n]|BOLD:AAB0100
Catocala irene|[1332]|QUNOE147-11|United States|California|658[0n]|BOLD:AAB0100
Catocala irene|[1333]|QUNOE197-12|United States|California|658[0n]|BOLD:AAB0100
Catocala irene|[1334]|QUNOE207-12|United States|California|658[0n]|BOLD:AAB0100
Catocala irene|[1335]|QUNOE219-12|United States|California|658[0n]|BOLD:AAB0100
Catocala faustina|[1336]|ABCNA019-06|United States|Utah|589[0n]|BOLD:AAB0100
Catocala faustina|[1337]|QUNO134-08|United States|Oregon|658[0n]|BOLD:AAB0100
Catocala faustina|[1338]|QUNOE222-12|United States|Utah|658[0n]|BOLD:AAB0100
Catocala irene|[1339]|ABCNA304-06|United States|Utah|658[0n]|BOLD:AAB0100
Catocala faustina|[1340]|QUNOE170-11|United States|California|615[0n]|BOLD:AAB0100
Catocala faustina|[1341]|QUNOE220-12|United States|California|658[0n]|BOLD:AAB0100
Catocala faustina|[1342]|QUNOE223-12|United States|Utah|658[0n]|BOLD:AAB0100
Catocala faustina|[1343]|QUNOE224-12|United States|Utah|658[0n]|BOLD:AAB0100
Catocala irene|[1344]|QUNOE225-12|United States|Utah|658[0n]|BOLD:AAB0100
Catocala irene|[1345]|QUNOE226-12|United States|Utah|658[0n]|BOLD:AAB0100
Catocala irene|[1346]|QUNOE227-12|United States|Utah|658[0n]|BOLD:AAB0100
Catocala faustina|[1347]|QUNOE277-12|United States|Washington|658[0n]|BOLD:AAB0100
Catocala faustina|[1348]|QUNOE508-12|United States|California|658[0n]|BOLD:AAB0100
Catocala meskei|[1349]|RDMAB171-05|Canada|Alberta|658[0n]|BOLD:AAB0100
Catocala meskei|[1350]|RDMAB163-05|Canada|Alberta|658[0n]|BOLD:AAB0100
Catocala meskei|[1351]|RDMAB162-05|Canada|Alberta|658[0n]|BOLD:AAB0100
Catocala meskei|[1352]|RDMAB160-05|Canada|Alberta|658[0n]|BOLD:AAB0100
Catocala meskei|[1353]|RDMAB542-06|Canada|Alberta|621[0n]|BOLD:AAB0100
Catocala luciana|[1354]|RDMAB176-05|Canada|Alberta|658[0n]|BOLD:AAB0100
Catocala luciana|[1355]|RDMAB177-05|Canada|Alberta|542[0n]|BOLD:AAB0100
Catocala parta|[1356]|RDMAB172-05|Canada|Alberta|658[0n]|BOLD:AAB0100
Catocala parta|[1357]|QUNOB298-09|United States|Wisconsin|658[0n]|BOLD:AAB0100
Catocala luciana|[1358]|QUNOB324-09|Canada|Saskatchewan|658[0n]|BOLD:AAB0100
Catocala luciana|[1359]|QUNOB325-09|Canada|Saskatchewan|658[0n]|BOLD:AAB0100
Catocala parta|[1360]|ABCNA433-07|United States|Iowa|524[0n]|BOLD:AAB0100
Catocala parta|[1361]|QUNO056-07|United States|Wisconsin|649[0n]|BOLD:AAB0100
Catocala meskei|[1362]|RDMAB156-05|Canada|Alberta|658[0n]|BOLD:AAB0100
Catocala parta|[1363]|QUNOB133-08|United States|Wisconsin|658[0n]|BOLD:AAB0100
Catocala meskei|[1364]|RDMAB161-05|Canada|Alberta|658[0n]|BOLD:AAB0100
Catocala parta|[1365]|QUNOB333-09|Canada|Saskatchewan|658[0n]|BOLD:AAB0100
Catocala luciana|[1366]|QUNOB322-09|Canada|Saskatchewan|658[0n]|BOLD:AAB0100
Catocala meskei|[1367]|QUNO779-08|United States|Wisconsin|658[0n]|BOLD:AAB0100
Catocala meskei|[1368]|QUNO778-08|United States|Wisconsin|658[0n]|BOLD:AAB0100
Catocala meskei|[1369]|QUNO777-08|United States|Wisconsin|658[0n]|BOLD:AAB0100
Catocala meskei|[1370]|RDMAB157-05|Canada|Alberta|658[0n]|BOLD:AAB0100
Catocala meskei|[1371]|QUNO047-07|United States|Wisconsin|653[0n]|BOLD:AAB0100
Catocala meskei|[1372]|ABCNA443-07|United States|South Dakota|593[0n]|BOLD:AAB0100
Catocala meskei|[1373]|ABCNA442-07|United States|South Dakota|593[0n]|BOLD:AAB0100
Catocala parta|[1374]|ABCNA172-06|United States|Utah|632[0n]|BOLD:AAB0100
Catocala meskei|[1375]|QUNO780-08|United States|Wisconsin|658[0n]|BOLD:AAB0100
Catocala parta|[1376]|QUNO057-07|United States|Wisconsin|658[0n]|BOLD:AAB0100
Catocala parta|[1377]|QUNOB128-08|United States|Wisconsin|658[0n]|BOLD:AAB0100
Catocala parta|[1378]|QUNOB326-09|Canada|Saskatchewan|658[0n]|BOLD:AAB0100
Catocala parta|[1379]|QUNOB330-09|Canada|Saskatchewan|658[0n]|BOLD:AAB0100
Catocala parta|[1380]|QUNOB331-09|Canada|Saskatchewan|658[0n]|BOLD:AAB0100
Catocala parta|[1381]|QUNOB332-09|Canada|Saskatchewan|658[0n]|BOLD:AAB0100
Catocala parta|[1382]|QUNOB335-09|Canada|Saskatchewan|658[0n]|BOLD:AAB0100
Catocala meskei|[1383]|QUNOB336-09|Canada|Saskatchewan|658[0n]|BOLD:AAB0100
Catocala meskei|[1384]|QUNOB337-09|Canada|Saskatchewan|658[0n]|BOLD:AAB0100
Catocala luciana|[1385]|QUNOB505-09|United States|Nebraska|658[0n]|BOLD:AAB0100
Catocala meskei|[1386]|QUNOC075-09|Canada|Alberta|658[0n]|BOLD:AAB0100
Catocala meskei|[1387]|QUNOC076-09|United States|Montana|658[0n]|BOLD:AAB0100
Catocala meskei|[1388]|RDMAB159-05|Canada|Alberta|531[0n]|BOLD:AAB0100
Catocala meskei|[1389]|QUNOB329-09|Canada|Saskatchewan|658[0n]|BOLD:AAB0100
Catocala meskei|[1390]|QUNOB327-09|Canada|Saskatchewan|658[0n]|BOLD:AAB0100
Catocala meskei|[1391]|RDMAB158-05|Canada|Alberta|658[0n]|BOLD:AAB0100
Catocala meskei|[1392]|RDMAB155-05|Canada|Alberta|605[2n]|BOLD:AAB0100
Catocala meskei|[1393]|QUNOC074-09|Canada|Alberta|641[0n]|BOLD:AAB0100
Catocala meskei|[1394]|QUNOC077-09|Canada|Alberta|658[0n]|BOLD:AAB0100
Catocala junctura|[1395]|QUNOE553-12|United States|Wyoming|658[0n]|BOLD:AAB0100
Catocala junctura|[1396]|QUNOB334-09|Canada|Saskatchewan|658[0n]|BOLD:AAB0100
Catocala junctura|[1397]|RDMAB178-05|Canada|Alberta|658[0n]|BOLD:AAB0100
Catocala junctura|[1398]|QUNO117-08|United States|Wyoming|592[0n]|BOLD:AAB0100

Catocala junctura|[1396]|QUNOB334-09|Canada|Saskatchewan|658[0n]|BOLD:AAB0100
Catocala junctura|[1397]|RDMAB178-05|Canada|Alberta|658[0n]|BOLD:AAB0100
Catocala junctura|[1398]|QUNO117-08|United States|Wyoming|592[0n]|BOLD:AAB0100
Catocala junctura|[1399]|QUNOE092-11|United States|Wyoming|658[0n]|BOLD:AAB0100
Catocala junctura|[1400]|QUNOE109-11|United States|Utah|658[0n]|BOLD:AAB0100
Catocala junctura|[1401]|QUNOE555-12|United States|Utah|658[0n]|BOLD:AAB0100
Catocala jessica|[1402]|ABCNA188-06|United States|Arizona|643[0n]|BOLD:AAB0100
Catocala jessica|[1403]|ABCNA191-06|United States|Utah|658[0n]|BOLD:AAB0100
Catocala jessica|[1404]|ABCNA042-06|United States|Arizona|658[0n]|BOLD:AAB0100
Catocala jessica|[1405]|HKONB491-09|United States|Texas|639[1n]|BOLD:AAB0100
Catocala jessica|[1406]|ABCNA190-06|United States|Arizona|658[0n]|BOLD:AAB0100
Catocala jessica|[1407]|ABCNA187-06|United States|Texas|614[0n]|BOLD:AAB0100
Catocala jessica|[1408]|ABCNA311-06|United States|Arizona|658[0n]|BOLD:AAB0100
Catocala jessica|[1409]|QUNOD129-10|United States|Texas|658[0n]|BOLD:AAB0100
Catocala jessica|[1410]|QUNOD130-10|United States|Texas|658[0n]|BOLD:AAB0100
Catocala jessica|[1411]|QUNOD153-10|United States|Texas|658[0n]|BOLD:AAB0100
Catocala jessica|[1412]|ABCNA041-06|United States|Arizona|610[0n]|BOLD:AAB0100
Catocala jessica|[1413]|ABCNA753-07|United States|Texas|628[1n]|BOLD:AAB0100
Catocala jessica|[1414]|QUNOE089-11|United States|Texas|658[0n]|BOLD:AAB0100
Catocala jessica|[1415]|QUNOE112-11|United States|Arizona|658[0n]|BOLD:AAB0100
Catocala jessica|[1416]|QUNOE552-12|United States|Utah|658[0n]|BOLD:AAB0100
Catocala junctura|[1417]|ABCNA455-07|United States|Utah|560[0n]|BOLD:AAB0100
Catocala junctura|[1418]|QUNOE548-12|United States|Utah|658[0n]|BOLD:AAB0100
Catocala junctura|[1419]|ABCNA047-06|United States|Utah|645[0n]|BOLD:AAB0100
Catocala junctura|[1420]|ABCNA310-06|United States|Utah|658[0n]|BOLD:AAB0100
Catocala junctura|[1421]|QUNOE539-12|United States|Utah|658[0n]|BOLD:AAB0100
Catocala junctura|[1422]|QUNOE540-12|United States|Utah|658[0n]|BOLD:AAB0100
Catocala junctura|[1423]|QUNOE541-12|United States|Utah|658[0n]|BOLD:AAB0100
Catocala junctura|[1424]|QUNOE544-12|United States|Utah|658[0n]|BOLD:AAB0100
Catocala junctura|[1425]|QUNOE546-12|United States|Utah|658[0n]|BOLD:AAB0100
Catocala junctura|[1426]|QUNOE547-12|United States|Utah|658[0n]|BOLD:AAB0100
Catocala jessica|[1427]|QUNOE549-12|United States|Utah|658[0n]|BOLD:AAB0100
Catocala jessica|[1428]|QUNOE550-12|United States|Utah|658[0n]|BOLD:AAB0100
Catocala jessica|[1429]|QUNOE111-11|United States|Arizona|658[0n]|BOLD:AAB0100
Catocala junctura|[1430]|ABCNA046-06|United States|Texas|589[0n]|BOLD:AAB0100
Catocala jessica|[1431]|HKONB490-09|United States|Texas|658[0n]|BOLD:AAB0100
Catocala junctura|[1432]|QUNOD131-10|United States|Texas|658[0n]|BOLD:AAB0100
Catocala junctura|[1433]|QUNOE136-11|United States|Arizona|658[0n]|BOLD:AAB0100
Catocala junctura|[1434]|QUNOD127-10|United States|Texas|658[0n]|BOLD:AAB0100
Catocala junctura|[1435]|ABCNA186-06|United States|Texas|658[0n]|BOLD:AAB0100
Catocala junctura|[1436]|ABCNA756-07|United States|Texas|654[0n]|BOLD:AAB0100
Catocala junctura|[1437]|ABCNA045-06|United States|Texas|614[0n]|BOLD:AAB0100
Catocala jessica|[1438]|QUNOE101-11|United States|Arizona|658[0n]|BOLD:AAB0100
Catocala junctura|[1439]|QUNOE110-11|United States|Utah|658[0n]|BOLD:AAB0100
Catocala jessica|[1440]|QUNOE099-11|United States|California|658[0n]|BOLD:AAB0100
Catocala jessica|[1441]|QUNOE169-11|United States|Arizona|658[0n]|BOLD:AAB0100
Catocala jessica|[1442]|QUNOE187-11|United States|Arizona|658[0n]|BOLD:AAB0100
Catocala junctura|[1443]|QUNOE132-11|United States|Arizona|658[0n]|BOLD:AAB0100
Catocala irene|[1444]|QUNOE542-12|United States|California|658[0n]|BOLD:AAB0100
Catocala junctura|[1445]|QUNOE131-11|United States|Arizona|658[0n]|BOLD:AAB0100
Catocala junctura|[1446]|QUNOE214-12|United States|California|658[0n]|BOLD:AAB0100
Catocala irene|[1447]|QUNOE203-12|United States|California|658[0n]|BOLD:AAB0100
Catocala irene|[1448]|QUNOE205-12|United States|California|658[0n]|BOLD:AAB0100
Catocala irene|[1449]|QUNOE206-12|United States|California|658[0n]|BOLD:AAB0100
Catocala irene|[1450]|QUNOE209-12|United States|California|658[0n]|BOLD:AAB0100
Catocala junctura|[1451]|QUNOE215-12|United States|California|658[0n]|BOLD:AAB0100
Catocala junctura|[1452]|QUNOE545-12|United States|Utah|658[0n]|BOLD:AAB0100
Catocala irene|[1453]|QUNOE192-12|United States|California|658[0n]|BOLD:AAB0100
Catocala junctura|[1454]|QUNOE196-12|United States|California|658[0n]|BOLD:AAB0100
Catocala irene|[1455]|QUNOE149-11|United States|California|658[0n]|BOLD:AAB0100
Catocala irene|[1456]|QUNOE191-12|United States|California|658[0n]|BOLD:AAB0100
Catocala irene|[1457]|QUNOE146-11|United States|Utah|658[0n]|BOLD:AAB0100
Catocala irene|[1458]|QUNOE148-11|United States|California|658[0n]|BOLD:AAB0100
Catocala junctura stretchi|[1459]|QUNOE135-11|United States|California|658[0n]|BOLD:AAB0100
Catocala irene|[1460]|QUNOE145-11|United States|Utah|658[0n]|BOLD:AAB0100
Catocala jessica|[1461]|QUNOE100-11|United States|California|658[0n]|BOLD:AAB0100
Catocala junctura|[1462]|QUNOE130-11|United States|California|658[0n]|BOLD:AAB0100
Catocala irene|[1463]|QUNOE091-11|United States|California|658[0n]|BOLD:AAB0100
Catocala junctura|[1464]|QUNOC294-10|United States|Arizona|658[0n]|BOLD:AAB0100
Catocala junctura|[1465]|QUNOD128-10|United States|Texas|658[0n]|BOLD:AAB0100
Catocala junctura|[1466]|QUNOD126-10|United States|Texas|658[0n]|BOLD:AAB0100
Catocala junctura|[1467]|LPOKC603-09|United States|Oklahoma|658[0n]|BOLD:AAB0100
Catocala junctura|[1468]|QUNOB303-09|United States|Wyoming|658[0n]|BOLD:AAB0100
Catocala junctura|[1469]|QUNOB301-09|United States|Wyoming|658[0n]|BOLD:AAB0100
Catocala junctura|[1470]|QUNO816-08|United States|Wyoming|658[0n]|BOLD:AAB0100
Catocala junctura|[1471]|ABCNA043-06|United States|California|655[0n]|BOLD:AAB0100
Catocala junctura|[1472]|QUNOE186-11|United States|Arizona|658[0n]|BOLD:AAB0100
Catocala irene|[1473]|QUNOE208-12|United States|California|658[0n]|BOLD:AAB0100
Catocala junctura|[1474]|ABCNA184-06|United States|Utah|658[0n]|BOLD:AAB0100
Catocala junctura|[1475]|ABCNA450-07|United States|Utah|592[0n]|BOLD:AAB0100
Catocala junctura|[1476]|ABCNA754-07|United States|Texas|592[1n]|BOLD:AAB0100
Catocala junctura|[1477]|ABCNA309-06|United States|Arizona|604[2n]|BOLD:AAB0100
Catocala electilis|[1478]|ABCNA746-07|Mexico||583[1n]|BOLD:AAB0100
Catocala junctura|[1479]|QUNO817-08|United States|Wyoming|658[0n]|BOLD:AAB0100
Catocala junctura|[1480]|ABCNA044-06|United States|Utah|604[0n]|BOLD:AAB0100
Catocala junctura|[1481]|ABCNA185-06|United States|Arizona|594[0n]|BOLD:AAB0100
Catocala electilis|[1482]|ABCNA747-07|Mexico||583[0n]|BOLD:AAB0100
Catocala junctura stretchi|[1483]|QUNOE133-11|United States|California|658[0n]|BOLD:AAB0100
Catocala junctura stretchi|[1484]|QUNOE134-11|United States|California|658[0n]|BOLD:AAB0100
Catocala junctura|[1485]|QUNOE551-12|United States|Utah|658[0n]|BOLD:AAB0100
Catocala junctura|[1486]|QUNOE554-12|United States|Wyoming|658[0n]|BOLD:AAB0100
Catocala junctura|[1487]|QUNOE556-12|United States|Utah|658[0n]|BOLD:AAB0100
Catocala marmorata|[1488]|ABCNA168-06|United States|Indiana|658[0n]|BOLD:AAB0100
Catocala marmorata|[1489]|ABCNA170-06|United States|Indiana|608[0n]|BOLD:AAB0100
Catocala marmorata|[1490]|QUNO619-08|United States|Indiana|658[0n]|BOLD:AAB0100
Catocala marmorata|[1491]|QUNO620-08|United States|Indiana|658[0n]|BOLD:AAB0100
Catocala marmorata|[1492]|QUNO623-08|United States|Indiana|658[0n]|BOLD:AAB0100
Catocala marmorata|[1493]|QUNO624-08|United States|Indiana|658[0n]|BOLD:AAB0100
Catocala marmorata|[1494]|ABCNA169-06|United States|Indiana|630[0n]|BOLD:AAB0100
Catocala marmorata|[1495]|QUNO621-08|United States|Indiana|658[0n]|BOLD:AAB0100
Catocala marmorata|[1496]|LNCC448-10|United States|North Carolina|658[0n]|BOLD:AAB0100
Catocala marmorata|[1497]|QUNO622-08|United States|Indiana|658[0n]|BOLD:AAB0100
Catocala marmorata|[1498]|LNCC449-10|United States|North Carolina|658[0n]|BOLD:AAB0100

Catocala marmorata|[1496]|LNCC448-10|United States|North Carolina|658[0n]|BOLD:AAB0100
Catocala marmorata|[1497]|QUNO622-08|United States|Indiana|658[0n]|BOLD:AAB0100
Catocala marmorata|[1498]|LNCC449-10|United States|North Carolina|658[0n]|BOLD:AAB0100
Catocala unijuga|[1499]|LBCD218-05|Canada|British Columbia|658[0n]|BOLD:AAB0100
Catocala unijuga|[1500]|RDLQB566-05|Canada|Quebec|658[0n]|BOLD:AAB0100
Catocala unijuga|[1501]|XAH340-05|Canada|Ontario|658[0n]|BOLD:AAB0100
Catocala unijuga|[1502]|MNBB573-05|Canada|New Brunswick|658[0n]|BOLD:AAB0100
Catocala unijuga|[1503]|ABCNA432-07|United States|Wisconsin|621[0n]|BOLD:AAB0100
Catocala unijuga|[1504]|DSCNI031-07|Canada|Manitoba|658[0n]|BOLD:AAB0100
Catocala unijuga|[1505]|QUNO085-07|United States|Wisconsin|658[0n]|BOLD:AAB0100
Catocala unijuga|[1506]|IMA002-07|United States|Massachusetts|658[0n]|BOLD:AAB0100
Catocala unijuga|[1507]|QUNOB131-08|United States|Wisconsin|658[0n]|BOLD:AAB0100
Catocala unijuga|[1508]|QUNOB132-08|United States|Wisconsin|658[0n]|BOLD:AAB0100
Catocala unijuga|[1509]|LPAB310-08|Canada|Alberta|658[0n]|BOLD:AAB0100
Catocala unijuga|[1510]|QUNOB328-09|Canada|Saskatchewan|658[0n]|BOLD:AAB0100
Catocala unijuga|[1511]|LPMNB312-09|Canada|Manitoba|658[0n]|BOLD:AAB0100
Catocala unijuga|[1512]|QUNOC033-09|United States|Wisconsin|658[0n]|BOLD:AAB0100
Catocala unijuga|[1513]|BBLPA321-10|Canada|Alberta|658[0n]|BOLD:AAB0100
Catocala unijuga|[1514]|BBLPA324-10|Canada|Alberta|658[0n]|BOLD:AAB0100
Catocala semirelicta hippolyta|[1515]|ABCNA001-06|United States|California|655[0n]|BOLD:AAB0100
Catocala semirelicta hippolyta|[1516]|ABCNA002-06|United States|California|658[0n]|BOLD:AAB0100
Catocala semirelicta hippolyta|[1517]|ABCNA003-06|United States|California|658[0n]|BOLD:AAB0100
Catocala semirelicta hippolyta|[1518]|ABCNA004-06|United States|California|658[0n]|BOLD:AAB0100
Catocala semirelicta hippolyta|[1519]|ABCNA005-06|United States|California|658[0n]|BOLD:AAB0100
Catocala semirelicta hippolyta|[1520]|ABCNA039-06|United States|California|658[0n]|BOLD:AAB0100
Catocala semirelicta hippolyta|[1521]|ABCNA040-06|United States|California|658[0n]|BOLD:AAB0100
Catocala semirelicta hippolyta|[1522]|QUNOE162-11|United States|California|658[0n]|BOLD:AAB0100
Catocala californica|[1523]|QUNO135-08|United States|California|568[5n]|BOLD:AAB0100
Catocala hermia|[1524]|QUNOE168-11|United States|Arizona|658[0n]|BOLD:AAB0100
Catocala hermia|[1525]|QUNO830-08|United States|Wyoming|658[0n]|BOLD:AAB0100
Catocala californica|[1526]|QUNOE077-11|United States|California|658[0n]|BOLD:AAB0100
Catocala hermia|[1527]|ABCNA306-06|United States|Utah|649[0n]|BOLD:AAB0100
Catocala hermia|[1528]|QUNO474-08|United States|Idaho|658[0n]|BOLD:AAB0100
Catocala hermia|[1529]|NAMUM364-09|United States|California|658[0n]|BOLD:AAB0100
Catocala hermia|[1530]|QUNOC064-09|United States|California|658[0n]|BOLD:AAB0100
Catocala hermia|[1531]|QUNOC065-09|United States|Idaho|658[0n]|BOLD:AAB0100
Catocala hermia|[1532]|QUNOD570-10|United States|California|658[0n]|BOLD:AAB0100
Catocala hermia|[1533]|QUNOD661-10|United States|California|658[0n]|BOLD:AAB0100
Catocala hermia|[1534]|QUNOE106-11|United States|Utah|658[0n]|BOLD:AAB0100
Catocala californica|[1535]|QUNOE137-11|United States|California|658[0n]|BOLD:AAB0100
Catocala californica|[1536]|QUNOE138-11|United States|California|658[0n]|BOLD:AAB0100
Catocala californica|[1537]|QUNOE141-11|United States|California|658[0n]|BOLD:AAB0100
Catocala faustina allusa|[1538]|RDMAB169-05|Canada|British Columbia|528[0n]|BOLD:AAB0100
Catocala briseis|[1539]|XAG218-05|Canada|Ontario|658[0n]|BOLD:AAB0100
Catocala briseis|[1540]|ABCNA177-06|United States|Wisconsin|630[0n]|BOLD:AAB0100
Catocala briseis|[1541]|QUNOD344-10|United States|Michigan|658[0n]|BOLD:AAB0100
Catocala briseis|[1542]|QUNOD347-10|United States|Minnesota|658[0n]|BOLD:AAB0100
Catocala briseis|[1543]|QUNOD348-10|United States|Minnesota|658[0n]|BOLD:AAB0100
Catocala briseis|[1544]|QUNOD373-10|United States|Wisconsin|658[0n]|BOLD:AAB0100
Catocala briseis|[1545]|QUNOD458-10|United States|Wisconsin|658[0n]|BOLD:AAB0100
Catocala briseis|[1546]|ABCNA031-06|United States|Wisconsin|655[0n]|BOLD:AAB0100
Catocala briseis|[1547]|RDMAB183-05|Canada|Alberta|658[0n]|BOLD:AAB0100
Catocala briseis|[1548]|RDLQB482-05|Canada|Quebec|658[0n]|BOLD:AAB0100
Catocala briseis|[1549]|ABCNA171-06|United States|Wisconsin|632[0n]|BOLD:AAB0100
Catocala briseis|[1550]|RDLQB568-05|Canada|Quebec|592[0n]|BOLD:AAB0100
Catocala briseis|[1551]|QUNO027-07|United States|Wisconsin|658[0n]|BOLD:AAB0100
Catocala briseis|[1552]|QUNOD345-10|United States|Minnesota|658[0n]|BOLD:AAB0100
Catocala briseis|[1553]|QUNOD459-10|United States|Wisconsin|658[0n]|BOLD:AAB0100
Catocala briseis|[1554]|QUNO281-08|United States|Wyoming|609[0n]|BOLD:AAB0100
Catocala semirelicta|[1555]|QUNO116-08|United States|Wyoming|658[0n]|BOLD:AAB0100
Catocala grotiana|[1556]|ABCNA180-06|United States|Arizona|658[0n]|BOLD:AAB0100
Catocala semirelicta|[1557]|ABCNA182-06|United States|Utah|628[0n]|BOLD:AAB0100
Catocala semirelicta|[1558]|ABCNA183-06|United States|Utah|621[0n]|BOLD:AAB0100
Catocala grotiana|[1559]|ABCNA179-06|United States|Arizona|620[0n]|BOLD:AAB0100
Catocala grotiana|[1560]|ABCNA032-06|United States|Arizona|604[0n]|BOLD:AAB0100
Catocala semirelicta|[1561]|ABCNA033-06|United States|Utah|601[0n]|BOLD:AAB0100
Catocala semirelicta|[1562]|ABCNA034-06|United States|Utah|600[0n]|BOLD:AAB0100
Catocala grotiana|[1563]|ABCNA178-06|United States|Arizona|597[0n]|BOLD:AAB0100
Catocala semirelicta|[1564]|ABCNA035-06|United States|Utah|593[0n]|BOLD:AAB0100
Catocala grotiana|[1565]|ABCNA440-07|United States|Arizona|595[0n]|BOLD:AAB0100
Catocala grotiana|[1566]|QUNO818-08|United States|Wyoming|658[0n]|BOLD:AAB0100
Catocala grotiana|[1567]|QUNO819-08|United States|Wyoming|658[0n]|BOLD:AAB0100
Catocala semirelicta|[1568]|QUNO821-08|United States|Wyoming|658[0n]|BOLD:AAB0100
Catocala semirelicta|[1569]|QUNO822-08|United States|Wyoming|658[0n]|BOLD:AAB0100
Catocala semirelicta|[1570]|QUNO823-08|United States|Wyoming|658[0n]|BOLD:AAB0100
Catocala semirelicta|[1571]|QUNO824-08|United States|Wyoming|658[0n]|BOLD:AAB0100
Catocala semirelicta|[1572]|QUNO825-08|United States|Wyoming|658[0n]|BOLD:AAB0100
Catocala semirelicta|[1573]|QUNO832-08|United States|Wyoming|658[0n]|BOLD:AAB0100
Catocala semirelicta|[1574]|QUNO835-08|United States|Wyoming|658[0n]|BOLD:AAB0100
Catocala grotiana|[1575]|QUNO837-08|United States|Wyoming|658[0n]|BOLD:AAB0100
Catocala semirelicta|[1576]|QUNO838-08|United States|Wyoming|658[0n]|BOLD:AAB0100
Catocala grotiana|[1577]|QUNOB300-09|United States|Wyoming|658[0n]|BOLD:AAB0100
Catocala semirelicta|[1578]|QUNOB302-09|United States|Wyoming|658[0n]|BOLD:AAB0100
Catocala grotiana|[1579]|QUNOB305-09|United States|Wyoming|658[0n]|BOLD:AAB0100
Catocala grotiana|[1580]|QUNOB307-09|United States|Wyoming|658[0n]|BOLD:AAB0100
Catocala grotiana|[1581]|RDNMJ286-11|United States|Arizona|658[0n]|BOLD:AAB0100
Catocala semirelicta|[1582]|JMMMB443-11|United States|California|658[0n]|BOLD:AAB0100
Catocala semirelicta|[1583]|QUNOE117-11|United States|California|658[0n]|BOLD:AAB0100
Catocala semirelicta|[1584]|QUNOE118-11|United States|California|658[0n]|BOLD:AAB0100
Catocala semirelicta|[1585]|QUNOE119-11|United States|California|658[0n]|BOLD:AAB0100
Catocala semirelicta|[1586]|QUNOE120-11|United States|California|658[0n]|BOLD:AAB0100
Catocala semirelicta|[1587]|QUNOE121-11|United States|Arizona|658[0n]|BOLD:AAB0100
Catocala semirelicta|[1588]|QUNOE122-11|United States|Arizona|658[0n]|BOLD:AAB0100
Catocala semirelicta|[1589]|QUNOE123-11|United States|Arizona|658[0n]|BOLD:AAB0100
Catocala semirelicta|[1590]|QUNOE124-11|United States|Arizona|658[0n]|BOLD:AAB0100
Catocala semirelicta|[1591]|QUNOE125-11|United States|Arizona|658[0n]|BOLD:AAB0100
Catocala semirelicta|[1592]|QUNOE126-11|United States|Arizona|658[0n]|BOLD:AAB0100
Catocala semirelicta|[1593]|QUNOE127-11|United States|Arizona|658[0n]|BOLD:AAB0100
Catocala semirelicta|[1594]|QUNOE128-11|United States|Arizona|658[0n]|BOLD:AAB0100
Catocala semirelicta|[1595]|QUNOE172-11|United States|California|658[0n]|BOLD:AAB0100
Catocala semirelicta|[1596]|QUNOE173-11|United States|California|658[0n]|BOLD:AAB0100
Catocala semirelicta|[1597]|QUNOE174-11|United States|Arizona|658[0n]|BOLD:AAB0100
Catocala semirelicta|[1598]|QUNOE175-11|United States|Arizona|658[0n]|BOLD:AAB0100

[illegible]
Catocala semirelicta|[1596]|QUNOE173-11|United States|California|658[0n]|BOLD:AAB0100
Catocala semirelicta|[1597]|QUNOE174-11|United States|Arizona|658[0n]|BOLD:AAB0100
Catocala semirelicta|[1598]|QUNOE175-11|United States|Arizona|658[0n]|BOLD:AAB0100
Catocala californica|[1599]|QUNOE198-12|United States|California|658[0n]|BOLD:AAB0100
Catocala californica|[1600]|QUNOE195-12|United States|California|658[0n]|BOLD:AAB0100
Catocala californica|[1601]|QUNOE166-11|United States|California|658[0n]|BOLD:AAB0100
Catocala californica|[1602]|QUNOE142-11|United States|California|658[0n]|BOLD:AAB0100
Catocala californica|[1603]|QUNOE139-11|United States|California|658[0n]|BOLD:AAB0100
Catocala hermia francisca|[1604]|QUNOE129-11|United States|California|658[0n]|BOLD:AAB0100
Catocala hermia|[1605]|QUNOE107-11|United States|Utah|658[0n]|BOLD:AAB0100
Catocala hermia|[1606]|QUNOE105-11|United States|Utah|658[0n]|BOLD:AAB0100
Catocala hermia|[1607]|QUNOE104-11|United States|Arizona|658[0n]|BOLD:AAB0100
Catocala hermia|[1608]|QUNOE103-11|United States|Arizona|658[0n]|BOLD:AAB0100
Catocala hermia|[1609]|QUNOE102-11|United States|California|658[0n]|BOLD:AAB0100
Catocala hermia|[1610]|QUNOE095-11|United States|Colorado|658[0n]|BOLD:AAB0100
Catocala hermia|[1611]|QUNOE093-11|United States|Wyoming|658[0n]|BOLD:AAB0100
Catocala californica|[1612]|LALPA707-10|Canada|British Columbia|658[0n]|BOLD:AAB0100
Catocala hermia|[1613]|QUNOD566-10|United States|Arizona|658[0n]|BOLD:AAB0100
Catocala hermia|[1614]|QUNOD450-10|Canada|Saskatchewan|658[0n]|BOLD:AAB0100
Catocala briseis|[1615]|LBCH6620-10|Canada|British Columbia|658[0n]|BOLD:AAB0100
Catocala californica|[1616]|QUNOC085-09|United States|California|658[0n]|BOLD:AAB0100
Catocala californica|[1617]|QUNOC084-09|United States|California|658[0n]|BOLD:AAB0100
Catocala californica|[1618]|QUNOC083-09|United States|Oregon|658[0n]|BOLD:AAB0100
Catocala californica|[1619]|QUNOC080-09|United States|Montana|658[0n]|BOLD:AAB0100
Catocala hermia|[1620]|QUNOC066-09|United States|Nevada|658[0n]|BOLD:AAB0100
Catocala hermia|[1621]|QUNOC057-09|United States|Idaho|658[0n]|BOLD:AAB0100
Catocala faustina allusa|[1622]|QUNOC048-09|United States|Oregon|658[0n]|BOLD:AAB0100
Catocala hermia|[1623]|QUNOB503-09|United States|Montana|658[0n]|BOLD:AAB0100
Catocala californica|[1624]|QUNOB379-09|United States|Montana|658[0n]|BOLD:AAB0100
Catocala hermia|[1625]|QUNOB323-09|Canada|Saskatchewan|658[0n]|BOLD:AAB0100
Catocala hermia|[1626]|QUNO834-08|United States|Wyoming|658[0n]|BOLD:AAB0100
Catocala hermia|[1627]|QUNO833-08|United States|Wyoming|658[0n]|BOLD:AAB0100
Catocala hermia|[1628]|QUNO828-08|United States|Wyoming|658[0n]|BOLD:AAB0100
Catocala hermia|[1629]|QUNO827-08|United States|Wyoming|658[0n]|BOLD:AAB0100
Catocala hermia|[1630]|QUNO826-08|United States|Wyoming|658[0n]|BOLD:AAB0100
Catocala hermia|[1631]|QUNO473-08|United States|Idaho|658[0n]|BOLD:AAB0100
Catocala hermia|[1632]|QUNO472-08|United States|Idaho|658[0n]|BOLD:AAB0100
Catocala hermia|[1633]|QUNO138-08|United States|Oregon|658[0n]|BOLD:AAB0100
Catocala californica|[1634]|QUNO136-08|United States|Oregon|658[0n]|BOLD:AAB0100
Catocala hermia|[1635]|ABCNA308-06|United States|Utah|658[0n]|BOLD:AAB0100
Catocala hermia|[1636]|ABCNA305-06|United States|Utah|658[0n]|BOLD:AAB0100
Catocala hermia francisca|[1637]|ABCNA026-06|United States|California|658[0n]|BOLD:AAB0100
Catocala hermia|[1638]|RDMAB686-06|Canada|Alberta|658[0n]|BOLD:AAB0100
Catocala hermia|[1639]|RDMAB181-05|Canada|Alberta|658[0n]|BOLD:AAB0100
Catocala hermia|[1640]|RDMAB179-05|Canada|Alberta|658[0n]|BOLD:AAB0100
Catocala californica|[1641]|RDMAB167-05|Canada|Alberta|658[0n]|BOLD:AAB0100
Catocala californica|[1642]|QUNOC081-09|United States|Oregon|658[1n]|BOLD:AAB0100
Catocala californica|[1643]|QUNOB306-09|United States|Wyoming|658[0n]|BOLD:AAB0100
Catocala hermia|[1644]|QUNO829-08|United States|Wyoming|658[0n]|BOLD:AAB0100
Catocala hermia|[1645]|QUNOC056-09|United States|Idaho|654[0n]|BOLD:AAB0100
Catocala hermia|[1646]|ABCNA175-06|United States|Utah|620[0n]|BOLD:AAB0100
Catocala hermia|[1647]|ABCNA024-06|United States|Utah|610[0n]|BOLD:AAB0100
Catocala hermia|[1648]|ABCNA022-06|United States|Utah|606[0n]|BOLD:AAB0100
Catocala faustina allusa|[1649]|ABCNA017-06|United States|Washington|593[0n]|BOLD:AAB0100
Catocala hermia|[1650]|ABCNA021-06|United States|Utah|602[0n]|BOLD:AAB0100
Catocala hermia|[1651]|ABCNA023-06|United States|Utah|604[0n]|BOLD:AAB0100
Catocala hermia francisca|[1652]|ABCNA027-06|United States|California|632[0n]|BOLD:AAB0100
Catocala faustina allusa|[1653]|QUNO133-08|United States|Washington|635[0n]|BOLD:AAB0100
Catocala faustina allusa|[1654]|QUNO137-08|United States|Washington|592[1n]|BOLD:AAB0100
Catocala faustina allusa|[1655]|QUNOC046-09|United States|Oregon|658[0n]|BOLD:AAB0100
Catocala faustina allusa|[1656]|QUNOC049-09|United States|Oregon|630[0n]|BOLD:AAB0100
Catocala faustina cleopatra|[1657]|QUNOC062-09|United States|California|658[0n]|BOLD:AAB0100
Catocala faustina cleopatra|[1658]|QUNOC063-09|United States|California|658[0n]|BOLD:AAB0100
Catocala faustina cleopatra|[1659]|QUNOE073-11|United States|California|658[0n]|BOLD:AAB0100
Catocala faustina cleopatra|[1660]|QUNOE074-11|United States|California|658[0n]|BOLD:AAB0100
Catocala faustina cleopatra|[1661]|QUNOE075-11|United States|California|658[0n]|BOLD:AAB0100
Catocala faustina cleopatra|[1662]|QUNOE113-11|United States|California|658[0n]|BOLD:AAB0100
Catocala faustina cleopatra|[1663]|QUNOE114-11|United States|California|658[0n]|BOLD:AAB0100
Catocala faustina cleopatra|[1664]|QUNOE115-11|United States|California|658[0n]|BOLD:AAB0100
Catocala faustina|[1665]|GMLC1195-12|United States|California|658[0n]|BOLD:AAB0100
Catocala californica|[1666]|QUNOE543-12|United States|Utah|658[0n]|BOLD:AAB0100
Catocala hermia|[1667]|ABCNA174-06|United States|Utah|632[0n]|BOLD:AAB0100
Catocala faustina allusa|[1668]|ABCNA016-06|United States|California|599[0n]|BOLD:AAB0100
Catocala hermia|[1669]|ABCNA176-06|United States|Utah|605[0n]|BOLD:AAB0100
Catocala hermia|[1670]|ABCNA307-06|United States|Utah|630[0n]|BOLD:AAB0100
Catocala faustina allusa|[1671]|QUNOC047-09|United States|California|658[0n]|BOLD:AAB0100
Catocala hermia|[1672]|QUNOC060-09|United States|Utah|658[0n]|BOLD:AAB0100
Catocala californica|[1673]|QUNOC082-09|United States|Oregon|658[0n]|BOLD:AAB0100
Catocala semirelicta|[1674]|QUNO836-08|United States|Wyoming|658[0n]|BOLD:AAB0100
Catocala briseis|[1675]|QUNO820-08|United States|Wyoming|658[0n]|BOLD:AAB0100
Catocala briseis|[1676]|QUNO028-07|United States|Wisconsin|658[0n]|BOLD:AAB0100
Catocala semirelicta|[1677]|RDMAB228-05|Canada|Alberta|658[0n]|BOLD:AAB0100
Catocala semirelicta|[1678]|RDMAB173-05|Canada|Alberta|658[0n]|BOLD:AAB0100
Catocala semirelicta|[1679]|RDMAB166-05|Canada|Alberta|658[0n]|BOLD:AAB0100
Catocala briseis|[1680]|QUNOC078-09|Canada|Alberta|641[0n]|BOLD:AAB0100
Catocala meskei|[1681]|QUNOC079-09|Canada|Alberta|658[0n]|BOLD:AAB0100
Catocala meskei|[1682]|QUNOC292-10|Canada|Alberta|658[0n]|BOLD:AAB0100
Catocala semirelicta|[1683]|QUNOD567-10|United States|California|658[0n]|BOLD:AAB0100
Catocala semirelicta|[1684]|QUNOE116-11|United States|California|658[0n]|BOLD:AAB0100
Catocala semirelicta|[1685]|QUNOE474-12|United States|California|658[0n]|BOLD:AAB0100
Catocala briseis|[1686]|QUNOD346-10|United States|Michigan|658[0n]|BOLD:AAB0100
Catocala semirelicta|[1687]|QUNOB304-09|United States|Wyoming|658[0n]|BOLD:AAB0100
Catocala briseis|[1688]|QUNOE094-11|United States|Wyoming|658[0n]|BOLD:AAB0100
Catocala briseis|[1689]|QUNOE217-12|United States|Wyoming|658[0n]|BOLD:AAB0100
Catocala hermia|[1690]|RDMAB180-05|Canada|Alberta|658[0n]|BOLD:AAB0100
Catocala californica|[1691]|ABCNA029-06|United States|California|608[0n]|BOLD:AAB0100
Catocala semirelicta|[1692]|ABCNA446-07|United States|Michigan|585[2n]|BOLD:AAB0100
Catocala briseis|[1693]|QUNO475-08|United States|Idaho|658[0n]|BOLD:AAB0100
Catocala briseis|[1694]|QUNOC058-09|United States|Idaho|658[0n]|BOLD:AAB0100
Catocala briseis|[1695]|QUNOC059-09|United States|Idaho|658[0n]|BOLD:AAB0100
Catocala briseis|[1696]|ABCNA934-08|United States|Wisconsin|609[0n]|BOLD:AAB0100
Catocala briseis|[1697]|BBLPA323-10|Canada|Saskatchewan|658[0n]|BOLD:AAB0100
Catocala briseis|[1698]|BBLPA334-10|Canada|Saskatchewan|658[0n]|BOLD:AAB0100

Catocala briseis|[1695]|QUNOC0[illegible]-09|United States|Idaho|658[0n]|BOLD:AAB0100
Catocala briseis|[1696]|ABCNA934-08|United States|Wisconsin|609[0n]|BOLD:AAB0100
Catocala briseis|[1697]|BBLPA323-10|Canada|Saskatchewan|658[0n]|BOLD:AAB0100
Catocala briseis|[1698]|BBLPA334-10|Canada|Saskatchewan|658[0n]|BOLD:AAB0100
Catocala semirelicta|[1699]|QUNOD454-10|United States|Wisconsin|658[0n]|BOLD:AAB0100
Catocala meskei|[1700]|QUNOD473-10|United States|Arizona|658[0n]|BOLD:AAB0100
Catocala semirelicta|[1701]|LBCH3449-10|Canada|British Columbia|658[0n]|BOLD:AAB0100
Catocala semirelicta|[1702]|QUNOD340-10|United States|Minnesota|658[0n]|BOLD:AAB0100
Catocala briseis|[1703]|QUNOB299-09|United States|Wisconsin|658[0n]|BOLD:AAB0100
Catocala briseis|[1704]|QUNOB504-09|United States|Montana|658[0n]|BOLD:AAB0100
Catocala californica|[1705]|QUNOE140-11|United States|California|658[0n]|BOLD:AAB0100
Catocala californica|[1706]|QUNOE143-11|United States|California|658[0n]|BOLD:AAB0100
Catocala semirelicta|[1707]|QUNO115-08|United States|Michigan|658[0n]|BOLD:AAB0100
Catocala briseis|[1708]|ABCNA933-08|United States|Wisconsin|657[0n]|BOLD:AAB0100
Catocala semirelicta|[1709]|QUNOD672-11|United States|Minnesota|658[0n]|BOLD:AAB0100
Catocala californica|[1710]|QUNOE076-11|United States|California|658[0n]|BOLD:AAB0100
Catocala semirelicta|[1711]|QUNO114-08|United States|Michigan|658[0n]|BOLD:AAB0100
Catocala semirelicta|[1712]|QUNO113-08|United States|Michigan|658[0n]|BOLD:AAB0100
Catocala briseis|[1713]|XAK523-07|Canada|Ontario|658[0n]|BOLD:AAB0100
Catocala briseis|[1714]|RDMAB182-05|Canada|Alberta|658[0n]|BOLD:AAB0100
Catocala semirelicta|[1715]|RDMAB175-05|Canada|Alberta|658[0n]|BOLD:AAB0100
Catocala semirelicta|[1716]|RDMAB165-05|Canada|Alberta|658[0n]|BOLD:AAB0100
Catocala briseis|[1717]|RDMAB690-06|Canada|Alberta|658[0n]|BOLD:AAB0100
Catocala briseis|[1718]|BBLPA322-10|Canada|Alberta|658[0n]|BOLD:AAB0100
Catocala californica|[1719]|ABCNA028-06|United States|California|658[1n]|BOLD:AAB0100
Catocala semirelicta|[1720]|RDNMK559-11|Canada|New Brunswick|606[0n]|BOLD:AAB0100
Catocala californica|[1721]|ABCNA030-06|United States|California|605[0n]|BOLD:AAB0100
Catocala semirelicta|[1722]|ABCNA037-06|United States|Wisconsin|625[0n]|BOLD:AAB0100
Catocala semirelicta|[1723]|QUNOD453-10|United States|Wisconsin|634[0n]|BOLD:AAB0100
Catocala semirelicta|[1724]|RDNMK560-11|Canada|New Brunswick|635[0n]|BOLD:AAB0100
Catocala californica|[1725]|QUNOE144-11|United States|California|658[0n]|BOLD:AAB0100
Catocala californica|[1726]|QUNOE200-12|United States|California|658[0n]|BOLD:AAB0100
Catocala californica|[1727]|QUNOE201-12|United States|California|658[0n]|BOLD:AAB0100
Catocala californica|[1728]|QUNOE202-12|United States|California|658[0n]|BOLD:AAB0100
Catocala semirelicta|[1729]|QUNOE216-12|United States|California|658[0n]|BOLD:AAB0100
Catocala semirelicta|[1730]|QUNOE408-12|United States|Michigan|658[0n]|BOLD:AAB0100
Catocala semirelicta|[1731]|QUNOE409-12|United States|Minnesota|658[0n]|BOLD:AAB0100
Catocala briseis clarissima|[1732]|QUNOE410-12|United States|Minnesota|658[0n]|BOLD:AAB0100
Catocala californica|[1733]|QUNOE475-12|United States|California|658[0n]|BOLD:AAB0100
Catocala briseis|[1734]|NOCNA089-14|United States|Michigan|658[0n]|BOLD:AAB0100
Catocala amatrix|[1735]|XAB645-04|Canada|Ontario|658[0n]|BOLD:AAB8332
Catocala amatrix|[1736]|ABCNA197-06|United States|Wisconsin|658[0n]|BOLD:AAB8332
Catocala amatrix|[1737]|ABCNA196-06|United States|Wisconsin|658[0n]|BOLD:AAB8332
Catocala amatrix|[1738]|ABCNA201-06|United States|Indiana|592[0n]|BOLD:AAB8332
Catocala amatrix|[1739]|QUNO486-08|United States|Texas|658[0n]|BOLD:AAB8332
Catocala amatrix|[1740]|QUNO584-08|United States|Indiana|658[0n]|BOLD:AAB8332
Catocala amatrix|[1741]|QUNO649-08|United States|Louisiana|658[0n]|BOLD:AAB8332
Catocala amatrix|[1742]|RDLQB466-05|Canada|Quebec|658[0n]|BOLD:AAB8332
Catocala amatrix|[1743]|ABCNA198-06|United States|Indiana|658[0n]|BOLD:AAB8332
Catocala amatrix|[1744]|ABCNA199-06|United States|Iowa|594[0n]|BOLD:AAB8332
Catocala amatrix|[1745]|ABCNA200-06|United States|Montana|658[0n]|BOLD:AAB8332
Catocala amatrix|[1746]|QUNO594-08|United States|Indiana|658[0n]|BOLD:AAB8332
Catocala amatrix|[1747]|QUNOB309-09|United States|Wisconsin|658[0n]|BOLD:AAB8332
Catocala amatrix|[1748]|QUNOC050-09|United States|Montana|658[0n]|BOLD:AAB8332
Catocala amatrix|[1749]|QUNOC051-09|United States|Montana|658[0n]|BOLD:AAB8332
Catocala amatrix|[1750]|QUNOC052-09|United States|Montana|658[0n]|BOLD:AAB8332
Catocala messalina|[1751]|ABCNA480-07|United States|Oklahoma|568[0n]|BOLD:AAB9035
Catocala messalina|[1752]|QUNOD205-10|United States|Texas|601[0n]|BOLD:AAB9035
Catocala messalina|[1753]|QUNOD204-10|United States|Texas|658[1n]|BOLD:AAB9035
Catocala messalina|[1754]|QUNOC095-09|United States|Texas|658[0n]|BOLD:AAB9035
Catocala messalina|[1755]|QUNOD203-10|United States|Texas|631[0n]|BOLD:AAB9035
Catocala messalina|[1756]|QUNOD206-10|United States|Texas|651[0n]|BOLD:AAB9035
Catocala messalina|[1757]|ABCNA336-06|United States|Oklahoma|595[5n]|BOLD:AAB9035
Catocala messalina|[1758]|QUNO015-07|United States|Florida|658[0n]|BOLD:AAB9035
Catocala messalina|[1759]|QUNO492-08|United States|Texas|658[0n]|BOLD:AAB9035
Catocala messalina|[1760]|QUNO495-08|United States|Texas|658[0n]|BOLD:AAB9035
Catocala messalina|[1761]|QUNO496-08|United States|Texas|658[0n]|BOLD:AAB9035
Catocala messalina|[1762]|QUNO497-08|United States|Texas|658[0n]|BOLD:AAB9035
Catocala messalina|[1763]|QUNO498-08|United States|Texas|658[0n]|BOLD:AAB9035
Catocala messalina|[1764]|QUNO499-08|United States|Texas|658[0n]|BOLD:AAB9035
Catocala messalina|[1765]|QUNO503-08|United States|Texas|658[0n]|BOLD:AAB9035
Catocala messalina|[1766]|QUNO504-08|United States|Texas|658[0n]|BOLD:AAB9035
Catocala messalina|[1767]|QUNO505-08|United States|Texas|658[0n]|BOLD:AAB9035
Catocala messalina|[1768]|LNCC756-11|United States|North Carolina|658[0n]|BOLD:AAB9035
Catocala similis|[1769]|QUNOB005-08|United States|Texas|658[0n]|BOLD:ABY7551
Catocala similis|[1770]|QUNOB013-08|United States|Texas|658[0n]|BOLD:ABY7551
Catocala similis|[1771]|ABCNA524-07|United States|Florida|568[0n]|BOLD:ABY7551
Catocala similis|[1772]|ABCNA525-07|United States|Florida|568[0n]|BOLD:ABY7551
Catocala similis|[1773]|QUNOB006-08|United States|Texas|658[0n]|BOLD:ABY7551
Catocala similis|[1774]|QUNOB014-08|United States|Texas|658[0n]|BOLD:ABY7551
Catocala similis|[1775]|LNCB439-07|United States|North Carolina|658[0n]|BOLD:ABY7551
Catocala similis|[1776]|LNCB440-07|United States|North Carolina|658[0n]|BOLD:ABY7551
Catocala similis|[1777]|LNCB441-07|United States|North Carolina|658[0n]|BOLD:ABY7551
Catocala similis|[1778]|QUNOB015-08|United States|Texas|658[0n]|BOLD:ABY7551
Catocala similis|[1779]|LOFLA236-06|United States|Florida|658[0n]|BOLD:ABY7551
Catocala similis|[1780]|ABCNA265-06|United States|Florida|658[0n]|BOLD:ABY7551
Catocala similis|[1781]|QUNO453-08|United States|Florida|658[0n]|BOLD:ABY7551
Catocala similis|[1782]|QUNO454-08|United States|Florida|658[0n]|BOLD:ABY7551
Catocala similis|[1783]|LSEU031-06|United States|Florida|576[2n]|BOLD:ABY7551
Catocala similis|[1784]|LSEU032-06|United States|Florida|537[2n]|BOLD:ABY7551
Catocala similis|[1785]|LOFLA533-06|United States|Florida|658[0n]|BOLD:ABY7551
Catocala similis|[1786]|ABCNA266-06|United States|Florida|658[0n]|BOLD:ABY7551
Catocala similis|[1787]|QUNO323-08|United States|Florida|658[0n]|BOLD:ABY7551
Catocala similis|[1788]|QUNO452-08|United States|Florida|658[0n]|BOLD:ABY7551
Catocala similis|[1789]|QUNO455-08|United States|Florida|658[0n]|BOLD:ABY7551
Catocala similis|[1790]|QUNO456-08|United States|Florida|658[0n]|BOLD:ABY7551
Catocala similis|[1791]|BBLOB1672-11|United States|Florida|658[0n]|BOLD:ABY7551
Catocala connubialis|[1792]|QUNOB065-08|United States|Texas|658[0n]|BOLD:ACE7219
Catocala connubialis|[1793]|ABCNA092-06|United States|Florida|656[0n]|BOLD:ACE7219
Catocala connubialis|[1794]|ABCNA346-06|United States|Kentucky|658[0n]|BOLD:ACE7219
Catocala connubialis|[1795]|QUNO315-08|United States|Florida|658[0n]|BOLD:ACE7219
Catocala connubialis|[1796]|QUNOB163-08|United States|Florida|658[0n]|BOLD:ACE7219
Catocala connubialis|[1797]|QUNOB164-08|United States|Florida|658[0n]|BOLD:ACE7219
Catocala connubialis|[1798]|QUNOB166-08|United States|Florida|658[0n]|BOLD:ACE7219

Catocala connubialis|[1795]|QUNO515-08|United States|Florida|658[0n]|BOLD:ACE7219
Catocala connubialis|[1796]|QUNOB163-08|United States|Florida|658[0n]|BOLD:ACE7219
Catocala connubialis|[1797]|QUNOB164-08|United States|Florida|658[0n]|BOLD:ACE7219
Catocala connubialis|[1798]|QUNOB166-08|United States|Florida|658[0n]|BOLD:ACE7219
Catocala connubialis|[1799]|LNCC052-10|United States|North Carolina|658[0n]|BOLD:ACE7219
Catocala connubialis|[1800]|LNCC110-10|United States|North Carolina|658[0n]|BOLD:ACE7219
Catocala connubialis|[1801]|QUNOB024-08|United States|Louisiana|658[0n]|BOLD:ACE7219
Catocala connubialis|[1802]|QUNOB165-08|United States|Florida|658[0n]|BOLD:ACE7219
Catocala connubialis|[1803]|QUNOB167-08|United States|Florida|658[0n]|BOLD:ACE7219
Catocala connubialis|[1804]|LNCC111-10|United States|North Carolina|658[0n]|BOLD:ACE7219
Catocala connubialis|[1805]|LNCC711-11|United States|North Carolina|658[0n]|BOLD:ACE7219
Catocala micronympha|[1806]|QUNO530-08|United States|Texas|658[0n]|BOLD:AAB3488
Catocala micronympha|[1807]|LPOKB969-09|United States|Oklahoma|658[0n]|BOLD:AAB3488
Catocala micronympha|[1808]|ABCNA281-06|United States|Florida|658[0n]|BOLD:AAB3488
Catocala micronympha|[1809]|ABCNA276-06|United States|Florida|658[0n]|BOLD:AAB3488
Catocala micronympha|[1810]|ABCNA578-07|United States|Florida|573[0n]|BOLD:AAB3488
Catocala micronympha|[1811]|ABCNA587-07|United States|Florida|564[0n]|BOLD:AAB3488
Catocala micronympha|[1812]|QUNO457-08|United States|Florida|658[0n]|BOLD:AAB3488
Catocala micronympha|[1813]|BBLOB1541-11|United States|Florida|658[0n]|BOLD:AAB3488
Catocala micronympha|[1814]|LSEU596-06|United States|Georgia|658[0n]|BOLD:AAB3488
Catocala micronympha|[1815]|ABCNA529-07|United States|Florida|560[0n]|BOLD:AAB3488
Catocala micronympha|[1816]|QUNO681-08|United States|Kentucky|658[0n]|BOLD:AAB3488
Catocala micronympha|[1817]|QUNO682-08|United States|Kentucky|658[0n]|BOLD:AAB3488
Catocala micronympha|[1818]|LPOKB993-09|United States|Oklahoma|658[0n]|BOLD:AAB3488
Catocala micronympha|[1819]|QUNOB099-08|United States|Louisiana|658[0n]|BOLD:AAB3488
Catocala micronympha|[1820]|QUNOB009-08|United States|Texas|658[0n]|BOLD:AAB3488
Catocala micronympha|[1821]|QUNOB007-08|United States|Texas|658[0n]|BOLD:AAB3488
Catocala micronympha|[1822]|QUNO735-08|United States|Kentucky|658[0n]|BOLD:AAB3488
Catocala micronympha|[1823]|QUNO388-08|United States|Florida|658[0n]|BOLD:AAB3488
Catocala micronympha|[1824]|QUNO381-08|United States|Florida|658[0n]|BOLD:AAB3488
Catocala micronympha|[1825]|ABCNA282-06|United States|Florida|658[0n]|BOLD:AAB3488
Catocala micronympha|[1826]|ABCNA278-06|United States|Florida|658[0n]|BOLD:AAB3488
Catocala micronympha|[1827]|ABCNA277-06|United States|Florida|658[0n]|BOLD:AAB3488
Catocala micronympha|[1828]|ABCNA091-06|United States|Florida|656[0n]|BOLD:AAB3488
Catocala micronympha|[1829]|LSEU597-06|United States|Georgia|658[0n]|BOLD:AAB3488
Catocala micronympha|[1830]|LOT526-04|United States|Tennessee|658[0n]|BOLD:AAB3488
Catocala micronympha|[1831]|LOT249-04|United States|Tennessee|609[0n]|BOLD:AAB3488
Catocala micronympha|[1832]|JRLAA025-09|United States|Alabama|632[0n]|BOLD:AAB3488
Catocala micronympha|[1833]|LNCC696-11|United States|North Carolina|658[0n]|BOLD:AAB3488
Catocala micronympha|[1834]|BBLOC194-11|United States|Florida|658[0n]|BOLD:AAB3488
Catocala innubens|[1835]|PHMO320-03|Canada|Ontario|639[0n]|BOLD:AAC3067
Catocala innubens|[1836]|QUNOB130-08|United States|Wisconsin|658[1n]|BOLD:AAC3067
Catocala innubens|[1837]|ABCNA108-06|United States|Wisconsin|658[0n]|BOLD:AAC3067
Catocala innubens|[1838]|ABCNA107-06|United States|Wisconsin|658[0n]|BOLD:AAC3067
Catocala innubens|[1839]|XAK294-06|Canada|Ontario|658[0n]|BOLD:AAC3067
Catocala innubens|[1840]|LGSM635-04|United States|Tennessee|609[0n]|BOLD:AAC3067
Catocala innubens|[1841]|LGSM737-04|United States|Tennessee|572[1n]|BOLD:AAC3067
Catocala innubens|[1842]|RDLQB473-05|Canada|Quebec|658[0n]|BOLD:AAC3067
Catocala innubens|[1843]|ABCNA109-06|United States|Wisconsin|658[0n]|BOLD:AAC3067
Catocala innubens|[1844]|QUNO632-08|United States|Mississippi|658[0n]|BOLD:AAC3067
Catocala innubens|[1845]|QUNOB204-08|United States|Indiana|658[0n]|BOLD:AAC3067
Catocala innubens|[1846]|QUNOB205-08|United States|Indiana|658[0n]|BOLD:AAC3067
Catocala umbrosa|[1847]|RDNMD486-06|United States|Florida|559[2n]|BOLD:AAB0903
Catocala ilia|[1848]|LOT237-04|United States|Tennessee|599[0n]|BOLD:AAB0903
Catocala ilia|[1849]|LOT235-04|United States|Tennessee|609[0n]|BOLD:AAB0903
Catocala ilia|[1850]|LPOKB981-09|United States|Oklahoma|658[0n]|BOLD:AAB0903
Catocala ilia|[1851]|QUNOB036-08|United States|Indiana|658[0n]|BOLD:AAB0903
Catocala ilia|[1852]|QUNO800-08|United States|Wisconsin|658[0n]|BOLD:AAB0903
Catocala ilia|[1853]|ABCNA160-06|United States|Wisconsin|658[0n]|BOLD:AAB0903
Catocala ilia|[1854]|RDNMB026-05|Canada|Ontario|658[0n]|BOLD:AAB0903
Catocala ilia|[1855]|LOT236-04|United States|Tennessee|658[0n]|BOLD:AAB0903
Catocala ilia|[1856]|RDLQB485-05|Canada|Quebec|658[0n]|BOLD:AAB0903
Catocala ilia|[1857]|RDLQB476-05|Canada|Quebec|658[0n]|BOLD:AAB0903
Catocala ilia|[1858]|ABCNA156-06|United States|Virginia|632[0n]|BOLD:AAB0903
Catocala ilia|[1859]|RDLQB471-05|Canada|Quebec|616[0n]|BOLD:AAB0903
Catocala ilia|[1860]|BBLPC575-09|Canada|Nova Scotia|638[0n]|BOLD:AAB0903
Catocala ilia|[1861]|BBLOB1131-11|United States|Florida|658[0n]|BOLD:AAB0903
Catocala umbrosa|[1862]|RDNMD489-06|United States|Florida|658[0n]|BOLD:AAB0903
Catocala umbrosa|[1863]|RDNMD488-06|United States|Florida|658[0n]|BOLD:AAB0903
Catocala umbrosa|[1864]|ABCNA166-06|United States|Texas|658[0n]|BOLD:AAB0903
Catocala ilia|[1865]|LOFLA239-06|United States|Florida|658[0n]|BOLD:AAB0903
Catocala umbrosa|[1866]|LOFLB119-06|United States|Florida|658[0n]|BOLD:AAB0903
Catocala umbrosa|[1867]|ABCNA164-06|United States|Florida|605[0n]|BOLD:AAB0903
Catocala umbrosa|[1868]|ABCNA167-06|United States|Florida|658[0n]|BOLD:AAB0903
Catocala umbrosa|[1869]|ABCNA427-07|United States|Florida|621[0n]|BOLD:AAB0903
Catocala umbrosa|[1870]|LPMNB308-09|Canada|Manitoba|658[0n]|BOLD:AAB0903
Catocala umbrosa|[1871]|QUNOD698-11|United States|North Carolina|658[0n]|BOLD:AAB0903
Catocala umbrosa|[1872]|BBLOB223-11|United States|Florida|658[0n]|BOLD:AAB0903
Catocala umbrosa|[1873]|QUNOB037-08|United States|Texas|658[1n]|BOLD:AAB0903
Catocala umbrosa|[1874]|QUNOB112-08|United States|Louisiana|658[0n]|BOLD:AAB0903
Catocala umbrosa|[1875]|BBLOB1523-11|United States|Florida|658[0n]|BOLD:AAB0903
Catocala umbrosa|[1876]|BBLOB1640-11|United States|Florida|658[0n]|BOLD:AAB0903
Catocala ilia|[1877]|RDNMB025-05|United States|Oregon|541[0n]|BOLD:AAB0903
Catocala ilia|[1878]|BBLOB1643-11|United States|Florida|658[2n]|BOLD:AAB0903
Catocala ilia|[1879]|ABCNA162-06|United States|Utah|608[0n]|BOLD:AAB0903
Catocala ilia|[1880]|LNCC1287-11|United States|North Carolina|658[0n]|BOLD:AAB0903
Catocala ilia|[1881]|CMAZA842-10|United States|Arizona|658[0n]|BOLD:AAB0903
Catocala pr. umbrosa|[1882]|LPOKD837-10|United States|Oklahoma|658[0n]|BOLD:AAB0903
Catocala umbrosa|[1883]|ABCNA940-08|United States|Florida|658[0n]|BOLD:AAB0903
Catocala ilia|[1884]|JBAZ146-09|United States|Arizona|658[0n]|BOLD:AAB0903
Catocala ilia|[1885]|RDNMD487-06|United States|Florida|658[0n]|BOLD:AAB0903
Catocala ilia|[1886]|LSUSA121-06|United States|Kentucky|658[0n]|BOLD:AAB0903
Catocala ilia|[1887]|LOT234-04|United States|Tennessee|609[0n]|BOLD:AAB0903
Catocala ilia|[1888]|QUNOB122-08|United States|Kentucky|658[0n]|BOLD:AAB0903
Catocala ilia|[1889]|QUNOB121-08|United States|Kentucky|658[0n]|BOLD:AAB0903
Catocala ilia|[1890]|ABCNA505-07|United States|Utah|568[0n]|BOLD:AAB0903
Catocala ilia|[1891]|BBLOC971-11|United States|Texas|622[0n]|BOLD:AAB0903
Catocala ilia|[1892]|LOCBE182-06|United States|California|629[1n]|BOLD:AAB0903
Catocala ilia|[1893]|ABCNA161-06|United States|Utah|608[0n]|BOLD:AAB0903
Catocala ilia|[1894]|ABCNA163-06|United States|Utah|608[0n]|BOLD:AAB0903
Catocala ilia|[1895]|ABCNA417-07|United States|Florida|621[0n]|BOLD:AAB0903
Catocala umbrosa|[1896]|ABCNA425-07|United States|Texas|621[0n]|BOLD:AAB0903
Catocala umbrosa|[1897]|QUNOB038-08|United States|Texas|658[0n]|BOLD:AAB0903
[illegible]

Catocala ilia|[1895]|ABCNA417-07|United States|Florida|621[0n]|BOLD:AAB0903
Catocala umbrosa|[1896]|ABCNA425-07|United States|Texas|621[0n]|BOLD:AAB0903
Catocala umbrosa|[1897]|QUNOB038-08|United States|Texas|658[0n]|BOLD:AAB0903
Catocala ilia|[1898]|ABCNA157-06|United States|Texas|617[0n]|BOLD:AAB0903
Catocala ilia|[1899]|ABCNA159-06|United States|Florida|608[0n]|BOLD:AAB0903
Catocala ilia|[1900]|ABCNA418-07|United States|Florida|621[0n]|BOLD:AAB0903
Catocala ilia|[1901]|BBLWU291-09|United States|Texas|658[0n]|BOLD:AAB0903
Catocala ilia|[1902]|QUNOD386-10|United States|Florida|658[0n]|BOLD:AAB0903
Catocala pr. umbrosa|[1903]|LNCC1288-11|United States|North Carolina|658[0n]|BOLD:AAB0903
Catocala cerogama|[1904]|RDLQB570-05|Canada|Quebec|571[0n]|BOLD:AAB3383
Catocala cerogama|[1905]|XAK249-06|Canada|Ontario|643[0n]|BOLD:AAB3383
Catocala cerogama|[1906]|XAG755-05|Canada|Ontario|636[0n]|BOLD:AAB3383
Catocala cerogama|[1907]|RDLQB480-05|Canada|Quebec|658[0n]|BOLD:AAB3383
Catocala cerogama|[1908]|RDLQB479-05|Canada|Quebec|658[0n]|BOLD:AAB3383
Catocala cerogama|[1909]|RDLQB478-05|Canada|Quebec|658[0n]|BOLD:AAB3383
Catocala cerogama|[1910]|XAH174-05|Canada|Ontario|658[0n]|BOLD:AAB3383
Catocala cerogama|[1911]|XAG871-05|Canada|Ontario|658[0n]|BOLD:AAB3383
Catocala cerogama|[1912]|XAG661-05|Canada|Ontario|658[0n]|BOLD:AAB3383
Catocala cerogama|[1913]|XAG310-05|Canada|Ontario|658[0n]|BOLD:AAB3383
Catocala cerogama|[1914]|RDLQB486-05|Canada|Quebec|658[0n]|BOLD:AAB3383
Catocala cerogama|[1915]|QUNO793-08|United States|Wisconsin|658[0n]|BOLD:AAB3383
Catocala cerogama|[1916]|QUNO794-08|United States|Wisconsin|658[0n]|BOLD:AAB3383
Catocala cerogama|[1917]|QUNO792-08|United States|Wisconsin|658[0n]|BOLD:AAB3383
Catocala cerogama|[1918]|QUNO791-08|United States|Wisconsin|658[0n]|BOLD:AAB3383
Catocala cerogama|[1919]|QUNO790-08|United States|Wisconsin|658[0n]|BOLD:AAB3383
Catocala cerogama|[1920]|QUNO031-07|United States|Wisconsin|658[0n]|BOLD:AAB3383
Catocala cerogama|[1921]|ABCNA194-06|United States|Wisconsin|658[0n]|BOLD:AAB3383
Catocala cerogama|[1922]|ABCNA193-06|United States|Wisconsin|658[0n]|BOLD:AAB3383
Catocala cerogama|[1923]|XAK270-06|Canada|Ontario|658[0n]|BOLD:AAB3383
Catocala cerogama|[1924]|RDLQB477-05|Canada|Quebec|658[0n]|BOLD:AAB3383
Catocala cerogama|[1925]|XAH293-05|Canada|Ontario|658[0n]|BOLD:AAB3383
Catocala cerogama|[1926]|RDLQB481-05|Canada|Quebec|658[0n]|BOLD:AAB3383
Catocala cerogama|[1927]|XAG422-05|Canada|Ontario|658[0n]|BOLD:AAB3383
Catocala cerogama|[1928]|LPSOD1070-09|Canada|Ontario|631[0n]|BOLD:AAB3383
Catocala relicta|[1929]|RDBBC377-05|Canada|British Columbia|594[13n]|
Catocala relicta|[1930]|XAD179-04|Canada|Ontario|564[0n]|BOLD:AAB3451
Catocala relicta|[1931]|RDLQB451-05|Canada|Quebec|591[0n]|BOLD:AAB3451
Catocala relicta|[1932]|XAH235-05|Canada|Ontario|623[0n]|BOLD:AAB3451
Catocala relicta|[1933]|PHMO333-03|Canada|Ontario|639[0n]|BOLD:AAB3451
Catocala relicta|[1934]|QUNO068-07|United States|Wisconsin|658[0n]|BOLD:AAB3451
Catocala relicta|[1935]|IMA003-07|United States|Massachusetts|658[0n]|BOLD:AAB3451
Catocala relicta|[1936]|XAG757-05|Canada|Ontario|658[1n]|BOLD:AAB3451
Catocala relicta|[1937]|XAB652-04|Canada|Ontario|658[0n]|BOLD:AAB3451
Catocala relicta|[1938]|XAG659-05|Canada|Ontario|650[0n]|BOLD:AAB3451
Catocala relicta|[1939]|XAH146-05|Canada|Ontario|658[0n]|BOLD:AAB3451
Catocala relicta|[1940]|XAH236-05|Canada|Ontario|658[0n]|BOLD:AAB3451
Catocala relicta|[1941]|XAH331-05|Canada|Ontario|658[0n]|BOLD:AAB3451
Catocala relicta|[1942]|XAH341-05|Canada|Ontario|658[0n]|BOLD:AAB3451
Catocala relicta|[1943]|RDLQB452-05|Canada|Quebec|658[0n]|BOLD:AAB3451
Catocala relicta|[1944]|ABCNA202-06|United States|Wisconsin|658[0n]|BOLD:AAB3451
Catocala relicta|[1945]|QUNO782-08|United States|Wisconsin|658[0n]|BOLD:AAB3451
Catocala relicta|[1946]|QUNO783-08|United States|Wisconsin|658[0n]|BOLD:AAB3451
Catocala relicta|[1947]|QUNOB308-09|United States|Wisconsin|658[0n]|BOLD:AAB3451
Catocala relicta|[1948]|LPMNB306-09|Canada|Manitoba|658[0n]|BOLD:AAB3451
Catocala relicta|[1949]|LPMNB307-09|Canada|Manitoba|658[0n]|BOLD:AAB3451
Catocala relicta|[1950]|LPMNB500-09|Canada|Manitoba|658[0n]|BOLD:AAB3451
Catocala relicta|[1951]|QUNOC053-09|United States|Montana|658[0n]|BOLD:AAB3451
Catocala relicta|[1952]|QUNOC055-09|United States|Montana|658[0n]|BOLD:AAB3451
Catocala relicta|[1953]|QUNOC319-10|United States|Idaho|658[0n]|BOLD:AAB3451
Catocala relicta|[1954]|BBLPA325-10|Canada|Saskatchewan|658[0n]|BOLD:AAB3451
Catocala relicta|[1955]|BBLPA326-10|Canada|Saskatchewan|658[0n]|BOLD:AAB3451
Catocala relicta|[1956]|QUNOE153-11|United States|California|658[0n]|BOLD:AAB3451
Catocala relicta|[1957]|CNRMD2428-12|Canada|Manitoba|632[0n]|BOLD:AAB3451
Catocala relicta|[1958]|CNRMG780-12|Canada|Manitoba|632[0n]|BOLD:AAB3451
Catocala cara|[1959]|QUNOD382-10|United States|Florida|658[0n]|BOLD:ABZ7619
Catocala cara|[1960]|QUNOD381-10|United States|Florida|604[0n]|BOLD:ABZ7619
Catocala cara|[1961]|QUNO156-08|United States|Florida|642[0n]|BOLD:ABZ7619
Catocala cara|[1962]|QUNO159-08|United States|Florida|658[0n]|BOLD:ABZ7619
Catocala cara|[1963]|QUNO160-08|United States|Florida|658[0n]|BOLD:ABZ7619
Catocala cara|[1964]|QUNOD383-10|United States|Florida|658[0n]|BOLD:ABZ7619
Catocala cara|[1965]|QUNOD384-10|United States|Florida|658[0n]|BOLD:ABZ7619
Catocala cara|[1966]|QUNO157-08|United States|Florida|658[0n]|BOLD:ABZ7619
Catocala cara|[1967]|QUNO158-08|United States|Florida|658[0n]|BOLD:ABZ7619
Catocala cara|[1968]|QUNOD385-10|United States|Florida|658[0n]|BOLD:ABZ7619
Catocala cara|[1969]|ABCNA051-06|United States|Wisconsin|610[0n]|BOLD:ABZ7619
Catocala cara|[1970]|PHMO353-03|Canada|Ontario|639[5n]|BOLD:ABZ7619
Catocala cara|[1971]|QUNO562-08|United States|Indiana|658[0n]|BOLD:ABZ7619
Catocala cara|[1972]|XAB465-04|Canada|Ontario|658[0n]|BOLD:ABZ7619
Catocala cara|[1973]|QUNO029-07|United States|Wisconsin|658[0n]|BOLD:ABZ7619
Catocala cara|[1974]|QUNO030-07|United States|Wisconsin|658[0n]|BOLD:ABZ7619
Catocala cara|[1975]|IMA001-07|United States|Massachusetts|658[0n]|BOLD:ABZ7619
Catocala cara|[1976]|XAH084-05|Canada|Ontario|621[0n]|BOLD:ABZ7619
Catocala cara|[1977]|ABCNA052-06|United States|Indiana|658[0n]|BOLD:ABZ7619
Catocala cara|[1978]|QUNO563-08|United States|Indiana|658[0n]|BOLD:ABZ7619
Catocala cara|[1979]|QUNOD528-10|United States|Wisconsin|658[0n]|BOLD:ABZ7619
Catocala cara|[1980]|QUNOD699-11|United States|Georgia|658[0n]|BOLD:ABZ7619
Catocala cara|[1981]|JSAUG1674-11|Canada|Ontario|658[0n]|BOLD:ABZ7619
Catocala carissima|[1982]|ABCNA349-06|United States|Florida|645[0n]|BOLD:ACF5142
Catocala carissima|[1983]|ABCNA360-06|United States|Florida|658[0n]|BOLD:ACF5142
Catocala carissima|[1984]|QUNO654-08|United States|Mississippi|658[0n]|BOLD:ACF5142
Catocala carissima|[1985]|ABCNA192-06|United States|Florida|658[0n]|BOLD:ACF5142
Catocala carissima|[1986]|ABCNA348-06|United States|Florida|658[0n]|BOLD:ACF5142
Catocala carissima|[1987]|ABCNA054-06|United States|Texas|658[0n]|BOLD:ACF5142
Catocala carissima|[1988]|ABCNA053-06|United States|Texas|658[0n]|BOLD:ACF5142
Catocala carissima|[1989]|ABCNA350-06|United States|Florida|636[0n]|BOLD:ACF5142
Catocala carissima|[1990]|ABCNA351-06|United States|Florida|658[0n]|BOLD:ACF5142
Catocala carissima|[1991]|QUNO161-08|United States|Florida|658[0n]|BOLD:ACF5142
Catocala carissima|[1992]|QUNO162-08|United States|Florida|658[0n]|BOLD:ACF5142
Catocala carissima|[1993]|QUNO163-08|United States|Florida|658[0n]|BOLD:ACF5142
Catocala carissima|[1994]|QUNO557-08|United States|Indiana|658[0n]|BOLD:ACF5142
Catocala carissima|[1995]|QUNO558-08|United States|Indiana|658[0n]|BOLD:ACF5142
Catocala carissima|[1996]|QUNO561-08|United States|Indiana|658[0n]|BOLD:ACF5142
Catocala carissima|[1997]|QUNOB203-08|United States|Indiana|658[0n]|BOLD:ACF5142

Catocala carissima|[1995]|QUNO558-08|United States|Indiana|658[0n]|BOLD:ACF5142
Catocala carissima|[1996]|QUNO561-08|United States|Indiana|658[0n]|BOLD:ACF5142
Catocala carissima|[1997]|QUNOB203-08|United States|Indiana|658[0n]|BOLD:ACF5142
Catocala carissima|[1998]|QUNOB550-09|United States|Indiana|658[0n]|BOLD:ACF5142
Catocala carissima|[1999]|LNCC1424-11|United States|North Carolina|658[0n]|BOLD:ACF5142
Catocala concumbens|[2000]|PHMNB275-04|Canada|New Brunswick|658[0n]|BOLD:AAB6567
Catocala concumbens|[2001]|PHMO313-03|Canada|Ontario|639[0n]|BOLD:AAB6567
Catocala concumbens|[2002]|XAH053-05|Canada|Ontario|658[0n]|BOLD:AAB6567
Catocala concumbens|[2003]|RDLQB565-05|Canada|Quebec|535[0n]|BOLD:AAB6567
Catocala concumbens|[2004]|PHMNB274-04|Canada|New Brunswick|658[0n]|BOLD:AAB6567
Catocala concumbens|[2005]|PHMNB273-04|Canada|New Brunswick|658[0n]|BOLD:AAB6567
Catocala concumbens|[2006]|ABCNA457-07|United States|Wisconsin|576[0n]|BOLD:AAB6567
Catocala concumbens|[2007]|QUNO799-08|United States|Wisconsin|658[0n]|BOLD:AAB6567
Catocala concumbens|[2008]|QUNOB175-08|United States|Wisconsin|658[0n]|BOLD:AAB6567
Catocala concumbens|[2009]|LNCC364-10|United States|North Carolina|658[0n]|BOLD:AAB6567
Catocala concumbens|[2010]|BBLEC457-09|Canada|New Brunswick|658[0n]|BOLD:AAB6567
Catocala concumbens|[2011]|QUNOB174-08|United States|Wisconsin|658[0n]|BOLD:AAB6567
Catocala concumbens|[2012]|QUNOB173-08|United States|Wisconsin|658[0n]|BOLD:AAB6567
Catocala concumbens|[2013]|QUNOB172-08|United States|Wisconsin|658[0n]|BOLD:AAB6567
Catocala concumbens|[2014]|RDLQF804-06|Canada|Quebec|657[0n]|BOLD:AAB6567
Catocala concumbens|[2015]|ABCNA195-06|United States|Wisconsin|658[0n]|BOLD:AAB6567
Catocala concumbens|[2016]|RDLQB491-05|Canada|Quebec|658[0n]|BOLD:AAB6567
Catocala concumbens|[2017]|RDLQB472-05|Canada|Quebec|658[0n]|BOLD:AAB6567
Catocala concumbens|[2018]|CNRMG781-12|Canada|Manitoba|633[0n]|BOLD:AAB6567
Catocala minuta|[2019]|ABCNA353-06|United States|Florida|658[0n]|BOLD:AAD7106
Catocala minuta|[2020]|XAK046-06|Canada|Ontario|658[0n]|BOLD:AAD7106
Catocala minuta|[2021]|ABCNA363-06|United States|Florida|658[0n]|BOLD:AAD7106
Catocala minuta|[2022]|QUNO048-07|United States|Wisconsin|658[0n]|BOLD:AAD7106
Catocala minuta|[2023]|QUNOD461-10|United States|Wisconsin|658[0n]|BOLD:AAD7106
Catocala minuta|[2024]|QUNOD462-10|United States|Wisconsin|658[0n]|BOLD:AAD7106
Catocala minuta|[2025]|ABCNA268-06|United States|Texas|658[0n]|BOLD:AAD7106
Catocala minuta|[2026]|ABCNA288-06|United States|Florida|658[0n]|BOLD:AAD7106
Catocala minuta|[2027]|LILLA352-11|United States|Illinois|658[0n]|BOLD:AAD7106
Catocala aholibah|[2028]|ABCNA302-06|United States|Utah|596[0n]|BOLD:AAE9541
Catocala aholibah|[2029]|ABCNA300-06|United States|Utah|658[0n]|BOLD:AAE9541
Catocala aholibah|[2030]|ABCNA301-06|United States|Utah|658[0n]|BOLD:AAE9541
Catocala aholibah|[2031]|RDNMJ287-11|United States|Arizona|658[0n]|BOLD:AAE9541
Catocala aholibah|[2032]|LTOLB1333-11|United States|California|658[0n]|BOLD:AAE9541
Catocala aholibah|[2033]|ABCNA303-06|United States|California|658[0n]|BOLD:AAE9541
Catocala aholibah|[2034]|QUNOE163-11|United States|California|658[0n]|BOLD:AAE9541
Catocala aholibah|[2035]|GMLC1053-12|United States|California|658[0n]|BOLD:AAE9541
Catocala herodias|[2036]|ABCNA071-06|United States|Oklahoma|649[0n]|BOLD:AAE4372
Catocala herodias|[2037]|ABCNA072-06|United States|Oklahoma|658[0n]|BOLD:AAE4372
Catocala herodias|[2038]|ABCNA338-06|United States|Oklahoma|658[0n]|BOLD:AAE4372
Catocala herodias|[2039]|QUNOD138-10|United States|Oklahoma|658[0n]|BOLD:AAE4372
Catocala herodias gerhardi|[2040]|QUNO009-07|United States|New Jersey|658[0n]|BOLD:AAE4372
Catocala herodias|[2041]|IMA006-07|United States|Massachusetts|659[0n]|BOLD:AAE4372
Catocala herodias gerhardi|[2042]|QUNOC324-10|United States|New Jersey|658[0n]|BOLD:AAE4372
Catocala ophelia|[2043]|ABCNA230-06|United States|California|658[0n]|BOLD:ABZ8153
Catocala ophelia|[2044]|ABCNA231-06|United States|California|658[0n]|BOLD:ABZ8153
Catocala ophelia|[2045]|ABCNA232-06|United States|California|658[0n]|BOLD:ABZ8153
Catocala ophelia|[2046]|QUNOE159-11|United States|California|658[0n]|BOLD:ABZ8153
Catocala violenta|[2047]|RDNMJ562-11|United States|Arizona|658[0n]|BOLD:AAF2061
Catocala violenta|[2048]|RDNMJ288-11|United States|Arizona|658[0n]|BOLD:AAF2061
Catocala violenta|[2049]|DMAZ251-10|United States|Arizona|658[0n]|BOLD:AAF2061
Catocala violenta|[2050]|CMAZA092-09|United States|Arizona|658[0n]|BOLD:AAF2061
Catocala violenta|[2051]|QUNOB562-09|United States|Arizona|658[0n]|BOLD:AAF2061
Catocala violenta|[2052]|ABCNA229-06|United States|Arizona|658[0n]|BOLD:AAF2061
Catocala violenta|[2053]|ABCNA228-06|United States|Arizona|658[0n]|BOLD:AAF2061
Catocala violenta|[2054]|QUNOE165-11|United States|Arizona|658[0n]|BOLD:AAF2061
Catocala verrilliana|[2055]|QUNOD152-10|United States|Texas|658[0n]|BOLD:ACF1300
Catocala verrilliana|[2056]|QUNOD027-10|United States|Texas|658[0n]|BOLD:ACF1300
Catocala verrilliana|[2057]|QUNOD026-10|United States|Texas|658[0n]|BOLD:ACF1300
Catocala verrilliana|[2058]|ABCNA917-08|United States|New Mexico|658[0n]|BOLD:ACF1300
Catocala verrilliana|[2059]|ABCNA916-08|United States|New Mexico|609[0n]|BOLD:ACF1300
Catocala verrilliana|[2060]|ABCNA919-08|United States|New Mexico|609[0n]|BOLD:ACF1300
Catocala verrilliana|[2061]|ABCNA921-08|United States|Texas|658[0n]|BOLD:ACF1300
Catocala verrilliana|[2062]|QUNOD208-10|United States|Texas|557[0n]|BOLD:ACF1300
Catocala verrilliana|[2063]|BBLSY925-09|United States|Texas|658[0n]|BOLD:ACF1301
Catocala verrilliana|[2064]|BBLSX759-09|United States|Texas|658[0n]|BOLD:ACF1301
Catocala verrilliana|[2065]|QUNOD209-10|United States|Texas|614[0n]|BOLD:ACF1301
Catocala verrilliana|[2066]|QUNOE158-11|United States|Utah|658[0n]|BOLD:AAA7092
Catocala verrilliana|[2067]|QUNOE157-11|United States|Utah|658[0n]|BOLD:AAA7092
Catocala verrilliana|[2068]|ABCNA495-07|United States|Utah|549[0n]|BOLD:AAA7092
Catocala verrilliana|[2069]|ABCNA920-08|United States|Utah|609[0n]|BOLD:AAA7092
Catocala verrilliana|[2070]|QUNOE183-11|United States|Arizona|658[0n]|BOLD:AAA7092
Catocala verrilliana|[2071]|QUNOD664-10|United States|Arizona|658[0n]|BOLD:AAA7092
Catocala verrilliana|[2072]|QUNOC040-09|United States|Arizona|610[0n]|BOLD:AAA7092
Catocala verrilliana|[2073]|QUNOE559-12|United States|Utah|658[0n]|BOLD:AAA7092
Catocala verrilliana|[2074]|QUNOE560-12|United States|Utah|658[0n]|BOLD:AAA7092
Catocala verrilliana|[2075]|QUNOE155-11|United States|Utah|658[0n]|BOLD:AAA7091
Catocala verrilliana|[2076]|QUNOE156-11|United States|Utah|658[0n]|BOLD:AAA7091
Catocala verrilliana|[2077]|LOCBE189-06|United States|California|658[0n]|BOLD:AAA7091
Catocala verrilliana|[2078]|LOCBE197-06|United States|California|645[0n]|BOLD:AAA7091
Catocala verrilliana|[2079]|LOCBE203-06|United States|California|626[1n]|BOLD:AAA7091
Catocala verrilliana|[2080]|LOCBE208-06|United States|California|658[1n]|BOLD:AAA7091
Catocala verrilliana|[2081]|QUNOE558-12|United States|California|658[0n]|BOLD:AAA7091
Catocala verrilliana|[2082]|QUNOE557-12|United States|California|658[0n]|BOLD:AAA7091
Catocala verrilliana|[2083]|QUNOE451-12|United States|California|658[0n]|BOLD:AAA7091
Catocala verrilliana|[2084]|ABCNA918-08|United States|California|658[0n]|BOLD:AAA7091
Catocala verrilliana|[2085]|LOCBE320-06|United States|California|658[0n]|BOLD:AAA7091
Catocala verrilliana|[2086]|LOCBE319-06|United States|California|658[0n]|BOLD:AAA7091
Catocala verrilliana|[2087]|LOCBE318-06|United States|California|658[0n]|BOLD:AAA7091
Catocala verrilliana|[2088]|LOCBE306-06|United States|California|658[0n]|BOLD:AAA7091
Catocala verrilliana|[2089]|LOCBE210-06|United States|California|658[0n]|BOLD:AAA7091
Catocala verrilliana|[2090]|LOCBE209-06|United States|California|658[0n]|BOLD:AAA7091
Catocala verrilliana|[2091]|LOCBE204-06|United States|California|658[0n]|BOLD:AAA7091
Catocala verrilliana|[2092]|LOCBE202-06|United States|California|658[0n]|BOLD:AAA7091
Catocala verrilliana|[2093]|LOCBE200-06|United States|California|658[0n]|BOLD:AAA7091
Catocala verrilliana|[2094]|LOCBE196-06|United States|California|658[0n]|BOLD:AAA7091
Catocala verrilliana|[2095]|LOCBE194-06|United States|California|658[0n]|BOLD:AAA7091
Catocala verrilliana|[2096]|LOCBE193-06|United States|California|658[0n]|BOLD:AAA7091
Catocala verrilliana|[2097]|LOCBE192-06|United States|California|658[0n]|BOLD:AAA7091

Catocala verrilliana|[2095]|LOCBE194-06|United States|California|658[0n]|BOLD:AAA7091
Catocala verrilliana|[2096]|LOCBE193-06|United States|California|658[0n]|BOLD:AAA7091
Catocala verrilliana|[2097]|LOCBE192-06|United States|California|658[0n]|BOLD:AAA7091
Catocala verrilliana|[2098]|LOCBE188-06|United States|California|658[0n]|BOLD:AAA7091
Catocala verrilliana|[2099]|LOCBE186-06|United States|California|658[0n]|BOLD:AAA7091
Catocala verrilliana|[2100]|LOCBD059-06|United States|California|658[0n]|BOLD:AAA7091
Catocala verrilliana|[2101]|LOCBD057-06|United States|California|658[0n]|BOLD:AAA7091
Catocala verrilliana|[2102]|LOCBC871-06|United States|California|658[0n]|BOLD:AAA7091
Catocala verrilliana|[2103]|LOCBC870-06|United States|California|658[0n]|BOLD:AAA7091
Catocala verrilliana|[2104]|LOCBC869-06|United States|California|658[0n]|BOLD:AAA7091
Catocala verrilliana|[2105]|LOCBC868-06|United States|California|658[0n]|BOLD:AAA7091
Catocala verrilliana|[2106]|LOCBE190-06|United States|California|621[0n]|BOLD:AAA7091
Catocala verrilliana|[2107]|LOCBE198-06|United States|California|636[1n]|BOLD:AAA7091
Catocala verrilliana|[2108]|LOCBE323-06|United States|California|594[0n]|BOLD:AAA7091
Catocala verrilliana|[2109]|LOCBE322-06|United States|California|594[0n]|BOLD:AAA7091
Catocala verrilliana|[2110]|LOCBE321-06|United States|California|594[0n]|BOLD:AAA7091
Catocala verrilliana|[2111]|LOCBE317-06|United States|California|594[0n]|BOLD:AAA7091
Catocala verrilliana|[2112]|LOCBE316-06|United States|California|594[0n]|BOLD:AAA7091
Catocala verrilliana|[2113]|LOCBE307-06|United States|California|594[0n]|BOLD:AAA7091
Catocala verrilliana|[2114]|LOCBE211-06|United States|California|594[0n]|BOLD:AAA7091
Catocala verrilliana|[2115]|LOCBE183-06|United States|California|593[0n]|BOLD:AAA7091
Catocala verrilliana|[2116]|LOCBE184-06|United States|California|580[0n]|BOLD:AAA7091
Catocala verrilliana|[2117]|LOCBE308-06|United States|California|590[0n]|BOLD:AAA7091
Catocala verrilliana|[2118]|ABCNA494-07|United States|California|568[0n]|BOLD:AAA7091
Catocala verrilliana|[2119]|ABCNA749-07|United States|California|593[0n]|BOLD:AAA7091
Catocala verrilliana|[2120]|LOCBD056-06|United States|California|658[0n]|BOLD:AAA7091
Catocala verrilliana|[2121]|LOCBE185-06|United States|California|655[0n]|BOLD:AAA7091
Catocala verrilliana|[2122]|QUNOE561-12|United States|California|658[0n]|BOLD:AAA7091
Catocala verrilliana|[2123]|QUNOC616-13|United States|California|658[0n]|BOLD:AAA7091
Catocala coccinata sinuosa|[2124]|QUNOB010-08|United States|Texas|658[0n]|BOLD:ABZ3836
Catocala coccinata sinuosa|[2125]|QUNOC007-09|United States|Texas|658[0n]|BOLD:ABZ3836
Catocala coccinata sinuosa|[2126]|ABCNA224-06|United States|Florida|517[0n]|BOLD:ABZ3836
Catocala coccinata sinuosa|[2127]|ABCNA227-06|United States|Florida|638[1n]|BOLD:ABZ3836
Catocala coccinata sinuosa|[2128]|MILEQ190-11|United States|Florida|658[0n]|BOLD:ABZ3836
Catocala coccinata sinuosa|[2129]|ABCNA906-08|United States|Florida|656[0n]|BOLD:ABZ3836
Catocala coccinata sinuosa|[2130]|QUNO035-07|United States|Florida|658[0n]|BOLD:ABZ3836
Catocala coccinata sinuosa|[2131]|ABCNA226-06|United States|Florida|658[0n]|BOLD:ABZ3836
Catocala coccinata sinuosa|[2132]|ABCNA225-06|United States|Florida|658[0n]|BOLD:ABZ3836
Catocala coccinata sinuosa|[2133]|QUNO036-07|United States|Florida|648[0n]|BOLD:ABZ3836
Catocala coccinata sinuosa|[2134]|QUNOD855-11|United States|Florida|605[2n]|BOLD:ABZ3836
Catocala coccinata sinuosa|[2135]|QUNOC628-13|United States|Georgia|658[0n]|BOLD:ABZ3836
Catocala coccinata|[2136]|QUNO717-08|United States|Kentucky|658[0n]|BOLD:ABZ3835
Catocala coccinata|[2137]|ABCNA496-07|United States|Virginia|568[0n]|BOLD:ABZ3835
Catocala coccinata|[2138]|LGSM494-04|United States|Tennessee|597[0n]|BOLD:ABZ3835
Catocala coccinata|[2139]|LPMNB313-09|Canada|Manitoba|603[0n]|BOLD:ABZ3835
Catocala coccinata|[2140]|LGSM493-04|United States|Tennessee|614[0n]|BOLD:ABZ3835
Catocala coccinata|[2141]|ABCNA489-07|United States|Kentucky|550[0n]|BOLD:ABZ3835
Catocala coccinata|[2142]|QUNO716-08|United States|Kentucky|658[0n]|BOLD:ABZ3835
Catocala coccinata|[2143]|LOT238-04|United States|Tennessee|609[0n]|BOLD:ABZ3835
Catocala coccinata|[2144]|XAJ962-06|Canada|Ontario|658[0n]|BOLD:ABZ3835
Catocala coccinata|[2145]|LSEU595-06|United States|Georgia|658[0n]|BOLD:ABZ3835
Catocala coccinata|[2146]|QUNO032-07|United States|Florida|658[0n]|BOLD:ABZ3835
Catocala coccinata|[2147]|QUNO033-07|United States|Florida|658[0n]|BOLD:ABZ3835
Catocala coccinata|[2148]|QUNO034-07|United States|Florida|658[0n]|BOLD:ABZ3835
Catocala coccinata|[2149]|ABCNA935-08|United States|Florida|655[0n]|BOLD:ABZ3835
Catocala coccinata|[2150]|QUNO714-08|United States|Kentucky|658[0n]|BOLD:ABZ3835
Catocala coccinata|[2151]|QUNO715-08|United States|Kentucky|658[0n]|BOLD:ABZ3835
Catocala coccinata|[2152]|QUNO781-08|United States|Wisconsin|658[0n]|BOLD:ABZ3835
Catocala coccinata|[2153]|QUNOB096-08|United States|Louisiana|658[0n]|BOLD:ABZ3835
Catocala coccinata|[2154]|LPMNB302-09|Canada|Manitoba|658[0n]|BOLD:ABZ3835
Catocala coccinata|[2155]|LPMNB303-09|Canada|Manitoba|658[0n]|BOLD:ABZ3835
Catocala coccinata|[2156]|LPMNB304-09|Canada|Manitoba|658[0n]|BOLD:ABZ3835
Catocala coccinata|[2157]|LPMNB305-09|Canada|Manitoba|658[0n]|BOLD:ABZ3835
Catocala coccinata|[2158]|LPMNB309-09|Canada|Manitoba|658[0n]|BOLD:ABZ3835
Catocala coccinata|[2159]|LPMNB310-09|Canada|Manitoba|658[0n]|BOLD:ABZ3835
Catocala coccinata|[2160]|LPMNB311-09|Canada|Manitoba|658[0n]|BOLD:ABZ3835
Catocala coccinata|[2161]|LPMNB501-09|Canada|Manitoba|658[0n]|BOLD:ABZ3835
Catocala coccinata|[2162]|LNCC114-10|United States|North Carolina|658[0n]|BOLD:ABZ3835
Catocala coccinata|[2163]|LNCC1277-11|United States|North Carolina|658[0n]|BOLD:ABZ3835
Catocala coccinata|[2164]|XAJ960-06|Canada|Ontario|658[0n]|BOLD:ABZ3835
Catocala coccinata|[2165]|XAJ961-06|Canada|Ontario|658[0n]|BOLD:ABZ3835
Catocala coccinata|[2166]|QUNOC629-13|United States|Georgia|658[0n]|BOLD:ABZ3835
Catocala frederici|[2167]|ABCNA924-08|United States|Texas|609[0n]|BOLD:AAB8293
Catocala frederici|[2168]|ABCNA926-08|United States|Texas|652[0n]|BOLD:AAB8293
Catocala frederici|[2169]|ABCNA923-08|United States|Texas|632[0n]|BOLD:AAB8293
Catocala frederici|[2170]|ABCNA205-06|United States|Texas|658[0n]|BOLD:AAB8293
Catocala frederici|[2171]|ABCNA206-06|United States|Texas|658[0n]|BOLD:AAB8293
Catocala frederici|[2172]|ABCNA207-06|United States|Texas|658[0n]|BOLD:AAB8293
Catocala frederici|[2173]|ABCNA208-06|United States|Texas|658[0n]|BOLD:AAB8293
Catocala frederici|[2174]|ABCNA325-06|United States|Texas|658[0n]|BOLD:AAB8293
Catocala frederici|[2175]|ABCNA925-08|United States|Texas|649[0n]|BOLD:AAB8293
Catocala frederici|[2176]|ABCNA928-08|United States|Texas|642[0n]|BOLD:AAB8293
Catocala frederici|[2177]|ABCNA326-06|United States|Texas|582[0n]|BOLD:AAB8293
Catocala frederici|[2178]|ABCNA324-06|United States|Texas|658[0n]|BOLD:AAB8293
Catocala frederici|[2179]|ABCNA927-08|United States|Texas|632[0n]|BOLD:AAB8293
Catocala frederici|[2180]|QUNO385-08|United States|Texas|658[0n]|BOLD:AAB8293
Catocala frederici|[2181]|HKONB555-09|United States|Texas|650[0n]|BOLD:AAB8293
Catocala frederici|[2182]|HKONB516-09|United States|Texas|658[0n]|BOLD:AAB8293
Catocala frederici|[2183]|BBLSW025-09|United States|Texas|658[0n]|BOLD:AAB8293
Catocala frederici|[2184]|BBLSY923-09|United States|Texas|658[0n]|BOLD:AAB8293
Catocala frederici|[2185]|QUNOD025-10|United States|Texas|658[0n]|BOLD:AAB8293
Catocala frederici|[2186]|QUNOD139-10|United States|Oklahoma|658[0n]|BOLD:ACF4087
Catocala frederici|[2187]|QUNOD140-10|United States|Oklahoma|658[0n]|BOLD:ACF4087
Catocala frederici|[2188]|QUNOD141-10|United States|Oklahoma|658[0n]|BOLD:ACF4087
Catocala frederici|[2189]|QUNOD142-10|United States|Oklahoma|658[0n]|BOLD:ACF4087
Catocala frederici|[2190]|QUNOD143-10|United States|Oklahoma|658[0n]|BOLD:ACF4087
Catocala frederici|[2191]|QUNOD144-10|United States|Oklahoma|658[0n]|BOLD:ACF4087
Catocala caesia|[2192]|ABCNA254-06|United States|Arizona|603[0n]|BOLD:AAI7365
Catocala caesia|[2193]|ABCNA502-07|United States|Arizona|568[0n]|BOLD:AAI7365
Catocala caesia|[2194]|QUNOD361-10|United States|Arizona|658[0n]|BOLD:AAI7365
Catocala benjamini mayhewi|[2195]|QUNOE519-12|United States|California|658[0n]|BOLD:ABZ6686
Catocala benjamini mayhewi|[2196]|QUNOE526-12|United States|California|658[0n]|BOLD:ABZ6686
Catocala benjamini mayhewi|[2197]|QUNOE527-12|United States|California|658[0n]|BOLD:ABZ6686

Catocala benjamini mayhewi|[2195]|QUNOE519-12|United States|California|658[0n]|BOLD:ABZ6686
Catocala benjamini mayhewi|[2196]|QUNOE526-12|United States|California|658[0n]|BOLD:ABZ6686
Catocala benjamini mayhewi|[2197]|QUNOE527-12|United States|California|658[0n]|BOLD:ABZ6686
Catocala benjamini mayhewi|[2198]|QUNOE528-12|United States|California|658[0n]|BOLD:ABZ6686
Catocala benjamini mayhewi|[2199]|QUNOD854-11|United States|California|658[0n]|BOLD:ABZ6686
Catocala benjamini mayhewi|[2200]|QUNOE450-12|United States|California|658[0n]|BOLD:ABZ6686
Catocala benjamini mayhewi|[2201]|QUNOD392-10|United States|California|658[0n]|BOLD:ABZ6686
Catocala benjamini mayhewi|[2202]|QUNOD469-10|United States|California|658[0n]|BOLD:ABZ6686
Catocala benjamini mayhewi|[2203]|QUNOE520-12|United States|California|658[0n]|BOLD:ABZ6686
Catocala benjamini mayhewi|[2204]|QUNOE521-12|United States|California|658[0n]|BOLD:ABZ6686
Catocala benjamini mayhewi|[2205]|QUNOE522-12|United States|California|658[0n]|BOLD:ABZ6686
Catocala benjamini mayhewi|[2206]|QUNOE523-12|United States|California|658[0n]|BOLD:ABZ6686
Catocala benjamini mayhewi|[2207]|QUNOE524-12|United States|California|658[0n]|BOLD:ABZ6686
Catocala benjamini mayhewi|[2208]|QUNOE525-12|United States|California|658[0n]|BOLD:ABZ6686
Catocala benjamini mayhewi|[2209]|QUNOE529-12|United States|California|658[0n]|BOLD:ABZ6686
Catocala benjamini mayhewi|[2210]|QUNOE530-12|United States|California|658[0n]|BOLD:ABZ6686
Catocala andromache|[2211]|ABCNA318-06|United States|California|658[0n]|BOLD:AAE4192
Catocala andromache|[2212]|ABCNA060-06|United States|California|658[0n]|BOLD:AAE4192
Catocala andromache wellsi|[2213]|ABCNA503-07|United States|California|568[0n]|BOLD:AAE4192
Catocala andromache|[2214]|ABCNA317-06|United States|California|658[0n]|BOLD:AAE4192
Catocala andromache|[2215]|ABCNA319-06|United States|California|658[0n]|BOLD:AAE4192
Catocala andromache|[2216]|QUNOE531-12|United States|California|658[0n]|BOLD:AAE4192
Catocala benjamini|[2217]|QUNOD831-11|United States|California|658[0n]|BOLD:ACE3896
Catocala benjamini|[2218]|RDNME048-07|United States|California|658[0n]|BOLD:ACE3896
Catocala benjamini|[2219]|QUNOE212-12|United States|California|658[0n]|BOLD:ACE3896
Catocala benjamini jumpi|[2220]|QUNOD662-10|United States|California|658[0n]|BOLD:ABZ6685
Catocala benjamini|[2221]|LOCBD055-06|United States|California|658[0n]|BOLD:ABZ6685
Catocala benjamini|[2222]|LOCBE324-06|United States|California|592[0n]|BOLD:ABZ6685
Catocala benjamini ute|[2223]|QUNOC067-09|United States|Utah|658[0n]|BOLD:ACE9750
Catocala benjamini ute|[2224]|QUNOC068-09|United States|Utah|658[0n]|BOLD:ACE9750
Catocala benjamini ute|[2225]|QUNOC069-09|United States|Utah|658[0n]|BOLD:ACE9750
Catocala benjamini ute|[2226]|QUNOE532-12|United States|Utah|658[0n]|BOLD:ACE9750
Catocala benjamini|[2227]|ABCNA321-06|United States|Utah|658[0n]|BOLD:ABZ6685
Catocala benjamini|[2228]|ABCNA320-06|United States|Utah|658[0n]|BOLD:ABZ6685
Catocala benjamini|[2229]|ABCNA062-06|United States|Utah|658[0n]|BOLD:ABZ6685
Catocala benjamini|[2230]|ABCNA322-06|United States|Utah|585[0n]|BOLD:ABZ6685
Catocala benjamini|[2231]|QUNOD565-10|United States|Arizona|658[0n]|BOLD:ABZ6685
Catocala benjamini|[2232]|QUNOE180-11|United States|Arizona|658[0n]|BOLD:ABZ6685
Catocala benjamini|[2233]|QUNOE181-11|United States|Arizona|658[0n]|BOLD:ABZ6685
Catocala benjamini|[2234]|QUNOE533-12|United States|Utah|658[0n]|BOLD:ABZ6685
Catocala|[2235]|ABCNA501-07|United States|California|568[0n]|BOLD:ACE9634
Catocala californiensis|[2236]|ABCNA252-06|United States|California|658[0n]|BOLD:ACE9634
Catocala californiensis|[2237]|ABCNA253-06|United States|California|658[0n]|BOLD:ACE9634
Catocala californiensis|[2238]|QUNO476-08|United States|California|658[0n]|BOLD:ACE9634
Catocala californiensis|[2239]|QUNOD835-11|United States|California|658[0n]|BOLD:ACE9634
Catocala californiensis|[2240]|QUNOE179-11|United States|California|658[0n]|BOLD:ACE9634
Catocala johnsoniana|[2241]|QUNOC038-09|United States|California|658[0n]|BOLD:ACE9634
Catocala johnsoniana|[2242]|QUNOB380-09|United States|California|658[0n]|BOLD:ACE9634
Catocala johnsoniana|[2243]|QUNOB554-09|United States|California|658[0n]|BOLD:ACE9634
Catocala johnsoniana|[2244]|QUNOC039-09|United States|California|658[0n]|BOLD:ACE9634
Catocala johnsoniana|[2245]|QUNOD527-10|United States|California|658[0n]|BOLD:ACE9634
Catocala johnsoniana|[2246]|QUNOE535-12|United States|Utah|658[0n]|BOLD:ACE9634
Catocala californiensis|[2247]|QUNOD468-10|United States|California|658[0n]|BOLD:ACE9636
Catocala californiensis|[2248]|QUNOD394-10|United States|California|658[0n]|BOLD:ACE9636
Catocala californiensis|[2249]|QUNOD522-10|United States|California|638[0n]|BOLD:ACE9636
Catocala californiensis|[2250]|QUNOE536-12|United States|California|658[0n]|BOLD:ACE9636
Catocala mcdunnoughi|[2251]|RDNME047-07|United States|California|658[0n]|BOLD:ABX6276
Catocala mcdunnoughi|[2252]|ABCNA203-06|United States|California|658[0n]|BOLD:ABX6276
Catocala mcdunnoughi|[2253]|ABCNA204-06|United States|California|658[0n]|BOLD:ABX6276
Catocala mcdunnoughi|[2254]|ABCNA331-06|United States|California|658[0n]|BOLD:ABX6276
Catocala mcdunnoughi|[2255]|QUNOD847-11|United States|California|658[0n]|BOLD:ABX6276
Catocala chelidonia|[2256]|ABCNA210-06|United States|Utah|592[0n]|BOLD:AAE4229
Catocala chelidonia|[2257]|ABCNA209-06|United States|Utah|658[0n]|BOLD:AAE4229
Catocala chelidonia|[2258]|ABCNA327-06|United States|Utah|651[0n]|BOLD:AAE4229
Catocala chelidonia|[2259]|ABCNA328-06|United States|Utah|632[0n]|BOLD:AAE4229
Catocala chelidonia|[2260]|QUNOE164-11|United States|Utah|658[0n]|BOLD:AAE4229
Catocala chelidonia|[2261]|ABCNA462-07|United States|Utah|593[0n]|BOLD:AAE4229
Catocala chelidonia|[2262]|BBLOD1321-11|United States|Arizona|658[0n]|BOLD:AAE4229
Catocala chelidonia|[2263]|BBLOE1481-12|United States|Arizona|658[0n]|BOLD:AAE4229
Catocala chelidonia|[2264]|BBLSY035-09|United States|Arizona|658[0n]|BOLD:AAE4229
Catocala chelidonia|[2265]|QUNOD474-10|United States|California|658[0n]|BOLD:AAE4229
Catocala chelidonia|[2266]|QUNOE509-12|United States|Arizona|658[0n]|BOLD:AAE4229
Catocala chelidonia|[2267]|QUNOD833-11|United States|California|658[0n]|BOLD:ABY3501
Catocala chelidonia|[2268]|QUNOD832-11|United States|California|593[0n]|BOLD:ABY3501
Catocala chelidonia|[2269]|QUNOD834-11|United States|California|648[0n]|BOLD:ABY3501
Catocala chelidonia|[2270]|QUNOD524-10|United States|California|658[0n]|BOLD:ABY3501
Catocala chelidonia|[2271]|QUNOD523-10|United States|California|658[0n]|BOLD:ABY3501
Catocala chelidonia|[2272]|QUNOD467-10|United States|California|658[0n]|BOLD:ABY3501
Catocala chelidonia|[2273]|QUNOD393-10|United States|California|658[0n]|BOLD:ABY3501
Catocala chelidonia|[2274]|QUNOD525-10|United States|California|622[0n]|BOLD:ABY3501
Catocala chelidonia|[2275]|QUNOD526-10|United States|California|658[0n]|BOLD:ABY3501
Catocala chelidonia occidentalis|[2276]|QUNOE537-12|United States|California|658[0n]|BOLD:ABY3501
Catocala delilah|[2277]|ABCNA057-06|United States|Florida|614[0n]|BOLD:AAC4717
Catocala delilah|[2278]|ABCNA314-06|United States|Florida|658[0n]|BOLD:AAC4717
Catocala delilah|[2279]|ABCNA354-06|United States|Florida|658[0n]|BOLD:AAC4717
Catocala delilah|[2280]|ABCNA315-06|United States|Florida|658[0n]|BOLD:AAC4717
Catocala delilah|[2281]|ABCNA313-06|United States|Florida|658[0n]|BOLD:AAC4717
Catocala delilah|[2282]|ABCNA056-06|United States|Florida|656[0n]|BOLD:AAC4717
Catocala delilah|[2283]|QUNOD135-10|United States|Oklahoma|658[0n]|BOLD:AAC4717
Catocala delilah|[2284]|QUNOD134-10|United States|Oklahoma|658[0n]|BOLD:AAC4717
Catocala delilah|[2285]|QUNOC073-09|United States|Oklahoma|658[0n]|BOLD:AAC4717
Catocala delilah|[2286]|QUNOC072-09|United States|Oklahoma|658[0n]|BOLD:AAC4717
Catocala delilah|[2287]|QUNOD136-10|United States|Oklahoma|658[0n]|BOLD:AAC4717
Catocala delilah|[2288]|QUNOD844-11|United States|Texas|658[0n]|BOLD:AAC4717
Catocala desdemona|[2289]|QUNOD137-10|United States|Oklahoma|658[0n]|BOLD:AAC4717
Catocala desdemona|[2290]|BBLOE1975-12|United States|Texas|658[0n]|BOLD:AAC4717
Catocala desdemona|[2291]|ABCNA058-06|United States|Texas|656[0n]|BOLD:AAC4717
Catocala desdemona|[2292]|CMAZA093-09|United States|Arizona|658[0n]|BOLD:AAC4717
Catocala desdemona|[2293]|HKONB519-09|United States|Texas|658[0n]|BOLD:AAC4717
Catocala desdemona|[2294]|ABCNA312-06|United States|Arizona|658[0n]|BOLD:AAC4717
Catocala desdemona|[2295]|ABCNA059-06|United States|Utah|658[0n]|BOLD:AAC4717
Catocala desdemona|[2296]|ABCNA316-06|United States|Arizona|642[0n]|BOLD:AAC4717
Catocala desdemona|[2297]|QUNOD022-10|United States|Texas|634[0n]|BOLD:AAC4717

[illegible]
Catocala desdemona|[2295]|ABCNA059-06|United States|Utah|658[0n]|BOLD:AAC4717
Catocala desdemona|[2296]|ABCNA316-06|United States|Arizona|642[0n]|BOLD:AAC4717
Catocala desdemona|[2297]|QUNOD022-10|United States|Texas|634[0n]|BOLD:AAC4717
Catocala desdemona|[2298]|QUNOD023-10|United States|Texas|658[0n]|BOLD:AAC4717
Catocala desdemona|[2299]|DMAZ255-10|United States|Arizona|658[0n]|BOLD:AAC4717
Catocala desdemona|[2300]|RDNMJ561-11|United States|Arizona|658[0n]|BOLD:AAC4717
Catocala desdemona|[2301]|QUNOE213-12|United States|Texas|658[0n]|BOLD:AAC4717
Catocala desdemona|[2302]|QUNOE534-12|United States|Utah|658[0n]|BOLD:AAC4717
Catocala gracilis|[2303]|ABCNA221-06|United States|Texas|572[3n]|BOLD:AAA6134
Catocala gracilis|[2304]|ABCNA066-06|United States|Texas|600[0n]|BOLD:AAA6134
Catocala gracilis|[2305]|QUNOB110-08|United States|Louisiana|658[0n]|BOLD:AAA6134
Catocala gracilis|[2306]|QUNOB095-08|United States|Louisiana|658[0n]|BOLD:AAA6134
Catocala gracilis|[2307]|ABCNA908-08|United States|Florida|658[0n]|BOLD:AAA6134
Catocala gracilis|[2308]|QUNO313-08|United States|Florida|658[0n]|BOLD:AAA6134
Catocala gracilis|[2309]|QUNO314-08|United States|Florida|658[0n]|BOLD:AAA6134
Catocala gracilis|[2310]|LNCC112-10|United States|North Carolina|658[0n]|BOLD:AAA6134
Catocala gracilis|[2311]|QUNO698-08|United States|Kentucky|658[1n]|BOLD:AAA6134
Catocala gracilis|[2312]|QUNO711-08|United States|Kentucky|658[0n]|BOLD:AAA6134
Catocala gracilis|[2313]|QUNO706-08|United States|Kentucky|658[0n]|BOLD:AAA6134
Catocala gracilis|[2314]|QUNO704-08|United States|Kentucky|658[0n]|BOLD:AAA6134
Catocala gracilis|[2315]|BBLOB1059-11|United States|Florida|658[0n]|BOLD:AAA6134
Catocala sordida|[2316]|RDLQB577-05|Canada|Quebec|539[0n]|BOLD:AAA6134
Catocala sordida|[2317]|LOT227-04|United States|Tennessee|609[0n]|BOLD:AAA6134
Catocala sordida|[2318]|LOT226-04|United States|Tennessee|609[0n]|BOLD:AAA6134
Catocala sordida|[2319]|LOT228-04|United States|Tennessee|609[0n]|BOLD:AAA6134
Catocala sordida|[2320]|RDLQB584-05|Canada|Quebec|658[0n]|BOLD:AAA6134
Catocala sordida|[2321]|RDLQB587-05|Canada|Quebec|658[0n]|BOLD:AAA6134
Catocala sordida|[2322]|QUNO079-07|United States|Wisconsin|658[0n]|BOLD:AAA6134
Catocala sordida|[2323]|TTMNB286-06|Canada|New Brunswick|600[1n]|BOLD:AAA6134
Catocala sordida|[2324]|RDLQB592-05|Canada|Quebec|540[1n]|BOLD:AAA6134
Catocala sordida|[2325]|TTMNB285-06|Canada|New Brunswick|613[0n]|BOLD:AAA6134
Catocala sordida|[2326]|LGSM767-04|United States|Tennessee|658[0n]|BOLD:AAA6134
Catocala sordida|[2327]|QUNO683-08|United States|Kentucky|658[0n]|BOLD:AAA6134
Catocala sordida|[2328]|QUNO692-08|United States|Kentucky|658[0n]|BOLD:AAA6134
Catocala sordida|[2329]|QUNO695-08|United States|Kentucky|658[0n]|BOLD:AAA6134
Catocala sordida|[2330]|QUNO696-08|United States|Kentucky|658[0n]|BOLD:AAA6134
Catocala sordida|[2331]|QUNO697-08|United States|Kentucky|658[0n]|BOLD:AAA6134
Catocala sordida|[2332]|BBLEC526-09|Canada|New Brunswick|644[0n]|BOLD:AAA6134
Catocala sordida|[2333]|BBLEC529-09|Canada|New Brunswick|648[0n]|BOLD:AAA6134
Catocala sordida|[2334]|RDLQB578-05|Canada|Quebec|658[0n]|BOLD:AAA6134
Catocala sordida|[2335]|RDLQB579-05|Canada|Quebec|658[0n]|BOLD:AAA6134
Catocala sordida|[2336]|RDLQB580-05|Canada|Quebec|658[0n]|BOLD:AAA6134
Catocala sordida|[2337]|RDLQB581-05|Canada|Quebec|658[0n]|BOLD:AAA6134
Catocala sordida|[2338]|RDLQB582-05|Canada|Quebec|658[0n]|BOLD:AAA6134
Catocala sordida|[2339]|RDLQB583-05|Canada|Quebec|658[0n]|BOLD:AAA6134
Catocala sordida|[2340]|RDLQB585-05|Canada|Quebec|658[0n]|BOLD:AAA6134
Catocala sordida|[2341]|RDLQB586-05|Canada|Quebec|658[0n]|BOLD:AAA6134
Catocala sordida|[2342]|RDLQB589-05|Canada|Quebec|658[0n]|BOLD:AAA6134
Catocala sordida|[2343]|RDLQB590-05|Canada|Quebec|658[0n]|BOLD:AAA6134
Catocala sordida|[2344]|RDLQB593-05|Canada|Quebec|658[0n]|BOLD:AAA6134
Catocala sordida|[2345]|RDLQB594-05|Canada|Quebec|658[0n]|BOLD:AAA6134
Catocala sordida|[2346]|RDLQB595-05|Canada|Quebec|658[0n]|BOLD:AAA6134
Catocala sordida|[2347]|RDLQB737-05|Canada|Quebec|658[0n]|BOLD:AAA6134
Catocala sordida|[2348]|TTMNB282-06|Canada|New Brunswick|658[0n]|BOLD:AAA6134
Catocala sordida|[2349]|TTMNB283-06|Canada|New Brunswick|658[0n]|BOLD:AAA6134
Catocala sordida|[2350]|TTMNB284-06|Canada|New Brunswick|658[0n]|BOLD:AAA6134
Catocala sordida|[2351]|TTMNB287-06|Canada|New Brunswick|658[0n]|BOLD:AAA6134
Catocala sordida|[2352]|RDLQF354-06|Canada|Quebec|658[0n]|BOLD:AAA6134
Catocala sordida|[2353]|RDLQF355-06|Canada|Quebec|658[0n]|BOLD:AAA6134
Catocala sordida|[2354]|RDLQF356-06|Canada|Quebec|658[0n]|BOLD:AAA6134
Catocala sordida|[2355]|BBLEC538-09|Canada|New Brunswick|658[0n]|BOLD:AAA6134
Catocala sordida|[2356]|BBLPC034-09|Canada|New Brunswick|647[0n]|BOLD:AAA6134
Catocala gracilis|[2357]|LGSM780-04|United States|Tennessee|658[0n]|BOLD:AAA6134
Catocala gracilis|[2358]|QUNO710-08|United States|Kentucky|658[0n]|BOLD:AAA6134
Catocala gracilis|[2359]|QUNO712-08|United States|Kentucky|658[0n]|BOLD:AAA6134
Catocala gracilis|[2360]|LGSM781-04|United States|North Carolina|658[0n]|BOLD:AAA6134
Catocala gracilis|[2361]|QUNO077-07|United States|Florida|658[0n]|BOLD:AAA6134
Catocala gracilis|[2362]|QUNOB088-08|United States|Texas|658[0n]|BOLD:AAA6134
Catocala gracilis|[2363]|QUNO705-08|United States|Kentucky|658[0n]|BOLD:AAA6134
Catocala gracilis|[2364]|QUNO702-08|United States|Kentucky|658[0n]|BOLD:AAA6134
Catocala gracilis|[2365]|QUNO701-08|United States|Kentucky|658[0n]|BOLD:AAA6134
Catocala gracilis|[2366]|QUNO700-08|United States|Kentucky|658[0n]|BOLD:AAA6134
Catocala gracilis|[2367]|QUNO707-08|United States|Kentucky|649[0n]|BOLD:AAA6134
Catocala gracilis|[2368]|QUNO708-08|United States|Kentucky|658[0n]|BOLD:AAA6134
Catocala gracilis|[2369]|QUNO709-08|United States|Kentucky|658[0n]|BOLD:AAA6134
Catocala gracilis|[2370]|QUNO713-08|United States|Kentucky|658[0n]|BOLD:AAA6134
Catocala gracilis|[2371]|QUNO541-08|United States|Louisiana|658[0n]|BOLD:AAA6134
Catocala gracilis|[2372]|QUNO544-08|United States|Louisiana|658[0n]|BOLD:AAA6134
Catocala gracilis|[2373]|QUNOB046-08|United States|Texas|658[0n]|BOLD:AAA6134
Catocala gracilis|[2374]|QUNOB100-08|United States|Louisiana|658[0n]|BOLD:AAA6134
Catocala gracilis|[2375]|ABCNA065-06|United States|Florida|658[0n]|BOLD:AAA6134
Catocala gracilis|[2376]|LNCC113-10|United States|North Carolina|658[0n]|BOLD:AAA6134
Catocala gracilis|[2377]|LNCC946-11|United States|North Carolina|658[0n]|BOLD:AAA6134
Catocala gracilis|[2378]|LNCC1281-11|United States|North Carolina|658[0n]|BOLD:AAA6134
Catocala sordida|[2379]|ABCNA881-08|United States|Kentucky|609[0n]|BOLD:AAA6134
Catocala sordida|[2380]|LOT224-04|United States|Tennessee|609[0n]|BOLD:AAA6134
Catocala sordida|[2381]|QUNO690-08|United States|Kentucky|658[0n]|BOLD:AAA6134
Catocala sordida|[2382]|QUNO689-08|United States|Kentucky|658[0n]|BOLD:AAA6134
Catocala sordida|[2383]|QUNO688-08|United States|Kentucky|658[0n]|BOLD:AAA6134
Catocala sordida|[2384]|QUNO686-08|United States|Kentucky|658[0n]|BOLD:AAA6134
Catocala sordida|[2385]|LGSMG598-07|United States|Tennessee|658[0n]|BOLD:AAA6134
Catocala sordida|[2386]|LOT225-04|United States|Tennessee|658[0n]|BOLD:AAA6134
Catocala sordida|[2387]|LGSM766-04|United States|Tennessee|658[0n]|BOLD:AAA6134
Catocala sordida|[2388]|QUNO684-08|United States|Kentucky|658[0n]|BOLD:AAA6134
Catocala sordida|[2389]|QUNO685-08|United States|Kentucky|658[0n]|BOLD:AAA6134
Catocala sordida|[2390]|RDLQF353-06|Canada|Quebec|658[0n]|BOLD:AAA6134
Catocala sordida|[2391]|RDLQF736-06|Canada|Quebec|658[0n]|BOLD:AAA6134
Catocala sordida|[2392]|QUNO687-08|United States|Kentucky|658[0n]|BOLD:AAA6134
Catocala sordida|[2393]|QUNO691-08|United States|Kentucky|658[0n]|BOLD:AAA6134
Catocala sordida|[2394]|QUNO693-08|United States|Kentucky|658[0n]|BOLD:AAA6134
Catocala sordida|[2395]|QUNO694-08|United States|Kentucky|658[0n]|BOLD:AAA6134
Catocala sordida|[2396]|QUNO699-08|United States|Kentucky|658[0n]|BOLD:AAA6134
Catocala sordida|[2397]|QUNO703-08|United States|Kentucky|658[0n]|BOLD:AAA6134

Catocala sordida|[2394]|QUNO[illegible]-08|United States|Kentucky|658[0n]|BOLD:AAA6134
Catocala sordida|[2395]|QUNO694-08|United States|Kentucky|658[0n]|BOLD:AAA6134
Catocala sordida|[2396]|QUNO699-08|United States|Kentucky|658[0n]|BOLD:AAA6134
Catocala sordida|[2397]|QUNO703-08|United States|Kentucky|658[0n]|BOLD:AAA6134
Catocala sordida|[2398]|LNCC1289-11|United States|North Carolina|658[0n]|BOLD:AAA6134
Catocala sordida|[2399]|LNCC1380-11|United States|North Carolina|658[0n]|BOLD:AAA6134
Catocala andromedae|[2400]|LOFLA219-06|United States|Florida|658[0n]|BOLD:AAA6877
Catocala andromedae|[2401]|LOFLA221-06|United States|Florida|658[0n]|BOLD:AAA6877
Catocala andromedae|[2402]|ABCNA067-06|United States|Texas|600[0n]|BOLD:AAA6877
Catocala louiseae n. sp.|[2403]|ABCNA340-06|United States|Texas|658[0n]|BOLD:AAA6877
Catocala louiseae n. sp.|[2404]|ABCNA237-06|United States|Texas|658[0n]|BOLD:AAA6877
Catocala louiseae n. sp.|[2405]|ABCNA068-06|United States|Texas|658[0n]|BOLD:AAA6877
Catocala louiseae n. sp.|[2406]|ABCNA773-07|United States|Texas|643[0n]|BOLD:AAA6877
Catocala louiseae n. sp.|[2407]|QUNO493-08|United States|Texas|658[0n]|BOLD:AAA6877
Catocala louiseae n. sp.|[2408]|QUNO510-08|United States|Texas|658[0n]|BOLD:AAA6877
Catocala louiseae n. sp.|[2409]|QUNO511-08|United States|Texas|658[0n]|BOLD:AAA6877
Catocala louiseae n. sp.|[2410]|QUNO512-08|United States|Texas|658[0n]|BOLD:AAA6877
Catocala louiseae n. sp.|[2411]|QUNO513-08|United States|Texas|658[0n]|BOLD:AAA6877
Catocala louiseae n. sp.|[2412]|QUNO514-08|United States|Texas|658[0n]|BOLD:AAA6877
Catocala louiseae n. sp.|[2413]|QUNO515-08|United States|Texas|658[0n]|BOLD:AAA6877
Catocala louiseae n. sp.|[2414]|QUNO516-08|United States|Texas|658[0n]|BOLD:AAA6877
Catocala louiseae n. sp.|[2415]|QUNO517-08|United States|Texas|658[0n]|BOLD:AAA6877
Catocala louiseae n. sp.|[2416]|QUNO518-08|United States|Texas|658[0n]|BOLD:AAA6877
Catocala louiseae n. sp.|[2417]|QUNO519-08|United States|Texas|658[0n]|BOLD:AAA6877
Catocala louiseae n. sp.|[2418]|QUNO520-08|United States|Texas|658[0n]|BOLD:AAA6877
Catocala louiseae n. sp.|[2419]|QUNO521-08|United States|Texas|658[0n]|BOLD:AAA6877
Catocala louiseae n. sp.|[2420]|QUNO522-08|United States|Texas|658[0n]|BOLD:AAA6877
Catocala louiseae n. sp.|[2421]|QUNO523-08|United States|Texas|658[0n]|BOLD:AAA6877
Catocala louiseae n. sp.|[2422]|QUNO542-08|United States|Louisiana|658[0n]|BOLD:AAA6877
Catocala louiseae n. sp.|[2423]|QUNOB002-08|United States|Texas|658[0n]|BOLD:AAA6877
Catocala louiseae n. sp.|[2424]|QUNOB008-08|United States|Texas|658[0n]|BOLD:AAA6877
Catocala louiseae n. sp.|[2425]|QUNOB011-08|United States|Texas|658[0n]|BOLD:AAA6877
Catocala louiseae n. sp.|[2426]|QUNOB052-08|United States|Texas|658[0n]|BOLD:AAA6877
Catocala louiseae n. sp.|[2427]|QUNOB556-09|United States|Louisiana|658[0n]|BOLD:AAA6877
Catocala louiseae n. sp.|[2428]|QUNOB557-09|United States|Louisiana|658[0n]|BOLD:AAA6877
Catocala andromedae|[2429]|QUNOB578-10|United States|Texas|647[0n]|BOLD:AAA6877
Catocala louiseae n. sp.|[2430]|QUNOB012-08|United States|Texas|609[0n]|BOLD:AAA6877
Catocala andromedae|[2431]|ABCNA611-07|United States|Florida|577[0n]|BOLD:AAA6877
Catocala andromedae|[2432]|QUNOB090-08|United States|Louisiana|658[0n]|BOLD:AAA6877
Catocala louiseae n. sp.|[2433]|QUNO500-08|United States|Texas|609[0n]|BOLD:AAA6877
Catocala andromedae|[2434]|ABCNA898-08|United States|Florida|609[0n]|BOLD:AAA6877
Catocala andromedae|[2435]|ABCNA897-08|United States|Florida|609[0n]|BOLD:AAA6877
Catocala andromedae|[2436]|LOT248-04|United States|Tennessee|609[0n]|BOLD:AAA6877
Catocala andromedae|[2437]|LGSM499-04|United States|Tennessee|609[0n]|BOLD:AAA6877
Catocala andromedae|[2438]|LOT247-04|United States|Tennessee|609[0n]|BOLD:AAA6877
Catocala andromedae|[2439]|ABCNA487-07|United States|Florida|568[0n]|BOLD:AAA6877
Catocala andromedae|[2440]|QUNO730-08|United States|Kentucky|658[0n]|BOLD:AAA6877
Catocala andromedae|[2441]|QUNO012-07|United States|Florida|648[0n]|BOLD:AAA6877
Catocala andromedae|[2442]|QUNO022-07|United States|Florida|658[0n]|BOLD:AAA6877
Catocala andromedae|[2443]|ABCNA899-08|United States|Florida|658[0n]|BOLD:AAA6877
Catocala andromedae|[2444]|LGSMG599-07|United States|North Carolina|658[0n]|BOLD:AAA6877
Catocala andromedae|[2445]|QUNO011-07|United States|Florida|658[0n]|BOLD:AAA6877
Catocala andromedae|[2446]|ABCNA246-06|United States|Florida|658[0n]|BOLD:AAA6877
Catocala andromedae|[2447]|ABCNA609-07|United States|Florida|658[0n]|BOLD:AAA6877
Catocala andromedae|[2448]|QUNO733-08|United States|Kentucky|658[0n]|BOLD:AAA6877
Catocala andromedae|[2449]|QUNOB044-08|United States|Texas|658[0n]|BOLD:AAA6877
Catocala andromedae|[2450]|ABCNA244-06|United States|Florida|658[0n]|BOLD:AAA6877
Catocala andromedae|[2451]|ABCNA245-06|United States|Florida|658[0n]|BOLD:AAA6877
Catocala andromedae|[2452]|QUNO731-08|United States|Kentucky|658[0n]|BOLD:AAA6877
Catocala andromedae|[2453]|QUNO732-08|United States|Kentucky|658[0n]|BOLD:AAA6877
Catocala andromedae|[2454]|LSEU598-06|United States|Georgia|658[0n]|BOLD:AAA6877
Catocala andromedae|[2455]|ABCNA243-06|United States|Florida|658[0n]|BOLD:AAA6877
Catocala andromedae|[2456]|ABCNA901-08|United States|Florida|658[0n]|BOLD:AAA6877
Catocala andromedae|[2457]|QUNO316-08|United States|Florida|658[0n]|BOLD:AAA6877
Catocala andromedae|[2458]|QUNOB091-08|United States|Louisiana|658[0n]|BOLD:AAA6877
Catocala andromedae|[2459]|NAMUM417-09|United States|Maryland|658[0n]|BOLD:AAA6877
Catocala andromedae|[2460]|LGSM500-04|United States|Tennessee|609[0n]|BOLD:AAA6877
Catocala andromedae|[2461]|QUNOB098-08|United States|Louisiana|658[0n]|BOLD:AAA6877
Catocala andromedae|[2462]|QUNOB087-08|United States|Texas|658[0n]|BOLD:AAA6877
Catocala andromedae|[2463]|QUNOB086-08|United States|Texas|658[0n]|BOLD:AAA6877
Catocala andromedae|[2464]|QUNOB085-08|United States|Texas|658[0n]|BOLD:AAA6877
Catocala andromedae|[2465]|QUNOB076-08|United States|Texas|658[0n]|BOLD:AAA6877
Catocala andromedae|[2466]|QUNOB074-08|United States|Texas|658[0n]|BOLD:AAA6877
Catocala andromedae|[2467]|QUNOB073-08|United States|Texas|658[0n]|BOLD:AAA6877
Catocala andromedae|[2468]|QUNOB060-08|United States|Texas|658[0n]|BOLD:AAA6877
Catocala andromedae|[2469]|QUNOB051-08|United States|Texas|658[0n]|BOLD:AAA6877
Catocala andromedae|[2470]|QUNOB050-08|United States|Texas|658[0n]|BOLD:AAA6877
Catocala andromedae|[2471]|QUNOB004-08|United States|Texas|658[0n]|BOLD:AAA6877
Catocala andromedae|[2472]|QUNO842-08|United States|Texas|658[0n]|BOLD:AAA6877
Catocala andromedae|[2473]|QUNO531-08|United States|Texas|658[0n]|BOLD:AAA6877
Catocala andromedae|[2474]|ABCNA900-08|United States|Florida|658[0n]|BOLD:AAA6877
Catocala andromedae|[2475]|QUNOB075-08|United States|Texas|658[0n]|BOLD:AAA6877
Catocala andromedae|[2476]|QUNO023-07|United States|Florida|658[0n]|BOLD:AAA6877
Catocala andromedae|[2477]|QUNOB084-08|United States|Texas|658[0n]|BOLD:AAA6877
Catocala andromedae|[2478]|QUNOB109-08|United States|Louisiana|658[0n]|BOLD:AAA6877
Catocala andromedae|[2479]|QUNOB378-09|United States|Texas|658[0n]|BOLD:AAA6877
Catocala andromedae|[2480]|QUNOB509-09|United States|Texas|658[0n]|BOLD:AAA6877
Catocala andromedae|[2481]|QUNOD688-11|United States|Massachusetts|658[0n]|BOLD:AAA6877
Catocala louiseae|[2482]|ABCNA069-06|United States|Florida|600[0n]|BOLD:AAA6877
Catocala louiseae|[2483]|ABCNA894-08|United States|Florida|621[0n]|BOLD:AAA6877
Catocala louiseae|[2484]|QUNO045-07|United States|Florida|658[0n]|BOLD:AAA6877
Catocala louiseae|[2485]|ABCNA339-06|United States|Florida|658[0n]|BOLD:AAA6877
Catocala louiseae|[2486]|ABCNA238-06|United States|Florida|658[0n]|BOLD:AAA6877
Catocala louiseae|[2487]|ABCNA239-06|United States|Florida|658[0n]|BOLD:AAA6877
Catocala louiseae|[2488]|QUNO046-07|United States|Florida|658[0n]|BOLD:AAA6877
Catocala louiseae|[2489]|ABCNA892-08|United States|Florida|658[0n]|BOLD:AAA6877
Catocala louiseae|[2490]|ABCNA893-08|United States|Florida|658[0n]|BOLD:AAA6877
Catocala louiseae|[2491]|ABCNA896-08|United States|Florida|658[0n]|BOLD:AAA6877
Catocala louiseae|[2492]|QUNO371-08|United States|Florida|658[0n]|BOLD:AAA6877
Catocala louiseae|[2493]|QUNOB555-09|United States|Florida|658[0n]|BOLD:AAA6877
Catocala louiseae|[2494]|QUNOC015-09|United States|Florida|658[0n]|BOLD:AAA6877
Catocala louiseae|[2495]|ABCNA895-08|United States|Florida|609[0n]|BOLD:AAA6877
Catocala louiseae|[2496]|ABCNA492-07|United States|Florida|544[0n]|BOLD:AAA6877
[illegible]

Catocala louiseae|[2494]|QUNOC015-09|United States|Florida|658[0n]|BOLD:AAA6877
Catocala louiseae|[2495]|ABCNA895-08|United States|Florida|609[0n]|BOLD:AAA6877
Catocala louiseae|[2496]|ABCNA492-07|United States|Florida|544[0n]|BOLD:AAA6877
Catocala louiseae|[2497]|ABCNA490-07|United States|Florida|568[0n]|BOLD:AAA6877
Catocala louiseae|[2498]|QUNO044-07|United States|Florida|656[0n]|BOLD:AAA6877
Catocala louiseae|[2499]|ABCNA612-07|United States|Florida|577[0n]|BOLD:AAA6877
Catocala louiseae|[2500]|ABCNA241-06|United States|Florida|658[0n]|BOLD:AAA6877
Catocala louiseae|[2501]|NOCNA052-14|United States|Florida|658[0n]|BOLD:AAA6877
Catocala louiseae|[2502]|NOCNA053-14|United States|Florida|658[0n]|BOLD:AAA6877
Catocala illecta|[2503]|LPOKB982-09|United States|Oklahoma|658[0n]|BOLD:AAE4366
Catocala illecta|[2504]|QUNO490-08|United States|Texas|658[0n]|BOLD:AAE4366
Catocala illecta|[2505]|QUNO489-08|United States|Texas|658[0n]|BOLD:AAE4366
Catocala illecta|[2506]|ABCNA465-07|United States|Missouri|621[0n]|BOLD:AAE4366
Catocala illecta|[2507]|QUNOD207-10|United States|Texas|623[0n]|BOLD:AAE4366
Catocala clintonii|[2508]|ABCNA516-07|United States|Florida|568[1n]|BOLD:ACE7289
Catocala clintonii|[2509]|BBLSX362-09|United States|Oklahoma|658[0n]|BOLD:ACE7289
Catocala clintonii|[2510]|QUNOE398-12|United States|Wisconsin|658[0n]|BOLD:ACE7289
Catocala clintonii|[2511]|QUNO449-08|United States|Florida|658[0n]|BOLD:AAC4282
Catocala clintonii|[2512]|QUNO450-08|United States|Florida|658[0n]|BOLD:AAC4282
Catocala clintonii|[2513]|QUNO447-08|United States|Florida|658[0n]|BOLD:AAC4282
Catocala clintonii|[2514]|QUNO448-08|United States|Florida|658[0n]|BOLD:AAC4282
Catocala clintonii|[2515]|QUNO451-08|United States|Florida|658[0n]|BOLD:AAC4282
Catocala clintonii|[2516]|ABCNA270-06|United States|Florida|658[0n]|BOLD:AAC4282
Catocala clintonii|[2517]|LNCB165-06|United States|North Carolina|658[0n]|BOLD:AAC4282
Catocala clintonii|[2518]|QUNO727-08|United States|Kentucky|658[0n]|BOLD:AAC4282
Catocala clintonii|[2519]|QUNOB561-09|United States|Texas|658[0n]|BOLD:AAC4282
Catocala clintonii|[2520]|LNCC947-11|United States|North Carolina|658[0n]|BOLD:AAC4282
Catocala clintonii|[2521]|ABCNA514-07|United States|Florida|534[0n]|BOLD:AAC4282
Catocala clintonii|[2522]|ABCNA515-07|United States|Florida|568[2n]|BOLD:AAC4282
Catocala clintonii|[2523]|CNCLB2463-14|Canada|Ontario|658[0n]|BOLD:AAC4282
Catocala clintonii|[2524]|CNCLB2464-14|Canada|Ontario|658[0n]|BOLD:AAC4282
Catocala abbreviatella|[2525]|QUNO019-07|United States|Wisconsin|658[0n]|BOLD:AAD0139
Catocala abbreviatella|[2526]|QUNO018-07|United States|Wisconsin|652[0n]|BOLD:AAD0139
Catocala abbreviatella|[2527]|QUNOB185-08|United States|Wisconsin|658[0n]|BOLD:AAD0139
Catocala abbreviatella|[2528]|ABCNA213-06|United States|Oklahoma|592[0n]|BOLD:AAD0139
Catocala abbreviatella|[2529]|ABCNA470-07|United States|Oklahoma|584[0n]|BOLD:AAD0139
Catocala abbreviatella|[2530]|ABCNA472-07|United States|Oklahoma|568[0n]|BOLD:AAD0139
Catocala abbreviatella|[2531]|QUNOB186-08|United States|Wisconsin|658[0n]|BOLD:AAD0139
Catocala abbreviatella|[2532]|QUNOB187-08|United States|Wisconsin|658[0n]|BOLD:AAD0139
Catocala abbreviatella|[2533]|QUNOB188-08|United States|Wisconsin|658[0n]|BOLD:AAD0139
Catocala amestris|[2534]|ABCNA335-06|United States|Florida|658[0n]|BOLD:AAD0142
Catocala amestris|[2535]|ABCNA334-06|United States|Florida|658[0n]|BOLD:AAD0142
Catocala amestris|[2536]|ABCNA216-06|United States|Florida|658[0n]|BOLD:AAD0142
Catocala amestris|[2537]|ABCNA477-07|United States|Florida|568[0n]|BOLD:AAD0142
Catocala amestris|[2538]|ABCNA476-07|United States|Oklahoma|568[0n]|BOLD:AAD0142
Catocala amestris|[2539]|ABCNA815-07|United States|Florida|590[0n]|BOLD:AAD0142
Catocala amestris|[2540]|QUNOB048-08|United States|Texas|658[0n]|BOLD:AAD0142
Catocala amestris|[2541]|QUNOB055-08|United States|Texas|658[0n]|BOLD:AAD0142
Catocala amestris|[2542]|QUNOB506-09|United States|Nebraska|658[0n]|BOLD:AAD0142
Catocala whitneyi|[2543]|QUNO088-07|United States|Wisconsin|658[0n]|BOLD:AAE3940
Catocala whitneyi|[2544]|QUNO087-07|United States|Wisconsin|658[0n]|BOLD:AAE3940
Catocala whitneyi|[2545]|QUNO086-07|United States|Wisconsin|658[0n]|BOLD:AAE3940
Catocala whitneyi|[2546]|QUNO089-07|United States|Wisconsin|530[0n]|BOLD:AAE3940
Catocala whitneyi|[2547]|QUNOB507-09|United States|Nebraska|658[0n]|BOLD:AAE3940
Catocala whitneyi|[2548]|QUNOE275-12|United States|Florida|658[0n]|BOLD:AAE3940
Catocala whitneyi|[2549]|QUNOE276-12|United States|Florida|658[0n]|BOLD:AAE3940
Catocala nuptialis|[2550]|QUNO568-08|United States|Indiana|658[0n]|BOLD:AAE9293
Catocala nuptialis|[2551]|ABCNA212-06|United States|Indiana|511[0n]|BOLD:AAE9293
Catocala nuptialis|[2552]|QUNO567-08|United States|Indiana|658[0n]|BOLD:AAE9293
Catocala nuptialis|[2553]|LPOKC608-09|United States|Oklahoma|658[0n]|BOLD:AAE9293
Catocala nuptialis|[2554]|QUNOE498-12|United States|Wisconsin|658[0n]|BOLD:AAE9293
Catocala miranda|[2555]|ABCNA361-06|United States|Florida|658[0n]|BOLD:AAC6526
Catocala miranda|[2556]|ABCNA362-06|United States|Florida|658[0n]|BOLD:AAC6526
Catocala miranda|[2557]|QUNOB157-08|United States|Florida|658[0n]|BOLD:AAC6526
Catocala miranda|[2558]|ABCNA233-06|United States|Virginia|658[0n]|BOLD:AAC6526
Catocala miranda|[2559]|ABCNA357-06|United States|Florida|658[0n]|BOLD:AAC6526
Catocala miranda|[2560]|ABCNA358-06|United States|Florida|580[2n]|BOLD:AAC6526
Catocala miranda|[2561]|ABCNA364-06|United States|Florida|658[0n]|BOLD:AAC6526
Catocala miranda|[2562]|QUNO310-08|United States|Florida|658[0n]|BOLD:AAC6526
Catocala miranda|[2563]|QUNOB156-08|United States|Florida|658[0n]|BOLD:AAC6526
Catocala miranda|[2564]|QUNOB158-08|United States|Florida|658[1n]|BOLD:AAC6526
Catocala mira|[2565]|ABCNA607-07|United States|Florida|577[0n]|BOLD:AAA7679
Catocala mira|[2566]|ABCNA605-07|United States|Florida|577[0n]|BOLD:AAA7679
Catocala mira|[2567]|ABCNA084-06|United States|Florida|658[0n]|BOLD:AAA7679
Catocala mira|[2568]|QUNO374-08|United States|Florida|658[0n]|BOLD:AAA7679
Catocala mira|[2569]|QUNO377-08|United States|Florida|658[0n]|BOLD:AAA7679
Catocala mira|[2570]|QUNO378-08|United States|Florida|658[0n]|BOLD:AAA7679
Catocala mira|[2571]|ABCNA257-06|United States|Texas|606[3n]|BOLD:AAA7679
Catocala mira|[2572]|QUNOB103-08|United States|Louisiana|658[0n]|BOLD:AAA7679
Catocala mira|[2573]|QUNOC001-09|United States|Florida|638[0n]|BOLD:AAA7679
Catocala mira|[2574]|ABCNA264-06|United States|Florida|658[0n]|BOLD:AAA7679
Catocala mira|[2575]|ABCNA261-06|United States|Florida|658[0n]|BOLD:AAA7679
Catocala mira|[2576]|ABCNA083-06|United States|Florida|656[0n]|BOLD:AAA7679
Catocala mira|[2577]|ABCNA571-07|United States|Florida|577[0n]|BOLD:AAA7679
Catocala mira|[2578]|QUNO458-08|United States|Florida|658[0n]|BOLD:AAA7679
Catocala mira|[2579]|QUNO459-08|United States|Florida|658[0n]|BOLD:AAA7679
Catocala mira|[2580]|QUNO460-08|United States|Florida|658[0n]|BOLD:AAA7679
Catocala mira|[2581]|QUNO461-08|United States|Florida|658[0n]|BOLD:AAA7679
Catocala mira|[2582]|QUNO375-08|United States|Florida|658[0n]|BOLD:AAA7679
Catocala mira|[2583]|QUNOC002-09|United States|Florida|658[0n]|BOLD:AAA7679
Catocala mira|[2584]|QUNO379-08|United States|Florida|650[0n]|BOLD:AAA7679
Catocala mira|[2585]|QUNO016-07|United States|Florida|658[0n]|BOLD:AAA7679
Catocala mira|[2586]|ABCNA603-07|United States|Florida|577[0n]|BOLD:AAA7679
Catocala mira|[2587]|ABCNA595-07|United States|Florida|572[0n]|BOLD:AAA7679
Catocala mira|[2588]|ABCNA604-07|United States|Florida|577[0n]|BOLD:AAA7679
Catocala mira|[2589]|ABCNA606-07|United States|Florida|577[0n]|BOLD:AAA7679
Catocala mira|[2590]|ABCNA608-07|United States|Florida|577[0n]|BOLD:AAA7679
Catocala mira|[2591]|QUNO324-08|United States|Florida|658[0n]|BOLD:AAA7679
Catocala mira|[2592]|QUNO376-08|United States|Florida|658[0n]|BOLD:AAA7679
Catocala mira|[2593]|QUNOB118-08|United States|Florida|658[0n]|BOLD:AAA7679
Catocala mira|[2594]|QUNOC003-09|United States|Florida|658[0n]|BOLD:AAA7679
Catocala mira|[2595]|QUNOC004-09|United States|Florida|658[0n]|BOLD:AAA7679
Catocala mira|[2596]|XAD392-04|Canada|Ontario|658[0n]|BOLD:AAA7679
[illegible]

Catocala mira|[2594]|QUNOC003-09|United States|Florida|658[0n]|BOLD:AAA7679
Catocala mira|[2595]|QUNOC004-09|United States|Florida|658[0n]|BOLD:AAA7679
Catocala mira|[2596]|XAD392-04|Canada|Ontario|658[0n]|BOLD:AAA7679
Catocala mira|[2597]|XAK432-06|Canada|Ontario|658[0n]|BOLD:AAA7679
Catocala mira|[2598]|XAK289-06|Canada|Ontario|658[0n]|BOLD:AAA7679
Catocala mira|[2599]|XAK288-06|Canada|Ontario|658[0n]|BOLD:AAA7679
Catocala mira|[2600]|XAK211-06|Canada|Ontario|658[0n]|BOLD:AAA7679
Catocala mira|[2601]|XAJ949-06|Canada|Ontario|658[0n]|BOLD:AAA7679
Catocala mira|[2602]|XAK515-07|Canada|Ontario|658[0n]|BOLD:AAA7679
Catocala mira|[2603]|XAD090-04|Canada|Ontario|571[0n]|BOLD:AAA7679
Catocala mira|[2604]|XAD345-04|Canada|Ontario|567[0n]|BOLD:AAA7679
Catocala mira|[2605]|HKONB479-09|United States|Wisconsin|627[1n]|BOLD:AAA7679
Catocala mira|[2606]|XAB679-04|Canada|Ontario|658[0n]|BOLD:AAA7679
Catocala mira|[2607]|XAG544-05|Canada|Ontario|658[0n]|BOLD:AAA7679
Catocala mira|[2608]|XAG800-05|Canada|Ontario|658[0n]|BOLD:AAA7679
Catocala mira|[2609]|XAK519-07|Canada|Ontario|658[0n]|BOLD:AAA7679
Catocala mira|[2610]|QUNO076-07|United States|Michigan|658[0n]|BOLD:AAA7679
Catocala mira|[2611]|LNCC225-10|United States|North Carolina|658[0n]|BOLD:AAA7679
Catocala mira|[2612]|QUNOC036-09|United States|Texas|658[0n]|BOLD:AAA7679
Catocala mira|[2613]|QUNOC632-13|United States|Kentucky|658[0n]|BOLD:AAA7679
Catocala praeclara|[2614]|RDLQF683-06|Canada|Quebec|619[0n]|BOLD:ABX6316
Catocala praeclara|[2615]|RDLQF351-06|Canada|Quebec|658[0n]|BOLD:ABX6316
Catocala praeclara|[2616]|RDLQF350-06|Canada|Quebec|658[0n]|BOLD:ABX6316
Catocala praeclara|[2617]|RDLQF349-06|Canada|Quebec|658[0n]|BOLD:ABX6316
Catocala praeclara|[2618]|RDLQF352-06|Canada|Quebec|658[0n]|BOLD:ABX6316
Catocala praeclara|[2619]|BBLEC535-09|Canada|New Brunswick|658[0n]|BOLD:ABX6316
Catocala praeclara|[2620]|QUNOE282-12|Canada|Quebec|658[0n]|BOLD:ABX6316
Catocala praeclara|[2621]|QUNOD827-11|United States|New Jersey|658[0n]|BOLD:ABX6316
Catocala praeclara|[2622]|QUNOD828-11|United States|New Jersey|658[0n]|BOLD:ABX6316
Catocala praeclara charlottae|[2623]|QUNO066-07|United States|Florida|658[0n]|BOLD:ABX6316
Catocala praeclara charlottae|[2624]|QUNOB184-08|United States|Florida|658[0n]|BOLD:ABX6316
Catocala praeclara charlottae|[2625]|QUNO671-08|United States|Kentucky|658[0n]|BOLD:ABX6316
Catocala praeclara|[2626]|QUNOD689-11|United States|New Hampshire|658[0n]|BOLD:ABX6316
Catocala praeclara charlottae|[2627]|QUNO665-08|United States|Kentucky|658[0n]|BOLD:ABX6316
Catocala praeclara charlottae|[2628]|QUNO668-08|United States|Kentucky|658[0n]|BOLD:ABX6316
Catocala praeclara|[2629]|QUNOD690-11|United States|New Hampshire|658[0n]|BOLD:ABX6316
Catocala praeclara charlottae|[2630]|QUNO663-08|United States|Kentucky|658[0n]|BOLD:ABX6316
Catocala praeclara charlottae|[2631]|QUNO662-08|United States|Kentucky|658[0n]|BOLD:ABX6316
Catocala praeclara charlottae|[2632]|QUNO661-08|United States|Kentucky|658[0n]|BOLD:ABX6316
Catocala praeclara charlottae|[2633]|QUNO067-07|United States|Florida|658[0n]|BOLD:ABX6316
Catocala praeclara charlottae|[2634]|QUNO065-07|United States|Florida|658[0n]|BOLD:ABX6316
Catocala praeclara charlottae|[2635]|QUNO064-07|United States|Florida|658[0n]|BOLD:ABX6316
Catocala praeclara charlottae|[2636]|QUNO063-07|United States|Florida|658[0n]|BOLD:ABX6316
Catocala praeclara charlottae|[2637]|ABCNA085-06|United States|Florida|658[0n]|BOLD:ABX6316
Catocala praeclara|[2638]|MILEP037-09|United States|North Carolina|658[0n]|BOLD:ABX6316
Catocala praeclara|[2639]|LNC098-05|United States|North Carolina|658[0n]|BOLD:ABX6316
Catocala praeclara|[2640]|LNC099-05|United States|North Carolina|658[0n]|BOLD:ABX6316
Catocala praeclara|[2641]|LNCB299-06|United States|North Carolina|658[0n]|BOLD:ABX6316
Catocala praeclara|[2642]|LNCB300-06|United States|North Carolina|658[0n]|BOLD:ABX6316
Catocala praeclara|[2643]|LNCB301-06|United States|North Carolina|658[0n]|BOLD:ABX6316
Catocala praeclara|[2644]|LNCB626-09|United States|North Carolina|658[0n]|BOLD:ABX6316
Catocala praeclara|[2645]|MILEP068-09|United States|North Carolina|658[0n]|BOLD:ABX6316
Catocala praeclara|[2646]|MILEP069-09|United States|North Carolina|658[0n]|BOLD:ABX6316
Catocala praeclara|[2647]|MILEP072-09|United States|North Carolina|658[0n]|BOLD:ABX6316
Catocala praeclara|[2648]|QUNOD691-11|United States|New Hampshire|621[0n]|BOLD:ABX6316
Catocala praeclara charlottae|[2649]|ABCNA086-06|United States|Louisiana|577[0n]|BOLD:ABX6316
Catocala praeclara charlottae|[2650]|ABCNA355-06|United States|Florida|563[0n]|BOLD:ABX6316
Catocala praeclara|[2651]|MILEP070-09|United States|North Carolina|658[0n]|BOLD:ABX6316
Catocala praeclara charlottae|[2652]|QUNO670-08|United States|Kentucky|658[0n]|BOLD:ABX6316
Catocala praeclara charlottae|[2653]|QUNO669-08|United States|Kentucky|658[0n]|BOLD:ABX6316
Catocala praeclara charlottae|[2654]|QUNO667-08|United States|Kentucky|658[0n]|BOLD:ABX6316
Catocala praeclara charlottae|[2655]|QUNO666-08|United States|Kentucky|658[0n]|BOLD:ABX6316
Catocala praeclara charlottae|[2656]|QUNO664-08|United States|Kentucky|658[0n]|BOLD:ABX6316
Catocala praeclara|[2657]|CONA005-05|United States||558[0n]|BOLD:ABX6316
Catocala praeclara|[2658]|CONA006-05|United States||565[1n]|BOLD:ABX6316
Catocala praeclara|[2659]|MILEP071-09|United States|North Carolina|632[0n]|BOLD:ABX6316
Catocala praeclara|[2660]|QUNOD809-11|United States|West Virginia|658[0n]|BOLD:ABX6316
Catocala praeclara|[2661]|QUNOD830-11|United States|New Jersey|658[0n]|BOLD:ABX6316
Catocala praeclara charlottae|[2662]|QUNO508-08|United States|Louisiana|658[0n]|BOLD:ABX6316
Catocala praeclara charlottae|[2663]|QUNOB023-08|United States|Louisiana|658[0n]|BOLD:ABX6316
Catocala praeclara charlottae|[2664]|CNCLB693-14|United States|Louisiana|658[0n]|BOLD:ABX6316
Catocala praeclara charlottae|[2665]|CNCLB1470-14|United States|Louisiana|649[0n]|BOLD:ABX6316
Catocala alabamae|[2666]|CONA011-05|United States||658[0n]|BOLD:ACE4888
Catocala praeclara|[2667]|RDMAB187-05|Canada|Alberta|658[0n]|BOLD:ACE4888
Catocala praeclara|[2668]|QUNO059-07|United States|Wisconsin|658[0n]|BOLD:ACE4888
Catocala praeclara|[2669]|QUNO060-07|United States|Wisconsin|658[0n]|BOLD:ACE4888
Catocala praeclara|[2670]|QUNO061-07|United States|Wisconsin|658[0n]|BOLD:ACE4888
Catocala praeclara|[2671]|QUNO062-07|United States|Wisconsin|658[0n]|BOLD:ACE4888
Catocala praeclara|[2672]|QUNOB319-09|Canada|Saskatchewan|658[0n]|BOLD:ACE4888
Catocala praeclara|[2673]|QUNOB320-09|Canada|Saskatchewan|658[0n]|BOLD:ACE4888
Catocala praeclara|[2674]|QUNOB321-09|Canada|Saskatchewan|658[0n]|BOLD:ACE4888
Catocala praeclara|[2675]|QUNOB341-09|United States|Ohio|658[0n]|BOLD:ACE4888
Catocala praeclara|[2676]|QUNOB342-09|United States|Ohio|658[0n]|BOLD:ACE4888
Catocala praeclara|[2677]|QUNOB343-09|United States|Ohio|658[0n]|BOLD:ACE4888
Catocala praeclara|[2678]|QUNOC042-09|United States|Ohio|658[0n]|BOLD:ACE4888
Catocala praeclara|[2679]|QUNOD466-10|United States|Wisconsin|658[0n]|BOLD:ACE4888
Catocala alabamae|[2680]|QUNO537-08|United States|Louisiana|658[0n]|BOLD:ACE4888
Catocala alabamae|[2681]|QUNO536-08|United States|Louisiana|658[0n]|BOLD:ACE4888
Catocala alabamae|[2682]|QUNO020-07|United States|Texas|658[0n]|BOLD:ACE4888
Catocala alabamae|[2683]|QUNOB105-08|United States|Louisiana|658[1n]|BOLD:ACE4888
Catocala alabamae|[2684]|QUNOD273-10|United States|Texas|658[0n]|BOLD:ACE4888
Catocala alabamae|[2685]|QUNOD210-10|United States|Texas|658[0n]|BOLD:ACE4888
Catocala alabamae|[2686]|QUNOB104-08|United States|Louisiana|658[0n]|BOLD:ACE4888
Catocala alabamae|[2687]|QUNOB047-08|United States|Texas|658[0n]|BOLD:ACE4888
Catocala alabamae|[2688]|QUNO535-08|United States|Louisiana|658[0n]|BOLD:ACE4888
Catocala alabamae|[2689]|QUNO509-08|United States|Texas|658[0n]|BOLD:ACE4888
Catocala alabamae|[2690]|QUNO488-08|United States|Texas|658[0n]|BOLD:ACE4888
Catocala alabamae|[2691]|QUNO021-07|United States|Texas|658[0n]|BOLD:ACE4888
Catocala alabamae|[2692]|ABCNA341-06|United States|Texas|658[0n]|BOLD:ACE4888
Catocala alabamae|[2693]|CONA003-05|United States||658[0n]|BOLD:ACE4888
Catocala alabamae|[2694]|CONA004-05|United States||580[0n]|BOLD:ACE4888
Catocala alabamae|[2695]|ABCNA088-06|United States|Oklahoma|600[0n]|BOLD:ACE4888
Catocala alabamae|[2696]|QUNOD274-10|United States|Texas|622[0n]|BOLD:ACE4888

Catocala alabamae|[2694]|CONA004-05|United States||580[0n]|BOLD:ACE4888
Catocala alabamae|[2695]|ABCNA088-06|United States|Oklahoma|600[0n]|BOLD:ACE4888
Catocala alabamae|[2696]|QUNOD274-10|United States|Texas|622[0n]|BOLD:ACE4888
Catocala alabamae|[2697]|QUNOD281-10|United States|Kentucky|658[0n]|BOLD:ACE4888
Catocala alabamae|[2698]|QUNOD282-10|United States|Kentucky|658[0n]|BOLD:ACE4888
Catocala alabamae|[2699]|QUNOD472-10|United States|Texas|658[0n]|BOLD:ACE4888
Catocala alabamae|[2700]|LNCB627-09|United States|North Carolina|658[0n]|BOLD:ACE4888
Catocala alabamae|[2701]|LNCB628-09|United States|North Carolina|658[0n]|BOLD:ACE4888
Catocala alabamae|[2702]|QUNOD284-10|United States|North Carolina|647[0n]|BOLD:ACE4888
Catocala alabamae|[2703]|ABCNA763-07|United States|Florida|641[0n]|BOLD:ACE4888
Catocala alabamae|[2704]|ABCNA359-06|United States|Florida|658[0n]|BOLD:ACE4888
Catocala alabamae|[2705]|QUNO380-08|United States|Florida|658[0n]|BOLD:ACE4888
Catocala alabamae|[2706]|ABCNA517-07|United States|Florida|568[0n]|BOLD:ACE4888
Catocala alabamae|[2707]|QUNOB162-08|United States|Florida|658[0n]|BOLD:ACE4888
Catocala alabamae|[2708]|ABCNA760-07|United States|Florida|641[0n]|BOLD:ACE4888
Catocala alabamae|[2709]|ABCNA761-07|United States|Florida|639[0n]|BOLD:ACE4888
Catocala alabamae|[2710]|ABCNA762-07|United States|Florida|645[0n]|BOLD:ACE4888
Catocala alabamae|[2711]|QUNOD692-11|United States|Ohio|658[0n]|BOLD:ACE4888
Catocala alabamae|[2712]|MILEP073-09|United States|North Carolina|658[0n]|BOLD:ACE4888
Catocala alabamae|[2713]|QUNOB159-08|United States|Florida|658[0n]|BOLD:ACE4888
Catocala alabamae|[2714]|QUNO435-08|United States|Florida|658[0n]|BOLD:ACE4888
Catocala alabamae|[2715]|ABCNA260-06|United States|Florida|658[0n]|BOLD:ACE4888
Catocala alabamae|[2716]|ABCNA089-06|United States|Florida|658[0n]|BOLD:ACE4888
Catocala alabamae|[2717]|ABCNA518-07|United States|Florida|568[0n]|BOLD:ACE4888
Catocala alabamae|[2718]|ABCNA808-07|United States|Florida|652[0n]|BOLD:ACE4888
Catocala alabamae|[2719]|PMG099-03|Canada|Ontario|617[0n]|BOLD:ACE4888
Catocala alabamae|[2720]|CONA009-05|United States||658[0n]|BOLD:ACE4888
Catocala alabamae|[2721]|CONA010-05|United States||658[0n]|BOLD:ACE4888
Catocala alabamae|[2722]|QUNOB160-08|United States|Florida|658[0n]|BOLD:ACE4888
Catocala alabamae|[2723]|CNCLB2465-14|Canada|Ontario|658[0n]|BOLD:ACE4888
Catocala orba|[2724]|ABCNA236-06|United States|Texas|658[0n]|BOLD:AAD0059
Catocala orba|[2725]|QUNO311-08|United States|Florida|658[0n]|BOLD:AAD0059
Catocala orba|[2726]|ABCNA366-06|United States|Florida|658[0n]|BOLD:AAD0059
Catocala orba|[2727]|ABCNA235-06|United States|Texas|658[0n]|BOLD:AAD0059
Catocala orba|[2728]|ABCNA234-06|United States|Florida|658[0n]|BOLD:AAD0059
Catocala orba|[2729]|QUNO312-08|United States|Florida|658[0n]|BOLD:AAD0059
Catocala orba|[2730]|QUNO538-08|United States|Louisiana|658[0n]|BOLD:AAD0059
Catocala orba|[2731]|QUNO539-08|United States|Louisiana|658[0n]|BOLD:AAD0059
Catocala orba|[2732]|QUNOB093-08|United States|Louisiana|658[0n]|BOLD:AAD0059
Catocala orba|[2733]|CNCLB2767-14|United States|North Carolina|658[0n]|BOLD:AAD0059
Catocala ultronia|[2734]|LNC075-05|United States|North Carolina|658[0n]|BOLD:AAA8724
Catocala ultronia|[2735]|RDLQF802-06|Canada|Quebec|658[0n]|BOLD:AAA8724
Catocala ultronia|[2736]|RDLQF738-06|Canada|Quebec|658[0n]|BOLD:AAA8724
Catocala ultronia|[2737]|RDLQB475-05|Canada|Quebec|658[0n]|BOLD:AAA8724
Catocala ultronia|[2738]|RDLQB474-05|Canada|Quebec|658[0n]|BOLD:AAA8724
Catocala ultronia|[2739]|LGSM497-04|United States|North Carolina|658[0n]|BOLD:AAA8724
Catocala ultronia|[2740]|XAD436-04|Canada|Ontario|580[0n]|BOLD:AAA8724
Catocala ultronia|[2741]|RDLQB567-05|Canada|Quebec|593[1n]|BOLD:AAA8724
Catocala ultronia|[2742]|XAK512-07|Canada|Ontario|658[0n]|BOLD:AAA8724
Catocala ultronia|[2743]|IMA004-07|United States|Massachusetts|658[0n]|BOLD:AAA8724
Catocala ultronia|[2744]|LNCC222-10|United States|North Carolina|658[0n]|BOLD:AAA8724
Catocala ultronia|[2745]|PHMO282-03|Canada|Ontario|639[0n]|BOLD:AAA8724
Catocala ultronia|[2746]|LGSMG600-07|United States|North Carolina|658[0n]|BOLD:AAA8724
Catocala ultronia|[2747]|QUNO446-08|United States|Florida|648[0n]|BOLD:AAA8724
Catocala ultronia|[2748]|QUNO439-08|United States|Florida|658[0n]|BOLD:AAA8724
Catocala ultronia|[2749]|QUNO440-08|United States|Florida|658[0n]|BOLD:AAA8724
Catocala ultronia|[2750]|QUNO441-08|United States|Florida|658[0n]|BOLD:AAA8724
Catocala ultronia|[2751]|QUNO442-08|United States|Florida|658[0n]|BOLD:AAA8724
Catocala ultronia|[2752]|QUNO443-08|United States|Florida|658[0n]|BOLD:AAA8724
Catocala ultronia|[2753]|QUNO444-08|United States|Florida|658[0n]|BOLD:AAA8724
Catocala ultronia|[2754]|QUNO445-08|United States|Florida|658[0n]|BOLD:AAA8724
Catocala ultronia|[2755]|LILLA564-11|United States|Illinois|658[0n]|BOLD:AAA8724
Catocala ultronia|[2756]|LILLA411-11|United States|Illinois|658[0n]|BOLD:AAA8724
Catocala ultronia|[2757]|JRLAA029-09|United States|Georgia|658[0n]|BOLD:AAA8724
Catocala ultronia|[2758]|QUNOB338-09|Canada|Saskatchewan|658[0n]|BOLD:AAA8724
Catocala ultronia|[2759]|QUNO815-08|United States|Wisconsin|658[0n]|BOLD:AAA8724
Catocala ultronia|[2760]|LTOL931-08|United States|Maryland|655[0n]|BOLD:AAA8724
Catocala ultronia|[2761]|QUNO084-07|United States|Wisconsin|658[0n]|BOLD:AAA8724
Catocala ultronia|[2762]|QUNO083-07|United States|Wisconsin|658[0n]|BOLD:AAA8724
Catocala ultronia|[2763]|XAK513-07|Canada|Ontario|658[0n]|BOLD:AAA8724
Catocala ultronia|[2764]|LGSMG601-07|United States|North Carolina|658[0n]|BOLD:AAA8724
Catocala ultronia|[2765]|RDLQF803-06|Canada|Quebec|658[0n]|BOLD:AAA8724
Catocala ultronia|[2766]|LNC076-05|United States|North Carolina|658[0n]|BOLD:AAA8724
Catocala ultronia|[2767]|XAG093-05|Canada|Ontario|658[0n]|BOLD:AAA8724
Catocala ultronia|[2768]|LGSM498-04|United States|North Carolina|658[0n]|BOLD:AAA8724
Catocala ultronia|[2769]|PHMO285-03|Canada|Ontario|639[0n]|BOLD:AAA8724
Catocala ultronia|[2770]|PHMO300-03|Canada|Ontario|639[0n]|BOLD:AAA8724
Catocala ultronia|[2771]|XAG174-05|Canada|Ontario|617[1n]|BOLD:AAA8724
Catocala ultronia|[2772]|LSEU697-06|United States|Georgia|658[0n]|BOLD:AAA8724
Catocala ultronia|[2773]|XAD574-04|Canada|Ontario|602[0n]|BOLD:AAA8724
Catocala ultronia|[2774]|XAG009-05|Canada|Ontario|629[0n]|BOLD:AAA8724
Catocala ultronia|[2775]|ABCNA497-07|United States|Florida|568[0n]|BOLD:AAA8724
Catocala ultronia|[2776]|ABCNA498-07|United States|Florida|568[0n]|BOLD:AAA8724
Catocala ultronia|[2777]|QUNO726-08|United States|Kentucky|658[0n]|BOLD:AAA8724
Catocala ultronia|[2778]|LPOKE266-11|United States|Oklahoma|658[0n]|BOLD:AAA8724
Catocala grisatra|[2779]|ABCNA356-06|United States|Florida|658[0n]|BOLD:AAE4385
Catocala grisatra|[2780]|ABCNA344-06|United States|Florida|658[0n]|BOLD:AAE4385
Catocala grisatra|[2781]|ABCNA259-06|United States|Florida|658[0n]|BOLD:AAE4385
Catocala grisatra|[2782]|ABCNA812-07|United States|Florida|648[0n]|BOLD:AAE4385
Catocala grisatra|[2783]|QUNOB508-09|United States|Florida|658[0n]|BOLD:AAE4385
Catocala crataegi|[2784]|QUNO013-07|United States|Florida|648[0n]|BOLD:AAI3786
Catocala crataegi|[2785]|QUNOD848-11|United States|Florida|658[0n]|BOLD:AAI3786
Catocala grynea|[2786]|XAK430-06|Canada|Ontario|658[0n]|BOLD:AAA9713
Catocala grynea|[2787]|XAK290-06|Canada|Ontario|658[0n]|BOLD:AAA9713
Catocala grynea|[2788]|XAK264-06|Canada|Ontario|658[0n]|BOLD:AAA9713
Catocala grynea|[2789]|XAG861-05|Canada|Ontario|658[0n]|BOLD:AAA9713
Catocala grynea|[2790]|XAG007-05|Canada|Ontario|658[0n]|BOLD:AAA9713
Catocala grynea|[2791]|MNBB068-05|Canada|New Brunswick|658[0n]|BOLD:AAA9713
Catocala grynea|[2792]|XAK522-07|Canada|Ontario|659[0n]|BOLD:AAA9713
Catocala grynea|[2793]|QUNO043-07|United States|Wisconsin|655[0n]|BOLD:AAA9713
Catocala grynea|[2794]|PHMO286-03|Canada|Ontario|639[0n]|BOLD:AAA9713
Catocala grynea|[2795]|XAK520-07|Canada|Ontario|658[0n]|BOLD:AAA9713
Catocala grynea|[2796]|XAC859-04|Canada|Ontario|599[1n]|BOLD:AAA9713

Catocala grynea|[2794]|PHMO286-03|Canada|Ontario|639[0n]|BOLD:AAA9713
Catocala grynea|[2795]|XAK520-07|Canada|Ontario|658[0n]|BOLD:AAA9713
Catocala grynea|[2796]|XAC859-04|Canada|Ontario|599[1n]|BOLD:AAA9713
Catocala grynea|[2797]|PHMO316-03|Canada|Ontario|639[0n]|BOLD:AAA9713
Catocala grynea|[2798]|XAC857-04|Canada|Ontario|591[0n]|BOLD:AAA9713
Catocala grynea|[2799]|XAD348-04|Canada|Ontario|565[0n]|BOLD:AAA9713
Catocala grynea|[2800]|XAK565-07|Canada|Ontario|581[1n]|BOLD:AAA9713
Catocala grynea|[2801]|ABCNA911-08|United States|Wisconsin|614[0n]|BOLD:AAA9713
Catocala grynea|[2802]|ABCNA912-08|United States|Florida|655[0n]|BOLD:AAA9713
Catocala grynea|[2803]|QUNO811-08|United States|Wisconsin|658[0n]|BOLD:AAA9713
Catocala grynea|[2804]|QUNO812-08|United States|Wisconsin|658[0n]|BOLD:AAA9713
Catocala grynea|[2805]|QUNO813-08|United States|Wisconsin|658[0n]|BOLD:AAA9713
Catocala grynea|[2806]|QUNO814-08|United States|Wisconsin|658[0n]|BOLD:AAA9713
Catocala grynea|[2807]|QUNOB102-08|United States|Louisiana|658[0n]|BOLD:AAA9713
Catocala grynea|[2808]|QUNOB101-08|United States|Louisiana|658[1n]|BOLD:AAA9713
Catocala grynea|[2809]|QUNOB061-08|United States|Texas|658[0n]|BOLD:AAA9713
Catocala grynea|[2810]|QUNOB092-08|United States|Louisiana|658[0n]|BOLD:AAA9713
Catocala grynea|[2811]|QUNOB094-08|United States|Louisiana|658[0n]|BOLD:AAA9713
Catocala grynea|[2812]|QUNOB106-08|United States|Louisiana|658[0n]|BOLD:AAA9713
Catocala grynea|[2813]|ABCNA256-06|United States|Texas|658[0n]|BOLD:AAA9713
Catocala grynea|[2814]|QUNOB107-08|United States|Louisiana|658[0n]|BOLD:AAA9713
Catocala lincolnana|[2815]|ABCNA251-06|United States|Texas|658[1n]|BOLD:ABY7952
Catocala lincolnana|[2816]|ABCNA598-07|United States|Florida|577[0n]|BOLD:ABY7952
Catocala lincolnana|[2817]|ABCNA370-06|United States|Florida|658[0n]|BOLD:ABY7952
Catocala lincolnana|[2818]|ABCNA372-06|United States|Florida|658[0n]|BOLD:ABY7952
Catocala lincolnana|[2819]|ABCNA373-06|United States|Florida|658[0n]|BOLD:ABY7952
Catocala lincolnana|[2820]|QUNO436-08|United States|Florida|658[0n]|BOLD:ABY7952
Catocala lincolnana|[2821]|QUNO437-08|United States|Florida|658[0n]|BOLD:ABY7952
Catocala lincolnana|[2822]|QUNO438-08|United States|Florida|658[0n]|BOLD:ABY7952
Catocala lincolnana|[2823]|QUNOB056-08|United States|Texas|658[0n]|BOLD:ABY7952
Catocala lincolnana|[2824]|QUNOB111-08|United States|Louisiana|658[0n]|BOLD:ABY7952
Catocala lincolnana|[2825]|LNCC053-10|United States|North Carolina|658[0n]|BOLD:ABY7952
Catocala pretiosa texarkana|[2826]|HKONB493-09|United States|Texas|658[0n]|BOLD:ACF4237
Catocala pretiosa texarkana|[2827]|QUNOD149-10|United States|Texas|658[0n]|BOLD:ACF4237
Catocala pretiosa texarkana|[2828]|QUNOD150-10|United States|Texas|658[0n]|BOLD:ACF4237
Catocala pretiosa|[2829]|ABCNA736-07|United States|New Jersey|615[1n]|BOLD:ACF3586
Catocala pretiosa|[2830]|ABCNA737-07|United States|New Jersey|606[0n]|BOLD:ACF3586
Catocala pretiosa|[2831]|LNCB673-09|United States|North Carolina|658[0n]|BOLD:ACF3586
Catocala pretiosa|[2832]|QUNOC325-10|United States|New Jersey|658[0n]|BOLD:ACF3586
Catocala pretiosa|[2833]|QUNOD696-11|United States|North Carolina|658[0n]|BOLD:ACF3586
Catocala pretiosa|[2834]|ABCNA368-06|United States|Florida|598[3n]|BOLD:ACF3586
Catocala pretiosa|[2835]|QUNOB295-09|United States|Florida|658[0n]|BOLD:ACF3586
Catocala pretiosa|[2836]|ABCNA600-07|United States|Florida|577[0n]|BOLD:ACF3586
Catocala pretiosa|[2837]|ABCNA367-06|United States|Florida|573[0n]|BOLD:ACF3586
Catocala pretiosa|[2838]|ABCNA599-07|United States|Florida|573[0n]|BOLD:ACF3586
Catocala pretiosa|[2839]|ABCNA510-07|United States|Florida|568[0n]|BOLD:ACF3586
Catocala pretiosa|[2840]|ABCNA508-07|United States|Florida|568[0n]|BOLD:ACF3586
Catocala pretiosa|[2841]|ABCNA507-07|United States|Florida|568[0n]|BOLD:ACF3586
Catocala pretiosa|[2842]|ABCNA509-07|United States|Florida|568[1n]|BOLD:ACF3586
Catocala pretiosa|[2843]|ABCNA075-06|United States|Florida|635[0n]|BOLD:ACF3586
Catocala pretiosa|[2844]|QUNO434-08|United States|Florida|658[1n]|BOLD:ACF3586
Catocala pretiosa|[2845]|ABCNA074-06|United States|Florida|607[0n]|BOLD:ACF3586
Catocala pretiosa|[2846]|ABCNA369-06|United States|Florida|631[0n]|BOLD:ACF3586
Catocala pretiosa|[2847]|QUNO322-08|United States|Florida|658[0n]|BOLD:ACF3586
Catocala pretiosa|[2848]|QUNO382-08|United States|Florida|658[0n]|BOLD:ACF3586
Catocala pretiosa|[2849]|QUNO383-08|United States|Florida|658[0n]|BOLD:ACF3586
Catocala pretiosa|[2850]|QUNOB293-09|United States|Florida|658[0n]|BOLD:ACF3586
Catocala pretiosa|[2851]|QUNOB294-09|United States|Florida|658[0n]|BOLD:ACF3586
Catocala pretiosa|[2852]|QUNOB296-09|United States|Florida|658[0n]|BOLD:ACF3586
Catocala pretiosa texarkana|[2853]|LSUSA191-06|United States|Kentucky|658[0n]|BOLD:ACF3586
Catocala pretiosa texarkana|[2854]|QUNOB067-08|United States|Texas|658[0n]|BOLD:ACF3586
Catocala pretiosa texarkana|[2855]|ABCNA250-06|United States|Texas|658[0n]|BOLD:ACF3586
Catocala pretiosa texarkana|[2856]|ABCNA247-06|United States|Texas|658[0n]|BOLD:ACF3586
Catocala pretiosa texarkana|[2857]|ABCNA076-06|United States|Texas|658[0n]|BOLD:ACF3586
Catocala pretiosa texarkana|[2858]|QUNO543-08|United States|Louisiana|658[0n]|BOLD:ACF3586
Catocala pretiosa texarkana|[2859]|QUNOB558-09|United States|Louisiana|658[0n]|BOLD:ACF3586
Catocala pretiosa texarkana|[2860]|QUNOB559-09|United States|Louisiana|658[0n]|BOLD:ACF3586
Catocala pretiosa texarkana|[2861]|LPOKB1001-09|United States|Oklahoma|658[0n]|BOLD:ACF3586
Catocala pretiosa|[2862]|QUNOD276-10|United States|Kentucky|658[0n]|BOLD:ACF3586
Catocala pretiosa|[2863]|QUNOD277-10|United States|Kentucky|658[0n]|BOLD:ACF3586
Catocala pretiosa|[2864]|QUNOD278-10|United States|Kentucky|658[0n]|BOLD:ACF3586
Catocala pretiosa texarkana|[2865]|QUNOD279-10|United States|Kentucky|658[0n]|BOLD:ACF3586
Catocala pretiosa texarkana|[2866]|QUNOD280-10|United States|Kentucky|658[0n]|BOLD:ACF3586
Catocala pretiosa texarkana|[2867]|QUNOD852-11|United States|Kentucky|658[0n]|BOLD:ACF3586
Catocala blandula|[2868]|QUNO677-08|United States|Kentucky|658[0n]|BOLD:ABZ4961
Catocala blandula manitoba|[2869]|RDMAB168-05|Canada|Alberta|658[0n]|BOLD:ABZ4961
Catocala blandula manitoba|[2870]|RDMAB185-05|Canada|Alberta|565[0n]|BOLD:ABZ4961
Catocala blandula manitoba|[2871]|RDMAB184-05|Canada|Alberta|559[1n]|BOLD:ABZ4961
Catocala blandula manitoba|[2872]|RDMAB186-05|Canada|Alberta|658[0n]|BOLD:ABZ4961
Catocala blandula|[2873]|ABCNA258-06|United States|Wisconsin|603[0n]|BOLD:ABZ4961
Catocala blandula|[2874]|HKONB480-09|United States|Wisconsin|658[0n]|BOLD:ABZ4961
Catocala blandula|[2875]|QUNOB129-08|United States|Wisconsin|658[0n]|BOLD:ABZ4961
Catocala blandula|[2876]|QUNO025-07|United States|Wisconsin|658[0n]|BOLD:ABZ4961
Catocala blandula|[2877]|HKONB478-09|United States|Wisconsin|630[0n]|BOLD:ABZ4961
Catocala blandula|[2878]|HKONB481-09|United States|Wisconsin|634[0n]|BOLD:ABZ4961
Catocala blandula|[2879]|HKONB482-09|United States|Wisconsin|658[0n]|BOLD:ABZ4961
Catocala blandula|[2880]|QUNO026-07|United States|Wisconsin|658[0n]|BOLD:ABZ4961
Catocala blandula|[2881]|HKONB483-09|United States|Wisconsin|658[0n]|BOLD:ABZ4961
Catocala blandula|[2882]|XAK521-07|Canada|Ontario|658[0n]|BOLD:ABZ4961
Catocala blandula|[2883]|LGSM459-04|United States|North Carolina|658[0n]|BOLD:ABZ4961
Catocala blandula|[2884]|QUNO467-08|United States|Tennessee|658[1n]|BOLD:ABZ4961
Catocala blandula|[2885]|RDLQF679-06|Canada|Quebec|637[0n]|BOLD:ABZ4961
Catocala blandula|[2886]|QUNO679-08|United States|Kentucky|658[0n]|BOLD:ABZ4961
Catocala blandula|[2887]|RDLQF805-06|Canada|Quebec|658[0n]|BOLD:ABZ4961
Catocala blandula|[2888]|RDLQF737-06|Canada|Quebec|658[0n]|BOLD:ABZ4961
Catocala blandula|[2889]|XAK431-06|Canada|Ontario|657[0n]|BOLD:ABZ4961
Catocala blandula|[2890]|LSEU696-06|United States|Georgia|658[0n]|BOLD:ABZ4961
Catocala blandula|[2891]|LSEU695-06|United States|Georgia|658[0n]|BOLD:ABZ4961
Catocala blandula|[2892]|LGSM458-04|United States|North Carolina|658[0n]|BOLD:ABZ4961
Catocala blandula|[2893]|RDLQF806-06|Canada|Quebec|592[0n]|BOLD:ABZ4961
Catocala blandula|[2894]|LGSMG602-07|United States|North Carolina|658[0n]|BOLD:ABZ4961
Catocala blandula|[2895]|LGSMG603-07|United States|North Carolina|658[0n]|BOLD:ABZ4961
Catocala blandula|[2896]|QUNO678-08|United States|Kentucky|658[0n]|BOLD:ABZ4961

Catocala blandula|[2894]|LGSMG602-07|United States|North Carolina|658[0n]|BOLD:ABZ4961
Catocala blandula|[2895]|LGSMG603-07|United States|North Carolina|658[0n]|BOLD:ABZ4961
Catocala blandula|[2896]|QUNO678-08|United States|Kentucky|658[0n]|BOLD:ABZ4961
Catocala blandula|[2897]|QUNO680-08|United States|Kentucky|658[0n]|BOLD:ABZ4961
Catocala blandula|[2898]|QUNO729-08|United States|Kentucky|658[0n]|BOLD:ABZ4961
Catocala blandula|[2899]|BBLPC078-09|Canada|New Brunswick|658[0n]|BOLD:ABZ4961
Catocala blandula|[2900]|BBLPC522-09|Canada|New Brunswick|658[0n]|BOLD:ABZ4961
Catocala grynea|[2901]|ABCNA910-08|United States|Florida|654[0n]|BOLD:ABZ2552
Catocala grynea|[2902]|ABCNA909-08|United States|Florida|653[0n]|BOLD:ABZ2552
Catocala grynea|[2903]|ABCNA602-07|United States|Florida|577[0n]|BOLD:ABZ2552
Catocala grynea|[2904]|ABCNA513-07|United States|Florida|550[1n]|BOLD:ABZ2552
Catocala grynea|[2905]|ABCNA511-07|United States|Florida|568[0n]|BOLD:ABZ2552
Catocala grynea|[2906]|ABCNA601-07|United States|Florida|577[0n]|BOLD:ABZ2552
Catocala grynea|[2907]|ABCNA913-08|United States|Wisconsin|658[0n]|BOLD:ABZ2552
Catocala grynea|[2908]|ABCNA936-08|United States|Florida|658[0n]|BOLD:ABZ2552
Catocala grynea|[2909]|ABCNA937-08|United States|Florida|655[0n]|BOLD:ABZ2552
Catocala grynea|[2910]|ABCNA938-08|United States|Florida|658[0n]|BOLD:ABZ2552
Catocala grynea|[2911]|ABCNA939-08|United States|Florida|649[0n]|BOLD:ABZ2552
Catocala grynea|[2912]|LGSM636-04|United States|Tennessee|609[0n]|BOLD:ABZ2552
Catocala grynea|[2913]|QUNO672-08|United States|Kentucky|658[0n]|BOLD:ABZ2552
Catocala grynea|[2914]|QUNO673-08|United States|Kentucky|658[0n]|BOLD:ABZ2552
Catocala grynea|[2915]|CNCLB2745-14|United States|North Carolina|658[0n]|BOLD:ABZ2552
Catocala dulciola|[2916]|QUNOB339-09|United States|Ohio|608[0n]|BOLD:AAE4437
Catocala dulciola|[2917]|QUNOB340-09|United States|Ohio|658[0n]|BOLD:AAE4437
Catocala dulciola|[2918]|QUNOC043-09|United States|Ohio|658[0n]|BOLD:AAE4437
Catocala dulciola|[2919]|ABCNA522-07|United States|Michigan|568[0n]|BOLD:AAE4437
Catocala dulciola|[2920]|QUNOC045-09|United States|Ohio|658[0n]|BOLD:AAE4437
Catocala crataegi|[2921]|QUNO037-07|United States|Wisconsin|658[0n]|BOLD:AAB2593
Catocala crataegi|[2922]|ABCNA078-06|United States|Wisconsin|609[0n]|BOLD:AAB2593
Catocala crataegi|[2923]|XAK516-07|Canada|Ontario|658[0n]|BOLD:AAB2593
Catocala crataegi|[2924]|QUNO038-07|United States|Michigan|658[0n]|BOLD:AAB2593
Catocala crataegi|[2925]|QUNO039-07|United States|Michigan|658[0n]|BOLD:AAB2593
Catocala crataegi|[2926]|QUNO040-07|United States|Michigan|658[0n]|BOLD:AAB2593
Catocala crataegi|[2927]|QUNO041-07|United States|Michigan|658[0n]|BOLD:AAB2593
Catocala crataegi|[2928]|QUNO042-07|United States|Michigan|658[0n]|BOLD:AAB2593
Catocala crataegi|[2929]|XAE622-04|Canada|Ontario|658[0n]|BOLD:AAB2593
Catocala crataegi|[2930]|MNBB001-05|Canada|New Brunswick|658[0n]|BOLD:AAB2593
Catocala crataegi|[2931]|XAC796-04|Canada|Ontario|658[0n]|BOLD:AAB2593
Catocala crataegi|[2932]|PMG098-03|Canada|Ontario|617[0n]|BOLD:AAB2593
Catocala crataegi|[2933]|XAJ977-06|Canada|Ontario|658[0n]|BOLD:AAB2593
Catocala crataegi|[2934]|XAK044-06|Canada|Ontario|658[0n]|BOLD:AAB2593
Catocala crataegi|[2935]|XAK045-06|Canada|Ontario|658[0n]|BOLD:AAB2593
Catocala crataegi|[2936]|BLTIB1056-08|Canada|Ontario|658[0n]|BOLD:AAB2593
Catocala crataegi|[2937]|PHSEP338-11|Canada|Ontario|657[0n]|BOLD:AAB2593
Catocala crataegi|[2938]|RDLQF682-06|Canada|Quebec|637[0n]|BOLD:AAB2593
Catocala crataegi|[2939]|RDLQF681-06|Canada|Quebec|637[0n]|BOLD:AAB2593
Catocala crataegi|[2940]|RDLQF680-06|Canada|Quebec|637[0n]|BOLD:AAB2593
Catocala crataegi|[2941]|MNBB574-05|Canada|New Brunswick|658[0n]|BOLD:AAB2593
Catocala crataegi|[2942]|RDLQB416-05|Canada|Quebec|658[0n]|BOLD:AAB2593
Catocala crataegi|[2943]|RDLQB732-05|Canada|Quebec|658[0n]|BOLD:AAB2593
Catocala crataegi|[2944]|XAK425-06|Canada|Ontario|658[0n]|BOLD:AAB2593
Catocala crataegi|[2945]|XAK456-06|Canada|Ontario|658[0n]|BOLD:AAB2593
Catocala crataegi|[2946]|RDLQG021-06|Canada|Quebec|658[0n]|BOLD:AAB2593
Catocala crataegi|[2947]|LNCC226-10|United States|North Carolina|658[0n]|BOLD:AAB2593
Catocala crataegi|[2948]|LNCC1202-11|United States|North Carolina|658[0n]|BOLD:AAB2593
Catocala crataegi|[2949]|QUNOC630-13|United States|Mississippi|613[0n]|BOLD:ABY6396
Catocala crataegi|[2950]|ABCNA249-06|United States|Texas|597[1n]|BOLD:ABY6396
Catocala crataegi|[2951]|ABCNA080-06|United States|Texas|607[0n]|BOLD:ABY6396
Catocala crataegi|[2952]|ABCNA081-06|United States|Texas|600[0n]|BOLD:ABY6396
Catocala crataegi|[2953]|ABCNA082-06|United States|Texas|658[0n]|BOLD:ABY6396
Catocala crataegi|[2954]|QUNO506-08|United States|Texas|658[0n]|BOLD:ABY6396
Catocala crataegi|[2955]|QUNO507-08|United States|Texas|658[0n]|BOLD:ABY6396
Catocala crataegi|[2956]|QUNOB066-08|United States|Texas|658[0n]|BOLD:ABY6396
Catocala crataegi|[2957]|QUNOB580-10|United States|Texas|658[0n]|BOLD:ABY6396
Catocala crataegi|[2958]|QUNOC631-13|United States|Mississippi|658[0n]|BOLD:ABY6396
Catocala aestivalia|[2959]|QUNO372-08|United States|Florida|658[0n]|BOLD:AAB3520
Catocala aestivalia|[2960]|QUNO429-08|United States|Florida|658[0n]|BOLD:AAB3520
Catocala aestivalia|[2961]|QUNO428-08|United States|Florida|658[0n]|BOLD:AAB3520
Catocala aestivalia|[2962]|QUNO427-08|United States|Florida|658[0n]|BOLD:AAB3520
Catocala aestivalia|[2963]|QUNO426-08|United States|Florida|658[0n]|BOLD:AAB3520
Catocala aestivalia|[2964]|QUNO320-08|United States|Florida|658[0n]|BOLD:AAB3520
Catocala aestivalia|[2965]|QUNO319-08|United States|Florida|658[0n]|BOLD:AAB3520
Catocala aestivalia|[2966]|QUNO317-08|United States|Florida|658[0n]|BOLD:AAB3520
Catocala aestivalia|[2967]|QUNO014-07|United States|Florida|658[0n]|BOLD:AAB3520
Catocala aestivalia|[2968]|QUNO425-08|United States|Florida|658[0n]|BOLD:AAB3520
Catocala aestivalia|[2969]|QUNO318-08|United States|Florida|658[0n]|BOLD:AAB3520
Catocala aestivalia|[2970]|ABCNA275-06|United States|Florida|608[0n]|BOLD:AAB3520
Catocala aestivalia|[2971]|ABCNA574-07|United States|Florida|577[0n]|BOLD:AAB3520
Catocala aestivalia|[2972]|ABCNA572-07|United States|Florida|577[0n]|BOLD:AAB3520
Catocala aestivalia|[2973]|ABCNA569-07|United States|Florida|577[0n]|BOLD:AAB3520
Catocala aestivalia|[2974]|ABCNA568-07|United States|Florida|577[0n]|BOLD:AAB3520
Catocala aestivalia|[2975]|ABCNA567-07|United States|Florida|577[0n]|BOLD:AAB3520
Catocala aestivalia|[2976]|ABCNA079-06|United States|Florida|582[0n]|BOLD:AAB3520
Catocala aestivalia|[2977]|ABCNA566-07|United States|Florida|577[0n]|BOLD:AAB3520
Catocala aestivalia|[2978]|ABCNA573-07|United States|Florida|577[0n]|BOLD:AAB3520
Catocala aestivalia|[2979]|ABCNA579-07|United States|Florida|577[0n]|BOLD:AAB3520
Catocala aestivalia|[2980]|ABCNA588-07|United States|Florida|577[0n]|BOLD:AAB3520
Catocala aestivalia|[2981]|ABCNA589-07|United States|Florida|577[0n]|BOLD:AAB3520
Catocala aestivalia|[2982]|QUNO464-08|United States|Florida|649[1n]|BOLD:AAB3520
Catocala aestivalia|[2983]|MILEQ181-11|United States|Florida|658[0n]|BOLD:AAB3520
Catocala aestivalia|[2984]|QUNOE210-12|United States|Florida|658[0n]|BOLD:AAB3520
Catocala aestivalia|[2985]|QUNOE607-14|United States|Florida|658[0n]|BOLD:AAB3520
Gonodontodes dispar|[2986]|GWOSX638-11|Cuba|Holguin|658[0n]|BOLD:ABW1373
Gonodontodes dispar|[2987]|GWOSX639-11|Cuba|Holguin|658[0n]|BOLD:ABW1373
Gonodontodes dispar|[2988]|CNCLB1631-14|United States|Florida|658[0n]|BOLD:ABW1373
Athyrma ganglio|[2989]|CNCLB1484-14|United States|Florida|307[0n]|
Athyrma ganglioDHJ02|[2990]|MHAUA546-05|Costa Rica|Guanacaste|658[0n]|BOLD:AAC7979
Athyrma ganglioDHJ02|[2991]|MHMXG253-07|Costa Rica|Guanacaste|658[0n]|BOLD:AAC7979
Athyrma ganglioDHJ02|[2992]|BLPDC269-09|Costa Rica|Guanacaste|658[0n]|BOLD:AAC7979
Athyrma ganglioDHJ02|[2993]|MHMYQ1684-12|Costa Rica||658[0n]|BOLD:AAC7979
Athyrma ganglioDHJ02|[2994]|MHAUA544-05|Costa Rica|Guanacaste|658[0n]|BOLD:AAC7979
Athyrma ganglioDHJ02|[2995]|MHAUA545-05|Costa Rica|Guanacaste|658[0n]|BOLD:AAC7979
Athyrma ganglioDHJ02|[2996]|MHMYN5269-14|Costa Rica||658[0n]|BOLD:AAC7979

[illegible]
Athyrma ganglioDHJ02|[2994]|MHAUA544-05|Costa Rica|Guanacaste|658[0n]|BOLD:AAC7979
Athyrma ganglioDHJ02|[2995]|MHAUA545-05|Costa Rica|Guanacaste|658[0n]|BOLD:AAC7979
Athyrma ganglioDHJ02|[2996]|MHMYN5269-14|Costa Rica||658[0n]|BOLD:AAC7979
Athyrma ganglioDHJ01|[2997]|MHMXD617-06|Costa Rica|Guanacaste|608[0n]|BOLD:AAC7979
Athyrma ganglioDHJ01|[2998]|MHAUA542-05|Costa Rica|Guanacaste|658[0n]|BOLD:AAC7979
Athyrma ganglio|[2999]|CNCLB1485-14|United States|Florida|658[0n]|BOLD:AAC7979
Athyrma ganglioDHJ01|[3000]|MHAUA543-05|Costa Rica|Guanacaste|658[0n]|BOLD:AAC7979
Athyrma ganglioDHJ01|[3001]|MHAUA541-05|Costa Rica|Guanacaste|658[0n]|BOLD:AAC7979
Athyrma ganglioDHJ01|[3002]|MHAUA547-05|Costa Rica|Guanacaste|658[0n]|BOLD:AAC7979
Athyrma ganglioDHJ01|[3003]|MHMYN140-11|Costa Rica||658[0n]|BOLD:AAC7979
Athyrma ganglioDHJ01|[3004]|MHMYQ1685-12|Costa Rica||658[0n]|BOLD:AAC7979
Athyrma ganglioDHJ01|[3005]|MHMYT219-13|Costa Rica||658[0n]|BOLD:AAC7979
Athyrma ganglioDHJ01|[3006]|MHMYN5270-14|Costa Rica||658[0n]|BOLD:AAC7979
Athyrma Poole01|[3007]|BLPEF6705-14|Costa Rica|Guanacaste|658[0n]|BOLD:AAK4103
Athyrma Poole01|[3008]|RDNML003-13|United States|Florida|658[0n]|BOLD:AAK4103
Athyrma Poole01|[3009]|BLPDZ212-11|Costa Rica|Guanacaste|658[0n]|BOLD:AAK4103
Athyrma Poole01|[3010]|BLPDJ439-09|Costa Rica|Guanacaste|658[0n]|BOLD:AAK4103
Athyrma Poole01|[3011]|BLPDY312-11|Costa Rica|Guanacaste|614[0n]|BOLD:AAK4103
Athyrma Poole01|[3012]|BLPEB027-11|Costa Rica|Guanacaste|632[0n]|BOLD:AAK4103
Athyrma Poole01|[3013]|CNCLB2243-14|Mexico||621[0n]|BOLD:AAK4103
Athyrma Poole01|[3014]|MHMYN6519-14|Costa Rica||658[0n]|BOLD:AAK4103
Lascoria aon|[3015]|LOCRC195-08|Costa Rica|Alajuela|658[0n]|BOLD:AAW9958
Ommatochila mundula|[3016]|RDNME637-08|United States|Tennessee|562[0n]|BOLD:AAC5684
Ommatochila mundula|[3017]|USLEP723-10|United States|Texas|627[0n]|BOLD:AAC5684
Ommatochila mundula|[3018]|LPOKA519-09|United States|Oklahoma|627[0n]|BOLD:AAC5684
Ommatochila mundula|[3019]|LPOKD270-09|United States|Oklahoma|657[0n]|BOLD:AAC5684
Ommatochila mundula|[3020]|LPOKD051-09|United States|Oklahoma|658[0n]|BOLD:AAC5684
Ommatochila mundula|[3021]|LPOKB007-09|United States|Oklahoma|658[0n]|BOLD:AAC5684
Ommatochila mundula|[3022]|LPOKA501-09|United States|Oklahoma|658[0n]|BOLD:AAC5684
Ommatochila mundula|[3023]|LPOKA404-09|United States|Oklahoma|658[0n]|BOLD:AAC5684
Ommatochila mundula|[3024]|HKONB333-09|United States|Texas|658[0n]|BOLD:AAC5684
Ommatochila mundula|[3025]|LPOKA312-08|United States|Oklahoma|658[0n]|BOLD:AAC5684
Ommatochila mundula|[3026]|LPOKA304-08|United States|Oklahoma|658[0n]|BOLD:AAC5684
Ommatochila mundula|[3027]|LPOKA263-08|United States|Oklahoma|658[0n]|BOLD:AAC5684
Ommatochila mundula|[3028]|RDNMG241-08|United States|Texas|658[0n]|BOLD:AAC5684
Ommatochila mundula|[3029]|LPOKB603-09|United States|Oklahoma|658[0n]|BOLD:AAC5684
Ommatochila mundula|[3030]|LPOKD417-09|United States|Oklahoma|649[0n]|BOLD:AAC5684
Ommatochila mundula|[3031]|LPOKE166-10|United States|Oklahoma|658[0n]|BOLD:AAC5684
Sigela sp. 5|[3032]|HKONS565-08|United States|Florida|655[0n]|BOLD:AAE9651
Sigela sp. 5|[3033]|RDNMD459-06|United States|Florida|617[0n]|BOLD:AAE9651
Sigela sp. 5|[3034]|MNAG601-08|United States|Florida|633[0n]|BOLD:AAE9651
Sigela sp. 5|[3035]|HKONB316-09|United States|Florida|658[0n]|BOLD:AAE9651
Sigela sp. 5|[3036]|RDNMK257-11|United States|Florida|658[0n]|BOLD:AAE9651
Sigela eoides|[3037]|HKONS654-08|United States|Florida|658[0n]|BOLD:AAJ6789
Sigela eoides|[3038]|RDNML304-13|United States|Florida|658[0n]|BOLD:AAJ6789
Sigela eoides|[3039]|CNCLB1773-14|United States|Florida|658[0n]|BOLD:AAJ6789
Euclidia cuspidea|[3040]|LGSMG583-07|United States|Tennessee|658[0n]|BOLD:AAB9610
Euclidia ardita|[3041]|NAMUM264-08|United States|California|658[0n]|BOLD:AAB9610
Euclidia cuspidea|[3042]|RDMAB133-05|Canada|Alberta|658[0n]|BOLD:AAB9610
Euclidia cuspidea|[3043]|LPMN466-08|Canada|Manitoba|658[0n]|BOLD:AAB9610
Euclidia cuspidea|[3044]|LPMN464-08|Canada|Manitoba|658[0n]|BOLD:AAB9610
Euclidia cuspidea|[3045]|LPMN201-08|Canada|Manitoba|658[0n]|BOLD:AAB9610
Euclidia cuspidea|[3046]|RDLQG276-06|Canada|Quebec|658[0n]|BOLD:AAB9610
Euclidia cuspidea|[3047]|LPMN465-08|Canada|Manitoba|658[0n]|BOLD:AAB9610
Euclidia cuspidea|[3048]|LPMN467-08|Canada|Manitoba|658[0n]|BOLD:AAB9610
Euclidia cuspidea|[3049]|BBLPB817-10|Canada|Saskatchewan|658[0n]|BOLD:AAB9610
Euclidia cuspidea|[3050]|BBLPB818-10|Canada|Saskatchewan|658[0n]|BOLD:AAB9610
Euclidia cuspidea|[3051]|CNWBA010-13|Canada|Alberta|619[0n]|BOLD:AAB9610
Euclidia cuspidea|[3052]|LPOKB123-09|United States|Oklahoma|658[0n]|BOLD:AAB9610
Euclidia cuspidea|[3053]|LGSM651-04|United States|Tennessee|658[0n]|BOLD:AAB9610
Euclidia cuspidea|[3054]|LGSM650-04|United States|Tennessee|658[0n]|BOLD:AAB9610
Euclidia cuspidea|[3055]|LGSMC908-05|United States|Tennessee|613[0n]|BOLD:AAB9610
Euclidia cuspidea|[3056]|TMNBB074-06|Canada|New Brunswick|658[0n]|BOLD:AAB9610
Euclidia cuspidea|[3057]|KPOEC108-08|Canada|Ontario|658[0n]|BOLD:AAB9610
Euclidia cuspidea|[3058]|KPOEC109-08|Canada|Ontario|658[0n]|BOLD:AAB9610
Euclidia cuspidea|[3059]|HKONB377-09|United States|Kentucky|658[0n]|BOLD:AAB9610
Euclidia cuspidea|[3060]|BBLOB1323-11|United States|Oklahoma|658[0n]|BOLD:AAB9610
Euclidia sp. 1|[3061]|QUNOC620-13|United States|Florida|658[0n]|BOLD:AAB9610
Caenurgia chloropha|[3062]|LPOKA362-08|United States|Oklahoma|658[0n]|BOLD:AAB6101
Caenurgia chloropha|[3063]|LSEU044-06|United States|Georgia|658[0n]|BOLD:AAB6101
Caenurgia chloropha|[3064]|LOFLA910-06|United States|Florida|656[0n]|BOLD:AAB6101
Caenurgia chloropha|[3065]|LOFLB480-06|United States|Florida|658[0n]|BOLD:AAB6101
Caenurgia chloropha|[3066]|ABNCC401-07|United States|Texas|652[0n]|BOLD:AAB6101
Caenurgia chloropha|[3067]|LSUSA020-06|United States|Kentucky|658[0n]|BOLD:AAB6101
Caenurgia chloropha|[3068]|LNC020-05|United States|North Carolina|658[0n]|BOLD:AAB6101
Caenurgia chloropha|[3069]|LGSM551-04|United States|Tennessee|614[0n]|BOLD:AAB6101
Caenurgia chloropha|[3070]|LNC019-05|United States|North Carolina|658[0n]|BOLD:AAB6101
Caenurgia chloropha|[3071]|LOFLC467-06|United States|Florida|658[0n]|BOLD:AAB6101
Caenurgia chloropha|[3072]|LPOKC768-09|United States|Oklahoma|658[0n]|BOLD:AAB6101
Caenurgia chloropha|[3073]|LGSM550-04|United States|Tennessee|541[0n]|BOLD:AAB6101
Caenurgia chloropha|[3074]|LOFLA891-06|United States|Florida|658[0n]|BOLD:AAB6101
Caenurgia chloropha|[3075]|LGSMG584-07|United States|Tennessee|658[0n]|BOLD:AAB6101
Caenurgia chloropha|[3076]|LOFLA904-06|United States|Florida|658[0n]|BOLD:AAB6101
Caenurgia chloropha|[3077]|LSEU043-06|United States|Georgia|605[0n]|BOLD:AAB6101
Caenurgia chloropha|[3078]|LOFLA905-06|United States|Florida|658[0n]|BOLD:AAB6101
Caenurgia chloropha|[3079]|LPOKA344-08|United States|Oklahoma|658[0n]|BOLD:AAB6101
Caenurgia chloropha|[3080]|LPOKA988-09|United States|Oklahoma|658[0n]|BOLD:AAB6101
Caenurgia chloropha|[3081]|BBLSU095-09|United States|Mississippi|658[0n]|BOLD:AAB6101
Caenurgia chloropha|[3082]|LSEU307-06|United States|Georgia|658[0n]|BOLD:AAB6101
Caenurgia chloropha|[3083]|BBLSU110-09|United States|Mississippi|658[0n]|BOLD:AAB6101
Caenurgia togataria|[3084]|LOCBD255-06|United States|California|658[0n]|BOLD:AAA9374
Caenurgia togataria|[3085]|LOCBD749-06|United States|California|663[0n]|BOLD:AAA9374
Caenurgia togataria|[3086]|LOCBD686-06|United States|California|658[0n]|BOLD:AAA9374
Caenurgia togataria|[3087]|GMLC003-09|United States|California|658[0n]|BOLD:AAA9374
Caenurgia togataria|[3088]|NAMUM372-09|United States|California|658[0n]|BOLD:AAA9374
Caenurgia togataria|[3089]|LOCBE113-06|United States|California|658[0n]|BOLD:AAA9374
Caenurgia togataria|[3090]|LOCBD687-06|United States|California|658[0n]|BOLD:AAA9374
Caenurgia togataria|[3091]|LOCBD685-06|United States|California|658[0n]|BOLD:AAA9374
Caenurgia togataria|[3092]|LOCBD283-06|United States|California|658[0n]|BOLD:AAA9374
Caenurgia togataria|[3093]|LOCBD279-06|United States|California|658[0n]|BOLD:AAA9374
Caenurgia togataria|[3094]|LOCBD262-06|United States|California|658[0n]|BOLD:AAA9374
Caenurgia togataria|[3095]|LOCBD256-06|United States|California|658[0n]|BOLD:AAA9374
[illegible]

Caenurgia togataria|[3093]|LOCBD279-06|United States|California|658[0n]|BOLD:AAA9374
Caenurgia togataria|[3094]|LOCBD262-06|United States|California|658[0n]|BOLD:AAA9374
Caenurgia togataria|[3095]|LOCBD256-06|United States|California|658[0n]|BOLD:AAA9374
Caenurgia togataria|[3096]|LOCBD230-06|United States|California|658[0n]|BOLD:AAA9374
Caenurgia togataria|[3097]|LOCBC419-06|United States|California|658[0n]|BOLD:AAA9374
Caenurgia togataria|[3098]|LOCBC415-06|United States|California|658[0n]|BOLD:AAA9374
Caenurgia togataria|[3099]|LOCBC157-06|United States|California|658[0n]|BOLD:AAA9374
Caenurgia togataria|[3100]|LOCBB654-06|United States|California|658[0n]|BOLD:AAA9374
Caenurgia togataria|[3101]|LOCBB307-06|United States|California|658[0n]|BOLD:AAA9374
Caenurgia togataria|[3102]|LOCBB306-06|United States|California|658[0n]|BOLD:AAA9374
Caenurgia togataria|[3103]|LOCBB305-06|United States|California|658[0n]|BOLD:AAA9374
Caenurgia togataria|[3104]|LOCBB304-06|United States|California|658[0n]|BOLD:AAA9374
Caenurgia togataria|[3105]|LOCBB303-06|United States|California|658[0n]|BOLD:AAA9374
Caenurgia togataria|[3106]|ABNCC420-07|United States|California|650[0n]|BOLD:AAA9374
Caenurgia togataria|[3107]|LOCBD688-06|United States|California|658[0n]|BOLD:AAA9374
Caenurgia togataria|[3108]|ABNCC421-07|United States|California|642[0n]|BOLD:AAA9374
Caenurgia togataria|[3109]|LOCBE112-06|United States|California|621[0n]|BOLD:AAA9374
Caenurgia togataria|[3110]|LOCBC062-06|United States|California|623[0n]|BOLD:AAA9374
Caenurgia togataria|[3111]|LOCBC061-06|United States|California|622[0n]|BOLD:AAA9374
Caenurgia togataria|[3112]|LOCBC797-06|United States|California|602[0n]|BOLD:AAA9374
Caenurgia togataria|[3113]|LOCBE362-06|United States|California|594[0n]|BOLD:AAA9374
Caenurgia togataria|[3114]|LOCBE412-06|United States|California|594[0n]|BOLD:AAA9374
Caenurgia togataria|[3115]|LOCBE413-06|United States|California|603[0n]|BOLD:AAA9374
Caenurgia togataria|[3116]|LOCBE414-06|United States|California|595[0n]|BOLD:AAA9374
Caenurgia togataria|[3117]|ABNCC422-07|United States|California|622[0n]|BOLD:AAA9374
Caenurgia togataria|[3118]|LOCBC798-06|United States|California|658[0n]|BOLD:AAA9374
Caenurgia togataria|[3119]|LOCBD284-06|United States|California|658[0n]|BOLD:AAA9374
Caenurgia togataria|[3120]|IAWL074-09|United States|California|658[0n]|BOLD:AAA9374
Caenurgia togataria|[3121]|IAWL075-09|United States|California|658[0n]|BOLD:AAA9374
Caenurgia togataria|[3122]|GMLC444-11|United States|California|658[0n]|BOLD:AAA9374
Caenurgia togataria|[3123]|GMLC457-11|United States|California|658[0n]|BOLD:AAA9374
Caenurgia togataria|[3124]|GMLC473-11|United States|California|658[0n]|BOLD:AAA9374
Caenurgia togataria|[3125]|GMLC474-11|United States|California|658[0n]|BOLD:AAA9374
Caenurgia togataria|[3126]|GMLC476-11|United States|California|658[0n]|BOLD:AAA9374
Caenurgia togataria|[3127]|GMLC853-12|United States|California|658[0n]|BOLD:AAA9374
Caenurgia togataria|[3128]|GMLC889-12|United States|California|658[0n]|BOLD:AAA9374
Caenurgia togataria|[3129]|GMLC1083-12|United States|California|658[0n]|BOLD:AAA9374
Caenurgia togataria|[3130]|GMLC1217-12|United States|California|658[0n]|BOLD:AAA9374
Celiptera valina|[3131]|ABNCC426-07|United States|Arkansas|650[1n]|BOLD:AAC9816
Celiptera valina|[3132]|IAWL184-09|United States|Arizona|658[0n]|BOLD:AAC9816
Celiptera valina|[3133]|IAWL181-09|United States|Arizona|658[0n]|BOLD:AAC9816
Celiptera valina|[3134]|IAWL182-09|United States|Arizona|658[0n]|BOLD:AAC9816
Celiptera valina|[3135]|IAWL183-09|United States|Arizona|658[0n]|BOLD:AAC9816
Celiptera valina|[3136]|IAWL186-09|United States|Arizona|658[0n]|BOLD:AAC9816
Celiptera valina|[3137]|IAWL185-09|United States|Arizona|658[0n]|BOLD:AAC9816
Celiptera valina|[3138]|IAWL190-09|United States|Arizona|658[0n]|BOLD:AAC9816
Celiptera valina|[3139]|RDNMH829-09|United States|Arizona|658[0n]|BOLD:AAC9816
Celiptera frustulum|[3140]|LNCB437-07|United States|North Carolina|658[0n]|BOLD:AAC2193
Celiptera frustulum|[3141]|LOCT090-05|United States|Connecticut|658[0n]|BOLD:AAC2193
Celiptera frustulum|[3142]|LPOKC625-09|United States|Oklahoma|658[0n]|BOLD:AAC2193
Celiptera frustulum|[3143]|CNPPI013-12|Canada|Ontario|639[0n]|BOLD:AAC2193
Celiptera frustulum|[3144]|LGSM726-04|United States|Tennessee|658[0n]|BOLD:AAC2193
Celiptera frustulum|[3145]|LPOKC752-09|United States|Oklahoma|658[0n]|BOLD:AAC2193
Celiptera frustulum|[3146]|LPSO875-08|Canada|Ontario|658[0n]|BOLD:AAC2193
Celiptera frustulum|[3147]|LPSO644-08|Canada|Ontario|658[0n]|BOLD:AAC2193
Celiptera frustulum|[3148]|LSUSA256-06|United States|Kentucky|658[0n]|BOLD:AAC2193
Celiptera frustulum|[3149]|ABNCC415-07|United States|Texas|643[2n]|BOLD:AAC2193
Celiptera frustulum|[3150]|LPSO885-08|Canada|Ontario|658[0n]|BOLD:AAC2193
Celiptera frustulum|[3151]|LILLB058-11|United States|Illinois|658[0n]|BOLD:AAC2193
Celiptera frustulum|[3152]|LGSM725-04|United States|Tennessee|658[0n]|BOLD:AAC2193
Celiptera frustulum|[3153]|ABNCC416-07|United States|Texas|594[0n]|BOLD:AAC2193
Celiptera frustulum|[3154]|CNPPJ1452-12|Canada|Ontario|644[0n]|BOLD:AAC2193
Celiptera frustulum|[3155]|LPOKA522-09|United States|Oklahoma|658[0n]|BOLD:AAC2193
Celiptera frustulum|[3156]|GMGSR073-13|United States|Tennessee|593[0n]|BOLD:AAC2193
Ptichodis herbarum|[3157]|LSEU067-06|United States|Georgia|598[1n]|BOLD:AAD2982
Ptichodis herbarum|[3158]|RDNMH947-09|United States|Florida|658[0n]|BOLD:AAD2982
Ptichodis herbarum|[3159]|LPOKA034-08|United States|Oklahoma|658[1n]|BOLD:AAD2982
Ptichodis herbarum|[3160]|PHFLO062-10|United States|Florida|634[0n]|BOLD:AAD2982
Ptichodis herbarum|[3161]|ABNCC418-07|United States|Texas|652[1n]|BOLD:AAD2982
Ptichodis herbarum|[3162]|LOFLA848-06|United States|Florida|658[0n]|BOLD:AAD2982
Ptichodis herbarum|[3163]|LOFLA850-06|United States|Florida|658[0n]|BOLD:AAD2982
Ptichodis herbarum|[3164]|LSEU359-06|United States|Georgia|658[0n]|BOLD:AAD2982
Ptichodis herbarum|[3165]|LGSMG591-07|United States|Tennessee|658[0n]|BOLD:AAD2982
Ptichodis herbarum|[3166]|BBLSX550-09|United States|Oklahoma|658[0n]|BOLD:AAD2982
Ptichodis herbarum|[3167]|MILEQ325-11|United States|Alabama|658[0n]|BOLD:AAD2982
Ptichodis herbarum|[3168]|MILEQ326-11|United States|Alabama|658[0n]|BOLD:AAD2982
Ptichodis herbarum|[3169]|LNCC1529-13|United States|North Carolina|658[0n]|BOLD:AAD2982
Ptichodis bucetum|[3170]|ABNCC053-07|United States|Utah|626[0n]|BOLD:AAF5365
Ptichodis bucetum|[3171]|NAMUM086-08|United States|Arizona|656[0n]|BOLD:AAF5365
Ptichodis sp.|[3172]|HKONB514-09|United States|Texas|658[0n]|BOLD:AAE0185
Ptichodis sp.|[3173]|RDNMG260-08|United States|Texas|658[0n]|BOLD:AAE0185
Ptichodis sp.|[3174]|HKONB536-09|United States|Texas|647[0n]|BOLD:AAE0185
Ptichodis sp.|[3175]|HKONB548-09|United States|Texas|647[0n]|BOLD:AAE0185
Ptichodis ovalis|[3176]|ABNCC014-07|United States|Arizona|634[0n]|BOLD:AAE0184
Ptichodis ovalis|[3177]|IAWL165-09|United States|Arizona|658[0n]|BOLD:AAE0184
Ptichodis ovalis|[3178]|CMAZA778-10|United States|Arizona|658[0n]|BOLD:AAE0184
Ptichodis ovalis|[3179]|RDNMJ508-11|United States|Arizona|658[0n]|BOLD:AAE0184
Ptichodis pacalis|[3180]|LOFLC324-06|United States|Florida|658[0n]|BOLD:AAF5367
Ptichodis pacalis|[3181]|RDNMH944-09|United States|Florida|658[0n]|BOLD:AAF5367
Ptichodis pacalis|[3182]|RDNMH945-09|United States|Florida|658[0n]|BOLD:AAF5367
Ptichodis pacalis|[3183]|BBLOB1295-11|United States|Florida|658[0n]|BOLD:AAF5367
Ptichodis bistrigata|[3184]|LNCC1587-13|United States|North Carolina|658[0n]|BOLD:AAL2327
Ptichodis bistrigata|[3185]|LSEU068-06|United States|Georgia|578[0n]|BOLD:AAL2327
Ptichodis bistrigata|[3186]|LNCC1465-13|United States|North Carolina|658[0n]|BOLD:AAL2327
Ptichodis bistrigata|[3187]|LNCC1586-13|United States|North Carolina|658[0n]|BOLD:AAL2327
Ptichodis bistrigata|[3188]|LNCC1588-13|United States|North Carolina|658[0n]|BOLD:AAL2327
Ptichodis vinculum|[3189]|LOFLA899-06|United States|Florida|658[0n]|BOLD:AAD8945
Ptichodis vinculum|[3190]|HKONS040-07|United States|Florida|658[0n]|BOLD:AAD8945
Ptichodis vinculum|[3191]|BBLOB817-11|United States|Florida|658[0n]|BOLD:AAD8945
Ptichodis vinculum|[3192]|BBLOB1298-11|United States|Florida|658[0n]|BOLD:AAD8945
Ptichodis vinculum|[3193]|LOFLC461-06|United States|Florida|658[0n]|BOLD:AAD8945
Ptichodis vinculum|[3194]|LOFLC458-06|United States|Florida|658[0n]|BOLD:AAD8945
Ptichodis vinculum|[3195]|LOFLC399-06|United States|Florida|658[0n]|BOLD:AAD8945
[illegible]

Ptichodis vinculum|[3193]|LOFLC461-06|United States|Florida|658[0n]|BOLD:AAD8945
Ptichodis vinculum|[3194]|LOFLC458-06|United States|Florida|658[0n]|BOLD:AAD8945
Ptichodis vinculum|[3195]|LOFLC399-06|United States|Florida|658[0n]|BOLD:AAD8945
Ptichodis vinculum|[3196]|LOFLA906-06|United States|Florida|658[0n]|BOLD:AAD8945
Ptichodis vinculum|[3197]|LOFLA889-06|United States|Florida|658[0n]|BOLD:AAD8945
Ptichodis vinculum|[3198]|ABNCC417-07|United States|Texas|639[0n]|BOLD:AAD8945
Ptichodis vinculum|[3199]|RDNMH952-09|United States|Florida|658[0n]|BOLD:AAD8945
Ptichodis vinculum|[3200]|BBLOB392-11|United States|Florida|658[0n]|BOLD:AAD8945
Ptichodis vinculum|[3201]|BBLOB1706-11|United States|Florida|658[0n]|BOLD:AAD8945
Ptichodis immunis|[3202]|LOCRE538-10|Costa Rica|San Jose|658[0n]|BOLD:AAA7258
Ptichodis immunis|[3203]|GWOSU366-11|Colombia||658[0n]|BOLD:AAA7258
Ptichodis immunis|[3204]|BLPAE506-06|Costa Rica|Guanacaste|658[0n]|BOLD:AAA7258
Ptichodis immunis|[3205]|LOCRE535-10|Costa Rica|San Jose|658[0n]|BOLD:AAA7258
Ptichodis immunis|[3206]|BLPCH040-08|Costa Rica|Guanacaste|658[0n]|BOLD:AAA7258
Ptichodis immunis|[3207]|MHAUC179-06|Costa Rica|Guanacaste|559[0n]|BOLD:AAA7258
Ptichodis immunis|[3208]|BLPBC852-07|Costa Rica|Alajuela|551[0n]|BOLD:AAA7258
Ptichodis immunis|[3209]|BLPDH750-09|Costa Rica|Alajuela|562[0n]|BOLD:AAA7258
Ptichodis immunis|[3210]|BLPCJ190-08|Costa Rica|Guanacaste|658[0n]|BOLD:AAA7258
Ptichodis immunis|[3211]|BLPAF653-07|Costa Rica|Guanacaste|658[0n]|BOLD:AAA7258
Ptichodis immunis|[3212]|BLPBC849-07|Costa Rica|Alajuela|631[1n]|BOLD:AAA7258
Ptichodis immunis|[3213]|BLPDH024-09|Costa Rica|Guanacaste|633[0n]|BOLD:AAA7258
Ptichodis immunis|[3214]|BLPDA708-09|Costa Rica|Guanacaste|632[0n]|BOLD:AAA7258
Ptichodis immunis|[3215]|LYNYM218-09|Mexico|Quintana Roo|628[0n]|BOLD:AAA7258
Ptichodis immunis|[3216]|BLPBC854-07|Costa Rica|Alajuela|645[0n]|BOLD:AAA7258
Ptichodis immunis|[3217]|BLPCJ558-08|Costa Rica|Guanacaste|658[0n]|BOLD:AAA7258
Ptichodis immunis|[3218]|BLPBD259-07|Costa Rica|Guanacaste|656[0n]|BOLD:AAA7258
Ptichodis immunis|[3219]|BLPAF652-07|Costa Rica|Guanacaste|658[0n]|BOLD:AAA7258
Ptichodis immunis|[3220]|LPYPB588-08|Mexico|Yucatan|658[1n]|BOLD:AAA7258
Ptichodis immunis|[3221]|BLPCK207-08|Costa Rica|Alajuela|658[0n]|BOLD:AAA7258
Ptichodis immunis|[3222]|BLPCK839-08|Costa Rica|Alajuela|658[0n]|BOLD:AAA7258
Ptichodis immunis|[3223]|BLPCK986-08|Costa Rica|Alajuela|658[0n]|BOLD:AAA7258
Ptichodis immunis|[3224]|BLPDB906-09|Costa Rica|Guanacaste|658[0n]|BOLD:AAA7258
Ptichodis immunis|[3225]|BLPDC664-09|Costa Rica|Alajuela|658[0n]|BOLD:AAA7258
Ptichodis immunis|[3226]|MHMXM227-07|Costa Rica|Guanacaste|658[0n]|BOLD:AAA7258
Ptichodis immunis|[3227]|BLPCA258-08|Costa Rica|Guanacaste|658[0n]|BOLD:AAA7258
Ptichodis immunis|[3228]|BLPCH038-08|Costa Rica|Guanacaste|658[0n]|BOLD:AAA7258
Ptichodis immunis|[3229]|BLPCJ090-08|Costa Rica|Guanacaste|658[0n]|BOLD:AAA7258
Ptichodis immunis|[3230]|BLPCA259-08|Costa Rica|Guanacaste|658[0n]|BOLD:AAA7258
Ptichodis immunis|[3231]|BLPCA871-08|Costa Rica|Guanacaste|658[0n]|BOLD:AAA7258
Ptichodis immunis|[3232]|BLPBD583-07|Costa Rica|Guanacaste|658[0n]|BOLD:AAA7258
Ptichodis immunis|[3233]|BLPBD257-07|Costa Rica|Guanacaste|658[0n]|BOLD:AAA7258
Ptichodis immunis|[3234]|BLPBB688-07|Costa Rica|Guanacaste|658[0n]|BOLD:AAA7258
Ptichodis immunis|[3235]|BLPBA011-07|Costa Rica|Guanacaste|658[0n]|BOLD:AAA7258
Ptichodis immunis|[3236]|BLPAG023-07|Costa Rica|Guanacaste|658[0n]|BOLD:AAA7258
Ptichodis immunis|[3237]|BLPAG022-07|Costa Rica|Guanacaste|658[0n]|BOLD:AAA7258
Ptichodis immunis|[3238]|BLPAF911-07|Costa Rica|Guanacaste|658[0n]|BOLD:AAA7258
Ptichodis immunis|[3239]|BLPAF656-07|Costa Rica|Guanacaste|658[0n]|BOLD:AAA7258
Ptichodis immunis|[3240]|BLPAC733-06|Costa Rica|Guanacaste|658[0n]|BOLD:AAA7258
Ptichodis immunis|[3241]|BLPAA290-06|Costa Rica|Guanacaste|658[0n]|BOLD:AAA7258
Ptichodis immunis|[3242]|BLPAA113-06|Costa Rica|Guanacaste|658[0n]|BOLD:AAA7258
Ptichodis immunis|[3243]|BLPAA095-06|Costa Rica|Guanacaste|658[0n]|BOLD:AAA7258
Ptichodis immunis|[3244]|BLPCK707-08|Costa Rica|Alajuela|658[0n]|BOLD:AAA7258
Ptichodis immunis|[3245]|BLPAB015-06|Costa Rica|Alajuela|658[0n]|BOLD:AAA7258
Ptichodis immunis|[3246]|BLPDH178-09|Costa Rica|Guanacaste|658[0n]|BOLD:AAA7258
Ptichodis immunis|[3247]|BLPCJ507-08|Costa Rica|Guanacaste|658[0n]|BOLD:AAA7258
Ptichodis immunis|[3248]|BLPAC326-06|Costa Rica|Guanacaste|622[0n]|BOLD:AAA7258
Ptichodis immunis|[3249]|LYHES456-09|Mexico|Yucatan|632[0n]|BOLD:AAA7258
Ptichodis immunis|[3250]|MXBLP165-11|Mexico|Jalisco|658[0n]|BOLD:AAA7258
Ptichodis immunis|[3251]|BLPCK206-08|Costa Rica|Alajuela|658[0n]|BOLD:AAA7258
Ptichodis immunis|[3252]|BLPCK209-08|Costa Rica|Alajuela|658[0n]|BOLD:AAA7258
Ptichodis immunis|[3253]|BLPCK702-08|Costa Rica|Alajuela|658[0n]|BOLD:AAA7258
Ptichodis immunis|[3254]|BLPCK706-08|Costa Rica|Alajuela|658[0n]|BOLD:AAA7258
Ptichodis immunis|[3255]|LYHES413-09|Mexico|Yucatan|658[0n]|BOLD:AAA7258
Ptichodis immunis|[3256]|LOCRC807-09|Costa Rica|Guanacaste|658[0n]|BOLD:AAA7258
Ptichodis immunis|[3257]|BLPDS458-10|Costa Rica|Alajuela|658[0n]|BOLD:AAA7258
Ptichodis immunis|[3258]|LOCRE536-10|Costa Rica|San Jose|658[0n]|BOLD:AAA7258
Ptichodis immunis|[3259]|BLPDD458-09|Costa Rica|Guanacaste|658[0n]|BOLD:AAA7258
Ptichodis immunis|[3260]|BLPDD459-09|Costa Rica|Guanacaste|658[0n]|BOLD:AAA7258
Ptichodis immunis|[3261]|BLPDE139-09|Costa Rica|Guanacaste|658[0n]|BOLD:AAA7258
Ptichodis immunis|[3262]|BLPDE313-09|Costa Rica|Guanacaste|658[0n]|BOLD:AAA7258
Ptichodis immunis|[3263]|BLPCI176-08|Costa Rica|Guanacaste|658[0n]|BOLD:AAA7258
Ptichodis immunis|[3264]|BLPCJ530-08|Costa Rica|Guanacaste|658[0n]|BOLD:AAA7258
Ptichodis immunis|[3265]|BLPCI605-08|Costa Rica|Guanacaste|658[0n]|BOLD:AAA7258
Ptichodis immunis|[3266]|BLPCK203-08|Costa Rica|Alajuela|658[0n]|BOLD:AAA7258
Ptichodis immunis|[3267]|LYPAP080-09|Mexico|Quintana Roo|658[0n]|BOLD:AAA7258
Ptichodis immunis|[3268]|LYNYM264-09|Mexico|Quintana Roo|658[0n]|BOLD:AAA7258
Ptichodis immunis|[3269]|LOCRE537-10|Costa Rica|San Jose|658[0n]|BOLD:AAA7258
Ptichodis immunis|[3270]|BLPED1747-12|Costa Rica|Guanacaste|658[0n]|BOLD:AAA7258
Ptichodis immunis|[3271]|BLPBE458-07|Costa Rica|Guanacaste|658[0n]|BOLD:AAA7258
Ptichodis immunis|[3272]|BLPBH746-07|Costa Rica|Guanacaste|658[0n]|BOLD:AAA7258
Ptichodis immunis|[3273]|CNCLB1650-14|United States|Florida|658[0n]|BOLD:AAA7258
Ptichodis immunis|[3274]|MHAUC180-06|Costa Rica|Guanacaste|571[0n]|BOLD:AAA7258
Ptichodis immunis|[3275]|BLPBD258-07|Costa Rica|Guanacaste|625[0n]|BOLD:AAA7258
Ptichodis immunis|[3276]|BLPDH179-09|Costa Rica|Guanacaste|658[1n]|BOLD:AAA7258
Ptichodis immunis|[3277]|LYHES191-09|Mexico|Yucatan|611[0n]|BOLD:AAA7258
Ptichodis immunis|[3278]|BLPAF655-07|Costa Rica|Guanacaste|658[0n]|BOLD:AAA7258
Ptichodis immunis|[3279]|BLPBA010-07|Costa Rica|Guanacaste|658[0n]|BOLD:AAA7258
Ptichodis immunis|[3280]|BLPBC848-07|Costa Rica|Alajuela|627[1n]|BOLD:AAA7258
Ptichodis immunis|[3281]|BLPBC730-07|Costa Rica|Guanacaste|621[0n]|BOLD:AAA7258
Ptichodis immunis|[3282]|BLPBC851-07|Costa Rica|Alajuela|630[0n]|BOLD:AAA7258
Ptichodis immunis|[3283]|BLPCK705-08|Costa Rica|Alajuela|658[0n]|BOLD:AAA7258
Ptichodis immunis|[3284]|BLPCK704-08|Costa Rica|Alajuela|658[0n]|BOLD:AAA7258
Ptichodis immunis|[3285]|BLPCK703-08|Costa Rica|Alajuela|658[0n]|BOLD:AAA7258
Ptichodis immunis|[3286]|BLPCK208-08|Costa Rica|Alajuela|658[0n]|BOLD:AAA7258
Ptichodis immunis|[3287]|BLPCJ909-08|Costa Rica|Guanacaste|658[0n]|BOLD:AAA7258
Ptichodis immunis|[3288]|BLPCI604-08|Costa Rica|Guanacaste|658[0n]|BOLD:AAA7258
Ptichodis immunis|[3289]|BLPBD582-07|Costa Rica|Guanacaste|658[0n]|BOLD:AAA7258
Ptichodis immunis|[3290]|BLPDB602-09|Costa Rica|Alajuela|603[0n]|BOLD:AAA7258
Ptichodis immunis|[3291]|BLPDC665-09|Costa Rica|Alajuela|658[0n]|BOLD:AAA7258
Ptichodis immunis|[3292]|BLPDD877-09|Costa Rica|Guanacaste|658[0n]|BOLD:AAA7258
Ptichodis immunis|[3293]|LYNYM214-09|Mexico|Quintana Roo|658[0n]|BOLD:AAA7258
Ptichodis immunis|[3294]|CNCLB2000-14|Guatemala||658[0n]|BOLD:AAA7258
Caenurgina erechtea|[3295]|LPSK201-08|Canada|Saskatchewan|658[0n]|BOLD:AAB0200

Ptichodis immunis|[3293]|LYNYM214-09|Mexico|Quintana Roo|658[0n]|BOLD:AAA7258
Ptichodis immunis|[3294]|CNCLB2000-14|Guatemala||658[0n]|BOLD:AAA7258
Caenurgina erechtea|[3295]|LPSK201-08|Canada|Saskatchewan|658[0n]|BOLD:AAB0200
Caenurgina erechtea|[3296]|LOWCE020-06|Canada|British Columbia|658[0n]|BOLD:AAB0200
Caenurgina erechtea|[3297]|LOWCE021-06|Canada|British Columbia|658[0n]|BOLD:AAB0200
Caenurgina erechtea|[3298]|JMMMB313-11|United States|California|658[0n]|BOLD:AAB0200
Caenurgina erechtea|[3299]|LOWCB594-05|Canada|British Columbia|658[0n]|BOLD:AAB0200
Caenurgina erechtea|[3300]|LOWCB597-05|Canada|British Columbia|627[0n]|BOLD:AAB0200
Caenurgina erechtea|[3301]|LOWCB606-05|Canada|British Columbia|599[0n]|BOLD:AAB0200
Caenurgina erechtea|[3302]|LOWCB600-05|Canada|British Columbia|591[2n]|BOLD:AAB0200
Caenurgina erechtea|[3303]|LOWCB598-05|Canada|British Columbia|610[0n]|BOLD:AAB0200
Caenurgina erechtea|[3304]|LOWCB601-05|Canada|British Columbia|603[0n]|BOLD:AAB0200
Caenurgina erechtea|[3305]|LOWCB603-05|Canada|British Columbia|592[0n]|BOLD:AAB0200
Caenurgina erechtea|[3306]|ABNCC402-07|United States|Texas|594[0n]|BOLD:AAB0200
Caenurgina erechtea|[3307]|LOWCE088-06|Canada|British Columbia|658[0n]|BOLD:AAB0200
Caenurgina erechtea|[3308]|ABNCC405-07|United States|Oklahoma|652[0n]|BOLD:AAB0200
Caenurgina erechtea|[3309]|LPSK523-08|Canada|Saskatchewan|658[0n]|BOLD:AAB0200
Caenurgina erechtea|[3310]|LPSK423-08|Canada|Saskatchewan|658[0n]|BOLD:AAB0200
Caenurgina erechtea|[3311]|ABNCC403-07|United States|Oklahoma|645[0n]|BOLD:AAB0200
Caenurgina erechtea|[3312]|LPSK635-08|Canada|Saskatchewan|629[0n]|BOLD:AAB0200
Caenurgina erechtea|[3313]|QUNOB288-09|United States|Indiana|658[0n]|BOLD:AAB0200
Caenurgina erechtea|[3314]|LPOKB995-09|United States|Oklahoma|658[0n]|BOLD:AAB0200
Caenurgina erechtea|[3315]|BBLSW032-09|United States|Texas|658[0n]|BOLD:AAB0200
Caenurgina erechtea|[3316]|BBLSX648-09|United States|Oklahoma|658[0n]|BOLD:AAB0200
Caenurgina erechtea|[3317]|BBLSX692-09|United States|Texas|658[0n]|BOLD:AAB0200
Caenurgina erechtea|[3318]|LALPA524-10|Canada|British Columbia|658[0n]|BOLD:AAB0200
Caenurgina erechtea|[3319]|ABNCC406-07|United States|Texas|640[0n]|BOLD:AAB0200
Caenurgina erechtea|[3320]|LPOKA964-09|United States|Oklahoma|658[0n]|BOLD:AAB0200
Caenurgina erechtea|[3321]|LPSK480-08|Canada|Saskatchewan|658[0n]|BOLD:AAB0200
Caenurgina erechtea|[3322]|BBLSX895-09|United States|Oklahoma|658[0n]|BOLD:AAB0200
Caenurgina erechtea|[3323]|RWWC473-11|United States|Washington|658[0n]|BOLD:AAB0200
Caenurgina erechtea|[3324]|RWWC748-11|United States|Washington|658[0n]|BOLD:AAB0200
Caenurgina erechtea|[3325]|LALPA1195-11|Canada|British Columbia|658[0n]|BOLD:AAB0200
Caenurgina erechtea|[3326]|LPSK395-08|Canada|Saskatchewan|658[0n]|BOLD:AAB0200
Caenurgina erechtea|[3327]|QUNOB287-09|United States|Indiana|658[0n]|BOLD:AAB0200
Caenurgina erechtea|[3328]|BBLOE1452-12|United States|Texas|658[0n]|BOLD:AAB0200
Caenurgina erechtea|[3329]|LOWCB602-05|Canada|British Columbia|658[64n]|BOLD:AAB0200
Caenurgina erechtea|[3330]|LOWCB604-05|Canada|British Columbia|658[135n]|
Caenurgina erechtea|[3331]|LPSK209-08|Canada|Saskatchewan|658[0n]|BOLD:AAB0200
Caenurgina erechtea|[3332]|BBLSX642-09|United States|Oklahoma|658[0n]|BOLD:AAB0200
Caenurgina erechtea|[3333]|BBLSX889-09|United States|Oklahoma|658[0n]|BOLD:AAB0200
Caenurgina erechtea|[3334]|LPOKE286-11|United States|Oklahoma|658[0n]|BOLD:AAB0200
Caenurgina erechtea|[3335]|CMAZA374-10|United States|Arizona|658[0n]|BOLD:AAB0200
Caenurgina erechtea|[3336]|LALPA576-10|Canada|British Columbia|658[0n]|BOLD:AAB0200
Caenurgina erechtea|[3337]|LALPA546-10|Canada|British Columbia|658[0n]|BOLD:AAB0200
Caenurgina erechtea|[3338]|LPOKC621-09|United States|Oklahoma|658[0n]|BOLD:AAB0200
Caenurgina erechtea|[3339]|BBLSX957-09|United States|Oklahoma|658[0n]|BOLD:AAB0200
Caenurgina erechtea|[3340]|BBLSX643-09|United States|Oklahoma|658[0n]|BOLD:AAB0200
Caenurgina erechtea|[3341]|BBLSW453-09|United States|Oklahoma|658[0n]|BOLD:AAB0200
Caenurgina erechtea|[3342]|LPMNB296-09|Canada|Manitoba|658[0n]|BOLD:AAB0200
Caenurgina erechtea|[3343]|LPSK557-08|Canada|Saskatchewan|658[0n]|BOLD:AAB0200
Caenurgina erechtea|[3344]|LPSK520-08|Canada|Saskatchewan|658[0n]|BOLD:AAB0200
Caenurgina erechtea|[3345]|LPSK424-08|Canada|Saskatchewan|658[0n]|BOLD:AAB0200
Caenurgina erechtea|[3346]|LPSK230-08|Canada|Saskatchewan|658[0n]|BOLD:AAB0200
Caenurgina erechtea|[3347]|LPSK202-08|Canada|Saskatchewan|658[0n]|BOLD:AAB0200
Caenurgina erechtea|[3348]|LOWCB595-05|Canada|British Columbia|658[0n]|BOLD:AAB0200
Caenurgina erechtea|[3349]|BBLOD896-11|United States|California|658[0n]|BOLD:AAB0200
Caenurgina erechtea|[3350]|LOWCB599-05|Canada|British Columbia|590[0n]|BOLD:AAB0200
Caenurgina erechtea|[3351]|LOWCB596-05|Canada|British Columbia|602[0n]|BOLD:AAB0200
Caenurgina erechtea|[3352]|LOWCB605-05|Canada|British Columbia|573[2n]|BOLD:AAB0200
Caenurgina erechtea|[3353]|GMLC1082-12|United States|California|632[0n]|BOLD:AAB0200
Caenurgina caerulea|[3354]|RDNMF105-08|United States|California|618[0n]|BOLD:ADA8489
Caenurgina caerulea|[3355]|RDNMF106-08|United States|California|658[0n]|BOLD:ADA8489
Caenurgina caerulea|[3356]|RDMAB1010-09|United States|California|658[0n]|BOLD:ADA8489
Caenurgina caerulea|[3357]|JMMMB312-11|United States|California|658[0n]|BOLD:ADA8489
Caenurgina crassiuscula|[3358]|LOWCB179-05|Canada|British Columbia|654[0n]|BOLD:ADA5642
Caenurgina crassiuscula|[3359]|LOWCE090-06|Canada|British Columbia|658[0n]|BOLD:ADA5642
Caenurgina crassiuscula|[3360]|RDNME520-08|Canada|Yukon Territory|658[0n]|BOLD:ADA5642
Caenurgina crassiuscula|[3361]|RDNME526-08|Canada|Yukon Territory|658[0n]|BOLD:ADA5642
Caenurgina crassiuscula|[3362]|BBLPB162-10|Canada|Saskatchewan|658[0n]|BOLD:ADA5642
Caenurgina crassiuscula|[3363]|LALPA845-11|Canada|British Columbia|658[0n]|BOLD:ADA5642
Caenurgina annexa|[3364]|LOWCE091-06|Canada|British Columbia|658[0n]|BOLD:AAE0528
Caenurgina annexa|[3365]|RDNMF107-08|Canada|Alberta|658[0n]|BOLD:AAE0528
Caenurgina annexa|[3366]|RDNMF363-08|Canada|Alberta|658[0n]|BOLD:AAE0528
Caenurgina annexa|[3367]|RDNMF364-08|Canada|Alberta|658[0n]|BOLD:AAE0528
Caenurgina annexa|[3368]|RDNMF365-08|Canada|Alberta|658[0n]|BOLD:AAE0528
Caenurgina annexa|[3369]|LALPA1093-11|Canada|British Columbia|658[0n]|BOLD:AAE0528
Caenurgina annexa|[3370]|LALPA1094-11|Canada|British Columbia|658[0n]|BOLD:AAE0528
Caenurgina crassiuscula|[3371]|CNPPE1051-12|Canada|Ontario|638[0n]|BOLD:AAA4171
Caenurgina crassiuscula|[3372]|MEC139-04|Canada|Quebec|649[0n]|BOLD:AAA4171
Caenurgina crassiuscula|[3373]|XAD291-04|Canada|Ontario|658[0n]|BOLD:AAA4171
Caenurgina crassiuscula|[3374]|XAG314-05|Canada|Ontario|658[0n]|BOLD:AAA4171
Caenurgina crassiuscula|[3375]|LGSMG585-07|United States|Tennessee|658[0n]|BOLD:AAA4171
Caenurgina crassiuscula|[3376]|BBLPC509-09|Canada|New Brunswick|658[0n]|BOLD:AAA4171
Caenurgina crassiuscula|[3377]|CNPPF1351-12|Canada|Ontario|642[0n]|BOLD:AAA4171
Caenurgina crassiuscula|[3378]|CNPPI015-12|Canada|Ontario|644[0n]|BOLD:AAA4171
Caenurgina crassiuscula|[3379]|CNPPE1034-12|Canada|Ontario|635[0n]|BOLD:AAA4171
Caenurgina crassiuscula|[3380]|CNPPE1024-12|Canada|Ontario|635[0n]|BOLD:AAA4171
Caenurgina crassiuscula|[3381]|XAC089-04|Canada|Ontario|591[0n]|BOLD:AAA4171
Caenurgina crassiuscula|[3382]|NCCH104-11|Canada|Ontario|655[0n]|BOLD:AAA4171
Caenurgina crassiuscula|[3383]|LGSM631-04|United States|Tennessee|609[0n]|BOLD:AAA4171
Caenurgina crassiuscula|[3384]|BBLEC736-09|Canada|Nova Scotia|655[1n]|BOLD:AAA4171
Caenurgina crassiuscula|[3385]|KPOEC107-08|Canada|Ontario|655[0n]|BOLD:AAA4171
Caenurgina crassiuscula|[3386]|BLTIB059-08|Canada|Ontario|658[0n]|BOLD:AAA4171
Caenurgina crassiuscula|[3387]|BLTIB717-08|Canada|Ontario|658[0n]|BOLD:AAA4171
Caenurgina crassiuscula|[3388]|BLTIB731-08|Canada|Ontario|658[0n]|BOLD:AAA4171
Caenurgina crassiuscula|[3389]|BLTIB732-08|Canada|Ontario|658[0n]|BOLD:AAA4171
Caenurgina crassiuscula|[3390]|BBLEC691-09|Canada|Nova Scotia|658[0n]|BOLD:AAA4171
Caenurgina crassiuscula|[3391]|LGSMG586-07|United States|Tennessee|658[0n]|BOLD:AAA4171
Caenurgina crassiuscula|[3392]|LGSMG587-07|United States|North Carolina|658[0n]|BOLD:AAA4171
Caenurgina crassiuscula|[3393]|TMNBB075-06|Canada|New Brunswick|658[0n]|BOLD:AAA4171
Caenurgina crassiuscula|[3394]|LNCNW111-06|United States|North Carolina|658[0n]|BOLD:AAA4171
Caenurgina crassiuscula|[3395]|XAH367-05|Canada|Ontario|655[0n]|BOLD:AAA4171

Caenurgina crassiuscula|[3393]|TMNBB075-06|Canada|New Brunswick|658[0n]|BOLD:AAA4171
Caenurgina crassiuscula|[3394]|LNCNW111-06|United States|North Carolina|658[0n]|BOLD:AAA4171
Caenurgina crassiuscula|[3395]|XAH367-05|Canada|Ontario|655[0n]|BOLD:AAA4171
Caenurgina crassiuscula|[3396]|XAH415-05|Canada|Ontario|657[0n]|BOLD:AAA4171
Caenurgina crassiuscula|[3397]|XAG092-05|Canada|Ontario|658[0n]|BOLD:AAA4171
Caenurgina crassiuscula|[3398]|XAH318-05|Canada|Ontario|657[0n]|BOLD:AAA4171
Caenurgina crassiuscula|[3399]|LGSMC909-05|United States|Tennessee|658[0n]|BOLD:AAA4171
Caenurgina crassiuscula|[3400]|XAF343-05|Canada|Ontario|658[0n]|BOLD:AAA4171
Caenurgina crassiuscula|[3401]|XAF325-05|Canada|Ontario|658[0n]|BOLD:AAA4171
Caenurgina crassiuscula|[3402]|XAD575-04|Canada|Ontario|658[0n]|BOLD:AAA4171
Caenurgina crassiuscula|[3403]|XAE153-04|Canada|Ontario|658[0n]|BOLD:AAA4171
Caenurgina crassiuscula|[3404]|XAC800-04|Canada|Ontario|658[0n]|BOLD:AAA4171
Caenurgina crassiuscula|[3405]|XAC070-04|Canada|Ontario|658[0n]|BOLD:AAA4171
Caenurgina crassiuscula|[3406]|XAC034-04|Canada|Ontario|658[0n]|BOLD:AAA4171
Caenurgina crassiuscula|[3407]|LGSM632-04|United States|Tennessee|658[0n]|BOLD:AAA4171
Caenurgina crassiuscula|[3408]|MEC140-04|Canada|Quebec|658[0n]|BOLD:AAA4171
Caenurgina crassiuscula|[3409]|XAK424-06|Canada|Ontario|658[0n]|BOLD:AAA4171
Caenurgina crassiuscula|[3410]|RDLQ423-07|Canada|Quebec|611[0n]|BOLD:AAA4171
Caenurgina crassiuscula|[3411]|XAD283-04|Canada|Ontario|591[0n]|BOLD:AAA4171
Caenurgina crassiuscula|[3412]|XAK225-06|Canada|Ontario|658[0n]|BOLD:AAA4171
Caenurgina crassiuscula|[3413]|ABNCC404-07|United States|Indiana|604[0n]|BOLD:AAA4171
Caenurgina crassiuscula|[3414]|RDLQ422-07|Canada|Quebec|606[4n]|BOLD:AAA4171
Caenurgina crassiuscula|[3415]|PMG096-03|Canada|Ontario|617[0n]|BOLD:AAA4171
Caenurgina crassiuscula|[3416]|TMG94-03|Canada|Ontario|639[0n]|BOLD:AAA4171
Caenurgina crassiuscula|[3417]|TMG95-03|Canada|Ontario|639[0n]|BOLD:AAA4171
Caenurgina crassiuscula|[3418]|RDLQ424-07|Canada|Quebec|593[3n]|BOLD:AAA4171
Caenurgina crassiuscula|[3419]|LGSMG299-07|United States|Tennessee|658[0n]|BOLD:AAA4171
Caenurgina crassiuscula|[3420]|LPSO651-08|Canada|Ontario|658[0n]|BOLD:AAA4171
Caenurgina crassiuscula|[3421]|LPSOB229-08|Canada|Ontario|658[0n]|BOLD:AAA4171
Caenurgina crassiuscula|[3422]|BBLPC357-09|Canada|New Brunswick|658[0n]|BOLD:AAA4171
Caenurgina crassiuscula|[3423]|LILLA316-11|United States|Illinois|658[0n]|BOLD:AAA4171
Caenurgina crassiuscula|[3424]|XAJ810-06|Canada|Ontario|658[0n]|BOLD:AAA4171
Caenurgina crassiuscula|[3425]|RBINA3548-13|Canada|Ontario|591[0n]|BOLD:AAA4171
Caenurgina crassiuscula|[3426]|LILLA896-11|United States|Illinois|658[0n]|BOLD:AAA4171
Caenurgina crassiuscula|[3427]|XAG677-05|Canada|Ontario|658[0n]|BOLD:AAA4171
Caenurgina crassiuscula|[3428]|KPOEC182-08|Canada|Ontario|658[0n]|BOLD:AAA4171
Caenurgina crassiuscula|[3429]|RBINA5362-13|Canada|Ontario|588[0n]|BOLD:AAA4171
Caenurgina crassiuscula|[3430]|BBLPC350-09|Canada|New Brunswick|655[0n]|BOLD:AAA4171
Caenurgina crassiuscula|[3431]|KPOEC077-08|Canada|Ontario|651[0n]|BOLD:AAA4171
Caenurgina crassiuscula|[3432]|KPOEC046-08|Canada|Ontario|646[0n]|BOLD:AAA4171
Caenurgina crassiuscula|[3433]|XAF304-05|Canada|Ontario|658[0n]|BOLD:AAA4171
Caenurgina crassiuscula|[3434]|LPSK233-08|Canada|Saskatchewan|658[0n]|BOLD:AAA4171
Caenurgina crassiuscula|[3435]|XAD510-04|Canada|Ontario|618[1n]|BOLD:AAA4171
Caenurgina crassiuscula|[3436]|BBLEC203-09|Canada|Nova Scotia|636[0n]|BOLD:AAA4171
Caenurgina crassiuscula|[3437]|XAB044-04|Canada|Ontario|617[2n]|BOLD:AAA4171
Caenurgina crassiuscula|[3438]|XAJ933-06|Canada|Ontario|658[0n]|BOLD:AAA4171
Caenurgina crassiuscula|[3439]|BBLPC384-09|Canada|New Brunswick|655[0n]|BOLD:AAA4171
Caenurgina crassiuscula|[3440]|LPMNB472-09|Canada|Manitoba|658[0n]|BOLD:AAA4171
Caenurgina crassiuscula|[3441]|LPSK507-08|Canada|Saskatchewan|658[0n]|BOLD:AAA4171
Caenurgina crassiuscula|[3442]|LPSK118-08|Canada|Saskatchewan|658[0n]|BOLD:AAA4171
Caenurgina crassiuscula|[3443]|LPSK117-08|Canada|Saskatchewan|658[0n]|BOLD:AAA4171
Caenurgina crassiuscula|[3444]|LPSK097-08|Canada|Saskatchewan|658[0n]|BOLD:AAA4171
Caenurgina crassiuscula|[3445]|LPSK045-08|Canada|Saskatchewan|658[0n]|BOLD:AAA4171
Caenurgina crassiuscula|[3446]|LPSK038-08|Canada|Saskatchewan|658[0n]|BOLD:AAA4171
Caenurgina crassiuscula|[3447]|LPSK035-08|Canada|Saskatchewan|658[0n]|BOLD:AAA4171
Caenurgina crassiuscula|[3448]|LPSK023-08|Canada|Saskatchewan|658[0n]|BOLD:AAA4171
Caenurgina crassiuscula|[3449]|LPSK021-08|Canada|Saskatchewan|658[0n]|BOLD:AAA4171
Caenurgina crassiuscula|[3450]|LPSK015-08|Canada|Saskatchewan|658[0n]|BOLD:AAA4171
Caenurgina crassiuscula|[3451]|LPSK009-08|Canada|Saskatchewan|658[0n]|BOLD:AAA4171
Caenurgina crassiuscula|[3452]|LPSK008-08|Canada|Saskatchewan|658[0n]|BOLD:AAA4171
Caenurgina crassiuscula|[3453]|KPOEC106-08|Canada|Ontario|658[0n]|BOLD:AAA4171
Caenurgina crassiuscula|[3454]|XAK461-06|Canada|Ontario|658[0n]|BOLD:AAA4171
Caenurgina crassiuscula|[3455]|XAJ839-06|Canada|Ontario|658[0n]|BOLD:AAA4171
Caenurgina crassiuscula|[3456]|TTMNB281-06|Canada|New Brunswick|658[0n]|BOLD:AAA4171
Caenurgina crassiuscula|[3457]|LMIS028-05|Canada|Ontario|658[0n]|BOLD:AAA4171
Caenurgina crassiuscula|[3458]|XAH449-05|Canada|Ontario|658[0n]|BOLD:AAA4171
Caenurgina crassiuscula|[3459]|MNBB466-05|Canada|New Brunswick|658[0n]|BOLD:AAA4171
Caenurgina crassiuscula|[3460]|MEC131-04|Canada|Quebec|658[0n]|BOLD:AAA4171
Caenurgina crassiuscula|[3461]|CNGRL410-13|Canada|Saskatchewan|588[0n]|BOLD:AAA4171
Caenurgina crassiuscula|[3462]|LPSK114-08|Canada|Saskatchewan|658[0n]|BOLD:AAA4171
Caenurgina crassiuscula|[3463]|LPSK003-08|Canada|Saskatchewan|569[2n]|BOLD:AAA4171
Caenurgina crassiuscula|[3464]|BLTIB661-08|Canada|Ontario|621[0n]|BOLD:AAA4171
Caenurgina crassiuscula|[3465]|RBINA5367-13|Canada|Ontario|559[0n]|BOLD:AAA4171
Caenurgina crassiuscula|[3466]|RBINA5369-13|Canada|Ontario|566[0n]|BOLD:AAA4171
Callistege sp.|[3467]|CMAZA520-10|United States|Arizona|658[0n]|BOLD:AAP6002
Callistege intercalaris|[3468]|ABNCC424-07|United States|Arizona|645[1n]|BOLD:AAJ0924
Callistege intercalaris|[3469]|CMAZA505-10|United States|Arizona|658[0n]|BOLD:AAJ0924
Callistege intercalaris|[3470]|QUNOD105-10|United States|Texas|658[0n]|BOLD:AAJ0924
Callistege intercalaris|[3471]|ABNCC423-07|United States|Arizona|635[0n]|BOLD:AAJ0924
Callistege intercalaris|[3472]|CMAZA523-10|United States|Arizona|658[0n]|BOLD:AAJ0924
Callistege intercalaris|[3473]|RDNMJ185-10|United States|New Mexico|658[0n]|BOLD:AAJ0924
Callistege diagonalis|[3474]|ABNCC425-07|United States|Arkansas|617[0n]|BOLD:ACF0240
Callistege diagonalis|[3475]|NAMUM071-08|United States|Arizona|657[0n]|BOLD:ACF0240
Callistege diagonalis|[3476]|RDNME074-07|United States|Arizona|658[0n]|BOLD:ACF0240
Callistege diagonalis|[3477]|IAWL152-09|United States|Arizona|648[0n]|BOLD:ACF0240
Callistege diagonalis|[3478]|IAWL153-09|United States|Arizona|658[0n]|BOLD:ACF0240
Callistege diagonalis|[3479]|IAWL154-09|United States|Arizona|658[0n]|BOLD:ACF0240
Callistege diagonalis|[3480]|IAWL155-09|United States|Arizona|658[0n]|BOLD:ACF0240
Callistege diagonalis|[3481]|CMAZA392-10|United States|Arizona|658[0n]|BOLD:ACF0240
Callistege diagonalis|[3482]|CMAZA507-10|United States|Arizona|658[0n]|BOLD:ACF0240
Callistege diagonalis|[3483]|CMAZA946-12|United States|Arizona|658[0n]|BOLD:ACF0240
Callistege triangula|[3484]|CNCLB849-14|United States|Arizona|658[0n]|BOLD:ACM4001
Callistege triangula|[3485]|CNCLB848-14|United States|Arizona|658[0n]|BOLD:ACM4001
Callistege triangula|[3486]|TML129-14|United States||543[0n]|BOLD:ACM4001
Doryodes bistrialis|[3487]|RDNMD295-06|United States|Florida|658[0n]|BOLD:AAD3663
Doryodes bistrialis|[3488]|RDNMD294-06|United States|Florida|658[0n]|BOLD:AAD3663
Doryodes bistrialis|[3489]|USLEP596-10|United States|Florida|658[0n]|BOLD:AAD3663
Doryodes bistrialis|[3490]|HKONS435-08|United States|Florida|658[1n]|BOLD:AAD3663
Doryodes bistrialis|[3491]|LNC052-05|United States|North Carolina|658[0n]|BOLD:AAD3663
Doryodes bistrialis|[3492]|LNC053-05|United States|North Carolina|658[0n]|BOLD:AAD3663
Doryodes bistrialis|[3493]|LNC932-06|United States|North Carolina|658[0n]|BOLD:AAD3663
Doryodes bistrialis|[3494]|LNCC1252-11|United States|North Carolina|658[0n]|BOLD:AAD3663
Doryodes latistriga|[3495]|LNCB861-09|United States|Alabama|658[0n]|BOLD:ABX5381

Doryodes bistrialis|[3493]|LNC932-06|United States|North Carolina|658[0n]|BOLD:AAD3663
Doryodes bistrialis|[3494]|LNCC1252-11|United States|North Carolina|658[0n]|BOLD:AAD3663
Doryodes latistriga|[3495]|LNCB861-09|United States|Alabama|658[0n]|BOLD:ABX5381
Doryodes latistriga|[3496]|HKONB279-09|United States|Texas|658[0n]|BOLD:ABX5381
Doryodes latistriga|[3497]|LNCB863-09|United States|Alabama|658[0n]|BOLD:ABX5381
Doryodes latistriga|[3498]|LNCB864-09|United States|Alabama|658[0n]|BOLD:ABX5381
Doryodes latistriga|[3499]|HKONB276-09|United States|Texas|658[0n]|BOLD:ABX5381
Doryodes latistriga|[3500]|HKONS451-08|United States|Florida|658[0n]|BOLD:ABX5381
Doryodes latistriga|[3501]|HKONS452-08|United States|Florida|658[0n]|BOLD:ABX5381
Doryodes latistriga|[3502]|HKONB277-09|United States|Texas|658[0n]|BOLD:ABX5381
Doryodes latistriga|[3503]|HKONB278-09|United States|Texas|658[0n]|BOLD:ABX5381
Doryodes latistriga|[3504]|LNCB862-09|United States|Alabama|658[0n]|BOLD:ABX5381
Doryodes latistriga|[3505]|CNCLB1273-14|United States|Louisiana|658[0n]|BOLD:ABX5381
Doryodes latistriga|[3506]|CNCLB1272-14|United States|Louisiana|658[0n]|BOLD:ABX5381
Doryodes latistriga|[3507]|CNCLB1277-14|United States|Louisiana|658[0n]|BOLD:ABX5381
Doryodes latistriga|[3508]|CNCLB1279-14|United States|Louisiana|658[0n]|BOLD:ABX5381
Doryodes tenuistriga|[3509]|HKONB489-09|United States|Texas|647[0n]|BOLD:AAI9811
Doryodes tenuistriga|[3510]|QUNOD013-10|United States|Texas|658[0n]|BOLD:AAI9811
Doryodes tenuistriga|[3511]|CNCLB1281-14|United States|Texas|658[0n]|BOLD:AAI9811
Doryodes broui|[3512]|HKONB282-09|United States|Texas|658[0n]|BOLD:ABY4587
Doryodes broui|[3513]|HKONB273-09|United States|Texas|658[0n]|BOLD:ABY4587
Doryodes broui|[3514]|HKONB283-09|United States|Texas|632[0n]|BOLD:ABY4587
Doryodes broui|[3515]|HKONB274-09|United States|Texas|612[0n]|BOLD:ABY4587
Doryodes broui|[3516]|CNCLB2620-14|United States|Mississippi|658[0n]|BOLD:ABY4587
Doryodes broui|[3517]|CNCLB2616-14|United States|Mississippi|658[0n]|BOLD:ABY4587
Doryodes broui|[3518]|CNCLB2625-14|United States|Mississippi|658[0n]|BOLD:ABY4587
Doryodes broui|[3519]|CNCLB1274-14|United States|Louisiana|658[0n]|BOLD:ABY4587
Doryodes broui|[3520]|CNCLB2491-14|United States|Mississippi|658[0n]|BOLD:ABY4587
Doryodes broui|[3521]|CNCLB2618-14|United States|Mississippi|658[0n]|BOLD:ABY4587
Doryodes broui|[3522]|CNCLB2623-14|United States|Mississippi|658[0n]|BOLD:ABY4587
Doryodes broui|[3523]|CNCLB2628-14|United States|Mississippi|658[0n]|BOLD:ABY4587
Doryodes reinecki|[3524]|HKONB271-09|United States|Texas|658[0n]|BOLD:ACE7230
Doryodes reinecki|[3525]|HKONB270-09|United States|Texas|658[0n]|BOLD:ACE7230
Doryodes reinecki|[3526]|LNCB867-09|United States|Alabama|658[0n]|BOLD:ACE7230
Doryodes reinecki|[3527]|LNCB868-09|United States|Alabama|658[0n]|BOLD:ACE7230
Doryodes reinecki|[3528]|LNCB865-09|United States|Alabama|658[0n]|BOLD:ACE7230
Doryodes reinecki|[3529]|LNCB866-09|United States|Alabama|621[0n]|BOLD:ACE7230
Doryodes reinecki|[3530]|CNCLB1275-14|United States|Louisiana|658[0n]|BOLD:ACE7230
Doryodes reinecki|[3531]|CNCLB1276-14|United States|Louisiana|658[0n]|BOLD:ACE7230
Doryodes reinecki|[3532]|CNCLB1278-14|United States|Louisiana|658[0n]|BOLD:ACE7230
Doryodes sp.|[3533]|HKONS114-08|United States|Florida|658[0n]|BOLD:ACE7229
Doryodes sp.|[3534]|HKONS453-08|United States|Florida|658[1n]|BOLD:ACE7229
Doryodes sp.|[3535]|HKONS454-08|United States|Florida|658[1n]|BOLD:ACE7229
Doryodes fusselli|[3536]|LNC523-06|United States|North Carolina|658[0n]|BOLD:AAC9851
Doryodes fusselli|[3537]|LNC524-06|United States|North Carolina|658[0n]|BOLD:AAC9851
Doryodes spadaria|[3538]|HKONS455-08|United States|Florida|658[0n]|
Doryodes spadaria|[3539]|HKONS456-08|United States|Florida|658[1n]|
Doryodes spadaria|[3540]|LNC522-06|United States|North Carolina|658[0n]|BOLD:ACE7232
Doryodes spadaria|[3541]|LNC521-06|United States|North Carolina|658[0n]|BOLD:ACE7232
Doryodes spadaria|[3542]|RDNMF524-08|United States|Maryland|609[0n]|BOLD:ACE7232
Doryodes spadaria|[3543]|MILEQ194-11|United States|Georgia|658[0n]|BOLD:ACE7232
Doryodes spadaria|[3544]|MILEQ195-11|United States|Georgia|658[0n]|BOLD:ACE7232
Doryodes spadaria|[3545]|MILEP294-10|United States|Georgia|658[0n]|BOLD:ACE7232
Doryodes spadaria|[3546]|MILEQ193-11|United States|Georgia|658[0n]|BOLD:ACE7232
Doryodes spadaria|[3547]|MILEP293-10|United States|Georgia|658[0n]|BOLD:ACE7232
Doryodes spadaria|[3548]|MILEP292-10|United States|Georgia|658[0n]|BOLD:ACE7232
Doryodes spadaria|[3549]|MILEP291-10|United States|Georgia|658[0n]|BOLD:ACE7232
Doryodes spadaria|[3550]|MILEP290-10|United States|Georgia|658[0n]|BOLD:ACE7232
Doryodes spadaria|[3551]|MILEP289-10|United States|Georgia|658[0n]|BOLD:ACE7232
Doryodes spadaria|[3552]|MILEP288-10|United States|Georgia|658[0n]|BOLD:ACE7232
Doryodes spadaria|[3553]|MILEP287-10|United States|Georgia|658[0n]|BOLD:ACE7232
Doryodes spadaria|[3554]|MILEP286-10|United States|Georgia|658[0n]|BOLD:ACE7232
Doryodes spadaria|[3555]|MILEQ192-11|United States|Georgia|658[0n]|BOLD:ACE7232
Doryodes spadaria|[3556]|MILEQ196-11|United States|Georgia|658[0n]|BOLD:ACE7232
Doryodes spadaria|[3557]|CNCLB2701-14|United States|North Carolina|658[0n]|BOLD:ACE7232
Euparthenos nubilis|[3558]|LSUSA155-06|United States|Kentucky|658[0n]|BOLD:AAB1002
Euparthenos nubilis|[3559]|LGSM634-04|United States|Tennessee|658[0n]|BOLD:AAB1002
Euparthenos nubilis|[3560]|LOT219-04|United States|Tennessee|609[0n]|BOLD:AAB1002
Euparthenos nubilis|[3561]|ABCNA620-07|United States|Kentucky|577[0n]|BOLD:AAB1002
Euparthenos nubilis|[3562]|ABCNA625-07|United States|Indiana|577[1n]|BOLD:AAB1002
Euparthenos nubilis|[3563]|QUNO093-07|United States|Wisconsin|658[0n]|BOLD:AAB1002
Euparthenos nubilis|[3564]|QUNO734-08|United States|Kentucky|658[0n]|BOLD:AAB1002
Euparthenos nubilis|[3565]|XAB190-04|Canada|Ontario|658[0n]|BOLD:AAB1002
Euparthenos nubilis|[3566]|LOTB275-05|United States|Tennessee|658[0n]|BOLD:AAB1002
Euparthenos nubilis|[3567]|LSUSA144-06|United States|Kentucky|658[0n]|BOLD:AAB1002
Euparthenos nubilis|[3568]|LGSMG577-07|United States|Tennessee|658[0n]|BOLD:AAB1002
Euparthenos nubilis|[3569]|LGSMG578-07|United States|North Carolina|658[0n]|BOLD:AAB1002
Euparthenos nubilis|[3570]|QUNO094-07|United States|Wisconsin|658[0n]|BOLD:AAB1002
Euparthenos nubilis|[3571]|LOCT120-05|United States|Connecticut|658[0n]|BOLD:AAB1002
Euparthenos nubilis|[3572]|LOTB486-05|United States|Tennessee|658[0n]|BOLD:AAB1002
Euparthenos nubilis|[3573]|LGSMC695-05|United States|Tennessee|658[0n]|BOLD:AAB1002
Euparthenos nubilis|[3574]|LGSMC694-05|United States|Tennessee|658[0n]|BOLD:AAB1002
Euparthenos nubilis|[3575]|LOT222-04|United States|Tennessee|609[0n]|BOLD:AAB1002
Euparthenos nubilis|[3576]|LOT221-04|United States|Tennessee|609[0n]|BOLD:AAB1002
Euparthenos nubilis|[3577]|LOT220-04|United States|Tennessee|609[0n]|BOLD:AAB1002
Euparthenos nubilis|[3578]|LOT223-04|United States|Tennessee|609[0n]|BOLD:AAB1002
Euparthenos nubilis|[3579]|LGSMC376-05|United States|Tennessee|560[0n]|BOLD:AAB1002
Euparthenos nubilis|[3580]|LGSMC377-05|United States|Tennessee|614[0n]|BOLD:AAB1002
Euparthenos nubilis|[3581]|ABCNA622-07|United States|Indiana|577[0n]|BOLD:AAB1002
Euparthenos nubilis|[3582]|ABCNA623-07|United States|West Virginia|577[0n]|BOLD:AAB1002
Euparthenos nubilis|[3583]|ABCNA624-07|United States|Arkansas|577[0n]|BOLD:AAB1002
Euparthenos nubilis|[3584]|QUNO805-08|United States|Wisconsin|658[0n]|BOLD:AAB1002
Euparthenos nubilis apache|[3585]|JBAZ147-09|United States|Arizona|658[0n]|BOLD:AAB1002
Euparthenos nubilis apache|[3586]|JBAZ144-09|United States|Arizona|658[0n]|BOLD:AAB1002
Euparthenos nubilis apache|[3587]|QUNOD145-10|United States|Arizona|658[0n]|BOLD:AAB1002
Euparthenos nubilis apache|[3588]|JBAZ148-09|United States|Arizona|658[0n]|BOLD:AAB1002
Euparthenos nubilis apache|[3589]|ABCNA626-07|United States|Arizona|577[0n]|BOLD:AAB1002
Euparthenos nubilis apache|[3590]|ABCNA627-07|United States|Arizona|577[0n]|BOLD:AAB1002
Euparthenos nubilis apache|[3591]|JBAZ145-09|United States|Arizona|634[0n]|BOLD:AAB1002
Euparthenos nubilis apache|[3592]|QUNOD146-10|United States|Arizona|658[0n]|BOLD:AAB1002
Euparthenos nubilis apache|[3593]|QUNOD147-10|United States|Arizona|658[0n]|BOLD:AAB1002
Euparthenos nubilis apache|[3594]|RDNMJ289-11|United States|Arizona|658[0n]|BOLD:AAB1002
Argyrostrotis erasa|[3595]|LNC742-06|United States|North Carolina|658[0n]|BOLD:AAD2062

[illegible]
Euparthenos nubilis apache|[3593]|QUNOD147-10|United States|Arizona|658[0n]|BOLD:AAB1002
Euparthenos nubilis apache|[3594]|RDNMJ289-11|United States|Arizona|658[0n]|BOLD:AAB1002
Argyrostrotis erasa|[3595]|LNC742-06|United States|North Carolina|658[0n]|BOLD:AAD2062
Argyrostrotis erasa|[3596]|HKONS427-08|United States|Florida|648[0n]|BOLD:AAD2062
Argyrostrotis erasa|[3597]|HKONS430-08|United States|Florida|658[2n]|BOLD:AAD2062
Argyrostrotis erasa|[3598]|LNC743-06|United States|North Carolina|658[0n]|BOLD:AAD2062
Argyrostrotis erasa|[3599]|LNCB314-06|United States|North Carolina|658[0n]|BOLD:AAD2062
Argyrostrotis erasa|[3600]|LNCB315-06|United States|North Carolina|658[0n]|BOLD:AAD2062
Argyrostrotis erasa|[3601]|RDNMH946-09|United States|Florida|658[0n]|BOLD:AAD2062
Argyrostrotis erasa|[3602]|ABNCC430-07|United States|Florida|608[0n]|BOLD:AAD2062
Argyrostrotis erasa|[3603]|HKONS428-08|United States|Florida|657[0n]|BOLD:AAD2062
Argyrostrotis erasa|[3604]|HKONS426-08|United States|Florida|658[0n]|BOLD:AAD2062
Argyrostrotis erasa|[3605]|HKONS429-08|United States|Florida|658[0n]|BOLD:AAD2062
Argyrostrotis erasa|[3606]|HKONS431-08|United States|Florida|658[0n]|BOLD:AAD2062
Argyrostrotis erasa|[3607]|ABNCC429-07|United States|Florida|608[0n]|BOLD:AAD2062
Argyrostrotis erasa|[3608]|USLEP641-10|United States|Florida|658[0n]|BOLD:AAD2062
Argyrostrotis sylvarum|[3609]|LSEU503-06|United States|Georgia|658[0n]|BOLD:AAC6640
Argyrostrotis sylvarum|[3610]|HKONS403-08|United States|Florida|658[0n]|BOLD:AAC6640
Argyrostrotis sylvarum|[3611]|HKONS405-08|United States|Florida|658[0n]|BOLD:AAC6640
Argyrostrotis sylvarum|[3612]|HKONS401-08|United States|Florida|658[0n]|BOLD:AAC6640
Argyrostrotis sylvarum|[3613]|HKONS402-08|United States|Florida|658[0n]|BOLD:AAC6640
Argyrostrotis sylvarum|[3614]|LSEU502-06|United States|Georgia|658[0n]|BOLD:AAC6640
Argyrostrotis sylvarum|[3615]|LNCB316-06|United States|North Carolina|658[0n]|BOLD:AAC6640
Argyrostrotis sylvarum|[3616]|HKONS404-08|United States|Florida|658[0n]|BOLD:AAC6640
Argyrostrotis sylvarum|[3617]|MILEQ219-11|United States|Alabama|658[0n]|BOLD:AAC6640
Argyrostrotis sylvarum|[3618]|MILEQ220-11|United States|Alabama|658[0n]|BOLD:AAC6640
Argyrostrotis sylvarum|[3619]|MILEQ221-11|United States|Alabama|658[0n]|BOLD:AAC6640
Argyrostrotis sylvarum|[3620]|LNC025-05|United States|North Carolina|658[0n]|BOLD:AAC6640
Argyrostrotis sylvarum|[3621]|LNC026-05|United States|North Carolina|658[0n]|BOLD:AAC6640
Argyrostrotis sylvarum|[3622]|RDNML055-13|United States|Louisiana|658[0n]|BOLD:AAC6640
Argyrostrotis flavistriaria|[3623]|LNCB312-06|United States|North Carolina|658[0n]|BOLD:AAC0230
Argyrostrotis flavistriaria|[3624]|LNCB313-06|United States|North Carolina|641[0n]|BOLD:AAC0230
Argyrostrotis flavistriaria|[3625]|LNC032-05|United States|North Carolina|658[0n]|BOLD:AAC0230
Argyrostrotis flavistriaria|[3626]|LNC033-05|United States|North Carolina|658[0n]|BOLD:AAC0230
Argyrostrotis flavistriaria|[3627]|LNCC1316-11|United States|North Carolina|658[0n]|BOLD:AAC0230
Argyrostrotis flavistriaria|[3628]|ABNCC419-07|United States|Texas|550[2n]|BOLD:AAC0230
Argyrostrotis flavistriaria|[3629]|RDNMH942-09|United States|Florida|658[0n]|BOLD:AAC0230
Argyrostrotis flavistriaria|[3630]|HKONS423-08|United States|Florida|658[0n]|BOLD:AAC0230
Argyrostrotis flavistriaria|[3631]|HKONS421-08|United States|Florida|658[0n]|BOLD:AAC0230
Argyrostrotis flavistriaria|[3632]|HKONS422-08|United States|Florida|658[0n]|BOLD:AAC0230
Argyrostrotis flavistriaria|[3633]|LSEU053-06|United States|Georgia|658[0n]|BOLD:AAC0230
Argyrostrotis flavistriaria|[3634]|LSEU054-06|United States|Georgia|658[0n]|BOLD:AAC0230
Argyrostrotis flavistriaria|[3635]|LSEU504-06|United States|Georgia|658[0n]|BOLD:AAC0230
Argyrostrotis flavistriaria|[3636]|HKONS419-08|United States|Florida|658[0n]|BOLD:AAC0230
Argyrostrotis flavistriaria|[3637]|HKONS420-08|United States|Florida|658[0n]|BOLD:AAC0230
Argyrostrotis flavistriaria|[3638]|HKONS424-08|United States|Florida|658[0n]|BOLD:AAC0230
Argyrostrotis flavistriaria|[3639]|HKONS425-08|United States|Florida|658[0n]|BOLD:AAC0230
Argyrostrotis flavistriaria|[3640]|RDNMH943-09|United States|Florida|658[0n]|BOLD:AAC0230
Argyrostrotis flavistriaria|[3641]|MILEQ222-11|United States|Alabama|658[0n]|BOLD:AAC0230
Argyrostrotis flavistriaria|[3642]|MILEQ255-11|United States|Alabama|658[0n]|BOLD:AAC0230
Gondysia similis|[3643]|MILEP378-10|United States|Alabama|658[0n]|BOLD:AAD9712
Gondysia similis|[3644]|HKONS395-08|United States|Florida|658[0n]|BOLD:AAD9712
Gondysia similis|[3645]|HKONS396-08|United States|Florida|658[0n]|BOLD:AAD9712
Gondysia similis|[3646]|MILEP377-10|United States|Alabama|658[0n]|BOLD:AAD9712
Gondysia similis|[3647]|MILEP379-10|United States|Alabama|658[0n]|BOLD:AAD9712
Gondysia similis|[3648]|LNC916-06|United States|North Carolina|658[0n]|BOLD:AAD9712
Gondysia similis|[3649]|LNC917-06|United States|North Carolina|658[0n]|BOLD:AAD9712
Gondysia similis|[3650]|LNCB077-06|United States|North Carolina|658[0n]|BOLD:AAD9712
Gondysia similis|[3651]|LNCC700-11|United States|North Carolina|658[0n]|BOLD:AAD9712
Gondysia similis|[3652]|LNCC821-11|United States|North Carolina|658[0n]|BOLD:AAD9712
Gondysia consobrina|[3653]|HKONS063-07|United States|Florida|632[1n]|BOLD:AAE8011
Gondysia consobrina|[3654]|HKONS064-07|United States|Florida|658[0n]|BOLD:AAE8011
Gondysia consobrina|[3655]|HKONS399-08|United States|Florida|658[1n]|BOLD:AAE8011
Gondysia consobrina|[3656]|HKONS400-08|United States|Florida|658[0n]|BOLD:AAE8011
Gondysia consobrina|[3657]|USLEP649-10|United States|Florida|658[0n]|BOLD:AAE8011
Gondysia smithii|[3658]|QUNOC379-10|United States|Texas|658[0n]|BOLD:AAC3013
Gondysia smithii|[3659]|LNCC847-11|United States|North Carolina|658[0n]|BOLD:AAC3013
Gondysia smithii|[3660]|LSEU066-06|United States|Georgia|590[1n]|BOLD:AAC3013
Gondysia smithii|[3661]|LNCB164-06|United States|North Carolina|658[0n]|BOLD:AAC3013
Gondysia smithii|[3662]|LNCB448-07|United States|North Carolina|658[0n]|BOLD:AAC3013
Gondysia smithii|[3663]|LNCC945-11|United States|North Carolina|658[0n]|BOLD:AAC3013
Gondysia smithii|[3664]|LNCC1677-13|United States|North Carolina|658[0n]|BOLD:AAC3013
Gondysia smithii|[3665]|LNCC1873-13|United States|North Carolina|658[0n]|BOLD:AAC3013
Gondysia telma|[3666]|ABNCC379-07|United States|Texas|595[0n]|BOLD:ABY4980
Gondysia telma|[3667]|USLEP1072-10|United States|Florida|658[0n]|BOLD:ABY4980
Gondysia telma|[3668]|LNCB801-09|United States|North Carolina|658[0n]|BOLD:ABY4980
Gondysia telma|[3669]|LNCB754-09|United States|North Carolina|658[0n]|BOLD:ABY4980
Gondysia telma|[3670]|MILEP028-09|United States|North Carolina|658[0n]|BOLD:ABY4980
Gondysia telma|[3671]|LNCB554-09|United States|North Carolina|658[0n]|BOLD:ABY4980
Gondysia telma|[3672]|LSUSA138-06|United States|Kentucky|658[0n]|BOLD:ABY4980
Gondysia telma|[3673]|HKONS398-08|United States|Florida|658[0n]|BOLD:ABY4980
Gondysia telma|[3674]|ABNCC380-07|United States|Indiana|610[0n]|BOLD:ABY4980
Gondysia telma|[3675]|HKONS397-08|United States|Florida|658[0n]|BOLD:ABY4980
Gondysia telma|[3676]|LNCC1265-11|United States|North Carolina|658[0n]|BOLD:ABY4980
Gondysia telma|[3677]|CNCLB1469-14|United States|Louisiana|658[0n]|BOLD:ABY4980
Argyrostrotis quadrifilaris|[3678]|LOFLA667-06|United States|Florida|658[0n]|BOLD:AAA9634
Argyrostrotis quadrifilaris|[3679]|LOFLA429-06|United States|Florida|658[0n]|BOLD:AAA9634
Argyrostrotis quadrifilaris|[3680]|LOFLC382-06|United States|Florida|658[0n]|BOLD:AAA9634
Argyrostrotis quadrifilaris|[3681]|USLEP533-10|United States|Florida|658[0n]|BOLD:AAA9634
Argyrostrotis quadrifilaris|[3682]|LOFLB248-06|United States|Florida|658[0n]|BOLD:AAA9634
Argyrostrotis quadrifilaris|[3683]|LSEU501-06|United States|Georgia|658[0n]|BOLD:AAA9634
Argyrostrotis quadrifilaris|[3684]|LOFLA530-06|United States|Florida|658[0n]|BOLD:AAA9634
Argyrostrotis quadrifilaris|[3685]|BBLOB823-11|United States|Florida|658[0n]|BOLD:AAA9634
Argyrostrotis quadrifilaris|[3686]|LNC921-06|United States|North Carolina|658[0n]|BOLD:AAA9634
Argyrostrotis quadrifilaris|[3687]|HKONS417-08|United States|Florida|621[1n]|BOLD:AAA9634
Argyrostrotis quadrifilaris|[3688]|LOFLC199-06|United States|Florida|632[0n]|BOLD:AAA9634
Argyrostrotis quadrifilaris|[3689]|LOFLB215-06|United States|Florida|674[0n]|BOLD:AAA9634
Argyrostrotis quadrifilaris|[3690]|LOFLB738-06|United States|Florida|658[0n]|BOLD:AAA9634
Argyrostrotis quadrifilaris|[3691]|LOFLB753-06|United States|Florida|658[0n]|BOLD:AAA9634
Argyrostrotis quadrifilaris|[3692]|USLEP600-10|United States|Florida|658[0n]|BOLD:AAA9634
Argyrostrotis quadrifilaris|[3693]|BBLOB263-11|United States|Florida|658[0n]|BOLD:AAA9634
Argyrostrotis quadrifilaris|[3694]|BBLOB820-11|United States|Florida|658[0n]|BOLD:AAA9634
Argyrostrotis quadrifilaris|[3695]|BBLOB827-11|United States|Florida|658[0n]|BOLD:AAA9634

Argyrostrotis quadrifilaris|[3692]|[illegible]|United States|Florida|658[0n]|BOLD:AAA9634
Argyrostrotis quadrifilaris|[3693]|BBLOB263-11|United States|Florida|658[0n]|BOLD:AAA9634
Argyrostrotis quadrifilaris|[3694]|BBLOB820-11|United States|Florida|658[0n]|BOLD:AAA9634
Argyrostrotis quadrifilaris|[3695]|BBLOB827-11|United States|Florida|658[0n]|BOLD:AAA9634
Argyrostrotis quadrifilaris|[3696]|BBLOB1283-11|United States|Florida|658[0n]|BOLD:AAA9634
Argyrostrotis quadrifilaris|[3697]|BBLOC106-11|United States|Florida|658[0n]|BOLD:AAA9634
Argyrostrotis quadrifilaris|[3698]|LOFLB728-06|United States|Florida|658[0n]|BOLD:AAA9634
Argyrostrotis quadrifilaris|[3699]|LOFLB729-06|United States|Florida|658[0n]|BOLD:AAA9634
Argyrostrotis quadrifilaris|[3700]|LOFLB712-06|United States|Florida|658[0n]|BOLD:AAA9634
Argyrostrotis quadrifilaris|[3701]|LOFLB714-06|United States|Florida|658[0n]|BOLD:AAA9634
Argyrostrotis quadrifilaris|[3702]|LOFLC217-06|United States|Florida|658[0n]|BOLD:AAA9634
Argyrostrotis quadrifilaris|[3703]|HKONS418-08|United States|Florida|658[0n]|BOLD:AAA9634
Argyrostrotis quadrifilaris|[3704]|LOFLB249-06|United States|Florida|658[0n]|BOLD:AAA9634
Argyrostrotis quadrifilaris|[3705]|LOFLB481-06|United States|Florida|658[0n]|BOLD:AAA9634
Argyrostrotis quadrifilaris|[3706]|LOFLB694-06|United States|Florida|658[0n]|BOLD:AAA9634
Argyrostrotis quadrifilaris|[3707]|LOFLB703-06|United States|Florida|658[0n]|BOLD:AAA9634
Argyrostrotis quadrifilaris|[3708]|LOFLB247-06|United States|Florida|658[0n]|BOLD:AAA9634
Argyrostrotis quadrifilaris|[3709]|LOFLB246-06|United States|Florida|658[0n]|BOLD:AAA9634
Argyrostrotis quadrifilaris|[3710]|LOFLB244-06|United States|Florida|658[0n]|BOLD:AAA9634
Argyrostrotis quadrifilaris|[3711]|LOFLB243-06|United States|Florida|658[0n]|BOLD:AAA9634
Argyrostrotis quadrifilaris|[3712]|LOFLB230-06|United States|Florida|658[0n]|BOLD:AAA9634
Argyrostrotis quadrifilaris|[3713]|LOFLB229-06|United States|Florida|658[0n]|BOLD:AAA9634
Argyrostrotis quadrifilaris|[3714]|LOFLA325-06|United States|Florida|658[0n]|BOLD:AAA9634
Argyrostrotis quadrifilaris|[3715]|LNC920-06|United States|North Carolina|658[0n]|BOLD:AAA9634
Argyrostrotis quadrifilaris|[3716]|LNC919-06|United States|North Carolina|658[0n]|BOLD:AAA9634
Argyrostrotis quadrifilaris|[3717]|LOFLB242-06|United States|Florida|658[0n]|BOLD:AAA9634
Argyrostrotis quadrifilaris|[3718]|LNC918-06|United States|North Carolina|658[0n]|BOLD:AAA9634
Argyrostrotis quadrifilaris|[3719]|LSEU055-06|United States|Florida|523[1n]|BOLD:AAA9634
Argyrostrotis quadrifilaris|[3720]|LOFLB245-06|United States|Florida|658[0n]|BOLD:AAA9634
Argyrostrotis quadrifilaris|[3721]|HKONS416-08|United States|Florida|636[0n]|BOLD:AAA9634
Argyrostrotis quadrifilaris|[3722]|BBLOC107-11|United States|Florida|658[0n]|BOLD:AAA9634
Argyrostrotis quadrifilaris|[3723]|BBLOC114-11|United States|Florida|658[0n]|BOLD:AAA9634
Cutina albopunctella|[3724]|ABNCC393-07|United States|Texas|597[0n]|BOLD:AAC3373
Cutina albopunctella|[3725]|HKONB303-09|United States|Texas|658[0n]|BOLD:AAC3373
Cutina albopunctella|[3726]|HKONB304-09|United States|Texas|658[0n]|BOLD:AAC3373
Cutina albopunctella|[3727]|HKONB445-09|United States|Indiana|658[0n]|BOLD:AAC3373
Cutina albopunctella|[3728]|LNCB869-09|United States|Alabama|658[0n]|BOLD:AAC3373
Cutina albopunctella|[3729]|LNC172-05|United States|North Carolina|658[0n]|BOLD:AAC3373
Cutina albopunctella|[3730]|LSEU135-06|United States|Florida|658[0n]|BOLD:AAC3373
Cutina albopunctella|[3731]|LSEU514-06|United States|Georgia|658[0n]|BOLD:AAC3373
Cutina albopunctella|[3732]|LOFLA349-06|United States|Florida|658[0n]|BOLD:AAC3373
Cutina albopunctella|[3733]|LOFLA339-06|United States|Florida|658[0n]|BOLD:AAC3373
Cutina albopunctella|[3734]|LSEU136-06|United States|Florida|658[0n]|BOLD:AAC3373
Cutina albopunctella|[3735]|LNC173-05|United States|North Carolina|658[0n]|BOLD:AAC3373
Cutina albopunctella|[3736]|RDNMK070-11|United States|Florida|625[0n]|BOLD:AAC3373
Cutina arcuata|[3737]|ABNCC397-07|United States|Texas|647[0n]|BOLD:ABZ8355
Cutina arcuata|[3738]|HKONB297-09|United States|Texas|618[0n]|BOLD:ABZ8355
Cutina arcuata|[3739]|ABNCC398-07|United States|Indiana|601[0n]|BOLD:ABZ8355
Cutina arcuata|[3740]|HKONB443-09|United States|Indiana|658[0n]|BOLD:ABZ8355
Cutina arcuata|[3741]|RDNMD349-06|United States|Florida|658[0n]|BOLD:AAE3224
Cutina arcuata|[3742]|RDNMK069-11|United States|Florida|658[0n]|BOLD:AAE3224
Cutina aluticolor|[3743]|LNCB204-06|United States|North Carolina|658[2n]|BOLD:AAD5710
Cutina aluticolor|[3744]|LNCB483-07|United States|North Carolina|658[0n]|BOLD:AAD5710
Cutina aluticolor|[3745]|ABNCC396-07|United States|Indiana|610[1n]|BOLD:AAD5710
Cutina aluticolor|[3746]|HKONB294-09|United States|Texas|658[0n]|BOLD:AAD5710
Cutina aluticolor|[3747]|HKONB295-09|United States|Texas|658[0n]|BOLD:AAD5710
Cutina aluticolor|[3748]|HKONB444-09|United States|Indiana|658[0n]|BOLD:AAD5710
Cutina aluticolor|[3749]|RDNMK068-11|United States|Florida|658[0n]|BOLD:AAD5710
Cutina distincta|[3750]|HKONB300-09|United States|Texas|658[0n]|BOLD:AAD3087
Cutina distincta|[3751]|ABNCC395-07|United States|Indiana|606[0n]|BOLD:AAD3087
Cutina distincta|[3752]|HKONB442-09|United States|Indiana|658[0n]|BOLD:AAD3087
Cutina distincta|[3753]|LNC199-05|United States|North Carolina|658[0n]|BOLD:AAD3087
Cutina distincta|[3754]|LNC844-06|United States|North Carolina|658[0n]|BOLD:AAD3087
Cutina distincta|[3755]|LNC845-06|United States|North Carolina|658[0n]|BOLD:AAD3087
Cutina distincta|[3756]|LNC846-06|United States|North Carolina|658[0n]|BOLD:AAD3087
Cutina distincta|[3757]|RDNMK071-11|United States|Florida|658[0n]|BOLD:AAD3087
Allotria elonympha|[3758]|LOT241-04|United States|Tennessee|658[0n]|BOLD:AAB0988
Allotria elonympha|[3759]|LGSMC356-05|United States|Tennessee|658[0n]|BOLD:AAB0988
Allotria elonympha|[3760]|HKONB258-09|United States|Texas|583[0n]|BOLD:AAB0988
Allotria elonympha|[3761]|QUNO017-07|United States|Florida|658[0n]|BOLD:AAB0988
Allotria elonympha|[3762]|ABCNA617-07|United States|Texas|577[0n]|BOLD:AAB0988
Allotria elonympha|[3763]|LSUSA249-06|United States|Kentucky|658[0n]|BOLD:AAB0988
Allotria elonympha|[3764]|LOTB087-05|United States|Tennessee|658[0n]|BOLD:AAB0988
Allotria elonympha|[3765]|ABCNA729-07|United States|Maryland|600[0n]|BOLD:AAB0988
Allotria elonympha|[3766]|LNC065-05|United States|North Carolina|658[0n]|BOLD:AAB0988
Allotria elonympha|[3767]|LNC066-05|United States|North Carolina|658[0n]|BOLD:AAB0988
Allotria elonympha|[3768]|LNCC666-11|United States|North Carolina|658[0n]|BOLD:AAB0988
Allotria elonympha|[3769]|LOT541-04|United States|Tennessee|611[1n]|BOLD:AAB0988
Allotria elonympha|[3770]|LOCT063-05|United States|Connecticut|658[0n]|BOLD:AAB0988
Allotria elonympha|[3771]|LOCT064-05|United States|Connecticut|658[0n]|BOLD:AAB0988
Allotria elonympha|[3772]|LSEU321-06|United States|Georgia|658[0n]|BOLD:AAB0988
Allotria elonympha|[3773]|LGSMG579-07|United States|Tennessee|658[0n]|BOLD:AAB0988
Allotria elonympha|[3774]|LGSMC359-05|United States|Tennessee|658[0n]|BOLD:AAB0988
Allotria elonympha|[3775]|LGSMC358-05|United States|Tennessee|658[0n]|BOLD:AAB0988
Allotria elonympha|[3776]|LGSMC357-05|United States|Tennessee|658[0n]|BOLD:AAB0988
Allotria elonympha|[3777]|LOT499-04|United States|Tennessee|658[0n]|BOLD:AAB0988
Allotria elonympha|[3778]|LOT246-04|United States|Tennessee|658[0n]|BOLD:AAB0988
Allotria elonympha|[3779]|LOT243-04|United States|Tennessee|658[0n]|BOLD:AAB0988
Allotria elonympha|[3780]|LOT242-04|United States|Tennessee|658[0n]|BOLD:AAB0988
Allotria elonympha|[3781]|LGSM425-04|United States|Tennessee|658[0n]|BOLD:AAB0988
Allotria elonympha|[3782]|LGSM424-04|United States|Tennessee|658[0n]|BOLD:AAB0988
Allotria elonympha|[3783]|LOT244-04|United States|Tennessee|597[0n]|BOLD:AAB0988
Allotria elonympha|[3784]|LOT245-04|United States|Tennessee|587[0n]|BOLD:AAB0988
Allotria elonympha|[3785]|LOTB088-05|United States|Tennessee|616[0n]|BOLD:AAB0988
Allotria elonympha|[3786]|LOTB089-05|United States|Tennessee|658[0n]|BOLD:AAB0988
Allotria elonympha|[3787]|LOTB090-05|United States|Tennessee|658[0n]|BOLD:AAB0988
Allotria elonympha|[3788]|LOTB322-05|United States|Tennessee|658[0n]|BOLD:AAB0988
Allotria elonympha|[3789]|LOTB488-05|United States|Tennessee|658[0n]|BOLD:AAB0988
Allotria elonympha|[3790]|BBLOC1033-11|United States|Texas|658[0n]|BOLD:AAB0988
Parallelia bistriaris|[3791]|XAB562-04|Canada|Ontario|658[0n]|BOLD:AAA8563
Parallelia bistriaris|[3792]|PHMO076-03|Canada|Ontario|639[0n]|BOLD:AAA8563
Parallelia bistriaris|[3793]|LGSMC819-05|United States|Tennessee|619[0n]|BOLD:AAA8563
Parallelia bistriaris|[3794]|BBLEC001-09|Canada|New Brunswick|658[0n]|BOLD:AAA8563
Parallelia bistriaris|[3795]|[illegible]

Parallelia bistriaris|[3792]|PHMO076-03|Canada|Ontario|639[0n]|BOLD:AAA8563
Parallelia bistriaris|[3793]|LGSMC819-05|United States|Tennessee|619[0n]|BOLD:AAA8563
Parallelia bistriaris|[3794]|BBLEC001-09|Canada|New Brunswick|658[0n]|BOLD:AAA8563
Parallelia bistriaris|[3795]|RDLQB253-05|Canada|Quebec|658[0n]|BOLD:AAA8563
Parallelia bistriaris|[3796]|TTMNB280-06|Canada|New Brunswick|658[0n]|BOLD:AAA8563
Parallelia bistriaris|[3797]|TMNBB069-06|Canada|New Brunswick|658[0n]|BOLD:AAA8563
Parallelia bistriaris|[3798]|TMNBB070-06|Canada|New Brunswick|658[0n]|BOLD:AAA8563
Parallelia bistriaris|[3799]|TMNBB071-06|Canada|New Brunswick|658[0n]|BOLD:AAA8563
Parallelia bistriaris|[3800]|TMNBB072-06|Canada|New Brunswick|658[0n]|BOLD:AAA8563
Parallelia bistriaris|[3801]|TMNBB073-06|Canada|New Brunswick|658[0n]|BOLD:AAA8563
Parallelia bistriaris|[3802]|XAJ680-06|Canada|Ontario|658[0n]|BOLD:AAA8563
Parallelia bistriaris|[3803]|RDLQF734-06|Canada|Quebec|658[0n]|BOLD:AAA8563
Parallelia bistriaris|[3804]|LPSO991-08|Canada|Ontario|658[0n]|BOLD:AAA8563
Parallelia bistriaris|[3805]|XAE628-04|Canada|Ontario|658[0n]|BOLD:AAA8563
Parallelia bistriaris|[3806]|MNBB054-05|Canada|New Brunswick|658[0n]|BOLD:AAA8563
Parallelia bistriaris|[3807]|MNBB201-05|Canada|New Brunswick|658[0n]|BOLD:AAA8563
Parallelia bistriaris|[3808]|XAG545-05|Canada|Ontario|658[0n]|BOLD:AAA8563
Parallelia bistriaris|[3809]|LPSOC307-08|Canada|Ontario|658[0n]|BOLD:AAA8563
Parallelia bistriaris|[3810]|BBLPE016-09|Canada|Nova Scotia|658[0n]|BOLD:AAA8563
Parallelia bistriaris|[3811]|LSEU305-06|United States|Georgia|658[0n]|BOLD:AAA8563
Parallelia bistriaris|[3812]|LGSMC820-05|United States|Tennessee|658[0n]|BOLD:AAA8563
Parallelia bistriaris|[3813]|LGSMG582-07|United States|Tennessee|658[0n]|BOLD:AAA8563
Parallelia bistriaris|[3814]|QUNOB189-08|United States|Indiana|658[0n]|BOLD:AAA8563
Parallelia bistriaris|[3815]|LGSMC822-05|United States|Tennessee|658[0n]|BOLD:AAA8563
Parallelia bistriaris|[3816]|LOTB338-05|United States|Tennessee|658[0n]|BOLD:AAA8563
Parallelia bistriaris|[3817]|LSUSA257-06|United States|Kentucky|658[0n]|BOLD:AAA8563
Parallelia bistriaris|[3818]|HKONS394-08|United States|Florida|658[0n]|BOLD:AAA8563
Parallelia bistriaris|[3819]|QUNOB286-09|United States|Indiana|658[0n]|BOLD:AAA8563
Parallelia bistriaris|[3820]|LPOKA449-09|United States|Oklahoma|658[0n]|BOLD:AAA8563
Parallelia bistriaris|[3821]|UDLEP082-09|United States|Maryland|630[0n]|BOLD:AAA8563
Parallelia bistriaris|[3822]|LNCB321-06|United States|North Carolina|658[0n]|BOLD:AAA8563
Parallelia bistriaris|[3823]|HKONS393-08|United States|Florida|658[0n]|BOLD:AAA8563
Parallelia bistriaris|[3824]|LNC069-05|United States|North Carolina|658[0n]|BOLD:AAA8563
Parallelia bistriaris|[3825]|LNC070-05|United States|North Carolina|658[0n]|BOLD:AAA8563
Parallelia bistriaris|[3826]|UDLEP089-09|United States|Maryland|658[0n]|BOLD:AAA8563
Parallelia bistriaris|[3827]|UDLEP093-09|United States|Maryland|631[0n]|BOLD:AAA8563
Parallelia bistriaris|[3828]|LNCB748-09|United States|North Carolina|621[0n]|BOLD:AAA8563
Parallelia bistriaris|[3829]|QUNOB082-08|United States|Texas|658[0n]|BOLD:AAA8563
Parallelia bistriaris|[3830]|PHMTV421-10|Canada|Ontario|658[0n]|BOLD:AAA8563
Parallelia bistriaris|[3831]|UDLEP057-09|United States|Maryland|658[0n]|BOLD:AAA8563
Parallelia bistriaris|[3832]|LOT250-04|United States|Tennessee|609[0n]|BOLD:AAA8563
Parallelia bistriaris|[3833]|LGSMC821-05|United States|Tennessee|658[0n]|BOLD:AAA8563
Parallelia bistriaris|[3834]|LGSMC368-05|United States|Tennessee|658[0n]|BOLD:AAA8563
Parallelia bistriaris|[3835]|LOT251-04|United States|Tennessee|658[0n]|BOLD:AAA8563
Parallelia bistriaris|[3836]|ABNCC391-07|United States|Texas|647[0n]|BOLD:AAA8563
Parallelia bistriaris|[3837]|UDLEP299-09|United States|Pennsylvania|658[0n]|BOLD:AAA8563
Parallelia bistriaris|[3838]|UDLEP319-09|United States|Pennsylvania|627[0n]|BOLD:AAA8563
Parallelia bistriaris|[3839]|JRLAA019-09|United States|Alabama|658[0n]|BOLD:AAA8563
Parallelia bistriaris|[3840]|LILLA786-11|United States|Illinois|658[0n]|BOLD:AAA8563
Argyrostrotis deleta|[3841]|HKONS412-08|United States|Florida|601[0n]|BOLD:AAC0229
Argyrostrotis deleta|[3842]|HKONS409-08|United States|Florida|658[0n]|BOLD:AAC0229
Argyrostrotis deleta|[3843]|LNC003-05|United States|North Carolina|658[0n]|BOLD:AAC0229
Argyrostrotis deleta|[3844]|LNC004-05|United States|North Carolina|658[0n]|BOLD:AAC0229
Argyrostrotis deleta|[3845]|LNCB141-06|United States|North Carolina|658[0n]|BOLD:AAC0229
Argyrostrotis deleta|[3846]|LNCB360-06|United States|North Carolina|658[0n]|BOLD:AAC0229
Argyrostrotis deleta|[3847]|LSEU500-06|United States|Georgia|639[0n]|BOLD:AAC0229
Argyrostrotis deleta|[3848]|HKONS413-08|United States|Florida|658[0n]|BOLD:AAC0229
Argyrostrotis deleta|[3849]|LSEU498-06|United States|Georgia|658[0n]|BOLD:AAC0229
Argyrostrotis deleta|[3850]|LSEU499-06|United States|Georgia|658[0n]|BOLD:AAC0229
Argyrostrotis deleta|[3851]|HKONS410-08|United States|Florida|658[0n]|BOLD:AAC0229
Argyrostrotis deleta|[3852]|HKONS411-08|United States|Florida|658[0n]|BOLD:AAC0229
Argyrostrotis deleta|[3853]|BBLOB1888-11|United States|Florida|658[0n]|BOLD:AAC0229
Argyrostrotis deleta|[3854]|BBLOB1897-11|United States|Florida|658[0n]|BOLD:AAC0229
Argyrostrotis deleta|[3855]|HKONS407-08|United States|Florida|635[0n]|BOLD:AAC0229
Argyrostrotis deleta|[3856]|HKONS406-08|United States|Florida|658[0n]|BOLD:AAC0229
Argyrostrotis deleta|[3857]|HKONS408-08|United States|Florida|658[0n]|BOLD:AAC0229
Argyrostrotis deleta|[3858]|USLEP919-10|United States|Florida|658[0n]|BOLD:AAC0229
Argyrostrotis deleta|[3859]|BBLOB1887-11|United States|Florida|658[0n]|BOLD:AAC0229
Argyrostrotis deleta|[3860]|MILEQ225-11|United States|Alabama|658[0n]|BOLD:AAC0229
Argyrostrotis anilis|[3861]|MNAG041-08|Canada|Manitoba|658[0n]|BOLD:AAC8698
Argyrostrotis anilis|[3862]|RDNMG1024-08|Canada|Ontario|658[0n]|BOLD:AAC8698
Argyrostrotis anilis|[3863]|RDNMG1025-08|Canada|Ontario|658[0n]|BOLD:AAC8698
Argyrostrotis anilis|[3864]|QUNOB517-09|United States|Kentucky|649[0n]|BOLD:AAC8698
Argyrostrotis anilis|[3865]|QUNOB519-09|United States|Kentucky|658[0n]|BOLD:AAC8698
Argyrostrotis anilis|[3866]|LNCC1632-13|United States|North Carolina|658[0n]|BOLD:AAC8698
Argyrostrotis anilis|[3867]|LNCC1631-13|United States|North Carolina|658[0n]|BOLD:AAC8698
Argyrostrotis anilis|[3868]|LNCC1630-13|United States|North Carolina|658[0n]|BOLD:AAC8698
Argyrostrotis anilis|[3869]|MILEP364-10|United States|Alabama|658[0n]|BOLD:AAC8698
Argyrostrotis anilis|[3870]|MILEP363-10|United States|Alabama|658[0n]|BOLD:AAC8698
Argyrostrotis anilis|[3871]|BBLSX110-09|United States|Oklahoma|658[0n]|BOLD:AAC8698
Argyrostrotis anilis|[3872]|BBLSX082-09|United States|Oklahoma|658[0n]|BOLD:AAC8698
Argyrostrotis anilis|[3873]|LPOKB1019-09|United States|Oklahoma|658[0n]|BOLD:AAC8698
Argyrostrotis anilis|[3874]|LPOKA064-08|United States|Oklahoma|658[0n]|BOLD:AAC8698
Argyrostrotis anilis|[3875]|RDNMD500-06|United States|Florida|658[0n]|BOLD:AAC8698
Argyrostrotis anilis|[3876]|ABNCC428-07|United States|Texas|606[0n]|BOLD:AAC8698
Argyrostrotis anilis|[3877]|LNAUT2656-14|United States|Massachusetts|615[0n]|BOLD:AAC8698
Argyrostrotis anilis|[3878]|LNAUT2657-14|United States|Massachusetts|658[0n]|BOLD:AAC8698
Argyrostrotis anilis|[3879]|LNAUT2658-14|United States|Massachusetts|658[0n]|BOLD:AAC8698
Ascalapha odorata|[3880]|DSCNI024-07|Canada|Manitoba|658[0n]|BOLD:AAA5595
Ascalapha odorata|[3881]|ABNCC310-07|United States|Texas|644[0n]|BOLD:AAA5595
Ascalapha odorata|[3882]|BBLOD179-11|United States|Texas|620[1n]|BOLD:AAA5595
Ascalapha odorata|[3883]|BBLOD183-11|United States|Texas|634[0n]|BOLD:AAA5595
Ascalapha odorata|[3884]|CMAZA848-10|United States|Arizona|658[0n]|BOLD:AAA5595
Ascalapha odorata|[3885]|QUNO487-08|United States|Texas|658[0n]|BOLD:AAA5595
Ascalapha odorata|[3886]|BBLOD176-11|United States|Texas|630[0n]|BOLD:AAA5595
Ascalapha odorata|[3887]|QUNOC009-09|United States|Texas|658[0n]|BOLD:AAA5595
Ascalapha odorata|[3888]|QUNOC008-09|United States|Texas|658[0n]|BOLD:AAA5595
Ascalapha odorata|[3889]|BBLOD1903-11|United States|Texas|658[0n]|BOLD:AAA5595
Isogona scindens|[3890]|HKONB025-08|United States|Texas|658[0n]|BOLD:AAA3992
Isogona scindens|[3891]|HKONB023-08|United States|Texas|658[0n]|BOLD:AAA3992
Isogona scindens|[3892]|HKONB024-08|United States|Texas|658[0n]|BOLD:AAA3992
Isogona scindens|[3893]|HKONB026-08|United States|Texas|658[0n]|BOLD:AAA3992
Isogona scindens|[3894]|HKONB027-08|United States|Texas|658[0n]|BOLD:AAA3992
[illegible]

Isogona scindens|[3892]|HKONB024-08|United States|Texas|658[0n]|BOLD:AAA3992
Isogona scindens|[3893]|HKONB026-08|United States|Texas|658[0n]|BOLD:AAA3992
Isogona scindens|[3894]|HKONB027-08|United States|Texas|658[0n]|BOLD:AAA3992
Isogona scindens|[3895]|HKONB497-09|United States|Texas|622[0n]|BOLD:AAA3992
Thysania zenobia|[3896]|HKONB001-08|United States|Texas|658[0n]|BOLD:AAB8451
Thysania zenobia|[3897]|QUNOD463-10|United States|Wisconsin|658[0n]|BOLD:AAB8451
Letis xylia|[3898]|BLPBG178-07|Costa Rica|Guanacaste|594[0n]|BOLD:AAB1499
Letis xylia|[3899]|MHAUF667-06|Costa Rica|Guanacaste|658[0n]|BOLD:AAB1499
Letis xylia|[3900]|BLPAF362-07|Costa Rica|Guanacaste|658[0n]|BOLD:AAB1499
Letis xylia|[3901]|BLPAE704-06|Costa Rica|Guanacaste|658[0n]|BOLD:AAB1499
Letis xylia|[3902]|MHAUF666-06|Costa Rica|Guanacaste|658[0n]|BOLD:AAB1499
Letis xylia|[3903]|BLPAF371-07|Costa Rica|Guanacaste|658[0n]|BOLD:AAB1499
Letis xylia|[3904]|BLPBG177-07|Costa Rica|Guanacaste|629[0n]|BOLD:AAB1499
Letis xylia|[3905]|BLPBG024-07|Costa Rica|Guanacaste|630[0n]|BOLD:AAB1499
Letis xylia|[3906]|BLPCO061-08|Costa Rica|Guanacaste|658[0n]|BOLD:AAB1499
Letis xylia|[3907]|BLPAF372-07|Costa Rica|Guanacaste|658[0n]|BOLD:AAB1499
Letis xylia|[3908]|BLPAG310-07|Costa Rica|Guanacaste|658[0n]|BOLD:AAB1499
Letis xylia|[3909]|BLPBB201-07|Costa Rica|Guanacaste|648[0n]|BOLD:AAB1499
Letis xylia|[3910]|BLPCD685-08|Costa Rica|Guanacaste|658[0n]|BOLD:AAB1499
Letis xylia|[3911]|BLPCD686-08|Costa Rica|Guanacaste|658[0n]|BOLD:AAB1499
Letis xylia|[3912]|BLPCD687-08|Costa Rica|Guanacaste|658[0n]|BOLD:AAB1499
Letis xylia|[3913]|BLPCD688-08|Costa Rica|Guanacaste|658[0n]|BOLD:AAB1499
Letis xylia|[3914]|BLPBB198-07|Costa Rica|Guanacaste|658[0n]|BOLD:AAB1499
Letis xylia|[3915]|BLPBB197-07|Costa Rica|Guanacaste|658[0n]|BOLD:AAB1499
Letis xylia|[3916]|BLPAG311-07|Costa Rica|Guanacaste|658[0n]|BOLD:AAB1499
Letis xylia|[3917]|BLPAF370-07|Costa Rica|Guanacaste|658[0n]|BOLD:AAB1499
Letis xylia|[3918]|BLPAF368-07|Costa Rica|Guanacaste|658[0n]|BOLD:AAB1499
Letis xylia|[3919]|BLPAF367-07|Costa Rica|Guanacaste|658[0n]|BOLD:AAB1499
Letis xylia|[3920]|BLPAF364-07|Costa Rica|Guanacaste|658[0n]|BOLD:AAB1499
Letis xylia|[3921]|BLPAF363-07|Costa Rica|Guanacaste|658[0n]|BOLD:AAB1499
Letis xylia|[3922]|BLPAF361-07|Costa Rica|Guanacaste|658[0n]|BOLD:AAB1499
Letis xylia|[3923]|BLPAF360-07|Costa Rica|Guanacaste|658[0n]|BOLD:AAB1499
Letis xylia|[3924]|BLPAF369-07|Costa Rica|Guanacaste|658[1n]|BOLD:AAB1499
Letis xylia|[3925]|BLPCO120-08|Costa Rica|Guanacaste|658[0n]|BOLD:AAB1499
Letis xylia|[3926]|MHAUF663-06|Costa Rica|Alajuela|658[1n]|BOLD:AAB1499
Letis xylia|[3927]|MHMYL3185-11|Costa Rica||658[0n]|BOLD:AAB1499
Letis xylia|[3928]|BLPCO060-08|Costa Rica|Guanacaste|658[0n]|BOLD:AAB1499
Letis xylia|[3929]|CNCLB1822-14|Guatemala||658[0n]|BOLD:AAB1499
Lesmone hinna|[3930]|ABNCC312-07|United States|Texas|629[1n]|BOLD:AAD1287
Lesmone hinna|[3931]|LOFLC333-06|United States|Florida|658[0n]|BOLD:AAD1287
Lesmone hinna|[3932]|HKONS048-07|United States|Florida|658[0n]|BOLD:AAD1287
Lesmone sp.|[3933]|LMEMB204-09|United States|Texas|658[0n]|BOLD:AAD1288
Lesmone sp.|[3934]|ABNCC313-07|United States|Texas|632[0n]|BOLD:AAD1288
Lesmone sp.|[3935]|LMEMB205-09|United States|Texas|658[0n]|BOLD:AAD1288
Lesmone sp.|[3936]|QUNOD843-11|United States|Texas|658[0n]|BOLD:AAD1288
Lesmone formularis|[3937]|LYNYM355-09|Mexico|Quintana Roo|576[1n]|BOLD:AAA3052
Lesmone formularis|[3938]|BLPCA453-08|Costa Rica|Guanacaste|658[2n]|BOLD:AAA3052
Lesmone formularis|[3939]|BLPBC842-07|Costa Rica|Alajuela|656[0n]|BOLD:AAA3052
Lesmone formularis|[3940]|MHMYH923-10|Costa Rica||658[0n]|BOLD:AAA3052
Lesmone formularis|[3941]|BLPDD654-09|Costa Rica|Guanacaste|658[0n]|BOLD:AAA3052
Lesmone formularis|[3942]|BLPDD652-09|Costa Rica|Guanacaste|658[0n]|BOLD:AAA3052
Lesmone formularis|[3943]|BLPDD651-09|Costa Rica|Guanacaste|658[0n]|BOLD:AAA3052
Lesmone formularis|[3944]|BLPDD647-09|Costa Rica|Guanacaste|658[0n]|BOLD:AAA3052
Lesmone formularis|[3945]|BLPDC650-09|Costa Rica|Alajuela|658[0n]|BOLD:AAA3052
Lesmone formularis|[3946]|BLPDC264-09|Costa Rica|Guanacaste|658[0n]|BOLD:AAA3052
Lesmone formularis|[3947]|BLPCK838-08|Costa Rica|Alajuela|658[0n]|BOLD:AAA3052
Lesmone formularis|[3948]|MHMXQ604-08|Costa Rica|Guanacaste|658[0n]|BOLD:AAA3052
Lesmone formularis|[3949]|BLPBC407-07|Costa Rica|Alajuela|658[0n]|BOLD:AAA3052
Lesmone formularis|[3950]|MHAUF423-06|Costa Rica|Alajuela|658[0n]|BOLD:AAA3052
Lesmone formularis|[3951]|MHAUF421-06|Costa Rica|Guanacaste|658[0n]|BOLD:AAA3052
Lesmone formularis|[3952]|MHAUF420-06|Costa Rica|Guanacaste|658[0n]|BOLD:AAA3052
Lesmone formularis|[3953]|MHAUF415-06|Costa Rica|Alajuela|658[0n]|BOLD:AAA3052
Lesmone formularis|[3954]|MHMYL3603-11|Costa Rica||658[0n]|BOLD:AAA3052
Lesmone formularis|[3955]|BLPCA454-08|Costa Rica|Guanacaste|658[0n]|BOLD:AAA3052
Lesmone formularis|[3956]|BLPDB404-09|Costa Rica|Alajuela|634[0n]|BOLD:AAA3052
Lesmone formularis|[3957]|BLPDB617-09|Costa Rica|Alajuela|577[0n]|BOLD:AAA3052
Lesmone formularis|[3958]|BLPDB622-09|Costa Rica|Alajuela|615[0n]|BOLD:AAA3052
Lesmone formularis|[3959]|BLPCC388-08|Costa Rica|Guanacaste|658[1n]|BOLD:AAA3052
Lesmone formularis|[3960]|MHMYL2965-11|Costa Rica||658[0n]|BOLD:AAA3052
Lesmone formularis|[3961]|BLPCI125-08|Costa Rica|Guanacaste|658[0n]|BOLD:AAA3052
Lesmone formularis|[3962]|BLPBC357-07|Costa Rica|Alajuela|659[0n]|BOLD:AAA3052
Lesmone formularis|[3963]|BLPAG399-07|Costa Rica|Guanacaste|658[0n]|BOLD:AAA3052
Lesmone formularis|[3964]|BLPAG376-07|Costa Rica|Guanacaste|658[0n]|BOLD:AAA3052
Lesmone formularis|[3965]|BLPAF854-07|Costa Rica|Guanacaste|658[0n]|BOLD:AAA3052
Lesmone formularis|[3966]|BLPAE698-06|Costa Rica|Guanacaste|658[0n]|BOLD:AAA3052
Lesmone formularis|[3967]|BLPAD053-06|Costa Rica|Guanacaste|658[0n]|BOLD:AAA3052
Lesmone formularis|[3968]|BLPAC481-06|Costa Rica|Guanacaste|658[0n]|BOLD:AAA3052
Lesmone formularis|[3969]|BLPAB691-06|Costa Rica|Guanacaste|658[0n]|BOLD:AAA3052
Lesmone formularis|[3970]|MHAUF425-06|Costa Rica|Alajuela|658[0n]|BOLD:AAA3052
Lesmone formularis|[3971]|MHAUF417-06|Costa Rica|Alajuela|658[0n]|BOLD:AAA3052
Lesmone formularis|[3972]|BLPAH312-07|Costa Rica|Guanacaste|658[1n]|BOLD:AAA3052
Lesmone formularis|[3973]|MHMXI213-07|Costa Rica|Guanacaste|632[0n]|BOLD:AAA3052
Lesmone formularis|[3974]|BLPBB686-07|Costa Rica|Guanacaste|633[0n]|BOLD:AAA3052
Lesmone formularis|[3975]|BLPBC356-07|Costa Rica|Alajuela|626[0n]|BOLD:AAA3052
Lesmone formularis|[3976]|BLPCE400-08|Costa Rica|Guanacaste|632[9n]|BOLD:AAA3052
Lesmone formularis|[3977]|BLPDA740-09|Costa Rica|Guanacaste|632[0n]|BOLD:AAA3052
Lesmone formularis|[3978]|LYHES253-09|Mexico|Quintana Roo|614[0n]|BOLD:AAA3052
Lesmone formularis|[3979]|MHMYC2355-09|Costa Rica|Guanacaste|658[0n]|BOLD:AAA3052
Lesmone formularis|[3980]|BLPCD423-08|Costa Rica|Guanacaste|658[0n]|BOLD:AAA3052
Lesmone formularis|[3981]|BLPCJ532-08|Costa Rica|Guanacaste|658[0n]|BOLD:AAA3052
Lesmone formularis|[3982]|BLPCC692-08|Costa Rica|Guanacaste|658[0n]|BOLD:AAA3052
Lesmone formularis|[3983]|BLPCD421-08|Costa Rica|Guanacaste|658[0n]|BOLD:AAA3052
Lesmone formularis|[3984]|BLPBE439-07|Costa Rica|Guanacaste|658[0n]|BOLD:AAA3052
Lesmone formularis|[3985]|BLPBE440-07|Costa Rica|Guanacaste|658[0n]|BOLD:AAA3052
Lesmone formularis|[3986]|BLPBC406-07|Costa Rica|Alajuela|658[0n]|BOLD:AAA3052
Lesmone formularis|[3987]|BLPBE438-07|Costa Rica|Guanacaste|658[0n]|BOLD:AAA3052
Lesmone formularis|[3988]|BLPAH316-07|Costa Rica|Guanacaste|658[0n]|BOLD:AAA3052
Lesmone formularis|[3989]|BLPBA219-07|Costa Rica|Guanacaste|658[0n]|BOLD:AAA3052
Lesmone formularis|[3990]|BLPAG421-07|Costa Rica|Guanacaste|658[0n]|BOLD:AAA3052
Lesmone formularis|[3991]|BLPAH311-07|Costa Rica|Guanacaste|658[0n]|BOLD:AAA3052
Lesmone formularis|[3992]|BLPCA452-08|Costa Rica|Guanacaste|658[0n]|BOLD:AAA3052
Lesmone formularis|[3993]|BLPCC469-08|Costa Rica|Guanacaste|658[0n]|BOLD:AAA3052
Lesmone formularis|[3994]|LPYPB520-08|Mexico|Quintana Roo|658[0n]|BOLD:AAA3052

Lesmone formularis|[3992]|BLPCA452-08|Costa Rica|Guanacaste|658[0n]|BOLD:AAA3052
Lesmone formularis|[3993]|BLPCC469-08|Costa Rica|Guanacaste|658[0n]|BOLD:AAA3052
Lesmone formularis|[3994]|LPYPB520-08|Mexico|Quintana Roo|658[0n]|BOLD:AAA3052
Lesmone formularis|[3995]|BLPCK120-08|Costa Rica|Alajuela|658[0n]|BOLD:AAA3052
Lesmone formularis|[3996]|BLPCK695-08|Costa Rica|Alajuela|658[0n]|BOLD:AAA3052
Lesmone formularis|[3997]|BLPCO140-08|Costa Rica|Guanacaste|658[0n]|BOLD:AAA3052
Lesmone formularis|[3998]|BLPDC228-09|Costa Rica|Guanacaste|658[0n]|BOLD:AAA3052
Lesmone formularis|[3999]|BLPDC265-09|Costa Rica|Guanacaste|658[0n]|BOLD:AAA3052
Lesmone formularis|[4000]|BLPDC266-09|Costa Rica|Guanacaste|658[0n]|BOLD:AAA3052
Lesmone formularis|[4001]|BLPDD648-09|Costa Rica|Guanacaste|658[0n]|BOLD:AAA3052
Lesmone formularis|[4002]|BLPDD649-09|Costa Rica|Guanacaste|658[0n]|BOLD:AAA3052
Lesmone formularis|[4003]|BLPDD653-09|Costa Rica|Guanacaste|658[0n]|BOLD:AAA3052
Lesmone formularis|[4004]|LYHES546-09|Mexico|Quintana Roo|658[0n]|BOLD:AAA3052
Lesmone formularis|[4005]|QUNOD009-10|United States|Texas|658[0n]|BOLD:AAA3052
Lesmone formularis|[4006]|GWORR718-10|Venezuela|Carabobo|658[0n]|BOLD:AAA3052
Lesmone formularis|[4007]|MHMYH626-10|Costa Rica||658[0n]|BOLD:AAA3052
Lesmone formularis|[4008]|LNOUB714-10|French Guiana||658[0n]|BOLD:AAA3052
Lesmone formularis|[4009]|BLPDU1168-11|Costa Rica|Guanacaste|658[0n]|BOLD:AAA3052
Lesmone formularis|[4010]|MHMYL3595-11|Costa Rica||658[0n]|BOLD:AAA3052
Lesmone formularis|[4011]|BLPEC681-11|Costa Rica|Alajuela|658[0n]|BOLD:AAA3052
Lesmone formularis|[4012]|GWOSU367-11|Nicaragua|Jinotega|658[0n]|BOLD:AAA3052
Lesmone formularis|[4013]|GWOSU368-11|Costa Rica|San Jose|658[0n]|BOLD:AAA3052
Lesmone formularis|[4014]|MHMYM097-11|Costa Rica||658[0n]|BOLD:AAA3052
Lesmone formularis|[4015]|MHMYM255-11|Costa Rica||658[0n]|BOLD:AAA3052
Lesmone formularis|[4016]|MHMYQ1665-12|Costa Rica||658[0n]|BOLD:AAA3052
Lesmone formularis|[4017]|BLPDB618-09|Costa Rica|Alajuela|645[0n]|BOLD:AAA3052
Lesmone formularis|[4018]|BLPCP486-08|Costa Rica|Guanacaste|645[0n]|BOLD:AAA3052
Lesmone formularis|[4019]|LYNYM175-09|Mexico|Yucatan|603[0n]|BOLD:AAA3052
Lesmone formularis|[4020]|BLPDE137-09|Costa Rica|Guanacaste|658[0n]|BOLD:AAA3052
Lesmone formularis|[4021]|BLPDE136-09|Costa Rica|Guanacaste|658[0n]|BOLD:AAA3052
Lesmone formularis|[4022]|BLPDD650-09|Costa Rica|Guanacaste|658[0n]|BOLD:AAA3052
Lesmone formularis|[4023]|BLPDC904-09|Costa Rica|Alajuela|658[0n]|BOLD:AAA3052
Lesmone formularis|[4024]|BLPDC651-09|Costa Rica|Alajuela|658[0n]|BOLD:AAA3052
Lesmone formularis|[4025]|BLPDC646-09|Costa Rica|Alajuela|658[0n]|BOLD:AAA3052
Lesmone formularis|[4026]|BLPDA332-09|Costa Rica|Guanacaste|658[0n]|BOLD:AAA3052
Lesmone formularis|[4027]|BLPCK694-08|Costa Rica|Alajuela|658[0n]|BOLD:AAA3052
Lesmone formularis|[4028]|BLPCJ509-08|Costa Rica|Guanacaste|658[0n]|BOLD:AAA3052
Lesmone formularis|[4029]|BLPCJ074-08|Costa Rica|Guanacaste|658[0n]|BOLD:AAA3052
Lesmone formularis|[4030]|BLPCJ073-08|Costa Rica|Guanacaste|658[0n]|BOLD:AAA3052
Lesmone formularis|[4031]|BLPCH589-08|Costa Rica|Guanacaste|658[0n]|BOLD:AAA3052
Lesmone formularis|[4032]|BLPCD422-08|Costa Rica|Guanacaste|658[0n]|BOLD:AAA3052
Lesmone formularis|[4033]|BLPCC472-08|Costa Rica|Guanacaste|658[0n]|BOLD:AAA3052
Lesmone formularis|[4034]|BLPCC471-08|Costa Rica|Guanacaste|658[0n]|BOLD:AAA3052
Lesmone formularis|[4035]|BLPCC470-08|Costa Rica|Guanacaste|658[0n]|BOLD:AAA3052
Lesmone formularis|[4036]|BLPCA873-08|Costa Rica|Guanacaste|658[0n]|BOLD:AAA3052
Lesmone formularis|[4037]|BLPBA220-07|Costa Rica|Guanacaste|658[0n]|BOLD:AAA3052
Lesmone formularis|[4038]|BLPAH315-07|Costa Rica|Guanacaste|658[0n]|BOLD:AAA3052
Lesmone formularis|[4039]|BLPAH314-07|Costa Rica|Guanacaste|658[0n]|BOLD:AAA3052
Lesmone formularis|[4040]|BLPAH313-07|Costa Rica|Guanacaste|658[0n]|BOLD:AAA3052
Lesmone formularis|[4041]|BLPAF859-07|Costa Rica|Guanacaste|658[0n]|BOLD:AAA3052
Lesmone formularis|[4042]|BLPAF856-07|Costa Rica|Guanacaste|658[0n]|BOLD:AAA3052
Lesmone formularis|[4043]|BLPAF855-07|Costa Rica|Guanacaste|658[0n]|BOLD:AAA3052
Lesmone formularis|[4044]|MHMXD729-06|Costa Rica|Alajuela|658[0n]|BOLD:AAA3052
Lesmone formularis|[4045]|BLPAE010-06|Costa Rica|Alajuela|658[0n]|BOLD:AAA3052
Lesmone formularis|[4046]|BLPAE004-06|Costa Rica|Alajuela|658[0n]|BOLD:AAA3052
Lesmone formularis|[4047]|BLPAC676-06|Costa Rica|Guanacaste|658[0n]|BOLD:AAA3052
Lesmone formularis|[4048]|BLPAC358-06|Costa Rica|Guanacaste|658[0n]|BOLD:AAA3052
Lesmone formularis|[4049]|BLPAC292-06|Costa Rica|Guanacaste|658[0n]|BOLD:AAA3052
Lesmone formularis|[4050]|BLPAC291-06|Costa Rica|Guanacaste|658[0n]|BOLD:AAA3052
Lesmone formularis|[4051]|BLPAA844-06|Costa Rica|Alajuela|658[0n]|BOLD:AAA3052
Lesmone formularis|[4052]|BLPAA225-06|Costa Rica|Guanacaste|658[0n]|BOLD:AAA3052
Lesmone formularis|[4053]|MHAUF424-06|Costa Rica|Alajuela|658[0n]|BOLD:AAA3052
Lesmone formularis|[4054]|MHAUF422-06|Costa Rica|Guanacaste|658[0n]|BOLD:AAA3052
Lesmone formularis|[4055]|MHAUF419-06|Costa Rica|Guanacaste|658[0n]|BOLD:AAA3052
Lesmone formularis|[4056]|MHAUF418-06|Costa Rica|Alajuela|658[0n]|BOLD:AAA3052
Lesmone formularis|[4057]|MHAUF414-06|Costa Rica|Alajuela|658[0n]|BOLD:AAA3052
Lesmone formularis|[4058]|MHAUC599-06|Costa Rica|Alajuela|658[0n]|BOLD:AAA3052
Lesmone formularis|[4059]|MHAUC594-06|Costa Rica|Alajuela|658[0n]|BOLD:AAA3052
Lesmone formularis|[4060]|BLPDA774-09|Costa Rica|Guanacaste|658[0n]|BOLD:AAA3052
Lesmone formularis|[4061]|BLPAB123-06|Costa Rica|Alajuela|658[1n]|BOLD:AAA3052
Lesmone formularis|[4062]|BLPBC843-07|Costa Rica|Alajuela|648[0n]|BOLD:AAA3052
Lesmone formularis|[4063]|BLPBC844-07|Costa Rica|Alajuela|646[0n]|BOLD:AAA3052
Lesmone formularis|[4064]|MHAUC595-06|Costa Rica|Guanacaste|563[1n]|BOLD:AAA3052
Lesmone formularis|[4065]|BLPCE401-08|Costa Rica|Guanacaste|658[27n]|BOLD:AAA3052
Lesmone formularis|[4066]|BLPDB621-09|Costa Rica|Alajuela|589[0n]|BOLD:AAA3052
Lesmone formularis|[4067]|BLPDB620-09|Costa Rica|Alajuela|602[0n]|BOLD:AAA3052
Lesmone formularis|[4068]|BLPDC456-09|Costa Rica|Alajuela|633[0n]|BOLD:AAA3052
Lesmone formularis|[4069]|BLPDE145-09|Costa Rica|Guanacaste|629[0n]|BOLD:AAA3052
Lesmone formularis|[4070]|BLPDE146-09|Costa Rica|Guanacaste|658[0n]|BOLD:AAA3052
Lesmone formularis|[4071]|BLPDE315-09|Costa Rica|Guanacaste|658[0n]|BOLD:AAA3052
Lesmone formularis|[4072]|LYHES232-09|Mexico|Yucatan|658[0n]|BOLD:AAA3052
Lesmone formularis|[4073]|QUNOD008-10|United States|Texas|658[0n]|BOLD:AAA3052
Lesmone formularis|[4074]|INCTA242-10|Brazil|Para|658[0n]|BOLD:AAA3052
Lesmone formularis|[4075]|LNOUB452-10|French Guiana||658[0n]|BOLD:AAA3052
Lesmone formularis|[4076]|BLPDY776-11|Costa Rica|Guanacaste|658[0n]|BOLD:AAA3052
Lesmone formularis|[4077]|MILEP975-11|French Guiana||658[0n]|BOLD:AAA3052
Lesmone formularis|[4078]|MHMYM043-11|Costa Rica||658[0n]|BOLD:AAA3052
Lesmone formularis|[4079]|CNCLB1955-14|Guatemala||658[0n]|BOLD:AAA3052
Metria bilineata|[4080]|ABNCC321-07|United States|Texas|650[0n]|BOLD:AAC2303
Metria bilineata|[4081]|ABNCC322-07|United States|Texas|647[0n]|BOLD:AAC2303
Metria bilineata|[4082]|HKONB138-08|United States|Texas|658[0n]|BOLD:AAC2303
Metria bilineata|[4083]|HKONB137-08|United States|Texas|658[0n]|BOLD:AAC2303
Metria bilineata|[4084]|HKONB135-08|United States|Texas|658[0n]|BOLD:AAC2303
Metria bilineata|[4085]|HKONB136-08|United States|Texas|658[0n]|BOLD:AAC2303
Metria bilineata|[4086]|HKONB139-08|United States|Texas|658[0n]|BOLD:AAC2303
Metria bilineata|[4087]|HKONB140-08|United States|Texas|658[0n]|BOLD:AAC2303
Metria amella|[4088]|LMEMB223-09|United States|Alabama|658[0n]|BOLD:AAB4949
Metria amella|[4089]|USLEP577-10|United States|Texas|658[0n]|BOLD:AAB4949
Metria amella|[4090]|LMEMB219-09|United States|Mississippi|658[0n]|BOLD:AAB4949
Metria amella|[4091]|LMEMB220-09|United States|Mississippi|658[0n]|BOLD:AAB4949
Metria amella|[4092]|BBLSW834-09|United States|Texas|658[0n]|BOLD:AAB4949
Metria amella|[4093]|LOFLB501-06|United States|Florida|658[0n]|BOLD:AAB4949
Metria amella|[4094]|LOFLC089-06|United States|Florida|658[0n]|BOLD:AAB4949

Metria amella|[4092]|BBLSW834-09|United States|Texas|658[0n]|BOLD:AAB4949
Metria amella|[4093]|LOFLB501-06|United States|Florida|658[0n]|BOLD:AAB4949
Metria amella|[4094]|LOFLC089-06|United States|Florida|658[0n]|BOLD:AAB4949
Metria amella|[4095]|BBLOB854-11|United States|Florida|658[0n]|BOLD:AAB4949
Metria amella|[4096]|ABNCC323-07|United States|Texas|643[0n]|BOLD:AAB4949
Metria amella|[4097]|LOFLB427-06|United States|Florida|658[0n]|BOLD:AAB4949
Metria amella|[4098]|BBLOB393-11|United States|Florida|658[0n]|BOLD:AAB4949
Metria amella|[4099]|LNCC1232-11|United States|North Carolina|658[0n]|BOLD:AAB4949
Metria amella|[4100]|USLEP574-10|United States|Florida|658[0n]|BOLD:AAB4949
Metria amella|[4101]|LOFLA537-06|United States|Florida|658[0n]|BOLD:AAB4949
Metria amella|[4102]|LNCB221-06|United States|North Carolina|658[0n]|BOLD:AAB4949
Metria amella|[4103]|LOFLB112-06|United States|Florida|658[0n]|BOLD:AAB4949
Metria amella|[4104]|LOFLB120-06|United States|Florida|658[0n]|BOLD:AAB4949
Metria amella|[4105]|HKONS534-08|United States|Florida|658[0n]|BOLD:AAB4949
Metria amella|[4106]|USLEP575-10|United States|Florida|658[0n]|BOLD:AAB4949
Metria amella|[4107]|USLEP573-10|United States|Florida|658[0n]|BOLD:AAB4949
Metria amella|[4108]|LOFLB216-06|United States|Florida|658[0n]|BOLD:AAB4949
Metria amella|[4109]|LOFLB441-06|United States|Florida|658[0n]|BOLD:AAB4949
Metria amella|[4110]|USLEP707-10|United States|Florida|658[0n]|BOLD:AAB4949
Metria amella|[4111]|BBLOB1542-11|United States|Florida|658[0n]|BOLD:AAB4949
Metria amella|[4112]|BBLOB1543-11|United States|Florida|658[0n]|BOLD:AAB4949
Metria amella|[4113]|LOFLA229-06|United States|Florida|658[0n]|BOLD:AAB4949
Metria amella|[4114]|BBLOB1535-11|United States|Florida|658[0n]|BOLD:AAB4949
Metria amella|[4115]|BBLSW840-09|United States|Texas|658[0n]|BOLD:AAB4949
Metria amella|[4116]|BBLSW839-09|United States|Texas|658[0n]|BOLD:AAB4949
Metria amella|[4117]|LMEMB222-09|United States|Alabama|625[0n]|BOLD:AAB4949
Metria amella|[4118]|BBLSW838-09|United States|Texas|658[0n]|BOLD:AAB4949
Metria amella|[4119]|JRLAA005-09|United States|Alabama|658[0n]|BOLD:AAB4949
Metria amella|[4120]|USLEP576-10|United States|Texas|658[0n]|BOLD:AAB4949
Metria amella|[4121]|LMEMB221-09|United States|Texas|658[0n]|BOLD:AAB4949
Metria amella|[4122]|LOFLA230-06|United States|Florida|658[0n]|BOLD:AAB4949
Metria amella|[4123]|LNCB219-06|United States|North Carolina|658[0n]|BOLD:AAB4949
Metria amella|[4124]|ABCNA582-07|United States|Florida|577[0n]|BOLD:AAB4949
Metria amella|[4125]|BBLOB814-11|United States|Florida|577[0n]|BOLD:AAB4949
Metria amella|[4126]|BBLOB1529-11|United States|Florida|658[0n]|BOLD:AAB4949
Metria amella|[4127]|BBLOB1624-11|United States|Florida|658[0n]|BOLD:AAB4949
Metria amella|[4128]|LOFLB504-06|United States|Florida|658[0n]|BOLD:AAB4949
Metria amella|[4129]|LNCB220-06|United States|North Carolina|658[0n]|BOLD:AAB4949
Metria amella|[4130]|USLEP1229-10|United States|Florida|658[0n]|BOLD:AAB4949
Metria amella|[4131]|BBLOB473-11|United States|Florida|658[0n]|BOLD:AAB4949
Metria amella|[4132]|BBLOB1531-11|United States|Florida|658[0n]|BOLD:AAB4949
Metria amella|[4133]|BBLOC193-11|United States|Florida|658[0n]|BOLD:AAB4949
Metria celia|[4134]|BLPCH052-08|Costa Rica|Guanacaste|658[0n]|BOLD:AAC6912
Metria celia|[4135]|BLPDM1495-10|Costa Rica|Guanacaste|658[0n]|BOLD:AAC6912
Metria celia|[4136]|BLPDM2137-10|Costa Rica|Guanacaste|658[0n]|BOLD:AAC6912
Metria celia|[4137]|QUNOD212-10|Ecuador|Napo|658[0n]|BOLD:AAC6912
Metria celia|[4138]|MHMXZ672-09|Costa Rica||658[0n]|BOLD:AAC6912
Metria celia|[4139]|BLPCG757-08|Costa Rica|Guanacaste|658[0n]|BOLD:AAC6912
Metria celia|[4140]|BLPCE615-08|Costa Rica|Guanacaste|658[0n]|BOLD:AAC6912
Metria celia|[4141]|BLPCC529-08|Costa Rica|Guanacaste|658[0n]|BOLD:AAC6912
Metria celia|[4142]|MHAUC838-06|Costa Rica|Guanacaste|603[0n]|BOLD:AAC6912
Metria celia|[4143]|MHAUC839-06|Costa Rica|Guanacaste|577[0n]|BOLD:AAC6912
Metria celia|[4144]|INCTA284-10|Brazil|Para|637[0n]|BOLD:AAC6912
Metria celia|[4145]|LNOUB720-10|French Guiana||658[0n]|BOLD:AAC6912
Metria celia|[4146]|BLPDY795-11|Costa Rica|Guanacaste|658[0n]|BOLD:AAC6912
Metria celia|[4147]|MILEP665-11|French Guiana||658[0n]|BOLD:AAC6912
Metria sp.|[4148]|MILEP988-11|French Guiana||658[0n]|BOLD:AAC6912
Metria sp.|[4149]|MILEP989-11|French Guiana||658[0n]|BOLD:AAC6912
Metria sp.|[4150]|MILEP990-11|French Guiana||658[0n]|BOLD:AAC6912
Metria sp.|[4151]|MILEQ124-11|French Guiana||658[0n]|BOLD:AAC6912
Metria celia|[4152]|GWOSZ779-11|Peru|Huanuco|658[0n]|BOLD:AAC6912
Metria celia|[4153]|GWOSZ780-11|Colombia||658[0n]|BOLD:AAC6912
Helia celita|[4154]|GWOSZ852-11|Peru|Huanuco|658[0n]|BOLD:AAC6912
Metria celia|[4155]|LNOUD1427-12|French Guiana||658[0n]|BOLD:AAC6912
Lesmone aenaria|[4156]|HKONB141-08|United States|Texas|658[0n]|BOLD:AAC0282
Lesmone aenaria|[4157]|QUNOC012-09|United States|Texas|643[0n]|BOLD:AAC0282
Lesmone aenaria|[4158]|RDNMH783-09|United States|Texas|658[0n]|BOLD:AAC0282
Lesmone detrahens|[4159]|BBLOC918-11|United States|Arkansas|658[0n]|BOLD:AAC0281
Lesmone detrahens|[4160]|LMEMB203-09|United States|Mississippi|658[0n]|BOLD:AAC0281
Lesmone detrahens|[4161]|LMEMB199-09|United States|Alabama|658[0n]|BOLD:AAC0281
Lesmone detrahens|[4162]|LMEMB201-09|United States|Alabama|658[0n]|BOLD:AAC0281
Lesmone detrahens|[4163]|LSUSA153-06|United States|Kentucky|658[0n]|BOLD:AAC0281
Lesmone detrahens|[4164]|BBLSW303-09|United States|Oklahoma|658[0n]|BOLD:AAC0281
Lesmone detrahens|[4165]|LNCB067-06|United States|North Carolina|658[0n]|BOLD:AAC0281
Lesmone detrahens|[4166]|LPOKB1030-09|United States|Oklahoma|658[0n]|BOLD:AAC0281
Lesmone detrahens|[4167]|BBLSX577-09|United States|Oklahoma|658[0n]|BOLD:AAC0281
Lesmone detrahens|[4168]|LNCB068-06|United States|North Carolina|656[0n]|BOLD:AAC0281
Lesmone detrahens|[4169]|LMEMB200-09|United States|Alabama|627[1n]|BOLD:AAC0281
Lesmone detrahens|[4170]|ABNCC311-07|United States|Texas|642[0n]|BOLD:AAC0281
Lesmone detrahens|[4171]|LPOKA256-08|United States|Oklahoma|658[0n]|BOLD:AAC0281
Lesmone detrahens|[4172]|LPOKA504-09|United States|Oklahoma|658[0n]|BOLD:AAC0281
Lesmone detrahens|[4173]|LMEMB202-09|United States|Mississippi|658[0n]|BOLD:AAC0281
Lesmone detrahens|[4174]|LMEMB206-09|United States|Mississippi|658[0n]|BOLD:AAC0281
Lesmone detrahens|[4175]|LPOKB1027-09|United States|Oklahoma|658[0n]|BOLD:AAC0281
Lesmone detrahens|[4176]|LILLA973-11|United States|Illinois|658[0n]|BOLD:AAC0281
Lesmone detrahens|[4177]|BBLOB1876-11|United States|Florida|658[0n]|BOLD:AAC0281
Lesmone detrahens|[4178]|BBLOC1046-11|United States|Arkansas|658[0n]|BOLD:AAC0281
Lesmone detrahens|[4179]|BBLOE1256-12|United States|Texas|658[0n]|BOLD:AAC0281
Lesmone griseipennis|[4180]|RDNMJ350-11|United States|Arizona|658[0n]|BOLD:AAC2996
Lesmone griseipennis|[4181]|LMEMB209-09|United States|Arizona|658[0n]|BOLD:AAC2996
Lesmone griseipennis|[4182]|IAWL156-09|United States|Arizona|658[0n]|BOLD:AAC2996
Lesmone griseipennis|[4183]|IAWL163-09|United States|Arizona|658[0n]|BOLD:AAC2996
Lesmone griseipennis|[4184]|RDNMJ113-10|United States|Arizona|658[0n]|BOLD:AAC2996
Lesmone griseipennis|[4185]|IAWL157-09|United States|Arizona|658[0n]|BOLD:AAC2996
Lesmone griseipennis|[4186]|CMAZA349-10|United States|Arizona|658[0n]|BOLD:AAC2996
Lesmone griseipennis|[4187]|ABNCC316-07|United States|Texas|655[0n]|BOLD:AAC2996
Lesmone griseipennis|[4188]|HKONB513-09|United States|Texas|658[0n]|BOLD:AAC2996
Lesmone griseipennis|[4189]|CMAZA110-09|United States|Arizona|658[0n]|BOLD:AAC2996
Lesmone griseipennis|[4190]|CMAZA788-10|United States|Arizona|658[0n]|BOLD:AAC2996
Lesmone griseipennis|[4191]|LMEMB207-09|United States|Arizona|658[0n]|BOLD:AAC2996
Lesmone griseipennis|[4192]|LMEMB210-09|United States|Arizona|658[0n]|BOLD:AAC2996
Lesmone griseipennis|[4193]|RDNMJ743-11|United States|Arizona|658[0n]|BOLD:AAC2996
Lesmone griseipennis|[4194]|ABNCC314-07|United States|Arizona|656[0n]|BOLD:AAC2996

Lesmone griseipennis|[4192]|LMEMB210-09|United States|Arizona|658[0n]|BOLD:AAC2996
Lesmone griseipennis|[4193]|RDNMJ743-11|United States|Arizona|658[0n]|BOLD:AAC2996
Lesmone griseipennis|[4194]|ABNCC314-07|United States|Arizona|656[0n]|BOLD:AAC2996
Lesmone griseipennis|[4195]|LMEMB208-09|United States|Arizona|658[0n]|BOLD:AAC2996
Lesmone griseipennis|[4196]|LMEMB211-09|United States|Arizona|658[0n]|BOLD:AAC2996
Lesmone griseipennis|[4197]|CMAZA041-09|United States|Arizona|658[0n]|BOLD:AAC2996
Lesmone griseipennis|[4198]|CMAZA805-10|United States|Arizona|658[0n]|BOLD:AAC2996
Lesmone griseipennis|[4199]|RDNMJ556-11|United States|Arizona|658[0n]|BOLD:AAC2996
Lesmone griseipennis|[4200]|IAWLB290-11|United States|Arizona|658[0n]|BOLD:AAC2996
Lesmone griseipennis|[4201]|CMAZA919-12|United States|Arizona|658[0n]|BOLD:AAC2996
Lesmone griseipennis|[4202]|CMAZA1019-12|United States|Arizona|658[0n]|BOLD:AAC2996
Epidromia lienaris|[4203]|MECB943-05|United States|Florida|590[0n]|BOLD:AAA3449
Epidromia lienaris|[4204]|RDNMF057-08|United States|Florida|658[0n]|BOLD:AAA3449
Epidromia lienaris|[4205]|RDNME850-08|United States|Arizona|658[0n]|BOLD:AAA3449
Epidromia lienaris|[4206]|IAWL061-09|United States|Arizona|658[0n]|BOLD:AAA3449
Epidromia rotundata|[4207]|LOFLA375-06|United States|Florida|658[0n]|BOLD:AAB1007
Epidromia rotundata|[4208]|HKONS030-07|United States|Florida|658[0n]|BOLD:AAB1007
Epidromia rotundata|[4209]|LOFLB213-06|United States|Florida|556[0n]|BOLD:AAB1007
Epidromia rotundata|[4210]|LOFLA242-06|United States|Florida|658[0n]|BOLD:AAB1007
Epidromia rotundata|[4211]|ABCNA583-07|United States|Florida|577[0n]|BOLD:AAB1007
Epidromia rotundata|[4212]|ABNCC376-07|United States|Florida|658[0n]|BOLD:AAB1007
Epidromia rotundata|[4213]|LOFLA689-06|United States|Florida|658[0n]|BOLD:AAB1007
Epidromia rotundata|[4214]|LOFLA527-06|United States|Florida|658[0n]|BOLD:AAB1007
Epidromia rotundata|[4215]|LOFLA368-06|United States|Florida|658[0n]|BOLD:AAB1007
Epidromia rotundata|[4216]|LOFLA321-06|United States|Florida|658[0n]|BOLD:AAB1007
Epidromia rotundata|[4217]|LOFLA307-06|United States|Florida|658[0n]|BOLD:AAB1007
Epidromia rotundata|[4218]|ABNCC377-07|United States|Florida|592[0n]|BOLD:AAB1007
Epidromia rotundata|[4219]|RDNME719-08|United States|Florida|658[0n]|BOLD:AAB1007
Epidromia rotundata|[4220]|LMEMB124-09|United States|Alabama|658[0n]|BOLD:AAB1007
Epidromia rotundata|[4221]|BBLOB453-11|United States|Florida|658[0n]|BOLD:AAB1007
Epidromia rotundata|[4222]|BBLOB478-11|United States|Florida|658[0n]|BOLD:AAB1007
Epidromia rotundata|[4223]|BBLOB637-11|United States|Florida|658[0n]|BOLD:AAB1007
Epidromia rotundata|[4224]|BBLOB1536-11|United States|Florida|658[0n]|BOLD:AAB1007
Epidromia rotundata|[4225]|BBLOB1546-11|United States|Florida|658[0n]|BOLD:AAB1007
Epidromia rotundata|[4226]|BBLOB1618-11|United States|Florida|658[0n]|BOLD:AAB1007
Epidromia rotundata|[4227]|BBLOB1619-11|United States|Florida|658[0n]|BOLD:AAB1007
Epidromia rotundata|[4228]|BBLOB1621-11|United States|Florida|658[0n]|BOLD:AAB1007
Epidromia rotundata|[4229]|BBLOB1622-11|United States|Florida|658[0n]|BOLD:AAB1007
Epidromia rotundata|[4230]|BBLOB1623-11|United States|Florida|658[0n]|BOLD:AAB1007
Epidromia rotundata|[4231]|BBLOB1663-11|United States|Florida|658[0n]|BOLD:AAB1007
Epidromia rotundata|[4232]|BBLOC187-11|United States|Florida|658[0n]|BOLD:AAB1007
Epidromia rotundata|[4233]|BBLOC189-11|United States|Florida|658[0n]|BOLD:AAB1007
Epidromia rotundata|[4234]|LNAUS3899-13|United States|Florida|658[0n]|BOLD:AAB1007
Epidromia pannosa|[4235]|CNCLB1479-14|United States|Florida|624[0n]|BOLD:AAB1008
Epidromia pannosa|[4236]|LNAUS3902-13|United States|Florida|658[0n]|BOLD:AAB1008
Epidromia pannosa|[4237]|LNAUS3901-13|United States|Florida|658[0n]|BOLD:AAB1008
Epidromia pannosa|[4238]|LNAUS3900-13|United States|Florida|658[0n]|BOLD:AAB1008
Epidromia pannosa|[4239]|LNAUS3898-13|United States|Florida|658[0n]|BOLD:AAB1008
Epidromia pannosa|[4240]|CNCLB1480-14|United States|Florida|618[0n]|BOLD:AAB1008
Toxonprucha pardalis|[4241]|LMEMB228-09|United States|Arizona|658[0n]|BOLD:AAD3095
Toxonprucha pardalis|[4242]|ABNCC327-07|United States|Arizona|654[0n]|BOLD:AAD3095
Toxonprucha pardalis|[4243]|ABNCC326-07|United States|Texas|648[0n]|BOLD:AAD3095
Toxonprucha pardalis|[4244]|ABNCC328-07|United States|Arizona|653[0n]|BOLD:AAD3095
Toxonprucha pardalis|[4245]|ABNCC325-07|United States|Texas|632[1n]|BOLD:AAD3095
Toxonprucha pardalis|[4246]|ABNCC522-07|United States|606[0n]|BOLD:AAD3095
Toxonprucha pardalis|[4247]|HKONB532-09|United States|Texas|658[0n]|BOLD:AAD3095
Toxonprucha pardalis|[4248]|RDNMJ732-11|United States|Arizona|658[0n]|BOLD:AAD3095
Toxonprucha pardalis|[4249]|LMEMB229-09|United States|Arizona|658[0n]|BOLD:AAD3095
Toxonprucha pardalis|[4250]|RDNMJ740-11|United States|Arizona|658[0n]|BOLD:AAD3095
Toxonprucha clientis|[4251]|RDNMH834-09|United States|Arizona|658[0n]|BOLD:AAD3094
Toxonprucha clientis|[4252]|BBLOC153-11|United States|Arizona|658[0n]|BOLD:AAD3094
Toxonprucha clientis|[4253]|LMEMB230-09|United States|Arizona|658[0n]|BOLD:AAD3094
Toxonprucha clientis|[4254]|HKONB280-09|United States|Texas|658[0n]|BOLD:AAD3094
Toxonprucha clientis|[4255]|HKONB044-08|United States|Texas|658[0n]|BOLD:AAD3094
Toxonprucha clientis|[4256]|CMAZA776-10|United States|Arizona|658[0n]|BOLD:AAD3094
Toxonprucha clientis|[4257]|HKONB281-09|United States|Texas|636[0n]|BOLD:AAD3094
Toxonprucha clientis|[4258]|BBLOE1762-12|United States|Arizona|636[0n]|BOLD:AAD3094
Toxonprucha strigalis|[4259]|RDNMJ742-11|United States|Arizona|658[0n]|BOLD:AAP5979
Toxonprucha strigalis|[4260]|RDNMJ481-11|United States|Arizona|658[0n]|BOLD:AAP5979
Toxonprucha strigalis|[4261]|CMAZA320-10|United States|Arizona|658[0n]|BOLD:AAP5979
Toxonprucha strigalis|[4262]|CMAZA1202-12|United States|Arizona|658[0n]|BOLD:AAP5979
Zaleops umbrina|[4263]|RDNMH837-09|United States|Arizona|658[0n]|BOLD:AAC2997
Zaleops umbrina|[4264]|CMAZA902-12|United States|Arizona|658[0n]|BOLD:AAC2997
Zaleops umbrina|[4265]|IAWL170-09|United States|Arizona|647[0n]|BOLD:AAC2997
Zaleops umbrina|[4266]|BBLOC612-11|United States|Arizona|658[0n]|BOLD:AAC2997
Zaleops umbrina|[4267]|ABNCC334-07|United States|Arizona|621[2n]|BOLD:AAC2997
Zaleops umbrina|[4268]|RDNMH836-09|United States|Arizona|658[0n]|BOLD:AAC2997
Zaleops umbrina|[4269]|BBLOD1326-11|United States|Arizona|658[0n]|BOLD:AAC2997
Zaleops umbrina|[4270]|RDNME046-07|United States|Arizona|658[0n]|BOLD:AAC2997
Zaleops umbrina|[4271]|IAWL171-09|United States|Arizona|658[0n]|BOLD:AAC2997
Zaleops umbrina|[4272]|CMAZA400-10|United States|Arizona|658[0n]|BOLD:AAC2997
Zaleops umbrina|[4273]|BBLOC230-11|United States|Arizona|658[0n]|BOLD:AAC2997
Zaleops umbrina|[4274]|BBLOD1325-11|United States|Arizona|658[0n]|BOLD:AAC2997
Zaleops umbrina|[4275]|CMAZA1066-12|United States|Arizona|658[0n]|BOLD:AAC2997
Zaleops umbrina|[4276]|HKONB260-09|United States|Texas|658[0n]|BOLD:AAC2997
Zaleops umbrina|[4277]|LTOLB1409-11|United States|Texas|658[0n]|BOLD:AAC2997
Zaleops umbrina|[4278]|HKONB259-09|United States|Texas|658[1n]|BOLD:AAC2997
Zaleops umbrina|[4279]|BBLOC625-11|United States|Arizona|658[0n]|BOLD:AAC2997
Zaleops umbrina|[4280]|HKONB562-09|United States|Texas|608[0n]|BOLD:AAC2997
Zaleops umbrina|[4281]|CNCLB1736-14|United States|New Mexico|658[0n]|BOLD:AAC2997
Coxina cinctipalpis|[4282]|HKONB002-08|United States|Texas|658[0n]|BOLD:AAC3634
Coxina cinctipalpis|[4283]|HKONB031-08|United States|Texas|658[0n]|BOLD:AAC3634
Coxina cinctipalpis|[4284]|HKONB037-08|United States|Texas|658[0n]|BOLD:AAC3634
Coxina cinctipalpis|[4285]|LPYPB251-08|Mexico|Quintana Roo|658[0n]|BOLD:AAC3634
Coxina cinctipalpis|[4286]|LPYPA349-08|Mexico|Yucatan|658[0n]|BOLD:AAC3634
Coxina cinctipalpis|[4287]|LPYPB253-08|Mexico|Quintana Roo|658[0n]|BOLD:AAC3634
Coxina cinctipalpis|[4288]|HKONB028-08|United States|Texas|658[0n]|BOLD:AAC3634
Coxina cinctipalpis|[4289]|HKONB029-08|United States|Texas|658[0n]|BOLD:AAC3634
Coxina cinctipalpis|[4290]|HKONB032-08|United States|Texas|658[0n]|BOLD:AAC3634
Coxina cinctipalpis|[4291]|HKONB033-08|United States|Texas|658[0n]|BOLD:AAC3634
Coxina cinctipalpis|[4292]|HKONB038-08|United States|Texas|658[0n]|BOLD:AAC3634
Coxina cinctipalpis|[4293]|LYNYM304-09|Mexico|Quintana Roo|614[0n]|BOLD:AAC3634
Coxina cinctipalpis|[4294]|HKONB030-08|United States|Texas|658[0n]|BOLD:AAC3634

[illegible]
Coxina cinctipalpis|[4292]|HKONB038-08|United States|Texas|658[0n]|BOLD:AAC3634
Coxina cinctipalpis|[4293]|LYNYM304-09|Mexico|Quintana Roo|614[0n]|BOLD:AAC3634
Coxina cinctipalpis|[4294]|HKONB030-08|United States|Texas|658[0n]|BOLD:AAC3634
Coxina cinctipalpis|[4295]|LPYPA064-08|Mexico|Campeche|658[0n]|BOLD:AAC3634
Coxina cinctipalpis|[4296]|LYPIE343-09|Mexico|Yucatan|658[0n]|BOLD:AAC3634
Coxina cinctipalpis|[4297]|RDNML044-13|United States|Florida|658[0n]|BOLD:AAC3634
Hemeroblemma opigena|[4298]|GWOSQ397-11|Nicaragua||658[0n]|BOLD:AAD6460
Hemeroblemma opigena|[4299]|RDNML344-13|United States|Florida|658[0n]|BOLD:AAD6460
Hemeroblemma opigena|[4300]|LOCRH059-11|Costa Rica|Limon|658[0n]|BOLD:AAD6460
Hemeroblemma opigena|[4301]|BLPDU1155-11|Costa Rica|Guanacaste|658[0n]|BOLD:AAD6460
Hemeroblemma opigena|[4302]|BLPDU286-11|Costa Rica|Guanacaste|658[0n]|BOLD:AAD6460
Hemeroblemma opigena|[4303]|GWOSH622-10|Venezuela|Carabobo|658[0n]|BOLD:AAD6460
Hemeroblemma opigena|[4304]|GWOSH621-10|Venezuela|Carabobo|658[0n]|BOLD:AAD6460
Hemeroblemma opigena|[4305]|GWOSH620-10|Brazil|Bahia|658[0n]|BOLD:AAD6460
Hemeroblemma opigena|[4306]|QUNOD052-10|Ecuador|Napo|658[0n]|BOLD:AAD6460
Hemeroblemma opigena|[4307]|BLPCO275-08|Costa Rica|Guanacaste|658[0n]|BOLD:AAD6460
Hemeroblemma opigena|[4308]|BLPCF902-08|Costa Rica|Alajuela|658[0n]|BOLD:AAD6460
Hemeroblemma opigena|[4309]|BLPCD667-08|Costa Rica|Guanacaste|658[0n]|BOLD:AAD6460
Hemeroblemma opigena|[4310]|BLPBB724-07|Costa Rica|Guanacaste|658[0n]|BOLD:AAD6460
Hemeroblemma opigena|[4311]|MHAUB431-05|Costa Rica|Guanacaste|658[0n]|BOLD:AAD6460
Hemeroblemma opigena|[4312]|LOCRH060-11|Costa Rica|Limon|608[0n]|BOLD:AAD6460
Hemeroblemma opigena|[4313]|BLPEB541-11|Costa Rica|Guanacaste|658[0n]|BOLD:AAD6460
Hemeroblemma opigena|[4314]|RDNML343-13|United States|Florida|658[0n]|BOLD:AAD6460
Hemeroblemma opigena|[4315]|BLPEF2388-13|Costa Rica|Guanacaste|658[0n]|BOLD:AAD6460
Hemeroblemma opigena|[4316]|BLPEF2396-13|Costa Rica|Guanacaste|658[0n]|BOLD:AAD6460
Hemeroblemma mexicana|[4317]|LYHES020-09|Mexico|Yucatan|658[0n]|BOLD:AAD4005
Hemeroblemma mexicana|[4318]|BLPDD490-09|Costa Rica|Guanacaste|658[0n]|BOLD:AAD4005
Hemeroblemma mexicana|[4319]|LPYPB107-08|Mexico|Quintana Roo|658[0n]|BOLD:AAD4005
Hemeroblemma mexicana|[4320]|MHAUF168-06|Costa Rica|Guanacaste|586[0n]|BOLD:AAD4005
Hemeroblemma mexicana|[4321]|BLPBB719-07|Costa Rica|Guanacaste|632[0n]|BOLD:AAD4005
Hemeroblemma mexicana|[4322]|BLPBD219-07|Costa Rica|Guanacaste|658[0n]|BOLD:AAD4005
Hemeroblemma mexicana|[4323]|BLPCD666-08|Costa Rica|Guanacaste|658[0n]|BOLD:AAD4005
Hemeroblemma mexicana|[4324]|MHMYH764-10|Costa Rica||658[0n]|BOLD:AAD4005
Hemeroblemma mexicana|[4325]|MHMYL3189-11|Costa Rica||658[0n]|BOLD:AAD4005
Hemeroblemma mexicana|[4326]|CNCLB1959-14|Guatemala||658[0n]|BOLD:AAD4005
Selenisa sueroides|[4327]|HKONS145-08|United States|Texas|658[0n]|BOLD:AAA5911
Selenisa sueroides|[4328]|RDNMD516-06|United States|Florida|658[0n]|BOLD:AAA5911
Selenisa sueroides|[4329]|HKONS053-07|United States|Florida|658[0n]|BOLD:AAA5911
Selenisa sueroides|[4330]|USLEP887-10|United States|Florida|658[0n]|BOLD:AAA5911
Obrima pyraloides|[4331]|LPYPB252-08|Mexico|Quintana Roo|658[0n]|BOLD:AAA8220
Obrima pyraloides|[4332]|LPYPB250-08|Mexico|Quintana Roo|658[0n]|BOLD:AAA8220
Obrima pyraloides|[4333]|LPYPB249-08|Mexico|Quintana Roo|658[0n]|BOLD:AAA8220
Obrima pyraloides|[4334]|LPYPB270-08|Mexico|Quintana Roo|658[0n]|BOLD:AAA8220
Obrima pyraloides|[4335]|LPYPC047-08|Mexico|Yucatan|658[0n]|BOLD:AAA8220
Obrima pyraloides|[4336]|LYPIE600-09|Mexico|Yucatan|658[0n]|BOLD:AAA8220
Obrima rinconada|[4337]|RDNMG250-08|United States|Texas|658[0n]|BOLD:AAC2725
Obrima rinconada|[4338]|HKONB510-09|United States|Texas|658[0n]|BOLD:AAC2725
Obrima rinconada|[4339]|IAWL031-09|United States|Arizona|658[0n]|BOLD:AAC2725
Obrima rinconada|[4340]|LMEMB119-09|United States|Arizona|658[0n]|BOLD:AAC2725
Obrima rinconada|[4341]|ABNCC002-07|United States|Arizona|630[1n]|BOLD:AAC2725
Obrima rinconada|[4342]|IAWL032-09|United States|Arizona|658[0n]|BOLD:AAC2725
Obrima rinconada|[4343]|ABNCC055-07|United States|Arizona|654[0n]|BOLD:AAC2725
Obrima rinconada|[4344]|IAWL033-09|United States|Arizona|658[0n]|BOLD:AAC2725
Obrima rinconada|[4345]|ABNCC056-07|United States|Arizona|631[0n]|BOLD:AAC2725
Obrima rinconada|[4346]|ABNCC057-07|United States|Arizona|639[0n]|BOLD:AAC2725
Obrima rinconada|[4347]|IAWL034-09|United States|Arizona|658[0n]|BOLD:AAC2725
Obrima rinconada|[4348]|CMAZA571-10|United States|Arizona|658[0n]|BOLD:AAC2725
Obrima rinconada|[4349]|RDNMH806-09|United States|Arizona|658[0n]|BOLD:AAC2725
Obrima rinconada|[4350]|CMAZA820-10|United States|Arizona|658[0n]|BOLD:AAC2725
Tyrissa multilinea|[4351]|HKONS259-08|United States|Texas|629[0n]|BOLD:AAD4775
Tyrissa multilinea|[4352]|HKONB087-08|United States|Texas|658[0n]|BOLD:AAD4775
Tyrissa multilinea|[4353]|HKONB088-08|United States|Texas|658[0n]|BOLD:AAD4775
Tyrissa multilinea|[4354]|BBLOD366-11|United States|Texas|658[0n]|BOLD:AAD4775
Tyrissa multilinea|[4355]|HKONB085-08|United States|Texas|658[0n]|BOLD:AAD4775
Tyrissa multilinea|[4356]|HKONB086-08|United States|Texas|658[0n]|BOLD:AAD4775
Tyrissa multilinea|[4357]|BBLOD342-11|United States|Texas|658[0n]|BOLD:AAD4775
Tyrissa multilinea|[4358]|BBLOD352-11|United States|Texas|658[0n]|BOLD:AAD4775
Tyrissa multilinea|[4359]|BBLOD368-11|United States|Texas|658[0n]|BOLD:AAD4775
Tyrissa multilinea|[4360]|CNCLB1784-14|United States|Florida|658[0n]|BOLD:AAD4775
Tyrissa multilinea|[4361]|CNCLB1785-14|United States|Florida|658[0n]|BOLD:AAD4775
Euclystis deterrima|[4362]|QUNOD159-10|United States|Texas|658[0n]|BOLD:AAB7437
Euclystis guerini|[4363]|BLPCA194-08|Costa Rica|Guanacaste|658[0n]|BOLD:AAA9512
Euclystis guerini|[4364]|BLPCD551-08|Costa Rica|Guanacaste|658[0n]|BOLD:AAA9512
Euclystis guerini|[4365]|MHAUA352-05|Costa Rica|Guanacaste|559[0n]|BOLD:AAA9512
Euclystis guerini|[4366]|MHMXZ760-09|Costa Rica||658[0n]|BOLD:AAA9512
Euclystis guerini|[4367]|MHAUG218-07|Costa Rica|Guanacaste|592[0n]|BOLD:AAA9512
Euclystis guerini|[4368]|LYNYM350-09|Mexico|Quintana Roo|621[0n]|BOLD:AAA9512
Euclystis guerini|[4369]|BLPDU508-11|Costa Rica|Guanacaste|658[0n]|BOLD:AAA9512
Euclystis guerini|[4370]|MHAUG216-07|Costa Rica|Guanacaste|632[0n]|BOLD:AAA9512
Euclystis guerini|[4371]|BLPDK1462-09|Costa Rica|Guanacaste|628[0n]|BOLD:AAA9512
Euclystis guerini|[4372]|BLPAC467-06|Costa Rica|Guanacaste|658[0n]|BOLD:AAA9512
Euclystis guerini|[4373]|BLPCD554-08|Costa Rica|Guanacaste|658[0n]|BOLD:AAA9512
Euclystis guerini|[4374]|MHAUG224-07|Costa Rica|Guanacaste|594[0n]|BOLD:AAA9512
Euclystis guerini|[4375]|MHAUG223-07|Costa Rica|Guanacaste|593[1n]|BOLD:AAA9512
Euclystis guerini|[4376]|LYNYM351-09|Mexico|Quintana Roo|626[0n]|BOLD:AAA9512
Euclystis guerini|[4377]|BLPEE6556-14|Costa Rica||658[0n]|BOLD:AAA9512
Euclystis guerini|[4378]|MHMYL3161-11|Costa Rica||658[0n]|BOLD:AAA9512
Euclystis guerini|[4379]|BLPEE6361-14|Costa Rica||658[0n]|BOLD:AAA9512
Euclystis guerini|[4380]|BLPDU228-11|Costa Rica|Guanacaste|658[0n]|BOLD:AAA9512
Euclystis guerini|[4381]|BLPDU496-11|Costa Rica|Guanacaste|658[0n]|BOLD:AAA9512
Euclystis guerini|[4382]|BLPDU1157-11|Costa Rica|Guanacaste|658[0n]|BOLD:AAA9512
Euclystis guerini|[4383]|BLPDV323-11|Costa Rica|Guanacaste|658[0n]|BOLD:AAA9512
Euclystis guerini|[4384]|BLPDV877-11|Costa Rica|Guanacaste|658[0n]|BOLD:AAA9512
Euclystis guerini|[4385]|BLPDV878-11|Costa Rica|Guanacaste|658[0n]|BOLD:AAA9512
Euclystis guerini|[4386]|BLPDW139-11|Costa Rica|Guanacaste|658[0n]|BOLD:AAA9512
Euclystis guerini|[4387]|BLPDW261-11|Costa Rica|Guanacaste|658[0n]|BOLD:AAA9512
Euclystis guerini|[4388]|BLPDK1698-09|Costa Rica|Guanacaste|658[0n]|BOLD:AAA9512
Euclystis guerini|[4389]|BCIGE374-10|Panama|Panama|658[0n]|BOLD:AAA9512
Euclystis guerini|[4390]|LPYPC066-08|Mexico|Yucatan|658[0n]|BOLD:AAA9512
Euclystis guerini|[4391]|LYPAP212-09|Mexico|Quintana Roo|658[0n]|BOLD:AAA9512
Euclystis guerini|[4392]|LYNYM216-09|Mexico|Quintana Roo|658[0n]|BOLD:AAA9512
Euclystis guerini|[4393]|BLPDK1461-09|Costa Rica|Guanacaste|658[0n]|BOLD:AAA9512
Euclystis guerini|[4394]|BLPCD552-08|Costa Rica|Guanacaste|658[0n]|BOLD:AAA9512

Euclystis guerini|[4391]|[illegible]
Euclystis guerini|[4392]|LYNYM216-09|Mexico|Quintana Roo|658[0n]|BOLD:AAA9512
Euclystis guerini|[4393]|BLPDK1461-09|Costa Rica|Guanacaste|658[0n]|BOLD:AAA9512
Euclystis guerini|[4394]|BLPCD552-08|Costa Rica|Guanacaste|658[0n]|BOLD:AAA9512
Euclystis guerini|[4395]|BLPCD556-08|Costa Rica|Guanacaste|658[0n]|BOLD:AAA9512
Euclystis guerini|[4396]|MHMXQ689-08|Costa Rica|Guanacaste|658[0n]|BOLD:AAA9512
Euclystis guerini|[4397]|BLPCB913-08|Costa Rica|Alajuela|658[0n]|BOLD:AAA9512
Euclystis guerini|[4398]|BLPCA896-08|Costa Rica|Guanacaste|658[0n]|BOLD:AAA9512
Euclystis guerini|[4399]|BLPCA234-08|Costa Rica|Guanacaste|658[0n]|BOLD:AAA9512
Euclystis guerini|[4400]|BLPBH394-07|Costa Rica|Guanacaste|658[0n]|BOLD:AAA9512
Euclystis guerini|[4401]|BLPAF402-07|Costa Rica|Guanacaste|658[0n]|BOLD:AAA9512
Euclystis guerini|[4402]|BLPAF401-07|Costa Rica|Guanacaste|658[0n]|BOLD:AAA9512
Euclystis guerini|[4403]|MHAUA355-05|Costa Rica|Alajuela|658[0n]|BOLD:AAA9512
Euclystis guerini|[4404]|MHMYL3391-11|Costa Rica||658[0n]|BOLD:AAA9512
Euclystis guerini|[4405]|MHAUA354-05|Costa Rica|Guanacaste|658[0n]|BOLD:AAA9512
Euclystis guerini|[4406]|MHAUA351-05|Costa Rica|Guanacaste|658[0n]|BOLD:AAA9512
Euclystis guerini|[4407]|MHAUA350-05|Costa Rica|Guanacaste|658[0n]|BOLD:AAA9512
Euclystis guerini|[4408]|BLPDU1156-11|Costa Rica|Guanacaste|658[0n]|BOLD:AAA9512
Euclystis guerini|[4409]|BLPEE6355-14|Costa Rica||658[0n]|BOLD:AAA9512
Euclystis guerini|[4410]|BLPEE6362-14|Costa Rica||658[0n]|BOLD:AAA9512
Euclystis guerini|[4411]|BLPEE6557-14|Costa Rica||658[0n]|BOLD:AAA9512
Euclystis guerini|[4412]|MHAUG217-07|Costa Rica|Guanacaste|613[0n]|BOLD:AAA9512
Euclystis guerini|[4413]|BLPCD555-08|Costa Rica|Guanacaste|658[0n]|BOLD:AAA9512
Euclystis guerini|[4414]|BLPBH395-07|Costa Rica|Guanacaste|658[0n]|BOLD:AAA9512
Euclystis guerini|[4415]|MHAUG222-07|Costa Rica|Guanacaste|607[1n]|BOLD:AAA9512
Euclystis guerini|[4416]|BLPAG002-07|Costa Rica|Guanacaste|658[0n]|BOLD:AAA9512
Euclystis guerini|[4417]|MHAUA353-05|Costa Rica|Guanacaste|658[0n]|BOLD:AAA9512
Euclystis guerini|[4418]|MHMXI271-07|Costa Rica|Guanacaste|642[0n]|BOLD:AAA9512
Euclystis guerini|[4419]|BLPCD557-08|Costa Rica|Guanacaste|658[0n]|BOLD:AAA9512
Euclystis guerini|[4420]|BLPCD558-08|Costa Rica|Guanacaste|658[0n]|BOLD:AAA9512
Euclystis guerini|[4421]|BLPCO633-08|Costa Rica|Guanacaste|658[0n]|BOLD:AAA9512
Euclystis guerini|[4422]|BLPDT1406-10|Costa Rica|Guanacaste|658[0n]|BOLD:AAA9512
Euclystis sp.|[4423]|CNCLB1830-14|Guatemala||658[0n]|BOLD:AAA9512
Allerastria albiciliatus|[4424]|RDNME066-07|United States|California|658[0n]|BOLD:AAE6099
Allerastria albiciliatus|[4425]|ABNCC037-07|United States|Utah|643[0n]|BOLD:AAE6099
Allerastria albiciliatus|[4426]|RDNME067-07|United States|California|658[0n]|BOLD:AAE6099
Allerastria albiciliatus|[4427]|BBLOC1545-11|United States|California|658[0n]|BOLD:AAE6099
Allerastria albiciliatus|[4428]|BBLOC1555-11|United States|California|658[0n]|BOLD:AAE6099
Allerastria albiciliatus|[4429]|BBLOC1597-11|United States|California|658[0n]|BOLD:AAE6099
Allerastria albiciliatus|[4430]|BBLOC1972-11|United States|California|658[0n]|BOLD:AAE6099
Allerastria albiciliatus|[4431]|BBLOC1980-11|United States|California|658[0n]|BOLD:AAE6099
Allerastria albiciliatus|[4432]|BBLSY968-09|United States|Arizona|658[0n]|BOLD:AAE6099
Allerastria albiciliatus|[4433]|BBLOC1291-11|United States|California|658[0n]|BOLD:AAE6099
Allerastria albiciliatus|[4434]|BBLOC1815-11|United States|California|622[0n]|BOLD:AAE6099
Allerastria albiciliatus|[4435]|NAMUM181-08|United States|California|658[0n]|BOLD:AAE6099
Allerastria albiciliatus|[4436]|RDNML207-13|United States|California|658[0n]|BOLD:AAE6099
Allerastria annae|[4437]|CNCLB837-14|United States|California|307[0n]|
Allerastria annae|[4438]|CNCLB838-14|United States|California|658[0n]|BOLD:ACM4226
Allerastria annae|[4439]|CNCLB836-14|United States|California|611[1n]|BOLD:ACM4226
Allerastria annae|[4440]|CNCLB839-14|United States|California|658[0n]|BOLD:ACM4226
Orodesma apicina|[4441]|CNCLB385-14|Mexico||682[0n]|BOLD:AAI2310
Orodesma apicina|[4442]|CNCLB386-14|Mexico|San Luis Potosi|682[0n]|BOLD:AAI2310
Orodesma apicina|[4443]|CNCLB387-14|Mexico|San Luis Potosi|682[0n]|BOLD:AAI2310
Boryzops purissima|[4444]|CNCLB1937-14|United States|Florida|658[0n]|BOLD:AAI8732
Boryzops purissima|[4445]|BLPED2256-12|Costa Rica|Guanacaste|658[0n]|BOLD:AAI8732
Boryzops purissima|[4446]|BLPCD568-08|Costa Rica|Guanacaste|658[0n]|BOLD:AAI8732
Boryzops purissima|[4447]|BLPCD567-08|Costa Rica|Guanacaste|658[0n]|BOLD:AAI8732
Boryzops purissima|[4448]|CNCLB1938-14|United States|Florida|407[0n]|
Boryzops purissima|[4449]|CNCLB1939-14|United States|Florida|658[0n]|BOLD:AAI8732
Boryzops purissima|[4450]|CNCLB1940-14|United States|Florida|658[0n]|BOLD:AAI8732
Heteranassa mima|[4451]|HKONS535-08|United States|Texas|609[0n]|BOLD:AAB5572
Heteranassa mima|[4452]|BBLSY110-09|United States|Texas|658[0n]|BOLD:AAB5572
Heteranassa mima|[4453]|QUNOC014-09|United States|Texas|658[0n]|BOLD:AAB5572
Heteranassa mima|[4454]|QUNOB530-09|United States|Texas|658[0n]|BOLD:AAB5572
Heteranassa mima|[4455]|ABNCC320-07|United States|Texas|646[0n]|BOLD:AAB5572
Heteranassa mima|[4456]|BBLOD1086-11|United States|Texas|658[0n]|BOLD:AAB5572
Heteranassa mima|[4457]|BBLOD1256-11|United States|Arizona|658[0n]|BOLD:AAB5572
Heteranassa mima|[4458]|BBLOC1647-11|United States|Arizona|658[0n]|BOLD:AAB5572
Heteranassa mima|[4459]|BBLOD1842-11|United States|Arizona|658[0n]|BOLD:AAB5572
Heteranassa mima|[4460]|IAWL174-09|United States|Arizona|658[0n]|BOLD:AAB5572
Heteranassa mima|[4461]|BBLOC902-11|United States|Arizona|658[0n]|BOLD:AAB5572
Heteranassa mima|[4462]|BBLOE1251-12|United States|Texas|658[0n]|BOLD:AAB5572
Heteranassa mima|[4463]|BBLOE1769-12|United States|New Mexico|658[0n]|BOLD:AAB5572
Heteranassa mima|[4464]|BBLOD1617-11|United States|Texas|658[0n]|BOLD:AAB5572
Heteranassa mima|[4465]|BBLOD1673-11|United States|Arizona|658[0n]|BOLD:AAB5572
Heteranassa mima|[4466]|BBLOC798-11|United States|Arizona|658[0n]|BOLD:AAB5572
Heteranassa mima|[4467]|BBLOC539-11|United States|Arizona|658[0n]|BOLD:AAB5572
Heteranassa mima|[4468]|BBLOC232-11|United States|Arizona|658[0n]|BOLD:AAB5572
Heteranassa mima|[4469]|IAWLB650-11|United States|Arizona|658[0n]|BOLD:AAB5572
Heteranassa mima|[4470]|BBLSY918-09|United States|Texas|658[0n]|BOLD:AAB5572
Heteranassa mima|[4471]|BBLSY296-09|United States|Arizona|658[0n]|BOLD:AAB5572
Heteranassa mima|[4472]|AWCLB199-10|United States|Arizona|658[0n]|BOLD:AAB5572
Heteranassa mima|[4473]|BBLOC1609-11|United States|Arizona|658[1n]|BOLD:AAB5572
Heteranassa mima|[4474]|BBLOD1327-11|United States|Arizona|658[0n]|BOLD:AAB5572
Heteranassa mima|[4475]|BBLOE1993-12|United States|Arizona|658[0n]|BOLD:AAB5572
Heteranassa mima|[4476]|BBLOC632-11|United States|Arizona|622[0n]|BOLD:AAB5572
Heteranassa mima|[4477]|BBLOC1616-11|United States|Arizona|658[0n]|BOLD:AAB5572
Heteranassa mima|[4478]|IAWL176-09|United States|Arizona|658[0n]|BOLD:AAB5572
Heteranassa mima|[4479]|BBLSY232-09|United States|Texas|658[0n]|BOLD:AAB5572
Heteranassa mima|[4480]|AWCLB184-10|United States|Arizona|658[0n]|BOLD:AAB5572
Heteranassa mima|[4481]|BBLOB582-11|United States|Arizona|658[0n]|BOLD:AAB5572
Heteranassa mima|[4482]|BBLOC1642-11|United States|Arizona|658[0n]|BOLD:AAB5572
Heteranassa mima|[4483]|BBLSY233-09|United States|Texas|658[6n]|BOLD:AAB5572
Heteranassa mima|[4484]|BBLOE1284-12|United States|Texas|658[0n]|BOLD:AAB5572
Heteranassa mima|[4485]|BBLOE1729-12|United States|Arizona|658[0n]|BOLD:AAB5572
Heteranassa mima|[4486]|BBLOC421-11|United States|Arizona|658[0n]|BOLD:AAB5572
Heteranassa mima|[4487]|BBLOD1324-11|United States|Arizona|658[0n]|BOLD:AAB5572
Heteranassa mima|[4488]|BBLOD1330-11|United States|Arizona|658[0n]|BOLD:AAB5572
Heteranassa mima|[4489]|BBLSY112-09|United States|Texas|658[0n]|BOLD:AAB5572
Heteranassa mima|[4490]|BBLSY221-09|United States|Texas|658[0n]|BOLD:AAB5572
Heteranassa mima|[4491]|BBLSY039-09|United States|Arizona|658[0n]|BOLD:AAB5572
Heteranassa mima|[4492]|BBLOC890-11|United States|Arizona|658[0n]|BOLD:AAB5572
Heteranassa mima|[4493]|AWCLB220-10|United States|Arizona|635[0n]|BOLD:AAB5572
[illegible]

Heteranassa mima|[4491]|BBLSY039-09|United States|Arizona|658[0n]|BOLD:AAB5572
Heteranassa mima|[4492]|BBLOC890-11|United States|Arizona|658[0n]|BOLD:AAB5572
Heteranassa mima|[4493]|AWCLB220-10|United States|Arizona|635[0n]|BOLD:AAB5572
Heteranassa mima|[4494]|LMEMB218-09|United States|Arizona|658[0n]|BOLD:AAB5572
Heteranassa mima|[4495]|BBLSY319-09|United States|Arizona|622[0n]|BOLD:AAB5572
Heteranassa mima|[4496]|CMAZA543-10|United States|Arizona|658[0n]|BOLD:AAB5572
Heteranassa mima|[4497]|AWCLB242-10|United States|Arizona|658[0n]|BOLD:AAB5572
Heteranassa mima|[4498]|AWCLB579-11|United States|Arizona|613[0n]|BOLD:AAB5572
Heteranassa mima|[4499]|IAWL168-09|United States|Arizona|658[0n]|BOLD:AAB5572
Heteranassa mima|[4500]|BBLOC228-11|United States|Arizona|658[0n]|BOLD:AAB5572
Heteranassa mima|[4501]|BBLOC1654-11|United States|Arizona|658[0n]|BOLD:AAB5572
Heteranassa mima|[4502]|BBLOC891-11|United States|Arizona|658[0n]|BOLD:AAB5572
Heteranassa mima|[4503]|BBLOB1571-11|United States|Arizona|658[0n]|BOLD:AAB5572
Heteranassa mima|[4504]|BBLSY040-09|United States|Arizona|658[0n]|BOLD:AAB5572
Heteranassa mima|[4505]|BBLOC809-11|United States|Arizona|658[0n]|BOLD:AAB5572
Heteranassa mima|[4506]|BBLSZ082-09|United States|Arizona|658[0n]|BOLD:AAB5572
Heteranassa mima|[4507]|BBLOD1677-11|United States|Arizona|658[0n]|BOLD:AAB5572
Heteranassa mima|[4508]|BBLOE1728-12|United States|Arizona|658[0n]|BOLD:AAB5572
Heteranassa mima|[4509]|BBLOC1992-11|United States|Texas|658[0n]|BOLD:AAB5572
Heteranassa mima|[4510]|IAWL166-09|United States|Arizona|658[0n]|BOLD:AAB5572
Heteranassa mima|[4511]|IAWL167-09|United States|Arizona|658[0n]|BOLD:AAB5572
Heteranassa mima|[4512]|IAWL172-09|United States|Arizona|658[0n]|BOLD:AAB5572
Heteranassa mima|[4513]|RDNMH850-09|United States|Arizona|574[0n]|BOLD:AAB5572
Heteranassa mima|[4514]|BBLOD1141-11|United States|Arizona|658[0n]|BOLD:AAB5572
Heteranassa mima|[4515]|BBLOD1671-11|United States|Arizona|658[0n]|BOLD:AAB5572
Heteranassa mima|[4516]|BBLOC537-11|United States|Arizona|658[0n]|BOLD:AAB5572
Heteranassa mima|[4517]|IAWLB649-11|United States|Arizona|658[1n]|BOLD:AAB5572
Heteranassa mima|[4518]|AWCLB295-10|United States|Arizona|658[0n]|BOLD:AAB5572
Heteranassa mima|[4519]|BBLOE1759-12|United States|Arizona|658[0n]|BOLD:AAB5572
Heteranassa mima|[4520]|AWCLB185-10|United States|Arizona|658[0n]|BOLD:AAB5572
Heteranassa mima|[4521]|BBLOD1860-11|United States|Arizona|658[0n]|BOLD:AAB5572
Heteranassa mima|[4522]|ABNCC031-07|United States|Arizona|634[0n]|BOLD:AAB5572
Heteranassa mima|[4523]|ABNCC319-07|United States|Texas|641[0n]|BOLD:AAB5572
Heteranassa mima|[4524]|RDNMH841-09|United States|Arizona|646[0n]|BOLD:AAB5572
Heteranassa mima|[4525]|BBLOD1695-11|United States|Arizona|658[0n]|BOLD:AAB5572
Heteranassa mima|[4526]|BBLOD1259-11|United States|Arizona|658[0n]|BOLD:AAB5572
Heteranassa mima|[4527]|BBLOE1445-12|United States|Texas|658[0n]|BOLD:AAB5572
Heteranassa mima|[4528]|BBLOE1311-12|United States|Arizona|658[0n]|BOLD:AAB5572
Heteranassa mima|[4529]|BBLOE1295-12|United States|Arizona|658[0n]|BOLD:AAB5572
Heteranassa mima|[4530]|BBLOE1280-12|United States|Texas|658[0n]|BOLD:AAB5572
Heteranassa mima|[4531]|BBLOD1882-11|United States|Arizona|658[0n]|BOLD:AAB5572
Heteranassa mima|[4532]|BBLOD1878-11|United States|Arizona|658[0n]|BOLD:AAB5572
Heteranassa mima|[4533]|BBLOD1877-11|United States|Arizona|658[0n]|BOLD:AAB5572
Heteranassa mima|[4534]|BBLOD1694-11|United States|Arizona|658[0n]|BOLD:AAB5572
Heteranassa mima|[4535]|BBLOD1693-11|United States|Arizona|658[0n]|BOLD:AAB5572
Heteranassa mima|[4536]|BBLOD1678-11|United States|Arizona|658[0n]|BOLD:AAB5572
Heteranassa mima|[4537]|BBLOD1676-11|United States|Arizona|658[0n]|BOLD:AAB5572
Heteranassa mima|[4538]|BBLOD1675-11|United States|Arizona|658[0n]|BOLD:AAB5572
Heteranassa mima|[4539]|BBLOD1670-11|United States|Arizona|658[0n]|BOLD:AAB5572
Heteranassa mima|[4540]|BBLOD1668-11|United States|Arizona|658[0n]|BOLD:AAB5572
Heteranassa mima|[4541]|BBLOD1667-11|United States|Arizona|658[0n]|BOLD:AAB5572
Heteranassa mima|[4542]|BBLOD1624-11|United States|Arizona|658[0n]|BOLD:AAB5572
Heteranassa mima|[4543]|BBLOD1615-11|United States|Texas|658[0n]|BOLD:AAB5572
Heteranassa mima|[4544]|BBLOD1611-11|United States|Texas|658[0n]|BOLD:AAB5572
Heteranassa mima|[4545]|BBLOD1329-11|United States|Arizona|658[0n]|BOLD:AAB5572
Heteranassa mima|[4546]|BBLOD1328-11|United States|Arizona|658[0n]|BOLD:AAB5572
Heteranassa mima|[4547]|BBLOD1323-11|United States|Arizona|658[0n]|BOLD:AAB5572
Heteranassa mima|[4548]|BBLOD1322-11|United States|Arizona|658[0n]|BOLD:AAB5572
Heteranassa mima|[4549]|BBLOD1261-11|United States|Arizona|658[0n]|BOLD:AAB5572
Heteranassa mima|[4550]|BBLOD1257-11|United States|Arizona|658[0n]|BOLD:AAB5572
Heteranassa mima|[4551]|BBLOD1103-11|United States|Texas|658[0n]|BOLD:AAB5572
Heteranassa mima|[4552]|BBLOD844-11|United States|Arizona|658[0n]|BOLD:AAB5572
Heteranassa mima|[4553]|BBLOC1657-11|United States|Arizona|658[0n]|BOLD:AAB5572
Heteranassa mima|[4554]|BBLOC1653-11|United States|Arizona|658[0n]|BOLD:AAB5572
Heteranassa mima|[4555]|BBLOC1652-11|United States|Arizona|658[0n]|BOLD:AAB5572
Heteranassa mima|[4556]|BBLOC1651-11|United States|Arizona|658[0n]|BOLD:AAB5572
Heteranassa mima|[4557]|BBLOC1644-11|United States|Arizona|658[0n]|BOLD:AAB5572
Heteranassa mima|[4558]|BBLOC1643-11|United States|Arizona|658[0n]|BOLD:AAB5572
Heteranassa mima|[4559]|BBLOC1583-11|United States|California|658[0n]|BOLD:AAB5572
Heteranassa mima|[4560]|BBLOC901-11|United States|Arizona|658[0n]|BOLD:AAB5572
Heteranassa mima|[4561]|BBLOC667-11|United States|Texas|658[0n]|BOLD:AAB5572
Heteranassa mima|[4562]|BBLOC556-11|United States|Arizona|658[0n]|BOLD:AAB5572
Heteranassa mima|[4563]|BBLOC555-11|United States|Arizona|658[0n]|BOLD:AAB5572
Heteranassa mima|[4564]|BBLOC554-11|United States|Arizona|658[0n]|BOLD:AAB5572
Heteranassa mima|[4565]|BBLOC414-11|United States|Arizona|658[0n]|BOLD:AAB5572
Heteranassa mima|[4566]|BBLOC393-11|United States|Arizona|658[0n]|BOLD:AAB5572
Heteranassa mima|[4567]|BBLOC392-11|United States|Arizona|658[0n]|BOLD:AAB5572
Heteranassa mima|[4568]|BBLOC275-11|United States|Arizona|658[0n]|BOLD:AAB5572
Heteranassa mima|[4569]|BBLOC269-11|United States|Arizona|658[0n]|BOLD:AAB5572
Heteranassa mima|[4570]|BBLOC260-11|United States|Arizona|658[0n]|BOLD:AAB5572
Heteranassa mima|[4571]|BBLOC257-11|United States|Arizona|658[0n]|BOLD:AAB5572
Heteranassa mima|[4572]|BBLOC174-11|United States|Arizona|658[0n]|BOLD:AAB5572
Heteranassa mima|[4573]|BBLOC160-11|United States|Arizona|658[0n]|BOLD:AAB5572
Heteranassa mima|[4574]|BBLOC149-11|United States|Arizona|658[0n]|BOLD:AAB5572
Heteranassa mima|[4575]|BBLOB1584-11|United States|Arizona|658[0n]|BOLD:AAB5572
Heteranassa mima|[4576]|IAWLB648-11|United States|Arizona|658[0n]|BOLD:AAB5572
Heteranassa mima|[4577]|RDNMJ690-11|United States|Arizona|658[0n]|BOLD:AAB5572
Heteranassa mima|[4578]|AWCLB399-10|United States|Arizona|658[0n]|BOLD:AAB5572
Heteranassa mima|[4579]|AWCLB351-10|United States|Arizona|658[0n]|BOLD:AAB5572
Heteranassa mima|[4580]|AWCLB308-10|United States|Arizona|658[0n]|BOLD:AAB5572
Heteranassa mima|[4581]|AWCLB248-10|United States|Arizona|658[0n]|BOLD:AAB5572
Heteranassa mima|[4582]|AWCLB247-10|United States|Arizona|658[0n]|BOLD:AAB5572
Heteranassa mima|[4583]|AWCLB246-10|United States|Arizona|658[0n]|BOLD:AAB5572
Heteranassa mima|[4584]|AWCLB236-10|United States|Arizona|658[0n]|BOLD:AAB5572
Heteranassa mima|[4585]|CMAZA777-10|United States|Arizona|658[0n]|BOLD:AAB5572
Heteranassa mima|[4586]|CMAZA316-10|United States|Arizona|658[0n]|BOLD:AAB5572
Heteranassa mima|[4587]|CMAZA315-10|United States|Arizona|658[0n]|BOLD:AAB5572
Heteranassa mima|[4588]|USLEP922-10|United States|New Mexico|658[0n]|BOLD:AAB5572
Heteranassa mima|[4589]|USLEP717-10|United States|Arizona|658[0n]|BOLD:AAB5572
Heteranassa mima|[4590]|USLEP716-10|United States|Arizona|658[0n]|BOLD:AAB5572
Heteranassa mima|[4591]|BBLSZ188-09|United States|Texas|658[0n]|BOLD:AAB5572
Heteranassa mima|[4592]|BBLSZ176-09|United States|Texas|658[0n]|BOLD:AAB5572
Heteranassa mima|[4593]|BBLSZ079-09|United States|Arizona|658[0n]|BOLD:AAB5572

Heteranassa mima|[4591]|BBLSZ188-09|United States|Texas|658[0n]|BOLD:AAB5572
Heteranassa mima|[4592]|BBLSZ176-09|United States|Texas|658[0n]|BOLD:AAB5572
Heteranassa mima|[4593]|BBLSZ079-09|United States|Arizona|658[0n]|BOLD:AAB5572
Heteranassa mima|[4594]|BBLSY985-09|United States|Arizona|658[0n]|BOLD:AAB5572
Heteranassa mima|[4595]|BBLSY916-09|United States|Texas|658[0n]|BOLD:AAB5572
Heteranassa mima|[4596]|BBLSY820-09|United States|Arizona|658[0n]|BOLD:AAB5572
Heteranassa mima|[4597]|BBLSX657-09|United States|Arizona|658[0n]|BOLD:AAB5572
Heteranassa mima|[4598]|BBLSY227-09|United States|Texas|658[0n]|BOLD:AAB5572
Heteranassa mima|[4599]|BBLSY111-09|United States|Texas|658[0n]|BOLD:AAB5572
Heteranassa mima|[4600]|BBLSW277-09|United States|Arizona|658[0n]|BOLD:AAB5572
Heteranassa mima|[4601]|BBLSW216-09|United States|Arizona|658[0n]|BOLD:AAB5572
Heteranassa mima|[4602]|BBLSW123-09|United States|Arizona|658[0n]|BOLD:AAB5572
Heteranassa mima|[4603]|BBLSW122-09|United States|Arizona|658[0n]|BOLD:AAB5572
Heteranassa mima|[4604]|BBLSW121-09|United States|Arizona|658[0n]|BOLD:AAB5572
Heteranassa mima|[4605]|BBLSW120-09|United States|Arizona|658[0n]|BOLD:AAB5572
Heteranassa mima|[4606]|LMEMB217-09|United States|Arizona|658[0n]|BOLD:AAB5572
Heteranassa mima|[4607]|LMEMB216-09|United States|Arizona|658[0n]|BOLD:AAB5572
Heteranassa mima|[4608]|LMEMB215-09|United States|Arizona|658[0n]|BOLD:AAB5572
Heteranassa mima|[4609]|IAWL178-09|United States|Arizona|658[0n]|BOLD:AAB5572
Heteranassa mima|[4610]|IAWL177-09|United States|Arizona|658[0n]|BOLD:AAB5572
Heteranassa mima|[4611]|IAWL175-09|United States|Arizona|658[0n]|BOLD:AAB5572
Heteranassa mima|[4612]|IAWL173-09|United States|Arizona|658[0n]|BOLD:AAB5572
Heteranassa mima|[4613]|RDNME086-07|United States|Arizona|658[0n]|BOLD:AAB5572
Heteranassa mima|[4614]|RDNME085-07|United States|Arizona|658[0n]|BOLD:AAB5572
Heteranassa mima|[4615]|BBLOC270-11|United States|Arizona|658[0n]|BOLD:AAB5572
Heteranassa mima|[4616]|BBLOD1136-11|United States|Arizona|658[0n]|BOLD:AAB5572
Heteranassa mima|[4617]|IAWL179-09|United States|Arizona|658[0n]|BOLD:AAB5572
Heteranassa mima|[4618]|AWCLB583-11|United States|Arizona|658[0n]|BOLD:AAB5572
Heteranassa mima|[4619]|USLEP779-10|United States|Arizona|658[0n]|BOLD:AAB5572
Heteranassa mima|[4620]|BBLOC1618-11|United States|Arizona|620[0n]|BOLD:AAB5572
Heteranassa mima|[4621]|AWCLB233-10|United States|Arizona|658[0n]|BOLD:AAB5572
Heteranassa mima|[4622]|BBLOC1610-11|United States|Arizona|620[0n]|BOLD:AAB5572
Heteranassa mima|[4623]|ABNCC032-07|United States|Arizona|599[0n]|BOLD:AAB5572
Heteranassa mima|[4624]|BBLSY315-09|United States|Arizona|631[0n]|BOLD:AAB5572
Heteranassa mima|[4625]|BBLSY314-09|United States|Arizona|626[0n]|BOLD:AAB5572
Heteranassa mima|[4626]|BBLOC235-11|United States|Arizona|621[0n]|BOLD:AAB5572
Heteranassa mima|[4627]|AWCLB303-10|United States|Arizona|618[0n]|BOLD:AAB5572
Heteranassa mima|[4628]|BBLOC1617-11|United States|Arizona|620[0n]|BOLD:AAB5572
Heteranassa mima|[4629]|BBLOD1616-11|United States|Texas|658[0n]|BOLD:AAB5572
Heteranassa mima|[4630]|BBLOD1281-11|United States|Texas|658[0n]|BOLD:AAB5572
Heteranassa mima|[4631]|BBLOD1696-11|United States|Arizona|658[0n]|BOLD:AAB5572
Heteranassa mima|[4632]|BBLOE1447-12|United States|Texas|658[0n]|BOLD:AAB5572
Heteranassa mima|[4633]|BBLOE1448-12|United States|Texas|658[0n]|BOLD:AAB5572
Heteranassa mima|[4634]|BBLOE1453-12|United States|Texas|658[0n]|BOLD:AAB5572
Heteranassa mima|[4635]|BBLOE1724-12|United States|Arizona|658[0n]|BOLD:AAB5572
Heteranassa mima|[4636]|BBLOE1736-12|United States|Arizona|658[0n]|BOLD:AAB5572
Heteranassa mima|[4637]|BBLOE1739-12|United States|Arizona|658[0n]|BOLD:AAB5572
Heteranassa mima|[4638]|BBLOE1746-12|United States|Texas|658[0n]|BOLD:AAB5572
Heteranassa mima|[4639]|BBLOE1758-12|United States|Arizona|658[0n]|BOLD:AAB5572
Heteranassa mima|[4640]|BBLOE1761-12|United States|Arizona|658[0n]|BOLD:AAB5572
Heteranassa mima|[4641]|BBLOE1765-12|United States|Arizona|658[0n]|BOLD:AAB5572
Heteranassa mima|[4642]|BBLOE1999-12|United States|Arizona|658[0n]|BOLD:AAB5572
Heteranassa mima|[4643]|CMAZA972-12|United States|Arizona|658[0n]|BOLD:AAB5572
Heteranassa mima|[4644]|CMAZA1003-12|United States|Arizona|658[0n]|BOLD:AAB5572
Heteranassa mima|[4645]|CMAZA1047-12|United States|Arizona|658[0n]|BOLD:AAB5572
Elousa albicans|[4646]|CNCLB1644-14|United States|Florida|658[0n]|BOLD:ACP4940
Coenipeta bibitrix|[4647]|MHAUB343-05|Costa Rica|Guanacaste|658[0n]|BOLD:AAA3313
Coenipeta bibitrix|[4648]|MHMXM248-07|Costa Rica|Guanacaste|658[0n]|BOLD:AAA3313
Coenipeta bibitrix|[4649]|BLPBH580-07|Costa Rica|Guanacaste|658[0n]|BOLD:AAA3313
Coenipeta bibitrix|[4650]|BLPBH579-07|Costa Rica|Guanacaste|658[0n]|BOLD:AAA3313
Coenipeta bibitrix|[4651]|BLPAF058-07|Costa Rica|Guanacaste|658[0n]|BOLD:AAA3313
Coenipeta bibitrix|[4652]|BLPBG100-07|Costa Rica|Guanacaste|637[1n]|BOLD:AAA3313
Coenipeta bibitrix|[4653]|MHMXM247-07|Costa Rica|Guanacaste|633[0n]|BOLD:AAA3313
Coenipeta bibitrix|[4654]|BLPDJ110-09|Costa Rica|Guanacaste|629[0n]|BOLD:AAA3313
Coenipeta bibitrix|[4655]|BLPDT1657-10|Costa Rica|Guanacaste|557[0n]|BOLD:AAA3313
Coenipeta bibitrix|[4656]|BLPDY164-11|Costa Rica|Guanacaste|658[0n]|BOLD:AAA3313
Coenipeta bibitrix|[4657]|BLPDU194-11|Costa Rica|Guanacaste|658[0n]|BOLD:AAA3313
Coenipeta bibitrix|[4658]|BLPAA178-06|Costa Rica|Guanacaste|658[4n]|BOLD:AAA3313
Coenipeta bibitrix|[4659]|BLPDT1658-10|Costa Rica|Guanacaste|658[1n]|BOLD:AAA3313
Coenipeta bibitrix|[4660]|BLPEF970-12|Costa Rica|Alajuela|643[0n]|BOLD:AAA3313
Coenipeta bibitrix|[4661]|BLPBH589-07|Costa Rica|Guanacaste|658[0n]|BOLD:AAA3313
Coenipeta bibitrix|[4662]|BLPDK1483-09|Costa Rica|Guanacaste|635[1n]|BOLD:AAA3313
Coenipeta bibitrix|[4663]|MHAUB351-05|Costa Rica|Guanacaste|658[0n]|BOLD:AAA3313
Coenipeta bibitrix|[4664]|BLPAD338-06|Costa Rica|Guanacaste|658[0n]|BOLD:AAA3313
Coenipeta bibitrix|[4665]|BLPCC453-08|Costa Rica|Guanacaste|658[0n]|BOLD:AAA3313
Coenipeta bibitrix|[4666]|BLPDI901-09|Costa Rica|Guanacaste|658[0n]|BOLD:AAA3313
Coenipeta bibitrix|[4667]|BLPDJ208-09|Costa Rica|Guanacaste|658[0n]|BOLD:AAA3313
Coenipeta bibitrix|[4668]|BLPDJ437-09|Costa Rica|Guanacaste|658[0n]|BOLD:AAA3313
Coenipeta bibitrix|[4669]|MHMYH953-10|Costa Rica||658[0n]|BOLD:AAA3313
Coenipeta bibitrix|[4670]|BLPDW206-11|Costa Rica|Guanacaste|658[0n]|BOLD:AAA3313
Coenipeta bibitrix|[4671]|BLPEF2329-13|Costa Rica|Guanacaste|658[0n]|BOLD:AAA3313
Coenipeta bibitrix|[4672]|BLPDU196-11|Costa Rica|Guanacaste|658[0n]|BOLD:AAA3313
Coenipeta bibitrix|[4673]|BLPDU487-11|Costa Rica|Guanacaste|658[1n]|BOLD:AAA3313
Coenipeta bibitrix|[4674]|BLPCC406-08|Costa Rica|Guanacaste|658[0n]|BOLD:AAA3313
Coenipeta bibitrix|[4675]|BLPEE1460-12|Costa Rica|Guanacaste|658[0n]|BOLD:AAA3313
Coenipeta bibitrix|[4676]|BLPEE1328-12|Costa Rica|Guanacaste|658[0n]|BOLD:AAA3313
Coenipeta bibitrix|[4677]|BLPED713-11|Costa Rica|Guanacaste|658[0n]|BOLD:AAA3313
Coenipeta bibitrix|[4678]|MHMYN070-11|Costa Rica||658[0n]|BOLD:AAA3313
Coenipeta bibitrix|[4679]|BLPDW1165-11|Costa Rica|Guanacaste|658[0n]|BOLD:AAA3313
Coenipeta bibitrix|[4680]|BLPDU1176-11|Costa Rica|Guanacaste|658[0n]|BOLD:AAA3313
Coenipeta bibitrix|[4681]|BLPDU812-11|Costa Rica|Guanacaste|658[0n]|BOLD:AAA3313
Coenipeta bibitrix|[4682]|BLPDU489-11|Costa Rica|Guanacaste|658[0n]|BOLD:AAA3313
Coenipeta bibitrix|[4683]|BLPDU200-11|Costa Rica|Guanacaste|658[0n]|BOLD:AAA3313
Coenipeta bibitrix|[4684]|BLPDU199-11|Costa Rica|Guanacaste|658[0n]|BOLD:AAA3313
Coenipeta bibitrix|[4685]|BLPDU198-11|Costa Rica|Guanacaste|658[0n]|BOLD:AAA3313
Coenipeta bibitrix|[4686]|BLPDU197-11|Costa Rica|Guanacaste|658[0n]|BOLD:AAA3313
Coenipeta bibitrix|[4687]|BLPDU195-11|Costa Rica|Guanacaste|658[0n]|BOLD:AAA3313
Coenipeta bibitrix|[4688]|BLPDT1656-10|Costa Rica|Guanacaste|658[0n]|BOLD:AAA3313
Coenipeta bibitrix|[4689]|BLPDT1433-10|Costa Rica|Guanacaste|658[0n]|BOLD:AAA3313
Coenipeta bibitrix|[4690]|MHMYH904-10|Costa Rica||658[0n]|BOLD:AAA3313
Coenipeta bibitrix|[4691]|MHMYH818-10|Costa Rica||658[0n]|BOLD:AAA3313
Coenipeta bibitrix|[4692]|BLPDM581-10|Costa Rica|Guanacaste|658[0n]|BOLD:AAA3313
Coenipeta bibitrix|[4693]|QUNOD178-10|United States|Texas|658[0n]|BOLD:AAA3313

Coenipeta bibitrix|[4691]|MHMYH818-10|Costa Rica||658[0n]|BOLD:AAA3313
Coenipeta bibitrix|[4692]|BLPDM581-10|Costa Rica|Guanacaste|658[0n]|BOLD:AAA3313
Coenipeta bibitrix|[4693]|QUNOD178-10|United States|Texas|658[0n]|BOLD:AAA3313
Coenipeta bibitrix|[4694]|BLPDL565-10|Costa Rica|Guanacaste|658[0n]|BOLD:AAA3313
Coenipeta bibitrix|[4695]|BLPDJ210-09|Costa Rica|Guanacaste|658[0n]|BOLD:AAA3313
Coenipeta bibitrix|[4696]|BLPDJ209-09|Costa Rica|Guanacaste|658[0n]|BOLD:AAA3313
Coenipeta bibitrix|[4697]|MHMXZ833-09|Costa Rica||658[0n]|BOLD:AAA3313
Coenipeta bibitrix|[4698]|MHMXZ832-09|Costa Rica||658[0n]|BOLD:AAA3313
Coenipeta bibitrix|[4699]|BLPDD872-09|Costa Rica|Guanacaste|658[0n]|BOLD:AAA3313
Coenipeta bibitrix|[4700]|BLPCO056-08|Costa Rica|Guanacaste|658[0n]|BOLD:AAA3313
Coenipeta bibitrix|[4701]|BLPCM341-08|Costa Rica|Guanacaste|658[0n]|BOLD:AAA3313
Coenipeta bibitrix|[4702]|BLPCM243-08|Costa Rica|Guanacaste|658[0n]|BOLD:AAA3313
Coenipeta bibitrix|[4703]|BLPCM120-08|Costa Rica|Guanacaste|658[0n]|BOLD:AAA3313
Coenipeta bibitrix|[4704]|LOCRC407-08|Costa Rica|Guanacaste|658[0n]|BOLD:AAA3313
Coenipeta bibitrix|[4705]|BLPCL478-08|Costa Rica|Guanacaste|658[0n]|BOLD:AAA3313
Coenipeta bibitrix|[4706]|LPYPB115-08|Mexico|Quintana Roo|658[0n]|BOLD:AAA3313
Coenipeta bibitrix|[4707]|LPYPB114-08|Mexico|Quintana Roo|658[0n]|BOLD:AAA3313
Coenipeta bibitrix|[4708]|LPYPB084-08|Mexico|Quintana Roo|658[0n]|BOLD:AAA3313
Coenipeta bibitrix|[4709]|BLPCE567-08|Costa Rica|Guanacaste|658[0n]|BOLD:AAA3313
Coenipeta bibitrix|[4710]|BLPCD613-08|Costa Rica|Guanacaste|658[0n]|BOLD:AAA3313
Coenipeta bibitrix|[4711]|BLPCA238-08|Costa Rica|Guanacaste|658[0n]|BOLD:AAA3313
Coenipeta bibitrix|[4712]|MHMXO379-08|Costa Rica|Guanacaste|658[0n]|BOLD:AAA3313
Coenipeta bibitrix|[4713]|MHMXO377-08|Costa Rica|Guanacaste|658[0n]|BOLD:AAA3313
Coenipeta bibitrix|[4714]|MHMXM251-07|Costa Rica|Guanacaste|658[0n]|BOLD:AAA3313
Coenipeta bibitrix|[4715]|MHMXM250-07|Costa Rica|Guanacaste|658[0n]|BOLD:AAA3313
Coenipeta bibitrix|[4716]|MHMXM249-07|Costa Rica|Guanacaste|658[0n]|BOLD:AAA3313
Coenipeta bibitrix|[4717]|BLPBH588-07|Costa Rica|Guanacaste|658[0n]|BOLD:AAA3313
Coenipeta bibitrix|[4718]|BLPBH584-07|Costa Rica|Guanacaste|658[0n]|BOLD:AAA3313
Coenipeta bibitrix|[4719]|BLPBH583-07|Costa Rica|Guanacaste|658[0n]|BOLD:AAA3313
Coenipeta bibitrix|[4720]|BLPBH582-07|Costa Rica|Guanacaste|658[0n]|BOLD:AAA3313
Coenipeta bibitrix|[4721]|BLPBH578-07|Costa Rica|Guanacaste|658[0n]|BOLD:AAA3313
Coenipeta bibitrix|[4722]|BLPBH577-07|Costa Rica|Guanacaste|658[0n]|BOLD:AAA3313
Coenipeta bibitrix|[4723]|BLPAG017-07|Costa Rica|Guanacaste|658[0n]|BOLD:AAA3313
Coenipeta bibitrix|[4724]|BLPAF136-07|Costa Rica|Guanacaste|658[0n]|BOLD:AAA3313
Coenipeta bibitrix|[4725]|BLPAF135-07|Costa Rica|Guanacaste|658[0n]|BOLD:AAA3313
Coenipeta bibitrix|[4726]|MHMXE283-06|Costa Rica|Guanacaste|658[0n]|BOLD:AAA3313
Coenipeta bibitrix|[4727]|MHMXD648-06|Costa Rica|Guanacaste|658[0n]|BOLD:AAA3313
Coenipeta bibitrix|[4728]|MHMXD618-06|Costa Rica|Guanacaste|658[0n]|BOLD:AAA3313
Coenipeta bibitrix|[4729]|BLPAE628-06|Costa Rica|Guanacaste|658[0n]|BOLD:AAA3313
Coenipeta bibitrix|[4730]|BLPAE626-06|Costa Rica|Guanacaste|658[0n]|BOLD:AAA3313
Coenipeta bibitrix|[4731]|BLPAE259-06|Costa Rica|Guanacaste|658[0n]|BOLD:AAA3313
Coenipeta bibitrix|[4732]|BLPAD774-06|Costa Rica|Alajuela|658[0n]|BOLD:AAA3313
Coenipeta bibitrix|[4733]|BLPAD773-06|Costa Rica|Alajuela|658[0n]|BOLD:AAA3313
Coenipeta bibitrix|[4734]|BLPAD342-06|Costa Rica|Guanacaste|658[0n]|BOLD:AAA3313
Coenipeta bibitrix|[4735]|MHAUB349-05|Costa Rica|Guanacaste|658[0n]|BOLD:AAA3313
Coenipeta bibitrix|[4736]|MHAUB348-05|Costa Rica|Guanacaste|658[0n]|BOLD:AAA3313
Coenipeta bibitrix|[4737]|MHAUB346-05|Costa Rica|Guanacaste|658[0n]|BOLD:AAA3313
Coenipeta bibitrix|[4738]|MHAUB344-05|Costa Rica|Guanacaste|658[0n]|BOLD:AAA3313
Coenipeta bibitrix|[4739]|MHAUB342-05|Costa Rica|Guanacaste|658[0n]|BOLD:AAA3313
Coenipeta bibitrix|[4740]|BLPEE054-12|Costa Rica|Guanacaste|658[0n]|BOLD:AAA3313
Coenipeta bibitrix|[4741]|MHAUB341-05|Costa Rica|Guanacaste|658[0n]|BOLD:AAA3313
Coenipeta bibitrix|[4742]|BLPBG096-07|Costa Rica|Guanacaste|658[0n]|BOLD:AAA3313
Coenipeta bibitrix|[4743]|BLPBG094-07|Costa Rica|Guanacaste|647[0n]|BOLD:AAA3313
Coenipeta bibitrix|[4744]|MHAUB350-05|Costa Rica|Guanacaste|658[0n]|BOLD:AAA3313
Coenipeta bibitrix|[4745]|BLPBF889-07|Costa Rica|Guanacaste|648[0n]|BOLD:AAA3313
Coenipeta bibitrix|[4746]|BLPBF890-07|Costa Rica|Guanacaste|647[0n]|BOLD:AAA3313
Coenipeta bibitrix|[4747]|BLPBG097-07|Costa Rica|Guanacaste|648[0n]|BOLD:AAA3313
Coenipeta bibitrix|[4748]|BLPBG098-07|Costa Rica|Guanacaste|650[0n]|BOLD:AAA3313
Coenipeta bibitrix|[4749]|MHMXO376-08|Costa Rica|Guanacaste|651[0n]|BOLD:AAA3313
Coenipeta bibitrix|[4750]|MHAUB345-05|Costa Rica|Guanacaste|658[0n]|BOLD:AAA3313
Coenipeta bibitrix|[4751]|BLPDT1659-10|Costa Rica|Guanacaste|613[0n]|BOLD:AAA3313
Coenipeta bibitrix|[4752]|MHAUB347-05|Costa Rica|Guanacaste|658[0n]|BOLD:AAA3313
Coenipeta bibitrix|[4753]|BLPAA313-06|Costa Rica|Guanacaste|612[0n]|BOLD:AAA3313
Coenipeta bibitrix|[4754]|LPYPB113-08|Mexico|Quintana Roo|609[0n]|BOLD:AAA3313
Coenipeta bibitrix|[4755]|BLPBF892-07|Costa Rica|Guanacaste|610[0n]|BOLD:AAA3313
Coenipeta bibitrix|[4756]|BLPBH586-07|Costa Rica|Guanacaste|621[0n]|BOLD:AAA3313
Coenipeta bibitrix|[4757]|BLPEE1462-12|Costa Rica|Guanacaste|626[0n]|BOLD:AAA3313
Coenipeta bibitrix|[4758]|MHMYS527-12|Costa Rica||658[0n]|BOLD:AAA3313
Coenipeta bibitrix|[4759]|MHMYS528-12|Costa Rica||658[0n]|BOLD:AAA3313
Coenipeta bibitrix|[4760]|BLPEF204-12|Costa Rica|Alajuela|658[0n]|BOLD:AAA3313
Coenipeta bibitrix|[4761]|MHMYS3000-13|Costa Rica|Guanacaste|658[0n]|BOLD:AAA3313
Coenipeta bibitrix|[4762]|MHMYS3007-13|Costa Rica|Guanacaste|658[0n]|BOLD:AAA3313
Coenipeta bibitrix|[4763]|MHMYS3008-13|Costa Rica|Guanacaste|658[0n]|BOLD:AAA3313
Coenipeta bibitrix|[4764]|MHMYS3009-13|Costa Rica|Guanacaste|658[0n]|BOLD:AAA3313
Coenipeta bibitrix|[4765]|MHMYS3206-13|Costa Rica|Guanacaste|658[0n]|BOLD:AAA3313
Focillidia texana|[4766]|HKONB039-08|United States|Texas|658[0n]|BOLD:AAC3317
Focillidia texana|[4767]|HKONB040-08|United States|Texas|658[0n]|BOLD:AAC3318
Focillidia texana|[4768]|HKONB041-08|United States|Texas|658[0n]|BOLD:AAC3318
Focillidia texana|[4769]|HKONB042-08|United States|Texas|658[0n]|BOLD:AAC3318
Focillidia texana|[4770]|HKONB043-08|United States|Texas|658[0n]|BOLD:AAC3318
Focillidia grenadensis|[4771]|CNCLB402-14|United States|Florida|682[0n]|BOLD:AAC3318
Focillidia grenadensis|[4772]|CNCLB401-14|United States|Florida|679[0n]|BOLD:AAC3318
Focillidia grenadensis|[4773]|CNCLB403-14|United States|Florida|682[0n]|BOLD:AAC3318
Coenipeta medina|[4774]|LYHES029-09|Mexico|Yucatan|561[0n]|BOLD:AAA3282
Coenipeta medina|[4775]|LYNYM165-09|Mexico|Yucatan|539[6n]|BOLD:AAA3282
Coenipeta medina|[4776]|LYHES052-09|Mexico|Yucatan|565[0n]|BOLD:AAA3282
Coenipeta medina|[4777]|LYHES057-09|Mexico|Yucatan|536[0n]|BOLD:AAA3282
Coenipeta medina|[4778]|LPYPB121-08|Mexico|Campeche|658[0n]|BOLD:AAA3282
Coenipeta medina|[4779]|MHAUB358-05|Costa Rica|Guanacaste|658[0n]|BOLD:AAA3282
Coenipeta medina|[4780]|MHAUB359-05|Costa Rica|Guanacaste|658[0n]|BOLD:AAA3282
Coenipeta medina|[4781]|MHAUB385-05|Costa Rica|Guanacaste|658[0n]|BOLD:AAA3282
Coenipeta medina|[4782]|MHAUB386-05|Costa Rica|Guanacaste|658[0n]|BOLD:AAA3282
Coenipeta medina|[4783]|LPYPB542-08|Mexico|Yucatan|658[0n]|BOLD:AAA3282
Coenipeta medina|[4784]|LYPAP062-09|Mexico|Quintana Roo|658[0n]|BOLD:AAA3282
Coenipeta medina|[4785]|LYHES010-09|Mexico|Yucatan|658[0n]|BOLD:AAA3282
Coenipeta medina|[4786]|LYNYM177-09|Mexico|Yucatan|658[0n]|BOLD:AAA3282
Coenipeta medina|[4787]|LYHES014-09|Mexico|Yucatan|658[0n]|BOLD:AAA3282
Coenipeta medina|[4788]|LYPIE851-09|Mexico|Yucatan|658[0n]|BOLD:AAA3282
Coenipeta medina|[4789]|MHMYN074-11|Costa Rica||658[0n]|BOLD:AAA3282
Coenipeta medina|[4790]|MHAUB356-05|Costa Rica|Guanacaste|532[0n]|BOLD:AAA3282
Coenipeta medina|[4791]|LYPAP636-09|Mexico|Yucatan|658[0n]|BOLD:AAA3282
Coenipeta medina|[4792]|LYHES009-09|Mexico|Yucatan|658[0n]|BOLD:AAA3282
Coenipeta medina|[4793]|LYPIE656-09|Mexico|Yucatan|617[0n]|BOLD:AAA3282

Coenipeta medina|[4791]|LYPAP636-09|Mexico|Yucatan|658[0n]|BOLD:AAA3282
Coenipeta medina|[4792]|LYHES009-09|Mexico|Yucatan|658[0n]|BOLD:AAA3282
Coenipeta medina|[4793]|LYPIE656-09|Mexico|Yucatan|617[0n]|BOLD:AAA3282
Coenipeta medina|[4794]|LYPIE892-09|Mexico|Yucatan|658[0n]|BOLD:AAA3282
Coenipeta medina|[4795]|MHAUB354-05|Costa Rica|Guanacaste|658[0n]|BOLD:AAA3282
Coenipeta medina|[4796]|LYNYM030-09|Mexico|Yucatan|658[0n]|BOLD:AAA3282
Coenipeta medina|[4797]|LYNYM019-09|Mexico|Yucatan|617[0n]|BOLD:AAA3282
Coenipeta medina|[4798]|LYPIE847-09|Mexico|Yucatan|658[0n]|BOLD:AAA3282
Coenipeta medina|[4799]|LYPIE654-09|Mexico|Yucatan|658[0n]|BOLD:AAA3282
Coenipeta medina|[4800]|LYPIE651-09|Mexico|Yucatan|658[0n]|BOLD:AAA3282
Coenipeta medina|[4801]|LYPIE634-09|Mexico|Yucatan|658[0n]|BOLD:AAA3282
Coenipeta medina|[4802]|LYHES030-09|Mexico|Yucatan|658[0n]|BOLD:AAA3282
Coenipeta medina|[4803]|LYHES011-09|Mexico|Yucatan|658[0n]|BOLD:AAA3282
Coenipeta medina|[4804]|LYNYM040-09|Mexico|Yucatan|658[0n]|BOLD:AAA3282
Coenipeta medina|[4805]|LYNYM032-09|Mexico|Yucatan|658[0n]|BOLD:AAA3282
Coenipeta medina|[4806]|LPYPB550-08|Mexico|Yucatan|658[0n]|BOLD:AAA3282
Coenipeta medina|[4807]|LPYPB549-08|Mexico|Yucatan|658[0n]|BOLD:AAA3282
Coenipeta medina|[4808]|LPYPB548-08|Mexico|Yucatan|658[0n]|BOLD:AAA3282
Coenipeta medina|[4809]|LPYPB539-08|Mexico|Yucatan|658[0n]|BOLD:AAA3282
Coenipeta medina|[4810]|LPYPB117-08|Mexico|Quintana Roo|658[0n]|BOLD:AAA3282
Coenipeta medina|[4811]|LPYPA267-08|Mexico|Quintana Roo|658[0n]|BOLD:AAA3282
Coenipeta medina|[4812]|MHMXD651-06|Costa Rica|Guanacaste|658[0n]|BOLD:AAA3282
Coenipeta medina|[4813]|MHMXD650-06|Costa Rica|Guanacaste|658[0n]|BOLD:AAA3282
Coenipeta medina|[4814]|MHAUB355-05|Costa Rica|Guanacaste|658[0n]|BOLD:AAA3282
Coenipeta medina|[4815]|LYPIE650-09|Mexico|Yucatan|594[0n]|BOLD:AAA3282
Coenipeta medina|[4816]|LYPIE857-09|Mexico|Yucatan|641[0n]|BOLD:AAA3282
Coenipeta medina|[4817]|LYPIE885-09|Mexico|Yucatan|658[0n]|BOLD:AAA3282
Coenipeta medina|[4818]|BLPDJ062-09|Costa Rica|Guanacaste|658[0n]|BOLD:AAA3282
Coenipeta medina|[4819]|MHMYN075-11|Costa Rica||658[0n]|BOLD:AAA3282
Coenipeta medina|[4820]|MHMYN077-11|Costa Rica||658[0n]|BOLD:AAA3282
Coenipeta medina|[4821]|MHAUB352-05|Costa Rica|Guanacaste|522[0n]|BOLD:AAA3282
Coenipeta medina|[4822]|LYHES023-09|Mexico|Yucatan|580[0n]|BOLD:AAA3282
Coenipeta medina|[4823]|LYHES022-09|Mexico|Yucatan|621[0n]|BOLD:AAA3282
Coenipeta medina|[4824]|MHMXD649-06|Costa Rica|Guanacaste|658[0n]|BOLD:AAA3282
Coenipeta medina|[4825]|LPYPB093-08|Mexico|Campeche|658[0n]|BOLD:AAA3282
Coenipeta medina|[4826]|LYNYM020-09|Mexico|Yucatan|658[0n]|BOLD:AAA3282
Coenipeta medina|[4827]|LYNYM021-09|Mexico|Yucatan|658[0n]|BOLD:AAA3282
Coenipeta medina|[4828]|LYNYM183-09|Mexico|Yucatan|657[0n]|BOLD:AAA3282
Coenipeta medina|[4829]|LYNYM187-09|Mexico|Yucatan|658[0n]|BOLD:AAA3282
Coenipeta medina|[4830]|LYPIE653-09|Mexico|Yucatan|656[0n]|BOLD:AAA3282
Coenipeta medina|[4831]|LYPIE858-09|Mexico|Yucatan|658[0n]|BOLD:AAA3282
Coenipeta medina|[4832]|LYPIE889-09|Mexico|Yucatan|658[0n]|BOLD:AAA3282
Coenipeta medina|[4833]|MHMYN073-11|Costa Rica||658[0n]|BOLD:AAA3282
Coenipeta medina|[4834]|LYHES008-09|Mexico|Yucatan|596[0n]|BOLD:AAA3282
Coenipeta medina|[4835]|LYNYM029-09|Mexico|Yucatan|621[1n]|BOLD:AAA3282
Coenipeta medina|[4836]|LPYPB546-08|Mexico|Yucatan|658[0n]|BOLD:AAA3282
Coenipeta medina|[4837]|LPYPB555-08|Mexico|Yucatan|658[0n]|BOLD:AAA3282
Coenipeta medina|[4838]|LYNYM025-09|Mexico|Yucatan|658[0n]|BOLD:AAA3282
Coenipeta medina|[4839]|LYNYM176-09|Mexico|Yucatan|658[0n]|BOLD:AAA3282
Coenipeta medina|[4840]|LYHES053-09|Mexico|Yucatan|658[0n]|BOLD:AAA3282
Coenipeta medina|[4841]|LYHES058-09|Mexico|Yucatan|658[0n]|BOLD:AAA3282
Coenipeta medina|[4842]|LYPIE631-09|Mexico|Yucatan|658[0n]|BOLD:AAA3282
Coenipeta medina|[4843]|LYPIE636-09|Mexico|Yucatan|658[0n]|BOLD:AAA3282
Coenipeta medina|[4844]|LYPIE865-09|Mexico|Yucatan|658[0n]|BOLD:AAA3282
Coenipeta medina|[4845]|LYPIE895-09|Mexico|Yucatan|658[0n]|BOLD:AAA3282
Coenipeta medina|[4846]|LPYPB541-08|Mexico|Yucatan|658[0n]|BOLD:AAA3282
Coenipeta medina|[4847]|LPYPB537-08|Mexico|Yucatan|658[0n]|BOLD:AAA3282
Coenipeta medina|[4848]|LPYPB536-08|Mexico|Yucatan|658[0n]|BOLD:AAA3282
Coenipeta medina|[4849]|LPYPB293-08|Mexico|Campeche|658[0n]|BOLD:AAA3282
Coenipeta medina|[4850]|LPYPB123-08|Mexico|Quintana Roo|658[0n]|BOLD:AAA3282
Coenipeta medina|[4851]|LPYPB120-08|Mexico|Quintana Roo|658[0n]|BOLD:AAA3282
Coenipeta medina|[4852]|LPYPB119-08|Mexico|Campeche|658[0n]|BOLD:AAA3282
Coenipeta medina|[4853]|LPYPB118-08|Mexico|Campeche|658[0n]|BOLD:AAA3282
Coenipeta medina|[4854]|LPYPB116-08|Mexico|Quintana Roo|658[0n]|BOLD:AAA3282
Coenipeta medina|[4855]|MHAUB357-05|Costa Rica|Guanacaste|658[0n]|BOLD:AAA3282
Coenipeta medina|[4856]|LYNYM015-09|Mexico|Yucatan|607[0n]|BOLD:AAA3282
Coenipeta medina|[4857]|LYHES050-09|Mexico|Yucatan|625[0n]|BOLD:AAA3282
Coenipeta medina|[4858]|LYNYM346-09|Mexico|Quintana Roo|626[0n]|BOLD:AAA3282
Coenipeta medina|[4859]|BLPCD385-08|Costa Rica|Guanacaste|658[0n]|BOLD:AAA3282
Coenipeta medina|[4860]|LPYPB552-08|Mexico|Yucatan|658[0n]|BOLD:AAA3282
Coenipeta medina|[4861]|LYNYM252-09|Mexico|Quintana Roo|658[0n]|BOLD:AAA3282
Coenipeta medina|[4862]|LYNYM258-09|Mexico|Quintana Roo|658[0n]|BOLD:AAA3282
Coenipeta medina|[4863]|LYNYM203-09|Mexico|Yucatan|623[0n]|BOLD:AAA3282
Coenipeta medina|[4864]|LYPIE638-09|Mexico|Yucatan|622[0n]|BOLD:AAA3282
Coenipeta medina|[4865]|MHMYN076-11|Costa Rica||658[0n]|BOLD:AAA3282
Coenipeta medina|[4866]|LYHES061-09|Mexico|Yucatan|658[0n]|BOLD:AAA3282
Coenipeta medina|[4867]|LYPIE890-09|Mexico|Yucatan|658[1n]|BOLD:AAA3282
Coenipeta medina|[4868]|LYNYM031-09|Mexico|Yucatan|658[1n]|BOLD:AAA3282
Coenipeta medina|[4869]|LPYPB545-08|Mexico|Yucatan|658[0n]|BOLD:AAA3282
Coenipeta medina|[4870]|LYPIE864-09|Mexico|Yucatan|658[0n]|BOLD:AAA3282
Coenipeta medina|[4871]|LYHES012-09|Mexico|Yucatan|658[0n]|BOLD:AAA3282
Coenipeta medina|[4872]|LYHES059-09|Mexico|Yucatan|658[0n]|BOLD:AAA3282
Coenipeta medina|[4873]|LYPIE644-09|Mexico|Yucatan|658[0n]|BOLD:AAA3282
Coenipeta medina|[4874]|LYPIE649-09|Mexico|Yucatan|658[0n]|BOLD:AAA3282
Coenipeta medina|[4875]|LYPIE658-09|Mexico|Yucatan|658[0n]|BOLD:AAA3282
Coenipeta medina|[4876]|LYPIE850-09|Mexico|Yucatan|658[0n]|BOLD:AAA3282
Coenipeta medina|[4877]|LYPIE911-09|Mexico|Yucatan|658[0n]|BOLD:AAA3282
Coenipeta medina|[4878]|LYPIE919-09|Mexico|Yucatan|658[0n]|BOLD:AAA3282
Coenipeta medina|[4879]|MHMYM2152-11|Costa Rica||658[0n]|BOLD:AAA3282
Coenipeta medina|[4880]|MHMYN072-11|Costa Rica||658[0n]|BOLD:AAA3282
Coenipeta medina|[4881]|LYNYM336-09|Mexico|Quintana Roo|658[0n]|BOLD:AAA3282
Coenipeta medina|[4882]|LYHES007-09|Mexico|Yucatan|658[0n]|BOLD:AAA3282
Coenipeta medina|[4883]|LPYPB553-08|Mexico|Yucatan|658[0n]|BOLD:AAA3282
Coenipeta medina|[4884]|LYNYM028-09|Mexico|Yucatan|658[0n]|BOLD:AAA3282
Coenipeta medina|[4885]|LPYPB547-08|Mexico|Yucatan|658[0n]|BOLD:AAA3282
Coenipeta medina|[4886]|LPYPB544-08|Mexico|Yucatan|658[0n]|BOLD:AAA3282
Coenipeta medina|[4887]|LPYPB543-08|Mexico|Yucatan|658[0n]|BOLD:AAA3282
Coenipeta medina|[4888]|LPYPB540-08|Mexico|Yucatan|658[0n]|BOLD:AAA3282
Coenipeta medina|[4889]|LPYPB538-08|Mexico|Yucatan|658[0n]|BOLD:AAA3282
Coenipeta medina|[4890]|LPYPB535-08|Mexico|Yucatan|658[0n]|BOLD:AAA3282
Coenipeta medina|[4891]|LPYPB294-08|Mexico|Campeche|658[0n]|BOLD:AAA3282
Coenipeta medina|[4892]|LPYPB122-08|Mexico|Quintana Roo|658[0n]|BOLD:AAA3282
Coenipeta medina|[4893]|MHAUB353-05|Costa Rica|Guanacaste|536[0n]|BOLD:AAA3282

[illegible]
Coenipeta medina|[4891]|LPYPB294-08|Mexico|Campeche|658[0n]|BOLD:AAA3282
Coenipeta medina|[4892]|LPYPB122-08|Mexico|Quintana Roo|658[0n]|BOLD:AAA3282
Coenipeta medina|[4893]|MHAUB353-05|Costa Rica|Guanacaste|536[0n]|BOLD:AAA3282
Coenipeta medina|[4894]|LYPIE652-09|Mexico|Yucatan|658[0n]|BOLD:AAA3282
Coenipeta medina|[4895]|LYPIE655-09|Mexico|Yucatan|620[0n]|BOLD:AAA3282
Coenipeta medina|[4896]|CNCLB2400-14|United States|Texas|658[0n]|BOLD:AAA3282
Lesmone fufius|[4897]|QUNOD180-10|United States|Texas|658[0n]|BOLD:AAI8034
Lesmone fufius|[4898]|CNCLB2409-14|United States|Texas|658[0n]|BOLD:AAI8034
Itomia opistographa|[4899]|HKONB009-08|United States|Texas|658[0n]|BOLD:AAB2564
Itomia opistographa|[4900]|QUNOB136-08|United States|Texas|658[0n]|BOLD:AAB2564
Itomia opistographa|[4901]|QUNOB137-08|United States|Texas|658[0n]|BOLD:AAB2564
Itomia opistographa|[4902]|HKONB008-08|United States|Texas|658[0n]|BOLD:AAB2564
Itomia opistographa|[4903]|HKONB010-08|United States|Texas|658[0n]|BOLD:AAB2564
Itomia opistographa|[4904]|CNCLB2459-14|United States|Texas|658[0n]|BOLD:AAB2564
Toxonprucha crudelis|[4905]|HKONB292-09|United States|Texas|658[0n]|BOLD:ACE4739
Toxonprucha crudelis|[4906]|HKONB533-09|United States|Texas|650[0n]|BOLD:ACE4739
Toxonprucha crudelis|[4907]|BBLSW084-09|United States|Texas|658[0n]|BOLD:ACE4739
Toxonprucha crudelis|[4908]|BBLSY143-09|United States|Texas|658[0n]|BOLD:ACE4739
Toxonprucha crudelis|[4909]|BBLOD534-11|United States|Texas|658[0n]|BOLD:AAC7419
Toxonprucha crudelis|[4910]|BBLOC1990-11|United States|Texas|658[0n]|BOLD:AAC7419
Toxonprucha crudelis|[4911]|BBLOC1575-11|United States|Texas|658[0n]|BOLD:AAC7419
Toxonprucha crudelis|[4912]|BBLSX793-09|United States|Texas|658[0n]|BOLD:AAC7419
Toxonprucha crudelis|[4913]|BBLSX182-09|United States|Texas|658[0n]|BOLD:AAC7419
Toxonprucha crudelis|[4914]|BBLSX179-09|United States|Texas|658[0n]|BOLD:AAC7419
Toxonprucha crudelis|[4915]|BBLSY147-09|United States|Texas|658[0n]|BOLD:AAC7419
Toxonprucha crudelis|[4916]|BBLSY146-09|United States|Texas|658[0n]|BOLD:AAC7419
Toxonprucha crudelis|[4917]|BBLSY144-09|United States|Texas|658[0n]|BOLD:AAC7419
Toxonprucha crudelis|[4918]|BBLSY142-09|United States|Texas|658[0n]|BOLD:AAC7419
Toxonprucha crudelis|[4919]|BBLSW075-09|United States|Texas|658[0n]|BOLD:AAC7419
Toxonprucha crudelis|[4920]|LMEMB234-09|United States|Texas|658[0n]|BOLD:AAC7419
Toxonprucha crudelis|[4921]|BBLSX235-09|United States|Texas|658[0n]|BOLD:AAC7419
Toxonprucha crudelis|[4922]|BBLSW733-09|United States|Texas|643[1n]|BOLD:AAC7419
Toxonprucha crudelis|[4923]|ABNCC331-07|United States|Texas|624[1n]|BOLD:AAC7419
Toxonprucha crudelis|[4924]|BBLOD110-11|United States|Texas|658[0n]|BOLD:AAC7419
Toxonprucha crudelis|[4925]|BBLOD168-11|United States|Texas|658[0n]|BOLD:AAC7419
Toxonprucha crudelis|[4926]|BBLOD186-11|United States|Texas|658[0n]|BOLD:AAC7419
Toxonprucha crudelis|[4927]|BBLOD349-11|United States|Texas|658[0n]|BOLD:AAC7419
Toxonprucha crudelis|[4928]|BBLOD357-11|United States|Texas|658[0n]|BOLD:AAC7419
Toxonprucha crudelis|[4929]|BBLOD535-11|United States|Texas|658[0n]|BOLD:AAC7419
Toxonprucha crudelis|[4930]|BBLOE1371-12|United States|Texas|658[0n]|BOLD:AAC7419
Toxonprucha crudelis|[4931]|IAWL159-09|United States|Arizona|658[0n]|BOLD:ABY9872
Toxonprucha crudelis|[4932]|RDNME071-07|United States|Arizona|658[0n]|BOLD:ABY9872
Toxonprucha crudelis|[4933]|LMEMB233-09|United States|Arizona|658[0n]|BOLD:ABY9872
Toxonprucha crudelis|[4934]|IAWL160-09|United States|Arizona|658[0n]|BOLD:ABY9872
Toxonprucha crudelis|[4935]|USLEP962-10|United States|Arizona|658[0n]|BOLD:ABY9872
Toxonprucha crudelis|[4936]|BBLOB581-11|United States|Arizona|658[0n]|BOLD:ABY9872
Toxonprucha crudelis|[4937]|BBLSY363-09|United States|Arizona|658[0n]|BOLD:ABY9872
Toxonprucha crudelis|[4938]|BBLOE1335-12|United States|Arizona|658[0n]|BOLD:ABY9872
Toxonprucha crudelis|[4939]|BBLOE1740-12|United States|Arizona|658[0n]|BOLD:ABY9872
Toxonprucha crudelis|[4940]|LMEMB231-09|United States|Arizona|658[0n]|BOLD:ABY9872
Toxonprucha crudelis|[4941]|LMEMB232-09|United States|Arizona|658[0n]|BOLD:ABY9872
Toxonprucha crudelis|[4942]|BBLSW233-09|United States|Arizona|658[0n]|BOLD:ABY9872
Toxonprucha crudelis|[4943]|BBLSX740-09|United States|Arizona|658[0n]|BOLD:ABY9872
Toxonprucha crudelis|[4944]|BBLSY330-09|United States|Arizona|658[0n]|BOLD:ABY9872
Toxonprucha crudelis|[4945]|RDNMK185-11|United States|Arizona|658[0n]|BOLD:ABY9872
Toxonprucha crudelis|[4946]|BBLOB588-11|United States|Arizona|658[0n]|BOLD:ABY9872
Toxonprucha crudelis|[4947]|BBLOB885-11|United States|Arizona|658[0n]|BOLD:ABY9872
Toxonprucha crudelis|[4948]|BBLOB1717-11|United States|Arizona|658[0n]|BOLD:ABY9872
Toxonprucha crudelis|[4949]|BBLOC447-11|United States|Arizona|658[0n]|BOLD:ABY9872
Toxonprucha crudelis|[4950]|BBLOD1794-11|United States|Arizona|658[0n]|BOLD:ABY9872
Toxonprucha crudelis|[4951]|CMAZA1076-12|United States|Arizona|658[0n]|BOLD:ABY9872
Toxonprucha psegmapteryx|[4952]|HKONB546-09|United States|Texas|658[0n]|BOLD:AAK1914
Toxonprucha n. sp. nr. volucris|[4953]|BBLSW036-09|United States|Texas|658[0n]|BOLD:AAH5260
Toxonprucha n. sp. nr. volucris|[4954]|BBLSY249-09|United States|Texas|658[0n]|BOLD:AAH5260
Toxonprucha n. sp. nr. volucris|[4955]|BBLSX831-09|United States|Texas|658[0n]|BOLD:AAH5260
Toxonprucha n. sp. nr. volucris|[4956]|BBLSY946-09|United States|Texas|658[0n]|BOLD:AAH5260
Toxonprucha repentis|[4957]|BBLSY752-09|United States|Arizona|658[0n]|BOLD:AAE0285
Toxonprucha repentis|[4958]|BBLOD1864-11|United States|Arizona|610[0n]|BOLD:AAE0285
Toxonprucha repentis|[4959]|RDNME072-07|United States|Arizona|658[0n]|BOLD:AAE0285
Toxonprucha repentis|[4960]|BBLOD1753-11|United States|California|645[0n]|BOLD:AAE0285
Toxonprucha repentis|[4961]|BBLSY835-09|United States|Arizona|658[0n]|BOLD:AAE0285
Toxonprucha repentis|[4962]|RDNMK186-11|United States|Arizona|658[0n]|BOLD:AAE0285
Toxonprucha repentis|[4963]|USLEP773-10|United States|Arizona|658[0n]|BOLD:AAE0285
Toxonprucha repentis|[4964]|BBLSY683-09|United States|Arizona|658[0n]|BOLD:AAE0285
Toxonprucha repentis|[4965]|BBLSW198-09|United States|Arizona|658[0n]|BOLD:AAE0285
Toxonprucha repentis|[4966]|RDNME073-07|United States|Arizona|658[0n]|BOLD:AAE0285
Toxonprucha repentis|[4967]|BBLOB1505-11|United States|Arizona|658[1n]|BOLD:AAE0285
Toxonprucha repentis|[4968]|BBLOE1316-12|United States|Arizona|658[0n]|BOLD:AAE0285
Toxonprucha repentis|[4969]|BBLOE1819-12|United States|Arizona|658[0n]|BOLD:AAE0285
Toxonprucha repentis|[4970]|BBLOE1866-12|United States|Arizona|658[0n]|BOLD:AAE0285
Toxonprucha repentis|[4971]|QUNOE455-12|United States|Texas|658[0n]|BOLD:AAE0285
Toxonprucha repentis|[4972]|CMAZA1105-12|United States|Arizona|658[0n]|BOLD:AAE0285
Toxonprucha volucris|[4973]|LOCBF1068-13|United States|California|546[0n]|BOLD:AAE0613
Toxonprucha volucris|[4974]|BBLOD1145-11|United States|Arizona|658[0n]|BOLD:AAE0613
Toxonprucha volucris|[4975]|BBLOC1578-11|United States|California|658[0n]|BOLD:AAE0613
Toxonprucha volucris|[4976]|CMAZA513-10|United States|Arizona|658[0n]|BOLD:AAE0613
Toxonprucha volucris|[4977]|BBLOB118-11|United States|Arizona|658[0n]|BOLD:AAE0613
Toxonprucha volucris|[4978]|BBLOB069-11|United States|Arizona|658[0n]|BOLD:AAE0613
Toxonprucha volucris|[4979]|USLEP983-10|United States|Arizona|658[0n]|BOLD:AAE0613
Toxonprucha volucris|[4980]|LMEMB226-09|United States|Arizona|658[0n]|BOLD:AAE0613
Toxonprucha volucris|[4981]|BBLOD1628-11|United States|Arizona|658[0n]|BOLD:AAE0613
Toxonprucha volucris|[4982]|USLEP980-10|United States|Arizona|658[0n]|BOLD:AAE0613
Toxonprucha volucris|[4983]|USLEP982-10|United States|Arizona|658[0n]|BOLD:AAE0613
Toxonprucha volucris|[4984]|BBLSY329-09|United States|Arizona|658[0n]|BOLD:AAE0613
Toxonprucha volucris|[4985]|BBLSY680-09|United States|Arizona|658[0n]|BOLD:AAE0613
Toxonprucha volucris|[4986]|BBLSY705-09|United States|Arizona|658[0n]|BOLD:AAE0613
Toxonprucha volucris|[4987]|USLEP979-10|United States|Arizona|658[0n]|BOLD:AAE0613
Toxonprucha volucris|[4988]|BBLOD738-11|United States|Arizona|658[0n]|BOLD:AAE0613
Toxonprucha volucris|[4989]|BBLOD851-11|United States|Arizona|658[0n]|BOLD:AAE0613
Toxonprucha volucris|[4990]|LMEMB225-09|United States|Arizona|658[0n]|BOLD:AAE0613
Toxonprucha volucris|[4991]|RDNMH833-09|United States|Arizona|658[0n]|BOLD:AAE0613
Toxonprucha volucris|[4992]|BBLOD1886-11|United States|Arizona|658[0n]|BOLD:AAE0613
Toxonprucha volucris|[4993]|BBLSY739-09|United States|Arizona|658[0n]|BOLD:AAE0613

[illegible]
Toxonprucha volucris|[4991]|RDNMH833-09|United States|Arizona|658[0n]|BOLD:AAE0613
Toxonprucha volucris|[4992]|BBLOD1886-11|United States|Arizona|658[0n]|BOLD:AAE0613
Toxonprucha volucris|[4993]|BBLSY739-09|United States|Arizona|658[0n]|BOLD:AAE0613
Toxonprucha volucris|[4994]|ABNCC329-07|United States|Texas|654[0n]|BOLD:AAE0613
Toxonprucha volucris|[4995]|USLEP794-10|United States|Arizona|627[0n]|BOLD:AAE0613
Toxonprucha volucris|[4996]|RDNMK107-11|United States|Arizona|658[0n]|BOLD:AAE0613
Toxonprucha volucris|[4997]|ABNCC330-07|United States|Texas|644[0n]|BOLD:AAE0613
Toxonprucha volucris|[4998]|LMEMB224-09|United States|Arizona|658[0n]|BOLD:AAE0613
Toxonprucha volucris|[4999]|BBLSY051-09|United States|Arizona|658[0n]|BOLD:AAE0613
Toxonprucha volucris|[5000]|BBLSY664-09|United States|Arizona|658[0n]|BOLD:AAE0613
Toxonprucha volucris|[5001]|RDNMJ709-11|United States|Arizona|658[0n]|BOLD:AAE0613
Toxonprucha volucris|[5002]|BBLOC783-11|United States|Arizona|658[0n]|BOLD:AAE0613
Toxonprucha volucris|[5003]|BBLOC1548-11|United States|California|658[0n]|BOLD:AAE0613
Toxonprucha volucris|[5004]|BBLOD1898-11|United States|Arizona|658[0n]|BOLD:AAE0613
Toxonprucha volucris|[5005]|LOCBF1120-13|United States|California|564[0n]|BOLD:AAE0613
Zale peruncta DHJ02|[5006]|BLPDZ650-11|Costa Rica|Alajuela|658[0n]|BOLD:AAB1416
Zale peruncta DHJ02|[5007]|BLPAB778-06|Costa Rica|Guanacaste|658[0n]|BOLD:AAB1416
Zale peruncta DHJ02|[5008]|BLPDE341-09|Costa Rica||658[0n]|BOLD:AAB1416
Zale peruncta DHJ02|[5009]|MHAUB816-05|Costa Rica|Guanacaste|595[0n]|BOLD:AAB1416
Zale peruncta DHJ02|[5010]|BLPDZ581-11|Costa Rica|Alajuela|632[0n]|BOLD:AAB1416
Zale peruncta|[5011]|HKONB492-09|United States|Texas|635[1n]|BOLD:AAB1416
Zale peruncta DHJ02|[5012]|BLPCK651-08|Costa Rica|Alajuela|658[0n]|BOLD:AAB1416
Zale peruncta DHJ02|[5013]|MHMXV136-08|Costa Rica||658[0n]|BOLD:AAB1416
Zale peruncta DHJ02|[5014]|BLPCD460-08|Costa Rica|Guanacaste|658[0n]|BOLD:AAB1416
Zale peruncta DHJ02|[5015]|MHMXV137-08|Costa Rica||658[0n]|BOLD:AAB1416
Zale peruncta DHJ02|[5016]|MHMYC2239-09|Costa Rica||658[0n]|BOLD:AAB1416
Zale peruncta DHJ02|[5017]|BLPDV1005-11|Costa Rica|Guanacaste|658[0n]|BOLD:AAB1416
Zale peruncta DHJ02|[5018]|MHMYL3236-11|Costa Rica||658[0n]|BOLD:AAB1416
Zale peruncta DHJ02|[5019]|MHMYS522-12|Costa Rica||658[0n]|BOLD:AAB1416
Zale peruncta|[5020]|GWORH201-09|Brazil|Rio Grande do Sul|658[0n]|BOLD:AAB1416
Zale peruncta|[5021]|LNOUE965-11|French Guiana||658[0n]|BOLD:AAB1416
Zale peruncta|[5022]|GWORH219-09|Brazil|Rio Grande do Sul|658[0n]|BOLD:AAB1416
Zale peruncta|[5023]|ARMOT717-12|Argentina|Misiones|658[0n]|BOLD:AAB1416
Zale peruncta|[5024]|ARMOT722-12|Argentina|Misiones|658[0n]|BOLD:AAB1416
Zale peruncta|[5025]|ARMOT727-12|Argentina|Misiones|658[0n]|BOLD:AAB1416
Zale peruncta|[5026]|MOTAR090-12|Argentina|Misiones|658[0n]|BOLD:AAB1416
Zale peruncta|[5027]|RDNML239-13|Puerto Rico||658[0n]|BOLD:AAB1416
Toxonprucha diffundens complex|[5028]|RDNMH403-09|United States|Florida|658[0n]|BOLD:AAI4900
Toxonprucha diffundens complex|[5029]|IAWL180-09|United States|Arizona|658[0n]|BOLD:AAC9857
Toxonprucha excavata|[5030]|CNCLB1727-14|Mexico|Nuevo Leon|307[0n]|
Toxonprucha excavata|[5031]|CMAZA533-10|United States|Arizona|658[0n]|BOLD:ACF4168
Toxonprucha excavata|[5032]|CNCLB900-14|United States|Arizona|658[0n]|BOLD:ACF4168
Toxonprucha diffundens complex|[5033]|HKONB075-08|United States|Texas|658[0n]|BOLD:ACF4168
Toxonprucha excavata|[5034]|BBLOD181-11|United States|Texas|620[0n]|BOLD:ACF4168
Toxonprucha excavata|[5035]|CNCLB898-14|United States|Texas|629[0n]|BOLD:ACF4168
Toxonprucha diffundens complex|[5036]|HKONB073-08|United States|Texas|658[0n]|BOLD:ACF4168
Toxonprucha diffundens complex|[5037]|HKONB078-08|United States|Texas|658[0n]|BOLD:ACF4168
Toxonprucha diffundens complex|[5038]|HKONB074-08|United States|Texas|658[0n]|BOLD:ACF4168
Toxonprucha diffundens complex|[5039]|HKONB072-08|United States|Texas|658[0n]|BOLD:ACF4168
Toxonprucha excavata|[5040]|CNCLB899-14|United States|Texas|658[0n]|BOLD:ACF4168
Toxonprucha excavata|[5041]|CNCLB1323-14|United States|Texas|658[0n]|BOLD:ACF4168
Toxonprucha diffundens complex|[5042]|HKONB079-08|United States|Texas|658[0n]|BOLD:ACF4168
Toxonprucha diffundens complex|[5043]|HKONB076-08|United States|Texas|658[0n]|BOLD:ACF4168
Toxonprucha diffundens complex|[5044]|HKONB077-08|United States|Texas|658[0n]|BOLD:ACF4168
Toxonprucha excavata|[5045]|CNCLB1726-14|Mexico|Nuevo Leon|578[0n]|BOLD:ACF4168
Toxonprucha excavata|[5046]|CNCLB2482-14|Dominica||658[0n]|BOLD:ACF4168
Bandelia angulata|[5047]|QUNOD167-10|United States|Arizona|658[0n]|BOLD:AAF0315
Bandelia angulata|[5048]|RDNME860-08|United States|Arizona|658[0n]|BOLD:AAF0315
Bandelia angulata|[5049]|IAWL022-09|United States|Arizona|658[1n]|BOLD:AAF0315
Bandelia angulata|[5050]|QUNOD168-10|United States|Arizona|579[0n]|BOLD:AAF0315
Bandelia angulata|[5051]|CMAZA581-10|United States|Arizona|658[0n]|BOLD:AAF0315
Bandelia angulata|[5052]|BBLOC159-11|United States|Arizona|658[0n]|BOLD:AAF0315
Euaontia clarki|[5053]|IAWL058-09|United States|Arizona|658[0n]|BOLD:AAF3752
Euaontia clarki|[5054]|AWCLB183-10|United States|Arizona|658[0n]|BOLD:AAF3752
Euaontia clarki|[5055]|AWCLB213-10|United States|Arizona|658[0n]|BOLD:AAF3752
Euaontia clarki|[5056]|IAWL059-09|United States|Arizona|658[0n]|BOLD:AAF3752
Euaontia clarki|[5057]|IAWL060-09|United States|Arizona|658[0n]|BOLD:AAF3752
Euaontia clarki|[5058]|AWCLB376-10|United States|Arizona|658[0n]|BOLD:AAF3752
Euaontia clarki|[5059]|AWCLB522-11|United States|Arizona|658[0n]|BOLD:AAF3752
Euaontia semirufa|[5060]|RDNMH879-09|United States|California|658[0n]|BOLD:AAF3752
Euaontia semirufa|[5061]|NAMUM149-08|United States|California|658[0n]|BOLD:AAF3752
Euaontia semirufa|[5062]|GBMIN20844-13|||671[0n]|BOLD:AAF3752
Ianius mosca|[5063]|ABNCC284-07|United States|Texas|594[0n]|BOLD:AAE6128
Ianius mosca|[5064]|ABNCC283-07|United States|Texas|599[0n]|BOLD:AAE6128
Ianius mosca|[5065]|RDNME990-08|United States|Texas|658[0n]|BOLD:AAE6128
Ianius mosca|[5066]|HKONB517-09|United States|Texas|658[0n]|BOLD:AAE6128
Ianius mosca|[5067]|UMSNH038-13|Mexico|Michoacan|658[0n]|BOLD:AAE6128
Mocis disseverans|[5068]|HKONS033-07|United States|Florida|658[0n]|BOLD:AAA9789
Mocis disseverans|[5069]|HKONS032-07|United States|Florida|658[0n]|BOLD:AAA9789
Mocis disseverans|[5070]|HKONS031-07|United States|Florida|658[0n]|BOLD:AAA9789
Mocis disseverans|[5071]|LOFLC293-06|United States|Florida|658[0n]|BOLD:AAA9789
Mocis disseverans|[5072]|HKONS034-07|United States|Florida|644[0n]|BOLD:AAA9789
Mocis disseverans|[5073]|HKONS035-07|United States|Florida|656[2n]|BOLD:AAA9789
Mocis disseverans|[5074]|ABNCC413-07|United States|Florida|600[1n]|BOLD:AAA9789
Mocis disseverans|[5075]|HKONS036-07|United States|Florida|656[2n]|BOLD:AAA9789
Mocis disseverans|[5076]|LNCB842-09|United States|Alabama|658[0n]|BOLD:AAA9789
Mocis latipes|[5077]|ABNCC407-07|United States|Texas|656[0n]|BOLD:AAA2438
Mocis latipes|[5078]|HKONS038-07|United States|Florida|658[0n]|BOLD:AAA2438
Mocis latipes|[5079]|ABNCC408-07|United States|Texas|653[0n]|BOLD:AAA2438
Mocis latipes|[5080]|MJMSL037-10|United States|Massachusetts|658[0n]|BOLD:AAA2438
Mocis latipes|[5081]|LNC518-06|United States|North Carolina|585[1n]|BOLD:AAA2438
Mocis latipes|[5082]|LNC422-05|United States|North Carolina|658[0n]|BOLD:AAA2438
Mocis latipes|[5083]|LOFLB491-06|United States|Florida|658[0n]|BOLD:AAA2438
Mocis latipes|[5084]|HKONS039-07|United States|Florida|658[0n]|BOLD:AAA2438
Mocis latipes|[5085]|BBLOD193-11|United States|Texas|658[0n]|BOLD:AAA2438
Mocis latipes|[5086]|BBLOD1092-11|United States|Texas|658[0n]|BOLD:AAA2438
Mocis marcida|[5087]|LSEU050-06|United States|Georgia|547[0n]|BOLD:ABZ1493
Mocis marcida|[5088]|ABNCC409-07|United States|Texas|606[0n]|BOLD:ABZ1493
Mocis marcida|[5089]|LNCB222-06|United States|North Carolina|658[0n]|BOLD:ABZ1493
Mocis marcida|[5090]|LOFLC193-06|United States|Florida|658[0n]|BOLD:ABZ1493
Mocis marcida|[5091]|LOFLC095-06|United States|Florida|658[0n]|BOLD:ABZ1493
Mocis marcida|[5092]|LOFLB802-06|United States|Florida|658[0n]|BOLD:ABZ1493
[illegible]

Mocis marcida|[5090]|LOFLC193-06|United States|Florida|658[0n]|BOLD:ABZ1493
Mocis marcida|[5091]|LOFLC095-06|United States|Florida|658[0n]|BOLD:ABZ1493
Mocis marcida|[5092]|LOFLB802-06|United States|Florida|658[0n]|BOLD:ABZ1493
Mocis marcida|[5093]|LOFLB795-06|United States|Florida|658[0n]|BOLD:ABZ1493
Mocis marcida|[5094]|LOFLB502-06|United States|Florida|658[0n]|BOLD:ABZ1493
Mocis marcida|[5095]|LOFLB132-06|United States|Florida|658[0n]|BOLD:ABZ1493
Mocis marcida|[5096]|LOFLA920-06|United States|Florida|658[0n]|BOLD:ABZ1493
Mocis marcida|[5097]|LOFLA366-06|United States|Florida|658[0n]|BOLD:ABZ1493
Mocis marcida|[5098]|LOFLA308-06|United States|Florida|658[0n]|BOLD:ABZ1493
Mocis marcida|[5099]|LSEU049-06|United States|Georgia|658[0n]|BOLD:ABZ1493
Mocis marcida|[5100]|LOFLB801-06|United States|Florida|658[0n]|BOLD:ABZ1493
Mocis marcida|[5101]|LOFLB407-06|United States|Florida|645[0n]|BOLD:ABZ1493
Mocis marcida|[5102]|ABNCC410-07|United States|Texas|640[0n]|BOLD:ABZ1493
Mocis marcida|[5103]|HKONS041-07|United States|Florida|658[0n]|BOLD:ABZ1493
Mocis marcida|[5104]|HKONS042-07|United States|Florida|658[0n]|BOLD:ABZ1493
Mocis marcida|[5105]|HKONS043-07|United States|Florida|658[0n]|BOLD:ABZ1493
Mocis marcida|[5106]|USLEP588-10|United States|Florida|658[0n]|BOLD:ABZ1493
Mocis marcida|[5107]|BBLOB1134-11|United States|Florida|658[0n]|BOLD:ABZ1493
Mocis marcida|[5108]|BBLOB1639-11|United States|Florida|658[0n]|BOLD:ABZ1493
Mocis marcida|[5109]|MILEQ333-11|United States|Alabama|658[0n]|BOLD:ABZ1493
Mocis marcida|[5110]|BBLOD1680-11|United States|Texas|658[0n]|BOLD:ABZ1493
Mocis marcida|[5111]|BBLOD1692-11|United States|Arizona|658[0n]|BOLD:ABZ1493
Mocis texana|[5112]|LGSMC818-05|United States|Tennessee|658[0n]|BOLD:AAA2439
Mocis texana|[5113]|ABNCC412-07|United States|Texas|652[0n]|BOLD:AAA2439
Mocis texana|[5114]|LILLB067-11|United States|Illinois|658[0n]|BOLD:AAA2439
Mocis texana|[5115]|LILLA891-11|United States|Illinois|658[0n]|BOLD:AAA2439
Mocis texana|[5116]|MILEP329-10|United States|Alabama|658[0n]|BOLD:AAA2439
Mocis texana|[5117]|USLEP808-10|United States|Texas|658[0n]|BOLD:AAA2439
Mocis texana|[5118]|USLEP807-10|United States|Texas|658[0n]|BOLD:AAA2439
Mocis texana|[5119]|USLEP806-10|United States|Texas|658[0n]|BOLD:AAA2439
Mocis texana|[5120]|USLEP805-10|United States|Texas|658[0n]|BOLD:AAA2439
Mocis texana|[5121]|JRLAA023-09|United States|Alabama|658[0n]|BOLD:AAA2439
Mocis texana|[5122]|JRLAA015-09|United States|Alabama|658[0n]|BOLD:AAA2439
Mocis texana|[5123]|BBLSX571-09|United States|Oklahoma|658[0n]|BOLD:AAA2439
Mocis texana|[5124]|LPOKB148-09|United States|Oklahoma|658[0n]|BOLD:AAA2439
Mocis texana|[5125]|LPOKB139-09|United States|Oklahoma|658[0n]|BOLD:AAA2439
Mocis texana|[5126]|LPOKB136-09|United States|Oklahoma|658[0n]|BOLD:AAA2439
Mocis texana|[5127]|LPOKA145-08|United States|Oklahoma|658[0n]|BOLD:AAA2439
Mocis texana|[5128]|LGSMG589-07|United States|Tennessee|658[0n]|BOLD:AAA2439
Mocis texana|[5129]|LSEU306-06|United States|Georgia|658[0n]|BOLD:AAA2439
Mocis texana|[5130]|LNCNW008-06|United States|North Carolina|658[0n]|BOLD:AAA2439
Mocis texana|[5131]|LNC272-05|United States|North Carolina|658[0n]|BOLD:AAA2439
Mocis texana|[5132]|LNC271-05|United States|North Carolina|658[0n]|BOLD:AAA2439
Mocis texana|[5133]|LGSMC817-05|United States|Tennessee|658[0n]|BOLD:AAA2439
Mocis texana|[5134]|LGSMC816-05|United States|Tennessee|658[0n]|BOLD:AAA2439
Mocis texana|[5135]|LGSMC815-05|United States|Tennessee|658[0n]|BOLD:AAA2439
Mocis texana|[5136]|LGSM648-04|United States|Tennessee|658[0n]|BOLD:AAA2439
Mocis texana|[5137]|LGSM647-04|United States|Tennessee|658[0n]|BOLD:AAA2439
Mocis texana|[5138]|ABNCC411-07|United States|Texas|606[0n]|BOLD:AAA2439
Mocis texana|[5139]|BBLSW482-09|United States|Oklahoma|633[0n]|BOLD:AAA2439
Mocis texana|[5140]|HESEP1374-12|Canada|Ontario|618[0n]|BOLD:AAA2439
Mocis cubana|[5141]|RDNML317-13|United States|Florida|622[0n]|BOLD:ACD9064
Mocis cubana|[5142]|RDNML316-13|United States|Florida|658[0n]|BOLD:ACJ8290
Mocis cubana|[5143]|LNAUS3912-13|United States|Florida|658[1n]|BOLD:ACJ8290
Spiloloma lunilinea|[5144]|ABNCC433-07|United States|Missouri|606[0n]|BOLD:AAC7835
Spiloloma lunilinea|[5145]|ABNCC432-07|United States|Oklahoma|594[0n]|BOLD:AAC7835
Spiloloma lunilinea|[5146]|HKONB255-09|United States|Louisiana|637[0n]|BOLD:AAC7835
Spiloloma lunilinea|[5147]|QUNOB291-09|United States|Indiana|658[0n]|BOLD:AAC7835
Spiloloma lunilinea|[5148]|QUNOB292-09|United States|Indiana|658[0n]|BOLD:AAC7835
Spiloloma lunilinea|[5149]|BBLSW441-09|United States|Oklahoma|658[0n]|BOLD:AAC7835
Spiloloma lunilinea|[5150]|LILLA805-11|United States|Illinois|658[0n]|BOLD:AAC7835
Spiloloma lunilinea|[5151]|LSUSA224-06|United States|Kentucky|658[0n]|BOLD:AAC7835
Spiloloma lunilinea|[5152]|QUNO577-08|United States|Indiana|658[0n]|BOLD:AAC7835
Spiloloma lunilinea|[5153]|QUNOB190-08|United States|Indiana|658[0n]|BOLD:AAC7835
Spiloloma lunilinea|[5154]|QUNOB290-09|United States|Indiana|658[0n]|BOLD:AAC7835
Spiloloma lunilinea|[5155]|ABNCC434-07|United States|Indiana|592[0n]|BOLD:AAC7835
Spiloloma lunilinea|[5156]|CNCLB2766-14|United States|North Carolina|658[0n]|BOLD:AAC7835
Achaea ablunaris|[5157]|GWOST442-11|Peru|Huanuco|658[0n]|BOLD:AAB7888
Achaea ablunaris|[5158]|LNAUS5593-13|Paraguay|Paraguari|564[0n]|BOLD:AAB7888
Achaea ablunaris|[5159]|LNAUS3524-13|Paraguay|Paraguari|658[0n]|BOLD:AAB7888
Achaea ablunaris|[5160]|LNAUS3525-13|Paraguay|Paraguari|658[0n]|BOLD:AAB7888
Achaea ablunaris|[5161]|LNAUS5595-13|Paraguay|Paraguari|658[0n]|BOLD:AAB7888
Achaea ablunarisDHJ01|[5162]|BLPCL005-08|Costa Rica|Alajuela|658[0n]|BOLD:AAB7888
Achaea ablunaris|[5163]|LOCRE603-10|Costa Rica|San Jose|658[0n]|BOLD:AAB7888
Achaea ablunaris|[5164]|LOCRE604-10|Costa Rica|San Jose|658[0n]|BOLD:AAB7888
Achaea ablunaris|[5165]|LNAUS3522-13|Paraguay|Alto Parana|658[0n]|BOLD:AAB7888
Achaea ablunaris|[5166]|LNAUS3523-13|Paraguay|Alto Parana|658[0n]|BOLD:AAB7888
Achaea ablunaris|[5167]|CNCLB1924-14|Brazil||658[0n]|BOLD:AAB7888
Achaea ablunarisDHJ02|[5168]|BLPBH722-07|Costa Rica|Guanacaste|658[1n]|BOLD:AAB7888
Achaea ablunarisDHJ02|[5169]|BLPCA535-08|Costa Rica|Guanacaste|632[0n]|BOLD:AAB7888
Achaea ablunarisDHJ02|[5170]|BLPAF712-07|Costa Rica|Guanacaste|631[0n]|BOLD:AAB7888
Achaea ablunarisDHJ02|[5171]|BLPCO840-08|Costa Rica|Guanacaste|658[0n]|BOLD:AAB7888
Achaea ablunarisDHJ02|[5172]|BLPCN884-08|Costa Rica|Guanacaste|658[0n]|BOLD:AAB7888
Achaea ablunarisDHJ02|[5173]|BLPCN637-08|Costa Rica|Guanacaste|658[0n]|BOLD:AAB7888
Achaea ablunarisDHJ02|[5174]|BLPCD651-08|Costa Rica|Guanacaste|658[0n]|BOLD:AAB7888
Achaea ablunarisDHJ02|[5175]|MHAUB330-05|Costa Rica|Guanacaste|541[1n]|BOLD:AAB7888
Achaea ablunarisDHJ02|[5176]|BLPCA919-08|Costa Rica|Guanacaste|658[0n]|BOLD:AAB7888
Achaea ablunarisDHJ02|[5177]|BLPBH666-07|Costa Rica|Guanacaste|658[0n]|BOLD:AAB7888
Achaea ablunarisDHJ02|[5178]|BLPBH478-07|Costa Rica|Guanacaste|658[0n]|BOLD:AAB7888
Achaea ablunarisDHJ02|[5179]|BLPCA536-08|Costa Rica|Guanacaste|626[3n]|BOLD:AAB7888
Achaea ablunaris|[5180]|LOCRC627-09|Costa Rica|Guanacaste|658[0n]|BOLD:AAB7888
Achaea ablunaris|[5181]|LOCRC630-09|Costa Rica|Alajuela|658[0n]|BOLD:AAB7888
Achaea ablunarisDHJ02|[5182]|MHMYL3162-11|Costa Rica||658[0n]|BOLD:AAB7888
Achaea ablunaris|[5183]|LOCRC629-09|Costa Rica|Guanacaste|658[0n]|BOLD:AAB7888
Achaea ablunarisDHJ02|[5184]|MHMYL3178-11|Costa Rica||658[0n]|BOLD:AAB7888
Achaea ablunaris|[5185]|CNCLB2233-14|Puerto Rico|Mayaguez|658[0n]|BOLD:AAB7888
Mimophisma forbesi|[5186]|CNCLB1655-14|United States|Florida|658[0n]|BOLD:ACP4536
Mimophisma delunaris|[5187]|GMARI007-14|Argentina|Misiones|531[0n]|
Mimophisma delunaris|[5188]|GMMCM001-15|Mexico|Jalisco|576[0n]|BOLD:AAB2848
Mimophisma delunaris|[5189]|GMART116-14|Argentina|Misiones|582[0n]|BOLD:AAB2848
Mimophisma delunaris|[5190]|GMMCL098-14|Mexico|Jalisco|582[0n]|BOLD:AAB2848
Mimophisma delunaris|[5191]|BLPCN044-08|Costa Rica|Guanacaste|658[0n]|BOLD:AAB2848
Mimophisma delunaris|[5192]|LYPIE862-09|Mexico|Yucatan|658[0n]|BOLD:AAB2848
[illegible]

Mimophisma delunaris|[5190]|GMMCL098-14|Mexico|Jalisco|582[0n]|BOLD:AAB2848
Mimophisma delunaris|[5191]|BLPCN044-08|Costa Rica|Guanacaste|658[0n]|BOLD:AAB2848
Mimophisma delunaris|[5192]|LYPIE862-09|Mexico|Yucatan|658[0n]|BOLD:AAB2848
Mimophisma delunaris|[5193]|MHMYL3163-11|Costa Rica||658[0n]|BOLD:AAB2848
Mimophisma delunaris|[5194]|MHAUB327-05|Costa Rica|Guanacaste|630[0n]|BOLD:AAB2848
Mimophisma delunaris|[5195]|BLPAG398-07|Costa Rica|Guanacaste|621[2n]|BOLD:AAB2848
Mimophisma delunaris|[5196]|BLPCA235-08|Costa Rica|Guanacaste|658[0n]|BOLD:AAB2848
Mimophisma delunaris|[5197]|BLPCD652-08|Costa Rica|Guanacaste|658[0n]|BOLD:AAB2848
Mimophisma delunaris|[5198]|MHMXZ758-09|Costa Rica||658[0n]|BOLD:AAB2848
Mimophisma delunaris|[5199]|LYPIE860-09|Mexico|Yucatan|658[0n]|BOLD:AAB2848
Mimophisma delunaris|[5200]|MHMYL3110-11|Costa Rica||658[0n]|BOLD:AAB2848
Mimophisma delunaris|[5201]|BLPED1749-12|Costa Rica|Guanacaste|658[0n]|BOLD:AAB2848
Mimophisma delunaris|[5202]|MHMXQ685-08|Costa Rica|Guanacaste|658[0n]|BOLD:AAB2848
Mimophisma delunaris|[5203]|HKONB003-08|United States|Texas|658[0n]|BOLD:AAB2848
Mimophisma delunaris|[5204]|MXBLP172-11|Mexico|Jalisco|658[0n]|BOLD:AAB2848
Mimophisma delunaris|[5205]|CNCLB1953-14|Guatemala||658[0n]|BOLD:AAB2848
Mimophisma delunaris|[5206]|GMMCO008-15|Mexico|Jalisco|559[1n]|BOLD:AAB2848
Mimophisma delunaris|[5207]|LYNYM047-09|Mexico|Yucatan|573[0n]|BOLD:AAB2848
Mimophisma delunaris|[5208]|MHAUG692-07|Costa Rica|Guanacaste|630[0n]|BOLD:AAB2848
Mimophisma delunaris|[5209]|BLPBH479-07|Costa Rica|Guanacaste|645[0n]|BOLD:AAB2848
Mimophisma delunaris|[5210]|LYNYM080-09|Mexico|Yucatan|611[0n]|BOLD:AAB2848
Mimophisma delunaris|[5211]|BLPEE6640-14|Costa Rica||658[1n]|BOLD:AAB2848
Mimophisma delunaris|[5212]|BLPEF4975-13|Costa Rica|Guanacaste|658[0n]|BOLD:AAB2848
Mimophisma delunaris|[5213]|LYNYM046-09|Mexico|Yucatan|658[4n]|BOLD:AAB2848
Mimophisma delunaris|[5214]|LYPIE635-09|Mexico|Yucatan|658[0n]|BOLD:AAB2848
Mimophisma delunaris|[5215]|LYPIE337-09|Mexico|Quintana Roo|658[0n]|BOLD:AAB2848
Mimophisma delunaris|[5216]|LYNYM312-09|Mexico|Quintana Roo|658[0n]|BOLD:AAB2848
Mimophisma delunaris|[5217]|LYNYM192-09|Mexico|Yucatan|658[0n]|BOLD:AAB2848
Mimophisma delunaris|[5218]|LYNYM191-09|Mexico|Yucatan|658[0n]|BOLD:AAB2848
Mimophisma delunaris|[5219]|LYNYM188-09|Mexico|Yucatan|658[0n]|BOLD:AAB2848
Mimophisma delunaris|[5220]|LYNYM182-09|Mexico|Yucatan|658[0n]|BOLD:AAB2848
Mimophisma delunaris|[5221]|LYNYM150-09|Mexico|Yucatan|658[0n]|BOLD:AAB2848
Mimophisma delunaris|[5222]|LYNYM147-09|Mexico|Yucatan|658[0n]|BOLD:AAB2848
Mimophisma delunaris|[5223]|LYNYM146-09|Mexico|Yucatan|658[0n]|BOLD:AAB2848
Mimophisma delunaris|[5224]|LYNYM062-09|Mexico|Yucatan|658[0n]|BOLD:AAB2848
Mimophisma delunaris|[5225]|LYNYM057-09|Mexico|Yucatan|658[0n]|BOLD:AAB2848
Mimophisma delunaris|[5226]|LYNYM052-09|Mexico|Yucatan|658[0n]|BOLD:AAB2848
Mimophisma delunaris|[5227]|LYNYM051-09|Mexico|Yucatan|658[0n]|BOLD:AAB2848
Mimophisma delunaris|[5228]|LYPAP609-09|Mexico|Yucatan|658[0n]|BOLD:AAB2848
Mimophisma delunaris|[5229]|HKONB007-08|United States|Texas|658[0n]|BOLD:AAB2848
Mimophisma delunaris|[5230]|HKONB006-08|United States|Texas|658[0n]|BOLD:AAB2848
Mimophisma delunaris|[5231]|HKONB005-08|United States|Texas|658[0n]|BOLD:AAB2848
Mimophisma delunaris|[5232]|HKONB004-08|United States|Texas|658[0n]|BOLD:AAB2848
Mimophisma delunaris|[5233]|LPYPB226-08|Mexico|Quintana Roo|658[0n]|BOLD:AAB2848
Mimophisma delunaris|[5234]|BLPCD650-08|Costa Rica|Guanacaste|658[0n]|BOLD:AAB2848
Mimophisma delunaris|[5235]|BLPCB415-08|Costa Rica|Alajuela|658[0n]|BOLD:AAB2848
Mimophisma delunaris|[5236]|BLPBB221-07|Costa Rica|Guanacaste|658[0n]|BOLD:AAB2848
Mimophisma delunaris|[5237]|LPMX264-07|Mexico|Campeche|658[0n]|BOLD:AAB2848
Mimophisma delunaris|[5238]|MHAUC214-06|Costa Rica|Guanacaste|658[0n]|BOLD:AAB2848
Mimophisma delunaris|[5239]|MHMXQ686-08|Costa Rica|Guanacaste|593[0n]|BOLD:AAB2848
Mimophisma delunaris|[5240]|MHAUB326-05|Costa Rica|Guanacaste|570[0n]|BOLD:AAB2848
Mimophisma delunaris|[5241]|LYNYM049-09|Mexico|Yucatan|622[0n]|BOLD:AAB2848
Mimophisma delunaris|[5242]|LYNYM058-09|Mexico|Yucatan|622[0n]|BOLD:AAB2848
Mimophisma delunaris|[5243]|LYPIE647-09|Mexico|Yucatan|623[0n]|BOLD:AAB2848
Mimophisma delunaris|[5244]|LYPIE881-09|Mexico|Yucatan|658[0n]|BOLD:AAB2848
Mimophisma delunaris|[5245]|LYPIE884-09|Mexico|Yucatan|658[0n]|BOLD:AAB2848
Mimophisma delunaris|[5246]|LYPIE913-09|Mexico|Yucatan|658[0n]|BOLD:AAB2848
Mimophisma delunaris|[5247]|MHMYL3225-11|Costa Rica||658[0n]|BOLD:AAB2848
Mimophisma delunaris|[5248]|MHMYN098-11|Costa Rica||658[0n]|BOLD:AAB2848
Mimophisma delunaris|[5249]|MHMYQ085-12|Costa Rica||658[0n]|BOLD:AAB2848
Mimophisma delunaris|[5250]|MHMYQ086-12|Costa Rica||658[0n]|BOLD:AAB2848
Mimophisma delunaris|[5251]|MHMYQ087-12|Costa Rica||658[0n]|BOLD:AAB2848
Mimophisma delunaris|[5252]|MHMYQ088-12|Costa Rica||658[0n]|BOLD:AAB2848
Mimophisma delunaris|[5253]|MHMYQ089-12|Costa Rica||658[0n]|BOLD:AAB2848
Mimophisma delunaris|[5254]|MHMYQ090-12|Costa Rica||658[0n]|BOLD:AAB2848
Mimophisma delunaris|[5255]|MHMYQ091-12|Costa Rica||658[0n]|BOLD:AAB2848
Mimophisma delunaris|[5256]|CQR228-13|Mexico|Quintana Roo|658[0n]|BOLD:AAB2848
Mimophisma delunaris|[5257]|CQR522-13|Mexico|Quintana Roo|658[0n]|BOLD:AAB2848
Mimophisma delunaris|[5258]|BLPEE6641-14|Costa Rica||658[0n]|BOLD:AAB2848
Mimophisma delunaris|[5259]|CNCLB1926-14|United States|Texas|658[0n]|BOLD:AAB2848
Mimophisma delunaris|[5260]|CNCLB1927-14|United States|Texas|658[0n]|BOLD:AAB2848
Mimophisma delunaris|[5261]|CNCLB1928-14|Guatemala||658[0n]|BOLD:AAB2848
Mimophisma delunaris|[5262]|GMMCO007-15|Mexico|Jalisco|583[0n]|BOLD:AAB2848
Mimophisma delunaris|[5263]|GMMCO009-15|Mexico|Jalisco|544[0n]|BOLD:AAB2848
Mimophisma delunaris|[5264]|GMARI006-14|Argentina|Misiones|576[1n]|BOLD:AAB2848
Mimophisma delunaris|[5265]|GMARR793-14|Argentina|Misiones|673[0n]|BOLD:AAB2848
Mimophisma delunaris|[5266]|ARMOT692-12|Argentina|Misiones|658[0n]|BOLD:AAB2848
Mimophisma delunaris|[5267]|ARMOT703-12|Argentina|Misiones|658[0n]|BOLD:AAB2848
Mimophisma delunaris|[5268]|ARMOT723-12|Argentina|Misiones|658[0n]|BOLD:AAB2848
Mimophisma delunaris|[5269]|ARMOT815-12|Argentina|Misiones|658[0n]|BOLD:AAB2848
Mimophisma delunaris|[5270]|MOTAR193-12|Argentina|Misiones|658[0n]|BOLD:AAB2848
Mimophisma delunaris|[5271]|LNAUS5592-13|Paraguay|Paraguari|658[0n]|BOLD:AAB2848
Mimophisma delunaris|[5272]|ARMOT697-12|Argentina|Misiones|658[0n]|BOLD:AAB2848
Mimophisma delunaris|[5273]|GMAGA033-15|Argentina|Misiones|658[0n]|BOLD:AAB2848
Mimophisma delunaris|[5274]|GMAGC485-15|Argentina|Misiones|582[0n]|BOLD:AAB2848
Melipotis indomita|[5275]|AWCLB620-11|United States|Arizona|631[0n]|BOLD:AAD4517
Melipotis indomita|[5276]|BBLOB595-11|United States|Arizona|658[0n]|BOLD:AAD4517
Melipotis indomita|[5277]|IAWLB533-11|United States|Arizona|658[0n]|BOLD:AAD4517
Melipotis indomita|[5278]|BBLOE1423-12|United States|Arizona|658[0n]|BOLD:AAD4517
Melipotis indomita|[5279]|BBLOE1440-12|United States|Texas|658[0n]|BOLD:AAD4517
Melipotis indomita|[5280]|QUNOB022-08|United States|Texas|658[0n]|BOLD:AAD4517
Melipotis indomita|[5281]|BBLOD889-11|United States|California|658[0n]|BOLD:AAD4517
Melipotis indomita|[5282]|BBLOD926-11|United States|Texas|658[0n]|BOLD:AAD4517
Melipotis indomita|[5283]|BBLOB1053-11|United States|Arizona|658[0n]|BOLD:AAD4517
Melipotis indomita|[5284]|BBLOD1288-11|United States|Texas|624[0n]|BOLD:AAD4517
Melipotis indomita|[5285]|BBLOD1270-11|United States|Texas|658[0n]|BOLD:AAD4517
Melipotis indomita|[5286]|BBLOD1277-11|United States|Texas|658[0n]|BOLD:AAD4517
Melipotis indomita|[5287]|RDNMG913-08|United States|Arizona|658[0n]|BOLD:AAD4517
Melipotis indomita|[5288]|LPOKD787-10|United States|Oklahoma|658[0n]|BOLD:AAD4517
Melipotis indomita|[5289]|BBLOD876-11|United States|California|658[0n]|BOLD:AAD4517
Melipotis indomita|[5290]|BBLOD882-11|United States|California|658[0n]|BOLD:AAD4517
Melipotis indomita|[5291]|BBLOD1269-11|United States|Texas|658[0n]|BOLD:AAD4517
Melipotis indomita|[5292]|BBLOD1278-11|United States|Texas|658[0n]|BOLD:AAD4517

Melipotis indomita|[5290]|BBLOD882-11|United States|California|658[0n]|BOLD:AAD4517
Melipotis indomita|[5291]|BBLOD1269-11|United States|Texas|658[0n]|BOLD:AAD4517
Melipotis indomita|[5292]|BBLOD1278-11|United States|Texas|658[0n]|BOLD:AAD4517
Melipotis indomita|[5293]|BBLOD1298-11|United States|Texas|658[0n]|BOLD:AAD4517
Melipotis indomita|[5294]|BBLOE1451-12|United States|Texas|658[0n]|BOLD:AAD4517
Melipotis indomita|[5295]|BBLSW343-09|United States|Arizona|658[0n]|BOLD:AAD4517
Melipotis indomita|[5296]|ABNCC265-07|United States|Texas|635[1n]|BOLD:AAD4517
Melipotis indomita|[5297]|IAWLB534-11|United States|Arizona|658[0n]|BOLD:AAD4517
Melipotis indomita|[5298]|USLEP297-10|United States|Texas|658[0n]|BOLD:AAD4517
Melipotis indomita|[5299]|RDNMG912-08|United States|Arizona|658[0n]|BOLD:AAD4517
Melipotis indomita|[5300]|IAWL067-09|United States|Arizona|658[0n]|BOLD:AAD4517
Melipotis indomita|[5301]|BBLSX679-09|United States|Arizona|658[0n]|BOLD:AAD4517
Melipotis indomita|[5302]|BBLOD883-11|United States|California|658[0n]|BOLD:AAD4517
Melipotis indomita|[5303]|BBLOD1658-11|United States|Texas|658[0n]|BOLD:AAD4517
Melipotis indomita|[5304]|BBLOE1449-12|United States|Texas|658[0n]|BOLD:AAD4517
Melipotis indomita|[5305]|LPOKC613-09|United States|Oklahoma|658[0n]|BOLD:AAD4517
Melipotis indomita|[5306]|ABNCC266-07|United States|Texas|636[0n]|BOLD:AAD4517
Melipotis indomita|[5307]|HKONB113-08|United States|Texas|658[0n]|BOLD:AAD4517
Melipotis indomita|[5308]|BBLSW525-09|United States|Oklahoma|658[0n]|BOLD:AAD4517
Melipotis indomita|[5309]|BBLOB594-11|United States|Arizona|658[0n]|BOLD:AAD4517
Melipotis indomita|[5310]|BBLOB610-11|United States|Texas|658[0n]|BOLD:AAD4517
Melipotis indomita|[5311]|BBLOB681-11|United States|Texas|658[0n]|BOLD:AAD4517
Melipotis indomita|[5312]|BBLOC223-11|United States|Arizona|658[0n]|BOLD:AAD4517
Melipotis indomita|[5313]|BBLOC224-11|United States|Arizona|658[0n]|BOLD:AAD4517
Melipotis indomita|[5314]|BBLOD194-11|United States|Texas|658[0n]|BOLD:AAD4517
Melipotis indomita|[5315]|BBLOD885-11|United States|California|658[0n]|BOLD:AAD4517
Melipotis indomita|[5316]|BBLOD1271-11|United States|Texas|658[0n]|BOLD:AAD4517
Melipotis indomita|[5317]|BBLOD1273-11|United States|Texas|658[0n]|BOLD:AAD4517
Melipotis indomita|[5318]|BBLOD1287-11|United States|Texas|658[0n]|BOLD:AAD4517
Melipotis indomita|[5319]|BBLOE1418-12|United States|Texas|658[0n]|BOLD:AAD4517
Melipotis indomita|[5320]|BBLOE1427-12|United States|Arizona|658[0n]|BOLD:AAD4517
Melipotis indomita|[5321]|BBLOE1464-12|United States|Texas|658[0n]|BOLD:AAD4517
Melipotis indomita|[5322]|LPOKD784-10|United States|Oklahoma|658[0n]|BOLD:AAD4517
Melipotis indomita|[5323]|BBLSX678-09|United States|Arizona|658[0n]|BOLD:AAD4517
Melipotis indomita|[5324]|BBLOC1413-11|United States|Texas|658[0n]|BOLD:AAD4517
Melipotis indomita|[5325]|BBLOD897-11|United States|California|658[0n]|BOLD:AAD4517
Melipotis indomita|[5326]|BBLOE1457-12|United States|Texas|658[0n]|BOLD:AAD4517
Melipotis indomita|[5327]|BBLOD1283-11|United States|Texas|658[0n]|BOLD:AAD4517
Melipotis indomita|[5328]|BBLOE1976-12|United States|Texas|658[0n]|BOLD:AAD4517
Melipotis indomita|[5329]|IAWL090-09|United States|Arizona|658[0n]|BOLD:AAD4517
Melipotis indomita|[5330]|IAWL092-09|United States|Arizona|658[0n]|BOLD:AAD4517
Melipotis indomita|[5331]|LPOKB153-09|United States|Oklahoma|658[0n]|BOLD:AAD4517
Melipotis indomita|[5332]|BBLSW704-09|United States|Texas|658[0n]|BOLD:AAD4517
Melipotis indomita|[5333]|BBLSW802-09|United States|Oklahoma|658[0n]|BOLD:AAD4517
Melipotis indomita|[5334]|BBLOC1029-11|United States|Texas|658[0n]|BOLD:AAD4517
Melipotis indomita|[5335]|BBLOD145-11|United States|Texas|658[0n]|BOLD:AAD4517
Melipotis indomita|[5336]|BBLOD1662-11|United States|Texas|658[0n]|BOLD:AAD4517
Melipotis indomita|[5337]|BBLOD1698-11|United States|Texas|658[0n]|BOLD:AAD4517
Melipotis indomita|[5338]|BBLOE1450-12|United States|Texas|658[0n]|BOLD:AAD4517
Melipotis indomita|[5339]|BBLOE1463-12|United States|Texas|658[0n]|BOLD:AAD4517
Melipotis indomita|[5340]|BBLOE1977-12|United States|Texas|658[0n]|BOLD:AAD4517
Melipotis indomita|[5341]|BBLOE1978-12|United States|Texas|658[0n]|BOLD:AAD4517
Melipotis indomita|[5342]|BBLOC609-11|United States|Arizona|658[0n]|BOLD:AAD4517
Melipotis indomita|[5343]|BBLOE1992-12|United States|Arizona|658[0n]|BOLD:AAD4517
Melipotis indomita|[5344]|CMAZA877-12|United States|Arizona|658[0n]|BOLD:AAD4517
Melipotis nigrobasis|[5345]|HKONB012-08|United States|Texas|658[0n]|BOLD:AAE9172
Melipotis nigrobasis|[5346]|HKONB013-08|United States|Texas|658[0n]|BOLD:AAE9172
Melipotis nigrobasis|[5347]|HKONB014-08|United States|Texas|658[0n]|BOLD:AAE9172
Melipotis nigrobasis|[5348]|QUNOB528-09|United States|Texas|658[0n]|BOLD:AAE9172
Melipotis cellaris|[5349]|HKONB115-08|United States|Texas|658[0n]|BOLD:AAA6973
Melipotis cellaris|[5350]|ABNCC267-07|United States|Texas|643[0n]|BOLD:AAA6973
Melipotis cellaris|[5351]|HKONB114-08|United States|Texas|658[0n]|BOLD:AAA6973
Melipotis cellaris|[5352]|ABNCC268-07|United States|Texas|652[0n]|BOLD:AAA6973
Melipotis cellaris|[5353]|QUNOB529-09|United States|Texas|658[0n]|BOLD:AAA6973
Melipotis cellaris|[5354]|LMEMB129-09|United States|Texas|658[0n]|BOLD:AAA6973
Melipotis cellaris|[5355]|LMEMB130-09|United States|Texas|658[0n]|BOLD:AAA6973
Melipotis januaris|[5356]|RDNML312-13|United States|Florida|658[0n]|BOLD:AAK7255
Melipotis januaris|[5357]|LOCRH104-11|Costa Rica|Limon|619[0n]|BOLD:AAK7255
Melipotis januaris|[5358]|LOCRH108-11|Costa Rica|Limon|637[0n]|BOLD:AAK7255
Melipotis januaris|[5359]|HKONB477-09|United States|Louisiana|658[0n]|BOLD:AAK7255
Melipotis januaris|[5360]|USLEP1114-10|United States|Florida|658[0n]|BOLD:AAK7255
Melipotis januaris|[5361]|GWOST456-11|Dominican Republic||658[0n]|BOLD:AAK7255
Melipotis januaris|[5362]|GWOST457-11|Dominican Republic||658[0n]|BOLD:AAK7255
Melipotis januaris|[5363]|RDNML313-13|United States|Florida|658[0n]|BOLD:AAK7255
Melipotis januaris|[5364]|LNAUS3917-13|United States|Florida|658[0n]|BOLD:AAK7255
Melipotis fasciolaris|[5365]|ABNCC264-07|United States|Arizona|649[0n]|BOLD:AAA8829
Melipotis fasciolaris|[5366]|HKONS351-08|United States|Florida|658[0n]|BOLD:AAA8829
Melipotis fasciolaris|[5367]|LPYPB269-08|Mexico|Quintana Roo|658[0n]|BOLD:AAA8829
Melipotis fasciolaris|[5368]|GWOSZ773-11|Peru|Tumbes|658[0n]|BOLD:AAA8829
Melipotis fasciolaris|[5369]|HKONB126-08|United States|Texas|658[0n]|BOLD:AAA8829
Melipotis fasciolaris|[5370]|HKONB127-08|United States|Texas|658[0n]|BOLD:AAA8829
Melipotis fasciolaris|[5371]|HKONB128-08|United States|Texas|658[0n]|BOLD:AAA8829
Melipotis fasciolaris|[5372]|LPYPB099-08|Mexico|Campeche|658[0n]|BOLD:AAA8829
Melipotis fasciolaris|[5373]|HKONB125-08|United States|Texas|658[0n]|BOLD:AAA8829
Melipotis fasciolaris|[5374]|HKONB129-08|United States|Texas|658[0n]|BOLD:AAA8829
Melipotis fasciolaris|[5375]|HKONB124-08|United States|Texas|658[0n]|BOLD:AAA8829
Melipotis fasciolaris|[5376]|GWOST448-11|Dominican Republic||658[0n]|BOLD:AAA8829
Melipotis fasciolaris|[5377]|GWOSZ772-11|Peru|Tumbes|658[0n]|BOLD:AAA8829
Melipotis fasciolaris|[5378]|RDNMD530-06|United States|Florida|658[0n]|BOLD:AAA8829
Melipotis fasciolaris|[5379]|ABNCC263-07|United States|Arizona|636[0n]|BOLD:AAA8829
Melipotis fasciolaris|[5380]|IAWL091-09|United States|Arizona|658[0n]|BOLD:AAA8829
Melipotis fasciolaris|[5381]|CMAZA943-12|United States|Arizona|658[0n]|BOLD:AAA8829
Bulia mexicana|[5382]|QUNOD006-10|United States|Texas|658[0n]|BOLD:AAI2789
Bulia deducta|[5383]|AWCLB589-11|United States|Arizona|561[3n]|BOLD:AAB5985
Bulia deducta|[5384]|LMEMB136-09|United States|Texas|658[0n]|BOLD:AAB5985
Bulia deducta|[5385]|LPOKC729-09|United States|Oklahoma|658[0n]|BOLD:AAB5985
Bulia deducta|[5386]|LPOKB1022-09|United States|Oklahoma|658[0n]|BOLD:AAB5985
Bulia deducta|[5387]|BBLOD924-11|United States|Texas|658[0n]|BOLD:AAB5985
Bulia deducta|[5388]|BBLOD1285-11|United States|Texas|658[0n]|BOLD:AAB5985
Bulia deducta|[5389]|BBLSW828-09|United States|Texas|658[0n]|BOLD:AAB5985
Bulia deducta|[5390]|IAWLB538-11|United States|Arizona|658[0n]|BOLD:AAB5985
Bulia deducta|[5391]|BBLOD1097-11|United States|Texas|658[0n]|BOLD:AAB5985
Bulia deducta|[5392]|BBLOE1390-12|United States|Texas|658[0n]|BOLD:AAB5985

Bulia deducta|[5390]|IAWLB538-11|United States|Arizona|658[0n]|BOLD:AAB5985
Bulia deducta|[5391]|BBLOD1097-11|United States|Texas|658[0n]|BOLD:AAB5985
Bulia deducta|[5392]|BBLOE1390-12|United States|Texas|658[0n]|BOLD:AAB5985
Bulia deducta|[5393]|LMEMB139-09|United States|Texas|658[0n]|BOLD:AAB5985
Bulia deducta|[5394]|BBLSW280-09|United States|Arizona|658[0n]|BOLD:AAB5985
Bulia deducta|[5395]|AWCLB615-11|United States|Arizona|617[0n]|BOLD:AAB5985
Bulia deducta|[5396]|AWCLB485-11|United States|Arizona|658[0n]|BOLD:AAB5985
Bulia deducta|[5397]|BBLSX767-09|United States|Texas|658[0n]|BOLD:AAB5985
Bulia deducta|[5398]|CMAZA348-10|United States|Arizona|658[0n]|BOLD:AAB5985
Bulia deducta|[5399]|AWCLB612-11|United States|Arizona|658[0n]|BOLD:AAB5985
Bulia deducta|[5400]|BBLOD877-11|United States|California|658[0n]|BOLD:AAB5985
Bulia deducta|[5401]|BBLOE1986-12|United States|Texas|658[0n]|BOLD:AAB5985
Bulia deducta|[5402]|BBLOB693-11|United States|Texas|658[0n]|BOLD:AAB5985
Bulia deducta|[5403]|ABNCC291-07|United States|Texas|653[0n]|BOLD:AAB5985
Bulia deducta|[5404]|BBLOD1612-11|United States|Texas|658[0n]|BOLD:AAB5985
Bulia deducta|[5405]|BBLOD1299-11|United States|Texas|658[0n]|BOLD:AAB5985
Bulia deducta|[5406]|BBLOD1286-11|United States|Texas|658[0n]|BOLD:AAB5985
Bulia deducta|[5407]|BBLOD1284-11|United States|Texas|658[0n]|BOLD:AAB5985
Bulia deducta|[5408]|BBLOD1279-11|United States|Texas|658[0n]|BOLD:AAB5985
Bulia deducta|[5409]|BBLOD1276-11|United States|Texas|658[0n]|BOLD:AAB5985
Bulia deducta|[5410]|BBLOD1275-11|United States|Texas|658[0n]|BOLD:AAB5985
Bulia deducta|[5411]|BBLOD1274-11|United States|Texas|658[0n]|BOLD:AAB5985
Bulia deducta|[5412]|BBLOD1272-11|United States|Texas|658[0n]|BOLD:AAB5985
Bulia deducta|[5413]|BBLOD1106-11|United States|Texas|658[0n]|BOLD:AAB5985
Bulia deducta|[5414]|BBLOD1102-11|United States|Texas|658[0n]|BOLD:AAB5985
Bulia deducta|[5415]|BBLOD1100-11|United States|Texas|658[0n]|BOLD:AAB5985
Bulia deducta|[5416]|BBLOD1098-11|United States|Texas|658[0n]|BOLD:AAB5985
Bulia deducta|[5417]|BBLOD1096-11|United States|Texas|658[0n]|BOLD:AAB5985
Bulia deducta|[5418]|BBLOD925-11|United States|Texas|658[0n]|BOLD:AAB5985
Bulia deducta|[5419]|BBLOD921-11|United States|Texas|658[0n]|BOLD:AAB5985
Bulia deducta|[5420]|BBLOD919-11|United States|Texas|658[0n]|BOLD:AAB5985
Bulia deducta|[5421]|BBLOD918-11|United States|Texas|658[0n]|BOLD:AAB5985
Bulia deducta|[5422]|BBLOD917-11|United States|Texas|658[0n]|BOLD:AAB5985
Bulia deducta|[5423]|BBLOD899-11|United States|California|658[0n]|BOLD:AAB5985
Bulia deducta|[5424]|BBLOD880-11|United States|California|658[0n]|BOLD:AAB5985
Bulia deducta|[5425]|BBLOD875-11|United States|California|658[0n]|BOLD:AAB5985
Bulia deducta|[5426]|BBLOD518-11|United States|Texas|658[0n]|BOLD:AAB5985
Bulia deducta|[5427]|BBLOD142-11|United States|Texas|658[0n]|BOLD:AAB5985
Bulia deducta|[5428]|BBLOD140-11|United States|Texas|658[0n]|BOLD:AAB5985
Bulia deducta|[5429]|BBLOD137-11|United States|Texas|658[0n]|BOLD:AAB5985
Bulia deducta|[5430]|BBLOC1995-11|United States|Texas|658[0n]|BOLD:AAB5985
Bulia deducta|[5431]|BBLOC1986-11|United States|Texas|658[0n]|BOLD:AAB5985
Bulia deducta|[5432]|BBLOC1649-11|United States|Arizona|658[0n]|BOLD:AAB5985
Bulia deducta|[5433]|BBLOC666-11|United States|Texas|658[0n]|BOLD:AAB5985
Bulia deducta|[5434]|BBLOC665-11|United States|Texas|658[0n]|BOLD:AAB5985
Bulia deducta|[5435]|BBLOC634-11|United States|Arizona|658[0n]|BOLD:AAB5985
Bulia deducta|[5436]|BBLOC633-11|United States|Arizona|658[0n]|BOLD:AAB5985
Bulia deducta|[5437]|BBLOC627-11|United States|Arizona|658[0n]|BOLD:AAB5985
Bulia deducta|[5438]|BBLOC626-11|United States|Arizona|658[0n]|BOLD:AAB5985
Bulia deducta|[5439]|BBLOC272-11|United States|Arizona|658[0n]|BOLD:AAB5985
Bulia deducta|[5440]|BBLOC234-11|United States|Arizona|658[0n]|BOLD:AAB5985
Bulia deducta|[5441]|BBLOC227-11|United States|Arizona|658[0n]|BOLD:AAB5985
Bulia deducta|[5442]|BBLOC226-11|United States|Arizona|658[0n]|BOLD:AAB5985
Bulia deducta|[5443]|BBLOC154-11|United States|Arizona|658[0n]|BOLD:AAB5985
Bulia deducta|[5444]|BBLOC152-11|United States|Arizona|658[0n]|BOLD:AAB5985
Bulia deducta|[5445]|BBLOC150-11|United States|Arizona|658[0n]|BOLD:AAB5985
Bulia deducta|[5446]|BBLOB1528-11|United States|Texas|658[0n]|BOLD:AAB5985
Bulia deducta|[5447]|BBLOB711-11|United States|Texas|658[0n]|BOLD:AAB5985
Bulia deducta|[5448]|BBLOB686-11|United States|Texas|658[0n]|BOLD:AAB5985
Bulia deducta|[5449]|BBLOB674-11|United States|Texas|658[0n]|BOLD:AAB5985
Bulia deducta|[5450]|BBLOB023-11|United States|Arizona|658[0n]|BOLD:AAB5985
Bulia deducta|[5451]|BBLOB019-11|United States|Arizona|658[0n]|BOLD:AAB5985
Bulia deducta|[5452]|BBLOB011-11|United States|Arizona|658[0n]|BOLD:AAB5985
Bulia deducta|[5453]|IAWLB541-11|United States|Arizona|658[0n]|BOLD:AAB5985
Bulia deducta|[5454]|IAWLB540-11|United States|Arizona|658[0n]|BOLD:AAB5985
Bulia deducta|[5455]|IAWLB539-11|United States|Arizona|658[0n]|BOLD:AAB5985
Bulia deducta|[5456]|IAWLB537-11|United States|Arizona|658[0n]|BOLD:AAB5985
Bulia deducta|[5457]|IAWLB536-11|United States|Arizona|658[0n]|BOLD:AAB5985
Bulia deducta|[5458]|LTOLB1362-11|United States|Texas|658[0n]|BOLD:AAB5985
Bulia deducta|[5459]|AWCLB618-11|United States|Arizona|658[0n]|BOLD:AAB5985
Bulia deducta|[5460]|AWCLB601-11|United States|Arizona|658[0n]|BOLD:AAB5985
Bulia deducta|[5461]|AWCLB600-11|United States|Arizona|658[0n]|BOLD:AAB5985
Bulia deducta|[5462]|AWCLB594-11|United States|Arizona|658[0n]|BOLD:AAB5985
Bulia deducta|[5463]|AWCLB568-11|United States|Arizona|658[0n]|BOLD:AAB5985
Bulia deducta|[5464]|AWCLB507-11|United States|Arizona|658[0n]|BOLD:AAB5985
Bulia deducta|[5465]|AWCLB492-11|United States|Arizona|658[0n]|BOLD:AAB5985
Bulia deducta|[5466]|AWCLB476-11|United States|Arizona|658[0n]|BOLD:AAB5985
Bulia deducta|[5467]|AWCLB414-10|United States|Arizona|658[0n]|BOLD:AAB5985
Bulia deducta|[5468]|AWCLB412-10|United States|Arizona|658[0n]|BOLD:AAB5985
Bulia deducta|[5469]|CMAZA796-10|United States|Arizona|658[0n]|BOLD:AAB5985
Bulia deducta|[5470]|USLEP1172-10|United States|Arizona|658[0n]|BOLD:AAB5985
Bulia deducta|[5471]|USLEP1171-10|United States|Arizona|658[0n]|BOLD:AAB5985
Bulia deducta|[5472]|USLEP1167-10|United States|Arizona|658[0n]|BOLD:AAB5985
Bulia deducta|[5473]|USLEP1166-10|United States|Texas|658[0n]|BOLD:AAB5985
Bulia deducta|[5474]|USLEP952-10|United States|Arizona|658[0n]|BOLD:AAB5985
Bulia deducta|[5475]|CMAZA281-09|United States|Arizona|658[0n]|BOLD:AAB5985
Bulia deducta|[5476]|BBLWU090-09|United States|Colorado|658[0n]|BOLD:AAB5985
Bulia deducta|[5477]|BBLSZ195-09|United States|Texas|658[0n]|BOLD:AAB5985
Bulia deducta|[5478]|BBLSZ179-09|United States|Texas|658[0n]|BOLD:AAB5985
Bulia deducta|[5479]|BBLSZ178-09|United States|Texas|658[0n]|BOLD:AAB5985
Bulia deducta|[5480]|BBLSZ177-09|United States|Texas|658[0n]|BOLD:AAB5985
Bulia deducta|[5481]|BBLSY917-09|United States|Texas|658[0n]|BOLD:AAB5985
Bulia deducta|[5482]|BBLSY915-09|United States|Texas|658[0n]|BOLD:AAB5985
Bulia deducta|[5483]|BBLSY914-09|United States|Texas|658[0n]|BOLD:AAB5985
Bulia deducta|[5484]|BBLSY909-09|United States|Texas|658[0n]|BOLD:AAB5985
Bulia deducta|[5485]|BBLSY906-09|United States|Texas|658[0n]|BOLD:AAB5985
Bulia deducta|[5486]|BBLSY838-09|United States|Arizona|658[0n]|BOLD:AAB5985
Bulia deducta|[5487]|BBLSY732-09|United States|Arizona|658[0n]|BOLD:AAB5985
Bulia deducta|[5488]|BBLSY676-09|United States|Arizona|658[0n]|BOLD:AAB5985
Bulia deducta|[5489]|BBLSY665-09|United States|Arizona|658[0n]|BOLD:AAB5985
Bulia deducta|[5490]|BBLSY294-09|United States|Arizona|658[0n]|BOLD:AAB5985
Bulia deducta|[5491]|BBLSX747-09|United States|Arizona|658[0n]|BOLD:AAB5985
Bulia deducta|[5492]|BBLSX183-09|United States|Texas|658[0n]|BOLD:AAB5985

Bulia deducta|[5490]|BBLSY294-09|United States|Arizona|658[0n]|BOLD:AAB5985
Bulia deducta|[5491]|BBLSX747-09|United States|Arizona|658[0n]|BOLD:AAB5985
Bulia deducta|[5492]|BBLSX183-09|United States|Texas|658[0n]|BOLD:AAB5985
Bulia deducta|[5493]|BBLSY230-09|United States|Texas|658[0n]|BOLD:AAB5985
Bulia deducta|[5494]|BBLSY125-09|United States|Texas|658[0n]|BOLD:AAB5985
Bulia deducta|[5495]|BBLSW324-09|United States|Arizona|658[0n]|BOLD:AAB5985
Bulia deducta|[5496]|BBLSW313-09|United States|Arizona|658[0n]|BOLD:AAB5985
Bulia deducta|[5497]|BBLSW080-09|United States|Texas|658[0n]|BOLD:AAB5985
Bulia deducta|[5498]|BBLSW057-09|United States|Texas|658[0n]|BOLD:AAB5985
Bulia deducta|[5499]|BBLSW056-09|United States|Texas|658[0n]|BOLD:AAB5985
Bulia deducta|[5500]|BBLSW031-09|United States|Texas|658[0n]|BOLD:AAB5985
Bulia deducta|[5501]|LPOKB131-09|United States|Oklahoma|658[0n]|BOLD:AAB5985
Bulia deducta|[5502]|LMEMB140-09|United States|Texas|658[0n]|BOLD:AAB5985
Bulia deducta|[5503]|IAWL114-09|United States|Arizona|658[0n]|BOLD:AAB5985
Bulia deducta|[5504]|IAWL111-09|United States|California|658[0n]|BOLD:AAB5985
Bulia deducta|[5505]|IAWL110-09|United States|Arizona|658[0n]|BOLD:AAB5985
Bulia deducta|[5506]|IAWL109-09|United States|Arizona|658[0n]|BOLD:AAB5985
Bulia deducta|[5507]|IAWL108-09|United States|Arizona|658[0n]|BOLD:AAB5985
Bulia deducta|[5508]|IAWL106-09|United States|Arizona|658[0n]|BOLD:AAB5985
Bulia deducta|[5509]|BBLOD1280-11|United States|Texas|658[0n]|BOLD:AAB5985
Bulia deducta|[5510]|BBLOD879-11|United States|California|658[0n]|BOLD:AAB5985
Bulia deducta|[5511]|BBLOC886-11|United States|Arizona|658[0n]|BOLD:AAB5985
Bulia deducta|[5512]|BBLOC1414-11|United States|Texas|648[0n]|BOLD:AAB5985
Bulia deducta|[5513]|BBLSY829-09|United States|Arizona|643[0n]|BOLD:AAB5985
Bulia deducta|[5514]|BBLSY295-09|United States|Arizona|642[0n]|BOLD:AAB5985
Bulia deducta|[5515]|LPOKB1023-09|United States|Oklahoma|658[0n]|BOLD:AAB5985
Bulia deducta|[5516]|BBLOD1081-11|United States|Texas|658[0n]|BOLD:AAB5985
Bulia deducta|[5517]|ABNCC289-07|United States|Texas|643[0n]|BOLD:AAB5985
Bulia deducta|[5518]|ABNCC290-07|United States|Texas|642[0n]|BOLD:AAB5985
Bulia deducta|[5519]|ABNCC287-07|United States|Texas|641[0n]|BOLD:AAB5985
Bulia deducta|[5520]|ABNCC288-07|United States|Texas|638[0n]|BOLD:AAB5985
Bulia deducta|[5521]|BBLSZ181-09|United States|Texas|648[0n]|BOLD:AAB5985
Bulia deducta|[5522]|LPOKD666-10|United States|Oklahoma|658[0n]|BOLD:AAB5985
Bulia deducta|[5523]|BBLOD127-11|United States|California|636[0n]|BOLD:AAB5985
Bulia deducta|[5524]|USLEP089-10|United States|Arizona|636[0n]|BOLD:AAB5985
Bulia deducta|[5525]|BBLSW146-09|United States|Arizona|638[0n]|BOLD:AAB5985
Bulia deducta|[5526]|USLEP1170-10|United States|Arizona|634[0n]|BOLD:AAB5985
Bulia deducta|[5527]|BBLOD1614-11|United States|Texas|581[3n]|BOLD:AAB5985
Bulia deducta|[5528]|BBLOD1664-11|United States|Texas|658[0n]|BOLD:AAB5985
Bulia deducta|[5529]|BBLOD1697-11|United States|Texas|658[0n]|BOLD:AAB5985
Bulia deducta|[5530]|BBLOE1425-12|United States|Arizona|658[0n]|BOLD:AAB5985
Bulia deducta|[5531]|BBLOE1441-12|United States|Texas|658[0n]|BOLD:AAB5985
Bulia deducta|[5532]|BBLOE1442-12|United States|Texas|658[0n]|BOLD:AAB5985
Bulia deducta|[5533]|BBLOE1456-12|United States|Texas|658[0n]|BOLD:AAB5985
Bulia deducta|[5534]|BBLOE1459-12|United States|Texas|658[0n]|BOLD:AAB5985
Bulia deducta|[5535]|BBLOE1460-12|United States|Texas|658[0n]|BOLD:AAB5985
Bulia deducta|[5536]|BBLOE1469-12|United States|Texas|658[0n]|BOLD:AAB5985
Bulia deducta|[5537]|BBLOE1707-12|United States|Texas|658[0n]|BOLD:AAB5985
Bulia deducta|[5538]|BBLOE1710-12|United States|Texas|658[0n]|BOLD:AAB5985
Bulia deducta|[5539]|BBLOE1984-12|United States|Texas|658[0n]|BOLD:AAB5985
Bulia deducta|[5540]|BBLOE1988-12|United States|Texas|658[0n]|BOLD:AAB5985
Bulia deducta|[5541]|BBLOE1991-12|United States|Texas|658[0n]|BOLD:AAB5985
Bulia deducta|[5542]|BBLOE2020-12|United States|Texas|658[0n]|BOLD:AAB5985
Bulia deducta|[5543]|BBLOE2021-12|United States|Texas|658[0n]|BOLD:AAB5985
Bulia deducta|[5544]|BBLOE2022-12|United States|Texas|658[0n]|BOLD:AAB5985
Bulia deducta|[5545]|CMAZA1021-12|United States|Arizona|658[0n]|BOLD:AAB5985
Bulia sp.|[5546]|AWCLB232-10|United States|Arizona|658[0n]|BOLD:AAB5986
Bulia sp.|[5547]|AWCLB433-10|United States|Arizona|658[0n]|BOLD:AAB5986
Bulia sp.|[5548]|IAWL112-09|United States|California|658[0n]|BOLD:AAB5986
Bulia sp.|[5549]|AWCLB264-10|United States|Arizona|658[0n]|BOLD:AAB5986
Bulia sp.|[5550]|AWCLB249-10|United States|Arizona|655[0n]|BOLD:AAB5986
Bulia sp.|[5551]|AWCLB261-10|United States|Arizona|658[0n]|BOLD:AAB5986
Bulia sp.|[5552]|AWCLB241-10|United States|Arizona|658[0n]|BOLD:AAB5986
Bulia sp.|[5553]|AWCLB235-10|United States|Arizona|658[0n]|BOLD:AAB5986
Bulia sp.|[5554]|AWCLB231-10|United States|Arizona|658[0n]|BOLD:AAB5986
Bulia sp.|[5555]|IAWL113-09|United States|Arizona|658[0n]|BOLD:AAB5986
Bulia sp.|[5556]|IAWL107-09|United States|California|658[0n]|BOLD:AAB5986
Bulia sp.|[5557]|AWCLB253-10|United States|Arizona|570[0n]|BOLD:AAB5986
Bulia sp.|[5558]|AWCLB588-11|United States|Arizona|658[0n]|BOLD:AAB5986
Bulia similaris|[5559]|BBLOD100-11|United States|Texas|658[0n]|BOLD:ACE8352
Bulia similaris|[5560]|BBLOD1078-11|United States|Texas|658[0n]|BOLD:ACE8352
Bulia similaris|[5561]|LMEMB137-09|United States|Texas|658[0n]|BOLD:ACE8352
Bulia similaris|[5562]|BBLOD525-11|United States|Texas|658[0n]|BOLD:ACE8352
Bulia similaris|[5563]|BBLOD149-11|United States|Texas|658[0n]|BOLD:ACE8352
Bulia similaris|[5564]|HKONB504-09|United States|Texas|658[0n]|BOLD:ACE8352
Bulia similaris|[5565]|LMEMB138-09|United States|Texas|621[0n]|BOLD:ACE8352
Bulia similaris|[5566]|BBLOD169-11|United States|Texas|658[0n]|BOLD:ACE8352
Bulia similaris|[5567]|BBLOC1987-11|United States|Texas|658[0n]|BOLD:ACE8352
Bulia similaris|[5568]|BBLOD175-11|United States|Texas|630[0n]|BOLD:ACE8352
Bulia similaris|[5569]|BBLOE2018-12|United States|Texas|658[0n]|BOLD:ACE8352
Bulia schausi|[5570]|LNAUS5598-13|Mexico|Sonora|658[0n]|BOLD:ACK9231
Bulia schausi|[5571]|CNCLB897-14|United States|Arizona|658[0n]|BOLD:ACK9231
Latebraria amphipyroides|[5572]|QUNOD176-10|United States|Texas|658[0n]|BOLD:AAC1011
Latebraria amphipyroides|[5573]|QUNOD177-10|United States|Texas|658[0n]|BOLD:AAC1011
Latebraria amphipyroides|[5574]|LYPIE616-09|Mexico|Campeche|658[0n]|BOLD:AAC1011
Latebraria amphipyroides|[5575]|LPYPC041-08|Mexico|Yucatan|658[0n]|BOLD:AAC1011
Latebraria amphipyroides|[5576]|LYPIE916-09|Mexico|Yucatan|658[1n]|BOLD:AAC1011
Latebraria amphipyroides|[5577]|MHAUA168-05|Costa Rica|Guanacaste|564[0n]|BOLD:AAC1011
Latebraria amphipyroides|[5578]|MHAUA169-05|Costa Rica|Guanacaste|568[0n]|BOLD:AAC1011
Latebraria amphipyroides|[5579]|MHAUA170-05|Costa Rica|Guanacaste|658[0n]|BOLD:AAC1011
Latebraria amphipyroides|[5580]|MHMXE368-06|Costa Rica|Guanacaste|658[0n]|BOLD:AAC1011
Latebraria amphipyroides|[5581]|LYPAP227-09|Mexico|Quintana Roo|658[0n]|BOLD:AAC1011
Latebraria amphipyroides|[5582]|LYPAP228-09|Mexico|Quintana Roo|658[0n]|BOLD:AAC1011
Latebraria amphipyroides|[5583]|LYHES091-09|Mexico|Yucatan|658[0n]|BOLD:AAC1011
Latebraria amphipyroides|[5584]|LYHES193-09|Mexico|Yucatan|658[0n]|BOLD:AAC1011
Latebraria amphipyroides|[5585]|LYPIE925-09|Mexico|Quintana Roo|658[0n]|BOLD:AAC1011
Latebraria amphipyroides|[5586]|MHMYO354-11|Costa Rica||658[0n]|BOLD:AAC1011
Melipotis perpendicularis|[5587]|QUNOD727-11|Dominican Republic||658[0n]|BOLD:AAB8726
Melipotis perpendicularis|[5588]|LYRIO176-09|Mexico|Yucatan|628[0n]|BOLD:AAB8726
Melipotis perpendicularis|[5589]|RDNMF502-08|Costa Rica|Alajuela|658[1n]|BOLD:AAB8726
Melipotis perpendicularis|[5590]|IAWL071-09|United States|Arizona|658[0n]|BOLD:AAB8726
Melipotis perpendicularis|[5591]|IAWL104-09|United States|Arizona|658[0n]|BOLD:AAB8726
Melipotis perpendicularis|[5592]|MXBLP237-11|Mexico|Jalisco|658[0n]|BOLD:AAB8726

[illegible]
Melipotis perpendicularis|[5590]|IAWL071-09|United States|Arizona|658[0n]|BOLD:AAB8726
Melipotis perpendicularis|[5591]|IAWL104-09|United States|Arizona|658[0n]|BOLD:AAB8726
Melipotis perpendicularis|[5592]|MXBLP237-11|Mexico|Jalisco|658[0n]|BOLD:AAB8726
Melipotis perpendicularis|[5593]|BBLOD502-11|United States|Texas|658[0n]|BOLD:AAB8726
Melipotis perpendicularis|[5594]|RDNMF501-08|United States|Arizona|658[0n]|BOLD:ABZ4611
Melipotis perpendicularis|[5595]|IAWL103-09|United States|Arizona|658[0n]|BOLD:ABZ4611
Melipotis perpendicularis|[5596]|IAWL077-09|United States|Arizona|658[0n]|BOLD:ABZ4611
Melipotis perpendicularis|[5597]|IAWL093-09|United States|Arizona|658[0n]|BOLD:ABZ4611
Melipotis perpendicularis|[5598]|QUNOD173-10|United States|Texas|658[0n]|BOLD:ABZ4611
Melipotis perpendicularis|[5599]|IAWL094-09|United States|Arizona|658[0n]|BOLD:ABZ4611
Melipotis perpendicularis|[5600]|IAWL095-09|United States|Arizona|658[0n]|BOLD:ABZ4611
Melipotis perpendicularis|[5601]|RDNMJ519-11|United States|Arizona|658[0n]|BOLD:ABZ4611
Melipotis perpendicularis|[5602]|IAWL105-09|United States|Arizona|658[0n]|BOLD:ABZ4611
Melipotis perpendicularis|[5603]|ABNCC324-07|United States|Arizona|627[2n]|BOLD:ABZ4611
Melipotis perpendicularis|[5604]|IAWL102-09|United States|Arizona|658[0n]|BOLD:ABZ4611
Melipotis perpendicularis|[5605]|IAWL101-09|United States|Arizona|658[0n]|BOLD:ABZ4611
Melipotis perpendicularis|[5606]|CMAZA033-09|United States|Arizona|658[0n]|BOLD:ABZ4611
Melipotis perpendicularis|[5607]|CMAZA885-12|United States|Arizona|658[0n]|BOLD:ABZ4611
Melipotis perpendicularis|[5608]|RDNMF503-08|United States|Florida|609[0n]|BOLD:ABZ4611
Melipotis perpendicularis|[5609]|RDNML354-13|United States|Florida|658[0n]|BOLD:ABZ4611
Melipotis perpendicularis|[5610]|RDNML355-13|United States|Florida|658[0n]|BOLD:ABZ4611
Melipotis perpendicularis|[5611]|CNCLB2992-14|United States|Florida|658[0n]|BOLD:ABZ4611
Helia agna|[5612]|ABNCC511-07|United States||616[0n]|BOLD:AAB8282
Helia agna|[5613]|ABNCC512-07|United States||542[0n]|BOLD:AAB8282
Helia agna|[5614]|HKONB065-08|United States|Texas|658[0n]|BOLD:AAB8282
Helia agna|[5615]|HKONB066-08|United States|Texas|658[0n]|BOLD:AAB8282
Helia agna|[5616]|HKONB067-08|United States|Texas|658[0n]|BOLD:AAB8282
Helia agna|[5617]|HKONB068-08|United States|Texas|658[0n]|BOLD:AAB8282
Helia agna|[5618]|HKONB069-08|United States|Texas|658[0n]|BOLD:AAB8282
Melipotis famelica|[5619]|HKONS185-08|United States|Texas|658[0n]|BOLD:AAB3373
Melipotis famelica|[5620]|HKONB011-08|United States|Texas|658[0n]|BOLD:AAB3373
Melipotis famelica|[5621]|HKONB123-08|United States|Texas|658[0n]|BOLD:AAB3373
Melipotis famelica|[5622]|BBLOD506-11|United States|Texas|658[0n]|BOLD:AAB3373
Melipotis contorta|[5623]|USLEP1230-10|United States|Florida|658[0n]|BOLD:AAL1704
Melipotis contorta|[5624]|BBLOB1263-11|United States|Florida|658[0n]|BOLD:AAL1704
Melipotis contorta|[5625]|USLEP1227-10|United States|Florida|658[0n]|BOLD:AAL1704
Melipotis contorta|[5626]|USLEP1228-10|United States|Florida|658[0n]|BOLD:AAL1704
Melipotis contorta|[5627]|RDNML314-13|United States|Florida|658[0n]|BOLD:AAL1704
Melipotis prolata|[5628]|USLEP294-10|United States|Florida|658[0n]|BOLD:AAN6806
Melipotis prolata|[5629]|RDNML315-13|United States|Florida|658[0n]|BOLD:AAN6806
Forsebia cinis|[5630]|RDNM755-05|United States|Arizona|502[0n]|BOLD:AAC0949
Forsebia cinis|[5631]|USLEP388-10|United States|Arizona|658[0n]|BOLD:AAC0949
Forsebia cinis|[5632]|BBLSY826-09|United States|Arizona|658[0n]|BOLD:AAC0949
Forsebia cinis|[5633]|AWCLB595-11|United States|Arizona|604[0n]|BOLD:AAC0949
Forsebia cinis|[5634]|LOCBB112-06|United States|California|658[0n]|BOLD:AAC0949
Forsebia cinis|[5635]|BBLSW314-09|United States|Arizona|580[0n]|BOLD:AAC0949
Forsebia cinis|[5636]|USLEP129-10|United States|Arizona|658[0n]|BOLD:AAC0949
Forsebia cinis|[5637]|BBLOC630-11|United States|Arizona|658[0n]|BOLD:AAC0949
Forsebia cinis|[5638]|BBLOD122-11|United States|California|658[0n]|BOLD:AAC0949
Forsebia cinis|[5639]|BBLOE1422-12|United States|Arizona|632[0n]|BOLD:AAC0949
Forsebia cinis|[5640]|BBLSW226-09|United States|Arizona|658[0n]|BOLD:AAC0949
Forsebia cinis|[5641]|AWCLB631-11|United States|Arizona|658[0n]|BOLD:AAC0949
Forsebia cinis|[5642]|LOCBF2314-13|United States|California|564[0n]|BOLD:AAC0949
Forsebia cinis|[5643]|LPOKA672-09|United States|Oklahoma|625[0n]|BOLD:AAC0949
Forsebia cinis|[5644]|BBLOC616-11|United States|Arizona|658[0n]|BOLD:AAC0949
Forsebia cinis|[5645]|BBLOC225-11|United States|Arizona|658[0n]|BOLD:AAC0949
Forsebia cinis|[5646]|BBLOC126-11|United States|California|658[0n]|BOLD:AAC0949
Forsebia cinis|[5647]|BBLOB784-11|United States|Arizona|658[0n]|BOLD:AAC0949
Forsebia cinis|[5648]|BBLOB569-11|United States|Arizona|658[0n]|BOLD:AAC0949
Forsebia cinis|[5649]|AWCLB598-11|United States|Arizona|658[0n]|BOLD:AAC0949
Forsebia cinis|[5650]|AWCLB495-11|United States|Arizona|658[0n]|BOLD:AAC0949
Forsebia cinis|[5651]|AWCLB323-10|United States|Arizona|658[0n]|BOLD:AAC0949
Forsebia cinis|[5652]|CMAZA396-10|United States|Arizona|658[0n]|BOLD:AAC0949
Forsebia cinis|[5653]|USLEP708-10|United States|Arizona|658[0n]|BOLD:AAC0949
Forsebia cinis|[5654]|BBLSZ068-09|United States|Arizona|658[0n]|BOLD:AAC0949
Forsebia cinis|[5655]|BBLSY663-09|United States|Arizona|658[0n]|BOLD:AAC0949
Forsebia cinis|[5656]|BBLSX681-09|United States|Arizona|658[0n]|BOLD:AAC0949
Forsebia cinis|[5657]|BBLSY047-09|United States|Arizona|658[0n]|BOLD:AAC0949
Forsebia cinis|[5658]|BBLSY044-09|United States|Arizona|658[0n]|BOLD:AAC0949
Forsebia cinis|[5659]|BBLSW193-09|United States|Arizona|658[0n]|BOLD:AAC0949
Forsebia cinis|[5660]|LMEMB135-09|United States|Arizona|658[0n]|BOLD:AAC0949
Forsebia cinis|[5661]|LMEMB134-09|United States|Arizona|658[0n]|BOLD:AAC0949
Forsebia cinis|[5662]|LMEMB132-09|United States|Arizona|658[0n]|BOLD:AAC0949
Forsebia cinis|[5663]|IAWL117-09|United States|Arizona|658[0n]|BOLD:AAC0949
Forsebia cinis|[5664]|IAWL115-09|United States|Arizona|658[0n]|BOLD:AAC0949
Forsebia cinis|[5665]|HKONB254-09|United States|Texas|658[0n]|BOLD:AAC0949
Forsebia cinis|[5666]|LOCBB113-06|United States|California|658[0n]|BOLD:AAC0949
Forsebia cinis|[5667]|AWCLB296-10|United States|Arizona|658[0n]|BOLD:AAC0949
Forsebia cinis|[5668]|AWCLB493-11|United States|Arizona|637[0n]|BOLD:AAC0949
Forsebia cinis|[5669]|ABNCC286-07|United States|Arizona|643[0n]|BOLD:AAC0949
Forsebia cinis|[5670]|BBLSY725-09|United States|Arizona|658[0n]|BOLD:AAC0949
Forsebia cinis|[5671]|BBLOB015-11|United States|Arizona|658[0n]|BOLD:AAC0949
Forsebia cinis|[5672]|BBLOC1641-11|United States|Arizona|622[0n]|BOLD:AAC0949
Forsebia cinis|[5673]|BBLOD123-11|United States|California|658[0n]|BOLD:AAC0949
Forsebia cinis|[5674]|BBLOD126-11|United States|California|658[0n]|BOLD:AAC0949
Forsebia cinis|[5675]|BBLOD129-11|United States|California|658[0n]|BOLD:AAC0949
Forsebia cinis|[5676]|BBLOE1421-12|United States|Arizona|658[0n]|BOLD:AAC0949
Forsebia cinis|[5677]|ABNCC285-07|United States|Arizona|610[1n]|BOLD:AAC0949
Forsebia cinis|[5678]|BBLSY043-09|United States|Arizona|658[0n]|BOLD:AAC0949
Forsebia cinis|[5679]|LOCBF2315-13|United States|California|552[0n]|BOLD:AAC0949
Forsebia cinis|[5680]|LOCBF2317-13|United States|California|603[0n]|BOLD:AAC0949
Forsebia cinis|[5681]|RDNM754-05|United States|Arizona|658[0n]|BOLD:AAC0949
Forsebia cinis|[5682]|AWCLB509-11|United States|Arizona|658[0n]|BOLD:AAC0949
Forsebia cinis|[5683]|AWCLB525-11|United States|Arizona|658[0n]|BOLD:AAC0949
Forsebia cinis|[5684]|AWCLB297-10|United States|Arizona|605[1n]|BOLD:AAC0949
Forsebia cinis|[5685]|BBLOE1426-12|United States|Arizona|658[0n]|BOLD:AAC0949
Forsebia cinis|[5686]|BBLOB016-11|United States|Arizona|658[0n]|BOLD:AAC0949
Forsebia cinis|[5687]|BBLSY628-09|United States|Arizona|579[0n]|BOLD:AAC0949
Forsebia cinis|[5688]|BBLOD124-11|United States|California|658[0n]|BOLD:AAC0949
Forsebia cinis|[5689]|BBLOB008-11|United States|Arizona|658[0n]|BOLD:AAC0949
Forsebia cinis|[5690]|AWCLB508-11|United States|Arizona|658[0n]|BOLD:AAC0949
Forsebia cinis|[5691]|USLEP1165-10|United States|Arizona|658[0n]|BOLD:AAC0949
Forsebia cinis|[5692]|BBLSZ069-09|United States|Arizona|658[0n]|BOLD:AAC0949

Forsebia cinis|[5689]|[illegible]|United States|Arizona|658[0n]|BOLD:AAC0949
Forsebia cinis|[5690]|AWCLB508-11|United States|Arizona|658[0n]|BOLD:AAC0949
Forsebia cinis|[5691]|USLEP1165-10|United States|Arizona|658[0n]|BOLD:AAC0949
Forsebia cinis|[5692]|BBLSZ069-09|United States|Arizona|658[0n]|BOLD:AAC0949
Forsebia cinis|[5693]|BBLSY965-09|United States|Arizona|658[0n]|BOLD:AAC0949
Forsebia cinis|[5694]|BBLSY730-09|United States|Arizona|658[0n]|BOLD:AAC0949
Forsebia cinis|[5695]|BBLSY726-09|United States|Arizona|658[0n]|BOLD:AAC0949
Forsebia cinis|[5696]|BBLSW318-09|United States|Arizona|658[0n]|BOLD:AAC0949
Forsebia cinis|[5697]|BBLSW190-09|United States|Arizona|658[0n]|BOLD:AAC0949
Forsebia cinis|[5698]|LOCBB109-06|United States|California|658[0n]|BOLD:AAC0949
Forsebia cinis|[5699]|BBLOE1735-12|United States|Arizona|658[0n]|BOLD:AAC0949
Forsebia cinis|[5700]|LOCBF2318-13|United States|California|603[0n]|BOLD:AAC0949
Forsebia cinis|[5701]|LOCBF2319-13|United States|California|603[0n]|BOLD:AAC0949
Drasteria hudsonica|[5702]|LPAB042-08|Canada|Alberta|658[0n]|BOLD:AAB3338
Drasteria hudsonica|[5703]|LPAB293-08|Canada|Alberta|658[0n]|BOLD:AAB3338
Drasteria hudsonica|[5704]|LPABB295-08|Canada|Alberta|658[0n]|BOLD:AAB3338
Drasteria hudsonica|[5705]|LPABB576-08|Canada|Alberta|658[0n]|BOLD:AAB3338
Drasteria hudsonica|[5706]|LPABC988-09|Canada|Alberta|658[0n]|BOLD:AAB3338
Drasteria hudsonica|[5707]|BBLPB327-10|Canada|Alberta|658[0n]|BOLD:AAB3338
Drasteria hudsonica|[5708]|BBLPB325-10|Canada|British Columbia|658[0n]|BOLD:AAB3338
Drasteria hudsonica|[5709]|LPABC642-09|Canada|Alberta|658[0n]|BOLD:AAB3338
Drasteria hudsonica|[5710]|BBLPB329-10|Canada|British Columbia|658[0n]|BOLD:AAB3338
Drasteria hudsonica|[5711]|LPABB280-08|Canada|Alberta|658[0n]|BOLD:AAB3338
Drasteria hudsonica|[5712]|BBLPB331-10|Canada|British Columbia|658[0n]|BOLD:AAB3338
Drasteria hudsonica|[5713]|LPMN899-08|Canada|Alberta|658[0n]|BOLD:AAB3338
Drasteria hudsonica|[5714]|LPAB043-08|Canada|Alberta|658[0n]|BOLD:AAB3338
Drasteria hudsonica|[5715]|LOWCE348-06|Canada|British Columbia|656[0n]|BOLD:AAB3338
Drasteria hudsonica|[5716]|BBLPB328-10|Canada|Alberta|658[0n]|BOLD:AAB3338
Drasteria hudsonica|[5717]|BBLPB326-10|Canada|British Columbia|658[0n]|BOLD:AAB3338
Drasteria hudsonica|[5718]|BBLPB324-10|Canada|British Columbia|658[0n]|BOLD:AAB3338
Drasteria hudsonica|[5719]|LBCG3441-09|Canada|British Columbia|658[0n]|BOLD:AAB3338
Drasteria hudsonica|[5720]|LPABB626-08|Canada|Alberta|658[0n]|BOLD:AAB3338
Drasteria hudsonica|[5721]|LPABB401-08|Canada|Alberta|658[0n]|BOLD:AAB3338
Drasteria hudsonica|[5722]|LPABB377-08|Canada|Alberta|658[0n]|BOLD:AAB3338
Drasteria hudsonica|[5723]|LPABB264-08|Canada|Alberta|658[0n]|BOLD:AAB3338
Drasteria hudsonica|[5724]|LPABB201-08|Canada|Alberta|658[0n]|BOLD:AAB3338
Drasteria hudsonica|[5725]|LPAB052-08|Canada|Alberta|658[0n]|BOLD:AAB3338
Drasteria hudsonica|[5726]|LOWCE087-06|Canada|British Columbia|658[0n]|BOLD:AAB3338
Drasteria hudsonica|[5727]|RDNM751-05|Canada|British Columbia|658[0n]|BOLD:AAB3338
Drasteria hudsonica|[5728]|LPMN952-08|Canada|Alberta|658[0n]|BOLD:AAB3338
Drasteria hudsonica|[5729]|LOWCE350-06|Canada|British Columbia|592[0n]|BOLD:AAB3338
Drasteria hudsonica|[5730]|LALPA848-11|Canada|British Columbia|658[0n]|BOLD:AAB3338
Drasteria hudsonica|[5731]|RDNM752-05|Canada|Saskatchewan|658[0n]|BOLD:ACJ8972
Drasteria hudsonica|[5732]|QUNOC659-13|Canada|Manitoba|658[0n]|BOLD:ACJ8972
Drasteria hudsonica|[5733]|LMEMB187-09|United States|Wyoming|657[0n]|BOLD:ACF5308
Drasteria hudsonica seposita|[5734]|ABNCC051-07|United States|Utah|635[0n]|BOLD:ACF5308
Drasteria hudsonica|[5735]|RDNM753-05|United States|Wyoming|658[0n]|BOLD:ACF5308
Drasteria hudsonica seposita|[5736]|ABNCC052-07|United States|Utah|650[0n]|BOLD:ACF5308
Drasteria hudsonica|[5737]|LMEMB189-09|United States|Wyoming|657[0n]|BOLD:ACF5308
Drasteria maculosa|[5738]|RDNM750-05|United States|Nevada|658[1n]|BOLD:ACF5308
Drasteria maculosa|[5739]|QUNOE218-12|United States|California|658[0n]|BOLD:ACF5308
Drasteria maculosa|[5740]|RDNMH763-09|United States|Nevada|658[0n]|BOLD:ACF5308
Drasteria maculosa|[5741]|CNCLB1341-14|United States|Nevada|658[0n]|BOLD:ACF5308
Drasteria grandirena|[5742]|ABNCC308-07|United States|Arkansas|612[0n]|BOLD:AAC3386
Drasteria grandirena|[5743]|LNC927-06|United States|North Carolina|658[0n]|BOLD:AAC3386
Drasteria grandirena|[5744]|LNC948-06|United States|North Carolina|605[0n]|BOLD:AAC3386
Drasteria grandirena|[5745]|HKONS432-08|United States|Florida|658[0n]|BOLD:AAC3386
Drasteria grandirena|[5746]|MMNA115-08|United States|Georgia|642[0n]|BOLD:AAC3386
Drasteria grandirena|[5747]|LMEMB194-09|United States|Alabama|612[0n]|BOLD:AAC3386
Drasteria grandirena|[5748]|MMNA114-08|United States|Georgia|658[0n]|BOLD:AAC3386
Drasteria grandirena|[5749]|HKONS433-08|United States|Florida|658[0n]|BOLD:AAC3386
Drasteria grandirena|[5750]|LMEMB193-09|United States|Alabama|621[0n]|BOLD:AAC3386
Drasteria grandirena|[5751]|LMEMB195-09|United States|Georgia|658[0n]|BOLD:AAC3386
Drasteria grandirena|[5752]|LNC947-06|United States|North Carolina|573[0n]|BOLD:AAC3386
Drasteria grandirena|[5753]|ABNCC309-07|United States|Texas|642[0n]|BOLD:AAC3386
Drasteria grandirena|[5754]|BBLCU191-09|United States|Michigan|658[0n]|BOLD:AAC3386
Drasteria grandirena|[5755]|BBLCU203-09|United States|Michigan|658[0n]|BOLD:AAC3386
Drasteria fumosa|[5756]|RDNMG282-08|United States|Texas|658[0n]|BOLD:AAA6830
Drasteria fumosa|[5757]|HKONB518-09|United States|Texas|656[0n]|BOLD:AAA6830
Drasteria fumosa|[5758]|LOCBD048-06|United States|California|658[0n]|BOLD:AAA6830
Drasteria fumosa|[5759]|LOCBD151-06|United States|California|658[0n]|BOLD:AAA6830
Drasteria fumosa|[5760]|LOCBD442-06|United States|California|657[0n]|BOLD:AAA6830
Drasteria fumosa|[5761]|LOCBD150-06|United States|California|658[0n]|BOLD:AAA6830
Drasteria fumosa|[5762]|LOCBE229-06|United States|California|648[0n]|BOLD:AAA6830
Drasteria fumosa|[5763]|ABNCC045-07|United States|Utah|605[1n]|BOLD:AAA6830
Drasteria fumosa|[5764]|LOCBE223-06|United States|California|658[0n]|BOLD:AAA6830
Drasteria fumosa|[5765]|LOCBE114-06|United States|California|658[0n]|BOLD:AAA6830
Drasteria fumosa|[5766]|LOCBD149-06|United States|California|658[0n]|BOLD:AAA6830
Drasteria fumosa|[5767]|LOCBD047-06|United States|California|658[0n]|BOLD:AAA6830
Drasteria fumosa|[5768]|LOCBE330-06|United States|California|658[0n]|BOLD:AAA6830
Drasteria fumosa|[5769]|LOCBC795-06|United States|California|622[0n]|BOLD:AAA6830
Drasteria fumosa|[5770]|LOCBE331-06|United States|California|568[0n]|BOLD:AAA6830
Drasteria fumosa|[5771]|LOCBE329-06|United States|California|593[0n]|BOLD:AAA6830
Drasteria fumosa|[5772]|LOCBE332-06|United States|California|584[0n]|BOLD:AAA6830
Drasteria fumosa|[5773]|LOCBE338-06|United States|California|658[0n]|BOLD:AAA6830
Drasteria fumosa|[5774]|LOCBE339-06|United States|California|595[0n]|BOLD:AAA6830
Drasteria fumosa|[5775]|ABNCC010-07|United States|California|610[0n]|BOLD:AAA6830
Drasteria fumosa|[5776]|JBAZ067-09|United States|California|658[0n]|BOLD:AAA6830
Drasteria fumosa|[5777]|LOCBE224-06|United States|California|658[0n]|BOLD:AAA6830
Drasteria fumosa|[5778]|LOCBE227-06|United States|California|646[0n]|BOLD:AAA6830
Drasteria fumosa|[5779]|LOCBE228-06|United States|California|617[1n]|BOLD:AAA6830
Drasteria fumosa|[5780]|LOCBD147-06|United States|California|658[0n]|BOLD:AAA6830
Drasteria fumosa|[5781]|ABNCC009-07|United States|California|632[1n]|BOLD:AAA6830
Drasteria fumosa|[5782]|LOCBE231-06|United States|California|621[0n]|BOLD:AAA6830
Drasteria fumosa|[5783]|ABNCC047-07|United States|Utah|638[0n]|BOLD:AAA6830
Drasteria fumosa|[5784]|LMEMB182-09|United States|Colorado|657[0n]|BOLD:AAA6830
Drasteria fumosa|[5785]|LOCBD152-06|United States|California|655[0n]|BOLD:AAA6830
Drasteria fumosa|[5786]|LOCBC792-06|United States|California|658[0n]|BOLD:AAA6830
Drasteria fumosa|[5787]|IAWL119-09|United States|California|658[0n]|BOLD:AAA6830
Drasteria fumosa|[5788]|ABNCC046-07|United States|Utah|651[0n]|BOLD:AAA6830
Drasteria fumosa|[5789]|LOCBE226-06|United States|California|646[0n]|BOLD:AAA6830
Drasteria fumosa|[5790]|LOCBE222-06|United States|California|647[0n]|BOLD:AAA6830
Drasteria fumosa|[5791]|IAWL120-09|United States|California|658[0n]|BOLD:AAA6830
Drasteria fumosa|[5792]|[illegible]

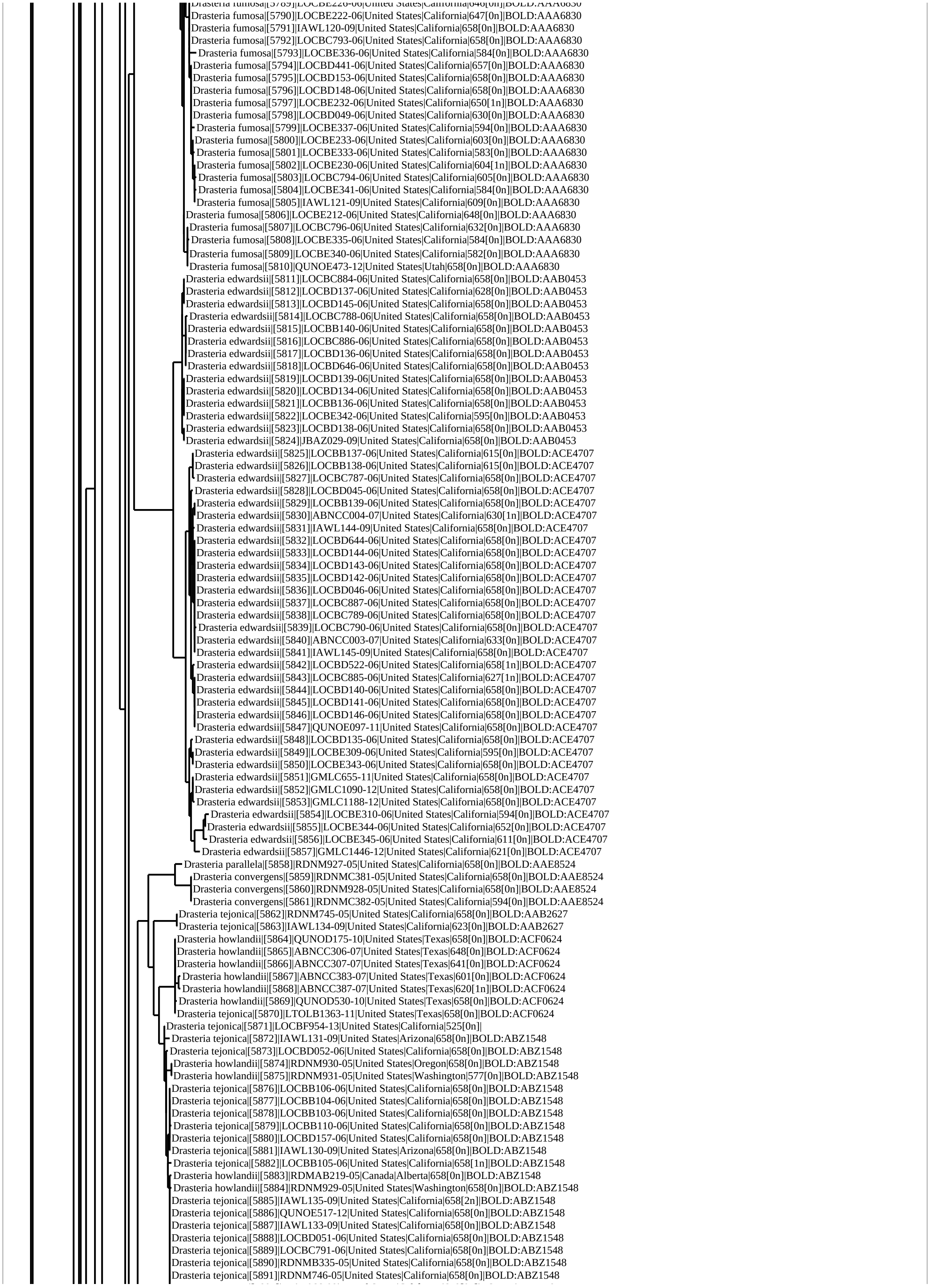
Drasteria fumosa|[5789]|LOCBE226-06|United States|California|646[0n]|BOLD:AAA6830
Drasteria fumosa|[5790]|LOCBE222-06|United States|California|647[0n]|BOLD:AAA6830
Drasteria fumosa|[5791]|IAWL120-09|United States|California|658[0n]|BOLD:AAA6830
Drasteria fumosa|[5792]|LOCBC793-06|United States|California|658[0n]|BOLD:AAA6830
Drasteria fumosa|[5793]|LOCBE336-06|United States|California|584[0n]|BOLD:AAA6830
Drasteria fumosa|[5794]|LOCBD441-06|United States|California|657[0n]|BOLD:AAA6830
Drasteria fumosa|[5795]|LOCBD153-06|United States|California|658[0n]|BOLD:AAA6830
Drasteria fumosa|[5796]|LOCBD148-06|United States|California|658[0n]|BOLD:AAA6830
Drasteria fumosa|[5797]|LOCBE232-06|United States|California|650[1n]|BOLD:AAA6830
Drasteria fumosa|[5798]|LOCBD049-06|United States|California|630[0n]|BOLD:AAA6830
Drasteria fumosa|[5799]|LOCBE337-06|United States|California|594[0n]|BOLD:AAA6830
Drasteria fumosa|[5800]|LOCBE233-06|United States|California|603[0n]|BOLD:AAA6830
Drasteria fumosa|[5801]|LOCBE333-06|United States|California|583[0n]|BOLD:AAA6830
Drasteria fumosa|[5802]|LOCBE230-06|United States|California|604[1n]|BOLD:AAA6830
Drasteria fumosa|[5803]|LOCBC794-06|United States|California|605[0n]|BOLD:AAA6830
Drasteria fumosa|[5804]|LOCBE341-06|United States|California|584[0n]|BOLD:AAA6830
Drasteria fumosa|[5805]|IAWL121-09|United States|California|609[0n]|BOLD:AAA6830
Drasteria fumosa|[5806]|LOCBE212-06|United States|California|648[0n]|BOLD:AAA6830
Drasteria fumosa|[5807]|LOCBC796-06|United States|California|632[0n]|BOLD:AAA6830
Drasteria fumosa|[5808]|LOCBE335-06|United States|California|584[0n]|BOLD:AAA6830
Drasteria fumosa|[5809]|LOCBE340-06|United States|California|582[0n]|BOLD:AAA6830
Drasteria fumosa|[5810]|QUNOE473-12|United States|Utah|658[0n]|BOLD:AAA6830
Drasteria edwardsii|[5811]|LOCBC884-06|United States|California|658[0n]|BOLD:AAB0453
Drasteria edwardsii|[5812]|LOCBD137-06|United States|California|628[0n]|BOLD:AAB0453
Drasteria edwardsii|[5813]|LOCBD145-06|United States|California|658[0n]|BOLD:AAB0453
Drasteria edwardsii|[5814]|LOCBC788-06|United States|California|658[0n]|BOLD:AAB0453
Drasteria edwardsii|[5815]|LOCBB140-06|United States|California|658[0n]|BOLD:AAB0453
Drasteria edwardsii|[5816]|LOCBC886-06|United States|California|658[0n]|BOLD:AAB0453
Drasteria edwardsii|[5817]|LOCBD136-06|United States|California|658[0n]|BOLD:AAB0453
Drasteria edwardsii|[5818]|LOCBD646-06|United States|California|658[0n]|BOLD:AAB0453
Drasteria edwardsii|[5819]|LOCBD139-06|United States|California|658[0n]|BOLD:AAB0453
Drasteria edwardsii|[5820]|LOCBD134-06|United States|California|658[0n]|BOLD:AAB0453
Drasteria edwardsii|[5821]|LOCBB136-06|United States|California|658[0n]|BOLD:AAB0453
Drasteria edwardsii|[5822]|LOCBE342-06|United States|California|595[0n]|BOLD:AAB0453
Drasteria edwardsii|[5823]|LOCBD138-06|United States|California|658[0n]|BOLD:AAB0453
Drasteria edwardsii|[5824]|JBAZ029-09|United States|California|658[0n]|BOLD:AAB0453
Drasteria edwardsii|[5825]|LOCBB137-06|United States|California|615[0n]|BOLD:ACE4707
Drasteria edwardsii|[5826]|LOCBB138-06|United States|California|615[0n]|BOLD:ACE4707
Drasteria edwardsii|[5827]|LOCBC787-06|United States|California|658[0n]|BOLD:ACE4707
Drasteria edwardsii|[5828]|LOCBD045-06|United States|California|658[0n]|BOLD:ACE4707
Drasteria edwardsii|[5829]|LOCBB139-06|United States|California|658[0n]|BOLD:ACE4707
Drasteria edwardsii|[5830]|ABNCC004-07|United States|California|630[1n]|BOLD:ACE4707
Drasteria edwardsii|[5831]|IAWL144-09|United States|California|658[0n]|BOLD:ACE4707
Drasteria edwardsii|[5832]|LOCBD644-06|United States|California|658[0n]|BOLD:ACE4707
Drasteria edwardsii|[5833]|LOCBD144-06|United States|California|658[0n]|BOLD:ACE4707
Drasteria edwardsii|[5834]|LOCBD143-06|United States|California|658[0n]|BOLD:ACE4707
Drasteria edwardsii|[5835]|LOCBD142-06|United States|California|658[0n]|BOLD:ACE4707
Drasteria edwardsii|[5836]|LOCBD046-06|United States|California|658[0n]|BOLD:ACE4707
Drasteria edwardsii|[5837]|LOCBC887-06|United States|California|658[0n]|BOLD:ACE4707
Drasteria edwardsii|[5838]|LOCBC789-06|United States|California|658[0n]|BOLD:ACE4707
Drasteria edwardsii|[5839]|LOCBC790-06|United States|California|658[0n]|BOLD:ACE4707
Drasteria edwardsii|[5840]|ABNCC003-07|United States|California|633[0n]|BOLD:ACE4707
Drasteria edwardsii|[5841]|IAWL145-09|United States|California|658[0n]|BOLD:ACE4707
Drasteria edwardsii|[5842]|LOCBD522-06|United States|California|658[1n]|BOLD:ACE4707
Drasteria edwardsii|[5843]|LOCBC885-06|United States|California|627[1n]|BOLD:ACE4707
Drasteria edwardsii|[5844]|LOCBD140-06|United States|California|658[0n]|BOLD:ACE4707
Drasteria edwardsii|[5845]|LOCBD141-06|United States|California|658[0n]|BOLD:ACE4707
Drasteria edwardsii|[5846]|LOCBD146-06|United States|California|658[0n]|BOLD:ACE4707
Drasteria edwardsii|[5847]|QUNOE097-11|United States|California|658[0n]|BOLD:ACE4707
Drasteria edwardsii|[5848]|LOCBD135-06|United States|California|658[0n]|BOLD:ACE4707
Drasteria edwardsii|[5849]|LOCBE309-06|United States|California|595[0n]|BOLD:ACE4707
Drasteria edwardsii|[5850]|LOCBE343-06|United States|California|658[0n]|BOLD:ACE4707
Drasteria edwardsii|[5851]|GMLC655-11|United States|California|658[0n]|BOLD:ACE4707
Drasteria edwardsii|[5852]|GMLC1090-12|United States|California|658[0n]|BOLD:ACE4707
Drasteria edwardsii|[5853]|GMLC1188-12|United States|California|658[0n]|BOLD:ACE4707
Drasteria edwardsii|[5854]|LOCBE310-06|United States|California|594[0n]|BOLD:ACE4707
Drasteria edwardsii|[5855]|LOCBE344-06|United States|California|652[0n]|BOLD:ACE4707
Drasteria edwardsii|[5856]|LOCBE345-06|United States|California|611[0n]|BOLD:ACE4707
Drasteria edwardsii|[5857]|GMLC1446-12|United States|California|621[0n]|BOLD:ACE4707
Drasteria parallela|[5858]|RDNM927-05|United States|California|658[0n]|BOLD:AAE8524
Drasteria convergens|[5859]|RDNMC381-05|United States|California|658[0n]|BOLD:AAE8524
Drasteria convergens|[5860]|RDNM928-05|United States|California|658[0n]|BOLD:AAE8524
Drasteria convergens|[5861]|RDNMC382-05|United States|California|594[0n]|BOLD:AAE8524
Drasteria tejonica|[5862]|RDNM745-05|United States|California|658[0n]|BOLD:AAB2627
Drasteria tejonica|[5863]|IAWL134-09|United States|California|623[0n]|BOLD:AAB2627
Drasteria howlandii|[5864]|QUNOD175-10|United States|Texas|658[0n]|BOLD:ACF0624
Drasteria howlandii|[5865]|ABNCC306-07|United States|Texas|648[0n]|BOLD:ACF0624
Drasteria howlandii|[5866]|ABNCC307-07|United States|Texas|641[0n]|BOLD:ACF0624
Drasteria howlandii|[5867]|ABNCC383-07|United States|Texas|601[0n]|BOLD:ACF0624
Drasteria howlandii|[5868]|ABNCC387-07|United States|Texas|620[1n]|BOLD:ACF0624
Drasteria howlandii|[5869]|QUNOD530-10|United States|Texas|658[0n]|BOLD:ACF0624
Drasteria tejonica|[5870]|LTOLB1363-11|United States|Texas|658[0n]|BOLD:ACF0624
Drasteria tejonica|[5871]|LOCBF954-13|United States|California|525[0n]|
Drasteria tejonica|[5872]|IAWL131-09|United States|Arizona|658[0n]|BOLD:ABZ1548
Drasteria tejonica|[5873]|LOCBD052-06|United States|California|658[0n]|BOLD:ABZ1548
Drasteria howlandii|[5874]|RDNM930-05|United States|Oregon|658[0n]|BOLD:ABZ1548
Drasteria howlandii|[5875]|RDNM931-05|United States|Washington|577[0n]|BOLD:ABZ1548
Drasteria tejonica|[5876]|LOCBB106-06|United States|California|658[0n]|BOLD:ABZ1548
Drasteria tejonica|[5877]|LOCBB104-06|United States|California|658[0n]|BOLD:ABZ1548
Drasteria tejonica|[5878]|LOCBB103-06|United States|California|658[0n]|BOLD:ABZ1548
Drasteria tejonica|[5879]|LOCBB110-06|United States|California|658[0n]|BOLD:ABZ1548
Drasteria tejonica|[5880]|LOCBD157-06|United States|California|658[0n]|BOLD:ABZ1548
Drasteria tejonica|[5881]|IAWL130-09|United States|Arizona|658[0n]|BOLD:ABZ1548
Drasteria tejonica|[5882]|LOCBB105-06|United States|California|658[1n]|BOLD:ABZ1548
Drasteria howlandii|[5883]|RDMAB219-05|Canada|Alberta|658[0n]|BOLD:ABZ1548
Drasteria howlandii|[5884]|RDNM929-05|United States|Washington|658[0n]|BOLD:ABZ1548
Drasteria tejonica|[5885]|IAWL135-09|United States|California|658[2n]|BOLD:ABZ1548
Drasteria tejonica|[5886]|QUNOE517-12|United States|California|658[0n]|BOLD:ABZ1548
Drasteria tejonica|[5887]|IAWL133-09|United States|California|658[0n]|BOLD:ABZ1548
Drasteria tejonica|[5888]|LOCBD051-06|United States|California|658[0n]|BOLD:ABZ1548
Drasteria tejonica|[5889]|LOCBC791-06|United States|California|658[0n]|BOLD:ABZ1548
Drasteria tejonica|[5890]|RDNMB335-05|United States|California|658[0n]|BOLD:ABZ1548
Drasteria tejonica|[5891]|RDNM746-05|United States|California|658[0n]|BOLD:ABZ1548
[illegible]

Drasteria tejonica|[5889]|LOCBC791-06|United States|California|658[0n]|BOLD:ABZ1548
Drasteria tejonica|[5890]|RDNMB335-05|United States|California|658[0n]|BOLD:ABZ1548
Drasteria tejonica|[5891]|RDNM746-05|United States|California|658[0n]|BOLD:ABZ1548
Drasteria tejonica|[5892]|JBAZ066-09|United States|California|658[0n]|BOLD:ABZ1548
Drasteria howlandii|[5893]|RDNMB338-05|United States|Oregon|631[0n]|BOLD:ABZ1548
Drasteria tejonica|[5894]|ABNCC013-07|United States|Arizona|635[1n]|BOLD:ABZ1548
Drasteria tejonica|[5895]|LOCBB107-06|United States|California|632[0n]|BOLD:ABZ1548
Drasteria tejonica|[5896]|LOCBE214-06|United States|California|647[3n]|BOLD:ABZ1548
Drasteria tejonica|[5897]|RDNMB336-05|United States|California|617[0n]|BOLD:ABZ1548
Drasteria tejonica|[5898]|LOCBE328-06|United States|California|595[0n]|BOLD:ABZ1548
Drasteria tejonica|[5899]|LOCBE334-06|United States|California|594[0n]|BOLD:ABZ1548
Drasteria tejonica|[5900]|RDNMB337-05|United States|Arizona|594[0n]|BOLD:ABZ1548
Drasteria tejonica|[5901]|LOCBB108-06|United States|California|618[0n]|BOLD:ABZ1548
Drasteria tejonica|[5902]|IAWL132-09|United States|Arizona|642[0n]|BOLD:ABZ1548
Drasteria tejonica|[5903]|LOCBF1324-13|United States|California|583[0n]|BOLD:ABZ1548
Drasteria tejonica|[5904]|LOCBF1326-13|United States|California|613[0n]|BOLD:ABZ1548
Drasteria pallescens|[5905]|LOCBB124-06|United States|California|658[0n]|BOLD:AAB4995
Drasteria pallescens|[5906]|IAWL076-09|United States|Arizona|658[0n]|BOLD:AAB4995
Drasteria pallescens|[5907]|BBLSX993-09|United States|Arizona|658[0n]|BOLD:AAB4995
Drasteria pallescens|[5908]|RDNMJ301-11|United States|Arizona|658[0n]|BOLD:AAB4995
Drasteria pallescens|[5909]|LOCBB115-06|United States|California|658[0n]|BOLD:AAB4995
Drasteria pallescens|[5910]|LOCBB123-06|United States|California|658[0n]|BOLD:AAB4995
Drasteria pallescens|[5911]|LOCBB125-06|United States|California|658[0n]|BOLD:AAB4995
Drasteria pallescens|[5912]|IAWL127-09|United States|California|658[0n]|BOLD:AAB4995
Drasteria pallescens|[5913]|LOCBD154-06|United States|California|658[0n]|BOLD:AAB4995
Drasteria pallescens|[5914]|LOCBB122-06|United States|California|658[0n]|BOLD:AAB4995
Drasteria pallescens|[5915]|LOCBB114-06|United States|California|658[0n]|BOLD:AAB4995
Drasteria pallescens|[5916]|ABNCC303-07|United States|Utah|632[1n]|BOLD:AAB4995
Drasteria pallescens|[5917]|IAWL128-09|United States|California|658[0n]|BOLD:AAB4995
Drasteria pallescens|[5918]|IAWL129-09|United States|Arizona|658[0n]|BOLD:AAB4995
Drasteria pallescens|[5919]|BBLSX994-09|United States|Arizona|658[0n]|BOLD:AAB4995
Drasteria pallescens|[5920]|BBLSY424-09|United States|Arizona|658[0n]|BOLD:AAB4995
Drasteria pallescens|[5921]|ABNCC012-07|United States|Utah|648[2n]|BOLD:AAB4995
Drasteria pallescens|[5922]|LMEMB183-09|United States|Arizona|657[0n]|BOLD:AAB4995
Drasteria pallescens|[5923]|ABNCC304-07|United States|Utah|630[1n]|BOLD:AAB4995
Drasteria pallescens|[5924]|LMEMB184-09|United States|Arizona|657[0n]|BOLD:AAB4995
Drasteria pallescens|[5925]|LMEMB185-09|United States|Arizona|657[0n]|BOLD:AAB4995
Drasteria pallescens|[5926]|BBLOB375-11|United States|Arizona|658[0n]|BOLD:AAB4995
Drasteria pallescens|[5927]|ABNCC302-07|United States|Texas|644[1n]|BOLD:AAB4995
Drasteria pallescens|[5928]|BBLSW727-09|United States|Texas|658[0n]|BOLD:AAB4995
Drasteria pallescens|[5929]|BBLOE1987-12|United States|Texas|658[0n]|BOLD:AAB4995
Drasteria pallescens|[5930]|LMEMB186-09|United States|Wyoming|657[0n]|BOLD:ACF2882
Drasteria pallescens|[5931]|RDNME949-08|United States|Texas|658[0n]|BOLD:ACF2882
Drasteria pallescens|[5932]|BBLSW718-09|United States|Texas|658[0n]|BOLD:ACF2882
Drasteria pallescens|[5933]|USLEP1063-10|United States|Colorado|658[0n]|BOLD:ACF2882
Drasteria pallescens|[5934]|USLEP1064-10|United States|Colorado|658[0n]|BOLD:ACF2882
Drasteria pallescens|[5935]|USLEP1065-10|United States|Colorado|658[0n]|BOLD:ACF2882
Drasteria pallescens|[5936]|BBLOE1416-12|United States|Texas|658[0n]|BOLD:ACF2882
Drasteria pallescens|[5937]|BBLOD922-11|United States|Texas|658[0n]|BOLD:ACF2882
Drasteria pallescens|[5938]|BBLOD878-11|United States|California|658[0n]|BOLD:ACF2882
Drasteria pallescens|[5939]|BBLOD1118-11|United States|Texas|658[0n]|BOLD:ACF2882
Drasteria pallescens|[5940]|BBLOE1709-12|United States|Texas|658[0n]|BOLD:ACF2882
Drasteria pallescens|[5941]|BBLOE1745-12|United States|Texas|658[0n]|BOLD:ACF2882
Drasteria pallescens|[5942]|BBLOE1996-12|United States|New Mexico|658[0n]|BOLD:ACF2882
Drasteria inepta|[5943]|HKONB554-09|United States|Texas|658[0n]|BOLD:ACF5113
Drasteria inepta|[5944]|LMEMB180-09|United States|Wyoming|658[0n]|BOLD:ACF5586
Drasteria inepta|[5945]|LMEMB181-09|United States|Wyoming|658[0n]|BOLD:ACF5586
Drasteria inepta|[5946]|BBLWU177-09|United States|Colorado|658[0n]|BOLD:ACF5586
Drasteria inepta|[5947]|BBLWU178-09|United States|Colorado|658[0n]|BOLD:ACF5586
Drasteria inepta|[5948]|BBLWU181-09|United States|Colorado|658[0n]|BOLD:ACF5586
Drasteria inepta|[5949]|ABNCC298-07|United States|Texas|640[0n]|BOLD:ACE8395
Drasteria inepta|[5950]|ABNCC299-07|United States|Texas|642[0n]|BOLD:ACE8395
Drasteria inepta|[5951]|QUNOD836-11|United States|Texas|658[0n]|BOLD:ACE8395
Drasteria inepta|[5952]|IAWL147-09|United States|Arizona|658[0n]|BOLD:ACE8396
Drasteria inepta|[5953]|ABNCC040-07|United States|Arizona|651[0n]|BOLD:AAC7458
Drasteria inepta|[5954]|CMAZA867-12|United States|Arizona|658[0n]|BOLD:AAC7458
Drasteria inepta|[5955]|ABNCC041-07|United States|Arizona|635[0n]|BOLD:AAC7458
Drasteria inepta|[5956]|IAWL146-09|United States|Arizona|658[0n]|BOLD:AAC7458
Drasteria inepta|[5957]|IAWL148-09|United States|Arizona|658[0n]|BOLD:AAC7458
Drasteria inepta|[5958]|QUNOD837-11|United States|Arizona|658[0n]|BOLD:AAC7458
Drasteria inepta|[5959]|CMAZA886-12|United States|Arizona|658[0n]|BOLD:AAC7458
Drasteria sabulosa|[5960]|LBCH5688-10|Canada|British Columbia|658[0n]|BOLD:AAA9524
Drasteria sabulosa|[5961]|LBCH5784-10|Canada|British Columbia|658[0n]|BOLD:AAA9524
Drasteria sabulosa|[5962]|LBCH5694-10|Canada|British Columbia|658[0n]|BOLD:AAA9524
Drasteria sabulosa|[5963]|LBCH5689-10|Canada|British Columbia|658[0n]|BOLD:AAA9524
Drasteria sabulosa|[5964]|LBCH5686-10|Canada|British Columbia|658[0n]|BOLD:AAA9524
Drasteria sabulosa|[5965]|LBCH5685-10|Canada|British Columbia|658[0n]|BOLD:AAA9524
Drasteria sabulosa|[5966]|LBCH5684-10|Canada|British Columbia|658[0n]|BOLD:AAA9524
Drasteria sabulosa|[5967]|LBCH5683-10|Canada|British Columbia|658[0n]|BOLD:AAA9524
Drasteria sabulosa|[5968]|LBCH5589-10|Canada|British Columbia|658[0n]|BOLD:AAA9524
Drasteria sabulosa|[5969]|LBCH5588-10|Canada|British Columbia|658[0n]|BOLD:AAA9524
Drasteria sabulosa|[5970]|LBCH5587-10|Canada|British Columbia|658[0n]|BOLD:AAA9524
Drasteria sabulosa|[5971]|LBCH5586-10|Canada|British Columbia|658[0n]|BOLD:AAA9524
Drasteria sabulosa|[5972]|LBCH5585-10|Canada|British Columbia|658[0n]|BOLD:AAA9524
Drasteria sabulosa|[5973]|LBCH5584-10|Canada|British Columbia|658[0n]|BOLD:AAA9524
Drasteria sabulosa|[5974]|LBCH5583-10|Canada|British Columbia|658[0n]|BOLD:AAA9524
Drasteria sabulosa|[5975]|LBCH5582-10|Canada|British Columbia|658[0n]|BOLD:AAA9524
Drasteria sabulosa|[5976]|LBCH5442-10|Canada|British Columbia|658[0n]|BOLD:AAA9524
Drasteria sabulosa|[5977]|LBCH5441-10|Canada|British Columbia|658[0n]|BOLD:AAA9524
Drasteria sabulosa|[5978]|LBCH5418-10|Canada|British Columbia|658[0n]|BOLD:AAA9524
Drasteria sabulosa|[5979]|LBCH5282-10|Canada|British Columbia|658[0n]|BOLD:AAA9524
Drasteria sabulosa|[5980]|LBCH5153-10|Canada|British Columbia|658[0n]|BOLD:AAA9524
Drasteria sabulosa|[5981]|LBCH5152-10|Canada|British Columbia|658[0n]|BOLD:AAA9524
Drasteria sabulosa|[5982]|LBCH5151-10|Canada|British Columbia|658[0n]|BOLD:AAA9524
Drasteria sabulosa|[5983]|LBCH5150-10|Canada|British Columbia|658[0n]|BOLD:AAA9524
Drasteria sabulosa|[5984]|LBCH5149-10|Canada|British Columbia|658[0n]|BOLD:AAA9524
Drasteria sabulosa|[5985]|LBCH5148-10|Canada|British Columbia|658[0n]|BOLD:AAA9524
Drasteria sabulosa|[5986]|LBCH5147-10|Canada|British Columbia|658[0n]|BOLD:AAA9524
Drasteria sabulosa|[5987]|LBCH5105-10|Canada|British Columbia|658[0n]|BOLD:AAA9524
Drasteria sabulosa|[5988]|LBCH5104-10|Canada|British Columbia|658[0n]|BOLD:AAA9524
Drasteria sabulosa|[5989]|LBCH5103-10|Canada|British Columbia|658[0n]|BOLD:AAA9524
Drasteria sabulosa|[5990]|LBCH5102-10|Canada|British Columbia|658[0n]|BOLD:AAA9524
Drasteria sabulosa|[5991]|LBCH5101-10|Canada|British Columbia|658[0n]|BOLD:AAA9524

Drasteria sabulosa|[5989]|LBCH5103-10|Canada|British Columbia|658[0n]|BOLD:AAA9524
Drasteria sabulosa|[5990]|LBCH5102-10|Canada|British Columbia|658[0n]|BOLD:AAA9524
Drasteria sabulosa|[5991]|LBCH5101-10|Canada|British Columbia|658[0n]|BOLD:AAA9524
Drasteria sabulosa|[5992]|LBCH5100-10|Canada|British Columbia|658[0n]|BOLD:AAA9524
Drasteria sabulosa|[5993]|LBCH5099-10|Canada|British Columbia|658[0n]|BOLD:AAA9524
Drasteria sabulosa|[5994]|LBCH5098-10|Canada|British Columbia|658[0n]|BOLD:AAA9524
Drasteria sabulosa|[5995]|RDNM921-05|United States|Oregon|658[0n]|BOLD:AAA9524
Drasteria sabulosa|[5996]|LBCH5687-10|Canada|British Columbia|658[0n]|BOLD:AAA9524
Drasteria sabulosa|[5997]|LBCH5146-10|Canada|British Columbia|658[0n]|BOLD:AAA9524
Drasteria sabulosa|[5998]|RDNM920-05|Canada|British Columbia|658[0n]|BOLD:AAA9524
Drasteria sabulosa|[5999]|RDNM922-05|United States|Washington|577[2n]|BOLD:AAA9524
Drasteria sabulosa|[6000]|LBCH5785-10|Canada|British Columbia|632[0n]|BOLD:AAA9524
Drasteria sabulosa|[6001]|LBCH5786-10|Canada|British Columbia|658[0n]|BOLD:AAA9524
Drasteria sabulosa|[6002]|LBCH5787-10|Canada|British Columbia|658[0n]|BOLD:AAA9524
Drasteria sabulosa|[6003]|LBCH5788-10|Canada|British Columbia|658[0n]|BOLD:AAA9524
Drasteria sabulosa|[6004]|LBCH5789-10|Canada|British Columbia|658[0n]|BOLD:AAA9524
Drasteria sabulosa|[6005]|LBCH5790-10|Canada|British Columbia|658[0n]|BOLD:AAA9524
Drasteria sabulosa|[6006]|LBCH5791-10|Canada|British Columbia|658[0n]|BOLD:AAA9524
Drasteria sabulosa|[6007]|LBCH5872-10|Canada|British Columbia|658[0n]|BOLD:AAA9524
Drasteria sabulosa|[6008]|LBCH5931-10|Canada|British Columbia|658[0n]|BOLD:AAA9524
Drasteria sabulosa|[6009]|LBCH6017-10|Canada|British Columbia|658[0n]|BOLD:AAA9524
Drasteria sabulosa|[6010]|LBCH6219-10|Canada|British Columbia|658[0n]|BOLD:AAA9524
Drasteria sabulosa|[6011]|QUNOD755-11|United States|Texas|658[0n]|BOLD:ACE6781
Drasteria sabulosa|[6012]|QUNOD756-11|United States|Texas|658[0n]|BOLD:ACE6781
Drasteria sabulosa|[6013]|ABNCC300-07|United States|Texas|645[0n]|BOLD:ACM4477
Drasteria sabulosa|[6014]|ABNCC301-07|United States|Texas|594[2n]|BOLD:ACM4477
Drasteria sabulosa|[6015]|QUNOD757-11|United States|Texas|658[0n]|BOLD:ACM4477
Drasteria sabulosa|[6016]|QUNOD758-11|United States|Texas|658[0n]|BOLD:ACM4477
Drasteria sabulosa|[6017]|ABNCC038-07|United States|Arizona|635[0n]|BOLD:ACM4476
Drasteria sabulosa|[6018]|ABNCC039-07|United States|Arizona|597[4n]|BOLD:ACM4476
Drasteria sabulosa|[6019]|ABNCC382-07|United States||594[0n]|BOLD:AAA9524
Drasteria sabulosa|[6020]|QUNOD759-11|United States|Texas|658[0n]|BOLD:AAA9524
Drasteria sabulosa|[6021]|QUNOD760-11|United States|Texas|658[0n]|BOLD:AAA9524
Drasteria biformata|[6022]|RDNMH759-09|United States|California|658[0n]|BOLD:ABX6605
Drasteria biformata|[6023]|RDNMH758-09|United States|California|658[0n]|BOLD:ABX6605
Drasteria biformata|[6024]|RDNMH760-09|United States|California|658[0n]|BOLD:ABX6605
Drasteria nr. biformata|[6025]|RDNMH761-09|United States|California|658[0n]|BOLD:ABX6605
Drasteria nr. biformata|[6026]|LOCBD053-06|United States|California|658[0n]|BOLD:ABX6605
Drasteria nr. biformata|[6027]|ABNCC021-07|United States|California|633[0n]|BOLD:ABX6605
Drasteria nr. biformata|[6028]|ABNCC022-07|United States|California|643[0n]|BOLD:ABX6605
Drasteria nr. biformata|[6029]|RDNMH762-09|United States|California|658[0n]|BOLD:ABX6605
Drasteria nr. biformata|[6030]|RDNMH764-09|United States|California|658[0n]|BOLD:ABX6605
Drasteria nr. biformata|[6031]|QUNOE514-12|United States|California|658[0n]|BOLD:ABX6605
Drasteria divergens|[6032]|LOCBC417-06|United States|California|658[0n]|BOLD:ABZ6755
Drasteria divergens|[6033]|RWWC538-11|United States|Washington|658[0n]|BOLD:ABZ6755
Drasteria divergens|[6034]|RDNM926-05|United States|Oregon|658[0n]|BOLD:ABZ6755
Drasteria divergens|[6035]|RDNM925-05|United States|California|658[0n]|BOLD:ABZ6755
Drasteria divergens|[6036]|RDNMC264-05|United States|California|658[0n]|BOLD:ABZ6755
Drasteria divergens|[6037]|IAWLB157-10|United States|California|658[0n]|BOLD:ABZ6755
Drasteria divergens|[6038]|IAWLB158-10|United States|California|658[0n]|BOLD:ABZ6755
Drasteria divergens|[6039]|LALPA1288-11|Canada|British Columbia|658[0n]|BOLD:ABZ6755
Drasteria divergens|[6040]|RDNMC265-05|United States|California|658[0n]|BOLD:ABZ6755
Drasteria divergens|[6041]|RDNMC263-05|United States|Washington|586[0n]|BOLD:ABZ6755
Drasteria divergens|[6042]|RDNMC262-05|United States|Washington|596[0n]|BOLD:ABZ6755
Drasteria divergens|[6043]|RDNMC268-05|United States|California|590[0n]|BOLD:ABZ6755
Drasteria divergens|[6044]|RDNMC380-05|United States|Oregon|580[0n]|BOLD:ABZ6755
Drasteria divergens|[6045]|LPMN951-08|Canada|Alberta|658[0n]|BOLD:ABZ6755
Drasteria divergens|[6046]|LPABB326-08|Canada|Alberta|658[0n]|BOLD:ABZ6755
Drasteria divergens|[6047]|QUNOE513-12|United States|Oregon|658[0n]|BOLD:ABZ6755
Drasteria divergens|[6048]|ABNCC049-07|United States|Utah|631[0n]|BOLD:ACM4330
Drasteria divergens|[6049]|ABNCC050-07|United States|Utah|647[0n]|BOLD:ACM4330
Drasteria divergens|[6050]|LPAB004-08|Canada|Alberta|658[0n]|BOLD:ACM4330
Drasteria divergens|[6051]|LOCBD156-06|United States|California|658[0n]|BOLD:ABZ6755
Drasteria divergens|[6052]|LOCBD643-06|United States|California|658[0n]|BOLD:ABZ6755
Drasteria divergens|[6053]|LOCBE213-06|United States|California|618[1n]|BOLD:ABZ6755
Drasteria divergens|[6054]|LOCBE219-06|United States|California|658[0n]|BOLD:ABZ6755
Drasteria divergens|[6055]|RDNMC378-05|United States|California|518[0n]|BOLD:ABZ6755
Drasteria divergens|[6056]|RDNMC377-05|United States|California|596[0n]|BOLD:ABZ6755
Drasteria divergens|[6057]|LOCBE220-06|United States|California|658[0n]|BOLD:ABZ6755
Drasteria divergens|[6058]|LOCBD155-06|United States|California|658[0n]|BOLD:ABZ6755
Drasteria divergens|[6059]|RDNM923-05|United States|Washington|570[2n]|BOLD:ABZ6755
Drasteria divergens|[6060]|LOCBE216-06|United States|California|648[0n]|BOLD:ABZ6755
Drasteria divergens|[6061]|LOCBE221-06|United States|California|658[0n]|BOLD:ABZ6755
Drasteria divergens|[6062]|LOCBE215-06|United States|California|658[0n]|BOLD:ABZ6755
Drasteria divergens|[6063]|LOCBD158-06|United States|California|658[0n]|BOLD:ABZ6755
Drasteria divergens|[6064]|RDNMC269-05|United States|Oregon|658[0n]|BOLD:ABZ6755
Drasteria divergens|[6065]|RDNM924-05|United States|Oregon|658[0n]|BOLD:ABZ6755
Drasteria divergens|[6066]|RDNMC267-05|Canada|British Columbia|597[1n]|BOLD:ABZ6755
Drasteria divergens|[6067]|RDNMC270-05|United States|Oregon|594[0n]|BOLD:ABZ6755
Drasteria divergens|[6068]|LOCBE327-06|United States|California|594[0n]|BOLD:ABZ6755
Drasteria divergens|[6069]|LOCBE217-06|United States|California|593[0n]|BOLD:ABZ6755
Drasteria divergens|[6070]|LBCH5792-10|Canada|British Columbia|658[0n]|BOLD:ABZ6755
Drasteria divergens|[6071]|ABNCC011-07|United States|California|616[0n]|BOLD:ABZ6755
Drasteria divergens|[6072]|LOCBD054-06|United States|California|658[0n]|BOLD:ABZ6755
Drasteria divergens|[6073]|LOCBE218-06|United States|California|658[0n]|BOLD:ABZ6755
Drasteria divergens|[6074]|QUNOE515-12|United States|California|658[0n]|BOLD:ABZ6755
Drasteria mirifica|[6075]|ABNCC292-07|United States|Texas|646[0n]|BOLD:ABZ6754
Drasteria mirifica|[6076]|RDNM747-05|United States|Nevada|658[0n]|BOLD:ABZ6754
Drasteria mirifica|[6077]|RDNM932-05|United States|Nevada|617[0n]|BOLD:ABZ6754
Drasteria mirifica|[6078]|LMEMB174-09|United States|Wyoming|505[0n]|BOLD:ABZ6754
Drasteria mirifica|[6079]|QUNOD174-10|United States|Texas|658[0n]|BOLD:ABZ6754
Drasteria mirifica|[6080]|QUNOD529-10|United States|Texas|658[0n]|BOLD:ABZ6754
Drasteria eubapta|[6081]|LOCBB099-06|United States|California|658[0n]|BOLD:ABZ6753
Drasteria eubapta|[6082]|LOCBB101-06|United States|California|658[0n]|BOLD:ABZ6753
Drasteria eubapta|[6083]|IAWL124-09|United States|Arizona|658[0n]|BOLD:ABZ6753
Drasteria eubapta|[6084]|IAWL125-09|United States|Arizona|658[0n]|BOLD:ABZ6753
Drasteria eubapta|[6085]|LOCBB100-06|United States|California|658[0n]|BOLD:ABZ6753
Drasteria eubapta|[6086]|IAWL126-09|United States|Arizona|658[0n]|BOLD:ABZ6753
Drasteria eubapta|[6087]|LOCBB098-06|United States|California|658[0n]|BOLD:ABZ6753
Drasteria eubapta|[6088]|LOCBB102-06|United States|California|658[0n]|BOLD:ABZ6753
Drasteria eubapta|[6089]|QUNOE511-12|United States|California|658[0n]|BOLD:ABZ6753
Drasteria hastingsii|[6090]|RDNM749-05|United States|Washington|658[0n]|BOLD:ACE6786
Drasteria hastingsii|[6091]|RDNMG546-08|United States|Washington|658[0n]|BOLD:ACE6786

Drasteria eubapta|[6089]|QUNOE511-12|United States|California|658[0n]|BOLD:ABZ6753
Drasteria hastingsii|[6090]|RDNM749-05|United States|Washington|658[0n]|BOLD:ACE6786
Drasteria hastingsii|[6091]|RDNMG546-08|United States|Washington|658[0n]|BOLD:ACE6786
Drasteria hastingsii|[6092]|RDNMG547-08|United States|Washington|658[0n]|BOLD:ACE6786
Drasteria hastingsii|[6093]|RDMAB1011-09|United States|Washington|635[0n]|BOLD:ACE6786
Drasteria hastingsii|[6094]|RDNMG545-08|United States|Washington|658[0n]|BOLD:ACE6786
Drasteria hastingsii|[6095]|RDNM748-05|United States|Washington|658[0n]|BOLD:ACE6786
Drasteria hastingsii|[6096]|RDMAB1012-09|United States|Washington|639[0n]|BOLD:ACE6786
Drasteria hastingsii|[6097]|RDMAB1013-09|United States|Washington|658[0n]|BOLD:ACE6786
Drasteria hastingsii|[6098]|QUNOE516-12|United States|California|658[0n]|BOLD:ACE6786
Drasteria walshi|[6099]|LNAUS5606-13|United States|Arizona|658[0n]|BOLD:ACM3960
Drasteria walshi|[6100]|CNCLB202-14|United States|Arizona|609[0n]|BOLD:ACM3960
Drasteria walshi|[6101]|CNCLB203-14|United States|Arizona|658[0n]|BOLD:ACM3960
Drasteria adumbrata alleni|[6102]|RDLQB252-05|Canada|Quebec|658[0n]|BOLD:AAB6890
Drasteria adumbrata alleni|[6103]|RDLQB249-05|Canada|Quebec|658[0n]|BOLD:AAB6890
Drasteria adumbrata alleni|[6104]|RDLQB248-05|Canada|Quebec|658[0n]|BOLD:AAB6890
Drasteria adumbrata alleni|[6105]|RDLQB251-05|Canada|Quebec|588[0n]|BOLD:AAB6890
Drasteria adumbrata alleni|[6106]|RDLQB250-05|Canada|Quebec|561[1n]|BOLD:AAB6890
Drasteria adumbrata|[6107]|ABNCC305-07|United States|Michigan|601[0n]|BOLD:AAB6890
Drasteria adumbrata|[6108]|LOWCE349-06|Canada|British Columbia|658[0n]|BOLD:AAB6890
Drasteria adumbrata|[6109]|RDMAB222-05|Canada|Manitoba|658[0n]|BOLD:AAB6890
Drasteria adumbrata|[6110]|RDMAB220-05|Canada|Alberta|658[0n]|BOLD:AAB6890
Drasteria adumbrata|[6111]|ABNCC007-07|United States|Utah|617[3n]|BOLD:AAB6890
Drasteria adumbrata|[6112]|ABNCC008-07|United States|Utah|635[0n]|BOLD:AAB6890
Drasteria adumbrata|[6113]|LMEMB192-09|United States|Wyoming|657[0n]|BOLD:AAB6890
Drasteria adumbrata|[6114]|LOWCE038-06|Canada|British Columbia|658[0n]|BOLD:AAB6890
Drasteria adumbrata|[6115]|LPMN948-08|Canada|Alberta|658[0n]|BOLD:AAB6890
Drasteria adumbrata|[6116]|LOWCE351-06|Canada|British Columbia|658[0n]|BOLD:AAB6890
Drasteria adumbrata|[6117]|LPAB002-08|Canada|Alberta|634[0n]|BOLD:AAB6890
Drasteria adumbrata|[6118]|LPAB003-08|Canada|Alberta|658[0n]|BOLD:AAB6890
Drasteria adumbrata|[6119]|LPAB053-08|Canada|Alberta|658[0n]|BOLD:AAB6890
Drasteria adumbrata|[6120]|LPABB254-08|Canada|Alberta|658[0n]|BOLD:AAB6890
Drasteria adumbrata|[6121]|LPABB360-08|Canada|Alberta|658[0n]|BOLD:AAB6890
Drasteria adumbrata|[6122]|LPABB363-08|Canada|Alberta|658[0n]|BOLD:AAB6890
Drasteria adumbrata|[6123]|LPABB368-08|Canada|Alberta|658[0n]|BOLD:AAB6890
Drasteria adumbrata|[6124]|LPABB518-08|Canada|Alberta|658[0n]|BOLD:AAB6890
Drasteria adumbrata|[6125]|LPABC823-09|Canada|Alberta|658[0n]|BOLD:AAB6890
Drasteria adumbrata|[6126]|LBCH5590-10|Canada|British Columbia|658[0n]|BOLD:AAB6890
Drasteria adumbrata|[6127]|IAWLB159-10|United States|California|658[0n]|BOLD:ACF4357
Drasteria adumbrata|[6128]|IAWLB160-10|United States|California|658[0n]|BOLD:ACF4357
Drasteria pulchra|[6129]|QUNOE518-12|United States|California|658[0n]|BOLD:ACA1687
Drasteria pulchra|[6130]|CNCLB204-14|United States|California|658[0n]|BOLD:ACA1687
Drasteria pulchra|[6131]|CNCLB205-14|United States|California|658[0n]|BOLD:ACA1687
Drasteria perplexa|[6132]|ABNCC005-07|United States|Utah|634[0n]|BOLD:AAE0308
Drasteria perplexa|[6133]|ABNCC006-07|United States|Utah|639[0n]|BOLD:AAE0308
Drasteria stretchii|[6134]|QUNOE510-12|United States|California|658[0n]|BOLD:AAE0308
Drasteria stretchii|[6135]|CNCLB1269-14|United States|Oregon|658[0n]|BOLD:AAE0308
Drasteria perplexa|[6136]|LMEMB190-09|United States|Utah|658[0n]|BOLD:AAE0308
Drasteria perplexa|[6137]|LMEMB191-09|United States|Utah|658[0n]|BOLD:AAE0308
Drasteria perplexa|[6138]|QUNOE096-11|United States|Utah|658[0n]|BOLD:AAE0308
Drasteria stretchii|[6139]|CNCLB1270-14|United States|Oregon|658[0n]|BOLD:AAE0308
Drasteria perplexa|[6140]|RDMAB094-05|Canada|Alberta|615[1n]|BOLD:AAE0308
Drasteria perplexa|[6141]|CNCLB1271-14|Canada|Alberta|658[0n]|BOLD:AAE0308
Drasteria ingeniculata|[6142]|ABNCC296-07|United States|Texas|641[0n]|BOLD:AAE8517
Drasteria ingeniculata|[6143]|ABNCC178-07|United States|Texas|594[0n]|BOLD:AAE8517
Drasteria ingeniculata|[6144]|ABNCC297-07|United States|Texas|642[0n]|BOLD:AAE8517
Drasteria ingeniculata|[6145]|ABNCC381-07|United States||650[0n]|BOLD:AAE8517
Drasteria ochracea|[6146]|LOCBD060-06|United States|California|658[0n]|BOLD:AAD3446
Drasteria ochracea|[6147]|LOCBD061-06|United States|California|658[0n]|BOLD:ACK5628
Drasteria ochracea|[6148]|ABNCC043-07|United States|California|642[0n]|BOLD:ACK5628
Drasteria ochracea|[6149]|LBCH5695-10|Canada|British Columbia|658[0n]|BOLD:ACK5628
Drasteria ochracea|[6150]|LOCBE346-06|United States|California|658[0n]|BOLD:ACK5628
Drasteria ochracea|[6151]|LOCBD062-06|United States|California|658[0n]|BOLD:ACK5628
Drasteria ochracea|[6152]|ABNCC042-07|United States|California|636[0n]|BOLD:ACK5628
Drasteria ochracea|[6153]|ABNCC044-07|United States|California|629[0n]|BOLD:ACK5628
Drasteria ochracea|[6154]|BBLOD497-11|United States|California|658[0n]|BOLD:ACK5628
Drasteria scrupulosa|[6155]|ABNCC025-07|United States|Utah|642[0n]|BOLD:AAE8069
Drasteria scrupulosa|[6156]|ABNCC026-07|United States|Utah|618[1n]|BOLD:AAE8069
Drasteria scrupulosa|[6157]|LTOLB1361-11|United States|California|658[0n]|BOLD:AAE8069
Drasteria scrupulosa|[6158]|IAWLB130-10|United States|California|658[0n]|BOLD:AAE8069
Drasteria scrupulosa|[6159]|ABNCC023-07|United States|California|633[0n]|BOLD:AAE8069
Drasteria scrupulosa|[6160]|ABNCC024-07|United States|California|634[0n]|BOLD:AAE8069
Drasteria scrupulosa|[6161]|QUNOE098-11|United States|California|658[0n]|BOLD:AAE8069
Drasteria petricola|[6162]|RDMAB224-05|Canada|Alberta|658[0n]|BOLD:AAE0309
Drasteria petricola|[6163]|LPABC649-09|Canada|Alberta|658[0n]|BOLD:AAE0309
Drasteria petricola|[6164]|RDMAB225-05|Canada|Alberta|631[0n]|BOLD:AAE0309
Drasteria petricola|[6165]|RDMAB226-05|Canada|Alberta|629[0n]|BOLD:AAE0309
Drasteria petricola|[6166]|RDMAB227-05|Canada|Alberta|525[1n]|BOLD:AAE0309
Drasteria petricola|[6167]|QUNOE512-12|Canada|Yukon Territory|658[0n]|BOLD:AAE0309
Drasteria occulta|[6168]|CNCLB854-14|United States|New Jersey|307[0n]|
Drasteria graphica|[6169]|LSUSA058-06|United States|Florida|658[0n]|BOLD:ACE6782
Drasteria graphica|[6170]|USLEP651-10|United States|Florida|658[0n]|BOLD:ACE6782
Drasteria graphica|[6171]|USLEP074-10|United States|Florida|658[0n]|BOLD:ACE6782
Drasteria graphica|[6172]|RDNMK043-11|United States|Florida|658[0n]|BOLD:ACE6782
Drasteria graphica|[6173]|QUNOE465-12|United States|Florida|658[0n]|BOLD:ACE6782
Drasteria occulta|[6174]|LNAUT867-14|United States|Massachusetts|658[0n]|BOLD:ACN8512
Drasteria occulta|[6175]|CNCLB1714-14|United States|New Jersey|658[0n]|BOLD:ACN8512
Drasteria graphica|[6176]|LSUSA057-06|United States|Florida|581[0n]|BOLD:ACE6784
Drasteria graphica|[6177]|LNAUT2633-14|United States|Massachusetts|658[0n]|BOLD:ACR7461
Drasteria graphica|[6178]|LNAUT2634-14|United States|Massachusetts|658[0n]|BOLD:ACR7461
Drasteria graphica|[6179]|LNAUT2635-14|United States|Massachusetts|658[0n]|BOLD:ACR7461
Drasteria graphica|[6180]|LNAUT2636-14|United States|Massachusetts|658[0n]|BOLD:ACR7461
Melipotis novanda|[6181]|IAWL143-09|United States|Arizona|658[0n]|BOLD:ACF4777
Melipotis agrotoides|[6182]|HKONB107-08|United States|Texas|658[0n]|BOLD:ACE3235
Melipotis agrotoides|[6183]|HKONB106-08|United States|Texas|658[0n]|BOLD:ACE3235
Melipotis agrotoides|[6184]|HKONB108-08|United States|Texas|658[0n]|BOLD:ACE3235
Melipotis agrotoides|[6185]|HKONB112-08|United States|Texas|658[0n]|BOLD:ACE3235
Melipotis agrotoides|[6186]|HKONB111-08|United States|Texas|658[0n]|BOLD:ACE3235
Melipotis agrotoides|[6187]|HKONB110-08|United States|Texas|658[0n]|BOLD:ACE3235
Melipotis agrotoides|[6188]|HKONB109-08|United States|Texas|658[0n]|BOLD:ACE3235
Melipotis agrotoides|[6189]|HKONB105-08|United States|Texas|658[0n]|BOLD:ACE3235
Melipotis agrotoides|[6190]|ABNCC279-07|United States|Texas|636[1n]|BOLD:ACE3235
Melipotis agrotoides|[6191]|ABNCC280-07|United States|Texas|646[0n]|BOLD:ACE3235

Melipotis agrotoides|[6189]|HKONB105-08|United States|Texas|658[0n]|BOLD:ACE3235
Melipotis agrotoides|[6190]|ABNCC279-07|United States|Texas|636[1n]|BOLD:ACE3235
Melipotis agrotoides|[6191]|ABNCC280-07|United States|Texas|646[0n]|BOLD:ACE3235
Melipotis agrotoides|[6192]|BBLOD505-11|United States|Texas|649[0n]|BOLD:ACE3235
Melipotis novanda|[6193]|CMAZA034-09|United States|Arizona|658[0n]|BOLD:AAE9170
Melipotis novanda|[6194]|IAWL070-09|United States|Arizona|658[0n]|BOLD:AAE9170
Melipotis novanda|[6195]|CMAZA228-09|United States|Arizona|658[0n]|BOLD:AAE9170
Melipotis novanda|[6196]|CMAZA171-09|United States|Arizona|658[0n]|BOLD:AAE9170
Melipotis novanda|[6197]|IAWL068-09|United States|Arizona|658[0n]|BOLD:AAE9170
Melipotis novanda|[6198]|ABNCC281-07|United States|Texas|650[0n]|BOLD:AAE9170
Melipotis novanda|[6199]|ABNCC282-07|United States|Texas|629[1n]|BOLD:AAE9170
Melipotis novanda|[6200]|CMAZA340-10|United States|Arizona|658[0n]|BOLD:AAE9170
Melipotis novanda|[6201]|CMAZA334-10|United States|Arizona|658[0n]|BOLD:AAE9170
Melipotis novanda|[6202]|IAWL142-09|United States|Arizona|658[0n]|BOLD:AAE9170
Melipotis novanda|[6203]|CMAZA276-09|United States|Arizona|637[0n]|BOLD:AAE9170
Melipotis novanda|[6204]|CMAZA880-12|United States|Arizona|658[0n]|BOLD:AAE9170
Melipotis jucunda|[6205]|BBLSZ183-09|United States|Texas|658[0n]|BOLD:AAB3598
Melipotis jucunda|[6206]|HKONB117-08|United States|Texas|658[0n]|BOLD:AAB3598
Melipotis jucunda|[6207]|HKONB118-08|United States|Texas|658[0n]|BOLD:AAB3598
Melipotis jucunda|[6208]|BBLSZ166-09|United States|Texas|658[0n]|BOLD:AAB3598
Melipotis jucunda|[6209]|BBLOD133-11|United States|Texas|658[0n]|BOLD:AAB3598
Melipotis jucunda|[6210]|BBLOD139-11|United States|Texas|658[0n]|BOLD:AAB3598
Melipotis jucunda|[6211]|BBLOC1417-11|United States|Texas|658[0n]|BOLD:AAB3598
Melipotis jucunda|[6212]|BBLOB676-11|United States|Texas|658[0n]|BOLD:AAB3598
Melipotis jucunda|[6213]|BBLSZ165-09|United States|Texas|658[0n]|BOLD:AAB3598
Melipotis jucunda|[6214]|BBLSY724-09|United States|Arizona|658[0n]|BOLD:AAB3598
Melipotis jucunda|[6215]|BBLSY109-09|United States|Texas|658[0n]|BOLD:AAB3598
Melipotis jucunda|[6216]|BBLSW005-09|United States|Oklahoma|658[0n]|BOLD:AAB3598
Melipotis jucunda|[6217]|IAWL137-09|United States|Arizona|658[0n]|BOLD:AAB3598
Melipotis jucunda|[6218]|HKONB472-09|United States|Texas|658[0n]|BOLD:AAB3598
Melipotis jucunda|[6219]|HKONB122-08|United States|Texas|658[0n]|BOLD:AAB3598
Melipotis jucunda|[6220]|HKONB120-08|United States|Texas|658[0n]|BOLD:AAB3598
Melipotis jucunda|[6221]|HKONB119-08|United States|Texas|658[0n]|BOLD:AAB3598
Melipotis jucunda|[6222]|HKONB116-08|United States|Texas|658[0n]|BOLD:AAB3598
Melipotis jucunda|[6223]|ABNCC278-07|United States|Indiana|636[0n]|BOLD:AAB3598
Melipotis jucunda|[6224]|BBLOD195-11|United States|Texas|633[0n]|BOLD:AAB3598
Melipotis jucunda|[6225]|BBLOD206-11|United States|Texas|658[0n]|BOLD:AAB3598
Melipotis jucunda|[6226]|BBLSW711-09|United States|Texas|658[0n]|BOLD:AAB3598
Melipotis jucunda|[6227]|LNC563-06|United States|North Carolina|590[11n]|
Melipotis jucunda|[6228]|HKONB121-08|United States|Texas|658[0n]|BOLD:AAB3598
Melipotis jucunda|[6229]|USLEP648-10|United States|Florida|658[0n]|BOLD:AAB3598
Melipotis jucunda|[6230]|BBLOB1544-11|United States|Florida|658[0n]|BOLD:AAB3598
Melipotis jucunda|[6231]|BBLSW030-09|United States|Texas|658[0n]|BOLD:AAB3598
Melipotis jucunda|[6232]|BBLSX104-09|United States|Oklahoma|658[0n]|BOLD:AAB3598
Melipotis jucunda|[6233]|USLEP701-10|United States|Arizona|658[0n]|BOLD:AAB3598
Melipotis jucunda|[6234]|LBCH5801-10|Canada|British Columbia|658[0n]|BOLD:AAB3598
Melipotis jucunda|[6235]|LTOL801-07|United States|Arizona|658[0n]|BOLD:AAB3598
Melipotis jucunda|[6236]|LPOKA636-09|United States|Oklahoma|658[0n]|BOLD:AAB3598
Melipotis jucunda|[6237]|BBLSZ167-09|United States|Texas|658[0n]|BOLD:AAB3598
Melipotis jucunda|[6238]|AWCLB597-11|United States|Arizona|658[0n]|BOLD:AAB3598
Melipotis jucunda|[6239]|AWCLB585-11|United States|Arizona|658[0n]|BOLD:AAB3598
Melipotis jucunda|[6240]|AWCLB586-11|United States|Arizona|658[0n]|BOLD:AAB3598
Melipotis jucunda|[6241]|AWCLB396-10|United States|Arizona|658[0n]|BOLD:AAB3598
Melipotis jucunda|[6242]|AWCLB343-10|United States|Arizona|658[0n]|BOLD:AAB3598
Melipotis jucunda|[6243]|LPABC962-09|Canada|Alberta|658[0n]|BOLD:AAB3598
Melipotis jucunda|[6244]|LPABB041-08|Canada|Alberta|658[0n]|BOLD:AAB3598
Melipotis jucunda|[6245]|LPAB013-08|Canada|Alberta|658[0n]|BOLD:AAB3598
Melipotis jucunda|[6246]|AWCLB441-10|United States|Arizona|658[0n]|BOLD:AAB3598
Melipotis jucunda|[6247]|ABNCC274-07|United States|Utah|645[1n]|BOLD:AAB3598
Melipotis jucunda|[6248]|AWCLB591-11|United States|Arizona|540[0n]|BOLD:AAB3598
Melipotis jucunda|[6249]|AWCLB596-11|United States|Arizona|658[0n]|BOLD:AAB3598
Melipotis jucunda|[6250]|AWCLB602-11|United States|Arizona|658[0n]|BOLD:AAB3598
Melipotis jucunda|[6251]|AWCLB599-11|United States|Arizona|658[0n]|BOLD:AAB3598
Melipotis jucunda|[6252]|AWCLB212-10|United States|Arizona|658[0n]|BOLD:AAB3598
Melipotis jucunda|[6253]|BBLSZ185-09|United States|Texas|658[0n]|BOLD:AAB3598
Melipotis jucunda|[6254]|BBLSY106-09|United States|Texas|658[0n]|BOLD:AAB3598
Melipotis jucunda|[6255]|NAMUM369-09|United States|Texas|658[0n]|BOLD:AAB3598
Melipotis jucunda|[6256]|BBLSZ182-09|United States|Texas|658[0n]|BOLD:AAB3598
Melipotis jucunda|[6257]|ABNCC275-07|United States|Texas|635[0n]|BOLD:AAB3598
Melipotis jucunda|[6258]|ABNCC276-07|United States|Texas|643[0n]|BOLD:AAB3598
Melipotis jucunda|[6259]|LALPA142-10|Canada|British Columbia|658[0n]|BOLD:AAB3598
Melipotis jucunda|[6260]|LALPA143-10|Canada|British Columbia|658[0n]|BOLD:AAB3598
Melipotis jucunda|[6261]|LALPA173-10|Canada|British Columbia|658[0n]|BOLD:AAB3598
Melipotis jucunda|[6262]|LALPA294-10|Canada|British Columbia|658[0n]|BOLD:AAB3598
Melipotis jucunda|[6263]|LALPA642-10|Canada|British Columbia|658[0n]|BOLD:AAB3598
Melipotis jucunda|[6264]|BBLOC669-11|United States|Texas|658[0n]|BOLD:AAB3598
Melipotis jucunda|[6265]|IAWL136-09|United States|Arizona|658[1n]|BOLD:AAB3598
Melipotis jucunda|[6266]|BBLSX748-09|United States|Arizona|658[0n]|BOLD:AAB3598
Melipotis jucunda|[6267]|BBLSW319-09|United States|Arizona|658[0n]|BOLD:AAB3598
Melipotis jucunda|[6268]|BBLSW317-09|United States|Arizona|658[0n]|BOLD:AAB3598
Melipotis jucunda|[6269]|BBLSW316-09|United States|Arizona|658[0n]|BOLD:AAB3598
Melipotis jucunda|[6270]|IAWL138-09|United States|Arizona|658[0n]|BOLD:AAB3598
Melipotis jucunda|[6271]|BBLSX677-09|United States|Arizona|658[0n]|BOLD:AAB3598
Melipotis jucunda|[6272]|BBLSX752-09|United States|Arizona|638[0n]|BOLD:AAB3598
Melipotis jucunda|[6273]|BBLSY668-09|United States|Arizona|658[0n]|BOLD:AAB3598
Melipotis jucunda|[6274]|BBLSY674-09|United States|Arizona|658[0n]|BOLD:AAB3598
Melipotis jucunda|[6275]|BBLSY718-09|United States|Arizona|658[0n]|BOLD:AAB3598
Melipotis jucunda|[6276]|USLEP706-10|United States|Arizona|658[0n]|BOLD:AAB3598
Melipotis jucunda|[6277]|AWCLB440-10|United States|Arizona|658[0n]|BOLD:AAB3598
Melipotis jucunda|[6278]|AWCLB497-11|United States|Arizona|658[0n]|BOLD:AAB3598
Melipotis jucunda|[6279]|BBLOB013-11|United States|Arizona|658[0n]|BOLD:AAB3598
Melipotis jucunda|[6280]|BBLOB014-11|United States|Arizona|658[0n]|BOLD:AAB3598
Melipotis jucunda|[6281]|BBLOB688-11|United States|Texas|658[0n]|BOLD:AAB3598
Melipotis jucunda|[6282]|BBLOB1056-11|United States|Arizona|658[0n]|BOLD:AAB3598
Melipotis jucunda|[6283]|BBLOB1574-11|United States|Arizona|658[0n]|BOLD:AAB3598
Melipotis jucunda|[6284]|BBLOD1665-11|United States|Arizona|658[0n]|BOLD:AAB3598
Melipotis jucunda|[6285]|BBLOE1420-12|United States|Arizona|658[0n]|BOLD:AAB3598
Melipotis jucunda|[6286]|BBLOE1424-12|United States|Arizona|658[0n]|BOLD:AAB3598
Melipotis jucunda|[6287]|BBLOE1465-12|United States|Texas|658[0n]|BOLD:AAB3598
Melipotis jucunda|[6288]|CMAZA890-12|United States|Arizona|658[0n]|BOLD:AAB3598
Melipotis jucunda|[6289]|CMAZA916-12|United States|Arizona|658[0n]|BOLD:AAB3598
Melipotis jucunda|[6290]|CNCLB1646-14|United States|Florida|658[0n]|BOLD:AAB3598
Cissusa indiscreta|[6291]|BDNME504-08|Canada|British Columbia|658[0n]|BOLD:AAB4232

Melipotis jucunda|[6288]|CMAZA[illegible]-12|United States|Arizona|658[0n]|BOLD:AAB3598
Melipotis jucunda|[6289]|CMAZA916-12|United States|Arizona|658[0n]|BOLD:AAB3598
Melipotis jucunda|[6290]|CNCLB1646-14|United States|Florida|658[0n]|BOLD:AAB3598
Cissusa indiscreta|[6291]|RDNMF504-08|Canada|British Columbia|658[0n]|BOLD:AAB4232
Cissusa indiscreta|[6292]|RDNMF507-08|Canada|British Columbia|609[0n]|BOLD:AAB4232
Cissusa indiscreta|[6293]|RDNMF506-08|Canada|British Columbia|658[0n]|BOLD:AAB4232
Cissusa indiscreta|[6294]|RDNMF508-08|United States|Oregon|658[0n]|BOLD:AAB4232
Cissusa indiscreta|[6295]|RDNMF505-08|United States|California|658[0n]|BOLD:AAB4232
Cissusa indiscreta|[6296]|JBAZ024-09|United States|California|658[0n]|BOLD:AAB4232
Cissusa indiscreta|[6297]|CGLCA025-10|United States|California|658[0n]|BOLD:AAB4232
Cissusa spadix|[6298]|USLEP591-10|United States|Florida|658[0n]|BOLD:AAB4232
Cissusa spadix|[6299]|USLEP288-10|United States|Florida|658[0n]|BOLD:AAB4232
Cissusa spadix|[6300]|USLEP287-10|United States|Florida|658[0n]|BOLD:AAB4232
Cissusa spadix|[6301]|LSUSA028-06|United States|Kentucky|658[0n]|BOLD:AAB4232
Cissusa spadix|[6302]|HKONS025-07|United States|Florida|658[0n]|BOLD:AAB4232
Cissusa spadix|[6303]|HKONS026-07|United States|Florida|646[0n]|BOLD:AAB4232
Cissusa spadix|[6304]|HKONS027-07|United States|Florida|658[0n]|BOLD:AAB4232
Cissusa spadix|[6305]|USLEP286-10|United States|Florida|658[0n]|BOLD:AAB4232
Cissusa spadix|[6306]|USLEP293-10|United States|Florida|658[0n]|BOLD:AAB4232
Cissusa spadix|[6307]|USLEP592-10|United States|Florida|658[0n]|BOLD:AAB4232
Cissusa spadix|[6308]|USLEP645-10|United States|Florida|658[0n]|BOLD:AAB4232
Cissusa spadix|[6309]|BBLSW789-09|United States|Oklahoma|658[0n]|BOLD:AAB4232
Cissusa spadix|[6310]|LOCT285-05|United States|Connecticut|658[0n]|BOLD:AAB4232
Cissusa spadix|[6311]|HKONB257-09|United States|Texas|658[0n]|BOLD:AAB4232
Cissusa spadix|[6312]|LPOKB277-09|United States|Oklahoma|658[0n]|BOLD:AAB4232
Cissusa spadix|[6313]|PMCSC003-11|United States||658[0n]|
Cissusa spadix|[6314]|IAWL080-09|United States|Arizona|658[0n]|BOLD:ABZ0006
Cissusa spadix|[6315]|CMAZA035-09|United States|Arizona|658[0n]|BOLD:ABZ0006
Cissusa spadix|[6316]|IAWL062-09|United States|Arizona|658[0n]|BOLD:ABZ0006
Cissusa spadix|[6317]|IAWL063-09|United States|Arizona|658[1n]|BOLD:ABZ0006
Cissusa spadix|[6318]|IAWL064-09|United States|Arizona|658[0n]|BOLD:ABZ0006
Cissusa spadix|[6319]|IAWL065-09|United States|Arizona|658[0n]|BOLD:ABZ0006
Cissusa spadix|[6320]|IAWL066-09|United States|Arizona|658[0n]|BOLD:ABZ0006
Cissusa spadix|[6321]|IAWL072-09|United States|Arizona|658[0n]|BOLD:ABZ0006
Cissusa spadix|[6322]|IAWL073-09|United States|Arizona|658[0n]|BOLD:ABZ0006
Cissusa spadix|[6323]|CMAZA055-09|United States|Arizona|658[0n]|BOLD:ABZ0006
Cissusa spadix|[6324]|ABCBF060-10|United States|Georgia|650[0n]|
Cissusa spadix|[6325]|USLEP289-10|United States|Florida|658[0n]|BOLD:ABZ0006
Cissusa spadix|[6326]|USLEP593-10|United States|Florida|658[0n]|BOLD:ABZ0006
Cissusa sp.|[6327]|HKONB535-09|United States|Texas|658[0n]|BOLD:ACE9553
Cissusa sp.|[6328]|BBLOD1074-11|United States|Texas|658[0n]|BOLD:ACE9553
Cissusa spadix|[6329]|LPOKB118-09|United States|Oklahoma|658[0n]|BOLD:ACE3472
Cissusa spadix|[6330]|RDNMJ184-10|United States|Georgia|658[0n]|BOLD:ACE3472
Cissusa spadix|[6331]|LPOKA915-09|United States|Oklahoma|658[0n]|BOLD:ACE9552
Cissusa spadix|[6332]|LPOKA996-09|United States|Oklahoma|658[0n]|BOLD:ACE9552
Cissusa spadix|[6333]|LPOKB158-09|United States|Oklahoma|658[0n]|BOLD:ACE9552
Cissusa spadix|[6334]|LPOKB229-09|United States|Oklahoma|658[0n]|BOLD:ACE9552
Cissusa spadix|[6335]|BBLSX908-09|United States|Arizona|658[0n]|BOLD:ACE9552
Cissusa spadix|[6336]|LPOKB343-09|United States|Oklahoma|658[0n]|BOLD:ACE9552
Cissusa spadix|[6337]|LPOKB255-09|United States|Oklahoma|658[0n]|BOLD:ACE9552
Cissusa spadix|[6338]|ABCBF061-10|United States|Georgia|658[0n]|
Cissusa spadix|[6339]|PMCSC001-11|United States||658[0n]|
Cissusa mucronata|[6340]|BBLOD1071-11|United States|Texas|658[0n]|BOLD:ACE8288
Cissusa mucronata|[6341]|BBLOD1301-11|United States|Texas|658[0n]|BOLD:ACE8288
Cissusa mucronata|[6342]|BBLOD1289-11|United States|Texas|658[0n]|BOLD:ACE8288
Cissusa mucronata|[6343]|BBLOD1290-11|United States|Texas|658[0n]|BOLD:ACE8288
Cissusa mucronata|[6344]|BBLOD141-11|United States|Texas|658[0n]|BOLD:ACE8288
Cissusa mucronata|[6345]|BBLOD138-11|United States|Texas|658[0n]|BOLD:ACE8288
Cissusa mucronata|[6346]|BBLOC1985-11|United States|Texas|658[0n]|BOLD:ACE8288
Cissusa mucronata|[6347]|BBLOD1302-11|United States|Texas|658[0n]|BOLD:ACE8288
Cissusa mucronata|[6348]|QUNOD107-10|United States|Texas|658[0n]|BOLD:ACE8288
Cissusa mucronata|[6349]|BBLOC1065-11|United States|Texas|658[0n]|BOLD:ACE8288
Cissusa mucronata|[6350]|BBLOD1303-11|United States|Texas|658[0n]|BOLD:ACE8288
Litocala sexsignata|[6351]|CGLCA150-10|United States|California|658[0n]|BOLD:AAK6707
Litocala sexsignata|[6352]|JBAZ033-09|United States|California|658[0n]|BOLD:AAK6707
Litocala sexsignata|[6353]|USLEP066-10|United States|Arizona|658[0n]|BOLD:AAK6707
Cissusa valens|[6354]|ABNCC272-07|United States|New Mexico|652[0n]|BOLD:AAC7639
Cissusa valens|[6355]|IAWL098-09|United States|Arizona|658[0n]|BOLD:AAC7639
Cissusa valens|[6356]|RDNME946-08|United States|Texas|658[0n]|BOLD:AAC7639
Cissusa valens|[6357]|ABNCC271-07|United States|Texas|594[0n]|BOLD:AAC7639
Cissusa valens|[6358]|HKONB531-09|United States|Texas|658[0n]|BOLD:AAC7639
Cissusa valens|[6359]|IAWL096-09|United States|Arizona|658[5n]|BOLD:AAC7639
Cissusa valens|[6360]|IAWL100-09|United States|Arizona|658[0n]|BOLD:AAC7639
Cissusa valens|[6361]|RDNMJ131-10|United States|Arizona|658[0n]|BOLD:AAC7639
Cissusa valens|[6362]|ABNCC027-07|United States|Arizona|642[0n]|BOLD:AAC7639
Cissusa valens|[6363]|ABNCC028-07|United States|Arizona|648[0n]|BOLD:AAC7639
Cissusa valens|[6364]|IAWL099-09|United States|Arizona|658[0n]|BOLD:AAC7639
Cissusa valens|[6365]|RDNMJ130-10|United States|Arizona|658[0n]|BOLD:AAC7639
Cissusa valens|[6366]|IAWLB575-11|United States|Arizona|658[0n]|BOLD:AAC7639
Phoberia ingenua|[6367]|LNC240-05|United States|North Carolina|593[0n]|BOLD:AAD3237
Phoberia ingenua|[6368]|RDNME953-08|United States|Georgia|658[0n]|BOLD:AAD3237
Phoberia ingenua|[6369]|RDNME368-07|United States|Florida|636[5n]|BOLD:AAD3237
Phoberia ingenua|[6370]|HKONS024-07|United States|Florida|658[0n]|BOLD:AAD3237
Phoberia ingenua|[6371]|LPOKA1017-09|United States|Oklahoma|658[0n]|BOLD:AAD3237
Phoberia ingenua|[6372]|RDNME550-08|United States|Colorado|658[0n]|BOLD:AAD3237
Phoberia ingenua|[6373]|RDNME551-08|United States|Colorado|658[0n]|BOLD:AAD3237
Phoberia ingenua|[6374]|LNCC1543-13|United States|North Carolina|658[0n]|BOLD:AAD3237
Phoberia atomaris|[6375]|PMCSC004-11|United States||658[0n]|
Phoberia atomaris|[6376]|GBGL11842-13|United States|Georgia|664[0n]|BOLD:AAA6714
Phoberia atomaris|[6377]|GBMIN20937-13|||671[0n]|BOLD:AAA6714
Phoberia atomaris|[6378]|HKONS019-07|United States|Florida|658[0n]|BOLD:AAA6714
Phoberia atomaris|[6379]|RDNME951-08|United States|Georgia|658[0n]|BOLD:AAA6714
Phoberia atomaris|[6380]|RDNME952-08|United States|Georgia|658[0n]|BOLD:AAA6714
Phoberia atomaris|[6381]|LSEU505-06|United States|Georgia|658[0n]|BOLD:AAA6714
Phoberia atomaris|[6382]|LPOKA983-09|United States|Oklahoma|622[0n]|BOLD:AAA6714
Phoberia atomaris|[6383]|HKONS021-07|United States|Florida|658[1n]|BOLD:AAA6714
Phoberia atomaris|[6384]|HKONS023-07|United States|Florida|627[2n]|BOLD:AAA6714
Phoberia atomaris|[6385]|HKONS017-07|United States|Florida|636[0n]|BOLD:AAA6714
Phoberia atomaris|[6386]|HKONS022-07|United States|Florida|658[1n]|BOLD:AAA6714
Phoberia atomaris|[6387]|HKONS020-07|United States|Florida|658[0n]|BOLD:AAA6714
Phoberia atomaris|[6388]|HKONS018-07|United States|Florida|658[1n]|BOLD:AAA6714
Phoberia atomaris|[6389]|RDNME365-07|United States|Florida|521[0n]|BOLD:AAA6714
Phoberia atomaris|[6390]|RDNME366-07|United States|Florida|634[3n]|BOLD:AAA6714
Phoberia atomaris|[6391]|PMCSC[illegible]-11|United States||658[0n]|

Phoberia atomaris|[6388]|HKONS018-07|United States|Florida|658[1n]|BOLD:AAA6714
Phoberia atomaris|[6389]|RDNME365-07|United States|Florida|521[0n]|BOLD:AAA6714
Phoberia atomaris|[6390]|RDNME366-07|United States|Florida|634[3n]|BOLD:AAA6714
Phoberia atomaris|[6391]|PMCSC002-11|United States||658[0n]|
Phoberia atomaris|[6392]|GBGL11843-13|United States|Georgia|664[0n]|BOLD:AAA6714
Phoberia atomaris|[6393]|LPOKA912-09|United States|Oklahoma|658[0n]|BOLD:AAA6714
Phoberia atomaris|[6394]|LNC726-06|United States|North Carolina|658[0n]|BOLD:AAA6714
Phoberia atomaris|[6395]|LNC727-06|United States|North Carolina|658[0n]|BOLD:AAA6714
Phoberia atomaris|[6396]|HKONS016-07|United States|Florida|658[0n]|BOLD:AAA6714
Phoberia atomaris|[6397]|RDLQ375-05|Canada|Quebec|658[0n]|BOLD:AAA6714
Phoberia atomaris|[6398]|RDLQ366-05|Canada|Quebec|658[0n]|BOLD:AAA6714
Phoberia atomaris|[6399]|RDLQ385-05|Canada|Quebec|579[0n]|BOLD:AAA6714
Phoberia atomaris|[6400]|RDLQ367-05|Canada|Quebec|658[0n]|BOLD:AAA6714
Phoberia atomaris|[6401]|MECB886-05|Canada|Quebec|658[0n]|BOLD:AAA6714
Phoberia atomaris|[6402]|RDLQ364-05|Canada|Quebec|573[0n]|BOLD:AAA6714
Phoberia atomaris|[6403]|RDLQ369-05|Canada|Quebec|658[0n]|BOLD:AAA6714
Phoberia atomaris|[6404]|LNAUT2648-14|United States|Massachusetts|658[0n]|BOLD:AAA6714
Phoberia atomaris|[6405]|MECB887-05|Canada|Quebec|658[0n]|BOLD:AAA6714
Phoberia atomaris|[6406]|RDLQ361-05|Canada|Quebec|658[0n]|BOLD:AAA6714
Phoberia atomaris|[6407]|RDLQ362-05|Canada|Quebec|658[0n]|BOLD:AAA6714
Phoberia atomaris|[6408]|RDLQ363-05|Canada|Quebec|658[0n]|BOLD:AAA6714
Phoberia atomaris|[6409]|RDLQ377-05|Canada|Quebec|658[0n]|BOLD:AAA6714
Phoberia atomaris|[6410]|RDLQ383-05|Canada|Quebec|658[0n]|BOLD:AAA6714
Phoberia atomaris|[6411]|RDLQ388-05|Canada|Quebec|658[0n]|BOLD:AAA6714
Phoberia atomaris|[6412]|HKONS045-07|United States|Florida|658[1n]|BOLD:AAA6714
Phoberia atomaris|[6413]|LPOKA973-09|United States|Oklahoma|658[0n]|BOLD:AAA6714
Phoberia atomaris|[6414]|LPOKA902-09|United States|Oklahoma|658[0n]|BOLD:AAA6714
Phoberia atomaris|[6415]|LPOKA952-09|United States|Oklahoma|658[0n]|BOLD:AAA6714
Phoberia atomaris|[6416]|LSEU048-06|United States|North Carolina|569[0n]|BOLD:AAA6714
Phoberia atomaris|[6417]|GMFRB194-14|United States|Virginia|549[0n]|BOLD:AAA6714
Phoberia atomaris|[6418]|RDLQ382-05|Canada|Quebec|658[0n]|BOLD:AAA6714
Phoberia atomaris|[6419]|RDLQ381-05|Canada|Quebec|658[0n]|BOLD:AAA6714
Phoberia atomaris|[6420]|RDLQ365-05|Canada|Quebec|658[0n]|BOLD:AAA6714
Phoberia atomaris|[6421]|RDLQ386-05|Canada|Quebec|658[0n]|BOLD:AAA6714
Phoberia atomaris|[6422]|RDLQ374-05|Canada|Quebec|658[0n]|BOLD:AAA6714
Phoberia atomaris|[6423]|RDLQ373-05|Canada|Quebec|658[0n]|BOLD:AAA6714
Phoberia atomaris|[6424]|RDLQ372-05|Canada|Quebec|658[0n]|BOLD:AAA6714
Phoberia atomaris|[6425]|RDLQ371-05|Canada|Quebec|658[0n]|BOLD:AAA6714
Phoberia atomaris|[6426]|RDLQ368-05|Canada|Quebec|658[0n]|BOLD:AAA6714
Phoberia atomaris|[6427]|RDLQ387-05|Canada|Quebec|602[0n]|BOLD:AAA6714
Phoberia atomaris|[6428]|RDLQ370-05|Canada|Quebec|638[0n]|BOLD:AAA6714
Phoberia atomaris|[6429]|RDLQ384-05|Canada|Quebec|579[0n]|BOLD:AAA6714
Phoberia atomaris|[6430]|MEC133-04|Canada|Quebec|600[0n]|BOLD:AAA6714
Phoberia atomaris|[6431]|LSEU046-06|United States|North Carolina|597[0n]|BOLD:AAA6714
Phoberia atomaris|[6432]|LSEU047-06|United States|North Carolina|597[0n]|BOLD:AAA6714
Phoberia atomaris|[6433]|LPOKA901-09|United States|Oklahoma|658[0n]|BOLD:AAA6714
Phoberia atomaris|[6434]|LPOKA945-09|United States|Oklahoma|658[0n]|BOLD:AAA6714
Phoberia atomaris|[6435]|LPOKA971-09|United States|Oklahoma|658[0n]|BOLD:AAA6714
Phoberia atomaris|[6436]|LNAUT2649-14|United States|Massachusetts|658[0n]|BOLD:AAA6714
Phoberia atomaris|[6437]|LNAUT2650-14|United States|Massachusetts|658[0n]|BOLD:AAA6714
Matigramma pulverilinea|[6438]|LPOKD189-09|United States|Oklahoma|658[0n]|BOLD:AAD3910
Matigramma pulverilinea|[6439]|ABNCC341-07|United States|Oklahoma|616[0n]|BOLD:AAD3910
Matigramma pulverilinea|[6440]|ABNCC340-07|United States|Oklahoma|651[1n]|BOLD:AAD3910
Matigramma pulverilinea|[6441]|LPOKA174-08|United States|Oklahoma|658[0n]|BOLD:AAD3910
Matigramma pulverilinea|[6442]|LPOKA415-09|United States|Oklahoma|658[0n]|BOLD:AAD3910
Matigramma pulverilinea|[6443]|LPOKB335-09|United States|Oklahoma|658[0n]|BOLD:AAD3910
Matigramma pulverilinea|[6444]|LPOKB1024-09|United States|Oklahoma|658[0n]|BOLD:AAD3910
Matigramma pulverilinea|[6445]|LPOKC809-09|United States|Oklahoma|658[0n]|BOLD:AAD3910
Matigramma pulverilinea|[6446]|BBLOC913-11|United States|Arkansas|658[0n]|BOLD:AAD3910
Matigramma obscurior|[6447]|ABNCC342-07|United States|Texas|641[0n]|BOLD:AAD8729
Matigramma obscurior|[6448]|ABNCC343-07|United States|Texas|633[0n]|BOLD:AAD8729
Matigramma obscurior|[6449]|HKONB130-08|United States|Texas|658[0n]|BOLD:AAD8729
Matigramma obscurior|[6450]|HKONB133-08|United States|Texas|658[0n]|BOLD:AAD8729
Matigramma obscurior|[6451]|HKONB131-08|United States|Texas|658[0n]|BOLD:AAD8729
Matigramma obscurior|[6452]|HKONB134-08|United States|Texas|658[0n]|BOLD:AAD8729
Matigramma rubrosuffusa|[6453]|LOCB370-06|United States|Arizona|658[0n]|BOLD:AAF1250
Matigramma rubrosuffusa|[6454]|LOCB367-06|United States|Arizona|658[0n]|BOLD:AAF1250
Matigramma rubrosuffusa|[6455]|BBLSZ194-09|United States|Texas|658[0n]|BOLD:AAF1250
Matigramma rubrosuffusa|[6456]|BBLSY229-09|United States|Texas|658[0n]|BOLD:AAF1250
Matigramma rubrosuffusa|[6457]|LOCB371-06|United States|Arizona|658[0n]|BOLD:AAF1250
Matigramma rubrosuffusa|[6458]|CMAZA333-10|United States|Arizona|658[0n]|BOLD:AAF1250
Matigramma rubrosuffusa|[6459]|QUNOD743-11|United States|Oklahoma|658[0n]|BOLD:AAF1250
Matigramma rubrosuffusa|[6460]|QUNOD744-11|United States|Texas|658[0n]|BOLD:AAF1250
Matigramma repentina|[6461]|RDNMH839-09|United States|Arizona|658[0n]|BOLD:AAB5574
Matigramma repentina|[6462]|RDNMH840-09|United States|Arizona|658[0n]|BOLD:AAB5574
Matigramma repentina|[6463]|QUNOD749-11|United States|Arizona|658[0n]|BOLD:AAB5574
Matigramma repentina|[6464]|ABNCC386-07|United States||638[0n]|BOLD:AAB5574
Matigramma repentina|[6465]|QUNOD748-11|United States|Arizona|658[0n]|BOLD:AAB5574
Matigramma repentina|[6466]|BBLOB679-11|United States|Texas|658[0n]|BOLD:AAB5574
Matigramma repentina|[6467]|BBLOB691-11|United States|Texas|658[0n]|BOLD:AAB5574
Matigramma emmilta|[6468]|RDNME868-08|United States|New Mexico|658[0n]|BOLD:AAC8911
Matigramma emmilta|[6469]|BBLSZ028-09|United States|Arizona|658[0n]|BOLD:AAC8911
Matigramma emmilta|[6470]|QUNOD746-11|United States|Texas|658[0n]|BOLD:AAC8911
Matigramma emmilta|[6471]|ABNCC336-07|United States|Texas|645[0n]|BOLD:AAC8911
Matigramma emmilta|[6472]|ABNCC335-07|United States|Texas|641[0n]|BOLD:AAC8911
Matigramma emmilta|[6473]|RDNMG277-08|United States|Texas|658[0n]|BOLD:AAC8911
Matigramma emmilta|[6474]|BBLOB694-11|United States|Texas|658[0n]|BOLD:AAC8911
Matigramma emmilta|[6475]|RDNME869-08|United States|New Mexico|658[0n]|BOLD:AAC8911
Matigramma emmilta|[6476]|BBLSY828-09|United States|Arizona|658[0n]|BOLD:AAC8911
Matigramma emmilta|[6477]|QUNOD745-11|United States|Texas|658[0n]|BOLD:AAC8911
Matigramma emmilta|[6478]|BBLOE1470-12|United States|Texas|658[0n]|BOLD:AAC8911
Matigramma emmilta|[6479]|BBLOC157-11|United States|Arizona|658[0n]|BOLD:AAC8911
Matigramma emmilta|[6480]|BBLOB585-11|United States|Arizona|658[0n]|BOLD:AAC8911
Matigramma emmilta|[6481]|BBLOC893-11|United States|Arizona|658[0n]|BOLD:AAC8911
Matigramma emmilta|[6482]|RDNMJ567-11|United States|Arizona|658[0n]|BOLD:AAC8911
Matigramma emmilta|[6483]|RDNME986-08|United States|New Mexico|658[0n]|BOLD:AAC8911
Matigramma emmilta|[6484]|BBLOB583-11|United States|Arizona|623[0n]|BOLD:AAC8911
Matigramma emmilta|[6485]|BBLOB584-11|United States|Arizona|627[0n]|BOLD:AAC8911
Matigramma emmilta|[6486]|BBLOB1581-11|United States|Arizona|658[0n]|BOLD:AAC8911
Matigramma emmilta|[6487]|BBLOC179-11|United States|Arizona|658[0n]|BOLD:AAC8911
Matigramma emmilta|[6488]|BBLSY964-09|United States|Arizona|658[0n]|BOLD:AAC8911
Matigramma emmilta|[6489]|BBLOC280-11|United States|Arizona|658[0n]|BOLD:AAC8911
Matigramma emmilta|[6490]|BBLOC229-11|United States|Arizona|658[0n]|BOLD:AAC8911
[illegible]

Matigramma emmilta|[6488]|BBLSY964-09|United States|Arizona|658[0n]|BOLD:AAC8911
Matigramma emmilta|[6489]|BBLOC280-11|United States|Arizona|658[0n]|BOLD:AAC8911
Matigramma emmilta|[6490]|BBLOC229-11|United States|Arizona|658[0n]|BOLD:AAC8911
Matigramma emmilta|[6491]|BBLOB587-11|United States|Arizona|658[0n]|BOLD:AAC8911
Matigramma emmilta|[6492]|QUNOD747-11|United States|Arizona|658[0n]|BOLD:AAC8911
Matigramma emmilta|[6493]|RDNMJ733-11|United States|Arizona|658[0n]|BOLD:AAC8911
Matigramma emmilta|[6494]|USLEP1017-10|United States|New Mexico|658[0n]|BOLD:AAC8911
Matigramma emmilta|[6495]|BBLSZ066-09|United States|Arizona|658[0n]|BOLD:AAC8911
Matigramma emmilta|[6496]|BBLSY975-09|United States|Arizona|658[0n]|BOLD:AAC8911
Matigramma emmilta|[6497]|BBLSY832-09|United States|Arizona|658[0n]|BOLD:AAC8911
Matigramma emmilta|[6498]|BBLSY299-09|United States|Arizona|658[0n]|BOLD:AAC8911
Matigramma emmilta|[6499]|BBLOC583-11|United States|Arizona|658[0n]|BOLD:AAC8911
Matigramma emmilta|[6500]|BBLOE1429-12|United States|Arizona|658[0n]|BOLD:AAC8911
Matigramma emmilta|[6501]|BBLOE1480-12|United States|Arizona|658[0n]|BOLD:AAC8911
Matigramma emmilta|[6502]|BBLSY046-09|United States|Arizona|658[0n]|BOLD:AAC8911
Matigramma emmilta|[6503]|BBLOC155-11|United States|Arizona|614[0n]|BOLD:AAC8911
Matigramma emmilta|[6504]|BBLOC177-11|United States|Arizona|658[0n]|BOLD:AAC8911
Matigramma emmilta|[6505]|LTOL1112-11|United States|Arizona|658[0n]|BOLD:AAC8911
Matigramma emmilta|[6506]|BBLOC629-11|United States|Arizona|658[0n]|BOLD:AAC8911
Matigramma emmilta|[6507]|RDNME867-08|United States|New Mexico|658[0n]|BOLD:AAC8911
Matigramma emmilta|[6508]|BBLSY038-09|United States|Arizona|658[0n]|BOLD:AAC8911
Matigramma emmilta|[6509]|BBLSY041-09|United States|Arizona|658[0n]|BOLD:AAC8911
Matigramma emmilta|[6510]|BBLSZ027-09|United States|Arizona|658[0n]|BOLD:AAC8911
Matigramma emmilta|[6511]|BBLOC628-11|United States|Arizona|658[0n]|BOLD:AAC8911
Matigramma emmilta|[6512]|BBLOC1646-11|United States|Arizona|658[0n]|BOLD:AAC8911
Matigramma emmilta|[6513]|RDNMD803-07|United States|Arizona|658[1n]|BOLD:AAC8911
Matigramma emmilta|[6514]|BBLSY298-09|United States|Arizona|658[0n]|BOLD:AAC8911
Matigramma emmilta|[6515]|BBLSY297-09|United States|Arizona|658[0n]|BOLD:AAC8911
Matigramma emmilta|[6516]|RDNME866-08|United States|New Mexico|658[0n]|BOLD:AAC8911
Matigramma emmilta|[6517]|BBLSY310-09|United States|Arizona|637[0n]|BOLD:AAC8911
Matigramma emmilta|[6518]|BBLSY300-09|United States|Arizona|638[0n]|BOLD:AAC8911
Matigramma emmilta|[6519]|BBLSY311-09|United States|Arizona|633[0n]|BOLD:AAC8911
Matigramma emmilta|[6520]|BBLSY360-09|United States|Arizona|622[0n]|BOLD:AAC8911
Matigramma emmilta|[6521]|BBLSY617-09|United States|Arizona|586[0n]|BOLD:AAC8911
Matigramma emmilta|[6522]|BBLSY831-09|United States|Arizona|658[0n]|BOLD:AAC8911
Matigramma emmilta|[6523]|USLEP092-10|United States|Arizona|658[0n]|BOLD:AAC8911
Matigramma emmilta|[6524]|USLEP1231-10|United States|Arizona|658[0n]|BOLD:AAC8911
Matigramma emmilta|[6525]|BBLOC178-11|United States|Arizona|658[0n]|BOLD:AAC8911
Matigramma emmilta|[6526]|BBLOC183-11|United States|Arizona|658[0n]|BOLD:AAC8911
Matigramma emmilta|[6527]|BBLOC255-11|United States|Arizona|658[0n]|BOLD:AAC8911
Matigramma emmilta|[6528]|BBLOC259-11|United States|Arizona|658[0n]|BOLD:AAC8911
Matigramma emmilta|[6529]|BBLOE1428-12|United States|Arizona|658[0n]|BOLD:AAC8911
Matigramma emmilta|[6530]|BBLOE1489-12|United States|New Mexico|658[0n]|BOLD:AAC8911
Matigramma emmilta|[6531]|BBLOE1490-12|United States|New Mexico|658[0n]|BOLD:AAC8911
Matigramma adoceta|[6532]|RDNMD802-07|United States|Arizona|658[0n]|BOLD:AAF1339
Matigramma adoceta|[6533]|RDNMG179-08|United States|California|658[0n]|BOLD:AAF1339
Matigramma adoceta|[6534]|RDNMG180-08|United States|Arizona|658[0n]|BOLD:AAF1339
Matigramma inopinata|[6535]|ABNCC338-07|United States|Texas|648[0n]|BOLD:AAD8733
Matigramma inopinata|[6536]|BBLOB1327-11|United States|Texas|658[0n]|BOLD:AAD8733
Matigramma inopinata|[6537]|BBLOE1468-12|United States|Texas|631[0n]|BOLD:AAD8733
Matigramma inopinata|[6538]|BBLOB614-11|United States|Texas|658[0n]|BOLD:AAD8733
Matigramma inopinata|[6539]|RDNME870-08|United States|New Mexico|658[0n]|BOLD:AAD8733
Matigramma inopinata|[6540]|RDNME865-08|United States|New Mexico|658[0n]|BOLD:AAD8733
Matigramma inopinata|[6541]|BBLSY219-09|United States|Texas|658[0n]|BOLD:AAD8733
Matigramma inopinata|[6542]|BBLOB718-11|United States|Texas|589[0n]|BOLD:AAD8733
Matigramma inopinata|[6543]|LPOKB250-09|United States|Oklahoma|646[0n]|BOLD:AAD8733
Matigramma inopinata|[6544]|ABNCC337-07|United States|Texas|594[0n]|BOLD:AAD8733
Matigramma inopinata|[6545]|BBLOE1990-12|United States|Texas|658[0n]|BOLD:AAD8733
Acritogramma metaleuca|[6546]|AWCLB394-10|United States|Arizona|658[0n]|BOLD:AAN7482
Acritogramma metaleuca|[6547]|AWCLB352-10|United States|Arizona|658[0n]|BOLD:AAN7482
Acritogramma metaleuca|[6548]|AWCLB228-10|United States|Arizona|658[0n]|BOLD:AAN7482
Acritogramma metaleuca|[6549]|AWCLB437-10|United States|Arizona|658[0n]|BOLD:AAN7482
Acritogramma metaleuca|[6550]|AWCLB484-11|United States|Arizona|658[0n]|BOLD:AAN7482
Eubolina impartialis|[6551]|BBLOD189-11|United States|Texas|658[0n]|BOLD:AAE4832
Eubolina impartialis|[6552]|BBLOC663-11|United States|Texas|658[0n]|BOLD:AAE4832
Eubolina impartialis|[6553]|BBLOC1418-11|United States|Texas|658[0n]|BOLD:AAE4832
Eubolina impartialis|[6554]|BBLOC1419-11|United States|Texas|658[0n]|BOLD:AAE4832
Eubolina impartialis|[6555]|BBLOC1998-11|United States|Texas|658[0n]|BOLD:AAE4832
Eubolina impartialis|[6556]|BBLOC1991-11|United States|Texas|658[0n]|BOLD:AAE4832
Eubolina impartialis|[6557]|HKONB060-08|United States|Texas|658[0n]|BOLD:AAE4832
Eubolina impartialis|[6558]|ABNCC374-07|United States|Texas|650[0n]|BOLD:AAE4832
Eubolina impartialis|[6559]|BBLOC670-11|United States|Texas|658[0n]|BOLD:AAE4832
Eubolina impartialis|[6560]|BBLOC602-11|United States|Texas|658[0n]|BOLD:AAE4832
Eubolina impartialis|[6561]|BBLSY220-09|United States|Texas|658[0n]|BOLD:AAE4832
Eubolina impartialis|[6562]|BBLOC388-11|United States|Texas|658[0n]|BOLD:AAE4832
Eubolina impartialis|[6563]|BBLOC1068-11|United States|Texas|633[0n]|BOLD:AAE4832
Eubolina impartialis|[6564]|BBLOC1988-11|United States|Texas|658[0n]|BOLD:AAE4832
Eubolina impartialis|[6565]|BBLOD152-11|United States|Texas|658[0n]|BOLD:AAE4832
Eubolina impartialis|[6566]|BBLOD172-11|United States|Texas|658[0n]|BOLD:AAE4832
Eubolina impartialis|[6567]|BBLOD524-11|United States|Texas|658[0n]|BOLD:AAE4832
Eubolina impartialis|[6568]|BBLOD533-11|United States|Texas|658[0n]|BOLD:AAE4832
Eubolina impartialis|[6569]|BBLOD1070-11|United States|Texas|658[0n]|BOLD:AAE4832
Eubolina impartialis|[6570]|BBLOD523-11|United States|Texas|658[0n]|BOLD:AAE4832
Eubolina impartialis|[6571]|BBLOC1067-11|United States|Texas|658[0n]|BOLD:AAE4832
Eubolina impartialis|[6572]|BBLOD508-11|United States|Texas|658[0n]|BOLD:AAE4832
Eubolina impartialis|[6573]|BBLOD513-11|United States|Texas|658[0n]|BOLD:AAE4832
Eubolina impartialis|[6574]|BBLOD516-11|United States|Texas|658[0n]|BOLD:AAE4832
Eubolina impartialis|[6575]|BBLOD1076-11|United States|Texas|658[0n]|BOLD:AAE4832
Eubolina impartialis|[6576]|ABNCC375-07|United States|Texas|655[0n]|BOLD:AAE4832
Eubolina impartialis|[6577]|BBLOC661-11|United States|Texas|658[0n]|BOLD:AAE4832
Eubolina impartialis|[6578]|BBLOD515-11|United States|Texas|658[0n]|BOLD:AAE4832
Eubolina impartialis|[6579]|BBLOD171-11|United States|Texas|658[0n]|BOLD:AAE4832
Eubolina impartialis|[6580]|BBLOC1984-11|United States|Texas|658[0n]|BOLD:AAE4832
Eubolina impartialis|[6581]|BBLOC1064-11|United States|Texas|658[0n]|BOLD:AAE4832
Eubolina impartialis|[6582]|BBLOC668-11|United States|Texas|658[0n]|BOLD:AAE4832
Eubolina impartialis|[6583]|BBLOC664-11|United States|Texas|658[0n]|BOLD:AAE4832
Eubolina impartialis|[6584]|BBLOC662-11|United States|Texas|658[0n]|BOLD:AAE4832
Eubolina impartialis|[6585]|HKONB059-08|United States|Texas|658[0n]|BOLD:AAE4832
Eubolina impartialis|[6586]|BBLOD517-11|United States|Texas|632[0n]|BOLD:AAE4832
Eubolina impartialis|[6587]|BBLOD521-11|United States|Texas|658[0n]|BOLD:AAE4832
Eubolina impartialis|[6588]|BBLOD530-11|United States|Texas|658[0n]|BOLD:AAE4832
Eubolina impartialis|[6589]|BBLOD1069-11|United States|Texas|658[0n]|BOLD:AAE4832
Eubolina impartialis|[6590]|BBLOD1072-11|United States|Texas|658[0n]|BOLD:AAE4832

Eubolina impartialis|[6588]|BBLOD530-11|United States|Texas|658[0n]|BOLD:AAE4832
Eubolina impartialis|[6589]|BBLOD1069-11|United States|Texas|658[0n]|BOLD:AAE4832
Eubolina impartialis|[6590]|BBLOD1072-11|United States|Texas|658[0n]|BOLD:AAE4832
Eubolina impartialis|[6591]|BBLOD1082-11|United States|Texas|658[0n]|BOLD:AAE4832
Eubolina impartialis|[6592]|BBLOD1084-11|United States|Texas|658[0n]|BOLD:AAE4832
Eubolina impartialis|[6593]|BBLOC1420-11|United States|Texas|658[0n]|BOLD:AAE4832
Eubolina impartialis|[6594]|BBLOD173-11|United States|Texas|658[0n]|BOLD:AAE4832
Eubolina impartialis|[6595]|BBLOD1080-11|United States|Texas|658[0n]|BOLD:AAE4832
Eubolina impartialis|[6596]|BBLOD522-11|United States|Texas|658[0n]|BOLD:AAE4832
Eubolina impartialis|[6597]|BBLOD528-11|United States|Texas|658[0n]|BOLD:AAE4832
Eubolina impartialis|[6598]|BBLOD101-11|United States|Texas|658[0n]|BOLD:AAE4832
Eubolina impartialis|[6599]|BBLOC1997-11|United States|Texas|658[0n]|BOLD:AAE4832
Eubolina impartialis|[6600]|BBLOD510-11|United States|Texas|658[0n]|BOLD:AAE4832
Eubolina impartialis|[6601]|BBLOD511-11|United States|Texas|658[0n]|BOLD:AAE4832
Eubolina impartialis|[6602]|BBLOD170-11|United States|Texas|658[0n]|BOLD:AAE4832
Eubolina impartialis|[6603]|BBLOD1077-11|United States|Texas|612[0n]|BOLD:AAE4832
Eubolina impartialis|[6604]|BBLOD512-11|United States|Texas|658[0n]|BOLD:AAE4832
Eubolina impartialis|[6605]|BBLOD520-11|United States|Texas|658[0n]|BOLD:AAE4832
Eubolina impartialis|[6606]|BBLOD153-11|United States|Texas|658[0n]|BOLD:AAE4832
Eubolina impartialis|[6607]|BBLOD097-11|United States|Texas|658[0n]|BOLD:AAE4832
Eubolina impartialis|[6608]|BBLOC1999-11|United States|Texas|658[0n]|BOLD:AAE4832
Eubolina impartialis|[6609]|BBLOC1996-11|United States|Texas|658[0n]|BOLD:AAE4832
Eubolina impartialis|[6610]|BBLOC1993-11|United States|Texas|658[0n]|BOLD:AAE4832
Eubolina impartialis|[6611]|BBLOC1572-11|United States|Texas|658[0n]|BOLD:AAE4832
Eubolina impartialis|[6612]|BBLOC1573-11|United States|Texas|620[0n]|BOLD:AAE4832
Eubolina impartialis|[6613]|BBLOD1066-11|United States|Texas|658[0n]|BOLD:AAE4832
Eubolina impartialis|[6614]|BBLOD1067-11|United States|Texas|658[0n]|BOLD:AAE4832
Eubolina impartialis|[6615]|BBLOD1083-11|United States|Texas|658[0n]|BOLD:AAE4832
Eubolina impartialis|[6616]|BBLOD150-11|United States|Texas|658[0n]|BOLD:AAE4832
Eubolina impartialis|[6617]|BBLOC1416-11|United States|Texas|658[0n]|BOLD:AAE4832
Eubolina impartialis|[6618]|BBLOC1570-11|United States|Texas|658[0n]|BOLD:AAE4832
Eubolina impartialis|[6619]|BBLOD1304-11|United States|Texas|658[0n]|BOLD:AAE4832
Acritogramma noctar|[6620]|CNCLB388-14|Mexico||682[0n]|BOLD:AAG5893
Acritogramma noctar|[6621]|CNCLB389-14|Mexico||682[0n]|BOLD:AAG5893
Acritogramma noctar|[6622]|LPYPB453-08|Mexico|Yucatan|658[0n]|BOLD:AAG5893
Acritogramma noctar|[6623]|BLPAE890-07|Costa Rica|Guanacaste|658[0n]|BOLD:AAG5893
Acritogramma noctar|[6624]|BLPAE889-07|Costa Rica|Guanacaste|658[0n]|BOLD:AAG5893
Acritogramma noctar|[6625]|LPYPB574-08|Mexico|Yucatan|635[0n]|BOLD:AAG5893
Acritogramma noctar|[6626]|LYRIO182-09|Mexico|Yucatan|658[0n]|BOLD:AAG5893
Acritogramma noctar|[6627]|CNCLB390-14|Mexico||682[0n]|BOLD:AAG5893
Acritogramma noctar|[6628]|CNCLB391-14|Mexico||682[0n]|BOLD:AAG5893
Acritogramma noctar|[6629]|CNCLB392-14|Mexico||682[0n]|BOLD:AAG5893
Melipotis acontioides|[6630]|BBLOD504-11|United States|Texas|658[0n]|BOLD:AAC4750
Melipotis acontioides|[6631]|HKONB100-08|United States|Texas|658[0n]|BOLD:AAC4750
Melipotis acontioides|[6632]|HKONB102-08|United States|Texas|658[0n]|BOLD:AAC4750
Melipotis acontioides|[6633]|BBLOC601-11|United States|Texas|622[0n]|BOLD:AAC4750
Melipotis acontioides|[6634]|BBLOB615-11|United States|Texas|658[0n]|BOLD:AAC4750
Melipotis acontioides|[6635]|BBLOD192-11|United States|Texas|658[0n]|BOLD:AAC4750
Melipotis acontioides|[6636]|LMEMB131-09|United States|Texas|658[0n]|BOLD:AAC4750
Melipotis acontioides|[6637]|IAWL141-09|United States|Arizona|658[0n]|BOLD:AAC4750
Melipotis acontioides|[6638]|HKONB103-08|United States|Texas|658[0n]|BOLD:AAC4750
Melipotis acontioides|[6639]|HKONB104-08|United States|Texas|658[0n]|BOLD:AAC4750
Melipotis acontioides|[6640]|IAWL139-09|United States|Arizona|658[0n]|BOLD:AAC4750
Melipotis acontioides|[6641]|IAWL140-09|United States|Arizona|658[0n]|BOLD:AAC4750
Melipotis acontioides|[6642]|BBLSW312-09|United States|Arizona|658[0n]|BOLD:AAC4750
Melipotis acontioides|[6643]|BBLOC605-11|United States|Texas|658[0n]|BOLD:AAC4750
Melipotis acontioides|[6644]|HKONB099-08|United States|Texas|658[0n]|BOLD:AAC4750
Melipotis acontioides|[6645]|HKONB101-08|United States|Texas|658[0n]|BOLD:AAC4750
Melipotis acontioides|[6646]|BBLOD503-11|United States|Texas|658[0n]|BOLD:AAC4750
Melipotis acontioides|[6647]|BBLOD507-11|United States|Texas|658[0n]|BOLD:AAC4750
Melipotis acontioides|[6648]|BBLOD1685-11|United States|Texas|633[0n]|BOLD:AAC4750
Dinumma deponens|[6649]|CNCLB2857-14|United States|North Carolina|658[0n]|BOLD:ACR9123
Bendisodes aeolia|[6650]|ABNCC317-07|United States|Texas|583[0n]|BOLD:AAB2072
Bendisodes aeolia|[6651]|ABNCC318-07|United States|Texas|644[0n]|BOLD:AAB2072
Bendisodes aeolia|[6652]|HKONB049-08|United States|Texas|658[0n]|BOLD:AAB2072
Bendisodes aeolia|[6653]|HKONB050-08|United States|Texas|658[0n]|BOLD:AAB2072
Bendisodes aeolia|[6654]|HKONB051-08|United States|Texas|658[0n]|BOLD:AAB2072
Bendisodes aeolia|[6655]|HKONB052-08|United States|Texas|658[0n]|BOLD:AAB2072
Bendisodes aeolia|[6656]|HKONB053-08|United States|Texas|658[0n]|BOLD:AAB2072
Ophisma tropicalis|[6657]|BLPBD239-07|Costa Rica|Guanacaste|637[0n]|BOLD:AAA4625
Ophisma tropicalis|[6658]|BLPAF494-07|Costa Rica|Guanacaste|658[0n]|BOLD:AAA4625
Ophisma tropicalis|[6659]|GWOST404-11|Argentina||658[0n]|BOLD:AAA4625
Ophisma tropicalis|[6660]|BLPDL024-10|Costa Rica|Guanacaste|658[0n]|BOLD:AAA4625
Ophisma tropicalis|[6661]|BLPCO121-08|Costa Rica|Guanacaste|658[0n]|BOLD:AAA4625
Ophisma tropicalis|[6662]|BLPCD653-08|Costa Rica|Guanacaste|658[0n]|BOLD:AAA4625
Ophisma tropicalis|[6663]|BLPAB886-06|Costa Rica|Guanacaste|658[0n]|BOLD:AAA4625
Ophisma tropicalis|[6664]|BLPAA911-06|Costa Rica|Alajuela|658[0n]|BOLD:AAA4625
Ophisma tropicalis|[6665]|BLPAC686-06|Costa Rica|Guanacaste|621[0n]|BOLD:AAA4625
Ophisma tropicalis|[6666]|LYNYM193-09|Mexico|Yucatan|637[0n]|BOLD:AAA4625
Ophisma tropicalis|[6667]|GWOST405-11|Bolivia||658[0n]|BOLD:AAA4625
Ophisma tropicalis|[6668]|RDNML252-13|Puerto Rico||658[0n]|BOLD:AAA4625
Ophisma tropicalis|[6669]|BLPAA349-06|Costa Rica|Guanacaste|658[3n]|BOLD:AAA4625
Ophisma tropicalis|[6670]|BLPAA916-06|Costa Rica|Alajuela|658[0n]|BOLD:AAA4625
Ophisma tropicalis|[6671]|BLPAC390-06|Costa Rica|Guanacaste|658[0n]|BOLD:AAA4625
Ophisma tropicalis|[6672]|BLPCD655-08|Costa Rica|Guanacaste|658[0n]|BOLD:AAA4625
Ophisma tropicalis|[6673]|BLPCD659-08|Costa Rica|Guanacaste|658[0n]|BOLD:AAA4625
Ophisma tropicalis|[6674]|QUNOD183-10|United States|Texas|658[0n]|BOLD:AAA4625
Ophisma tropicalis|[6675]|MILEP743-11|French Guiana||658[0n]|BOLD:AAA4625
Ophisma tropicalis|[6676]|LNOUD670-11|French Guiana||658[0n]|BOLD:AAA4625
Ophisma tropicalis|[6677]|BLPDB099-09|Costa Rica|Alajuela|658[0n]|BOLD:AAA4625
Ophisma tropicalis|[6678]|BLPCP252-08|Costa Rica|Guanacaste|658[0n]|BOLD:AAA4625
Ophisma tropicalis|[6679]|BLPCO843-08|Costa Rica|Guanacaste|658[0n]|BOLD:AAA4625
Ophisma tropicalis|[6680]|BLPCO625-08|Costa Rica|Guanacaste|658[0n]|BOLD:AAA4625
Ophisma tropicalis|[6681]|BLPCO623-08|Costa Rica|Guanacaste|658[0n]|BOLD:AAA4625
Ophisma tropicalis|[6682]|BLPCO277-08|Costa Rica|Guanacaste|658[0n]|BOLD:AAA4625
Ophisma tropicalis|[6683]|BLPCO124-08|Costa Rica|Guanacaste|658[0n]|BOLD:AAA4625
Ophisma tropicalis|[6684]|BLPCO040-08|Costa Rica|Guanacaste|658[0n]|BOLD:AAA4625
Ophisma tropicalis|[6685]|BLPCO039-08|Costa Rica|Guanacaste|658[0n]|BOLD:AAA4625
Ophisma tropicalis|[6686]|BLPCL019-08|Costa Rica|Alajuela|658[0n]|BOLD:AAA4625
Ophisma tropicalis|[6687]|BLPCK858-08|Costa Rica|Alajuela|658[0n]|BOLD:AAA4625
Ophisma tropicalis|[6688]|BLPCK857-08|Costa Rica|Alajuela|658[0n]|BOLD:AAA4625
Ophisma tropicalis|[6689]|BLPCK856-08|Costa Rica|Alajuela|658[0n]|BOLD:AAA4625
Ophisma tropicalis|[6690]|LPYPB106-08|Mexico|Campeche|658[0n]|BOLD:AAA4625

Ophisma tropicalis|[6688]|BLPCK857-08|Costa Rica|Alajuela|658[0n]|BOLD:AAA4625
Ophisma tropicalis|[6689]|BLPCK856-08|Costa Rica|Alajuela|658[0n]|BOLD:AAA4625
Ophisma tropicalis|[6690]|LPYPB106-08|Mexico|Campeche|658[0n]|BOLD:AAA4625
Ophisma tropicalis|[6691]|LOCRC027-08|Costa Rica|Alajuela|657[0n]|BOLD:AAA4625
Ophisma tropicalis|[6692]|BLPCJ104-08|Costa Rica|Guanacaste|658[0n]|BOLD:AAA4625
Ophisma tropicalis|[6693]|BLPCJ103-08|Costa Rica|Guanacaste|658[0n]|BOLD:AAA4625
Ophisma tropicalis|[6694]|BLPCG755-08|Costa Rica|Guanacaste|658[0n]|BOLD:AAA4625
Ophisma tropicalis|[6695]|BLPCG754-08|Costa Rica|Guanacaste|658[0n]|BOLD:AAA4625
Ophisma tropicalis|[6696]|BLPCH641-08|Costa Rica|Guanacaste|658[0n]|BOLD:AAA4625
Ophisma tropicalis|[6697]|BLPCH640-08|Costa Rica|Guanacaste|658[0n]|BOLD:AAA4625
Ophisma tropicalis|[6698]|BLPCE870-08|Costa Rica|Alajuela|658[0n]|BOLD:AAA4625
Ophisma tropicalis|[6699]|BLPCE366-08|Costa Rica|Guanacaste|658[0n]|BOLD:AAA4625
Ophisma tropicalis|[6700]|BLPCD661-08|Costa Rica|Guanacaste|658[0n]|BOLD:AAA4625
Ophisma tropicalis|[6701]|BLPCD658-08|Costa Rica|Guanacaste|658[0n]|BOLD:AAA4625
Ophisma tropicalis|[6702]|BLPCD657-08|Costa Rica|Guanacaste|658[0n]|BOLD:AAA4625
Ophisma tropicalis|[6703]|BLPCD656-08|Costa Rica|Guanacaste|658[0n]|BOLD:AAA4625
Ophisma tropicalis|[6704]|BLPCD654-08|Costa Rica|Guanacaste|658[0n]|BOLD:AAA4625
Ophisma tropicalis|[6705]|BLPCC523-08|Costa Rica|Guanacaste|658[0n]|BOLD:AAA4625
Ophisma tropicalis|[6706]|BLPCC436-08|Costa Rica|Guanacaste|658[0n]|BOLD:AAA4625
Ophisma tropicalis|[6707]|BLPCB414-08|Costa Rica|Alajuela|658[0n]|BOLD:AAA4625
Ophisma tropicalis|[6708]|BLPCA930-08|Costa Rica|Guanacaste|658[0n]|BOLD:AAA4625
Ophisma tropicalis|[6709]|MHMXM197-07|Costa Rica|Alajuela|658[0n]|BOLD:AAA4625
Ophisma tropicalis|[6710]|MHMXM091-07|Costa Rica|Alajuela|658[0n]|BOLD:AAA4625
Ophisma tropicalis|[6711]|BLPBH477-07|Costa Rica|Guanacaste|658[0n]|BOLD:AAA4625
Ophisma tropicalis|[6712]|BLPBG848-07|Costa Rica|Guanacaste|658[0n]|BOLD:AAA4625
Ophisma tropicalis|[6713]|BLPBF019-07|Costa Rica|Guanacaste|658[0n]|BOLD:AAA4625
Ophisma tropicalis|[6714]|BLPBD152-07|Costa Rica|Alajuela|658[0n]|BOLD:AAA4625
Ophisma tropicalis|[6715]|BLPBC393-07|Costa Rica|Alajuela|658[0n]|BOLD:AAA4625
Ophisma tropicalis|[6716]|BLPAF868-07|Costa Rica|Guanacaste|658[0n]|BOLD:AAA4625
Ophisma tropicalis|[6717]|BLPAF516-07|Costa Rica|Guanacaste|658[0n]|BOLD:AAA4625
Ophisma tropicalis|[6718]|BLPAE415-06|Costa Rica|Guanacaste|658[0n]|BOLD:AAA4625
Ophisma tropicalis|[6719]|BLPAC392-06|Costa Rica|Guanacaste|658[0n]|BOLD:AAA4625
Ophisma tropicalis|[6720]|BLPAC391-06|Costa Rica|Guanacaste|658[0n]|BOLD:AAA4625
Ophisma tropicalis|[6721]|BLPAC389-06|Costa Rica|Guanacaste|658[0n]|BOLD:AAA4625
Ophisma tropicalis|[6722]|BLPAB885-06|Costa Rica|Guanacaste|658[0n]|BOLD:AAA4625
Ophisma tropicalis|[6723]|BLPAB884-06|Costa Rica|Guanacaste|658[0n]|BOLD:AAA4625
Ophisma tropicalis|[6724]|BLPAB883-06|Costa Rica|Guanacaste|658[0n]|BOLD:AAA4625
Ophisma tropicalis|[6725]|BLPAB387-06|Costa Rica|Guanacaste|658[0n]|BOLD:AAA4625
Ophisma tropicalis|[6726]|BLPAB386-06|Costa Rica|Guanacaste|658[0n]|BOLD:AAA4625
Ophisma tropicalis|[6727]|BLPAB385-06|Costa Rica|Guanacaste|658[0n]|BOLD:AAA4625
Ophisma tropicalis|[6728]|BLPAA915-06|Costa Rica|Alajuela|658[0n]|BOLD:AAA4625
Ophisma tropicalis|[6729]|BLPAA910-06|Costa Rica|Alajuela|658[0n]|BOLD:AAA4625
Ophisma tropicalis|[6730]|BLPAA909-06|Costa Rica|Alajuela|658[0n]|BOLD:AAA4625
Ophisma tropicalis|[6731]|BLPAA901-06|Costa Rica|Alajuela|658[0n]|BOLD:AAA4625
Ophisma tropicalis|[6732]|MHAUB340-05|Costa Rica|Guanacaste|658[0n]|BOLD:AAA4625
Ophisma tropicalis|[6733]|MHAUB338-05|Costa Rica|Guanacaste|658[0n]|BOLD:AAA4625
Ophisma tropicalis|[6734]|BLPAA828-06|Costa Rica|Alajuela|658[0n]|BOLD:AAA4625
Ophisma tropicalis|[6735]|BLPBF017-07|Costa Rica|Guanacaste|645[0n]|BOLD:AAA4625
Ophisma tropicalis|[6736]|LYNYM144-09|Mexico|Yucatan|628[0n]|BOLD:AAA4625
Ophisma tropicalis|[6737]|LYNYM161-09|Mexico|Yucatan|658[0n]|BOLD:AAA4625
Ophisma tropicalis|[6738]|LYPIE882-09|Mexico|Yucatan|658[0n]|BOLD:AAA4625
Ophisma tropicalis|[6739]|BLPDK1699-09|Costa Rica|Guanacaste|658[0n]|BOLD:AAA4625
Ophisma tropicalis|[6740]|BLPDM591-10|Costa Rica|Guanacaste|658[0n]|BOLD:AAA4625
Ophisma tropicalis|[6741]|LEMMZ021-10|Brazil|Parana|658[0n]|BOLD:AAA4625
Ophisma tropicalis|[6742]|LEMMZ113-10|Brazil|Parana|658[0n]|BOLD:AAA4625
Ophisma tropicalis|[6743]|LEMMZ114-10|Brazil|Parana|658[0n]|BOLD:AAA4625
Ophisma tropicalis|[6744]|LEMMZ115-10|Brazil|Parana|658[0n]|BOLD:AAA4625
Ophisma tropicalis|[6745]|LEMMZ116-10|Brazil|Parana|658[0n]|BOLD:AAA4625
Ophisma tropicalis|[6746]|BLPDU238-11|Costa Rica|Guanacaste|658[0n]|BOLD:AAA4625
Ophisma tropicalis|[6747]|BLPDU239-11|Costa Rica|Guanacaste|658[0n]|BOLD:AAA4625
Ophisma tropicalis|[6748]|BLPDU499-11|Costa Rica|Guanacaste|658[0n]|BOLD:AAA4625
Ophisma tropicalis|[6749]|BLPDX493-11|Costa Rica|Guanacaste|658[0n]|BOLD:AAA4625
Ophisma tropicalis|[6750]|BLPDZ090-11|Costa Rica|Guanacaste|658[0n]|BOLD:AAA4625
Ophisma tropicalis|[6751]|GWOSQ386-11|Nicaragua||658[0n]|BOLD:AAA4625
Ophisma tropicalis|[6752]|MHMYN100-11|Costa Rica||658[0n]|BOLD:AAA4625
Ophisma tropicalis|[6753]|GWOST406-11|Peru||658[0n]|BOLD:AAA4625
Ophisma tropicalis|[6754]|MHMYO351-11|Costa Rica||658[0n]|BOLD:AAA4625
Ophisma tropicalis|[6755]|ARMOT148-12|Argentina|Misiones|658[0n]|BOLD:AAA4625
Ophisma tropicalis|[6756]|ARMOT684-12|Argentina|Misiones|658[0n]|BOLD:AAA4625
Ophisma tropicalis|[6757]|ARMOT701-12|Argentina|Misiones|658[0n]|BOLD:AAA4625
Ophisma tropicalis|[6758]|ARMOT711-12|Argentina|Misiones|658[0n]|BOLD:AAA4625
Ophisma tropicalis|[6759]|MOTAR184-12|Argentina|Misiones|658[0n]|BOLD:AAA4625
Ophisma tropicalis|[6760]|BLPEF3132-13|Costa Rica|Guanacaste|658[0n]|BOLD:AAA4625
Ophisma tropicalis|[6761]|MHMYS3020-13|Costa Rica|Guanacaste|658[0n]|BOLD:AAA4625
Ledaea perditalis|[6762]|LOCT232-05|United States|Connecticut|658[0n]|BOLD:AAB5246
Ledaea perditalis|[6763]|LOCT233-05|United States|Connecticut|658[0n]|BOLD:AAB5246
Ledaea perditalis|[6764]|UDLEP083-09|United States|Maryland|633[0n]|BOLD:AAB5246
Ledaea perditalis|[6765]|LOFLB701-06|United States|Florida|658[0n]|BOLD:AAB5246
Ledaea perditalis|[6766]|LOFLB278-06|United States|Florida|658[0n]|BOLD:AAB5246
Ledaea perditalis|[6767]|LOFLB698-06|United States|Florida|658[0n]|BOLD:AAB5246
Ledaea perditalis|[6768]|LOFLB708-06|United States|Florida|658[0n]|BOLD:AAB5246
Ledaea perditalis|[6769]|LOFLB719-06|United States|Florida|658[0n]|BOLD:AAB5246
Ledaea perditalis|[6770]|LOFLB739-06|United States|Florida|658[0n]|BOLD:AAB5246
Ledaea perditalis|[6771]|LOFLB762-06|United States|Florida|658[0n]|BOLD:AAB5246
Ledaea perditalis|[6772]|LOCT295-05|United States|Connecticut|658[0n]|BOLD:AAB5246
Ledaea perditalis|[6773]|LNCB346-06|United States|North Carolina|658[0n]|BOLD:AAB5246
Ledaea perditalis|[6774]|UDLEP039-09|United States|Maryland|658[0n]|BOLD:AAB5246
Ledaea perditalis|[6775]|UDLEP041-09|United States|Maryland|658[0n]|BOLD:AAB5246
Ledaea perditalis|[6776]|UDLEP096-09|United States|Maryland|658[0n]|BOLD:AAB5246
Ledaea perditalis|[6777]|UDLEP101-09|United States|Maryland|658[0n]|BOLD:AAB5246
Ledaea perditalis|[6778]|UDLEP107-09|United States|Maryland|658[0n]|BOLD:AAB5246
Ledaea perditalis|[6779]|UDLEP114-09|United States|Maryland|658[0n]|BOLD:AAB5246
Ledaea perditalis|[6780]|XAJ866-06|Canada|Ontario|658[0n]|BOLD:AAB5246
Ledaea perditalis|[6781]|LILLA290-11|United States|Illinois|658[0n]|BOLD:AAB5246
Ledaea perditalis|[6782]|LMEMB042-09|United States|Mississippi|641[0n]|BOLD:AAB5246
Ledaea perditalis|[6783]|ABNCC207-07|United States|Indiana|634[0n]|BOLD:AAB5246
Ledaea perditalis|[6784]|LMEMB046-09|United States|Mississippi|658[0n]|BOLD:AAB5246
Ledaea perditalis|[6785]|LMEMB044-09|United States|Louisiana|658[0n]|BOLD:AAB5246
Ledaea perditalis|[6786]|LPSO604-08|Canada|Ontario|658[0n]|BOLD:AAB5246
Ledaea perditalis|[6787]|LNCB345-06|United States|North Carolina|658[0n]|BOLD:AAB5246
Ledaea perditalis|[6788]|XAE608-04|Canada|Ontario|658[0n]|BOLD:AAB5246
Ledaea perditalis|[6789]|ABNCC206-07|United States|Texas|612[1n]|BOLD:AAB5246
Ledaea perditalis|[6790]|LPOKB310-09|United States|Oklahoma|646[0n]|BOLD:AAB5246

Ledaea perditalis|[6788]|XAE608-04|Canada|Ontario|658[0n]|BOLD:AAB5246
Ledaea perditalis|[6789]|ABNCC206-07|United States|Texas|612[1n]|BOLD:AAB5246
Ledaea perditalis|[6790]|LPOKB310-09|United States|Oklahoma|646[0n]|BOLD:AAB5246
Ledaea perditalis|[6791]|LNCC842-11|United States|North Carolina|658[0n]|BOLD:AAB5246
Ledaea perditalis|[6792]|MILEQ226-11|United States|Alabama|658[0n]|BOLD:AAB5246
Pangrapta decoralis|[6793]|MILEP083-09|United States|North Carolina|658[0n]|BOLD:ABY8422
Pangrapta decoralis|[6794]|LNCB399-06|United States|North Carolina|658[0n]|BOLD:ABY8422
Pangrapta decoralis|[6795]|LNCB391-06|United States|North Carolina|658[0n]|BOLD:ABY8422
Pangrapta decoralis|[6796]|LNC336-05|United States|North Carolina|536[0n]|BOLD:ABY8422
Pangrapta decoralis|[6797]|LNCB394-06|United States|North Carolina|658[0n]|BOLD:ABY8422
Pangrapta decoralis|[6798]|LNCB396-06|United States|North Carolina|658[0n]|BOLD:ABY8422
Pangrapta decoralis|[6799]|LNCB542-09|United States|North Carolina|658[0n]|BOLD:ABY8422
Pangrapta decoralis|[6800]|LNCB544-09|United States|North Carolina|658[0n]|BOLD:ABY8422
Pangrapta decoralis|[6801]|LNCB611-09|United States|North Carolina|658[0n]|BOLD:ABY8422
Pangrapta decoralis|[6802]|LNCB612-09|United States|North Carolina|658[0n]|BOLD:ABY8422
Pangrapta decoralis|[6803]|LNCB633-09|United States|North Carolina|658[0n]|BOLD:ABY8422
Pangrapta decoralis|[6804]|LNCB706-09|United States|North Carolina|658[0n]|BOLD:ABY8422
Pangrapta decoralis|[6805]|LNCB768-09|United States|North Carolina|658[0n]|BOLD:ABY8422
Pangrapta decoralis|[6806]|MILEP085-09|United States|North Carolina|658[0n]|BOLD:ABY8422
Pangrapta decoralis|[6807]|LNCC554-11|United States|North Carolina|658[0n]|BOLD:ABY8422
Pangrapta decoralis|[6808]|LNCB609-09|United States|North Carolina|658[0n]|BOLD:ABY8422
Pangrapta decoralis|[6809]|LNCB617-09|United States|North Carolina|658[0n]|BOLD:ABY8422
Pangrapta decoralis|[6810]|LNCB607-09|United States|North Carolina|658[0n]|BOLD:ABY8422
Pangrapta decoralis|[6811]|LNCB608-09|United States|North Carolina|658[0n]|BOLD:ABY8422
Pangrapta decoralis|[6812]|LNCB606-09|United States|North Carolina|658[0n]|BOLD:ABY8422
Pangrapta decoralis|[6813]|LNCB591-09|United States|North Carolina|658[0n]|BOLD:ABY8422
Pangrapta decoralis|[6814]|LNCB578-09|United States|North Carolina|658[0n]|BOLD:ABY8422
Pangrapta decoralis|[6815]|LNCB565-09|United States|North Carolina|658[0n]|BOLD:ABY8422
Pangrapta decoralis|[6816]|LNCB389-06|United States|North Carolina|658[0n]|BOLD:ABY8422
Pangrapta decoralis|[6817]|LNCB385-06|United States|North Carolina|658[0n]|BOLD:ABY8422
Pangrapta decoralis|[6818]|LNCB384-06|United States|North Carolina|658[0n]|BOLD:ABY8422
Pangrapta decoralis|[6819]|LOFLA911-06|United States|Florida|658[0n]|BOLD:ABY8422
Pangrapta decoralis|[6820]|LNC815-06|United States|North Carolina|658[0n]|BOLD:ABY8422
Pangrapta decoralis|[6821]|LNC334-05|United States|North Carolina|658[0n]|BOLD:ABY8422
Pangrapta decoralis|[6822]|LNCB577-09|United States|North Carolina|658[0n]|BOLD:ABY8422
Pangrapta decoralis|[6823]|MILEP086-09|United States|North Carolina|658[0n]|BOLD:ABY8422
Pangrapta decoralis|[6824]|LNCB605-09|United States|North Carolina|658[0n]|BOLD:ABY8422
Pangrapta decoralis|[6825]|MILEP043-09|United States|North Carolina|658[0n]|BOLD:ABY8422
Pangrapta decoralis|[6826]|MILEP082-09|United States|North Carolina|658[0n]|BOLD:ABY8422
Pangrapta decoralis|[6827]|LNCB560-09|United States|North Carolina|635[0n]|BOLD:ABY8422
Pangrapta decoralis|[6828]|MILEP084-09|United States|North Carolina|634[0n]|BOLD:ABY8422
Pangrapta decoralis|[6829]|BBLOB912-11|United States|Florida|658[0n]|BOLD:ABY8422
Pangrapta decoralis|[6830]|BBLOB437-11|United States|Florida|658[0n]|BOLD:ACE8667
Pangrapta decoralis|[6831]|LNC813-06|United States|North Carolina|658[0n]|BOLD:ACE8667
Pangrapta decoralis|[6832]|LNCB393-06|United States|North Carolina|658[0n]|BOLD:ACE8667
Pangrapta decoralis|[6833]|LNC814-06|United States|North Carolina|656[0n]|BOLD:ACE8667
Pangrapta decoralis|[6834]|LNCB401-06|United States|North Carolina|658[0n]|BOLD:ACE8667
Pangrapta decoralis|[6835]|LNCB400-06|United States|North Carolina|658[0n]|BOLD:ACE8667
Pangrapta decoralis|[6836]|LNCB398-06|United States|North Carolina|658[0n]|BOLD:ACE8667
Pangrapta decoralis|[6837]|LNCB397-06|United States|North Carolina|658[0n]|BOLD:ACE8667
Pangrapta decoralis|[6838]|LNCB395-06|United States|North Carolina|658[0n]|BOLD:ACE8667
Pangrapta decoralis|[6839]|LNCB392-06|United States|North Carolina|658[0n]|BOLD:ACE8667
Pangrapta decoralis|[6840]|LNCB390-06|United States|North Carolina|658[0n]|BOLD:ACE8667
Pangrapta decoralis|[6841]|LNC337-05|United States|North Carolina|569[0n]|BOLD:ACE8667
Pangrapta decoralis|[6842]|LNCB562-09|United States|North Carolina|634[0n]|BOLD:ACE8667
Pangrapta decoralis|[6843]|LNCB704-09|United States|North Carolina|658[0n]|BOLD:ACE8667
Pangrapta decoralis|[6844]|LNCB705-09|United States|North Carolina|658[0n]|BOLD:ACE8667
Pangrapta decoralis|[6845]|LNCB614-09|United States|North Carolina|658[0n]|BOLD:ACE8667
Pangrapta decoralis|[6846]|LNCB613-09|United States|North Carolina|658[0n]|BOLD:ACE8667
Pangrapta decoralis|[6847]|LNCB569-09|United States|North Carolina|658[0n]|BOLD:ACE8667
Pangrapta decoralis|[6848]|LNCB615-09|United States|North Carolina|638[0n]|BOLD:ACE8667
Pangrapta decoralis|[6849]|LNCB616-09|United States|North Carolina|658[0n]|BOLD:ACE8667
Pangrapta decoralis|[6850]|LNCB610-09|United States|North Carolina|658[0n]|BOLD:ACE8667
Pangrapta decoralis|[6851]|LNCC003-10|United States|North Carolina|658[0n]|BOLD:ACE8667
Pangrapta decoralis|[6852]|LOFLA277-06|United States|Florida|658[0n]|BOLD:ACE8667
Pangrapta decoralis|[6853]|LOFLB689-06|United States|Florida|658[0n]|BOLD:ACE8667
Pangrapta decoralis|[6854]|LNCB821-09|United States|Alabama|658[0n]|BOLD:ACE8667
Pangrapta decoralis|[6855]|LOFLA168-06|United States|Florida|658[0n]|BOLD:ACE8667
Pangrapta decoralis|[6856]|LNCB566-09|United States|North Carolina|658[0n]|BOLD:ACE8667
Pangrapta decoralis|[6857]|LNCB817-09|United States|Alabama|658[0n]|BOLD:ACE8667
Pangrapta decoralis|[6858]|LNCB818-09|United States|Alabama|658[0n]|BOLD:ACE8667
Pangrapta decoralis|[6859]|LNCB819-09|United States|Alabama|658[0n]|BOLD:ACE8667
Pangrapta decoralis|[6860]|LNCB820-09|United States|Alabama|658[0n]|BOLD:ACE8667
Pangrapta decoralis|[6861]|LNCB822-09|United States|Alabama|658[0n]|BOLD:ACE8667
Pangrapta decoralis|[6862]|MILEP331-10|United States|Alabama|658[0n]|BOLD:ACE8667
Pangrapta decoralis|[6863]|MILEP332-10|United States|Alabama|658[0n]|BOLD:ACE8667
Pangrapta decoralis|[6864]|MILEP333-10|United States|Alabama|658[0n]|BOLD:ACE8667
Pangrapta decoralis|[6865]|MILEP334-10|United States|Alabama|658[0n]|BOLD:ACE8667
Pangrapta decoralis|[6866]|BBLOB1857-11|United States|Florida|658[0n]|BOLD:ACE8667
Pangrapta decoralis|[6867]|LOFLA571-06|United States|Florida|658[0n]|BOLD:ACE8667
Pangrapta decoralis|[6868]|LOFLB691-06|United States|Florida|658[0n]|BOLD:ACE8667
Pangrapta decoralis|[6869]|LOFLA608-06|United States|Florida|658[0n]|BOLD:ACE8667
Pangrapta decoralis|[6870]|LNCB816-09|United States|Alabama|632[0n]|BOLD:ACE8667
Pangrapta decoralis|[6871]|BBLOC102-11|United States|Florida|658[0n]|BOLD:ACE8667
Pangrapta decoralis|[6872]|LNCC004-10|United States|North Carolina|658[0n]|BOLD:AAA3086
Pangrapta decoralis|[6873]|LNCB540-09|United States|North Carolina|621[0n]|BOLD:AAA3086
Pangrapta decoralis|[6874]|LNCB568-09|United States|North Carolina|658[0n]|BOLD:AAA3086
Pangrapta decoralis|[6875]|LNCB713-09|United States|North Carolina|658[0n]|BOLD:AAA3086
Pangrapta decoralis|[6876]|LNCB363-06|United States|North Carolina|658[0n]|BOLD:AAA3086
Pangrapta decoralis|[6877]|LNCB546-09|United States|North Carolina|658[0n]|BOLD:AAA3086
Pangrapta decoralis|[6878]|LNCB666-09|United States|North Carolina|658[0n]|BOLD:AAA3086
Pangrapta decoralis|[6879]|LNCC009-10|United States|North Carolina|658[0n]|BOLD:AAA3086
Pangrapta decoralis|[6880]|LGSM742-04|United States|Tennessee|658[0n]|BOLD:AAA3086
Pangrapta decoralis|[6881]|MILEP016-09|United States|North Carolina|658[0n]|BOLD:AAA3086
Pangrapta decoralis|[6882]|MILEP019-09|United States|North Carolina|658[0n]|BOLD:AAA3086
Pangrapta decoralis|[6883]|MILEP025-09|United States|North Carolina|658[0n]|BOLD:AAA3086
Pangrapta decoralis|[6884]|LNCC007-10|United States|North Carolina|658[0n]|BOLD:AAA3086
Pangrapta decoralis|[6885]|LTOLB014-08|United States|Maryland|658[0n]|BOLD:AAA3086
Pangrapta decoralis|[6886]|LNCB541-09|United States|North Carolina|658[0n]|BOLD:AAA3086
Pangrapta decoralis|[6887]|LNCC087-10|United States|North Carolina|658[0n]|BOLD:AAA3086
Pangrapta decoralis|[6888]|LNCB548-09|United States|North Carolina|658[0n]|BOLD:AAA3086
Pangrapta decoralis|[6889]|LNCB567-09|United States|North Carolina|658[0n]|BOLD:AAA3086
Pangrapta decoralis|[6890]|LNCB717-09|United States|North Carolina|658[0n]|BOLD:AAA3086

[illegible]
Pangrapta decoralis|[6888]|LNCB548-09|United States|North Carolina|658[0n]|BOLD:AAA3086
Pangrapta decoralis|[6889]|LNCB567-09|United States|North Carolina|658[0n]|BOLD:AAA3086
Pangrapta decoralis|[6890]|LNCB717-09|United States|North Carolina|658[0n]|BOLD:AAA3086
Pangrapta decoralis|[6891]|LNCC558-11|United States|North Carolina|658[0n]|BOLD:AAA3086
Pangrapta decoralis|[6892]|MILEP022-09|United States|North Carolina|658[0n]|BOLD:AAA3086
Pangrapta decoralis|[6893]|LNCB579-09|United States|North Carolina|658[0n]|BOLD:AAA3086
Pangrapta decoralis|[6894]|LNCC559-11|United States|North Carolina|658[0n]|BOLD:AAA3086
Pangrapta decoralis|[6895]|LNCC753-11|United States|North Carolina|658[0n]|BOLD:AAA3086
Pangrapta decoralis|[6896]|LNCC005-10|United States|North Carolina|658[0n]|BOLD:AAA3086
Pangrapta decoralis|[6897]|LNCC008-10|United States|North Carolina|658[0n]|BOLD:AAA3086
Pangrapta decoralis|[6898]|MILEP023-09|United States|North Carolina|658[0n]|BOLD:AAA3086
Pangrapta decoralis|[6899]|MILEP024-09|United States|North Carolina|658[0n]|BOLD:AAA3086
Pangrapta decoralis|[6900]|MILEP018-09|United States|North Carolina|658[0n]|BOLD:AAA3086
Pangrapta decoralis|[6901]|MILEP015-09|United States|North Carolina|658[0n]|BOLD:AAA3086
Pangrapta decoralis|[6902]|LNCB718-09|United States|North Carolina|658[0n]|BOLD:AAA3086
Pangrapta decoralis|[6903]|LNCB716-09|United States|North Carolina|658[0n]|BOLD:AAA3086
Pangrapta decoralis|[6904]|LNCB715-09|United States|North Carolina|658[0n]|BOLD:AAA3086
Pangrapta decoralis|[6905]|LNCB564-09|United States|North Carolina|658[0n]|BOLD:AAA3086
Pangrapta decoralis|[6906]|LNCB563-09|United States|North Carolina|658[0n]|BOLD:AAA3086
Pangrapta decoralis|[6907]|LNCB547-09|United States|North Carolina|658[0n]|BOLD:AAA3086
Pangrapta decoralis|[6908]|LNCB543-09|United States|North Carolina|658[0n]|BOLD:AAA3086
Pangrapta decoralis|[6909]|LNCB539-09|United States|North Carolina|658[0n]|BOLD:AAA3086
Pangrapta decoralis|[6910]|LNCB362-06|United States|North Carolina|658[0n]|BOLD:AAA3086
Pangrapta decoralis|[6911]|LNC301-05|United States|North Carolina|658[0n]|BOLD:AAA3086
Pangrapta decoralis|[6912]|MILEP021-09|United States|North Carolina|614[0n]|BOLD:AAA3086
Pangrapta decoralis|[6913]|LNCC754-11|United States|North Carolina|658[0n]|BOLD:AAA3086
Pangrapta decoralis|[6914]|LNCB665-09|United States|North Carolina|658[0n]|BOLD:AAA3086
Pangrapta decoralis|[6915]|LNCB549-09|United States|North Carolina|658[0n]|BOLD:AAA3086
Pangrapta decoralis|[6916]|LNCC751-11|United States|North Carolina|658[0n]|BOLD:AAA3086
Pangrapta decoralis|[6917]|LNC300-05|United States|North Carolina|658[0n]|BOLD:AAA3086
Pangrapta decoralis|[6918]|LNCB712-09|United States|North Carolina|658[0n]|BOLD:AAA3086
Pangrapta decoralis|[6919]|MILEP026-09|United States|North Carolina|658[0n]|BOLD:AAA3086
Pangrapta decoralis|[6920]|MILEP031-09|United States|North Carolina|658[0n]|BOLD:AAA3086
Pangrapta decoralis|[6921]|LNCC755-11|United States|North Carolina|658[0n]|BOLD:AAA3086
Pangrapta decoralis|[6922]|LOT467-04|United States|Tennessee|658[0n]|BOLD:AAA3086
Pangrapta decoralis|[6923]|LNCB387-06|United States|North Carolina|658[0n]|BOLD:AAA3086
Pangrapta decoralis|[6924]|LOTB119-05|United States|Tennessee|658[0n]|BOLD:AAA3086
Pangrapta decoralis|[6925]|LOTB121-05|United States|Tennessee|658[0n]|BOLD:AAA3086
Pangrapta decoralis|[6926]|LNCB361-06|United States|North Carolina|658[0n]|BOLD:AAA3086
Pangrapta decoralis|[6927]|LSEU350-06|United States|Georgia|658[0n]|BOLD:AAA3086
Pangrapta decoralis|[6928]|LOTB456-05|United States|Tennessee|658[0n]|BOLD:AAA3086
Pangrapta decoralis|[6929]|LOTB158-05|United States|Tennessee|658[0n]|BOLD:AAA3086
Pangrapta decoralis|[6930]|LOTB120-05|United States|Tennessee|658[0n]|BOLD:AAA3086
Pangrapta decoralis|[6931]|LGSMC347-05|United States|Tennessee|658[0n]|BOLD:AAA3086
Pangrapta decoralis|[6932]|LGSMC346-05|United States|Tennessee|658[0n]|BOLD:AAA3086
Pangrapta decoralis|[6933]|LGSMC345-05|United States|Tennessee|658[0n]|BOLD:AAA3086
Pangrapta decoralis|[6934]|LGSMC344-05|United States|Tennessee|658[0n]|BOLD:AAA3086
Pangrapta decoralis|[6935]|LGSMC343-05|United States|Tennessee|658[0n]|BOLD:AAA3086
Pangrapta decoralis|[6936]|LOT193-04|United States|Tennessee|658[0n]|BOLD:AAA3086
Pangrapta decoralis|[6937]|LSEU080-06|United States|North Carolina|658[0n]|BOLD:AAA3086
Pangrapta decoralis|[6938]|LOTB535-05|United States|Tennessee|658[2n]|BOLD:AAA3086
Pangrapta decoralis|[6939]|LOT196-04|United States|Tennessee|609[0n]|BOLD:AAA3086
Pangrapta decoralis|[6940]|LOTB421-05|United States|Tennessee|636[0n]|BOLD:AAA3086
Pangrapta decoralis|[6941]|LSEU351-06|United States|Georgia|634[0n]|BOLD:AAA3086
Pangrapta decoralis|[6942]|LNCB388-06|United States|North Carolina|628[0n]|BOLD:AAA3086
Pangrapta decoralis|[6943]|LGSMG551-07|United States|North Carolina|657[0n]|BOLD:AAA3086
Pangrapta decoralis|[6944]|LGSMG552-07|United States|Tennessee|658[0n]|BOLD:AAA3086
Pangrapta decoralis|[6945]|LNCC088-10|United States|North Carolina|658[0n]|BOLD:AAA3086
Pangrapta decoralis|[6946]|LNCB386-06|United States|North Carolina|658[0n]|BOLD:AAA3086
Pangrapta decoralis|[6947]|LMEMB037-09|United States|Alabama|658[0n]|BOLD:AAA3086
Pangrapta decoralis|[6948]|LNCC089-10|United States|North Carolina|658[0n]|BOLD:AAA3086
Pangrapta decoralis|[6949]|BBLEC647-09|Canada|Nova Scotia|658[0n]|BOLD:AAA3086
Pangrapta decoralis|[6950]|BBLPC580-09|Canada|Nova Scotia|658[0n]|BOLD:AAA3086
Pangrapta decoralis|[6951]|BBLPC143-09|Canada|Nova Scotia|658[0n]|BOLD:AAA3086
Pangrapta decoralis|[6952]|BBLEC623-09|Canada|Nova Scotia|658[0n]|BOLD:AAA3086
Pangrapta decoralis|[6953]|BBLPE057-09|Canada|Nova Scotia|658[0n]|BOLD:AAA3086
Pangrapta decoralis|[6954]|BBLPE093-09|Canada|Nova Scotia|658[0n]|BOLD:AAA3086
Pangrapta decoralis|[6955]|BBLPE160-09|Canada|Nova Scotia|658[0n]|BOLD:AAA3086
Pangrapta decoralis|[6956]|MILEP029-09|United States|North Carolina|658[0n]|BOLD:AAA3086
Pangrapta decoralis|[6957]|LNCC006-10|United States|North Carolina|658[0n]|BOLD:AAA3086
Pangrapta decoralis|[6958]|LNCB618-09|United States|North Carolina|658[0n]|BOLD:AAA3086
Pangrapta decoralis|[6959]|LNCB656-09|United States|North Carolina|658[0n]|BOLD:AAA3086
Pangrapta decoralis|[6960]|MILEP020-09|United States|North Carolina|658[0n]|BOLD:AAA3086
Pangrapta decoralis|[6961]|MILEP030-09|United States|North Carolina|658[0n]|BOLD:AAA3086
Pangrapta decoralis|[6962]|LOFLA435-06|United States|Florida|658[0n]|BOLD:AAA3086
Pangrapta decoralis|[6963]|LNCB580-09|United States|North Carolina|658[0n]|BOLD:AAA3086
Pangrapta decoralis|[6964]|MILEP080-09|United States|North Carolina|658[0n]|BOLD:AAA3086
Pangrapta decoralis|[6965]|MILEP081-09|United States|North Carolina|658[0n]|BOLD:AAA3086
Pangrapta decoralis|[6966]|LNCC556-11|United States|North Carolina|658[0n]|BOLD:AAA3086
Pangrapta decoralis|[6967]|LNCC560-11|United States|North Carolina|658[0n]|BOLD:AAA3086
Pangrapta decoralis|[6968]|ABNCC205-07|United States|Texas|595[2n]|BOLD:AAA3086
Pangrapta decoralis|[6969]|ABNCC204-07|United States|Texas|638[0n]|BOLD:AAA3086
Pangrapta decoralis|[6970]|LPOKA084-08|United States|Oklahoma|658[0n]|BOLD:AAA3086
Pangrapta decoralis|[6971]|LMEMB039-09|United States|Louisiana|658[0n]|BOLD:AAA3086
Pangrapta decoralis|[6972]|LMEMB038-09|United States|Mississippi|658[0n]|BOLD:AAA3086
Pangrapta decoralis|[6973]|LNCB664-09|United States|North Carolina|658[0n]|BOLD:AAA3086
Pangrapta decoralis|[6974]|LNCC086-10|United States|North Carolina|658[0n]|BOLD:AAA3086
Pangrapta decoralis|[6975]|LGSM757-04|United States|Tennessee|658[0n]|BOLD:AAA3086
Pangrapta decoralis|[6976]|LOT194-04|United States|Tennessee|609[0n]|BOLD:AAA3086
Pangrapta decoralis|[6977]|LOT195-04|United States|Tennessee|609[0n]|BOLD:AAA3086
Pangrapta decoralis|[6978]|LSEU404-06|United States|Georgia|658[0n]|BOLD:AAA3086
Pangrapta decoralis|[6979]|LNCB545-09|United States|North Carolina|658[0n]|BOLD:AAA3086
Pangrapta decoralis|[6980]|LNCB561-09|United States|North Carolina|658[0n]|BOLD:AAA3086
Pangrapta decoralis|[6981]|LNCB655-09|United States|North Carolina|658[0n]|BOLD:AAA3086
Pangrapta decoralis|[6982]|LNCB714-09|United States|North Carolina|658[0n]|BOLD:AAA3086
Pangrapta decoralis|[6983]|LNCB719-09|United States|North Carolina|658[0n]|BOLD:AAA3086
Pangrapta decoralis|[6984]|LNCB720-09|United States|North Carolina|658[0n]|BOLD:AAA3086
Pangrapta decoralis|[6985]|MILEP027-09|United States|North Carolina|658[0n]|BOLD:AAA3086
Pangrapta decoralis|[6986]|LNCC301-10|United States|North Carolina|658[0n]|BOLD:AAA3086
Pangrapta decoralis|[6987]|LNCC555-11|United States|North Carolina|658[0n]|BOLD:AAA3086
Pangrapta decoralis|[6988]|LNCC557-11|United States|North Carolina|658[0n]|BOLD:AAA3086
Pangrapta decoralis|[6989]|LNCC752-11|United States|North Carolina|658[0n]|BOLD:AAA3086
Pangrapta decoralis|[6990]|LNCC300-10|United States|North Carolina|658[0n]|BOLD:AAA3086

Pangrapta decoralis|[698[illegible]]|LNCC5[illegible]-11|United States|North Carolina|658[0n]|BOLD:AAA3086
Pangrapta decoralis|[6988]|LNCC557-11|United States|North Carolina|658[0n]|BOLD:AAA3086
Pangrapta decoralis|[6989]|LNCC752-11|United States|North Carolina|658[0n]|BOLD:AAA3086
Pangrapta decoralis|[6990]|LNCC300-10|United States|North Carolina|658[0n]|BOLD:AAA3086
Pangrapta decoralis|[6991]|LNCC268-10|United States|North Carolina|658[0n]|BOLD:AAA3086
Pangrapta decoralis|[6992]|LGSM741-04|United States|Tennessee|658[0n]|BOLD:AAA3086
Pangrapta decoralis|[6993]|LNCC302-10|United States|North Carolina|658[0n]|BOLD:AAA3086
Pangrapta decoralis|[6994]|LNCC327-10|United States|North Carolina|658[0n]|BOLD:AAA3086
Pangrapta decoralis|[6995]|LNCC328-10|United States|North Carolina|658[0n]|BOLD:AAA3086
Pangrapta decoralis|[6996]|RDNMK272-11|Canada|Ontario|658[0n]|BOLD:AAA3086
Pangrapta decoralis|[6997]|LNCC1069-11|United States|North Carolina|658[0n]|BOLD:AAA3086
Pangrapta decoralis|[6998]|LMEMB041-09|United States|Mississippi|658[0n]|BOLD:AAA3086
Pangrapta decoralis|[6999]|LOFLA165-06|United States|Florida|658[0n]|BOLD:AAA3086
Pangrapta decoralis|[7000]|LOFLA182-06|United States|Florida|658[0n]|BOLD:AAA3086
Pangrapta decoralis|[7001]|BBLOB895-11|United States|Florida|658[0n]|BOLD:AAA3086
Pangrapta decoralis|[7002]|MILEP330-10|United States|Alabama|658[0n]|BOLD:AAA3086
Pangrapta decoralis|[7003]|LMEMB040-09|United States|Mississippi|633[0n]|BOLD:AAA3086
Pangrapta decoralis|[7004]|BBLOC024-11|United States|Florida|658[0n]|BOLD:AAA3086
Pangrapta decoralis|[7005]|HPPPE1560-13|Canada|Nova Scotia|542[0n]|BOLD:AAA3086
Pangrapta decoralis|[7006]|HPPPD1649-13|Canada|Nova Scotia|547[0n]|BOLD:AAA3086
Pangrapta decoralis|[7007]|HPPPE1565-13|Canada|Nova Scotia|555[0n]|BOLD:ACF1497
Pangrapta decoralis|[7008]|HPPPE1575-13|Canada|Nova Scotia|548[0n]|BOLD:ACF1497
Pangrapta decoralis|[7009]|HPPPE1574-13|Canada|Nova Scotia|542[0n]|BOLD:ACF1497
Pangrapta decoralis|[7010]|PHMNB152-04|Canada|New Brunswick|658[1n]|BOLD:ACF1497
Pangrapta decoralis|[7011]|TMNBB065-06|Canada|New Brunswick|658[0n]|BOLD:ACF1497
Pangrapta decoralis|[7012]|RDNMK307-11|Canada|Ontario|658[0n]|BOLD:ACF1497
Pangrapta decoralis|[7013]|RDLQG832-06|Canada|Quebec|658[0n]|BOLD:ACF1497
Pangrapta decoralis|[7014]|RDLQF495-06|Canada|Quebec|658[0n]|BOLD:ACF1497
Pangrapta decoralis|[7015]|RDLQF494-06|Canada|Quebec|658[0n]|BOLD:ACF1497
Pangrapta decoralis|[7016]|RDLQF493-06|Canada|Quebec|658[0n]|BOLD:ACF1497
Pangrapta decoralis|[7017]|RDLQB373-05|Canada|Quebec|658[0n]|BOLD:ACF1497
Pangrapta decoralis|[7018]|RDLQB372-05|Canada|Quebec|658[0n]|BOLD:ACF1497
Pangrapta decoralis|[7019]|BBLPB891-10|Canada|Ontario|633[0n]|BOLD:ACF1497
Pangrapta decoralis|[7020]|RDNMK273-11|Canada|Ontario|658[0n]|BOLD:ACF1497
Pangrapta decoralis|[7021]|RDNML368-13|Canada|Ontario|658[0n]|BOLD:ACF1497
Pangrapta decoralis|[7022]|HPPPE1567-13|Canada|Nova Scotia|546[0n]|BOLD:ACF1497
Pangrapta decoralis|[7023]|HPPPE1566-13|Canada|Nova Scotia|557[0n]|BOLD:ACF1497
Pangrapta decoralis|[7024]|BBLPE092-09|Canada|Nova Scotia|658[0n]|BOLD:ACF1497
Pangrapta decoralis|[7025]|BBLPE046-09|Canada|Nova Scotia|658[0n]|BOLD:ACF1497
Pangrapta decoralis|[7026]|BBLEC930-09|Canada|Nova Scotia|658[0n]|BOLD:ACF1497
Pangrapta decoralis|[7027]|BBLEC633-09|Canada|Nova Scotia|658[0n]|BOLD:ACF1497
Pangrapta decoralis|[7028]|BBLEC627-09|Canada|Nova Scotia|658[0n]|BOLD:ACF1497
Pangrapta decoralis|[7029]|HPPPE1564-13|Canada|Nova Scotia|524[0n]|BOLD:ACF1497
Pangrapta decoralis|[7030]|HPPPD1650-13|Canada|Nova Scotia|549[0n]|BOLD:ACF1497
Panula inconstans|[7031]|LYPAP598-09|Mexico|Quintana Roo|532[0n]|
Panula inconstans|[7032]|BLPBH771-07|Costa Rica|Guanacaste|658[0n]|BOLD:AAA6972
Panula inconstans|[7033]|BLPDJ207-09|Costa Rica|Guanacaste|654[0n]|BOLD:AAA6972
Panula inconstans|[7034]|MHAUC194-06|Costa Rica|Guanacaste|615[0n]|BOLD:AAA6972
Panula inconstans|[7035]|BLPBB214-07|Costa Rica|Guanacaste|658[0n]|BOLD:AAA6972
Panula inconstans|[7036]|BLPBB252-07|Costa Rica|Guanacaste|658[0n]|BOLD:AAA6972
Panula inconstans|[7037]|BLPAF926-07|Costa Rica|Guanacaste|658[0n]|BOLD:AAA6972
Panula inconstans|[7038]|BLPAF689-07|Costa Rica|Guanacaste|658[0n]|BOLD:AAA6972
Panula inconstans|[7039]|BLPAF466-07|Costa Rica|Guanacaste|658[0n]|BOLD:AAA6972
Panula inconstans|[7040]|BLPAF465-07|Costa Rica|Guanacaste|658[0n]|BOLD:AAA6972
Panula inconstans|[7041]|MHAUC193-06|Costa Rica|Guanacaste|583[0n]|BOLD:AAA6972
Panula inconstans|[7042]|MHAUC189-06|Costa Rica|Guanacaste|566[0n]|BOLD:AAA6972
Panula inconstans|[7043]|MHAUC188-06|Costa Rica|Guanacaste|542[0n]|BOLD:AAA6972
Panula inconstans|[7044]|MHAUC190-06|Costa Rica|Guanacaste|552[0n]|BOLD:AAA6972
Panula inconstans|[7045]|MHAUC191-06|Costa Rica|Guanacaste|572[0n]|BOLD:AAA6972
Panula inconstans|[7046]|BLPBB287-07|Costa Rica|Guanacaste|635[0n]|BOLD:AAA6972
Panula inconstans|[7047]|BLPDI658-09|Costa Rica|Guanacaste|658[0n]|BOLD:AAA6972
Panula inconstans|[7048]|BLPDJ214-09|Costa Rica|Guanacaste|658[0n]|BOLD:AAA6972
Panula inconstans|[7049]|BLPBH763-07|Costa Rica|Guanacaste|537[0n]|BOLD:AAA6972
Panula inconstans|[7050]|BLPAF558-07|Costa Rica|Guanacaste|658[0n]|BOLD:AAA6972
Panula inconstans|[7051]|BLPDG610-09|Costa Rica|Guanacaste|615[0n]|BOLD:AAA6972
Panula inconstans|[7052]|BLPAF677-07|Costa Rica|Guanacaste|658[0n]|BOLD:AAA6972
Panula inconstans|[7053]|BLPAF556-07|Costa Rica|Guanacaste|658[0n]|BOLD:AAA6972
Panula inconstans|[7054]|BLPAF555-07|Costa Rica|Guanacaste|658[0n]|BOLD:AAA6972
Panula inconstans|[7055]|BLPAF464-07|Costa Rica|Guanacaste|658[0n]|BOLD:AAA6972
Panula inconstans|[7056]|MHAUC192-06|Costa Rica|Guanacaste|552[0n]|BOLD:AAA6972
Panula inconstans|[7057]|BLPBB290-07|Costa Rica|Guanacaste|622[0n]|BOLD:AAA6972
Panula inconstans|[7058]|BLPEE718-12|Costa Rica|Guanacaste|658[0n]|BOLD:AAA6972
Panula inconstans|[7059]|BLPEE1677-12|Costa Rica|Guanacaste|658[0n]|BOLD:AAA6972
Panula inconstans|[7060]|BLPAF939-07|Costa Rica|Guanacaste|658[0n]|BOLD:AAA6972
Panula inconstans|[7061]|CQR093-13|Mexico|Quintana Roo|658[0n]|BOLD:AAA6972
Panula inconstans|[7062]|CQR094-13|Mexico|Quintana Roo|658[0n]|BOLD:AAA6972
Panula inconstans|[7063]|CNCLB382-14|United States|Arizona|682[0n]|BOLD:AAA6972
Panula inconstans|[7064]|BLPBB266-07|Costa Rica|Guanacaste|658[0n]|BOLD:AAA6972
Panula inconstans|[7065]|BLPAF925-07|Costa Rica|Guanacaste|658[0n]|BOLD:AAA6972
Panula inconstans|[7066]|BLPAF557-07|Costa Rica|Guanacaste|658[0n]|BOLD:AAA6972
Panula inconstans|[7067]|BLPAF554-07|Costa Rica|Guanacaste|658[0n]|BOLD:AAA6972
Panula inconstans|[7068]|BLPAF467-07|Costa Rica|Guanacaste|658[0n]|BOLD:AAA6972
Panula inconstans|[7069]|BLPAF463-07|Costa Rica|Guanacaste|658[0n]|BOLD:AAA6972
Panula inconstans|[7070]|BLPAE814-06|Costa Rica|Guanacaste|658[0n]|BOLD:AAA6972
Panula inconstans|[7071]|BLPAE508-06|Costa Rica|Guanacaste|658[0n]|BOLD:AAA6972
Panula inconstans|[7072]|BLPDA806-09|Costa Rica|Guanacaste|658[0n]|BOLD:AAA6972
Panula inconstans|[7073]|BLPAE507-06|Costa Rica|Guanacaste|658[0n]|BOLD:AAA6972
Panula inconstans|[7074]|BLPAF553-07|Costa Rica|Guanacaste|630[0n]|BOLD:AAA6972
Panula inconstans|[7075]|BLPDA746-09|Costa Rica|Guanacaste|632[0n]|BOLD:AAA6972
Panula inconstans|[7076]|BLPDA747-09|Costa Rica|Guanacaste|632[0n]|BOLD:AAA6972
Panula inconstans|[7077]|BLPDG609-09|Costa Rica|Guanacaste|633[0n]|BOLD:AAA6972
Panula inconstans|[7078]|BLPDH028-09|Costa Rica|Guanacaste|633[0n]|BOLD:AAA6972
Panula inconstans|[7079]|CNCLB1643-14|United States|Florida|658[0n]|BOLD:AAA6972
Panula inconstans|[7080]|BLPCA851-08|Costa Rica|Guanacaste|658[0n]|BOLD:AAA6972
Panula inconstans|[7081]|BLPDG415-09|Costa Rica|Guanacaste|658[0n]|BOLD:AAA6972
Panula inconstans|[7082]|BLPCA473-08|Costa Rica|Guanacaste|658[0n]|BOLD:AAA6972
Panula inconstans|[7083]|BLPCA474-08|Costa Rica|Guanacaste|658[0n]|BOLD:AAA6972
Panula inconstans|[7084]|BLPEE1458-12|Costa Rica|Guanacaste|658[0n]|BOLD:AAA6972
Panula inconstans|[7085]|CNCLB1645-14|United States|Florida|658[0n]|BOLD:AAA6972
Pseudanthracia coracias|[7086]|HKONS362-08|United States|Florida|658[0n]|BOLD:AAB9734
Pseudanthracia coracias|[7087]|HKONS363-08|United States|Florida|658[0n]|BOLD:AAB9734
Pseudanthracia coracias|[7088]|ABNCC199-07|United States|Florida|653[2n]|BOLD:AAB9734
Pseudanthracia coracias|[7089]|ABNCC198-07|United States|Florida|650[0n]|BOLD:AAB9734
[illegible]

Pseudanthracia coracias|[7087]|HKONS365-08|United States|Florida|658[0n]|BOLD:AAB9734
Pseudanthracia coracias|[7088]|ABNCC199-07|United States|Florida|653[2n]|BOLD:AAB9734
Pseudanthracia coracias|[7089]|ABNCC198-07|United States|Florida|650[0n]|BOLD:AAB9734
Pseudanthracia coracias|[7090]|RDNMD519-06|United States|Florida|658[0n]|BOLD:AAB9734
Pseudanthracia coracias|[7091]|LOFLA521-06|United States|Florida|658[0n]|BOLD:AAB9734
Pseudanthracia coracias|[7092]|LOFLA520-06|United States|Florida|657[0n]|BOLD:AAB9734
Pseudanthracia coracias|[7093]|ABNCC197-07|United States|Florida|644[0n]|BOLD:AAB9734
Pseudanthracia coracias|[7094]|RDNMD598-06|United States|Florida|614[0n]|BOLD:AAB9734
Pseudanthracia coracias|[7095]|LOFLA540-06|United States|Florida|610[0n]|BOLD:AAB9734
Pseudanthracia coracias|[7096]|ABNCC048-07|United States|Florida|618[0n]|BOLD:AAB9734
Pseudanthracia coracias|[7097]|ABNCC344-07|United States|Florida|611[1n]|BOLD:AAB9734
Pseudanthracia coracias|[7098]|HKONS364-08|United States|Florida|658[0n]|BOLD:AAB9734
Pseudanthracia coracias|[7099]|HKONS367-08|United States|Florida|658[0n]|BOLD:AAB9734
Pseudanthracia coracias|[7100]|HKONB272-09|United States|Texas|658[0n]|BOLD:AAB9734
Pseudanthracia coracias|[7101]|LNCB807-09|United States|North Carolina|658[0n]|BOLD:AAB9734
Pseudanthracia coracias|[7102]|BBLOB225-11|United States|Florida|658[0n]|BOLD:AAB9734
Pseudanthracia coracias|[7103]|BBLOB249-11|United States|Florida|658[0n]|BOLD:AAB9734
Pseudanthracia coracias|[7104]|BBLOB1072-11|United States|Florida|658[0n]|BOLD:AAB9734
Pseudanthracia coracias|[7105]|BBLOB1292-11|United States|Florida|658[0n]|BOLD:AAB9734
Pseudanthracia coracias|[7106]|BBLOB1592-11|United States|Florida|658[0n]|BOLD:AAB9734
Pseudanthracia coracias|[7107]|BBLOE1493-12|United States|Florida|658[0n]|BOLD:AAB9734
Zale horrida|[7108]|RDLQG308-06|Canada|Quebec|658[0n]|BOLD:AAB2002
Zale horrida|[7109]|RDLQG305-06|Canada|Quebec|658[0n]|BOLD:AAB2002
Zale horrida|[7110]|RDLQG304-06|Canada|Quebec|658[0n]|BOLD:AAB2002
Zale horrida|[7111]|RDLQF500-06|Canada|Quebec|658[0n]|BOLD:AAB2002
Zale horrida|[7112]|LOCT231-05|United States|Connecticut|658[0n]|BOLD:AAB2002
Zale horrida|[7113]|PHMO138-03|Canada|Ontario|639[0n]|BOLD:AAB2002
Zale horrida|[7114]|PHMO140-03|Canada|Ontario|639[0n]|BOLD:AAB2002
Zale horrida|[7115]|XAJ403-06|Canada|Ontario|658[0n]|BOLD:AAB2002
Zale horrida|[7116]|XAJ578-06|Canada|Ontario|658[0n]|BOLD:AAB2002
Zale horrida|[7117]|XAJ683-06|Canada|Ontario|658[0n]|BOLD:AAB2002
Zale horrida|[7118]|RDLQG306-06|Canada|Quebec|583[0n]|BOLD:AAB2002
Zale horrida|[7119]|LPSOC324-08|Canada|Ontario|657[0n]|BOLD:AAB2002
Zale horrida|[7120]|HKONS014-07|United States|Florida|658[2n]|BOLD:AAB2002
Zale horrida|[7121]|HKONS049-07|United States|Florida|658[1n]|BOLD:AAB2002
Zale horrida|[7122]|HKONS013-07|United States|Florida|658[1n]|BOLD:AAB2002
Zale horrida|[7123]|MILEP369-10|United States|Alabama|658[0n]|BOLD:AAB2002
Zale horrida|[7124]|LNC949-06|United States|North Carolina|601[0n]|BOLD:AAB2002
Zale horrida|[7125]|QUNOB077-08|United States|Texas|658[0n]|BOLD:AAB2002
Zale horrida|[7126]|ABNCC372-07|United States|Texas|626[0n]|BOLD:AAB2002
Zale horrida|[7127]|QUNOB062-08|United States|Texas|658[0n]|BOLD:AAB2002
Zale horrida|[7128]|QUNOB081-08|United States|Texas|658[0n]|BOLD:AAB2002
Zale horrida|[7129]|QUNOB083-08|United States|Texas|658[0n]|BOLD:AAB2002
Zale horrida|[7130]|QUNOB526-09|United States|Texas|658[0n]|BOLD:AAB2002
Zale horrida|[7131]|XAE348-04|Canada|Ontario|658[0n]|BOLD:AAB2002
Zale horrida|[7132]|RDLQB551-05|Canada|Quebec|658[0n]|BOLD:AAB2002
Zale horrida|[7133]|RDLQF499-06|Canada|Quebec|658[0n]|BOLD:AAB2002
Zale horrida|[7134]|RDLQG307-06|Canada|Quebec|658[0n]|BOLD:AAB2002
Zale horrida|[7135]|MILEP038-09|United States|North Carolina|658[0n]|BOLD:AAB2002
Zale horrida|[7136]|LSEU734-06|United States|Georgia|658[0n]|BOLD:AAB2002
Zale horrida|[7137]|LILLA015-11|United States|Illinois|658[0n]|BOLD:AAB2002
Zale nr. termina|[7138]|HKONB558-09|United States|Texas|658[0n]|BOLD:AAD9320
Zale termina|[7139]|ABCNA715-07|United States|Arizona|552[0n]|BOLD:AAE4656
Zale termina|[7140]|ABNCC348-07|United States|Utah|628[0n]|BOLD:AAE4656
Zale termina|[7141]|IAWL188-09|United States|California|658[0n]|BOLD:AAE4656
Zale termina|[7142]|LOCBC619-06|United States|California|605[0n]|BOLD:AAE4656
Zale termina|[7143]|JMMMB177-11|United States|California|639[0n]|BOLD:AAE4656
Zale termina|[7144]|LTOL1150-11|United States|California|658[0n]|BOLD:AAE4656
Zale termina|[7145]|RDNMK268-11|United States|California|658[0n]|BOLD:AAE4656
Zale edusina|[7146]|BBLSY235-09|United States|Texas|658[0n]|BOLD:AAF5142
Zale edusina|[7147]|HKONB263-09|United States|Texas|658[0n]|BOLD:AAF5142
Zale edusina|[7148]|BBLOD1073-11|United States|Texas|658[0n]|BOLD:AAF5142
Zale edusina|[7149]|BBLSW833-09|United States|Texas|658[0n]|BOLD:AAF5142
Zale edusina|[7150]|BBLSY103-09|United States|Texas|658[0n]|BOLD:AAF5142
Zale edusina|[7151]|BBLSX047-09|United States|Texas|658[0n]|BOLD:AAF5142
Zale edusina|[7152]|BBLSZ184-09|United States|Texas|658[0n]|BOLD:AAF5142
Zale edusina|[7153]|ABNCC349-07|United States|Texas|593[0n]|BOLD:AAF5142
Zale edusina|[7154]|HKONB264-09|United States|Texas|634[0n]|BOLD:AAF5142
Zale edusina|[7155]|BBLSX046-09|United States|Texas|658[0n]|BOLD:AAF5142
Zale edusina|[7156]|BBLSY104-09|United States|Texas|658[0n]|BOLD:AAF5142
Zale edusina|[7157]|BBLSY212-09|United States|Texas|658[0n]|BOLD:AAF5142
Zale edusina|[7158]|BBLSY238-09|United States|Texas|658[0n]|BOLD:AAF5142
Zale edusina|[7159]|BBLOC1066-11|United States|Texas|658[0n]|BOLD:AAF5142
Zale edusina|[7160]|BBLOD1087-11|United States|Texas|658[0n]|BOLD:AAF5142
Zale edusina|[7161]|BBLSW832-09|United States|Texas|653[0n]|BOLD:AAF5142
Zale edusina|[7162]|BBLOD1297-11|United States|Texas|658[0n]|BOLD:AAF5142
Zale chisosensis|[7163]|HKONB505-09|United States|Texas|658[0n]|BOLD:AAD9319
Zale chisosensis|[7164]|HKONS658-08|United States|Arizona|524[0n]|BOLD:AAD9319
Zale chisosensis|[7165]|HKONB503-09|United States|Texas|658[0n]|BOLD:AAD9319
Zale chisosensis|[7166]|HKONB506-09|United States|Texas|658[0n]|BOLD:AAD9319
Zale chisosensis|[7167]|HKONB557-09|United States|Texas|658[0n]|BOLD:AAD9319
Zale smithi|[7168]|RDNML353-13|United States|Florida|658[0n]|BOLD:AAB1414
Zale declarans|[7169]|LOFLB725-06|United States|Florida|658[0n]|BOLD:AAA6008
Zale declarans|[7170]|LOFLB734-06|United States|Florida|658[0n]|BOLD:AAA6008
Zale declarans|[7171]|LOFLB735-06|United States|Florida|658[0n]|BOLD:AAA6008
Zale declarans|[7172]|LOFLC372-06|United States|Florida|658[0n]|BOLD:AAA6008
Zale declarans|[7173]|LOFLB240-06|United States|Florida|658[0n]|BOLD:AAA6008
Zale declarans|[7174]|LOFLB233-06|United States|Florida|658[0n]|BOLD:AAA6008
Zale declarans|[7175]|LOFLC187-06|United States|Florida|657[0n]|BOLD:AAA6008
Zale declarans|[7176]|LOFLC367-06|United States|Florida|658[0n]|BOLD:AAA6008
Zale declarans|[7177]|LOFLC436-06|United States|Florida|658[0n]|BOLD:AAA6008
Zale declarans|[7178]|USLEP405-10|United States|Florida|658[0n]|BOLD:AAA6008
Zale declarans|[7179]|LOFLC294-06|United States|Florida|641[0n]|BOLD:AAA6008
Zale declarans|[7180]|LOFLA902-06|United States|Florida|658[0n]|BOLD:AAA6008
Zale declarans|[7181]|LOFLC363-06|United States|Florida|658[0n]|BOLD:AAA6008
Zale declarans|[7182]|LOFLC369-06|United States|Florida|658[0n]|BOLD:AAA6008
Zale declarans|[7183]|LOFLC374-06|United States|Florida|658[0n]|BOLD:AAA6008
Zale declarans|[7184]|LOFLC376-06|United States|Florida|658[0n]|BOLD:AAA6008
Zale declarans|[7185]|LOFLB234-06|United States|Florida|658[0n]|BOLD:AAA6008
Zale declarans|[7186]|LOFLC397-06|United States|Florida|658[0n]|BOLD:AAA6008
Zale declarans|[7187]|LOFLB235-06|United States|Florida|658[0n]|BOLD:AAA6008
Zale declarans|[7188]|LOFLB731-06|United States|Florida|658[0n]|BOLD:AAA6008
Zale declarans|[7189]|LOFLC094-06|United States|Florida|658[0n]|BOLD:AAA6008
[illegible]

Zale declarans|[7187]|LOFLB235-06|United States|Florida|658[0n]|BOLD:AAA6008
Zale declarans|[7188]|LOFLB731-06|United States|Florida|658[0n]|BOLD:AAA6008
Zale declarans|[7189]|LOFLC094-06|United States|Florida|658[0n]|BOLD:AAA6008
Zale declarans|[7190]|LOFLB736-06|United States|Florida|658[0n]|BOLD:AAA6008
Zale declarans|[7191]|LOFLC198-06|United States|Florida|656[0n]|BOLD:AAA6008
Zale declarans|[7192]|LOFLC398-06|United States|Florida|658[0n]|BOLD:AAA6008
Zale declarans|[7193]|LOFLB114-06|United States|Florida|674[0n]|BOLD:AAA6008
Zale declarans|[7194]|LOFLC434-06|United States|Florida|658[0n]|BOLD:AAA6008
Zale declarans|[7195]|LOFLC185-06|United States|Florida|658[0n]|BOLD:AAA6008
Zale declarans|[7196]|LOFLA542-06|United States|Florida|658[0n]|BOLD:AAA6008
Zale declarans|[7197]|LOFLC124-06|United States|Florida|653[0n]|BOLD:AAA6008
Zale declarans|[7198]|LOFLC379-06|United States|Florida|658[0n]|BOLD:AAA6008
Zale declarans|[7199]|LOFLC438-06|United States|Florida|658[0n]|BOLD:AAA6008
Zale declarans|[7200]|LOFLB219-06|United States|Florida|658[0n]|BOLD:AAA6008
Zale declarans|[7201]|LOFLB254-06|United States|Florida|658[0n]|BOLD:AAA6008
Zale declarans|[7202]|LOFLC181-06|United States|Florida|651[0n]|BOLD:AAA6008
Zale declarans|[7203]|LOFLB717-06|United States|Florida|658[0n]|BOLD:AAA6008
Zale declarans|[7204]|LOFLB231-06|United States|Florida|658[0n]|BOLD:AAA6008
Zale declarans|[7205]|LOFLA544-06|United States|Florida|658[0n]|BOLD:AAA6008
Zale declarans|[7206]|LOFLC402-06|United States|Florida|641[0n]|BOLD:AAA6008
Zale declarans|[7207]|LOFLC450-06|United States|Florida|658[0n]|BOLD:AAA6008
Zale declarans|[7208]|USLEP585-10|United States|Florida|658[0n]|BOLD:AAA6008
Zale declarans|[7209]|LOFLB238-06|United States|Florida|658[0n]|BOLD:AAA6008
Zale declarans|[7210]|USLEP582-10|United States|Florida|658[0n]|BOLD:AAA6008
Zale declarans|[7211]|USLEP583-10|United States|Florida|658[0n]|BOLD:AAA6008
Zale declarans|[7212]|USLEP626-10|United States|Florida|658[0n]|BOLD:AAA6008
Zale declarans|[7213]|USLEP628-10|United States|Florida|658[0n]|BOLD:AAA6008
Zale declarans|[7214]|HKONS091-07|United States|Florida|653[1n]|BOLD:AAA6008
Zale declarans|[7215]|LOFLB255-06|United States|Florida|658[0n]|BOLD:AAA6008
Zale declarans|[7216]|LOFLC373-06|United States|Florida|658[0n]|BOLD:AAA6008
Zale declarans|[7217]|ABNCC347-07|United States|Florida|645[0n]|BOLD:AAA6008
Zale declarans|[7218]|LOFLB237-06|United States|Florida|658[0n]|BOLD:AAA6008
Zale declarans|[7219]|LOFLC378-06|United States|Florida|658[0n]|BOLD:AAA6008
Zale declarans|[7220]|LOFLC380-06|United States|Florida|658[0n]|BOLD:AAA6008
Zale declarans|[7221]|LOFLC448-06|United States|Florida|658[0n]|BOLD:AAA6008
Zale declarans|[7222]|LOFLC463-06|United States|Florida|658[0n]|BOLD:AAA6008
Zale declarans|[7223]|LOFLC319-06|United States|Florida|658[0n]|BOLD:AAA6008
Zale declarans|[7224]|LOFLC375-06|United States|Florida|658[0n]|BOLD:AAA6008
Zale declarans|[7225]|LOFLC464-06|United States|Florida|658[0n]|BOLD:AAA6008
Zale declarans|[7226]|LOFLC315-06|United States|Florida|658[0n]|BOLD:AAA6008
Zale declarans|[7227]|LOFLB810-06|United States|Florida|655[0n]|BOLD:AAA6008
Zale declarans|[7228]|LOFLB732-06|United States|Florida|658[0n]|BOLD:AAA6008
Zale declarans|[7229]|LOFLB724-06|United States|Florida|658[0n]|BOLD:AAA6008
Zale declarans|[7230]|LOFLB722-06|United States|Florida|658[0n]|BOLD:AAA6008
Zale declarans|[7231]|LOFLB239-06|United States|Florida|658[0n]|BOLD:AAA6008
Zale declarans|[7232]|LOFLB236-06|United States|Florida|658[0n]|BOLD:AAA6008
Zale declarans|[7233]|LOFLB228-06|United States|Florida|658[0n]|BOLD:AAA6008
Zale declarans|[7234]|LOFLB111-06|United States|Florida|658[0n]|BOLD:AAA6008
Zale declarans|[7235]|LOFLA382-06|United States|Florida|658[0n]|BOLD:AAA6008
Zale declarans|[7236]|ABNCC346-07|United States|Florida|610[1n]|BOLD:AAA6008
Zale declarans|[7237]|USLEP899-10|United States|Florida|636[0n]|BOLD:AAA6008
Zale sabena|[7238]|QUNOB372-09|United States|Texas|658[0n]|BOLD:AAJ9525
Zale sabena|[7239]|RDNMJ759-11|United States|Arizona|658[0n]|BOLD:AAJ9525
Zale insuda|[7240]|HKONB556-09|United States|Texas|658[0n]|BOLD:AAD2427
Zale insuda|[7241]|IAWL161-09|United States|Arizona|658[0n]|BOLD:AAD2427
Zale insuda|[7242]|CNCLB1738-14|United States|New Mexico|658[0n]|BOLD:AAD2427
Zale insuda|[7243]|QUNOD839-11|United States|Texas|658[0n]|BOLD:AAD2427
Zale insuda|[7244]|RDNMJ330-11|United States|Arizona|658[0n]|BOLD:AAD2427
Zale insuda|[7245]|ABNCC352-07|United States|Texas|647[0n]|BOLD:AAD2427
Zale insuda|[7246]|ABNCC353-07|United States|Texas|638[0n]|BOLD:AAD2427
Zale insuda|[7247]|CMAZA817-10|United States|Arizona|658[0n]|BOLD:AAD2427
Zale insuda|[7248]|RDNMJ302-11|United States|Arizona|658[0n]|BOLD:AAD2427
Zale insuda|[7249]|RDNMJ563-11|United States|Arizona|658[0n]|BOLD:AAD2427
Zale insuda|[7250]|IAWL187-09|United States|Arizona|658[0n]|BOLD:AAD2427
Zale insuda|[7251]|IAWL162-09|United States|Arizona|658[0n]|BOLD:AAD2427
Zale insuda|[7252]|RDNMJ290-11|United States|Arizona|658[0n]|BOLD:AAD2427
Zale insuda|[7253]|RDNMJ736-11|United States|Arizona|658[0n]|BOLD:AAD2427
Zale insuda|[7254]|QUNOD838-11|United States|Texas|658[0n]|BOLD:AAD2427
Zale insuda|[7255]|BBLOD1672-11|United States|Arizona|658[0n]|BOLD:AAD2427
Zale insuda|[7256]|RDNMJ675-11|United States|Arizona|658[0n]|BOLD:AAD2427
Zale insuda|[7257]|CMAZA887-12|United States|Arizona|658[0n]|BOLD:AAD2427
Zale insuda|[7258]|CNCLB1739-14|United States|New Mexico|658[0n]|BOLD:AAD2427
Zale insuda|[7259]|CMAZA873-12|United States|Arizona|658[0n]|BOLD:AAD2427
Zale insuda|[7260]|RDNMJ730-11|United States|Arizona|658[0n]|BOLD:AAD2427
Zale insuda|[7261]|HKONB508-09|United States|Texas|658[0n]|BOLD:AAD2427
Zale insuda|[7262]|HKONB507-09|United States|Texas|658[0n]|BOLD:AAD2427
Zale insuda|[7263]|RDNMJ677-11|United States|Arizona|658[0n]|BOLD:AAD2427
Zale insuda|[7264]|CNCLB1740-14|United States|New Mexico|658[0n]|BOLD:AAD2427
Zale perculta|[7265]|RDNMD590-06|United States|Florida|658[1n]|BOLD:AAF6786
Zale perculta|[7266]|HKONS349-08|United States|Florida|658[0n]|BOLD:AAF6786
Zale perculta|[7267]|HKONS350-08|United States|Florida|658[0n]|BOLD:AAF6786
Zale lunifera|[7268]|RDNMG189-08|United States|New York|658[1n]|BOLD:AAB1413
Zale lunifera|[7269]|HKONS011-07|United States|Florida|654[0n]|BOLD:AAB1413
Zale lunifera|[7270]|LNCC575-11|United States|North Carolina|658[0n]|BOLD:AAB1413
Zale lunifera|[7271]|LNCC574-11|United States|North Carolina|658[0n]|BOLD:AAB1413
Zale lunifera|[7272]|RDNMG219-08|United States|New York|658[0n]|BOLD:AAB1413
Zale lunifera|[7273]|RDNMG218-08|United States|New York|658[0n]|BOLD:AAB1413
Zale lunifera|[7274]|RDNMD499-06|United States|Florida|658[0n]|BOLD:AAB1413
Zale lunifera|[7275]|LNC642-06|United States|North Carolina|658[0n]|BOLD:AAB1413
Zale lunifera|[7276]|LNC641-06|United States|North Carolina|658[0n]|BOLD:AAB1413
Zale lunifera|[7277]|LNC643-06|United States|North Carolina|658[0n]|BOLD:AAB1413
Zale lunifera|[7278]|LNCC652-11|United States|North Carolina|632[0n]|BOLD:AAB1413
Zale lunifera|[7279]|HKONS012-07|United States|Florida|657[0n]|BOLD:AAB1413
Zale lunifera|[7280]|LNCC653-11|United States|North Carolina|658[0n]|BOLD:AAB1413
Zale lunifera|[7281]|RDNMK056-11|United States|Florida|658[0n]|BOLD:AAB1413
Zale lunifera|[7282]|RDNMK057-11|United States|Florida|658[0n]|BOLD:AAB1413
Zale intenta|[7283]|RDLQH062-06|Canada|Quebec|658[0n]|BOLD:AAB1413
Zale intenta|[7284]|RDLQG303-06|Canada|Quebec|658[0n]|BOLD:AAB1413
Zale intenta|[7285]|RDLQH063-06|Canada|Quebec|659[0n]|BOLD:AAB1413
Zale intenta|[7286]|QUNOD357-10|United States|Minnesota|658[0n]|BOLD:AAB1413
Zale intenta|[7287]|RDNMI164-10|United States|Georgia|658[0n]|BOLD:AAB1413
Zale intenta|[7288]|LTOLB015-08|United States|West Virginia|658[0n]|BOLD:AAB1413
Zale intenta|[7289]|TMNBB067-06|Canada|New Brunswick|658[0n]|BOLD:AAB1413

Zale intenta|[7287]|RDNMI164-10|United States|Georgia|658[0n]|BOLD:AAB1413
Zale intenta|[7288]|LTOLB015-08|United States|West Virginia|658[0n]|BOLD:AAB1413
Zale intenta|[7289]|TMNBB067-06|Canada|New Brunswick|658[0n]|BOLD:AAB1413
Zale intenta|[7290]|TTMNB277-06|Canada|New Brunswick|658[0n]|BOLD:AAB1413
Zale intenta|[7291]|LOCT234-05|United States|Connecticut|658[0n]|BOLD:AAB1413
Zale intenta|[7292]|LNCB950-10|United States|North Carolina|658[0n]|BOLD:AAB1413
Zale intenta|[7293]|ABNCC366-07|United States|Wisconsin|603[0n]|BOLD:AAB1413
Zale intenta|[7294]|LNC790-06|United States|Tennessee|658[0n]|BOLD:AAB1413
Zale intenta|[7295]|LNC791-06|United States|Tennessee|658[0n]|BOLD:AAB1413
Zale intenta|[7296]|LSEU320-06|United States|Georgia|658[0n]|BOLD:AAB1413
Zale intenta|[7297]|LNCC589-11|United States|North Carolina|658[0n]|BOLD:AAB1413
Zale intenta|[7298]|LNCC590-11|United States|North Carolina|658[0n]|BOLD:AAB1413
Zale intenta|[7299]|LTOL1147-11|United States|West Virginia|658[0n]|BOLD:AAB1413
Zale lunata|[7300]|CNPPA3913-12|Canada|Ontario|602[0n]|BOLD:AAB8831
Zale lunata|[7301]|LNCC438-10|United States|North Carolina|658[0n]|BOLD:AAB8831
Zale lunata|[7302]|LNCC437-10|United States|North Carolina|658[0n]|BOLD:AAB8831
Zale lunata|[7303]|CNPPA3912-12|Canada|Ontario|602[0n]|BOLD:AAB8831
Zale lunata|[7304]|LNCC440-10|United States|North Carolina|658[0n]|BOLD:AAB8831
Zale lunata|[7305]|CNPPA3916-12|Canada|Ontario|606[0n]|BOLD:AAB8831
Zale lunata|[7306]|CNPPA3914-12|Canada|Ontario|606[0n]|BOLD:AAB8831
Zale lunata|[7307]|CNPPA3909-12|Canada|Ontario|593[0n]|BOLD:AAB8831
Zale lunata|[7308]|CNPPA3911-12|Canada|Ontario|602[0n]|BOLD:AAB8831
Zale lunata|[7309]|CNPPA3910-12|Canada|Ontario|602[0n]|BOLD:AAB8831
Zale lunata|[7310]|CNPPA3904-12|Canada|Ontario|602[0n]|BOLD:AAB8831
Zale lunata|[7311]|HKONS009-07|United States|Florida|658[1n]|BOLD:AAB8831
Zale lunata|[7312]|LNCB749-09|United States|North Carolina|658[0n]|BOLD:AAB8831
Zale lunata|[7313]|BBLOE2015-12|United States|Florida|658[0n]|BOLD:AAB8831
Zale lunata|[7314]|LNCC1248-11|United States|North Carolina|658[0n]|BOLD:AAB8831
Zale lunata|[7315]|BBLOC656-11|United States|Arkansas|658[0n]|BOLD:AAB8831
Zale lunata|[7316]|LNCC1231-11|United States|North Carolina|658[0n]|BOLD:AAB8831
Zale lunata|[7317]|LILLB059-11|United States|Illinois|658[0n]|BOLD:AAB8831
Zale lunata|[7318]|LILLA761-11|United States|Illinois|658[0n]|BOLD:AAB8831
Zale lunata|[7319]|LILLA755-11|United States|Illinois|658[0n]|BOLD:AAB8831
Zale lunata|[7320]|LNCC467-10|United States|North Carolina|658[0n]|BOLD:AAB8831
Zale lunata|[7321]|LNCC466-10|United States|North Carolina|658[0n]|BOLD:AAB8831
Zale lunata|[7322]|LNCC442-10|United States|North Carolina|658[0n]|BOLD:AAB8831
Zale lunata|[7323]|LNCC439-10|United States|North Carolina|658[0n]|BOLD:AAB8831
Zale lunata|[7324]|LNCC436-10|United States|North Carolina|658[0n]|BOLD:AAB8831
Zale lunata|[7325]|USLEP584-10|United States|Florida|658[0n]|BOLD:AAB8831
Zale lunata|[7326]|LPOKC609-09|United States|Oklahoma|658[0n]|BOLD:AAB8831
Zale lunata|[7327]|LPOKB368-09|United States|Oklahoma|658[0n]|BOLD:AAB8831
Zale lunata|[7328]|UDLEP238-09|United States|Delaware|658[0n]|BOLD:AAB8831
Zale lunata|[7329]|QUNOB524-09|United States|Texas|658[0n]|BOLD:AAB8831
Zale lunata|[7330]|BLTIB243-08|Canada|Ontario|658[0n]|BOLD:AAB8831
Zale lunata|[7331]|QUNOB049-08|United States|Texas|658[0n]|BOLD:AAB8831
Zale lunata|[7332]|RDLQH030-06|Canada|Quebec|658[0n]|BOLD:AAB8831
Zale lunata|[7333]|LNC496-06|United States|North Carolina|658[0n]|BOLD:AAB8831
Zale lunata|[7334]|XAH650-05|Canada|Ontario|658[0n]|BOLD:AAB8831
Zale lunata|[7335]|LOT263-04|United States|Tennessee|658[1n]|BOLD:AAB8831
Zale lunata|[7336]|UDLEP098-09|United States|Maryland|630[0n]|BOLD:AAB8831
Zale lunata|[7337]|LGSMG566-07|United States|North Carolina|646[0n]|BOLD:AAB8831
Zale lunata|[7338]|LGSMG686-07|United States|Tennessee|646[0n]|BOLD:AAB8831
Zale lunata|[7339]|HKONS467-08|United States|Florida|576[0n]|BOLD:AAB8831
Zale lunata|[7340]|RDLQB465-05|Canada|Quebec|550[3n]|BOLD:AAB8831
Zale lunata|[7341]|UDLEP056-09|United States|Maryland|598[0n]|BOLD:AAB8831
Zale lunata|[7342]|LNCC441-10|United States|North Carolina|640[0n]|BOLD:AAB8831
Zale lunata|[7343]|CNPPE1022-12|Canada|Ontario|631[0n]|BOLD:AAB8831
Zale lunata|[7344]|QUNOB525-09|United States|Texas|658[0n]|BOLD:AAB8832
Zale lunata|[7345]|LNC497-06|United States|North Carolina|606[2n]|BOLD:AAB8832
Zale lunata|[7346]|BBLOB816-11|United States|Florida|600[1n]|BOLD:AAB8832
Zale lunata|[7347]|RDNMC284-05|United States|Florida|606[0n]|BOLD:AAB8832
Zale lunata|[7348]|HKONS010-07|United States|Florida|621[0n]|BOLD:AAB8832
Zale lunata|[7349]|HKONS468-08|United States|Florida|579[0n]|BOLD:AAB8832
Zale lunata|[7350]|BBLOB851-11|United States|Florida|658[0n]|BOLD:AAB8832
Zale lunata|[7351]|BBLOC1409-11|United States|California|658[0n]|BOLD:AAB8832
Zale lunata|[7352]|LALPA090-10|Canada|British Columbia|658[0n]|BOLD:AAB8832
Zale lunata|[7353]|DUNLP362-08|Canada|British Columbia|658[0n]|BOLD:AAB8832
Zale lunata|[7354]|LALPA615-10|Canada|British Columbia|658[0n]|BOLD:AAB8832
Zale lunata|[7355]|LALPA095-10|Canada|British Columbia|658[0n]|BOLD:AAB8832
Zale lunata|[7356]|GMLC1373-12|United States|California|622[0n]|BOLD:AAB8832
Zale phaeocapna|[7357]|RDLQH088-06|Canada|Quebec|658[0n]|BOLD:AAE4850
Zale phaeocapna|[7358]|HKONS470-08|United States|Florida|544[41n]|
Zale phaeocapna|[7359]|HKONS471-08|United States|Florida|658[0n]|BOLD:AAE4850
Zale phaeocapna|[7360]|RDNMG928-08|Canada|Ontario|658[0n]|BOLD:AAE4850
Zale colorado|[7361]|HKONB539-09|United States|Texas|658[0n]|BOLD:ACF5273
Zale colorado|[7362]|HKONB509-09|United States|Texas|658[0n]|BOLD:ACF5273
Zale colorado|[7363]|HKONB540-09|United States|Texas|647[0n]|BOLD:ACF5273
Zale colorado|[7364]|HKONB538-09|United States|Texas|642[0n]|BOLD:ACF5273
Zale colorado|[7365]|HKONB543-09|United States|Texas|622[0n]|BOLD:ACF5273
Zale colorado|[7366]|HKONB544-09|United States|Texas|658[0n]|BOLD:ACF5273
Zale colorado|[7367]|USLEP084-10|United States|Colorado|658[0n]|BOLD:AAB1411
Zale colorado|[7368]|USLEP083-10|United States|Colorado|658[0n]|BOLD:AAB1411
Zale colorado|[7369]|USLEP085-10|United States|Colorado|636[0n]|BOLD:AAB1411
Zale colorado|[7370]|USLEP1168-10|United States|Colorado|658[0n]|BOLD:AAB1411
Zale colorado|[7371]|LMEMB237-09|United States|New Mexico|658[0n]|BOLD:AAB1411
Zale colorado|[7372]|RDNMC610-06|United States|New Mexico|658[0n]|BOLD:AAB1411
Zale colorado|[7373]|LMEMB235-09|United States|New Mexico|658[0n]|BOLD:AAB1411
Zale colorado|[7374]|BBLSY413-09|United States|Arizona|658[0n]|BOLD:AAB1411
Zale colorado|[7375]|LMEMB236-09|United States|New Mexico|658[0n]|BOLD:AAB1411
Zale colorado|[7376]|RDNMK265-11|United States|Utah|658[0n]|BOLD:AAB1411
Zale colorado|[7377]|RDNMK266-11|United States|Colorado|658[0n]|BOLD:ACF5275
Zale colorado|[7378]|RDNMK267-11|United States|Colorado|655[0n]|BOLD:ACF5275
Zale colorado|[7379]|RDNMD651-06|United States|New Mexico|658[1n]|BOLD:ACF5275
Zale colorado|[7380]|RDNMK269-11|United States|Colorado|624[0n]|BOLD:ACF5275
Zale n. sp. 4|[7381]|ABNCC368-07|United States|Arizona|624[1n]|BOLD:ABY6417
Zale n. sp. 4|[7382]|ABNCC369-07|United States|Arizona|605[0n]|BOLD:ABY6417
Zale n. sp. 4|[7383]|RDNMJ670-11|United States|Arizona|658[0n]|BOLD:ABY6417
Zale n. sp. 4|[7384]|IAWLB618-11|United States|Arizona|658[0n]|BOLD:ABY6417
Zale n. sp. 4|[7385]|RDNMK270-11|United States|Arizona|658[0n]|BOLD:ABY6417
Zale calycanthata|[7386]|LSEU037-06|United States|Georgia|658[0n]|BOLD:ACF5274
Zale calycanthata|[7387]|LSEU497-06|United States|Georgia|658[0n]|BOLD:ACF5274
Zale calycanthata|[7388]|HKONS001-07|United States|Florida|658[0n]|BOLD:ACF5274
Zale calycanthata|[7389]|HKONS462-08|United States|Florida|609[0n]|BOLD:ACF5274

Zale calycanthata|[7387]|LSEU497-06|United States|Georgia|658[0n]|BOLD:ACF5274
Zale calycanthata|[7388]|HKONS001-07|United States|Florida|658[0n]|BOLD:ACF5274
Zale calycanthata|[7389]|HKONS462-08|United States|Florida|609[0n]|BOLD:ACF5274
Zale calycanthata|[7390]|CNCLB634-14|Canada|Ontario|658[0n]|BOLD:ACF5274
Zale calycanthata|[7391]|CNCLB635-14|Canada|Ontario|658[0n]|BOLD:ACF5274
Zale calycanthata|[7392]|CNCLB636-14|Canada|Ontario|658[0n]|BOLD:ACF5274
Zale rubi|[7393]|ABNCC029-07|United States|California|630[0n]|BOLD:AAH8426
Zale rubi|[7394]|ABNCC030-07|United States|California|634[0n]|BOLD:AAH8426
Zale rubi|[7395]|JMMMB305-11|United States|California|658[0n]|BOLD:AAH8426
Zale rubiata|[7396]|ABNCC365-07|United States|Texas|611[0n]|BOLD:AAE4693
Zale rubiata|[7397]|HKONS105-08|United States|Texas|658[0n]|BOLD:AAE4693
Zale rubiata|[7398]|IAWL169-09|United States|Arizona|658[0n]|BOLD:AAE4693
Zale rubiata|[7399]|IAWL189-09|United States|Arizona|658[0n]|BOLD:AAE4693
Zale rubiata|[7400]|CMAZA311-10|United States|Arizona|658[0n]|BOLD:AAE4693
Zale rubiata|[7401]|BBLOB1550-11|United States|Arizona|658[0n]|BOLD:AAE4693
Zale rubiata|[7402]|USLEP389-10|United States|Colorado|658[0n]|BOLD:AAE4693
Zale rubiata|[7403]|CNCLB1737-14|United States|New Mexico|658[0n]|BOLD:AAE4693
Zale unilineata|[7404]|TTMNB279-06|Canada|New Brunswick|658[0n]|BOLD:AAC1852
Zale unilineata|[7405]|LGSMC917-05|United States|Tennessee|658[0n]|BOLD:AAC1852
Zale unilineata|[7406]|LOCT102-05|United States|Connecticut|658[0n]|BOLD:AAC1852
Zale unilineata|[7407]|TTMNB278-06|Canada|New Brunswick|658[0n]|BOLD:AAC1852
Zale unilineata|[7408]|XAJ465-06|Canada|Ontario|658[0n]|BOLD:AAC1852
Zale unilineata|[7409]|LOCT101-05|United States|Connecticut|658[0n]|BOLD:AAC1852
Zale unilineata|[7410]|XAJ485-06|Canada|Ontario|658[0n]|BOLD:AAC1852
Zale unilineata|[7411]|XAJ422-06|Canada|Ontario|658[0n]|BOLD:AAC1852
Zale unilineata|[7412]|XAD587-05|Canada|Ontario|658[0n]|BOLD:AAC1852
Zale unilineata|[7413]|LSEU034-06|United States|North Carolina|583[0n]|BOLD:AAC1852
Zale unilineata|[7414]|PMG168-03|Canada|Ontario|617[0n]|BOLD:AAC1852
Zale unilineata|[7415]|TMG93-03|Canada|Ontario|639[0n]|BOLD:AAC1852
Zale unilineata|[7416]|LSEU035-06|United States|North Carolina|590[0n]|BOLD:AAC1852
Zale unilineata|[7417]|GMGSK025-12|United States|Tennessee|602[0n]|BOLD:AAC1852
Zale undularis|[7418]|QUNOB149-08|United States|Kentucky|658[0n]|BOLD:AAC5348
Zale undularis|[7419]|RDLQH117-06|Canada|Quebec|658[0n]|BOLD:AAC5348
Zale undularis|[7420]|MMNA125-08|United States|North Carolina|658[0n]|BOLD:AAC5348
Zale undularis|[7421]|PHMO137-03|Canada|Ontario|639[0n]|BOLD:AAC5348
Zale undularis|[7422]|LGSMC433-05|United States|Tennessee|617[0n]|BOLD:AAC5348
Zale undularis|[7423]|XAD588-05|Canada|Ontario|658[0n]|BOLD:AAC5348
Zale undularis|[7424]|CNCLB2882-14|United States|North Carolina|658[0n]|BOLD:AAC5348
Zale undularis|[7425]|LOCT080-05|United States|Connecticut|658[0n]|BOLD:AAC5348
Zale undularis|[7426]|LOCT103-05|United States|Connecticut|658[0n]|BOLD:AAC5348
Zale undularis|[7427]|LSUSA184-06|United States|Kentucky|658[0n]|BOLD:AAC5348
Zale undularis|[7428]|QUNOB147-08|United States|Kentucky|658[0n]|BOLD:AAC5348
Zale undularis|[7429]|QUNOB148-08|United States|Kentucky|658[0n]|BOLD:AAC5348
Zale undularis|[7430]|CNCLB2883-14|United States|North Carolina|658[0n]|BOLD:AAC5348
Zale aeruginosa|[7431]|LGSMG567-07|United States|North Carolina|658[0n]|BOLD:AAB4323
Zale aeruginosa|[7432]|TMNBB066-06|Canada|New Brunswick|656[0n]|BOLD:AAB4323
Zale aeruginosa|[7433]|TTMNB055-06|Canada|New Brunswick|599[0n]|BOLD:AAB4323
Zale aeruginosa|[7434]|RDLQG298-06|Canada|Quebec|658[0n]|BOLD:AAB4323
Zale aeruginosa|[7435]|QUNOD358-10|United States|Minnesota|658[0n]|BOLD:AAB4323
Zale aeruginosa|[7436]|LOT270-04|United States|Tennessee|658[0n]|BOLD:AAB4323
Zale aeruginosa|[7437]|LOT271-04|United States|Tennessee|658[0n]|BOLD:AAB4323
Zale aeruginosa|[7438]|LGSMC914-05|United States|Tennessee|658[0n]|BOLD:AAB4323
Zale aeruginosa|[7439]|LGSMC915-05|United States|Tennessee|658[0n]|BOLD:AAB4323
Zale aeruginosa|[7440]|LSEU317-06|United States|Georgia|658[0n]|BOLD:AAB4323
Zale aeruginosa|[7441]|LSEU318-06|United States|Georgia|658[0n]|BOLD:AAB4323
Zale aeruginosa|[7442]|LGSMG569-07|United States|Tennessee|658[0n]|BOLD:AAB4323
Zale aeruginosa|[7443]|QUNOB143-08|United States|Kentucky|658[0n]|BOLD:AAB4323
Zale aeruginosa|[7444]|LGSM480-04|United States|Tennessee|658[0n]|BOLD:AAB4323
Zale aeruginosa|[7445]|LGSM481-04|United States|Tennessee|658[0n]|BOLD:AAB4323
Zale aeruginosa|[7446]|QUNOB144-08|United States|Kentucky|658[0n]|BOLD:AAB4323
Zale aeruginosa|[7447]|LNC951-06|United States|North Carolina|658[0n]|BOLD:AAB4323
Zale aeruginosa|[7448]|LOFLB241-06|United States|Florida|658[0n]|BOLD:AAB4323
Zale aeruginosa|[7449]|LOFLB134-06|United States|Florida|658[0n]|BOLD:AAB4323
Zale aeruginosa|[7450]|LNC779-06|United States|North Carolina|658[0n]|BOLD:AAB4323
Zale aeruginosa|[7451]|LGSM762-04|United States|Tennessee|571[0n]|BOLD:AAB4323
Zale aeruginosa|[7452]|LOFLA847-06|United States|Florida|608[0n]|BOLD:AAB4323
Zale aeruginosa|[7453]|ABCNA560-07|United States|Kentucky|568[0n]|BOLD:AAB4323
Zale aeruginosa|[7454]|QUNO502-08|United States|Texas|658[0n]|BOLD:AAB4323
Zale aeruginosa|[7455]|LNCC669-11|United States|North Carolina|658[0n]|BOLD:AAB4323
Zale aeruginosa|[7456]|BBLOB1701-11|United States|Florida|658[0n]|BOLD:AAB4323
Zale minerea|[7457]|LOCT081-05|United States|Connecticut|617[0n]|BOLD:AAA4887
Zale minerea|[7458]|HKONS003-07|United States|Florida|642[0n]|BOLD:ACF0362
Zale minerea|[7459]|RDLQG208-06|Canada|Quebec|658[0n]|BOLD:ABY9488
Zale minerea|[7460]|LPMN325-08|Canada|Manitoba|658[0n]|BOLD:ABY9488
Zale minerea|[7461]|LOWCE247-06|Canada|British Columbia|658[0n]|BOLD:ABY9488
Zale minerea|[7462]|RDNMD473-06|United States|Colorado|658[0n]|BOLD:ABY9488
Zale minerea|[7463]|RDNMD474-06|United States|Colorado|658[0n]|BOLD:ABY9488
Zale minerea|[7464]|LOWCB608-05|Canada|British Columbia|572[0n]|BOLD:ABY9488
Zale minerea|[7465]|LOWCB609-05|Canada|British Columbia|584[0n]|BOLD:ABY9488
Zale minerea|[7466]|LOWCB610-05|Canada|British Columbia|584[1n]|BOLD:ABY9488
Zale minerea|[7467]|LOWCB611-05|Canada|British Columbia|585[0n]|BOLD:ABY9488
Zale minerea|[7468]|LBCS029-07|Canada|British Columbia|658[0n]|BOLD:ABY9488
Zale minerea|[7469]|LBCS023-07|Canada|British Columbia|658[0n]|BOLD:ABY9488
Zale minerea|[7470]|LALPA073-10|Canada|British Columbia|658[0n]|BOLD:ABY9488
Zale minerea|[7471]|LALPA172-10|Canada|British Columbia|658[0n]|BOLD:ABY9488
Zale minerea|[7472]|LOT536-04|United States|Tennessee|529[0n]|BOLD:AAA4886
Zale minerea|[7473]|LGSMC896-05|United States|Tennessee|658[0n]|BOLD:AAA4886
Zale minerea|[7474]|LNCB332-06|United States|North Carolina|658[0n]|BOLD:AAA4886
Zale minerea|[7475]|LGSMC428-05|United States|Tennessee|658[0n]|BOLD:AAA4886
Zale minerea|[7476]|LSEU315-06|United States|Georgia|658[0n]|BOLD:AAA4886
Zale minerea|[7477]|LSEU316-06|United States|Georgia|658[0n]|BOLD:AAA4886
Zale minerea|[7478]|LNCB331-06|United States|North Carolina|658[0n]|BOLD:AAA4886
Zale minerea|[7479]|LNCC274-10|United States|North Carolina|658[0n]|BOLD:AAA4886
Zale minerea|[7480]|BBLOC1667-11|United States|Texas|658[0n]|BOLD:AAA4886
Zale minerea|[7481]|XAJ348-06|Canada|Ontario|656[0n]|BOLD:AAA4886
Zale minerea|[7482]|XAB218-04|Canada|Ontario|658[0n]|BOLD:AAA4886
Zale minerea|[7483]|LGSMC895-05|United States|Tennessee|658[0n]|BOLD:AAA4886
Zale minerea|[7484]|LGSMC897-05|United States|Tennessee|658[0n]|BOLD:AAA4886
Zale minerea|[7485]|LSUSA032-06|United States|Kentucky|658[0n]|BOLD:AAA4886
Zale minerea|[7486]|XAJ543-06|Canada|Ontario|658[0n]|BOLD:AAA4886
Zale minerea|[7487]|LPMN175-08|Canada|Manitoba|658[0n]|BOLD:AAA4886
Zale minerea|[7488]|BLTIB066-08|Canada|Ontario|658[0n]|BOLD:AAA4886
Zale minerea|[7489]|XAB016-04|Canada|Ontario|605[0n]|BOLD:AAA4886

Zale minerea|[7487]|LPMN175-08|Canada|Manitoba|658[0n]|BOLD:AAA4886
Zale minerea|[7488]|BLTIB066-08|Canada|Ontario|658[0n]|BOLD:AAA4886
Zale minerea|[7489]|XAB016-04|Canada|Ontario|605[0n]|BOLD:AAA4886
Zale minerea|[7490]|QUNOB145-08|United States|Kentucky|658[0n]|BOLD:AAA4886
Zale minerea|[7491]|XAK122-06|Canada|Ontario|658[0n]|BOLD:AAA4886
Zale minerea|[7492]|XAJ461-06|Canada|Ontario|658[0n]|BOLD:AAA4886
Zale minerea|[7493]|XAJ290-06|Canada|Ontario|658[0n]|BOLD:AAA4886
Zale minerea|[7494]|TTMNB275-06|Canada|New Brunswick|658[0n]|BOLD:AAA4886
Zale minerea|[7495]|XAE229-04|Canada|Ontario|658[0n]|BOLD:AAA4886
Zale minerea|[7496]|LOT272-04|United States|Tennessee|658[0n]|BOLD:AAA4886
Zale minerea|[7497]|LOT264-04|United States|Tennessee|658[0n]|BOLD:AAA4886
Zale minerea|[7498]|LGSM445-04|United States|North Carolina|658[0n]|BOLD:AAA4886
Zale minerea|[7499]|LGSM444-04|United States|Tennessee|658[0n]|BOLD:AAA4886
Zale minerea|[7500]|LOCT082-05|United States|Connecticut|624[0n]|BOLD:AAA4886
Zale minerea|[7501]|XAB050-04|Canada|Ontario|624[0n]|BOLD:AAA4886
Zale minerea|[7502]|PHMO089-03|Canada|Ontario|639[2n]|BOLD:AAA4886
Zale minerea|[7503]|LOTB305-05|United States|Tennessee|632[0n]|BOLD:AAA4886
Zale minerea|[7504]|XAB029-04|Canada|Ontario|573[0n]|BOLD:AAA4886
Zale minerea|[7505]|XAE494-04|Canada|Ontario|575[0n]|BOLD:AAA4886
Zale minerea|[7506]|LOTB110-05|United States|Tennessee|614[0n]|BOLD:AAA4886
Zale minerea|[7507]|RDLQG207-06|Canada|Quebec|583[3n]|BOLD:AAA4886
Zale minerea|[7508]|RDLQG209-06|Canada|Quebec|658[0n]|BOLD:AAA4886
Zale minerea|[7509]|RDLQG211-06|Canada|Quebec|658[0n]|BOLD:AAA4886
Zale minerea|[7510]|RDLQH061-06|Canada|Quebec|658[0n]|BOLD:AAA4886
Zale minerea|[7511]|QUNOB146-08|United States|Kentucky|658[0n]|BOLD:AAA4886
Zale minerea|[7512]|BBLCU015-09|United States|Michigan|658[0n]|BOLD:AAA4886
Zale minerea|[7513]|LGSMC430-05|United States|Tennessee|658[0n]|BOLD:AAA4886
Zale minerea|[7514]|RDLQH057-06|Canada|Quebec|658[0n]|BOLD:AAA4886
Zale minerea|[7515]|RDLQH059-06|Canada|Quebec|658[0n]|BOLD:AAA4886
Zale minerea|[7516]|LGSMC427-05|United States|Tennessee|658[0n]|BOLD:AAA4886
Zale minerea|[7517]|LGSMC426-05|United States|Tennessee|658[0n]|BOLD:AAA4886
Zale minerea|[7518]|LGSMC425-05|United States|Tennessee|658[0n]|BOLD:AAA4886
Zale minerea|[7519]|LGSMC429-05|United States|Tennessee|658[0n]|BOLD:AAA4886
Zale minerea|[7520]|LGSMC431-05|United States|Tennessee|658[0n]|BOLD:AAA4886
Zale minerea|[7521]|RDLQG210-06|Canada|Quebec|658[0n]|BOLD:AAA4886
Zale minerea|[7522]|XAJ466-06|Canada|Ontario|656[0n]|BOLD:AAA4886
Zale minerea|[7523]|RDLQH060-06|Canada|Quebec|658[0n]|BOLD:AAA4886
Zale minerea|[7524]|KPOEC149-08|Canada|Ontario|658[0n]|BOLD:AAA4886
Zale minerea|[7525]|RDLQH080-06|Canada|Quebec|658[0n]|BOLD:AAA4886
Zale minerea|[7526]|RDLQH058-06|Canada|Quebec|658[0n]|BOLD:AAA4886
Zale minerea|[7527]|RDLQG206-06|Canada|Quebec|658[0n]|BOLD:AAA4886
Zale minerea|[7528]|LSEU314-06|United States|Georgia|658[0n]|BOLD:AAA4886
Zale minerea|[7529]|XAJ460-06|Canada|Ontario|658[0n]|BOLD:AAA4886
Zale minerea|[7530]|XAJ277-06|Canada|Ontario|658[0n]|BOLD:AAA4886
Zale minerea|[7531]|TTMNB276-06|Canada|New Brunswick|658[0n]|BOLD:AAA4886
Zale minerea|[7532]|RDLQH056-06|Canada|Quebec|658[0n]|BOLD:AAA4886
Zale minerea|[7533]|XAE364-04|Canada|Ontario|658[0n]|BOLD:AAA4886
Zale minerea|[7534]|XAE207-04|Canada|Ontario|658[0n]|BOLD:AAA4886
Zale minerea|[7535]|LOT273-04|United States|Tennessee|658[0n]|BOLD:AAA4886
Zale minerea|[7536]|XAE502-04|Canada|Ontario|575[2n]|BOLD:AAA4886
Zale minerea|[7537]|HPPPB174-13|Canada|Nova Scotia|595[0n]|BOLD:AAA4886
Zale galbanata|[7538]|RDLQF735-06|Canada|Quebec|658[0n]|BOLD:AAA9400
Zale galbanata|[7539]|XAJ720-06|Canada|Ontario|658[0n]|BOLD:AAA9400
Zale galbanata|[7540]|RDLQF689-06|Canada|Quebec|637[0n]|BOLD:AAA9400
Zale galbanata|[7541]|RDLQG066-06|Canada|Quebec|592[0n]|BOLD:AAA9400
Zale galbanata|[7542]|LNCC849-11|United States|North Carolina|658[0n]|BOLD:AAA9400
Zale galbanata|[7543]|RDLQH075-06|Canada|Quebec|658[0n]|BOLD:AAA9400
Zale galbanata|[7544]|RDLQF677-06|Canada|Quebec|609[0n]|BOLD:AAA9400
Zale galbanata|[7545]|ABCNA576-07|United States|Florida|577[1n]|BOLD:AAA9400
Zale galbanata|[7546]|ABCNA581-07|United States|Florida|577[0n]|BOLD:AAA9400
Zale galbanata|[7547]|LOCT108-05|United States|Connecticut|616[0n]|BOLD:AAA9400
Zale galbanata|[7548]|LPOKD076-09|United States|Oklahoma|658[0n]|BOLD:AAA9400
Zale galbanata|[7549]|LNCB949-10|United States|North Carolina|658[0n]|BOLD:AAA9400
Zale galbanata|[7550]|LPOKB353-09|United States|Oklahoma|658[0n]|BOLD:AAA9400
Zale galbanata|[7551]|BBLSU115-09|United States|Mississippi|658[0n]|BOLD:AAA9400
Zale galbanata|[7552]|LPOKB186-09|United States|Oklahoma|658[0n]|BOLD:AAA9400
Zale galbanata|[7553]|LPOKB238-09|United States|Oklahoma|658[0n]|BOLD:AAA9400
Zale galbanata|[7554]|XAJ816-06|Canada|Ontario|658[0n]|BOLD:AAA9400
Zale galbanata|[7555]|XAJ319-06|Canada|Ontario|658[0n]|BOLD:AAA9400
Zale galbanata|[7556]|LGSMC916-05|United States|Tennessee|658[0n]|BOLD:AAA9400
Zale galbanata|[7557]|MNBB025-05|Canada|New Brunswick|658[0n]|BOLD:AAA9400
Zale galbanata|[7558]|XAE580-04|Canada|Ontario|658[0n]|BOLD:AAA9400
Zale galbanata|[7559]|XAE236-04|Canada|Ontario|658[0n]|BOLD:AAA9400
Zale galbanata|[7560]|XAC717-04|Canada|Ontario|658[0n]|BOLD:AAA9400
Zale galbanata|[7561]|XAB137-04|Canada|Ontario|658[0n]|BOLD:AAA9400
Zale galbanata|[7562]|PHMO093-03|Canada|Ontario|639[0n]|BOLD:AAA9400
Zale galbanata|[7563]|PMG166-03|Canada|Ontario|617[0n]|BOLD:AAA9400
Zale galbanata|[7564]|PHMO126-03|Canada|Ontario|639[0n]|BOLD:AAA9400
Zale galbanata|[7565]|RDLQF676-06|Canada|Quebec|609[0n]|BOLD:AAA9400
Zale galbanata|[7566]|ABCNA577-07|United States|Florida|577[0n]|BOLD:AAA9400
Zale galbanata|[7567]|ABCNA580-07|United States|Florida|577[0n]|BOLD:AAA9400
Zale galbanata|[7568]|ABCNA584-07|United States|Florida|577[0n]|BOLD:AAA9400
Zale galbanata|[7569]|LPSO237-08|Canada|Ontario|658[0n]|BOLD:AAA9400
Zale galbanata|[7570]|LPSO887-08|Canada|Ontario|658[0n]|BOLD:AAA9400
Zale galbanata|[7571]|QUNOB063-08|United States|Texas|658[0n]|BOLD:AAA9400
Zale galbanata|[7572]|QUNOB527-09|United States|Texas|658[0n]|BOLD:AAA9400
Zale galbanata|[7573]|LNCC848-11|United States|North Carolina|658[0n]|BOLD:AAA9400
Zale galbanata|[7574]|BBLOB1557-11|United States|Arizona|658[0n]|BOLD:AAA9400
Zale galbanata|[7575]|BBLSX282-09|United States|Oklahoma|658[0n]|BOLD:AAA9400
Zale galbanata|[7576]|RDLQF678-06|Canada|Quebec|637[0n]|BOLD:AAA9400
Zale galbanata|[7577]|LGSMG568-07|United States|Tennessee|658[0n]|BOLD:AAA9400
Zale galbanata|[7578]|RDLQF834-06|Canada|Quebec|658[0n]|BOLD:AAA9400
Zale galbanata|[7579]|XAK116-06|Canada|Ontario|658[0n]|BOLD:AAA9400
Zale galbanata|[7580]|XAJ268-06|Canada|Ontario|658[0n]|BOLD:AAA9400
Zale galbanata|[7581]|UDLEP318-09|United States|Pennsylvania|623[0n]|BOLD:AAA9400
Zale galbanata|[7582]|LPOKB246-09|United States|Oklahoma|628[0n]|BOLD:AAA9400
Zale galbanata|[7583]|LNCC1688-13|United States|North Carolina|658[0n]|BOLD:AAA9400
Zale bethunei|[7584]|LTOL1149-11|United States|Maryland|658[0n]|BOLD:ABY8160
Zale bethunei|[7585]|QUNOB142-08|United States|Kentucky|658[0n]|BOLD:ABY8160
Zale bethunei|[7586]|QUNOB140-08|United States|Kentucky|658[0n]|BOLD:ABY8160
Zale bethunei|[7587]|QUNOB141-08|United States|Kentucky|658[0n]|BOLD:ABY8160
Zale bethunei|[7588]|QUNOB139-08|United States|Kentucky|658[0n]|BOLD:ABY8160
Zale bethunei|[7589]|QUNOB138-08|United States|Kentucky|658[0n]|BOLD:ABY8160

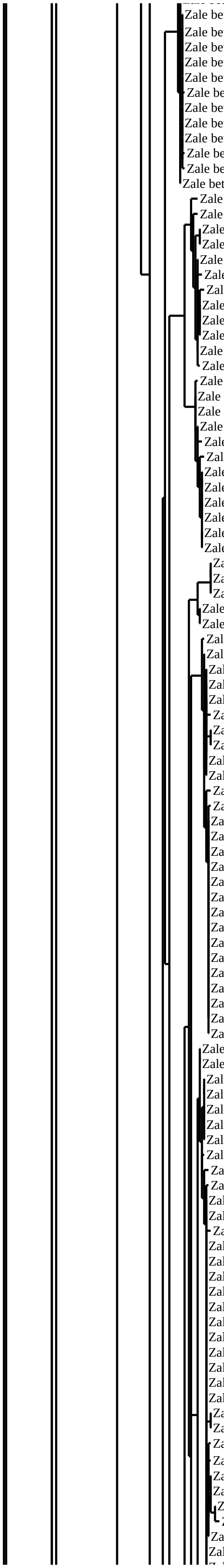

[illegible]
Zale bethunei|[7587]|QUNOB141-08|United States|Kentucky|658[0n]|BOLD:ABY8160
Zale bethunei|[7588]|QUNOB139-08|United States|Kentucky|658[0n]|BOLD:ABY8160
Zale bethunei|[7589]|QUNOB138-08|United States|Kentucky|658[0n]|BOLD:ABY8160
Zale bethunei|[7590]|LGSMG572-07|United States|Tennessee|658[0n]|BOLD:ABY8160
Zale bethunei|[7591]|LGSMG571-07|United States|Tennessee|658[0n]|BOLD:ABY8160
Zale bethunei|[7592]|LSEU626-06|United States|Georgia|658[0n]|BOLD:ABY8160
Zale bethunei|[7593]|LSEU627-06|United States|Georgia|658[0n]|BOLD:ABY8160
Zale bethunei|[7594]|LOT269-04|United States|Tennessee|658[0n]|BOLD:ABY8160
Zale bethunei|[7595]|LGSM548-04|United States|Tennessee|658[0n]|BOLD:ABY8160
Zale bethunei|[7596]|LGSM549-04|United States|Tennessee|582[0n]|BOLD:ABY8160
Zale bethunei|[7597]|LNCC1670-13|United States|North Carolina|658[0n]|BOLD:ABY8160
Zale bethunei|[7598]|LNCC1671-13|United States|North Carolina|658[0n]|BOLD:ABY8160
Zale buchholzi|[7599]|LNCC153-10|United States|North Carolina|658[0n]|BOLD:ABY9489
Zale buchholzi|[7600]|LNCC654-11|United States|North Carolina|658[0n]|BOLD:ABY9489
Zale buchholzi|[7601]|QUNOC011-09|United States|Louisiana|658[0n]|BOLD:ABY9489
Zale buchholzi|[7602]|BBLSU117-09|United States|Mississippi|658[0n]|BOLD:ABY9489
Zale buchholzi|[7603]|LNC644-06|United States|North Carolina|658[0n]|BOLD:ABY9489
Zale buchholzi|[7604]|HKONS002-07|United States|Florida|646[0n]|BOLD:ABY9489
Zale buchholzi|[7605]|LNC950-06|United States|North Carolina|658[0n]|BOLD:ABY9489
Zale buchholzi|[7606]|HKONS097-08|United States|Florida|658[0n]|
Zale buchholzi|[7607]|HKONS108-08|United States|Florida|658[0n]|BOLD:ABY9489
Zale buchholzi|[7608]|HKONS472-08|United States|Florida|658[0n]|BOLD:ABY9489
Zale buchholzi|[7609]|LNCC187-10|United States|North Carolina|658[0n]|BOLD:ABY9489
Zale buchholzi|[7610]|LNCC1228-11|United States|North Carolina|658[0n]|BOLD:ABY9489
Zale submediana|[7611]|RDNMG381-08|Canada|Ontario|658[0n]|BOLD:ABY9489
Zale submediana|[7612]|QUNOC327-10|United States|New Jersey|658[0n]|BOLD:ABY9489
Zale submediana|[7613]|CNCLB633-14|Canada|Ontario|658[0n]|BOLD:ABY9489
Zale submediana|[7614]|QUNOD351-10|United States|Wisconsin|658[0n]|BOLD:ABY9489
Zale submediana|[7615]|CNCLA5269-13|Canada|Ontario|658[0n]|BOLD:ABY9489
Zale submediana|[7616]|QUNOD350-10|United States|Wisconsin|658[0n]|BOLD:ABY9489
Zale submediana|[7617]|RDLQG294-06|Canada|Quebec|658[0n]|BOLD:ABY9489
Zale submediana|[7618]|LPSOB304-08|Canada|Ontario|658[0n]|BOLD:ABY9489
Zale submediana|[7619]|QUNOD349-10|United States|Minnesota|658[0n]|BOLD:ABY9489
Zale submediana|[7620]|QUNOD352-10|United States|Wisconsin|658[0n]|BOLD:ABY9489
Zale submediana|[7621]|PSAT141-10|Canada|Ontario|658[0n]|BOLD:ABY9489
Zale submediana|[7622]|CNCLB638-14|Canada|Ontario|658[0n]|BOLD:ABY9489
Zale pr. n. sp. 1|[7623]|LNCC1463-13|United States|North Carolina|658[0n]|BOLD:ABY8158
Zale pr. n. sp. 1|[7624]|LNCC1464-13|United States|North Carolina|658[0n]|BOLD:ABY8158
Zale pr. n. sp. 1|[7625]|CNCLA5270-13|Canada|Ontario|658[0n]|BOLD:ABY8158
Zale n. sp. 2|[7626]|CNCLB1433-14|United States|North Carolina|658[0n]|BOLD:ABY8158
Zale n. sp. 2|[7627]|CNCLB1434-14|United States|North Carolina|658[0n]|BOLD:ABY8158
Zale helata|[7628]|XAB034-04|Canada|Ontario|658[0n]|BOLD:ABY8158
Zale helata|[7629]|XAJ579-06|Canada|Ontario|658[0n]|BOLD:ABY8158
Zale helata|[7630]|XAE311-04|Canada|Ontario|619[0n]|BOLD:ABY8158
Zale helata|[7631]|RDLQG301-06|Canada|Quebec|658[0n]|BOLD:ABY8158
Zale helata|[7632]|RDLQG300-06|Canada|Quebec|658[0n]|BOLD:ABY8158
Zale helata|[7633]|RDLQF497-06|Canada|Quebec|658[0n]|BOLD:ABY8158
Zale helata|[7634]|ABNCC356-07|United States|Wisconsin|628[2n]|BOLD:ABY8158
Zale helata|[7635]|QUNOD356-10|United States|Minnesota|658[0n]|BOLD:ABY8158
Zale helata|[7636]|QUNOD363-10|United States|Minnesota|658[0n]|BOLD:ABY8158
Zale helata|[7637]|QUNOD365-10|United States|Minnesota|658[0n]|BOLD:ABY8158
Zale helata|[7638]|RDLQF498-06|Canada|Quebec|658[0n]|BOLD:ABY8158
Zale helata|[7639]|XAE310-04|Canada|Ontario|658[0n]|BOLD:ABY8158
Zale helata|[7640]|RDLQH118-06|Canada|Quebec|615[1n]|BOLD:ABY8158
Zale helata|[7641]|XAJ541-06|Canada|Ontario|658[0n]|BOLD:ABY8158
Zale helata|[7642]|RDLQB550-05|Canada|Quebec|658[0n]|BOLD:ABY8158
Zale helata|[7643]|LOTB216-05|United States|Tennessee|658[0n]|BOLD:ABY8158
Zale helata|[7644]|LOTB149-05|United States|Tennessee|658[0n]|BOLD:ABY8158
Zale helata|[7645]|LOTB141-05|United States|Tennessee|658[0n]|BOLD:ABY8158
Zale helata|[7646]|XAJ619-06|Canada|Ontario|643[0n]|BOLD:ABY8158
Zale helata|[7647]|RDNM744-05|Canada|Ontario|608[0n]|BOLD:ABY8158
Zale helata|[7648]|RDLQG205-06|Canada|Quebec|595[0n]|BOLD:ABY8158
Zale helata|[7649]|RDLQG299-06|Canada|Quebec|593[0n]|BOLD:ABY8158
Zale helata|[7650]|LPSOB210-08|Canada|Ontario|658[0n]|BOLD:ABY8158
Zale helata|[7651]|RDNMG064-08|Canada|Ontario|658[0n]|BOLD:ABY8158
Zale helata|[7652]|RDNMG065-08|Canada|Ontario|658[0n]|BOLD:ABY8158
Zale helata|[7653]|QUNOD364-10|United States|Minnesota|658[0n]|BOLD:ABY8158
Zale helata|[7654]|RDNMJ190-10|Canada|Ontario|658[0n]|BOLD:ABY8158
Zale duplicata|[7655]|XAD612-05|Canada|Ontario|658[0n]|BOLD:ABY8158
Zale duplicata|[7656]|XAD646-05|Canada|Ontario|658[0n]|BOLD:ABY8158
Zale duplicata|[7657]|RDLQH042-06|Canada|Quebec|657[0n]|BOLD:ABY8158
Zale duplicata|[7658]|RDLQH077-06|Canada|Quebec|658[0n]|BOLD:ABY8158
Zale duplicata|[7659]|XAJ349-06|Canada|Ontario|658[0n]|BOLD:ABY8158
Zale duplicata|[7660]|XAJ353-06|Canada|Ontario|658[0n]|BOLD:ABY8158
Zale duplicata|[7661]|RDLQH078-06|Canada|Quebec|658[0n]|BOLD:ABY8158
Zale duplicata|[7662]|XAF370-05|Canada|Ontario|658[0n]|BOLD:ABY8158
Zale duplicata|[7663]|RDLQH055-06|Canada|Quebec|658[0n]|BOLD:ABY8158
Zale duplicata|[7664]|XAB045-04|Canada|Ontario|658[1n]|BOLD:ABY8158
Zale duplicata|[7665]|RDLQH052-06|Canada|Quebec|649[0n]|BOLD:ABY8158
Zale duplicata|[7666]|RDLQH051-06|Canada|Quebec|649[0n]|BOLD:ABY8158
Zale duplicata|[7667]|RDLQH103-06|Canada|Quebec|658[0n]|BOLD:ABY8158
Zale duplicata|[7668]|RDLQH049-06|Canada|Quebec|658[0n]|BOLD:ABY8158
Zale duplicata|[7669]|RDLQH048-06|Canada|Quebec|658[0n]|BOLD:ABY8158
Zale duplicata|[7670]|RDLQH047-06|Canada|Quebec|658[0n]|BOLD:ABY8158
Zale duplicata|[7671]|RDLQH046-06|Canada|Quebec|658[0n]|BOLD:ABY8158
Zale duplicata|[7672]|RDLQH043-06|Canada|Quebec|658[0n]|BOLD:ABY8158
Zale duplicata|[7673]|RDLQH041-06|Canada|Quebec|658[0n]|BOLD:ABY8158
Zale duplicata|[7674]|RDLQG302-06|Canada|Quebec|658[0n]|BOLD:ABY8158
Zale duplicata|[7675]|RDLQG296-06|Canada|Quebec|658[0n]|BOLD:ABY8158
Zale duplicata|[7676]|RDLQG220-06|Canada|Quebec|658[0n]|BOLD:ABY8158
Zale duplicata|[7677]|XAF332-05|Canada|Ontario|658[0n]|BOLD:ABY8158
Zale duplicata|[7678]|XAE171-04|Canada|Ontario|658[0n]|BOLD:ABY8158
Zale duplicata|[7679]|XAB030-04|Canada|Ontario|649[0n]|BOLD:ABY8158
Zale duplicata|[7680]|XAE161-04|Canada|Ontario|658[0n]|BOLD:ABY8158
Zale duplicata|[7681]|RDLQH044-06|Canada|Quebec|658[0n]|BOLD:ABY8158
Zale duplicata|[7682]|RDLQH045-06|Canada|Quebec|654[0n]|BOLD:ABY8158
Zale duplicata|[7683]|PMG167-03|Canada|Ontario|617[0n]|BOLD:ABY8158
Zale duplicata|[7684]|PHMO366-03|Canada|Ontario|639[0n]|BOLD:ABY8158
Zale duplicata|[7685]|XAB028-04|Canada|Ontario|573[0n]|BOLD:ABY8158
Zale duplicata|[7686]|RDLQG204-06|Canada|Quebec|593[1n]|BOLD:ABY8158
Zale duplicata|[7687]|RDLQH050-06|Canada|Quebec|654[0n]|BOLD:ABY8158
Zale duplicata|[7688]|RDLQH053-06|Canada|Quebec|658[0n]|BOLD:ABY8158
[illegible]

Zale duplicata|[7686]|RDLQG204-06|Canada|Quebec|593[1n]|BOLD:ABY8158
Zale duplicata|[7687]|RDLQH050-06|Canada|Quebec|654[0n]|BOLD:ABY8158
Zale duplicata|[7688]|RDLQH053-06|Canada|Quebec|658[0n]|BOLD:ABY8158
Zale duplicata|[7689]|RDLQH054-06|Canada|Quebec|658[0n]|BOLD:ABY8158
Zale duplicata|[7690]|RDLQH079-06|Canada|Quebec|658[0n]|BOLD:ABY8158
Zale duplicata|[7691]|KPOEC170-08|Canada|Ontario|658[0n]|BOLD:ABY8158
Zale duplicata|[7692]|RDNMG383-08|Canada|Ontario|658[0n]|BOLD:ABY8158
Zale duplicata|[7693]|QUNOD354-10|United States|Minnesota|658[0n]|BOLD:ABY8158
Zale duplicata|[7694]|QUNOD359-10|United States|Minnesota|658[0n]|BOLD:ABY8158
Zale duplicata|[7695]|RDNMG358-08|Canada|British Columbia|658[0n]|BOLD:ABY8158
Zale duplicata|[7696]|LOWCB607-05|Canada|British Columbia|658[0n]|BOLD:ABY8158
Zale duplicata|[7697]|RDLQG295-06|Canada|Quebec|593[0n]|BOLD:ABY8158
Zale duplicata|[7698]|RDLQG297-06|Canada|Quebec|658[0n]|BOLD:ABY8158
Zale duplicata|[7699]|RDNMG174-08|Canada|New Brunswick|658[0n]|BOLD:ABY8158
Zale duplicata|[7700]|RDNMJ797-11|Canada|Quebec|658[0n]|BOLD:ABY8158
Zale duplicata|[7701]|LALPA1120-11|Canada|British Columbia|658[0n]|BOLD:ABY8158
Zale duplicata|[7702]|RDNMG359-08|Canada|Alberta|658[0n]|BOLD:ABY8158
Zale duplicata|[7703]|QUNOD355-10|United States|Minnesota|658[0n]|BOLD:ABY8158
Zale duplicata|[7704]|QUNOD353-10|United States|Minnesota|658[0n]|BOLD:ABY8158
Zale duplicata|[7705]|RDNMG173-08|Canada|New Brunswick|658[0n]|BOLD:ABY8158
Zale duplicata|[7706]|CNPAB351-13|Canada|Saskatchewan|628[0n]|BOLD:ABY8158
Zale metatoides|[7707]|PSAT140-10|Canada|Ontario|658[0n]|BOLD:ABY8158
Zale metatoides|[7708]|CNCLB1493-14|Canada|Ontario|658[0n]|BOLD:ABY8158
Zale metatoides|[7709]|LNCC1672-13|United States|North Carolina|658[4n]|BOLD:ABY8158
Zale metatoides|[7710]|LNCC1689-13|United States|North Carolina|658[3n]|BOLD:ABY8158
Zale metatoides|[7711]|RDNMD476-06|United States|Colorado|658[0n]|BOLD:ABY8158
Zale metatoides|[7712]|RDNMD477-06|United States|Colorado|658[0n]|BOLD:ABY8158
Zale metatoides|[7713]|LNCC546-11|United States|North Carolina|658[0n]|BOLD:ABY8158
Zale metatoides|[7714]|QUNOD401-10|United States|New Jersey|658[0n]|BOLD:ABY8158
Zale metatoides|[7715]|RDNMG063-08|Canada|Ontario|658[0n]|BOLD:ABY8158
Zale metatoides|[7716]|BBLCU188-09|United States|Michigan|658[0n]|BOLD:ABY8158
Zale metatoides|[7717]|RDNMG382-08|Canada|Ontario|658[0n]|BOLD:ABY8158
Zale metatoides|[7718]|LGSMC899-05|United States|Tennessee|658[0n]|BOLD:ABY8158
Zale metatoides|[7719]|HKONS450-08|United States|Florida|577[0n]|BOLD:ABY8158
Zale metatoides|[7720]|CNCLB1496-14|Canada|Ontario|617[0n]|BOLD:ABY8158
Zale pr. n. sp. 3|[7721]|LNCB698-09|United States|North Carolina|658[0n]|BOLD:ACF0361
Zale pr. n. sp. 3|[7722]|QUNOD181-10|United States|Texas|658[0n]|BOLD:ACF0361
Zale pr. n. sp. 3|[7723]|QUNOD182-10|United States|Texas|658[0n]|BOLD:ACF0361
Zale pr. n. sp. 3|[7724]|LNCC919-11|United States|North Carolina|658[0n]|BOLD:ACF0361
Zale pr. n. sp. 2|[7725]|LGSMC825-05|United States|Tennessee|658[0n]|BOLD:ACF0361
Zale pr. n. sp. 2|[7726]|LGSMC824-05|United States|Tennessee|658[0n]|BOLD:ACF0361
Zale pr. n. sp. 2|[7727]|LOTB097-05|United States|Tennessee|658[0n]|BOLD:ACF0361
Zale pr. n. sp. 2|[7728]|LSEU311-06|United States|Georgia|658[0n]|BOLD:ACF0361
Zale pr. n. sp. 2|[7729]|LGSMC823-05|United States|Tennessee|658[0n]|BOLD:ACF0361
Zale pr. n. sp. 2|[7730]|LGSMC826-05|United States|Tennessee|658[0n]|BOLD:ACF0361
Zale pr. n. sp. 2|[7731]|LNCC1227-11|United States|North Carolina|658[0n]|BOLD:ACF0361
Zale metatoides|[7732]|LOTB455-05|United States|Tennessee|620[0n]|BOLD:ACF0360
Zale metatoides|[7733]|LSEU308-06|United States|Georgia|658[0n]|BOLD:ACF0360
Zale metatoides|[7734]|LOTB098-05|United States|Tennessee|658[0n]|BOLD:ACF0360
Zale metatoides|[7735]|LGSMC900-05|United States|Tennessee|658[0n]|BOLD:ACF0360
Zale metatoides|[7736]|LOTB096-05|United States|Tennessee|658[0n]|BOLD:ACF0360
Zale metatoides|[7737]|LOTB099-05|United States|Tennessee|612[0n]|BOLD:ACF0360
Zale metatoides|[7738]|LSEU319-06|United States|Georgia|658[0n]|BOLD:ACF0360
Zale curema|[7739]|MILEP014-09|United States|North Carolina|658[0n]|BOLD:ACF0360
Zale curema|[7740]|HKONS106-08|United States|Texas|658[0n]|BOLD:ACF0360
Zale curema|[7741]|LNCC1693-13|United States|North Carolina|658[0n]|BOLD:ACF0360
Zale curema|[7742]|CNCLB2896-14|United States|North Carolina|658[0n]|BOLD:ACF0360
Zale curema|[7743]|CNCLB2897-14|United States|North Carolina|658[0n]|BOLD:ACF0360
Zale n. sp. nr. obliqua|[7744]|LNCC1266-11|United States|North Carolina|658[0n]|BOLD:AAC2470
Zale n. sp. nr. obliqua|[7745]|LNCC655-11|United States|North Carolina|658[0n]|BOLD:AAC2470
Zale n. sp. nr. obliqua|[7746]|QUNOD400-10|United States||658[0n]|BOLD:AAC2470
Zale n. sp. nr. obliqua|[7747]|LNC067-05|United States|North Carolina|658[0n]|BOLD:AAC2470
Zale n. sp. nr. obliqua|[7748]|LNC068-05|United States|North Carolina|624[0n]|BOLD:AAC2470
Zale n. sp. nr. obliqua|[7749]|LNCC670-11|United States|North Carolina|633[0n]|BOLD:AAC2470
Zale n. sp. nr. obliqua|[7750]|LNCC742-11|United States|North Carolina|658[0n]|BOLD:AAC2470
Zale n. sp. nr. obliqua|[7751]|LNCC1267-11|United States|North Carolina|658[0n]|BOLD:AAC2470
Zale n. sp. nr. obliqua|[7752]|LNCC1268-11|United States|North Carolina|658[0n]|BOLD:AAC2470
Zale n. sp. nr. obliqua|[7753]|LNCC1269-11|United States|North Carolina|658[0n]|BOLD:AAC2470
Zale obliqua|[7754]|HKONS461-08|United States|Texas|606[0n]|BOLD:ACE3777
Zale obliqua|[7755]|HKONS441-08|United States|Florida|658[1n]|BOLD:ACE3777
Zale obliqua|[7756]|HKONS437-08|United States|Florida|658[0n]|BOLD:ACE3777
Zale obliqua|[7757]|HKONS465-08|United States|Florida|598[0n]|BOLD:ACE3777
Zale obliqua|[7758]|HKONS439-08|United States|Florida|658[1n]|BOLD:ACE3777
Zale obliqua|[7759]|QUNOB021-08|United States|Texas|658[0n]|BOLD:ACE3777
Zale obliqua|[7760]|BBLCU190-09|United States|Michigan|658[0n]|BOLD:ACE3777
Zale obliqua|[7761]|HKONS440-08|United States|Florida|658[0n]|BOLD:ACE3777
Zale obliqua|[7762]|HKONS438-08|United States|Florida|658[0n]|BOLD:ACE3777
Zale obliqua|[7763]|HKONS004-07|United States|Florida|658[0n]|BOLD:ACE3777
Zale obliqua|[7764]|LSEU495-06|United States|Georgia|658[0n]|BOLD:ACE3777
Zale obliqua|[7765]|HKONS460-08|United States|Texas|609[0n]|BOLD:ACE3777
Zale obliqua|[7766]|HKONS005-07|United States|Florida|658[0n]|BOLD:ACE3777
Zale obliqua|[7767]|HKONS463-08|United States|Florida|609[0n]|BOLD:ACE3777
Zale obliqua|[7768]|HKONS006-07|United States|Florida|648[0n]|BOLD:ACE3777
Zale obliqua|[7769]|ABNCC358-07|United States|Texas|593[0n]|BOLD:ACE3777
Zale obliqua|[7770]|HKONS464-08|United States|Florida|568[0n]|BOLD:ACE3777
Zale obliqua|[7771]|MILEP337-10|United States|Alabama|621[0n]|BOLD:ACE3777
Zale obliqua|[7772]|CNCLA5272-13|United States|Louisiana|658[0n]|BOLD:ACE3777
Zale squamularis|[7773]|LSEU496-06|United States|Georgia|658[0n]|BOLD:ABY9490
Zale squamularis|[7774]|HKONS183-08|United States|Florida|658[0n]|BOLD:ABY9490
Zale squamularis|[7775]|LGSMG570-07|United States|Tennessee|658[0n]|BOLD:ABY9490
Zale squamularis|[7776]|LOTB203-05|United States|Tennessee|658[0n]|BOLD:ABY9490
Zale squamularis|[7777]|LGSMC918-05|United States|Tennessee|658[0n]|BOLD:ABY9490
Zale squamularis|[7778]|LGSM668-04|United States|Tennessee|658[0n]|BOLD:ABY9490
Zale squamularis|[7779]|LOTB147-05|United States|Tennessee|658[0n]|BOLD:ABY9490
Zale squamularis|[7780]|LOT268-04|United States|Tennessee|658[2n]|BOLD:ABY9490
Zale squamularis|[7781]|HKONS458-08|United States|Kentucky|609[0n]|BOLD:ABY9490
Zale squamularis|[7782]|HKONS459-08|United States|Kentucky|609[0n]|BOLD:ABY9490
Zale squamularis|[7783]|QUNOC010-09|United States|Louisiana|658[0n]|BOLD:ABY9490
Zale squamularis|[7784]|CNCLA5273-13|United States|Louisiana|658[0n]|BOLD:ABY9490
Zale metata|[7785]|LOFLA227-06|United States|Florida|658[0n]|BOLD:ACF0359
Zale metata|[7786]|HKONS096-08|United States|Florida|658[0n]|BOLD:ACF0359
Zale metata|[7787]|HKONS098-08|United States|Florida|658[0n]|BOLD:ACF0359
Zale metata|[7788]|RDNMD592-06|United States|Florida|658[0n]|BOLD:ACF0359
[illegible]

Zale metata|[7786]|HKONS096-08|United States|Florida|658[0n]|BOLD:ACF0359
Zale metata|[7787]|HKONS098-08|United States|Florida|658[0n]|BOLD:ACF0359
Zale metata|[7788]|RDNMD592-06|United States|Florida|658[0n]|BOLD:ACF0359
Zale metata|[7789]|LOFLB017-06|United States|Florida|658[0n]|BOLD:ACF0359
Zale metata|[7790]|LOFLA852-06|United States|Florida|658[0n]|BOLD:ACF0359
Zale metata|[7791]|RDNMD593-06|United States|Florida|658[0n]|BOLD:ACF0359
Zale metata|[7792]|RDNMH101-09|United States|Georgia|658[0n]|BOLD:ACF0359
Zale metata|[7793]|RDNMH102-09|United States|Georgia|658[0n]|BOLD:ACF0359
Zale metata|[7794]|RDNMH271-09|United States|Georgia|658[0n]|BOLD:ACF0359
Zale metata|[7795]|RDNMD481-06|United States|Colorado|658[0n]|BOLD:ACF0359
Zale metata|[7796]|CNCLA5271-13|United States|Florida|658[0n]|BOLD:ACF0359
Zale metata|[7797]|LOFLB251-06|United States|Florida|658[0n]|BOLD:ACF0359
Zale metata|[7798]|HKONS095-08|United States|Florida|658[0n]|BOLD:ACF0359
Zale metata|[7799]|HKONS099-08|United States|Florida|658[0n]|BOLD:ACF0359
Zale metata|[7800]|HKONS100-08|United States|Florida|658[0n]|BOLD:ACF0359
Zale metata|[7801]|LOTB148-05|United States|Tennessee|658[0n]|BOLD:ACF0359
Zale metata|[7802]|LGSMG575-07|United States|Tennessee|656[0n]|BOLD:ACF0359
Zale metata|[7803]|HKONS142-08|United States|Kentucky|658[2n]|BOLD:ACF0359
Zale metata|[7804]|LGSMG573-07|United States|Tennessee|658[0n]|BOLD:ACF0359
Zale metata|[7805]|LSEU394-06|United States|Georgia|658[0n]|BOLD:ACF0359
Zale metata|[7806]|LSEU310-06|United States|Georgia|658[0n]|BOLD:ACF0359
Zale metata|[7807]|LSEU309-06|United States|Georgia|658[0n]|BOLD:ACF0359
Zale metata|[7808]|LOT266-04|United States|Tennessee|658[0n]|BOLD:ACF0359
Zale metata|[7809]|LOT265-04|United States|Tennessee|658[0n]|BOLD:ACF0359
Zale metata|[7810]|LGSM528-04|United States|Tennessee|658[0n]|BOLD:ACF0359
Zale metata|[7811]|LGSM527-04|United States|Tennessee|658[0n]|BOLD:ACF0359
Zale metata|[7812]|LGSMC898-05|United States|Tennessee|615[0n]|BOLD:ACF0359
Zale metata|[7813]|LGSMG574-07|United States|Tennessee|636[0n]|BOLD:ACF0359
Zale metata|[7814]|LGSMG576-07|United States|Tennessee|658[0n]|BOLD:ACF0359
Zale metata|[7815]|QUNOB150-08|United States|Kentucky|658[0n]|BOLD:ACF0359
Zale metata|[7816]|QUNOB151-08|United States|Kentucky|658[0n]|BOLD:ACF0359
Zale metata|[7817]|QUNOB152-08|United States|Kentucky|658[0n]|BOLD:ACF0359
Zale metata|[7818]|QUNOB153-08|United States|Kentucky|658[0n]|BOLD:ACF0359
Zale metata|[7819]|QUNOB154-08|United States|Kentucky|658[0n]|BOLD:ACF0359
Zale metata|[7820]|QUNOB155-08|United States|Kentucky|658[0n]|BOLD:ACF0359
Zale metata|[7821]|CNCLB2893-14|United States|North Carolina|658[0n]|BOLD:ACF0359
Zale metata|[7822]|LOT267-04|United States|Tennessee|658[0n]|BOLD:ACF0359
Zale metata|[7823]|HKONS444-08|United States|Florida|658[0n]|BOLD:ACF0359
Zale metata|[7824]|HKONS445-08|United States|Florida|581[1n]|BOLD:ACF0359
Zale metata|[7825]|HKONS446-08|United States|Florida|658[0n]|BOLD:ACF0359
Zale metata|[7826]|HKONS447-08|United States|Florida|658[0n]|BOLD:ACF0359
Zale metata|[7827]|HKONS448-08|United States|Florida|658[0n]|BOLD:ACF0359
Zale metata|[7828]|LTOL1146-11|United States|Maryland|658[0n]|BOLD:ACF0359
Zale metata|[7829]|LNCC1570-13|United States|North Carolina|658[0n]|BOLD:ACF0359
Zale metata|[7830]|LNCC1692-13|United States|North Carolina|658[0n]|BOLD:ACF0359
Zale metata|[7831]|CNCLB2926-14|United States|North Carolina|658[0n]|BOLD:ACF0359
Zale confusa|[7832]|HKONS103-08|United States|Texas|658[0n]|BOLD:ABY8159
Zale confusa|[7833]|HKONS443-08|United States|Florida|658[0n]|BOLD:ABY8159
Zale confusa|[7834]|HKONS104-08|United States|Texas|658[0n]|BOLD:ABY8159
Zale confusa|[7835]|HKONS102-08|United States|Texas|658[0n]|BOLD:ABY8159
Zale confusa|[7836]|HKONS101-08|United States|Texas|658[0n]|BOLD:ABY8159
Zale confusa|[7837]|HKONS449-08|United States|Florida|547[0n]|BOLD:ABY8159
Zale confusa|[7838]|LNCC392-10|United States|North Carolina|658[0n]|BOLD:ABY8159
Zale confusa|[7839]|LNCC671-11|United States|North Carolina|658[0n]|BOLD:ABY8159
Zale confusa|[7840]|LNCC1369-11|United States|North Carolina|658[0n]|BOLD:ABY8159
Zale confusa|[7841]|LNCC1691-13|United States|North Carolina|658[0n]|BOLD:ABY8159
Zale confusa|[7842]|CNCLB2924-14|United States|North Carolina|658[0n]|BOLD:ABY8159
Zale confusa|[7843]|HKONS442-08|United States|Florida|658[0n]|BOLD:ABY8159
Zale confusa|[7844]|LNCC1690-13|United States|North Carolina|658[0n]|BOLD:ABY8159
Zale confusa|[7845]|CNCLB2923-14|United States|North Carolina|658[0n]|BOLD:ABY8159
Zale confusa|[7846]|CNCLB2927-14|United States|North Carolina|658[0n]|BOLD:ABY8159
Zale confusa|[7847]|HKONS143-08|United States|Florida|658[0n]|BOLD:ABY8159
Zale confusa|[7848]|HKONS144-08|United States|Florida|658[0n]|BOLD:ABY8159
Zale confusa|[7849]|CNCLB2929-14|United States|North Carolina|658[0n]|BOLD:ABY8159
Zale fictilis|[7850]|QUNOB119-08|United States|Texas|658[0n]|BOLD:AAC0149
Zale fictilis|[7851]|RDNML358-13|United States|Florida|658[0n]|BOLD:AAC0149
Zale fictilis|[7852]|HKONS007-07|United States|Florida|658[0n]|BOLD:AAC0149
Zale fictilis|[7853]|HKONS008-07|United States|Florida|658[1n]|BOLD:AAC0149
Zale fictilis|[7854]|RDNML359-13|United States|Florida|658[0n]|BOLD:AAC0149
Zale exhausta|[7855]|BLPDK528-09|Costa Rica|Guanacaste|658[0n]|BOLD:AAC7176
Zale exhausta|[7856]|ARMOT729-12|Argentina|Misiones|658[0n]|BOLD:AAC7176
Zale exhausta|[7857]|LOCRC571-09|Costa Rica|Alajuela|658[0n]|BOLD:AAC7176
Zale exhausta|[7858]|MHAUB827-05|Costa Rica|Guanacaste|658[0n]|BOLD:AAC7176
Zale exhausta|[7859]|BLPCO123-08|Costa Rica|Guanacaste|658[0n]|BOLD:AAC7176
Zale exhausta|[7860]|BLPDC508-09|Costa Rica|Guanacaste|658[0n]|BOLD:AAC7176
Zale exhausta|[7861]|MHMYL3243-11|Costa Rica||658[0n]|BOLD:AAC7176
Zale exhausta|[7862]|BLPCG255-08|Costa Rica|Alajuela|658[0n]|BOLD:AAC7176
Zale exhausta|[7863]|BLPCH055-08|Costa Rica|Guanacaste|658[0n]|BOLD:AAC7176
Zale exhausta|[7864]|BLPCH053-08|Costa Rica|Guanacaste|658[0n]|BOLD:AAC7176
Zale exhausta|[7865]|BLPAF411-07|Costa Rica|Guanacaste|658[0n]|BOLD:AAC7176
Zale exhausta|[7866]|BLPBB299-07|Costa Rica|Guanacaste|658[0n]|BOLD:AAC7176
Zale exhausta|[7867]|GMART114-14|Argentina|Misiones|588[0n]|BOLD:AAC7176
Zale exhausta|[7868]|CNCLB1789-14|United States|Florida|658[0n]|BOLD:AAC7176
Zale exhausta|[7869]|MOTAR736-12|Argentina|Misiones|658[0n]|BOLD:AAC7176
Zale exhausta|[7870]|ARMOT855-12|Argentina|Misiones|658[0n]|BOLD:AAC7176
Zale exhausta|[7871]|ARMOT159-12|Argentina|Misiones|658[0n]|BOLD:AAC7176
Zale exhausta|[7872]|INCTB016-10|Brazil|Maranhao|658[0n]|BOLD:AAC7176
Zale exhausta|[7873]|INCTA813-10|Brazil|Maranhao|658[0n]|BOLD:AAC7176
Zale exhausta|[7874]|GMART113-14|Argentina|Misiones|585[0n]|BOLD:AAC7176
Zale exhausta|[7875]|GMARU209-14|Argentina|Misiones|588[0n]|BOLD:AAC7176
Zale exhausta|[7876]|CNCLB1790-14|United States|Florida|597[0n]|BOLD:AAC7176
Zale obsita|[7877]|CNCLB396-14|Brazil||315[0n]|
Zale sp.|[7878]|CNCLB2523-14|United States|Texas|658[0n]|BOLD:ABY4634
Zale n. sp. 5|[7879]|RDNML345-13|United States|Florida|658[0n]|BOLD:ACD9041
Zale n. sp. 5|[7880]|RDNML346-13|United States|Florida|658[0n]|BOLD:ACD9041
Zale n. sp. 5|[7881]|CNCLB1652-14|United States|Florida|658[0n]|BOLD:ACD9041
Zale strigimacula|[7882]|BLPCC437-08|Costa Rica|Guanacaste|658[0n]|BOLD:AAD0033
Zale strigimacula|[7883]|MHMXZ595-09|Costa Rica||658[0n]|BOLD:AAD0033
Zale strigimacula|[7884]|BLPDC503-09|Costa Rica|Guanacaste|658[0n]|BOLD:AAD0033
Zale strigimacula|[7885]|BLPDU237-11|Costa Rica|Guanacaste|658[0n]|BOLD:AAD0033
Zale strigimacula|[7886]|MHMYL3244-11|Costa Rica||658[0n]|BOLD:AAD0033
Zale strigimacula|[7887]|CNCLB1961-14|Guatemala||658[0n]|BOLD:AAD0033
Zale strigimacula|[7888]|BLPEE6069-14|Costa Rica||658[0n]|BOLD:AAD0033

Zale strigimacula|[7886]|MHMYL3244-11|Costa Rica||658[0n]|BOLD:AAD0033
Zale strigimacula|[7887]|CNCLB1961-14|Guatemala||658[0n]|BOLD:AAD0033
Zale strigimacula|[7888]|BLPEE6069-14|Costa Rica||658[0n]|BOLD:AAD0033
Zale strigimacula|[7889]|LEMMZ160-10|Brazil|Parana|658[0n]|BOLD:AAD0033
Zale strigimacula|[7890]|GWOSN469-11|Venezuela|Carabobo|658[0n]|BOLD:AAD0033
Zale strigimacula|[7891]|CNCLB1962-14|Guatemala||658[0n]|BOLD:AAD0033
Zale strigimacula|[7892]|CNCLB1715-14|United States|Texas|658[0n]|BOLD:AAD0033
Zale strigimacula|[7893]|BLPEE6068-14|Costa Rica||658[0n]|BOLD:AAD0033
Zale strigimacula|[7894]|BLPDU504-11|Costa Rica|Guanacaste|658[0n]|BOLD:AAD0033
Zale strigimacula|[7895]|BLPDU502-11|Costa Rica|Guanacaste|658[0n]|BOLD:AAD0033
Zale strigimacula|[7896]|LPYPB222-08|Mexico|Quintana Roo|658[0n]|BOLD:AAD0033
Zale strigimacula|[7897]|MHMYM2155-11|Costa Rica||658[0n]|BOLD:AAD0033
Zale strigimacula|[7898]|HKONB256-09|United States|Texas|658[0n]|BOLD:AAD0033
Zale strigimacula|[7899]|BLPCH636-08|Costa Rica|Guanacaste|658[0n]|BOLD:AAD0033
Zale strigimacula|[7900]|BLPCC720-08|Costa Rica|Guanacaste|658[0n]|BOLD:AAD0033
Zale strigimacula|[7901]|HKONS107-08|United States|Texas|658[0n]|BOLD:AAD0033
Zale strigimacula|[7902]|BLPAF918-07|Costa Rica|Guanacaste|658[0n]|BOLD:AAD0033
Zale strigimacula|[7903]|MXBLP224-11|Mexico|Jalisco|658[0n]|BOLD:AAD0033
Zale strigimacula|[7904]|CNCLB1964-14|Guatemala||658[0n]|BOLD:AAD0033
Zale strigimacula|[7905]|MHMYL3167-11|Costa Rica||658[0n]|BOLD:AAD0033
Zale strigimacula|[7906]|MXBLP418-15|Mexico|Jalisco|638[0n]|BOLD:AAD0033
Lymantria dispar dispar|[7907]|SMTPB8373-13|Canada|Ontario|504[0n]|
Lymantria dispar dispar|[7908]|LYMMK015-09|Canada|British Columbia|658[0n]|BOLD:AAA2052
Lymantria dispar dispar|[7909]|LYMMK013-09|Canada|British Columbia|658[0n]|BOLD:AAA2052
Lymantria dispar dispar|[7910]|LYMMK014-09|Canada|British Columbia|658[0n]|BOLD:AAA2052
Lymantria dispar|[7911]|LBCH7981-10|Canada|British Columbia|658[0n]|BOLD:AAA2052
Lymantria dispar dispar|[7912]|LPSOD1058-09|Canada|Ontario|632[0n]|BOLD:AAA2052
Lymantria dispar dispar|[7913]|CNPPD2599-12|Canada|Ontario|613[0n]|BOLD:AAA2052
Lymantria dispar dispar|[7914]|SMTPB8374-13|Canada|Ontario|534[0n]|BOLD:AAA2052
Lymantria dispar dispar|[7915]|LYMAN015-08|United States|Virginia|605[0n]|BOLD:AAA2052
Lymantria dispar dispar|[7916]|LYMAN017-08|United States|Maryland|612[0n]|BOLD:AAA2052
Lymantria dispar dispar|[7917]|TTMNB260-06|Canada|New Brunswick|658[0n]|BOLD:AAA2052
Lymantria dispar dispar|[7918]|SMTPB8372-13|Canada|Ontario|552[0n]|BOLD:AAA2052
Lymantria dispar dispar|[7919]|TTMNB259-06|Canada|New Brunswick|658[0n]|BOLD:AAA2052
Lymantria dispar dispar|[7920]|LYMAN018-08|United States|Maryland|636[0n]|BOLD:AAA2052
Lymantria dispar dispar|[7921]|GBGL1515-06|Canada|Ontario|624[0n]|BOLD:AAA2052
Lymantria dispar dispar|[7922]|GBGL1602-06|United States||621[1n]|BOLD:AAA2052
Lymantria dispar dispar|[7923]|LYMMK162-09|United States|West Virginia|658[0n]|BOLD:AAA2052
Lymantria dispar dispar|[7924]|LYMMK161-09|United States|West Virginia|658[0n]|BOLD:AAA2052
Lymantria dispar dispar|[7925]|LYMMK160-09|United States|West Virginia|658[0n]|BOLD:AAA2052
Lymantria dispar dispar|[7926]|LYMMK159-09|United States|Massachusetts|658[0n]|BOLD:AAA2052
Lymantria dispar dispar|[7927]|LYMMK158-09|United States|Massachusetts|658[0n]|BOLD:AAA2052
Lymantria dispar dispar|[7928]|LYMMK157-09|United States|Massachusetts|658[0n]|BOLD:AAA2052
Lymantria dispar dispar|[7929]|LYMMK156-09|United States|New York|658[0n]|BOLD:AAA2052
Lymantria dispar dispar|[7930]|LYMMK155-09|United States|New York|658[0n]|BOLD:AAA2052
Lymantria dispar dispar|[7931]|LYMMK154-09|United States|New York|658[0n]|BOLD:AAA2052
Lymantria dispar dispar|[7932]|LYMMK153-09|United States|New York|658[0n]|BOLD:AAA2052
Lymantria dispar dispar|[7933]|LYMMK152-09|United States|New York|658[0n]|BOLD:AAA2052
Lymantria dispar dispar|[7934]|LYMMK151-09|United States|New York|658[0n]|BOLD:AAA2052
Lymantria dispar dispar|[7935]|LYMMK150-09|United States|New York|658[0n]|BOLD:AAA2052
Lymantria dispar dispar|[7936]|LYMMK149-09|United States|New York|658[0n]|BOLD:AAA2052
Lymantria dispar dispar|[7937]|LYMMK148-09|United States|New York|658[0n]|BOLD:AAA2052
Lymantria dispar dispar|[7938]|LYMMK147-09|United States|New York|658[0n]|BOLD:AAA2052
Lymantria dispar dispar|[7939]|LYMMK146-09|United States|New York|658[0n]|BOLD:AAA2052
Lymantria dispar dispar|[7940]|LYMMK145-09|United States|New York|658[0n]|BOLD:AAA2052
Lymantria dispar dispar|[7941]|LYMMK144-09|United States|New York|658[0n]|BOLD:AAA2052
Lymantria dispar dispar|[7942]|LYMMK143-09|United States|New York|658[0n]|BOLD:AAA2052
Lymantria dispar dispar|[7943]|LYMMK142-09|United States|New York|658[0n]|BOLD:AAA2052
Lymantria dispar dispar|[7944]|LYMMK141-09|United States|Connecticut|658[0n]|BOLD:AAA2052
Lymantria dispar dispar|[7945]|LYMMK140-09|United States|Connecticut|658[0n]|BOLD:AAA2052
Lymantria dispar dispar|[7946]|LYMMK139-09|United States|Connecticut|658[0n]|BOLD:AAA2052
Lymantria dispar dispar|[7947]|LYMMK138-09|United States|Connecticut|658[0n]|BOLD:AAA2052
Lymantria dispar dispar|[7948]|LYMMK137-09|United States|Connecticut|658[0n]|BOLD:AAA2052
Lymantria dispar dispar|[7949]|LYMMK136-09|United States|Connecticut|658[0n]|BOLD:AAA2052
Lymantria dispar dispar|[7950]|LYMMK135-09|United States|North Carolina|658[0n]|BOLD:AAA2052
Lymantria dispar dispar|[7951]|LYMMK134-09|United States|North Carolina|658[0n]|BOLD:AAA2052
Lymantria dispar dispar|[7952]|LYMMK133-09|United States|North Carolina|658[0n]|BOLD:AAA2052
Lymantria dispar dispar|[7953]|BLTIB901-08|Canada|Ontario|658[0n]|BOLD:AAA2052
Lymantria dispar dispar|[7954]|BLTIB844-08|Canada|Ontario|658[0n]|BOLD:AAA2052
Lymantria dispar dispar|[7955]|BLTIB831-08|Canada|Ontario|658[0n]|BOLD:AAA2052
Lymantria dispar dispar|[7956]|ABCNA929-08|United States|Michigan|658[0n]|BOLD:AAA2052
Lymantria dispar dispar|[7957]|TMNBD447-07|Canada|New Brunswick|658[0n]|BOLD:AAA2052
Lymantria dispar dispar|[7958]|TMNBD446-07|Canada|New Brunswick|658[0n]|BOLD:AAA2052
Lymantria dispar dispar|[7959]|TMNBD445-07|Canada|New Brunswick|658[0n]|BOLD:AAA2052
Lymantria dispar dispar|[7960]|LMHRG036-06|Canada|British Columbia|658[0n]|BOLD:AAA2052
Lymantria dispar dispar|[7961]|LMHRG035-06|Canada|British Columbia|658[0n]|BOLD:AAA2052
Lymantria dispar dispar|[7962]|LMHRG033-06|Canada|British Columbia|658[0n]|BOLD:AAA2052
Lymantria dispar dispar|[7963]|LMHRG032-06|Canada|British Columbia|658[0n]|BOLD:AAA2052
Lymantria dispar dispar|[7964]|LMHRG031-06|Canada|British Columbia|658[0n]|BOLD:AAA2052
Lymantria dispar dispar|[7965]|LMHRG030-06|Canada|British Columbia|658[0n]|BOLD:AAA2052
Lymantria dispar dispar|[7966]|LMHRG029-06|Canada|British Columbia|658[0n]|BOLD:AAA2052
Lymantria dispar dispar|[7967]|LMHRG027-06|Canada|British Columbia|658[0n]|BOLD:AAA2052
Lymantria dispar dispar|[7968]|LMHRG025-06|Canada|British Columbia|658[0n]|BOLD:AAA2052
Lymantria dispar dispar|[7969]|LMHRG023-06|Canada|British Columbia|658[0n]|BOLD:AAA2052
Lymantria dispar dispar|[7970]|LMHRG022-06|Canada|British Columbia|658[0n]|BOLD:AAA2052
Lymantria dispar dispar|[7971]|LMHRG021-06|Canada|British Columbia|658[0n]|BOLD:AAA2052
Lymantria dispar dispar|[7972]|LMHRG020-06|Canada|British Columbia|658[0n]|BOLD:AAA2052
Lymantria dispar dispar|[7973]|LMHRG019-06|Canada|British Columbia|658[0n]|BOLD:AAA2052
Lymantria dispar dispar|[7974]|LMHRG016-06|Canada|British Columbia|658[0n]|BOLD:AAA2052
Lymantria dispar dispar|[7975]|LMHRG015-06|Canada|British Columbia|658[0n]|BOLD:AAA2052
Lymantria dispar dispar|[7976]|LMHRG014-06|Canada|British Columbia|658[0n]|BOLD:AAA2052
Lymantria dispar dispar|[7977]|LMHRG012-06|Canada|British Columbia|658[0n]|BOLD:AAA2052
Lymantria dispar dispar|[7978]|LMHRG010-06|Canada|British Columbia|658[0n]|BOLD:AAA2052
Lymantria dispar dispar|[7979]|LMHRG009-06|Canada|British Columbia|658[0n]|BOLD:AAA2052
Lymantria dispar dispar|[7980]|LMHRG007-06|Canada|British Columbia|658[0n]|BOLD:AAA2052
Lymantria dispar dispar|[7981]|LMHRG006-06|Canada|British Columbia|658[0n]|BOLD:AAA2052
Lymantria dispar dispar|[7982]|LMHRG004-06|Canada|British Columbia|658[0n]|BOLD:AAA2052
Lymantria dispar dispar|[7983]|LMHRG002-06|Canada|British Columbia|658[0n]|BOLD:AAA2052
Lymantria dispar dispar|[7984]|LMHRG001-06|Canada|British Columbia|658[0n]|BOLD:AAA2052
Lymantria dispar dispar|[7985]|XAK268-06|Canada|Ontario|658[0n]|BOLD:AAA2052
Lymantria dispar dispar|[7986]|XAK267-06|Canada|Ontario|658[0n]|BOLD:AAA2052
Lymantria dispar dispar|[7987]|XAG348-05|Canada|Ontario|658[0n]|BOLD:AAA2052
Lymantria dispar dispar|[7988]|XAG308-05|Canada|Ontario|658[0n]|BOLD:AAA2052

Lymantria dispar dispar|[7986]|XAK267-06|Canada|Ontario|658[0n]|BOLD:AAA2052
Lymantria dispar dispar|[7987]|XAG348-05|Canada|Ontario|658[0n]|BOLD:AAA2052
Lymantria dispar dispar|[7988]|XAG308-05|Canada|Ontario|658[0n]|BOLD:AAA2052
Lymantria dispar dispar|[7989]|XAG307-05|Canada|Ontario|658[0n]|BOLD:AAA2052
Lymantria dispar dispar|[7990]|XAG277-05|Canada|Ontario|658[0n]|BOLD:AAA2052
Lymantria dispar dispar|[7991]|XAG235-05|Canada|Ontario|658[0n]|BOLD:AAA2052
Lymantria dispar dispar|[7992]|XAG216-05|Canada|Ontario|658[0n]|BOLD:AAA2052
Lymantria dispar dispar|[7993]|XAG215-05|Canada|Ontario|658[0n]|BOLD:AAA2052
Lymantria dispar dispar|[7994]|XAG214-05|Canada|Ontario|658[0n]|BOLD:AAA2052
Lymantria dispar dispar|[7995]|XAG177-05|Canada|Ontario|658[0n]|BOLD:AAA2052
Lymantria dispar dispar|[7996]|XAG005-05|Canada|Ontario|658[0n]|BOLD:AAA2052
Lymantria dispar dispar|[7997]|LMHRG008-06|Canada|British Columbia|658[0n]|BOLD:AAA2052
Lymantria dispar dispar|[7998]|LMHRG024-06|Canada|British Columbia|658[0n]|BOLD:AAA2052
Lymantria dispar dispar|[7999]|LMHRG026-06|Canada|British Columbia|658[0n]|BOLD:AAA2052
Lymantria dispar dispar|[8000]|XAG658-05|Canada|Ontario|658[3n]|BOLD:AAA2052
Lymantria dispar dispar|[8001]|GBGL1535-06|Canada|Ontario|621[0n]|BOLD:AAA2052
Lymantria dispar dispar|[8002]|LMHRG011-06|Canada|British Columbia|638[0n]|BOLD:AAA2052
Lymantria dispar dispar|[8003]|TMNBD448-07|Canada|New Brunswick|646[0n]|BOLD:AAA2052
Lymantria dispar dispar|[8004]|XAG217-05|Canada|Ontario|626[1n]|BOLD:AAA2052
Lymantria dispar dispar|[8005]|GBGL1520-06|Canada|Ontario|624[0n]|BOLD:AAA2052
Lymantria dispar dispar|[8006]|BLTIB832-08|Canada|Ontario|645[0n]|BOLD:AAA2052
Lymantria dispar dispar|[8007]|LYMAN016-08|United States|Maryland|648[0n]|BOLD:AAA2052
Lymantria dispar dispar|[8008]|LPSOD1060-09|Canada|Ontario|616[0n]|BOLD:AAA2052
Lymantria dispar dispar|[8009]|NAGEO059-09|United States|Maryland|658[0n]|
Lymantria dispar dispar|[8010]|AHLEP040-10|United States|Pennsylvania|656[0n]|BOLD:AAA2052
Lymantria dispar dispar|[8011]|GBGL1579-06|United States|Virginia|624[0n]|BOLD:AAA2052
Lymantria dispar dispar|[8012]|CNPPB2148-12|Canada|Ontario|590[0n]|BOLD:AAA2052
Lymantria dispar dispar|[8013]|CNPPC1921-12|Canada|Ontario|624[0n]|BOLD:AAA2052
Lymantria dispar dispar|[8014]|CNPPD2600-12|Canada|Ontario|630[0n]|BOLD:AAA2052
Lymantria dispar dispar|[8015]|SMTPB8370-13|Canada|Ontario|552[0n]|BOLD:AAA2052
Lymantria dispar dispar|[8016]|SMTPB8371-13|Canada|Ontario|552[0n]|BOLD:AAA2052
Lymantria dispar dispar|[8017]|SMTPB8375-13|Canada|Ontario|552[0n]|BOLD:AAA2052
Euproctis similis|[8018]|RFELP034-08|Russia|Primorsky Krai|658[0n]|BOLD:AAC3712
Euproctis similis|[8019]|LEFIC264-10|Finland|Uusimaa|658[0n]|BOLD:AAC3712
Euproctis similis|[8020]|CGUKA283-09|United Kingdom|England|637[0n]|BOLD:AAC3712
Euproctis similis|[8021]|CGUKA696-09|United Kingdom||658[0n]|BOLD:AAC3712
Euproctis similis|[8022]|CGUKA710-09|United Kingdom||658[0n]|BOLD:AAC3712
Euproctis similis|[8023]|CGUKB318-09|United Kingdom|England|658[0n]|BOLD:AAC3712
Euproctis similis|[8024]|CGUKB901-09|United Kingdom|Wales|658[0n]|BOLD:AAC3712
Euproctis similis|[8025]|CGUKD553-09|United Kingdom|England|641[0n]|BOLD:AAC3712
Euproctis similis|[8026]|LEFIC263-10|Finland|Uusimaa|658[0n]|BOLD:AAC3712
Euproctis similis|[8027]|FBLMW374-10|Germany|Bavaria|658[0n]|BOLD:AAC3712
Euproctis similis|[8028]|LEFIK943-10|Finland||658[0n]|BOLD:AAC3712
Euproctis similis|[8029]|CGUKC168-09|United Kingdom|England|658[0n]|BOLD:AAC3712
Euproctis similis|[8030]|GWORO669-09|Germany|Bavaria|658[0n]|BOLD:AAC3712
Euproctis similis|[8031]|CGUKB617-09|United Kingdom|England|658[0n]|BOLD:AAC3712
Euproctis similis|[8032]|PHLAA249-09|Austria|Styria|655[0n]|BOLD:AAC3712
Euproctis similis|[8033]|NLLEA759-12|Netherlands|South Holland|658[0n]|BOLD:AAC3712
Euproctis similis|[8034]|NLLEA813-12|Netherlands|South Holland|658[0n]|BOLD:AAC3712
Euproctis chrysorrhoea|[8035]|BTM083-10|Spain|Balearic Islands|658[0n]|BOLD:ABY5435
Euproctis chrysorrhoea|[8036]|CGUKC451-09|United Kingdom|England|658[0n]|BOLD:ABY5435
Euproctis chrysorrhoea|[8037]|BTM001-10|Spain|Andalusia|658[0n]|BOLD:ABY5435
Euproctis chrysorrhoea|[8038]|BTM023-10|Spain|La Rioja|658[0n]|BOLD:ABY5435
Euproctis chrysorrhoea|[8039]|BTM032-10|Spain|La Rioja|658[0n]|BOLD:ABY5435
Euproctis chrysorrhoea|[8040]|BTM039-10|Spain|Aragon|658[0n]|BOLD:ABY5435
Euproctis chrysorrhoea|[8041]|BTM040-10|Spain|Aragon|658[0n]|BOLD:ABY5435
Euproctis chrysorrhoea|[8042]|BTM048-10|Spain|Aragon|658[0n]|BOLD:ABY5435
Euproctis chrysorrhoea|[8043]|BTM092-10|Spain|Castilla y Leon|658[0n]|BOLD:ABY5435
Euproctis chrysorrhoea|[8044]|IBLAO564-12|Spain|Catalonia|658[0n]|BOLD:ABY5435
Euproctis chrysorrhoea|[8045]|IBLAO565-12|Spain|Catalonia|658[0n]|BOLD:ABY5435
Euproctis chrysorrhoea|[8046]|BTM057-10|Romania|Botosani|658[0n]|BOLD:AAD5030
Euproctis chrysorrhoea|[8047]|BTM058-10|Romania|Botosani|658[0n]|BOLD:AAD5030
Euproctis chrysorrhoea|[8048]|NLLEA782-12|Netherlands|South Holland|658[0n]|BOLD:AAD5030
Euproctis chrysorrhoea|[8049]|BTM011-10|Spain|Andalusia|658[0n]|BOLD:AAD5030
Euproctis chrysorrhoea|[8050]|BTM014-10|Spain|Andalusia|658[0n]|BOLD:AAD5030
Euproctis chrysorrhoea|[8051]|GWORU379-10|Italy|Calabria|658[0n]|BOLD:AAD5030
Euproctis chrysorrhoea|[8052]|GWORZ097-10|Italy|Basilicata|658[0n]|BOLD:AAD5030
Euproctis chrysorrhoea|[8053]|PHLSA637-11|Italy||658[0n]|BOLD:AAD5030
Euproctis chrysorrhoea|[8054]|FBLMU524-09|Germany|Bavaria|617[0n]|BOLD:AAD5030
Euproctis chrysorrhoea|[8055]|BTM085-10|Spain|Balearic Islands|658[0n]|BOLD:AAD5030
Euproctis chrysorrhoea|[8056]|BTM084-10|Spain|Balearic Islands|658[0n]|BOLD:AAD5030
Euproctis chrysorrhoea|[8057]|BTM082-10|Spain|Balearic Islands|658[0n]|BOLD:AAD5030
Euproctis chrysorrhoea|[8058]|BTM081-10|Spain|Balearic Islands|658[0n]|BOLD:AAD5030
Euproctis chrysorrhoea|[8059]|BTM080-10|Spain|Balearic Islands|658[0n]|BOLD:AAD5030
Euproctis chrysorrhoea|[8060]|BTM079-10|Spain|Balearic Islands|658[0n]|BOLD:AAD5030
Euproctis chrysorrhoea|[8061]|BTM078-10|Spain|Balearic Islands|658[0n]|BOLD:AAD5030
Euproctis chrysorrhoea|[8062]|BTM077-10|Spain|Balearic Islands|658[0n]|BOLD:AAD5030
Euproctis chrysorrhoea|[8063]|BTM076-10|Spain|Balearic Islands|658[0n]|BOLD:AAD5030
Euproctis chrysorrhoea|[8064]|BTM056-10|Romania|Botosani|658[0n]|BOLD:AAD5030
Euproctis chrysorrhoea|[8065]|BTM055-10|Romania|Botosani|658[0n]|BOLD:AAD5030
Euproctis chrysorrhoea|[8066]|BTM052-10|Romania|Botosani|658[0n]|BOLD:AAD5030
Euproctis chrysorrhoea|[8067]|BTM051-10|Romania|Botosani|658[0n]|BOLD:AAD5030
Euproctis chrysorrhoea|[8068]|BTM050-10|Romania|Botosani|658[0n]|BOLD:AAD5030
Euproctis chrysorrhoea|[8069]|BTM049-10|Romania|Botosani|658[0n]|BOLD:AAD5030
Euproctis chrysorrhoea|[8070]|BTM027-10|Spain|La Rioja|658[0n]|BOLD:AAD5030
Euproctis chrysorrhoea|[8071]|BTM026-10|Spain|La Rioja|658[0n]|BOLD:AAD5030
Euproctis chrysorrhoea|[8072]|GWOR4215-09|Germany|Bavaria|658[0n]|BOLD:AAD5030
Euproctis chrysorrhoea|[8073]|GWOSP624-11|Tunisia||658[0n]|BOLD:AAD5030
Euproctis chrysorrhoea|[8074]|CGUKA293-09|United Kingdom|England|658[0n]|BOLD:AAD5030
Euproctis chrysorrhoea|[8075]|GWOR4168-09|Germany|Bavaria|658[0n]|BOLD:AAD5030
Euproctis chrysorrhoea|[8076]|BTM053-10|Romania|Botosani|658[0n]|BOLD:AAD5030
Euproctis chrysorrhoea|[8077]|BTM054-10|Romania|Botosani|658[0n]|BOLD:AAD5030
Euproctis chrysorrhoea|[8078]|NLLEA383-12|Netherlands|South Holland|658[0n]|BOLD:AAD5030
Euproctis chrysorrhoea|[8079]|NLLEA797-12|Netherlands|South Holland|658[0n]|BOLD:AAD5030
Euproctis chrysorrhoea|[8080]|GBLGC217-12|Germany|Bavaria|658[0n]|BOLD:AAD5030
Euproctis chrysorrhoea|[8081]|GBLGC264-12|Germany|Bavaria|658[0n]|BOLD:AAD5030
Euproctis chrysorrhoea|[8082]|GBLAB617-13|Germany|Brandenburg|658[0n]|BOLD:AAD5030
Azeta n. sp. 1|[8083]|RDNMH472-09|United States|Louisiana|658[0n]|BOLD:AAE4555
Azeta n. sp. 1|[8084]|LPYPB638-08|Mexico|Campeche|658[0n]|BOLD:AAE4555
Azeta n. sp. 1|[8085]|LPMX454-07|Mexico|Campeche|658[0n]|BOLD:AAE4555
Azeta n. sp. 1|[8086]|LYPAP082-09|Mexico|Quintana Roo|625[0n]|BOLD:AAE4555
Azeta n. sp. 1|[8087]|LYHES192-09|Mexico|Yucatan|658[0n]|BOLD:AAE4555
Azeta n. sp. 1|[8088]|COR037-13|Mexico|Quintana Roo|658[0n]|BOLD:AAE4555

Azeta n. sp. 1|[8086]|LYPAP082-09|Mexico|Quintana Roo|625[0n]|BOLD:AAE4555
Azeta n. sp. 1|[8087]|LYHES192-09|Mexico|Yucatan|658[0n]|BOLD:AAE4555
Azeta n. sp. 1|[8088]|CQR037-13|Mexico|Quintana Roo|658[0n]|BOLD:AAE4555
Azeta repugnalis|[8089]|BBLOB790-11|United States|Florida|658[0n]|BOLD:AAC5832
Azeta repugnalis|[8090]|GWOSU370-11|Dominican Republic||658[0n]|BOLD:AAC5832
Azeta repugnalis|[8091]|GWOSU371-11|Dominican Republic||658[0n]|BOLD:AAC5832
Azeta repugnalis|[8092]|GWOSU372-11|Dominican Republic||658[0n]|BOLD:AAC5832
Azeta repugnalis|[8093]|LYPIE588-09|Mexico|Yucatan|658[0n]|BOLD:AAC5832
Azeta repugnalis|[8094]|LYHES506-09|Mexico|Yucatan|658[0n]|BOLD:AAC5832
Azeta repugnalis|[8095]|LYNYM273-09|Mexico|Quintana Roo|658[0n]|BOLD:AAC5832
Azeta repugnalis|[8096]|LYNYM174-09|Mexico|Yucatan|618[0n]|BOLD:AAC5832
Azeta repugnalis|[8097]|LYHES223-09|Mexico|Yucatan|624[0n]|BOLD:AAC5832
Azeta repugnalis|[8098]|LYHES239-09|Mexico|Yucatan|580[0n]|BOLD:AAC5832
Azeta repugnalis|[8099]|LYPIE571-09|Mexico|Yucatan|545[0n]|BOLD:AAC5832
Azeta repugnalis|[8100]|GMMCE148-14|Mexico|Jalisco|525[0n]|BOLD:AAC5832
Azeta repugnalis|[8101]|CNCLB2244-14|Mexico||658[0n]|BOLD:AAC5832
Azeta n. sp. 2|[8102]|RDNMH824-09|United States|Arizona|658[0n]|BOLD:AAD4060
Azeta schausi|[8103]|QUNOD172-10|United States|Arizona|658[0n]|BOLD:AAE4450
Azeta schausi|[8104]|RDNMH825-09|United States|Arizona|658[0n]|BOLD:AAE4450
Azeta schausi|[8105]|LMEMB120-09|United States|Arizona|658[0n]|BOLD:AAE4450
Azeta schausi|[8106]|CNCLB2286-14|United States|Arizona|540[0n]|BOLD:AAE4450
Amolita obliqua|[8107]|LOFLA615-06|United States|Florida|658[0n]|BOLD:AAC4468
Amolita obliqua|[8108]|LOFLA442-06|United States|Florida|658[0n]|BOLD:AAC4468
Amolita obliqua|[8109]|LPOKD313-09|United States|Oklahoma|658[0n]|BOLD:AAC4468
Amolita obliqua|[8110]|LNC249-05|United States|North Carolina|658[0n]|BOLD:AAC4468
Amolita obliqua|[8111]|LNC250-05|United States|North Carolina|658[0n]|BOLD:AAC4468
Amolita obliqua|[8112]|LOFLA627-06|United States|Florida|658[0n]|BOLD:AAC4468
Amolita obliqua|[8113]|LOFLB361-06|United States|Florida|658[0n]|BOLD:AAC4468
Amolita obliqua|[8114]|LOFLC024-06|United States|Florida|658[0n]|BOLD:AAC4468
Amolita obliqua|[8115]|LOFLC307-06|United States|Florida|658[0n]|BOLD:AAC4468
Amolita obliqua|[8116]|LOFLC456-06|United States|Florida|658[0n]|BOLD:AAC4468
Amolita obliqua|[8117]|LOFLD021-07|United States|Florida|658[0n]|BOLD:AAC4468
Amolita obliqua|[8118]|HKONS336-08|United States|Florida|658[0n]|BOLD:AAC4468
Amolita obliqua|[8119]|PHFLO079-10|United States|Florida|658[0n]|BOLD:AAC4468
Amolita obliqua|[8120]|RDNMJ010-10|United States|Florida|658[0n]|BOLD:AAC4468
Amolita obliqua|[8121]|RDNMJ011-10|United States|Florida|658[0n]|BOLD:AAC4468
Amolita obliqua|[8122]|LNCC1211-11|United States|North Carolina|658[0n]|BOLD:AAC4468
Amolita obliqua|[8123]|LNCC1212-11|United States|North Carolina|658[0n]|BOLD:AAC4468
Amolita obliqua|[8124]|BBLOB949-11|United States|Florida|658[0n]|BOLD:AAC4468
Amolita obliqua|[8125]|BBLOB1259-11|United States|Florida|658[0n]|BOLD:AAC4468
Amolita obliqua|[8126]|MILEQ273-11|United States|Alabama|658[0n]|BOLD:AAC4468
Amolita fessa|[8127]|LOFLA346-06|United States|Florida|658[0n]|BOLD:AAC1871
Amolita fessa|[8128]|LOFLB853-06|United States|Florida|658[0n]|BOLD:AAC1871
Amolita fessa|[8129]|HKONS050-07|United States|Florida|658[0n]|BOLD:AAC1871
Amolita fessa|[8130]|HKONS051-07|United States|Florida|654[1n]|BOLD:AAC1871
Amolita fessa|[8131]|HKONS052-07|United States|Florida|658[1n]|BOLD:AAC1871
Amolita fessa|[8132]|PMG089-03|Canada|Ontario|617[0n]|BOLD:AAC1870
Amolita fessa|[8133]|BLTIB438-08|Canada|Ontario|656[2n]|BOLD:AAC1870
Amolita fessa|[8134]|XAJ901-06|Canada|Ontario|658[0n]|BOLD:AAC1870
Amolita fessa|[8135]|BLTIB471-08|Canada|Ontario|658[0n]|BOLD:AAC1870
Amolita fessa|[8136]|RDLQB424-05|Canada|Quebec|658[0n]|BOLD:AAC1870
Amolita fessa|[8137]|XAJ897-06|Canada|Ontario|658[0n]|BOLD:AAC1870
Amolita fessa|[8138]|XAK020-06|Canada|Ontario|658[0n]|BOLD:AAC1870
Amolita fessa|[8139]|BLTIB502-08|Canada|Ontario|658[0n]|BOLD:AAC1870
Amolita fessa|[8140]|LILLA823-11|United States|Illinois|658[0n]|BOLD:ABA2088
Amolita obliqua|[8141]|MILEQ274-11|United States|Alabama|658[0n]|BOLD:ABA2088
Hypena lividalis|[8142]|IBLAO897-12|Spain||658[0n]|BOLD:AAE1121
Hypena lividalis|[8143]|GWOSD237-10|Ethiopia|Oromia|658[0n]|BOLD:AAE1121
Hypena lividalis|[8144]|GWORU305-10|Italy|Calabria|658[0n]|BOLD:AAE1121
Hypena abjuralis|[8145]|QUNOD192-10|United States|Texas|658[0n]|BOLD:AAE1121
Hypena lividalis|[8146]|GWOSW470-11|Taiwan||625[0n]|BOLD:AAE1121
Hypena lividalis|[8147]|IBLAO948-12|Spain||658[0n]|BOLD:AAE1121
Amolita roseola|[8148]|LNC190-05|United States|North Carolina|658[0n]|BOLD:AAE2742
Amolita roseola|[8149]|LNC191-05|United States|North Carolina|658[0n]|BOLD:AAE2742
Amolita roseola|[8150]|HKONS627-08|United States|Florida|658[0n]|BOLD:AAE2742
Amolita roseola|[8151]|HKONS628-08|United States|Florida|658[0n]|BOLD:AAE2742
Amolita roseola|[8152]|HKONS629-08|United States|Florida|658[0n]|BOLD:ACP5235
Amolita roseola|[8153]|CNCLB1677-14|United States|Florida|658[0n]|BOLD:ACP5235
Amolita fratercula|[8154]|CMAZA620-10|United States|Arizona|658[0n]|BOLD:AAJ4600
Amolita fratercula|[8155]|RDNMH812-09|United States|Arizona|658[0n]|BOLD:AAJ4600
Amolita fratercula|[8156]|CMAZA786-10|United States|Arizona|658[0n]|BOLD:AAJ4600
Amolita fratercula|[8157]|LTOL1063-11|United States|Arizona|658[0n]|BOLD:AAJ4600
Amolita fratercula|[8158]|CNCLB206-14|United States|Arizona|658[0n]|BOLD:AAJ4600
Amolita delicata|[8159]|LNAUT944-14|United States|New Mexico|658[0n]|BOLD:ACN8603
Amolita delicata|[8160]|LNAUT943-14|United States|New Mexico|658[0n]|BOLD:ACN8603
Amolita delicata|[8161]|LNAUT942-14|United States|New Mexico|658[0n]|BOLD:ACN8603
Amolita delicata|[8162]|CNCLB2117-14|United States|New Mexico|582[0n]|BOLD:ACN8603
Amolita delicata|[8163]|CNCLB2118-14|United States|New Mexico|658[0n]|BOLD:ACN8603
Metalectra nr. cinctus|[8164]|QUNOD214-10|United States|Texas|658[0n]|BOLD:AAK7653
Metalectra edilis|[8165]|IAWL046-09|United States|Arizona|658[0n]|BOLD:AAD4059
Metalectra edilis|[8166]|IAWL047-09|United States|Arizona|658[0n]|BOLD:AAD4059
Metalectra edilis|[8167]|RDNMG437-08|United States|Arizona|658[0n]|BOLD:AAD4059
Metalectra edilis|[8168]|RDNMG436-08|United States|Arizona|658[0n]|BOLD:AAD4059
Metalectra edilis|[8169]|ABNCC217-07|United States|New Mexico|648[0n]|BOLD:AAD4059
Metalectra edilis|[8170]|HKONB498-09|United States|Arizona|538[0n]|BOLD:AAD4059
Metalectra edilis|[8171]|RDNMH827-09|United States|Arizona|618[0n]|BOLD:AAD4059
Metalectra edilis|[8172]|CMAZA606-10|United States|Arizona|658[0n]|BOLD:AAD4059
Metalectra cinctus|[8173]|QUNOD285-10|United States|Arizona|658[0n]|BOLD:ACF1032
Metalectra cinctus|[8174]|RDNMH041-09|United States|Arizona|658[0n]|BOLD:ACF1032
Metalectra cinctus|[8175]|PSAT127-10|United States|Arizona|658[0n]|BOLD:ACF1032
Metalectra sp. nr. edilis|[8176]|QUNOD232-10|United States|Texas|658[0n]|BOLD:ABZ5201
Metalectra sp. nr. edilis|[8177]|QUNOD739-11|United States|Texas|658[0n]|BOLD:ABZ5201
Metalectra diabolica|[8178]|HKONB299-09|United States|Texas|658[0n]|BOLD:AAD1209
Metalectra diabolica|[8179]|LPOKA295-08|United States|Oklahoma|598[0n]|BOLD:AAD1209
Metalectra diabolica|[8180]|HKONB305-09|United States|Louisiana|658[0n]|BOLD:AAD1209
Metalectra diabolica|[8181]|HKONB179-08|United States|Texas|658[0n]|BOLD:AAD1209
Metalectra diabolica|[8182]|HKONS146-08|United States|Florida|624[0n]|BOLD:AAD1209
Metalectra diabolica|[8183]|HKONS147-08|United States|Florida|614[0n]|BOLD:AAD1209
Metalectra diabolica|[8184]|ABNCC222-07|United States|Texas|650[0n]|BOLD:AAD1209
Metalectra diabolica|[8185]|LPOKB414-09|United States|Oklahoma|658[0n]|BOLD:AAD1209
Metalectra diabolica|[8186]|BBLSW987-09|United States|Texas|658[0n]|BOLD:AAD1209
Metalectra diabolica|[8187]|LPOKD027-09|United States|Oklahoma|658[0n]|BOLD:AAD1209
Metalectra diabolica|[8188]|LPOKD848-10|United States|Oklahoma|658[0n]|BOLD:AAD1209

Metalectra diabolica|[8186]|BBLSW987-09|United States|Texas|658[0n]|BOLD:AAD1209
Metalectra diabolica|[8187]|LPOKD027-09|United States|Oklahoma|658[0n]|BOLD:AAD1209
Metalectra diabolica|[8188]|LPOKD848-10|United States|Oklahoma|658[0n]|BOLD:AAD1209
Metalectra diabolica|[8189]|LILLA512-11|United States|Illinois|658[0n]|BOLD:AAD1209
Metalectra tantillus|[8190]|RDNMD521-06|United States|Florida|658[2n]|BOLD:AAC4497
Metalectra tantillus|[8191]|RDNME598-08|United States|Florida|658[0n]|BOLD:AAC4497
Metalectra tantillus|[8192]|MILEQ266-11|United States|Alabama|658[0n]|BOLD:AAC4497
Metalectra tantillus|[8193]|HKONS247-08|United States|Florida|658[0n]|BOLD:AAC4497
Metalectra tantillus|[8194]|HKONS246-08|United States|Florida|658[0n]|BOLD:AAC4497
Metalectra tantillus|[8195]|HKONS089-07|United States|Florida|658[0n]|BOLD:AAC4497
Metalectra tantillus|[8196]|LNCB008-06|United States|North Carolina|658[0n]|BOLD:AAC4497
Metalectra tantillus|[8197]|LNCB007-06|United States|North Carolina|658[0n]|BOLD:AAC4497
Metalectra tantillus|[8198]|LNC060-05|United States|North Carolina|626[0n]|BOLD:AAC4497
Metalectra tantillus|[8199]|HKONB296-09|United States|Texas|658[0n]|BOLD:AAC4497
Metalectra tantillus|[8200]|LMEMB052-09|United States|Alabama|658[0n]|BOLD:AAC4497
Metalectra tantillus|[8201]|LMEMB053-09|United States|Mississippi|504[2n]|
Metalectra tantillus|[8202]|MILEQ267-11|United States|Alabama|658[0n]|BOLD:AAC4497
Metalectra sp.|[8203]|RDNME578-08|United States|Florida|658[0n]|BOLD:AAE9529
Metalectra sp.|[8204]|RDNMJ001-10|United States|Florida|658[0n]|BOLD:AAE9529
Metalectra sp.|[8205]|BBLOB446-11|United States|Florida|658[0n]|BOLD:AAE9529
Homocerynea cleoriformis|[8206]|PSAT118-10|United States|Arizona|658[0n]|BOLD:AAX1167
Metalectra geminicincta|[8207]|HKONS242-08|United States|Florida|658[0n]|BOLD:AAH3004
Metalectra geminicincta|[8208]|HKONS243-08|United States|Florida|658[0n]|BOLD:AAH3004
Metalectra geminicincta|[8209]|RDNML054-13|United States|Florida|658[0n]|BOLD:AAH3004
Metalectra discalis|[8210]|RDLQF599-06|Canada|Quebec|658[0n]|BOLD:AAA8207
Metalectra discalis|[8211]|RDLQF598-06|Canada|Quebec|658[0n]|BOLD:AAA8207
Metalectra discalis|[8212]|RDLQF597-06|Canada|Quebec|658[0n]|BOLD:AAA8207
Metalectra discalis|[8213]|RDLQF595-06|Canada|Quebec|658[0n]|BOLD:AAA8207
Metalectra discalis|[8214]|RDLQF524-06|Canada|Quebec|658[0n]|BOLD:AAA8207
Metalectra discalis|[8215]|RDLQF523-06|Canada|Quebec|658[0n]|BOLD:AAA8207
Metalectra discalis|[8216]|RDLQF420-06|Canada|Quebec|658[0n]|BOLD:AAA8207
Metalectra discalis|[8217]|XAJ849-06|Canada|Ontario|658[0n]|BOLD:AAA8207
Metalectra discalis|[8218]|RDLQF570-06|Canada|Quebec|627[0n]|BOLD:AAA8207
Metalectra discalis|[8219]|RDNMD223-06|Canada|Quebec|611[0n]|BOLD:AAA8207
Metalectra discalis|[8220]|RDNMD224-06|Canada|Quebec|600[0n]|BOLD:AAA8207
Metalectra discalis|[8221]|RDLQF752-06|Canada|Quebec|595[1n]|BOLD:AAA8207
Metalectra discalis|[8222]|RDLQG032-06|Canada|Quebec|658[0n]|BOLD:AAA8207
Metalectra discalis|[8223]|RDLQF751-06|Canada|Quebec|658[0n]|BOLD:AAA8207
Metalectra discalis|[8224]|RDLQF750-06|Canada|Quebec|658[0n]|BOLD:AAA8207
Metalectra discalis|[8225]|RDLQG079-06|Canada|Quebec|629[0n]|BOLD:AAA8207
Metalectra discalis|[8226]|LGSMG553-07|United States|Tennessee|658[0n]|BOLD:AAA8207
Metalectra discalis|[8227]|LPOKC772-09|United States|Oklahoma|658[0n]|BOLD:AAA8207
Metalectra discalis|[8228]|RDLQF568-06|Canada|Quebec|658[0n]|BOLD:AAA8207
Metalectra discalis|[8229]|BBLSW556-09|United States|Oklahoma|636[0n]|BOLD:AAA8207
Metalectra discalis|[8230]|RDLQG080-06|Canada|Quebec|625[0n]|BOLD:AAA8207
Metalectra discalis|[8231]|LPOKD081-09|United States|Oklahoma|614[0n]|BOLD:AAA8207
Metalectra discalis|[8232]|LPOKD846-10|United States|Oklahoma|658[0n]|BOLD:AAA8207
Metalectra discalis|[8233]|RDNME574-08|United States|Indiana|658[0n]|BOLD:AAA8207
Metalectra discalis|[8234]|LPOKC806-09|United States|Oklahoma|658[0n]|BOLD:AAA8207
Metalectra discalis|[8235]|LMEMB058-09|United States|Mississippi|658[0n]|BOLD:AAA8207
Metalectra discalis|[8236]|LMEMB057-09|United States|Alabama|658[0n]|BOLD:AAA8207
Metalectra discalis|[8237]|LMEMB056-09|United States|Mississippi|658[0n]|BOLD:AAA8207
Metalectra discalis|[8238]|LMEMB055-09|United States|Mississippi|658[0n]|BOLD:AAA8207
Metalectra discalis|[8239]|LPOKA517-09|United States|Oklahoma|658[0n]|BOLD:AAA8207
Metalectra discalis|[8240]|HKONB289-09|United States|Texas|658[0n]|BOLD:AAA8207
Metalectra discalis|[8241]|RDNME576-08|United States|Indiana|658[0n]|BOLD:AAA8207
Metalectra discalis|[8242]|RDLQF571-06|Canada|Quebec|658[0n]|BOLD:AAA8207
Metalectra discalis|[8243]|LGSM556-04|United States|North Carolina|607[0n]|BOLD:AAA8207
Metalectra discalis|[8244]|LOFLB013-06|United States|Florida|658[0n]|BOLD:AAA8207
Metalectra discalis|[8245]|LPOKA481-09|United States|Oklahoma|658[0n]|BOLD:AAA8207
Metalectra discalis|[8246]|BBLSU050-09|United States|Arkansas|658[0n]|BOLD:AAA8207
Metalectra discalis|[8247]|LPOKC769-09|United States|Oklahoma|658[0n]|BOLD:AAA8207
Metalectra discalis|[8248]|LPOKA330-08|United States|Oklahoma|658[0n]|BOLD:AAA8207
Metalectra discalis|[8249]|RDNME581-08|United States|Florida|658[0n]|BOLD:AAA8207
Metalectra discalis|[8250]|RDNME580-08|United States|Florida|658[0n]|BOLD:AAA8207
Metalectra discalis|[8251]|HKONS337-08|United States|Florida|658[0n]|BOLD:AAA8207
Metalectra discalis|[8252]|LOFLA874-06|United States|Florida|658[0n]|BOLD:AAA8207
Metalectra discalis|[8253]|LOFLA547-06|United States|Florida|658[0n]|BOLD:AAA8207
Metalectra discalis|[8254]|LPOKA381-09|United States|Oklahoma|602[0n]|BOLD:AAA8207
Metalectra discalis|[8255]|LGSM557-04|United States|Tennessee|589[0n]|BOLD:AAA8207
Metalectra discalis|[8256]|LNCB766-09|United States|North Carolina|632[0n]|BOLD:AAA8207
Metalectra discalis|[8257]|LPOKD403-09|United States|Oklahoma|658[0n]|BOLD:AAA8207
Metalectra discalis|[8258]|RDNMH954-09|United States|Florida|658[0n]|BOLD:AAA8207
Metalectra discalis|[8259]|LPOKE114-10|United States|Oklahoma|658[0n]|BOLD:AAA8207
Metalectra discalis|[8260]|LNCC643-11|United States|North Carolina|658[0n]|BOLD:AAA8207
Metalectra discalis|[8261]|LILLA341-11|United States|Illinois|658[0n]|BOLD:AAA8207
Metalectra discalis|[8262]|LILLA372-11|United States|Illinois|658[0n]|BOLD:AAA8207
Metalectra quadrisignata|[8263]|RDLQF569-06|Canada|Quebec|658[0n]|BOLD:AAB4859
Metalectra quadrisignata|[8264]|HKONS340-08|United States|Florida|658[0n]|BOLD:AAB4859
Metalectra quadrisignata|[8265]|RDLQF596-06|Canada|Quebec|658[0n]|BOLD:AAB4859
Metalectra quadrisignata|[8266]|RDLQB080-05|Canada|Quebec|658[0n]|BOLD:AAB4859
Metalectra quadrisignata|[8267]|RDLQB079-05|Canada|Quebec|658[0n]|BOLD:AAB4859
Metalectra quadrisignata|[8268]|RDLQF594-06|Canada|Quebec|649[0n]|BOLD:AAB4859
Metalectra quadrisignata|[8269]|RDNMD225-06|Canada|Quebec|610[0n]|BOLD:AAB4859
Metalectra quadrisignata|[8270]|RDLQG078-06|Canada|Quebec|633[0n]|BOLD:AAB4859
Metalectra quadrisignata|[8271]|RDNME575-08|United States|Indiana|658[0n]|BOLD:AAB4859
Metalectra quadrisignata|[8272]|RDNME577-08|United States|Michigan|658[0n]|BOLD:AAB4859
Metalectra quadrisignata|[8273]|LPOKB370-09|United States|Oklahoma|658[0n]|BOLD:AAB4859
Metalectra quadrisignata|[8274]|LMEMB062-09|United States|Louisiana|658[0n]|BOLD:AAB4859
Metalectra quadrisignata|[8275]|HKONB293-09|United States|Texas|658[0n]|BOLD:AAB4859
Metalectra quadrisignata|[8276]|RDNME573-08|United States|Indiana|658[0n]|BOLD:AAB4859
Metalectra quadrisignata|[8277]|LGSMC856-05|United States|Tennessee|658[0n]|BOLD:AAB4859
Metalectra quadrisignata|[8278]|LNC855-06|United States|North Carolina|658[0n]|BOLD:AAB4859
Metalectra quadrisignata|[8279]|RDNMH955-09|United States|Florida|571[0n]|BOLD:AAB4859
Metalectra quadrisignata|[8280]|MILEQ268-11|United States|Alabama|658[0n]|BOLD:AAB4859
Metalectra quadrisignata|[8281]|MILEQ269-11|United States|Alabama|658[0n]|BOLD:AAB4859
Metalectra quadrisignata|[8282]|MILEQ270-11|United States|Alabama|658[0n]|BOLD:AAB4859
Metalectra quadrisignata|[8283]|MILEQ271-11|United States|Alabama|658[0n]|BOLD:AAB4859
Metalectra quadrisignata|[8284]|LNCB771-09|United States|North Carolina|658[0n]|BOLD:AAB4859
Metalectra quadrisignata|[8285]|MILEQ272-11|United States|Alabama|658[0n]|BOLD:AAB4859
Metalectra quadrisignata|[8286]|LNCB852-09|United States|Georgia|658[0n]|BOLD:AAB4859
Metalectra quadrisignata|[8287]|LNCB770-09|United States|North Carolina|658[0n]|BOLD:AAB4859

[illegible]
Metalectra quadrisignata|[8286]|LNCB852-09|United States|Georgia|658[0n]|BOLD:AAB4859
Metalectra quadrisignata|[8287]|LNCB770-09|United States|North Carolina|658[0n]|BOLD:AAB4859
Metalectra quadrisignata|[8288]|LPOKB703-09|United States|Oklahoma|658[0n]|BOLD:AAB4859
Metalectra quadrisignata|[8289]|RDNME579-08|United States|Florida|658[0n]|BOLD:AAB4859
Metalectra quadrisignata|[8290]|HKONS339-08|United States|Florida|658[0n]|BOLD:AAB4859
Metalectra quadrisignata|[8291]|LNCC1884-13|United States|North Carolina|634[0n]|BOLD:AAB4859
Metalectra nr. bigallis|[8292]|RDNMH090-09|United States|Arizona|658[0n]|BOLD:AAE4420
Metalectra nr. bigallis|[8293]|RDNMH628-09|United States|Arizona|658[0n]|BOLD:AAE4420
Metalectra bigallis|[8294]|RDNMJ707-11|United States|Arizona|658[0n]|BOLD:AAE4419
Metalectra bigallis|[8295]|ABNCC218-07|United States|Arizona|647[0n]|BOLD:AAE4419
Metalectra bigallis|[8296]|ABNCC219-07|United States|Arizona|638[0n]|BOLD:AAE4419
Metalectra bigallis|[8297]|RDNMH826-09|United States|Arizona|658[0n]|BOLD:AAE4419
Metalectra bigallis|[8298]|QUNOE460-12|United States|Arizona|658[0n]|BOLD:AAE4419
Metalectra nr. discalis|[8299]|HKONB061-08|United States|Texas|658[0n]|BOLD:AAD6720
Metalectra nr. discalis|[8300]|HKONB063-08|United States|Texas|658[0n]|BOLD:AAD6720
Metalectra nr. discalis|[8301]|HKONB064-08|United States|Texas|658[0n]|BOLD:AAD6720
Metalectra nr. discalis|[8302]|HKONB062-08|United States|Texas|658[0n]|BOLD:AAD6720
Metalectra nr. discalis|[8303]|HKONB291-09|United States|Texas|658[0n]|BOLD:AAD6720
Metalectra nr. discalis|[8304]|HKONS300-08|United States|Texas|658[0n]|BOLD:AAD6720
Metalectra nr. discalis|[8305]|RDNMH043-09|United States|Texas|658[0n]|BOLD:AAD6720
Metalectra nr. discalis|[8306]|CNCLB1324-14|United States|Texas|658[0n]|BOLD:AAD6720
Parascotia fuliginaria|[8307]|IBLAO877-12|Spain||658[0n]|BOLD:AAC4237
Parascotia fuliginaria|[8308]|FBLMV251-09|Germany|Bavaria|658[0n]|BOLD:AAC4237
Parascotia fuliginaria|[8309]|TTNFS040-09|Serbia||658[0n]|BOLD:AAC4237
Parascotia fuliginaria|[8310]|LEFIA358-10|Finland|South Karelia|658[0n]|BOLD:AAC4237
Parascotia fuliginaria|[8311]|LEFIA357-10|Finland|South Karelia|658[0n]|BOLD:AAC4237
Parascotia fuliginaria|[8312]|GWORK529-09|Germany|Bavaria|658[0n]|BOLD:AAC4237
Parascotia fuliginaria|[8313]|GWORK467-09|Germany|Bavaria|658[0n]|BOLD:AAC4237
Parascotia fuliginaria|[8314]|CGUKC085-09|United Kingdom|England|658[0n]|BOLD:AAC4237
Parascotia fuliginaria|[8315]|GWORE2022-09|Germany|Bavaria|632[0n]|BOLD:AAC4237
Parascotia fuliginaria|[8316]|LEFIA1279-10|Finland|Uusimaa|669[1n]|BOLD:AAC4237
Parascotia fuliginaria|[8317]|GSCMW1364-10|Austria|Upper Austria|658[0n]|BOLD:AAC4237
Parascotia fuliginaria|[8318]|FBLMZ414-12|Germany|Bavaria|658[0n]|BOLD:AAC4237
Parascotia fuliginaria|[8319]|GWOTL419-13|Germany|Bavaria|658[0n]|BOLD:AAC4237
Parascotia fuliginaria|[8320]|GBLAB702-13|Germany|Brandenburg|658[0n]|BOLD:AAC4237
Parascotia fuliginaria|[8321]|FBLMW099-10|Germany|Bavaria|658[0n]|BOLD:AAC4237
Parascotia fuliginaria|[8322]|CGUKB378-09|United Kingdom|England|658[0n]|BOLD:AAC4237
Parascotia fuliginaria|[8323]|GWOR3941-09|Germany|Bavaria|658[0n]|BOLD:AAC4237
Parascotia fuliginaria|[8324]|LENOA012-11|France|Haute Normandie|658[0n]|BOLD:AAC4237
Parascotia fuliginaria|[8325]|IBLAO762-12|Spain||658[0n]|BOLD:AAC4237
Parascotia fuliginaria|[8326]|LEATB687-13|Italy|South Tyrol|658[0n]|BOLD:AAC4237
Mycterophora longipalpata|[8327]|RDNME564-08|United States|California|658[0n]|BOLD:AAD9555
Mycterophora longipalpata|[8328]|LBCG530-08|Canada|British Columbia|658[0n]|BOLD:AAD9555
Mycterophora longipalpata|[8329]|LOWCC738-05|Canada|British Columbia|658[0n]|BOLD:AAD9555
Mycterophora longipalpata|[8330]|RDNMF729-08|Canada|British Columbia|576[0n]|BOLD:AAD9555
Mycterophora longipalpata|[8331]|LBCG1135-09|Canada|British Columbia|658[0n]|BOLD:AAD9555
Mycterophora longipalpata|[8332]|LBCH6192-10|Canada|British Columbia|658[0n]|BOLD:AAD9555
Mycterophora longipalpata|[8333]|LBCH6257-10|Canada|British Columbia|658[0n]|BOLD:AAD9555
Mycterophora longipalpata|[8334]|LBCH6687-10|Canada|British Columbia|658[0n]|BOLD:AAD9555
Mycterophora longipalpata|[8335]|LBCH6723-10|Canada|British Columbia|658[0n]|BOLD:AAD9555
Mycterophora longipalpata|[8336]|LBCH7362-10|Canada|British Columbia|658[0n]|BOLD:AAD9555
Mycterophora longipalpata|[8337]|LBCH7832-10|Canada|British Columbia|636[0n]|BOLD:AAD9555
Mycterophora longipalpata|[8338]|JMMMB307-11|United States|California|658[0n]|BOLD:AAD9555
Mycterophora longipalpata|[8339]|GMLC1222-12|United States|California|658[0n]|BOLD:AAD9555
Mycterophora longipalpata|[8340]|JMMMB309-11|United States|California|658[0n]|BOLD:AAD9555
Mycterophora longipalpata|[8341]|JMMMB520-13|United States|California|591[0n]|BOLD:AAD9555
Mycterophora inexplicata|[8342]|RDNMF374-08|Canada|Alberta|658[0n]|BOLD:ABY9282
Mycterophora inexplicata|[8343]|RDNMF375-08|Canada|Alberta|658[0n]|BOLD:ABY9282
Mycterophora inexplicata|[8344]|RDNMF825-08|Canada|Alberta|658[0n]|BOLD:ABY9282
Mycterophora inexplicata|[8345]|LBCH4785-10|Canada|British Columbia|658[0n]|BOLD:ABY9282
Mycterophora inexplicata|[8346]|SSPAA2597-13|Canada|Saskatchewan|658[0n]|BOLD:ABY9282
Mycterophora geometriformis|[8347]|NAMUM367-09|United States|California|658[0n]|BOLD:ABY7377
Mycterophora geometriformis|[8348]|RDNMH219-09|United States|California|636[0n]|BOLD:ABY7377
Mycterophora rubricans|[8349]|LTOLB140-08|United States|California|658[0n]|BOLD:ABY7377
Mycterophora rubricans|[8350]|JMMMB308-11|United States|California|658[0n]|BOLD:ABY7377
Mycterophora sp.|[8351]|NAMUM163-08|United States|California|658[0n]|BOLD:AAD9556
Mycterophora sp.|[8352]|LBCC807-05|Canada|British Columbia|658[0n]|BOLD:ABX5596
Mycterophora sp.|[8353]|LBCD437-05|Canada|British Columbia|658[0n]|BOLD:ABX5596
Mycterophora sp.|[8354]|BBLPD884-10|Canada|British Columbia|658[0n]|BOLD:ABX5596
Mycterophora monticola|[8355]|TML127-14|United States||599[0n]|BOLD:ACO1373
Metalectra albilinea|[8356]|LOFLA822-06|United States|Florida|658[0n]|BOLD:AAD1230
Metalectra albilinea|[8357]|RDNMD221-06|United States|Florida|596[0n]|BOLD:AAD1230
Metalectra albilinea|[8358]|RDNMD222-06|United States|Florida|602[0n]|BOLD:AAD1230
Metalectra albilinea|[8359]|LOFLA712-06|United States|Florida|658[0n]|BOLD:AAD1230
Metalectra albilinea|[8360]|HKONS274-08|United States|Florida|658[0n]|BOLD:AAD1230
Metalectra albilinea|[8361]|HKONS275-08|United States|Florida|658[0n]|BOLD:AAD1230
Metalectra albilinea|[8362]|HKONS276-08|United States|Florida|658[0n]|BOLD:AAD1230
Metalectra albilinea|[8363]|HKONS277-08|United States|Florida|658[0n]|BOLD:AAD1230
Metalectra albilinea|[8364]|BBLOB1750-11|United States|Florida|658[0n]|BOLD:AAD1230
Metalectra miserulata|[8365]|RDNMD219-06|United States|Colorado|609[0n]|BOLD:AAC4678
Metalectra miserulata|[8366]|RDNMD220-06|United States|Colorado|590[0n]|BOLD:AAC4678
Metalectra miserulata|[8367]|RDNME582-08|United States|Colorado|658[0n]|BOLD:AAC4678
Metalectra miserulata|[8368]|HKONB547-09|United States|Texas|646[0n]|BOLD:ACE4543
Metalectra miserulata|[8369]|HKONB551-09|United States|Texas|658[0n]|BOLD:ACE4543
Metalectra miserulata|[8370]|HKONB520-09|United States|Texas|658[0n]|BOLD:ACE4543
Metalectra miserulata|[8371]|HKONB552-09|United States|Texas|639[0n]|BOLD:ACE4543
Metalectra miserulata|[8372]|HKONB553-09|United States|Texas|658[0n]|BOLD:ACE4543
Metalectra miserulata|[8373]|ABNCC227-07|United States|Texas|647[4n]|BOLD:ABZ3664
Metalectra miserulata|[8374]|IAWL684-09|United States|Texas|658[1n]|BOLD:ABZ3664
Metalectra miserulata|[8375]|ABNCC228-07|United States|Texas|594[0n]|BOLD:ABZ3664
Metalectra miserulata|[8376]|HKONB298-09|United States|Texas|658[0n]|BOLD:ABZ3664
Metalectra miserulata|[8377]|IAWL683-09|United States|Texas|658[0n]|BOLD:ABZ3664
Metalectra miserulata|[8378]|IAWL685-09|United States|Texas|658[0n]|BOLD:ABZ3664
Metalectra miserulata|[8379]|RDNMJ704-11|United States|Arizona|658[0n]|BOLD:ABZ3663
Metalectra miserulata|[8380]|PSAT130-10|United States|Arizona|597[0n]|BOLD:ABZ3663
Metalectra miserulata|[8381]|CMAZA597-10|United States|Arizona|658[0n]|BOLD:ABZ3663
Metalectra miserulata|[8382]|RDNMJ741-11|United States|Arizona|658[0n]|BOLD:ABZ3663
Metalectra miserulata|[8383]|QUNOE462-12|United States|Arizona|658[0n]|BOLD:ABZ3663
Metalectra miserulata|[8384]|CMAZA1224-12|United States|Arizona|658[0n]|BOLD:ABZ3663
Metalectra richardsi|[8385]|LMEMB063-09|United States|Tennessee|658[0n]|BOLD:ACF5141
Metalectra richardsi|[8386]|LMEMB067-09|United States|Alabama|572[0n]|BOLD:AAB4858
Metalectra richardsi|[8387]|LGSM769-04|United States|Tennessee|658[0n]|BOLD:AAB4858
[illegible]

Metalectra richardsi|[8385]|LMEMB063-09|United States|Tennessee|658[0n]|BOLD:ACF3141
Metalectra richardsi|[8386]|LMEMB067-09|United States|Alabama|572[0n]|BOLD:AAB4858
Metalectra richardsi|[8387]|LGSM769-04|United States|Tennessee|658[0n]|BOLD:AAB4858
Metalectra richardsi|[8388]|RDNMD215-06|United States|North Carolina|597[0n]|BOLD:AAB4858
Metalectra richardsi|[8389]|LGSMC846-05|United States|Tennessee|593[0n]|BOLD:AAB4858
Metalectra richardsi|[8390]|LGSMG556-07|United States|Tennessee|658[0n]|BOLD:AAB4858
Metalectra richardsi|[8391]|LGSMG555-07|United States|Tennessee|658[0n]|BOLD:AAB4858
Metalectra richardsi|[8392]|LGSMG554-07|United States|Tennessee|658[0n]|BOLD:AAB4858
Metalectra richardsi|[8393]|LNC152-05|United States|North Carolina|658[0n]|BOLD:AAB4858
Metalectra richardsi|[8394]|LGSMC845-05|United States|Tennessee|658[0n]|BOLD:AAB4858
Metalectra richardsi|[8395]|LGSMC844-05|United States|Tennessee|658[0n]|BOLD:AAB4858
Metalectra richardsi|[8396]|LGSMC842-05|United States|Tennessee|658[0n]|BOLD:AAB4858
Metalectra richardsi|[8397]|LGSM768-04|United States|Tennessee|658[0n]|BOLD:AAB4858
Metalectra richardsi|[8398]|LGSMC843-05|United States|Tennessee|658[0n]|BOLD:AAB4858
Metalectra richardsi|[8399]|LGSM740-04|United States|Tennessee|590[0n]|BOLD:AAB4858
Metalectra richardsi|[8400]|HKONB301-09|United States|Louisiana|658[0n]|BOLD:AAB4858
Metalectra richardsi|[8401]|HKONB302-09|United States|Texas|658[0n]|BOLD:AAB4858
Metalectra richardsi|[8402]|ABNCC225-07|United States|Texas|594[0n]|BOLD:AAB4858
Metalectra richardsi|[8403]|LOTB208-05|United States|Tennessee|658[0n]|BOLD:AAB4858
Metalectra richardsi|[8404]|LMEMB065-09|United States|Alabama|619[0n]|BOLD:AAB4858
Metalectra richardsi|[8405]|LMEMB066-09|United States|Alabama|658[0n]|BOLD:AAB4858
Metalectra richardsi|[8406]|CNCLB2933-14|United States|North Carolina|658[0n]|BOLD:AAB4858
Dyspyralis noloides|[8407]|QUNOD190-10|United States|Texas|658[0n]|BOLD:ABZ5294
Coxina hadenoides|[8408]|CNCLB2006-14|Guatemala||658[0n]|BOLD:AAB5573
Coxina hadenoides|[8409]|CNCLB2431-14|United States|Texas|658[0n]|BOLD:AAB5573
Coxina hadenoides|[8410]|HKONB054-08|United States|Texas|658[0n]|BOLD:AAB5573
Coxina hadenoides|[8411]|HKONB055-08|United States|Texas|658[0n]|BOLD:AAB5573
Coxina hadenoides|[8412]|HKONB056-08|United States|Texas|658[0n]|BOLD:AAB5573
Coxina hadenoides|[8413]|QUNOC013-09|United States|Texas|658[0n]|BOLD:AAB5573
Coxina hadenoides|[8414]|CNCLB2432-14|United States|Texas|658[0n]|BOLD:AAB5573
Kakopoda progenies|[8415]|LYPAP647-09|Mexico|Yucatan|658[0n]|BOLD:AAF3406
Kakopoda progenies|[8416]|LYPAP035-09|Mexico|Yucatan|658[0n]|BOLD:AAF3406
Kakopoda progenies|[8417]|QUNOD179-10|United States|Texas|658[0n]|BOLD:AAF3406
Redectis pygmaea|[8418]|LNCB742-09|United States|North Carolina|658[0n]|BOLD:AAD4876
Redectis pygmaea|[8419]|HKONS160-08|United States|Florida|658[0n]|BOLD:AAD4876
Redectis pygmaea|[8420]|LNC374-05|United States|North Carolina|531[0n]|BOLD:AAD4876
Redectis pygmaea|[8421]|LNCC710-11|United States|North Carolina|658[0n]|BOLD:AAD4876
Redectis pygmaea|[8422]|BBLOE215-11|United States|Texas|658[0n]|BOLD:AAD4876
Redectis pygmaea|[8423]|LGSM730-04|United States|Tennessee|658[0n]|BOLD:AAD4876
Redectis pygmaea|[8424]|LNC375-05|United States|North Carolina|522[0n]|BOLD:AAD4876
Redectis pygmaea|[8425]|BBLOE290-11|United States|Texas|658[0n]|BOLD:AAD4876
Oruza albocostaliata|[8426]|LSUSA134-06|United States|Kentucky|658[0n]|BOLD:AAD3234
Oruza albocostaliata|[8427]|LOFLA037-06|United States|Florida|658[0n]|BOLD:AAD3234
Oruza albocostaliata|[8428]|LNCB159-06|United States|North Carolina|658[0n]|BOLD:AAD3234
Oruza albocostaliata|[8429]|LOT357-04|United States|Tennessee|658[0n]|BOLD:AAD3234
Oruza albocostaliata|[8430]|LOT356-04|United States|Tennessee|658[0n]|BOLD:AAD3234
Oruza albocostaliata|[8431]|LNCB170-06|United States|North Carolina|658[0n]|BOLD:AAD3234
Oruza albocostaliata|[8432]|LNCB381-06|United States|North Carolina|658[0n]|BOLD:AAD3234
Oruza albocostaliata|[8433]|LNCC038-10|United States|North Carolina|658[0n]|BOLD:AAD3234
Oruza albocostata|[8434]|NAMUM047-08|United States|Arizona|657[0n]|BOLD:AAI1921
Oruza albocostata|[8435]|RDNME877-08|United States|Arizona|658[0n]|BOLD:AAI1921
Oruza albocostata|[8436]|CMAZA598-10|United States|Arizona|658[0n]|BOLD:AAI1921
Eudocima tyrannus|[8437]|QUNOB608-10|Myanmar|Kachin|617[0n]|BOLD:AAF1407
Eudocima tyrannus|[8438]|JMZCA142-09|Russia|Primorsky Krai|658[0n]|BOLD:AAF1407
Eudocima tyrannus|[8439]|QUNOD553-10|China||658[0n]|BOLD:AAF1407
Eudocima tyrannus|[8440]|QUNOD634-10|China||658[0n]|BOLD:AAF1407
Eudocima tyrannus|[8441]|GBMIN20928-13|||671[0n]|BOLD:AAF1407
Eudocima apta|[8442]|GWOSH632-10|Brazil|Rio Grande do Sul|658[0n]|BOLD:AAC6829
Eudocima apta|[8443]|BLPDV063-11|Costa Rica|Guanacaste|658[0n]|BOLD:AAC6829
Eudocima apta|[8444]|BLPDV062-11|Costa Rica|Guanacaste|658[0n]|BOLD:AAC6829
Eudocima apta|[8445]|LEMMZ001-10|Brazil|Parana|658[0n]|BOLD:AAC6829
Eudocima apta|[8446]|BLPDV061-11|Costa Rica|Guanacaste|658[0n]|BOLD:AAC6829
Eudocima apta|[8447]|MHAUB573-05|Costa Rica|Guanacaste|658[0n]|BOLD:AAC6829
Eudocima apta|[8448]|GWORA2664-09|Ecuador||658[0n]|BOLD:AAC6829
Eudocima apta|[8449]|ABCNA891-08|United States|Arizona|567[0n]|BOLD:AAC6829
Eudocima apta|[8450]|BLPAB410-06|Costa Rica|Guanacaste|658[0n]|BOLD:AAC6829
Eudocima apta|[8451]|BLPAD073-06|Costa Rica|Guanacaste|658[0n]|BOLD:AAC6829
Eudocima apta|[8452]|QUNO091-07|United States|Florida|658[0n]|BOLD:AAC6829
Eudocima apta|[8453]|BLPDO1010-10|Costa Rica|Alajuela|658[0n]|BOLD:AAC6829
Eudocima apta|[8454]|MHMYL3566-11|Costa Rica||658[0n]|BOLD:AAC6829
Eudocima apta|[8455]|BLPEC507-11|Costa Rica|Alajuela|658[0n]|BOLD:AAC6829
Eudocima serpentifera|[8456]|MHAUB577-05|Costa Rica|Guanacaste|658[0n]|BOLD:AAD0912
Eudocima serpentifera|[8457]|MHAUB578-05|Costa Rica|Guanacaste|658[0n]|BOLD:AAD0912
Eudocima serpentifera|[8458]|MHAUC252-06|Costa Rica|Alajuela|658[0n]|BOLD:AAD0912
Eudocima serpentifera|[8459]|MHAUF492-06|Costa Rica|Alajuela|658[0n]|BOLD:AAD0912
Eudocima serpentifera|[8460]|MHMXE367-06|Costa Rica|Alajuela|658[0n]|BOLD:AAD0912
Eudocima serpentifera|[8461]|MHMXE684-07|Costa Rica|Alajuela|658[0n]|BOLD:AAD0912
Eudocima serpentifera|[8462]|BLPCA161-08|Costa Rica|Guanacaste|658[0n]|BOLD:AAD0912
Eudocima serpentifera|[8463]|LYNYM142-09|Mexico|Yucatan|658[0n]|BOLD:AAD0912
Eudocima serpentifera|[8464]|BLPDV064-11|Costa Rica|Guanacaste|658[0n]|BOLD:AAD0912
Eudocima serpentifera|[8465]|MHMYS2805-13|Costa Rica|Guanacaste|658[0n]|BOLD:AAD0912
Calyptra canadensis|[8466]|XAJ831-06|Canada|Ontario|658[0n]|BOLD:ABY4439
Calyptra canadensis|[8467]|BLTIB568-08|Canada|Ontario|658[0n]|BOLD:ABY4439
Calyptra canadensis|[8468]|JMZCA159-09|United States||624[0n]|BOLD:AAD2635
Calyptra canadensis|[8469]|JMZCA160-09|United States||614[0n]|BOLD:AAD2635
Calyptra canadensis|[8470]|XAD756-05|Canada|Ontario|658[0n]|BOLD:AAD2635
Calyptra canadensis|[8471]|BLTIB708-08|Canada|Ontario|658[0n]|BOLD:AAD2635
Calyptra canadensis|[8472]|XAK005-06|Canada|Ontario|658[0n]|BOLD:AAD2635
Calyptra canadensis|[8473]|RDLQH115-06|Canada|Quebec|658[0n]|BOLD:AAD2635
Calyptra canadensis|[8474]|BLTIB738-08|Canada|Ontario|658[0n]|BOLD:AAD2635
Calyptra canadensis|[8475]|LNCC2082-14|United States|North Carolina|658[0n]|BOLD:AAD2635
Epitausa copperyiDHJ02|[8476]|MHMXN678-07|Costa Rica|Alajuela|658[0n]|BOLD:AAB9787
Epitausa copperyiDHJ02|[8477]|MHMYM570-11|Costa Rica||632[0n]|BOLD:AAB9787
Epitausa coppryi|[8478]|GWOST438-11|Colombia||658[0n]|BOLD:AAB9787
Epitausa copperyiDHJ02|[8479]|MHMYT084-13|Costa Rica||658[0n]|BOLD:AAB9787
Epitausa coppryi|[8480]|GWOSI949-10|Mexico|Oaxaca|658[0n]|BOLD:AAB9787
Epitausa coppryi|[8481]|GWOSU527-11|Mexico|Oaxaca|658[0n]|BOLD:AAB9787
Epitausa coppryi|[8482]|GWOTK244-12|Mexico|Oaxaca|658[0n]|BOLD:AAB9787
Epitausa coppryi|[8483]|CNCLB2447-14|United States|Texas|658[0n]|BOLD:AAB9787
Antiblemma rufinans|[8484]|RDNML306-13|United States|Florida|627[0n]|BOLD:AAX5075
Antiblemma rufinans|[8485]|RDNML307-13|United States|Florida|658[0n]|BOLD:AAX5075
Massala obvertensDHJ02|[8486]|BLPCB912-08|Costa Rica|Alajuela|658[0n]|BOLD:AAB5725
Massala obvertensDHJ02|[8487]|BLPDV042-11|Costa Rica|Guanacaste|635[0n]|BOLD:AAB5725

Antiblemma rufinans|[8485]|RDNML307-13|United States|Florida|658[0n]|BOLD:AAX5075
Massala obvertensDHJ02|[8486]|BLPCB912-08|Costa Rica|Alajuela|658[0n]|BOLD:AAB5725
Massala obvertensDHJ02|[8487]|BLPDV042-11|Costa Rica|Guanacaste|635[0n]|BOLD:AAB5725
Massala obvertensDHJ02|[8488]|BLPBH742-07|Costa Rica|Guanacaste|614[0n]|BOLD:AAB5725
Massala obvertens|[8489]|LOCRB802-08|Costa Rica|Alajuela|613[0n]|BOLD:AAB5725
Massala obvertensDHJ02|[8490]|MHAUA651-05|Costa Rica|Guanacaste|560[0n]|BOLD:AAB5725
Massala obvertensDHJ02|[8491]|BLPDU511-11|Costa Rica|Guanacaste|658[0n]|BOLD:AAB5725
Massala obvertensDHJ02|[8492]|MHAUA649-05|Costa Rica|Guanacaste|567[1n]|BOLD:AAB5725
Massala obvertensDHJ02|[8493]|MHAUA650-05|Costa Rica|Guanacaste|578[0n]|BOLD:AAB5725
Massala obvertensDHJ02|[8494]|BLPCP111-08|Costa Rica|Guanacaste|658[0n]|BOLD:AAB5725
Massala obvertensDHJ02|[8495]|BLPEE6846-14|Costa Rica||658[0n]|BOLD:AAB5725
Massala obvertensDHJ02|[8496]|BLPCA469-08|Costa Rica|Guanacaste|658[0n]|BOLD:AAB5725
Massala obvertensDHJ02|[8497]|MHAUA652-05|Costa Rica|Guanacaste|586[0n]|BOLD:AAB5725
Massala obvertensDHJ02|[8498]|BLPDV039-11|Costa Rica|Guanacaste|658[0n]|BOLD:AAB5725
Massala obvertensDHJ02|[8499]|BLPDU847-11|Costa Rica|Guanacaste|658[0n]|BOLD:AAB5725
Massala obvertensDHJ02|[8500]|BLPDV036-11|Costa Rica|Guanacaste|658[0n]|BOLD:AAB5725
Massala obvertensDHJ02|[8501]|BLPDV037-11|Costa Rica|Guanacaste|658[0n]|BOLD:AAB5725
Massala obvertensDHJ02|[8502]|BLPDV038-11|Costa Rica|Guanacaste|658[0n]|BOLD:AAB5725
Massala obvertensDHJ02|[8503]|BLPDV328-11|Costa Rica|Guanacaste|658[0n]|BOLD:AAB5725
Massala obvertensDHJ02|[8504]|BLPDV337-11|Costa Rica|Guanacaste|658[0n]|BOLD:AAB5725
Massala obvertensDHJ02|[8505]|MHMYL3638-11|Costa Rica||658[0n]|BOLD:AAB5725
Massala obvertensDHJ02|[8506]|MHMYQ1752-12|Costa Rica||658[0n]|BOLD:AAB5725
Massala obvertensDHJ02|[8507]|MHMYQ1753-12|Costa Rica||658[0n]|BOLD:AAB5725
Massala obvertens|[8508]|ARMOT856-12|Argentina|Misiones|658[0n]|BOLD:AAB5725
Massala obvertensDHJ02|[8509]|MHMYT228-13|Costa Rica||658[0n]|BOLD:AAB5725
Massala obvertensDHJ02|[8510]|MHMYT1215-13|Costa Rica||658[0n]|BOLD:AAB5725
Massala obvertensDHJ02|[8511]|BLPEE6848-14|Costa Rica||658[0n]|BOLD:AAB5725
Massala obvertensDHJ02|[8512]|BLPEE6849-14|Costa Rica||658[0n]|BOLD:AAB5725
Massala obvertens|[8513]|LYHES248-09|Mexico|Yucatan|658[0n]|BOLD:AAB5725
Massala obvertens|[8514]|INCTA949-10|Brazil|Maranhao|658[0n]|BOLD:AAB5725
Massala obvertensDHJ02|[8515]|BLPCO304-08|Costa Rica|Guanacaste|658[0n]|BOLD:AAB5725
Massala obvertensDHJ02|[8516]|BLPCD635-08|Costa Rica|Guanacaste|658[0n]|BOLD:AAB5725
Massala obvertensDHJ02|[8517]|BLPCD634-08|Costa Rica|Guanacaste|658[0n]|BOLD:AAB5725
Massala obvertens|[8518]|LOCRB801-08|Costa Rica|Alajuela|658[0n]|BOLD:AAB5725
Massala obvertensDHJ02|[8519]|BLPAF711-07|Costa Rica|Guanacaste|658[0n]|BOLD:AAB5725
Massala obvertensDHJ02|[8520]|MHAUF795-06|Costa Rica|Guanacaste|658[0n]|BOLD:AAB5725
Massala obvertensDHJ02|[8521]|MHAUA653-05|Costa Rica|Guanacaste|658[0n]|BOLD:AAB5725
Massala obvertensDHJ02|[8522]|BLPAE421-06|Costa Rica|Guanacaste|649[5n]|BOLD:AAB5725
Massala obvertensDHJ02|[8523]|MHAUF794-06|Costa Rica|Guanacaste|637[0n]|BOLD:AAB5725
Massala obvertensDHJ02|[8524]|BLPDV040-11|Costa Rica|Guanacaste|615[0n]|BOLD:AAB5725
Massala obvertensDHJ02|[8525]|BLPDV041-11|Costa Rica|Guanacaste|628[0n]|BOLD:AAB5725
Massala obvertens|[8526]|LMEMB125-09|Puerto Rico||658[0n]|BOLD:AAB5725
Massala obvertens|[8527]|BBLOB442-11|United States|Florida|658[0n]|BOLD:AAB5725
Massala obvertens|[8528]|BBLOB635-11|United States|Florida|658[0n]|BOLD:AAB5725
Massala obvertensDHJ02|[8529]|MHMYT229-13|Costa Rica||658[0n]|BOLD:AAB5725
Massala obvertensDHJ02|[8530]|BLPEE6850-14|Costa Rica||658[0n]|BOLD:AAB5725
Hemeroplanis trilineosa|[8531]|HKONB441-09|United States|Texas|658[0n]|BOLD:AAD3905
Hemeroplanis parallela|[8532]|QUNOD197-10|United States|Texas|658[0n]|BOLD:AAD3905
Hemeroplanis parallela|[8533]|QUNOD115-10|United States|Texas|658[0n]|BOLD:AAD3905
Hemeroplanis parallela|[8534]|QUNOD196-10|United States|Texas|658[0n]|BOLD:AAD3905
Hemeroplanis trilineosa|[8535]|QUNOD742-11|United States|Texas|658[0n]|BOLD:AAD3905
Hemeroplanis trilineosa|[8536]|ABNCC151-07|United States|Texas|594[0n]|BOLD:AAD3905
Hemeroplanis parallela|[8537]|LMEMB026-09|United States|Arizona|627[0n]|BOLD:AAD3905
Hemeroplanis parallela|[8538]|LMEMB027-09|United States|Arizona|658[0n]|BOLD:AAD3905
Hemeroplanis parallela|[8539]|LMEMB028-09|United States|Arizona|658[0n]|BOLD:AAD3905
Hemeroplanis parallela|[8540]|CMAZA001-09|United States|Arizona|658[0n]|BOLD:AAD3905
Hemeroplanis parallela|[8541]|RDNMJ512-11|United States|Arizona|658[0n]|BOLD:AAD3905
Hemeroplanis parallela|[8542]|IAWLB633-11|United States|Arizona|658[0n]|BOLD:AAD3905
Hemeroplanis parallela|[8543]|QUNOE453-12|United States|Arizona|658[0n]|BOLD:AAD3905
Hemeroplanis immaculalis|[8544]|RDNMJ097-10|United States|Oklahoma|658[0n]|BOLD:AAU3567
Hemeroplanis sp.|[8545]|LGSMC672-05|United States|Tennessee|658[0n]|BOLD:AAD3904
Hemeroplanis sp.|[8546]|LGSMC673-05|United States|Tennessee|658[0n]|BOLD:AAD3904
Hemeroplanis sp.|[8547]|LGSMG580-07|United States|Tennessee|658[0n]|BOLD:AAD3904
Hemeroplanis sp.|[8548]|LGSMG581-07|United States|Tennessee|658[0n]|BOLD:AAD3904
Hemeroplanis habitalis|[8549]|LMEM103-09|United States|Mississippi|658[0n]|BOLD:AAA9264
Hemeroplanis habitalis|[8550]|LOFLA163-06|United States|Florida|658[0n]|BOLD:AAA9264
Hemeroplanis habitalis|[8551]|LOFLB280-06|United States|Florida|658[0n]|BOLD:AAA9264
Hemeroplanis habitalis|[8552]|LOFLB422-06|United States|Florida|640[0n]|BOLD:AAA9264
Hemeroplanis habitalis|[8553]|LOFLB287-06|United States|Florida|658[0n]|BOLD:AAA9264
Hemeroplanis habitalis|[8554]|LOFLC119-06|United States|Florida|654[0n]|BOLD:AAA9264
Hemeroplanis habitalis|[8555]|LOFLB286-06|United States|Florida|658[0n]|BOLD:AAA9264
Hemeroplanis habitalis|[8556]|LOFLC454-06|United States|Florida|658[0n]|BOLD:AAA9264
Hemeroplanis habitalis|[8557]|LOFLC478-06|United States|Florida|658[0n]|BOLD:AAA9264
Hemeroplanis habitalis|[8558]|HKONS653-08|United States|Florida|658[0n]|BOLD:AAA9264
Hemeroplanis habitalis|[8559]|LOFLC441-06|United States|Florida|658[0n]|BOLD:AAA9264
Hemeroplanis habitalis|[8560]|LOFLC107-06|United States|Florida|658[0n]|BOLD:AAA9264
Hemeroplanis habitalis|[8561]|LOFLB782-06|United States|Florida|658[0n]|BOLD:AAA9264
Hemeroplanis habitalis|[8562]|LOFLB704-06|United States|Florida|658[0n]|BOLD:AAA9264
Hemeroplanis habitalis|[8563]|LOFLB541-06|United States|Florida|658[0n]|BOLD:AAA9264
Hemeroplanis habitalis|[8564]|LOFLB527-06|United States|Florida|658[0n]|BOLD:AAA9264
Hemeroplanis habitalis|[8565]|LOFLA933-06|United States|Florida|658[0n]|BOLD:AAA9264
Hemeroplanis habitalis|[8566]|LOFLA875-06|United States|Florida|658[0n]|BOLD:AAA9264
Hemeroplanis habitalis|[8567]|LOFLA873-06|United States|Florida|658[0n]|BOLD:AAA9264
Hemeroplanis habitalis|[8568]|LOFLA872-06|United States|Florida|658[0n]|BOLD:AAA9264
Hemeroplanis habitalis|[8569]|LOFLA612-06|United States|Florida|658[0n]|BOLD:AAA9264
Hemeroplanis habitalis|[8570]|LOFLA570-06|United States|Florida|658[0n]|BOLD:AAA9264
Hemeroplanis habitalis|[8571]|LOFLA564-06|United States|Florida|658[0n]|BOLD:AAA9264
Hemeroplanis habitalis|[8572]|LOFLA352-06|United States|Florida|658[0n]|BOLD:AAA9264
Hemeroplanis habitalis|[8573]|LOFLA351-06|United States|Florida|658[0n]|BOLD:AAA9264
Hemeroplanis habitalis|[8574]|LOFLD005-07|United States|Florida|636[2n]|BOLD:AAA9264
Hemeroplanis habitalis|[8575]|MNAB222-07|United States|Florida|637[0n]|BOLD:AAA9264
Hemeroplanis habitalis|[8576]|ABNCC118-07|United States|Texas|642[0n]|BOLD:AAA9264
Hemeroplanis habitalis|[8577]|ABNCC154-07|United States|Texas|593[0n]|BOLD:AAA9264
Hemeroplanis habitalis|[8578]|BBLSX490-09|United States|Oklahoma|658[0n]|BOLD:AAA9264
Hemeroplanis habitalis|[8579]|USLEP1003-10|United States|Florida|658[0n]|BOLD:AAA9264
Hemeroplanis habitalis|[8580]|USLEP1004-10|United States|Florida|658[0n]|BOLD:AAA9264
Hemeroplanis habitalis|[8581]|USLEP1195-10|United States|Florida|658[0n]|BOLD:AAA9264
Hemeroplanis habitalis|[8582]|USLEP1274-10|United States|Florida|658[0n]|BOLD:AAA9264
Hemeroplanis habitalis|[8583]|LNCC896-11|United States|North Carolina|658[0n]|BOLD:AAA9264
Hemeroplanis habitalis|[8584]|LNCC898-11|United States|North Carolina|658[0n]|BOLD:AAA9264
Hemeroplanis habitalis|[8585]|BBLOB1194-11|United States|Florida|658[0n]|BOLD:AAA9264
Hemeroplanis habitalis|[8586]|LOFLD455-07|United States|Florida|609[0n]|BOLD:AAA9264
Hemeroplanis habitalis|[8587]|LMEM102-09|United States|Mississippi|658[0n]|BOLD:AAA9264

Hemeroplanis habitalis|[8585]|BBLOB1194-11|United States|Florida|658[0n]|BOLD:AAA9264
Hemeroplanis habitalis|[8586]|LOFLD455-07|United States|Florida|609[0n]|BOLD:AAA9264
Hemeroplanis habitalis|[8587]|LMEM102-09|United States|Mississippi|658[0n]|BOLD:AAA9264
Hemeroplanis habitalis|[8588]|LMEM100-09|United States|Alabama|658[0n]|BOLD:AAA9264
Hemeroplanis habitalis|[8589]|LOFLB781-06|United States|Florida|658[0n]|BOLD:AAA9264
Hemeroplanis habitalis|[8590]|LOFLA567-06|United States|Florida|658[0n]|BOLD:AAA9264
Hemeroplanis habitalis|[8591]|LSEU184-06|United States|Georgia|658[0n]|BOLD:AAA9264
Hemeroplanis habitalis|[8592]|LOFLB288-06|United States|Florida|658[0n]|BOLD:AAA9264
Hemeroplanis habitalis|[8593]|LNC527-06|United States|North Carolina|630[0n]|BOLD:AAA9264
Hemeroplanis habitalis|[8594]|LNCC703-11|United States|North Carolina|636[0n]|BOLD:AAA9264
Hemeroplanis habitalis|[8595]|LNCC747-11|United States|North Carolina|658[0n]|BOLD:AAA9264
Hemeroplanis habitalis|[8596]|LNCC897-11|United States|North Carolina|658[0n]|BOLD:AAA9264
Hemeroplanis habitalis|[8597]|MILEQ282-11|United States|Alabama|658[0n]|BOLD:AAA9264
Hemeroplanis sp.|[8598]|NAMUM255-08|United States|California|658[0n]|BOLD:ABY9897
Hemeroplanis historialis|[8599]|LOCBD570-06|United States|California|658[0n]|BOLD:AAB7998
Hemeroplanis historialis|[8600]|BBLOC1827-11|United States|California|658[0n]|BOLD:AAB7998
Hemeroplanis historialis|[8601]|LOCBE303-06|United States|California|658[0n]|BOLD:AAB7998
Hemeroplanis historialis|[8602]|LOCBB337-06|United States|California|658[0n]|BOLD:AAB7998
Hemeroplanis historialis|[8603]|LOCBD571-06|United States|California|658[0n]|BOLD:AAB7998
Hemeroplanis historialis|[8604]|LOCBC478-06|United States|California|658[0n]|BOLD:AAB7998
Hemeroplanis historialis|[8605]|LOCBC482-06|United States|California|658[0n]|BOLD:AAB7998
Hemeroplanis historialis|[8606]|LOCBD568-06|United States|California|658[0n]|BOLD:AAB7998
Hemeroplanis historialis|[8607]|LOCB441-06|United States|California|658[0n]|BOLD:AAB7998
Hemeroplanis historialis|[8608]|LOCBC217-06|United States|California|658[0n]|BOLD:AAB7998
Hemeroplanis historialis|[8609]|LOCBC592-06|United States|California|658[0n]|BOLD:AAB7998
Hemeroplanis historialis|[8610]|LOCBD085-06|United States|California|658[0n]|BOLD:AAB7998
Hemeroplanis historialis|[8611]|LOCBD087-06|United States|California|658[0n]|BOLD:AAB7998
Hemeroplanis historialis|[8612]|LOCBD459-06|United States|California|657[0n]|BOLD:AAB7998
Hemeroplanis historialis|[8613]|LOCBD569-06|United States|California|658[0n]|BOLD:AAB7998
Hemeroplanis historialis|[8614]|LOCBD669-06|United States|California|658[0n]|BOLD:AAB7998
Hemeroplanis historialis|[8615]|IAWL023-09|United States|Arizona|658[0n]|BOLD:AAB7998
Hemeroplanis historialis|[8616]|LOCBD668-06|United States|California|658[1n]|BOLD:AAB7998
Hemeroplanis historialis|[8617]|LOCBD086-06|United States|California|658[0n]|BOLD:AAB7998
Hemeroplanis historialis|[8618]|LOCBB740-06|United States|California|615[0n]|BOLD:AAB7998
Hemeroplanis historialis|[8619]|LOCBC219-06|United States|California|658[0n]|BOLD:AAB7998
Hemeroplanis historialis|[8620]|LOCBC687-06|United States|California|658[0n]|BOLD:AAB7998
Hemeroplanis historialis|[8621]|LOCBC690-06|United States|California|658[0n]|BOLD:AAB7998
Hemeroplanis historialis|[8622]|LOCBB096-06|United States|California|658[0n]|BOLD:AAB7998
Hemeroplanis historialis|[8623]|LOCBE136-06|United States|California|658[0n]|BOLD:AAB7998
Hemeroplanis historialis|[8624]|BBLOD574-11|United States|California|658[0n]|BOLD:AAB7998
Hemeroplanis historialis|[8625]|BBLOE1816-12|United States|Arizona|658[0n]|BOLD:AAB7998
Hemeroplanis historialis|[8626]|QUNOE454-12|United States|Texas|658[0n]|BOLD:AAB7998
Hemeroplanis rectalis|[8627]|HKONB527-09|United States|Texas|658[1n]|BOLD:ACE7495
Hemeroplanis rectalis|[8628]|RDNMG245-08|United States|Texas|658[0n]|BOLD:ACE7495
Hemeroplanis rectalis|[8629]|RDNMG246-08|United States|Texas|658[0n]|BOLD:ACE7495
Hemeroplanis rectalis|[8630]|RDNMG251-08|United States|Texas|658[0n]|BOLD:ACE7495
Hemeroplanis rectalis|[8631]|HKONB528-09|United States|Texas|658[0n]|BOLD:ACE7495
Hemeroplanis rectalis|[8632]|HKONB529-09|United States|Texas|658[0n]|BOLD:ACE7495
Hemeroplanis rectalis|[8633]|ABNCC129-07|United States|Texas|648[0n]|BOLD:ACE7495
Hemeroplanis rectalis|[8634]|QUNOD113-10|United States|Texas|658[0n]|BOLD:ACE7495
Hemeroplanis rectalis|[8635]|QUNOD112-10|United States|Texas|658[0n]|BOLD:ACE7495
Hemeroplanis rectalis|[8636]|QUNOD114-10|United States|Texas|658[0n]|BOLD:ACE7495
Hemeroplanis rectalis|[8637]|CMAZA613-10|United States|Arizona|658[0n]|BOLD:AAB7998
Hemeroplanis rectalis|[8638]|CMAZA412-10|United States|Arizona|658[0n]|BOLD:AAB7998
Hemeroplanis rectalis|[8639]|ABNCC127-07|United States|Arizona|591[1n]|BOLD:AAB7998
Hemeroplanis rectalis|[8640]|LMEMB024-09|United States|Arizona|632[0n]|BOLD:AAB7998
Hemeroplanis rectalis|[8641]|CMAZA460-10|United States|Arizona|658[0n]|BOLD:AAB7998
Hemeroplanis rectalis|[8642]|BBLOB574-11|United States|Arizona|658[0n]|BOLD:AAB7998
Hemeroplanis rectalis|[8643]|CMAZA1112-12|United States|Arizona|658[0n]|BOLD:AAB7998
Hemeroplanis sp.|[8644]|ABNCC126-07|United States|Arizona|641[0n]|BOLD:ACE7494
Hemeroplanis sp.|[8645]|RDNML045-13|United States|New Mexico|658[0n]|BOLD:ACE7494
Hemeroplanis incusalis|[8646]|LOCBF1062-13|United States|California|543[0n]|BOLD:AAB7998
Hemeroplanis incusalis|[8647]|LOCBF1882-13|United States|California|615[0n]|BOLD:AAB7998
Hemeroplanis incusalis|[8648]|BBLSY074-09|United States|Arizona|658[0n]|BOLD:AAB7998
Hemeroplanis incusalis|[8649]|BBLSY844-09|United States|Arizona|658[0n]|BOLD:AAB7998
Hemeroplanis incusalis|[8650]|BBLOB122-11|United States|Arizona|658[0n]|BOLD:AAB7998
Hemeroplanis incusalis|[8651]|BBLOE1332-12|United States|Arizona|658[0n]|BOLD:AAB7998
Hemeroplanis incusalis|[8652]|CMAZA720-10|United States|Arizona|658[0n]|BOLD:AAB7998
Hemeroplanis incusalis|[8653]|LOCBF1135-13|United States|California|592[0n]|BOLD:AAB7998
Hemeroplanis incusalis|[8654]|BBLOE1934-12|United States|Arizona|658[0n]|BOLD:AAB7998
Hemeroplanis incusalis|[8655]|BBLOC504-11|United States|Arizona|621[0n]|BOLD:AAB7998
Hemeroplanis incusalis|[8656]|BBLOE1341-12|United States|Arizona|658[0n]|BOLD:AAB7998
Hemeroplanis incusalis|[8657]|BBLSZ032-09|United States|Arizona|658[0n]|BOLD:AAB7998
Hemeroplanis incusalis|[8658]|BBLSY967-09|United States|Arizona|658[0n]|BOLD:AAB7998
Hemeroplanis incusalis|[8659]|BBLSY076-09|United States|Arizona|658[0n]|BOLD:AAB7998
Hemeroplanis incusalis|[8660]|BBLSY069-09|United States|Arizona|658[0n]|BOLD:AAB7998
Hemeroplanis incusalis|[8661]|BBLSY057-09|United States|Arizona|658[0n]|BOLD:AAB7998
Hemeroplanis incusalis|[8662]|BBLSZ087-09|United States|Arizona|658[0n]|BOLD:AAB7998
Hemeroplanis incusalis|[8663]|BBLOB1576-11|United States|Arizona|658[0n]|BOLD:AAB7998
Hemeroplanis incusalis|[8664]|BBLOE1653-12|United States|Arizona|658[0n]|BOLD:AAB7998
Hemeroplanis incusalis|[8665]|BBLOE1766-12|United States|Arizona|658[0n]|BOLD:AAB7998
Hemeroplanis incusalis|[8666]|LOCBF1197-13|United States|California|610[0n]|BOLD:AAB7998
Hemeroplanis incusalis|[8667]|LOCBF1089-13|United States|California|600[0n]|BOLD:AAB7998
Hemeroplanis incusalis|[8668]|BBLOB152-11|United States|Arizona|658[0n]|BOLD:AAB7998
Hemeroplanis incusalis|[8669]|CMAZA422-10|United States|Arizona|658[0n]|BOLD:AAB7998
Hemeroplanis incusalis|[8670]|BBLOB1583-11|United States|Arizona|658[0n]|BOLD:AAB7998
Hemeroplanis incusalis|[8671]|LOCBF1119-13|United States|California|573[0n]|BOLD:AAB7998
Hemeroplanis incusalis|[8672]|BBLOB364-11|United States|Arizona|658[0n]|BOLD:AAB7998
Hemeroplanis incusalis|[8673]|QUNOD195-10|United States|Arizona|658[0n]|BOLD:AAB7998
Hemeroplanis incusalis|[8674]|BBLSY060-09|United States|Arizona|658[0n]|BOLD:AAB7998
Hemeroplanis incusalis|[8675]|QUNOE463-12|United States|Utah|658[0n]|BOLD:AAB7998
Hemeroplanis incusalis|[8676]|BBLSZ031-09|United States|Arizona|621[0n]|BOLD:AAB7998
Hemeroplanis incusalis|[8677]|AWCLB516-11|United States|Arizona|658[0n]|BOLD:AAB7998
Hemeroplanis incusalis|[8678]|IAWL024-09|United States|Arizona|658[20n]|
Hemeroplanis incusalis|[8679]|BBLOE1315-12|United States|Arizona|658[0n]|BOLD:AAB7998
Hemeroplanis incusalis|[8680]|BBLOE1317-12|United States|Arizona|658[0n]|BOLD:AAB7998
Hemeroplanis incusalis|[8681]|BBLOC788-11|United States|Arizona|658[0n]|BOLD:AAB7998
Hemeroplanis incusalis|[8682]|BBLOC889-11|United States|Arizona|658[0n]|BOLD:AAB7998
Hemeroplanis incusalis|[8683]|BBLOC536-11|United States|Arizona|658[0n]|BOLD:AAB7998
Hemeroplanis incusalis|[8684]|BBLOC553-11|United States|Arizona|658[0n]|BOLD:AAB7998
Hemeroplanis incusalis|[8685]|BBLOB147-11|United States|Arizona|658[0n]|BOLD:AAB7998
Hemeroplanis incusalis|[8686]|BBLOB149-11|United States|Arizona|658[0n]|BOLD:AAB7998
Hemeroplanis incusalis|[8687]|CMAZA367-10|United States|Arizona|658[0n]|BOLD:AAB7998

Hemeroplanis incusalis|[8685]|BBLOB147-11|United States|Arizona|658[0n]|BOLD:AAB7998
Hemeroplanis incusalis|[8686]|BBLOB149-11|United States|Arizona|658[0n]|BOLD:AAB7998
Hemeroplanis incusalis|[8687]|CMAZA367-10|United States|Arizona|658[0n]|BOLD:AAB7998
Hemeroplanis incusalis|[8688]|AWCLB309-10|United States|Arizona|658[0n]|BOLD:AAB7998
Hemeroplanis incusalis|[8689]|BBLSZ073-09|United States|Arizona|658[0n]|BOLD:AAB7998
Hemeroplanis incusalis|[8690]|USLEP1169-10|United States|Arizona|658[0n]|BOLD:AAB7998
Hemeroplanis incusalis|[8691]|BBLSZ020-09|United States|Arizona|658[0n]|BOLD:AAB7998
Hemeroplanis incusalis|[8692]|BBLSY840-09|United States|Arizona|658[0n]|BOLD:AAB7998
Hemeroplanis incusalis|[8693]|BBLSY821-09|United States|Arizona|658[0n]|BOLD:AAB7998
Hemeroplanis incusalis|[8694]|BBLSY083-09|United States|Arizona|658[0n]|BOLD:AAB7998
Hemeroplanis incusalis|[8695]|BBLSY072-09|United States|Arizona|658[0n]|BOLD:AAB7998
Hemeroplanis incusalis|[8696]|BBLSY062-09|United States|Arizona|658[0n]|BOLD:AAB7998
Hemeroplanis incusalis|[8697]|BBLSY061-09|United States|Arizona|658[0n]|BOLD:AAB7998
Hemeroplanis incusalis|[8698]|BBLSY056-09|United States|Arizona|658[0n]|BOLD:AAB7998
Hemeroplanis incusalis|[8699]|IAWL021-09|United States|Arizona|658[0n]|BOLD:AAB7998
Hemeroplanis incusalis|[8700]|BBLSY834-09|United States|Arizona|658[0n]|BOLD:AAB7998
Hemeroplanis incusalis|[8701]|IAWL015-09|United States|Arizona|577[4n]|BOLD:AAB7998
Hemeroplanis incusalis|[8702]|LMEMB017-09|United States|Arizona|641[0n]|BOLD:AAB7998
Hemeroplanis incusalis|[8703]|LMEMB019-09|United States|Arizona|634[0n]|BOLD:AAB7998
Hemeroplanis incusalis|[8704]|BBLSY611-09|United States|Arizona|589[0n]|BOLD:AAB7998
Hemeroplanis incusalis|[8705]|BBLSY629-09|United States|Arizona|589[0n]|BOLD:AAB7998
Hemeroplanis incusalis|[8706]|BBLOC1824-11|United States|California|582[0n]|BOLD:AAB7998
Hemeroplanis incusalis|[8707]|BBLOE1325-12|United States|Arizona|658[0n]|BOLD:AAB7998
Hemeroplanis incusalis|[8708]|BBLOB1561-11|United States|Arizona|658[0n]|BOLD:AAB7998
Hemeroplanis incusalis|[8709]|CMAZA1097-12|United States|Arizona|658[0n]|BOLD:AAB7998
Hemeroplanis incusalis|[8710]|LOCBF1086-13|United States|California|600[0n]|BOLD:AAB7998
Hemeroplanis incusalis|[8711]|LOCBF1088-13|United States|California|610[0n]|BOLD:AAB7998
Hemeroplanis incusalis|[8712]|AWCLB515-11|United States|Arizona|658[0n]|BOLD:AAB7998
Hemeroplanis incusalis|[8713]|LOCBF1881-13|United States|California|567[0n]|BOLD:AAB7998
Hemeroplanis incusalis|[8714]|LOCBF1883-13|United States|California|607[0n]|BOLD:AAB7998
Hemeroplanis incusalis|[8715]|LOCBF1884-13|United States|California|609[0n]|BOLD:AAB7998
Hemeroplanis incusalis|[8716]|BBLOE1302-12|United States|Arizona|658[0n]|BOLD:AAB7998
Hemeroplanis incusalis|[8717]|LOCBF1090-13|United States|California|581[0n]|BOLD:AAB7998
Hemeroplanis incusalis|[8718]|BBLOE1347-12|United States|Arizona|618[1n]|BOLD:AAB7998
Hemeroplanis incusalis|[8719]|CMAZA420-10|United States|Arizona|658[0n]|BOLD:AAB7998
Hemeroplanis incusalis|[8720]|BBLSY684-09|United States|Arizona|658[0n]|BOLD:AAB7998
Hemeroplanis incusalis|[8721]|LOCBF979-13|United States|California|546[1n]|BOLD:AAB7998
Hemeroplanis incusalis|[8722]|LMEMB021-09|United States|Arizona|625[0n]|BOLD:AAB7998
Hemeroplanis incusalis|[8723]|IAWL627-09|United States|Arizona|619[0n]|BOLD:AAB7998
Hemeroplanis incusalis|[8724]|BBLOE1352-12|United States|Arizona|658[0n]|BOLD:AAB7998
Hemeroplanis incusalis|[8725]|AWCLB514-11|United States|Arizona|658[0n]|BOLD:AAB7998
Hemeroplanis incusalis|[8726]|BBLOC424-11|United States|Arizona|658[0n]|BOLD:AAB7998
Hemeroplanis incusalis|[8727]|BBLOC520-11|United States|Arizona|658[0n]|BOLD:AAB7998
Hemeroplanis incusalis|[8728]|BBLOE1346-12|United States|Arizona|658[0n]|BOLD:AAB7998
Hemeroplanis incusalis|[8729]|BBLOE1700-12|United States|Arizona|658[0n]|BOLD:AAB7998
Hemeroplanis incusalis|[8730]|BBLOE1301-12|United States|Arizona|658[0n]|BOLD:AAB7998
Hemeroplanis incusalis|[8731]|BBLOE1303-12|United States|Arizona|658[0n]|BOLD:AAB7998
Hemeroplanis incusalis|[8732]|BBLOE1328-12|United States|Arizona|658[0n]|BOLD:AAB7998
Hemeroplanis incusalis|[8733]|BBLOE1329-12|United States|Arizona|658[0n]|BOLD:AAB7998
Hemeroplanis incusalis|[8734]|BBLOE1704-12|United States|Arizona|658[0n]|BOLD:AAB7998
Hemeroplanis incusalis|[8735]|BBLOE1764-12|United States|Arizona|658[0n]|BOLD:AAB7998
Hemeroplanis incusalis|[8736]|BBLOB1579-11|United States|Arizona|658[0n]|BOLD:AAB7998
Hemeroplanis incusalis|[8737]|BBLOB1586-11|United States|Arizona|658[0n]|BOLD:AAB7998
Hemeroplanis incusalis|[8738]|BBLOE1768-12|United States|Arizona|658[0n]|BOLD:AAB7998
Hemeroplanis incusalis|[8739]|BBLOB114-11|United States|Arizona|658[0n]|BOLD:AAB7998
Hemeroplanis incusalis|[8740]|AWCLB518-11|United States|Arizona|658[0n]|BOLD:AAB7998
Hemeroplanis incusalis|[8741]|QUNOD194-10|United States|Arizona|658[0n]|BOLD:AAB7998
Hemeroplanis incusalis|[8742]|BBLSY952-09|United States|Arizona|658[0n]|BOLD:AAB7998
Hemeroplanis incusalis|[8743]|BBLSY846-09|United States|Arizona|658[0n]|BOLD:AAB7998
Hemeroplanis incusalis|[8744]|BBLSY740-09|United States|Arizona|658[0n]|BOLD:AAB7998
Hemeroplanis incusalis|[8745]|BBLSY712-09|United States|Arizona|658[0n]|BOLD:AAB7998
Hemeroplanis incusalis|[8746]|BBLSY697-09|United States|Arizona|658[0n]|BOLD:AAB7998
Hemeroplanis incusalis|[8747]|BBLSY071-09|United States|Arizona|658[0n]|BOLD:AAB7998
Hemeroplanis incusalis|[8748]|BBLSY055-09|United States|Arizona|658[0n]|BOLD:AAB7998
Hemeroplanis incusalis|[8749]|BBLSY842-09|United States|Arizona|658[0n]|BOLD:AAB7998
Hemeroplanis incusalis|[8750]|IAWL026-09|United States|Arizona|658[0n]|BOLD:AAB7998
Hemeroplanis incusalis|[8751]|BBLSZ018-09|United States|Arizona|658[0n]|BOLD:AAB7998
Hemeroplanis incusalis|[8752]|AWCLB512-11|United States|Arizona|658[0n]|BOLD:AAB7998
Hemeroplanis incusalis|[8753]|LMEMB018-09|United States|Arizona|633[0n]|BOLD:AAB7998
Hemeroplanis incusalis|[8754]|LMEMB020-09|United States|Arizona|632[0n]|BOLD:AAB7998
Hemeroplanis incusalis|[8755]|BBLSY058-09|United States|Arizona|644[0n]|BOLD:AAB7998
Hemeroplanis incusalis|[8756]|BBLSY075-09|United States|Arizona|658[0n]|BOLD:AAB7998
Hemeroplanis incusalis|[8757]|BBLSY622-09|United States|Arizona|589[0n]|BOLD:AAB7998
Hemeroplanis incusalis|[8758]|BBLSY073-09|United States|Arizona|658[0n]|BOLD:AAB7998
Hemeroplanis incusalis|[8759]|BBLOB1585-11|United States|Arizona|658[0n]|BOLD:AAB7998
Hemeroplanis incusalis|[8760]|BBLOE1318-12|United States|Arizona|658[0n]|BOLD:AAB7998
Hemeroplanis incusalis|[8761]|IAWL025-09|United States|California|658[0n]|BOLD:AAB7998
Hemeroplanis incusalis|[8762]|BBLOE1334-12|United States|Arizona|658[0n]|BOLD:AAB7998
Hemeroplanis incusalis|[8763]|BBLSY977-09|United States|Arizona|658[0n]|BOLD:AAB7998
Hemeroplanis incusalis|[8764]|BBLSY762-09|United States|Arizona|658[0n]|BOLD:AAB7998
Hemeroplanis incusalis|[8765]|BBLSY755-09|United States|Arizona|658[0n]|BOLD:AAB7998
Hemeroplanis incusalis|[8766]|BBLSY068-09|United States|Arizona|658[0n]|BOLD:AAB7998
Hemeroplanis incusalis|[8767]|LOCBF1087-13|United States|California|541[0n]|BOLD:AAB7998
Hemeroplanis incusalis|[8768]|LOCBF1118-13|United States|California|600[0n]|BOLD:AAB7998
Hemeroplanis incusalis|[8769]|LOCBF1885-13|United States|California|615[0n]|BOLD:AAB7998
Hemeroplanis sp.|[8770]|LMEMB023-09|United States|Arizona|634[0n]|BOLD:AAC1412
Hemeroplanis sp.|[8771]|LMEMB025-09|United States|Arizona|633[0n]|BOLD:AAC1412
Hemeroplanis reversalis|[8772]|LOCRC656-09|Costa Rica|Guanacaste|658[0n]|BOLD:AAC1412
Hemeroplanis reversalis|[8773]|BLPCA500-08|Costa Rica|Guanacaste|632[0n]|BOLD:AAC1412
Hemeroplanis reversalis|[8774]|BLPEF5683-13|Costa Rica||630[0n]|BOLD:AAC1412
Hemeroplanis reversalis|[8775]|BLPEE1452-12|Costa Rica|Guanacaste|658[0n]|BOLD:AAC1412
Hemeroplanis reversalis|[8776]|BLPEF5682-13|Costa Rica||658[0n]|BOLD:AAC1412
Hemeroplanis reversalis|[8777]|BLPEE1327-12|Costa Rica|Guanacaste|658[0n]|BOLD:AAC1412
Hemeroplanis reversalis|[8778]|BLPEE1451-12|Costa Rica|Guanacaste|658[0n]|BOLD:AAC1412
Hemeroplanis reversalis|[8779]|QUNOD193-10|United States|Texas|658[0n]|BOLD:AAC1412
Hemeroplanis reversalis|[8780]|BLPED712-11|Costa Rica|Guanacaste|658[0n]|BOLD:AAC1412
Hemeroplanis reversalis|[8781]|BLPDJ205-09|Costa Rica|Guanacaste|658[0n]|BOLD:AAC1412
Hemeroplanis reversalis|[8782]|BLPDJ206-09|Costa Rica|Guanacaste|658[0n]|BOLD:AAC1412
Hemeroplanis reversalis|[8783]|BLPDL392-10|Costa Rica|Guanacaste|658[0n]|BOLD:AAC1412
Hemeroplanis reversalis|[8784]|LOCRC658-09|Costa Rica|Guanacaste|658[0n]|BOLD:AAC1412
Hemeroplanis reversalis|[8785]|LOCRC657-09|Costa Rica|Guanacaste|658[0n]|BOLD:AAC1412
Hemeroplanis reversalis|[8786]|LOCRC655-09|Costa Rica|Guanacaste|658[0n]|BOLD:AAC1412
Hemeroplanis reversalis|[8787]|LOCRC654-09|Costa Rica|Guanacaste|655[0n]|BOLD:AAC1412

Hemeroplanis reversalis|[8785]|LOCRC657-09|Costa Rica|Guanacaste|658[0n]|BOLD:AAC1412
Hemeroplanis reversalis|[8786]|LOCRC655-09|Costa Rica|Guanacaste|658[0n]|BOLD:AAC1412
Hemeroplanis reversalis|[8787]|LOCRC654-09|Costa Rica|Guanacaste|655[0n]|BOLD:AAC1412
Hemeroplanis reversalis|[8788]|LOCRC653-09|Costa Rica|Guanacaste|658[0n]|BOLD:AAC1412
Hemeroplanis reversalis|[8789]|BLPDE160-09|Costa Rica|Guanacaste|658[0n]|BOLD:AAC1412
Hemeroplanis reversalis|[8790]|BLPDE149-09|Costa Rica|Guanacaste|658[0n]|BOLD:AAC1412
Hemeroplanis reversalis|[8791]|BLPDD871-09|Costa Rica|Guanacaste|658[0n]|BOLD:AAC1412
Hemeroplanis reversalis|[8792]|BLPCM426-08|Costa Rica|Guanacaste|658[0n]|BOLD:AAC1412
Hemeroplanis reversalis|[8793]|BLPCA284-08|Costa Rica|Guanacaste|658[0n]|BOLD:AAC1412
Hemeroplanis reversalis|[8794]|BLPCA283-08|Costa Rica|Guanacaste|658[0n]|BOLD:AAC1412
Hemeroplanis reversalis|[8795]|BLPAG261-07|Costa Rica|Guanacaste|658[0n]|BOLD:AAC1412
Hemeroplanis reversalis|[8796]|MHAUB871-05|Costa Rica|Guanacaste|658[0n]|BOLD:AAC1412
Hemeroplanis reversalis|[8797]|MHAUB870-05|Costa Rica|Guanacaste|590[0n]|BOLD:AAC1412
Hemeroplanis reversalis|[8798]|BLPDL455-10|Costa Rica|Guanacaste|627[0n]|BOLD:AAC1412
Hemeroplanis reversalis|[8799]|CNCLB1963-14|Guatemala||658[0n]|BOLD:AAC1412
Hormoschista latipalpis|[8800]|RDNME693-08|United States|Florida|658[0n]|BOLD:ABY8653
Hormoschista latipalpis|[8801]|HKONS176-08|United States|Florida|658[0n]|BOLD:ABY8653
Hormoschista latipalpis|[8802]|HKONS177-08|United States|Florida|658[0n]|BOLD:ABY8653
Hormoschista latipalpis|[8803]|LNC609-06|United States|North Carolina|643[0n]|BOLD:ABY8653
Hormoschista latipalpis|[8804]|RDNME692-08|United States|Florida|658[0n]|BOLD:ABY8653
Hormoschista latipalpis|[8805]|RDNMJ034-10|United States|Florida|658[0n]|BOLD:ABY8653
Hormoschista latipalpis|[8806]|LNCC1527-13|United States|North Carolina|658[0n]|BOLD:ABY8653
Syllectra erycata|[8807]|RDNML341-13|United States|Florida|658[0n]|BOLD:AAF2579
Syllectra erycata|[8808]|RDNML342-13|United States|Florida|604[0n]|BOLD:AAF2579
Syllectra erycata|[8809]|LNAUS5617-13|United States|Florida|658[0n]|BOLD:AAF2579
Syllectra erycata|[8810]|LNAUS5618-13|United States|Florida|658[0n]|BOLD:AAF2579
Panopoda rigida|[8811]|CMAZA821-10|United States|Arizona|658[0n]|BOLD:AAA9611
Panopoda rigida|[8812]|RDNMJ552-11|United States|Arizona|658[0n]|BOLD:AAA9611
Panopoda rigida|[8813]|JBAZ132-09|United States|Arizona|658[0n]|BOLD:AAA9611
Panopoda rigida|[8814]|IAWL086-09|United States|Arizona|658[0n]|BOLD:AAA9611
Panopoda rigida|[8815]|IAWL083-09|United States|Arizona|658[0n]|BOLD:AAA9611
Panopoda rigida|[8816]|IAWL081-09|United States|Arizona|658[0n]|BOLD:AAA9611
Panopoda rigida|[8817]|IAWL082-09|United States|Arizona|658[0n]|BOLD:AAA9611
Panopoda rigida|[8818]|IAWL079-09|United States|Arizona|658[0n]|BOLD:AAA9611
Panopoda rigida|[8819]|IAWL078-09|United States|Arizona|658[0n]|BOLD:AAA9611
Panopoda rigida|[8820]|IAWL084-09|United States|Arizona|658[0n]|BOLD:AAA9611
Panopoda rigida|[8821]|IAWL085-09|United States|Arizona|621[0n]|BOLD:AAA9611
Panopoda rigida|[8822]|LTOLB757-11|United States|Arizona|658[0n]|BOLD:AAA9611
Panopoda rigida|[8823]|CMAZA864-12|United States|Arizona|658[0n]|BOLD:AAA9611
Panopoda repanda|[8824]|USLEP475-10|United States|Florida|658[0n]|BOLD:AAA7227
Panopoda repanda|[8825]|USLEP474-10|United States|Florida|658[0n]|BOLD:AAA7227
Panopoda repanda|[8826]|USLEP473-10|United States|Florida|658[0n]|BOLD:AAA7227
Panopoda repanda|[8827]|USLEP472-10|United States|Florida|658[0n]|BOLD:AAA7227
Panopoda repanda|[8828]|USLEP471-10|United States|Florida|658[0n]|BOLD:AAA7227
Panopoda repanda|[8829]|USLEP470-10|United States|Florida|658[0n]|BOLD:AAA7227
Panopoda repanda|[8830]|USLEP469-10|United States|Florida|658[0n]|BOLD:AAA7227
Panopoda repanda|[8831]|USLEP468-10|United States|Florida|658[0n]|BOLD:AAA7227
Panopoda repanda|[8832]|USLEP467-10|United States|Florida|658[0n]|BOLD:AAA7227
Panopoda repanda|[8833]|ABNCC260-07|United States|Florida|657[0n]|BOLD:AAA7227
Panopoda repanda|[8834]|LOFLC459-06|United States|Florida|657[0n]|BOLD:AAA7227
Panopoda repanda|[8835]|LOFLC396-06|United States|Florida|657[0n]|BOLD:AAA7227
Panopoda repanda|[8836]|LOFLC368-06|United States|Florida|658[0n]|BOLD:AAA7227
Panopoda repanda|[8837]|LOFLB499-06|United States|Florida|657[0n]|BOLD:AAA7227
Panopoda repanda|[8838]|LOFLB495-06|United States|Florida|657[0n]|BOLD:AAA7227
Panopoda repanda|[8839]|LSEU051-06|United States|Georgia|603[1n]|BOLD:AAA7227
Panopoda repanda|[8840]|LSEU052-06|United States|Georgia|585[0n]|BOLD:AAA7227
Panopoda repanda|[8841]|ABNCC259-07|United States|Florida|633[0n]|BOLD:AAA7227
Panopoda repanda|[8842]|USLEP750-10|United States|Florida|636[0n]|BOLD:AAA7227
Panopoda repanda|[8843]|BBLOB404-11|United States|Florida|658[0n]|BOLD:AAA7227
Panopoda repanda|[8844]|BBLOB810-11|United States|Florida|658[0n]|BOLD:AAA7227
Panopoda repanda|[8845]|BBLOB811-11|United States|Florida|658[0n]|BOLD:AAA7227
Panopoda repanda|[8846]|BBLOB1290-11|United States|Florida|658[0n]|BOLD:AAA7227
Panopoda repanda|[8847]|BBLOB1310-11|United States|Florida|658[0n]|BOLD:AAA7227
Panopoda repanda|[8848]|BBLOC191-11|United States|Florida|658[0n]|BOLD:AAA7227
Panopoda rufimargo|[8849]|BBLSZ111-09|United States|Oklahoma|658[0n]|BOLD:AAA7227
Panopoda rufimargo|[8850]|LOFLB811-06|United States|Florida|656[0n]|BOLD:AAA7227
Panopoda rufimargo|[8851]|BBLOC1663-11|United States|Texas|658[0n]|BOLD:AAA7227
Panopoda rufimargo|[8852]|BBLSZ112-09|United States|Oklahoma|658[0n]|BOLD:AAA7227
Panopoda rufimargo|[8853]|BBLSZ101-09|United States|Oklahoma|658[0n]|BOLD:AAA7227
Panopoda rufimargo|[8854]|LMEMB128-09|United States|Georgia|658[0n]|BOLD:AAA7227
Panopoda rufimargo|[8855]|LMEMB126-09|United States|Mississippi|658[0n]|BOLD:AAA7227
Panopoda rufimargo|[8856]|NAMUM418-09|United States|Maryland|658[0n]|BOLD:AAA7227
Panopoda rufimargo|[8857]|LSUSA225-06|United States|Kentucky|657[0n]|BOLD:AAA7227
Panopoda rufimargo|[8858]|LOFLB218-06|United States|Florida|657[0n]|BOLD:AAA7227
Panopoda rufimargo|[8859]|LOFLA360-06|United States|Florida|657[0n]|BOLD:AAA7227
Panopoda rufimargo|[8860]|LOFLA225-06|United States|Florida|657[0n]|BOLD:AAA7227
Panopoda rufimargo|[8861]|BBLOC1669-11|United States|Texas|658[0n]|BOLD:AAA7227
Panopoda rufimargo|[8862]|LGSMG561-07|United States|North Carolina|656[0n]|BOLD:AAA7227
Panopoda rufimargo|[8863]|LOT258-04|United States|Tennessee|658[0n]|BOLD:AAA7227
Panopoda rufimargo|[8864]|LOT255-04|United States|Tennessee|658[0n]|BOLD:AAA7227
Panopoda rufimargo|[8865]|PHMO163-03|Canada|Ontario|639[0n]|BOLD:AAA7227
Panopoda rufimargo|[8866]|LGSM473-04|United States|Tennessee|619[1n]|BOLD:AAA7227
Panopoda rufimargo|[8867]|LOT259-04|United States|Tennessee|658[0n]|BOLD:AAA7227
Panopoda rufimargo|[8868]|LOT260-04|United States|Tennessee|630[0n]|BOLD:AAA7227
Panopoda rufimargo|[8869]|LOT261-04|United States|Tennessee|658[0n]|BOLD:AAA7227
Panopoda rufimargo|[8870]|LGSMG562-07|United States|Tennessee|658[0n]|BOLD:AAA7227
Panopoda rufimargo|[8871]|RDLQF932-06|Canada|Quebec|658[0n]|BOLD:AAA7227
Panopoda rufimargo|[8872]|LPOKB989-09|United States|Oklahoma|658[0n]|BOLD:AAA7227
Panopoda rufimargo|[8873]|BBLSZ120-09|United States|Oklahoma|658[0n]|BOLD:AAA7227
Panopoda rufimargo|[8874]|LOFLA211-06|United States|Florida|656[0n]|BOLD:AAA7227
Panopoda rufimargo|[8875]|LNCB089-06|United States|North Carolina|656[0n]|BOLD:AAA7227
Panopoda rufimargo|[8876]|LNCB055-06|United States|North Carolina|656[0n]|BOLD:AAA7227
Panopoda rufimargo|[8877]|LOFLA106-06|United States|Florida|657[0n]|BOLD:AAA7227
Panopoda rufimargo|[8878]|LOFLA517-06|United States|Florida|657[0n]|BOLD:AAA7227
Panopoda rufimargo|[8879]|LOFLA541-06|United States|Florida|657[0n]|BOLD:AAA7227
Panopoda rufimargo|[8880]|LOFLA672-06|United States|Florida|657[0n]|BOLD:AAA7227
Panopoda rufimargo|[8881]|LOFLB016-06|United States|Florida|657[0n]|BOLD:AAA7227
Panopoda rufimargo|[8882]|LOT257-04|United States|Tennessee|658[0n]|BOLD:AAA7227
Panopoda rufimargo|[8883]|LMEMB127-09|United States|Mississippi|658[0n]|BOLD:AAA7227
Panopoda rufimargo|[8884]|JRLAA020-09|United States|Alabama|658[0n]|BOLD:AAA7227
Panopoda rufimargo|[8885]|BBLSZ119-09|United States|Oklahoma|658[0n]|BOLD:AAA7227
Panopoda rufimargo|[8886]|LOFLB737-06|United States|Florida|656[0n]|BOLD:AAA7227
Panopoda rufimargo|[8887]|OUNOB520-09|United States|Kentucky|658[0n]|BOLD:AAA7227

Panopoda rufimargo|[8884]|[illegible]|United States|[illegible]|BOLD:AAA7227
Panopoda rufimargo|[8885]|BBLSZ119-09|United States|Oklahoma|658[0n]|BOLD:AAA7227
Panopoda rufimargo|[8886]|LOFLB737-06|United States|Florida|656[0n]|BOLD:AAA7227
Panopoda rufimargo|[8887]|QUNOB520-09|United States|Kentucky|658[0n]|BOLD:AAA7227
Panopoda rufimargo|[8888]|BBLSY871-09|United States|Oklahoma|658[0n]|BOLD:AAA7227
Panopoda rufimargo|[8889]|LOT262-04|United States|Tennessee|658[0n]|BOLD:AAA7227
Panopoda rufimargo|[8890]|BBLSZ121-09|United States|Oklahoma|658[0n]|BOLD:AAA7227
Panopoda rufimargo|[8891]|LILLA811-11|United States|Illinois|658[0n]|BOLD:AAA7227
Panopoda rufimargo|[8892]|USLEP1068-10|United States|Arkansas|648[0n]|BOLD:AAA7227
Panopoda rufimargo|[8893]|LOFLB207-06|United States|Florida|656[0n]|BOLD:AAA7227
Panopoda rufimargo|[8894]|LOFLC468-06|United States|Florida|657[0n]|BOLD:AAA7227
Panopoda rufimargo|[8895]|LOFLA538-06|United States|Florida|657[0n]|BOLD:AAA7227
Panopoda rufimargo|[8896]|LOFLA220-06|United States|Florida|657[0n]|BOLD:AAA7227
Panopoda rufimargo|[8897]|LOFLA217-06|United States|Florida|657[0n]|BOLD:AAA7227
Panopoda rufimargo|[8898]|LOFLA107-06|United States|Florida|657[0n]|BOLD:AAA7227
Panopoda rufimargo|[8899]|LOCT048-05|United States|Connecticut|658[0n]|BOLD:AAA7227
Panopoda rufimargo|[8900]|LOT256-04|United States|Tennessee|658[0n]|BOLD:AAA7227
Panopoda rufimargo|[8901]|LGSM472-04|United States|Tennessee|657[0n]|BOLD:AAA7227
Panopoda rufimargo|[8902]|LOT254-04|United States|Tennessee|658[1n]|BOLD:AAA7227
Panopoda rufimargo|[8903]|LOTB300-05|United States|Tennessee|648[0n]|BOLD:AAA7227
Panopoda rufimargo|[8904]|PHMO107-03|Canada|Ontario|639[0n]|BOLD:AAA7227
Panopoda rufimargo|[8905]|LOT560-04|United States|Tennessee|545[0n]|BOLD:AAA7227
Panopoda rufimargo|[8906]|ABNCC257-07|United States|Texas|600[0n]|BOLD:AAA7227
Panopoda rufimargo|[8907]|BBLOC1668-11|United States|Texas|658[0n]|BOLD:AAA7227
Panopoda repanda|[8908]|LNC519-06|United States|North Carolina|657[0n]|BOLD:AAA7227
Panopoda repanda|[8909]|LNC241-05|United States|North Carolina|658[0n]|BOLD:AAA7227
Panopoda repanda|[8910]|LOFLA363-06|United States|Florida|657[0n]|BOLD:AAA7227
Panopoda repanda|[8911]|LOFLA369-06|United States|Florida|657[0n]|BOLD:AAA7227
Panopoda repanda|[8912]|LOFLA532-06|United States|Florida|657[0n]|BOLD:AAA7227
Panopoda repanda|[8913]|LOFLA885-06|United States|Florida|657[0n]|BOLD:AAA7227
Panopoda repanda|[8914]|LOFLB004-06|United States|Florida|657[0n]|BOLD:AAA7227
Panopoda repanda|[8915]|LOFLA362-06|United States|Florida|657[0n]|BOLD:AAA7227
Panopoda repanda|[8916]|LOFLA361-06|United States|Florida|657[0n]|BOLD:AAA7227
Panopoda repanda|[8917]|LOFLA359-06|United States|Florida|657[0n]|BOLD:AAA7227
Panopoda repanda|[8918]|LOFLA212-06|United States|Florida|657[0n]|BOLD:AAA7227
Panopoda repanda|[8919]|BBLOE1720-12|United States|Florida|658[0n]|BOLD:AAA7227
Panopoda carneicosta|[8920]|BBLSW672-09|United States|Oklahoma|658[0n]|BOLD:AAA9857
Panopoda carneicosta|[8921]|BBLOC1665-11|United States|Texas|658[0n]|BOLD:AAA9857
Panopoda carneicosta|[8922]|LPOKC622-09|United States|Oklahoma|658[0n]|BOLD:AAA9857
Panopoda carneicosta|[8923]|LPOKC616-09|United States|Oklahoma|658[0n]|BOLD:AAA9857
Panopoda carneicosta|[8924]|BBLSX572-09|United States|Oklahoma|658[0n]|BOLD:AAA9857
Panopoda carneicosta|[8925]|BBLSW530-09|United States|Oklahoma|658[0n]|BOLD:AAA9857
Panopoda carneicosta|[8926]|BBLSW529-09|United States|Oklahoma|658[0n]|BOLD:AAA9857
Panopoda carneicosta|[8927]|BBLSW483-09|United States|Oklahoma|658[0n]|BOLD:AAA9857
Panopoda carneicosta|[8928]|LPOKA526-09|United States|Oklahoma|658[0n]|BOLD:AAA9857
Panopoda carneicosta|[8929]|BBLSX112-09|United States|Oklahoma|658[0n]|BOLD:AAA9857
Panopoda carneicosta|[8930]|LPOKB974-09|United States|Oklahoma|658[0n]|BOLD:AAA9857
Panopoda carneicosta|[8931]|LPOKC628-09|United States|Oklahoma|631[0n]|BOLD:AAA9857
Panopoda carneicosta|[8932]|BBLSY858-09|United States|Oklahoma|658[0n]|BOLD:AAA9857
Panopoda carneicosta|[8933]|USLEP1069-10|United States|Arkansas|658[0n]|BOLD:AAA9857
Panopoda carneicosta|[8934]|BBLOC875-11|United States|Arkansas|658[0n]|BOLD:AAA9857
Panopoda carneicosta|[8935]|LOFLB130-06|United States|Florida|658[0n]|BOLD:AAA9857
Panopoda carneicosta|[8936]|LOFLB133-06|United States|Florida|658[0n]|BOLD:AAA9857
Panopoda carneicosta|[8937]|LOFLB803-06|United States|Florida|658[0n]|BOLD:AAA9857
Panopoda carneicosta|[8938]|LOFLB807-06|United States|Florida|658[0n]|BOLD:AAA9857
Panopoda carneicosta|[8939]|HKONS352-08|United States|Florida|658[0n]|BOLD:AAA9857
Panopoda carneicosta|[8940]|BBLOE2024-12|United States|Texas|658[0n]|BOLD:AAA9857
Panopoda carneicosta|[8941]|USLEP1070-10|United States|Arkansas|658[0n]|BOLD:AAA9857
Panopoda carneicosta|[8942]|LGSM516-04|United States|Tennessee|658[0n]|BOLD:AAA9857
Panopoda carneicosta|[8943]|LOT253-04|United States|Tennessee|658[0n]|BOLD:AAA9857
Panopoda carneicosta|[8944]|IAWL029-09|United States|Virginia|658[0n]|BOLD:AAA9857
Panopoda carneicosta|[8945]|LOFLC371-06|United States|Florida|658[0n]|BOLD:AAA9857
Panopoda carneicosta|[8946]|LOFLC435-06|United States|Florida|658[0n]|BOLD:AAA9857
Panopoda carneicosta|[8947]|LOFLB137-06|United States|Florida|658[0n]|BOLD:AAA9857
Panopoda carneicosta|[8948]|LOFLB498-06|United States|Florida|658[0n]|BOLD:AAA9857
Panopoda carneicosta|[8949]|LOFLC365-06|United States|Florida|658[0n]|BOLD:AAA9857
Panopoda carneicosta|[8950]|LOFLB214-06|United States|Florida|658[0n]|BOLD:AAA9857
Panopoda carneicosta|[8951]|LOFLB217-06|United States|Florida|658[0n]|BOLD:AAA9857
Panopoda carneicosta|[8952]|LOFLB493-06|United States|Florida|658[0n]|BOLD:AAA9857
Panopoda carneicosta|[8953]|LOFLB721-06|United States|Florida|658[0n]|BOLD:AAA9857
Panopoda carneicosta|[8954]|LOFLC092-06|United States|Florida|658[0n]|BOLD:AAA9857
Panopoda carneicosta|[8955]|LOFLC362-06|United States|Florida|658[0n]|BOLD:AAA9857
Panopoda carneicosta|[8956]|LOFLC460-06|United States|Florida|657[0n]|BOLD:AAA9857
Panopoda carneicosta|[8957]|RDNMJ036-10|United States|Florida|658[0n]|BOLD:AAA9857
Panopoda carneicosta|[8958]|XAJ811-06|Canada|Ontario|658[0n]|BOLD:AAA9857
Panopoda carneicosta|[8959]|LGSMG563-07|United States|North Carolina|658[0n]|BOLD:AAA9857
Panopoda carneicosta|[8960]|RDLQ753-07|Canada|Quebec|658[0n]|BOLD:AAA9857
Panopoda carneicosta|[8961]|LSUSA149-06|United States|Kentucky|658[0n]|BOLD:AAA9857
Panopoda carneicosta|[8962]|LSUSA129-06|United States|Kentucky|658[0n]|BOLD:AAA9857
Panopoda carneicosta|[8963]|LNCB090-06|United States|North Carolina|658[0n]|BOLD:AAA9857
Panopoda carneicosta|[8964]|BBLOC874-11|United States|Arkansas|658[0n]|BOLD:AAA9857
Panopoda carneicosta|[8965]|LGSM515-04|United States|Tennessee|658[0n]|BOLD:AAA9857
Panopoda carneicosta|[8966]|LOT252-04|United States|Tennessee|658[0n]|BOLD:AAA9857
Panopoda carneicosta|[8967]|PMG147-03|Canada|Ontario|617[0n]|BOLD:AAA9857
Panopoda carneicosta|[8968]|XAJ809-06|Canada|Ontario|632[0n]|BOLD:AAA9857
Panopoda carneicosta|[8969]|GMGSV012-13|United States|Tennessee|614[0n]|BOLD:AAA9857
Ephyrodes cacata|[8970]|MHAUF150-06|Costa Rica|Guanacaste|658[0n]|BOLD:AAB1377
Ephyrodes cacata|[8971]|BLPBB647-07|Costa Rica|Guanacaste|613[0n]|BOLD:AAB1377
Ephyrodes cacata|[8972]|LYPAP583-09|Mexico|Quintana Roo|658[0n]|BOLD:AAB1377
Ephyrodes cacata|[8973]|LYHES001-09|Mexico|Quintana Roo|658[0n]|BOLD:AAB1377
Ephyrodes cacata|[8974]|BLPDD461-09|Costa Rica|Guanacaste|658[0n]|BOLD:AAB1377
Ephyrodes cacata|[8975]|HKONS055-07|United States|Florida|658[0n]|BOLD:AAB1377
Ephyrodes cacata|[8976]|HKONS054-07|United States|Florida|658[0n]|BOLD:AAB1377
Ephyrodes cacata|[8977]|MHAUA155-05|Costa Rica|Guanacaste|658[0n]|BOLD:AAB1377
Ephyrodes cacata|[8978]|LYPIE574-09|Mexico|Yucatan|630[0n]|BOLD:AAB1377
Ephyrodes cacata|[8979]|RDNMI038-10|United States|Florida|658[0n]|BOLD:AAB1377
Aglaonice otignatha|[8980]|CNCLB782-14|United States|Florida|658[0n]|BOLD:ACM4203
Aglaonice otignatha|[8981]|CNCLB833-14|United States|Florida|658[0n]|BOLD:ACM4203
Colobochyla interpuncta|[8982]|RDNMH087-09|United States|Arizona|658[0n]|BOLD:AAB9363
Colobochyla interpuncta|[8983]|RDNMH637-09|United States|Arizona|658[0n]|BOLD:AAB9363
Colobochyla interpuncta|[8984]|LNC324-05|United States|North Carolina|658[0n]|BOLD:AAB9362
Colobochyla interpuncta|[8985]|LNCB269-06|United States|North Carolina|658[0n]|BOLD:AAB9362
Colobochyla interpuncta|[8986]|USLEP646-10|United States|Florida|658[0n]|BOLD:AAB9362
Colobochyla interpuncta|[8987]|ABNCC111-07|United States|Texas|635[0n]|BOLD:AAB9362

[illegible]
Colobochyla interpuncta|[8985]|LNCB269-06|United States|North Carolina|658[0n]|BOLD:AAB9362
Colobochyla interpuncta|[8986]|USLEP646-10|United States|Florida|658[0n]|BOLD:AAB9362
Colobochyla interpuncta|[8987]|ABNCC111-07|United States|Texas|635[0n]|BOLD:AAB9362
Colobochyla interpuncta|[8988]|LPOKB215-09|United States|Oklahoma|658[0n]|BOLD:AAB9362
Colobochyla interpuncta|[8989]|LPOKB681-09|United States|Oklahoma|658[0n]|BOLD:AAB9362
Colobochyla interpuncta|[8990]|BBLSW676-09|United States|Oklahoma|658[0n]|BOLD:AAB9362
Colobochyla interpuncta|[8991]|ABNCC109-07|United States|Florida|642[0n]|BOLD:AAB9362
Colobochyla interpuncta|[8992]|ABNCC110-07|United States|Florida|649[0n]|BOLD:AAB9362
Colobochyla interpuncta|[8993]|RDLQG931-06|Canada|Quebec|645[0n]|BOLD:AAB9362
Colobochyla interpuncta|[8994]|LILLA768-11|United States|Illinois|658[0n]|BOLD:AAB9362
Colobochyla interpuncta|[8995]|RDLQF383-06|Canada|Quebec|659[0n]|BOLD:AAB9362
Colobochyla interpuncta|[8996]|XAJ633-06|Canada|Ontario|658[0n]|BOLD:AAB9362
Colobochyla interpuncta|[8997]|RDLQG685-06|Canada|Quebec|646[0n]|BOLD:AAB9362
Colobochyla interpuncta|[8998]|HKONS595-08|United States|Florida|658[0n]|BOLD:AAB9362
Colobochyla interpuncta|[8999]|LMEM345-09|United States|Mississippi|658[0n]|BOLD:AAB9362
Colobochyla interpuncta|[9000]|LMEM347-09|United States|Alabama|658[0n]|BOLD:AAB9362
Colobochyla interpuncta|[9001]|LMEM348-09|United States|Mississippi|658[0n]|BOLD:AAB9362
Colobochyla interpuncta|[9002]|LILLA065-11|United States|Illinois|658[0n]|BOLD:AAB9362
Colobochyla interpuncta|[9003]|LILLA853-11|United States|Illinois|658[0n]|BOLD:AAB9362
Antiblemma concinnula|[9004]|HKONS129-08|United States|Florida|658[0n]|BOLD:AAC2502
Antiblemma concinnula|[9005]|RDNMK025-11|United States|Florida|658[0n]|BOLD:AAC2502
Antiblemma concinnula|[9006]|LOFLD487-07|United States|Florida|658[1n]|BOLD:AAC2502
Antiblemma concinnula|[9007]|LOFLD484-07|United States|Florida|658[0n]|BOLD:AAC2502
Antiblemma concinnula|[9008]|RDNMK026-11|United States|Florida|658[0n]|BOLD:AAC2502
Antiblemma concinnula|[9009]|LOFLB859-06|United States|Florida|658[0n]|BOLD:AAC2502
Antiblemma concinnula|[9010]|LOFLB539-06|United States|Florida|658[0n]|BOLD:AAC2502
Antiblemma concinnula|[9011]|LOFLB458-06|United States|Florida|658[0n]|BOLD:AAC2502
Antiblemma concinnula|[9012]|LOFLB386-06|United States|Florida|658[0n]|BOLD:AAC2502
Antiblemma concinnula|[9013]|LOFLB189-06|United States|Florida|658[0n]|BOLD:AAC2502
Antiblemma concinnula|[9014]|LOFLB101-06|United States|Florida|658[0n]|BOLD:AAC2502
Antiblemma concinnula|[9015]|LOFLB154-06|United States|Florida|641[0n]|BOLD:AAC2502
Antiblemma concinnula|[9016]|ABNCC194-07|United States|Florida|637[1n]|BOLD:AAC2502
Antiblemma concinnula|[9017]|LOFLD010-07|United States|Florida|657[0n]|BOLD:AAC2502
Antiblemma concinnula|[9018]|RDNMK027-11|United States|Florida|658[0n]|BOLD:AAC2502
Antiblemma concinnula|[9019]|RDNMK713-11|United States|Florida|658[0n]|BOLD:AAC2502
Antiblemma concinnula|[9020]|BBLOB1470-11|United States|Florida|658[0n]|BOLD:AAC2502
Antiblemma filaria|[9021]|BBLOB557-11|United States|Arizona|658[0n]|BOLD:ABZ9064
Antiblemma filaria|[9022]|RDNML308-13|United States|Florida|658[0n]|BOLD:ABZ9064
Antiblemma filaria|[9023]|RDNML309-13|United States|Florida|658[0n]|BOLD:ABZ9064
Antiblemma sp.|[9024]|RDNML310-13|United States|Florida|658[0n]|BOLD:ACD8952
Antiblemma sp.|[9025]|RDNML311-13|United States|Florida|658[0n]|BOLD:ACD8952
Tathorhynchus exsiccata|[9026]|RDNMI192-12|United States|Wyoming|601[0n]|BOLD:AAD0612
Tathorhynchus exsiccata|[9027]|LMEMB014-09|United States|Kansas|658[0n]|BOLD:AAD0612
Tathorhynchus exsiccata|[9028]|IAWL011-09|United States|Arizona|658[0n]|BOLD:AAD0612
Tathorhynchus exsiccata|[9029]|RDNMF625-08|United States|Missouri|658[0n]|BOLD:AAD0612
Tathorhynchus exsiccata|[9030]|RDNMF623-08|United States|Missouri|658[0n]|BOLD:AAD0612
Tathorhynchus exsiccata|[9031]|IAWL004-09|United States|Arizona|619[0n]|BOLD:AAD0612
Tathorhynchus exsiccata|[9032]|IAWL012-09|United States|Arizona|646[0n]|BOLD:AAD0612
Tathorhynchus exsiccata|[9033]|BBLSW038-09|United States|Texas|658[0n]|BOLD:AAD0612
Tathorhynchus exsiccata|[9034]|CMAZA168-09|United States|Arizona|658[0n]|BOLD:AAD0612
Tathorhynchus exsiccata|[9035]|BBLSW759-09|United States|Texas|658[0n]|BOLD:AAD0612
Tathorhynchus exsiccata|[9036]|LMEMB015-09|United States|Kansas|658[0n]|BOLD:AAD0612
Tathorhynchus exsiccata|[9037]|IAWL014-09|United States|Arizona|658[0n]|BOLD:AAD0612
Tathorhynchus exsiccata|[9038]|IAWL005-09|United States|Arizona|658[0n]|BOLD:AAD0612
Tathorhynchus exsiccata|[9039]|RDNMF624-08|United States|Missouri|658[0n]|BOLD:AAD0612
Tathorhynchus exsiccata|[9040]|BBLSY248-09|United States|Texas|658[0n]|BOLD:AAD0612
Tathorhynchus exsiccata|[9041]|RDNMJ705-11|United States|Arizona|658[0n]|BOLD:AAD0612
Tathorhynchus exsiccata|[9042]|LTOL1143-11|United States|Arizona|658[0n]|BOLD:AAD0612
Tathorhynchus exsiccata|[9043]|RDNMI195-12|Canada|Manitoba|621[0n]|BOLD:AAD0612
Tathorhynchus exsiccata|[9044]|GWOSU642-11|Ethiopia||655[0n]|BOLD:AAD0612
Tathorhynchus exsiccata|[9045]|LEUAE1151-14|United Arab Emirates|Fujairah|658[0n]|BOLD:AAD0612
Tathorhynchus exsiccata|[9046]|GWOSD346-10|Ethiopia|Oromia|658[0n]|BOLD:AAD0612
Tathorhynchus exsiccata|[9047]|QUNOE458-12|United States|Texas|658[0n]|BOLD:AAD0612
Tathorhynchus exsiccata|[9048]|LEUAE1152-14|United Arab Emirates|Ras al-Khaymah|658[0n]|BOLD:AAD0612
Tathorhynchus exsiccata|[9049]|LEUAE1153-14|United Arab Emirates|Fujairah|658[0n]|BOLD:AAD0612
Hemeroplanis punitalis|[9050]|IAWL048-09|United States|Arizona|658[0n]|BOLD:AAB1107
Hemeroplanis punitalis|[9051]|RDNME997-08|United States|Arizona|658[0n]|BOLD:AAB1107
Hemeroplanis punitalis|[9052]|LMEMB030-09|United States|Arizona|658[0n]|BOLD:AAB1107
Hemeroplanis nr. scopulepes|[9053]|HKONB331-09|United States|Texas|658[0n]|BOLD:AAB1106
Hemeroplanis nr. scopulepes|[9054]|QUNOD111-10|United States|Texas|658[0n]|BOLD:AAB1106
Hemeroplanis scopulepes group|[9055]|LOFLB709-06|United States|Florida|658[0n]|BOLD:AAB1106
Hemeroplanis scopulepes group|[9056]|LOFLC205-06|United States|Florida|656[0n]|BOLD:AAB1106
Hemeroplanis scopulepes group|[9057]|BBLOB444-11|United States|Florida|658[0n]|BOLD:AAB1106
Hemeroplanis scopulepes group|[9058]|BBLOB497-11|United States|Florida|658[0n]|BOLD:AAB1106
Hemeroplanis scopulepes group|[9059]|BBLOB639-11|United States|Florida|658[0n]|BOLD:AAB1106
Hemeroplanis scopulepes group|[9060]|BBLOB926-11|United States|Florida|658[0n]|BOLD:AAB1106
Hemeroplanis scopulepes group|[9061]|CNCLB1649-14|United States|Florida|658[0n]|BOLD:AAB1106
Hemeroplanis scopulepes|[9062]|LNCC250-10|United States|North Carolina|658[0n]|BOLD:AAB1105
Hemeroplanis scopulepes|[9063]|LMEMB033-09|United States|Mississippi|658[0n]|BOLD:AAB1105
Hemeroplanis scopulepes|[9064]|LMEMB029-09|United States|Mississippi|658[0n]|BOLD:AAB1105
Hemeroplanis scopulepes|[9065]|LMEMB034-09|United States|Mississippi|632[0n]|BOLD:AAB1105
Hemeroplanis scopulepes|[9066]|LNCC667-11|United States|North Carolina|658[0n]|BOLD:AAB1105
Hemeroplanis scopulepes|[9067]|LNCC1378-11|United States|North Carolina|658[0n]|BOLD:AAB1105
Hemeroplanis scopulepes|[9068]|MILEQ287-11|United States|Alabama|658[0n]|BOLD:AAB1105
Hemeroplanis scopulepes|[9069]|LOFLB109-06|United States|Florida|674[0n]|BOLD:AAB1105
Hemeroplanis scopulepes|[9070]|HKONS652-08|United States|Florida|658[0n]|BOLD:AAB1105
Hemeroplanis scopulepes|[9071]|RDNME713-08|United States|Florida|658[0n]|BOLD:AAB1105
Hemeroplanis scopulepes|[9072]|LOFLB521-06|United States|Florida|658[0n]|BOLD:AAB1105
Hemeroplanis scopulepes|[9073]|RDNME715-08|United States|Florida|658[0n]|BOLD:AAB1105
Hemeroplanis scopulepes|[9074]|LMEMB032-09|United States|Mississippi|658[0n]|BOLD:AAB1105
Hemeroplanis scopulepes|[9075]|LNCB653-09|United States|North Carolina|658[0n]|BOLD:AAB1105
Hemeroplanis scopulepes|[9076]|RDNML052-13|United States|Florida|658[0n]|BOLD:AAB1105
Hemeroplanis scopulepes|[9077]|LOFLB482-06|United States|Florida|674[0n]|BOLD:AAB1105
Hemeroplanis scopulepes|[9078]|HKONS334-08|United States|Florida|658[0n]|BOLD:AAB1105
Hemeroplanis scopulepes|[9079]|RDNME714-08|United States|Florida|658[0n]|BOLD:AAB1105
Hemeroplanis scopulepes|[9080]|CNCLB1651-14|United States|Florida|658[0n]|BOLD:AAB1105
Schrankia|[9081]|IAWL890-09|United States|Arizona|631[4n]|BOLD:AAH4894
Schrankia macula|[9082]|LNC445-05|United States|North Carolina|658[0n]|BOLD:AAB2278
Schrankia macula|[9083]|BBLOB1378-11|United States|Florida|658[0n]|BOLD:AAB2278
Schrankia macula|[9084]|LPOKB921-09|United States|Oklahoma|658[0n]|BOLD:AAB2278
Schrankia macula|[9085]|MNAB369-07|United States|Florida|658[0n]|BOLD:AAB2278
Schrankia macula|[9086]|BBLOB1026-11|United States|Florida|658[0n]|BOLD:AAB2278
[illegible]

Schrankia macula|[9084]|LPOKB921-09|United States|Oklahoma|658[0n]|BOLD:AAB2278
Schrankia macula|[9085]|MNAB369-07|United States|Florida|658[0n]|BOLD:AAB2278
Schrankia macula|[9086]|BBLOB1026-11|United States|Florida|658[0n]|BOLD:AAB2278
Schrankia macula|[9087]|MNAB398-07|United States|Florida|656[0n]|BOLD:AAB2278
Schrankia macula|[9088]|LOFLD274-07|United States|Florida|658[0n]|BOLD:AAB2278
Schrankia macula|[9089]|RDNMG191-08|United States|New York|658[0n]|BOLD:AAB2278
Schrankia macula|[9090]|LNCC421-10|United States|North Carolina|658[0n]|BOLD:AAB2278
Schrankia macula|[9091]|LNCC1842-13|United States|North Carolina|658[0n]|BOLD:AAB2278
Schrankia macula|[9092]|LMEM360-09|United States|Alabama|658[0n]|BOLD:AAB2278
Schrankia macula|[9093]|LMEM361-09|United States|Alabama|658[0n]|BOLD:AAB2278
Schrankia macula|[9094]|LPOKB016-09|United States|Oklahoma|658[0n]|BOLD:AAB2278
Schrankia macula|[9095]|LPOKB017-09|United States|Oklahoma|658[0n]|BOLD:AAB2278
Schrankia macula|[9096]|LPOKB039-09|United States|Oklahoma|658[0n]|BOLD:AAB2278
Schrankia macula|[9097]|LPOKB523-09|United States|Oklahoma|658[0n]|BOLD:AAB2278
Schrankia macula|[9098]|LPOKB595-09|United States|Oklahoma|658[0n]|BOLD:AAB2278
Schrankia macula|[9099]|LPOKB636-09|United States|Oklahoma|658[0n]|BOLD:AAB2278
Schrankia macula|[9100]|LPOKD545-09|United States|Oklahoma|658[0n]|BOLD:AAB2278
Schrankia macula|[9101]|USLEP222-10|United States|Florida|658[0n]|BOLD:AAB2278
Schrankia macula|[9102]|USLEP240-10|United States|Florida|658[0n]|BOLD:AAB2278
Schrankia macula|[9103]|USLEP242-10|United States|Florida|658[0n]|BOLD:AAB2278
Schrankia macula|[9104]|BBLOE395-11|United States|Florida|658[0n]|BOLD:AAB2278
Schrankia macula|[9105]|BBLOE398-11|United States|Florida|658[0n]|BOLD:AAB2278
Schrankia macula|[9106]|RDNMD538-06|United States|Florida|658[0n]|BOLD:AAB2278
Schrankia macula|[9107]|RDNMD537-06|United States|Florida|658[0n]|BOLD:AAB2278
Schrankia macula|[9108]|LOFLB938-06|United States|Florida|658[0n]|BOLD:AAB2278
Schrankia macula|[9109]|LOFLB337-06|United States|Florida|658[0n]|BOLD:AAB2278
Schrankia macula|[9110]|LOFLA760-06|United States|Florida|658[0n]|BOLD:AAB2278
Schrankia macula|[9111]|LOFLA648-06|United States|Florida|658[0n]|BOLD:AAB2278
Schrankia macula|[9112]|LSEU217-06|United States|North Carolina|658[0n]|BOLD:AAB2278
Schrankia macula|[9113]|LGSMD418-05|United States|Tennessee|658[0n]|BOLD:AAB2278
Schrankia macula|[9114]|LNC585-06|United States|North Carolina|623[1n]|BOLD:AAB2278
Schrankia macula|[9115]|MNAB243-07|United States|Florida|632[0n]|BOLD:AAB2278
Schrankia macula|[9116]|LOFLD030-07|United States|Florida|658[0n]|BOLD:AAB2278
Schrankia macula|[9117]|LOFLD399-07|United States|Florida|658[0n]|BOLD:AAB2278
Schrankia macula|[9118]|LOFLD410-07|United States|Florida|658[0n]|BOLD:AAB2278
Schrankia macula|[9119]|HKONS163-08|United States|Florida|658[0n]|BOLD:AAB2278
Schrankia macula|[9120]|RDNME638-08|United States|Florida|658[0n]|BOLD:AAB2278
Schrankia macula|[9121]|LMEM359-09|United States|Mississippi|658[0n]|BOLD:AAB2278
Schrankia macula|[9122]|LNCC1843-13|United States|North Carolina|658[0n]|BOLD:AAB2278
Baniana minor|[9123]|RDNME859-08|United States|Arizona|658[0n]|BOLD:AAE2247
Baniana minor|[9124]|BLPED1555-12|Costa Rica|Guanacaste|658[0n]|BOLD:AAE2247
Baniana minor|[9125]|BLPCI937-08|Costa Rica|Guanacaste|658[0n]|BOLD:AAE2247
Baniana minor|[9126]|BLPCH686-08|Costa Rica|Guanacaste|658[0n]|BOLD:AAE2247
Baniana minor|[9127]|BLPBH694-07|Costa Rica|Guanacaste|658[0n]|BOLD:AAE2247
Baniana minor|[9128]|BLPBA378-07|Costa Rica|Guanacaste|658[0n]|BOLD:AAE2247
Baniana minor|[9129]|BLPDC420-09|Costa Rica|Alajuela|640[0n]|BOLD:AAE2247
Baniana minor|[9130]|BLPED1693-12|Costa Rica|Guanacaste|658[0n]|BOLD:AAE2247
Baniana minor|[9131]|BLPED1820-12|Costa Rica|Guanacaste|658[0n]|BOLD:AAE2247
Baniana minor|[9132]|BLPEF285-12|Costa Rica|Guanacaste|658[0n]|BOLD:AAE2247
Deinopa angitia|[9133]|LPYPA882-08|Mexico|Campeche|658[0n]|BOLD:AAG5888
Deinopa angitia|[9134]|RDNME609-08|United States|Texas|648[0n]|BOLD:AAG5888
Deinopa angitia|[9135]|LPYPB725-08|Mexico|Yucatan|536[0n]|BOLD:AAG5888
Deinopa angitia|[9136]|LYPAP306-09|Mexico|Yucatan|554[0n]|BOLD:AAG5888
Deinopa angitia|[9137]|LYHES436-09|Mexico|Yucatan|658[0n]|BOLD:AAG5888
Glympis concors|[9138]|BLPCJ465-08|Costa Rica|Guanacaste|658[0n]|BOLD:AAW3174
Glympis concors|[9139]|CNCLB1972-14|United States|Texas|658[0n]|BOLD:AAW3174
Phyprosopus callitrichoides|[9140]|LOFLB840-06|United States|Florida|674[0n]|BOLD:ACF2162
Phyprosopus callitrichoides|[9141]|LMEMB086-09|United States|Louisiana|658[0n]|BOLD:ACF2162
Phyprosopus callitrichoides|[9142]|LMEMB089-09|United States|Mississippi|658[0n]|BOLD:ACF2162
Phyprosopus callitrichoides|[9143]|HKONS358-08|United States|Florida|658[0n]|BOLD:ACF2162
Phyprosopus callitrichoides|[9144]|HKONS359-08|United States|Florida|658[0n]|BOLD:ACF2162
Phyprosopus callitrichoides|[9145]|LOFLC408-06|United States|Florida|658[0n]|BOLD:ACF2162
Phyprosopus callitrichoides|[9146]|HKONS357-08|United States|Florida|658[0n]|BOLD:ACF2162
Phyprosopus callitrichoides|[9147]|LOFLB849-06|United States|Florida|658[0n]|BOLD:ACF2162
Phyprosopus callitrichoides|[9148]|LOFLB767-06|United States|Florida|658[0n]|BOLD:ACF2162
Phyprosopus callitrichoides|[9149]|LOFLB755-06|United States|Florida|658[0n]|BOLD:ACF2162
Phyprosopus callitrichoides|[9150]|LOFLB384-06|United States|Florida|658[0n]|BOLD:ACF2162
Phyprosopus callitrichoides|[9151]|LOFLB277-06|United States|Florida|658[0n]|BOLD:ACF2162
Phyprosopus callitrichoides|[9152]|LOFLB203-06|United States|Florida|658[0n]|BOLD:ACF2162
Phyprosopus callitrichoides|[9153]|LOFLA543-06|United States|Florida|658[0n]|BOLD:ACF2162
Phyprosopus callitrichoides|[9154]|LNC510-06|United States|North Carolina|658[0n]|BOLD:ACF2162
Phyprosopus callitrichoides|[9155]|LNC042-05|United States|North Carolina|658[0n]|BOLD:ACF2162
Phyprosopus callitrichoides|[9156]|LGSMC911-05|United States|Tennessee|658[0n]|BOLD:ACF2162
Phyprosopus callitrichoides|[9157]|ABNCC241-07|United States|Texas|641[0n]|BOLD:ACF2162
Phyprosopus callitrichoides|[9158]|LMEMB088-09|United States|Mississippi|622[0n]|BOLD:ACF2162
Phyprosopus callitrichoides|[9159]|LMEMB090-09|United States|Alabama|658[0n]|BOLD:ACF2162
Phyprosopus callitrichoides|[9160]|USLEP093-10|United States|Florida|658[0n]|BOLD:ACF2162
Phyprosopus callitrichoides|[9161]|USLEP595-10|United States|Florida|658[0n]|BOLD:ACF2162
Phyprosopus callitrichoides|[9162]|RDNMK018-11|United States|Florida|658[0n]|BOLD:ACF2162
Phyprosopus callitrichoides|[9163]|USLEP953-10|United States|Texas|658[0n]|BOLD:ACF2161
Phyprosopus callitrichoides|[9164]|BBLSX056-09|United States|Texas|658[0n]|BOLD:ACF2161
Phyprosopus callitrichoides|[9165]|BBLSX044-09|United States|Texas|658[0n]|BOLD:ACF2161
Phyprosopus callitrichoides|[9166]|ABNCC240-07|United States|Texas|642[0n]|BOLD:ACF2161
Phyprosopus callitrichoides|[9167]|USLEP954-10|United States|Texas|653[0n]|BOLD:ACF2161
Phyprosopus callitrichoides|[9168]|USLEP955-10|United States|Texas|658[0n]|BOLD:ACF2161
Phyprosopus callitrichoides|[9169]|USLEP956-10|United States|Texas|658[0n]|BOLD:ACF2161
Phyprosopus callitrichoides|[9170]|LPOKE292-11|United States|Oklahoma|658[0n]|BOLD:ACF2161
Scoliopteryx libatrix|[9171]|RDLQH065-06|Canada|Quebec|658[0n]|BOLD:AAB0295
Scoliopteryx libatrix|[9172]|RDLQG268-06|Canada|Quebec|658[0n]|BOLD:AAB0295
Scoliopteryx libatrix|[9173]|RDLQH066-06|Canada|Quebec|658[0n]|BOLD:AAB0295
Scoliopteryx libatrix|[9174]|LOWCE112-06|Canada|British Columbia|658[0n]|BOLD:AAB0295
Scoliopteryx libatrix|[9175]|LMIS013-05|Canada|Ontario|658[0n]|BOLD:AAB0295
Scoliopteryx libatrix|[9176]|LBCA029-05|Canada|British Columbia|658[0n]|BOLD:AAB0295
Scoliopteryx libatrix|[9177]|XAF435-05|Canada|Ontario|658[0n]|BOLD:AAB0295
Scoliopteryx libatrix|[9178]|RDLQH064-06|Canada|Quebec|646[0n]|BOLD:AAB0295
Scoliopteryx libatrix|[9179]|RDLQH067-06|Canada|Quebec|648[0n]|BOLD:AAB0295
Scoliopteryx libatrix|[9180]|RDLQH068-06|Canada|Quebec|646[0n]|BOLD:AAB0295
Scoliopteryx libatrix|[9181]|LBCS019-07|Canada|British Columbia|658[0n]|BOLD:AAB0295
Scoliopteryx libatrix|[9182]|LBCS117-07|Canada|British Columbia|658[0n]|BOLD:AAB0295
Scoliopteryx libatrix|[9183]|BLTIB565-08|Canada|Ontario|658[0n]|BOLD:AAB0295
Scoliopteryx libatrix|[9184]|HKONB376-09|United States|Indiana|658[0n]|BOLD:AAB0295
Scoliopteryx libatrix|[9185]|BBLPC635-09|Canada|Newfoundland and Labrador|658[0n]|BOLD:AAB0295
Scoliopteryx libatrix|[9186]|BBLPB500-10|Canada|Ontario|658[0n]|BOLD:AAB0295

Scoliopteryx libatrix|[9184]|HKONB376-09|United States|Indiana|658[0n]|BOLD:AAB0295
Scoliopteryx libatrix|[9185]|BBLPC635-09|Canada|Newfoundland and Labrador|658[0n]|BOLD:AAB0295
Scoliopteryx libatrix|[9186]|BBLPB500-10|Canada|Ontario|658[0n]|BOLD:AAB0295
Scoliopteryx libatrix|[9187]|BBLPB501-10|Canada|Saskatchewan|658[0n]|BOLD:AAB0295
Scoliopteryx libatrix|[9188]|BBLPB502-10|Canada|British Columbia|658[0n]|BOLD:AAB0295
Scoliopteryx libatrix|[9189]|LOWCB624-05|Canada|British Columbia|608[0n]|BOLD:ACE7769
Scoliopteryx libatrix|[9190]|ABKWR042-07|United States|Alaska|658[0n]|BOLD:ACE7769
Scoliopteryx libatrix|[9191]|LBCH5277-10|Canada|British Columbia|658[0n]|BOLD:ACE7769
Scoliopteryx libatrix|[9192]|LBCH5341-10|Canada|British Columbia|658[0n]|BOLD:ACE7769
Scoliopteryx libatrix|[9193]|LBCG463-08|Canada|British Columbia|658[0n]|BOLD:ACE7769
Scoliopteryx libatrix|[9194]|LMEMB117-09|United States|Idaho|658[0n]|BOLD:ACE7769
Scoliopteryx libatrix|[9195]|UAMIC1217-13|United States|Alaska|658[0n]|BOLD:ACE7769
Anomis illita|[9196]|HKONS378-08|United States|Florida|658[0n]|BOLD:AAA3356
Anomis illita|[9197]|HKONS046-07|United States|Florida|658[0n]|BOLD:AAA3356
Anomis illita|[9198]|RDNMF608-08|United States|Florida|658[0n]|BOLD:AAA3356
Alabama argillacea|[9199]|RDNMF609-08|United States|Missouri|614[0n]|BOLD:AAJ2486
Alabama argillacea|[9200]|RDNMF610-08|United States|Mississippi|658[0n]|BOLD:AAJ2486
Anomis luridula|[9201]|RDNMH142-09|United States|Florida|658[0n]|BOLD:AAA2708
Anomis luridula|[9202]|RDNMH143-09|United States|Florida|658[0n]|BOLD:AAA2708
Anomis texana|[9203]|LNAUS3541-13|United States|Texas|658[0n]|BOLD:AAC4326
Anomis texana|[9204]|LNAUS3540-13|United States|Florida|527[0n]|BOLD:AAC4326
Anomis catagellas|[9205]|QUNOD170-10|United States|Texas|621[0n]|BOLD:AAC4326
Anomis texana|[9206]|MHAUG668-07|Costa Rica|Alajuela|630[0n]|BOLD:AAC4326
Anomis texana|[9207]|MHAUA310-05|Costa Rica|Alajuela|606[0n]|BOLD:AAC4326
Anomis texana|[9208]|MHAUA296-05|Costa Rica|Alajuela|606[0n]|BOLD:AAC4326
Anomis texana|[9209]|MHAUG318-07|Costa Rica|Alajuela|658[0n]|BOLD:AAC4326
Anomis texana|[9210]|MHAUG317-07|Costa Rica|Alajuela|658[0n]|BOLD:AAC4326
Anomis texana|[9211]|MHAUA297-05|Costa Rica|Alajuela|658[0n]|BOLD:AAC4326
Anomis texana|[9212]|MHAUA286-05|Costa Rica|Guanacaste|658[0n]|BOLD:AAC4326
Anomis texana|[9213]|MHAUA311-05|Costa Rica|Alajuela|593[0n]|BOLD:AAC4326
Anomis texana|[9214]|BLPBD268-07|Costa Rica|Guanacaste|646[0n]|BOLD:AAC4326
Anomis texana|[9215]|BLPBD269-07|Costa Rica|Guanacaste|658[0n]|BOLD:AAC4326
Anomis texana|[9216]|LNAUS3537-13|United States|Texas|658[0n]|BOLD:AAC4326
Anomis texana|[9217]|LNAUS3538-13|United States|Texas|658[0n]|BOLD:AAC4326
Anomis texana|[9218]|LNAUS3539-13|United States|Texas|658[0n]|BOLD:AAC4326
Anomis texana|[9219]|MHMYL3450-11|Costa Rica||658[0n]|BOLD:AAC4326
Anomis texana|[9220]|MHMXI437-07|Costa Rica|Alajuela|634[0n]|BOLD:AAC4326
Anomis texana|[9221]|BLPBH743-07|Costa Rica|Guanacaste|658[0n]|BOLD:AAC4326
Anomis texana|[9222]|MHMYN5605-14|Costa Rica||658[0n]|BOLD:AAC4326
Anomis texana|[9223]|QUNOD016-10|United States|Texas|658[0n]|BOLD:AAA2709
Anomis sp.|[9224]|LPYPB436-08|Mexico|Campeche|651[0n]|BOLD:AAA2709
Anomis sp.|[9225]|LYPAP571-09|Mexico|Quintana Roo|658[0n]|BOLD:AAA2709
Anomis sp.|[9226]|LYPAP566-09|Mexico|Quintana Roo|658[0n]|BOLD:AAA2709
Anomis sp.|[9227]|LPYPB966-08|Mexico|Campeche|658[0n]|BOLD:AAA2709
Anomis sp.|[9228]|CQR439-13|Mexico|Quintana Roo|658[0n]|BOLD:AAA2709
Anomis sp.|[9229]|CNCLB2245-14|Mexico||658[0n]|BOLD:AAA2709
Anomis gentilis|[9230]|MHAUG662-07|Costa Rica|Guanacaste|636[0n]|BOLD:AAB4534
Anomis gentilis|[9231]|MHMYN2260-13|Costa Rica||658[0n]|BOLD:AAB4534
Anomis gentilis|[9232]|BLPCH559-08|Costa Rica|Guanacaste|658[0n]|BOLD:AAB4534
Anomis gentilis|[9233]|MHAUG670-07|Costa Rica|Guanacaste|658[0n]|BOLD:AAB4534
Anomis gentilis|[9234]|MHMXI215-07|Costa Rica|Guanacaste|612[0n]|BOLD:AAB4534
Anomis gentilis|[9235]|MHMYQ048-12|Costa Rica||634[0n]|BOLD:AAB4534
Anomis gentilis|[9236]|BLPEF6125-13|Costa Rica||632[0n]|BOLD:AAB4534
Anomis gentilis|[9237]|BLPEF6127-13|Costa Rica||658[0n]|BOLD:AAB4534
Anomis gentilis|[9238]|BLPAA128-06|Costa Rica|Guanacaste|658[0n]|BOLD:AAB4534
Anomis gentilis|[9239]|MHAUG671-07|Costa Rica|Guanacaste|594[0n]|BOLD:AAB4534
Anomis gentilis|[9240]|MHAUG104-07|Costa Rica|Guanacaste|658[0n]|BOLD:AAB4534
Anomis gentilis|[9241]|MHAUG659-07|Costa Rica|Guanacaste|632[0n]|BOLD:AAB4534
Anomis gentilis|[9242]|MHAUG665-07|Costa Rica|Guanacaste|636[0n]|BOLD:AAB4534
Anomis gentilis|[9243]|BLPCB009-08|Costa Rica|Guanacaste|658[0n]|BOLD:AAB4534
Anomis gentilis|[9244]|BLPEF6126-13|Costa Rica||658[0n]|BOLD:AAB4534
Anomis gentilis|[9245]|BLPEF6124-13|Costa Rica||658[0n]|BOLD:AAB4534
Anomis gentilis|[9246]|BLPEF6110-13|Costa Rica||658[0n]|BOLD:AAB4534
Anomis gentilis|[9247]|BLPCI714-08|Costa Rica|Guanacaste|658[0n]|BOLD:AAB4534
Anomis gentilis|[9248]|BLPCH560-08|Costa Rica|Guanacaste|658[0n]|BOLD:AAB4534
Anomis gentilis|[9249]|MHMXM152-07|Costa Rica|Guanacaste|658[0n]|BOLD:AAB4534
Anomis gentilis|[9250]|MHMXM151-07|Costa Rica|Guanacaste|658[0n]|BOLD:AAB4534
Anomis gentilis|[9251]|BLPBD314-07|Costa Rica|Guanacaste|658[0n]|BOLD:AAB4534
Anomis gentilis|[9252]|MHAUG667-07|Costa Rica|Alajuela|658[0n]|BOLD:AAB4534
Anomis gentilis|[9253]|MHAUG663-07|Costa Rica|Guanacaste|658[0n]|BOLD:AAB4534
Anomis gentilis|[9254]|MHAUG661-07|Costa Rica|Guanacaste|658[0n]|BOLD:AAB4534
Anomis gentilis|[9255]|MHAUG660-07|Costa Rica|Guanacaste|658[0n]|BOLD:AAB4534
Anomis gentilis|[9256]|MHAUG105-07|Costa Rica|Alajuela|599[0n]|BOLD:AAB4534
Anomis gentilis|[9257]|MHAUG666-07|Costa Rica|Guanacaste|647[0n]|BOLD:AAB4534
Anomis gentilis|[9258]|MHAUG664-07|Costa Rica|Guanacaste|618[0n]|BOLD:AAB4534
Anomis gentilis|[9259]|CNCLB1929-14|United States|Texas|407[0n]|BOLD:AAB4534
Anomis gentilis|[9260]|CNCLB2424-14|United States|Texas|658[0n]|BOLD:AAB4534
Anomis editrix|[9261]|MHAUG109-07|Costa Rica|Guanacaste|658[0n]|BOLD:AAA9135
Anomis editrix|[9262]|MHAUC260-06|Costa Rica|Alajuela|658[0n]|BOLD:AAA9135
Anomis editrix|[9263]|MHAUC261-06|Costa Rica|Alajuela|658[0n]|BOLD:AAA9135
Anomis editrix|[9264]|MHAUA283-05|Costa Rica|Guanacaste|658[0n]|BOLD:AAA9135
Anomis editrix|[9265]|MHAUG658-07|Costa Rica|Guanacaste|658[0n]|BOLD:AAA9135
Anomis editrix|[9266]|BLPDQ430-10|Costa Rica|Alajuela|658[0n]|BOLD:AAA9135
Anomis editrix|[9267]|MHAUA272-05|Costa Rica|Guanacaste|658[0n]|BOLD:AAA9135
Anomis editrix|[9268]|MHAUA278-05|Costa Rica|Alajuela|658[0n]|BOLD:AAA9135
Anomis editrix|[9269]|MHAUA276-05|Costa Rica|Guanacaste|618[0n]|BOLD:AAA9135
Anomis editrix|[9270]|BLPBC735-07|Costa Rica|Guanacaste|625[0n]|BOLD:AAA9135
Anomis editrix|[9271]|MHAUA282-05|Costa Rica|Alajuela|612[0n]|BOLD:AAA9135
Anomis editrix|[9272]|MXBLP171-11|Mexico|Jalisco|658[0n]|BOLD:AAA9135
Anomis editrix|[9273]|MHMYN181-11|Costa Rica||658[0n]|BOLD:AAA9135
Anomis editrix|[9274]|MHMXE316-06|Costa Rica|Guanacaste|634[0n]|BOLD:AAA9135
Anomis editrix|[9275]|MHAUG657-07|Costa Rica|Guanacaste|655[0n]|BOLD:AAA9135
Anomis editrix|[9276]|MHMXI225-07|Costa Rica|Guanacaste|659[0n]|BOLD:AAA9135
Anomis editrix|[9277]|MXBLP169-11|Mexico|Jalisco|658[0n]|BOLD:AAA9135
Anomis editrix|[9278]|LOCRC023-08|Costa Rica|Alajuela|658[0n]|BOLD:AAA9135
Anomis editrix|[9279]|LOCRC220-08|Costa Rica|Alajuela|658[0n]|BOLD:AAA9135
Anomis editrix|[9280]|MHMYL3078-11|Costa Rica||658[0n]|BOLD:AAA9135
Anomis editrix|[9281]|MHMYM108-11|Costa Rica||658[0n]|BOLD:AAA9135
Anomis editrix|[9282]|MHMYN182-11|Costa Rica||658[0n]|BOLD:AAA9135
Anomis editrix|[9283]|MHMYQ050-12|Costa Rica||658[0n]|BOLD:AAA9135
Anomis editrix|[9284]|MHMXZ429-09|Costa Rica||658[0n]|BOLD:AAA9135
Anomis editrix|[9285]|BLPDK1702-09|Costa Rica|Guanacaste|658[0n]|BOLD:AAA9135
Anomis editrix|[9286]|MHMXO302-08|Costa Rica|Alajuela|658[0n]|BOLD:AAA9135

Anomis editrix|[9284]|MHMXZ429-09|Costa Rica||658[0n]|BOLD:AAA9135
Anomis editrix|[9285]|BLPDK1702-09|Costa Rica|Guanacaste|658[0n]|BOLD:AAA9135
Anomis editrix|[9286]|MHMXO302-08|Costa Rica|Alajuela|658[0n]|BOLD:AAA9135
Anomis editrix|[9287]|BLPCC417-08|Costa Rica|Guanacaste|658[0n]|BOLD:AAA9135
Anomis editrix|[9288]|MHAUG655-07|Costa Rica|Guanacaste|658[0n]|BOLD:AAA9135
Anomis editrix|[9289]|BLPBD249-07|Costa Rica|Guanacaste|658[0n]|BOLD:AAA9135
Anomis editrix|[9290]|MHMXE884-07|Costa Rica|Guanacaste|658[0n]|BOLD:AAA9135
Anomis editrix|[9291]|MHMXI174-07|Costa Rica|Guanacaste|658[0n]|BOLD:AAA9135
Anomis editrix|[9292]|MHAUC259-06|Costa Rica|Alajuela|658[0n]|BOLD:AAA9135
Anomis editrix|[9293]|MHAUC262-06|Costa Rica|Alajuela|658[0n]|BOLD:AAA9135
Anomis editrix|[9294]|MHAUC257-06|Costa Rica|Guanacaste|658[0n]|BOLD:AAA9135
Anomis editrix|[9295]|MHAUC258-06|Costa Rica|Guanacaste|658[0n]|BOLD:AAA9135
Anomis editrix|[9296]|MHAUA284-05|Costa Rica|Alajuela|658[0n]|BOLD:AAA9135
Anomis editrix|[9297]|MHAUA281-05|Costa Rica|Guanacaste|658[0n]|BOLD:AAA9135
Anomis editrix|[9298]|MHAUA280-05|Costa Rica|Guanacaste|658[0n]|BOLD:AAA9135
Anomis editrix|[9299]|MHAUA279-05|Costa Rica|Guanacaste|658[0n]|BOLD:AAA9135
Anomis editrix|[9300]|MHAUA277-05|Costa Rica|Alajuela|658[0n]|BOLD:AAA9135
Anomis editrix|[9301]|MHAUA275-05|Costa Rica|Guanacaste|658[0n]|BOLD:AAA9135
Anomis editrix|[9302]|MHAUA273-05|Costa Rica|Guanacaste|658[0n]|BOLD:AAA9135
Anomis editrix|[9303]|MHAUA271-05|Costa Rica|Alajuela|658[0n]|BOLD:AAA9135
Anomis editrix|[9304]|MHAUG656-07|Costa Rica|Guanacaste|642[0n]|BOLD:AAA9135
Anomis editrix|[9305]|MHMXZ430-09|Costa Rica||658[0n]|BOLD:AAA9135
Anomis editrix|[9306]|QUNOD171-10|United States|Texas|658[0n]|BOLD:AAA9135
Anomis editrix|[9307]|RDNML147-13|United States|North Carolina|658[0n]|BOLD:AAA9135
Anomis editrix|[9308]|NOCNA079-14|United States|Florida|658[0n]|BOLD:AAA9135
Anomis flava|[9309]|HKONS377-08|United States|Florida|658[0n]|BOLD:AAB5675
Anomis flava|[9310]|RDNMI056-10|United States|Florida|658[0n]|BOLD:AAB5675
Anomis erosa|[9311]|HKONS382-08|United States|Florida|658[0n]|BOLD:AAC1739
Anomis erosa|[9312]|RDNMF621-08|United States|Missouri|609[0n]|BOLD:AAC1739
Anomis erosa|[9313]|LMEMB116-09|United States|Mississippi|658[0n]|BOLD:AAC1739
Anomis erosa|[9314]|HKONS384-08|United States|Florida|658[0n]|BOLD:AAC1739
Anomis erosa|[9315]|HKONS381-08|United States|Florida|658[0n]|BOLD:AAC1739
Anomis erosa|[9316]|HKONS380-08|United States|Florida|658[0n]|BOLD:AAC1739
Anomis erosa|[9317]|HKONS047-07|United States|Florida|656[0n]|BOLD:AAC1739
Anomis erosa|[9318]|RDNMF620-08|United States|Missouri|640[0n]|BOLD:AAC1739
Anomis erosa|[9319]|RDNMI057-10|United States|Florida|658[0n]|BOLD:AAC1739
Anomis erosa|[9320]|RDNMI058-10|United States|Florida|658[0n]|BOLD:AAC1739
Anomis erosa|[9321]|HKONS385-08|United States|Florida|658[0n]|BOLD:AAC1739
Anomis erosa|[9322]|HKONS379-08|United States|Florida|658[0n]|BOLD:AAC1739
Anomis erosa|[9323]|HKONS383-08|United States|Florida|658[0n]|BOLD:AAC1739
Anomis erosa|[9324]|HKONS386-08|United States|Florida|658[0n]|BOLD:AAC1739
Anomis erosa|[9325]|RDNMI059-10|United States|Florida|658[0n]|BOLD:AAC1739
Anomis privata|[9326]|CNCLB444-14|Japan||682[0n]|BOLD:AAR3287
Anomis privata|[9327]|CNCLB447-14|United States|Virginia|682[0n]|BOLD:AAR3287
Anomis privata|[9328]|CNCLB446-14|United States|Virginia|682[0n]|BOLD:AAR3287
Anomis privata|[9329]|LNCC1484-13|United States|North Carolina|658[0n]|BOLD:AAR3287
Anomis privata|[9330]|PMANL2636-13|United States|Virginia|658[0n]|BOLD:AAR3287
Anomis privata|[9331]|PMANL2637-13|United States|Maryland|658[0n]|BOLD:AAR3287
Anomis privata|[9332]|PMANL2638-13|United States|Maryland|658[0n]|BOLD:AAR3287
Anomis privata|[9333]|CNCLB2637-14|United States|North Carolina|658[0n]|BOLD:AAR3287
Macristis schausi|[9334]|HKONB342-09|United States|Texas|658[0n]|BOLD:AAE8635
Macristis schausi|[9335]|HKONB341-09|United States|Texas|658[0n]|BOLD:AAE8635
Macristis schausi|[9336]|HKONB343-09|United States|Texas|658[0n]|BOLD:AAE8635
Macristis schausi|[9337]|HKONB344-09|United States|Texas|658[0n]|BOLD:AAE8635
Redectis vitrea|[9338]|LNCNW107-06|United States|North Carolina|658[4n]|BOLD:AAC7139
Redectis vitrea|[9339]|LMEM327-09|United States|Mississippi|658[0n]|BOLD:AAC7139
Redectis vitrea|[9340]|HKONS161-08|United States|Florida|658[0n]|BOLD:AAC7139
Redectis vitrea|[9341]|RDNMD507-06|United States|Florida|658[0n]|BOLD:AAC7139
Redectis vitrea|[9342]|LSEU779-06|United States|Missouri|658[0n]|BOLD:AAC7139
Redectis vitrea|[9343]|LNCNW101-06|United States|North Carolina|658[0n]|BOLD:AAC7139
Redectis vitrea|[9344]|LNC253-05|United States|North Carolina|658[0n]|BOLD:AAC7139
Redectis vitrea|[9345]|LNC252-05|United States|North Carolina|658[0n]|BOLD:AAC7139
Redectis vitrea|[9346]|LPOKA412-09|United States|Oklahoma|631[0n]|BOLD:AAC7139
Redectis vitrea|[9347]|LMEM328-09|United States|Alabama|658[0n]|BOLD:AAC7139
Redectis vitrea|[9348]|LMEM332-09|United States|Mississippi|658[0n]|BOLD:AAC7139
Redectis vitrea|[9349]|LPOKB082-09|United States|Oklahoma|658[0n]|BOLD:AAC7139
Redectis vitrea|[9350]|BBLPA624-10|Canada|Ontario|658[0n]|BOLD:AAC7139
Redectis vitrea|[9351]|LILLA137-11|United States|Illinois|658[0n]|BOLD:AAC7139
Redectis vitrea|[9352]|LILLA454-11|United States|Illinois|658[0n]|BOLD:AAC7139
Redectis vitrea|[9353]|MILEQ332-11|United States|Alabama|658[0n]|BOLD:AAC7139
Macristis geminipunctalis|[9354]|CNCLB1653-14|United States|Florida|658[0n]|BOLD:ACP5069
Erebidae|[9355]|SYC2316-14|French Polynesia||658[0n]|BOLD:AAD1694
Erebidae|[9356]|SYC2314-14|French Polynesia||658[0n]|BOLD:AAD1694
Erebidae|[9357]|SYC3901-14|French Polynesia||658[0n]|BOLD:AAD1694
Erebidae|[9358]|SYC5128-14|French Polynesia||658[0n]|BOLD:AAD1694
Simplicia cornicalis|[9359]|IMLR982-11|Australia|Queensland|658[0n]|BOLD:AAD1694
Simplicia cornicalis|[9360]|NSWHM202-11|Australia|New South Wales|658[0n]|BOLD:AAD1694
Simplicia cornicalis|[9361]|ANICE615-10|Australia|Queensland|658[0n]|BOLD:AAD1694
Simplicia cornicalis|[9362]|GWORY874-10|Australia|Queensland|658[0n]|BOLD:AAD1694
Simplicia cornicalis|[9363]|GWORY480-10|Australia|Queensland|658[0n]|BOLD:AAD1694
Simplicia cornicalis|[9364]|GWORY479-10|Australia|Queensland|658[0n]|BOLD:AAD1694
Simplicia cornicalis|[9365]|LLISA152-06|Australia|New South Wales|658[0n]|BOLD:AAD1694
Simplicia cornicalis|[9366]|LCANA043-06|Australia|Australian Capital Territory|658[0n]|BOLD:AAD1694
Simplicia cornicalis|[9367]|LOQC206-05|Australia|Queensland|658[0n]|BOLD:AAD1694
Simplicia cornicalis|[9368]|LPNGA348-05|Papua New Guinea|Madang|604[0n]|BOLD:AAD1694
Simplicia cornicalis|[9369]|GWORX170-10|Australia|Queensland|658[0n]|BOLD:AAD1694
Simplicia cornicalis|[9370]|ANICE616-10|Australia|New South Wales|609[0n]|BOLD:AAD1694
Simplicia cornicalis|[9371]|GWOSW661-11|Thailand|Phangnga|658[0n]|BOLD:AAD1694
Simplicia cornicalis|[9372]|GWOSW594-11|Thailand|Phangnga|658[0n]|BOLD:AAD1694
Simplicia cornicalis|[9373]|BBLOB472-11|United States|Florida|658[0n]|BOLD:AAD1694
Simplicia cornicalis|[9374]|RDNMH478-09|United States|Louisiana|658[0n]|BOLD:AAD1694
Simplicia cornicalis|[9375]|RDNMH477-09|United States|Louisiana|658[0n]|BOLD:AAD1694
Simplicia cornicalis|[9376]|HKONB375-09|United States|Florida|658[0n]|BOLD:AAD1694
Simplicia cornicalis|[9377]|RDNME572-08|United States|Florida|658[0n]|BOLD:AAD1694
Simplicia cornicalis|[9378]|LEPMY401-13|Malaysia|Pahang|603[0n]|BOLD:AAD1694
Simplicia cornicalis|[9379]|GBGL16217-14|||640[0n]|BOLD:AAD1694
Simplicia cornicalis|[9380]|GBGL16218-14|||640[0n]|BOLD:AAD1694
Zanclognatha jacchusalis|[9381]|CNSLR431-13|Canada|Ontario|513[0n]|
Zanclognatha jacchusalis|[9382]|CNSLR430-13|Canada|Ontario|528[0n]|
Zanclognatha jacchusalis|[9383]|CNSLQ013-13|Canada|Ontario|513[0n]|
Zanclognatha sp. 1|[9384]|BBLPB978-10|Canada|Alberta|658[0n]|BOLD:AAA5206
Zanclognatha sp. 1|[9385]|BBLPB981-10|Canada|Alberta|658[0n]|BOLD:AAA5206
Zanclognatha jacchusalis|[9386]|CNPEP083-14|Canada|Prince Edward Island|518[2n]|

Zanclognatha sp. 1|[9384]|BBLPB978-10|Canada|Alberta|658[0n]|BOLD:AAA5206
Zanclognatha sp. 1|[9385]|BBLPB981-10|Canada|Alberta|658[0n]|BOLD:AAA5206
Zanclognatha jacchusalis|[9386]|CNPEP083-14|Canada|Prince Edward Island|518[2n]|
Zanclognatha jacchusalis|[9387]|CNPEF081-14|Canada|Prince Edward Island|515[0n]|
Zanclognatha jacchusalis|[9388]|CNPEP079-14|Canada|Prince Edward Island|518[0n]|
Zanclognatha jacchusalis|[9389]|CNPEP068-14|Canada|Prince Edward Island|532[0n]|
Zanclognatha jacchusalis|[9390]|CNPEQ040-14|Canada|Prince Edward Island|532[0n]|
Zanclognatha jacchusalis|[9391]|CNPEQ041-14|Canada|Prince Edward Island|532[0n]|
Zanclognatha jacchusalis|[9392]|CNKJF026-14|Canada|Nova Scotia|528[0n]|
Zanclognatha dentata|[9393]|LGSMC890-05|United States|Tennessee|658[0n]|BOLD:AAA5206
Zanclognatha dentata|[9394]|ABNCC114-07|United States|Wisconsin|640[1n]|BOLD:AAA5206
Zanclognatha dentata|[9395]|RDNMD901-07|United States|Massachusetts|650[0n]|BOLD:AAA5206
Zanclognatha dentata|[9396]|RDLQF533-06|Canada|Quebec|645[0n]|BOLD:AAA5206
Zanclognatha dentata|[9397]|ABNCC116-07|United States|Wisconsin|616[1n]|BOLD:AAA5206
Zanclognatha dentata|[9398]|BBLPC014-09|Canada|New Brunswick|658[0n]|BOLD:AAA5206
Zanclognatha dentata|[9399]|BLTIB802-08|Canada|Ontario|658[0n]|BOLD:AAA5206
Zanclognatha dentata|[9400]|LGSMG479-07|United States|Tennessee|658[0n]|BOLD:AAA5206
Zanclognatha dentata|[9401]|RDLQG027-06|Canada|Quebec|658[0n]|BOLD:AAA5206
Zanclognatha dentata|[9402]|RDLQF342-06|Canada|Quebec|658[0n]|BOLD:AAA5206
Zanclognatha dentata|[9403]|TMNBB049-06|Canada|New Brunswick|658[0n]|BOLD:AAA5206
Zanclognatha dentata|[9404]|TMNBB048-06|Canada|New Brunswick|658[0n]|BOLD:AAA5206
Zanclognatha dentata|[9405]|HKONB416-09|United States|Kentucky|658[0n]|BOLD:AAA5206
Zanclognatha dentata|[9406]|HKONB415-09|United States|Kentucky|658[0n]|BOLD:AAA5206
Zanclognatha dentata|[9407]|PHMO226-03|Canada|Ontario|639[0n]|BOLD:AAA5206
Zanclognatha dentata|[9408]|LOT167-04|United States|Tennessee|609[0n]|BOLD:AAA5206
Zanclognatha dentata|[9409]|RDLQF415-06|Canada|Quebec|635[0n]|BOLD:AAA5206
Zanclognatha dentata|[9410]|RDNMK489-11|United States|Connecticut|658[0n]|BOLD:AAA5206
Zanclognatha theralis|[9411]|RDNMD334-06|United States|North Carolina|658[0n]|BOLD:AAA5206
Zanclognatha theralis|[9412]|LNC469-05|United States|North Carolina|658[0n]|BOLD:AAA5206
Zanclognatha theralis|[9413]|LNCB733-09|United States|North Carolina|656[0n]|BOLD:AAA5206
Zanclognatha theralis|[9414]|MILEP009-09|United States|North Carolina|658[0n]|BOLD:AAA5206
Zanclognatha theralis|[9415]|LNCB734-09|United States|North Carolina|658[0n]|BOLD:AAA5206
Zanclognatha theralis|[9416]|LNCB781-09|United States|North Carolina|658[0n]|BOLD:AAA5206
Zanclognatha theralis|[9417]|LNCC948-11|United States|North Carolina|658[0n]|BOLD:AAA5206
Zanclognatha theralis|[9418]|MILEP017-09|United States|North Carolina|658[0n]|BOLD:AAA5206
Zanclognatha theralis|[9419]|LNCB779-09|United States|North Carolina|658[0n]|BOLD:AAA5206
Zanclognatha theralis|[9420]|LNCC925-11|United States|North Carolina|658[0n]|BOLD:AAA5206
Zanclognatha theralis|[9421]|LNCC1274-11|United States|North Carolina|658[0n]|BOLD:AAA5206
Zanclognatha atrilineella|[9422]|HKONS332-08|United States|Florida|658[0n]|BOLD:AAA5206
Zanclognatha atrilineella|[9423]|LSEU216-06|United States|Georgia|658[0n]|BOLD:AAA5206
Zanclognatha atrilineella|[9424]|LMEM183-09|United States|Alabama|620[0n]|BOLD:AAA5206
Zanclognatha atrilineella|[9425]|LMEM184-09|United States|Mississippi|621[0n]|BOLD:AAA5206
Zanclognatha atrilineella|[9426]|LMEM185-09|United States|Mississippi|658[0n]|BOLD:AAA5206
Zanclognatha atrilineella|[9427]|LMEM186-09|United States|Mississippi|658[0n]|BOLD:AAA5206
Zanclognatha atrilineella|[9428]|LMEM187-09|United States|Mississippi|658[0n]|BOLD:AAA5206
Zanclognatha atrilineella|[9429]|LNCB598-09|United States|North Carolina|658[0n]|BOLD:AAA5206
Zanclognatha atrilineella|[9430]|MILEP012-09|United States|North Carolina|658[0n]|BOLD:AAA5206
Zanclognatha martha|[9431]|LNCC092-10|United States|North Carolina|658[0n]|BOLD:AAA5206
Zanclognatha martha|[9432]|LNCC093-10|United States|North Carolina|658[0n]|BOLD:AAA5206
Zanclognatha martha|[9433]|LNCC1698-13|United States|North Carolina|658[0n]|BOLD:AAA5206
Zanclognatha sp.|[9434]|CNLMC045-14|Canada|Quebec|528[0n]|BOLD:AAA5206
Zanclognatha minoralis sp. 1|[9435]|HKONB352-09|United States|Texas|658[0n]|BOLD:AAA5206
Zanclognatha theralis|[9436]|LMEM170-09|United States|Mississippi|658[0n]|BOLD:AAA5206
Zanclognatha minoralis sp. 3|[9437]|HKONS260-08|United States|Florida|658[0n]|BOLD:AAA5206
Zanclognatha sp. 1|[9438]|HKONB346-09|United States|Texas|658[0n]|BOLD:AAA5206
Zanclognatha sp. 1|[9439]|HKONB347-09|United States|Texas|658[0n]|BOLD:AAA5206
Zanclognatha sp. 1|[9440]|HKONB348-09|United States|Texas|658[0n]|BOLD:AAA5206
Zanclognatha minoralis sp. 1|[9441]|HKONB351-09|United States|Texas|658[0n]|BOLD:AAA5206
Zanclognatha theralis|[9442]|LGSM532-04|United States|North Carolina|658[0n]|BOLD:AAA5206
Zanclognatha theralis|[9443]|RDLQG680-06|Canada|Quebec|658[0n]|BOLD:AAA5206
Zanclognatha minoralis sp. 1|[9444]|HKONS265-08|United States|Florida|658[0n]|BOLD:AAA5206
Zanclognatha theralis|[9445]|RDNMD335-06|United States|North Carolina|658[0n]|BOLD:AAA5206
Zanclognatha theralis|[9446]|ABNCC479-07|United States||583[0n]|BOLD:AAA5206
Zanclognatha theralis|[9447]|TTMNB025-06|Canada|New Brunswick|656[0n]|BOLD:AAA5206
Zanclognatha theralis|[9448]|BBLEC556-09|Canada|Nova Scotia|658[0n]|BOLD:AAA5206
Zanclognatha theralis|[9449]|BBLEC750-09|Canada|Nova Scotia|632[0n]|BOLD:AAA5206
Zanclognatha theralis|[9450]|RDLQB257-05|Canada|Quebec|658[0n]|BOLD:AAA5206
Zanclognatha theralis|[9451]|ABNCC478-07|United States||572[0n]|BOLD:AAA5206
Zanclognatha theralis|[9452]|LGSM531-04|United States|North Carolina|658[0n]|BOLD:AAA5206
Zanclognatha theralis|[9453]|LNC466-05|United States|North Carolina|581[1n]|BOLD:AAA5206
Zanclognatha theralis|[9454]|LNC468-05|United States|North Carolina|549[0n]|BOLD:AAA5206
Zanclognatha theralis|[9455]|RDLQH144-06|Canada|Quebec|606[5n]|BOLD:AAA5206
Zanclognatha theralis|[9456]|RDLQH146-06|Canada|Quebec|599[3n]|BOLD:AAA5206
Zanclognatha theralis|[9457]|LNC465-05|United States|North Carolina|569[0n]|BOLD:AAA5206
Zanclognatha theralis|[9458]|LNC467-05|United States|North Carolina|573[0n]|BOLD:AAA5206
Zanclognatha theralis|[9459]|RDLQH149-06|Canada|Quebec|595[0n]|BOLD:AAA5206
Zanclognatha sp.|[9460]|LGSMG482-07|United States|North Carolina|658[0n]|BOLD:AAA5206
Zanclognatha sp.|[9461]|LGSMG483-07|United States|North Carolina|658[0n]|BOLD:AAA5206
Zanclognatha theralis|[9462]|LNCC1554-13|United States|North Carolina|658[0n]|BOLD:AAA5206
Zanclognatha theralis|[9463]|MILEP033-09|United States|North Carolina|658[0n]|BOLD:AAA5206
Zanclognatha theralis|[9464]|MILEP032-09|United States|North Carolina|658[0n]|BOLD:AAA5206
Zanclognatha theralis|[9465]|MILEP035-09|United States|North Carolina|658[0n]|BOLD:AAA5206
Zanclognatha theralis|[9466]|LNC457-05|United States|North Carolina|658[0n]|BOLD:AAA5206
Zanclognatha theralis|[9467]|MILEP010-09|United States|North Carolina|658[0n]|BOLD:AAA5206
Zanclognatha theralis|[9468]|LNCC1879-13|United States|North Carolina|658[0n]|BOLD:AAA5206
Zanclognatha theralis|[9469]|LNCC1887-13|United States|North Carolina|658[0n]|BOLD:AAA5206
Zanclognatha theralis|[9470]|LMEM165-09|United States|Tennessee|609[0n]|BOLD:AAA5206
Zanclognatha martha|[9471]|ABNCC071-07|United States|Texas|638[0n]|BOLD:AAA5206
Zanclognatha theralis|[9472]|LNCB849-09|United States|Georgia|658[0n]|BOLD:AAA5206
Zanclognatha minoralis sp. 3|[9473]|HKONS261-08|United States|Florida|658[0n]|BOLD:AAA5206
Zanclognatha theralis|[9474]|LNCB735-09|United States|North Carolina|658[0n]|BOLD:AAA5206
Zanclognatha minoralis sp. 3|[9475]|HKONS262-08|United States|Florida|658[0n]|BOLD:AAA5206
Zanclognatha minoralis sp. 3|[9476]|HKONS264-08|United States|Florida|658[0n]|BOLD:AAA5206
Zanclognatha minoralis sp. 3|[9477]|HKONS263-08|United States|Florida|658[0n]|BOLD:AAA5206
Zanclognatha sp.|[9478]|HKONS255-08|United States|Florida|658[0n]|BOLD:AAA5206
Zanclognatha minoralis sp. 3|[9479]|HKONS304-08|United States|Florida|658[0n]|BOLD:AAA5206
Zanclognatha theralis|[9480]|LMEM172-09|United States|Georgia|650[0n]|BOLD:AAA5206
Zanclognatha minoralis sp. 1|[9481]|HKONS087-07|United States|Florida|658[0n]|BOLD:AAA5206
Zanclognatha minoralis|[9482]|LMEM176-09|United States|Georgia|658[0n]|BOLD:AAA5206
Zanclognatha theralis|[9483]|LNC470-05|United States|North Carolina|658[0n]|BOLD:AAA5206
Zanclognatha theralis|[9484]|RDNMD329-06|United States|North Carolina|658[0n]|BOLD:AAA5206
Zanclognatha theralis|[9485]|RDNMD330-06|United States|North Carolina|658[0n]|BOLD:AAA5206
Zanclognatha theralis|[9486]|LNCB803-09|United States|North Carolina|658[0n]|BOLD:AAA5206

[illegible]
Zanclognatha theralis|[9484]|RDNMD329-06|United States|North Carolina|658[0n]|BOLD:AAA5206
Zanclognatha theralis|[9485]|RDNMD330-06|United States|North Carolina|658[0n]|BOLD:AAA5206
Zanclognatha theralis|[9486]|LNCB803-09|United States|North Carolina|658[0n]|BOLD:AAA5206
Zanclognatha theralis|[9487]|LMEM171-09|United States|Georgia|658[0n]|BOLD:AAA5206
Zanclognatha theralis|[9488]|RDLQH148-06|Canada|Quebec|631[0n]|BOLD:AAA5206
Zanclognatha theralis|[9489]|RDLQB256-05|Canada|Quebec|570[0n]|BOLD:AAA5206
Zanclognatha minoralis|[9490]|RDNMD511-06|United States|Florida|658[0n]|BOLD:AAA5206
Zanclognatha minoralis|[9491]|LMEM175-09|United States|Georgia|618[0n]|BOLD:AAA5206
Zanclognatha minoralis|[9492]|LMEM177-09|United States|Georgia|601[0n]|BOLD:AAA5206
Zanclognatha theralis|[9493]|BBLPE174-09|Canada|Nova Scotia|658[0n]|BOLD:AAA5206
Zanclognatha minoralis|[9494]|RDNMK042-11|United States|Florida|658[0n]|BOLD:AAA5206
Zanclognatha martha|[9495]|LNCC1847-13|United States|North Carolina|658[0n]|BOLD:AAA5206
Zanclognatha sp.|[9496]|RDLQF412-06|Canada|Quebec|654[0n]|BOLD:AAA5206
Zanclognatha dentata|[9497]|RDLQF579-06|Canada|Quebec|649[0n]|BOLD:AAA5206
Zanclognatha dentata|[9498]|RDNMD899-07|United States|Rhode Island|655[0n]|BOLD:AAA5206
Zanclognatha dentata|[9499]|RDNMD900-07|United States|Rhode Island|655[0n]|BOLD:AAA5206
Zanclognatha sp.|[9500]|RDLQF537-06|Canada|Quebec|658[0n]|BOLD:AAA5206
Zanclognatha dentata|[9501]|RDLQF580-06|Canada|Quebec|658[0n]|BOLD:AAA5206
Zanclognatha dentata|[9502]|RDLQF782-06|Canada|Quebec|658[0n]|BOLD:AAA5206
Zanclognatha dentata|[9503]|RDLQF837-06|Canada|Quebec|658[0n]|BOLD:AAA5206
Zanclognatha sp.|[9504]|RDLQG026-06|Canada|Quebec|658[0n]|BOLD:AAA5206
Zanclognatha sp.|[9505]|RDLQG167-06|Canada|Quebec|658[0n]|BOLD:AAA5206
Zanclognatha sp.|[9506]|RDLQG171-06|Canada|Quebec|658[0n]|BOLD:AAA5206
Zanclognatha dentata|[9507]|RDLQG345-06|Canada|Quebec|658[0n]|BOLD:AAA5206
Zanclognatha sp.|[9508]|RDLQF417-06|Canada|Quebec|658[0n]|BOLD:AAA5206
Zanclognatha sp.|[9509]|RDLQF534-06|Canada|Quebec|658[0n]|BOLD:AAA5206
Zanclognatha dentata|[9510]|RDLQF343-06|Canada|Quebec|658[0n]|BOLD:AAA5206
Zanclognatha sp.|[9511]|RDLQF408-06|Canada|Quebec|658[0n]|BOLD:AAA5206
Zanclognatha sp.|[9512]|RDLQF339-06|Canada|Quebec|658[0n]|BOLD:AAA5206
Zanclognatha sp.|[9513]|RDLQF340-06|Canada|Quebec|658[0n]|BOLD:AAA5206
Zanclognatha sp.|[9514]|RDNMD902-07|United States|Rhode Island|655[0n]|BOLD:AAA5206
Zanclognatha sp.|[9515]|BLTIB804-08|Canada|Ontario|658[0n]|BOLD:AAA5206
Zanclognatha sp.|[9516]|RDLQF338-06|Canada|Quebec|658[0n]|BOLD:AAA5206
Zanclognatha sp.|[9517]|RDLQF335-06|Canada|Quebec|658[0n]|BOLD:AAA5206
Zanclognatha sp.|[9518]|RDLQF331-06|Canada|Quebec|658[0n]|BOLD:AAA5206
Zanclognatha sp.|[9519]|RDLQF330-06|Canada|Quebec|658[0n]|BOLD:AAA5206
Zanclognatha sp.|[9520]|XAJ983-06|Canada|Ontario|658[0n]|BOLD:AAA5206
Zanclognatha sp.|[9521]|XAJ876-06|Canada|Ontario|658[0n]|BOLD:AAA5206
Zanclognatha sp.|[9522]|TMNBB051-06|Canada|New Brunswick|658[0n]|BOLD:AAA5206
Zanclognatha sp.|[9523]|MNBB565-05|Canada|New Brunswick|658[0n]|BOLD:AAA5206
Zanclognatha sp.|[9524]|RDLQF337-06|Canada|Quebec|658[0n]|BOLD:AAA5206
Zanclognatha sp.|[9525]|RDLQF406-06|Canada|Quebec|658[1n]|BOLD:AAA5206
Zanclognatha dentata|[9526]|RDLQF419-06|Canada|Quebec|658[0n]|BOLD:AAA5206
Zanclognatha sp.|[9527]|RDLQF341-06|Canada|Quebec|658[0n]|BOLD:AAA5206
Zanclognatha sp.|[9528]|RDLQF333-06|Canada|Quebec|631[0n]|BOLD:AAA5206
Zanclognatha dentata|[9529]|RDLQF332-06|Canada|Quebec|632[0n]|BOLD:AAA5206
Zanclognatha dentata|[9530]|PHMO223-03|Canada|Ontario|639[0n]|BOLD:AAA5206
Zanclognatha sp.|[9531]|BLGSM091-09|Canada|Ontario|630[0n]|BOLD:AAA5206
Zanclognatha dentata|[9532]|RDLQF705-06|Canada|Quebec|637[0n]|BOLD:AAA5206
Zanclognatha sp.|[9533]|XAG192-05|Canada|Ontario|587[0n]|BOLD:AAA5206
Zanclognatha sp.|[9534]|RDLQF336-06|Canada|Quebec|622[0n]|BOLD:AAA5206
Zanclognatha sp.|[9535]|RDLQF416-06|Canada|Quebec|598[0n]|BOLD:AAA5206
Zanclognatha sp.|[9536]|RDLQF783-06|Canada|Quebec|589[2n]|BOLD:AAA5206
Zanclognatha sp.|[9537]|BBLEC067-09|Canada|New Brunswick|621[0n]|BOLD:AAA5206
Zanclognatha sp.|[9538]|BBLEC058-09|Canada|New Brunswick|658[0n]|BOLD:AAA5206
Zanclognatha sp.|[9539]|BBLPC516-09|Canada|New Brunswick|658[0n]|BOLD:AAA5206
Zanclognatha sp.|[9540]|BBLPC546-09|Canada|New Brunswick|658[0n]|BOLD:AAA5206
Zanclognatha sp.|[9541]|BBLPC616-09|Canada|Nova Scotia|655[0n]|BOLD:AAA5206
Zanclognatha sp.|[9542]|LNCC1699-13|United States|North Carolina|658[0n]|BOLD:AAA5206
Zanclognatha sp.|[9543]|CNLMF1056-14|Canada|Quebec|552[1n]|BOLD:AAA5206
Zanclognatha sp.|[9544]|LGSMG484-07|United States|Tennessee|658[0n]|BOLD:AAA5206
Zanclognatha|[9545]|LOTB144-05|United States|Tennessee|658[0n]|BOLD:AAA5206
Zanclognatha minoralis|[9546]|LMEM178-09|United States|Mississippi|658[0n]|BOLD:AAA5206
Zanclognatha theralis|[9547]|BBLEC234-09|Canada|Nova Scotia|658[0n]|BOLD:AAA5206
Zanclognatha|[9548]|LOTB127-05|United States|Tennessee|614[0n]|BOLD:AAA5206
Zanclognatha minoralis|[9549]|RDNMD331-06|United States|Florida|652[0n]|BOLD:AAA5206
Zanclognatha minoralis|[9550]|RDNMD332-06|United States|Florida|583[10n]|BOLD:AAA5206
Zanclognatha theralis|[9551]|LMEM173-09|United States|Alabama|658[0n]|BOLD:AAA5206
Zanclognatha theralis|[9552]|LNCB780-09|United States|North Carolina|658[0n]|BOLD:AAA5206
Zanclognatha theralis|[9553]|LNCC1799-13|United States|North Carolina|658[0n]|BOLD:AAA5206
Zanclognatha theralis|[9554]|CNCLB2781-14|United States|North Carolina|658[0n]|BOLD:AAA5206
Zanclognatha jacchusalis|[9555]|CNSLQ018-13|Canada|Ontario|513[0n]|
Zanclognatha cruralis|[9556]|CNPEO1399-14|Canada|Prince Edward Island|516[0n]|
Zanclognatha cruralis|[9557]|CNKOE2205-14|Canada|New Brunswick|528[0n]|BOLD:AAA5206
Zanclognatha cruralis|[9558]|CNKOE2200-14|Canada|New Brunswick|537[0n]|BOLD:AAA5206
Zanclognatha cruralis|[9559]|RDLQF536-06|Canada|Quebec|658[0n]|BOLD:AAA5206
Zanclognatha cruralis|[9560]|RDLQF437-06|Canada|Quebec|659[0n]|BOLD:AAA5206
Zanclognatha cruralis|[9561]|RDLQG471-06|Canada|Quebec|658[0n]|BOLD:AAA5206
Zanclognatha cruralis|[9562]|RDLQF436-06|Canada|Quebec|658[0n]|BOLD:AAA5206
Zanclognatha|[9563]|LOTB165-05|United States|Tennessee|658[0n]|BOLD:AAA5206
Zanclognatha cruralis|[9564]|XAC295-04|Canada|Ontario|658[0n]|BOLD:AAA5206
Zanclognatha sp.|[9565]|MMNA105-08|United States|North Carolina|658[0n]|BOLD:AAA5206
Zanclognatha cruralis|[9566]|CNSLP1123-13|Canada|Ontario|613[0n]|BOLD:AAA5206
Zanclognatha cruralis|[9567]|CNSLP1121-13|Canada|Ontario|613[0n]|BOLD:AAA5206
Zanclognatha cruralis|[9568]|CNSLO007-13|Canada|Ontario|588[0n]|BOLD:AAA5206
Zanclognatha cruralis|[9569]|CNSLO013-13|Canada|Ontario|573[0n]|BOLD:AAA5206
Zanclognatha cruralis|[9570]|CNBRD027-14|Canada|Nova Scotia|576[0n]|BOLD:AAA5206
Zanclognatha cruralis|[9571]|CNKOE2202-14|Canada|New Brunswick|564[0n]|BOLD:AAA5206
Zanclognatha cruralis|[9572]|CNKOE2210-14|Canada|New Brunswick|564[0n]|BOLD:AAA5206
Zanclognatha cruralis|[9573]|CNKOP1606-14|Canada|New Brunswick|562[0n]|BOLD:AAA5206
Zanclognatha marcidilinea|[9574]|GMGSV006-13|United States|Tennessee|523[0n]|
Zanclognatha jacchusalis|[9575]|CNKJF027-14|Canada|Nova Scotia|519[0n]|
Zanclognatha jacchusalis|[9576]|CNPEP069-14|Canada|Prince Edward Island|518[0n]|
Zanclognatha jacchusalis|[9577]|CNKJF011-14|Canada|Nova Scotia|528[0n]|
Zanclognatha jacchusalis|[9578]|BBLEC914-09|Canada|Nova Scotia|658[0n]|BOLD:AAA5206
Zanclognatha|[9579]|CNSLQ029-13|Canada|Ontario|537[0n]|BOLD:AAA5206
Zanclognatha|[9580]|CNSLQ039-13|Canada|Ontario|576[0n]|BOLD:AAA5206
Zanclognatha jacchusalis|[9581]|BBLPE144-09|Canada|Nova Scotia|658[0n]|BOLD:AAA5206
Zanclognatha jacchusalis|[9582]|BBLPE131-09|Canada|Nova Scotia|658[0n]|BOLD:AAA5206
Zanclognatha jacchusalis|[9583]|BBLPE098-09|Canada|Nova Scotia|658[0n]|BOLD:AAA5206
Zanclognatha jacchusalis|[9584]|BBLEC553-09|Canada|Nova Scotia|658[0n]|BOLD:AAA5206
Zanclognatha jacchusalis|[9585]|BBLEC552-09|Canada|Nova Scotia|658[0n]|BOLD:AAA5206
Zanclognatha jacchusalis|[9586]|BBLEC255-09|Canada|Nova Scotia|658[0n]|BOLD:AAA5206

Zanclognatha jacchusalis|[9583]|BBLE[illegible]-09|Canada|Nova Scotia|658[0n]|BOLD:AAA5206
Zanclognatha jacchusalis|[9584]|BBLEC553-09|Canada|Nova Scotia|658[0n]|BOLD:AAA5206
Zanclognatha jacchusalis|[9585]|BBLEC552-09|Canada|Nova Scotia|658[0n]|BOLD:AAA5206
Zanclognatha jacchusalis|[9586]|BBLEC255-09|Canada|Nova Scotia|658[0n]|BOLD:AAA5206
Zanclognatha jacchusalis|[9587]|BBLEC207-09|Canada|Nova Scotia|658[0n]|BOLD:AAA5206
Zanclognatha jacchusalis|[9588]|RDLQF641-06|Canada|Quebec|637[0n]|BOLD:AAA5206
Zanclognatha|[9589]|CNPEP071-14|Canada|Prince Edward Island|563[0n]|BOLD:AAA5206
Zanclognatha cruralis|[9590]|CNPEP078-14|Canada|Prince Edward Island|554[0n]|BOLD:AAA5206
Zanclognatha|[9591]|CNKJN160-14|Canada|Nova Scotia|528[0n]|BOLD:AAA5206
Zanclognatha jacchusalis|[9592]|RDLQF586-06|Canada|Quebec|658[0n]|BOLD:AAA5206
Zanclognatha jacchusalis|[9593]|LGSM534-04|United States|Tennessee|658[0n]|BOLD:AAA5206
Zanclognatha jacchusalis|[9594]|LGSMG497-07|United States|Tennessee|658[0n]|BOLD:AAA5206
Zanclognatha jacchusalis|[9595]|RDLQG175-06|Canada|Quebec|658[0n]|BOLD:AAA5206
Zanclognatha jacchusalis|[9596]|RDLQF769-06|Canada|Quebec|658[0n]|BOLD:AAA5206
Zanclognatha jacchusalis|[9597]|RDLQF640-06|Canada|Quebec|637[0n]|BOLD:AAA5206
Zanclognatha jacchusalis|[9598]|CNSLG300-12|Canada|Ontario|606[0n]|BOLD:AAA5206
Zanclognatha jacchusalis|[9599]|XAC836-04|Canada|Ontario|658[0n]|BOLD:AAA5206
Zanclognatha jacchusalis|[9600]|XAG164-05|Canada|Ontario|658[0n]|BOLD:AAA5206
Zanclognatha jacchusalis|[9601]|CNSLQ020-13|Canada|Ontario|561[0n]|BOLD:AAA5206
Zanclognatha jacchusalis|[9602]|RDLQG174-06|Canada|Quebec|658[0n]|BOLD:AAA5206
Zanclognatha jacchusalis|[9603]|LGSMG498-07|United States|North Carolina|655[0n]|BOLD:AAA5206
Zanclognatha jacchusalis|[9604]|LGSM533-04|United States|North Carolina|658[0n]|BOLD:AAA5206
Zanclognatha jacchusalis|[9605]|LSEU732-06|United States|Georgia|658[0n]|BOLD:AAA5206
Zanclognatha jacchusalis|[9606]|LNCC1012-11|United States|North Carolina|658[0n]|BOLD:AAA5206
Zanclognatha jacchusalis|[9607]|QUNOE470-12|United States|Kentucky|658[1n]|BOLD:AAA5206
Zanclognatha jacchusalis|[9608]|RDNMF778-08|Canada|British Columbia|658[0n]|BOLD:AAA5206
Zanclognatha jacchusalis|[9609]|LBCS240-07|Canada|British Columbia|658[0n]|BOLD:AAA5206
Zanclognatha jacchusalis|[9610]|RDNMF779-08|Canada|British Columbia|658[0n]|BOLD:AAA5206
Zanclognatha jacchusalis|[9611]|RDNMF780-08|Canada|British Columbia|658[0n]|BOLD:AAA5206
Zanclognatha jacchusalis|[9612]|LALPA491-10|Canada|British Columbia|658[0n]|BOLD:AAA5206
Zanclognatha jacchusalis|[9613]|LALPA549-10|Canada|British Columbia|658[0n]|BOLD:AAA5206
Zanclognatha jacchusalis|[9614]|LALPA557-10|Canada|British Columbia|658[0n]|BOLD:AAA5206
Zanclognatha jacchusalis|[9615]|LNCC1013-11|United States|North Carolina|658[0n]|BOLD:AAA5206
Zanclognatha jacchusalis|[9616]|LNCC1011-11|United States|North Carolina|658[0n]|BOLD:AAA5206
Zanclognatha jacchusalis|[9617]|RDNMH1036-09|United States|North Carolina|658[0n]|BOLD:AAA5206
Zanclognatha jacchusalis|[9618]|RDNMF100-08|United States|Rhode Island|658[0n]|BOLD:AAA5206
Zanclognatha jacchusalis|[9619]|RDNMF099-08|Canada|Ontario|658[0n]|BOLD:AAA5206
Zanclognatha jacchusalis|[9620]|LSEU733-06|United States|Georgia|658[0n]|BOLD:AAA5206
Zanclognatha jacchusalis|[9621]|XAD001-04|Canada|Ontario|658[0n]|BOLD:AAA5206
Zanclognatha jacchusalis|[9622]|RDLQF422-06|Canada|Quebec|658[0n]|BOLD:AAA5206
Zanclognatha jacchusalis|[9623]|RDLQF539-06|Canada|Quebec|658[0n]|BOLD:AAA5206
Zanclognatha jacchusalis|[9624]|RDLQF766-06|Canada|Quebec|658[0n]|BOLD:AAA5206
Zanclognatha jacchusalis|[9625]|RDLQF638-06|Canada|Quebec|600[0n]|BOLD:AAA5206
Zanclognatha jacchusalis|[9626]|RDLQG089-06|Canada|Quebec|621[0n]|BOLD:AAA5206
Zanclognatha jacchusalis|[9627]|RDLQF636-06|Canada|Quebec|637[0n]|BOLD:AAA5206
Zanclognatha jacchusalis|[9628]|RDLQF635-06|Canada|Quebec|622[0n]|BOLD:AAA5206
Zanclognatha jacchusalis|[9629]|LOT197-04|United States|Tennessee|609[0n]|BOLD:AAA5206
Zanclognatha jacchusalis|[9630]|RDLQG090-06|Canada|Quebec|592[0n]|BOLD:AAA5206
Zanclognatha jacchusalis|[9631]|LNCC1010-11|United States|North Carolina|658[0n]|BOLD:AAA5206
Zanclognatha jacchusalis|[9632]|RDLQF645-06|Canada|Quebec|637[0n]|BOLD:AAA5206
Zanclognatha jacchusalis|[9633]|XAE630-04|Canada|Ontario|658[0n]|BOLD:AAA5206
Zanclognatha jacchusalis|[9634]|XAD011-04|Canada|Ontario|658[0n]|BOLD:AAA5206
Zanclognatha jacchusalis|[9635]|XAD007-04|Canada|Ontario|554[0n]|BOLD:AAA5206
Zanclognatha jacchusalis|[9636]|RDNMF096-08|Canada|New Brunswick|628[0n]|BOLD:AAA5206
Zanclognatha jacchusalis|[9637]|RDLQF764-06|Canada|Quebec|658[0n]|BOLD:AAA5206
Zanclognatha jacchusalis|[9638]|RDLQF644-06|Canada|Quebec|643[0n]|BOLD:AAA5206
Zanclognatha jacchusalis|[9639]|RDLQF688-06|Canada|Quebec|637[0n]|BOLD:AAA5206
Zanclognatha jacchusalis|[9640]|RDLQF647-06|Canada|Quebec|637[0n]|BOLD:AAA5206
Zanclognatha jacchusalis|[9641]|RDLQF637-06|Canada|Quebec|602[3n]|BOLD:AAA5206
Zanclognatha jacchusalis|[9642]|RDLQF763-06|Canada|Quebec|621[0n]|BOLD:AAA5206
Zanclognatha jacchusalis|[9643]|ABNCC070-07|United States|Wisconsin|631[0n]|BOLD:AAA5206
Zanclognatha jacchusalis|[9644]|BLTIB920-08|Canada|Ontario|658[0n]|BOLD:AAA5206
Zanclognatha jacchusalis|[9645]|LOT192-04|United States|Tennessee|609[0n]|BOLD:AAA5206
Zanclognatha jacchusalis|[9646]|LOT154-04|United States|Tennessee|609[0n]|BOLD:AAA5206
Zanclognatha jacchusalis|[9647]|LOT151-04|United States|Tennessee|609[0n]|BOLD:AAA5206
Zanclognatha jacchusalis|[9648]|CNSLQ017-13|Canada|Ontario|585[0n]|BOLD:AAA5206
Zanclognatha jacchusalis|[9649]|CNSLR435-13|Canada|Ontario|583[0n]|BOLD:AAA5206
Zanclognatha jacchusalis|[9650]|LOT157-04|United States|Tennessee|572[0n]|BOLD:AAA5206
Zanclognatha jacchusalis|[9651]|XAC856-04|Canada|Ontario|591[0n]|BOLD:AAA5206
Zanclognatha jacchusalis|[9652]|CNROP339-13|Canada|Ontario|568[0n]|BOLD:AAA5206
Zanclognatha jacchusalis|[9653]|CNROP347-13|Canada|Ontario|576[0n]|BOLD:AAA5206
Zanclognatha protumnusalis|[9654]|RDLQG804-06|Canada|Quebec|653[0n]|BOLD:AAA5206
Zanclognatha sp.|[9655]|LNCC1014-11|United States|North Carolina|658[0n]|BOLD:AAA5206
Zanclognatha sp.|[9656]|LGSM712-04|United States|North Carolina|658[0n]|BOLD:AAA5206
Zanclognatha sp.|[9657]|LGSMG489-07|United States|Tennessee|658[0n]|BOLD:AAA5206
Zanclognatha sp.|[9658]|LNCC1015-11|United States|North Carolina|658[0n]|BOLD:AAA5206
Zanclognatha cruralis|[9659]|PHJUN4022-11|Canada|Ontario|658[0n]|BOLD:AAA5206
Zanclognatha cruralis|[9660]|PHJUN4021-11|Canada|Ontario|658[0n]|BOLD:AAA5206
Zanclognatha cruralis|[9661]|RDNMH1037-09|United States|North Carolina|658[0n]|BOLD:AAA5206
Zanclognatha cruralis|[9662]|LPSOC136-08|Canada|Ontario|658[0n]|BOLD:AAA5206
Zanclognatha cruralis|[9663]|LGSMG488-07|United States|North Carolina|658[0n]|BOLD:AAA5206
Zanclognatha cruralis|[9664]|RDLQG035-06|Canada|Quebec|658[0n]|BOLD:AAA5206
Zanclognatha cruralis|[9665]|RDLQF919-06|Canada|Quebec|658[0n]|BOLD:AAA5206
Zanclognatha cruralis|[9666]|RDLQF916-06|Canada|Quebec|658[0n]|BOLD:AAA5206
Zanclognatha cruralis|[9667]|RDLQF915-06|Canada|Quebec|658[0n]|BOLD:AAA5206
Zanclognatha cruralis|[9668]|LOTB174-05|United States|Tennessee|658[0n]|BOLD:AAA5206
Zanclognatha cruralis|[9669]|XAC293-04|Canada|Ontario|658[0n]|BOLD:AAA5206
Zanclognatha cruralis|[9670]|XAB621-04|Canada|Ontario|658[0n]|BOLD:AAA5206
Zanclognatha cruralis|[9671]|ABNCC075-07|United States|Wisconsin|635[0n]|BOLD:AAA5206
Zanclognatha cruralis|[9672]|CNSLO012-13|Canada|Ontario|543[0n]|BOLD:AAA5206
Zanclognatha cruralis|[9673]|BLTIB574-08|Canada|Ontario|631[0n]|
Zanclognatha cruralis|[9674]|CNSLD557-12|Canada|Ontario|632[0n]|BOLD:AAA5206
Zanclognatha cruralis|[9675]|CNSLE003-12|Canada|Ontario|632[0n]|BOLD:AAA5206
Zanclognatha cruralis|[9676]|CNSLD230-12|Canada|Ontario|632[0n]|BOLD:AAA5206
Zanclognatha cruralis|[9677]|CNSLO005-13|Canada|Ontario|546[0n]|BOLD:AAA5206
Zanclognatha cruralis|[9678]|CNSLN111-13|Canada|Ontario|546[0n]|BOLD:AAA5206
Zanclognatha cruralis|[9679]|CNROO664-13|Canada|Ontario|546[0n]|BOLD:AAA5206
Zanclognatha cruralis|[9680]|CNROE067-13|Canada|Ontario|561[0n]|BOLD:AAA5206
Zanclognatha cruralis|[9681]|RBINA3050-13|Canada|Ontario|606[0n]|BOLD:AAA5206
Zanclognatha cruralis|[9682]|RBINA5370-13|Canada|Ontario|577[0n]|BOLD:AAA5206
Zanclognatha cruralis|[9683]|CNROF613-13|Canada|Ontario|570[0n]|BOLD:AAA5206
Zanclognatha cruralis|[9684]|CNSLO014-13|Canada|Ontario|573[0n]|BOLD:AAA5206
Zanclognatha cruralis|[9685]|CNSLO011-13|Canada|Ontario|573[0n]|BOLD:AAA5206
Zanclognatha cruralis|[9686]|[illegible]

Zanclognatha cruralis|[9683]|CNROF615-13|Canada|Ontario|570[0n]|BOLD:AAA5206
Zanclognatha cruralis|[9684]|CNSLO014-13|Canada|Ontario|573[0n]|BOLD:AAA5206
Zanclognatha cruralis|[9685]|CNSLO011-13|Canada|Ontario|573[0n]|BOLD:AAA5206
Zanclognatha cruralis|[9686]|CNSLO015-13|Canada|Ontario|584[0n]|BOLD:AAA5206
Zanclognatha cruralis|[9687]|CNSLO009-13|Canada|Ontario|585[0n]|BOLD:AAA5206
Zanclognatha cruralis|[9688]|CNROF135-13|Canada|Ontario|576[0n]|BOLD:AAA5206
Zanclognatha cruralis|[9689]|CNSLO010-13|Canada|Ontario|567[0n]|BOLD:AAA5206
Zanclognatha cruralis|[9690]|CNSLO006-13|Canada|Ontario|576[0n]|BOLD:AAA5206
Zanclognatha cruralis|[9691]|CNSLO023-13|Canada|Ontario|577[0n]|BOLD:AAA5206
Zanclognatha cruralis|[9692]|CNROO667-13|Canada|Ontario|580[0n]|BOLD:AAA5206
Zanclognatha cruralis|[9693]|CNGBM424-14|Canada|Ontario|537[0n]|BOLD:AAA5206
Zanclognatha obscuripennis|[9694]|LOTB162-05|United States|Tennessee|658[0n]|BOLD:AAA5206
Zanclognatha obscuripennis|[9695]|LOTB100-05|United States|Tennessee|658[0n]|BOLD:AAA5206
Zanclognatha obscuripennis|[9696]|LOTB103-05|United States|Tennessee|658[0n]|BOLD:AAA5206
Zanclognatha obscuripennis|[9697]|LGSM713-04|United States|Tennessee|658[0n]|BOLD:AAA5206
Zanclognatha obscuripennis|[9698]|LGSMC414-05|United States|Tennessee|658[0n]|BOLD:AAA5206
Zanclognatha obscuripennis|[9699]|LGSMC418-05|United States|Tennessee|658[0n]|BOLD:AAA5206
Zanclognatha obscuripennis|[9700]|LOTB164-05|United States|Tennessee|658[0n]|BOLD:AAA5206
Zanclognatha obscuripennis|[9701]|LOTB166-05|United States|Tennessee|658[0n]|BOLD:AAA5206
Zanclognatha obscuripennis|[9702]|LOTB168-05|United States|Tennessee|658[0n]|BOLD:AAA5206
Zanclognatha obscuripennis|[9703]|LOTB171-05|United States|Tennessee|658[0n]|BOLD:AAA5206
Zanclognatha obscuripennis|[9704]|LGSM639-04|United States|Tennessee|609[0n]|BOLD:AAA5206
Zanclognatha obscuripennis|[9705]|LGSMC415-05|United States|Tennessee|658[0n]|BOLD:AAA5206
Zanclognatha obscuripennis|[9706]|LGSMC837-05|United States|Tennessee|658[0n]|BOLD:AAA5206
Zanclognatha obscuripennis|[9707]|LOTB101-05|United States|Tennessee|658[0n]|BOLD:AAA5206
Zanclognatha obscuripennis|[9708]|LOTB105-05|United States|Tennessee|658[0n]|BOLD:AAA5206
Zanclognatha obscuripennis|[9709]|LOTB106-05|United States|Tennessee|658[0n]|BOLD:AAA5206
Zanclognatha obscuripennis|[9710]|LOTB170-05|United States|Tennessee|658[0n]|BOLD:AAA5206
Zanclognatha obscuripennis|[9711]|LOTB172-05|United States|Tennessee|658[0n]|BOLD:AAA5206
Zanclognatha obscuripennis|[9712]|RDNMD501-06|United States|Florida|658[0n]|BOLD:AAA5206
Zanclognatha obscuripennis|[9713]|RDNMH149-09|United States|Florida|638[0n]|BOLD:AAA5206
Zanclognatha obscuripennis|[9714]|LGSMC416-05|United States|Tennessee|658[0n]|BOLD:AAA5206
Zanclognatha obscuripennis|[9715]|LOTB102-05|United States|Tennessee|617[0n]|BOLD:AAA5206
Zanclognatha obscuripennis|[9716]|LNCC1818-13|United States|North Carolina|658[0n]|BOLD:AAA5206
Zanclognatha obscuripennis|[9717]|LNCC1819-13|United States|North Carolina|658[0n]|BOLD:AAA5206
Zanclognatha obscuripennis|[9718]|LNCC1214-11|United States|North Carolina|658[0n]|BOLD:AAA5206
Zanclognatha obscuripennis|[9719]|LNCC1545-13|United States|North Carolina|658[0n]|BOLD:AAA5206
Zanclognatha obscuripennis|[9720]|LNCB538-09|United States|North Carolina|658[0n]|BOLD:AAA5206
Zanclognatha obscuripennis|[9721]|LNCB537-09|United States|North Carolina|658[0n]|BOLD:AAA5206
Zanclognatha obscuripennis|[9722]|HKONS327-08|United States|Florida|658[0n]|BOLD:AAA5206
Zanclognatha obscuripennis|[9723]|LNCB203-06|United States|North Carolina|658[0n]|BOLD:AAA5206
Zanclognatha obscuripennis|[9724]|LNC833-06|United States|North Carolina|658[0n]|BOLD:AAA5206
Zanclognatha obscuripennis|[9725]|LNC830-06|United States|North Carolina|658[0n]|BOLD:AAA5206
Zanclognatha obscuripennis|[9726]|LGSMC877-05|United States|Tennessee|658[0n]|BOLD:AAA5206
Zanclognatha obscuripennis|[9727]|LGSMC417-05|United States|Tennessee|658[0n]|BOLD:AAA5206
Zanclognatha obscuripennis|[9728]|LGSMC413-05|United States|Tennessee|658[0n]|BOLD:AAA5206
Zanclognatha obscuripennis|[9729]|LOT169-04|United States|Tennessee|609[0n]|BOLD:AAA5206
Zanclognatha obscuripennis|[9730]|ABNCC067-07|United States|Florida|598[1n]|BOLD:AAA5206
Zanclognatha obscuripennis|[9731]|ABNCC068-07|United States|Florida|593[0n]|BOLD:AAA5206
Zanclognatha obscuripennis|[9732]|LNCB848-09|United States|Alabama|658[0n]|BOLD:AAA5206
Zanclognatha obscuripennis|[9733]|MILEP346-10|United States|Alabama|658[0n]|BOLD:AAA5206
Zanclognatha obscuripennis|[9734]|LNCC959-11|United States|North Carolina|658[0n]|BOLD:AAA5206
Zanclognatha obscuripennis|[9735]|LNCC1896-13|United States|North Carolina|658[0n]|BOLD:AAA5206
Zanclognatha jacchusalis|[9736]|BBLEC254-09|Canada|Nova Scotia|624[0n]|BOLD:AAA5206
Zanclognatha jacchusalis|[9737]|CNPEQ043-14|Canada|Prince Edward Island|540[0n]|BOLD:AAA5206
Zanclognatha jacchusalis|[9738]|RDLQF702-06|Canada|Quebec|637[0n]|BOLD:AAA5206
Zanclognatha jacchusalis|[9739]|RDNMJ348-11|Canada|Quebec|658[0n]|BOLD:AAA5206
Zanclognatha jacchusalis|[9740]|BBLPE137-09|Canada|Nova Scotia|658[0n]|BOLD:AAA5206
Zanclognatha jacchusalis|[9741]|BBLPE108-09|Canada|Nova Scotia|658[0n]|BOLD:AAA5206
Zanclognatha jacchusalis|[9742]|BBLPE107-09|Canada|Nova Scotia|658[0n]|BOLD:AAA5206
Zanclognatha jacchusalis|[9743]|BBLPE106-09|Canada|Nova Scotia|658[0n]|BOLD:AAA5206
Zanclognatha jacchusalis|[9744]|BBLPE105-09|Canada|Nova Scotia|658[0n]|BOLD:AAA5206
Zanclognatha jacchusalis|[9745]|BBLPE090-09|Canada|Nova Scotia|658[0n]|BOLD:AAA5206
Zanclognatha jacchusalis|[9746]|BBLPE084-09|Canada|Nova Scotia|658[0n]|BOLD:AAA5206
Zanclognatha jacchusalis|[9747]|BBLPE073-09|Canada|Nova Scotia|658[0n]|BOLD:AAA5206
Zanclognatha jacchusalis|[9748]|BBLPE072-09|Canada|Nova Scotia|658[0n]|BOLD:AAA5206
Zanclognatha jacchusalis|[9749]|BBLPE055-09|Canada|Nova Scotia|658[0n]|BOLD:AAA5206
Zanclognatha jacchusalis|[9750]|BBLPE032-09|Canada|Nova Scotia|658[0n]|BOLD:AAA5206
Zanclognatha jacchusalis|[9751]|BBLEC889-09|Canada|Nova Scotia|658[0n]|BOLD:AAA5206
Zanclognatha jacchusalis|[9752]|BBLEC227-09|Canada|Nova Scotia|658[0n]|BOLD:AAA5206
Zanclognatha jacchusalis|[9753]|BBLEC141-09|Canada|Nova Scotia|658[0n]|BOLD:AAA5206
Zanclognatha jacchusalis|[9754]|RDLQF770-06|Canada|Quebec|658[0n]|BOLD:AAA5206
Zanclognatha jacchusalis|[9755]|RDLQF768-06|Canada|Quebec|658[0n]|BOLD:AAA5206
Zanclognatha jacchusalis|[9756]|RDLQF574-06|Canada|Quebec|658[0n]|BOLD:AAA5206
Zanclognatha jacchusalis|[9757]|RDLQF443-06|Canada|Quebec|658[0n]|BOLD:AAA5206
Zanclognatha jacchusalis|[9758]|BBLEC292-09|Canada|Nova Scotia|658[0n]|BOLD:AAA5206
Zanclognatha jacchusalis|[9759]|RDLQF423-06|Canada|Quebec|658[0n]|BOLD:AAA5206
Zanclognatha jacchusalis|[9760]|TMNBB047-06|Canada|New Brunswick|658[0n]|BOLD:AAA5206
Zanclognatha jacchusalis|[9761]|RDLQB418-05|Canada|Quebec|658[0n]|BOLD:AAA5206
Zanclognatha jacchusalis|[9762]|BBLEC948-09|Canada|Nova Scotia|644[0n]|BOLD:AAA5206
Zanclognatha jacchusalis|[9763]|CNPEP066-14|Canada|Prince Edward Island|537[0n]|BOLD:AAA5206
Zanclognatha jacchusalis|[9764]|CNFDF379-14|Canada|New Brunswick|546[0n]|BOLD:AAA5206
Zanclognatha jacchusalis|[9765]|CNPEP074-14|Canada|Prince Edward Island|550[0n]|BOLD:AAA5206
Zanclognatha jacchusalis|[9766]|CNROH651-13|Canada|Ontario|558[0n]|BOLD:AAA5206
Zanclognatha jacchusalis|[9767]|RDLQF704-06|Canada|Quebec|637[0n]|BOLD:AAA5206
Zanclognatha jacchusalis|[9768]|RDLQF703-06|Canada|Quebec|637[0n]|BOLD:AAA5206
Zanclognatha jacchusalis|[9769]|RDLQF687-06|Canada|Quebec|637[0n]|BOLD:AAA5206
Zanclognatha jacchusalis|[9770]|RDLQF643-06|Canada|Quebec|637[0n]|BOLD:AAA5206
Zanclognatha jacchusalis|[9771]|CNKJF035-14|Canada|Nova Scotia|555[0n]|BOLD:AAA5206
Zanclognatha jacchusalis|[9772]|CNPEF075-14|Canada|Prince Edward Island|557[0n]|BOLD:AAA5206
Zanclognatha jacchusalis|[9773]|RDLQF584-06|Canada|Quebec|622[1n]|BOLD:AAA5206
Zanclognatha jacchusalis|[9774]|CNKOF128-14|Canada|New Brunswick|552[0n]|BOLD:AAA5206
Zanclognatha jacchusalis|[9775]|RDLQF585-06|Canada|Quebec|637[0n]|BOLD:AAA5206
Zanclognatha jacchusalis|[9776]|BBLEC195-09|Canada|Nova Scotia|621[0n]|BOLD:AAA5206
Zanclognatha jacchusalis|[9777]|CNSLH302-12|Canada|Ontario|606[0n]|BOLD:AAA5206
Zanclognatha jacchusalis|[9778]|RDLQF642-06|Canada|Quebec|637[0n]|BOLD:AAA5206
Zanclognatha jacchusalis|[9779]|CNSLP1124-13|Canada|Ontario|601[0n]|BOLD:AAA5206
Zanclognatha jacchusalis|[9780]|SSPAC3399-13|Canada|Saskatchewan|600[0n]|BOLD:AAA5206
Zanclognatha jacchusalis|[9781]|RDLQF701-06|Canada|Quebec|637[0n]|BOLD:AAA5206
Zanclognatha jacchusalis|[9782]|CNGBF1756-14|Canada|Ontario|578[0n]|BOLD:AAA5206
Zanclognatha jacchusalis|[9783]|CNGBF1760-14|Canada|Ontario|575[0n]|BOLD:AAA5206
Zanclognatha jacchusalis|[9784]|CNSLQ037-13|Canada|Ontario|564[0n]|BOLD:AAA5206
Zanclognatha jacchusalis|[9785]|RDLQF771-06|Canada|Quebec|585[3n]|BOLD:AAA5206
[illegible]

Zanclognatha jacchusalis|[9783]|CNGBF1760-14|Canada|Ontario|575[0n]|BOLD:AAA5206
Zanclognatha jacchusalis|[9784]|CNSLQ037-13|Canada|Ontario|564[0n]|BOLD:AAA5206
Zanclognatha jacchusalis|[9785]|RDLQF771-06|Canada|Quebec|585[3n]|BOLD:AAA5206
Zanclognatha jacchusalis|[9786]|CNGBF1776-14|Canada|Ontario|576[1n]|BOLD:AAA5206
Zanclognatha jacchusalis|[9787]|CNPEF080-14|Canada|Prince Edward Island|568[0n]|BOLD:AAA5206
Zanclognatha jacchusalis|[9788]|CNPEP075-14|Canada|Prince Edward Island|576[0n]|BOLD:AAA5206
Zanclognatha jacchusalis|[9789]|CNBRN830-14|Canada|Nova Scotia|576[0n]|BOLD:AAA5206
Zanclognatha jacchusalis|[9790]|CNKJN155-14|Canada|Nova Scotia|564[0n]|BOLD:AAA5206
Zanclognatha jacchusalis|[9791]|TMNBB052-06|Canada|New Brunswick|658[0n]|BOLD:AAA5206
Zanclognatha jacchusalis|[9792]|SSPAC074-13|Canada|Saskatchewan|528[0n]|BOLD:AAA5206
Zanclognatha theralis|[9793]|RDNMD333-06|United States|Florida|595[0n]|BOLD:AAA5206
Zanclognatha jacchusalis|[9794]|BBLEC548-09|Canada|Nova Scotia|658[0n]|BOLD:AAA5206
Zanclognatha jacchusalis|[9795]|BBLPB993-10|Canada|Alberta|658[0n]|BOLD:AAA5206
Zanclognatha jacchusalis|[9796]|CNGBN1744-14|Canada|Ontario|537[0n]|BOLD:AAA5206
Zanclognatha jacchusalis|[9797]|LOWCB630-05|Canada|British Columbia|601[1n]|BOLD:AAA5206
Zanclognatha jacchusalis|[9798]|RDNMF098-08|Canada|Alberta|658[0n]|BOLD:AAA5206
Zanclognatha jacchusalis|[9799]|CNSLQ014-13|Canada|Ontario|540[0n]|BOLD:AAA5206
Zanclognatha jacchusalis|[9800]|CNRMF3887-12|Canada|Manitoba|614[0n]|BOLD:AAA5206
Zanclognatha jacchusalis|[9801]|CNBPO524-13|Canada|Ontario|594[0n]|BOLD:AAA5206
Zanclognatha jacchusalis|[9802]|ALLEP432-13|Canada|Ontario|658[0n]|BOLD:AAA5206
Zanclognatha jacchusalis|[9803]|BBLPD782-10|Canada|British Columbia|658[0n]|BOLD:AAA5206
Zanclognatha jacchusalis|[9804]|BBLPD774-10|Canada|British Columbia|658[0n]|BOLD:AAA5206
Zanclognatha jacchusalis|[9805]|BBLPB980-10|Canada|Alberta|658[0n]|BOLD:AAA5206
Zanclognatha jacchusalis|[9806]|BBLPB446-10|Canada|Alberta|658[0n]|BOLD:AAA5206
Zanclognatha jacchusalis|[9807]|BBLPB146-10|Canada|British Columbia|658[0n]|BOLD:AAA5206
Zanclognatha jacchusalis|[9808]|BBLPB145-10|Canada|British Columbia|658[0n]|BOLD:AAA5206
Zanclognatha jacchusalis|[9809]|BBLPB144-10|Canada|British Columbia|658[0n]|BOLD:AAA5206
Zanclognatha jacchusalis|[9810]|BBLPB143-10|Canada|British Columbia|658[0n]|BOLD:AAA5206
Zanclognatha jacchusalis|[9811]|BBLPB142-10|Canada|British Columbia|658[0n]|BOLD:AAA5206
Zanclognatha jacchusalis|[9812]|BBLPA864-10|Canada|British Columbia|658[0n]|BOLD:AAA5206
Zanclognatha jacchusalis|[9813]|RDNMF097-08|Canada|British Columbia|658[0n]|BOLD:AAA5206
Zanclognatha jacchusalis|[9814]|RDNME671-08|Canada|Ontario|658[0n]|BOLD:AAA5206
Zanclognatha jacchusalis|[9815]|RDLQF767-06|Canada|Quebec|658[0n]|BOLD:AAA5206
Zanclognatha jacchusalis|[9816]|BBLPB992-10|Canada|Alberta|658[0n]|BOLD:AAA5206
Zanclognatha jacchusalis|[9817]|LOWCB638-05|Canada|British Columbia|658[0n]|BOLD:AAA5206
Zanclognatha jacchusalis|[9818]|LOWCB634-05|Canada|British Columbia|658[0n]|BOLD:AAA5206
Zanclognatha jacchusalis|[9819]|CNWBG1062-13|Canada|Alberta|543[0n]|BOLD:AAA5206
Zanclognatha jacchusalis|[9820]|CNGBF1759-14|Canada|Ontario|549[0n]|BOLD:AAA5206
Zanclognatha jacchusalis|[9821]|RDLQF646-06|Canada|Quebec|637[0n]|BOLD:AAA5206
Zanclognatha jacchusalis|[9822]|RDLQF639-06|Canada|Quebec|637[0n]|BOLD:AAA5206
Zanclognatha jacchusalis|[9823]|CNSLS036-13|Canada|Ontario|592[2n]|BOLD:AAA5206
Zanclognatha jacchusalis|[9824]|CNGBN1745-14|Canada|Ontario|555[0n]|BOLD:AAA5206
Zanclognatha jacchusalis|[9825]|CNRMF3883-12|Canada|Manitoba|602[0n]|BOLD:AAA5206
Zanclognatha jacchusalis|[9826]|CNRME1850-12|Canada|Manitoba|621[0n]|BOLD:AAA5206
Zanclognatha jacchusalis|[9827]|CNRMF529-12|Canada|Manitoba|618[0n]|BOLD:AAA5206
Zanclognatha jacchusalis|[9828]|CNBPF456-13|Canada|Ontario|614[0n]|BOLD:AAA5206
Zanclognatha jacchusalis|[9829]|CNRME5007-12|Canada|Manitoba|614[0n]|BOLD:AAA5206
Zanclognatha jacchusalis|[9830]|CNRMF3755-12|Canada|Manitoba|634[0n]|BOLD:AAA5206
Zanclognatha jacchusalis|[9831]|CNLMP1943-14|Canada|Quebec|600[0n]|BOLD:AAA5206
Zanclognatha jacchusalis|[9832]|BBLPB976-10|Canada|Alberta|658[0n]|BOLD:AAA5206
Zanclognatha jacchusalis|[9833]|SSPAC3394-13|Canada|Saskatchewan|592[0n]|BOLD:AAA5206
Zanclognatha jacchusalis|[9834]|CNSLG302-12|Canada|Ontario|597[0n]|BOLD:AAA5206
Zanclognatha jacchusalis|[9835]|CNGBN1743-14|Canada|Ontario|561[0n]|BOLD:AAA5206
Zanclognatha jacchusalis|[9836]|CNWBG3117-13|Canada|Alberta|577[0n]|BOLD:AAA5206
Zanclognatha jacchusalis|[9837]|CNRMF528-12|Canada|Manitoba|632[0n]|BOLD:AAA5206
Zanclognatha jacchusalis|[9838]|CNRME4992-12|Canada|Manitoba|631[0n]|BOLD:AAA5206
Zanclognatha jacchusalis|[9839]|CNEID3351-12|Canada|Alberta|633[0n]|BOLD:AAA5206
Zanclognatha jacchusalis|[9840]|CNRMF3748-12|Canada|Manitoba|632[0n]|BOLD:AAA5206
Zanclognatha jacchusalis|[9841]|CNGBN1747-14|Canada|Ontario|591[0n]|BOLD:AAA5206
Zanclognatha jacchusalis|[9842]|CNROG080-13|Canada|Ontario|607[0n]|BOLD:AAA5206
Zanclognatha jacchusalis|[9843]|CNROG069-13|Canada|Ontario|607[0n]|BOLD:AAA5206
Zanclognatha jacchusalis|[9844]|SSPAC3401-13|Canada|Saskatchewan|601[0n]|BOLD:AAA5206
Zanclognatha jacchusalis|[9845]|SSPAC6843-13|Canada|Saskatchewan|594[0n]|BOLD:AAA5206
Zanclognatha jacchusalis|[9846]|CNSLS024-13|Canada|Ontario|579[0n]|BOLD:AAA5206
Zanclognatha jacchusalis|[9847]|CNGBN1751-14|Canada|Ontario|588[0n]|BOLD:AAA5206
Zanclognatha jacchusalis|[9848]|CNGBF1778-14|Canada|Ontario|590[0n]|BOLD:AAA5206
Zanclognatha jacchusalis|[9849]|CNGBF1767-14|Canada|Ontario|590[0n]|BOLD:AAA5206
Zanclognatha jacchusalis|[9850]|CNGBF1765-14|Canada|Ontario|590[0n]|BOLD:AAA5206
Zanclognatha jacchusalis|[9851]|CNROG083-13|Canada|Ontario|604[0n]|BOLD:AAA5206
Zanclognatha jacchusalis|[9852]|SSPAC8348-13|Canada|Saskatchewan|589[0n]|BOLD:AAA5206
Zanclognatha jacchusalis|[9853]|CNWBG3063-13|Canada|Alberta|592[0n]|BOLD:AAA5206
Zanclognatha jacchusalis|[9854]|CNSLQ011-13|Canada|Ontario|531[0n]|BOLD:AAA5206
Zanclognatha jacchusalis|[9855]|CNGBF1779-14|Canada|Ontario|566[0n]|BOLD:AAA5206
Zanclognatha jacchusalis|[9856]|SSPAC6829-13|Canada|Saskatchewan|567[0n]|BOLD:AAA5206
Zanclognatha jacchusalis|[9857]|SSPAC6825-13|Canada|Saskatchewan|567[0n]|BOLD:AAA5206
Zanclognatha jacchusalis|[9858]|SSPAC6799-13|Canada|Saskatchewan|567[0n]|BOLD:AAA5206
Zanclognatha jacchusalis|[9859]|CNSLR433-13|Canada|Ontario|573[0n]|BOLD:AAA5206
Zanclognatha jacchusalis|[9860]|CNROR004-13|Canada|Ontario|588[0n]|BOLD:AAA5206
Zanclognatha jacchusalis|[9861]|SSPAC6841-13|Canada|Saskatchewan|582[0n]|BOLD:AAA5206
Zanclognatha jacchusalis|[9862]|SSPAC6842-13|Canada|Saskatchewan|577[0n]|BOLD:AAA5206
Zanclognatha jacchusalis|[9863]|SSPAC6837-13|Canada|Saskatchewan|576[0n]|BOLD:AAA5206
Zanclognatha jacchusalis|[9864]|SSPAC6832-13|Canada|Saskatchewan|576[0n]|BOLD:AAA5206
Zanclognatha jacchusalis|[9865]|SSPAC6828-13|Canada|Saskatchewan|576[0n]|BOLD:AAA5206
Zanclognatha jacchusalis|[9866]|SSPAC6816-13|Canada|Saskatchewan|576[0n]|BOLD:AAA5206
Zanclognatha jacchusalis|[9867]|SSPAC6811-13|Canada|Saskatchewan|576[0n]|BOLD:AAA5206
Zanclognatha jacchusalis|[9868]|SSPAC3393-13|Canada|Saskatchewan|584[0n]|BOLD:AAA5206
Zanclognatha jacchusalis|[9869]|CNWBG3115-13|Canada|Alberta|570[0n]|BOLD:AAA5206
Zanclognatha jacchusalis|[9870]|CNWBG1034-13|Canada|Alberta|558[0n]|BOLD:AAA5206
Zanclognatha jacchusalis|[9871]|CNSLF933-12|Canada|Ontario|591[0n]|BOLD:AAA5206
Zanclognatha jacchusalis|[9872]|LOWCB635-05|Canada|British Columbia|585[0n]|BOLD:AAA5206
Zanclognatha jacchusalis|[9873]|LOWCB636-05|Canada|British Columbia|585[2n]|BOLD:AAA5206
Zanclognatha jacchusalis|[9874]|LOWCB633-05|Canada|British Columbia|584[1n]|BOLD:AAA5206
Zanclognatha jacchusalis|[9875]|SSPAC8334-13|Canada|Saskatchewan|545[0n]|BOLD:AAA5206
Zanclognatha jacchusalis|[9876]|CNROQ382-13|Canada|Ontario|576[0n]|BOLD:AAA5206
Zanclognatha jacchusalis|[9877]|CNGBF1762-14|Canada|Ontario|576[0n]|BOLD:AAA5206
Zanclognatha jacchusalis|[9878]|CNGBF1764-14|Canada|Ontario|576[0n]|BOLD:AAA5206
Zanclognatha jacchusalis|[9879]|CNKJF016-14|Canada|Nova Scotia|567[0n]|BOLD:AAA5206
Zanclognatha jacchusalis|[9880]|CNLMP1963-14|Canada|Quebec|594[0n]|BOLD:AAA5206
Zanclognatha jacchusalis|[9881]|CNLMP1986-14|Canada|Quebec|591[0n]|BOLD:AAA5206
Zanclognatha obscuripennis|[9882]|LMEM200-09|United States|Alabama|658[0n]|BOLD:AAA5206
Zanclognatha obscuripennis|[9883]|LMEM189-09|United States|Mississippi|658[0n]|BOLD:AAA5206
Zanclognatha obscuripennis|[9884]|LPOKA399-09|United States|Oklahoma|658[0n]|BOLD:AAA5206
Zanclognatha obscuripennis|[9885]|LNCB205-06|United States|North Carolina|658[0n]|BOLD:AAA5206

Zanclognatha obscuripennis|[9883]|LMEM189-09|United States|Mississippi|658[0n]|BOLD:AAA5206
Zanclognatha obscuripennis|[9884]|LPOKA399-09|United States|Oklahoma|658[0n]|BOLD:AAA5206
Zanclognatha obscuripennis|[9885]|LNCB205-06|United States|North Carolina|658[0n]|BOLD:AAA5206
Zanclognatha obscuripennis|[9886]|LSEU360-06|United States|Georgia|658[0n]|BOLD:AAA5206
Zanclognatha obscuripennis|[9887]|LOTB169-05|United States|Tennessee|658[0n]|BOLD:AAA5206
Zanclognatha obscuripennis|[9888]|LOTB104-05|United States|Tennessee|658[0n]|BOLD:AAA5206
Zanclognatha obscuripennis|[9889]|LOT168-04|United States|Tennessee|609[0n]|BOLD:AAA5206
Zanclognatha obscuripennis|[9890]|LMEM202-09|United States|Alabama|623[0n]|BOLD:AAA5206
Zanclognatha obscuripennis|[9891]|LMEM205-09|United States|Mississippi|658[0n]|BOLD:AAA5206
Zanclognatha obscuripennis|[9892]|LGSMC841-05|United States|Tennessee|658[0n]|BOLD:AAA5206
Zanclognatha obscuripennis|[9893]|ABNCC072-07|United States|Texas|636[0n]|BOLD:AAA5206
Zanclognatha obscuripennis|[9894]|LMEM190-09|United States|Mississippi|658[0n]|BOLD:AAA5206
Zanclognatha obscuripennis|[9895]|LMEM201-09|United States|Alabama|658[0n]|BOLD:AAA5206
Zanclognatha obscuripennis|[9896]|LILLA147-11|United States|Illinois|658[0n]|BOLD:AAA5206
Zanclognatha obscuripennis|[9897]|LNCC1213-11|United States|North Carolina|658[0n]|BOLD:AAA5206
Zanclognatha protumnusalis|[9898]|LGSM568-04|United States|Tennessee|658[0n]|BOLD:AAA5206
Zanclognatha protumnusalis|[9899]|LGSMC884-05|United States|Tennessee|658[0n]|BOLD:AAA5206
Zanclognatha sp.|[9900]|LGSMG491-07|United States|Tennessee|658[0n]|BOLD:AAA5206
Zanclognatha sp.|[9901]|LGSMG492-07|United States|Tennessee|658[0n]|BOLD:AAA5206
Zanclognatha sp.|[9902]|LGSMG494-07|United States|North Carolina|658[0n]|BOLD:AAA5206
Zanclognatha sp.|[9903]|LGSMG496-07|United States|North Carolina|658[0n]|BOLD:AAA5206
Zanclognatha minoralis sp. 2|[9904]|HKONS254-08|United States|Florida|658[0n]|BOLD:AAA5206
Zanclognatha sp.|[9905]|HKONS256-08|United States|Florida|653[0n]|BOLD:AAA5206
Zanclognatha minoralis sp. 2|[9906]|HKONS257-08|United States|Florida|658[0n]|BOLD:AAA5206
Zanclognatha sp.|[9907]|LMEM180-09|United States|Alabama|658[0n]|BOLD:AAA5206
Zanclognatha sp.|[9908]|MILEP340-10|United States|Alabama|641[0n]|BOLD:AAA5206
Zanclognatha minoralis sp. 2|[9909]|HKONS073-07|United States|Florida|658[0n]|BOLD:AAA5206
Zanclognatha minoralis sp. 2|[9910]|HKONB354-09|United States|Texas|658[0n]|BOLD:AAA5206
Zanclognatha minoralis sp. 2|[9911]|HKONB355-09|United States|Texas|658[0n]|BOLD:AAA5206
Zanclognatha sp.|[9912]|CNCLB526-14|United States|Louisiana|658[0n]|BOLD:AAA5206
Zanclognatha theralis|[9913]|CNCLB3005-14|United States|North Carolina|658[0n]|BOLD:AAA5206
Zanclognatha marcidilinea|[9914]|LNCC942-11|United States|North Carolina|658[0n]|BOLD:AAA5206
Zanclognatha protumnusalis|[9915]|LOTB337-05|United States|Tennessee|658[0n]|BOLD:AAA5206
Zanclognatha marcidilinea|[9916]|XAG122-05|Canada|Ontario|658[0n]|BOLD:AAA5206
Zanclognatha protumnusalis|[9917]|RDLQF442-06|Canada|Quebec|658[0n]|BOLD:AAA5206
Zanclognatha protumnusalis|[9918]|BLTIB575-08|Canada|Ontario|644[0n]|BOLD:AAA5206
Zanclognatha protumnusalis|[9919]|RDMAB965-09|Canada|Ontario|618[0n]|BOLD:AAA5206
Zanclognatha protumnusalis|[9920]|RDLQB709-05|Canada|Quebec|606[3n]|BOLD:AAA5206
Zanclognatha protumnusalis|[9921]|LNCC1166-11|United States|North Carolina|658[0n]|BOLD:AAA5206
Zanclognatha protumnusalis|[9922]|RDLQG909-06|Canada|Quebec|658[0n]|BOLD:AAA5206
Zanclognatha protumnusalis|[9923]|RDLQG609-06|Canada|Quebec|658[0n]|BOLD:AAA5206
Zanclognatha protumnusalis|[9924]|RDLQG597-06|Canada|Quebec|658[0n]|BOLD:AAA5206
Zanclognatha protumnusalis|[9925]|RDLQG028-06|Canada|Quebec|658[0n]|BOLD:AAA5206
Zanclognatha protumnusalis|[9926]|RDLQF560-06|Canada|Quebec|658[0n]|BOLD:AAA5206
Zanclognatha protumnusalis|[9927]|RDLQF345-06|Canada|Quebec|658[0n]|BOLD:AAA5206
Zanclognatha protumnusalis|[9928]|TMNBB053-06|Canada|New Brunswick|658[0n]|BOLD:AAA5206
Zanclognatha protumnusalis|[9929]|RDLQG045-06|Canada|Quebec|658[0n]|BOLD:AAA5206
Zanclognatha protumnusalis|[9930]|RDLQB688-05|Canada|Quebec|658[0n]|BOLD:AAA5206
Zanclognatha protumnusalis|[9931]|MNBB080-05|Canada|New Brunswick|658[0n]|BOLD:AAA5206
Zanclognatha protumnusalis|[9932]|PHMO196-03|Canada|Ontario|639[0n]|BOLD:AAA5206
Zanclognatha protumnusalis|[9933]|ABNCC069-07|United States|Wisconsin|641[0n]|BOLD:AAA5206
Zanclognatha protumnusalis|[9934]|LGSMG487-07|United States|Tennessee|658[0n]|BOLD:AAA5206
Zanclognatha protumnusalis|[9935]|LGSMG495-07|United States|North Carolina|658[0n]|BOLD:AAA5206
Zanclognatha protumnusalis|[9936]|BLTIB692-08|Canada|Ontario|658[0n]|
Zanclognatha protumnusalis|[9937]|BBLPC513-09|Canada|New Brunswick|658[0n]|BOLD:AAA5206
Zanclognatha protumnusalis|[9938]|BBLPE153-09|Canada|Nova Scotia|658[0n]|BOLD:AAA5206
Zanclognatha protumnusalis|[9939]|LNCC1164-11|United States|North Carolina|658[0n]|BOLD:AAA5206
Zanclognatha protumnusalis|[9940]|LNCC1165-11|United States|North Carolina|658[0n]|BOLD:AAA5206
Zanclognatha protumnusalis|[9941]|RDLQG774-06|Canada|Quebec|658[0n]|BOLD:AAA5206
Zanclognatha protumnusalis|[9942]|ABNCC115-07|United States|Wisconsin|643[0n]|BOLD:AAA5206
Zanclognatha protumnusalis|[9943]|RDLQF410-06|Canada|Quebec|658[0n]|BOLD:AAA5206
Zanclognatha protumnusalis|[9944]|RDLQF413-06|Canada|Quebec|658[0n]|BOLD:AAA5206
Zanclognatha protumnusalis|[9945]|RDLQF344-06|Canada|Quebec|658[0n]|BOLD:AAA5206
Zanclognatha protumnusalis|[9946]|RDLQF409-06|Canada|Quebec|658[0n]|BOLD:AAA5206
Zanclognatha protumnusalis|[9947]|RDLQF765-06|Canada|Quebec|658[0n]|BOLD:AAA5206
Zanclognatha protumnusalis|[9948]|RDLQF780-06|Canada|Quebec|658[0n]|BOLD:AAA5206
Zanclognatha protumnusalis|[9949]|RDLQF784-06|Canada|Quebec|658[0n]|BOLD:AAA5206
Zanclognatha protumnusalis|[9950]|RDLQF785-06|Canada|Quebec|658[0n]|BOLD:AAA5206
Zanclognatha protumnusalis|[9951]|RDLQG029-06|Canada|Quebec|658[0n]|BOLD:AAA5206
Zanclognatha protumnusalis|[9952]|RDLQG048-06|Canada|Quebec|658[0n]|BOLD:AAA5206
Zanclognatha protumnusalis|[9953]|RDLQG169-06|Canada|Quebec|658[0n]|BOLD:AAA5206
Zanclognatha protumnusalis|[9954]|RDLQG170-06|Canada|Quebec|658[0n]|BOLD:AAA5206
Zanclognatha protumnusalis|[9955]|RDLQG679-06|Canada|Quebec|658[0n]|BOLD:AAA5206
Zanclognatha protumnusalis|[9956]|RDLQG790-06|Canada|Quebec|658[0n]|BOLD:AAA5206
Zanclognatha protumnusalis|[9957]|RDLQG845-06|Canada|Quebec|658[0n]|BOLD:AAA5206
Zanclognatha protumnusalis|[9958]|BLTIB757-08|Canada|Ontario|658[0n]|BOLD:AAA5206
Zanclognatha protumnusalis|[9959]|BLTIB1138-08|Canada|Ontario|658[0n]|BOLD:AAA5206
Zanclognatha protumnusalis|[9960]|BBLEC743-09|Canada|Nova Scotia|658[0n]|BOLD:AAA5206
Zanclognatha protumnusalis|[9961]|BBLPC464-09|Canada|New Brunswick|658[0n]|BOLD:AAA5206
Zanclognatha protumnusalis|[9962]|RDLQF578-06|Canada|Quebec|658[0n]|BOLD:AAA5206
Zanclognatha protumnusalis|[9963]|XAK066-06|Canada|Ontario|658[0n]|BOLD:AAA5206
Zanclognatha protumnusalis|[9964]|XAH218-05|Canada|Ontario|658[0n]|BOLD:AAA5206
Zanclognatha protumnusalis|[9965]|XAG720-05|Canada|Ontario|658[0n]|BOLD:AAA5206
Zanclognatha protumnusalis|[9966]|XAG595-05|Canada|Ontario|658[0n]|BOLD:AAA5206
Zanclognatha protumnusalis|[9967]|XAG331-05|Canada|Ontario|658[0n]|BOLD:AAA5206
Zanclognatha protumnusalis|[9968]|XAG323-05|Canada|Ontario|658[0n]|BOLD:AAA5206
Zanclognatha protumnusalis|[9969]|XAG299-05|Canada|Ontario|658[0n]|BOLD:AAA5206
Zanclognatha protumnusalis|[9970]|XAG099-05|Canada|Ontario|658[0n]|BOLD:AAA5206
Zanclognatha protumnusalis|[9971]|XAD689-05|Canada|Ontario|658[0n]|BOLD:AAA5206
Zanclognatha protumnusalis|[9972]|MNBB029-05|Canada|New Brunswick|658[0n]|BOLD:AAA5206
Zanclognatha protumnusalis|[9973]|XAE604-04|Canada|Ontario|658[0n]|BOLD:AAA5206
Zanclognatha protumnusalis|[9974]|XAC045-04|Canada|Ontario|658[0n]|BOLD:AAA5206
Zanclognatha protumnusalis|[9975]|TMNBB050-06|Canada|New Brunswick|658[0n]|BOLD:AAA5206
Zanclognatha protumnusalis|[9976]|RDLQG789-06|Canada|Quebec|658[0n]|BOLD:AAA5206
Zanclognatha protumnusalis|[9977]|RDLQF407-06|Canada|Quebec|658[0n]|BOLD:AAA5206
Zanclognatha protumnusalis|[9978]|XAC666-04|Canada|Ontario|658[0n]|BOLD:AAA5206
Zanclognatha protumnusalis|[9979]|RDLQF414-06|Canada|Quebec|621[0n]|BOLD:AAA5206
Zanclognatha protumnusalis|[9980]|HEJUL264-12|Canada|Ontario|625[0n]|BOLD:AAA5206
Zanclognatha protumnusalis|[9981]|PMG169-03|Canada|Ontario|617[0n]|BOLD:AAA5206
Zanclognatha protumnusalis|[9982]|XAH227-05|Canada|Ontario|627[0n]|BOLD:AAA5206
Zanclognatha protumnusalis|[9983]|RDMAB964-09|Canada|Ontario|622[0n]|BOLD:AAA5206
Zanclognatha protumnusalis|[9984]|RDLQG117-06|Canada|Quebec|606[2n]|BOLD:AAA5206
Zanclognatha protumnusalis|[9985]|RDLQF781-06|Canada|Quebec|597[1n]|BOLD:AAA5206

Zanclognatha protumnusalis|[9983]|RDMAB964-09|Canada|Ontario|622[0n]|BOLD:AAA5206
Zanclognatha protumnusalis|[9984]|RDLQG117-06|Canada|Quebec|606[2n]|BOLD:AAA5206
Zanclognatha protumnusalis|[9985]|RDLQF781-06|Canada|Quebec|597[1n]|BOLD:AAA5206
Zanclognatha protumnusalis|[9986]|RDLQF418-06|Canada|Quebec|621[0n]|BOLD:AAA5206
Zanclognatha protumnusalis|[9987]|XAE548-04|Canada|Ontario|586[1n]|BOLD:AAA5206
Zanclognatha protumnusalis|[9988]|CNSLP1127-13|Canada|Ontario|612[0n]|BOLD:AAA5206
Zanclognatha protumnusalis|[9989]|RDLQF411-06|Canada|Quebec|658[0n]|BOLD:AAA5206
Zanclognatha protumnusalis|[9990]|RDLQG168-06|Canada|Quebec|594[2n]|BOLD:AAA5206
Zanclognatha protumnusalis|[9991]|CNSLF928-12|Canada|Ontario|603[0n]|BOLD:AAA5206
Zanclognatha protumnusalis|[9992]|CNPEF083-14|Canada|Prince Edward Island|568[0n]|BOLD:AAA5206
Zanclognatha protumnusalis|[9993]|CNLMO2428-14|Canada|Quebec|552[1n]|BOLD:AAA5206
Zanclognatha marcidilinea|[9994]|HKONS136-08|United States|Florida|658[0n]|BOLD:AAA5206
Zanclognatha marcidilinea|[9995]|RDNMH147-09|United States|Florida|658[0n]|BOLD:AAA5206
Zanclognatha marcidilinea|[9996]|RDNMH148-09|United States|Florida|658[0n]|BOLD:AAA5206
Zanclognatha marcidilinea|[9997]|USLEP619-10|United States|Florida|658[0n]|BOLD:AAA5206
Zanclognatha marcidilinea|[9998]|LNCC1862-13|United States|North Carolina|654[1n]|BOLD:AAA5206
Zanclognatha marcidilinea|[9999]|LNCB765-09|United States|North Carolina|655[0n]|BOLD:AAA5206
Zanclognatha marcidilinea|[10000]|LNCC1863-13|United States|North Carolina|658[0n]|BOLD:AAA5206
Zanclognatha marcidilinea|[10001]|LNCC863-11|United States|North Carolina|658[0n]|BOLD:AAA5206
Zanclognatha marcidilinea|[10002]|LILLA934-11|United States|Illinois|658[0n]|BOLD:AAA5206
Zanclognatha marcidilinea|[10003]|RDNMH1038-09|United States|North Carolina|658[0n]|BOLD:AAA5206
Zanclognatha marcidilinea|[10004]|HKONB338-09|United States|Texas|658[0n]|BOLD:AAA5206
Zanclognatha marcidilinea|[10005]|LNC838-06|United States|North Carolina|658[0n]|BOLD:AAA5206
Zanclognatha marcidilinea|[10006]|LOTB173-05|United States|Tennessee|658[0n]|BOLD:AAA5206
Zanclognatha marcidilinea|[10007]|LILLA798-11|United States|Illinois|658[0n]|BOLD:AAA5206
Zanclognatha marcidilinea|[10008]|LOTB161-05|United States|Tennessee|658[0n]|BOLD:AAA5206
Zanclognatha marcidilinea|[10009]|LGSMC840-05|United States|Tennessee|658[0n]|BOLD:AAA5206
Zanclognatha marcidilinea|[10010]|LMEM196-09|United States|Tennessee|658[0n]|BOLD:AAA5206
Zanclognatha marcidilinea|[10011]|LGSMC838-05|United States|Tennessee|658[0n]|BOLD:AAA5206
Zanclognatha marcidilinea|[10012]|CNSLF930-12|Canada|Ontario|564[0n]|BOLD:AAA5206
Zanclognatha marcidilinea|[10013]|LNCC354-10|United States|North Carolina|658[0n]|BOLD:AAA5206
Zanclognatha marcidilinea|[10014]|MILEQ283-11|United States|Alabama|658[0n]|BOLD:AAA5206
Zanclognatha marcidilinea|[10015]|LNCC1016-11|United States|North Carolina|658[0n]|BOLD:AAA5206
Zanclognatha marcidilinea|[10016]|RDNMJ347-11|Canada|Quebec|658[0n]|BOLD:AAA5206
Zanclognatha marcidilinea|[10017]|RDNMJ346-11|Canada|Quebec|658[0n]|BOLD:AAA5206
Zanclognatha marcidilinea|[10018]|BLTIB470-08|Canada|Ontario|658[0n]|BOLD:AAA5206
Zanclognatha marcidilinea|[10019]|HKONS268-08|United States|Florida|656[0n]|BOLD:AAA5206
Zanclognatha marcidilinea|[10020]|RDLQF257-06|Canada|Quebec|658[0n]|BOLD:AAA5206
Zanclognatha marcidilinea|[10021]|LOCT256-05|United States|Connecticut|658[0n]|BOLD:AAA5206
Zanclognatha marcidilinea|[10022]|LILLA998-11|United States|Illinois|658[0n]|BOLD:AAA5206
Zanclognatha marcidilinea|[10023]|LGSMC883-05|United States|Tennessee|658[0n]|BOLD:AAA5206
Zanclognatha marcidilinea|[10024]|LGSMC839-05|United States|Tennessee|658[0n]|BOLD:AAA5206
Zanclognatha marcidilinea|[10025]|ABNCC077-07|United States|Virginia|638[0n]|BOLD:AAA5206
Zanclognatha marcidilinea|[10026]|ABNCC076-07|United States|Virginia|635[0n]|BOLD:AAA5206
Zanclognatha marcidilinea|[10027]|PHMO221-03|Canada|Ontario|639[0n]|BOLD:AAA5206
Zanclognatha marcidilinea|[10028]|BLGSM006-09|Canada|Ontario|644[0n]|BOLD:AAA5206
Zanclognatha marcidilinea|[10029]|RDLQH139-06|Canada|Quebec|636[0n]|BOLD:AAA5206
Zanclognatha marcidilinea|[10030]|XAG135-05|Canada|Ontario|636[0n]|BOLD:AAA5206
Zanclognatha marcidilinea|[10031]|HKONS135-08|United States|Florida|621[0n]|BOLD:AAA5206
Zanclognatha marcidilinea|[10032]|CNSLR027-13|Canada|Ontario|576[0n]|BOLD:AAA5206
Zanclognatha jacchusalis|[10033]|BLTIB1137-08|Canada|Ontario|656[0n]|BOLD:AAA5206
Zanclognatha jacchusalis|[10034]|XAH594-05|Canada|Ontario|658[0n]|BOLD:AAA5206
Zanclognatha jacchusalis|[10035]|RDLQF258-06|Canada|Quebec|658[0n]|BOLD:AAA5206
Zanclognatha jacchusalis|[10036]|RDLQG911-06|Canada|Quebec|658[0n]|BOLD:AAA5206
Zanclognatha jacchusalis|[10037]|BLTIB768-08|Canada|Ontario|658[0n]|BOLD:AAA5206
Zanclognatha jacchusalis|[10038]|BLTIB1098-08|Canada|Ontario|658[0n]|BOLD:AAA5206
Zanclognatha jacchusalis|[10039]|BLTIB1099-08|Canada|Ontario|658[0n]|BOLD:AAA5206
Zanclognatha jacchusalis|[10040]|XAE637-04|Canada|Ontario|658[0n]|BOLD:AAA5206
Zanclognatha jacchusalis|[10041]|BBLPB977-10|Canada|Alberta|658[0n]|BOLD:AAA5206
Zanclognatha jacchusalis|[10042]|SSPAC6804-13|Canada|Saskatchewan|567[0n]|BOLD:AAA5206
Zanclognatha theralis|[10043]|LMEM174-09|United States|Mississippi|658[0n]|BOLD:AAA5206
Zanclognatha minoralis|[10044]|LMEM179-09|United States|Mississippi|615[0n]|BOLD:AAA5206
Zanclognatha jacchusalis|[10045]|LPSOD929-09|Canada|Ontario|658[0n]|BOLD:AAA5206
Zanclognatha sp.|[10046]|LGSMG493-07|United States|North Carolina|658[0n]|BOLD:AAA5206
Zanclognatha sp.|[10047]|LGSMC885-05|United States|Tennessee|658[0n]|BOLD:AAA5206
Zanclognatha|[10048]|BLTIB572-08|Canada|Ontario|609[2n]|BOLD:AAA5206
Zanclognatha jacchusalis|[10049]|RDLQH140-06|Canada|Quebec|615[0n]|BOLD:AAA5206
Zanclognatha|[10050]|CNRMF3876-12|Canada|Manitoba|602[0n]|BOLD:AAA5206
Zanclognatha|[10051]|CNEID3353-12|Canada|Alberta|636[0n]|BOLD:AAA5206
Zanclognatha jacchusalis|[10052]|XAC025-04|Canada|Ontario|582[0n]|BOLD:AAA5206
Zanclognatha jacchusalis|[10053]|MNBB027-05|Canada|New Brunswick|589[0n]|BOLD:AAA5206
Zanclognatha jacchusalis|[10054]|RDLQH141-06|Canada|Quebec|597[2n]|BOLD:AAA5206
Zanclognatha|[10055]|CNSLP058-13|Canada|Ontario|573[0n]|BOLD:AAA5206
Zanclognatha|[10056]|CNLMG005-14|Canada|Quebec|561[2n]|BOLD:AAA5206
Zanclognatha minoralis sp. 2|[10057]|HKONS258-08|United States|Florida|648[0n]|BOLD:AAA5206
Zanclognatha theralis|[10058]|MILEP034-09|United States|North Carolina|658[0n]|BOLD:AAA5206
Zanclognatha theralis|[10059]|CNCLB3006-14|United States|North Carolina|658[0n]|BOLD:AAA5206
Zanclognatha pedipilalis|[10060]|RDLQF917-06|Canada|Quebec|658[0n]|BOLD:ABZ2935
Zanclognatha pedipilalis|[10061]|RDLQG436-06|Canada|Quebec|658[0n]|BOLD:ABZ2935
Zanclognatha pedipilalis|[10062]|RDLQG437-06|Canada|Quebec|658[0n]|BOLD:ABZ2935
Zanclognatha pedipilalis|[10063]|PHMNB420-04|Canada|New Brunswick|658[0n]|BOLD:ABZ2935
Zanclognatha pedipilalis|[10064]|BLTIB442-08|Canada|Ontario|658[0n]|BOLD:ABZ2935
Zanclognatha pedipilalis|[10065]|RDLQF424-06|Canada|Quebec|658[0n]|BOLD:ABZ2935
Zanclognatha pedipilalis|[10066]|RDLQF918-06|Canada|Quebec|658[0n]|BOLD:ABZ2935
Zanclognatha pedipilalis|[10067]|RDLQF920-06|Canada|Quebec|658[0n]|BOLD:ABZ2935
Zanclognatha pedipilalis|[10068]|RDLQF921-06|Canada|Quebec|658[0n]|BOLD:ABZ2935
Zanclognatha pedipilalis|[10069]|RDLQF922-06|Canada|Quebec|658[0n]|BOLD:ABZ2935
Zanclognatha pedipilalis|[10070]|RDLQF438-06|Canada|Quebec|658[0n]|BOLD:ABZ2935
Zanclognatha pedipilalis|[10071]|RDLQF923-06|Canada|Quebec|658[0n]|BOLD:ABZ2935
Zanclognatha pedipilalis|[10072]|LPSOB799-08|Canada|Ontario|658[0n]|BOLD:ABZ2935
Zanclognatha pedipilalis|[10073]|BLTIB267-08|Canada|Ontario|658[0n]|BOLD:ABZ2935
Zanclognatha pedipilalis|[10074]|XAB520-04|Canada|Ontario|658[0n]|BOLD:ABZ2935
Zanclognatha pedipilalis|[10075]|XAC610-04|Canada|Ontario|582[0n]|BOLD:ABZ2935
Zanclognatha pedipilalis|[10076]|BLTIB345-08|Canada|Ontario|643[0n]|BOLD:ABZ2935
Zanclognatha pedipilalis|[10077]|BLTIB440-08|Canada|Ontario|658[0n]|BOLD:ABZ2935
Zanclognatha pedipilalis|[10078]|CNCLB2951-14|United States|North Carolina|658[0n]|BOLD:ABZ2935
Zanclognatha pedipilalis|[10079]|LOCT257-05|United States|Connecticut|658[0n]|BOLD:ABZ2935
Zanclognatha pedipilalis|[10080]|LGSM535-04|United States|North Carolina|658[0n]|BOLD:ABZ2935
Zanclognatha pedipilalis|[10081]|LGSMD675-05|United States|North Carolina|658[0n]|BOLD:ABZ2935
Zanclognatha pedipilalis|[10082]|LGSM536-04|United States|North Carolina|658[0n]|BOLD:ABZ2935
Zanclognatha pedipilalis|[10083]|LGSMC946-05|United States|Tennessee|658[2n]|BOLD:ABZ2935
Zanclognatha pedipilalis|[10084]|LNCB528-07|United States|North Carolina|658[0n]|BOLD:ABZ2935
Zanclognatha pedipilalis|[10085]|LGSMG485-07|United States|Tennessee|658[0n]|BOLD:ABZ2935

Zanclognatha pedipilalis|[10083]|LGSMC946-05|United States|Tennessee|658[2n]|BOLD:ABZ2935
Zanclognatha pedipilalis|[10084]|LNCB528-07|United States|North Carolina|658[0n]|BOLD:ABZ2935
Zanclognatha pedipilalis|[10085]|LGSMG485-07|United States|Tennessee|658[0n]|BOLD:ABZ2935
Zanclognatha pedipilalis|[10086]|LGSMG486-07|United States|Tennessee|658[0n]|BOLD:ABZ2935
Zanclognatha pedipilalis|[10087]|LPSO563-08|Canada|Ontario|658[0n]|BOLD:ABZ2935
Zanclognatha pedipilalis|[10088]|LPSO565-08|Canada|Ontario|658[0n]|BOLD:ABZ2935
Zanclognatha pedipilalis|[10089]|LPSO571-08|Canada|Ontario|658[0n]|BOLD:ABZ2935
Zanclognatha pedipilalis|[10090]|LNCC352-10|United States|North Carolina|658[0n]|BOLD:ABZ2935
Zanclognatha pedipilalis|[10091]|LNCC353-10|United States|North Carolina|658[0n]|BOLD:ABZ2935
Zanclognatha pedipilalis|[10092]|CNCLB2783-14|United States|North Carolina|658[0n]|BOLD:ABZ2935
Zanclognatha pedipilalis|[10093]|CNCLB2952-14|United States|North Carolina|658[0n]|BOLD:ABZ2935
Zanclognatha laevigata|[10094]|RDLQG198-06|Canada|Quebec|658[0n]|BOLD:ACH2253
Zanclognatha laevigata|[10095]|GMGSH415-12|United States|Tennessee|673[0n]|BOLD:ACH2253
Chytolita sp. 2|[10096]|LGSM547-04|United States|Tennessee|658[0n]|BOLD:ABZ6822
Chytolita sp. 2|[10097]|LGSMG499-07|United States|Tennessee|631[0n]|BOLD:ABZ6822
Chytolita|[10098]|CNEIB1524-12|Canada|Alberta|661[0n]|BOLD:ABZ6822
Chytolita|[10099]|SSEIA4402-13|Canada|Alberta|658[0n]|BOLD:ABZ6822
Chytolita sp. 1|[10100]|QUNOD679-11|United States|Minnesota|658[0n]|BOLD:AAA2868
Chytolita sp. 1|[10101]|MNAE841-13|Canada|Ontario|658[0n]|BOLD:AAA2868
Chytolita sp. 1|[10102]|BBLPA641-10|Canada|Ontario|658[0n]|BOLD:AAA2868
Chytolita sp. 1|[10103]|RDNML262-13|Canada|Ontario|658[0n]|BOLD:AAA2868
Chytolita sp. 1|[10104]|RDNML263-13|Canada|Ontario|658[0n]|BOLD:AAA2868
Chytolita sp. 1|[10105]|LGSMC913-05|United States|Tennessee|658[0n]|BOLD:AAA2868
Chytolita sp. 1|[10106]|LNC799-06|United States|North Carolina|658[0n]|BOLD:AAA2868
Chytolita sp. 1|[10107]|LGSMG500-07|United States|Tennessee|658[0n]|BOLD:AAA2868
Chytolita sp. 1|[10108]|HKONS267-08|United States|Florida|658[0n]|BOLD:AAA2868
Chytolita sp. 1|[10109]|LMEM207-09|United States|Mississippi|658[0n]|BOLD:AAA2868
Chytolita sp. 1|[10110]|LMEM209-09|United States|Mississippi|658[0n]|BOLD:AAA2868
Chytolita sp. 1|[10111]|LMEM212-09|United States|Mississippi|658[0n]|BOLD:AAA2868
Chytolita sp. 1|[10112]|LPOKB325-09|United States|Oklahoma|658[0n]|BOLD:AAA2868
Chytolita sp. 1|[10113]|LNCB531-09|United States|North Carolina|658[0n]|BOLD:AAA2868
Chytolita sp. 1|[10114]|LNCB532-09|United States|North Carolina|658[0n]|BOLD:AAA2868
Chytolita sp. 1|[10115]|MILEP013-09|United States|North Carolina|658[0n]|BOLD:AAA2868
Chytolita sp. 1|[10116]|LNCB769-09|United States|North Carolina|658[0n]|BOLD:AAA2868
Chytolita sp. 1|[10117]|LNCB782-09|United States|North Carolina|658[0n]|BOLD:AAA2868
Chytolita sp. 1|[10118]|LNCB783-09|United States|North Carolina|658[0n]|BOLD:AAA2868
Chytolita sp. 1|[10119]|LILLA091-11|United States|Illinois|658[0n]|BOLD:AAA2868
Chytolita sp. 1|[10120]|LILLA102-11|United States|Illinois|658[0n]|BOLD:AAA2868
Chytolita sp. 1|[10121]|LNCC958-11|United States|North Carolina|658[0n]|BOLD:AAA2868
Chytolita sp. 1|[10122]|LNCC1955-14|United States|North Carolina|658[0n]|BOLD:AAA2868
Chytolita sp. 1|[10123]|RDLQF538-06|Canada|Quebec|658[0n]|BOLD:AAA2868
Chytolita sp. 1|[10124]|RDLQF924-06|Canada|Quebec|658[0n]|BOLD:AAA2868
Chytolita sp. 1|[10125]|LPSOC151-08|Canada|Ontario|658[0n]|BOLD:AAA2868
Chytolita sp. 1|[10126]|LPSOC156-08|Canada|Ontario|658[0n]|BOLD:AAA2868
Chytolita sp. 1|[10127]|LPSOB810-08|Canada|Ontario|658[0n]|BOLD:AAA2868
Chytolita sp. 1|[10128]|LILLA655-11|United States|Illinois|658[0n]|BOLD:AAA2868
Chytolita petrealis|[10129]|CNCLB1458-14|United States|Louisiana|658[0n]|BOLD:AAA2868
Chytolita morbidalis|[10130]|LPSOB669-08|Canada|Ontario|647[0n]|BOLD:AAA2868
Chytolita morbidalis|[10131]|LPSOB647-08|Canada|Ontario|647[0n]|BOLD:AAA2868
Chytolita morbidalis|[10132]|LPSOB965-08|Canada|Ontario|658[0n]|BOLD:AAA2868
Chytolita morbidalis|[10133]|BLTIB246-08|Canada|Ontario|658[0n]|BOLD:AAA2868
Chytolita morbidalis|[10134]|LMEM214-09|United States|Alabama|658[0n]|BOLD:AAA2868
Chytolita morbidalis|[10135]|LPSOB956-08|Canada|Ontario|658[0n]|BOLD:AAA2868
Chytolita morbidalis|[10136]|LPSOB655-08|Canada|Ontario|658[0n]|BOLD:AAA2868
Chytolita morbidalis|[10137]|LPSOB627-08|Canada|Ontario|658[0n]|BOLD:AAA2868
Chytolita morbidalis|[10138]|LPSOC234-08|Canada|Ontario|658[0n]|BOLD:AAA2868
Chytolita morbidalis|[10139]|LPSOC148-08|Canada|Ontario|658[0n]|BOLD:AAA2868
Chytolita morbidalis|[10140]|LPSOC039-08|Canada|Ontario|658[0n]|BOLD:AAA2868
Chytolita morbidalis|[10141]|LPSOC038-08|Canada|Ontario|658[0n]|BOLD:AAA2868
Chytolita morbidalis|[10142]|XAI058-05|Canada|Ontario|658[0n]|BOLD:AAA2868
Chytolita morbidalis|[10143]|LGSMC439-05|United States|Tennessee|658[0n]|BOLD:AAA2868
Chytolita morbidalis|[10144]|LGSMC438-05|United States|Tennessee|658[0n]|BOLD:AAA2868
Chytolita morbidalis|[10145]|LPSOC240-08|Canada|Ontario|658[0n]|BOLD:AAA2868
Chytolita morbidalis|[10146]|RDLQF219-06|Canada|Quebec|658[0n]|BOLD:AAA2868
Chytolita morbidalis|[10147]|RDLQF213-06|Canada|Quebec|658[0n]|BOLD:AAA2868
Chytolita morbidalis|[10148]|CNSLN114-13|Canada|Ontario|546[0n]|BOLD:AAA2868
Chytolita morbidalis|[10149]|CNROE070-13|Canada|Ontario|576[0n]|BOLD:AAA2868
Chytolita morbidalis|[10150]|BLTIB253-08|Canada|Ontario|658[0n]|BOLD:AAA2868
Chytolita morbidalis|[10151]|LMEM213-09|United States|West Virginia|658[0n]|BOLD:AAA2868
Chytolita morbidalis|[10152]|RDLQF216-06|Canada|Quebec|655[0n]|BOLD:AAA2868
Chytolita morbidalis|[10153]|LNCB202-06|United States|North Carolina|658[0n]|BOLD:AAA2868
Chytolita morbidalis|[10154]|LOCT071-05|United States|Connecticut|658[0n]|BOLD:AAA2868
Chytolita morbidalis|[10155]|LGSMC435-05|United States|Tennessee|658[0n]|BOLD:AAA2868
Chytolita morbidalis|[10156]|LGSMC434-05|United States|Tennessee|658[0n]|BOLD:AAA2868
Chytolita morbidalis|[10157]|RDLQF212-06|Canada|Quebec|658[0n]|BOLD:AAA2868
Chytolita morbidalis|[10158]|CNSLO008-13|Canada|Ontario|583[0n]|BOLD:AAA2868
Chytolita morbidalis|[10159]|CNROO669-13|Canada|Ontario|580[0n]|BOLD:AAA2868
Chytolita morbidalis|[10160]|RDLQF214-06|Canada|Quebec|658[0n]|BOLD:AAA2868
Chytolita morbidalis|[10161]|LMEM215-09|United States|Alabama|658[0n]|BOLD:AAA2868
Chytolita morbidalis|[10162]|CNWBB131-13|Canada|Alberta|555[0n]|BOLD:AAA2868
Chytolita morbidalis|[10163]|RDNML267-13|Canada|Ontario|658[0n]|BOLD:AAA2868
Chytolita morbidalis|[10164]|RDLQF222-06|Canada|Quebec|658[0n]|BOLD:AAA2868
Chytolita morbidalis|[10165]|RDLQG439-06|Canada|Quebec|658[0n]|BOLD:AAA2868
Chytolita morbidalis|[10166]|LPSOB668-08|Canada|Ontario|645[0n]|BOLD:AAA2868
Chytolita morbidalis|[10167]|RDNML265-13|Canada|Ontario|614[0n]|BOLD:AAA2868
Chytolita morbidalis|[10168]|PHMNB224-04|Canada|New Brunswick|580[0n]|BOLD:AAA2868
Chytolita morbidalis|[10169]|LPMN122-08|Canada|Manitoba|654[0n]|BOLD:AAA2868
Chytolita morbidalis|[10170]|LPMN385-08|Canada|Manitoba|658[0n]|BOLD:AAA2868
Chytolita morbidalis|[10171]|LPMN613-08|Canada|Manitoba|658[0n]|BOLD:AAA2868
Chytolita morbidalis|[10172]|LPMN801-08|Canada|Manitoba|658[0n]|BOLD:AAA2868
Chytolita morbidalis|[10173]|LPMN848-08|Canada|Manitoba|657[0n]|BOLD:AAA2868
Chytolita morbidalis|[10174]|RDLQG440-06|Canada|Quebec|658[0n]|BOLD:AAA2868
Chytolita morbidalis|[10175]|RDNM911-05|Canada|Ontario|658[0n]|BOLD:AAA2868
Chytolita morbidalis|[10176]|RDLQG247-06|Canada|Quebec|658[0n]|BOLD:AAA2868
Chytolita morbidalis|[10177]|LPSOB659-08|Canada|Ontario|646[0n]|BOLD:AAA2868
Chytolita morbidalis|[10178]|BBLPD459-10|Canada|Ontario|658[0n]|BOLD:AAA2868
Chytolita morbidalis|[10179]|BBLPD437-10|Canada|Ontario|658[0n]|BOLD:AAA2868
Chytolita morbidalis|[10180]|BBLPD150-10|Canada|Saskatchewan|658[0n]|BOLD:AAA2868
Chytolita morbidalis|[10181]|BBLPC147-09|Canada|Nova Scotia|658[0n]|BOLD:AAA2868
Chytolita morbidalis|[10182]|LPMN475-08|Canada|Manitoba|658[0n]|BOLD:AAA2868
Chytolita morbidalis|[10183]|LPMN474-08|Canada|Manitoba|658[0n]|BOLD:AAA2868
Chytolita morbidalis|[10184]|LPMN471-08|Canada|Manitoba|658[0n]|BOLD:AAA2868
Chytolita morbidalis|[10185]|LPMN415-08|Canada|Manitoba|655[0n]|BOLD:AAA2868

[illegible]
Chytolita morbidalis|[10183]|LPMN474-08|Canada|Manitoba|658[0n]|BOLD:AAA2868
Chytolita morbidalis|[10184]|LPMN471-08|Canada|Manitoba|658[0n]|BOLD:AAA2868
Chytolita morbidalis|[10185]|LPMN415-08|Canada|Manitoba|655[0n]|BOLD:AAA2868
Chytolita morbidalis|[10186]|LPSOC165-08|Canada|Ontario|658[0n]|BOLD:AAA2868
Chytolita morbidalis|[10187]|RDLQF217-06|Canada|Quebec|658[0n]|BOLD:AAA2868
Chytolita morbidalis|[10188]|LOCT072-05|United States|Connecticut|658[0n]|BOLD:AAA2868
Chytolita morbidalis|[10189]|RDLQG441-06|Canada|Quebec|621[0n]|BOLD:AAA2868
Chytolita morbidalis|[10190]|CNRMD2065-12|Canada|Manitoba|634[0n]|BOLD:AAA2868
Chytolita morbidalis|[10191]|CNRMD2066-12|Canada|Manitoba|633[0n]|BOLD:AAA2868
Chytolita morbidalis|[10192]|CNEIC3025-12|Canada|Alberta|631[0n]|BOLD:AAA2868
Chytolita morbidalis|[10193]|CNRME4983-12|Canada|Manitoba|613[0n]|BOLD:AAA2868
Chytolita morbidalis|[10194]|RDLQG248-06|Canada|Quebec|607[0n]|BOLD:AAA2868
Chytolita morbidalis|[10195]|CNWBB135-13|Canada|Alberta|582[0n]|BOLD:AAA2868
Chytolita morbidalis|[10196]|CNCLA2588-13|Canada|Ontario|658[0n]|BOLD:AAA2868
Chytolita morbidalis|[10197]|RDLQF440-06|Canada|Quebec|639[0n]|BOLD:AAA2868
Chytolita morbidalis|[10198]|CNSLN112-13|Canada|Ontario|573[0n]|BOLD:AAA2868
Chytolita morbidalis|[10199]|LPMN354-08|Canada|Manitoba|658[0n]|BOLD:AAA2868
Chytolita morbidalis|[10200]|QUNOB534-09|United States|Kentucky|658[0n]|BOLD:AAA2868
Chytolita morbidalis|[10201]|LOTB159-05|United States|Tennessee|658[0n]|BOLD:AAA2868
Chytolita morbidalis|[10202]|LMEM210-09|United States|Alabama|621[0n]|BOLD:AAA2868
Chytolita morbidalis|[10203]|RDNML266-13|Canada|Ontario|602[1n]|BOLD:AAA2868
Chytolita morbidalis|[10204]|RWWB859-10|United States|Washington|634[0n]|BOLD:AAA2868
Chytolita morbidalis|[10205]|RWWB845-10|United States|Washington|634[0n]|BOLD:AAA2868
Chytolita morbidalis|[10206]|BBLPD119-10|Canada|Saskatchewan|658[0n]|BOLD:AAA2868
Chytolita morbidalis|[10207]|RWWC278-11|United States|Washington|658[0n]|BOLD:AAA2868
Chytolita morbidalis|[10208]|RWWC410-11|United States|Washington|658[0n]|BOLD:AAA2868
Chytolita morbidalis|[10209]|RDNML264-13|Canada|Ontario|658[0n]|BOLD:AAA2868
Chytolita morbidalis|[10210]|RWWB849-10|United States|Washington|658[0n]|BOLD:AAA2868
Chytolita morbidalis|[10211]|RWWB773-10|United States|Washington|658[0n]|BOLD:AAA2868
Chytolita morbidalis|[10212]|RWWB740-10|United States|Washington|658[0n]|BOLD:AAA2868
Chytolita morbidalis|[10213]|RWWA739-09|United States|Washington|658[0n]|BOLD:AAA2868
Chytolita morbidalis|[10214]|RWWA513-09|United States|Washington|658[0n]|BOLD:AAA2868
Chytolita morbidalis|[10215]|RWWA394-09|United States|Washington|658[0n]|BOLD:AAA2868
Chytolita morbidalis|[10216]|RWWA233-09|United States|Washington|658[0n]|BOLD:AAA2868
Chytolita morbidalis|[10217]|LPMN655-08|Canada|Manitoba|658[0n]|BOLD:AAA2868
Chytolita morbidalis|[10218]|RDLQF925-06|Canada|Quebec|658[0n]|BOLD:AAA2868
Chytolita morbidalis|[10219]|RDLQF848-06|Canada|Quebec|658[0n]|BOLD:AAA2868
Chytolita morbidalis|[10220]|RDLQF220-06|Canada|Quebec|658[0n]|BOLD:AAA2868
Chytolita morbidalis|[10221]|RDLQF218-06|Canada|Quebec|658[0n]|BOLD:AAA2868
Chytolita morbidalis|[10222]|RDLQF215-06|Canada|Quebec|658[0n]|BOLD:AAA2868
Chytolita morbidalis|[10223]|RDNM909-05|Canada|British Columbia|658[0n]|BOLD:AAA2868
Chytolita morbidalis|[10224]|LOWCC895-05|Canada|British Columbia|523[1n]|BOLD:AAA2868
Chytolita morbidalis|[10225]|RWWA920-09|United States|Washington|615[0n]|BOLD:AAA2868
Chytolita morbidalis|[10226]|RDNM910-05|Canada|British Columbia|573[0n]|BOLD:AAA2868
Chytolita morbidalis|[10227]|SSPAC082-13|Canada|Saskatchewan|583[0n]|BOLD:AAA2868
Chytolita morbidalis|[10228]|LPSOD882-09|Canada|Ontario|658[0n]|BOLD:AAA2868
Chytolita morbidalis|[10229]|LPMN824-08|Canada|Manitoba|658[0n]|BOLD:AAA2868
Chytolita morbidalis|[10230]|LPMN821-08|Canada|Manitoba|658[0n]|BOLD:AAA2868
Chytolita morbidalis|[10231]|LPMN163-08|Canada|Manitoba|658[0n]|BOLD:AAA2868
Chytolita morbidalis|[10232]|LPSOB660-08|Canada|Ontario|658[0n]|BOLD:AAA2868
Chytolita morbidalis|[10233]|RDLQF441-06|Canada|Quebec|658[0n]|BOLD:AAA2868
Chytolita morbidalis|[10234]|RDLQB242-05|Canada|Quebec|658[0n]|BOLD:AAA2868
Chytolita morbidalis|[10235]|LPMN470-08|Canada|Manitoba|658[0n]|BOLD:AAA2868
Chytolita morbidalis|[10236]|RDLQF439-06|Canada|Quebec|621[0n]|BOLD:AAA2868
Chytolita morbidalis|[10237]|CNRMD2069-12|Canada|Manitoba|636[0n]|BOLD:AAA2868
Chytolita morbidalis|[10238]|CNRMD2110-12|Canada|Manitoba|636[0n]|BOLD:AAA2868
Chytolita morbidalis|[10239]|RDLQF221-06|Canada|Quebec|658[0n]|BOLD:AAA2868
Chytolita morbidalis|[10240]|LGSMC437-05|United States|Tennessee|658[0n]|BOLD:AAA2868
Chytolita morbidalis|[10241]|RDLQH137-06|Canada|Quebec|589[1n]|BOLD:AAA2868
Chytolita morbidalis|[10242]|LGSMC436-05|United States|Tennessee|658[0n]|BOLD:AAA2868
Chytolita morbidalis|[10243]|LOTB160-05|United States|Tennessee|658[0n]|BOLD:AAA2868
Chytolita morbidalis|[10244]|LOTB163-05|United States|Tennessee|658[0n]|BOLD:AAA2868
Chytolita morbidalis|[10245]|LOTB167-05|United States|Tennessee|658[0n]|BOLD:AAA2868
Chytolita morbidalis|[10246]|LOTB175-05|United States|Tennessee|658[0n]|BOLD:AAA2868
Chytolita morbidalis|[10247]|LMEM211-09|United States|Alabama|658[0n]|BOLD:AAA2868
Chytolita morbidalis|[10248]|CNCLA2586-13|Canada|Ontario|658[0n]|BOLD:AAA2868
Chytolita morbidalis|[10249]|CNCLA2587-13|Canada|Ontario|658[0n]|BOLD:AAA2868
Chytolita morbidalis|[10250]|CNCLB2785-14|United States|North Carolina|658[0n]|BOLD:AAA2868
Zanclognatha lituralis|[10251]|LGSMC891-05|United States|Tennessee|658[0n]|BOLD:AAB3774
Zanclognatha lituralis|[10252]|HKONB378-09|United States|Kentucky|658[0n]|BOLD:AAB3774
Zanclognatha lituralis|[10253]|RDLQH151-06|Canada|Quebec|566[3n]|BOLD:AAB3774
Zanclognatha lituralis|[10254]|HKONB450-09|United States|Kentucky|658[0n]|BOLD:AAB3774
Zanclognatha lituralis|[10255]|LNCC1019-11|United States|North Carolina|658[0n]|BOLD:AAB3774
Zanclognatha lituralis|[10256]|CNSLE373-12|Canada|Ontario|613[0n]|BOLD:AAB3774
Zanclognatha lituralis|[10257]|CNSLE372-12|Canada|Ontario|618[0n]|BOLD:AAB3774
Zanclognatha lituralis|[10258]|LSEU710-06|United States|Georgia|658[0n]|BOLD:AAB3774
Zanclognatha lituralis|[10259]|LNCC1020-11|United States|North Carolina|658[0n]|BOLD:AAB3774
Zanclognatha lituralis|[10260]|LNCC1018-11|United States|North Carolina|658[0n]|BOLD:AAB3774
Zanclognatha lituralis|[10261]|LNCC1017-11|United States|North Carolina|658[0n]|BOLD:AAB3774
Zanclognatha lituralis|[10262]|LSEU731-06|United States|Georgia|658[0n]|BOLD:AAB3774
Zanclognatha lituralis|[10263]|LOTB128-05|United States|Tennessee|658[0n]|BOLD:AAB3774
Zanclognatha lituralis|[10264]|LSEU709-06|United States|Georgia|658[1n]|BOLD:AAB3774
Zanclognatha lituralis|[10265]|CNSLO022-13|Canada|Ontario|549[0n]|BOLD:AAB3774
Zanclognatha lituralis|[10266]|CNSLP1131-13|Canada|Ontario|598[0n]|BOLD:AAB3774
Zanclognatha lituralis|[10267]|RDLQF535-06|Canada|Quebec|658[0n]|BOLD:AAB3774
Zanclognatha lituralis|[10268]|CNSLF929-12|Canada|Ontario|561[0n]|BOLD:AAB3774
Zanclognatha lituralis|[10269]|CNSLF931-12|Canada|Ontario|576[0n]|BOLD:AAB3774
Zanclognatha lituralis|[10270]|CNSLO018-13|Canada|Ontario|564[0n]|BOLD:AAB3774
Zanclognatha lituralis|[10271]|CNSLP1126-13|Canada|Ontario|612[0n]|BOLD:AAB3774
Zanclognatha lituralis|[10272]|CNSLP1132-13|Canada|Ontario|612[0n]|BOLD:AAB3774
Zanclognatha lituralis|[10273]|CNSLP1137-13|Canada|Ontario|613[0n]|BOLD:AAB3774
Zanclognatha lituralis|[10274]|LNCC1553-13|United States|North Carolina|658[0n]|BOLD:AAB3774
Zanclognatha nr. lituralis|[10275]|RDLQH150-06|Canada|Quebec|609[4n]|BOLD:ABX5357
Zanclognatha nr. lituralis|[10276]|BLTIB415-08|Canada|Ontario|658[0n]|BOLD:ABX5357
Zanclognatha nr. lituralis|[10277]|PHJUN3397-11|Canada|Ontario|658[0n]|BOLD:ABX5357
Zanclognatha nr. lituralis|[10278]|LNCC1555-13|United States|North Carolina|658[0n]|BOLD:ABX5357
Zanclognatha nr. lituralis|[10279]|LNCB778-09|United States|North Carolina|658[0n]|BOLD:ABX5357
Zanclognatha nr. lituralis|[10280]|MILEQ265-11|United States|Alabama|629[0n]|BOLD:ABX5357
Zanclognatha nr. lituralis|[10281]|LNC946-06|United States|North Carolina|656[0n]|BOLD:ABX5357
Zanclognatha nr. lituralis|[10282]|LNCB758-09|United States|North Carolina|658[0n]|BOLD:ABX5357
Zanclognatha nr. lituralis|[10283]|MILEP008-09|United States|North Carolina|658[0n]|BOLD:ABX5357
Zanclognatha nr. lituralis|[10284]|RDNMH640-09|United States|Louisiana|658[0n]|BOLD:ABX5357
Zanclognatha nr. lituralis|[10285]|LMEM168-09|United States|Alabama|658[0n]|BOLD:ABX5357

[illegible]
Zanclognatha nr. lituralis|[10283]|MILEP008-09|United States|North Carolina|658[0n]|BOLD:ABX5357
Zanclognatha nr. lituralis|[10284]|RDNMH640-09|United States|Louisiana|658[0n]|BOLD:ABX5357
Zanclognatha nr. lituralis|[10285]|LMEM168-09|United States|Alabama|658[0n]|BOLD:ABX5357
Zanclognatha nr. lituralis|[10286]|LMEM167-09|United States|Alabama|658[0n]|BOLD:ABX5357
Zanclognatha nr. lituralis|[10287]|LMEM166-09|United States|Alabama|658[0n]|BOLD:ABX5357
Zanclognatha nr. lituralis|[10288]|HKONB350-09|United States|Texas|658[0n]|BOLD:ABX5357
Zanclognatha nr. lituralis|[10289]|HKONB349-09|United States|Texas|658[0n]|BOLD:ABX5357
Zanclognatha nr. lituralis|[10290]|HKONS266-08|United States|Florida|658[0n]|BOLD:ABX5357
Zanclognatha nr. lituralis|[10291]|LGSMG481-07|United States|Tennessee|658[0n]|BOLD:ABX5357
Zanclognatha nr. lituralis|[10292]|LGSMG480-07|United States|Tennessee|658[0n]|BOLD:ABX5357
Zanclognatha nr. lituralis|[10293]|LNC459-05|United States|North Carolina|658[0n]|BOLD:ABX5357
Zanclognatha nr. lituralis|[10294]|LMEM169-09|United States|Alabama|658[0n]|BOLD:ABX5357
Zanclognatha nr. lituralis|[10295]|RDNMH479-09|United States|Louisiana|658[0n]|BOLD:ABX5357
Zanclognatha nr. lituralis|[10296]|RDNMH480-09|United States|Louisiana|658[0n]|BOLD:ABX5357
Zanclognatha nr. lituralis|[10297]|LNC458-05|United States|North Carolina|658[0n]|BOLD:ABX5357
Zanclognatha nr. lituralis|[10298]|HKONS365-08|United States|Florida|658[0n]|BOLD:ABX5357
Zanclognatha nr. lituralis|[10299]|LNC945-06|United States|North Carolina|658[0n]|BOLD:ABX5357
Zanclognatha nr. lituralis|[10300]|LNCB777-09|United States|North Carolina|658[0n]|BOLD:ABX5357
Zanclognatha nr. lituralis|[10301]|LNCC1339-11|United States|North Carolina|658[0n]|BOLD:ABX5357
Zanclognatha nr. lituralis|[10302]|LNCC1365-11|United States|North Carolina|658[0n]|BOLD:ABX5357
Zanclognatha nr. lituralis|[10303]|LNCC1492-13|United States|North Carolina|658[0n]|BOLD:ABX5357
Zanclognatha nr. lituralis|[10304]|LNCC1820-13|United States|North Carolina|658[0n]|BOLD:ABX5357
Zanclognatha nr. lituralis|[10305]|LNCC1821-13|United States|North Carolina|658[0n]|BOLD:ABX5357
Zanclognatha nr. lituralis|[10306]|LNCC1822-13|United States|North Carolina|658[0n]|BOLD:ABX5357
Zanclognatha nr. lituralis|[10307]|LNCC1823-13|United States|North Carolina|658[0n]|BOLD:ABX5357
Zanclognatha nr. lituralis|[10308]|LNCC1888-13|United States|North Carolina|658[0n]|BOLD:ABX5357
Zanclognatha nr. lituralis|[10309]|CNCLB2934-14|United States|North Carolina|658[0n]|BOLD:ABX5357
Zanclognatha laevigata|[10310]|ALLEP024-13|Canada|Ontario|658[0n]|BOLD:ABZ7767
Zanclognatha laevigata|[10311]|HEJUL2093-12|Canada|Ontario|658[0n]|BOLD:ABZ7767
Zanclognatha laevigata|[10312]|RDLQG034-06|Canada|Quebec|658[0n]|BOLD:ABZ7767
Zanclognatha laevigata|[10313]|RDLQG033-06|Canada|Quebec|658[0n]|BOLD:ABZ7767
Zanclognatha laevigata|[10314]|RDLQF747-06|Canada|Quebec|658[0n]|BOLD:ABZ7767
Zanclognatha laevigata|[10315]|RDLQF745-06|Canada|Quebec|658[0n]|BOLD:ABZ7767
Zanclognatha laevigata|[10316]|RDLQF706-06|Canada|Quebec|617[0n]|BOLD:ABZ7767
Zanclognatha laevigata|[10317]|RDLQF717-06|Canada|Quebec|637[0n]|BOLD:ABZ7767
Zanclognatha laevigata|[10318]|RDLQF716-06|Canada|Quebec|637[0n]|BOLD:ABZ7767
Zanclognatha laevigata|[10319]|RDLQF715-06|Canada|Quebec|637[0n]|BOLD:ABZ7767
Zanclognatha laevigata|[10320]|RDLQF632-06|Canada|Quebec|637[0n]|BOLD:ABZ7767
Zanclognatha laevigata|[10321]|RDLQF631-06|Canada|Quebec|637[0n]|BOLD:ABZ7767
Zanclognatha laevigata|[10322]|RDLQF630-06|Canada|Quebec|637[0n]|BOLD:ABZ7767
Zanclognatha laevigata|[10323]|CNROP338-13|Canada|Ontario|583[0n]|BOLD:ABZ7767
Zanclognatha laevigata|[10324]|CNROG063-13|Canada|Ontario|606[0n]|BOLD:ABZ7767
Zanclognatha laevigata|[10325]|RDLQG077-06|Canada|Quebec|621[0n]|BOLD:ABZ7767
Zanclognatha laevigata|[10326]|CNSLQ016-13|Canada|Ontario|561[0n]|BOLD:ABZ7767
Zanclognatha laevigata|[10327]|XAD088-04|Canada|Ontario|580[0n]|BOLD:ABZ7767
Zanclognatha laevigata|[10328]|CNSLQ026-13|Canada|Ontario|552[0n]|BOLD:ABZ7767
Zanclognatha laevigata|[10329]|CNROG074-13|Canada|Ontario|603[0n]|BOLD:ABZ7767
Zanclognatha laevigata|[10330]|CNROP342-13|Canada|Ontario|594[0n]|BOLD:ABZ7767
Zanclognatha laevigata|[10331]|RDLQF530-06|Canada|Quebec|658[0n]|BOLD:ABZ7767
Zanclognatha laevigata|[10332]|RDLQF743-06|Canada|Quebec|658[0n]|BOLD:ABZ7767
Zanclognatha laevigata|[10333]|XAE636-04|Canada|Ontario|658[1n]|BOLD:ABZ7767
Zanclognatha laevigata|[10334]|IAWLB382-11|United States|Virginia|572[0n]|BOLD:ABZ7767
Zanclognatha laevigata|[10335]|IAWLB383-11|United States|Virginia|658[0n]|BOLD:ABZ7767
Zanclognatha laevigata|[10336]|LNCC1023-11|United States|North Carolina|658[0n]|BOLD:ABZ7767
Zanclognatha laevigata|[10337]|LNCC1022-11|United States|North Carolina|658[0n]|BOLD:ABZ7767
Zanclognatha laevigata|[10338]|LNCC1021-11|United States|North Carolina|658[0n]|BOLD:ABZ7767
Zanclognatha laevigata|[10339]|LMEM181-09|United States|Tennessee|658[0n]|BOLD:ABZ7767
Zanclognatha laevigata|[10340]|LGSMG490-07|United States|North Carolina|658[0n]|BOLD:ABZ7767
Zanclognatha laevigata|[10341]|RDLQG076-06|Canada|Quebec|658[0n]|BOLD:ABZ7767
Zanclognatha laevigata|[10342]|RDLQF744-06|Canada|Quebec|658[0n]|BOLD:ABZ7767
Zanclognatha laevigata|[10343]|LGSM042-04|United States|North Carolina|658[0n]|BOLD:ABZ7767
Zanclognatha laevigata|[10344]|BBLEC182-09|Canada|Nova Scotia|658[0n]|BOLD:ABZ7767
Zanclognatha laevigata|[10345]|LGSM041-04|United States|North Carolina|658[0n]|BOLD:ABZ7767
Zanclognatha laevigata|[10346]|PAJUL1136-12|Canada|Ontario|591[0n]|BOLD:ABZ7767
Zanclognatha laevigata|[10347]|ALLEP335-13|Canada|Ontario|630[0n]|BOLD:ABZ7767
Zanclognatha laevigata|[10348]|CNROG060-13|Canada|Ontario|603[0n]|BOLD:ABZ7767
Zanclognatha laevigata|[10349]|LOT153-04|United States|Tennessee|609[0n]|BOLD:ABZ7767
Zanclognatha laevigata|[10350]|CNLMF1041-14|Canada|Quebec|561[0n]|BOLD:ABZ7767
Zanclognatha laevigata|[10351]|CNSLQ019-13|Canada|Ontario|577[0n]|BOLD:ABZ7767
Zanclognatha laevigata|[10352]|LSEU716-06|United States|Georgia|624[1n]|BOLD:ABZ7767
Zanclognatha laevigata|[10353]|CNLMG1088-14|Canada|Quebec|564[0n]|BOLD:ABZ7767
Zanclognatha laevigata|[10354]|CNLMP1947-14|Canada|Quebec|588[0n]|BOLD:ABZ7767
Zanclognatha laevigata|[10355]|CNROP345-13|Canada|Ontario|592[0n]|BOLD:ABZ7767
Zanclognatha laevigata|[10356]|RDLQG197-06|Canada|Quebec|658[0n]|BOLD:ABZ7767
Zanclognatha laevigata|[10357]|RDLQF746-06|Canada|Quebec|658[0n]|BOLD:ABZ7767
Zanclognatha laevigata|[10358]|LSEU715-06|United States|Georgia|658[0n]|BOLD:ABZ7767
Zanclognatha laevigata|[10359]|LSEU714-06|United States|Georgia|658[0n]|BOLD:ABZ7767
Zanclognatha laevigata|[10360]|LOT149-04|United States|Tennessee|609[0n]|BOLD:ABZ7767
Zanclognatha laevigata|[10361]|ABNCC066-07|United States|West Virginia|640[0n]|BOLD:ABZ7767
Zanclognatha laevigata|[10362]|LMEM182-09|United States|Tennessee|658[0n]|BOLD:ABZ7767
Zanclognatha laevigata|[10363]|ALLEP267-13|Canada|Ontario|658[0n]|BOLD:ABZ7767
Zanclognatha laevigata|[10364]|IAWLB385-11|United States|Virginia|658[0n]|BOLD:ABZ7767
Zanclognatha laevigata|[10365]|ALLEP400-13|Canada|Ontario|658[0n]|BOLD:ABZ7767
Zanclognatha laevigata|[10366]|CNCLB3009-14|United States|North Carolina|658[0n]|BOLD:ABZ7767
Palthis asopialis|[10367]|HKONB210-09|United States|Texas|658[0n]|BOLD:ACE8240
Palthis asopialis|[10368]|HKONB211-09|United States|Texas|658[0n]|BOLD:ACE8240
Palthis asopialis|[10369]|LOFLD282-07|United States|Florida|658[0n]|BOLD:AAB6251
Palthis asopialis|[10370]|LPOKA640-09|United States|Oklahoma|636[0n]|BOLD:AAB6251
Palthis asopialis|[10371]|LOT161-04|United States|Tennessee|609[0n]|BOLD:AAB6251
Palthis asopialis|[10372]|LGSM554-04|United States|Tennessee|658[0n]|BOLD:AAB6251
Palthis asopialis|[10373]|LOTB112-05|United States|Tennessee|658[0n]|BOLD:AAB6251
Palthis asopialis|[10374]|BBLPA750-10|Canada|Ontario|658[0n]|BOLD:AAB6251
Palthis asopialis|[10375]|LPSOD1061-09|Canada|Ontario|658[0n]|BOLD:AAB6251
Palthis asopialis|[10376]|HKONS343-08|United States|Florida|658[0n]|BOLD:AAB6251
Palthis asopialis|[10377]|LGSMC851-05|United States|Tennessee|658[0n]|BOLD:AAB6251
Palthis asopialis|[10378]|LGSMC384-05|United States|Tennessee|658[0n]|BOLD:AAB6251
Palthis asopialis|[10379]|LNC582-06|United States|North Carolina|649[0n]|BOLD:AAB6251
Palthis asopialis|[10380]|LTOLB018-08|United States|Maryland|647[0n]|BOLD:AAB6251
Palthis asopialis|[10381]|BBLSW578-09|United States|Oklahoma|658[0n]|BOLD:AAB6251
Palthis asopialis|[10382]|BBLSW599-09|United States|Oklahoma|658[0n]|BOLD:AAB6251
Palthis asopialis|[10383]|BBLSX378-09|United States|Oklahoma|658[0n]|BOLD:AAB6251
Palthis asopialis|[10384]|BBLSX381-09|United States|Oklahoma|658[0n]|BOLD:AAB6251
[illegible]

Palthis asopialis|[10382]|BBLSW599-09|United States|Oklahoma|658[0n]|BOLD:AAB6251
Palthis asopialis|[10383]|BBLSX378-09|United States|Oklahoma|658[0n]|BOLD:AAB6251
Palthis asopialis|[10384]|BBLSX381-09|United States|Oklahoma|658[0n]|BOLD:AAB6251
Palthis asopialis|[10385]|BBLSX410-09|United States|Oklahoma|658[0n]|BOLD:AAB6251
Palthis asopialis|[10386]|MJMSL108-10|United States|Massachusetts|658[0n]|BOLD:AAB6251
Palthis asopialis|[10387]|BBLPA623-10|Canada|Ontario|658[0n]|BOLD:AAB6251
Palthis asopialis|[10388]|BBLOB1457-11|United States|Florida|658[0n]|BOLD:AAB6251
Palthis asopialis|[10389]|LNCNW109-06|United States|North Carolina|658[1n]|BOLD:AAB6251
Palthis asopialis|[10390]|BBLOC779-11|United States|Arkansas|658[0n]|BOLD:AAB6251
Palthis asopialis|[10391]|BBLSX162-09|United States|Oklahoma|658[0n]|BOLD:AAB6251
Palthis asopialis|[10392]|HKONS342-08|United States|Florida|658[0n]|BOLD:AAB6251
Palthis asopialis|[10393]|LGSMG520-07|United States|Tennessee|658[0n]|BOLD:AAB6251
Palthis asopialis|[10394]|LOFLA636-06|United States|Florida|658[0n]|BOLD:AAB6251
Palthis asopialis|[10395]|LGSM555-04|United States|Tennessee|658[0n]|BOLD:AAB6251
Palthis asopialis|[10396]|LGSMC852-05|United States|Tennessee|658[0n]|BOLD:AAB6251
Palthis asopialis|[10397]|BBLSW596-09|United States|Oklahoma|653[0n]|BOLD:AAB6251
Palthis asopialis|[10398]|BBLSW624-09|United States|Oklahoma|658[0n]|BOLD:AAB6251
Palthis asopialis|[10399]|BBLSW625-09|United States|Oklahoma|658[0n]|BOLD:AAB6251
Palthis asopialis|[10400]|BBLSX099-09|United States|Oklahoma|658[0n]|BOLD:AAB6251
Palthis asopialis|[10401]|BBLSX398-09|United States|Oklahoma|658[0n]|BOLD:AAB6251
Palthis asopialis|[10402]|BBLSX400-09|United States|Oklahoma|658[0n]|BOLD:AAB6251
Palthis asopialis|[10403]|LILLA871-11|United States|Illinois|658[0n]|BOLD:AAB6251
Palthis asopialis|[10404]|BBLOC727-11|United States|Arkansas|658[0n]|BOLD:AAB6251
Palthis asopialis|[10405]|BBLOC828-11|United States|Arkansas|658[0n]|BOLD:AAB6251
Palthis angulalis|[10406]|RWWA437-09|United States|Washington|658[0n]|BOLD:AAA3933
Palthis angulalis|[10407]|RDLQG740-06|Canada|Quebec|658[0n]|BOLD:AAA3933
Palthis angulalis|[10408]|BBLSX916-09|United States|Arizona|658[0n]|BOLD:AAA3933
Palthis angulalis|[10409]|LILLA073-11|United States|Illinois|658[0n]|BOLD:AAA3933
Palthis angulalis|[10410]|BBLPD755-10|Canada|British Columbia|658[0n]|BOLD:AAA3933
Palthis angulalis|[10411]|RDLQG566-06|Canada|Quebec|658[0n]|BOLD:AAA3933
Palthis angulalis|[10412]|BBLPD992-10|Canada|Alberta|658[0n]|BOLD:AAA3933
Palthis angulalis|[10413]|IAWL003-09|United States|Washington|595[1n]|BOLD:AAA3933
Palthis angulalis|[10414]|LALPA190-10|Canada|British Columbia|658[0n]|BOLD:AAA3933
Palthis angulalis|[10415]|LALPA249-10|Canada|British Columbia|658[0n]|BOLD:AAA3933
Palthis angulalis|[10416]|LHLEP330-06|Canada|British Columbia|657[0n]|BOLD:AAA3933
Palthis angulalis|[10417]|LHLEP331-06|Canada|British Columbia|657[0n]|BOLD:AAA3933
Palthis angulalis|[10418]|LHLEP333-06|Canada|British Columbia|657[0n]|BOLD:AAA3933
Palthis angulalis|[10419]|LHLEP332-06|Canada|British Columbia|657[0n]|BOLD:AAA3933
Palthis angulalis|[10420]|LBCA531-05|Canada|British Columbia|658[0n]|BOLD:AAA3933
Palthis angulalis|[10421]|PMG146-03|Canada|Ontario|617[0n]|BOLD:AAA3933
Palthis angulalis|[10422]|LBCA530-05|Canada|British Columbia|658[0n]|BOLD:AAA3933
Palthis angulalis|[10423]|RWWA572-09|United States|Washington|658[0n]|BOLD:AAA3933
Palthis angulalis|[10424]|RWWA573-09|United States|Washington|658[0n]|BOLD:AAA3933
Palthis angulalis|[10425]|RWWA974-09|United States|Washington|658[0n]|BOLD:AAA3933
Palthis angulalis|[10426]|RWWB108-09|United States|Washington|658[0n]|BOLD:AAA3933
Palthis angulalis|[10427]|RWWB898-10|United States|Washington|658[0n]|BOLD:AAA3933
Palthis angulalis|[10428]|LALPA243-10|Canada|British Columbia|658[0n]|BOLD:AAA3933
Palthis angulalis|[10429]|RWWC476-11|United States|Washington|658[0n]|BOLD:AAA3933
Palthis angulalis|[10430]|XAK276-06|Canada|Ontario|576[0n]|BOLD:AAA3933
Palthis angulalis|[10431]|XAD002-04|Canada|Ontario|510[0n]|BOLD:AAA3933
Palthis angulalis|[10432]|BBLSX534-09|United States|Oklahoma|658[0n]|BOLD:AAA3933
Palthis angulalis|[10433]|LILLA790-11|United States|Illinois|658[0n]|BOLD:AAA3933
Palthis angulalis|[10434]|LGSM423-04|United States|North Carolina|658[3n]|BOLD:AAA3933
Palthis angulalis|[10435]|XAE102-04|Canada|Ontario|658[0n]|BOLD:AAA3933
Palthis angulalis|[10436]|RDLQG615-06|Canada|Quebec|655[0n]|BOLD:AAA3933
Palthis angulalis|[10437]|LGSM422-04|United States|North Carolina|658[2n]|BOLD:AAA3933
Palthis angulalis|[10438]|LPOKA459-09|United States|Oklahoma|658[0n]|BOLD:AAA3933
Palthis angulalis|[10439]|HKONS320-08|United States|Florida|658[0n]|BOLD:AAA3933
Palthis angulalis|[10440]|PHAUG1516-11|Canada|Ontario|658[0n]|BOLD:AAA3933
Palthis angulalis|[10441]|BBLOC104-11|United States|Florida|658[0n]|BOLD:AAA3933
Palthis angulalis|[10442]|BBLPB892-10|Canada|Manitoba|658[0n]|BOLD:AAA3933
Palthis angulalis|[10443]|BBLPE505-09|Canada|Newfoundland and Labrador|658[0n]|BOLD:AAA3933
Palthis angulalis|[10444]|BBLPC850-09|Canada|Newfoundland and Labrador|658[0n]|BOLD:AAA3933
Palthis angulalis|[10445]|BBLPC735-09|Canada|Newfoundland and Labrador|658[0n]|BOLD:AAA3933
Palthis angulalis|[10446]|BBLPC687-09|Canada|Newfoundland and Labrador|658[0n]|BOLD:AAA3933
Palthis angulalis|[10447]|BBLEC867-09|Canada|Newfoundland and Labrador|658[0n]|BOLD:AAA3933
Palthis angulalis|[10448]|BBLEC636-09|Canada|Nova Scotia|658[0n]|BOLD:AAA3933
Palthis angulalis|[10449]|BBLSX594-09|United States|Oklahoma|658[0n]|BOLD:AAA3933
Palthis angulalis|[10450]|BBLSX489-09|United States|Oklahoma|658[0n]|BOLD:AAA3933
Palthis angulalis|[10451]|BBLSX379-09|United States|Oklahoma|658[0n]|BOLD:AAA3933
Palthis angulalis|[10452]|LPOKB194-09|United States|Oklahoma|658[0n]|BOLD:AAA3933
Palthis angulalis|[10453]|LPOKA1031-09|United States|Oklahoma|658[0n]|BOLD:AAA3933
Palthis angulalis|[10454]|LPSOD1065-09|Canada|Ontario|658[0n]|BOLD:AAA3933
Palthis angulalis|[10455]|UDLEP158-09|United States|Pennsylvania|658[0n]|BOLD:AAA3933
Palthis angulalis|[10456]|BLTIB1073-08|Canada|Ontario|658[0n]|BOLD:AAA3933
Palthis angulalis|[10457]|BLTIB1016-08|Canada|Ontario|658[0n]|BOLD:AAA3933
Palthis angulalis|[10458]|BLTIB969-08|Canada|Ontario|658[0n]|BOLD:AAA3933
Palthis angulalis|[10459]|BLTIB965-08|Canada|Ontario|658[0n]|BOLD:AAA3933
Palthis angulalis|[10460]|BLTIB745-08|Canada|Ontario|658[0n]|BOLD:AAA3933
Palthis angulalis|[10461]|BLTIB425-08|Canada|Ontario|658[0n]|BOLD:AAA3933
Palthis angulalis|[10462]|LPSOC445-08|Canada|Ontario|658[0n]|BOLD:AAA3933
Palthis angulalis|[10463]|LPSOC056-08|Canada|Ontario|658[0n]|BOLD:AAA3933
Palthis angulalis|[10464]|LPSO284-08|Canada|Ontario|658[0n]|BOLD:AAA3933
Palthis angulalis|[10465]|LPSO273-08|Canada|Ontario|658[0n]|BOLD:AAA3933
Palthis angulalis|[10466]|LPSO085-08|Canada|Ontario|658[0n]|BOLD:AAA3933
Palthis angulalis|[10467]|HKONS321-08|United States|Florida|658[0n]|BOLD:AAA3933
Palthis angulalis|[10468]|XAK554-07|Canada|Ontario|658[0n]|BOLD:AAA3933
Palthis angulalis|[10469]|RDLQG820-06|Canada|Quebec|658[0n]|BOLD:AAA3933
Palthis angulalis|[10470]|RDLQG809-06|Canada|Quebec|658[0n]|BOLD:AAA3933
Palthis angulalis|[10471]|RDLQG756-06|Canada|Quebec|658[0n]|BOLD:AAA3933
Palthis angulalis|[10472]|RDLQG594-06|Canada|Quebec|658[0n]|BOLD:AAA3933
Palthis angulalis|[10473]|RDLQG279-06|Canada|Quebec|658[0n]|BOLD:AAA3933
Palthis angulalis|[10474]|RDLQG254-06|Canada|Quebec|658[0n]|BOLD:AAA3933
Palthis angulalis|[10475]|RDLQG041-06|Canada|Quebec|658[0n]|BOLD:AAA3933
Palthis angulalis|[10476]|RDLQG040-06|Canada|Quebec|658[0n]|BOLD:AAA3933
Palthis angulalis|[10477]|RDLQF727-06|Canada|Quebec|658[0n]|BOLD:AAA3933
Palthis angulalis|[10478]|RDLQF492-06|Canada|Quebec|658[0n]|BOLD:AAA3933
Palthis angulalis|[10479]|RDLQF445-06|Canada|Quebec|658[0n]|BOLD:AAA3933
Palthis angulalis|[10480]|RDLQF444-06|Canada|Quebec|658[0n]|BOLD:AAA3933
Palthis angulalis|[10481]|LNCB268-06|United States|North Carolina|658[0n]|BOLD:AAA3933
Palthis angulalis|[10482]|LNCNW096-06|United States|North Carolina|658[0n]|BOLD:AAA3933
Palthis angulalis|[10483]|TMNBC180-06|Canada|New Brunswick|658[0n]|BOLD:AAA3933
Palthis angulalis|[10484]|LOFLA008-06|United States|Florida|658[0n]|BOLD:AAA3933
[illegible]

Palthis angulalis|[10482]|LNCNW096-06|United States|North Carolina|658[0n]|BOLD:AAA3933
Palthis angulalis|[10483]|TMNBC180-06|Canada|New Brunswick|658[0n]|BOLD:AAA3933
Palthis angulalis|[10484]|LOFLA008-06|United States|Florida|658[0n]|BOLD:AAA3933
Palthis angulalis|[10485]|LNC839-06|United States|North Carolina|658[0n]|BOLD:AAA3933
Palthis angulalis|[10486]|TTMNB270-06|Canada|New Brunswick|658[0n]|BOLD:AAA3933
Palthis angulalis|[10487]|LSUSA096-06|United States|Kentucky|658[0n]|BOLD:AAA3933
Palthis angulalis|[10488]|XAG980-05|Canada|Ontario|658[0n]|BOLD:AAA3933
Palthis angulalis|[10489]|RDMAB021-05|Canada|Alberta|658[0n]|BOLD:AAA3933
Palthis angulalis|[10490]|LGSMD248-05|United States|Tennessee|658[0n]|BOLD:AAA3933
Palthis angulalis|[10491]|LOCT329-05|United States|Connecticut|658[0n]|BOLD:AAA3933
Palthis angulalis|[10492]|LOCT328-05|United States|Connecticut|658[0n]|BOLD:AAA3933
Palthis angulalis|[10493]|LOCT296-05|United States|Connecticut|658[0n]|BOLD:AAA3933
Palthis angulalis|[10494]|LOCT116-05|United States|Connecticut|658[0n]|BOLD:AAA3933
Palthis angulalis|[10495]|LOCT115-05|United States|Connecticut|658[0n]|BOLD:AAA3933
Palthis angulalis|[10496]|LOTB118-05|United States|Tennessee|658[0n]|BOLD:AAA3933
Palthis angulalis|[10497]|LOTB117-05|United States|Tennessee|658[0n]|BOLD:AAA3933
Palthis angulalis|[10498]|LOTB116-05|United States|Tennessee|658[0n]|BOLD:AAA3933
Palthis angulalis|[10499]|LOTB115-05|United States|Tennessee|658[0n]|BOLD:AAA3933
Palthis angulalis|[10500]|LOTB114-05|United States|Tennessee|658[0n]|BOLD:AAA3933
Palthis angulalis|[10501]|LOTB113-05|United States|Tennessee|658[0n]|BOLD:AAA3933
Palthis angulalis|[10502]|LGSMC849-05|United States|Tennessee|658[0n]|BOLD:AAA3933
Palthis angulalis|[10503]|PHMNB485-04|Canada|New Brunswick|658[0n]|BOLD:AAA3933
Palthis angulalis|[10504]|PHMNB186-04|Canada|New Brunswick|658[0n]|BOLD:AAA3933
Palthis angulalis|[10505]|MEC715-04|Canada|Quebec|658[0n]|BOLD:AAA3933
Palthis angulalis|[10506]|XAE223-04|Canada|Ontario|658[0n]|BOLD:AAA3933
Palthis angulalis|[10507]|LOT164-04|United States|Tennessee|609[0n]|BOLD:AAA3933
Palthis angulalis|[10508]|LOT163-04|United States|Tennessee|609[0n]|BOLD:AAA3933
Palthis angulalis|[10509]|LOT162-04|United States|Tennessee|609[0n]|BOLD:AAA3933
Palthis angulalis|[10510]|LGSMC850-05|United States|Tennessee|613[0n]|BOLD:AAA3933
Palthis angulalis|[10511]|XAB301-04|Canada|Ontario|613[0n]|BOLD:AAA3933
Palthis angulalis|[10512]|LOT199-04|United States|Tennessee|609[0n]|BOLD:AAA3933
Palthis angulalis|[10513]|XAD295-04|Canada|Ontario|559[0n]|BOLD:AAA3933
Palthis angulalis|[10514]|LPSOB591-08|Canada|Ontario|621[0n]|BOLD:AAA3933
Palthis angulalis|[10515]|LPSOC164-08|Canada|Ontario|646[0n]|BOLD:AAA3933
Palthis angulalis|[10516]|PHMNB062-03|Canada|New Brunswick|639[0n]|BOLD:AAA3933
Palthis angulalis|[10517]|RDMAB020-05|Canada|Alberta|631[0n]|BOLD:AAA3933
Palthis angulalis|[10518]|RDLQB680-05|Canada|Quebec|552[1n]|BOLD:AAA3933
Palthis angulalis|[10519]|LOWCB640-05|Canada|British Columbia|577[1n]|BOLD:AAA3933
Palthis angulalis|[10520]|XAK611-07|Canada|Ontario|603[1n]|BOLD:AAA3933
Palthis angulalis|[10521]|UDLEP086-09|United States|Maryland|606[0n]|BOLD:AAA3933
Palthis angulalis|[10522]|UDLEP170-09|United States|Delaware|608[0n]|BOLD:AAA3933
Palthis angulalis|[10523]|CNJAB067-12|Canada|Alberta|631[0n]|BOLD:AAA3933
Palthis angulalis|[10524]|CNPPG815-12|Canada|Ontario|627[0n]|BOLD:AAA3933
Palthis angulalis|[10525]|RBINA5361-13|Canada|Ontario|575[0n]|BOLD:AAA3933
Idia majoralis|[10526]|ABCNA932-08|United States|Wisconsin|658[0n]|BOLD:AAD8378
Idia majoralis|[10527]|LMEM154-09|United States|Mississippi|624[0n]|BOLD:AAD8378
Idia majoralis|[10528]|LMEM153-09|United States|Mississippi|658[0n]|BOLD:AAD8378
Idia majoralis|[10529]|LMEM155-09|United States|Alabama|658[0n]|BOLD:AAD8378
Idia majoralis|[10530]|LMEM156-09|United States|Mississippi|658[0n]|BOLD:AAD8378
Idia majoralis|[10531]|LPOKD420-09|United States|Oklahoma|658[0n]|BOLD:AAD8378
Idia americalis|[10532]|LBCA884-05|Canada|British Columbia|658[0n]|BOLD:AAA2087
Idia americalis|[10533]|LBCA885-05|Canada|British Columbia|603[0n]|BOLD:AAA2087
Idia americalis|[10534]|LOCBD169-06|United States|California|658[0n]|BOLD:AAA2087
Idia americalis|[10535]|LOCBE298-06|United States|California|596[0n]|BOLD:AAA2087
Idia americalis|[10536]|RDNM915-05|United States|Oregon|658[0n]|BOLD:AAA2087
Idia americalis|[10537]|LOCBD170-06|United States|California|658[0n]|BOLD:AAA2087
Idia americalis|[10538]|LOCBD697-06|United States|California|658[0n]|BOLD:AAA2087
Idia americalis|[10539]|GMLC1091-12|United States|California|658[0n]|BOLD:AAA2087
Idia americalis|[10540]|RDNM917-05|Canada|Ontario|578[0n]|BOLD:AAA2087
Idia americalis|[10541]|BLTIB458-08|Canada|Ontario|658[0n]|BOLD:AAA2087
Idia americalis|[10542]|LOWCB658-05|Canada|British Columbia|593[2n]|BOLD:AAA2087
Idia americalis|[10543]|XAK316-06|Canada|Ontario|658[0n]|BOLD:AAA2087
Idia americalis|[10544]|LOWCD867-06|Canada|British Columbia|605[0n]|BOLD:AAA2087
Idia americalis|[10545]|RDNMD539-06|United States|Florida|656[0n]|BOLD:AAA2087
Idia americalis|[10546]|BBLOC1174-11|United States|Texas|636[0n]|BOLD:AAA2087
Idia americalis|[10547]|RDLQF772-06|Canada|Quebec|658[0n]|BOLD:AAA2087
Idia americalis|[10548]|LMEM120-09|United States|Alabama|658[0n]|BOLD:AAA2087
Idia americalis|[10549]|LMEM121-09|United States|Alabama|658[0n]|BOLD:AAA2087
Idia americalis|[10550]|RDLQF725-06|Canada|Quebec|654[0n]|BOLD:AAA2087
Idia americalis|[10551]|LOWCD874-06|Canada|British Columbia|591[0n]|BOLD:AAA2087
Idia americalis|[10552]|LPABC663-09|Canada|Alberta|658[0n]|BOLD:AAA2087
Idia americalis|[10553]|BBLOC1191-11|United States|Texas|658[0n]|BOLD:AAA2087
Idia americalis|[10554]|BBLOC1186-11|United States|Texas|658[0n]|BOLD:AAA2087
Idia americalis|[10555]|BBLOC1182-11|United States|Texas|658[0n]|BOLD:AAA2087
Idia americalis|[10556]|BBLOC724-11|United States|Arkansas|658[0n]|BOLD:AAA2087
Idia americalis|[10557]|BBLOB921-11|United States|Florida|658[0n]|BOLD:AAA2087
Idia americalis|[10558]|MJMSL161-10|United States|Massachusetts|658[0n]|BOLD:AAA2087
Idia americalis|[10559]|MJMSL160-10|United States|Massachusetts|658[0n]|BOLD:AAA2087
Idia americalis|[10560]|MJMSL159-10|United States|Massachusetts|658[0n]|BOLD:AAA2087
Idia americalis|[10561]|PHFLO076-10|United States|Florida|658[0n]|BOLD:AAA2087
Idia americalis|[10562]|LBCH4439-10|Canada|British Columbia|658[0n]|BOLD:AAA2087
Idia americalis|[10563]|LBCH4051-10|Canada|British Columbia|658[0n]|BOLD:AAA2087
Idia americalis|[10564]|LBCH351-10|Canada|British Columbia|658[0n]|BOLD:AAA2087
Idia americalis|[10565]|LBCH093-10|Canada|British Columbia|658[0n]|BOLD:AAA2087
Idia americalis|[10566]|BBLSX125-09|United States|Oklahoma|658[0n]|BOLD:AAA2087
Idia americalis|[10567]|BBLSW597-09|United States|Oklahoma|658[0n]|BOLD:AAA2087
Idia americalis|[10568]|LMEM124-09|United States|Mississippi|658[0n]|BOLD:AAA2087
Idia americalis|[10569]|LMEM123-09|United States|Mississippi|658[0n]|BOLD:AAA2087
Idia americalis|[10570]|LMEM122-09|United States|Mississippi|658[0n]|BOLD:AAA2087
Idia americalis|[10571]|IAWL001-09|United States|Virginia|658[0n]|BOLD:AAA2087
Idia americalis|[10572]|BLTIB1037-08|Canada|Ontario|658[0n]|BOLD:AAA2087
Idia americalis|[10573]|LPSOC397-08|Canada|Ontario|658[0n]|BOLD:AAA2087
Idia americalis|[10574]|LPSOC233-08|Canada|Ontario|658[0n]|BOLD:AAA2087
Idia americalis|[10575]|LGSMG465-07|United States|Tennessee|658[0n]|BOLD:AAA2087
Idia americalis|[10576]|MNAD087-07|Canada|Ontario|658[0n]|BOLD:AAA2087
Idia americalis|[10577]|MNAD043-07|Canada|Quebec|658[0n]|BOLD:AAA2087
Idia americalis|[10578]|RDLQG936-06|Canada|Quebec|658[0n]|BOLD:AAA2087
Idia americalis|[10579]|RDLQG934-06|Canada|Quebec|658[0n]|BOLD:AAA2087
Idia americalis|[10580]|RDLQG612-06|Canada|Quebec|658[0n]|BOLD:AAA2087
Idia americalis|[10581]|RDLQG607-06|Canada|Quebec|658[0n]|BOLD:AAA2087
Idia americalis|[10582]|RDLQG497-06|Canada|Quebec|658[0n]|BOLD:AAA2087
Idia americalis|[10583]|RDLQG475-06|Canada|Quebec|658[0n]|BOLD:AAA2087
Idia americalis|[10584]|RDLQG474-06|Canada|Quebec|658[0n]|BOLD:AAA2087

Idia americalis|[10582]|RDLQG497-06|Canada|Quebec|658[0n]|BOLD:AAA2087
Idia americalis|[10583]|RDLQG475-06|Canada|Quebec|658[0n]|BOLD:AAA2087
Idia americalis|[10584]|RDLQG474-06|Canada|Quebec|658[0n]|BOLD:AAA2087
Idia americalis|[10585]|RDLQG473-06|Canada|Quebec|658[0n]|BOLD:AAA2087
Idia americalis|[10586]|RDLQG472-06|Canada|Quebec|658[0n]|BOLD:AAA2087
Idia americalis|[10587]|RDLQG263-06|Canada|Quebec|658[0n]|BOLD:AAA2087
Idia americalis|[10588]|RDLQG199-06|Canada|Quebec|658[0n]|BOLD:AAA2087
Idia americalis|[10589]|RDLQG091-06|Canada|Quebec|658[0n]|BOLD:AAA2087
Idia americalis|[10590]|RDLQF724-06|Canada|Quebec|658[0n]|BOLD:AAA2087
Idia americalis|[10591]|RDLQF723-06|Canada|Quebec|658[0n]|BOLD:AAA2087
Idia americalis|[10592]|RDLQF605-06|Canada|Quebec|658[0n]|BOLD:AAA2087
Idia americalis|[10593]|RDLQF572-06|Canada|Quebec|658[0n]|BOLD:AAA2087
Idia americalis|[10594]|RDLQF540-06|Canada|Quebec|658[0n]|BOLD:AAA2087
Idia americalis|[10595]|RDLQF447-06|Canada|Quebec|658[0n]|BOLD:AAA2087
Idia americalis|[10596]|RDLQF381-06|Canada|Quebec|658[0n]|BOLD:AAA2087
Idia americalis|[10597]|RDLQF380-06|Canada|Quebec|658[0n]|BOLD:AAA2087
Idia americalis|[10598]|LOWCB647-05|Canada|British Columbia|658[0n]|BOLD:AAA2087
Idia americalis|[10599]|XAH486-05|Canada|Ontario|658[0n]|BOLD:AAA2087
Idia americalis|[10600]|LBCB741-05|Canada|British Columbia|658[0n]|BOLD:AAA2087
Idia americalis|[10601]|RDNM914-05|Canada|Ontario|658[0n]|BOLD:AAA2087
Idia americalis|[10602]|PHMNB333-04|Canada|New Brunswick|658[0n]|BOLD:AAA2087
Idia americalis|[10603]|RDLQF573-06|Canada|Quebec|658[1n]|BOLD:AAA2087
Idia americalis|[10604]|RDLQG478-06|Canada|Quebec|640[0n]|BOLD:AAA2087
Idia americalis|[10605]|RDLQG496-06|Canada|Quebec|644[1n]|BOLD:AAA2087
Idia americalis|[10606]|BBLOC1198-11|United States|Texas|642[0n]|BOLD:AAA2087
Idia americalis|[10607]|BBLOC1203-11|United States|California|658[0n]|BOLD:AAA2087
Idia americalis|[10608]|BBLOC1626-11|United States|Texas|658[0n]|BOLD:AAA2087
Idia americalis|[10609]|LOWCB655-05|Canada|British Columbia|601[0n]|BOLD:AAA2087
Idia americalis|[10610]|LOWCB654-05|Canada|British Columbia|601[0n]|BOLD:AAA2087
Idia americalis|[10611]|LOWCB641-05|Canada|British Columbia|608[1n]|BOLD:AAA2087
Idia americalis|[10612]|LOWCD866-06|Canada|British Columbia|596[0n]|BOLD:AAA2087
Idia americalis|[10613]|LOWCD864-06|Canada|British Columbia|597[0n]|BOLD:AAA2087
Idia americalis|[10614]|LOWCB646-05|Canada|British Columbia|565[2n]|BOLD:AAA2087
Idia americalis|[10615]|LOWCB653-05|Canada|British Columbia|658[0n]|BOLD:AAA2087
Idia americalis|[10616]|LOWCB659-05|Canada|British Columbia|658[0n]|BOLD:AAA2087
Idia americalis|[10617]|LOWCD870-06|Canada|British Columbia|658[0n]|BOLD:AAA2087
Idia americalis|[10618]|RDMAB006-05|Canada|Alberta|658[0n]|BOLD:AAA2087
Idia americalis|[10619]|RDMAB074-05|Canada|Alberta|658[0n]|BOLD:AAA2087
Idia americalis|[10620]|RDNM916-05|Canada|British Columbia|658[0n]|BOLD:AAA2087
Idia americalis|[10621]|BBLPB340-10|Canada|Alberta|658[0n]|BOLD:AAA2087
Idia americalis|[10622]|LNCNW071-06|United States|North Carolina|658[0n]|BOLD:AAA2087
Idia americalis|[10623]|PHMNB009-03|Canada|New Brunswick|639[0n]|BOLD:AAA2087
Idia americalis|[10624]|BBLEC026-09|Canada|New Brunswick|658[0n]|BOLD:AAA2087
Idia americalis|[10625]|LSEU368-06|United States|Georgia|658[0n]|BOLD:AAA2087
Idia americalis|[10626]|ABCNA590-07|United States|Florida|577[0n]|BOLD:AAA2087
Idia americalis|[10627]|LGSMC857-05|United States|Tennessee|612[0n]|BOLD:AAA2087
Idia americalis|[10628]|MILEQ262-11|United States|Alabama|632[0n]|BOLD:AAA2087
Idia americalis|[10629]|PMG121-03|Canada|Ontario|617[0n]|BOLD:AAA2087
Idia americalis|[10630]|LOTB259-05|United States|Tennessee|658[0n]|BOLD:AAA2087
Idia americalis|[10631]|MNBB469-05|Canada|New Brunswick|658[0n]|BOLD:AAA2087
Idia americalis|[10632]|LOTB258-05|United States|Tennessee|658[0n]|BOLD:AAA2087
Idia americalis|[10633]|LGSMC336-05|United States|Tennessee|658[0n]|BOLD:AAA2087
Idia americalis|[10634]|LGSMC335-05|United States|Tennessee|658[0n]|BOLD:AAA2087
Idia americalis|[10635]|LGSMC334-05|United States|Tennessee|658[0n]|BOLD:AAA2087
Idia americalis|[10636]|PHMNB617-04|Canada|New Brunswick|658[0n]|BOLD:AAA2087
Idia americalis|[10637]|RDLQG935-06|Canada|Quebec|658[0n]|BOLD:AAA2087
Idia americalis|[10638]|LOT287-04|United States|Tennessee|658[0n]|BOLD:AAA2087
Idia americalis|[10639]|LOT286-04|United States|Tennessee|658[0n]|BOLD:AAA2087
Idia americalis|[10640]|LOT285-04|United States|Tennessee|658[0n]|BOLD:AAA2087
Idia americalis|[10641]|LGSM553-04|United States|North Carolina|658[0n]|BOLD:AAA2087
Idia americalis|[10642]|LGSM552-04|United States|Tennessee|658[0n]|BOLD:AAA2087
Idia americalis|[10643]|PHMNB109-04|Canada|New Brunswick|573[4n]|BOLD:AAA2087
Idia americalis|[10644]|IAWL002-09|United States|Texas|556[0n]|BOLD:AAA2087
Idia americalis|[10645]|LILLA445-11|United States|Illinois|658[0n]|BOLD:AAA2087
Idia americalis|[10646]|BBLOC822-11|United States|Texas|658[0n]|BOLD:AAA2087
Idia americalis|[10647]|MILEQ292-11|United States|Alabama|658[0n]|BOLD:AAA2087
Idia americalis|[10648]|MILEQ328-11|United States|Alabama|658[0n]|BOLD:AAA2087
Idia americalis|[10649]|RDLQF604-06|Canada|Quebec|658[0n]|BOLD:AAA2087
Idia americalis|[10650]|RDLQG705-06|Canada|Quebec|658[0n]|BOLD:AAA2087
Idia americalis|[10651]|RDLQG780-06|Canada|Quebec|655[0n]|BOLD:AAA2087
Idia americalis|[10652]|LGSMG466-07|United States|North Carolina|658[0n]|BOLD:AAA2087
Idia americalis|[10653]|LPSOC224-08|Canada|Ontario|658[0n]|BOLD:AAA2087
Idia americalis|[10654]|LPOKB266-09|United States|Oklahoma|658[0n]|BOLD:AAA2087
Idia americalis|[10655]|LPOKB344-09|United States|Oklahoma|658[0n]|BOLD:AAA2087
Idia americalis|[10656]|BBLSX161-09|United States|Oklahoma|658[0n]|BOLD:AAA2087
Idia americalis|[10657]|LNC303-05|United States|North Carolina|658[0n]|BOLD:AAA2087
Idia americalis|[10658]|LSEU213-06|United States|North Carolina|658[0n]|BOLD:AAA2087
Idia americalis|[10659]|LNC508-06|United States|North Carolina|658[0n]|BOLD:AAA2087
Idia americalis|[10660]|LNCB274-06|United States|North Carolina|658[0n]|BOLD:AAA2087
Idia americalis|[10661]|BBLCU232-09|United States|Michigan|658[0n]|BOLD:AAA2087
Idia americalis|[10662]|BBLCU321-09|United States|Michigan|658[0n]|BOLD:AAA2087
Idia americalis|[10663]|LNCB860-09|United States|Alabama|658[0n]|BOLD:AAA2087
Idia americalis|[10664]|BBLEC181-09|Canada|Nova Scotia|658[0n]|BOLD:AAA2087
Idia americalis|[10665]|MILEQ329-11|United States|Alabama|658[0n]|BOLD:AAA2087
Idia americalis|[10666]|BBLOC1106-11|United States|Texas|658[0n]|BOLD:AAA2087
Idia americalis|[10667]|BBLOC1197-11|United States|Texas|658[0n]|BOLD:AAA2087
Idia americalis|[10668]|BBLOD1234-11|United States|Oklahoma|658[0n]|BOLD:AAA2087
Idia americalis|[10669]|IAWL013-09|United States|Texas|614[0n]|BOLD:AAA2087
Idia americalis|[10670]|XAH044-05|Canada|Ontario|658[0n]|BOLD:AAA2087
Idia americalis|[10671]|XAH306-05|Canada|Ontario|658[0n]|BOLD:AAA2087
Idia americalis|[10672]|TTMNB261-06|Canada|New Brunswick|658[0n]|BOLD:AAA2087
Idia americalis|[10673]|XAH286-05|Canada|Ontario|658[0n]|BOLD:AAA2087
Idia americalis|[10674]|XAH301-05|Canada|Ontario|658[0n]|BOLD:AAA2087
Idia americalis|[10675]|TTMNB262-06|Canada|New Brunswick|658[0n]|BOLD:AAA2087
Idia americalis|[10676]|RDLQG092-06|Canada|Quebec|658[0n]|BOLD:AAA2087
Idia americalis|[10677]|XAG297-05|Canada|Ontario|658[0n]|BOLD:AAA2087
Idia americalis|[10678]|XAF740-05|Canada|Ontario|658[0n]|BOLD:AAA2087
Idia americalis|[10679]|LOCT400-05|United States|Connecticut|658[0n]|BOLD:AAA2087
Idia americalis|[10680]|LOCT312-05|United States|Connecticut|658[0n]|BOLD:AAA2087
Idia americalis|[10681]|LOCT308-05|United States|Connecticut|658[0n]|BOLD:AAA2087
Idia americalis|[10682]|XAC463-04|Canada|Ontario|658[0n]|BOLD:AAA2087
Idia americalis|[10683]|XAC041-04|Canada|Ontario|658[0n]|BOLD:AAA2087
Idia americalis|[10684]|XAB192-04|Canada|Ontario|658[0n]|BOLD:AAA2087

Idia americalis|[10682]|XAC463-04|Canada|Ontario|658[0n]|BOLD:AAA2087
Idia americalis|[10683]|XAC041-04|Canada|Ontario|658[0n]|BOLD:AAA2087
Idia americalis|[10684]|XAB192-04|Canada|Ontario|658[0n]|BOLD:AAA2087
Idia americalis|[10685]|LOCT096-05|United States|Connecticut|658[0n]|BOLD:AAA2087
Idia americalis|[10686]|RDLQG488-06|Canada|Quebec|658[0n]|BOLD:AAA2087
Idia americalis|[10687]|RDLQG549-06|Canada|Quebec|658[0n]|BOLD:AAA2087
Idia americalis|[10688]|LPSOC227-08|Canada|Ontario|658[0n]|BOLD:AAA2087
Idia americalis|[10689]|LPABB308-08|Canada|Alberta|658[0n]|BOLD:AAA2087
Idia|[10690]|HKONB420-09|United States|Indiana|658[0n]|BOLD:AAA2087
Idia americalis|[10691]|LPMNB337-09|Canada|Manitoba|658[0n]|BOLD:AAA2087
Idia americalis|[10692]|LPMNB355-09|Canada|Manitoba|658[0n]|BOLD:AAA2087
Idia americalis|[10693]|LPMNB406-09|Canada|Manitoba|658[0n]|BOLD:AAA2087
Idia americalis|[10694]|LPMNB415-09|Canada|Manitoba|658[0n]|BOLD:AAA2087
Idia americalis|[10695]|LPMNB539-09|Canada|Manitoba|658[0n]|BOLD:AAA2087
Idia americalis|[10696]|LPOKC815-09|United States|Oklahoma|658[0n]|BOLD:AAA2087
Idia americalis|[10697]|MJMSL044-10|United States|Massachusetts|658[0n]|BOLD:AAA2087
Idia americalis|[10698]|MJMSL106-10|United States|Massachusetts|658[0n]|BOLD:AAA2087
Idia americalis|[10699]|MJMSL162-10|United States|Massachusetts|658[0n]|BOLD:AAA2087
Idia americalis|[10700]|JSAUG484-11|Canada|Ontario|658[0n]|BOLD:AAA2087
Idia americalis|[10701]|HEJUL247-12|Canada|Ontario|618[0n]|BOLD:AAA2087
Idia americalis|[10702]|LOWCB644-05|Canada|British Columbia|573[3n]|BOLD:AAA2087
Idia americalis|[10703]|BBLPB418-10|Canada|Alberta|658[0n]|BOLD:AAA2087
Idia americalis|[10704]|BBLEC755-09|Canada|Nova Scotia|614[0n]|BOLD:AAA2087
Idia americalis|[10705]|RDLQG540-06|Canada|Quebec|658[0n]|BOLD:AAA2087
Idia americalis|[10706]|RDLQG544-06|Canada|Quebec|658[0n]|BOLD:AAA2087
Idia americalis|[10707]|RDLQG545-06|Canada|Quebec|658[0n]|BOLD:AAA2087
Idia americalis|[10708]|RDLQG546-06|Canada|Quebec|658[0n]|BOLD:AAA2087
Idia americalis|[10709]|BBLEC713-09|Canada|Nova Scotia|658[0n]|BOLD:AAA2087
Idia americalis|[10710]|BBLPC045-09|Canada|New Brunswick|658[0n]|BOLD:AAA2087
Idia americalis|[10711]|RDNMJ805-11|Canada|Quebec|658[0n]|BOLD:AAA2087
Idia americalis|[10712]|LOWCB652-05|Canada|British Columbia|573[0n]|BOLD:AAA2087
Idia americalis|[10713]|RDNM912-05|Canada|British Columbia|546[3n]|BOLD:AAA2087
Idia americalis|[10714]|RDLQG610-06|Canada|Quebec|658[0n]|BOLD:AAA2087
Idia americalis|[10715]|RDLQG894-06|Canada|Quebec|658[0n]|BOLD:AAA2087
Idia americalis|[10716]|RDLQG899-06|Canada|Quebec|634[0n]|BOLD:AAA2087
Idia americalis|[10717]|MNAF867-08|Canada|Manitoba|658[0n]|BOLD:AAA2087
Idia americalis|[10718]|LOWCE842-06|Canada|British Columbia|615[0n]|BOLD:AAA2087
Idia americalis|[10719]|LOWCB651-05|Canada|British Columbia|601[1n]|BOLD:AAA2087
Idia americalis|[10720]|BBLPC047-09|Canada|New Brunswick|658[0n]|BOLD:AAA2087
Idia americalis|[10721]|LOWCB649-05|Canada|British Columbia|601[0n]|BOLD:AAA2087
Idia americalis|[10722]|LOWCB645-05|Canada|British Columbia|599[1n]|BOLD:AAA2087
Idia americalis|[10723]|LBCH4653-10|Canada|British Columbia|658[0n]|BOLD:AAA2087
Idia americalis|[10724]|LOWCD875-06|Canada|British Columbia|590[0n]|BOLD:AAA2087
Idia americalis|[10725]|LOWCB643-05|Canada|British Columbia|592[2n]|BOLD:AAA2087
Idia americalis|[10726]|BBLEC324-09|Canada|Nova Scotia|632[0n]|BOLD:AAA2087
Idia americalis|[10727]|RDNM913-05|Canada|British Columbia|535[2n]|BOLD:AAA2087
Idia americalis|[10728]|LOWCE861-06|Canada|British Columbia|616[0n]|BOLD:AAA2087
Idia americalis|[10729]|LOWCD872-06|Canada|British Columbia|617[0n]|BOLD:AAA2087
Idia americalis|[10730]|LOWCD871-06|Canada|British Columbia|616[0n]|BOLD:AAA2087
Idia americalis|[10731]|BBLEC334-09|Canada|Nova Scotia|658[0n]|BOLD:AAA2087
Idia americalis|[10732]|LOWCB642-05|Canada|British Columbia|658[0n]|BOLD:AAA2087
Idia americalis|[10733]|LOWCB648-05|Canada|British Columbia|601[2n]|BOLD:AAA2087
Idia americalis|[10734]|LOWCD865-06|Canada|British Columbia|605[0n]|BOLD:AAA2087
Idia americalis|[10735]|LOWCD869-06|Canada|British Columbia|608[0n]|BOLD:AAA2087
Idia americalis|[10736]|LBCH3226-10|Canada|British Columbia|658[0n]|BOLD:AAA2087
Idia americalis|[10737]|LOWCE834-06|Canada|British Columbia|656[0n]|BOLD:AAA2087
Idia americalis|[10738]|LPMN056-08|Canada|Manitoba|658[0n]|BOLD:AAA2087
Idia americalis|[10739]|BBLPE139-09|Canada|Nova Scotia|658[0n]|BOLD:AAA2087
Idia americalis|[10740]|LILLA183-11|United States|Illinois|658[0n]|BOLD:AAA2087
Idia americalis|[10741]|RDNMJ804-11|Canada|Quebec|658[0n]|BOLD:AAA2087
Idia americalis|[10742]|BBLPB159-10|Canada|Alberta|658[0n]|BOLD:AAA2087
Idia americalis|[10743]|BBLPE184-09|Canada|Nova Scotia|658[0n]|BOLD:AAA2087
Idia americalis|[10744]|BBLPE155-09|Canada|Nova Scotia|658[0n]|BOLD:AAA2087
Idia americalis|[10745]|BBLPC044-09|Canada|New Brunswick|658[0n]|BOLD:AAA2087
Idia americalis|[10746]|LPABC870-09|Canada|Alberta|658[0n]|BOLD:AAA2087
Idia americalis|[10747]|LPABC666-09|Canada|Alberta|658[0n]|BOLD:AAA2087
Idia americalis|[10748]|LPMNB523-09|Canada|Manitoba|658[0n]|BOLD:AAA2087
Idia americalis|[10749]|LPMNB513-09|Canada|Manitoba|658[0n]|BOLD:AAA2087
Idia americalis|[10750]|LPVIA081-08|Canada|British Columbia|658[0n]|BOLD:AAA2087
Idia americalis|[10751]|LPAB817-08|Canada|Alberta|658[0n]|BOLD:AAA2087
Idia americalis|[10752]|LPMN665-08|Canada|Manitoba|658[0n]|BOLD:AAA2087
Idia americalis|[10753]|LPMN447-08|Canada|Manitoba|658[0n]|BOLD:AAA2087
Idia americalis|[10754]|LPMN353-08|Canada|Manitoba|658[0n]|BOLD:AAA2087
Idia americalis|[10755]|LPMN352-08|Canada|Manitoba|658[0n]|BOLD:AAA2087
Idia americalis|[10756]|LPSK057-08|Canada|Saskatchewan|658[0n]|BOLD:AAA2087
Idia americalis|[10757]|RDLQG678-06|Canada|Quebec|658[0n]|BOLD:AAA2087
Idia americalis|[10758]|RDLQG547-06|Canada|Quebec|658[0n]|BOLD:AAA2087
Idia americalis|[10759]|RDLQG542-06|Canada|Quebec|658[0n]|BOLD:AAA2087
Idia americalis|[10760]|TMNBB043-06|Canada|New Brunswick|658[0n]|BOLD:AAA2087
Idia americalis|[10761]|LOWCD868-06|Canada|British Columbia|658[0n]|BOLD:AAA2087
Idia americalis|[10762]|RDLQB243-05|Canada|Quebec|658[0n]|BOLD:AAA2087
Idia americalis|[10763]|LPMN370-08|Canada|Manitoba|658[0n]|BOLD:AAA2087
Idia americalis|[10764]|LOWCB657-05|Canada|British Columbia|658[0n]|BOLD:AAA2087
Idia americalis|[10765]|LOWCB656-05|Canada|British Columbia|658[0n]|BOLD:AAA2087
Idia americalis|[10766]|RDNM918-05|Canada|British Columbia|658[0n]|BOLD:AAA2087
Idia americalis|[10767]|LOWCB650-05|Canada|British Columbia|564[1n]|BOLD:AAA2087
Idia americalis|[10768]|SSEIA7725-13|Canada|Alberta|577[0n]|BOLD:AAA2087
Idia lubricalis complex|[10769]|CMAZA897-12|United States|Arizona|658[0n]|BOLD:ABW6352
Idia lubricalis complex|[10770]|RDNMG249-08|United States|Texas|658[0n]|BOLD:ACF3661
Idia lubricalis|[10771]|LMEM151-09|United States|Louisiana|658[0n]|BOLD:ACF3661
Idia lubricalis|[10772]|LNCC808-11|United States|North Carolina|658[0n]|BOLD:ACF3661
Idia lubricalis|[10773]|LBCC768-05|Canada|British Columbia|658[0n]|BOLD:ACF3661
Idia lubricalis|[10774]|XAJ842-06|Canada|Ontario|658[0n]|BOLD:ACF3661
Idia lubricalis|[10775]|LNCC1155-11|United States|North Carolina|658[0n]|BOLD:ACF3661
Idia lubricalis complex|[10776]|MNAE835-13|Canada|Ontario|656[0n]|BOLD:ACF3661
Idia lubricalis|[10777]|RDLQF710-06|Canada|Quebec|637[0n]|BOLD:ACF3661
Idia lubricalis|[10778]|RDLQF593-06|Canada|Quebec|658[0n]|BOLD:ACF3661
Idia lubricalis complex|[10779]|MNAE844-13|Canada|Ontario|658[0n]|BOLD:ACF3661
Idia lubricalis complex|[10780]|MNAE845-13|Canada|Ontario|658[0n]|BOLD:ACF3661
Idia lubricalis complex|[10781]|MNAE846-13|Canada|Ontario|658[0n]|BOLD:ACF3661
Idia lubricalis complex|[10782]|MNAE848-13|Canada|Ontario|658[0n]|BOLD:ACF3661
Idia sp.|[10783]|MNAE853-13|United States|Florida|603[0n]|BOLD:ACF3661
Idia lubricalis|[10784]|LMEM150-09|United States|Alabama|551[0n]|BOLD:ACF4864

Idia lubricalis complex|[10782]|MNAE848-13|Canada|Ontario|658[0n]|BOLD:ACF3661
Idia sp.|[10783]|MNAE853-13|United States|Florida|603[0n]|BOLD:ACF3661
Idia lubricalis|[10784]|LMEM150-09|United States|Alabama|551[0n]|BOLD:ACF4864
Idia lubricalis|[10785]|LPOKD029-09|United States|Oklahoma|658[0n]|BOLD:ACF4864
Idia lubricalis|[10786]|MILEQ291-11|United States|Alabama|658[0n]|BOLD:ACF4864
Idia lubricalis|[10787]|LNCB266-06|United States|North Carolina|658[0n]|BOLD:ACF4864
Idia lubricalis|[10788]|LNCB267-06|United States|North Carolina|658[0n]|BOLD:ACF4864
Idia lubricalis|[10789]|RDNMD454-06|United States|Florida|658[0n]|BOLD:ACF4864
Idia lubricalis|[10790]|RDNMD579-06|United States|Florida|658[0n]|BOLD:ACF4864
Idia lubricalis|[10791]|RDNMD581-06|United States|Florida|658[0n]|BOLD:ACF4864
Idia lubricalis|[10792]|LNCC835-11|United States|North Carolina|658[0n]|BOLD:ACF4864
Idia lubricalis|[10793]|LNCC836-11|United States|North Carolina|658[0n]|BOLD:ACF4864
Idia lubricalis|[10794]|LNCC843-11|United States|North Carolina|658[0n]|BOLD:ACF4864
Idia lubricalis|[10795]|LNCC940-11|United States|North Carolina|658[0n]|BOLD:ACF4864
Idia sp.|[10796]|MILEQ330-11|United States|Alabama|658[0n]|BOLD:ACF4864
Idia lubricalis|[10797]|LNCC1882-13|United States|North Carolina|658[0n]|BOLD:ACF4864
Idia lubricalis|[10798]|LNCC1883-13|United States|North Carolina|658[0n]|BOLD:ACF4864
Idia occidentalis|[10799]|HKONB345-09|United States|Texas|658[0n]|BOLD:ABY9095
Idia lubricalis complex|[10800]|LMEM152-09|United States|Mississippi|658[0n]|BOLD:ABY9095
Idia lubricalis|[10801]|RDLQF726-06|Canada|Quebec|658[0n]|BOLD:ACF4863
Idia lubricalis|[10802]|RDLQG070-06|Canada|Quebec|658[0n]|BOLD:ACF4863
Idia denticulalis|[10803]|RDLQF600-06|Canada|Quebec|658[0n]|BOLD:ACF4863
Idia denticulalis|[10804]|RDLQG081-06|Canada|Quebec|632[0n]|BOLD:ACF4863
Idia denticulalis|[10805]|ABNCC062-07|United States|Texas|636[0n]|BOLD:ACF4863
Idia lubricalis|[10806]|RDLQF709-06|Canada|Quebec|637[0n]|BOLD:ACF3662
Idia lubricalis|[10807]|LGSMG477-07|United States|North Carolina|618[0n]|BOLD:ACF3662
Idia lubricalis|[10808]|MILEQ264-11|United States|Alabama|634[0n]|BOLD:ACF3662
Idia lubricalis complex|[10809]|RDNMG248-08|United States|Texas|658[0n]|BOLD:ACX3309
Idia sp.|[10810]|QUNOD014-10|United States|Texas|658[0n]|BOLD:AAA2230
Idia lubricalis complex|[10811]|LBCH6242-10|Canada|British Columbia|658[0n]|BOLD:ACE7853
Idia lubricalis complex|[10812]|LBCH6234-10|Canada|British Columbia|658[0n]|BOLD:ACE7853
Idia lubricalis complex|[10813]|LBCG1107-09|Canada|British Columbia|658[0n]|BOLD:ACE7853
Idia lubricalis complex|[10814]|LBCH7267-10|Canada|British Columbia|634[0n]|BOLD:ACE7853
Idia lubricalis complex|[10815]|LBCH7268-10|Canada|British Columbia|635[0n]|BOLD:ACE7853
Idia lubricalis|[10816]|RDNMF841-08|United States|Arizona|658[0n]|BOLD:ACF3664
Idia lubricalis|[10817]|CMAZA1217-12|United States|Arizona|658[0n]|BOLD:ACF3664
Idia lubricalis|[10818]|BBLSX504-09|United States|Oklahoma|658[0n]|BOLD:ABY9096
Idia lubricalis|[10819]|BBLSX517-09|United States|Arizona|658[0n]|
Idia lubricalis|[10820]|HKONB470-09|United States|Texas|658[0n]|BOLD:ABY9096
Idia lubricalis|[10821]|BBLSW470-09|United States|Oklahoma|658[0n]|BOLD:ABY9096
Idia lubricalis|[10822]|BBLSX443-09|United States|Oklahoma|658[0n]|BOLD:ABY9096
Idia lubricalis|[10823]|BBLSZ186-09|United States|Texas|658[0n]|BOLD:ABY9096
Idia lubricalis|[10824]|LPOKC784-09|United States|Oklahoma|658[0n]|BOLD:ABY9096
Idia denticulalis|[10825]|RDLQG085-06|Canada|Quebec|624[0n]|BOLD:ABY9096
Idia denticulalis|[10826]|RDLQF729-06|Canada|Quebec|658[1n]|BOLD:ABY9096
Idia denticulalis|[10827]|RDLQG086-06|Canada|Quebec|658[1n]|BOLD:ABY9096
Idia denticulalis|[10828]|RDLQG087-06|Canada|Quebec|658[1n]|BOLD:ABY9096
Idia denticulalis|[10829]|RDLQG083-06|Canada|Quebec|624[0n]|BOLD:ABY9096
Idia denticulalis|[10830]|RDLQG082-06|Canada|Quebec|630[0n]|BOLD:ABY9096
Idia denticulalis|[10831]|RDLQF602-06|Canada|Quebec|658[0n]|BOLD:ABY9096
Idia denticulalis|[10832]|RDLQF601-06|Canada|Quebec|658[0n]|BOLD:ABY9096
Idia denticulalis|[10833]|LGSM658-04|United States|North Carolina|658[0n]|BOLD:ABY9096
Idia denticulalis|[10834]|RDLQG084-06|Canada|Quebec|653[1n]|BOLD:ABY9096
Idia denticulalis|[10835]|RDLQG088-06|Canada|Quebec|632[0n]|BOLD:ABY9096
Idia lubricalis|[10836]|LNCC941-11|United States|North Carolina|658[0n]|BOLD:ABY9096
Idia lubricalis|[10837]|RDNMD456-06|United States|Florida|658[0n]|BOLD:ABY9096
Idia lubricalis|[10838]|RDNMD576-06|United States|Florida|658[0n]|BOLD:ABY9096
Idia lubricalis|[10839]|BBLSX399-09|United States|Oklahoma|658[0n]|BOLD:ABY9096
Idia lubricalis|[10840]|BBLSX424-09|United States|Oklahoma|658[0n]|BOLD:ABY9096
Idia lubricalis|[10841]|MILEQ261-11|United States|Alabama|658[0n]|BOLD:ABY9096
Idia lubricalis|[10842]|MILEQ290-11|United States|Alabama|658[0n]|BOLD:ABY9096
Idia lubricalis complex|[10843]|MNAE849-13|Canada|Ontario|658[0n]|BOLD:ABY9096
Idia lubricalis|[10844]|RDLQ109-05|Canada|Quebec|658[0n]|BOLD:ABY9096
Idia denticulalis|[10845]|RDLQF603-06|Canada|Quebec|658[0n]|BOLD:ABY9096
Idia lubricalis|[10846]|RDLQG067-06|Canada|Quebec|658[0n]|BOLD:ABY9096
Idia lubricalis|[10847]|RDLQG075-06|Canada|Quebec|658[0n]|BOLD:ABY9096
Idia lubricalis complex|[10848]|MNAE834-13|Canada|Ontario|658[0n]|BOLD:ABY9096
Idia lubricalis complex|[10849]|MNAE836-13|Canada|Ontario|658[0n]|BOLD:ABY9096
Idia lubricalis complex|[10850]|MNAE842-13|Canada|Ontario|658[0n]|BOLD:ABY9096
Idia lubricalis|[10851]|MNBB627-05|Canada|New Brunswick|658[0n]|BOLD:ABY9096
Idia lubricalis|[10852]|TMNBC559-06|Canada|New Brunswick|658[0n]|BOLD:ABY9096
Idia lubricalis complex|[10853]|MNAE843-13|Canada|Ontario|658[0n]|BOLD:ABY9096
Idia denticulalis|[10854]|LMDH193-11|United States|Minnesota|658[0n]|BOLD:ABY9096
Idia denticulalis|[10855]|LMEM157-09|United States|Mississippi|621[0n]|BOLD:ABY9096
Idia lubricalis|[10856]|RDNMD580-06|United States|Florida|656[0n]|BOLD:ABY9096
Idia lubricalis|[10857]|MILEQ263-11|United States|Alabama|630[0n]|BOLD:ABY9096
Idia lubricalis|[10858]|RDLQG069-06|Canada|Quebec|630[0n]|BOLD:ABY9096
Idia denticulalis|[10859]|ABNCC061-07|United States|Texas|646[0n]|BOLD:ABY9096
Idia lubricalis complex|[10860]|MNAE840-13|Canada|Ontario|658[0n]|BOLD:ABY9096
Idia lubricalis complex|[10861]|MNAE831-13|Canada|Ontario|658[0n]|BOLD:ABY9096
Idia lubricalis|[10862]|MILEQ289-11|United States|Alabama|658[0n]|BOLD:ABY9096
Idia lubricalis|[10863]|LNCC943-11|United States|North Carolina|658[0n]|BOLD:ABY9096
Idia lubricalis|[10864]|LNCC273-10|United States|North Carolina|658[0n]|BOLD:ABY9096
Idia lubricalis|[10865]|LNCC055-10|United States|North Carolina|658[0n]|BOLD:ABY9096
Idia lubricalis|[10866]|BBLPE573-09|Canada|Nova Scotia|658[0n]|BOLD:ABY9096
Idia lubricalis|[10867]|LNCB625-09|United States|North Carolina|658[0n]|BOLD:ABY9096
Idia denticulalis|[10868]|HKONB469-09|United States|Texas|658[0n]|BOLD:ABY9096
Idia denticulalis|[10869]|HKONB468-09|United States|Texas|658[0n]|BOLD:ABY9096
Idia lubricalis|[10870]|LPOKA192-08|United States|Oklahoma|658[0n]|BOLD:ABY9096
Idia denticulalis|[10871]|HKONS079-07|United States|Florida|658[0n]|BOLD:ABY9096
Idia lubricalis|[10872]|RDNMD578-06|United States|Florida|658[0n]|BOLD:ABY9096
Idia lubricalis|[10873]|RDNMD575-06|United States|Florida|658[0n]|BOLD:ABY9096
Idia lubricalis|[10874]|RDLQG074-06|Canada|Quebec|658[0n]|BOLD:ABY9096
Idia lubricalis|[10875]|RDLQG073-06|Canada|Quebec|658[0n]|BOLD:ABY9096
Idia lubricalis|[10876]|RDLQG072-06|Canada|Quebec|658[0n]|BOLD:ABY9096
Idia lubricalis|[10877]|RDLQG071-06|Canada|Quebec|658[0n]|BOLD:ABY9096
Idia lubricalis|[10878]|RDLQG068-06|Canada|Quebec|658[0n]|BOLD:ABY9096
Idia lubricalis|[10879]|RDLQF749-06|Canada|Quebec|658[0n]|BOLD:ABY9096
Idia lubricalis|[10880]|RDLQF748-06|Canada|Quebec|658[0n]|BOLD:ABY9096
Idia lubricalis|[10881]|RDLQF591-06|Canada|Quebec|658[0n]|BOLD:ABY9096
Idia lubricalis|[10882]|RDLQF590-06|Canada|Quebec|658[0n]|BOLD:ABY9096
Idia lubricalis|[10883]|RDLQF589-06|Canada|Quebec|658[0n]|BOLD:ABY9096
Idia lubricalis|[10884]|RDLQF577-06|Canada|Quebec|658[0n]|BOLD:ABY9096

[illegible]
Idia lubricalis|[10882]|RDLQF590-06|Canada|Quebec|658[0n]|BOLD:ABY9096
Idia lubricalis|[10883]|RDLQF589-06|Canada|Quebec|658[0n]|BOLD:ABY9096
Idia lubricalis|[10884]|RDLQF577-06|Canada|Quebec|658[0n]|BOLD:ABY9096
Idia lubricalis|[10885]|RDLQF576-06|Canada|Quebec|658[0n]|BOLD:ABY9096
Idia lubricalis|[10886]|RDLQF565-06|Canada|Quebec|658[0n]|BOLD:ABY9096
Idia lubricalis|[10887]|RDLQF564-06|Canada|Quebec|658[0n]|BOLD:ABY9096
Idia lubricalis|[10888]|RDLQF532-06|Canada|Quebec|658[0n]|BOLD:ABY9096
Idia lubricalis|[10889]|RDNMD455-06|United States|Florida|658[0n]|BOLD:ABY9096
Idia lubricalis|[10890]|TTMNB098-06|Canada|New Brunswick|658[0n]|BOLD:ABY9096
Idia lubricalis|[10891]|LNC578-06|United States|North Carolina|658[0n]|BOLD:ABY9096
Idia lubricalis|[10892]|LNC542-06|United States|North Carolina|658[0n]|BOLD:ABY9096
Idia denticulalis|[10893]|GMGSF406-12|United States|Tennessee|625[0n]|BOLD:ABY9096
Idia lubricalis|[10894]|RDLQ108-05|Canada|Quebec|658[0n]|BOLD:ABY9096
Idia lubricalis|[10895]|LGSM756-04|United States|Tennessee|658[0n]|BOLD:ABY9096
Idia lubricalis|[10896]|XAJ821-06|Canada|Ontario|658[0n]|BOLD:ABY9096
Idia lubricalis|[10897]|ABNCC063-07|United States|Florida|595[0n]|BOLD:ABY9096
Idia lubricalis|[10898]|ABNCC064-07|United States|Florida|637[0n]|BOLD:ABY9096
Idia lubricalis complex|[10899]|PHSEP342-11|Canada|Ontario|652[0n]|BOLD:ABY9096
Idia lubricalis complex|[10900]|PHSEP348-11|Canada|Ontario|652[0n]|BOLD:ABY9096
Idia lubricalis|[10901]|LNCC1223-11|United States|North Carolina|658[0n]|BOLD:ABY9096
Idia lubricalis complex|[10902]|MNAE850-13|Canada|Quebec|658[0n]|BOLD:ABY9096
Idia lubricalis complex|[10903]|MNAE851-13|Canada|Quebec|658[0n]|BOLD:ABY9096
Idia lubricalis|[10904]|LNCC1550-13|United States|North Carolina|658[0n]|BOLD:ABY9096
Idia lubricalis|[10905]|LNCC1551-13|United States|North Carolina|658[0n]|BOLD:ABY9096
Idia lubricalis|[10906]|RDLQF588-06|Canada|Quebec|658[2n]|BOLD:AAA2230
Idia lubricalis|[10907]|PHSEP351-11|Canada|Ontario|652[0n]|BOLD:AAA2230
Idia lubricalis|[10908]|RDLQF076-06|Canada|Quebec|658[0n]|BOLD:AAA2230
Idia lubricalis|[10909]|RDLQF075-06|Canada|Quebec|658[0n]|BOLD:AAA2230
Idia lubricalis|[10910]|PHSEP345-11|Canada|Ontario|652[0n]|BOLD:AAA2230
Idia lubricalis|[10911]|RDLQF592-06|Canada|Quebec|658[0n]|BOLD:AAA2230
Idia lubricalis complex|[10912]|MNAE832-13|Canada|Ontario|639[0n]|BOLD:AAA2230
Idia lubricalis complex|[10913]|MNAE833-13|Canada|Ontario|658[0n]|BOLD:AAA2230
Idia lubricalis complex|[10914]|MNAE837-13|Canada|Ontario|658[0n]|BOLD:AAA2230
Idia lubricalis complex|[10915]|MNAE838-13|Canada|Ontario|658[0n]|BOLD:AAA2230
Idia lubricalis complex|[10916]|MNAE839-13|Canada|Ontario|658[0n]|BOLD:AAA2230
Idia lubricalis complex|[10917]|MNAE847-13|Canada|Ontario|658[0n]|BOLD:AAA2230
Idia lubricalis complex|[10918]|USLEP321-10|United States|Florida|658[0n]|BOLD:AAA2230
Idia sp.|[10919]|MILEP004-09|United States|North Carolina|658[0n]|BOLD:AAA2230
Idia lubricalis|[10920]|LNCC1861-13|United States|North Carolina|658[0n]|BOLD:AAA2230
Idia lubricalis complex|[10921]|BBLSW461-09|United States|Oklahoma|658[0n]|BOLD:AAA2230
Idia lubricalis|[10922]|RDMAB512-06|Canada|Alberta|658[0n]|BOLD:AAA2230
Idia lubricalis|[10923]|RDMAB513-06|Canada|Alberta|658[0n]|BOLD:AAA2230
Idia lubricalis|[10924]|RDMAB514-06|Canada|Alberta|658[0n]|BOLD:AAA2230
Idia lubricalis|[10925]|HKONB471-09|United States|Texas|658[0n]|BOLD:AAA2230
Idia lubricalis complex|[10926]|LPOKB315-09|United States|Oklahoma|658[0n]|BOLD:AAA2230
Idia lubricalis|[10927]|LNCC1272-11|United States|North Carolina|658[0n]|BOLD:AAA2230
Idia lubricalis|[10928]|LNCC809-11|United States|North Carolina|658[0n]|BOLD:AAA2230
Idia sp.|[10929]|LNCB756-09|United States|North Carolina|655[0n]|BOLD:AAA2230
Idia lubricalis complex|[10930]|LPOKC797-09|United States|Oklahoma|658[0n]|BOLD:AAA2230
Idia lubricalis complex|[10931]|LPOKA874-09|United States|Oklahoma|658[0n]|BOLD:AAA2230
Idia lubricalis complex|[10932]|LPOKA306-08|United States|Oklahoma|658[0n]|BOLD:AAA2230
Idia lubricalis complex|[10933]|HKONS150-08|United States|Kentucky|658[0n]|BOLD:AAA2230
Idia sp.|[10934]|MNAE852-13|United States|Florida|658[0n]|BOLD:AAA2230
Idia sp.|[10935]|MNAE856-13|United States|Florida|638[0n]|BOLD:AAA2230
Idia lubricalis|[10936]|LNCC1897-13|United States|North Carolina|658[0n]|BOLD:AAA2230
Idia immaculalis|[10937]|LMEM161-09|United States|Wyoming|658[0n]|BOLD:ACF2450
Idia immaculalis|[10938]|RDNMG565-08|United States|Wyoming|658[0n]|BOLD:ACF2450
Idia immaculalis|[10939]|LMEM159-09|United States|Wyoming|658[0n]|BOLD:ACF2450
Idia immaculalis|[10940]|LMEM158-09|United States|Wyoming|658[0n]|BOLD:ACF2450
Idia immaculalis|[10941]|RDMAB508-06|Canada|Alberta|658[0n]|BOLD:ACF2450
Idia immaculalis|[10942]|RDMAB509-06|Canada|Alberta|622[1n]|BOLD:ACF2450
Idia immaculalis|[10943]|LPSK497-08|Canada|Saskatchewan|658[0n]|BOLD:ACF2450
Idia immaculalis|[10944]|RDNMG563-08|United States|Oregon|658[0n]|BOLD:ACF2450
Idia immaculalis|[10945]|RDNMG564-08|United States|Wyoming|658[0n]|BOLD:ACF2450
Idia immaculalis|[10946]|LMEM160-09|United States|Wyoming|658[0n]|BOLD:ACF2450
Idia immaculalis|[10947]|LMEM162-09|United States|Wyoming|658[0n]|BOLD:ACF2450
Idia immaculalis|[10948]|QUNOD185-10|United States|Texas|658[0n]|BOLD:ACF2450
Idia occidentalis|[10949]|RDMAB510-06|Canada|Alberta|632[0n]|BOLD:ABZ4706
Idia occidentalis|[10950]|RDMAB511-06|Canada|Alberta|656[0n]|BOLD:ABZ4706
Idia occidentalis|[10951]|RDNMG110-08|United States|Colorado|658[0n]|BOLD:ABZ4706
Idia occidentalis|[10952]|RDNMG111-08|United States|Colorado|658[0n]|BOLD:ABZ4706
Idia occidentalis|[10953]|LBCH7408-10|Canada|British Columbia|658[0n]|BOLD:ABZ4706
Idia occidentalis|[10954]|LBCH7407-10|Canada|British Columbia|658[0n]|BOLD:ABZ4706
Idia occidentalis|[10955]|LBCH7324-10|Canada|British Columbia|658[0n]|BOLD:ABZ4706
Idia occidentalis|[10956]|LBCH6243-10|Canada|British Columbia|658[0n]|BOLD:ABZ4706
Idia occidentalis|[10957]|LBCH6135-10|Canada|British Columbia|658[0n]|BOLD:ABZ4706
Idia occidentalis|[10958]|RDNMD797-07|United States|California|658[0n]|BOLD:ABZ4706
Idia occidentalis|[10959]|LBCH7447-10|Canada|British Columbia|638[0n]|BOLD:ABZ4706
Idia occidentalis|[10960]|LBCH7448-10|Canada|British Columbia|658[0n]|BOLD:ABZ4706
Idia occidentalis|[10961]|LBCH7491-10|Canada|British Columbia|658[0n]|BOLD:ABZ4706
Idia occidentalis|[10962]|RDNMF866-08|United States|Arizona|658[0n]|BOLD:ACF3663
Idia sp.|[10963]|QUNOE472-12|United States|Arizona|658[0n]|BOLD:ACF3663
Idia gopheri|[10964]|LNAUS4339-13|United States|Florida|542[1n]|BOLD:ACJ6470
Idia gopheri|[10965]|LNAUS4338-13|United States|Florida|658[0n]|BOLD:ACJ6470
Idia gopheri|[10966]|LNAUS4337-13|United States|Florida|658[0n]|BOLD:ACJ6470
Idia gopheri|[10967]|LNAUS4341-13|United States|Florida|611[0n]|BOLD:ACJ6470
Idia aemula|[10968]|MNAF866-08|Canada|Manitoba|658[0n]|BOLD:AAA2229
Idia aemula|[10969]|MEC612-04|Canada|Quebec|615[4n]|BOLD:AAA2229
Idia aemula|[10970]|RDLQF085-06|Canada|Quebec|595[1n]|BOLD:AAA2229
Idia aemula|[10971]|RDLQF084-06|Canada|Quebec|658[0n]|BOLD:AAA2229
Idia aemula|[10972]|RDLQF086-06|Canada|Quebec|658[0n]|BOLD:AAA2229
Idia aemula|[10973]|RDLQF081-06|Canada|Quebec|658[0n]|BOLD:AAA2229
Idia aemula|[10974]|RDLQF082-06|Canada|Quebec|658[0n]|BOLD:AAA2229
Idia aemula|[10975]|RDLQF091-06|Canada|Quebec|658[0n]|BOLD:AAA2229
Idia aemula|[10976]|RDLQG755-06|Canada|Quebec|658[0n]|BOLD:AAA2229
Idia aemula|[10977]|RDLQG764-06|Canada|Quebec|658[0n]|BOLD:AAA2229
Idia aemula|[10978]|RDLQG884-06|Canada|Quebec|658[0n]|BOLD:AAA2229
Idia aemula|[10979]|RDLQF732-06|Canada|Quebec|658[0n]|BOLD:AAA2229
Idia aemula|[10980]|RDLQG573-06|Canada|Quebec|658[0n]|BOLD:AAA2229
Idia aemula|[10981]|RDLQH019-06|Canada|Quebec|658[0n]|BOLD:AAA2229
Idia aemula|[10982]|MECB135-04|Canada|Quebec|658[0n]|BOLD:AAA2229
Idia aemula|[10983]|BBLPD054-10|Canada|Alberta|658[0n]|BOLD:AAA2229
[illegible]

Idia aemula|[10981]|RDLQH019-06|Canada|Quebec|658[0n]|BOLD:AAA2229
Idia aemula|[10982]|MECB135-04|Canada|Quebec|658[0n]|BOLD:AAA2229
Idia aemula|[10983]|BBLPD054-10|Canada|Alberta|658[0n]|BOLD:AAA2229
Idia aemula|[10984]|BBLPB940-10|Canada|Alberta|658[0n]|BOLD:AAA2229
Idia aemula|[10985]|BBLPB939-10|Canada|Alberta|658[0n]|BOLD:AAA2229
Idia aemula|[10986]|BBLPB938-10|Canada|Alberta|658[0n]|BOLD:AAA2229
Idia aemula|[10987]|BBLPB937-10|Canada|Alberta|658[0n]|BOLD:AAA2229
Idia aemula|[10988]|BBLPB934-10|Canada|Alberta|658[0n]|BOLD:AAA2229
Idia aemula|[10989]|MJMSL158-10|United States|Massachusetts|658[0n]|BOLD:AAA2229
Idia aemula|[10990]|BBLPE614-09|Canada|Nova Scotia|658[0n]|BOLD:AAA2229
Idia aemula|[10991]|RDLQH020-06|Canada|Quebec|658[0n]|BOLD:AAA2229
Idia aemula|[10992]|RDLQG787-06|Canada|Quebec|658[0n]|BOLD:AAA2229
Idia aemula|[10993]|RDLQG677-06|Canada|Quebec|658[0n]|BOLD:AAA2229
Idia aemula|[10994]|RDLQF792-06|Canada|Quebec|658[0n]|BOLD:AAA2229
Idia aemula|[10995]|RDLQF789-06|Canada|Quebec|658[0n]|BOLD:AAA2229
Idia aemula|[10996]|RDLQF731-06|Canada|Quebec|658[0n]|BOLD:AAA2229
Idia aemula|[10997]|RDLQF730-06|Canada|Quebec|658[0n]|BOLD:AAA2229
Idia aemula|[10998]|RDLQF608-06|Canada|Quebec|658[0n]|BOLD:AAA2229
Idia aemula|[10999]|RDLQF606-06|Canada|Quebec|658[0n]|BOLD:AAA2229
Idia aemula|[11000]|XAJ875-06|Canada|Ontario|658[0n]|BOLD:AAA2229
Idia aemula|[11001]|TTMNB266-06|Canada|New Brunswick|658[0n]|BOLD:AAA2229
Idia aemula|[11002]|XAC833-04|Canada|Ontario|658[0n]|BOLD:AAA2229
Idia aemula|[11003]|XAE606-04|Canada|Ontario|658[0n]|BOLD:AAA2229
Idia aemula|[11004]|RDLQF790-06|Canada|Quebec|616[0n]|BOLD:AAA2229
Idia aemula|[11005]|RDLQG602-06|Canada|Quebec|632[0n]|BOLD:AAA2229
Idia aemula|[11006]|BBLPD055-10|Canada|Alberta|658[0n]|BOLD:AAA2229
Idia aemula|[11007]|BBLPD056-10|Canada|Alberta|658[0n]|BOLD:AAA2229
Idia aemula|[11008]|LNCC1004-11|United States|North Carolina|658[0n]|BOLD:AAA2229
Idia aemula|[11009]|LMEM125-09|United States|Mississippi|658[0n]|BOLD:AAA2229
Idia aemula|[11010]|LNCC1636-13|United States|North Carolina|658[0n]|BOLD:AAA2229
Idia aemula|[11011]|LPOKD456-09|United States|Oklahoma|658[0n]|BOLD:AAA2229
Idia aemula|[11012]|BBLSX141-09|United States|Oklahoma|658[0n]|BOLD:AAA2229
Idia aemula|[11013]|LPOKD823-10|United States|Oklahoma|658[0n]|BOLD:AAA2229
Idia aemula|[11014]|BBLSW554-09|United States|Oklahoma|591[0n]|BOLD:AAA2229
Idia aemula|[11015]|BBLSW545-09|United States|Oklahoma|658[0n]|BOLD:AAA2229
Idia aemula|[11016]|LPOKC290-09|United States|Oklahoma|658[0n]|BOLD:AAA2229
Idia aemula|[11017]|LMEM129-09|United States|Mississippi|658[0n]|BOLD:AAA2229
Idia aemula|[11018]|LMEM128-09|United States|Mississippi|658[0n]|BOLD:AAA2229
Idia aemula|[11019]|LPOKA634-09|United States|Oklahoma|658[0n]|BOLD:AAA2229
Idia aemula|[11020]|LPOKA411-09|United States|Oklahoma|658[0n]|BOLD:AAA2229
Idia aemula|[11021]|HKONB419-09|United States|Indiana|658[0n]|BOLD:AAA2229
Idia aemula|[11022]|LPOKA356-08|United States|Oklahoma|658[0n]|BOLD:AAA2229
Idia aemula|[11023]|LPOKA490-09|United States|Oklahoma|658[0n]|BOLD:AAA2229
Idia aemula|[11024]|BBLSW532-09|United States|Oklahoma|633[0n]|BOLD:AAA2229
Idia aemula|[11025]|BBLSW548-09|United States|Oklahoma|638[0n]|BOLD:AAA2229
Idia aemula|[11026]|BBLSW565-09|United States|Oklahoma|658[0n]|BOLD:AAA2229
Idia aemula|[11027]|BBLSW568-09|United States|Oklahoma|658[0n]|BOLD:AAA2229
Idia aemula|[11028]|BBLSW598-09|United States|Oklahoma|658[0n]|BOLD:AAA2229
Idia aemula|[11029]|BBLSW614-09|United States|Oklahoma|658[0n]|BOLD:AAA2229
Idia aemula|[11030]|BBLSW627-09|United States|Oklahoma|658[0n]|BOLD:AAA2229
Idia aemula|[11031]|BBLSW657-09|United States|Oklahoma|658[0n]|BOLD:AAA2229
Idia aemula|[11032]|BBLSW667-09|United States|Oklahoma|658[0n]|BOLD:AAA2229
Idia aemula|[11033]|BBLSW993-09|United States|Texas|658[0n]|BOLD:AAA2229
Idia aemula|[11034]|BBLSX149-09|United States|Oklahoma|658[0n]|BOLD:AAA2229
Idia aemula|[11035]|BBLSX288-09|United States|Oklahoma|658[0n]|BOLD:AAA2229
Idia aemula|[11036]|BBLSX327-09|United States|Oklahoma|658[0n]|BOLD:AAA2229
Idia aemula|[11037]|BBLSX335-09|United States|Oklahoma|658[0n]|BOLD:AAA2229
Idia aemula|[11038]|BBLSX452-09|United States|Oklahoma|658[0n]|BOLD:AAA2229
Idia aemula|[11039]|BBLSY883-09|United States|Oklahoma|658[0n]|BOLD:AAA2229
Idia aemula|[11040]|BBLSY886-09|United States|Oklahoma|658[0n]|BOLD:AAA2229
Idia aemula|[11041]|BBLSZ131-09|United States|Oklahoma|658[0n]|BOLD:AAA2229
Idia aemula|[11042]|LPOKD304-09|United States|Oklahoma|658[0n]|BOLD:AAA2229
Idia aemula|[11043]|LPOKE296-11|United States|Oklahoma|658[0n]|BOLD:AAA2229
Idia aemula|[11044]|BBLOC844-11|United States|Arkansas|658[0n]|BOLD:AAA2229
Idia aemula|[11045]|BBLOC1245-11|United States|Arkansas|658[0n]|BOLD:AAA2229
Idia aemula|[11046]|BBLOD1583-11|United States|Oklahoma|658[0n]|BOLD:AAA2229
Idia aemula|[11047]|HKONB452-09|United States|Kentucky|658[0n]|BOLD:AAA2229
Idia aemula|[11048]|BBLOC1144-11|United States|Arkansas|658[0n]|BOLD:AAA2229
Idia aemula|[11049]|LNCC1003-11|United States|North Carolina|658[0n]|BOLD:AAA2229
Idia aemula|[11050]|LNCC1002-11|United States|North Carolina|658[0n]|BOLD:AAA2229
Idia aemula|[11051]|LILLA057-11|United States|Illinois|658[0n]|BOLD:AAA2229
Idia aemula|[11052]|BBLPB935-10|Canada|Alberta|658[0n]|BOLD:AAA2229
Idia aemula|[11053]|BBLSX528-09|United States|Oklahoma|658[0n]|BOLD:AAA2229
Idia aemula|[11054]|BBLSX351-09|United States|Oklahoma|658[0n]|BOLD:AAA2229
Idia aemula|[11055]|BBLSW535-09|United States|Oklahoma|658[0n]|BOLD:AAA2229
Idia aemula|[11056]|LPOKA035-08|United States|Oklahoma|658[0n]|BOLD:AAA2229
Idia aemula|[11057]|RDLQF788-06|Canada|Quebec|658[0n]|BOLD:AAA2229
Idia aemula|[11058]|LOTB132-05|United States|Tennessee|658[0n]|BOLD:AAA2229
Idia aemula|[11059]|LOT279-04|United States|Tennessee|658[0n]|BOLD:AAA2229
Idia aemula|[11060]|RDLQF079-06|Canada|Quebec|658[0n]|BOLD:AAA2229
Idia aemula|[11061]|RDLQG096-06|Canada|Quebec|632[0n]|BOLD:AAA2229
Idia aemula|[11062]|LNCC1638-13|United States|North Carolina|658[0n]|BOLD:AAA2229
Idia nr. aemula|[11063]|HKONB523-09|United States|Texas|658[0n]|BOLD:AAA2229
Idia nr. aemula|[11064]|HKONS282-08|United States|Texas|658[0n]|BOLD:AAA2229
Idia nr. aemula|[11065]|HKONB537-09|United States|Texas|658[0n]|BOLD:AAA2229
Idia aemula|[11066]|HKONB413-09|United States|Indiana|658[0n]|BOLD:AAA2229
Idia aemula|[11067]|RDLQG797-06|Canada|Quebec|658[0n]|BOLD:AAA2229
Idia aemula|[11068]|RDLQG933-06|Canada|Quebec|658[0n]|BOLD:AAA2229
Idia aemula|[11069]|XAB486-04|Canada|Ontario|658[0n]|BOLD:AAA2229
Idia aemula|[11070]|RDLQB417-05|Canada|Quebec|658[0n]|BOLD:AAA2229
Idia aemula|[11071]|BBLPE118-09|Canada|Nova Scotia|658[0n]|BOLD:AAA2229
Idia aemula|[11072]|BBLPC565-09|Canada|Nova Scotia|658[0n]|BOLD:AAA2229
Idia aemula|[11073]|BBLPC197-09|Canada|Nova Scotia|658[0n]|BOLD:AAA2229
Idia aemula|[11074]|RDLQF609-06|Canada|Quebec|658[0n]|BOLD:AAA2229
Idia aemula|[11075]|BBLPE281-09|Canada|Nova Scotia|648[0n]|BOLD:AAA2229
Idia aemula|[11076]|BBLPB936-10|Canada|Alberta|658[0n]|BOLD:AAA2229
Idia aemula|[11077]|TTMNB267-06|Canada|New Brunswick|658[0n]|BOLD:AAA2229
Idia concisa|[11078]|BBLPC437-09|Canada|New Brunswick|658[0n]|BOLD:AAA2229
Idia concisa|[11079]|TTMNB263-06|Canada|New Brunswick|656[0n]|BOLD:AAA2229
Idia concisa|[11080]|BBLPC170-09|Canada|Nova Scotia|658[0n]|BOLD:AAA2229
Idia concisa|[11081]|RDMAB008-05|Canada|Alberta|658[0n]|BOLD:AAA2229
Idia concisa|[11082]|RDLQH021-06|Canada|Quebec|658[0n]|BOLD:AAA2229
Idia concisa|[11083]|BBLEC310-09|Canada|Nova Scotia|658[0n]|BOLD:AAA2229
[illegible]

Idia concisa|[11081]|RDMAB008-05|Canada|Alberta|658[0n]|BOLD:AAA2229
Idia concisa|[11082]|RDLQH021-06|Canada|Quebec|658[0n]|BOLD:AAA2229
Idia concisa|[11083]|BBLEC310-09|Canada|Nova Scotia|658[0n]|BOLD:AAA2229
Idia concisa|[11084]|BBLPC157-09|Canada|Nova Scotia|658[2n]|BOLD:AAA2229
Idia concisa|[11085]|LBCH4856-10|Canada|British Columbia|658[0n]|BOLD:AAA2229
Idia concisa|[11086]|LBCH4857-10|Canada|British Columbia|658[0n]|BOLD:AAA2229
Idia concisa|[11087]|LBCH4858-10|Canada|British Columbia|658[0n]|BOLD:AAA2229
Idia concisa|[11088]|LBCH4859-10|Canada|British Columbia|658[0n]|BOLD:AAA2229
Idia concisa|[11089]|LBCH4781-10|Canada|British Columbia|658[0n]|BOLD:AAA2229
Idia concisa|[11090]|LBCH4854-10|Canada|British Columbia|658[0n]|BOLD:AAA2229
Idia concisa|[11091]|LBCH3225-10|Canada|British Columbia|658[0n]|BOLD:AAA2229
Idia concisa|[11092]|LBCH3825-10|Canada|British Columbia|658[0n]|BOLD:AAA2229
Idia concisa|[11093]|LBCH4081-10|Canada|British Columbia|658[0n]|BOLD:AAA2229
Idia concisa|[11094]|LBCH4487-10|Canada|British Columbia|658[0n]|BOLD:AAA2229
Idia concisa|[11095]|LBCH895-10|Canada|British Columbia|658[0n]|BOLD:AAA2229
Idia concisa|[11096]|LBCH3082-10|Canada|British Columbia|658[0n]|BOLD:AAA2229
Idia concisa|[11097]|BBLPE112-09|Canada|Nova Scotia|658[0n]|BOLD:AAA2229
Idia concisa|[11098]|LBCH202-10|Canada|British Columbia|658[0n]|BOLD:AAA2229
Idia concisa|[11099]|LBCH321-10|Canada|British Columbia|658[0n]|BOLD:AAA2229
Idia concisa|[11100]|LBCH448-10|Canada|British Columbia|658[0n]|BOLD:AAA2229
Idia concisa|[11101]|LBCH606-10|Canada|British Columbia|658[0n]|BOLD:AAA2229
Idia concisa|[11102]|LBCH744-10|Canada|British Columbia|658[0n]|BOLD:AAA2229
Idia concisa|[11103]|BBLPE070-09|Canada|Nova Scotia|658[0n]|BOLD:AAA2229
Idia concisa|[11104]|BBLPE091-09|Canada|Nova Scotia|658[0n]|BOLD:AAA2229
Idia concisa|[11105]|BBLPC997-09|Canada|Nova Scotia|658[0n]|BOLD:AAA2229
Idia concisa|[11106]|BBLPE067-09|Canada|Nova Scotia|658[0n]|BOLD:AAA2229
Idia concisa|[11107]|BBLEC369-09|Canada|Newfoundland and Labrador|658[0n]|BOLD:AAA2229
Idia concisa|[11108]|BBLEC582-09|Canada|Nova Scotia|658[0n]|BOLD:AAA2229
Idia concisa|[11109]|BBLEC632-09|Canada|Nova Scotia|658[0n]|BOLD:AAA2229
Idia concisa|[11110]|BBLEC648-09|Canada|Nova Scotia|658[0n]|BOLD:AAA2229
Idia concisa|[11111]|BBLEC659-09|Canada|Nova Scotia|658[0n]|BOLD:AAA2229
Idia concisa|[11112]|BBLEC661-09|Canada|Nova Scotia|715[0n]|BOLD:AAA2229
Idia concisa|[11113]|BBLPC586-09|Canada|Nova Scotia|658[0n]|BOLD:AAA2229
Idia concisa|[11114]|BBLPC617-09|Canada|Nova Scotia|658[0n]|BOLD:AAA2229
Idia concisa|[11115]|BBLEC104-09|Canada|Nova Scotia|658[0n]|BOLD:AAA2229
Idia concisa|[11116]|BBLEC308-09|Canada|Nova Scotia|658[0n]|BOLD:AAA2229
Idia concisa|[11117]|BLTIB435-08|Canada|Ontario|658[0n]|BOLD:AAA2229
Idia concisa|[11118]|BLTIB1103-08|Canada|Ontario|658[0n]|BOLD:AAA2229
Idia concisa|[11119]|RDLQG827-06|Canada|Quebec|658[0n]|BOLD:AAA2229
Idia concisa|[11120]|RDLQG830-06|Canada|Quebec|658[0n]|BOLD:AAA2229
Idia concisa|[11121]|RDLQG823-06|Canada|Quebec|658[0n]|BOLD:AAA2229
Idia concisa|[11122]|RDLQG825-06|Canada|Quebec|658[0n]|BOLD:AAA2229
Idia concisa|[11123]|RDLQG784-06|Canada|Quebec|658[0n]|BOLD:AAA2229
Idia concisa|[11124]|RDLQG794-06|Canada|Quebec|658[0n]|BOLD:AAA2229
Idia concisa|[11125]|RDLQG752-06|Canada|Quebec|658[0n]|BOLD:AAA2229
Idia concisa|[11126]|RDLQB412-05|Canada|Quebec|658[0n]|BOLD:AAA2229
Idia concisa|[11127]|MNBB051-05|Canada|New Brunswick|658[0n]|BOLD:AAA2229
Idia concisa|[11128]|XAE402-04|Canada|Ontario|658[0n]|BOLD:AAA2229
Idia concisa|[11129]|XAE329-04|Canada|Ontario|658[0n]|BOLD:AAA2229
Idia concisa|[11130]|XAC608-04|Canada|Ontario|658[0n]|BOLD:AAA2229
Idia concisa|[11131]|XAB121-04|Canada|Ontario|658[0n]|BOLD:AAA2229
Idia concisa|[11132]|XAB099-04|Canada|Ontario|658[0n]|BOLD:AAA2229
Idia concisa|[11133]|LBCG2246-09|Canada|British Columbia|624[0n]|BOLD:AAA2229
Idia concisa|[11134]|XAC467-04|Canada|Ontario|658[0n]|BOLD:AAA2229
Idia concisa|[11135]|RDLQG676-06|Canada|Quebec|658[0n]|BOLD:AAA2229
Idia concisa|[11136]|BBLEC300-09|Canada|Nova Scotia|627[0n]|BOLD:AAA2229
Idia concisa|[11137]|BBLEC658-09|Canada|Nova Scotia|658[0n]|BOLD:AAA2229
Idia concisa|[11138]|BBLPC160-09|Canada|Nova Scotia|658[0n]|BOLD:AAA2229
Idia concisa|[11139]|XAK543-07|Canada|Ontario|658[1n]|BOLD:AAA2229
Idia concisa|[11140]|RDLQG791-06|Canada|Quebec|639[0n]|BOLD:AAA2229
Idia concisa|[11141]|BBLPC989-09|Canada|Nova Scotia|633[0n]|
Idia concisa|[11142]|BBLPE159-09|Canada|Nova Scotia|636[0n]|BOLD:AAA2229
Idia concisa|[11143]|BLTIB670-08|Canada|Ontario|658[0n]|BOLD:AAA2229
Idia concisa|[11144]|XAC860-04|Canada|Ontario|598[1n]|BOLD:AAA2229
Idia concisa|[11145]|LBCH081-10|Canada|British Columbia|643[0n]|BOLD:AAA2229
Idia concisa|[11146]|LBCH4860-10|Canada|British Columbia|658[0n]|BOLD:AAA2229
Idia concisa|[11147]|LOWCD873-06|Canada|British Columbia|600[0n]|BOLD:AAA2229
Idia concisa|[11148]|LBCH4861-10|Canada|British Columbia|658[0n]|BOLD:AAA2229
Idia concisa|[11149]|RDLQG783-06|Canada|Quebec|658[0n]|BOLD:AAA2229
Idia concisa|[11150]|BBLPD602-10|Canada|British Columbia|658[0n]|BOLD:AAA2229
Idia concisa|[11151]|BBLPC557-09|Canada|New Brunswick|658[0n]|BOLD:AAA2229
Idia concisa|[11152]|MNBB034-05|Canada|New Brunswick|614[1n]|BOLD:AAA2229
Idia concisa|[11153]|LBCH3489-10|Canada|British Columbia|658[0n]|BOLD:AAA2229
Idia concisa|[11154]|LBCH4855-10|Canada|British Columbia|658[0n]|BOLD:AAA2229
Idia concisa|[11155]|LBCG798-09|Canada|British Columbia|658[0n]|BOLD:AAA2229
Idia concisa|[11156]|BBLPC491-09|Canada|New Brunswick|658[0n]|BOLD:AAA2229
Idia concisa|[11157]|BBLPC581-09|Canada|Nova Scotia|658[0n]|BOLD:AAA2229
Idia concisa|[11158]|BBLPC762-09|Canada|Newfoundland and Labrador|658[0n]|BOLD:AAA2229
Idia concisa|[11159]|BLTIB1127-08|Canada|Ontario|658[0n]|BOLD:AAA2229
Idia concisa|[11160]|BLTIB560-08|Canada|Ontario|658[0n]|
Idia concisa|[11161]|BLTIB354-08|Canada|Ontario|658[0n]|BOLD:AAA2229
Idia concisa|[11162]|RDLQG914-06|Canada|Quebec|658[0n]|BOLD:AAA2229
Idia concisa|[11163]|LOWCE860-06|Canada|British Columbia|658[0n]|BOLD:AAA2229
Idia concisa|[11164]|LOWCE795-06|Canada|British Columbia|658[0n]|BOLD:AAA2229
Idia concisa|[11165]|TTMNB265-06|Canada|New Brunswick|658[0n]|BOLD:AAA2229
Idia concisa|[11166]|TTMNB264-06|Canada|New Brunswick|658[0n]|BOLD:AAA2229
Idia concisa|[11167]|XAE589-04|Canada|Ontario|658[0n]|BOLD:AAA2229
Idia concisa|[11168]|BLTIB608-08|Canada|Ontario|658[1n]|BOLD:AAA2229
Idia concisa|[11169]|PHMNB255-04|Canada|New Brunswick|609[0n]|BOLD:AAA2229
Idia concisa|[11170]|PHMNB073-03|Canada|New Brunswick|639[0n]|BOLD:AAA2229
Idia concisa|[11171]|BLTIB573-08|Canada|Ontario|631[0n]|BOLD:AAA2229
Idia concisa|[11172]|PHMNB106-04|Canada|New Brunswick|616[2n]|BOLD:AAA2229
Idia concisa|[11173]|PMG122-03|Canada|Ontario|617[0n]|BOLD:AAA2229
Idia concisa|[11174]|BBLPE048-09|Canada|Nova Scotia|658[0n]|BOLD:AAA2229
Idia concisa|[11175]|LILLA603-11|United States|Illinois|658[1n]|BOLD:AAA2229
Idia concisa|[11176]|LOWCE801-06|Canada|British Columbia|658[0n]|BOLD:AAA2229
Idia concisa|[11177]|XAE615-04|Canada|Ontario|658[0n]|BOLD:AAA2229
Idia concisa|[11178]|LOWCC918-05|Canada|British Columbia|552[0n]|BOLD:AAA2229
Idia concisa|[11179]|LOWCB639-05|Canada|British Columbia|658[0n]|BOLD:AAA2229
Idia concisa|[11180]|LBCG1822-09|Canada|British Columbia|658[0n]|BOLD:AAA2229
Idia concisa|[11181]|LBCD410-05|Canada|British Columbia|658[0n]|BOLD:AAA2229
Idia concisa|[11182]|LBCG909-09|Canada|British Columbia|658[0n]|BOLD:AAA2229
Idia concisa|[11183]|LPABC242-09|Canada|Alberta|658[1n]|BOLD:AAA2229

Idia concisa|[11181]|LBCD410-05|Canada|British Columbia|658[0n]|BOLD:AAA2229
Idia concisa|[11182]|LBCG909-09|Canada|British Columbia|658[0n]|BOLD:AAA2229
Idia concisa|[11183]|LPABC242-09|Canada|Alberta|658[1n]|BOLD:AAA2229
Idia concisa|[11184]|BBLEC740-09|Canada|Nova Scotia|658[0n]|BOLD:AAA2229
Idia concisa|[11185]|LILLA154-11|United States|Illinois|658[0n]|BOLD:AAA2229
Idia concisa|[11186]|BBLPD603-10|Canada|British Columbia|658[0n]|BOLD:AAA2229
Idia concisa|[11187]|LBCH1481-10|Canada|British Columbia|658[0n]|BOLD:AAA2229
Idia concisa|[11188]|LBCH1480-10|Canada|British Columbia|658[0n]|BOLD:AAA2229
Idia concisa|[11189]|LBCG1997-09|Canada|British Columbia|658[0n]|BOLD:AAA2229
Idia concisa|[11190]|LBCG895-09|Canada|British Columbia|658[0n]|BOLD:AAA2229
Idia concisa|[11191]|LBCG589-09|Canada|British Columbia|658[0n]|BOLD:AAA2229
Idia concisa|[11192]|MNAG088-08|Canada|Manitoba|658[0n]|BOLD:AAA2229
Idia concisa|[11193]|LBCD409-05|Canada|British Columbia|658[0n]|BOLD:AAA2229
Idia concisa|[11194]|LBCD408-05|Canada|British Columbia|658[0n]|BOLD:AAA2229
Idia concisa|[11195]|XAC023-04|Canada|Ontario|599[0n]|BOLD:AAA2229
Idia concisa|[11196]|RDMAB007-05|Canada|Alberta|592[0n]|BOLD:AAA2229
Idia concisa|[11197]|SSPAB033-13|Canada|Saskatchewan|574[0n]|BOLD:AAA2229
Idia aemula|[11198]|MNAF485-08|Canada|Manitoba|658[1n]|BOLD:AAA2229
Idia aemula|[11199]|ABNCC099-07|United States|Kentucky|649[2n]|BOLD:AAA2229
Idia aemula|[11200]|PHSEP350-11|Canada|Ontario|652[0n]|BOLD:AAA2229
Idia aemula|[11201]|HKONB449-09|United States|Kentucky|658[0n]|BOLD:AAA2229
Idia aemula|[11202]|LNCC1005-11|United States|North Carolina|658[0n]|BOLD:AAA2229
Idia aemula|[11203]|LNCC1635-13|United States|North Carolina|658[0n]|BOLD:AAA2229
Idia aemula|[11204]|LOT485-04|United States|Tennessee|658[0n]|BOLD:AAA2229
Idia aemula|[11205]|LPOKA010-08|United States|Oklahoma|658[0n]|BOLD:AAA2229
Idia aemula|[11206]|RDLQG584-06|Canada|Quebec|658[0n]|BOLD:AAA2229
Idia aemula|[11207]|RDLQF791-06|Canada|Quebec|658[0n]|BOLD:AAA2229
Idia aemula|[11208]|RDLQF787-06|Canada|Quebec|658[0n]|BOLD:AAA2229
Idia aemula|[11209]|RDLQF607-06|Canada|Quebec|658[0n]|BOLD:AAA2229
Idia aemula|[11210]|XAJ860-06|Canada|Ontario|658[0n]|BOLD:AAA2229
Idia aemula|[11211]|RDLQF090-06|Canada|Quebec|658[0n]|BOLD:AAA2229
Idia aemula|[11212]|ABNCC100-07|United States|Virginia|618[4n]|BOLD:AAA2229
Idia aemula|[11213]|HKONB451-09|United States|Kentucky|658[0n]|BOLD:AAA2229
Idia aemula|[11214]|LNCC1001-11|United States|North Carolina|658[0n]|BOLD:AAA2229
Idia aemula|[11215]|RDLQF077-06|Canada|Quebec|658[0n]|BOLD:AAA2229
Idia aemula|[11216]|RDLQF088-06|Canada|Quebec|658[0n]|BOLD:AAA2229
Idia aemula|[11217]|LNCC1637-13|United States|North Carolina|627[0n]|BOLD:AAA2229
Idia aemula|[11218]|BBLPF076-10|Canada|Alberta|658[0n]|BOLD:AAA2229
Idia aemula|[11219]|BBLPD107-10|Canada|British Columbia|658[0n]|BOLD:AAA2229
Idia aemula|[11220]|LOWCC514-05|Canada|British Columbia|658[0n]|BOLD:AAA2229
Idia aemula|[11221]|LPMNB405-09|Canada|Manitoba|636[0n]|BOLD:AAA2229
Idia aemula|[11222]|CNCLB648-14|United States|Colorado|658[0n]|BOLD:AAA2229
Idia aemula|[11223]|CNCLB650-14|United States|Colorado|611[0n]|BOLD:AAA2229
Idia aemula|[11224]|CNCLB651-14|United States|California|658[0n]|BOLD:AAA2229
Idia aemula|[11225]|LOTB130-05|United States|Tennessee|658[0n]|BOLD:AAA2229
Idia aemula|[11226]|MJMSL100-10|United States|Massachusetts|658[0n]|BOLD:AAA2229
Idia aemula|[11227]|MJMSL104-10|United States|Massachusetts|658[0n]|BOLD:AAA2229
Idia aemula|[11228]|LGSMC814-05|United States|Tennessee|658[0n]|BOLD:AAA2229
Idia aemula|[11229]|XAH215-05|Canada|Ontario|649[0n]|BOLD:AAA2229
Idia aemula|[11230]|LOCT117-05|United States|Connecticut|658[0n]|BOLD:AAA2229
Idia aemula|[11231]|LGSMD242-05|United States|Tennessee|658[0n]|BOLD:AAA2229
Idia aemula|[11232]|XAH593-05|Canada|Ontario|658[0n]|BOLD:AAA2229
Idia aemula|[11233]|LNC304-05|United States|North Carolina|658[0n]|BOLD:AAA2229
Idia aemula|[11234]|XAH713-05|Canada|Ontario|658[0n]|BOLD:AAA2229
Idia aemula|[11235]|XAH714-05|Canada|Ontario|658[0n]|BOLD:AAA2229
Idia aemula|[11236]|RDLQF080-06|Canada|Quebec|658[0n]|BOLD:AAA2229
Idia aemula|[11237]|LOFLA041-06|United States|Florida|658[0n]|BOLD:AAA2229
Idia aemula|[11238]|LOFLA142-06|United States|Florida|658[0n]|BOLD:AAA2229
Idia aemula|[11239]|LOFLA144-06|United States|Florida|658[0n]|BOLD:AAA2229
Idia aemula|[11240]|LSEU363-06|United States|Georgia|658[0n]|BOLD:AAA2229
Idia aemula|[11241]|LSEU364-06|United States|Georgia|658[0n]|BOLD:AAA2229
Idia aemula|[11242]|RDLQF379-06|Canada|Quebec|658[0n]|BOLD:AAA2229
Idia aemula|[11243]|LGSMG467-07|United States|North Carolina|658[0n]|BOLD:AAA2229
Idia aemula|[11244]|LGSMG468-07|United States|Tennessee|658[0n]|BOLD:AAA2229
Idia aemula|[11245]|LGSMG469-07|United States|Tennessee|658[0n]|BOLD:AAA2229
Idia aemula|[11246]|HKONS335-08|United States|Florida|658[0n]|BOLD:AAA2229
Idia aemula|[11247]|RDNME584-08|United States|Florida|658[0n]|BOLD:AAA2229
Idia aemula|[11248]|XAH420-05|Canada|Ontario|658[0n]|BOLD:AAA2229
Idia aemula|[11249]|XAH426-05|Canada|Ontario|658[0n]|BOLD:AAA2229
Idia aemula|[11250]|XAH568-05|Canada|Ontario|658[0n]|BOLD:AAA2229
Idia aemula|[11251]|XAH575-05|Canada|Ontario|658[0n]|BOLD:AAA2229
Idia aemula|[11252]|XAH576-05|Canada|Ontario|658[0n]|BOLD:AAA2229
Idia aemula|[11253]|XAH583-05|Canada|Ontario|658[0n]|BOLD:AAA2229
Idia aemula|[11254]|LPSK135-08|Canada|Saskatchewan|658[0n]|BOLD:AAA2229
Idia aemula|[11255]|LPSK258-08|Canada|Saskatchewan|658[0n]|BOLD:AAA2229
Idia aemula|[11256]|LGSMC813-05|United States|Tennessee|658[0n]|BOLD:AAA2229
Idia aemula|[11257]|LOTB129-05|United States|Tennessee|658[0n]|BOLD:AAA2229
Idia aemula|[11258]|LNCB711-09|United States|North Carolina|658[0n]|BOLD:AAA2229
Idia aemula|[11259]|BBLCU031-09|United States|Michigan|658[0n]|BOLD:AAA2229
Idia aemula|[11260]|LOTB131-05|United States|Tennessee|658[0n]|BOLD:AAA2229
Idia aemula|[11261]|LOTB133-05|United States|Tennessee|658[0n]|BOLD:AAA2229
Idia aemula|[11262]|BBLCU034-09|United States|Michigan|658[0n]|BOLD:AAA2229
Idia aemula|[11263]|BBLCU054-09|United States|Michigan|658[0n]|BOLD:AAA2229
Idia aemula|[11264]|BBLCU066-09|United States|Michigan|658[0n]|BOLD:AAA2229
Idia aemula|[11265]|BBLCU128-09|United States|Michigan|658[0n]|BOLD:AAA2229
Idia aemula|[11266]|BBLCU139-09|United States|Michigan|658[0n]|BOLD:AAA2229
Idia aemula|[11267]|BBLCU140-09|United States|Michigan|658[0n]|BOLD:AAA2229
Idia aemula|[11268]|BBLCU247-09|United States|Michigan|658[0n]|BOLD:AAA2229
Idia aemula|[11269]|BBLCU322-09|United States|Michigan|658[0n]|BOLD:AAA2229
Idia aemula|[11270]|XAH327-05|Canada|Ontario|658[0n]|BOLD:AAA2229
Idia aemula|[11271]|XAH393-05|Canada|Ontario|658[0n]|BOLD:AAA2229
Idia aemula|[11272]|XAH478-05|Canada|Ontario|658[0n]|BOLD:AAA2229
Idia aemula|[11273]|XAH490-05|Canada|Ontario|658[0n]|BOLD:AAA2229
Idia aemula|[11274]|LGSMC812-05|United States|Tennessee|658[0n]|BOLD:AAA2229
Idia aemula|[11275]|LGSMC811-05|United States|Tennessee|658[0n]|BOLD:AAA2229
Idia aemula|[11276]|XAD499-04|Canada|Ontario|658[0n]|BOLD:AAA2229
Idia aemula|[11277]|XAE332-04|Canada|Ontario|658[0n]|BOLD:AAA2229
Idia aemula|[11278]|XAC831-04|Canada|Ontario|658[0n]|BOLD:AAA2229
Idia aemula|[11279]|LOT284-04|United States|Tennessee|658[0n]|BOLD:AAA2229
Idia aemula|[11280]|LOT283-04|United States|Tennessee|658[0n]|BOLD:AAA2229
Idia aemula|[11281]|LOT282-04|United States|Tennessee|658[0n]|BOLD:AAA2229
Idia aemula|[11282]|LOT281-04|United States|Tennessee|658[0n]|BOLD:AAA2229
Idia aemula|[11283]|LOT280-04|United States|Tennessee|658[0n]|BOLD:AAA2229

Idia aemula|[11281]|LOT282-04|United States|Tennessee|658[0n]|BOLD:AAA2229
Idia aemula|[11282]|LOT281-04|United States|Tennessee|658[0n]|BOLD:AAA2229
Idia aemula|[11283]|LOT280-04|United States|Tennessee|658[0n]|BOLD:AAA2229
Idia aemula|[11284]|LGSM642-04|United States|Tennessee|658[0n]|BOLD:AAA2229
Idia aemula|[11285]|XAB659-04|Canada|Ontario|658[1n]|BOLD:AAA2229
Idia aemula|[11286]|RDLQF092-06|Canada|Quebec|658[0n]|BOLD:AAA2229
Idia aemula|[11287]|LOCT303-05|United States|Connecticut|658[0n]|BOLD:AAA2229
Idia aemula|[11288]|LNCNW072-06|United States|North Carolina|658[0n]|BOLD:AAA2229
Idia aemula|[11289]|XAH715-05|Canada|Ontario|597[0n]|BOLD:AAA2229
Idia aemula|[11290]|LOCT397-05|United States|Connecticut|608[0n]|BOLD:AAA2229
Idia aemula|[11291]|LNCB015-06|United States|North Carolina|608[0n]|BOLD:AAA2229
Idia aemula|[11292]|RDMAB493-06|Canada|Alberta|612[3n]|BOLD:AAA2229
Idia aemula|[11293]|RDMAB492-06|Canada|Alberta|658[0n]|BOLD:AAA2229
Idia aemula|[11294]|LMEM127-09|United States|South Carolina|536[0n]|BOLD:AAA2229
Idia aemula|[11295]|MILEP005-09|United States|North Carolina|643[0n]|BOLD:AAA2229
Idia aemula|[11296]|BBLCU351-09|United States|Michigan|658[0n]|BOLD:AAA2229
Idia aemula|[11297]|RDNMI037-10|United States|Colorado|658[0n]|BOLD:AAA2229
Idia aemula|[11298]|MJMSL036-10|United States|Massachusetts|658[0n]|BOLD:AAA2229
Idia aemula|[11299]|MJMSL101-10|United States|Massachusetts|658[0n]|BOLD:AAA2229
Idia aemula|[11300]|MJMSL102-10|United States|Massachusetts|658[0n]|BOLD:AAA2229
Idia aemula|[11301]|MJMSL103-10|United States|Massachusetts|658[0n]|BOLD:AAA2229
Idia aemula|[11302]|MJMSL105-10|United States|Massachusetts|658[0n]|BOLD:AAA2229
Idia aemula|[11303]|MJMSL156-10|United States|Massachusetts|658[0n]|BOLD:AAA2229
Idia aemula|[11304]|MJMSL157-10|United States|Massachusetts|658[0n]|BOLD:AAA2229
Idia aemula|[11305]|LILLA548-11|United States|Illinois|658[0n]|BOLD:AAA2229
Idia aemula|[11306]|LPSK157-08|Canada|Saskatchewan|658[0n]|BOLD:AAA2229
Idia sp.|[11307]|BBLOC1343-11|United States|California|658[0n]|BOLD:AAA2229
Idia sp.|[11308]|CNCLB645-14|United States|Washington|658[0n]|BOLD:AAA2229
Idia sp.|[11309]|CNCLB646-14|United States|Washington|611[0n]|BOLD:AAA2229
Idia sp.|[11310]|CNCLB647-14|United States|Washington|658[0n]|BOLD:AAA2229
Idia suffusalis|[11311]|ABNCC017-07|United States|Arizona|612[0n]|BOLD:AAA2229
Idia suffusalis|[11312]|ABNCC016-07|United States|Arizona|616[0n]|BOLD:AAA2229
Idia suffusalis|[11313]|RDNMF842-08|United States|Arizona|658[0n]|BOLD:AAA2229
Idia suffusalis|[11314]|CNCLB643-14|United States|Arizona|658[0n]|BOLD:AAA2229
Idia suffusalis|[11315]|CNCLB644-14|United States|Arizona|658[0n]|BOLD:AAA2229
Idia suffusalis|[11316]|CNCLB652-14|United States|Arizona|658[0n]|BOLD:AAA2229
Idia parvulalis|[11317]|RDNME895-08|United States|Arizona|658[0n]|BOLD:AAH5660
Idia parvulalis|[11318]|CMAZA164-09|United States|Arizona|658[0n]|BOLD:AAH5660
Idia parvulalis|[11319]|MNAE858-13|United States|Arizona|658[0n]|BOLD:AAH5660
Idia parvulalis|[11320]|MNAE859-13|United States|Arizona|658[0n]|BOLD:AAH5660
Idia sp.|[11321]|MNAE860-13|United States|Arizona|658[0n]|BOLD:ACF4833
Idia sp.|[11322]|MNAE857-13|United States|Arizona|658[0n]|BOLD:ACF4833
Idia lubricalis complex|[11323]|CNCLB2285-14|United States|Arizona|658[0n]|BOLD:ACF4833
Idia forbesii|[11324]|LMEM142-09|United States|Tennessee|658[0n]|BOLD:AAB5884
Idia forbesii|[11325]|LMEM140-09|United States|Tennessee|658[0n]|BOLD:AAB5884
Idia forbesii|[11326]|ABNCC059-07|United States|Virginia|636[0n]|BOLD:AAB5884
Idia forbesii|[11327]|LMEM141-09|United States|Tennessee|658[0n]|BOLD:AAB5884
Idia forbesii|[11328]|LNCC1522-13|United States|North Carolina|658[0n]|BOLD:AAB5884
Idia forbesii|[11329]|HKONS302-08|United States|Florida|658[0n]|BOLD:AAB5885
Idia forbesii|[11330]|HKONS303-08|United States|Florida|658[0n]|BOLD:AAB5885
Idia forbesii|[11331]|MILEQ277-11|United States|Alabama|658[0n]|BOLD:AAB5885
Idia forbesii|[11332]|RDLQF660-06|Canada|Quebec|639[0n]|BOLD:ABZ1974
Idia forbesii|[11333]|RDLQG105-06|Canada|Quebec|632[0n]|BOLD:ABZ1974
Idia forbesii|[11334]|RDLQF659-06|Canada|Quebec|642[0n]|BOLD:ABZ1974
Idia forbesii|[11335]|RDLQG122-06|Canada|Quebec|656[0n]|BOLD:ABZ1974
Idia forbesii|[11336]|RDLQF610-06|Canada|Quebec|658[0n]|BOLD:ABZ1974
Idia forbesii|[11337]|RDLQF661-06|Canada|Quebec|620[0n]|BOLD:ABZ1974
Idia forbesii|[11338]|RDLQG123-06|Canada|Quebec|636[0n]|BOLD:ABZ1974
Idia forbesii|[11339]|LMEM143-09|United States|Mississippi|658[0n]|BOLD:AAB5882
Idia forbesii|[11340]|LPOKA030-08|United States|Oklahoma|658[0n]|BOLD:AAB5882
Idia forbesii|[11341]|LGSMG473-07|United States|Tennessee|658[0n]|BOLD:AAB5882
Idia forbesii|[11342]|LGSMG472-07|United States|Tennessee|658[0n]|BOLD:AAB5882
Idia forbesii|[11343]|LGSMG471-07|United States|Tennessee|658[0n]|BOLD:AAB5882
Idia forbesii|[11344]|LGSM759-04|United States|Tennessee|658[0n]|BOLD:AAB5882
Idia forbesii|[11345]|LGSM682-04|United States|Tennessee|658[0n]|BOLD:AAB5882
Idia forbesii|[11346]|ABNCC060-07|United States|Arkansas|633[0n]|BOLD:AAB5882
Idia forbesii|[11347]|LMEM139-09|United States|Arkansas|575[0n]|BOLD:AAB5882
Idia forbesii|[11348]|LNCC1567-13|United States|North Carolina|658[0n]|BOLD:AAB5882
Idia forbesii|[11349]|LNCC1568-13|United States|North Carolina|658[0n]|BOLD:AAB5882
Idia forbesii|[11350]|LNCC1634-13|United States|North Carolina|658[0n]|BOLD:AAB5882
Idia terrebralis|[11351]|RDNMJ554-11|United States|Arizona|658[0n]|BOLD:AAT9794
Idia terrebralis|[11352]|RDNMJ819-11|United States|Arizona|658[0n]|BOLD:AAT9794
Idia terrebralis|[11353]|IAWLB292-11|United States|Arizona|658[0n]|BOLD:AAT9794
Idia laurentii|[11354]|LGSM683-04|United States|North Carolina|658[0n]|BOLD:AAC0879
Idia scobialis|[11355]|XAJ859-06|Canada|Ontario|658[0n]|BOLD:AAC0879
Idia scobialis|[11356]|XAJ862-06|Canada|Ontario|658[0n]|BOLD:AAC0879
Idia scobialis|[11357]|QUNOB552-09|United States|Kentucky|658[0n]|BOLD:AAC0879
Idia scobialis|[11358]|LNCC837-11|United States|North Carolina|658[0n]|BOLD:AAC0879
Idia scobialis|[11359]|LNCC838-11|United States|North Carolina|658[0n]|BOLD:AAC0879
Idia scobialis|[11360]|LNCC839-11|United States|North Carolina|658[0n]|BOLD:AAC0879
Idia scobialis|[11361]|LNCC840-11|United States|North Carolina|658[0n]|BOLD:AAC0879
Idia scobialis|[11362]|LNCC1208-11|United States|North Carolina|658[0n]|BOLD:AAC0879
Idia scobialis|[11363]|LNCC1209-11|United States|North Carolina|658[0n]|BOLD:AAC0879
Idia scobialis|[11364]|LNCC1210-11|United States|North Carolina|658[0n]|BOLD:AAC0879
Idia scobialis|[11365]|LNCC1412-11|United States|North Carolina|658[0n]|BOLD:AAC0879
Idia laurentii|[11366]|LSEU711-06|United States|Georgia|658[0n]|BOLD:AAC0879
Idia laurentii|[11367]|LSEU712-06|United States|Georgia|658[0n]|BOLD:AAC0879
Idia laurentii|[11368]|LGSMG476-07|United States|North Carolina|658[0n]|BOLD:AAC0879
Idia laurentii|[11369]|HKONS149-08|United States|Kentucky|657[0n]|BOLD:AAC0879
Idia laurentii|[11370]|HKONS151-08|United States|Kentucky|658[0n]|BOLD:AAC0879
Idia laurentii|[11371]|LNCC996-11|United States|North Carolina|658[0n]|BOLD:AAC0879
Idia laurentii|[11372]|LSEU713-06|United States|Georgia|658[0n]|BOLD:AAC0879
Idia laurentii|[11373]|LNCC205-10|United States|North Carolina|658[0n]|BOLD:AAC0879
Idia laurentii|[11374]|LNCC206-10|United States|North Carolina|658[0n]|BOLD:AAC0879
Idia laurentii|[11375]|LNCC207-10|United States|North Carolina|658[0n]|BOLD:AAC0879
Idia laurentii|[11376]|LNCC254-10|United States|North Carolina|658[0n]|BOLD:AAC0879
Idia laurentii|[11377]|LNCC255-10|United States|North Carolina|658[0n]|BOLD:AAC0879
Idia laurentii|[11378]|LNCC990-11|United States|North Carolina|658[0n]|BOLD:AAC0879
Idia laurentii|[11379]|LNCC991-11|United States|North Carolina|658[0n]|BOLD:AAC0879
Idia laurentii|[11380]|LNCC992-11|United States|North Carolina|658[0n]|BOLD:AAC0879
Idia laurentii|[11381]|LNCC993-11|United States|North Carolina|658[0n]|BOLD:AAC0879
Idia laurentii|[11382]|LNCC994-11|United States|North Carolina|658[0n]|BOLD:AAC0879
Idia laurentii|[11383]|LNCC995-11|United States|North Carolina|658[0n]|BOLD:AAC0879

Idia laurentii|[11381]|LNCC993-11|United States|North Carolina|658[0n]|BOLD:AAC0879
Idia laurentii|[11382]|LNCC994-11|United States|North Carolina|658[0n]|BOLD:AAC0879
Idia laurentii|[11383]|LNCC995-11|United States|North Carolina|658[0n]|BOLD:AAC0879
Idia laurentii|[11384]|CNCLB2648-14|United States|North Carolina|658[0n]|BOLD:AAC0879
Idia diminuendis|[11385]|LOFLC012-06|United States|Florida|674[0n]|BOLD:ACE3112
Idia diminuendis|[11386]|MNAB109-07|United States|Florida|603[0n]|BOLD:ACE3112
Idia diminuendis|[11387]|BBLSW594-09|United States|Oklahoma|656[0n]|BOLD:ACE4385
Idia diminuendis|[11388]|BBLSW555-09|United States|Oklahoma|658[0n]|BOLD:ACE4385
Idia diminuendis|[11389]|LNCB022-06|United States|North Carolina|629[1n]|BOLD:ACE4385
Idia diminuendis|[11390]|BBLSW641-09|United States|Oklahoma|658[0n]|BOLD:ACE4385
Idia diminuendis|[11391]|LNCB021-06|United States|North Carolina|617[0n]|BOLD:ACE4385
Idia diminuendis|[11392]|LPOKB483-09|United States|Oklahoma|658[0n]|BOLD:ACE4385
Idia diminuendis|[11393]|BBLSW593-09|United States|Oklahoma|658[0n]|BOLD:ACE4385
Idia diminuendis|[11394]|BBLSW615-09|United States|Oklahoma|658[0n]|BOLD:ACE4385
Idia diminuendis|[11395]|BBLSX130-09|United States|Oklahoma|658[0n]|BOLD:ACE4385
Idia diminuendis|[11396]|BBLSX136-09|United States|Oklahoma|658[0n]|BOLD:ACE4385
Idia diminuendis|[11397]|LGSMG475-07|United States|Tennessee|658[0n]|BOLD:AAB5887
Idia diminuendis|[11398]|MECB117-04|Canada|Quebec|658[0n]|BOLD:AAB5887
Idia diminuendis|[11399]|LGSMC831-05|United States|Tennessee|658[0n]|BOLD:AAB5887
Idia diminuendis|[11400]|LOTB157-05|United States|Tennessee|658[0n]|BOLD:AAB5887
Idia diminuendis|[11401]|LGSMC834-05|United States|Tennessee|658[0n]|BOLD:AAB5887
Idia diminuendis|[11402]|LMEM147-09|United States|Alabama|658[0n]|BOLD:AAB5887
Idia diminuendis|[11403]|LMEM148-09|United States|Alabama|658[0n]|BOLD:AAB5887
Idia diminuendis|[11404]|LGSMC833-05|United States|Tennessee|658[0n]|BOLD:AAB5887
Idia diminuendis|[11405]|CNCLB2787-14|United States|North Carolina|658[0n]|BOLD:AAB5887
Idia diminuendis|[11406]|MILEQ216-11|United States|Georgia|658[0n]|BOLD:AAB5887
Idia diminuendis|[11407]|LMEM145-09|United States|Alabama|658[0n]|BOLD:AAB5887
Idia sp.|[11408]|MILEQ279-11|United States|Alabama|658[0n]|BOLD:AAB5887
Idia sp.|[11409]|MILEQ281-11|United States|Alabama|658[0n]|BOLD:AAB5887
Idia diminuendis|[11410]|HKONS338-08|United States|Florida|658[0n]|BOLD:AAB5887
Idia sp.|[11411]|MILEQ280-11|United States|Alabama|658[0n]|BOLD:AAB5887
Idia diminuendis|[11412]|LGSMC830-05|United States|Tennessee|658[0n]|BOLD:AAB5887
Idia diminuendis|[11413]|LGSMC832-05|United States|Tennessee|658[0n]|BOLD:AAB5887
Idia diminuendis|[11414]|LGSMC835-05|United States|Tennessee|658[0n]|BOLD:AAB5887
Idia diminuendis|[11415]|QUNOC092-09|United States|Kentucky|658[0n]|BOLD:AAB5887
Idia diminuendis|[11416]|QUNOC093-09|United States|Kentucky|658[0n]|BOLD:AAB5887
Idia diminuendis|[11417]|CNCLB2872-14|United States|North Carolina|658[0n]|BOLD:AAB5887
Idia lubricalis|[11418]|LNCB700-09|United States|North Carolina|658[0n]|BOLD:AAA5904
Idia lubricalis|[11419]|LNCB701-09|United States|North Carolina|658[0n]|BOLD:AAA5904
Idia julia|[11420]|LNCB020-06|United States|North Carolina|615[0n]|BOLD:AAB5894
Idia julia|[11421]|USLEP1279-10|United States|Arkansas|658[0n]|BOLD:AAB5894
Idia julia|[11422]|LGSM506-04|United States|North Carolina|658[0n]|BOLD:AAB5894
Idia julia|[11423]|HKONS341-08|United States|Florida|658[0n]|BOLD:AAB5894
Idia julia|[11424]|LMEM136-09|United States|Tennessee|658[0n]|BOLD:AAB5894
Idia julia|[11425]|LNCB805-09|United States|North Carolina|624[0n]|BOLD:AAB5894
Idia julia|[11426]|LGSM505-04|United States|North Carolina|658[0n]|BOLD:AAB5894
Idia julia|[11427]|LNCB018-06|United States|North Carolina|658[0n]|BOLD:AAB5894
Idia julia|[11428]|LNCB019-06|United States|North Carolina|658[0n]|BOLD:AAB5894
Idia julia|[11429]|LGSMG474-07|United States|Tennessee|658[0n]|BOLD:AAB5894
Idia julia|[11430]|LMEM135-09|United States|Tennessee|658[0n]|BOLD:AAB5894
Idia julia|[11431]|LNCC708-11|United States|North Carolina|658[0n]|BOLD:AAB5894
Idia julia|[11432]|LNCC709-11|United States|North Carolina|658[0n]|BOLD:AAB5894
Idia julia|[11433]|LNCC810-11|United States|North Carolina|658[0n]|BOLD:AAB5894
Idia julia|[11434]|LNCC811-11|United States|North Carolina|658[0n]|BOLD:AAB5894
Idia julia|[11435]|LNCC832-11|United States|North Carolina|658[0n]|BOLD:AAB5894
Idia julia|[11436]|LNCC899-11|United States|North Carolina|658[0n]|BOLD:AAB5894
Idia julia|[11437]|LNCC902-11|United States|North Carolina|658[0n]|BOLD:AAB5894
Idia julia|[11438]|MILEQ215-11|United States|Georgia|658[0n]|BOLD:AAB5894
Idia julia|[11439]|LSEU212-06|United States|Georgia|658[0n]|BOLD:AAB5894
Idia julia|[11440]|LPOKA383-09|United States|Oklahoma|658[0n]|BOLD:AAB5894
Idia julia|[11441]|LPOKA389-09|United States|Oklahoma|658[0n]|BOLD:AAB5894
Idia julia|[11442]|LPOKA498-09|United States|Oklahoma|658[0n]|BOLD:AAB5894
Idia julia|[11443]|LMEM137-09|United States|Alabama|658[0n]|BOLD:AAB5894
Idia julia|[11444]|LMEM138-09|United States|Mississippi|658[0n]|BOLD:AAB5894
Idia julia|[11445]|BBLSW442-09|United States|Oklahoma|658[0n]|BOLD:AAB5894
Idia julia|[11446]|BBLSW604-09|United States|Oklahoma|658[0n]|BOLD:AAB5894
Idia julia|[11447]|BBLSX152-09|United States|Oklahoma|658[0n]|BOLD:AAB5894
Idia julia|[11448]|BBLSX385-09|United States|Oklahoma|658[0n]|BOLD:AAB5894
Idia julia|[11449]|BBLSX465-09|United States|Oklahoma|658[0n]|BOLD:AAB5894
Idia julia|[11450]|LPOKD424-09|United States|Oklahoma|658[0n]|BOLD:AAB5894
Idia julia|[11451]|LNCC830-11|United States|North Carolina|658[0n]|BOLD:AAB5894
Idia julia|[11452]|LNCC831-11|United States|North Carolina|658[0n]|BOLD:AAB5894
Idia julia|[11453]|MILEQ217-11|United States|Georgia|658[0n]|BOLD:AAB5894
Idia julia|[11454]|RDLQG106-06|Canada|Quebec|587[0n]|BOLD:AAA3327
Idia julia|[11455]|RDLQG929-06|Canada|Quebec|658[0n]|BOLD:AAA3327
Idia julia|[11456]|RDLQG682-06|Canada|Quebec|658[0n]|BOLD:AAA3327
Idia julia|[11457]|MNAF433-08|Canada|Manitoba|649[0n]|BOLD:AAA3327
Idia julia|[11458]|LPMNB350-09|Canada|Manitoba|658[0n]|BOLD:AAA3327
Idia julia|[11459]|LPMNB364-09|Canada|Manitoba|658[0n]|BOLD:AAA3327
Idia julia|[11460]|LPMNB392-09|Canada|Manitoba|658[0n]|BOLD:AAA3327
Idia julia|[11461]|LPMNB403-09|Canada|Manitoba|658[0n]|BOLD:AAA3327
Idia julia|[11462]|LPMNB478-09|Canada|Manitoba|658[0n]|BOLD:AAA3327
Idia julia|[11463]|CNSLG301-12|Canada|Ontario|606[0n]|BOLD:AAA3327
Idia julia|[11464]|CNSLQ035-13|Canada|Ontario|543[0n]|BOLD:AAA3327
Idia sp.|[11465]|LMEM130-09|United States|Alabama|658[0n]|BOLD:AAA3328
Idia sp.|[11466]|LMEM131-09|United States|Alabama|658[0n]|BOLD:AAA3328
Idia sp.|[11467]|LPOKA798-09|United States|Oklahoma|658[0n]|BOLD:ACE8590
Idia sp.|[11468]|BBUSA529-09|United States|Oklahoma|658[0n]|BOLD:ACE8590
Idia rotundalis|[11469]|RDLQG101-06|Canada|Quebec|614[0n]|BOLD:ACE4734
Idia rotundalis|[11470]|BBLEC959-09|Canada|Nova Scotia|636[0n]|BOLD:ACE4734
Idia rotundalis|[11471]|RDLQG103-06|Canada|Quebec|614[0n]|BOLD:ACE4734
Idia rotundalis|[11472]|RDLQG102-06|Canada|Quebec|614[0n]|BOLD:ACE4734
Idia rotundalis|[11473]|CNSLS026-13|Canada|Ontario|570[0n]|BOLD:ACE4734
Idia rotundalis|[11474]|RDLQG100-06|Canada|Quebec|620[1n]|BOLD:ACE4734
Idia rotundalis|[11475]|CNSLQ034-13|Canada|Ontario|525[0n]|BOLD:ACE4734
Idia rotundalis|[11476]|CNSLH034-12|Canada|Ontario|637[0n]|BOLD:ACE4734
Idia rotundalis|[11477]|RDLQB710-05|Canada|Quebec|587[1n]|BOLD:ACE4734
Idia rotundalis|[11478]|BBLEC973-09|Canada|Nova Scotia|632[0n]|BOLD:ACE4734
Idia rotundalis|[11479]|RDLQG099-06|Canada|Quebec|632[0n]|BOLD:ACE4734
Idia rotundalis|[11480]|LNCC998-11|United States|North Carolina|658[0n]|BOLD:ACE4734
Idia rotundalis|[11481]|TMNBD514-07|Canada|New Brunswick|658[0n]|BOLD:ACE4734
Idia rotundalis|[11482]|TMNBD513-07|Canada|New Brunswick|656[0n]|BOLD:ACE4734
Idia rotundalis|[11483]|BBLEC057-09|Canada|New Brunswick|658[0n]|BOLD:ACE4734

[illegible]
Idia rotundalis|[11481]|TMNBD514-07|Canada|New Brunswick|658[0n]|BOLD:ACE4734
Idia rotundalis|[11482]|TMNBD513-07|Canada|New Brunswick|656[0n]|BOLD:ACE4734
Idia rotundalis|[11483]|BBLEC057-09|Canada|New Brunswick|658[0n]|BOLD:ACE4734
Idia rotundalis|[11484]|BBLEC546-09|Canada|Nova Scotia|658[0n]|BOLD:ACE4734
Idia rotundalis|[11485]|BBLEC963-09|Canada|Nova Scotia|658[0n]|BOLD:ACE4734
Idia rotundalis|[11486]|BBLEC986-09|Canada|Nova Scotia|658[0n]|BOLD:ACE4734
Idia rotundalis|[11487]|RDLQF793-06|Canada|Quebec|658[0n]|BOLD:ACE4734
Idia rotundalis|[11488]|RDLQG907-06|Canada|Quebec|658[0n]|BOLD:ACE4734
Idia rotundalis|[11489]|BBLEC990-09|Canada|Nova Scotia|658[0n]|BOLD:ACE4734
Idia rotundalis|[11490]|RDLQG684-06|Canada|Quebec|658[0n]|BOLD:ACE4734
Idia rotundalis|[11491]|BBLEC902-09|Canada|Nova Scotia|658[0n]|BOLD:ACE4734
Idia rotundalis|[11492]|RDLQF384-06|Canada|Quebec|658[0n]|BOLD:ACE4734
Idia rotundalis|[11493]|BBLEC557-09|Canada|Nova Scotia|656[0n]|BOLD:ACE4734
Idia rotundalis|[11494]|LNCC997-11|United States|North Carolina|658[0n]|BOLD:ACE4734
Idia rotundalis|[11495]|LNCC999-11|United States|North Carolina|658[0n]|BOLD:ACE4734
Idia rotundalis|[11496]|LNCC256-10|United States|North Carolina|658[0n]|BOLD:ACE4734
Idia rotundalis|[11497]|LNCC257-10|United States|North Carolina|658[0n]|BOLD:ACE4734
Idia rotundalis|[11498]|TMNBD515-07|Canada|New Brunswick|658[0n]|BOLD:ACE4734
Idia rotundalis|[11499]|BBLPE597-09|Canada|Nova Scotia|658[0n]|BOLD:ACE4734
Idia rotundalis|[11500]|RDLQH013-06|Canada|Quebec|658[0n]|BOLD:ACE4734
Idia rotundalis|[11501]|RDLQH014-06|Canada|Quebec|658[0n]|BOLD:ACE4734
Idia rotundalis|[11502]|RDLQG906-06|Canada|Quebec|658[0n]|BOLD:ACE4734
Idia rotundalis|[11503]|RDLQG905-06|Canada|Quebec|658[0n]|BOLD:ACE4734
Idia rotundalis|[11504]|RDLQG683-06|Canada|Quebec|658[0n]|BOLD:ACE4734
Idia rotundalis|[11505]|RDLQG681-06|Canada|Quebec|658[0n]|BOLD:ACE4734
Idia rotundalis|[11506]|RDLQG608-06|Canada|Quebec|658[0n]|BOLD:ACE4734
Idia rotundalis|[11507]|RDLQG599-06|Canada|Quebec|658[0n]|BOLD:ACE4734
Idia rotundalis|[11508]|RDLQG564-06|Canada|Quebec|658[0n]|BOLD:ACE4734
Idia rotundalis|[11509]|RDLQG165-06|Canada|Quebec|658[0n]|BOLD:ACE4734
Idia rotundalis|[11510]|RDLQG097-06|Canada|Quebec|658[0n]|BOLD:ACE4734
Idia rotundalis|[11511]|RDLQF794-06|Canada|Quebec|658[0n]|BOLD:ACE4734
Idia rotundalis|[11512]|MNBB692-05|Canada|New Brunswick|658[0n]|BOLD:ACE4734
Idia rotundalis|[11513]|RDLQG098-06|Canada|Quebec|650[0n]|BOLD:ACE4734
Idia rotundalis|[11514]|TMNBB044-06|Canada|New Brunswick|658[0n]|BOLD:ACE4734
Idia rotundalis|[11515]|RDLQF714-06|Canada|Quebec|637[0n]|BOLD:ACE4734
Idia rotundalis|[11516]|RDLQF713-06|Canada|Quebec|637[0n]|BOLD:ACE4734
Idia rotundalis|[11517]|RDLQF658-06|Canada|Quebec|637[0n]|BOLD:ACE4734
Idia rotundalis|[11518]|RDLQF657-06|Canada|Quebec|637[0n]|BOLD:ACE4734
Idia rotundalis|[11519]|RDLQF656-06|Canada|Quebec|637[0n]|BOLD:ACE4734
Idia rotundalis|[11520]|RDLQF654-06|Canada|Quebec|637[0n]|BOLD:ACE4734
Idia rotundalis|[11521]|RDLQG598-06|Canada|Quebec|643[0n]|BOLD:ACE4734
Idia rotundalis|[11522]|BBLEC246-09|Canada|Nova Scotia|658[0n]|BOLD:ACE4734
Idia rotundalis|[11523]|RDLQF655-06|Canada|Quebec|637[0n]|BOLD:ACE4734
Idia rotundalis|[11524]|BBLEC233-09|Canada|Nova Scotia|658[0n]|BOLD:ACE4734
Idia rotundalis|[11525]|BBLEC551-09|Canada|Nova Scotia|658[0n]|BOLD:ACE4734
Idia rotundalis|[11526]|BBLEC763-09|Canada|Nova Scotia|658[0n]|BOLD:ACE4734
Idia rotundalis|[11527]|BBLEC992-09|Canada|Nova Scotia|658[0n]|BOLD:ACE4734
Idia rotundalis|[11528]|BBLPC533-09|Canada|New Brunswick|658[0n]|BOLD:ACE4734
Idia rotundalis|[11529]|BBLPE596-09|Canada|Nova Scotia|658[0n]|BOLD:ACE4734
Idia rotundalis|[11530]|LNCC1000-11|United States|North Carolina|658[0n]|BOLD:ACE4734
Idia rotundalis|[11531]|BBLPC386-09|Canada|New Brunswick|658[0n]|BOLD:ACE4734
Idia rotundalis|[11532]|BBLEC925-09|Canada|Nova Scotia|658[0n]|BOLD:ACE4734
Idia rotundalis|[11533]|BBLEC910-09|Canada|Nova Scotia|658[0n]|BOLD:ACE4734
Idia rotundalis|[11534]|BBLEC720-09|Canada|Nova Scotia|658[0n]|BOLD:ACE4734
Idia rotundalis|[11535]|BBLEC936-09|Canada|Nova Scotia|649[0n]|BOLD:ACE4734
Idia rotundalis|[11536]|BBLEC994-09|Canada|Nova Scotia|658[0n]|BOLD:ACE4734
Idia rotundalis|[11537]|BBLPE561-09|Canada|Nova Scotia|658[0n]|BOLD:ACE4734
Idia rotundalis|[11538]|CNSLQ036-13|Canada|Ontario|549[0n]|BOLD:ACE4734
Idia rotundalis|[11539]|HPPPE1571-13|Canada|Nova Scotia|584[0n]|BOLD:ACE4734
Idia rotundalis|[11540]|HPPPE1572-13|Canada|Nova Scotia|584[0n]|BOLD:ACE4734
Idia rotundalis|[11541]|LMIS070-06|Canada|Ontario|658[0n]|BOLD:AAA3326
Idia rotundalis|[11542]|LPOKA114-08|United States|Oklahoma|658[0n]|BOLD:AAA3326
Idia rotundalis|[11543]|MILEQ278-11|United States|Alabama|636[0n]|BOLD:AAA3326
Idia rotundalis|[11544]|CNSLH303-12|Canada|Ontario|603[0n]|BOLD:AAA3326
Idia rotundalis|[11545]|LOFLC018-06|United States|Florida|674[0n]|BOLD:AAA3326
Idia rotundalis|[11546]|LOFLC272-06|United States|Florida|632[0n]|BOLD:AAA3326
Idia rotundalis|[11547]|LNCB737-09|United States|North Carolina|616[0n]|BOLD:AAA3326
Idia rotundalis|[11548]|LNCB739-09|United States|North Carolina|658[0n]|BOLD:AAA3326
Idia rotundalis|[11549]|LNCB736-09|United States|North Carolina|658[0n]|BOLD:AAA3326
Idia rotundalis|[11550]|LNCB738-09|United States|North Carolina|658[0n]|BOLD:AAA3326
Idia rotundalis|[11551]|LNCB740-09|United States|North Carolina|658[0n]|BOLD:AAA3326
Idia rotundalis|[11552]|HKONS291-08|United States|Florida|658[0n]|BOLD:AAA3326
Idia rotundalis|[11553]|LNCC706-11|United States|North Carolina|658[0n]|BOLD:AAA3326
Idia rotundalis|[11554]|LNCC707-11|United States|North Carolina|658[0n]|BOLD:AAA3326
Idia rotundalis|[11555]|LNCC833-11|United States|North Carolina|658[0n]|BOLD:AAA3326
Idia rotundalis|[11556]|LNCC834-11|United States|North Carolina|658[0n]|BOLD:AAA3326
Idia rotundalis|[11557]|LNC841-06|United States|North Carolina|658[0n]|BOLD:AAA3326
Idia rotundalis|[11558]|LNCC814-11|United States|North Carolina|658[0n]|BOLD:AAA3326
Idia rotundalis|[11559]|LNCC815-11|United States|North Carolina|658[0n]|BOLD:AAA3326
Idia rotundalis|[11560]|LNC840-06|United States|North Carolina|658[0n]|BOLD:AAA3326
Idia rotundalis|[11561]|LNCC812-11|United States|North Carolina|658[0n]|BOLD:AAA3326
Idia rotundalis|[11562]|LNCC900-11|United States|North Carolina|658[0n]|BOLD:AAA3326
Idia rotundalis|[11563]|LNCC813-11|United States|North Carolina|658[0n]|BOLD:AAA3326
Idia rotundalis|[11564]|LNCC704-11|United States|North Carolina|658[0n]|BOLD:AAA3326
Idia rotundalis|[11565]|LNCC705-11|United States|North Carolina|658[0n]|BOLD:AAA3326
Idia rotundalis|[11566]|LNCC901-11|United States|North Carolina|658[0n]|BOLD:AAA3326
Idia rotundalis|[11567]|HKONS344-08|United States|Florida|658[0n]|BOLD:AAA3326
Idia rotundalis|[11568]|LMEM134-09|United States|Alabama|658[0n]|BOLD:AAA3326
Idia rotundalis|[11569]|LMEM133-09|United States|Alabama|658[0n]|BOLD:AAA3326
Idia rotundalis|[11570]|HKONS289-08|United States|Florida|658[0n]|BOLD:AAA3326
Idia rotundalis|[11571]|LOFLA650-06|United States|Florida|658[0n]|BOLD:AAA3326
Idia rotundalis|[11572]|HKONS290-08|United States|Florida|658[0n]|BOLD:AAA3326
Idia rotundalis|[11573]|LOFLB271-06|United States|Florida|622[0n]|BOLD:AAA3326
Idia rotundalis|[11574]|LOFLB303-06|United States|Florida|658[0n]|BOLD:AAA3326
Idia rotundalis|[11575]|LOFLB555-06|United States|Florida|658[0n]|BOLD:AAA3326
Idia rotundalis|[11576]|LOFLB844-06|United States|Florida|658[0n]|BOLD:AAA3326
Idia rotundalis|[11577]|LOFLB867-06|United States|Florida|658[0n]|BOLD:AAA3326
Idia rotundalis|[11578]|LOFLB888-06|United States|Florida|658[0n]|BOLD:AAA3326
Idia rotundalis|[11579]|LOFLC347-06|United States|Florida|658[0n]|BOLD:AAA3326
Idia rotundalis|[11580]|MILEP336-10|United States|Alabama|658[0n]|BOLD:AAA3326
Idia rotundalis|[11581]|MILEQ214-11|United States|Georgia|658[0n]|BOLD:AAA3326
Idia rotundalis|[11582]|LPOKA482-09|United States|Oklahoma|633[0n]|BOLD:AAA3326
Idia rotundalis|[11583]|LPOKA050-08|United States|Oklahoma|658[0n]|BOLD:AAA3326

[illegible]
Idia rotundalis|[11581]|MILEQ214-11|United States|Georgia|658[0n]|BOLD:AAA3326
Idia rotundalis|[11582]|LPOKA482-09|United States|Oklahoma|633[0n]|BOLD:AAA3326
Idia rotundalis|[11583]|LPOKA050-08|United States|Oklahoma|658[0n]|BOLD:AAA3326
Idia rotundalis|[11584]|LPOKA003-08|United States|Oklahoma|658[0n]|BOLD:AAA3326
Idia rotundalis|[11585]|LPOKD277-09|United States|Oklahoma|657[0n]|BOLD:AAA3326
Idia rotundalis|[11586]|LPOKD360-09|United States|Oklahoma|658[0n]|BOLD:AAA3326
Idia rotundalis|[11587]|HKONS292-08|United States|Florida|658[0n]|BOLD:AAA3326
Idia rotundalis|[11588]|MILEQ275-11|United States|Alabama|627[0n]|BOLD:AAA3326
Idia rotundalis|[11589]|MILEQ276-11|United States|Alabama|658[0n]|BOLD:AAA3326
Idia rotundalis|[11590]|CNSLQ027-13|Canada|Ontario|537[0n]|BOLD:AAA3326
Idia rotundalis|[11591]|LNCC036-10|United States|North Carolina|658[0n]|BOLD:AAA3326
Idia rotundalis|[11592]|LNCC035-10|United States|North Carolina|658[0n]|BOLD:AAA3326
Idia rotundalis|[11593]|LNCC281-10|United States|North Carolina|658[0n]|BOLD:AAA3326
Idia rotundalis|[11594]|XAC718-04|Canada|Ontario|658[0n]|BOLD:AAA3326
Idia rotundalis|[11595]|RDLQB543-05|Canada|Quebec|658[0n]|BOLD:AAA3326
Idia rotundalis|[11596]|CNSLH035-12|Canada|Ontario|634[0n]|BOLD:AAA3326
Idia rotundalis|[11597]|CNSLR432-13|Canada|Ontario|555[0n]|BOLD:AAA3326
Idia rotundalis|[11598]|CNSLQ031-13|Canada|Ontario|543[0n]|BOLD:AAA3326
Idia rotundalis|[11599]|LGSMC365-05|United States|Tennessee|615[0n]|BOLD:AAA3326
Idia rotundalis|[11600]|LMEM132-09|United States|Mississippi|658[0n]|BOLD:AAA3326
Idia rotundalis|[11601]|LGSM539-04|United States|Tennessee|658[0n]|BOLD:AAA3326
Idia rotundalis|[11602]|LGSMG470-07|United States|Tennessee|658[0n]|BOLD:AAA3326
Idia rotundalis|[11603]|CNCLB2784-14|United States|North Carolina|658[0n]|BOLD:AAA3326
Idia rotundalis|[11604]|LGSMC367-05|United States|Tennessee|658[0n]|BOLD:AAA3326
Idia rotundalis|[11605]|LGSMC366-05|United States|Tennessee|658[0n]|BOLD:AAA3326
Idia rotundalis|[11606]|LOTB185-05|United States|Tennessee|658[0n]|BOLD:AAA3326
Idia rotundalis|[11607]|LOTB186-05|United States|Tennessee|658[0n]|BOLD:AAA3326
Idia rotundalis|[11608]|LOTB187-05|United States|Tennessee|658[0n]|BOLD:AAA3326
Idia rotundalis|[11609]|LGSM540-04|United States|Tennessee|658[0n]|BOLD:AAA3326
Idia rotundalis|[11610]|LGSMC364-05|United States|Tennessee|658[0n]|BOLD:AAA3326
Idia rotundalis|[11611]|CNCLB2968-14|United States|North Carolina|658[0n]|BOLD:AAA3326
Bleptina hydrillalis|[11612]|RDNMG235-08|Dominican Republic||647[0n]|BOLD:AAI8822
Bleptina n. sp. 1|[11613]|RDNML050-13|United States|Florida|658[0n]|BOLD:ACD3830
Bleptina minimalis|[11614]|QUNOD015-10|United States|Texas|658[0n]|BOLD:AAJ8274
Bleptina n. sp. 2|[11615]|RDNMI051-10|United States|Florida|658[0n]|BOLD:AAI8821
Bleptina n. sp. 2|[11616]|RDNMI182-10|United States|Florida|658[0n]|BOLD:AAI8821
Bleptina n. sp. 2|[11617]|HKONS059-07|United States|Florida|657[0n]|BOLD:AAI8821
Bleptina n. sp. 2|[11618]|PSAT113-10|United States|Florida|658[0n]|BOLD:AAI8821
Bleptina n. sp. 2|[11619]|RDNMK076-11|United States|Florida|658[0n]|BOLD:AAI8821
Bleptina n. sp. 2|[11620]|RDNML049-13|United States|Florida|658[0n]|BOLD:AAI8821
Bleptina n. sp. 6|[11621]|RDNMH093-09|United States|Texas|658[0n]|BOLD:AAA9242
Bleptina n. sp. 6|[11622]|HKONB180-08|United States|Texas|658[0n]|BOLD:AAA9242
Bleptina n. sp. 6|[11623]|HKONB182-08|United States|Texas|658[0n]|BOLD:AAA9242
Bleptina n. sp. 6|[11624]|HKONB181-08|United States|Texas|658[0n]|BOLD:AAA9242
Bleptina n. sp. 6|[11625]|HKONB183-08|United States|Texas|658[0n]|BOLD:AAA9242
Bleptina n. sp. 6|[11626]|HKONB184-08|United States|Texas|658[0n]|BOLD:AAA9242
Bleptina n. sp. 6|[11627]|HKONB185-08|United States|Texas|658[0n]|BOLD:AAA9242
Bleptina n. sp. 6|[11628]|HKONB339-09|United States|Texas|658[0n]|BOLD:AAA9242
Bleptina n. sp. 6|[11629]|HKONB340-09|United States|Texas|658[0n]|BOLD:AAA9242
Bleptina n. sp. 7|[11630]|RDNME968-08|United States|Arizona|643[1n]|BOLD:AAA9244
Bleptina n. sp. 7|[11631]|CMAZA366-10|United States|Arizona|658[0n]|BOLD:AAA9244
Bleptina caradrinalis|[11632]|LMEM259-09|United States|Tennessee|632[1n]|BOLD:AAA2867
Bleptina caradrinalis|[11633]|XAC658-04|Canada|Ontario|658[0n]|BOLD:AAA2867
Bleptina caradrinalis|[11634]|RDLQB241-05|Canada|Quebec|658[0n]|BOLD:AAA2867
Bleptina caradrinalis|[11635]|LALPA408-10|Canada|British Columbia|658[0n]|BOLD:AAA2867
Bleptina caradrinalis|[11636]|LALPA434-10|Canada|British Columbia|658[0n]|BOLD:AAA2867
Bleptina caradrinalis|[11637]|LALPA497-10|Canada|British Columbia|658[0n]|BOLD:AAA2867
Bleptina caradrinalis|[11638]|LALPA536-10|Canada|British Columbia|658[0n]|BOLD:AAA2867
Bleptina caradrinalis|[11639]|BBLPD859-10|Canada|British Columbia|658[0n]|BOLD:AAA2867
Bleptina caradrinalis|[11640]|LALPA1214-11|Canada|British Columbia|658[0n]|BOLD:AAA2867
Bleptina caradrinalis|[11641]|LPSK132-08|Canada|Saskatchewan|658[0n]|BOLD:AAA2867
Bleptina caradrinalis|[11642]|RWWB130-09|United States|Washington|658[0n]|BOLD:AAA2867
Bleptina caradrinalis|[11643]|RDLQG359-06|Canada|Quebec|658[0n]|BOLD:AAA2867
Bleptina caradrinalis|[11644]|RDLQG920-06|Canada|Quebec|658[0n]|BOLD:AAA2867
Bleptina caradrinalis|[11645]|LPSK047-08|Canada|Saskatchewan|658[0n]|BOLD:AAA2867
Bleptina caradrinalis|[11646]|LPSK050-08|Canada|Saskatchewan|658[0n]|BOLD:AAA2867
Bleptina caradrinalis|[11647]|LPMN097-08|Canada|Manitoba|658[0n]|BOLD:AAA2867
Bleptina caradrinalis|[11648]|LPMN116-08|Canada|Manitoba|658[0n]|BOLD:AAA2867
Bleptina caradrinalis|[11649]|RDLQF433-06|Canada|Quebec|658[0n]|BOLD:AAA2867
Bleptina caradrinalis|[11650]|RDLQF435-06|Canada|Quebec|658[0n]|BOLD:AAA2867
Bleptina caradrinalis|[11651]|TMNBB060-06|Canada|New Brunswick|658[0n]|BOLD:AAA2867
Bleptina caradrinalis|[11652]|RDLQF432-06|Canada|Quebec|658[0n]|BOLD:AAA2867
Bleptina caradrinalis|[11653]|TMNBB057-06|Canada|New Brunswick|658[0n]|BOLD:AAA2867
Bleptina caradrinalis|[11654]|TMNBB059-06|Canada|New Brunswick|658[0n]|BOLD:AAA2867
Bleptina caradrinalis|[11655]|TMNBB056-06|Canada|New Brunswick|658[0n]|BOLD:AAA2867
Bleptina caradrinalis|[11656]|TMNBB055-06|Canada|New Brunswick|658[0n]|BOLD:AAA2867
Bleptina caradrinalis|[11657]|TMNBB054-06|Canada|New Brunswick|658[0n]|BOLD:AAA2867
Bleptina caradrinalis|[11658]|TTMNB268-06|Canada|New Brunswick|658[0n]|BOLD:AAA2867
Bleptina caradrinalis|[11659]|TTMNB096-06|Canada|New Brunswick|658[0n]|BOLD:AAA2867
Bleptina caradrinalis|[11660]|TTMNB092-06|Canada|New Brunswick|657[0n]|BOLD:AAA2867
Bleptina caradrinalis|[11661]|RDLQB259-05|Canada|Quebec|658[0n]|BOLD:AAA2867
Bleptina caradrinalis|[11662]|MNBB005-05|Canada|New Brunswick|658[0n]|BOLD:AAA2867
Bleptina caradrinalis|[11663]|PHMNB215-04|Canada|New Brunswick|658[0n]|BOLD:AAA2867
Bleptina caradrinalis|[11664]|PHMNB157-04|Canada|New Brunswick|658[0n]|BOLD:AAA2867
Bleptina caradrinalis|[11665]|RDNMF102-08|Canada|Saskatchewan|658[0n]|BOLD:AAA2867
Bleptina caradrinalis|[11666]|TMNBB058-06|Canada|New Brunswick|658[0n]|BOLD:AAA2867
Bleptina caradrinalis|[11667]|CNPPJ1454-12|Canada|Ontario|636[0n]|BOLD:AAA2867
Bleptina caradrinalis|[11668]|LPMN092-08|Canada|Manitoba|647[0n]|BOLD:AAA2867
Bleptina caradrinalis|[11669]|PHMO201-03|Canada|Ontario|639[0n]|BOLD:AAA2867
Bleptina caradrinalis|[11670]|TTMNB269-06|Canada|New Brunswick|658[0n]|BOLD:AAA2867
Bleptina caradrinalis|[11671]|RDMAB131-05|Canada|Alberta|631[0n]|BOLD:AAA2867
Bleptina caradrinalis|[11672]|XAC840-04|Canada|Ontario|615[1n]|BOLD:AAA2867
Bleptina caradrinalis|[11673]|RDMAB049-05|Canada|Alberta|638[0n]|BOLD:AAA2867
Bleptina caradrinalis|[11674]|RDLQG196-06|Canada|Quebec|583[0n]|BOLD:AAA2867
Bleptina caradrinalis|[11675]|LALPA726-10|Canada|British Columbia|630[0n]|BOLD:AAA2867
Bleptina caradrinalis|[11676]|PHMNB193-04|Canada|New Brunswick|658[0n]|BOLD:AAA2867
Bleptina caradrinalis|[11677]|HPPPE1568-13|Canada|Nova Scotia|552[0n]|BOLD:AAA2867
Bleptina caradrinalis|[11678]|HKONB530-09|United States|Texas|658[0n]|BOLD:AAA2867
Bleptina caradrinalis|[11679]|LPOKA258-08|United States|Oklahoma|658[0n]|BOLD:AAA2867
Bleptina caradrinalis|[11680]|BBLSW650-09|United States|Oklahoma|658[0n]|BOLD:AAA2867
Bleptina caradrinalis|[11681]|BBLSX395-09|United States|Oklahoma|658[0n]|BOLD:AAA2867
Bleptina caradrinalis|[11682]|USLEP605-10|United States|Florida|658[0n]|BOLD:AAA2867
[illegible]

Bleptina caradrinalis|[11680]|BBLSW650-09|United States|Oklahoma|658[0n]|BOLD:AAA2867
Bleptina caradrinalis|[11681]|BBLSX395-09|United States|Oklahoma|658[0n]|BOLD:AAA2867
Bleptina caradrinalis|[11682]|USLEP605-10|United States|Florida|658[0n]|BOLD:AAA2867
Bleptina caradrinalis|[11683]|ABNCC020-07|United States|Utah|634[1n]|BOLD:AAA2867
Bleptina caradrinalis|[11684]|LGSMC371-05|United States|Tennessee|658[0n]|BOLD:AAA2867
Bleptina caradrinalis|[11685]|LGSMC372-05|United States|Tennessee|658[0n]|BOLD:AAA2867
Bleptina caradrinalis|[11686]|LNCNW067-06|United States|North Carolina|658[0n]|BOLD:AAA2867
Bleptina caradrinalis|[11687]|HKONB447-09|United States|Indiana|658[0n]|BOLD:AAA2867
Bleptina caradrinalis|[11688]|LMEM263-09|United States|Tennessee|658[0n]|BOLD:AAA2867
Bleptina caradrinalis|[11689]|LMEM268-09|United States|Alabama|658[0n]|BOLD:AAA2867
Bleptina caradrinalis|[11690]|BBLSX157-09|United States|Oklahoma|658[0n]|BOLD:AAA2867
Bleptina caradrinalis|[11691]|BBLPA660-10|Canada|Ontario|658[0n]|BOLD:AAA2867
Bleptina caradrinalis|[11692]|LOFLA864-06|United States|Florida|655[0n]|BOLD:AAA2867
Bleptina caradrinalis|[11693]|LILLA219-11|United States|Illinois|658[0n]|BOLD:AAA2867
Bleptina caradrinalis|[11694]|USLEP624-10|United States|Florida|658[0n]|BOLD:AAA2867
Bleptina caradrinalis|[11695]|BBLSU001-09|United States|Arkansas|658[0n]|BOLD:AAA2867
Bleptina caradrinalis|[11696]|BBLSY853-09|United States|Oklahoma|658[0n]|BOLD:AAA2867
Bleptina caradrinalis|[11697]|BBLSX506-09|United States|Oklahoma|658[0n]|BOLD:AAA2867
Bleptina caradrinalis|[11698]|BBLSX417-09|United States|Oklahoma|658[0n]|BOLD:AAA2867
Bleptina caradrinalis|[11699]|BBLSW679-09|United States|Oklahoma|658[0n]|BOLD:AAA2867
Bleptina caradrinalis|[11700]|LPOKB734-09|United States|Oklahoma|658[0n]|BOLD:AAA2867
Bleptina caradrinalis|[11701]|LMEM267-09|United States|Mississippi|658[0n]|BOLD:AAA2867
Bleptina caradrinalis|[11702]|LMEM261-09|United States|Alabama|658[0n]|BOLD:AAA2867
Bleptina caradrinalis|[11703]|LMEM260-09|United States|Georgia|658[0n]|BOLD:AAA2867
Bleptina caradrinalis|[11704]|LMEM258-09|United States|Mississippi|658[0n]|BOLD:AAA2867
Bleptina caradrinalis|[11705]|LPSO689-08|Canada|Ontario|658[0n]|BOLD:AAA2867
Bleptina caradrinalis|[11706]|HKONS333-08|United States|Florida|658[0n]|BOLD:AAA2867
Bleptina caradrinalis|[11707]|LOFLB842-06|United States|Florida|658[0n]|BOLD:AAA2867
Bleptina caradrinalis|[11708]|LOFLA737-06|United States|Florida|658[0n]|BOLD:AAA2867
Bleptina caradrinalis|[11709]|LOFLA032-06|United States|Florida|658[0n]|BOLD:AAA2867
Bleptina caradrinalis|[11710]|LGSM537-04|United States|Tennessee|658[0n]|BOLD:AAA2867
Bleptina caradrinalis|[11711]|BBLSX075-09|United States|Oklahoma|658[0n]|BOLD:AAA2867
Bleptina caradrinalis|[11712]|LMEM257-09|United States|Louisiana|633[0n]|BOLD:AAA2867
Bleptina caradrinalis|[11713]|ABNCC090-07|United States|Florida|634[0n]|BOLD:AAA2867
Bleptina caradrinalis|[11714]|BBLOE1139-12|United States|Texas|633[0n]|BOLD:AAA2867
Bleptina caradrinalis|[11715]|CNPPD2357-12|Canada|Ontario|630[0n]|BOLD:AAA2867
Bleptina caradrinalis|[11716]|LMEM266-09|United States|Alabama|658[0n]|BOLD:AAA2867
Bleptina caradrinalis|[11717]|ABNCC089-07|United States|Florida|638[1n]|BOLD:AAA2867
Bleptina caradrinalis|[11718]|XAC722-04|Canada|Ontario|658[0n]|BOLD:AAA2867
Bleptina caradrinalis|[11719]|BBLSW497-09|United States|Oklahoma|630[0n]|BOLD:AAA2867
Bleptina caradrinalis|[11720]|BBLSW795-09|United States|Oklahoma|636[0n]|BOLD:AAA2867
Bleptina caradrinalis|[11721]|LOCT069-05|United States|Connecticut|616[0n]|BOLD:AAA2867
Bleptina caradrinalis|[11722]|LGSMC370-05|United States|Tennessee|616[0n]|BOLD:AAA2867
Bleptina caradrinalis|[11723]|BBLSW510-09|United States|Oklahoma|634[0n]|BOLD:AAA2867
Bleptina caradrinalis|[11724]|XAC662-04|Canada|Ontario|581[0n]|BOLD:AAA2867
Bleptina caradrinalis|[11725]|RDLQG173-06|Canada|Quebec|658[0n]|BOLD:AAA2867
Bleptina caradrinalis|[11726]|XAB186-04|Canada|Ontario|572[0n]|BOLD:AAA2867
Bleptina caradrinalis|[11727]|BBLSW558-09|United States|Oklahoma|623[0n]|BOLD:AAA2867
Bleptina caradrinalis|[11728]|LPOKE592-12|United States|Oklahoma|632[0n]|BOLD:AAA2867
Bleptina caradrinalis|[11729]|LOFLA399-06|United States|Florida|658[0n]|BOLD:AAA2867
Bleptina caradrinalis|[11730]|LNC535-06|United States||658[0n]|BOLD:AAA2867
Bleptina caradrinalis|[11731]|LNC174-05|United States|North Carolina|658[0n]|BOLD:AAA2867
Bleptina caradrinalis|[11732]|LOTB249-05|United States|Tennessee|658[0n]|BOLD:AAA2867
Bleptina caradrinalis|[11733]|LGSMC374-05|United States|Tennessee|658[0n]|BOLD:AAA2867
Bleptina caradrinalis|[11734]|LMEM264-09|United States|Mississippi|658[0n]|BOLD:AAA2867
Bleptina caradrinalis|[11735]|LGSMC375-05|United States|Tennessee|658[0n]|BOLD:AAA2867
Bleptina caradrinalis|[11736]|LGSMC373-05|United States|Tennessee|658[0n]|BOLD:AAA2867
Bleptina caradrinalis|[11737]|XAE558-04|Canada|Ontario|658[0n]|BOLD:AAA2867
Bleptina caradrinalis|[11738]|XAC460-04|Canada|Ontario|658[0n]|BOLD:AAA2867
Bleptina caradrinalis|[11739]|XAC047-04|Canada|Ontario|658[0n]|BOLD:AAA2867
Bleptina caradrinalis|[11740]|LOFLA574-06|United States|Florida|658[0n]|BOLD:AAA2867
Bleptina caradrinalis|[11741]|BBLSW566-09|United States|Oklahoma|658[0n]|BOLD:AAA2867
Bleptina caradrinalis|[11742]|LOFLA400-06|United States|Florida|658[0n]|BOLD:AAA2867
Bleptina caradrinalis|[11743]|LOFLA408-06|United States|Florida|658[0n]|BOLD:AAA2867
Bleptina caradrinalis|[11744]|LOFLA425-06|United States|Florida|658[0n]|BOLD:AAA2867
Bleptina caradrinalis|[11745]|LOFLA559-06|United States|Florida|658[0n]|BOLD:AAA2867
Bleptina caradrinalis|[11746]|LOFLA846-06|United States|Florida|658[0n]|BOLD:AAA2867
Bleptina caradrinalis|[11747]|LOFLA865-06|United States|Florida|658[0n]|BOLD:AAA2867
Bleptina caradrinalis|[11748]|LOFLC316-06|United States|Florida|658[0n]|BOLD:AAA2867
Bleptina caradrinalis|[11749]|LOFLC394-06|United States|Florida|658[0n]|BOLD:AAA2867
Bleptina caradrinalis|[11750]|RDLQF434-06|Canada|Quebec|658[0n]|BOLD:AAA2867
Bleptina caradrinalis|[11751]|RDLQF821-06|Canada|Quebec|658[0n]|BOLD:AAA2867
Bleptina caradrinalis|[11752]|LGSMG511-07|United States|Tennessee|658[0n]|BOLD:AAA2867
Bleptina caradrinalis|[11753]|HKONB446-09|United States|Indiana|658[0n]|BOLD:AAA2867
Bleptina caradrinalis|[11754]|LMEM262-09|United States|Mississippi|658[0n]|BOLD:AAA2867
Bleptina caradrinalis|[11755]|LMEM265-09|United States|Mississippi|658[0n]|BOLD:AAA2867
Bleptina caradrinalis|[11756]|BBLSW543-09|United States|Oklahoma|658[0n]|BOLD:AAA2867
Bleptina caradrinalis|[11757]|BBLSW635-09|United States|Oklahoma|658[0n]|BOLD:AAA2867
Bleptina caradrinalis|[11758]|BBLSW797-09|United States|Oklahoma|658[0n]|BOLD:AAA2867
Bleptina caradrinalis|[11759]|BBLSX069-09|United States|Texas|658[0n]|BOLD:AAA2867
Bleptina caradrinalis|[11760]|BBLSX076-09|United States|Oklahoma|658[0n]|BOLD:AAA2867
Bleptina caradrinalis|[11761]|BBLSX078-09|United States|Oklahoma|658[0n]|BOLD:AAA2867
Bleptina caradrinalis|[11762]|BBLSX079-09|United States|Oklahoma|658[0n]|BOLD:AAA2867
Bleptina caradrinalis|[11763]|BBLSX091-09|United States|Oklahoma|658[0n]|BOLD:AAA2867
Bleptina caradrinalis|[11764]|BBLSX144-09|United States|Oklahoma|658[0n]|BOLD:AAA2867
Bleptina caradrinalis|[11765]|BBLSX290-09|United States|Oklahoma|658[0n]|BOLD:AAA2867
Bleptina caradrinalis|[11766]|BBLSX302-09|United States|Oklahoma|658[0n]|BOLD:AAA2867
Bleptina caradrinalis|[11767]|BBLSX371-09|United States|Oklahoma|658[0n]|BOLD:AAA2867
Bleptina caradrinalis|[11768]|BBLSX375-09|United States|Oklahoma|658[0n]|BOLD:AAA2867
Bleptina caradrinalis|[11769]|BBLSX380-09|United States|Oklahoma|658[0n]|BOLD:AAA2867
Bleptina caradrinalis|[11770]|BBLSX393-09|United States|Oklahoma|658[0n]|BOLD:AAA2867
Bleptina caradrinalis|[11771]|BBLSX474-09|United States|Oklahoma|658[0n]|BOLD:AAA2867
Bleptina caradrinalis|[11772]|BBLSX476-09|United States|Oklahoma|658[0n]|BOLD:AAA2867
Bleptina caradrinalis|[11773]|BBLSX500-09|United States|Oklahoma|658[0n]|BOLD:AAA2867
Bleptina caradrinalis|[11774]|BBLSX518-09|United States|Oklahoma|658[0n]|BOLD:AAA2867
Bleptina caradrinalis|[11775]|BBLSX912-09|United States|Arizona|658[0n]|BOLD:AAA2867
Bleptina caradrinalis|[11776]|BBLSX917-09|United States|Arizona|658[0n]|BOLD:AAA2867
Bleptina caradrinalis|[11777]|BBLSX958-09|United States|Oklahoma|658[0n]|BOLD:AAA2867
Bleptina caradrinalis|[11778]|BBLSX969-09|United States|Oklahoma|658[0n]|BOLD:AAA2867
Bleptina caradrinalis|[11779]|BBLSU028-09|United States|Arkansas|658[0n]|BOLD:AAA2867
Bleptina caradrinalis|[11780]|MILEP341-10|United States|Alabama|658[0n]|BOLD:AAA2867
Bleptina caradrinalis|[11781]|MILEP380-10|United States|Alabama|658[0n]|BOLD:AAA2867
Bleptina caradrinalis|[11782]|LNCC350-10|United States|North Carolina|658[0n]|BOLD:AAA2867
[illegible]

Bleptina caradrinalis|[11780]|MILEP341-10|United States|Alabama|658[0n]|BOLD:AAA2867
Bleptina caradrinalis|[11781]|MILEP380-10|United States|Alabama|658[0n]|BOLD:AAA2867
Bleptina caradrinalis|[11782]|LNCC350-10|United States|North Carolina|658[0n]|BOLD:AAA2867
Bleptina caradrinalis|[11783]|LNCC459-10|United States|North Carolina|658[0n]|BOLD:AAA2867
Bleptina caradrinalis|[11784]|LILLA727-11|United States|Illinois|658[0n]|BOLD:AAA2867
Bleptina caradrinalis|[11785]|BBLOC1178-11|United States|Texas|658[0n]|BOLD:AAA2867
Bleptina caradrinalis|[11786]|BBLOE1691-12|United States|Oklahoma|658[0n]|BOLD:AAA2867
Bleptina caradrinalis|[11787]|BBLOE1733-12|United States|Florida|658[0n]|BOLD:AAA2867
Bleptina caradrinalis|[11788]|BBLOE1734-12|United States|Florida|658[0n]|BOLD:AAA2867
Bleptina caradrinalis|[11789]|LNCC1642-13|United States|North Carolina|658[0n]|BOLD:AAA2867
Bleptina n. sp. 5|[11790]|RDNML047-13|United States|Florida|658[0n]|BOLD:AAH4580
Bleptina n. sp. 5|[11791]|RDNML046-13|United States|Florida|658[0n]|BOLD:AAH4580
Bleptina n. sp. 5|[11792]|BBLOE1365-12|United States|Florida|658[0n]|BOLD:AAH4580
Bleptina n. sp. 5|[11793]|IBAH025-08|Bahamas|Exuma|658[0n]|BOLD:AAH4580
Bleptina n. sp. 5|[11794]|BBLOE1362-12|United States|Florida|658[0n]|BOLD:AAH4580
Bleptina n. sp. 5|[11795]|BBLOE1364-12|United States|Florida|658[0n]|BOLD:AAH4580
Bleptina n. sp. 5|[11796]|BBLOE1367-12|United States|Florida|658[0n]|BOLD:AAH4580
Bleptina n. sp. 5|[11797]|RDNML048-13|United States|Florida|658[0n]|BOLD:AAH4580
Bleptina n. sp. 5|[11798]|RDNML051-13|United States|Florida|658[0n]|BOLD:AAH4580
Bleptina sp.|[11799]|CNCLB2731-14|United States|Florida|658[0n]|BOLD:AAH4580
Bleptina sp.|[11800]|CNCLB2733-14|United States|Florida|658[0n]|BOLD:AAH4580
Spargaloma sexpunctata|[11801]|RDMAB048-05|Canada|Alberta|658[0n]|BOLD:AAB3581
Spargaloma sexpunctata|[11802]|LBCH5642-10|Canada|British Columbia|658[0n]|BOLD:AAB3581
Spargaloma sexpunctata|[11803]|RDLQF847-06|Canada|Quebec|658[0n]|BOLD:AAB3581
Spargaloma sexpunctata|[11804]|TMNBB064-06|Canada|New Brunswick|658[0n]|BOLD:AAB3581
Spargaloma sexpunctata|[11805]|LPMN065-08|Canada|Manitoba|658[0n]|BOLD:AAB3581
Spargaloma sexpunctata|[11806]|LBCC489-05|Canada|British Columbia|658[0n]|BOLD:AAB3581
Spargaloma sexpunctata|[11807]|LBCB644-05|Canada|British Columbia|658[0n]|BOLD:AAB3581
Spargaloma sexpunctata|[11808]|LOCT255-05|United States|Connecticut|658[0n]|BOLD:AAB3581
Spargaloma sexpunctata|[11809]|LOCT254-05|United States|Connecticut|658[0n]|BOLD:AAB3581
Spargaloma sexpunctata|[11810]|XAC607-04|Canada|Ontario|658[0n]|BOLD:AAB3581
Spargaloma sexpunctata|[11811]|LBCA675-05|Canada|British Columbia|654[0n]|BOLD:AAB3581
Spargaloma sexpunctata|[11812]|LBCA130-05|Canada|British Columbia|658[0n]|BOLD:AAB3581
Spargaloma sexpunctata|[11813]|LBCB267-05|Canada|British Columbia|658[0n]|BOLD:AAB3581
Spargaloma sexpunctata|[11814]|LOWCB162-05|Canada|British Columbia|658[0n]|BOLD:AAB3581
Spargaloma sexpunctata|[11815]|LOWCB163-05|Canada|British Columbia|564[0n]|BOLD:AAB3581
Spargaloma sexpunctata|[11816]|LOWCB631-05|Canada|British Columbia|548[0n]|BOLD:AAB3581
Spargaloma sexpunctata|[11817]|RDLQG358-06|Canada|Quebec|658[0n]|BOLD:AAB3581
Spargaloma sexpunctata|[11818]|LPSOB517-08|Canada|Ontario|658[0n]|BOLD:AAB3581
Spargaloma sexpunctata|[11819]|LBCH5776-10|Canada|British Columbia|658[0n]|BOLD:AAB3581
Spargaloma sexpunctata|[11820]|BBLPA981-10|Canada|British Columbia|658[0n]|BOLD:AAB3581
Spargaloma sexpunctata|[11821]|LGSMC692-05|United States|Tennessee|658[0n]|BOLD:ABY7017
Spargaloma sexpunctata|[11822]|LPOKB696-09|United States|Oklahoma|658[0n]|BOLD:ABY7017
Spargaloma sexpunctata|[11823]|LPOKB300-09|United States|Oklahoma|658[0n]|BOLD:ABY7017
Spargaloma sexpunctata|[11824]|ABNCC190-07|United States|Indiana|635[0n]|BOLD:ABY7017
Spargaloma sexpunctata|[11825]|ABNCC189-07|United States|Indiana|591[0n]|BOLD:ABY7017
Spargaloma sexpunctata|[11826]|LPOKA484-09|United States|Oklahoma|597[0n]|BOLD:ABY7017
Spargaloma sexpunctata|[11827]|LPOKB732-09|United States|Oklahoma|617[0n]|BOLD:ABY7017
Spargaloma sexpunctata|[11828]|LPOKB776-09|United States|Oklahoma|658[0n]|BOLD:ABY7017
Spargaloma sexpunctata|[11829]|LPOKC748-09|United States|Oklahoma|658[0n]|BOLD:ABY7017
Spargaloma sexpunctata|[11830]|LNCC141-10|United States|North Carolina|658[0n]|BOLD:ABY7017
Spargaloma sexpunctata|[11831]|CNCLB2891-14|United States|North Carolina|658[0n]|BOLD:ABY7017
Tetanolita floridana|[11832]|ABNCC087-07|United States|Florida|617[0n]|BOLD:AAA9814
Tetanolita floridana|[11833]|USLEP532-10|United States|Florida|658[0n]|BOLD:AAA9814
Tetanolita floridana|[11834]|LGSMC927-05|United States|Tennessee|609[0n]|BOLD:AAA9814
Tetanolita floridana|[11835]|LGSMC928-05|United States|Tennessee|658[2n]|BOLD:AAA9814
Tetanolita floridana|[11836]|ABNCC088-07|United States|Florida|640[0n]|BOLD:AAA9814
Tetanolita floridana|[11837]|LGSMC926-05|United States|Tennessee|658[0n]|BOLD:AAA9814
Tetanolita floridana|[11838]|LOFLA017-06|United States|Florida|658[0n]|BOLD:AAA9814
Tetanolita floridana|[11839]|LOFLB076-06|United States|Florida|658[0n]|BOLD:AAA9814
Tetanolita floridana|[11840]|LMEM245-09|United States|Mississippi|658[0n]|BOLD:AAA9814
Tetanolita floridana|[11841]|LMEM247-09|United States|Mississippi|658[0n]|BOLD:AAA9814
Tetanolita floridana|[11842]|LMEM248-09|United States|Alabama|658[0n]|BOLD:AAA9814
Tetanolita floridana|[11843]|LMEM249-09|United States|Mississippi|658[0n]|BOLD:AAA9814
Tetanolita floridana|[11844]|LMEM250-09|United States|Alabama|658[0n]|BOLD:AAA9814
Tetanolita floridana|[11845]|LMEM251-09|United States|Alabama|658[0n]|BOLD:AAA9814
Tetanolita floridana|[11846]|LMEM252-09|United States|Alabama|658[0n]|BOLD:AAA9814
Tetanolita floridana|[11847]|LMEM254-09|United States|Mississippi|658[0n]|BOLD:AAA9814
Tetanolita floridana|[11848]|LMEM255-09|United States|Mississippi|658[0n]|BOLD:AAA9814
Tetanolita floridana|[11849]|LPOKB621-09|United States|Oklahoma|658[0n]|BOLD:AAA9814
Tetanolita floridana|[11850]|BBLSX332-09|United States|Oklahoma|658[0n]|BOLD:AAA9814
Tetanolita floridana|[11851]|BBLSU010-09|United States|Arkansas|658[0n]|BOLD:AAA9814
Tetanolita floridana|[11852]|USLEP611-10|United States|Florida|658[0n]|BOLD:AAA9814
Tetanolita floridana|[11853]|USLEP916-10|United States|Florida|658[0n]|BOLD:AAA9814
Tetanolita floridana|[11854]|USLEP950-10|United States|Florida|658[0n]|BOLD:AAA9814
Tetanolita floridana|[11855]|BBLOB1243-11|United States|Florida|658[0n]|BOLD:AAA9814
Tetanolita floridana|[11856]|MILEQ331-11|United States|Alabama|658[0n]|BOLD:AAA9814
Tetanolita sp.|[11857]|HKONS279-08|United States|Texas|658[0n]|BOLD:AAB7241
Tetanolita sp.|[11858]|LILLA855-11|United States|Illinois|658[0n]|BOLD:AAB7241
Tetanolita sp.|[11859]|LOCT244-05|United States|Connecticut|658[0n]|BOLD:AAB7241
Tetanolita sp.|[11860]|LOCT245-05|United States|Connecticut|658[0n]|BOLD:AAB7241
Tetanolita sp.|[11861]|LNCNW099-06|United States|North Carolina|658[0n]|BOLD:AAB7241
Tetanolita sp.|[11862]|QUNOB521-09|United States|Kentucky|658[0n]|BOLD:AAB7241
Tetanolita sp.|[11863]|LILLA178-11|United States|Illinois|658[0n]|BOLD:AAB7241
Tetanolita sp.|[11864]|IAWLB398-11|United States|Virginia|658[0n]|BOLD:AAB7241
Tetanolita sp.|[11865]|LPOKD459-09|United States|Oklahoma|658[0n]|BOLD:AAB7241
Tetanolita sp.|[11866]|QUNOE464-12|United States|Utah|658[0n]|BOLD:AAB7241
Tetanolita palligera|[11867]|RDNMF736-08|Canada|British Columbia|609[0n]|BOLD:ACF1365
Tetanolita palligera|[11868]|NAMUM207-08|United States|California|658[0n]|BOLD:ACF1365
Tetanolita palligera|[11869]|GMLC274-11|United States|California|658[0n]|BOLD:ACF1365
Tetanolita palligera|[11870]|GMLC1007-12|United States|California|658[0n]|BOLD:ACF1365
Tetanolita palligera|[11871]|LOCBB226-06|United States|California|658[0n]|BOLD:ACF1365
Tetanolita palligera|[11872]|BBLOD773-11|United States|California|658[0n]|BOLD:ACF1365
Tetanolita palligera|[11873]|LOCBC147-06|United States|California|658[0n]|BOLD:ACF1365
Tetanolita palligera|[11874]|LOCBC146-06|United States|California|658[0n]|BOLD:ACF1365
Tetanolita palligera|[11875]|LOCBC145-06|United States|California|658[0n]|BOLD:ACF1365
Tetanolita palligera|[11876]|LOCBB583-06|United States|California|658[0n]|BOLD:ACF1365
Tetanolita palligera|[11877]|LOCBB227-06|United States|California|658[0n]|BOLD:ACF1365
Tetanolita palligera|[11878]|LOCBB225-06|United States|California|658[0n]|BOLD:ACF1365
Tetanolita palligera|[11879]|LOCBB224-06|United States|California|658[0n]|BOLD:ACF1365
Tetanolita palligera|[11880]|LOCBB223-06|United States|California|658[0n]|BOLD:ACF1365
Tetanolita palligera|[11881]|LOCBB222-06|United States|California|658[0n]|BOLD:ACF1365
Tetanolita palligera|[11882]|LOCBF458-13|United States|California|600[0n]|BOLD:ACF1365

Tetanolita palligera|[11880]|LOCBB223-06|United States|California|658[0n]|BOLD:ACF1365
Tetanolita palligera|[11881]|LOCBB222-06|United States|California|658[0n]|BOLD:ACF1365
Tetanolita palligera|[11882]|LOCBF458-13|United States|California|600[0n]|BOLD:ACF1365
Phalaenostola eumelusalis|[11883]|BLTIB1068-08|Canada|Ontario|656[0n]|BOLD:AAB8468
Phalaenostola eumelusalis|[11884]|LGSMC945-05|United States|Tennessee|658[0n]|BOLD:AAB8468
Phalaenostola eumelusalis|[11885]|XAG442-05|Canada|Ontario|658[0n]|BOLD:AAB8468
Phalaenostola eumelusalis|[11886]|BLTIB674-08|Canada|Ontario|658[0n]|BOLD:AAB8468
Phalaenostola eumelusalis|[11887]|BLTIB677-08|Canada|Ontario|658[0n]|BOLD:AAB8468
Phalaenostola eumelusalis|[11888]|BLTIB793-08|Canada|Ontario|658[0n]|BOLD:AAB8468
Phalaenostola eumelusalis|[11889]|BLTIB823-08|Canada|Ontario|658[0n]|BOLD:AAB8468
Phalaenostola eumelusalis|[11890]|BLTIB888-08|Canada|Ontario|658[0n]|BOLD:AAB8468
Phalaenostola eumelusalis|[11891]|BLTIB1069-08|Canada|Ontario|658[0n]|BOLD:AAB8468
Phalaenostola eumelusalis|[11892]|BLTIB1070-08|Canada|Ontario|658[0n]|BOLD:AAB8468
Phalaenostola eumelusalis|[11893]|RDLQF633-06|Canada|Quebec|606[3n]|BOLD:AAB8468
Phalaenostola eumelusalis|[11894]|RDLQF634-06|Canada|Quebec|643[0n]|BOLD:AAB8468
Phalaenostola eumelusalis|[11895]|RDLQG932-06|Canada|Quebec|658[0n]|BOLD:AAB8468
Phalaenostola eumelusalis|[11896]|BLTIB659-08|Canada|Ontario|644[0n]|BOLD:AAB8468
Phalaenostola eumelusalis|[11897]|LNCC1153-11|United States|North Carolina|658[0n]|BOLD:AAB8468
Phalaenostola eumelusalis|[11898]|LOCT246-05|United States|Connecticut|658[0n]|BOLD:AAB8467
Phalaenostola eumelusalis|[11899]|HKONB379-09|United States|Indiana|658[0n]|BOLD:AAB8467
Phalaenostola eumelusalis|[11900]|LPOKC794-09|United States|Oklahoma|658[0n]|BOLD:AAB8467
Phalaenostola eumelusalis|[11901]|LILLA138-11|United States|Illinois|658[0n]|BOLD:AAB8467
Phalaenostola eumelusalis|[11902]|LILLA550-11|United States|Illinois|658[0n]|BOLD:AAB8467
Phalaenostola eumelusalis|[11903]|LILLA645-11|United States|Illinois|658[0n]|BOLD:AAB8467
Phalaenostola eumelusalis|[11904]|LNCC1489-13|United States|North Carolina|658[0n]|BOLD:AAB8467
Phalaenostola eumelusalis|[11905]|LNCC1490-13|United States|North Carolina|658[0n]|BOLD:AAB8467
Phalaenostola larentioides|[11906]|RDLQG485-06|Canada|Quebec|658[0n]|BOLD:AAA7226
Phalaenostola larentioides|[11907]|RDLQG778-06|Canada|Quebec|658[0n]|BOLD:AAA7226
Phalaenostola larentioides|[11908]|BLTIB576-08|Canada|Ontario|643[1n]|BOLD:AAA7226
Phalaenostola larentioides|[11909]|BLTIB795-08|Canada|Ontario|658[0n]|BOLD:AAA7226
Phalaenostola larentioides|[11910]|PMG149-03|Canada|Ontario|617[0n]|BOLD:AAA7226
Phalaenostola larentioides|[11911]|RDLQG798-06|Canada|Quebec|658[0n]|BOLD:AAA7226
Phalaenostola larentioides|[11912]|RDLQG918-06|Canada|Quebec|658[0n]|BOLD:AAA7226
Phalaenostola larentioides|[11913]|RDLQG916-06|Canada|Quebec|658[0n]|BOLD:AAA7226
Phalaenostola larentioides|[11914]|RDLQG762-06|Canada|Quebec|658[0n]|BOLD:AAA7226
Phalaenostola larentioides|[11915]|RDLQG477-06|Canada|Quebec|618[0n]|BOLD:AAA7226
Phalaenostola larentioides|[11916]|RDLQG486-06|Canada|Quebec|658[0n]|BOLD:AAA7226
Phalaenostola larentioides|[11917]|RDLQG095-06|Canada|Quebec|658[0n]|BOLD:AAA7226
Phalaenostola larentioides|[11918]|RDLQB719-05|Canada|Quebec|658[0n]|BOLD:AAA7226
Phalaenostola larentioides|[11919]|RDLQB718-05|Canada|Quebec|658[0n]|BOLD:AAA7226
Phalaenostola larentioides|[11920]|RDLQG481-06|Canada|Quebec|643[0n]|BOLD:AAA7226
Phalaenostola larentioides|[11921]|RDLQG482-06|Canada|Quebec|642[1n]|BOLD:AAA7226
Phalaenostola larentioides|[11922]|RDLQG758-06|Canada|Quebec|658[1n]|BOLD:AAA7226
Phalaenostola larentioides|[11923]|RDLQG763-06|Canada|Quebec|658[0n]|BOLD:AAA7226
Phalaenostola larentioides|[11924]|RDLQG890-06|Canada|Quebec|658[0n]|BOLD:AAA7226
Phalaenostola larentioides|[11925]|RDLQG904-06|Canada|Quebec|658[0n]|BOLD:AAA7226
Phalaenostola larentioides|[11926]|RDLQG919-06|Canada|Quebec|658[0n]|BOLD:AAA7226
Phalaenostola larentioides|[11927]|BLTIB887-08|Canada|Ontario|658[0n]|BOLD:AAA7226
Phalaenostola larentioides|[11928]|BLTIB974-08|Canada|Ontario|658[0n]|BOLD:AAA7226
Phalaenostola larentioides|[11929]|LPOKB405-09|United States|Oklahoma|658[0n]|BOLD:AAA7226
Phalaenostola larentioides|[11930]|BBLCU284-09|United States|Illinois|658[0n]|BOLD:AAA7226
Phalaenostola larentioides|[11931]|RDNMH639-09|United States|Louisiana|658[0n]|BOLD:AAA7226
Phalaenostola larentioides|[11932]|LOT188-04|United States|Tennessee|609[0n]|BOLD:AAA7226
Phalaenostola larentioides|[11933]|LPOKA216-08|United States|Oklahoma|658[0n]|BOLD:AAA7226
Phalaenostola larentioides|[11934]|HKONS346-08|United States|Florida|658[0n]|BOLD:AAA7226
Phalaenostola larentioides|[11935]|HKONS285-08|United States|Florida|658[0n]|BOLD:AAA7226
Phalaenostola larentioides|[11936]|LGSMG508-07|United States|North Carolina|658[0n]|BOLD:AAA7226
Phalaenostola larentioides|[11937]|LGSMG507-07|United States|Tennessee|658[0n]|BOLD:AAA7226
Phalaenostola larentioides|[11938]|RDLQG761-06|Canada|Quebec|658[0n]|BOLD:AAA7226
Phalaenostola larentioides|[11939]|LNCB147-06|United States|North Carolina|658[0n]|BOLD:AAA7226
Phalaenostola larentioides|[11940]|LGSMC386-05|United States|Tennessee|658[0n]|BOLD:AAA7226
Phalaenostola larentioides|[11941]|LGSMC385-05|United States|Tennessee|658[0n]|BOLD:AAA7226
Phalaenostola larentioides|[11942]|LGSM660-04|United States|Tennessee|658[0n]|BOLD:AAA7226
Phalaenostola larentioides|[11943]|ABNCC086-07|United States|Florida|627[0n]|BOLD:AAA7226
Phalaenostola larentioides|[11944]|BLTIB836-08|Canada|Ontario|658[0n]|BOLD:AAA7226
Phalaenostola larentioides|[11945]|LPOKA255-08|United States|Oklahoma|658[0n]|BOLD:AAA7226
Phalaenostola larentioides|[11946]|LPOKA466-09|United States|Oklahoma|658[0n]|BOLD:AAA7226
Phalaenostola larentioides|[11947]|LPOKA540-09|United States|Oklahoma|658[0n]|BOLD:AAA7226
Phalaenostola larentioides|[11948]|LMEM233-09|United States|Kansas|658[0n]|BOLD:AAA7226
Phalaenostola larentioides|[11949]|LMEM234-09|United States|Kansas|658[0n]|BOLD:AAA7226
Phalaenostola larentioides|[11950]|LMEM236-09|United States|Kansas|658[0n]|BOLD:AAA7226
Phalaenostola larentioides|[11951]|LMEM237-09|United States|Kansas|658[0n]|BOLD:AAA7226
Phalaenostola larentioides|[11952]|LMEM238-09|United States|Mississippi|658[0n]|BOLD:AAA7226
Phalaenostola larentioides|[11953]|LMEM239-09|United States|Tennessee|658[0n]|BOLD:AAA7226
Phalaenostola larentioides|[11954]|LMEM240-09|United States|Alabama|658[0n]|BOLD:AAA7226
Phalaenostola larentioides|[11955]|LPOKB050-09|United States|Oklahoma|658[0n]|BOLD:AAA7226
Phalaenostola larentioides|[11956]|LPOKB743-09|United States|Oklahoma|658[0n]|BOLD:AAA7226
Phalaenostola larentioides|[11957]|LPOKB772-09|United States|Oklahoma|658[0n]|BOLD:AAA7226
Phalaenostola larentioides|[11958]|BBLSU013-09|United States|Arkansas|658[0n]|BOLD:AAA7226
Phalaenostola larentioides|[11959]|BBLCU283-09|United States|Illinois|658[0n]|BOLD:AAA7226
Phalaenostola larentioides|[11960]|LNCC094-10|United States|North Carolina|658[0n]|BOLD:AAA7226
Phalaenostola larentioides|[11961]|LILLA568-11|United States|Illinois|658[0n]|BOLD:AAA7226
Phalaenostola larentioides|[11962]|LILLA650-11|United States|Illinois|658[0n]|BOLD:AAA7226
Phalaenostola larentioides|[11963]|IAWLB392-11|United States|Virginia|658[0n]|BOLD:AAA7226
Phalaenostola larentioides|[11964]|CNPPD2369-12|Canada|Ontario|632[0n]|BOLD:AAA7226
Phalaenostola larentioides|[11965]|LPOKA142-08|United States|Oklahoma|658[0n]|BOLD:AAA7226
Phalaenostola larentioides|[11966]|LNCC095-10|United States|North Carolina|658[0n]|BOLD:AAA7226
Phalaenostola larentioides|[11967]|LPOKC810-09|United States|Oklahoma|658[0n]|BOLD:AAA7226
Phalaenostola larentioides|[11968]|LPOKA087-08|United States|Oklahoma|658[0n]|BOLD:AAA7226
Phalaenostola larentioides|[11969]|LNCB148-06|United States|North Carolina|658[0n]|BOLD:AAA7226
Phalaenostola larentioides|[11970]|CNPPH809-12|Canada|Ontario|640[0n]|BOLD:AAA7226
Phalaenostola metonalis|[11971]|RDLQG109-06|Canada|Quebec|618[0n]|BOLD:ACF3696
Phalaenostola metonalis|[11972]|BLTIB638-08|Canada|Ontario|644[0n]|BOLD:ACF3696
Phalaenostola metonalis|[11973]|CNPPH812-12|Canada|Ontario|664[0n]|BOLD:ACF3696
Phalaenostola metonalis|[11974]|RDLQG116-06|Canada|Quebec|612[2n]|BOLD:AAA7565
Phalaenostola metonalis|[11975]|RDLQG111-06|Canada|Quebec|638[0n]|BOLD:AAA7565
Phalaenostola metonalis|[11976]|RDLQG114-06|Canada|Quebec|612[2n]|BOLD:AAA7565
Phalaenostola metonalis|[11977]|RDLQG112-06|Canada|Quebec|597[1n]|BOLD:AAA7565
Phalaenostola metonalis|[11978]|RDLQG108-06|Canada|Quebec|617[0n]|BOLD:AAA7565
Phalaenostola metonalis|[11979]|RDLQG176-06|Canada|Quebec|607[0n]|BOLD:AAA7565
Phalaenostola metonalis|[11980]|BBLEC109-09|Canada|Nova Scotia|658[0n]|BOLD:AAA7565
Phalaenostola metonalis|[11981]|BBLEC608-09|Canada|Nova Scotia|658[0n]|BOLD:AAA7565
Phalaenostola metonalis|[11982]|CNRME749-12|Canada|Manitoba|633[0n]|BOLD:AAA7565

Phalaenostola metonalis|[11980]|BBLEC109-09|Canada|Nova Scotia|658[0n]|BOLD:AAA7565
Phalaenostola metonalis|[11981]|BBLEC608-09|Canada|Nova Scotia|658[0n]|BOLD:AAA7565
Phalaenostola metonalis|[11982]|CNRME749-12|Canada|Manitoba|633[0n]|BOLD:AAA7565
Phalaenostola metonalis|[11983]|CNEID3321-12|Canada|Alberta|634[0n]|BOLD:AAA7565
Phalaenostola metonalis|[11984]|CNEID3336-12|Canada|Alberta|634[0n]|BOLD:AAA7565
Phalaenostola metonalis|[11985]|CNRME1812-12|Canada|Manitoba|635[0n]|BOLD:AAA7565
Phalaenostola metonalis|[11986]|CNRME2777-12|Canada|Manitoba|633[0n]|BOLD:AAA7565
Phalaenostola metonalis|[11987]|CNRME2765-12|Canada|Manitoba|633[0n]|BOLD:AAA7565
Phalaenostola metonalis|[11988]|LPMN384-08|Canada|Manitoba|632[0n]|BOLD:AAA7565
Phalaenostola metonalis|[11989]|RDLQG113-06|Canada|Quebec|631[0n]|BOLD:AAA7565
Phalaenostola metonalis|[11990]|LPMN593-08|Canada|Manitoba|658[0n]|BOLD:AAA7565
Phalaenostola metonalis|[11991]|RDLQG110-06|Canada|Quebec|657[0n]|BOLD:AAA7565
Phalaenostola metonalis|[11992]|LPMN591-08|Canada|Manitoba|658[0n]|BOLD:AAA7565
Phalaenostola metonalis|[11993]|LPMN601-08|Canada|Manitoba|658[0n]|BOLD:AAA7565
Phalaenostola metonalis|[11994]|LPMNB255-09|Canada|Manitoba|658[0n]|BOLD:AAA7565
Phalaenostola metonalis|[11995]|BBLEC380-09|Canada|Newfoundland and Labrador|658[0n]|BOLD:AAA7565
Phalaenostola metonalis|[11996]|BBLPB974-10|Canada|Alberta|658[0n]|BOLD:AAA7565
Phalaenostola metonalis|[11997]|BBLPB975-10|Canada|Alberta|658[0n]|BOLD:AAA7565
Phalaenostola metonalis|[11998]|BBLPB985-10|Canada|Alberta|658[0n]|BOLD:AAA7565
Phalaenostola metonalis|[11999]|BBLPB986-10|Canada|Alberta|658[0n]|BOLD:AAA7565
Phalaenostola metonalis|[12000]|BBLPB991-10|Canada|Alberta|658[0n]|BOLD:AAA7565
Phalaenostola metonalis|[12001]|RDNM563-05|United States|California|577[1n]|BOLD:AAA7565
Phalaenostola metonalis|[12002]|RDLQG115-06|Canada|Quebec|618[0n]|BOLD:AAA7565
Phalaenostola metonalis|[12003]|CNEID3326-12|Canada|Alberta|641[0n]|BOLD:AAA7565
Phalaenostola metonalis|[12004]|CNEID3349-12|Canada|Alberta|638[0n]|BOLD:AAA7565
Phalaenostola metonalis|[12005]|CNEID3354-12|Canada|Alberta|636[0n]|BOLD:AAA7565
Phalaenostola metonalis|[12006]|LOWCB629-05|Canada|British Columbia|658[0n]|BOLD:AAA7565
Phalaenostola metonalis|[12007]|LBCB693-05|Canada|British Columbia|658[0n]|BOLD:AAA7565
Phalaenostola metonalis|[12008]|CNEID3341-12|Canada|Alberta|640[0n]|BOLD:AAA7565
Phalaenostola metonalis|[12009]|CNEID3352-12|Canada|Alberta|634[0n]|BOLD:AAA7565
Phalaenostola metonalis|[12010]|CNEID3324-12|Canada|Alberta|634[0n]|BOLD:AAA7565
Phalaenostola metonalis|[12011]|CNEIE021-12|Canada|Alberta|630[0n]|BOLD:AAA7565
Phalaenostola metonalis|[12012]|BBLPD615-10|Canada|British Columbia|658[0n]|BOLD:AAA7565
Phalaenostola metonalis|[12013]|BBLPD614-10|Canada|British Columbia|658[0n]|BOLD:AAA7565
Phalaenostola metonalis|[12014]|BBLPD613-10|Canada|British Columbia|658[0n]|BOLD:AAA7565
Phalaenostola metonalis|[12015]|LNCC324-10|United States|North Carolina|658[0n]|BOLD:AAA7565
Phalaenostola metonalis|[12016]|LNCC323-10|United States|North Carolina|658[0n]|BOLD:AAA7565
Phalaenostola metonalis|[12017]|LNCC322-10|United States|North Carolina|658[0n]|BOLD:AAA7565
Phalaenostola metonalis|[12018]|LNCC321-10|United States|North Carolina|658[0n]|BOLD:AAA7565
Phalaenostola metonalis|[12019]|BBLPC317-09|Canada|Newfoundland and Labrador|658[0n]|BOLD:AAA7565
Phalaenostola metonalis|[12020]|BBLPC298-09|Canada|Newfoundland and Labrador|658[0n]|BOLD:AAA7565
Phalaenostola metonalis|[12021]|BBLPC150-09|Canada|Nova Scotia|658[0n]|BOLD:AAA7565
Phalaenostola metonalis|[12022]|LPABB508-08|Canada|Alberta|658[0n]|BOLD:AAA7565
Phalaenostola metonalis|[12023]|LPMN653-08|Canada|Manitoba|658[0n]|BOLD:AAA7565
Phalaenostola metonalis|[12024]|LPMN647-08|Canada|Manitoba|658[0n]|BOLD:AAA7565
Phalaenostola metonalis|[12025]|LPMN610-08|Canada|Manitoba|658[0n]|BOLD:AAA7565
Phalaenostola metonalis|[12026]|LPMN525-08|Canada|Manitoba|658[0n]|BOLD:AAA7565
Phalaenostola metonalis|[12027]|MMNA104-08|United States|North Carolina|658[0n]|BOLD:AAA7565
Phalaenostola metonalis|[12028]|LGSMG506-07|United States|North Carolina|658[0n]|BOLD:AAA7565
Phalaenostola metonalis|[12029]|LGSMG505-07|United States|Tennessee|658[0n]|BOLD:AAA7565
Phalaenostola metonalis|[12030]|RDLQF581-06|Canada|Quebec|658[0n]|BOLD:AAA7565
Phalaenostola metonalis|[12031]|LOWCB628-05|Canada|British Columbia|658[0n]|BOLD:AAA7565
Phalaenostola metonalis|[12032]|LOWCB627-05|Canada|British Columbia|658[0n]|BOLD:AAA7565
Phalaenostola metonalis|[12033]|LOWCB177-05|Canada|British Columbia|658[0n]|BOLD:AAA7565
Phalaenostola metonalis|[12034]|LOWCB167-05|Canada|British Columbia|658[0n]|BOLD:AAA7565
Phalaenostola metonalis|[12035]|LOWCB166-05|Canada|British Columbia|658[0n]|BOLD:AAA7565
Phalaenostola metonalis|[12036]|LOWCB165-05|Canada|British Columbia|658[0n]|BOLD:AAA7565
Phalaenostola metonalis|[12037]|LBCA524-05|Canada|British Columbia|658[0n]|BOLD:AAA7565
Phalaenostola metonalis|[12038]|LBCA423-05|Canada|British Columbia|658[0n]|BOLD:AAA7565
Phalaenostola metonalis|[12039]|LBCA401-05|Canada|British Columbia|658[0n]|BOLD:AAA7565
Phalaenostola metonalis|[12040]|LGSMC397-05|United States|Tennessee|658[0n]|BOLD:AAA7565
Phalaenostola metonalis|[12041]|LGSMC396-05|United States|Tennessee|658[0n]|BOLD:AAA7565
Phalaenostola metonalis|[12042]|LGSMC395-05|United States|Tennessee|658[0n]|BOLD:AAA7565
Phalaenostola metonalis|[12043]|LGSMC394-05|United States|Tennessee|658[0n]|BOLD:AAA7565
Phalaenostola metonalis|[12044]|LGSM502-04|United States|Tennessee|658[0n]|BOLD:AAA7565
Phalaenostola metonalis|[12045]|LGSM501-04|United States|Tennessee|658[0n]|BOLD:AAA7565
Phalaenostola metonalis|[12046]|LGSMC398-05|United States|Tennessee|619[0n]|BOLD:AAA7565
Phalaenostola metonalis|[12047]|LPAB082-08|Canada|Alberta|582[0n]|BOLD:AAA7565
Phalaenostola metonalis|[12048]|SSPAA2611-13|Canada|Saskatchewan|604[0n]|BOLD:AAA7565
Macrochilo santerivalis|[12049]|LNCB145-06|United States|North Carolina|658[0n]|BOLD:AAJ1440
Macrochilo santerivalis|[12050]|LNCB146-06|United States|North Carolina|658[0n]|BOLD:AAJ1440
Macrochilo louisiana|[12051]|RDLQG937-06|Canada|Quebec|658[0n]|BOLD:AAC0807
Macrochilo louisiana|[12052]|RDLQG781-06|Canada|Quebec|658[0n]|BOLD:AAC0807
Macrochilo louisiana|[12053]|RDLQG625-06|Canada|Quebec|658[0n]|BOLD:AAC0807
Macrochilo louisiana|[12054]|RDLQB405-05|Canada|Quebec|581[0n]|BOLD:AAC0807
Macrochilo louisiana|[12055]|RDLQH154-06|Canada|Quebec|606[0n]|BOLD:AAC0807
Macrochilo louisiana|[12056]|LOFLB765-06|United States|Florida|658[0n]|BOLD:AAC0807
Macrochilo louisiana|[12057]|LOFLB673-06|United States|Florida|658[0n]|BOLD:AAC0807
Macrochilo louisiana|[12058]|LOFLA447-06|United States|Florida|656[0n]|BOLD:AAC0807
Macrochilo louisiana|[12059]|LOFLB372-06|United States|Florida|658[0n]|BOLD:AAC0807
Macrochilo louisiana|[12060]|LOFLB298-06|United States|Florida|658[0n]|BOLD:AAC0807
Macrochilo louisiana|[12061]|LOFLB679-06|United States|Florida|635[0n]|BOLD:AAC0807
Macrochilo louisiana|[12062]|HKONS293-08|United States|Florida|658[0n]|BOLD:AAC0807
Macrochilo louisiana|[12063]|LOFLB866-06|United States|Florida|658[0n]|BOLD:AAC0807
Macrochilo louisiana|[12064]|CNPPE1048-12|Canada|Ontario|616[0n]|BOLD:AAC0807
Macrochilo louisiana|[12065]|LNCC1526-13|United States|North Carolina|658[0n]|BOLD:AAC0807
Macrochilo louisiana|[12066]|LNCC1815-13|United States|North Carolina|658[0n]|BOLD:AAC0807
Macrochilo louisiana|[12067]|LNC255-05|United States|North Carolina|658[0n]|BOLD:AAC0807
Macrochilo louisiana|[12068]|LNCC1816-13|United States|North Carolina|658[0n]|BOLD:AAC0807
Macrochilo louisiana|[12069]|LNCC1889-13|United States|North Carolina|658[0n]|BOLD:AAC0807
Macrochilo bivittata|[12070]|RDNMF781-08|Canada|Alberta|658[0n]|BOLD:AAD6368
Macrochilo bivittata|[12071]|RDNME947-08|Canada|Alberta|658[0n]|BOLD:AAD6368
Macrochilo bivittata|[12072]|RDNME948-08|Canada|Ontario|658[0n]|BOLD:AAD6368
Macrochilo bivittata|[12073]|RDNMF782-08|Canada|Alberta|658[0n]|BOLD:AAD6368
Macrochilo bivittata|[12074]|RDNMF783-08|Canada|Ontario|658[0n]|BOLD:AAD6368
Macrochilo bivittata|[12075]|RDNMF784-08|Canada|Alberta|658[0n]|BOLD:AAD6368
Macrochilo bivittata|[12076]|QUNOD680-11|United States|Minnesota|658[0n]|BOLD:AAD6368
Macrochilo|[12077]|MILEP078-09|United States|North Carolina|658[0n]|BOLD:ABZ5098
Macrochilo|[12078]|MILEP079-09|United States|North Carolina|658[0n]|BOLD:ABZ5098
Macrochilo absorptalis|[12079]|XAJ776-06|Canada|Ontario|636[0n]|BOLD:ACF4191
Macrochilo absorptalis|[12080]|RDLQG910-06|Canada|Quebec|653[0n]|BOLD:ACF4191
Macrochilo absorptalis|[12081]|RDLQB399-05|Canada|Quebec|658[0n]|BOLD:AAB3885
Macrochilo absorptalis|[12082]|BLTIB743-08|Canada|Ontario|657[0n]|BOLD:AAB3885

Macrochilo absorptalis|[12080]|RDLQG910-06|Canada|Quebec|653[0n]|BOLD:ACF4191
Macrochilo absorptalis|[12081]|RDLQB399-05|Canada|Quebec|658[0n]|BOLD:AAB3885
Macrochilo absorptalis|[12082]|BLTIB743-08|Canada|Ontario|657[0n]|BOLD:AAB3885
Macrochilo absorptalis|[12083]|RDLQG779-06|Canada|Quebec|658[0n]|BOLD:AAB3885
Macrochilo absorptalis|[12084]|RDLQF427-06|Canada|Quebec|658[0n]|BOLD:AAB3885
Macrochilo absorptalis|[12085]|RDLQF559-06|Canada|Quebec|658[0n]|BOLD:AAB3885
Macrochilo absorptalis|[12086]|RDLQG166-06|Canada|Quebec|658[0n]|BOLD:AAB3885
Macrochilo absorptalis|[12087]|RDLQG588-06|Canada|Quebec|658[0n]|BOLD:AAB3885
Macrochilo absorptalis|[12088]|RDLQG902-06|Canada|Quebec|658[0n]|BOLD:AAB3885
Macrochilo absorptalis|[12089]|RDLQG903-06|Canada|Quebec|658[0n]|BOLD:AAB3885
Macrochilo absorptalis|[12090]|ABNCC078-07|United States|Wisconsin|625[0n]|BOLD:AAB3885
Macrochilo absorptalis|[12091]|BLTIB357-08|Canada|Ontario|632[0n]|BOLD:AAB3885
Macrochilo absorptalis|[12092]|BLTIB1074-08|Canada|Ontario|658[0n]|BOLD:AAB3885
Macrochilo absorptalis|[12093]|LNCC1151-11|United States|North Carolina|658[0n]|BOLD:AAB3885
Macrochilo absorptalis|[12094]|LNCC1027-11|United States|North Carolina|658[0n]|BOLD:AAB3885
Macrochilo absorptalis|[12095]|RDLQG938-06|Canada|Quebec|658[0n]|BOLD:AAB3885
Macrochilo absorptalis|[12096]|RDLQG924-06|Canada|Quebec|658[0n]|BOLD:AAB3885
Macrochilo absorptalis|[12097]|RDLQG667-06|Canada|Quebec|658[0n]|BOLD:AAB3885
Macrochilo absorptalis|[12098]|PHMNB672-04|Canada|New Brunswick|658[0n]|BOLD:AAB3885
Macrochilo absorptalis|[12099]|PHMNB216-04|Canada|New Brunswick|658[0n]|BOLD:AAB3885
Macrochilo absorptalis|[12100]|XAJ898-06|Canada|Ontario|658[0n]|BOLD:AAB3885
Macrochilo absorptalis|[12101]|RDLQG668-06|Canada|Quebec|658[0n]|BOLD:AAB3885
Macrochilo absorptalis|[12102]|BLTIB446-08|Canada|Ontario|658[0n]|BOLD:AAB3885
Macrochilo absorptalis|[12103]|PHMO255-03|Canada|Ontario|639[0n]|BOLD:AAB3885
Macrochilo absorptalis|[12104]|BLTIB630-08|Canada|Ontario|635[0n]|BOLD:AAB3885
Macrochilo absorptalis|[12105]|LNCC1152-11|United States|North Carolina|632[0n]|BOLD:AAB3885
Macrochilo hypocritalis|[12106]|LPOKE233-10|United States|Oklahoma|658[0n]|BOLD:AAB8540
Macrochilo hypocritalis|[12107]|LGSM758-04|United States|Tennessee|658[0n]|BOLD:AAB8540
Macrochilo hypocritalis|[12108]|LMEM226-09|United States|Mississippi|658[0n]|BOLD:AAB8540
Macrochilo hypocritalis|[12109]|LGSMG502-07|United States|Tennessee|658[0n]|BOLD:AAB8540
Macrochilo hypocritalis|[12110]|LGSM470-04|United States|Tennessee|658[0n]|BOLD:AAB8540
Macrochilo hypocritalis|[12111]|LGSM471-04|United States|Tennessee|606[1n]|BOLD:AAB8540
Macrochilo hypocritalis|[12112]|LMEM221-09|United States|Mississippi|658[0n]|BOLD:AAB8540
Macrochilo hypocritalis|[12113]|LMEM223-09|United States|Alabama|658[0n]|BOLD:AAB8540
Macrochilo hypocritalis|[12114]|LILLA693-11|United States|Illinois|658[0n]|BOLD:AAB8540
Macrochilo hypocritalis|[12115]|LGSMG501-07|United States|Tennessee|658[0n]|BOLD:AAB8540
Macrochilo hypocritalis|[12116]|HKONS286-08|United States|Florida|658[0n]|BOLD:AAB8540
Macrochilo hypocritalis|[12117]|LMEM220-09|United States|Tennessee|658[0n]|BOLD:AAB8540
Macrochilo hypocritalis|[12118]|LMEM225-09|United States|Texas|658[0n]|BOLD:AAB8540
Macrochilo hypocritalis|[12119]|LMEM224-09|United States|Alabama|658[0n]|BOLD:AAB8540
Macrochilo hypocritalis|[12120]|LILLA746-11|United States|Illinois|658[0n]|BOLD:AAB8540
Macrochilo hypocritalis|[12121]|LNC180-05|United States|North Carolina|658[0n]|BOLD:AAB8540
Macrochilo hypocritalis|[12122]|HKONS287-08|United States|Florida|658[0n]|BOLD:AAB8540
Macrochilo hypocritalis|[12123]|ABNCC079-07|United States|Wisconsin|620[0n]|BOLD:AAB8540
Macrochilo hypocritalis|[12124]|LOFLC322-06|United States|Florida|658[0n]|BOLD:AAB8540
Macrochilo hypocritalis|[12125]|RDNMJ012-10|United States|Florida|658[0n]|BOLD:AAB8540
Macrochilo hypocritalis|[12126]|BBLOB1174-11|United States|Florida|658[0n]|BOLD:AAB8540
Macrochilo hypocritalis|[12127]|LNC179-05|United States|North Carolina|658[0n]|BOLD:AAB8540
Macrochilo hypocritalis|[12128]|RDNMD348-06|United States|Florida|574[0n]|BOLD:AAB8540
Macrochilo hypocritalis|[12129]|LNCC850-11|United States|North Carolina|658[0n]|BOLD:AAB8540
Macrochilo hypocritalis|[12130]|MILEQ198-11|United States|Georgia|658[0n]|BOLD:AAB8540
Macrochilo hypocritalis|[12131]|MILEQ199-11|United States|Georgia|631[0n]|BOLD:AAB8540
Phalaenostola hanhami|[12132]|RDNMF605-08|Canada|Alberta|658[0n]|BOLD:AAD3686
Phalaenostola hanhami|[12133]|RDNMF606-08|Canada|Alberta|658[0n]|BOLD:AAD3686
Phalaenostola hanhami|[12134]|RDNMF103-08|Canada|British Columbia|658[0n]|BOLD:AAD3686
Phalaenostola hanhami|[12135]|RDNMF101-08|Canada|Ontario|658[0n]|BOLD:AAD3686
Phalaenostola hanhami|[12136]|RDNMF604-08|Canada|Alberta|609[0n]|BOLD:AAD3686
Phalaenostola hanhami|[12137]|RDNMF104-08|Canada|Ontario|658[0n]|BOLD:AAD3686
Phalaenostola hanhami|[12138]|RDNMF523-08|Canada|Manitoba|658[0n]|BOLD:AAD3686
Phalaenostola hanhami|[12139]|QUNOE405-12|United States|Minnesota|658[0n]|BOLD:AAD3686
Phalaenostola hanhami|[12140]|QUNOE406-12|United States|Minnesota|658[0n]|BOLD:AAD3686
Macrochilo litophora sp. 2|[12141]|LNCC1491-13|United States|North Carolina|658[0n]|BOLD:AAC6075
Macrochilo litophora sp. 2|[12142]|XAB489-04|Canada|Ontario|658[0n]|BOLD:AAC6075
Macrochilo litophora sp. 2|[12143]|ABNCC080-07|United States|Wisconsin|632[0n]|BOLD:AAC6075
Macrochilo litophora sp. 2|[12144]|LILLA230-11|United States|Illinois|658[0n]|BOLD:AAC6075
Macrochilo litophora sp. 2|[12145]|LNCC1840-13|United States|North Carolina|658[0n]|BOLD:AAC6075
Macrochilo litophora|[12146]|LMEM218-09|United States|Mississippi|623[0n]|BOLD:AAC6076
Macrochilo litophora|[12147]|LNCC413-10|United States|North Carolina|658[0n]|BOLD:AAC6076
Macrochilo litophora|[12148]|MILEP006-09|United States|North Carolina|658[0n]|BOLD:AAC6076
Macrochilo litophora|[12149]|LMEM219-09|United States|Tennessee|658[0n]|BOLD:AAC6076
Macrochilo litophora|[12150]|LMEM217-09|United States|Mississippi|658[0n]|BOLD:AAC6076
Macrochilo litophora|[12151]|HKONB464-09|United States|Indiana|658[0n]|BOLD:AAC6076
Macrochilo litophora|[12152]|LGSMG503-07|United States|Tennessee|658[0n]|BOLD:AAC6076
Macrochilo litophora|[12153]|LGSMC924-05|United States|Tennessee|658[0n]|BOLD:AAC6076
Macrochilo litophora|[12154]|HKONB466-09|United States|Texas|648[0n]|BOLD:AAC6076
Macrochilo litophora|[12155]|LNCC1841-13|United States|North Carolina|648[0n]|BOLD:AAC6076
Macrochilo litophora|[12156]|LILLA265-11|United States|Illinois|658[0n]|BOLD:AAC6076
Macrochilo litophora|[12157]|LILLA730-11|United States|Illinois|658[0n]|BOLD:AAC6076
Macrochilo litophora|[12158]|LNCC864-11|United States|North Carolina|658[0n]|BOLD:AAC6076
Macrochilo litophora|[12159]|CNCLB1258-14|United States|North Carolina|658[0n]|BOLD:AAC6076
Macrochilo orciferalis|[12160]|MNAF487-08|Canada|Manitoba|658[0n]|BOLD:AAB1327
Macrochilo orciferalis|[12161]|RDLQG913-06|Canada|Quebec|658[0n]|BOLD:AAB1327
Macrochilo orciferalis|[12162]|RDLQG672-06|Canada|Quebec|658[0n]|BOLD:AAB1327
Macrochilo orciferalis|[12163]|RDLQG671-06|Canada|Quebec|658[0n]|BOLD:AAB1327
Macrochilo orciferalis|[12164]|RDLQG670-06|Canada|Quebec|658[0n]|BOLD:AAB1327
Macrochilo orciferalis|[12165]|RDLQG669-06|Canada|Quebec|658[0n]|BOLD:AAB1327
Macrochilo orciferalis|[12166]|RDLQG172-06|Canada|Quebec|658[0n]|BOLD:AAB1327
Macrochilo orciferalis|[12167]|RDLQB463-05|Canada|Quebec|658[0n]|BOLD:AAB1327
Macrochilo orciferalis|[12168]|XAB004-04|Canada|Ontario|565[0n]|BOLD:AAB1327
Macrochilo orciferalis|[12169]|RDLQH155-06|Canada|Quebec|600[0n]|BOLD:AAB1327
Macrochilo orciferalis|[12170]|RDLQH156-06|Canada|Quebec|594[4n]|BOLD:AAB1327
Macrochilo orciferalis|[12171]|ABNCC083-07|United States|Wisconsin|636[0n]|BOLD:AAB1327
Macrochilo orciferalis|[12172]|MNAF865-08|Canada|Manitoba|658[0n]|BOLD:AAB1327
Macrochilo orciferalis|[12173]|LMEM232-09|United States|Louisiana|658[0n]|BOLD:AAB1328
Macrochilo orciferalis|[12174]|LMEM229-09|United States|Louisiana|658[0n]|BOLD:AAB1328
Macrochilo orciferalis|[12175]|LMEM230-09|United States|Louisiana|658[0n]|BOLD:AAB1328
Macrochilo orciferalis|[12176]|LMEM231-09|United States|Louisiana|658[0n]|BOLD:AAB1328
Macrochilo orciferalis|[12177]|HKONS345-08|United States|Florida|658[0n]|BOLD:AAB1328
Macrochilo orciferalis|[12178]|LNCNW093-06|United States|North Carolina|658[0n]|BOLD:AAB1328
Macrochilo orciferalis|[12179]|LILLA703-11|United States|Illinois|658[0n]|BOLD:AAB1328
Macrochilo orciferalis|[12180]|LMEM228-09|United States|Alabama|658[0n]|BOLD:AAB1328
Macrochilo orciferalis sp. 2|[12181]|HKONB462-09|United States|Texas|658[0n]|BOLD:AAB1328
Macrochilo orciferalis|[12182]|LPSO759-08|Canada|Ontario|656[0n]|BOLD:AAB1328

Macrochilo orciferalis|[12179]|[illegible]|BOLD:AAB1328
Macrochilo orciferalis|[12180]|LMEM228-09|United States|Alabama|658[0n]|BOLD:AAB1328
Macrochilo orciferalis sp. 2|[12181]|HKONB462-09|United States|Texas|658[0n]|BOLD:AAB1328
Macrochilo orciferalis|[12182]|LPSO759-08|Canada|Ontario|656[0n]|BOLD:AAB1328
Macrochilo orciferalis|[12183]|HKONS284-08|United States|Florida|658[0n]|BOLD:AAB1328
Macrochilo orciferalis|[12184]|HKONS283-08|United States|Florida|658[0n]|BOLD:AAB1328
Macrochilo orciferalis|[12185]|LGSMG504-07|United States|Tennessee|658[0n]|BOLD:AAB1328
Macrochilo orciferalis|[12186]|LSEU773-06|United States|Missouri|658[0n]|BOLD:AAB1328
Macrochilo orciferalis|[12187]|LSEU772-06|United States|Missouri|658[0n]|BOLD:AAB1328
Macrochilo orciferalis|[12188]|LNC323-05|United States|North Carolina|658[0n]|BOLD:AAB1328
Macrochilo orciferalis|[12189]|LOCT266-05|United States|Connecticut|658[0n]|BOLD:AAB1328
Macrochilo orciferalis|[12190]|LOCT265-05|United States|Connecticut|658[0n]|BOLD:AAB1328
Macrochilo orciferalis|[12191]|LNC322-05|United States|North Carolina|658[0n]|BOLD:AAB1328
Macrochilo orciferalis|[12192]|CNPPJ1464-12|Canada|Ontario|644[0n]|BOLD:AAB1328
Macrochilo orciferalis|[12193]|CNCLB2918-14|United States|North Carolina|658[0n]|BOLD:AAB1328
Phlyctaina irrigualis|[12194]|RDNMD502-06|United States|Florida|658[0n]|BOLD:AAE0446
Phlyctaina irrigualis|[12195]|RDNMD524-06|United States|Florida|658[0n]|BOLD:AAE0446
Phlyctaina irrigualis|[12196]|HKONS061-07|United States|Florida|658[0n]|BOLD:AAE0446
Phlyctaina irrigualis|[12197]|HKONS088-07|United States|Florida|657[0n]|BOLD:AAE0446
Phlyctaina sp. 1|[12198]|RDNME612-08|United States|Texas|658[0n]|BOLD:AAI1677
Phlyctaina sp. 1|[12199]|RDNME700-08|United States|Texas|658[0n]|BOLD:AAI1677
Phlyctaina sp. 1|[12200]|QUNOD186-10|United States|Texas|658[0n]|BOLD:AAI1677
Hypenula caminalis|[12201]|IAWL036-09|United States|Arizona|658[0n]|BOLD:ACF2365
Hypenula caminalis|[12202]|CMAZA383-10|United States|Arizona|658[0n]|BOLD:ACF2365
Hypenula caminalis|[12203]|HKONB526-09|United States|Texas|647[0n]|BOLD:ABY4908
Hypenula caminalis|[12204]|RDNMG240-08|United States|Texas|658[0n]|BOLD:ABY4908
Hypenula caminalis|[12205]|RDNMG239-08|United States|Texas|658[0n]|BOLD:ABY4908
Hypenula caminalis|[12206]|HKONB524-09|United States|Texas|658[0n]|BOLD:ABY4908
Hypenula caminalis|[12207]|HKONB525-09|United States|Texas|645[0n]|BOLD:ABY4908
Hypenula caminalis|[12208]|BBLSW713-09|United States|Texas|658[0n]|BOLD:ABY4908
Hypenula caminalis|[12209]|BBLSY113-09|United States|Texas|658[0n]|BOLD:ABY4908
Hypenula caminalis|[12210]|BBLSY114-09|United States|Texas|658[0n]|BOLD:ABY4908
Hypenula caminalis|[12211]|BBLSY145-09|United States|Texas|658[0n]|BOLD:ABY4908
Hypenula caminalis|[12212]|BBLSY164-09|United States|Texas|658[0n]|BOLD:ABY4908
Hypenula caminalis|[12213]|BBLSY165-09|United States|Texas|658[0n]|BOLD:ABY4908
Hypenula caminalis|[12214]|BBLSY228-09|United States|Texas|658[0n]|BOLD:ABY4908
Hypenula caminalis|[12215]|BBLSY237-09|United States|Texas|658[0n]|BOLD:ABY4908
Hypenula caminalis|[12216]|BBLSY239-09|United States|Texas|658[0n]|BOLD:ABY4908
Hypenula caminalis|[12217]|BBLSX763-09|United States|Texas|658[0n]|BOLD:ABY4908
Hypenula caminalis|[12218]|ABNCC097-07|United States|Arizona|646[0n]|BOLD:ABY4908
Hypenula caminalis|[12219]|ABNCC098-07|United States|Arizona|642[0n]|BOLD:ABY4908
Hypenula caminalis|[12220]|CMAZA807-10|United States|Arizona|658[0n]|BOLD:ABY4908
Hypenula deleona|[12221]|CNCLB1735-14|Cuba||658[0n]|BOLD:ACP7267
Hypenula cacuminalis|[12222]|BBLOB455-11|United States|Florida|658[0n]|BOLD:AAB3233
Hypenula cacuminalis|[12223]|LOFLC211-06|United States|Florida|658[0n]|BOLD:AAB3233
Hypenula cacuminalis|[12224]|RDNMD515-06|United States|Florida|658[3n]|BOLD:AAB3233
Hypenula cacuminalis|[12225]|HKONS361-08|United States|Florida|658[0n]|BOLD:AAB3233
Hypenula cacuminalis|[12226]|LNCB858-09|United States|Georgia|658[0n]|BOLD:AAB3233
Hypenula cacuminalis|[12227]|HKONS436-08|United States|Florida|658[0n]|BOLD:AAB3233
Hypenula cacuminalis|[12228]|LNCB857-09|United States|Georgia|658[0n]|BOLD:AAB3233
Hypenula cacuminalis|[12229]|LSEU214-06|United States|Florida|658[0n]|BOLD:AAB3233
Hypenula cacuminalis|[12230]|LSEU215-06|United States|Florida|658[0n]|BOLD:AAB3233
Hypenula cacuminalis|[12231]|LOFLA271-06|United States|Florida|658[0n]|BOLD:AAB3233
Hypenula cacuminalis|[12232]|LOFLA393-06|United States|Florida|658[0n]|BOLD:AAB3233
Hypenula cacuminalis|[12233]|LOFLB232-06|United States|Florida|658[0n]|BOLD:AAB3233
Hypenula cacuminalis|[12234]|LOFLC204-06|United States|Florida|656[0n]|BOLD:AAB3233
Hypenula cacuminalis|[12235]|LOFLC216-06|United States|Florida|658[0n]|BOLD:AAB3233
Hypenula cacuminalis|[12236]|LOFLC395-06|United States|Florida|658[0n]|BOLD:AAB3233
Hypenula cacuminalis|[12237]|RDNMD514-06|United States|Florida|658[0n]|BOLD:AAB3233
Hypenula cacuminalis|[12238]|HKONS288-08|United States|Florida|658[0n]|BOLD:AAB3233
Hypenula cacuminalis|[12239]|RDNME694-08|United States|Florida|658[0n]|BOLD:AAB3233
Hypenula cacuminalis|[12240]|RDNME695-08|United States|Florida|658[0n]|BOLD:AAB3233
Hypenula cacuminalis|[12241]|BBLOB231-11|United States|Florida|658[0n]|BOLD:AAB3233
Hypenula cacuminalis|[12242]|BBLOB824-11|United States|Florida|658[0n]|BOLD:AAB3233
Hypenula cacuminalis|[12243]|BBLOB826-11|United States|Florida|658[0n]|BOLD:AAB3233
Hypenula cacuminalis|[12244]|BBLOC111-11|United States|Florida|658[0n]|BOLD:AAB3233
Hypenula cacuminalis|[12245]|BBLOC118-11|United States|Florida|658[0n]|BOLD:AAB3233
Hypenula cacuminalis|[12246]|BBLOC122-11|United States|Florida|658[0n]|BOLD:AAB3233
Hypenula cacuminalis|[12247]|BBLOE2006-12|United States|Florida|658[0n]|BOLD:AAB3233
Hypenula cacuminalis|[12248]|BBLOE2010-12|United States|Florida|658[0n]|BOLD:AAB3233
Hypenula cacuminalis|[12249]|BBLOE2012-12|United States|Florida|658[0n]|BOLD:AAB3233
Hypenula cacuminalis|[12250]|ABNCC094-07|United States|Florida|601[0n]|BOLD:AAB3233
Hypenula cacuminalis|[12251]|LNCB859-09|United States|Georgia|658[0n]|BOLD:AAB3233
Hypenula cacuminalis|[12252]|CNCLB2487-14|United States|Florida|658[0n]|BOLD:AAB3233
Renia sp. 2|[12253]|HKONB080-08|United States|Texas|658[0n]|BOLD:AAA3875
Renia sp. 2|[12254]|HKONB083-08|United States|Texas|658[0n]|BOLD:AAA3875
Renia sp. 2|[12255]|HKONB081-08|United States|Texas|658[0n]|BOLD:AAA3875
Renia sp. 2|[12256]|HKONB082-08|United States|Texas|658[0n]|BOLD:AAA3875
Renia sp. 2|[12257]|HKONB084-08|United States|Texas|658[0n]|BOLD:AAA3875
Renia rigida|[12258]|RDNMD172-06|United States|Colorado|603[0n]|BOLD:AAC9630
Renia rigida|[12259]|RDNMD173-06|United States|Colorado|595[0n]|BOLD:AAC9630
Renia sp. 1|[12260]|IAWL164-09|United States|Arizona|658[0n]|BOLD:AAC9570
Renia sp. 1|[12261]|RDNME884-08|United States|Arizona|658[0n]|BOLD:AAC9570
Renia sp. 1|[12262]|RDNME885-08|United States|Arizona|658[0n]|BOLD:AAC9570
Renia sp. 1|[12263]|RDNME887-08|United States|Arizona|658[0n]|BOLD:AAC9570
Renia sp. 1|[12264]|IAWL158-09|United States|Arizona|658[0n]|BOLD:AAC9570
Renia sp. 1|[12265]|CMAZA539-10|United States|Arizona|658[0n]|BOLD:AAC9570
Tetanolita mynesalis|[12266]|LOFLB459-06|United States|Florida|658[0n]|BOLD:AAA9813
Tetanolita mynesalis|[12267]|LOFLB301-06|United States|Florida|626[4n]|BOLD:AAA9813
Tetanolita mynesalis|[12268]|BBLSU039-09|United States|Arkansas|658[0n]|BOLD:AAA9813
Tetanolita mynesalis|[12269]|LOFLB546-06|United States|Florida|658[0n]|BOLD:AAA9813
Tetanolita mynesalis|[12270]|LOFLB861-06|United States|Florida|658[0n]|BOLD:AAA9813
Tetanolita mynesalis|[12271]|LPOKA420-09|United States|Oklahoma|658[0n]|BOLD:AAA9813
Tetanolita mynesalis|[12272]|LMEM243-09|United States|Alabama|658[0n]|BOLD:AAA9813
Tetanolita mynesalis|[12273]|BBLOC721-11|United States|Arkansas|658[0n]|BOLD:AAA9813
Tetanolita mynesalis|[12274]|LGSMG509-07|United States|Tennessee|658[0n]|BOLD:AAA9813
Tetanolita mynesalis|[12275]|LPOKA254-08|United States|Oklahoma|658[0n]|BOLD:AAA9813
Tetanolita mynesalis|[12276]|LNC610-06|United States|North Carolina|658[1n]|BOLD:AAA9813
Tetanolita mynesalis|[12277]|LOFLC346-06|United States|Florida|658[0n]|BOLD:AAA9813
Tetanolita mynesalis|[12278]|LOFLB175-06|United States|Florida|658[0n]|BOLD:AAA9813
Tetanolita mynesalis|[12279]|LOFLA619-06|United States|Florida|658[0n]|BOLD:AAA9813
Tetanolita mynesalis|[12280]|LNC611-06|United States|North Carolina|658[0n]|BOLD:AAA9813
Tetanolita mynesalis|[12281]|LPOKB614-09|United States|Oklahoma|562[0n]|BOLD:AAA9813
[illegible]

Tetanolita mynesalis|[12279]|LOFLA619-06|United States|Florida|658[0n]|BOLD:AAA9813
Tetanolita mynesalis|[12280]|LNC611-06|United States|North Carolina|658[0n]|BOLD:AAA9813
Tetanolita mynesalis|[12281]|LPOKB614-09|United States|Oklahoma|562[0n]|BOLD:AAA9813
Tetanolita mynesalis|[12282]|LPOKD423-09|United States|Oklahoma|658[0n]|BOLD:AAA9813
Tetanolita mynesalis|[12283]|USLEP762-10|United States|Texas|658[0n]|BOLD:AAA9813
Tetanolita mynesalis|[12284]|USLEP777-10|United States|Arkansas|658[0n]|BOLD:AAA9813
Tetanolita mynesalis|[12285]|MJMSL115-10|United States|Massachusetts|658[0n]|BOLD:AAA9813
Tetanolita mynesalis|[12286]|LILLA745-11|United States|Illinois|658[0n]|BOLD:AAA9813
Tetanolita mynesalis|[12287]|LILLB062-11|United States|Illinois|658[0n]|BOLD:AAA9813
Tetanolita mynesalis|[12288]|BBLOC046-11|United States|Florida|658[0n]|BOLD:AAA9813
Tetanolita mynesalis|[12289]|LMEM246-09|United States|Texas|658[0n]|BOLD:AAA9813
Tetanolita mynesalis|[12290]|LPOKB337-09|United States|Oklahoma|658[0n]|BOLD:AAA9813
Tetanolita mynesalis|[12291]|LMEM242-09|United States|Mississippi|658[0n]|BOLD:AAA9813
Tetanolita mynesalis|[12292]|LMEM244-09|United States|Alabama|658[0n]|BOLD:AAA9813
Tetanolita mynesalis|[12293]|BBLOC1105-11|United States|Texas|658[0n]|BOLD:AAA9813
Tetanolita mynesalis|[12294]|LMEM241-09|United States|Alabama|658[0n]|BOLD:AAA9813
Tetanolita mynesalis|[12295]|HKONS392-08|United States|Florida|658[0n]|BOLD:AAA9813
Tetanolita mynesalis|[12296]|HKONS391-08|United States|Florida|658[0n]|BOLD:AAA9813
Tetanolita mynesalis|[12297]|LOFLD028-07|United States|Florida|658[0n]|BOLD:AAA9813
Tetanolita mynesalis|[12298]|LNCB383-06|United States|North Carolina|658[0n]|BOLD:AAA9813
Tetanolita mynesalis|[12299]|LNCB279-06|United States|North Carolina|658[0n]|BOLD:AAA9813
Tetanolita mynesalis|[12300]|LOFLB884-06|United States|Florida|658[0n]|BOLD:AAA9813
Tetanolita mynesalis|[12301]|LOFLB190-06|United States|Florida|658[0n]|BOLD:AAA9813
Tetanolita mynesalis|[12302]|LNC584-06|United States|North Carolina|658[0n]|BOLD:AAA9813
Tetanolita mynesalis|[12303]|LGSMC488-05|United States|Tennessee|658[0n]|BOLD:AAA9813
Tetanolita mynesalis|[12304]|LGSM760-04|United States|North Carolina|658[0n]|BOLD:AAA9813
Tetanolita mynesalis|[12305]|LGSMC489-05|United States|Tennessee|601[0n]|BOLD:AAA9813
Tetanolita mynesalis|[12306]|LGSMC487-05|United States|Tennessee|616[0n]|BOLD:AAA9813
Tetanolita mynesalis|[12307]|LOFLB300-06|United States|Florida|585[0n]|BOLD:AAA9813
Tetanolita mynesalis|[12308]|LGSMG510-07|United States|Tennessee|625[0n]|BOLD:AAA9813
Tetanolita mynesalis|[12309]|LPOKA582-09|United States|Oklahoma|636[0n]|BOLD:AAA9813
Tetanolita mynesalis|[12310]|BBLOE1387-12|United States|Arkansas|658[1n]|BOLD:AAA9813
Renia mortualis|[12311]|LNAUS5622-13|United States|Arizona|658[0n]|BOLD:ACK9007
Renia mortualis|[12312]|LNAUS5623-13|United States|Arizona|658[0n]|BOLD:ACK9007
Renia mortualis|[12313]|LNAUS5624-13|United States|Arizona|658[0n]|BOLD:ACK9007
Renia mortualis|[12314]|LNAUS5625-13|United States|Arizona|658[0n]|BOLD:ACK9007
Renia mortualis|[12315]|LNAUS5626-13|United States|Arizona|658[0n]|BOLD:ACK9007
Renia mortualis|[12316]|CNCLB414-14|United States|Arizona|682[0n]|BOLD:ACK9007
Renia mortualis|[12317]|CNCLB415-14|United States|Arizona|682[0n]|BOLD:ACK9007
Renia flavipunctalis|[12318]|LNCC1373-11|United States|North Carolina|658[0n]|BOLD:AAA7213
Renia flavipunctalis|[12319]|LNCC1379-11|United States|North Carolina|658[0n]|BOLD:AAA7213
Renia flavipunctalis|[12320]|RDNMD184-06|Canada|Quebec|658[0n]|BOLD:AAA7213
Renia flavipunctalis|[12321]|RDLQF628-06|Canada|Quebec|658[0n]|BOLD:AAA7213
Renia flavipunctalis|[12322]|LPMN1005-09|Canada|Manitoba|658[0n]|BOLD:AAA7213
Renia flavipunctalis|[12323]|RDLQF795-06|Canada|Quebec|658[0n]|BOLD:AAA7213
Renia flavipunctalis|[12324]|RDLQF627-06|Canada|Quebec|658[0n]|BOLD:AAA7213
Renia flavipunctalis|[12325]|RDLQF575-06|Canada|Quebec|658[0n]|BOLD:AAA7213
Renia flavipunctalis|[12326]|RDLQF719-06|Canada|Quebec|642[0n]|BOLD:AAA7213
Renia flavipunctalis|[12327]|RDLQB714-05|Canada|Quebec|593[0n]|BOLD:AAA7213
Renia flavipunctalis|[12328]|RDLQF721-06|Canada|Quebec|639[0n]|BOLD:AAA7213
Renia flavipunctalis|[12329]|RDLQF796-06|Canada|Quebec|658[0n]|BOLD:AAA7213
Renia flavipunctalis|[12330]|RDLQF797-06|Canada|Quebec|658[0n]|BOLD:AAA7213
Renia flavipunctalis|[12331]|RDLQG094-06|Canada|Quebec|658[0n]|BOLD:AAA7213
Renia flavipunctalis|[12332]|LPMN1000-09|Canada|Manitoba|658[0n]|BOLD:AAA7213
Renia flavipunctalis|[12333]|LPMNB397-09|Canada|Manitoba|658[0n]|BOLD:AAA7213
Renia flavipunctalis|[12334]|LPMNB559-09|Canada|Manitoba|658[0n]|BOLD:AAA7213
Renia flavipunctalis|[12335]|RDNMD185-06|Canada|Quebec|654[0n]|BOLD:AAA7213
Renia flavipunctalis|[12336]|RDLQF624-06|Canada|Quebec|658[0n]|BOLD:AAA7213
Renia flavipunctalis|[12337]|RDLQF623-06|Canada|Quebec|658[0n]|BOLD:AAA7213
Renia flavipunctalis|[12338]|RDLQF626-06|Canada|Quebec|649[0n]|BOLD:AAA7213
Renia flavipunctalis|[12339]|RDLQF629-06|Canada|Quebec|643[0n]|BOLD:AAA7213
Renia flavipunctalis|[12340]|RDLQF720-06|Canada|Quebec|637[0n]|BOLD:AAA7213
Renia flavipunctalis|[12341]|RDLQF722-06|Canada|Quebec|642[0n]|BOLD:AAA7213
Renia flavipunctalis|[12342]|MNAF483-08|Canada|Manitoba|658[0n]|BOLD:AAA7213
Renia flavipunctalis|[12343]|LPMNB340-09|Canada|Manitoba|658[0n]|BOLD:AAA7213
Renia flavipunctalis|[12344]|LPMNB342-09|Canada|Manitoba|658[0n]|BOLD:AAA7213
Renia flavipunctalis|[12345]|LPMNB343-09|Canada|Manitoba|658[0n]|BOLD:AAA7213
Renia flavipunctalis|[12346]|LPMNB360-09|Canada|Manitoba|658[0n]|BOLD:AAA7213
Renia flavipunctalis|[12347]|LPMNB368-09|Canada|Manitoba|658[0n]|BOLD:AAA7213
Renia flavipunctalis|[12348]|LPMNB378-09|Canada|Manitoba|658[0n]|BOLD:AAA7213
Renia flavipunctalis|[12349]|LPMNB484-09|Canada|Manitoba|658[0n]|BOLD:AAA7213
Renia flavipunctalis|[12350]|LPMNB562-09|Canada|Manitoba|658[0n]|BOLD:AAA7213
Renia flavipunctalis|[12351]|RDLQF718-06|Canada|Quebec|644[0n]|BOLD:AAA7213
Renia flavipunctalis|[12352]|LPMNB351-09|Canada|Manitoba|658[0n]|BOLD:AAA7213
Renia flavipunctalis|[12353]|LPMNB377-09|Canada|Manitoba|658[0n]|BOLD:AAA7213
Renia flavipunctalis|[12354]|LPMNB384-09|Canada|Manitoba|658[0n]|BOLD:AAA7213
Renia flavipunctalis|[12355]|LPMNB413-09|Canada|Manitoba|658[0n]|BOLD:AAA7213
Renia flavipunctalis|[12356]|LPMNB336-09|Canada|Manitoba|658[0n]|BOLD:AAA7213
Renia flavipunctalis|[12357]|LNCC1425-11|United States|North Carolina|658[0n]|BOLD:AAA7213
Renia flavipunctalis|[12358]|RDLQF622-06|Canada|Quebec|658[0n]|BOLD:AAA7213
Renia flavipunctalis|[12359]|PMG158-03|Canada|Ontario|617[0n]|BOLD:AAA7213
Renia flavipunctalis|[12360]|CNPPF1347-12|Canada|Ontario|639[0n]|BOLD:AAA7213
Renia flavipunctalis|[12361]|CNPPG804-12|Canada|Ontario|646[0n]|BOLD:AAA7213
Renia flavipunctalis|[12362]|XAK324-06|Canada|Ontario|658[0n]|BOLD:AAA7213
Renia flavipunctalis|[12363]|RDNMD180-06|United States|Florida|594[0n]|BOLD:AAA7213
Renia flavipunctalis|[12364]|RDNMD181-06|United States|Florida|553[1n]|BOLD:AAA7213
Renia flavipunctalis|[12365]|RDNMD183-06|United States|Florida|579[1n]|BOLD:AAA7213
Renia flavipunctalis|[12366]|RDNMD548-06|United States|Florida|658[0n]|BOLD:AAA7213
Renia flavipunctalis|[12367]|RDNMD549-06|United States|Florida|658[1n]|BOLD:AAA7213
Renia flavipunctalis|[12368]|RDNME372-07|United States|Florida|658[1n]|BOLD:AAA7213
Renia flavipunctalis|[12369]|HKONS326-08|United States|Florida|658[0n]|BOLD:AAA7213
Renia flavipunctalis|[12370]|LNCC132-10|United States|North Carolina|658[0n]|BOLD:AAA7213
Renia flavipunctalis|[12371]|LNCC133-10|United States|North Carolina|658[0n]|BOLD:AAA7213
Renia flavipunctalis|[12372]|RDLQF820-06|Canada|Quebec|658[0n]|BOLD:AAA7213
Renia flavipunctalis|[12373]|MNAF484-08|Canada|Manitoba|658[0n]|BOLD:AAA7213
Renia flavipunctalis|[12374]|LILLA552-11|United States|Illinois|658[0n]|BOLD:AAA7213
Renia flavipunctalis|[12375]|CNPPG807-12|Canada|Ontario|643[0n]|BOLD:AAA7213
Renia pulverosalis|[12376]|QUNOD029-10|United States|Texas|658[0n]|BOLD:AAE7340
Renia pulverosalis|[12377]|CNCLB410-14|United States|Colorado|682[0n]|BOLD:AAE7340
Renia pulverosalis|[12378]|RDNMD203-06|United States|Colorado|602[0n]|BOLD:AAE7340
Renia pulverosalis|[12379]|CNCLB411-14|United States|Colorado|597[1n]|BOLD:AAE7340
Renia pulverosalis|[12380]|CNCLB412-14|United States|Colorado|675[0n]|BOLD:AAE7340
Renia hutsoni|[12381]|RDNML230-13|United States|Arizona|658[0n]|BOLD:AAE7341
[illegible]

Renia pulverosalis|[12379]|CNCLB411-14|United States|Colorado|597[1n]|BOLD:AAE7340
Renia pulverosalis|[12380]|CNCLB412-14|United States|Colorado|675[0n]|BOLD:AAE7340
Renia hutsoni|[12381]|RDNML230-13|United States|Arizona|658[0n]|BOLD:AAE7341
Renia hutsoni|[12382]|RDNMH842-09|United States|Arizona|658[0n]|BOLD:AAE7341
Renia hutsoni|[12383]|RDNMJ298-11|United States|Arizona|658[0n]|BOLD:AAE7341
Renia hutsoni|[12384]|IAWLB291-11|United States|Arizona|658[0n]|BOLD:AAE7341
Renia hutsoni|[12385]|RDNMJ534-11|United States|Arizona|658[0n]|BOLD:AAE7341
Renia hutsoni|[12386]|RDNMD801-07|United States|Arizona|658[0n]|BOLD:AAE7341
Renia hutsoni|[12387]|RDNMJ296-11|United States|Arizona|658[0n]|BOLD:AAE7341
Renia hutsoni|[12388]|RDNMJ536-11|United States|Arizona|658[0n]|BOLD:AAE7341
Renia hutsoni|[12389]|QUNOE461-12|United States|Arizona|658[0n]|BOLD:AAE7341
Renia hutsoni|[12390]|RDNMJ293-11|United States|Arizona|658[0n]|BOLD:AAE7341
Renia hutsoni|[12391]|ABNCC101-07|United States|Utah|644[0n]|BOLD:AAE7341
Renia hutsoni|[12392]|RDNMJ700-11|United States|Arizona|658[0n]|BOLD:AAE7341
Renia hutsoni|[12393]|CNCLB413-14|United States|Colorado|682[0n]|BOLD:AAE7341
Renia sobrialis|[12394]|TMNBB061-06|Canada|New Brunswick|656[0n]|BOLD:AAA8622
Renia sobrialis|[12395]|RDLQF619-06|Canada|Quebec|658[0n]|BOLD:AAA8622
Renia sobrialis|[12396]|RDLQF583-06|Canada|Quebec|658[0n]|BOLD:AAA8622
Renia sobrialis|[12397]|RDLQF582-06|Canada|Quebec|658[0n]|BOLD:AAA8622
Renia sobrialis|[12398]|RDLQF392-06|Canada|Quebec|658[0n]|BOLD:AAA8622
Renia sobrialis|[12399]|RDLQF390-06|Canada|Quebec|658[0n]|BOLD:AAA8622
Renia sobrialis|[12400]|RDLQF389-06|Canada|Quebec|658[0n]|BOLD:AAA8622
Renia sobrialis|[12401]|RDLQF388-06|Canada|Quebec|658[0n]|BOLD:AAA8622
Renia sobrialis|[12402]|RDLQF387-06|Canada|Quebec|658[0n]|BOLD:AAA8622
Renia sobrialis|[12403]|XAG258-05|Canada|Ontario|658[0n]|BOLD:AAA8622
Renia sobrialis|[12404]|PHMO180-03|Canada|Ontario|639[0n]|BOLD:AAA8622
Renia sobrialis|[12405]|RDLQG156-06|Canada|Quebec|640[0n]|BOLD:AAA8622
Renia sobrialis|[12406]|RDLQG155-06|Canada|Quebec|638[0n]|BOLD:AAA8622
Renia sobrialis|[12407]|RDNMD188-06|Canada|Quebec|583[0n]|BOLD:AAA8622
Renia sobrialis|[12408]|RDLQF093-06|Canada|Quebec|622[0n]|BOLD:AAA8622
Renia sobrialis|[12409]|RDLQG157-06|Canada|Quebec|632[0n]|BOLD:AAA8622
Renia sobrialis|[12410]|RDLQF708-06|Canada|Quebec|611[0n]|BOLD:AAA8622
Renia sobrialis|[12411]|RDLQG154-06|Canada|Quebec|642[0n]|BOLD:AAA8622
Renia sobrialis|[12412]|RDLQG153-06|Canada|Quebec|643[0n]|BOLD:AAA8622
Renia sobrialis|[12413]|RDLQG158-06|Canada|Quebec|594[0n]|BOLD:AAA8622
Renia sobrialis|[12414]|RDLQG159-06|Canada|Quebec|658[0n]|BOLD:AAA8622
Renia sobrialis|[12415]|RDLQG160-06|Canada|Quebec|658[0n]|BOLD:AAA8622
Renia sobrialis|[12416]|RDLQG161-06|Canada|Quebec|658[0n]|BOLD:AAA8622
Renia sobrialis|[12417]|RDLQG162-06|Canada|Quebec|658[0n]|BOLD:AAA8622
Renia sobrialis|[12418]|RDLQG163-06|Canada|Quebec|658[0n]|BOLD:AAA8622
Renia sobrialis|[12419]|RDLQG164-06|Canada|Quebec|658[0n]|BOLD:AAA8622
Renia sobrialis|[12420]|BBLEC975-09|Canada|Nova Scotia|658[0n]|BOLD:AAA8622
Renia sobrialis|[12421]|BBLPC539-09|Canada|New Brunswick|658[0n]|BOLD:AAA8622
Renia sobrialis|[12422]|LNCC351-10|United States|North Carolina|658[0n]|BOLD:AAA8622
Renia sobrialis|[12423]|LNCC1154-11|United States|North Carolina|658[0n]|BOLD:AAA8622
Renia sobrialis|[12424]|QUNOE466-12|United States|Connecticut|658[0n]|BOLD:AAA8622
Renia sobrialis|[12425]|LNCB012-06|United States|North Carolina|658[0n]|BOLD:AAA8623
Renia sobrialis|[12426]|LNCC895-11|United States|North Carolina|658[0n]|BOLD:AAA8623
Renia sobrialis|[12427]|RDLQF094-06|Canada|Quebec|658[0n]|BOLD:AAA8623
Renia sobrialis|[12428]|LNC586-06|United States|North Carolina|657[0n]|BOLD:AAA8623
Renia sobrialis|[12429]|LNCC894-11|United States|North Carolina|658[0n]|BOLD:AAA8623
Renia sobrialis|[12430]|LNCC893-11|United States|North Carolina|658[0n]|BOLD:AAA8623
Renia sobrialis|[12431]|LNCC892-11|United States|North Carolina|658[0n]|BOLD:AAA8623
Renia sobrialis|[12432]|LMEM282-09|United States|Alabama|658[0n]|BOLD:AAA8623
Renia sobrialis|[12433]|HKONS072-07|United States|Florida|658[0n]|BOLD:AAA8623
Renia sobrialis|[12434]|LSEU402-06|United States|Georgia|658[0n]|BOLD:AAA8623
Renia sobrialis|[12435]|LNC587-06|United States|North Carolina|658[0n]|BOLD:AAA8623
Renia sobrialis|[12436]|LMEM300-09|United States|Alabama|658[0n]|BOLD:AAA8623
Renia sobrialis|[12437]|LMEM304-09|United States|Mississippi|638[0n]|BOLD:AAA8623
Renia sobrialis|[12438]|LMEM303-09|United States|Mississippi|638[0n]|BOLD:AAA8623
Renia sobrialis|[12439]|LMEM301-09|United States|Alabama|629[0n]|BOLD:AAA8623
Renia sobrialis|[12440]|LNCB013-06|United States|North Carolina|617[0n]|BOLD:AAA8623
Renia sobrialis|[12441]|HPPPK941-13|Canada|Nova Scotia|588[0n]|BOLD:AAA8623
Renia sobrialis|[12442]|HPPPK942-13|Canada|Nova Scotia|588[0n]|BOLD:AAA8623
Renia sobrialis|[12443]|HPPPK943-13|Canada|Nova Scotia|592[0n]|BOLD:AAA8623
Renia sobrialis|[12444]|LNCC1709-13|United States|North Carolina|658[0n]|BOLD:AAA8623
Renia adspergillus|[12445]|LGSMG512-07|United States|Tennessee|658[0n]|BOLD:AAA6692
Renia adspergillus|[12446]|HKONS311-08|United States|Florida|658[1n]|BOLD:AAA6692
Renia adspergillus|[12447]|LPOKB377-09|United States|Oklahoma|614[0n]|BOLD:AAA6692
Renia adspergillus|[12448]|LPOKB328-09|United States|Oklahoma|614[0n]|BOLD:AAA6692
Renia adspergillus|[12449]|BBLSX455-09|United States|Oklahoma|658[0n]|BOLD:AAA6692
Renia adspergillus|[12450]|LNCB726-09|United States|North Carolina|633[0n]|BOLD:AAA6692
Renia adspergillus|[12451]|BBLSX382-09|United States|Oklahoma|632[0n]|BOLD:AAA6692
Renia adspergillus|[12452]|HKONS319-08|United States|Florida|658[1n]|BOLD:AAA6692
Renia adspergillus|[12453]|HKONS313-08|United States|Florida|658[1n]|BOLD:AAA6692
Renia adspergillus|[12454]|HKONS307-08|United States|Florida|658[1n]|BOLD:AAA6692
Renia adspergillus|[12455]|LPOKB729-09|United States|Oklahoma|658[0n]|BOLD:AAA6692
Renia adspergillus|[12456]|USLEP799-10|United States|Arkansas|658[0n]|BOLD:AAA6692
Renia adspergillus|[12457]|LNCB763-09|United States|North Carolina|658[0n]|BOLD:AAA6692
Renia adspergillus|[12458]|BBLSX578-09|United States|Oklahoma|658[0n]|BOLD:AAA6692
Renia adspergillus|[12459]|BBLSX419-09|United States|Oklahoma|658[0n]|BOLD:AAA6692
Renia adspergillus|[12460]|BBLSW668-09|United States|Oklahoma|658[0n]|BOLD:AAA6692
Renia adspergillus|[12461]|BBLSW645-09|United States|Oklahoma|658[0n]|BOLD:AAA6692
Renia adspergillus|[12462]|LNCB536-09|United States|North Carolina|658[0n]|BOLD:AAA6692
Renia adspergillus|[12463]|LNCB535-09|United States|North Carolina|658[0n]|BOLD:AAA6692
Renia adspergillus|[12464]|LNCB534-09|United States|North Carolina|658[0n]|BOLD:AAA6692
Renia adspergillus|[12465]|LNCB533-09|United States|North Carolina|658[0n]|BOLD:AAA6692
Renia adspergillus|[12466]|LPOKB100-09|United States|Oklahoma|658[0n]|BOLD:AAA6692
Renia adspergillus|[12467]|LPOKA655-09|United States|Oklahoma|658[0n]|BOLD:AAA6692
Renia adspergillus|[12468]|LPOKA503-09|United States|Oklahoma|658[0n]|BOLD:AAA6692
Renia adspergillus|[12469]|LPOKA419-09|United States|Oklahoma|658[0n]|BOLD:AAA6692
Renia adspergillus|[12470]|LPOKA386-09|United States|Oklahoma|658[0n]|BOLD:AAA6692
Renia adspergillus|[12471]|LPOKA317-08|United States|Oklahoma|658[0n]|BOLD:AAA6692
Renia adspergillus|[12472]|LPOKA272-08|United States|Oklahoma|658[0n]|BOLD:AAA6692
Renia adspergillus|[12473]|HKONS318-08|United States|Florida|658[0n]|BOLD:AAA6692
Renia adspergillus|[12474]|HKONS316-08|United States|Florida|658[0n]|BOLD:AAA6692
Renia adspergillus|[12475]|HKONS312-08|United States|Florida|658[0n]|BOLD:AAA6692
Renia adspergillus|[12476]|HKONS310-08|United States|Florida|658[0n]|BOLD:AAA6692
Renia adspergillus|[12477]|HKONS308-08|United States|Florida|658[0n]|BOLD:AAA6692
Renia adspergillus|[12478]|HKONS306-08|United States|Florida|658[0n]|BOLD:AAA6692
Renia adspergillus|[12479]|HKONS273-08|United States|Florida|658[0n]|BOLD:AAA6692
Renia adspergillus|[12480]|LSUSA198-06|United States|Kentucky|658[0n]|BOLD:AAA6692
Renia adspergillus|[12481]|LGSMC942-05|United States|Tennessee|658[0n]|BOLD:AAA6692

Renia adspergillus|[12479]|HKONS273-08|United States|Florida|658[0n]|BOLD:AAA6692
Renia adspergillus|[12480]|LSUSA198-06|United States|Kentucky|658[0n]|BOLD:AAA6692
Renia adspergillus|[12481]|LGSMC942-05|United States|Tennessee|658[0n]|BOLD:AAA6692
Renia adspergillus|[12482]|LGSMC666-05|United States|Tennessee|658[0n]|BOLD:AAA6692
Renia adspergillus|[12483]|LNC595-06|United States|North Carolina|658[0n]|BOLD:AAA6692
Renia adspergillus|[12484]|LNCB724-09|United States|North Carolina|649[0n]|BOLD:AAA6692
Renia adspergillus|[12485]|LNCB725-09|United States|North Carolina|535[0n]|BOLD:AAA6692
Renia adspergillus|[12486]|LNCB762-09|United States|North Carolina|658[0n]|BOLD:AAA6692
Renia adspergillus|[12487]|LPOKD771-10|United States|Oklahoma|639[0n]|BOLD:AAA6692
Renia adspergillus|[12488]|LTOLB759-11|United States|Maryland|658[0n]|BOLD:AAA6692
Renia adspergillus|[12489]|LTOLB760-11|United States|Maryland|658[0n]|BOLD:AAA6692
Renia adspergillus|[12490]|LILLA201-11|United States|Illinois|658[0n]|BOLD:AAA6692
Renia adspergillus|[12491]|LNCC1875-13|United States|North Carolina|658[0n]|BOLD:AAA6692
Renia adspergillus|[12492]|BLTIB360-08|Canada|Ontario|609[2n]|BOLD:AAA6692
Renia adspergillus|[12493]|HKONS314-08|United States|Florida|658[2n]|BOLD:AAA6692
Renia adspergillus|[12494]|HKONS272-08|United States|Florida|658[0n]|BOLD:AAA6692
Renia adspergillus|[12495]|HKONS315-08|United States|Florida|658[0n]|BOLD:AAA6692
Renia adspergillus|[12496]|HKONS317-08|United States|Florida|658[0n]|BOLD:AAA6692
Renia adspergillus|[12497]|RDLQG193-06|Canada|Quebec|601[0n]|BOLD:AAA6692
Renia adspergillus|[12498]|RDLQF525-06|Canada|Quebec|658[0n]|BOLD:AAA6692
Renia adspergillus|[12499]|RDLQG195-06|Canada|Quebec|658[0n]|BOLD:AAA6692
Renia adspergillus|[12500]|RDLQG194-06|Canada|Quebec|658[0n]|BOLD:AAA6692
Renia adspergillus|[12501]|RDLQG192-06|Canada|Quebec|658[0n]|BOLD:AAA6692
Renia adspergillus|[12502]|RDLQG019-06|Canada|Quebec|658[0n]|BOLD:AAA6692
Renia adspergillus|[12503]|RDLQF527-06|Canada|Quebec|658[0n]|BOLD:AAA6692
Renia adspergillus|[12504]|RDLQF526-06|Canada|Quebec|658[0n]|BOLD:AAA6692
Renia adspergillus|[12505]|RDLQF074-06|Canada|Quebec|658[0n]|BOLD:AAA6692
Renia adspergillus|[12506]|RDLQG489-06|Canada|Quebec|636[1n]|BOLD:AAA6692
Renia adspergillus|[12507]|RDLQG922-06|Canada|Quebec|658[0n]|BOLD:AAA6692
Renia adspergillus|[12508]|RDLQG923-06|Canada|Quebec|658[0n]|BOLD:AAA6692
Renia adspergillus|[12509]|LPOKA434-09|United States|Oklahoma|658[0n]|BOLD:AAA6692
Renia adspergillus|[12510]|LMEM296-09|United States|Mississippi|658[0n]|BOLD:AAA6692
Renia adspergillus|[12511]|LNCC1881-13|United States|North Carolina|658[0n]|BOLD:AAA6692
Renia discoloralis|[12512]|RDNME708-08|United States|Florida|658[6n]|BOLD:AAA6434
Renia discoloralis|[12513]|RDNMD550-06|United States|Florida|658[8n]|
Renia discoloralis|[12514]|LNC605-06|United States|North Carolina|658[0n]|BOLD:AAA6434
Renia discoloralis|[12515]|LNCB693-09|United States|North Carolina|632[0n]|BOLD:AAA6434
Renia discoloralis|[12516]|LNCB694-09|United States|North Carolina|658[0n]|BOLD:AAA6434
Renia discoloralis|[12517]|LMEM290-09|United States|Alabama|658[0n]|BOLD:AAA6434
Renia discoloralis|[12518]|LNCB196-06|United States|North Carolina|658[0n]|BOLD:AAA6434
Renia discoloralis|[12519]|LNCB832-09|United States|Alabama|649[0n]|BOLD:AAA6434
Renia discoloralis|[12520]|LNCC462-10|United States|North Carolina|658[0n]|BOLD:AAA6434
Renia discoloralis|[12521]|MILEQ288-11|United States|Alabama|658[0n]|BOLD:AAA6434
Renia discoloralis|[12522]|LNCB707-09|United States|North Carolina|658[0n]|BOLD:AAA6434
Renia discoloralis|[12523]|LNCB800-09|United States|North Carolina|658[0n]|BOLD:AAA6434
Renia discoloralis|[12524]|LNCB814-09|United States|North Carolina|614[0n]|BOLD:AAA6434
Renia discoloralis|[12525]|LNCB798-09|United States|North Carolina|614[0n]|BOLD:AAA6434
Renia discoloralis|[12526]|LNCB692-09|United States|North Carolina|658[0n]|BOLD:AAA6434
Renia discoloralis|[12527]|LNCB695-09|United States|North Carolina|658[0n]|BOLD:AAA6434
Renia discoloralis|[12528]|MILEP003-09|United States|North Carolina|658[0n]|BOLD:AAA6434
Renia discoloralis|[12529]|MILEP039-09|United States|North Carolina|658[0n]|BOLD:AAA6434
Renia discoloralis|[12530]|MILEP040-09|United States|North Carolina|658[0n]|BOLD:AAA6434
Renia discoloralis|[12531]|LNCB753-09|United States|North Carolina|658[0n]|BOLD:AAA6434
Renia discoloralis|[12532]|LMEM289-09|United States|Alabama|658[0n]|BOLD:AAA6434
Renia discoloralis|[12533]|LNCB637-09|United States|North Carolina|658[0n]|BOLD:AAA6434
Renia discoloralis|[12534]|HKONS356-08|United States|Florida|658[0n]|BOLD:AAA6434
Renia discoloralis|[12535]|RDNME709-08|United States|Florida|658[0n]|BOLD:AAA6434
Renia discoloralis|[12536]|HKONS354-08|United States|Florida|658[0n]|BOLD:AAA6434
Renia discoloralis|[12537]|HKONS355-08|United States|Florida|658[0n]|BOLD:AAA6434
Renia discoloralis|[12538]|LNCB326-06|United States|North Carolina|658[0n]|BOLD:AAA6434
Renia discoloralis|[12539]|LNCB462-07|United States|North Carolina|658[0n]|BOLD:AAA6434
Renia discoloralis|[12540]|LNCB229-06|United States|North Carolina|658[0n]|BOLD:AAA6434
Renia discoloralis|[12541]|LNCB230-06|United States|North Carolina|658[0n]|BOLD:AAA6434
Renia discoloralis|[12542]|LNCB198-06|United States|North Carolina|658[0n]|BOLD:AAA6434
Renia discoloralis|[12543]|LNCB197-06|United States|North Carolina|658[0n]|BOLD:AAA6434
Renia discoloralis|[12544]|LNCB195-06|United States|North Carolina|658[0n]|BOLD:AAA6434
Renia discoloralis|[12545]|LNCB194-06|United States|North Carolina|658[0n]|BOLD:AAA6434
Renia discoloralis|[12546]|LNCB193-06|United States|North Carolina|658[0n]|BOLD:AAA6434
Renia discoloralis|[12547]|LOFLA316-06|United States|Florida|658[0n]|BOLD:AAA6434
Renia discoloralis|[12548]|RDNMD174-06|United States|Florida|658[0n]|BOLD:AAA6434
Renia discoloralis|[12549]|LNC603-06|United States|North Carolina|658[0n]|BOLD:AAA6434
Renia discoloralis|[12550]|LNC602-06|United States|North Carolina|657[0n]|BOLD:AAA6434
Renia discoloralis|[12551]|LNCB691-09|United States|North Carolina|658[1n]|BOLD:AAA6434
Renia discoloralis|[12552]|RDNME369-07|United States|Florida|623[0n]|BOLD:AAA6434
Renia discoloralis|[12553]|RDNMD175-06|United States|Florida|601[0n]|BOLD:AAA6434
Renia discoloralis|[12554]|RDNMD176-06|United States|Florida|608[0n]|BOLD:AAA6434
Renia discoloralis|[12555]|RDNMD177-06|United States|Florida|588[0n]|BOLD:AAA6434
Renia discoloralis|[12556]|LNCB750-09|United States|North Carolina|631[0n]|BOLD:AAA6434
Renia discoloralis|[12557]|LNCB799-09|United States|North Carolina|631[0n]|BOLD:AAA6434
Renia discoloralis|[12558]|LNCB797-09|United States|North Carolina|658[0n]|BOLD:AAA6434
Renia discoloralis|[12559]|LNCC130-10|United States|North Carolina|658[0n]|BOLD:AAA6434
Renia discoloralis|[12560]|LNCC131-10|United States|North Carolina|658[0n]|BOLD:AAA6434
Renia discoloralis|[12561]|LNCC1493-13|United States|North Carolina|658[0n]|BOLD:AAA6434
Renia discoloralis|[12562]|LNCC1372-11|United States|North Carolina|658[0n]|BOLD:AAA6434
Renia discoloralis|[12563]|LNCC1371-11|United States|North Carolina|658[0n]|BOLD:AAA6434
Renia discoloralis|[12564]|LNCB553-09|United States|North Carolina|658[0n]|BOLD:AAA6434
Renia discoloralis|[12565]|LGSMG539-07|United States|Tennessee|658[0n]|BOLD:AAA6434
Renia discoloralis|[12566]|LGSMG518-07|United States|Tennessee|658[0n]|BOLD:AAA6434
Renia discoloralis|[12567]|LGSMG517-07|United States|Tennessee|658[0n]|BOLD:AAA6434
Renia discoloralis|[12568]|LNCNW112-06|United States|North Carolina|658[0n]|BOLD:AAA6434
Renia discoloralis|[12569]|LNC604-06|United States|North Carolina|658[0n]|BOLD:AAA6434
Renia discoloralis|[12570]|LNC601-06|United States|North Carolina|658[0n]|BOLD:AAA6434
Renia discoloralis|[12571]|LGSM718-04|United States|Tennessee|658[0n]|BOLD:AAA6434
Renia discoloralis|[12572]|LNCC1370-11|United States|North Carolina|658[1n]|BOLD:AAA6434
Renia discoloralis|[12573]|LOT503-04|United States|Tennessee|658[0n]|BOLD:AAA6434
Renia discoloralis|[12574]|LOT156-04|United States|Tennessee|609[0n]|BOLD:AAA6434
Renia discoloralis|[12575]|LTOLB758-11|United States|Maryland|658[0n]|BOLD:AAA6434
Renia discoloralis|[12576]|LOT190-04|United States|Tennessee|609[0n]|BOLD:AAA6434
Renia discoloralis|[12577]|LOT158-04|United States|Tennessee|609[0n]|BOLD:AAA6434
Renia discoloralis|[12578]|LOT155-04|United States|Tennessee|609[0n]|BOLD:AAA6434
Renia discoloralis|[12579]|LOT152-04|United States|Tennessee|609[0n]|BOLD:AAA6434
Renia discoloralis|[12580]|CNSLH301-12|Canada|Ontario|603[0n]|BOLD:AAA6434
Renia discoloralis|[12581]|CNSLR428-13|Canada|Ontario|582[0n]|BOLD:AAA6434

Renia discoloralis|[12579]|LOT152-04|United States|Tennessee|609[0n]|BOLD:AAA6434
Renia discoloralis|[12580]|CNSLH301-12|Canada|Ontario|603[0n]|BOLD:AAA6434
Renia discoloralis|[12581]|CNSLR428-13|Canada|Ontario|582[0n]|BOLD:AAA6434
Renia discoloralis|[12582]|LGSM717-04|United States|Tennessee|658[0n]|BOLD:AAA6434
Renia discoloralis|[12583]|CNCLB1541-14|Canada|Ontario|562[0n]|BOLD:AAA6434
Renia discoloralis|[12584]|LNCC1374-11|United States|North Carolina|658[0n]|BOLD:AAA6434
Renia discoloralis|[12585]|CNCLB1542-14|Canada|Ontario|658[0n]|BOLD:AAA6434
Renia nemoralis|[12586]|LNC592-06|United States|North Carolina|658[0n]|BOLD:AAC4231
Renia nemoralis|[12587]|LNC591-06|United States|North Carolina|658[0n]|BOLD:AAC4231
Renia nemoralis|[12588]|LNCB788-09|United States|North Carolina|658[0n]|BOLD:AAC4231
Renia nemoralis|[12589]|LGSMG516-07|United States|North Carolina|658[0n]|BOLD:AAC4231
Renia nemoralis|[12590]|LNCB751-09|United States|North Carolina|658[0n]|BOLD:AAC4231
Renia nemoralis|[12591]|LNCB815-09|United States|North Carolina|640[0n]|BOLD:AAC4231
Renia nemoralis|[12592]|LNCB824-09|United States|Alabama|658[0n]|BOLD:AAC4231
Renia nemoralis|[12593]|LNCB463-07|United States|North Carolina|658[0n]|BOLD:AAC4231
Renia nemoralis|[12594]|MILEP001-09|United States|North Carolina|658[0n]|BOLD:AAC4231
Renia nemoralis|[12595]|MILEP002-09|United States|North Carolina|658[0n]|BOLD:AAC4231
Renia nemoralis|[12596]|LNCB752-09|United States|North Carolina|658[0n]|BOLD:AAC4231
Renia nemoralis|[12597]|LNCB823-09|United States|Alabama|658[0n]|BOLD:AAC4231
Renia nemoralis|[12598]|LNCC846-11|United States|North Carolina|658[0n]|BOLD:AAC4231
Renia salusalis|[12599]|RDNMD189-06|United States|Florida|608[0n]|BOLD:ACE9348
Renia salusalis|[12600]|RDNMD167-06|United States|Florida|570[1n]|BOLD:ACE9348
Renia salusalis|[12601]|RDNMD209-06|United States|Florida|616[0n]|BOLD:ACE9348
Renia salusalis|[12602]|RDNMD194-06|United States|Florida|612[0n]|BOLD:ACE9348
Renia salusalis|[12603]|RDNMD191-06|United States|Florida|608[0n]|BOLD:ACE9348
Renia salusalis|[12604]|RDNMD204-06|United States|Florida|608[0n]|BOLD:ACE9348
Renia salusalis|[12605]|USLEP528-10|United States|Florida|658[0n]|BOLD:ACE9348
Renia salusalis|[12606]|RDNMD190-06|United States|Florida|658[0n]|BOLD:ACE9348
Renia salusalis|[12607]|USLEP524-10|United States|Florida|658[0n]|BOLD:ACE9348
Renia salusalis|[12608]|USLEP527-10|United States|Florida|658[0n]|BOLD:ACE9348
Renia salusalis|[12609]|LMEM286-09|United States|Alabama|658[0n]|BOLD:ACE9348
Renia salusalis|[12610]|HKONS331-08|United States|Florida|658[0n]|BOLD:ACE9348
Renia salusalis|[12611]|HKONS328-08|United States|Florida|658[0n]|BOLD:ACE9348
Renia salusalis|[12612]|RDNMD164-06|United States|Florida|585[0n]|BOLD:ACE9348
Renia salusalis|[12613]|RDNMD169-06|United States|Florida|607[0n]|BOLD:ACE9348
Renia salusalis|[12614]|RDNMD196-06|United States|Florida|616[0n]|BOLD:ACE9348
Renia salusalis|[12615]|RDNMD205-06|United States|Florida|616[0n]|BOLD:ACE9348
Renia salusalis|[12616]|RDNMD208-06|United States|Florida|619[0n]|BOLD:ACE9348
Renia salusalis|[12617]|RDNMD210-06|United States|Florida|601[0n]|BOLD:ACE9348
Renia salusalis|[12618]|RDNMD206-06|United States|Florida|598[0n]|BOLD:ACE9348
Renia salusalis|[12619]|RDNMD207-06|United States|Florida|590[0n]|BOLD:ACE9348
Renia salusalis|[12620]|USLEP529-10|United States|Florida|658[0n]|BOLD:ACE9348
Renia salusalis|[12621]|USLEP530-10|United States|Florida|658[0n]|BOLD:ACE9348
Renia salusalis|[12622]|HKONB465-09|United States|Texas|658[0n]|BOLD:ABZ6573
Renia salusalis|[12623]|LMEM287-09|United States|Mississippi|658[0n]|BOLD:ABZ6573
Renia salusalis|[12624]|BBLSX159-09|United States|Oklahoma|658[0n]|BOLD:ABZ6573
Renia salusalis|[12625]|LMEM292-09|United States|Alabama|658[0n]|BOLD:ABZ6573
Renia salusalis|[12626]|QUNOB522-09|United States|Kentucky|658[0n]|BOLD:ABZ6573
Renia salusalis|[12627]|LNCC1839-13|United States|North Carolina|658[0n]|BOLD:ABZ6573
Renia salusalis|[12628]|LNC593-06|United States|North Carolina|656[0n]|BOLD:ABZ6573
Renia salusalis|[12629]|LNCC1876-13|United States|North Carolina|658[0n]|BOLD:ABZ6573
Renia salusalis|[12630]|LNCC1877-13|United States|North Carolina|658[0n]|BOLD:ABZ6573
Renia fraternalis|[12631]|LNC588-06|United States|North Carolina|658[0n]|BOLD:AAA6252
Renia fraternalis|[12632]|LOFLA611-06|United States|Florida|658[0n]|BOLD:AAA6252
Renia fraternalis|[12633]|HKONB335-09|United States|Texas|658[0n]|BOLD:AAA6252
Renia fraternalis|[12634]|LNC589-06|United States|North Carolina|658[0n]|BOLD:AAA6252
Renia fraternalis|[12635]|LGSM665-04|United States|Tennessee|658[0n]|BOLD:AAA6252
Renia fraternalis|[12636]|LNC594-06|United States|North Carolina|658[0n]|BOLD:AAA6252
Renia fraternalis|[12637]|RDNMD200-06|United States|Florida|658[0n]|BOLD:AAA6252
Renia fraternalis|[12638]|LNC935-06|United States|North Carolina|658[0n]|BOLD:AAA6252
Renia fraternalis|[12639]|LNC936-06|United States|North Carolina|658[0n]|BOLD:AAA6252
Renia fraternalis|[12640]|LOFLA020-06|United States|Florida|658[0n]|BOLD:AAA6252
Renia fraternalis|[12641]|LOFLB281-06|United States|Florida|658[0n]|BOLD:AAA6252
Renia fraternalis|[12642]|LOFLB761-06|United States|Florida|658[0n]|BOLD:AAA6252
Renia fraternalis|[12643]|LOFLB790-06|United States|Florida|658[0n]|BOLD:AAA6252
Renia fraternalis|[12644]|LOFLB839-06|United States|Florida|658[0n]|BOLD:AAA6252
Renia fraternalis|[12645]|LNCB595-09|United States|North Carolina|658[0n]|BOLD:AAA6252
Renia fraternalis|[12646]|LNCB838-09|United States|Alabama|658[0n]|BOLD:AAA6252
Renia fraternalis|[12647]|USLEP1005-10|United States|Florida|658[0n]|BOLD:AAA6252
Renia fraternalis|[12648]|LNCC1639-13|United States|North Carolina|658[0n]|BOLD:AAA6252
Renia fraternalis|[12649]|USLEP1275-10|United States|Florida|658[0n]|BOLD:AAA6252
Renia fraternalis|[12650]|RDNMD198-06|United States|Florida|609[0n]|BOLD:AAA6252
Renia fraternalis|[12651]|LMEM295-09|United States|Mississippi|658[0n]|BOLD:AAA6252
Renia fraternalis|[12652]|LNCB652-09|United States|North Carolina|658[0n]|BOLD:AAA6252
Renia fraternalis|[12653]|RDNMD545-06|United States|Florida|658[0n]|BOLD:AAA6252
Renia fraternalis|[12654]|RDNMD547-06|United States|Florida|658[0n]|BOLD:AAA6252
Renia fraternalis|[12655]|RDNMD543-06|United States|Florida|658[0n]|BOLD:AAA6252
Renia fraternalis|[12656]|RDNMD544-06|United States|Florida|658[0n]|BOLD:AAA6252
Renia fraternalis|[12657]|RDNMD202-06|United States|Florida|658[0n]|BOLD:AAA6252
Renia fraternalis|[12658]|RDNMD201-06|United States|Florida|658[0n]|BOLD:AAA6252
Renia fraternalis|[12659]|RDNMD166-06|United States|Florida|658[0n]|BOLD:AAA6252
Renia fraternalis|[12660]|LGSMC921-05|United States|Tennessee|658[0n]|BOLD:AAA6252
Renia fraternalis|[12661]|LGSMC665-05|United States|Tennessee|658[0n]|BOLD:AAA6252
Renia fraternalis|[12662]|LGSMC664-05|United States|Tennessee|658[0n]|BOLD:AAA6252
Renia fraternalis|[12663]|LGSMC455-05|United States|Tennessee|658[0n]|BOLD:AAA6252
Renia fraternalis|[12664]|LGSMC454-05|United States|Tennessee|658[0n]|BOLD:AAA6252
Renia fraternalis|[12665]|LGSMC452-05|United States|Tennessee|658[0n]|BOLD:AAA6252
Renia fraternalis|[12666]|LGSMC451-05|United States|Tennessee|658[0n]|BOLD:AAA6252
Renia fraternalis|[12667]|LGSMC453-05|United States|Tennessee|658[0n]|BOLD:AAA6252
Renia fraternalis|[12668]|LOFLC383-06|United States|Florida|658[0n]|BOLD:AAA6252
Renia fraternalis|[12669]|RDNMD168-06|United States|Florida|604[0n]|BOLD:AAA6252
Renia fraternalis|[12670]|RDNMD165-06|United States|Florida|605[0n]|BOLD:AAA6252
Renia fraternalis|[12671]|RDNMD193-06|United States|Florida|617[0n]|BOLD:AAA6252
Renia fraternalis|[12672]|LOFLB296-06|United States|Florida|658[0n]|BOLD:AAA6252
Renia fraternalis|[12673]|LOFLD461-07|United States|Florida|609[0n]|BOLD:AAA6252
Renia fraternalis|[12674]|HKONB336-09|United States|Texas|658[0n]|BOLD:AAA6252
Renia fraternalis|[12675]|HKONB337-09|United States|Texas|658[0n]|BOLD:AAA6252
Renia fraternalis|[12676]|LMEM293-09|United States|Alabama|658[0n]|BOLD:AAA6252
Renia fraternalis|[12677]|LMEM294-09|United States|Alabama|658[0n]|BOLD:AAA6252
Renia fraternalis|[12678]|LOFLB789-06|United States|Florida|658[0n]|BOLD:AAA6252
Renia fraternalis|[12679]|LOFLB836-06|United States|Florida|658[0n]|BOLD:AAA6252
Renia fraternalis|[12680]|LOFLC342-06|United States|Florida|658[0n]|BOLD:AAA6252
Renia fraternalis|[12681]|LOFLC406-06|United States|Florida|658[0n]|BOLD:AAA6252

Renia fraternalis|[12679]|LOFLB836-06|United States|Florida|658[0n]|BOLD:AAA6252
Renia fraternalis|[12680]|LOFLC342-06|United States|Florida|658[0n]|BOLD:AAA6252
Renia fraternalis|[12681]|LOFLC406-06|United States|Florida|658[0n]|BOLD:AAA6252
Renia fraternalis|[12682]|LOFLB705-06|United States|Florida|658[0n]|BOLD:AAA6252
Renia fraternalis|[12683]|LOFLB757-06|United States|Florida|658[0n]|BOLD:AAA6252
Renia fraternalis|[12684]|LOFLB299-06|United States|Florida|658[0n]|BOLD:AAA6252
Renia fraternalis|[12685]|LOFLB385-06|United States|Florida|658[0n]|BOLD:AAA6252
Renia fraternalis|[12686]|LOFLA569-06|United States|Florida|658[0n]|BOLD:AAA6252
Renia fraternalis|[12687]|LOFLA616-06|United States|Florida|658[0n]|BOLD:AAA6252
Renia fraternalis|[12688]|LOFLA030-06|United States|Florida|658[0n]|BOLD:AAA6252
Renia fraternalis|[12689]|LOFLA154-06|United States|Florida|658[0n]|BOLD:AAA6252
Renia fraternalis|[12690]|LNCB671-09|United States|North Carolina|658[0n]|BOLD:AAA6252
Renia fraternalis|[12691]|LNCB672-09|United States|North Carolina|658[0n]|BOLD:AAA6252
Renia fraternalis|[12692]|LNCB825-09|United States|Alabama|658[0n]|BOLD:AAA6252
Renia fraternalis|[12693]|LNCB839-09|United States|Alabama|658[0n]|BOLD:AAA6252
Renia fraternalis|[12694]|USLEP525-10|United States|Florida|658[0n]|BOLD:AAA6252
Renia fraternalis|[12695]|USLEP526-10|United States|Florida|658[0n]|BOLD:AAA6252
Renia fraternalis|[12696]|USLEP613-10|United States|Florida|658[0n]|BOLD:AAA6252
Renia fraternalis|[12697]|USLEP798-10|United States|Florida|658[0n]|BOLD:AAA6252
Renia fraternalis|[12698]|LNCC1640-13|United States|North Carolina|658[0n]|BOLD:AAA6252
Renia fraternalis|[12699]|LNCC1878-13|United States|North Carolina|658[0n]|BOLD:AAA6252
Renia factiosalis|[12700]|HKONB353-09|United States|Texas|658[0n]|BOLD:ACE4873
Renia factiosalis|[12701]|HKONB414-09|United States|Indiana|658[0n]|BOLD:ACE4873
Renia factiosalis|[12702]|LNCC701-11|United States|North Carolina|658[0n]|BOLD:ACE4873
Renia factiosalis|[12703]|LNCB764-09|United States|North Carolina|658[0n]|BOLD:ACE4873
Renia factiosalis|[12704]|HKONS270-08|United States|Florida|658[0n]|BOLD:ACE4873
Renia factiosalis|[12705]|LNCB273-06|United States|North Carolina|658[0n]|BOLD:ACE4873
Renia factiosalis|[12706]|LNCB272-06|United States|North Carolina|658[0n]|BOLD:ACE4873
Renia factiosalis|[12707]|HKONS269-08|United States|Florida|626[0n]|BOLD:ACE4873
Renia factiosalis|[12708]|CNCLB1293-14|United States|North Carolina|658[0n]|BOLD:ACE4873
Renia factiosalis|[12709]|CNCLB1294-14|United States|North Carolina|658[0n]|BOLD:ACE4873
Renia factiosalis|[12710]|MILEQ286-11|United States|Alabama|658[0n]|BOLD:AAA8624
Renia factiosalis|[12711]|RDLQF612-06|Canada|Quebec|658[0n]|BOLD:AAA8624
Renia factiosalis|[12712]|RDNMD186-06|Canada|Quebec|658[0n]|BOLD:AAA8624
Renia factiosalis|[12713]|HKONS271-08|United States|Florida|658[0n]|BOLD:AAA8624
Renia factiosalis|[12714]|LNCC252-10|United States|North Carolina|658[0n]|BOLD:AAA8624
Renia factiosalis|[12715]|LNCC253-10|United States|North Carolina|658[0n]|BOLD:AAA8624
Renia factiosalis|[12716]|RDLQF778-06|Canada|Quebec|658[0n]|BOLD:AAA8624
Renia factiosalis|[12717]|LGSMG513-07|United States|Tennessee|658[0n]|BOLD:AAA8624
Renia factiosalis|[12718]|LGSMG514-07|United States|North Carolina|658[0n]|BOLD:AAA8624
Renia factiosalis|[12719]|LGSMG515-07|United States|North Carolina|658[0n]|BOLD:AAA8624
Renia factiosalis|[12720]|IAWL044-09|United States|Virginia|658[0n]|BOLD:AAA8624
Renia factiosalis|[12721]|MJMSL141-10|United States|Massachusetts|658[0n]|BOLD:AAA8624
Renia factiosalis|[12722]|RDLQF776-06|Canada|Quebec|658[0n]|BOLD:AAA8624
Renia factiosalis|[12723]|RDLQF777-06|Canada|Quebec|658[0n]|BOLD:AAA8624
Renia factiosalis|[12724]|RDLQF774-06|Canada|Quebec|658[0n]|BOLD:AAA8624
Renia factiosalis|[12725]|RDLQF775-06|Canada|Quebec|658[0n]|BOLD:AAA8624
Renia factiosalis|[12726]|RDLQF728-06|Canada|Quebec|658[0n]|BOLD:AAA8624
Renia factiosalis|[12727]|RDLQF773-06|Canada|Quebec|658[0n]|BOLD:AAA8624
Renia factiosalis|[12728]|RDLQF621-06|Canada|Quebec|658[0n]|BOLD:AAA8624
Renia factiosalis|[12729]|RDLQF620-06|Canada|Quebec|658[0n]|BOLD:AAA8624
Renia factiosalis|[12730]|RDLQF617-06|Canada|Quebec|658[0n]|BOLD:AAA8624
Renia factiosalis|[12731]|RDLQF616-06|Canada|Quebec|658[0n]|BOLD:AAA8624
Renia factiosalis|[12732]|RDLQF615-06|Canada|Quebec|658[0n]|BOLD:AAA8624
Renia factiosalis|[12733]|RDLQF614-06|Canada|Quebec|658[0n]|BOLD:AAA8624
Renia factiosalis|[12734]|RDLQF611-06|Canada|Quebec|658[0n]|BOLD:AAA8624
Renia factiosalis|[12735]|LSEU762-06|United States|Georgia|658[0n]|BOLD:AAA8624
Renia factiosalis|[12736]|RDNMD187-06|Canada|Quebec|658[0n]|BOLD:AAA8624
Renia factiosalis|[12737]|XAG121-05|Canada|Ontario|658[0n]|BOLD:AAA8624
Renia factiosalis|[12738]|LGSM751-04|United States|North Carolina|658[0n]|BOLD:AAA8624
Renia factiosalis|[12739]|RDLQF618-06|Canada|Quebec|658[1n]|BOLD:AAA8624
Renia factiosalis|[12740]|RDLQF613-06|Canada|Quebec|658[1n]|BOLD:AAA8624
Renia factiosalis|[12741]|LMEM302-09|United States|Tennessee|636[0n]|BOLD:AAA8624
Renia factiosalis|[12742]|CNSLH037-12|Canada|Ontario|631[0n]|BOLD:AAA8624
Renia factiosalis|[12743]|CNSLR429-13|Canada|Ontario|588[0n]|BOLD:AAA8624
Renia factiosalis|[12744]|CNSLR434-13|Canada|Ontario|577[0n]|BOLD:AAA8624
Renia factiosalis|[12745]|CNCLB3007-14|United States|North Carolina|658[0n]|BOLD:AAA8624
Renia factiosalis|[12746]|CNCLB3008-14|United States|North Carolina|658[0n]|BOLD:AAA8624
Bleptina n. sp. 3|[12747]|HKONB090-08|United States|Texas|658[0n]|BOLD:AAA9243
Bleptina inferior|[12748]|HKONB332-09|United States|Texas|658[0n]|BOLD:AAA2866
Bleptina inferior|[12749]|HKONB456-09|United States|Texas|658[0n]|BOLD:AAA2866
Bleptina inferior|[12750]|BBLSW944-09|United States|Texas|640[0n]|BOLD:AAA2866
Bleptina inferior|[12751]|BBLSW952-09|United States|Texas|658[0n]|BOLD:AAA2866
Bleptina inferior|[12752]|LOFLA876-06|United States|Florida|658[0n]|BOLD:AAA2866
Bleptina inferior|[12753]|RDNMD546-06|United States|Florida|658[0n]|BOLD:AAA2866
Bleptina inferior|[12754]|LOFLA410-06|United States|Florida|658[0n]|BOLD:AAA2866
Bleptina inferior|[12755]|MILEP053-09|United States|North Carolina|658[0n]|BOLD:AAA2866
Bleptina inferior|[12756]|LOFLA935-06|United States|Florida|653[0n]|BOLD:AAA2866
Bleptina inferior|[12757]|LOFLA264-06|United States|Florida|658[0n]|BOLD:AAA2866
Bleptina inferior|[12758]|LOFLB777-06|United States|Florida|630[0n]|BOLD:AAA2866
Bleptina inferior|[12759]|RDNMD195-06|United States|Florida|606[0n]|BOLD:AAA2866
Bleptina inferior|[12760]|RDNMD192-06|United States|Florida|602[0n]|BOLD:AAA2866
Bleptina inferior|[12761]|LOFLA407-06|United States|Florida|658[0n]|BOLD:AAA2866
Bleptina inferior|[12762]|LOFLD265-07|United States|Florida|593[0n]|BOLD:AAA2866
Bleptina inferior|[12763]|LOFLB835-06|United States|Florida|658[0n]|BOLD:AAA2866
Bleptina inferior|[12764]|LNCB669-09|United States|North Carolina|622[0n]|BOLD:AAA2866
Bleptina inferior|[12765]|MILEP059-09|United States|North Carolina|630[0n]|BOLD:AAA2866
Bleptina inferior|[12766]|LOFLA059-06|United States|Florida|658[0n]|BOLD:AAA2866
Bleptina inferior|[12767]|LOFLB779-06|United States|Florida|669[0n]|BOLD:AAA2866
Bleptina inferior|[12768]|USLEP604-10|United States|Florida|658[0n]|BOLD:AAA2866
Bleptina inferior|[12769]|LNCB836-09|United States|Alabama|658[0n]|BOLD:AAA2866
Bleptina inferior|[12770]|MILEP067-09|United States|North Carolina|658[0n]|BOLD:AAA2866
Bleptina inferior|[12771]|MILEP066-09|United States|North Carolina|658[0n]|BOLD:AAA2866
Bleptina inferior|[12772]|MILEP065-09|United States|North Carolina|658[0n]|BOLD:AAA2866
Bleptina inferior|[12773]|MILEP064-09|United States|North Carolina|658[0n]|BOLD:AAA2866
Bleptina inferior|[12774]|MILEP062-09|United States|North Carolina|658[0n]|BOLD:AAA2866
Bleptina inferior|[12775]|MILEP061-09|United States|North Carolina|658[0n]|BOLD:AAA2866
Bleptina inferior|[12776]|MILEP060-09|United States|North Carolina|658[0n]|BOLD:AAA2866
Bleptina inferior|[12777]|MILEP058-09|United States|North Carolina|658[0n]|BOLD:AAA2866
Bleptina inferior|[12778]|MILEP057-09|United States|North Carolina|658[0n]|BOLD:AAA2866
Bleptina inferior|[12779]|MILEP056-09|United States|North Carolina|658[0n]|BOLD:AAA2866
Bleptina inferior|[12780]|MILEP055-09|United States|North Carolina|658[0n]|BOLD:AAA2866
Bleptina inferior|[12781]|LNCB670-09|United States|North Carolina|658[0n]|BOLD:AAA2866

[illegible]
Bleptina inferior|[12779]|MILEP056-09|United States|North Carolina|658[0n]|BOLD:AAA2866
Bleptina inferior|[12780]|MILEP055-09|United States|North Carolina|658[0n]|BOLD:AAA2866
Bleptina inferior|[12781]|LNCB670-09|United States|North Carolina|658[0n]|BOLD:AAA2866
Bleptina inferior|[12782]|LNCB668-09|United States|North Carolina|658[0n]|BOLD:AAA2866
Bleptina inferior|[12783]|LNCB667-09|United States|North Carolina|658[0n]|BOLD:AAA2866
Bleptina inferior|[12784]|LNCB658-09|United States|North Carolina|658[0n]|BOLD:AAA2866
Bleptina inferior|[12785]|LNCB641-09|United States|North Carolina|658[0n]|BOLD:AAA2866
Bleptina inferior|[12786]|LNCB640-09|United States|North Carolina|658[0n]|BOLD:AAA2866
Bleptina inferior|[12787]|LNCB639-09|United States|North Carolina|658[0n]|BOLD:AAA2866
Bleptina inferior|[12788]|LMEM274-09|United States|Georgia|658[0n]|BOLD:AAA2866
Bleptina inferior|[12789]|LMEM269-09|United States|Mississippi|658[0n]|BOLD:AAA2866
Bleptina inferior|[12790]|HKONS330-08|United States|Florida|658[0n]|BOLD:AAA2866
Bleptina inferior|[12791]|HKONS329-08|United States|Florida|658[0n]|BOLD:AAA2866
Bleptina inferior|[12792]|LOFLD046-07|United States|Florida|658[0n]|BOLD:AAA2866
Bleptina inferior|[12793]|LNCB490-07|United States|North Carolina|658[0n]|BOLD:AAA2866
Bleptina inferior|[12794]|LOFLC479-06|United States|Florida|658[0n]|BOLD:AAA2866
Bleptina inferior|[12795]|LOFLC326-06|United States|Florida|658[0n]|BOLD:AAA2866
Bleptina inferior|[12796]|LOFLC309-06|United States|Florida|658[0n]|BOLD:AAA2866
Bleptina inferior|[12797]|LOFLC232-06|United States|Florida|658[0n]|BOLD:AAA2866
Bleptina inferior|[12798]|LOFLC230-06|United States|Florida|658[0n]|BOLD:AAA2866
Bleptina inferior|[12799]|LOFLC226-06|United States|Florida|658[0n]|BOLD:AAA2866
Bleptina inferior|[12800]|LOFLC223-06|United States|Florida|658[0n]|BOLD:AAA2866
Bleptina inferior|[12801]|LOFLC210-06|United States|Florida|658[0n]|BOLD:AAA2866
Bleptina inferior|[12802]|LOFLB841-06|United States|Florida|658[0n]|BOLD:AAA2866
Bleptina inferior|[12803]|LOFLB837-06|United States|Florida|658[0n]|BOLD:AAA2866
Bleptina inferior|[12804]|LOFLB785-06|United States|Florida|658[0n]|BOLD:AAA2866
Bleptina inferior|[12805]|LOFLB775-06|United States|Florida|658[0n]|BOLD:AAA2866
Bleptina inferior|[12806]|LOFLB773-06|United States|Florida|658[0n]|BOLD:AAA2866
Bleptina inferior|[12807]|LOFLB769-06|United States|Florida|658[0n]|BOLD:AAA2866
Bleptina inferior|[12808]|LOFLB759-06|United States|Florida|658[0n]|BOLD:AAA2866
Bleptina inferior|[12809]|LOFLB756-06|United States|Florida|658[0n]|BOLD:AAA2866
Bleptina inferior|[12810]|LOFLB754-06|United States|Florida|658[0n]|BOLD:AAA2866
Bleptina inferior|[12811]|LOFLB750-06|United States|Florida|658[0n]|BOLD:AAA2866
Bleptina inferior|[12812]|LOFLB699-06|United States|Florida|658[0n]|BOLD:AAA2866
Bleptina inferior|[12813]|LOFLB697-06|United States|Florida|658[0n]|BOLD:AAA2866
Bleptina inferior|[12814]|LOFLB695-06|United States|Florida|658[0n]|BOLD:AAA2866
Bleptina inferior|[12815]|LOFLB535-06|United States|Florida|658[0n]|BOLD:AAA2866
Bleptina inferior|[12816]|LOFLB515-06|United States|Florida|658[0n]|BOLD:AAA2866
Bleptina inferior|[12817]|LOFLB393-06|United States|Florida|658[0n]|BOLD:AAA2866
Bleptina inferior|[12818]|LOFLB284-06|United States|Florida|658[0n]|BOLD:AAA2866
Bleptina inferior|[12819]|LOFLB181-06|United States|Florida|658[0n]|BOLD:AAA2866
Bleptina inferior|[12820]|LOFLA877-06|United States|Florida|658[0n]|BOLD:AAA2866
Bleptina inferior|[12821]|LOFLA869-06|United States|Florida|658[0n]|BOLD:AAA2866
Bleptina inferior|[12822]|LOFLA868-06|United States|Florida|658[0n]|BOLD:AAA2866
Bleptina inferior|[12823]|LOFLA867-06|United States|Florida|658[0n]|BOLD:AAA2866
Bleptina inferior|[12824]|LOFLA866-06|United States|Florida|658[0n]|BOLD:AAA2866
Bleptina inferior|[12825]|LOFLA863-06|United States|Florida|658[0n]|BOLD:AAA2866
Bleptina inferior|[12826]|LOFLA862-06|United States|Florida|658[0n]|BOLD:AAA2866
Bleptina inferior|[12827]|LOFLA861-06|United States|Florida|658[0n]|BOLD:AAA2866
Bleptina inferior|[12828]|LOFLA859-06|United States|Florida|658[0n]|BOLD:AAA2866
Bleptina inferior|[12829]|LOFLA686-06|United States|Florida|658[0n]|BOLD:AAA2866
Bleptina inferior|[12830]|LOFLA668-06|United States|Florida|658[0n]|BOLD:AAA2866
Bleptina inferior|[12831]|LOFLA618-06|United States|Florida|658[0n]|BOLD:AAA2866
Bleptina inferior|[12832]|LOFLA591-06|United States|Florida|658[0n]|BOLD:AAA2866
Bleptina inferior|[12833]|LOFLA568-06|United States|Florida|658[0n]|BOLD:AAA2866
Bleptina inferior|[12834]|LOFLA565-06|United States|Florida|658[0n]|BOLD:AAA2866
Bleptina inferior|[12835]|LOFLA548-06|United States|Florida|658[0n]|BOLD:AAA2866
Bleptina inferior|[12836]|LOFLA441-06|United States|Florida|658[0n]|BOLD:AAA2866
Bleptina inferior|[12837]|LOFLA431-06|United States|Florida|658[0n]|BOLD:AAA2866
Bleptina inferior|[12838]|LOFLA430-06|United States|Florida|658[0n]|BOLD:AAA2866
Bleptina inferior|[12839]|LOFLA428-06|United States|Florida|658[0n]|BOLD:AAA2866
Bleptina inferior|[12840]|LOFLA426-06|United States|Florida|658[0n]|BOLD:AAA2866
Bleptina inferior|[12841]|LOFLA424-06|United States|Florida|658[0n]|BOLD:AAA2866
Bleptina inferior|[12842]|LOFLA422-06|United States|Florida|658[0n]|BOLD:AAA2866
Bleptina inferior|[12843]|LOFLA417-06|United States|Florida|658[0n]|BOLD:AAA2866
Bleptina inferior|[12844]|LOFLA416-06|United States|Florida|658[0n]|BOLD:AAA2866
Bleptina inferior|[12845]|LOFLA414-06|United States|Florida|658[0n]|BOLD:AAA2866
Bleptina inferior|[12846]|LOFLA412-06|United States|Florida|658[0n]|BOLD:AAA2866
Bleptina inferior|[12847]|LOFLA411-06|United States|Florida|658[0n]|BOLD:AAA2866
Bleptina inferior|[12848]|LOFLA409-06|United States|Florida|658[0n]|BOLD:AAA2866
Bleptina inferior|[12849]|LOFLA406-06|United States|Florida|658[0n]|BOLD:AAA2866
Bleptina inferior|[12850]|LOFLA404-06|United States|Florida|658[0n]|BOLD:AAA2866
Bleptina inferior|[12851]|LOFLA403-06|United States|Florida|658[0n]|BOLD:AAA2866
Bleptina inferior|[12852]|LOFLA402-06|United States|Florida|658[0n]|BOLD:AAA2866
Bleptina inferior|[12853]|LOFLA401-06|United States|Florida|658[0n]|BOLD:AAA2866
Bleptina inferior|[12854]|LOFLA395-06|United States|Florida|658[0n]|BOLD:AAA2866
Bleptina inferior|[12855]|LOFLA320-06|United States|Florida|658[0n]|BOLD:AAA2866
Bleptina inferior|[12856]|LOFLA197-06|United States|Florida|658[0n]|BOLD:AAA2866
Bleptina inferior|[12857]|LOFLA166-06|United States|Florida|658[0n]|BOLD:AAA2866
Bleptina inferior|[12858]|LOFLA160-06|United States|Florida|658[0n]|BOLD:AAA2866
Bleptina inferior|[12859]|LOFLA159-06|United States|Florida|658[0n]|BOLD:AAA2866
Bleptina inferior|[12860]|LOFLA156-06|United States|Florida|658[0n]|BOLD:AAA2866
Bleptina inferior|[12861]|LOFLA146-06|United States|Florida|658[0n]|BOLD:AAA2866
Bleptina inferior|[12862]|LOFLA139-06|United States|Florida|658[0n]|BOLD:AAA2866
Bleptina inferior|[12863]|LOFLA136-06|United States|Florida|658[0n]|BOLD:AAA2866
Bleptina inferior|[12864]|LOFLA135-06|United States|Florida|658[0n]|BOLD:AAA2866
Bleptina inferior|[12865]|LOFLA134-06|United States|Florida|658[0n]|BOLD:AAA2866
Bleptina inferior|[12866]|LOFLA058-06|United States|Florida|658[0n]|BOLD:AAA2866
Bleptina inferior|[12867]|LOFLA057-06|United States|Florida|658[0n]|BOLD:AAA2866
Bleptina inferior|[12868]|LOFLA034-06|United States|Florida|658[0n]|BOLD:AAA2866
Bleptina inferior|[12869]|LOFLA007-06|United States|Florida|658[0n]|BOLD:AAA2866
Bleptina inferior|[12870]|LNC821-06|United States|North Carolina|658[0n]|BOLD:AAA2866
Bleptina inferior|[12871]|LNC536-06|United States|North Carolina|658[0n]|BOLD:AAA2866
Bleptina inferior|[12872]|MILEP063-09|United States|North Carolina|658[0n]|BOLD:AAA2866
Bleptina inferior|[12873]|LOFLA602-06|United States|Florida|658[0n]|BOLD:AAA2866
Bleptina inferior|[12874]|LOFLB784-06|United States|Florida|658[0n]|BOLD:AAA2866
Bleptina inferior|[12875]|LOFLA670-06|United States|Florida|658[0n]|BOLD:AAA2866
Bleptina inferior|[12876]|USLEP1278-10|United States|Florida|658[0n]|BOLD:AAA2866
Bleptina inferior|[12877]|LNCC077-10|United States|North Carolina|658[0n]|BOLD:AAA2866
Bleptina inferior|[12878]|LNCC078-10|United States|North Carolina|658[0n]|BOLD:AAA2866
Bleptina inferior|[12879]|LNCC079-10|United States|North Carolina|658[0n]|BOLD:AAA2866
Bleptina inferior|[12880]|LNCC080-10|United States|North Carolina|658[0n]|BOLD:AAA2866
Bleptina inferior|[12881]|LNCC081-10|United States|North Carolina|658[0n]|BOLD:AAA2866

Bleptina inferior|[12878]|LNCC078-10|United States|North Carolina|658[0n]|BOLD:AAA2866
Bleptina inferior|[12879]|LNCC079-10|United States|North Carolina|658[0n]|BOLD:AAA2866
Bleptina inferior|[12880]|LNCC080-10|United States|North Carolina|658[0n]|BOLD:AAA2866
Bleptina inferior|[12881]|LNCC081-10|United States|North Carolina|658[0n]|BOLD:AAA2866
Bleptina inferior|[12882]|LNCC082-10|United States|North Carolina|658[0n]|BOLD:AAA2866
Bleptina inferior|[12883]|RDNMJ035-10|United States|Florida|658[0n]|BOLD:AAA2866
Bleptina inferior|[12884]|LNCC457-10|United States|North Carolina|658[0n]|BOLD:AAA2866
Bleptina inferior|[12885]|LNCC458-10|United States|North Carolina|658[0n]|BOLD:AAA2866
Bleptina inferior|[12886]|BBLOB271-11|United States|Florida|658[0n]|BOLD:AAA2866
Bleptina inferior|[12887]|BBLOB385-11|United States|Florida|658[0n]|BOLD:AAA2866
Bleptina inferior|[12888]|LNCC1215-11|United States|North Carolina|658[0n]|BOLD:AAA2866
Bleptina inferior|[12889]|LOFLA405-06|United States|Florida|658[0n]|BOLD:AAA2866
Bleptina inferior|[12890]|LOFLB188-06|United States|Florida|658[0n]|BOLD:AAA2866
Bleptina inferior|[12891]|MILEP054-09|United States|North Carolina|658[0n]|BOLD:AAA2866
Bleptina inferior|[12892]|BBLOB1877-11|United States|Florida|658[0n]|BOLD:AAA2866
Bleptina flaviguttalis|[12893]|RDNMJ681-11|United States|Arizona|658[0n]|BOLD:AAD9144
Bleptina flaviguttalis|[12894]|RDNMJ547-11|United States|Arizona|658[0n]|BOLD:AAD9144
Bleptina flaviguttalis|[12895]|RDNME908-08|United States|Arizona|658[0n]|BOLD:AAD9144
Bleptina flaviguttalis|[12896]|RDNMJ349-11|United States|Arizona|658[0n]|BOLD:AAD9144
Bleptina flaviguttalis|[12897]|RDNMJ537-11|United States|Arizona|658[0n]|BOLD:AAD9144
Bleptina flaviguttalis|[12898]|CMAZA1106-12|United States|Arizona|658[0n]|BOLD:AAD9144
Bleptina n. sp. 4|[12899]|RDNMG238-08|United States|Texas|658[0n]|BOLD:ABZ3942
Bleptina n. sp. 4|[12900]|RDNMG329-08|United States|Texas|658[0n]|BOLD:ABZ3942
Bleptina n. sp. 4|[12901]|HKONB521-09|United States|Texas|628[0n]|BOLD:ABZ3942
Bleptina n. sp. 4|[12902]|HKONB565-09|United States|Texas|658[0n]|BOLD:ABZ3942
Bleptina n. sp. 4|[12903]|HKONB522-09|United States|Texas|637[0n]|BOLD:ABZ3942
Bleptina n. sp. 4|[12904]|RDNMG330-08|United States|Texas|658[0n]|BOLD:ABZ3942
Bleptina n. sp. 4|[12905]|RDNMG236-08|United States|Texas|658[0n]|BOLD:ABZ3942
Bleptina n. sp. 4|[12906]|RDNMG237-08|United States|Texas|658[0n]|BOLD:ABZ3942
Bleptina n. sp. 4|[12907]|HKONB566-09|United States|Texas|658[0n]|BOLD:ABZ3942
Bleptina n. sp. 4|[12908]|HKONB567-09|United States|Texas|633[0n]|BOLD:ABZ3942
Bleptina n. sp. 4|[12909]|ABNCC091-07|United States|Oklahoma|632[0n]|BOLD:ABZ3942
Bleptina n. sp. 4|[12910]|ABNCC092-07|United States|Oklahoma|584[1n]|BOLD:ABZ3942
Bleptina n. sp. 4|[12911]|BBLSY209-09|United States|Texas|658[0n]|BOLD:ABZ3942
Bleptina sangamonia|[12912]|LMEM271-09|United States|Georgia|658[0n]|BOLD:AAA2869
Bleptina sangamonia|[12913]|LNCC1584-13|United States|North Carolina|658[0n]|BOLD:AAA2869
Bleptina sangamonia|[12914]|LNCC1220-11|United States|North Carolina|658[0n]|BOLD:AAA2869
Bleptina sangamonia|[12915]|LNCC1219-11|United States|North Carolina|658[0n]|BOLD:AAA2869
Bleptina sangamonia|[12916]|LNCC1218-11|United States|North Carolina|658[0n]|BOLD:AAA2869
Bleptina sangamonia|[12917]|LNCC1217-11|United States|North Carolina|658[0n]|BOLD:AAA2869
Bleptina sangamonia|[12918]|LNCC1216-11|United States|North Carolina|658[0n]|BOLD:AAA2869
Bleptina sangamonia|[12919]|LNCC936-11|United States|North Carolina|658[0n]|BOLD:AAA2869
Bleptina sangamonia|[12920]|LNCC935-11|United States|North Carolina|658[0n]|BOLD:AAA2869
Bleptina sangamonia|[12921]|LNCC934-11|United States|North Carolina|658[0n]|BOLD:AAA2869
Bleptina sangamonia|[12922]|LNCC933-11|United States|North Carolina|658[0n]|BOLD:AAA2869
Bleptina sangamonia|[12923]|BBLSX551-09|United States|Oklahoma|658[0n]|BOLD:AAA2869
Bleptina sangamonia|[12924]|BBLSX151-09|United States|Oklahoma|658[0n]|BOLD:AAA2869
Bleptina sangamonia|[12925]|LNCB559-09|United States|North Carolina|658[0n]|BOLD:AAA2869
Bleptina sangamonia|[12926]|LMEM285-09|United States|Tennessee|658[0n]|BOLD:AAA2869
Bleptina sangamonia|[12927]|LMEM284-09|United States|Alabama|658[0n]|BOLD:AAA2869
Bleptina sangamonia|[12928]|LMEM283-09|United States|Alabama|658[0n]|BOLD:AAA2869
Bleptina sangamonia|[12929]|LMEM281-09|United States|Tennessee|658[0n]|BOLD:AAA2869
Bleptina sangamonia|[12930]|LMEM280-09|United States|Tennessee|658[0n]|BOLD:AAA2869
Bleptina sangamonia|[12931]|LMEM279-09|United States|Tennessee|658[0n]|BOLD:AAA2869
Bleptina sangamonia|[12932]|LMEM278-09|United States|Tennessee|658[0n]|BOLD:AAA2869
Bleptina sangamonia|[12933]|LMEM277-09|United States|Tennessee|658[0n]|BOLD:AAA2869
Bleptina sangamonia|[12934]|LMEM276-09|United States|Tennessee|658[0n]|BOLD:AAA2869
Bleptina sangamonia|[12935]|LMEM275-09|United States|Tennessee|658[0n]|BOLD:AAA2869
Bleptina sangamonia|[12936]|LMEM273-09|United States|Mississippi|658[0n]|BOLD:AAA2869
Bleptina sangamonia|[12937]|LMEM272-09|United States|Mississippi|658[0n]|BOLD:AAA2869
Bleptina sangamonia|[12938]|LMEM270-09|United States|Georgia|658[0n]|BOLD:AAA2869
Bleptina sangamonia|[12939]|HKONB372-09|United States|Louisiana|658[0n]|BOLD:AAA2869
Bleptina sangamonia|[12940]|HKONB371-09|United States|Texas|658[0n]|BOLD:AAA2869
Bleptina sangamonia|[12941]|HKONB370-09|United States|Louisiana|658[0n]|BOLD:AAA2869
Bleptina sangamonia|[12942]|HKONB369-09|United States|Texas|658[0n]|BOLD:AAA2869
Bleptina sangamonia|[12943]|LGSMC667-05|United States|Tennessee|658[0n]|BOLD:AAA2869
Bleptina sangamonia|[12944]|LGSM538-04|United States|Tennessee|658[0n]|BOLD:AAA2869
Bleptina sangamonia|[12945]|LGSMC668-05|United States|Tennessee|658[0n]|BOLD:AAA2869
Bleptina sangamonia|[12946]|LGSMC669-05|United States|Tennessee|590[0n]|BOLD:AAA2869
Bleptina sangamonia|[12947]|RDNMF967-08|United States|Texas|609[0n]|BOLD:AAA2869
Bleptina sangamonia|[12948]|RDNMF969-08|United States|Texas|609[0n]|BOLD:AAA2869
Bleptina sangamonia|[12949]|LNCC1585-13|United States|North Carolina|658[0n]|BOLD:AAA2869
Bleptina sangamonia|[12950]|LNCC1641-13|United States|North Carolina|658[0n]|BOLD:AAA2869
Phalaenophana pyramusalis|[12951]|LPMN473-08|Canada|Manitoba|658[0n]|BOLD:AAA5643
Phalaenophana pyramusalis|[12952]|LOCT395-05|United States|Connecticut|601[0n]|BOLD:AAA5643
Phalaenophana pyramusalis|[12953]|LOCT396-05|United States|Connecticut|600[0n]|BOLD:AAA5643
Phalaenophana pyramusalis|[12954]|PHJUN3375-11|Canada|Ontario|658[0n]|BOLD:AAA5643
Phalaenophana pyramusalis|[12955]|RDLQG107-06|Canada|Quebec|616[0n]|BOLD:AAA5643
Phalaenophana pyramusalis|[12956]|LILLA665-11|United States|Illinois|658[0n]|BOLD:AAA5643
Phalaenophana pyramusalis|[12957]|LGSMC654-05|United States|Tennessee|613[0n]|BOLD:AAA5643
Phalaenophana pyramusalis|[12958]|LOCT111-05|United States|Connecticut|658[0n]|BOLD:AAA5643
Phalaenophana pyramusalis|[12959]|LGSMG478-07|United States|North Carolina|658[0n]|BOLD:AAA5643
Phalaenophana pyramusalis|[12960]|LGSMD370-05|United States|Tennessee|658[0n]|BOLD:AAA5643
Phalaenophana pyramusalis|[12961]|LNCB487-07|United States|North Carolina|654[0n]|BOLD:AAA5643
Phalaenophana pyramusalis|[12962]|LNCB486-07|United States|North Carolina|658[0n]|BOLD:AAA5643
Phalaenophana pyramusalis|[12963]|LOCT110-05|United States|Connecticut|658[0n]|BOLD:AAA5643
Phalaenophana pyramusalis|[12964]|LPSO949-08|Canada|Ontario|658[0n]|BOLD:AAA5643
Phalaenophana pyramusalis|[12965]|HKONB334-09|United States|Texas|658[0n]|BOLD:AAA5643
Phalaenophana pyramusalis|[12966]|LMEM253-09|United States|Tennessee|658[0n]|BOLD:AAA5643
Phalaenophana pyramusalis|[12967]|LPOKB169-09|United States|Oklahoma|658[0n]|BOLD:AAA5643
Phalaenophana pyramusalis|[12968]|LPOKB195-09|United States|Oklahoma|658[0n]|BOLD:AAA5643
Phalaenophana pyramusalis|[12969]|LNCB632-09|United States|North Carolina|658[0n]|BOLD:AAA5643
Phalaenophana pyramusalis|[12970]|LPOKD454-09|United States|Oklahoma|658[0n]|BOLD:AAA5643
Phalaenophana pyramusalis|[12971]|LILLA032-11|United States|Illinois|658[0n]|BOLD:AAA5643
Phalaenophana pyramusalis|[12972]|LPSO907-08|Canada|Ontario|658[0n]|BOLD:AAA5643
Phalaenophana pyramusalis|[12973]|PHJUN3372-11|Canada|Ontario|658[0n]|BOLD:AAA5643
Phalaenophana pyramusalis|[12974]|LPSO944-08|Canada|Ontario|658[0n]|BOLD:AAA5643
Phalaenophana pyramusalis|[12975]|BLTIB541-08|Canada|Ontario|658[0n]|BOLD:AAA5643
Phalaenophana pyramusalis|[12976]|LPSO908-08|Canada|Ontario|658[0n]|BOLD:AAA5643
Phalaenophana pyramusalis|[12977]|LPSO941-08|Canada|Ontario|658[0n]|BOLD:AAA5643
Phalaenophana pyramusalis|[12978]|RDLQG420-06|Canada|Quebec|658[0n]|BOLD:AAA5643
Phalaenophana pyramusalis|[12979]|RDLQG043-06|Canada|Quebec|658[0n]|BOLD:AAA5643
Phalaenophana pyramusalis|[12980]|TMNBB046-06|Canada|New Brunswick|658[0n]|BOLD:AAA5643
Phalaenophana pyramusalis|[12981]|[illegible]|United States|Connecticut|658[0n]|BOLD:AAA5643

Phalaenophana pyramusalis|[12978]|RDLQG420-06|Canada|Quebec|658[0n]|BOLD:AAA5643
Phalaenophana pyramusalis|[12979]|RDLQG043-06|Canada|Quebec|658[0n]|BOLD:AAA5643
Phalaenophana pyramusalis|[12980]|TMNBB046-06|Canada|New Brunswick|658[0n]|BOLD:AAA5643
Phalaenophana pyramusalis|[12981]|LOCT309-05|United States|Connecticut|658[0n]|BOLD:AAA5643
Phalaenophana pyramusalis|[12982]|LOCT243-05|United States|Connecticut|658[0n]|BOLD:AAA5643
Phalaenophana pyramusalis|[12983]|PHMNB490-04|Canada|New Brunswick|658[0n]|BOLD:AAA5643
Phalaenophana pyramusalis|[12984]|RDLQG421-06|Canada|Quebec|658[1n]|BOLD:AAA5643
Phalaenophana pyramusalis|[12985]|RDLQG422-06|Canada|Quebec|658[0n]|BOLD:AAA5643
Phalaenophana pyramusalis|[12986]|RDLQG424-06|Canada|Quebec|658[0n]|BOLD:AAA5643
Phalaenophana pyramusalis|[12987]|RDLQG429-06|Canada|Quebec|658[0n]|BOLD:AAA5643
Phalaenophana pyramusalis|[12988]|LPSO672-08|Canada|Ontario|658[0n]|BOLD:AAA5643
Phalaenophana pyramusalis|[12989]|LPSO702-08|Canada|Ontario|658[0n]|BOLD:AAA5643
Phalaenophana pyramusalis|[12990]|LPSO893-08|Canada|Ontario|658[0n]|BOLD:AAA5643
Phalaenophana pyramusalis|[12991]|LPSO896-08|Canada|Ontario|658[0n]|BOLD:AAA5643
Phalaenophana pyramusalis|[12992]|BLTIB643-08|Canada|Ontario|658[0n]|BOLD:AAA5643
Phalaenophana pyramusalis|[12993]|TMNBB045-06|Canada|New Brunswick|622[0n]|BOLD:AAA5643
Phalaenophana pyramusalis|[12994]|LPSO193-08|Canada|Ontario|658[0n]|BOLD:AAA5643
Phalaenophana pyramusalis|[12995]|LPSO320-08|Canada|Ontario|658[0n]|BOLD:AAA5643
Phalaenophana pyramusalis|[12996]|LPSO385-08|Canada|Ontario|658[0n]|BOLD:AAA5643
Phalaenophana pyramusalis|[12997]|LPSO699-08|Canada|Ontario|658[0n]|BOLD:AAA5643
Phalaenophana pyramusalis|[12998]|LOCT242-05|United States|Connecticut|658[0n]|BOLD:AAA5643
Phalaenophana pyramusalis|[12999]|LOCT307-05|United States|Connecticut|658[0n]|BOLD:AAA5643
Phalaenophana pyramusalis|[13000]|LPSO902-08|Canada|Ontario|658[0n]|BOLD:AAA5643
Phalaenophana pyramusalis|[13001]|BLTIB700-08|Canada|Ontario|658[0n]|BOLD:AAA5643
Phalaenophana pyramusalis|[13002]|CNRMD2092-12|Canada|Manitoba|634[0n]|BOLD:AAA5643
Phalaenophana pyramusalis|[13003]|CNRMD2096-12|Canada|Manitoba|633[0n]|BOLD:AAA5643
Phalaenophana pyramusalis|[13004]|CNRMD2115-12|Canada|Manitoba|633[0n]|BOLD:AAA5643
Phalaenophana pyramusalis|[13005]|CNRME1804-12|Canada|Manitoba|632[0n]|BOLD:AAA5643
Phalaenophana pyramusalis|[13006]|CNRMD2419-12|Canada|Manitoba|614[0n]|BOLD:AAA5643
Phalaenophana pyramusalis|[13007]|CNRME1792-12|Canada|Manitoba|618[0n]|BOLD:AAA5643
Phalaenophana pyramusalis|[13008]|BBLPD149-10|Canada|Saskatchewan|658[0n]|BOLD:AAA5643
Phalaenophana pyramusalis|[13009]|CNRME710-12|Canada|Manitoba|631[0n]|BOLD:AAA5643
Phalaenophana pyramusalis|[13010]|SSEIA4407-13|Canada|Alberta|608[0n]|BOLD:AAA5643
Phalaenophana pyramusalis|[13011]|LPSO391-08|Canada|Ontario|637[0n]|BOLD:AAA5643
Phalaenophana pyramusalis|[13012]|RDLQH153-06|Canada|Quebec|616[3n]|BOLD:AAA5643
Phalaenophana pyramusalis|[13013]|SSEIA7726-13|Canada|Alberta|582[0n]|BOLD:AAA5643
Phalaenophana pyramusalis|[13014]|LPSOD820-09|Canada|Ontario|658[0n]|BOLD:AAA5643
Phalaenophana pyramusalis|[13015]|LPSOD817-09|Canada|Ontario|658[0n]|BOLD:AAA5643
Phalaenophana pyramusalis|[13016]|LPSOD791-09|Canada|Ontario|658[0n]|BOLD:AAA5643
Phalaenophana pyramusalis|[13017]|LPSOD604-09|Canada|Ontario|658[0n]|BOLD:AAA5643
Phalaenophana pyramusalis|[13018]|LPSOD533-09|Canada|Ontario|658[0n]|BOLD:AAA5643
Phalaenophana pyramusalis|[13019]|RDLQG811-06|Canada|Quebec|658[0n]|BOLD:AAA5643
Phalaenophana pyramusalis|[13020]|RDLQG427-06|Canada|Quebec|658[0n]|BOLD:AAA5643
Phalaenophana pyramusalis|[13021]|RDLQG426-06|Canada|Quebec|658[0n]|BOLD:AAA5643
Phalaenophana pyramusalis|[13022]|RDLQG425-06|Canada|Quebec|658[0n]|BOLD:AAA5643
Phalaenophana pyramusalis|[13023]|RDLQG423-06|Canada|Quebec|658[0n]|BOLD:AAA5643
Phalaenophana pyramusalis|[13024]|TTMNB546-06|Canada|New Brunswick|658[0n]|BOLD:AAA5643
Phalaenophana pyramusalis|[13025]|XAF810-05|Canada|Ontario|658[0n]|BOLD:AAA5643
Phalaenophana pyramusalis|[13026]|RDLQG430-06|Canada|Quebec|658[0n]|BOLD:AAA5643
Phalaenophana pyramusalis|[13027]|PHMNB671-04|Canada|New Brunswick|658[0n]|BOLD:AAA5643
Phalaenophana pyramusalis|[13028]|LPSOB657-08|Canada|Ontario|658[1n]|BOLD:AAA5643
Phalaenophana pyramusalis|[13029]|BBLPE047-09|Canada|Nova Scotia|658[0n]|BOLD:AAA5643
Phalaenophana pyramusalis|[13030]|RDLQG428-06|Canada|Quebec|632[0n]|BOLD:AAA5643
Phalaenophana pyramusalis|[13031]|LPSOB828-08|Canada|Ontario|658[0n]|BOLD:AAA5643
Phalaenophana pyramusalis|[13032]|BBLPD439-10|Canada|Ontario|658[0n]|BOLD:AAA5643
Phalaenophana pyramusalis|[13033]|RDLQH138-06|Canada|Quebec|633[0n]|BOLD:AAA5643
Phalaenophana pyramusalis|[13034]|CNPAL1084-13|Canada|Saskatchewan|581[0n]|BOLD:AAA5643
Phalaenophana extremalis|[13035]|LNAUS5642-13|United States|New Mexico|658[0n]|BOLD:ACK9172
Phalaenophana extremalis|[13036]|LNAUS5643-13|United States|New Mexico|658[0n]|BOLD:ACK9172
Phalaenophana extremalis|[13037]|LNAUS5644-13|United States|New Mexico|658[0n]|BOLD:ACK9172
Phalaenophana extremalis|[13038]|LNAUS5645-13|United States|New Mexico|658[0n]|BOLD:ACK9172
Phalaenophana extremalis|[13039]|LNAUS5646-13|United States|New Mexico|658[0n]|BOLD:ACK9172
Lascoria orneodalis|[13040]|CNCLB1799-14|United States|Florida|658[0n]|BOLD:AAB4990
Lascoria sp.|[13041]|RDNMD526-06|United States|Florida|658[0n]|BOLD:AAB4991
Lascoria sp.|[13042]|HKONS058-07|United States|Florida|655[0n]|BOLD:AAB4991
Lascoria sp.|[13043]|HKONS133-08|United States|Florida|657[0n]|BOLD:AAB4991
Lascoria sp.|[13044]|HKONS134-08|United States|Florida|658[0n]|BOLD:AAB4991
Lascoria sp.|[13045]|RDNMJ040-10|United States|Florida|658[0n]|BOLD:AAB4991
Lascoria ambigualis|[13046]|RDLQG261-06|Canada|Quebec|658[0n]|BOLD:AAA4458
Lascoria ambigualis|[13047]|RDLQG226-06|Canada|Quebec|658[0n]|BOLD:AAA4458
Lascoria ambigualis|[13048]|RDLQG586-06|Canada|Quebec|658[0n]|BOLD:AAA4458
Lascoria ambigualis|[13049]|RDLQG576-06|Canada|Quebec|658[0n]|BOLD:AAA4458
Lascoria ambigualis|[13050]|RDLQG227-06|Canada|Quebec|658[0n]|BOLD:AAA4458
Lascoria ambigualis|[13051]|RDLQG259-06|Canada|Quebec|658[0n]|BOLD:AAA4458
Lascoria ambigualis|[13052]|RDLQG260-06|Canada|Quebec|658[0n]|BOLD:AAA4458
Lascoria ambigualis|[13053]|XAE238-04|Canada|Ontario|570[3n]|BOLD:AAA4458
Lascoria ambigualis|[13054]|RDLQG258-06|Canada|Quebec|616[0n]|BOLD:AAA4458
Lascoria ambigualis|[13055]|RDLQG280-06|Canada|Quebec|601[0n]|BOLD:AAA4458
Lascoria ambigualis|[13056]|RDLQG585-06|Canada|Quebec|658[0n]|BOLD:AAA4458
Lascoria ambigualis|[13057]|RDLQG587-06|Canada|Quebec|658[0n]|BOLD:AAA4458
Lascoria ambigualis|[13058]|RDLQG601-06|Canada|Quebec|647[0n]|BOLD:AAA4458
Lascoria ambigualis|[13059]|LGSMC422-05|United States|Tennessee|658[0n]|BOLD:AAA4458
Lascoria ambigualis|[13060]|LNCB264-06|United States|North Carolina|658[0n]|BOLD:AAA4458
Lascoria ambigualis|[13061]|LNCB217-06|United States|North Carolina|658[0n]|BOLD:AAA4458
Lascoria ambigualis|[13062]|LPOKB283-09|United States|Oklahoma|658[0n]|BOLD:AAA4458
Lascoria ambigualis|[13063]|LPSO287-08|Canada|Ontario|658[0n]|BOLD:AAA4458
Lascoria ambigualis|[13064]|BLTIB013-08|Canada|Ontario|658[0n]|BOLD:AAA4458
Lascoria ambigualis|[13065]|BLTIB082-08|Canada|Ontario|658[0n]|BOLD:AAA4458
Lascoria ambigualis|[13066]|BBLSX296-09|United States|Oklahoma|658[0n]|BOLD:AAA4458
Lascoria ambigualis|[13067]|LNCB160-06|United States|North Carolina|658[0n]|BOLD:AAA4458
Lascoria ambigualis|[13068]|LNCB265-06|United States|North Carolina|632[0n]|BOLD:AAA4458
Lascoria ambigualis|[13069]|IAWL045-09|United States|Virginia|658[0n]|BOLD:AAA4458
Lascoria ambigualis|[13070]|LMEM309-09|United States|Louisiana|658[0n]|BOLD:AAA4458
Lascoria ambigualis|[13071]|BBLSX090-09|United States|Oklahoma|658[0n]|BOLD:AAA4458
Lascoria ambigualis|[13072]|BBLSX552-09|United States|Oklahoma|658[0n]|BOLD:AAA4458
Lascoria ambigualis|[13073]|LILLA443-11|United States|Illinois|658[0n]|BOLD:AAA4458
Lascoria ambigualis|[13074]|BBLSX919-09|United States|Arizona|658[0n]|BOLD:AAA4458
Lascoria ambigualis|[13075]|LPSO947-08|Canada|Ontario|657[0n]|BOLD:AAA4458
Lascoria ambigualis|[13076]|XAJ631-06|Canada|Ontario|658[0n]|BOLD:AAA4458
Lascoria ambigualis|[13077]|IAWL593-09|United States|Virginia|658[0n]|BOLD:AAA4458
Lascoria ambigualis|[13078]|LPOKA941-09|United States|Oklahoma|658[0n]|BOLD:AAA4458
Lascoria ambigualis|[13079]|LPOKA994-09|United States|Oklahoma|658[0n]|BOLD:AAA4458
Lascoria ambigualis|[13080]|BLTIB056-08|Canada|Ontario|658[0n]|BOLD:AAA4458
[illegible]

Lascoria ambigualis|[13078]|LPOKA941-09|United States|Oklahoma|658[0n]|BOLD:AAA4458
Lascoria ambigualis|[13079]|LPOKA994-09|United States|Oklahoma|658[0n]|BOLD:AAA4458
Lascoria ambigualis|[13080]|BLTIB056-08|Canada|Ontario|658[0n]|BOLD:AAA4458
Lascoria ambigualis|[13081]|BLTIB062-08|Canada|Ontario|658[0n]|BOLD:AAA4458
Lascoria ambigualis|[13082]|LPSO300-08|Canada|Ontario|658[0n]|BOLD:AAA4458
Lascoria ambigualis|[13083]|LPSO463-08|Canada|Ontario|658[0n]|BOLD:AAA4458
Lascoria ambigualis|[13084]|LPSO700-08|Canada|Ontario|658[0n]|BOLD:AAA4458
Lascoria ambigualis|[13085]|LPSO946-08|Canada|Ontario|658[0n]|BOLD:AAA4458
Lascoria ambigualis|[13086]|BLTIB074-08|Canada|Ontario|658[0n]|BOLD:AAA4458
Lascoria ambigualis|[13087]|BLTIB120-08|Canada|Ontario|658[0n]|BOLD:AAA4458
Lascoria ambigualis|[13088]|BLTIB154-08|Canada|Ontario|658[0n]|BOLD:AAA4458
Lascoria ambigualis|[13089]|BLTIB159-08|Canada|Ontario|658[0n]|BOLD:AAA4458
Lascoria ambigualis|[13090]|BLTIB164-08|Canada|Ontario|658[0n]|BOLD:AAA4458
Lascoria ambigualis|[13091]|BLTIB181-08|Canada|Ontario|658[0n]|BOLD:AAA4458
Lascoria ambigualis|[13092]|BLTIB182-08|Canada|Ontario|658[0n]|BOLD:AAA4458
Lascoria ambigualis|[13093]|BLTIB183-08|Canada|Ontario|658[0n]|BOLD:AAA4458
Lascoria ambigualis|[13094]|BLTIB185-08|Canada|Ontario|658[0n]|BOLD:AAA4458
Lascoria ambigualis|[13095]|BLTIB187-08|Canada|Ontario|658[0n]|BOLD:AAA4458
Lascoria ambigualis|[13096]|LPOKA292-08|United States|Oklahoma|658[0n]|BOLD:AAA4458
Lascoria ambigualis|[13097]|LPOKA435-09|United States|Oklahoma|658[0n]|BOLD:AAA4458
Lascoria ambigualis|[13098]|BLTIB230-08|Canada|Ontario|658[0n]|BOLD:AAA4458
Lascoria ambigualis|[13099]|BLTIB257-08|Canada|Ontario|658[0n]|BOLD:AAA4458
Lascoria ambigualis|[13100]|BLTIB949-08|Canada|Ontario|658[0n]|BOLD:AAA4458
Lascoria ambigualis|[13101]|LPOKA267-08|United States|Oklahoma|658[0n]|BOLD:AAA4458
Lascoria ambigualis|[13102]|LPOKA591-09|United States|Oklahoma|658[0n]|BOLD:AAA4458
Lascoria ambigualis|[13103]|LPOKA923-09|United States|Oklahoma|658[0n]|BOLD:AAA4458
Lascoria ambigualis|[13104]|LMEM306-09|United States|Mississippi|658[0n]|BOLD:AAA4458
Lascoria ambigualis|[13105]|LMEM307-09|United States|Mississippi|658[0n]|BOLD:AAA4458
Lascoria ambigualis|[13106]|LPOKC344-09|United States|Oklahoma|658[0n]|BOLD:AAA4458
Lascoria ambigualis|[13107]|BBLSX083-09|United States|Oklahoma|658[0n]|BOLD:AAA4458
Lascoria ambigualis|[13108]|BBLSX092-09|United States|Oklahoma|658[0n]|BOLD:AAA4458
Lascoria ambigualis|[13109]|BBLSX523-09|United States|Oklahoma|658[0n]|BOLD:AAA4458
Lascoria ambigualis|[13110]|LMEM308-09|United States|Georgia|658[0n]|BOLD:AAA4458
Lascoria ambigualis|[13111]|LPOKB583-09|United States|Oklahoma|658[0n]|BOLD:AAA4458
Lascoria ambigualis|[13112]|LILLA772-11|United States|Illinois|658[0n]|BOLD:AAA4458
Lascoria ambigualis|[13113]|LPSO299-08|Canada|Ontario|658[0n]|BOLD:AAA4458
Lascoria ambigualis|[13114]|LPSO298-08|Canada|Ontario|658[0n]|BOLD:AAA4458
Lascoria ambigualis|[13115]|LPSO110-08|Canada|Ontario|658[0n]|BOLD:AAA4458
Lascoria ambigualis|[13116]|LPSO109-08|Canada|Ontario|658[0n]|BOLD:AAA4458
Lascoria ambigualis|[13117]|LPSO098-08|Canada|Ontario|658[0n]|BOLD:AAA4458
Lascoria ambigualis|[13118]|LPSO082-08|Canada|Ontario|656[0n]|BOLD:AAA4458
Lascoria ambigualis|[13119]|LPSO061-08|Canada|Ontario|658[0n]|BOLD:AAA4458
Lascoria ambigualis|[13120]|XAJ358-06|Canada|Ontario|658[0n]|BOLD:AAA4458
Lascoria ambigualis|[13121]|XAJ333-06|Canada|Ontario|658[0n]|BOLD:AAA4458
Lascoria ambigualis|[13122]|LOCT113-05|United States|Connecticut|658[0n]|BOLD:AAA4458
Lascoria ambigualis|[13123]|LOCT112-05|United States|Connecticut|658[0n]|BOLD:AAA4458
Lascoria ambigualis|[13124]|XAE245-04|Canada|Ontario|658[0n]|BOLD:AAA4458
Lascoria ambigualis|[13125]|LGSM763-04|United States|Tennessee|658[0n]|BOLD:AAA4458
Lascoria ambigualis|[13126]|LGSMC925-05|United States|Tennessee|658[0n]|BOLD:AAA4458
Lascoria ambigualis|[13127]|BLGSM062-09|Canada|Ontario|658[0n]|BOLD:AAA4458
Lascoria ambigualis|[13128]|LPOKD279-09|United States|Oklahoma|642[0n]|BOLD:AAA4458
Lascoria ambigualis|[13129]|TMG92-03|Canada|Ontario|639[0n]|BOLD:AAA4458
Lascoria ambigualis|[13130]|BLTIB078-08|Canada|Ontario|577[1n]|BOLD:AAA4458
Lascoria ambigualis|[13131]|ABCNA591-07|United States|Florida|577[0n]|BOLD:AAA4458
Lascoria ambigualis|[13132]|PMG125-03|Canada|Ontario|617[0n]|BOLD:AAA4458
Lascoria ambigualis|[13133]|BLTIB118-08|Canada|Ontario|609[0n]|BOLD:AAA4458
Lascoria ambigualis|[13134]|BLTIB204-08|Canada|Ontario|609[0n]|BOLD:AAA4458
Lascoria ambigualis|[13135]|BLTIB206-08|Canada|Ontario|614[0n]|BOLD:AAA4458
Lascoria ambigualis|[13136]|BLTIB122-08|Canada|Ontario|541[2n]|
Lascoria ambigualis|[13137]|LMEM305-09|United States|Mississippi|636[0n]|BOLD:AAA4458
Lascoria ambigualis|[13138]|CNPPB1407-12|Canada|Ontario|601[0n]|BOLD:AAA4458
Lascoria ambigualis|[13139]|RDLQF391-06|Canada|Quebec|658[0n]|BOLD:AAA4458
Lascoria ambigualis|[13140]|RDLQG575-06|Canada|Quebec|643[0n]|BOLD:AAA4458
Lascoria ambigualis|[13141]|RDNMD527-06|United States|Florida|658[0n]|BOLD:AAA4458
Lascoria ambigualis|[13142]|RDNMD528-06|United States|Florida|658[0n]|BOLD:AAA4458
Lascoria ambigualis|[13143]|BBLOB1473-11|United States|Florida|658[0n]|BOLD:AAA4458
Lascoria ambigualis|[13144]|CNCLB1802-14|United States|Florida|658[0n]|BOLD:AAA4458
Lascoria alucitalis|[13145]|LOFLB200-06|United States|Florida|656[0n]|BOLD:AAB2758
Lascoria alucitalis|[13146]|RDNMD525-06|United States|Florida|658[0n]|BOLD:AAB2758
Lascoria alucitalis|[13147]|CNCLB1800-14|United States|Florida|658[0n]|BOLD:AAB2758
Lascoria alucitalis|[13148]|CNCLB1801-14|United States|Florida|658[0n]|BOLD:AAB2758
Lascoria alucitalis|[13149]|CNCLB1803-14|United States|Florida|658[0n]|BOLD:AAB2758
Aristaria theroalis|[13150]|HKONS174-08|United States|Florida|658[1n]|BOLD:AAC6354
Aristaria theroalis|[13151]|HKONS175-08|United States|Florida|658[0n]|BOLD:AAC6354
Rejectaria albisinuata|[13152]|ABNCC102-07|United States|Arizona|648[0n]|BOLD:AAD4853
Rejectaria albisinuata|[13153]|ABNCC103-07|United States|Arizona|640[0n]|BOLD:AAD4853
Rejectaria albisinuata|[13154]|LMEM319-09|United States|New Mexico|658[0n]|BOLD:AAD4853
Rejectaria albisinuata|[13155]|LMEM320-09|United States|New Mexico|658[0n]|BOLD:AAD4853
Rejectaria albisinuata|[13156]|LMEM321-09|United States|New Mexico|658[0n]|BOLD:AAD4853
Rejectaria albisinuata|[13157]|LMEM322-09|United States|New Mexico|658[0n]|BOLD:AAD4853
Rejectaria albisinuata|[13158]|LMEM323-09|United States|New Mexico|658[0n]|BOLD:AAD4853
Physula albipunctilla|[13159]|HKONS128-08|United States|Florida|658[0n]|BOLD:AAH9380
Physula albipunctilla|[13160]|RDNMD531-06|United States|Florida|658[1n]|BOLD:AAH9380
Physula albipunctilla|[13161]|CNCLB3015-14|United States|Florida|658[0n]|BOLD:AAH9380
Bleptina sp.|[13162]|RDNMF971-08|United States|Texas|658[0n]|BOLD:AAI4654
Bleptina sp.|[13163]|RDNMF972-08|United States|Texas|658[0n]|BOLD:AAI4655
Bleptina sp. 5|[13164]|QUNOD187-10|United States|Texas|621[0n]|BOLD:AAI4655
Euclystis insana|[13165]|MXBLP160-11|Mexico|Jalisco|658[0n]|BOLD:AAD5580
Lygephila victoria|[13166]|LOWCB613-05|Canada|British Columbia|511[0n]|BOLD:AAB4508
Lygephila victoria|[13167]|LOWCB623-05|Canada|British Columbia|506[0n]|BOLD:AAB4508
Lygephila victoria|[13168]|LBCD323-05|Canada|British Columbia|658[0n]|BOLD:AAB4508
Lygephila victoria|[13169]|LBCD318-05|Canada|British Columbia|658[0n]|BOLD:AAB4508
Lygephila victoria|[13170]|LOWCD452-06|Canada|British Columbia|598[0n]|BOLD:AAB4508
Lygephila victoria|[13171]|LOWCB620-05|Canada|British Columbia|610[1n]|BOLD:AAB4508
Lygephila victoria|[13172]|LOWCB619-05|Canada|British Columbia|589[2n]|BOLD:AAB4508
Lygephila victoria|[13173]|LOWCB615-05|Canada|British Columbia|608[2n]|BOLD:AAB4508
Lygephila victoria|[13174]|LOWCB622-05|Canada|British Columbia|608[0n]|BOLD:AAB4508
Lygephila victoria|[13175]|LOWCB614-05|Canada|British Columbia|608[1n]|BOLD:AAB4508
Lygephila victoria|[13176]|LOWCB616-05|Canada|British Columbia|605[1n]|BOLD:AAB4508
Lygephila victoria|[13177]|LOWCB618-05|Canada|British Columbia|604[0n]|BOLD:AAB4508
Lygephila victoria|[13178]|LOWCB617-05|Canada|British Columbia|533[0n]|BOLD:AAB4508
Lygephila victoria|[13179]|LOWCB612-05|Canada|British Columbia|532[1n]|BOLD:AAB4508
Lygephila victoria|[13180]|LOWCB621-05|Canada|British Columbia|510[1n]|BOLD:AAB4508

Lygephila victoria|[13178]|LOWCB617-05|Canada|British Columbia|533[0n]|BOLD:AAB4508
Lygephila victoria|[13179]|LOWCB612-05|Canada|British Columbia|532[1n]|BOLD:AAB4508
Lygephila victoria|[13180]|LOWCB621-05|Canada|British Columbia|510[1n]|BOLD:AAB4508
Lygephila victoria|[13181]|LOWCD451-06|Canada|British Columbia|605[0n]|BOLD:AAB4508
Lygephila victoria|[13182]|LOWCD453-06|Canada|British Columbia|603[0n]|BOLD:AAB4508
Lygephila victoria|[13183]|LOWCD454-06|Canada|British Columbia|608[0n]|BOLD:AAB4508
Lygephila victoria|[13184]|LOWCD455-06|Canada|British Columbia|658[0n]|BOLD:AAB4508
Lygephila victoria|[13185]|LOWCD456-06|Canada|British Columbia|658[0n]|BOLD:AAB4508
Lygephila victoria|[13186]|LOWCD457-06|Canada|British Columbia|658[0n]|BOLD:AAB4508
Lygephila victoria|[13187]|LBCH6622-10|Canada|British Columbia|658[0n]|BOLD:AAB4508
Lygephila victoria|[13188]|BBLPB315-10|Canada|British Columbia|658[0n]|BOLD:AAB4508
Lygephila victoria|[13189]|ABNCC254-07|United States|Arizona|608[1n]|BOLD:AAB4509
Lygephila victoria|[13190]|RDNML370-13|United States|New Mexico|658[0n]|BOLD:AAB4509
Parahypenodes quadralis|[13191]|RDLQF663-06|Canada|Quebec|637[0n]|BOLD:AAC8389
Parahypenodes quadralis|[13192]|RDLQF662-06|Canada|Quebec|637[0n]|BOLD:AAC8389
Parahypenodes quadralis|[13193]|RDLQG119-06|Canada|Quebec|632[6n]|BOLD:AAC8389
Parahypenodes quadralis|[13194]|RDLQG120-06|Canada|Quebec|621[2n]|BOLD:AAC8389
Parahypenodes quadralis|[13195]|HKONS621-08|United States|Florida|658[0n]|BOLD:AAC8389
Parahypenodes quadralis|[13196]|HKONS622-08|United States|Florida|658[0n]|BOLD:AAC8389
Parahypenodes quadralis|[13197]|RDLQG118-06|Canada|Quebec|625[0n]|BOLD:AAC8390
Parahypenodes quadralis|[13198]|RDLQG121-06|Canada|Quebec|614[0n]|BOLD:AAC8390
Parahypenodes quadralis|[13199]|BBLEC117-09|Canada|Nova Scotia|658[0n]|BOLD:AAC8390
Hypocala andremona|[13200]|LGSMG564-07|United States|North Carolina|658[0n]|BOLD:AAA6609
Hypocala andremona|[13201]|QUNO479-08|United States|Louisiana|658[0n]|BOLD:AAA6609
Hypocala andremona|[13202]|IAWL149-09|United States|Arizona|658[0n]|BOLD:AAA6609
Hypocala andremona|[13203]|LMEMB196-09|United States|Texas|658[0n]|BOLD:AAA6609
Goniapteryx servia|[13204]|ABNCC249-07|United States|Texas|639[0n]|BOLD:AAB1756
Goniapteryx servia|[13205]|ABNCC250-07|United States|Texas|656[0n]|BOLD:AAB1756
Goniapteryx servia|[13206]|BBLSX051-09|United States|Texas|658[0n]|BOLD:AAB1756
Hypsoropha monilis|[13207]|LGSMC369-05|United States|Tennessee|616[0n]|BOLD:AAB8628
Hypsoropha monilis|[13208]|LOTB064-05|United States|Tennessee|656[0n]|BOLD:AAB8628
Hypsoropha monilis|[13209]|LNC747-06|United States|North Carolina|658[0n]|BOLD:AAB8628
Hypsoropha monilis|[13210]|LSEU513-06|United States|Georgia|658[0n]|BOLD:AAB8628
Hypsoropha monilis|[13211]|HKONS029-07|United States|Florida|658[0n]|BOLD:AAB8628
Hypsoropha monilis|[13212]|LNC215-05|United States|North Carolina|658[0n]|BOLD:AAB8628
Hypsoropha monilis|[13213]|LMEMB097-09|United States|Mississippi|658[0n]|BOLD:AAB8628
Hypsoropha monilis|[13214]|LMEMB098-09|United States|Alabama|658[0n]|BOLD:AAB8628
Hypsoropha monilis|[13215]|LMEMB100-09|United States|Georgia|658[0n]|BOLD:AAB8628
Hypsoropha monilis|[13216]|LSEU343-06|United States|Georgia|658[0n]|BOLD:AAB8628
Hypsoropha monilis|[13217]|LNC746-06|United States|North Carolina|658[0n]|BOLD:AAB8628
Hypsoropha monilis|[13218]|LSUSA035-06|United States|Kentucky|658[0n]|BOLD:AAB8628
Hypsoropha monilis|[13219]|LMEMB099-09|United States|Mississippi|658[0n]|BOLD:AAB8628
Hypsoropha monilis|[13220]|ABNCC242-07|United States|Texas|644[0n]|BOLD:AAB8628
Hypsoropha monilis|[13221]|ABNCC243-07|United States|Texas|641[0n]|BOLD:AAB8628
Hypsoropha monilis|[13222]|LGSMC907-05|United States|Tennessee|612[0n]|BOLD:AAB8628
Hypsoropha monilis|[13223]|HKONS028-07|United States|Florida|658[1n]|BOLD:AAB8628
Hypsoropha monilis|[13224]|BBLSW478-09|United States|Oklahoma|631[0n]|BOLD:AAB8628
Hypsoropha monilis|[13225]|BBLSW479-09|United States|Oklahoma|658[0n]|BOLD:AAB8628
Hypsoropha monilis|[13226]|BBLSW480-09|United States|Oklahoma|658[0n]|BOLD:AAB8628
Hypsoropha monilis|[13227]|BBLSX072-09|United States|Oklahoma|658[0n]|BOLD:AAB8628
Hypsoropha monilis|[13228]|BBLSX520-09|United States|Oklahoma|658[0n]|BOLD:AAB8628
Hypsoropha monilis|[13229]|BBLSX573-09|United States|Oklahoma|658[0n]|BOLD:AAB8628
Hypsoropha hormos|[13230]|ABNCC244-07|United States|Texas|644[0n]|BOLD:AAB8640
Hypsoropha hormos|[13231]|BBLSW491-09|United States|Oklahoma|632[0n]|BOLD:AAB8640
Hypsoropha hormos|[13232]|LMEMB094-09|United States|Mississippi|658[0n]|BOLD:AAB8640
Hypsoropha hormos|[13233]|ABNCC245-07|United States|Texas|569[0n]|BOLD:AAB8640
Hypsoropha hormos|[13234]|BBLSW557-09|United States|Oklahoma|643[0n]|BOLD:AAB8640
Hypsoropha hormos|[13235]|BBLSW559-09|United States|Oklahoma|631[0n]|BOLD:AAB8640
Hypsoropha hormos|[13236]|LPOKB784-09|United States|Oklahoma|658[0n]|BOLD:AAB8640
Hypsoropha hormos|[13237]|BBLSW564-09|United States|Oklahoma|636[0n]|BOLD:AAB8640
Hypsoropha hormos|[13238]|BBLSY867-09|United States|Oklahoma|658[0n]|BOLD:AAB8640
Hypsoropha hormos|[13239]|LGSMC878-05|United States|Tennessee|616[0n]|BOLD:AAB8640
Hypsoropha hormos|[13240]|LOTB339-05|United States|Tennessee|658[0n]|BOLD:AAB8640
Hypsoropha hormos|[13241]|LMEMB095-09|United States|Mississippi|658[0n]|BOLD:AAB8640
Hypsoropha hormos|[13242]|RDNMD352-06|United States|Florida|658[0n]|BOLD:AAB8640
Hypsoropha hormos|[13243]|LSUSA165-06|United States|Kentucky|658[0n]|BOLD:AAB8640
Hypsoropha hormos|[13244]|LNC922-06|United States|North Carolina|658[0n]|BOLD:AAB8640
Hypsoropha hormos|[13245]|LOFLA606-06|United States|Florida|658[0n]|BOLD:AAB8640
Hypsoropha hormos|[13246]|LGSMG560-07|United States|Tennessee|658[0n]|BOLD:AAB8640
Hypsoropha hormos|[13247]|LOFLA558-06|United States|Florida|658[0n]|BOLD:AAB8640
Hypsoropha hormos|[13248]|LNC216-05|United States|North Carolina|658[0n]|BOLD:AAB8640
Hypsoropha hormos|[13249]|LNCB329-06|United States|North Carolina|658[0n]|BOLD:AAB8640
Hypsoropha hormos|[13250]|LMEMB092-09|United States|Alabama|658[0n]|BOLD:AAB8640
Hypsoropha hormos|[13251]|BBLSY869-09|United States|Oklahoma|658[0n]|BOLD:AAB8640
Hypsoropha hormos|[13252]|LMEMB091-09|United States|Mississippi|658[0n]|BOLD:AAB8640
Hypsoropha hormos|[13253]|LILLA064-11|United States|Illinois|658[0n]|BOLD:AAB8640
Hypsoropha hormos|[13254]|LILLA702-11|United States|Illinois|658[0n]|BOLD:AAB8640
Hypsoropha baja|[13255]|CNCLB426-14|Mexico||679[0n]|BOLD:ACM4634
Hypsoropha baja|[13256]|CNCLB427-14|Mexico|Baja California|682[0n]|BOLD:ACM4634
Aon noctuiformis|[13257]|RDNMH032-09|United States|Texas|658[0n]|BOLD:AAJ1423
Aon noctuiformis|[13258]|QUNOD104-10|United States|Texas|658[0n]|BOLD:AAJ1423
Psammathodoxa cochlidioides|[13259]|CNCLB2422-14|United States|Texas|658[0n]|BOLD:AAK0141
Hyperstrotia pr. villificans|[13260]|LGSMG625-07|United States|Tennessee|658[0n]|BOLD:AAI2234
Hyperstrotia nana|[13261]|LGSMG627-07|United States|Tennessee|657[0n]|BOLD:ACE3004
Hyperstrotia nana|[13262]|LSEU584-06|United States|Georgia|658[0n]|BOLD:ACE3004
Hyperstrotia nana|[13263]|LNCB376-06|United States|North Carolina|658[0n]|BOLD:ACE3004
Hyperstrotia nana|[13264]|LNCB261-06|United States|North Carolina|658[0n]|BOLD:ACE3004
Hyperstrotia nana|[13265]|LSEU374-06|United States|Georgia|658[0n]|BOLD:ACE3004
Hyperstrotia nana|[13266]|LSEU372-06|United States|Georgia|658[0n]|BOLD:ACE3004
Hyperstrotia nana|[13267]|LGSMD243-05|United States|Tennessee|658[0n]|BOLD:ACE3004
Hyperstrotia nana|[13268]|LGSMC644-05|United States|Tennessee|658[0n]|BOLD:ACE3004
Hyperstrotia nana|[13269]|LGSM715-04|United States|Tennessee|658[0n]|BOLD:ACE3004
Hyperstrotia nana|[13270]|LGSM727-04|United States|Tennessee|658[0n]|BOLD:ACE3004
Hyperstrotia nana|[13271]|RDNME373-07|United States|Florida|602[0n]|BOLD:ACE3004
Hyperstrotia nana|[13272]|LPOKB406-09|United States|Oklahoma|658[0n]|BOLD:ACE3004
Hyperstrotia nana|[13273]|LPOKB666-09|United States|Oklahoma|658[0n]|BOLD:ACE3004
Hyperstrotia nana|[13274]|LPOKB778-09|United States|Oklahoma|658[0n]|BOLD:ACE3004
Hyperstrotia nana|[13275]|LNCB590-09|United States|North Carolina|658[0n]|BOLD:ACE3004
Hyperstrotia nana|[13276]|LPOKC281-09|United States|Oklahoma|658[0n]|BOLD:ACE3004
Hyperstrotia nana|[13277]|LPOKC430-09|United States|Oklahoma|658[0n]|BOLD:ACE3004
Hyperstrotia nana|[13278]|LILLA274-11|United States|Illinois|658[0n]|BOLD:ACE3004
Hyperstrotia nana|[13279]|LILLA691-11|United States|Illinois|658[0n]|BOLD:ACE3004
Hyperstrotia nana|[13280]|LNCC678-11|United States|North Carolina|658[0n]|BOLD:ACE3004

Hyperstrotia nana|[13278]|LILLA274-11|United States|Illinois|658[0n]|BOLD:ACE3004
Hyperstrotia nana|[13279]|LILLA691-11|United States|Illinois|658[0n]|BOLD:ACE3004
Hyperstrotia nana|[13280]|LNCC678-11|United States|North Carolina|658[0n]|BOLD:ACE3004
Hyperstrotia secta|[13281]|LSEU373-06|United States|Georgia|658[0n]|BOLD:ACF1580
Hyperstrotia secta|[13282]|LSEU371-06|United States|Georgia|658[0n]|BOLD:ACF1580
Hyperstrotia secta|[13283]|LGSM736-04|United States|Tennessee|658[0n]|BOLD:ACF1580
Hyperstrotia secta|[13284]|LSUSA233-06|United States|Kentucky|658[0n]|BOLD:ACF1580
Hyperstrotia secta|[13285]|LSEU601-06|United States|Georgia|658[0n]|BOLD:ACF1580
Hyperstrotia secta|[13286]|LNCB484-07|United States|North Carolina|658[0n]|BOLD:ACF1580
Hyperstrotia secta|[13287]|LNCB485-07|United States|North Carolina|658[0n]|BOLD:ACF1580
Hyperstrotia secta|[13288]|LGSMG628-07|United States|Tennessee|657[0n]|BOLD:ACF1580
Hyperstrotia secta|[13289]|LMEMB430-09|United States|Mississippi|658[0n]|BOLD:ACF1580
Hyperstrotia secta|[13290]|LMEMB435-09|United States|Alabama|658[0n]|BOLD:ACF1580
Hyperstrotia secta|[13291]|LMEMB436-09|United States|Alabama|658[0n]|BOLD:ACF1580
Hyperstrotia nana|[13292]|LGSM714-04|United States|Tennessee|658[0n]|BOLD:ACE6422
Hyperstrotia nana|[13293]|LGSMC460-05|United States|Tennessee|658[0n]|BOLD:ACE6422
Hyperstrotia nana|[13294]|LGSMC645-05|United States|Tennessee|658[0n]|BOLD:ACE6422
Hyperstrotia nana|[13295]|LGSMC646-05|United States|Tennessee|658[0n]|BOLD:ACE6422
Hyperstrotia nana|[13296]|LOTB139-05|United States|Tennessee|658[0n]|BOLD:ACE6422
Hyperstrotia nana|[13297]|LOTB220-05|United States|Tennessee|658[0n]|BOLD:ACE6422
Hyperstrotia nana|[13298]|LOTB284-05|United States|Tennessee|658[0n]|BOLD:ACE6422
Hyperstrotia nana|[13299]|LGSMG626-07|United States|Tennessee|658[0n]|BOLD:ACE6422
Hyperstrotia nana|[13300]|MNAF486-08|Canada|Manitoba|658[0n]|BOLD:ACE6422
Hyperstrotia nana|[13301]|HKONS223-08|United States|Florida|658[1n]|BOLD:ACE6422
Hyperstrotia nana|[13302]|LOT363-04|United States|Tennessee|658[0n]|BOLD:ACE6422
Hyperstrotia nana|[13303]|LGSMC459-05|United States|Tennessee|658[0n]|BOLD:ACE6422
Hyperstrotia nana|[13304]|LILLA657-11|United States|Illinois|658[0n]|BOLD:ACE6422
Hyperstrotia nana|[13305]|RDLQC460-06|Canada|Quebec|658[0n]|BOLD:ACE6422
Hyperstrotia nana|[13306]|RDLQG882-06|Canada|Quebec|658[0n]|BOLD:ACE6422
Hyperstrotia nana|[13307]|RDNMK653-11|Canada|Ontario|658[0n]|BOLD:ACE6422
Hyperstrotia pervertens|[13308]|TTMNB297-06|Canada|New Brunswick|602[0n]|BOLD:ACE3003
Hyperstrotia pervertens|[13309]|RDLQG748-06|Canada|Quebec|658[0n]|BOLD:ACE3003
Hyperstrotia pervertens|[13310]|RDLQH009-06|Canada|Quebec|658[0n]|BOLD:ACE3003
Hyperstrotia pervertens|[13311]|RDLQH008-06|Canada|Quebec|658[0n]|BOLD:ACE3003
Hyperstrotia pervertens|[13312]|RDLQH007-06|Canada|Quebec|658[0n]|BOLD:ACE3003
Hyperstrotia pervertens|[13313]|RDLQH006-06|Canada|Quebec|658[0n]|BOLD:ACE3003
Hyperstrotia pervertens|[13314]|RDLQH005-06|Canada|Quebec|658[0n]|BOLD:ACE3003
Hyperstrotia pervertens|[13315]|RDLQH004-06|Canada|Quebec|658[0n]|BOLD:ACE3003
Hyperstrotia pervertens|[13316]|RDLQH003-06|Canada|Quebec|658[0n]|BOLD:ACE3003
Hyperstrotia pervertens|[13317]|RDLQH001-06|Canada|Quebec|656[0n]|BOLD:ACE3003
Hyperstrotia pervertens|[13318]|RDLQG940-06|Canada|Quebec|658[0n]|BOLD:ACE3003
Hyperstrotia pervertens|[13319]|RDLQG939-06|Canada|Quebec|658[0n]|BOLD:ACE3003
Hyperstrotia pervertens|[13320]|RDLQG927-06|Canada|Quebec|658[0n]|BOLD:ACE3003
Hyperstrotia pervertens|[13321]|RDLQG786-06|Canada|Quebec|658[0n]|BOLD:ACE3003
Hyperstrotia pervertens|[13322]|RDLQG747-06|Canada|Quebec|658[0n]|BOLD:ACE3003
Hyperstrotia pervertens|[13323]|RDLQG713-06|Canada|Quebec|658[0n]|BOLD:ACE3003
Hyperstrotia pervertens|[13324]|RDLQG712-06|Canada|Quebec|658[0n]|BOLD:ACE3003
Hyperstrotia pervertens|[13325]|RDLQG582-06|Canada|Quebec|658[0n]|BOLD:ACE3003
Hyperstrotia pervertens|[13326]|TMNBC528-06|Canada|New Brunswick|658[0n]|BOLD:ACE3003
Hyperstrotia pervertens|[13327]|TMNBC519-06|Canada|New Brunswick|658[0n]|BOLD:ACE3003
Hyperstrotia pervertens|[13328]|XAK079-06|Canada|Ontario|658[0n]|BOLD:ACE3003
Hyperstrotia pervertens|[13329]|RDLQD890-06|Canada|Quebec|658[0n]|BOLD:ACE3003
Hyperstrotia pervertens|[13330]|RDLQD889-06|Canada|Quebec|658[0n]|BOLD:ACE3003
Hyperstrotia pervertens|[13331]|TTMNB296-06|Canada|New Brunswick|617[1n]|BOLD:ACE3003
Hyperstrotia pervertens|[13332]|RDLQH002-06|Canada|Quebec|649[0n]|BOLD:ACE3003
Hyperstrotia pervertens|[13333]|RDLQH010-06|Canada|Quebec|654[0n]|BOLD:ACE3003
Hyperstrotia pervertens|[13334]|RDLQH011-06|Canada|Quebec|658[0n]|BOLD:ACE3003
Hyperstrotia pervertens|[13335]|RDLQH012-06|Canada|Quebec|658[0n]|BOLD:ACE3003
Hyperstrotia pervertens|[13336]|UDLEP292-09|United States|Pennsylvania|658[0n]|BOLD:ACE3003
Hyperstrotia pervertens|[13337]|BBLPC618-09|Canada|Nova Scotia|658[0n]|BOLD:ACE3003
Hyperstrotia pervertens|[13338]|LNCC1073-11|United States|North Carolina|658[0n]|BOLD:ACE3003
Hyperstrotia pervertens|[13339]|LGSMC462-05|United States|Tennessee|658[0n]|BOLD:ACE3003
Hyperstrotia pervertens|[13340]|LNCC1410-11|United States|North Carolina|658[0n]|BOLD:ACE3003
Hyperstrotia flaviguttata|[13341]|LMEMB432-09|United States|Alabama|658[0n]|BOLD:ACE3005
Hyperstrotia flaviguttata|[13342]|LMEMB429-09|United States|Alabama|658[0n]|BOLD:ACE3005
Hyperstrotia flaviguttata|[13343]|LMEMB431-09|United States|Alabama|658[0n]|BOLD:ACE3005
Hyperstrotia flaviguttata|[13344]|LSEU183-06|United States|Georgia|658[0n]|BOLD:ACE3005
Hyperstrotia flaviguttata|[13345]|LNCB260-06|United States|North Carolina|658[0n]|BOLD:ACE3005
Hyperstrotia flaviguttata|[13346]|LMEMB428-09|United States|Alabama|658[0n]|BOLD:ACE3005
Hyperstrotia flaviguttata|[13347]|LPOKB965-09|United States|Oklahoma|658[0n]|BOLD:ACE3005
Hyperstrotia flaviguttata|[13348]|LNCB636-09|United States|North Carolina|658[0n]|BOLD:ACE3005
Hyperstrotia flaviguttata|[13349]|LPOKC494-09|United States|Oklahoma|658[0n]|BOLD:ACE3005
Hyperstrotia flaviguttata|[13350]|MILEP044-09|United States|North Carolina|658[0n]|BOLD:ACE3005
Hyperstrotia flaviguttata|[13351]|LNCC126-10|United States|North Carolina|658[0n]|BOLD:ACE3005
Hyperstrotia flaviguttata|[13352]|LNCC127-10|United States|North Carolina|658[0n]|BOLD:ACE3005
Hyperstrotia flaviguttata|[13353]|LNCC128-10|United States|North Carolina|658[0n]|BOLD:ACE3005
Hyperstrotia n. sp.|[13354]|LOFLB312-06|United States|Florida|620[0n]|BOLD:AAB0296
Hyperstrotia n. sp.|[13355]|MILEP049-09|United States|North Carolina|658[0n]|BOLD:AAB0296
Hyperstrotia n. sp.|[13356]|LSEU182-06|United States|Georgia|658[0n]|BOLD:AAB0296
Hyperstrotia n. sp.|[13357]|LMEMB443-09|United States|Georgia|658[0n]|BOLD:AAB0296
Hyperstrotia n. sp.|[13358]|LMEMB439-09|United States|Georgia|658[0n]|BOLD:AAB0296
Hyperstrotia n. sp.|[13359]|LSEU181-06|United States|Georgia|658[0n]|BOLD:AAB0296
Hyperstrotia n. sp.|[13360]|LSEU180-06|United States|Georgia|658[0n]|BOLD:AAB0296
Hyperstrotia n. sp.|[13361]|LOFLB905-06|United States|Florida|654[0n]|BOLD:AAB0296
Hyperstrotia n. sp.|[13362]|LOFLB329-06|United States|Florida|658[0n]|BOLD:AAB0296
Hyperstrotia n. sp.|[13363]|LOFLB592-06|United States|Florida|658[0n]|BOLD:AAB0296
Hyperstrotia n. sp.|[13364]|LOFLB602-06|United States|Florida|658[0n]|BOLD:AAB0296
Hyperstrotia n. sp.|[13365]|LOFLB613-06|United States|Florida|658[0n]|BOLD:AAB0296
Hyperstrotia n. sp.|[13366]|LOFLB637-06|United States|Florida|658[0n]|BOLD:AAB0296
Hyperstrotia n. sp.|[13367]|LOFLB930-06|United States|Florida|658[0n]|BOLD:AAB0296
Hyperstrotia n. sp.|[13368]|LOFLC064-06|United States|Florida|658[0n]|BOLD:AAB0296
Hyperstrotia n. sp.|[13369]|LOFLD525-07|United States|Florida|658[0n]|BOLD:AAB0296
Hyperstrotia n. sp.|[13370]|CNCLB1321-14|United States|Florida|658[0n]|BOLD:AAB0296
Hyperstrotia aetheria|[13371]|LOFLB081-06|United States|Florida|634[1n]|BOLD:AAB0296
Hyperstrotia aetheria|[13372]|RDNMK055-11|United States|Florida|658[0n]|BOLD:AAB0296
Hyperstrotia aetheria|[13373]|RDNMD529-06|United States|Florida|658[1n]|BOLD:AAB0296
Hyperstrotia aetheria|[13374]|LOFLA462-06|United States|Florida|658[0n]|BOLD:AAB0296
Hyperstrotia aetheria|[13375]|LOFLA499-06|United States|Florida|658[0n]|BOLD:AAB0296
Hyperstrotia aetheria|[13376]|LOFLA655-06|United States|Florida|658[0n]|BOLD:AAB0296
Hyperstrotia aetheria|[13377]|LOFLA773-06|United States|Florida|658[0n]|BOLD:AAB0296
Hyperstrotia aetheria|[13378]|LOFLD295-07|United States|Florida|658[0n]|BOLD:AAB0296
Hyperstrotia aetheria|[13379]|LOFLD331-07|United States|Florida|658[0n]|BOLD:AAB0296
Hyperstrotia aetheria|[13380]|LOFLD419-07|United States|Florida|658[0n]|BOLD:AAB0296

Hyperstrotia aetheria|[13378]|LOFLD295-07|United States|Florida|658[0n]|BOLD:AAB0296
Hyperstrotia aetheria|[13379]|LOFLD331-07|United States|Florida|658[0n]|BOLD:AAB0296
Hyperstrotia aetheria|[13380]|LOFLD419-07|United States|Florida|658[0n]|BOLD:AAB0296
Hyperstrotia aetheria|[13381]|HKONB438-09|United States|Louisiana|658[0n]|BOLD:AAB0296
Hyperstrotia aetheria|[13382]|LMEMB441-09|United States|Alabama|658[0n]|BOLD:AAB0296
Hyperstrotia aetheria|[13383]|LMEMB442-09|United States|Georgia|658[0n]|BOLD:AAB0296
Hyperstrotia aetheria|[13384]|RDNMD463-06|United States|Florida|658[0n]|BOLD:AAB0296
Hyperstrotia aetheria|[13385]|MNAC291-07|United States|Florida|658[0n]|BOLD:AAB0296
Hyperstrotia aetheria|[13386]|LOFLA790-06|United States|Florida|658[0n]|BOLD:AAB0296
Hyperstrotia aetheria|[13387]|LOFLB071-06|United States|Florida|658[0n]|BOLD:AAB0296
Hyperstrotia aetheria|[13388]|LOFLB080-06|United States|Florida|658[0n]|BOLD:AAB0296
Hyperstrotia aetheria|[13389]|LOFLC032-06|United States|Florida|658[0n]|BOLD:AAB0296
Hyperstrotia aetheria|[13390]|LNCB635-09|United States|North Carolina|658[0n]|BOLD:AAB0296
Hyperstrotia aetheria|[13391]|MILEP047-09|United States|North Carolina|658[0n]|BOLD:AAB0296
Hyperstrotia aetheria|[13392]|MILEP048-09|United States|North Carolina|658[0n]|BOLD:AAB0296
Hyperstrotia aetheria|[13393]|MILEP050-09|United States|North Carolina|658[0n]|BOLD:AAB0296
Hyperstrotia aetheria|[13394]|BBLOB1193-11|United States|Florida|658[0n]|BOLD:AAB0296
Hyperstrotia aetheria|[13395]|CNCLB1322-14|United States|Florida|658[0n]|BOLD:AAB0296
Dyspyralis nigellus|[13396]|LILLA441-11|United States|Illinois|658[0n]|BOLD:AAC3111
Dyspyralis nigellus|[13397]|RDLQB681-05|Canada|Quebec|542[0n]|BOLD:AAC3111
Dyspyralis nigellus|[13398]|PMG108-03|Canada|Ontario|617[0n]|BOLD:AAC3111
Dyspyralis nigellus|[13399]|LNCC1880-13|United States|North Carolina|658[0n]|BOLD:AAC3111
Dyspyralis nigellus|[13400]|HKONS348-08|United States|Michigan|658[0n]|BOLD:AAC3111
Dyspyralis nigellus|[13401]|ABCNA931-08|United States|Michigan|658[0n]|BOLD:AAC3111
Dyspyralis nigellus|[13402]|RDLQG590-06|Canada|Quebec|658[0n]|BOLD:AAC3111
Dyspyralis nigellus|[13403]|RDLQG589-06|Canada|Quebec|658[0n]|BOLD:AAC3111
Dyspyralis nigellus|[13404]|RDLQG583-06|Canada|Quebec|658[0n]|BOLD:AAC3111
Dyspyralis nigellus|[13405]|RDLQF786-06|Canada|Quebec|658[0n]|BOLD:AAC3111
Dyspyralis nigellus|[13406]|LGSM655-04|United States|North Carolina|658[0n]|BOLD:AAC3111
Dyspyralis nigellus|[13407]|PHMO265-03|Canada|Ontario|639[4n]|BOLD:AAC3111
Dyspyralis nigellus|[13408]|RDLQF673-06|Canada|Quebec|637[0n]|BOLD:AAC3111
Dyspyralis nigellus|[13409]|RDLQF674-06|Canada|Quebec|637[0n]|BOLD:AAC3111
Dyspyralis nigellus|[13410]|CNKJF023-14|Canada|Nova Scotia|588[0n]|BOLD:AAC3111
Dyspyralis new sp. 3|[13411]|HKONS322-08|United States|Florida|658[0n]|BOLD:AAD7219
Dyspyralis new sp. 3|[13412]|HKONS323-08|United States|Florida|658[0n]|BOLD:AAD7219
Dyspyralis new sp. 3|[13413]|HKONS324-08|United States|Florida|658[0n]|BOLD:AAD7219
Dyspyralis new sp. 1|[13414]|HKONS162-08|United States|Florida|658[0n]|BOLD:AAD7218
Dyspyralis new sp. 1|[13415]|RDNME597-08|United States|Florida|658[0n]|BOLD:AAD7218
Dyspyralis new sp. 2|[13416]|HKONS325-08|United States|Florida|658[0n]|BOLD:AAD7220
Dyspyralis new sp. 2|[13417]|HKONB360-09|United States|Texas|658[0n]|BOLD:AAD7220
Dyspyralis puncticosta|[13418]|RDLQH160-07|Canada|Quebec|632[0n]|BOLD:AAE8238
Dyspyralis puncticosta|[13419]|RDLQF675-06|Canada|Quebec|611[0n]|BOLD:AAE8238
Dyspyralis puncticosta|[13420]|LGSMG532-07|United States|Tennessee|658[0n]|BOLD:AAE8238
Dyspyralis puncticosta|[13421]|LNCC137-10|United States|North Carolina|658[0n]|BOLD:AAE8238
Dyspyralis puncticosta|[13422]|CNGBF1516-14|Canada|Ontario|660[0n]|BOLD:AAE8238
Dyspyralis puncticosta|[13423]|CNGBF1520-14|Canada|Ontario|660[0n]|BOLD:AAE8238
Dyspyralis illocata|[13424]|CNSLP767-13|Canada|Ontario|538[0n]|
Dyspyralis illocata|[13425]|CNLMH718-14|Canada|Quebec|525[0n]|BOLD:AAB6432
Dyspyralis illocata|[13426]|GMGSH281-12|United States|Tennessee|636[0n]|BOLD:AAB6432
Dyspyralis illocata|[13427]|HKONS605-08|United States|Florida|658[0n]|BOLD:AAB6432
Dyspyralis illocata|[13428]|HKONB433-09|United States|Kentucky|658[0n]|BOLD:AAB6432
Dyspyralis illocata|[13429]|GMGSM003-13|United States|Tennessee|619[0n]|BOLD:AAB6432
Dyspyralis illocata|[13430]|CNKJO060-14|Canada|Nova Scotia|564[0n]|BOLD:AAB6432
Dyspyralis illocata|[13431]|LGSM716-04|United States|Tennessee|658[0n]|BOLD:AAB6432
Dyspyralis illocata|[13432]|LGSM732-04|United States|North Carolina|658[0n]|BOLD:AAB6432
Dyspyralis illocata|[13433]|LGSMG530-07|United States|Tennessee|658[0n]|BOLD:AAB6432
Dyspyralis illocata|[13434]|LGSMG531-07|United States|Tennessee|658[0n]|BOLD:AAB6432
Dyspyralis illocata|[13435]|LGSMG528-07|United States|Tennessee|658[0n]|BOLD:AAB6432
Dyspyralis illocata|[13436]|LGSMG529-07|United States|Tennessee|658[0n]|BOLD:AAB6432
Dyspyralis illocata|[13437]|LNCC880-11|United States|North Carolina|658[0n]|BOLD:AAB6432
Dyspyralis illocata|[13438]|GMGSE443-12|United States|Tennessee|633[0n]|BOLD:AAB6432
Dyspyralis illocata|[13439]|GMGSO023-13|United States|Tennessee|621[0n]|BOLD:AAB6432
Dyspyralis illocata|[13440]|CNSLF315-12|Canada|Ontario|616[0n]|BOLD:AAB6432
Dyspyralis illocata|[13441]|RDLQF733-06|Canada|Quebec|658[0n]|BOLD:AAB6432
Dyspyralis illocata|[13442]|RDLQD888-06|Canada|Quebec|658[0n]|BOLD:AAB6432
Dyspyralis illocata|[13443]|CNBPH395-13|Canada|Ontario|630[0n]|BOLD:AAB6432
Dyspyralis illocata|[13444]|CNSLF325-12|Canada|Ontario|634[0n]|BOLD:AAB6432
Dyspyralis illocata|[13445]|CNSLF321-12|Canada|Ontario|634[0n]|BOLD:AAB6432
Dyspyralis illocata|[13446]|CNSLF318-12|Canada|Ontario|634[0n]|BOLD:AAB6432
Dyspyralis illocata|[13447]|CNSLF293-12|Canada|Ontario|634[0n]|BOLD:AAB6432
Dyspyralis illocata|[13448]|CNBPG508-13|Canada|Ontario|621[0n]|BOLD:AAB6432
Dyspyralis illocata|[13449]|CNSLF005-12|Canada|Ontario|632[0n]|BOLD:AAB6432
Dyspyralis illocata|[13450]|CNSLE011-12|Canada|Ontario|631[0n]|BOLD:AAB6432
Dyspyralis illocata|[13451]|CNLMP1971-14|Canada|Quebec|591[0n]|BOLD:AAB6432
Dyspyralis illocata|[13452]|RDLQF669-06|Canada|Quebec|644[0n]|BOLD:AAB6432
Dyspyralis illocata|[13453]|CNLMG467-14|Canada|Quebec|564[0n]|BOLD:AAB6432
Dyspyralis illocata|[13454]|RDLQF666-06|Canada|Quebec|644[0n]|BOLD:AAB6432
Dyspyralis illocata|[13455]|RDLQF664-06|Canada|Quebec|643[1n]|BOLD:AAB6432
Dyspyralis illocata|[13456]|CNSLP745-13|Canada|Ontario|567[0n]|BOLD:AAB6432
Dyspyralis illocata|[13457]|RDLQF672-06|Canada|Quebec|624[0n]|BOLD:AAB6432
Dyspyralis illocata|[13458]|RDLQF671-06|Canada|Quebec|638[0n]|BOLD:AAB6432
Dyspyralis illocata|[13459]|RDLQF670-06|Canada|Quebec|638[0n]|BOLD:AAB6432
Dyspyralis illocata|[13460]|RDLQF668-06|Canada|Quebec|637[0n]|BOLD:AAB6432
Dyspyralis illocata|[13461]|RDLQF667-06|Canada|Quebec|637[0n]|BOLD:AAB6432
Dyspyralis illocata|[13462]|ABNCC538-07|United States||610[0n]|BOLD:AAB6432
Dyspyralis illocata|[13463]|RDLQF665-06|Canada|Quebec|631[0n]|BOLD:AAB6432
Dyspyralis illocata|[13464]|CNBPN593-13|Canada|Ontario|587[0n]|BOLD:AAB6432
Dyspyralis illocata|[13465]|PAJUL1145-12|Canada|Ontario|591[0n]|BOLD:AAB6432
Dyspyralis illocata|[13466]|CNROP366-13|Canada|Ontario|590[0n]|BOLD:AAB6432
Dyspyralis illocata|[13467]|CNLMP1954-14|Canada|Quebec|603[0n]|BOLD:AAB6432
Dyspyralis illocata|[13468]|CNLMP1980-14|Canada|Quebec|588[0n]|BOLD:AAB6432
Abablemma bilineata|[13469]|HKONB071-08|United States|Texas|658[0n]|BOLD:AAX5505
Abablemma grandimacula|[13470]|CNCLB438-14|Guatemala||675[0n]|BOLD:ACE7013
Abablemma brimleyana|[13471]|HKONB363-09|United States|Texas|658[0n]|BOLD:ABX6040
Abablemma brimleyana|[13472]|RDNMD461-06|United States|Florida|658[0n]|BOLD:AAC7336
Abablemma brimleyana|[13473]|LOFLA350-06|United States|Florida|658[0n]|BOLD:AAC7336
Abablemma brimleyana|[13474]|LNC153-05|United States|North Carolina|658[0n]|BOLD:AAC7336
Abablemma brimleyana|[13475]|LGSM558-04|United States|Tennessee|615[0n]|BOLD:AAC7336
Abablemma brimleyana|[13476]|ABNCC119-07|United States|Florida|592[1n]|BOLD:AAC7336
Abablemma brimleyana|[13477]|LNC251-05|United States|North Carolina|554[22n]|
Abablemma brimleyana|[13478]|LMEM368-09|United States|Mississippi|658[0n]|BOLD:AAC7336
Abablemma brimleyana|[13479]|LMEM371-09|United States|Tennessee|658[0n]|BOLD:AAC7336
Abablemma brimleyana|[13480]|LMEM372-09|United States|Mississippi|658[0n]|BOLD:AAC7336

[illegible]
Ablablemma brimleyana|[13478]|LMEM368-09|United States|Mississippi|658[0n]|BOLD:AAC7336
Ablablemma brimleyana|[13479]|LMEM371-09|United States|Tennessee|658[0n]|BOLD:AAC7336
Ablablemma brimleyana|[13480]|LMEM372-09|United States|Mississippi|658[0n]|BOLD:AAC7336
Ablablemma duomaculata|[13481]|CNCLB439-14|United States|Texas|682[0n]|BOLD:AAJ4592
Ablablemma duomaculata|[13482]|CNCLB440-14|United States|Texas|682[0n]|BOLD:AAJ4592
Ablablemma duomaculata|[13483]|CNCLB442-14|United States|Texas|682[0n]|BOLD:AAJ4592
Ablablemma nr. brimleyana|[13484]|HKONB089-08|United States|Texas|658[0n]|BOLD:AAJ4592
Ablablemma duomaculata|[13485]|CNCLB443-14|United States|Texas|682[0n]|BOLD:AAJ4592
Carteris lineata|[13486]|MHMXD735-06|Costa Rica|Alajuela|658[3n]|BOLD:AAB2254
Carteris lineata|[13487]|MHMXD739-06|Costa Rica|Alajuela|658[0n]|BOLD:AAB2254
Carteris lineata|[13488]|BLPBD294-07|Costa Rica|Guanacaste|658[0n]|BOLD:AAB2254
Carteris lineata|[13489]|LYPAP006-09|Mexico|Quintana Roo|658[0n]|BOLD:AAB2254
Carteris lineata|[13490]|LYNYM111-09|Mexico|Yucatan|605[0n]|BOLD:AAB2254
Carteris lineata|[13491]|MHMXD641-06|Costa Rica|Alajuela|658[0n]|BOLD:AAB2254
Carteris lineata|[13492]|BLPBC107-07|Costa Rica|Guanacaste|631[0n]|BOLD:AAB2254
Carteris lineata|[13493]|MHMXD738-06|Costa Rica|Alajuela|658[0n]|BOLD:AAB2254
Carteris lineata|[13494]|BLPDC286-09|Costa Rica|Guanacaste|658[0n]|BOLD:AAB2254
Carteris lineata|[13495]|MHMXD685-06|Costa Rica|Alajuela|658[0n]|BOLD:AAB2254
Carteris lineata|[13496]|MHMXD640-06|Costa Rica|Alajuela|658[0n]|BOLD:AAB2254
Carteris lineata|[13497]|MHMXD639-06|Costa Rica|Alajuela|658[0n]|BOLD:AAB2254
Carteris lineata|[13498]|MHMXD638-06|Costa Rica|Alajuela|658[0n]|BOLD:AAB2254
Carteris lineata|[13499]|MHMXD637-06|Costa Rica|Alajuela|658[0n]|BOLD:AAB2254
Carteris lineata|[13500]|BLPAC727-06|Costa Rica|Guanacaste|658[0n]|BOLD:AAB2254
Carteris lineata|[13501]|MHMXA791-06|Costa Rica|Guanacaste|658[0n]|BOLD:AAB2254
Carteris lineata|[13502]|MHMXD642-06|Costa Rica|Alajuela|658[0n]|BOLD:AAB2254
Carteris lineata|[13503]|MHMXD643-06|Costa Rica|Alajuela|658[0n]|BOLD:AAB2254
Carteris lineata|[13504]|MHMXD736-06|Costa Rica|Alajuela|658[0n]|BOLD:AAB2254
Carteris lineata|[13505]|MHMXD737-06|Costa Rica|Alajuela|658[0n]|BOLD:AAB2254
Carteris lineata|[13506]|BLPBA142-07|Costa Rica|Guanacaste|658[0n]|BOLD:AAB2254
Carteris lineata|[13507]|BLPBD295-07|Costa Rica|Guanacaste|658[0n]|BOLD:AAB2254
Carteris lineata|[13508]|BLPCH574-08|Costa Rica|Guanacaste|658[0n]|BOLD:AAB2254
Carteris lineata|[13509]|BLPCG939-08|Costa Rica|Guanacaste|658[0n]|BOLD:AAB2254
Carteris lineata|[13510]|BLPCK221-08|Costa Rica|Alajuela|658[0n]|BOLD:AAB2254
Carteris lineata|[13511]|BLPDG331-09|Costa Rica|Guanacaste|658[0n]|BOLD:AAB2254
Carteris lineata|[13512]|MHMYC2200-09|Costa Rica|Guanacaste|658[0n]|BOLD:AAB2254
Carteris lineata|[13513]|QUNOD007-10|United States|Texas|658[0n]|BOLD:AAB2254
Carteris lineata|[13514]|BLPDZ688-11|Costa Rica|Alajuela|658[0n]|BOLD:AAB2254
Carteris lineata|[13515]|BLPEA320-11|Costa Rica||658[0n]|BOLD:AAB2254
Carteris oculatalis|[13516]|RDNML335-13|United States|Florida|658[0n]|BOLD:AAD5856
Carteris oculatalis|[13517]|RDNML336-13|United States|Florida|658[0n]|BOLD:AAD5856
Prosoparia perfuscaria|[13518]|RDNMD871-07|United States|Arizona|644[1n]|BOLD:AAE9999
Prosoparia perfuscaria|[13519]|RDNMD872-07|United States|Arizona|655[0n]|BOLD:AAE9999
Prosoparia perfuscaria|[13520]|IAWL037-09|United States|Arizona|551[2n]|BOLD:AAE9999
Prosoparia perfuscaria|[13521]|CMAZA491-10|United States|Arizona|658[0n]|BOLD:AAE9999
Prosoparia perfuscaria|[13522]|CMAZA492-10|United States|Arizona|658[0n]|BOLD:AAE9999
Prosoparia perfuscaria|[13523]|RDNMK356-11|United States|New Mexico|658[0n]|BOLD:AAE9999
Prosoparia perfuscaria|[13524]|QUNOC378-10|United States|Texas|658[0n]|BOLD:AAE9999
Prosoparia perfuscaria|[13525]|RDNMK357-11|United States|New Mexico|658[0n]|BOLD:AAE9999
Prosoparia perfuscaria|[13526]|RDNMK355-11|United States|New Mexico|658[0n]|BOLD:AAE9999
Prosoparia perfuscaria|[13527]|RDNMK354-11|United States|New Mexico|658[0n]|BOLD:AAE9999
Prosoparia perfuscaria|[13528]|RDNMK358-11|United States|New Mexico|658[0n]|BOLD:AAE9999
Prosoparia perfuscaria|[13529]|RDNMK359-11|United States|New Mexico|658[0n]|BOLD:AAE9999
Prosoparia anormalis|[13530]|IAWL589-09|United States|Arizona|639[0n]|BOLD:AAH4820
Prosoparia anormalis|[13531]|RDNMD870-07|United States|Arizona|655[0n]|BOLD:AAH4820
Prosoparia anormalis|[13532]|IAWL588-09|United States|Arizona|658[0n]|BOLD:AAH4820
Prosoparia anormalis|[13533]|BBLOE1194-12|United States|Arizona|658[0n]|BOLD:AAH4820
Prosoparia floridana|[13534]|RDNMD517-06|United States|Florida|658[0n]|BOLD:AAC9733
Prosoparia floridana|[13535]|RDNMD518-06|United States|Florida|658[0n]|BOLD:AAC9733
Prosoparia floridana|[13536]|RDNMD337-06|United States|Florida|658[0n]|BOLD:AAC9733
Prosoparia floridana|[13537]|HKONS305-08|United States|Florida|658[0n]|BOLD:AAC9733
Prosoparia floridana|[13538]|HKONS593-08|United States|Florida|658[0n]|BOLD:AAC9733
Prosoparia floridana|[13539]|RDNME1022-08|United States|Florida|658[0n]|BOLD:AAC9733
Prosoparia floridana|[13540]|LOFLB766-06|United States|Florida|658[0n]|BOLD:AAC9733
Prosoparia floridana|[13541]|LOFLB125-06|United States|Florida|658[0n]|BOLD:AAC9733
Prosoparia floridana|[13542]|LOFLB387-06|United States|Florida|658[0n]|BOLD:AAC9733
Prosoparia floridana|[13543]|LOFLB702-06|United States|Florida|658[0n]|BOLD:AAC9733
Prosoparia floridana|[13544]|LOFLB710-06|United States|Florida|658[0n]|BOLD:AAC9733
Prosoparia floridana|[13545]|BBLOB1602-11|United States|Florida|658[0n]|BOLD:AAC9733
Prosoparia floridana|[13546]|BBLOB1603-11|United States|Florida|658[0n]|BOLD:AAC9733
Prosoparia floridana|[13547]|BBLOB1604-11|United States|Florida|658[0n]|BOLD:AAC9733
Prosoparia floridana|[13548]|BBLOB1606-11|United States|Florida|658[0n]|BOLD:AAC9733
Prosoparia floridana|[13549]|BBLOB1765-11|United States|Florida|658[0n]|BOLD:AAC9733
Prosoparia floridana|[13550]|BBLOB1780-11|United States|Florida|658[0n]|BOLD:AAC9733
Prosoparia floridana|[13551]|BBLOB1782-11|United States|Florida|658[0n]|BOLD:AAC9733
Prosoparia floridana|[13552]|BBLOB1802-11|United States|Florida|658[0n]|BOLD:AAC9733
Prosoparia floridana|[13553]|BBLOB236-11|United States|Florida|658[0n]|BOLD:AAC9733
Prosoparia floridana|[13554]|BBLOC108-11|United States|Florida|658[0n]|BOLD:AAC9733
Prosoparia floridana|[13555]|BBLOC109-11|United States|Florida|658[0n]|BOLD:AAC9733
Prosoparia floridana|[13556]|BBLOE2011-12|United States|Florida|658[0n]|BOLD:AAC9733
Prosoparia floridana|[13557]|BBLOE2013-12|United States|Florida|658[0n]|BOLD:AAC9733
Janseodes melanospila|[13558]|ANIAD1951-11|Australia|Northern Territory|658[0n]|BOLD:AAI2780
Janseodes melanospila|[13559]|ANIAD1952-11|Australia|Northern Territory|658[0n]|BOLD:AAI2780
Janseodes melanospila|[13560]|BLPDG601-09|Costa Rica|Guanacaste|633[0n]|BOLD:AAI2780
Janseodes melanospila|[13561]|BLPED947-11|Costa Rica|Guanacaste|658[0n]|BOLD:AAI2780
Janseodes melanospila|[13562]|RDNML339-13|United States|Florida|658[0n]|BOLD:AAI2780
Janseodes melanospila|[13563]|CNCLB1949-14|United States|Florida|658[0n]|BOLD:AAI2780
Arugisa lutea|[13564]|LOFLB358-06|United States|Florida|658[0n]|BOLD:AAB7185
Arugisa lutea|[13565]|LOFLB560-06|United States|Florida|658[0n]|BOLD:AAB7185
Arugisa lutea|[13566]|LMEMB071-09|United States|Mississippi|658[0n]|BOLD:AAB7185
Arugisa lutea|[13567]|LPOKD292-09|United States|Oklahoma|657[0n]|BOLD:AAB7185
Arugisa lutea|[13568]|LSEU578-06|United States|Georgia|658[0n]|BOLD:AAB7185
Arugisa lutea|[13569]|LOFLC473-06|United States|Florida|658[0n]|BOLD:AAB7185
Arugisa lutea|[13570]|LOFLB366-06|United States|Florida|658[0n]|BOLD:AAB7185
Arugisa lutea|[13571]|LNC283-05|United States|North Carolina|658[0n]|BOLD:AAB7185
Arugisa lutea|[13572]|LNC282-05|United States|North Carolina|658[0n]|BOLD:AAB7185
Arugisa lutea|[13573]|ABNCC229-07|United States|Texas|641[0n]|BOLD:AAB7185
Arugisa lutea|[13574]|ABNCC230-07|United States|Texas|595[0n]|BOLD:AAB7185
Arugisa lutea|[13575]|LGSMG557-07|United States|Tennessee|658[0n]|BOLD:AAB7185
Arugisa lutea|[13576]|LPOKA261-08|United States|Oklahoma|658[0n]|BOLD:AAB7185
Arugisa lutea|[13577]|LPOKA270-08|United States|Oklahoma|658[0n]|BOLD:AAB7185
Arugisa lutea|[13578]|LPOKA271-08|United States|Oklahoma|658[0n]|BOLD:AAB7185
Arugisa lutea|[13579]|LPOKA310-08|United States|Oklahoma|658[0n]|BOLD:AAB7185
Arugisa lutea|[13580]|LMEMB068-09|United States|Mississippi|658[0n]|BOLD:AAB7185

Arugisa lutea|[13577]|LPOKA270-08|United States|Oklahoma|658[0n]|BOLD:AAB7185
Arugisa lutea|[13578]|LPOKA271-08|United States|Oklahoma|658[0n]|BOLD:AAB7185
Arugisa lutea|[13579]|LPOKA310-08|United States|Oklahoma|658[0n]|BOLD:AAB7185
Arugisa lutea|[13580]|LMEMB068-09|United States|Mississippi|658[0n]|BOLD:AAB7185
Arugisa lutea|[13581]|LMEMB069-09|United States|Mississippi|658[0n]|BOLD:AAB7185
Arugisa lutea|[13582]|LMEMB070-09|United States|Mississippi|658[0n]|BOLD:AAB7185
Arugisa lutea|[13583]|LILLA791-11|United States|Illinois|658[0n]|BOLD:AAB7185
Arugisa latiorella|[13584]|ABNCC231-07|United States|Texas|619[0n]|BOLD:ACE5679
Arugisa latiorella|[13585]|ABNCC232-07|United States|Texas|623[1n]|BOLD:ACE5679
Arugisa latiorella|[13586]|LILLA778-11|United States|Illinois|658[0n]|BOLD:ACE5679
Arugisa latiorella|[13587]|RDNMD522-06|United States|Florida|658[0n]|BOLD:AAE0283
Arugisa latiorella|[13588]|LOFLD049-07|United States|Florida|658[0n]|BOLD:AAE0283
Arugisa latiorella|[13589]|RDNME728-08|United States|Florida|609[0n]|BOLD:AAE0283
Arugisa latiorella|[13590]|RDNMK054-11|United States|Florida|658[0n]|BOLD:AAE0283
Arugisa latiorella|[13591]|BBLOB443-11|United States|Florida|658[0n]|BOLD:AAE0283
Phobolosia nr. anfracta3|[13592]|IAWL838-09|United States|California|658[0n]|BOLD:AAH4884
Phobolosia nr. anfracta1|[13593]|RDNMG271-08|United States|Texas|658[0n]|BOLD:AAE0325
Phobolosia nr. anfracta1|[13594]|HKONB545-09|United States|Texas|633[0n]|BOLD:AAE0325
Phobolosia nr. anfracta2|[13595]|LOCBD701-06|United States|California|658[0n]|BOLD:ACE9390
Phobolosia nr. anfracta2|[13596]|LOCBD700-06|United States|California|658[0n]|BOLD:ACE9390
Phobolosia nr. anfracta2|[13597]|LOCBD573-06|United States|California|658[0n]|BOLD:ACE9390
Phobolosia nr. anfracta2|[13598]|LOCBD572-06|United States|California|658[0n]|BOLD:ACE9390
Phobolosia nr. anfracta2|[13599]|LOCBD498-06|United States|California|657[0n]|BOLD:ACE9390
Phobolosia nr. anfracta2|[13600]|LOCBD496-06|United States|California|657[0n]|BOLD:ACE9390
Phobolosia nr. anfracta2|[13601]|LOCBD404-06|United States|California|658[0n]|BOLD:ACE9390
Phobolosia nr. anfracta2|[13602]|LOCBD403-06|United States|California|658[0n]|BOLD:ACE9390
Phobolosia nr. anfracta2|[13603]|LOCBD402-06|United States|California|658[0n]|BOLD:ACE9390
Phobolosia nr. anfracta2|[13604]|LOCBD314-06|United States|California|658[0n]|BOLD:ACE9390
Phobolosia nr. anfracta2|[13605]|LOCBC384-06|United States|California|658[0n]|BOLD:ACE9390
Phobolosia nr. anfracta2|[13606]|LOCBD698-06|United States|California|634[0n]|BOLD:ACE9390
Phobolosia nr. anfracta2|[13607]|LOCBD699-06|United States|California|644[0n]|BOLD:ACE9390
Phobolosia nr. anfracta2|[13608]|LOCBD705-06|United States|California|644[0n]|BOLD:ACE9390
Phobolosia nr. anfracta2|[13609]|RDNME087-07|United States|California|658[1n]|BOLD:AAE0324
Phobolosia nr. anfracta2|[13610]|RDNMD798-07|United States|California|658[0n]|BOLD:AAE0324
Phobolosia nr. anfracta2|[13611]|RDNME088-07|United States|California|658[1n]|BOLD:AAE0324
Phobolosia anfracta|[13612]|LBCH6722-10|Canada|British Columbia|658[0n]|BOLD:AAL6037
Phobolosia anfracta|[13613]|LBCH7700-10|Canada|British Columbia|658[0n]|BOLD:AAL6037
Phobolosia anfracta|[13614]|JMMMB126-11|United States|California|621[0n]|BOLD:AAL6037
Nigetia formosalis|[13615]|LPOKB040-09|United States|Oklahoma|658[0n]|BOLD:AAC1717
Nigetia formosalis|[13616]|LPOKB459-09|United States|Oklahoma|658[0n]|BOLD:AAC1717
Nigetia formosalis|[13617]|BBLSW623-09|United States|Oklahoma|658[0n]|BOLD:AAC1717
Nigetia formosalis|[13618]|BBLSX391-09|United States|Oklahoma|658[0n]|BOLD:AAC1717
Nigetia formosalis|[13619]|LPOKD055-09|United States|Oklahoma|658[0n]|BOLD:AAC1717
Nigetia formosalis|[13620]|HKONS166-08|United States|Florida|658[0n]|BOLD:AAC1716
Nigetia formosalis|[13621]|LNC312-05|United States|North Carolina|658[0n]|BOLD:AAC1716
Nigetia formosalis|[13622]|LMEM373-09|United States|Tennessee|611[0n]|BOLD:AAC1716
Nigetia formosalis|[13623]|LMEM374-09|United States|Tennessee|658[0n]|BOLD:AAC1716
Nigetia formosalis|[13624]|LNCB731-09|United States|North Carolina|658[0n]|BOLD:AAC1716
Nigetia formosalis|[13625]|LNC313-05|United States|North Carolina|658[0n]|BOLD:AAC1716
Nigetia formosalis|[13626]|LNCB732-09|United States|North Carolina|658[1n]|BOLD:AAC1716
Nigetia formosalis|[13627]|ABNCC121-07|United States|Florida|638[0n]|BOLD:AAC1716
Nigetia formosalis|[13628]|ABNCC123-07|United States|Florida|608[1n]|BOLD:AAC1716
Nigetia formosalis|[13629]|LMEM377-09|United States|Alabama|658[0n]|BOLD:AAC1716
Nigetia formosalis|[13630]|LSUSA202-06|United States|Kentucky|658[0n]|BOLD:AAC1716
Nigetia formosalis|[13631]|LSUSA182-06|United States|Kentucky|658[0n]|BOLD:AAC1716
Nigetia formosalis|[13632]|BLTIB874-08|Canada|Ontario|658[0n]|BOLD:AAC1716
Nigetia formosalis|[13633]|LILLA318-11|United States|Illinois|658[0n]|BOLD:AAC1716
Nigetia formosalis|[13634]|LILLA799-11|United States|Illinois|658[0n]|BOLD:AAC1716
Pharga pallens|[13635]|QUNOD157-10|United States|Arizona|658[0n]|BOLD:AAE6225
Pharga pallens|[13636]|RDNME888-08|United States|Arizona|658[0n]|BOLD:AAE6225
Pharga pallens|[13637]|IAWL054-09|United States|Arizona|658[0n]|BOLD:AAE6225
Pharga pallens|[13638]|QUNOD158-10|United States|Arizona|658[0n]|BOLD:AAE6225
Pseudorgyia versuta|[13639]|ABNCC233-07|United States|Texas|648[0n]|BOLD:AAH8241
Pseudorgyia versuta|[13640]|RDNMG259-08|United States|Texas|658[0n]|BOLD:AAH8241
Pseudorgyia russula|[13641]|BBLSW145-09|United States|Arizona|658[0n]|BOLD:AAC5248
Pseudorgyia russula|[13642]|CMAZA008-09|United States|Arizona|658[0n]|BOLD:AAC5248
Pseudorgyia russula|[13643]|CMAZA037-09|United States|Arizona|658[0n]|BOLD:AAC5248
Pseudorgyia russula|[13644]|CMAZA142-09|United States|Arizona|658[0n]|BOLD:AAC5248
Pseudorgyia russula|[13645]|CMAZA353-10|United States|Arizona|658[0n]|BOLD:AAC5248
Pseudorgyia russula|[13646]|IAWL006-09|United States|Arizona|658[0n]|BOLD:AAC5248
Pseudorgyia russula|[13647]|IAWL007-09|United States|Arizona|658[0n]|BOLD:AAC5248
Pseudorgyia russula|[13648]|RDNMK103-11|United States|Arizona|658[0n]|BOLD:AAC5248
Pseudorgyia russula|[13649]|RDNMD800-07|United States|Arizona|658[0n]|BOLD:AAC5248
Pseudorgyia russula|[13650]|IAWL008-09|United States|Arizona|658[1n]|BOLD:AAC5248
Pseudorgyia russula|[13651]|IAWL009-09|United States|Arizona|658[0n]|BOLD:AAC5248
Pseudorgyia russula|[13652]|RDNMJ688-11|United States|Arizona|658[0n]|BOLD:AAC5248
Pseudorgyia russula|[13653]|LMEMB075-09|United States|Arizona|658[0n]|BOLD:AAC5248
Pseudorgyia russula|[13654]|LMEMB074-09|United States|Arizona|658[0n]|BOLD:AAC5248
Pseudorgyia russula|[13655]|HKONB499-09|United States|Arizona|645[0n]|BOLD:AAC5248
Pseudorgyia russula|[13656]|LMEMB073-09|United States|Arizona|622[0n]|BOLD:AAC5248
Pseudorgyia russula|[13657]|LMEMB076-09|United States|Arizona|630[0n]|BOLD:AAC5248
Pseudorgyia russula|[13658]|LMEMB077-09|United States|Arizona|658[0n]|BOLD:AAC5248
Pseudorgyia russula|[13659]|IAWLB584-11|United States|Arizona|571[0n]|BOLD:AAC5248
Pseudorgyia russula|[13660]|BBLOB1580-11|United States|Arizona|658[0n]|BOLD:AAC5248
Palpidia pallidior|[13661]|BBLOB494-11|United States|Florida|658[0n]|BOLD:AAX2010
Palpidia pallidior|[13662]|BBLOB638-11|United States|Florida|658[0n]|BOLD:AAX2010
Palpidia pallidior|[13663]|BBLOB475-11|United States|Florida|658[0n]|BOLD:AAX2010
Palpidia pallidior|[13664]|BBLOB491-11|United States|Florida|658[0n]|BOLD:AAX2010
Palpidia pallidior|[13665]|BBLOE1718-12|United States|Florida|658[0n]|BOLD:AAX2010
Palpidia pallidior|[13666]|LOFLD052-07|United States|Florida|658[0n]|BOLD:AAX2010
Palpidia pallidior|[13667]|BBLOB1545-11|United States|Florida|658[0n]|BOLD:AAX2010
Palpidia pallidior|[13668]|NOCNA081-14|United States|Florida|658[0n]|BOLD:AAX2010
Rhosologia porrecta|[13669]|BLPDB608-09|Costa Rica|Alajuela|633[0n]|BOLD:ABZ1202
Rhosologia porrecta|[13670]|BLPCM133-08|Costa Rica|Guanacaste|658[0n]|BOLD:ABZ1202
Rhosologia porrecta|[13671]|BLPBG499-07|Costa Rica|Guanacaste|658[0n]|BOLD:ABZ1202
Rhosologia porrecta|[13672]|BLPBC169-07|Costa Rica|Guanacaste|658[0n]|BOLD:ABZ1202
Rhosologia porrecta|[13673]|BLPCL342-08|Costa Rica|Guanacaste|658[0n]|BOLD:ABZ1202
Rhosologia porrecta|[13674]|BLPDT1641-10|Costa Rica|Guanacaste|658[0n]|BOLD:ABZ1202
Rhosologia porrecta|[13675]|BLPDG061-09|Costa Rica|Guanacaste|658[0n]|BOLD:ABZ1202
Rhosologia porrecta|[13676]|MHAUC712-06|Costa Rica|Guanacaste|585[1n]|BOLD:ABZ1202
Rhosologia porrecta|[13677]|BLPED1152-12|Costa Rica|Guanacaste|658[0n]|BOLD:ABZ1202
Rhosologia porrecta|[13678]|BLPED1153-12|Costa Rica|Guanacaste|658[0n]|BOLD:ABZ1202
Gabara stygialis|[13679]|CMAZA540-10|United States|Arizona|658[0n]|BOLD:AAF5186
[illegible]

Rhosologia porrecta|[13677]|BLPED1152-12|Costa Rica|Guanacaste|658[0n]|BOLD:ABZ1202
Rhosologia porrecta|[13678]|BLPED1153-12|Costa Rica|Guanacaste|658[0n]|BOLD:ABZ1202
Gabara stygialis|[13679]|CMAZA540-10|United States|Arizona|658[0n]|BOLD:AAF5186
Gabara stygialis|[13680]|CMAZA551-10|United States|Arizona|658[0n]|BOLD:AAF5186
Gabara stygialis|[13681]|HKONB501-09|United States|Arizona|589[0n]|BOLD:AAF5186
Gabara stygialis|[13682]|IAWLB287-11|United States|Arizona|658[0n]|BOLD:AAF5186
Gabara stygialis|[13683]|NAMUM070-08|United States|Arizona|656[0n]|BOLD:AAF5186
Gabara stygialis|[13684]|RDNMJ299-11|United States|Arizona|658[0n]|BOLD:AAF5186
Gabara stygialis|[13685]|RDNMJ725-11|United States|Arizona|658[0n]|BOLD:AAF5186
Gabara stygialis|[13686]|RDNME696-08|United States|Arizona|658[0n]|BOLD:AAF5186
Gabara stygialis|[13687]|IAWLB286-11|United States|Arizona|658[0n]|BOLD:AAF5186
Gabara stygialis|[13688]|IAWLB289-11|United States|Arizona|658[0n]|BOLD:AAF5186
Scolecocampa atriluna|[13689]|ABNCC237-07|United States|Arizona|635[0n]|BOLD:AAI0775
Scolecocampa atriluna|[13690]|ABNCC236-07|United States|Arizona|648[0n]|BOLD:AAI0775
Scolecocampa atriluna|[13691]|CMAZA818-10|United States|Arizona|658[0n]|BOLD:AAI0775
Scolecocampa atriluna|[13692]|RDNMJ511-11|United States|Arizona|658[0n]|BOLD:AAI0775
Gabara obscura|[13693]|RDNME861-08|United States|Arizona|658[0n]|BOLD:AAE6265
Gabara obscura|[13694]|HKONB502-09|United States|Arizona|658[0n]|BOLD:AAE6265
Gabara obscura|[13695]|IAWL028-09|United States|Arizona|658[0n]|BOLD:AAE6265
Gabara obscura|[13696]|LMEMB084-09|United States|Arizona|658[0n]|BOLD:AAE6265
Gabara obscura|[13697]|LMEMB083-09|United States|Arizona|658[0n]|BOLD:AAE6265
Gabara obscura|[13698]|CMAZA521-10|United States|Arizona|641[0n]|BOLD:AAE6265
Gabara obscura|[13699]|CMAZA541-10|United States|Arizona|658[0n]|BOLD:AAE6265
Gabara obscura|[13700]|IAWLB288-11|United States|Arizona|658[0n]|BOLD:AAE6265
Scolecocampa liburna|[13701]|LPOKA193-08|United States|Oklahoma|658[0n]|BOLD:AAC1567
Scolecocampa liburna|[13702]|LPOKB374-09|United States|Oklahoma|658[0n]|BOLD:AAC1567
Scolecocampa liburna|[13703]|LOFLA546-06|United States|Florida|658[0n]|BOLD:AAC1567
Scolecocampa liburna|[13704]|BBLOB504-11|United States|Florida|658[0n]|BOLD:AAC1567
Scolecocampa liburna|[13705]|LSUSA226-06|United States|Kentucky|606[0n]|BOLD:AAC1567
Scolecocampa liburna|[13706]|LOFLA534-06|United States|Florida|658[0n]|BOLD:AAC1567
Scolecocampa liburna|[13707]|LOFLC117-06|United States|Florida|652[0n]|BOLD:AAC1567
Scolecocampa liburna|[13708]|LGSMG559-07|United States|North Carolina|658[0n]|BOLD:AAC1567
Scolecocampa liburna|[13709]|LTOLB762-11|United States|Maryland|658[0n]|BOLD:AAC1567
Scolecocampa liburna|[13710]|LILLA674-11|United States|Illinois|658[0n]|BOLD:AAC1567
Scolecocampa liburna|[13711]|LOT187-04|United States|Tennessee|609[0n]|BOLD:AAC1567
Scolecocampa liburna|[13712]|RDNMD787-07|United States|North Carolina|658[0n]|BOLD:AAC1567
Scolecocampa liburna|[13713]|LGSM415-04|United States|Tennessee|658[0n]|BOLD:AAC1567
Scolecocampa liburna|[13714]|LNCC1402-11|United States|North Carolina|658[0n]|BOLD:AAC1567
Scolecocampa liburna|[13715]|LPOKD243-09|United States|Oklahoma|657[0n]|BOLD:AAC1567
Scolecocampa liburna|[13716]|MILEQ257-11|United States|Alabama|631[0n]|BOLD:AAC1567
Scolecocampa liburna|[13717]|LNC244-05|United States|North Carolina|602[0n]|BOLD:AAC1567
Scolecocampa liburna|[13718]|LNCC1253-11|United States|North Carolina|658[0n]|BOLD:AAC1567
Scolecocampa liburna|[13719]|LNCC1254-11|United States|North Carolina|658[0n]|BOLD:AAC1567
Scolecocampa liburna|[13720]|LNCC1403-11|United States|North Carolina|658[0n]|BOLD:AAC1567
Scolecocampa liburna|[13721]|MILEQ256-11|United States|Alabama|658[0n]|BOLD:AAC1567
Scolecocampa liburna|[13722]|LGSM414-04|United States|North Carolina|658[0n]|BOLD:AAC1567
Scolecocampa liburna|[13723]|LNC109-05|United States|North Carolina|658[0n]|BOLD:AAC1567
Scolecocampa liburna|[13724]|MILEQ258-11|United States|Alabama|658[0n]|BOLD:AAC1567
Scolecocampa liburna|[13725]|ABNCC235-07|United States|Texas|633[0n]|BOLD:AAC1567
Scolecocampa liburna|[13726]|BBLSX106-09|United States|Oklahoma|658[0n]|BOLD:AAC1567
Scolecocampa liburna|[13727]|BBLSX107-09|United States|Oklahoma|658[0n]|BOLD:AAC1567
Scolecocampa liburna|[13728]|BBLOB655-11|United States|Florida|658[0n]|BOLD:AAC1567
Scolecocampa liburna|[13729]|MILEQ259-11|United States|Alabama|658[0n]|BOLD:AAC1567
Gabara gigantea|[13730]|CNCLB436-14|Mexico|Coahuila|674[1n]|BOLD:ACM4511
Gabara subnivosella|[13731]|LOFLA871-06|United States|Florida|658[0n]|BOLD:AAB8349
Gabara sp.|[13732]|QUNOD389-10|United States|Florida|658[0n]|BOLD:ACG7387
Gabara subnivosella|[13733]|ABNCC239-07|United States|Oklahoma|635[0n]|BOLD:ACE7401
Gabara sp. 4|[13734]|QUNOD842-11|United States|Oklahoma|658[0n]|BOLD:ACE7401
Gabara subnivosella|[13735]|LNCB412-06|United States|North Carolina|658[0n]|BOLD:ABX6126
Gabara sp.|[13736]|LNCC1335-11|United States|North Carolina|658[0n]|BOLD:ABX6126
Gabara pulverosalis|[13737]|RDNME668-08|United States|Florida|658[0n]|BOLD:ACF5316
Gabara distema|[13738]|LNCC806-11|United States|North Carolina|658[0n]|BOLD:ACF5316
Gabara distema|[13739]|LNCC793-11|United States|North Carolina|658[0n]|BOLD:ACF5316
Gabara pulverosalis|[13740]|LNCC885-11|United States|North Carolina|658[0n]|BOLD:ACF5316
Gabara pulverosalis|[13741]|LNCC886-11|United States|North Carolina|658[0n]|BOLD:ACF5316
Gabara pulverosalis|[13742]|LNCC887-11|United States|North Carolina|658[0n]|BOLD:ACF5316
Gabara pulverosalis|[13743]|LNCB416-06|United States|North Carolina|658[0n]|BOLD:ACF5316
Gabara pulverosalis|[13744]|LNCB404-06|United States|North Carolina|658[0n]|BOLD:ACF5316
Gabara pulverosalis|[13745]|LNCB276-06|United States|North Carolina|658[0n]|BOLD:ACF5316
Gabara pulverosalis|[13746]|LNC203-05|United States|North Carolina|658[0n]|BOLD:ACF5316
Gabara pulverosalis|[13747]|LNC202-05|United States|North Carolina|658[0n]|BOLD:ACF5316
Gabara pulverosalis|[13748]|LNCB417-06|United States|North Carolina|658[0n]|BOLD:ACF5316
Gabara pulverosalis|[13749]|LNCB418-06|United States|North Carolina|658[0n]|BOLD:ACF5316
Gabara pulverosalis|[13750]|LNCB419-06|United States|North Carolina|658[0n]|BOLD:ACF5316
Gabara pulverosalis|[13751]|LNCB420-06|United States|North Carolina|658[0n]|BOLD:ACF5316
Gabara pulverosalis|[13752]|LNCB421-06|United States|North Carolina|658[0n]|BOLD:ACF5316
Gabara distema|[13753]|LNCC790-11|United States|North Carolina|658[0n]|BOLD:ACF5316
Gabara distema|[13754]|LNCC791-11|United States|North Carolina|658[0n]|BOLD:ACF5316
Gabara distema|[13755]|LNCC792-11|United States|North Carolina|658[0n]|BOLD:ACF5316
Gabara pulverosalis|[13756]|LNCC888-11|United States|North Carolina|658[0n]|BOLD:ACF5316
Gabara pulverosalis|[13757]|MILEP074-09|United States|North Carolina|658[0n]|BOLD:ACF5316
Gabara pulverosalis|[13758]|MILEP075-09|United States|North Carolina|623[0n]|BOLD:ACF5316
Gabara pulverosalis|[13759]|HKONB288-09|United States|Louisiana|658[0n]|BOLD:ACF5316
Gabara pulverosalis|[13760]|LNCB277-06|United States|North Carolina|658[0n]|BOLD:ACF5316
Gabara sp.|[13761]|LNCB657-09|United States|North Carolina|658[0n]|BOLD:ACF5316
Gabara pulverosalis|[13762]|HKONS559-08|United States|Florida|658[0n]|BOLD:ACF5316
Gabara pulverosalis|[13763]|MILEP076-09|United States|North Carolina|658[0n]|BOLD:ACF5316
Gabara pulverosalis|[13764]|MILEP077-09|United States|North Carolina|658[0n]|BOLD:ACF5316
Gabara pulverosalis|[13765]|LNCC067-10|United States|North Carolina|658[0n]|BOLD:ACF5316
Gabara pulverosalis|[13766]|BBLOB282-11|United States|Florida|658[0n]|BOLD:ACF5316
Gabara pulverosalis|[13767]|BBLOB830-11|United States|Florida|658[0n]|BOLD:ACF5316
Gabara pulverosalis|[13768]|BBLOB1315-11|United States|Florida|658[0n]|BOLD:ACF5316
Gabara pulverosalis|[13769]|BBLOB1614-11|United States|Florida|658[0n]|BOLD:ACF5316
Gabara pulverosalis|[13770]|BBLOE1789-12|United States|Florida|629[0n]|BOLD:ACF5316
Gabara subnivosella|[13771]|LNCC823-11|United States|North Carolina|658[0n]|BOLD:AAB6666
Gabara sp.|[13772]|LNCC014-10|United States|North Carolina|658[0n]|BOLD:AAB6666
Gabara sp.|[13773]|LNCC013-10|United States|North Carolina|658[0n]|BOLD:AAB6666
Gabara distema|[13774]|LNCB426-06|United States|North Carolina|658[0n]|BOLD:AAB6666
Gabara subnivosella|[13775]|LNCB415-06|United States|North Carolina|658[0n]|BOLD:AAB6666
Gabara subnivosella|[13776]|LNCB414-06|United States|North Carolina|658[0n]|BOLD:AAB6666
Gabara subnivosella|[13777]|LNCB411-06|United States|North Carolina|658[0n]|BOLD:AAB6666
Gabara subnivosella|[13778]|LNCB409-06|United States|North Carolina|658[0n]|BOLD:AAB6666
Gabara subnivosella|[13779]|LNCB405-06|United States|North Carolina|658[0n]|BOLD:AAB6666

Gabara subnivosella|[13777]|LNCB411-06|United States|North Carolina|658[0n]|BOLD:AAB6666
Gabara subnivosella|[13778]|LNCB409-06|United States|North Carolina|658[0n]|BOLD:AAB6666
Gabara subnivosella|[13779]|LNCB405-06|United States|North Carolina|658[0n]|BOLD:AAB6666
Gabara subnivosella|[13780]|LNCB403-06|United States|North Carolina|658[0n]|BOLD:AAB6666
Gabara subnivosella|[13781]|LNCB402-06|United States|North Carolina|658[0n]|BOLD:AAB6666
Gabara distema|[13782]|LNCB245-06|United States|North Carolina|658[0n]|BOLD:AAB6666
Gabara sp.|[13783]|LNCC1332-11|United States|North Carolina|658[0n]|BOLD:AAB6666
Gabara distema|[13784]|LNCC789-11|United States|North Carolina|658[0n]|BOLD:AAB6666
Gabara distema|[13785]|LNCC797-11|United States|North Carolina|658[0n]|BOLD:AAB6666
Gabara distema|[13786]|LNCC798-11|United States|North Carolina|658[0n]|BOLD:AAB6666
Gabara distema|[13787]|LNCC800-11|United States|North Carolina|658[0n]|BOLD:AAB6666
Gabara distema|[13788]|LNCC802-11|United States|North Carolina|658[0n]|BOLD:AAB6666
Gabara subnivosella|[13789]|LNC367-05|United States|North Carolina|556[0n]|BOLD:AAB6666
Gabara subnivosella|[13790]|LNC368-05|United States|North Carolina|565[2n]|BOLD:AAB6666
Gabara distema|[13791]|LNCC065-10|United States|North Carolina|658[0n]|BOLD:AAB6666
Gabara distema|[13792]|LNCC066-10|United States|North Carolina|658[0n]|BOLD:AAB6666
Gabara distema|[13793]|LNCC085-10|United States|North Carolina|658[0n]|BOLD:AAB6666
Gabara subnivosella|[13794]|LNCB413-06|United States|North Carolina|658[0n]|BOLD:AAB6666
Gabara distema|[13795]|LNCB246-06|United States|North Carolina|658[0n]|BOLD:AAB6666
Gabara subnivosella|[13796]|LNCB406-06|United States|North Carolina|658[0n]|BOLD:AAB6666
Gabara sp.|[13797]|LNCC746-11|United States|North Carolina|658[0n]|BOLD:AAB6666
Gabara sp.|[13798]|LNCC1336-11|United States|North Carolina|658[0n]|BOLD:AAB6666
Gabara subnivosella|[13799]|LNCB407-06|United States|North Carolina|658[0n]|BOLD:AAB6666
Gabara subnivosella|[13800]|LNCB408-06|United States|North Carolina|658[0n]|BOLD:AAB6666
Gabara subnivosella|[13801]|LNCB410-06|United States|North Carolina|658[0n]|BOLD:AAB6666
Gabara subnivosella|[13802]|LNCC822-11|United States|North Carolina|658[0n]|BOLD:AAB6666
Gabara sp.|[13803]|LNCC1331-11|United States|North Carolina|658[0n]|BOLD:AAB6666
Gabara sp.|[13804]|LNCC1333-11|United States|North Carolina|658[0n]|BOLD:AAB6666
Gabara sp.|[13805]|LNCC1334-11|United States|North Carolina|658[0n]|BOLD:AAB6666
Gabara distema|[13806]|LNCB425-06|United States|North Carolina|649[0n]|BOLD:AAB6666
Gabara distema|[13807]|LNC370-05|United States|North Carolina|558[0n]|BOLD:AAB6666
Gabara distema|[13808]|LNC371-05|United States|North Carolina|562[0n]|BOLD:AAB6666
Gabara distema|[13809]|LNCB422-06|United States|North Carolina|658[0n]|BOLD:AAB6666
Gabara distema|[13810]|LNCB423-06|United States|North Carolina|658[0n]|BOLD:AAB6666
Gabara distema|[13811]|LNCB424-06|United States|North Carolina|658[0n]|BOLD:AAB6666
Gabara distema|[13812]|LNCB428-06|United States|North Carolina|658[0n]|BOLD:AAB6666
Gabara distema|[13813]|LNCB429-06|United States|North Carolina|658[0n]|BOLD:AAB6666
Gabara distema|[13814]|LNCB430-06|United States|North Carolina|658[0n]|BOLD:AAB6666
Gabara distema|[13815]|LNCB434-06|United States|North Carolina|658[0n]|BOLD:AAB6666
Gabara distema|[13816]|LNCC786-11|United States|North Carolina|658[0n]|BOLD:AAB6666
Gabara distema|[13817]|LNCC787-11|United States|North Carolina|658[0n]|BOLD:AAB6666
Gabara distema|[13818]|LNCC788-11|United States|North Carolina|658[0n]|BOLD:AAB6666
Gabara distema|[13819]|LNCC794-11|United States|North Carolina|658[0n]|BOLD:AAB6666
Gabara distema|[13820]|LNCC795-11|United States|North Carolina|658[0n]|BOLD:AAB6666
Gabara distema|[13821]|LNCC796-11|United States|North Carolina|655[0n]|BOLD:AAB6666
Gabara distema|[13822]|LNCC799-11|United States|North Carolina|658[0n]|BOLD:AAB6666
Gabara distema|[13823]|LNCC801-11|United States|North Carolina|658[0n]|BOLD:AAB6666
Gabara distema|[13824]|LNCC803-11|United States|North Carolina|658[0n]|BOLD:AAB6666
Gabara distema|[13825]|LNCC804-11|United States|North Carolina|658[0n]|BOLD:AAB6666
Gabara distema|[13826]|LNCC805-11|United States|North Carolina|658[0n]|BOLD:AAB6666
Gabara distema|[13827]|LNCC890-11|United States|North Carolina|658[0n]|BOLD:AAB6666
Gabara distema|[13828]|LNCC891-11|United States|North Carolina|658[0n]|BOLD:AAB6666
Gabara distema|[13829]|LNCC1315-11|United States|North Carolina|658[0n]|BOLD:AAB6666
Gabara distema|[13830]|CNCLB2913-14|United States|North Carolina|658[0n]|BOLD:AAB6666
Gabara distema|[13831]|LNCB427-06|United States|North Carolina|656[0n]|BOLD:AAB6666
Gabara distema|[13832]|LNCB431-06|United States|North Carolina|658[0n]|BOLD:AAB6666
Gabara distema|[13833]|LNCB432-06|United States|North Carolina|657[0n]|BOLD:AAB6666
Gabara sp.|[13834]|LNCB853-09|United States|Georgia|658[0n]|BOLD:AAB6666
Gabara sp.|[13835]|LNCB854-09|United States|Georgia|658[0n]|BOLD:AAB6666
Gabara sp.|[13836]|LNCB855-09|United States|Georgia|658[0n]|BOLD:AAB6666
Gabara sp.|[13837]|LNCB856-09|United States|Georgia|658[0n]|BOLD:AAB6666
Gabara sp.|[13838]|MILEQ197-11|United States|Georgia|658[0n]|BOLD:AAB6666
Gabara distema|[13839]|HKONS415-08|United States|Florida|658[0n]|BOLD:AAB6666
Gabara distema|[13840]|LNCB433-06|United States|North Carolina|658[0n]|BOLD:AAB6666
Gabara distema|[13841]|LNCC889-11|United States|North Carolina|658[0n]|BOLD:AAB6666
Gabara distema|[13842]|HKONS414-08|United States|Florida|658[0n]|BOLD:AAB6666
Gabara distema|[13843]|CNCLB2915-14|United States|North Carolina|658[0n]|BOLD:AAB6666
Gabara distema|[13844]|LNCC844-11|United States|North Carolina|658[0n]|BOLD:AAB6666
Gabara distema|[13845]|LNCC845-11|United States|North Carolina|658[0n]|BOLD:AAB6666
Gabara distema|[13846]|LNCC937-11|United States|North Carolina|658[0n]|BOLD:AAB6666
Gabara distema|[13847]|LNCC1221-11|United States|North Carolina|658[0n]|BOLD:AAB6666
Gabara distema|[13848]|LNCC1222-11|United States|North Carolina|658[0n]|BOLD:AAB6666
Gabara distema|[13849]|LNCC1313-11|United States|North Carolina|658[0n]|BOLD:AAB6666
Gabara distema|[13850]|LNCC1314-11|United States|North Carolina|658[0n]|BOLD:AAB6666
Gabara distema|[13851]|LNCC1337-11|United States|North Carolina|658[0n]|BOLD:AAB6666
Gabara distema|[13852]|RDNML158-13|United States|North Carolina|658[0n]|BOLD:AAB6666
Gabara distema|[13853]|CNCLB2914-14|United States|North Carolina|658[0n]|BOLD:AAB6666
Gabara distema|[13854]|CNCLB2922-14|United States|North Carolina|658[0n]|BOLD:AAB6666
Phytometra orgiae|[13855]|BBLSW965-09|United States|Texas|658[0n]|BOLD:AAE0480
Phytometra orgiae|[13856]|HKONB368-09|United States|Texas|658[0n]|BOLD:AAE0480
Phytometra orgiae|[13857]|ABNCC201-07|United States|Texas|548[0n]|BOLD:AAE0480
Phytometra orgiae|[13858]|ABNCC200-07|United States|Texas|635[0n]|BOLD:AAE0480
Phytometra orgiae|[13859]|IAWL030-09|United States|Texas|614[0n]|BOLD:AAE0480
Phytometra orgiae|[13860]|LMEMB036-09|United States|Texas|658[0n]|BOLD:AAE0480
Phytometra orgiae|[13861]|BBLSW042-09|United States|Texas|658[0n]|BOLD:AAE0480
Phytometra orgiae|[13862]|BBLSW043-09|United States|Texas|658[0n]|BOLD:AAE0480
Phytometra orgiae|[13863]|BBLSW045-09|United States|Texas|658[0n]|BOLD:AAE0480
Phytometra orgiae|[13864]|BBLSW137-09|United States|Texas|658[0n]|BOLD:AAE0480
Phytometra orgiae|[13865]|BBLSW138-09|United States|Texas|658[0n]|BOLD:AAE0480
Phytometra orgiae|[13866]|BBLSY180-09|United States|Texas|658[0n]|BOLD:AAE0480
Phytometra orgiae|[13867]|BBLSY181-09|United States|Texas|658[0n]|BOLD:AAE0480
Phytometra orgiae|[13868]|BBLSX186-09|United States|Texas|658[0n]|BOLD:AAE0480
Phytometra orgiae|[13869]|BBLSX221-09|United States|Texas|658[0n]|BOLD:AAE0480
Phytometra orgiae|[13870]|BBLSX264-09|United States|Texas|658[0n]|BOLD:AAE0480
Phytometra orgiae|[13871]|BBLSY284-09|United States|Texas|658[0n]|BOLD:AAE0480
Phytometra orgiae|[13872]|BBLSZ160-09|United States|Texas|658[0n]|BOLD:AAE0480
Phytometra apicata|[13873]|QUNOE457-12|United States|Arizona|658[0n]|BOLD:ABV5431
Phytometra obliqualis|[13874]|CMAZA1148-12|United States|Arizona|658[0n]|BOLD:AAH5364
Phytometra obliqualis|[13875]|IAWLB377-11|United States|Arizona|629[0n]|BOLD:AAH5364
Phytometra obliqualis|[13876]|BBLSX786-09|United States|Texas|658[0n]|BOLD:AAH5364
Phytometra obliqualis|[13877]|QUNOE452-12|United States|Texas|658[0n]|BOLD:AAH5364
Phytometra obliqualis|[13878]|IAWLB376-11|United States|Arizona|658[0n]|BOLD:AAH5364
Phytometra obliqualis|[13879]|CMAZA1188-12|United States|Arizona|658[0n]|BOLD:AAH5364

Phytometra obliqualis|[13877]|QUNOE452-12|United States|Texas|658[0n]|BOLD:AAH5364
Phytometra obliqualis|[13878]|IAWLB376-11|United States|Arizona|658[0n]|BOLD:AAH5364
Phytometra obliqualis|[13879]|CMAZA1188-12|United States|Arizona|658[0n]|BOLD:AAH5364
Phytometra ernestinana|[13880]|RDNME723-08|United States|Florida|658[0n]|BOLD:AAD6965
Phytometra ernestinana|[13881]|LPOKD343-09|United States|Oklahoma|658[0n]|BOLD:AAD6965
Phytometra ernestinana|[13882]|LPOKB245-09|United States|Oklahoma|658[0n]|BOLD:AAD6965
Phytometra ernestinana|[13883]|LMEM111-09|United States|Texas|658[0n]|BOLD:AAD6965
Phytometra ernestinana|[13884]|HKONS594-08|United States|Florida|658[0n]|BOLD:AAD6965
Phytometra ernestinana|[13885]|ABNCC191-07|United States|Mississippi|583[0n]|BOLD:AAD6965
Phytometra ernestinana|[13886]|LMEM110-09|United States|Texas|633[0n]|BOLD:AAD6965
Phytometra ernestinana|[13887]|BBLOB648-11|United States|Florida|658[0n]|BOLD:AAD6965
Phytometra rhodarialis sp. 2|[13888]|ABNCC192-07|United States|Texas|635[0n]|BOLD:AAC3524
Phytometra rhodarialis sp. 2|[13889]|ABNCC193-07|United States|Missouri|606[0n]|BOLD:AAC3524
Phytometra rhodarialis sp. 2|[13890]|HKONS585-08|United States|Florida|656[0n]|BOLD:AAC3524
Phytometra rhodarialis sp. 2|[13891]|HKONB366-09|United States|Texas|658[0n]|BOLD:AAC3524
Phytometra rhodarialis sp. 2|[13892]|HKONB367-09|United States|Louisiana|658[0n]|BOLD:AAC3524
Phytometra rhodarialis sp. 2|[13893]|LMEM112-09|United States|Alabama|658[0n]|BOLD:AAC3524
Phytometra rhodarialis sp. 2|[13894]|LMEM113-09|United States|Alabama|658[0n]|BOLD:AAC3524
Phytometra rhodarialis sp. 2|[13895]|LMEM114-09|United States|Alabama|658[0n]|BOLD:AAC3524
Phytometra rhodarialis sp. 2|[13896]|LMEM115-09|United States|Alabama|658[0n]|BOLD:AAC3524
Phytometra rhodarialis|[13897]|LNCB847-09|United States|Alabama|658[0n]|BOLD:AAC0499
Phytometra rhodarialis|[13898]|HKONS589-08|United States|Florida|658[0n]|BOLD:AAC0499
Phytometra rhodarialis|[13899]|LSEU178-06|United States|Georgia|658[0n]|BOLD:AAC0499
Phytometra rhodarialis|[13900]|HKONS586-08|United States|Florida|630[0n]|BOLD:AAC0499
Phytometra rhodarialis|[13901]|HKONS587-08|United States|Florida|609[0n]|BOLD:AAC0499
Phytometra rhodarialis|[13902]|HKONS590-08|United States|Florida|618[0n]|BOLD:AAC0499
Phytometra rhodarialis|[13903]|HKONS592-08|United States|Florida|658[0n]|BOLD:AAC0499
Phytometra rhodarialis|[13904]|LMEM116-09|United States|Mississippi|658[0n]|BOLD:AAC0499
Phytometra rhodarialis|[13905]|LMEM117-09|United States|Mississippi|658[0n]|BOLD:AAC0499
Phytometra rhodarialis|[13906]|BBLOB1890-11|United States|Florida|658[0n]|BOLD:AAC0499
Phytometra rhodarialis|[13907]|LOFLB864-06|United States|Florida|674[0n]|BOLD:AAC0499
Phytometra rhodarialis|[13908]|HKONS588-08|United States|Florida|658[2n]|BOLD:AAC0499
Phytometra rhodarialis|[13909]|HKONS591-08|United States|Florida|658[0n]|BOLD:AAC0499
Phytometra rhodarialis|[13910]|RDNME722-08|United States|Florida|658[0n]|BOLD:AAC0499
Phytometra rhodarialis|[13911]|HKONB374-09|United States|Texas|658[0n]|BOLD:AAC0499
Phytometra rhodarialis|[13912]|LNCB845-09|United States|Alabama|658[0n]|BOLD:AAC0499
Phytometra rhodarialis|[13913]|BBLOC036-11|United States|Florida|658[0n]|BOLD:AAC0499
Phytometra rhodarialis|[13914]|LNAUT2624-14|United States|Massachusetts|658[0n]|BOLD:AAC0499
Phytometra rhodarialis|[13915]|LNCB846-09|United States|Alabama|658[0n]|BOLD:AAC0499
Phytometra rhodarialis|[13916]|LNCB844-09|United States|Alabama|658[0n]|BOLD:AAC0499
Phytometra rhodarialis|[13917]|LMEM119-09|United States|Mississippi|658[0n]|BOLD:AAC0499
Phytometra rhodarialis|[13918]|LMEM118-09|United States|Mississippi|658[0n]|BOLD:AAC0499
Phytometra rhodarialis|[13919]|HKONB373-09|United States|Louisiana|658[0n]|BOLD:AAC0499
Phytometra rhodarialis|[13920]|LNCB374-06|United States|North Carolina|658[0n]|BOLD:AAC0499
Phytometra rhodarialis|[13921]|LNC064-05|United States|North Carolina|658[0n]|BOLD:AAC0499
Phytometra rhodarialis|[13922]|LNC063-05|United States|North Carolina|658[0n]|BOLD:AAC0499
Phytometra rhodarialis|[13923]|LNAUT2625-14|United States|Massachusetts|658[0n]|BOLD:AAC0499
Gonodonta sinaldus|[13924]|HKONB020-08|United States|Texas|658[0n]|BOLD:ABZ0328
Gonodonta sinaldus|[13925]|HKONB021-08|United States|Texas|658[0n]|BOLD:ABZ0328
Gonodonta sinaldus|[13926]|HKONB018-08|United States|Texas|658[0n]|BOLD:ABZ0328
Gonodonta sinaldus|[13927]|HKONB019-08|United States|Texas|658[0n]|BOLD:ABZ0328
Gonodonta sinaldus|[13928]|HKONB022-08|United States|Texas|658[0n]|BOLD:ABZ0328
Gonodonta pyrgo|[13929]|LMEMB115-09|United States|Alabama|658[0n]|BOLD:AAA3286
Gonodonta pyrgo|[13930]|QUNOD165-10|United States|Texas|658[0n]|BOLD:AAA3286
Gonodonta pyrgo|[13931]|QUNOD166-10|United States|Texas|658[0n]|BOLD:AAA3286
Gonodonta sicheas|[13932]|LYPAP613-09|Mexico|Yucatan|610[1n]|BOLD:AAA4300
Gonodonta sicheas|[13933]|MHAUA018-05|Costa Rica|Guanacaste|573[1n]|BOLD:AAA4300
Gonodonta sicheas|[13934]|LYHES247-09|Mexico|Yucatan|636[0n]|BOLD:AAA4300
Gonodonta sicheas|[13935]|LPMX049-07|Mexico|Campeche|636[0n]|BOLD:AAA4300
Gonodonta sicheas|[13936]|MHAUA012-05|Costa Rica|Guanacaste|658[0n]|BOLD:AAA4300
Gonodonta sicheas|[13937]|MHAUA015-05|Costa Rica|Guanacaste|658[0n]|BOLD:AAA4300
Gonodonta sicheas|[13938]|MHAUA017-05|Costa Rica|Alajuela|658[0n]|BOLD:AAA4300
Gonodonta sicheas|[13939]|MHAUC306-06|Costa Rica|Alajuela|658[0n]|BOLD:AAA4300
Gonodonta sicheas|[13940]|BLPAC300-06|Costa Rica|Guanacaste|658[0n]|BOLD:AAA4300
Gonodonta sicheas|[13941]|BLPAF419-07|Costa Rica|Guanacaste|658[0n]|BOLD:AAA4300
Gonodonta sicheas|[13942]|BLPBA266-07|Costa Rica|Guanacaste|658[0n]|BOLD:AAA4300
Gonodonta sicheas|[13943]|MHMXO288-08|Costa Rica|Alajuela|658[0n]|BOLD:AAA4300
Gonodonta sicheas|[13944]|MHMXO450-08|Costa Rica|Guanacaste|658[0n]|BOLD:AAA4300
Gonodonta sicheas|[13945]|BLPCC476-08|Costa Rica|Guanacaste|658[0n]|BOLD:AAA4300
Gonodonta sicheas|[13946]|BLPCD546-08|Costa Rica|Guanacaste|658[0n]|BOLD:AAA4300
Gonodonta sicheas|[13947]|BLPCD548-08|Costa Rica|Guanacaste|658[0n]|BOLD:AAA4300
Gonodonta sicheas|[13948]|BLPCD549-08|Costa Rica|Guanacaste|658[0n]|BOLD:AAA4300
Gonodonta sicheas|[13949]|BLPCE821-08|Costa Rica|Alajuela|658[0n]|BOLD:AAA4300
Gonodonta sicheas|[13950]|BLPCI622-08|Costa Rica|Guanacaste|658[0n]|BOLD:AAA4300
Gonodonta sicheas|[13951]|LPYPA382-08|Mexico|Campeche|658[0n]|BOLD:AAA4300
Gonodonta sicheas|[13952]|BLPCP238-08|Costa Rica|Guanacaste|658[0n]|BOLD:AAA4300
Gonodonta sicheas|[13953]|MHMXZ200-09|Costa Rica||658[0n]|BOLD:AAA4300
Gonodonta sicheas|[13954]|MHMXZ201-09|Costa Rica||658[0n]|BOLD:AAA4300
Gonodonta sicheas|[13955]|LYHES242-09|Mexico|Yucatan|658[0n]|BOLD:AAA4300
Gonodonta sicheas|[13956]|BLPDQ436-10|Costa Rica|Alajuela|658[0n]|BOLD:AAA4300
Gonodonta sicheas|[13957]|BLPDR503-10|Costa Rica|Alajuela|658[0n]|BOLD:AAA4300
Gonodonta sicheas|[13958]|LEMMZ094-10|Brazil|Parana|658[0n]|BOLD:AAA4300
Gonodonta sicheas|[13959]|LOCRE810-10|Costa Rica|Limon|658[0n]|BOLD:AAA4300
Gonodonta sicheas|[13960]|LOCRE811-10|Costa Rica|Limon|658[0n]|BOLD:AAA4300
Gonodonta sicheas|[13961]|BLPDV362-11|Costa Rica|Guanacaste|658[0n]|BOLD:AAA4300
Gonodonta sicheas|[13962]|BLPDV901-11|Costa Rica|Guanacaste|658[0n]|BOLD:AAA4300
Gonodonta sicheas|[13963]|BLPDW237-11|Costa Rica|Guanacaste|658[0n]|BOLD:AAA4300
Gonodonta sicheas|[13964]|BLPCD543-08|Costa Rica|Guanacaste|658[0n]|BOLD:AAA4300
Gonodonta sicheas|[13965]|MHAUC071-05|Costa Rica|Alajuela|658[0n]|BOLD:AAA4300
Gonodonta sicheas|[13966]|BLPCC478-08|Costa Rica|Guanacaste|658[0n]|BOLD:AAA4300
Gonodonta sicheas|[13967]|BLPCJ843-08|Costa Rica|Guanacaste|658[0n]|BOLD:AAA4300
Gonodonta sicheas|[13968]|MHMXZ199-09|Costa Rica||658[0n]|BOLD:AAA4300
Gonodonta sicheas|[13969]|MHMXZ198-09|Costa Rica||658[0n]|BOLD:AAA4300
Gonodonta sicheas|[13970]|MHMXZ197-09|Costa Rica||658[0n]|BOLD:AAA4300
Gonodonta sicheas|[13971]|MHMXZ196-09|Costa Rica||658[0n]|BOLD:AAA4300
Gonodonta sicheas|[13972]|MHMXZ195-09|Costa Rica||658[0n]|BOLD:AAA4300
Gonodonta sicheas|[13973]|MHMXZ194-09|Costa Rica||658[0n]|BOLD:AAA4300
Gonodonta sicheas|[13974]|MHMXZ193-09|Costa Rica||658[0n]|BOLD:AAA4300
Gonodonta sicheas|[13975]|MHMXZ192-09|Costa Rica||658[0n]|BOLD:AAA4300
Gonodonta sicheas|[13976]|LMEMB113-09|Puerto Rico||658[0n]|BOLD:AAA4300
Gonodonta sicheas|[13977]|LMEMB112-09|Puerto Rico||658[0n]|BOLD:AAA4300
Gonodonta sicheas|[13978]|LMEMB111-09|Puerto Rico||658[0n]|BOLD:AAA4300
Gonodonta sicheas|[13979]|LMEMB110-09|Puerto Rico||658[0n]|BOLD:AAA4300

Gonodonta sicheas|[13977]|LMEMB112-09|Puerto Rico||658[0n]|BOLD:AAA4300
Gonodonta sicheas|[13978]|LMEMB111-09|Puerto Rico||658[0n]|BOLD:AAA4300
Gonodonta sicheas|[13979]|LMEMB110-09|Puerto Rico||658[0n]|BOLD:AAA4300
Gonodonta sicheas|[13980]|BLPCF884-08|Costa Rica|Alajuela|658[0n]|BOLD:AAA4300
Gonodonta sicheas|[13981]|BLPCF879-08|Costa Rica|Alajuela|658[0n]|BOLD:AAA4300
Gonodonta sicheas|[13982]|BLPCE869-08|Costa Rica|Alajuela|658[0n]|BOLD:AAA4300
Gonodonta sicheas|[13983]|BLPCD550-08|Costa Rica|Guanacaste|658[0n]|BOLD:AAA4300
Gonodonta sicheas|[13984]|BLPCD547-08|Costa Rica|Guanacaste|658[0n]|BOLD:AAA4300
Gonodonta sicheas|[13985]|BLPCD545-08|Costa Rica|Guanacaste|658[0n]|BOLD:AAA4300
Gonodonta sicheas|[13986]|BLPCD544-08|Costa Rica|Guanacaste|658[0n]|BOLD:AAA4300
Gonodonta sicheas|[13987]|BLPCC477-08|Costa Rica|Guanacaste|658[0n]|BOLD:AAA4300
Gonodonta sicheas|[13988]|BLPCC475-08|Costa Rica|Guanacaste|658[0n]|BOLD:AAA4300
Gonodonta sicheas|[13989]|MHMXO451-08|Costa Rica|Guanacaste|658[0n]|BOLD:AAA4300
Gonodonta sicheas|[13990]|BLPBA667-07|Costa Rica|Guanacaste|658[0n]|BOLD:AAA4300
Gonodonta sicheas|[13991]|BLPAF420-07|Costa Rica|Guanacaste|658[0n]|BOLD:AAA4300
Gonodonta sicheas|[13992]|BLPAC303-06|Costa Rica|Guanacaste|658[0n]|BOLD:AAA4300
Gonodonta sicheas|[13993]|MHAUC308-06|Costa Rica|Alajuela|658[0n]|BOLD:AAA4300
Gonodonta sicheas|[13994]|MHAUC305-06|Costa Rica|Alajuela|658[0n]|BOLD:AAA4300
Gonodonta sicheas|[13995]|MHAUC073-05|Costa Rica|Alajuela|658[0n]|BOLD:AAA4300
Gonodonta sicheas|[13996]|MHAUC072-05|Costa Rica|Guanacaste|658[0n]|BOLD:AAA4300
Gonodonta sicheas|[13997]|MHAUC070-05|Costa Rica|Alajuela|658[0n]|BOLD:AAA4300
Gonodonta sicheas|[13998]|MHAUA020-05|Costa Rica|Alajuela|658[0n]|BOLD:AAA4300
Gonodonta sicheas|[13999]|MHAUA019-05|Costa Rica|Alajuela|658[0n]|BOLD:AAA4300
Gonodonta sicheas|[14000]|MHAUA016-05|Costa Rica|Alajuela|658[0n]|BOLD:AAA4300
Gonodonta sicheas|[14001]|MHAUA014-05|Costa Rica|Guanacaste|658[0n]|BOLD:AAA4300
Gonodonta sicheas|[14002]|MHAUA013-05|Costa Rica|Guanacaste|658[0n]|BOLD:AAA4300
Gonodonta sicheas|[14003]|MHAUA011-05|Costa Rica|Guanacaste|658[0n]|BOLD:AAA4300
Gonodonta sicheas|[14004]|MHAUA010-05|Costa Rica|Guanacaste|658[0n]|BOLD:AAA4300
Gonodonta sicheas|[14005]|MHAUA009-05|Costa Rica|Guanacaste|658[0n]|BOLD:AAA4300
Gonodonta sicheas|[14006]|MHAUA008-05|Costa Rica|Guanacaste|658[0n]|BOLD:AAA4300
Gonodonta sicheas|[14007]|MHAUA007-05|Costa Rica|Guanacaste|658[0n]|BOLD:AAA4300
Gonodonta sicheas|[14008]|MHAUA006-05|Costa Rica|Guanacaste|658[0n]|BOLD:AAA4300
Gonodonta sicheas|[14009]|JMZCA151-09|Ecuador||658[0n]|BOLD:AAA4300
Gonodonta sicheas|[14010]|JMZCA152-09|Ecuador||658[0n]|BOLD:AAA4300
Gonodonta sicheas|[14011]|BLPDA325-09|Costa Rica|Guanacaste|642[0n]|BOLD:AAA4300
Gonodonta sicheas|[14012]|BLPAG388-07|Costa Rica|Guanacaste|621[0n]|BOLD:AAA4300
Gonodonta sicheas|[14013]|BLPDK355-09|Costa Rica|Guanacaste|658[0n]|BOLD:AAA4300
Gonodonta sicheas|[14014]|BLPDK1695-09|Costa Rica|Guanacaste|658[0n]|BOLD:AAA4300
Gonodonta sicheas|[14015]|BLPDL019-10|Costa Rica|Guanacaste|658[0n]|BOLD:AAA4300
Gonodonta sicheas|[14016]|BLPDL020-10|Costa Rica|Guanacaste|658[0n]|BOLD:AAA4300
Gonodonta sicheas|[14017]|BLPDL1167-10|Costa Rica|Alajuela|658[0n]|BOLD:AAA4300
Gonodonta sicheas|[14018]|QUNOD164-10|United States|Texas|658[0n]|BOLD:AAA4300
Gonodonta sicheas|[14019]|LEMMZ012-10|Brazil|Parana|658[0n]|BOLD:AAA4300
Gonodonta sicheas|[14020]|LNOUC210-10|French Guiana||658[0n]|BOLD:AAA4300
Gonodonta sicheas|[14021]|LEMMZ100-10|Brazil|Parana|658[0n]|BOLD:AAA4300
Gonodonta sicheas|[14022]|LOCRE812-10|Costa Rica|Limon|658[0n]|BOLD:AAA4300
Gonodonta sicheas|[14023]|BLPDU539-11|Costa Rica|Guanacaste|658[0n]|BOLD:AAA4300
Gonodonta sicheas|[14024]|BLPDU540-11|Costa Rica|Guanacaste|658[0n]|BOLD:AAA4300
Gonodonta sicheas|[14025]|BLPDV023-11|Costa Rica|Guanacaste|658[0n]|BOLD:AAA4300
Gonodonta sicheas|[14026]|BLPDV024-11|Costa Rica|Guanacaste|658[0n]|BOLD:AAA4300
Gonodonta sicheas|[14027]|MHMYL3496-11|Costa Rica||658[0n]|BOLD:AAA4300
Gonodonta sicheas|[14028]|GWOSX602-11|Venezuela|Carabobo|658[0n]|BOLD:AAA4300
Gonodonta sicheas|[14029]|GWOSX659-11|Guatemala||658[0n]|BOLD:AAA4300
Gonodonta sicheas|[14030]|BLPEF2311-13|Costa Rica|Guanacaste|658[0n]|BOLD:AAA4300
Gonodonta nutrix|[14031]|JMZCA138-09|United States|Florida|658[0n]|BOLD:AAA3287
Gonodonta nutrix|[14032]|JMZCA139-09|United States|Florida|658[0n]|BOLD:AAA3287
Gonodonta bidens|[14033]|LYHES002-09|Mexico|Quintana Roo|544[0n]|BOLD:AAA4308
Gonodonta bidens|[14034]|MHAUA437-05|Costa Rica|Guanacaste|630[0n]|BOLD:AAA4308
Gonodonta bidens|[14035]|MHAUA440-05|Costa Rica|Alajuela|658[0n]|BOLD:AAA4308
Gonodonta bidens|[14036]|MHAUG708-07|Costa Rica|Alajuela|658[0n]|BOLD:AAA4308
Gonodonta bidens|[14037]|MHAUG710-07|Costa Rica|Alajuela|658[0n]|BOLD:AAA4308
Gonodonta bidens|[14038]|MHAUG711-07|Costa Rica|Alajuela|658[0n]|BOLD:AAA4308
Gonodonta bidens|[14039]|MHMYC2147-09|Costa Rica|Alajuela|658[0n]|BOLD:AAA4308
Gonodonta bidens|[14040]|BLPDV020-11|Costa Rica|Guanacaste|658[0n]|BOLD:AAA4308
Gonodonta bidens|[14041]|MHAUA431-05|Costa Rica|Alajuela|658[0n]|BOLD:AAA4308
Gonodonta bidens|[14042]|MHAUA430-05|Costa Rica|Guanacaste|658[0n]|BOLD:AAA4308
Gonodonta bidens|[14043]|MHAUA096-05|Costa Rica|Guanacaste|658[0n]|BOLD:AAA4308
Gonodonta bidens|[14044]|MHAUA432-05|Costa Rica|Alajuela|621[0n]|BOLD:AAA4308
Gonodonta bidens|[14045]|MHAUA433-05|Costa Rica|Guanacaste|658[0n]|BOLD:AAA4308
Gonodonta bidens|[14046]|MHAUA434-05|Costa Rica|Alajuela|658[0n]|BOLD:AAA4308
Gonodonta bidens|[14047]|MHAUA435-05|Costa Rica|Alajuela|658[0n]|BOLD:AAA4308
Gonodonta bidens|[14048]|MHAUA436-05|Costa Rica|Alajuela|658[0n]|BOLD:AAA4308
Gonodonta bidens|[14049]|MHAUA438-05|Costa Rica|Guanacaste|658[0n]|BOLD:AAA4308
Gonodonta bidens|[14050]|MHAUA439-05|Costa Rica|Alajuela|658[0n]|BOLD:AAA4308
Gonodonta bidens|[14051]|MHAUA441-05|Costa Rica|Alajuela|658[0n]|BOLD:AAA4308
Gonodonta bidens|[14052]|MHAUA442-05|Costa Rica|Alajuela|658[0n]|BOLD:AAA4308
Gonodonta bidens|[14053]|MHAUA443-05|Costa Rica|Guanacaste|658[0n]|BOLD:AAA4308
Gonodonta bidens|[14054]|MHAUA444-05|Costa Rica|Alajuela|658[0n]|BOLD:AAA4308
Gonodonta bidens|[14055]|MHAUA445-05|Costa Rica|Guanacaste|658[0n]|BOLD:AAA4308
Gonodonta bidens|[14056]|MHAUA446-05|Costa Rica|Guanacaste|658[0n]|BOLD:AAA4308
Gonodonta bidens|[14057]|MHAUA447-05|Costa Rica|Guanacaste|658[0n]|BOLD:AAA4308
Gonodonta bidens|[14058]|MHAUG709-07|Costa Rica|Alajuela|658[0n]|BOLD:AAA4308
Gonodonta bidens|[14059]|MHMXM136-07|Costa Rica|Guanacaste|658[0n]|BOLD:AAA4308
Gonodonta bidens|[14060]|MHMXM137-07|Costa Rica|Guanacaste|658[0n]|BOLD:AAA4308
Gonodonta bidens|[14061]|MHMXO277-08|Costa Rica|Guanacaste|658[0n]|BOLD:AAA4308
Gonodonta bidens|[14062]|MHMXO279-08|Costa Rica|Guanacaste|658[0n]|BOLD:AAA4308
Gonodonta bidens|[14063]|MHMXO285-08|Costa Rica|Guanacaste|658[0n]|BOLD:AAA4308
Gonodonta bidens|[14064]|BLPCA185-08|Costa Rica|Guanacaste|658[0n]|BOLD:AAA4308
Gonodonta bidens|[14065]|BLPCG782-08|Costa Rica|Guanacaste|658[0n]|BOLD:AAA4308
Gonodonta bidens|[14066]|MHMXZ220-09|Costa Rica||658[0n]|BOLD:AAA4308
Gonodonta bidens|[14067]|MHMXZ221-09|Costa Rica||658[0n]|BOLD:AAA4308
Gonodonta bidens|[14068]|MHMXZ222-09|Costa Rica||658[0n]|BOLD:AAA4308
Gonodonta bidens|[14069]|MHMXZ223-09|Costa Rica||658[0n]|BOLD:AAA4308
Gonodonta bidens|[14070]|MHMYC2148-09|Costa Rica|Guanacaste|658[0n]|BOLD:AAA4308
Gonodonta bidens|[14071]|BLPDK520-09|Costa Rica|Guanacaste|658[0n]|BOLD:AAA4308
Gonodonta bidens|[14072]|MHMYH605-10|Costa Rica||658[0n]|BOLD:AAA4308
Gonodonta bidens|[14073]|MHMYH606-10|Costa Rica||658[0n]|BOLD:AAA4308
Gonodonta bidens|[14074]|MHMYH607-10|Costa Rica||658[0n]|BOLD:AAA4308
Gonodonta bidens|[14075]|MHMYH940-10|Costa Rica||658[0n]|BOLD:AAA4308
Gonodonta bidens|[14076]|LOCRE808-10|Costa Rica|Limon|658[0n]|BOLD:AAA4308
Gonodonta bidens|[14077]|BLPDV019-11|Costa Rica|Guanacaste|658[0n]|BOLD:AAA4308
Gonodonta bidens|[14078]|MHMYO169-11|Costa Rica||658[0n]|BOLD:AAA4308
Gonodonta bidens|[14079]|BLPEF2412-12|Costa Rica|Guanacaste|658[0n]|BOLD:AAA4308

[illegible]
Gonodonta bidens|[14077]|BLPDV019-11|Costa Rica|Guanacaste|658[0n]|BOLD:AAA4308
Gonodonta bidens|[14078]|MHMYO169-11|Costa Rica||658[0n]|BOLD:AAA4308
Gonodonta bidens|[14079]|BLPED2412-12|Costa Rica|Guanacaste|658[0n]|BOLD:AAA4308
Gonodonta fulvangula|[14080]|MHMXZ255-09|Costa Rica||658[0n]|BOLD:AAA4308
Gonodonta fulvangula|[14081]|MHAUA044-05|Costa Rica|Guanacaste|658[0n]|BOLD:AAA4308
Gonodonta fulvangula|[14082]|MHAUG617-07|Costa Rica|Guanacaste|658[0n]|BOLD:AAA4308
Gonodonta fulvangula|[14083]|GWOSS020-11|Cuba|Holguin|658[0n]|BOLD:AAA4308
Gonodonta fulvangula|[14084]|MHAUG647-07|Costa Rica|Guanacaste|594[0n]|BOLD:AAA4308
Gonodonta fulvangula|[14085]|MHAUG642-07|Costa Rica|Guanacaste|593[0n]|BOLD:AAA4308
Gonodonta fulvangula|[14086]|MHAUG630-07|Costa Rica|Guanacaste|593[0n]|BOLD:AAA4308
Gonodonta fulvangula|[14087]|BLPDL1461-10|Costa Rica|Alajuela|631[0n]|BOLD:AAA4308
Gonodonta fulvangula|[14088]|MHMXZ249-09|Costa Rica||658[0n]|BOLD:AAA4308
Gonodonta fulvangula|[14089]|MHMXZ245-09|Costa Rica||658[0n]|BOLD:AAA4308
Gonodonta fulvangula|[14090]|MHMXZ244-09|Costa Rica||658[0n]|BOLD:AAA4308
Gonodonta fulvangula|[14091]|MHMXZ241-09|Costa Rica||658[0n]|BOLD:AAA4308
Gonodonta fulvangula|[14092]|LPYPA454-08|Mexico|Campeche|658[0n]|BOLD:AAA4308
Gonodonta fulvangula|[14093]|MHAUG639-07|Costa Rica|Guanacaste|658[0n]|BOLD:AAA4308
Gonodonta fulvangula|[14094]|MHAUG628-07|Costa Rica|Alajuela|658[0n]|BOLD:AAA4308
Gonodonta fulvangula|[14095]|MHAUG626-07|Costa Rica|Alajuela|658[0n]|BOLD:AAA4308
Gonodonta fulvangula|[14096]|MHAUG616-07|Costa Rica|Alajuela|658[0n]|BOLD:AAA4308
Gonodonta fulvangula|[14097]|MHAUG615-07|Costa Rica|Guanacaste|658[0n]|BOLD:AAA4308
Gonodonta fulvangula|[14098]|MHAUG614-07|Costa Rica|Alajuela|658[0n]|BOLD:AAA4308
Gonodonta fulvangula|[14099]|MHAUG608-07|Costa Rica|Alajuela|658[0n]|BOLD:AAA4308
Gonodonta fulvangula|[14100]|MHAUG607-07|Costa Rica|Guanacaste|658[0n]|BOLD:AAA4308
Gonodonta fulvangula|[14101]|MHAUC282-06|Costa Rica|Alajuela|658[0n]|BOLD:AAA4308
Gonodonta fulvangula|[14102]|MHAUA043-05|Costa Rica|Guanacaste|658[0n]|BOLD:AAA4308
Gonodonta fulvangula|[14103]|MHAUA040-05|Costa Rica|Guanacaste|658[0n]|BOLD:AAA4308
Gonodonta fulvangula|[14104]|MHAUA037-05|Costa Rica|Guanacaste|658[0n]|BOLD:AAA4308
Gonodonta fulvangula|[14105]|MHAUA036-05|Costa Rica|Guanacaste|658[0n]|BOLD:AAA4308
Gonodonta fulvangula|[14106]|MHAUA035-05|Costa Rica|Alajuela|658[0n]|BOLD:AAA4308
Gonodonta fulvangula|[14107]|MHAUG621-07|Costa Rica|Guanacaste|648[0n]|BOLD:AAA4308
Gonodonta fulvangula|[14108]|MHMXZ242-09|Costa Rica||658[0n]|BOLD:AAA4308
Gonodonta fulvangula|[14109]|MHAUA038-05|Costa Rica|Guanacaste|658[0n]|BOLD:AAA4308
Gonodonta fulvangula|[14110]|MHAUA046-05|Costa Rica|Alajuela|658[0n]|BOLD:AAA4308
Gonodonta fulvangula|[14111]|MHAUG609-07|Costa Rica|Alajuela|658[0n]|BOLD:AAA4308
Gonodonta fulvangula|[14112]|MHAUG612-07|Costa Rica|Alajuela|658[0n]|BOLD:AAA4308
Gonodonta fulvangula|[14113]|MHMXO283-08|Costa Rica|Guanacaste|658[0n]|BOLD:AAA4308
Gonodonta fulvangula|[14114]|MHMXO452-08|Costa Rica|Guanacaste|658[0n]|BOLD:AAA4308
Gonodonta fulvangula|[14115]|MHMXZ246-09|Costa Rica||658[0n]|BOLD:AAA4308
Gonodonta fulvangula|[14116]|INCTA015-10|Brazil|Para|658[0n]|BOLD:AAA4308
Gonodonta fulvangula|[14117]|MHMYH615-10|Costa Rica||658[0n]|BOLD:AAA4308
Gonodonta fulvangula|[14118]|JMZCA150-09|Ecuador||658[0n]|BOLD:AAA4308
Gonodonta fulvangula|[14119]|BLPDL1460-10|Costa Rica|Alajuela|658[0n]|BOLD:AAA4308
Gonodonta fulvangula|[14120]|LOCRE820-10|Costa Rica|Limon|658[0n]|BOLD:AAA4308
Gonodonta fulvangula|[14121]|LNOUF742-11|French Guiana||658[0n]|BOLD:AAA4308
Gonodonta fulvangula|[14122]|MILEP711-11|French Guiana||658[0n]|BOLD:AAA4308
Gonodonta fulvangula|[14123]|MHMYN064-11|Costa Rica||658[0n]|BOLD:AAA4308
Gonodonta fulvangula|[14124]|MHMYN065-11|Costa Rica||658[0n]|BOLD:AAA4308
Gonodonta fulvangula|[14125]|MHMYO163-11|Costa Rica||658[0n]|BOLD:AAA4308
Gonodonta fulvangula|[14126]|MHMYO164-11|Costa Rica||658[0n]|BOLD:AAA4308
Gonodonta fulvangula|[14127]|MHMYO165-11|Costa Rica||658[0n]|BOLD:AAA4308
Gonodonta fulvangula|[14128]|MHMYQ017-12|Costa Rica||658[0n]|BOLD:AAA4308
Gonodonta fulvangula|[14129]|MHMYQ018-12|Costa Rica||658[0n]|BOLD:AAA4308
Gonodonta fulvangula|[14130]|MHMYQ019-12|Costa Rica||658[0n]|BOLD:AAA4308
Gonodonta fulvangula|[14131]|MHMYQ1636-12|Costa Rica||658[0n]|BOLD:AAA4308
Gonodonta fulvangula|[14132]|MHMYT166-13|Costa Rica||634[0n]|BOLD:AAA4308
Gonodonta fulvangula|[14133]|MHAUG634-07|Costa Rica|Guanacaste|544[0n]|BOLD:AAA4308
Gonodonta fulvangula|[14134]|MHMXZ254-09|Costa Rica||658[0n]|BOLD:AAA4308
Gonodonta fulvangula|[14135]|MHMYH859-10|Costa Rica||658[0n]|BOLD:AAA4308
Gonodonta fulvangula|[14136]|MHAUG631-07|Costa Rica|Guanacaste|608[0n]|BOLD:AAA4308
Gonodonta fulvangula|[14137]|MHAUG643-07|Costa Rica|Guanacaste|594[0n]|BOLD:AAA4308
Gonodonta fulvangula|[14138]|MHAUG638-07|Costa Rica|Guanacaste|593[0n]|BOLD:AAA4308
Gonodonta fulvangula|[14139]|MHAUG632-07|Costa Rica|Alajuela|593[0n]|BOLD:AAA4308
Gonodonta fulvangula|[14140]|MHAUG606-07|Costa Rica|Alajuela|658[0n]|BOLD:AAA4308
Gonodonta fulvangula|[14141]|MHAUG624-07|Costa Rica|Guanacaste|595[0n]|BOLD:AAA4308
Gonodonta fulvangula|[14142]|MHMXZ248-09|Costa Rica||658[0n]|BOLD:AAA4308
Gonodonta fulvangula|[14143]|MHMXZ250-09|Costa Rica||658[0n]|BOLD:AAA4308
Gonodonta fulvangula|[14144]|MHMXZ251-09|Costa Rica||658[0n]|BOLD:AAA4308
Gonodonta fulvangula|[14145]|MHMXZ252-09|Costa Rica||658[0n]|BOLD:AAA4308
Gonodonta fulvangula|[14146]|MHMXZ253-09|Costa Rica||658[0n]|BOLD:AAA4308
Gonodonta fulvangula|[14147]|QUNOD162-10|United States|Texas|658[0n]|BOLD:AAA4308
Gonodonta fulvangula|[14148]|QUNOD231-10|Ecuador|Napo|658[0n]|BOLD:AAA4308
Gonodonta fulvangula|[14149]|MHMYH616-10|Costa Rica||658[0n]|BOLD:AAA4308
Gonodonta fulvangula|[14150]|MHAUG623-07|Costa Rica|Guanacaste|658[0n]|BOLD:AAA4308
Gonodonta fulvangula|[14151]|MHAUG625-07|Costa Rica|Guanacaste|658[0n]|BOLD:AAA4308
Gonodonta fulvangula|[14152]|MHAUG627-07|Costa Rica|Alajuela|658[0n]|BOLD:AAA4308
Gonodonta fulvangula|[14153]|MHAUG629-07|Costa Rica|Alajuela|658[0n]|BOLD:AAA4308
Gonodonta fulvangula|[14154]|MHAUG635-07|Costa Rica|Guanacaste|658[0n]|BOLD:AAA4308
Gonodonta fulvangula|[14155]|MHAUG650-07|Costa Rica|Guanacaste|658[0n]|BOLD:AAA4308
Gonodonta fulvangula|[14156]|MHMXZ243-09|Costa Rica||658[0n]|BOLD:AAA4308
Gonodonta fulvangula|[14157]|MHMXZ247-09|Costa Rica||658[0n]|BOLD:AAA4308
Gonodonta fulvangula|[14158]|MHAUG620-07|Costa Rica|Guanacaste|658[0n]|BOLD:AAA4308
Gonodonta fulvangula|[14159]|MHAUG622-07|Costa Rica|Guanacaste|658[0n]|BOLD:AAA4308
Gonodonta fulvangula|[14160]|MHAUG619-07|Costa Rica|Alajuela|658[0n]|BOLD:AAA4308
Gonodonta fulvangula|[14161]|MHAUG613-07|Costa Rica|Alajuela|658[0n]|BOLD:AAA4308
Gonodonta fulvangula|[14162]|MHAUG611-07|Costa Rica|Alajuela|658[0n]|BOLD:AAA4308
Gonodonta fulvangula|[14163]|MHAUG605-07|Costa Rica|Alajuela|658[0n]|BOLD:AAA4308
Gonodonta fulvangula|[14164]|LPMX186-07|Mexico|Campeche|658[0n]|BOLD:AAA4308
Gonodonta fulvangula|[14165]|MHAUA048-05|Costa Rica|Guanacaste|658[0n]|BOLD:AAA4308
Gonodonta fulvangula|[14166]|MHAUA047-05|Costa Rica|Guanacaste|658[0n]|BOLD:AAA4308
Gonodonta fulvangula|[14167]|MHAUA045-05|Costa Rica|Guanacaste|658[0n]|BOLD:AAA4308
Gonodonta fulvangula|[14168]|MHAUA042-05|Costa Rica|Alajuela|658[0n]|BOLD:AAA4308
Gonodonta fulvangula|[14169]|MHAUA041-05|Costa Rica|Alajuela|658[0n]|BOLD:AAA4308
Gonodonta fulvangula|[14170]|MHAUA039-05|Costa Rica|Guanacaste|658[0n]|BOLD:AAA4308
Gonodonta fulvangula|[14171]|MHAUA034-05|Costa Rica|Alajuela|658[0n]|BOLD:AAA4308
Gonodonta fulvangula|[14172]|MHAUG610-07|Costa Rica|Guanacaste|621[0n]|BOLD:AAA4308
Gonodonta fulvangula|[14173]|LTOLB009-08|Costa Rica|Guanacaste|648[0n]|BOLD:AAA4308
Gonodonta fulvangula|[14174]|BLPDY791-11|Costa Rica|Guanacaste|658[0n]|BOLD:AAA4308
Gonodonta fulvangula|[14175]|MHMYH617-10|Costa Rica||658[0n]|BOLD:AAA4308
Gonodonta fulvangula|[14176]|MILEP710-11|French Guiana||658[0n]|BOLD:AAA4308
Gonodonta fulvangula|[14177]|MILEP1004-11|French Guiana||658[0n]|BOLD:AAA4308
Gonodonta fulvangula|[14178]|MILEP1005-11|French Guiana||658[0n]|BOLD:AAA4308
Gonodonta fulvangula|[14179]|GWOSZ818-11|Peru|Huanuco|658[0n]|BOLD:AAA4308

Gonodonta fulvangula|[14176]|MILEP710-11|French Guiana||658[0n]|BOLD:AAA4308
Gonodonta fulvangula|[14177]|MILEP1004-11|French Guiana||658[0n]|BOLD:AAA4308
Gonodonta fulvangula|[14178]|MILEP1005-11|French Guiana||658[0n]|BOLD:AAA4308
Gonodonta fulvangula|[14179]|GWOSZ818-11|Peru|Huanuco|658[0n]|BOLD:AAA4308
Gonodonta fulvangula|[14180]|GWOSZ819-11|Peru|Huanuco|658[0n]|BOLD:AAA4308
Gonodonta fulvangula|[14181]|MHMYQ020-12|Costa Rica||658[0n]|BOLD:AAA4308
Gonodonta fulvangula|[14182]|BLPEF3131-13|Costa Rica|Guanacaste|658[0n]|BOLD:AAA4308
Gonodonta fulvangula|[14183]|BLPEE5408-14|Costa Rica|Guanacaste|658[0n]|BOLD:AAA4308
Gonodonta fulvangula|[14184]|BLPEE8001-14|Costa Rica||658[0n]|BOLD:AAA4308
Gonodonta unica|[14185]|RDNML305-13|United States|Florida|658[0n]|BOLD:AAA4308
Gonodonta unica|[14186]|LNAUS3903-13|United States|Florida|658[0n]|BOLD:AAA4308
Gonodonta unica|[14187]|LNAUS3904-13|United States|Florida|658[0n]|BOLD:AAA4308
Gonodonta incurva|[14188]|MHMYH939-10|Costa Rica||571[0n]|BOLD:AAA4308
Gonodonta incurva|[14189]|MHMYH938-10|Costa Rica||658[0n]|BOLD:AAA4308
Gonodonta incurva|[14190]|BLPDW227-11|Costa Rica|Guanacaste|658[0n]|BOLD:AAA4308
Gonodonta incurva|[14191]|LOCRI787-11|Costa Rica|Cartago|658[0n]|BOLD:AAA4308
Gonodonta incurva|[14192]|BLPEE8064-14|Costa Rica||658[0n]|BOLD:AAA4308
Gonodonta incurva|[14193]|LMEMB109-09|Puerto Rico||658[0n]|BOLD:AAA4308
Gonodonta incurva|[14194]|MHMXZ211-09|Costa Rica||658[0n]|BOLD:AAA4308
Gonodonta incurva|[14195]|CNCLB1931-14|Puerto Rico|Mayaguez|658[0n]|BOLD:AAA4308
Gonodonta incurva|[14196]|BLPEE8002-14|Costa Rica||658[0n]|BOLD:AAA4308
Gonodonta incurva|[14197]|BLPEE8061-14|Costa Rica||658[0n]|BOLD:AAA4308
Gonodonta incurva|[14198]|BLPEE8062-14|Costa Rica||658[0n]|BOLD:AAA4308
Gonodonta incurva|[14199]|BLPEE8063-14|Costa Rica||658[0n]|BOLD:AAA4308
Gonodonta incurva|[14200]|BLPEE8068-14|Costa Rica||658[0n]|BOLD:AAA4308
Gonodonta incurva|[14201]|BLPEE8070-14|Costa Rica||658[0n]|BOLD:AAA4308
Gonodonta incurva|[14202]|BLPEE8065-14|Costa Rica||658[0n]|BOLD:AAA4308
Gonodonta incurva|[14203]|BLPEE8066-14|Costa Rica||658[0n]|BOLD:AAA4308
Gonodonta incurva|[14204]|BLPEE8071-14|Costa Rica||658[0n]|BOLD:AAA4308
Gonodonta incurva|[14205]|BLPEE8072-14|Costa Rica||658[0n]|BOLD:AAA4308
Gonodonta incurva|[14206]|MHMXO282-08|Costa Rica|Guanacaste|658[0n]|BOLD:AAA4308
Gonodonta incurva|[14207]|MHMXZ203-09|Costa Rica||658[0n]|BOLD:AAA4308
Gonodonta incurva|[14208]|MHMXZ213-09|Costa Rica||658[0n]|BOLD:AAA4308
Gonodonta incurva|[14209]|MHMXZ215-09|Costa Rica||658[0n]|BOLD:AAA4308
Gonodonta incurva|[14210]|LOCRI788-11|Costa Rica|Cartago|658[0n]|BOLD:AAA4308
Gonodonta incurva|[14211]|MHMYT164-13|Costa Rica||658[0n]|BOLD:AAA4308
Gonodonta incurva|[14212]|BLPEF6170-13|Costa Rica||658[0n]|BOLD:AAA4308
Gonodonta incurva|[14213]|MHMYS3208-13|Costa Rica|Guanacaste|658[0n]|BOLD:AAA4308
Gonodonta incurva|[14214]|BLPDV017-11|Costa Rica|Guanacaste|658[0n]|BOLD:AAA4308
Gonodonta incurva|[14215]|BLPDV364-11|Costa Rica|Guanacaste|658[0n]|BOLD:AAA4308
Gonodonta incurva|[14216]|MHMYL3076-11|Costa Rica||658[0n]|BOLD:AAA4308
Gonodonta incurva|[14217]|MHMYM215-11|Costa Rica||658[0n]|BOLD:AAA4308
Gonodonta incurva|[14218]|MHMYT165-13|Costa Rica||658[0n]|BOLD:AAA4308
Gonodonta incurva|[14219]|BLPEF4973-13|Costa Rica|Guanacaste|658[0n]|BOLD:AAA4308
Gonodonta incurva|[14220]|MHMXZ208-09|Costa Rica||658[0n]|BOLD:AAA4308
Gonodonta incurva|[14221]|MHMXZ209-09|Costa Rica||658[0n]|BOLD:AAA4308
Gonodonta incurva|[14222]|MHMYF735-10|Costa Rica|Alajuela|658[0n]|BOLD:AAA4308
Gonodonta incurva|[14223]|MHMYH619-10|Costa Rica||658[0n]|BOLD:AAA4308
Gonodonta incurva|[14224]|MHMXZ210-09|Costa Rica||658[0n]|BOLD:AAA4308
Gonodonta incurva|[14225]|MHMXZ212-09|Costa Rica||658[0n]|BOLD:AAA4308
Gonodonta incurva|[14226]|MHMYH620-10|Costa Rica||658[0n]|BOLD:AAA4308
Gonodonta incurva|[14227]|MHMYH621-10|Costa Rica||658[0n]|BOLD:AAA4308
Gonodonta incurva|[14228]|MHMXZ204-09|Costa Rica||658[0n]|BOLD:AAA4308
Gonodonta incurva|[14229]|MHMXZ205-09|Costa Rica||658[0n]|BOLD:AAA4308
Gonodonta incurva|[14230]|MHMXZ206-09|Costa Rica||658[0n]|BOLD:AAA4308
Gonodonta incurva|[14231]|MHMXZ207-09|Costa Rica||658[0n]|BOLD:AAA4308
Gonodonta incurva|[14232]|MHAUC281-06|Costa Rica|Alajuela|658[0n]|BOLD:AAA4308
Gonodonta incurva|[14233]|MHMXM138-07|Costa Rica|Guanacaste|658[0n]|BOLD:AAA4308
Gonodonta incurva|[14234]|MHAUA077-05|Costa Rica|Alajuela|658[0n]|BOLD:AAA4308
Gonodonta incurva|[14235]|MHAUA078-05|Costa Rica|Guanacaste|658[0n]|BOLD:AAA4308
Gonodonta incurva|[14236]|MHAUA076-05|Costa Rica|Guanacaste|658[0n]|BOLD:AAA4308
Gonodonta incurva|[14237]|MHAUA075-05|Costa Rica|Guanacaste|658[0n]|BOLD:AAA4308
Gonodonta incurva|[14238]|MHAUA074-05|Costa Rica|Alajuela|658[0n]|BOLD:AAA4308
Gonodonta incurva|[14239]|MHAUA073-05|Costa Rica|Alajuela|658[0n]|BOLD:AAA4308
Gonodonta incurva|[14240]|MHAUA072-05|Costa Rica|Alajuela|658[0n]|BOLD:AAA4308
Gonodonta incurva|[14241]|MHAUA071-05|Costa Rica|Alajuela|658[0n]|BOLD:AAA4308
Gonodonta incurva|[14242]|MHAUA070-05|Costa Rica|Alajuela|658[0n]|BOLD:AAA4308
Gonodonta incurva|[14243]|MHAUA069-05|Costa Rica|Alajuela|658[0n]|BOLD:AAA4308
Gonodonta incurva|[14244]|MHAUA068-05|Costa Rica|Alajuela|658[0n]|BOLD:AAA4308
Gonodonta incurva|[14245]|MHAUA067-05|Costa Rica|Alajuela|658[0n]|BOLD:AAA4308
Gonodonta incurva|[14246]|MHAUA066-05|Costa Rica|Guanacaste|658[0n]|BOLD:AAA4308
Gonodonta incurva|[14247]|MHAUA065-05|Costa Rica|Guanacaste|658[0n]|BOLD:AAA4308
Gonodonta incurva|[14248]|MHMXZ214-09|Costa Rica||634[0n]|BOLD:AAA4308
Gonodonta incurva|[14249]|BLPDK354-09|Costa Rica|Guanacaste|634[0n]|BOLD:AAA4308
Gonodonta incurva|[14250]|BLPDK1458-09|Costa Rica|Guanacaste|658[1n]|BOLD:AAA4308
Gonodonta incurva|[14251]|MHMYF734-10|Costa Rica|Alajuela|658[0n]|BOLD:AAA4308
Gonodonta incurva|[14252]|BLPEE8073-14|Costa Rica||658[0n]|BOLD:AAA4308
Gonodonta incurva|[14253]|BLPEE8074-14|Costa Rica||658[0n]|BOLD:AAA4308
Gonodonta incurva|[14254]|BLPEE8173-14|Costa Rica||658[0n]|BOLD:AAA4308
Gonodonta incurva|[14255]|BLPEE8174-14|Costa Rica||658[0n]|BOLD:AAA4308
Gonodonta incurva|[14256]|BLPEE8176-14|Costa Rica||658[0n]|BOLD:AAA4308
Gonodonta incurva|[14257]|CNCLB1932-14|Mexico|Chiapas|658[0n]|BOLD:AAA4308
Gonodonta nitidimacula|[14258]|LPYPB260-08|Mexico|Quintana Roo|609[0n]|BOLD:AAA4308
Gonodonta nitidimacula|[14259]|LPMX063-07|Mexico|Campeche|632[0n]|BOLD:AAA4308
Gonodonta nitidimacula|[14260]|MHAUA394-05|Costa Rica|Guanacaste|658[0n]|BOLD:AAA4308
Gonodonta nitidimacula|[14261]|MHAUA395-05|Costa Rica|Guanacaste|658[0n]|BOLD:AAA4308
Gonodonta nitidimacula|[14262]|MHAUA396-05|Costa Rica|Guanacaste|658[0n]|BOLD:AAA4308
Gonodonta nitidimacula|[14263]|LPYPB317-08|Mexico|Campeche|658[0n]|BOLD:AAA4308
Gonodonta nitidimacula|[14264]|LPYPB702-08|Mexico|Campeche|658[0n]|BOLD:AAA4308
Gonodonta nitidimacula|[14265]|LPYPB710-08|Mexico|Campeche|658[0n]|BOLD:AAA4308
Gonodonta nitidimacula|[14266]|GWOSS022-11|Cuba|Holguin|658[0n]|BOLD:AAA4308
Gonodonta nitidimacula|[14267]|CQR127-13|Mexico|Quintana Roo|601[0n]|BOLD:AAA4308
Gonodonta nitidimacula|[14268]|LPMX016-07|Mexico|Campeche|647[2n]|BOLD:AAA4308
Gonodonta nitidimacula|[14269]|MHAUA401-05|Costa Rica|Guanacaste|658[0n]|BOLD:AAA4308
Gonodonta nitidimacula|[14270]|MHAUA403-05|Costa Rica|Guanacaste|658[0n]|BOLD:AAA4308
Gonodonta nitidimacula|[14271]|MHMXI359-07|Costa Rica|Guanacaste|658[0n]|BOLD:AAA4308
Gonodonta nitidimacula|[14272]|MHMXO281-08|Costa Rica|Guanacaste|658[0n]|BOLD:AAA4308
Gonodonta nitidimacula|[14273]|LPMX715-08|Mexico|Campeche|658[0n]|BOLD:AAA4308
Gonodonta nitidimacula|[14274]|LPYPB141-08|Mexico|Campeche|658[0n]|BOLD:AAA4308
Gonodonta nitidimacula|[14275]|LPYPB262-08|Mexico|Quintana Roo|658[0n]|BOLD:AAA4308
Gonodonta nitidimacula|[14276]|LPYPB335-08|Mexico|Campeche|658[0n]|BOLD:AAA4308
Gonodonta nitidimacula|[14277]|GWOSS021-11|Cuba|Holguin|658[0n]|BOLD:AAA4308
Gonodonta nitidimacula|[14278]|MHMYT251-13|Costa Rica||658[0n]|BOLD:AAA4308
Gonodonta nitidimacula|[14279]|CQR638-13|Mexico|Quintana Roo|658[0n]|BOLD:AAA4308

Gonodonta nitidimacula|[14276]|LPYPB335-08|Mexico|Campeche|658[0n]|BOLD:AAA4308
Gonodonta nitidimacula|[14277]|GWOSS021-11|Cuba|Holguin|658[0n]|BOLD:AAA4308
Gonodonta nitidimacula|[14278]|MHMYT251-13|Costa Rica||658[0n]|BOLD:AAA4308
Gonodonta nitidimacula|[14279]|CQR638-13|Mexico|Quintana Roo|658[0n]|BOLD:AAA4308
Gonodonta nitidimacula|[14280]|CQR662-13|Mexico|Quintana Roo|658[0n]|BOLD:AAA4308
Gonodonta nitidimacula|[14281]|MHAUA393-05|Costa Rica|Guanacaste|658[0n]|BOLD:AAA4308
Gonodonta nitidimacula|[14282]|MHAUA397-05|Costa Rica|Guanacaste|658[0n]|BOLD:AAA4308
Gonodonta nitidimacula|[14283]|MHMYS3207-13|Costa Rica|Guanacaste|658[0n]|BOLD:AAA4308
Gonodonta nitidimacula|[14284]|MHAUA402-05|Costa Rica|Guanacaste|658[0n]|BOLD:AAA4308
Gonodonta nitidimacula|[14285]|CQR209-13|Mexico|Quintana Roo|628[0n]|BOLD:AAA4308
Gonodonta nitidimacula|[14286]|MHAUA400-05|Costa Rica|Guanacaste|658[0n]|BOLD:AAA4308
Gonodonta nitidimacula|[14287]|LPYPB316-08|Mexico|Campeche|658[0n]|BOLD:AAA4308
Gonodonta nitidimacula|[14288]|MHAUA398-05|Costa Rica|Guanacaste|658[0n]|BOLD:AAA4308
Gonodonta nitidimacula|[14289]|MHAUA399-05|Costa Rica|Guanacaste|658[0n]|BOLD:AAA4308
Gonodonta nitidimacula|[14290]|BLPEE8188-14|Costa Rica||658[0n]|BOLD:AAA4308
Gonodonta nitidimacula|[14291]|CNCLB1933-14|Puerto Rico|Mayaguez|658[0n]|BOLD:AAA4308
Anticarsia gemmatalis|[14292]|LNC484-06|United States|North Carolina|658[0n]|BOLD:AAA6923
Anticarsia gemmatalis|[14293]|LSEU045-06|United States|Georgia|658[0n]|BOLD:AAA6923
Anticarsia gemmatalis|[14294]|HKONS044-07|United States|Florida|658[2n]|BOLD:AAA6923
Anticarsia gemmatalis|[14295]|HKONS388-08|United States|Florida|658[0n]|BOLD:AAA6923
Anticarsia gemmatalis|[14296]|LNC447-05|United States|North Carolina|658[0n]|BOLD:AAA6923
Anticarsia gemmatalis|[14297]|HKONS389-08|United States|Florida|658[0n]|BOLD:AAA6923
Anticarsia gemmatalis|[14298]|LPOKE267-11|United States|Oklahoma|658[0n]|BOLD:AAA6923
Anticarsia gemmatalis|[14299]|LPOKE268-11|United States|Oklahoma|658[0n]|BOLD:AAA6923
Epitausa prona|[14300]|CNCLB2249-14|Mexico||658[0n]|BOLD:AAD1849
Epitausa prona|[14301]|MHAUC869-06|Costa Rica|Alajuela|658[0n]|BOLD:AAD1849
Epitausa prona|[14302]|BLPBD245-07|Costa Rica|Guanacaste|642[0n]|BOLD:AAD1849
Epitausa prona|[14303]|BLPCA885-08|Costa Rica|Guanacaste|658[0n]|BOLD:AAD1849
Epitausa prona|[14304]|BLPCA886-08|Costa Rica|Guanacaste|658[0n]|BOLD:AAD1849
Epitausa prona|[14305]|BLPCH652-08|Costa Rica|Guanacaste|658[0n]|BOLD:AAD1849
Epitausa prona|[14306]|BLPCH653-08|Costa Rica|Guanacaste|658[0n]|BOLD:AAD1849
Epitausa prona|[14307]|BLPCH654-08|Costa Rica|Guanacaste|658[0n]|BOLD:AAD1849
Epitausa prona|[14308]|BLPCO653-08|Costa Rica|Guanacaste|658[0n]|BOLD:AAD1849
Epitausa prona|[14309]|ARMOT143-12|Argentina|Misiones|658[0n]|BOLD:AAD1849
Epitausa prona|[14310]|CNCLB2418-14|United States|Texas|658[0n]|BOLD:AAD1849
Herminocala stigmaphiles|[14311]|RDNMG253-08|United States|Texas|658[0n]|BOLD:AAI1855
Herminocala stigmaphiles|[14312]|HKONB559-09|United States|Texas|636[0n]|BOLD:AAI1855
Herminocala stigmaphiles|[14313]|HKONB560-09|United States|Texas|625[0n]|BOLD:AAI1855
Herminocala stigmaphiles|[14314]|HKONB561-09|United States|Texas|645[0n]|BOLD:AAI1855
Herminocala stigmaphiles|[14315]|QUNOD032-10|United States|Texas|658[0n]|BOLD:AAI1855
Nychioptera|[14316]|RDNME658-08|United States|Texas|609[0n]|BOLD:AAH3260
Nychioptera|[14317]|RDNME613-08|United States|Texas|609[0n]|BOLD:AAH3260
Nychioptera|[14318]|QUNOD156-10|United States|Texas|658[0n]|BOLD:AAH3260
Nychioptera accola|[14319]|ABNCC018-07|United States|Arizona|635[0n]|BOLD:AAG5849
Nychioptera accola|[14320]|RDNMH821-09|United States|Arizona|658[0n]|BOLD:AAG5849
Nychioptera opada|[14321]|RDNMJ565-11|United States|Arizona|658[0n]|BOLD:AAV2237
Nychioptera opada|[14322]|RDNMJ667-11|United States|Arizona|658[0n]|BOLD:AAV2237
Nychioptera noctuidalis|[14323]|RDNMJ303-11|United States|Arizona|658[0n]|BOLD:AAH4762
Nychioptera noctuidalis|[14324]|IAWL349-09|United States|Arizona|658[0n]|BOLD:AAH4762
Nychioptera noctuidalis|[14325]|RDNMH820-09|United States|Arizona|658[0n]|BOLD:AAH4762
Nychioptera noctuidalis|[14326]|IAWL350-09|United States|Arizona|658[0n]|BOLD:AAH4762
Nychioptera noctuidalis|[14327]|QUNOD199-10|United States|Arizona|658[0n]|BOLD:AAH4762
Nychioptera noctuidalis|[14328]|RDNMJ548-11|United States|Arizona|658[0n]|BOLD:AAH4762
Nychioptera noctuidalis|[14329]|RDNMJ568-11|United States|Arizona|658[0n]|BOLD:AAH4762
Nychioptera noctuidalis|[14330]|RDNMJ674-11|United States|Arizona|658[0n]|BOLD:AAH4762
Nychioptera noctuidalis|[14331]|RDNMJ689-11|United States|Arizona|658[0n]|BOLD:AAH4762
Nychioptera noctuidalis|[14332]|CMAZA1182-12|United States|Arizona|658[0n]|BOLD:AAH4762
Nychioptera noctuidalis|[14333]|CMAZA1245-12|United States|Arizona|658[0n]|BOLD:AAH4762
Oxycilla basipallida|[14334]|QUNOD189-10|United States|Arizona|658[0n]|BOLD:AAK5884
Oxycilla basipallida|[14335]|CNCLB2458-14|United States|Arizona|658[0n]|BOLD:AAK5884
Isogona snowi|[14336]|HKONB285-09|United States|Texas|658[0n]|BOLD:ABZ0950
Isogona snowi|[14337]|HKONB091-08|United States|Texas|658[0n]|BOLD:ABZ0950
Isogona snowi|[14338]|HKONB284-09|United States|Texas|658[0n]|BOLD:ABZ0950
Isogona snowi|[14339]|HKONB057-08|United States|Texas|658[0n]|BOLD:ABZ0950
Isogona snowi|[14340]|HKONB286-09|United States|Texas|658[0n]|BOLD:ABZ0950
Isogona snowi|[14341]|HKONB058-08|United States|Texas|658[0n]|BOLD:ABZ0950
Isogona snowi|[14342]|HKONB287-09|United States|Texas|658[0n]|BOLD:ABZ0950
Hypena minualis|[14343]|RDNMD533-06|United States|Florida|658[0n]|BOLD:AAE1423
Hypena minualis|[14344]|ABNCC124-07|United States|Texas|642[0n]|BOLD:AAE1423
Hypena minualis|[14345]|LPOKB013-09|United States|Oklahoma|658[0n]|BOLD:AAE1423
Hypena minualis|[14346]|LPOKB552-09|United States|Oklahoma|658[0n]|BOLD:AAE1423
Hypena variabilis|[14347]|LOCRF634-11|Costa Rica|Cartago|658[0n]|BOLD:AAB3042
Hypena variabilis|[14348]|LOCRA364-07|Costa Rica|Alajuela|658[0n]|BOLD:AAB3042
Hypena variabilis|[14349]|LOCRA365-07|Costa Rica|Alajuela|658[0n]|BOLD:AAB3042
Hypena variabilis|[14350]|CNCLB1254-14|United States|Louisiana|307[0n]|
Hypena tenebrosa|[14351]|RDNML338-13|United States|Florida|658[0n]|BOLD:AAD2712
Hypena tenebrosa|[14352]|CNCLB423-14|United States|Texas|682[0n]|BOLD:AAD2712
Hypena tenebrosa|[14353]|CNCLB1253-14|United States|Louisiana|658[0n]|BOLD:AAD2712
Hypena californica|[14354]|LOWCE528-06|Canada|British Columbia|572[0n]|BOLD:AAB4369
Hypena californica|[14355]|LHLEP343-06|Canada|British Columbia|655[0n]|BOLD:AAB4369
Hypena californica|[14356]|GMLC1049-12|United States|California|658[0n]|BOLD:AAB4369
Hypena californica|[14357]|LALPA026-10|Canada|British Columbia|658[0n]|BOLD:AAB4369
Hypena californica|[14358]|LOPN068-06|United States|Oregon|658[0n]|BOLD:AAB4369
Hypena californica|[14359]|LHLEP342-06|Canada|British Columbia|657[0n]|BOLD:AAB4369
Hypena californica|[14360]|LHLEP341-06|Canada|British Columbia|657[0n]|BOLD:AAB4369
Hypena californica|[14361]|LHLEP344-06|Canada|British Columbia|657[0n]|BOLD:AAB4369
Hypena californica|[14362]|LHLEP345-06|Canada|British Columbia|657[0n]|BOLD:AAB4369
Hypena californica|[14363]|RWWA742-09|United States|Washington|658[0n]|BOLD:AAB4369
Hypena californica|[14364]|LALPA429-10|Canada|British Columbia|658[0n]|BOLD:AAB4369
Hypena californica|[14365]|RWWC895-12|United States|Washington|658[0n]|BOLD:AAB4369
Hypena californica|[14366]|LOPN067-06|United States|Oregon|508[0n]|BOLD:AAB4369
Hypena californica|[14367]|LHLEP655-06|Canada|British Columbia|658[0n]|BOLD:AAB4369
Hypena californica|[14368]|LHLEP654-06|Canada|British Columbia|658[0n]|BOLD:AAB4369
Hypena californica|[14369]|LOWCE503-06|Canada|British Columbia|658[0n]|BOLD:AAB4369
Hypena californica|[14370]|LOWCE502-06|Canada|British Columbia|658[0n]|BOLD:AAB4369
Hypena californica|[14371]|LOWCE481-06|Canada|British Columbia|658[0n]|BOLD:AAB4369
Hypena californica|[14372]|LOWCE385-06|Canada|British Columbia|599[0n]|BOLD:AAB4369
Hypena californica|[14373]|LOWCE504-06|Canada|British Columbia|606[0n]|BOLD:AAB4369
Hypena californica|[14374]|ABKWR161-07|United States|Alaska|630[0n]|BOLD:AAB4369
Hypena californica|[14375]|LOWCE400-06|Canada|British Columbia|615[0n]|BOLD:AAB4369
Hypena californica|[14376]|LOWCB626-05|Canada|British Columbia|606[0n]|BOLD:AAB4369
Hypena californica|[14377]|LOWCE510-06|Canada|British Columbia|611[0n]|BOLD:AAB4369
Hypena californica|[14378]|LOWCB625-05|Canada|British Columbia|612[0n]|BOLD:AAB4369
[illegible]

Hypena californica|[14376]|LOWCB626-05|Canada|British Columbia|606[0n]|BOLD:AAB4369
Hypena californica|[14377]|LOWCE510-06|Canada|British Columbia|611[0n]|BOLD:AAB4369
Hypena californica|[14378]|LOWCB625-05|Canada|British Columbia|612[0n]|BOLD:AAB4369
Hypena californica|[14379]|LOWCE399-06|Canada|British Columbia|617[0n]|BOLD:AAB4369
Hypena californica|[14380]|LOWCE505-06|Canada|British Columbia|601[0n]|BOLD:AAB4369
Hypena californica|[14381]|LOWCE509-06|Canada|British Columbia|610[0n]|BOLD:AAB4369
Hypena californica|[14382]|LBCF007-07|Canada|British Columbia|618[0n]|BOLD:AAB4369
Hypena californica|[14383]|LALPA409-10|Canada|British Columbia|658[0n]|BOLD:AAB4369
Hypena californica|[14384]|CNCLB1627-14|Canada|British Columbia|658[0n]|BOLD:AAB4369
Hypena vetustalis|[14385]|LPMX540-08|Mexico|Campeche|658[0n]|BOLD:AAD1573
Hypena vetustalis|[14386]|CNCLB1252-14|United States|Louisiana|658[0n]|BOLD:AAD1573
Hypena vetustalis|[14387]|RDNML337-13|United States|Florida|658[0n]|BOLD:AAD1573
Hypena vetustalis|[14388]|RDNML042-13|United States|Florida|658[0n]|BOLD:AAD1573
Hypena vetustalis|[14389]|MXBLP156-11|Mexico|Jalisco|658[0n]|BOLD:AAD1573
Hypena vetustalis|[14390]|BLPBE337-07|Costa Rica|Guanacaste|658[0n]|BOLD:AAD1573
Hypena vetustalis|[14391]|BLPBD244-07|Costa Rica|Guanacaste|658[0n]|BOLD:AAD1573
Hypena vetustalis|[14392]|BLPAF685-07|Costa Rica|Guanacaste|658[0n]|BOLD:AAD1573
Hypena vetustalis|[14393]|MHMXE277-06|Costa Rica|Alajuela|658[0n]|BOLD:AAD1573
Hypena vetustalis|[14394]|MHMXE276-06|Costa Rica|Alajuela|658[0n]|BOLD:AAD1573
Hypena vetustalis|[14395]|BLPAC366-06|Costa Rica|Guanacaste|658[0n]|BOLD:AAD1573
Hypena vetustalis|[14396]|BLPAA481-06|Costa Rica|Guanacaste|658[0n]|BOLD:AAD1573
Hypena vetustalis|[14397]|MHAUG691-07|Costa Rica|Guanacaste|618[0n]|BOLD:AAD1573
Hypena vetustalis|[14398]|BLPEG1334-14|Costa Rica|Guanacaste|658[0n]|BOLD:AAD1573
Hypena vetustalis|[14399]|CNCLB1807-14|Puerto Rico|Mayaguez|658[0n]|BOLD:AAD1573
Hypena edictalis|[14400]|RDLQH129-06|Canada|Quebec|617[2n]|BOLD:ACI5170
Hypena edictalis|[14401]|LPABC680-09|Canada|Alberta|658[0n]|BOLD:AAB3069
Hypena edictalis|[14402]|LGSMG542-07|United States|North Carolina|658[0n]|BOLD:AAB3069
Hypena edictalis|[14403]|LSEU692-06|United States|Georgia|658[0n]|BOLD:AAB3069
Hypena edictalis|[14404]|LGSMG543-07|United States|North Carolina|658[2n]|BOLD:AAB3069
Hypena edictalis|[14405]|BBLPB437-10|Canada|Alberta|658[0n]|BOLD:AAB3069
Hypena edictalis|[14406]|BBLPB742-10|Canada|Alberta|658[0n]|BOLD:AAB3069
Hypena edictalis|[14407]|LMEM998-09|United States|North Carolina|658[0n]|BOLD:AAB3069
Hypena edictalis|[14408]|LMEMB001-09|United States|Alabama|658[0n]|BOLD:AAB3069
Hypena edictalis|[14409]|LMEM997-09|United States|North Carolina|658[0n]|BOLD:AAB3069
Hypena edictalis|[14410]|LMEM995-09|United States|North Carolina|658[0n]|BOLD:AAB3069
Hypena edictalis|[14411]|LPMNB262-09|Canada|Manitoba|658[0n]|BOLD:AAB3069
Hypena edictalis|[14412]|LPMNB261-09|Canada|Manitoba|658[0n]|BOLD:AAB3069
Hypena edictalis|[14413]|QUNOB511-09|United States|Kentucky|658[0n]|BOLD:AAB3069
Hypena edictalis|[14414]|LGSMG545-07|United States|North Carolina|657[0n]|BOLD:AAB3069
Hypena edictalis|[14415]|LGSMG544-07|United States|Tennessee|658[0n]|BOLD:AAB3069
Hypena edictalis|[14416]|LSEU694-06|United States|Georgia|658[0n]|BOLD:AAB3069
Hypena edictalis|[14417]|LSEU693-06|United States|Georgia|658[0n]|BOLD:AAB3069
Hypena edictalis|[14418]|LGSM711-04|United States|North Carolina|658[0n]|BOLD:AAB3069
Hypena edictalis|[14419]|LPMNB260-09|Canada|Manitoba|613[0n]|BOLD:AAB3069
Hypena edictalis|[14420]|QUNOB510-09|United States|Kentucky|658[0n]|BOLD:AAB3069
Hypena edictalis|[14421]|QUNOB512-09|United States|Kentucky|658[0n]|BOLD:AAB3069
Hypena edictalis|[14422]|LOT150-04|United States|Tennessee|609[0n]|BOLD:AAB3069
Hypena edictalis|[14423]|LGSMG546-07|United States|North Carolina|643[0n]|BOLD:AAB3069
Hypena edictalis|[14424]|LGSM710-04|United States|North Carolina|615[2n]|BOLD:AAB3069
Hypena edictalis|[14425]|RDLQH130-06|Canada|Quebec|606[5n]|BOLD:AAB3069
Hypena edictalis|[14426]|RDLQH131-06|Canada|Quebec|605[0n]|BOLD:AAB3069
Hypena edictalis|[14427]|LMEM996-09|United States|North Carolina|630[0n]|BOLD:AAB3069
Hypena edictalis|[14428]|CNEIE1810-12|Canada|Alberta|603[0n]|BOLD:AAB3069
Hypena edictalis|[14429]|CNEIF2114-12|Canada|Alberta|591[0n]|BOLD:AAB3069
Hypena edictalis|[14430]|CNEIE1806-12|Canada|Alberta|582[0n]|BOLD:AAB3069
Hypena edictalis|[14431]|SSPAB4516-13|Canada|Saskatchewan|591[0n]|BOLD:AAB3069
Hypena atomaria|[14432]|SSEIB12906-13|Canada|Alberta|576[0n]|BOLD:ACF1119
Hypena atomaria|[14433]|BLTIB272-08|Canada|Ontario|658[1n]|BOLD:ACF1119
Hypena atomaria|[14434]|SSEIB12891-13|Canada|Alberta|658[0n]|BOLD:ACF1119
Hypena atomaria|[14435]|RDNMG592-08|Canada|Ontario|658[0n]|BOLD:ACF1119
Hypena atomaria|[14436]|RDMAB960-09|Canada|Alberta|622[1n]|BOLD:ACF1119
Hypena atomaria|[14437]|SSEIB12903-13|Canada|Alberta|579[0n]|BOLD:ACF1119
Hypena atomaria|[14438]|LMDH148-11|United States|Minnesota|658[0n]|BOLD:ACF1119
Hypena atomaria|[14439]|SSEIA7721-13|Canada|Alberta|624[0n]|BOLD:ACF1119
Hypena atomaria|[14440]|SSEIB12898-13|Canada|Alberta|591[0n]|BOLD:ACF1119
Hypena atomaria|[14441]|RDMAB958-09|Canada|Alberta|625[0n]|BOLD:ACF1119
Hypena atomaria|[14442]|SSEIB12913-13|Canada|Alberta|552[0n]|BOLD:ACF1119
Hypena atomaria|[14443]|SSEIB12902-13|Canada|Alberta|552[0n]|BOLD:ACF1119
Hypena atomaria|[14444]|RDLQH126-06|Canada|Quebec|600[0n]|BOLD:ACF1119
Hypena atomaria|[14445]|RDMAB959-09|Canada|Alberta|584[0n]|BOLD:ACF1119
Hypena atomaria|[14446]|SSEIB12914-13|Canada|Alberta|579[0n]|BOLD:ACF1119
Hypena ramstadtii|[14447]|RDNMI046-10|United States|Florida|658[0n]|BOLD:AAY4916
Hypena ramstadtii|[14448]|LNCC1817-13|United States|North Carolina|658[0n]|BOLD:AAY4916
Hypena heuloa|[14449]|RDNMJ352-11|United States|Arizona|658[0n]|BOLD:AAX1801
Hypena vega|[14450]|RDNMJ353-11|United States|Arizona|658[0n]|BOLD:AAV6583
Hypena vega|[14451]|RDNMJ731-11|United States|Arizona|658[0n]|BOLD:AAV6583
Hypena degesalis|[14452]|HKONS178-08|United States|Florida|658[0n]|BOLD:AAC9452
Hypena degesalis|[14453]|HKONS180-08|United States|Florida|658[0n]|BOLD:AAC9452
Hypena degesalis|[14454]|RDNMD534-06|United States|Florida|658[0n]|BOLD:AAC9452
Hypena degesalis|[14455]|HKONS179-08|United States|Florida|657[0n]|BOLD:AAC9452
Hypena degesalis|[14456]|HKONS181-08|United States|Florida|658[0n]|BOLD:AAC9452
Hypena degesalis|[14457]|HKONS651-08|United States|Florida|658[0n]|BOLD:AAC9452
Hypena porrectalis|[14458]|CNCLB1629-14|United States|Florida|658[0n]|BOLD:AAB3497
Hypena porrectalis|[14459]|CNCLB1630-14|United States|Florida|658[0n]|BOLD:AAB3497
Hypena decorata|[14460]|RDNMF108-08|United States|California|640[0n]|BOLD:AAX1808
Hypena sp.|[14461]|LMIS026-05|Canada|Ontario|658[0n]|BOLD:AAB1352
Hypena humuli|[14462]|RDLQF378-06|Canada|Quebec|641[0n]|BOLD:AAB1353
Hypena humuli|[14463]|ABNCC145-07|United States|Wisconsin|592[1n]|BOLD:AAB1353
Hypena humuli|[14464]|LMEMB003-09|United States|North Carolina|658[1n]|BOLD:AAB1349
Hypena humuli|[14465]|LMEMB002-09|United States|North Carolina|658[0n]|BOLD:AAB1349
Hypena humuli|[14466]|LGSMC485-05|United States|Tennessee|617[0n]|BOLD:AAB1349
Hypena humuli|[14467]|PMG119-03|Canada|Ontario|617[0n]|BOLD:AAB1349
Hypena humuli|[14468]|RDLQF251-06|Canada|Quebec|634[0n]|BOLD:AAB1349
Hypena humuli|[14469]|RDLQF830-06|Canada|Quebec|658[0n]|BOLD:AAB1349
Hypena humuli|[14470]|KPOEC081-08|Canada|Ontario|658[0n]|BOLD:AAB1349
Hypena humuli|[14471]|KPOEC187-08|Canada|Ontario|658[0n]|BOLD:AAB1349
Hypena humuli|[14472]|HKONB410-09|United States|Kentucky|658[0n]|BOLD:AAB1349
Hypena humuli|[14473]|BBLPB152-10|Canada|Saskatchewan|658[0n]|BOLD:AAB1349
Hypena humuli|[14474]|LBCA051-05|Canada|British Columbia|658[0n]|BOLD:AAB1350
Hypena humuli|[14475]|LBCA047-05|Canada|British Columbia|658[0n]|BOLD:AAB1350
Hypena humuli|[14476]|LBCA048-05|Canada|British Columbia|658[0n]|BOLD:AAB1350
Hypena humuli|[14477]|LBCA049-05|Canada|British Columbia|658[0n]|BOLD:AAB1350
Hypena humuli|[14478]|LBCA050-05|Canada|British Columbia|658[0n]|BOLD:AAB1350

Hypena humuli|[14476]|LBCA048-05|Canada|British Columbia|658[0n]|BOLD:AAB1350
Hypena humuli|[14477]|LBCA049-05|Canada|British Columbia|658[0n]|BOLD:AAB1350
Hypena humuli|[14478]|LBCA050-05|Canada|British Columbia|658[0n]|BOLD:AAB1350
Hypena humuli|[14479]|LBCA516-05|Canada|British Columbia|658[0n]|BOLD:AAB1350
Hypena humuli|[14480]|LOWCB178-05|Canada|British Columbia|658[0n]|BOLD:AAB1350
Hypena humuli|[14481]|LOPN069-06|United States|Oregon|657[0n]|BOLD:AAB1350
Hypena humuli|[14482]|LBCA052-05|Canada|British Columbia|658[0n]|BOLD:AAB1350
Hypena humuli|[14483]|LBCA377-05|Canada|British Columbia|658[0n]|BOLD:AAB1350
Hypena humuli|[14484]|LBCA398-05|Canada|British Columbia|658[0n]|BOLD:AAB1350
Hypena humuli|[14485]|LBCG041-08|Canada|British Columbia|658[0n]|BOLD:AAB1350
Hypena humuli|[14486]|LBCA378-05|Canada|British Columbia|658[0n]|BOLD:ABX4983
Hypena humuli|[14487]|LOPN070-06|United States|Oregon|658[0n]|BOLD:ABX4983
Hypena humuli|[14488]|LBCA400-05|Canada|British Columbia|658[0n]|BOLD:ABX4983
Hypena humuli|[14489]|LOWCE380-06|Canada|British Columbia|658[0n]|BOLD:ABX4983
Hypena humuli|[14490]|LBCS473-07|Canada|British Columbia|658[0n]|BOLD:ABX4983
Hypena humuli|[14491]|LBCH1420-10|Canada|British Columbia|658[0n]|BOLD:ABX4983
Hypena humuli|[14492]|BBLPB153-10|Canada|British Columbia|658[0n]|BOLD:ABX4983
Hypena humuli|[14493]|LALPA1110-11|Canada|British Columbia|658[0n]|BOLD:ABX4983
Hypena subidalis|[14494]|LPYPB086-08|Mexico|Campeche|658[0n]|BOLD:AAA8284
Hypena subidalis|[14495]|BLPCI711-08|Costa Rica|Guanacaste|658[0n]|BOLD:AAA8284
Hypena subidalis|[14496]|BLPAA127-06|Costa Rica|Guanacaste|658[2n]|BOLD:AAA8284
Hypena subidalis|[14497]|BLPAA126-06|Costa Rica|Guanacaste|658[2n]|BOLD:AAA8284
Hypena subidalis|[14498]|BLPAA177-06|Costa Rica|Guanacaste|658[5n]|BOLD:AAA8284
Hypena subidalis|[14499]|BLPCI713-08|Costa Rica|Guanacaste|658[0n]|BOLD:AAA8284
Hypena subidalis|[14500]|LPYPB085-08|Mexico|Campeche|658[0n]|BOLD:AAA8284
Hypena subidalis|[14501]|LPYPB091-08|Mexico|Campeche|658[0n]|BOLD:AAA8284
Hypena subidalis|[14502]|BLPCK217-08|Costa Rica|Alajuela|658[0n]|BOLD:AAA8284
Hypena subidalis|[14503]|BLPCK722-08|Costa Rica|Alajuela|658[0n]|BOLD:AAA8284
Hypena subidalis|[14504]|BLPCK786-08|Costa Rica|Alajuela|658[0n]|BOLD:AAA8284
Hypena subidalis|[14505]|BLPDE325-09|Costa Rica|Guanacaste|658[0n]|BOLD:AAA8284
Hypena subidalis|[14506]|BLPDE326-09|Costa Rica|Guanacaste|658[0n]|BOLD:AAA8284
Hypena subidalis|[14507]|LYNYM268-09|Mexico|Quintana Roo|658[0n]|BOLD:AAA8284
Hypena subidalis|[14508]|LYHES250-09|Mexico|Quintana Roo|658[0n]|BOLD:AAA8284
Hypena subidalis|[14509]|BLPCJ478-08|Costa Rica|Guanacaste|658[0n]|BOLD:AAA8284
Hypena subidalis|[14510]|BLPCI712-08|Costa Rica|Guanacaste|658[0n]|BOLD:AAA8284
Hypena subidalis|[14511]|IBOLG174-08|Costa Rica|Alajuela|657[0n]|BOLD:AAA8284
Hypena subidalis|[14512]|BLPCJ477-08|Costa Rica|Guanacaste|658[0n]|BOLD:AAA8284
Hypena subidalis|[14513]|BLPBD289-07|Costa Rica|Guanacaste|658[0n]|BOLD:AAA8284
Hypena subidalis|[14514]|BLPBE067-07|Costa Rica|Guanacaste|658[0n]|BOLD:AAA8284
Hypena subidalis|[14515]|BLPBD287-07|Costa Rica|Guanacaste|658[0n]|BOLD:AAA8284
Hypena subidalis|[14516]|BLPBD288-07|Costa Rica|Guanacaste|658[0n]|BOLD:AAA8284
Hypena subidalis|[14517]|BLPDE323-09|Costa Rica|Guanacaste|658[0n]|BOLD:AAA8284
Hypena subidalis|[14518]|BLPDE324-09|Costa Rica|Guanacaste|658[0n]|BOLD:AAA8284
Hypena subidalis|[14519]|BLPBC817-07|Costa Rica|Alajuela|658[0n]|BOLD:AAA8284
Hypena subidalis|[14520]|BLPBD286-07|Costa Rica|Guanacaste|658[0n]|BOLD:AAA8284
Hypena subidalis|[14521]|BLPBC816-07|Costa Rica|Alajuela|658[0n]|BOLD:AAA8284
Hypena subidalis|[14522]|BLPBC815-07|Costa Rica|Alajuela|658[0n]|BOLD:AAA8284
Hypena subidalis|[14523]|BLPBC629-07|Costa Rica|Guanacaste|658[0n]|BOLD:AAA8284
Hypena subidalis|[14524]|BLPBC628-07|Costa Rica|Guanacaste|658[0n]|BOLD:AAA8284
Hypena subidalis|[14525]|BLPAD045-06|Costa Rica|Guanacaste|658[0n]|BOLD:AAA8284
Hypena subidalis|[14526]|BLPAC489-06|Costa Rica|Guanacaste|658[0n]|BOLD:AAA8284
Hypena subidalis|[14527]|BLPAA263-06|Costa Rica|Guanacaste|658[0n]|BOLD:AAA8284
Hypena subidalis|[14528]|BLPAA181-06|Costa Rica|Guanacaste|658[0n]|BOLD:AAA8284
Hypena subidalis|[14529]|BLPAA171-06|Costa Rica|Guanacaste|658[0n]|BOLD:AAA8284
Hypena subidalis|[14530]|BLPBD285-07|Costa Rica|Guanacaste|658[0n]|BOLD:AAA8284
Hypena subidalis|[14531]|BLPBB774-07|Costa Rica|Guanacaste|646[0n]|BOLD:AAA8284
Hypena subidalis|[14532]|BLPBC813-07|Costa Rica|Alajuela|643[0n]|BOLD:AAA8284
Hypena subidalis|[14533]|BLPBC814-07|Costa Rica|Alajuela|612[0n]|BOLD:AAA8284
Hypena subidalis|[14534]|BLPBC818-07|Costa Rica|Alajuela|648[0n]|BOLD:AAA8284
Hypena subidalis|[14535]|BLPBC819-07|Costa Rica|Alajuela|647[0n]|BOLD:AAA8284
Hypena subidalis|[14536]|LYNYM329-09|Mexico|Quintana Roo|577[0n]|BOLD:AAA8284
Hypena subidalis|[14537]|BLPDZ422-11|Costa Rica|Alajuela|658[0n]|BOLD:AAA8284
Hypena scabra|[14538]|LPSOC325-08|Canada|Ontario|648[0n]|BOLD:AAA4222
Hypena scabra|[14539]|LILLA505-11|United States|Illinois|658[0n]|BOLD:AAA4222
Hypena scabra|[14540]|CNPPA3957-12|Canada|Ontario|577[0n]|BOLD:AAA4222
Hypena scabra|[14541]|UDLEP062-09|United States|Maryland|658[0n]|BOLD:AAA4222
Hypena scabra|[14542]|LPSOC347-08|Canada|Ontario|656[0n]|BOLD:AAA4222
Hypena scabra|[14543]|SMTPD113-13|Canada|Ontario|567[0n]|BOLD:AAA4222
Hypena scabra|[14544]|RDLQF210-06|Canada|Quebec|658[0n]|BOLD:AAA4222
Hypena scabra|[14545]|RDLQF554-06|Canada|Quebec|658[0n]|BOLD:AAA4222
Hypena scabra|[14546]|RDLQG062-06|Canada|Quebec|658[0n]|BOLD:AAA4222
Hypena scabra|[14547]|RDLQH024-06|Canada|Quebec|656[0n]|BOLD:AAA4222
Hypena scabra|[14548]|RDLQH027-06|Canada|Quebec|656[0n]|BOLD:AAA4222
Hypena scabra|[14549]|LTOL155-06|United States|Maryland|657[0n]|BOLD:AAA4222
Hypena scabra|[14550]|LPSOC349-08|Canada|Ontario|658[0n]|BOLD:AAA4222
Hypena scabra|[14551]|LPSOC391-08|Canada|Ontario|658[0n]|BOLD:AAA4222
Hypena scabra|[14552]|LPSOC393-08|Canada|Ontario|658[0n]|BOLD:AAA4222
Hypena scabra|[14553]|BLTIB871-08|Canada|Ontario|658[0n]|BOLD:AAA4222
Hypena scabra|[14554]|BLTIB923-08|Canada|Ontario|658[0n]|BOLD:AAA4222
Hypena scabra|[14555]|LPOKA337-08|United States|Oklahoma|658[0n]|BOLD:AAA4222
Hypena scabra|[14556]|LPOKA347-08|United States|Oklahoma|658[0n]|BOLD:AAA4222
Hypena scabra|[14557]|BLGSM007-09|Canada|Ontario|658[0n]|BOLD:AAA4222
Hypena scabra|[14558]|LGSMG550-07|United States|North Carolina|658[0n]|BOLD:AAA4222
Hypena scabra|[14559]|LPSOC348-08|Canada|Ontario|658[0n]|BOLD:AAA4222
Hypena scabra|[14560]|RDLQF201-06|Canada|Quebec|658[0n]|BOLD:AAA4222
Hypena scabra|[14561]|RDLQF202-06|Canada|Quebec|658[0n]|BOLD:AAA4222
Hypena scabra|[14562]|XAH877-05|Canada|Ontario|658[0n]|BOLD:AAA4222
Hypena scabra|[14563]|XAH846-05|Canada|Ontario|658[0n]|BOLD:AAA4222
Hypena scabra|[14564]|XAH840-05|Canada|Ontario|658[0n]|BOLD:AAA4222
Hypena scabra|[14565]|XAH825-05|Canada|Ontario|658[0n]|BOLD:AAA4222
Hypena scabra|[14566]|XAH821-05|Canada|Ontario|658[0n]|BOLD:AAA4222
Hypena scabra|[14567]|XAH666-05|Canada|Ontario|658[0n]|BOLD:AAA4222
Hypena scabra|[14568]|LGSMD676-05|United States|North Carolina|658[0n]|BOLD:AAA4222
Hypena scabra|[14569]|XAB792-05|Canada|Ontario|658[0n]|BOLD:AAA4222
Hypena scabra|[14570]|XAD465-04|Canada|Ontario|658[0n]|BOLD:AAA4222
Hypena scabra|[14571]|XAE151-04|Canada|Ontario|658[0n]|BOLD:AAA4222
Hypena scabra|[14572]|XAB543-04|Canada|Ontario|658[0n]|BOLD:AAA4222
Hypena scabra|[14573]|LOT511-04|United States|Tennessee|658[0n]|BOLD:AAA4222
Hypena scabra|[14574]|LOT166-04|United States|Tennessee|658[0n]|BOLD:AAA4222
Hypena scabra|[14575]|XAH874-05|Canada|Ontario|658[0n]|BOLD:AAA4222
Hypena scabra|[14576]|PHMNB270-04|Canada|New Brunswick|658[0n]|BOLD:AAA4222
Hypena scabra|[14577]|LPSOC374-08|Canada|Ontario|652[0n]|BOLD:AAA4222
Hypena scabra|[14578]|LPOKA665-09|United States|Oklahoma|637[0n]|BOLD:AAA4222

Hypena scabra|[14576]|PHMNB270-04|Canada|New Brunswick|658[0n]|BOLD:AAA4222
Hypena scabra|[14577]|LPSOC374-08|Canada|Ontario|652[0n]|BOLD:AAA4222
Hypena scabra|[14578]|LPOKA665-09|United States|Oklahoma|637[0n]|BOLD:AAA4222
Hypena scabra|[14579]|LPOKA578-09|United States|Oklahoma|658[0n]|BOLD:AAA4222
Hypena scabra|[14580]|UDLEP058-09|United States|Maryland|658[0n]|BOLD:AAA4222
Hypena scabra|[14581]|UDLEP067-09|United States|Maryland|658[0n]|BOLD:AAA4222
Hypena scabra|[14582]|UDLEP087-09|United States|Maryland|658[0n]|BOLD:AAA4222
Hypena scabra|[14583]|UDLEP207-09|United States|Delaware|658[0n]|BOLD:AAA4222
Hypena scabra|[14584]|UDLEP212-09|United States|Delaware|658[0n]|BOLD:AAA4222
Hypena scabra|[14585]|UDLEP230-09|United States|Delaware|658[0n]|BOLD:AAA4222
Hypena scabra|[14586]|UDLEP247-09|United States|Pennsylvania|658[0n]|BOLD:AAA4222
Hypena scabra|[14587]|UDLEP266-09|United States|Delaware|658[0n]|BOLD:AAA4222
Hypena scabra|[14588]|UDLEP274-09|United States|Delaware|658[0n]|BOLD:AAA4222
Hypena scabra|[14589]|UDLEP276-09|United States|Delaware|658[0n]|BOLD:AAA4222
Hypena scabra|[14590]|UDLEP282-09|United States|Delaware|658[0n]|BOLD:AAA4222
Hypena scabra|[14591]|LPOKA875-09|United States|Oklahoma|658[0n]|BOLD:AAA4222
Hypena scabra|[14592]|LPOKA883-09|United States|Oklahoma|658[0n]|BOLD:AAA4222
Hypena scabra|[14593]|LPOKA1025-09|United States|Oklahoma|658[0n]|BOLD:AAA4222
Hypena scabra|[14594]|LMEMB004-09|United States|Mississippi|658[0n]|BOLD:AAA4222
Hypena scabra|[14595]|LMEMB005-09|United States|Texas|658[0n]|BOLD:AAA4222
Hypena scabra|[14596]|LMEMB006-09|United States|Mississippi|658[0n]|BOLD:AAA4222
Hypena scabra|[14597]|MECD360-06|United States|Maryland|658[0n]|BOLD:AAA4222
Hypena scabra|[14598]|XAK282-06|Canada|Ontario|658[0n]|BOLD:AAA4222
Hypena scabra|[14599]|LMEMB007-09|United States|Mississippi|658[0n]|BOLD:AAA4222
Hypena scabra|[14600]|LMEMB008-09|United States|Mississippi|658[0n]|BOLD:AAA4222
Hypena scabra|[14601]|BBLSX366-09|United States|Oklahoma|658[0n]|BOLD:AAA4222
Hypena scabra|[14602]|BBLSX423-09|United States|Oklahoma|658[0n]|BOLD:AAA4222
Hypena scabra|[14603]|USLEP1222-10|United States|Arkansas|658[0n]|BOLD:AAA4222
Hypena scabra|[14604]|USLEP1224-10|United States|Arkansas|658[0n]|BOLD:AAA4222
Hypena scabra|[14605]|LILLA922-11|United States|Illinois|658[0n]|BOLD:AAA4222
Hypena scabra|[14606]|IAWLB386-11|United States|Virginia|658[0n]|BOLD:AAA4222
Hypena scabra|[14607]|BBLOD1339-11|United States|Oklahoma|658[0n]|BOLD:AAA4222
Hypena scabra|[14608]|ABCBF176-12|United States|Texas|658[0n]|
Hypena scabra|[14609]|BLTIB870-08|Canada|Ontario|658[0n]|BOLD:AAA4222
Hypena scabra|[14610]|UDLEP280-09|United States|Delaware|658[0n]|BOLD:AAA4222
Hypena scabra|[14611]|LPOKB682-09|United States|Oklahoma|658[0n]|BOLD:AAA4222
Hypena scabra|[14612]|CNPPA3959-12|Canada|Ontario|606[0n]|BOLD:AAA4222
Hypena scabra|[14613]|CNPPA3926-12|Canada|Ontario|606[0n]|BOLD:AAA4222
Hypena scabra|[14614]|BLTIB842-08|Canada|Ontario|658[0n]|BOLD:AAA4222
Hypena scabra|[14615]|UDLEP278-09|United States|Delaware|658[0n]|BOLD:AAA4222
Hypena scabra|[14616]|LOT165-04|United States|Tennessee|609[0n]|BOLD:AAA4222
Hypena scabra|[14617]|LNC648-06|United States|North Carolina|621[0n]|BOLD:AAA4222
Hypena scabra|[14618]|XAB684-04|Canada|Ontario|642[0n]|BOLD:AAA4222
Hypena scabra|[14619]|LGSMG548-07|United States|Tennessee|640[0n]|BOLD:AAA4222
Hypena scabra|[14620]|PHMO206-03|Canada|Ontario|639[3n]|BOLD:AAA4222
Hypena scabra|[14621]|UDLEP306-09|United States|Pennsylvania|631[0n]|BOLD:AAA4222
Hypena scabra|[14622]|XAD269-04|Canada|Ontario|595[0n]|BOLD:AAA4222
Hypena scabra|[14623]|XAD005-04|Canada|Ontario|554[0n]|BOLD:AAA4222
Hypena scabra|[14624]|PHMNB288-04|Canada|New Brunswick|605[0n]|BOLD:AAA4222
Hypena scabra|[14625]|PHMNB277-04|Canada|New Brunswick|594[10n]|BOLD:AAA4222
Hypena scabra|[14626]|UDLEP029-09|United States|Maryland|586[0n]|BOLD:AAA4222
Hypena scabra|[14627]|LPOKA870-09|United States|Oklahoma|608[0n]|BOLD:AAA4222
Hypena scabra|[14628]|CNPPA3960-12|Canada|Ontario|598[0n]|BOLD:AAA4222
Hypena scabra|[14629]|CNPPA3972-12|Canada|Ontario|592[0n]|BOLD:AAA4222
Hypena scabra|[14630]|SMTPD1569-13|Canada|Ontario|576[0n]|BOLD:AAA4222
Hypena scabra|[14631]|XAH753-05|Canada|Ontario|658[0n]|BOLD:AAA4222
Hypena scabra|[14632]|LGSM746-04|United States|Tennessee|658[0n]|BOLD:AAA4222
Hypena scabra|[14633]|CNBPK387-13|Canada|Ontario|568[0n]|BOLD:AAA4222
Hypena scabra|[14634]|RDLQH025-06|Canada|Quebec|658[0n]|BOLD:AAA4222
Hypena scabra|[14635]|ABNCC147-07|United States|Wisconsin|651[0n]|BOLD:AAA4222
Hypena scabra|[14636]|XAB793-05|Canada|Ontario|658[0n]|BOLD:AAA4222
Hypena scabra|[14637]|MNAF372-08|Canada|Manitoba|658[0n]|BOLD:AAA4222
Hypena scabra|[14638]|BBLSX649-09|United States|Oklahoma|658[0n]|BOLD:AAA4222
Hypena scabra|[14639]|IAWLB270-11|United States|Virginia|658[0n]|BOLD:AAA4222
Hypena scabra|[14640]|LPOKA876-09|United States|Oklahoma|658[0n]|BOLD:AAA4222
Hypena scabra|[14641]|LPOKA877-09|United States|Oklahoma|658[0n]|BOLD:AAA4222
Hypena scabra|[14642]|BLTIB512-08|Canada|Ontario|658[0n]|BOLD:AAA4222
Hypena scabra|[14643]|BLTIB1012-08|Canada|Ontario|658[0n]|BOLD:AAA4222
Hypena scabra|[14644]|LPSOC373-08|Canada|Ontario|656[0n]|BOLD:AAA4222
Hypena scabra|[14645]|LPOKA211-08|United States|Oklahoma|658[0n]|BOLD:AAA4222
Hypena scabra|[14646]|RDLQH026-06|Canada|Quebec|658[0n]|BOLD:AAA4222
Hypena scabra|[14647]|LPSOC372-08|Canada|Ontario|656[0n]|BOLD:AAA4222
Hypena scabra|[14648]|XAJ160-06|Canada|Ontario|658[0n]|BOLD:AAA4222
Hypena scabra|[14649]|LNCB108-06|United States|North Carolina|658[0n]|BOLD:AAA4222
Hypena scabra|[14650]|XAH875-05|Canada|Ontario|658[0n]|BOLD:AAA4222
Hypena scabra|[14651]|XAH842-05|Canada|Ontario|658[0n]|BOLD:AAA4222
Hypena scabra|[14652]|XAH838-05|Canada|Ontario|658[0n]|BOLD:AAA4222
Hypena scabra|[14653]|XAH801-05|Canada|Ontario|658[0n]|BOLD:AAA4222
Hypena scabra|[14654]|XAH737-05|Canada|Ontario|658[0n]|BOLD:AAA4222
Hypena scabra|[14655]|PHMNB294-04|Canada|New Brunswick|658[0n]|BOLD:AAA4222
Hypena scabra|[14656]|XAB541-04|Canada|Ontario|658[0n]|BOLD:AAA4222
Hypena scabra|[14657]|LOT492-04|United States|Tennessee|658[0n]|BOLD:AAA4222
Hypena scabra|[14658]|CNPPA3958-12|Canada|Ontario|606[0n]|BOLD:AAA4222
Hypena scabra|[14659]|CNPPI010-12|Canada|Ontario|637[0n]|BOLD:AAA4222
Hypena scabra|[14660]|CNRMC1489-12|Canada|Manitoba|634[0n]|BOLD:AAA4222
Hypena scabra|[14661]|CNPPI011-12|Canada|Ontario|640[0n]|BOLD:AAA4222
Hypena scabra|[14662]|LPSOC364-08|Canada|Ontario|653[0n]|BOLD:AAA4222
Hypena scabra|[14663]|LOT189-04|United States|Tennessee|609[0n]|BOLD:AAA4222
Hypena scabra|[14664]|PHMNB036-03|Canada|New Brunswick|639[0n]|BOLD:AAA4222
Hypena scabra|[14665]|PHMO075-03|Canada|Ontario|639[0n]|BOLD:AAA4222
Hypena scabra|[14666]|PHMNB279-04|Canada|New Brunswick|573[0n]|BOLD:AAA4222
Hypena scabra|[14667]|XAH711-05|Canada|Ontario|622[0n]|BOLD:AAA4222
Hypena scabra|[14668]|XAB524-04|Canada|Ontario|582[1n]|BOLD:AAA4222
Hypena scabra|[14669]|XAB668-04|Canada|Ontario|616[0n]|BOLD:AAA4222
Hypena scabra|[14670]|ABNCC148-07|United States|Indiana|594[0n]|BOLD:AAA4222
Hypena scabra|[14671]|LGSMG549-07|United States|North Carolina|609[0n]|BOLD:AAA4222
Hypena scabra|[14672]|UDLEP049-09|United States|Maryland|612[0n]|BOLD:AAA4222
Hypena scabra|[14673]|CNPPE1038-12|Canada|Ontario|635[0n]|BOLD:AAA4222
Hypena scabra|[14674]|CNPPI014-12|Canada|Ontario|635[0n]|BOLD:AAA4222
Hypena scabra|[14675]|CNPPI016-12|Canada|Ontario|640[0n]|BOLD:AAA4222
Hypena scabra|[14676]|CNPPA3961-12|Canada|Ontario|593[0n]|BOLD:AAA4222
Hypena scabra|[14677]|CNPPB1406-12|Canada|Ontario|603[0n]|BOLD:AAA4222
Hypena scabra|[14678]|CNRMG779-12|Canada|Manitoba|622[0n]|BOLD:AAA4222

Hypena scabra|[14676]|CNPPA3961-12|Canada|Ontario|593[0n]|BOLD:AAA4222
Hypena scabra|[14677]|CNPPB1406-12|Canada|Ontario|603[0n]|BOLD:AAA4222
Hypena scabra|[14678]|CNRMG779-12|Canada|Manitoba|622[0n]|BOLD:AAA4222
Hypena scabra|[14679]|SMTPB19950-13|Canada|Ontario|573[0n]|BOLD:AAA4222
Hypena scabra|[14680]|CNSLT032-13|Canada|Ontario|582[0n]|BOLD:AAA4222
Hypena scabra|[14681]|SMTPD1570-13|Canada|Ontario|606[0n]|BOLD:AAA4222
Hypena baltimoralis|[14682]|RDLQG350-06|Canada|Quebec|658[0n]|BOLD:AAA2330
Hypena baltimoralis|[14683]|RDLQG356-06|Canada|Quebec|658[0n]|BOLD:AAA2330
Hypena baltimoralis|[14684]|XAC124-04|Canada|Ontario|554[2n]|BOLD:AAA2330
Hypena baltimoralis|[14685]|LOCT352-05|United States|Connecticut|658[0n]|BOLD:AAA2330
Hypena baltimoralis|[14686]|LSEU362-06|United States|Georgia|605[1n]|BOLD:AAA2330
Hypena baltimoralis|[14687]|LOTB233-05|United States|Tennessee|540[1n]|BOLD:AAA2330
Hypena baltimoralis|[14688]|JRLAA006-09|United States|Alabama|658[0n]|BOLD:AAA2330
Hypena baltimoralis|[14689]|HKONS573-08|United States|Florida|658[0n]|BOLD:AAA2330
Hypena baltimoralis|[14690]|RDLQF880-06|Canada|Quebec|587[3n]|BOLD:AAA2330
Hypena baltimoralis|[14691]|BBLEC641-09|Canada|Nova Scotia|658[0n]|BOLD:AAA2330
Hypena baltimoralis|[14692]|MECD361-06|United States|Maryland|656[0n]|BOLD:AAA2330
Hypena baltimoralis|[14693]|TTMNB271-06|Canada|New Brunswick|658[0n]|BOLD:AAA2330
Hypena baltimoralis|[14694]|LMDH136-11|United States|Minnesota|658[0n]|BOLD:AAA2330
Hypena baltimoralis|[14695]|RDLQF326-06|Canada|Quebec|658[0n]|BOLD:AAA2330
Hypena baltimoralis|[14696]|PHMNB179-04|Canada|New Brunswick|505[0n]|BOLD:AAA2330
Hypena baltimoralis|[14697]|PHMO312-03|Canada|Ontario|639[0n]|BOLD:AAA2330
Hypena baltimoralis|[14698]|RDLQF888-06|Canada|Quebec|625[0n]|BOLD:AAA2330
Hypena baltimoralis|[14699]|PHMO321-03|Canada|Ontario|639[0n]|BOLD:AAA2330
Hypena baltimoralis|[14700]|TTMNB088-06|Canada|New Brunswick|656[0n]|BOLD:AAA2330
Hypena baltimoralis|[14701]|RDLQF486-06|Canada|Quebec|646[0n]|BOLD:AAA2330
Hypena baltimoralis|[14702]|RDLQF760-06|Canada|Quebec|658[0n]|BOLD:AAA2330
Hypena baltimoralis|[14703]|RDLQG238-06|Canada|Quebec|658[0n]|BOLD:AAA2330
Hypena baltimoralis|[14704]|RDLQG352-06|Canada|Quebec|658[0n]|BOLD:AAA2330
Hypena baltimoralis|[14705]|RDLQF490-06|Canada|Quebec|658[0n]|BOLD:AAA2330
Hypena baltimoralis|[14706]|RDLQF484-06|Canada|Quebec|658[0n]|BOLD:AAA2330
Hypena baltimoralis|[14707]|RDLQF483-06|Canada|Quebec|658[0n]|BOLD:AAA2330
Hypena baltimoralis|[14708]|RDLQF431-06|Canada|Quebec|658[0n]|BOLD:AAA2330
Hypena baltimoralis|[14709]|RDLQF405-06|Canada|Quebec|658[0n]|BOLD:AAA2330
Hypena baltimoralis|[14710]|RDLQF399-06|Canada|Quebec|658[0n]|BOLD:AAA2330
Hypena baltimoralis|[14711]|RDLQF397-06|Canada|Quebec|658[0n]|BOLD:AAA2330
Hypena baltimoralis|[14712]|RDLQF396-06|Canada|Quebec|658[0n]|BOLD:AAA2330
Hypena baltimoralis|[14713]|RDLQF393-06|Canada|Quebec|658[0n]|BOLD:AAA2330
Hypena baltimoralis|[14714]|RDLQF325-06|Canada|Quebec|658[0n]|BOLD:AAA2330
Hypena baltimoralis|[14715]|RDLQF316-06|Canada|Quebec|658[0n]|BOLD:AAA2330
Hypena baltimoralis|[14716]|RDLQF196-06|Canada|Quebec|658[0n]|BOLD:AAA2330
Hypena baltimoralis|[14717]|RDLQF195-06|Canada|Quebec|658[0n]|BOLD:AAA2330
Hypena baltimoralis|[14718]|TTMNB274-06|Canada|New Brunswick|658[0n]|BOLD:AAA2330
Hypena baltimoralis|[14719]|TTMNB089-06|Canada|New Brunswick|657[1n]|BOLD:AAA2330
Hypena baltimoralis|[14720]|LOTB226-05|United States|Tennessee|658[0n]|BOLD:AAA2330
Hypena baltimoralis|[14721]|RDLQB771-05|Canada|Quebec|617[0n]|BOLD:AAA2330
Hypena baltimoralis|[14722]|RDLQF753-06|Canada|Quebec|576[2n]|BOLD:AAA2330
Hypena baltimoralis|[14723]|RDLQF758-06|Canada|Quebec|621[0n]|BOLD:AAA2330
Hypena baltimoralis|[14724]|BBLPC138-09|Canada|Nova Scotia|628[0n]|BOLD:AAA2330
Hypena baltimoralis|[14725]|GMGSX011-13|United States|Tennessee|562[0n]|BOLD:AAA2330
Hypena baltimoralis|[14726]|PHMNB108-04|Canada|New Brunswick|626[4n]|BOLD:AAA2330
Hypena baltimoralis|[14727]|RDLQF426-06|Canada|Quebec|645[0n]|BOLD:AAA2330
Hypena baltimoralis|[14728]|RDLQF485-06|Canada|Quebec|618[1n]|BOLD:AAA2330
Hypena baltimoralis|[14729]|PHMNB048-03|Canada|New Brunswick|639[0n]|BOLD:AAA2330
Hypena baltimoralis|[14730]|RDLQF402-06|Canada|Quebec|637[0n]|BOLD:AAA2330
Hypena baltimoralis|[14731]|RDLQF197-06|Canada|Quebec|658[0n]|BOLD:AAA2330
Hypena baltimoralis|[14732]|TTMNB272-06|Canada|New Brunswick|608[0n]|BOLD:AAA2330
Hypena baltimoralis|[14733]|RDLQG223-06|Canada|Quebec|608[0n]|BOLD:AAA2330
Hypena baltimoralis|[14734]|LOCT327-05|United States|Connecticut|658[0n]|BOLD:AAA2330
Hypena baltimoralis|[14735]|RDLQG061-06|Canada|Quebec|655[0n]|BOLD:AAA2330
Hypena baltimoralis|[14736]|UDLEP333-09|United States|Pennsylvania|623[0n]|BOLD:AAA2330
Hypena baltimoralis|[14737]|RDLQF319-06|Canada|Quebec|620[0n]|BOLD:AAA2330
Hypena baltimoralis|[14738]|RDLQF896-06|Canada|Quebec|594[0n]|BOLD:AAA2330
Hypena baltimoralis|[14739]|RDLQG240-06|Canada|Quebec|658[0n]|BOLD:AAA2330
Hypena baltimoralis|[14740]|RDLQG245-06|Canada|Quebec|594[0n]|BOLD:AAA2330
Hypena baltimoralis|[14741]|RDLQG241-06|Canada|Quebec|594[0n]|BOLD:AAA2330
Hypena baltimoralis|[14742]|RDLQF889-06|Canada|Quebec|593[0n]|BOLD:AAA2330
Hypena baltimoralis|[14743]|RDLQF429-06|Canada|Quebec|658[0n]|BOLD:AAA2330
Hypena baltimoralis|[14744]|RDLQF481-06|Canada|Quebec|658[0n]|BOLD:AAA2330
Hypena baltimoralis|[14745]|RDLQF403-06|Canada|Quebec|658[0n]|BOLD:AAA2330
Hypena baltimoralis|[14746]|RDLQF404-06|Canada|Quebec|658[0n]|BOLD:AAA2330
Hypena baltimoralis|[14747]|RDLQF395-06|Canada|Quebec|658[0n]|BOLD:AAA2330
Hypena baltimoralis|[14748]|RDLQF398-06|Canada|Quebec|658[0n]|BOLD:AAA2330
Hypena baltimoralis|[14749]|RDLQF327-06|Canada|Quebec|658[0n]|BOLD:AAA2330
Hypena baltimoralis|[14750]|RDLQF394-06|Canada|Quebec|658[0n]|BOLD:AAA2330
Hypena baltimoralis|[14751]|RDLQF318-06|Canada|Quebec|658[0n]|BOLD:AAA2330
Hypena baltimoralis|[14752]|RDLQF323-06|Canada|Quebec|658[0n]|BOLD:AAA2330
Hypena baltimoralis|[14753]|RDLQF227-06|Canada|Quebec|658[0n]|BOLD:AAA2330
Hypena baltimoralis|[14754]|RDLQF315-06|Canada|Quebec|658[0n]|BOLD:AAA2330
Hypena baltimoralis|[14755]|RDLQF754-06|Canada|Quebec|659[0n]|BOLD:AAA2330
Hypena baltimoralis|[14756]|RDLQF194-06|Canada|Quebec|658[0n]|BOLD:AAA2330
Hypena baltimoralis|[14757]|XAK280-06|Canada|Ontario|658[0n]|BOLD:AAA2330
Hypena baltimoralis|[14758]|TMNBB063-06|Canada|New Brunswick|658[0n]|BOLD:AAA2330
Hypena baltimoralis|[14759]|TMNBB062-06|Canada|New Brunswick|658[0n]|BOLD:AAA2330
Hypena baltimoralis|[14760]|TTMNB547-06|Canada|New Brunswick|658[0n]|BOLD:AAA2330
Hypena baltimoralis|[14761]|XAG797-05|Canada|Ontario|658[0n]|BOLD:AAA2330
Hypena baltimoralis|[14762]|LOCT304-05|United States|Connecticut|658[0n]|BOLD:AAA2330
Hypena baltimoralis|[14763]|LOCT274-05|United States|Connecticut|658[0n]|BOLD:AAA2330
Hypena baltimoralis|[14764]|LOCT273-05|United States|Connecticut|658[0n]|BOLD:AAA2330
Hypena baltimoralis|[14765]|RDLQG141-06|Canada|Quebec|658[3n]|BOLD:AAA2330
Hypena baltimoralis|[14766]|PHMNB097-04|Canada|New Brunswick|590[0n]|BOLD:AAA2330
Hypena baltimoralis|[14767]|LOTB222-05|United States|Tennessee|658[0n]|BOLD:AAA2330
Hypena baltimoralis|[14768]|RDLQF890-06|Canada|Quebec|584[0n]|BOLD:AAA2330
Hypena baltimoralis|[14769]|RDLQF762-06|Canada|Quebec|605[3n]|BOLD:AAA2330
Hypena baltimoralis|[14770]|RDLQF314-06|Canada|Quebec|658[0n]|BOLD:AAA2330
Hypena baltimoralis|[14771]|BBLPE171-09|Canada|Nova Scotia|658[0n]|BOLD:AAA2330
Hypena baltimoralis|[14772]|RDLQF482-06|Canada|Quebec|658[0n]|BOLD:AAA2330
Hypena baltimoralis|[14773]|RDLQF489-06|Canada|Quebec|658[0n]|BOLD:AAA2330
Hypena baltimoralis|[14774]|RDLQF491-06|Canada|Quebec|658[0n]|BOLD:AAA2330
Hypena baltimoralis|[14775]|RDLQF755-06|Canada|Quebec|658[0n]|BOLD:AAA2330
Hypena baltimoralis|[14776]|RDLQF759-06|Canada|Quebec|658[0n]|BOLD:AAA2330
Hypena baltimoralis|[14777]|RDLQF851-06|Canada|Quebec|658[0n]|BOLD:AAA2330
Hypena baltimoralis|[14778]|RDLQF893-06|Canada|Quebec|658[0n]|BOLD:AAA2330

[illegible]
Hypena baltimoralis|[14776]|RDLQF759-06|Canada|Quebec|658[0n]|BOLD:AAA2330
Hypena baltimoralis|[14777]|RDLQF851-06|Canada|Quebec|658[0n]|BOLD:AAA2330
Hypena baltimoralis|[14778]|RDLQF893-06|Canada|Quebec|658[0n]|BOLD:AAA2330
Hypena baltimoralis|[14779]|RDLQF894-06|Canada|Quebec|658[0n]|BOLD:AAA2330
Hypena baltimoralis|[14780]|RDLQG273-06|Canada|Quebec|658[0n]|BOLD:AAA2330
Hypena baltimoralis|[14781]|RDLQG309-06|Canada|Quebec|658[0n]|BOLD:AAA2330
Hypena baltimoralis|[14782]|RDLQF895-06|Canada|Quebec|658[0n]|BOLD:AAA2330
Hypena baltimoralis|[14783]|RDLQF897-06|Canada|Quebec|658[0n]|BOLD:AAA2330
Hypena baltimoralis|[14784]|RDLQG216-06|Canada|Quebec|658[0n]|BOLD:AAA2330
Hypena baltimoralis|[14785]|RDLQG239-06|Canada|Quebec|658[0n]|BOLD:AAA2330
Hypena baltimoralis|[14786]|RDLQG348-06|Canada|Quebec|658[0n]|BOLD:AAA2330
Hypena baltimoralis|[14787]|RDLQG357-06|Canada|Quebec|658[0n]|BOLD:AAA2330
Hypena baltimoralis|[14788]|BBLEC883-09|Canada|Nova Scotia|658[0n]|BOLD:AAA2330
Hypena baltimoralis|[14789]|BBLPC179-09|Canada|Nova Scotia|658[0n]|BOLD:AAA2330
Hypena baltimoralis|[14790]|BBLCU043-09|United States|Michigan|658[0n]|BOLD:AAA2330
Hypena baltimoralis|[14791]|BBLEC103-09|Canada|Nova Scotia|658[0n]|BOLD:AAA2330
Hypena baltimoralis|[14792]|LPSOC139-08|Canada|Ontario|658[0n]|BOLD:AAA2330
Hypena baltimoralis|[14793]|UDLEP240-09|United States|Delaware|658[0n]|BOLD:AAA2330
Hypena baltimoralis|[14794]|BBLPC443-09|Canada|New Brunswick|658[0n]|BOLD:AAA2330
Hypena baltimoralis|[14795]|BBLPE257-09|Canada|Nova Scotia|658[0n]|BOLD:AAA2330
Hypena baltimoralis|[14796]|TTMNB273-06|Canada|New Brunswick|578[0n]|BOLD:AAA2330
Hypena baltimoralis|[14797]|RDLQG311-06|Canada|Quebec|594[0n]|BOLD:AAA2330
Hypena baltimoralis|[14798]|RDLQG244-06|Canada|Quebec|582[0n]|BOLD:AAA2330
Hypena baltimoralis|[14799]|LGSM640-04|United States|Tennessee|609[0n]|BOLD:AAA2330
Hypena baltimoralis|[14800]|LOT342-04|United States|Tennessee|658[0n]|BOLD:AAA2330
Hypena baltimoralis|[14801]|LGSMC423-05|United States|Tennessee|658[0n]|BOLD:AAA2330
Hypena baltimoralis|[14802]|LGSMC424-05|United States|Tennessee|658[0n]|BOLD:AAA2330
Hypena baltimoralis|[14803]|LOTB215-05|United States|Tennessee|658[0n]|BOLD:AAA2330
Hypena baltimoralis|[14804]|LOTB229-05|United States|Tennessee|658[0n]|BOLD:AAA2330
Hypena baltimoralis|[14805]|LOTB230-05|United States|Tennessee|658[0n]|BOLD:AAA2330
Hypena baltimoralis|[14806]|LOTB231-05|United States|Tennessee|658[0n]|BOLD:AAA2330
Hypena baltimoralis|[14807]|LOTB232-05|United States|Tennessee|658[0n]|BOLD:AAA2330
Hypena baltimoralis|[14808]|LSUSA241-06|United States|Kentucky|658[0n]|BOLD:AAA2330
Hypena baltimoralis|[14809]|RDLQF245-06|Canada|Quebec|658[0n]|BOLD:AAA2330
Hypena baltimoralis|[14810]|RDLQF324-06|Canada|Quebec|658[0n]|BOLD:AAA2330
Hypena baltimoralis|[14811]|RDLQF329-06|Canada|Quebec|658[0n]|BOLD:AAA2330
Hypena baltimoralis|[14812]|RDLQF756-06|Canada|Quebec|658[0n]|BOLD:AAA2330
Hypena baltimoralis|[14813]|RDLQG347-06|Canada|Quebec|658[0n]|BOLD:AAA2330
Hypena baltimoralis|[14814]|HKONS574-08|United States|Florida|658[0n]|BOLD:AAA2330
Hypena baltimoralis|[14815]|LPOKD373-09|United States|Oklahoma|658[0n]|BOLD:AAA2330
Hypena baltimoralis|[14816]|XAD300-04|Canada|Ontario|567[1n]|BOLD:AAA2330
Hypena baltimoralis|[14817]|RDLQF320-06|Canada|Quebec|567[0n]|BOLD:AAA2330
Hypena baltimoralis|[14818]|IAWLB384-11|United States|Virginia|658[0n]|BOLD:AAA2330
Hypena baltimoralis|[14819]|LOTB223-05|United States|Tennessee|658[0n]|BOLD:AAA2330
Hypena baltimoralis|[14820]|RDLQF192-06|Canada|Quebec|632[0n]|BOLD:AAA2330
Hypena baltimoralis|[14821]|LGSMG536-07|United States|North Carolina|658[0n]|BOLD:AAA2330
Hypena baltimoralis|[14822]|RDLQF891-06|Canada|Quebec|582[0n]|BOLD:AAA2330
Hypena baltimoralis|[14823]|LOT341-04|United States|Tennessee|658[1n]|BOLD:AAA2330
Hypena baltimoralis|[14824]|LNC306-05|United States|North Carolina|614[0n]|BOLD:AAA2330
Hypena baltimoralis|[14825]|RDLQF401-06|Canada|Quebec|616[0n]|BOLD:AAA2330
Hypena baltimoralis|[14826]|LOTB227-05|United States|Tennessee|620[0n]|BOLD:AAA2330
Hypena baltimoralis|[14827]|RDLQF400-06|Canada|Quebec|630[1n]|BOLD:AAA2330
Hypena baltimoralis|[14828]|XAJ509-06|Canada|Ontario|643[0n]|BOLD:AAA2330
Hypena baltimoralis|[14829]|RDLQF757-06|Canada|Quebec|575[6n]|
Hypena baltimoralis|[14830]|RDLQF761-06|Canada|Quebec|594[3n]|BOLD:AAA2330
Hypena baltimoralis|[14831]|RDLQG242-06|Canada|Quebec|590[0n]|BOLD:AAA2330
Hypena baltimoralis|[14832]|RDLQG312-06|Canada|Quebec|583[0n]|BOLD:AAA2330
Hypena baltimoralis|[14833]|LGSMC650-05|United States|Tennessee|658[0n]|BOLD:AAA2330
Hypena baltimoralis|[14834]|LOTB214-05|United States|Tennessee|658[0n]|BOLD:AAA2330
Hypena baltimoralis|[14835]|LOTB221-05|United States|Tennessee|658[0n]|BOLD:AAA2330
Hypena baltimoralis|[14836]|LOTB225-05|United States|Tennessee|658[0n]|BOLD:AAA2330
Hypena baltimoralis|[14837]|LOTB228-05|United States|Tennessee|658[0n]|BOLD:AAA2330
Hypena baltimoralis|[14838]|LOTB234-05|United States|Tennessee|658[0n]|BOLD:AAA2330
Hypena baltimoralis|[14839]|XAH297-05|Canada|Ontario|658[0n]|BOLD:AAA2330
Hypena baltimoralis|[14840]|LNC571-06|United States|North Carolina|658[0n]|BOLD:AAA2330
Hypena baltimoralis|[14841]|LOFLA549-06|United States|Florida|658[0n]|BOLD:AAA2330
Hypena baltimoralis|[14842]|RDLQF193-06|Canada|Quebec|658[0n]|BOLD:AAA2330
Hypena baltimoralis|[14843]|RDLQF317-06|Canada|Quebec|658[0n]|BOLD:AAA2330
Hypena baltimoralis|[14844]|RDLQF430-06|Canada|Quebec|658[0n]|BOLD:AAA2330
Hypena baltimoralis|[14845]|RDLQF487-06|Canada|Quebec|658[0n]|BOLD:AAA2330
Hypena baltimoralis|[14846]|RDLQF488-06|Canada|Quebec|658[0n]|BOLD:AAA2330
Hypena baltimoralis|[14847]|RDLQG349-06|Canada|Quebec|658[0n]|BOLD:AAA2330
Hypena baltimoralis|[14848]|RDLQG351-06|Canada|Quebec|658[0n]|BOLD:AAA2330
Hypena baltimoralis|[14849]|BLTIB956-08|Canada|Ontario|658[0n]|BOLD:AAA2330
Hypena baltimoralis|[14850]|UDLEP309-09|United States|Pennsylvania|658[0n]|BOLD:AAA2330
Hypena baltimoralis|[14851]|BBLEC888-09|Canada|Nova Scotia|658[0n]|BOLD:AAA2330
Hypena baltimoralis|[14852]|BBLPE151-09|Canada|Nova Scotia|658[0n]|BOLD:AAA2330
Hypena baltimoralis|[14853]|HKONS572-08|United States|Florida|658[0n]|BOLD:AAA2330
Hypena baltimoralis|[14854]|LPSO660-08|Canada|Ontario|656[0n]|BOLD:AAA2330
Hypena baltimoralis|[14855]|LILLA026-11|United States|Illinois|658[0n]|BOLD:AAA2330
Hypena baltimoralis|[14856]|UDLEP272-09|United States|Delaware|658[0n]|BOLD:AAA2330
Hypena baltimoralis|[14857]|PHMNB361-04|Canada|New Brunswick|658[0n]|BOLD:AAA2330
Hypena baltimoralis|[14858]|LOT524-04|United States|Tennessee|658[0n]|BOLD:AAA2330
Hypena baltimoralis|[14859]|LGSMC469-05|United States|Tennessee|617[0n]|BOLD:AAA2330
Hypena baltimoralis|[14860]|GMGSY007-13|United States|Tennessee|633[0n]|BOLD:AAA2330
Hypena baltimoralis|[14861]|LGSMC651-05|United States|Tennessee|611[0n]|BOLD:AAA2330
Hypena baltimoralis|[14862]|GMGAA045-13|United States|Tennessee|633[0n]|BOLD:AAA2330
Hypena eductalis|[14863]|LOCT230-05|United States|Connecticut|658[0n]|BOLD:AAB6002
Hypena eductalis|[14864]|LGSMC655-05|United States|Tennessee|658[0n]|BOLD:AAB6002
Hypena eductalis|[14865]|LGSMG547-07|United States|Tennessee|610[0n]|BOLD:AAB6002
Hypena eductalis|[14866]|HKONS074-07|United States|Florida|658[1n]|BOLD:AAB6002
Hypena eductalis|[14867]|HKONS576-08|United States|Florida|658[0n]|BOLD:AAB6002
Hypena eductalis|[14868]|RDLQF909-06|Canada|Quebec|658[0n]|BOLD:AAB6003
Hypena eductalis|[14869]|ABNCC143-07|United States|Wisconsin|629[1n]|BOLD:AAB6003
Hypena eductalis|[14870]|RDLQF448-06|Canada|Quebec|658[0n]|BOLD:AAB6003
Hypena eductalis|[14871]|RDLQF908-06|Canada|Quebec|658[0n]|BOLD:AAB6003
Hypena eductalis|[14872]|RDLQF496-06|Canada|Quebec|658[0n]|BOLD:AAB6003
Hypena eductalis|[14873]|RDLQF386-06|Canada|Quebec|658[0n]|BOLD:AAB6003
Hypena eductalis|[14874]|RDLQF385-06|Canada|Quebec|648[0n]|BOLD:AAB6003
Hypena eductalis|[14875]|RDLQG604-06|Canada|Quebec|621[0n]|BOLD:AAB6003
Hypena eductalis|[14876]|ABNCC144-07|United States|Wisconsin|594[0n]|BOLD:AAB6003
Hypena eductalis|[14877]|BBLPC923-09|Canada|Newfoundland and Labrador|658[0n]|BOLD:AAB6003
Hypena eductalis|[14878]|BBLPE097-09|Canada|Nova Scotia|658[0n]|BOLD:AAB6003

Hypena eductalis|[14875]|RDLQG604-06|Canada|Quebec|621[0n]|BOLD:AAB6003
Hypena eductalis|[14876]|ABNCC144-07|United States|Wisconsin|594[0n]|BOLD:AAB6003
Hypena eductalis|[14877]|BBLPC923-09|Canada|Newfoundland and Labrador|658[0n]|BOLD:AAB6003
Hypena eductalis|[14878]|BBLPE097-09|Canada|Nova Scotia|658[0n]|BOLD:AAB6003
Hypena eductalis|[14879]|BBLPC814-09|Canada|Newfoundland and Labrador|658[0n]|BOLD:AAB6003
Hypena eductalis|[14880]|BBLPC783-09|Canada|Newfoundland and Labrador|658[0n]|BOLD:AAB6003
Hypena eductalis|[14881]|RDLQG315-06|Canada|Quebec|658[0n]|BOLD:AAB6003
Hypena eductalis|[14882]|BBLPE002-09|Canada|Nova Scotia|622[0n]|BOLD:AAB6003
Hypena eductalis|[14883]|BBLPE255-09|Canada|Nova Scotia|635[0n]|BOLD:AAB6003
Hypena bijugalis|[14884]|RDLQB255-05|Canada|Quebec|658[0n]|BOLD:AAA8814
Hypena bijugalis|[14885]|JSJUL1658-11|Canada|Ontario|658[0n]|BOLD:AAA8814
Hypena bijugalis|[14886]|XAJ717-06|Canada|Ontario|658[0n]|BOLD:AAA8814
Hypena bijugalis|[14887]|XAJ516-06|Canada|Ontario|658[0n]|BOLD:AAA8814
Hypena bijugalis|[14888]|XAJ394-06|Canada|Ontario|658[0n]|BOLD:AAA8814
Hypena bijugalis|[14889]|RDLQH124-06|Canada|Quebec|588[0n]|BOLD:AAA8814
Hypena bijugalis|[14890]|CNEIB1519-12|Canada|Alberta|625[0n]|BOLD:AAA8814
Hypena bijugalis|[14891]|SSPAC3390-13|Canada|Saskatchewan|600[0n]|BOLD:AAA8814
Hypena bijugalis|[14892]|LOTB224-05|United States|Tennessee|658[0n]|BOLD:AAA8814
Hypena bijugalis|[14893]|LNC843-06|United States|North Carolina|658[0n]|BOLD:AAA8814
Hypena bijugalis|[14894]|UDLEP092-09|United States|Maryland|658[0n]|BOLD:AAA8814
Hypena bijugalis|[14895]|LMEM977-09|United States|Mississippi|658[1n]|BOLD:AAA8814
Hypena bijugalis|[14896]|LGSM755-04|United States|Tennessee|658[0n]|BOLD:AAA8814
Hypena bijugalis|[14897]|LNCB449-07|United States|North Carolina|658[0n]|BOLD:AAA8814
Hypena bijugalis|[14898]|LNCB450-07|United States|North Carolina|658[0n]|BOLD:AAA8814
Hypena bijugalis|[14899]|HKONS569-08|United States|Florida|658[0n]|BOLD:AAA8814
Hypena bijugalis|[14900]|LPSO692-08|Canada|Ontario|658[0n]|BOLD:AAA8814
Hypena bijugalis|[14901]|LPSO748-08|Canada|Ontario|658[0n]|BOLD:AAA8814
Hypena bijugalis|[14902]|LMEM979-09|United States|Mississippi|658[0n]|BOLD:AAA8814
Hypena bijugalis|[14903]|LPOKB276-09|United States|Oklahoma|658[1n]|BOLD:AAA8814
Hypena bijugalis|[14904]|LPSO661-08|Canada|Ontario|616[0n]|BOLD:AAA8814
Hypena bijugalis|[14905]|LPOKC363-09|United States|Oklahoma|658[0n]|BOLD:AAA8814
Hypena bijugalis|[14906]|BBLCU250-09|United States|Michigan|658[0n]|BOLD:AAA8814
Hypena bijugalis|[14907]|LPOKB210-09|United States|Oklahoma|658[0n]|BOLD:AAA8814
Hypena bijugalis|[14908]|LMEM978-09|United States|Mississippi|658[0n]|BOLD:AAA8814
Hypena bijugalis|[14909]|LPOKA117-08|United States|Oklahoma|658[0n]|BOLD:AAA8814
Hypena bijugalis|[14910]|LPSO890-08|Canada|Ontario|658[0n]|BOLD:AAA8814
Hypena bijugalis|[14911]|LPSO470-08|Canada|Ontario|656[0n]|BOLD:AAA8814
Hypena bijugalis|[14912]|LPSO281-08|Canada|Ontario|658[0n]|BOLD:AAA8814
Hypena bijugalis|[14913]|LPSO189-08|Canada|Ontario|658[0n]|BOLD:AAA8814
Hypena bijugalis|[14914]|LPSO063-08|Canada|Ontario|658[0n]|BOLD:AAA8814
Hypena bijugalis|[14915]|HKONS568-08|United States|Florida|658[0n]|BOLD:AAA8814
Hypena bijugalis|[14916]|LBCS474-07|Canada|British Columbia|658[0n]|BOLD:AAA8814
Hypena bijugalis|[14917]|RDNMD597-06|United States|Florida|658[0n]|BOLD:AAA8814
Hypena bijugalis|[14918]|LSEU397-06|United States|Georgia|658[0n]|BOLD:AAA8814
Hypena bijugalis|[14919]|XAG303-05|Canada|Ontario|658[0n]|BOLD:AAA8814
Hypena bijugalis|[14920]|LOT160-04|United States|Tennessee|570[0n]|BOLD:AAA8814
Hypena bijugalis|[14921]|CNPPE1062-12|Canada|Ontario|632[0n]|BOLD:AAA8814
Hypena bijugalis|[14922]|RDLQB240-05|Canada|Quebec|603[0n]|BOLD:AAA8814
Hypena bijugalis|[14923]|ABNCC136-07|United States|Texas|595[0n]|BOLD:AAA8814
Hypena bijugalis|[14924]|XAK488-07|Canada|Ontario|575[0n]|BOLD:AAA8814
Hypena bijugalis|[14925]|XAK489-07|Canada|Ontario|575[0n]|BOLD:AAA8814
Hypena bijugalis|[14926]|LMEM976-09|United States|Alabama|632[0n]|BOLD:AAA8814
Hypena bijugalis|[14927]|CNPPH816-12|Canada|Ontario|644[0n]|BOLD:AAA8814
Hypena bijugalis|[14928]|SMTPD633-13|Canada|Ontario|552[0n]|BOLD:AAA8814
Hypena madefactalis|[14929]|HKONS578-08|United States|Florida|658[0n]|BOLD:ACE2873
Hypena madefactalis|[14930]|LMEM994-09|United States|Mississippi|658[0n]|BOLD:ACE2873
Hypena madefactalis|[14931]|LGSMC947-05|United States|Tennessee|606[1n]|BOLD:ACE2873
Hypena madefactalis|[14932]|LGSMG540-07|United States|Tennessee|658[0n]|BOLD:ACE2873
Hypena madefactalis|[14933]|LSEU643-06|United States|Georgia|658[0n]|BOLD:ACE2873
Hypena madefactalis|[14934]|LSEU459-06|United States|Georgia|658[0n]|BOLD:ACE2873
Hypena madefactalis|[14935]|LSEU401-06|United States|Georgia|658[0n]|BOLD:ACE2873
Hypena madefactalis|[14936]|LGSM748-04|United States|Tennessee|658[0n]|BOLD:ACE2873
Hypena madefactalis|[14937]|HKONS577-08|United States|Florida|658[1n]|BOLD:ACE2873
Hypena madefactalis|[14938]|HKONS579-08|United States|Florida|658[0n]|BOLD:ACE2873
Hypena madefactalis|[14939]|UDLEP228-09|United States|Delaware|658[0n]|BOLD:ACE2873
Hypena madefactalis|[14940]|LMEM990-09|United States|Alabama|658[0n]|BOLD:ACE2873
Hypena madefactalis|[14941]|LMEM991-09|United States|Alabama|658[0n]|BOLD:ACE2873
Hypena madefactalis|[14942]|LMEM992-09|United States|Tennessee|658[0n]|BOLD:ACE2873
Hypena madefactalis|[14943]|LMEM993-09|United States|Mississippi|658[0n]|BOLD:ACE2873
Hypena madefactalis|[14944]|BBLSZ123-09|United States|Oklahoma|658[0n]|BOLD:ACE2873
Hypena madefactalis|[14945]|LILLA117-11|United States|Illinois|658[0n]|BOLD:ACE2873
Hypena madefactalis|[14946]|XAC851-04|Canada|Ontario|592[0n]|BOLD:ACE2873
Hypena madefactalis|[14947]|RDLQF707-06|Canada|Quebec|637[0n]|BOLD:ACE2873
Hypena madefactalis|[14948]|LPSO898-08|Canada|Ontario|658[0n]|BOLD:ACE2873
Hypena madefactalis|[14949]|LPSO693-08|Canada|Ontario|658[0n]|BOLD:ACE2873
Hypena madefactalis|[14950]|LPSO678-08|Canada|Ontario|658[0n]|BOLD:ACE2873
Hypena madefactalis|[14951]|LPSO666-08|Canada|Ontario|658[0n]|BOLD:ACE2873
Hypena madefactalis|[14952]|LPSO650-08|Canada|Ontario|658[0n]|BOLD:ACE2873
Hypena madefactalis|[14953]|LPSO560-08|Canada|Ontario|658[0n]|BOLD:ACE2873
Hypena madefactalis|[14954]|LPSO546-08|Canada|Ontario|658[0n]|BOLD:ACE2873
Hypena madefactalis|[14955]|LPSO542-08|Canada|Ontario|658[0n]|BOLD:ACE2873
Hypena madefactalis|[14956]|LPSO261-08|Canada|Ontario|658[0n]|BOLD:ACE2873
Hypena madefactalis|[14957]|RDLQG020-06|Canada|Quebec|658[0n]|BOLD:ACE2873
Hypena madefactalis|[14958]|RDLQF907-06|Canada|Quebec|658[0n]|BOLD:ACE2873
Hypena madefactalis|[14959]|RDLQF200-06|Canada|Quebec|658[0n]|BOLD:ACE2873
Hypena madefactalis|[14960]|LPSO081-08|Canada|Ontario|654[0n]|BOLD:ACE2873
Hypena madefactalis|[14961]|BBLPA651-10|Canada|Ontario|658[0n]|BOLD:ACE2873
Hypena madefactalis|[14962]|LPSO652-08|Canada|Ontario|646[0n]|BOLD:ACE2873
Hypena madefactalis|[14963]|CNPPC1890-12|Canada|Ontario|633[0n]|BOLD:ACE2873
Hypena madefactalis|[14964]|CNPPE406-12|Canada|Ontario|633[0n]|BOLD:ACE2873
Hypena madefactalis|[14965]|CNPPE2271-12|Canada|Ontario|639[0n]|BOLD:ACE2873
Hypena madefactalis|[14966]|CNPPG855-12|Canada|Ontario|640[0n]|BOLD:ACE2873
Hypena madefactalis|[14967]|LNCC266-10|United States|North Carolina|658[0n]|BOLD:ACE2873
Hypena madefactalis|[14968]|LPSO860-08|Canada|Ontario|658[0n]|BOLD:ACE2873
Hypena madefactalis|[14969]|LPSO642-08|Canada|Ontario|658[0n]|BOLD:ACE2873
Hypena madefactalis|[14970]|LPSO357-08|Canada|Ontario|658[0n]|BOLD:ACE2873
Hypena madefactalis|[14971]|LPSO048-08|Canada|Ontario|658[0n]|BOLD:ACE2873
Hypena madefactalis|[14972]|RDLQF906-06|Canada|Quebec|658[0n]|BOLD:ACE2873
Hypena madefactalis|[14973]|RDLQF905-06|Canada|Quebec|658[0n]|BOLD:ACE2873
Hypena madefactalis|[14974]|RDLQF903-06|Canada|Quebec|658[0n]|BOLD:ACE2873
Hypena madefactalis|[14975]|RDLQF904-06|Canada|Quebec|658[1n]|BOLD:ACE2873
Hypena madefactalis|[14976]|LPSO044-08|Canada|Ontario|646[0n]|BOLD:ACE2873
Hypena madefactalis|[14977]|CNPPF1405-12|Canada|Ontario|642[0n]|BOLD:ACE2873
[illegible]

Hypena madefactalis|[14975]|RDLQF904-06|Canada|Quebec|658[1n]|BOLD:ACE2873
Hypena madefactalis|[14976]|LPSO044-08|Canada|Ontario|646[0n]|BOLD:ACE2873
Hypena madefactalis|[14977]|CNPPF1405-12|Canada|Ontario|642[0n]|BOLD:ACE2873
Hypena madefactalis|[14978]|CNPPF1392-12|Canada|Ontario|640[0n]|BOLD:ACE2873
Hypena madefactalis|[14979]|LPSO659-08|Canada|Ontario|630[0n]|BOLD:ACE2873
Hypena madefactalis|[14980]|CNPPG861-12|Canada|Ontario|646[0n]|BOLD:ACE2873
Hypena madefactalis|[14981]|CNPPE2275-12|Canada|Ontario|636[0n]|BOLD:ACE2873
Hypena madefactalis|[14982]|LPSO665-08|Canada|Ontario|632[0n]|BOLD:ACE2873
Hypena madefactalis|[14983]|RDLQH121-06|Canada|Quebec|618[0n]|BOLD:ACE2873
Hypena madefactalis|[14984]|RDLQH122-06|Canada|Quebec|582[0n]|BOLD:ACE2873
Hypena madefactalis|[14985]|CNPPH1084-12|Canada|Ontario|641[0n]|BOLD:ACE2873
Hypena madefactalis|[14986]|CNPPF1478-12|Canada|Ontario|619[0n]|BOLD:ACE2873
Hypena madefactalis|[14987]|CNPPF1479-12|Canada|Ontario|609[0n]|BOLD:ACE2873
Hypena abalienalis|[14988]|LHLEP035-06|Canada|British Columbia|658[0n]|BOLD:ABY9634
Hypena abalienalis|[14989]|LBCS138-07|Canada|British Columbia|658[0n]|BOLD:ABY9634
Hypena abalienalis|[14990]|LBCS139-07|Canada|British Columbia|658[0n]|BOLD:ABY9634
Hypena abalienalis|[14991]|LBCS140-07|Canada|British Columbia|658[0n]|BOLD:ABY9634
Hypena abalienalis|[14992]|LPVIA608-08|Canada|British Columbia|658[0n]|BOLD:ABY9634
Hypena abalienalis|[14993]|LPVIA631-08|Canada|British Columbia|658[0n]|BOLD:ABY9634
Hypena abalienalis|[14994]|RDLQF198-06|Canada|Quebec|658[0n]|BOLD:ABY9634
Hypena abalienalis|[14995]|RDLQF284-06|Canada|Quebec|658[0n]|BOLD:ABY9634
Hypena abalienalis|[14996]|LPMN204-08|Canada|Manitoba|658[0n]|BOLD:ABY9634
Hypena abalienalis|[14997]|HKONS567-08|United States|Florida|658[0n]|BOLD:ABY9634
Hypena abalienalis|[14998]|RDLQG346-06|Canada|Quebec|658[0n]|BOLD:ABY9634
Hypena abalienalis|[14999]|RDLQF779-06|Canada|Quebec|658[0n]|BOLD:ABY9634
Hypena abalienalis|[15000]|RDLQF199-06|Canada|Quebec|658[0n]|BOLD:ABY9634
Hypena abalienalis|[15001]|RDLQB833-05|Canada|Quebec|658[0n]|BOLD:ABY9634
Hypena abalienalis|[15002]|LPSO658-08|Canada|Ontario|646[0n]|BOLD:ABY9634
Hypena abalienalis|[15003]|RDLQG246-06|Canada|Quebec|608[0n]|BOLD:ABY9634
Hypena abalienalis|[15004]|XAC595-04|Canada|Ontario|616[0n]|BOLD:ABY9634
Hypena abalienalis|[15005]|XAC622-04|Canada|Ontario|617[0n]|BOLD:ABY9634
Hypena abalienalis|[15006]|HKONS566-08|United States|Florida|609[0n]|BOLD:ABY9634
Hypena abalienalis|[15007]|LMEM988-09|United States|Mississippi|618[0n]|BOLD:ABY9634
Hypena abalienalis|[15008]|LPOKC754-09|United States|Oklahoma|658[0n]|BOLD:ABY9634
Hypena abalienalis|[15009]|LPOKD040-09|United States|Oklahoma|658[0n]|BOLD:ABY9634
Hypena abalienalis|[15010]|CNCLB2773-14|United States|North Carolina|658[0n]|BOLD:ABY9634
Hypena abalienalis|[15011]|CNCLB2779-14|United States|North Carolina|658[0n]|BOLD:ABY9634
Hypena palparia|[15012]|LGSM545-04|United States|Tennessee|517[0n]|BOLD:ABY9635
Hypena palparia|[15013]|LGSMC944-05|United States|Tennessee|615[0n]|BOLD:ABY9635
Hypena palparia|[15014]|LGSMC943-05|United States|Tennessee|658[1n]|BOLD:ABY9635
Hypena palparia|[15015]|LGSM546-04|United States|Tennessee|658[0n]|BOLD:ABY9635
Hypena palparia|[15016]|LMEM975-09|United States|Alabama|635[0n]|BOLD:ABY9635
Hypena palparia|[15017]|LMEM983-09|United States|Mississippi|658[0n]|BOLD:ABY9635
Hypena palparia|[15018]|LMEM982-09|United States|Mississippi|620[1n]|BOLD:ABY9635
Hypena palparia|[15019]|RDLQF226-06|Canada|Quebec|658[0n]|BOLD:ABY9635
Hypena palparia|[15020]|LSUSA132-06|United States|Kentucky|658[0n]|BOLD:ABY9635
Hypena palparia|[15021]|LMEM984-09|United States|Mississippi|629[0n]|BOLD:ABY9635
Hypena palparia|[15022]|RDNMJ646-11|Canada|New Brunswick|658[0n]|BOLD:ABY9635
Hypena palparia|[15023]|BBLCU224-09|United States|Michigan|658[0n]|BOLD:ABY9635
Hypena palparia|[15024]|LPSOC344-08|Canada|Ontario|658[0n]|BOLD:ABY9635
Hypena palparia|[15025]|RDLQF902-06|Canada|Quebec|658[0n]|BOLD:ABY9635
Hypena palparia|[15026]|RDLQF900-06|Canada|Quebec|658[0n]|BOLD:ABY9635
Hypena palparia|[15027]|RDLQF899-06|Canada|Quebec|658[0n]|BOLD:ABY9635
Hypena palparia|[15028]|RDLQF528-06|Canada|Quebec|658[0n]|BOLD:ABY9635
Hypena palparia|[15029]|RDLQH125-06|Canada|Quebec|611[3n]|BOLD:ABY9635
Hypena palparia|[15030]|RDNMJ648-11|Canada|Ontario|619[0n]|BOLD:ABY9635
Hypena palparia|[15031]|RDNMJ650-11|Canada|Ontario|658[0n]|BOLD:ABY9635
Hypena palparia|[15032]|RDNMJ651-11|Canada|Ontario|658[0n]|BOLD:ABY9635
Hypena palparia|[15033]|CNRME1824-12|Canada|Manitoba|613[0n]|BOLD:ABY9635
Hypena palparia|[15034]|CNRME1857-12|Canada|Manitoba|605[0n]|BOLD:ABY9635
Hypena palparia|[15035]|CNRME1825-12|Canada|Manitoba|606[0n]|BOLD:ABY9635
Hypena palparia|[15036]|CNRME1810-12|Canada|Manitoba|612[0n]|BOLD:ABY9635
Hypena palparia|[15037]|CNRME1835-12|Canada|Manitoba|630[0n]|BOLD:ABY9635
Hypena palparia|[15038]|CNRME1849-12|Canada|Manitoba|605[0n]|BOLD:ABY9635
Hypena palparia|[15039]|LPMN181-08|Canada|Manitoba|658[0n]|BOLD:ABY9635
Hypena palparia|[15040]|RDNMJ649-11|Canada|New Brunswick|658[0n]|BOLD:ABY9635
Hypena palparia|[15041]|CNRME1791-12|Canada|Manitoba|628[0n]|BOLD:ABY9635
Hypena palparia|[15042]|CNRME5003-12|Canada|Manitoba|629[0n]|BOLD:ABY9635
Hypena palparia|[15043]|CNRME1813-12|Canada|Manitoba|619[0n]|BOLD:ABY9635
Hypena palparia|[15044]|CNRMF3751-12|Canada|Manitoba|630[0n]|BOLD:ABY9635
Hypena palparia|[15045]|CNRME4996-12|Canada|Manitoba|630[0n]|BOLD:ABY9635
Hypena palparia|[15046]|CNRME1854-12|Canada|Manitoba|630[0n]|BOLD:ABY9635
Hypena palparia|[15047]|RDLQB239-05|Canada|Quebec|574[1n]|BOLD:ABY9635
Hypena palparia|[15048]|RDNMJ645-11|Canada|New Brunswick|658[0n]|BOLD:ABY9635
Hypena palparia|[15049]|CNRME042-12|Canada|Manitoba|629[0n]|BOLD:ABY9635
Hypena palparia|[15050]|CNRME1802-12|Canada|Manitoba|629[0n]|BOLD:ABY9635
Hypena palparia|[15051]|CNRME1822-12|Canada|Manitoba|629[0n]|BOLD:ABY9635
Hypena palparia|[15052]|CNRME1832-12|Canada|Manitoba|630[0n]|BOLD:ABY9635
Hypena palparia|[15053]|CNRME1816-12|Canada|Manitoba|630[0n]|BOLD:ABY9635
Hypena palparia|[15054]|RDNME437-08|Canada|Alberta|658[0n]|BOLD:ABY9635
Hypena palparia|[15055]|LGSMG534-07|United States|Tennessee|656[0n]|BOLD:ABY9635
Hypena palparia|[15056]|LPSOB823-08|Canada|Ontario|658[0n]|BOLD:ABY9635
Hypena palparia|[15057]|BBLPE145-09|Canada|Nova Scotia|658[0n]|BOLD:ABY9635
Hypena palparia|[15058]|BBLPB208-10|Canada|Ontario|658[0n]|BOLD:ABY9635
Hypena palparia|[15059]|RDNME436-08|Canada|Alberta|658[0n]|BOLD:ABY9635
Hypena palparia|[15060]|RDNME435-08|Canada|Alberta|658[0n]|BOLD:ABY9635
Hypena palparia|[15061]|LBCS213-07|Canada|British Columbia|658[0n]|BOLD:ABY9635
Hypena palparia|[15062]|RDLQG435-06|Canada|Quebec|658[0n]|BOLD:ABY9635
Hypena palparia|[15063]|RDLQG360-06|Canada|Quebec|658[0n]|BOLD:ABY9635
Hypena palparia|[15064]|RDLQF901-06|Canada|Quebec|658[0n]|BOLD:ABY9635
Hypena palparia|[15065]|RDLQF451-06|Canada|Quebec|658[0n]|BOLD:ABY9635
Hypena palparia|[15066]|RDLQB244-05|Canada|Quebec|658[0n]|BOLD:ABY9635
Hypena palparia|[15067]|CNRME4984-12|Canada|Manitoba|634[0n]|BOLD:ABY9635
Hypena palparia|[15068]|CNRME4979-12|Canada|Manitoba|634[0n]|BOLD:ABY9635
Hypena palparia|[15069]|CNRME049-12|Canada|Manitoba|635[0n]|BOLD:ABY9635
Hypena palparia|[15070]|CNRME1826-12|Canada|Manitoba|622[0n]|BOLD:ABY9635
Hypena palparia|[15071]|BBLPD754-10|Canada|British Columbia|628[0n]|BOLD:ABY9635
Hypena palparia|[15072]|RDNMJ647-11|Canada|New Brunswick|613[0n]|BOLD:ABY9635
Hypena palparia|[15073]|CNRME047-12|Canada|Manitoba|631[0n]|BOLD:ABY9635
Hypena palparia|[15074]|CNRME5002-12|Canada|Manitoba|632[0n]|BOLD:ABY9635
Hypena palparia|[15075]|CNRMF3752-12|Canada|Manitoba|632[0n]|BOLD:ABY9635
Hypena palparia|[15076]|CNRMF3753-12|Canada|Manitoba|635[0n]|BOLD:ABY9635
Hypena palparia|[15077]|CNRMF3885-12|Canada|Manitoba|633[0n]|BOLD:ABY9635
[illegible]

Hypena palparia|[15075]|CNRMF3752-12|Canada|Manitoba|632[0n]|BOLD:ABY9635
Hypena palparia|[15076]|CNRMF3753-12|Canada|Manitoba|635[0n]|BOLD:ABY9635
Hypena palparia|[15077]|CNRMF3885-12|Canada|Manitoba|633[0n]|BOLD:ABY9635
Hypena deceptalis|[15078]|XAC847-04|Canada|Ontario|593[1n]|BOLD:AAA8145
Hypena deceptalis|[15079]|XAG975-05|Canada|Ontario|658[0n]|BOLD:AAA8145
Hypena deceptalis|[15080]|RDLQG253-06|Canada|Quebec|658[1n]|BOLD:AAA8145
Hypena deceptalis|[15081]|RDLQG036-06|Canada|Quebec|658[0n]|BOLD:AAA8145
Hypena deceptalis|[15082]|RDLQF914-06|Canada|Quebec|658[0n]|BOLD:AAA8145
Hypena deceptalis|[15083]|RDLQF912-06|Canada|Quebec|658[0n]|BOLD:AAA8145
Hypena deceptalis|[15084]|RDLQF911-06|Canada|Quebec|658[0n]|BOLD:AAA8145
Hypena deceptalis|[15085]|RDLQF742-06|Canada|Quebec|658[0n]|BOLD:AAA8145
Hypena deceptalis|[15086]|RDLQF741-06|Canada|Quebec|658[0n]|BOLD:AAA8145
Hypena deceptalis|[15087]|RDLQF740-06|Canada|Quebec|658[0n]|BOLD:AAA8145
Hypena deceptalis|[15088]|RDLQF739-06|Canada|Quebec|658[0n]|BOLD:AAA8145
Hypena deceptalis|[15089]|RDLQF425-06|Canada|Quebec|658[0n]|BOLD:AAA8145
Hypena deceptalis|[15090]|RDLQG140-06|Canada|Quebec|631[0n]|BOLD:AAA8145
Hypena deceptalis|[15091]|RDLQG250-06|Canada|Quebec|658[1n]|BOLD:AAA8145
Hypena deceptalis|[15092]|RDLQG251-06|Canada|Quebec|658[0n]|BOLD:AAA8145
Hypena deceptalis|[15093]|RDLQG354-06|Canada|Quebec|658[0n]|BOLD:AAA8145
Hypena deceptalis|[15094]|RDLQG355-06|Canada|Quebec|658[0n]|BOLD:AAA8145
Hypena deceptalis|[15095]|HKONS575-08|United States|Florida|609[0n]|BOLD:AAA8145
Hypena deceptalis|[15096]|LGSMC652-05|United States|Tennessee|614[0n]|BOLD:AAA8145
Hypena deceptalis|[15097]|QUNOB516-09|United States|Kentucky|658[0n]|BOLD:AAA8145
Hypena deceptalis|[15098]|RDLQF913-06|Canada|Quebec|658[0n]|BOLD:AAA8145
Hypena deceptalis|[15099]|RDLQF910-06|Canada|Quebec|658[0n]|BOLD:AAA8145
Hypena deceptalis|[15100]|RDLQF567-06|Canada|Quebec|658[0n]|BOLD:AAA8145
Hypena deceptalis|[15101]|RDLQF529-06|Canada|Quebec|658[0n]|BOLD:AAA8145
Hypena deceptalis|[15102]|RDLQF205-06|Canada|Quebec|658[0n]|BOLD:AAA8145
Hypena deceptalis|[15103]|XAC155-04|Canada|Ontario|658[0n]|BOLD:AAA8145
Hypena deceptalis|[15104]|RDLQG353-06|Canada|Quebec|649[0n]|BOLD:AAA8145
Hypena deceptalis|[15105]|XAE546-04|Canada|Ontario|565[1n]|BOLD:AAA8145
Hypena deceptalis|[15106]|XAD296-04|Canada|Ontario|544[0n]|BOLD:AAA8145
Hypena deceptalis|[15107]|RDLQG249-06|Canada|Quebec|614[0n]|BOLD:AAA8145
Hypena deceptalis|[15108]|RDLQG252-06|Canada|Quebec|590[2n]|BOLD:AAA8145
Hypena deceptalis|[15109]|RDLQH119-06|Canada|Quebec|602[1n]|BOLD:AAA8145
Hypena deceptalis|[15110]|LMEM989-09|United States|Mississippi|658[0n]|BOLD:AAA8145
Hypena deceptalis|[15111]|LGSMG537-07|United States|Tennessee|658[0n]|BOLD:AAA8145
Hypena deceptalis|[15112]|LGSMG541-07|United States|Tennessee|658[0n]|BOLD:AAA8145
Hypena deceptalis|[15113]|MMNA098-08|United States|North Carolina|658[0n]|BOLD:AAA8145
Hypena deceptalis|[15114]|LGSMG538-07|United States|Tennessee|658[0n]|BOLD:AAA8145
Hypena deceptalis|[15115]|LSEU361-06|United States|Georgia|658[0n]|BOLD:AAA8145
Hypena deceptalis|[15116]|LGSMC421-05|United States|Tennessee|658[0n]|BOLD:AAA8145
Hypena deceptalis|[15117]|LGSM659-04|United States|North Carolina|658[0n]|BOLD:AAA8145
Hypena deceptalis|[15118]|LGSMC420-05|United States|Tennessee|568[0n]|BOLD:AAA8145
Hypena deceptalis|[15119]|QUNOB514-09|United States|Kentucky|658[0n]|BOLD:AAA8145
Hypena deceptalis|[15120]|QUNOB515-09|United States|Kentucky|658[0n]|BOLD:AAA8145
Hypena deceptalis|[15121]|QUNOB518-09|United States|Kentucky|658[0n]|BOLD:AAA8145
Hypena deceptalis|[15122]|LNCC259-10|United States|North Carolina|658[0n]|BOLD:AAA8145
Hypena deceptalis|[15123]|LNCC1466-13|United States|North Carolina|658[0n]|BOLD:AAA8145
Hypena deceptalis|[15124]|LNCC1467-13|United States|North Carolina|658[0n]|BOLD:AAA8145
Hypena sordidula|[15125]|LOT159-04|United States|Tennessee|658[0n]|BOLD:ACF0234
Hypena sordidula|[15126]|HKONB418-09|United States|Indiana|658[0n]|BOLD:ACF0234
Hypena sordidula|[15127]|LGSMC660-05|United States|Tennessee|620[0n]|BOLD:ACF0234
Hypena sordidula|[15128]|RDLQH128-06|Canada|Quebec|605[1n]|BOLD:ACF0234
Hypena sordidula|[15129]|ABCNA556-07|United States|Kentucky|568[0n]|BOLD:ACF0234
Hypena sordidula|[15130]|LPSO657-08|Canada|Ontario|564[0n]|BOLD:ACF0234
Hypena sordidula|[15131]|LPSO839-08|Canada|Ontario|658[0n]|BOLD:ACF0234
Hypena sordidula|[15132]|LPSO950-08|Canada|Ontario|658[0n]|BOLD:ACF0234
Hypena sordidula|[15133]|LPSOC331-08|Canada|Ontario|658[0n]|BOLD:ACF0234
Hypena sordidula|[15134]|QUNOB523-09|United States|Kentucky|658[0n]|BOLD:ACF0234
Hypena sordidula|[15135]|CNCLB2895-14|United States|North Carolina|658[0n]|BOLD:ACF0234
Hypena sordidula|[15136]|CNCLB2898-14|United States|North Carolina|658[0n]|BOLD:ACF0234
Hypena manalis|[15137]|RDLQH135-06|Canada|Quebec|577[2n]|BOLD:AAB5993
Hypena manalis|[15138]|LPSOC375-08|Canada|Ontario|653[0n]|BOLD:AAB5993
Hypena manalis|[15139]|LGSMG535-07|United States|Tennessee|658[0n]|BOLD:AAB5993
Hypena manalis|[15140]|LNCB461-07|United States|North Carolina|658[0n]|BOLD:AAB5993
Hypena manalis|[15141]|LNCNW116-06|United States|North Carolina|658[0n]|BOLD:AAB5993
Hypena manalis|[15142]|LNC852-06|United States|North Carolina|658[0n]|BOLD:AAB5993
Hypena manalis|[15143]|LGSM734-04|United States|Tennessee|658[0n]|BOLD:AAB5993
Hypena manalis|[15144]|LGSM733-04|United States|Tennessee|658[0n]|BOLD:AAB5993
Hypena manalis|[15145]|LGSMC985-05|United States|Tennessee|599[0n]|BOLD:AAB5993
Hypena manalis|[15146]|HKONS570-08|United States|Florida|609[0n]|BOLD:AAB5993
Hypena manalis|[15147]|HKONS571-08|United States|Florida|658[0n]|BOLD:AAB5993
Hypena manalis|[15148]|LPSO078-08|Canada|Ontario|658[0n]|BOLD:AAB5993
Hypena manalis|[15149]|LPSO573-08|Canada|Ontario|658[0n]|BOLD:AAB5993
Hypena manalis|[15150]|LPSO574-08|Canada|Ontario|658[0n]|BOLD:AAB5993
Hypena manalis|[15151]|LPSO834-08|Canada|Ontario|658[0n]|BOLD:AAB5993
Hypena manalis|[15152]|LPSO891-08|Canada|Ontario|658[0n]|BOLD:AAB5993
Hypena manalis|[15153]|LPSOB037-08|Canada|Ontario|658[0n]|BOLD:AAB5993
Hypena manalis|[15154]|LPOKA068-08|United States|Oklahoma|658[0n]|BOLD:AAB5993
Hypena manalis|[15155]|LPOKA078-08|United States|Oklahoma|658[0n]|BOLD:AAB5993
Hypena manalis|[15156]|BLTIB457-08|Canada|Ontario|658[0n]|BOLD:AAB5993
Hypena manalis|[15157]|BBLSX529-09|United States|Oklahoma|658[0n]|BOLD:AAB5993
Hypena manalis|[15158]|LILLA671-11|United States|Illinois|658[0n]|BOLD:AAB5993
Hypena appalachiensis|[15159]|ABNCC131-07|United States|Kentucky|612[5n]|BOLD:AAE5906
Hypena appalachiensis|[15160]|QUNO720-08|United States|Kentucky|658[0n]|BOLD:AAE5906
Hypena appalachiensis|[15161]|QUNO721-08|United States|Kentucky|658[0n]|BOLD:AAE5906
Hypena appalachiensis|[15162]|QUNO728-08|United States|Kentucky|658[0n]|BOLD:AAE5906
Hypena appalachiensis|[15163]|CNCLB2932-14|United States|North Carolina|658[0n]|BOLD:AAE5906
Sigela basipunctaria|[15164]|HKONS130-08|United States|Florida|658[0n]|BOLD:AAE9652
Sigela basipunctaria|[15165]|HKONS347-08|United States|Florida|658[0n]|BOLD:AAE9652
Sigela basipunctaria|[15166]|CNCLB1765-14|United States|Florida|658[0n]|BOLD:AAE9652
Sigela basipunctaria|[15167]|CNCLB1766-14|United States|Florida|658[0n]|BOLD:AAE9652
Sigela nr. basipunctaria|[15168]|CNCLB1770-14|United States|Florida|307[0n]|
Sigela nr. basipunctaria|[15169]|CNCLB1767-14|United States|Florida|658[0n]|BOLD:AAE9653
Sigela nr. basipunctaria|[15170]|HKONB364-09|United States|Texas|658[0n]|BOLD:AAE9653
Sigela nr. basipunctaria|[15171]|HKONB365-09|United States|Texas|658[0n]|BOLD:AAE9653
Sigela nr. basipunctaria|[15172]|CNCLB1768-14|United States|Florida|658[0n]|BOLD:AAE9653
Sigela nr. basipunctaria|[15173]|CNCLB1769-14|United States|Florida|658[0n]|BOLD:AAE9653
Sigela nr. basipunctaria|[15174]|CNCLB1771-14|United States|Florida|658[0n]|BOLD:AAE9653
Sigela sp. 2|[15175]|RDNMH400-09|United States|Florida|658[0n]|BOLD:AAC6450
Sigela sp. 2|[15176]|HKONS607-08|United States|Florida|658[0n]|BOLD:AAC6450
Sigela sp. 2|[15177]|HKONS249-08|United States|Florida|658[0n]|BOLD:AAC6450

Sigela sp. 2|[15175]|RDNMH400-09|United States|Florida|658[0n]|BOLD:AAC6450
Sigela sp. 2|[15176]|HKONS607-08|United States|Florida|658[0n]|BOLD:AAC6450
Sigela sp. 2|[15177]|HKONS249-08|United States|Florida|658[0n]|BOLD:AAC6450
Sigela sp. 2|[15178]|RDNMH401-09|United States|Florida|633[0n]|BOLD:AAC6450
Sigela sp. 2|[15179]|RDNMH402-09|United States|Florida|658[0n]|BOLD:AAC6450
Sigela sp. 2|[15180]|RDNML356-13|United States|Florida|658[0n]|BOLD:AAC6450
Sigela sp. 2|[15181]|HKONS252-08|United States|Florida|658[0n]|BOLD:AAC6450
Sigela sp. 2|[15182]|HKONB361-09|United States|Texas|658[0n]|BOLD:AAC6450
Sigela sp. 2|[15183]|HKONB362-09|United States|Louisiana|658[0n]|BOLD:AAC6450
Sigela sp. 2|[15184]|RDNML357-13|United States|Florida|658[0n]|BOLD:AAC6450
Sigela sp. 2|[15185]|HKONS250-08|United States|Florida|658[0n]|BOLD:AAC6450
Sigela sp. 2|[15186]|HKONS251-08|United States|Florida|658[0n]|BOLD:AAC6450
Sigela sp. 2|[15187]|CNCLB1292-14|United States|Louisiana|658[0n]|BOLD:AAC6450
Sigela penumbrata|[15188]|CNCLB1772-14|United States|Florida|307[0n]|
Sigela penumbrata|[15189]|HKONS615-08|United States|Florida|658[0n]|BOLD:AAF4076
Sigela penumbrata|[15190]|HKONS614-08|United States|Florida|609[0n]|BOLD:AAF4076
Sigela penumbrata|[15191]|HKONS630-08|United States|Florida|609[0n]|BOLD:AAF4076
Sigela penumbrata|[15192]|HKONS613-08|United States|Florida|658[0n]|BOLD:AAF4076
Sigela penumbrata|[15193]|CNCLB2369-14|United States|Florida|658[0n]|BOLD:AAF4076
Sigela sp. 4|[15194]|HKONB358-09|United States|Texas|658[0n]|BOLD:AAE1159
Sigela sp. 4|[15195]|HKONB356-09|United States|Texas|658[0n]|BOLD:AAE1159
Sigela sp. 4|[15196]|HKONS619-08|United States|Texas|609[0n]|BOLD:AAE1159
Sigela sp. 4|[15197]|HKONB357-09|United States|Texas|658[0n]|BOLD:AAE1159
Sigela sp. 4|[15198]|HKONB359-09|United States|Louisiana|658[0n]|BOLD:AAE1159
Sigela sp. 4|[15199]|LPOKE064-10|United States|Oklahoma|658[0n]|BOLD:AAE1159
Sigela sp. 1|[15200]|HKONS620-08|United States|Texas|609[0n]|BOLD:AAH5071
Sigela sp. 1|[15201]|RDNMH094-09|United States|Texas|658[0n]|BOLD:AAH5071
Sigela sp. 1|[15202]|BBUSA125-09|United States|Texas|658[0n]|BOLD:AAH5071
Sigela sp. 1|[15203]|USLEP547-10|United States|Colorado|658[0n]|BOLD:AAH5071
Sigela sp. 6|[15204]|QUNOD840-11|United States|Texas|658[0n]|BOLD:ACE5035
Sigela sp. 6|[15205]|QUNOD841-11|United States|Texas|658[0n]|BOLD:ACE5035
Sigela brauneata|[15206]|RDNMD347-06|United States|Florida|604[0n]|BOLD:AAB7659
Sigela brauneata|[15207]|RDNMD457-06|United States|Florida|658[0n]|BOLD:AAB7659
Sigela brauneata|[15208]|RDNMD458-06|United States|Florida|658[0n]|BOLD:AAB7659
Sigela brauneata|[15209]|LNC155-05|United States|North Carolina|658[0n]|BOLD:AAB7659
Sigela brauneata|[15210]|LGSMD417-05|United States|Tennessee|658[0n]|BOLD:AAB7659
Sigela brauneata|[15211]|LGSMD414-05|United States|Tennessee|658[0n]|BOLD:AAB7659
Sigela brauneata|[15212]|LGSMD413-05|United States|Tennessee|658[0n]|BOLD:AAB7659
Sigela brauneata|[15213]|LGSMD412-05|United States|Tennessee|658[0n]|BOLD:AAB7659
Sigela brauneata|[15214]|LGSMD411-05|United States|Tennessee|658[0n]|BOLD:AAB7659
Sigela brauneata|[15215]|LGSMD410-05|United States|Tennessee|658[0n]|BOLD:AAB7659
Sigela brauneata|[15216]|LGSMD409-05|United States|Tennessee|658[0n]|BOLD:AAB7659
Sigela brauneata|[15217]|LGSMC675-05|United States|Tennessee|658[0n]|BOLD:AAB7659
Sigela brauneata|[15218]|LGSM731-04|United States|Tennessee|658[0n]|BOLD:AAB7659
Sigela brauneata|[15219]|LGSMD416-05|United States|Tennessee|617[0n]|BOLD:AAB7659
Sigela brauneata|[15220]|LGSMD415-05|United States|Tennessee|617[0n]|BOLD:AAB7659
Sigela brauneata|[15221]|ABNCC117-07|United States|Tennessee|575[6n]|
Sigela brauneata|[15222]|MNAA768-07|United States|Tennessee|636[0n]|BOLD:AAB7659
Sigela brauneata|[15223]|HKONS165-08|United States|Florida|658[0n]|BOLD:AAB7659
Sigela brauneata|[15224]|CNCLB2920-14|United States|North Carolina|658[0n]|BOLD:AAB7659
Cecharismena nectarea|[15225]|LOFLB460-06|United States|Florida|658[0n]|BOLD:AAA8251
Cecharismena nectarea|[15226]|RDNME594-08|United States|Florida|658[0n]|BOLD:AAA8251
Isogona natatrix|[15227]|HKONB035-08|United States|Texas|658[0n]|BOLD:AAB1119
Isogona natatrix|[15228]|HKONB036-08|United States|Texas|658[0n]|BOLD:AAB1119
Isogona natatrix|[15229]|HKONB034-08|United States|Texas|658[0n]|BOLD:AAB1119
Isogona natatrix|[15230]|HKONB495-09|United States|Texas|658[0n]|BOLD:AAB1119
Isogona punctipennis|[15231]|IAWL049-09|United States|Arizona|658[0n]|BOLD:AAI8205
Isogona punctipennis|[15232]|RDNMH807-09|United States|Arizona|658[0n]|BOLD:AAI8205
Isogona texana|[15233]|HKONB267-09|United States|Texas|658[0n]|BOLD:AAI8207
Isogona texana|[15234]|HKONB268-09|United States|Texas|658[0n]|BOLD:AAI8207
Isogona tenuis|[15235]|HKONS360-08|United States|Florida|658[0n]|BOLD:AAB5798
Isogona tenuis|[15236]|RDNMK058-11|United States|Florida|658[0n]|BOLD:AAB5798
Isogona tenuis|[15237]|ABNCC209-07|United States|Texas|641[0n]|BOLD:AAB5798
Isogona tenuis|[15238]|RDNME665-08|United States|Texas|658[0n]|BOLD:AAB5798
Isogona tenuis|[15239]|HKONB045-08|United States|Texas|658[0n]|BOLD:AAB5798
Isogona tenuis|[15240]|HKONB047-08|United States|Texas|658[0n]|BOLD:AAB5798
Isogona tenuis|[15241]|HKONB048-08|United States|Texas|658[0n]|BOLD:AAB5798
Isogona tenuis|[15242]|HKONB046-08|United States|Texas|658[0n]|BOLD:AAB5798
Isogona tenuis|[15243]|PSAT152-10|United States|Texas|658[0n]|BOLD:AAB5798
Isogona tenuis|[15244]|ABNCC210-07|United States|Texas|652[0n]|BOLD:AAB5798
Isogona tenuis|[15245]|ABNCC211-07|United States|Texas|633[0n]|BOLD:AAB5798
Isogona tenuis|[15246]|ABNCC212-07|United States|Texas|620[0n]|BOLD:AAB5798
Isogona tenuis|[15247]|USLEP957-10|United States|Texas|658[0n]|BOLD:AAB5798
Isogona tenuis|[15248]|LNCC1225-11|United States|North Carolina|658[0n]|BOLD:AAB5798
Isogona tenuis|[15249]|BBLOC1177-11|United States|Texas|658[0n]|BOLD:AAB5798
Isogona tenuis|[15250]|LGSMC923-05|United States|Tennessee|658[1n]|BOLD:AAB5798
Isogona tenuis|[15251]|LGSM544-04|United States|Tennessee|658[0n]|BOLD:AAB5798
Isogona tenuis|[15252]|LGSMC922-05|United States|Tennessee|658[0n]|BOLD:AAB5798
Isogona tenuis|[15253]|LMEMB049-09|United States|Louisiana|658[0n]|BOLD:AAB5798
Isogona tenuis|[15254]|LILLA145-11|United States|Illinois|658[0n]|BOLD:AAB5798
Isogona tenuis|[15255]|LMEMB047-09|United States|Alabama|658[0n]|BOLD:AAB5798
Isogona tenuis|[15256]|LMEMB048-09|United States|Alabama|658[0n]|BOLD:AAB5798
Isogona tenuis|[15257]|LMEMB050-09|United States|Louisiana|658[0n]|BOLD:AAB5798
Isogona tenuis|[15258]|LMEMB051-09|United States|Mississippi|658[0n]|BOLD:AAB5798
Isogona tenuis|[15259]|LILLA323-11|United States|Illinois|658[0n]|BOLD:AAB5798
Isogona tenuis|[15260]|LILLA373-11|United States|Illinois|658[0n]|BOLD:AAB5798
Isogona tenuis|[15261]|LGSM543-04|United States|Tennessee|658[0n]|BOLD:AAB5798
Isogona tenuis|[15262]|ABNCC214-07|United States|Texas|656[0n]|BOLD:AAB5798
Isogona tenuis|[15263]|BBLOE1254-12|United States|Texas|658[0n]|BOLD:AAB5798
Isogona segura|[15264]|BBLOD1850-11|United States|Arizona|658[0n]|BOLD:AAD5048
Isogona segura|[15265]|IAWL038-09|United States|Arizona|658[0n]|BOLD:AAD5048
Isogona segura|[15266]|HKONB496-09|United States|Arizona|644[0n]|BOLD:AAD5048
Isogona segura|[15267]|IAWL039-09|United States|Arizona|658[0n]|BOLD:AAD5048
Isogona segura|[15268]|IAWL040-09|United States|Arizona|658[0n]|BOLD:AAD5048
Isogona segura|[15269]|IAWL041-09|United States|Arizona|658[0n]|BOLD:AAD5048
Isogona segura|[15270]|IAWL050-09|United States|Arizona|658[0n]|BOLD:AAD5048
Isogona segura|[15271]|RDNMH771-09|United States|Arizona|658[0n]|BOLD:AAD5048
Isogona segura|[15272]|RDNMH808-09|United States|Arizona|658[0n]|BOLD:AAD5048
Isogona segura|[15273]|BBLOE1730-12|United States|Arizona|658[0n]|BOLD:AAD5048
Cecharismena jalapena|[15274]|QUNOD011-10|United States|Texas|658[0n]|BOLD:AAA2894
Cecharismena jalapena|[15275]|LYPIE512-09|Mexico|Yucatan|631[0n]|BOLD:AAA2894
Cecharismena jalapena|[15276]|LPYPC043-08|Mexico|Yucatan|658[0n]|BOLD:AAA2894
Cecharismena jalapenaDHJ02|[15277]|BLPDK833-09|Costa Rica|Guanacaste|613[0n]|BOLD:AAA2894

Cecharismena jalapena|[15275]|LYPIE512-09|Mexico|Yucatan|631[0n]|BOLD:AAA2894
Cecharismena jalapena|[15276]|LPYPC043-08|Mexico|Yucatan|658[0n]|BOLD:AAA2894
Cecharismena jalapenaDHJ02|[15277]|BLPDK833-09|Costa Rica|Guanacaste|613[0n]|BOLD:AAA2894
Cecharismena jalapena|[15278]|QUNOD001-10|United States|Texas|658[0n]|BOLD:AAA2894
Cecharismena jalapena|[15279]|CQR571-13|Mexico|Quintana Roo|658[0n]|BOLD:AAA2894
Cecharismena cara|[15280]|CNCLB905-14|United States|Florida|658[0n]|BOLD:ACM3987
Cecharismena anartoides|[15281]|MHMXC570-06|Costa Rica|Guanacaste|658[1n]|BOLD:AAB8320
Cecharismena anartoides|[15282]|MHAUA567-05|Costa Rica|Guanacaste|658[0n]|BOLD:AAB8320
Cecharismena anartoides|[15283]|MHAUA568-05|Costa Rica|Guanacaste|658[0n]|BOLD:AAB8320
Cecharismena anartoides|[15284]|MHAUA569-05|Costa Rica|Guanacaste|658[0n]|BOLD:AAB8320
Cecharismena anartoides|[15285]|MHAUA570-05|Costa Rica|Alajuela|658[0n]|BOLD:AAB8320
Cecharismena anartoides|[15286]|MHAUF170-06|Costa Rica|Guanacaste|658[0n]|BOLD:AAB8320
Cecharismena anartoides|[15287]|MHAUG118-07|Costa Rica|Alajuela|658[0n]|BOLD:AAB8320
Cecharismena anartoides|[15288]|MHMXO401-08|Costa Rica|Alajuela|658[0n]|BOLD:AAB8320
Cecharismena anartoides|[15289]|BLPCB440-08|Costa Rica|Alajuela|658[0n]|BOLD:AAB8320
Cecharismena anartoides|[15290]|BLPCB885-08|Costa Rica|Alajuela|658[0n]|BOLD:AAB8320
Cecharismena anartoides|[15291]|BLPCB886-08|Costa Rica|Alajuela|658[0n]|BOLD:AAB8320
Cecharismena anartoides|[15292]|MHMXQ846-08|Costa Rica||658[0n]|BOLD:AAB8320
Cecharismena anartoides|[15293]|LPMX878-08|Mexico|Campeche|658[0n]|BOLD:AAB8320
Cecharismena anartoides|[15294]|LPYPA219-08|Mexico|Yucatan|658[0n]|BOLD:AAB8320
Cecharismena anartoides|[15295]|BLPDC769-09|Costa Rica|Alajuela|658[0n]|BOLD:AAB8320
Cecharismena anartoides|[15296]|MHMYE1160-09|Costa Rica|Alajuela|658[0n]|BOLD:AAB8320
Cecharismena anartoides|[15297]|BLPDL1164-10|Costa Rica|Alajuela|658[0n]|BOLD:AAB8320
Cecharismena anartoides|[15298]|BLPDT080-10|Costa Rica|Alajuela|658[0n]|BOLD:AAB8320
Cecharismena anartoides|[15299]|BLPDW625-11|Costa Rica|Guanacaste|658[0n]|BOLD:AAB8320
Cecharismena anartoides|[15300]|LOCRI231-11|Costa Rica|Limon|658[0n]|BOLD:AAB8320
Cecharismena anartoides|[15301]|MHMYL3320-11|Costa Rica||658[0n]|BOLD:AAB8320
Cecharismena anartoides|[15302]|MHMYN179-11|Costa Rica||658[0n]|BOLD:AAB8320
Cecharismena anartoides|[15303]|MHMYN180-11|Costa Rica||658[0n]|BOLD:AAB8320
Cecharismena anartoides|[15304]|MHMYS6265-14|Costa Rica||658[0n]|BOLD:AAB8320
Cecharismena anartoides|[15305]|MHMYS6367-14|Costa Rica||658[0n]|BOLD:AAB8320
Cecharismena anartoides|[15306]|MHMYS6368-14|Costa Rica||658[0n]|BOLD:AAB8320
Cecharismena anartoides|[15307]|MHMYS6372-14|Costa Rica||658[0n]|BOLD:AAB8320
Proroblemma testa|[15308]|USLEP359-10|United States|Florida|658[0n]|BOLD:AAH7694
Proroblemma testa|[15309]|USLEP325-10|United States|Florida|658[0n]|BOLD:AAH7694
Proroblemma testa|[15310]|USLEP346-10|United States|Florida|658[0n]|BOLD:AAH7694
Proroblemma testa|[15311]|LOFLD384-07|United States|Florida|658[0n]|BOLD:AAH7694
Proroblemma testa|[15312]|USLEP323-10|United States|Florida|658[0n]|BOLD:AAH7694
Proroblemma testa|[15313]|USLEP324-10|United States|Florida|658[0n]|BOLD:AAH7694
Proroblemma testa|[15314]|USLEP347-10|United States|Florida|658[0n]|BOLD:AAH7694
Proroblemma testa|[15315]|USLEP333-10|United States|Florida|632[0n]|BOLD:AAH7694
Proroblemma testa|[15316]|USLEP823-10|United States|Florida|658[0n]|BOLD:AAH7694
Proroblemma testa|[15317]|USLEP1086-10|United States|Florida|658[1n]|BOLD:AAH7694
Mursa phtisialis|[15318]|BLPCJ157-08|Costa Rica|Guanacaste|658[0n]|BOLD:AAG2981
Mursa phtisialis|[15319]|IBOLG025-08|Costa Rica|Alajuela|657[0n]|BOLD:AAG2981
Mursa phtisialis|[15320]|LPYPB244-08|Mexico|Campeche|577[0n]|BOLD:AAG2981
Mursa phtisialis|[15321]|CNCLB1251-14|United States|Louisiana|658[0n]|BOLD:AAG2981
Mursa phtisialis|[15322]|CNCLB2240-14|Puerto Rico|Mayaguez|658[0n]|BOLD:AAG2981
Oxycilla mitographa|[15323]|HKONS169-08|United States|Florida|658[0n]|BOLD:AAC5999
Oxycilla mitographa|[15324]|LMEM343-09|United States|Mississippi|633[0n]|BOLD:AAC5999
Oxycilla mitographa|[15325]|HKONB460-09|United States|Louisiana|658[0n]|BOLD:AAC5999
Oxycilla mitographa|[15326]|LNCB680-09|United States|North Carolina|658[0n]|BOLD:AAC5999
Oxycilla mitographa|[15327]|LNCB681-09|United States|North Carolina|658[0n]|BOLD:AAC5999
Oxycilla mitographa|[15328]|LNCB476-07|United States|North Carolina|658[0n]|BOLD:AAC5999
Oxycilla mitographa|[15329]|LNCB477-07|United States|North Carolina|658[0n]|BOLD:AAC5999
Oxycilla mitographa|[15330]|LMEM341-09|United States|Alabama|658[0n]|BOLD:AAC5999
Oxycilla mitographa|[15331]|LMEM342-09|United States|Alabama|658[0n]|BOLD:AAC5999
Oxycilla mitographa|[15332]|LNCB679-09|United States|North Carolina|658[0n]|BOLD:AAC5999
Oxycilla mitographa|[15333]|LNCC932-11|United States|North Carolina|658[0n]|BOLD:AAC5999
Plusiodonta amado|[15334]|RDNMH576-09|United States|Arizona|658[0n]|BOLD:AAY3011
Plusiodonta compressipalpis|[15335]|IAWL051-09|United States|Arizona|658[0n]|BOLD:AAB7158
Plusiodonta compressipalpis|[15336]|IAWL052-09|United States|Arizona|658[0n]|BOLD:AAB7158
Plusiodonta compressipalpis|[15337]|IAWL053-09|United States|Arizona|658[0n]|BOLD:AAB7158
Plusiodonta compressipalpis|[15338]|RDNMH577-09|United States|Arizona|658[0n]|BOLD:AAB7158
Plusiodonta compressipalpis|[15339]|RDNMH578-09|United States|Arizona|658[0n]|BOLD:AAB7158
Plusiodonta compressipalpis|[15340]|BBLOC910-11|United States|Arkansas|658[0n]|BOLD:AAB7156
Plusiodonta compressipalpis|[15341]|BBLOC1286-11|United States|Arkansas|658[0n]|BOLD:AAB7156
Plusiodonta compressipalpis|[15342]|BBLSW537-09|United States|Oklahoma|634[0n]|BOLD:AAB7156
Plusiodonta compressipalpis|[15343]|BBLSX177-09|United States|Oklahoma|658[0n]|BOLD:AAB7156
Plusiodonta compressipalpis|[15344]|BBLOE1373-12|United States|Arkansas|658[0n]|BOLD:AAB7156
Plusiodonta compressipalpis|[15345]|IAWL035-09|United States|Texas|658[0n]|BOLD:AAB7156
Plusiodonta compressipalpis|[15346]|BBLOC917-11|United States|Arkansas|658[0n]|BOLD:AAB7156
Plusiodonta compressipalpis|[15347]|BBLOC904-11|United States|Arkansas|658[0n]|BOLD:AAB7156
Plusiodonta compressipalpis|[15348]|LMEMB101-09|United States|Alabama|658[0n]|BOLD:AAB7156
Plusiodonta compressipalpis|[15349]|BBLOC836-11|United States|Arkansas|658[0n]|BOLD:AAB7156
Plusiodonta compressipalpis|[15350]|BBLSX397-09|United States|Oklahoma|658[0n]|BOLD:AAB7156
Plusiodonta compressipalpis|[15351]|QUNOC094-09|United States|Kentucky|658[0n]|BOLD:AAB7156
Plusiodonta compressipalpis|[15352]|BBLOC1057-11|United States|Arkansas|658[0n]|BOLD:AAB7156
Plusiodonta compressipalpis|[15353]|BBLOC908-11|United States|Arkansas|658[0n]|BOLD:AAB7156
Plusiodonta compressipalpis|[15354]|BBLOC907-11|United States|Arkansas|658[0n]|BOLD:AAB7156
Plusiodonta compressipalpis|[15355]|BBLOC906-11|United States|Arkansas|658[0n]|BOLD:AAB7156
Plusiodonta compressipalpis|[15356]|BBLOC839-11|United States|Arkansas|658[0n]|BOLD:AAB7156
Plusiodonta compressipalpis|[15357]|LILLA863-11|United States|Illinois|658[0n]|BOLD:AAB7156
Plusiodonta compressipalpis|[15358]|BBLPA655-10|Canada|Ontario|658[0n]|BOLD:AAB7156
Plusiodonta compressipalpis|[15359]|BBLSY919-09|United States|Texas|658[0n]|BOLD:AAB7156
Plusiodonta compressipalpis|[15360]|BBLSX275-09|United States|Texas|658[0n]|BOLD:AAB7156
Plusiodonta compressipalpis|[15361]|BBLSY088-09|United States|Oklahoma|658[0n]|BOLD:AAB7156
Plusiodonta compressipalpis|[15362]|BBLSW994-09|United States|Texas|658[0n]|BOLD:AAB7156
Plusiodonta compressipalpis|[15363]|BBLSW468-09|United States|Oklahoma|658[0n]|BOLD:AAB7156
Plusiodonta compressipalpis|[15364]|LMEMB103-09|United States|Mississippi|658[0n]|BOLD:AAB7156
Plusiodonta compressipalpis|[15365]|LMEMB102-09|United States|Alabama|658[0n]|BOLD:AAB7156
Plusiodonta compressipalpis|[15366]|HKONB290-09|United States|Texas|658[0n]|BOLD:AAB7156
Plusiodonta compressipalpis|[15367]|LMEMB104-09|United States|Mississippi|658[0n]|BOLD:AAB7156
Plusiodonta compressipalpis|[15368]|BBLOC919-11|United States|Arkansas|658[0n]|BOLD:AAB7156
Plusiodonta compressipalpis|[15369]|LSUSA090-06|United States|Kentucky|580[0n]|BOLD:AAB7156
Plusiodonta compressipalpis|[15370]|HKONS387-08|United States|Florida|631[0n]|BOLD:AAB7156
Plusiodonta compressipalpis|[15371]|JMZCA070-08|United States|Florida|622[0n]|BOLD:AAB7156
Plusiodonta compressipalpis|[15372]|RDLQH116-06|Canada|Quebec|601[0n]|BOLD:AAB7156
Plusiodonta compressipalpis|[15373]|LPOKB254-09|United States|Oklahoma|629[0n]|BOLD:AAB7156
Plusiodonta compressipalpis|[15374]|BBLSW857-09|United States|Texas|646[0n]|BOLD:AAB7156
Plusiodonta compressipalpis|[15375]|BBLSW663-09|United States|Oklahoma|658[0n]|BOLD:AAB7156
Plusiodonta compressipalpis|[15376]|MILEQ260-11|United States|Alabama|658[0n]|BOLD:AAB7156
Plusiodonta compressipalpis|[15377]|BBLOC1238-11|United States|Arkansas|658[0n]|BOLD:AAB7156

[illegible]
Plusiodonta compressipalpis|[15375]|BBLSW663-09|United States|Oklahoma|658[0n]|BOLD:AAB7156
Plusiodonta compressipalpis|[15376]|MILEQ260-11|United States|Alabama|658[0n]|BOLD:AAB7156
Plusiodonta compressipalpis|[15377]|BBLOC1238-11|United States|Arkansas|658[0n]|BOLD:AAB7156
Plusiodonta compressipalpis|[15378]|BBLOC1672-11|United States|Texas|658[0n]|BOLD:AAB7156
Plusiodonta compressipalpis|[15379]|BBLOD531-11|United States|Texas|658[0n]|BOLD:AAB7156
Plusiodonta compressipalpis|[15380]|BBLOD1305-11|United States|Texas|658[0n]|BOLD:AAB7156
Plusiodonta compressipalpis|[15381]|BBLOE1376-12|United States|Arkansas|658[0n]|BOLD:AAB7156
Plusiodonta compressipalpis|[15382]|USLEP086-10|United States|Arkansas|658[0n]|BOLD:AAB7156
Plusiodonta compressipalpis|[15383]|BBLOE1608-12|United States|Arkansas|658[0n]|BOLD:AAB7156
Eublemma minima|[15384]|LPOKB077-09|United States|Oklahoma|658[0n]|BOLD:ACE8521
Eublemma minima|[15385]|LPOKB501-09|United States|Oklahoma|658[0n]|BOLD:ACE8521
Eublemma minima|[15386]|LPOKB627-09|United States|Oklahoma|658[0n]|BOLD:ACE8521
Eublemma minima|[15387]|CMAZA127-09|United States|Arizona|658[0n]|BOLD:ACE8521
Eublemma minima|[15388]|CMAZA419-10|United States|Arizona|658[0n]|BOLD:ACE8521
Eublemma minima|[15389]|LOCBE059-06|United States|California|658[0n]|BOLD:ACE8521
Eublemma minima|[15390]|LOCBE022-06|United States|California|658[0n]|BOLD:ACE8521
Eublemma minima|[15391]|LOCBD971-06|United States|California|658[0n]|BOLD:ACE8521
Eublemma minima|[15392]|LOCBD968-06|United States|California|658[0n]|BOLD:ACE8521
Eublemma minima|[15393]|LOCBC746-06|United States|California|658[0n]|BOLD:ACE8521
Eublemma minima|[15394]|LOCBE176-06|United States|California|643[0n]|BOLD:ACE8521
Eublemma minima|[15395]|HKONB269-09|United States|Texas|658[0n]|BOLD:ACE8521
Eublemma minima|[15396]|GMLC275-11|United States|California|658[0n]|BOLD:ACE8521
Eublemma minima|[15397]|LOFLD443-07|United States|Florida|658[0n]|BOLD:AAC4233
Eublemma minima|[15398]|LNC441-05|United States|North Carolina|658[0n]|BOLD:AAC4233
Eublemma minima|[15399]|LOFLB590-06|United States|Florida|632[0n]|BOLD:AAC4233
Eublemma minima|[15400]|LOFLA719-06|United States|Florida|658[0n]|BOLD:AAC4233
Eublemma minima|[15401]|LSEU375-06|United States|Georgia|658[0n]|BOLD:AAC4233
Eublemma minima|[15402]|RDNMD506-06|United States|Florida|658[0n]|BOLD:AAC4233
Eublemma minima|[15403]|ZYCRM660-11|United States|South Carolina|658[0n]|BOLD:AAC4233
Eublemma recta|[15404]|CNCLB2111-14|United States|Florida|658[0n]|BOLD:AAF8322
Eublemma recta|[15405]|IAWL553-09|United States|Arizona|658[0n]|BOLD:AAF8322
Eublemma recta|[15406]|HKONB329-09|United States|Texas|658[0n]|BOLD:AAF8322
Eublemma recta|[15407]|HKONB070-08|United States|Texas|658[0n]|BOLD:AAF8322
Eublemma recta|[15408]|IAWL552-09|United States|Arizona|658[1n]|BOLD:AAF8322
Eublemma recta|[15409]|RDNMF707-08|United States|Arizona|650[0n]|BOLD:AAF8322
Eublemma recta|[15410]|IAWL409-09|United States|Arizona|593[0n]|BOLD:AAF8322
Eublemma recta|[15411]|CNCLB2112-14|United States|Florida|658[0n]|BOLD:AAF8322
Eublemma cinnamomea|[15412]|CNCLB1943-14|United States|Florida|658[0n]|BOLD:AAG5894
Eublemma cinnamomea|[15413]|LPYPB501-08|Mexico|Quintana Roo|658[0n]|BOLD:AAG5894
Eublemma cinnamomea|[15414]|LPYPB875-08|Mexico|Quintana Roo|658[0n]|BOLD:AAG5894
Eublemma cinnamomea|[15415]|LOCRI382-11|Costa Rica|Limon|658[0n]|BOLD:AAG5894
Eublemma cinnamomea|[15416]|RDNML348-13|United States|Florida|658[0n]|BOLD:AAG5894
Eublemma cinnamomea|[15417]|CNCLB1942-14|United States|Florida|658[0n]|BOLD:AAG5894
Eublemma cinnamomea|[15418]|CNCLB1945-14|United States|Florida|658[0n]|BOLD:AAG5894
Eublemma cinnamomea|[15419]|CNCLB1946-14|United States|Florida|658[0n]|BOLD:AAG5894
Eublemma cinnamomea|[15420]|CNCLB1647-14|United States|Florida|658[0n]|BOLD:ACP3799
Eublemma cinnamomea|[15421]|CNCLB1944-14|United States|Florida|658[0n]|BOLD:ACP3799
Eublemma cinnamomea|[15422]|CNCLB2113-14|United States|Florida|658[0n]|BOLD:ACP3799
Eublemma cinnamomea|[15423]|RDNML347-13|United States|Florida|658[0n]|BOLD:ACP3799
Eublemma cinnamomea|[15424]|CNCLB1941-14|United States|Florida|658[0n]|BOLD:ACP3799
Eublemma cinnamomea|[15425]|CNCLB2114-14|United States|Florida|658[0n]|BOLD:ACP3799
Dasyblemma straminea|[15426]|RDNMJ318-11|United States|California|627[1n]|BOLD:AAV6674
Dasyblemma straminea|[15427]|HKONB563-09|United States|Texas|644[0n]|BOLD:AAC3069
Dasyblemma straminea|[15428]|RDNMG264-08|United States|Texas|658[0n]|BOLD:AAC3069
Dasyblemma straminea|[15429]|QUNOE459-12|United States|Texas|658[0n]|BOLD:AAC3069
Oxycilla malaca|[15430]|LGSM773-04|United States|North Carolina|658[0n]|BOLD:AAC6000
Oxycilla malaca|[15431]|LGSMC393-05|United States|Tennessee|658[0n]|BOLD:AAC6000
Oxycilla malaca|[15432]|LMEM338-09|United States|Mississippi|658[0n]|BOLD:AAC6000
Oxycilla malaca|[15433]|LNCB678-09|United States|North Carolina|658[0n]|BOLD:AAC6000
Oxycilla malaca|[15434]|QUNOD741-11|United States|Mississippi|658[0n]|BOLD:AAC6000
Oxycilla tripla|[15435]|CMAZA589-10|United States|Arizona|658[0n]|BOLD:AAG5975
Oxycilla tripla|[15436]|IAWL042-09|United States|Arizona|658[0n]|BOLD:AAG5975
Oxycilla tripla|[15437]|RDNMH809-09|United States|Arizona|658[0n]|BOLD:AAG5975
Oxycilla tripla|[15438]|CMAZA013-09|United States|Arizona|658[0n]|BOLD:AAG5975
Oxycilla tripla|[15439]|QUNOD188-10|United States|Arizona|658[0n]|BOLD:AAG5975
Oxycilla tripla|[15440]|CMAZA599-10|United States|Arizona|658[0n]|BOLD:AAG5975
Oxycilla tripla|[15441]|LTOLB763-11|United States|Arizona|658[0n]|BOLD:AAG5975
Oxycilla tripla|[15442]|CMAZA1101-12|United States|Arizona|658[0n]|BOLD:AAG5975
Oxycilla ondo|[15443]|RDNMH810-09|United States|Arizona|658[0n]|BOLD:AAI5466
Oxycilla ondo|[15444]|RDNMH811-09|United States|Arizona|658[0n]|BOLD:AAI5466
Oxycilla ondo|[15445]|RDNMJ710-11|United States|Arizona|658[0n]|BOLD:AAI5466
Zelicodes linearis|[15446]|IAWL346-09|United States|Arizona|658[0n]|BOLD:AAG5976
Zelicodes linearis|[15447]|ABNCC108-07|United States|Arizona|650[0n]|BOLD:AAG5976
Zelicodes linearis|[15448]|RDNMG013-08|United States|Arizona|658[0n]|BOLD:AAG5976
Zelicodes linearis|[15449]|RDNMK649-11|United States|Arizona|658[0n]|BOLD:AAG5976
Zelicodes linearis|[15450]|IAWL043-09|United States|Arizona|658[0n]|BOLD:AAG5976
Zelicodes linearis|[15451]|IAWL849-09|United States|Arizona|658[0n]|BOLD:AAG5976
Zelicodes linearis|[15452]|CMAZA604-10|United States|Arizona|639[0n]|BOLD:AAG5976
Zelicodes linearis|[15453]|CMAZA1179-12|United States|Arizona|658[0n]|BOLD:AAG5976
Rivula pusilla|[15454]|RDNMD460-06|United States|Florida|658[0n]|BOLD:ACX4832
Rivula pusilla|[15455]|RDNMD509-06|United States|Florida|658[0n]|BOLD:ACX4832
Rivula pusilla|[15456]|HKONS167-08|United States|Florida|658[0n]|BOLD:ACX4832
Rivula pusilla|[15457]|RDNMD510-06|United States|Florida|652[0n]|BOLD:AAB2943
Rivula pusilla|[15458]|HKONS168-08|United States|Florida|656[0n]|BOLD:AAB2943
Rivula stepheni|[15459]|LNC345-05|United States|North Carolina|658[0n]|BOLD:AAX8877
Rivula stepheni|[15460]|CNCLB1255-14|United States|North Carolina|658[0n]|BOLD:AAX8877
Rivula propinqualis|[15461]|LNC348-05|United States|North Carolina|502[0n]|BOLD:AAA4282
Rivula propinqualis|[15462]|LGSMG523-07|United States|North Carolina|658[0n]|BOLD:AAA4282
Rivula propinqualis|[15463]|LGSMG524-07|United States|North Carolina|658[0n]|BOLD:AAA4282
Rivula propinqualis|[15464]|LILLA453-11|United States|Illinois|658[0n]|BOLD:AAA4282
Rivula propinqualis|[15465]|LPSOB874-08|Canada|Ontario|658[1n]|BOLD:AAA4282
Rivula propinqualis|[15466]|BLTIB434-08|Canada|Ontario|658[0n]|BOLD:AAA4282
Rivula propinqualis|[15467]|LPSOB875-08|Canada|Ontario|658[0n]|BOLD:AAA4282
Rivula propinqualis|[15468]|LPSOB873-08|Canada|Ontario|658[0n]|BOLD:AAA4282
Rivula propinqualis|[15469]|PMG159-03|Canada|Ontario|617[0n]|BOLD:AAA4282
Rivula propinqualis|[15470]|LPSOB675-08|Canada|Ontario|646[0n]|BOLD:AAA4282
Rivula propinqualis|[15471]|BLGSM053-09|Canada|Ontario|612[0n]|BOLD:AAA4282
Rivula propinqualis|[15472]|LNCC303-10|United States|North Carolina|658[0n]|BOLD:AAA4282
Rivula propinqualis|[15473]|LNCC304-10|United States|North Carolina|658[0n]|BOLD:AAA4282
Rivula propinqualis|[15474]|LNCC305-10|United States|North Carolina|658[0n]|BOLD:AAA4282
Rivula propinqualis|[15475]|JSAUG482-11|Canada|Ontario|658[0n]|BOLD:AAA4282
Rivula propinqualis|[15476]|LGSMD371-05|United States|Tennessee|658[0n]|BOLD:AAA4282
Rivula propinqualis|[15477]|LGSM754-04|United States|North Carolina|658[0n]|BOLD:AAA4282

Rivula propinqualis|[15474]|[illegible]|United States|North Carolina|658[0n]|BOLD:AAA4282
Rivula propinqualis|[15475]|JSAUG482-11|Canada|Ontario|658[0n]|BOLD:AAA4282
Rivula propinqualis|[15476]|LGSMD371-05|United States|Tennessee|658[0n]|BOLD:AAA4282
Rivula propinqualis|[15477]|LGSM754-04|United States|North Carolina|658[0n]|BOLD:AAA4282
Rivula propinqualis|[15478]|LGSMG525-07|United States|Tennessee|658[0n]|BOLD:AAA4282
Rivula propinqualis|[15479]|LALPA559-10|Canada|British Columbia|658[0n]|BOLD:AAA4282
Rivula propinqualis|[15480]|RDLQE417-06|Canada|Quebec|657[0n]|BOLD:AAA4282
Rivula propinqualis|[15481]|LMIS048-06|Canada|Ontario|658[0n]|BOLD:AAA4282
Rivula propinqualis|[15482]|TTMNB513-06|Canada|New Brunswick|658[0n]|BOLD:AAA4282
Rivula propinqualis|[15483]|RDLQG605-06|Canada|Quebec|658[0n]|BOLD:AAA4282
Rivula propinqualis|[15484]|RDLQG555-06|Canada|Quebec|655[0n]|BOLD:AAA4282
Rivula propinqualis|[15485]|RDLQG493-06|Canada|Quebec|654[0n]|BOLD:AAA4282
Rivula propinqualis|[15486]|RDLQE342-06|Canada|Quebec|656[0n]|BOLD:AAA4282
Rivula propinqualis|[15487]|RDLQG042-06|Canada|Quebec|603[0n]|BOLD:AAA4282
Rivula propinqualis|[15488]|RDLQG552-06|Canada|Quebec|658[0n]|BOLD:AAA4282
Rivula propinqualis|[15489]|RDLQG553-06|Canada|Quebec|658[0n]|BOLD:AAA4282
Rivula propinqualis|[15490]|RDLQG554-06|Canada|Quebec|658[0n]|BOLD:AAA4282
Rivula propinqualis|[15491]|RDLQG570-06|Canada|Quebec|658[0n]|BOLD:AAA4282
Rivula propinqualis|[15492]|RDLQG571-06|Canada|Quebec|658[0n]|BOLD:AAA4282
Rivula propinqualis|[15493]|RDLQG695-06|Canada|Quebec|658[0n]|BOLD:AAA4282
Rivula propinqualis|[15494]|RDLQG696-06|Canada|Quebec|658[0n]|BOLD:AAA4282
Rivula propinqualis|[15495]|RDLQG697-06|Canada|Quebec|658[0n]|BOLD:AAA4282
Rivula propinqualis|[15496]|BLTIB211-08|Canada|Ontario|658[0n]|BOLD:AAA4282
Rivula propinqualis|[15497]|BLTIB803-08|Canada|Ontario|658[0n]|BOLD:AAA4282
Rivula propinqualis|[15498]|BBLPC731-09|Canada|Newfoundland and Labrador|658[0n]|BOLD:AAA4282
Rivula propinqualis|[15499]|BBLPB973-10|Canada|Alberta|658[0n]|BOLD:AAA4282
Rivula propinqualis|[15500]|JSAUG510-11|Canada|Ontario|658[0n]|BOLD:AAA4282
Rivula propinqualis|[15501]|RBINA3987-13|Canada|Ontario|561[0n]|BOLD:AAA4282
Rivula propinqualis|[15502]|PHMO143-03|Canada|Ontario|639[0n]|BOLD:AAA4282
Rivula propinqualis|[15503]|XAF461-05|Canada|Ontario|564[0n]|BOLD:AAA4282
Rivula propinqualis|[15504]|BLTIB1026-08|Canada|Ontario|658[0n]|BOLD:AAA4282
Rivula propinqualis|[15505]|BLGSM010-09|Canada|Ontario|658[0n]|BOLD:AAA4282
Rivula propinqualis|[15506]|PHAUG1522-11|Canada|Ontario|658[0n]|BOLD:AAA4282
Rivula propinqualis|[15507]|RDLQD886-06|Canada|Quebec|658[0n]|BOLD:AAA4282
Rivula propinqualis|[15508]|LPSOC253-08|Canada|Ontario|656[0n]|BOLD:AAA4282
Rivula propinqualis|[15509]|XAJ650-06|Canada|Ontario|656[0n]|BOLD:AAA4282
Rivula propinqualis|[15510]|BLTIB883-08|Canada|Ontario|658[0n]|BOLD:AAA4282
Rivula propinqualis|[15511]|MEC584-04|Canada|Quebec|655[1n]|BOLD:AAA4282
Rivula propinqualis|[15512]|LPMNB253-09|Canada|Manitoba|658[0n]|BOLD:AAA4282
Rivula propinqualis|[15513]|LPMNB237-09|Canada|Manitoba|658[0n]|BOLD:AAA4282
Rivula propinqualis|[15514]|LPMNB235-09|Canada|Manitoba|658[0n]|BOLD:AAA4282
Rivula propinqualis|[15515]|LPABB234-08|Canada|Alberta|658[0n]|BOLD:AAA4282
Rivula propinqualis|[15516]|BLTIB1007-08|Canada|Ontario|658[0n]|BOLD:AAA4282
Rivula propinqualis|[15517]|BLTIB172-08|Canada|Ontario|658[0n]|BOLD:AAA4282
Rivula propinqualis|[15518]|LPMN875-08|Canada|Alberta|658[0n]|BOLD:AAA4282
Rivula propinqualis|[15519]|LPSOC252-08|Canada|Ontario|658[0n]|BOLD:AAA4282
Rivula propinqualis|[15520]|LPSOC251-08|Canada|Ontario|658[0n]|BOLD:AAA4282
Rivula propinqualis|[15521]|LPSOC066-08|Canada|Ontario|658[0n]|BOLD:AAA4282
Rivula propinqualis|[15522]|LPSOB043-08|Canada|Ontario|658[0n]|BOLD:AAA4282
Rivula propinqualis|[15523]|LPSOB032-08|Canada|Ontario|658[0n]|BOLD:AAA4282
Rivula propinqualis|[15524]|LPSO762-08|Canada|Ontario|658[0n]|BOLD:AAA4282
Rivula propinqualis|[15525]|LPSO593-08|Canada|Ontario|658[0n]|BOLD:AAA4282
Rivula propinqualis|[15526]|LNCNW068-06|United States|North Carolina|658[0n]|BOLD:AAA4282
Rivula propinqualis|[15527]|RDLQD887-06|Canada|Quebec|658[0n]|BOLD:AAA4282
Rivula propinqualis|[15528]|RDLQB690-05|Canada|Quebec|658[0n]|BOLD:AAA4282
Rivula propinqualis|[15529]|XAG953-05|Canada|Ontario|658[0n]|BOLD:AAA4282
Rivula propinqualis|[15530]|XAG921-05|Canada|Ontario|658[0n]|BOLD:AAA4282
Rivula propinqualis|[15531]|XAG045-05|Canada|Ontario|658[0n]|BOLD:AAA4282
Rivula propinqualis|[15532]|XAE317-04|Canada|Ontario|658[0n]|BOLD:AAA4282
Rivula propinqualis|[15533]|BLTIB305-08|Canada|Ontario|650[0n]|BOLD:AAA4282
Rivula propinqualis|[15534]|LPMNB453-09|Canada|Manitoba|658[0n]|BOLD:AAA4282
Rivula propinqualis|[15535]|LPMNB516-09|Canada|Manitoba|658[0n]|BOLD:AAA4282
Rivula propinqualis|[15536]|LPMNB544-09|Canada|Manitoba|658[0n]|BOLD:AAA4282
Rivula propinqualis|[15537]|PHMTV435-10|Canada|Ontario|658[0n]|BOLD:AAA4282
Rivula propinqualis|[15538]|PHMTV439-10|Canada|Ontario|658[0n]|BOLD:AAA4282
Rivula propinqualis|[15539]|PHMTV441-10|Canada|Ontario|658[0n]|BOLD:AAA4282
Rivula propinqualis|[15540]|BBLPD116-10|Canada|Saskatchewan|658[0n]|BOLD:AAA4282
Rivula propinqualis|[15541]|BBLPD974-10|Canada|Alberta|658[0n]|BOLD:AAA4282
Rivula propinqualis|[15542]|BBLPD975-10|Canada|Alberta|658[0n]|BOLD:AAA4282
Rivula propinqualis|[15543]|LPABB173-08|Canada|Alberta|638[0n]|BOLD:AAA4282
Rivula propinqualis|[15544]|LPSOD1005-09|Canada|Ontario|621[0n]|BOLD:AAA4282
Rivula propinqualis|[15545]|XAD389-04|Canada|Ontario|582[0n]|BOLD:AAA4282
Rivula propinqualis|[15546]|BLTIB308-08|Canada|Ontario|658[1n]|BOLD:AAA4282
Rivula propinqualis|[15547]|XAD730-05|Canada|Ontario|524[2n]|BOLD:AAA4282
Rivula propinqualis|[15548]|LNCNW069-06|United States|North Carolina|629[0n]|BOLD:AAA4282
Rivula propinqualis|[15549]|LNC349-05|United States|North Carolina|576[0n]|BOLD:AAA4282
Rivula propinqualis|[15550]|MEC585-04|Canada|Quebec|587[0n]|BOLD:AAA4282
Rivula propinqualis|[15551]|LPMNB410-09|Canada|Manitoba|597[0n]|BOLD:AAA4282
Rivula propinqualis|[15552]|SSPAA6093-13|Canada|Saskatchewan|606[0n]|BOLD:AAA4282
Rivula propinqualis|[15553]|SSPAC3415-13|Canada|Saskatchewan|601[0n]|BOLD:AAA4282
Rivula propinqualis|[15554]|LGSMG522-07|United States|Tennessee|654[0n]|BOLD:AAA4282
Rivula propinqualis|[15555]|LPOKC197-09|United States|Oklahoma|658[0n]|BOLD:AAA4282
Rivula propinqualis|[15556]|LPOKD031-09|United States|Oklahoma|658[0n]|BOLD:AAA4282
Rivula propinqualis|[15557]|CNCLB1256-14|United States|North Carolina|658[0n]|BOLD:AAA4282
Rivula propinqualis|[15558]|CNCLB2981-14|United States|North Carolina|658[0n]|BOLD:AAA4282
Rivula propinqualis|[15559]|ABNCC107-07|United States|Indiana|653[2n]|BOLD:ABZ7274
Rivula propinqualis|[15560]|LTOLB065-08|United States|Maryland|656[0n]|BOLD:ABZ7274
Rivula propinqualis|[15561]|LPOKA455-09|United States|Oklahoma|658[0n]|BOLD:ABZ7274
Rivula propinqualis|[15562]|LPOKA423-09|United States|Oklahoma|658[0n]|BOLD:ABZ7274
Rivula propinqualis|[15563]|HKONS164-08|United States|Florida|658[0n]|BOLD:ABZ7274
Rivula propinqualis|[15564]|HKONS060-07|United States|Florida|658[0n]|BOLD:ABZ7274
Rivula propinqualis|[15565]|LNC346-05|United States|North Carolina|658[0n]|BOLD:ABZ7274
Rivula propinqualis|[15566]|LNC347-05|United States|North Carolina|545[0n]|BOLD:ABZ7274
Rivula propinqualis|[15567]|LMEM334-09|United States|Mississippi|638[0n]|BOLD:ABZ7274
Rivula propinqualis|[15568]|LMEM337-09|United States|Mississippi|658[0n]|BOLD:ABZ7274
Rivula propinqualis|[15569]|LPOKB742-09|United States|Oklahoma|658[0n]|BOLD:ABZ7274
Rivula propinqualis|[15570]|LNCB634-09|United States|North Carolina|658[0n]|BOLD:ABZ7274
Rivula propinqualis|[15571]|LNCB728-09|United States|North Carolina|658[0n]|BOLD:ABZ7274
Rivula propinqualis|[15572]|LNCC414-10|United States|North Carolina|658[0n]|BOLD:ABZ7274
Rivula propinqualis|[15573]|LNCC1411-11|United States|North Carolina|658[0n]|BOLD:ABZ7274
Rivula propinqualis|[15574]|CNCLB1257-14|United States|North Carolina|658[0n]|BOLD:ABZ7274
Rivula propinqualis|[15575]|CNCLB2982-14|United States|North Carolina|658[0n]|BOLD:ABZ7274
Rivula propinqualis|[15576]|CNCLB2983-14|United States|North Carolina|658[0n]|BOLD:ABZ7274
[illegible]|[15577]|[illegible]|United States|Florida|658[0n]|BOLD:[illegible]

Rivula propinqualis|[15574]|CNCLB1257-14|United States|North Carolina|658[0n]|BOLD:ABZ7274
Rivula propinqualis|[15575]|CNCLB2982-14|United States|North Carolina|658[0n]|BOLD:ABZ7274
Rivula propinqualis|[15576]|CNCLB2983-14|United States|North Carolina|658[0n]|BOLD:ABZ7274
Manbuta pyraliformis|[15577]|BBLOB793-11|United States|Florida|658[0n]|BOLD:ABA1152
Manbuta pyraliformis|[15578]|BBLOB788-11|United States|Florida|658[0n]|BOLD:ABA1152
Manbuta pyraliformis|[15579]|CNCLB993-14|United States|Florida|629[0n]|BOLD:ABA1152
Manbuta pyraliformis|[15580]|CNCLB994-14|United States|Florida|658[0n]|BOLD:ABA1152
Eulepidotis persimilis|[15581]|CNCLB1976-14|Guatemala||658[0n]|BOLD:AAD4925
Eulepidotis persimilis|[15582]|CNCLB1978-14|Guatemala||658[0n]|BOLD:AAD4925
Eulepidotis persimilis|[15583]|LOCRE559-10|Costa Rica|San Jose|658[0n]|BOLD:AAD4925
Eulepidotis persimilis|[15584]|GWOSS468-11|Honduras|Atlantida|658[0n]|BOLD:AAD4925
Eulepidotis persimilis|[15585]|GWOSS555-11|Honduras|Atlantida|658[0n]|BOLD:AAD4925
Eulepidotis persimilis|[15586]|CNCLB1977-14|Guatemala||658[0n]|BOLD:AAD4925
Eulepidotis persimilis|[15587]|CNCLB1979-14|Mexico||658[0n]|BOLD:AAD4925
Eulepidotis persimilis|[15588]|CNCLB1980-14|Guatemala||658[0n]|BOLD:AAD4925
Eulepidotis electa|[15589]|MHMXZ767-09|Costa Rica||658[0n]|BOLD:AAB2540
Eulepidotis electa|[15590]|BLPCI598-08|Costa Rica|Guanacaste|658[0n]|BOLD:AAB2540
Eulepidotis electa|[15591]|CNCLB1981-14|Guatemala||658[0n]|BOLD:AAB2540
Eulepidotis electa|[15592]|MHMYL3446-11|Costa Rica||658[0n]|BOLD:AAB2540
Eulepidotis electa|[15593]|BLPDV992-11|Costa Rica|Guanacaste|658[0n]|BOLD:AAB2540
Eulepidotis electa|[15594]|BLPDV991-11|Costa Rica|Guanacaste|658[0n]|BOLD:AAB2540
Eulepidotis electa|[15595]|BLPDV990-11|Costa Rica|Guanacaste|658[0n]|BOLD:AAB2540
Eulepidotis electa|[15596]|BLPDV988-11|Costa Rica|Guanacaste|658[0n]|BOLD:AAB2540
Eulepidotis electa|[15597]|BLPDV987-11|Costa Rica|Guanacaste|658[0n]|BOLD:AAB2540
Eulepidotis electa|[15598]|BLPDV984-11|Costa Rica|Guanacaste|658[0n]|BOLD:AAB2540
Eulepidotis electa|[15599]|BLPDV341-11|Costa Rica|Guanacaste|658[0n]|BOLD:AAB2540
Eulepidotis electa|[15600]|BLPDV340-11|Costa Rica|Guanacaste|658[0n]|BOLD:AAB2540
Eulepidotis electa|[15601]|LOCRE543-10|Costa Rica|San Jose|658[0n]|BOLD:AAB2540
Eulepidotis electa|[15602]|LOCRE542-10|Costa Rica|San Jose|658[0n]|BOLD:AAB2540
Eulepidotis electa|[15603]|BLPDK1886-09|Costa Rica|Guanacaste|658[0n]|BOLD:AAB2540
Eulepidotis electa|[15604]|BLPDK1313-09|Costa Rica|Guanacaste|658[0n]|BOLD:AAB2540
Eulepidotis electa|[15605]|MHMXZ768-09|Costa Rica||658[0n]|BOLD:AAB2540
Eulepidotis electa|[15606]|BLPDG520-09|Costa Rica|Guanacaste|658[0n]|BOLD:AAB2540
Eulepidotis electa|[15607]|BLPCD419-08|Costa Rica|Guanacaste|658[0n]|BOLD:AAB2540
Eulepidotis electa|[15608]|BLPCD418-08|Costa Rica|Guanacaste|658[0n]|BOLD:AAB2540
Eulepidotis electa|[15609]|BLPCA221-08|Costa Rica|Guanacaste|658[0n]|BOLD:AAB2540
Eulepidotis electa|[15610]|BLPBH512-07|Costa Rica|Guanacaste|658[0n]|BOLD:AAB2540
Eulepidotis electa|[15611]|BLPBH511-07|Costa Rica|Guanacaste|658[0n]|BOLD:AAB2540
Eulepidotis electa|[15612]|BLPBH510-07|Costa Rica|Guanacaste|658[0n]|BOLD:AAB2540
Eulepidotis electa|[15613]|BLPAH275-07|Costa Rica|Guanacaste|658[0n]|BOLD:AAB2540
Eulepidotis electa|[15614]|BLPAH273-07|Costa Rica|Guanacaste|658[0n]|BOLD:AAB2540
Eulepidotis electa|[15615]|MHAUB751-05|Costa Rica|Guanacaste|658[0n]|BOLD:AAB2540
Eulepidotis electa|[15616]|MHAUB749-05|Costa Rica|Guanacaste|658[0n]|BOLD:AAB2540
Eulepidotis electa|[15617]|MHAUB748-05|Costa Rica|Guanacaste|658[0n]|BOLD:AAB2540
Eulepidotis electa|[15618]|MHAUB300-05|Costa Rica|Guanacaste|658[0n]|BOLD:AAB2540
Eulepidotis electa|[15619]|BLPDU789-11|Costa Rica|Guanacaste|658[1n]|BOLD:AAB2540
Eulepidotis electa|[15620]|BLPDK1487-09|Costa Rica|Guanacaste|628[1n]|BOLD:AAB2540
Eulepidotis electa|[15621]|BLPDM011-10|Costa Rica|Guanacaste|636[0n]|BOLD:AAB2540
Eulepidotis electa|[15622]|MHAUB755-05|Costa Rica|Guanacaste|522[0n]|BOLD:AAB2540
Eulepidotis electa|[15623]|BLPDX154-11|Costa Rica|Guanacaste|658[0n]|BOLD:AAB2540
Eulepidotis electa|[15624]|CNCLB1982-14|Guatemala||658[0n]|BOLD:AAB2540
Eulepidotis electa|[15625]|CNCLB1983-14|Guatemala||658[0n]|BOLD:AAB2540
Eulepidotis electa|[15626]|BLPCA223-08|Costa Rica|Guanacaste|658[0n]|BOLD:AAB2540
Eulepidotis electa|[15627]|MHMXZ765-09|Costa Rica||658[0n]|BOLD:AAB2540
Eulepidotis electa|[15628]|MHMXE203-06|Costa Rica|Guanacaste|658[0n]|BOLD:AAB2540
Eulepidotis electa|[15629]|BLPCF441-08|Costa Rica|Alajuela|658[0n]|BOLD:AAB2540
Eulepidotis electa|[15630]|MHMXZ766-09|Costa Rica||658[0n]|BOLD:AAB2540
Eulepidotis electa|[15631]|BLPAH272-07|Costa Rica|Guanacaste|658[0n]|BOLD:AAB2540
Eulepidotis electa|[15632]|CNCLB1984-14|Brazil|Distrito Federal|658[0n]|BOLD:AAB2540
Eulepidotis sp.|[15633]|CNCLB1985-14|Guatemala||658[0n]|BOLD:AAP4402
Eulepidotis rectimargo|[15634]|LOCRH185-11|Costa Rica|Limon|657[0n]|BOLD:AAA5135
Eulepidotis rectimargo|[15635]|MHAUB303-05|Costa Rica|Guanacaste|658[0n]|BOLD:AAA5135
Eulepidotis rectimargo|[15636]|MHAUB305-05|Costa Rica|Guanacaste|658[0n]|BOLD:AAA5135
Eulepidotis rectimargo|[15637]|BLPBH500-07|Costa Rica|Guanacaste|658[0n]|BOLD:AAA5135
Eulepidotis rectimargo|[15638]|BLPAE850-07|Costa Rica|Guanacaste|658[0n]|BOLD:AAA5135
Eulepidotis rectimargo|[15639]|BLPAH270-07|Costa Rica|Guanacaste|658[0n]|BOLD:AAA5135
Eulepidotis rectimargo|[15640]|MHAUG510-07|Costa Rica|Guanacaste|599[1n]|BOLD:AAA5135
Eulepidotis rectimargo|[15641]|MHAUG501-07|Costa Rica|Guanacaste|593[0n]|BOLD:AAA5135
Eulepidotis rectimargo|[15642]|MHAUG508-07|Costa Rica|Guanacaste|578[0n]|BOLD:AAA5135
Eulepidotis rectimargo|[15643]|MHAUG511-07|Costa Rica|Guanacaste|594[0n]|BOLD:AAA5135
Eulepidotis rectimargo|[15644]|BLPDG608-09|Costa Rica|Guanacaste|633[0n]|BOLD:AAA5135
Eulepidotis rectimargo|[15645]|MHAUB304-05|Costa Rica|Guanacaste|658[0n]|BOLD:AAA5135
Eulepidotis rectimargo|[15646]|BLPAG402-07|Costa Rica|Guanacaste|658[0n]|BOLD:AAA5135
Eulepidotis rectimargo|[15647]|MHAUG504-07|Costa Rica|Guanacaste|593[0n]|BOLD:AAA5135
Eulepidotis rectimargo|[15648]|MHAUG515-07|Costa Rica|Guanacaste|593[0n]|BOLD:AAA5135
Eulepidotis rectimargo|[15649]|MHAUG517-07|Costa Rica|Guanacaste|599[0n]|BOLD:AAA5135
Eulepidotis rectimargo|[15650]|MHAUG514-07|Costa Rica|Guanacaste|599[2n]|BOLD:AAA5135
Eulepidotis rectimargo|[15651]|MHAUG518-07|Costa Rica|Guanacaste|593[0n]|BOLD:AAA5135
Eulepidotis rectimargo|[15652]|MHAUG519-07|Costa Rica|Guanacaste|594[0n]|BOLD:AAA5135
Eulepidotis rectimargo|[15653]|GWOSS092-11|Peru|Huanuco|658[0n]|BOLD:AAA5135
Eulepidotis rectimargo|[15654]|GWOSS093-11|Peru|Huanuco|658[0n]|BOLD:AAA5135
Eulepidotis rectimargo|[15655]|MHAUG502-07|Costa Rica|Guanacaste|593[0n]|BOLD:AAA5135
Eulepidotis rectimargo|[15656]|LEPPA220-12|Argentina|Entre Rios|658[0n]|BOLD:AAA5135
Eulepidotis rectimargo|[15657]|BLPCC460-08|Costa Rica|Guanacaste|658[0n]|BOLD:AAA5135
Eulepidotis rectimargo|[15658]|BLPDK670-09|Costa Rica|Guanacaste|658[0n]|BOLD:AAA5135
Eulepidotis rectimargo|[15659]|BLPDV989-11|Costa Rica|Guanacaste|658[0n]|BOLD:AAA5135
Eulepidotis rectimargo|[15660]|BLPDU791-11|Costa Rica|Guanacaste|658[0n]|BOLD:AAA5135
Eulepidotis rectimargo|[15661]|LOCRE560-10|Costa Rica|San Jose|658[0n]|BOLD:AAA5135
Eulepidotis rectimargo|[15662]|BLPDW224-11|Costa Rica|Guanacaste|658[0n]|BOLD:AAA5135
Eulepidotis rectimargo|[15663]|MHAUC200-06|Costa Rica|Guanacaste|658[0n]|BOLD:AAA5135
Eulepidotis rectimargo|[15664]|BLPAE852-07|Costa Rica|Guanacaste|658[0n]|BOLD:AAA5135
Eulepidotis rectimargo|[15665]|MHMYC2227-09|Costa Rica|Guanacaste|658[0n]|BOLD:AAA5135
Eulepidotis rectimargo|[15666]|LOCRA735-07|Costa Rica|Alajuela|658[0n]|BOLD:AAA5135
Eulepidotis rectimargo|[15667]|MHMXQ585-08|Costa Rica|Guanacaste|656[0n]|BOLD:AAA5135
Eulepidotis rectimargo|[15668]|CNCLB1987-14|Guatemala||658[0n]|BOLD:AAA5135
Eulepidotis rectimargo|[15669]|GWOSS562-11|Honduras|Atlantida|658[0n]|BOLD:AAA5135
Eulepidotis rectimargo|[15670]|BLPDW1179-11|Costa Rica|Guanacaste|658[0n]|BOLD:AAA5135
Eulepidotis rectimargo|[15671]|BLPDV983-11|Costa Rica|Guanacaste|658[0n]|BOLD:AAA5135
Eulepidotis rectimargo|[15672]|BLPDU790-11|Costa Rica|Guanacaste|658[0n]|BOLD:AAA5135
Eulepidotis rectimargo|[15673]|MHMXZ764-09|Costa Rica||658[0n]|BOLD:AAA5135
Eulepidotis rectimargo|[15674]|MHMXZ763-09|Costa Rica||658[0n]|BOLD:AAA5135
Eulepidotis rectimargo|[15675]|MHMXZ762-09|Costa Rica||658[0n]|BOLD:AAA5135
Eulepidotis rectimargo|[15676]|LPYPC070-08|Mexico|Yucatan|658[0n]|BOLD:AAA5135
[illegible]

Eulepidotis rectimargo|[15674]|MHMXZ763-09|Costa Rica||658[0n]|BOLD:AAA5135
Eulepidotis rectimargo|[15675]|MHMXZ762-09|Costa Rica||658[0n]|BOLD:AAA5135
Eulepidotis rectimargo|[15676]|LPYPC070-08|Mexico|Yucatan|658[0n]|BOLD:AAA5135
Eulepidotis rectimargo|[15677]|MHMXQ586-08|Costa Rica|Guanacaste|658[0n]|BOLD:AAA5135
Eulepidotis rectimargo|[15678]|BLPCA222-08|Costa Rica|Guanacaste|658[0n]|BOLD:AAA5135
Eulepidotis rectimargo|[15679]|BLPBH502-07|Costa Rica|Guanacaste|658[0n]|BOLD:AAA5135
Eulepidotis rectimargo|[15680]|BLPBH501-07|Costa Rica|Guanacaste|658[0n]|BOLD:AAA5135
Eulepidotis rectimargo|[15681]|BLPBH498-07|Costa Rica|Guanacaste|658[0n]|BOLD:AAA5135
Eulepidotis rectimargo|[15682]|BLPBH497-07|Costa Rica|Guanacaste|658[0n]|BOLD:AAA5135
Eulepidotis rectimargo|[15683]|BLPBG809-07|Costa Rica|Guanacaste|658[0n]|BOLD:AAA5135
Eulepidotis rectimargo|[15684]|BLPBF907-07|Costa Rica|Guanacaste|658[0n]|BOLD:AAA5135
Eulepidotis rectimargo|[15685]|BLPAH271-07|Costa Rica|Guanacaste|658[0n]|BOLD:AAA5135
Eulepidotis rectimargo|[15686]|BLPAF424-07|Costa Rica|Guanacaste|658[0n]|BOLD:AAA5135
Eulepidotis rectimargo|[15687]|BLPAE851-07|Costa Rica|Guanacaste|658[0n]|BOLD:AAA5135
Eulepidotis rectimargo|[15688]|BLPAE611-06|Costa Rica|Guanacaste|658[0n]|BOLD:AAA5135
Eulepidotis rectimargo|[15689]|BLPAE609-06|Costa Rica|Guanacaste|658[0n]|BOLD:AAA5135
Eulepidotis rectimargo|[15690]|BLPAE446-06|Costa Rica|Guanacaste|658[0n]|BOLD:AAA5135
Eulepidotis rectimargo|[15691]|BLPAC175-06|Costa Rica|Guanacaste|658[0n]|BOLD:AAA5135
Eulepidotis rectimargo|[15692]|MHAUC915-06|Costa Rica|Guanacaste|658[0n]|BOLD:AAA5135
Eulepidotis rectimargo|[15693]|MHAUC202-06|Costa Rica|Guanacaste|658[0n]|BOLD:AAA5135
Eulepidotis rectimargo|[15694]|MHAUC201-06|Costa Rica|Guanacaste|658[0n]|BOLD:AAA5135
Eulepidotis rectimargo|[15695]|MHAUC198-06|Costa Rica|Guanacaste|658[0n]|BOLD:AAA5135
Eulepidotis rectimargo|[15696]|MHAUB302-05|Costa Rica|Guanacaste|658[0n]|BOLD:AAA5135
Eulepidotis rectimargo|[15697]|MHAUB301-05|Costa Rica|Guanacaste|658[0n]|BOLD:AAA5135
Eulepidotis rectimargo|[15698]|MHAUB299-05|Costa Rica|Guanacaste|658[0n]|BOLD:AAA5135
Eulepidotis rectimargo|[15699]|MHAUB297-05|Costa Rica|Guanacaste|658[0n]|BOLD:AAA5135
Eulepidotis rectimargo|[15700]|MHAUC196-06|Costa Rica|Guanacaste|658[0n]|BOLD:AAA5135
Eulepidotis rectimargo|[15701]|MHAUG520-07|Costa Rica|Guanacaste|545[0n]|BOLD:AAA5135
Eulepidotis rectimargo|[15702]|MHAUG503-07|Costa Rica|Guanacaste|598[0n]|BOLD:AAA5135
Eulepidotis rectimargo|[15703]|MHAUG506-07|Costa Rica|Guanacaste|594[0n]|BOLD:AAA5135
Eulepidotis rectimargo|[15704]|MHAUG505-07|Costa Rica|Guanacaste|594[0n]|BOLD:AAA5135
Eulepidotis rectimargo|[15705]|MHAUG507-07|Costa Rica|Guanacaste|592[0n]|BOLD:AAA5135
Eulepidotis rectimargo|[15706]|GBGL10148-12|||608[0n]|BOLD:AAA5135
Eulepidotis rectimargo|[15707]|MHAUC199-06|Costa Rica|Guanacaste|658[0n]|BOLD:AAA5135
Eulepidotis rectimargo|[15708]|CNCLB1988-14|Guatemala||658[0n]|BOLD:AAA5135
Eulepidotis rectimargo|[15709]|CNCLB1989-14|Guatemala||658[0n]|BOLD:AAA5135
Eulepidotis rectimargo|[15710]|BLPAE849-07|Costa Rica|Guanacaste|658[0n]|BOLD:AAA5135
Eulepidotis rectimargo|[15711]|BLPAF423-07|Costa Rica|Guanacaste|658[0n]|BOLD:AAA5135
Eulepidotis rectimargo|[15712]|BLPAE610-06|Costa Rica|Guanacaste|658[0n]|BOLD:AAA5135
Eulepidotis rectimargo|[15713]|BLPAF422-07|Costa Rica|Guanacaste|658[0n]|BOLD:AAA5135
Eulepidotis rectimargo|[15714]|MHAUB296-05|Costa Rica|Guanacaste|658[0n]|BOLD:AAA5135
Eulepidotis rectimargo|[15715]|MHAUB298-05|Costa Rica|Guanacaste|658[0n]|BOLD:AAA5135
Eulepidotis rectimargo|[15716]|MHAUC197-06|Costa Rica|Guanacaste|658[0n]|BOLD:AAA5135
Eulepidotis rectimargo|[15717]|BLPAE445-06|Costa Rica|Guanacaste|658[0n]|BOLD:AAA5135
Eulepidotis rectimargo|[15718]|BLPAH274-07|Costa Rica|Guanacaste|658[0n]|BOLD:AAA5135
Eulepidotis rectimargo|[15719]|BLPBH499-07|Costa Rica|Guanacaste|658[0n]|BOLD:AAA5135
Eulepidotis rectimargo|[15720]|LOCRA734-07|Costa Rica|Alajuela|658[0n]|BOLD:AAA5135
Eulepidotis rectimargo|[15721]|BLPCG806-08|Costa Rica|Guanacaste|658[0n]|BOLD:AAA5135
Eulepidotis rectimargo|[15722]|BLPCI599-08|Costa Rica|Guanacaste|658[0n]|BOLD:AAA5135
Eulepidotis rectimargo|[15723]|BLPDV985-11|Costa Rica|Guanacaste|658[0n]|BOLD:AAA5135
Eulepidotis rectimargo|[15724]|BLPDV986-11|Costa Rica|Guanacaste|658[0n]|BOLD:AAA5135
Eulepidotis rectimargo|[15725]|GWOSU565-11|Mexico|Oaxaca|658[0n]|BOLD:AAA5135
Eulepidotis rectimargo|[15726]|CNCLB1986-14|Guatemala||658[0n]|BOLD:AAA5135
Eulepidotis rectimargo|[15727]|CNCLB1990-14|Guatemala||658[0n]|BOLD:AAA5135
Eulepidotis dominicata|[15728]|MILEQ089-11|Ecuador|Orellana|658[0n]|BOLD:AAL4364
Eulepidotis dominicata|[15729]|MILEQ090-11|Ecuador|Orellana|658[0n]|BOLD:AAL4364
Eulepidotis dominicata|[15730]|LOCRI641-11|Costa Rica|Cartago|658[0n]|BOLD:AAL4364
Eulepidotis dominicata|[15731]|GWOSS080-11|Peru|Huanuco|658[0n]|BOLD:AAL4364
Eulepidotis dominicata|[15732]|LNOUF148-11|French Guiana||658[0n]|BOLD:AAL4364
Eulepidotis dominicata|[15733]|MILEP928-11|Ecuador|Sucumbios|658[0n]|BOLD:AAL4364
Eulepidotis dominicata|[15734]|LNOUD757-11|French Guiana||658[0n]|BOLD:AAL4364
Eulepidotis dominicata|[15735]|GWOSS081-11|Peru|Huanuco|658[0n]|BOLD:AAL4364
Eulepidotis dominicata|[15736]|LNOUB022-10|French Guiana||658[0n]|BOLD:AAL4364
Eulepidotis dominicata|[15737]|GWOST471-11|Peru|Huanuco|658[0n]|BOLD:AAL4364
Eulepidotis dominicata|[15738]|MILEP927-11|Ecuador|Sucumbios|658[0n]|BOLD:AAL4364
Eulepidotis dominicata|[15739]|GWOST387-11|Peru|Huanuco|658[0n]|BOLD:AAL4364
Eulepidotis dominicata|[15740]|GWOST385-11|Peru|Huanuco|658[0n]|BOLD:AAL4364
Eulepidotis dominicata|[15741]|GWOSS079-11|Peru|Huanuco|658[0n]|BOLD:AAL4364
Eulepidotis dominicata|[15742]|LOCRA736-07|Costa Rica|Alajuela|658[0n]|BOLD:AAL4364
Eulepidotis dominicata|[15743]|RDNML191-13|United States|Texas|658[0n]|BOLD:AAL4364
Eulepidotis micca|[15744]|LOCRE555-10|Costa Rica|San Jose|658[0n]|BOLD:AAD9949
Eulepidotis micca|[15745]|LOCRE557-10|Costa Rica|San Jose|658[0n]|BOLD:AAD9949
Eulepidotis micca|[15746]|LOCRE556-10|Costa Rica|San Jose|658[0n]|BOLD:AAD9949
Eulepidotis micca|[15747]|LOCRE554-10|Costa Rica|San Jose|658[0n]|BOLD:AAD9949
Eulepidotis micca|[15748]|LOCRH183-11|Costa Rica|Limon|657[0n]|BOLD:AAD9949
Eulepidotis micca|[15749]|QUNOE456-12|United States|Texas|658[0n]|BOLD:AAD9949
Eulepidotis micca|[15750]|CNCLB1995-14|Mexico||658[0n]|BOLD:AAD9949
Eulepidotis micca|[15751]|CNCLB1996-14|Mexico||658[0n]|BOLD:AAD9949
Metallata absumens|[15752]|LOFLA545-06|United States|Florida|658[0n]|BOLD:AAC8148
Metallata absumens|[15753]|HKONS090-07|United States|Florida|658[1n]|BOLD:AAC8148
Metallata absumens|[15754]|HKONS434-08|United States|Florida|640[0n]|BOLD:AAC8148
Renodes curviluna|[15755]|MHAUA674-05|Costa Rica|Guanacaste|526[0n]|BOLD:AAB0630
Renodes curviluna|[15756]|MHAUA673-05|Costa Rica|Guanacaste|522[0n]|BOLD:AAB0630
Renodes curviluna|[15757]|MHAUA667-05|Costa Rica|Guanacaste|520[0n]|BOLD:AAB0630
Renodes curviluna|[15758]|MHMYQ1530-12|Costa Rica||658[0n]|BOLD:AAB0630
Renodes curviluna|[15759]|MHMYH924-10|Costa Rica||658[0n]|BOLD:AAB0630
Renodes curviluna|[15760]|MHMXZ869-09|Costa Rica||658[0n]|BOLD:AAB0630
Renodes curviluna|[15761]|MHMXZ870-09|Costa Rica||658[0n]|BOLD:AAB0630
Renodes curviluna|[15762]|MHMXZ871-09|Costa Rica||658[0n]|BOLD:AAB0630
Renodes curviluna|[15763]|MHMYT274-13|Costa Rica||658[0n]|BOLD:AAB0630
Renodes curviluna|[15764]|MHMYT281-13|Costa Rica||658[0n]|BOLD:AAB0630
Renodes curviluna|[15765]|BLPEE6609-14|Costa Rica||658[0n]|BOLD:AAB0630
Renodes curviluna|[15766]|MHMYC2202-09|Costa Rica|Alajuela|658[0n]|BOLD:AAB0630
Renodes curviluna|[15767]|MHMYC2206-09|Costa Rica|Alajuela|658[0n]|BOLD:AAB0630
Renodes curviluna|[15768]|MHMXU515-08|||658[0n]|BOLD:AAB0630
Renodes curviluna|[15769]|BLPDC276-09|Costa Rica|Guanacaste|658[0n]|BOLD:AAB0630
Renodes curviluna|[15770]|MHMYC2209-09|Costa Rica|Alajuela|658[0n]|BOLD:AAB0630
Renodes curviluna|[15771]|MHMXZ559-09|Costa Rica||658[1n]|BOLD:AAB0630
Renodes curviluna|[15772]|MHMYF824-10|Costa Rica|Alajuela|658[0n]|BOLD:AAB0630
Renodes curviluna|[15773]|MHMYL3144-11|Costa Rica||658[0n]|BOLD:AAB0630
Renodes curviluna|[15774]|MHMYS1407-12|Costa Rica||658[0n]|BOLD:AAB0630
Renodes curviluna|[15775]|MHMYS1408-12|Costa Rica||658[0n]|BOLD:AAB0630
Renodes curviluna|[15776]|BLPEE6567-14|Costa Rica||658[0n]|BOLD:AAB0630

Renodes curviluna|[15774]|MHMYS1407-12|Costa Rica||658[0n]|BOLD:AAB0630
Renodes curviluna|[15775]|MHMYS1408-12|Costa Rica||658[0n]|BOLD:AAB0630
Renodes curviluna|[15776]|BLPEE6567-14|Costa Rica||658[0n]|BOLD:AAB0630
Renodes curviluna|[15777]|BLPEE6573-14|Costa Rica||658[0n]|BOLD:AAB0630
Renodes curviluna|[15778]|MHMXZ872-09|Costa Rica||658[0n]|BOLD:AAB0630
Renodes curviluna|[15779]|MHMYC2204-09|Costa Rica|Alajuela|658[0n]|BOLD:AAB0630
Renodes curviluna|[15780]|MHMYC2210-09|Costa Rica|Alajuela|658[0n]|BOLD:AAB0630
Renodes curviluna|[15781]|MHMYF823-10|Costa Rica|Alajuela|658[0n]|BOLD:AAB0630
Renodes curviluna|[15782]|MHMYC2207-09|Costa Rica|Alajuela|658[0n]|BOLD:AAB0630
Renodes curviluna|[15783]|MHMYC2208-09|Costa Rica|Alajuela|658[0n]|BOLD:AAB0630
Renodes curviluna|[15784]|MHMYM237-11|Costa Rica||658[0n]|BOLD:AAB0630
Renodes curviluna|[15785]|BLPED351-11|Costa Rica|Guanacaste|658[0n]|BOLD:AAB0630
Renodes curviluna|[15786]|BLPEE6581-14|Costa Rica||658[0n]|BOLD:AAB0630
Renodes curviluna|[15787]|MHMXZ868-09|Costa Rica||658[0n]|BOLD:AAB0630
Renodes curviluna|[15788]|MHMXZ867-09|Costa Rica||658[0n]|BOLD:AAB0630
Renodes curviluna|[15789]|MHMXZ866-09|Costa Rica||658[0n]|BOLD:AAB0630
Renodes curviluna|[15790]|MHMXZ865-09|Costa Rica||658[0n]|BOLD:AAB0630
Renodes curviluna|[15791]|MHMXV079-08|Costa Rica||658[0n]|BOLD:AAB0630
Renodes curviluna|[15792]|MHMXV078-08|Costa Rica||658[0n]|BOLD:AAB0630
Renodes curviluna|[15793]|MHMXV077-08|Costa Rica||658[0n]|BOLD:AAB0630
Renodes curviluna|[15794]|BLPCO694-08|Costa Rica|Guanacaste|658[0n]|BOLD:AAB0630
Renodes curviluna|[15795]|MHMXU517-08|||658[0n]|BOLD:AAB0630
Renodes curviluna|[15796]|MHMXU466-08|||658[0n]|BOLD:AAB0630
Renodes curviluna|[15797]|BLPCD583-08|Costa Rica|Guanacaste|658[0n]|BOLD:AAB0630
Renodes curviluna|[15798]|MHMXU514-08|||658[0n]|BOLD:AAB0630
Renodes curviluna|[15799]|MHAUA666-05|Costa Rica|Guanacaste|570[0n]|BOLD:AAB0630
Renodes curviluna|[15800]|BLPEE6610-14|Costa Rica||647[2n]|BOLD:AAB0630
Goniocarsia electrica|[15801]|LOCRF114-10|Costa Rica|Cartago|658[0n]|BOLD:AAD4785
Goniocarsia electrica|[15802]|LOCRF115-10|Costa Rica|Cartago|658[0n]|BOLD:AAD4785
Goniocarsia electrica|[15803]|LOCRF117-10|Costa Rica|Cartago|658[0n]|BOLD:AAD4785
Goniocarsia electrica|[15804]|LOCRF118-10|Costa Rica|Cartago|658[0n]|BOLD:AAD4785
Goniocarsia electrica|[15805]|LOCRG143-11|Costa Rica|Cartago|658[0n]|BOLD:AAD4785
Goniocarsia electrica|[15806]|LOCRG144-11|Costa Rica|Cartago|658[0n]|BOLD:AAD4785
Goniocarsia electrica|[15807]|LOCRG145-11|Costa Rica|Cartago|658[0n]|BOLD:AAD4785
Goniocarsia electrica|[15808]|LOCRG146-11|Costa Rica|Cartago|658[0n]|BOLD:AAD4785
Goniocarsia electrica|[15809]|LOCRG147-11|Costa Rica|Cartago|658[0n]|BOLD:AAD4785
Goniocarsia electrica|[15810]|LOCRG148-11|Costa Rica|Cartago|658[0n]|BOLD:AAD4785
Goniocarsia electrica|[15811]|LOCRF112-10|Costa Rica|Cartago|658[0n]|BOLD:AAD4785
Goniocarsia electrica|[15812]|LOCRF113-10|Costa Rica|Cartago|658[0n]|BOLD:AAD4785
Goniocarsia electrica|[15813]|LOCRA256-06|Costa Rica|San Jose|658[0n]|BOLD:AAD4785
Goniocarsia electrica|[15814]|LOCRA469-07|Costa Rica|Alajuela|658[0n]|BOLD:AAD4785
Goniocarsia electrica|[15815]|LOCRG509-11|Costa Rica|Cartago|658[0n]|BOLD:AAD4785
Goniocarsia electrica|[15816]|LOCRI804-11|Costa Rica|Cartago|658[0n]|BOLD:AAD4785
Goniocarsia electrica|[15817]|LOCRA257-06|Costa Rica|San Jose|658[0n]|BOLD:AAD4786
Goniocarsia electrica|[15818]|LOCRB569-08|Costa Rica|Alajuela|658[0n]|BOLD:AAD4786
Goniocarsia electrica|[15819]|LOCRG510-11|Costa Rica|Cartago|658[0n]|BOLD:AAD4786
Goniocarsia electrica|[15820]|LOCRB570-08|Costa Rica|Alajuela|619[2n]|BOLD:AAD4786
Goniocarsia electrica|[15821]|LOCRB571-08|Costa Rica|Alajuela|658[0n]|BOLD:AAD4786
Goniocarsia electrica|[15822]|LOCRB887-08|Costa Rica|Alajuela|658[0n]|BOLD:AAD4786
Goniocarsia electrica|[15823]|BLPDP730-10|Costa Rica|Alajuela|658[0n]|BOLD:AAD4786
Goniocarsia electrica|[15824]|BLPDX149-11|Costa Rica|Guanacaste|658[0n]|BOLD:AAD4786
Goniocarsia electrica|[15825]|BLPDX169-11|Costa Rica|Guanacaste|658[0n]|BOLD:AAD4786
Goniocarsia electrica|[15826]|LOCRG511-11|Costa Rica|Cartago|658[0n]|BOLD:AAD4786
Goniocarsia electrica|[15827]|CNCLB2417-14|United States|Texas|658[0n]|BOLD:ACR9131
Eulepidotis striaepuncta|[15828]|LNAUT1091-14|Cuba||658[0n]|BOLD:ACN7446
Eulepidotis striaepuncta|[15829]|LNAUT1092-14|Cuba|Pinar del Rio|658[0n]|BOLD:ACN7446
Eulepidotis striaepuncta|[15830]|LNAUT1095-14|Cuba|Pinar del Rio|658[0n]|BOLD:ACN7446
Eulepidotis striaepuncta|[15831]|LNAUT1096-14|Cuba|Pinar del Rio|658[0n]|BOLD:ACN7446
Eulepidotis addens|[15832]|LNOUB199-10|French Guiana||658[0n]|BOLD:AAL5783
Eulepidotis addens|[15833]|LNOUC033-10|French Guiana||645[0n]|BOLD:AAL5783
Eulepidotis addens|[15834]|LNOUC669-10|French Guiana|Cayenne|658[0n]|BOLD:AAL5783
Eulepidotis addens|[15835]|LNOUD1091-12|French Guiana||658[1n]|BOLD:AAL5783
Eulepidotis addens|[15836]|LNOUB911-10|French Guiana||658[0n]|BOLD:AAL5783
Eulepidotis addens|[15837]|LNOUB859-10|French Guiana||658[0n]|BOLD:AAL5783
Eulepidotis addens|[15838]|LNOUB628-10|French Guiana||658[0n]|BOLD:AAL5783
Eulepidotis addens|[15839]|LNOUB574-10|French Guiana||658[0n]|BOLD:AAL5783
Eulepidotis addens|[15840]|LNOUB198-10|French Guiana||658[0n]|BOLD:AAL5783
Eulepidotis addens|[15841]|LNOUA174-10|French Guiana||658[0n]|BOLD:AAL5783
Eulepidotis addens|[15842]|LNOUB581-10|French Guiana||658[0n]|BOLD:AAL5783
Eulepidotis addens|[15843]|LNOUB053-10|French Guiana||658[1n]|BOLD:AAL5783
Eulepidotis addens|[15844]|LNOUB950-10|French Guiana||658[1n]|BOLD:AAL5783
Eulepidotis addens|[15845]|LNOUD1506-12|French Guiana||658[0n]|BOLD:AAL5783
Eulepidotis addens|[15846]|LNAUT1094-14|Dominican Republic|La Vega|658[0n]|BOLD:ACE9114
Eulepidotis addens|[15847]|BLPCE603-08|Costa Rica|Guanacaste|658[0n]|BOLD:ACE9114
Eulepidotis addens|[15848]|MHMXZ797-09|Costa Rica||658[0n]|BOLD:ACE9114
Eulepidotis addens|[15849]|MHMXZ799-09|Costa Rica||658[0n]|BOLD:ACE9114
Eulepidotis addens|[15850]|MHMXV050-08|Costa Rica||658[0n]|BOLD:ACE9114
Eulepidotis addens|[15851]|MHMXV049-08|Costa Rica||658[0n]|BOLD:ACE9114
Eulepidotis addens|[15852]|MHMXU454-08|||658[0n]|BOLD:ACE9114
Eulepidotis addens|[15853]|MHMXU453-08|||658[0n]|BOLD:ACE9114
Eulepidotis addens|[15854]|MHMXU451-08|||658[0n]|BOLD:ACE9114
Eulepidotis addens|[15855]|MHMXQ642-08|Costa Rica|Alajuela|658[0n]|BOLD:ACE9114
Eulepidotis addens|[15856]|MHAUB807-05|Costa Rica|Guanacaste|595[0n]|BOLD:ACE9114
Eulepidotis addens|[15857]|MHAUB809-05|Costa Rica|Guanacaste|533[0n]|BOLD:ACE9114
Eulepidotis addens|[15858]|MHAUB805-05|Costa Rica|Guanacaste|529[0n]|BOLD:ACE9114
Eulepidotis addens|[15859]|MHAUB810-05|Costa Rica|Guanacaste|552[0n]|BOLD:ACE9114
Eulepidotis addens|[15860]|RDNML234-13|Puerto Rico||658[0n]|BOLD:ACE9114
Eulepidotis addens|[15861]|LNAUT1093-14|Dominican Republic|La Vega|658[0n]|BOLD:ACE9114
Eulepidotis addens|[15862]|CNCLB1997-14|Puerto Rico|Mayaguez|658[0n]|BOLD:ACE9114
Ipnista sp.|[15863]|RDNML043-13|United States|New Mexico|658[0n]|BOLD:ACD3740
Ipnista sp.|[15864]|CNCLB2472-14|United States|New Mexico|658[0n]|BOLD:ACD3740
Melanomma auricinctaria|[15865]|RDLQE915-06|Canada|Quebec|509[0n]|BOLD:AAC4698
Melanomma auricinctaria|[15866]|RDLQE914-06|Canada|Quebec|507[0n]|BOLD:AAC4698
Melanomma auricinctaria|[15867]|RDLQE913-06|Canada|Quebec|501[0n]|BOLD:AAC4698
Melanomma auricinctaria|[15868]|LNCB552-09|United States|North Carolina|658[0n]|BOLD:AAC4698
Melanomma auricinctaria|[15869]|ABNCC113-07|United States|Florida|628[0n]|BOLD:AAC4698
Melanomma auricinctaria|[15870]|HKONS596-08|United States|Florida|657[0n]|BOLD:AAC4698
Melanomma auricinctaria|[15871]|LGSMG146-07|United States|Tennessee|658[0n]|BOLD:AAC4698
Melanomma auricinctaria|[15872]|LGSMG526-07|United States|Tennessee|658[0n]|BOLD:AAC4698
Melanomma auricinctaria|[15873]|LPOKB781-09|United States|Oklahoma|658[0n]|BOLD:AAC4698
Melanomma auricinctaria|[15874]|LNCB602-09|United States|North Carolina|658[0n]|BOLD:AAC4698
Melanomma auricinctaria|[15875]|LPOKC288-09|United States|Oklahoma|658[0n]|BOLD:AAC4698
Melanomma auricinctaria|[15876]|LPOKD355-09|United States|Oklahoma|658[0n]|BOLD:AAC4698

Melanomma auricinctaria|[15874]|LNCB602-09|United States|North Carolina|658[0n]|BOLD:AAC4698
Melanomma auricinctaria|[15875]|LPOKC288-09|United States|Oklahoma|658[0n]|BOLD:AAC4698
Melanomma auricinctaria|[15876]|LPOKD355-09|United States|Oklahoma|658[0n]|BOLD:AAC4698
Melanomma auricinctaria|[15877]|LILLA494-11|United States|Illinois|658[0n]|BOLD:AAC4698
Melanomma auricinctaria|[15878]|LILLA721-11|United States|Illinois|658[0n]|BOLD:AAC4698
Araeopteron vilhelmina|[15879]|CNCLB1707-14|United States|Florida|658[0n]|BOLD:ABA1145
Coenobela sp.|[15880]|QUNOB373-09|United States|Texas|658[0n]|BOLD:AAG6563
Leucoma salicis|[15881]|IBLAO562-12|Spain|Catalonia|658[0n]|BOLD:AAA5528
Leucoma salicis|[15882]|PHLAF319-11|Macedonia||658[0n]|BOLD:AAA5528
Leucoma salicis|[15883]|LEATB298-13|Italy|South Tyrol|622[0n]|BOLD:AAA5528
Leucoma salicis|[15884]|GWORE2042-09|Germany|Bavaria|632[1n]|BOLD:AAA5528
Leucoma salicis|[15885]|GWORZ093-10|Italy|Basilicata|658[0n]|BOLD:AAA5528
Leucoma salicis|[15886]|GWOSN720-11|Germany|Bavaria|658[0n]|BOLD:AAA5528
Leucoma salicis|[15887]|IBLAO563-12|Spain|Catalonia|658[0n]|BOLD:AAA5528
Leucoma salicis|[15888]|LEATC572-13|Italy|South Tyrol|658[0n]|BOLD:AAA5528
Leucoma salicis|[15889]|BBLPE553-09|Canada|Newfoundland and Labrador|639[0n]|BOLD:AAA5528
Leucoma salicis|[15890]|PHMNB083-03|Canada|New Brunswick|639[0n]|BOLD:AAA5528
Leucoma salicis|[15891]|BLTIB547-08|Canada|Ontario|656[0n]|BOLD:AAA5528
Leucoma salicis|[15892]|LBCD265-05|Canada|British Columbia|656[0n]|BOLD:AAA5528
Leucoma salicis|[15893]|RDLQF828-06|Canada|Quebec|658[0n]|BOLD:AAA5528
Leucoma salicis|[15894]|LBCC404-05|Canada|British Columbia|658[0n]|BOLD:AAA5528
Leucoma salicis|[15895]|LBCC040-05|Canada|British Columbia|658[0n]|BOLD:AAA5528
Leucoma salicis|[15896]|LBCC039-05|Canada|British Columbia|658[0n]|BOLD:AAA5528
Leucoma salicis|[15897]|LBCC038-05|Canada|British Columbia|658[0n]|BOLD:AAA5528
Leucoma salicis|[15898]|LBCC037-05|Canada|British Columbia|658[0n]|BOLD:AAA5528
Leucoma salicis|[15899]|LBCC036-05|Canada|British Columbia|658[0n]|BOLD:AAA5528
Leucoma salicis|[15900]|LBCC035-05|Canada|British Columbia|658[0n]|BOLD:AAA5528
Leucoma salicis|[15901]|LBCC034-05|Canada|British Columbia|658[0n]|BOLD:AAA5528
Leucoma salicis|[15902]|LBCB146-05|Canada|British Columbia|658[0n]|BOLD:AAA5528
Leucoma salicis|[15903]|LBCA574-05|Canada|British Columbia|658[0n]|BOLD:AAA5528
Leucoma salicis|[15904]|MNBB069-05|Canada|New Brunswick|658[0n]|BOLD:AAA5528
Leucoma salicis|[15905]|LPVIB872-08|Canada|British Columbia|632[0n]|BOLD:AAA5528
Leucoma salicis|[15906]|CGUKD416-09|United Kingdom|England|616[0n]|BOLD:AAA5528
Leucoma salicis|[15907]|LPVIB883-08|Canada|British Columbia|635[0n]|BOLD:AAA5528
Leucoma salicis|[15908]|LPVIA094-08|Canada|British Columbia|637[0n]|BOLD:AAA5528
Leucoma salicis|[15909]|LBCD266-05|Canada|British Columbia|637[0n]|BOLD:AAA5528
Leucoma salicis|[15910]|TMNBD450-07|Canada|New Brunswick|646[0n]|BOLD:AAA5528
Leucoma salicis|[15911]|CGUKB317-09|United Kingdom|England|642[0n]|BOLD:AAA5528
Leucoma salicis|[15912]|CNGIK029-13|Canada|British Columbia|607[0n]|BOLD:AAA5528
Leucoma salicis|[15913]|LPVIA002-08|Canada|British Columbia|658[0n]|BOLD:AAA5528
Leucoma salicis|[15914]|LPVIB839-08|Canada|British Columbia|658[0n]|BOLD:AAA5528
Leucoma salicis|[15915]|LPABB079-08|Canada|Alberta|658[0n]|BOLD:AAA5528
Leucoma salicis|[15916]|LPABB081-08|Canada|Alberta|658[0n]|BOLD:AAA5528
Leucoma salicis|[15917]|LPABB043-08|Canada|Alberta|658[0n]|BOLD:AAA5528
Leucoma salicis|[15918]|LPABB044-08|Canada|Alberta|658[0n]|BOLD:AAA5528
Leucoma salicis|[15919]|LBCC747-05|Canada|British Columbia|658[0n]|BOLD:AAA5528
Leucoma salicis|[15920]|LBCD033-05|Canada|British Columbia|658[0n]|BOLD:AAA5528
Leucoma salicis|[15921]|LBCD034-05|Canada|British Columbia|658[0n]|BOLD:AAA5528
Leucoma salicis|[15922]|LBCD035-05|Canada|British Columbia|658[0n]|BOLD:AAA5528
Leucoma salicis|[15923]|LBCD036-05|Canada|British Columbia|658[0n]|BOLD:AAA5528
Leucoma salicis|[15924]|LBCD037-05|Canada|British Columbia|658[0n]|BOLD:AAA5528
Leucoma salicis|[15925]|LBCD109-05|Canada|British Columbia|658[0n]|BOLD:AAA5528
Leucoma salicis|[15926]|LBCD110-05|Canada|British Columbia|658[0n]|BOLD:AAA5528
Leucoma salicis|[15927]|LBCD111-05|Canada|British Columbia|658[0n]|BOLD:AAA5528
Leucoma salicis|[15928]|LBCD267-05|Canada|British Columbia|658[0n]|BOLD:AAA5528
Leucoma salicis|[15929]|LBCC405-05|Canada|British Columbia|658[0n]|BOLD:AAA5528
Leucoma salicis|[15930]|LBCC406-05|Canada|British Columbia|658[0n]|BOLD:AAA5528
Leucoma salicis|[15931]|RDLQB423-05|Canada|Quebec|658[0n]|BOLD:AAA5528
Leucoma salicis|[15932]|RDLQB430-05|Canada|Quebec|658[0n]|BOLD:AAA5528
Leucoma salicis|[15933]|LBCC407-05|Canada|British Columbia|658[0n]|BOLD:AAA5528
Leucoma salicis|[15934]|LBCC408-05|Canada|British Columbia|658[0n]|BOLD:AAA5528
Leucoma salicis|[15935]|RDLQB431-05|Canada|Quebec|658[0n]|BOLD:AAA5528
Leucoma salicis|[15936]|LMH002-06|Canada|British Columbia|658[0n]|BOLD:AAA5528
Leucoma salicis|[15937]|TMNBD449-07|Canada|New Brunswick|658[0n]|BOLD:AAA5528
Leucoma salicis|[15938]|DUNLP003-08|Canada|British Columbia|658[0n]|BOLD:AAA5528
Leucoma salicis|[15939]|DUNLP004-08|Canada|British Columbia|658[0n]|BOLD:AAA5528
Leucoma salicis|[15940]|BLTIB531-08|Canada|Ontario|658[0n]|BOLD:AAA5528
Leucoma salicis|[15941]|LPABB019-08|Canada|Alberta|658[0n]|BOLD:AAA5528
Leucoma salicis|[15942]|LPABB037-08|Canada|Alberta|658[0n]|BOLD:AAA5528
Leucoma salicis|[15943]|LPABB383-08|Canada|Alberta|658[0n]|BOLD:AAA5528
Leucoma salicis|[15944]|CGUKA272-09|United Kingdom|England|658[0n]|BOLD:AAA5528
Leucoma salicis|[15945]|CGUKA733-09|United Kingdom||658[0n]|BOLD:AAA5528
Leucoma salicis|[15946]|LPABC683-09|Canada|Alberta|658[0n]|BOLD:AAA5528
Leucoma salicis|[15947]|LPABC911-09|Canada|Alberta|658[0n]|BOLD:AAA5528
Leucoma salicis|[15948]|BBLPC642-09|Canada|Newfoundland and Labrador|658[0n]|BOLD:AAA5528
Leucoma salicis|[15949]|BBLPC688-09|Canada|Newfoundland and Labrador|658[0n]|BOLD:AAA5528
Leucoma salicis|[15950]|BBLPC689-09|Canada|Newfoundland and Labrador|658[0n]|BOLD:AAA5528
Leucoma salicis|[15951]|LBCC409-05|Canada|British Columbia|658[0n]|BOLD:AAA5528
Leucoma salicis|[15952]|LBCC555-05|Canada|British Columbia|658[0n]|BOLD:AAA5528
Leucoma salicis|[15953]|LBCH225-10|Canada|British Columbia|658[0n]|BOLD:AAA5528
Leucoma salicis|[15954]|LBCH3452-10|Canada|British Columbia|658[0n]|BOLD:AAA5528
Leucoma salicis|[15955]|LALPA273-10|Canada|British Columbia|658[0n]|BOLD:AAA5528
Leucoma salicis|[15956]|BBLPA367-10|Canada|British Columbia|658[0n]|BOLD:AAA5528
Leucoma salicis|[15957]|BBLPA368-10|Canada|British Columbia|658[0n]|BOLD:AAA5528
Leucoma salicis|[15958]|BBLPA369-10|Canada|British Columbia|658[0n]|BOLD:AAA5528
Leucoma salicis|[15959]|BBLPA370-10|Canada|British Columbia|658[0n]|BOLD:AAA5528
Leucoma salicis|[15960]|BBLPA371-10|Canada|British Columbia|658[0n]|BOLD:AAA5528
Leucoma salicis|[15961]|JMMMB438-11|United States|California|658[0n]|BOLD:AAA5528
Leucoma salicis|[15962]|LALPA890-11|Canada|British Columbia|658[0n]|BOLD:AAA5528
Leucoma salicis|[15963]|LEATG193-14|Austria|Tirol|658[0n]|BOLD:AAA5528
Leucoma salicis|[15964]|LEATG194-14|Austria|Tirol|658[0n]|BOLD:AAA5528
Leucoma salicis|[15965]|LNAUT2725-14|United States|Montana|658[0n]|BOLD:AAA5528
Leucoma salicis|[15966]|LNAUT2726-14|United States|Montana|658[0n]|BOLD:AAA5528
Leucoma salicis|[15967]|LBCD268-05|Canada|British Columbia|658[0n]|BOLD:AAA5528
Leucoma salicis|[15968]|LBCD269-05|Canada|British Columbia|658[0n]|BOLD:AAA5528
Leucoma salicis|[15969]|ABOLA449-14|Austria|Tirol|658[0n]|BOLD:AAA5528
Leucoma salicis|[15970]|FBLMU523-09|Germany|Bavaria|658[0n]|BOLD:AAA5528
Leucoma salicis|[15971]|LEFIA1203-10|Finland|Aland Islands|670[0n]|BOLD:AAA5528
Leucoma salicis|[15972]|LEFIA839-10|Finland|Aland Islands|658[0n]|BOLD:AAA5528
Leucoma salicis|[15973]|LEFIA894-10|Finland|Aland Islands|658[0n]|BOLD:AAA5528
Leucoma salicis|[15974]|LEFIF546-10|Finland||658[0n]|BOLD:AAA5528
Leucoma salicis|[15975]|GBLAF449-14|Germany|Brandenburg|658[0n]|BOLD:AAA5528
Leucoma salicis|[15976]|LON1055-12|Norway|Akershus|658[0n]|BOLD:AAA5528

Leucoma salicis|[15974]|LEFIF546-10|Finland||658[0n]|BOLD:AAA5528
Leucoma salicis|[15975]|GBLAF449-14|Germany|Brandenburg|658[0n]|BOLD:AAA5528
Leucoma salicis|[15976]|LON1055-12|Norway|Akershus|658[0n]|BOLD:AAA5528
Leucoma salicis|[15977]|HOWAN033-15|China||658[0n]|BOLD:AAA5528
Dasychira cinnamomea|[15978]|ABCNA930-08|United States|Michigan|658[0n]|BOLD:AAB8421
Dasychira cinnamomea|[15979]|HKONS673-08|United States|Michigan|658[0n]|BOLD:AAB8421
Dasychira cinnamomea|[15980]|CNCLB1295-14|United States|Connecticut|658[0n]|BOLD:AAB8421
Dasychira cinnamomea|[15981]|CNCLB1297-14|United States|Connecticut|658[0n]|BOLD:AAB8421
Dasychira sp.|[15982]|LNCC1653-13|United States|North Carolina|658[0n]|BOLD:AAB8421
Dasychira tephra|[15983]|LNC544-06|United States||622[3n]|BOLD:AAB8421
Dasychira tephra|[15984]|LNC543-06|United States||622[2n]|BOLD:AAB8421
Dasychira tephra|[15985]|LNCC1263-11|United States|North Carolina|658[0n]|BOLD:AAB8421
Dasychira tephra|[15986]|LNCC816-11|United States|North Carolina|658[0n]|BOLD:AAB8421
Dasychira tephra|[15987]|LNCB512-07|United States|North Carolina|658[0n]|BOLD:AAB8421
Dasychira tephra|[15988]|LSUSA213-06|United States|Kentucky|658[0n]|BOLD:AAB8421
Dasychira tephra|[15989]|LNCC1651-13|United States|North Carolina|648[0n]|BOLD:AAB8421
Dasychira tephra|[15990]|LNCC1656-13|United States|North Carolina|658[0n]|BOLD:AAB8421
Dasychira tephra|[15991]|LNCC1659-13|United States|North Carolina|658[0n]|BOLD:AAB8421
Dasychira tephra|[15992]|LNCC1660-13|United States|North Carolina|658[0n]|BOLD:AAB8421
Dasychira tephra|[15993]|LNCC1867-13|United States|North Carolina|658[0n]|BOLD:AAB8421
Dasychira tephra|[15994]|LNCC1874-13|United States|North Carolina|658[0n]|BOLD:AAB8421
Dasychira tephra|[15995]|LNCB513-07|United States|North Carolina|658[0n]|BOLD:AAB8421
Dasychira dorsipennata|[15996]|RDLQB692-05|Canada|Quebec|658[0n]|BOLD:AAB8421
Dasychira dorsipennata|[15997]|JSSEP1056-11|Canada|Ontario|609[0n]|BOLD:AAB8421
Dasychira tephra|[15998]|LNCC698-11|United States|North Carolina|658[0n]|BOLD:AAB8421
Dasychira tephra|[15999]|UDLEP084-09|United States|Maryland|633[0n]|BOLD:AAB8421
Dasychira tephra|[16000]|HKONS670-08|United States|Florida|632[0n]|BOLD:AAB8421
Dasychira tephra|[16001]|HKONS669-08|United States|Florida|658[0n]|BOLD:AAB8421
Dasychira tephra|[16002]|HKONS671-08|United States|Florida|658[0n]|BOLD:AAB8421
Dasychira tephra|[16003]|HKONS678-08|United States|Florida|658[0n]|BOLD:AAB8421
Dasychira tephra|[16004]|LPOKD276-09|United States|Oklahoma|657[0n]|BOLD:AAB8421
Dasychira tephra|[16005]|LPOKD819-10|United States|Oklahoma|658[0n]|BOLD:AAB8421
Dasychira tephra|[16006]|BBLOB1537-11|United States|Florida|658[0n]|BOLD:AAB8421
Dasychira tephra|[16007]|BBLOC120-11|United States|Florida|658[0n]|BOLD:AAB8421
Dasychira tephra|[16008]|LNCB511-07|United States|North Carolina|658[0n]|BOLD:AAB8421
Dasychira tephra|[16009]|LNCC939-11|United States|North Carolina|658[0n]|BOLD:AAB8421
Dasychira tephra|[16010]|LNCC1885-13|United States|North Carolina|658[0n]|BOLD:AAB8421
Dasychira tephra|[16011]|LNCC1865-13|United States|North Carolina|658[0n]|BOLD:AAB8421
Dasychira tephra|[16012]|LNCC1866-13|United States|North Carolina|658[0n]|BOLD:AAB8421
Dasychira tephra|[16013]|LNCC1886-13|United States|North Carolina|658[0n]|BOLD:AAB8421
Dasychira tephra|[16014]|LNCB460-07|United States|North Carolina|658[0n]|BOLD:AAB8421
Dasychira dorsipennata|[16015]|CNCLB1300-14|Canada|Saskatchewan|658[0n]|BOLD:AAB8421
Dasychira sp.|[16016]|HKONS066-07|United States|Florida|658[1n]|BOLD:AAI5807
Dasychira leucophaea|[16017]|HKONS370-08|United States|Florida|658[0n]|BOLD:AAL1246
Dasychira leucophaea|[16018]|BBLOB239-11|United States|Florida|658[0n]|BOLD:AAL1246
Dasychira leucophaea|[16019]|CNCLB1305-14|United States|South Carolina|658[0n]|BOLD:AAL1246
Dasychira leucophaea|[16020]|CNCLB1306-14|United States|South Carolina|658[0n]|BOLD:AAL1246
Dasychira leucophaea|[16021]|CNCLB1308-14|United States|Florida|658[1n]|BOLD:AAL1246
Dasychira obliquata|[16022]|CNCLB1309-14|United States|Rhode Island|483[1n]|
Dasychira obliquata|[16023]|LGSM614-04|United States|Tennessee|601[7n]|BOLD:AAB8420
Dasychira obliquata|[16024]|LSEU718-06|United States|Georgia|658[0n]|BOLD:AAB8420
Dasychira obliquata|[16025]|LGSMG127-07|United States|North Carolina|658[0n]|BOLD:AAB8420
Dasychira obliquata|[16026]|LGSMG128-07|United States|Tennessee|658[0n]|BOLD:AAB8420
Dasychira obliquata|[16027]|LNCC237-10|United States|North Carolina|658[0n]|BOLD:AAB8420
Dasychira obliquata|[16028]|LNCC238-10|United States|North Carolina|658[0n]|BOLD:AAB8420
Dasychira obliquata|[16029]|LNCC983-11|United States|North Carolina|658[0n]|BOLD:AAB8420
Dasychira obliquata|[16030]|LNCC984-11|United States|North Carolina|658[0n]|BOLD:AAB8420
Dasychira obliquata|[16031]|LNCC987-11|United States|North Carolina|658[0n]|BOLD:AAB8420
Dasychira obliquata|[16032]|LNCC1169-11|United States|North Carolina|658[0n]|BOLD:AAB8420
Dasychira obliquata|[16033]|LNCC1171-11|United States|North Carolina|658[0n]|BOLD:AAB8420
Dasychira obliquata|[16034]|LNCC1285-11|United States|North Carolina|658[0n]|BOLD:AAB8420
Dasychira obliquata|[16035]|LNCC1286-11|United States|North Carolina|658[0n]|BOLD:AAB8420
Dasychira obliquata|[16036]|LNCC1344-11|United States|North Carolina|658[0n]|BOLD:AAB8420
Dasychira obliquata|[16037]|LNCC1375-11|United States|North Carolina|658[0n]|BOLD:AAB8420
Dasychira obliquata|[16038]|LNCC1377-11|United States|North Carolina|658[0n]|BOLD:AAB8420
Dasychira obliquata|[16039]|PHMO293-03|Canada|Ontario|639[2n]|BOLD:AAB8420
Dasychira obliquata|[16040]|CNCLB1310-14|United States|New Jersey|658[0n]|BOLD:AAB8420
Dasychira obliquata|[16041]|RDLQB829-05|Canada|Quebec|658[0n]|BOLD:AAB8420
Dasychira obliquata|[16042]|LSEU607-06|United States|Georgia|658[0n]|BOLD:AAB8420
Dasychira obliquata|[16043]|RDLQB828-05|Canada|Quebec|615[0n]|BOLD:AAB8420
Dasychira obliquata|[16044]|LNCC1376-11|United States|North Carolina|658[0n]|BOLD:AAB8420
Dasychira obliquata|[16045]|HKONB383-09|United States|Kentucky|658[0n]|BOLD:AAB8420
Dasychira obliquata|[16046]|LGSMG129-07|United States|North Carolina|658[0n]|BOLD:AAB8420
Dasychira obliquata|[16047]|LGSM616-04|United States|Tennessee|609[0n]|BOLD:AAB8420
Dasychira obliquata|[16048]|LOT124-04|United States|Tennessee|609[0n]|BOLD:AAB8420
Dasychira obliquata|[16049]|LSEU719-06|United States|Georgia|658[0n]|BOLD:AAB8420
Dasychira obliquata|[16050]|RDLQF809-06|Canada|Quebec|658[0n]|BOLD:AAB8420
Dasychira obliquata|[16051]|LGSMG126-07|United States|North Carolina|658[0n]|BOLD:AAB8420
Dasychira obliquata|[16052]|RDNMF631-08|Canada|Ontario|658[0n]|BOLD:AAB8420
Dasychira obliquata|[16053]|RDNMF632-08|Canada|Ontario|658[0n]|BOLD:AAB8420
Dasychira obliquata|[16054]|RDNML069-13|Canada|Ontario|658[0n]|BOLD:AAB8420
Dasychira obliquata|[16055]|CNCLB1312-14|Canada|Ontario|658[0n]|BOLD:AAB8420
Dasychira obliquata|[16056]|CNCLB1313-14|Canada|Ontario|658[0n]|BOLD:AAB8420
Dasychira dominickaria|[16057]|HKONS369-08|United States|Texas|658[0n]|BOLD:AAL1267
Dasychira mescalera|[16058]|RDNMJ171-10|United States|New Mexico|658[0n]|BOLD:ACE7279
Dasychira vagans|[16059]|LOPN111-06|United States|Oregon|578[1n]|BOLD:ACE7279
Dasychira vagans|[16060]|LOPN110-06|United States|Oregon|571[0n]|BOLD:ACE7279
Dasychira vagans|[16061]|LPMN922-08|Canada|Alberta|654[0n]|BOLD:ACE7279
Dasychira vagans|[16062]|LOPN112-06|United States|Oregon|553[12n]|
Dasychira vagans|[16063]|LOPN113-06|United States|Oregon|571[0n]|BOLD:ACE7279
Dasychira vagans|[16064]|LPABB076-08|Canada|Alberta|658[0n]|BOLD:ACE7279
Dasychira vagans|[16065]|LPABB503-08|Canada|Alberta|658[0n]|BOLD:ACE7279
Dasychira vagans|[16066]|LPABC841-09|Canada|Alberta|658[0n]|BOLD:ACE7279
Dasychira vagans|[16067]|RDLQ754-07|Canada|Quebec|658[0n]|BOLD:ACE7279
Dasychira vagans|[16068]|RDLQG370-06|Canada|Quebec|658[0n]|BOLD:ACE7279
Dasychira vagans|[16069]|RDLQG369-06|Canada|Quebec|658[0n]|BOLD:ACE7279
Dasychira vagans|[16070]|XAE448-04|Canada|Ontario|573[0n]|BOLD:ACE7279
Dasychira vagans|[16071]|RDNMF633-08|Canada|Ontario|652[0n]|BOLD:ACE7279
Dasychira vagans|[16072]|BBLPB637-10|Canada|British Columbia|658[0n]|BOLD:ACE7279
Dasychira vagans|[16073]|BBLPB636-10|Canada|British Columbia|658[0n]|BOLD:ACE7279
Dasychira vagans|[16074]|LBCD015-05|Canada|British Columbia|658[0n]|BOLD:ACE7279
Dasychira vagans|[16075]|LBCD014-05|Canada|British Columbia|658[0n]|BOLD:ACE7279
Dasychira vagans|[16076]|LBCC019-05|Canada|British Columbia|658[0n]|BOLD:ACE7279

[illegible]
Dasychira vagans|[16074]|LBCD015-05|Canada|British Columbia|658[0n]|BOLD:ACE7279
Dasychira vagans|[16075]|LBCD014-05|Canada|British Columbia|658[0n]|BOLD:ACE7279
Dasychira vagans|[16076]|LBCC019-05|Canada|British Columbia|658[0n]|BOLD:ACE7279
Dasychira vagans|[16077]|LBCC015-05|Canada|British Columbia|658[0n]|BOLD:ACE7279
Dasychira vagans|[16078]|LBCB202-05|Canada|British Columbia|658[0n]|BOLD:ACE7279
Dasychira vagans|[16079]|LBCA798-05|Canada|British Columbia|658[0n]|BOLD:ACE7279
Dasychira vagans|[16080]|LOWCB661-05|Canada|British Columbia|571[0n]|BOLD:ACE7279
Dasychira vagans|[16081]|RDNMJ656-11|Canada|Alberta|642[0n]|BOLD:ACE7279
Dasychira basiflava|[16082]|LNCB211-06|United States|North Carolina|658[0n]|BOLD:ACE7279
Dasychira basiflava|[16083]|LNCB213-06|United States|North Carolina|658[0n]|BOLD:ACE7279
Dasychira meridionalis|[16084]|LNCC1531-13|United States|North Carolina|658[0n]|BOLD:ACE7279
Dasychira atrivenosa|[16085]|HKONB405-09|United States|Texas|658[0n]|BOLD:ACE7279
Dasychira basiflava|[16086]|LGSM615-04|United States|Tennessee|658[1n]|BOLD:ACE7279
Dasychira basiflava|[16087]|LOT554-04|United States|Tennessee|595[0n]|BOLD:ACE7279
Dasychira basiflava|[16088]|LSEU720-06|United States|Georgia|658[0n]|BOLD:ACE7279
Dasychira basiflava|[16089]|HKONS746-08|United States|Tennessee|658[0n]|BOLD:ACE7279
Dasychira basiflava|[16090]|HKONS732-08|United States|Florida|658[0n]|BOLD:ACE7279
Dasychira basiflava|[16091]|HKONS068-07|United States|Florida|658[1n]|BOLD:ACE7279
Dasychira basiflava|[16092]|HKONS069-07|United States|Florida|658[0n]|BOLD:ACE7279
Dasychira basiflava|[16093]|HKONS731-08|United States|Florida|658[0n]|BOLD:ACE7279
Dasychira basiflava|[16094]|HKONS733-08|United States|Florida|658[0n]|BOLD:ACE7279
Dasychira meridionalis|[16095]|MILEQ294-11|United States|Alabama|658[0n]|BOLD:ACE7279
Dasychira basiflava|[16096]|LSEU721-06|United States|Georgia|658[0n]|BOLD:ACE7279
Dasychira meridionalis|[16097]|HKONB261-09|United States|Texas|638[0n]|BOLD:ACE7279
Dasychira basiflava|[16098]|HKONS738-08|United States|Texas|658[0n]|BOLD:ACE7279
Dasychira basiflava|[16099]|LNCC117-10|United States|North Carolina|658[0n]|BOLD:ACE7279
Dasychira basiflava|[16100]|HKONB394-09|United States|Kentucky|658[0n]|BOLD:ACE7279
Dasychira basiflava|[16101]|LNCC118-10|United States|North Carolina|658[0n]|BOLD:ACE7279
Dasychira basiflava|[16102]|LNCC119-10|United States|North Carolina|658[0n]|BOLD:ACE7279
Dasychira basiflava|[16103]|LNCC1173-11|United States|North Carolina|658[0n]|BOLD:ACE7279
Dasychira atrivenosa|[16104]|LNC550-06|United States||626[7n]|BOLD:ACE7279
Dasychira atrivenosa|[16105]|LNCB330-06|United States|North Carolina|658[0n]|BOLD:ACE7279
Dasychira meridionalis|[16106]|LNCC1260-11|United States|North Carolina|658[0n]|BOLD:ACE7279
Dasychira basiflava|[16107]|LNCC1655-13|United States|North Carolina|658[0n]|BOLD:ACE7279
Dasychira basiflava|[16108]|HKONS740-08|United States|Texas|627[0n]|BOLD:ACE7279
Dasychira basiflava|[16109]|LNCC985-11|United States|North Carolina|658[0n]|BOLD:ACE7279
Dasychira basiflava|[16110]|HKONS741-08|United States|Texas|658[0n]|BOLD:ACE7279
Dasychira sp.|[16111]|LNCC986-11|United States|North Carolina|658[0n]|BOLD:ACE7279
Dasychira basiflava|[16112]|LNCC1172-11|United States|North Carolina|658[0n]|BOLD:ACE7279
Dasychira matheri|[16113]|CNCLA5253-13|United States|Mississippi|658[0n]|BOLD:ACE7279
Dasychira basiflava|[16114]|HKONS748-08|United States|Virginia|605[0n]|BOLD:ACE7279
Dasychira basiflava|[16115]|LTOL618-07|United States|Maryland|633[0n]|BOLD:ACE7279
Dasychira basiflava|[16116]|LNCC988-11|United States|North Carolina|658[0n]|BOLD:ACE7279
Dasychira basiflava|[16117]|HKONS739-08|United States|Texas|658[0n]|BOLD:ACE7279
Dasychira basiflava|[16118]|HKONS745-08|United States|Arkansas|658[0n]|BOLD:ACE7279
Dasychira atrivenosa|[16119]|LNCC1534-13|United States|North Carolina|658[0n]|BOLD:ACE7279
Dasychira atrivenosa|[16120]|LNCC1533-13|United States|North Carolina|658[0n]|BOLD:ACE7279
Dasychira sp.|[16121]|LNCC1661-13|United States|North Carolina|631[0n]|BOLD:ACE7279
Dasychira meridionalis|[16122]|HKONB262-09|United States|Texas|658[0n]|BOLD:ACE7279
Dasychira mescalera|[16123]|BBLSY108-09|United States|Texas|658[0n]|BOLD:ACE7279
Dasychira mescalera|[16124]|BBLSY913-09|United States|Texas|658[0n]|BOLD:ACE7279
Dasychira mescalera|[16125]|BBLSZ170-09|United States|Texas|658[0n]|BOLD:ACE7279
Dasychira mescalera|[16126]|BBLSZ171-09|United States|Texas|658[0n]|BOLD:ACE7279
Dasychira mescalera|[16127]|BBLSZ197-09|United States|Texas|658[0n]|BOLD:ACE7279
Dasychira meridionalis pallorosa|[16128]|CNCLB1298-14|United States|Texas|658[0n]|BOLD:ACE7279
Dasychira mescalera|[16129]|BBLSY926-09|United States|Texas|658[0n]|BOLD:ACE7279
Dasychira mescalera|[16130]|BBLSZ168-09|United States|Texas|658[0n]|BOLD:ACE7279
Dasychira mescalera|[16131]|BBLSZ169-09|United States|Texas|658[0n]|BOLD:ACE7279
Dasychira meridionalis pallorosa|[16132]|CNCLB1299-14|United States|Texas|658[0n]|BOLD:ACE7279
Dasychira mescalera|[16133]|CNCLB1301-14|United States|Texas|658[0n]|BOLD:ACE7279
Dasychira mescalera|[16134]|CNCLB1302-14|United States|Texas|658[0n]|BOLD:ACE7279
Dasychira mescalera|[16135]|CNCLB1303-14|United States|Texas|658[0n]|BOLD:ACE7279
Dasychira mescalera|[16136]|CNCLB1304-14|United States|Texas|658[0n]|BOLD:ACE7279
Dasychira atrivenosa|[16137]|LNC434-05|United States|North Carolina|658[0n]|BOLD:AAE3358
Dasychira tephra|[16138]|LNCC116-10|United States|North Carolina|658[0n]|BOLD:ABY8872
Dasychira meridionalis|[16139]|HKONS737-08|United States|Florida|658[0n]|BOLD:AAA7428
Dasychira meridionalis|[16140]|HKONS724-08|United States|Florida|658[0n]|BOLD:AAA7428
Dasychira atrivenosa|[16141]|HKONS056-07|United States|Florida|658[0n]|BOLD:AAA7428
Dasychira meridionalis|[16142]|HKONS699-08|United States|Florida|620[0n]|BOLD:AAA7428
Dasychira meridionalis|[16143]|HKONS695-08|United States|Florida|658[0n]|BOLD:AAA7428
Dasychira meridionalis|[16144]|HKONS691-08|United States|Florida|658[0n]|BOLD:AAA7428
Dasychira meridionalis|[16145]|HKONS084-07|United States|Florida|656[2n]|BOLD:AAA7428
Dasychira meridionalis|[16146]|HKONS664-08|United States|Florida|658[0n]|BOLD:AAA7428
Dasychira meridionalis|[16147]|HKONS702-08|United States|Florida|658[0n]|BOLD:AAA7428
Dasychira meridionalis|[16148]|HKONS705-08|United States|Florida|658[0n]|BOLD:AAA7428
Dasychira meridionalis|[16149]|HKONS721-08|United States|Florida|658[0n]|BOLD:AAA7428
Dasychira meridionalis|[16150]|HKONS067-07|United States|Florida|658[0n]|BOLD:AAA7428
Dasychira meridionalis|[16151]|HKONS679-08|United States|Florida|658[0n]|BOLD:AAA7428
Dasychira meridionalis|[16152]|HKONS681-08|United States|Florida|658[0n]|BOLD:AAA7428
Dasychira meridionalis|[16153]|HKONS683-08|United States|Florida|658[0n]|BOLD:AAA7428
Dasychira meridionalis|[16154]|HKONS684-08|United States|Florida|658[0n]|BOLD:AAA7428
Dasychira meridionalis|[16155]|HKONS685-08|United States|Florida|658[0n]|BOLD:AAA7428
Dasychira meridionalis|[16156]|HKONS686-08|United States|Florida|658[0n]|BOLD:AAA7428
Dasychira meridionalis|[16157]|HKONS687-08|United States|Florida|658[0n]|BOLD:AAA7428
Dasychira meridionalis|[16158]|HKONS688-08|United States|Florida|658[0n]|BOLD:AAA7428
Dasychira meridionalis|[16159]|HKONS689-08|United States|Florida|658[0n]|BOLD:AAA7428
Dasychira meridionalis|[16160]|HKONS690-08|United States|Florida|658[0n]|BOLD:AAA7428
Dasychira meridionalis|[16161]|HKONS693-08|United States|Florida|658[0n]|BOLD:AAA7428
Dasychira meridionalis|[16162]|HKONS696-08|United States|Florida|658[0n]|BOLD:AAA7428
Dasychira meridionalis|[16163]|HKONS698-08|United States|Florida|658[0n]|BOLD:AAA7428
Dasychira meridionalis|[16164]|HKONS736-08|United States|Florida|658[0n]|BOLD:AAA7428
Dasychira meridionalis|[16165]|HKONS722-08|United States|Florida|658[0n]|BOLD:AAA7428
Dasychira meridionalis|[16166]|HKONS727-08|United States|Florida|658[0n]|BOLD:AAA7428
Dasychira meridionalis|[16167]|HKONS728-08|United States|Florida|658[0n]|BOLD:AAA7428
Dasychira meridionalis|[16168]|HKONS729-08|United States|Florida|658[0n]|BOLD:AAA7428
Dasychira meridionalis|[16169]|HKONS730-08|United States|Florida|658[0n]|BOLD:AAA7428
Dasychira meridionalis|[16170]|HKONS734-08|United States|Florida|658[0n]|BOLD:AAA7428
Dasychira meridionalis|[16171]|HKONS735-08|United States|Florida|658[0n]|BOLD:AAA7428
Dasychira meridionalis|[16172]|HKONS743-08|United States|Florida|658[0n]|BOLD:AAA7428
Dasychira meridionalis|[16173]|HKONS680-08|United States|Florida|658[0n]|BOLD:AAA7428
Dasychira meridionalis|[16174]|HKONS706-08|United States|Florida|658[0n]|BOLD:AAA7428
Dasychira meridionalis|[16175]|HKONS744-08|United States|Florida|658[0n]|BOLD:AAA7428
Dasychira meridionalis|[16176]|HKONS666-08|United States|Florida|658[0n]|BOLD:AAA7428

Dasychira meridionalis|[16173]|HKONS[illegible]-08|United States|Florida|658[0n]|BOLD:AAA7428
Dasychira meridionalis|[16174]|HKONS706-08|United States|Florida|658[0n]|BOLD:AAA7428
Dasychira meridionalis|[16175]|HKONS744-08|United States|Florida|658[0n]|BOLD:AAA7428
Dasychira meridionalis|[16176]|HKONS666-08|United States|Florida|658[0n]|BOLD:AAA7428
Dasychira meridionalis|[16177]|HKONS701-08|United States|Florida|620[0n]|BOLD:AAA7428
Dasychira meridionalis|[16178]|HKONS663-08|United States|Florida|658[0n]|BOLD:AAA7428
Dasychira meridionalis|[16179]|HKONS665-08|United States|Florida|658[0n]|BOLD:AAA7428
Dasychira meridionalis|[16180]|HKONS668-08|United States|Florida|658[0n]|BOLD:AAA7428
Dasychira meridionalis|[16181]|HKONS682-08|United States|Florida|658[0n]|BOLD:AAA7428
Dasychira meridionalis|[16182]|HKONS692-08|United States|Florida|658[0n]|BOLD:AAA7428
Dasychira meridionalis|[16183]|HKONS694-08|United States|Florida|658[0n]|BOLD:AAA7428
Dasychira meridionalis|[16184]|HKONS697-08|United States|Florida|658[0n]|BOLD:AAA7428
Dasychira meridionalis|[16185]|HKONS723-08|United States|Florida|658[0n]|BOLD:AAA7428
Dasychira meridionalis|[16186]|HKONS726-08|United States|Florida|658[0n]|BOLD:AAA7428
Dasychira meridionalis|[16187]|LOFLA661-06|United States|Florida|658[0n]|BOLD:AAA7428
Dasychira meridionalis|[16188]|HKONS667-08|United States|Florida|658[0n]|BOLD:AAA7428
Dasychira meridionalis|[16189]|LNCB214-06|United States|North Carolina|658[0n]|BOLD:AAA7428
Dasychira meridionalis|[16190]|LNCB212-06|United States|North Carolina|658[0n]|BOLD:AAA7428
Dasychira meridionalis|[16191]|LNCC817-11|United States|North Carolina|658[0n]|BOLD:AAA7428
Dasychira meridionalis|[16192]|LNCC1530-13|United States|North Carolina|658[0n]|BOLD:AAA7428
Dasychira meridionalis|[16193]|LNCC1532-13|United States|North Carolina|658[0n]|BOLD:AAA7428
Dasychira meridionalis|[16194]|LNCC1868-13|United States|North Carolina|658[0n]|BOLD:AAA7428
Dasychira sp.|[16195]|USLEP594-10|United States|Florida|658[0n]|BOLD:ACE7277
Dasychira leucophaea|[16196]|CNCLB1307-14|United States|Florida|658[0n]|BOLD:ACE7277
Dasychira plagiata|[16197]|RDLQG142-06|Canada|Quebec|658[2n]|BOLD:AAB2274
Dasychira plagiata|[16198]|RDLQB698-05|Canada|Quebec|658[0n]|BOLD:AAB2274
Dasychira plagiata|[16199]|XAK010-06|Canada|Ontario|658[0n]|BOLD:AAB2274
Dasychira plagiata|[16200]|RDNMF634-08|Canada|Ontario|658[0n]|BOLD:AAB2274
Dasychira plagiata|[16201]|XAB003-04|Canada|Ontario|658[0n]|BOLD:AAB2274
Dasychira plagiata|[16202]|XAG097-05|Canada|Ontario|658[0n]|BOLD:AAB2274
Dasychira plagiata|[16203]|XAG179-05|Canada|Ontario|658[0n]|BOLD:AAB2274
Dasychira plagiata|[16204]|LOTB201-05|United States|Tennessee|658[0n]|BOLD:AAB2274
Dasychira plagiata|[16205]|LGSMG124-07|United States|Tennessee|658[0n]|BOLD:AAB2274
Dasychira plagiata|[16206]|PHMO239-03|Canada|Ontario|639[0n]|BOLD:AAB2274
Dasychira plagiata|[16207]|XAG403-05|Canada|Ontario|658[0n]|BOLD:AAB2274
Dasychira plagiata|[16208]|RDNML066-13|Canada|Ontario|658[0n]|BOLD:AAB2274
Dasychira plagiata|[16209]|LNCC1657-13|United States|North Carolina|658[0n]|BOLD:AAB2274
Dasychira plagiata|[16210]|LOTB325-05|United States|Tennessee|658[0n]|BOLD:AAB2274
Dasychira plagiata|[16211]|LGSMG125-07|United States|Tennessee|658[0n]|BOLD:AAB2274
Dasychira plagiata|[16212]|XAG161-05|Canada|Ontario|658[0n]|BOLD:AAB2274
Dasychira plagiata|[16213]|LNCC1649-13|United States|North Carolina|658[0n]|BOLD:AAB2274
Dasychira plagiata|[16214]|RDNML067-13|Canada|Nova Scotia|658[0n]|BOLD:AAB2274
Dasychira plagiata|[16215]|XAK261-06|Canada|Ontario|658[0n]|BOLD:AAB2274
Dasychira plagiata|[16216]|XAG163-05|Canada|Ontario|658[0n]|BOLD:AAB2274
Dasychira plagiata|[16217]|MNBB059-05|Canada|New Brunswick|658[0n]|BOLD:AAB2274
Dasychira plagiata|[16218]|PHMO001-03|Canada|Ontario|639[0n]|BOLD:AAB2274
Dasychira plagiata|[16219]|XAG162-05|Canada|Ontario|658[0n]|BOLD:AAB2274
Dasychira plagiata|[16220]|RDLQF463-06|Canada|Quebec|658[0n]|BOLD:AAB2274
Dasychira plagiata|[16221]|XAK015-06|Canada|Ontario|658[0n]|BOLD:AAB2274
Dasychira plagiata|[16222]|XAG114-05|Canada|Ontario|658[0n]|BOLD:AAB2274
Dasychira plagiata|[16223]|MNBB002-05|Canada|New Brunswick|658[0n]|BOLD:AAB2274
Dasychira plagiata|[16224]|XAB485-04|Canada|Ontario|658[0n]|BOLD:AAB2274
Dasychira plagiata|[16225]|PHMO237-03|Canada|Ontario|639[0n]|BOLD:AAB2274
Dasychira plagiata|[16226]|PHMO391-03|Canada|Ontario|639[0n]|BOLD:AAB2274
Dasychira plagiata|[16227]|CNBPD665-13|Canada|Ontario|621[0n]|BOLD:AAB2274
Dasychira plagiata|[16228]|LNCC1654-13|United States|North Carolina|658[0n]|BOLD:AAB2274
Dasychira plagiata|[16229]|LNCC1731-13|United States|North Carolina|658[0n]|BOLD:AAB2274
Dasychira plagiata|[16230]|LNCC1732-13|United States|North Carolina|658[0n]|BOLD:AAB2274
Dasychira plagiata|[16231]|LNCC1652-13|United States|North Carolina|658[0n]|BOLD:AAB2274
Dasychira plagiata|[16232]|LNCC1170-11|United States|North Carolina|658[0n]|BOLD:AAB2274
Dasychira plagiata|[16233]|LNCC1650-13|United States|North Carolina|658[0n]|BOLD:AAB2274
Dasychira plagiata|[16234]|LNCC1733-13|United States|North Carolina|658[0n]|BOLD:AAB2274
Dasychira grisefacta|[16235]|RDNMJ798-11|Canada|Quebec|658[0n]|BOLD:ABZ5347
Dasychira grisefacta|[16236]|RDNMJ799-11|Canada|Quebec|658[0n]|BOLD:ABZ5347
Dasychira grisefacta ella|[16237]|LPVIA113-08|Canada|British Columbia|658[0n]|BOLD:ABZ5347
Dasychira grisefacta|[16238]|LBCB617-05|Canada|British Columbia|639[1n]|BOLD:ABZ5347
Dasychira grisefacta|[16239]|LBCD252-05|Canada|British Columbia|658[0n]|BOLD:ABZ5347
Dasychira grisefacta|[16240]|CNCLA2655-13|United States|Colorado|658[0n]|BOLD:ABZ5347
Dasychira grisefacta|[16241]|RDNMJ409-11|Canada|Quebec|658[0n]|BOLD:ABZ5347
Dasychira grisefacta|[16242]|LBCH506-10|Canada|British Columbia|658[0n]|BOLD:ABZ5347
Dasychira grisefacta|[16243]|LBCH801-10|Canada|British Columbia|658[0n]|BOLD:ABZ5347
Dasychira grisefacta|[16244]|LPAB219-08|Canada|Alberta|656[1n]|BOLD:ABZ5347
Dasychira grisefacta|[16245]|LBCD324-05|Canada|British Columbia|642[0n]|BOLD:ABZ5347
Dasychira grisefacta|[16246]|DUNLP142-08|Canada|British Columbia|658[0n]|BOLD:ABZ5347
Dasychira grisefacta|[16247]|LBCD100-05|Canada|British Columbia|658[0n]|BOLD:ABZ5347
Dasychira grisefacta|[16248]|LBCB183-05|Canada|British Columbia|613[0n]|BOLD:ABZ5347
Dasychira grisefacta|[16249]|SSJAC2079-13|Canada|Alberta|589[0n]|BOLD:ABZ5347
Dasychira grisefacta|[16250]|LPABC077-09|Canada|Alberta|658[1n]|BOLD:ABZ5347
Dasychira grisefacta|[16251]|LOWCE828-06|Canada|British Columbia|658[0n]|BOLD:ABZ5347
Dasychira grisefacta ella|[16252]|LMH018-06|Canada|British Columbia|656[0n]|BOLD:ABZ5347
Dasychira grisefacta|[16253]|RDLQB199-05|Canada|Quebec|658[0n]|BOLD:ABZ5347
Dasychira grisefacta|[16254]|LBCD281-05|Canada|British Columbia|658[0n]|BOLD:ABZ5347
Dasychira grisefacta|[16255]|LBCD272-05|Canada|British Columbia|658[0n]|BOLD:ABZ5347
Dasychira grisefacta|[16256]|RDNMK656-11|Canada|British Columbia|618[0n]|BOLD:ABZ5347
Dasychira grisefacta|[16257]|LBCC870-05|Canada|British Columbia|658[0n]|BOLD:ABZ5347
Dasychira grisefacta|[16258]|LBCB203-05|Canada|British Columbia|658[0n]|BOLD:ABZ5347
Dasychira grisefacta|[16259]|LBCB148-05|Canada|British Columbia|658[0n]|BOLD:ABZ5347
Dasychira pinicola|[16260]|RDNML068-13|Canada|Ontario|658[0n]|BOLD:ABZ5347
Dasychira pinicola|[16261]|RDNML065-13|Canada|Ontario|658[0n]|BOLD:ABZ5347
Dasychira grisefacta|[16262]|LBCG2301-09|Canada|British Columbia|658[0n]|BOLD:ABZ5347
Dasychira grisefacta|[16263]|LBCD251-05|Canada|British Columbia|645[0n]|BOLD:ABZ5347
Dasychira grisefacta|[16264]|LOPN184-06|United States|Oregon|504[1n]|BOLD:ABZ5347
Dasychira grisefacta|[16265]|LPABC092-09|Canada|Alberta|606[2n]|BOLD:ABZ5347
Dasychira grisefacta|[16266]|CNCLB1315-14|Canada|Manitoba|658[0n]|BOLD:ABZ5347
Dasychira grisefacta ella|[16267]|LMH019-06|Canada|British Columbia|658[0n]|BOLD:ABZ5347
Dasychira grisefacta|[16268]|RDLQF859-06|Canada|Quebec|658[0n]|BOLD:ABZ5347
Dasychira grisefacta|[16269]|LPMN964-08|Canada|Alberta|658[0n]|BOLD:ABZ5347
Dasychira grisefacta|[16270]|LPVIB264-08|Canada|British Columbia|658[0n]|BOLD:ABZ5347
Dasychira grisefacta|[16271]|LPVIB265-08|Canada|British Columbia|658[0n]|BOLD:ABZ5347
Dasychira grisefacta|[16272]|LPVIB448-08|Canada|British Columbia|658[0n]|BOLD:ABZ5347
Dasychira grisefacta|[16273]|LPVIB848-08|Canada|British Columbia|658[0n]|BOLD:ABZ5347
Dasychira grisefacta|[16274]|LPABC071-09|Canada|Alberta|658[0n]|BOLD:ABZ5347
Dasychira grisefacta|[16275]|LPABC343-09|Canada|Alberta|658[0n]|BOLD:ABZ5347
Dasychira grisefacta|[16276]|LBCH116-10|Canada|British Columbia|658[0n]|BOLD:ABZ5347

Dasychira grisefacta|[16273]|LPVIB848-08|Canada|British Columbia|658[0n]|BOLD:ABZ5347
Dasychira grisefacta|[16274]|LPABC071-09|Canada|Alberta|658[0n]|BOLD:ABZ5347
Dasychira grisefacta|[16275]|LPABC343-09|Canada|Alberta|658[0n]|BOLD:ABZ5347
Dasychira grisefacta|[16276]|LBCH116-10|Canada|British Columbia|658[0n]|BOLD:ABZ5347
Dasychira grisefacta|[16277]|LBCH222-10|Canada|British Columbia|658[0n]|BOLD:ABZ5347
Dasychira grisefacta|[16278]|LBCH698-10|Canada|British Columbia|658[0n]|BOLD:ABZ5347
Dasychira grisefacta|[16279]|LBCH2129-10|Canada|British Columbia|658[0n]|BOLD:ABZ5347
Dasychira grisefacta|[16280]|BBLPB641-10|Canada|British Columbia|658[0n]|BOLD:ABZ5347
Dasychira grisefacta|[16281]|RDNMJ408-11|Canada|Quebec|658[0n]|BOLD:ABZ5347
Dasychira grisefacta|[16282]|LALPA840-11|Canada|British Columbia|658[0n]|BOLD:ABZ5347
Dasychira grisefacta|[16283]|RDNMK657-11|Canada|British Columbia|658[0n]|BOLD:ABZ5347
Dasychira grisefacta|[16284]|CNCLB1316-14|Canada|Alberta|658[0n]|BOLD:ABZ5347
Dasychira grisefacta|[16285]|CNCLB1317-14|Canada|Alberta|658[0n]|BOLD:ABZ5347
Dasychira manto|[16286]|BBLOB1087-11|United States|Florida|658[0n]|BOLD:AAB8426
Dasychira manto|[16287]|LOWCE857-06|Canada|British Columbia|658[0n]|BOLD:AAB8426
Dasychira manto|[16288]|LNC797-06|United States|North Carolina|658[0n]|BOLD:AAB8426
Dasychira manto|[16289]|LNC796-06|United States|North Carolina|658[0n]|BOLD:AAB8426
Dasychira manto|[16290]|LNC798-06|United States|North Carolina|658[0n]|BOLD:AAB8426
Dasychira manto|[16291]|LOWCE848-06|Canada|British Columbia|658[0n]|BOLD:AAB8426
Dasychira manto|[16292]|LOWCE856-06|Canada|British Columbia|658[0n]|BOLD:AAB8426
Dasychira manto|[16293]|LOFLA681-06|United States|Florida|658[0n]|BOLD:AAB8426
Dasychira manto|[16294]|MILEQ201-11|United States|Georgia|658[0n]|BOLD:AAB8426
Dasychira manto|[16295]|HKONS659-08|United States|Florida|616[0n]|BOLD:AAB8426
Dasychira manto|[16296]|LOFLA334-06|United States|Florida|658[0n]|BOLD:AAB8426
Dasychira manto|[16297]|MILEQ200-11|United States|Georgia|658[0n]|BOLD:AAB8426
Dasychira manto|[16298]|LOFLA551-06|United States|Florida|658[0n]|BOLD:AAB8426
Dasychira manto|[16299]|HKONS661-08|United States|Florida|658[0n]|BOLD:AAB8426
Dasychira manto|[16300]|HKONS662-08|United States|Florida|658[0n]|BOLD:AAB8426
Dasychira manto|[16301]|LGSM613-04|United States|Tennessee|609[0n]|BOLD:AAB8426
Dasychira manto|[16302]|LGSM612-04|United States|Tennessee|658[0n]|BOLD:AAB8426
Dasychira manto|[16303]|LOTB335-05|United States|Tennessee|623[0n]|BOLD:AAB8426
Dasychira manto|[16304]|LSUSA071-06|United States|Kentucky|658[0n]|BOLD:AAB8426
Dasychira manto|[16305]|LNCC1486-13|United States|North Carolina|658[0n]|BOLD:AAB8426
Dasychira manto|[16306]|LNCC1487-13|United States|North Carolina|658[0n]|BOLD:AAB8426
Dasychira manto|[16307]|LNCC1658-13|United States|North Carolina|658[0n]|BOLD:AAB8426
Dasychira manto|[16308]|HKONS660-08|United States|Florida|658[0n]|BOLD:AAB8426
Dasychira manto|[16309]|LNCC1662-13|United States|North Carolina|658[0n]|BOLD:AAB8426
Dasychira pinicola|[16310]|CNCLB1318-14|United States|Michigan|658[0n]|BOLD:ACO0761
Dasychira pinicola|[16311]|CNCLB1320-14|United States|Minnesota|658[0n]|BOLD:ACO0761
Orgyia antiqua|[16312]|LBCH2462-10|Canada|British Columbia|658[0n]|BOLD:AAA6432
Orgyia antiqua|[16313]|GBGL1507-06|||624[0n]|BOLD:AAA6432
Orgyia antiqua|[16314]|ABKWR040-07|United States|Alaska|632[0n]|BOLD:AAA6432
Orgyia antiqua|[16315]|RDNME228-07|Canada|Alberta|658[0n]|BOLD:AAA6432
Orgyia antiqua nova|[16316]|RDLQF824-06|Canada|Quebec|658[0n]|BOLD:AAA6432
Orgyia antiqua nova|[16317]|RDLQF548-06|Canada|Quebec|658[0n]|BOLD:AAA6432
Orgyia antiqua|[16318]|RWWB416-09|United States|Washington|658[0n]|BOLD:AAA6432
Orgyia antiqua|[16319]|RWWC819-11|United States|Washington|658[0n]|BOLD:AAA6432
Orgyia antiqua|[16320]|RWWC837-12|United States|Washington|658[0n]|BOLD:AAA6432
Orgyia antiqua|[16321]|RWWC853-12|United States|Washington|658[0n]|BOLD:AAA6432
Orgyia antiqua|[16322]|RWWC856-12|United States|Washington|658[0n]|BOLD:AAA6432
Orgyia antiqua|[16323]|RWWC878-12|United States|Washington|658[0n]|BOLD:AAA6432
Orgyia antiqua|[16324]|SSEIC059-13|Canada|Alberta|597[0n]|BOLD:AAA6432
Orgyia antiqua|[16325]|CNMIF1134-14|Canada|Quebec|582[0n]|BOLD:AAA6432
Orgyia antiqua|[16326]|LEFIE512-10|Finland||658[0n]|BOLD:AAA6432
Orgyia antiqua|[16327]|GMGRE2831-13|Germany|Bavaria|591[0n]|BOLD:AAA6432
Orgyia antiqua|[16328]|FBLMV290-09|Germany|Bavaria|658[0n]|BOLD:AAA6432
Orgyia antiqua|[16329]|LON654-09|Norway||639[0n]|BOLD:AAA6432
Orgyia antiqua|[16330]|LEFIF538-10|Finland||658[0n]|BOLD:AAA6432
Orgyia antiqua|[16331]|LEATG203-14|Austria|Tirol|658[0n]|BOLD:AAA6432
Orgyia antiqua|[16332]|LEATG254-14|Austria|Tirol|658[0n]|BOLD:AAA6432
Orgyia antiqua|[16333]|IBLAO1097-14|Spain|La Rioja|658[0n]|BOLD:AAA6432
Orgyia antiqua|[16334]|GBLAA340-14|Germany|Schleswig-Holstein|658[0n]|BOLD:AAA6432
Orgyia antiqua|[16335]|LEATC478-13|Austria|Vorarlberg|658[0n]|BOLD:AAA6432
Orgyia antiqua|[16336]|IBLAO1096-14|Spain|La Rioja|658[0n]|BOLD:AAA6432
Orgyia antiqua|[16337]|GWOTL128-13|Germany|Saarland|658[0n]|BOLD:AAA6432
Orgyia antiqua|[16338]|GBLGC179-12|Germany|Bavaria|658[0n]|BOLD:AAA6432
Orgyia antiqua|[16339]|GWORZ091-10|Italy|Basilicata|658[0n]|BOLD:AAA6432
Orgyia antiqua|[16340]|LEFIC718-10|Finland|Finland Proper|658[0n]|BOLD:AAA6432
Orgyia antiqua|[16341]|FBLMV499-09|Germany|Bavaria|658[0n]|BOLD:AAA6432
Orgyia antiqua|[16342]|GWORK474-09|Germany|Bavaria|656[0n]|BOLD:AAA6432
Orgyia antiqua|[16343]|GWOR3971-09|Germany|Bavaria|658[0n]|BOLD:AAA6432
Orgyia antiqua|[16344]|GWOR3845-09|Germany|Bavaria|658[0n]|BOLD:AAA6432
Orgyia antiqua|[16345]|CGUKB669-09|United Kingdom|England|658[0n]|BOLD:AAA6432
Orgyia antiqua|[16346]|CGUKA292-09|United Kingdom|England|658[0n]|BOLD:AAA6432
Orgyia antiqua|[16347]|CGUKA113-09|United Kingdom|England|658[0n]|BOLD:AAA6432
Orgyia antiqua|[16348]|RDNME396-08|Denmark||658[0n]|BOLD:AAA6432
Orgyia antiqua|[16349]|NORIN012-13|Norway|Akershus|634[0n]|BOLD:AAA6432
Orgyia antiqua|[16350]|LASTS027-14|Austria|Tirol|658[0n]|BOLD:AAA6432
Orgyia falcata|[16351]|CMAZA915-12|United States|Arizona|658[0n]|BOLD:ABW3021
Orgyia detrita|[16352]|LNCB236-06|United States|North Carolina|658[0n]|BOLD:AAD1755
Orgyia detrita|[16353]|HKONS707-08|United States|Florida|658[0n]|BOLD:AAD1755
Orgyia detrita|[16354]|HKONS708-08|United States|Florida|658[0n]|BOLD:AAD1755
Orgyia detrita|[16355]|LNCB237-06|United States|North Carolina|658[0n]|BOLD:AAD1755
Orgyia detrita|[16356]|LNCB238-06|United States|North Carolina|658[0n]|BOLD:AAD1755
Orgyia detrita|[16357]|LNCB239-06|United States|North Carolina|658[0n]|BOLD:AAD1755
Orgyia detrita|[16358]|HKONS710-08|United States|Florida|658[0n]|BOLD:AAD1755
Orgyia detrita|[16359]|HKONS711-08|United States|Florida|658[0n]|BOLD:AAD1755
Orgyia leucostigma|[16360]|LPOKA552-09|United States|Oklahoma|658[0n]|BOLD:AAA6431
Orgyia leucostigma intermedia|[16361]|LSUSA200-06|United States|Kentucky|658[0n]|BOLD:AAA6431
Orgyia leucostigma|[16362]|LPOKA660-09|United States|Oklahoma|658[0n]|BOLD:AAA6431
Orgyia leucostigma|[16363]|LPOKA1033-09|United States|Oklahoma|658[0n]|BOLD:AAA6431
Orgyia leucostigma|[16364]|LPOKB373-09|United States|Oklahoma|658[0n]|BOLD:AAA6431
Orgyia leucostigma|[16365]|LPOKC320-09|United States|Oklahoma|658[0n]|BOLD:AAA6431
Orgyia leucostigma|[16366]|LPOKC796-09|United States|Oklahoma|658[0n]|BOLD:AAA6431
Orgyia leucostigma intermedia|[16367]|BBLSU032-09|United States|Arkansas|658[0n]|BOLD:AAA6431
Orgyia leucostigma|[16368]|BBLOE1377-12|United States|Arkansas|658[0n]|BOLD:AAA6431
Orgyia leucostigma|[16369]|TMNBB040-06|Canada|New Brunswick|658[0n]|BOLD:AAA6431
Orgyia leucostigma|[16370]|XAH784-05|Canada|Ontario|658[0n]|BOLD:AAA6431
Orgyia leucostigma|[16371]|XAH597-05|Canada|Ontario|658[0n]|BOLD:AAA6431
Orgyia leucostigma|[16372]|XAH579-05|Canada|Ontario|658[0n]|BOLD:AAA6431
Orgyia leucostigma|[16373]|XAG846-05|Canada|Ontario|658[0n]|BOLD:AAA6431
Orgyia leucostigma|[16374]|XAG765-05|Canada|Ontario|658[0n]|BOLD:AAA6431
Orgyia leucostigma|[16375]|XAG648-05|Canada|Ontario|657[0n]|BOLD:AAA6431
[illegible]

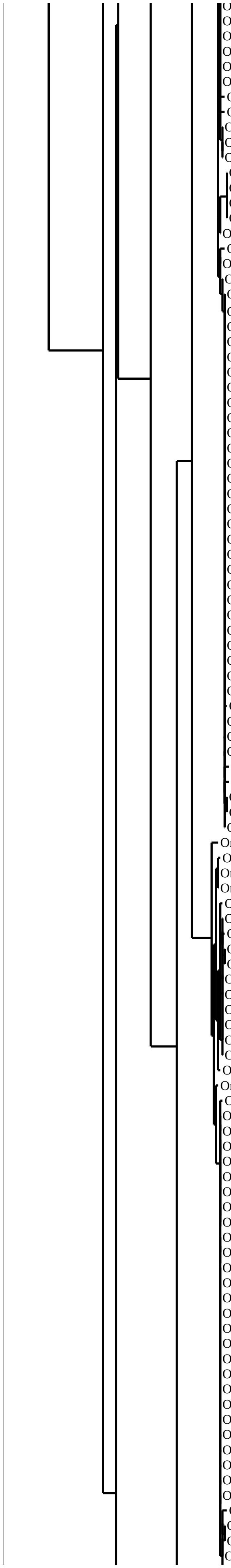

Orgyia leucostigma|[16373]|XAG846-05|Canada|Ontario|658[0n]|BOLD:AAA6431
Orgyia leucostigma|[16374]|XAG765-05|Canada|Ontario|658[0n]|BOLD:AAA6431
Orgyia leucostigma|[16375]|XAG648-05|Canada|Ontario|657[0n]|BOLD:AAA6431
Orgyia leucostigma|[16376]|MNBB081-05|Canada|New Brunswick|658[0n]|BOLD:AAA6431
Orgyia leucostigma|[16377]|XAB403-04|Canada|Ontario|658[0n]|BOLD:AAA6431
Orgyia leucostigma|[16378]|XAB214-04|Canada|Ontario|658[0n]|BOLD:AAA6431
Orgyia leucostigma|[16379]|XAB449-04|Canada|Ontario|658[0n]|BOLD:AAA6431
Orgyia leucostigma|[16380]|XAB418-04|Canada|Ontario|658[0n]|BOLD:AAA6431
Orgyia leucostigma|[16381]|TMNBD444-07|Canada|New Brunswick|646[0n]|BOLD:AAA6431
Orgyia leucostigma|[16382]|PHMO309-03|Canada|Ontario|639[0n]|BOLD:AAA6431
Orgyia leucostigma|[16383]|CNPPD2598-12|Canada|Ontario|615[0n]|BOLD:AAA6431
Orgyia leucostigma|[16384]|XAB642-04|Canada|Ontario|658[0n]|BOLD:AAA6431
Orgyia leucostigma|[16385]|XAG350-05|Canada|Ontario|658[0n]|BOLD:AAA6431
Orgyia leucostigma|[16386]|TMNBD441-07|Canada|New Brunswick|658[0n]|BOLD:AAA6431
Orgyia leucostigma|[16387]|TMNBD442-07|Canada|New Brunswick|658[0n]|BOLD:AAA6431
Orgyia leucostigma|[16388]|HKONS672-08|United States|Florida|658[0n]|BOLD:AAA6431
Orgyia leucostigma|[16389]|RDNMH870-09|Canada|Nova Scotia|658[0n]|BOLD:AAA6431
Orgyia leucostigma|[16390]|RDNMH871-09|Canada|Nova Scotia|658[0n]|BOLD:AAA6431
Orgyia leucostigma|[16391]|XAB464-04|Canada|Ontario|658[0n]|BOLD:AAA6431
Orgyia leucostigma intermedia|[16392]|RDLQF248-06|Canada|Quebec|655[0n]|BOLD:AAA6431
Orgyia leucostigma|[16393]|LNCB137-06|United States|North Carolina|656[0n]|BOLD:AAA6431
Orgyia leucostigma|[16394]|MJMSL153-10|United States|Massachusetts|658[0n]|BOLD:AAA6431
Orgyia leucostigma|[16395]|MJMSL043-10|United States|Massachusetts|658[0n]|BOLD:AAA6431
Orgyia leucostigma|[16396]|MJMSL038-10|United States|Massachusetts|658[0n]|BOLD:AAA6431
Orgyia leucostigma|[16397]|UDLEP336-09|United States|Delaware|658[0n]|BOLD:AAA6431
Orgyia leucostigma|[16398]|UDLEP002-09|United States|Delaware|658[0n]|BOLD:AAA6431
Orgyia leucostigma|[16399]|LTOLB016-08|United States|West Virginia|658[0n]|BOLD:AAA6431
Orgyia leucostigma|[16400]|HKONS675-08|United States|Florida|658[0n]|BOLD:AAA6431
Orgyia leucostigma|[16401]|HKONS674-08|United States|Florida|658[0n]|BOLD:AAA6431
Orgyia leucostigma|[16402]|TMNBD440-07|Canada|New Brunswick|658[0n]|BOLD:AAA6431
Orgyia leucostigma intermedia|[16403]|RDLQF249-06|Canada|Quebec|658[0n]|BOLD:AAA6431
Orgyia leucostigma|[16404]|XAK017-06|Canada|Ontario|658[0n]|BOLD:AAA6431
Orgyia leucostigma|[16405]|LNCNW086-06|United States|North Carolina|658[0n]|BOLD:AAA6431
Orgyia leucostigma|[16406]|LNCB136-06|United States|North Carolina|658[0n]|BOLD:AAA6431
Orgyia leucostigma|[16407]|TMNBB041-06|Canada|New Brunswick|658[0n]|BOLD:AAA6431
Orgyia leucostigma|[16408]|TMNBB039-06|Canada|New Brunswick|658[0n]|BOLD:AAA6431
Orgyia leucostigma|[16409]|TTMNB258-06|Canada|New Brunswick|658[0n]|BOLD:AAA6431
Orgyia leucostigma intermedia|[16410]|RDLQB513-05|Canada|Quebec|658[0n]|BOLD:AAA6431
Orgyia leucostigma|[16411]|XAH599-05|Canada|Ontario|658[0n]|BOLD:AAA6431
Orgyia leucostigma|[16412]|XAH578-05|Canada|Ontario|658[0n]|BOLD:AAA6431
Orgyia leucostigma|[16413]|XAH482-05|Canada|Ontario|658[0n]|BOLD:AAA6431
Orgyia leucostigma|[16414]|XAH279-05|Canada|Ontario|658[0n]|BOLD:AAA6431
Orgyia leucostigma|[16415]|XAH269-05|Canada|Ontario|658[0n]|BOLD:AAA6431
Orgyia leucostigma|[16416]|XAH085-05|Canada|Ontario|658[0n]|BOLD:AAA6431
Orgyia leucostigma|[16417]|XAG647-05|Canada|Ontario|658[0n]|BOLD:AAA6431
Orgyia leucostigma|[16418]|XAG610-05|Canada|Ontario|658[0n]|BOLD:AAA6431
Orgyia leucostigma|[16419]|XAG316-05|Canada|Ontario|658[0n]|BOLD:AAA6431
Orgyia leucostigma|[16420]|CNSLD283-12|Canada|Ontario|639[0n]|BOLD:AAA6431
Orgyia leucostigma|[16421]|LPOKA638-09|United States|Oklahoma|639[0n]|BOLD:AAA6431
Orgyia leucostigma|[16422]|TMNBD443-07|Canada|New Brunswick|648[0n]|BOLD:AAA6431
Orgyia leucostigma|[16423]|XAK615-07|Canada|Ontario|604[2n]|BOLD:AAA6431
Orgyia leucostigma|[16424]|PHMO311-03|Canada|Ontario|639[0n]|BOLD:AAA6431
Orgyia leucostigma|[16425]|LPOKA656-09|United States|Oklahoma|593[0n]|BOLD:AAA6431
Orgyia leucostigma|[16426]|UDLEP050-09|United States|Maryland|634[0n]|BOLD:AAA6431
Orgyia leucostigma|[16427]|CNPPE2351-12|Canada|Ontario|624[0n]|BOLD:AAA6431
Orgyia definita|[16428]|XAG923-05|Canada|Ontario|658[0n]|BOLD:AAA7686
Orgyia definita|[16429]|LNCB134-06|United States|North Carolina|658[0n]|BOLD:AAA7686
Orgyia definita|[16430]|LNCB233-06|United States|North Carolina|658[0n]|BOLD:AAA7686
Orgyia definita|[16431]|LNCB359-06|United States|North Carolina|654[0n]|BOLD:AAA7686
Orgyia definita|[16432]|LOT509-04|United States|Tennessee|658[0n]|BOLD:AAA7686
Orgyia definita|[16433]|LGSM610-04|United States|Tennessee|658[0n]|BOLD:AAA7686
Orgyia definita|[16434]|LOT558-04|United States|Tennessee|563[0n]|BOLD:AAA7686
Orgyia definita|[16435]|LOT540-04|United States|Tennessee|511[0n]|BOLD:AAA7686
Orgyia definita|[16436]|XAB406-04|Canada|Ontario|658[0n]|BOLD:AAA7686
Orgyia definita|[16437]|LNCNW087-06|United States|North Carolina|658[0n]|BOLD:AAA7686
Orgyia definita|[16438]|LGSMG130-07|United States|North Carolina|658[0n]|BOLD:AAA7686
Orgyia definita|[16439]|LGSMG131-07|United States|Tennessee|658[0n]|BOLD:AAA7686
Orgyia definita|[16440]|LGSMG132-07|United States|Tennessee|658[0n]|BOLD:AAA7686
Orgyia definita|[16441]|HKONB265-09|United States|Texas|658[0n]|BOLD:AAA7686
Orgyia definita|[16442]|HKONB266-09|United States|Texas|658[0n]|BOLD:AAA7686
Orgyia definita|[16443]|LILLB068-11|United States|Illinois|658[0n]|BOLD:AAA7686
Orgyia definita|[16444]|XAH749-05|Canada|Ontario|658[0n]|BOLD:AAA7686
Orgyia definita|[16445]|XAH554-05|Canada|Ontario|658[0n]|BOLD:AAA7686
Orgyia definita|[16446]|XAH456-05|Canada|Ontario|658[0n]|BOLD:AAA7686
Orgyia definita|[16447]|XAH515-05|Canada|Ontario|658[0n]|BOLD:AAA7686
Orgyia definita|[16448]|XAH553-05|Canada|Ontario|658[0n]|BOLD:AAA7686
Orgyia definita|[16449]|XAH598-05|Canada|Ontario|658[0n]|BOLD:AAA7686
Orgyia definita|[16450]|XAH611-05|Canada|Ontario|658[0n]|BOLD:AAA7686
Orgyia definita|[16451]|XAH717-05|Canada|Ontario|658[0n]|BOLD:AAA7686
Orgyia definita|[16452]|RDLQF250-06|Canada|Quebec|658[0n]|BOLD:AAA7686
Orgyia definita|[16453]|RDLQF887-06|Canada|Quebec|658[0n]|BOLD:AAA7686
Orgyia definita|[16454]|XAH527-05|Canada|Ontario|658[0n]|BOLD:AAA7686
Orgyia definita|[16455]|XAH528-05|Canada|Ontario|658[0n]|BOLD:AAA7686
Orgyia definita|[16456]|AHLEP025-10|United States|Pennsylvania|658[0n]|BOLD:AAA7686
Orgyia definita|[16457]|XAH414-05|Canada|Ontario|658[0n]|BOLD:AAA7686
Orgyia definita|[16458]|XAH413-05|Canada|Ontario|658[0n]|BOLD:AAA7686
Orgyia definita|[16459]|XAH347-05|Canada|Ontario|658[0n]|BOLD:AAA7686
Orgyia definita|[16460]|XAH298-05|Canada|Ontario|658[0n]|BOLD:AAA7686
Orgyia definita|[16461]|XAH178-05|Canada|Ontario|658[0n]|BOLD:AAA7686
Orgyia definita|[16462]|XAH038-05|Canada|Ontario|658[0n]|BOLD:AAA7686
Orgyia definita|[16463]|XAB651-04|Canada|Ontario|658[0n]|BOLD:AAA7686
Orgyia definita|[16464]|XAB644-04|Canada|Ontario|658[0n]|BOLD:AAA7686
Orgyia definita|[16465]|XAB451-04|Canada|Ontario|658[0n]|BOLD:AAA7686
Orgyia definita|[16466]|XAB427-04|Canada|Ontario|658[0n]|BOLD:AAA7686
Orgyia definita|[16467]|XAB404-04|Canada|Ontario|658[0n]|BOLD:AAA7686
Orgyia definita|[16468]|XAB405-04|Canada|Ontario|614[0n]|BOLD:AAA7686
Orgyia definita|[16469]|XAH457-05|Canada|Ontario|646[0n]|BOLD:AAA7686
Orgyia definita|[16470]|XAB420-04|Canada|Ontario|616[0n]|BOLD:AAA7686
Orgyia definita|[16471]|PHMO308-03|Canada|Ontario|639[0n]|BOLD:AAA7686
Orgyia definita|[16472]|LGSM611-04|United States|Tennessee|548[20n]|
Orgyia definita|[16473]|XAG972-05|Canada|Ontario|633[0n]|BOLD:AAA7686
Orgyia definita|[16474]|RDLQB506-05|Canada|Quebec|591[0n]|BOLD:AAA7686
Orgyia definita|[16475]|CNSLG091-12|Canada|Ontario|622[0n]|BOLD:AAA7686

Orgyia definita|[16473]|XAG972-05|Canada|Ontario|633[0n]|BOLD:AAA7686
Orgyia definita|[16474]|RDLQB506-05|Canada|Quebec|591[0n]|BOLD:AAA7686
Orgyia definita|[16475]|CNSLG091-12|Canada|Ontario|622[0n]|BOLD:AAA7686
Orgyia definita|[16476]|CNSLO888-13|Canada|Ontario|612[0n]|BOLD:AAA7686
Orgyia leuschneri|[16477]|LNAUS4137-13|United States|Arizona|658[0n]|BOLD:AAW8993
Orgyia leuschneri|[16478]|LNAUS4138-13|United States|Arizona|658[0n]|BOLD:AAW8993
Orgyia leuschneri|[16479]|LNAUS4139-13|United States|Arizona|658[0n]|BOLD:AAW8993
Orgyia leuschneri|[16480]|LNAUS4140-13|United States|Arizona|658[0n]|BOLD:AAW8993
Orgyia leuschneri|[16481]|LNAUS4141-13|United States|Arizona|658[0n]|BOLD:AAW8993
Orgyia pseudotsugata|[16482]|RDNMJ264-11|United States|Arizona|658[0n]|BOLD:AAT8892
Orgyia pseudotsugata|[16483]|RDNMJ695-11|United States|Arizona|658[0n]|BOLD:AAT8892
Orgyia pseudotsugata|[16484]|RDNMJ696-11|United States|Arizona|658[0n]|BOLD:AAT8892
Orgyia pseudotsugata|[16485]|DUNLP143-08|Canada|British Columbia|621[6n]|
Orgyia pseudotsugata|[16486]|LBCH7204-10|Canada|British Columbia|658[0n]|BOLD:AAE5045
Orgyia pseudotsugata|[16487]|LBCH6142-10|Canada|British Columbia|658[0n]|BOLD:AAE5045
Orgyia pseudotsugata|[16488]|LBCH6143-10|Canada|British Columbia|658[0n]|BOLD:AAE5045
Orgyia pseudotsugata|[16489]|LBCH6151-10|Canada|British Columbia|658[0n]|BOLD:AAE5045
Orgyia pseudotsugata|[16490]|LBCH6152-10|Canada|British Columbia|658[0n]|BOLD:AAE5045
Orgyia pseudotsugata|[16491]|LBCH6263-10|Canada|British Columbia|658[0n]|BOLD:AAE5045
Orgyia pseudotsugata|[16492]|LBCH6264-10|Canada|British Columbia|658[0n]|BOLD:AAE5045
Orgyia pseudotsugata|[16493]|LBCH6265-10|Canada|British Columbia|658[0n]|BOLD:AAE5045
Orgyia pseudotsugata|[16494]|LBCH6268-10|Canada|British Columbia|658[0n]|BOLD:AAE5045
Orgyia pseudotsugata|[16495]|LBCH7075-10|Canada|British Columbia|658[0n]|BOLD:AAE5045
Orgyia pseudotsugata|[16496]|LBCH7080-10|Canada|British Columbia|658[0n]|BOLD:AAE5045
Orgyia pseudotsugata|[16497]|LBCH7082-10|Canada|British Columbia|658[0n]|BOLD:AAE5045
Orgyia pseudotsugata|[16498]|LBCH7166-10|Canada|British Columbia|658[0n]|BOLD:AAE5045
Orgyia pseudotsugata|[16499]|LBCH7203-10|Canada|British Columbia|658[0n]|BOLD:AAE5045
Orgyia pseudotsugata|[16500]|LBCH7273-10|Canada|British Columbia|658[0n]|BOLD:AAE5045
Orgyia pseudotsugata|[16501]|LBCH7275-10|Canada|British Columbia|658[0n]|BOLD:AAE5045
Orgyia pseudotsugata|[16502]|LBCH7427-10|Canada|British Columbia|658[0n]|BOLD:AAE5045
Orgyia pseudotsugata|[16503]|LBCH7471-10|Canada|British Columbia|658[0n]|BOLD:AAE5045
Orgyia pseudotsugata|[16504]|LBCH7515-10|Canada|British Columbia|658[0n]|BOLD:AAE5045
Orgyia pseudotsugata|[16505]|LBCH7165-10|Canada|British Columbia|658[0n]|BOLD:AAE5045
Orgyia pseudotsugata|[16506]|LBCH7206-10|Canada|British Columbia|658[0n]|BOLD:AAE5045
Orgyia pseudotsugata|[16507]|LBCH6149-10|Canada|British Columbia|658[0n]|BOLD:AAE5045
Orgyia pseudotsugata|[16508]|LBCH7208-10|Canada|British Columbia|658[0n]|BOLD:AAE5045
Orgyia pseudotsugata|[16509]|LBCH6147-10|Canada|British Columbia|658[0n]|BOLD:AAE5045
Orgyia pseudotsugata|[16510]|LBCH6150-10|Canada|British Columbia|658[0n]|BOLD:AAE5045
Orgyia pseudotsugata|[16511]|LBCH6224-10|Canada|British Columbia|658[0n]|BOLD:AAE5045
Orgyia pseudotsugata|[16512]|LBCH6266-10|Canada|British Columbia|658[0n]|BOLD:AAE5045
Orgyia pseudotsugata|[16513]|LBCH6267-10|Canada|British Columbia|658[0n]|BOLD:AAE5045
Orgyia pseudotsugata|[16514]|LBCH6269-10|Canada|British Columbia|658[0n]|BOLD:AAE5045
Orgyia pseudotsugata|[16515]|LBCH7076-10|Canada|British Columbia|657[0n]|BOLD:AAE5045
Orgyia pseudotsugata|[16516]|LBCH7077-10|Canada|British Columbia|658[0n]|BOLD:AAE5045
Orgyia pseudotsugata|[16517]|LBCH7079-10|Canada|British Columbia|658[0n]|BOLD:AAE5045
Orgyia pseudotsugata|[16518]|LBCH7081-10|Canada|British Columbia|658[0n]|BOLD:AAE5045
Orgyia pseudotsugata|[16519]|LBCH7161-10|Canada|British Columbia|658[0n]|BOLD:AAE5045
Orgyia pseudotsugata|[16520]|LBCH7163-10|Canada|British Columbia|658[0n]|BOLD:AAE5045
Orgyia pseudotsugata|[16521]|LBCH7164-10|Canada|British Columbia|658[0n]|BOLD:AAE5045
Orgyia pseudotsugata|[16522]|LBCH7167-10|Canada|British Columbia|658[0n]|BOLD:AAE5045
Orgyia pseudotsugata|[16523]|LBCH7202-10|Canada|British Columbia|658[0n]|BOLD:AAE5045
Orgyia pseudotsugata|[16524]|LBCH7205-10|Canada|British Columbia|658[0n]|BOLD:AAE5045
Orgyia pseudotsugata|[16525]|LBCH7272-10|Canada|British Columbia|658[0n]|BOLD:AAE5045
Orgyia pseudotsugata|[16526]|LBCH7276-10|Canada|British Columbia|658[0n]|BOLD:AAE5045
Orgyia pseudotsugata|[16527]|LBCH7277-10|Canada|British Columbia|658[0n]|BOLD:AAE5045
Orgyia pseudotsugata|[16528]|LBCH7278-10|Canada|British Columbia|658[0n]|BOLD:AAE5045
Orgyia pseudotsugata|[16529]|LBCH7391-10|Canada|British Columbia|658[0n]|BOLD:AAE5045
Orgyia pseudotsugata|[16530]|LBCH6270-10|Canada|British Columbia|658[0n]|BOLD:AAE5045
Orgyia pseudotsugata|[16531]|LBCH7590-10|Canada|British Columbia|658[0n]|BOLD:AAE5045
Orgyia pseudotsugata|[16532]|LBCH7499-10|Canada|British Columbia|658[0n]|BOLD:AAE5045
Orgyia pseudotsugata|[16533]|LBCH7498-10|Canada|British Columbia|658[0n]|BOLD:AAE5045
Orgyia pseudotsugata|[16534]|LBCH7497-10|Canada|British Columbia|658[0n]|BOLD:AAE5045
Orgyia pseudotsugata|[16535]|LBCH7496-10|Canada|British Columbia|658[0n]|BOLD:AAE5045
Orgyia pseudotsugata|[16536]|LBCH7495-10|Canada|British Columbia|658[0n]|BOLD:AAE5045
Orgyia pseudotsugata|[16537]|LBCH7494-10|Canada|British Columbia|658[0n]|BOLD:AAE5045
Orgyia pseudotsugata|[16538]|LBCH7493-10|Canada|British Columbia|658[0n]|BOLD:AAE5045
Orgyia pseudotsugata|[16539]|LBCH7492-10|Canada|British Columbia|658[0n]|BOLD:AAE5045
Orgyia pseudotsugata|[16540]|LBCH7371-10|Canada|British Columbia|658[0n]|BOLD:AAE5045
Orgyia pseudotsugata|[16541]|LBCH7279-10|Canada|British Columbia|658[0n]|BOLD:AAE5045
Orgyia pseudotsugata|[16542]|LBCH7207-10|Canada|British Columbia|658[0n]|BOLD:AAE5045
Orgyia pseudotsugata|[16543]|LBCH7201-10|Canada|British Columbia|658[0n]|BOLD:AAE5045
Orgyia pseudotsugata|[16544]|LBCH7078-10|Canada|British Columbia|658[0n]|BOLD:AAE5045
Orgyia pseudotsugata|[16545]|LBCH7028-10|Canada|British Columbia|658[0n]|BOLD:AAE5045
Orgyia pseudotsugata|[16546]|LBCH7027-10|Canada|British Columbia|658[0n]|BOLD:AAE5045
Orgyia pseudotsugata|[16547]|LBCH7026-10|Canada|British Columbia|658[0n]|BOLD:AAE5045
Orgyia pseudotsugata|[16548]|LBCH7025-10|Canada|British Columbia|658[0n]|BOLD:AAE5045
Orgyia pseudotsugata|[16549]|LBCH7024-10|Canada|British Columbia|658[0n]|BOLD:AAE5045
Orgyia pseudotsugata|[16550]|LBCH7023-10|Canada|British Columbia|658[0n]|BOLD:AAE5045
Orgyia pseudotsugata|[16551]|LBCH7022-10|Canada|British Columbia|658[0n]|BOLD:AAE5045
Orgyia pseudotsugata|[16552]|LBCH7021-10|Canada|British Columbia|658[0n]|BOLD:AAE5045
Orgyia pseudotsugata|[16553]|LBCH6953-10|Canada|British Columbia|658[0n]|BOLD:AAE5045
Orgyia pseudotsugata|[16554]|LBCH6871-10|Canada|British Columbia|658[0n]|BOLD:AAE5045
Orgyia pseudotsugata|[16555]|LBCH6870-10|Canada|British Columbia|658[0n]|BOLD:AAE5045
Orgyia pseudotsugata|[16556]|LBCH6869-10|Canada|British Columbia|658[0n]|BOLD:AAE5045
Orgyia pseudotsugata|[16557]|LBCH6868-10|Canada|British Columbia|658[0n]|BOLD:AAE5045
Orgyia pseudotsugata|[16558]|LBCH6867-10|Canada|British Columbia|658[0n]|BOLD:AAE5045
Orgyia pseudotsugata|[16559]|LBCH6866-10|Canada|British Columbia|658[0n]|BOLD:AAE5045
Orgyia pseudotsugata|[16560]|LBCH6865-10|Canada|British Columbia|658[0n]|BOLD:AAE5045
Orgyia pseudotsugata|[16561]|LBCH6864-10|Canada|British Columbia|658[0n]|BOLD:AAE5045
Orgyia pseudotsugata|[16562]|LBCH6837-10|Canada|British Columbia|658[0n]|BOLD:AAE5045
Orgyia pseudotsugata|[16563]|LBCH6773-10|Canada|British Columbia|658[0n]|BOLD:AAE5045
Orgyia pseudotsugata|[16564]|LBCH6771-10|Canada|British Columbia|658[0n]|BOLD:AAE5045
Orgyia pseudotsugata|[16565]|LBCH6770-10|Canada|British Columbia|658[0n]|BOLD:AAE5045
Orgyia pseudotsugata|[16566]|LBCH6769-10|Canada|British Columbia|658[0n]|BOLD:AAE5045
Orgyia pseudotsugata|[16567]|LBCH6768-10|Canada|British Columbia|658[0n]|BOLD:AAE5045
Orgyia pseudotsugata|[16568]|LBCH6767-10|Canada|British Columbia|658[0n]|BOLD:AAE5045
Orgyia pseudotsugata|[16569]|LBCH6766-10|Canada|British Columbia|658[0n]|BOLD:AAE5045
Orgyia pseudotsugata|[16570]|LBCH6602-10|Canada|British Columbia|658[0n]|BOLD:AAE5045
Orgyia pseudotsugata|[16571]|LBCH6601-10|Canada|British Columbia|658[0n]|BOLD:AAE5045
Orgyia pseudotsugata|[16572]|LBCH6600-10|Canada|British Columbia|658[0n]|BOLD:AAE5045
Orgyia pseudotsugata|[16573]|LBCH6599-10|Canada|British Columbia|658[0n]|BOLD:AAE5045
Orgyia pseudotsugata|[16574]|LBCH6598-10|Canada|British Columbia|658[0n]|BOLD:AAE5045
Orgyia pseudotsugata|[16575]|LBCH6597-10|Canada|British Columbia|658[0n]|BOLD:AAE5045

Orgyia pseudotsugata|[16573]|LBCH6599-10|Canada|British Columbia|658[0n]|BOLD:AAE5045
Orgyia pseudotsugata|[16574]|LBCH6598-10|Canada|British Columbia|658[0n]|BOLD:AAE5045
Orgyia pseudotsugata|[16575]|LBCH6597-10|Canada|British Columbia|658[0n]|BOLD:AAE5045
Orgyia pseudotsugata|[16576]|LBCH6596-10|Canada|British Columbia|658[0n]|BOLD:AAE5045
Orgyia pseudotsugata|[16577]|LBCH6595-10|Canada|British Columbia|658[0n]|BOLD:AAE5045
Orgyia pseudotsugata|[16578]|LBCH6521-10|Canada|British Columbia|658[0n]|BOLD:AAE5045
Orgyia pseudotsugata|[16579]|LBCH6520-10|Canada|British Columbia|658[0n]|BOLD:AAE5045
Orgyia pseudotsugata|[16580]|LBCH6519-10|Canada|British Columbia|658[0n]|BOLD:AAE5045
Orgyia pseudotsugata|[16581]|LBCH6518-10|Canada|British Columbia|658[0n]|BOLD:AAE5045
Orgyia pseudotsugata|[16582]|LBCH6517-10|Canada|British Columbia|658[0n]|BOLD:AAE5045
Orgyia pseudotsugata|[16583]|LBCH6516-10|Canada|British Columbia|658[0n]|BOLD:AAE5045
Orgyia pseudotsugata|[16584]|LBCH6515-10|Canada|British Columbia|658[0n]|BOLD:AAE5045
Orgyia pseudotsugata|[16585]|LBCH6514-10|Canada|British Columbia|658[0n]|BOLD:AAE5045
Orgyia pseudotsugata|[16586]|LBCH6408-10|Canada|British Columbia|658[0n]|BOLD:AAE5045
Orgyia pseudotsugata|[16587]|LBCH6407-10|Canada|British Columbia|658[0n]|BOLD:AAE5045
Orgyia pseudotsugata|[16588]|LBCH6406-10|Canada|British Columbia|658[0n]|BOLD:AAE5045
Orgyia pseudotsugata|[16589]|LBCH6405-10|Canada|British Columbia|658[0n]|BOLD:AAE5045
Orgyia pseudotsugata|[16590]|LBCH6404-10|Canada|British Columbia|658[0n]|BOLD:AAE5045
Orgyia pseudotsugata|[16591]|LBCH6403-10|Canada|British Columbia|658[0n]|BOLD:AAE5045
Orgyia pseudotsugata|[16592]|LBCH6402-10|Canada|British Columbia|658[0n]|BOLD:AAE5045
Orgyia pseudotsugata|[16593]|LBCH6401-10|Canada|British Columbia|658[0n]|BOLD:AAE5045
Orgyia pseudotsugata|[16594]|LBCH6353-10|Canada|British Columbia|658[0n]|BOLD:AAE5045
Orgyia pseudotsugata|[16595]|LBCH6352-10|Canada|British Columbia|658[0n]|BOLD:AAE5045
Orgyia pseudotsugata|[16596]|LBCH6351-10|Canada|British Columbia|658[0n]|BOLD:AAE5045
Orgyia pseudotsugata|[16597]|LBCH6350-10|Canada|British Columbia|658[0n]|BOLD:AAE5045
Orgyia pseudotsugata|[16598]|LBCH6349-10|Canada|British Columbia|658[0n]|BOLD:AAE5045
Orgyia pseudotsugata|[16599]|LBCH6348-10|Canada|British Columbia|658[0n]|BOLD:AAE5045
Orgyia pseudotsugata|[16600]|LBCH6347-10|Canada|British Columbia|658[0n]|BOLD:AAE5045
Orgyia pseudotsugata|[16601]|LBCH6346-10|Canada|British Columbia|658[0n]|BOLD:AAE5045
Orgyia pseudotsugata|[16602]|LBCH6148-10|Canada|British Columbia|658[0n]|BOLD:AAE5045
Orgyia pseudotsugata|[16603]|LBCH6146-10|Canada|British Columbia|658[0n]|BOLD:AAE5045
Orgyia pseudotsugata|[16604]|LBCH6145-10|Canada|British Columbia|658[0n]|BOLD:AAE5045
Orgyia pseudotsugata|[16605]|LBCH6141-10|Canada|British Columbia|658[0n]|BOLD:AAE5045
Orgyia pseudotsugata|[16606]|LBCH7162-10|Canada|British Columbia|639[0n]|BOLD:AAE5045
Orgyia pseudotsugata|[16607]|LBCH7160-10|Canada|British Columbia|636[0n]|BOLD:AAE5045
Orgyia pseudotsugata|[16608]|LBCH6772-10|Canada|British Columbia|645[0n]|BOLD:AAE5045
Orgyia pseudotsugata|[16609]|LBCH7274-10|Canada|British Columbia|629[0n]|BOLD:AAE5045
Orgyia pseudotsugata|[16610]|LBCH7591-10|Canada|British Columbia|641[0n]|BOLD:AAE5045
Orgyia pseudotsugata|[16611]|LBCH7592-10|Canada|British Columbia|658[0n]|BOLD:AAE5045
Orgyia pseudotsugata|[16612]|LBCH7593-10|Canada|British Columbia|658[0n]|BOLD:AAE5045
Orgyia pseudotsugata|[16613]|LBCH7594-10|Canada|British Columbia|658[0n]|BOLD:AAE5045
Orgyia pseudotsugata|[16614]|LBCH7595-10|Canada|British Columbia|658[0n]|BOLD:AAE5045
Orgyia pseudotsugata|[16615]|LBCH7596-10|Canada|British Columbia|658[0n]|BOLD:AAE5045
Orgyia pseudotsugata|[16616]|LBCH7597-10|Canada|British Columbia|658[0n]|BOLD:AAE5045
Orgyia pseudotsugata|[16617]|LBCH7728-10|Canada|British Columbia|658[0n]|BOLD:AAE5045
Orgyia pseudotsugata|[16618]|LBCH7729-10|Canada|British Columbia|658[0n]|BOLD:AAE5045
Orgyia pseudotsugata|[16619]|LBCH7730-10|Canada|British Columbia|658[0n]|BOLD:AAE5045
Orgyia pseudotsugata|[16620]|LBCH7731-10|Canada|British Columbia|658[0n]|BOLD:AAE5045
Orgyia pseudotsugata|[16621]|LBCH7732-10|Canada|British Columbia|658[0n]|BOLD:AAE5045
Orgyia pseudotsugata|[16622]|LBCH7733-10|Canada|British Columbia|658[0n]|BOLD:AAE5045
Orgyia pseudotsugata|[16623]|LBCH7734-10|Canada|British Columbia|658[0n]|BOLD:AAE5045
Orgyia pseudotsugata|[16624]|LBCH7735-10|Canada|British Columbia|658[0n]|BOLD:AAE5045
Orgyia pseudotsugata|[16625]|LBCH7839-10|Canada|British Columbia|658[0n]|BOLD:AAE5045
Orgyia pseudotsugata|[16626]|LBCH7840-10|Canada|British Columbia|658[0n]|BOLD:AAE5045
Orgyia pseudotsugata|[16627]|LBCH7841-10|Canada|British Columbia|658[0n]|BOLD:AAE5045
Orgyia pseudotsugata|[16628]|LBCH7842-10|Canada|British Columbia|658[0n]|BOLD:AAE5045
Orgyia pseudotsugata|[16629]|LBCH7843-10|Canada|British Columbia|658[0n]|BOLD:AAE5045
Orgyia pseudotsugata|[16630]|LBCH7844-10|Canada|British Columbia|658[0n]|BOLD:AAE5045
Orgyia pseudotsugata|[16631]|LBCH7845-10|Canada|British Columbia|658[0n]|BOLD:AAE5045
Orgyia pseudotsugata|[16632]|LBCH7846-10|Canada|British Columbia|658[0n]|BOLD:AAE5045
Orgyia pseudotsugata|[16633]|MNAD418-07|Canada|British Columbia|658[0n]|BOLD:AAE5045
Orgyia pseudotsugata|[16634]|LALPA805-10|Canada|British Columbia|658[0n]|BOLD:AAE5045
Orgyia pseudotsugata|[16635]|LALPA1329-12|Canada|British Columbia|601[0n]|BOLD:AAE5045
Orgyia magna|[16636]|LOCBF393-13|United States|California|623[0n]|BOLD:AAF1087
Orgyia magna|[16637]|LOCBF2601-13|United States|California|658[0n]|BOLD:AAF1087
Orgyia magna|[16638]|LOCBF2627-13|United States|California|658[0n]|BOLD:AAF1087
Orgyia magna|[16639]|CNCLB430-14|United States|California|682[0n]|BOLD:AAF1087
Orgyia magna|[16640]|LOCBF422-13|United States|California|628[0n]|BOLD:AAF1087
Orgyia magna|[16641]|LOCBF392-13|United States|California|630[0n]|BOLD:AAF1087
Orgyia magna|[16642]|LNAUT268-14|United States|California|658[0n]|BOLD:AAF1087
Orgyia magna|[16643]|LNAUT269-14|United States|California|628[0n]|BOLD:AAF1087
Orgyia magna|[16644]|LOCBF4101-14|United States|California|573[0n]|BOLD:AAF1087
Orgyia cana|[16645]|LOPN176-06|United States|Oregon|506[3n]|BOLD:AAF1087
Orgyia cana|[16646]|LOPN175-06|United States|Oregon|515[5n]|BOLD:AAF1087
Orgyia cana|[16647]|LOPN177-06|United States|Oregon|595[2n]|BOLD:AAF1087
Orgyia cana|[16648]|LNAUS4336-13|United States|Idaho|658[0n]|BOLD:AAF1087
Orgyia sp.|[16649]|LOCBF395-13|United States|California|631[0n]|BOLD:AAF1087
Orgyia vetusta|[16650]|CGLCA094-10|United States|California|658[0n]|BOLD:AAF1087
Orgyia vetusta|[16651]|GBGL1582-06|United States|California|657[1n]|BOLD:AAF1087
Orgyia vetusta|[16652]|GBGL1583-06|United States|California|657[1n]|BOLD:AAF1087
Orgyia sp.|[16653]|LOCBF1336-13|United States|California|562[0n]|BOLD:AAF1087
Orgyia sp.|[16654]|LOCBF1337-13|United States|California|588[0n]|BOLD:AAF1087
Orgyia vetusta|[16655]|GMLC902-12|United States|California|658[0n]|BOLD:AAF1087
Orgyia sp.|[16656]|LOCBF1339-13|United States|California|610[0n]|BOLD:AAF1087
Orgyia cana|[16657]|LNAUS4142-13|United States|Idaho|658[0n]|BOLD:AAF1087
Orgyia cana|[16658]|LNAUS4333-13|United States|Idaho|658[0n]|BOLD:AAF1087
Orgyia cana|[16659]|LNAUS4334-13|United States|Idaho|658[0n]|BOLD:AAF1087
Orgyia cana|[16660]|LNAUS4335-13|United States|Idaho|658[0n]|BOLD:AAF1087
Orgyia on-manzanita|[16661]|LOPN179-06|United States|California|658[0n]|BOLD:AAF1087
Orgyia on-manzanita|[16662]|LOCBE115-06|United States|California|658[0n]|BOLD:AAF1087
Orgyia vetusta|[16663]|GMLC900-12|United States|California|658[0n]|BOLD:AAF1087
Orgyia vetusta|[16664]|GMLC917-12|United States|California|658[0n]|BOLD:AAF1087
Orgyia vetusta|[16665]|GMLC1052-12|United States|California|658[0n]|BOLD:AAF1087
Orgyia cana|[16666]|CNCLB429-14|United States|California|676[0n]|BOLD:AAF1087
Orgyia sp.|[16667]|LOCBF1340-13|United States|California|612[0n]|BOLD:AAF1087
Orgyia sp.|[16668]|LOCBF1338-13|United States|California|610[0n]|BOLD:AAF1087
Orgyia sp.|[16669]|LOCBF396-13|United States|California|628[0n]|BOLD:AAF1087
Orgyia on-manzanita|[16670]|LOCBD006-06|United States|California|658[0n]|BOLD:AAF1087
Orgyia sp.|[16671]|LOCBF4102-14|United States|California|600[0n]|BOLD:AAF1087
Orgyia vetusta|[16672]|GMLC944-12|United States|California|658[0n]|BOLD:AAF1087
Orgyia cana|[16673]|CNCLB1437-14|United States|Idaho|658[0n]|BOLD:AAF1087
