# Supplementary material for: Probing planetary biodiversity with DNA barcodes: The Noctuoidea of North America
Source: PLoS One. 2017 Jun 1;12(6):e0178548. doi: 10.1371/journal.pone.0178548 (PMC5453547; doi:10.1371/journal.pone.0178548)
Supplement: S6 Tree — NJ tree based on sequence variation in the barcode region of the cytochrome c oxidase I gene for North American species in the family Noctuidae-1. (PDF) [file pone.0178548.s019.pdf]

BOLD TaxonID Tree

Title	:	Tree Result - Search (12132 records)
Date	:	27-April-2016
Data Type	:	Nucleotide
Distance Model	:	Kimura 2 Parameter
Marker	:	COI-5P

Label	:	Process ID
Label	:	Taxon
Label	:	Country
Label	:	Province/State
Label	:	Sequence Length
Label	:	Barcode Cluster (BIN)

Sequence Count	:	12132
Species count	:	1094
Genus count	:	189
Family count	:	1
Unidentified	:	0

BIN Count	:	1056

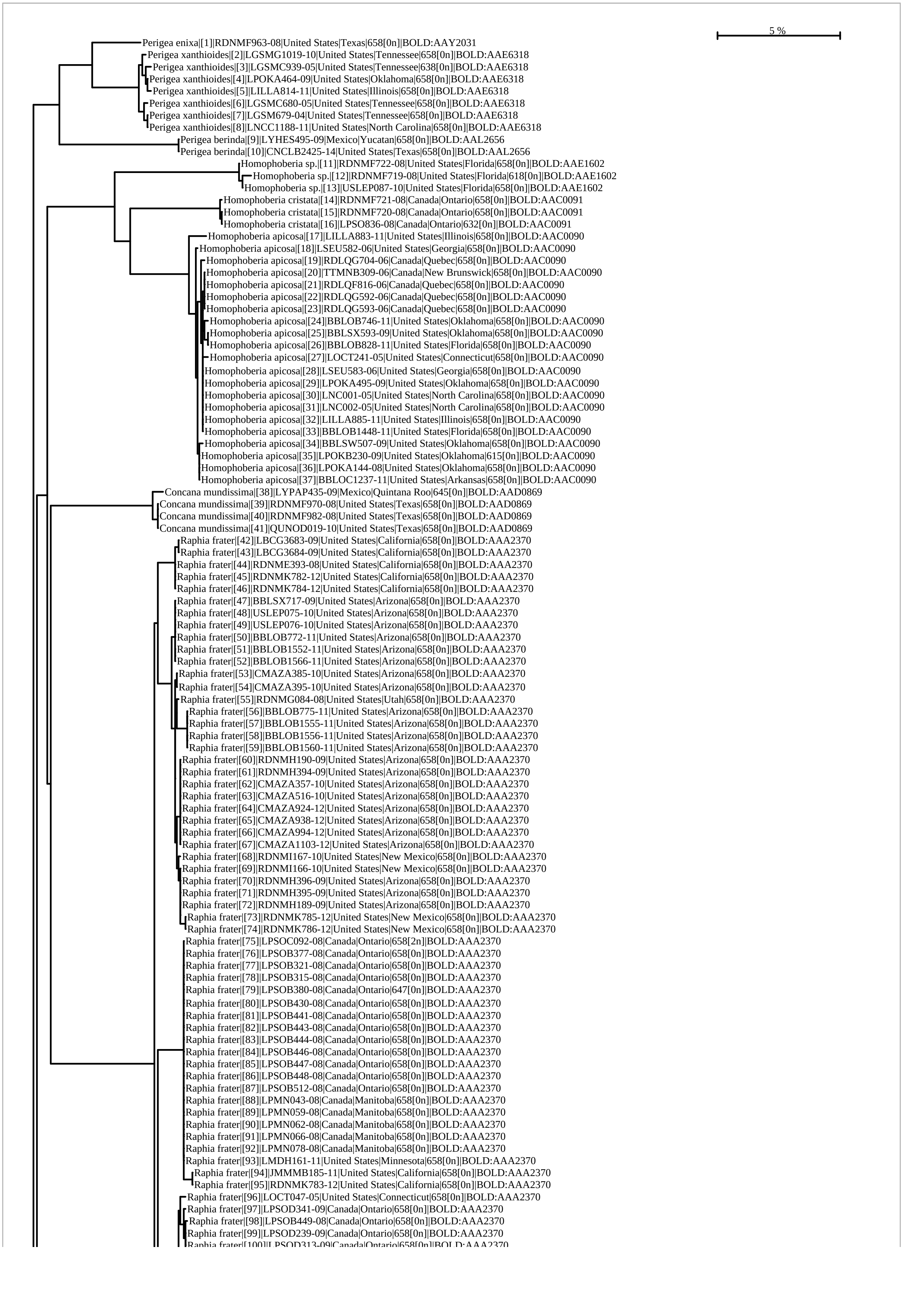
5 %
Perigea enixa|[1]|RDNMF963-08|United States|Texas|658[0n]|BOLD:AAY2031
Perigea xanthioides|[2]|LGSMG1019-10|United States|Tennessee|658[0n]|BOLD:AAE6318
Perigea xanthioides|[3]|LGSMC939-05|United States|Tennessee|638[0n]|BOLD:AAE6318
Perigea xanthioides|[4]|LPOKA464-09|United States|Oklahoma|658[0n]|BOLD:AAE6318
Perigea xanthioides|[5]|LILLA814-11|United States|Illinois|658[0n]|BOLD:AAE6318
Perigea xanthioides|[6]|LGSMC680-05|United States|Tennessee|658[0n]|BOLD:AAE6318
Perigea xanthioides|[7]|LGSM679-04|United States|Tennessee|658[0n]|BOLD:AAE6318
Perigea xanthioides|[8]|LNCC1188-11|United States|North Carolina|658[0n]|BOLD:AAE6318
Perigea berinda|[9]|LYHES495-09|Mexico|Yucatan|658[0n]|BOLD:AAL2656
Perigea berinda|[10]|CNCLB2425-14|United States|Texas|658[0n]|BOLD:AAL2656
Homophoberia sp.|[11]|RDNMF722-08|United States|Florida|658[0n]|BOLD:AAE1602
Homophoberia sp.|[12]|RDNMF719-08|United States|Florida|618[0n]|BOLD:AAE1602
Homophoberia sp.|[13]|USLEP087-10|United States|Florida|658[0n]|BOLD:AAE1602
Homophoberia cristata|[14]|RDNMF721-08|Canada|Ontario|658[0n]|BOLD:AAC0091
Homophoberia cristata|[15]|RDNMF720-08|Canada|Ontario|658[0n]|BOLD:AAC0091
Homophoberia cristata|[16]|LPSO836-08|Canada|Ontario|632[0n]|BOLD:AAC0091
Homophoberia apicosa|[17]|LILLA883-11|United States|Illinois|658[0n]|BOLD:AAC0090
Homophoberia apicosa|[18]|LSEU582-06|United States|Georgia|658[0n]|BOLD:AAC0090
Homophoberia apicosa|[19]|RDLQG704-06|Canada|Quebec|658[0n]|BOLD:AAC0090
Homophoberia apicosa|[20]|TTMNB309-06|Canada|New Brunswick|658[0n]|BOLD:AAC0090
Homophoberia apicosa|[21]|RDLQF816-06|Canada|Quebec|658[0n]|BOLD:AAC0090
Homophoberia apicosa|[22]|RDLQG592-06|Canada|Quebec|658[0n]|BOLD:AAC0090
Homophoberia apicosa|[23]|RDLQG593-06|Canada|Quebec|658[0n]|BOLD:AAC0090
Homophoberia apicosa|[24]|BBLOB746-11|United States|Oklahoma|658[0n]|BOLD:AAC0090
Homophoberia apicosa|[25]|BBLSX593-09|United States|Oklahoma|658[0n]|BOLD:AAC0090
Homophoberia apicosa|[26]|BBLOB828-11|United States|Florida|658[0n]|BOLD:AAC0090
Homophoberia apicosa|[27]|LOCT241-05|United States|Connecticut|658[0n]|BOLD:AAC0090
Homophoberia apicosa|[28]|LSEU583-06|United States|Georgia|658[0n]|BOLD:AAC0090
Homophoberia apicosa|[29]|LPOKA495-09|United States|Oklahoma|658[0n]|BOLD:AAC0090
Homophoberia apicosa|[30]|LNC001-05|United States|North Carolina|658[0n]|BOLD:AAC0090
Homophoberia apicosa|[31]|LNC002-05|United States|North Carolina|658[0n]|BOLD:AAC0090
Homophoberia apicosa|[32]|LILLA885-11|United States|Illinois|658[0n]|BOLD:AAC0090
Homophoberia apicosa|[33]|BBLOB1448-11|United States|Florida|658[0n]|BOLD:AAC0090
Homophoberia apicosa|[34]|BBLSW507-09|United States|Oklahoma|658[0n]|BOLD:AAC0090
Homophoberia apicosa|[35]|LPOKB230-09|United States|Oklahoma|615[0n]|BOLD:AAC0090
Homophoberia apicosa|[36]|LPOKA144-08|United States|Oklahoma|658[0n]|BOLD:AAC0090
Homophoberia apicosa|[37]|BBLOC1237-11|United States|Arkansas|658[0n]|BOLD:AAC0090
Concana mundissima|[38]|LYPAP435-09|Mexico|Quintana Roo|645[0n]|BOLD:AAD0869
Concana mundissima|[39]|RDNMF970-08|United States|Texas|658[0n]|BOLD:AAD0869
Concana mundissima|[40]|RDNMF982-08|United States|Texas|658[0n]|BOLD:AAD0869
Concana mundissima|[41]|QUNOD019-10|United States|Texas|658[0n]|BOLD:AAD0869
Raphia frater|[42]|LBCG3683-09|United States|California|658[0n]|BOLD:AAA2370
Raphia frater|[43]|LBCG3684-09|United States|California|658[0n]|BOLD:AAA2370
Raphia frater|[44]|RDNME393-08|United States|California|658[0n]|BOLD:AAA2370
Raphia frater|[45]|RDNMK782-12|United States|California|658[0n]|BOLD:AAA2370
Raphia frater|[46]|RDNMK784-12|United States|California|658[0n]|BOLD:AAA2370
Raphia frater|[47]|BBLSX717-09|United States|Arizona|658[0n]|BOLD:AAA2370
Raphia frater|[48]|USLEP075-10|United States|Arizona|658[0n]|BOLD:AAA2370
Raphia frater|[49]|USLEP076-10|United States|Arizona|658[0n]|BOLD:AAA2370
Raphia frater|[50]|BBLOB772-11|United States|Arizona|658[0n]|BOLD:AAA2370
Raphia frater|[51]|BBLOB1552-11|United States|Arizona|658[0n]|BOLD:AAA2370
Raphia frater|[52]|BBLOB1566-11|United States|Arizona|658[0n]|BOLD:AAA2370
Raphia frater|[53]|CMAZA385-10|United States|Arizona|658[0n]|BOLD:AAA2370
Raphia frater|[54]|CMAZA395-10|United States|Arizona|658[0n]|BOLD:AAA2370
Raphia frater|[55]|RDNMG084-08|United States|Utah|658[0n]|BOLD:AAA2370
Raphia frater|[56]|BBLOB775-11|United States|Arizona|658[0n]|BOLD:AAA2370
Raphia frater|[57]|BBLOB1555-11|United States|Arizona|658[0n]|BOLD:AAA2370
Raphia frater|[58]|BBLOB1556-11|United States|Arizona|658[0n]|BOLD:AAA2370
Raphia frater|[59]|BBLOB1560-11|United States|Arizona|658[0n]|BOLD:AAA2370
Raphia frater|[60]|RDNMH190-09|United States|Arizona|658[0n]|BOLD:AAA2370
Raphia frater|[61]|RDNMH394-09|United States|Arizona|658[0n]|BOLD:AAA2370
Raphia frater|[62]|CMAZA357-10|United States|Arizona|658[0n]|BOLD:AAA2370
Raphia frater|[63]|CMAZA516-10|United States|Arizona|658[0n]|BOLD:AAA2370
Raphia frater|[64]|CMAZA924-12|United States|Arizona|658[0n]|BOLD:AAA2370
Raphia frater|[65]|CMAZA938-12|United States|Arizona|658[0n]|BOLD:AAA2370
Raphia frater|[66]|CMAZA994-12|United States|Arizona|658[0n]|BOLD:AAA2370
Raphia frater|[67]|CMAZA1103-12|United States|Arizona|658[0n]|BOLD:AAA2370
Raphia frater|[68]|RDNMI167-10|United States|New Mexico|658[0n]|BOLD:AAA2370
Raphia frater|[69]|RDNMI166-10|United States|New Mexico|658[0n]|BOLD:AAA2370
Raphia frater|[70]|RDNMH396-09|United States|Arizona|658[0n]|BOLD:AAA2370
Raphia frater|[71]|RDNMH395-09|United States|Arizona|658[0n]|BOLD:AAA2370
Raphia frater|[72]|RDNMH189-09|United States|Arizona|658[0n]|BOLD:AAA2370
Raphia frater|[73]|RDNMK785-12|United States|New Mexico|658[0n]|BOLD:AAA2370
Raphia frater|[74]|RDNMK786-12|United States|New Mexico|658[0n]|BOLD:AAA2370
Raphia frater|[75]|LPSOC092-08|Canada|Ontario|658[2n]|BOLD:AAA2370
Raphia frater|[76]|LPSOB377-08|Canada|Ontario|658[0n]|BOLD:AAA2370
Raphia frater|[77]|LPSOB321-08|Canada|Ontario|658[0n]|BOLD:AAA2370
Raphia frater|[78]|LPSOB315-08|Canada|Ontario|658[0n]|BOLD:AAA2370
Raphia frater|[79]|LPSOB380-08|Canada|Ontario|647[0n]|BOLD:AAA2370
Raphia frater|[80]|LPSOB430-08|Canada|Ontario|658[0n]|BOLD:AAA2370
Raphia frater|[81]|LPSOB441-08|Canada|Ontario|658[0n]|BOLD:AAA2370
Raphia frater|[82]|LPSOB443-08|Canada|Ontario|658[0n]|BOLD:AAA2370
Raphia frater|[83]|LPSOB444-08|Canada|Ontario|658[0n]|BOLD:AAA2370
Raphia frater|[84]|LPSOB446-08|Canada|Ontario|658[0n]|BOLD:AAA2370
Raphia frater|[85]|LPSOB447-08|Canada|Ontario|658[0n]|BOLD:AAA2370
Raphia frater|[86]|LPSOB448-08|Canada|Ontario|658[0n]|BOLD:AAA2370
Raphia frater|[87]|LPSOB512-08|Canada|Ontario|658[0n]|BOLD:AAA2370
Raphia frater|[88]|LPMN043-08|Canada|Manitoba|658[0n]|BOLD:AAA2370
Raphia frater|[89]|LPMN059-08|Canada|Manitoba|658[0n]|BOLD:AAA2370
Raphia frater|[90]|LPMN062-08|Canada|Manitoba|658[0n]|BOLD:AAA2370
Raphia frater|[91]|LPMN066-08|Canada|Manitoba|658[0n]|BOLD:AAA2370
Raphia frater|[92]|LPMN078-08|Canada|Manitoba|658[0n]|BOLD:AAA2370
Raphia frater|[93]|LMDH161-11|United States|Minnesota|658[0n]|BOLD:AAA2370
Raphia frater|[94]|JMMMB185-11|United States|California|658[0n]|BOLD:AAA2370
Raphia frater|[95]|RDNMK783-12|United States|California|658[0n]|BOLD:AAA2370
Raphia frater|[96]|LOCT047-05|United States|Connecticut|658[0n]|BOLD:AAA2370
Raphia frater|[97]|LPSOD341-09|Canada|Ontario|658[0n]|BOLD:AAA2370
Raphia frater|[98]|LPSOB449-08|Canada|Ontario|658[0n]|BOLD:AAA2370
Raphia frater|[99]|LPSOD239-09|Canada|Ontario|658[0n]|BOLD:AAA2370
Raphia frater|[100]|LPSOD313-09|Canada|Ontario|658[0n]|BOLD:AAA2370

[illegible]
Raphia frater|[98]|LPSOB449-08|Canada|Ontario|658[0n]|BOLD:AAA2370
Raphia frater|[99]|LPSOD239-09|Canada|Ontario|658[0n]|BOLD:AAA2370
Raphia frater|[100]|LPSOD313-09|Canada|Ontario|658[0n]|BOLD:AAA2370
Raphia frater|[101]|BLTIB363-08|Canada|Ontario|658[0n]|BOLD:AAA2370
Raphia frater|[102]|LPSOB613-08|Canada|Ontario|658[0n]|BOLD:AAA2370
Raphia frater|[103]|LPSOB378-08|Canada|Ontario|658[0n]|BOLD:AAA2370
Raphia frater|[104]|KPOEC071-08|Canada|Ontario|658[0n]|BOLD:AAA2370
Raphia frater|[105]|TMNBB149-06|Canada|New Brunswick|658[0n]|BOLD:AAA2370
Raphia frater|[106]|RDLQB702-05|Canada|Quebec|658[0n]|BOLD:AAA2370
Raphia frater|[107]|MNBB301-05|Canada|New Brunswick|658[0n]|BOLD:AAA2370
Raphia frater|[108]|LOCT225-05|United States|Connecticut|658[0n]|BOLD:AAA2370
Raphia frater|[109]|XAE454-04|Canada|Ontario|658[0n]|BOLD:AAA2370
Raphia frater|[110]|XAC187-04|Canada|Ontario|658[0n]|BOLD:AAA2370
Raphia frater|[111]|XAB356-04|Canada|Ontario|658[0n]|BOLD:AAA2370
Raphia frater|[112]|XAE456-04|Canada|Ontario|649[0n]|BOLD:AAA2370
Raphia frater|[113]|XAD590-05|Canada|Ontario|617[0n]|BOLD:AAA2370
Raphia frater|[114]|XAB142-04|Canada|Ontario|617[0n]|BOLD:AAA2370
Raphia frater|[115]|PMG157-03|Canada|Ontario|617[0n]|BOLD:AAA2370
Raphia frater|[116]|RDNMB345-05|Canada|Ontario|605[0n]|BOLD:AAA2370
Raphia frater|[117]|LPSOD845-09|Canada|Ontario|658[0n]|BOLD:AAA2370
Raphia frater|[118]|BBLPC236-09|Canada|Nova Scotia|658[0n]|BOLD:AAA2370
Raphia frater|[119]|BBLEC635-09|Canada|Nova Scotia|658[0n]|BOLD:AAA2370
Raphia frater|[120]|BLTIB716-08|Canada|Ontario|658[0n]|BOLD:AAA2370
Raphia frater|[121]|BLTIB332-08|Canada|Ontario|658[0n]|BOLD:AAA2370
Raphia frater|[122]|TMNBB151-06|Canada|New Brunswick|658[0n]|BOLD:AAA2370
Raphia frater|[123]|TMNBB148-06|Canada|New Brunswick|658[0n]|BOLD:AAA2370
Raphia frater|[124]|TTMNB317-06|Canada|New Brunswick|658[0n]|BOLD:AAA2370
Raphia frater|[125]|TTMNB315-06|Canada|New Brunswick|658[0n]|BOLD:AAA2370
Raphia frater|[126]|MNBB324-05|Canada|New Brunswick|658[0n]|BOLD:AAA2370
Raphia frater|[127]|MNBB300-05|Canada|New Brunswick|658[0n]|BOLD:AAA2370
Raphia frater|[128]|MNBB181-05|Canada|New Brunswick|658[0n]|BOLD:AAA2370
Raphia frater|[129]|PHMNB602-04|Canada|New Brunswick|658[0n]|BOLD:AAA2370
Raphia frater|[130]|XAB596-04|Canada|Ontario|658[0n]|BOLD:AAA2370
Raphia frater|[131]|XAB567-04|Canada|Ontario|658[0n]|BOLD:AAA2370
Raphia frater|[132]|XAB321-04|Canada|Ontario|658[0n]|BOLD:AAA2370
Raphia frater|[133]|XAC288-04|Canada|Ontario|658[0n]|BOLD:AAA2370
Raphia frater|[134]|BBLEC189-09|Canada|Nova Scotia|646[0n]|BOLD:AAA2370
Raphia frater|[135]|BBLPC232-09|Canada|Nova Scotia|658[0n]|BOLD:AAA2370
Raphia frater|[136]|XAB256-04|Canada|Ontario|658[0n]|BOLD:AAA2370
Raphia frater|[137]|PHMNB771-05|Canada|New Brunswick|617[0n]|BOLD:AAA2370
Raphia frater|[138]|XAE086-04|Canada|Ontario|606[0n]|BOLD:AAA2370
Raphia frater|[139]|PHMNB045-03|Canada|New Brunswick|639[0n]|BOLD:AAA2370
Raphia frater|[140]|XAF655-05|Canada|Ontario|567[0n]|BOLD:AAA2370
Raphia frater|[141]|PHMNB153-04|Canada|New Brunswick|574[4n]|BOLD:AAA2370
Raphia frater|[142]|PHMNB101-04|Canada|New Brunswick|570[0n]|BOLD:AAA2370
Raphia frater|[143]|PHMNB156-04|Canada|New Brunswick|655[2n]|BOLD:AAA2370
Raphia frater|[144]|MNBB182-05|Canada|New Brunswick|577[0n]|BOLD:AAA2370
Raphia frater|[145]|TTMNB316-06|Canada|New Brunswick|601[0n]|BOLD:AAA2370
Raphia frater|[146]|PHMNB601-04|Canada|New Brunswick|658[0n]|BOLD:AAA2370
Raphia frater|[147]|PHMNB752-05|Canada|New Brunswick|658[0n]|BOLD:AAA2370
Raphia frater|[148]|MNBB396-05|Canada|New Brunswick|658[0n]|BOLD:AAA2370
Raphia frater|[149]|MNBB617-05|Canada|New Brunswick|658[0n]|BOLD:AAA2370
Raphia frater|[150]|BBLEC188-09|Canada|Nova Scotia|658[0n]|BOLD:AAA2370
Raphia frater|[151]|BBLPC518-09|Canada|New Brunswick|658[0n]|BOLD:AAA2370
Raphia frater|[152]|LTOLB137-08|United States|Maryland|658[0n]|BOLD:AAA2370
Raphia frater|[153]|LEFIA1337-10|United States|Maryland|670[0n]|BOLD:AAA2370
Raphia frater|[154]|RDNMC737-06|United States|Texas|582[0n]|BOLD:AAA2370
Raphia frater|[155]|RDNMK773-12|United States|Texas|658[0n]|BOLD:AAA2370
Raphia frater|[156]|RDNMK794-12|United States|Oregon|658[0n]|BOLD:AAA2370
Raphia frater|[157]|LPAB005-08|Canada|Alberta|658[0n]|BOLD:AAA2370
Raphia frater|[158]|LPABB490-08|Canada|Alberta|658[0n]|BOLD:AAA2370
Raphia frater|[159]|LPABB606-08|Canada|Alberta|658[0n]|BOLD:AAA2370
Raphia frater|[160]|LBCD284-05|Canada|British Columbia|646[0n]|BOLD:AAA2370
Raphia frater|[161]|RDNMB348-05|United States|Oregon|658[0n]|BOLD:AAA2370
Raphia frater|[162]|RDMAB252-05|Canada|Alberta|658[0n]|BOLD:AAA2370
Raphia frater|[163]|LPABB006-08|Canada|Alberta|658[0n]|BOLD:AAA2370
Raphia frater|[164]|LPABB574-08|Canada|Alberta|658[0n]|BOLD:AAA2370
Raphia frater|[165]|RDNMK797-12|United States|Washington|658[0n]|BOLD:AAA2370
Raphia frater|[166]|LBCB200-05|Canada|British Columbia|658[0n]|BOLD:AAA2370
Raphia frater|[167]|LBCA807-05|Canada|British Columbia|658[0n]|BOLD:AAA2370
Raphia frater|[168]|LBCA802-05|Canada|British Columbia|658[0n]|BOLD:AAA2370
Raphia frater|[169]|LBCC428-05|Canada|British Columbia|658[1n]|BOLD:AAA2370
Raphia frater|[170]|BBLPB777-10|Canada|British Columbia|658[0n]|BOLD:AAA2370
Raphia frater|[171]|LBCA801-05|Canada|British Columbia|658[1n]|BOLD:AAA2370
Raphia frater|[172]|LOWCC389-05|Canada|British Columbia|658[1n]|BOLD:AAA2370
Raphia frater|[173]|LPVIA594-08|Canada|British Columbia|658[0n]|BOLD:AAA2370
Raphia frater|[174]|LPVIA593-08|Canada|British Columbia|658[0n]|BOLD:AAA2370
Raphia frater|[175]|LHLEP190-06|Canada|British Columbia|658[0n]|BOLD:AAA2370
Raphia frater|[176]|LOWCC386-05|Canada|British Columbia|658[0n]|BOLD:AAA2370
Raphia frater|[177]|LOWCB144-05|Canada|British Columbia|658[0n]|BOLD:AAA2370
Raphia frater|[178]|LBCC761-05|Canada|British Columbia|658[0n]|BOLD:AAA2370
Raphia frater|[179]|LBCC005-05|Canada|British Columbia|658[0n]|BOLD:AAA2370
Raphia frater|[180]|LBCB205-05|Canada|British Columbia|658[0n]|BOLD:AAA2370
Raphia frater|[181]|LBCA803-05|Canada|British Columbia|658[0n]|BOLD:AAA2370
Raphia frater|[182]|LBCA079-05|Canada|British Columbia|658[0n]|BOLD:AAA2370
Raphia frater|[183]|LOWCC387-05|Canada|British Columbia|658[1n]|BOLD:AAA2370
Raphia frater|[184]|LOWCC392-05|Canada|British Columbia|658[1n]|BOLD:AAA2370
Raphia frater|[185]|LOWCC393-05|Canada|British Columbia|658[1n]|BOLD:AAA2370
Raphia frater|[186]|LOWCC383-05|Canada|British Columbia|658[1n]|BOLD:AAA2370
Raphia frater|[187]|LBCC453-05|Canada|British Columbia|658[1n]|BOLD:AAA2370
Raphia frater|[188]|LPABB375-08|Canada|Alberta|658[1n]|BOLD:AAA2370
Raphia frater|[189]|LALPA881-11|Canada|British Columbia|658[0n]|BOLD:AAA2370
Raphia frater|[190]|RDNMK798-12|United States|Oregon|658[0n]|BOLD:AAA2370
Raphia frater|[191]|BBLPC134-09|Canada|Nova Scotia|633[0n]|BOLD:AAA2370
Raphia frater|[192]|BBLPC159-09|Canada|Nova Scotia|658[0n]|BOLD:AAA2370
Raphia frater|[193]|LILLA035-11|United States|Illinois|658[0n]|BOLD:AAA2370
Raphia frater|[194]|BBLCU029-09|United States|Michigan|658[0n]|BOLD:AAA2370
Raphia frater|[195]|LPSOB605-08|Canada|Ontario|658[0n]|BOLD:AAA2370
Raphia frater|[196]|LPSOB318-08|Canada|Ontario|658[0n]|BOLD:AAA2370
Raphia frater|[197]|LPSOB607-08|Canada|Ontario|658[0n]|BOLD:AAA2370
Raphia frater|[198]|LPSOB612-08|Canada|Ontario|658[0n]|BOLD:AAA2370
Raphia frater|[199]|KPOEC168-08|Canada|Ontario|658[0n]|BOLD:AAA2370

Raphia frater|[197]|LPSOB607-08|Canada|Ontario|658[0n]|BOLD:AAA2370
Raphia frater|[198]|LPSOB612-08|Canada|Ontario|658[0n]|BOLD:AAA2370
Raphia frater|[199]|KPOEC168-08|Canada|Ontario|658[0n]|BOLD:AAA2370
Raphia frater|[200]|LPSOB450-08|Canada|Ontario|658[0n]|BOLD:AAA2370
Raphia frater|[201]|LPSOB445-08|Canada|Ontario|658[0n]|BOLD:AAA2370
Raphia frater|[202]|LPSOB442-08|Canada|Ontario|658[0n]|BOLD:AAA2370
Raphia frater|[203]|LPSOB312-08|Canada|Ontario|658[0n]|BOLD:AAA2370
Raphia frater|[204]|TMNBB147-06|Canada|New Brunswick|658[0n]|BOLD:AAA2370
Raphia frater|[205]|RDLQB653-05|Canada|Quebec|658[0n]|BOLD:AAA2370
Raphia frater|[206]|XAE445-04|Canada|Ontario|658[0n]|BOLD:AAA2370
Raphia frater|[207]|XAE435-04|Canada|Ontario|658[0n]|BOLD:AAA2370
Raphia frater|[208]|XAB399-04|Canada|Ontario|658[0n]|BOLD:AAA2370
Raphia frater|[209]|XAB153-04|Canada|Ontario|658[0n]|BOLD:AAA2370
Raphia frater|[210]|LPSOB319-08|Canada|Ontario|658[0n]|BOLD:AAA2370
Raphia frater|[211]|TMNBB145-06|Canada|New Brunswick|658[0n]|BOLD:AAA2370
Raphia frater|[212]|TMG111-03|Canada|Ontario|639[0n]|BOLD:AAA2370
Raphia frater|[213]|XAB108-04|Canada|Ontario|567[0n]|BOLD:AAA2370
Raphia frater|[214]|XAE458-04|Canada|Ontario|613[0n]|BOLD:AAA2370
Raphia frater|[215]|PHMNB015-03|Canada|New Brunswick|639[0n]|BOLD:AAA2370
Raphia frater|[216]|MNBB249-05|Canada|New Brunswick|576[5n]|BOLD:AAA2370
Raphia frater|[217]|MNBB325-05|Canada|New Brunswick|658[0n]|BOLD:AAA2370
Raphia frater|[218]|TMNBB146-06|Canada|New Brunswick|658[0n]|BOLD:AAA2370
Raphia frater|[219]|TMNBB150-06|Canada|New Brunswick|658[0n]|BOLD:AAA2370
Raphia frater|[220]|BBLEC626-09|Canada|Nova Scotia|658[0n]|BOLD:AAA2370
Raphia frater|[221]|BBLEC640-09|Canada|Nova Scotia|658[0n]|BOLD:AAA2370
Raphia frater|[222]|BBLPC186-09|Canada|Nova Scotia|658[0n]|BOLD:AAA2370
Raphia frater|[223]|BBLPC228-09|Canada|Nova Scotia|658[0n]|BOLD:AAA2370
Raphia frater|[224]|BBLPC245-09|Canada|Nova Scotia|658[0n]|BOLD:AAA2370
Raphia frater|[225]|BBLPC246-09|Canada|Nova Scotia|658[0n]|BOLD:AAA2370
Raphia frater|[226]|LPSO846-08|Canada|Ontario|658[0n]|BOLD:AAA2370
Raphia frater|[227]|BBLPB763-10|Canada|Saskatchewan|658[0n]|BOLD:AAA2370
Raphia frater|[228]|BBLOE1374-12|United States|Arkansas|658[0n]|BOLD:AAA2370
Raphia frater|[229]|RDNMK789-12|United States|New Mexico|658[0n]|BOLD:AAA2370
Raphia frater|[230]|RDNMK788-12|United States|New Mexico|658[0n]|BOLD:AAA2370
Raphia frater|[231]|RDNMK614-11|United States|New Mexico|658[0n]|BOLD:AAA2370
Raphia frater|[232]|BBLSY559-09|United States|New Mexico|658[0n]|BOLD:AAA2370
Raphia frater|[233]|RDNMK790-12|United States|New Mexico|658[0n]|BOLD:AAA2370
Raphia frater|[234]|RDNMK791-12|United States|New Mexico|658[0n]|BOLD:AAA2370
Raphia frater|[235]|RDNMK792-12|United States|New Mexico|658[0n]|BOLD:AAA2370
Raphia frater|[236]|LPOKA189-08|United States|Oklahoma|658[0n]|BOLD:AAA2370
Raphia frater|[237]|LPOKD687-10|United States|Oklahoma|658[0n]|BOLD:AAA2370
Raphia frater|[238]|LPOKA238-08|United States|Oklahoma|658[0n]|BOLD:AAA2370
Raphia frater|[239]|LPOKB212-09|United States|Oklahoma|658[0n]|BOLD:AAA2370
Raphia frater|[240]|RDNMH863-09|United States|Kansas|658[0n]|BOLD:AAA2370
Raphia frater|[241]|LPOKA1024-09|United States|Oklahoma|658[0n]|BOLD:AAA2370
Raphia frater|[242]|LPOKC330-09|United States|Oklahoma|658[0n]|BOLD:AAA2370
Raphia frater|[243]|RDNMH862-09|United States|Kansas|658[0n]|BOLD:AAA2370
Raphia frater|[244]|LPOKB388-09|United States|Oklahoma|658[0n]|BOLD:AAA2370
Raphia frater|[245]|LPOKC365-09|United States|Oklahoma|658[0n]|BOLD:AAA2370
Raphia frater|[246]|LPOKB130-09|United States|Oklahoma|658[0n]|BOLD:AAA2370
Raphia frater|[247]|RDNMH861-09|United States|Kansas|658[0n]|BOLD:AAA2370
Raphia frater|[248]|RDNMH864-09|United States|Kansas|658[0n]|BOLD:AAA2370
Raphia frater|[249]|RDNMK774-12|United States|Texas|658[2n]|BOLD:AAA2370
Raphia frater|[250]|LPOKB782-09|United States|Oklahoma|658[0n]|BOLD:AAA2370
Raphia frater|[251]|RDNMC736-06|United States|Oklahoma|658[0n]|BOLD:AAA2370
Raphia frater|[252]|RDNMB349-05|Canada|Alberta|578[0n]|BOLD:AAA2370
Raphia frater|[253]|BBLSX580-09|United States|Oklahoma|658[0n]|BOLD:AAA2370
Raphia frater|[254]|RDNMG083-08|United States|Colorado|658[0n]|BOLD:AAA2370
Raphia frater|[255]|LPOKB371-09|United States|Oklahoma|658[0n]|BOLD:AAA2370
Raphia frater|[256]|LPOKC739-09|United States|Oklahoma|658[0n]|BOLD:AAA2370
Raphia frater|[257]|LPOKC749-09|United States|Oklahoma|658[0n]|BOLD:AAA2370
Raphia frater|[258]|RDNMH865-09|United States|Kansas|658[0n]|BOLD:AAA2370
Raphia frater|[259]|RDNMK610-11|United States|New Mexico|658[0n]|BOLD:AAA2370
Raphia frater|[260]|RDNMK787-12|United States|Colorado|658[0n]|BOLD:AAA2370
Raphia frater|[261]|BBLSY555-09|United States|New Mexico|658[0n]|BOLD:AAA2370
Raphia frater|[262]|BBLSY564-09|United States|New Mexico|658[0n]|BOLD:AAA2370
Raphia frater|[263]|RDNMH860-09|United States|Kansas|658[0n]|BOLD:AAA2370
Raphia frater|[264]|BBLPB779-10|Canada|Saskatchewan|658[0n]|BOLD:AAA2370
Raphia frater|[265]|BBLPB780-10|Canada|Saskatchewan|658[0n]|BOLD:AAA2370
Raphia frater|[266]|LPSOD583-09|Canada|Ontario|658[0n]|BOLD:AAA2370
Raphia frater|[267]|LPSOD839-09|Canada|Ontario|658[0n]|BOLD:AAA2370
Raphia frater|[268]|BBLPB775-10|Canada|Alberta|658[0n]|BOLD:AAA2370
Raphia frater|[269]|BBLPB776-10|Canada|Alberta|658[0n]|BOLD:AAA2370
Raphia frater|[270]|BBLPB783-10|Canada|Saskatchewan|658[0n]|BOLD:AAA2370
Raphia frater|[271]|LMDH139-11|United States|Minnesota|658[0n]|BOLD:AAA2370
Raphia frater|[272]|BBLPB781-10|Canada|Saskatchewan|658[0n]|BOLD:AAA2370
Raphia frater|[273]|BBLPB782-10|Canada|Saskatchewan|658[0n]|BOLD:AAA2370
Raphia frater|[274]|LPMN329-08|Canada|Manitoba|658[0n]|BOLD:AAA2370
Raphia frater|[275]|LPMN632-08|Canada|Manitoba|658[0n]|BOLD:AAA2370
Raphia frater|[276]|LPSOD879-09|Canada|Ontario|658[0n]|BOLD:AAA2370
Raphia frater|[277]|BBLPB774-10|Canada|Alberta|658[0n]|BOLD:AAA2370
Raphia frater|[278]|LPMN324-08|Canada|Manitoba|658[0n]|BOLD:AAA2370
Raphia frater|[279]|LPMN317-08|Canada|Manitoba|658[0n]|BOLD:AAA2370
Raphia frater|[280]|LPMN273-08|Canada|Manitoba|658[0n]|BOLD:AAA2370
Raphia frater|[281]|LPMN271-08|Canada|Manitoba|658[0n]|BOLD:AAA2370
Raphia frater|[282]|LPMN038-08|Canada|Manitoba|658[0n]|BOLD:AAA2370
Raphia frater|[283]|LPMN029-08|Canada|Manitoba|658[0n]|BOLD:AAA2370
Raphia frater|[284]|RDMAB536-06|Canada|Alberta|658[0n]|BOLD:AAA2370
Raphia frater|[285]|RDMAB370-05|Canada|Alberta|658[0n]|BOLD:AAA2370
Raphia frater|[286]|RDMAB269-05|Canada|Alberta|658[0n]|BOLD:AAA2370
Raphia frater|[287]|RDMAB268-05|Canada|Alberta|658[0n]|BOLD:AAA2370
Raphia frater|[288]|RDMAB262-05|Canada|Alberta|658[0n]|BOLD:AAA2370
Raphia frater|[289]|RDMAB257-05|Canada|Alberta|658[0n]|BOLD:AAA2370
Raphia frater|[290]|LPMN348-08|Canada|Manitoba|658[0n]|BOLD:AAA2370
Raphia frater|[291]|LPABB612-08|Canada|Alberta|658[0n]|BOLD:AAA2370
Raphia frater|[292]|LPSOD465-09|Canada|Ontario|624[0n]|BOLD:AAA2370
Raphia frater|[293]|LMDH180-11|United States|Minnesota|658[0n]|BOLD:AAA2370
Raphia frater|[294]|RDNMB346-05|Canada|British Columbia|597[0n]|BOLD:AAA2370
Raphia frater|[295]|RDNMB347-05|United States|Nevada|608[0n]|BOLD:AAA2370
Raphia frater|[296]|RDNMB344-05|Canada|Alberta|605[0n]|BOLD:AAA2370
Raphia frater|[297]|LOWCC385-05|Canada|British Columbia|634[1n]|BOLD:AAA2370
Raphia frater|[298]|LOWCC390-05|Canada|British Columbia|658[1n]|BOLD:AAA2370
Raphia frater|[299]|RDNMK795-12|United States|Washington|658[0n]|BOLD:AAA2370

Raphia frater|[296]|[illegible]|Canada|Alberta|[illegible]|BOLD:AAA2370
Raphia frater|[297]|LOWCC385-05|Canada|British Columbia|634[1n]|BOLD:AAA2370
Raphia frater|[298]|LOWCC390-05|Canada|British Columbia|658[1n]|BOLD:AAA2370
Raphia frater|[299]|RDNMK795-12|United States|Washington|658[0n]|BOLD:AAA2370
Raphia frater|[300]|RDNMK796-12|United States|Washington|658[0n]|BOLD:AAA2370
Raphia frater|[301]|LBCC760-05|Canada|British Columbia|658[0n]|BOLD:AAA2370
Raphia frater|[302]|LBCH5603-10|Canada|British Columbia|658[0n]|BOLD:AAA2370
Raphia frater|[303]|RDNMK799-12|United States|Washington|658[0n]|BOLD:AAA2370
Panthea apanthea|[304]|MMNA005-08|United States|Arizona|658[0n]|BOLD:AAE7008
Panthea apanthea|[305]|CNCLB828-14|United States|Arizona|658[0n]|BOLD:AAE7008
Panthea furcilla|[306]|RDMAB193-05|United States|North Carolina|510[1n]|BOLD:AAA3812
Panthea furcilla|[307]|RDMAB209-05|United States|Pennsylvania|529[0n]|BOLD:AAA3812
Panthea acronyctoides|[308]|RDMAB206-05|United States|Wyoming|559[0n]|BOLD:AAA3812
Panthea acronyctoides|[309]|TTMNB313-06|Canada|New Brunswick|573[1n]|BOLD:AAA3812
Panthea acronyctoides|[310]|LSEU088-06|United States|North Carolina|578[0n]|BOLD:AAA3812
Panthea virginarius|[311]|LOWCC207-05|Canada|British Columbia|590[0n]|BOLD:AAA3812
Panthea virginarius|[312]|LOWCC208-05|Canada|British Columbia|595[0n]|BOLD:AAA3812
Panthea virginarius|[313]|LOWCC209-05|Canada|British Columbia|599[0n]|BOLD:AAA3812
Panthea acronyctoides|[314]|LGSM431-04|United States|Tennessee|658[0n]|BOLD:AAA3812
Panthea acronyctoides|[315]|LOT349-04|United States|Tennessee|658[0n]|BOLD:AAA3812
Panthea acronyctoides|[316]|LOT352-04|United States|Tennessee|658[0n]|BOLD:AAA3812
Panthea acronyctoides|[317]|LGSMC827-05|United States|Tennessee|658[0n]|BOLD:AAA3812
Panthea acronyctoides|[318]|LGSMC829-05|United States|Tennessee|658[0n]|BOLD:AAA3812
Panthea acronyctoides|[319]|TMG104-03|Canada|Ontario|639[0n]|BOLD:AAA3812
Panthea acronyctoides|[320]|LOT557-04|United States|Tennessee|616[0n]|BOLD:AAA3812
Panthea acronyctoides|[321]|LOT350-04|United States|Tennessee|615[0n]|BOLD:AAA3812
Panthea acronyctoides|[322]|TMNBB130-06|Canada|New Brunswick|656[0n]|BOLD:AAA3812
Panthea virginarius|[323]|LOWCC375-05|Canada|British Columbia|615[0n]|BOLD:AAA3812
Panthea acronyctoides|[324]|TTMNB021-06|Canada|New Brunswick|658[0n]|BOLD:AAA3812
Panthea acronyctoides|[325]|TTMNB022-06|Canada|New Brunswick|658[0n]|BOLD:AAA3812
Panthea acronyctoides|[326]|TTMNB084-06|Canada|New Brunswick|658[0n]|BOLD:AAA3812
Panthea acronyctoides|[327]|TMNBB125-06|Canada|New Brunswick|656[0n]|BOLD:AAA3812
Panthea virginarius|[328]|LBCB152-05|Canada|British Columbia|658[0n]|BOLD:AAA3812
Panthea virginarius|[329]|LBCC399-05|Canada|British Columbia|658[0n]|BOLD:AAA3812
Panthea acronyctoides|[330]|PHMNB031-03|Canada|New Brunswick|639[0n]|BOLD:AAA3812
Panthea virginarius|[331]|LBCA787-05|Canada|British Columbia|658[0n]|BOLD:AAA3812
Panthea virginarius|[332]|LBCA789-05|Canada|British Columbia|658[0n]|BOLD:AAA3812
Panthea acronyctoides|[333]|MNBB298-05|Canada|New Brunswick|658[0n]|BOLD:AAA3812
Panthea acronyctoides|[334]|MNBB334-05|Canada|New Brunswick|658[0n]|BOLD:AAA3812
Panthea acronyctoides|[335]|MNBB335-05|Canada|New Brunswick|658[0n]|BOLD:AAA3812
Panthea acronyctoides|[336]|MNBB457-05|Canada|New Brunswick|658[0n]|BOLD:AAA3812
Panthea virginarius|[337]|LBCB054-05|Canada|British Columbia|658[0n]|BOLD:AAA3812
Panthea virginarius|[338]|LBCB057-05|Canada|British Columbia|658[0n]|BOLD:AAA3812
Panthea virginarius|[339]|LBCB058-05|Canada|British Columbia|658[0n]|BOLD:AAA3812
Panthea virginarius|[340]|LBCB169-05|Canada|British Columbia|658[0n]|BOLD:AAA3812
Panthea virginarius|[341]|LBCB172-05|Canada|British Columbia|658[0n]|BOLD:AAA3812
Panthea virginarius|[342]|LBCB173-05|Canada|British Columbia|658[0n]|BOLD:AAA3812
Panthea virginarius|[343]|LBCB174-05|Canada|British Columbia|658[0n]|BOLD:AAA3812
Panthea virginarius|[344]|LBCB175-05|Canada|British Columbia|658[0n]|BOLD:AAA3812
Panthea virginarius|[345]|LBCB178-05|Canada|British Columbia|658[0n]|BOLD:AAA3812
Panthea virginarius|[346]|LBCC343-05|Canada|British Columbia|658[0n]|BOLD:AAA3812
Panthea virginarius|[347]|LBCC393-05|Canada|British Columbia|658[0n]|BOLD:AAA3812
Panthea virginarius|[348]|LBCC743-05|Canada|British Columbia|658[0n]|BOLD:AAA3812
Panthea virginarius|[349]|LBCC745-05|Canada|British Columbia|658[0n]|BOLD:AAA3812
Panthea acronyctoides|[350]|RDMAB191-05|Canada|Nova Scotia|658[0n]|BOLD:AAA3812
Panthea acronyctoides|[351]|RDMAB202-05|Canada|Alberta|658[0n]|BOLD:AAA3812
Panthea acronyctoides|[352]|RDMAB203-05|Canada|Alberta|658[0n]|BOLD:AAA3812
Panthea acronyctoides|[353]|RDMAB207-05|United States|Wyoming|658[0n]|BOLD:AAA3812
Panthea acronyctoides|[354]|TTMNB083-06|Canada|New Brunswick|657[0n]|BOLD:AAA3812
Panthea acronyctoides|[355]|TMNBB124-06|Canada|New Brunswick|658[0n]|BOLD:AAA3812
Panthea acronyctoides|[356]|TMNBB126-06|Canada|New Brunswick|658[0n]|BOLD:AAA3812
Panthea acronyctoides|[357]|TMNBB127-06|Canada|New Brunswick|658[0n]|BOLD:AAA3812
Panthea acronyctoides|[358]|TMNBB128-06|Canada|New Brunswick|658[0n]|BOLD:AAA3812
Panthea acronyctoides|[359]|TMNBB129-06|Canada|New Brunswick|658[0n]|BOLD:AAA3812
Panthea acronyctoides|[360]|TMNBB131-06|Canada|New Brunswick|658[0n]|BOLD:AAA3812
Panthea acronyctoides|[361]|TMNBB132-06|Canada|New Brunswick|658[0n]|BOLD:AAA3812
Panthea acronyctoides|[362]|TMNBB133-06|Canada|New Brunswick|658[0n]|BOLD:AAA3812
Panthea acronyctoides|[363]|TMNBB134-06|Canada|New Brunswick|658[0n]|BOLD:AAA3812
Panthea acronyctoides|[364]|TMNBB135-06|Canada|New Brunswick|658[0n]|BOLD:AAA3812
Panthea acronyctoides|[365]|TMNBB136-06|Canada|New Brunswick|658[0n]|BOLD:AAA3812
Panthea acronyctoides|[366]|XAK013-06|Canada|Ontario|658[0n]|BOLD:AAA3812
Panthea acronyctoides|[367]|RDLQ440-07|Canada|Quebec|658[0n]|BOLD:AAA3812
Panthea acronyctoides|[368]|LPMN341-08|Canada|Manitoba|658[0n]|BOLD:AAA3812
Panthea acronyctoides|[369]|LPMN637-08|Canada|Manitoba|658[0n]|BOLD:AAA3812
Panthea acronyctoides|[370]|LPMN726-08|Canada|Manitoba|658[0n]|BOLD:AAA3812
Panthea acronyctoides|[371]|BLTIB833-08|Canada|Ontario|658[0n]|BOLD:AAA3812
Panthea virginarius|[372]|RDNMH005-09|United States|Utah|658[0n]|BOLD:AAA3812
Panthea acronyctoides|[373]|LPSOD865-09|Canada|Ontario|658[0n]|BOLD:AAA3812
Panthea acronyctoides|[374]|BBLEC723-09|Canada|Nova Scotia|658[0n]|BOLD:AAA3812
Panthea acronyctoides|[375]|BBLPC247-09|Canada|Nova Scotia|658[0n]|BOLD:AAA3812
Panthea acronyctoides|[376]|BBLPC356-09|Canada|New Brunswick|658[0n]|BOLD:AAA3812
Panthea acronyctoides|[377]|BBLPC750-09|Canada|Newfoundland and Labrador|658[0n]|BOLD:AAA3812
Panthea acronyctoides|[378]|BBLPE022-09|Canada|Nova Scotia|658[0n]|BOLD:AAA3812
Panthea acronyctoides|[379]|BBLPE555-09|Canada|Newfoundland and Labrador|658[0n]|BOLD:AAA3812
Panthea virginarius|[380]|LBCH220-10|Canada|British Columbia|658[0n]|BOLD:AAA3812
Panthea virginarius|[381]|LBCH647-10|Canada|British Columbia|658[0n]|BOLD:AAA3812
Panthea virginarius|[382]|LBCH5556-10|Canada|British Columbia|658[0n]|BOLD:AAA3812
Panthea sp.|[383]|HKONB404-09|United States|Kentucky|658[0n]|BOLD:AAA3812
Panthea acronyctoides|[384]|LPSOD527-09|Canada|Ontario|658[0n]|BOLD:AAA3812
Panthea virginarius|[385]|LBCH5911-10|Canada|British Columbia|658[0n]|BOLD:AAA3812
Panthea virginarius|[386]|LALPA364-10|Canada|British Columbia|658[0n]|BOLD:AAA3812
Panthea virginarius|[387]|LBCA748-05|Canada|British Columbia|658[0n]|BOLD:AAA3812
Panthea virginarius|[388]|LBCA755-05|Canada|British Columbia|658[0n]|BOLD:AAA3812
Panthea virginarius|[389]|LBCA760-05|Canada|British Columbia|658[0n]|BOLD:AAA3812
Panthea virginarius|[390]|LBCA784-05|Canada|British Columbia|658[0n]|BOLD:AAA3812
Panthea acronyctoides|[391]|TMNBB122-06|Canada|New Brunswick|658[0n]|BOLD:AAA3812
Panthea acronyctoides|[392]|TMNBB123-06|Canada|New Brunswick|658[0n]|BOLD:AAA3812
Panthea acronyctoides|[393]|TTMNB314-06|Canada|New Brunswick|658[0n]|BOLD:AAA3812
Panthea acronyctoides|[394]|TMNBB121-06|Canada|New Brunswick|658[0n]|BOLD:AAA3812
Panthea acronyctoides|[395]|LGSMC828-05|United States|Tennessee|658[0n]|BOLD:AAA3812
Panthea acronyctoides|[396]|MNBB042-05|Canada|New Brunswick|658[0n]|BOLD:AAA3812
Panthea acronyctoides|[397]|PHMNB735-05|Canada|New Brunswick|658[0n]|BOLD:AAA3812
Panthea acronyctoides|[398]|PHMNB597-04|Canada|New Brunswick|658[0n]|BOLD:AAA3812

Panthea acronyctoides|[396]|MNBB042-05|Canada|New Brunswick|658[0n]|BOLD:AAA3812
Panthea acronyctoides|[397]|PHMNB735-05|Canada|New Brunswick|658[0n]|BOLD:AAA3812
Panthea acronyctoides|[398]|PHMNB597-04|Canada|New Brunswick|658[0n]|BOLD:AAA3812
Panthea acronyctoides|[399]|XAE282-04|Canada|Ontario|658[0n]|BOLD:AAA3812
Panthea acronyctoides|[400]|XAC721-04|Canada|Ontario|658[0n]|BOLD:AAA3812
Panthea acronyctoides|[401]|LOT351-04|United States|Tennessee|658[0n]|BOLD:AAA3812
Panthea acronyctoides|[402]|LOT348-04|United States|Tennessee|658[0n]|BOLD:AAA3812
Panthea acronyctoides|[403]|LGSM430-04|United States|Tennessee|658[0n]|BOLD:AAA3812
Panthea virginarius|[404]|LBCC398-05|Canada|British Columbia|658[1n]|BOLD:AAA3812
Panthea acronyctoides|[405]|BBLPB286-10|Canada|British Columbia|658[0n]|BOLD:AAA3812
Panthea acronyctoides|[406]|BBLPB285-10|Canada|Ontario|658[0n]|BOLD:AAA3812
Panthea acronyctoides|[407]|BBLPB647-10|Canada|Ontario|658[0n]|BOLD:AAA3812
Panthea virginarius|[408]|RDNMJ378-11|United States|California|658[0n]|BOLD:AAA3812
Panthea virginarius|[409]|LALPA970-11|Canada|British Columbia|658[0n]|BOLD:AAA3812
Panthea furcilla|[410]|XAG490-05|Canada|Ontario|535[0n]|BOLD:AAA3812
Panthea virginarius|[411]|LOWCC378-05|Canada|British Columbia|502[0n]|BOLD:AAA3812
Panthea virginarius|[412]|LOWCC376-05|Canada|British Columbia|564[0n]|BOLD:AAA3812
Panthea virginarius|[413]|LOWCC381-05|Canada|British Columbia|557[0n]|BOLD:AAA3812
Panthea virginarius|[414]|LBCA781-05|Canada|British Columbia|537[0n]|BOLD:AAA3812
Panthea virginarius|[415]|LOWCC382-05|Canada|British Columbia|550[0n]|BOLD:AAA3812
Panthea furcilla|[416]|RDMAB196-05|Canada|Alberta|571[1n]|BOLD:AAA3812
Panthea furcilla|[417]|RDMAB087-05|United States|Florida|553[0n]|BOLD:AAA3812
Panthea furcilla|[418]|XAB075-04|Canada|Ontario|605[0n]|BOLD:AAA3812
Panthea furcilla|[419]|LNCB529-07|United States|North Carolina|656[0n]|BOLD:AAA3812
Panthea furcilla|[420]|LNCB459-07|United States|North Carolina|658[0n]|BOLD:AAA3812
Panthea furcilla|[421]|RDNMD483-06|United States|Florida|658[0n]|BOLD:AAA3812
Panthea furcilla|[422]|LNC079-05|United States|North Carolina|658[0n]|BOLD:AAA3812
Panthea furcilla|[423]|RDMAB088-05|United States|Florida|658[0n]|BOLD:AAA3812
Panthea furcilla|[424]|RDNMD482-06|United States|Florida|658[2n]|BOLD:AAA3812
Panthea furcilla|[425]|HKONS517-08|United States|Florida|658[2n]|BOLD:AAA3812
Panthea furcilla|[426]|HKONS518-08|United States|Florida|658[0n]|BOLD:AAA3812
Panthea furcilla|[427]|LOTB495-05|United States|Tennessee|658[0n]|BOLD:AAA3812
Panthea furcilla|[428]|LOTB494-05|United States|Tennessee|658[0n]|BOLD:AAA3812
Panthea furcilla|[429]|LOTB493-05|United States|Tennessee|658[0n]|BOLD:AAA3812
Panthea furcilla|[430]|LOTB347-05|United States|Tennessee|658[0n]|BOLD:AAA3812
Panthea furcilla|[431]|LGSMC836-05|United States|Tennessee|658[0n]|BOLD:AAA3812
Panthea furcilla|[432]|LOTB496-05|United States|Tennessee|658[2n]|BOLD:AAA3812
Panthea furcilla|[433]|LOTB497-05|United States|Tennessee|658[0n]|BOLD:AAA3812
Panthea furcilla|[434]|XAB111-04|Canada|Ontario|658[0n]|BOLD:AAA3812
Panthea furcilla|[435]|XAK002-06|Canada|Ontario|632[0n]|BOLD:AAA3812
Panthea furcilla|[436]|RDMAB194-05|United States|Georgia|658[0n]|BOLD:AAA3812
Panthea furcilla|[437]|KPOEC143-08|Canada|Ontario|658[0n]|BOLD:AAA3812
Panthea furcilla|[438]|XAJ972-06|Canada|Ontario|658[0n]|BOLD:AAA3812
Panthea furcilla|[439]|TMNBB139-06|Canada|New Brunswick|658[0n]|BOLD:AAA3812
Panthea furcilla|[440]|XAG094-05|Canada|Ontario|658[0n]|BOLD:AAA3812
Panthea furcilla|[441]|MNBB072-05|Canada|New Brunswick|658[0n]|BOLD:AAA3812
Panthea furcilla|[442]|XAE462-04|Canada|Ontario|658[0n]|BOLD:AAA3812
Panthea furcilla|[443]|XAC044-04|Canada|Ontario|658[0n]|BOLD:AAA3812
Panthea furcilla|[444]|XAB209-04|Canada|Ontario|658[0n]|BOLD:AAA3812
Panthea furcilla|[445]|XAB181-04|Canada|Ontario|658[0n]|BOLD:AAA3812
Panthea furcilla|[446]|XAB083-04|Canada|Ontario|658[0n]|BOLD:AAA3812
Panthea furcilla|[447]|XAD240-04|Canada|Ontario|658[0n]|BOLD:AAA3812
Panthea furcilla|[448]|TMG105-03|Canada|Ontario|639[0n]|BOLD:AAA3812
Panthea furcilla|[449]|TMG106-03|Canada|Ontario|639[0n]|BOLD:AAA3812
Panthea furcilla|[450]|BLTIB585-08|Canada|Ontario|639[0n]|BOLD:AAA3812
Panthea furcilla|[451]|BBLCU011-09|United States|Michigan|658[0n]|BOLD:AAA3812
Panthea furcilla|[452]|BBLCU301-09|United States|Michigan|658[0n]|BOLD:AAA3812
Panthea furcilla|[453]|BBLCU302-09|United States|Michigan|658[0n]|BOLD:AAA3812
Panthea virginarius|[454]|LBCA761-05|Canada|British Columbia|549[0n]|BOLD:AAA3812
Panthea acronyctoides|[455]|RDMAB204-05|Canada|Alberta|580[0n]|BOLD:AAA3812
Panthea virginarius|[456]|LBCC380-05|Canada|British Columbia|516[0n]|BOLD:AAA3812
Panthea virginarius|[457]|LOWCC374-05|Canada|British Columbia|584[1n]|BOLD:AAA3812
Panthea virginarius|[458]|LOWCC379-05|Canada|British Columbia|557[0n]|BOLD:AAA3812
Panthea furcilla|[459]|RDMAB188-05|Canada|Nova Scotia|583[0n]|BOLD:AAA3812
Panthea furcilla|[460]|XAC858-04|Canada|Ontario|594[0n]|BOLD:AAA3812
Panthea furcilla|[461]|RDMAB197-05|Canada|Alberta|628[0n]|BOLD:AAA3812
Panthea furcilla|[462]|TMNBB140-06|Canada|New Brunswick|658[0n]|BOLD:AAA3812
Panthea furcilla|[463]|TMNBB141-06|Canada|New Brunswick|658[0n]|BOLD:AAA3812
Panthea furcilla|[464]|TMNBB144-06|Canada|New Brunswick|656[0n]|BOLD:AAA3812
Panthea furcilla|[465]|XAK417-06|Canada|Ontario|658[0n]|BOLD:AAA3812
Panthea furcilla|[466]|TMNBB142-06|Canada|New Brunswick|658[0n]|BOLD:AAA3812
Panthea furcilla|[467]|TMNBB138-06|Canada|New Brunswick|658[0n]|BOLD:AAA3812
Panthea furcilla|[468]|TTMNB045-06|Canada|New Brunswick|658[0n]|BOLD:AAA3812
Panthea furcilla|[469]|RDMAB198-05|Canada|Alberta|658[0n]|BOLD:AAA3812
Panthea furcilla|[470]|RDMAB195-05|Canada|Alberta|658[0n]|BOLD:AAA3812
Panthea furcilla|[471]|RDMAB190-05|Canada|Nova Scotia|658[0n]|BOLD:AAA3812
Panthea furcilla|[472]|RDMAB189-05|Canada|Nova Scotia|658[0n]|BOLD:AAA3812
Panthea furcilla|[473]|MNBB199-05|Canada|New Brunswick|658[0n]|BOLD:AAA3812
Panthea furcilla|[474]|MNBB646-05|Canada|New Brunswick|658[0n]|BOLD:AAA3812
Panthea furcilla|[475]|MNBB041-05|Canada|New Brunswick|658[0n]|BOLD:AAA3812
Panthea furcilla|[476]|XAC071-04|Canada|Ontario|658[0n]|BOLD:AAA3812
Panthea furcilla|[477]|MNBB333-05|Canada|New Brunswick|658[0n]|BOLD:AAA3812
Panthea furcilla|[478]|PMG148-03|Canada|Ontario|617[0n]|BOLD:AAA3812
Panthea furcilla|[479]|XAD576-04|Canada|Ontario|614[0n]|BOLD:AAA3812
Panthea furcilla|[480]|MNBB332-05|Canada|New Brunswick|658[0n]|BOLD:AAA3812
Panthea furcilla|[481]|RDLQB092-05|Canada|Quebec|590[0n]|BOLD:AAA3812
Panthea furcilla|[482]|LPABC037-09|Canada|Alberta|634[0n]|BOLD:AAA3812
Panthea furcilla|[483]|TMNBB137-06|Canada|New Brunswick|658[0n]|BOLD:AAA3812
Panthea furcilla|[484]|TMNBB143-06|Canada|New Brunswick|658[0n]|BOLD:AAA3812
Panthea furcilla|[485]|LPABC082-09|Canada|Alberta|658[1n]|BOLD:AAA3812
Panthea furcilla|[486]|BBLEC178-09|Canada|Nova Scotia|658[0n]|BOLD:AAA3812
Panthea furcilla|[487]|BBLEC200-09|Canada|Nova Scotia|658[0n]|BOLD:AAA3812
Panthea greyi|[488]|RDMAB729-06|United States|Arizona|556[0n]|BOLD:AAA3812
Panthea greyi|[489]|RDMAB728-06|United States|Arizona|658[0n]|BOLD:AAA3812
Panthea greyi|[490]|MMNA008-08|United States|Arizona|658[0n]|BOLD:AAA3812
Panthea virginarius|[491]|LPABC902-09|Canada|Alberta|658[0n]|BOLD:AAA3812
Panthea virginarius|[492]|LPMNB550-09|Canada|Alberta|658[0n]|BOLD:AAA3812
Panthea virginarius|[493]|LBCB887-05|Canada|British Columbia|617[0n]|BOLD:AAA3812
Panthea virginarius|[494]|LBCA431-05|Canada|British Columbia|632[0n]|BOLD:AAA3812
Panthea virginarius|[495]|LBCA590-05|Canada|British Columbia|645[0n]|BOLD:AAA3812
Panthea virginarius|[496]|LBCA591-05|Canada|British Columbia|654[0n]|BOLD:AAA3812
Panthea virginarius|[497]|LBCD262-05|Canada|British Columbia|656[0n]|BOLD:AAA3812
Panthea virginarius|[498]|[illegible]

Panthea virginarius|[495]|LBCA590-05|Canada|British Columbia|643[0n]|BOLD:AAA3812
Panthea virginarius|[496]|LBCA591-05|Canada|British Columbia|654[0n]|BOLD:AAA3812
Panthea virginarius|[497]|LBCD262-05|Canada|British Columbia|656[0n]|BOLD:AAA3812
Panthea virginarius|[498]|LBCC912-05|Canada|British Columbia|658[0n]|BOLD:AAA3812
Panthea virginarius|[499]|LBCD255-05|Canada|British Columbia|658[0n]|BOLD:AAA3812
Panthea virginarius|[500]|LBCD256-05|Canada|British Columbia|646[0n]|BOLD:AAA3812
Panthea virginarius|[501]|BBLPB649-10|Canada|British Columbia|658[0n]|BOLD:AAA3812
Panthea virginarius|[502]|LALPA376-10|Canada|British Columbia|658[0n]|BOLD:AAA3812
Panthea virginarius|[503]|LALPA354-10|Canada|British Columbia|658[0n]|BOLD:AAA3812
Panthea virginarius|[504]|RWWB628-10|United States|Washington|658[0n]|BOLD:AAA3812
Panthea virginarius|[505]|LBCH5692-10|Canada|British Columbia|658[0n]|BOLD:AAA3812
Panthea virginarius|[506]|LBCH5208-10|Canada|British Columbia|658[0n]|BOLD:AAA3812
Panthea virginarius|[507]|LBCH807-10|Canada|British Columbia|658[0n]|BOLD:AAA3812
Panthea virginarius|[508]|LBCH486-10|Canada|British Columbia|658[0n]|BOLD:AAA3812
Panthea virginarius|[509]|LBCH115-10|Canada|British Columbia|658[0n]|BOLD:AAA3812
Panthea virginarius|[510]|LBCH112-10|Canada|British Columbia|658[0n]|BOLD:AAA3812
Panthea virginarius|[511]|LBCH012-10|Canada|British Columbia|658[0n]|BOLD:AAA3812
Panthea virginarius|[512]|RWWA714-09|United States|Washington|658[0n]|BOLD:AAA3812
Panthea virginarius|[513]|RWWA432-09|United States|Washington|658[0n]|BOLD:AAA3812
Panthea virginarius|[514]|RWWA395-09|United States|Washington|658[0n]|BOLD:AAA3812
Panthea virginarius|[515]|RWWA289-09|United States|Washington|658[0n]|BOLD:AAA3812
Panthea virginarius|[516]|RWWA285-09|United States|Washington|658[0n]|BOLD:AAA3812
Panthea virginarius|[517]|RWWA256-09|United States|Washington|658[0n]|BOLD:AAA3812
Panthea virginarius|[518]|RWWA154-09|United States|Washington|658[0n]|BOLD:AAA3812
Panthea virginarius|[519]|LPABC919-09|Canada|Alberta|658[0n]|BOLD:AAA3812
Panthea virginarius|[520]|LPABC340-09|Canada|Alberta|658[0n]|BOLD:AAA3812
Panthea virginarius|[521]|LPABC083-09|Canada|Alberta|658[0n]|BOLD:AAA3812
Panthea virginarius|[522]|LPABC033-09|Canada|Alberta|658[0n]|BOLD:AAA3812
Panthea virginarius|[523]|LPABB317-08|Canada|Alberta|658[0n]|BOLD:AAA3812
Panthea virginarius|[524]|DUNLP180-08|Canada|British Columbia|658[0n]|BOLD:AAA3812
Panthea virginarius|[525]|CDIBC006-07|Canada|British Columbia|658[0n]|BOLD:AAA3812
Panthea virginarius|[526]|LMH045-06|Canada|British Columbia|656[0n]|BOLD:AAA3812
Panthea virginarius|[527]|RDMAB205-05|Canada|Alberta|658[0n]|BOLD:AAA3812
Panthea virginarius|[528]|RDMAB201-05|Canada|Alberta|658[0n]|BOLD:AAA3812
Panthea virginarius|[529]|RDMAB200-05|Canada|Alberta|658[0n]|BOLD:AAA3812
Panthea virginarius|[530]|RDMAB199-05|Canada|Alberta|658[0n]|BOLD:AAA3812
Panthea virginarius|[531]|RDMAB192-05|United States|California|658[0n]|BOLD:AAA3812
Panthea virginarius|[532]|LOWCC372-05|Canada|British Columbia|658[0n]|BOLD:AAA3812
Panthea virginarius|[533]|LBCD260-05|Canada|British Columbia|658[0n]|BOLD:AAA3812
Panthea virginarius|[534]|LBCD259-05|Canada|British Columbia|658[0n]|BOLD:AAA3812
Panthea virginarius|[535]|LBCD257-05|Canada|British Columbia|658[0n]|BOLD:AAA3812
Panthea virginarius|[536]|LBCD254-05|Canada|British Columbia|658[0n]|BOLD:AAA3812
Panthea virginarius|[537]|LBCC915-05|Canada|British Columbia|658[0n]|BOLD:AAA3812
Panthea virginarius|[538]|LBCC914-05|Canada|British Columbia|658[0n]|BOLD:AAA3812
Panthea virginarius|[539]|LBCC742-05|Canada|British Columbia|658[0n]|BOLD:AAA3812
Panthea virginarius|[540]|LBCC732-05|Canada|British Columbia|658[0n]|BOLD:AAA3812
Panthea virginarius|[541]|LBCC403-05|Canada|British Columbia|658[0n]|BOLD:AAA3812
Panthea virginarius|[542]|LBCC402-05|Canada|British Columbia|658[0n]|BOLD:AAA3812
Panthea virginarius|[543]|LBCC401-05|Canada|British Columbia|658[0n]|BOLD:AAA3812
Panthea virginarius|[544]|LBCC400-05|Canada|British Columbia|658[0n]|BOLD:AAA3812
Panthea virginarius|[545]|LBCC397-05|Canada|British Columbia|658[0n]|BOLD:AAA3812
Panthea virginarius|[546]|LBCC396-05|Canada|British Columbia|658[0n]|BOLD:AAA3812
Panthea virginarius|[547]|LBCC394-05|Canada|British Columbia|658[0n]|BOLD:AAA3812
Panthea virginarius|[548]|LBCB886-05|Canada|British Columbia|658[0n]|BOLD:AAA3812
Panthea virginarius|[549]|LBCB880-05|Canada|British Columbia|658[0n]|BOLD:AAA3812
Panthea virginarius|[550]|LBCB171-05|Canada|British Columbia|658[0n]|BOLD:AAA3812
Panthea virginarius|[551]|LBCB168-05|Canada|British Columbia|658[0n]|BOLD:AAA3812
Panthea virginarius|[552]|LBCB167-05|Canada|British Columbia|658[0n]|BOLD:AAA3812
Panthea virginarius|[553]|LBCB151-05|Canada|British Columbia|658[0n]|BOLD:AAA3812
Panthea virginarius|[554]|LBCB150-05|Canada|British Columbia|658[0n]|BOLD:AAA3812
Panthea virginarius|[555]|LBCB056-05|Canada|British Columbia|658[0n]|BOLD:AAA3812
Panthea virginarius|[556]|LBCB055-05|Canada|British Columbia|658[0n]|BOLD:AAA3812
Panthea virginarius|[557]|LBCB053-05|Canada|British Columbia|658[0n]|BOLD:AAA3812
Panthea virginarius|[558]|LBCB052-05|Canada|British Columbia|658[0n]|BOLD:AAA3812
Panthea virginarius|[559]|LBCB051-05|Canada|British Columbia|658[0n]|BOLD:AAA3812
Panthea virginarius|[560]|LBCB050-05|Canada|British Columbia|658[0n]|BOLD:AAA3812
Panthea virginarius|[561]|LBCB049-05|Canada|British Columbia|658[0n]|BOLD:AAA3812
Panthea virginarius|[562]|LBCA796-05|Canada|British Columbia|658[0n]|BOLD:AAA3812
Panthea virginarius|[563]|LBCA788-05|Canada|British Columbia|658[0n]|BOLD:AAA3812
Panthea virginarius|[564]|LBCA786-05|Canada|British Columbia|658[0n]|BOLD:AAA3812
Panthea virginarius|[565]|LBCA785-05|Canada|British Columbia|658[0n]|BOLD:AAA3812
Panthea virginarius|[566]|LBCA783-05|Canada|British Columbia|658[0n]|BOLD:AAA3812
Panthea virginarius|[567]|LBCA782-05|Canada|British Columbia|658[0n]|BOLD:AAA3812
Panthea virginarius|[568]|LBCA763-05|Canada|British Columbia|658[0n]|BOLD:AAA3812
Panthea virginarius|[569]|LBCA762-05|Canada|British Columbia|658[0n]|BOLD:AAA3812
Panthea virginarius|[570]|LBCA759-05|Canada|British Columbia|658[0n]|BOLD:AAA3812
Panthea virginarius|[571]|LBCA758-05|Canada|British Columbia|658[0n]|BOLD:AAA3812
Panthea virginarius|[572]|LBCC744-05|Canada|British Columbia|658[0n]|BOLD:AAA3812
Panthea virginarius|[573]|LBCA749-05|Canada|British Columbia|658[0n]|BOLD:AAA3812
Panthea virginarius|[574]|LBCA211-05|Canada|British Columbia|658[0n]|BOLD:AAA3812
Panthea virginarius|[575]|LBCA076-05|Canada|British Columbia|658[0n]|BOLD:AAA3812
Panthea virginarius|[576]|LBCA074-05|Canada|British Columbia|658[0n]|BOLD:AAA3812
Panthea virginarius|[577]|RWWA682-09|United States|Washington|658[0n]|BOLD:AAA3812
Panthea virginarius|[578]|LBCA757-05|Canada|British Columbia|644[0n]|BOLD:AAA3812
Panthea virginarius|[579]|LOWCC380-05|Canada|British Columbia|639[0n]|BOLD:AAA3812
Panthea virginarius|[580]|LOWCC373-05|Canada|British Columbia|582[0n]|BOLD:AAA3812
Panthea virginarius|[581]|LMH022-06|Canada|British Columbia|649[1n]|BOLD:AAA3812
Panthea virginarius|[582]|LBCC754-05|Canada|British Columbia|616[0n]|BOLD:AAA3812
Panthea virginarius|[583]|JMMMB370-11|United States|California|658[0n]|BOLD:AAA3812
Panthea virginarius|[584]|LALPA828-11|Canada|British Columbia|658[0n]|BOLD:AAA3812
Panthea virginarius|[585]|LALPA898-11|Canada|British Columbia|658[0n]|BOLD:AAA3812
Panthea virginarius|[586]|LALPA1181-11|Canada|British Columbia|658[0n]|BOLD:AAA3812
Panthea virginarius|[587]|GMNCN006-13|United States|Washington|613[0n]|BOLD:AAA3812
Panthea gigantea|[588]|DUNLP179-08|Canada|British Columbia|633[1n]|BOLD:ACE5933
Panthea gigantea|[589]|RDNMG588-08|Canada|British Columbia|658[0n]|BOLD:ACE5933
Panthea gigantea|[590]|RDNMH006-09|United States|Idaho|658[0n]|BOLD:ACE5933
Panthea gigantea|[591]|RDNMK696-11|United States|Utah|658[0n]|BOLD:AAD3314
Panthea gigantea|[592]|RDNMK695-11|United States|California|658[0n]|BOLD:AAD3314
Panthea gigantea|[593]|RDNMK670-11|United States|California|658[0n]|BOLD:AAD3314
Panthea gigantea|[594]|RDNMK669-11|United States|California|658[0n]|BOLD:AAD3314
Panthea gigantea|[595]|RDNMK666-11|United States||658[0n]|BOLD:AAD3314
Panthea gigantea|[596]|RDNMH007-09|United States|California|658[0n]|BOLD:AAD3314
Panthea gigantea|[597]|RDNMK697-11|United States|Wyoming|658[0n]|BOLD:AAD3314

Panthea gigantea|[595]|RDNMK666-11|United States||658[0n]|BOLD:AAD3314
Panthea gigantea|[596]|RDNMH007-09|United States|California|658[0n]|BOLD:AAD3314
Panthea gigantea|[597]|RDNMK697-11|United States|Wyoming|658[0n]|BOLD:AAD3314
Panthea gigantea|[598]|RDNMK693-11|United States|Utah|658[0n]|BOLD:AAD3314
Panthea gigantea|[599]|RDNMH009-09|United States|New Mexico|658[0n]|BOLD:AAD3314
Panthea gigantea|[600]|RDNMH010-09|United States|New Mexico|658[0n]|BOLD:AAD3314
Panthea gigantea|[601]|RDNMH011-09|United States|New Mexico|658[0n]|BOLD:AAD3314
Panthea gigantea|[602]|RDMAB730-06|United States|Arizona|658[0n]|BOLD:AAD3314
Panthea gigantea|[603]|RDNMH001-09|United States|Arizona|649[0n]|BOLD:AAD3314
Panthea gigantea|[604]|RDNMK694-11|United States|Arizona|658[0n]|BOLD:AAD3314
Panthea gigantea|[605]|RDNMH008-09|United States|Arizona|658[0n]|BOLD:AAD3314
Panthea gigantea|[606]|CMAZA347-10|United States|Arizona|658[0n]|BOLD:AAD3314
Panthea gigantea|[607]|CMAZA883-12|United States|Arizona|658[0n]|BOLD:AAD3314
Panthea judyae|[608]|AWCLB564-11|United States|Arizona|658[0n]|BOLD:AAE7009
Panthea judyae|[609]|CNCLB829-14|United States|Arizona|658[0n]|BOLD:AAE7009
Panthea judyae|[610]|MMNA007-08|United States|Arizona|658[0n]|BOLD:AAE7009
Panthea judyae|[611]|RDNMH004-09|United States|Arizona|658[0n]|BOLD:AAE7009
Panthea judyae|[612]|CNCLB830-14|United States|Arizona|658[0n]|BOLD:AAE7009
Panthea judyae|[613]|MMNA006-08|United States|New Mexico|658[0n]|BOLD:AAE7009
Panthea judyae|[614]|RDNMH003-09|United States|New Mexico|658[0n]|BOLD:AAE7009
Panthea judyae|[615]|CNCLB832-14|United States|New Mexico|658[0n]|BOLD:AAE7009
Charadra dispulsa|[616]|RDNME194-07|United States|Texas|617[3n]|BOLD:AAW6849
Charadra dispulsa|[617]|RDNMK761-12|United States|Texas|658[0n]|BOLD:AAW6849
Charadra dispulsa|[618]|RDNMK762-12|United States|Texas|658[0n]|BOLD:AAW6849
Charadra dispulsa|[619]|RDNMK775-12|United States|Texas|658[0n]|BOLD:AAW6849
Charadra moneta|[620]|RDNMH853-09|United States|New Mexico|658[0n]|BOLD:ABY4848
Charadra moneta|[621]|RDNMH854-09|United States|New Mexico|658[0n]|BOLD:ABY4848
Charadra moneta|[622]|RDNMH855-09|United States|New Mexico|627[0n]|BOLD:ABY4848
Charadra moneta|[623]|PSAT142-10|United States|New Mexico|658[0n]|BOLD:ABY4848
Charadra deridens|[624]|RDNMC590-06|Canada|Ontario|656[0n]|BOLD:AAB0661
Charadra deridens|[625]|PHMNB758-05|Canada|New Brunswick|658[0n]|BOLD:AAB0661
Charadra deridens|[626]|MNBB283-05|Canada|New Brunswick|658[0n]|BOLD:AAB0661
Charadra deridens|[627]|LSUSA185-06|United States|Kentucky|658[0n]|BOLD:AAB0661
Charadra deridens|[628]|RDLQF852-06|Canada|Quebec|658[0n]|BOLD:AAB0661
Charadra deridens|[629]|LPSOD311-09|Canada|Ontario|658[0n]|BOLD:AAB0661
Charadra deridens|[630]|BBLCU303-09|United States|Michigan|658[0n]|BOLD:AAB0661
Charadra deridens|[631]|LOT185-04|United States|Tennessee|609[0n]|BOLD:AAB0661
Charadra deridens|[632]|MMNA124-08|United States|North Carolina|638[0n]|BOLD:AAB0661
Charadra deridens|[633]|LPSO864-08|Canada|Ontario|658[0n]|BOLD:AAB0661
Charadra deridens|[634]|MILEP373-10|United States|Alabama|658[0n]|BOLD:AAB0661
Charadra deridens|[635]|LNCC339-10|United States|North Carolina|658[0n]|BOLD:AAB0661
Charadra deridens|[636]|XAE353-04|Canada|Ontario|658[0n]|BOLD:AAB0661
Charadra deridens|[637]|TMG110-03|Canada|Ontario|639[0n]|BOLD:AAB0661
Charadra deridens|[638]|LSEU086-06|United States|Florida|549[0n]|BOLD:AAB0661
Charadra deridens|[639]|BBLWU095-09|United States|Colorado|658[0n]|BOLD:AAB0661
Charadra deridens|[640]|RDNMH859-09|United States|Kansas|658[0n]|BOLD:AAB0661
Charadra deridens|[641]|NCCH111-11|Canada|Ontario|658[0n]|BOLD:AAB0661
Charadra deridens|[642]|LGSM625-04|United States|North Carolina|609[0n]|BOLD:AAB0661
Charadra deridens|[643]|LGSM626-04|United States|North Carolina|609[0n]|BOLD:AAB0661
Charadra deridens|[644]|MNBB070-05|Canada|New Brunswick|658[0n]|BOLD:AAB0661
Charadra deridens|[645]|RDNMH856-09|United States|Kansas|658[0n]|BOLD:AAB0661
Charadra deridens|[646]|RDNMH857-09|United States|Kansas|658[0n]|BOLD:AAB0661
Charadra deridens|[647]|LOTB491-05|United States|Tennessee|658[2n]|BOLD:AAB0661
Charadra deridens|[648]|LPOKD719-10|United States|Oklahoma|658[0n]|BOLD:AAB0661
Charadra deridens|[649]|LOTB492-05|United States|Tennessee|658[0n]|BOLD:AAB0661
Charadra deridens|[650]|LOTB489-05|United States|Tennessee|658[0n]|BOLD:AAB0661
Charadra deridens|[651]|XAE410-04|Canada|Ontario|658[0n]|BOLD:AAB0661
Charadra deridens|[652]|TMG109-03|Canada|Ontario|639[0n]|BOLD:AAB0661
Charadra deridens|[653]|TMG108-03|Canada|Ontario|639[0n]|BOLD:AAB0661
Charadra deridens|[654]|PMG101-03|Canada|Ontario|617[0n]|BOLD:AAB0661
Charadra deridens|[655]|LOTB490-05|United States|Tennessee|614[2n]|BOLD:AAB0661
Charadra deridens|[656]|RDLQB560-05|Canada|Quebec|658[0n]|BOLD:AAB0661
Charadra deridens|[657]|LSEU087-06|United States|North Carolina|658[0n]|BOLD:AAB0661
Charadra deridens|[658]|LSUSA008-06|United States|Kentucky|658[0n]|BOLD:AAB0661
Charadra deridens|[659]|LGSMG643-07|United States|North Carolina|658[0n]|BOLD:AAB0661
Charadra deridens|[660]|LPSO337-08|Canada|Ontario|658[0n]|BOLD:AAB0661
Charadra deridens|[661]|LPSOC084-08|Canada|Ontario|658[0n]|BOLD:AAB0661
Charadra deridens|[662]|RDNMH858-09|United States|Kansas|658[0n]|BOLD:AAB0661
Charadra deridens|[663]|BBLOC1666-11|United States|Texas|658[0n]|BOLD:AAB0661
Charadra deridens|[664]|LNCC1673-13|United States|North Carolina|658[0n]|BOLD:AAB0661
Pseudopanthea palata|[665]|CMAZA312-10|United States|Arizona|658[0n]|BOLD:AAH8209
Pseudopanthea palata|[666]|CMAZA401-10|United States|Arizona|658[0n]|BOLD:AAH8209
Pseudopanthea palata|[667]|RDNMJ520-11|United States|Arizona|658[0n]|BOLD:AAH8209
Pseudopanthea palata|[668]|RDNMJ546-11|United States|Arizona|658[0n]|BOLD:AAH8209
Pseudopanthea palata|[669]|RDNMJ673-11|United States|Arizona|658[0n]|BOLD:AAH8209
Pseudopanthea palata|[670]|IAWLB620-11|United States|Arizona|658[0n]|BOLD:AAH8209
Pseudopanthea palata|[671]|NAMUM089-08|United States|Arizona|658[0n]|BOLD:AAH8209
Pseudopanthea palata|[672]|CMAZA328-10|United States|Arizona|658[0n]|BOLD:AAH8209
Pseudopanthea palata|[673]|CMAZA891-12|United States|Arizona|658[0n]|BOLD:AAH8209
Pseudopanthea palata|[674]|RDNME984-08|United States|Texas|658[0n]|BOLD:ACF0914
Pseudopanthea palata|[675]|RDNMK776-12|United States|Texas|658[0n]|BOLD:ACF0914
Pseudopanthea palata|[676]|RDNMK777-12|United States|Texas|658[0n]|BOLD:ACF0914
Charadra tapa|[677]|RDNMK678-11|Mexico|Sonora|658[0n]|BOLD:AAI4186
Charadra sp.|[678]|RDNML022-13|Mexico|Baja California Sur|658[0n]|BOLD:AAI4186
Charadra franclemonti|[679]|LNAUS5587-13|United States|Arizona|638[0n]|BOLD:AAI4186
Charadra franclemonti|[680]|LNAUS5589-13|United States|Arizona|658[0n]|BOLD:AAI4186
Charadra franclemonti|[681]|LNAUS5590-13|United States|Arizona|638[0n]|BOLD:AAI4186
Charadra franclemonti|[682]|NAMUM049-08|United States|Arizona|657[0n]|BOLD:AAI4186
Charadra franclemonti|[683]|LNAUS5588-13|United States|Arizona|658[0n]|BOLD:AAI4186
Charadra franclemonti|[684]|RDNMH443-09|United States|Arizona|658[0n]|BOLD:AAI4186
Charadra franclemonti|[685]|RDNMH442-09|United States|Arizona|658[0n]|BOLD:AAI4186
Charadra franclemonti|[686]|RDNMH441-09|United States|Arizona|658[0n]|BOLD:AAI4186
Charadra franclemonti|[687]|RDNMH444-09|United States|Arizona|658[0n]|BOLD:AAI4186
Charadra franclemonti|[688]|RDNMH445-09|United States|Arizona|658[0n]|BOLD:AAI4186
Charadra franclemonti|[689]|RDNMJ685-11|United States|Arizona|658[0n]|BOLD:AAI4186
Charadra franclemonti|[690]|CNCLB773-14|United States|Arizona|658[0n]|BOLD:AAI4186
Charadra franclemonti|[691]|CNCLB774-14|United States|Arizona|658[0n]|BOLD:AAI4186
Charadra franclemonti|[692]|CNCLB775-14|United States|Arizona|658[0n]|BOLD:AAI4186
Charadra franclemonti|[693]|CNCLB776-14|United States|Arizona|658[0n]|BOLD:AAI4186
Charadra franclemonti|[694]|CNCLB777-14|United States|Arizona|658[0n]|BOLD:AAI4186
Colocasia flavicornis|[695]|RDNMB342-05|Canada|Ontario|597[0n]|BOLD:ACF0763
Colocasia flavicornis|[696]|RDNMB341-05|Canada|Ontario|585[0n]|BOLD:ACF0763
Colocasia flavicornis|[697]|[illegible]|Canada|New Brunswick|658[0n]|BOLD:ACF0763

Charadra franclemonti|[694]|CNCLB777-14|United States|Arizona|658[0n]|BOLD:AAI4186
Colocasia flavicornis|[695]|RDNMB342-05|Canada|Ontario|597[0n]|BOLD:ACF0763
Colocasia flavicornis|[696]|RDNMB341-05|Canada|Ontario|585[0n]|BOLD:ACF0763
Colocasia flavicornis|[697]|RDNMH623-09|Canada|New Brunswick|658[0n]|BOLD:ACF0763
Colocasia flavicornis|[698]|RDNMH622-09|Canada|New Brunswick|658[0n]|BOLD:ACF0763
Colocasia flavicornis|[699]|RDNMH612-09|Canada|Ontario|658[0n]|BOLD:ACF0763
Colocasia flavicornis|[700]|RDNMH611-09|Canada|Ontario|658[0n]|BOLD:ACF0763
Colocasia flavicornis|[701]|RDNMH610-09|Canada|Ontario|658[0n]|BOLD:ACF0763
Colocasia flavicornis|[702]|NAMUM355-08|United States|Maryland|658[0n]|BOLD:ACF0763
Colocasia flavicornis|[703]|NAMUM354-08|United States|Maryland|658[0n]|BOLD:ACF0763
Colocasia flavicornis|[704]|NAMUM353-08|United States|Maryland|658[0n]|BOLD:ACF0763
Colocasia flavicornis|[705]|RDNMB339-05|Canada|Ontario|658[0n]|BOLD:ACF0763
Colocasia flavicornis|[706]|RDNMH621-09|Canada|New Brunswick|658[0n]|BOLD:ACF0763
Colocasia flavicornis|[707]|BBLPB638-10|Canada|Manitoba|658[0n]|BOLD:ACF0763
Colocasia flavicornis|[708]|LNCC585-11|United States|North Carolina|658[0n]|BOLD:ACF0763
Colocasia flavicornis|[709]|LNCC586-11|United States|North Carolina|658[0n]|BOLD:ACF0763
Colocasia flavicornis|[710]|LNCC672-11|United States|North Carolina|658[0n]|BOLD:ACF0763
Colocasia propinquilinea|[711]|RDLQG272-06|Canada|Quebec|658[0n]|BOLD:ACF0764
Colocasia propinquilinea|[712]|LSEU313-06|United States|Georgia|658[0n]|BOLD:ACF0764
Colocasia propinquilinea|[713]|LGSMC392-05|United States|Tennessee|658[0n]|BOLD:ACF0764
Colocasia propinquilinea|[714]|KPOEC167-08|Canada|Ontario|658[0n]|BOLD:ACF0764
Colocasia propinquilinea|[715]|RDNMH614-09|Canada|Ontario|658[0n]|BOLD:AAA8275
Colocasia propinquilinea|[716]|LOT329-04|United States|Tennessee|658[0n]|BOLD:AAA8275
Colocasia propinquilinea|[717]|BBLCU201-09|United States|Michigan|658[0n]|BOLD:AAA8275
Colocasia propinquilinea|[718]|XAE279-04|Canada|Ontario|658[0n]|BOLD:AAA8275
Colocasia propinquilinea|[719]|LGSM446-04|United States|North Carolina|658[0n]|BOLD:AAA8275
Colocasia propinquilinea|[720]|LGSMC903-05|United States|Tennessee|658[0n]|BOLD:AAA8275
Colocasia propinquilinea|[721]|LNCC967-11|United States|North Carolina|658[0n]|BOLD:AAA8275
Colocasia propinquilinea|[722]|LOT331-04|United States|Tennessee|658[0n]|BOLD:AAA8275
Colocasia propinquilinea|[723]|LOT512-04|United States|Tennessee|658[0n]|BOLD:AAA8275
Colocasia propinquilinea|[724]|LGSMG641-07|United States|Tennessee|658[0n]|BOLD:AAA8275
Colocasia propinquilinea|[725]|LGSM447-04|United States|North Carolina|658[0n]|BOLD:AAA8275
Colocasia propinquilinea|[726]|PMG102-03|Canada|Ontario|617[0n]|BOLD:AAA8275
Colocasia propinquilinea|[727]|LOTB326-05|United States|Tennessee|658[0n]|BOLD:AAA8275
Colocasia propinquilinea|[728]|LSEU763-06|United States|Georgia|658[0n]|BOLD:AAA8275
Colocasia propinquilinea|[729]|LOT330-04|United States|Tennessee|658[0n]|BOLD:AAA8275
Colocasia propinquilinea|[730]|LOT535-04|United States|Tennessee|658[0n]|BOLD:AAA8275
Colocasia propinquilinea|[731]|LGSMG642-07|United States|North Carolina|658[0n]|BOLD:AAA8275
Colocasia propinquilinea|[732]|RDMAB211-05|Canada|Alberta|658[0n]|BOLD:AAA8275
Colocasia propinquilinea|[733]|RDMAB212-05|Canada|Alberta|658[0n]|BOLD:AAA8275
Colocasia propinquilinea|[734]|BBLCU014-09|United States|Michigan|658[0n]|BOLD:AAA8275
Colocasia propinquilinea|[735]|LNCC1008-11|United States|North Carolina|658[0n]|BOLD:AAA8275
Colocasia propinquilinea|[736]|LNCC1009-11|United States|North Carolina|658[0n]|BOLD:AAA8275
Colocasia propinquilinea|[737]|XAB338-04|Canada|Ontario|658[0n]|BOLD:AAA8275
Colocasia propinquilinea|[738]|XAF651-05|Canada|Ontario|658[0n]|BOLD:AAA8275
Colocasia propinquilinea|[739]|LPSOC217-08|Canada|Ontario|656[0n]|BOLD:AAA8275
Colocasia propinquilinea|[740]|RDNMH615-09|Canada|Ontario|658[0n]|BOLD:AAA8275
Colocasia propinquilinea|[741]|LPSOC032-08|Canada|Ontario|658[0n]|BOLD:AAA8275
Colocasia propinquilinea|[742]|XAE312-04|Canada|Ontario|658[0n]|BOLD:AAA8275
Colocasia propinquilinea|[743]|LOT328-04|United States|Tennessee|658[0n]|BOLD:AAA8275
Colocasia propinquilinea|[744]|BLTIB070-08|Canada|Ontario|658[0n]|BOLD:AAA8275
Colocasia propinquilinea|[745]|CNCLB1472-14|Canada|Ontario|658[0n]|BOLD:AAA8275
Colocasia propinquilinea|[746]|LOT332-04|United States|Tennessee|658[0n]|BOLD:AAA8275
Colocasia propinquilinea|[747]|LOCT055-05|United States|Connecticut|656[0n]|BOLD:AAA8275
Colocasia propinquilinea|[748]|LOCT054-05|United States|Connecticut|637[0n]|BOLD:AAA8275
Colocasia propinquilinea|[749]|LOCT056-05|United States|Connecticut|658[0n]|BOLD:AAA8275
Colocasia propinquilinea|[750]|RDNMH618-09|Canada|New Brunswick|658[0n]|BOLD:AAA8275
Colocasia propinquilinea|[751]|TMG107-03|Canada|Ontario|639[0n]|BOLD:AAA8275
Colocasia propinquilinea|[752]|XAB276-04|Canada|Ontario|658[0n]|BOLD:AAA8275
Colocasia propinquilinea|[753]|XAB287-04|Canada|Ontario|658[0n]|BOLD:AAA8275
Colocasia propinquilinea|[754]|XAF652-05|Canada|Ontario|658[0n]|BOLD:AAA8275
Colocasia propinquilinea|[755]|RDLQG271-06|Canada|Quebec|658[0n]|BOLD:AAA8275
Colocasia propinquilinea|[756]|XAF550-05|Canada|Ontario|658[0n]|BOLD:AAA8275
Colocasia propinquilinea|[757]|XAB226-04|Canada|Ontario|658[0n]|BOLD:AAA8275
Colocasia propinquilinea|[758]|RDLQB701-05|Canada|Quebec|658[0n]|BOLD:AAA8275
Colocasia propinquilinea|[759]|BBLCU186-09|United States|Michigan|658[0n]|BOLD:AAA8275
Colocasia propinquilinea|[760]|BBLCU187-09|United States|Michigan|658[0n]|BOLD:AAA8275
Colocasia propinquilinea|[761]|CNCLB1471-14|Canada|Ontario|658[0n]|BOLD:AAA8275
Colocasia propinquilinea|[762]|RDNMH613-09|Canada|Ontario|658[0n]|BOLD:AAA8275
Colocasia propinquilinea|[763]|RDNMB340-05|Canada|Quebec|658[0n]|BOLD:AAA8275
Colocasia propinquilinea|[764]|CNCLB1473-14|Canada|Ontario|658[0n]|BOLD:AAA8275
Lichnoptera decora|[765]|RDNMH988-09|United States|Arizona|407[0n]|
Lichnoptera decora|[766]|RDNMK840-12|United States|Arizona|584[0n]|BOLD:ABW1737
Meleneta antennata|[767]|NAMUM075-08|United States|Arizona|657[0n]|BOLD:AAK7202
Meleneta antennata|[768]|RDNMI130-10|United States|Arizona|658[1n]|BOLD:AAK7202
Meleneta antennata|[769]|RDNMJ691-11|United States|Arizona|658[0n]|BOLD:AAK7202
Meleneta antennata|[770]|CNCLB1648-14|United States|Utah|658[0n]|BOLD:AAK7202
Marimatha nigrofimbria|[771]|LGSMD244-05|United States|Tennessee|658[0n]|BOLD:AAB1670
Marimatha nigrofimbria|[772]|RDNMH030-09|United States|Texas|658[0n]|BOLD:AAB1670
Marimatha nigrofimbria|[773]|BBLSX422-09|United States|Oklahoma|658[0n]|BOLD:AAB1670
Marimatha nigrofimbria|[774]|USLEP1176-10|United States|Arkansas|658[0n]|BOLD:AAB1670
Marimatha nigrofimbria|[775]|LMEMB450-09|United States|Alabama|658[0n]|BOLD:AAB1670
Marimatha nigrofimbria|[776]|BBLSX354-09|United States|Oklahoma|658[0n]|BOLD:AAB1670
Marimatha nigrofimbria|[777]|BBLSU008-09|United States|Arkansas|658[0n]|BOLD:AAB1670
Marimatha nigrofimbria|[778]|BBLOB914-11|United States|Florida|658[0n]|BOLD:AAB1670
Marimatha nigrofimbria|[779]|BBLOC853-11|United States|Arkansas|658[0n]|BOLD:AAB1670
Marimatha nigrofimbria|[780]|LNC182-05|United States|North Carolina|658[0n]|BOLD:AAB1670
Marimatha nigrofimbria|[781]|LGSMG630-07|United States|North Carolina|658[0n]|BOLD:AAB1670
Marimatha nigrofimbria|[782]|BBLOB950-11|United States|Florida|624[0n]|BOLD:AAB1670
Marimatha nigrofimbria|[783]|BBLSU059-09|United States|Arkansas|658[0n]|BOLD:AAB1670
Marimatha nigrofimbria|[784]|BBLOD080-11|United States|Texas|658[0n]|BOLD:AAB1670
Marimatha nigrofimbria|[785]|LOFLC274-06|United States|Florida|658[0n]|BOLD:AAB1670
Marimatha nigrofimbria|[786]|BBLSX353-09|United States|Oklahoma|658[0n]|BOLD:AAB1670
Marimatha nigrofimbria|[787]|BBLSU084-09|United States|Mississippi|658[0n]|BOLD:AAB1670
Marimatha nigrofimbria|[788]|LMEMB452-09|United States|Mississippi|658[0n]|BOLD:AAB1670
Marimatha nigrofimbria|[789]|LNC181-05|United States|North Carolina|658[0n]|BOLD:AAB1670
Marimatha nigrofimbria|[790]|LGSMC458-05|United States|Tennessee|613[0n]|BOLD:AAB1670
Marimatha nigrofimbria|[791]|LOT216-04|United States|Tennessee|609[0n]|BOLD:AAB1670
Marimatha nigrofimbria|[792]|LOFLA701-06|United States|Florida|658[0n]|BOLD:AAB1670
Marimatha nigrofimbria|[793]|LMEMB453-09|United States|Mississippi|658[0n]|BOLD:AAB1670
Marimatha nigrofimbria|[794]|BBLOC1455-11|United States|Texas|658[0n]|BOLD:AAB1670
Marimatha nigrofimbria|[795]|LOFLB169-06|United States|Florida|658[0n]|BOLD:AAB1670
Marimatha nigrofimbria|[796]|LILLA257-11|United States|Illinois|658[0n]|BOLD:AAB1670

Marimatha nigrofimbria|[794]|BBLOC1455-11|United States|Texas|658[0n]|BOLD:AAB1670
Marimatha nigrofimbria|[795]|LOFLB169-06|United States|Florida|658[0n]|BOLD:AAB1670
Marimatha nigrofimbria|[796]|LILLA257-11|United States|Illinois|658[0n]|BOLD:AAB1670
Marimatha nigrofimbria|[797]|LGSM443-04|United States|North Carolina|658[1n]|BOLD:AAB1670
Marimatha nigrofimbria|[798]|BBLOB1428-11|United States|Florida|658[0n]|BOLD:AAB1670
Marimatha nigrofimbria|[799]|BBLSU052-09|United States|Arkansas|658[0n]|BOLD:AAB1670
Marimatha nigrofimbria|[800]|BBLSU016-09|United States|Arkansas|658[0n]|BOLD:AAB1670
Marimatha nigrofimbria|[801]|BBLSX705-09|United States|Texas|658[0n]|BOLD:AAB1670
Marimatha nigrofimbria|[802]|BBLSX458-09|United States|Oklahoma|658[0n]|BOLD:AAB1670
Marimatha nigrofimbria|[803]|BBLSX356-09|United States|Oklahoma|658[0n]|BOLD:AAB1670
Marimatha nigrofimbria|[804]|BBLSX355-09|United States|Oklahoma|658[0n]|BOLD:AAB1670
Marimatha nigrofimbria|[805]|BBLSX171-09|United States|Oklahoma|658[0n]|BOLD:AAB1670
Marimatha nigrofimbria|[806]|BBLSX142-09|United States|Oklahoma|658[0n]|BOLD:AAB1670
Marimatha nigrofimbria|[807]|LPOKB708-09|United States|Oklahoma|658[0n]|BOLD:AAB1670
Marimatha nigrofimbria|[808]|LMEMB456-09|United States|Tennessee|658[0n]|BOLD:AAB1670
Marimatha nigrofimbria|[809]|LMEMB455-09|United States|Louisiana|658[0n]|BOLD:AAB1670
Marimatha nigrofimbria|[810]|LMEMB454-09|United States|Alabama|658[0n]|BOLD:AAB1670
Marimatha nigrofimbria|[811]|LMEMB451-09|United States|Alabama|658[0n]|BOLD:AAB1670
Marimatha nigrofimbria|[812]|LOFLD051-07|United States|Florida|658[0n]|BOLD:AAB1670
Marimatha nigrofimbria|[813]|LOFLD047-07|United States|Florida|658[0n]|BOLD:AAB1670
Marimatha nigrofimbria|[814]|LGSMG629-07|United States|Tennessee|658[0n]|BOLD:AAB1670
Marimatha nigrofimbria|[815]|LOFLC245-06|United States|Florida|658[0n]|BOLD:AAB1670
Marimatha nigrofimbria|[816]|LOFLB552-06|United States|Florida|658[0n]|BOLD:AAB1670
Marimatha nigrofimbria|[817]|LOFLB536-06|United States|Florida|658[0n]|BOLD:AAB1670
Marimatha nigrofimbria|[818]|LOFLB168-06|United States|Florida|658[0n]|BOLD:AAB1670
Marimatha nigrofimbria|[819]|LOFLA710-06|United States|Florida|658[0n]|BOLD:AAB1670
Marimatha nigrofimbria|[820]|LGSMC681-05|United States|Tennessee|658[0n]|BOLD:AAB1670
Marimatha nigrofimbria|[821]|LGSM442-04|United States|North Carolina|658[0n]|BOLD:AAB1670
Marimatha nigrofimbria|[822]|LGSMC457-05|United States|Tennessee|618[0n]|BOLD:AAB1670
Marimatha nigrofimbria|[823]|RDNMH773-09|United States|Texas|646[0n]|BOLD:AAB1670
Marimatha nigrofimbria|[824]|LILLA056-11|United States|Illinois|658[0n]|BOLD:AAB1670
Marimatha nigrofimbria|[825]|BBLOB1886-11|United States|Florida|658[0n]|BOLD:AAB1670
Marimatha nigrofimbria|[826]|BBLOC830-11|United States|Arkansas|658[0n]|BOLD:AAB1670
Marimatha nigrofimbria|[827]|MILEQ335-11|United States|Alabama|658[0n]|BOLD:AAB1670
Marimatha nigrofimbria|[828]|BBLOE1138-12|United States|Texas|658[0n]|BOLD:AAB1670
Marimatha nigrofimbria|[829]|BBLOE1147-12|United States|Texas|658[0n]|BOLD:AAB1670
Marimatha piscimala|[830]|HKONB164-08|United States|Texas|658[0n]|BOLD:AAD5203
Marimatha piscimala|[831]|HKONB165-08|United States|Texas|658[0n]|BOLD:AAD5203
Marimatha piscimala|[832]|RDNMF961-08|United States|Texas|658[0n]|BOLD:AAD5203
Marimatha piscimala|[833]|RDNMG1021-08|United States|Arizona|658[0n]|BOLD:AAD5203
Marimatha piscimala|[834]|RDNMH028-09|United States|Texas|658[0n]|BOLD:AAD5203
Marimatha piscimala|[835]|RDNMH031-09|United States|Texas|658[0n]|BOLD:AAD5203
Marimatha piscimala|[836]|RDNMH775-09|United States|Texas|635[0n]|BOLD:AAD5203
Marimatha piscimala|[837]|RDNMF962-08|United States|Texas|658[1n]|BOLD:AAD5203
Marimatha piscimala|[838]|RDNMH774-09|United States|Texas|658[0n]|BOLD:AAD5203
Marimatha piscimala|[839]|RDNMH848-09|United States|Arizona|658[0n]|BOLD:AAD5203
Marimatha tripuncta|[840]|RDNMG230-08|Dominican Republic||609[0n]|BOLD:ACP4167
Marimatha tripuncta|[841]|RDNMG229-08|Cuba||550[0n]|BOLD:AAD3751
Marimatha tripuncta|[842]|RDNMG231-08|Cuba||549[0n]|BOLD:AAD3751
Marimatha tripuncta|[843]|LOFLC423-06|United States|Florida|658[0n]|BOLD:AAD3751
Marimatha tripuncta|[844]|LOFLC013-06|United States|Florida|658[0n]|BOLD:AAD3751
Marimatha tripuncta|[845]|LOFLD002-07|United States|Florida|639[1n]|BOLD:AAD3751
Marimatha tripuncta|[846]|LOFLD003-07|United States|Florida|636[1n]|BOLD:AAD3751
Marimatha tripuncta|[847]|BBLSX704-09|United States|Texas|658[0n]|BOLD:AAD3751
Marimatha quadrata|[848]|RDNMH026-09|United States|Texas|658[0n]|BOLD:AAD5632
Marimatha quadrata|[849]|RDNMH027-09|United States|Texas|658[0n]|BOLD:AAD5632
Marimatha quadrata|[850]|RDNMH025-09|United States|Texas|658[0n]|BOLD:AAD5632
Marimatha quadrata|[851]|RDNMH029-09|United States|Texas|658[0n]|BOLD:AAD5632
Marimatha quadrata|[852]|RDNMH772-09|United States|Texas|658[0n]|BOLD:AAD5632
Marimatha quadrata|[853]|RDNMH572-09|United States|Arizona|658[0n]|BOLD:ACF0462
Marimatha quadrata|[854]|BBLOE1818-12|United States|Texas|658[0n]|BOLD:ACF0462
Marimatha squala|[855]|RDNMJ687-11|United States|Arizona|658[0n]|BOLD:AAC2638
Marimatha squala|[856]|RDNMG589-08|United States|Arizona|640[0n]|BOLD:AAC2638
Marimatha squala|[857]|RDNMH569-09|United States|Arizona|658[0n]|BOLD:AAC2638
Marimatha squala|[858]|RDNMH570-09|United States|Arizona|658[0n]|BOLD:AAC2638
Marimatha squala|[859]|CMAZA1172-12|United States|Arizona|658[0n]|BOLD:AAC2638
Abrostola ovalis|[860]|LGSM463-04|United States|North Carolina|619[0n]|BOLD:AAE9121
Abrostola ovalis|[861]|LOT218-04|United States|Tennessee|609[0n]|BOLD:AAE9121
Abrostola ovalis|[862]|LGSM462-04|United States|North Carolina|614[0n]|BOLD:AAE9121
Abrostola ovalis|[863]|RDLQB562-05|Canada|Quebec|658[0n]|BOLD:AAE9121
Abrostola urentis|[864]|RDNM841-05|Canada|British Columbia|568[2n]|BOLD:AAC4871
Abrostola urentis|[865]|RDLQF206-06|Canada|Quebec|658[0n]|BOLD:AAC4871
Abrostola urentis|[866]|XAK335-06|Canada|Ontario|658[0n]|BOLD:AAC4871
Abrostola urentis|[867]|XAJ540-06|Canada|Ontario|658[0n]|BOLD:AAC4871
Abrostola urentis|[868]|RDLQB831-05|Canada|Quebec|658[0n]|BOLD:AAC4871
Abrostola urentis|[869]|XAG317-05|Canada|Ontario|658[0n]|BOLD:AAC4871
Abrostola urentis|[870]|RDNM840-05|Canada|Ontario|658[0n]|BOLD:AAC4871
Abrostola urentis|[871]|LOCT263-05|United States|Connecticut|658[0n]|BOLD:AAC4871
Abrostola urentis|[872]|RDNM839-05|Canada|Ontario|588[0n]|BOLD:AAC4871
Abrostola urentis|[873]|RDLQG055-06|Canada|Quebec|608[0n]|BOLD:AAC4871
Abrostola urentis|[874]|LPSOB817-08|Canada|Ontario|658[0n]|BOLD:AAC4871
Abrostola urentis|[875]|SSPAB7605-13|Canada|Saskatchewan|564[0n]|BOLD:AAC4871
Abrostola parvula|[876]|RDNMG291-08|United States|Arizona|658[0n]|BOLD:AAJ5426
Abrostola parvula|[877]|RDNMJ138-10|United States|Nevada|658[0n]|BOLD:AAJ5426
Abrostola parvula|[878]|RDNMJ139-10|United States|Nevada|658[0n]|BOLD:AAJ5426
Abrostola microvalis|[879]|NOCNA042-14|United States|Oklahoma|658[0n]|BOLD:ACM6941
Abrostola microvalis|[880]|NOCNA043-14|United States|Texas|658[0n]|BOLD:ACM6941
Abrostola microvalis|[881]|CNCLB288-14|United States|Texas|658[0n]|BOLD:ACM6941
Abrostola microvalis|[882]|CNCLB289-14|United States|Texas|658[0n]|BOLD:ACM6941
Abrostola microvalis|[883]|CNCLB290-14|United States|Texas|658[0n]|BOLD:ACM6941
Abrostola microvalis|[884]|CNCLB291-14|United States|Texas|658[0n]|BOLD:ACM6941
Abrostola microvalis|[885]|CNCLB292-14|United States|Texas|658[0n]|BOLD:ACM6941
Amiana sp.|[886]|RDNMH583-09|United States|Arizona|658[0n]|BOLD:AAK8794
Amiana niama|[887]|ABNCC033-07|United States|Arizona|632[1n]|BOLD:AAF3103
Amiana niama|[888]|NAMUM087-08|United States|Arizona|657[0n]|BOLD:AAF3103
Amiana niama|[889]|RDNME925-08|United States|Arizona|658[0n]|BOLD:AAF3103
Amiana niama|[890]|RDNMJ739-11|United States|Arizona|658[0n]|BOLD:AAF3103
Amiana niama|[891]|RDNMJ747-11|United States|Arizona|658[0n]|BOLD:AAF3103
Maliattha concinnimacula|[892]|TMNBB116-06|Canada|New Brunswick|658[0n]|BOLD:AAB4249
Maliattha concinnimacula|[893]|MEC388-04|Canada|Quebec|658[0n]|BOLD:AAB4249
Maliattha concinnimacula|[894]|PHMNB706-04|Canada|New Brunswick|658[0n]|BOLD:AAB4249
Maliattha concinnimacula|[895]|RDLQG528-06|Canada|Quebec|658[0n]|BOLD:AAB4249

Maliattha concinnimacula|[893]|MEC388-04|Canada|Quebec|658[0n]|BOLD:AAB4249
Maliattha concinnimacula|[894]|PHMNB706-04|Canada|New Brunswick|658[0n]|BOLD:AAB4249
Maliattha concinnimacula|[895]|RDLQG528-06|Canada|Quebec|658[0n]|BOLD:AAB4249
Maliattha concinnimacula|[896]|RDLQG529-06|Canada|Quebec|658[0n]|BOLD:AAB4249
Maliattha concinnimacula|[897]|RDLQG530-06|Canada|Quebec|658[0n]|BOLD:AAB4249
Maliattha concinnimacula|[898]|MEC713-04|Canada|Quebec|658[0n]|BOLD:AAB4249
Maliattha concinnimacula|[899]|PHMNB603-04|Canada|New Brunswick|658[0n]|BOLD:AAB4249
Maliattha concinnimacula|[900]|PHMNB494-04|Canada|New Brunswick|658[0n]|BOLD:AAB4249
Maliattha concinnimacula|[901]|TMNBB117-06|Canada|New Brunswick|658[0n]|BOLD:AAB4249
Maliattha concinnimacula|[902]|LGSMC663-05|United States|Tennessee|658[0n]|BOLD:AAB4249
Maliattha concinnimacula|[903]|TMNBB115-06|Canada|New Brunswick|658[0n]|BOLD:AAB4249
Maliattha concinnimacula|[904]|LSEU649-06|United States|Georgia|658[0n]|BOLD:AAB4249
Maliattha concinnimacula|[905]|RDLQG824-06|Canada|Quebec|658[0n]|BOLD:AAB4249
Maliattha concinnimacula|[906]|RDNMB256-05|Canada|Ontario|658[0n]|BOLD:AAB4249
Maliattha concinnimacula|[907]|RDLQG812-06|Canada|Quebec|658[0n]|BOLD:AAB4249
Maliattha concinnimacula|[908]|LPSOC196-08|Canada|Ontario|658[0n]|BOLD:AAB4249
Maliattha concinnimacula|[909]|LPSOC198-08|Canada|Ontario|658[0n]|BOLD:AAB4249
Maliattha concinnimacula|[910]|LPSOC199-08|Canada|Ontario|658[0n]|BOLD:AAB4249
Maliattha concinnimacula|[911]|LPSOC223-08|Canada|Ontario|658[0n]|BOLD:AAB4249
Maliattha concinnimacula|[912]|LPSOC267-08|Canada|Ontario|658[0n]|BOLD:AAB4249
Maliattha concinnimacula|[913]|LPSOB750-08|Canada|Ontario|658[0n]|BOLD:AAB4249
Maliattha concinnimacula|[914]|LMEMB449-09|United States|Texas|658[0n]|BOLD:AAB4249
Maliattha concinnimacula|[915]|BBLSX539-09|United States|Oklahoma|658[0n]|BOLD:AAB4249
Maliattha synochitis|[916]|LPSO767-08|Canada|Ontario|609[0n]|BOLD:AAA4972
Maliattha synochitis|[917]|LPSO716-08|Canada|Ontario|658[0n]|BOLD:AAA4972
Maliattha synochitis|[918]|BBLSX386-09|United States|Oklahoma|658[0n]|BOLD:AAA4972
Maliattha synochitis|[919]|BBLSX510-09|United States|Oklahoma|658[0n]|BOLD:AAA4972
Maliattha synochitis|[920]|BBLSX672-09|United States|Oklahoma|658[0n]|BOLD:AAA4972
Maliattha synochitis|[921]|RDLQG843-06|Canada|Quebec|658[0n]|BOLD:AAA4972
Maliattha synochitis|[922]|TTMNB305-06|Canada|New Brunswick|658[1n]|BOLD:AAA4972
Maliattha synochitis|[923]|BLTIB484-08|Canada|Ontario|656[0n]|BOLD:AAA4972
Maliattha synochitis|[924]|PHMO177-03|Canada|Ontario|639[0n]|BOLD:AAA4972
Maliattha synochitis|[925]|PHMNB344-04|Canada|New Brunswick|658[0n]|BOLD:AAA4972
Maliattha synochitis|[926]|XAG294-05|Canada|Ontario|658[0n]|BOLD:AAA4972
Maliattha synochitis|[927]|TTMNB302-06|Canada|New Brunswick|657[0n]|BOLD:AAA4972
Maliattha synochitis|[928]|LNC853-06|United States|North Carolina|658[0n]|BOLD:AAA4972
Maliattha synochitis|[929]|RDLQG518-06|Canada|Quebec|658[0n]|BOLD:AAA4972
Maliattha synochitis|[930]|RDLQG688-06|Canada|Quebec|658[0n]|BOLD:AAA4972
Maliattha synochitis|[931]|BLTIB995-08|Canada|Ontario|658[0n]|BOLD:AAA4972
Maliattha synochitis|[932]|BBLSW574-09|United States|Oklahoma|658[0n]|BOLD:AAA4972
Maliattha synochitis|[933]|BBLSX456-09|United States|Oklahoma|658[0n]|BOLD:AAA4972
Maliattha synochitis|[934]|BBLSX553-09|United States|Oklahoma|658[0n]|BOLD:AAA4972
Maliattha synochitis|[935]|BBLEC307-09|Canada|Nova Scotia|658[0n]|BOLD:AAA4972
Maliattha synochitis|[936]|BBLEC607-09|Canada|Nova Scotia|658[0n]|BOLD:AAA4972
Maliattha synochitis|[937]|BBLPC260-09|Canada|Nova Scotia|658[0n]|BOLD:AAA4972
Maliattha synochitis|[938]|BBLPE040-09|Canada|Nova Scotia|658[0n]|BOLD:AAA4972
Maliattha synochitis|[939]|LGSM782-04|United States|North Carolina|658[0n]|BOLD:AAA4972
Maliattha synochitis|[940]|BBLSX472-09|United States|Oklahoma|658[0n]|BOLD:AAA4972
Maliattha synochitis|[941]|BLTIB250-08|Canada|Ontario|615[1n]|BOLD:AAA4972
Maliattha synochitis|[942]|LOCT275-05|United States|Connecticut|658[0n]|BOLD:AAA4972
Maliattha synochitis|[943]|LOCT276-05|United States|Connecticut|658[0n]|BOLD:AAA4972
Maliattha synochitis|[944]|RDLQG517-06|Canada|Quebec|658[0n]|BOLD:AAA4972
Maliattha synochitis|[945]|BBLSW592-09|United States|Oklahoma|658[0n]|BOLD:AAA4972
Maliattha synochitis|[946]|PHMNB059-03|Canada|New Brunswick|639[0n]|BOLD:AAA4972
Maliattha synochitis|[947]|PHMNB393-04|Canada|New Brunswick|658[0n]|BOLD:AAA4972
Maliattha synochitis|[948]|PHMNB397-04|Canada|New Brunswick|658[0n]|BOLD:AAA4972
Maliattha synochitis|[949]|PHMNB489-04|Canada|New Brunswick|658[0n]|BOLD:AAA4972
Maliattha synochitis|[950]|MNBB209-05|Canada|New Brunswick|658[0n]|BOLD:AAA4972
Maliattha synochitis|[951]|TMNBB114-06|Canada|New Brunswick|658[0n]|BOLD:AAA4972
Maliattha synochitis|[952]|LPSO392-08|Canada|Ontario|658[0n]|BOLD:AAA4972
Maliattha synochitis|[953]|BBLSX346-09|United States|Oklahoma|658[0n]|BOLD:AAA4972
Maliattha synochitis|[954]|BBLSX475-09|United States|Oklahoma|658[0n]|BOLD:AAA4972
Maliattha synochitis|[955]|LPSO766-08|Canada|Ontario|609[0n]|BOLD:AAA4972
Maliattha synochitis|[956]|LPSO768-08|Canada|Ontario|609[0n]|BOLD:AAA4972
Maliattha synochitis|[957]|XAE380-04|Canada|Ontario|520[2n]|BOLD:AAA4972
Maliattha synochitis|[958]|TTMNB304-06|Canada|New Brunswick|658[4n]|BOLD:AAA4972
Maliattha synochitis|[959]|RDLQG831-06|Canada|Quebec|658[0n]|BOLD:AAA4972
Maliattha synochitis|[960]|RDLQG834-06|Canada|Quebec|658[0n]|BOLD:AAA4972
Maliattha synochitis|[961]|RDLQG835-06|Canada|Quebec|658[0n]|BOLD:AAA4972
Maliattha synochitis|[962]|LPSO205-08|Canada|Ontario|658[0n]|BOLD:AAA4972
Maliattha synochitis|[963]|LPSO626-08|Canada|Ontario|658[0n]|BOLD:AAA4972
Maliattha synochitis|[964]|LPSO911-08|Canada|Ontario|658[0n]|BOLD:AAA4972
Maliattha synochitis|[965]|BLTIB274-08|Canada|Ontario|657[0n]|BOLD:AAA4972
Maliattha synochitis|[966]|BLTIB347-08|Canada|Ontario|658[0n]|BOLD:AAA4972
Maliattha synochitis|[967]|BLTIB361-08|Canada|Ontario|658[0n]|BOLD:AAA4972
Maliattha synochitis|[968]|BLTIB487-08|Canada|Ontario|658[0n]|BOLD:AAA4972
Maliattha synochitis|[969]|BLTIB493-08|Canada|Ontario|658[0n]|BOLD:AAA4972
Maliattha synochitis|[970]|BLTIB930-08|Canada|Ontario|658[0n]|BOLD:AAA4972
Maliattha synochitis|[971]|BLGSM048-09|Canada|Ontario|658[0n]|BOLD:AAA4972
Maliattha synochitis|[972]|LPSOD299-09|Canada|Ontario|658[0n]|BOLD:AAA4972
Maliattha synochitis|[973]|LPSO931-08|Canada|Ontario|658[0n]|BOLD:AAA4972
Maliattha synochitis|[974]|LPSOB576-08|Canada|Ontario|658[0n]|BOLD:AAA4972
Maliattha synochitis|[975]|LPSOB603-08|Canada|Ontario|658[0n]|BOLD:AAA4972
Maliattha synochitis|[976]|LPSOB914-08|Canada|Ontario|658[0n]|BOLD:AAA4972
Maliattha synochitis|[977]|BLTIB157-08|Canada|Ontario|658[0n]|BOLD:AAA4972
Maliattha synochitis|[978]|BLTIB244-08|Canada|Ontario|658[0n]|BOLD:AAA4972
Maliattha synochitis|[979]|LPSO453-08|Canada|Ontario|658[0n]|BOLD:AAA4972
Maliattha synochitis|[980]|LPSO625-08|Canada|Ontario|658[0n]|BOLD:AAA4972
Maliattha synochitis|[981]|BLTIB261-08|Canada|Ontario|658[0n]|BOLD:AAA4972
Maliattha synochitis|[982]|BLTIB262-08|Canada|Ontario|658[0n]|BOLD:AAA4972
Maliattha synochitis|[983]|LPOKB412-09|United States|Oklahoma|658[0n]|BOLD:AAA4972
Maliattha synochitis|[984]|LPOKB747-09|United States|Oklahoma|658[0n]|BOLD:AAA4972
Maliattha synochitis|[985]|BBUSA672-09|United States|Oklahoma|658[0n]|BOLD:AAA4972
Maliattha synochitis|[986]|BBLSW010-09|United States|Oklahoma|658[0n]|BOLD:AAA4972
Maliattha synochitis|[987]|BBLSW452-09|United States|Oklahoma|658[0n]|BOLD:AAA4972
Maliattha synochitis|[988]|BBLSW458-09|United States|Oklahoma|658[0n]|BOLD:AAA4972
Maliattha synochitis|[989]|BBLSX137-09|United States|Oklahoma|658[0n]|BOLD:AAA4972
Maliattha synochitis|[990]|BBLSX372-09|United States|Oklahoma|658[0n]|BOLD:AAA4972
Maliattha synochitis|[991]|BBLSX412-09|United States|Oklahoma|658[0n]|BOLD:AAA4972
Maliattha synochitis|[992]|BBLSX446-09|United States|Oklahoma|658[0n]|BOLD:AAA4972
Maliattha synochitis|[993]|BBLSX466-09|United States|Oklahoma|658[0n]|BOLD:AAA4972
Maliattha synochitis|[994]|BBLSX479-09|United States|Oklahoma|658[0n]|BOLD:AAA4972
Maliattha synochitis|[995]|BBLSW459-09|United States|Oklahoma|658[0n]|BOLD:AAA4972

Maliattha synochitis|[993]|BBLSX466-09|United States|Oklahoma|658[0n]|BOLD:AAA4972
Maliattha synochitis|[994]|BBLSX479-09|United States|Oklahoma|658[0n]|BOLD:AAA4972
Maliattha synochitis|[995]|BBLSW459-09|United States|Oklahoma|658[0n]|BOLD:AAA4972
Maliattha synochitis|[996]|RDLQG728-06|Canada|Quebec|658[0n]|BOLD:AAA4972
Maliattha synochitis|[997]|LSEU648-06|United States|Georgia|658[0n]|BOLD:AAA4972
Maliattha synochitis|[998]|TMNBB480-06|Canada|New Brunswick|658[0n]|BOLD:AAA4972
Maliattha synochitis|[999]|LSUSA136-06|United States|Kentucky|658[0n]|BOLD:AAA4972
Maliattha synochitis|[1000]|RDLQB777-05|Canada|Quebec|658[0n]|BOLD:AAA4972
Maliattha synochitis|[1001]|XAD728-05|Canada|Ontario|658[0n]|BOLD:AAA4972
Maliattha synochitis|[1002]|XAF792-05|Canada|Ontario|658[0n]|BOLD:AAA4972
Maliattha synochitis|[1003]|XAF706-05|Canada|Ontario|658[0n]|BOLD:AAA4972
Maliattha synochitis|[1004]|XAF507-05|Canada|Ontario|658[0n]|BOLD:AAA4972
Maliattha synochitis|[1005]|XAD677-05|Canada|Ontario|658[0n]|BOLD:AAA4972
Maliattha synochitis|[1006]|PHMNB664-04|Canada|New Brunswick|658[0n]|BOLD:AAA4972
Maliattha synochitis|[1007]|PHMNB550-04|Canada|New Brunswick|658[0n]|BOLD:AAA4972
Maliattha synochitis|[1008]|PHMNB398-04|Canada|New Brunswick|658[0n]|BOLD:AAA4972
Maliattha synochitis|[1009]|MEC712-04|Canada|Quebec|658[0n]|BOLD:AAA4972
Maliattha synochitis|[1010]|XAB375-04|Canada|Ontario|658[0n]|BOLD:AAA4972
Maliattha synochitis|[1011]|LPSOB915-08|Canada|Ontario|658[0n]|BOLD:AAA4972
Maliattha synochitis|[1012]|XAE437-04|Canada|Ontario|615[0n]|BOLD:AAA4972
Maliattha synochitis|[1013]|PHMNB657-04|Canada|New Brunswick|624[0n]|BOLD:AAA4972
Maliattha synochitis|[1014]|BLTIB604-08|Canada|Ontario|634[0n]|BOLD:AAA4972
Maliattha synochitis|[1015]|BLTIB605-08|Canada|Ontario|635[0n]|BOLD:AAA4972
Maliattha synochitis|[1016]|BBLSX124-09|United States|Oklahoma|635[0n]|BOLD:AAA4972
Maliattha synochitis|[1017]|BBLSX584-09|United States|Oklahoma|658[0n]|BOLD:AAA4972
Maliattha synochitis|[1018]|PHMTV434-10|Canada|Ontario|658[0n]|BOLD:AAA4972
Maliattha synochitis|[1019]|LILLA082-11|United States|Illinois|658[0n]|BOLD:AAA4972
Maliattha synochitis|[1020]|LILLA188-11|United States|Illinois|658[0n]|BOLD:AAA4972
Maliattha synochitis|[1021]|PHJUN3374-11|Canada|Ontario|658[0n]|BOLD:AAA4972
Maliattha synochitis|[1022]|CNPPA2520-12|Canada|Ontario|598[0n]|BOLD:AAA4972
Lithacodia new sp. 2|[1023]|RDNMG288-08|United States|Arizona|610[0n]|BOLD:AAF6556
Lithacodia new sp. 2|[1024]|RDNME920-08|United States|Arizona|658[0n]|BOLD:AAF6556
Lithacodia new sp. 2|[1025]|PSAT129-10|United States|Arizona|658[0n]|BOLD:AAF6556
Lithacodia new sp. 4|[1026]|RDNME921-08|United States|Arizona|658[0n]|BOLD:AAF6557
Lithacodia new sp. 4|[1027]|RDNMJ541-11|United States|Arizona|658[0n]|BOLD:AAF6557
Lithacodia new sp. 4|[1028]|RDNMJ542-11|United States|Arizona|658[0n]|BOLD:AAF6557
Lithacodia new sp. 4|[1029]|RDNMJ551-11|United States|Arizona|658[0n]|BOLD:AAF6557
Lithacodia new sp. 4|[1030]|RDNMJ672-11|United States|Arizona|658[0n]|BOLD:AAF6557
Lithacodia new sp. 4|[1031]|RDNMJ694-11|United States|Arizona|658[0n]|BOLD:AAF6557
Lithacodia new sp. 4|[1032]|RDNMJ827-11|United States|Arizona|658[0n]|BOLD:AAF6557
Ozarba propera|[1033]|BBUSA209-09|United States|Texas|658[0n]|BOLD:AAE5333
Ozarba propera|[1034]|NAMUM078-08|United States|Arizona|657[0n]|BOLD:AAE5333
Ozarba propera|[1035]|BBUSA219-09|United States|Texas|658[0n]|BOLD:AAE5333
Ozarba propera|[1036]|BBUSA232-09|United States|Texas|658[0n]|BOLD:AAE5333
Ozarba propera|[1037]|BBUSA236-09|United States|Texas|658[0n]|BOLD:AAE5333
Ozarba propera|[1038]|BBLSX244-09|United States|Texas|658[0n]|BOLD:AAE5333
Ozarba propera|[1039]|RDNMK168-11|United States|Arizona|658[0n]|BOLD:AAE5333
Ozarba propera|[1040]|BBLOB1936-11|United States|Arizona|658[0n]|BOLD:AAE5333
Ozarba propera|[1041]|CMAZA1246-12|United States|Arizona|658[0n]|BOLD:AAE5333
Ozarba propera|[1042]|RDNMH835-09|United States|Arizona|658[0n]|BOLD:AAE5333
Ozarba propera|[1043]|RDNMK169-11|United States|Arizona|658[0n]|BOLD:AAE5333
Ozarba propera|[1044]|BBLOC301-11|United States|Arizona|658[0n]|BOLD:AAE5333
Ozarba propera|[1045]|BBLOE1870-12|United States|Arizona|658[0n]|BOLD:AAE5333
Ozarba propera|[1046]|MNAD164-07|United States|Arizona|658[0n]|BOLD:AAE5333
Ozarba propera|[1047]|MNAD163-07|United States|Arizona|658[0n]|BOLD:AAE5333
Ozarba propera|[1048]|RDNMK167-11|United States|Arizona|658[0n]|BOLD:AAE5333
Ozarba propera|[1049]|BBLOC302-11|United States|Arizona|658[0n]|BOLD:AAE5333
Ozarba propera|[1050]|BBLOC304-11|United States|Arizona|658[0n]|BOLD:AAE5333
Ozarba propera|[1051]|BBLOC309-11|United States|Arizona|658[0n]|BOLD:AAE5333
Ozarba propera|[1052]|CMAZA1268-12|United States|Arizona|658[0n]|BOLD:AAE5333
Deltote bellicula|[1053]|LNCB368-06|United States|North Carolina|658[0n]|BOLD:AAB6909
Deltote bellicula|[1054]|LNCB369-06|United States|North Carolina|658[0n]|BOLD:AAB6909
Deltote bellicula|[1055]|LNCB370-06|United States|North Carolina|658[0n]|BOLD:AAB6909
Deltote bellicula|[1056]|RDLQE263-06|Canada|Quebec|656[0n]|BOLD:AAB6909
Deltote bellicula|[1057]|RDLQE264-06|Canada|Quebec|657[0n]|BOLD:AAB6909
Deltote bellicula|[1058]|RDLQG673-06|Canada|Quebec|658[0n]|BOLD:AAB6909
Deltote bellicula|[1059]|RDLQG897-06|Canada|Quebec|658[0n]|BOLD:AAB6909
Deltote bellicula|[1060]|RDLQG896-06|Canada|Quebec|658[0n]|BOLD:AAB6909
Deltote bellicula|[1061]|RDLQE262-06|Canada|Quebec|658[0n]|BOLD:AAB6909
Deltote bellicula|[1062]|RDLQB811-05|Canada|Quebec|658[0n]|BOLD:AAB6909
Deltote bellicula|[1063]|RDLQG898-06|Canada|Quebec|658[0n]|BOLD:AAB6909
Deltote bellicula|[1064]|XAJ873-06|Canada|Ontario|658[0n]|BOLD:AAB6909
Deltote bellicula|[1065]|XAD784-05|Canada|Ontario|658[0n]|BOLD:AAB6909
Deltote bellicula|[1066]|RDLQG700-06|Canada|Quebec|658[0n]|BOLD:AAB6909
Deltote bellicula|[1067]|LPOKA177-08|United States|Oklahoma|658[0n]|BOLD:AAB6909
Deltote bellicula|[1068]|LPOKA451-09|United States|Oklahoma|624[0n]|BOLD:AAB6909
Deltote bellicula|[1069]|LPOKA477-09|United States|Oklahoma|658[0n]|BOLD:AAB6909
Deltote bellicula|[1070]|LPOKB404-09|United States|Oklahoma|658[0n]|BOLD:AAB6909
Deltote bellicula|[1071]|LPOKC551-09|United States|Oklahoma|658[0n]|BOLD:AAB6909
Lithacodia musta|[1072]|LGSM483-04|United States|Tennessee|658[0n]|BOLD:AAC9715
Lithacodia musta|[1073]|LPOKA515-09|United States|Oklahoma|648[0n]|BOLD:AAC9715
Lithacodia musta|[1074]|UDLEP314-09|United States|Pennsylvania|590[0n]|BOLD:AAC9715
Lithacodia musta|[1075]|LPOKC262-09|United States|Oklahoma|658[0n]|BOLD:AAC9715
Lithacodia musta|[1076]|LGSM482-04|United States|Tennessee|615[0n]|BOLD:AAC9715
Lithacodia musta|[1077]|LGSMC662-05|United States|Tennessee|658[0n]|BOLD:AAC9715
Lithacodia musta|[1078]|LSUSA205-06|United States|Kentucky|658[0n]|BOLD:AAC9715
Lithacodia musta|[1079]|LGSMG634-07|United States|North Carolina|658[0n]|BOLD:AAC9715
Lithacodia musta|[1080]|LPOKA471-09|United States|Oklahoma|658[0n]|BOLD:AAC9715
Lithacodia musta|[1081]|LPOKC486-09|United States|Oklahoma|658[0n]|BOLD:AAC9715
Lithacodia musta|[1082]|LNCC208-10|United States|North Carolina|658[0n]|BOLD:AAC9715
Lithacodia musta|[1083]|LILLA213-11|United States|Illinois|658[0n]|BOLD:AAC9715
Lithacodia musta group|[1084]|RDNMF964-08|United States|Texas|658[0n]|BOLD:AAJ2080
Lithacodia musta group|[1085]|RDNMF965-08|United States|Arizona|658[0n]|BOLD:AAJ2080
Lithacodia phya|[1086]|RDNMH582-09|United States|Arizona|658[0n]|BOLD:AAX6374
Lithacodia phya|[1087]|RDNMK908-13|Mexico|Sonora|658[0n]|BOLD:AAX6374
Argillophora furcilla|[1088]|LSEU640-06|United States|Georgia|658[0n]|BOLD:AAC6378
Argillophora furcilla|[1089]|LSEU642-06|United States|Georgia|658[0n]|BOLD:AAC6378
Argillophora furcilla|[1090]|LSEU641-06|United States|Georgia|658[0n]|BOLD:AAC6378
Argillophora furcilla|[1091]|LSEU061-06|United States|Georgia|585[0n]|BOLD:AAC6378
Argillophora furcilla|[1092]|LSEU062-06|United States|Georgia|658[0n]|BOLD:AAC6378
Argillophora furcilla|[1093]|LSUSA189-06|United States|Kentucky|658[0n]|BOLD:AAC6378
Argillophora furcilla|[1094]|RDNMD771-07|United States|Georgia|658[0n]|BOLD:AAC6378

Argillophora furcilla|[1092]|LSEU062-06|United States|Georgia|658[0n]|BOLD:AAC6378
Argillophora furcilla|[1093]|LSUSA189-06|United States|Kentucky|658[0n]|BOLD:AAC6378
Argillophora furcilla|[1094]|RDNMD771-07|United States|Georgia|658[0n]|BOLD:AAC6378
Argillophora furcilla|[1095]|HKONS481-08|United States|Florida|658[0n]|BOLD:AAC6378
Argillophora furcilla|[1096]|HKONS482-08|United States|Florida|658[0n]|BOLD:AAC6378
Lithacodia new sp. 1|[1097]|HKONS484-08|United States|Florida|658[0n]|BOLD:AAE6640
Lithacodia new sp. 1|[1098]|LNC818-06|United States|North Carolina|658[0n]|BOLD:AAE6640
Lithacodia new sp. 1|[1099]|LNC817-06|United States|North Carolina|658[0n]|BOLD:AAE6640
Lithacodia new sp. 1|[1100]|HKONS485-08|United States|Florida|609[0n]|BOLD:AAE6640
Lithacodia new sp. 1|[1101]|RDNMH510-09|United States|Louisiana|658[0n]|BOLD:AAE6640
Lithacodia new sp. 1|[1102]|LNCC010-10|United States|North Carolina|658[0n]|BOLD:AAE6640
Lithacodia new sp. 1|[1103]|LNCC011-10|United States|North Carolina|658[0n]|BOLD:AAE6640
Lithacodia new sp. 1|[1104]|LNCC012-10|United States|North Carolina|658[0n]|BOLD:AAE6640
Lithacodia new sp. 3|[1105]|LNCB603-09|United States|North Carolina|658[0n]|BOLD:ABY4370
Lithacodia new sp. 3|[1106]|LNCB604-09|United States|North Carolina|658[0n]|BOLD:ABY4370
Lithacodia new sp. 3|[1107]|LNCB806-09|United States|North Carolina|658[0n]|BOLD:ABY4370
Lithacodia new sp. 3|[1108]|LNCC931-11|United States|North Carolina|658[0n]|BOLD:ABY4370
Capis archaia|[1109]|RDLQ026-05|Canada|Quebec|658[0n]|BOLD:AAC6122
Capis archaia|[1110]|RDLQB401-05|Canada|Quebec|658[0n]|BOLD:AAC6122
Capis archaia|[1111]|RDLQB403-05|Canada|Quebec|658[0n]|BOLD:AAC6122
Capis archaia|[1112]|RDLQB410-05|Canada|Quebec|658[0n]|BOLD:AAC6122
Capis archaia|[1113]|RDLQB400-05|Canada|Quebec|658[0n]|BOLD:AAC6122
Capis archaia|[1114]|RDLQB402-05|Canada|Quebec|658[0n]|BOLD:AAC6122
Capis archaia|[1115]|RDLQB409-05|Canada|Quebec|658[0n]|BOLD:AAC6122
Capis archaia|[1116]|RDLQG565-06|Canada|Quebec|658[0n]|BOLD:AAC6122
Capis archaia|[1117]|RDLQG846-06|Canada|Quebec|658[0n]|BOLD:AAC6122
Capis archaia|[1118]|RDLQG921-06|Canada|Quebec|656[0n]|BOLD:AAC6122
Capis curvata|[1119]|RDLQB544-05|Canada|Quebec|510[0n]|BOLD:AAB7154
Capis curvata|[1120]|BLTIB599-08|Canada|Ontario|642[0n]|BOLD:AAB7154
Capis curvata|[1121]|RDLQG614-06|Canada|Quebec|658[0n]|BOLD:AAB7154
Capis curvata|[1122]|RDLQG665-06|Canada|Quebec|658[0n]|BOLD:AAB7154
Capis curvata|[1123]|RDLQG708-06|Canada|Quebec|658[0n]|BOLD:AAB7154
Capis curvata|[1124]|RDLQB266-05|Canada|Quebec|658[0n]|BOLD:AAB7154
Capis curvata|[1125]|RDLQB545-05|Canada|Quebec|658[0n]|BOLD:AAB7154
Capis curvata|[1126]|RDLQG664-06|Canada|Quebec|658[0n]|BOLD:AAB7154
Capis curvata|[1127]|RDLQG666-06|Canada|Quebec|658[0n]|BOLD:AAB7154
Capis curvata|[1128]|RDLQG701-06|Canada|Quebec|658[0n]|BOLD:AAB7154
Capis curvata|[1129]|RDLQG707-06|Canada|Quebec|658[0n]|BOLD:AAB7154
Capis curvata|[1130]|RDLQG744-06|Canada|Quebec|658[0n]|BOLD:AAB7154
Capis curvata|[1131]|RDLQG822-06|Canada|Quebec|658[0n]|BOLD:AAB7154
Capis curvata|[1132]|LPSOB293-08|Canada|Ontario|658[0n]|BOLD:AAB7154
Capis curvata|[1133]|LPMN704-08|Canada|Manitoba|658[0n]|BOLD:AAB7154
Capis curvata|[1134]|MECB076-04|Canada|Quebec|658[0n]|BOLD:AAB7154
Capis curvata|[1135]|PHMNB391-04|Canada|New Brunswick|658[0n]|BOLD:AAB7154
Capis curvata|[1136]|BLTIB1061-08|Canada|Ontario|658[0n]|BOLD:AAB7154
Protodeltote muscosula|[1137]|LPOKA478-09|United States|Oklahoma|658[0n]|BOLD:AAA4259
Protodeltote muscosula|[1138]|LGSMC465-05|United States|Tennessee|658[0n]|BOLD:AAA4259
Protodeltote muscosula|[1139]|LGSMD373-05|United States|Tennessee|658[0n]|BOLD:AAA4259
Protodeltote muscosula|[1140]|LNC819-06|United States|North Carolina|658[0n]|BOLD:AAA4259
Protodeltote muscosula|[1141]|LGSMG633-07|United States|Tennessee|658[0n]|BOLD:AAA4259
Protodeltote muscosula|[1142]|LSEU581-06|United States|Georgia|658[0n]|BOLD:AAA4259
Protodeltote muscosula|[1143]|LGSMC466-05|United States|Tennessee|658[0n]|BOLD:AAA4259
Protodeltote muscosula|[1144]|LGSMC464-05|United States|Tennessee|658[0n]|BOLD:AAA4259
Protodeltote muscosula|[1145]|MNBB050-05|Canada|New Brunswick|658[0n]|BOLD:AAA4259
Protodeltote muscosula|[1146]|LOTB248-05|United States|Tennessee|658[0n]|BOLD:AAA4259
Protodeltote muscosula|[1147]|LPOKD385-09|United States|Oklahoma|658[0n]|BOLD:AAA4259
Protodeltote muscosula|[1148]|LNCC360-10|United States|North Carolina|658[0n]|BOLD:AAA4259
Protodeltote muscosula|[1149]|RDLQG710-06|Canada|Quebec|658[0n]|BOLD:AAA4259
Protodeltote muscosula|[1150]|BBLPE282-09|Canada|Nova Scotia|647[0n]|BOLD:AAA4259
Protodeltote muscosula|[1151]|BLTIB491-08|Canada|Ontario|658[0n]|BOLD:AAA4259
Protodeltote muscosula|[1152]|BLTIB1050-08|Canada|Ontario|632[0n]|BOLD:AAA4259
Protodeltote muscosula|[1153]|BLTIB850-08|Canada|Ontario|641[0n]|BOLD:AAA4259
Protodeltote muscosula|[1154]|BBLPC169-09|Canada|Nova Scotia|655[0n]|BOLD:AAA4259
Protodeltote muscosula|[1155]|BLTIB1121-08|Canada|Ontario|656[0n]|BOLD:AAA4259
Protodeltote muscosula|[1156]|LILLA637-11|United States|Illinois|658[0n]|BOLD:AAA4259
Protodeltote muscosula|[1157]|PHMO296-03|Canada|Ontario|639[0n]|BOLD:AAA4259
Protodeltote muscosula|[1158]|XAC598-04|Canada|Ontario|658[0n]|BOLD:AAA4259
Protodeltote muscosula|[1159]|XAB205-04|Canada|Ontario|658[0n]|BOLD:AAA4259
Protodeltote muscosula|[1160]|XAD153-04|Canada|Ontario|593[0n]|BOLD:AAA4259
Protodeltote muscosula|[1161]|RDLQH023-06|Canada|Quebec|655[0n]|BOLD:AAA4259
Protodeltote muscosula|[1162]|BLTIB993-08|Canada|Ontario|658[0n]|BOLD:AAA4259
Protodeltote muscosula|[1163]|XAG242-05|Canada|Ontario|658[0n]|BOLD:AAA4259
Protodeltote muscosula|[1164]|BLTIB1120-08|Canada|Ontario|606[0n]|BOLD:AAA4259
Protodeltote muscosula|[1165]|RDLQG926-06|Canada|Quebec|658[0n]|BOLD:AAA4259
Protodeltote muscosula|[1166]|BLTIB399-08|Canada|Ontario|658[0n]|BOLD:AAA4259
Protodeltote muscosula|[1167]|LMDH168-11|United States|Minnesota|658[0n]|BOLD:AAA4259
Protodeltote muscosula|[1168]|BBLPE050-09|Canada|Nova Scotia|658[0n]|BOLD:AAA4259
Protodeltote muscosula|[1169]|BBLPC177-09|Canada|Nova Scotia|658[0n]|BOLD:AAA4259
Protodeltote muscosula|[1170]|BBLEC210-09|Canada|Nova Scotia|658[0n]|BOLD:AAA4259
Protodeltote muscosula|[1171]|LPOKC813-09|United States|Oklahoma|658[0n]|BOLD:AAA4259
Protodeltote muscosula|[1172]|LPOKC578-09|United States|Oklahoma|658[0n]|BOLD:AAA4259
Protodeltote muscosula|[1173]|UDLEP152-09|United States|Pennsylvania|658[0n]|BOLD:AAA4259
Protodeltote muscosula|[1174]|BLTIB454-08|Canada|Ontario|658[0n]|BOLD:AAA4259
Protodeltote muscosula|[1175]|BLTIB236-08|Canada|Ontario|658[0n]|BOLD:AAA4259
Protodeltote muscosula|[1176]|LPSOB833-08|Canada|Ontario|658[0n]|BOLD:AAA4259
Protodeltote muscosula|[1177]|LPSOC399-08|Canada|Ontario|658[0n]|BOLD:AAA4259
Protodeltote muscosula|[1178]|LPSOB045-08|Canada|Ontario|658[0n]|BOLD:AAA4259
Protodeltote muscosula|[1179]|LPSO968-08|Canada|Ontario|658[0n]|BOLD:AAA4259
Protodeltote muscosula|[1180]|LGSMG632-07|United States|North Carolina|658[0n]|BOLD:AAA4259
Protodeltote muscosula|[1181]|LGSMG631-07|United States|North Carolina|658[0n]|BOLD:AAA4259
Protodeltote muscosula|[1182]|RDLQG915-06|Canada|Quebec|658[0n]|BOLD:AAA4259
Protodeltote muscosula|[1183]|RDLQG792-06|Canada|Quebec|655[0n]|BOLD:AAA4259
Protodeltote muscosula|[1184]|RDLQG745-06|Canada|Quebec|658[0n]|BOLD:AAA4259
Protodeltote muscosula|[1185]|RDLQG711-06|Canada|Quebec|658[0n]|BOLD:AAA4259
Protodeltote muscosula|[1186]|RDLQG709-06|Canada|Quebec|658[0n]|BOLD:AAA4259
Protodeltote muscosula|[1187]|XAK185-06|Canada|Ontario|658[0n]|BOLD:AAA4259
Protodeltote muscosula|[1188]|XAJ864-06|Canada|Ontario|658[0n]|BOLD:AAA4259
Protodeltote muscosula|[1189]|TMNBB109-06|Canada|New Brunswick|658[0n]|BOLD:AAA4259
Protodeltote muscosula|[1190]|TMNBB108-06|Canada|New Brunswick|658[0n]|BOLD:AAA4259
Protodeltote muscosula|[1191]|TMNBB107-06|Canada|New Brunswick|658[0n]|BOLD:AAA4259
Protodeltote muscosula|[1192]|TMNBB106-06|Canada|New Brunswick|658[0n]|BOLD:AAA4259
Protodeltote muscosula|[1193]|TMNBB105-06|Canada|New Brunswick|658[0n]|BOLD:AAA4259
Protodeltote muscosula|[1194]|LNC820-06|United States|North Carolina|658[0n]|BOLD:AAA4259

[illegible]
Protodeltote muscosula|[1192]|TMNBB106-06|Canada|New Brunswick|658[0n]|BOLD:AAA4259
Protodeltote muscosula|[1193]|TMNBB105-06|Canada|New Brunswick|658[0n]|BOLD:AAA4259
Protodeltote muscosula|[1194]|LNC820-06|United States|North Carolina|658[0n]|BOLD:AAA4259
Protodeltote muscosula|[1195]|TTMNB299-06|Canada|New Brunswick|658[0n]|BOLD:AAA4259
Protodeltote muscosula|[1196]|TTMNB298-06|Canada|New Brunswick|658[0n]|BOLD:AAA4259
Protodeltote muscosula|[1197]|TTMNB028-06|Canada|New Brunswick|658[0n]|BOLD:AAA4259
Protodeltote muscosula|[1198]|MNBB414-05|Canada|New Brunswick|658[0n]|BOLD:AAA4259
Protodeltote muscosula|[1199]|MNBB208-05|Canada|New Brunswick|658[0n]|BOLD:AAA4259
Protodeltote muscosula|[1200]|LGSMD372-05|United States|Tennessee|658[0n]|BOLD:AAA4259
Protodeltote muscosula|[1201]|XAF811-05|Canada|Ontario|658[0n]|BOLD:AAA4259
Protodeltote muscosula|[1202]|LOTB146-05|United States|Tennessee|658[0n]|BOLD:AAA4259
Protodeltote muscosula|[1203]|LOTB145-05|United States|Tennessee|658[0n]|BOLD:AAA4259
Protodeltote muscosula|[1204]|LGSMC468-05|United States|Tennessee|658[0n]|BOLD:AAA4259
Protodeltote muscosula|[1205]|LGSMC463-05|United States|Tennessee|658[0n]|BOLD:AAA4259
Protodeltote muscosula|[1206]|PHMNB644-04|Canada|New Brunswick|658[0n]|BOLD:AAA4259
Protodeltote muscosula|[1207]|PHMNB399-04|Canada|New Brunswick|658[0n]|BOLD:AAA4259
Protodeltote muscosula|[1208]|PHMNB385-04|Canada|New Brunswick|658[0n]|BOLD:AAA4259
Protodeltote muscosula|[1209]|PHMNB334-04|Canada|New Brunswick|658[0n]|BOLD:AAA4259
Protodeltote muscosula|[1210]|MECB138-04|Canada|Quebec|658[0n]|BOLD:AAA4259
Protodeltote muscosula|[1211]|XAC657-04|Canada|Ontario|658[0n]|BOLD:AAA4259
Protodeltote muscosula|[1212]|PHMNB147-04|Canada|New Brunswick|658[0n]|BOLD:AAA4259
Protodeltote muscosula|[1213]|XAC615-04|Canada|Ontario|658[0n]|BOLD:AAA4259
Protodeltote muscosula|[1214]|XAC605-04|Canada|Ontario|658[0n]|BOLD:AAA4259
Protodeltote muscosula|[1215]|XAC040-04|Canada|Ontario|658[0n]|BOLD:AAA4259
Protodeltote muscosula|[1216]|XAB134-04|Canada|Ontario|658[0n]|BOLD:AAA4259
Protodeltote muscosula|[1217]|LGSM439-04|United States|North Carolina|658[0n]|BOLD:AAA4259
Protodeltote muscosula|[1218]|LGSMC467-05|United States|Tennessee|658[0n]|BOLD:AAA4259
Protodeltote muscosula|[1219]|LGSM438-04|United States|North Carolina|658[0n]|BOLD:AAA4259
Protodeltote muscosula|[1220]|PHMNB521-04|Canada|New Brunswick|658[0n]|BOLD:AAA4259
Protodeltote muscosula|[1221]|PHMNB100-04|Canada|New Brunswick|658[0n]|BOLD:AAA4259
Protodeltote muscosula|[1222]|XAD390-04|Canada|Ontario|592[0n]|BOLD:AAA4259
Protodeltote muscosula|[1223]|PHMNB041-03|Canada|New Brunswick|639[0n]|BOLD:AAA4259
Protodeltote muscosula|[1224]|PHMO175-03|Canada|Ontario|639[0n]|BOLD:AAA4259
Protodeltote muscosula|[1225]|PHMO134-03|Canada|Ontario|639[0n]|BOLD:AAA4259
Protodeltote muscosula|[1226]|XAD281-04|Canada|Ontario|602[0n]|BOLD:AAA4259
Protodeltote muscosula|[1227]|TTMNB031-06|Canada|New Brunswick|622[0n]|BOLD:AAA4259
Protodeltote muscosula|[1228]|RDLQF650-06|Canada|Quebec|595[0n]|BOLD:AAA4259
Protodeltote muscosula|[1229]|LPSOC401-08|Canada|Ontario|634[0n]|BOLD:AAA4259
Protodeltote muscosula|[1230]|LMDH192-11|United States|Minnesota|658[0n]|BOLD:AAA4259
Protodeltote muscosula|[1231]|LILLA192-11|United States|Illinois|658[0n]|BOLD:AAA4259
Protodeltote muscosula|[1232]|LILLA565-11|United States|Illinois|658[0n]|BOLD:AAA4259
Protodeltote muscosula|[1233]|PHJUN4025-11|Canada|Ontario|658[0n]|BOLD:AAA4259
Protodeltote albidula|[1234]|BBLPE470-09|Canada|Newfoundland and Labrador|658[0n]|BOLD:AAA2331
Protodeltote albidula|[1235]|TTMNB301-06|Canada|New Brunswick|658[0n]|BOLD:AAA2331
Protodeltote albidula|[1236]|TTMNB303-06|Canada|New Brunswick|658[1n]|BOLD:AAA2331
Protodeltote albidula|[1237]|TMNBB110-06|Canada|New Brunswick|658[0n]|BOLD:AAA2331
Protodeltote albidula|[1238]|TMNBB112-06|Canada|New Brunswick|658[0n]|BOLD:AAA2331
Protodeltote albidula|[1239]|TMNBB113-06|Canada|New Brunswick|658[0n]|BOLD:AAA2331
Protodeltote albidula|[1240]|XAJ782-06|Canada|Ontario|658[0n]|BOLD:AAA2331
Protodeltote albidula|[1241]|RDLQG559-06|Canada|Quebec|658[0n]|BOLD:AAA2331
Protodeltote albidula|[1242]|LHLEP244-06|Canada|British Columbia|658[0n]|BOLD:AAA2331
Protodeltote albidula|[1243]|RDLQG802-06|Canada|Quebec|658[0n]|BOLD:AAA2331
Protodeltote albidula|[1244]|LHLEP248-06|Canada|British Columbia|655[0n]|BOLD:AAA2331
Protodeltote albidula|[1245]|LHLEP246-06|Canada|British Columbia|658[0n]|BOLD:AAA2331
Protodeltote albidula|[1246]|XAB576-04|Canada|Ontario|658[0n]|BOLD:AAA2331
Protodeltote albidula|[1247]|LHLEP051-06|Canada|British Columbia|649[0n]|BOLD:AAA2331
Protodeltote albidula|[1248]|BLGSM041-09|Canada|Ontario|658[0n]|BOLD:AAA2331
Protodeltote albidula|[1249]|BLGSM040-09|Canada|Ontario|658[0n]|BOLD:AAA2331
Protodeltote albidula|[1250]|BLTIB741-08|Canada|Ontario|658[0n]|BOLD:AAA2331
Protodeltote albidula|[1251]|BLTIB248-08|Canada|Ontario|658[0n]|BOLD:AAA2331
Protodeltote albidula|[1252]|LBCS755-07|Canada|British Columbia|658[0n]|BOLD:AAA2331
Protodeltote albidula|[1253]|LHLEP245-06|Canada|British Columbia|658[0n]|BOLD:AAA2331
Protodeltote albidula|[1254]|LHLEP243-06|Canada|British Columbia|658[0n]|BOLD:AAA2331
Protodeltote albidula|[1255]|LHLEP242-06|Canada|British Columbia|658[0n]|BOLD:AAA2331
Protodeltote albidula|[1256]|LHLEP241-06|Canada|British Columbia|658[0n]|BOLD:AAA2331
Protodeltote albidula|[1257]|LHLEP240-06|Canada|British Columbia|658[0n]|BOLD:AAA2331
Protodeltote albidula|[1258]|LHLEP239-06|Canada|British Columbia|658[0n]|BOLD:AAA2331
Protodeltote albidula|[1259]|LHLEP237-06|Canada|British Columbia|658[0n]|BOLD:AAA2331
Protodeltote albidula|[1260]|LHLEP088-06|Canada|British Columbia|658[0n]|BOLD:AAA2331
Protodeltote albidula|[1261]|LHLEP086-06|Canada|British Columbia|658[0n]|BOLD:AAA2331
Protodeltote albidula|[1262]|RDLQG847-06|Canada|Quebec|658[0n]|BOLD:AAA2331
Protodeltote albidula|[1263]|XAF516-05|Canada|Ontario|658[0n]|BOLD:AAA2331
Protodeltote albidula|[1264]|XAB614-04|Canada|Ontario|658[0n]|BOLD:AAA2331
Protodeltote albidula|[1265]|XAB613-04|Canada|Ontario|658[0n]|BOLD:AAA2331
Protodeltote albidula|[1266]|LPMN604-08|Canada|Manitoba|658[0n]|BOLD:AAA2331
Protodeltote albidula|[1267]|PMG128-03|Canada|Ontario|617[0n]|BOLD:AAA2331
Protodeltote albidula|[1268]|XAB577-04|Canada|Ontario|658[0n]|BOLD:AAA2331
Protodeltote albidula|[1269]|XAC609-04|Canada|Ontario|561[0n]|BOLD:AAA2331
Protodeltote albidula|[1270]|BLTIB406-08|Canada|Ontario|658[0n]|BOLD:AAA2331
Protodeltote albidula|[1271]|BLGSM051-09|Canada|Ontario|632[0n]|BOLD:AAA2331
Protodeltote albidula|[1272]|LALPA224-10|Canada|British Columbia|635[0n]|BOLD:AAA2331
Protodeltote albidula|[1273]|LALPA225-10|Canada|British Columbia|658[0n]|BOLD:AAA2331
Protodeltote albidula|[1274]|BBLPD144-10|Canada|Saskatchewan|658[0n]|BOLD:AAA2331
Protodeltote albidula|[1275]|XAB132-04|Canada|Ontario|658[0n]|BOLD:AAA2331
Protodeltote albidula|[1276]|XAB149-04|Canada|Ontario|658[0n]|BOLD:AAA2331
Protodeltote albidula|[1277]|XAF719-05|Canada|Ontario|615[0n]|BOLD:AAA2331
Protodeltote albidula|[1278]|BLTIB672-08|Canada|Ontario|658[0n]|BOLD:AAA2331
Protodeltote albidula|[1279]|BLGSM049-09|Canada|Ontario|658[0n]|BOLD:AAA2331
Protodeltote albidula|[1280]|LPMN559-08|Canada|Manitoba|658[0n]|BOLD:AAA2331
Protodeltote albidula|[1281]|BLTIB520-08|Canada|Ontario|658[0n]|BOLD:AAA2331
Protodeltote albidula|[1282]|RDLQG900-06|Canada|Quebec|658[0n]|BOLD:AAA2331
Protodeltote albidula|[1283]|RDLQG908-06|Canada|Quebec|658[0n]|BOLD:AAA2331
Protodeltote albidula|[1284]|RDLQG799-06|Canada|Quebec|658[0n]|BOLD:AAA2331
Protodeltote albidula|[1285]|RDLQG803-06|Canada|Quebec|658[0n]|BOLD:AAA2331
Protodeltote albidula|[1286]|RDLQG777-06|Canada|Quebec|658[0n]|BOLD:AAA2331
Protodeltote albidula|[1287]|RDLQG596-06|Canada|Quebec|658[0n]|BOLD:AAA2331
Protodeltote albidula|[1288]|RDLQG595-06|Canada|Quebec|658[0n]|BOLD:AAA2331
Protodeltote albidula|[1289]|RDLQG572-06|Canada|Quebec|658[0n]|BOLD:AAA2331
Protodeltote albidula|[1290]|RDLQF363-06|Canada|Quebec|658[0n]|BOLD:AAA2331
Protodeltote albidula|[1291]|XAK076-06|Canada|Ontario|658[0n]|BOLD:AAA2331
Protodeltote albidula|[1292]|XAG265-05|Canada|Ontario|658[0n]|BOLD:AAA2331
Protodeltote albidula|[1293]|XAG119-05|Canada|Ontario|658[0n]|BOLD:AAA2331

Protodeltote albidula|[1291]|XAK076-06|Canada|Ontario|658[0n]|BOLD:AAA2331
Protodeltote albidula|[1292]|XAG265-05|Canada|Ontario|658[0n]|BOLD:AAA2331
Protodeltote albidula|[1293]|XAG119-05|Canada|Ontario|658[0n]|BOLD:AAA2331
Protodeltote albidula|[1294]|XAF588-05|Canada|Ontario|658[0n]|BOLD:AAA2331
Protodeltote albidula|[1295]|XAC604-04|Canada|Ontario|658[0n]|BOLD:AAA2331
Protodeltote albidula|[1296]|XAC020-04|Canada|Ontario|592[0n]|BOLD:AAA2331
Protodeltote albidula|[1297]|RDLQG901-06|Canada|Quebec|658[0n]|BOLD:AAA2331
Protodeltote albidula|[1298]|BLGSM016-09|Canada|Ontario|632[0n]|BOLD:AAA2331
Protodeltote albidula|[1299]|JSJUN2060-11|Canada|Ontario|658[0n]|BOLD:AAA2331
Protodeltote albidula|[1300]|SSEIB12894-13|Canada|Alberta|564[0n]|BOLD:AAA2331
Protodeltote albidula|[1301]|BBLPD777-10|Canada|British Columbia|658[0n]|BOLD:AAA2331
Protodeltote albidula|[1302]|LBCA066-05|Canada|British Columbia|658[0n]|BOLD:AAA2331
Protodeltote albidula|[1303]|LBCA006-05|Canada|British Columbia|658[0n]|BOLD:AAA2331
Protodeltote albidula|[1304]|LBCA057-05|Canada|British Columbia|658[0n]|BOLD:AAA2331
Protodeltote albidula|[1305]|LBCA409-05|Canada|British Columbia|658[0n]|BOLD:AAA2331
Protodeltote albidula|[1306]|LBCA411-05|Canada|British Columbia|658[0n]|BOLD:AAA2331
Protodeltote albidula|[1307]|LBCA413-05|Canada|British Columbia|658[0n]|BOLD:AAA2331
Protodeltote albidula|[1308]|LBCA518-05|Canada|British Columbia|658[0n]|BOLD:AAA2331
Protodeltote albidula|[1309]|LBCA521-05|Canada|British Columbia|658[0n]|BOLD:AAA2331
Protodeltote albidula|[1310]|LBCA895-05|Canada|British Columbia|658[1n]|BOLD:AAA2331
Protodeltote albidula|[1311]|LOWCB852-05|Canada|British Columbia|658[0n]|BOLD:AAA2331
Protodeltote albidula|[1312]|BBLPD776-10|Canada|British Columbia|658[0n]|BOLD:AAA2331
Protodeltote albidula|[1313]|BBLPD775-10|Canada|British Columbia|658[0n]|BOLD:AAA2331
Protodeltote albidula|[1314]|BBLPD628-10|Canada|British Columbia|658[0n]|BOLD:AAA2331
Protodeltote albidula|[1315]|BBLPD624-10|Canada|British Columbia|658[0n]|BOLD:AAA2331
Protodeltote albidula|[1316]|BBLPD622-10|Canada|British Columbia|658[0n]|BOLD:AAA2331
Protodeltote albidula|[1317]|BBLPD440-10|Canada|Ontario|658[0n]|BOLD:AAA2331
Protodeltote albidula|[1318]|BBLPD438-10|Canada|Ontario|658[0n]|BOLD:AAA2331
Protodeltote albidula|[1319]|BBLPD432-10|Canada|Ontario|658[0n]|BOLD:AAA2331
Protodeltote albidula|[1320]|BBLPD200-10|Canada|British Columbia|658[0n]|BOLD:AAA2331
Protodeltote albidula|[1321]|BBLPD199-10|Canada|British Columbia|658[0n]|BOLD:AAA2331
Protodeltote albidula|[1322]|BBLPD198-10|Canada|British Columbia|658[0n]|BOLD:AAA2331
Protodeltote albidula|[1323]|BBLPD197-10|Canada|British Columbia|658[0n]|BOLD:AAA2331
Protodeltote albidula|[1324]|BBLPD153-10|Canada|Saskatchewan|658[0n]|BOLD:AAA2331
Protodeltote albidula|[1325]|BBLPD152-10|Canada|Saskatchewan|658[0n]|BOLD:AAA2331
Protodeltote albidula|[1326]|BBLPD151-10|Canada|Saskatchewan|658[0n]|BOLD:AAA2331
Protodeltote albidula|[1327]|BBLPD148-10|Canada|Saskatchewan|658[0n]|BOLD:AAA2331
Protodeltote albidula|[1328]|BBLPD146-10|Canada|Saskatchewan|658[0n]|BOLD:AAA2331
Protodeltote albidula|[1329]|BBLPD145-10|Canada|Saskatchewan|658[0n]|BOLD:AAA2331
Protodeltote albidula|[1330]|BBLPB984-10|Canada|Alberta|658[0n]|BOLD:AAA2331
Protodeltote albidula|[1331]|BBLPB982-10|Canada|Alberta|658[0n]|BOLD:AAA2331
Protodeltote albidula|[1332]|RWWB805-10|United States|Washington|658[0n]|BOLD:AAA2331
Protodeltote albidula|[1333]|BBLPE473-09|Canada|Newfoundland and Labrador|658[0n]|BOLD:AAA2331
Protodeltote albidula|[1334]|BBLPE401-09|Canada|Newfoundland and Labrador|658[0n]|BOLD:AAA2331
Protodeltote albidula|[1335]|BBLPE156-09|Canada|Nova Scotia|658[0n]|BOLD:AAA2331
Protodeltote albidula|[1336]|BBLPC821-09|Canada|Newfoundland and Labrador|658[0n]|BOLD:AAA2331
Protodeltote albidula|[1337]|BBLPC782-09|Canada|Newfoundland and Labrador|658[0n]|BOLD:AAA2331
Protodeltote albidula|[1338]|BBLPC738-09|Canada|Newfoundland and Labrador|658[0n]|BOLD:AAA2331
Protodeltote albidula|[1339]|BBLPC737-09|Canada|Newfoundland and Labrador|658[0n]|BOLD:AAA2331
Protodeltote albidula|[1340]|BBLPC721-09|Canada|Newfoundland and Labrador|658[0n]|BOLD:AAA2331
Protodeltote albidula|[1341]|BBLPC659-09|Canada|Newfoundland and Labrador|658[0n]|BOLD:AAA2331
Protodeltote albidula|[1342]|BBLPC614-09|Canada|Nova Scotia|658[0n]|BOLD:AAA2331
Protodeltote albidula|[1343]|BBLPC302-09|Canada|Newfoundland and Labrador|658[0n]|BOLD:AAA2331
Protodeltote albidula|[1344]|RWWA603-09|United States|Washington|658[0n]|BOLD:AAA2331
Protodeltote albidula|[1345]|RWWA520-09|United States|Washington|658[0n]|BOLD:AAA2331
Protodeltote albidula|[1346]|RWWA359-09|United States|Washington|658[0n]|BOLD:AAA2331
Protodeltote albidula|[1347]|RWWA223-09|United States|Washington|658[0n]|BOLD:AAA2331
Protodeltote albidula|[1348]|LPSOD692-09|Canada|Ontario|658[0n]|BOLD:AAA2331
Protodeltote albidula|[1349]|LPSOD618-09|Canada|Ontario|658[0n]|BOLD:AAA2331
Protodeltote albidula|[1350]|LPABB279-08|Canada|Alberta|658[0n]|BOLD:AAA2331
Protodeltote albidula|[1351]|LPABB272-08|Canada|Alberta|658[0n]|BOLD:AAA2331
Protodeltote albidula|[1352]|LPABB232-08|Canada|Alberta|658[0n]|BOLD:AAA2331
Protodeltote albidula|[1353]|LBCW029-08|Canada|British Columbia|658[0n]|BOLD:AAA2331
Protodeltote albidula|[1354]|LBCW028-08|Canada|British Columbia|658[0n]|BOLD:AAA2331
Protodeltote albidula|[1355]|LPMN616-08|Canada|Manitoba|658[0n]|BOLD:AAA2331
Protodeltote albidula|[1356]|LPMN524-08|Canada|Manitoba|658[0n]|BOLD:AAA2331
Protodeltote albidula|[1357]|LPMN492-08|Canada|Manitoba|658[0n]|BOLD:AAA2331
Protodeltote albidula|[1358]|LPMN491-08|Canada|Manitoba|658[0n]|BOLD:AAA2331
Protodeltote albidula|[1359]|LPMN489-08|Canada|Manitoba|658[0n]|BOLD:AAA2331
Protodeltote albidula|[1360]|LHLEP247-06|Canada|British Columbia|658[0n]|BOLD:AAA2331
Protodeltote albidula|[1361]|LHLEP238-06|Canada|British Columbia|658[0n]|BOLD:AAA2331
Protodeltote albidula|[1362]|LHLEP087-06|Canada|British Columbia|658[0n]|BOLD:AAA2331
Protodeltote albidula|[1363]|RDLQG675-06|Canada|Quebec|658[0n]|BOLD:AAA2331
Protodeltote albidula|[1364]|RDLQG674-06|Canada|Quebec|658[0n]|BOLD:AAA2331
Protodeltote albidula|[1365]|RDLQG532-06|Canada|Quebec|658[0n]|BOLD:AAA2331
Protodeltote albidula|[1366]|RDLQG531-06|Canada|Quebec|658[0n]|BOLD:AAA2331
Protodeltote albidula|[1367]|RDLQG434-06|Canada|Quebec|658[0n]|BOLD:AAA2331
Protodeltote albidula|[1368]|RDLQG433-06|Canada|Quebec|658[0n]|BOLD:AAA2331
Protodeltote albidula|[1369]|RDLQG432-06|Canada|Quebec|658[0n]|BOLD:AAA2331
Protodeltote albidula|[1370]|RDLQG431-06|Canada|Quebec|658[0n]|BOLD:AAA2331
Protodeltote albidula|[1371]|TMNBB111-06|Canada|New Brunswick|658[0n]|BOLD:AAA2331
Protodeltote albidula|[1372]|LOWCE338-06|Canada|British Columbia|658[0n]|BOLD:AAA2331
Protodeltote albidula|[1373]|RDLQB258-05|Canada|Quebec|658[0n]|BOLD:AAA2331
Protodeltote albidula|[1374]|LOWCB180-05|Canada|British Columbia|658[0n]|BOLD:AAA2331
Protodeltote albidula|[1375]|LOWCB164-05|Canada|British Columbia|658[0n]|BOLD:AAA2331
Protodeltote albidula|[1376]|LBCD608-05|Canada|British Columbia|658[0n]|BOLD:AAA2331
Protodeltote albidula|[1377]|LBCD607-05|Canada|British Columbia|658[0n]|BOLD:AAA2331
Protodeltote albidula|[1378]|LBCD606-05|Canada|British Columbia|658[0n]|BOLD:AAA2331
Protodeltote albidula|[1379]|LBCD605-05|Canada|British Columbia|658[0n]|BOLD:AAA2331
Protodeltote albidula|[1380]|LBCD604-05|Canada|British Columbia|658[0n]|BOLD:AAA2331
Protodeltote albidula|[1381]|LBCD603-05|Canada|British Columbia|658[0n]|BOLD:AAA2331
Protodeltote albidula|[1382]|LBCD602-05|Canada|British Columbia|658[0n]|BOLD:AAA2331
Protodeltote albidula|[1383]|LBCD581-05|Canada|British Columbia|658[0n]|BOLD:AAA2331
Protodeltote albidula|[1384]|LBCA897-05|Canada|British Columbia|658[0n]|BOLD:AAA2331
Protodeltote albidula|[1385]|LBCA529-05|Canada|British Columbia|658[0n]|BOLD:AAA2331
Protodeltote albidula|[1386]|LBCA527-05|Canada|British Columbia|658[0n]|BOLD:AAA2331
Protodeltote albidula|[1387]|LBCA526-05|Canada|British Columbia|658[0n]|BOLD:AAA2331
Protodeltote albidula|[1388]|LBCA525-05|Canada|British Columbia|658[0n]|BOLD:AAA2331
Protodeltote albidula|[1389]|LBCA523-05|Canada|British Columbia|658[0n]|BOLD:AAA2331
Protodeltote albidula|[1390]|LBCA522-05|Canada|British Columbia|658[0n]|BOLD:AAA2331
Protodeltote albidula|[1391]|LBCA520-05|Canada|British Columbia|658[0n]|BOLD:AAA2331
Protodeltote albidula|[1392]|LBCA425-05|Canada|British Columbia|658[0n]|BOLD:AAA2331
Protodeltote albidula|[1393]|LBCA422-05|Canada|British Columbia|658[0n]|BOLD:AAA2331

Protodeltote albidula|[1390]|LBCA522-05|Canada|British Columbia|658[0n]|BOLD:AAA2331
Protodeltote albidula|[1391]|LBCA520-05|Canada|British Columbia|658[0n]|BOLD:AAA2331
Protodeltote albidula|[1392]|LBCA425-05|Canada|British Columbia|658[0n]|BOLD:AAA2331
Protodeltote albidula|[1393]|LBCA422-05|Canada|British Columbia|658[0n]|BOLD:AAA2331
Protodeltote albidula|[1394]|LBCA421-05|Canada|British Columbia|658[0n]|BOLD:AAA2331
Protodeltote albidula|[1395]|LBCA420-05|Canada|British Columbia|658[0n]|BOLD:AAA2331
Protodeltote albidula|[1396]|LBCA419-05|Canada|British Columbia|658[0n]|BOLD:AAA2331
Protodeltote albidula|[1397]|LBCA418-05|Canada|British Columbia|658[0n]|BOLD:AAA2331
Protodeltote albidula|[1398]|LBCA417-05|Canada|British Columbia|658[0n]|BOLD:AAA2331
Protodeltote albidula|[1399]|LBCA416-05|Canada|British Columbia|658[0n]|BOLD:AAA2331
Protodeltote albidula|[1400]|LBCA415-05|Canada|British Columbia|658[0n]|BOLD:AAA2331
Protodeltote albidula|[1401]|LBCA414-05|Canada|British Columbia|658[0n]|BOLD:AAA2331
Protodeltote albidula|[1402]|LBCA412-05|Canada|British Columbia|658[0n]|BOLD:AAA2331
Protodeltote albidula|[1403]|LBCA410-05|Canada|British Columbia|658[0n]|BOLD:AAA2331
Protodeltote albidula|[1404]|LBCA134-05|Canada|British Columbia|658[0n]|BOLD:AAA2331
Protodeltote albidula|[1405]|LBCA071-05|Canada|British Columbia|658[0n]|BOLD:AAA2331
Protodeltote albidula|[1406]|LBCA069-05|Canada|British Columbia|658[0n]|BOLD:AAA2331
Protodeltote albidula|[1407]|LBCA068-05|Canada|British Columbia|658[0n]|BOLD:AAA2331
Protodeltote albidula|[1408]|LBCA067-05|Canada|British Columbia|658[0n]|BOLD:AAA2331
Protodeltote albidula|[1409]|LBCA065-05|Canada|British Columbia|658[0n]|BOLD:AAA2331
Protodeltote albidula|[1410]|LBCA064-05|Canada|British Columbia|658[0n]|BOLD:AAA2331
Protodeltote albidula|[1411]|LBCA063-05|Canada|British Columbia|658[0n]|BOLD:AAA2331
Protodeltote albidula|[1412]|LBCA062-05|Canada|British Columbia|658[0n]|BOLD:AAA2331
Protodeltote albidula|[1413]|LBCA061-05|Canada|British Columbia|658[0n]|BOLD:AAA2331
Protodeltote albidula|[1414]|LBCA060-05|Canada|British Columbia|658[0n]|BOLD:AAA2331
Protodeltote albidula|[1415]|LBCA059-05|Canada|British Columbia|658[0n]|BOLD:AAA2331
Protodeltote albidula|[1416]|LBCA058-05|Canada|British Columbia|658[0n]|BOLD:AAA2331
Protodeltote albidula|[1417]|LBCA056-05|Canada|British Columbia|658[0n]|BOLD:AAA2331
Protodeltote albidula|[1418]|LBCA055-05|Canada|British Columbia|658[0n]|BOLD:AAA2331
Protodeltote albidula|[1419]|LBCA054-05|Canada|British Columbia|658[0n]|BOLD:AAA2331
Protodeltote albidula|[1420]|LBCA010-05|Canada|British Columbia|658[0n]|BOLD:AAA2331
Protodeltote albidula|[1421]|LBCA009-05|Canada|British Columbia|658[0n]|BOLD:AAA2331
Protodeltote albidula|[1422]|LBCA008-05|Canada|British Columbia|658[0n]|BOLD:AAA2331
Protodeltote albidula|[1423]|LBCA007-05|Canada|British Columbia|658[0n]|BOLD:AAA2331
Protodeltote albidula|[1424]|LBCA005-05|Canada|British Columbia|658[0n]|BOLD:AAA2331
Protodeltote albidula|[1425]|LBCA004-05|Canada|British Columbia|658[0n]|BOLD:AAA2331
Protodeltote albidula|[1426]|CNRME1852-12|Canada|Manitoba|636[0n]|BOLD:AAA2331
Protodeltote albidula|[1427]|LBCA003-05|Canada|British Columbia|658[0n]|BOLD:AAA2331
Protodeltote albidula|[1428]|TTMNB300-06|Canada|New Brunswick|658[1n]|BOLD:AAA2331
Protodeltote albidula|[1429]|LOWCC870-05|Canada|British Columbia|658[0n]|BOLD:AAA2331
Protodeltote albidula|[1430]|CNRMD2099-12|Canada|Manitoba|614[0n]|BOLD:AAA2331
Protodeltote albidula|[1431]|LOWCB853-05|Canada|British Columbia|560[0n]|BOLD:AAA2331
Protodeltote albidula|[1432]|LBCA894-05|Canada|British Columbia|615[0n]|BOLD:AAA2331
Protodeltote albidula|[1433]|LOWCB849-05|Canada|British Columbia|574[0n]|BOLD:AAA2331
Protodeltote albidula|[1434]|LOWCB850-05|Canada|British Columbia|574[0n]|BOLD:AAA2331
Protodeltote albidula|[1435]|LOWCB851-05|Canada|British Columbia|592[0n]|BOLD:AAA2331
Protodeltote albidula|[1436]|RWWB868-10|United States|Washington|632[0n]|BOLD:AAA2331
Protodeltote albidula|[1437]|BBLPD626-10|Canada|British Columbia|639[0n]|BOLD:AAA2331
Protodeltote albidula|[1438]|CNRME2762-12|Canada|Manitoba|621[0n]|BOLD:AAA2331
Protodeltote albidula|[1439]|CNRME2823-12|Canada|Manitoba|637[0n]|BOLD:AAA2331
Protodeltote albidula|[1440]|SSEIB11414-13|Canada|Alberta|597[0n]|BOLD:AAA2331
Protodeltote albidula|[1441]|LPMN622-08|Canada|Manitoba|658[0n]|BOLD:AAA2331
Protodeltote albidula|[1442]|LPMN657-08|Canada|Manitoba|658[0n]|BOLD:AAA2331
Protodeltote albidula|[1443]|BBLPB983-10|Canada|Alberta|658[0n]|BOLD:AAA2331
Protodeltote albidula|[1444]|BBLPB989-10|Canada|Alberta|658[0n]|BOLD:AAA2331
Protodeltote albidula|[1445]|BBLPB990-10|Canada|Alberta|658[0n]|BOLD:AAA2331
Protodeltote albidula|[1446]|BBLPD143-10|Canada|Saskatchewan|658[0n]|BOLD:AAA2331
Protodeltote albidula|[1447]|BBLPD147-10|Canada|Saskatchewan|658[0n]|BOLD:AAA2331
Protodeltote albidula|[1448]|BBLPD154-10|Canada|Saskatchewan|658[0n]|BOLD:AAA2331
Protodeltote albidula|[1449]|SSEIB11415-13|Canada|Alberta|570[0n]|BOLD:AAA2331
Protodeltote albidula|[1450]|SSEIB12907-13|Canada|Alberta|564[0n]|BOLD:AAA2331
Protodeltote albidula|[1451]|SSEIB12908-13|Canada|Alberta|564[0n]|BOLD:AAA2331
Phoenicophanta bicolor|[1452]|CMAZA1259-12|United States|Arizona|633[0n]|BOLD:ABW6351
Phoenicophanta modestula|[1453]|CNCLB2151-14|United States|Arizona|658[0n]|BOLD:AAK3779
Phoenicophanta modestula|[1454]|CNCLB2152-14|United States|Arizona|658[0n]|BOLD:AAK3779
Phoenicophanta modestula|[1455]|CNCLB2153-14|United States|Arizona|658[0n]|BOLD:AAK3779
Notioplusia illustrata|[1456]|MHMYH731-10|Costa Rica||658[0n]|BOLD:AAF2716
Notioplusia illustrata|[1457]|MHMYO199-11|Costa Rica||658[0n]|BOLD:AAF2716
Notioplusia illustrata|[1458]|MHMYQ1710-12|Costa Rica||658[0n]|BOLD:AAF2716
Notioplusia illustrata|[1459]|MHMYQ1713-12|Costa Rica||658[0n]|BOLD:AAF2716
Notioplusia illustrata|[1460]|MHMYO200-11|Costa Rica||658[0n]|BOLD:AAF2716
Notioplusia illustrata|[1461]|MHMYQ1711-12|Costa Rica||658[0n]|BOLD:AAF2716
Notioplusia illustrata|[1462]|MHMYQ1712-12|Costa Rica||658[0n]|BOLD:AAF2716
Notioplusia illustrata|[1463]|NOCJB061-08|Saint Kitts and Nevis||593[0n]|BOLD:AAF2716
Notioplusia illustrata|[1464]|MHMYM2360-11|Costa Rica||658[0n]|BOLD:AAF2716
Notioplusia illustrata|[1465]|GWORX842-10|Australia|Queensland|658[0n]|BOLD:AAF2716
Notioplusia illustrata|[1466]|HKONS094-07|United States|Florida|658[1n]|BOLD:AAF2716
Notioplusia illustrata|[1467]|NOCJB062-08|Cuba||640[0n]|BOLD:AAF2716
Notioplusia illustrata|[1468]|MHMYQ1714-12|Costa Rica||620[0n]|BOLD:AAF2716
Rachiplusia ou|[1469]|BBLSW439-09|United States|Oklahoma|658[0n]|BOLD:AAE7504
Rachiplusia ou|[1470]|LPOKE571-12|United States|Oklahoma|590[0n]|BOLD:AAE7504
Rachiplusia ou|[1471]|RDNMG534-08|United States|Arizona|658[0n]|BOLD:AAE7504
Rachiplusia ou|[1472]|BLPEE5746-14|Costa Rica||658[0n]|BOLD:AAE7504
Rachiplusia ou|[1473]|RDNMF443-08|Canada|Ontario|609[0n]|BOLD:AAE7504
Rachiplusia ou|[1474]|BLPBH780-07|Costa Rica|Guanacaste|632[0n]|BOLD:AAE7504
Rachiplusia nu|[1475]|NOCJB064-08|Peru|Amazonas|658[0n]|BOLD:AAE7504
Rachiplusia ou|[1476]|LPOKB290-09|United States|Oklahoma|658[0n]|BOLD:AAE7504
Rachiplusia ou|[1477]|LILLA724-11|United States|Illinois|658[0n]|BOLD:AAE7504
Rachiplusia ou|[1478]|BLPEE5750-14|Costa Rica||658[0n]|BOLD:AAE7504
Rachiplusia ou|[1479]|RDNMF651-08|United States|Missouri|658[0n]|BOLD:AAE7504
Rachiplusia ou|[1480]|NOCJB065-08|Guatemala|Quezaltenango|658[0n]|BOLD:AAE7504
Rachiplusia ou|[1481]|CMAZA393-10|United States|Arizona|658[0n]|BOLD:AAE7504
Rachiplusia ou|[1482]|LILLB011-11|United States|Illinois|658[0n]|BOLD:AAE7504
Rachiplusia ou|[1483]|MHMYT338-13|Costa Rica||658[0n]|BOLD:AAE7504
Rachiplusia ou|[1484]|BLPEE5751-14|Costa Rica||658[0n]|BOLD:AAE7504
Autoplusia egenoides|[1485]|LOCRE177-10|Costa Rica|Cartago|658[0n]|BOLD:AAF4115
Autoplusia egenoides|[1486]|LOCRA570-07|Costa Rica|Alajuela|658[0n]|BOLD:AAF4115
Autoplusia egenoides|[1487]|LOCRA571-07|Costa Rica|Alajuela|657[0n]|BOLD:AAF4115
Autoplusia egenoides|[1488]|NOCJB056-08|Panama|Chiriqui|646[0n]|BOLD:AAF4115
Autoplusia egenoides|[1489]|GMLC1221-12|United States|California|658[0n]|BOLD:AAF4115
Autoplusia olivacea|[1490]|CNCLB293-14|United States|California|658[0n]|BOLD:AAF4115
Autoplusia egena egena|[1491]|NOCJB055-08|Bolivia|Tarija|658[0n]|BOLD:AAJ9168
Autoplusia egena|[1492]|MHMXZ404-09|Costa Rica||658[0n]|BOLD:AAJ9168

Autoplusia olivacea|[1490]|CNCLB293-14|United States|California|658[0n]|BOLD:AAF4115
Autoplusia egena egena|[1491]|NOCJB055-08|Bolivia|Tarija|658[0n]|BOLD:AAJ9168
Autoplusia egena|[1492]|MHMXZ404-09|Costa Rica||658[0n]|BOLD:AAJ9168
Autoplusia egena|[1493]|CNCLB1935-14|United States|Texas|658[1n]|BOLD:AAJ9168
Autoplusia egena|[1494]|CNCLB2399-14|United States|Texas|658[0n]|BOLD:AAJ9168
Enigmogramma basigera|[1495]|LOT325-04|United States|Tennessee|658[0n]|BOLD:ACF0954
Enigmogramma basigera|[1496]|LOFLA685-06|United States|Florida|658[0n]|BOLD:ACF0954
Enigmogramma basigera|[1497]|MHMXZ931-09|Costa Rica||658[0n]|BOLD:ACF0954
Enigmogramma antillea|[1498]|RDNML008-13|United States|Florida|658[0n]|BOLD:ACD3793
Enigmogramma antillea|[1499]|RDNML287-13|United States|Florida|658[0n]|BOLD:ACD3793
Enigmogramma antillea|[1500]|RDNML288-13|United States|Florida|658[0n]|BOLD:ACD3793
Trichoplusia ni|[1501]|BBLSW843-09|United States|Texas|657[0n]|BOLD:AAC3409
Trichoplusia ni|[1502]|AWCLB109-10|United States|Arizona|657[0n]|BOLD:AAC3409
Trichoplusia ni|[1503]|LTOL544-07|United States|Maryland|654[0n]|BOLD:AAC3409
Trichoplusia ni|[1504]|LBCA607-05|Canada|British Columbia|618[0n]|BOLD:AAC3409
Trichoplusia ni|[1505]|PHMO357-03|Canada|Ontario|639[0n]|BOLD:AAC3409
Trichoplusia ni|[1506]|XAD373-04|Canada|Ontario|658[0n]|BOLD:AAC3409
Trichoplusia ni|[1507]|RDLQB835-05|Canada|Quebec|658[0n]|BOLD:AAC3409
Trichoplusia ni|[1508]|NAGEO043-09|United States|Maryland|658[0n]|
Trichoplusia ni|[1509]|JMMMB414-11|United States|California|658[0n]|BOLD:AAC3409
Trichoplusia ni|[1510]|LBCA806-05|Canada|British Columbia|658[0n]|BOLD:AAC3409
Trichoplusia ni|[1511]|RDLQB754-05|Canada|Quebec|617[0n]|BOLD:AAC3409
Trichoplusia ni|[1512]|RDLQB834-05|Canada|Quebec|620[1n]|BOLD:AAC3409
Trichoplusia ni|[1513]|LPOKA528-09|United States|Oklahoma|658[0n]|BOLD:AAC3409
Trichoplusia ni|[1514]|LPOKB294-09|United States|Oklahoma|658[0n]|BOLD:AAC3409
Trichoplusia ni|[1515]|BBLSW325-09|United States|Arizona|658[0n]|BOLD:AAC3409
Trichoplusia ni|[1516]|BBLSX680-09|United States|Arizona|658[0n]|BOLD:AAC3409
Trichoplusia ni|[1517]|CMAZA172-09|United States|Arizona|658[0n]|BOLD:AAC3409
Trichoplusia ni|[1518]|LPOKD621-09|United States|Oklahoma|658[0n]|BOLD:AAC3409
Trichoplusia ni|[1519]|AWCLB402-10|United States|Arizona|658[0n]|BOLD:AAC3409
Trichoplusia ni|[1520]|AWCLB413-10|United States|Arizona|658[0n]|BOLD:AAC3409
Trichoplusia ni|[1521]|AWCLB415-10|United States|Arizona|658[0n]|BOLD:AAC3409
Trichoplusia ni|[1522]|AWCLB418-10|United States|Arizona|658[0n]|BOLD:AAC3409
Trichoplusia ni|[1523]|AWCLB448-10|United States|Arizona|658[0n]|BOLD:AAC3409
Trichoplusia ni|[1524]|BBLPB456-10|Canada|Ontario|658[0n]|BOLD:AAC3409
Trichoplusia ni|[1525]|LILLA566-11|United States|Illinois|658[0n]|BOLD:AAC3409
Trichoplusia ni|[1526]|BBLOC614-11|United States|Arizona|658[0n]|BOLD:AAC3409
Trichoplusia ni|[1527]|GMLC1200-12|United States|California|658[0n]|BOLD:AAC3409
Trichoplusia ni|[1528]|LOCBF2599-13|United States|California|658[0n]|BOLD:AAC3409
Trichoplusia ni|[1529]|LOCBF2603-13|United States|California|658[0n]|BOLD:AAC3409
Argyrogramma verruca|[1530]|LNC850-06|United States|North Carolina|658[0n]|BOLD:AAC5989
Argyrogramma verruca|[1531]|LPOKA597-09|United States|Oklahoma|658[0n]|BOLD:AAC5989
Argyrogramma verruca|[1532]|LNCB072-06|United States|North Carolina|658[0n]|BOLD:AAC5989
Argyrogramma verruca|[1533]|RDNMK050-11|United States|Florida|658[0n]|BOLD:AAC5989
Chrysodeixis chalcites|[1534]|LEUAE895-12|United Arab Emirates|Fujairah|658[0n]|BOLD:AAB3384
Chrysodeixis chalcites|[1535]|CNCLA570-13|United States|Hawaii|658[0n]|BOLD:AAB3384
Chrysodeixis chalcites|[1536]|GWORR870-10|Spain|Murcia|658[0n]|BOLD:AAB3384
Chrysodeixis chalcites|[1537]|GWOSZ718-11|Cameroon|Sud-Ouest|658[0n]|BOLD:AAB3384
Chrysodeixis chalcites|[1538]|LEUAE348-12|United Arab Emirates|Fujairah|658[0n]|BOLD:AAB3384
Chrysodeixis chalcites|[1539]|LMHRG044-06|Canada|British Columbia|613[0n]|BOLD:AAB3384
Chrysodeixis chalcites|[1540]|LMHRG045-06|Canada|British Columbia|635[0n]|BOLD:AAB3384
Chrysodeixis chalcites|[1541]|PHLPM056-11|Portugal|Madeira|658[0n]|BOLD:AAB3384
Chrysodeixis chalcites|[1542]|GWOSP064-11|Zimbabwe||658[0n]|BOLD:AAB3384
Chrysodeixis chalcites|[1543]|TZBCA102-06|Canada|Ontario|658[0n]|BOLD:AAB3384
Chrysodeixis chalcites|[1544]|RDNMH238-09|Canada|Ontario|658[0n]|BOLD:AAB3384
Chrysodeixis chalcites|[1545]|GWOSA330-10|Italy|Calabria|658[0n]|BOLD:AAB3384
Chrysodeixis chalcites|[1546]|RDNMK153-11|Canada|Ontario|658[0n]|BOLD:AAB3384
Chrysodeixis chalcites|[1547]|RDNMK154-11|Canada|Ontario|658[0n]|BOLD:AAB3384
Chrysodeixis chalcites|[1548]|RDNMK155-11|Canada|Ontario|658[0n]|BOLD:AAB3384
Chrysodeixis chalcites|[1549]|RDNMK156-11|Canada|Ontario|658[0n]|BOLD:AAB3384
Chrysodeixis chalcites|[1550]|RDNMK227-11|Portugal||658[0n]|BOLD:AAB3384
Chrysodeixis chalcites|[1551]|BCMI041-11|Israel||658[0n]|BOLD:AAB3384
Chrysodeixis chalcites|[1552]|BCMI449-11|Israel||658[0n]|BOLD:AAB3384
Chrysodeixis chalcites|[1553]|GWOTF359-12|Italy|Basilicata|658[0n]|BOLD:AAB3384
Chrysodeixis chalcites|[1554]|LEUAE349-12|United Arab Emirates|Fujairah|658[0n]|BOLD:AAB3384
Chrysodeixis chalcites|[1555]|TRLEP109-13|Turkey|Mersin|658[0n]|BOLD:AAB3384
Chrysodeixis chalcites|[1556]|GBLAC219-13|Germany|Bavaria|658[0n]|BOLD:AAB3384
Chrysodeixis chalcites|[1557]|IBLAO1037-14|Spain|Murcia|658[0n]|BOLD:AAB3384
Chrysodeixis includens|[1558]|BLPAF765-07|Costa Rica|Guanacaste|658[0n]|BOLD:AAA6794
Chrysodeixis includens|[1559]|GBGL12676-13|Brazil||658[0n]|BOLD:AAA6794
Chrysodeixis includens|[1560]|LGSMG611-07|United States|North Carolina|658[0n]|BOLD:AAA6794
Chrysodeixis includens|[1561]|NOCJB053-08|Bolivia|Tarija|609[0n]|BOLD:AAA6794
Chrysodeixis includens|[1562]|LOCRA264-06|Costa Rica|San Jose|628[0n]|BOLD:AAA6794
Chrysodeixis includens|[1563]|ECPD519-14|Argentina|Formosa|658[0n]|BOLD:AAA6794
Chrysodeixis includens|[1564]|GBGL12675-13|Brazil||658[0n]|BOLD:AAA6794
Chrysodeixis includens|[1565]|GBGL12674-13|Brazil||658[0n]|BOLD:AAA6794
Chrysodeixis includens|[1566]|GBGL12673-13|Brazil||658[0n]|BOLD:AAA6794
Chrysodeixis includens|[1567]|GBGL12671-13|Brazil||658[0n]|BOLD:AAA6794
Chrysodeixis includens|[1568]|GBGL12670-13|Brazil||658[0n]|BOLD:AAA6794
Chrysodeixis includens|[1569]|GBGL12669-13|Brazil||658[0n]|BOLD:AAA6794
Chrysodeixis includens|[1570]|GBGL12668-13|Brazil||658[0n]|BOLD:AAA6794
Chrysodeixis includens|[1571]|GBGL12667-13|Brazil||658[0n]|BOLD:AAA6794
Chrysodeixis includens|[1572]|GBGL12666-13|Brazil||658[0n]|BOLD:AAA6794
Chrysodeixis includens|[1573]|GBGL12665-13|Brazil||658[0n]|BOLD:AAA6794
Chrysodeixis includens|[1574]|LNAUP107-13|Peru||658[0n]|BOLD:AAA6794
Chrysodeixis includens|[1575]|LNAUP106-13|Peru||658[0n]|BOLD:AAA6794
Chrysodeixis includens|[1576]|MOTAR206-12|Argentina|Entre Rios|658[0n]|BOLD:AAA6794
Chrysodeixis includens|[1577]|MOTAR197-12|Argentina|Misiones|658[0n]|BOLD:AAA6794
Chrysodeixis includens|[1578]|MOTAR192-12|Argentina|Misiones|658[0n]|BOLD:AAA6794
Chrysodeixis includens|[1579]|ARMOT187-12|Argentina|Entre Rios|658[0n]|BOLD:AAA6794
Chrysodeixis includens|[1580]|MHMYO207-11|Costa Rica||658[0n]|BOLD:AAA6794
Chrysodeixis includens|[1581]|MHMYM006-11|Costa Rica||658[0n]|BOLD:AAA6794
Chrysodeixis includens|[1582]|MHMYL3411-11|Costa Rica||658[0n]|BOLD:AAA6794
Chrysodeixis includens|[1583]|MHMYL3283-11|Costa Rica||658[0n]|BOLD:AAA6794
Chrysodeixis includens|[1584]|MHMYL3282-11|Costa Rica||658[0n]|BOLD:AAA6794
Chrysodeixis includens|[1585]|MHMYL3281-11|Costa Rica||658[0n]|BOLD:AAA6794
Chrysodeixis includens|[1586]|MHMYL3269-11|Costa Rica||658[0n]|BOLD:AAA6794
Chrysodeixis includens|[1587]|LILLB064-11|United States|Illinois|658[0n]|BOLD:AAA6794
Chrysodeixis includens|[1588]|LILLA958-11|United States|Illinois|658[0n]|BOLD:AAA6794
Chrysodeixis includens|[1589]|LILLA916-11|United States|Illinois|658[0n]|BOLD:AAA6794
Chrysodeixis includens|[1590]|BLPDV009-11|Costa Rica|Guanacaste|658[0n]|BOLD:AAA6794
Chrysodeixis includens|[1591]|BLPDV008-11|Costa Rica|Guanacaste|658[0n]|BOLD:AAA6794
Chrysodeixis includens|[1592]|BLPDU802-11|Costa Rica|Guanacaste|658[0n]|BOLD:AAA6794

Chrysodeixis includens|[1589]|[illegible]|United States|Illinois|658[0n]|BOLD:AAA6794
Chrysodeixis includens|[1590]|BLPDV009-11|Costa Rica|Guanacaste|658[0n]|BOLD:AAA6794
Chrysodeixis includens|[1591]|BLPDV008-11|Costa Rica|Guanacaste|658[0n]|BOLD:AAA6794
Chrysodeixis includens|[1592]|BLPDU802-11|Costa Rica|Guanacaste|658[0n]|BOLD:AAA6794
Chrysodeixis includens|[1593]|BLPDU520-11|Costa Rica|Guanacaste|658[0n]|BOLD:AAA6794
Chrysodeixis includens|[1594]|BLPDU206-11|Costa Rica|Guanacaste|658[0n]|BOLD:AAA6794
Chrysodeixis includens|[1595]|BLPDU205-11|Costa Rica|Guanacaste|658[0n]|BOLD:AAA6794
Chrysodeixis includens|[1596]|LEMMZ375-10|Brazil|Parana|658[0n]|BOLD:AAA6794
Chrysodeixis includens|[1597]|LEMMZ374-10|Brazil|Parana|658[0n]|BOLD:AAA6794
Chrysodeixis includens|[1598]|LEMMZ138-10|Brazil|Parana|658[0n]|BOLD:AAA6794
Chrysodeixis includens|[1599]|LEMMZ136-10|Brazil|Parana|658[0n]|BOLD:AAA6794
Chrysodeixis includens|[1600]|LEMMZ135-10|Brazil|Parana|658[0n]|BOLD:AAA6794
Chrysodeixis includens|[1601]|MHMYH813-10|Costa Rica||658[0n]|BOLD:AAA6794
Chrysodeixis includens|[1602]|MHMYH812-10|Costa Rica||658[0n]|BOLD:AAA6794
Chrysodeixis includens|[1603]|LPOKD449-09|United States|Oklahoma|658[0n]|BOLD:AAA6794
Chrysodeixis includens|[1604]|LPOKD432-09|United States|Oklahoma|658[0n]|BOLD:AAA6794
Chrysodeixis includens|[1605]|LPOKD427-09|United States|Oklahoma|658[0n]|BOLD:AAA6794
Chrysodeixis includens|[1606]|LPOKD407-09|United States|Oklahoma|658[0n]|BOLD:AAA6794
Chrysodeixis includens|[1607]|MHMYC2167-09|Costa Rica|Alajuela|658[0n]|BOLD:AAA6794
Chrysodeixis includens|[1608]|LPOKA1032-09|United States|Oklahoma|658[0n]|BOLD:AAA6794
Chrysodeixis includens|[1609]|LPOKA567-09|United States|Oklahoma|658[0n]|BOLD:AAA6794
Chrysodeixis includens|[1610]|LPYPB275-08|Mexico|Quintana Roo|658[0n]|BOLD:AAA6794
Chrysodeixis includens|[1611]|BLPCD637-08|Costa Rica|Guanacaste|658[0n]|BOLD:AAA6794
Chrysodeixis includens|[1612]|BLPCC493-08|Costa Rica|Guanacaste|658[0n]|BOLD:AAA6794
Chrysodeixis includens|[1613]|MHMXM175-07|Costa Rica|Alajuela|658[0n]|BOLD:AAA6794
Chrysodeixis includens|[1614]|MHMXM174-07|Costa Rica|Alajuela|658[0n]|BOLD:AAA6794
Chrysodeixis includens|[1615]|MHMXM173-07|Costa Rica|Alajuela|658[0n]|BOLD:AAA6794
Chrysodeixis includens|[1616]|MHMXM172-07|Costa Rica|Alajuela|658[0n]|BOLD:AAA6794
Chrysodeixis includens|[1617]|MHMXM171-07|Costa Rica|Alajuela|658[0n]|BOLD:AAA6794
Chrysodeixis includens|[1618]|MHMXM170-07|Costa Rica|Alajuela|658[0n]|BOLD:AAA6794
Chrysodeixis includens|[1619]|MHMXM101-07|Costa Rica|Alajuela|658[0n]|BOLD:AAA6794
Chrysodeixis includens|[1620]|LGSMG613-07|United States|North Carolina|658[0n]|BOLD:AAA6794
Chrysodeixis includens|[1621]|LGSMG612-07|United States|North Carolina|658[0n]|BOLD:AAA6794
Chrysodeixis includens|[1622]|MHAUG682-07|Costa Rica|Alajuela|658[0n]|BOLD:AAA6794
Chrysodeixis includens|[1623]|MHAUG681-07|Costa Rica|Alajuela|658[0n]|BOLD:AAA6794
Chrysodeixis includens|[1624]|MHAUG679-07|Costa Rica|Alajuela|658[0n]|BOLD:AAA6794
Chrysodeixis includens|[1625]|MHAUG678-07|Costa Rica|Alajuela|658[0n]|BOLD:AAA6794
Chrysodeixis includens|[1626]|MHAUG100-07|Costa Rica|Guanacaste|658[0n]|BOLD:AAA6794
Chrysodeixis includens|[1627]|BLPAF924-07|Costa Rica|Guanacaste|658[0n]|BOLD:AAA6794
Chrysodeixis includens|[1628]|MHMXE344-06|Costa Rica|Guanacaste|658[0n]|BOLD:AAA6794
Chrysodeixis includens|[1629]|LOFLB770-06|United States|Florida|658[0n]|BOLD:AAA6794
Chrysodeixis includens|[1630]|BLPAB093-06|Costa Rica|Alajuela|658[0n]|BOLD:AAA6794
Chrysodeixis includens|[1631]|BLPAB057-06|Costa Rica|Alajuela|658[0n]|BOLD:AAA6794
Chrysodeixis includens|[1632]|BLPAB056-06|Costa Rica|Alajuela|658[0n]|BOLD:AAA6794
Chrysodeixis includens|[1633]|BLPAA848-06|Costa Rica|Alajuela|658[0n]|BOLD:AAA6794
Chrysodeixis includens|[1634]|BLPAA727-06|Costa Rica|Alajuela|658[0n]|BOLD:AAA6794
Chrysodeixis includens|[1635]|LNCB071-06|United States|North Carolina|658[0n]|BOLD:AAA6794
Chrysodeixis includens|[1636]|MHMXA818-06|Costa Rica|Guanacaste|658[0n]|BOLD:AAA6794
Chrysodeixis includens|[1637]|MHMXA782-06|Costa Rica|Guanacaste|658[0n]|BOLD:AAA6794
Chrysodeixis includens|[1638]|LNC849-06|United States|North Carolina|658[0n]|BOLD:AAA6794
Chrysodeixis includens|[1639]|BLPAA312-06|Costa Rica|Guanacaste|658[0n]|BOLD:AAA6794
Chrysodeixis includens|[1640]|MHAUB705-05|Costa Rica|Guanacaste|658[0n]|BOLD:AAA6794
Chrysodeixis includens|[1641]|MHAUB704-05|Costa Rica|Alajuela|658[0n]|BOLD:AAA6794
Chrysodeixis includens|[1642]|MHAUB703-05|Costa Rica|Alajuela|658[0n]|BOLD:AAA6794
Chrysodeixis includens|[1643]|MHAUB702-05|Costa Rica|Alajuela|658[0n]|BOLD:AAA6794
Chrysodeixis includens|[1644]|MHAUB701-05|Costa Rica|Alajuela|658[0n]|BOLD:AAA6794
Chrysodeixis includens|[1645]|MHAUB699-05|Costa Rica|Alajuela|658[0n]|BOLD:AAA6794
Chrysodeixis includens|[1646]|MHAUB698-05|Costa Rica|Guanacaste|658[0n]|BOLD:AAA6794
Chrysodeixis includens|[1647]|MHAUB697-05|Costa Rica|Alajuela|658[0n]|BOLD:AAA6794
Chrysodeixis includens|[1648]|MHAUB696-05|Costa Rica|Alajuela|658[0n]|BOLD:AAA6794
Chrysodeixis includens|[1649]|XAH608-05|Canada|Ontario|658[0n]|BOLD:AAA6794
Chrysodeixis includens|[1650]|LEMMZ137-10|Brazil|Parana|658[0n]|BOLD:AAA6794
Chrysodeixis includens|[1651]|XAB463-04|Canada|Ontario|658[0n]|BOLD:AAA6794
Chrysodeixis includens|[1652]|MHMYL3084-11|Costa Rica||658[0n]|BOLD:AAA6794
Chrysodeixis includens|[1653]|BLPDU207-11|Costa Rica|Guanacaste|658[0n]|BOLD:AAA6794
Chrysodeixis includens|[1654]|GBGL12672-13|Brazil||658[0n]|BOLD:AAA6794
Chrysodeixis includens|[1655]|ARMOT129-12|Argentina|Entre Rios|658[0n]|BOLD:AAA6794
Chrysodeixis includens|[1656]|MHMYM085-11|Costa Rica||658[0n]|BOLD:AAA6794
Chrysodeixis includens|[1657]|LPOKA628-09|United States|Oklahoma|658[0n]|BOLD:AAA6794
Chrysodeixis includens|[1658]|MHMYL3504-11|Costa Rica||658[0n]|BOLD:AAA6794
Chrysodeixis includens|[1659]|LPOKA343-08|United States|Oklahoma|658[0n]|BOLD:AAA6794
Chrysodeixis includens|[1660]|BLPDK1887-09|Costa Rica|Guanacaste|653[0n]|BOLD:AAA6794
Chrysodeixis includens|[1661]|MHAUG680-07|Costa Rica|Alajuela|630[0n]|BOLD:AAA6794
Chrysodeixis includens|[1662]|LPOKA661-09|United States|Oklahoma|637[0n]|BOLD:AAA6794
Chrysodeixis includens|[1663]|MHAUG683-07|Costa Rica|Alajuela|637[0n]|BOLD:AAA6794
Chrysodeixis includens|[1664]|NOCJB052-08|Peru|Amazonas|647[0n]|BOLD:AAA6794
Chrysodeixis includens|[1665]|MHMXI418-07|Costa Rica|Alajuela|644[0n]|BOLD:AAA6794
Chrysodeixis includens|[1666]|RDLQ426-07|Canada|Quebec|643[0n]|BOLD:AAA6794
Chrysodeixis includens|[1667]|MHMYM140-11|Costa Rica||632[0n]|BOLD:AAA6794
Chrysodeixis includens|[1668]|LEPPA1129-14|Argentina|Tucuman|602[0n]|BOLD:AAA6794
Chrysodeixis includens|[1669]|LEPPA1141-14|Argentina|Tucuman|658[0n]|BOLD:AAA6794
Ctenoplusia oxygramma|[1670]|RDNMF629-08|United States|Maryland|658[0n]|BOLD:AAA9797
Ctenoplusia oxygramma|[1671]|MHMYH814-10|Costa Rica||658[0n]|BOLD:AAA9797
Ctenoplusia oxygramma|[1672]|BLPEE5738-14|Costa Rica||658[0n]|BOLD:AAA9797
Ctenoplusia oxygramma|[1673]|MHMXI242-07|Costa Rica|Guanacaste|659[0n]|BOLD:AAA9797
Ctenoplusia oxygramma|[1674]|BLPEE5758-14|Costa Rica||658[0n]|BOLD:AAA9797
Ctenoplusia oxygramma|[1675]|BLPEE5754-14|Costa Rica||658[0n]|BOLD:AAA9797
Ctenoplusia oxygramma|[1676]|BLPEE5753-14|Costa Rica||658[0n]|BOLD:AAA9797
Ctenoplusia oxygramma|[1677]|BLPEE5752-14|Costa Rica||658[0n]|BOLD:AAA9797
Ctenoplusia oxygramma|[1678]|BLPEE5743-14|Costa Rica||658[0n]|BOLD:AAA9797
Ctenoplusia oxygramma|[1679]|BLPEE5741-14|Costa Rica||658[0n]|BOLD:AAA9797
Ctenoplusia oxygramma|[1680]|BLPEE5739-14|Costa Rica||658[0n]|BOLD:AAA9797
Ctenoplusia oxygramma|[1681]|BLPEE5736-14|Costa Rica||658[0n]|BOLD:AAA9797
Ctenoplusia oxygramma|[1682]|BLPEE5733-14|Costa Rica||658[0n]|BOLD:AAA9797
Ctenoplusia oxygramma|[1683]|BLPEE5732-14|Costa Rica||658[0n]|BOLD:AAA9797
Ctenoplusia oxygramma|[1684]|BLPEF6144-13|Costa Rica||658[0n]|BOLD:AAA9797
Ctenoplusia oxygramma|[1685]|MHMYT340-13|Costa Rica||658[0n]|BOLD:AAA9797
Ctenoplusia oxygramma|[1686]|MHMYT339-13|Costa Rica||658[0n]|BOLD:AAA9797
Ctenoplusia oxygramma|[1687]|BLPEF814-12|Costa Rica|Guanacaste|658[0n]|BOLD:AAA9797
Ctenoplusia oxygramma|[1688]|MHMYQ1724-12|Costa Rica||658[0n]|BOLD:AAA9797
Ctenoplusia oxygramma|[1689]|MHMYQ1723-12|Costa Rica||658[0n]|BOLD:AAA9797
Ctenoplusia oxygramma|[1690]|MHMYQ1715-12|Costa Rica||658[0n]|BOLD:AAA9797
Ctenoplusia oxygramma|[1691]|MHMYQ032-12|Costa Rica||658[0n]|BOLD:AAA9797

Ctenoplusia oxygramma|[1689]|MHMYQ1723-12|Costa Rica||658[0n]|BOLD:AAA9797
Ctenoplusia oxygramma|[1690]|MHMYQ1715-12|Costa Rica||658[0n]|BOLD:AAA9797
Ctenoplusia oxygramma|[1691]|MHMYQ032-12|Costa Rica||658[0n]|BOLD:AAA9797
Ctenoplusia oxygramma|[1692]|MHMYQ031-12|Costa Rica||658[0n]|BOLD:AAA9797
Ctenoplusia oxygramma|[1693]|MHMYQ030-12|Costa Rica||658[0n]|BOLD:AAA9797
Ctenoplusia oxygramma|[1694]|MHMYQ029-12|Costa Rica||658[0n]|BOLD:AAA9797
Ctenoplusia oxygramma|[1695]|LNCC1242-11|United States|North Carolina|658[0n]|BOLD:AAA9797
Ctenoplusia oxygramma|[1696]|MHMYN062-11|Costa Rica||658[0n]|BOLD:AAA9797
Ctenoplusia oxygramma|[1697]|MHMYN061-11|Costa Rica||658[0n]|BOLD:AAA9797
Ctenoplusia oxygramma|[1698]|MHMYN060-11|Costa Rica||658[0n]|BOLD:AAA9797
Ctenoplusia oxygramma|[1699]|MHMYN059-11|Costa Rica||658[0n]|BOLD:AAA9797
Ctenoplusia oxygramma|[1700]|MHMYM039-11|Costa Rica||658[0n]|BOLD:AAA9797
Ctenoplusia oxygramma|[1701]|MHMYM038-11|Costa Rica||658[0n]|BOLD:AAA9797
Ctenoplusia oxygramma|[1702]|MHMYM037-11|Costa Rica||658[0n]|BOLD:AAA9797
Ctenoplusia oxygramma|[1703]|MHMYM036-11|Costa Rica||658[0n]|BOLD:AAA9797
Ctenoplusia oxygramma|[1704]|MHMYM035-11|Costa Rica||658[0n]|BOLD:AAA9797
Ctenoplusia oxygramma|[1705]|MHMYM034-11|Costa Rica||658[0n]|BOLD:AAA9797
Ctenoplusia oxygramma|[1706]|MHMYM031-11|Costa Rica||658[0n]|BOLD:AAA9797
Ctenoplusia oxygramma|[1707]|MHMYM030-11|Costa Rica||658[0n]|BOLD:AAA9797
Ctenoplusia oxygramma|[1708]|BLPEC407-11|Costa Rica|Alajuela|658[0n]|BOLD:AAA9797
Ctenoplusia oxygramma|[1709]|MHMYL2921-11|Costa Rica||658[0n]|BOLD:AAA9797
Ctenoplusia oxygramma|[1710]|LILLA884-11|United States|Illinois|658[0n]|BOLD:AAA9797
Ctenoplusia oxygramma|[1711]|LILLA879-11|United States|Illinois|658[0n]|BOLD:AAA9797
Ctenoplusia oxygramma|[1712]|MHMYH1067-10|Costa Rica||658[0n]|BOLD:AAA9797
Ctenoplusia oxygramma|[1713]|MHMYA1161-09|Costa Rica||658[0n]|BOLD:AAA9797
Ctenoplusia oxygramma|[1714]|MHMXZ468-09|Costa Rica||658[0n]|BOLD:AAA9797
Ctenoplusia oxygramma|[1715]|MHMXZ466-09|Costa Rica||658[0n]|BOLD:AAA9797
Ctenoplusia oxygramma|[1716]|LPOKA609-09|United States|Oklahoma|658[0n]|BOLD:AAA9797
Ctenoplusia oxygramma|[1717]|BLPCK719-08|Costa Rica|Alajuela|658[0n]|BOLD:AAA9797
Ctenoplusia oxygramma|[1718]|BLPCK670-08|Costa Rica|Alajuela|658[0n]|BOLD:AAA9797
Ctenoplusia oxygramma|[1719]|NOCJB037-08|Peru|Amazonas|658[0n]|BOLD:AAA9797
Ctenoplusia oxygramma|[1720]|RDNMF628-08|Canada|New Brunswick|658[0n]|BOLD:AAA9797
Ctenoplusia oxygramma|[1721]|RDNMF626-08|United States|Florida|658[0n]|BOLD:AAA9797
Ctenoplusia oxygramma|[1722]|BLPCD638-08|Costa Rica|Guanacaste|658[0n]|BOLD:AAA9797
Ctenoplusia oxygramma|[1723]|MHMXO329-08|Costa Rica|Guanacaste|658[0n]|BOLD:AAA9797
Ctenoplusia oxygramma|[1724]|MHMXO328-08|Costa Rica|Guanacaste|655[0n]|BOLD:AAA9797
Ctenoplusia oxygramma|[1725]|MHMXM092-07|Costa Rica|Guanacaste|658[0n]|BOLD:AAA9797
Ctenoplusia oxygramma|[1726]|MHAUG707-07|Costa Rica|Guanacaste|658[0n]|BOLD:AAA9797
Ctenoplusia oxygramma|[1727]|MHMXI299-07|Costa Rica|Guanacaste|658[0n]|BOLD:AAA9797
Ctenoplusia oxygramma|[1728]|BLPAG716-07|Costa Rica|Guanacaste|658[0n]|BOLD:AAA9797
Ctenoplusia oxygramma|[1729]|LOCRA211-06|Costa Rica|San Jose|658[0n]|BOLD:AAA9797
Ctenoplusia oxygramma|[1730]|BLPAC732-06|Costa Rica|Guanacaste|658[0n]|BOLD:AAA9797
Ctenoplusia oxygramma|[1731]|MHAUA499-05|Costa Rica|Guanacaste|658[0n]|BOLD:AAA9797
Ctenoplusia oxygramma|[1732]|MHAUA498-05|Costa Rica|Guanacaste|658[0n]|BOLD:AAA9797
Ctenoplusia oxygramma|[1733]|MHAUA496-05|Costa Rica|Guanacaste|658[0n]|BOLD:AAA9797
Ctenoplusia oxygramma|[1734]|MHAUA495-05|Costa Rica|Guanacaste|658[0n]|BOLD:AAA9797
Ctenoplusia oxygramma|[1735]|MHAUA494-05|Costa Rica|Guanacaste|658[0n]|BOLD:AAA9797
Ctenoplusia oxygramma|[1736]|MHAUA492-05|Costa Rica|Guanacaste|658[0n]|BOLD:AAA9797
Ctenoplusia oxygramma|[1737]|MHAUA491-05|Costa Rica|Guanacaste|658[0n]|BOLD:AAA9797
Ctenoplusia oxygramma|[1738]|MHAUA490-05|Costa Rica|Guanacaste|658[0n]|BOLD:AAA9797
Ctenoplusia oxygramma|[1739]|MHAUA489-05|Costa Rica|Guanacaste|658[0n]|BOLD:AAA9797
Ctenoplusia oxygramma|[1740]|RDNMF627-08|Canada|New Brunswick|658[0n]|BOLD:AAA9797
Ctenoplusia oxygramma|[1741]|MHAUA493-05|Costa Rica|Guanacaste|658[0n]|BOLD:AAA9797
Ctenoplusia oxygramma|[1742]|MHAUA497-05|Costa Rica|Guanacaste|658[0n]|BOLD:AAA9797
Ctenoplusia oxygramma|[1743]|GWOST430-11|Peru|Huanuco|658[0n]|BOLD:AAA9797
Ctenoplusia oxygramma|[1744]|RDNMF630-08|United States|Mississippi|609[0n]|BOLD:AAA9797
Ctenoplusia oxygramma|[1745]|NOCJB038-08|Dominican Republic||658[0n]|BOLD:AAA9797
Ctenoplusia oxygramma|[1746]|MHMYQ1716-12|Costa Rica||630[0n]|BOLD:AAA9797
Ctenoplusia oxygramma|[1747]|MHMYQ1721-12|Costa Rica||630[0n]|BOLD:AAA9797
Ctenoplusia oxygramma|[1748]|MHMYQ1455-12|Costa Rica||658[0n]|BOLD:AAA9797
Ctenoplusia oxygramma|[1749]|MHMYQ1722-12|Costa Rica||616[1n]|BOLD:AAA9797
Ctenoplusia oxygramma|[1750]|RDNMK073-11|United States|Florida|658[0n]|BOLD:AAA9797
Ctenoplusia oxygramma|[1751]|MHMYQ1720-12|Costa Rica||658[0n]|BOLD:AAA9797
Ctenoplusia oxygramma|[1752]|MHMXM169-07|Costa Rica|Guanacaste|658[0n]|BOLD:AAA9797
Ctenoplusia oxygramma|[1753]|MHMXZ467-09|Costa Rica||658[0n]|BOLD:AAA9797
Ctenoplusia oxygramma|[1754]|BLPEE5745-14|Costa Rica||658[0n]|BOLD:AAA9797
Ctenoplusia oxygramma|[1755]|MHMYN6520-14|Costa Rica||658[0n]|BOLD:AAA9797
Ctenoplusia oxygramma|[1756]|MHMYN6521-14|Costa Rica||658[0n]|BOLD:AAA9797
Ctenoplusia oxygramma|[1757]|KSLEP036-14|Canada|Ontario|658[0n]|BOLD:AAA9797
Euchalcia borealis|[1758]|RDNM847-05|Canada|British Columbia|599[0n]|BOLD:AAX7948
Euchalcia albavitta|[1759]|RDNME982-08|United States|Arizona|658[0n]|BOLD:AAX7936
Plusia putnami|[1760]|RDLQB013-05|Canada|Quebec|573[0n]|BOLD:AAA5394
Plusia putnami|[1761]|RDNM856-05|Canada|New Brunswick|504[0n]|BOLD:AAA5394
Plusia putnami|[1762]|RDLQ293-05|Canada|Quebec|622[0n]|BOLD:AAA5394
Plusia putnami|[1763]|RDLQF098-06|Canada|Quebec|621[8n]|BOLD:AAA5394
Plusia putnami|[1764]|RDNM863-05|Canada|British Columbia|577[0n]|BOLD:AAA5394
Plusia putnami|[1765]|RDLQB014-05|Canada|Quebec|556[0n]|BOLD:AAA5394
Plusia putnami|[1766]|RDNM859-05|Canada|Ontario|658[0n]|BOLD:AAA5394
Plusia putnami|[1767]|BBLPC366-09|Canada|New Brunswick|654[0n]|BOLD:AAA5394
Plusia putnami|[1768]|LCHQ312-08|Canada|Manitoba|658[0n]|BOLD:AAA5394
Plusia putnami|[1769]|LCHP756-07|Canada|Manitoba|657[0n]|BOLD:AAA5394
Plusia putnami|[1770]|LCHP746-07|Canada|Manitoba|655[0n]|BOLD:AAA5394
Plusia putnami|[1771]|RDLQF109-06|Canada|Quebec|657[0n]|BOLD:AAA5394
Plusia putnami|[1772]|RDLQF107-06|Canada|Quebec|657[0n]|BOLD:AAA5394
Plusia putnami|[1773]|RDLQF106-06|Canada|Quebec|657[0n]|BOLD:AAA5394
Plusia putnami|[1774]|RDLQF105-06|Canada|Quebec|657[0n]|BOLD:AAA5394
Plusia putnami|[1775]|RDLQF104-06|Canada|Quebec|657[0n]|BOLD:AAA5394
Plusia putnami|[1776]|RDLQF101-06|Canada|Quebec|657[0n]|BOLD:AAA5394
Plusia putnami|[1777]|RDNM860-05|Canada|Alberta|658[0n]|BOLD:AAA5394
Plusia putnami|[1778]|RDNM857-05|Canada|Ontario|658[0n]|BOLD:AAA5394
Plusia putnami|[1779]|RDNM852-05|Canada|British Columbia|658[0n]|BOLD:AAA5394
Plusia putnami|[1780]|RDNM850-05|Canada|British Columbia|658[0n]|BOLD:AAA5394
Plusia putnami|[1781]|RDLQ298-05|Canada|Quebec|657[0n]|BOLD:AAA5394
Plusia putnami|[1782]|RDLQ220-05|Canada|Quebec|657[0n]|BOLD:AAA5394
Plusia putnami|[1783]|RDLQ119-05|Canada|Quebec|657[0n]|BOLD:AAA5394
Plusia putnami|[1784]|PHMNB683-04|Canada|New Brunswick|657[0n]|BOLD:AAA5394
Plusia putnami|[1785]|PHMNB466-04|Canada|New Brunswick|657[0n]|BOLD:AAA5394
Plusia putnami|[1786]|PHMNB465-04|Canada|New Brunswick|657[0n]|BOLD:AAA5394
Plusia putnami|[1787]|PHMNB320-04|Canada|New Brunswick|657[0n]|BOLD:AAA5394
Plusia putnami|[1788]|RDLQF110-06|Canada|Quebec|619[0n]|BOLD:AAA5394
Plusia putnami|[1789]|LCHQ714-08|Canada|Manitoba|658[0n]|BOLD:AAA5394
Plusia putnami|[1790]|LCHQ715-08|Canada|Manitoba|657[0n]|BOLD:AAA5394
Plusia putnami|[1791]|LCHQ716-08|Canada|Manitoba|658[0n]|BOLD:AAA5394

Plusia putnami|[1788]|RDLQF110-06|Canada|Quebec|619[0n]|BOLD:AAA5394
Plusia putnami|[1789]|LCHQ714-08|Canada|Manitoba|658[0n]|BOLD:AAA5394
Plusia putnami|[1790]|LCHQ715-08|Canada|Manitoba|657[0n]|BOLD:AAA5394
Plusia putnami|[1791]|LCHQ716-08|Canada|Manitoba|658[0n]|BOLD:AAA5394
Plusia putnami|[1792]|LCHQ920-08|Canada|Manitoba|658[0n]|BOLD:AAA5394
Plusia putnami|[1793]|LPMN209-08|Canada|Manitoba|658[0n]|BOLD:AAA5394
Plusia putnami|[1794]|LPABB676-08|Canada|Alberta|658[0n]|BOLD:AAA5394
Plusia putnami|[1795]|BBLPC180-09|Canada|Nova Scotia|658[0n]|BOLD:AAA5394
Plusia putnami|[1796]|BBLPE082-09|Canada|Nova Scotia|658[0n]|BOLD:AAA5394
Plusia magnimacula|[1797]|RDLQ027-05|Canada|Quebec|508[0n]|BOLD:ACZ9793
Plusia magnimacula|[1798]|RDLQ285-05|Canada|Quebec|573[0n]|BOLD:ACZ9793
Plusia magnimacula|[1799]|RDLQF097-06|Canada|Quebec|598[0n]|BOLD:ACZ9793
Plusia magnimacula|[1800]|RDLQF095-06|Canada|Quebec|598[0n]|BOLD:ACZ9793
Plusia magnimacula|[1801]|RDLQF099-06|Canada|Quebec|620[0n]|BOLD:ACZ9793
Plusia magnimacula|[1802]|RDLQ284-05|Canada|Quebec|600[0n]|BOLD:ACZ9793
Plusia magnimacula|[1803]|RDLQ295-05|Canada|Quebec|603[0n]|BOLD:ACZ9793
Plusia magnimacula|[1804]|LCHP831-07|Canada|Manitoba|658[0n]|BOLD:ACZ9793
Plusia magnimacula|[1805]|MECB359-05|Canada|Quebec|574[0n]|BOLD:ACZ9793
Plusia magnimacula|[1806]|MECB360-05|Canada|Quebec|583[0n]|BOLD:ACZ9793
Plusia magnimacula|[1807]|MECB358-05|Canada|Quebec|595[0n]|BOLD:ACZ9793
Plusia magnimacula|[1808]|RDLQ300-05|Canada|Quebec|597[0n]|BOLD:ACZ9793
Plusia magnimacula|[1809]|RDLQ294-05|Canada|Quebec|598[0n]|BOLD:ACZ9793
Plusia magnimacula|[1810]|RDNM858-05|Canada|New Brunswick|585[0n]|BOLD:ACZ9793
Plusia magnimacula|[1811]|RDLQB767-05|Canada|Quebec|616[0n]|BOLD:ACZ9793
Plusia magnimacula|[1812]|RDNM864-05|Canada|Ontario|611[0n]|BOLD:ACZ9793
Plusia magnimacula|[1813]|RDLQB768-05|Canada|Quebec|658[0n]|BOLD:ACZ9793
Plusia magnimacula|[1814]|RDLQB015-05|Canada|Quebec|657[0n]|BOLD:ACZ9793
Plusia magnimacula|[1815]|RDNM867-05|Canada|Ontario|658[0n]|BOLD:ACZ9793
Plusia magnimacula|[1816]|RDNM866-05|Canada|Ontario|658[0n]|BOLD:ACZ9793
Plusia magnimacula|[1817]|RDNM865-05|Canada|Ontario|658[0n]|BOLD:ACZ9793
Plusia magnimacula|[1818]|MECB357-05|Canada|Quebec|658[0n]|BOLD:ACZ9793
Plusia magnimacula|[1819]|RDLQF096-06|Canada|Quebec|657[0n]|BOLD:ACZ9793
Plusia magnimacula|[1820]|RDLQF102-06|Canada|Quebec|657[0n]|BOLD:ACZ9793
Plusia magnimacula|[1821]|RDLQF103-06|Canada|Quebec|657[0n]|BOLD:ACZ9793
Plusia magnimacula|[1822]|RDLQF111-06|Canada|Quebec|657[0n]|BOLD:ACZ9793
Plusia magnimacula|[1823]|LPSOB228-08|Canada|Ontario|658[0n]|BOLD:ACZ9793
Plusia contexta|[1824]|RDLQB723-05|Canada|Quebec|599[0n]|BOLD:AAC3534
Plusia contexta|[1825]|XAE283-04|Canada|Ontario|609[3n]|BOLD:AAC3534
Plusia contexta|[1826]|RDLQB762-05|Canada|Quebec|617[0n]|BOLD:AAC3534
Plusia contexta|[1827]|RDNMB600-05|Canada|Ontario|658[0n]|BOLD:AAC3534
Plusia contexta|[1828]|RDNMB601-05|Canada|Ontario|658[0n]|BOLD:AAC3534
Plusia contexta|[1829]|RDNMB602-05|Canada|Ontario|658[0n]|BOLD:AAC3534
Plusia contexta|[1830]|RDLQB540-05|Canada|Quebec|658[0n]|BOLD:AAC3534
Plusia contexta|[1831]|XAJ499-06|Canada|Ontario|657[0n]|BOLD:AAC3534
Plusia contexta|[1832]|RDLQF937-06|Canada|Quebec|658[0n]|BOLD:AAC3534
Plusia contexta|[1833]|LPSO882-08|Canada|Ontario|658[0n]|BOLD:AAC3534
Plusia contexta|[1834]|LPSOC118-08|Canada|Ontario|658[0n]|BOLD:AAC3534
Plusia contexta|[1835]|LPSOC120-08|Canada|Ontario|654[0n]|BOLD:AAC3534
Plusia venusta|[1836]|XAJ827-06|Canada|Ontario|657[0n]|BOLD:ACZ8852
Plusia venusta|[1837]|PHMO319-03|Canada|Ontario|639[0n]|BOLD:ACZ8852
Plusia venusta|[1838]|RDNMB597-05|Canada|Ontario|658[0n]|BOLD:ACZ8852
Plusia venusta|[1839]|RDNMB598-05|Canada|Saskatchewan|658[0n]|BOLD:ACZ8852
Plusia venusta|[1840]|RDNMB599-05|Canada|Ontario|658[0n]|BOLD:ACZ8852
Plusia venusta|[1841]|LHLEP123-06|Canada|British Columbia|658[0n]|BOLD:ACZ8852
Plusia nichollae|[1842]|RDNM855-05|Canada|British Columbia|571[0n]|BOLD:AAB2458
Plusia nichollae|[1843]|RDNM854-05|Canada|British Columbia|570[0n]|BOLD:AAB2458
Plusia nichollae|[1844]|RDNM862-05|Canada|British Columbia|554[0n]|BOLD:AAB2458
Plusia nichollae|[1845]|LBCH488-10|Canada|British Columbia|658[0n]|BOLD:AAB2458
Plusia nichollae|[1846]|RWWB057-09|United States|Washington|619[0n]|BOLD:AAB2458
Plusia nichollae|[1847]|RDNM853-05|Canada|British Columbia|596[1n]|BOLD:AAB2458
Plusia nichollae|[1848]|RWWA484-09|United States|Washington|658[0n]|BOLD:AAB2458
Plusia nichollae|[1849]|RDNM851-05|United States|Washington|594[0n]|BOLD:AAB2458
Plusia nichollae|[1850]|RWWA549-09|United States|Washington|625[0n]|BOLD:AAB2458
Plusia nichollae|[1851]|LBCW026-08|Canada|British Columbia|658[0n]|BOLD:AAB2458
Plusia nichollae|[1852]|LBCW027-08|Canada|British Columbia|658[0n]|BOLD:AAB2458
Plusia nichollae|[1853]|RWWA005-09|United States|Washington|658[0n]|BOLD:AAB2458
Plusia nichollae|[1854]|RWWA140-09|United States|Washington|658[0n]|BOLD:AAB2458
Plusia nichollae|[1855]|RWWA181-09|United States|Washington|656[0n]|BOLD:AAB2458
Plusia nichollae|[1856]|RWWA184-09|United States|Washington|658[0n]|BOLD:AAB2458
Plusia nichollae|[1857]|RWWA436-09|United States|Washington|658[0n]|BOLD:AAB2458
Plusia nichollae|[1858]|RWWB211-09|United States|Washington|658[0n]|BOLD:AAB2458
Plusia nichollae|[1859]|RWWB293-09|United States|Washington|658[0n]|BOLD:AAB2458
Plusia nichollae|[1860]|RWWB317-09|United States|Washington|658[0n]|BOLD:AAB2458
Plusia nichollae|[1861]|RWWC081-10|United States|Washington|658[0n]|BOLD:AAB2458
Plusia nichollae|[1862]|RWWC308-11|United States|Washington|658[0n]|BOLD:AAB2458
Plusia nichollae|[1863]|RDNM861-05|United States|Washington|658[0n]|BOLD:AAB2458
Plusia nichollae|[1864]|LBCG001-08|Canada|British Columbia|658[0n]|BOLD:AAB2458
Plusia nichollae|[1865]|RWWC807-11|United States|Washington|658[0n]|BOLD:AAB2458
Mouralia tinctoides|[1866]|HKONS112-08|United States|Florida|658[0n]|BOLD:AAF3149
Mouralia tinctoides|[1867]|NOCJB026-08|Paraguay|Presidente Hayes|658[0n]|BOLD:AAF3149
Exyra ridingsii|[1868]|LNCB354-06|United States|North Carolina|658[0n]|BOLD:AAC8139
Exyra ridingsii|[1869]|HKONS487-08|United States|Florida|658[0n]|BOLD:AAC8139
Exyra ridingsii|[1870]|LNCB356-06|United States|North Carolina|658[0n]|BOLD:AAC8139
Exyra ridingsii|[1871]|LNCB357-06|United States|North Carolina|658[0n]|BOLD:AAC8139
Exyra ridingsii|[1872]|LNCB355-06|United States|North Carolina|658[0n]|BOLD:AAC8139
Exyra ridingsii|[1873]|LNCB358-06|United States|North Carolina|658[0n]|BOLD:AAC8139
Exyra ridingsii|[1874]|HKONS486-08|United States|Florida|658[1n]|BOLD:AAC8139
Exyra ridingsii|[1875]|LSEU077-06|United States|Florida|574[1n]|BOLD:AAC8139
Exyra ridingsii|[1876]|HKONS488-08|United States|Florida|658[0n]|BOLD:AAC8139
Exyra semicrocea|[1877]|ABNCC465-07|United States|Florida|645[0n]|BOLD:AAD7703
Exyra semicrocea|[1878]|HKONS489-08|United States|Florida|658[0n]|BOLD:AAD7703
Exyra semicrocea|[1879]|HKONS490-08|United States|Florida|658[0n]|BOLD:AAD7703
Exyra semicrocea|[1880]|LOFLA343-06|United States|Florida|658[0n]|BOLD:AAD7703
Exyra semicrocea|[1881]|LNCB353-06|United States|North Carolina|658[0n]|BOLD:AAD7703
Exyra semicrocea|[1882]|HKONS491-08|United States|Florida|658[0n]|BOLD:AAD7703
Exyra fax|[1883]|HKONS492-08|United States|Florida|658[0n]|BOLD:ABX5283
Exyra fax|[1884]|HKONS493-08|United States|Florida|658[0n]|BOLD:ABX5283
Exyra fax|[1885]|HKONS494-08|United States|Florida|658[2n]|BOLD:ABX5283
Exyra fax|[1886]|LNC011-05|United States|North Carolina|658[0n]|BOLD:AAD1241
Exyra fax|[1887]|LNCB375-06|United States|North Carolina|658[0n]|BOLD:AAD1241
Exyra fax|[1888]|RDLQB267-05|Canada|Quebec|658[0n]|BOLD:ACF4632
Exyra fax|[1889]|RDNMH150-09|Canada|Quebec|658[0n]|BOLD:ACF4632
Exyra fax|[1890]|RDNMH151-09|Canada|Quebec|658[0n]|BOLD:ACF4632

Exyra fax|[1888]|RDLQB267-05|Canada|Quebec|658[0n]|BOLD:ACF4632
Exyra fax|[1889]|RDNMH150-09|Canada|Quebec|658[0n]|BOLD:ACF4632
Exyra fax|[1890]|RDNMH151-09|Canada|Quebec|658[0n]|BOLD:ACF4632
Diachrysia balluca|[1891]|XAG250-05|Canada|Ontario|658[0n]|BOLD:AAD0732
Diachrysia balluca|[1892]|PHMO275-03|Canada|Ontario|639[2n]|BOLD:AAD0732
Diachrysia balluca|[1893]|RDLQB830-05|Canada|Quebec|634[0n]|BOLD:AAD0732
Diachrysia balluca|[1894]|MECD403-06|Canada|Quebec|658[0n]|BOLD:AAD0732
Diachrysia balluca|[1895]|LPMNB449-09|Canada|Manitoba|658[0n]|BOLD:AAD0732
Diachrysia balluca|[1896]|LPMNB568-09|Canada|Manitoba|658[0n]|BOLD:AAD0732
Diachrysia balluca|[1897]|RDLQB422-05|Canada|Quebec|658[4n]|BOLD:AAD0732
Diachrysia balluca|[1898]|BBLEC957-09|Canada|Nova Scotia|658[3n]|BOLD:AAD0732
Polychrysia morigera|[1899]|RDNMD339-06|United States|Oregon|657[0n]|BOLD:AAE1534
Polychrysia morigera|[1900]|RDNMD338-06|United States|Oregon|657[0n]|BOLD:AAE1534
Polychrysia morigera|[1901]|RDNMD340-06|United States|Oregon|657[0n]|BOLD:AAE1534
Polychrysia morigera|[1902]|RDNME604-08|United States|Colorado|658[0n]|BOLD:ACV2832
Polychrysia morigera|[1903]|RDNMD341-06|United States|North Carolina|658[0n]|BOLD:ACV2832
Polychrysia morigera|[1904]|LNCC1025-11|United States|North Carolina|658[0n]|BOLD:ACV2832
Polychrysia morigera|[1905]|LNCC1026-11|United States|North Carolina|658[0n]|BOLD:ACV2832
Polychrysia esmeralda|[1906]|RDMAB086-05|Canada|Alberta|639[0n]|BOLD:AAF0891
Polychrysia esmeralda|[1907]|RDNMD361-06|Canada|Alberta|658[0n]|BOLD:AAF0891
Polychrysia esmeralda|[1908]|ABKWR057-07|United States|Alaska|658[0n]|BOLD:AAF0891
Polychrysia esmeralda|[1909]|UAMIC1162-13|United States|Alaska|658[0n]|BOLD:AAF0891
Polychrysia esmeralda|[1910]|CNWBG3088-13|Canada|Alberta|658[0n]|BOLD:AAF0891
Allagrapha aerea|[1911]|XAE557-04|Canada|Ontario|658[4n]|BOLD:AAB0752
Allagrapha aerea|[1912]|XAE564-04|Canada|Ontario|658[0n]|BOLD:AAB0752
Allagrapha aerea|[1913]|XAD152-04|Canada|Ontario|658[0n]|BOLD:AAB0752
Allagrapha aerea|[1914]|XAC459-04|Canada|Ontario|658[0n]|BOLD:AAB0752
Allagrapha aerea|[1915]|XAB466-04|Canada|Ontario|658[0n]|BOLD:AAB0752
Allagrapha aerea|[1916]|LOT510-04|United States|Tennessee|658[0n]|BOLD:AAB0752
Allagrapha aerea|[1917]|LGSM449-04|United States|Tennessee|658[0n]|BOLD:AAB0752
Allagrapha aerea|[1918]|LGSM448-04|United States|Tennessee|658[0n]|BOLD:AAB0752
Allagrapha aerea|[1919]|PHMO344-03|Canada|Ontario|640[1n]|BOLD:AAB0752
Allagrapha aerea|[1920]|XAD154-04|Canada|Ontario|576[0n]|BOLD:AAB0752
Allagrapha aerea|[1921]|PMG088-03|Canada|Ontario|617[0n]|BOLD:AAB0752
Allagrapha aerea|[1922]|PHMO114-03|Canada|Ontario|639[0n]|BOLD:AAB0752
Allagrapha aerea|[1923]|XAD293-04|Canada|Ontario|597[0n]|BOLD:AAB0752
Allagrapha aerea|[1924]|XAD388-04|Canada|Ontario|658[0n]|BOLD:AAB0752
Allagrapha aerea|[1925]|XAH278-05|Canada|Ontario|658[0n]|BOLD:AAB0752
Allagrapha aerea|[1926]|XAH418-05|Canada|Ontario|658[0n]|BOLD:AAB0752
Allagrapha aerea|[1927]|RDLQB558-05|Canada|Quebec|658[0n]|BOLD:AAB0752
Allagrapha aerea|[1928]|RDLQB720-05|Canada|Quebec|658[0n]|BOLD:AAB0752
Allagrapha aerea|[1929]|LSUSA018-06|United States|Kentucky|658[0n]|BOLD:AAB0752
Allagrapha aerea|[1930]|XAJ538-06|Canada|Ontario|658[0n]|BOLD:AAB0752
Allagrapha aerea|[1931]|LSUSA142-06|United States|Kentucky|658[0n]|BOLD:AAB0752
Allagrapha aerea|[1932]|XAK160-06|Canada|Ontario|658[0n]|BOLD:AAB0752
Allagrapha aerea|[1933]|RDLQF273-06|Canada|Quebec|658[0n]|BOLD:AAB0752
Allagrapha aerea|[1934]|LGSMG609-07|United States|North Carolina|658[0n]|BOLD:AAB0752
Allagrapha aerea|[1935]|LGSMG610-07|United States|North Carolina|658[0n]|BOLD:AAB0752
Allagrapha aerea|[1936]|LPSOB099-08|Canada|Ontario|658[0n]|BOLD:AAB0752
Allagrapha aerea|[1937]|LPSOC034-08|Canada|Ontario|658[0n]|BOLD:AAB0752
Allagrapha aerea|[1938]|LPSOC314-08|Canada|Ontario|658[0n]|BOLD:AAB0752
Allagrapha aerea|[1939]|LPSOC361-08|Canada|Ontario|656[0n]|BOLD:AAB0752
Allagrapha aerea|[1940]|BLTIB218-08|Canada|Ontario|658[0n]|BOLD:AAB0752
Allagrapha aerea|[1941]|LPOKA417-09|United States|Oklahoma|658[0n]|BOLD:AAB0752
Allagrapha aerea|[1942]|LPOKB390-09|United States|Oklahoma|658[0n]|BOLD:AAB0752
Allagrapha aerea|[1943]|BBLSW440-09|United States|Oklahoma|658[0n]|BOLD:AAB0752
Allagrapha aerea|[1944]|LNCC347-10|United States|North Carolina|658[0n]|BOLD:AAB0752
Allagrapha aerea|[1945]|LILLA111-11|United States|Illinois|658[0n]|BOLD:AAB0752
Allagrapha aerea|[1946]|LILLA765-11|United States|Illinois|658[0n]|BOLD:AAB0752
Diachrysia aereoides|[1947]|RDLQF813-06|Canada|Quebec|658[1n]|BOLD:AAB3241
Diachrysia aereoides|[1948]|LOWCE830-06|Canada|British Columbia|658[0n]|BOLD:AAB3241
Diachrysia aereoides|[1949]|BLTIB843-08|Canada|Ontario|658[1n]|BOLD:AAB3241
Diachrysia aereoides|[1950]|PMG107-03|Canada|Ontario|617[0n]|BOLD:AAB3241
Diachrysia aereoides|[1951]|BBLEC266-09|Canada|Nova Scotia|658[0n]|BOLD:AAB3241
Diachrysia aereoides|[1952]|RDLQB059-05|Canada|Quebec|658[0n]|BOLD:AAB3241
Diachrysia aereoides|[1953]|BBLPC541-09|Canada|New Brunswick|638[0n]|BOLD:AAB3241
Diachrysia aereoides|[1954]|XAK223-06|Canada|Ontario|658[0n]|BOLD:AAB3241
Diachrysia aereoides|[1955]|BBLEC321-09|Canada|Nova Scotia|658[0n]|BOLD:AAB3241
Diachrysia aereoides|[1956]|XAC728-04|Canada|Ontario|658[0n]|BOLD:AAB3241
Diachrysia aereoides|[1957]|BBLPE023-09|Canada|Nova Scotia|658[1n]|BOLD:AAB3241
Diachrysia aereoides|[1958]|BLTIB724-08|Canada|Ontario|658[1n]|BOLD:AAB3241
Diachrysia aereoides|[1959]|TMG96-03|Canada|Ontario|639[0n]|BOLD:AAB3241
Diachrysia aereoides|[1960]|LGSM036-04|United States|North Carolina|658[0n]|BOLD:AAB3241
Diachrysia aereoides|[1961]|LGSM037-04|United States|North Carolina|658[0n]|BOLD:AAB3241
Diachrysia aereoides|[1962]|XAC458-04|Canada|Ontario|658[0n]|BOLD:AAB3241
Diachrysia aereoides|[1963]|XAC704-04|Canada|Ontario|658[0n]|BOLD:AAB3241
Diachrysia aereoides|[1964]|PHMNB528-04|Canada|New Brunswick|658[0n]|BOLD:AAB3241
Diachrysia aereoides|[1965]|XAJ830-06|Canada|Ontario|658[0n]|BOLD:AAB3241
Diachrysia aereoides|[1966]|RDLQF814-06|Canada|Quebec|658[0n]|BOLD:AAB3241
Diachrysia aereoides|[1967]|LGSMG608-07|United States|North Carolina|658[0n]|BOLD:AAB3241
Diachrysia aereoides|[1968]|BBLPC604-09|Canada|Nova Scotia|658[0n]|BOLD:AAB3241
Diachrysia aereoides|[1969]|BBLPE017-09|Canada|Nova Scotia|658[0n]|BOLD:AAB3241
Diachrysia aereoides|[1970]|BBLPE052-09|Canada|Nova Scotia|658[0n]|BOLD:AAB3241
Diachrysia aereoides|[1971]|LNCC1190-11|United States|North Carolina|658[0n]|BOLD:AAB3241
Diachrysia aereoides|[1972]|LNCC1191-11|United States|North Carolina|658[0n]|BOLD:AAB3241
Diachrysia aereoides|[1973]|LNCC1192-11|United States|North Carolina|658[0n]|BOLD:AAB3241
Diachrysia aereoides|[1974]|PHJUN3996-11|Canada|Ontario|658[0n]|BOLD:AAB3241
Diachrysia aereoides|[1975]|XAC793-04|Canada|Ontario|658[0n]|BOLD:AAB3241
Diachrysia aereoides|[1976]|CNRMF3754-12|Canada|Manitoba|634[0n]|BOLD:AAB3241
Chrysanympha formosa|[1977]|CNLMF1038-14|Canada|Quebec|564[0n]|BOLD:AAB6600
Chrysanympha formosa|[1978]|RDLQB605-05|Canada|Quebec|536[0n]|BOLD:AAB6600
Chrysanympha formosa|[1979]|QUNOB531-09|United States|Kentucky|658[0n]|BOLD:AAB6600
Chrysanympha formosa|[1980]|RDLQB261-05|Canada|Quebec|658[0n]|BOLD:AAB6600
Chrysanympha formosa|[1981]|RDLQB260-05|Canada|Quebec|658[0n]|BOLD:AAB6600
Chrysanympha formosa|[1982]|LGSMC657-05|United States|Tennessee|656[0n]|BOLD:AAB6600
Chrysanympha formosa|[1983]|TMNBB077-06|Canada|New Brunswick|657[1n]|BOLD:AAB6600
Chrysanympha formosa|[1984]|TMNBB078-06|Canada|New Brunswick|657[0n]|BOLD:AAB6600
Chrysanympha formosa|[1985]|TMNBB079-06|Canada|New Brunswick|657[0n]|BOLD:AAB6600
Chrysanympha formosa|[1986]|LSEU398-06|United States|Georgia|657[0n]|BOLD:AAB6600
Chrysanympha formosa|[1987]|BLTIB391-08|Canada|Ontario|657[0n]|BOLD:AAB6600
Chrysanympha formosa|[1988]|QUNOB532-09|United States|Kentucky|658[0n]|BOLD:AAB6600
Chrysanympha formosa|[1989]|BBLPE054-09|Canada|Nova Scotia|658[0n]|BOLD:AAB6600
[illegible]

Chrysanympha formosa|[1987]|BLTIB391-08|Canada|Ontario|657[0n]|BOLD:AAB6600
Chrysanympha formosa|[1988]|QUNOB532-09|United States|Kentucky|658[0n]|BOLD:AAB6600
Chrysanympha formosa|[1989]|BBLPE054-09|Canada|Nova Scotia|658[0n]|BOLD:AAB6600
Chrysanympha formosa|[1990]|RDLQB262-05|Canada|Quebec|658[0n]|BOLD:AAB6600
Chrysanympha formosa|[1991]|TMNBB080-06|Canada|New Brunswick|657[0n]|BOLD:AAB6600
Chrysanympha formosa|[1992]|RDLQF933-06|Canada|Quebec|657[0n]|BOLD:AAB6600
Chrysanympha formosa|[1993]|BBLPE078-09|Canada|Nova Scotia|658[0n]|BOLD:AAB6600
Chrysanympha formosa|[1994]|BBLPE115-09|Canada|Nova Scotia|658[0n]|BOLD:AAB6600
Chrysanympha formosa|[1995]|BBLPE280-09|Canada|Nova Scotia|658[0n]|BOLD:AAB6600
Chrysanympha formosa|[1996]|BBLPE328-09|Canada|Newfoundland and Labrador|658[0n]|BOLD:AAB6600
Chrysanympha formosa|[1997]|LNCC1187-11|United States|North Carolina|658[0n]|BOLD:AAB6600
Chrysanympha formosa|[1998]|CNLMF1039-14|Canada|Quebec|588[0n]|BOLD:AAB6600
Chrysanympha formosa|[1999]|CNLMO1682-14|Canada|Quebec|588[0n]|BOLD:AAB6600
Chrysanympha formosa|[2000]|CNLMP2000-14|Canada|Quebec|582[0n]|BOLD:AAB6600
Autographa precationis|[2001]|XAJ545-06|Canada|Ontario|656[10n]|
Autographa precationis|[2002]|XAG875-05|Canada|Ontario|658[0n]|BOLD:AAA3836
Autographa precationis|[2003]|BLTIB847-08|Canada|Ontario|658[0n]|BOLD:AAA3836
Autographa precationis|[2004]|XAB458-04|Canada|Ontario|658[0n]|BOLD:AAA3836
Autographa precationis|[2005]|SMTPD5185-13|Canada|Ontario|606[0n]|BOLD:AAA3836
Autographa precationis|[2006]|MNAC550-07|United States|Maryland|658[0n]|BOLD:AAA3836
Autographa precationis|[2007]|LOT531-04|United States|Tennessee|658[0n]|BOLD:AAA3836
Autographa precationis|[2008]|LOCT239-05|United States|Connecticut|658[0n]|BOLD:AAA3836
Autographa precationis|[2009]|XAJ266-06|Canada|Ontario|658[0n]|BOLD:AAA3836
Autographa precationis|[2010]|XAH034-05|Canada|Ontario|658[1n]|BOLD:AAA3836
Autographa precationis|[2011]|XAJ427-06|Canada|Ontario|658[0n]|BOLD:AAA3836
Autographa precationis|[2012]|XAB665-04|Canada|Ontario|658[0n]|BOLD:AAA3836
Autographa precationis|[2013]|XAD464-04|Canada|Ontario|570[0n]|BOLD:AAA3836
Autographa precationis|[2014]|BLTIB902-08|Canada|Ontario|658[0n]|BOLD:AAA3836
Autographa precationis|[2015]|BLTIB776-08|Canada|Ontario|658[0n]|BOLD:AAA3836
Autographa precationis|[2016]|LPSOB337-08|Canada|Ontario|658[0n]|BOLD:AAA3836
Autographa precationis|[2017]|LPSOB247-08|Canada|Ontario|658[0n]|BOLD:AAA3836
Autographa precationis|[2018]|LPSOB233-08|Canada|Ontario|658[0n]|BOLD:AAA3836
Autographa precationis|[2019]|LPSOC359-08|Canada|Ontario|658[0n]|BOLD:AAA3836
Autographa precationis|[2020]|LPSOC350-08|Canada|Ontario|658[0n]|BOLD:AAA3836
Autographa precationis|[2021]|LPSOC329-08|Canada|Ontario|658[0n]|BOLD:AAA3836
Autographa precationis|[2022]|LPSO371-08|Canada|Ontario|658[0n]|BOLD:AAA3836
Autographa precationis|[2023]|LPSO040-08|Canada|Ontario|658[0n]|BOLD:AAA3836
Autographa precationis|[2024]|LPSO039-08|Canada|Ontario|658[0n]|BOLD:AAA3836
Autographa precationis|[2025]|MMNA100-08|United States|North Carolina|658[0n]|BOLD:AAA3836
Autographa precationis|[2026]|LGSMG689-07|United States|Tennessee|658[0n]|BOLD:AAA3836
Autographa precationis|[2027]|MNAC549-07|United States|Maryland|658[0n]|BOLD:AAA3836
Autographa precationis|[2028]|XAJ674-06|Canada|Ontario|658[0n]|BOLD:AAA3836
Autographa precationis|[2029]|XAJ484-06|Canada|Ontario|658[0n]|BOLD:AAA3836
Autographa precationis|[2030]|XAJ450-06|Canada|Ontario|658[0n]|BOLD:AAA3836
Autographa precationis|[2031]|LMIS046-06|Canada|Ontario|658[0n]|BOLD:AAA3836
Autographa precationis|[2032]|RDLQB510-05|Canada|Quebec|658[0n]|BOLD:AAA3836
Autographa precationis|[2033]|XAH552-05|Canada|Ontario|658[0n]|BOLD:AAA3836
Autographa precationis|[2034]|XAH410-05|Canada|Ontario|658[0n]|BOLD:AAA3836
Autographa precationis|[2035]|XAH409-05|Canada|Ontario|658[0n]|BOLD:AAA3836
Autographa precationis|[2036]|XAH408-05|Canada|Ontario|658[0n]|BOLD:AAA3836
Autographa precationis|[2037]|XAH371-05|Canada|Ontario|658[0n]|BOLD:AAA3836
Autographa precationis|[2038]|XAH370-05|Canada|Ontario|658[0n]|BOLD:AAA3836
Autographa precationis|[2039]|XAH206-05|Canada|Ontario|658[0n]|BOLD:AAA3836
Autographa precationis|[2040]|XAH027-05|Canada|Ontario|658[0n]|BOLD:AAA3836
Autographa precationis|[2041]|XAG105-05|Canada|Ontario|658[0n]|BOLD:AAA3836
Autographa precationis|[2042]|PHMNB365-04|Canada|New Brunswick|658[0n]|BOLD:AAA3836
Autographa precationis|[2043]|XAD431-04|Canada|Ontario|658[0n]|BOLD:AAA3836
Autographa precationis|[2044]|XAD382-04|Canada|Ontario|658[0n]|BOLD:AAA3836
Autographa precationis|[2045]|XAD363-04|Canada|Ontario|658[0n]|BOLD:AAA3836
Autographa precationis|[2046]|XAG204-05|Canada|Ontario|658[1n]|BOLD:AAA3836
Autographa precationis|[2047]|UDLEP065-09|United States|Maryland|633[0n]|BOLD:AAA3836
Autographa precationis|[2048]|XAH300-05|Canada|Ontario|617[0n]|BOLD:AAA3836
Autographa precationis|[2049]|XAB676-04|Canada|Ontario|573[0n]|BOLD:AAA3836
Autographa precationis|[2050]|XAD263-04|Canada|Ontario|578[0n]|BOLD:AAA3836
Autographa precationis|[2051]|XAH205-05|Canada|Ontario|658[0n]|BOLD:AAA3836
Autographa precationis|[2052]|XAD432-04|Canada|Ontario|554[0n]|BOLD:AAA3836
Autographa precationis|[2053]|PHMO351-03|Canada|Ontario|639[0n]|BOLD:AAA3836
Autographa precationis|[2054]|XAD368-04|Canada|Ontario|551[0n]|BOLD:AAA3836
Autographa precationis|[2055]|XAG534-05|Canada|Ontario|566[1n]|BOLD:AAA3836
Autographa precationis|[2056]|UDLEP324-09|United States|Pennsylvania|605[0n]|BOLD:AAA3836
Autographa precationis|[2057]|BBLPC748-09|Canada|Newfoundland and Labrador|658[0n]|BOLD:AAA3836
Autographa precationis|[2058]|BBLOB1116-11|United States|West Virginia|658[0n]|BOLD:AAA3836
Autographa precationis|[2059]|JSOCT032-11|Canada|Ontario|658[0n]|BOLD:AAA3836
Autographa precationis|[2060]|PHMNB280-04|Canada|New Brunswick|658[0n]|BOLD:AAA3836
Autographa precationis|[2061]|UDLEP325-09|United States|Pennsylvania|658[0n]|BOLD:AAA3836
Autographa precationis|[2062]|BLTIB1021-08|Canada|Ontario|658[0n]|BOLD:AAA3836
Autographa precationis|[2063]|BLTIB861-08|Canada|Ontario|658[0n]|BOLD:AAA3836
Autographa precationis|[2064]|BLTIB785-08|Canada|Ontario|658[0n]|BOLD:AAA3836
Autographa precationis|[2065]|LPSOB362-08|Canada|Ontario|658[0n]|BOLD:AAA3836
Autographa precationis|[2066]|LPSOB324-08|Canada|Ontario|658[0n]|BOLD:AAA3836
Autographa precationis|[2067]|LPSOC360-08|Canada|Ontario|655[0n]|BOLD:AAA3836
Autographa precationis|[2068]|LPSO918-08|Canada|Ontario|658[0n]|BOLD:AAA3836
Autographa precationis|[2069]|XAJ494-06|Canada|Ontario|658[0n]|BOLD:AAA3836
Autographa precationis|[2070]|RDLQB559-05|Canada|Quebec|658[0n]|BOLD:AAA3836
Autographa precationis|[2071]|XAH704-05|Canada|Ontario|658[0n]|BOLD:AAA3836
Autographa precationis|[2072]|XAH685-05|Canada|Ontario|658[0n]|BOLD:AAA3836
Autographa precationis|[2073]|XAH317-05|Canada|Ontario|658[0n]|BOLD:AAA3836
Autographa precationis|[2074]|XAG877-05|Canada|Ontario|658[0n]|BOLD:AAA3836
Autographa precationis|[2075]|XAG613-05|Canada|Ontario|658[0n]|BOLD:AAA3836
Autographa precationis|[2076]|XAG085-05|Canada|Ontario|658[0n]|BOLD:AAA3836
Autographa precationis|[2077]|LOCT238-05|United States|Connecticut|658[0n]|BOLD:AAA3836
Autographa precationis|[2078]|PHMNB696-04|Canada|New Brunswick|658[0n]|BOLD:AAA3836
Autographa precationis|[2079]|PHMNB468-04|Canada|New Brunswick|658[0n]|BOLD:AAA3836
Autographa precationis|[2080]|XAB674-04|Canada|Ontario|658[0n]|BOLD:AAA3836
Autographa precationis|[2081]|XAB648-04|Canada|Ontario|658[0n]|BOLD:AAA3836
Autographa precationis|[2082]|BLTIB736-08|Canada|Ontario|641[4n]|BOLD:AAA3836
Autographa precationis|[2083]|LGSM774-04|United States|North Carolina|658[0n]|BOLD:AAA3836
Autographa precationis|[2084]|XAJ544-06|Canada|Ontario|658[0n]|BOLD:AAA3836
Autographa precationis|[2085]|XAG313-05|Canada|Ontario|658[0n]|BOLD:AAA3836
Autographa precationis|[2086]|UDLEP156-09|United States|Pennsylvania|658[0n]|BOLD:AAA3836
Autographa precationis|[2087]|XAG549-05|Canada|Ontario|658[1n]|BOLD:AAA3836
Autographa precationis|[2088]|CNPPB1404-12|Canada|Ontario|599[0n]|BOLD:AAA3836
Autographa precationis|[2089]|BLGSM078-09|Canada|Ontario|615[0n]|BOLD:AAA3836

Autographa precationis|[2087]|XAG549-05|Canada|Ontario|658[1n]|BOLD:AAA3836
Autographa precationis|[2088]|CNPPB1404-12|Canada|Ontario|599[0n]|BOLD:AAA3836
Autographa precationis|[2089]|BLGSM078-09|Canada|Ontario|615[0n]|BOLD:AAA3836
Autographa precationis|[2090]|CNPPD2355-12|Canada|Ontario|632[0n]|BOLD:AAA3836
Autographa precationis|[2091]|XAH088-05|Canada|Ontario|632[1n]|BOLD:AAA3836
Autographa precationis|[2092]|PHMO332-03|Canada|Ontario|639[0n]|BOLD:AAA3836
Autographa precationis|[2093]|XAD241-04|Canada|Ontario|592[0n]|BOLD:AAA3836
Autographa precationis|[2094]|XAD266-04|Canada|Ontario|593[0n]|BOLD:AAA3836
Autographa precationis|[2095]|XAD268-04|Canada|Ontario|602[0n]|BOLD:AAA3836
Autographa precationis|[2096]|XAD289-04|Canada|Ontario|587[0n]|BOLD:AAA3836
Autographa precationis|[2097]|XAD482-04|Canada|Ontario|586[0n]|BOLD:AAA3836
Autographa precationis|[2098]|XAK590-07|Canada|Ontario|592[1n]|BOLD:AAA3836
Autographa precationis|[2099]|SMTPD4322-13|Canada|Ontario|603[0n]|BOLD:AAA3836
Autographa precationis|[2100]|XAH628-05|Canada|Ontario|658[0n]|BOLD:AAA3836
Autographa precationis|[2101]|XAD485-04|Canada|Ontario|589[0n]|BOLD:AAA3836
Autographa precationis|[2102]|PHMNB299-04|Canada|New Brunswick|658[8n]|BOLD:AAA3836
Autographa precationis|[2103]|XAH132-05|Canada|Ontario|658[0n]|BOLD:AAA3836
Autographa precationis|[2104]|LOCT240-05|United States|Connecticut|658[0n]|BOLD:AAA3836
Autographa precationis|[2105]|SMTPD5501-13|Canada|Ontario|614[0n]|BOLD:AAA3836
Autographa precationis|[2106]|BLTIB1130-08|Canada|Ontario|603[1n]|BOLD:AAA3836
Autographa precationis|[2107]|LPSOB452-08|Canada|Ontario|658[0n]|BOLD:AAA3836
Autographa precationis|[2108]|BLTIB737-08|Canada|Ontario|658[0n]|
Autographa precationis|[2109]|SMTPD5769-13|Canada|Ontario|614[0n]|BOLD:AAA3836
Autographa rubidus|[2110]|LOWCC851-05|Canada|British Columbia|658[5n]|BOLD:AAD5970
Autographa rubidus|[2111]|LOWCE119-06|Canada|British Columbia|658[0n]|BOLD:AAD5970
Autographa rubidus|[2112]|LOWCE121-06|Canada|British Columbia|658[0n]|BOLD:AAD5970
Autographa rubidus|[2113]|RDLQG264-06|Canada|Quebec|658[0n]|BOLD:AAD5970
Autographa rubidus|[2114]|BBLPB313-10|Canada|Ontario|658[0n]|BOLD:AAD5970
Autographa rubidus|[2115]|LOWCE120-06|Canada|British Columbia|658[0n]|BOLD:AAD5970
Autographa rubidus|[2116]|LPSOD326-09|Canada|Ontario|658[0n]|BOLD:AAD5970
Autographa rubidus|[2117]|BBLPB311-10|Canada|Ontario|658[0n]|BOLD:AAD5970
Autographa rubidus|[2118]|BBLPB312-10|Canada|Ontario|658[0n]|BOLD:AAD5970
Autographa rubidus|[2119]|BBLPB314-10|Canada|Saskatchewan|658[0n]|BOLD:AAD5970
Autographa rubidus|[2120]|CNRMD2062-12|Canada|Manitoba|636[0n]|BOLD:AAD5970
Autographa californica|[2121]|GMLC047-09|United States|California|658[0n]|BOLD:AAB2628
Autographa californica|[2122]|GMLC1295-12|United States|California|632[0n]|BOLD:AAB2628
Autographa californica|[2123]|LOCB737-06|United States|California|611[0n]|BOLD:AAB2628
Autographa californica|[2124]|LBCW053-08|Canada|British Columbia|658[0n]|BOLD:AAB2628
Autographa californica|[2125]|LALPA343-10|Canada|British Columbia|658[0n]|BOLD:AAB2628
Autographa californica|[2126]|GMLC434-11|United States|California|658[0n]|BOLD:AAB2628
Autographa californica|[2127]|CGLCA026-10|United States|California|658[0n]|BOLD:AAB2628
Autographa californica|[2128]|BBLSW323-09|United States|Arizona|658[0n]|BOLD:AAB2628
Autographa californica|[2129]|BBLSW329-09|United States|Arizona|658[0n]|BOLD:AAB2628
Autographa californica|[2130]|RWWB380-09|United States|Washington|658[0n]|BOLD:AAB2628
Autographa californica|[2131]|LPOKC624-09|United States|Oklahoma|658[0n]|BOLD:AAB2628
Autographa californica|[2132]|RWWB659-10|United States|Washington|658[0n]|BOLD:AAB2628
Autographa californica|[2133]|LALPA453-10|Canada|British Columbia|658[0n]|BOLD:AAB2628
Autographa californica|[2134]|CMAZA899-12|United States|Arizona|658[0n]|BOLD:AAB2628
Autographa californica|[2135]|RWWB456-10|United States|Washington|658[0n]|BOLD:AAB2628
Autographa californica|[2136]|LBCH5279-10|Canada|British Columbia|658[0n]|BOLD:AAB2628
Autographa californica|[2137]|AWCLB306-10|United States|Arizona|658[0n]|BOLD:AAB2628
Autographa californica|[2138]|LALPA634-10|Canada|British Columbia|658[0n]|BOLD:AAB2628
Autographa californica|[2139]|LALPA788-10|Canada|British Columbia|658[0n]|BOLD:AAB2628
Autographa californica|[2140]|RWWC077-10|United States|Washington|658[0n]|BOLD:AAB2628
Autographa californica|[2141]|JMMMB400-11|United States|California|658[0n]|BOLD:AAB2628
Autographa californica|[2142]|GMLC442-11|United States|California|658[0n]|BOLD:AAB2628
Autographa californica|[2143]|GMLC446-11|United States|California|658[0n]|BOLD:AAB2628
Autographa californica|[2144]|CMAZA903-12|United States|Arizona|658[0n]|BOLD:AAB2628
Autographa californica|[2145]|BBLSW332-09|United States|Arizona|658[0n]|BOLD:AAB2628
Autographa californica|[2146]|BBLSX746-09|United States|Arizona|658[0n]|BOLD:AAB2628
Autographa californica|[2147]|BBLSW330-09|United States|Arizona|658[0n]|BOLD:AAB2628
Autographa californica|[2148]|BBLSW331-09|United States|Arizona|658[0n]|BOLD:AAB2628
Autographa californica|[2149]|GMLC029-09|United States|California|658[0n]|BOLD:AAB2628
Autographa californica|[2150]|LPABC039-09|Canada|Alberta|658[0n]|BOLD:AAB2628
Autographa californica|[2151]|LBCS186-07|Canada|British Columbia|658[0n]|BOLD:AAB2628
Autographa californica|[2152]|LBCS681-07|Canada|British Columbia|658[0n]|BOLD:AAB2628
Autographa californica|[2153]|LHLEP021-06|Canada|British Columbia|658[0n]|BOLD:AAB2628
Autographa californica|[2154]|LHLEP280-06|Canada|British Columbia|658[0n]|BOLD:AAB2628
Autographa californica|[2155]|LOCBB659-06|United States|California|658[0n]|BOLD:AAB2628
Autographa californica|[2156]|LOCBB660-06|United States|California|658[0n]|BOLD:AAB2628
Autographa californica|[2157]|RWWC866-12|United States|Washington|658[0n]|BOLD:AAB2628
Autographa californica|[2158]|LOCBB658-06|United States|California|658[0n]|BOLD:AAB2628
Autographa californica|[2159]|LOCBB277-06|United States|California|658[0n]|BOLD:AAB2628
Autographa californica|[2160]|LOCBB276-06|United States|California|658[0n]|BOLD:AAB2628
Autographa californica|[2161]|LOCBB275-06|United States|California|658[0n]|BOLD:AAB2628
Autographa californica|[2162]|LOCBB274-06|United States|California|658[0n]|BOLD:AAB2628
Autographa californica|[2163]|LOCBB273-06|United States|California|658[0n]|BOLD:AAB2628
Autographa californica|[2164]|LOCB429-06|United States|California|658[0n]|BOLD:AAB2628
Autographa californica|[2165]|LOWCE084-06|Canada|British Columbia|658[0n]|BOLD:AAB2628
Autographa californica|[2166]|LBCA805-05|Canada|British Columbia|658[0n]|BOLD:AAB2628
Autographa californica|[2167]|RWWA006-09|United States|Washington|635[0n]|BOLD:AAB2628
Autographa californica|[2168]|LBCA075-05|Canada|British Columbia|658[0n]|BOLD:AAB2628
Autographa californica|[2169]|CGLCA142-10|United States|California|658[0n]|BOLD:AAB2628
Autographa californica|[2170]|RWWB434-10|United States|Washington|634[0n]|BOLD:AAB2628
Autographa californica|[2171]|RDNMH183-09|Canada|British Columbia|639[0n]|BOLD:AAB2628
Autographa californica|[2172]|RWWB857-10|United States|Washington|635[0n]|BOLD:AAB2628
Autographa californica|[2173]|GMLC1293-12|United States|California|635[0n]|BOLD:AAB2628
Autographa californica|[2174]|GMLC1296-12|United States|California|629[0n]|BOLD:AAB2628
Autographa californica|[2175]|LOCBF281-13|United States|California|640[0n]|BOLD:AAB2628
Autographa gamma|[2176]|CGUKA006-09|United Kingdom|England|632[1n]|BOLD:AAB4345
Autographa gamma|[2177]|TRLEP113-13|Turkey|Mersin|601[0n]|BOLD:AAB4345
Autographa gamma|[2178]|NORIN148-13|Norway|Akershus|644[0n]|BOLD:AAB4345
Autographa gamma|[2179]|GBMIN38640-13|||641[0n]|BOLD:AAB4345
Autographa gamma|[2180]|GBMIN38591-13|||641[0n]|BOLD:AAB4345
Autographa gamma|[2181]|GBMIN38590-13|||641[0n]|BOLD:AAB4345
Autographa gamma|[2182]|LEATC657-13|Italy|South Tyrol|630[0n]|BOLD:AAB4345
Autographa gamma|[2183]|LON202-08|Sweden|Kalmar|608[0n]|BOLD:AAB4345
Autographa gamma|[2184]|LEFIA1013-10|Finland|Uusimaa|609[0n]|BOLD:AAB4345
Autographa gamma|[2185]|GWORO846-09|Germany|Bavaria|658[0n]|BOLD:AAB4345
Autographa gamma|[2186]|LON024-08|Norway|Hedmark|657[1n]|BOLD:AAB4345
Autographa gamma|[2187]|CGUKA990-09|United Kingdom|England|589[0n]|BOLD:AAB4345
Autographa gamma|[2188]|NLLEA1040-12|Netherlands|South Holland|658[0n]|BOLD:AAB4345

Autographa gamma|[2186]|LON024-08|Norway|Hedmark|657[1n]|BOLD:AAB4345
Autographa gamma|[2187]|CGUKA990-09|United Kingdom|England|589[0n]|BOLD:AAB4345
Autographa gamma|[2188]|NLLEA1040-12|Netherlands|South Holland|658[0n]|BOLD:AAB4345
Autographa gamma|[2189]|NLLEA379-12|Netherlands|South Holland|658[0n]|BOLD:AAB4345
Autographa gamma|[2190]|GWOSZ251-11|Italy|Trentino-Alto Adige|658[0n]|BOLD:AAB4345
Autographa gamma|[2191]|BCMI444-11|Israel||658[0n]|BOLD:AAB4345
Autographa gamma|[2192]|BCMI442-11|Israel||658[0n]|BOLD:AAB4345
Autographa gamma|[2193]|BCMI037-11|Israel||658[0n]|BOLD:AAB4345
Autographa gamma|[2194]|GWOSN407-11|China|Qinghai|658[0n]|BOLD:AAB4345
Autographa gamma|[2195]|IBLAO088-11|Spain|Murcia|658[0n]|BOLD:AAB4345
Autographa gamma|[2196]|GWOSA324-10|Italy|Basilicata|658[0n]|BOLD:AAB4345
Autographa gamma|[2197]|LEFIF656-10|Finland||658[0n]|BOLD:AAB4345
Autographa gamma|[2198]|LEFIF177-10|Finland||658[0n]|BOLD:AAB4345
Autographa gamma|[2199]|LEFIC671-10|Finland|Finland Proper|658[0n]|BOLD:AAB4345
Autographa gamma|[2200]|LEFIC580-10|Finland|Finland Proper|658[0n]|BOLD:AAB4345
Autographa gamma|[2201]|GWORR706-10|Germany|Bavaria|658[0n]|BOLD:AAB4345
Autographa gamma|[2202]|GWORR705-10|Germany|Bavaria|658[0n]|BOLD:AAB4345
Autographa gamma|[2203]|GWORR389-10|Italy|Calabria|658[0n]|BOLD:AAB4345
Autographa gamma|[2204]|FBLMV212-09|Germany|Bavaria|658[0n]|BOLD:AAB4345
Autographa gamma|[2205]|GWORK570-09|Germany|Bavaria|658[0n]|BOLD:AAB4345
Autographa gamma|[2206]|GWORK237-09|Germany|Bavaria|658[0n]|BOLD:AAB4345
Autographa gamma|[2207]|CGUKB065-09|United Kingdom|England|658[0n]|BOLD:AAB4345
Autographa gamma|[2208]|CGUKA730-09|United Kingdom||658[0n]|BOLD:AAB4345
Autographa gamma|[2209]|CGUKA586-09|United Kingdom|England|658[0n]|BOLD:AAB4345
Autographa gamma|[2210]|CGUKA439-09|United Kingdom|England|658[0n]|BOLD:AAB4345
Autographa gamma|[2211]|TTNFS046-09|Serbia||658[0n]|BOLD:AAB4345
Autographa gamma|[2212]|LON311-08|Norway|Vest-Agder|657[0n]|BOLD:AAB4345
Autographa gamma|[2213]|RDNMB077-05|Denmark||658[0n]|BOLD:AAB4345
Autographa gamma|[2214]|CGUKA646-09|United Kingdom|England|637[0n]|BOLD:AAB4345
Autographa gamma|[2215]|CGUKB310-09|United Kingdom|England|634[0n]|BOLD:AAB4345
Autographa gamma|[2216]|NLLEA1055-12|Netherlands|South Holland|658[0n]|BOLD:AAB4345
Autographa gamma|[2217]|IBLAO710-12|Spain|Murcia|658[0n]|BOLD:AAB4345
Autographa gamma|[2218]|ZMUCG332-12|Greenland||658[0n]|BOLD:AAB4345
Autographa gamma|[2219]|GBLGC332-12|Germany|Bavaria|658[0n]|BOLD:AAB4345
Autographa gamma|[2220]|PHLAV348-12|Austria|Vorarlberg|658[0n]|BOLD:AAB4345
Autographa gamma|[2221]|LEUAE882-12|United Arab Emirates|Fujairah|658[0n]|BOLD:AAB4345
Autographa gamma|[2222]|GBLAB705-13|Germany|Brandenburg|658[0n]|BOLD:AAB4345
Autographa gamma|[2223]|LEATA310-13|Austria|Tirol|658[0n]|BOLD:AAB4345
Autographa gamma|[2224]|LEATB708-13|Italy|South Tyrol|658[0n]|BOLD:AAB4345
Autographa gamma|[2225]|LEATC263-13|Italy|South Tyrol|658[0n]|BOLD:AAB4345
Autographa gamma|[2226]|LEATD249-13|Austria|Tirol|658[0n]|BOLD:AAB4345
Autographa gamma|[2227]|GBLAC325-13|Germany|Thuringia|658[0n]|BOLD:AAB4345
Autographa gamma|[2228]|GBLAC612-13|Germany|Bavaria|658[0n]|BOLD:AAB4345
Autographa gamma|[2229]|GBLAC930-13|Germany|Bavaria|658[0n]|BOLD:AAB4345
Autographa gamma|[2230]|GBLAA200-14|Germany|Schleswig-Holstein|658[0n]|BOLD:AAB4345
Autographa flagellum|[2231]|RDLQ755-07|Canada|Quebec|658[0n]|BOLD:AAC2492
Autographa flagellum|[2232]|PHMO115-03|Canada|Ontario|639[0n]|BOLD:AAC2492
Autographa flagellum|[2233]|LPMN758-08|Canada|Manitoba|609[0n]|BOLD:AAC2492
Autographa flagellum|[2234]|BBLPC187-09|Canada|Nova Scotia|658[0n]|BOLD:AAC2492
Autographa flagellum|[2235]|PHMNB723-05|Canada|New Brunswick|658[0n]|BOLD:AAC2492
Autographa flagellum|[2236]|BBLEC354-09|Canada|Newfoundland and Labrador|658[0n]|BOLD:AAC2492
Autographa flagellum|[2237]|BBLEC389-09|Canada|Newfoundland and Labrador|658[0n]|BOLD:AAC2492
Autographa flagellum|[2238]|BBLPC577-09|Canada|Nova Scotia|658[0n]|BOLD:AAC2492
Autographa flagellum|[2239]|BBLPC650-09|Canada|Newfoundland and Labrador|658[0n]|BOLD:AAC2492
Autographa flagellum|[2240]|BBLPC902-09|Canada|Newfoundland and Labrador|658[0n]|BOLD:AAC2492
Autographa flagellum|[2241]|BBLPE009-09|Canada|Nova Scotia|658[0n]|BOLD:AAC2492
Autographa flagellum|[2242]|BBLPE373-09|Canada|Newfoundland and Labrador|658[0n]|BOLD:AAC2492
Autographa flagellum|[2243]|BBLPE411-09|Canada|Newfoundland and Labrador|658[0n]|BOLD:AAC2492
Autographa pasiphaeia|[2244]|GMLC1442-12|United States|California|658[0n]|BOLD:ACD0446
Autographa pasiphaeia|[2245]|GMLC1445-12|United States|California|658[0n]|BOLD:ACD0446
Autographa pasiphaeia|[2246]|LNAUS3518-13|United States|California|658[0n]|BOLD:ACD0446
Autographa metallica|[2247]|RDNME606-08|United States|California|658[0n]|BOLD:ABX6057
Autographa metallica|[2248]|RDNMF377-08|Canada|British Columbia|658[0n]|BOLD:ABX6057
Autographa metallica|[2249]|RDNMF376-08|Canada|British Columbia|658[0n]|BOLD:ABX6057
Autographa metallica|[2250]|RDNMF378-08|Canada|British Columbia|658[0n]|BOLD:ABX6057
Autographa metallica|[2251]|LPAB063-08|Canada|Alberta|658[0n]|BOLD:ABX6057
Autographa metallica|[2252]|LBCG2840-09|Canada|British Columbia|658[0n]|BOLD:ABX6057
Autographa metallica|[2253]|LBCH1499-10|Canada|British Columbia|658[0n]|BOLD:ABX6057
Autographa metallica|[2254]|LALPA1149-11|Canada|British Columbia|658[0n]|BOLD:ABX6057
Autographa metallica|[2255]|LALPA1274-11|Canada|British Columbia|658[0n]|BOLD:ABX6057
Autographa metallica|[2256]|LALPA1325-12|Canada|British Columbia|629[0n]|BOLD:ABX6057
Autographa bimaculata|[2257]|CNWBG3049-13|Canada|Alberta|563[1n]|BOLD:AAC5751
Autographa bimaculata|[2258]|CNWBG3084-13|Canada|Alberta|604[1n]|BOLD:AAC5751
Autographa bimaculata|[2259]|CNWBC130-13|Canada|Alberta|571[0n]|BOLD:AAC5751
Autographa bimaculata|[2260]|CNWBC133-13|Canada|Alberta|582[0n]|BOLD:AAC5751
Autographa bimaculata|[2261]|CNWBG3067-13|Canada|Alberta|608[0n]|BOLD:AAC5751
Autographa bimaculata|[2262]|CNWBG3122-13|Canada|Alberta|587[2n]|BOLD:AAC5751
Autographa bimaculata|[2263]|CNWBG3082-13|Canada|Alberta|591[0n]|BOLD:AAC5751
Autographa bimaculata|[2264]|LOWCD297-06|Canada|British Columbia|558[0n]|BOLD:AAC5751
Autographa bimaculata|[2265]|BBLPE117-09|Canada|Nova Scotia|658[0n]|BOLD:AAC5751
Autographa bimaculata|[2266]|BBLPB515-10|Canada|Alberta|658[0n]|BOLD:AAC5751
Autographa bimaculata|[2267]|RDLQB624-05|Canada|Quebec|584[0n]|BOLD:AAC5751
Autographa bimaculata|[2268]|BBLPC588-09|Canada|Nova Scotia|658[0n]|BOLD:AAC5751
Autographa bimaculata|[2269]|BBLEC313-09|Canada|Nova Scotia|658[0n]|BOLD:AAC5751
Autographa bimaculata|[2270]|LPMNB507-09|Canada|Manitoba|658[0n]|BOLD:AAC5751
Autographa bimaculata|[2271]|RDNMG1005-08|Canada|New Brunswick|658[0n]|BOLD:AAC5751
Autographa bimaculata|[2272]|RDNMG1004-08|Canada|New Brunswick|658[0n]|BOLD:AAC5751
Autographa bimaculata|[2273]|LBCD311-05|Canada|British Columbia|639[0n]|BOLD:AAC5751
Autographa bimaculata|[2274]|LPMNB512-09|Canada|Manitoba|609[0n]|BOLD:AAC5751
Autographa bimaculata|[2275]|BBLPC991-09|Canada|Nova Scotia|623[0n]|
Autographa bimaculata|[2276]|BBLPB514-10|Canada|Alberta|658[0n]|BOLD:AAC5751
Autographa bimaculata|[2277]|CNWBG3161-13|Canada|Alberta|628[0n]|BOLD:AAC5751
Autographa bimaculata|[2278]|CNWBH1367-13|Canada|Alberta|576[0n]|BOLD:AAC5751
Autographa pseudogamma|[2279]|RDNMG611-08|Canada|New Brunswick|658[0n]|BOLD:AAD2600
Autographa pseudogamma|[2280]|LPABC086-09|Canada|Alberta|658[0n]|BOLD:AAD2600
Autographa pseudogamma|[2281]|CNWBG3121-13|Canada|Alberta|571[0n]|BOLD:AAD2600
Autographa pseudogamma|[2282]|UAMIC1145-13|United States|Alaska|658[0n]|BOLD:AAD2600
Autographa pseudogamma|[2283]|BBLPE486-09|Canada|Newfoundland and Labrador|658[0n]|BOLD:AAD2600
Autographa pseudogamma|[2284]|LPABB190-08|Canada|Alberta|658[0n]|BOLD:AAD2600
Autographa pseudogamma|[2285]|NAMUM316-08|United States|California|658[0n]|BOLD:AAD2600
Autographa pseudogamma|[2286]|RDNMG631-08|United States|California|658[0n]|BOLD:AAD2600
Autographa pseudogamma|[2287]|RDNMG612-08|Canada|New Brunswick|658[0n]|BOLD:AAD2600
Autographa pseudogamma|[2288]|SSWLD5794-13|Canada|Alberta|554[0n]|BOLD:AAD2600

[illegible]
Autographa pseudogamma|[2286]|RDNMG631-08|United States|California|658[0n]|BOLD:AAD2600
Autographa pseudogamma|[2287]|RDNMG612-08|Canada|New Brunswick|658[0n]|BOLD:AAD2600
Autographa pseudogamma|[2288]|SSWLD5794-13|Canada|Alberta|554[0n]|BOLD:AAD2600
Autographa pseudogamma|[2289]|CNWBG3109-13|Canada|Alberta|611[0n]|BOLD:AAD2600
Autographa pseudogamma|[2290]|LPABC007-09|Canada|Alberta|636[1n]|BOLD:AAD2600
Autographa pseudogamma|[2291]|SSWLD6404-13|Canada|Alberta|544[0n]|BOLD:AAD2600
Autographa sansoni|[2292]|LBCH2957-10|Canada|British Columbia|658[0n]|BOLD:AAC1557
Autographa sansoni|[2293]|SSWLD6385-13|Canada|Alberta|534[1n]|BOLD:AAC1557
Autographa sansoni|[2294]|SSWLD6412-13|Canada|Alberta|576[9n]|BOLD:AAC1557
Autographa sansoni|[2295]|SSWLD7215-13|Canada|Alberta|591[0n]|BOLD:AAC1557
Autographa sansoni|[2296]|LBCA042-05|Canada|British Columbia|658[0n]|BOLD:AAC1557
Autographa sansoni|[2297]|SSWLD6401-13|Canada|Alberta|562[0n]|BOLD:AAC1557
Autographa sansoni|[2298]|LBCH2238-10|Canada|British Columbia|658[0n]|BOLD:AAC1557
Autographa sansoni|[2299]|LBCH1759-10|Canada|British Columbia|658[0n]|BOLD:AAC1557
Autographa sansoni|[2300]|LBCG3028-09|Canada|British Columbia|658[0n]|BOLD:AAC1557
Autographa sansoni|[2301]|LBCG3027-09|Canada|British Columbia|658[0n]|BOLD:AAC1557
Autographa sansoni|[2302]|LBCG3025-09|Canada|British Columbia|658[0n]|BOLD:AAC1557
Autographa sansoni|[2303]|LBCG2833-09|Canada|British Columbia|658[0n]|BOLD:AAC1557
Autographa sansoni|[2304]|LBCG2541-09|Canada|British Columbia|658[0n]|BOLD:AAC1557
Autographa sansoni|[2305]|LBCG1885-09|Canada|British Columbia|658[0n]|BOLD:AAC1557
Autographa sansoni|[2306]|LOWCE195-06|Canada|British Columbia|658[0n]|BOLD:AAC1557
Autographa sansoni|[2307]|RDMAB527-06|Canada|Alberta|658[0n]|BOLD:AAC1557
Autographa sansoni|[2308]|LBCC562-05|Canada|British Columbia|658[0n]|BOLD:AAC1557
Autographa sansoni|[2309]|LBCA036-05|Canada|British Columbia|658[0n]|BOLD:AAC1557
Autographa sansoni|[2310]|SSWLA3860-13|Canada|Alberta|592[0n]|BOLD:AAC1557
Autographa sansoni|[2311]|LBCG3026-09|Canada|British Columbia|658[0n]|BOLD:AAC1557
Autographa sansoni|[2312]|CNWLB640-12|Canada|Alberta|614[0n]|BOLD:AAC1557
Autographa sansoni|[2313]|SSWLA3861-13|Canada|Alberta|607[0n]|BOLD:AAC1557
Autographa sansoni|[2314]|SSWLD5795-13|Canada|Alberta|591[0n]|BOLD:AAC1557
Autographa sansoni|[2315]|SSWLD6400-13|Canada|Alberta|589[0n]|BOLD:AAC1557
Autographa sansoni|[2316]|SSWLD7217-13|Canada|Alberta|619[0n]|BOLD:AAC1557
Autographa sansoni|[2317]|LOWCD134-06|Canada|British Columbia|658[0n]|BOLD:AAC1557
Autographa sansoni|[2318]|SSWLD7219-13|Canada|Alberta|597[0n]|BOLD:AAC1557
Autographa ampla|[2319]|LOWCD123-06|Canada|British Columbia|569[0n]|BOLD:AAA8729
Autographa ampla|[2320]|BBLEC773-09|Canada|Newfoundland and Labrador|658[0n]|BOLD:AAA8729
Autographa ampla|[2321]|CNEIC3019-12|Canada|Alberta|611[0n]|BOLD:AAA8729
Autographa ampla|[2322]|CNEIC3029-12|Canada|Alberta|634[0n]|BOLD:AAA8729
Autographa ampla|[2323]|RDLQB029-05|Canada|Quebec|602[1n]|BOLD:AAA8729
Autographa ampla|[2324]|BBLPC227-09|Canada|Nova Scotia|658[0n]|BOLD:AAA8729
Autographa ampla|[2325]|BBLPC132-09|Canada|Nova Scotia|621[0n]|BOLD:AAA8729
Autographa ampla|[2326]|RDLQB032-05|Canada|Quebec|658[0n]|BOLD:AAA8729
Autographa ampla|[2327]|LOWCB589-05|Canada|British Columbia|592[0n]|BOLD:AAA8729
Autographa ampla|[2328]|LOWCB590-05|Canada|British Columbia|611[0n]|BOLD:AAA8729
Autographa ampla|[2329]|LOWCD171-06|Canada|British Columbia|599[0n]|BOLD:AAA8729
Autographa ampla|[2330]|PHMO131-03|Canada|Ontario|639[0n]|BOLD:AAA8729
Autographa ampla|[2331]|RDLQB028-05|Canada|Quebec|658[0n]|BOLD:AAA8729
Autographa ampla|[2332]|RDLQB030-05|Canada|Quebec|658[0n]|BOLD:AAA8729
Autographa ampla|[2333]|BBLPC233-09|Canada|Nova Scotia|658[0n]|BOLD:AAA8729
Autographa ampla|[2334]|LHLEP121-06|Canada|British Columbia|617[0n]|BOLD:AAA8729
Autographa ampla|[2335]|BBLPC243-09|Canada|Nova Scotia|658[0n]|BOLD:AAA8729
Autographa ampla|[2336]|LBCH118-10|Canada|British Columbia|658[0n]|BOLD:AAA8729
Autographa ampla|[2337]|LBCH914-10|Canada|British Columbia|658[0n]|BOLD:AAA8729
Autographa ampla|[2338]|CNEIC3027-12|Canada|Alberta|634[0n]|BOLD:AAA8729
Autographa ampla|[2339]|CNWLE2401-12|Canada|Alberta|632[0n]|BOLD:AAA8729
Autographa ampla|[2340]|CNWLE2406-12|Canada|Alberta|614[0n]|BOLD:AAA8729
Autographa ampla|[2341]|LBCA610-05|Canada|British Columbia|633[0n]|BOLD:AAA8729
Autographa ampla|[2342]|XAE315-04|Canada|Ontario|658[0n]|BOLD:AAA8729
Autographa ampla|[2343]|LBCA077-05|Canada|British Columbia|658[0n]|BOLD:AAA8729
Autographa ampla|[2344]|LBCC289-05|Canada|British Columbia|658[0n]|BOLD:AAA8729
Autographa ampla|[2345]|LBCC641-05|Canada|British Columbia|658[0n]|BOLD:AAA8729
Autographa ampla|[2346]|LBCD031-05|Canada|British Columbia|658[0n]|BOLD:AAA8729
Autographa ampla|[2347]|LBCD451-05|Canada|British Columbia|658[0n]|BOLD:AAA8729
Autographa ampla|[2348]|RDLQB031-05|Canada|Quebec|658[0n]|BOLD:AAA8729
Autographa ampla|[2349]|TMNBB082-06|Canada|New Brunswick|658[0n]|BOLD:AAA8729
Autographa ampla|[2350]|LHLEP120-06|Canada|British Columbia|658[0n]|BOLD:AAA8729
Autographa ampla|[2351]|LHLEP122-06|Canada|British Columbia|658[0n]|BOLD:AAA8729
Autographa ampla|[2352]|LPVIA612-08|Canada|British Columbia|658[0n]|BOLD:AAA8729
Autographa ampla|[2353]|LPABB397-08|Canada|Alberta|658[0n]|BOLD:AAA8729
Autographa ampla|[2354]|LPABC978-09|Canada|Alberta|658[0n]|BOLD:AAA8729
Autographa ampla|[2355]|RWWA296-09|United States|Washington|658[0n]|BOLD:AAA8729
Autographa ampla|[2356]|RWWA378-09|United States|Washington|658[0n]|BOLD:AAA8729
Autographa ampla|[2357]|RWWA454-09|United States|Washington|658[0n]|BOLD:AAA8729
Autographa ampla|[2358]|RWWA660-09|United States|Washington|658[0n]|BOLD:AAA8729
Autographa ampla|[2359]|RWWA895-09|United States|Washington|658[0n]|BOLD:AAA8729
Autographa ampla|[2360]|LBCH006-10|Canada|British Columbia|658[0n]|BOLD:AAA8729
Autographa ampla|[2361]|LBCH230-10|Canada|British Columbia|658[0n]|BOLD:AAA8729
Autographa ampla|[2362]|LBCH491-10|Canada|British Columbia|658[0n]|BOLD:AAA8729
Autographa ampla|[2363]|LBCH651-10|Canada|British Columbia|658[0n]|BOLD:AAA8729
Autographa ampla|[2364]|LBCH809-10|Canada|British Columbia|658[0n]|BOLD:AAA8729
Autographa ampla|[2365]|LBCH3066-10|Canada|British Columbia|658[0n]|BOLD:AAA8729
Autographa ampla|[2366]|LBCH3332-10|Canada|British Columbia|658[0n]|BOLD:AAA8729
Autographa ampla|[2367]|LBCH3451-10|Canada|British Columbia|658[0n]|BOLD:AAA8729
Autographa ampla|[2368]|LBCH3781-10|Canada|British Columbia|658[0n]|BOLD:AAA8729
Autographa ampla|[2369]|LBCH4403-10|Canada|British Columbia|658[0n]|BOLD:AAA8729
Autographa ampla|[2370]|LALPA843-11|Canada|British Columbia|658[0n]|BOLD:AAA8729
Autographa ampla|[2371]|LALPA954-11|Canada|British Columbia|658[0n]|BOLD:AAA8729
Autographa ampla|[2372]|LALPA1289-11|Canada|British Columbia|658[0n]|BOLD:AAA8729
Autographa ampla|[2373]|CNEID3329-12|Canada|Alberta|633[0n]|BOLD:AAA8729
Autographa ampla|[2374]|LBCB904-05|Canada|British Columbia|636[0n]|BOLD:AAA8729
Autographa ampla|[2375]|SSPAA2606-13|Canada|Saskatchewan|582[0n]|BOLD:AAA8729
Autographa speciosa|[2376]|RDNMG613-08|United States|Oregon|658[0n]|BOLD:ABZ0513
Autographa speciosa|[2377]|RDNMF416-08|United States|Oregon|658[0n]|BOLD:ABZ0513
Autographa speciosa|[2378]|RDNMF417-08|United States|Oregon|658[0n]|BOLD:ABZ0513
Autographa speciosa|[2379]|RDNMG614-08|United States|Oregon|649[0n]|BOLD:ABZ0513
Autographa v-alba|[2380]|RDNMF001-08|United States|Utah|658[0n]|BOLD:ABZ0514
Autographa v-alba|[2381]|RDNMG615-08|United States|Washington|658[0n]|BOLD:ABZ0514
Autographa v-alba|[2382]|RDNMG616-08|United States|Washington|658[0n]|BOLD:ABZ0514
Autographa v-alba|[2383]|RDNMG617-08|United States|Washington|658[0n]|BOLD:ABZ0514
Autographa v-alba|[2384]|RDNMG675-08|United States|Utah|658[0n]|BOLD:ABZ0514
Autographa v-alba|[2385]|LPMN900-08|Canada|Alberta|658[0n]|BOLD:ABZ0514
Autographa v-alba|[2386]|LPABC073-09|Canada|Alberta|658[0n]|BOLD:ABZ0514
Autographa flavida|[2387]|RDNME593-08|United States|Colorado|658[0n]|BOLD:ABU7112

Autographa v-alba|[2385]|LPMN900-08|Canada|Alberta|658[0n]|BOLD:ABZ0514
Autographa v-alba|[2386]|LPABC073-09|Canada|Alberta|658[0n]|BOLD:ABZ0514
Autographa flavida|[2387]|RDNME593-08|United States|Colorado|658[0n]|BOLD:ABU7112
Autographa buraetica|[2388]|RDNMF084-08|Canada|Yukon Territory|658[0n]|BOLD:AAA3990
Autographa buraetica|[2389]|ABKWR131-07|United States|Alaska|632[0n]|BOLD:AAA3990
Autographa buraetica|[2390]|UAMIC555-13|United States|Alaska|624[0n]|BOLD:AAA3990
Autographa buraetica|[2391]|UAMIC556-13|United States|Alaska|603[0n]|BOLD:AAA3990
Autographa mappa|[2392]|BBLPB330-10|Canada|British Columbia|658[0n]|BOLD:AAA9921
Autographa mappa|[2393]|BBLEC274-09|Canada|Nova Scotia|658[0n]|BOLD:AAA9921
Autographa mappa|[2394]|LOWCD176-06|Canada|British Columbia|569[1n]|BOLD:AAA9921
Autographa mappa|[2395]|LPABB036-08|Canada|Alberta|658[0n]|BOLD:AAA9921
Autographa mappa|[2396]|LPSOC281-08|Canada|Ontario|658[0n]|BOLD:AAA9921
Autographa mappa|[2397]|RDLQF855-06|Canada|Quebec|658[0n]|BOLD:AAA9921
Autographa mappa|[2398]|RDLQF854-06|Canada|Quebec|658[0n]|BOLD:AAA9921
Autographa mappa|[2399]|RDLQB009-05|Canada|Quebec|658[0n]|BOLD:AAA9921
Autographa mappa|[2400]|RDLQB008-05|Canada|Quebec|658[0n]|BOLD:AAA9921
Autographa mappa|[2401]|RDLQB007-05|Canada|Quebec|658[0n]|BOLD:AAA9921
Autographa mappa|[2402]|RDLQB006-05|Canada|Quebec|658[0n]|BOLD:AAA9921
Autographa mappa|[2403]|RDLQB005-05|Canada|Quebec|658[0n]|BOLD:AAA9921
Autographa mappa|[2404]|LBCB549-05|Canada|British Columbia|658[0n]|BOLD:AAA9921
Autographa mappa|[2405]|LBCA035-05|Canada|British Columbia|658[0n]|BOLD:AAA9921
Autographa mappa|[2406]|LBCA034-05|Canada|British Columbia|658[0n]|BOLD:AAA9921
Autographa mappa|[2407]|LBCB921-05|Canada|British Columbia|636[0n]|BOLD:AAA9921
Autographa mappa|[2408]|LBCB920-05|Canada|British Columbia|636[0n]|BOLD:AAA9921
Autographa mappa|[2409]|RWWA252-09|United States|Washington|658[0n]|BOLD:AAA9921
Autographa mappa|[2410]|RWWA261-09|United States|Washington|658[0n]|BOLD:AAA9921
Autographa mappa|[2411]|RWWA487-09|United States|Washington|658[0n]|BOLD:AAA9921
Autographa mappa|[2412]|BBLEC269-09|Canada|Nova Scotia|633[0n]|BOLD:AAA9921
Autographa mappa|[2413]|RDLQB010-05|Canada|Quebec|595[1n]|BOLD:AAA9921
Autographa mappa|[2414]|BBLEC370-09|Canada|Newfoundland and Labrador|615[0n]|BOLD:AAA9921
Autographa mappa|[2415]|BBLEC102-09|Canada|Nova Scotia|636[0n]|BOLD:AAA9921
Autographa mappa|[2416]|BBLEC373-09|Canada|Newfoundland and Labrador|637[0n]|BOLD:AAA9921
Autographa mappa|[2417]|BBLPE120-09|Canada|Nova Scotia|632[0n]|BOLD:AAA9921
Autographa mappa|[2418]|LPABC941-09|Canada|Alberta|658[0n]|BOLD:AAA9921
Autographa mappa|[2419]|RWWA362-09|United States|Washington|658[0n]|BOLD:AAA9921
Autographa mappa|[2420]|RWWA478-09|United States|Washington|658[0n]|BOLD:AAA9921
Autographa mappa|[2421]|BBLEC352-09|Canada|Newfoundland and Labrador|658[0n]|BOLD:AAA9921
Autographa mappa|[2422]|BBLEC372-09|Canada|Newfoundland and Labrador|658[0n]|BOLD:AAA9921
Autographa mappa|[2423]|BBLPC213-09|Canada|Nova Scotia|658[0n]|BOLD:AAA9921
Autographa mappa|[2424]|BBLPC221-09|Canada|Nova Scotia|658[0n]|BOLD:AAA9921
Autographa mappa|[2425]|BBLPE491-09|Canada|Newfoundland and Labrador|658[0n]|BOLD:AAA9921
Autographa mappa|[2426]|BBLPE538-09|Canada|Newfoundland and Labrador|658[0n]|BOLD:AAA9921
Autographa mappa|[2427]|LBCH094-10|Canada|British Columbia|658[0n]|BOLD:AAA9921
Autographa mappa|[2428]|RWWB825-10|United States|Washington|658[0n]|BOLD:AAA9921
Autographa mappa|[2429]|BBLPB277-10|Canada|British Columbia|658[0n]|BOLD:AAA9921
Autographa mappa|[2430]|BBLPB278-10|Canada|Ontario|658[0n]|BOLD:AAA9921
Autographa mappa|[2431]|RWWC250-11|United States|Washington|658[0n]|BOLD:AAA9921
Autographa mappa|[2432]|SSPAB7604-13|Canada|Saskatchewan|605[0n]|BOLD:AAA9921
Autographa corusca|[2433]|CNCLB296-14|United States|California|307[0n]|
Autographa corusca|[2434]|CNCLB297-14|United States|California|550[0n]|BOLD:ABZ0515
Autographa corusca|[2435]|CNCLB298-14|United States|California|550[0n]|BOLD:ABZ0515
Autographa corusca|[2436]|LBCH4650-10|Canada|British Columbia|636[0n]|BOLD:ABZ0515
Autographa corusca|[2437]|LOPN143-06|United States|Oregon|526[2n]|BOLD:ABZ0515
Autographa corusca|[2438]|LHLEP277-06|Canada|British Columbia|658[0n]|BOLD:ABZ0515
Autographa corusca|[2439]|LHLEP278-06|Canada|British Columbia|658[0n]|BOLD:ABZ0515
Autographa corusca|[2440]|LHLEP279-06|Canada|British Columbia|658[0n]|BOLD:ABZ0515
Autographa corusca|[2441]|LBCS261-07|Canada|British Columbia|658[0n]|BOLD:ABZ0515
Autographa corusca|[2442]|LBCS262-07|Canada|British Columbia|658[0n]|BOLD:ABZ0515
Autographa corusca|[2443]|RDNMF089-08|Canada|British Columbia|658[0n]|BOLD:ABZ0515
Autographa corusca|[2444]|LPVIB068-08|Canada|British Columbia|658[0n]|BOLD:ABZ0515
Autographa corusca|[2445]|RWWA637-09|United States|Washington|658[0n]|BOLD:ABZ0515
Autographa corusca|[2446]|RWWA687-09|United States|Washington|658[0n]|BOLD:ABZ0515
Autographa corusca|[2447]|RWWA695-09|United States|Washington|658[0n]|BOLD:ABZ0515
Autographa corusca|[2448]|RWWA701-09|United States|Washington|658[0n]|BOLD:ABZ0515
Autographa corusca|[2449]|RWWA730-09|United States|Washington|658[0n]|BOLD:ABZ0515
Autographa corusca|[2450]|LBCH120-10|Canada|British Columbia|658[0n]|BOLD:ABZ0515
Autographa corusca|[2451]|LBCH1302-10|Canada|British Columbia|658[0n]|BOLD:ABZ0515
Autographa corusca|[2452]|LBCH4679-10|Canada|British Columbia|658[0n]|BOLD:ABZ0515
Autographa corusca|[2453]|LALPA633-10|Canada|British Columbia|658[0n]|BOLD:ABZ0515
Autographa corusca|[2454]|LALPA661-10|Canada|British Columbia|658[0n]|BOLD:ABZ0515
Autographa corusca|[2455]|LALPA662-10|Canada|British Columbia|658[0n]|BOLD:ABZ0515
Autographa corusca|[2456]|LALPA687-10|Canada|British Columbia|658[0n]|BOLD:ABZ0515
Autographa corusca|[2457]|RWWC066-10|United States|Washington|658[0n]|BOLD:ABZ0515
Autographa corusca|[2458]|RWWC431-11|United States|Washington|658[0n]|BOLD:ABZ0515
Autographa corusca|[2459]|RWWC474-11|United States|Washington|658[0n]|BOLD:ABZ0515
Autographa corusca|[2460]|RWWC530-11|United States|Washington|658[0n]|BOLD:ABZ0515
Autographa corusca|[2461]|CNCLB299-14|United States|California|550[0n]|BOLD:ABZ0515
Autographa corusca|[2462]|CNCLB300-14|||307[0n]|
Megalographa biloba|[2463]|LILLA626-11|United States|Illinois|658[0n]|BOLD:AAD7518
Megalographa biloba|[2464]|BBLSU015-09|United States|Arkansas|658[0n]|BOLD:AAD7518
Megalographa biloba|[2465]|PMG131-03|Canada|Ontario|617[0n]|BOLD:AAD7518
Megalographa biloba|[2466]|RDNMD582-06|United States|Florida|658[0n]|BOLD:AAD7518
Megalographa biloba|[2467]|CMAZA229-09|United States|Arizona|658[0n]|BOLD:AAD7518
Megalographa biloba|[2468]|GMLC903-12|United States|California|658[0n]|BOLD:AAD7518
Megalographa biloba|[2469]|GMLC1228-12|United States|California|658[0n]|BOLD:AAD7518
Megalographa biloba|[2470]|GMLC1326-12|United States|California|628[0n]|BOLD:AAD7518
Megalographa biloba|[2471]|TMG97-03|Canada|Ontario|639[0n]|BOLD:AAD7518
Megalographa biloba|[2472]|LOCBE357-06|United States|California|589[0n]|BOLD:AAD7518
Megalographa biloba|[2473]|MJMSL039-10|United States|Massachusetts|658[0n]|BOLD:AAD7518
Megalographa biloba|[2474]|LILLA029-11|United States|Illinois|658[0n]|BOLD:AAD7518
Megalographa biloba|[2475]|LPOKA1011-09|United States|Oklahoma|658[0n]|BOLD:AAD7518
Megalographa biloba|[2476]|SMTPD2612-13|Canada|Ontario|558[0n]|BOLD:AAD7518
Syngrapha epigaea|[2477]|LALPA1320-12|Canada|British Columbia|599[0n]|BOLD:AAC8825
Syngrapha epigaea|[2478]|CNWBG3099-13|Canada|Alberta|587[0n]|BOLD:AAC8825
Syngrapha epigaea|[2479]|BBLPB310-10|Canada|British Columbia|658[0n]|BOLD:AAC8825
Syngrapha epigaea|[2480]|RDLQB619-05|Canada|Quebec|585[0n]|BOLD:AAC8825
Syngrapha epigaea|[2481]|RDLQB617-05|Canada|Quebec|585[0n]|BOLD:AAC8825
Syngrapha epigaea|[2482]|RDLQB618-05|Canada|Quebec|592[0n]|BOLD:AAC8825
Syngrapha epigaea|[2483]|LOWCB588-05|Canada|British Columbia|611[0n]|BOLD:AAC8825
Syngrapha epigaea|[2484]|BBLPC995-09|Canada|Nova Scotia|599[0n]|BOLD:AAC8825
Syngrapha epigaea|[2485]|RDLQB620-05|Canada|Quebec|643[0n]|BOLD:AAC8825
Syngrapha epigaea|[2486]|RDLQB841-05|Canada|Quebec|658[0n]|BOLD:AAC8825
Syngrapha epigaea|[2487]|BBLEC524-09|Canada|New Brunswick|658[0n]|BOLD:AAC8825

Syngrapha epigaea|[2484]|BBLPC593-09|Canada|Nova Scotia|[illegible][0n]|BOLD:AAC8825
Syngrapha epigaea|[2485]|RDLQB620-05|Canada|Quebec|643[0n]|BOLD:AAC8825
Syngrapha epigaea|[2486]|RDLQB841-05|Canada|Quebec|658[0n]|BOLD:AAC8825
Syngrapha epigaea|[2487]|BBLEC524-09|Canada|New Brunswick|658[0n]|BOLD:AAC8825
Syngrapha epigaea|[2488]|BBLPE080-09|Canada|Nova Scotia|658[0n]|BOLD:AAC8825
Syngrapha epigaea|[2489]|LBCH3782-10|Canada|British Columbia|658[0n]|BOLD:AAC8825
Syngrapha epigaea|[2490]|LALPA695-10|Canada|British Columbia|658[0n]|BOLD:AAC8825
Syngrapha epigaea|[2491]|LALPA708-10|Canada|British Columbia|658[0n]|BOLD:AAC8825
Syngrapha epigaea|[2492]|SSJAE1895-13|Canada|Alberta|614[0n]|BOLD:AAC8825
Syngrapha epigaea|[2493]|CNMIG908-14|Canada|Quebec|588[0n]|BOLD:AAC8825
Syngrapha epigaea|[2494]|CNTNG185-14|Canada|Newfoundland and Labrador|588[0n]|BOLD:AAC8825
Syngrapha epigaea|[2495]|CNTNH1260-14|Canada|Newfoundland and Labrador|540[2n]|BOLD:AAC8825
Syngrapha microgamma|[2496]|RDLQB004-05|Canada|Quebec|537[0n]|BOLD:ABY6569
Syngrapha microgamma|[2497]|LCH322-04|Canada|Manitoba|658[0n]|BOLD:ABY6569
Syngrapha microgamma|[2498]|LCH321-04|Canada|Manitoba|658[0n]|BOLD:ABY6569
Syngrapha microgamma|[2499]|LCH320-04|Canada|Manitoba|658[0n]|BOLD:ABY6569
Syngrapha microgamma|[2500]|RDLQB001-05|Canada|Quebec|526[1n]|BOLD:ABY6569
Syngrapha microgamma|[2501]|RDLQB003-05|Canada|Quebec|658[0n]|BOLD:ABY6569
Syngrapha microgamma|[2502]|LCHP820-07|Canada|Manitoba|658[0n]|BOLD:ABY6569
Syngrapha microgamma|[2503]|LCHP832-07|Canada|Manitoba|651[0n]|BOLD:ABY6569
Syngrapha montana|[2504]|LCH318-04|Canada|Manitoba|658[0n]|BOLD:ABZ6945
Syngrapha montana|[2505]|RDLQG368-06|Canada|Quebec|658[0n]|BOLD:ABZ6945
Syngrapha montana|[2506]|RDNMG623-08|Canada|Quebec|658[0n]|BOLD:ABZ6945
Syngrapha montana|[2507]|RDNMG624-08|Canada|New Brunswick|658[0n]|BOLD:ABZ6945
Syngrapha angulidens|[2508]|LSEU784-06|United States|Colorado|658[0n]|BOLD:ABY6571
Syngrapha angulidens|[2509]|LBCG874-09|Canada|British Columbia|630[0n]|BOLD:ABY6571
Syngrapha angulidens|[2510]|LBCH1508-10|Canada|British Columbia|639[0n]|BOLD:ABY6571
Syngrapha angulidens|[2511]|LBCG601-09|Canada|British Columbia|658[0n]|BOLD:ABY6571
Syngrapha angulidens|[2512]|LBCH1573-10|Canada|British Columbia|658[0n]|BOLD:ABY6571
Syngrapha angulidens|[2513]|LBCC559-05|Canada|British Columbia|658[0n]|BOLD:ABY6571
Syngrapha angulidens|[2514]|LBCD453-05|Canada|British Columbia|658[0n]|BOLD:ABY6571
Syngrapha angulidens|[2515]|LBCG2060-09|Canada|British Columbia|658[0n]|BOLD:ABY6571
Syngrapha angulidens|[2516]|LBCG2067-09|Canada|British Columbia|658[0n]|BOLD:ABY6571
Syngrapha angulidens|[2517]|LBCH1105-10|Canada|British Columbia|658[0n]|BOLD:ABY6571
Syngrapha angulidens|[2518]|LBCH1446-10|Canada|British Columbia|658[0n]|BOLD:ABY6571
Syngrapha angulidens|[2519]|LBCH1447-10|Canada|British Columbia|658[0n]|BOLD:ABY6571
Syngrapha angulidens|[2520]|LBCH1507-10|Canada|British Columbia|658[0n]|BOLD:ABY6571
Syngrapha angulidens|[2521]|LBCH1510-10|Canada|British Columbia|658[0n]|BOLD:ABY6571
Syngrapha angulidens|[2522]|LBCH1511-10|Canada|British Columbia|658[0n]|BOLD:ABY6571
Syngrapha angulidens|[2523]|LBCH1512-10|Canada|British Columbia|658[0n]|BOLD:ABY6571
Syngrapha angulidens|[2524]|LBCH1513-10|Canada|British Columbia|658[0n]|BOLD:ABY6571
Syngrapha angulidens|[2525]|LBCH1514-10|Canada|British Columbia|658[0n]|BOLD:ABY6571
Syngrapha angulidens|[2526]|LBCH1515-10|Canada|British Columbia|658[0n]|BOLD:ABY6571
Syngrapha angulidens|[2527]|LBCG1020-09|Canada|British Columbia|658[0n]|BOLD:ABY6571
Syngrapha angulidens|[2528]|LBCG2059-09|Canada|British Columbia|658[0n]|BOLD:ABY6571
Syngrapha angulidens|[2529]|LBCH1516-10|Canada|British Columbia|658[0n]|BOLD:ABY6571
Syngrapha angulidens|[2530]|LBCH1569-10|Canada|British Columbia|658[0n]|BOLD:ABY6571
Syngrapha angulidens|[2531]|LBCH1570-10|Canada|British Columbia|658[0n]|BOLD:ABY6571
Syngrapha angulidens|[2532]|LBCH1571-10|Canada|British Columbia|658[0n]|BOLD:ABY6571
Syngrapha angulidens|[2533]|LBCH1572-10|Canada|British Columbia|658[0n]|BOLD:ABY6571
Syngrapha angulidens|[2534]|LBCH1574-10|Canada|British Columbia|658[0n]|BOLD:ABY6571
Syngrapha angulidens|[2535]|LBCH1575-10|Canada|British Columbia|658[0n]|BOLD:ABY6571
Syngrapha angulidens|[2536]|LBCH1576-10|Canada|British Columbia|658[0n]|BOLD:ABY6571
Syngrapha angulidens|[2537]|LBCH1682-10|Canada|British Columbia|658[0n]|BOLD:ABY6571
Syngrapha angulidens|[2538]|LBCH2112-10|Canada|British Columbia|658[0n]|BOLD:ABY6571
Syngrapha angulidens|[2539]|LBCH2113-10|Canada|British Columbia|658[0n]|BOLD:ABY6571
Syngrapha angulidens|[2540]|LBCH2115-10|Canada|British Columbia|658[0n]|BOLD:ABY6571
Syngrapha angulidens|[2541]|LBCH2116-10|Canada|British Columbia|658[0n]|BOLD:ABY6571
Syngrapha angulidens|[2542]|LBCH2117-10|Canada|British Columbia|658[0n]|BOLD:ABY6571
Syngrapha angulidens|[2543]|LBCH2118-10|Canada|British Columbia|658[0n]|BOLD:ABY6571
Syngrapha angulidens|[2544]|LBCH2119-10|Canada|British Columbia|658[0n]|BOLD:ABY6571
Syngrapha angulidens|[2545]|LPABB205-08|Canada|Alberta|658[0n]|BOLD:ABY6571
Syngrapha angulidens|[2546]|LBCG632-09|Canada|British Columbia|658[0n]|BOLD:ABY6571
Syngrapha angulidens|[2547]|LBCG884-09|Canada|British Columbia|658[0n]|BOLD:ABY6571
Syngrapha angulidens|[2548]|LPABC835-09|Canada|Alberta|658[0n]|BOLD:ABY6571
Syngrapha angulidens|[2549]|LBCG738-09|Canada|British Columbia|658[0n]|BOLD:ABY6571
Syngrapha angulidens|[2550]|LBCG765-09|Canada|British Columbia|658[0n]|BOLD:ABY6571
Syngrapha angulidens|[2551]|LBCD462-05|Canada|British Columbia|658[0n]|BOLD:ABY6571
Syngrapha angulidens|[2552]|LBCD141-05|Canada|British Columbia|658[0n]|BOLD:ABY6571
Syngrapha angulidens|[2553]|LBCD140-05|Canada|British Columbia|658[0n]|BOLD:ABY6571
Syngrapha angulidens|[2554]|LBCC873-05|Canada|British Columbia|658[0n]|BOLD:ABY6571
Syngrapha angulidens|[2555]|LBCC872-05|Canada|British Columbia|658[0n]|BOLD:ABY6571
Syngrapha angulidens|[2556]|LBCC871-05|Canada|British Columbia|658[0n]|BOLD:ABY6571
Syngrapha angulidens|[2557]|LBCC561-05|Canada|British Columbia|658[0n]|BOLD:ABY6571
Syngrapha angulidens|[2558]|LBCC560-05|Canada|British Columbia|658[0n]|BOLD:ABY6571
Syngrapha angulidens|[2559]|LBCC558-05|Canada|British Columbia|658[0n]|BOLD:ABY6571
Syngrapha angulidens|[2560]|DUNLP183-08|Canada|British Columbia|639[0n]|BOLD:ABY6571
Syngrapha angulidens|[2561]|LBCD215-05|Canada|British Columbia|643[0n]|BOLD:ABY6571
Syngrapha angulidens|[2562]|LBCD423-05|Canada|British Columbia|650[0n]|BOLD:ABY6571
Syngrapha angulidens|[2563]|LBCH1509-10|Canada|British Columbia|634[0n]|BOLD:ABY6571
Syngrapha angulidens|[2564]|LBCH2245-10|Canada|British Columbia|658[0n]|BOLD:ABY6571
Syngrapha celsa|[2565]|DUNLP184-08|Canada|British Columbia|627[0n]|BOLD:ABY6570
Syngrapha celsa|[2566]|LALPA989-11|Canada|British Columbia|658[0n]|BOLD:ABY6570
Syngrapha celsa|[2567]|LALPA797-10|Canada|British Columbia|658[0n]|BOLD:ABY6570
Syngrapha celsa|[2568]|LBCH3455-10|Canada|British Columbia|658[0n]|BOLD:ABY6570
Syngrapha celsa|[2569]|LBCH3069-10|Canada|British Columbia|658[0n]|BOLD:ABY6570
Syngrapha celsa|[2570]|LBCH2090-10|Canada|British Columbia|658[0n]|BOLD:ABY6570
Syngrapha celsa|[2571]|LBCH1518-10|Canada|British Columbia|658[0n]|BOLD:ABY6570
Syngrapha celsa|[2572]|LBCH804-10|Canada|British Columbia|658[0n]|BOLD:ABY6570
Syngrapha celsa|[2573]|LBCH009-10|Canada|British Columbia|658[0n]|BOLD:ABY6570
Syngrapha celsa|[2574]|LBCG2058-09|Canada|British Columbia|658[0n]|BOLD:ABY6570
Syngrapha celsa|[2575]|LBCG861-09|Canada|British Columbia|658[0n]|BOLD:ABY6570
Syngrapha celsa|[2576]|LPVIB070-08|Canada|British Columbia|658[0n]|BOLD:ABY6570
Syngrapha celsa|[2577]|LBCS680-07|Canada|British Columbia|658[0n]|BOLD:ABY6570
Syngrapha celsa|[2578]|LBCS446-07|Canada|British Columbia|658[0n]|BOLD:ABY6570
Syngrapha celsa|[2579]|LHLEP266-06|Canada|British Columbia|658[0n]|BOLD:ABY6570
Syngrapha celsa|[2580]|LHLEP265-06|Canada|British Columbia|658[0n]|BOLD:ABY6570
Syngrapha celsa|[2581]|LBCD445-05|Canada|British Columbia|658[0n]|BOLD:ABY6570
Syngrapha celsa|[2582]|LBCD139-05|Canada|British Columbia|658[0n]|BOLD:ABY6570
Syngrapha celsa|[2583]|LALPA1007-11|Canada|British Columbia|658[1n]|BOLD:ABY6570
Syngrapha celsa|[2584]|LALPA1281-11|Canada|British Columbia|658[0n]|BOLD:ABY6570
Syngrapha celsa|[2585]|LALPA1282-11|Canada|British Columbia|658[0n]|BOLD:ABY6570
Syngrapha celsa|[2586]|LALPA1295-11|Canada|British Columbia|658[0n]|BOLD:ABY6570

Syngrapha celsa|[2584]|LALPA1281-11|Canada|British Columbia|658[0n]|BOLD:ABY6570
Syngrapha celsa|[2585]|LALPA1282-11|Canada|British Columbia|658[0n]|BOLD:ABY6570
Syngrapha celsa|[2586]|LALPA1295-11|Canada|British Columbia|658[0n]|BOLD:ABY6570
Syngrapha celsa|[2587]|LALPA1298-11|Canada|British Columbia|658[0n]|BOLD:ABY6570
Syngrapha celsa|[2588]|LALPA1319-12|Canada|British Columbia|621[0n]|BOLD:ABY6570
Syngrapha celsa|[2589]|LALPA1328-12|Canada|British Columbia|621[0n]|BOLD:ABY6570
Syngrapha rectangula group|[2590]|LHLEP281-06|Canada|British Columbia|658[0n]|BOLD:AAA4309
Syngrapha rectangula group|[2591]|BBLPB537-10|Canada|British Columbia|658[0n]|BOLD:AAA4309
Syngrapha rectangula|[2592]|BBLPE036-09|Canada|Nova Scotia|658[0n]|BOLD:AAA4309
Syngrapha abstrusa|[2593]|BBLPB534-10|Canada|Alberta|658[0n]|BOLD:AAA4309
Syngrapha rectangula|[2594]|LHLEP377-06|Canada|British Columbia|658[0n]|BOLD:AAA4309
Syngrapha rectangula|[2595]|LHLEP378-06|Canada|British Columbia|658[0n]|BOLD:AAA4309
Syngrapha rectangula|[2596]|LBCS400-07|Canada|British Columbia|658[0n]|BOLD:AAA4309
Syngrapha rectangula|[2597]|LBCS445-07|Canada|British Columbia|658[0n]|BOLD:AAA4309
Syngrapha rectangula|[2598]|BBLPC082-09|Canada|New Brunswick|627[0n]|BOLD:AAA4309
Syngrapha cryptica|[2599]|RDNMG621-08|Canada|Ontario|639[0n]|BOLD:AAA4309
Syngrapha cryptica|[2600]|RDNMG622-08|Canada|Ontario|658[0n]|BOLD:AAA4309
Syngrapha rectangula|[2601]|RDNMD346-06|United States|Tennessee|656[0n]|BOLD:AAA4309
Syngrapha rectangula|[2602]|TTMNB294-06|Canada|New Brunswick|656[0n]|BOLD:AAA4309
Syngrapha rectangula|[2603]|TTMNB033-06|Canada|New Brunswick|654[0n]|BOLD:AAA4309
Syngrapha abstrusa|[2604]|XAB246-04|Canada|Ontario|658[1n]|BOLD:AAA4309
Syngrapha abstrusa|[2605]|PHMNB367-04|Canada|New Brunswick|658[0n]|BOLD:AAA4309
Syngrapha rectangula group|[2606]|PHMNB212-04|Canada|New Brunswick|658[0n]|BOLD:AAA4309
Syngrapha rectangula group|[2607]|TTMNB289-06|Canada|New Brunswick|658[1n]|BOLD:AAA4309
Syngrapha rectangula group|[2608]|BBLPB536-10|Canada|British Columbia|636[0n]|BOLD:AAA4309
Syngrapha rectangula group|[2609]|BBLPB540-10|Canada|British Columbia|658[0n]|BOLD:AAA4309
Syngrapha rectangula group|[2610]|BBLPB541-10|Canada|British Columbia|658[0n]|BOLD:AAA4309
Syngrapha rectangula group|[2611]|LGSM664-04|United States|Tennessee|590[0n]|BOLD:AAA4309
Syngrapha cryptica|[2612]|BBLPE053-09|Canada|Nova Scotia|658[0n]|BOLD:AAA4309
Syngrapha cryptica|[2613]|BBLPE015-09|Canada|Nova Scotia|658[0n]|BOLD:AAA4309
Syngrapha alias|[2614]|BBLPC994-09|Canada|Nova Scotia|658[0n]|BOLD:AAA4309
Syngrapha rectangula group|[2615]|BBLPC811-09|Canada|Newfoundland and Labrador|658[0n]|BOLD:AAA4309
Syngrapha rectangula group|[2616]|BBLPC212-09|Canada|Nova Scotia|658[0n]|BOLD:AAA4309
Syngrapha rectangula group|[2617]|BBLPC207-09|Canada|Nova Scotia|658[0n]|BOLD:AAA4309
Syngrapha rectangula group|[2618]|BBLPC139-09|Canada|Nova Scotia|658[0n]|BOLD:AAA4309
Syngrapha rectangula|[2619]|BBLPC097-09|Canada|New Brunswick|658[0n]|BOLD:AAA4309
Syngrapha rectangula|[2620]|BBLEC071-09|Canada|New Brunswick|658[0n]|BOLD:AAA4309
Syngrapha rectangula|[2621]|RWWA978-09|United States|Washington|658[0n]|BOLD:AAA4309
Syngrapha rectangula|[2622]|RWWA836-09|United States|Washington|658[0n]|BOLD:AAA4309
Syngrapha cryptica|[2623]|RDNMF415-08|Canada|Ontario|658[0n]|BOLD:AAA4309
Syngrapha cryptica|[2624]|RDNMF414-08|Canada|Ontario|658[0n]|BOLD:AAA4309
Syngrapha rectangula|[2625]|LGSMG617-07|United States|Tennessee|658[0n]|BOLD:AAA4309
Syngrapha rectangula group|[2626]|LGSMG615-07|United States|North Carolina|658[0n]|BOLD:AAA4309
Syngrapha abstrusa|[2627]|RDLQF518-06|Canada|Quebec|658[0n]|BOLD:AAA4309
Syngrapha abstrusa|[2628]|RDLQF517-06|Canada|Quebec|658[0n]|BOLD:AAA4309
Syngrapha rectangula|[2629]|RDLQF366-06|Canada|Quebec|658[0n]|BOLD:AAA4309
Syngrapha abstrusa|[2630]|XAK179-06|Canada|Ontario|658[0n]|BOLD:AAA4309
Syngrapha rectangula group|[2631]|TMNBB084-06|Canada|New Brunswick|658[0n]|BOLD:AAA4309
Syngrapha rectangula|[2632]|TTMNB295-06|Canada|New Brunswick|658[0n]|BOLD:AAA4309
Syngrapha abstrusa|[2633]|RDLQB791-05|Canada|Quebec|658[0n]|BOLD:AAA4309
Syngrapha rectangula|[2634]|RDLQB435-05|Canada|Quebec|658[0n]|BOLD:AAA4309
Syngrapha abstrusa|[2635]|RDLQB026-05|Canada|Quebec|658[0n]|BOLD:AAA4309
Syngrapha abstrusa|[2636]|RDLQB025-05|Canada|Quebec|658[0n]|BOLD:AAA4309
Syngrapha abstrusa|[2637]|RDLQB021-05|Canada|Quebec|658[0n]|BOLD:AAA4309
Syngrapha abstrusa|[2638]|RDLQB020-05|Canada|Quebec|658[0n]|BOLD:AAA4309
Syngrapha abstrusa|[2639]|RDLQB018-05|Canada|Quebec|658[0n]|BOLD:AAA4309
Syngrapha rectangula|[2640]|RDLQB012-05|Canada|Quebec|658[0n]|BOLD:AAA4309
Syngrapha rectangula|[2641]|RDLQB011-05|Canada|Quebec|658[0n]|BOLD:AAA4309
Syngrapha alias|[2642]|XAG212-05|Canada|Ontario|658[0n]|BOLD:AAA4309
Syngrapha rectangula group|[2643]|PHMNB366-04|Canada|New Brunswick|658[0n]|BOLD:AAA4309
Syngrapha rectangula group|[2644]|XAC600-04|Canada|Ontario|658[0n]|BOLD:AAA4309
Syngrapha cryptica|[2645]|BBLPC066-09|Canada|New Brunswick|643[0n]|BOLD:AAA4309
Syngrapha rectangula group|[2646]|XAB089-04|Canada|Ontario|658[0n]|BOLD:AAA4309
Syngrapha rectangula|[2647]|RDLQB623-05|Canada|Quebec|586[0n]|BOLD:AAA4309
Syngrapha rectangula|[2648]|BBLEC063-09|Canada|New Brunswick|625[0n]|BOLD:AAA4309
Syngrapha rectangula group|[2649]|PHMO116-03|Canada|Ontario|639[0n]|BOLD:AAA4309
Syngrapha abstrusa|[2650]|RDLQB792-05|Canada|Quebec|617[0n]|BOLD:AAA4309
Syngrapha abstrusa|[2651]|XAE391-04|Canada|Ontario|617[0n]|BOLD:AAA4309
Syngrapha abstrusa|[2652]|XAC630-04|Canada|Ontario|616[0n]|BOLD:AAA4309
Syngrapha rectangula|[2653]|RDNMC335-05|United States|Tennessee|523[0n]|BOLD:AAA4309
Syngrapha rectangula|[2654]|LPVIA628-08|Canada|British Columbia|635[0n]|BOLD:AAA4309
Syngrapha rectangula group|[2655]|BBLPC578-09|Canada|Nova Scotia|648[0n]|BOLD:AAA4309
Syngrapha rectangula|[2656]|BBLPE083-09|Canada|Nova Scotia|647[0n]|BOLD:AAA4309
Syngrapha rectangula|[2657]|BBLPE087-09|Canada|Nova Scotia|658[0n]|BOLD:AAA4309
Syngrapha cryptica|[2658]|BBLPE261-09|Canada|Nova Scotia|658[0n]|BOLD:AAA4309
Syngrapha rectangula|[2659]|LALPA510-10|Canada|British Columbia|658[0n]|BOLD:AAA4309
Syngrapha rectangula|[2660]|LALPA624-10|Canada|British Columbia|658[0n]|BOLD:AAA4309
Syngrapha rectangula group|[2661]|LALPA635-10|Canada|British Columbia|658[0n]|BOLD:AAA4309
Syngrapha rectangula|[2662]|LALPA639-10|Canada|British Columbia|658[0n]|BOLD:AAA4309
Syngrapha rectangula|[2663]|LALPA644-10|Canada|British Columbia|658[0n]|BOLD:AAA4309
Syngrapha rectangula|[2664]|LALPA660-10|Canada|British Columbia|658[0n]|BOLD:AAA4309
Syngrapha rectangula|[2665]|RWWC582-11|United States|Washington|658[0n]|BOLD:AAA4309
Syngrapha rectangula|[2666]|RWWC672-11|United States|Washington|658[0n]|BOLD:AAA4309
Syngrapha rectangula|[2667]|BBLEC273-09|Canada|Nova Scotia|658[0n]|BOLD:AAA4309
Syngrapha rectangula group|[2668]|BBLEC493-09|Canada|New Brunswick|652[0n]|BOLD:AAA4309
Syngrapha rectangula group|[2669]|BBLPC691-09|Canada|Newfoundland and Labrador|658[0n]|BOLD:AAA4309
Syngrapha rectangula group|[2670]|BBLPC318-09|Canada|Newfoundland and Labrador|658[0n]|BOLD:AAA4309
Syngrapha rectangula group|[2671]|RWWB010-09|United States|Washington|658[0n]|BOLD:AAA4309
Syngrapha rectangula group|[2672]|RWWA968-09|United States|Washington|658[0n]|BOLD:AAA4309
Syngrapha alias|[2673]|RDLQF367-06|Canada|Quebec|658[0n]|BOLD:AAA4309
Syngrapha alias|[2674]|RDLQB037-05|Canada|Quebec|658[0n]|BOLD:AAA4309
Syngrapha rectangula group|[2675]|BBLPC376-09|Canada|New Brunswick|640[0n]|BOLD:AAA4309
Syngrapha alias|[2676]|RDLQB622-05|Canada|Quebec|642[0n]|BOLD:AAA4309
Syngrapha rectangula|[2677]|BBLPE256-09|Canada|Nova Scotia|643[0n]|BOLD:AAA4309
Syngrapha rectangula group|[2678]|PHMO113-03|Canada|Ontario|639[5n]|BOLD:AAA4309
Syngrapha abstrusa|[2679]|RDLQB027-05|Canada|Quebec|658[0n]|BOLD:AAA4309
Syngrapha rectangula group|[2680]|LBCD291-05|Canada|British Columbia|633[0n]|BOLD:AAA4309
Syngrapha rectangula group|[2681]|BBLPC140-09|Canada|Nova Scotia|637[0n]|BOLD:AAA4309
Syngrapha rectangula group|[2682]|RWWA817-09|United States|Washington|658[0n]|BOLD:AAA4309
Syngrapha rectangula|[2683]|LALPA842-11|Canada|British Columbia|658[0n]|BOLD:AAA4309
Syngrapha rectangula group|[2684]|BBLPB535-10|Canada|British Columbia|658[0n]|BOLD:AAA4309
Syngrapha alias|[2685]|LBCH4046-10|Canada|British Columbia|658[0n]|BOLD:AAA4309
Syngrapha alias|[2686]|LBCH3780-10|Canada|British Columbia|658[0n]|BOLD:AAA4309

Syngrapha rectangula|[2683]|[illegible]|Canada|British Columbia|[illegible]|BOLD:AAA4309
Syngrapha rectangula group|[2684]|BBLPB535-10|Canada|British Columbia|658[0n]|BOLD:AAA4309
Syngrapha alias|[2685]|LBCH4046-10|Canada|British Columbia|658[0n]|BOLD:AAA4309
Syngrapha alias|[2686]|LBCH3780-10|Canada|British Columbia|658[0n]|BOLD:AAA4309
Syngrapha rectangula group|[2687]|LBCH2111-10|Canada|British Columbia|658[0n]|BOLD:AAA4309
Syngrapha rectangula group|[2688]|LBCH916-10|Canada|British Columbia|658[0n]|BOLD:AAA4309
Syngrapha rectangula|[2689]|LBCH654-10|Canada|British Columbia|658[0n]|BOLD:AAA4309
Syngrapha rectangula group|[2690]|LBCH231-10|Canada|British Columbia|658[0n]|BOLD:AAA4309
Syngrapha rectangula group|[2691]|LBCH121-10|Canada|British Columbia|658[0n]|BOLD:AAA4309
Syngrapha rectangula group|[2692]|BBLEC634-09|Canada|Nova Scotia|658[0n]|BOLD:AAA4309
Syngrapha rectangula group|[2693]|RWWB254-09|United States|Washington|658[0n]|BOLD:AAA4309
Syngrapha rectangula group|[2694]|RWWB161-09|United States|Washington|658[0n]|BOLD:AAA4309
Syngrapha rectangula group|[2695]|RWWA986-09|United States|Washington|658[0n]|BOLD:AAA4309
Syngrapha rectangula|[2696]|RWWA896-09|United States|Washington|658[0n]|BOLD:AAA4309
Syngrapha rectangula group|[2697]|RWWA870-09|United States|Washington|658[0n]|BOLD:AAA4309
Syngrapha rectangula group|[2698]|RWWA796-09|United States|Washington|658[0n]|BOLD:AAA4309
Syngrapha rectangula group|[2699]|RWWA793-09|United States|Washington|658[0n]|BOLD:AAA4309
Syngrapha rectangula group|[2700]|RWWA791-09|United States|Washington|658[0n]|BOLD:AAA4309
Syngrapha rectangula group|[2701]|RWWA782-09|United States|Washington|658[0n]|BOLD:AAA4309
Syngrapha rectangula group|[2702]|RWWA771-09|United States|Washington|658[0n]|BOLD:AAA4309
Syngrapha rectangula group|[2703]|RWWA717-09|United States|Washington|658[0n]|BOLD:AAA4309
Syngrapha alias|[2704]|RWWA565-09|United States|Washington|658[0n]|BOLD:AAA4309
Syngrapha rectangula|[2705]|LBCG2061-09|Canada|British Columbia|658[0n]|BOLD:AAA4309
Syngrapha rectangula group|[2706]|LPMN210-08|Canada|Manitoba|658[0n]|BOLD:AAA4309
Syngrapha abstrusa|[2707]|RDLQF519-06|Canada|Quebec|658[0n]|BOLD:AAA4309
Syngrapha abstrusa|[2708]|XAK162-06|Canada|Ontario|658[0n]|BOLD:AAA4309
Syngrapha rectangula group|[2709]|XAJ724-06|Canada|Ontario|658[0n]|BOLD:AAA4309
Syngrapha abstrusa|[2710]|RDLQB024-05|Canada|Quebec|658[0n]|BOLD:AAA4309
Syngrapha abstrusa|[2711]|RDLQB023-05|Canada|Quebec|658[0n]|BOLD:AAA4309
Syngrapha abstrusa|[2712]|RDLQB022-05|Canada|Quebec|658[0n]|BOLD:AAA4309
Syngrapha abstrusa|[2713]|RDLQB019-05|Canada|Quebec|658[0n]|BOLD:AAA4309
Syngrapha abstrusa|[2714]|RDLQB017-05|Canada|Quebec|658[0n]|BOLD:AAA4309
Syngrapha abstrusa|[2715]|RDLQB016-05|Canada|Quebec|658[0n]|BOLD:AAA4309
Syngrapha rectangula group|[2716]|LBCD465-05|Canada|British Columbia|658[0n]|BOLD:AAA4309
Syngrapha rectangula group|[2717]|LBCC439-05|Canada|British Columbia|658[0n]|BOLD:AAA4309
Syngrapha rectangula group|[2718]|PHMNB700-04|Canada|New Brunswick|658[0n]|BOLD:AAA4309
Syngrapha rectangula group|[2719]|LCH585-04|Canada|Manitoba|658[0n]|BOLD:AAA4309
Syngrapha rectangula group|[2720]|RDBBC610-05|Canada|British Columbia|658[1n]|BOLD:AAA4309
Syngrapha rectangula group|[2721]|LBCD448-05|Canada|British Columbia|658[0n]|BOLD:AAA4309
Syngrapha rectangula group|[2722]|LBCD459-05|Canada|British Columbia|650[0n]|BOLD:AAA4309
Syngrapha rectangula group|[2723]|BBLPE270-09|Canada|Nova Scotia|510[0n]|BOLD:AAA4309
Syngrapha rectangula group|[2724]|LBCH802-10|Canada|British Columbia|639[0n]|BOLD:AAA4309
Syngrapha rectangula group|[2725]|BBLPB538-10|Canada|British Columbia|658[0n]|BOLD:AAA4309
Syngrapha rectangula group|[2726]|BBLPB539-10|Canada|British Columbia|658[0n]|BOLD:AAA4309
Syngrapha rectangula|[2727]|RWWC532-11|United States|Washington|658[0n]|BOLD:AAA4309
Syngrapha rectangula group|[2728]|RWWC552-11|United States|Washington|658[0n]|BOLD:AAA4309
Syngrapha rectangula group|[2729]|SSWLD7926-13|Canada|Alberta|543[0n]|BOLD:AAA4309
Syngrapha rectangula group|[2730]|CNWBG3160-13|Canada|Alberta|576[0n]|BOLD:AAA4309
Syngrapha rectangula group|[2731]|SSWLD7974-13|Canada|Alberta|564[0n]|BOLD:AAA4309
Syngrapha rectangula group|[2732]|CNWBG3136-13|Canada|Alberta|537[2n]|BOLD:AAA4309
Syngrapha rectangula group|[2733]|CNWBG3064-13|Canada|Alberta|551[0n]|BOLD:AAA4309
Syngrapha alias|[2734]|BBLEC110-09|Canada|Nova Scotia|625[0n]|BOLD:AAA4309
Syngrapha rectangula group|[2735]|SSJAC211-13|Canada|Alberta|573[0n]|BOLD:AAA4309
Syngrapha rectangula group|[2736]|SSPAA5673-13|Canada|Saskatchewan|592[0n]|BOLD:AAA4309
Syngrapha alias|[2737]|LBCD446-05|Canada|British Columbia|658[0n]|BOLD:AAA4309
Syngrapha alias|[2738]|LOWCD153-06|Canada|British Columbia|658[0n]|BOLD:AAA4309
Syngrapha rectangula|[2739]|DUNLP185-08|Canada|British Columbia|658[0n]|BOLD:AAA4309
Syngrapha rectangula group|[2740]|LBCD447-05|Canada|British Columbia|658[0n]|BOLD:AAA4309
Syngrapha rectangula|[2741]|LOWCD187-06|Canada|British Columbia|658[0n]|BOLD:AAA4309
Syngrapha rectangula|[2742]|LBCG882-09|Canada|British Columbia|658[0n]|BOLD:AAA4309
Syngrapha rectangula|[2743]|LBCG2062-09|Canada|British Columbia|658[0n]|BOLD:AAA4309
Syngrapha rectangula|[2744]|LBCH010-10|Canada|British Columbia|658[0n]|BOLD:AAA4309
Syngrapha rectangula|[2745]|LBCH3068-10|Canada|British Columbia|658[0n]|BOLD:AAA4309
Syngrapha rectangula|[2746]|LBCH3331-10|Canada|British Columbia|658[0n]|BOLD:AAA4309
Syngrapha rectangula|[2747]|LBCH3779-10|Canada|British Columbia|658[0n]|BOLD:AAA4309
Syngrapha alias|[2748]|LOWCD151-06|Canada|British Columbia|614[0n]|BOLD:AAA4309
Syngrapha alias|[2749]|DUNLP182-08|Canada|British Columbia|622[0n]|BOLD:AAA4309
Syngrapha alias|[2750]|LPABB675-08|Canada|Alberta|658[0n]|BOLD:AAA4309
Syngrapha rectangula group|[2751]|BBLPB533-10|Canada|Alberta|658[0n]|BOLD:AAA4309
Syngrapha rectangula group|[2752]|SSBAD6407-13|Canada|Alberta|577[0n]|BOLD:AAA4309
Syngrapha rectangula group|[2753]|CNWBG3108-13|Canada|Alberta|575[0n]|BOLD:AAA4309
Syngrapha rectangula group|[2754]|CNWBG3152-13|Canada|Alberta|613[0n]|BOLD:AAA4309
Syngrapha rectangula group|[2755]|CNPAN051-13|Canada|Saskatchewan|605[5n]|BOLD:AAA4309
Anagrapha falcifera|[2756]|LBCH5172-10|Canada|British Columbia|658[0n]|BOLD:AAA6337
Anagrapha falcifera|[2757]|LBCH5211-10|Canada|British Columbia|658[0n]|BOLD:AAA6337
Anagrapha falcifera|[2758]|RDLQB726-05|Canada|Quebec|564[0n]|BOLD:AAA6337
Anagrapha falcifera|[2759]|XAD430-04|Canada|Ontario|592[0n]|BOLD:AAA6337
Anagrapha falcifera|[2760]|CNGRL426-13|Canada|Saskatchewan|579[0n]|BOLD:AAA6337
Anagrapha falcifera|[2761]|XAD303-04|Canada|Ontario|594[1n]|BOLD:AAA6337
Anagrapha falcifera|[2762]|XAD360-04|Canada|Ontario|582[0n]|BOLD:AAA6337
Anagrapha falcifera|[2763]|LOWCB592-05|Canada|British Columbia|575[1n]|BOLD:AAA6337
Anagrapha falcifera|[2764]|CNGRD1025-12|Canada|Saskatchewan|627[0n]|BOLD:AAA6337
Anagrapha falcifera|[2765]|LOWCB593-05|Canada|British Columbia|605[2n]|BOLD:AAA6337
Anagrapha falcifera|[2766]|PHMO325-03|Canada|Ontario|639[5n]|BOLD:AAA6337
Anagrapha falcifera|[2767]|LOWCB591-05|Canada|British Columbia|585[0n]|BOLD:AAA6337
Anagrapha falcifera|[2768]|CNGRJ072-13|Canada|Saskatchewan|588[0n]|BOLD:AAA6337
Anagrapha falcifera|[2769]|RDLQB730-05|Canada|Quebec|592[0n]|BOLD:AAA6337
Anagrapha falcifera|[2770]|CNBPD280-12|Canada|Ontario|630[0n]|BOLD:AAA6337
Anagrapha falcifera|[2771]|XAJ487-06|Canada|Ontario|649[0n]|BOLD:AAA6337
Anagrapha falcifera|[2772]|LPSOB390-08|Canada|Ontario|658[0n]|BOLD:AAA6337
Anagrapha falcifera|[2773]|LPSK534-08|Canada|Saskatchewan|658[0n]|BOLD:AAA6337
Anagrapha falcifera|[2774]|XAF700-05|Canada|Ontario|658[0n]|BOLD:AAA6337
Anagrapha falcifera|[2775]|LPOKA653-09|United States|Oklahoma|634[0n]|BOLD:AAA6337
Anagrapha falcifera|[2776]|LPSK415-08|Canada|Saskatchewan|658[0n]|BOLD:AAA6337
Anagrapha falcifera|[2777]|LPSOB721-08|Canada|Ontario|640[0n]|BOLD:AAA6337
Anagrapha falcifera|[2778]|XAJ489-06|Canada|Ontario|649[0n]|BOLD:AAA6337
Anagrapha falcifera|[2779]|XAD591-05|Canada|Ontario|658[0n]|BOLD:AAA6337
Anagrapha falcifera|[2780]|LPSOC333-08|Canada|Ontario|658[0n]|BOLD:AAA6337
Anagrapha falcifera|[2781]|LPSOB217-08|Canada|Ontario|658[0n]|BOLD:AAA6337
Anagrapha falcifera|[2782]|LPSOB234-08|Canada|Ontario|658[0n]|BOLD:AAA6337
Anagrapha falcifera|[2783]|LPSOB276-08|Canada|Ontario|658[0n]|BOLD:AAA6337
Anagrapha falcifera|[2784]|LPSOB371-08|Canada|Ontario|657[0n]|BOLD:AAA6337
Anagrapha falcifera|[2785]|LPSOB404-08|Canada|Ontario|658[0n]|BOLD:AAA6337

Anagrapha falcifera|[2783]|LPSOB276-08|Canada|Ontario|658[0n]|BOLD:AAA6337
Anagrapha falcifera|[2784]|LPSOB371-08|Canada|Ontario|657[0n]|BOLD:AAA6337
Anagrapha falcifera|[2785]|LPSOB404-08|Canada|Ontario|658[0n]|BOLD:AAA6337
Anagrapha falcifera|[2786]|LPSOB464-08|Canada|Ontario|658[0n]|BOLD:AAA6337
Anagrapha falcifera|[2787]|LPSK048-08|Canada|Saskatchewan|658[0n]|BOLD:AAA6337
Anagrapha falcifera|[2788]|LPSK498-08|Canada|Saskatchewan|658[0n]|BOLD:AAA6337
Anagrapha falcifera|[2789]|LPSK513-08|Canada|Saskatchewan|658[0n]|BOLD:AAA6337
Anagrapha falcifera|[2790]|LPSK526-08|Canada|Saskatchewan|658[0n]|BOLD:AAA6337
Anagrapha falcifera|[2791]|LPSK532-08|Canada|Saskatchewan|658[0n]|BOLD:AAA6337
Anagrapha falcifera|[2792]|LPSK533-08|Canada|Saskatchewan|658[0n]|BOLD:AAA6337
Anagrapha falcifera|[2793]|LPSK538-08|Canada|Saskatchewan|658[0n]|BOLD:AAA6337
Anagrapha falcifera|[2794]|LPSK541-08|Canada|Saskatchewan|658[0n]|BOLD:AAA6337
Anagrapha falcifera|[2795]|LPSK559-08|Canada|Saskatchewan|658[0n]|BOLD:AAA6337
Anagrapha falcifera|[2796]|LPSK569-08|Canada|Saskatchewan|658[0n]|BOLD:AAA6337
Anagrapha falcifera|[2797]|LPSK591-08|Canada|Saskatchewan|658[0n]|BOLD:AAA6337
Anagrapha falcifera|[2798]|BLTIB462-08|Canada|Ontario|658[0n]|BOLD:AAA6337
Anagrapha falcifera|[2799]|BLTIB752-08|Canada|Ontario|658[0n]|BOLD:AAA6337
Anagrapha falcifera|[2800]|BLTIB915-08|Canada|Ontario|658[0n]|BOLD:AAA6337
Anagrapha falcifera|[2801]|BLTIB1003-08|Canada|Ontario|658[0n]|BOLD:AAA6337
Anagrapha falcifera|[2802]|LBCG039-08|Canada|British Columbia|658[0n]|BOLD:AAA6337
Anagrapha falcifera|[2803]|LPSOC121-08|Canada|Ontario|658[0n]|BOLD:AAA6337
Anagrapha falcifera|[2804]|LPOKA364-08|United States|Oklahoma|658[0n]|BOLD:AAA6337
Anagrapha falcifera|[2805]|LPSOD636-09|Canada|Ontario|658[0n]|BOLD:AAA6337
Anagrapha falcifera|[2806]|LPSOD742-09|Canada|Ontario|658[0n]|BOLD:AAA6337
Anagrapha falcifera|[2807]|LPSOD1046-09|Canada|Ontario|658[0n]|BOLD:AAA6337
Anagrapha falcifera|[2808]|LPOKB160-09|United States|Oklahoma|658[0n]|BOLD:AAA6337
Anagrapha falcifera|[2809]|BBLCU105-09|United States|Kansas|658[0n]|BOLD:AAA6337
Anagrapha falcifera|[2810]|XAK247-06|Canada|Ontario|658[0n]|BOLD:AAA6337
Anagrapha falcifera|[2811]|XAK246-06|Canada|Ontario|658[0n]|BOLD:AAA6337
Anagrapha falcifera|[2812]|XAJ498-06|Canada|Ontario|658[0n]|BOLD:AAA6337
Anagrapha falcifera|[2813]|XAJ412-06|Canada|Ontario|658[0n]|BOLD:AAA6337
Anagrapha falcifera|[2814]|TTMNB288-06|Canada|New Brunswick|658[0n]|BOLD:AAA6337
Anagrapha falcifera|[2815]|XAH835-05|Canada|Ontario|658[0n]|BOLD:AAA6337
Anagrapha falcifera|[2816]|MNBB528-05|Canada|New Brunswick|658[0n]|BOLD:AAA6337
Anagrapha falcifera|[2817]|XAJ506-06|Canada|Ontario|658[0n]|BOLD:AAA6337
Anagrapha falcifera|[2818]|XAC667-04|Canada|Ontario|658[0n]|BOLD:AAA6337
Anagrapha falcifera|[2819]|LOCT114-05|United States|Connecticut|658[0n]|BOLD:AAA6337
Anagrapha falcifera|[2820]|LPSOB691-08|Canada|Ontario|645[0n]|BOLD:AAA6337
Anagrapha falcifera|[2821]|LPOKA900-09|United States|Oklahoma|658[0n]|BOLD:AAA6337
Anagrapha falcifera|[2822]|LNCC1859-13|United States|North Carolina|658[0n]|BOLD:AAA6337
Syngrapha u-aureum|[2823]|LCH590-04|Canada|Manitoba|591[0n]|BOLD:ABX6351
Syngrapha u-aureum|[2824]|LCH457-04|Canada|Manitoba|617[0n]|BOLD:ABX6351
Syngrapha u-aureum|[2825]|LCHIP141-07|Canada|Manitoba|651[0n]|BOLD:ABX6351
Syngrapha u-aureum|[2826]|MHLEP137-07|Canada|Manitoba|644[0n]|BOLD:ABX6351
Syngrapha u-aureum|[2827]|LCH324-04|Canada|Manitoba|658[1n]|BOLD:ABX6351
Syngrapha u-aureum|[2828]|LCH458-04|Canada|Manitoba|658[0n]|BOLD:ABX6351
Syngrapha u-aureum|[2829]|LCH460-04|Canada|Manitoba|658[0n]|BOLD:ABX6351
Syngrapha u-aureum|[2830]|MHLEP136-07|Canada|Manitoba|658[0n]|BOLD:ABX6351
Syngrapha u-aureum|[2831]|LCHP772-07|Canada|Manitoba|658[0n]|BOLD:ABX6351
Syngrapha u-aureum|[2832]|LCHQ029-07|Canada|Manitoba|658[0n]|BOLD:ABX6351
Syngrapha u-aureum|[2833]|LCHQ127-07|Canada|Manitoba|657[0n]|BOLD:ABX6351
Syngrapha u-aureum|[2834]|LCHQ381-08|Canada|Manitoba|658[0n]|BOLD:ABX6351
Syngrapha surena|[2835]|LOWCB123-05|Canada|British Columbia|635[0n]|BOLD:ABX6351
Syngrapha surena|[2836]|RDNMF412-08|Canada|New Brunswick|658[0n]|BOLD:ABX6351
Syngrapha surena|[2837]|RDNMF413-08|Canada|New Brunswick|658[0n]|BOLD:ABX6351
Syngrapha surena|[2838]|RDNMG1007-08|Canada|New Brunswick|658[0n]|BOLD:ABX6351
Syngrapha surena|[2839]|RDNMG1008-08|Canada|New Brunswick|658[0n]|BOLD:ABX6351
Syngrapha interrogationis|[2840]|LCH516-04|Canada|Manitoba|586[0n]|BOLD:AAB3481
Syngrapha interrogationis|[2841]|LCHIP231-07|Canada|Manitoba|651[0n]|BOLD:AAB3481
Syngrapha interrogationis|[2842]|LCHIP137-07|Canada|Manitoba|651[0n]|BOLD:AAB3481
Syngrapha interrogationis|[2843]|LCH448-04|Canada|Manitoba|658[0n]|BOLD:AAB3481
Syngrapha interrogationis|[2844]|LCH323-04|Canada|Manitoba|658[0n]|BOLD:AAB3481
Syngrapha interrogationis|[2845]|LCH325-04|Canada|Manitoba|658[1n]|BOLD:AAB3481
Syngrapha interrogationis|[2846]|LCHP959-07|Canada|Manitoba|658[0n]|BOLD:AAB3481
Syngrapha interrogationis|[2847]|LBCH2790-10|Canada|British Columbia|658[0n]|BOLD:AAB3481
Syngrapha altera|[2848]|RDLQ432-07|Canada|Quebec|658[0n]|BOLD:AAB4820
Syngrapha altera|[2849]|BBLEC350-09|Canada|Newfoundland and Labrador|627[0n]|BOLD:AAB4820
Syngrapha altera|[2850]|BBLEC361-09|Canada|Newfoundland and Labrador|658[0n]|BOLD:AAB4820
Syngrapha altera|[2851]|RDNMD261-06|Canada|New Brunswick|658[0n]|BOLD:AAB4820
Syngrapha altera|[2852]|TTMNB290-06|Canada|New Brunswick|658[0n]|BOLD:AAB4820
Syngrapha altera|[2853]|BBLPE124-09|Canada|Nova Scotia|658[0n]|BOLD:AAB4820
Syngrapha altera|[2854]|RDLQB044-05|Canada|Quebec|658[0n]|BOLD:AAB4820
Syngrapha altera|[2855]|RDNMD262-06|Canada|New Brunswick|658[0n]|BOLD:AAB4820
Syngrapha altera|[2856]|RDNMD260-06|Canada|New Brunswick|658[0n]|BOLD:AAB4820
Syngrapha altera|[2857]|RDLQB042-05|Canada|Quebec|658[0n]|BOLD:AAB4820
Syngrapha altera|[2858]|RDLQB041-05|Canada|Quebec|658[0n]|BOLD:AAB4820
Syngrapha altera|[2859]|RDLQB040-05|Canada|Quebec|658[0n]|BOLD:AAB4820
Syngrapha altera|[2860]|RDLQB039-05|Canada|Quebec|658[0n]|BOLD:AAB4820
Syngrapha altera|[2861]|RDLQB036-05|Canada|Quebec|658[0n]|BOLD:AAB4820
Syngrapha altera|[2862]|RDLQB035-05|Canada|Quebec|658[0n]|BOLD:AAB4820
Syngrapha altera|[2863]|RDLQB034-05|Canada|Quebec|658[0n]|BOLD:AAB4820
Syngrapha altera|[2864]|RDLQB033-05|Canada|Quebec|658[0n]|BOLD:AAB4820
Syngrapha altera|[2865]|RDLQ430-07|Canada|Quebec|636[0n]|BOLD:AAB4820
Syngrapha altera|[2866]|RDLQ431-07|Canada|Quebec|658[0n]|BOLD:AAB4820
Syngrapha altera|[2867]|BBLPE344-09|Canada|Newfoundland and Labrador|658[0n]|BOLD:AAB4820
Syngrapha altera|[2868]|BBLPE517-09|Canada|Newfoundland and Labrador|658[0n]|BOLD:AAB4820
Syngrapha altera|[2869]|BBLPE536-09|Canada|Newfoundland and Labrador|658[0n]|BOLD:AAB4820
Syngrapha selecta|[2870]|RDNMD255-06|Canada|New Brunswick|627[0n]|BOLD:ABZ3678
Syngrapha selecta|[2871]|RDNMD254-06|Canada|New Brunswick|627[0n]|BOLD:ABZ3678
Syngrapha selecta|[2872]|LCH517-04|Canada|Manitoba|590[0n]|BOLD:ABZ3678
Syngrapha selecta|[2873]|LCH520-04|Canada|Manitoba|658[0n]|BOLD:ABZ3678
Syngrapha selecta|[2874]|DSCNI030-07|Canada|Manitoba|658[0n]|BOLD:ABZ3678
Syngrapha selecta|[2875]|LCHQ634-08|Canada|Manitoba|658[0n]|BOLD:ABZ3678
Syngrapha selecta|[2876]|BBLPC039-09|Canada|New Brunswick|602[0n]|BOLD:ABZ3678
Syngrapha selecta|[2877]|LBCH3777-10|Canada|British Columbia|658[0n]|BOLD:ABZ3678
Syngrapha octoscripta|[2878]|TTMNB292-06|Canada|New Brunswick|621[1n]|BOLD:AAA3949
Syngrapha octoscripta|[2879]|LCHQ706-08|Canada|Manitoba|658[0n]|BOLD:AAA3949
Syngrapha octoscripta|[2880]|TTMNB291-06|Canada|New Brunswick|612[4n]|BOLD:AAA3949
Syngrapha octoscripta|[2881]|LCHQ597-08|Canada|Manitoba|658[0n]|BOLD:AAA3949
Syngrapha octoscripta|[2882]|LCHQ126-07|Canada|Manitoba|653[0n]|BOLD:AAA3949
Syngrapha octoscripta|[2883]|RDLQ428-07|Canada|Quebec|587[1n]|BOLD:AAA3949
Syngrapha octoscripta|[2884]|BBLEC437-09|Canada|New Brunswick|658[0n]|BOLD:AAA3949
Syngrapha octoscripta|[2885]|RDLQ427-07|Canada|Quebec|630[0n]|BOLD:AAA3949

Syngrapha octoscripta|[2882]|LCHQ126-07|Canada|Manitoba|653[0n]|BOLD:AAA3949
Syngrapha octoscripta|[2883]|RDLQ428-07|Canada|Quebec|587[1n]|BOLD:AAA3949
Syngrapha octoscripta|[2884]|BBLEC437-09|Canada|New Brunswick|658[0n]|BOLD:AAA3949
Syngrapha octoscripta|[2885]|RDLQ427-07|Canada|Quebec|620[0n]|BOLD:AAA3949
Syngrapha octoscripta|[2886]|BBLPC346-09|Canada|New Brunswick|656[0n]|BOLD:AAA3949
Syngrapha octoscripta|[2887]|LCHQ773-08|Canada|Manitoba|656[0n]|BOLD:AAA3949
Syngrapha octoscripta|[2888]|LCHQ568-08|Canada|Manitoba|656[0n]|BOLD:AAA3949
Syngrapha octoscripta|[2889]|LCHIP078-07|Canada|Manitoba|651[0n]|BOLD:AAA3949
Syngrapha octoscripta|[2890]|LCHQ547-08|Canada|Manitoba|659[0n]|BOLD:AAA3949
Syngrapha octoscripta|[2891]|BBLPC380-09|Canada|New Brunswick|658[0n]|BOLD:AAA3949
Syngrapha octoscripta|[2892]|BBLPC379-09|Canada|New Brunswick|658[0n]|BOLD:AAA3949
Syngrapha octoscripta|[2893]|BBLPC377-09|Canada|New Brunswick|658[0n]|BOLD:AAA3949
Syngrapha octoscripta|[2894]|BBLPC373-09|Canada|New Brunswick|658[0n]|BOLD:AAA3949
Syngrapha octoscripta|[2895]|BBLPC364-09|Canada|New Brunswick|658[0n]|BOLD:AAA3949
Syngrapha octoscripta|[2896]|BBLPC362-09|Canada|New Brunswick|658[0n]|BOLD:AAA3949
Syngrapha octoscripta|[2897]|BBLPC361-09|Canada|New Brunswick|658[0n]|BOLD:AAA3949
Syngrapha octoscripta|[2898]|BBLPC359-09|Canada|New Brunswick|658[0n]|BOLD:AAA3949
Syngrapha octoscripta|[2899]|BBLPC354-09|Canada|New Brunswick|658[0n]|BOLD:AAA3949
Syngrapha octoscripta|[2900]|BBLPC351-09|Canada|New Brunswick|658[0n]|BOLD:AAA3949
Syngrapha octoscripta|[2901]|BBLPC349-09|Canada|New Brunswick|658[0n]|BOLD:AAA3949
Syngrapha octoscripta|[2902]|BBLPC121-09|Canada|New Brunswick|658[0n]|BOLD:AAA3949
Syngrapha octoscripta|[2903]|BBLPC115-09|Canada|New Brunswick|658[0n]|BOLD:AAA3949
Syngrapha octoscripta|[2904]|BBLPC007-09|Canada|New Brunswick|658[0n]|BOLD:AAA3949
Syngrapha octoscripta|[2905]|BBLEC525-09|Canada|New Brunswick|658[0n]|BOLD:AAA3949
Syngrapha octoscripta|[2906]|BBLEC513-09|Canada|New Brunswick|658[0n]|BOLD:AAA3949
Syngrapha octoscripta|[2907]|BBLEC510-09|Canada|New Brunswick|658[0n]|BOLD:AAA3949
Syngrapha octoscripta|[2908]|BBLEC499-09|Canada|New Brunswick|658[0n]|BOLD:AAA3949
Syngrapha octoscripta|[2909]|BBLEC492-09|Canada|New Brunswick|658[0n]|BOLD:AAA3949
Syngrapha octoscripta|[2910]|BBLEC481-09|Canada|New Brunswick|658[0n]|BOLD:AAA3949
Syngrapha octoscripta|[2911]|BBLEC480-09|Canada|New Brunswick|658[0n]|BOLD:AAA3949
Syngrapha octoscripta|[2912]|BBLEC471-09|Canada|New Brunswick|658[0n]|BOLD:AAA3949
Syngrapha octoscripta|[2913]|BBLEC463-09|Canada|New Brunswick|658[0n]|BOLD:AAA3949
Syngrapha octoscripta|[2914]|BBLEC449-09|Canada|New Brunswick|658[0n]|BOLD:AAA3949
Syngrapha octoscripta|[2915]|BBLEC445-09|Canada|New Brunswick|658[0n]|BOLD:AAA3949
Syngrapha octoscripta|[2916]|BBLEC442-09|Canada|New Brunswick|658[0n]|BOLD:AAA3949
Syngrapha octoscripta|[2917]|BBLEC436-09|Canada|New Brunswick|658[0n]|BOLD:AAA3949
Syngrapha octoscripta|[2918]|BBLEC426-09|Canada|New Brunswick|658[0n]|BOLD:AAA3949
Syngrapha octoscripta|[2919]|BBLEC425-09|Canada|New Brunswick|658[0n]|BOLD:AAA3949
Syngrapha octoscripta|[2920]|BBLEC092-09|Canada|Nova Scotia|658[0n]|BOLD:AAA3949
Syngrapha octoscripta|[2921]|BBLEC046-09|Canada|New Brunswick|658[0n]|BOLD:AAA3949
Syngrapha octoscripta|[2922]|BBLEC024-09|Canada|New Brunswick|658[0n]|BOLD:AAA3949
Syngrapha octoscripta|[2923]|LPABC373-09|Canada|Alberta|658[0n]|BOLD:AAA3949
Syngrapha octoscripta|[2924]|LCHQ922-08|Canada|Manitoba|658[0n]|BOLD:AAA3949
Syngrapha octoscripta|[2925]|LCHQ921-08|Canada|Manitoba|658[0n]|BOLD:AAA3949
Syngrapha octoscripta|[2926]|LCHQ765-08|Canada|Manitoba|658[0n]|BOLD:AAA3949
Syngrapha octoscripta|[2927]|LCHQ726-08|Canada|Manitoba|658[0n]|BOLD:AAA3949
Syngrapha octoscripta|[2928]|LCHQ722-08|Canada|Manitoba|658[0n]|BOLD:AAA3949
Syngrapha octoscripta|[2929]|LCHQ713-08|Canada|Manitoba|658[0n]|BOLD:AAA3949
Syngrapha octoscripta|[2930]|LCHQ703-08|Canada|Manitoba|658[0n]|BOLD:AAA3949
Syngrapha octoscripta|[2931]|LCHQ660-08|Canada|Manitoba|658[0n]|BOLD:AAA3949
Syngrapha octoscripta|[2932]|LCHQ653-08|Canada|Manitoba|658[0n]|BOLD:AAA3949
Syngrapha octoscripta|[2933]|LCHQ646-08|Canada|Manitoba|658[0n]|BOLD:AAA3949
Syngrapha octoscripta|[2934]|LCHQ567-08|Canada|Manitoba|658[0n]|BOLD:AAA3949
Syngrapha octoscripta|[2935]|LCHQ566-08|Canada|Manitoba|658[0n]|BOLD:AAA3949
Syngrapha octoscripta|[2936]|LCHQ565-08|Canada|Manitoba|658[0n]|BOLD:AAA3949
Syngrapha octoscripta|[2937]|LCHQ564-08|Canada|Manitoba|658[0n]|BOLD:AAA3949
Syngrapha octoscripta|[2938]|LCHQ557-08|Canada|Manitoba|658[0n]|BOLD:AAA3949
Syngrapha octoscripta|[2939]|LCHQ556-08|Canada|Manitoba|658[0n]|BOLD:AAA3949
Syngrapha octoscripta|[2940]|LCHQ554-08|Canada|Manitoba|658[0n]|BOLD:AAA3949
Syngrapha octoscripta|[2941]|LCHQ430-08|Canada|Manitoba|658[0n]|BOLD:AAA3949
Syngrapha octoscripta|[2942]|LCHQ121-07|Canada|Manitoba|658[0n]|BOLD:AAA3949
Syngrapha octoscripta|[2943]|LCHQ119-07|Canada|Manitoba|658[0n]|BOLD:AAA3949
Syngrapha octoscripta|[2944]|LCHP958-07|Canada|Manitoba|658[0n]|BOLD:AAA3949
Syngrapha octoscripta|[2945]|LCHP957-07|Canada|Manitoba|658[0n]|BOLD:AAA3949
Syngrapha octoscripta|[2946]|LCHP952-07|Canada|Manitoba|658[0n]|BOLD:AAA3949
Syngrapha octoscripta|[2947]|LCHP942-07|Canada|Manitoba|658[0n]|BOLD:AAA3949
Syngrapha octoscripta|[2948]|LCHP941-07|Canada|Manitoba|658[0n]|BOLD:AAA3949
Syngrapha octoscripta|[2949]|LCHP940-07|Canada|Manitoba|655[0n]|BOLD:AAA3949
Syngrapha octoscripta|[2950]|LCHP869-07|Canada|Manitoba|658[0n]|BOLD:AAA3949
Syngrapha octoscripta|[2951]|LCHP833-07|Canada|Manitoba|658[0n]|BOLD:AAA3949
Syngrapha octoscripta|[2952]|LCHP824-07|Canada|Manitoba|658[0n]|BOLD:AAA3949
Syngrapha octoscripta|[2953]|LCHP817-07|Canada|Manitoba|658[0n]|BOLD:AAA3949
Syngrapha octoscripta|[2954]|LCHP666-07|Canada|Manitoba|658[0n]|BOLD:AAA3949
Syngrapha octoscripta|[2955]|TMNBB081-06|Canada|New Brunswick|658[0n]|BOLD:AAA3949
Syngrapha octoscripta|[2956]|RDNMD259-06|Canada|New Brunswick|658[0n]|BOLD:AAA3949
Syngrapha octoscripta|[2957]|RDLQB043-05|Canada|Quebec|658[0n]|BOLD:AAA3949
Syngrapha octoscripta|[2958]|RDLQB038-05|Canada|Quebec|658[0n]|BOLD:AAA3949
Syngrapha octoscripta|[2959]|LCH515-04|Canada|Manitoba|658[0n]|BOLD:AAA3949
Syngrapha octoscripta|[2960]|BBLPC105-09|Canada|New Brunswick|636[0n]|BOLD:AAA3949
Syngrapha octoscripta|[2961]|RDNMD258-06|Canada|New Brunswick|632[0n]|BOLD:AAA3949
Syngrapha octoscripta|[2962]|BBLEC039-09|Canada|New Brunswick|639[0n]|BOLD:AAA3949
Syngrapha octoscripta|[2963]|BBLEC479-09|Canada|New Brunswick|636[0n]|BOLD:AAA3949
Syngrapha octoscripta|[2964]|TTMNB293-06|Canada|New Brunswick|628[2n]|BOLD:AAA3949
Syngrapha octoscripta|[2965]|RDLQ429-07|Canada|Quebec|606[0n]|BOLD:AAA3949
Syngrapha octoscripta|[2966]|LCHQ783-08|Canada|Manitoba|647[0n]|BOLD:AAA3949
Syngrapha octoscripta|[2967]|BBLEC523-09|Canada|New Brunswick|638[0n]|BOLD:AAA3949
Syngrapha octoscripta|[2968]|BBLPC405-09|Canada|New Brunswick|632[0n]|BOLD:AAA3949
Syngrapha octoscripta|[2969]|BBLPC414-09|Canada|New Brunswick|658[0n]|BOLD:AAA3949
Syngrapha octoscripta|[2970]|BBLPC416-09|Canada|New Brunswick|658[0n]|BOLD:AAA3949
Syngrapha octoscripta|[2971]|LBCH1517-10|Canada|British Columbia|658[0n]|BOLD:AAA3949
Syngrapha octoscripta|[2972]|LBCH2114-10|Canada|British Columbia|658[0n]|BOLD:AAA3949
Syngrapha octoscripta|[2973]|LBCH4429-10|Canada|British Columbia|658[0n]|BOLD:AAA3949
Syngrapha octoscripta|[2974]|BBLPB532-10|Canada|Alberta|658[0n]|BOLD:AAA3949
Syngrapha viridisigma|[2975]|LOWCC366-05|Canada|British Columbia|520[1n]|BOLD:AAA8795
Syngrapha viridisigma|[2976]|LOWCC363-05|Canada|British Columbia|509[1n]|BOLD:AAA8795
Syngrapha viridisigma|[2977]|CNWBG3071-13|Canada|Alberta|551[0n]|BOLD:AAA8795
Syngrapha viridisigma|[2978]|CNWBG3130-13|Canada|Alberta|540[0n]|BOLD:AAA8795
Syngrapha viridisigma|[2979]|UAMIC531-13|United States|Alaska|633[0n]|BOLD:AAA8795
Syngrapha viridisigma|[2980]|LOWCC368-05|Canada|British Columbia|582[0n]|BOLD:AAA8795
Syngrapha viridisigma|[2981]|LOWCC362-05|Canada|British Columbia|546[0n]|BOLD:AAA8795
Syngrapha viridisigma|[2982]|CNWBG3110-13|Canada|Alberta|604[1n]|BOLD:AAA8795
Syngrapha viridisigma|[2983]|CNWBG3137-13|Canada|Alberta|534[0n]|BOLD:AAA8795
Syngrapha viridisigma|[2984]|LCHIP207-07|Canada|Manitoba|651[0n]|BOLD:AAA8795

[illegible]
Syngrapha viridisigma|[2982]|CNWBG3110-13|Canada|Alberta|604[1n]|BOLD:AAA8795
Syngrapha viridisigma|[2983]|CNWBG3137-13|Canada|Alberta|534[0n]|BOLD:AAA8795
Syngrapha viridisigma|[2984]|LCHIP207-07|Canada|Manitoba|651[0n]|BOLD:AAA8795
Syngrapha viridisigma|[2985]|LCHIP105-07|Canada|Manitoba|651[0n]|BOLD:AAA8795
Syngrapha viridisigma|[2986]|CNJAE969-12|Canada|Alberta|627[0n]|BOLD:AAA8795
Syngrapha viridisigma|[2987]|RDNMD256-06|Canada|New Brunswick|627[0n]|BOLD:AAA8795
Syngrapha viridisigma|[2988]|LOWCC361-05|Canada|British Columbia|617[0n]|BOLD:AAA8795
Syngrapha viridisigma|[2989]|LOWCC364-05|Canada|British Columbia|568[0n]|BOLD:AAA8795
Syngrapha viridisigma|[2990]|LCHIP201-07|Canada|Manitoba|628[0n]|BOLD:AAA8795
Syngrapha viridisigma|[2991]|LOWCC370-05|Canada|British Columbia|579[0n]|BOLD:AAA8795
Syngrapha viridisigma|[2992]|BBLEC450-09|Canada|New Brunswick|658[0n]|BOLD:AAA8795
Syngrapha viridisigma|[2993]|BBLEC461-09|Canada|New Brunswick|658[0n]|BOLD:AAA8795
Syngrapha viridisigma|[2994]|BBLEC491-09|Canada|New Brunswick|658[0n]|BOLD:AAA8795
Syngrapha viridisigma|[2995]|BBLPC375-09|Canada|New Brunswick|658[0n]|BOLD:AAA8795
Syngrapha viridisigma|[2996]|BBLPC406-09|Canada|New Brunswick|658[0n]|BOLD:AAA8795
Syngrapha viridisigma|[2997]|BBLPC439-09|Canada|New Brunswick|658[0n]|BOLD:AAA8795
Syngrapha viridisigma|[2998]|BBLPE027-09|Canada|Nova Scotia|658[0n]|BOLD:AAA8795
Syngrapha viridisigma|[2999]|BBLPE134-09|Canada|Nova Scotia|658[0n]|BOLD:AAA8795
Syngrapha viridisigma|[3000]|LBCH913-10|Canada|British Columbia|658[0n]|BOLD:AAA8795
Syngrapha viridisigma|[3001]|LBCH1504-10|Canada|British Columbia|658[0n]|BOLD:AAA8795
Syngrapha viridisigma|[3002]|LBCH3065-10|Canada|British Columbia|658[0n]|BOLD:AAA8795
Syngrapha viridisigma|[3003]|LBCH3453-10|Canada|British Columbia|658[0n]|BOLD:AAA8795
Syngrapha viridisigma|[3004]|LBCH3454-10|Canada|British Columbia|658[0n]|BOLD:AAA8795
Syngrapha viridisigma|[3005]|LBCH3778-10|Canada|British Columbia|658[0n]|BOLD:AAA8795
Syngrapha viridisigma|[3006]|LBCH4117-10|Canada|British Columbia|658[0n]|BOLD:AAA8795
Syngrapha viridisigma|[3007]|LBCH4118-10|Canada|British Columbia|658[0n]|BOLD:AAA8795
Syngrapha viridisigma|[3008]|LBCH4404-10|Canada|British Columbia|658[0n]|BOLD:AAA8795
Syngrapha viridisigma|[3009]|LBCH4651-10|Canada|British Columbia|658[0n]|BOLD:AAA8795
Syngrapha viridisigma|[3010]|LBCH4665-10|Canada|British Columbia|658[0n]|BOLD:AAA8795
Syngrapha viridisigma|[3011]|LALPA609-10|Canada|British Columbia|658[0n]|BOLD:AAA8795
Syngrapha viridisigma|[3012]|LALPA689-10|Canada|British Columbia|658[0n]|BOLD:AAA8795
Syngrapha viridisigma|[3013]|LALPA716-10|Canada|British Columbia|658[0n]|BOLD:AAA8795
Syngrapha viridisigma|[3014]|LALPA727-10|Canada|British Columbia|658[0n]|BOLD:AAA8795
Syngrapha viridisigma|[3015]|LALPA753-10|Canada|British Columbia|658[0n]|BOLD:AAA8795
Syngrapha viridisigma|[3016]|RWWC085-10|United States|Washington|658[0n]|BOLD:AAA8795
Syngrapha viridisigma|[3017]|BBLPB679-10|Canada|Alberta|658[0n]|BOLD:AAA8795
Syngrapha viridisigma|[3018]|BBLPB694-10|Canada|Alberta|658[0n]|BOLD:AAA8795
Syngrapha viridisigma|[3019]|BBLPB759-10|Canada|Alberta|658[0n]|BOLD:AAA8795
Syngrapha viridisigma|[3020]|LALPA1299-11|Canada|British Columbia|658[0n]|BOLD:AAA8795
Syngrapha viridisigma|[3021]|BBLEC447-09|Canada|New Brunswick|658[0n]|BOLD:AAA8795
Syngrapha viridisigma|[3022]|LPSOD1003-09|Canada|Ontario|658[0n]|BOLD:AAA8795
Syngrapha viridisigma|[3023]|LPSOD977-09|Canada|Ontario|658[0n]|BOLD:AAA8795
Syngrapha viridisigma|[3024]|LPSOD969-09|Canada|Ontario|658[0n]|BOLD:AAA8795
Syngrapha viridisigma|[3025]|DUNLP186-08|Canada|British Columbia|658[0n]|BOLD:AAA8795
Syngrapha viridisigma|[3026]|LCHQ678-08|Canada|Manitoba|658[0n]|BOLD:AAA8795
Syngrapha viridisigma|[3027]|ABKWR062-07|United States|Alaska|658[0n]|BOLD:AAA8795
Syngrapha viridisigma|[3028]|TMNBB083-06|Canada|New Brunswick|658[0n]|BOLD:AAA8795
Syngrapha viridisigma|[3029]|LOWCD149-06|Canada|British Columbia|658[0n]|BOLD:AAA8795
Syngrapha viridisigma|[3030]|RDLQB885-05|Canada|Quebec|658[0n]|BOLD:AAA8795
Syngrapha viridisigma|[3031]|RDNMK117-11|United States|Colorado|658[0n]|BOLD:AAA8795
Syngrapha viridisigma|[3032]|CNWBG3086-13|Canada|Alberta|604[0n]|BOLD:AAA8795
Syngrapha viridisigma|[3033]|LOWCC367-05|Canada|British Columbia|553[0n]|BOLD:AAA8795
Syngrapha viridisigma|[3034]|BBLPE165-09|Canada|Nova Scotia|631[0n]|BOLD:AAA8795
Syngrapha viridisigma|[3035]|LCH519-04|Canada|Manitoba|584[0n]|BOLD:AAA8795
Syngrapha viridisigma|[3036]|RDNMD257-06|Canada|New Brunswick|630[0n]|BOLD:AAA8795
Syngrapha viridisigma|[3037]|BBLEC465-09|Canada|New Brunswick|634[0n]|BOLD:AAA8795
Syngrapha viridisigma|[3038]|LOWCC369-05|Canada|British Columbia|555[0n]|BOLD:AAA8795
Syngrapha viridisigma|[3039]|LOWCC365-05|Canada|British Columbia|584[0n]|BOLD:AAA8795
Syngrapha viridisigma|[3040]|LOWCD127-06|Canada|British Columbia|616[0n]|BOLD:AAA8795
Syngrapha viridisigma|[3041]|UAMIC530-13|United States|Alaska|632[0n]|BOLD:AAA8795
Syngrapha viridisigma|[3042]|CNWBG3056-13|Canada|Alberta|587[0n]|BOLD:AAA8795
Syngrapha viridisigma|[3043]|CNWBG3100-13|Canada|Alberta|602[0n]|BOLD:AAA8795
Syngrapha viridisigma|[3044]|CNWBG3119-13|Canada|Alberta|589[0n]|BOLD:AAA8795
Syngrapha viridisigma|[3045]|CNWBG3141-13|Canada|Alberta|588[0n]|BOLD:AAA8795
Syngrapha viridisigma|[3046]|CNWBG3169-13|Canada|Alberta|588[0n]|BOLD:AAA8795
Syngrapha ignea|[3047]|LSEU783-06|United States|Colorado|658[0n]|BOLD:ABZ2814
Syngrapha ignea|[3048]|LOWCD145-06|Canada|British Columbia|603[0n]|BOLD:ABZ2814
Syngrapha ignea|[3049]|LOWCE079-06|Canada|British Columbia|658[0n]|BOLD:ABZ2814
Syngrapha ignea|[3050]|LOWCE080-06|Canada|British Columbia|658[0n]|BOLD:ABZ2814
Syngrapha ignea|[3051]|RDNME527-08|Canada|Yukon Territory|658[0n]|BOLD:ABZ2814
Syngrapha ignea|[3052]|JSYKA292-10|Canada|Yukon Territory|658[0n]|BOLD:ABZ2814
Syngrapha alticola|[3053]|MHLEP114-07|Canada|Manitoba|658[0n]|BOLD:ABY8699
Syngrapha alticola|[3054]|LCH523-04|Canada|Manitoba|584[0n]|BOLD:ABY8699
Syngrapha alticola|[3055]|LCHP020-07|Canada|Manitoba|644[0n]|BOLD:ABY8699
Syngrapha alticola|[3056]|LCHQ110-07|Canada|Manitoba|658[0n]|BOLD:ABY8699
Syngrapha parilis|[3057]|RDNMF739-08|Canada|Nunavut|609[0n]|BOLD:AAD7310
Syngrapha parilis|[3058]|GRAFW331-11|Greenland||658[0n]|BOLD:AAD7310
Syngrapha parilis|[3059]|GRAFW330-11|Greenland||658[0n]|BOLD:AAD7310
Syngrapha parilis|[3060]|GRAFW093-11|Greenland||658[0n]|BOLD:AAD7310
Syngrapha parilis|[3061]|GRAFW060-11|Greenland||658[0n]|BOLD:AAD7310
Syngrapha parilis|[3062]|GRAFW059-11|Greenland||658[0n]|BOLD:AAD7310
Syngrapha parilis|[3063]|GRAFW058-11|Greenland||658[0n]|BOLD:AAD7310
Syngrapha parilis|[3064]|GRAFW057-11|Greenland||658[0n]|BOLD:AAD7310
Syngrapha parilis|[3065]|GRAFW056-11|Greenland||658[0n]|BOLD:AAD7310
Syngrapha parilis|[3066]|LEFIL495-10|Finland||658[0n]|BOLD:AAD7310
Syngrapha parilis|[3067]|LEFII100-10|Finland|Lapland|658[0n]|BOLD:AAD7310
Syngrapha parilis|[3068]|LEFIF285-10|Finland||658[0n]|BOLD:AAD7310
Syngrapha parilis|[3069]|LEFIF284-10|Finland||658[0n]|BOLD:AAD7310
Syngrapha parilis|[3070]|LEFIF283-10|Finland||658[0n]|BOLD:AAD7310
Syngrapha parilis|[3071]|RDNMF740-08|Canada|Yukon Territory|658[0n]|BOLD:AAD7310
Syngrapha parilis|[3072]|RDNME518-08|Canada|Yukon Territory|658[0n]|BOLD:AAD7310
Syngrapha parilis|[3073]|GRAFW1028-12|Greenland||658[0n]|BOLD:AAD7310
Syngrapha parilis|[3074]|GRAFW1140-12|Greenland||658[0n]|BOLD:AAD7310
Syngrapha orophila|[3075]|RDNMF357-08|Canada|Alberta|658[0n]|BOLD:AAD2335
Syngrapha orophila|[3076]|LBCG739-09|Canada|British Columbia|658[0n]|BOLD:AAD2335
Syngrapha orophila|[3077]|RDNMF355-08|Canada|Alberta|658[0n]|BOLD:AAD2335
Syngrapha orophila|[3078]|RDNMF356-08|Canada|Alberta|658[0n]|BOLD:AAD2335
Syngrapha orophila|[3079]|LBCG764-09|Canada|British Columbia|658[0n]|BOLD:AAD2335
Syngrapha orophila|[3080]|LBCG2837-09|Canada|British Columbia|658[0n]|BOLD:AAD2335
Syngrapha orophila|[3081]|LBCH1093-10|Canada|British Columbia|658[0n]|BOLD:AAD2335
Syngrapha orophila|[3082]|LBCH1161-10|Canada|British Columbia|658[0n]|BOLD:AAD2335
Syngrapha orophila|[3083]|LBCH1974-10|Canada|British Columbia|658[0n]|BOLD:AAD2335
[illegible]

Syngrapha orophila|[3081]|LBCH1093-10|Canada|British Columbia|658[0n]|BOLD:AAD2335
Syngrapha orophila|[3082]|LBCH1161-10|Canada|British Columbia|658[0n]|BOLD:AAD2335
Syngrapha orophila|[3083]|LBCH1974-10|Canada|British Columbia|658[0n]|BOLD:AAD2335
Syngrapha orophila|[3084]|LBCH6625-10|Canada|British Columbia|658[0n]|BOLD:AAD2335
Syngrapha diasema|[3085]|LCH329-04|Canada|Manitoba|658[0n]|BOLD:AAA6513
Syngrapha diasema|[3086]|LCHIP055-07|Canada|Manitoba|650[0n]|BOLD:AAA6513
Syngrapha diasema|[3087]|LCH337-04|Canada|Manitoba|658[0n]|BOLD:AAA6513
Syngrapha diasema|[3088]|LCHP841-07|Canada|Manitoba|631[0n]|BOLD:AAA6513
Syngrapha diasema|[3089]|LCHP578-07|Canada|Manitoba|658[0n]|BOLD:AAA6513
Syngrapha diasema|[3090]|LCH338-04|Canada|Manitoba|658[0n]|BOLD:AAA6513
Syngrapha diasema|[3091]|LCH333-04|Canada|Manitoba|658[0n]|BOLD:AAA6513
Syngrapha diasema|[3092]|LCH443-04|Canada|Manitoba|658[0n]|BOLD:AAA6513
Syngrapha diasema|[3093]|LCH450-04|Canada|Manitoba|658[0n]|BOLD:AAA6513
Syngrapha diasema|[3094]|LCH454-04|Canada|Manitoba|658[0n]|BOLD:AAA6513
Syngrapha diasema|[3095]|LCHP577-07|Canada|Manitoba|654[0n]|BOLD:AAA6513
Syngrapha diasema|[3096]|LCH335-04|Canada|Manitoba|658[0n]|BOLD:AAA6513
Syngrapha diasema|[3097]|LCHP478-07|Canada|Manitoba|647[0n]|BOLD:AAA6513
Syngrapha diasema|[3098]|LCHP903-07|Canada|Manitoba|644[0n]|BOLD:AAA6513
Syngrapha diasema|[3099]|JGLL047-10|Canada|Manitoba|658[1n]|BOLD:AAA6513
Syngrapha diasema|[3100]|CHLEP222-09|Canada|Manitoba|658[0n]|BOLD:AAA6513
Syngrapha diasema|[3101]|CHLEP213-09|Canada|Manitoba|658[0n]|BOLD:AAA6513
Syngrapha diasema|[3102]|LCHQ909-08|Canada|Manitoba|658[0n]|BOLD:AAA6513
Syngrapha diasema|[3103]|LCHQ590-08|Canada|Manitoba|655[0n]|BOLD:AAA6513
Syngrapha diasema|[3104]|LCHQ482-08|Canada|Manitoba|657[0n]|BOLD:AAA6513
Syngrapha diasema|[3105]|LCHQ191-07|Canada|Manitoba|658[0n]|BOLD:AAA6513
Syngrapha diasema|[3106]|LCHQ035-07|Canada|Manitoba|657[0n]|BOLD:AAA6513
Syngrapha diasema|[3107]|LCHP896-07|Canada|Manitoba|658[0n]|BOLD:AAA6513
Syngrapha diasema|[3108]|LCHP834-07|Canada|Manitoba|657[0n]|BOLD:AAA6513
Syngrapha diasema|[3109]|LCHP789-07|Canada|Manitoba|658[0n]|BOLD:AAA6513
Syngrapha diasema|[3110]|LCHP755-07|Canada|Manitoba|658[0n]|BOLD:AAA6513
Syngrapha diasema|[3111]|LCHP626-07|Canada|Manitoba|658[0n]|BOLD:AAA6513
Syngrapha diasema|[3112]|LCHP553-07|Canada|Manitoba|657[0n]|BOLD:AAA6513
Syngrapha diasema|[3113]|LCHP539-07|Canada|Manitoba|657[0n]|BOLD:AAA6513
Syngrapha diasema|[3114]|LCHP493-07|Canada|Manitoba|657[0n]|BOLD:AAA6513
Syngrapha diasema|[3115]|LCHP419-07|Canada|Manitoba|657[0n]|BOLD:AAA6513
Syngrapha diasema|[3116]|MHLEP138-07|Canada|Manitoba|658[0n]|BOLD:AAA6513
Syngrapha diasema|[3117]|LCH524-04|Canada|Manitoba|658[0n]|BOLD:AAA6513
Syngrapha diasema|[3118]|LCH459-04|Canada|Manitoba|658[0n]|BOLD:AAA6513
Syngrapha diasema|[3119]|LCH455-04|Canada|Manitoba|658[0n]|BOLD:AAA6513
Syngrapha diasema|[3120]|LCH453-04|Canada|Manitoba|658[0n]|BOLD:AAA6513
Syngrapha diasema|[3121]|LCH452-04|Canada|Manitoba|658[0n]|BOLD:AAA6513
Syngrapha diasema|[3122]|LCH451-04|Canada|Manitoba|658[0n]|BOLD:AAA6513
Syngrapha diasema|[3123]|LCH449-04|Canada|Manitoba|658[0n]|BOLD:AAA6513
Syngrapha diasema|[3124]|LCH445-04|Canada|Manitoba|658[0n]|BOLD:AAA6513
Syngrapha diasema|[3125]|LCH444-04|Canada|Manitoba|658[0n]|BOLD:AAA6513
Syngrapha diasema|[3126]|LCH340-04|Canada|Manitoba|658[0n]|BOLD:AAA6513
Syngrapha diasema|[3127]|LCH339-04|Canada|Manitoba|658[0n]|BOLD:AAA6513
Syngrapha diasema|[3128]|LCH336-04|Canada|Manitoba|658[0n]|BOLD:AAA6513
Syngrapha diasema|[3129]|LCH332-04|Canada|Manitoba|658[0n]|BOLD:AAA6513
Syngrapha diasema|[3130]|LCH331-04|Canada|Manitoba|658[0n]|BOLD:AAA6513
Syngrapha diasema|[3131]|LCH330-04|Canada|Manitoba|658[0n]|BOLD:AAA6513
Syngrapha diasema|[3132]|LCH328-04|Canada|Manitoba|658[0n]|BOLD:AAA6513
Syngrapha diasema|[3133]|LCH327-04|Canada|Manitoba|658[0n]|BOLD:AAA6513
Syngrapha diasema|[3134]|LCH341-04|Canada|Manitoba|658[1n]|BOLD:AAA6513
Syngrapha diasema|[3135]|LCH446-04|Canada|Manitoba|658[0n]|BOLD:AAA6513
Syngrapha diasema|[3136]|LCH334-04|Canada|Manitoba|658[2n]|BOLD:AAA6513
Syngrapha diasema|[3137]|LCHP889-07|Canada|Manitoba|636[0n]|BOLD:AAA6513
Syngrapha diasema|[3138]|LCHP890-07|Canada|Manitoba|636[0n]|BOLD:AAA6513
Syngrapha diasema|[3139]|LCHP901-07|Canada|Manitoba|645[0n]|BOLD:AAA6513
Syngrapha diasema|[3140]|LCHP902-07|Canada|Manitoba|658[0n]|BOLD:AAA6513
Syngrapha diasema|[3141]|LEFID882-10|Finland||647[0n]|BOLD:AAA6513
Syngrapha diasema|[3142]|LEFIF282-10|Finland||658[0n]|BOLD:AAA6513
Syngrapha diasema|[3143]|LEFII099-10|Finland|Lapland|658[0n]|BOLD:AAA6513
Syngrapha borea|[3144]|RDNMF358-08|Canada|British Columbia|658[0n]|BOLD:AAE2536
Syngrapha borea|[3145]|RDNMF359-08|Canada|Alberta|658[0n]|BOLD:AAE2536
Syngrapha borea|[3146]|RDNMF360-08|Canada|Yukon Territory|658[0n]|BOLD:AAE2536
Syngrapha borea|[3147]|RDNMF361-08|Canada|British Columbia|658[0n]|BOLD:AAE2536
Syngrapha borea|[3148]|RDNMF362-08|Canada|British Columbia|658[0n]|BOLD:AAE2536
Syngrapha borea|[3149]|ZMUCG327-12|Greenland||658[0n]|BOLD:AAE2536
Syngrapha borea|[3150]|ZMUCG328-12|Greenland||658[0n]|BOLD:AAE2536
Syngrapha sackenii|[3151]|CNCLB2080-14|United States|Colorado|658[0n]|BOLD:AAE2536
Syngrapha sackenii|[3152]|CNCLB2081-14|United States|Utah|658[0n]|BOLD:AAE2536
Syngrapha sackenii|[3153]|CNCLB2082-14|United States|Colorado|658[0n]|BOLD:AAE2536
Syngrapha sackenii|[3154]|CNCLB2083-14|United States|Colorado|658[0n]|BOLD:AAE2536
Pseudeva palligera|[3155]|RDNMF444-08|Canada|British Columbia|658[0n]|BOLD:AAD5210
Pseudeva palligera|[3156]|RDMAB984-09|Canada|Alberta|627[0n]|BOLD:AAD5210
Pseudeva palligera|[3157]|RDMAB985-09|Canada|British Columbia|644[0n]|BOLD:AAD5210
Pseudeva palligera|[3158]|RDMAB986-09|Canada|British Columbia|658[0n]|BOLD:AAD5210
Pseudeva palligera|[3159]|LPABC876-09|Canada|Alberta|658[0n]|BOLD:AAD5210
Pseudeva palligera|[3160]|LPABC877-09|Canada|Alberta|658[0n]|BOLD:AAD5210
Pseudeva palligera|[3161]|LPABC969-09|Canada|Alberta|658[0n]|BOLD:AAD5210
Pseudeva purpurigera|[3162]|RDLQG143-06|Canada|Quebec|613[1n]|BOLD:AAD7521
Pseudeva purpurigera|[3163]|BLTIB703-08|Canada|Ontario|658[0n]|BOLD:AAD7521
Pseudeva purpurigera|[3164]|RDNMG936-08|Canada|Ontario|658[0n]|BOLD:AAD7521
Pseudeva purpurigera|[3165]|RDNMG937-08|Canada|Ontario|658[0n]|BOLD:AAD7521
Pseudeva purpurigera|[3166]|LPMNB553-09|Canada|Manitoba|658[0n]|BOLD:AAD7521
Pseudeva purpurigera|[3167]|RDLQF815-06|Canada|Quebec|658[0n]|BOLD:AAD7521
Pseudeva purpurigera|[3168]|CNWLF2014-12|Canada|Alberta|658[0n]|BOLD:AAD7521
Eosphoropteryx thyatyroides|[3169]|LPMNB327-09|Canada|Manitoba|600[0n]|BOLD:AAD4019
Eosphoropteryx thyatyroides|[3170]|LALPA421-10|Canada|British Columbia|658[0n]|BOLD:AAD4019
Eosphoropteryx thyatyroides|[3171]|LBCC753-05|Canada|British Columbia|658[0n]|BOLD:AAD4019
Eosphoropteryx thyatyroides|[3172]|LALPA424-10|Canada|British Columbia|658[0n]|BOLD:AAD4019
Eosphoropteryx thyatyroides|[3173]|SSWLD7967-13|Canada|Alberta|573[0n]|BOLD:AAD4019
Eosphoropteryx thyatyroides|[3174]|LPMNB353-09|Canada|Manitoba|658[0n]|BOLD:AAD4019
Eosphoropteryx thyatyroides|[3175]|CNPEH029-14|Canada|Prince Edward Island|562[0n]|BOLD:AAD4019
Eosphoropteryx thyatyroides|[3176]|XAG913-05|Canada|Ontario|658[0n]|BOLD:AAD4019
Eosphoropteryx thyatyroides|[3177]|RDLQ425-07|Canada|Quebec|645[0n]|BOLD:AAD4019
Eosphoropteryx thyatyroides|[3178]|LGSMG606-07|United States|North Carolina|658[0n]|BOLD:AAD4019
Eosphoropteryx thyatyroides|[3179]|LGSMG607-07|United States|North Carolina|658[0n]|BOLD:AAD4019
Eosphoropteryx thyatyroides|[3180]|LNCC1024-11|United States|North Carolina|658[0n]|BOLD:AAD4019
Eosphoropteryx thyatyroides|[3181]|CNCLB2630-14|United States|North Carolina|658[0n]|BOLD:AAD4019
Cropia ruthaea|[3182]|CNCLB2419-14|United States|Texas|658[0n]|BOLD:ACR9407
Schinia walsinghamii|[3183]|LBCH7779-10|Canada|British Columbia|658[0n]|BOLD:AAC2308

Eosphoropteryx thyatyroides|[3181]|CNCLB2630-14|United States|North Carolina|658[0n]|BOLD:AAD4019
Cropia ruthaea|[3182]|CNCLB2419-14|United States|Texas|658[0n]|BOLD:ACR9407
Schinia walsinghami|[3183]|LBCH7779-10|Canada|British Columbia|658[0n]|BOLD:AAC2308
Schinia walsinghami|[3184]|LBCH7778-10|Canada|British Columbia|658[0n]|BOLD:AAC2308
Schinia walsinghami|[3185]|LBCH7777-10|Canada|British Columbia|658[0n]|BOLD:AAC2308
Schinia walsinghami|[3186]|LBCH7776-10|Canada|British Columbia|658[0n]|BOLD:AAC2308
Schinia walsinghami|[3187]|LBCH7775-10|Canada|British Columbia|658[0n]|BOLD:AAC2308
Schinia walsinghami|[3188]|LBCH7624-10|Canada|British Columbia|658[0n]|BOLD:AAC2308
Schinia walsinghami|[3189]|LBCH7622-10|Canada|British Columbia|658[0n]|BOLD:AAC2308
Schinia walsinghami|[3190]|LBCH7621-10|Canada|British Columbia|658[0n]|BOLD:AAC2308
Schinia walsinghami|[3191]|LBCH7620-10|Canada|British Columbia|658[0n]|BOLD:AAC2308
Schinia walsinghami|[3192]|LBCH7619-10|Canada|British Columbia|658[0n]|BOLD:AAC2308
Schinia walsinghami|[3193]|LBCH7047-10|Canada|British Columbia|658[0n]|BOLD:AAC2308
Schinia walsinghami|[3194]|LBCH6631-10|Canada|British Columbia|658[0n]|BOLD:AAC2308
Schinia walsinghami|[3195]|LBCH6555-10|Canada|British Columbia|658[0n]|BOLD:AAC2308
Schinia walsinghami|[3196]|LBCH6430-10|Canada|British Columbia|658[0n]|BOLD:AAC2308
Schinia walsinghami|[3197]|LBCH6429-10|Canada|British Columbia|658[0n]|BOLD:AAC2308
Schinia walsinghami|[3198]|HELNA239-06|United States|Nevada|656[0n]|BOLD:AAC2308
Schinia walsinghami|[3199]|LBCH6427-10|Canada|British Columbia|658[0n]|BOLD:AAC2308
Schinia walsinghami|[3200]|LBCH6425-10|Canada|British Columbia|658[0n]|BOLD:AAC2308
Schinia walsinghami|[3201]|LBCH6388-10|Canada|British Columbia|658[0n]|BOLD:AAC2308
Schinia walsinghami|[3202]|LBCH6390-10|Canada|British Columbia|658[0n]|BOLD:AAC2308
Schinia walsinghami|[3203]|LBCH6432-10|Canada|British Columbia|658[0n]|BOLD:AAC2308
Schinia walsinghami|[3204]|LBCH7625-10|Canada|British Columbia|658[0n]|BOLD:AAC2308
Schinia walsinghami|[3205]|LBCH7773-10|Canada|British Columbia|658[0n]|BOLD:AAC2308
Schinia walsinghami|[3206]|LBCH6428-10|Canada|British Columbia|658[0n]|BOLD:AAC2308
Schinia walsinghami|[3207]|LBCH7772-10|Canada|British Columbia|658[0n]|BOLD:AAC2308
Schinia walsinghami|[3208]|LBCH6431-10|Canada|British Columbia|658[0n]|BOLD:AAC2308
Schinia walsinghami|[3209]|LBCH7623-10|Canada|British Columbia|658[0n]|BOLD:AAC2308
Schinia walsinghami|[3210]|LBCH7774-10|Canada|British Columbia|658[0n]|BOLD:AAC2308
Schinia walsinghami|[3211]|LBCH6426-10|Canada|British Columbia|658[0n]|BOLD:AAC2308
Schinia walsinghami|[3212]|LBCH7626-10|Canada|British Columbia|658[0n]|BOLD:AAC2308
Schinia psamathea|[3213]|HELNA906-10|United States|Florida|658[0n]|BOLD:AAC2308
Schinia psamathea|[3214]|HELNA365-09|United States|Alabama|656[0n]|BOLD:AAC2308
Schinia psamathea|[3215]|HELNA907-10|United States|Alabama|658[0n]|BOLD:AAC2308
Schinia psamathea|[3216]|HELNA908-10|United States|Georgia|658[0n]|BOLD:AAC2308
Schinia psamathea|[3217]|HELNA1015-10|United States|Florida|658[0n]|BOLD:AAC2308
Schinia walsinghami|[3218]|HELNA897-10|United States|Colorado|658[0n]|BOLD:AAC2308
Schinia psamathea|[3219]|HELNA303-09|United States||658[0n]|BOLD:AAC2308
Schinia psamathea|[3220]|HELNA909-10|United States|Florida|658[0n]|BOLD:AAC2308
Schinia walsinghami|[3221]|HELNA238-06|United States|Utah|656[0n]|BOLD:AAC2308
Schinia walsinghami|[3222]|HELNA905-10|United States|Colorado|658[0n]|BOLD:AAC2308
Schinia walsinghami|[3223]|JBAZ152-09|United States|Arizona|658[0n]|BOLD:AAC2308
Schinia walsinghami|[3224]|HELNA117-06|United States|Colorado|657[0n]|BOLD:AAC2308
Schinia poguei|[3225]|CNCLB122-14|United States|New Mexico|658[0n]|BOLD:AAC2308
Schinia poguei|[3226]|CNCLB123-14|United States|New Mexico|658[0n]|BOLD:AAC2308
Schinia separata|[3227]|RDNMB219-05|United States|Washington|658[2n]|BOLD:ACE3505
Schinia separata|[3228]|RDNMC448-05|Canada|British Columbia|539[0n]|BOLD:ACE3505
Schinia separata|[3229]|RDNMC445-05|United States|Washington|565[1n]|BOLD:ACE3505
Schinia separata|[3230]|LBCH7695-10|Canada|British Columbia|658[0n]|BOLD:ACE3505
Schinia separata|[3231]|LBCH7771-10|Canada|British Columbia|658[0n]|BOLD:ACE3505
Schinia separata|[3232]|LBCH6554-10|Canada|British Columbia|658[0n]|BOLD:ACE3505
Schinia separata|[3233]|LBCH6630-10|Canada|British Columbia|658[0n]|BOLD:ACE3505
Schinia separata|[3234]|HELNA006-06|United States|Montana|657[0n]|BOLD:ACE3505
Schinia separata|[3235]|LBCH6389-10|Canada|British Columbia|658[0n]|BOLD:ACE3505
Schinia separata|[3236]|LBCH7879-10|Canada|British Columbia|658[0n]|BOLD:ACE3505
Schinia separata|[3237]|RDNMC447-05|United States|Oregon|592[0n]|BOLD:ACE3505
Schinia separata|[3238]|RDNMC449-05|United States|Washington|594[0n]|BOLD:ACE3505
Schinia separata|[3239]|HELNA005-06|United States|Montana|658[0n]|BOLD:ACE3505
Schinia separata|[3240]|HELNA007-06|United States|Montana|658[0n]|BOLD:ACE3505
Schinia separata|[3241]|HELNA008-06|United States|Montana|658[0n]|BOLD:ACE3505
Schinia separata|[3242]|HELNA009-06|United States|Wyoming|658[0n]|BOLD:ACE3505
Schinia separata|[3243]|HELNA010-06|United States|Wyoming|658[0n]|BOLD:ACE3505
Schinia separata|[3244]|HELNA835-10|United States|Oregon|658[0n]|BOLD:ACE3505
Schinia acutilinea|[3245]|RDNMC444-05|United States|Wyoming|553[0n]|BOLD:AAB4314
Schinia acutilinea|[3246]|HELNA836-10|Canada|Alberta|658[0n]|BOLD:AAB4314
Schinia acutilinea|[3247]|RDMAB550-06|Canada|Alberta|657[0n]|BOLD:AAB4314
Schinia acutilinea|[3248]|RDNMB217-05|Canada|Alberta|658[0n]|BOLD:AAB4314
Schinia acutilinea|[3249]|RDNMC439-05|Canada|Alberta|592[0n]|BOLD:AAB4314
Schinia acutilinea|[3250]|RDNMC441-05|Canada|Alberta|589[0n]|BOLD:AAB4314
Schinia acutilinea|[3251]|RDMAB549-06|Canada|Alberta|658[0n]|BOLD:AAB4314
Schinia acutilinea|[3252]|HELNA837-10|Canada|Alberta|658[0n]|BOLD:AAB4314
Schinia acutilinea|[3253]|RDNMB218-05|Canada|Alberta|658[0n]|BOLD:ACF4896
Schinia acutilinea|[3254]|HELNA242-06|United States|Montana|622[0n]|BOLD:ACF4896
Schinia acutilinea|[3255]|HELNA246-06|United States|Montana|658[0n]|BOLD:ACF4896
Schinia acutilinea|[3256]|HELNA244-06|United States|Utah|658[0n]|BOLD:ACF4896
Schinia acutilinea|[3257]|RDNMC438-05|Canada|Alberta|602[0n]|BOLD:ACF4896
Schinia acutilinea|[3258]|HELNA001-06|United States|Colorado|658[0n]|BOLD:ACF4896
Schinia acutilinea|[3259]|HELNA004-06|United States|Colorado|658[0n]|BOLD:ACF4896
Schinia acutilinea|[3260]|HELNA280-09|United States|Utah|658[0n]|BOLD:ACF4896
Schinia acutilinea|[3261]|HELNA243-06|United States|New Mexico|658[0n]|BOLD:ACF4896
Schinia acutilinea|[3262]|HELNA003-06|United States|Colorado|658[0n]|BOLD:ACF4896
Schinia acutilinea|[3263]|HELNA245-06|United States|Montana|654[0n]|BOLD:ACF4896
Schinia acutilinea|[3264]|HELNA002-06|United States|Colorado|656[0n]|BOLD:ACF4896
Schinia acutilinea|[3265]|RDNMC440-05|Canada|Alberta|597[0n]|BOLD:ACF4896
Schinia acutilinea|[3266]|RDNMC443-05|Canada|Alberta|599[0n]|BOLD:ACF4896
Schinia acutilinea|[3267]|RDNMC442-05|Canada|Alberta|599[0n]|BOLD:ACF4896
Schinia acutilinea|[3268]|HELNA277-09|United States|Utah|658[0n]|BOLD:ACF4896
Schinia acutilinea|[3269]|HELNA850-10|United States|Colorado|658[0n]|BOLD:ACF4896
Schinia separata|[3270]|JBAZ158-09|United States|California|645[0n]|BOLD:ACF2418
Schinia separata|[3271]|RDNMC446-05|United States|California|589[0n]|BOLD:ACF2418
Schinia separata|[3272]|HELNA922-10|United States|California|658[0n]|BOLD:ACF2418
Schinia separata|[3273]|HELNA923-10|United States|California|658[0n]|BOLD:ACF2418
Schinia separata|[3274]|HELNA924-10|United States|California|658[0n]|BOLD:ACF2418
Schinia regia|[3275]|CNCLB124-14|United States|Texas|658[0n]|BOLD:AAC9845
Schinia regina|[3276]|HELNA085-06|United States|Colorado|658[0n]|BOLD:AAC9845
Schinia regina|[3277]|HELNA285-09|United States|New Mexico|658[0n]|BOLD:AAC9845
Schinia regina|[3278]|LSEU071-06|United States|Texas|584[0n]|BOLD:AAC9845
Schinia regina|[3279]|HELNA086-06|United States|Colorado|658[0n]|BOLD:AAC9845
Schinia regina|[3280]|HELNA084-06|United States|Colorado|658[0n]|BOLD:AAC9845
Schinia regina|[3281]|HELNA227-06|United States|Colorado|658[0n]|BOLD:AAC9845
Schinia regina|[3282]|HELNA228-06|United States|Colorado|658[0n]|BOLD:AAC9845

Schinia regina|[3280]|HELNA084-06|United States|Colorado|658[0n]|BOLD:AAC9845
Schinia regina|[3281]|HELNA227-06|United States|Colorado|658[0n]|BOLD:AAC9845
Schinia regina|[3282]|HELNA228-06|United States|Colorado|658[0n]|BOLD:AAC9845
Schinia regia|[3283]|CNCLB125-14|United States|Texas|658[0n]|BOLD:AAC9845
Schinia villosa|[3284]|HELNA207-06|United States|Colorado|658[0n]|BOLD:AAE3854
Schinia villosa|[3285]|RDMAB1028-09|United States|Colorado|652[0n]|BOLD:AAE3854
Schinia villosa|[3286]|RDMAB1029-09|United States|Colorado|649[0n]|BOLD:AAE3854
Schinia villosa|[3287]|HELNA173-06|United States|Colorado|640[2n]|BOLD:AAE3854
Schinia villosa|[3288]|RDMAB1030-09|United States|Colorado|658[0n]|BOLD:AAE3854
Schinia sp.|[3289]|HELNA172-06|United States|Montana|658[0n]|BOLD:ACE6404
Schinia vacciniae|[3290]|NAMUM330-08|United States|California|658[0n]|BOLD:ACE6404
Schinia vacciniae|[3291]|GBGL5872-09|||722[0n]|BOLD:ACE6404
Schinia sexata|[3292]|HELNA843-10|Canada|Manitoba|658[0n]|BOLD:ACE6404
Schinia sexata|[3293]|HELNA844-10|Canada|Manitoba|658[0n]|BOLD:ACE6404
Schinia sexata|[3294]|HELNA1012-10|Canada|Manitoba|658[0n]|BOLD:ACE6404
Schinia intermontana|[3295]|CNCLB3113-15|United States|Oregon|658[0n]|BOLD:ACE6404
Schinia oculata|[3296]|HELNA211-06|United States|Nevada|658[0n]|BOLD:AAF2077
Schinia oculata|[3297]|JBAZ164-09|United States|Arizona|658[0n]|BOLD:AAF2077
Schinia oculata|[3298]|CMAZA009-09|United States|Arizona|658[0n]|BOLD:AAF2077
Schinia oculata|[3299]|CMAZA1138-12|United States|Arizona|658[0n]|BOLD:AAF2077
Schinia oculata|[3300]|JBAZ163-09|United States|Arizona|658[0n]|BOLD:AAF2077
Schinia oculata|[3301]|CMAZA1150-12|United States|Arizona|658[0n]|BOLD:AAF2077
Schinia oculata|[3302]|CMAZA1212-12|United States|Arizona|658[0n]|BOLD:AAF2077
Schinia albafascia|[3303]|HELNA012-06|United States|Colorado|658[0n]|BOLD:AAE9289
Schinia albafascia|[3304]|HELNA011-06|United States|Colorado|648[0n]|BOLD:AAE9289
Schinia albafascia|[3305]|HELNA013-06|United States|New Mexico|656[0n]|BOLD:AAE9289
Schinia brunnea|[3306]|HELNA925-10|United States|California|641[0n]|BOLD:AAE9289
Schinia brunnea|[3307]|HELNA926-10|United States|California|658[0n]|BOLD:AAE9289
Schinia brunnea|[3308]|HELNA927-10|United States|California|658[0n]|BOLD:AAE9289
Schinia brunnea|[3309]|HELNA1016-10|United States|California|658[0n]|BOLD:AAE9289
Schinia albafascia|[3310]|NAMUM138-08|United States|California|658[0n]|BOLD:AAE9289
Schinia albafascia|[3311]|LTOLB1294-11|United States|California|658[0n]|BOLD:AAE9289
Schinia ferrisi|[3312]|LNAUP103-13|United States|New Mexico|658[0n]|BOLD:AAE9289
Schinia ferrisi|[3313]|LNAUP105-13|United States|New Mexico|658[0n]|BOLD:AAE9289
Schinia ferrisi|[3314]|LNAUP104-13|United States|New Mexico|658[0n]|BOLD:AAE9289
Schinia ferrisi|[3315]|HELNA350-09|United States|New Mexico|658[0n]|BOLD:AAE9289
Schinia ferrisi|[3316]|HELNA351-09|United States|New Mexico|658[0n]|BOLD:AAE9289
Schinia brunnea|[3317]|HELNA900-10|United States|California|658[0n]|BOLD:AAE9289
Schinia ferrisi|[3318]|HELNA352-09|United States|Arizona|635[0n]|BOLD:AAE9289
Schinia brunnea|[3319]|HELNA898-10|United States|California|658[0n]|BOLD:AAE9289
Schinia brunnea|[3320]|HELNA899-10|United States|California|658[0n]|BOLD:AAE9289
Schinia erosa|[3321]|CNCLB114-14|United States|California|658[0n]|BOLD:AAE9289
Schinia erosa|[3322]|CNCLB115-14|United States|California|658[0n]|BOLD:AAE9289
Schinia argentifascia|[3323]|HELNA864-10|United States|California|658[0n]|BOLD:ABX6166
Schinia argentifascia|[3324]|HELNA863-10|United States|California|658[0n]|BOLD:ABX6166
Schinia argentifascia|[3325]|HELNA919-10|United States|California|658[0n]|BOLD:ABX6166
Schinia argentifascia|[3326]|HELNA862-10|United States|California|658[0n]|BOLD:ABX6166
Schinia argentifascia|[3327]|HELNA920-10|United States|California|624[0n]|BOLD:ABX6166
Schinia argentifascia|[3328]|HELNA921-10|United States|California|634[0n]|BOLD:ABX6166
Schinia argentifascia|[3329]|CMAZA106-09|United States|Arizona|658[0n]|BOLD:ABX6166
Schinia argentifascia|[3330]|CMAZA1053-12|United States|Arizona|658[0n]|BOLD:ABX6166
Schinia argentifascia|[3331]|HELNA901-10|United States|Arizona|658[0n]|BOLD:ABX6166
Schinia argentifascia|[3332]|JBAZ165-09|United States|Arizona|658[0n]|BOLD:ABX6166
Schinia argentifascia|[3333]|JBAZ166-09|United States|Arizona|658[0n]|BOLD:ABX6166
Schinia argentifascia|[3334]|HELNA902-10|United States|Arizona|658[0n]|BOLD:ABX6166
Schinia argentifascia|[3335]|HELNA903-10|United States|Arizona|658[0n]|BOLD:ABX6166
Schinia argentifascia|[3336]|HELNA904-10|United States|Arizona|658[0n]|BOLD:ABX6166
Schinia argentifascia|[3337]|CMAZA1086-12|United States|Arizona|658[0n]|BOLD:ABX6166
Schinia argentifascia|[3338]|CMAZA1159-12|United States|Arizona|658[0n]|BOLD:ABX6166
Schinia chrysellus|[3339]|HELNA019-06|United States|Colorado|658[0n]|BOLD:AAD6853
Schinia chrysellus|[3340]|LPOKA396-09|United States|Oklahoma|658[0n]|BOLD:AAD6853
Schinia chrysellus|[3341]|LTOLB1297-11|United States|Kansas|658[0n]|BOLD:AAD6853
Schinia chrysellus|[3342]|LTOLB1298-11|United States|Kansas|658[0n]|BOLD:AAD6853
Schinia chrysellus|[3343]|LPOKA215-08|United States|Oklahoma|658[0n]|BOLD:AAD6853
Schinia chrysellus|[3344]|LPOKA167-08|United States|Oklahoma|658[0n]|BOLD:AAD6853
Schinia chrysellus|[3345]|HELNA018-06|United States|Colorado|654[0n]|BOLD:AAD6853
Schinia chrysellus|[3346]|LPOKA524-09|United States|Oklahoma|632[0n]|BOLD:AAD6853
Schinia chrysellus|[3347]|BBLOE1629-12|United States|Oklahoma|658[0n]|BOLD:AAD6853
Schinia arefacta|[3348]|LNAUT2064-14|United States|Florida|658[0n]|BOLD:ACR1846
Schinia arefacta|[3349]|CNCLB2189-14|United States|Florida|371[0n]|
Schinia obscurata|[3350]|HELNA170-06|United States|Oklahoma|584[26n]|
Schinia obscurata|[3351]|RDLQB415-05|Canada|Quebec|658[0n]|BOLD:AAB9588
Schinia obscurata|[3352]|HELNA171-06|United States|Oklahoma|658[0n]|BOLD:AAB9588
Schinia obscurata|[3353]|XAE570-04|Canada|Ontario|625[0n]|BOLD:AAB9588
Schinia obscurata|[3354]|HKONS497-08|United States|Florida|658[0n]|BOLD:AAB9588
Schinia obscurata|[3355]|HELNA938-10|United States|Maryland|658[0n]|BOLD:AAB9588
Schinia sp.|[3356]|HELNA941-10|United States|Maryland|658[0n]|BOLD:AAB9588
Schinia lynx|[3357]|HELNA499-09|United States|Maryland|658[0n]|BOLD:AAB9588
Schinia lynx|[3358]|RDNMF430-08|United States|Florida|658[0n]|BOLD:AAB9588
Schinia lynx|[3359]|LNCC1509-13|United States|North Carolina|658[0n]|BOLD:AAB9588
Schinia lynx|[3360]|HELNA340-09|United States|Maryland|603[0n]|BOLD:AAB9588
Schinia lynx|[3361]|RDNMF431-08|United States|Florida|658[0n]|BOLD:AAB9588
Schinia lynx|[3362]|BBLOE1636-12|United States|Oklahoma|658[0n]|BOLD:AAB9588
Schinia lynx|[3363]|HELNA500-09|United States|Maryland|658[0n]|BOLD:AAB9588
Schinia lynx|[3364]|HELNA502-09|United States|Maryland|658[0n]|BOLD:AAB9588
Schinia lynx|[3365]|LNCC1837-13|United States|North Carolina|658[0n]|BOLD:AAB9588
Schinia lynx|[3366]|HKONS499-08|United States|Florida|658[0n]|BOLD:AAB9588
Schinia lynx|[3367]|HELNA501-09|United States|Maryland|658[0n]|BOLD:AAB9588
Schinia lynx|[3368]|HELNA519-09|United States|Maryland|658[0n]|BOLD:AAB9588
Schinia lynx|[3369]|HELNA339-09|United States|Maryland|658[0n]|BOLD:AAB9588
Schinia lynx|[3370]|HELNA293-09|United States|Georgia|658[0n]|BOLD:AAB9588
Schinia lynx|[3371]|LPOKA475-09|United States|Oklahoma|658[0n]|BOLD:AAB9588
Schinia lynx|[3372]|RDNMF428-08|United States|Texas|658[0n]|BOLD:AAB9588
Schinia lynx|[3373]|RDNMF427-08|United States|Arizona|658[0n]|BOLD:AAB9588
Schinia lynx|[3374]|HKONS498-08|United States|Florida|658[0n]|BOLD:AAB9588
Schinia lynx|[3375]|LPOKD275-09|United States|Oklahoma|641[0n]|BOLD:AAB9588
Schinia lynx|[3376]|HELNA939-10|United States|Maryland|658[0n]|BOLD:AAB9588
Schinia lynx|[3377]|CNCLB1265-14|United States|Arizona|658[0n]|BOLD:AAB9588
Schinia coercita|[3378]|HELNA028-06|United States|Colorado|658[0n]|BOLD:AAD6834
Schinia coercita|[3379]|HELNA029-06|United States|New Mexico|658[1n]|BOLD:AAD6834
Schinia coercita|[3380]|HELNA032-06|United States|Oklahoma|658[0n]|BOLD:AAD6834
Schinia coercita|[3381]|HELNA031-06|United States|Oklahoma|658[0n]|BOLD:AAD6834
Schinia coercita|[3382]|HELNA145-06|United States|Colorado|658[0n]|BOLD:AAD6834

Schinia coercita|[3380]|HELNA032-06|United States|Oklahoma|658[0n]|BOLD:AAD6834
Schinia coercita|[3381]|HELNA031-06|United States|Oklahoma|658[0n]|BOLD:AAD6834
Schinia coercita|[3382]|HELNA145-06|United States|Colorado|658[0n]|BOLD:AAD6834
Schinia unimacula|[3383]|HELNA851-10|United States|Wyoming|658[0n]|BOLD:AAD6896
Schinia unimacula|[3384]|RDNMG449-08|United States|Colorado|658[0n]|BOLD:AAD6896
Schinia unimacula|[3385]|HELNA847-10|United States|Colorado|658[0n]|BOLD:AAD6896
Schinia unimacula|[3386]|HELNA241-06|United States|Nevada|658[0n]|BOLD:AAD6896
Schinia unimacula|[3387]|HELNA232-06|United States|Colorado|658[0n]|BOLD:AAD6896
Schinia unimacula|[3388]|HELNA208-06|United States|Colorado|658[0n]|BOLD:AAD6896
Schinia unimacula|[3389]|HELNA240-06|United States|Colorado|658[0n]|BOLD:AAD6896
Schinia unimacula|[3390]|HELNA541-09|United States|New Mexico|658[0n]|BOLD:AAD6896
Schinia unimacula|[3391]|HELNA935-10|United States|California|658[0n]|BOLD:AAD6896
Schinia unimacula|[3392]|HELNA1017-10|United States|New Mexico|658[0n]|BOLD:AAD6896
Schinia maculata|[3393]|LNAUS4065-13|United States|Texas|407[1n]|
Schinia maculata|[3394]|CNCLB2193-14|United States|Texas|658[0n]|BOLD:ACR5533
Schinia maculata|[3395]|CNCLB2405-14|United States|Texas|658[0n]|BOLD:ACR5533
Schinia scissoides|[3396]|MMNA123-08|United States|Georgia|658[0n]|BOLD:AAB9589
Schinia scissoides|[3397]|LSEU075-06|United States|Georgia|603[0n]|BOLD:AAB9589
Schinia scissoides|[3398]|HELNA301-09|United States|Georgia|658[0n]|BOLD:AAB9589
Schinia scissoides|[3399]|PSAT139-10|United States|Florida|658[0n]|BOLD:AAB9589
Schinia carolinensis|[3400]|LNCC807-11|United States|North Carolina|658[0n]|BOLD:ABU6098
Schinia thoreaui|[3401]|RDNMF396-08|United States|Mississippi|616[0n]|BOLD:AAD3713
Schinia thoreaui|[3402]|NAMUM145-08|United States|Kansas|657[0n]|BOLD:AAD3713
Schinia thoreaui|[3403]|RDNMF397-08|United States|Mississippi|658[0n]|BOLD:AAD3713
Schinia thoreaui|[3404]|LPOKA041-08|United States|Oklahoma|658[0n]|BOLD:AAD3713
Schinia thoreaui|[3405]|LPOKA137-08|United States|Oklahoma|658[0n]|BOLD:AAD3713
Schinia thoreaui|[3406]|LPOKA474-09|United States|Oklahoma|658[0n]|BOLD:AAD3713
Schinia thoreaui|[3407]|LPOKD020-09|United States|Oklahoma|658[0n]|BOLD:AAD3713
Schinia thoreaui|[3408]|LPOKD192-09|United States|Oklahoma|658[0n]|BOLD:AAD3713
Schinia thoreaui|[3409]|LPOKA461-09|United States|Oklahoma|658[0n]|BOLD:AAD3713
Schinia thoreaui|[3410]|LPOKD188-09|United States|Oklahoma|658[0n]|BOLD:AAD3713
Schinia thoreaui|[3411]|LPOKD295-09|United States|Oklahoma|658[0n]|BOLD:AAD3713
Schinia sanguinea|[3412]|HELNA093-06|United States|Oklahoma|658[0n]|BOLD:ABX6641
Schinia sanguinea|[3413]|HELNA148-06|United States|Arkansas|658[0n]|BOLD:ABX6641
Schinia sanguinea|[3414]|HELNA094-06|United States|Oklahoma|658[0n]|BOLD:ABX6641
Schinia sanguinea|[3415]|LNCB808-09|United States|North Carolina|658[0n]|BOLD:ABX6641
Schinia sanguinea|[3416]|LNCB809-09|United States|North Carolina|658[1n]|BOLD:ABX6641
Schinia sanguinea|[3417]|HELNA146-06|United States|Montana|658[0n]|BOLD:ABX6641
Schinia sanguinea|[3418]|HELNA147-06|United States|Oklahoma|610[0n]|BOLD:ABX6641
Schinia sanguinea|[3419]|CNCLB110-14|United States|Florida|658[0n]|BOLD:ABX6641
Schinia tertia|[3420]|HELNA111-06|United States|New Mexico|658[0n]|BOLD:AAC2241
Schinia tertia|[3421]|HELNA167-06|United States|Utah|658[0n]|BOLD:AAC2241
Schinia tertia|[3422]|HELNA107-06|United States|Colorado|658[0n]|BOLD:AAC2241
Schinia tertia|[3423]|HELNA110-06|United States|New Mexico|656[0n]|BOLD:AAC2241
Schinia tertia|[3424]|HELNA113-06|United States|Oklahoma|658[0n]|BOLD:AAC2241
Schinia tertia|[3425]|HELNA106-06|United States|Colorado|658[0n]|BOLD:AAC2241
Schinia tertia|[3426]|HELNA168-06|United States|Oklahoma|614[1n]|BOLD:AAC2241
Schinia tertia|[3427]|LPOKA534-09|United States|Oklahoma|658[0n]|BOLD:AAC2241
Schinia tertia|[3428]|HELNA108-06|United States|Colorado|658[0n]|BOLD:AAC2241
Schinia tertia|[3429]|HELNA109-06|United States|New Mexico|658[0n]|BOLD:AAC2241
Schinia tertia|[3430]|HELNA112-06|United States|Oklahoma|658[0n]|BOLD:AAC2241
Schinia tertia|[3431]|HELNA276-09|United States|Utah|658[0n]|BOLD:AAC2241
Schinia nundina|[3432]|HELNA237-06|United States|Arkansas|658[0n]|BOLD:AAD0906
Schinia nundina|[3433]|HELNA515-09|United States|Maryland|658[0n]|BOLD:AAD0906
Schinia nundina|[3434]|HELNA081-06|United States|Oklahoma|658[0n]|BOLD:AAD0906
Schinia nundina|[3435]|HELNA516-09|United States|Maryland|658[0n]|BOLD:AAD0906
Schinia nundina|[3436]|HELNA236-06|United States|Arkansas|658[0n]|BOLD:AAD0906
Schinia nundina|[3437]|HELNA297-09|United States|Georgia|658[0n]|BOLD:AAD0906
Schinia nundina|[3438]|HELNA517-09|United States|Maryland|658[0n]|BOLD:AAD0906
Schinia spinosae|[3439]|HELNA270-09|United States|Connecticut|658[0n]|BOLD:AAE9316
Schinia spinosae|[3440]|HELNA530-09|United States|Maryland|658[0n]|BOLD:AAE9316
Schinia spinosae|[3441]|HELNA531-09|United States|Maryland|658[0n]|BOLD:AAE9316
Schinia spinosae|[3442]|HELNA532-09|United States|Maryland|658[0n]|BOLD:AAE9316
Schinia subspinosae|[3443]|LNAUS3932-13|United States|Alabama|658[0n]|BOLD:ACJ9329
Schinia subspinosae|[3444]|CNCLB130-14|United States|Alabama|603[0n]|BOLD:ACJ9329
Schinia biundulata|[3445]|HELNA346-09|United States|Utah|658[0n]|BOLD:AAE3874
Schinia biundulata|[3446]|HELNA348-09|United States|Utah|658[0n]|BOLD:AAE3874
Schinia biundulata|[3447]|NAMUM379-09|United States|California|658[0n]|BOLD:AAE3874
Schinia biundulata|[3448]|NAMUM378-09|United States|California|658[0n]|BOLD:AAE3874
Schinia biundulata|[3449]|HELNA347-09|United States|Utah|658[0n]|BOLD:AAE3874
Schinia biundulata|[3450]|HELNA349-09|United States|Utah|658[0n]|BOLD:AAE3874
Schinia sordidus|[3451]|HELNA184-06|United States|Mississippi|658[0n]|BOLD:ACE8548
Schinia sordidus|[3452]|HELNA105-06|United States|Colorado|658[0n]|BOLD:ACE8548
Schinia sordidus|[3453]|HELNA185-06|United States|Oklahoma|592[1n]|BOLD:ACE8548
Schinia sordidus|[3454]|HELNA182-06|United States|Oklahoma|584[12n]|
Schinia sordidus|[3455]|HELNA212-06|United States|Kansas|658[0n]|BOLD:ACE8548
Schinia sordidus|[3456]|HELNA506-09|United States|North Carolina|614[0n]|BOLD:AAB9590
Schinia sordidus|[3457]|HELNA302-09|United States|Georgia|658[2n]|BOLD:AAB9590
Schinia sordidus|[3458]|HELNA503-09|United States|North Carolina|658[0n]|BOLD:AAB9590
Schinia sordidus|[3459]|HELNA299-09|United States|Georgia|658[0n]|BOLD:AAB9590
Schinia sordidus|[3460]|LNCB730-09|United States|North Carolina|650[1n]|BOLD:AAB9590
Schinia sordidus|[3461]|LNCB812-09|United States|North Carolina|658[0n]|BOLD:AAB9590
Schinia sordidus|[3462]|RDNML156-13|United States|North Carolina|658[0n]|BOLD:AAB9590
Schinia sordidus|[3463]|LNCC1510-13|United States|North Carolina|658[0n]|BOLD:AAB9590
Schinia sordidus|[3464]|LNCC1511-13|United States|North Carolina|658[0n]|BOLD:AAB9590
Schinia bimatris|[3465]|RDNME199-07|Canada|Manitoba|658[0n]|BOLD:AAD3739
Schinia bimatris|[3466]|RDNME197-07|United States|Mississippi|616[3n]|BOLD:AAD3739
Schinia bimatris|[3467]|RDNME195-07|United States|Mississippi|658[0n]|BOLD:AAD3739
Schinia bimatris|[3468]|RDNME196-07|United States|Mississippi|658[0n]|BOLD:AAD3739
Schinia bimatris|[3469]|RDNME198-07|United States|Mississippi|658[0n]|BOLD:AAD3739
Schinia bimatris|[3470]|RDNME200-07|Canada|Manitoba|592[0n]|BOLD:AAD3739
Schinia bimatris|[3471]|RDNME205-07|Canada|Manitoba|614[0n]|BOLD:AAD3739
Schinia grandimedia|[3472]|HELNA052-06|United States|Colorado|658[0n]|BOLD:AAC3314
Schinia grandimedia|[3473]|HELNA218-06|United States|Colorado|658[0n]|BOLD:AAC3314
Schinia sp.|[3474]|CNCLB126-14|United States|Texas|584[0n]|BOLD:ACM4323
Schinia rivulosa|[3475]|LSUSA024-06|United States|Kentucky|657[0n]|BOLD:AAB5917
Schinia rivulosa|[3476]|BBLOD1449-11|United States|Oklahoma|658[0n]|BOLD:AAB5917
Schinia rivulosa|[3477]|HELNA341-09|United States|Maryland|658[0n]|BOLD:AAB5917
Schinia rivulosa|[3478]|HELNA342-09|United States|Maryland|634[1n]|BOLD:AAB5917
Schinia rivulosa|[3479]|BBLOD1561-11|United States|Oklahoma|658[0n]|BOLD:AAB5917
Schinia rivulosa|[3480]|BBLOD1335-11|United States|Oklahoma|658[0n]|BOLD:AAB5917
Schinia rivulosa|[3481]|LPOKD012-09|United States|Oklahoma|658[0n]|BOLD:AAB5917

Schinia rivulosa|[3479]|BBLOD1561-11|United States|Oklahoma|658[0n]|BOLD:AAB5917
Schinia rivulosa|[3480]|BBLOD1335-11|United States|Oklahoma|658[0n]|BOLD:AAB5917
Schinia rivulosa|[3481]|LPOKD012-09|United States|Oklahoma|658[0n]|BOLD:AAB5917
Schinia rivulosa|[3482]|LPOKA185-08|United States|Oklahoma|658[0n]|BOLD:AAB5917
Schinia rivulosa|[3483]|LPOKA073-08|United States|Oklahoma|658[0n]|BOLD:AAB5917
Schinia rivulosa|[3484]|RDLQB839-05|Canada|Quebec|658[0n]|BOLD:AAB5917
Schinia rivulosa|[3485]|BBLOD1574-11|United States|Oklahoma|658[0n]|BOLD:AAB5917
Schinia rivulosa|[3486]|BBLOE1695-12|United States|Oklahoma|658[0n]|BOLD:AAB5917
Schinia rivulosa|[3487]|LPOKA131-08|United States|Oklahoma|658[0n]|BOLD:AAB5917
Schinia rivulosa|[3488]|HELNA092-06|United States|Colorado|658[0n]|BOLD:AAB5917
Schinia rivulosa|[3489]|LSUSA086-06|United States|Illinois|658[0n]|BOLD:AAB5917
Schinia rivulosa|[3490]|LPOKA488-09|United States|Oklahoma|658[0n]|BOLD:AAB5917
Schinia rivulosa|[3491]|HELNA535-09|United States|Maryland|658[0n]|BOLD:AAB5917
Schinia rivulosa|[3492]|HELNA533-09|United States|Maryland|658[0n]|BOLD:AAB5917
Schinia rivulosa|[3493]|LPOKA173-08|United States|Oklahoma|658[0n]|BOLD:AAB5917
Schinia rivulosa|[3494]|LPOKA151-08|United States|Oklahoma|658[0n]|BOLD:AAB5917
Schinia rivulosa|[3495]|LPOKA063-08|United States|Oklahoma|658[0n]|BOLD:AAB5917
Schinia rivulosa|[3496]|LPOKA040-08|United States|Oklahoma|658[0n]|BOLD:AAB5917
Schinia rivulosa|[3497]|HELNA252-06|United States|Kansas|658[0n]|BOLD:AAB5917
Schinia rivulosa|[3498]|HELNA091-06|United States|Colorado|658[0n]|BOLD:AAB5917
Schinia rivulosa|[3499]|HELNA090-06|United States|Colorado|658[0n]|BOLD:AAB5917
Schinia rivulosa|[3500]|LPOKA007-08|United States|Oklahoma|658[0n]|BOLD:AAB5917
Schinia rivulosa|[3501]|LGSMG931-10|United States|North Carolina|640[0n]|BOLD:AAB5917
Schinia rivulosa|[3502]|LNCC359-10|United States|North Carolina|658[0n]|BOLD:AAB5917
Schinia rivulosa|[3503]|LILLA804-11|United States|Illinois|658[0n]|BOLD:AAB5917
Schinia rivulosa|[3504]|LILLA940-11|United States|Illinois|658[0n]|BOLD:AAB5917
Schinia rivulosa|[3505]|BBLOD1445-11|United States|Oklahoma|658[0n]|BOLD:AAB5917
Schinia rivulosa|[3506]|BBLOD1591-11|United States|Oklahoma|658[0n]|BOLD:AAB5917
Schinia rivulosa|[3507]|LGSMG930-10|United States|North Carolina|658[0n]|BOLD:AAB5917
Schinia rivulosa|[3508]|BBLOE1696-12|United States|Oklahoma|658[0n]|BOLD:AAB5917
Schinia accessa|[3509]|CMAZA1096-12|United States|Arizona|658[0n]|BOLD:ABW6340
Schinia gracilenta|[3510]|BBLOD1580-11|United States|Oklahoma|611[0n]|BOLD:AAB4299
Schinia gracilenta|[3511]|BBLOD1556-11|United States|Oklahoma|610[0n]|BOLD:AAB4299
Schinia gracilenta|[3512]|LPOKA469-09|United States|Oklahoma|658[0n]|BOLD:AAB4299
Schinia gracilenta|[3513]|LPOKA446-09|United States|Oklahoma|658[0n]|BOLD:AAB4299
Schinia gracilenta|[3514]|LPOKA180-08|United States|Oklahoma|658[0n]|BOLD:AAB4299
Schinia gracilenta|[3515]|LPOKA156-08|United States|Oklahoma|658[0n]|BOLD:AAB4299
Schinia gracilenta|[3516]|LPOKA150-08|United States|Oklahoma|658[0n]|BOLD:AAB4299
Schinia gracilenta|[3517]|LPOKA138-08|United States|Oklahoma|658[0n]|BOLD:AAB4299
Schinia gracilenta|[3518]|LPOKA107-08|United States|Oklahoma|658[0n]|BOLD:AAB4299
Schinia gracilenta|[3519]|LPOKA058-08|United States|Oklahoma|658[0n]|BOLD:AAB4299
Schinia gracilenta|[3520]|LPOKA044-08|United States|Oklahoma|658[0n]|BOLD:AAB4299
Schinia gracilenta|[3521]|LPOKA043-08|United States|Oklahoma|658[0n]|BOLD:AAB4299
Schinia gracilenta|[3522]|LPOKA021-08|United States|Oklahoma|658[0n]|BOLD:AAB4299
Schinia gracilenta|[3523]|HELNA263-06|United States|Kansas|658[0n]|BOLD:AAB4299
Schinia gracilenta|[3524]|HELNA262-06|United States|Oklahoma|658[0n]|BOLD:AAB4299
Schinia gracilenta|[3525]|HELNA047-06|United States|Colorado|658[0n]|BOLD:AAB4299
Schinia gracilenta|[3526]|LPOKA104-08|United States|Oklahoma|656[0n]|BOLD:AAB4299
Schinia gracilenta|[3527]|LPOKA536-09|United States|Oklahoma|658[0n]|BOLD:AAB4299
Schinia gracilenta|[3528]|LPOKD158-09|United States|Oklahoma|658[0n]|BOLD:AAB4299
Schinia gracilenta|[3529]|BBLOD1566-11|United States|Oklahoma|658[0n]|BOLD:AAB4299
Schinia gracilenta|[3530]|BBLOD1587-11|United States|Oklahoma|658[0n]|BOLD:AAB4299
Schinia gracilenta|[3531]|HELNA543-09|United States|Maryland|658[0n]|BOLD:AAB4299
Schinia gracilenta|[3532]|HELNA544-09|United States|Maryland|596[0n]|BOLD:AAB4299
Schinia gracilenta|[3533]|LSUSA108-06|United States|Illinois|658[0n]|BOLD:AAB4299
Schinia gracilenta|[3534]|LPOKA077-08|United States|Oklahoma|658[0n]|BOLD:AAB4299
Schinia gracilenta|[3535]|HELNA294-09|United States|Georgia|658[0n]|BOLD:AAB4299
Schinia gracilenta|[3536]|HELNA542-09|United States|Maryland|658[0n]|BOLD:AAB4299
Schinia gracilenta|[3537]|BBLOD1589-11|United States|Oklahoma|658[0n]|BOLD:AAB4299
Schinia gracilenta|[3538]|LNAUP167-13|United States|Maryland|658[0n]|BOLD:AAB4299
Schinia coercita|[3539]|HELNA030-06|United States|New Mexico|658[0n]|BOLD:AAD6835
Schinia coercita|[3540]|HELNA540-09|United States|New Mexico|658[0n]|BOLD:AAD6835
Schinia crenilinea|[3541]|CNCLB2190-14|United States|Arkansas|307[0n]|
Schinia crenilinea|[3542]|CNCLB2191-14|United States|Arkansas|307[0n]|
Schinia crenilinea|[3543]|CNCLB2192-14|United States|Texas|658[0n]|BOLD:ACR9032
Schinia crenilinea|[3544]|CNCLB2404-14|United States|Texas|604[1n]|BOLD:ACR9032
Schinia diffusa|[3545]|HELNA040-06|United States|Colorado|658[0n]|BOLD:AAE3918
Schinia diffusa|[3546]|HELNA039-06|United States|Colorado|658[0n]|BOLD:AAE3918
Schinia diffusa|[3547]|HELNA230-06|United States|Colorado|650[0n]|BOLD:AAE3918
Schinia diffusa|[3548]|HELNA231-06|United States|Colorado|658[0n]|BOLD:AAE3918
Schinia arcigera|[3549]|LSEU070-06|United States|North Carolina|555[0n]|BOLD:AAB0117
Schinia arcigera|[3550]|LNCB785-09|United States|North Carolina|658[1n]|BOLD:AAB0117
Schinia arcigera|[3551]|LSUSA088-06|United States|Kentucky|658[0n]|BOLD:AAB0117
Schinia arcigera|[3552]|HELNA165-06|United States|Arkansas|612[1n]|BOLD:AAB0117
Schinia arcigera|[3553]|XAG712-05|Canada|Ontario|658[0n]|BOLD:AAB0117
Schinia arcigera|[3554]|XAI046-05|Canada|Ontario|658[0n]|BOLD:AAB0117
Schinia arcigera|[3555]|LNC433-05|United States|North Carolina|658[0n]|BOLD:AAB0117
Schinia arcigera|[3556]|LSUSA025-06|United States|Kentucky|657[0n]|BOLD:AAB0117
Schinia arcigera|[3557]|LPOKA269-08|United States|Oklahoma|658[0n]|BOLD:AAB0117
Schinia arcigera|[3558]|XAG674-05|Canada|Ontario|624[3n]|BOLD:AAB0117
Schinia arcigera|[3559]|PHMO310-03|Canada|Ontario|639[0n]|BOLD:AAB0117
Schinia arcigera|[3560]|PHMO318-03|Canada|Ontario|639[0n]|BOLD:AAB0117
Schinia arcigera|[3561]|LNC432-05|United States|North Carolina|658[0n]|BOLD:AAB0117
Schinia arcigera|[3562]|HELNA300-09|United States|Georgia|658[0n]|BOLD:AAB0117
Schinia arcigera|[3563]|XAH055-05|Canada|Ontario|658[0n]|BOLD:AAB0117
Schinia arcigera|[3564]|XAG021-05|Canada|Ontario|658[0n]|BOLD:AAB0117
Schinia arcigera|[3565]|XAH041-05|Canada|Ontario|658[2n]|BOLD:AAB0117
Schinia arcigera|[3566]|XAH086-05|Canada|Ontario|635[0n]|BOLD:AAB0117
Schinia arcigera|[3567]|LSEU069-06|United States|Georgia|605[0n]|BOLD:AAB0117
Schinia arcigera|[3568]|HELNA130-06|United States|Tennessee|658[0n]|BOLD:AAB0117
Schinia arcigera|[3569]|LPOKA557-09|United States|Oklahoma|658[0n]|BOLD:AAB0117
Schinia arcigera|[3570]|HELNA335-09|United States|Georgia|658[0n]|BOLD:AAB0117
Schinia arcigera|[3571]|HELNA336-09|United States|Georgia|658[0n]|BOLD:AAB0117
Schinia arcigera|[3572]|HELNA337-09|United States|Maryland|658[0n]|BOLD:AAB0117
Schinia arcigera|[3573]|LPOKD245-09|United States|Oklahoma|657[0n]|BOLD:AAB0117
Schinia arcigera|[3574]|HELNA166-06|United States|Kansas|658[0n]|BOLD:AAB0117
Schinia arcigera|[3575]|HELNA131-06|United States|Tennessee|658[0n]|BOLD:AAB0117
Schinia arcigera|[3576]|HELNA338-09|United States|Maryland|636[0n]|BOLD:AAB0117
Schinia arcigera|[3577]|LILLA816-11|United States|Illinois|658[0n]|BOLD:AAB0117
Schinia ferricasta|[3578]|HELNA282-09|United States|Utah|658[0n]|BOLD:ACE9276
Schinia ferricasta|[3579]|CMAZA611-10|United States|Arizona|658[0n]|BOLD:ACE9276
Schinia ferricasta|[3580]|JBAZ172-09|United States|Arizona|658[0n]|BOLD:ACE9276
Schinia ferricasta|[3581]|JBAZ171-09|United States|Arizona|658[0n]|BOLD:ACE9276

Schinia ferricasta|[3578]|HELNA282-09|United States|Utah|658[0n]|BOLD:ACE9276
Schinia ferricasta|[3579]|CMAZA611-10|United States|Arizona|658[0n]|BOLD:ACE9276
Schinia ferricasta|[3580]|JBAZ172-09|United States|Arizona|658[0n]|BOLD:ACE9276
Schinia ferricasta|[3581]|JBAZ171-09|United States|Arizona|658[0n]|BOLD:ACE9276
Schinia ferricasta|[3582]|JBAZ170-09|United States|Arizona|658[0n]|BOLD:ACE9276
Schinia ferricasta|[3583]|JBAZ169-09|United States|Arizona|639[0n]|BOLD:ACE9276
Schinia ferricasta|[3584]|HELNA164-06|United States|Utah|619[2n]|BOLD:ACE9276
Schinia ferricasta|[3585]|CMAZA584-10|United States|Arizona|658[0n]|BOLD:ACE9276
Schinia ferricasta|[3586]|HELNA932-10|United States|Arizona|658[0n]|BOLD:ACE9276
Schinia ferricasta|[3587]|HELNA933-10|United States|Arizona|658[0n]|BOLD:ACE9276
Schinia ferricasta|[3588]|CMAZA1078-12|United States|Arizona|658[0n]|BOLD:ACE9276
Schinia ferricasta|[3589]|CMAZA1116-12|United States|Arizona|658[0n]|BOLD:ACE9276
Schinia ferricasta|[3590]|CMAZA1124-12|United States|Arizona|658[0n]|BOLD:ACE9276
Schinia sara|[3591]|HELNA225-06|United States|Colorado|658[0n]|BOLD:AAC9841
Schinia sara|[3592]|HELNA226-06|United States|Oklahoma|658[0n]|BOLD:AAC9841
Schinia sara|[3593]|JBAZ161-09|United States|Arizona|658[0n]|BOLD:AAC9841
Schinia n. sp. 2|[3594]|HELNA283-09|United States|Utah|658[0n]|BOLD:ABX6394
Schinia n. sp. 2|[3595]|HELNA928-10|United States|California|658[0n]|BOLD:ABX6394
Schinia n. sp. 2|[3596]|HELNA929-10|United States|California|658[0n]|BOLD:ABX6394
Schinia n. sp. 2|[3597]|HELNA930-10|United States|California|658[0n]|BOLD:ABX6394
Schinia n. sp. 2|[3598]|HELNA931-10|United States|California|658[0n]|BOLD:ABX6394
Schinia trifascia|[3599]|LSUSA056-06|United States|Florida|535[0n]|BOLD:AAC4627
Schinia trifascia|[3600]|LSUSA089-06|United States|Kentucky|658[0n]|BOLD:AAC4627
Schinia trifascia|[3601]|HELNA548-09|United States|Maryland|658[0n]|BOLD:AAC4627
Schinia trifascia|[3602]|LNC435-05|United States|North Carolina|658[0n]|BOLD:AAC4627
Schinia trifascia|[3603]|LNC580-06|United States|North Carolina|658[0n]|BOLD:AAC4627
Schinia trifascia|[3604]|LNC581-06|United States|North Carolina|658[0n]|BOLD:AAC4627
Schinia trifascia|[3605]|HELNA343-09|United States|Virginia|658[0n]|BOLD:AAC4627
Schinia trifascia|[3606]|HELNA344-09|United States|Florida|658[0n]|BOLD:AAC4627
Schinia trifascia|[3607]|HELNA345-09|United States|Florida|658[0n]|BOLD:AAC4627
Schinia trifascia|[3608]|LGSMG932-10|United States|Tennessee|658[0n]|BOLD:AAC4627
Schinia trifascia|[3609]|LGSMG933-10|United States|Tennessee|658[0n]|BOLD:AAC4627
Schinia trifascia|[3610]|HELNA890-10|Canada|Ontario|658[0n]|BOLD:AAC4627
Schinia trifascia|[3611]|HELNA891-10|Canada|Ontario|658[0n]|BOLD:AAC4627
Schinia trifascia|[3612]|HELNA892-10|Canada|Ontario|658[0n]|BOLD:AAC4627
Schinia trifascia|[3613]|LNC436-05|United States|North Carolina|626[0n]|BOLD:AAC4627
Schinia trifascia|[3614]|HELNA893-10|Canada|Ontario|562[0n]|BOLD:AAC4627
Schinia trifascia|[3615]|LILLA976-11|United States|Illinois|658[0n]|BOLD:AAC4627
Schinia parmeliana|[3616]|LPOKE237-10|United States|Oklahoma|658[0n]|BOLD:AAH0494
Schinia parmeliana|[3617]|HELNA176-06|United States|Arkansas|658[0n]|BOLD:AAH0494
Schinia parmeliana|[3618]|LNAUS3949-13|United States|South Carolina|658[1n]|BOLD:AAH0494
Schinia wiklei|[3619]|CNCLB3081-15|United States|Utah|658[0n]|BOLD:ACR9485
Schinia wiklei|[3620]|CNCLB2539-14|United States|Utah|658[0n]|BOLD:ACR9485
Schinia wiklei|[3621]|CNCLB3082-15|United States|Utah|658[0n]|BOLD:ACR9485
Schinia grandimedia|[3622]|HELNA051-06|United States|Kansas|658[15n]|
Schinia grandimedia|[3623]|HELNA219-06|United States|Colorado|658[0n]|BOLD:AAC3313
Schinia grandimedia|[3624]|HELNA220-06|United States|Colorado|658[0n]|BOLD:AAC3313
Schinia grandimedia|[3625]|HELNA054-06|United States|Oklahoma|658[0n]|BOLD:AAC3313
Schinia grandimedia|[3626]|HELNA053-06|United States|Colorado|658[0n]|BOLD:AAC3313
Schinia grandimedia|[3627]|HELNA050-06|United States|Nebraska|658[0n]|BOLD:AAC3313
Schinia grandimedia|[3628]|HELNA049-06|United States|Colorado|658[0n]|BOLD:AAC3313
Schinia grandimedia|[3629]|HELNA048-06|United States|Colorado|658[0n]|BOLD:AAC3313
Schinia grandimedia|[3630]|HELNA221-06|United States|Colorado|658[0n]|BOLD:AAC3313
Schinia grandimedia|[3631]|HELNA222-06|United States|Arizona|658[0n]|BOLD:AAC3313
Schinia grandimedia|[3632]|CMAZA1089-12|United States|Arizona|658[0n]|BOLD:AAC3313
Schinia n. sp. 1|[3633]|HELNA546-09|United States|New Mexico|658[0n]|BOLD:ABX6151
Schinia n. sp. 1|[3634]|LNAUP087-13|United States|New Mexico|658[0n]|BOLD:ABX6151
Schinia n. sp. 1|[3635]|HELNA082-06|United States|New Mexico|658[0n]|BOLD:ABX6151
Schinia n. sp. 1|[3636]|HELNA083-06|United States|New Mexico|658[0n]|BOLD:ABX6151
Schinia n. sp. 1|[3637]|HELNA545-09|United States|New Mexico|658[0n]|BOLD:ABX6151
Schinia n. sp. 1|[3638]|HELNA547-09|United States|New Mexico|658[0n]|BOLD:ABX6151
Schinia n. sp. 1|[3639]|HELNA951-10|United States|New Mexico|658[0n]|BOLD:ABX6151
Schinia n. sp. 1|[3640]|HELNA952-10|United States|New Mexico|658[0n]|BOLD:ABX6151
Schinia n. sp. 1|[3641]|LNAUP088-13|United States|New Mexico|658[0n]|BOLD:ABX6151
Schinia n. sp. 1|[3642]|LNAUP089-13|United States|New Mexico|658[0n]|BOLD:ABX6151
Schinia n. sp. 1|[3643]|LNAUP090-13|United States|New Mexico|658[0n]|BOLD:ABX6151
Schinia n. sp. 1|[3644]|LNAUP091-13|United States|New Mexico|658[0n]|BOLD:ABX6151
Schinia cumatilis|[3645]|HELNA036-06|United States|New Mexico|658[1n]|BOLD:ABZ3209
Schinia cumatilis|[3646]|RDMAB502-06|Canada|Alberta|658[0n]|BOLD:ABZ3209
Schinia cumatilis|[3647]|HELNA034-06|United States|Colorado|658[0n]|BOLD:ABZ3209
Schinia cumatilis|[3648]|HELNA033-06|United States|Colorado|658[0n]|BOLD:ABZ3209
Schinia cumatilis|[3649]|HELNA035-06|United States|Nebraska|658[0n]|BOLD:ABZ3209
Schinia cumatilis|[3650]|HELNA197-06|United States|Colorado|658[0n]|BOLD:ABZ3209
Schinia cumatilis|[3651]|HELNA198-06|United States|New Mexico|658[0n]|BOLD:ABZ3209
Schinia illustra|[3652]|HELNA060-06|United States|Colorado|658[0n]|BOLD:AAF2442
Schinia illustra|[3653]|HELNA061-06|United States|Wyoming|658[0n]|BOLD:AAF2442
Schinia illustra|[3654]|HELNA224-06|United States|Colorado|658[0n]|BOLD:AAF2442
Schinia reniformis|[3655]|HELNA848-10|United States|Colorado|658[0n]|BOLD:AAD6816
Schinia reniformis|[3656]|HELNA204-06|United States|Colorado|658[0n]|BOLD:AAD6816
Schinia reniformis|[3657]|HELNA087-06|United States|Colorado|658[0n]|BOLD:AAD6816
Schinia reniformis|[3658]|HELNA089-06|United States|New Mexico|656[0n]|BOLD:AAD6816
Schinia reniformis|[3659]|HELNA205-06|United States|Colorado|658[0n]|BOLD:AAD6816
Schinia reniformis|[3660]|HELNA206-06|United States|Colorado|658[0n]|BOLD:AAD6816
Schinia reniformis|[3661]|HELNA088-06|United States|Colorado|658[0n]|BOLD:AAD6816
Schinia reniformis|[3662]|HELNA849-10|United States|Colorado|658[0n]|BOLD:AAD6816
Schinia reniformis|[3663]|HELNA852-10|United States|Colorado|658[0n]|BOLD:AAD6816
Schinia new sp.|[3664]|HELNA068-06|United States|New Mexico|658[0n]|BOLD:ABX5869
Schinia new sp.|[3665]|HELNA127-06|United States|New Mexico|658[0n]|BOLD:ABX5869
Schinia new sp.|[3666]|HELNA128-06|United States|New Mexico|658[0n]|BOLD:ABX5869
Schinia new sp.|[3667]|HELNA202-06|United States|Colorado|658[0n]|BOLD:ABX5869
Schinia new sp.|[3668]|HELNA203-06|United States|New Mexico|656[0n]|BOLD:ABX5869
Schinia hulstia|[3669]|HELNA056-06|United States|Colorado|656[4n]|BOLD:ABX5869
Schinia hulstia|[3670]|HELNA057-06|United States|Colorado|658[0n]|BOLD:ABX5869
Schinia hulstia|[3671]|HELNA058-06|United States|Colorado|658[0n]|BOLD:ABX5869
Schinia hulstia|[3672]|HELNA059-06|United States|Oklahoma|658[0n]|BOLD:ABX5869
Schinia hulstia|[3673]|JBAZ156-09|United States|Arizona|658[0n]|BOLD:ABX5869
Schinia hulstia|[3674]|JBAZ157-09|United States|Arizona|658[0n]|BOLD:ABX5869
Schinia hulstia|[3675]|CMAZA1110-12|United States|Arizona|658[0n]|BOLD:ABX5869
Schinia saturata|[3676]|HELNA944-10|United States|Maryland|573[0n]|BOLD:AAC2307
Schinia saturata|[3677]|LSUSA066-06|United States|Florida|658[0n]|BOLD:AAC2307
Schinia saturata|[3678]|HELNA512-09|United States|Maryland|658[0n]|BOLD:AAC2307
Schinia saturata|[3679]|HELNA513-09|United States|Maryland|658[0n]|BOLD:AAC2307
Schinia saturata|[3680]|HELNA514-09|United States|Maryland|658[1n]|BOLD:AAC2307

Schinia saturata|[3678]|HELNA512-09|United States|Maryland|658[0n]|BOLD:AAC2307
Schinia saturata|[3679]|HELNA513-09|United States|Maryland|658[0n]|BOLD:AAC2307
Schinia saturata|[3680]|HELNA514-09|United States|Maryland|658[1n]|BOLD:AAC2307
Schinia saturata|[3681]|RDNMD504-06|United States|Florida|658[0n]|BOLD:AAC2307
Schinia saturata|[3682]|LSUSA065-06|United States|Florida|658[0n]|BOLD:AAC2307
Schinia saturata|[3683]|HELNA945-10|United States|Maryland|658[0n]|BOLD:AAC2307
Schinia saturata|[3684]|HELNA096-06|United States|Colorado|658[0n]|BOLD:AAC2307
Schinia saturata|[3685]|HELNA095-06|United States|Colorado|658[0n]|BOLD:AAC2307
Schinia saturata|[3686]|HELNA264-06|United States|Oklahoma|658[0n]|BOLD:AAC2307
Schinia saturata|[3687]|HELNA265-06|United States|Colorado|658[0n]|BOLD:AAC2307
Schinia saturata|[3688]|HELNA957-10|United States|Colorado|658[0n]|BOLD:AAC2307
Schinia saturata|[3689]|HELNA958-10|United States|Colorado|658[0n]|BOLD:AAC2307
Schinia saturata|[3690]|HELNA959-10|United States|Colorado|658[0n]|BOLD:AAC2307
Schinia saturata|[3691]|HELNA860-10|United States|Arizona|574[0n]|BOLD:AAC2307
Schinia saturata|[3692]|HELNA098-06|United States|Colorado|658[0n]|BOLD:AAC2307
Schinia saturata|[3693]|HELNA097-06|United States|Colorado|658[0n]|BOLD:AAC2307
Schinia saturata|[3694]|CMAZA973-12|United States|Arizona|658[0n]|BOLD:AAC2307
Schinia saturata|[3695]|HELNA859-10|United States|Arizona|658[0n]|BOLD:AAC2307
Schinia saturata|[3696]|HELNA861-10|United States|Arizona|658[0n]|BOLD:AAC2307
Schinia saturata|[3697]|CMAZA978-12|United States|Arizona|658[0n]|BOLD:AAC2307
Schinia saturata|[3698]|CMAZA1070-12|United States|Arizona|658[0n]|BOLD:AAC2307
Schinia buta|[3699]|LNAUS4058-13|United States|California|658[0n]|BOLD:ACJ9328
Schinia buta|[3700]|LNAUS4059-13|United States|California|658[0n]|BOLD:ACJ9328
Schinia buta|[3701]|CNCLB104-14|United States|California|658[0n]|BOLD:ACJ9328
Schinia buta|[3702]|CNCLB105-14|United States|California|525[0n]|
Schinia ciliata|[3703]|HELNA016-06|United States|New Mexico|658[0n]|BOLD:ABX6651
Schinia ciliata|[3704]|HELNA020-06|United States|Colorado|658[0n]|BOLD:ABX6651
Schinia ciliata|[3705]|HELNA015-06|United States|New Mexico|658[0n]|BOLD:ABX6651
Schinia ciliata|[3706]|HELNA017-06|United States|New Mexico|658[0n]|BOLD:ABX6651
Schinia ciliata|[3707]|HELNA021-06|United States|Colorado|655[0n]|BOLD:ABX6651
Schinia ciliata|[3708]|HELNA286-09|United States|New Mexico|658[0n]|BOLD:ABX6651
Schinia velaris|[3709]|HELNA838-10|United States|California|658[0n]|BOLD:ABZ0046
Schinia chryselloides|[3710]|HELNA199-06|United States|Colorado|658[0n]|BOLD:ABZ3554
Schinia chryselloides|[3711]|HELNA200-06|United States|Colorado|658[0n]|BOLD:ABZ3554
Schinia chryselloides|[3712]|HELNA201-06|United States|Colorado|658[0n]|BOLD:ABZ3554
Schinia chryselloides|[3713]|HELNA333-09|United States|Colorado|658[0n]|BOLD:ABZ3554
Schinia chryselloides|[3714]|HELNA334-09|United States|Colorado|658[0n]|BOLD:ABZ3554
Schinia niveicosta|[3715]|HELNA080-06|United States|California|658[0n]|BOLD:ABX6773
Schinia niveicosta|[3716]|HELNA078-06|United States|California|658[0n]|BOLD:ABX6773
Schinia niveicosta|[3717]|HELNA077-06|United States|California|658[0n]|BOLD:ABX6773
Schinia niveicosta|[3718]|HELNA079-06|United States|California|658[0n]|BOLD:ABX6773
Schinia niveicosta|[3719]|JBAZ188-09|United States|California|658[0n]|BOLD:ABX6773
Schinia niveicosta|[3720]|JBAZ189-09|United States|California|658[0n]|BOLD:ABX6773
Schinia niveicosta|[3721]|AWCLB422-10|United States|Arizona|658[0n]|BOLD:ABX6773
Schinia niveicosta|[3722]|AWCLB462-10|United States|Arizona|658[0n]|BOLD:ABX6773
Schinia alencis|[3723]|HELNA014-06|United States|Colorado|656[0n]|BOLD:AAF2397
Schinia alencis|[3724]|HELNA209-06|United States|Colorado|658[0n]|BOLD:AAF2397
Schinia alencis|[3725]|HELNA210-06|United States|Oklahoma|658[0n]|BOLD:AAF2397
Schinia ciliata|[3726]|CNCLB3079-15|United States|California|658[1n]|BOLD:ACW8347
Schinia ciliata|[3727]|CNCLB3080-15|United States|Arizona|658[0n]|BOLD:ACW8347
Schinia simplex|[3728]|HELNA295-09|United States|South Dakota|658[0n]|BOLD:AAI0818
Schinia simplex|[3729]|RDNME615-08|United States|Colorado|658[0n]|BOLD:AAI0818
Schinia simplex|[3730]|HELNA880-10|United States|Colorado|658[0n]|BOLD:AAI0818
Schinia tobia|[3731]|NAMUM182-08|United States|California|648[0n]|BOLD:AAE3938
Schinia tobia|[3732]|NAMUM180-08|United States|California|658[0n]|BOLD:AAE3938
Schinia tobia|[3733]|NAMUM179-08|United States|California|646[0n]|BOLD:AAE3938
Schinia tobia|[3734]|NAMUM183-08|United States|California|646[0n]|BOLD:AAE3938
Schinia tobia|[3735]|JBAZ167-09|United States|Arizona|658[0n]|BOLD:AAE3938
Schinia roseitincta|[3736]|RDNMF419-08|United States|Texas|620[0n]|BOLD:AAF2082
Schinia roseitincta|[3737]|RDNMF420-08|United States|Texas|621[0n]|BOLD:AAF2082
Schinia roseitincta|[3738]|RDNMF421-08|United States|New Mexico|640[2n]|BOLD:AAF2082
Schinia roseitincta|[3739]|LNAUP094-13|United States|New Mexico|658[1n]|BOLD:AAF2082
Schinia graefiana|[3740]|JBAZ149-09|United States|California|658[0n]|BOLD:AAJ6413
Schinia graefiana|[3741]|HELNA973-10|United States|California|658[0n]|BOLD:AAJ6413
Schinia biforma|[3742]|HELNA177-06|United States|New Mexico|658[0n]|BOLD:AAC3287
Schinia biforma|[3743]|HELNA253-06|United States|Colorado|658[0n]|BOLD:AAC3287
Schinia biforma|[3744]|HELNA178-06|United States|Texas|658[0n]|BOLD:AAC3287
Schinia biforma|[3745]|HELNA254-06|United States|Texas|658[0n]|BOLD:AAC3287
Schinia sanrafaeli|[3746]|HELNA364-09|United States|Utah|658[0n]|BOLD:AAC3287
Schinia sanrafaeli|[3747]|HELNA195-06|United States|Utah|557[7n]|BOLD:AAC3287
Schinia volupia|[3748]|HELNA121-06|United States|New Mexico|656[0n]|BOLD:AAC3287
Schinia volupia|[3749]|HELNA288-09|United States|New Mexico|658[0n]|BOLD:AAC3287
Schinia volupia|[3750]|HELNA122-06|United States|New Mexico|657[0n]|BOLD:AAC3287
Schinia volupia|[3751]|HELNA292-09|United States|New Mexico|658[0n]|BOLD:AAC3287
Schinia volupia|[3752]|HELNA361-09|United States|New Mexico|658[0n]|BOLD:AAC3287
Schinia volupia|[3753]|HELNA193-06|United States|Texas|621[1n]|BOLD:AAC3287
Schinia volupia|[3754]|HELNA192-06|United States|Colorado|657[0n]|BOLD:AAC3287
Schinia volupia|[3755]|HELNA125-06|United States|Colorado|657[0n]|BOLD:AAC3287
Schinia volupia|[3756]|HELNA190-06|United States|New Mexico|606[1n]|BOLD:AAC3287
Schinia fulleri|[3757]|HELNA186-06|United States|Florida|657[0n]|BOLD:AAC3287
Schinia volupia|[3758]|HELNA188-06|United States|Colorado|657[0n]|BOLD:AAC3287
Schinia volupia|[3759]|HELNA126-06|United States|Colorado|657[0n]|BOLD:AAC3287
Schinia volupia|[3760]|HELNA124-06|United States|Colorado|657[0n]|BOLD:AAC3287
Schinia volupia|[3761]|HELNA123-06|United States|Colorado|657[0n]|BOLD:AAC3287
Schinia miniana|[3762]|HELNA155-06|United States|New Mexico|609[0n]|BOLD:AAC3287
Schinia volupia|[3763]|HELNA189-06|United States|Colorado|656[0n]|BOLD:AAC3287
Schinia volupia|[3764]|HELNA191-06|United States|New Mexico|656[0n]|BOLD:AAC3287
Schinia volupia|[3765]|HELNA194-06|United States|Colorado|656[0n]|BOLD:AAC3287
Schinia volupia|[3766]|HELNA256-06|United States|Colorado|657[0n]|BOLD:AAC3287
Schinia volupia|[3767]|HELNA258-06|United States|Colorado|657[0n]|BOLD:AAC3287
Schinia fulleri|[3768]|HELNA296-09|United States|Florida|658[0n]|BOLD:AAC3287
Schinia miniana|[3769]|HELNA305-09|United States|Texas|658[0n]|BOLD:AAC3287
Schinia sanrafaeli|[3770]|HELNA362-09|United States|New Mexico|658[0n]|BOLD:AAC3287
Schinia volupia|[3771]|HELNA396-09|United States|Louisiana|658[0n]|BOLD:AAC3287
Schinia volupia|[3772]|TAMIC937-10|United States|Texas|658[0n]|BOLD:AAC3287
Schinia miniana|[3773]|CMAZA1176-12|United States|Arizona|658[0n]|BOLD:AAC3287
Schinia miniana|[3774]|HELNA157-06|United States|Nevada|621[0n]|BOLD:AAC3287
Schinia miniana|[3775]|HELNA158-06|United States|Nevada|658[0n]|BOLD:AAC3287
Schinia miniana|[3776]|HELNA156-06|United States|Nevada|658[0n]|BOLD:AAC3287
Schinia miniana|[3777]|HELNA159-06|United States|Arizona|632[0n]|BOLD:AAC3287
Schinia sanrafaeli|[3778]|HELNA196-06|United States|Utah|658[0n]|BOLD:AAC3287
Schinia volupia|[3779]|HELNA363-09|United States|Utah|658[0n]|BOLD:AAC3287
Schinia miniana|[3780]|CMAZA1177-12|United States|Arizona|658[0n]|BOLD:AAC3287

Schinia miniana|[3777]|HELNA159-06|United States|Arizona|652[0n]|BOLD:AAC3287
Schinia sanrafaeli|[3778]|HELNA196-06|United States|Utah|658[0n]|BOLD:AAC3287
Schinia volupia|[3779]|HELNA363-09|United States|Utah|658[0n]|BOLD:AAC3287
Schinia miniana|[3780]|CMAZA1177-12|United States|Arizona|658[0n]|BOLD:AAC3287
Schinia antonio|[3781]|USLEP509-10|United States|Texas|658[0n]|BOLD:AAN6812
Schinia antonio|[3782]|CNCLB100-14|United States|Texas|658[0n]|BOLD:AAN6812
Schinia sexplagiata|[3783]|HELNA100-06|United States|Colorado|658[0n]|BOLD:AAI0819
Schinia sexplagiata|[3784]|HELNA215-06|United States|Colorado|658[0n]|BOLD:AAI0819
Schinia meadi|[3785]|RDMAB523-06|Canada|Alberta|658[0n]|BOLD:AAF2436
Schinia meadi|[3786]|HELNA233-06|United States|Colorado|616[0n]|BOLD:AAF2436
Schinia meadi|[3787]|HELNA234-06|United States|Colorado|658[0n]|BOLD:AAF2436
Schinia aurantiaca|[3788]|NAMUM252-08|United States|Arizona|658[0n]|BOLD:AAJ6403
Schinia aurantiaca|[3789]|HELNA968-10|United States|California|658[0n]|BOLD:AAJ6403
Schinia olivacea|[3790]|NOCNA036-14|United States|Texas|658[0n]|BOLD:ACM4481
Schinia olivacea|[3791]|NOCNA035-14|United States|Texas|658[0n]|BOLD:ACM4481
Schinia olivacea|[3792]|CNCLB118-14|United States|Texas|658[0n]|BOLD:ACM4481
Schinia olivacea|[3793]|CNCLB119-14|United States|Texas|658[0n]|BOLD:ACM4481
Schinia snowi|[3794]|HELNA213-06|United States|Texas|658[0n]|BOLD:AAE9321
Schinia snowi|[3795]|HELNA354-09|United States|Oklahoma|658[0n]|BOLD:AAE9321
Schinia snowi|[3796]|HELNA353-09|United States|Oklahoma|658[0n]|BOLD:AAE9321
Schinia snowi|[3797]|HELNA355-09|United States|Colorado|658[0n]|BOLD:AAE9321
Heliolonche carolus|[3798]|HELNA135-06|United States|Arizona|656[7n]|
Heliolonche carolus|[3799]|HELNA134-06|United States|Arizona|656[4n]|BOLD:AAF1522
Heliolonche carolus|[3800]|RDNME987-08|United States|Arizona|658[0n]|BOLD:AAF1522
Heliolonche modicella|[3801]|NAMUM328-08|United States|California|658[0n]|BOLD:AAI6735
Heliolonche modicella|[3802]|GBGL5921-09|||683[0n]|BOLD:AAI6735
Schinia macneilli|[3803]|HELNA970-10|United States|California|658[0n]|BOLD:AAO0093
Heliolonche pictipennis|[3804]|NAMUM361-08|United States|California|658[0n]|BOLD:AAE3142
Heliolonche pictipennis|[3805]|NAMUM362-08|United States|California|658[0n]|BOLD:AAE3142
Heliolonche pictipennis|[3806]|HELNA376-09|United States|Utah|658[0n]|BOLD:AAE3142
Heliolonche pictipennis|[3807]|HELNA377-09|United States|Utah|658[0n]|BOLD:AAE3142
Heliolonche celeris|[3808]|LTOLB1264-11|United States|California|658[0n]|BOLD:AAY5324
Heliolonche celeris|[3809]|LTOLB1265-11|United States|California|658[0n]|BOLD:AAY5324
Heliolonche celeris|[3810]|LTOLB1266-11|United States|California|658[0n]|BOLD:AAY5324
Heliolonche celeris|[3811]|LTOLB1267-11|United States|California|658[0n]|BOLD:AAY5324
Heliolonche joaquinensis|[3812]|CNCLB2181-14|United States|California|658[0n]|BOLD:AAY5324
Schinia persimilis|[3813]|RDNME247-07|Canada|Alberta|616[1n]|BOLD:AAI0752
Schinia persimilis|[3814]|RDNME359-07|Canada|Yukon Territory|658[0n]|BOLD:AAI0752
Schinia persimilis|[3815]|LTOLB1304-11|United States|Colorado|658[0n]|BOLD:AAI0752
Schinia persimilis|[3816]|LTOLB1305-11|United States|Colorado|658[0n]|BOLD:AAI0752
Schinia verna|[3817]|RDNMF776-08|Canada|Alberta|658[0n]|BOLD:AAF2456
Schinia verna|[3818]|RDNMF777-08|Canada|Alberta|658[0n]|BOLD:AAF2456
Schinia honesta|[3819]|RDNMF049-08|Canada|British Columbia|658[0n]|BOLD:AAF2456
Schinia honesta|[3820]|RDMAB1027-09|Canada|Alberta|658[0n]|BOLD:AAF2456
Schinia honesta|[3821]|HELNA271-09|United States|California|658[0n]|BOLD:AAF2456
Schinia citrinellus|[3822]|CMAZA593-10|United States|Arizona|658[0n]|BOLD:AAD3752
Schinia citrinellus|[3823]|HELNA538-09|United States|New Mexico|658[0n]|BOLD:AAD3752
Schinia citrinellus|[3824]|HELNA287-09|United States|New Mexico|658[1n]|BOLD:AAD3752
Schinia citrinellus|[3825]|HELNA223-06|United States|Texas|658[0n]|BOLD:AAD3752
Schinia citrinellus|[3826]|HELNA229-06|United States|Texas|658[0n]|BOLD:AAD3752
Schinia citrinellus|[3827]|HELNA027-06|United States|Colorado|658[0n]|BOLD:AAD3752
Schinia citrinellus|[3828]|HELNA537-09|United States|New Mexico|658[0n]|BOLD:AAD3752
Schinia citrinellus|[3829]|BBLSW068-09|United States|Texas|658[0n]|BOLD:AAD3752
Schinia citrinellus|[3830]|HELNA026-06|United States|Colorado|658[0n]|BOLD:AAD3752
Schinia citrinellus|[3831]|BBLSX234-09|United States|Texas|658[0n]|BOLD:AAD3752
Schinia citrinellus|[3832]|RDNMK024-11|United States|Arizona|658[0n]|BOLD:AAD3752
Schinia suetus|[3833]|NAMUM329-08|United States|California|658[0n]|BOLD:ACE9042
Schinia suetus|[3834]|NAMUM331-08|United States|California|658[0n]|BOLD:ACE9042
Schinia suetus|[3835]|NAMUM332-08|United States|California|658[0n]|BOLD:ACE9042
Schinia suetus martini|[3836]|HELNA174-06|United States|Washington|642[2n]|BOLD:ACE9042
Schinia suetus|[3837]|HELNA281-09|United States|Utah|658[0n]|BOLD:ACE9042
Schinia suetus|[3838]|HELNA374-09|United States|Utah|658[0n]|BOLD:ACE9042
Schinia suetus|[3839]|HELNA375-09|United States|Nevada|658[0n]|BOLD:ACE9042
Schinia suetus californicus|[3840]|JBAZ154-09|United States|California|658[0n]|BOLD:AAC4714
Schinia suetus californicus|[3841]|NAMUM337-08|United States|California|658[0n]|BOLD:AAC4714
Schinia suetus californicus|[3842]|HELNA882-10|United States|California|658[0n]|BOLD:AAC4714
Schinia suetus californicus|[3843]|RDNMG627-08|United States|California|658[0n]|BOLD:AAC4714
Schinia suetus californicus|[3844]|RDNMG628-08|United States|California|658[0n]|BOLD:AAC4714
Schinia suetus californicus|[3845]|CGLCA125-10|United States|California|658[0n]|BOLD:AAC4714
Schinia suetus californicus|[3846]|CGLCA126-10|United States|California|658[0n]|BOLD:AAC4714
Schinia suetus californicus|[3847]|HELNA881-10|United States|California|658[0n]|BOLD:AAC4714
Schinia suetus californicus|[3848]|HELNA883-10|United States|California|658[0n]|BOLD:AAC4714
Schinia suetus californicus|[3849]|RDNMF197-08|United States|California|658[0n]|BOLD:AAC4714
Schinia suetus californicus|[3850]|HELNA884-10|United States|California|658[0n]|BOLD:AAC4714
Schinia jaguarina|[3851]|HELNA161-06|United States|Montana|658[0n]|BOLD:AAC6459
Schinia jaguarina|[3852]|HELNA066-06|United States|Wyoming|658[0n]|BOLD:AAC6459
Schinia jaguarina|[3853]|HELNA162-06|United States|Colorado|658[0n]|BOLD:AAC6459
Schinia jaguarina|[3854]|HELNA247-06|United States|Montana|658[0n]|BOLD:AAC6459
Schinia jaguarina|[3855]|HELNA248-06|United States|Montana|658[0n]|BOLD:AAC6459
Schinia jaguarina|[3856]|HELNA536-09|United States|New Mexico|658[0n]|BOLD:AAC6459
Schinia jaguarina|[3857]|HELNA855-10|United States|Kansas|658[0n]|BOLD:AAC6459
Schinia jaguarina|[3858]|HELNA067-06|United States|Kansas|658[0n]|BOLD:AAC6459
Schinia jaguarina|[3859]|HELNA249-06|United States|Montana|658[0n]|BOLD:AAC6459
Schinia jaguarina|[3860]|LTOLB1301-11|United States|Colorado|658[0n]|BOLD:AAC6459
Schinia hardwickorum|[3861]|CNCLB1264-14|United States|Colorado|658[0n]|BOLD:ACO0765
Schinia anabelleae|[3862]|HELNA934-10|United States|North Carolina|658[0n]|BOLD:AAJ6531
Schinia anabelleae|[3863]|RDNML153-13|United States|North Carolina|658[0n]|BOLD:AAJ6531
Schinia anabelleae|[3864]|HELNA163-06|United States|Oklahoma|610[1n]|BOLD:AAJ6531
Schinia anabelleae|[3865]|CNCLB1267-14|United States|Illinois|658[0n]|BOLD:AAJ6531
Schinia luxa|[3866]|NAMUM322-08|United States|Georgia|658[0n]|BOLD:AAD3753
Schinia luxa|[3867]|BBLOC453-11|United States|Arizona|658[0n]|BOLD:AAD3753
Schinia luxa|[3868]|HELNA878-10|United States|Arizona|658[0n]|BOLD:AAD3753
Schinia luxa|[3869]|RDNMK175-11|United States|Arizona|658[0n]|BOLD:AAD3753
Schinia luxa|[3870]|BBLOC446-11|United States|Arizona|658[0n]|BOLD:AAD3753
Schinia luxa|[3871]|CMAZA794-10|United States|Arizona|658[0n]|BOLD:AAD3753
Schinia luxa|[3872]|RDNMH589-09|United States|Arizona|658[0n]|BOLD:AAD3753
Schinia luxa|[3873]|BBLOC557-11|United States|Arizona|658[0n]|BOLD:AAD3753
Schinia luxa|[3874]|CMAZA1114-12|United States|Arizona|658[0n]|BOLD:AAD3753
Schinia luxa|[3875]|BBLOC552-11|United States|Arizona|658[0n]|BOLD:AAD3753
Schinia luxa|[3876]|RDNMH849-09|United States|Arizona|658[0n]|BOLD:AAD3753
Schinia luxa|[3877]|HELNA879-10|United States|Arizona|658[0n]|BOLD:AAD3753
Schinia luxa|[3878]|NAMUM323-08|United States|Georgia|648[0n]|BOLD:AAD3753
Schinia luxa|[3879]|JBAZ168-09|United States|Arizona|658[0n]|BOLD:AAD3753

Schinia luxa|[3877]|HELNA879-10|United States|Arizona|658[0n]|BOLD:AAD3753
Schinia luxa|[3878]|NAMUM323-08|United States|Georgia|648[0n]|BOLD:AAD3753
Schinia luxa|[3879]|JBAZ168-09|United States|Arizona|658[0n]|BOLD:AAD3753
Schinia luxa|[3880]|HELNA876-10|United States|Arizona|658[0n]|BOLD:AAD3753
Schinia luxa|[3881]|RDNMK328-11|United States|New Mexico|658[0n]|BOLD:AAD3753
Schinia luxa|[3882]|BBLOC394-11|United States|Arizona|658[0n]|BOLD:AAD3753
Schinia luxa|[3883]|BBLOC452-11|United States|Arizona|658[0n]|BOLD:AAD3753
Schinia luxa|[3884]|BBLOC514-11|United States|Arizona|658[0n]|BOLD:AAD3753
Schinia luxa|[3885]|LNAUP101-13|United States|New Mexico|658[0n]|BOLD:AAD3753
Schinia luxa|[3886]|BBLOC523-11|United States|Arizona|658[0n]|BOLD:AAD3753
Schinia luxa|[3887]|HELNA304-09|United States|Texas|658[0n]|BOLD:AAD3753
Schinia luxa|[3888]|HELNA877-10|United States|Arizona|658[0n]|BOLD:AAD3753
Schinia luxa|[3889]|LNAUP102-13|United States|New Mexico|658[0n]|BOLD:AAD3753
Schinia carrizoensis|[3890]|HELNA129-06|United States|California|658[0n]|BOLD:AAD0799
Schinia carrizoensis|[3891]|HELNA966-10|United States|California|658[0n]|BOLD:AAD0799
Schinia carrizoensis|[3892]|HELNA967-10|United States|California|658[0n]|BOLD:AAD0799
Schinia deserticola|[3893]|HELNA023-06|United States|California|658[0n]|BOLD:AAD0798
Schinia deserticola|[3894]|JBAZ192-09|United States|California|658[0n]|BOLD:AAD0798
Schinia deserticola|[3895]|LOCBF275-13|United States|California|624[0n]|BOLD:AAD0798
Schinia deserticola|[3896]|HELNA025-06|United States|California|658[0n]|BOLD:AAD0798
Schinia deserticola|[3897]|HELNA858-10|United States|California|658[0n]|BOLD:AAD0798
Schinia deserticola|[3898]|HELNA857-10|United States|California|658[0n]|BOLD:AAD0798
Schinia deserticola|[3899]|HELNA856-10|United States|California|658[0n]|BOLD:AAD0798
Schinia deserticola|[3900]|JBAZ193-09|United States|California|658[0n]|BOLD:AAD0798
Schinia deserticola|[3901]|HELNA024-06|United States|California|658[0n]|BOLD:AAD0798
Schinia deserticola|[3902]|LOCBF276-13|United States|California|628[0n]|BOLD:AAD0798
Schinia deserticola|[3903]|LOCBF1006-13|United States|California|600[0n]|BOLD:AAD0798
Schinia cupes|[3904]|HELNA268-06|United States|Colorado|658[0n]|BOLD:AAE9292
Schinia cupes|[3905]|LTOLB1299-11|United States|Georgia|658[0n]|BOLD:AAE9292
Schinia cupes|[3906]|HELNA267-06|United States|Oklahoma|658[0n]|BOLD:AAE9292
Schinia cupes|[3907]|HELNA037-06|United States|Oklahoma|658[0n]|BOLD:AAE9292
Schinia cupes|[3908]|HELNA038-06|United States|Colorado|658[0n]|BOLD:AAE9292
Schinia cupes|[3909]|BBLSW054-09|United States|Texas|658[0n]|BOLD:AAE9292
Schinia cupes|[3910]|LTOLB1300-11|United States|Georgia|658[0n]|BOLD:AAE9292
Schinia crotchii|[3911]|CNCLB112-14|United States|California|624[0n]|BOLD:ACM4046
Schinia crotchii|[3912]|CNCLB113-14|United States|California|658[0n]|BOLD:ACM4046
Schinia edwardsii|[3913]|LNAUS4077-13|United States|Oregon|658[0n]|BOLD:ACJ8415
Schinia edwardsii|[3914]|TML163-14|United States||658[0n]|BOLD:ACJ8415
Schinia edwardsii|[3915]|CNCLB1579-14|United States|Washington|307[0n]|
Schinia angulilinea|[3916]|CNCLB3087-15|United States|Arizona|658[0n]|BOLD:ACW8658
Schinia scarletina|[3917]|JBAZ150-09|United States|California|658[0n]|BOLD:AAJ6507
Schinia florida|[3918]|XAD016-04|Canada|Ontario|520[0n]|BOLD:AAB6844
Schinia florida|[3919]|XAI054-05|Canada|Ontario|658[0n]|BOLD:AAB6844
Schinia florida|[3920]|HELNA152-06|Canada|Manitoba|658[0n]|BOLD:AAB6844
Schinia florida|[3921]|BLTIB971-08|Canada|Ontario|658[0n]|BOLD:AAB6844
Schinia florida|[3922]|HELNA154-06|United States|Nebraska|658[0n]|BOLD:AAB6844
Schinia florida|[3923]|HELNA119-06|United States|Oklahoma|658[0n]|BOLD:AAB6844
Schinia florida|[3924]|HELNA120-06|United States|Oklahoma|658[0n]|BOLD:AAB6844
Schinia florida|[3925]|HELNA118-06|United States|Oklahoma|658[0n]|BOLD:AAB6844
Schinia florida|[3926]|HELNA153-06|United States|Oklahoma|614[0n]|BOLD:AAB6844
Schinia florida|[3927]|PMG160-03|Canada|Ontario|617[0n]|BOLD:AAB6844
Schinia florida|[3928]|XAD006-04|Canada|Ontario|658[0n]|BOLD:AAB6844
Schinia florida|[3929]|XAD004-04|Canada|Ontario|658[0n]|BOLD:AAB6844
Schinia florida|[3930]|JBAZ159-09|Canada|Ontario|658[0n]|BOLD:AAB6844
Schinia florida|[3931]|LGSM434-04|United States|North Carolina|658[0n]|BOLD:AAB6844
Schinia florida|[3932]|LGSM435-04|United States|North Carolina|658[0n]|BOLD:AAB6844
Schinia florida|[3933]|HELNA886-10|Canada|New Brunswick|658[0n]|BOLD:AAB6844
Schinia florida|[3934]|HELNA887-10|Canada|New Brunswick|658[0n]|BOLD:AAB6844
Schinia florida|[3935]|HELNA151-06|Canada|Nova Scotia|658[0n]|BOLD:AAB6844
Schinia florida|[3936]|PHMNB722-05|Canada|New Brunswick|658[0n]|BOLD:AAB6844
Schinia florida|[3937]|HELNA885-10|Canada|New Brunswick|658[0n]|BOLD:AAB6844
Schinia florida|[3938]|HELNA214-06|Canada|Nova Scotia|634[0n]|BOLD:AAB6844
Schinia florida|[3939]|BBLPC972-09|Canada|Newfoundland and Labrador|658[0n]|BOLD:AAB6844
Schinia florida|[3940]|HELNA888-10|Canada|New Brunswick|658[0n]|BOLD:AAB6844
Schinia felicitata|[3941]|NAMUM374-09|United States|California|658[0n]|BOLD:AAD0894
Schinia felicitata|[3942]|JBAZ181-09|United States|California|658[0n]|BOLD:AAD0894
Schinia felicitata|[3943]|JBAZ194-09|United States|California|658[0n]|BOLD:AAD0894
Schinia felicitata|[3944]|HELNA914-10|United States|California|658[0n]|BOLD:AAD0894
Schinia felicitata|[3945]|LOCBF982-13|United States|California|580[0n]|BOLD:AAD0894
Schinia felicitata|[3946]|JBAZ185-09|United States|California|658[0n]|BOLD:AAD0894
Schinia felicitata|[3947]|JBAZ187-09|United States|California|658[0n]|BOLD:AAD0894
Schinia felicitata|[3948]|HELNA913-10|United States|California|658[0n]|BOLD:AAD0894
Schinia felicitata|[3949]|NAMUM427-09|United States|Arizona|658[0n]|BOLD:AAD0894
Schinia felicitata|[3950]|JBAZ186-09|United States|California|658[0n]|BOLD:AAD0894
Schinia felicitata|[3951]|JBAZ182-09|United States|California|658[0n]|BOLD:AAD0894
Schinia lynda|[3952]|HELNA069-06|United States|Oregon|658[0n]|BOLD:AAD0894
Schinia lynda|[3953]|HELNA070-06|United States|Oregon|658[0n]|BOLD:AAD0894
Schinia lynda|[3954]|HELNA150-06|United States|Oregon|658[0n]|BOLD:AAD0894
Schinia lynda|[3955]|CNCLB116-14|United States|Oregon|658[0n]|BOLD:AAD0894
Schinia lynda|[3956]|CNCLB117-14|United States|Oregon|658[0n]|BOLD:AAD0894
Schinia perminuta|[3957]|HELNA969-10|United States|California|658[0n]|BOLD:AAO0098
Schinia perminuta|[3958]|LNAUS3944-13|United States|Oregon|658[0n]|BOLD:AAO0098
Schinia perminuta|[3959]|LNAUS3945-13|United States|Oregon|658[0n]|BOLD:AAO0098
Schinia perminuta|[3960]|CNCLB1577-14|United States|Washington|658[0n]|BOLD:AAO0098
Schinia gaurae|[3961]|LSEU073-06|United States|Florida|546[2n]|BOLD:AAD6868
Schinia gaurae|[3962]|LSEU074-06|United States|Florida|551[1n]|BOLD:AAD6868
Schinia gaurae|[3963]|HELNA284-09|United States|New Mexico|658[0n]|BOLD:AAD6868
Schinia gaurae|[3964]|LPOKE032-10|United States|Oklahoma|658[0n]|BOLD:AAD6868
Schinia gaurae|[3965]|HELNA045-06|United States|Colorado|657[5n]|BOLD:AAD6868
Schinia gaurae|[3966]|HELNA046-06|United States|Colorado|658[5n]|BOLD:AAD6868
Schinia gaurae|[3967]|CMAZA537-10|United States|Arizona|658[0n]|BOLD:AAD6868
Schinia gaurae|[3968]|CMAZA404-10|United States|Arizona|658[0n]|BOLD:AAD6868
Schinia gaurae|[3969]|CMAZA803-10|United States|Arizona|658[0n]|BOLD:AAD6868
Schinia gaurae|[3970]|CMAZA1074-12|United States|Arizona|658[0n]|BOLD:AAD6868
Schinia mexicana|[3971]|LNAUS4054-13|United States|Arizona|658[0n]|BOLD:ACC9172
Schinia mexicana|[3972]|LNAUS4055-13|United States|Arizona|658[0n]|BOLD:ACC9172
Schinia mexicana|[3973]|LNAUS4056-13|United States|Arizona|658[0n]|BOLD:ACC9172
Schinia mexicana|[3974]|LNAUS4057-13|United States|Arizona|658[0n]|BOLD:ACC9172
Schinia carminatra|[3975]|CNCLB108-14|United States|Colorado|658[0n]|BOLD:ACM4423
Schinia zuni|[3976]|HELNA979-10|United States|Arizona|658[0n]|BOLD:AAO0101
Schinia zuni|[3977]|LNAUP173-13|United States|Arizona|658[0n]|BOLD:AAO0101
Schinia zuni|[3978]|LNAUP174-13|United States|Arizona|658[0n]|BOLD:AAO0101
[illegible]

Schinia zuni|[3976]|HELNA979-10|United States|Arizona|658[0n]|BOLD:AAO0101
Schinia zuni|[3977]|LNAUP173-13|United States|Arizona|658[0n]|BOLD:AAO0101
Schinia zuni|[3978]|LNAUP174-13|United States|Arizona|658[0n]|BOLD:AAO0101
Schinia zuni|[3979]|LNAUP172-13|United States|Arizona|658[0n]|BOLD:AAO0101
Schinia zuni|[3980]|HELNA912-10|United States|Arizona|658[0n]|BOLD:AAO0101
Schinia zuni|[3981]|HELNA910-10|United States|Arizona|658[0n]|BOLD:AAO0101
Schinia zuni|[3982]|HELNA911-10|United States|Arizona|658[0n]|BOLD:AAO0101
Schinia zuni|[3983]|LNAUP175-13|United States|Arizona|658[0n]|BOLD:AAO0101
Schinia lucens|[3984]|HELNA235-06|United States|Nebraska|658[0n]|BOLD:AAI0692
Schinia lucens|[3985]|RDMAB1031-09|United States|Colorado|658[0n]|BOLD:AAI0692
Schinia lucens|[3986]|CNCLB3085-15|United States|Arizona|658[0n]|BOLD:AAI0692
Schinia pulchripennis|[3987]|HELNA841-10|United States|California|658[0n]|BOLD:AAP6202
Schinia pulchripennis|[3988]|HELNA842-10|United States|California|658[0n]|BOLD:AAP6202
Schinia pulchripennis|[3989]|HELNA839-10|United States|California|658[0n]|BOLD:AAP6202
Schinia pulchripennis|[3990]|HELNA840-10|United States|California|658[0n]|BOLD:AAP6202
Schinia pulchripennis|[3991]|HELNA889-10|United States|California|658[0n]|BOLD:AAP6202
Schinia hanga|[3992]|HELNA055-06|United States|Colorado|658[0n]|BOLD:AAI0693
Schinia hanga|[3993]|HELNA266-06|United States|Oklahoma|609[0n]|BOLD:AAI0693
Schinia petulans|[3994]|HELNA175-06|United States|Florida|605[0n]|BOLD:AAD3714
Schinia petulans|[3995]|CNCLB120-14|United States|Florida|658[0n]|BOLD:AAD3714
Schinia petulans|[3996]|CNCLB121-14|United States|Florida|658[0n]|BOLD:AAD3714
Schinia bicuspida|[3997]|HELNA250-06|United States|Colorado|658[0n]|BOLD:AAI0688
Schinia bicuspida|[3998]|HELNA251-06|United States|Colorado|658[0n]|BOLD:AAI0688
Schinia mortua|[3999]|HELNA072-06|United States|Colorado|658[0n]|BOLD:AAE3962
Schinia mortua|[4000]|HELNA073-06|United States|Colorado|658[0n]|BOLD:AAE3962
Schinia mortua|[4001]|HELNA075-06|United States|New Mexico|658[0n]|BOLD:AAE3962
Schinia mortua|[4002]|HELNA071-06|United States|Colorado|658[0n]|BOLD:AAE3962
Schinia mortua|[4003]|HELNA076-06|United States|New Mexico|658[0n]|BOLD:AAE3962
Schinia mortua|[4004]|LTOLB1302-11|United States|Kansas|658[0n]|BOLD:AAE3962
Schinia mortua|[4005]|LTOLB1303-11|United States|Kansas|658[0n]|BOLD:AAE3962
Schinia mortua|[4006]|BBLOD849-11|United States|Arizona|658[0n]|BOLD:AAE3962
Schinia mortua|[4007]|BBLOD1704-11|United States|California|658[0n]|BOLD:AAE3962
Schinia varix|[4008]|HELNA255-06|United States|Oklahoma|604[0n]|BOLD:AAI0823
Schinia varix|[4009]|HELNA257-06|United States|Oklahoma|608[0n]|BOLD:AAI0823
Schinia siren|[4010]|HELNA101-06|United States|Colorado|658[0n]|BOLD:AAC9828
Schinia siren|[4011]|HELNA261-06|United States|Colorado|658[0n]|BOLD:AAC9828
Schinia siren|[4012]|HELNA102-06|United States|Colorado|658[0n]|BOLD:AAC9828
Schinia siren|[4013]|HELNA260-06|United States|Kansas|658[0n]|BOLD:AAC9828
Schinia siren|[4014]|HELNA259-06|United States|Oklahoma|658[0n]|BOLD:AAC9828
Schinia siren|[4015]|HELNA103-06|United States|Colorado|658[0n]|BOLD:AAC9828
Schinia siren|[4016]|HELNA104-06|United States|Colorado|658[0n]|BOLD:AAC9828
Schinia siren|[4017]|LTOLB1308-11|United States|Kansas|658[0n]|BOLD:AAC9828
Schinia siren|[4018]|LNCB786-09|United States|North Carolina|658[0n]|BOLD:AAC9828
Schinia siren|[4019]|LSEU076-06|United States|Georgia|590[0n]|BOLD:AAC9828
Schinia siren|[4020]|HELNA504-09|United States|North Carolina|658[0n]|BOLD:AAC9828
Schinia siren|[4021]|RDNML151-13|United States|North Carolina|658[0n]|BOLD:AAC9828
Schinia septentrionalis|[4022]|RDNMG626-08|United States|Massachusetts|649[0n]|BOLD:AAD6937
Schinia septentrionalis|[4023]|HELNA269-09|United States|Connecticut|658[0n]|BOLD:AAD6937
Schinia septentrionalis|[4024]|HELNA272-09|United States|Kentucky|526[3n]|BOLD:AAD6937
Schinia septentrionalis|[4025]|RDNMG625-08|United States|Massachusetts|658[0n]|BOLD:AAD6937
Schinia septentrionalis|[4026]|HELNA894-10|United States|Massachusetts|658[0n]|BOLD:AAD6937
Schinia septentrionalis|[4027]|HELNA895-10|United States|Massachusetts|658[0n]|BOLD:AAD6937
Schinia septentrionalis|[4028]|HELNA896-10|United States|Massachusetts|658[0n]|BOLD:AAD6937
Schinia septentrionalis|[4029]|HELNA1014-10|United States|Massachusetts|658[0n]|BOLD:AAD6937
Schinia septentrionalis|[4030]|HELNA099-06|United States|Colorado|658[0n]|BOLD:AAD6937
Schinia septentrionalis|[4031]|HELNA273-09|United States|Colorado|658[0n]|BOLD:AAD6937
Schinia septentrionalis|[4032]|LNAUP086-13|United States|New Mexico|658[0n]|BOLD:AAD6937
Schinia ultima|[4033]|LTOLB1311-11|United States|Kansas|658[1n]|BOLD:AAF2108
Schinia ultima|[4034]|HELNA114-06|United States|Colorado|658[0n]|BOLD:AAF2108
Schinia ultima|[4035]|HELNA115-06|United States|Colorado|658[0n]|BOLD:AAF2108
Schinia ultima|[4036]|HELNA116-06|United States|Colorado|658[0n]|BOLD:AAF2108
Schinia ultima|[4037]|LTOLB1309-11|United States|Kansas|658[0n]|BOLD:AAF2108
Schinia ultima|[4038]|LTOLB1310-11|United States|Kansas|658[0n]|BOLD:AAF2108
Schinia ultima|[4039]|LTOLB1312-11|United States|Kansas|658[0n]|BOLD:AAF2108
Schinia rufipenna|[4040]|CNCLB128-14|United States|Florida|658[0n]|BOLD:ACM4204
Schinia immaculata|[4041]|HELNA915-10|United States|Utah|658[0n]|BOLD:AAE3971
Schinia ligeae|[4042]|NAMUM380-09|United States|California|658[0n]|BOLD:AAE3971
Schinia ligeae|[4043]|NAMUM395-09|United States|California|658[0n]|BOLD:AAE3971
Schinia immaculata|[4044]|HELNA063-06|United States|Arizona|658[0n]|BOLD:AAE3971
Schinia immaculata|[4045]|HELNA062-06|United States|Arizona|658[0n]|BOLD:AAE3971
Schinia ligeae|[4046]|NAMUM363-08|United States|Nevada|658[0n]|BOLD:AAE3971
Schinia ligeae|[4047]|HELNA217-06|United States|Nevada|658[0n]|BOLD:AAE3971
Schinia ligeae|[4048]|CNCLB3083-15|United States|Arizona|658[0n]|BOLD:AAE3971
Schinia nubila|[4049]|CNCLB2975-14|United States|North Carolina|658[0n]|BOLD:AAC9829
Schinia nubila|[4050]|HELNA074-06|United States|Colorado|656[0n]|BOLD:AAC9829
Schinia nubila|[4051]|LNC530-06|United States||575[6n]|BOLD:AAC9829
Schinia nubila|[4052]|LNC512-06|United States|North Carolina|632[0n]|BOLD:AAC9829
Schinia nubila|[4053]|HELNA505-09|United States|North Carolina|658[0n]|BOLD:AAC9829
Schinia nubila|[4054]|LNC531-06|United States|North Carolina|605[0n]|BOLD:AAC9829
Schinia nubila|[4055]|HELNA298-09|United States|Georgia|658[1n]|BOLD:AAC9829
Schinia nubila|[4056]|HELNA946-10|United States|Maryland|658[0n]|BOLD:AAC9829
Schinia nubila|[4057]|RDNML152-13|United States|North Carolina|658[0n]|BOLD:AAC9829
Schinia nubila|[4058]|RDNML154-13|United States|North Carolina|658[0n]|BOLD:AAC9829
Schinia nubila|[4059]|RDNML155-13|United States|North Carolina|658[0n]|BOLD:AAC9829
Schinia nubila|[4060]|CNCLB2976-14|United States|North Carolina|658[0n]|BOLD:AAC9829
Schinia jaegeri|[4061]|JBAZ180-09|United States|California|658[0n]|BOLD:AAE9323
Schinia jaegeri|[4062]|JBAZ177-09|United States|California|658[0n]|BOLD:AAE9323
Schinia jaegeri|[4063]|HELNA916-10|United States|California|658[0n]|BOLD:AAE9323
Schinia jaegeri|[4064]|JBAZ179-09|United States|California|658[0n]|BOLD:AAE9323
Schinia jaegeri|[4065]|HELNA917-10|United States|California|658[0n]|BOLD:AAE9323
Schinia jaegeri|[4066]|JBAZ178-09|United States|California|658[0n]|BOLD:AAE9323
Schinia jaegeri|[4067]|HELNA918-10|United States|California|658[0n]|BOLD:AAE9323
Schinia jaegeri|[4068]|HELNA865-10|United States|California|658[0n]|BOLD:AAE9323
Schinia jaegeri|[4069]|HELNA866-10|United States|California|658[0n]|BOLD:AAE9323
Schinia jaegeri|[4070]|HELNA1013-10|United States|California|658[0n]|BOLD:AAE9323
Schinia errans|[4071]|HELNA042-06|United States|Colorado|658[0n]|BOLD:AAD0797
Schinia errans|[4072]|RDNMK009-11|United States|Arizona|658[0n]|BOLD:AAD0797
Schinia errans|[4073]|CMAZA769-10|United States|Arizona|658[0n]|BOLD:AAD0797
Schinia errans|[4074]|HELNA041-06|United States|Colorado|658[0n]|BOLD:AAD0797
Schinia errans|[4075]|HELNA043-06|United States|Colorado|658[0n]|BOLD:AAD0797
Schinia errans|[4076]|HELNA044-06|United States|New Mexico|658[0n]|BOLD:AAD0797
Schinia errans|[4077]|JBAZ173-09|United States|Arizona|658[0n]|BOLD:AAD0797
Schinia errans|[4078]|JBAZ174-09|United States|Arizona|658[0n]|BOLD:AAD0797

Schinia errans|[4076]|HELNA044-06|United States|New Mexico|658[0n]|BOLD:AAD0797
Schinia errans|[4077]|JBAZ173-09|United States|Arizona|658[0n]|BOLD:AAD0797
Schinia errans|[4078]|JBAZ174-09|United States|Arizona|658[0n]|BOLD:AAD0797
Schinia errans|[4079]|JBAZ175-09|United States|Arizona|658[0n]|BOLD:AAD0797
Schinia errans|[4080]|JBAZ176-09|United States|Arizona|658[0n]|BOLD:AAD0797
Schinia errans|[4081]|HELNA274-09|United States|Oklahoma|658[0n]|BOLD:AAD0797
Schinia errans|[4082]|CMAZA585-10|United States|Arizona|658[0n]|BOLD:AAD0797
Schinia errans|[4083]|CMAZA586-10|United States|Arizona|658[0n]|BOLD:AAD0797
Schinia errans|[4084]|CMAZA762-10|United States|Arizona|658[0n]|BOLD:AAD0797
Schinia errans|[4085]|RDNMK010-11|United States|Arizona|658[0n]|BOLD:AAD0797
Schinia errans|[4086]|CMAZA1133-12|United States|Arizona|658[0n]|BOLD:AAD0797
Schinia errans|[4087]|CMAZA1161-12|United States|Arizona|658[0n]|BOLD:AAD0797
Schinia tuberculum|[4088]|LNCB810-09|United States|North Carolina|658[2n]|BOLD:AAC9830
Schinia tuberculum|[4089]|LNCB811-09|United States|North Carolina|658[1n]|BOLD:AAC9830
Schinia tuberculum|[4090]|HELNA940-10|United States|North Carolina|658[0n]|BOLD:AAC9830
Schinia tuberculum|[4091]|LSUSA064-06|United States|Florida|549[0n]|BOLD:AAC9830
Schinia tuberculum|[4092]|LNAUP171-13|United States|North Carolina|658[0n]|BOLD:AAC9830
Schinia tuberculum|[4093]|LNAUP170-13|United States|North Carolina|658[0n]|BOLD:AAC9830
Schinia tuberculum|[4094]|LNAUP169-13|United States|North Carolina|658[0n]|BOLD:AAC9830
Schinia tuberculum|[4095]|HELNA943-10|United States|North Carolina|658[0n]|BOLD:AAC9830
Schinia tuberculum|[4096]|HELNA942-10|United States|North Carolina|658[0n]|BOLD:AAC9830
Schinia tuberculum|[4097]|HELNA511-09|United States|North Carolina|658[0n]|BOLD:AAC9830
Schinia tuberculum|[4098]|HELNA509-09|United States|North Carolina|658[0n]|BOLD:AAC9830
Schinia tuberculum|[4099]|HELNA508-09|United States|North Carolina|658[0n]|BOLD:AAC9830
Schinia tuberculum|[4100]|HELNA507-09|United States|North Carolina|658[0n]|BOLD:AAC9830
Schinia tuberculum|[4101]|RDNMD505-06|United States|Florida|658[0n]|BOLD:AAC9830
Schinia tuberculum|[4102]|LSUSA054-06|United States|Florida|658[0n]|BOLD:AAC9830
Schinia tuberculum|[4103]|LNAUP168-13|United States|North Carolina|658[0n]|BOLD:AAC9830
Schinia tuberculum|[4104]|HELNA510-09|United States|North Carolina|658[0n]|BOLD:AAC9830
Schinia tuberculum|[4105]|CNCLB3111-15|United States|Florida|245[1n]|
Schinia tuberculum|[4106]|CNCLB3131-15|United States|Florida|658[0n]|BOLD:AAC9830
Schinia intrabilis|[4107]|AWCLB453-10|United States|Arizona|658[0n]|BOLD:AAH5362
Schinia intrabilis|[4108]|AWCLB391-10|United States|Arizona|658[0n]|BOLD:AAH5362
Schinia intrabilis|[4109]|AWCLB405-10|United States|Arizona|658[0n]|BOLD:AAH5362
Schinia intrabilis|[4110]|AWCLB406-10|United States|Arizona|658[0n]|BOLD:AAH5362
Schinia intrabilis|[4111]|AWCLB454-10|United States|Arizona|658[0n]|BOLD:AAH5362
Schinia intrabilis|[4112]|AWCLB221-10|United States|Arizona|658[0n]|BOLD:AAH5362
Schinia intrabilis|[4113]|AWCLB390-10|United States|Arizona|658[0n]|BOLD:AAH5362
Schinia intrabilis|[4114]|AWCLB401-10|United States|Arizona|658[0n]|BOLD:AAH5362
Schinia intrabilis|[4115]|AWCLB407-10|United States|Arizona|658[0n]|BOLD:AAH5362
Schinia intrabilis|[4116]|AWCLB223-10|United States|Arizona|569[0n]|BOLD:AAH5362
Schinia intrabilis|[4117]|AWCLB392-10|United States|Arizona|658[0n]|BOLD:AAH5362
Schinia intrabilis|[4118]|AWCLB222-10|United States|Arizona|647[0n]|BOLD:AAH5362
Schinia intrabilis|[4119]|AWCLB240-10|United States|Arizona|658[0n]|BOLD:AAH5362
Schinia intrabilis|[4120]|CGLCA133-10|United States|California|608[0n]|BOLD:AAH5362
Schinia intrabilis|[4121]|AWCLB250-10|United States|Arizona|658[0n]|BOLD:AAH5362
Schinia intrabilis|[4122]|AWCLB347-10|United States|Arizona|658[0n]|BOLD:AAH5362
Schinia intrabilis|[4123]|AWCLB400-10|United States|Arizona|658[0n]|BOLD:AAH5362
Schinia intrabilis|[4124]|AWCLB404-10|United States|Arizona|658[0n]|BOLD:AAH5362
Schinia intrabilis|[4125]|AWCLB291-10|United States|Arizona|658[0n]|BOLD:AAH5362
Schinia intrabilis|[4126]|AWCLB229-10|United States|Arizona|658[0n]|BOLD:AAH5362
Schinia intrabilis|[4127]|AWCLB224-10|United States|Arizona|658[0n]|BOLD:AAH5362
Schinia intrabilis|[4128]|BBLSX750-09|United States|Arizona|658[0n]|BOLD:AAH5362
Schinia intrabilis|[4129]|HELNA065-06|United States|Arizona|658[0n]|BOLD:AAH5362
Schinia intrabilis|[4130]|HELNA064-06|United States|California|658[0n]|BOLD:AAH5362
Schinia intrabilis|[4131]|AWCLB299-10|United States|Arizona|658[0n]|BOLD:AAH5362
Schinia intrabilis|[4132]|AWCLB344-10|United States|Arizona|658[0n]|BOLD:AAH5362
Schinia intrabilis|[4133]|AWCLB345-10|United States|Arizona|658[0n]|BOLD:AAH5362
Schinia intrabilis|[4134]|AWCLB346-10|United States|Arizona|658[0n]|BOLD:AAH5362
Schinia intrabilis|[4135]|AWCLB389-10|United States|Arizona|658[0n]|BOLD:AAH5362
Schinia intrabilis|[4136]|AWCLB395-10|United States|Arizona|658[0n]|BOLD:AAH5362
Schinia intrabilis|[4137]|AWCLB398-10|United States|Arizona|658[0n]|BOLD:AAH5362
Schinia intrabilis|[4138]|AWCLB408-10|United States|Arizona|658[0n]|BOLD:AAH5362
Schinia intrabilis|[4139]|AWCLB409-10|United States|Arizona|658[0n]|BOLD:AAH5362
Schinia intrabilis|[4140]|AWCLB423-10|United States|Arizona|658[0n]|BOLD:AAH5362
Schinia intrabilis|[4141]|AWCLB428-10|United States|Arizona|658[0n]|BOLD:AAH5362
Schinia intrabilis|[4142]|AWCLB452-10|United States|Arizona|658[0n]|BOLD:AAH5362
Schinia intrabilis|[4143]|AWCLB577-11|United States|Arizona|658[0n]|BOLD:AAH5362
Schinia mitis|[4144]|HELNA216-06|United States|Oklahoma|658[0n]|BOLD:AAJ6511
Schinia mitis|[4145]|CNCLB2940-14|United States|North Carolina|658[0n]|BOLD:AAJ6511
Schinia mitis|[4146]|CNCLB2941-14|United States|North Carolina|658[0n]|BOLD:AAJ6511
Schinia amblys|[4147]|CNCLB3088-15|United States|Arizona|658[0n]|BOLD:ACW9944
Schinia dobla|[4148]|CNCLB106-14|United States|California|658[0n]|BOLD:ACM4013
Schinia dobla|[4149]|CNCLB107-14|United States|California|658[0n]|BOLD:ACM4013
Schinia amaryllis|[4150]|JBAZ151-09|United States|California|658[0n]|BOLD:AAJ6401
Schinia amaryllis|[4151]|HELNA971-10|United States|California|658[0n]|BOLD:AAJ6401
Schinia amaryllis|[4152]|CNCLB3084-15|United States|California|658[0n]|BOLD:AAJ6401
Schinia meskeana|[4153]|HELNA275-09|United States|Kansas|658[0n]|BOLD:AAD0806
Schinia meskeana|[4154]|HELNA367-09|United States|Colorado|658[0n]|BOLD:AAD0806
Schinia meskeana|[4155]|CNCLB3125-15|United States|Texas|658[0n]|BOLD:AAD0806
Schinia bina|[4156]|CNCLB3124-15|United States|Florida|658[0n]|BOLD:AAD0806
Schinia bina|[4157]|CNCLB3121-15|United States|Florida|407[0n]|
Schinia bina|[4158]|CNCLB3130-15|United States|Ohio|658[0n]|BOLD:AAD0806
Schinia bina|[4159]|CNCLB3132-15|United States|Missouri|407[0n]|
Schinia scalena n. sp.|[4160]|CNCLB3107-15|Canada|Saskatchewan|658[0n]|BOLD:ABY8607
Schinia scalena n. sp.|[4161]|CNCLB3127-15|Canada|Saskatchewan|658[0n]|BOLD:ABY8607
Schinia scalena n. sp.|[4162]|HELNA366-09|United States|Montana|658[0n]|BOLD:ABY8607
Schinia scalena n. sp.|[4163]|CNCLB3128-15|Canada|Saskatchewan|658[0n]|BOLD:ABY8607
Schinia apera|[4164]|CNCLB3108-15|Mexico||658[0n]|BOLD:ACW8729
Schinia meskeana|[4165]|CNCLB3126-15|United States|Texas|658[0n]|BOLD:AAD0806
Schinia meskeana|[4166]|CNCLB3133-15|Mexico|Nuevo Leon|658[0n]|BOLD:AAD0806
Schinia meskeana|[4167]|CNCLB3118-15|United States|Texas|658[0n]|BOLD:AAD0806
Schinia meskeana|[4168]|CNCLB3109-15|Mexico|Durango|658[0n]|BOLD:AAD0806
Schinia meskeana|[4169]|LPOKC819-09|United States|Oklahoma|658[0n]|BOLD:AAD0806
Schinia meskeana|[4170]|RDNMF386-08|United States|Texas|641[0n]|BOLD:AAD0806
Schinia meskeana|[4171]|CNCLB3119-15|Mexico|Chihuahua|263[0n]|
Schinia meskeana|[4172]|CNCLB3120-15|Mexico|Nuevo Leon|307[0n]|
Schinia meskeana|[4173]|CNCLB3134-15|Mexico|Durango|307[0n]|
Schinia bina|[4174]|CNCLB3114-15|United States|Florida|658[0n]|BOLD:AAD0806
Schinia bina|[4175]|CNCLB3129-15|United States|Ohio|307[1n]|
Schinia bina|[4176]|CNCLB3110-15|United States|Florida|658[0n]|BOLD:AAD0806
Schinia bina|[4177]|RDNMF387-08|United States|Florida|658[0n]|BOLD:AAD0806
[illegible]

Schinia bina|[4175]|CNCLB3129-15|United States|Ohio|307[1n]|
Schinia bina|[4176]|CNCLB3110-15|United States|Florida|658[0n]|BOLD:AAD0806
Schinia bina|[4177]|RDNMF387-08|United States|Florida|658[0n]|BOLD:AAD0806
Schinia bina|[4178]|RDNMF384-08|United States|Florida|658[0n]|BOLD:AAD0806
Schinia bina|[4179]|RDNMF385-08|United States|Florida|640[0n]|BOLD:AAD0806
Schinia bina|[4180]|CNCLB3117-15|United States|Florida|307[0n]|
Schinia bina|[4181]|CNCLB3135-15|United States|Florida|658[0n]|BOLD:AAD0806
Schinia avemensis|[4182]|RDMAB091-05|Canada|Manitoba|602[0n]|BOLD:AAD0775
Schinia avemensis|[4183]|HELNA179-06|Canada|Manitoba|658[0n]|BOLD:AAD0775
Schinia avemensis|[4184]|RDNMB496-05|Canada|Alberta|616[0n]|BOLD:AAD0775
Schinia avemensis|[4185]|RDMAB092-05|Canada|Alberta|639[0n]|BOLD:AAD0775
Schinia avemensis|[4186]|RDNMB115-05|Canada|Saskatchewan|658[0n]|BOLD:AAD0775
Schinia avemensis|[4187]|HELNA180-06|Canada|Alberta|658[0n]|BOLD:AAD0775
Schinia avemensis|[4188]|RDMAB093-05|United States|Colorado|624[0n]|BOLD:AAD0775
Schinia avemensis|[4189]|HELNA181-06|United States|Colorado|658[0n]|BOLD:AAD0775
Schinia avemensis|[4190]|HELNA833-10|United States|Colorado|658[0n]|BOLD:AAD0775
Schinia avemensis|[4191]|HELNA834-10|United States|Colorado|658[0n]|BOLD:AAD0775
Prothrinax luteomedia|[4192]|BBLOC541-11|United States|Arizona|658[0n]|BOLD:AAK9902
Prothrinax luteomedia|[4193]|RDNMD810-07|United States|Arizona|658[0n]|BOLD:AAK9902
Prothrinax luteomedia|[4194]|AWCLB006-10|United States|Arizona|658[0n]|BOLD:AAK9902
Prothrinax luteomedia|[4195]|RDNMJ172-10|United States|Arizona|658[0n]|BOLD:AAK9902
Prothrinax luteomedia|[4196]|BBLOC397-11|United States|Arizona|658[0n]|BOLD:AAK9902
Prothrinax luteomedia|[4197]|RDNML215-13|United States|Arizona|658[0n]|BOLD:AAK9902
Ozarba nebula|[4198]|RDNMD508-06|United States|Florida|658[1n]|BOLD:AAE4326
Ozarba nebula|[4199]|NAMUM098-08|United States|Texas|658[0n]|BOLD:AAE4326
Ozarba nebula|[4200]|LPOKA252-08|United States|Oklahoma|658[0n]|BOLD:AAE4326
Ozarba nebula|[4201]|LPOKA443-09|United States|Oklahoma|658[0n]|BOLD:AAE4326
Ozarba nebula|[4202]|LPOKA584-09|United States|Oklahoma|658[0n]|BOLD:AAE4326
Ozarba nebula|[4203]|LPOKD235-09|United States|Oklahoma|657[0n]|BOLD:AAE4326
Ozarba sp. 1|[4204]|RDNME667-08|United States|Texas|609[0n]|BOLD:AAE4330
Ozarba sp. 2|[4205]|BBLSZ159-09|United States|Texas|658[0n]|BOLD:AAG6036
Ozarba sp. 2|[4206]|RDNMJ480-11|United States|Arizona|658[0n]|BOLD:AAG6036
Ozarba sp. 2|[4207]|RDNMJ676-11|United States|Arizona|658[0n]|BOLD:AAG6036
Ozarba sp. 2|[4208]|RDNMH845-09|United States|Arizona|574[0n]|BOLD:AAG6036
Ozarba sp. 2|[4209]|BBLSX862-09|United States|Texas|658[0n]|BOLD:AAG6036
Ozarba sp. 2|[4210]|USLEP774-10|United States|Texas|658[0n]|BOLD:AAG6036
Ozarba sp. 2|[4211]|CMAZA614-10|United States|Arizona|658[0n]|BOLD:AAG6036
Ozarba sp. 2|[4212]|USLEP961-10|United States|Texas|658[0n]|BOLD:AAG6036
Ozarba sp. 2|[4213]|USLEP356-10|United States|Texas|634[0n]|BOLD:AAG6036
Ozarba sp. 2|[4214]|USLEP377-10|United States|Texas|658[0n]|BOLD:AAG6036
Ozarba sp. 2|[4215]|CMAZA615-10|United States|Arizona|658[0n]|BOLD:AAG6036
Ozarba sp. 2|[4216]|CMAZA532-10|United States|Arizona|658[0n]|BOLD:AAG6036
Ozarba sp. 2|[4217]|RDNMJ669-11|United States|Arizona|658[0n]|BOLD:AAG6036
Ozarba sp. 2|[4218]|RDNMK330-11|United States|New Mexico|658[0n]|BOLD:AAG6036
Ozarba aeria|[4219]|LNCC037-10|United States|North Carolina|658[0n]|BOLD:AAD3618
Ozarba aeria|[4220]|RDNMK331-11|United States|Ohio|658[0n]|BOLD:AAD3618
Ozarba aeria|[4221]|LPOKB663-09|United States|Oklahoma|658[0n]|BOLD:AAD3618
Ozarba aeria|[4222]|LNCC1273-11|United States|North Carolina|658[0n]|BOLD:AAD3618
Ozarba aeria|[4223]|LILLA399-11|United States|Illinois|658[0n]|BOLD:AAD3618
Ozarba aeria|[4224]|LPOKD152-09|United States|Oklahoma|658[0n]|BOLD:AAD3618
Ozarba aeria|[4225]|LPOKA425-09|United States|Oklahoma|658[0n]|BOLD:AAD3618
Ozarba aeria|[4226]|LPOKA162-08|United States|Oklahoma|658[0n]|BOLD:AAD3618
Ozarba aeria|[4227]|LPOKE583-12|United States|Oklahoma|632[0n]|BOLD:AAD3618
Ozarba aeria|[4228]|LPOKA483-09|United States|Oklahoma|632[0n]|BOLD:AAD3618
Ozarba aeria|[4229]|LPOKB196-09|United States|Oklahoma|658[0n]|BOLD:AAD3618
Ozarba aeria|[4230]|LPOKB674-09|United States|Oklahoma|658[0n]|BOLD:AAD3618
Ozarba aeria|[4231]|LNCB654-09|United States|North Carolina|658[0n]|BOLD:AAD3618
Ozarba aeria|[4232]|LPOKC900-09|United States|Oklahoma|636[0n]|BOLD:AAD3618
Ozarba aeria|[4233]|LPOKE599-12|United States|Oklahoma|632[0n]|BOLD:AAD3618
Micrathetis tecnion|[4234]|BLPDY277-11|Costa Rica|Guanacaste|658[0n]|BOLD:AAB3442
Micrathetis tecnion|[4235]|BLPDK109-09|Costa Rica|Guanacaste|658[0n]|BOLD:AAB3442
Micrathetis tecnion|[4236]|BLPDL199-10|Costa Rica|Alajuela|658[0n]|BOLD:AAB3442
Micrathetis tecnion|[4237]|BLPEF4842-13|Costa Rica|Guanacaste|658[0n]|BOLD:AAB3442
Micrathetis tecnion|[4238]|BLPCK816-08|Costa Rica|Alajuela|658[0n]|BOLD:AAB3442
Micrathetis tecnion|[4239]|BLPDG211-09|Costa Rica|Guanacaste|658[0n]|BOLD:AAB3442
Micrathetis tecnion|[4240]|BLPDK2002-09|Costa Rica|Alajuela|658[0n]|BOLD:AAB3442
Micrathetis tecnion|[4241]|BLPDM551-10|Costa Rica|Guanacaste|658[0n]|BOLD:AAB3442
Micrathetis tecnion|[4242]|BLPDS635-10|Costa Rica|Alajuela|658[0n]|BOLD:AAB3442
Micrathetis tecnion|[4243]|BLPDX1149-11|Costa Rica|Guanacaste|658[0n]|BOLD:AAB3442
Micrathetis tecnion|[4244]|BLPEC425-11|Costa Rica|Alajuela|658[0n]|BOLD:AAB3442
Micrathetis tecnion|[4245]|BLPED992-12|Costa Rica|Guanacaste|658[0n]|BOLD:AAB3442
Micrathetis tecnion|[4246]|BLPED1537-12|Costa Rica|Guanacaste|658[0n]|BOLD:AAB3442
Micrathetis tecnion|[4247]|BLPED1635-12|Costa Rica|Guanacaste|658[0n]|BOLD:AAB3442
Micrathetis tecnion|[4248]|BLPED1638-12|Costa Rica|Guanacaste|658[0n]|BOLD:AAB3442
Micrathetis tecnion|[4249]|BLPED1791-12|Costa Rica|Guanacaste|658[0n]|BOLD:AAB3442
Micrathetis tecnion|[4250]|BLPED1792-12|Costa Rica|Guanacaste|658[0n]|BOLD:AAB3442
Micrathetis tecnion|[4251]|BLPED1796-12|Costa Rica|Guanacaste|658[0n]|BOLD:AAB3442
Micrathetis tecnion|[4252]|LPYPB492-08|Mexico|Quintana Roo|658[0n]|BOLD:AAB3442
Micrathetis tecnion|[4253]|BLPDG163-09|Costa Rica|Guanacaste|658[0n]|BOLD:AAB3442
Micrathetis tecnion|[4254]|BLPDR735-10|Costa Rica|Alajuela|658[0n]|BOLD:AAB3442
Micrathetis tecnion|[4255]|BLPDH415-09|Costa Rica|Alajuela|658[0n]|BOLD:AAB3442
Micrathetis tecnion|[4256]|BLPDH403-09|Costa Rica|Alajuela|658[0n]|BOLD:AAB3442
Micrathetis tecnion|[4257]|BLPDL1520-10|Costa Rica|Alajuela|638[0n]|BOLD:AAB3442
Micrathetis tecnion|[4258]|BLPDX901-11|Costa Rica|Guanacaste|658[0n]|BOLD:AAB3442
Micrathetis tecnion|[4259]|BLPDX1148-11|Costa Rica|Guanacaste|658[0n]|BOLD:AAB3442
Micrathetis tecnion|[4260]|BLPEF1980-13|Costa Rica|Guanacaste|658[0n]|BOLD:AAB3442
Micrathetis tecnion|[4261]|BLPED1795-12|Costa Rica|Guanacaste|658[0n]|BOLD:AAB3442
Micrathetis tecnion|[4262]|BLPDG089-09|Costa Rica|Guanacaste|658[1n]|BOLD:AAB3442
Micrathetis tecnion|[4263]|BLPDL1519-10|Costa Rica|Alajuela|658[1n]|BOLD:AAB3442
Micrathetis tecnion|[4264]|BLPDF930-09|Costa Rica|Guanacaste|658[1n]|BOLD:AAB3442
Micrathetis tecnion|[4265]|BLPED1950-12|Costa Rica|Guanacaste|658[0n]|BOLD:AAB3442
Micrathetis tecnion|[4266]|BLPED1794-12|Costa Rica|Guanacaste|658[0n]|BOLD:AAB3442
Micrathetis tecnion|[4267]|BLPED1793-12|Costa Rica|Guanacaste|658[0n]|BOLD:AAB3442
Micrathetis tecnion|[4268]|BLPED1790-12|Costa Rica|Guanacaste|658[0n]|BOLD:AAB3442
Micrathetis tecnion|[4269]|BLPED1789-12|Costa Rica|Guanacaste|658[0n]|BOLD:AAB3442
Micrathetis tecnion|[4270]|BLPED1033-12|Costa Rica|Guanacaste|658[0n]|BOLD:AAB3442
Micrathetis tecnion|[4271]|BLPDY266-11|Costa Rica|Guanacaste|658[0n]|BOLD:AAB3442
Micrathetis tecnion|[4272]|BLPDY248-11|Costa Rica|Guanacaste|658[0n]|BOLD:AAB3442
Micrathetis tecnion|[4273]|BLPDX1207-11|Costa Rica|Guanacaste|658[0n]|BOLD:AAB3442
Micrathetis tecnion|[4274]|BLPDX1198-11|Costa Rica|Guanacaste|658[0n]|BOLD:AAB3442
Micrathetis tecnion|[4275]|BLPDX1137-11|Costa Rica|Guanacaste|658[0n]|BOLD:AAB3442
Micrathetis tecnion|[4276]|BLPDL1254-10|Costa Rica|Alajuela|658[0n]|BOLD:AAB3442
Micrathetis tecnion|[4277]|BLPDL1253-10|Costa Rica|Alajuela|658[0n]|BOLD:AAB3442

Micrathetis tecnion|[4275]|BLPDX1137-11|Costa Rica|Guanacaste|658[0n]|BOLD:AAB3442
Micrathetis tecnion|[4276]|BLPDL1254-10|Costa Rica|Alajuela|658[0n]|BOLD:AAB3442
Micrathetis tecnion|[4277]|BLPDL1253-10|Costa Rica|Alajuela|658[0n]|BOLD:AAB3442
Micrathetis tecnion|[4278]|BLPDK2328-09|Costa Rica|Alajuela|658[0n]|BOLD:AAB3442
Micrathetis tecnion|[4279]|BLPDK2309-09|Costa Rica|Alajuela|658[0n]|BOLD:AAB3442
Micrathetis tecnion|[4280]|BLPDG294-09|Costa Rica|Guanacaste|658[0n]|BOLD:AAB3442
Micrathetis tecnion|[4281]|BLPDG168-09|Costa Rica|Guanacaste|658[0n]|BOLD:AAB3442
Micrathetis tecnion|[4282]|BLPCF729-08|Costa Rica|Alajuela|658[0n]|BOLD:AAB3442
Micrathetis tecnion|[4283]|BLPCJ440-08|Costa Rica|Guanacaste|658[1n]|BOLD:AAB3442
Micrathetis tecnion|[4284]|LPYPA890-08|Mexico|Campeche|658[0n]|BOLD:AAB3442
Micrathetis tecnion|[4285]|RDNMG804-08|United States|Texas|643[0n]|BOLD:AAB3442
Micrathetis tecnion|[4286]|BLPDK243-09|Costa Rica|Guanacaste|658[0n]|BOLD:AAB3442
Micrathetis tecnion|[4287]|BLPDL1252-10|Costa Rica|Alajuela|634[0n]|BOLD:AAB3442
Micrathetis tecnion|[4288]|BLPDH645-09|Costa Rica|Alajuela|658[0n]|BOLD:AAB3442
Micrathetis tecnion|[4289]|BLPDK2003-09|Costa Rica|Alajuela|658[0n]|BOLD:AAB3442
Micrathetis tecnion|[4290]|BLPDS797-10|Costa Rica|Alajuela|658[0n]|BOLD:AAB3442
Micrathetis tecnion|[4291]|BLPED1951-12|Costa Rica|Guanacaste|658[0n]|BOLD:AAB3442
Micrathetis tecnion|[4292]|BLPED1952-12|Costa Rica|Guanacaste|658[0n]|BOLD:AAB3442
Micrathetis tecnion|[4293]|BLPED1953-12|Costa Rica|Guanacaste|658[0n]|BOLD:AAB3442
Micrathetis tecnion|[4294]|BLPED1954-12|Costa Rica|Guanacaste|658[0n]|BOLD:AAB3442
Micrathetis tecnion|[4295]|BLPED1955-12|Costa Rica|Guanacaste|658[0n]|BOLD:AAB3442
Micrathetis tecnion|[4296]|BLPED2185-12|Costa Rica|Guanacaste|658[0n]|BOLD:AAB3442
Micrathetis tecnion|[4297]|BLPEF2472-13|Costa Rica|Guanacaste|658[0n]|BOLD:AAB3442
Micrathetis tecnion|[4298]|BLPEF2477-13|Costa Rica|Guanacaste|658[0n]|BOLD:AAB3442
Micrathetis tecnion|[4299]|BLPEF2883-13|Costa Rica|Guanacaste|658[0n]|BOLD:AAB3442
Micrathetis tecnion|[4300]|BLPEE2810-14|Costa Rica|Guanacaste|658[0n]|BOLD:AAB3442
Micrathetis tecnion|[4301]|BLPEE4340-14|Costa Rica|Guanacaste|658[0n]|BOLD:AAB3442
Micrathetis tecnion|[4302]|BLPEE5280-14|Costa Rica|Guanacaste|658[0n]|BOLD:AAB3442
Micrathetis tecnion|[4303]|BLPEE5281-14|Costa Rica|Guanacaste|658[0n]|BOLD:AAB3442
Pyrrhia aurantiago|[4304]|RDNMF495-08|United States|Florida|641[0n]|BOLD:AAF5886
Pyrrhia aurantiago|[4305]|HELNA518-09|United States|Florida|599[0n]|BOLD:AAF5886
Derrima stellata|[4306]|LNAUT381-14|United States|Florida|658[0n]|BOLD:ACN8459
Derrima stellata|[4307]|LNAUT380-14|United States|Florida|658[0n]|BOLD:ACN8459
Derrima stellata|[4308]|LNAUT382-14|United States|Florida|658[0n]|BOLD:ACN8459
Derrima nr. stellata|[4309]|NOCNA004-14|United States|Oklahoma|658[0n]|BOLD:ACN8643
Derrima nr. stellata|[4310]|NOCNA005-14|United States|Oklahoma|610[0n]|BOLD:ACN8643
Derrima nr. stellata|[4311]|LNAUP154-13|United States|Virginia|658[0n]|BOLD:ACD0198
Derrima nr. stellata|[4312]|LNAUT385-14|United States|Virginia|658[0n]|BOLD:ACD0198
Cryphia fascia|[4313]|RDNME044-07|United States|California|658[0n]|BOLD:AAH4510
Cryphia fascia|[4314]|LOCBD741-06|United States|California|663[0n]|BOLD:ABA9743
Cryphia fascia|[4315]|RDNMH314-09|United States|California|626[0n]|BOLD:ABA9743
Cryphia pallidioides|[4316]|RDNMJ401-11|United States|Arizona|658[0n]|BOLD:ACE9979
Cryphia olivacea|[4317]|LBCH7346-10|Canada|British Columbia|658[0n]|BOLD:ACE9980
Cryphia olivacea|[4318]|LBCH6137-10|Canada|British Columbia|658[0n]|BOLD:ACE9980
Cryphia olivacea|[4319]|LBCH7338-10|Canada|British Columbia|658[0n]|BOLD:ACE9980
Cryphia olivacea|[4320]|LBCH7343-10|Canada|British Columbia|658[0n]|BOLD:ACE9980
Cryphia olivacea|[4321]|LBCH7344-10|Canada|British Columbia|658[0n]|BOLD:ACE9980
Cryphia olivacea|[4322]|LBCH7345-10|Canada|British Columbia|658[0n]|BOLD:ACE9980
Cryphia olivacea|[4323]|LBCH7347-10|Canada|British Columbia|658[0n]|BOLD:ACE9980
Cryphia olivacea|[4324]|LBCH7348-10|Canada|British Columbia|658[0n]|BOLD:ACE9980
Cryphia olivacea|[4325]|LBCH7373-10|Canada|British Columbia|658[0n]|BOLD:ACE9980
Cryphia olivacea|[4326]|LBCH7446-10|Canada|British Columbia|658[0n]|BOLD:ACE9980
Cryphia olivacea|[4327]|LBCH7093-10|Canada|British Columbia|658[0n]|BOLD:ACE9980
Cryphia olivacea|[4328]|LBCH7349-10|Canada|British Columbia|628[0n]|BOLD:ACE9980
Cryphia olivacea|[4329]|LBCH6237-10|Canada|British Columbia|658[0n]|BOLD:ACE9980
Cryphia olivacea|[4330]|LBCH7350-10|Canada|British Columbia|658[0n]|BOLD:ACE9980
Cryphia olivacea|[4331]|LBCH7478-10|Canada|British Columbia|658[0n]|BOLD:ACE9980
Cryphia olivacea|[4332]|ABNCC019-07|United States|Utah|638[1n]|BOLD:ACE9978
Cryphia flavidior|[4333]|RDNMH316-09|United States|Oregon|658[0n]|BOLD:AAE9413
Cryphia olivacea|[4334]|RDNMD495-06|United States|Colorado|658[0n]|BOLD:AAE9413
Cryphia olivacea|[4335]|LBCH6532-10|Canada|British Columbia|658[0n]|BOLD:AAE9413
Cryphia olivacea|[4336]|RDNMK092-11|United States|Colorado|658[0n]|BOLD:AAE9413
Cryphia olivacea|[4337]|RDNMD350-06|United States|Colorado|658[0n]|BOLD:AAE9413
Cryphia olivacea|[4338]|RDNMJ634-11|United States|Colorado|658[0n]|BOLD:AAE9413
Cryphia olivacea|[4339]|RDNMK093-11|United States|Colorado|658[0n]|BOLD:AAE9413
Cryphia olivacea|[4340]|RDNMK094-11|United States|Colorado|658[0n]|BOLD:AAE9413
Cryphia olivacea|[4341]|RDNMK095-11|United States|Colorado|658[0n]|BOLD:AAE9413
Cryphia cuerva|[4342]|RDNMH317-09|United States|Oregon|658[0n]|BOLD:AAC1995
Cryphia cuerva|[4343]|RDNME775-08|Canada|Alberta|658[0n]|BOLD:AAC1995
Cryphia cuerva|[4344]|LBCH6458-10|Canada|British Columbia|658[0n]|BOLD:AAC1995
Cryphia cuerva|[4345]|LBCG2861-09|Canada|British Columbia|658[0n]|BOLD:AAC1995
Cryphia cuerva|[4346]|LBCG2856-09|Canada|British Columbia|658[0n]|BOLD:AAC1995
Cryphia cuerva|[4347]|LBCH6462-10|Canada|British Columbia|658[1n]|BOLD:AAC1995
Cryphia cuerva|[4348]|LOWCC355-05|Canada|British Columbia|658[0n]|BOLD:AAC1995
Cryphia cuerva|[4349]|LOWCB181-05|Canada|British Columbia|658[0n]|BOLD:AAC1995
Cryphia cuerva|[4350]|LOWCC356-05|Canada|British Columbia|580[0n]|BOLD:AAC1995
Cryphia cuerva|[4351]|LOWCC357-05|Canada|British Columbia|520[0n]|
Cryphia cuerva|[4352]|LOWCC358-05|Canada|British Columbia|658[0n]|BOLD:AAC1995
Cryphia cuerva|[4353]|LOWCC359-05|Canada|British Columbia|658[0n]|BOLD:AAC1995
Cryphia cuerva|[4354]|LOWCC360-05|Canada|British Columbia|658[0n]|BOLD:AAC1995
Cryphia cuerva|[4355]|LBCG2858-09|Canada|British Columbia|658[0n]|BOLD:AAC1995
Cryphia cuerva|[4356]|LOWCB182-05|Canada|British Columbia|613[0n]|BOLD:AAC1995
Cryphia cuerva|[4357]|LBCG2857-09|Canada|British Columbia|658[0n]|BOLD:AAC1995
Cryphia cuerva|[4358]|LBCG2859-09|Canada|British Columbia|658[0n]|BOLD:AAC1995
Cryphia cuerva|[4359]|LBCG2860-09|Canada|British Columbia|658[0n]|BOLD:AAC1995
Cryphia cuerva|[4360]|RDNMH315-09|United States|Washington|658[0n]|BOLD:AAC1995
Cryphia cuerva|[4361]|LBCH6726-10|Canada|British Columbia|658[0n]|BOLD:AAC1995
Cryphia cuerva|[4362]|LBCH6925-10|Canada|British Columbia|658[0n]|BOLD:AAC1995
Cryphia cuerva|[4363]|LBCH6964-10|Canada|British Columbia|658[0n]|BOLD:AAC1995
Cryphia cuerva|[4364]|LBCH7601-10|Canada|British Columbia|658[0n]|BOLD:AAC1995
Cryphia cuerva|[4365]|LBCH7602-10|Canada|British Columbia|658[0n]|BOLD:AAC1995
Cryphia cuerva|[4366]|LBCH7603-10|Canada|British Columbia|658[0n]|BOLD:AAC1995
Cryphia cuerva|[4367]|LBCH7604-10|Canada|British Columbia|658[0n]|BOLD:AAC1995
Cryphia cuerva|[4368]|LBCH7605-10|Canada|British Columbia|658[0n]|BOLD:AAC1995
Cryphia cuerva|[4369]|LBCH7606-10|Canada|British Columbia|658[0n]|BOLD:AAC1995
Cryphia cuerva|[4370]|LBCH7607-10|Canada|British Columbia|658[0n]|BOLD:AAC1995
Cryphia cuerva|[4371]|LBCH7608-10|Canada|British Columbia|658[0n]|BOLD:AAC1995
Cryphia cuerva|[4372]|LBCH7762-10|Canada|British Columbia|658[0n]|BOLD:AAC1995
Cryphia cuerva|[4373]|LBCH7763-10|Canada|British Columbia|658[0n]|BOLD:AAC1995
Cryphia cuerva|[4374]|LBCH7764-10|Canada|British Columbia|658[0n]|BOLD:AAC1995
Cryphia cuerva|[4375]|LBCH7765-10|Canada|British Columbia|658[0n]|BOLD:AAC1995
Cryphia cuerva|[4376]|LBCH7766-10|Canada|British Columbia|658[0n]|BOLD:AAC1995
[illegible]

Cryphia cuerva|[4374]|LBCH7764-10|Canada|British Columbia|658[0n]|BOLD:AAC1995
Cryphia cuerva|[4375]|LBCH7765-10|Canada|British Columbia|658[0n]|BOLD:AAC1995
Cryphia cuerva|[4376]|LBCH7766-10|Canada|British Columbia|658[0n]|BOLD:AAC1995
Cryphia cuerva|[4377]|LBCH7768-10|Canada|British Columbia|658[0n]|BOLD:AAC1995
Cryphia cuerva|[4378]|LBCH7825-10|Canada|British Columbia|658[0n]|BOLD:AAC1995
Cryphia oaklandiae|[4379]|LNAUS3530-13|United States|California|658[0n]|BOLD:AAC1995
Cryphia cuerva|[4380]|NAMUM125-08|United States|California|658[0n]|BOLD:AAC1995
Cryphia cuerva|[4381]|RDNMB258-05|United States|Oregon|582[0n]|BOLD:AAC1995
Cryphia cuerva|[4382]|RDNMB257-05|United States|Oregon|574[0n]|BOLD:AAC1995
Cryphia flavidior|[4383]|RDNMH318-09|United States|Oregon|658[0n]|BOLD:AAC1995
Cryphia oaklandiae|[4384]|LNAUS3528-13|United States|California|658[0n]|BOLD:AAC1995
Cryphia oaklandiae|[4385]|LNAUS3527-13|United States|California|588[0n]|BOLD:AAC1995
Cryphia cuerva|[4386]|NAMUM142-08|United States|California|658[0n]|BOLD:AAC1995
Cryphia oaklandiae|[4387]|LNAUS3531-13|United States|California|658[0n]|BOLD:AAC1995
Gloanna grisescens|[4388]|CNCLB364-14|United States|Arizona|658[0n]|BOLD:ADA1590
Acopa carina|[4389]|TXLEP006-14|United States|Texas|564[0n]|BOLD:AAY9428
Acopa carina|[4390]|TXLEP003-14|United States|Texas|588[0n]|BOLD:AAY9428
Acopa carina|[4391]|TXLEP002-14|United States|Texas|588[0n]|BOLD:AAY9428
Acopa carina|[4392]|TXLEP004-14|United States|Texas|591[0n]|BOLD:AAY9428
Acopa carina|[4393]|TXLEP005-14|United States|Texas|588[0n]|BOLD:AAY9428
Acopa carina|[4394]|RDNMK127-11|United States|Texas|658[0n]|BOLD:AAY9428
Acopa carina|[4395]|TXLEP007-14|United States|Texas|588[0n]|BOLD:AAY9428
Acopa perpallida|[4396]|RDNMF639-08|United States|Arizona|658[0n]|BOLD:AAD5594
Acopa perpallida|[4397]|RDNMF635-08|United States|Arizona|658[5n]|BOLD:AAD5594
Acopa perpallida|[4398]|RDNMF637-08|United States|Arizona|658[0n]|BOLD:AAD5594
Acopa perpallida|[4399]|RDNMF638-08|United States|Arizona|658[0n]|BOLD:AAD5594
Acopa perpallida|[4400]|RDNMF640-08|United States|Arizona|658[0n]|BOLD:AAD5594
Acopa perpallida|[4401]|RDNMF636-08|United States|New Mexico|658[0n]|BOLD:ACE4350
Acopa perpallida|[4402]|RDNMJ181-10|United States|New Mexico|658[0n]|BOLD:ACE4350
Acopa perpallida|[4403]|RDNMJ180-10|United States|New Mexico|658[0n]|BOLD:ACE4350
Acopa perpallida|[4404]|CNCLB1694-14|United States|New Mexico|658[0n]|BOLD:ACE4350
Gloanna hecate|[4405]|CNCLB220-14|United States|Texas|658[0n]|BOLD:ACM4638
Cryphia sarepta|[4406]|RDNMD353-06|United States|Texas|658[0n]|BOLD:AAJ5975
Cryphia sarepta|[4407]|RDNMJ397-11|United States|Arizona|658[0n]|BOLD:AAV7936
Eviridemas minuta|[4408]|CNCLB2128-14|United States|Texas|658[0n]|BOLD:ACR5274
Eviridemas minuta|[4409]|CNCLB2416-14|United States|Texas|658[0n]|BOLD:ACR5274
Melaporphyria immortua|[4410]|NOCNA056-14|United States|Minnesota|658[0n]|BOLD:ACM6731
Melaporphyria immortua|[4411]|NOCNA012-14|United States|Minnesota|658[0n]|BOLD:ACM6731
Melaporphyria immortua|[4412]|CNCLB1086-14|Canada|Saskatchewan|658[0n]|BOLD:ACM6731
Melaporphyria immortua|[4413]|LNAUT396-14|United States|Connecticut|658[0n]|BOLD:ACM6731
Melaporphyria immortua|[4414]|LNAUT397-14|United States|Connecticut|658[0n]|BOLD:ACM6731
Eutricopis nr. nexilis|[4415]|NAMUM334-08|United States|California|658[0n]|BOLD:AAC8109
Eutricopis nr. nexilis|[4416]|NAMUM335-08|United States|California|658[0n]|BOLD:AAC8109
Eutricopis nexilis|[4417]|RDNMG536-08|Canada|Alberta|658[0n]|BOLD:AAC8108
Eutricopis nexilis|[4418]|RDNMG537-08|Canada|Alberta|658[0n]|BOLD:AAC8108
Eutricopis nexilis|[4419]|RDNMF052-08|Canada|Alberta|658[0n]|BOLD:AAC8108
Eutricopis nexilis|[4420]|RDNMG535-08|Canada|Alberta|658[0n]|BOLD:AAC8108
Eutricopis nexilis|[4421]|RDNMG538-08|Canada|Alberta|658[0n]|BOLD:AAC8108
Eutricopis nexilis|[4422]|HELNA528-09|United States|Wyoming|658[0n]|BOLD:AAC8108
Eutricopis nexilis|[4423]|HELNA867-10|Canada|British Columbia|658[0n]|BOLD:AAC8108
Eutricopis nexilis|[4424]|HELNA868-10|Canada|British Columbia|658[0n]|BOLD:AAC8108
Eutricopis nexilis|[4425]|HELNA869-10|Canada|British Columbia|658[0n]|BOLD:AAC8108
Baptarma felicita|[4426]|HELNA978-10|United States|California|658[0n]|BOLD:ABU7055
Narinosus searsi|[4427]|HELNA310-09|United States|California|658[0n]|BOLD:AAB6430
Narinosus searsi|[4428]|HELNA318-09|United States|California|658[0n]|BOLD:AAB6430
Narinosus searsi|[4429]|HELNA312-09|United States|California|658[0n]|BOLD:AAB6430
Narinosus searsi|[4430]|HELNA316-09|United States|California|658[0n]|BOLD:AAB6430
Narinosus searsi|[4431]|HELNA308-09|United States|California|658[0n]|BOLD:AAB6430
Narinosus searsi|[4432]|HELNA309-09|United States|California|658[0n]|BOLD:AAB6430
Narinosus searsi|[4433]|HELNA319-09|United States|California|658[0n]|BOLD:AAB6430
Narinosus searsi|[4434]|HELNA311-09|United States|California|632[0n]|BOLD:AAB6430
Narinosus searsi|[4435]|HELNA313-09|United States|California|658[0n]|BOLD:AAB6430
Narinosus searsi|[4436]|HELNA314-09|United States|California|658[0n]|BOLD:AAB6430
Narinosus searsi|[4437]|HELNA315-09|United States|California|658[0n]|BOLD:AAB6430
Narinosus searsi|[4438]|HELNA317-09|United States|California|658[0n]|BOLD:AAB6430
Narinosus searsi|[4439]|HELNA373-09|United States|California|658[0n]|BOLD:AAB6430
Microhelia angelica|[4440]|HELNA378-09|United States|California|658[0n]|BOLD:AAC2677
Microhelia angelica|[4441]|HELNA321-09|United States|California|658[0n]|BOLD:AAC2675
Microhelia angelica|[4442]|CNCLB1268-14|United States|California|658[0n]|BOLD:AAC2675
Microhelia angelica|[4443]|HELNA323-09|United States|California|658[0n]|BOLD:AAC2675
Microhelia angelica|[4444]|HELNA320-09|United States|California|658[0n]|BOLD:AAC2675
Microhelia angelica|[4445]|HELNA322-09|United States|California|658[0n]|BOLD:AAC2675
Microhelia angelica|[4446]|CNCLB1441-14|United States|Washington|658[0n]|BOLD:AAC2675
Heliothodes diminutivus|[4447]|CNCLB1618-14|United States|California|568[0n]|BOLD:AAB6428
Heliothodes diminutivus|[4448]|CNCLB1617-14|United States|California|658[0n]|BOLD:AAB6428
Heliothodes diminutivus|[4449]|CNCLB1619-14|United States|California|658[0n]|BOLD:AAB6428
Heliothodes diminutivus|[4450]|NAMUM338-08|United States|California|658[0n]|BOLD:AAB6428
Heliothodes diminutivus|[4451]|NAMUM405-09|United States|California|658[0n]|BOLD:AAB6428
Heliothodes diminutivus|[4452]|NAMUM425-09|United States|California|658[0n]|BOLD:AAB6428
Heliothodes diminutivus|[4453]|NAMUM426-09|United States|California|658[0n]|BOLD:AAB6428
Heliothodes diminutivus|[4454]|HELNA326-09|United States|California|658[0n]|BOLD:AAB6428
Heliothodes diminutivus|[4455]|HELNA332-09|United States|California|658[0n]|BOLD:AAB6428
Heliothodes diminutivus|[4456]|CGLCA130-10|United States|California|658[0n]|BOLD:AAB6428
Heliothodes diminutivus|[4457]|CNCLB1620-14|United States|California|658[0n]|BOLD:AAB6428
Heliothodes diminutivus|[4458]|CNCLB1621-14|United States|California|658[0n]|BOLD:AAB6428
Heliothodes diminutivus|[4459]|CNCLB1623-14|United States|California|658[0n]|BOLD:AAB6428
Heliothodes sp.|[4460]|CNCLB1622-14|United States|California|555[3n]|BOLD:AAB6427
Heliothodes diminutivus|[4461]|NAMUM333-08|United States|California|658[0n]|BOLD:AAB6427
Heliothodes diminutivus|[4462]|NAMUM336-08|United States|California|658[0n]|BOLD:AAB6427
Heliothodes diminutivus|[4463]|HELNA324-09|United States|California|658[0n]|BOLD:AAB6427
Heliothodes diminutivus|[4464]|HELNA325-09|United States|California|658[0n]|BOLD:AAB6427
Heliothodes diminutivus|[4465]|CNCLB1449-14|United States|Washington|585[0n]|BOLD:AAB6427
Heliothodes diminutivus|[4466]|CGLCA129-10|United States|California|658[0n]|BOLD:AAB6427
Heliothodes baldwini|[4467]|CNCLB2535-14|United States|California|658[0n]|BOLD:AAB6427
Heliothodes diminutivus|[4468]|HELNA328-09|United States|California|658[0n]|BOLD:ABZ1309
Heliothodes diminutivus|[4469]|HELNA327-09|United States|California|658[0n]|BOLD:ABZ1309
Heliothodes diminutivus|[4470]|HELNA329-09|United States|California|658[1n]|BOLD:ABZ1309
Heliothodes diminutivus|[4471]|HELNA331-09|United States|California|658[0n]|BOLD:ABZ1309
Heliothodes puniceus|[4472]|HELNA307-09|United States|Oregon|658[0n]|BOLD:ABZ1310
Heliothodes puniceus|[4473]|HELNA330-09|United States|Oregon|658[0n]|BOLD:ABZ1310
Heliothodes puniceus|[4474]|CNCLB1624-14|United States|Oregon|658[0n]|BOLD:ABZ1310
Heliothodes maritimus|[4475]|HELNA976-10|United States|California|658[0n]|BOLD:ABY5234
Heliothodes maritimus|[4476]|CNCLB2536-14|United States|California|658[0n]|BOLD:ABY5234

[illegible]
Heliothodes puniceus|[4474]|CNCLB1624-14|United States|Oregon|658[0n]|BOLD:ABZ1310
Heliothodes maritinus|[4475]|HELNA976-10|United States|California|658[0n]|BOLD:ABY5234
Heliothodes maritinus|[4476]|CNCLB2536-14|United States|California|658[0n]|BOLD:ABY5234
Heliothodes diminutivus|[4477]|CGLCA127-10|United States|California|658[0n]|BOLD:ABX4832
Heliothodes diminutivus|[4478]|CGLCA128-10|United States|California|658[0n]|BOLD:ABX4832
Heliothodes diminutivus|[4479]|CGLCA131-10|United States|California|658[0n]|BOLD:ABX4832
Heliothodes diminutivus|[4480]|CGLCA132-10|United States|California|658[0n]|BOLD:ABX4832
Heliothodes rubicundus|[4481]|CNCLB2537-14|United States|Oregon|658[0n]|BOLD:ABX4832
Heliothodes diminutivus|[4482]|CNCLB1578-14|United States|Washington|599[0n]|BOLD:ABX4832
Heliothodes diminutivus|[4483]|CNCLB1575-14|United States|Washington|658[0n]|BOLD:ABX4832
Heliothodes diminutivus|[4484]|CNCLB1576-14|United States|Washington|658[0n]|BOLD:ABX4832
Heliothodes diminutivus|[4485]|CNCLB1574-14|United States|Washington|621[0n]|BOLD:ABX4832
Heliothodes diminutivus|[4486]|CNCLB2533-14|United States|California|658[0n]|BOLD:ABX4832
Heliothodes hemizonianus|[4487]|CNCLB2534-14|United States|California|658[0n]|BOLD:ABX4832
Heliothodes hemizonianus|[4488]|CNCLB2538-14|United States|California|307[0n]|
Pyrrhia exprimens|[4489]|LHLEP189-06|Canada|British Columbia|658[5n]|BOLD:AAB3280
Pyrrhia exprimens|[4490]|HELNA278-09|United States|Colorado|658[0n]|BOLD:AAB3280
Pyrrhia exprimens|[4491]|RDNMC037-05|United States|Oregon|658[0n]|BOLD:AAB3280
Pyrrhia exprimens|[4492]|HELNA853-10|United States|Oregon|658[0n]|BOLD:AAB3280
Pyrrhia exprimens|[4493]|RWWC965-12|United States|Washington|658[0n]|BOLD:AAB3280
Pyrrhia exprimens|[4494]|LGSM406-04|United States|Tennessee|658[0n]|BOLD:AAB3280
Pyrrhia exprimens|[4495]|HELNA290-09|United States|North Carolina|614[0n]|BOLD:AAB3280
Pyrrhia exprimens|[4496]|RDLQB216-05|Canada|Quebec|658[0n]|BOLD:AAB3280
Pyrrhia exprimens|[4497]|RDLQB214-05|Canada|Quebec|658[0n]|BOLD:AAB3280
Pyrrhia exprimens|[4498]|RDLQB215-05|Canada|Quebec|679[0n]|BOLD:AAB3280
Pyrrhia exprimens|[4499]|LOT495-04|United States|Tennessee|658[0n]|BOLD:AAB3280
Pyrrhia exprimens|[4500]|LBCH2960-10|Canada|British Columbia|658[0n]|BOLD:AAB3280
Pyrrhia exprimens|[4501]|HELNA289-09|United States|Tennessee|658[0n]|BOLD:AAB3280
Pyrrhia exprimens|[4502]|LPSOC096-08|Canada|Ontario|658[0n]|BOLD:AAB3280
Pyrrhia exprimens|[4503]|LHLEP271-06|Canada|British Columbia|658[0n]|BOLD:AAB3280
Pyrrhia exprimens|[4504]|RDLQB799-05|Canada|Quebec|658[0n]|BOLD:AAB3280
Pyrrhia exprimens|[4505]|LGSMC810-05|United States|Tennessee|658[0n]|BOLD:AAB3280
Pyrrhia exprimens|[4506]|LGSMC809-05|United States|Tennessee|658[0n]|BOLD:AAB3280
Pyrrhia exprimens|[4507]|LGSMC808-05|United States|Tennessee|658[0n]|BOLD:AAB3280
Pyrrhia exprimens|[4508]|LGSMC807-05|United States|Tennessee|658[0n]|BOLD:AAB3280
Pyrrhia exprimens|[4509]|LOT500-04|United States|Tennessee|658[0n]|BOLD:AAB3280
Pyrrhia exprimens|[4510]|LGSM407-04|United States|North Carolina|658[0n]|BOLD:AAB3280
Pyrrhia exprimens|[4511]|LGSMC387-05|United States|Tennessee|619[0n]|BOLD:AAB3280
Pyrrhia exprimens|[4512]|RDLQB798-05|Canada|Quebec|533[0n]|BOLD:AAB3280
Pyrrhia exprimens|[4513]|LSEU072-06|United States|North Carolina|596[0n]|BOLD:AAB3280
Pyrrhia exprimens|[4514]|BBLPB496-10|Canada|Saskatchewan|658[0n]|BOLD:AAB3280
Pyrrhia exprimens|[4515]|BBLPB499-10|Canada|Ontario|658[0n]|BOLD:AAB3280
Pyrrhia exprimens|[4516]|CNCLB2645-14|United States|North Carolina|658[0n]|BOLD:AAB3280
Pyrrhia cilisca|[4517]|LGSMG925-10|United States|Tennessee|658[0n]|BOLD:AAC7528
Pyrrhia cilisca|[4518]|CNCLB2885-14|United States|North Carolina|632[0n]|BOLD:AAC7528
Pyrrhia cilisca|[4519]|LGSM405-04|United States|Tennessee|658[0n]|BOLD:AAC7528
Pyrrhia cilisca|[4520]|RDNMC036-05|Canada|Ontario|658[0n]|BOLD:AAC7528
Pyrrhia cilisca|[4521]|RDLQ747-07|Canada|Quebec|657[0n]|BOLD:AAC7528
Pyrrhia cilisca|[4522]|RDNMG823-08|Canada|Ontario|658[0n]|BOLD:AAC7528
Pyrrhia cilisca|[4523]|LTOLB142-08|United States|Georgia|658[0n]|BOLD:AAC7528
Pyrrhia cilisca|[4524]|NAMUM319-08|United States|Georgia|658[0n]|BOLD:AAC7528
Pyrrhia cilisca|[4525]|CNCLB2886-14|United States|North Carolina|658[0n]|BOLD:AAC7528
Chamaeclea basiochrea|[4526]|NAMUM114-08|United States|Texas|658[0n]|BOLD:AAI7205
Chamaeclea basiochrea|[4527]|NAMUM115-08|United States|Texas|658[0n]|BOLD:AAI7205
Chamaeclea pernana|[4528]|RDNMH823-09|United States|Arizona|658[0n]|BOLD:AAI7206
Chamaeclea pernana|[4529]|RDNMJ392-11|United States|Arizona|658[0n]|BOLD:AAI7206
Chamaeclea pernana|[4530]|RDNMJ531-11|United States|Arizona|658[0n]|BOLD:AAI7206
Chamaeclea pernana|[4531]|NAMUM073-08|United States|Arizona|657[0n]|BOLD:AAI7206
Chamaeclea pernana|[4532]|RDNMK190-11|United States|Arizona|658[0n]|BOLD:AAI7206
Chamaeclea|[4533]|RDNMK341-11|United States|New Mexico|658[0n]|BOLD:AAZ8757
Chamaeclea|[4534]|RDNMK340-11|United States|New Mexico|658[0n]|BOLD:AAZ8757
Chamaeclea|[4535]|RDNMK342-11|United States|New Mexico|658[0n]|BOLD:AAZ8757
Aleptinoides ochrea|[4536]|RDNMK343-11|United States|New Mexico|658[0n]|BOLD:AAZ9512
Hemioslaria pima|[4537]|RDNME898-08|United States|Arizona|658[0n]|BOLD:AAI6822
Hemioslaria pima|[4538]|JBAZ117-09|United States|Arizona|658[0n]|BOLD:AAI6822
Heminocloa mirabilis|[4539]|RDNMD808-07|United States|Arizona|658[0n]|BOLD:AAI6829
Heminocloa mirabilis|[4540]|AWCL004-09|United States|Arizona|658[0n]|BOLD:AAI6829
Thurberiphaga diffusa|[4541]|RDNMD818-07|United States|Arizona|656[0n]|BOLD:AAH4919
Thurberiphaga diffusa|[4542]|CMAZA792-10|United States|Arizona|658[0n]|BOLD:AAH4919
Thurberiphaga diffusa|[4543]|AWCL005-09|United States|Arizona|658[0n]|BOLD:AAH4919
Thurberiphaga diffusa|[4544]|AWCL070-09|United States|Arizona|658[0n]|BOLD:AAH4919
Thurberiphaga diffusa|[4545]|DMAZ148-10|United States|Arizona|658[0n]|BOLD:AAH4919
Thurberiphaga diffusa|[4546]|CMAZA982-12|United States|Arizona|658[0n]|BOLD:AAH4919
Azenia virida|[4547]|CNCLB949-14|United States|California|658[0n]|BOLD:ACM4690
Azenia virida|[4548]|CNCLB950-14|United States|California|658[0n]|BOLD:ACM4690
Psectrotarsia suavis|[4549]|CMAZA557-10|United States|Arizona|658[0n]|BOLD:AAE8165
Psectrotarsia suavis|[4550]|AWCL069-09|United States|Arizona|658[0n]|BOLD:AAE8165
Psectrotarsia suavis|[4551]|AWCL068-09|United States|Arizona|658[0n]|BOLD:AAE8165
Psectrotarsia suavis|[4552]|RDNMK104-11|United States|Arizona|658[0n]|BOLD:AAE8165
Psectrotarsia suavis|[4553]|HELNA140-06|United States|Oklahoma|658[0n]|BOLD:AAE8165
Psectrotarsia suavis|[4554]|HELNA141-06|United States|New Mexico|658[0n]|BOLD:AAE8165
Psectrotarsia suavis|[4555]|AWCL067-09|United States|Arizona|658[0n]|BOLD:AAE8165
Psectrotarsia suavis|[4556]|HELNA369-09|United States|Colorado|658[0n]|BOLD:AAE8165
Psectrotarsia suavis|[4557]|QUNOD106-10|United States|Texas|658[0n]|BOLD:AAE8165
Psectrotarsia suavis|[4558]|LTOLB1259-11|United States|Kansas|658[0n]|BOLD:AAE8165
Psectrotarsia suavis|[4559]|LTOLB1260-11|United States|Kansas|658[0n]|BOLD:AAE8165
Psectrotarsia suavis|[4560]|LNAUP153-13|United States|Colorado|658[0n]|BOLD:AAE8165
Psectrotarsia hebardi|[4561]|LNAUT391-14|United States|New Jersey|658[0n]|BOLD:ACM4611
Psectrotarsia hebardi|[4562]|CNCLB131-14|United States|Ohio|658[0n]|BOLD:ACM4611
Psectrotarsia hebardi|[4563]|LNAUT392-14|United States|Ohio|658[0n]|BOLD:ACM4611
Balsa malana|[4564]|XAB313-04|Canada|Ontario|658[0n]|BOLD:AAB2064
Balsa malana|[4565]|XAF696-05|Canada|Ontario|658[0n]|BOLD:AAB2064
Balsa malana|[4566]|XAG211-05|Canada|Ontario|658[0n]|BOLD:AAB2064
Balsa malana|[4567]|XAI027-05|Canada|Ontario|658[0n]|BOLD:AAB2064
Balsa malana|[4568]|LPSOC400-08|Canada|Ontario|630[0n]|BOLD:AAB2064
Balsa malana|[4569]|LPSOC410-08|Canada|Ontario|658[0n]|BOLD:AAB2064
Balsa malana|[4570]|XAK108-06|Canada|Ontario|658[0n]|BOLD:AAB2064
Balsa malana|[4571]|PHMO368-03|Canada|Ontario|639[0n]|BOLD:AAB2064
Balsa malana|[4572]|RDLQ497-07|Canada|Quebec|599[0n]|BOLD:AAB2064
Balsa malana|[4573]|KPOEC134-08|Canada|Ontario|658[0n]|BOLD:AAB2064
Balsa tristrigella|[4574]|LPSOC405-08|Canada|Ontario|658[0n]|BOLD:AAA6646
Balsa tristrigella|[4575]|PHMNB640-04|Canada|New Brunswick|658[0n]|BOLD:AAA6646

Balsa malana|[4573]|KPOEC134-08|Canada|Ontario|658[0n]|BOLD:AAB2064
Balsa tristrigella|[4574]|LPSOC405-08|Canada|Ontario|658[0n]|BOLD:AAA6646
Balsa tristrigella|[4575]|PHMNB640-04|Canada|New Brunswick|658[0n]|BOLD:AAA6646
Balsa tristrigella|[4576]|BLTIB265-08|Canada|Ontario|627[0n]|BOLD:AAA6646
Balsa tristrigella|[4577]|MECC186-06|Canada|Quebec|658[0n]|BOLD:AAA6646
Balsa tristrigella|[4578]|PMG095-03|Canada|Ontario|617[0n]|BOLD:AAA6646
Balsa tristrigella|[4579]|PHMO081-03|Canada|Ontario|639[0n]|BOLD:AAA6646
Balsa tristrigella|[4580]|XAJ630-06|Canada|Ontario|658[0n]|BOLD:AAA6646
Balsa tristrigella|[4581]|XAJ699-06|Canada|Ontario|658[0n]|BOLD:AAA6646
Balsa tristrigella|[4582]|XAJ700-06|Canada|Ontario|658[0n]|BOLD:AAA6646
Balsa tristrigella|[4583]|XAK107-06|Canada|Ontario|658[0n]|BOLD:AAA6646
Balsa tristrigella|[4584]|LPSOC176-08|Canada|Ontario|658[0n]|BOLD:AAA6646
Balsa tristrigella|[4585]|LPSOC177-08|Canada|Ontario|658[0n]|BOLD:AAA6646
Balsa tristrigella|[4586]|PHMNB653-04|Canada|New Brunswick|658[0n]|BOLD:AAA6646
Balsa tristrigella|[4587]|RDLQG691-06|Canada|Quebec|658[0n]|BOLD:AAA6646
Balsa tristrigella|[4588]|RDLQG730-06|Canada|Quebec|614[0n]|BOLD:AAA6646
Balsa tristrigella|[4589]|PHMNB175-04|Canada|New Brunswick|658[1n]|BOLD:AAA6646
Balsa tristrigella|[4590]|LPSOC245-08|Canada|Ontario|658[0n]|BOLD:AAA6646
Balsa tristrigella|[4591]|LPSOC244-08|Canada|Ontario|658[0n]|BOLD:AAA6646
Balsa tristrigella|[4592]|LPSOC243-08|Canada|Ontario|658[0n]|BOLD:AAA6646
Balsa tristrigella|[4593]|LPSOC181-08|Canada|Ontario|658[0n]|BOLD:AAA6646
Balsa tristrigella|[4594]|LPSOC163-08|Canada|Ontario|658[0n]|BOLD:AAA6646
Balsa tristrigella|[4595]|LPSOC161-08|Canada|Ontario|658[0n]|BOLD:AAA6646
Balsa tristrigella|[4596]|LPSOC062-08|Canada|Ontario|658[0n]|BOLD:AAA6646
Balsa tristrigella|[4597]|RDLQG725-06|Canada|Quebec|658[0n]|BOLD:AAA6646
Balsa tristrigella|[4598]|RDLQG706-06|Canada|Quebec|658[0n]|BOLD:AAA6646
Balsa tristrigella|[4599]|RDLQG702-06|Canada|Quebec|658[0n]|BOLD:AAA6646
Balsa tristrigella|[4600]|RDLQG519-06|Canada|Quebec|658[0n]|BOLD:AAA6646
Balsa tristrigella|[4601]|RDLQG262-06|Canada|Quebec|658[0n]|BOLD:AAA6646
Balsa tristrigella|[4602]|XAK192-06|Canada|Ontario|658[0n]|BOLD:AAA6646
Balsa tristrigella|[4603]|LSEU079-06|United States|North Carolina|658[0n]|BOLD:AAA6646
Balsa tristrigella|[4604]|PHMNB646-04|Canada|New Brunswick|658[0n]|BOLD:AAA6646
Balsa tristrigella|[4605]|PHMNB642-04|Canada|New Brunswick|658[0n]|BOLD:AAA6646
Balsa tristrigella|[4606]|PHMNB638-04|Canada|New Brunswick|658[0n]|BOLD:AAA6646
Balsa tristrigella|[4607]|PHMNB637-04|Canada|New Brunswick|658[0n]|BOLD:AAA6646
Balsa tristrigella|[4608]|PHMNB636-04|Canada|New Brunswick|658[0n]|BOLD:AAA6646
Balsa tristrigella|[4609]|PHMNB620-04|Canada|New Brunswick|658[0n]|BOLD:AAA6646
Balsa tristrigella|[4610]|PHMNB555-04|Canada|New Brunswick|658[0n]|BOLD:AAA6646
Balsa tristrigella|[4611]|PHMNB394-04|Canada|New Brunswick|658[0n]|BOLD:AAA6646
Balsa tristrigella|[4612]|XAB615-04|Canada|Ontario|658[0n]|BOLD:AAA6646
Balsa tristrigella|[4613]|XAB357-04|Canada|Ontario|658[0n]|BOLD:AAA6646
Balsa tristrigella|[4614]|XAB305-04|Canada|Ontario|658[0n]|BOLD:AAA6646
Balsa tristrigella|[4615]|LPSOC242-08|Canada|Ontario|656[0n]|BOLD:AAA6646
Balsa tristrigella|[4616]|XAJ614-06|Canada|Ontario|658[0n]|BOLD:AAA6646
Balsa tristrigella|[4617]|XAJ705-06|Canada|Ontario|658[0n]|BOLD:AAA6646
Balsa tristrigella|[4618]|XAK181-06|Canada|Ontario|658[0n]|BOLD:AAA6646
Balsa tristrigella|[4619]|XAK189-06|Canada|Ontario|658[0n]|BOLD:AAA6646
Balsa tristrigella|[4620]|LPSOC054-08|Canada|Ontario|658[0n]|BOLD:AAA6646
Balsa tristrigella|[4621]|LPSOC158-08|Canada|Ontario|658[0n]|BOLD:AAA6646
Balsa tristrigella|[4622]|LPSOC159-08|Canada|Ontario|658[0n]|BOLD:AAA6646
Balsa tristrigella|[4623]|LPSOC160-08|Canada|Ontario|658[0n]|BOLD:AAA6646
Balsa tristrigella|[4624]|XAB583-04|Canada|Ontario|658[0n]|BOLD:AAA6646
Balsa tristrigella|[4625]|XAC027-04|Canada|Ontario|658[0n]|BOLD:AAA6646
Balsa tristrigella|[4626]|LPSOC402-08|Canada|Ontario|658[0n]|BOLD:AAA6646
Balsa tristrigella|[4627]|LPSOC407-08|Canada|Ontario|658[0n]|BOLD:AAA6646
Balsa tristrigella|[4628]|LPSOB744-08|Canada|Ontario|658[0n]|BOLD:AAA6646
Balsa tristrigella|[4629]|LILLA140-11|United States|Illinois|658[0n]|BOLD:AAA6646
Balsa tristrigella|[4630]|MECC002-06|Canada|Quebec|658[0n]|BOLD:AAA6646
Balsa tristrigella|[4631]|RDLQG520-06|Canada|Quebec|658[0n]|BOLD:AAA6646
Balsa tristrigella|[4632]|RDLQG724-06|Canada|Quebec|658[0n]|BOLD:AAA6646
Balsa tristrigella|[4633]|RDLQG732-06|Canada|Quebec|658[0n]|BOLD:AAA6646
Balsa tristrigella|[4634]|LPSOC241-08|Canada|Ontario|658[0n]|BOLD:AAA6646
Balsa tristrigella|[4635]|BBLCU156-09|United States|Michigan|658[0n]|BOLD:AAA6646
Balsa tristrigella|[4636]|PHMNB392-04|Canada|New Brunswick|658[0n]|BOLD:AAA6646
Balsa tristrigella|[4637]|LGSMC674-05|United States|Tennessee|658[0n]|BOLD:AAA6646
Balsa tristrigella|[4638]|LILLA204-11|United States|Illinois|658[0n]|BOLD:AAA6646
Balsa labecula|[4639]|XAI026-05|Canada|Ontario|658[0n]|BOLD:AAB2063
Balsa labecula|[4640]|LNCC203-10|United States|North Carolina|658[0n]|BOLD:AAB2063
Balsa labecula|[4641]|LGSMG1001-10|United States|Tennessee|658[0n]|BOLD:AAB2063
Balsa labecula|[4642]|LNCC1067-11|United States|North Carolina|658[0n]|BOLD:AAB2063
Balsa labecula|[4643]|XAC043-04|Canada|Ontario|658[0n]|BOLD:AAB2063
Balsa labecula|[4644]|XAC457-04|Canada|Ontario|658[0n]|BOLD:AAB2063
Balsa labecula|[4645]|XAC719-04|Canada|Ontario|658[0n]|BOLD:AAB2063
Balsa labecula|[4646]|XAC838-04|Canada|Ontario|658[0n]|BOLD:AAB2063
Balsa labecula|[4647]|XAE581-04|Canada|Ontario|658[0n]|BOLD:AAB2063
Balsa labecula|[4648]|MNBB064-05|Canada|New Brunswick|658[0n]|BOLD:AAB2063
Balsa labecula|[4649]|LGSMC477-05|United States|Tennessee|658[0n]|BOLD:AAB2063
Balsa labecula|[4650]|LGSMC697-05|United States|Tennessee|658[0n]|BOLD:AAB2063
Balsa labecula|[4651]|RDLQB394-05|Canada|Quebec|658[0n]|BOLD:AAB2063
Balsa labecula|[4652]|RDLQF479-06|Canada|Quebec|658[0n]|BOLD:AAB2063
Balsa labecula|[4653]|RDLQF480-06|Canada|Quebec|658[0n]|BOLD:AAB2063
Balsa labecula|[4654]|RDLQG511-06|Canada|Quebec|658[0n]|BOLD:AAB2063
Balsa labecula|[4655]|RDLQG782-06|Canada|Quebec|658[0n]|BOLD:AAB2063
Balsa labecula|[4656]|RDLQG793-06|Canada|Quebec|658[0n]|BOLD:AAB2063
Balsa labecula|[4657]|MMNA101-08|United States|North Carolina|658[0n]|BOLD:AAB2063
Balsa labecula|[4658]|LPSOC060-08|Canada|Ontario|658[0n]|BOLD:AAB2063
Balsa labecula|[4659]|LPSOC443-08|Canada|Ontario|658[0n]|BOLD:AAB2063
Balsa labecula|[4660]|LSEU625-06|United States|Georgia|658[0n]|BOLD:AAB2063
Balsa labecula|[4661]|RDLQE879-06|Canada|Quebec|658[0n]|BOLD:AAB2063
Balsa labecula|[4662]|MNBB345-05|Canada|New Brunswick|658[0n]|BOLD:AAB2063
Balsa labecula|[4663]|LGSM772-04|United States|North Carolina|658[0n]|BOLD:AAB2063
Balsa labecula|[4664]|PHMNB194-04|Canada|New Brunswick|658[0n]|BOLD:AAB2063
Balsa labecula|[4665]|XAB308-04|Canada|Ontario|658[0n]|BOLD:AAB2063
Balsa labecula|[4666]|PMG094-03|Canada|Ontario|617[0n]|BOLD:AAB2063
Balsa labecula|[4667]|TMG118-03|Canada|Ontario|639[0n]|BOLD:AAB2063
Balsa labecula|[4668]|LSUSA174-06|United States|Kentucky|658[0n]|BOLD:AAB2063
Balsa labecula|[4669]|RDLQG690-06|Canada|Quebec|658[0n]|BOLD:AAB2063
Balsa labecula|[4670]|LPSOB347-08|Canada|Ontario|658[0n]|BOLD:AAB2063
Balsa labecula|[4671]|LNCC204-10|United States|North Carolina|658[0n]|BOLD:AAB2063
Balsa labecula|[4672]|RDLQF382-06|Canada|Quebec|658[1n]|BOLD:AAB2063
Balsa labecula|[4673]|LPOKB716-09|United States|Oklahoma|658[0n]|BOLD:AAB2063
Balsa labecula|[4674]|MNBB058-05|Canada|New Brunswick|658[0n]|BOLD:AAB2063
Balsa labecula|[4675]|RDLQG321-06|Canada|Quebec|658[0n]|BOLD:AAB2063

Balsa labecula|[4672]|[illegible]|Canada|Quebec|658[1n]|BOLD:AAB2063
Balsa labecula|[4673]|LPOKB716-09|United States|Oklahoma|658[0n]|BOLD:AAB2063
Balsa labecula|[4674]|MNBB058-05|Canada|New Brunswick|658[0n]|BOLD:AAB2063
Balsa labecula|[4675]|RDLQG321-06|Canada|Quebec|658[0n]|BOLD:AAB2063
Balsa labecula|[4676]|RDLQF478-06|Canada|Quebec|658[0n]|BOLD:AAB2063
Balsa labecula|[4677]|PHMNB624-04|Canada|New Brunswick|658[0n]|BOLD:AAB2063
Balsa labecula|[4678]|LOT551-04|United States|Tennessee|596[0n]|BOLD:AAB2063
Balsa labecula|[4679]|LILLA241-11|United States|Illinois|658[0n]|BOLD:AAB2063
Balsa labecula|[4680]|LNCC1167-11|United States|North Carolina|658[0n]|BOLD:AAB2063
Calophasia lunula|[4681]|BLTIB069-08|Canada|Ontario|548[0n]|BOLD:AAB0052
Calophasia lunula|[4682]|GWORL453-09|Germany|Bavaria|658[0n]|BOLD:AAB0052
Calophasia lunula|[4683]|PHLAA309-09|France|Provence-Alpes-Cote d'Azur|658[0n]|BOLD:AAB0052
Calophasia lunula|[4684]|NLLEA1301-14|Netherlands|South Holland|658[0n]|BOLD:AAB0052
Calophasia lunula|[4685]|NLLEA1302-14|Netherlands|South Holland|658[0n]|BOLD:AAB0052
Calophasia lunula|[4686]|LEFIF666-10|Finland||658[0n]|BOLD:AAB0052
Calophasia lunula|[4687]|LEFIA624-10|Finland|South Karelia|638[1n]|BOLD:AAB0052
Calophasia lunula|[4688]|XAG084-05|Canada|Ontario|617[0n]|BOLD:AAB0052
Calophasia lunula|[4689]|CGUKA347-09|United Kingdom|England|641[0n]|BOLD:AAB0052
Calophasia lunula|[4690]|LENOA502-11|France|Haute Normandie|658[0n]|BOLD:AAB0052
Calophasia lunula|[4691]|PHLAF414-11|Macedonia||658[0n]|BOLD:AAB0052
Calophasia lunula|[4692]|GWORZ277-10|Italy|Basilicata|658[0n]|BOLD:AAB0052
Calophasia lunula|[4693]|LENOA503-11|France|Haute Normandie|658[0n]|BOLD:AAB0052
Calophasia lunula|[4694]|CGUKB178-09|United Kingdom|England|658[0n]|BOLD:AAB0052
Calophasia lunula|[4695]|LEFIF665-10|Finland||658[0n]|BOLD:AAB0052
Calophasia lunula|[4696]|RDLQ607-07|Canada|Quebec|658[0n]|BOLD:AAB0052
Calophasia lunula|[4697]|CGUKA461-09|United Kingdom|England|658[0n]|BOLD:AAB0052
Calophasia lunula|[4698]|XAJ599-06|Canada|Ontario|658[0n]|BOLD:AAB0052
Calophasia lunula|[4699]|RDLQF881-06|Canada|Quebec|658[0n]|BOLD:AAB0052
Calophasia lunula|[4700]|GBLAB347-13|Germany|Rhineland-Palatinate|658[0n]|BOLD:AAB0052
Calophasia lunula|[4701]|GBLAB712-13|Germany|Brandenburg|658[0n]|BOLD:AAB0052
Calophasia lunula|[4702]|LEATA335-13|Austria|Tirol|658[0n]|BOLD:AAB0052
Calophasia lunula|[4703]|LEATG138-14|Austria|Tirol|658[0n]|BOLD:AAB0052
Calophasia lunula|[4704]|LEATG139-14|Austria|Tirol|658[0n]|BOLD:AAB0052
Calophasia lunula|[4705]|GBLAA920-14|Germany|Rhineland-Palatinate|658[0n]|BOLD:AAB0052
Calophasia lunula|[4706]|GBLAA921-14|Germany|North Rhine-Westphalia|658[0n]|BOLD:AAB0052
Calophasia lunula|[4707]|GBLAA922-14|Germany|North Rhine-Westphalia|658[0n]|BOLD:AAB0052
Calophasia lunula|[4708]|LASTS143-14|Italy|South Tyrol|658[0n]|BOLD:AAB0052
Calophasia lunula|[4709]|LASTS144-14|Italy|South Tyrol|658[0n]|BOLD:AAB0052
Calophasia lunula|[4710]|XAJ238-06|Canada|Ontario|658[0n]|BOLD:AAB0052
Calophasia lunula|[4711]|XAJ245-06|Canada|Ontario|658[0n]|BOLD:AAB0052
Calophasia lunula|[4712]|XAG945-05|Canada|Ontario|658[0n]|BOLD:AAB0052
Calophasia lunula|[4713]|XAG884-05|Canada|Ontario|658[0n]|BOLD:AAB0052
Calophasia lunula|[4714]|XAG260-05|Canada|Ontario|658[0n]|BOLD:AAB0052
Calophasia lunula|[4715]|XAG106-05|Canada|Ontario|658[0n]|BOLD:AAB0052
Calophasia lunula|[4716]|XAG020-05|Canada|Ontario|658[0n]|BOLD:AAB0052
Calophasia lunula|[4717]|XAF699-05|Canada|Ontario|658[0n]|BOLD:AAB0052
Calophasia lunula|[4718]|XAG312-05|Canada|Ontario|658[0n]|BOLD:AAB0052
Calophasia lunula|[4719]|XAH602-05|Canada|Ontario|658[0n]|BOLD:AAB0052
Calophasia lunula|[4720]|TTMNB388-06|Canada|New Brunswick|658[0n]|BOLD:AAB0052
Calophasia lunula|[4721]|TTMNB389-06|Canada|New Brunswick|658[0n]|BOLD:AAB0052
Calophasia lunula|[4722]|TTMNB390-06|Canada|New Brunswick|658[0n]|BOLD:AAB0052
Calophasia lunula|[4723]|TTMNB391-06|Canada|New Brunswick|658[0n]|BOLD:AAB0052
Calophasia lunula|[4724]|XAJ237-06|Canada|Ontario|658[0n]|BOLD:AAB0052
Calophasia lunula|[4725]|XAJ260-06|Canada|Ontario|658[0n]|BOLD:AAB0052
Calophasia lunula|[4726]|PMG097-03|Canada|Ontario|617[0n]|BOLD:AAB0052
Calophasia lunula|[4727]|TMG124-03|Canada|Ontario|639[0n]|BOLD:AAB0052
Calophasia lunula|[4728]|XAD013-04|Canada|Ontario|614[0n]|BOLD:AAB0052
Calophasia lunula|[4729]|XAG763-05|Canada|Ontario|658[0n]|BOLD:AAB0052
Calophasia lunula|[4730]|KPOEC079-08|Canada|Ontario|640[0n]|BOLD:AAB0052
Calophasia lunula|[4731]|ABOLA190-14|Austria||658[0n]|BOLD:AAB0052
Ponometia septuosa|[4732]|HKONB251-09|United States|Texas|634[0n]|BOLD:AAD8629
Ponometia phecolisca|[4733]|RDNME065-07|United States|Arizona|658[0n]|BOLD:AAE9641
Ponometia phecolisca|[4734]|BBLSY161-09|United States|Texas|658[0n]|BOLD:AAE9641
Ponometia phecolisca|[4735]|BBLSW940-09|United States|Texas|658[0n]|BOLD:AAE9641
Ponometia phecolisca|[4736]|BBLSW861-09|United States|Texas|658[0n]|BOLD:AAE9641
Ponometia phecolisca|[4737]|RDNMH692-09|United States|Arizona|658[0n]|BOLD:AAE9641
Ponometia phecolisca|[4738]|RDNME064-07|United States|Arizona|658[0n]|BOLD:AAE9641
Ponometia phecolisca|[4739]|MNAD162-07|United States|Arizona|658[0n]|BOLD:AAE9641
Ponometia phecolisca|[4740]|BBLSY274-09|United States|Texas|658[0n]|BOLD:AAE9641
Ponometia phecolisca|[4741]|BBLSX834-09|United States|Texas|658[0n]|BOLD:AAE9641
Ponometia phecolisca|[4742]|USLEP1181-10|United States|Texas|658[0n]|BOLD:AAE9641
Ponometia phecolisca|[4743]|USLEP1182-10|United States|Texas|658[0n]|BOLD:AAE9641
Ponometia phecolisca|[4744]|CMAZA640-10|United States|Arizona|658[0n]|BOLD:AAE9641
Ponometia phecolisca|[4745]|PSAT123-10|United States|Arizona|658[0n]|BOLD:AAE9641
Ponometia phecolisca|[4746]|LPOKE225-10|United States|Oklahoma|658[0n]|BOLD:AAE9641
Ponometia elegantula|[4747]|RDNME070-07|United States|California|601[0n]|BOLD:AAC6158
Ponometia elegantula|[4748]|RDNMB263-05|United States|Oregon|597[0n]|BOLD:AAC6158
Ponometia elegantula|[4749]|RDNME913-08|United States|Arizona|658[0n]|BOLD:AAC6158
Ponometia elegantula|[4750]|BBLSY082-09|United States|Arizona|658[0n]|BOLD:AAC6158
Ponometia elegantula|[4751]|AWCLB205-10|United States|Arizona|658[0n]|BOLD:AAC6158
Ponometia elegantula|[4752]|AWCLB654-11|United States|Arizona|658[0n]|BOLD:AAC6158
Ponometia elegantula|[4753]|BBLOB049-11|United States|Arizona|658[0n]|BOLD:AAC6158
Ponometia elegantula|[4754]|BBLOB088-11|United States|Arizona|658[0n]|BOLD:AAC6158
Ponometia elegantula|[4755]|BBLOC1163-11|United States|California|658[0n]|BOLD:AAC6158
Ponometia elegantula|[4756]|RDNMH686-09|United States|California|622[0n]|BOLD:AAC6158
Ponometia elegantula|[4757]|RDNMB267-05|Canada|Alberta|593[0n]|BOLD:AAC6158
Ponometia elegantula|[4758]|RDNMB264-05|United States|Nevada|658[0n]|BOLD:AAC6158
Ponometia elegantula|[4759]|RDNMB265-05|Canada|Alberta|658[0n]|BOLD:AAC6158
Ponometia elegantula|[4760]|RDNME911-08|United States|Arizona|658[0n]|BOLD:AAC6158
Ponometia elegantula|[4761]|USLEP386-10|United States|Arizona|658[0n]|BOLD:AAC6158
Ponometia elegantula|[4762]|AWCLB181-10|United States|Arizona|658[0n]|BOLD:AAC6158
Ponometia elegantula|[4763]|AWCLB209-10|United States|Arizona|658[0n]|BOLD:AAC6158
Ponometia elegantula|[4764]|BBLOC1453-11|United States|California|658[0n]|BOLD:AAC6158
Ponometia elegantula|[4765]|BBLOC1845-11|United States|California|658[0n]|BOLD:AAC6158
Ponometia elegantula|[4766]|BBLOC1889-11|United States|California|658[0n]|BOLD:AAC6158
Ponometia elegantula|[4767]|RDNMB268-05|United States|Arizona|594[0n]|BOLD:AAC6158
Ponometia elegantula|[4768]|BBLOC1905-11|United States|California|658[0n]|BOLD:AAC6158
Ponometia elegantula|[4769]|RDNMB266-05|United States|Arizona|558[0n]|BOLD:AAC6158
Ponometia elegantula|[4770]|BBLOC1160-11|United States|California|658[0n]|BOLD:AAC6158
Ponometia elegantula|[4771]|BBLOC1840-11|United States|California|658[0n]|BOLD:AAC6158
Ponometia elegantula|[4772]|BBLOE1893-12|United States|Arizona|658[0n]|BOLD:AAC6158
Ponometia elegantula|[4773]|RDNME912-08|United States|Arizona|658[0n]|BOLD:AAC6158
Ponometia elegantula|[4774]|BBLOE808-12|United States|Texas|658[0n]|BOLD:AAC6158

Ponometia elegantula|[4772]|BBLOE1893-12|United States|Arizona|658[0n]|BOLD:AAC6158
Ponometia elegantula|[4773]|RDNME912-08|United States|Arizona|658[0n]|BOLD:AAC6158
Ponometia elegantula|[4774]|BBLOE808-12|United States|Texas|658[0n]|BOLD:AAC6158
Ponometia elegantula|[4775]|BBLOE1548-12|United States|New Mexico|658[0n]|BOLD:AAC6158
Ponometia elegantula|[4776]|BBLOE1899-12|United States|New Mexico|658[0n]|BOLD:AAC6158
Ponometia clausula|[4777]|RDNMD804-07|United States|Arizona|658[0n]|BOLD:AAH5345
Ponometia clausula|[4778]|BBLSY079-09|United States|Arizona|658[0n]|BOLD:AAH5345
Ponometia exigua|[4779]|HKONB319-09|United States|Texas|658[0n]|BOLD:AAF1707
Ponometia exigua|[4780]|HKONS560-08|United States|Florida|658[0n]|BOLD:AAF1707
Ponometia exigua|[4781]|BLPBB255-07|Costa Rica|Guanacaste|658[0n]|BOLD:AAF1707
Ponometia exigua|[4782]|HKONS561-08|United States|Florida|648[0n]|BOLD:AAF1707
Ponometia exigua|[4783]|HKONB318-09|United States|Texas|658[0n]|BOLD:AAF1707
Ponometia exigua|[4784]|BLPDI905-09|Costa Rica|Guanacaste|658[0n]|BOLD:AAF1707
Ponometia exigua|[4785]|BLPDL464-10|Costa Rica|Guanacaste|658[0n]|BOLD:AAF1707
Ponometia venustula complex|[4786]|BBLOB700-11|United States|Texas|658[0n]|BOLD:AAH9818
Ponometia venustula complex|[4787]|BBLOB670-11|United States|Texas|658[0n]|BOLD:AAH9818
Ponometia venustula complex|[4788]|BBLOB622-11|United States|Texas|658[0n]|BOLD:AAH9818
Ponometia venustula complex|[4789]|CMAZA730-10|United States|Arizona|658[0n]|BOLD:AAH9818
Ponometia venustula complex|[4790]|CMAZA452-10|United States|Arizona|658[0n]|BOLD:AAH9818
Ponometia venustula complex|[4791]|RDNMH680-09|United States|Arizona|658[0n]|BOLD:AAH9818
Ponometia venustula complex|[4792]|RDNMH679-09|United States|Arizona|658[0n]|BOLD:AAH9818
Ponometia venustula complex|[4793]|BBLOB701-11|United States|Texas|658[0n]|BOLD:AAH9818
Ponometia venustula complex|[4794]|CMAZA456-10|United States|Arizona|658[0n]|BOLD:AAH9818
Ponometia venustula complex|[4795]|BBLOE1821-12|United States|Arizona|658[0n]|BOLD:AAH9818
Ponometia venustula complex|[4796]|BBLOE1850-12|United States|Texas|658[0n]|BOLD:AAH9818
Ponometia heonyx|[4797]|LNAUS5355-13|United States|Texas|658[0n]|BOLD:ABA1150
Ponometia heonyx|[4798]|BBLOE1830-12|United States|Texas|658[0n]|BOLD:ABA1150
Ponometia heonyx|[4799]|BBLOE1825-12|United States|Texas|658[0n]|BOLD:ABA1150
Ponometia heonyx|[4800]|BBLOE1513-12|United States|Texas|658[0n]|BOLD:ABA1150
Ponometia heonyx|[4801]|BBLOB623-11|United States|Texas|658[0n]|BOLD:ABA1150
Ponometia heonyx|[4802]|LNAUS5357-13|United States|Texas|507[1n]|BOLD:ABA1150
Ponometia heonyx|[4803]|LNAUS5358-13|United States|Texas|658[0n]|BOLD:ABA1150
Ponometia heonyx|[4804]|BBLOE1852-12|United States|Texas|658[0n]|BOLD:ABA1147
Ponometia heonyx|[4805]|BBLOB618-11|United States|Texas|658[0n]|BOLD:ABA1147
Ponometia heonyx|[4806]|NOCNA018-14|United States|Texas|658[0n]|BOLD:ABA1147
Ponometia heonyx|[4807]|CNCLB234-14|United States|Arizona|658[0n]|BOLD:ABA1147
Ponometia venustula|[4808]|BBLOB713-11|United States|Texas|658[0n]|BOLD:ABA1148
Ponometia venustula|[4809]|BBLOB619-11|United States|Texas|658[0n]|BOLD:ABA1148
Ponometia venustula|[4810]|BBLOE1509-12|United States|Texas|658[0n]|BOLD:ABA1148
Ponometia venustula|[4811]|BBLOE1510-12|United States|Texas|658[0n]|BOLD:ABA1148
Ponometia venustula|[4812]|BBLOE1617-12|United States|Texas|658[0n]|BOLD:ABA1148
Ponometia venustula|[4813]|BBLOE1861-12|United States|Texas|658[0n]|BOLD:ABA1148
Ponometia venustula|[4814]|BBLOE1515-12|United States|Texas|658[0n]|BOLD:ABA1148
Ponometia venustula complex|[4815]|CNCLB909-14|United States|Arizona|658[0n]|BOLD:ABA1148
Ponometia venustula complex|[4816]|CNCLB907-14|United States|Arizona|658[0n]|BOLD:ABA1148
Ponometia venustula complex|[4817]|CNCLB908-14|United States|Arizona|658[0n]|BOLD:ABA1148
Ponometia venustula complex|[4818]|CNCLB910-14|United States|Arizona|605[0n]|BOLD:ABA1148
Ponometia nr. fasciatella|[4819]|BBLOC490-11|United States|Arizona|658[0n]|BOLD:ABA1189
Ponometia nr. fasciatella|[4820]|BBLOB1938-11|United States|Arizona|658[0n]|BOLD:ABA1189
Ponometia nr. fasciatella|[4821]|CNCLB913-14|United States|Arizona|658[0n]|BOLD:ABA1189
Ponometia nr. fasciatella|[4822]|HKONS557-08|United States|Florida|658[0n]|BOLD:AAD9106
Ponometia nr. fasciatella|[4823]|HKONS556-08|United States|Florida|658[0n]|BOLD:AAD9106
Ponometia nr. fasciatella|[4824]|HKONS555-08|United States|Florida|658[0n]|BOLD:AAD9106
Ponometia nr. fasciatella|[4825]|HKONS553-08|United States|Florida|658[0n]|BOLD:AAD9106
Ponometia nr. fasciatella|[4826]|HKONS554-08|United States|Florida|658[0n]|BOLD:AAD9106
Ponometia nr. fasciatella|[4827]|HKONS558-08|United States|Florida|658[1n]|BOLD:AAD9106
Ponometia nr. fasciatella|[4828]|BBLSW878-09|United States|Texas|658[0n]|BOLD:AAD9106
Ponometia nr. fasciatella|[4829]|LPOKD089-09|United States|Oklahoma|658[0n]|BOLD:AAD9106
Ponometia fasciatella|[4830]|IAWL351-09|United States|Arizona|571[1n]|BOLD:AAE0826
Ponometia fasciatella|[4831]|RDNMH704-09|United States|Arizona|658[0n]|BOLD:AAE0826
Ponometia fasciatella|[4832]|BBLOB1942-11|United States|Arizona|658[0n]|BOLD:AAE0826
Ponometia fasciatella|[4833]|BBLOE1576-12|United States|Arizona|658[0n]|BOLD:AAE0826
Ponometia fasciatella|[4834]|BBLOC797-11|United States|Arizona|658[0n]|BOLD:AAE0826
Ponometia fasciatella|[4835]|BBLOB1940-11|United States|Arizona|658[0n]|BOLD:AAE0826
Ponometia fasciatella|[4836]|CMAZA746-10|United States|Arizona|658[0n]|BOLD:AAE0826
Ponometia fasciatella|[4837]|CMAZA470-10|United States|Arizona|658[0n]|BOLD:AAE0826
Ponometia fasciatella|[4838]|IAWL875-09|United States|Arizona|658[0n]|BOLD:AAE0826
Ponometia fasciatella|[4839]|IAWL782-09|United States|Arizona|658[0n]|BOLD:AAE0826
Ponometia fasciatella|[4840]|IAWL592-09|United States|Arizona|658[0n]|BOLD:AAE0826
Ponometia fasciatella|[4841]|RDNMF710-08|United States|Arizona|618[1n]|BOLD:AAE0826
Ponometia fasciatella|[4842]|IAWL577-09|United States|Arizona|658[0n]|BOLD:AAE0826
Ponometia fasciatella|[4843]|RDNMG019-08|United States|Arizona|658[0n]|BOLD:AAE0826
Ponometia fasciatella|[4844]|RDNMF709-08|United States|Arizona|640[0n]|BOLD:AAE0826
Ponometia fasciatella|[4845]|RDNMF711-08|United States|Arizona|639[0n]|BOLD:AAE0826
Ponometia fasciatella|[4846]|CNCLB915-14|United States|Arizona|658[0n]|BOLD:AAE0826
Ponometia fasciatella|[4847]|CNCLB916-14|United States|Arizona|658[0n]|BOLD:AAE0826
Ponometia hutsoni|[4848]|RDNME069-07|United States|California|658[0n]|BOLD:AAI7167
Ponometia hutsoni|[4849]|NAMUM140-08|United States|California|658[0n]|BOLD:AAI7167
Ponometia hutsoni|[4850]|PSAT126-10|United States|Arizona|658[0n]|BOLD:AAI7167
Ponometia hutsoni|[4851]|LOCBF2409-13|United States|California|658[0n]|BOLD:AAI7167
Ponometia acutus|[4852]|LOCBC693-06|United States|California|658[0n]|BOLD:AAD1210
Ponometia acutus|[4853]|LOCBE144-06|United States|California|658[0n]|BOLD:AAD1210
Ponometia acutus|[4854]|RDNMD805-07|United States|California|658[0n]|BOLD:AAD1210
Ponometia acutus|[4855]|CGLCA048-10|United States|California|658[0n]|BOLD:AAD1210
Ponometia acutus|[4856]|LOCBB759-06|United States|California|658[0n]|BOLD:AAD1210
Ponometia acutus|[4857]|CGLCA049-10|United States|California|658[0n]|BOLD:AAD1210
Ponometia acutus|[4858]|BBLSW207-09|United States|Arizona|636[0n]|BOLD:AAD1210
Ponometia acutus|[4859]|BBLOC1903-11|United States|California|658[0n]|BOLD:AAD1210
Ponometia acutus|[4860]|LOCBB638-06|United States|California|658[0n]|BOLD:AAD1210
Ponometia acutus|[4861]|BBLSY756-09|United States|Arizona|658[0n]|BOLD:AAD1210
Ponometia acutus|[4862]|BBLSY958-09|United States|Arizona|658[0n]|BOLD:AAD1210
Ponometia acutus|[4863]|BBLSZ054-09|United States|Arizona|658[0n]|BOLD:AAD1210
Ponometia acutus|[4864]|BBLOC1590-11|United States|California|658[0n]|BOLD:AAD1210
Ponometia acutus|[4865]|BBLOC1911-11|United States|California|658[0n]|BOLD:AAD1210
Ponometia acutus|[4866]|BBLOC1981-11|United States|California|658[0n]|BOLD:AAD1210
Ponometia acutus|[4867]|BBLOC1913-11|United States|California|658[0n]|BOLD:AAD1210
Ponometia acutus|[4868]|BBLOC1902-11|United States|California|658[0n]|BOLD:AAD1210
Ponometia acutus|[4869]|BBLSZ091-09|United States|Arizona|658[0n]|BOLD:AAD1210
Ponometia acutus|[4870]|BBLSZ037-09|United States|Arizona|658[0n]|BOLD:AAD1210
Ponometia acutus|[4871]|BBLSY714-09|United States|Arizona|658[0n]|BOLD:AAD1210
Ponometia acutus|[4872]|BBLSW220-09|United States|Arizona|658[0n]|BOLD:AAD1210
Ponometia acutus|[4873]|LOCBD028-06|United States|California|658[0n]|BOLD:AAD1210
Ponometia acutus|[4874]|LOCBB755-06|United States|California|658[0n]|BOLD:AAD1210

Ponometia acutus|[4871]|[illegible]|United States|Arizona|[illegible]|BOLD:AAD1210
Ponometia acutus|[4872]|BBLSW220-09|United States|Arizona|658[0n]|BOLD:AAD1210
Ponometia acutus|[4873]|LOCBD028-06|United States|California|658[0n]|BOLD:AAD1210
Ponometia acutus|[4874]|LOCBB755-06|United States|California|658[0n]|BOLD:AAD1210
Ponometia acutus|[4875]|LOCBF1108-13|United States|California|592[0n]|BOLD:AAD1210
Ponometia acutus|[4876]|LOCBB769-06|United States|California|612[0n]|BOLD:AAD1210
Ponometia acutus|[4877]|LOCBC097-06|United States|California|616[0n]|BOLD:AAD1210
Ponometia acutus|[4878]|LOCBF2410-13|United States|California|561[2n]|BOLD:AAD1210
Ponometia acutus|[4879]|LOCBF2411-13|United States|California|577[0n]|BOLD:AAD1210
Ponometia huita|[4880]|NOCNA008-14|United States|Texas|658[0n]|BOLD:AAT9394
Ponometia huita|[4881]|CNCLB236-14|United States|Arizona|658[0n]|BOLD:AAT9394
Ponometia huita|[4882]|CNCLB235-14|United States|Arizona|658[0n]|BOLD:AAT9394
Ponometia huita|[4883]|RDNMJ555-11|United States|Arizona|658[0n]|BOLD:AAT9394
Ponometia huita|[4884]|RDNMJ543-11|United States|Arizona|658[0n]|BOLD:AAT9394
Ponometia huita|[4885]|RDNMJ509-11|United States|Arizona|658[0n]|BOLD:AAT9394
Ponometia huita|[4886]|CNCLB237-14|United States|Texas|658[0n]|BOLD:AAT9394
Ponometia huita|[4887]|CNCLB238-14|United States|Arizona|658[0n]|BOLD:AAT9394
Ponometia binocula|[4888]|RDNMG214-08|United States|Ohio|649[1n]|BOLD:AAE1793
Ponometia binocula|[4889]|RDNMG215-08|United States|Ohio|648[0n]|BOLD:AAE1793
Ponometia binocula|[4890]|RDNMG770-08|United States|Ohio|643[1n]|BOLD:AAE1793
Ponometia binocula|[4891]|RDNMG771-08|United States|Ohio|643[1n]|BOLD:AAE1793
Ponometia binocula|[4892]|LPOKE059-10|United States|Oklahoma|658[0n]|BOLD:AAE1793
Ponometia binocula|[4893]|LPOKE176-10|United States|Oklahoma|658[0n]|BOLD:AAE1793
Ponometia sp.|[4894]|CNCLB911-14|United States|Texas|614[0n]|BOLD:ACK3820
Ponometia sp.|[4895]|CNCLB912-14|United States|Texas|658[0n]|BOLD:ACK3820
Ponometia erastrioides|[4896]|LPOKA012-08|United States|Oklahoma|657[0n]|BOLD:AAA8108
Ponometia erastrioides|[4897]|LGSM739-04|United States|Tennessee|658[0n]|BOLD:AAA8108
Ponometia erastrioides|[4898]|XAG200-05|Canada|Ontario|658[0n]|BOLD:AAA8108
Ponometia erastrioides|[4899]|XAH258-05|Canada|Ontario|658[0n]|BOLD:AAA8108
Ponometia erastrioides|[4900]|XAG460-05|Canada|Ontario|658[0n]|BOLD:AAA8108
Ponometia erastrioides|[4901]|LPOKB466-09|United States|Oklahoma|658[0n]|BOLD:AAA8108
Ponometia erastrioides|[4902]|RDLQG759-06|Canada|Quebec|658[0n]|BOLD:AAA8108
Ponometia erastrioides|[4903]|BBLSX401-09|United States|Oklahoma|658[0n]|BOLD:AAA8108
Ponometia erastrioides|[4904]|BBLSU060-09|United States|Arkansas|658[0n]|BOLD:AAA8108
Ponometia erastrioides|[4905]|BBLSW575-09|United States|Oklahoma|658[0n]|BOLD:AAA8108
Ponometia erastrioides|[4906]|BBLSW469-09|United States|Oklahoma|658[0n]|BOLD:AAA8108
Ponometia erastrioides|[4907]|LPOKB960-09|United States|Oklahoma|658[0n]|BOLD:AAA8108
Ponometia erastrioides|[4908]|LPOKA826-09|United States|Oklahoma|658[0n]|BOLD:AAA8108
Ponometia erastrioides|[4909]|LPOKA260-08|United States|Oklahoma|658[0n]|BOLD:AAA8108
Ponometia erastrioides|[4910]|BLTIB1009-08|Canada|Ontario|658[0n]|BOLD:AAA8108
Ponometia erastrioides|[4911]|BLTIB758-08|Canada|Ontario|658[0n]|BOLD:AAA8108
Ponometia erastrioides|[4912]|LPOKA111-08|United States|Oklahoma|658[0n]|BOLD:AAA8108
Ponometia erastrioides|[4913]|RDLQH022-06|Canada|Quebec|658[0n]|BOLD:AAA8108
Ponometia erastrioides|[4914]|RDLQG850-06|Canada|Quebec|656[0n]|BOLD:AAA8108
Ponometia erastrioides|[4915]|RDLQG801-06|Canada|Quebec|658[0n]|BOLD:AAA8108
Ponometia erastrioides|[4916]|RDLQG770-06|Canada|Quebec|658[0n]|BOLD:AAA8108
Ponometia erastrioides|[4917]|RDLQG767-06|Canada|Quebec|658[0n]|BOLD:AAA8108
Ponometia erastrioides|[4918]|RDLQG757-06|Canada|Quebec|658[0n]|BOLD:AAA8108
Ponometia erastrioides|[4919]|RDLQG729-06|Canada|Quebec|658[0n]|BOLD:AAA8108
Ponometia erastrioides|[4920]|RDLQG720-06|Canada|Quebec|658[0n]|BOLD:AAA8108
Ponometia erastrioides|[4921]|RDLQG687-06|Canada|Quebec|658[0n]|BOLD:AAA8108
Ponometia erastrioides|[4922]|RDLQG558-06|Canada|Quebec|658[0n]|BOLD:AAA8108
Ponometia erastrioides|[4923]|RDLQG539-06|Canada|Quebec|658[0n]|BOLD:AAA8108
Ponometia erastrioides|[4924]|LSUSA099-06|United States|Kentucky|658[0n]|BOLD:AAA8108
Ponometia erastrioides|[4925]|XAH025-05|Canada|Ontario|658[0n]|BOLD:AAA8108
Ponometia erastrioides|[4926]|XAG959-05|Canada|Ontario|658[0n]|BOLD:AAA8108
Ponometia erastrioides|[4927]|XAG568-05|Canada|Ontario|658[0n]|BOLD:AAA8108
Ponometia erastrioides|[4928]|XAD729-05|Canada|Ontario|658[0n]|BOLD:AAA8108
Ponometia erastrioides|[4929]|XAE582-04|Canada|Ontario|658[0n]|BOLD:AAA8108
Ponometia erastrioides|[4930]|XAC661-04|Canada|Ontario|658[0n]|BOLD:AAA8108
Ponometia erastrioides|[4931]|PHMO097-03|Canada|Ontario|658[0n]|BOLD:AAA8108
Ponometia erastrioides|[4932]|RDLQD892-06|Canada|Quebec|656[0n]|BOLD:AAA8108
Ponometia erastrioides|[4933]|TMNBB620-06|Canada|New Brunswick|658[0n]|BOLD:AAA8108
Ponometia erastrioides|[4934]|XAC478-04|Canada|Ontario|597[0n]|BOLD:AAA8108
Ponometia erastrioides|[4935]|PMG163-03|Canada|Ontario|617[0n]|BOLD:AAA8108
Ponometia erastrioides|[4936]|TMG103-03|Canada|Ontario|639[0n]|BOLD:AAA8108
Ponometia erastrioides|[4937]|LPOKB735-09|United States|Oklahoma|634[0n]|BOLD:AAA8108
Ponometia erastrioides|[4938]|XAF551-05|Canada|Ontario|658[0n]|BOLD:AAA8108
Ponometia erastrioides|[4939]|LPOKD102-09|United States|Oklahoma|658[0n]|BOLD:AAA8108
Ponometia erastrioides|[4940]|RDLQD891-06|Canada|Quebec|657[0n]|BOLD:AAA8108
Ponometia erastrioides|[4941]|XAG904-05|Canada|Ontario|658[0n]|BOLD:AAA8108
Ponometia erastrioides|[4942]|XAF775-05|Canada|Ontario|658[0n]|BOLD:AAA8108
Ponometia erastrioides|[4943]|XAC024-04|Canada|Ontario|599[0n]|BOLD:AAA8108
Ponometia erastrioides|[4944]|XAD015-04|Canada|Ontario|620[0n]|BOLD:AAA8108
Ponometia erastrioides|[4945]|BLTIB600-08|Canada|Ontario|638[0n]|BOLD:AAA8108
Ponometia erastrioides|[4946]|BBLSX299-09|United States|Oklahoma|658[0n]|BOLD:AAA8108
Ponometia erastrioides|[4947]|BBLSX414-09|United States|Oklahoma|658[0n]|BOLD:AAA8108
Ponometia erastrioides|[4948]|BBLCU279-09|United States|Illinois|658[0n]|BOLD:AAA8108
Ponometia erastrioides|[4949]|LILLA297-11|United States|Illinois|658[0n]|BOLD:AAA8108
Ponometia erastrioides|[4950]|LILLA406-11|United States|Illinois|658[0n]|BOLD:AAA8108
Ponometia tripartita|[4951]|RDNMJ703-11|United States|Arizona|658[0n]|BOLD:AAL0748
Ponometia tripartita|[4952]|RDNME919-08|United States|Arizona|658[0n]|BOLD:AAL0748
Ponometia tripartita|[4953]|CMAZA602-10|United States|Arizona|658[0n]|BOLD:AAL0748
Ponometia tripartita|[4954]|RDNMK004-11|United States|Arizona|658[0n]|BOLD:AAL0748
Ponometia tripartita|[4955]|IAWLB307-11|United States|Arizona|658[0n]|BOLD:AAL0748
Ponometia dorneri|[4956]|RDNMH682-09|United States|New Mexico|658[0n]|BOLD:AAK0371
Ponometia dorneri|[4957]|RDNMH683-09|United States|New Mexico|658[0n]|BOLD:AAK0371
Ponometia dorneri|[4958]|BBLOB612-11|United States|Texas|658[0n]|BOLD:AAK0371
Ponometia sutrix|[4959]|RDNME927-08|United States|New Mexico|658[0n]|BOLD:AAF0286
Ponometia sutrix|[4960]|RDMAB543-06|Canada|Alberta|603[1n]|BOLD:AAF0286
Ponometia sutrix|[4961]|RDMAB544-06|United States|Nevada|617[0n]|BOLD:AAF0286
Ponometia sutrix|[4962]|RDNME955-08|United States|Colorado|658[0n]|BOLD:AAF0286
Ponometia sp.|[4963]|RDNME068-07|United States|Arizona|658[0n]|BOLD:ACQ0395
Ponometia sp.|[4964]|RDNMJ502-11|United States|Arizona|658[0n]|BOLD:ACQ0395
Ponometia candefacta|[4965]|LOFLC271-06|United States|Florida|636[0n]|BOLD:AAA5642
Ponometia candefacta|[4966]|RDLQB751-05|Canada|Quebec|658[0n]|BOLD:AAA5642
Ponometia candefacta|[4967]|LOFLB928-06|United States|Florida|658[0n]|BOLD:AAA5642
Ponometia candefacta|[4968]|RDLQG561-06|Canada|Quebec|658[0n]|BOLD:AAA5642
Ponometia candefacta|[4969]|BBLSX647-09|United States|Oklahoma|658[0n]|BOLD:AAA5642
Ponometia candefacta|[4970]|RDLQG889-06|Canada|Quebec|658[0n]|BOLD:AAA5642
Ponometia candefacta|[4971]|LOFLC060-06|United States|Florida|658[0n]|BOLD:AAA5642
Ponometia candefacta|[4972]|RDLQG796-06|Canada|Quebec|658[0n]|BOLD:AAA5642
Ponometia candefacta|[4973]|USLEP1183-10|United States|Texas|658[0n]|BOLD:AAA5642

Ponometia candefacta|[4971]|LOFLC060-06|United States|Florida|658[0n]|BOLD:AAA5642
Ponometia candefacta|[4972]|RDLQG796-06|Canada|Quebec|658[0n]|BOLD:AAA5642
Ponometia candefacta|[4973]|USLEP1183-10|United States|Texas|658[0n]|BOLD:AAA5642
Ponometia candefacta|[4974]|LPOKB228-09|United States|Oklahoma|658[0n]|BOLD:AAA5642
Ponometia candefacta|[4975]|XAK193-06|Canada|Ontario|643[0n]|BOLD:AAA5642
Ponometia candefacta|[4976]|XAK194-06|Canada|Ontario|658[0n]|BOLD:AAA5642
Ponometia candefacta|[4977]|RDLQG844-06|Canada|Quebec|658[0n]|BOLD:AAA5642
Ponometia candefacta|[4978]|LOCBD027-06|United States|California|658[0n]|BOLD:AAA5642
Ponometia candefacta|[4979]|LOCBE151-06|United States|California|638[0n]|BOLD:AAA5642
Ponometia candefacta|[4980]|XAH231-05|Canada|Ontario|658[0n]|BOLD:AAA5642
Ponometia candefacta|[4981]|XAK077-06|Canada|Ontario|658[0n]|BOLD:AAA5642
Ponometia candefacta|[4982]|LOCBB770-06|United States|California|612[0n]|BOLD:AAA5642
Ponometia candefacta|[4983]|LPOKC339-09|United States|Oklahoma|658[0n]|BOLD:AAA5642
Ponometia candefacta|[4984]|XAH259-05|Canada|Ontario|614[0n]|BOLD:AAA5642
Ponometia candefacta|[4985]|XAJ283-06|Canada|Ontario|658[0n]|BOLD:AAA5642
Ponometia candefacta|[4986]|LOCBC682-06|United States|California|658[0n]|BOLD:AAA5642
Ponometia candefacta|[4987]|XAH232-05|Canada|Ontario|658[0n]|BOLD:AAA5642
Ponometia candefacta|[4988]|RDLQE358-06|Canada|Quebec|657[0n]|BOLD:AAA5642
Ponometia candefacta|[4989]|LOFLB910-06|United States|Florida|658[0n]|BOLD:AAA5642
Ponometia candefacta|[4990]|CMAZA761-10|United States|Arizona|658[0n]|BOLD:AAA5642
Ponometia candefacta|[4991]|BBLOD1745-11|United States|Texas|625[0n]|BOLD:AAA5642
Ponometia candefacta|[4992]|LOFLA816-06|United States|Florida|656[0n]|BOLD:AAA5642
Ponometia candefacta|[4993]|LOCBB335-06|United States|California|656[0n]|BOLD:AAA5642
Ponometia candefacta|[4994]|LPOKC445-09|United States|Oklahoma|658[0n]|BOLD:AAA5642
Ponometia candefacta|[4995]|LPOKC382-09|United States|Oklahoma|658[0n]|BOLD:AAA5642
Ponometia candefacta|[4996]|UDLEP073-09|United States|Delaware|658[0n]|BOLD:AAA5642
Ponometia candefacta|[4997]|BLGSM004-09|Canada|Ontario|658[0n]|BOLD:AAA5642
Ponometia candefacta|[4998]|BLTIB1034-08|Canada|Ontario|658[0n]|BOLD:AAA5642
Ponometia candefacta|[4999]|LPOKA165-08|United States|Oklahoma|658[0n]|BOLD:AAA5642
Ponometia candefacta|[5000]|LPOKA076-08|United States|Oklahoma|658[0n]|BOLD:AAA5642
Ponometia candefacta|[5001]|RDNMG435-08|United States|Florida|658[0n]|BOLD:AAA5642
Ponometia candefacta|[5002]|RDLQG888-06|Canada|Quebec|658[0n]|BOLD:AAA5642
Ponometia candefacta|[5003]|RDLQG887-06|Canada|Quebec|658[0n]|BOLD:AAA5642
Ponometia candefacta|[5004]|RDLQG842-06|Canada|Quebec|658[0n]|BOLD:AAA5642
Ponometia candefacta|[5005]|RDLQG563-06|Canada|Quebec|658[0n]|BOLD:AAA5642
Ponometia candefacta|[5006]|RDLQG560-06|Canada|Quebec|658[0n]|BOLD:AAA5642
Ponometia candefacta|[5007]|RDLQG550-06|Canada|Quebec|658[0n]|BOLD:AAA5642
Ponometia candefacta|[5008]|LOCBE162-06|United States|California|658[0n]|BOLD:AAA5642
Ponometia candefacta|[5009]|LOCBE028-06|United States|California|658[0n]|BOLD:AAA5642
Ponometia candefacta|[5010]|LOCBD042-06|United States|California|658[0n]|BOLD:AAA5642
Ponometia candefacta|[5011]|LNCNW100-06|United States|North Carolina|658[0n]|BOLD:AAA5642
Ponometia candefacta|[5012]|LOCBB639-06|United States|California|658[0n]|BOLD:AAA5642
Ponometia candefacta|[5013]|LOFLC238-06|United States|Florida|658[0n]|BOLD:AAA5642
Ponometia candefacta|[5014]|LOFLB295-06|United States|Florida|658[0n]|BOLD:AAA5642
Ponometia candefacta|[5015]|LOCBB336-06|United States|California|658[0n]|BOLD:AAA5642
Ponometia candefacta|[5016]|LOCBB334-06|United States|California|658[0n]|BOLD:AAA5642
Ponometia candefacta|[5017]|LOCBB333-06|United States|California|658[0n]|BOLD:AAA5642
Ponometia candefacta|[5018]|LOCBB332-06|United States|California|658[0n]|BOLD:AAA5642
Ponometia candefacta|[5019]|LOCBB331-06|United States|California|658[0n]|BOLD:AAA5642
Ponometia candefacta|[5020]|LSUSA098-06|United States|Kentucky|658[0n]|BOLD:AAA5642
Ponometia candefacta|[5021]|LPOKB257-09|United States|Oklahoma|658[0n]|BOLD:AAA5642
Ponometia candefacta|[5022]|RDLQE357-06|Canada|Quebec|658[0n]|BOLD:AAA5642
Ponometia candefacta|[5023]|RDLQE207-06|Canada|Quebec|657[0n]|BOLD:AAA5642
Ponometia candefacta|[5024]|RDLQD893-06|Canada|Quebec|657[0n]|BOLD:AAA5642
Ponometia candefacta|[5025]|XAG792-05|Canada|Ontario|658[0n]|BOLD:AAA5642
Ponometia candefacta|[5026]|RDLQG562-06|Canada|Quebec|658[0n]|BOLD:AAA5642
Ponometia candefacta|[5027]|PHMO062-03|Canada|Ontario|639[0n]|BOLD:AAA5642
Ponometia candefacta|[5028]|PHMO057-03|Canada|Ontario|639[0n]|BOLD:AAA5642
Ponometia candefacta|[5029]|RDNMB261-05|Canada|Ontario|601[0n]|BOLD:AAA5642
Ponometia candefacta|[5030]|RDNMB262-05|United States|Oregon|596[0n]|BOLD:AAA5642
Ponometia candefacta|[5031]|XAG937-05|Canada|Ontario|617[0n]|BOLD:AAA5642
Ponometia candefacta|[5032]|XAJ520-06|Canada|Ontario|657[0n]|BOLD:AAA5642
Ponometia candefacta|[5033]|LOCBD919-06|United States|California|646[0n]|BOLD:AAA5642
Ponometia candefacta|[5034]|BBLOB2000-11|United States|Arizona|658[0n]|BOLD:AAA5642
Ponometia candefacta|[5035]|BBLOE1283-12|United States|Texas|658[0n]|BOLD:AAA5642
Ponometia candefacta|[5036]|LOFLB549-06|United States|Florida|658[0n]|BOLD:AAA5642
Ponometia candefacta|[5037]|LOFLC087-06|United States|Florida|658[0n]|BOLD:AAA5642
Ponometia candefacta|[5038]|USLEP1113-10|United States|Colorado|658[0n]|BOLD:AAA5642
Ponometia candefacta|[5039]|CMAZA417-10|United States|Arizona|658[0n]|BOLD:AAA5642
Ponometia candefacta|[5040]|CMAZA1181-12|United States|Arizona|658[0n]|BOLD:AAA5642
Ponometia semiflava|[5041]|RDNME620-08|United States|Colorado|658[0n]|BOLD:AAC8096
Ponometia semiflava|[5042]|RDMAB033-05|Canada|Alberta|623[0n]|BOLD:AAC8096
Ponometia semiflava|[5043]|RDMAB516-06|Canada|Alberta|655[0n]|BOLD:AAC8096
Ponometia semiflava|[5044]|BBLWU021-09|United States|Colorado|658[0n]|BOLD:AAC8096
Ponometia semiflava|[5045]|LNCC548-11|United States|North Carolina|658[0n]|BOLD:AAC8096
Ponometia semiflava|[5046]|LNC525-06|United States|North Carolina|658[0n]|BOLD:AAC8096
Ponometia semiflava|[5047]|LNC526-06|United States|North Carolina|658[0n]|BOLD:AAC8096
Ponometia semiflava|[5048]|LOFLC037-06|United States|Florida|658[0n]|BOLD:AAC8096
Ponometia semiflava|[5049]|RDNMH914-09|United States|Florida|658[0n]|BOLD:AAC8096
Ponometia semiflava|[5050]|LPOKC527-09|United States|Oklahoma|658[0n]|BOLD:AAC8096
Ponometia semiflava|[5051]|USLEP070-10|United States|Colorado|658[0n]|BOLD:AAC8096
Ponometia semiflava|[5052]|RDNMK332-11|United States|New Mexico|658[0n]|BOLD:AAC8096
Ponometia alata|[5053]|IAWL913-09|United States|Arizona|658[0n]|BOLD:AAH4900
Ponometia alata|[5054]|RDNMG010-08|United States|Arizona|658[0n]|BOLD:AAH4905
Ponometia alata|[5055]|IAWL933-09|United States|Arizona|658[0n]|BOLD:AAH4905
Ponometia alata|[5056]|IAWL934-09|United States|Arizona|658[0n]|BOLD:AAH4905
Ponometia alata|[5057]|IAWL935-09|United States|Arizona|658[0n]|BOLD:AAH4905
Ponometia virginalis|[5058]|CMAZA737-10|United States|Arizona|658[0n]|BOLD:AAE1792
Ponometia virginalis|[5059]|CMAZA740-10|United States|Arizona|658[0n]|BOLD:AAE1792
Ponometia virginalis|[5060]|CMAZA600-10|United States|Arizona|658[0n]|BOLD:AAE1792
Ponometia virginalis|[5061]|RDMAB494-06|Canada|Alberta|658[0n]|BOLD:AAE1792
Ponometia virginalis|[5062]|RDMAB054-05|Canada|Alberta|643[0n]|BOLD:AAE1792
Ponometia virginalis|[5063]|RDNMG450-08|United States|Colorado|658[0n]|BOLD:AAE1792
Ponometia virginalis|[5064]|RDNMG505-08|United States|Colorado|658[0n]|BOLD:AAE1792
Ponometia virginalis|[5065]|CNCLB240-14|United States|Arizona|658[0n]|BOLD:AAE1792
Ponometia bicolorata complex|[5066]|CMAZA763-10|United States|Arizona|658[0n]|BOLD:AAC6681
Ponometia bicolorata complex|[5067]|BBLSY187-09|United States|Texas|658[0n]|BOLD:ACF5229
Ponometia bicolorata complex|[5068]|HKONB437-09|United States|Texas|658[0n]|BOLD:ACF5229
Ponometia bicolorata complex|[5069]|BBLSW134-09|United States|Texas|658[0n]|BOLD:ACF5229
Ponometia bicolorata complex|[5070]|BBLSY189-09|United States|Texas|658[0n]|BOLD:ACF5229
Ponometia bicolorata complex|[5071]|BBLSY190-09|United States|Texas|658[0n]|BOLD:ACF5229
Ponometia bicolorata complex|[5072]|BBLSX856-09|United States|Texas|658[0n]|BOLD:ACF5229
Ponometia bicolorata complex|[5073]|RDNMK334-11|United States|New Mexico|658[0n]|BOLD:ACF5229

Ponometia bicolorata complex|[5070]|BBLSY189-09|United States|Texas|658[0n]|BOLD:ACF5229
Ponometia bicolorata complex|[5071]|BBLSY190-09|United States|Texas|658[0n]|BOLD:ACF5229
Ponometia bicolorata complex|[5072]|BBLSX856-09|United States|Texas|658[0n]|BOLD:ACF5229
Ponometia bicolorata complex|[5073]|RDNMK324-11|United States|New Mexico|658[0n]|BOLD:ACF5229
Ponometia bicolorata complex|[5074]|RDNMK335-11|United States|New Mexico|658[0n]|BOLD:ACF5229
Ponometia bicolorata complex|[5075]|RDNME918-08|United States|Arizona|658[0n]|BOLD:AAY3487
Ponometia bicolorata|[5076]|RDNMG014-08|United States|Arizona|649[0n]|BOLD:AAC8097
Ponometia bicolorata|[5077]|HKONS211-08|United States|Texas|648[0n]|BOLD:AAC8097
Ponometia bicolorata|[5078]|HKONS210-08|United States|Texas|658[2n]|BOLD:AAC8097
Ponometia bicolorata|[5079]|RDNME041-07|United States|Arizona|658[0n]|BOLD:AAC8097
Ponometia bicolorata|[5080]|RDNME914-08|United States|Arizona|658[0n]|BOLD:AAC8097
Ponometia bicolorata|[5081]|RDNME916-08|United States|Arizona|658[0n]|BOLD:AAC8097
Ponometia bicolorata|[5082]|RDNME917-08|United States|Arizona|658[0n]|BOLD:AAC8097
Ponometia bicolorata|[5083]|RDNMH705-09|United States|Arizona|658[0n]|BOLD:AAC8097
Ponometia bicolorata|[5084]|RDNMK325-11|United States|New Mexico|658[0n]|BOLD:AAC8097
Ponometia bicolorata|[5085]|CMAZA1136-12|United States|Arizona|658[0n]|BOLD:AAC8097
Ponometia bicolorata|[5086]|CMAZA1166-12|United States|Arizona|658[0n]|BOLD:AAC8097
Ponometia bicolorata|[5087]|TML134-14|United States||658[0n]|BOLD:AAC8097
Ponometia libedis|[5088]|CMAZA693-10|United States|Arizona|658[0n]|BOLD:AAH5267
Ponometia libedis|[5089]|CMAZA749-10|United States|Arizona|658[0n]|BOLD:AAH5267
Ponometia libedis|[5090]|BBLSY263-09|United States|Texas|658[0n]|BOLD:AAH5267
Ponometia libedis|[5091]|RDNMK001-11|United States|Arizona|658[0n]|BOLD:AAH5267
Ponometia libedis|[5092]|RDNME964-08|United States|New Mexico|658[0n]|BOLD:AAH5267
Ponometia libedis|[5093]|BBLSW071-09|United States|Texas|658[0n]|BOLD:AAH5267
Ponometia libedis|[5094]|BBLSY191-09|United States|Texas|658[0n]|BOLD:AAH5267
Ponometia libedis|[5095]|BBLSY192-09|United States|Texas|658[0n]|BOLD:AAH5267
Ponometia libedis|[5096]|BBLSY259-09|United States|Texas|658[0n]|BOLD:AAH5267
Ponometia libedis|[5097]|BBLSY265-09|United States|Texas|658[0n]|BOLD:AAH5267
Ponometia libedis|[5098]|BBLSY271-09|United States|Texas|658[0n]|BOLD:AAH5267
Ponometia libedis|[5099]|BBLSY277-09|United States|Texas|658[0n]|BOLD:AAH5267
Ponometia libedis|[5100]|BBLSX818-09|United States|Texas|658[0n]|BOLD:AAH5267
Ponometia libedis|[5101]|BBLSX832-09|United States|Texas|658[0n]|BOLD:AAH5267
Ponometia libedis|[5102]|BBLSZ153-09|United States|Texas|658[0n]|BOLD:AAH5267
Ponometia libedis|[5103]|CMAZA481-10|United States|Arizona|658[0n]|BOLD:AAH5267
Ponometia libedis|[5104]|CMAZA482-10|United States|Arizona|658[0n]|BOLD:AAH5267
Ponometia libedis|[5105]|CMAZA701-10|United States|Arizona|658[0n]|BOLD:AAH5267
Ponometia libedis|[5106]|CMAZA765-10|United States|Arizona|658[0n]|BOLD:AAH5267
Ponometia libedis|[5107]|RDNMK005-11|United States|Arizona|658[0n]|BOLD:AAH5267
Ponometia libedis|[5108]|BBLOB705-11|United States|Texas|658[0n]|BOLD:AAH5267
Ponometia libedis|[5109]|BBLOC1476-11|United States|Texas|658[0n]|BOLD:AAH5267
Ponometia libedis|[5110]|BBLOE1272-12|United States|Texas|658[0n]|BOLD:AAH5267
Ponometia libedis|[5111]|BBLOE1546-12|United States|Texas|658[0n]|BOLD:AAH5267
Ponometia nannodes|[5112]|RDNMK016-11|United States|Arizona|658[0n]|BOLD:AAH9815
Ponometia nannodes|[5113]|NAMUM044-08|United States|Arizona|657[0n]|BOLD:AAH9815
Ponometia nannodes|[5114]|BBLOE1502-12|United States|Texas|658[0n]|BOLD:AAH9815
Ponometia nannodes|[5115]|BBLOE1167-12|United States|Texas|658[0n]|BOLD:AAH9815
Ponometia nannodes|[5116]|BBLOE1540-12|United States|Texas|658[0n]|BOLD:AAH9815
Ponometia nannodes|[5117]|RDNMK351-11|United States|New Mexico|658[0n]|BOLD:AAH9815
Ponometia nannodes|[5118]|RDNMK352-11|United States|New Mexico|658[0n]|BOLD:AAH9815
Ponometia nannodes|[5119]|BBLOE1541-12|United States|Texas|658[0n]|BOLD:AAH9815
Ponometia nannodes|[5120]|BBLOE1543-12|United States|Texas|620[0n]|BOLD:AAH9815
Ponometia nannodes|[5121]|BBLOE1590-12|United States|Texas|658[0n]|BOLD:AAH9815
Ponometia nannodes|[5122]|BBLOE1288-12|United States|Texas|658[0n]|BOLD:AAH9815
Ponometia nannodes|[5123]|RDNMJ686-11|United States|Arizona|658[0n]|BOLD:AAH9815
Ponometia nannodes|[5124]|RDNMH910-09|United States|Arizona|658[0n]|BOLD:AAH9815
Ponometia nannodes|[5125]|BBLOE1273-12|United States|Texas|658[0n]|BOLD:AAH9815
Ponometia nannodes|[5126]|BBLOE1274-12|United States|Texas|658[0n]|BOLD:AAH9815
Ponometia nannodes|[5127]|BBLOE1503-12|United States|Texas|658[0n]|BOLD:AAH9815
Ponometia nannodes|[5128]|BBLOE1539-12|United States|Texas|658[0n]|BOLD:AAH9815
Ponometia nannodes|[5129]|BBLOE1592-12|United States|Texas|658[0n]|BOLD:AAH9815
Ponometia nannodes|[5130]|BBLOE1661-12|United States|Texas|658[0n]|BOLD:AAH9815
Ponometia nannodes|[5131]|BBLOE1501-12|United States|Texas|658[0n]|BOLD:AAH9815
Ponometia nannodes|[5132]|RDNMH909-09|United States|Arizona|650[0n]|BOLD:AAH9815
Ponometia nannodes|[5133]|CMAZA454-10|United States|Arizona|658[0n]|BOLD:AAH9815
Ponometia nannodes|[5134]|CMAZA477-10|United States|Arizona|658[0n]|BOLD:AAH9815
Ponometia nannodes|[5135]|RDNMK184-11|United States|Arizona|658[0n]|BOLD:AAH9815
Ponometia nannodes|[5136]|CMAZA1185-12|United States|Arizona|658[0n]|BOLD:AAH9815
Ponometia nannodes|[5137]|CMAZA1225-12|United States|Arizona|658[0n]|BOLD:AAH9815
Ponometia nannodes|[5138]|CMAZA1248-12|United States|Arizona|658[0n]|BOLD:AAH9815
Ponometia megocula|[5139]|NAMUM251-08|United States|Arizona|658[0n]|BOLD:AAL0747
Ponometia altera|[5140]|RDNMJ305-11|United States|Arizona|658[0n]|BOLD:AAJ7278
Ponometia altera|[5141]|RDNMC595-06|United States|Arizona|658[0n]|BOLD:AAJ7278
Ponometia altera|[5142]|RDNMK017-11|United States|Arizona|658[0n]|BOLD:AAJ7278
Ponometia altera|[5143]|CMAZA700-10|United States|Arizona|658[0n]|BOLD:AAJ7278
Ponometia altera|[5144]|RDNMK003-11|United States|Arizona|658[0n]|BOLD:AAJ7278
Ponometia altera|[5145]|BBLOE1829-12|United States|Texas|658[0n]|BOLD:AAJ7278
Ponometia cuta|[5146]|BBLOE1511-12|United States|Texas|658[2n]|BOLD:ABZ4846
Ponometia cuta|[5147]|BBLOB672-11|United States|Texas|658[0n]|BOLD:ABZ4846
Ponometia cuta|[5148]|BBLOB621-11|United States|Texas|658[0n]|BOLD:ABZ4846
Ponometia cuta|[5149]|RDNME619-08|United States|New Mexico|658[0n]|BOLD:ABZ4846
Ponometia cuta|[5150]|BBLOB671-11|United States|Texas|658[0n]|BOLD:ABZ4846
Ponometia cuta|[5151]|BBLOE1837-12|United States|Texas|658[0n]|BOLD:ABZ4846
Ponometia cuta|[5152]|BBLOE1851-12|United States|Texas|658[0n]|BOLD:ABZ4846
Ponometia cuta|[5153]|BBLOE1854-12|United States|Texas|658[0n]|BOLD:ABZ4846
Ponometia pulchra|[5154]|CNCLB460-14|United States|Texas|550[0n]|BOLD:ABA1149
Ponometia macdunnoughi|[5155]|NAMUM377-09|United States|California|658[0n]|BOLD:ABA1149
Ponometia pulchra|[5156]|CNCLB459-14|United States|Texas|658[0n]|BOLD:ABA1149
Ponometia macdunnoughi|[5157]|RDNMH687-09|United States|Nevada|658[0n]|BOLD:ABA1149
Ponometia macdunnoughi|[5158]|NAMUM381-09|United States|California|658[0n]|BOLD:ABA1149
Ponometia macdunnoughi|[5159]|CNCLB1555-14|United States|California|658[0n]|BOLD:ABA1149
Ponometia albitermen|[5160]|CNCLB233-14|United States|Texas|658[0n]|BOLD:ACM4240
Ponometia parvula|[5161]|LNC933-06|United States|North Carolina|658[0n]|BOLD:AAH9817
Ponometia parvula|[5162]|LNC316-05|United States|North Carolina|658[0n]|BOLD:AAH9817
Ponometia parvula|[5163]|MILEP352-10|United States|Alabama|658[0n]|BOLD:AAH9817
Ponometia tortricina|[5164]|RDNMF126-08|Canada|British Columbia|658[0n]|BOLD:AAE1800
Ponometia tortricina|[5165]|RDNMF127-08|United States|Oregon|658[0n]|BOLD:AAE1800
Ponometia tortricina|[5166]|RDNMF128-08|Canada|Alberta|658[0n]|BOLD:AAE1800
Ponometia tortricina|[5167]|RDNMF125-08|Canada|Manitoba|658[0n]|BOLD:AAE1800
Ponometia tortricina|[5168]|RDNMF129-08|Canada|Alberta|658[1n]|BOLD:AAE1800
Ponometia fumata|[5169]|LNAUS5362-13|United States|Oregon|658[0n]|BOLD:ACK3440
Ponometia fumata|[5170]|LNAUS5359-13|United States|Oregon|658[0n]|BOLD:ACK3440
Ponometia fumata|[5171]|LNAUS5360-13|United States|Oregon|658[0n]|BOLD:ACK3440
Ponometia fumata|[5172]|LNAUS5361-13|United States|Oregon|658[0n]|BOLD:ACK3440

Ponometia fumata|[5170]|LNAUS5359-13|United States|Oregon|658[0n]|BOLD:ACK3440
Ponometia fumata|[5171]|LNAUS5360-13|United States|Oregon|658[0n]|BOLD:ACK3440
Ponometia fumata|[5172]|LNAUS5361-13|United States|Oregon|658[0n]|BOLD:ACK3440
Ponometia fumata|[5173]|LNAUS5363-13|United States|Oregon|658[0n]|BOLD:ACK3440
Ponometia nigra|[5174]|CNCLB3167-15|Canada|British Columbia|658[0n]|BOLD:ACM6855
Ponometia nigra|[5175]|CNCLB454-14|Canada|British Columbia|658[0n]|BOLD:ACM6855
Ponometia nigra|[5176]|CNCLB3201-15|United States|California|658[0n]|BOLD:ACM6855
Protoschinia nuchalis|[5177]|RDNMF216-08|United States|Washington|652[0n]|BOLD:AAD3664
Protoschinia nuchalis|[5178]|RDNMF219-08|United States|Washington|658[0n]|BOLD:AAD3664
Protoschinia nuchalis|[5179]|RDNMF217-08|United States|Washington|658[0n]|BOLD:AAD3664
Protoschinia nuchalis|[5180]|RDNMF218-08|United States|Washington|658[0n]|BOLD:AAD3664
Protoschinia nuchalis|[5181]|RDNMF220-08|Canada|British Columbia|595[0n]|BOLD:AAD3664
Protoschinia nuchalis|[5182]|HELNA279-09|United States|Colorado|658[0n]|BOLD:AAD3664
Protoschinia nuchalis|[5183]|HELNA368-09|United States|Colorado|658[0n]|BOLD:AAD3664
Protoschinia nuchalis|[5184]|LNAUP099-13|United States|New Mexico|658[0n]|BOLD:AAD3664
Protoschinia nuchalis|[5185]|LNAUP100-13|United States|New Mexico|658[0n]|BOLD:AAD3664
Heliothis proruptus|[5186]|NAMUM368-09|United States|California|658[0n]|BOLD:AAE6903
Heliothis proruptus|[5187]|HELNA381-09|United States|California|658[0n]|BOLD:AAE6903
Heliothis proruptus|[5188]|HELNA380-09|United States|California|658[0n]|BOLD:AAE6903
Heliothis proruptus|[5189]|HELNA975-10|United States|California|658[0n]|BOLD:AAE6903
Heliothis borealis|[5190]|RDNMF043-08|Canada|Alberta|658[0n]|BOLD:AAE6907
Heliothis borealis|[5191]|RDMAB1024-09|Canada|Alberta|658[0n]|BOLD:AAE6907
Heliothis borealis|[5192]|RDMAB1025-09|Canada|Alberta|635[0n]|BOLD:AAE6907
Heliothis borealis|[5193]|RDMAB1026-09|Canada|Alberta|593[0n]|BOLD:AAE6907
Heliothis belladonna|[5194]|HELNA379-09|United States|Nevada|658[0n]|BOLD:ABZ0916
Heliothis belladonna|[5195]|HELNA974-10|United States|California|611[0n]|BOLD:ABZ0916
Heliothis phloxiphaga|[5196]|BBLCU099-09|United States|Kansas|658[0n]|BOLD:AAC5164
Heliothis phloxiphaga|[5197]|HELNA854-10|United States|Wyoming|658[0n]|BOLD:AAC5164
Heliothis phloxiphaga|[5198]|GMLC1103-12|United States|California|658[0n]|BOLD:AAC5164
Heliothis phloxiphaga|[5199]|GMLC1029-12|United States|California|658[0n]|BOLD:AAC5164
Heliothis phloxiphaga|[5200]|PBCAG977-11|United States|California|658[0n]|BOLD:AAC5164
Heliothis phloxiphaga|[5201]|LOCBD647-06|United States|California|658[0n]|BOLD:AAC5164
Heliothis phloxiphaga|[5202]|BBLOD338-11|United States|California|658[0n]|BOLD:AAC5164
Heliothis phloxiphaga|[5203]|BBLOC1433-11|United States|California|658[0n]|BOLD:AAC5164
Heliothis phloxiphaga|[5204]|PBCAG979-11|United States|California|658[0n]|BOLD:AAC5164
Heliothis phloxiphaga|[5205]|PBCAG978-11|United States|California|658[0n]|BOLD:AAC5164
Heliothis phloxiphaga|[5206]|HELNA371-09|United States|Colorado|658[0n]|BOLD:AAC5164
Heliothis phloxiphaga|[5207]|HELNA370-09|United States|Colorado|658[0n]|BOLD:AAC5164
Heliothis phloxiphaga|[5208]|LOCBE358-06|United States|California|658[0n]|BOLD:AAC5164
Heliothis phloxiphaga|[5209]|LOCBE085-06|United States|California|658[0n]|BOLD:AAC5164
Heliothis phloxiphaga|[5210]|LOCBC489-06|United States|California|658[0n]|BOLD:AAC5164
Heliothis phloxiphaga|[5211]|HELNA137-06|United States|Colorado|658[0n]|BOLD:AAC5164
Heliothis phloxiphaga|[5212]|LHLEP236-06|Canada|British Columbia|658[0n]|BOLD:AAC5164
Heliothis phloxiphaga|[5213]|HELNA136-06|United States|Oklahoma|658[0n]|BOLD:AAC5164
Heliothis phloxiphaga|[5214]|CMAZA1034-12|United States|Arizona|658[0n]|BOLD:AAC5164
Heliothis phloxiphaga|[5215]|GMLC960-12|United States|California|658[0n]|BOLD:AAC5164
Heliothis phloxiphaga|[5216]|GMLC1009-12|United States|California|658[0n]|BOLD:AAC5164
Heliothis phloxiphaga|[5217]|GMLC1211-12|United States|California|658[0n]|BOLD:AAC5164
Heliothis ononis|[5218]|RDNMF042-08|Canada|Alberta|658[0n]|BOLD:AAE6902
Heliothis ononis|[5219]|RDNMG603-08|Canada|Saskatchewan|658[0n]|BOLD:AAE6902
Heliothis ononis|[5220]|RDNMG604-08|Canada|Saskatchewan|658[0n]|BOLD:AAE6902
Heliothis ononis|[5221]|HELNA846-10|Canada|Saskatchewan|658[0n]|BOLD:AAE6902
Heliothis acesias|[5222]|RDLQB539-05|Canada|Quebec|658[0n]|BOLD:ABY8163
Heliothis acesias|[5223]|RDLQB721-05|Canada|Quebec|658[0n]|BOLD:ABY8163
Heliothis acesias|[5224]|LNAUP144-13|United States|Indiana|658[0n]|BOLD:ABY8163
Heliothis acesias|[5225]|RDLQ007-05|Canada|Quebec|609[0n]|BOLD:ABY8164
Heliothis acesias|[5226]|RDLQ008-05|Canada|Quebec|600[0n]|BOLD:ABY8164
Heliothis acesias|[5227]|RDLQB733-05|Canada|Quebec|658[0n]|BOLD:ABY8164
Heliothis acesias|[5228]|RDLQF883-06|Canada|Quebec|658[0n]|BOLD:ABY8164
Heliothis acesias|[5229]|LNAUP143-13|United States|Indiana|658[0n]|BOLD:ABY8164
Heliothis acesias|[5230]|LNAUP145-13|United States|Indiana|658[0n]|BOLD:ABY8164
Heliothis oregonica|[5231]|NAMUM327-08|United States|California|658[0n]|BOLD:AAD0196
Heliothis oregonica|[5232]|LPABB554-08|Canada|Alberta|658[0n]|BOLD:AAD0196
Heliothis oregonica|[5233]|LOWCE094-06|Canada|British Columbia|658[0n]|BOLD:AAD0196
Heliothis oregonica|[5234]|LOWCE093-06|Canada|British Columbia|658[0n]|BOLD:AAD0196
Heliothis oregonica|[5235]|RDNMF050-08|Canada|Alberta|658[0n]|BOLD:AAD0196
Heliothis oregonica|[5236]|LOWCE085-06|Canada|British Columbia|658[0n]|BOLD:AAD0196
Heliothis oregonica|[5237]|LOWCE086-06|Canada|British Columbia|658[0n]|BOLD:AAD0196
Heliothis oregonica|[5238]|LNAUP147-13|United States|South Dakota|658[0n]|BOLD:AAD0196
Heliothis australis|[5239]|CNCLB1081-14|United States|Arizona|658[0n]|BOLD:ACD0020
Heliothis australis|[5240]|LNAUP148-13|United States|New Mexico|658[0n]|BOLD:ACD0020
Heliothis australis|[5241]|LNAUS4088-13|United States|New Mexico|658[0n]|BOLD:ACD0020
Heliothis australis|[5242]|CNCLB1079-14|United States|New Mexico|658[0n]|BOLD:ACD0020
Heliothis australis|[5243]|CNCLB1080-14|United States|New Mexico|658[0n]|BOLD:ACD0020
Heliothis australis|[5244]|LNAUS4092-13|Mexico|Chiapas|658[0n]|BOLD:ACD0020
Heliothis australis|[5245]|CNCLB1082-14|Mexico||658[0n]|BOLD:ACD0020
Heliothis australis|[5246]|CNCLB1083-14|Mexico||658[0n]|BOLD:ACD0020
Heliocheilus toralis|[5247]|HELNA871-10|United States|Arizona|658[0n]|BOLD:AAD6252
Heliocheilus toralis|[5248]|NAMUM321-08|United States|Georgia|658[0n]|BOLD:AAD6252
Heliocheilus toralis|[5249]|CMAZA1109-12|United States|Arizona|658[0n]|BOLD:AAD6252
Heliocheilus toralis|[5250]|HELNA521-09|United States|New Mexico|658[0n]|BOLD:AAD6252
Heliocheilus toralis|[5251]|HELNA874-10|United States|Arizona|658[0n]|BOLD:AAD6252
Heliocheilus toralis|[5252]|HELNA873-10|United States|Arizona|658[0n]|BOLD:AAD6252
Heliocheilus toralis|[5253]|RDNMH818-09|United States|Arizona|658[0n]|BOLD:AAD6252
Heliocheilus toralis|[5254]|HELNA520-09|United States|New Mexico|658[0n]|BOLD:AAD6252
Heliocheilus toralis|[5255]|HELNA291-09|United States|Texas|658[0n]|BOLD:AAD6252
Heliocheilus toralis|[5256]|NAMUM320-08|United States|Georgia|658[0n]|BOLD:AAD6252
Heliocheilus toralis|[5257]|HELNA872-10|United States|Arizona|581[0n]|BOLD:AAD6252
Heliocheilus toralis|[5258]|CMAZA1113-12|United States|Arizona|658[0n]|BOLD:AAD6252
Heliocheilus toralis|[5259]|LNAUP095-13|United States|New Mexico|658[0n]|BOLD:AAD6252
Heliocheilus toralis|[5260]|LNAUP096-13|United States|New Mexico|658[0n]|BOLD:AAD6252
Heliocheilus toralis|[5261]|LNAUP097-13|United States|New Mexico|658[0n]|BOLD:AAD6252
Heliocheilus toralis|[5262]|LNAUP098-13|United States|New Mexico|658[0n]|BOLD:AAD6252
Heliocheilus lupatus|[5263]|HELNA937-10|United States|Maryland|658[0n]|BOLD:AAF1452
Heliocheilus lupatus|[5264]|HELNA936-10|United States|Maryland|642[0n]|BOLD:AAF1452
Heliocheilus lupatus|[5265]|HELNA496-09|United States|Maryland|658[0n]|BOLD:AAF1452
Heliocheilus lupatus|[5266]|HELNA497-09|United States|Maryland|658[0n]|BOLD:AAF1452
Heliocheilus lupatus|[5267]|HELNA498-09|United States|Maryland|658[0n]|BOLD:AAF1452
Heliocheilus lupatus|[5268]|HELNA1018-10|United States|Maryland|658[0n]|BOLD:AAF1452
Heliocheilus lupatus|[5269]|HELNA1019-10|United States|Maryland|658[0n]|BOLD:AAF1452
Heliocheilus lupatus|[5270]|LNCC944-11|United States|North Carolina|658[0n]|BOLD:AAF1452
Heliocheilus paradoxus|[5271]|BBLSX206-09|United States|Texas|658[0n]|BOLD:AAC7180

Heliocheilus lupatus|[5269]|HELNA1019-10|United States|Maryland|658[0n]|BOLD:AAF1452
Heliocheilus lupatus|[5270]|LNCC944-11|United States|North Carolina|658[0n]|BOLD:AAF1452
Heliocheilus paradoxus|[5271]|BBLSX206-09|United States|Texas|658[0n]|BOLD:AAC7180
Heliocheilus paradoxus|[5272]|RDNMG601-08|United States|Arizona|649[0n]|BOLD:AAC7180
Heliocheilus paradoxus|[5273]|BBLSW112-09|United States|Texas|658[0n]|BOLD:AAC7180
Heliocheilus paradoxus|[5274]|HELNA949-10|United States|New Mexico|658[0n]|BOLD:AAC7180
Heliocheilus paradoxus|[5275]|CMAZA1118-12|United States|Arizona|658[0n]|BOLD:AAC7180
Heliocheilus paradoxus|[5276]|CMAZA1006-12|United States|Arizona|658[0n]|BOLD:AAC7180
Heliocheilus paradoxus|[5277]|HELNA372-09|United States|Colorado|658[0n]|BOLD:AAC7180
Heliocheilus paradoxus|[5278]|CMAZA1126-12|United States|Arizona|658[0n]|BOLD:AAC7180
Heliocheilus paradoxus|[5279]|CMAZA1117-12|United States|Arizona|658[0n]|BOLD:AAC7180
Heliocheilus paradoxus|[5280]|HELNA132-06|United States|Colorado|658[0n]|BOLD:AAC7180
Heliocheilus paradoxus|[5281]|HELNA133-06|United States|Colorado|658[0n]|BOLD:AAC7180
Heliocheilus paradoxus|[5282]|RDNMG412-08|United States|Colorado|658[0n]|BOLD:AAC7180
Heliocheilus paradoxus|[5283]|HELNA948-10|United States|New Mexico|658[0n]|BOLD:AAC7180
Heliocheilus paradoxus|[5284]|BBLSX228-09|United States|Texas|658[0n]|BOLD:AAC7180
Heliocheilus paradoxus|[5285]|HELNA523-09|United States|New Mexico|658[0n]|BOLD:AAC7180
Heliocheilus paradoxus|[5286]|HELNA522-09|United States|New Mexico|658[0n]|BOLD:AAC7180
Heliocheilus paradoxus|[5287]|RDNMG602-08|United States|Arizona|658[0n]|BOLD:AAC7180
Heliocheilus paradoxus|[5288]|RDNMG600-08|United States|Arizona|658[0n]|BOLD:AAC7180
Heliocheilus paradoxus|[5289]|CMAZA780-10|United States|Arizona|639[0n]|BOLD:AAC7180
Heliocheilus paradoxus|[5290]|LNAUP092-13|United States|New Mexico|658[0n]|BOLD:AAC7180
Heliocheilus julia|[5291]|DMAZ029-09|United States|Arizona|658[0n]|BOLD:AAH4917
Heliocheilus julia|[5292]|LNAUP149-13|United States|Arizona|658[0n]|BOLD:AAH4917
Heliocheilus julia|[5293]|AWCL062-09|United States|Arizona|658[0n]|BOLD:AAH4917
Heliocheilus julia|[5294]|AWCL063-09|United States|Arizona|658[0n]|BOLD:AAH4917
Heliocheilus julia|[5295]|HELNA395-09|United States|Arizona|658[0n]|BOLD:AAH4917
Heliocheilus julia|[5296]|LNAUP150-13|United States|Arizona|658[0n]|BOLD:AAH4917
Heliocheilus julia|[5297]|LNAUP151-13|United States|Arizona|658[0n]|BOLD:AAH4917
Heliocheilus julia|[5298]|HELNA875-10|United States|Arizona|658[0n]|BOLD:AAH4917
Heliocheilus julia|[5299]|AWCL064-09|United States|Arizona|658[0n]|BOLD:AAH4917
Heliocheilus julia|[5300]|RDNMH819-09|United States|Arizona|658[0n]|BOLD:AAH4917
Heliocheilus julia|[5301]|CMAZA576-10|United States|Arizona|658[0n]|BOLD:AAH4917
Heliocheilus julia|[5302]|LNAUP152-13|United States|Arizona|658[0n]|BOLD:AAH4917
Chloridea subflexa|[5303]|LNAUP126-13|Peru||658[0n]|BOLD:AAE4433
Chloridea subflexa|[5304]|LNAUP129-13|Peru||658[0n]|BOLD:AAE4433
Chloridea subflexa|[5305]|CMAZA1000-12|United States|Arizona|658[0n]|BOLD:AAE4433
Chloridea subflexa|[5306]|HELNA870-10|United States|Colorado|658[0n]|BOLD:AAE4433
Chloridea subflexa|[5307]|LPOKD352-09|United States|Oklahoma|658[0n]|BOLD:AAE4433
Chloridea subflexa|[5308]|GBGL5910-09|||722[0n]|BOLD:AAE4433
Chloridea subflexa|[5309]|CMAZA1039-12|United States|Arizona|658[0n]|BOLD:AAE4433
Chloridea subflexa|[5310]|CMAZA999-12|United States|Arizona|658[0n]|BOLD:AAE4433
Chloridea subflexa|[5311]|CMAZA564-10|United States|Arizona|658[0n]|BOLD:AAE4433
Chloridea subflexa|[5312]|BLPBH617-07|Costa Rica|Guanacaste|658[0n]|BOLD:AAE4433
Chloridea subflexa|[5313]|HELNA139-06|United States|Colorado|658[0n]|BOLD:AAE4433
Chloridea subflexa|[5314]|HELNA138-06|United States|Colorado|658[0n]|BOLD:AAE4433
Chloridea subflexa|[5315]|BLPDC429-09|Costa Rica|Alajuela|618[0n]|BOLD:AAE4433
Chloridea subflexa|[5316]|LNAUP162-13|United States|Oklahoma|658[0n]|BOLD:AAE4433
Chloridea virescens|[5317]|HELNA527-09|United States|North Carolina|658[0n]|BOLD:AAB2018
Chloridea virescens|[5318]|MHMXF823-07|Costa Rica|Guanacaste|658[0n]|BOLD:AAB2018
Chloridea virescens|[5319]|LNAUP141-13|United States|Maryland|646[0n]|BOLD:AAB2018
Chloridea virescens|[5320]|LOT347-04|United States|Tennessee|658[0n]|BOLD:AAB2018
Chloridea virescens|[5321]|MHMXF825-07|Costa Rica|Guanacaste|658[0n]|BOLD:AAB2018
Chloridea virescens|[5322]|MHMXI286-07|Costa Rica|Guanacaste|631[0n]|BOLD:AAB2018
Chloridea virescens|[5323]|LNAUP123-13|Peru||658[0n]|BOLD:AAB2018
Chloridea virescens|[5324]|LNAUP117-13|Peru||658[0n]|BOLD:AAB2018
Chloridea virescens|[5325]|LTOL1273-12|United States|Hawaii|658[0n]|BOLD:AAB2018
Chloridea virescens|[5326]|LTOL1271-12|United States|Hawaii|658[0n]|BOLD:AAB2018
Chloridea virescens|[5327]|LTOL1270-12|United States|Hawaii|658[0n]|BOLD:AAB2018
Chloridea virescens|[5328]|BBLOD1268-11|United States|Arizona|658[0n]|BOLD:AAB2018
Chloridea virescens|[5329]|HELNA981-10|Mexico||658[0n]|BOLD:AAB2018
Chloridea virescens|[5330]|HELNA953-10|United States|North Carolina|658[0n]|BOLD:AAB2018
Chloridea virescens|[5331]|BBLSW309-09|United States|Arizona|658[0n]|BOLD:AAB2018
Chloridea virescens|[5332]|LYPAP588-09|Mexico|Yucatan|658[0n]|BOLD:AAB2018
Chloridea virescens|[5333]|HELNA526-09|United States|Maryland|658[0n]|BOLD:AAB2018
Chloridea virescens|[5334]|MHMXM179-07|Costa Rica|Alajuela|658[0n]|BOLD:AAB2018
Chloridea virescens|[5335]|MHMXI287-07|Costa Rica|Guanacaste|658[0n]|BOLD:AAB2018
Chloridea virescens|[5336]|MHMXD612-06|Costa Rica|Guanacaste|658[0n]|BOLD:AAB2018
Chloridea virescens|[5337]|MHMXD611-06|Costa Rica|Guanacaste|658[0n]|BOLD:AAB2018
Chloridea virescens|[5338]|MHAUF164-06|Costa Rica|Guanacaste|658[0n]|BOLD:AAB2018
Chloridea virescens|[5339]|MHMXI283-07|Costa Rica|Guanacaste|562[0n]|BOLD:AAB2018
Chloridea virescens|[5340]|MHMXI285-07|Costa Rica|Guanacaste|631[0n]|BOLD:AAB2018
Chloridea virescens|[5341]|MHMXI284-07|Costa Rica|Guanacaste|658[0n]|BOLD:AAB2018
Chloridea virescens|[5342]|MHAUG091-07|Costa Rica|Guanacaste|658[0n]|BOLD:AAB2018
Chloridea virescens|[5343]|MHMXF824-07|Costa Rica|Guanacaste|658[0n]|BOLD:AAB2018
Chloridea virescens|[5344]|MHMXF822-07|Costa Rica|Guanacaste|658[0n]|BOLD:AAB2018
Chloridea virescens|[5345]|MHAUC156-06|Costa Rica|Guanacaste|518[0n]|BOLD:AAB2018
Chloridea virescens|[5346]|BLPAC670-06|Costa Rica|Guanacaste|621[0n]|BOLD:AAB2018
Chloridea virescens|[5347]|LNAUP142-13|United States|Maryland|563[0n]|BOLD:AAB2018
Chloridea virescens|[5348]|MHAUC160-06|Costa Rica|Guanacaste|658[0n]|BOLD:AAB2018
Chloridea virescens|[5349]|MHAUC155-06|Costa Rica|Guanacaste|658[0n]|BOLD:AAB2018
Chloridea virescens|[5350]|MHMXD610-06|Costa Rica|Guanacaste|658[1n]|BOLD:AAB2018
Chloridea virescens|[5351]|MHMXM180-07|Costa Rica|Guanacaste|576[0n]|BOLD:AAB2018
Chloridea virescens|[5352]|MHMXM181-07|Costa Rica|Guanacaste|658[0n]|BOLD:AAB2018
Chloridea virescens|[5353]|MHMYT1258-13|Costa Rica||658[0n]|BOLD:AAB2018
Chloridea virescens|[5354]|BLPEE6303-14|Costa Rica||658[0n]|BOLD:AAB2018
Helicoverpa zea|[5355]|RWWC1290-13|United States|Washington|595[0n]|BOLD:ABZ5815
Helicoverpa zea|[5356]|ECPD047-14|Argentina|Cordoba|585[2n]|BOLD:ABZ5815
Helicoverpa zea|[5357]|BBLOE1411-12|United States|Texas|658[0n]|BOLD:ABZ5815
Helicoverpa zea|[5358]|GBGL11177-12|||669[0n]|BOLD:ABZ5815
Helicoverpa zea|[5359]|GBMIN22010-13|||658[0n]|BOLD:ABZ5815
Helicoverpa zea|[5360]|GBMIN22016-13|||658[0n]|BOLD:ABZ5815
Helicoverpa zea|[5361]|GBMIN38598-13|United States||641[0n]|BOLD:ABZ5815
Helicoverpa zea|[5362]|GBMIN38647-13|United States||641[0n]|BOLD:ABZ5815
Helicoverpa zea|[5363]|GBMIN38648-13|United States||641[0n]|BOLD:ABZ5815
Helicoverpa zea|[5364]|HELNA142-06|United States|Kansas|658[0n]|BOLD:ABZ5815
Helicoverpa zea|[5365]|BBLSX113-09|United States|Oklahoma|634[0n]|BOLD:ABZ5815
Helicoverpa zea|[5366]|BBLOB677-11|United States|Texas|658[0n]|BOLD:ABZ5815
Helicoverpa zea|[5367]|LOCBB205-06|United States|California|658[0n]|BOLD:ABZ5815
Helicoverpa zea|[5368]|LPOKA497-09|United States|Oklahoma|630[0n]|BOLD:ABZ5815
Helicoverpa zea|[5369]|HELNA525-09|United States|Maryland|630[0n]|BOLD:ABZ5815
Helicoverpa zea|[5370]|LOCBB208-06|United States|California|653[0n]|BOLD:ABZ5815
Helicoverpa zea|[5371]|LOCBB662-06|United States|California|658[0n]|BOLD:ABZ5815

Helicoverpa zea|[5369]|HELNA525-09|United States|Maryland|630[0n]|BOLD:ABZ5815
Helicoverpa zea|[5370]|LOCBB208-06|United States|California|653[0n]|BOLD:ABZ5815
Helicoverpa zea|[5371]|LOCBB662-06|United States|California|658[0n]|BOLD:ABZ5815
Helicoverpa zea|[5372]|BBLSW307-09|United States|Arizona|658[0n]|BOLD:ABZ5815
Helicoverpa zea|[5373]|HELNA990-10|Dominican Republic||658[0n]|BOLD:ABZ5815
Helicoverpa zea|[5374]|GBGL5900-09|||722[0n]|BOLD:ABZ5815
Helicoverpa zea|[5375]|LOCBF2621-13|United States|California|658[0n]|BOLD:ABZ5815
Helicoverpa zea|[5376]|LNAUP166-13|United States|Virginia|658[0n]|BOLD:ABZ5815
Helicoverpa zea|[5377]|LNAUP165-13|United States|California|658[0n]|BOLD:ABZ5815
Helicoverpa zea|[5378]|LNAUP164-13|United States|Maryland|658[0n]|BOLD:ABZ5815
Helicoverpa zea|[5379]|LNAUP163-13|United States|New Mexico|658[0n]|BOLD:ABZ5815
Helicoverpa zea|[5380]|LNAUP115-13|Peru||658[0n]|BOLD:ABZ5815
Helicoverpa zea|[5381]|LNAUP114-13|Peru||658[0n]|BOLD:ABZ5815
Helicoverpa zea|[5382]|ARMOT146-12|Argentina|Entre Rios|658[0n]|BOLD:ABZ5815
Helicoverpa zea|[5383]|ARMOT137-12|Argentina|Entre Rios|658[0n]|BOLD:ABZ5815
Helicoverpa zea|[5384]|ARMOT121-12|Argentina|Entre Rios|658[0n]|BOLD:ABZ5815
Helicoverpa zea|[5385]|ARMOT103-12|Argentina|Entre Rios|658[0n]|BOLD:ABZ5815
Helicoverpa zea|[5386]|GMLC1240-12|United States|California|658[0n]|BOLD:ABZ5815
Helicoverpa zea|[5387]|CMAZA1041-12|United States|Arizona|658[0n]|BOLD:ABZ5815
Helicoverpa zea|[5388]|CMAZA1010-12|United States|Arizona|658[0n]|BOLD:ABZ5815
Helicoverpa zea|[5389]|BBLOE1989-12|United States|Texas|658[0n]|BOLD:ABZ5815
Helicoverpa zea|[5390]|BBLOE1985-12|United States|Texas|658[0n]|BOLD:ABZ5815
Helicoverpa zea|[5391]|BBLOE1466-12|United States|Texas|658[0n]|BOLD:ABZ5815
Helicoverpa zea|[5392]|BBLOE1462-12|United States|Texas|658[0n]|BOLD:ABZ5815
Helicoverpa zea|[5393]|BBLOD1647-11|United States|Oklahoma|658[0n]|BOLD:ABZ5815
Helicoverpa zea|[5394]|BBLOD898-11|United States|California|658[0n]|BOLD:ABZ5815
Helicoverpa zea|[5395]|BBLOD143-11|United States|Texas|658[0n]|BOLD:ABZ5815
Helicoverpa zea|[5396]|BBLOC877-11|United States|Arkansas|658[0n]|BOLD:ABZ5815
Helicoverpa zea|[5397]|BBLOB690-11|United States|Texas|658[0n]|BOLD:ABZ5815
Helicoverpa zea|[5398]|BBLOB682-11|United States|Texas|658[0n]|BOLD:ABZ5815
Helicoverpa zea|[5399]|BBLOB611-11|United States|Texas|658[0n]|BOLD:ABZ5815
Helicoverpa zea|[5400]|LEPPA146-11|Argentina|Buenos Aires|658[0n]|BOLD:ABZ5815
Helicoverpa zea|[5401]|MHMYN079-11|Costa Rica||658[0n]|BOLD:ABZ5815
Helicoverpa zea|[5402]|LILLB060-11|United States|Illinois|658[0n]|BOLD:ABZ5815
Helicoverpa zea|[5403]|AWCLB510-11|United States|Arizona|658[0n]|BOLD:ABZ5815
Helicoverpa zea|[5404]|AWCLB498-11|United States|Arizona|658[0n]|BOLD:ABZ5815
Helicoverpa zea|[5405]|AWCLB496-11|United States|Arizona|658[0n]|BOLD:ABZ5815
Helicoverpa zea|[5406]|HELNA1001-10|Trinidad and Tobago||658[0n]|BOLD:ABZ5815
Helicoverpa zea|[5407]|HELNA1000-10|Dominican Republic||658[0n]|BOLD:ABZ5815
Helicoverpa zea|[5408]|HELNA998-10|Guatemala||658[0n]|BOLD:ABZ5815
Helicoverpa zea|[5409]|HELNA997-10|Dominican Republic||658[0n]|BOLD:ABZ5815
Helicoverpa zea|[5410]|LEMMZ095-10|Brazil|Parana|658[0n]|BOLD:ABZ5815
Helicoverpa zea|[5411]|LEMMZ015-10|Brazil|Parana|658[0n]|BOLD:ABZ5815
Helicoverpa zea|[5412]|USLEP387-10|United States|Texas|658[0n]|BOLD:ABZ5815
Helicoverpa zea|[5413]|LGSMG929-10|United States|North Carolina|658[0n]|BOLD:ABZ5815
Helicoverpa zea|[5414]|LGSMG928-10|United States|North Carolina|658[0n]|BOLD:ABZ5815
Helicoverpa zea|[5415]|LGSMG927-10|United States|North Carolina|658[0n]|BOLD:ABZ5815
Helicoverpa zea|[5416]|LGSMG926-10|United States|Tennessee|658[0n]|BOLD:ABZ5815
Helicoverpa zea|[5417]|CMAZA286-09|United States|Arizona|658[0n]|BOLD:ABZ5815
Helicoverpa zea|[5418]|BBLCU378-09|United States|Kansas|658[0n]|BOLD:ABZ5815
Helicoverpa zea|[5419]|BBLSX745-09|United States|Arizona|658[0n]|BOLD:ABZ5815
Helicoverpa zea|[5420]|BBLSX682-09|United States|Arizona|658[0n]|BOLD:ABZ5815
Helicoverpa zea|[5421]|BBLSW320-09|United States|Arizona|658[0n]|BOLD:ABZ5815
Helicoverpa zea|[5422]|BBLSW310-09|United States|Arizona|658[0n]|BOLD:ABZ5815
Helicoverpa zea|[5423]|BBLSW308-09|United States|Arizona|658[0n]|BOLD:ABZ5815
Helicoverpa zea|[5424]|BBLSW306-09|United States|Arizona|658[0n]|BOLD:ABZ5815
Helicoverpa zea|[5425]|BBLSW305-09|United States|Arizona|658[0n]|BOLD:ABZ5815
Helicoverpa zea|[5426]|BBLSW304-09|United States|Arizona|658[0n]|BOLD:ABZ5815
Helicoverpa zea|[5427]|HELNA399-09|United States|Oklahoma|658[0n]|BOLD:ABZ5815
Helicoverpa zea|[5428]|HELNA397-09|United States|Oklahoma|658[0n]|BOLD:ABZ5815
Helicoverpa zea|[5429]|HELNA360-09|United States|Maryland|658[0n]|BOLD:ABZ5815
Helicoverpa zea|[5430]|HELNA359-09|United States|Maryland|658[0n]|BOLD:ABZ5815
Helicoverpa zea|[5431]|HELNA358-09|United States|Maryland|658[0n]|BOLD:ABZ5815
Helicoverpa zea|[5432]|LPOKA1023-09|United States|Oklahoma|658[0n]|BOLD:ABZ5815
Helicoverpa zea|[5433]|LPOKA658-09|United States|Oklahoma|658[0n]|BOLD:ABZ5815
Helicoverpa zea|[5434]|LPOKA619-09|United States|Oklahoma|658[0n]|BOLD:ABZ5815
Helicoverpa zea|[5435]|LPOKA615-09|United States|Oklahoma|658[0n]|BOLD:ABZ5815
Helicoverpa zea|[5436]|LPOKA612-09|United States|Oklahoma|658[0n]|BOLD:ABZ5815
Helicoverpa zea|[5437]|LPOKA592-09|United States|Oklahoma|658[0n]|BOLD:ABZ5815
Helicoverpa zea|[5438]|LPOKA559-09|United States|Oklahoma|658[0n]|BOLD:ABZ5815
Helicoverpa zea|[5439]|LPOKA543-09|United States|Oklahoma|658[0n]|BOLD:ABZ5815
Helicoverpa zea|[5440]|LPOKA512-09|United States|Oklahoma|658[0n]|BOLD:ABZ5815
Helicoverpa zea|[5441]|LPOKA510-09|United States|Oklahoma|658[0n]|BOLD:ABZ5815
Helicoverpa zea|[5442]|LPOKA372-08|United States|Oklahoma|658[0n]|BOLD:ABZ5815
Helicoverpa zea|[5443]|LPOKA355-08|United States|Oklahoma|658[0n]|BOLD:ABZ5815
Helicoverpa zea|[5444]|LTOLB085-08|United States|Maryland|658[0n]|BOLD:ABZ5815
Helicoverpa zea|[5445]|LTOLB084-08|United States|Maryland|658[0n]|BOLD:ABZ5815
Helicoverpa zea|[5446]|LTOL934-08|United States|Mississippi|655[0n]|BOLD:ABZ5815
Helicoverpa zea|[5447]|NSBUG170-07|Canada|Nova Scotia|657[0n]|BOLD:ABZ5815
Helicoverpa zea|[5448]|MHMXM182-07|Costa Rica|Alajuela|658[0n]|BOLD:ABZ5815
Helicoverpa zea|[5449]|BLPAB899-06|Costa Rica|Guanacaste|658[0n]|BOLD:ABZ5815
Helicoverpa zea|[5450]|BLPAA705-06|Costa Rica|Guanacaste|658[0n]|BOLD:ABZ5815
Helicoverpa zea|[5451]|LOCB543-06|United States|California|658[0n]|BOLD:ABZ5815
Helicoverpa zea|[5452]|LOCB435-06|United States|California|658[0n]|BOLD:ABZ5815
Helicoverpa zea|[5453]|HELNA144-06|United States|New Mexico|658[0n]|BOLD:ABZ5815
Helicoverpa zea|[5454]|HELNA143-06|United States|Kansas|658[0n]|BOLD:ABZ5815
Helicoverpa zea|[5455]|LNC570-06|United States|North Carolina|658[0n]|BOLD:ABZ5815
Helicoverpa zea|[5456]|XAH731-05|Canada|Ontario|658[0n]|BOLD:ABZ5815
Helicoverpa zea|[5457]|XAH369-05|Canada|Ontario|658[0n]|BOLD:ABZ5815
Helicoverpa zea|[5458]|LOT346-04|United States|Tennessee|658[0n]|BOLD:ABZ5815
Helicoverpa zea|[5459]|LOCBB862-06|United States|California|656[0n]|BOLD:ABZ5815
Helicoverpa zea|[5460]|LOCB428-06|United States|California|656[0n]|BOLD:ABZ5815
Helicoverpa zea|[5461]|HELNA398-09|United States|Oklahoma|658[0n]|BOLD:ABZ5815
Helicoverpa zea|[5462]|LPOKA673-09|United States|Oklahoma|647[0n]|BOLD:ABZ5815
Helicoverpa zea|[5463]|LPOKA623-09|United States|Oklahoma|593[0n]|BOLD:ABZ5815
Helicoverpa zea|[5464]|LOCB491-06|United States|California|658[0n]|BOLD:ABZ5815
Helicoverpa zea|[5465]|LOCBB661-06|United States|California|658[0n]|BOLD:ABZ5815
Helicoverpa zea|[5466]|LOCBB663-06|United States|California|658[0n]|BOLD:ABZ5815
Helicoverpa zea|[5467]|LEMMZ096-10|Brazil|Parana|658[0n]|BOLD:ABZ5815
Helicoverpa zea|[5468]|LPOKA286-08|United States|Oklahoma|607[0n]|BOLD:ABZ5815
Helicoverpa zea|[5469]|LPOKA545-09|United States|Oklahoma|658[0n]|BOLD:ABZ5815
Helicoverpa zea|[5470]|MHMXM183-07|Costa Rica|Alajuela|644[0n]|BOLD:ABZ5815

Helicoverpa zea|[5468]|LPOKA286-08|United States|Oklahoma|607[0n]|BOLD:ABZ5815
Helicoverpa zea|[5469]|LPOKA545-09|United States|Oklahoma|658[0n]|BOLD:ABZ5815
Helicoverpa zea|[5470]|MHMXM183-07|Costa Rica|Alajuela|644[0n]|BOLD:ABZ5815
Helicoverpa zea|[5471]|LOCB546-06|United States|California|606[0n]|BOLD:ABZ5815
Helicoverpa zea|[5472]|LOCBC087-06|United States|California|625[0n]|BOLD:ABZ5815
Helicoverpa zea|[5473]|PHMO346-03|Canada|Ontario|639[0n]|BOLD:ABZ5815
Helicoverpa zea|[5474]|LOCB548-06|United States|California|623[0n]|BOLD:ABZ5815
Helicoverpa zea|[5475]|LOCBC088-06|United States|California|599[0n]|BOLD:ABZ5815
Helicoverpa zea|[5476]|LOCBC156-06|United States|California|610[0n]|BOLD:ABZ5815
Helicoverpa zea|[5477]|XAD496-04|Canada|Ontario|573[0n]|BOLD:ABZ5815
Helicoverpa zea|[5478]|LPOKA590-09|United States|Oklahoma|617[0n]|BOLD:ABZ5815
Helicoverpa zea|[5479]|LOCBB206-06|United States|California|658[0n]|BOLD:ABZ5815
Helicoverpa zea|[5480]|LOCB544-06|United States|California|608[0n]|BOLD:ABZ5815
Helicoverpa zea|[5481]|XAD468-04|Canada|Ontario|572[0n]|BOLD:ABZ5815
Helicoverpa zea|[5482]|LOCB547-06|United States|California|604[0n]|BOLD:ABZ5815
Helicoverpa zea|[5483]|LOCBB207-06|United States|California|649[0n]|BOLD:ABZ5815
Helicoverpa zea|[5484]|LPOKA588-09|United States|Oklahoma|614[0n]|BOLD:ABZ5815
Helicoverpa zea|[5485]|HELNA999-10|Dominican Republic||603[0n]|BOLD:ABZ5815
Helicoverpa zea|[5486]|LILLA849-11|United States|Illinois|627[0n]|BOLD:ABZ5815
Helicoverpa zea|[5487]|MHAUF283-06|Costa Rica|Guanacaste|638[0n]|BOLD:ABZ5815
Helicoverpa zea|[5488]|GBGL12593-13|||1000[2n]|
Helicoverpa zea|[5489]|GBGL15330-14|||655[0n]|BOLD:ABZ5815
Helicoverpa zea|[5490]|GBGL15331-14|||655[0n]|BOLD:ABZ5815
Helicoverpa zea|[5491]|GBGL15332-14|||655[0n]|BOLD:ABZ5815
Emarginea dulcinea|[5492]|RDNMC359-05|United States|Arizona|546[0n]|BOLD:AAX7625
Emarginea dulcinea|[5493]|CMAZA1180-12|United States|Arizona|658[0n]|BOLD:AAX7625
Emarginea dulcinea|[5494]|LNAUT773-14|United States|Arizona|658[0n]|BOLD:AAX7625
Emarginea dulcinea|[5495]|LNAUT774-14|United States|Arizona|658[0n]|BOLD:AAX7625
Emarginea percara|[5496]|LOCBF1138-13|United States|California|582[0n]|BOLD:AAA9304
Emarginea percara|[5497]|LNAUT775-14|United States|Arizona|658[0n]|BOLD:AAA9304
Emarginea percara|[5498]|CMAZA323-10|United States|Arizona|658[0n]|BOLD:AAA9304
Emarginea percara|[5499]|CMAZA697-10|United States|Arizona|649[0n]|BOLD:AAA9304
Emarginea percara|[5500]|BBLSW116-09|United States|Arizona|658[0n]|BOLD:AAA9304
Emarginea percara|[5501]|BBLSW117-09|United States|Arizona|658[0n]|BOLD:AAA9304
Emarginea percara|[5502]|BBLSW118-09|United States|Arizona|658[0n]|BOLD:AAA9304
Emarginea percara|[5503]|BBLOB1502-11|United States|Arizona|658[0n]|BOLD:AAA9304
Emarginea percara|[5504]|BBLOD762-11|United States|Arizona|658[0n]|BOLD:AAA9304
Emarginea percara|[5505]|BBLOD1915-11|United States|Arizona|658[0n]|BOLD:AAA9304
Emarginea percara|[5506]|TML137-14|United States||621[0n]|BOLD:AAA9304
Emarginea percara|[5507]|CMAZA1228-12|United States|Arizona|658[0n]|BOLD:AAA9304
Emarginea percara|[5508]|BBLSW115-09|United States|Arizona|658[0n]|BOLD:AAA9304
Emarginea percara|[5509]|LPOKD026-09|United States|Oklahoma|658[0n]|BOLD:AAA9304
Emarginea percara|[5510]|USLEP670-10|United States|Texas|658[0n]|BOLD:AAA9304
Emarginea percara|[5511]|LNAUT777-14|United States|Mississippi|658[0n]|BOLD:AAA9304
Emarginea percara|[5512]|BBLSX274-09|United States|Texas|658[0n]|BOLD:AAA9304
Emarginea percara|[5513]|LOCBF1121-13|United States|California|600[0n]|BOLD:AAA9304
Emarginea percara|[5514]|CNCLB244-14|United States|Nevada|658[0n]|BOLD:AAA9304
Emarginea percara|[5515]|BBLSX052-09|United States|Texas|658[0n]|BOLD:AAA9304
Emarginea percara|[5516]|USLEP671-10|United States|Texas|658[0n]|BOLD:AAA9304
Emarginea percara|[5517]|USLEP672-10|United States|Texas|658[0n]|BOLD:AAA9304
Emarginea percara|[5518]|USLEP674-10|United States|Texas|658[0n]|BOLD:AAA9304
Emarginea percara|[5519]|BBLOB663-11|United States|Oklahoma|658[0n]|BOLD:AAA9304
Emarginea percara|[5520]|BBLOB666-11|United States|Oklahoma|658[0n]|BOLD:AAA9304
Emarginea percara|[5521]|BBLOB667-11|United States|Oklahoma|658[0n]|BOLD:AAA9304
Emarginea percara|[5522]|BBLOB668-11|United States|Oklahoma|658[0n]|BOLD:AAA9304
Emarginea percara|[5523]|CMAZA747-10|United States|Arizona|658[0n]|BOLD:AAA9304
Emarginea percara|[5524]|RDNMC564-06|United States|New Mexico|658[0n]|BOLD:AAA9304
Emarginea percara|[5525]|BBLSW854-09|United States|Texas|652[0n]|BOLD:AAA9304
Emarginea percara|[5526]|BBLSW855-09|United States|Texas|658[0n]|BOLD:AAA9304
Emarginea percara|[5527]|BBLSX053-09|United States|Texas|658[0n]|BOLD:AAA9304
Emarginea percara|[5528]|BBLSX054-09|United States|Texas|658[0n]|BOLD:AAA9304
Emarginea percara|[5529]|BBLSX055-09|United States|Texas|658[0n]|BOLD:AAA9304
Emarginea percara|[5530]|BBLSX345-09|United States|Oklahoma|658[0n]|BOLD:AAA9304
Emarginea percara|[5531]|BBLSX814-09|United States|Texas|658[0n]|BOLD:AAA9304
Emarginea percara|[5532]|USLEP673-10|United States|Texas|658[0n]|BOLD:AAA9304
Emarginea percara|[5533]|CMAZA467-10|United States|Arizona|658[0n]|BOLD:AAA9304
Emarginea percara|[5534]|CMAZA474-10|United States|Arizona|658[0n]|BOLD:AAA9304
Emarginea percara|[5535]|LNAUT776-14|United States|Mississippi|658[0n]|BOLD:AAA9304
Emarginea percara|[5536]|LNAUT778-14|United States|Mississippi|658[0n]|BOLD:AAA9304
Emarginea percara|[5537]|LNAUT779-14|United States|Mississippi|658[0n]|BOLD:AAA9304
Emarginea percara|[5538]|LNAUT780-14|United States|Mississippi|658[0n]|BOLD:AAA9304
Emarginea percara|[5539]|CNCLB2406-14|United States|Texas|658[0n]|BOLD:AAA9304
Emarginea percara|[5540]|CNCLB2407-14|United States|Texas|658[0n]|BOLD:AAA9304
Brachionycha borealis|[5541]|MEC123-04|Canada|Quebec|578[11n]|BOLD:AAE8262
Brachionycha borealis|[5542]|RDNMG530-08|Canada|British Columbia|658[0n]|BOLD:AAE8262
Brachionycha borealis|[5543]|RDMAB982-09|Canada|Alberta|655[0n]|BOLD:AAE8262
Brachionycha borealis|[5544]|RDMAB983-09|Canada|Alberta|635[0n]|BOLD:AAE8262
Catabena lineolata|[5545]|HKONB174-08|United States|Texas|658[0n]|BOLD:AAE4319
Catabena lineolata|[5546]|RDMAB684-06|Canada|Alberta|632[0n]|BOLD:AAE4319
Catabena lineolata|[5547]|HKONB173-08|United States|Texas|658[0n]|BOLD:AAE4319
Catabena lineolata|[5548]|BBLSY193-09|United States|Texas|658[0n]|BOLD:AAE4319
Catabena lineolata|[5549]|RDLQ600-07|Canada|Quebec|594[0n]|BOLD:AAE4319
Catabena lineolata|[5550]|CNCLA5289-13|Canada|Ontario|658[0n]|BOLD:AAE4319
Catabena lineolata|[5551]|CNCLA5290-13|Canada|Ontario|658[0n]|BOLD:AAE4319
Catabena nr. lineolata|[5552]|CNCLA5288-13|United States|Arizona|658[0n]|BOLD:ABZ3964
Catabena nr. lineolata|[5553]|NAMUM156-08|United States|California|621[0n]|BOLD:ABZ3964
Catabena nr. lineolata|[5554]|CGLCA022-10|United States|California|658[0n]|BOLD:ABZ3964
Catabena nr. lineolata|[5555]|GMLC246-11|United States|California|658[0n]|BOLD:ABZ3964
Catabena nr. lineolata|[5556]|GMLC840-12|United States|California|658[0n]|BOLD:ABZ3964
Catabena nr. lineolata|[5557]|GMLC1412-12|United States|California|614[0n]|BOLD:ABZ3964
Catabena nr. lineolata|[5558]|GMLC1003-12|United States|California|658[0n]|BOLD:ABZ3964
Catabena nr. lineolata|[5559]|GMLC915-12|United States|California|658[0n]|BOLD:ABZ3964
Catabena nr. lineolata|[5560]|CNCLA5291-13|United States|California|658[0n]|BOLD:ABZ3964
Feralia major|[5561]|LSEU130-06|United States|Georgia|658[0n]|BOLD:AAA9672
Feralia major|[5562]|RDNM159-05|United States|Louisiana|658[0n]|BOLD:AAA9672
Feralia major|[5563]|RDNMC558-06|United States|Georgia|658[0n]|BOLD:AAA9672
Feralia major|[5564]|RDNMC559-06|United States|Georgia|561[0n]|BOLD:AAA9672
Feralia major|[5565]|RDNM158-05|United States|Louisiana|658[0n]|BOLD:AAA9672
Feralia major|[5566]|NAMUM407-09|United States|Texas|658[0n]|BOLD:AAA9672
Feralia major|[5567]|NAMUM408-09|United States|Texas|658[0n]|BOLD:AAA9672
Feralia deceptiva|[5568]|LBCF002-07|Canada|British Columbia|654[0n]|BOLD:AAA9672
Feralia deceptiva|[5569]|LBCF003-07|Canada|British Columbia|658[0n]|BOLD:AAA9672
Feralia deceptiva|[5570]|LBCF001-07|Canada|British Columbia|630[0n]|BOLD:AAA9672

[illegible]
Feralia deceptiva|[5568]|LBCF002-07|Canada|British Columbia|654[0n]|BOLD:AAA9672
Feralia deceptiva|[5569]|LBCF003-07|Canada|British Columbia|658[0n]|BOLD:AAA9672
Feralia deceptiva|[5570]|LBCF001-07|Canada|British Columbia|630[0n]|BOLD:AAA9672
Feralia deceptiva|[5571]|LBCF008-07|Canada|British Columbia|658[1n]|BOLD:AAA9672
Feralia deceptiva|[5572]|DUNLP155-08|Canada|British Columbia|658[0n]|BOLD:AAA9672
Feralia deceptiva|[5573]|LALPA038-10|Canada|British Columbia|658[0n]|BOLD:AAA9672
Feralia deceptiva|[5574]|LALPA047-10|Canada|British Columbia|658[0n]|BOLD:AAA9672
Feralia major|[5575]|RDNMC561-06|United States|Georgia|658[1n]|BOLD:AAA9672
Feralia major|[5576]|RDNM156-05|Canada|Alberta|658[0n]|BOLD:AAA9672
Feralia major|[5577]|LSEU129-06|United States|Georgia|658[0n]|BOLD:AAA9672
Feralia major|[5578]|XAJ095-06|Canada|Ontario|658[0n]|BOLD:AAA9672
Feralia major|[5579]|RDNM157-05|Canada|Ontario|658[0n]|BOLD:AAA9672
Feralia major|[5580]|RDLQH099-06|Canada|Quebec|658[0n]|BOLD:AAA9672
Feralia major|[5581]|XAJ094-06|Canada|Ontario|658[0n]|BOLD:AAA9672
Feralia major|[5582]|XAF318-05|Canada|Ontario|658[0n]|BOLD:AAA9672
Feralia major|[5583]|PHMO022-03|Canada|Ontario|639[0n]|BOLD:AAA9672
Feralia major|[5584]|PHMO019-03|Canada|Ontario|639[0n]|BOLD:AAA9672
Feralia major|[5585]|TMG121-03|Canada|Ontario|639[0n]|BOLD:AAA9672
Feralia major|[5586]|PMG116-03|Canada|Ontario|617[0n]|BOLD:AAA9672
Feralia major|[5587]|PHMO020-03|Canada|Ontario|639[0n]|BOLD:AAA9672
Feralia major|[5588]|XAJ115-06|Canada|Ontario|607[0n]|BOLD:AAA9672
Feralia major|[5589]|XAJ117-06|Canada|Ontario|658[0n]|BOLD:AAA9672
Feralia major|[5590]|XAJ200-06|Canada|Ontario|658[0n]|BOLD:AAA9672
Feralia major|[5591]|XAJ098-06|Canada|Ontario|658[0n]|BOLD:AAA9672
Feralia major|[5592]|XAJ099-06|Canada|Ontario|658[0n]|BOLD:AAA9672
Feralia major|[5593]|XAJ118-06|Canada|Ontario|658[0n]|BOLD:AAA9672
Feralia major|[5594]|XAJ121-06|Canada|Ontario|658[0n]|BOLD:AAA9672
Feralia major|[5595]|XAJ164-06|Canada|Ontario|658[0n]|BOLD:AAA9672
Feralia major|[5596]|RDLQH100-06|Canada|Quebec|658[0n]|BOLD:AAA9672
Feralia major|[5597]|RDLQH101-06|Canada|Quebec|658[0n]|BOLD:AAA9672
Feralia jocosa|[5598]|RDLQH102-06|Canada|Quebec|658[0n]|BOLD:AAA9672
Feralia jocosa|[5599]|XAJ183-06|Canada|Ontario|658[0n]|BOLD:AAA9672
Feralia jocosa|[5600]|XAJ119-06|Canada|Ontario|658[0n]|BOLD:AAA9672
Feralia jocosa|[5601]|XAJ114-06|Canada|Ontario|658[0n]|BOLD:AAA9672
Feralia jocosa|[5602]|XAJ053-06|Canada|Ontario|658[0n]|BOLD:AAA9672
Feralia jocosa|[5603]|TMNBB285-06|Canada|New Brunswick|658[0n]|BOLD:AAA9672
Feralia jocosa|[5604]|TMNBB283-06|Canada|New Brunswick|658[0n]|BOLD:AAA9672
Feralia jocosa|[5605]|XAF428-05|Canada|Ontario|658[0n]|BOLD:AAA9672
Feralia jocosa|[5606]|XAF322-05|Canada|Ontario|658[0n]|BOLD:AAA9672
Feralia jocosa|[5607]|RDLQ003-05|Canada|Quebec|658[0n]|BOLD:AAA9672
Feralia jocosa|[5608]|TMNBB284-06|Canada|New Brunswick|658[0n]|BOLD:AAA9672
Feralia jocosa|[5609]|PMG115-03|Canada|Ontario|617[0n]|BOLD:AAA9672
Feralia jocosa|[5610]|RDLQ001-05|Canada|Quebec|552[0n]|BOLD:AAA9672
Feralia jocosa|[5611]|RDLQ002-05|Canada|Quebec|567[0n]|BOLD:AAA9672
Feralia jocosa|[5612]|RDLQH110-06|Canada|Quebec|605[0n]|BOLD:AAA9672
Feralia jocosa|[5613]|TMNBB282-06|Canada|New Brunswick|658[0n]|BOLD:AAA9672
Feralia jocosa|[5614]|TMNBB286-06|Canada|New Brunswick|658[0n]|BOLD:AAA9672
Feralia jocosa|[5615]|TMNBB287-06|Canada|New Brunswick|656[0n]|BOLD:AAA9672
Feralia jocosa|[5616]|XAJ188-06|Canada|Ontario|658[0n]|BOLD:AAA9672
Feralia jocosa|[5617]|XAJ184-06|Canada|Ontario|658[0n]|BOLD:AAA9672
Feralia jocosa|[5618]|XAJ182-06|Canada|Ontario|658[0n]|BOLD:AAA9672
Feralia jocosa|[5619]|XAF276-05|Canada|Ontario|658[0n]|BOLD:AAA9672
Feralia jocosa|[5620]|PHMO033-03|Canada|Ontario|639[0n]|BOLD:AAA9672
Feralia jocosa|[5621]|PHMO012-03|Canada|Ontario|639[0n]|BOLD:AAA9672
Feralia jocosa|[5622]|XAJ054-06|Canada|Ontario|592[0n]|BOLD:AAA9672
Feralia jocosa|[5623]|DUNLP161-08|Canada|British Columbia|627[0n]|BOLD:AAA9672
Feralia jocosa|[5624]|XAJ116-06|Canada|Ontario|658[0n]|BOLD:AAA9672
Feralia jocosa|[5625]|XAJ228-06|Canada|Ontario|658[0n]|BOLD:AAA9672
Feralia jocosa|[5626]|DUNLP162-08|Canada|British Columbia|641[0n]|BOLD:AAA9672
Feralia jocosa|[5627]|DUNLP163-08|Canada|British Columbia|639[1n]|BOLD:AAA9672
Feralia jocosa|[5628]|LMDH024-11|United States|Minnesota|658[0n]|BOLD:AAA9672
Feralia jocosa|[5629]|XAJ231-06|Canada|Ontario|658[0n]|BOLD:AAA9672
Feralia jocosa|[5630]|XAJ181-06|Canada|Ontario|658[0n]|BOLD:AAA9672
Feralia jocosa|[5631]|XAJ180-06|Canada|Ontario|658[0n]|BOLD:AAA9672
Feralia jocosa|[5632]|XAF344-05|Canada|Ontario|658[0n]|BOLD:AAA9672
Feralia jocosa|[5633]|PHMO028-03|Canada|Ontario|639[0n]|BOLD:AAA9672
Feralia jocosa|[5634]|LGSMG702-07|United States|Tennessee|622[0n]|BOLD:AAA9672
Feralia jocosa|[5635]|LNCC1476-13|United States|North Carolina|658[0n]|BOLD:AAA9672
Feralia comstocki|[5636]|LPSOB329-08|Canada|Ontario|657[0n]|BOLD:AAB2071
Feralia comstocki|[5637]|RWWA027-09|United States|Washington|658[0n]|BOLD:AAB2071
Feralia comstocki|[5638]|LALPA059-10|Canada|British Columbia|658[0n]|BOLD:AAB2071
Feralia comstocki|[5639]|XAF425-05|Canada|Ontario|658[0n]|BOLD:AAB2071
Feralia comstocki|[5640]|XAD613-05|Canada|Ontario|658[0n]|BOLD:AAB2071
Feralia comstocki|[5641]|LPSOB469-08|Canada|Ontario|658[0n]|BOLD:AAB2071
Feralia comstocki|[5642]|LSUSA026-06|United States|Kentucky|658[0n]|BOLD:AAB2071
Feralia comstocki|[5643]|RDLQ601-07|Canada|Quebec|606[0n]|BOLD:AAB2071
Feralia comstocki|[5644]|XAD586-05|Canada|Ontario|658[0n]|BOLD:AAB2071
Feralia comstocki|[5645]|TTMNB387-06|Canada|New Brunswick|658[0n]|BOLD:AAB2071
Feralia comstocki|[5646]|RDLQ602-07|Canada|Quebec|616[0n]|BOLD:AAB2071
Feralia comstocki|[5647]|LGSMC448-05|United States|North Carolina|658[0n]|BOLD:AAB2071
Feralia comstocki|[5648]|DUNLP158-08|Canada|British Columbia|658[0n]|BOLD:AAB2071
Feralia comstocki|[5649]|DUNLP157-08|Canada|British Columbia|658[0n]|BOLD:AAB2071
Feralia comstocki|[5650]|TMNBB290-06|Canada|New Brunswick|658[0n]|BOLD:AAB2071
Feralia comstocki|[5651]|TMNBB289-06|Canada|New Brunswick|658[0n]|BOLD:AAB2071
Feralia comstocki|[5652]|TMNBB288-06|Canada|New Brunswick|658[0n]|BOLD:AAB2071
Feralia comstocki|[5653]|TTMNB385-06|Canada|New Brunswick|658[0n]|BOLD:AAB2071
Feralia comstocki|[5654]|LMIS017-05|Canada|Ontario|658[0n]|BOLD:AAB2071
Feralia comstocki|[5655]|LGSMC447-05|United States|North Carolina|658[0n]|BOLD:AAB2071
Feralia comstocki|[5656]|XAB027-04|Canada|Ontario|658[0n]|BOLD:AAB2071
Feralia comstocki|[5657]|LGSMC449-05|United States|North Carolina|658[0n]|BOLD:AAB2071
Feralia comstocki|[5658]|TMNBB291-06|Canada|New Brunswick|658[0n]|BOLD:AAB2071
Feralia comstocki|[5659]|TTMNB386-06|Canada|New Brunswick|658[0n]|BOLD:AAB2071
Feralia comstocki|[5660]|TMG122-03|Canada|Ontario|639[0n]|BOLD:AAB2071
Feralia comstocki|[5661]|PMG114-03|Canada|Ontario|617[0n]|BOLD:AAB2071
Feralia comstocki|[5662]|LGSMC446-05|United States|North Carolina|583[0n]|BOLD:AAB2071
Feralia comstocki|[5663]|LOWCE405-06|Canada|British Columbia|608[0n]|BOLD:AAB2071
Feralia comstocki|[5664]|XAJ244-06|Canada|Ontario|633[0n]|BOLD:AAB2071
Feralia comstocki|[5665]|DUNLP159-08|Canada|British Columbia|627[1n]|BOLD:AAB2071
Feralia comstocki|[5666]|DUNLP160-08|Canada|British Columbia|658[0n]|BOLD:AAB2071
Feralia comstocki|[5667]|LPSOB241-08|Canada|Ontario|658[0n]|BOLD:AAB2071
Feralia comstocki|[5668]|RWWA216-09|United States|Washington|658[0n]|BOLD:AAB2071
Feralia comstocki|[5669]|LALPA060-10|Canada|British Columbia|658[0n]|BOLD:AAB2071

Feralia comstocki|[5667]|LPSOB241-08|Canada|Ontario|658[0n]|BOLD:AAB2071
Feralia comstocki|[5668]|RWWA216-09|United States|Washington|658[0n]|BOLD:AAB2071
Feralia comstocki|[5669]|LALPA060-10|Canada|British Columbia|658[0n]|BOLD:AAB2071
Feralia comstocki|[5670]|LALPA061-10|Canada|British Columbia|658[0n]|BOLD:AAB2071
Feralia comstocki|[5671]|RWWB634-10|United States|Washington|658[0n]|BOLD:AAB2071
Feralia februalis|[5672]|CGLCA121-10|United States|California|658[0n]|BOLD:ACO5629
Feralia februalis|[5673]|NAMUM294-08|United States|California|658[0n]|BOLD:AAC0869
Feralia februalis|[5674]|LOCB846-06|United States|California|658[0n]|BOLD:AAC0869
Feralia februalis|[5675]|LOCB730-06|United States|California|658[0n]|BOLD:AAC0869
Feralia februalis|[5676]|LOCB732-06|United States|California|658[0n]|BOLD:AAC0869
Feralia februalis|[5677]|CGLCA122-10|United States|California|658[0n]|BOLD:AAC0869
Feralia februalis|[5678]|CGLCA120-10|United States|California|658[0n]|BOLD:AAC0869
Feralia februalis|[5679]|CGLCA123-10|United States|California|658[0n]|BOLD:AAC0869
Feralia februalis|[5680]|LOCB733-06|United States|California|658[0n]|BOLD:AAC0869
Feralia februalis|[5681]|LOCB842-06|United States|California|656[0n]|BOLD:AAC0869
Feralia februalis|[5682]|LOCB844-06|United States|California|658[0n]|BOLD:AAC0869
Feralia februalis|[5683]|LOCB841-06|United States|California|658[0n]|BOLD:AAC0869
Feralia februalis|[5684]|LOCB825-06|United States|California|658[0n]|BOLD:AAC0869
Feralia februalis|[5685]|LOCB824-06|United States|California|658[0n]|BOLD:AAC0869
Feralia februalis|[5686]|LOCB731-06|United States|California|658[0n]|BOLD:AAC0869
Feralia februalis|[5687]|LOCB843-06|United States|California|658[0n]|BOLD:AAC0869
Feralia februalis|[5688]|LOCB845-06|United States|California|658[0n]|BOLD:AAC0869
Feralia februalis|[5689]|LOCB914-06|United States|California|658[0n]|BOLD:AAC0869
Feralia februalis|[5690]|CGLCA124-10|United States|California|658[0n]|BOLD:AAC0869
Feralia februalis|[5691]|LOCBF285-13|United States|California|611[4n]|BOLD:AAC0869
Feralia meadowsi|[5692]|TML136-14|United States||621[0n]|BOLD:ACO5832
Amyna amplificans|[5693]|RDNML197-13|United States|Arizona|658[0n]|BOLD:AAB1231
Amyna amplificans|[5694]|CNCLB2260-14|Guatemala|San Marcos|658[0n]|BOLD:AAB1231
Cydosia nobilitella|[5695]|LNAUT2932-14|United States|Florida|370[0n]|
Tripudia goyanensis|[5696]|CNCLB1709-14|United States|Florida|658[0n]|BOLD:ACP4040
Tripudia flavofasciata complex|[5697]|BBUSA238-09|United States|Texas|658[0n]|BOLD:AAH5092
Tripudia flavofasciata complex|[5698]|BBUSA252-09|United States|Texas|658[0n]|BOLD:AAH5092
Tripudia flavofasciata complex|[5699]|PSAT128-10|United States|Arizona|591[0n]|BOLD:AAV0491
Tripudia flavofasciata complex|[5700]|MNAE480-11|United States|Arizona|658[0n]|BOLD:AAV0491
Tripudia flavofasciata complex|[5701]|MNAG605-08|United States|Arizona|649[0n]|BOLD:AAI3867
Tripudia flavofasciata complex|[5702]|MNAG606-08|United States|Arizona|658[0n]|BOLD:AAI3867
Tripudia flavofasciata complex|[5703]|LPOKA109-08|United States|Oklahoma|658[0n]|BOLD:AAF6886
Tripudia flavofasciata complex|[5704]|LPOKA164-08|United States|Oklahoma|658[0n]|BOLD:AAF6886
Tripudia flavofasciata complex|[5705]|LPOKB098-09|United States|Oklahoma|658[0n]|BOLD:AAF6886
Tripudia flavofasciata complex|[5706]|LPOKD131-09|United States|Oklahoma|658[0n]|BOLD:AAF6886
Tripudia flavofasciata complex|[5707]|LPOKD213-09|United States|Oklahoma|657[0n]|BOLD:AAF6886
Tripudia flavofasciata complex|[5708]|LPOKD255-09|United States|Oklahoma|656[0n]|BOLD:AAF6886
Tripudia flavofasciata complex|[5709]|LPOKD257-09|United States|Oklahoma|657[0n]|BOLD:AAF6886
Tripudia flavofasciata complex|[5710]|LPOKD271-09|United States|Oklahoma|657[0n]|BOLD:AAF6886
Tripudia flavofasciata complex|[5711]|LNCC1844-13|United States|North Carolina|658[1n]|BOLD:AAF6886
Tripudia flavofasciata complex|[5712]|HKONS191-08|United States|Florida|630[1n]|BOLD:AAE7958
Tripudia flavofasciata complex|[5713]|LPOKD145-09|United States|Oklahoma|658[0n]|BOLD:AAE7958
Tripudia flavofasciata complex|[5714]|LPOKD036-09|United States|Oklahoma|658[0n]|BOLD:AAE7958
Tripudia flavofasciata complex|[5715]|HKONS190-08|United States|Florida|658[0n]|BOLD:AAE7958
Tripudia flavofasciata complex|[5716]|HKONS192-08|United States|Florida|658[0n]|BOLD:AAE7958
Tripudia flavofasciata complex|[5717]|HKONS200-08|United States|Florida|658[0n]|BOLD:AAE7958
Tripudia flavofasciata complex|[5718]|HKONS201-08|United States|Florida|658[0n]|BOLD:AAE7958
Tripudia flavofasciata complex|[5719]|HKONS202-08|United States|Florida|658[0n]|BOLD:AAE7958
Tripudia flavofasciata complex|[5720]|HKONS203-08|United States|Florida|658[0n]|BOLD:AAE7958
Tripudia flavofasciata complex|[5721]|RDNMH474-09|United States|Louisiana|658[0n]|BOLD:AAE7958
Tripudia flavofasciata complex|[5722]|LNCC1271-11|United States|North Carolina|658[0n]|BOLD:AAE7958
Tripudia versutus|[5723]|RDNMH473-09|United States|Louisiana|658[0n]|BOLD:AAE7958
Tripudia versutus|[5724]|CNCLB1105-14|United States|Louisiana|606[1n]|BOLD:AAE7958
Tripudia versutus|[5725]|CNCLB1107-14|United States|Mississippi|550[0n]|BOLD:AAE7958
Tripudia quadrifera|[5726]|BBLSW889-09|United States|Texas|658[0n]|BOLD:AAH5046
Tripudia quadrifera|[5727]|BBUSA018-09|United States|Texas|658[0n]|BOLD:AAH5046
Tripudia quadrifera|[5728]|USLEP234-10|United States|Texas|658[0n]|BOLD:AAH5046
Tripudia quadrifera|[5729]|BBLSW888-09|United States|Texas|658[0n]|BOLD:AAH5046
Tripudia quadrifera|[5730]|BBLSW891-09|United States|Texas|658[0n]|BOLD:AAH5046
Tripudia quadrifera|[5731]|BBLSW905-09|United States|Texas|658[0n]|BOLD:AAH5046
Tripudia quadrifera|[5732]|BBLSW988-09|United States|Texas|658[0n]|BOLD:AAH5046
Tripudia quadrifera|[5733]|USLEP216-10|United States|Texas|658[0n]|BOLD:AAH5046
Tripudia quadrifera|[5734]|USLEP217-10|United States|Texas|658[0n]|BOLD:AAH5046
Tripudia quadrifera|[5735]|HKONB162-08|United States|Texas|658[0n]|BOLD:AAH5046
Tripudia quadrifera|[5736]|BBUSA040-09|United States|Texas|658[0n]|BOLD:AAH5046
Tripudia quadrifera|[5737]|BBUSA066-09|United States|Texas|658[0n]|BOLD:AAH5046
Tripudia quadrifera|[5738]|BBLSW882-09|United States|Texas|658[0n]|BOLD:AAH5046
Tripudia quadrifera|[5739]|USLEP874-10|United States|Texas|658[0n]|BOLD:AAH5046
Tripudia quadrifera|[5740]|USLEP910-10|United States|Texas|658[0n]|BOLD:AAH5046
Tripudia quadrifera|[5741]|USLEP911-10|United States|Texas|658[0n]|BOLD:AAH5046
Tripudia quadrifera|[5742]|RDNMK333-11|United States|New Mexico|658[0n]|BOLD:AAH5046
Tripudia quadrifera|[5743]|RDNMK353-11|United States|New Mexico|658[0n]|BOLD:AAH5046
Tripudia paraplesia|[5744]|LPYPA888-08|Mexico|Campeche|658[0n]|BOLD:AAF6827
Tripudia rectangula|[5745]|MNAC446-07|United States|Florida|658[0n]|BOLD:AAD2720
Tripudia rectangula|[5746]|LPOKC404-09|United States|Oklahoma|658[0n]|BOLD:AAD2720
Tripudia rectangula|[5747]|LPOKD744-10|United States|Oklahoma|658[0n]|BOLD:AAD2720
Tripudia rectangula|[5748]|BBLOC1539-11|United States|Texas|658[0n]|BOLD:AAD2720
Tripudia rectangula|[5749]|LNCB684-09|United States|North Carolina|658[0n]|BOLD:AAD2720
Tripudia rectangula|[5750]|LPOKC207-09|United States|Oklahoma|658[0n]|BOLD:AAD2720
Tripudia rectangula|[5751]|RDNMH476-09|United States|Louisiana|658[0n]|BOLD:AAD2720
Tripudia rectangula|[5752]|RDNMH475-09|United States|Louisiana|658[0n]|BOLD:AAD2720
Tripudia rectangula|[5753]|LPOKA248-08|United States|Oklahoma|658[0n]|BOLD:AAD2720
Tripudia rectangula|[5754]|LOFLA477-06|United States|Florida|658[0n]|BOLD:AAD2720
Tripudia rectangula|[5755]|LPOKC397-09|United States|Oklahoma|631[0n]|BOLD:AAD2720
Tripudia rectangula|[5756]|LPOKC427-09|United States|Oklahoma|658[0n]|BOLD:AAD2720
Tripudia rectangula|[5757]|LPOKD278-09|United States|Oklahoma|657[0n]|BOLD:AAD2720
Tripudia rectangula|[5758]|LPOKD379-09|United States|Oklahoma|658[0n]|BOLD:AAD2720
Tripudia rectangula|[5759]|LPOKD825-10|United States|Oklahoma|658[0n]|BOLD:AAD2720
Tripudia rectangula|[5760]|LNCC1270-11|United States|North Carolina|658[0n]|BOLD:AAD2720
Tripudia rectangula|[5761]|CNCLB1455-14|United States|Louisiana|658[1n]|BOLD:AAD2720
Tripudia grapholithoides|[5762]|LNAUS3549-13|United States Virgin Islands||658[0n]|BOLD:ABZ9822
Tripudia lamina|[5763]|RDNML222-13|Puerto Rico||658[0n]|BOLD:AAB7486
Tripudia lamina|[5764]|CNCLB2378-14|United States|Florida|658[0n]|BOLD:AAB7486
Tripudia balteata|[5765]|BBLOB1732-11|United States|Arizona|658[0n]|BOLD:AAP6021
Tripudia balteata|[5766]|BBLOB056-11|United States|Arizona|658[0n]|BOLD:AAP6021
Tripudia balteata|[5767]|BBLOB034-11|United States|Arizona|658[0n]|BOLD:AAP6021
Tripudia balteata|[5768]|CMAZA709-10|United States|Arizona|658[0n]|BOLD:AAP6021
Tripudia balteata|[5769]|CMAZA708-10|United States|Arizona|658[0n]|BOLD:AAP6021

[illegible]
Tripudia balteata|[5767]|BBLOB034-11|United States|Arizona|658[0n]|BOLD:AAP6021
Tripudia balteata|[5768]|CMAZA709-10|United States|Arizona|658[0n]|BOLD:AAP6021
Tripudia balteata|[5769]|CMAZA708-10|United States|Arizona|658[0n]|BOLD:AAP6021
Tripudia balteata|[5770]|CMAZA680-10|United States|Arizona|658[0n]|BOLD:AAP6021
Tripudia balteata|[5771]|BBLOC1888-11|United States|California|658[1n]|BOLD:AAP6021
Tripudia balteata|[5772]|BBLOE1528-12|United States|Arizona|658[0n]|BOLD:AAP6021
Tripudia balteata|[5773]|CMAZA1235-12|United States|Arizona|658[0n]|BOLD:AAP6021
Tripudia munna|[5774]|CNCLB1109-14|United States|California|550[0n]|BOLD:AAP6021
Tripudia limbatus|[5775]|CMAZA685-10|United States|Arizona|658[0n]|BOLD:AAH5665
Tripudia limbatus|[5776]|CMAZA183-09|United States|Arizona|658[0n]|BOLD:AAH5665
Tripudia limbatus|[5777]|CMAZA686-10|United States|Arizona|658[0n]|BOLD:AAH5665
Tripudia limbatus|[5778]|CMAZA1195-12|United States|Arizona|658[0n]|BOLD:AAH5665
Tripudia damozela complex|[5779]|RDNME663-08|United States|Texas|658[1n]|BOLD:AAF6826
Tripudia damozela|[5780]|RDNMH632-09|United States|Texas|658[0n]|BOLD:AAF6828
Tripudia damozela complex|[5781]|HKONS195-08|United States|Florida|658[1n]|BOLD:AAD7574
Tripudia damozela complex|[5782]|HKONS193-08|United States|Florida|646[0n]|BOLD:AAD7574
Tripudia damozela complex|[5783]|HKONS194-08|United States|Florida|658[0n]|BOLD:AAD7574
Tripudia damozela complex|[5784]|HKONS197-08|United States|Florida|658[0n]|BOLD:AAD7574
Tripudia damozela complex|[5785]|HKONS198-08|United States|Florida|658[0n]|BOLD:AAD7574
Tripudia damozela complex|[5786]|HKONS199-08|United States|Florida|658[0n]|BOLD:AAD7574
Tripudia inquaesita|[5787]|BBLOB960-11|United States|Arizona|658[0n]|BOLD:AAH4888
Tripudia inquaesita|[5788]|IAWL855-09|United States|Arizona|658[0n]|BOLD:AAH4888
Tripudia inquaesita|[5789]|CMAZA441-10|United States|Arizona|658[0n]|BOLD:AAH4888
Tripudia inquaesita|[5790]|USLEP269-10|United States|Arizona|658[0n]|BOLD:AAH4888
Tripudia inquaesita|[5791]|USLEP1108-10|United States|Arizona|658[0n]|BOLD:AAH4888
Tripudia inquaesita|[5792]|RDNME926-08|United States|Arizona|658[0n]|BOLD:AAH4888
Tripudia inquaesita|[5793]|USLEP268-10|United States|Arizona|658[0n]|BOLD:AAH4888
Tripudia inquaesita|[5794]|CMAZA428-10|United States|Arizona|658[0n]|BOLD:AAH4888
Tripudia inquaesita|[5795]|CMAZA1200-12|United States|Arizona|658[0n]|BOLD:AAH4888
Tripudia luxuriosa complex|[5796]|BBUSA112-09|United States|Texas|658[0n]|BOLD:AAH5067
Tripudia luxuriosa complex|[5797]|BBUSA203-09|United States|Texas|658[0n]|BOLD:AAH5067
Tripudia luxuriosa complex|[5798]|BBUSA231-09|United States|Texas|658[0n]|BOLD:AAH5067
Tripudia luxuriosa complex|[5799]|BBLSW094-09|United States|Texas|636[0n]|BOLD:AAH5270
Tripudia luxuriosa complex|[5800]|BBLSW095-09|United States|Texas|658[0n]|BOLD:AAH5271
Tripudia luxuriosa complex|[5801]|CMAZA1264-12|United States|Arizona|658[0n]|BOLD:AAH4862
Tripudia luxuriosa complex|[5802]|CMAZA424-10|United States|Arizona|658[0n]|BOLD:AAH4862
Tripudia luxuriosa complex|[5803]|IAWL739-09|United States|Arizona|658[0n]|BOLD:AAH4862
Tripudia luxuriosa complex|[5804]|CMAZA071-09|United States|Arizona|658[0n]|BOLD:AAH4862
Tripudia luxuriosa complex|[5805]|CMAZA423-10|United States|Arizona|658[0n]|BOLD:AAH4862
Tripudia luxuriosa complex|[5806]|RDNMK334-11|United States|New Mexico|658[0n]|BOLD:AAH4862
Tripudia luxuriosa complex|[5807]|BBLOB306-11|United States|Arizona|658[0n]|BOLD:AAH4862
Tripudia luxuriosa complex|[5808]|CMAZA1269-12|United States|Arizona|658[0n]|BOLD:AAH4862
Tripudia dimidata complex|[5809]|CMAZA647-10|United States|Arizona|658[0n]|BOLD:AAP5987
Tripudia dimidata complex|[5810]|CMAZA413-10|United States|Arizona|638[0n]|BOLD:AAP5987
Tripudia dimidata complex|[5811]|BBLOC1893-11|United States|California|658[0n]|BOLD:AAP5987
Tripudia dimidata complex|[5812]|IAWL619-09|United States|Arizona|569[0n]|BOLD:AAH4826
Tripudia dimidata complex|[5813]|IAWL738-09|United States|Arizona|658[0n]|BOLD:AAH4861
Tripudia dimidata complex|[5814]|PSAT124-10|United States|Arizona|658[0n]|BOLD:AAH4861
Tripudia luda|[5815]|CMAZA089-09|United States|Arizona|658[0n]|BOLD:AAH5643
Tripudia luda|[5816]|CMAZA205-09|United States|Arizona|658[0n]|BOLD:AAH5643
Tripudia luda|[5817]|CNCLB1454-14|United States|Arizona|658[0n]|BOLD:AAH5643
Tripudia sp.|[5818]|CNCLB1678-14|United States|Florida|658[0n]|BOLD:ACP5031
Tripudia sp.|[5819]|CNCLB1679-14|United States|Florida|658[0n]|BOLD:ACP5031
Tripudia sp.|[5820]|CNCLB2379-14|United States|Florida|658[0n]|BOLD:ACP5031
Micrathetis n. sp.|[5821]|RDNMG644-08|United States|Arizona|658[0n]|BOLD:AAB0867
Micrathetis n. sp.|[5822]|RDNMG016-08|United States|Arizona|658[0n]|BOLD:AAB0867
Micrathetis n. sp.|[5823]|CMAZA484-10|United States|Arizona|658[0n]|BOLD:AAB0867
Micrathetis n. sp.|[5824]|RDNMK339-11|United States|Arizona|658[0n]|BOLD:AAB0867
Micrathetis n. sp.|[5825]|RDNMG645-08|United States|Arizona|658[0n]|BOLD:AAB0867
Micrathetis n. sp.|[5826]|CMAZA1149-12|United States|Arizona|658[0n]|BOLD:AAB0867
Cobubatha lixiva|[5827]|RDNMJ550-11|United States|Arizona|658[0n]|BOLD:AAV8038
Cobubatha lixiva|[5828]|RDNMG114-08|United States|Arizona|658[0n]|BOLD:AAF4384
Cobubatha lixiva|[5829]|CMAZA767-10|United States|Arizona|658[0n]|BOLD:AAF4384
Cobubatha lixiva|[5830]|RDNMG289-08|United States|Arizona|658[0n]|BOLD:AAF4384
Cobubatha lixiva|[5831]|RDNMG333-08|United States|Arizona|648[0n]|BOLD:AAF4384
Cobubatha lixiva|[5832]|IAWLB654-11|United States|Arizona|640[0n]|BOLD:AAF4384
Cobubatha lixiva|[5833]|BBLOC327-11|United States|Arizona|658[0n]|BOLD:AAF4384
Cobubatha orthozona|[5834]|CMAZA011-09|United States|Arizona|658[0n]|BOLD:AAH5609
Cobubatha orthozona|[5835]|BBLOB1982-11|United States|Arizona|658[0n]|BOLD:AAH5609
Cobubatha orthozona|[5836]|BBLOC303-11|United States|Arizona|658[0n]|BOLD:AAH5609
Cobubatha orthozona|[5837]|RDNMG115-08|United States|Arizona|642[0n]|BOLD:AAH5609
Cobubatha orthozona|[5838]|RDNMG332-08|United States|Arizona|658[0n]|BOLD:AAH5609
Cobubatha orthozona|[5839]|CMAZA176-09|United States|Arizona|658[0n]|BOLD:AAH5609
Cobubatha orthozona|[5840]|RDNMJ510-11|United States|Arizona|658[0n]|BOLD:AAH5609
Cobubatha orthozona|[5841]|BBLOB1720-11|United States|Arizona|658[0n]|BOLD:AAH5609
Cobubatha orthozona|[5842]|BBLOB1983-11|United States|Arizona|658[0n]|BOLD:AAH5609
Cobubatha orthozona|[5843]|BBLOE1185-12|United States|Arizona|658[0n]|BOLD:AAH5609
Cobubatha orthozona|[5844]|BBLOE1187-12|United States|Arizona|658[0n]|BOLD:AAH5609
Cobubatha orthozona|[5845]|BBLOE1189-12|United States|Arizona|658[0n]|BOLD:AAH5609
Cobubatha orthozona|[5846]|BBLOB1731-11|United States|Arizona|658[0n]|BOLD:AAH5609
Cobubatha orthozona|[5847]|BBLOE1876-12|United States|Arizona|658[0n]|BOLD:AAH5609
Cobubatha orthozona|[5848]|BBLOB084-11|United States|Arizona|658[0n]|BOLD:AAH5609
Cobubatha orthozona|[5849]|RDNMK006-11|United States|Arizona|658[0n]|BOLD:AAH5609
Cobubatha orthozona|[5850]|BBLOE1886-12|United States|Arizona|658[0n]|BOLD:AAH5609
Cobubatha orthozona|[5851]|BBLOE1892-12|United States|Arizona|658[0n]|BOLD:AAH5609
Cobubatha sp. 1 nr. orthozona|[5852]|RDNME928-08|United States|Arizona|609[0n]|BOLD:AAD7346
Cobubatha sp. 1 nr. orthozona|[5853]|RDNMG331-08|United States|Arizona|640[0n]|BOLD:AAD7346
Cobubatha sp. 2 nr. orthozona|[5854]|RDNME930-08|United States|Arizona|658[0n]|BOLD:AAD7347
Cobubatha sp. 2 nr. orthozona|[5855]|TML160-14|United States||658[0n]|BOLD:AAD7347
Cobubatha ipilla|[5856]|RDNML199-13|United States|Arizona|658[0n]|BOLD:ACD9252
Cobubatha ipilla|[5857]|RDNML224-13|Mexico|Sonora|658[0n]|BOLD:ACD9252
Cobubatha ipilla|[5858]|CNCLB2427-14|United States|Texas|658[0n]|BOLD:ACD9252
Cobubatha metaspilaris|[5859]|RDNML212-13|United States|Arizona|658[0n]|BOLD:ACD9177
Cobubatha metaspilaris|[5860]|CNCLB1641-14|United States|Florida|658[0n]|BOLD:ACD9177
Cobubatha megaplaga|[5861]|RDNME610-08|United States|Texas|658[0n]|BOLD:AAD7345
Cobubatha megaplaga|[5862]|RDNME699-08|United States|Texas|658[0n]|BOLD:AAD7345
Cobubatha megaplaga|[5863]|RDNMH635-09|United States|Texas|658[0n]|BOLD:AAD7345
Cobubatha megaplaga|[5864]|CNCLB2426-14|United States|Texas|658[0n]|BOLD:AAD7345
Cobubatha dividua|[5865]|BBLOC1452-11|United States|California|658[0n]|BOLD:AAE2548
Cobubatha dividua|[5866]|BBLOB1975-11|United States|Arizona|658[0n]|BOLD:AAE2548
Cobubatha dividua|[5867]|BBLOB1950-11|United States|Arizona|658[0n]|BOLD:AAE2548
Cobubatha dividua|[5868]|CMAZA698-10|United States|Arizona|658[0n]|BOLD:AAE2548

Cobubatha dividua|[5866]|BBLOB1975-11|United States|Arizona|658[0n]|BOLD:AAE2548
Cobubatha dividua|[5867]|BBLOB1950-11|United States|Arizona|658[0n]|BOLD:AAE2548
Cobubatha dividua|[5868]|CMAZA698-10|United States|Arizona|658[0n]|BOLD:AAE2548
Cobubatha dividua|[5869]|CMAZA445-10|United States|Arizona|658[0n]|BOLD:AAE2548
Cobubatha dividua|[5870]|LPOKC892-09|United States|Oklahoma|658[0n]|BOLD:AAE2548
Cobubatha dividua|[5871]|LPOKD117-09|United States|Oklahoma|658[0n]|BOLD:AAE2548
Cobubatha dividua|[5872]|BBLSX815-09|United States|Texas|658[0n]|BOLD:AAE2548
Cobubatha dividua|[5873]|BBLSW070-09|United States|Texas|658[0n]|BOLD:AAE2548
Cobubatha dividua|[5874]|LPOKA203-08|United States|Oklahoma|658[0n]|BOLD:AAE2548
Cobubatha dividua|[5875]|RDNMF745-08|United States|California|658[0n]|BOLD:AAE2548
Cobubatha dividua|[5876]|RDNME923-08|United States|Arizona|658[0n]|BOLD:AAE2548
Cobubatha dividua|[5877]|RDNMF743-08|United States|California|658[0n]|BOLD:AAE2548
Cobubatha dividua|[5878]|RDNMF744-08|United States|California|652[0n]|BOLD:AAE2548
Cobubatha dividua|[5879]|BBLOB040-11|United States|Arizona|604[0n]|BOLD:AAE2548
Cobubatha dividua|[5880]|BBLOC1737-11|United States|California|622[0n]|BOLD:AAE2548
Cobubatha dividua|[5881]|BBLOC1741-11|United States|California|658[0n]|BOLD:AAE2548
Cobubatha dividua|[5882]|BBLOC1843-11|United States|California|658[0n]|BOLD:AAE2548
Cobubatha dividua|[5883]|BBLOC1891-11|United States|California|658[0n]|BOLD:AAE2548
Cobubatha dividua|[5884]|BBLOC1892-11|United States|California|658[0n]|BOLD:AAE2548
Cobubatha dividua|[5885]|BBLOC1900-11|United States|California|658[0n]|BOLD:AAE2548
Cobubatha dividua|[5886]|BBLOC1904-11|United States|California|658[0n]|BOLD:AAE2548
Cobubatha dividua|[5887]|BBLOD1947-11|United States|California|658[0n]|BOLD:AAE2548
Cobubatha dividua|[5888]|BBLOE1184-12|United States|Arizona|658[0n]|BOLD:AAE2548
Cobubatha dividua|[5889]|BBLOE1191-12|United States|Arizona|658[0n]|BOLD:AAE2548
Tripudia chihuahua|[5890]|CNCLB1097-14|United States|Texas|550[0n]|BOLD:ACM6712
Tripudia chihuahua|[5891]|CNCLB1096-14|United States|Texas|550[0n]|BOLD:ACM6712
Tripudia chihuahua|[5892]|CNCLB1099-14|United States|Texas|658[0n]|BOLD:ACM6712
Cobubatha numaDHJ01|[5893]|RDNME924-08|United States|Arizona|658[0n]|BOLD:AAC2036
Cobubatha numa|[5894]|CNCLB1786-14|United States|Texas|307[0n]|
Cobubatha numa|[5895]|QUNOD109-10|United States|Texas|658[0n]|BOLD:AAJ4041
Cobubatha numa|[5896]|CNCLB2429-14|United States|Texas|658[0n]|BOLD:AAJ4041
Cobubatha numa|[5897]|LNAUT2933-14|United States|Texas|658[0n]|BOLD:AAJ4041
Policocnemis ungulatus|[5898]|RDNMC721-06|United States|New Mexico|658[0n]|BOLD:AAY2765
Oxycnemis fusimacula|[5899]|RDNMD443-06|United States|California|658[0n]|BOLD:AAE5222
Oxycnemis fusimacula|[5900]|RDNMD444-06|United States|California|658[0n]|BOLD:AAE5222
Oxycnemis fusimacula|[5901]|NAMUM249-08|United States|Arizona|658[0n]|BOLD:AAE5222
Oxycnemis fusimacula|[5902]|RDNMG292-08|United States|Arizona|658[1n]|BOLD:AAE5222
Oxycnemis fusimacula|[5903]|BBLSY635-09|United States|Arizona|658[0n]|BOLD:AAE5222
Oxycnemis fusimacula|[5904]|BBLSZ093-09|United States|Arizona|658[0n]|BOLD:AAE5222
Oxycnemis fusimacula|[5905]|BBLSY974-09|United States|Arizona|658[0n]|BOLD:AAE5222
Oxycnemis fusimacula|[5906]|USLEP100-10|United States|Arizona|658[0n]|BOLD:AAE5222
Oxycnemis fusimacula|[5907]|BBLSY666-09|United States|Arizona|658[0n]|BOLD:AAE5222
Oxycnemis fusimacula|[5908]|BBLSZ088-09|United States|Arizona|658[0n]|BOLD:AAE5222
Oxycnemis fusimacula|[5909]|USLEP102-10|United States|Arizona|658[0n]|BOLD:AAE5222
Oxycnemis fusimacula|[5910]|USLEP101-10|United States|Arizona|658[0n]|BOLD:AAE5222
Oxycnemis fusimacula|[5911]|BBLSZ085-09|United States|Arizona|658[0n]|BOLD:AAE5222
Oxycnemis fusimacula|[5912]|BBLSY963-09|United States|Arizona|658[0n]|BOLD:AAE5222
Oxycnemis fusimacula|[5913]|BBLSY708-09|United States|Arizona|658[0n]|BOLD:AAE5222
Oxycnemis fusimacula|[5914]|BBLSY702-09|United States|Arizona|658[0n]|BOLD:AAE5222
Oxycnemis fusimacula|[5915]|BBLSY678-09|United States|Arizona|658[0n]|BOLD:AAE5222
Oxycnemis fusimacula|[5916]|BBLSY616-09|United States|Arizona|658[0n]|BOLD:AAE5222
Oxycnemis fusimacula|[5917]|USLEP099-10|United States|Arizona|658[0n]|BOLD:AAE5222
Oxycnemis fusimacula|[5918]|USLEP103-10|United States|Arizona|658[0n]|BOLD:AAE5222
Oxycnemis fusimacula|[5919]|BBLSZ092-09|United States|Arizona|658[0n]|BOLD:AAE5222
Oxycnemis fusimacula|[5920]|BBLSY690-09|United States|Arizona|658[0n]|BOLD:AAE5222
Oxycnemis fusimacula|[5921]|BBLSY606-09|United States|Arizona|589[0n]|BOLD:AAE5222
Oxycnemis fusimacula|[5922]|USLEP974-10|United States|Arizona|658[0n]|BOLD:AAE5222
Oxycnemis fusimacula|[5923]|CMAZA319-10|United States|Arizona|658[0n]|BOLD:AAE5222
Oxycnemis advena|[5924]|RDNMJ107-10|United States|Arizona|658[0n]|BOLD:AAK5883
Oxycnemis advena|[5925]|RDNMG293-08|United States|Arizona|658[0n]|BOLD:AAK5883
Oxycnemis advena|[5926]|CMAZA497-10|United States|Arizona|658[0n]|BOLD:AAK5883
Oxycnemis advena|[5927]|BBLOD809-11|United States|Arizona|658[0n]|BOLD:AAK5883
Oxycnemis advena|[5928]|BBLOD1895-11|United States|Arizona|658[0n]|BOLD:AAK5883
Oxycnemis advena|[5929]|CNCLA5285-13|United States|Arizona|658[0n]|BOLD:AAK5883
Pseudocopivaleria sonoma|[5930]|NAMUM246-08|United States|California|658[0n]|BOLD:AAH8266
Pseudocopivaleria sonoma|[5931]|NAMUM247-08|United States|California|658[0n]|BOLD:AAH8266
Psaphida grandis|[5932]|LSEU103-06|United States|North Carolina|658[0n]|BOLD:AAH7903
Psaphida grandis|[5933]|LGSMG695-07|United States|Tennessee|658[0n]|BOLD:AAH7903
Psaphida thaxterianus|[5934]|RDNMF460-08|United States|Alabama|658[0n]|BOLD:AAX8246
Psaphida styracis|[5935]|NAMUM113-08|United States|Texas|658[0n]|BOLD:AAD4668
Psaphida styracis|[5936]|NAMUM350-08|United States|West Virginia|658[0n]|BOLD:AAD4668
Psaphida styracis|[5937]|NAMUM351-08|United States|West Virginia|658[0n]|BOLD:AAD4668
Psaphida styracis|[5938]|RDLQ593-07|Canada|Quebec|596[0n]|BOLD:AAD4668
Psaphida styracis|[5939]|RDLQ594-07|Canada|Ontario|593[0n]|BOLD:AAD4668
Psaphida styracis|[5940]|RDNMH481-09|United States|Louisiana|658[0n]|BOLD:AAD4668
Psaphida styracis|[5941]|RDNMH482-09|United States|Louisiana|658[0n]|BOLD:AAD4668
Psaphida styracis|[5942]|LNCC1541-13|United States|North Carolina|658[0n]|BOLD:AAD4668
Psaphida styracis|[5943]|LNCC1542-13|United States|North Carolina|658[0n]|BOLD:AAD4668
Provia argentata|[5944]|RDNMC693-06|United States|California|658[0n]|BOLD:AAE8195
Provia argentata|[5945]|RDNMC694-06|United States|California|658[0n]|BOLD:AAE8195
Provia argentata|[5946]|RDNMD811-07|United States|California|658[0n]|BOLD:AAE8195
Provia argentata|[5947]|RDNMD812-07|United States|California|658[0n]|BOLD:AAE8195
Pseudocopivaleria anaverta|[5948]|RDNMD827-07|United States|California|658[0n]|BOLD:AAE0280
Pseudocopivaleria anaverta|[5949]|RDNME092-07|United States|California|658[0n]|BOLD:AAE0280
Pseudocopivaleria anaverta|[5950]|RDNME093-07|United States|California|658[0n]|BOLD:AAE0280
Pseudocopivaleria anaverta|[5951]|NAMUM200-08|United States|California|657[0n]|BOLD:AAE0280
Pseudocopivaleria anaverta|[5952]|NAMUM201-08|United States|California|646[0n]|BOLD:AAE0280
Copivaleria grotei|[5953]|LPOKB165-09|United States|Oklahoma|658[0n]|BOLD:ACE8379
Copivaleria grotei|[5954]|LPOKB159-09|United States|Oklahoma|658[0n]|BOLD:ACE8379
Copivaleria grotei|[5955]|LPOKB183-09|United States|Oklahoma|658[0n]|BOLD:ACE8379
Copivaleria grotei|[5956]|LPOKB178-09|United States|Oklahoma|658[0n]|BOLD:ACE8379
Copivaleria grotei|[5957]|LPOKB174-09|United States|Oklahoma|658[0n]|BOLD:ACE8379
Copivaleria grotei|[5958]|LPOKB121-09|United States|Oklahoma|658[0n]|BOLD:ACE8379
Copivaleria grotei|[5959]|LPOKB203-09|United States|Oklahoma|621[0n]|BOLD:ACE8379
Copivaleria grotei|[5960]|LPOKB171-09|United States|Oklahoma|658[0n]|BOLD:ACE8379
Copivaleria grotei|[5961]|LPOKA888-09|United States|Oklahoma|658[0n]|BOLD:ACE8379
Copivaleria grotei|[5962]|LPOKB164-09|United States|Oklahoma|658[0n]|BOLD:ACE8379
Copivaleria grotei|[5963]|LPOKB170-09|United States|Oklahoma|658[0n]|BOLD:ACE8379
Copivaleria grotei|[5964]|LPOKB176-09|United States|Oklahoma|658[0n]|BOLD:ACE8379
Copivaleria grotei|[5965]|LPOKB208-09|United States|Oklahoma|658[0n]|BOLD:ACE8379
Copivaleria grotei|[5966]|LPOKB249-09|United States|Oklahoma|658[0n]|BOLD:ACE8379
Copivaleria grotei|[5967]|LSUSA037-06|United States|Kentucky|644[0n]|BOLD:ACE8378
Copivaleria grotei|[5968]|LPOKB199-09|United States|Oklahoma|658[0n]|BOLD:ACE8378

Copivaleria grotei|[5965]|LPOKB208-09|United States|Oklahoma|658[0n]|BOLD:ACE8379
Copivaleria grotei|[5966]|LPOKB249-09|United States|Oklahoma|658[0n]|BOLD:ACE8379
Copivaleria grotei|[5967]|LSUSA037-06|United States|Kentucky|644[0n]|BOLD:ACE8378
Copivaleria grotei|[5968]|LPOKB199-09|United States|Oklahoma|658[0n]|BOLD:ACE8378
Copivaleria grotei|[5969]|XAC082-04|Canada|Ontario|564[0n]|BOLD:AAA6394
Copivaleria grotei|[5970]|XAF380-05|Canada|Ontario|604[0n]|BOLD:AAA6394
Copivaleria grotei|[5971]|XAJ242-06|Canada|Ontario|610[0n]|BOLD:AAA6394
Copivaleria grotei|[5972]|XAF410-05|Canada|Ontario|575[0n]|BOLD:AAA6394
Copivaleria grotei|[5973]|XAC083-04|Canada|Ontario|580[0n]|BOLD:AAA6394
Copivaleria grotei|[5974]|TMG123-03|Canada|Ontario|639[0n]|BOLD:AAA6394
Copivaleria grotei|[5975]|PMG104-03|Canada|Ontario|617[0n]|BOLD:AAA6394
Copivaleria grotei|[5976]|XAC086-04|Canada|Ontario|658[0n]|BOLD:AAA6394
Copivaleria grotei|[5977]|XAC122-04|Canada|Ontario|658[0n]|BOLD:AAA6394
Copivaleria grotei|[5978]|XAF089-05|Canada|Ontario|658[0n]|BOLD:AAA6394
Copivaleria grotei|[5979]|XAF277-05|Canada|Ontario|658[0n]|BOLD:AAA6394
Copivaleria grotei|[5980]|XAF334-05|Canada|Ontario|658[0n]|BOLD:AAA6394
Copivaleria grotei|[5981]|XAF422-05|Canada|Ontario|658[0n]|BOLD:AAA6394
Copivaleria grotei|[5982]|XAF239-05|Canada|Ontario|658[0n]|BOLD:AAA6394
Copivaleria grotei|[5983]|XAJ058-06|Canada|Ontario|658[0n]|BOLD:AAA6394
Copivaleria grotei|[5984]|RDLQH109-06|Canada|Quebec|589[0n]|BOLD:AAA6394
Copivaleria grotei|[5985]|XAJ102-06|Canada|Ontario|658[0n]|BOLD:AAA6394
Copivaleria grotei|[5986]|XAJ060-06|Canada|Ontario|658[0n]|BOLD:AAA6394
Copivaleria grotei|[5987]|XAF424-05|Canada|Ontario|658[0n]|BOLD:AAA6394
Copivaleria grotei|[5988]|XAF384-05|Canada|Ontario|658[0n]|BOLD:AAA6394
Copivaleria grotei|[5989]|XAF383-05|Canada|Ontario|658[0n]|BOLD:AAA6394
Copivaleria grotei|[5990]|XAF330-05|Canada|Ontario|658[0n]|BOLD:AAA6394
Copivaleria grotei|[5991]|XAF306-05|Canada|Ontario|658[0n]|BOLD:AAA6394
Copivaleria grotei|[5992]|XAF305-05|Canada|Ontario|658[0n]|BOLD:AAA6394
Copivaleria grotei|[5993]|XAF283-05|Canada|Ontario|658[0n]|BOLD:AAA6394
Copivaleria grotei|[5994]|XAF275-05|Canada|Ontario|658[0n]|BOLD:AAA6394
Copivaleria grotei|[5995]|XAF203-05|Canada|Ontario|658[0n]|BOLD:AAA6394
Copivaleria grotei|[5996]|XAF158-05|Canada|Ontario|658[0n]|BOLD:AAA6394
Copivaleria grotei|[5997]|XAE003-04|Canada|Ontario|658[0n]|BOLD:AAA6394
Copivaleria grotei|[5998]|XAC097-04|Canada|Ontario|658[0n]|BOLD:AAA6394
Copivaleria grotei|[5999]|XAC087-04|Canada|Ontario|658[0n]|BOLD:AAA6394
Copivaleria grotei|[6000]|XAC085-04|Canada|Ontario|658[0n]|BOLD:AAA6394
Copivaleria grotei|[6001]|XAC084-04|Canada|Ontario|658[0n]|BOLD:AAA6394
Copivaleria grotei|[6002]|XAC080-04|Canada|Ontario|658[0n]|BOLD:AAA6394
Copivaleria grotei|[6003]|MEC122-04|Canada|Quebec|658[0n]|BOLD:AAA6394
Copivaleria grotei|[6004]|XAC091-04|Canada|Ontario|606[1n]|BOLD:AAA6394
Copivaleria grotei|[6005]|XAD684-05|Canada|Ontario|636[0n]|BOLD:AAA6394
Copivaleria grotei|[6006]|RDLQH108-06|Canada|Quebec|653[0n]|BOLD:AAA6394
Copivaleria grotei|[6007]|CNCLB1349-14|United States|North Carolina|658[0n]|BOLD:AAA6394
Copivaleria grotei|[6008]|LSEU110-06|United States|North Carolina|658[0n]|BOLD:AAA6394
Copivaleria grotei|[6009]|XAJ059-06|Canada|Ontario|596[0n]|BOLD:AAA6394
Copivaleria grotei|[6010]|XAJ169-06|Canada|Ontario|596[0n]|BOLD:AAA6394
Copivaleria grotei|[6011]|XAC081-04|Canada|Ontario|587[0n]|BOLD:AAA6394
Copivaleria grotei|[6012]|CNCLB1348-14|United States|North Carolina|658[0n]|BOLD:AAA6394
Copivaleria grotei|[6013]|CNCLB1388-14|United States|North Carolina|658[0n]|BOLD:AAA6394
Psaphida rolandi|[6014]|LPOKA898-09|United States|Oklahoma|658[0n]|BOLD:ACZ8211
Psaphida rolandi|[6015]|LPOKA911-09|United States|Oklahoma|658[0n]|BOLD:ACZ8211
Psaphida rolandi|[6016]|LPOKA948-09|United States|Oklahoma|658[0n]|BOLD:ACZ8211
Psaphida rolandi|[6017]|MEC130-04|Canada|Quebec|658[0n]|BOLD:AAD4664
Psaphida rolandi|[6018]|RDLQ597-07|Canada|Ontario|592[0n]|BOLD:AAD4664
Psaphida rolandi|[6019]|NAMUM352-08|United States|West Virginia|658[0n]|BOLD:AAD4664
Psaphida rolandi|[6020]|LSEU106-06|United States|North Carolina|658[0n]|BOLD:AAD4664
Psaphida rolandi|[6021]|LNCC571-11|United States|North Carolina|658[0n]|BOLD:AAD4664
Psaphida resumens|[6022]|RDNME293-07|United States|Florida|658[0n]|BOLD:ABX6106
Psaphida resumens|[6023]|USLEP752-10|United States|Florida|658[0n]|BOLD:ABX6106
Psaphida resumens|[6024]|USLEP753-10|United States|Florida|641[0n]|BOLD:ABX6106
Psaphida resumens|[6025]|USLEP754-10|United States|Florida|658[0n]|BOLD:ABX6106
Psaphida resumens|[6026]|USLEP755-10|United States|Florida|658[0n]|BOLD:ABX6106
Psaphida resumens|[6027]|USLEP756-10|United States|Florida|658[0n]|BOLD:ABX6106
Psaphida resumens|[6028]|USLEP1154-10|United States|Florida|658[0n]|BOLD:ABX6106
Psaphida resumens|[6029]|RDLQ595-07|Canada|Ontario|591[0n]|BOLD:AAC7169
Psaphida resumens|[6030]|RDLQ596-07|Canada|Quebec|581[0n]|BOLD:AAC7169
Psaphida resumens|[6031]|LPOKA936-09|United States|Oklahoma|658[0n]|BOLD:AAC7169
Psaphida resumens|[6032]|LSEU108-06|United States|North Carolina|658[0n]|BOLD:AAC7169
Psaphida resumens|[6033]|LTOLB136-08|United States|West Virginia|658[0n]|BOLD:AAC7169
Psaphida resumens|[6034]|LSEU109-06|United States|North Carolina|658[0n]|BOLD:AAC7169
Psaphida resumens|[6035]|LNC758-06|United States|North Carolina|658[0n]|BOLD:AAC7169
Psaphida resumens|[6036]|NAMUM416-09|United States|Maryland|658[0n]|BOLD:AAC7169
Psaphida resumens|[6037]|LSEU508-06|United States|Georgia|658[0n]|BOLD:AAC7169
Psaphida resumens|[6038]|LNCC572-11|United States|North Carolina|658[0n]|BOLD:AAC7169
Psaphida resumens|[6039]|LNCC573-11|United States|North Carolina|658[0n]|BOLD:AAC7169
Psaphida resumens|[6040]|LSEU107-06|United States|North Carolina|658[1n]|BOLD:AAC7169
Psaphida resumens|[6041]|CNCLB1378-14|United States|North Carolina|658[0n]|BOLD:AAC7169
Psaphida damalis|[6042]|LNAUS5312-13|United States|California|658[0n]|BOLD:ACK3405
Psaphida damalis|[6043]|LNAUS5313-13|United States|California|407[0n]|
Psaphida damalis|[6044]|CNCLB349-14|United States|California|658[0n]|BOLD:ACK3405
Psaphida damalis|[6045]|CNCLB350-14|United States|California|658[0n]|BOLD:ACK3405
Psaphida damalis|[6046]|CNCLB351-14|United States|California|658[0n]|BOLD:ACK3405
Psaphida electilis|[6047]|RDNMH646-09|Canada|Ontario|658[0n]|BOLD:AAE0827
Psaphida electilis|[6048]|LSEU102-06|United States|North Carolina|658[0n]|BOLD:AAE0827
Psaphida electilis|[6049]|CNCLA5250-13|United States|Georgia|658[0n]|BOLD:AAE0827
Psaphida electilis|[6050]|CNCLA5251-13|United States|Georgia|658[0n]|BOLD:AAE0827
Psaphida electilis|[6051]|CNCLA5252-13|United States|Georgia|658[0n]|BOLD:AAE0827
Psaphida electilis|[6052]|LPOKA1008-09|United States|Oklahoma|658[0n]|BOLD:AAE0827
Psaphida electilis|[6053]|XAF329-05|Canada|Ontario|572[0n]|BOLD:AAE0827
Psaphida electilis|[6054]|RDLQH105-06|Canada|Quebec|658[0n]|BOLD:AAE0827
Psaphida electilis|[6055]|CNCLB1389-14|United States|North Carolina|658[0n]|BOLD:AAE0827
Psaphida electilis|[6056]|CNCLB1427-14|United States|North Carolina|658[0n]|BOLD:AAE0827
Psaphidinae|[6057]|RDNMH038-09|United States|Texas|658[0n]|BOLD:AAG1048
Oxycnemis grandimacula|[6058]|HKONB219-09|United States|Texas|640[0n]|BOLD:AAI5464
Oxycnemis grandimacula|[6059]|RDNM814-05|United States|Texas|658[0n]|BOLD:AAI5464
Oxycnemis grandimacula|[6060]|HKONB220-09|United States|Texas|658[0n]|BOLD:AAI5464
Oxycnemis grandimacula|[6061]|AWCLB328-10|United States|Arizona|658[0n]|BOLD:AAI5464
Unciella primula|[6062]|RDNMC689-06|United States|California|658[0n]|BOLD:AAI1673
Unciella primula|[6063]|RDNMC690-06|United States|California|658[0n]|BOLD:AAI1673
Unciella primula|[6064]|LOCBF282-13|United States|California|664[0n]|BOLD:AAI1673
Crimona pallimedia|[6065]|NAMUM250-08|United States|Arizona|658[0n]|BOLD:AAR5103
Triocnemis saporis|[6066]|CGLCA047-10|United States|California|658[0n]|BOLD:AAH8242
Triocnemis saporis|[6067]|CGLCA044-10|United States|California|658[0n]|BOLD:AAH8242

Crimona pallimedia|[6065]|NAMUM250-08|United States|Arizona|658[0n]|BOLD:AAR5103
Triocnemis saporis|[6066]|CGLCA047-10|United States|California|658[0n]|BOLD:AAH8242
Triocnemis saporis|[6067]|CGLCA044-10|United States|California|658[0n]|BOLD:AAH8242
Triocnemis saporis|[6068]|RDNME616-08|United States|California|658[0n]|BOLD:AAH8242
Triocnemis saporis|[6069]|RDNME630-08|United States|Colorado|658[0n]|BOLD:AAH8242
Triocnemis saporis|[6070]|AWCLB393-10|United States|Arizona|658[0n]|BOLD:AAH8242
Triocnemis saporis|[6071]|RDNMK015-11|United States|Arizona|658[0n]|BOLD:AAH8242
Triocnemis saporis|[6072]|BBLOB374-11|United States|Arizona|658[0n]|BOLD:AAH8242
Unciella flagrantis|[6073]|CNCLB1566-14|United States|Nevada|658[0n]|BOLD:ACO7959
Copibryophila sp.|[6074]|HKONB171-08|United States|Texas|658[0n]|BOLD:AAI9394
Copibryophila sp.|[6075]|HKONB172-08|United States|Texas|658[0n]|BOLD:AAI9394
Copibryophila angelica|[6076]|LOCBF3595-14|United States|California|528[0n]|
Copibryophila angelica|[6077]|LOCBF2333-13|United States|California|543[0n]|
Copibryophila angelica|[6078]|LOCBF3465-14|United States|California|528[0n]|BOLD:ABW8301
Copibryophila angelica|[6079]|LOCBF3594-14|United States|California|588[0n]|BOLD:ABW8301
Copibryophila angelica|[6080]|LOCBF3479-14|United States|California|588[0n]|BOLD:ABW8301
Copibryophila angelica|[6081]|LOCBF1352-13|United States|California|610[0n]|BOLD:ABW8301
Copibryophila angelica|[6082]|LOCBF2560-13|United States|California|610[0n]|BOLD:ABW8301
Copibryophila angelica|[6083]|LOCBF2334-13|United States|California|610[0n]|BOLD:ABW8301
Copibryophila angelica|[6084]|LOCBF2577-13|United States|California|606[0n]|BOLD:ABW8301
Copibryophila angelica|[6085]|LOCBF1342-13|United States|California|658[0n]|BOLD:ABW8301
Copibryophila angelica|[6086]|BBLOD695-11|United States|California|658[0n]|BOLD:ABW8301
Copibryophila angelica|[6087]|BBLOD775-11|United States|California|658[0n]|BOLD:ABW8301
Copibryophila angelica|[6088]|LOCBF1341-13|United States|California|658[0n]|BOLD:ABW8301
Copibryophila angelica|[6089]|LOCBF1351-13|United States|California|658[0n]|BOLD:ABW8301
Copibryophila angelica|[6090]|LOCBF3478-14|United States|California|579[0n]|BOLD:ABW8301
Copibryophila angelica|[6091]|LOCBF3593-14|United States|California|591[0n]|BOLD:ABW8301
Copibryophila angelica|[6092]|LOCBF3596-14|United States|California|591[0n]|BOLD:ABW8301
Copibryophila angelica|[6093]|LOCBF1353-13|United States|California|600[0n]|BOLD:ABW8301
Copibryophila angelica|[6094]|LOCBF3797-14|United States|California|588[0n]|BOLD:ABW8301
Callopistria floridensis|[6095]|LPOKA326-08|United States|Oklahoma|658[0n]|BOLD:AAA4051
Callopistria floridensis|[6096]|LPOKA650-09|United States|Oklahoma|639[0n]|BOLD:AAA4051
Callopistria floridensis|[6097]|LPOKD141-09|United States|Oklahoma|658[0n]|BOLD:AAA4051
Callopistria floridensis|[6098]|LPOKD272-09|United States|Oklahoma|657[0n]|BOLD:AAA4051
Callopistria floridensis|[6099]|BBLOB439-11|United States|Florida|658[0n]|BOLD:AAA4051
Callopistria floridensis|[6100]|RDNMH1021-09|United States|Texas|658[0n]|BOLD:AAA4051
Callopistria floridensis|[6101]|RDNMI049-10|United States|Florida|658[0n]|BOLD:AAA4051
Callopistria floridensis|[6102]|LPOKA071-08|United States|Oklahoma|658[0n]|BOLD:AAA4051
Callopistria floridensis|[6103]|LPOKA547-09|United States|Oklahoma|658[0n]|BOLD:AAA4051
Callopistria floridensis|[6104]|LPOKD288-09|United States|Oklahoma|657[0n]|BOLD:AAA4051
Callopistria floridensis|[6105]|BBLOB471-11|United States|Florida|658[0n]|BOLD:AAA4051
Callopistria mollissima|[6106]|LPSOC183-08|Canada|Ontario|656[0n]|BOLD:AAB0829
Callopistria mollissima|[6107]|LSEU403-06|United States|Georgia|658[0n]|BOLD:AAB0829
Callopistria mollissima|[6108]|LGSM436-04|United States|Tennessee|574[1n]|BOLD:AAB0829
Callopistria mollissima|[6109]|LGSM437-04|United States|Tennessee|658[0n]|BOLD:AAB0829
Callopistria mollissima|[6110]|LOT360-04|United States|Tennessee|649[0n]|BOLD:AAB0829
Callopistria mollissima|[6111]|LOTB304-05|United States|Tennessee|658[0n]|BOLD:AAB0829
Callopistria mollissima|[6112]|LSEU646-06|United States|Georgia|658[0n]|BOLD:AAB0829
Callopistria mollissima|[6113]|BBLSW626-09|United States|Oklahoma|658[0n]|BOLD:AAB0829
Callopistria mollissima|[6114]|BBLSX970-09|United States|Oklahoma|642[0n]|BOLD:AAB0829
Callopistria mollissima|[6115]|LPOKC745-09|United States|Oklahoma|658[0n]|BOLD:AAB0829
Callopistria mollissima|[6116]|LNC009-05|United States|North Carolina|624[0n]|BOLD:AAB0829
Callopistria mollissima|[6117]|LNCB142-06|United States|North Carolina|658[0n]|BOLD:AAB0829
Callopistria mollissima|[6118]|LNCB143-06|United States|North Carolina|658[0n]|BOLD:AAB0829
Callopistria mollissima|[6119]|LNCB271-06|United States|North Carolina|658[0n]|BOLD:AAB0829
Callopistria mollissima|[6120]|LOCT119-05|United States|Connecticut|658[0n]|BOLD:AAB0829
Callopistria mollissima|[6121]|BBLSW791-09|United States|Oklahoma|658[0n]|BOLD:AAB0829
Callopistria mollissima|[6122]|BBLEC260-09|Canada|Nova Scotia|633[0n]|BOLD:AAB0829
Callopistria mollissima|[6123]|BBLPC594-09|Canada|Nova Scotia|633[0n]|BOLD:AAB0829
Callopistria mollissima|[6124]|MECD363-06|Canada|Quebec|658[0n]|BOLD:AAB0829
Callopistria mollissima|[6125]|PHMNB760-05|Canada|New Brunswick|658[0n]|BOLD:AAB0829
Callopistria mollissima|[6126]|PHMNB169-04|Canada|New Brunswick|658[0n]|BOLD:AAB0829
Callopistria mollissima|[6127]|MEC811-04|Canada|Quebec|658[0n]|BOLD:AAB0829
Callopistria mollissima|[6128]|MEC714-04|Canada|Quebec|658[0n]|BOLD:AAB0829
Callopistria mollissima|[6129]|XAC716-04|Canada|Ontario|658[0n]|BOLD:AAB0829
Callopistria mollissima|[6130]|PHMNB035-03|Canada|New Brunswick|639[0n]|BOLD:AAB0829
Callopistria mollissima|[6131]|PHMNB164-04|Canada|New Brunswick|561[1n]|BOLD:AAB0829
Callopistria mollissima|[6132]|MECD364-06|Canada|Quebec|656[0n]|BOLD:AAB0829
Callopistria mollissima|[6133]|TTMNB026-06|Canada|New Brunswick|658[0n]|BOLD:AAB0829
Callopistria mollissima|[6134]|LSEU645-06|United States|Georgia|658[0n]|BOLD:AAB0829
Callopistria mollissima|[6135]|MNAC729-07|Canada|Quebec|656[0n]|BOLD:AAB0829
Callopistria mollissima|[6136]|MNAC853-07|Canada|Quebec|658[0n]|BOLD:AAB0829
Callopistria mollissima|[6137]|BBLPE041-09|Canada|Nova Scotia|658[0n]|BOLD:AAB0829
Callopistria mollissima|[6138]|BBLPE074-09|Canada|Nova Scotia|658[0n]|BOLD:AAB0829
Callopistria mollissima|[6139]|BBLPE127-09|Canada|Nova Scotia|658[0n]|BOLD:AAB0829
Callopistria mollissima|[6140]|LNCC265-10|United States|North Carolina|658[0n]|BOLD:AAB0829
Callopistria mollissima|[6141]|LNCC345-10|United States|North Carolina|658[0n]|BOLD:AAB0829
Callopistria granitosa|[6142]|LNC010-05|United States|North Carolina|658[0n]|BOLD:AAE7590
Callopistria granitosa|[6143]|LNC930-06|United States|North Carolina|658[0n]|BOLD:AAE7590
Callopistria granitosa|[6144]|LNCB270-06|United States|North Carolina|658[0n]|BOLD:AAE7590
Callopistria granitosa|[6145]|HKONB427-09|United States|Texas|658[0n]|BOLD:AAE7590
Callopistria granitosa|[6146]|BBLOB894-11|United States|Florida|658[0n]|BOLD:AAE7590
Callopistria cordata|[6147]|BBLPE037-09|Canada|Nova Scotia|658[0n]|BOLD:AAA7113
Callopistria cordata|[6148]|BBLPC258-09|Canada|Nova Scotia|658[0n]|BOLD:AAA7113
Callopistria cordata|[6149]|BBLPE183-09|Canada|Nova Scotia|639[0n]|BOLD:AAA7113
Callopistria cordata|[6150]|BBLPE157-09|Canada|Nova Scotia|658[0n]|BOLD:AAA7113
Callopistria cordata|[6151]|BBLEC100-09|Canada|Nova Scotia|658[0n]|BOLD:AAA7113
Callopistria cordata|[6152]|TTMNB032-06|Canada|New Brunswick|656[0n]|BOLD:AAA7113
Callopistria cordata|[6153]|MECD365-06|Canada|Quebec|656[0n]|BOLD:AAA7113
Callopistria cordata|[6154]|BBLPE142-09|Canada|Nova Scotia|658[0n]|BOLD:AAA7113
Callopistria cordata|[6155]|BBLPE138-09|Canada|Nova Scotia|658[0n]|BOLD:AAA7113
Callopistria cordata|[6156]|BBLPE069-09|Canada|Nova Scotia|658[0n]|BOLD:AAA7113
Callopistria cordata|[6157]|BBLPE064-09|Canada|Nova Scotia|658[0n]|BOLD:AAA7113
Callopistria cordata|[6158]|BBLPE042-09|Canada|Nova Scotia|658[0n]|BOLD:AAA7113
Callopistria cordata|[6159]|BBLPE034-09|Canada|Nova Scotia|658[0n]|BOLD:AAA7113
Callopistria cordata|[6160]|BBLPC195-09|Canada|Nova Scotia|658[0n]|BOLD:AAA7113
Callopistria cordata|[6161]|BBLPC194-09|Canada|Nova Scotia|658[0n]|BOLD:AAA7113
Callopistria cordata|[6162]|BBLPC146-09|Canada|Nova Scotia|658[0n]|BOLD:AAA7113
Callopistria cordata|[6163]|BBLEC678-09|Canada|Nova Scotia|658[0n]|BOLD:AAA7113
Callopistria cordata|[6164]|BBLEC643-09|Canada|Nova Scotia|658[0n]|BOLD:AAA7113
Callopistria cordata|[6165]|BBLEC518-09|Canada|New Brunswick|658[0n]|BOLD:AAA7113
Callopistria cordata|[6166]|BBLEC115-09|Canada|Nova Scotia|658[0n]|BOLD:AAA7113
Callopistria cordata|[6167]|BBLCH041-09|United States|Michigan|658[0n]|BOLD:AAA7113

Callopistria cordata|[6164]|BBLEC643-09|Canada|Nova Scotia|658[0n]|BOLD:AAA7113
Callopistria cordata|[6165]|BBLEC518-09|Canada|New Brunswick|658[0n]|BOLD:AAA7113
Callopistria cordata|[6166]|BBLEC115-09|Canada|Nova Scotia|658[0n]|BOLD:AAA7113
Callopistria cordata|[6167]|BBLCU041-09|United States|Michigan|658[0n]|BOLD:AAA7113
Callopistria cordata|[6168]|MNAC786-07|Canada|Quebec|658[0n]|BOLD:AAA7113
Callopistria cordata|[6169]|RDLQF938-06|Canada|Quebec|658[0n]|BOLD:AAA7113
Callopistria cordata|[6170]|RDLQF521-06|Canada|Quebec|658[0n]|BOLD:AAA7113
Callopistria cordata|[6171]|TMNBB207-06|Canada|New Brunswick|658[0n]|BOLD:AAA7113
Callopistria cordata|[6172]|TMNBB206-06|Canada|New Brunswick|658[0n]|BOLD:AAA7113
Callopistria cordata|[6173]|TMNBB205-06|Canada|New Brunswick|658[0n]|BOLD:AAA7113
Callopistria cordata|[6174]|TMNBB203-06|Canada|New Brunswick|658[0n]|BOLD:AAA7113
Callopistria cordata|[6175]|TMNBB202-06|Canada|New Brunswick|658[0n]|BOLD:AAA7113
Callopistria cordata|[6176]|TMNBB201-06|Canada|New Brunswick|658[0n]|BOLD:AAA7113
Callopistria cordata|[6177]|TMNBB200-06|Canada|New Brunswick|658[0n]|BOLD:AAA7113
Callopistria cordata|[6178]|TMNBB199-06|Canada|New Brunswick|658[0n]|BOLD:AAA7113
Callopistria cordata|[6179]|TMNBB198-06|Canada|New Brunswick|658[0n]|BOLD:AAA7113
Callopistria cordata|[6180]|TMNBB197-06|Canada|New Brunswick|658[0n]|BOLD:AAA7113
Callopistria cordata|[6181]|TMNBB196-06|Canada|New Brunswick|658[0n]|BOLD:AAA7113
Callopistria cordata|[6182]|TTMNB036-06|Canada|New Brunswick|658[0n]|BOLD:AAA7113
Callopistria cordata|[6183]|BBLPE130-09|Canada|Nova Scotia|658[0n]|BOLD:AAA7113
Callopistria cordata|[6184]|RDLQB264-05|Canada|Quebec|658[0n]|BOLD:AAA7113
Callopistria cordata|[6185]|PHMNB054-03|Canada|New Brunswick|639[0n]|BOLD:AAA7113
Callopistria cordata|[6186]|PHMNB051-03|Canada|New Brunswick|639[0n]|BOLD:AAA7113
Callopistria cordata|[6187]|TMNBB204-06|Canada|New Brunswick|632[0n]|BOLD:AAA7113
Callopistria cordata|[6188]|PHMNB241-04|Canada|New Brunswick|609[0n]|BOLD:AAA7113
Callopistria cordata|[6189]|TTMNB369-06|Canada|New Brunswick|610[0n]|BOLD:AAA7113
Callopistria cordata|[6190]|RDLQG522-06|Canada|Quebec|621[0n]|BOLD:AAA7113
Callopistria cordata|[6191]|BBLPE113-09|Canada|Nova Scotia|637[0n]|BOLD:AAA7113
Callopistria cordata|[6192]|BBLPE185-09|Canada|Nova Scotia|636[0n]|BOLD:AAA7113
Callopistria cordata|[6193]|BBLPE260-09|Canada|Nova Scotia|658[0n]|BOLD:AAA7113
Callopistria cordata|[6194]|LOFLA031-06|United States|Florida|656[0n]|BOLD:AAA7114
Callopistria cordata|[6195]|LOFLC299-06|United States|Florida|658[0n]|BOLD:AAA7114
Callopistria cordata|[6196]|LOTB188-05|United States|Tennessee|658[0n]|BOLD:AAA7114
Callopistria cordata|[6197]|LNC043-05|United States|North Carolina|615[0n]|BOLD:AAA7114
Callopistria cordata|[6198]|LNC044-05|United States|North Carolina|658[0n]|BOLD:AAA7114
Callopistria cordata|[6199]|LNCB311-06|United States|North Carolina|658[0n]|BOLD:AAA7114
Callopistria cordata|[6200]|BBLOC095-11|United States|Florida|658[0n]|BOLD:AAA7114
Callopistria jamaicensis|[6201]|CNCLB2004-14|Puerto Rico|Mayaguez|658[0n]|BOLD:ACR1988
Condica begallo|[6202]|RDNMD939-07|United States|Arizona|655[0n]|BOLD:AAJ7229
Condica begallo|[6203]|CMAZA764-10|United States|Arizona|658[0n]|BOLD:AAJ7229
Condica begallo|[6204]|RDNMK101-11|United States|Arizona|658[0n]|BOLD:AAJ7229
Condica egestis|[6205]|NAMUM082-08|United States|Arizona|655[0n]|BOLD:AAX8948
Condica egestis|[6206]|RDNML213-13|United States|Arizona|627[0n]|BOLD:AAX8948
Condica sp.|[6207]|BBLWU203-09|United States|Colorado|658[0n]|BOLD:AAH5442
Condica sp.|[6208]|LNAUT810-14|United States|New Mexico|658[0n]|BOLD:AAH5442
Ogdoconta fergusoni|[6209]|HKONS240-08|United States|Florida|653[0n]|BOLD:AAA7222
Ogdoconta rufipenna|[6210]|IAWLB358-11|United States|Arizona|658[0n]|BOLD:AAZ0851
Ogdoconta tacna|[6211]|HKONB324-09|United States|Texas|658[0n]|BOLD:AAF4601
Ogdoconta tacna|[6212]|BBLSW816-09|United States|Texas|658[0n]|BOLD:AAF4601
Ogdoconta tacna|[6213]|HKONB323-09|United States|Texas|658[0n]|BOLD:AAF4601
Ogdoconta tacna|[6214]|HKONB564-09|United States|Texas|648[0n]|BOLD:AAF4601
Ogdoconta tacna|[6215]|BBLSW924-09|United States|Texas|658[0n]|BOLD:AAF4601
Ogdoconta margareta|[6216]|CNCLB1443-14|United States|Arizona|658[0n]|BOLD:AAX5158
Ogdoconta satana|[6217]|RDNMJ345-11|United States|New Mexico|658[0n]|BOLD:ABZ4216
Ogdoconta cinereola|[6218]|LNC528-06|United States|North Carolina|590[5n]|BOLD:AAB2383
Ogdoconta cinereola|[6219]|RDNMD542-06|United States|Florida|658[0n]|BOLD:AAB2383
Ogdoconta cinereola|[6220]|LGSM765-04|United States|Tennessee|658[0n]|BOLD:AAB2383
Ogdoconta cinereola|[6221]|PHMO348-03|Canada|Ontario|639[2n]|BOLD:AAB2383
Ogdoconta cinereola|[6222]|XAB329-04|Canada|Ontario|658[0n]|BOLD:AAB2383
Ogdoconta cinereola|[6223]|LSEU647-06|United States|Georgia|658[0n]|BOLD:AAB2383
Ogdoconta cinereola|[6224]|RDLQF926-06|Canada|Quebec|658[0n]|BOLD:AAB2383
Ogdoconta cinereola|[6225]|LPOKA146-08|United States|Oklahoma|658[0n]|BOLD:AAB2383
Ogdoconta cinereola|[6226]|BBLSW501-09|United States|Oklahoma|658[0n]|BOLD:AAB2383
Ogdoconta cinereola|[6227]|LNC529-06|United States||560[0n]|BOLD:AAB2383
Ogdoconta cinereola|[6228]|LPOKA550-09|United States|Oklahoma|658[0n]|BOLD:AAB2383
Ogdoconta cinereola|[6229]|BLTIB153-08|Canada|Ontario|658[0n]|BOLD:AAB2383
Ogdoconta cinereola|[6230]|LILLA435-11|United States|Illinois|658[0n]|BOLD:AAB2383
Ogdoconta cinereola|[6231]|LPOKB320-09|United States|Oklahoma|632[0n]|BOLD:AAB2383
Ogdoconta cinereola|[6232]|LOFLC254-06|United States|Florida|636[0n]|BOLD:AAB2383
Ogdoconta cinereola|[6233]|LNCB523-07|United States|North Carolina|658[0n]|BOLD:AAB2383
Ogdoconta cinereola|[6234]|RDLQF927-06|Canada|Quebec|658[0n]|BOLD:AAB2383
Ogdoconta cinereola|[6235]|RDLQB735-05|Canada|Quebec|658[0n]|BOLD:AAB2383
Ogdoconta cinereola|[6236]|XAG916-05|Canada|Ontario|658[0n]|BOLD:AAB2383
Ogdoconta cinereola|[6237]|XAG820-05|Canada|Ontario|658[0n]|BOLD:AAB2383
Ogdoconta cinereola|[6238]|XAB515-04|Canada|Ontario|658[0n]|BOLD:AAB2383
Ogdoconta cinereola|[6239]|RDNME906-08|United States|Arizona|658[0n]|BOLD:AAB2383
Ogdoconta cinereola|[6240]|LPSO322-08|Canada|Ontario|658[0n]|BOLD:AAB2383
Ogdoconta cinereola|[6241]|LPOKA057-08|United States|Oklahoma|658[0n]|BOLD:AAB2383
Ogdoconta cinereola|[6242]|LPOKA176-08|United States|Oklahoma|658[0n]|BOLD:AAB2383
Ogdoconta cinereola|[6243]|LILLA809-11|United States|Illinois|658[0n]|BOLD:AAB2383
Ogdoconta cinereola|[6244]|XAG318-05|Canada|Ontario|658[0n]|BOLD:AAB2383
Ogdoconta cinereola|[6245]|XAF762-05|Canada|Ontario|658[0n]|BOLD:AAB2383
Ogdoconta cinereola|[6246]|XAD298-04|Canada|Ontario|592[0n]|BOLD:AAB2383
Ogdoconta cinereola|[6247]|RDLQF558-06|Canada|Quebec|658[0n]|BOLD:AAB2383
Ogdoconta cinereola|[6248]|BBLOD923-11|United States|Texas|658[0n]|BOLD:AAB2383
Ogdoconta moreno|[6249]|RDNME043-07|United States|Arizona|658[0n]|BOLD:AAI3343
Ogdoconta moreno|[6250]|NAMUM090-08|United States|Arizona|657[0n]|BOLD:AAI3343
Ogdoconta moreno|[6251]|CMAZA1122-12|United States|Arizona|658[0n]|BOLD:AAI3343
Ogdoconta altura|[6252]|CNCLB231-14|United States|Texas|658[0n]|BOLD:AAH5330
Ogdoconta altura|[6253]|CNCLB1695-14|United States|Texas|658[0n]|BOLD:AAH5330
Ogdoconta sexta|[6254]|NOCNA046-14|United States|Texas|658[0n]|BOLD:AAH5330
Ogdoconta altura|[6255]|BBLSX276-09|United States|Texas|658[0n]|BOLD:AAH5330
Ogdoconta altura|[6256]|BBLSW986-09|United States|Texas|658[0n]|BOLD:AAH5330
Ogdoconta altura|[6257]|BBLSX035-09|United States|Texas|633[0n]|BOLD:AAH5330
Ogdoconta altura|[6258]|BBLSW928-09|United States|Texas|647[0n]|BOLD:AAH5330
Ogdoconta altura|[6259]|BBLSX009-09|United States|Texas|632[0n]|BOLD:AAH5330
Ogdoconta altura|[6260]|BBLSW906-09|United States|Texas|606[0n]|BOLD:AAH5330
Ogdoconta altura|[6261]|CNCLB2401-14|United States|Texas|604[0n]|BOLD:AAH5330
Ruacodes tela|[6262]|BBLOC156-11|United States|Arizona|658[0n]|BOLD:AAE7663
Ruacodes tela|[6263]|RDNMG112-08|United States|Arizona|658[0n]|BOLD:AAE7663
Ruacodes tela|[6264]|RDNMD817-07|United States|Arizona|658[0n]|BOLD:AAE7663
Ruacodes tela|[6265]|RDNMJ801-11|United States|New Mexico|658[0n]|BOLD:AAE7663
Ruacodes tela|[6266]|BBLOC417-11|United States|Arizona|658[0n]|BOLD:AAE7663

Ruacodes tela|[6264]|RDNMD817-07|United States|Arizona|658[0n]|BOLD:AAE7663
Ruacodes tela|[6265]|RDNMJ801-11|United States|New Mexico|658[0n]|BOLD:AAE7663
Ruacodes tela|[6266]|BBLOC417-11|United States|Arizona|658[0n]|BOLD:AAE7663
Ruacodes tela|[6267]|IAWL961-09|United States|Arizona|658[0n]|BOLD:AAE7663
Ruacodes tela|[6268]|RDNMG904-08|United States|Arizona|658[0n]|BOLD:AAE7663
Ruacodes tela|[6269]|IAWL963-09|United States|Arizona|658[0n]|BOLD:AAE7663
Ruacodes tela|[6270]|IAWLB295-11|United States|Arizona|658[0n]|BOLD:AAE7663
Ruacodes tela|[6271]|IAWLB301-11|United States|Arizona|658[0n]|BOLD:AAE7663
Ruacodes tela|[6272]|BBLOC176-11|United States|Arizona|658[0n]|BOLD:AAE7663
Ruacodes tela|[6273]|BBLOC444-11|United States|Arizona|658[0n]|BOLD:AAE7663
Ruacodes tela|[6274]|IAWL960-09|United States|Arizona|658[0n]|BOLD:AAE7663
Ruacodes tela|[6275]|IAWL962-09|United States|Arizona|658[0n]|BOLD:AAE7663
Ruacodes tela|[6276]|NAMUM077-08|United States|Arizona|657[0n]|BOLD:AAE7663
Ruacodes tela|[6277]|BBLOC559-11|United States|Arizona|658[0n]|BOLD:AAE7663
Ruacodes tela|[6278]|BBLOE1738-12|United States|Arizona|658[0n]|BOLD:AAE7663
Ruacodes tela|[6279]|CMAZA1203-12|United States|Arizona|658[0n]|BOLD:AAE7663
Nocloa duplicatus|[6280]|LNAUS5330-13|United States|Texas|658[0n]|BOLD:ACK3357
Nocloa duplicatus|[6281]|LNAUS5331-13|United States|Nebraska|658[0n]|BOLD:ACK3357
Nocloa duplicatus|[6282]|LNAUS5332-13|United States|Nebraska|658[0n]|BOLD:ACK3357
Paramiana perissa|[6283]|RDNME603-08|United States|New Mexico|658[0n]|BOLD:AAX2518
Oslaria viridifera|[6284]|AWCL085-09|United States|Arizona|658[0n]|BOLD:AAD6022
Oslaria viridifera|[6285]|RDNMK263-11|United States|Texas|658[0n]|BOLD:AAD6022
Oslaria viridifera|[6286]|RDNMK264-11|United States|Texas|658[0n]|BOLD:AAD6022
Oslaria viridifera|[6287]|QUNOD031-10|United States|Texas|658[0n]|BOLD:AAD6022
Oslaria viridifera|[6288]|BBLOC279-11|United States|Arizona|658[0n]|BOLD:AAD6022
Oslaria viridifera|[6289]|BBLOC558-11|United States|Arizona|658[0n]|BOLD:AAD6022
Oslaria viridifera|[6290]|BBLOC262-11|United States|Arizona|658[0n]|BOLD:AAD6022
Oslaria viridifera|[6291]|BBLOC897-11|United States|Arizona|658[0n]|BOLD:AAD6022
Oslaria viridifera|[6292]|BBLOC1656-11|United States|Arizona|658[0n]|BOLD:AAD6022
Oslaria viridifera|[6293]|BBLOC808-11|United States|Arizona|658[0n]|BOLD:AAD6022
Oslaria viridifera|[6294]|BBLOC898-11|United States|Arizona|658[0n]|BOLD:AAD6022
Oslaria viridifera|[6295]|BBLOC884-11|United States|Arizona|658[0n]|BOLD:AAD6022
Oslaria viridifera|[6296]|BBLOC812-11|United States|Arizona|658[0n]|BOLD:AAD6022
Oslaria viridifera|[6297]|BBLOC538-11|United States|Arizona|658[0n]|BOLD:AAD6022
Oslaria viridifera|[6298]|BBLOC534-11|United States|Arizona|658[0n]|BOLD:AAD6022
Oslaria viridifera|[6299]|BBLOC395-11|United States|Arizona|658[0n]|BOLD:AAD6022
Oslaria viridifera|[6300]|BBLOC277-11|United States|Arizona|658[0n]|BOLD:AAD6022
Oslaria viridifera|[6301]|BBLOC274-11|United States|Arizona|658[0n]|BOLD:AAD6022
Oslaria viridifera|[6302]|BBLOC264-11|United States|Arizona|658[0n]|BOLD:AAD6022
Oslaria viridifera|[6303]|BBLOC263-11|United States|Arizona|658[0n]|BOLD:AAD6022
Oslaria viridifera|[6304]|BBLOC261-11|United States|Arizona|658[0n]|BOLD:AAD6022
Oslaria viridifera|[6305]|BBLOC258-11|United States|Arizona|658[0n]|BOLD:AAD6022
Oslaria viridifera|[6306]|BBLOC184-11|United States|Arizona|658[0n]|BOLD:AAD6022
Oslaria viridifera|[6307]|CMAZA570-10|United States|Arizona|658[0n]|BOLD:AAD6022
Oslaria viridifera|[6308]|RDNMH815-09|United States|Arizona|658[0n]|BOLD:AAD6022
Oslaria viridifera|[6309]|AWCL084-09|United States|Arizona|658[0n]|BOLD:AAD6022
Oslaria viridifera|[6310]|AWCL083-09|United States|Arizona|658[0n]|BOLD:AAD6022
Oslaria viridifera|[6311]|IAWL017-09|United States|Arizona|658[0n]|BOLD:AAD6022
Oslaria viridifera|[6312]|NAMUM093-08|United States|Arizona|658[0n]|BOLD:AAD6022
Oslaria viridifera|[6313]|RDNMD930-07|United States|Arizona|655[0n]|BOLD:AAD6022
Oslaria viridifera|[6314]|RDNMD929-07|United States|Arizona|655[0n]|BOLD:AAD6022
Oslaria viridifera|[6315]|RDNMD815-07|United States|Arizona|658[0n]|BOLD:AAD6022
Oslaria viridifera|[6316]|BBLOC1607-11|United States|Arizona|620[0n]|BOLD:AAD6022
Oslaria viridifera|[6317]|BBLOC1650-11|United States|Arizona|658[0n]|BOLD:AAD6022
Oslaria viridifera|[6318]|BBLOC1655-11|United States|Arizona|658[0n]|BOLD:AAD6022
Oslaria viridifera|[6319]|BBLOC1662-11|United States|Arizona|658[0n]|BOLD:AAD6022
Oslaria viridifera|[6320]|CMAZA968-12|United States|Arizona|658[0n]|BOLD:AAD6022
Oslaria viridifera|[6321]|CMAZA975-12|United States|Arizona|658[0n]|BOLD:AAD6022
Oslaria viridifera|[6322]|CMAZA1026-12|United States|Arizona|658[0n]|BOLD:AAD6022
Nocloa nanata|[6323]|CMAZA768-10|United States|Arizona|658[0n]|BOLD:AAP6027
Nocloa nanata|[6324]|CMAZA1111-12|United States|Arizona|658[0n]|BOLD:AAP6027
Nocloa cordova|[6325]|LNAUS5325-13|United States|Arizona|658[0n]|BOLD:ACK3420
Nocloa cordova|[6326]|LNAUS5326-13|United States|Arizona|658[0n]|BOLD:ACK3420
Nocloa cordova|[6327]|CNCLB213-14|United States|Arizona|658[0n]|BOLD:ACK3420
Nocloa cordova|[6328]|CNCLB214-14|United States|Arizona|658[0n]|BOLD:ACK3420
Oslaria pura|[6329]|RDNMH816-09|United States|Arizona|658[0n]|BOLD:AAI1795
Oslaria pura|[6330]|RDNME907-08|United States|Arizona|658[0n]|BOLD:AAI1795
Oslaria pura|[6331]|RDNMJ679-11|United States|Arizona|658[0n]|BOLD:AAI1795
Nocloa plagiata|[6332]|NAMUM056-08|United States|Arizona|656[0n]|BOLD:AAF3581
Nocloa plagiata|[6333]|QUNOD030-10|United States|Texas|627[0n]|BOLD:AAF3581
Nocloa plagiata|[6334]|RDNMH813-09|United States|Arizona|658[0n]|BOLD:AAF3581
Nocloa plagiata|[6335]|CMAZA1072-12|United States|Arizona|658[0n]|BOLD:AAF3581
Nocloa plagiata|[6336]|RDNML214-13|United States|Arizona|658[0n]|BOLD:AAF3581
Nocloa pallens|[6337]|CGLCA064-10|United States|California|658[0n]|BOLD:AAG4587
Nocloa pallens|[6338]|RDNMD442-06|United States|California|658[0n]|BOLD:AAG4587
Nocloa pallens|[6339]|JBAZ063-09|United States|California|658[0n]|BOLD:AAG4587
Nocloa pallens|[6340]|BBLSW264-09|United States|California|658[0n]|BOLD:AAG4587
Nocloa pallens|[6341]|USLEP510-10|United States|Arizona|658[0n]|BOLD:AAG4587
Nocloa rivulosa|[6342]|LOCBF209-13|United States|California|600[0n]|BOLD:AAG5838
Nocloa rivulosa|[6343]|LOCBF210-13|United States|California|595[0n]|BOLD:AAG5838
Nocloa rivulosa|[6344]|LOCBB180-06|United States|California|658[0n]|BOLD:AAG5838
Nocloa rivulosa|[6345]|LOCBB182-06|United States|California|658[0n]|BOLD:AAG5838
Nocloa rivulosa|[6346]|LOCBB181-06|United States|California|658[0n]|BOLD:AAG5838
Nocloa rivulosa|[6347]|LOCBB179-06|United States|California|658[0n]|BOLD:AAG5838
Nocloa rivulosa|[6348]|LOCBC494-06|United States|California|658[0n]|BOLD:AAG5838
Nocloa rivulosa|[6349]|LOCBC495-06|United States|California|658[0n]|BOLD:AAG5838
Nocloa rivulosa|[6350]|RDNMG018-08|United States|Arizona|658[0n]|BOLD:AAG5838
Nocloa rivulosa|[6351]|LOCBF279-13|United States|California|629[0n]|BOLD:AAG5838
Nocloa rivulosa|[6352]|LOCBF280-13|United States|California|628[0n]|BOLD:AAG5838
Nocloa aliaga|[6353]|RDNMG583-08|United States|Arizona|658[0n]|BOLD:AAF3576
Nocloa aliaga|[6354]|RDNMH585-09|United States|Arizona|658[0n]|BOLD:AAF3576
Nocloa aliaga|[6355]|RDNMG584-08|United States|Arizona|658[0n]|BOLD:AAF3576
Nocloa aliaga|[6356]|BBLOC1612-11|United States|Arizona|658[0n]|BOLD:AAF3576
Nocloa alcandra|[6357]|LNAUS5335-13|United States|Arizona|658[0n]|BOLD:AAK8260
Nocloa alcandra|[6358]|LNAUS5336-13|United States|Arizona|658[0n]|BOLD:AAK8260
Nocloa alcandra|[6359]|RDNMH814-09|United States|Arizona|658[0n]|BOLD:AAK8260
Nocloa alcandra|[6360]|LNAUS5338-13|United States|Arizona|658[0n]|BOLD:AAK8260
Nocloa alcandra|[6361]|LNAUS5337-13|United States|Arizona|658[0n]|BOLD:AAK8260
Nocloa alcandra|[6362]|LNAUS5339-13|United States|Arizona|658[0n]|BOLD:AAK8260
Nocloa alcandra|[6363]|LNAUS5340-13|United States|Arizona|658[0n]|BOLD:AAK8260
Paramiana sp.|[6364]|RDNMJ765-11|United States|New Mexico|658[0n]|BOLD:AAT9244
Paramiana sp.|[6365]|IAWLB558-11|United States|Arizona|658[0n]|BOLD:AAT9244
[illegible]

Noctua alcandra|[6363]|LNAUS5340-13|United States|Arizona|658[0n]|BOLD:AAK8260
Paramiana sp.|[6364]|RDNMJ765-11|United States|New Mexico|658[0n]|BOLD:AAT9244
Paramiana sp.|[6365]|IAWLB558-11|United States|Arizona|658[0n]|BOLD:AAT9244
Paramiana n. sp. 2|[6366]|RDNMG004-08|United States|Arizona|658[0n]|BOLD:AAE6879
Paramiana marina|[6367]|RDNMG011-08|United States|Arizona|649[0n]|BOLD:AAI6354
Paramiana n. sp. 1|[6368]|RDNMG005-08|United States|Arizona|658[0n]|BOLD:AAE6880
Paramiana n. sp. 4|[6369]|RDNMG007-08|United States|Arizona|649[0n]|BOLD:AAE6881
Paramiana n. sp. 4|[6370]|CMAZA974-12|United States|Arizona|658[0n]|BOLD:AAE6881
Paramiana n. sp. 3|[6371]|RDNMG012-08|United States|New Mexico|658[0n]|BOLD:AAI6355
Paramiana n. sp. 3|[6372]|RDNMH634-09|United States|Texas|658[0n]|BOLD:AAI6355
Paramiana n. sp. 3|[6373]|RDNMK179-11|United States|Arizona|658[0n]|BOLD:AAI6355
Paramiana n. sp. 3|[6374]|RDNMK180-11|United States|Arizona|658[0n]|BOLD:AAI6355
Paramiana smaragdina|[6375]|RDNMG006-08|United States|Arizona|658[0n]|BOLD:AAI6356
Paramiana smaragdina|[6376]|RDNME910-08|United States|Arizona|658[0n]|BOLD:AAI6356
Paramiana smaragdina|[6377]|CMAZA1099-12|United States|Arizona|658[0n]|BOLD:AAI6356
Paramiana smaragdina|[6378]|CMAZA1102-12|United States|Arizona|658[0n]|BOLD:AAI6356
Euamiana contrasta|[6379]|BBLOC619-11|United States|Arizona|658[0n]|BOLD:ABA5662
Euamiana contrasta|[6380]|BBLOC895-11|United States|Arizona|658[0n]|BOLD:ABA5662
Euamiana dissimilis|[6381]|CNCLB208-14|United States|Arizona|658[0n]|BOLD:ACM4122
Euamiana dissimilis|[6382]|CNCLB209-14|United States|Arizona|658[0n]|BOLD:ACM4122
Lythrodes tripuncta|[6383]|CMAZA981-12|United States|Arizona|658[0n]|BOLD:ABW2948
Lythrodes tripuncta|[6384]|CMAZA1093-12|United States|Arizona|658[0n]|BOLD:ABW2948
Redingtonia alba|[6385]|RDNME963-08|United States|New Mexico|658[0n]|BOLD:AAY0623
Lythrodes venatus|[6386]|LNAUS5321-13|United States|New Mexico|658[0n]|BOLD:AAY9523
Lythrodes venatus|[6387]|LNAUS5319-13|United States|New Mexico|658[0n]|BOLD:AAY9523
Lythrodes venatus|[6388]|CNCLB217-14|United States|New Mexico|658[0n]|BOLD:AAY9523
Lythrodes radiatus|[6389]|RDNMK189-11|United States|Arizona|658[0n]|BOLD:AAY9523
Lythrodes venatus|[6390]|LNAUS5320-13|United States|New Mexico|637[0n]|BOLD:AAY9523
Lythrodes radiatus|[6391]|CNCLB1452-14|United States|Arizona|658[0n]|BOLD:AAY9523
Lythrodes radiatus|[6392]|CNCLB1453-14|United States|Arizona|658[0n]|BOLD:ACO8999
Amphipyra tragopoginis|[6393]|GWOSN915-11|Germany|Bavaria|658[0n]|BOLD:AAB3277
Amphipyra tragopoginis|[6394]|GWORZ528-10|Germany|Bavaria|633[0n]|
Amphipyra tragopoginis|[6395]|LEATD405-13|Austria|Tirol|658[0n]|BOLD:AAB3277
Amphipyra tragopoginis|[6396]|LEATD122-13|Austria|Tirol|658[0n]|BOLD:AAB3277
Amphipyra tragopoginis|[6397]|LEATB734-13|Italy|South Tyrol|658[0n]|BOLD:AAB3277
Amphipyra tragopoginis|[6398]|PHLAH731-12|Austria|Vorarlberg|658[0n]|BOLD:AAB3277
Amphipyra tragopoginis|[6399]|IBLAO442-12|Spain|Catalonia|658[0n]|BOLD:AAB3277
Amphipyra tragopoginis|[6400]|PHLSA584-11|Italy||658[0n]|BOLD:AAB3277
Amphipyra tragopoginis|[6401]|GWOSA244-10|Italy|Basilicata|658[0n]|BOLD:AAB3277
Amphipyra tragopoginis|[6402]|GWORR896-10|Spain|Murcia|658[0n]|BOLD:AAB3277
Amphipyra tragopoginis|[6403]|LEATD555-13|Italy|South Tyrol|637[0n]|BOLD:AAB3277
Amphipyra tragopoginis|[6404]|CGUKA984-09|United Kingdom|England|631[0n]|BOLD:AAB3277
Amphipyra tragopoginis|[6405]|CGUKA985-09|United Kingdom|England|615[0n]|BOLD:AAB3277
Amphipyra tragopoginis|[6406]|LENOA420-11|France|Haute Normandie|658[0n]|BOLD:AAB3277
Amphipyra tragopoginis|[6407]|PHMO343-03|Canada|Ontario|639[1n]|BOLD:AAB3277
Amphipyra tragopoginis|[6408]|XAD156-04|Canada|Ontario|586[0n]|BOLD:AAB3277
Amphipyra tragopoginis|[6409]|RDLQG219-06|Canada|Quebec|658[0n]|BOLD:AAB3277
Amphipyra tragopoginis|[6410]|LBCW059-08|Canada|British Columbia|658[0n]|BOLD:AAB3277
Amphipyra tragopoginis|[6411]|CGUKB283-09|United Kingdom|England|624[0n]|BOLD:AAB3277
Amphipyra tragopoginis|[6412]|LEFIC716-10|Finland|Finland Proper|658[0n]|BOLD:AAB3277
Amphipyra tragopoginis|[6413]|CGUKC186-09|United Kingdom|England|641[0n]|BOLD:AAB3277
Amphipyra tragopoginis|[6414]|LEFIA616-10|Finland|South Karelia|658[0n]|BOLD:AAB3277
Amphipyra tragopoginis|[6415]|LALPA1279-11|Canada|British Columbia|658[0n]|BOLD:AAB3277
Amphipyra tragopoginis|[6416]|CGUKA509-09|United Kingdom|England|603[0n]|BOLD:AAB3277
Amphipyra tragopoginis|[6417]|LALPA770-10|Canada|British Columbia|658[0n]|BOLD:AAB3277
Amphipyra tragopoginis|[6418]|LENOA421-11|France|Haute Normandie|658[0n]|BOLD:AAB3277
Amphipyra tragopoginis|[6419]|LNCC1175-11|United States|North Carolina|658[0n]|BOLD:AAB3277
Amphipyra tragopoginis|[6420]|LNCC1178-11|United States|North Carolina|658[0n]|BOLD:AAB3277
Amphipyra tragopoginis|[6421]|GBLAB308-13|Germany|Saarland|658[0n]|BOLD:AAB3277
Amphipyra tragopoginis|[6422]|GBLAB720-13|Germany|Brandenburg|658[0n]|BOLD:AAB3277
Amphipyra tragopoginis|[6423]|NORIN072-13|Norway|Akershus|656[0n]|BOLD:AAB3277
Amphipyra tragopoginis|[6424]|GBLAF534-14|Germany|Brandenburg|658[0n]|BOLD:AAB3277
Amphipyra tragopoginis|[6425]|LALPA759-10|Canada|British Columbia|658[0n]|BOLD:AAB3277
Amphipyra tragopoginis|[6426]|LALPA760-10|Canada|British Columbia|658[0n]|BOLD:AAB3277
Amphipyra tragopoginis|[6427]|PHMO276-03|Canada|Ontario|639[0n]|BOLD:AAB3277
Amphipyra tragopoginis|[6428]|LOWCB917-05|Canada|British Columbia|658[0n]|BOLD:AAB3277
Amphipyra tragopoginis|[6429]|LOWCB919-05|Canada|British Columbia|658[0n]|BOLD:AAB3277
Amphipyra tragopoginis|[6430]|LPVIB231-08|Canada|British Columbia|658[0n]|BOLD:AAB3277
Amphipyra tragopoginis|[6431]|LPVIB508-08|Canada|British Columbia|658[0n]|BOLD:AAB3277
Amphipyra tragopoginis|[6432]|TTNFS067-09|Serbia||658[0n]|BOLD:AAB3277
Amphipyra tragopoginis|[6433]|CGUKC104-09|United Kingdom|England|658[0n]|BOLD:AAB3277
Amphipyra tragopoginis|[6434]|CGUKC452-09|United Kingdom|England|658[0n]|BOLD:AAB3277
Amphipyra tragopoginis|[6435]|CGUKD468-09|United Kingdom|England|658[0n]|BOLD:AAB3277
Amphipyra tragopoginis|[6436]|GWORL395-09|Germany|Bavaria|658[0n]|BOLD:AAB3277
Amphipyra tragopoginis|[6437]|LEFIA617-10|Finland|South Karelia|658[0n]|BOLD:AAB3277
Amphipyra tragopoginis|[6438]|LBCH1503-10|Canada|British Columbia|658[0n]|BOLD:AAB3277
Amphipyra tragopoginis|[6439]|GWORZ527-10|Germany|Bavaria|658[0n]|BOLD:AAB3277
Amphipyra tragopoginis|[6440]|LBCH7951-10|Canada|British Columbia|658[0n]|BOLD:AAB3277
Amphipyra tragopoginis|[6441]|LALPA600-10|Canada|British Columbia|658[0n]|BOLD:AAB3277
Amphipyra tragopoginis|[6442]|CNCLB2631-14|United States|North Carolina|658[0n]|BOLD:AAB3277
Amphipyra tragopoginis|[6443]|NGAAB055-14|Canada|British Columbia|588[0n]|BOLD:AAB3277
Amphipyra tragopoginis|[6444]|NGAAB056-14|Canada|British Columbia|582[0n]|BOLD:AAB3277
Amphipyra tragopoginis|[6445]|LOWCB916-05|Canada|British Columbia|658[0n]|BOLD:AAB3277
Amphipyra tragopoginis|[6446]|LOWCB918-05|Canada|British Columbia|658[0n]|BOLD:AAB3277
Amphipyra tragopoginis|[6447]|LBCG630-09|Canada|British Columbia|658[0n]|BOLD:AAB3277
Amphipyra tragopoginis|[6448]|LALPA802-10|Canada|British Columbia|658[0n]|BOLD:AAB3277
Amphipyra tragopoginis|[6449]|LNCC1177-11|United States|North Carolina|658[0n]|BOLD:AAB3277
Amphipyra tragopoginis|[6450]|LALPA1345-12|Canada|British Columbia|658[0n]|BOLD:AAB3277
Amphipyra tragopoginis|[6451]|ABOLA729-14|Austria||658[0n]|BOLD:AAB3277
Eusceptis flavifrimbriata|[6452]|RDNME685-08|United States|Texas|658[0n]|BOLD:AAC7926
Supralathosea obtusa|[6453]|RDNME883-08|United States|Arizona|658[0n]|BOLD:AAH9892
Supralathosea obtusa|[6454]|IAWLB528-11|United States|Arizona|658[0n]|BOLD:AAH9892
Anycteola fotelloides|[6455]|BBLOE1856-12|United States|Texas|658[0n]|BOLD:ABW8668
Anycteola fotelloides|[6456]|RDNMK885-13|Mexico|Sonora|658[0n]|BOLD:ABW8668
Amphipyra pyramidoides|[6457]|RDNML012-13|United States|Florida|658[0n]|BOLD:AAA8525
Amphipyra pyramidoides|[6458]|XAH469-05|Canada|Ontario|658[0n]|BOLD:AAA8525
Amphipyra pyramidoides|[6459]|JSJUL2387-11|Canada|Ontario|658[0n]|BOLD:AAA8525
Amphipyra pyramidoides|[6460]|RDLQB845-05|Canada|Quebec|658[0n]|BOLD:AAA8525
Amphipyra pyramidoides|[6461]|LOPN089-06|United States|Oregon|658[0n]|BOLD:AAA8525
Amphipyra pyramidoides|[6462]|TTMNB511-06|Canada|New Brunswick|654[0n]|BOLD:AAA8525
Amphipyra pyramidoides|[6463]|LOPN090-06|United States|Oregon|658[0n]|BOLD:AAA8525
Amphipyra pyramidoides|[6464]|LOPN087-06|United States|Oregon|658[0n]|BOLD:AAA8525
Amphipyra pyramidoides|[6465]|LOWCC835-05|Canada|British Columbia|658[0n]|BOLD:AAA8525

Amphipyra pyramidoides|[6463]|LOPN090-06|United States|Oregon|658[0n]|BOLD:AAA8525
Amphipyra pyramidoides|[6464]|LOPN087-06|United States|Oregon|658[0n]|BOLD:AAA8525
Amphipyra pyramidoides|[6465]|LOWCC835-05|Canada|British Columbia|658[0n]|BOLD:AAA8525
Amphipyra pyramidoides|[6466]|LOPN088-06|United States|Oregon|658[0n]|BOLD:AAA8525
Amphipyra pyramidoides|[6467]|LALPA1230-11|Canada|British Columbia|658[0n]|BOLD:AAA8525
Amphipyra pyramidoides|[6468]|LALPA1287-11|Canada|British Columbia|658[0n]|BOLD:AAA8525
Amphipyra pyramidoides|[6469]|XAB417-04|Canada|Ontario|658[0n]|BOLD:AAA8525
Amphipyra pyramidoides|[6470]|XAG175-05|Canada|Ontario|621[0n]|BOLD:AAA8525
Amphipyra pyramidoides|[6471]|XAB646-04|Canada|Ontario|658[0n]|BOLD:AAA8525
Amphipyra pyramidoides|[6472]|XAD097-04|Canada|Ontario|658[0n]|BOLD:AAA8525
Amphipyra pyramidoides|[6473]|XAH337-05|Canada|Ontario|658[0n]|BOLD:AAA8525
Amphipyra pyramidoides|[6474]|BBLEC482-09|Canada|New Brunswick|658[0n]|BOLD:AAA8525
Amphipyra pyramidoides|[6475]|LPOKD614-09|United States|Oklahoma|658[0n]|BOLD:AAA8525
Amphipyra pyramidoides|[6476]|JSJUL2388-11|Canada|Ontario|658[0n]|BOLD:AAA8525
Amphipyra pyramidoides|[6477]|TMNBB209-06|Canada|New Brunswick|656[0n]|BOLD:AAA8525
Amphipyra pyramidoides|[6478]|RDLQB484-05|Canada|Quebec|658[0n]|BOLD:AAA8525
Amphipyra pyramidoides|[6479]|TMNBB210-06|Canada|New Brunswick|658[0n]|BOLD:AAA8525
Amphipyra pyramidoides|[6480]|XAK428-06|Canada|Ontario|658[0n]|BOLD:AAA8525
Amphipyra pyramidoides|[6481]|XAK429-06|Canada|Ontario|658[0n]|BOLD:AAA8525
Amphipyra pyramidoides|[6482]|LTOLB046-08|United States|Maryland|658[0n]|BOLD:AAA8525
Amphipyra pyramidoides|[6483]|LGSMG1000-10|United States|North Carolina|658[0n]|BOLD:AAA8525
Amphipyra pyramidoides|[6484]|MJMSL152-10|United States|Massachusetts|658[0n]|BOLD:AAA8525
Amphipyra pyramidoides|[6485]|XAB459-04|Canada|Ontario|640[0n]|BOLD:AAA8525
Amphipyra pyramidoides|[6486]|XAH099-05|Canada|Ontario|658[0n]|BOLD:AAA8525
Amphipyra pyramidoides|[6487]|TTMNB512-06|Canada|New Brunswick|656[0n]|BOLD:AAA8525
Amphipyra pyramidoides|[6488]|XAK426-06|Canada|Ontario|658[0n]|BOLD:AAA8525
Amphipyra pyramidoides|[6489]|LNCNW117-06|United States|North Carolina|658[0n]|BOLD:AAA8525
Amphipyra pyramidoides|[6490]|TMNBB208-06|Canada|New Brunswick|658[0n]|BOLD:AAA8525
Amphipyra pyramidoides|[6491]|RDNMB966-05|Canada|Ontario|658[0n]|BOLD:AAA8525
Amphipyra pyramidoides|[6492]|XAH518-05|Canada|Ontario|658[0n]|BOLD:AAA8525
Amphipyra pyramidoides|[6493]|XAH090-05|Canada|Ontario|658[0n]|BOLD:AAA8525
Amphipyra pyramidoides|[6494]|LNC028-05|United States|North Carolina|658[0n]|BOLD:AAA8525
Amphipyra pyramidoides|[6495]|XAG219-05|Canada|Ontario|658[0n]|BOLD:AAA8525
Amphipyra pyramidoides|[6496]|XAD466-04|Canada|Ontario|658[0n]|BOLD:AAA8525
Amphipyra pyramidoides|[6497]|XAD102-04|Canada|Ontario|658[0n]|BOLD:AAA8525
Amphipyra pyramidoides|[6498]|XAB542-04|Canada|Ontario|658[0n]|BOLD:AAA8525
Amphipyra pyramidoides|[6499]|XAB410-04|Canada|Ontario|658[0n]|BOLD:AAA8525
Amphipyra pyramidoides|[6500]|XAD437-04|Canada|Ontario|565[0n]|BOLD:AAA8525
Amphipyra pyramidoides|[6501]|LNC027-05|United States|North Carolina|658[0n]|BOLD:AAA8525
Amphipyra pyramidoides|[6502]|XAH264-05|Canada|Ontario|604[0n]|BOLD:AAA8525
Amphipyra pyramidoides|[6503]|BBLEC486-09|Canada|New Brunswick|639[0n]|BOLD:AAA8525
Amphipyra pyramidoides|[6504]|BBLEC487-09|Canada|New Brunswick|658[0n]|BOLD:AAA8525
Amphipyra pyramidoides|[6505]|BBLEC505-09|Canada|New Brunswick|658[0n]|BOLD:AAA8525
Amphipyra pyramidoides|[6506]|LPOKD637-09|United States|Oklahoma|658[0n]|BOLD:AAA8525
Amphipyra pyramidoides|[6507]|AHLEP086-10|United States|Pennsylvania|658[0n]|BOLD:AAA8525
Amphipyra pyramidoides|[6508]|MJMSL040-10|United States|Massachusetts|658[0n]|BOLD:AAA8525
Amphipyra pyramidoides|[6509]|MJMSL084-10|United States|Massachusetts|658[0n]|BOLD:AAA8525
Amphipyra pyramidoides|[6510]|LILLA567-11|United States|Illinois|658[0n]|BOLD:AAA8525
Amphipyra pyramidoides|[6511]|CNSLS022-13|Canada|Ontario|622[0n]|BOLD:AAA8525
Euamiana torniplaga|[6512]|RDNMH822-09|United States|Arizona|658[0n]|BOLD:AAJ0885
Euamiana torniplaga|[6513]|RDNME900-08|United States|Arizona|658[0n]|BOLD:AAJ0885
Euamiana torniplaga|[6514]|RDNMJ291-11|United States|Arizona|658[0n]|BOLD:AAJ0885
Euamiana torniplaga|[6515]|RDNMJ294-11|United States|Arizona|658[0n]|BOLD:AAJ0885
Euamiana torniplaga|[6516]|RDNMJ698-11|United States|Arizona|658[0n]|BOLD:AAJ0885
Euamiana n. sp.|[6517]|RDNMF966-08|United States|Texas|658[0n]|BOLD:AAE6878
Euamiana n. sp.|[6518]|RDNMJ564-11|United States|Arizona|658[0n]|BOLD:AAE6878
Euamiana n. sp.|[6519]|RDNMG003-08|United States|Arizona|658[0n]|BOLD:AAE6878
Euamiana n. sp.|[6520]|RDNMG184-08|United States|Arizona|658[0n]|BOLD:AAE6878
Euamiana n. sp.|[6521]|CNCLB210-14|United States|Arizona|658[0n]|BOLD:AAE6878
Acontia chea|[6522]|RDNME932-08|United States|Arizona|658[0n]|BOLD:AAJ3070
Acontia chea|[6523]|RDNMG342-08|United States|Arizona|658[0n]|BOLD:AAJ3070
Acontia sp.|[6524]|BBLOC1989-11|United States|Texas|658[0n]|BOLD:ABW8305
Acontia jaliscana|[6525]|RDNMH1024-09|United States|Texas|658[0n]|BOLD:AAJ3037
Acontia jaliscana|[6526]|RDNMK941-13|Mexico|Sonora|658[0n]|BOLD:AAJ3037
Acontia behrii|[6527]|RDNME052-07|United States|Arizona|658[0n]|BOLD:AAJ2954
Acontia behrii|[6528]|JBAZ162-09|United States|Arizona|658[0n]|BOLD:AAJ2954
Acontia cretata|[6529]|HKONB188-08|United States|Texas|658[0n]|BOLD:AAD4844
Acontia cretata|[6530]|RDNMG340-08|United States|Arizona|658[0n]|BOLD:AAD4844
Acontia cretata|[6531]|RDNME965-08|United States|New Mexico|580[0n]|BOLD:AAD4844
Acontia cretata|[6532]|RDNMK007-11|United States|Arizona|658[0n]|BOLD:AAD4844
Acontia cretata|[6533]|BBLSX821-09|United States|Texas|658[0n]|BOLD:AAD4844
Acontia cretata|[6534]|BBLSY162-09|United States|Texas|658[0n]|BOLD:AAD4844
Acontia cretata|[6535]|RDNMG341-08|United States|Arizona|658[0n]|BOLD:AAD4844
Acontia cretata|[6536]|NAMUM043-08|United States|Arizona|657[0n]|BOLD:AAD4844
Acontia cretata|[6537]|RDNMG009-08|United States|Arizona|649[0n]|BOLD:AAD4844
Acontia cretata|[6538]|RDNMK012-11|United States|Arizona|658[0n]|BOLD:AAD4844
Acontia cretata|[6539]|IAWLB304-11|United States|Arizona|658[0n]|BOLD:AAD4844
Acontia cretata|[6540]|RDNME931-08|United States|Arizona|658[0n]|BOLD:AAD4844
Acontia cretata|[6541]|BBLOE1231-12|United States|California|658[0n]|BOLD:AAD4844
Acontia cretata|[6542]|BBLSW357-09|United States|Arizona|658[0n]|BOLD:AAD4844
Acontia coquillettii|[6543]|CNCLB450-14|United States|California|540[0n]|BOLD:AAD4844
Acontia coquillettii|[6544]|CNCLB451-14|United States|California|540[0n]|BOLD:AAD4844
Acontia coquillettii|[6545]|CNCLB453-14|United States|California|540[0n]|BOLD:AAD4844
Spragueia dama|[6546]|HKONB202-09|United States|Texas|658[0n]|BOLD:AAB3399
Spragueia dama|[6547]|HKONB205-09|United States|Texas|658[0n]|BOLD:AAB3399
Spragueia dama|[6548]|HKONB204-09|United States|Texas|647[0n]|BOLD:AAB3399
Spragueia dama|[6549]|LSUSA085-06|United States|Kentucky|658[0n]|BOLD:AAB3399
Spragueia dama|[6550]|MNAC698-07|United States|Maryland|645[0n]|BOLD:AAB3399
Spragueia dama|[6551]|MNAC711-07|United States|Maryland|647[0n]|BOLD:AAB3399
Spragueia dama|[6552]|LPOKD298-09|United States|Oklahoma|658[0n]|BOLD:AAB3399
Spragueia dama|[6553]|LPOKD367-09|United States|Oklahoma|658[0n]|BOLD:AAB3399
Spragueia cleta|[6554]|RDNMH691-09|United States|Arizona|658[0n]|BOLD:AAJ3510
Spragueia obatra|[6555]|NAMUM095-08|United States|Arizona|658[0n]|BOLD:AAJ3539
Spragueia obatra|[6556]|RDNMJ553-11|United States|Arizona|658[0n]|BOLD:AAJ3539
Spragueia obatra|[6557]|RDNMJ566-11|United States|Arizona|658[0n]|BOLD:AAJ3539
Spragueia obatra|[6558]|CMAZA1220-12|United States|Arizona|658[0n]|BOLD:AAJ3539
Spragueia onagrus|[6559]|LNC541-06|United States|North Carolina|658[0n]|BOLD:ACE6651
Spragueia onagrus|[6560]|LNC823-06|United States|North Carolina|658[0n]|BOLD:ACE6651
Spragueia onagrus|[6561]|PHFLO082-10|United States|Florida|658[0n]|BOLD:ACE6651
Spragueia onagrus|[6562]|LOFLC266-06|United States|Florida|658[0n]|BOLD:ACE6651
Spragueia onagrus|[6563]|PHFLO091-10|United States|Florida|658[0n]|BOLD:ACE6651
Spragueia onagrus|[6564]|PHFLO093-10|United States|Florida|658[0n]|BOLD:ACE6651
[illegible]

Spragueia onagrus|[6562]|LOFLC266-06|United States|Florida|658[0n]|BOLD:ACE6651
Spragueia onagrus|[6563]|PHFLO091-10|United States|Florida|658[0n]|BOLD:ACE6651
Spragueia onagrus|[6564]|PHFLO093-10|United States|Florida|658[0n]|BOLD:ACE6651
Spragueia onagrus|[6565]|LOFLC062-06|United States|Florida|658[0n]|BOLD:ACE6651
Spragueia onagrus|[6566]|LOFLC249-06|United States|Florida|658[0n]|BOLD:ACE6651
Spragueia onagrus|[6567]|PHFLO089-10|United States|Florida|658[0n]|BOLD:ACE6651
Spragueia onagrus|[6568]|PHFLO090-10|United States|Florida|658[0n]|BOLD:ACE6651
Spragueia onagrus|[6569]|LOFLC250-06|United States|Florida|658[0n]|BOLD:ACE6651
Spragueia onagrus|[6570]|LOFLD011-07|United States|Florida|658[0n]|BOLD:ACE6651
Spragueia onagrus|[6571]|PHFLO092-10|United States|Florida|658[0n]|BOLD:ACE6651
Spragueia onagrus|[6572]|PHFLO094-10|United States|Florida|658[0n]|BOLD:ACE6651
Spragueia jaguaralis|[6573]|RDNMH688-09|United States|Arizona|658[0n]|BOLD:ABY5481
Spragueia jaguaralis|[6574]|HKONB201-09|United States|Texas|658[0n]|BOLD:ABY5481
Spragueia jaguaralis|[6575]|BBLSY267-09|United States|Texas|658[0n]|BOLD:ABY5481
Spragueia jaguaralis|[6576]|RDNMH690-09|United States|Arizona|658[0n]|BOLD:ABY5481
Spragueia jaguaralis|[6577]|RDNMH689-09|United States|Arizona|658[0n]|BOLD:ABY5481
Spragueia jaguaralis|[6578]|BBUSA767-09|United States|Texas|658[0n]|BOLD:ABY5481
Spragueia jaguaralis|[6579]|MNAD161-07|United States|Arizona|658[0n]|BOLD:ABY5481
Spragueia jaguaralis|[6580]|BBUSA246-09|United States|Texas|658[1n]|BOLD:ABY5481
Spragueia jaguaralis|[6581]|RDNMH735-09|United States|Arizona|629[0n]|BOLD:ABY5481
Spragueia jaguaralis|[6582]|BBLSX685-09|United States|Texas|658[0n]|BOLD:ABY5481
Spragueia jaguaralis|[6583]|BBLSX777-09|United States|Texas|658[0n]|BOLD:ABY5481
Spragueia jaguaralis|[6584]|BBLSX811-09|United States|Texas|658[0n]|BOLD:ABY5481
Spragueia jaguaralis|[6585]|BBLSX854-09|United States|Texas|658[0n]|BOLD:ABY5481
Spragueia jaguaralis|[6586]|BBLSX857-09|United States|Texas|658[0n]|BOLD:ABY5481
Spragueia jaguaralis|[6587]|BBLSZ152-09|United States|Texas|658[0n]|BOLD:ABY5481
Spragueia jaguaralis|[6588]|RDNMJ570-11|United States|Arizona|658[0n]|BOLD:ABY5481
Spragueia leo|[6589]|LNC822-06|United States|North Carolina|658[0n]|BOLD:AAB8175
Spragueia leo|[6590]|LPOKA514-09|United States|Oklahoma|658[0n]|BOLD:AAB8175
Spragueia leo|[6591]|MNAI271-09|United States|Maryland|658[0n]|BOLD:AAB8175
Spragueia leo|[6592]|LNCNW102-06|United States|North Carolina|658[0n]|BOLD:AAB8175
Spragueia leo|[6593]|LSEU579-06|United States|Georgia|658[0n]|BOLD:AAB8175
Spragueia leo|[6594]|BBLPA741-10|Canada|Ontario|658[0n]|BOLD:AAB8175
Spragueia leo|[6595]|LSEU580-06|United States|Georgia|658[0n]|BOLD:AAB8175
Spragueia leo|[6596]|LNCB275-06|United States|North Carolina|658[0n]|BOLD:AAB8175
Spragueia leo|[6597]|LSUSA230-06|United States|Kentucky|658[0n]|BOLD:AAB8175
Spragueia leo|[6598]|MECD352-06|United States|Maryland|658[0n]|BOLD:AAB8175
Spragueia leo|[6599]|MECD351-06|United States|Maryland|658[0n]|BOLD:AAB8175
Spragueia leo|[6600]|LGSM487-04|United States|North Carolina|658[0n]|BOLD:AAB8175
Spragueia leo|[6601]|LGSM486-04|United States|North Carolina|614[0n]|BOLD:AAB8175
Spragueia leo|[6602]|LGSMG640-07|United States|Tennessee|654[0n]|BOLD:AAB8175
Spragueia leo|[6603]|LPOKA277-08|United States|Oklahoma|658[0n]|BOLD:AAB8175
Spragueia leo|[6604]|LPOKA279-08|United States|Oklahoma|658[0n]|BOLD:AAB8175
Spragueia leo|[6605]|LPOKA836-09|United States|Oklahoma|658[0n]|BOLD:AAB8175
Spragueia leo|[6606]|LPOKA855-09|United States|Oklahoma|658[0n]|BOLD:AAB8175
Spragueia leo|[6607]|LPOKC277-09|United States|Oklahoma|658[0n]|BOLD:AAB8175
Spragueia leo|[6608]|LILLA782-11|United States|Illinois|658[0n]|BOLD:AAB8175
Spragueia leo|[6609]|LILLA874-11|United States|Illinois|658[0n]|BOLD:AAB8175
Spragueia leo|[6610]|LILLA987-11|United States|Illinois|658[0n]|BOLD:AAB8175
Spragueia guttata|[6611]|HKONB194-09|United States|Texas|658[0n]|BOLD:AAB8175
Spragueia guttata|[6612]|HKONB195-09|United States|Texas|658[0n]|BOLD:AAB8175
Spragueia guttata|[6613]|BBLOD1192-11|United States|Texas|658[0n]|BOLD:AAB8175
Spragueia funeralis|[6614]|RDNMH693-09|United States|Arizona|658[0n]|BOLD:AAI0233
Spragueia funeralis|[6615]|RDNMH694-09|United States|Arizona|658[0n]|BOLD:AAI0233
Spragueia funeralis|[6616]|CMAZA493-10|United States|Arizona|658[0n]|BOLD:AAI0233
Spragueia funeralis|[6617]|CMAZA494-10|United States|Arizona|658[0n]|BOLD:AAI0233
Spragueia funeralis|[6618]|CMAZA692-10|United States|Arizona|658[0n]|BOLD:AAI0233
Spragueia funeralis|[6619]|CMAZA695-10|United States|Arizona|658[0n]|BOLD:AAI0233
Spragueia funeralis|[6620]|CMAZA1229-12|United States|Arizona|658[0n]|BOLD:AAI0233
Spragueia magnifica|[6621]|RDNMG279-08|United States|Texas|658[0n]|BOLD:AAJ3536
Spragueia magnifica|[6622]|BBLOC489-11|United States|Arizona|658[0n]|BOLD:AAJ3536
Spragueia magnifica|[6623]|BBLOC1602-11|United States|Arizona|658[0n]|BOLD:AAJ3536
Spragueia magnifica|[6624]|BBLOE1575-12|United States|Arizona|658[0n]|BOLD:AAJ3536
Spragueia magnifica|[6625]|CMAZA1251-12|United States|Arizona|658[0n]|BOLD:AAJ3536
Spragueia apicalis|[6626]|LGSM488-04|United States|Tennessee|610[0n]|BOLD:AAB8767
Spragueia apicalis|[6627]|LGSM678-04|United States|Tennessee|658[0n]|BOLD:AAB8767
Spragueia apicalis|[6628]|LPOKE324-11|United States|Oklahoma|658[0n]|BOLD:AAB8767
Spragueia perstructana|[6629]|LMEMB731-09|Puerto Rico||658[3n]|BOLD:AAB7093
Spragueia perstructana|[6630]|CNCLB1330-14|United States|Arizona|658[0n]|BOLD:AAB7093
Spragueia perstructana|[6631]|CNCLB1332-14|United States|Arizona|658[0n]|BOLD:AAB7093
Spragueia perstructana|[6632]|BLPDU002-11|Costa Rica|Guanacaste|658[0n]|BOLD:AAB7093
Spragueia perstructana|[6633]|BLPDL1605-10|Costa Rica|Guanacaste|658[0n]|BOLD:AAB7093
Spragueia perstructana|[6634]|BLPDU050-11|Costa Rica|Guanacaste|658[0n]|BOLD:AAB7093
Spragueia perstructana|[6635]|BLPDK1070-09|Costa Rica|Guanacaste|622[0n]|BOLD:AAB7093
Spragueia perstructana|[6636]|BLPDK960-09|Costa Rica|Guanacaste|658[0n]|BOLD:AAB7093
Spragueia perstructana|[6637]|BLPDK1026-09|Costa Rica|Guanacaste|658[0n]|BOLD:AAB7093
Spragueia perstructana|[6638]|MHMYS2877-13|Costa Rica|Guanacaste|658[0n]|BOLD:AAB7093
Spragueia perstructana|[6639]|BLPEG1899-14|Costa Rica|Guanacaste|658[0n]|BOLD:AAB7093
Spragueia perstructana|[6640]|BLPCI738-08|Costa Rica|Guanacaste|658[0n]|BOLD:AAB7093
Spragueia perstructana|[6641]|BLPDK271-09|Costa Rica|Guanacaste|658[0n]|BOLD:AAB7093
Spragueia perstructana|[6642]|BLPDK872-09|Costa Rica|Guanacaste|658[0n]|BOLD:AAB7093
Spragueia perstructana|[6643]|BLPDK903-09|Costa Rica|Guanacaste|658[0n]|BOLD:AAB7093
Spragueia perstructana|[6644]|BLPDK970-09|Costa Rica|Guanacaste|658[0n]|BOLD:AAB7093
Spragueia perstructana|[6645]|BLPDK1022-09|Costa Rica|Guanacaste|658[0n]|BOLD:AAB7093
Spragueia perstructana|[6646]|BLPDK1023-09|Costa Rica|Guanacaste|658[0n]|BOLD:AAB7093
Spragueia perstructana|[6647]|BLPDK1024-09|Costa Rica|Guanacaste|658[0n]|BOLD:AAB7093
Spragueia perstructana|[6648]|BLPDK1025-09|Costa Rica|Guanacaste|658[0n]|BOLD:AAB7093
Spragueia perstructana|[6649]|BLPDK1027-09|Costa Rica|Guanacaste|658[0n]|BOLD:AAB7093
Spragueia perstructana|[6650]|BLPDK1028-09|Costa Rica|Guanacaste|658[0n]|BOLD:AAB7093
Spragueia perstructana|[6651]|BLPDK1029-09|Costa Rica|Guanacaste|658[0n]|BOLD:AAB7093
Spragueia perstructana|[6652]|BLPDK1074-09|Costa Rica|Guanacaste|658[0n]|BOLD:AAB7093
Spragueia perstructana|[6653]|BLPDK1076-09|Costa Rica|Guanacaste|658[0n]|BOLD:AAB7093
Spragueia perstructana|[6654]|BLPDU010-11|Costa Rica|Guanacaste|658[0n]|BOLD:AAB7093
Spragueia perstructana|[6655]|BLPDU039-11|Costa Rica|Guanacaste|658[0n]|BOLD:AAB7093
Spragueia perstructana|[6656]|BLPDU047-11|Costa Rica|Guanacaste|658[0n]|BOLD:AAB7093
Spragueia perstructana|[6657]|BLPDU049-11|Costa Rica|Guanacaste|658[0n]|BOLD:AAB7093
Spragueia perstructana|[6658]|BLPDU069-11|Costa Rica|Guanacaste|658[0n]|BOLD:AAB7093
Spragueia perstructana|[6659]|BLPDU075-11|Costa Rica|Guanacaste|658[0n]|BOLD:AAB7093
Spragueia perstructana|[6660]|BLPDU098-11|Costa Rica|Guanacaste|658[0n]|BOLD:AAB7093
Spragueia perstructana|[6661]|BLPDU103-11|Costa Rica|Guanacaste|658[0n]|BOLD:AAB7093
Spragueia perstructana|[6662]|BLPDX1200-11|Costa Rica|Guanacaste|658[0n]|BOLD:AAB7093
Spragueia perstructana|[6663]|BLPED809-11|Costa Rica|Guanacaste|658[0n]|BOLD:AAB7093
Spragueia perstructana|[6664]|BLPEF2840-13|Costa Rica|Guanacaste|658[0n]|BOLD:AAB7093

[illegible]
Spragueia perstructana|[6662]|BLPDX1200-11|Costa Rica|Guanacaste|658[0n]|BOLD:AAB7093
Spragueia perstructana|[6663]|BLPED809-11|Costa Rica|Guanacaste|658[0n]|BOLD:AAB7093
Spragueia perstructana|[6664]|BLPEF2840-13|Costa Rica|Guanacaste|658[0n]|BOLD:AAB7093
Spragueia perstructana|[6665]|BLPEG2228-14|Costa Rica|Guanacaste|658[0n]|BOLD:AAB7093
Spragueia margana|[6666]|BLPDM1772-10|Costa Rica|Guanacaste|658[0n]|BOLD:AAD4819
Spragueia margana|[6667]|BLPDU091-11|Costa Rica|Guanacaste|658[0n]|BOLD:AAD4819
Spragueia margana|[6668]|BLPDU006-11|Costa Rica|Guanacaste|658[1n]|BOLD:AAD4819
Spragueia margana|[6669]|BLPDK1060-09|Costa Rica|Guanacaste|658[0n]|BOLD:AAD4819
Spragueia margana|[6670]|BLPDK1031-09|Costa Rica|Guanacaste|658[0n]|BOLD:AAD4819
Spragueia margana|[6671]|BLPDK1030-09|Costa Rica|Guanacaste|658[0n]|BOLD:AAD4819
Spragueia margana|[6672]|BLPDJ384-09|Costa Rica|Guanacaste|658[0n]|BOLD:AAD4819
Spragueia margana|[6673]|HKONB198-09|United States|Texas|658[0n]|BOLD:AAD4819
Spragueia margana|[6674]|BLPAF720-07|Costa Rica|Guanacaste|658[0n]|BOLD:AAD4819
Spragueia margana|[6675]|MHMXF758-07|Costa Rica|Guanacaste|621[0n]|BOLD:AAD4819
Spragueia margana|[6676]|BLPDK1077-09|Costa Rica|Guanacaste|658[0n]|BOLD:AAD4819
Spragueia margana|[6677]|BLPDM164-10|Costa Rica|Guanacaste|658[0n]|BOLD:AAD4819
Spragueia margana|[6678]|BLPDT1889-10|Costa Rica|Guanacaste|658[0n]|BOLD:AAD4819
Spragueia margana|[6679]|BLPDU061-11|Costa Rica|Guanacaste|658[0n]|BOLD:AAD4819
Spragueia margana|[6680]|BLPDU089-11|Costa Rica|Guanacaste|658[0n]|BOLD:AAD4819
Spragueia margana|[6681]|LOCRI386-11|Costa Rica|Limon|658[0n]|BOLD:AAD4819
Spragueia margana|[6682]|BLPED820-11|Costa Rica|Guanacaste|658[0n]|BOLD:AAD4819
Spragueia margana|[6683]|BLPEF2407-13|Costa Rica|Guanacaste|658[0n]|BOLD:AAD4819
Spragueia margana|[6684]|BLPEF2408-13|Costa Rica|Guanacaste|658[0n]|BOLD:AAD4819
Spragueia margana|[6685]|BLPEF2409-13|Costa Rica|Guanacaste|658[0n]|BOLD:AAD4819
Spragueia margana|[6686]|BLPEF2410-13|Costa Rica|Guanacaste|658[0n]|BOLD:AAD4819
Spragueia margana|[6687]|BLPEF4863-13|Costa Rica|Guanacaste|658[0n]|BOLD:AAD4819
Spragueia margana|[6688]|CNCLB1331-14|United States|Texas|658[0n]|BOLD:AAD4819
Spragueia margana|[6689]|CNCLB1333-14|United States|Texas|658[0n]|BOLD:AAD4819
Spragueia margana|[6690]|BLPEG2016-14|Costa Rica|Guanacaste|658[0n]|BOLD:AAD4819
Spragueia margana|[6691]|BLPEF6335-14|Costa Rica|Guanacaste|658[0n]|BOLD:AAD4819
Spragueia margana|[6692]|BLPEF6336-14|Costa Rica|Guanacaste|658[0n]|BOLD:AAD4819
Spragueia margana|[6693]|BLPEF6348-14|Costa Rica|Guanacaste|658[0n]|BOLD:AAD4819
Tarache augustipennis|[6694]|LPABB114-08|Canada|Alberta|658[0n]|BOLD:AAC9149
Tarache augustipennis|[6695]|LPABB108-08|Canada|Alberta|658[0n]|BOLD:AAC9149
Tarache augustipennis|[6696]|LPABB100-08|Canada|Alberta|658[0n]|BOLD:AAC9149
Tarache augustipennis|[6697]|LPSK417-08|Canada|Saskatchewan|658[0n]|BOLD:AAC9149
Tarache augustipennis|[6698]|LPSK069-08|Canada|Saskatchewan|658[0n]|BOLD:AAC9149
Tarache augustipennis|[6699]|RDMAB127-05|Canada|Alberta|658[0n]|BOLD:AAC9149
Tarache augustipennis|[6700]|RDNMB260-05|Canada|Alberta|658[0n]|BOLD:AAC9149
Tarache augustipennis|[6701]|RDNMB259-05|Canada|Alberta|658[0n]|BOLD:AAC9149
Tarache augustipennis|[6702]|LPABC245-09|Canada|Alberta|621[2n]|BOLD:AAC9149
Tarache augustipennis|[6703]|BBLCU107-09|United States|Kansas|658[0n]|BOLD:AAC9149
Tarache augustipennis|[6704]|BBLCU386-09|United States|Kansas|658[0n]|BOLD:AAC9149
Tarache augustipennis|[6705]|USLEP1276-10|United States|Colorado|658[0n]|BOLD:AAC9149
Tarache sedata|[6706]|HKONB187-08|United States|Texas|658[0n]|BOLD:AAH5367
Tarache sedata|[6707]|RDNMH787-09|United States|Texas|658[0n]|BOLD:AAH5367
Tarache sedata|[6708]|BBLSX859-09|United States|Texas|658[0n]|BOLD:AAH5367
Tarache sedata|[6709]|CMAZA739-10|United States|Arizona|658[0n]|BOLD:AAH5367
Tarache major|[6710]|RDNME089-07|United States|California|658[0n]|BOLD:AAD6261
Tarache major|[6711]|RDNME090-07|United States|California|658[0n]|BOLD:AAD6261
Tarache major|[6712]|LPMN881-08|Canada|Alberta|658[0n]|BOLD:AAD6261
Tarache major|[6713]|LPAB031-08|Canada|Alberta|658[0n]|BOLD:AAD6261
Tarache major|[6714]|LPABB224-08|Canada|Alberta|658[0n]|BOLD:AAD6261
Tarache major|[6715]|LPABB429-08|Canada|Alberta|658[0n]|BOLD:AAD6261
Tarache major|[6716]|JMMMB121-11|United States|California|658[0n]|BOLD:AAD6261
Tarache major|[6717]|JMMMB157-11|United States|California|658[0n]|BOLD:AAD6261
Tarache lanceolata|[6718]|RDNME957-08|United States|New Mexico|658[0n]|BOLD:AAF7461
Tarache lanceolata|[6719]|RDNME958-08|United States|New Mexico|658[0n]|BOLD:AAF7461
Tarache lanceolata|[6720]|RDNME960-08|United States|New Mexico|658[0n]|BOLD:AAF7461
Tarache lanceolata|[6721]|RDNMH892-09|United States|Texas|658[0n]|BOLD:AAF7461
Tarache lanceolata|[6722]|CMAZA732-10|United States|Arizona|658[0n]|BOLD:AAF7461
Tarache lanceolata|[6723]|IAWLB308-11|United States|Arizona|658[0n]|BOLD:AAF7461
Tarache huachuca|[6724]|RDNMG176-08|United States|Arizona|658[0n]|BOLD:AAE3211
Tarache huachuca|[6725]|RDNMG175-08|United States|Arizona|658[0n]|BOLD:AAE3211
Tarache huachuca|[6726]|RDNME935-08|United States|Arizona|658[0n]|BOLD:AAE3211
Tarache huachuca|[6727]|RDNME872-08|United States|Arizona|658[0n]|BOLD:AAE3211
Tarache huachuca|[6728]|RDNME922-08|United States|Arizona|609[0n]|BOLD:AAE3211
Tarache huachuca|[6729]|RDNMG177-08|United States|Arizona|609[0n]|BOLD:AAE3211
Tarache huachuca|[6730]|RDNMJ549-11|United States|Arizona|658[0n]|BOLD:AAE3211
Tarache huachuca|[6731]|IAWLB305-11|United States|Arizona|658[0n]|BOLD:AAE3211
Tarache huachuca|[6732]|IAWLB306-11|United States|Arizona|658[0n]|BOLD:AAE3211
Tarache huachuca|[6733]|IAWLB361-11|United States|Arizona|658[0n]|BOLD:AAE3211
Tarache axendra|[6734]|RDNMH888-09|United States|Arizona|658[0n]|BOLD:AAH5586
Tarache axendra|[6735]|RDNMH890-09|United States|Arizona|658[0n]|BOLD:AAH5586
Tarache axendra|[6736]|RDNMH889-09|United States|Arizona|658[0n]|BOLD:AAH5586
Tarache axendra|[6737]|RDNMH887-09|United States|Arizona|658[0n]|BOLD:AAH5586
Tarache axendra|[6738]|RDNMH700-09|United States|Arizona|621[0n]|BOLD:AAH5586
Tarache axendra|[6739]|RDNMH891-09|United States|Arizona|658[0n]|BOLD:AAH5586
Tarache bilimeki|[6740]|RDNME863-08|United States|Arizona|658[0n]|BOLD:AAF7385
Tarache bilimeki|[6741]|RDNME873-08|United States|Arizona|658[0n]|BOLD:AAF7385
Tarache bilimeki|[6742]|RDNMG178-08|United States|Arizona|595[0n]|BOLD:AAF7385
Tarache bilimeki|[6743]|CMAZA122-09|United States|Arizona|658[0n]|BOLD:AAF7385
Tarache acerba|[6744]|CNCLB466-14|United States|California|550[0n]|BOLD:ACQ0843
Tarache lucasi|[6745]|RDNME062-07|United States|Arizona|658[0n]|BOLD:AAD6257
Tarache lucasi|[6746]|RDNME063-07|United States|Arizona|658[0n]|BOLD:AAD6257
Tarache lucasi|[6747]|RDNMG256-08|United States|Texas|658[0n]|BOLD:AAD6257
Tarache lucasi|[6748]|RDNMG255-08|United States|Texas|658[0n]|BOLD:AAD6257
Tarache lucasi|[6749]|RDNMH785-09|United States|Texas|658[0n]|BOLD:AAD6257
Tarache lucasi|[6750]|BBLOB613-11|United States|Texas|658[0n]|BOLD:AAD6257
Tarache idella|[6751]|RDNMG183-08|United States|Arizona|658[0n]|BOLD:AAK4870
Tarache idella|[6752]|RDNMJ505-11|United States|Arizona|658[0n]|BOLD:AAK4870
Tarache idella|[6753]|CMAZA1091-12|United States|Arizona|658[0n]|BOLD:AAK4870
Pseudalypia crotchii|[6754]|LNAUS5373-13|United States|California|658[0n]|BOLD:ACK3791
Pseudalypia crotchii|[6755]|TML135-14|United States||615[0n]|BOLD:ACK3791
Tarache albifusa|[6756]|RDNMG187-08|United States|Arizona|658[0n]|BOLD:AAC1970
Tarache albifusa|[6757]|RDNMH904-09|United States|Arizona|658[0n]|BOLD:AAC1970
Tarache albifusa|[6758]|RDNMG320-08|United States|Arizona|658[0n]|BOLD:AAC1970
Tarache albifusa|[6759]|RDNMH906-09|United States|Arizona|658[0n]|BOLD:AAC1970
Tarache areli|[6760]|RDNME054-07|United States|California|658[0n]|BOLD:AAF7593
Tarache areli|[6761]|RDNMG323-08|United States|California|658[0n]|BOLD:AAF7593
Tarache areli|[6762]|RDNMG324-08|United States|Colorado|658[1n]|BOLD:AAF7593
Tarache areli|[6763]|IAWLB309-11|United States|Arizona|658[0n]|BOLD:AAF7593

Tarache areli|[6761]|RDNMG323-08|United States|California|658[0n]|BOLD:AAF7593
Tarache areli|[6762]|RDNMG324-08|United States|Colorado|658[1n]|BOLD:AAF7593
Tarache areli|[6763]|IAWLB309-11|United States|Arizona|658[0n]|BOLD:AAF7593
Tarache areli|[6764]|IAWLB310-11|United States|Arizona|658[0n]|BOLD:AAF7593
Tarache toddi|[6765]|RDNME956-08|United States|New Mexico|658[0n]|BOLD:AAC1972
Tarache toddi|[6766]|RDNMG321-08|United States|Nevada|658[0n]|BOLD:AAC1972
Tarache toddi|[6767]|RDNMG322-08|United States|Oregon|658[0n]|BOLD:AAC1972
Tarache toddi|[6768]|RDNMH907-09|United States|Colorado|658[0n]|BOLD:AAC1972
Tarache geminocula|[6769]|TAMIC939-10|United States|Texas|658[0n]|BOLD:AAC1969
Tarache geminocula|[6770]|RDNMG325-08|United States|Arizona|658[1n]|BOLD:AAC1969
Tarache geminocula|[6771]|RDNMG319-08|United States|Arizona|658[0n]|BOLD:AAC1969
Tarache geminocula|[6772]|RDNMG384-08|United States|Arizona|658[0n]|BOLD:AAC1969
Tarache geminocula|[6773]|CMAZA752-10|United States|Arizona|658[0n]|BOLD:AAC1969
Tarache geminocula|[6774]|RDNME053-07|United States|Arizona|658[0n]|BOLD:AAC1969
Tarache geminocula|[6775]|RDNME936-08|United States|Arizona|658[0n]|BOLD:AAC1969
Tarache geminocula|[6776]|IAWLB362-11|United States|Arizona|658[0n]|BOLD:AAC1969
Tarache areloides|[6777]|RDNML216-13|United States|New Mexico|658[0n]|BOLD:ACD9418
Tarache areloides|[6778]|LNAUS3878-13|United States|New Mexico|658[0n]|BOLD:ACD9418
Tarache areloides|[6779]|LNAUS3879-13|United States|New Mexico|658[0n]|BOLD:ACD9418
Tarache areloides|[6780]|LNAUS3880-13|United States|New Mexico|658[0n]|BOLD:ACD9418
Tarache areloides|[6781]|LNAUS3881-13|United States|New Mexico|658[0n]|BOLD:ACD9418
Tarache areloides|[6782]|LNAUS3882-13|United States|New Mexico|658[0n]|BOLD:ACD9418
Tarache cora|[6783]|RDNMG337-08|United States|Arizona|658[0n]|BOLD:AAJ3044
Tarache cora|[6784]|RDNMH695-09|United States|Arizona|658[0n]|BOLD:AAJ3044
Tarache expolita|[6785]|HKONB175-08|United States|Texas|658[0n]|BOLD:AAF7392
Tarache expolita|[6786]|IAWLB312-11|United States|Arizona|658[0n]|BOLD:AAF7392
Tarache expolita|[6787]|RDNME059-07|United States|Arizona|658[0n]|BOLD:AAF7392
Tarache expolita|[6788]|RDNMK011-11|United States|Arizona|658[0n]|BOLD:AAF7392
Tarache expolita|[6789]|BBLOE1814-12|United States|Arizona|658[0n]|BOLD:AAF7392
Tarache expolita|[6790]|IAWLB313-11|United States|Arizona|658[0n]|BOLD:AAF7392
Tarache expolita|[6791]|RDNMJ693-11|United States|Arizona|658[0n]|BOLD:AAF7392
Tarache expolita|[6792]|RDNME058-07|United States|Arizona|658[0n]|BOLD:AAF7392
Tarache expolita|[6793]|CMAZA1139-12|United States|Arizona|658[0n]|BOLD:AAF7392
Tarache bella|[6794]|RDNMG339-08|United States|Arizona|658[1n]|BOLD:AAJ3062
Tarache bella|[6795]|NAMUM424-09|United States|Arizona|658[0n]|BOLD:AAJ3062
Tarache n. sp. 1|[6796]|RDNMH1022-09|United States|Texas|658[0n]|BOLD:ACE5042
Tarache n. sp. 1|[6797]|RDNMK299-11|United States|Texas|658[0n]|BOLD:ACE5042
Tarache arida|[6798]|RDNMG338-08|United States|Arizona|648[0n]|BOLD:AAH5344
Tarache arida|[6799]|BBLSY077-09|United States|Arizona|658[0n]|BOLD:AAH5344
Tarache arida|[6800]|RDNMK002-11|United States|Arizona|658[0n]|BOLD:AAH5344
Tarache arida|[6801]|NOCNA009-14|United States|Texas|658[0n]|BOLD:AAH5344
Tarache n. sp. 2|[6802]|RDNMH1023-09|United States|Texas|658[0n]|BOLD:AAI2756
Tarache terminimaculata|[6803]|HKONS495-08|United States|Florida|658[4n]|BOLD:AAE7399
Tarache terminimaculata|[6804]|LNCB341-06|United States|North Carolina|658[0n]|BOLD:AAE7399
Tarache terminimaculata|[6805]|LNC006-05|United States|North Carolina|658[0n]|BOLD:AAE7399
Tarache terminimaculata|[6806]|LNCB340-06|United States|North Carolina|658[0n]|BOLD:AAE7399
Tarache terminimaculata|[6807]|HKONS496-08|United States|Florida|658[0n]|BOLD:AAE7399
Tarache terminimaculata|[6808]|UDLEP075-09|United States|Delaware|658[0n]|BOLD:AAE7399
Tarache terminimaculata|[6809]|UDLEP125-09|United States|Delaware|658[0n]|BOLD:AAE7399
Tarache terminimaculata|[6810]|UDLEP186-09|United States|Delaware|658[0n]|BOLD:AAE7399
Tarache terminimaculata|[6811]|UDLEP187-09|United States|Delaware|658[0n]|BOLD:AAE7399
Tarache dacia|[6812]|LPYPB898-08|Mexico|Quintana Roo|658[0n]|BOLD:AAC1971
Tarache dacia|[6813]|BLPCI724-08|Costa Rica|Guanacaste|658[0n]|BOLD:AAC1971
Tarache dacia|[6814]|MHMYC2182-09|Costa Rica|Guanacaste|658[0n]|BOLD:AAC1971
Tarache dacia|[6815]|MHMYC2183-09|Costa Rica|Guanacaste|658[0n]|BOLD:AAC1971
Tarache dacia|[6816]|LOCRE513-10|Costa Rica|San Jose|658[0n]|BOLD:AAC1971
Tarache dacia|[6817]|LOCRE514-10|Costa Rica|San Jose|658[0n]|BOLD:AAC1971
Tarache dacia|[6818]|BLPED100-11|Costa Rica|Guanacaste|658[0n]|BOLD:AAC1971
Tarache dacia|[6819]|BLPCJ048-08|Costa Rica|Guanacaste|658[0n]|BOLD:AAC1971
Tarache dacia|[6820]|LYPAP313-09|Mexico|Quintana Roo|612[5n]|BOLD:AAC1971
Tarache dacia|[6821]|LYPIE214-09|Mexico|Quintana Roo|658[0n]|BOLD:AAC1971
Tarache dacia|[6822]|BLPEE144-12|Costa Rica|Guanacaste|658[0n]|BOLD:AAC1971
Tarache dacia|[6823]|BLPEE145-12|Costa Rica|Guanacaste|658[0n]|BOLD:AAC1971
Tarache lactipennis|[6824]|LNAUS3870-13|United States|Texas|658[0n]|BOLD:ACJ8245
Tarache lactipennis|[6825]|LNAUS3869-13|United States|Texas|658[0n]|BOLD:ACJ8245
Tarache lactipennis|[6826]|LNAUS3871-13|United States|Texas|608[0n]|BOLD:ACJ8245
Tarache tetragona|[6827]|HKONB207-09|United States|Texas|658[0n]|BOLD:AAD2497
Tarache tetragona|[6828]|HKONB208-09|United States|Texas|658[0n]|BOLD:AAD2497
Tarache tetragona|[6829]|LPYPC068-08|Mexico|Yucatan|658[0n]|BOLD:AAD2497
Tarache tetragona|[6830]|HKONB206-09|United States|Texas|658[0n]|BOLD:AAD2497
Tarache tetragona|[6831]|HKONB209-09|United States|Texas|658[0n]|BOLD:AAD2497
Tarache tetragona|[6832]|RDNMG233-08|Dominican Republic||658[0n]|BOLD:AAD2497
Tarache tetragona|[6833]|RDNMG232-08|Dominican Republic||649[0n]|BOLD:AAD2497
Tarache tetragona|[6834]|RDNMH908-09|Cuba|Granma|658[0n]|BOLD:AAD2497
Tarache tetragona|[6835]|RDNMH912-09|United States|Texas|658[0n]|BOLD:AAD2497
Tarache quadriplaga|[6836]|CMAZA489-10|United States|Arizona|658[0n]|BOLD:AAD6026
Tarache quadriplaga|[6837]|BBLOD1111-11|United States|Texas|658[0n]|BOLD:AAD6026
Tarache quadriplaga|[6838]|BBLSX824-09|United States|Texas|658[0n]|BOLD:AAD6026
Tarache quadriplaga|[6839]|BBLSZ138-09|United States|Texas|658[0n]|BOLD:AAD6026
Tarache quadriplaga|[6840]|BBLOC1920-11|United States|Texas|658[1n]|BOLD:AAD6026
Tarache quadriplaga|[6841]|HKONB176-08|United States|Texas|658[0n]|BOLD:AAD6026
Tarache quadriplaga|[6842]|BBLSX058-09|United States|Texas|658[0n]|BOLD:AAD6026
Tarache quadriplaga|[6843]|BBLSX200-09|United States|Texas|658[0n]|BOLD:AAD6026
Tarache quadriplaga|[6844]|BBLSX784-09|United States|Texas|658[0n]|BOLD:AAD6026
Tarache quadriplaga|[6845]|BBLSX801-09|United States|Texas|658[0n]|BOLD:AAD6026
Tarache quadriplaga|[6846]|BBLSY938-09|United States|Texas|658[0n]|BOLD:AAD6026
Tarache quadriplaga|[6847]|BBLOD346-11|United States|Texas|658[0n]|BOLD:AAD6026
Tarache quadriplaga|[6848]|BBLOD1007-11|United States|Texas|658[0n]|BOLD:AAD6026
Tarache quadriplaga|[6849]|BBLOD1731-11|United States|Texas|658[0n]|BOLD:AAD6026
Tarache quadriplaga|[6850]|BBLSY937-09|United States|Texas|658[0n]|BOLD:AAD6026
Tarache quadriplaga|[6851]|RDNMH698-09|United States|Arizona|619[0n]|BOLD:AAD6026
Tarache quadriplaga|[6852]|RDNMH697-09|United States|Arizona|658[0n]|BOLD:AAD6026
Tarache quadriplaga|[6853]|RDNMH699-09|United States|Arizona|658[0n]|BOLD:AAD6026
Tarache quadriplaga|[6854]|RDNMH702-09|United States|Arizona|658[0n]|BOLD:AAD6026
Tarache quadriplaga|[6855]|CMAZA378-10|United States|Arizona|658[0n]|BOLD:AAD6026
Tarache quadriplaga|[6856]|RDNME060-07|United States|Arizona|658[0n]|BOLD:AAD6026
Tarache quadriplaga|[6857]|RDNME061-07|United States|Arizona|658[0n]|BOLD:AAD6026
Tarache quadriplaga|[6858]|IAWLB311-11|United States|Arizona|658[0n]|BOLD:AAD6026
Tarache quadriplaga|[6859]|BBLSY254-09|United States|Texas|630[0n]|BOLD:AAD6026
Tarache quadriplaga|[6860]|BBLOB1898-11|United States|Texas|658[0n]|BOLD:AAD6026
Tarache quadriplaga|[6861]|BBLOB620-11|United States|Texas|658[0n]|BOLD:AAD6026
Tarache quadriplaga|[6862]|BBLSZ140-09|United States|Texas|658[0n]|BOLD:AAD6026
Tarache quadriplaga|[6863]|BBLSY939-09|United States|Texas|658[0n]|BOLD:AAD6026

[illegible]
Tarache quadriplaga|[6861]|BBLOB620-11|United States|Texas|658[0n]|BOLD:AAD6026
Tarache quadriplaga|[6862]|BBLSZ140-09|United States|Texas|658[0n]|BOLD:AAD6026
Tarache quadriplaga|[6863]|BBLSY939-09|United States|Texas|658[0n]|BOLD:AAD6026
Tarache quadriplaga|[6864]|BBLSY936-09|United States|Texas|658[0n]|BOLD:AAD6026
Tarache quadriplaga|[6865]|BBLSX835-09|United States|Texas|658[0n]|BOLD:AAD6026
Tarache quadriplaga|[6866]|BBLSX826-09|United States|Texas|658[0n]|BOLD:AAD6026
Tarache quadriplaga|[6867]|BBLSX803-09|United States|Texas|658[0n]|BOLD:AAD6026
Tarache quadriplaga|[6868]|BBLSY160-09|United States|Texas|658[0n]|BOLD:AAD6026
Tarache quadriplaga|[6869]|BBLSW870-09|United States|Texas|658[0n]|BOLD:AAD6026
Tarache quadriplaga|[6870]|BBLSY256-09|United States|Texas|658[0n]|BOLD:AAD6026
Tarache quadriplaga|[6871]|BBLSW871-09|United States|Texas|642[0n]|BOLD:AAD6026
Tarache quadriplaga|[6872]|BBLOC1479-11|United States|Texas|658[0n]|BOLD:AAD6026
Tarache quadriplaga|[6873]|BBLOD104-11|United States|Texas|658[0n]|BOLD:AAD6026
Tarache quadriplaga|[6874]|BBLOD105-11|United States|Texas|658[0n]|BOLD:AAD6026
Tarache quadriplaga|[6875]|BBLOD360-11|United States|Texas|658[0n]|BOLD:AAD6026
Tarache quadriplaga|[6876]|BBLOD1120-11|United States|Texas|658[0n]|BOLD:AAD6026
Tarache quadriplaga|[6877]|BBLOE1171-12|United States|Texas|658[0n]|BOLD:AAD6026
Tarache quadriplaga|[6878]|BBLOE1290-12|United States|Texas|658[0n]|BOLD:AAD6026
Tarache quadriplaga|[6879]|BBLOE1666-12|United States|Texas|658[0n]|BOLD:AAD6026
Tarache delecta|[6880]|LPOKB686-09|United States|Oklahoma|658[0n]|BOLD:ACF5455
Tarache delecta|[6881]|LNAUS3883-13|United States|Maryland|658[0n]|BOLD:ACF5455
Tarache delecta|[6882]|LNAUS3885-13|United States|Maryland|658[0n]|BOLD:ACF5455
Tarache delecta|[6883]|LNAUS3884-13|United States|Maryland|658[0n]|BOLD:ACF5455
Tarache delecta|[6884]|LNAUS3886-13|United States|Maryland|658[0n]|BOLD:ACF5455
Tarache delecta|[6885]|LNAUS3887-13|United States|Maryland|658[0n]|BOLD:ACF5455
Tarache abdominalis|[6886]|LPOKA925-09|United States|Oklahoma|658[0n]|BOLD:AAD6237
Tarache flavipennis|[6887]|LPOKC352-09|United States|Oklahoma|658[0n]|BOLD:AAD6237
Tarache abdominalis|[6888]|LNAUS3562-13|United States|Texas|658[0n]|BOLD:AAD6237
Tarache abdominalis|[6889]|LNAUS3563-13|United States|Texas|658[0n]|BOLD:AAD6237
Tarache abdominalis|[6890]|LNAUS3565-13|United States|Texas|658[0n]|BOLD:AAD6237
Tarache abdominalis|[6891]|LPOKA231-08|United States|Oklahoma|658[0n]|BOLD:AAD6237
Tarache abdominalis|[6892]|LPOKB471-09|United States|Oklahoma|658[0n]|BOLD:AAD6237
Tarache abdominalis|[6893]|LNAUS3567-13|United States|Texas|658[0n]|BOLD:AAD6237
Tarache flavipennis|[6894]|RDNMH706-09|United States|California|658[0n]|BOLD:ACE2865
Tarache flavipennis|[6895]|RDNMH701-09|United States|California|658[0n]|BOLD:ACE2865
Tarache knowltoni|[6896]|LNAUS3568-13|United States|Washington|658[0n]|BOLD:ACE2865
Tarache knowltoni|[6897]|LNAUS3569-13|United States|Washington|658[0n]|BOLD:ACE2865
Tarache knowltoni|[6898]|LNAUS3570-13|United States|Washington|658[0n]|BOLD:ACE2865
Tarache assimilis|[6899]|CNCLB468-14|United States|California|658[0n]|BOLD:ACE2865
Tarache assimilis|[6900]|CNCLB471-14|United States|California|658[0n]|BOLD:ACE2865
Tarache assimilis|[6901]|CNCLB472-14|United States|California|550[2n]|BOLD:ACE2865
Tarache lagunae|[6902]|LNAUS3864-13|United States|California|658[0n]|BOLD:ACE2865
Tarache lagunae|[6903]|CNCLB1559-14|United States|California|648[0n]|BOLD:ACE2865
Tarache apela|[6904]|BBLOB1939-11|United States|Arizona|658[0n]|BOLD:AAC1973
Tarache apela|[6905]|RDNMH905-09|United States|Arizona|658[0n]|BOLD:AAC1973
Tarache apela|[6906]|BBLOB1970-11|United States|Arizona|658[0n]|BOLD:AAC1973
Tarache apela|[6907]|BBLOC697-11|United States|Arizona|658[0n]|BOLD:AAC1973
Tarache apela|[6908]|RDNMH734-09|United States|Arizona|658[0n]|BOLD:AAC1973
Tarache apela|[6909]|RDNMH703-09|United States|Arizona|658[0n]|BOLD:AAC1973
Tarache apela|[6910]|RDNMH696-09|United States|Arizona|658[0n]|BOLD:AAC1973
Tarache apela|[6911]|BBLOC791-11|United States|Arizona|627[0n]|BOLD:AAC1973
Tarache apela|[6912]|RDNMH678-09|United States|Arizona|658[0n]|BOLD:AAC1973
Tarache apela|[6913]|BBLOE1820-12|United States|Arizona|658[0n]|BOLD:AAC1973
Tarache sutor|[6914]|RDNME934-08|United States|Arizona|658[0n]|BOLD:AAJ5622
Tarache tenuicula|[6915]|BBLOD003-11|United States|Texas|658[0n]|BOLD:AAJ5622
Tarache tenuicula|[6916]|CNCLB2122-14|United States|Texas|540[0n]|BOLD:AAJ5622
Tarache tenuicula|[6917]|CNCLB2123-14|United States|Texas|362[0n]|
Tarache aprica|[6918]|USLEP843-10|United States|Texas|658[0n]|BOLD:AAB8647
Tarache aprica|[6919]|HKONB199-09|United States|Texas|658[0n]|BOLD:AAB8647
Tarache aprica|[6920]|LPOKC744-09|United States|Oklahoma|658[0n]|BOLD:AAB8647
Tarache aprica|[6921]|USLEP844-10|United States|Texas|658[0n]|BOLD:AAB8647
Tarache aprica|[6922]|USLEP842-10|United States|Texas|658[0n]|BOLD:AAB8647
Tarache aprica|[6923]|USLEP841-10|United States|Texas|658[0n]|BOLD:AAB8647
Tarache aprica|[6924]|USLEP840-10|United States|Texas|658[0n]|BOLD:AAB8647
Tarache aprica|[6925]|USLEP839-10|United States|Texas|658[0n]|BOLD:AAB8647
Tarache aprica|[6926]|LPOKD305-09|United States|Oklahoma|658[0n]|BOLD:AAB8647
Tarache aprica|[6927]|BBLSX822-09|United States|Texas|658[0n]|BOLD:AAB8647
Tarache aprica|[6928]|BBLSX802-09|United States|Texas|658[0n]|BOLD:AAB8647
Tarache aprica|[6929]|BBLSX779-09|United States|Texas|658[0n]|BOLD:AAB8647
Tarache aprica|[6930]|BBLSX765-09|United States|Texas|658[0n]|BOLD:AAB8647
Tarache aprica|[6931]|BBLSX702-09|United States|Texas|658[0n]|BOLD:AAB8647
Tarache aprica|[6932]|BBLSW934-09|United States|Texas|658[0n]|BOLD:AAB8647
Tarache aprica|[6933]|BBLSW860-09|United States|Texas|658[0n]|BOLD:AAB8647
Tarache aprica|[6934]|RDNMH786-09|United States|Texas|658[0n]|BOLD:AAB8647
Tarache aprica|[6935]|LPOKB731-09|United States|Oklahoma|658[0n]|BOLD:AAB8647
Tarache aprica|[6936]|HKONB200-09|United States|Texas|658[0n]|BOLD:AAB8647
Tarache aprica|[6937]|LTOLB139-08|United States|Georgia|658[0n]|BOLD:AAB8647
Tarache aprica|[6938]|LSUSA164-06|United States|Kentucky|658[0n]|BOLD:AAB8647
Tarache aprica|[6939]|LOFLC215-06|United States|Florida|658[0n]|BOLD:AAB8647
Tarache aprica|[6940]|LOFLB902-06|United States|Florida|658[0n]|BOLD:AAB8647
Tarache aprica|[6941]|LOFLB846-06|United States|Florida|658[0n]|BOLD:AAB8647
Tarache aprica|[6942]|LOFLB838-06|United States|Florida|658[0n]|BOLD:AAB8647
Tarache aprica|[6943]|LOFLB454-06|United States|Florida|658[0n]|BOLD:AAB8647
Tarache aprica|[6944]|LSUSA084-06|United States|Kentucky|658[0n]|BOLD:AAB8647
Tarache aprica|[6945]|LOFLB886-06|United States|Florida|658[0n]|BOLD:AAB8647
Tarache aprica|[6946]|LPOKA744-09|United States|Oklahoma|614[0n]|BOLD:AAB8647
Tarache aprica|[6947]|MNAC699-07|United States|Maryland|645[1n]|BOLD:AAB8647
Tarache aprica|[6948]|LSUSA076-06|United States|Kentucky|594[0n]|BOLD:AAB8647
Tarache aprica|[6949]|USLEP845-10|United States|Texas|646[0n]|BOLD:AAB8647
Tarache aprica|[6950]|LILLA984-11|United States|Illinois|658[0n]|BOLD:AAB8647
Tarache aprica|[6951]|LILLB018-11|United States|Illinois|658[0n]|BOLD:AAB8647
Tarache aprica|[6952]|BBLOD1308-11|United States|Texas|658[0n]|BOLD:AAB8647
Tarache aprica|[6953]|CNCLB2403-14|United States|Texas|658[0n]|BOLD:AAB8647
Azenia procida|[6954]|RDNMK513-11|United States|Arizona|658[0n]|BOLD:AAK4041
Azenia procida|[6955]|RDNMK515-11|United States|Arizona|627[0n]|BOLD:AAK4041
Azenia procida|[6956]|RDNMK514-11|United States|Arizona|658[0n]|BOLD:AAK4041
Azenia procida|[6957]|RDNMK516-11|United States|Arizona|628[0n]|BOLD:AAK4041
Azenia procida|[6958]|RDNMK512-11|United States|Arizona|658[0n]|BOLD:AAK4041
Azenia procida|[6959]|RDNMD376-06|United States|Arizona|658[0n]|BOLD:AAK4041
Azenia procida|[6960]|RDNMK561-11|United States|Arizona|658[0n]|BOLD:AAK4041
Azenia edentata|[6961]|RDNMK170-11|United States|Arizona|658[0n]|BOLD:AAF3947
Azenia edentata|[6962]|QUNOD120-10|United States|Texas|658[0n]|BOLD:AAF3947

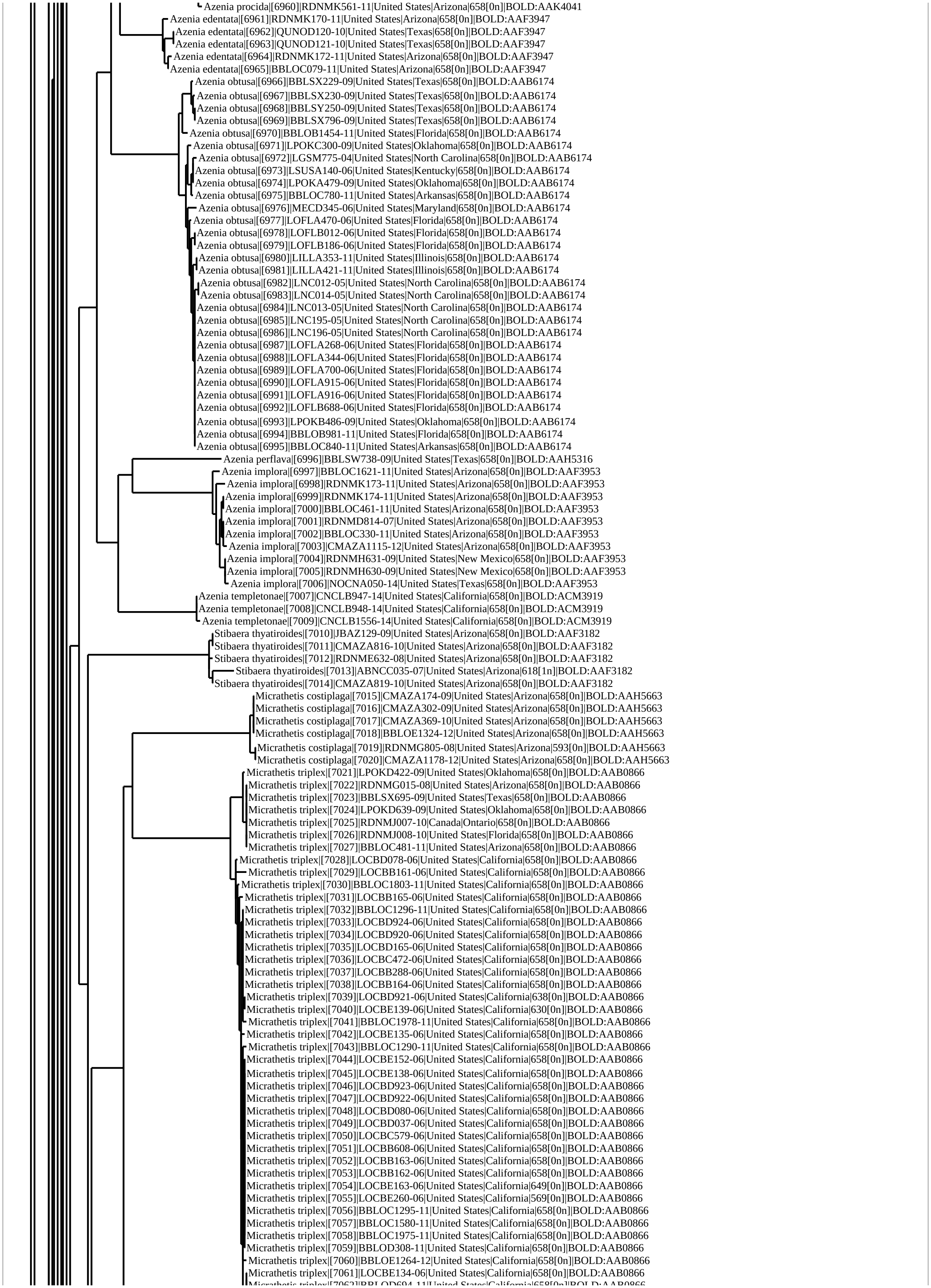

Azenia procida|[6960]|RDNMK561-11|United States|Arizona|658[0n]|BOLD:AAK4041
Azenia edentata|[6961]|RDNMK170-11|United States|Arizona|658[0n]|BOLD:AAF3947
Azenia edentata|[6962]|QUNOD120-10|United States|Texas|658[0n]|BOLD:AAF3947
Azenia edentata|[6963]|QUNOD121-10|United States|Texas|658[0n]|BOLD:AAF3947
Azenia edentata|[6964]|RDNMK172-11|United States|Arizona|658[0n]|BOLD:AAF3947
Azenia edentata|[6965]|BBLOC079-11|United States|Arizona|658[0n]|BOLD:AAF3947
Azenia obtusa|[6966]|BBLSX229-09|United States|Texas|658[0n]|BOLD:AAB6174
Azenia obtusa|[6967]|BBLSX230-09|United States|Texas|658[0n]|BOLD:AAB6174
Azenia obtusa|[6968]|BBLSY250-09|United States|Texas|658[0n]|BOLD:AAB6174
Azenia obtusa|[6969]|BBLSX796-09|United States|Texas|658[0n]|BOLD:AAB6174
Azenia obtusa|[6970]|BBLOB1454-11|United States|Florida|658[0n]|BOLD:AAB6174
Azenia obtusa|[6971]|LPOKC300-09|United States|Oklahoma|658[0n]|BOLD:AAB6174
Azenia obtusa|[6972]|LGSM775-04|United States|North Carolina|658[0n]|BOLD:AAB6174
Azenia obtusa|[6973]|LSUSA140-06|United States|Kentucky|658[0n]|BOLD:AAB6174
Azenia obtusa|[6974]|LPOKA479-09|United States|Oklahoma|658[0n]|BOLD:AAB6174
Azenia obtusa|[6975]|BBLOC780-11|United States|Arkansas|658[0n]|BOLD:AAB6174
Azenia obtusa|[6976]|MECD345-06|United States|Maryland|658[0n]|BOLD:AAB6174
Azenia obtusa|[6977]|LOFLA470-06|United States|Florida|658[0n]|BOLD:AAB6174
Azenia obtusa|[6978]|LOFLB012-06|United States|Florida|658[0n]|BOLD:AAB6174
Azenia obtusa|[6979]|LOFLB186-06|United States|Florida|658[0n]|BOLD:AAB6174
Azenia obtusa|[6980]|LILLA353-11|United States|Illinois|658[0n]|BOLD:AAB6174
Azenia obtusa|[6981]|LILLA421-11|United States|Illinois|658[0n]|BOLD:AAB6174
Azenia obtusa|[6982]|LNC012-05|United States|North Carolina|658[0n]|BOLD:AAB6174
Azenia obtusa|[6983]|LNC014-05|United States|North Carolina|658[0n]|BOLD:AAB6174
Azenia obtusa|[6984]|LNC013-05|United States|North Carolina|658[0n]|BOLD:AAB6174
Azenia obtusa|[6985]|LNC195-05|United States|North Carolina|658[0n]|BOLD:AAB6174
Azenia obtusa|[6986]|LNC196-05|United States|North Carolina|658[0n]|BOLD:AAB6174
Azenia obtusa|[6987]|LOFLA268-06|United States|Florida|658[0n]|BOLD:AAB6174
Azenia obtusa|[6988]|LOFLA344-06|United States|Florida|658[0n]|BOLD:AAB6174
Azenia obtusa|[6989]|LOFLA700-06|United States|Florida|658[0n]|BOLD:AAB6174
Azenia obtusa|[6990]|LOFLA915-06|United States|Florida|658[0n]|BOLD:AAB6174
Azenia obtusa|[6991]|LOFLA916-06|United States|Florida|658[0n]|BOLD:AAB6174
Azenia obtusa|[6992]|LOFLB688-06|United States|Florida|658[0n]|BOLD:AAB6174
Azenia obtusa|[6993]|LPOKB486-09|United States|Oklahoma|658[0n]|BOLD:AAB6174
Azenia obtusa|[6994]|BBLOB981-11|United States|Florida|658[0n]|BOLD:AAB6174
Azenia obtusa|[6995]|BBLOC840-11|United States|Arkansas|658[0n]|BOLD:AAB6174
Azenia perflava|[6996]|BBLSW738-09|United States|Texas|658[0n]|BOLD:AAH5316
Azenia implora|[6997]|BBLOC1621-11|United States|Arizona|658[0n]|BOLD:AAF3953
Azenia implora|[6998]|RDNMK173-11|United States|Arizona|658[0n]|BOLD:AAF3953
Azenia implora|[6999]|RDNMK174-11|United States|Arizona|658[0n]|BOLD:AAF3953
Azenia implora|[7000]|BBLOC461-11|United States|Arizona|658[0n]|BOLD:AAF3953
Azenia implora|[7001]|RDNMD814-07|United States|Arizona|658[0n]|BOLD:AAF3953
Azenia implora|[7002]|BBLOC330-11|United States|Arizona|658[0n]|BOLD:AAF3953
Azenia implora|[7003]|CMAZA1115-12|United States|Arizona|658[0n]|BOLD:AAF3953
Azenia implora|[7004]|RDNMH631-09|United States|New Mexico|658[0n]|BOLD:AAF3953
Azenia implora|[7005]|RDNMH630-09|United States|New Mexico|658[0n]|BOLD:AAF3953
Azenia implora|[7006]|NOCNA050-14|United States|Texas|658[0n]|BOLD:AAF3953
Azenia templetonae|[7007]|CNCLB947-14|United States|California|658[0n]|BOLD:ACM3919
Azenia templetonae|[7008]|CNCLB948-14|United States|California|658[0n]|BOLD:ACM3919
Azenia templetonae|[7009]|CNCLB1556-14|United States|California|658[0n]|BOLD:ACM3919
Stibaera thyatiroides|[7010]|JBAZ129-09|United States|Arizona|658[0n]|BOLD:AAF3182
Stibaera thyatiroides|[7011]|CMAZA816-10|United States|Arizona|658[0n]|BOLD:AAF3182
Stibaera thyatiroides|[7012]|RDNME632-08|United States|Arizona|658[0n]|BOLD:AAF3182
Stibaera thyatiroides|[7013]|ABNCC035-07|United States|Arizona|618[1n]|BOLD:AAF3182
Stibaera thyatiroides|[7014]|CMAZA819-10|United States|Arizona|658[0n]|BOLD:AAF3182
Micrathetis costiplaga|[7015]|CMAZA174-09|United States|Arizona|658[0n]|BOLD:AAH5663
Micrathetis costiplaga|[7016]|CMAZA302-09|United States|Arizona|658[0n]|BOLD:AAH5663
Micrathetis costiplaga|[7017]|CMAZA369-10|United States|Arizona|658[0n]|BOLD:AAH5663
Micrathetis costiplaga|[7018]|BBLOE1324-12|United States|Arizona|658[0n]|BOLD:AAH5663
Micrathetis costiplaga|[7019]|RDNMG805-08|United States|Arizona|593[0n]|BOLD:AAH5663
Micrathetis costiplaga|[7020]|CMAZA1178-12|United States|Arizona|658[0n]|BOLD:AAH5663
Micrathetis triplex|[7021]|LPOKD422-09|United States|Oklahoma|658[0n]|BOLD:AAB0866
Micrathetis triplex|[7022]|RDNMG015-08|United States|Arizona|658[0n]|BOLD:AAB0866
Micrathetis triplex|[7023]|BBLSX695-09|United States|Texas|658[0n]|BOLD:AAB0866
Micrathetis triplex|[7024]|LPOKD639-09|United States|Oklahoma|658[0n]|BOLD:AAB0866
Micrathetis triplex|[7025]|RDNMJ007-10|Canada|Ontario|658[0n]|BOLD:AAB0866
Micrathetis triplex|[7026]|RDNMJ008-10|United States|Florida|658[0n]|BOLD:AAB0866
Micrathetis triplex|[7027]|BBLOC481-11|United States|Arizona|658[0n]|BOLD:AAB0866
Micrathetis triplex|[7028]|LOCBD078-06|United States|California|658[0n]|BOLD:AAB0866
Micrathetis triplex|[7029]|LOCBB161-06|United States|California|658[0n]|BOLD:AAB0866
Micrathetis triplex|[7030]|BBLOC1803-11|United States|California|658[0n]|BOLD:AAB0866
Micrathetis triplex|[7031]|LOCBB165-06|United States|California|658[0n]|BOLD:AAB0866
Micrathetis triplex|[7032]|BBLOC1296-11|United States|California|658[0n]|BOLD:AAB0866
Micrathetis triplex|[7033]|LOCBD924-06|United States|California|658[0n]|BOLD:AAB0866
Micrathetis triplex|[7034]|LOCBD920-06|United States|California|658[0n]|BOLD:AAB0866
Micrathetis triplex|[7035]|LOCBD165-06|United States|California|658[0n]|BOLD:AAB0866
Micrathetis triplex|[7036]|LOCBC472-06|United States|California|658[0n]|BOLD:AAB0866
Micrathetis triplex|[7037]|LOCBB288-06|United States|California|658[0n]|BOLD:AAB0866
Micrathetis triplex|[7038]|LOCBB164-06|United States|California|658[0n]|BOLD:AAB0866
Micrathetis triplex|[7039]|LOCBD921-06|United States|California|638[0n]|BOLD:AAB0866
Micrathetis triplex|[7040]|LOCBE139-06|United States|California|630[0n]|BOLD:AAB0866
Micrathetis triplex|[7041]|BBLOC1978-11|United States|California|658[0n]|BOLD:AAB0866
Micrathetis triplex|[7042]|LOCBE135-06|United States|California|658[0n]|BOLD:AAB0866
Micrathetis triplex|[7043]|BBLOC1290-11|United States|California|658[0n]|BOLD:AAB0866
Micrathetis triplex|[7044]|LOCBE152-06|United States|California|658[0n]|BOLD:AAB0866
Micrathetis triplex|[7045]|LOCBE138-06|United States|California|658[0n]|BOLD:AAB0866
Micrathetis triplex|[7046]|LOCBD923-06|United States|California|658[0n]|BOLD:AAB0866
Micrathetis triplex|[7047]|LOCBD922-06|United States|California|658[0n]|BOLD:AAB0866
Micrathetis triplex|[7048]|LOCBD080-06|United States|California|658[0n]|BOLD:AAB0866
Micrathetis triplex|[7049]|LOCBD037-06|United States|California|658[0n]|BOLD:AAB0866
Micrathetis triplex|[7050]|LOCBC579-06|United States|California|658[0n]|BOLD:AAB0866
Micrathetis triplex|[7051]|LOCBB608-06|United States|California|658[0n]|BOLD:AAB0866
Micrathetis triplex|[7052]|LOCBB163-06|United States|California|658[0n]|BOLD:AAB0866
Micrathetis triplex|[7053]|LOCBB162-06|United States|California|658[0n]|BOLD:AAB0866
Micrathetis triplex|[7054]|LOCBE163-06|United States|California|649[0n]|BOLD:AAB0866
Micrathetis triplex|[7055]|LOCBE260-06|United States|California|569[0n]|BOLD:AAB0866
Micrathetis triplex|[7056]|BBLOC1295-11|United States|California|658[0n]|BOLD:AAB0866
Micrathetis triplex|[7057]|BBLOC1580-11|United States|California|658[0n]|BOLD:AAB0866
Micrathetis triplex|[7058]|BBLOC1975-11|United States|California|658[0n]|BOLD:AAB0866
Micrathetis triplex|[7059]|BBLOD308-11|United States|California|658[0n]|BOLD:AAB0866
Micrathetis triplex|[7060]|BBLOE1264-12|United States|California|658[0n]|BOLD:AAB0866
Micrathetis triplex|[7061]|LOCBE134-06|United States|California|658[0n]|BOLD:AAB0866
Micrathetis triplex|[7062]|BBLOD694-11|United States|California|658[0n]|BOLD:AAB0866

Micrathetis triplex|[illegible]
Micrathetis triplex|[7060]|BBLOE1264-12|United States|California|658[0n]|BOLD:AAB0866
Micrathetis triplex|[7061]|LOCBE134-06|United States|California|658[0n]|BOLD:AAB0866
Micrathetis triplex|[7062]|BBLOD694-11|United States|California|658[0n]|BOLD:AAB0866
Micrathetis triplex|[7063]|LOCBF2623-13|United States|California|658[0n]|BOLD:AAB0866
Fotella sp.|[7064]|RDNMJ852-11|United States|Arizona|658[0n]|BOLD:AAY4015
Fotella notalis|[7065]|USLEP978-10|United States|Arizona|658[0n]|BOLD:AAH5343
Fotella notalis|[7066]|USLEP796-10|United States|Arizona|658[0n]|BOLD:AAH5343
Fotella notalis|[7067]|BBLOD1848-11|United States|Arizona|609[0n]|BOLD:AAH5343
Fotella notalis|[7068]|BBLOC1304-11|United States|California|658[0n]|BOLD:AAH5343
Fotella notalis|[7069]|BBLSY054-09|United States|Arizona|658[0n]|BOLD:AAH5343
Fotella notalis|[7070]|BBLOC1294-11|United States|California|658[0n]|BOLD:AAH5343
Fotella notalis|[7071]|BBLOC1162-11|United States|California|632[0n]|BOLD:AAH5343
Fotella notalis|[7072]|BBLOC1552-11|United States|California|620[0n]|BOLD:AAH5343
Fotella notalis|[7073]|BBLOC1589-11|United States|California|658[0n]|BOLD:AAH5343
Fotella notalis|[7074]|BBLOC1825-11|United States|California|658[0n]|BOLD:AAH5343
Fotella notalis|[7075]|BBLOC1826-11|United States|California|658[0n]|BOLD:AAH5343
Fotella notalis|[7076]|BBLOE1877-12|United States|Arizona|658[0n]|BOLD:AAH5343
Fotella new sp. 1|[7077]|NOCNA019-14|United States|Texas|658[0n]|BOLD:AAH5343
Cropia connecta|[7078]|BLPCO126-08|Costa Rica|Guanacaste|658[0n]|BOLD:AAA5899
Cropia connecta|[7079]|BLPBH527-07|Costa Rica|Guanacaste|658[0n]|BOLD:AAA5899
Cropia connecta|[7080]|BLPAE687-06|Costa Rica|Guanacaste|658[0n]|BOLD:AAA5899
Cropia connecta|[7081]|BLPCD489-08|Costa Rica|Guanacaste|658[0n]|BOLD:AAA5899
Cropia connecta|[7082]|BLPCC450-08|Costa Rica|Guanacaste|658[0n]|BOLD:AAA5899
Cropia connecta|[7083]|MHMXE190-06|Costa Rica|Guanacaste|658[0n]|BOLD:AAA5899
Cropia connecta|[7084]|BLPCA195-08|Costa Rica|Guanacaste|658[0n]|BOLD:AAA5899
Cropia connecta|[7085]|BLPDK1107-09|Costa Rica|Guanacaste|658[0n]|BOLD:AAA5899
Cropia connecta|[7086]|MHAUA821-05|Costa Rica|Guanacaste|658[0n]|BOLD:AAA5899
Cropia connecta|[7087]|BLPEF4968-13|Costa Rica|Guanacaste|658[0n]|BOLD:AAA5899
Cropia connecta|[7088]|BLPEF4969-13|Costa Rica|Guanacaste|658[0n]|BOLD:AAA5899
Cropia connecta|[7089]|BLPAE634-06|Costa Rica|Guanacaste|658[0n]|BOLD:AAA5899
Cropia connecta|[7090]|MHAUA827-05|Costa Rica|Guanacaste|658[0n]|BOLD:AAA5899
Cropia connecta|[7091]|BLPEE8228-14|Costa Rica||658[0n]|BOLD:AAA5899
Cropia connecta|[7092]|BLPEE2474-14|Costa Rica|Guanacaste|658[0n]|BOLD:AAA5899
Cropia connecta|[7093]|MHAUA829-05|Costa Rica|Guanacaste|658[0n]|BOLD:AAA5899
Cropia connecta|[7094]|BLPAG048-07|Costa Rica|Guanacaste|658[0n]|BOLD:AAA5899
Cropia connecta|[7095]|BLPAE635-06|Costa Rica|Guanacaste|658[1n]|BOLD:AAA5899
Cropia connecta|[7096]|BLPCO829-08|Costa Rica|Guanacaste|658[0n]|BOLD:AAA5899
Cropia connecta|[7097]|MHMYS3114-13|Costa Rica|Guanacaste|658[0n]|BOLD:AAA5899
Cropia connecta|[7098]|BLPAE812-06|Costa Rica|Guanacaste|658[0n]|BOLD:AAA5899
Cropia connecta|[7099]|CNCLA5292-13|United States|Florida|599[0n]|BOLD:AAA5899
Cropia connecta|[7100]|MHAUA826-05|Costa Rica|Guanacaste|658[0n]|BOLD:AAA5899
Cropia connecta|[7101]|BLPDU230-11|Costa Rica|Guanacaste|658[0n]|BOLD:AAA5899
Cropia connecta|[7102]|BLPDK1479-09|Costa Rica|Guanacaste|658[0n]|BOLD:AAA5899
Cropia connecta|[7103]|BLPDU834-11|Costa Rica|Guanacaste|658[0n]|BOLD:AAA5899
Cropia connecta|[7104]|MHMXZ918-09|Costa Rica||658[0n]|BOLD:AAA5899
Cropia connecta|[7105]|BLPCL463-08|Costa Rica|Guanacaste|658[0n]|BOLD:AAA5899
Cropia connecta|[7106]|BLPBG513-07|Costa Rica|Guanacaste|658[0n]|BOLD:AAA5899
Cropia connecta|[7107]|MHMXE191-06|Costa Rica|Guanacaste|658[0n]|BOLD:AAA5899
Cropia connecta|[7108]|BLPAE636-06|Costa Rica|Guanacaste|658[0n]|BOLD:AAA5899
Cropia connecta|[7109]|MHAUA825-05|Costa Rica|Guanacaste|658[0n]|BOLD:AAA5899
Cropia connecta|[7110]|MHAUA824-05|Costa Rica|Guanacaste|658[0n]|BOLD:AAA5899
Cropia connecta|[7111]|MHAUA823-05|Costa Rica|Guanacaste|658[0n]|BOLD:AAA5899
Cropia connecta|[7112]|MHAUA822-05|Costa Rica|Guanacaste|658[0n]|BOLD:AAA5899
Cropia connecta|[7113]|BLPCL467-08|Costa Rica|Guanacaste|658[0n]|BOLD:AAA5899
Cropia connecta|[7114]|BLPDJ063-09|Costa Rica|Guanacaste|654[0n]|BOLD:AAA5899
Cropia connecta|[7115]|MHMYH748-10|Costa Rica||658[0n]|BOLD:AAA5899
Cropia connecta|[7116]|BLPEE8234-14|Costa Rica||658[0n]|BOLD:AAA5899
Cropia connecta|[7117]|BLPAE633-06|Costa Rica|Guanacaste|658[0n]|BOLD:AAA5899
Cropia connecta|[7118]|BLPAE638-06|Costa Rica|Guanacaste|658[0n]|BOLD:AAA5899
Cropia connecta|[7119]|BLPBH729-07|Costa Rica|Guanacaste|638[0n]|BOLD:AAA5899
Cropia connecta|[7120]|BLPCC710-08|Costa Rica|Guanacaste|658[0n]|BOLD:AAA5899
Cropia connecta|[7121]|BLPAF755-07|Costa Rica|Guanacaste|658[0n]|BOLD:AAA5899
Cropia connecta|[7122]|MHMXO347-08|Costa Rica|Guanacaste|658[0n]|BOLD:AAA5899
Cropia connecta|[7123]|MHMXO346-08|Costa Rica|Guanacaste|658[0n]|BOLD:AAA5899
Cropia connecta|[7124]|MHMXM246-07|Costa Rica|Guanacaste|647[0n]|BOLD:AAA5899
Cropia connecta|[7125]|BLPCA240-08|Costa Rica|Guanacaste|658[0n]|BOLD:AAA5899
Cropia connecta|[7126]|MHMXO353-08|Costa Rica|Guanacaste|658[0n]|BOLD:AAA5899
Cropia connecta|[7127]|BLPCA552-08|Costa Rica|Guanacaste|658[0n]|BOLD:AAA5899
Cropia connecta|[7128]|BLPDU514-11|Costa Rica|Guanacaste|658[0n]|BOLD:AAA5899
Cropia connecta|[7129]|MHMYS3113-13|Costa Rica|Guanacaste|658[0n]|BOLD:AAA5899
Cropia connecta|[7130]|MHMXZ919-09|Costa Rica||658[0n]|BOLD:AAA5899
Cropia connecta|[7131]|BLPDJ212-09|Costa Rica|Guanacaste|658[0n]|BOLD:AAA5899
Cropia connecta|[7132]|BLPCA241-08|Costa Rica|Guanacaste|658[0n]|BOLD:AAA5899
Cropia connecta|[7133]|BLPCO661-08|Costa Rica|Guanacaste|658[0n]|BOLD:AAA5899
Cropia connecta|[7134]|BLPCA239-08|Costa Rica|Guanacaste|658[0n]|BOLD:AAA5899
Cropia connecta|[7135]|BLPCA209-08|Costa Rica|Guanacaste|658[0n]|BOLD:AAA5899
Cropia connecta|[7136]|BLPBH726-07|Costa Rica|Guanacaste|658[0n]|BOLD:AAA5899
Cropia connecta|[7137]|BLPBH526-07|Costa Rica|Guanacaste|658[0n]|BOLD:AAA5899
Cropia connecta|[7138]|BLPBH524-07|Costa Rica|Guanacaste|658[0n]|BOLD:AAA5899
Cropia connecta|[7139]|BLPBH523-07|Costa Rica|Guanacaste|658[0n]|BOLD:AAA5899
Cropia connecta|[7140]|MHMXI309-07|Costa Rica|Guanacaste|658[0n]|BOLD:AAA5899
Cropia connecta|[7141]|BLPAE637-06|Costa Rica|Guanacaste|658[0n]|BOLD:AAA5899
Cropia connecta|[7142]|MHAUA828-05|Costa Rica|Guanacaste|658[0n]|BOLD:AAA5899
Cropia connecta|[7143]|MHAUG719-07|Costa Rica|Guanacaste|646[0n]|BOLD:AAA5899
Cropia connecta|[7144]|MHAUA820-05|Costa Rica|Guanacaste|658[0n]|BOLD:AAA5899
Cropia connecta|[7145]|MHMXZ755-09|Costa Rica||658[0n]|BOLD:AAA5899
Cropia connecta|[7146]|BLPEE2643-14|Costa Rica|Guanacaste|658[0n]|BOLD:AAA5899
Cropia connecta|[7147]|BLPBH728-07|Costa Rica|Guanacaste|658[0n]|BOLD:AAA5899
Cropia connecta|[7148]|BLPBH525-07|Costa Rica|Guanacaste|658[0n]|BOLD:AAA5899
Cropia connecta|[7149]|MHMXO352-08|Costa Rica|Guanacaste|653[0n]|BOLD:AAA5899
Cropia connecta|[7150]|BLPCD490-08|Costa Rica|Guanacaste|658[0n]|BOLD:AAA5899
Cropia connecta|[7151]|BLPBH730-07|Costa Rica|Guanacaste|657[0n]|BOLD:AAA5899
Cropia connecta|[7152]|BLPDI663-09|Costa Rica|Guanacaste|658[0n]|BOLD:AAA5899
Cropia connecta|[7153]|BLPBH727-07|Costa Rica|Guanacaste|658[0n]|BOLD:AAA5899
Cropia connecta|[7154]|CNCLB2420-14|United States|Texas|658[0n]|BOLD:AAA5899
Cropia connecta|[7155]|CNCLB2421-14|United States|Texas|658[0n]|BOLD:AAA5899
Dolocucullia minor|[7156]|QUNOD119-10|United States|Texas|658[0n]|BOLD:AAL2121
Dolocucullia dentilinea|[7157]|NAMUM045-08|United States|Arizona|657[0n]|BOLD:AAF5239
Dolocucullia dentilinea|[7158]|RDNME996-08|United States|Arizona|658[0n]|BOLD:AAF5239
Dolocucullia dentilinea|[7159]|RDNMJ523-11|United States|Arizona|658[0n]|BOLD:AAF5239
Dolocucullia dentilinea|[7160]|RDNMJ722-11|United States|Arizona|658[0n]|BOLD:AAF5239
Dolocucullia dentilinea|[7161]|RWWC337-11|United States|Washington|658[0n]|BOLD:AAF5240

Dolocucullia dentilinea|[7159]|RDNMJ523-11|United States|Arizona|658[0n]|BOLD:AAF5239
Dolocucullia dentilinea|[7160]|RDNMJ722-11|United States|Arizona|658[0n]|BOLD:AAF5239
Dolocucullia dentilinea|[7161]|RWWC337-11|United States|Washington|658[0n]|BOLD:AAF5240
Dolocucullia dentilinea|[7162]|RWWC314-11|United States|Washington|658[0n]|BOLD:AAF5240
Dolocucullia dentilinea|[7163]|RWWB041-09|United States|Washington|658[0n]|BOLD:AAF5240
Dolocucullia dentilinea|[7164]|RWWC432-11|United States|Washington|651[0n]|BOLD:AAF5240
Dolocucullia dentilinea|[7165]|RWWC460-11|United States|Washington|658[0n]|BOLD:AAF5240
Dolocucullia dentilinea|[7166]|GMLC957-12|United States|California|658[0n]|BOLD:AAF5240
Dolocucullia dentilinea|[7167]|GMLC1430-12|United States|California|621[0n]|BOLD:AAF5240
Emariannia cucullidea|[7168]|NOCNA038-14|United States|Texas|658[0n]|BOLD:ACN5215
Emariannia cucullidea|[7169]|NOCNA037-14|United States|Texas|658[0n]|BOLD:ACN5215
Emariannia cucullidea|[7170]|CNCLB341-14|United States|Texas|540[0n]|BOLD:ACN5215
Noctuidae|[7171]|CNCLB347-14|United States|California|658[0n]|BOLD:ACM6890
Opsigalea blanchardi|[7172]|BBLSW757-09|United States|Texas|658[0n]|BOLD:AAH5317
Cucullia styx|[7173]|RDNML221-13|United States|Arizona|658[0n]|BOLD:ACD9385
Cucullia speyeri|[7174]|RDNMG492-08|Canada|Alberta|658[0n]|BOLD:AAD3289
Cucullia speyeri|[7175]|XAD580-04|Canada|Ontario|601[1n]|BOLD:AAD3289
Cucullia speyeri|[7176]|RDNMD796-07|United States|Wyoming|657[0n]|BOLD:AAD3289
Cucullia speyeri|[7177]|RDLQH159-07|Canada|Quebec|658[0n]|BOLD:AAD3289
Cucullia speyeri|[7178]|RDNMF044-08|Canada|Alberta|658[0n]|BOLD:AAD3289
Cucullia speyeri|[7179]|LPSK535-08|Canada|Saskatchewan|658[0n]|BOLD:AAD3289
Cucullia speyeri|[7180]|RDMAB1015-09|Canada|Alberta|658[0n]|BOLD:AAD3289
Cucullia speyeri|[7181]|CNGRF018-12|Canada|Saskatchewan|629[0n]|BOLD:AAD3289
Cucullia lethe|[7182]|CMAZA870-12|United States|Arizona|658[0n]|BOLD:ABW6353
Cucullia lethe|[7183]|CMAZA925-12|United States|Arizona|658[0n]|BOLD:ABW6353
Cucullia lethe|[7184]|CMAZA1056-12|United States|Arizona|658[0n]|BOLD:ABW6353
Cucullia lethe|[7185]|CNCLB245-14|United States|Texas|658[0n]|BOLD:ABW6353
Cucullia laetifica|[7186]|RDNMG275-08|United States|Texas|658[0n]|BOLD:ACF3177
Cucullia laetifica|[7187]|LPOKE281-11|United States|Oklahoma|658[0n]|BOLD:ACF3177
Cucullia charon|[7188]|RDNME993-08|United States|New Mexico|658[0n]|BOLD:AAH5649
Cucullia charon|[7189]|CMAZA109-09|United States|Arizona|658[0n]|BOLD:AAH5649
Cucullia charon|[7190]|IAWLB642-11|United States|Arizona|658[0n]|BOLD:AAH5649
Cucullia charon|[7191]|CMAZA910-12|United States|Arizona|658[0n]|BOLD:AAH5649
Cucullia charon|[7192]|CMAZA936-12|United States|Arizona|658[0n]|BOLD:AAH5649
Cucullia dorsalis|[7193]|NAMUM277-08|United States|California|658[0n]|BOLD:ABZ3749
Cucullia dorsalis|[7194]|RDNMG491-08|Canada|Alberta|658[0n]|BOLD:ABZ3749
Cucullia dorsalis|[7195]|RDMAB1014-09|United States|Washington|658[0n]|BOLD:ABZ3749
Cucullia dorsalis|[7196]|IAWLB143-10|United States|California|658[0n]|BOLD:ABZ3749
Cucullia dorsalis|[7197]|IAWLB144-10|United States|California|658[0n]|BOLD:ABZ3749
Cucullia eccissica|[7198]|LNAUS3486-13|United States|Arizona|658[0n]|BOLD:ACI5310
Cucullia eccissica|[7199]|LNAUS3487-13|United States|Arizona|407[0n]|BOLD:ACI5310
Cucullia alfarata|[7200]|CNCLB317-14|United States|Arkansas|658[0n]|BOLD:ACM6713
Cucullia alfarata|[7201]|CNCLB315-14|United States|Arkansas|658[0n]|BOLD:ACM6713
Cucullia alfarata|[7202]|CNCLB316-14|United States|Arkansas|658[0n]|BOLD:ACM6713
Cucullia alfarata|[7203]|CNCLB318-14|United States|Tennessee|658[0n]|BOLD:ACM6713
Cucullia alfarata|[7204]|CNCLB319-14|United States|Arkansas|658[0n]|BOLD:ACM6713
Cucullia dammersi|[7205]|JBAZ042-09|United States|California|658[0n]|BOLD:ABX5223
Cucullia dammersi|[7206]|CNCLB335-14|United States|California|658[0n]|BOLD:ABX5223
Cucullia dammersi|[7207]|CNCLB336-14|United States|California|658[0n]|BOLD:ABX5223
Cucullia pulla|[7208]|RDNMF054-08|Canada|British Columbia|658[0n]|BOLD:AAE3239
Cucullia pulla|[7209]|RDNMG490-08|United States|Washington|638[0n]|BOLD:AAE3239
Cucullia pulla|[7210]|RDNMG523-08|United States|Wyoming|658[0n]|BOLD:AAE3239
Cucullia pulla|[7211]|RDNMG524-08|Canada|British Columbia|658[0n]|BOLD:AAE3239
Cucullia sp.|[7212]|RDNME995-08|United States|New Mexico|658[0n]|BOLD:AAE3240
Cucullia dammersi|[7213]|CNCLB337-14|United States|California|658[1n]|BOLD:AAE3240
Cucullia strigata|[7214]|RDNMF144-08|Canada|British Columbia|658[0n]|BOLD:AAE3238
Cucullia strigata|[7215]|RDNMF145-08|Canada|British Columbia|640[0n]|BOLD:AAE3238
Cucullia strigata|[7216]|RDNMF146-08|United States|Wyoming|658[0n]|BOLD:AAE3238
Cucullia strigata|[7217]|RDNMF147-08|Canada|British Columbia|658[0n]|BOLD:AAE3238
Cucullia strigata|[7218]|RDNMF148-08|Canada|British Columbia|658[0n]|BOLD:AAE3238
Cucullia albida|[7219]|RDNMF616-08|Canada|Saskatchewan|652[0n]|BOLD:AAE3238
Cucullia albida|[7220]|RDNMF617-08|Canada|Saskatchewan|658[0n]|BOLD:AAE3238
Cucullia albida|[7221]|RDNMF618-08|Canada|Saskatchewan|658[0n]|BOLD:AAE3238
Cucullia comstocki|[7222]|IAWLB480-11|United States|Arizona|658[0n]|BOLD:ACF1385
Cucullia comstocki|[7223]|JBAZ044-09|United States|California|658[0n]|BOLD:ACF1385
Cucullia comstocki|[7224]|LNAUS3491-13|United States|California|658[0n]|BOLD:ACF1385
Cucullia comstocki|[7225]|LNAUS3492-13|United States|California|658[0n]|BOLD:ACF1385
Cucullia comstocki|[7226]|CNCLB331-14|United States|California|550[0n]|BOLD:ACF1385
Cucullia comstocki|[7227]|LNAUS3493-13|United States|California|658[0n]|BOLD:ACF1385
Cucullia comstocki|[7228]|CNCLB333-14|United States|California|658[0n]|BOLD:ACF1385
Cucullia comstocki|[7229]|CNCLB334-14|United States|California|658[2n]|BOLD:ACF1385
Cucullia comstocki|[7230]|TML156-14|United States||565[0n]|BOLD:ACF1385
Cucullia serraticornis|[7231]|GMLC1294-12|United States|California|615[0n]|BOLD:ACF1383
Cucullia serraticornis|[7232]|GMLC1331-12|United States|California|612[0n]|BOLD:ACF1383
Cucullia serraticornis|[7233]|GMLC1332-12|United States|California|615[0n]|BOLD:ACF1383
Cucullia serraticornis|[7234]|GMLC1329-12|United States|California|607[0n]|BOLD:ACF1383
Cucullia serraticornis|[7235]|LOCBC497-06|United States|California|658[0n]|BOLD:ACF1383
Cucullia serraticornis|[7236]|LOCBC498-06|United States|California|658[0n]|BOLD:ACF1383
Cucullia serraticornis|[7237]|CGLCA067-10|United States|California|658[0n]|BOLD:ACF1383
Cucullia serraticornis|[7238]|GMLC218-11|United States|California|658[0n]|BOLD:ACF1383
Cucullia serraticornis|[7239]|IAWLB481-11|United States|Arizona|658[0n]|BOLD:ACF1383
Cucullia serraticornis|[7240]|IAWLB477-11|United States|Arizona|658[0n]|BOLD:ACF1383
Cucullia serraticornis|[7241]|IAWLB478-11|United States|Arizona|658[0n]|BOLD:ACF1383
Cucullia serraticornis|[7242]|IAWLB479-11|United States|Arizona|658[0n]|BOLD:ACF1383
Cucullia serraticornis|[7243]|CNCLB343-14|United States|Arizona|658[0n]|BOLD:ACF1383
Cucullia serraticornis|[7244]|CNCLB344-14|United States|Arizona|658[0n]|BOLD:ACF1383
Cucullia serraticornis|[7245]|CNCLB346-14|United States|California|550[0n]|BOLD:ACF1383
Cucullia serraticornis|[7246]|TML159-14|United States||513[0n]|
Cucullia umbratica|[7247]|RDNMC457-05|Germany||484[1n]|
Cucullia umbratica|[7248]|LEFIA1077-10|Finland|Finland Proper|670[2n]|BOLD:AAC0849
Cucullia umbratica|[7249]|LEFIC782-10|Finland|Finland Proper|658[0n]|BOLD:AAC0849
Cucullia umbratica|[7250]|LEATD164-13|Austria|Tirol|658[0n]|BOLD:AAC0849
Cucullia umbratica|[7251]|IBLAO993-14|Spain||658[0n]|BOLD:AAC0849
Cucullia umbratica|[7252]|LEFID693-10|Finland|Aland Islands|658[0n]|BOLD:AAC0849
Cucullia umbratica|[7253]|LEFIG210-10|Finland||658[0n]|BOLD:AAC0849
Cucullia umbratica|[7254]|FBLMW121-10|Germany|Bavaria|658[0n]|BOLD:AAC0849
Cucullia umbratica|[7255]|GBLAC1077-13|Germany|Bavaria|658[0n]|BOLD:AAC0849
Cucullia umbratica|[7256]|GWORL407-09|Germany|Bavaria|658[0n]|BOLD:AAC0849
Cucullia umbratica|[7257]|FBLMT863-09|Germany|Bavaria|631[0n]|BOLD:AAC0849
Cucullia umbratica|[7258]|CGUKB270-09|United Kingdom|England|596[1n]|BOLD:AAC0849
Cucullia umbratica|[7259]|GWORK219-09|Germany|Bavaria|658[0n]|BOLD:AAC0849
Cucullia umbratica|[7260]|GWORK217-09|Germany|Bavaria|658[0n]|BOLD:AAC0849
[illegible]

Cucullia umbratica|[7258]|CGUKB270-09|United Kingdom|England|596[1n]|BOLD:AAC0849
Cucullia umbratica|[7259]|GWORK219-09|Germany|Bavaria|658[0n]|BOLD:AAC0849
Cucullia umbratica|[7260]|GWORK217-09|Germany|Bavaria|658[0n]|BOLD:AAC0849
Cucullia umbratica|[7261]|CGUKD207-09|United Kingdom|England|658[0n]|BOLD:AAC0849
Cucullia umbratica|[7262]|CGUKB951-09|United Kingdom|Wales|658[0n]|BOLD:AAC0849
Cucullia umbratica|[7263]|TTNFS058-09|Serbia||658[0n]|BOLD:AAC0849
Cucullia umbratica|[7264]|GWORK428-09|Germany|Bavaria|653[0n]|BOLD:AAC0849
Cucullia umbratica|[7265]|RDNMC456-05|Canada|Quebec|600[0n]|BOLD:AAC0849
Cucullia umbratica|[7266]|GWORL452-09|Germany|Bavaria|638[0n]|BOLD:AAC0849
Cucullia umbratica|[7267]|GWORZ273-10|Italy|Basilicata|658[0n]|BOLD:AAC0849
Cucullia umbratica|[7268]|PHLAB1142-10|Italy|Abruzzi|658[0n]|BOLD:AAC0849
Cucullia umbratica|[7269]|PHLAF440-11|Macedonia||658[0n]|BOLD:AAC0849
Cucullia umbratica|[7270]|GWOSZ131-11|Italy|Trentino-Alto Adige|658[0n]|BOLD:AAC0849
Cucullia umbratica|[7271]|GBLGC312-12|Germany|Bavaria|658[0n]|BOLD:AAC0849
Cucullia umbratica|[7272]|GBLGC314-12|Germany|Bavaria|658[0n]|BOLD:AAC0849
Cucullia umbratica|[7273]|PHLAV301-12|Austria|Vorarlberg|658[0n]|BOLD:AAC0849
Cucullia umbratica|[7274]|LEATB451-13|Austria|Tirol|658[0n]|BOLD:AAC0849
Cucullia umbratica|[7275]|IBLAO994-14|Spain||658[0n]|BOLD:AAC0849
Cucullia umbratica|[7276]|GBLAD443-14|Germany|Bavaria|658[0n]|BOLD:AAC0849
Cucullia heinrichi|[7277]|IAWLB482-11|United States|Arizona|658[0n]|BOLD:AAI5740
Cucullia heinrichi|[7278]|CNCLB328-14|United States|California|658[0n]|BOLD:AAI5740
Cucullia heinrichi|[7279]|CNCLB329-14|United States|California|550[0n]|BOLD:AAI5740
Cucullia heinrichi|[7280]|JBAZ043-09|United States|California|658[0n]|BOLD:AAI5740
Cucullia heinrichi|[7281]|CNCLB327-14|United States|California|550[0n]|BOLD:AAI5740
Cucullia heinrichi|[7282]|CNCLB325-14|United States|California|658[0n]|BOLD:AAI5740
Cucullia heinrichi|[7283]|CNCLB326-14|United States|Nevada|658[0n]|BOLD:AAI5740
Cucullia heinrichi|[7284]|TML158-14|United States||658[0n]|BOLD:AAI5740
Cucullia intermedia|[7285]|LPSK517-08|Canada|Saskatchewan|658[0n]|BOLD:AAB0275
Cucullia intermedia|[7286]|PHMNB435-04|Canada|New Brunswick|658[0n]|BOLD:AAB0275
Cucullia intermedia|[7287]|RDLQ616-07|Canada|Quebec|658[0n]|BOLD:AAB0275
Cucullia intermedia|[7288]|RDLQ615-07|Canada|Quebec|658[0n]|BOLD:AAB0275
Cucullia intermedia|[7289]|RDLQ614-07|Canada|Quebec|658[0n]|BOLD:AAB0275
Cucullia intermedia|[7290]|XAJ291-06|Canada|Ontario|658[0n]|BOLD:AAB0275
Cucullia intermedia|[7291]|TTMNB392-06|Canada|New Brunswick|658[0n]|BOLD:AAB0275
Cucullia intermedia|[7292]|XAH196-05|Canada|Ontario|658[0n]|BOLD:AAB0275
Cucullia intermedia|[7293]|XAH049-05|Canada|Ontario|658[0n]|BOLD:AAB0275
Cucullia intermedia|[7294]|XAD641-05|Canada|Ontario|658[0n]|BOLD:AAB0275
Cucullia intermedia|[7295]|XAD639-05|Canada|Ontario|658[0n]|BOLD:AAB0275
Cucullia intermedia|[7296]|XAC184-04|Canada|Ontario|658[0n]|BOLD:AAB0275
Cucullia intermedia|[7297]|XAC183-04|Canada|Ontario|658[0n]|BOLD:AAB0275
Cucullia intermedia|[7298]|XAB588-04|Canada|Ontario|658[0n]|BOLD:AAB0275
Cucullia intermedia|[7299]|XAB067-04|Canada|Ontario|658[0n]|BOLD:AAB0275
Cucullia intermedia|[7300]|XAB018-04|Canada|Ontario|658[0n]|BOLD:AAB0275
Cucullia intermedia|[7301]|XAK154-06|Canada|Ontario|617[0n]|BOLD:AAB0275
Cucullia intermedia|[7302]|XAC290-04|Canada|Ontario|584[0n]|BOLD:AAB0275
Cucullia intermedia|[7303]|XAH263-05|Canada|Ontario|658[0n]|BOLD:AAB0275
Cucullia intermedia|[7304]|TMG126-03|Canada|Ontario|639[0n]|BOLD:AAB0275
Cucullia intermedia|[7305]|TMG125-03|Canada|Ontario|639[0n]|BOLD:AAB0275
Cucullia intermedia|[7306]|XAJ490-06|Canada|Ontario|653[0n]|BOLD:AAB0275
Cucullia intermedia|[7307]|PMG106-03|Canada|Ontario|617[0n]|BOLD:AAB0275
Cucullia intermedia|[7308]|XAH240-05|Canada|Ontario|617[0n]|BOLD:AAB0275
Cucullia intermedia|[7309]|RDLQ617-07|Canada|Quebec|623[0n]|BOLD:AAB0275
Cucullia intermedia|[7310]|KPOEC171-08|Canada|Ontario|658[0n]|BOLD:AAB0275
Cucullia intermedia|[7311]|LBCG337-08|Canada|British Columbia|658[0n]|BOLD:AAB0275
Cucullia intermedia|[7312]|LPMN817-08|Canada|Manitoba|658[0n]|BOLD:AAB0275
Cucullia intermedia|[7313]|LPSK543-08|Canada|Saskatchewan|658[0n]|BOLD:AAB0275
Cucullia intermedia|[7314]|BLTIB034-08|Canada|Ontario|658[0n]|BOLD:AAB0275
Cucullia intermedia|[7315]|BLTIB954-08|Canada|Ontario|658[0n]|BOLD:AAB0275
Cucullia intermedia|[7316]|BLGSM074-09|Canada|Ontario|658[0n]|BOLD:AAB0275
Cucullia intermedia|[7317]|LBCG2491-09|Canada|British Columbia|658[0n]|BOLD:AAB0275
Cucullia intermedia|[7318]|LALPA891-11|Canada|British Columbia|658[0n]|BOLD:AAB0275
Cucullia luna|[7319]|RDNM899-05|United States|Nevada|658[0n]|BOLD:AAE3253
Cucullia luna|[7320]|RDNM900-05|Canada|Alberta|540[0n]|BOLD:AAE3253
Cucullia luna|[7321]|RDNMG486-08|Canada|Saskatchewan|658[0n]|BOLD:AAE3253
Cucullia luna|[7322]|RDNMG487-08|Canada|Manitoba|658[0n]|BOLD:AAE3253
Cucullia luna|[7323]|RDNMG488-08|United States|Nevada|658[0n]|BOLD:AAE3253
Cucullia eulepis|[7324]|RDNMC691-06|United States|California|658[0n]|BOLD:AAD2766
Cucullia eulepis|[7325]|LOCBB313-06|United States|California|658[0n]|BOLD:AAD2766
Cucullia eulepis|[7326]|LOCBB314-06|United States|California|658[0n]|BOLD:AAD2766
Cucullia eulepis|[7327]|LOCBB315-06|United States|California|658[0n]|BOLD:AAD2766
Cucullia eulepis|[7328]|LOCBB316-06|United States|California|658[0n]|BOLD:AAD2766
Cucullia eulepis|[7329]|LOCBD465-06|United States|California|657[0n]|BOLD:AAD2766
Cucullia eulepis|[7330]|LOCBD722-06|United States|California|663[0n]|BOLD:AAD2766
Cucullia eulepis|[7331]|CGLCA043-10|United States|California|658[0n]|BOLD:AAD2766
Cucullia eulepis|[7332]|LBCH6122-10|Canada|British Columbia|658[0n]|BOLD:AAD2766
Cucullia basipuncta|[7333]|CNCLB321-14|United States|California|658[0n]|BOLD:ACM6834
Cucullia basipuncta|[7334]|CNCLB320-14|United States|California|658[0n]|BOLD:ACM6834
Cucullia basipuncta|[7335]|CNCLB322-14|United States|California|658[0n]|BOLD:ACM6834
Cucullia basipuncta|[7336]|CNCLB324-14|United States|California|658[0n]|BOLD:ACM6834
Cucullia mcdunnoughi|[7337]|RDNMF132-08|Canada|British Columbia|657[1n]|BOLD:AAE3252
Cucullia mcdunnoughi|[7338]|RDNMF133-08|Canada|British Columbia|658[0n]|BOLD:AAE3252
Cucullia mcdunnoughi|[7339]|RDNMF130-08|United States|Washington|658[0n]|BOLD:AAE3252
Cucullia mcdunnoughi|[7340]|RDNMF131-08|United States|California|658[0n]|BOLD:AAE3252
Cucullia mcdunnoughi|[7341]|RDNMF134-08|United States|Washington|658[0n]|BOLD:AAE3252
Cucullia cuculliоides|[7342]|RDNME045-07|United States|California|658[1n]|BOLD:AAI9893
Cucullia cucullioides|[7343]|RDNME992-08|United States|Arizona|658[0n]|BOLD:AAI9893
Cucullia oribac|[7344]|NAMUM048-08|United States|Arizona|657[0n]|BOLD:AAR4840
Cucullia oribac|[7345]|RDNML220-13|United States|New Mexico|658[0n]|BOLD:AAR4840
Cucullia lilacina|[7346]|CMAZA544-10|United States|Arizona|658[0n]|BOLD:ABZ3443
Cucullia lilacina|[7347]|CMAZA785-10|United States|Arizona|658[0n]|BOLD:ABZ3443
Cucullia eucaena|[7348]|RDNMH788-09|United States|Arizona|658[0n]|BOLD:ABY8875
Cucullia convexipennis|[7349]|LGSM451-04|United States|North Carolina|658[0n]|BOLD:AAD2762
Cucullia convexipennis|[7350]|MNBB371-05|Canada|New Brunswick|658[0n]|BOLD:AAD2762
Cucullia convexipennis|[7351]|RDLQ609-07|Canada|Quebec|630[0n]|BOLD:AAD2762
Cucullia convexipennis|[7352]|LGSM450-04|United States|North Carolina|658[0n]|BOLD:AAD2762
Cucullia convexipennis|[7353]|LGSM643-04|United States|North Carolina|658[0n]|BOLD:AAD2762
Cucullia convexipennis|[7354]|RDLQ610-07|Canada|Quebec|658[0n]|BOLD:AAD2762
Cucullia convexipennis|[7355]|LTOL933-08|United States|Texas|655[0n]|BOLD:AAD2762
Cucullia convexipennis|[7356]|CNCLB2807-14|United States|North Carolina|658[0n]|BOLD:AAD2762
Cucullia convexipennis|[7357]|CNCLB2813-14|United States|North Carolina|658[0n]|BOLD:AAD2762
Cucullia montanae|[7358]|RDNMF150-08|United States|Washington|658[1n]|BOLD:ACF2438
Cucullia montanae|[7359]|RDNMF151-08|United States|Washington|641[0n]|BOLD:ACF2438
Cucullia montanae|[7360]|RDNMF152-08|United States|Washington|640[0n]|BOLD:ACF2438

Cucullia montanae|[7358]|RDNMF150-08|United States|Washington|658[1n]|BOLD:ACF2438
Cucullia montanae|[7359]|RDNMF151-08|United States|Washington|641[0n]|BOLD:ACF2438
Cucullia montanae|[7360]|RDNMF152-08|United States|Washington|640[0n]|BOLD:ACF2438
Cucullia montanae|[7361]|RDNMH789-09|United States|Arizona|658[0n]|BOLD:ACF2438
Cucullia montanae|[7362]|RWWA811-09|United States|Washington|658[0n]|BOLD:ACF2438
Cucullia montanae|[7363]|RWWB792-10|United States|Washington|658[0n]|BOLD:ACF2438
Cucullia montanae|[7364]|RWWB937-10|United States|Washington|658[0n]|BOLD:ACF2438
Cucullia montanae|[7365]|RWWC346-11|United States|Washington|658[0n]|BOLD:ACF2438
Cucullia montanae|[7366]|RWWC952-12|United States|Washington|658[0n]|BOLD:ACF2438
Cucullia montanae|[7367]|CNCLA544-13|United States|Arizona|658[0n]|BOLD:ACF2438
Cucullia omissa|[7368]|RDLQB263-05|Canada|Quebec|658[0n]|BOLD:ABZ8006
Cucullia omissa|[7369]|RDNMG483-08|Canada|New Brunswick|658[0n]|BOLD:ABZ8006
Cucullia omissa|[7370]|CNJAC988-12|Canada|Alberta|658[0n]|BOLD:ABZ8006
Cucullia asteroides|[7371]|RBINA707-13|Canada|Ontario|567[0n]|BOLD:AAB9406
Cucullia asteroides|[7372]|RDLQ611-07|Canada|Quebec|658[0n]|BOLD:AAB9406
Cucullia asteroides|[7373]|XAH373-05|Canada|Ontario|658[0n]|BOLD:AAB9406
Cucullia asteroides|[7374]|XAG874-05|Canada|Ontario|658[0n]|BOLD:AAB9406
Cucullia asteroides|[7375]|XAG015-05|Canada|Ontario|658[0n]|BOLD:AAB9406
Cucullia asteroides|[7376]|XAD371-04|Canada|Ontario|658[0n]|BOLD:AAB9406
Cucullia asteroides|[7377]|XAE242-04|Canada|Ontario|658[0n]|BOLD:AAB9406
Cucullia asteroides|[7378]|XAG761-05|Canada|Ontario|658[1n]|BOLD:AAB9406
Cucullia asteroides|[7379]|XAH221-05|Canada|Ontario|621[0n]|BOLD:AAB9406
Cucullia asteroides|[7380]|PHMO304-03|Canada|Ontario|639[1n]|BOLD:AAB9406
Cucullia asteroides|[7381]|XAE594-04|Canada|Ontario|563[0n]|BOLD:AAB9406
Cucullia asteroides|[7382]|XAD294-04|Canada|Ontario|583[0n]|BOLD:AAB9406
Cucullia asteroides|[7383]|XAD178-04|Canada|Ontario|574[0n]|BOLD:AAB9406
Cucullia asteroides|[7384]|XAD395-04|Canada|Ontario|584[0n]|BOLD:AAB9406
Cucullia asteroides|[7385]|XAH002-05|Canada|Ontario|620[0n]|BOLD:AAB9406
Cucullia asteroides|[7386]|RDLQ612-07|Canada|Quebec|641[0n]|BOLD:AAB9406
Cucullia asteroides|[7387]|RDLQ613-07|Canada|Quebec|658[0n]|BOLD:AAB9406
Cucullia asteroides|[7388]|BLGSM012-09|Canada|Ontario|658[0n]|BOLD:AAB9406
Cucullia asteroides|[7389]|LGSMG1026-10|United States|North Carolina|658[0n]|BOLD:AAB9406
Cucullia asteroides|[7390]|LGSMG1027-10|United States|North Carolina|658[0n]|BOLD:AAB9406
Cucullia asteroides|[7391]|LGSMG1028-10|United States|North Carolina|658[0n]|BOLD:AAB9406
Cucullia asteroides|[7392]|RBINA709-13|Canada|Ontario|600[0n]|BOLD:AAB9406
Cucullia asteroides|[7393]|RBINA5364-13|Canada|Ontario|566[0n]|BOLD:AAB9406
Cucullia asteroides|[7394]|RBINA5366-13|Canada|Ontario|581[0n]|BOLD:AAB9406
Cucullia postera|[7395]|RDLQ608-07|Canada|Quebec|616[0n]|BOLD:ABZ8007
Cucullia postera|[7396]|RDNMG480-08|Canada|New Brunswick|658[0n]|BOLD:ABZ8007
Cucullia postera|[7397]|RDNMG481-08|Canada|New Brunswick|658[0n]|BOLD:ABZ8007
Cucullia postera|[7398]|XAG253-05|Canada|Ontario|658[0n]|BOLD:ABZ8007
Cucullia postera|[7399]|XAC046-04|Canada|Ontario|658[0n]|BOLD:ABZ8007
Cucullia postera|[7400]|XAC533-04|Canada|Ontario|658[0n]|BOLD:ABZ8007
Cucullia postera|[7401]|MNBB168-05|Canada|New Brunswick|658[0n]|BOLD:ABZ8007
Cucullia postera|[7402]|RDNMG482-08|Canada|New Brunswick|658[0n]|BOLD:ABZ8007
Cucullia florea|[7403]|PHMNB185-04|Canada|New Brunswick|609[0n]|BOLD:ABZ8007
Cucullia florea|[7404]|LGSMG1025-10|United States|North Carolina|658[0n]|BOLD:ABZ8007
Cucullia florea|[7405]|LOWCC838-05|Canada|British Columbia|658[0n]|BOLD:ABZ8007
Cucullia florea|[7406]|PHMNB769-05|Canada|New Brunswick|658[0n]|BOLD:ABZ8007
Cucullia florea|[7407]|RDNMG484-08|Canada|New Brunswick|658[0n]|BOLD:ABZ8007
Cucullia florea|[7408]|BBLPE495-09|Canada|Newfoundland and Labrador|658[0n]|BOLD:ABZ8007
Cucullia florea|[7409]|SSWLD7213-13|Canada|Alberta|622[0n]|BOLD:ABZ8007
Cucullia similaris|[7410]|RDNMF141-08|United States|Oregon|658[0n]|BOLD:ABZ8007
Cucullia similaris|[7411]|RDNMF140-08|United States|Wyoming|658[0n]|BOLD:ABZ8007
Cucullia similaris|[7412]|RDNMF142-08|Canada|British Columbia|645[0n]|BOLD:ABZ8007
Cucullia similaris|[7413]|RDNMF143-08|Canada|British Columbia|658[0n]|BOLD:ABZ8007
Cucullia sp.|[7414]|RDNMF149-08|Canada|British Columbia|658[0n]|BOLD:ABZ8007
Cucullia florea|[7415]|RDNMG485-08|Canada|New Brunswick|658[0n]|BOLD:ABZ8007
Cucullia florea|[7416]|PHMO168-03|Canada|Ontario|639[0n]|BOLD:ABZ8007
Cucullia florea|[7417]|LOWCD760-06|Canada|British Columbia|657[0n]|BOLD:ABZ8007
Cucullia florea|[7418]|BLGSM023-09|Canada|Ontario|658[0n]|BOLD:ABZ8007
Cucullia postera|[7419]|CNCLB2823-14|United States|North Carolina|658[0n]|BOLD:ABZ8007
Cucullia postera|[7420]|CNCLB2825-14|United States|North Carolina|658[0n]|BOLD:ABZ8007
Cucullia antipoda group|[7421]|RDNMC431-05|United States|California|656[0n]|BOLD:AAB2265
Cucullia antipoda group|[7422]|RDNM906-05|United States|California|658[0n]|BOLD:AAB2265
Cucullia antipoda group|[7423]|RDNMC258-05|United States|Colorado|520[0n]|BOLD:ACF1320
Cucullia antipoda group|[7424]|RDNMC260-05|United States|Utah|561[0n]|BOLD:ACF1320
Cucullia antipoda group|[7425]|RDNMC430-05|United States|Oregon|531[0n]|BOLD:AAB2265
Cucullia antipoda group|[7426]|RDNMC425-05|United States|Washington|539[0n]|BOLD:AAB2265
Cucullia antipoda group|[7427]|RDNMC429-05|United States|Washington|506[0n]|BOLD:AAB2265
Cucullia antipoda group|[7428]|RDNMC435-05|United States|Washington|539[1n]|BOLD:AAB2265
Cucullia antipoda group|[7429]|RDNM904-05|Canada|Alberta|571[0n]|BOLD:AAB2265
Cucullia antipoda group|[7430]|RDNMC261-05|United States|Wyoming|581[0n]|BOLD:AAB2265
Cucullia antipoda group|[7431]|RDNMC437-05|Canada|Alberta|577[1n]|BOLD:AAB2265
Cucullia antipoda group|[7432]|RDNMB351-05|United States|California|658[0n]|BOLD:ABY5327
Cucullia eurekae|[7433]|RDNMB353-05|United States|California|658[0n]|BOLD:ABY5329
Cucullia eurekae|[7434]|RDNM901-05|United States|Oregon|658[0n]|BOLD:ABY5329
Cucullia eurekae|[7435]|RDNM902-05|United States|Oregon|658[0n]|BOLD:ABY5329
Cucullia eurekae|[7436]|RDNMB354-05|United States|California|658[0n]|BOLD:ABY5329
Cucullia antipoda group|[7437]|RDNMC420-05|United States|Wyoming|590[0n]|BOLD:ABY5329
Cucullia astigma|[7438]|RDNMC259-05|United States|Colorado|601[0n]|BOLD:ACE6994
Cucullia astigma|[7439]|RDNMD438-06|United States|California|656[0n]|BOLD:ACE6994
Cucullia astigma|[7440]|RDNMD439-06|United States|California|658[0n]|BOLD:ACE6994
Cucullia astigma|[7441]|RDNMB352-05|United States|California|590[0n]|BOLD:ACE6994
Cucullia astigma|[7442]|RDNMD440-06|United States|California|656[0n]|BOLD:ACE6994
Cucullia astigma|[7443]|RDNME094-07|United States|California|658[0n]|BOLD:ACE6994
Cucullia antipoda group|[7444]|RDNMC427-05|United States|California|569[0n]|BOLD:ABY5329
Cucullia antipoda group|[7445]|RDNMC423-05|United States|Oregon|521[3n]|BOLD:ABY5329
Cucullia antipoda group|[7446]|RDNMB350-05|United States|California|658[0n]|BOLD:ABY5329
Cucullia antipoda group|[7447]|RDNM905-05|United States|California|658[0n]|BOLD:ABY5329
Cucullia antipoda group|[7448]|RDNMC432-05|United States|Oregon|565[0n]|BOLD:ABY5329
Cucullia antipoda group|[7449]|RDNMC428-05|United States|Oregon|563[0n]|BOLD:ABY5329
Cucullia antipoda group|[7450]|RDNMC433-05|United States|Oregon|564[0n]|BOLD:ABY5329
Cucullia antipoda group|[7451]|RDNMC417-05|Canada|British Columbia|557[0n]|BOLD:ABY5329
Cucullia antipoda group|[7452]|RDNMC422-05|Canada|British Columbia|559[0n]|BOLD:ABY5329
Cucullia antipoda group|[7453]|LBCG339-08|Canada|British Columbia|658[0n]|BOLD:ABY5329
Cucullia antipoda group|[7454]|LBCH5547-10|Canada|British Columbia|658[0n]|BOLD:ABY5329
Cucullia antipoda group|[7455]|RDNMC418-05|United States|Washington|513[0n]|BOLD:ABY5329
Cucullia antipoda group|[7456]|RDNMC416-05|United States|Washington|540[3n]|BOLD:ABY5329
Cucullia antipoda group|[7457]|RDNMB356-05|United States|Oregon|563[0n]|BOLD:ABY5329
Cucullia antipoda group|[7458]|RDNMC419-05|United States|Wyoming|591[0n]|BOLD:ABY5329
Cucullia antipoda group|[7459]|RDNMC436-05|United States|Washington|512[0n]|BOLD:ABY5329
[illegible]

Cucullia antipoda group|[7457]|RDNMB356-05|United States|Oregon|[illegible]|BOLD:ABY5329
Cucullia antipoda group|[7458]|RDNMC419-05|United States|Wyoming|591[0n]|BOLD:ABY5329
Cucullia antipoda group|[7459]|RDNMC436-05|United States|Washington|512[0n]|BOLD:ABY5329
Cucullia antipoda group|[7460]|RDNMD441-06|United States|California|658[0n]|BOLD:ABY5329
Cucullia antipoda group|[7461]|RDNM908-05|United States|California|529[2n]|BOLD:ABY5329
Cucullia antipoda group|[7462]|IAWLB145-10|United States|California|574[0n]|BOLD:ABY5329
Cucullia antipoda group|[7463]|JMMMB355-11|United States|California|658[0n]|BOLD:ABY5329
Cucullia antipoda group|[7464]|CGLCA028-10|United States|California|658[0n]|BOLD:ABY5329
Cucullia antipoda group|[7465]|LNAUS3490-13|United States|California|658[0n]|BOLD:ABY5329
Cucullia incresa|[7466]|CGLCA033-10|United States|California|658[0n]|BOLD:AAL6665
Cucullia incresa|[7467]|CNCLB3091-15|United States|California|658[0n]|BOLD:AAL6665
Pleromella opter|[7468]|RDNME091-07|United States|California|658[0n]|BOLD:AAE4862
Pleromella opter|[7469]|RDNMD813-07|United States|California|658[0n]|BOLD:AAE4862
Pleromella opter|[7470]|CGLCA066-10|United States|California|658[0n]|BOLD:AAE4862
Pleromella opter|[7471]|NAMUM157-08|United States|California|658[0n]|BOLD:AAE4862
Pleromella opter|[7472]|NAMUM158-08|United States|California|627[0n]|BOLD:AAE4862
Pleromella opter|[7473]|JMMMB335-11|United States|California|658[0n]|BOLD:AAE4862
Apsaphida eremna|[7474]|RDNME889-08|United States|Arizona|658[0n]|BOLD:AAF5477
Apsaphida eremna|[7475]|RDNME890-08|United States|Arizona|658[0n]|BOLD:AAF5477
Apsaphida eremna|[7476]|NAMUM076-08|United States|Arizona|657[0n]|BOLD:AAF5477
Apsaphida eremna|[7477]|IAWLB625-11|United States|Arizona|658[0n]|BOLD:AAF5477
Paratrachea viridescens|[7478]|RDNMD594-06|United States|Arizona|658[1n]|BOLD:AAX1239
Paratrachea viridescens|[7479]|IAWLB297-11|United States|Arizona|658[0n]|BOLD:AAX1239
Paratrachea viridescens|[7480]|IAWLB518-11|United States|Arizona|658[0n]|BOLD:AAX1239
Paratrachea viridescens|[7481]|IAWLB519-11|United States|Arizona|658[0n]|BOLD:AAX1239
Viridemas galena|[7482]|LNAUS5304-13|United States|Arizona|658[0n]|BOLD:ACK3496
Viridemas galena|[7483]|CNCLB211-14|United States|Arizona|658[0n]|BOLD:ACK3496
Viridemas galena|[7484]|LNAUS5303-13|United States|Arizona|658[0n]|BOLD:ACK3496
Viridemas galena|[7485]|LNAUS5375-13|United States|Arizona|658[0n]|BOLD:ACK3496
Viridemas galena|[7486]|LNAUS5306-13|United States||658[0n]|BOLD:ACK3496
Viridemas galena|[7487]|LNAUS5305-13|United States|Arizona|658[0n]|BOLD:ACK3496
Viridemas galena|[7488]|LNAUS5302-13|United States|Arizona|658[0n]|BOLD:ACK3496
Viridemas galena|[7489]|LNAUS5301-13|United States|Arizona|658[0n]|BOLD:ACK3496
Viridemas galena|[7490]|CNCLB212-14|United States|Arizona|658[0n]|BOLD:ACK3496
Annaphila danistica|[7491]|CNCLB368-14|Canada|British Columbia|658[4n]|BOLD:ACM6780
Annaphila danistica|[7492]|CNCLB367-14|United States|California|658[0n]|BOLD:ACM6780
Annaphila hennei|[7493]|CNCLB2132-14|United States|California|540[0n]|BOLD:ACM6780
Annaphila hennei|[7494]|TML145-14|United States||407[0n]|
Annaphila hennei|[7495]|CNCLB2133-14|United States|California|305[0n]|BOLD:ACM6780
Annaphila mera|[7496]|TML149-14|United States||658[0n]|BOLD:ACO2222
Annaphila mera sp. eremia|[7497]|CNCLB3097-15|United States|California|658[0n]|BOLD:ACO2222
Fota armata|[7498]|BBLSW781-09|United States|New Mexico|638[0n]|BOLD:AAH5319
Fota armata|[7499]|RDNMK887-13|Mexico|Sonora|658[0n]|BOLD:AAH5319
Fota armata|[7500]|CNCLA7384-13|United States|New Mexico|658[0n]|BOLD:AAH5319
Fota armata|[7501]|LNAUS5051-13|United States|Arizona|658[0n]|BOLD:AAH5319
Fota armata|[7502]|LNAUS5053-13|United States|Arizona|658[0n]|BOLD:AAH5319
Fota minorata|[7503]|LNAUS5058-13|United States|Arizona|658[0n]|BOLD:ACK3613
Annaphila depicta|[7504]|CNCLB1059-14|United States|California|572[3n]|BOLD:ACM6882
Annaphila depicta|[7505]|CNCLB1061-14|United States|California|658[0n]|BOLD:ACM6882
Annaphila depicta|[7506]|CNCLB1062-14|United States|California|658[0n]|BOLD:ACM6882
Annaphila depicta|[7507]|CNCLB1060-14|United States|California|618[0n]|BOLD:ACM6882
Annaphila depicta|[7508]|CNCLB1063-14|United States|California|658[0n]|BOLD:ACM6882
Annaphila depicta|[7509]|LNAUT539-14|United States|Oregon|658[0n]|BOLD:ACM6882
Annaphila depicta|[7510]|TML142-14|United States||658[0n]|BOLD:ACM6882
Annaphila evansi|[7511]|CNCLB199-14|United States|California|658[0n]|BOLD:ACM4074
Annaphila evansi|[7512]|CNCLB200-14|United States|California|658[0n]|BOLD:ACM4074
Annaphila superba|[7513]|CNCLB1058-14|United States|California|312[0n]|
Annaphila superba|[7514]|CNCLB1056-14|United States|California|550[0n]|BOLD:ACQ7188
Annaphila superba|[7515]|CNCLB2136-14|United States|California|658[0n]|BOLD:ACQ7188
Annaphila superba|[7516]|CNCLB2135-14|United States|California|658[0n]|BOLD:ACQ7188
Annaphila spila|[7517]|TML144-14|United States|California|407[1n]|
Annaphila spila|[7518]|TML148-14|United States||407[0n]|
Annaphila spila|[7519]|CNCLB1642-14|United States|California|407[0n]|
Annaphila superba|[7520]|CNCLB2137-14|United States|California|337[0n]|BOLD:ACQ7188
Annaphila spila|[7521]|CNCLB3098-15|United States|California|658[0n]|BOLD:ACQ7188
Annaphila lithosina|[7522]|RDNMC360-05|United States|California|603[0n]|BOLD:AAR3428
Annaphila miona|[7523]|CNCLB1051-14|United States|California|550[0n]|BOLD:ACM6703
Annaphila miona|[7524]|CNCLB1050-14|United States|California|658[0n]|BOLD:ACM6703
Annaphila casta|[7525]|CNCLB1053-14|United States|Oregon|658[0n]|BOLD:ACM6703
Annaphila casta|[7526]|LNAUT532-14|United States|Oregon|658[0n]|BOLD:ACM6703
Annaphila casta|[7527]|LNAUT533-14|United States|Oregon|658[0n]|BOLD:ACM6703
Annaphila decia|[7528]|LNAUT543-14|United States|California|550[0n]|BOLD:ACN8829
Annaphila sp. nr. decia|[7529]|RDNMF352-08|Canada|British Columbia|658[0n]|BOLD:AAR3429
Annaphila diva|[7530]|NAMUM254-08|United States|California|658[0n]|BOLD:AAF0525
Annaphila diva|[7531]|LALPA058-10|Canada|British Columbia|658[0n]|BOLD:AAF0525
Annaphila diva|[7532]|LALPA081-10|Canada|British Columbia|658[0n]|BOLD:AAF0525
Annaphila diva|[7533]|RDNMF786-08|Canada|British Columbia|658[0n]|BOLD:AAF0525
Annaphila diva|[7534]|RDNMF787-08|Canada|British Columbia|658[0n]|BOLD:AAF0525
Annaphila diva|[7535]|LALPA082-10|Canada|British Columbia|658[0n]|BOLD:AAF0525
Annaphila scurlockorum|[7536]|CNCLB1808-14|United States|California|658[0n]|BOLD:ACM4165
Annaphila abdita|[7537]|TML139-14|United States||658[0n]|BOLD:ACM4165
Annaphila scurlockorum|[7538]|CNCLB201-14|United States|California|658[0n]|BOLD:ACM4165
Annaphila scurlockorum|[7539]|CNCLB2107-14|United States|California|658[0n]|BOLD:ACM4165
Annaphila arvalis|[7540]|CNCLB2531-14|United States|Washington|658[0n]|BOLD:ACR9063
Annaphila arvalis|[7541]|CNCLB2532-14|United States|California|658[0n]|BOLD:ACR9063
Annaphila ida|[7542]|CNCLB377-14|United States|California|658[5n]|BOLD:ACM6789
Annaphila ida|[7543]|TML146-14|United States||658[0n]|BOLD:ACM6789
Annaphila ida|[7544]|CNCLB2106-14|United States|California|658[0n]|BOLD:ACM6789
Annaphila astrologa|[7545]|LNAUT515-14|United States|California|550[0n]|BOLD:ACQ0420
Annaphila divinula|[7546]|CNCLB1046-14|United States|California|658[0n]|BOLD:ACM6790
Annaphila divinula|[7547]|TML143-14|United States||658[0n]|BOLD:ACM6790
Annaphila vivianae|[7548]|RDNML367-13|United States|California|653[0n]|BOLD:ACD9131
Annaphila vivianae|[7549]|LNAUT512-14|United States|California|658[0n]|BOLD:ACD9131
Annaphila pseudoastrologa|[7550]|CNCLB372-14|United States|California|540[0n]|BOLD:ACD9131
Annaphila pseudoastrologa|[7551]|CNCLB371-14|United States|California|540[0n]|BOLD:ACD9131
Annaphila pseudoastrologa|[7552]|CNCLB373-14|United States|California|540[1n]|BOLD:ACD9131
Annaphila pseudoastrologa|[7553]|LNAUT516-14|United States|California|540[0n]|BOLD:ACD9131
Annaphila olgae|[7554]|CNCLB3176-15|United States|California|658[0n]|BOLD:ACO2223
Cirrhophanus triangulifer|[7555]|LPOKD349-09|United States|Oklahoma|658[0n]|BOLD:AAE9149
Cirrhophanus triangulifer|[7556]|LSEU140-06|United States|Tennessee|528[1n]|BOLD:AAE9149
Cirrhophanus triangulifer|[7557]|NAMUM346-08|United States|Maryland|658[0n]|BOLD:AAE9149
Cirrhophanus triangulifer|[7558]|NAMUM347-08|United States|Maryland|658[0n]|BOLD:AAE9149
Cirrhophanus triangulifer|[7559]|NAMUM348-08|United States|Maryland|658[0n]|BOLD:AAE9149

[illegible]
Cirrhophanus triangulifer|[7557]|NAMUM346-08|United States|Maryland|658[0n]|BOLD:AAE9149
Cirrhophanus triangulifer|[7558]|NAMUM347-08|United States|Maryland|658[0n]|BOLD:AAE9149
Cirrhophanus triangulifer|[7559]|NAMUM348-08|United States|Maryland|658[0n]|BOLD:AAE9149
Cirrhophanus triangulifer|[7560]|LILLB038-11|United States|Illinois|658[0n]|BOLD:AAE9149
Amyna bullula|[7561]|LSEU219-06|United States|Georgia|658[0n]|BOLD:AAA8980
Amyna bullula|[7562]|LSUSA070-06|United States|Kentucky|658[0n]|BOLD:AAA8980
Amyna stricta|[7563]|LPOKE294-11|United States|Oklahoma|658[0n]|BOLD:AAA8978
Amyna stricta|[7564]|RDNMD520-06|United States|Florida|658[0n]|BOLD:AAA8978
Amyna stricta|[7565]|LOFLC228-06|United States|Florida|658[0n]|BOLD:AAA8978
Amyna stricta|[7566]|LOFLB201-06|United States|Florida|658[0n]|BOLD:AAA8978
Amyna stricta|[7567]|LOFLB196-06|United States|Florida|658[0n]|BOLD:AAA8978
Amyna stricta|[7568]|LSEU218-06|United States|Georgia|658[0n]|BOLD:AAA8978
Amyna stricta|[7569]|LPOKA599-09|United States|Oklahoma|637[0n]|BOLD:AAA8978
Amyna stricta|[7570]|LPOKA618-09|United States|Oklahoma|658[0n]|BOLD:AAA8978
Amyna stricta|[7571]|LPOKD247-09|United States|Oklahoma|657[0n]|BOLD:AAA8978
Amyna stricta|[7572]|CMAZA115-09|United States|Arizona|658[0n]|BOLD:AAA8978
Amyna stricta|[7573]|RDNMK089-11|United States|Florida|658[0n]|BOLD:AAA8978
Amyna stricta|[7574]|LPOKE297-11|United States|Oklahoma|658[0n]|BOLD:AAA8978
Amyna stricta|[7575]|CMAZA959-12|United States|Arizona|658[0n]|BOLD:AAA8978
Amyna stricta|[7576]|CMAZA1157-12|United States|Arizona|658[0n]|BOLD:AAA8978
Cerathosia tricolor|[7577]|RDNMK484-11|United States|Texas|601[0n]|BOLD:AAH5350
Cerathosia tricolor|[7578]|BBLSX829-09|United States|Texas|658[0n]|BOLD:AAH5350
Cerathosia tricolor|[7579]|USLEP537-10|United States|Texas|658[0n]|BOLD:AAH5350
Cerathosia tricolor|[7580]|USLEP538-10|United States|Texas|658[0n]|BOLD:AAH5350
Cerathosia tricolor|[7581]|USLEP539-10|United States|Texas|658[0n]|BOLD:AAH5350
Cerathosia tricolor|[7582]|USLEP540-10|United States|Texas|658[0n]|BOLD:AAH5350
Cerathosia tricolor|[7583]|USLEP541-10|United States|Texas|658[0n]|BOLD:AAH5350
Cerathosia tricolor|[7584]|BBLSY216-09|United States|Texas|658[0n]|BOLD:AAH5350
Cerathosia tricolor|[7585]|BBLSY217-09|United States|Texas|658[0n]|BOLD:AAH5350
Cerathosia tricolor|[7586]|BBLOC1415-11|United States|Texas|658[0n]|BOLD:AAH5350
Acronicta funeralis|[7587]|PHMNB187-04|Canada|New Brunswick|524[0n]|BOLD:AAB7001
Acronicta funeralis|[7588]|PHMNB155-04|Canada|New Brunswick|653[1n]|BOLD:AAB7001
Acronicta funeralis|[7589]|PHMNB070-03|Canada|New Brunswick|639[0n]|BOLD:AAB7001
Acronicta funeralis|[7590]|PHMNB002-03|Canada|New Brunswick|639[0n]|BOLD:AAB7001
Acronicta funeralis|[7591]|RDNMJ083-10|United States|Texas|658[0n]|BOLD:AAB7001
Acronicta funeralis|[7592]|RDNMI017-10|United States|California|658[0n]|BOLD:AAB7001
Acronicta funeralis|[7593]|UDLEP252-09|United States|Pennsylvania|658[0n]|BOLD:AAB7001
Acronicta funeralis|[7594]|LPABC804-09|Canada|Alberta|658[0n]|BOLD:AAB7001
Acronicta funeralis|[7595]|LPABB388-08|Canada|Alberta|658[0n]|BOLD:AAB7001
Acronicta funeralis|[7596]|RDLQF512-06|Canada|Quebec|658[0n]|BOLD:AAB7001
Acronicta funeralis|[7597]|RDLQF511-06|Canada|Quebec|658[0n]|BOLD:AAB7001
Acronicta funeralis|[7598]|RDLQF510-06|Canada|Quebec|658[0n]|BOLD:AAB7001
Acronicta funeralis|[7599]|TMNBB156-06|Canada|New Brunswick|658[0n]|BOLD:AAB7001
Acronicta funeralis|[7600]|TMNBB155-06|Canada|New Brunswick|658[0n]|BOLD:AAB7001
Acronicta funeralis|[7601]|RDLQB159-05|Canada|Quebec|658[0n]|BOLD:AAB7001
Acronicta funeralis|[7602]|RDLQB158-05|Canada|Quebec|658[0n]|BOLD:AAB7001
Acronicta funeralis|[7603]|MNBB245-05|Canada|New Brunswick|658[0n]|BOLD:AAB7001
Acronicta funeralis|[7604]|LOTB311-05|United States|Tennessee|658[0n]|BOLD:AAB7001
Acronicta funeralis|[7605]|PHMNB770-05|Canada|New Brunswick|658[0n]|BOLD:AAB7001
Acronicta funeralis|[7606]|PHMNB695-04|Canada|New Brunswick|658[0n]|BOLD:AAB7001
Acronicta funeralis|[7607]|PHMNB578-04|Canada|New Brunswick|658[0n]|BOLD:AAB7001
Acronicta funeralis|[7608]|PHMNB246-04|Canada|New Brunswick|658[0n]|BOLD:AAB7001
Acronicta funeralis|[7609]|RDNMJ621-11|United States|Texas|623[0n]|BOLD:AAB7001
Acronicta funeralis|[7610]|RDNMJ622-11|United States|Texas|622[0n]|BOLD:AAB7001
Acronicta funeralis|[7611]|CNCLB1430-14|United States|North Carolina|658[0n]|BOLD:AAB7001
Acronicta funeralis|[7612]|CNCLB2579-14|United States|North Carolina|601[0n]|BOLD:AAB7001
Acronicta funeralis|[7613]|CNCLB2581-14|United States|North Carolina|618[0n]|BOLD:AAB7001
Narthecophora pulverea|[7614]|BBLOC407-11|United States|Arizona|658[0n]|BOLD:ABA1195
Narthecophora pulverea|[7615]|BBLOC448-11|United States|Arizona|658[0n]|BOLD:ABA1195
Narthecophora pulverea|[7616]|BBLOC415-11|United States|Arizona|658[0n]|BOLD:ABA1195
Narthecophora pulverea|[7617]|BBLOC085-11|United States|Arizona|658[0n]|BOLD:ABA1195
Narthecophora pulverea|[7618]|BBLOC457-11|United States|Arizona|614[0n]|BOLD:ABA1195
Narthecophora pulverea|[7619]|BBLOC349-11|United States|Arizona|614[0n]|BOLD:ABA1195
Narthecophora pulverea|[7620]|BBLOC329-11|United States|Arizona|614[0n]|BOLD:ABA1195
Narthecophora pulverea|[7621]|BBLOC435-11|United States|Arizona|658[3n]|BOLD:ABA1195
Narthecophora pulverea|[7622]|BBLOC158-11|United States|Arizona|658[0n]|BOLD:ABA1195
Narthecophora pulverea|[7623]|BBLOC348-11|United States|Arizona|658[0n]|BOLD:ABA1195
Narthecophora pulverea|[7624]|BBLOC236-11|United States|Arizona|658[0n]|BOLD:ABA1195
Narthecophora pulverea|[7625]|BBLOC473-11|United States|Arizona|621[0n]|BOLD:ABA1195
Narthecophora pulverea|[7626]|BBLOC080-11|United States|Arizona|658[0n]|BOLD:ABA1195
Narthecophora pulverea|[7627]|BBLOC090-11|United States|Arizona|658[0n]|BOLD:ABA1195
Narthecophora pulverea|[7628]|BBLOC469-11|United States|Arizona|658[0n]|BOLD:ABA1195
Narthecophora pulverea|[7629]|BBLOC467-11|United States|Arizona|658[0n]|BOLD:ABA1195
Narthecophora pulverea|[7630]|BBLOC468-11|United States|Arizona|658[0n]|BOLD:ABA1195
Narthecophora pulverea|[7631]|BBLOC470-11|United States|Arizona|658[0n]|BOLD:ABA1195
Narthecophora pulverea|[7632]|BBLOC475-11|United States|Arizona|658[0n]|BOLD:ABA1195
Narthecophora pulverea|[7633]|BBLOC478-11|United States|Arizona|658[0n]|BOLD:ABA1195
Narthecophora pulverea|[7634]|BBLOC479-11|United States|Arizona|658[0n]|BOLD:ABA1195
Narthecophora pulverea|[7635]|BBLOC480-11|United States|Arizona|658[0n]|BOLD:ABA1195
Narthecophora pulverea|[7636]|BBLOC496-11|United States|Arizona|658[0n]|BOLD:ABA1195
Narthecophora pulverea|[7637]|BBLOC497-11|United States|Arizona|658[0n]|BOLD:ABA1195
Narthecophora pulverea|[7638]|BBLOC515-11|United States|Arizona|658[0n]|BOLD:ABA1195
Narthecophora pulverea|[7639]|BBLOC516-11|United States|Arizona|658[0n]|BOLD:ABA1195
Narthecophora pulverea|[7640]|BBLOC529-11|United States|Arizona|658[0n]|BOLD:ABA1195
Narthecophora pulverea|[7641]|BBLOC544-11|United States|Arizona|658[0n]|BOLD:ABA1195
Narthecophora pulverea|[7642]|BBLOC545-11|United States|Arizona|658[0n]|BOLD:ABA1195
Narthecophora pulverea|[7643]|BBLOC546-11|United States|Arizona|658[0n]|BOLD:ABA1195
Narthecophora pulverea|[7644]|BBLOC805-11|United States|Arizona|658[0n]|BOLD:ABA1195
Narthecophora pulverea|[7645]|BBLOC896-11|United States|Arizona|658[0n]|BOLD:ABA1195
Narthecophora pulverea|[7646]|BBLOC903-11|United States|Arizona|658[0n]|BOLD:ABA1195
Narthecophora pulverea|[7647]|BBLOC1521-11|United States|Arizona|658[0n]|BOLD:ABA1195
Narthecophora pulverea|[7648]|BBLOC1600-11|United States|Arizona|658[0n]|BOLD:ABA1195
Narthecophora pulverea|[7649]|BBLOC1601-11|United States|Arizona|658[0n]|BOLD:ABA1195
Narthecophora pulverea|[7650]|BBLOC1603-11|United States|Arizona|658[0n]|BOLD:ABA1195
Narthecophora pulverea|[7651]|BBLOC1605-11|United States|Arizona|658[0n]|BOLD:ABA1195
Narthecophora pulverea|[7652]|BBLOC1658-11|United States|Arizona|658[0n]|BOLD:ABA1195
Narthecophora pulverea|[7653]|BBLOC437-11|United States|Arizona|658[0n]|BOLD:ABA1195
Narthecophora pulverea|[7654]|BBLOC458-11|United States|Arizona|658[0n]|BOLD:ABA1195
Narthecophora pulverea|[7655]|BBLOC459-11|United States|Arizona|658[0n]|BOLD:ABA1195
Narthecophora pulverea|[7656]|BBLOC460-11|United States|Arizona|658[0n]|BOLD:ABA1195
Narthecophora pulverea|[7657]|BBLOC463-11|United States|Arizona|658[0n]|BOLD:ABA1195
Narthecophora pulverea|[7658]|BBLOC466-11|United States|Arizona|658[0n]|BOLD:ABA1195
[illegible]

Narthecophora pulverea|[7656]|BBLOC460-11|United States|Arizona|658[0n]|BOLD:ABA1195
Narthecophora pulverea|[7657]|BBLOC463-11|United States|Arizona|658[0n]|BOLD:ABA1195
Narthecophora pulverea|[7658]|BBLOC466-11|United States|Arizona|658[0n]|BOLD:ABA1195
Narthecophora pulverea|[7659]|BBLOC429-11|United States|Arizona|658[0n]|BOLD:ABA1195
Narthecophora pulverea|[7660]|BBLOC436-11|United States|Arizona|658[0n]|BOLD:ABA1195
Narthecophora pulverea|[7661]|BBLOC418-11|United States|Arizona|658[0n]|BOLD:ABA1195
Narthecophora pulverea|[7662]|BBLOC419-11|United States|Arizona|658[0n]|BOLD:ABA1195
Narthecophora pulverea|[7663]|BBLOC403-11|United States|Arizona|658[0n]|BOLD:ABA1195
Narthecophora pulverea|[7664]|BBLOC413-11|United States|Arizona|658[0n]|BOLD:ABA1195
Narthecophora pulverea|[7665]|BBLOC400-11|United States|Arizona|658[0n]|BOLD:ABA1195
Narthecophora pulverea|[7666]|BBLOC391-11|United States|Arizona|658[0n]|BOLD:ABA1195
Narthecophora pulverea|[7667]|BBLOC333-11|United States|Arizona|658[0n]|BOLD:ABA1195
Narthecophora pulverea|[7668]|BBLOC332-11|United States|Arizona|658[0n]|BOLD:ABA1195
Narthecophora pulverea|[7669]|BBLOC233-11|United States|Arizona|658[0n]|BOLD:ABA1195
Narthecophora pulverea|[7670]|BBLOC089-11|United States|Arizona|658[0n]|BOLD:ABA1195
Narthecophora pulverea|[7671]|BBLOC086-11|United States|Arizona|658[0n]|BOLD:ABA1195
Narthecophora pulverea|[7672]|BBLOC084-11|United States|Arizona|658[0n]|BOLD:ABA1195
Narthecophora pulverea|[7673]|BBLOC083-11|United States|Arizona|658[0n]|BOLD:ABA1195
Narthecophora pulverea|[7674]|BBLOC487-11|United States|Arizona|658[0n]|BOLD:ABA1195
Narthecophora pulverea|[7675]|BBLOC340-11|United States|Arizona|658[0n]|BOLD:ABA1195
Narthecophora pulverea|[7676]|BBLOC450-11|United States|Arizona|658[0n]|BOLD:ABA1195
Narthecophora pulverea|[7677]|BBLOC346-11|United States|Arizona|658[0n]|BOLD:ABA1195
Narthecophora pulverea|[7678]|BBLOC792-11|United States|Arizona|658[0n]|BOLD:ABA1195
Narthecophora pulverea|[7679]|BBLOC1606-11|United States|Arizona|620[0n]|BOLD:ABA1195
Narthecophora pulverea|[7680]|BBLOC1659-11|United States|Arizona|658[0n]|BOLD:ABA1195
Chrysoecia thoracica|[7681]|CMAZA1135-12|United States|Arizona|634[0n]|BOLD:ABW6380
Chrysoecia thoracica|[7682]|RDNMK936-13|Mexico|Sonora|658[0n]|BOLD:ABW6380
Chalcopasta fulgens|[7683]|NAMUM422-09|United States|Texas|658[0n]|BOLD:AAW6635
Chalcopasta howardi|[7684]|NAMUM105-08|United States|Texas|658[0n]|BOLD:AAH5615
Chalcopasta howardi|[7685]|RDNME904-08|United States|Arizona|658[0n]|BOLD:AAH5615
Chalcopasta howardi|[7686]|CMAZA025-09|United States|Arizona|658[0n]|BOLD:AAH5615
Basilodes pepita|[7687]|LPOKA405-09|United States|Oklahoma|636[0n]|BOLD:ACE7173
Basilodes pepita|[7688]|HKONS093-07|United States|Florida|656[0n]|BOLD:ACE7173
Basilodes pepita|[7689]|LPOKA208-08|United States|Oklahoma|658[0n]|BOLD:ACE7173
Basilodes pepita|[7690]|LPOKA225-08|United States|Oklahoma|658[0n]|BOLD:ACE7173
Basilodes pepita|[7691]|LNCC258-10|United States|North Carolina|658[0n]|BOLD:ACE7173
Basilodes pepita|[7692]|LNCC1256-11|United States|North Carolina|658[0n]|BOLD:ACE7173
Basilodes chrysopis|[7693]|CMAZA568-10|United States|Arizona|658[0n]|BOLD:AAF0293
Basilodes chrysopis|[7694]|NAMUM054-08|United States|Arizona|657[0n]|BOLD:AAF0293
Basilodes chrysopis|[7695]|RDNME057-07|United States|Arizona|658[0n]|BOLD:AAF0293
Basilodes chrysopis|[7696]|NAMUM370-09|United States|Texas|658[0n]|BOLD:AAF0293
Basilodes chrysopis|[7697]|RDNMH446-09|United States|Arizona|658[0n]|BOLD:AAF0293
Basilodes chrysopis|[7698]|NAMUM371-09|United States|Texas|658[0n]|BOLD:AAF0293
Basilodes chrysopis|[7699]|CMAZA876-12|United States|Arizona|658[0n]|BOLD:AAF0293
Basilodes chrysopis|[7700]|CMAZA967-12|United States|Arizona|658[0n]|BOLD:AAF0293
Basilodes straminea|[7701]|CMAZA1011-12|United States|Arizona|658[0n]|BOLD:AAF0293
Stiria sulphurea|[7702]|RDNME903-08|United States|Arizona|658[0n]|BOLD:ABU6135
Stiria satana|[7703]|RDNMD806-07|United States|Arizona|656[0n]|BOLD:ABX6335
Stiria satana|[7704]|RDNMJ402-11|United States|Arizona|658[0n]|BOLD:ABX6335
Stiria satana|[7705]|RDNMJ403-11|United States|Arizona|658[0n]|BOLD:ABX6335
Stiria consuela|[7706]|NAMUM253-08|United States|Arizona|658[1n]|BOLD:ACE8450
Stiria consuela|[7707]|RDNME905-08|United States|Arizona|658[0n]|BOLD:ACE8450
Stiria consuela|[7708]|USLEP381-10|United States|Arizona|658[0n]|BOLD:ACE8450
Stiria consuela|[7709]|USLEP382-10|United States|Arizona|658[0n]|BOLD:ACE8450
Stiria consuela|[7710]|RDNMJ098-10|United States|Nevada|658[0n]|BOLD:ACE8450
Stiria rugifrons|[7711]|RDNMG635-08|United States|Indiana|658[0n]|BOLD:AAC8472
Stiria rugifrons|[7712]|LTOLB126-08|United States|Texas|658[0n]|BOLD:AAC8472
Stiria rugifrons|[7713]|RDNMB117-05|Canada|Alberta|616[0n]|BOLD:AAC8472
Stiria rugifrons|[7714]|LPOKA166-08|United States|Oklahoma|658[0n]|BOLD:AAC8472
Stiria rugifrons|[7715]|RDMAB945-09|Canada|Alberta|639[0n]|BOLD:AAC8472
Stiria rugifrons|[7716]|RDMAB946-09|Canada|Alberta|625[0n]|BOLD:AAC8472
Stiria rugifrons|[7717]|RDMAB947-09|Canada|Alberta|656[0n]|BOLD:AAC8472
Stiria rugifrons|[7718]|LPOKA097-08|United States|Oklahoma|658[1n]|BOLD:ABZ3456
Stiria rugifrons|[7719]|LPOKD109-09|United States|Oklahoma|658[0n]|BOLD:ABZ3456
Stiria rugifrons|[7720]|CMAZA795-10|United States|Arizona|658[0n]|BOLD:ABZ3456
Stiria intermixta|[7721]|CMAZA931-12|United States|Arizona|658[0n]|BOLD:ACF4926
Stiria intermixta|[7722]|CMAZA932-12|United States|Arizona|658[0n]|BOLD:ACF4926
Stiria intermixta|[7723]|CMAZA993-12|United States|Arizona|658[0n]|BOLD:ACF4926
Stiria intermixta|[7724]|RDNME902-08|United States|Arizona|658[0n]|BOLD:ACF4926
Stiria intermixta|[7725]|JBAZ122-09|United States|Arizona|658[0n]|BOLD:ACF4926
Stiria intermixta|[7726]|CMAZA1007-12|United States|Arizona|658[0n]|BOLD:ACF4926
Stiria intermixta|[7727]|JBAZ121-09|United States|Arizona|658[0n]|BOLD:ACF4926
Stiria intermixta|[7728]|CMAZA1061-12|United States|Arizona|658[0n]|BOLD:ACF4926
Stiria dyari|[7729]|LNAUS3501-13|United States|Arizona|658[0n]|BOLD:ACI5683
Stiria dyari|[7730]|LNAUS3504-13|United States|Arizona|658[0n]|BOLD:ACI5683
Stiria blanchardi|[7731]|LNAUS3505-13|United States|New Mexico|658[0n]|BOLD:ACI6921
Stiria blanchardi|[7732]|LNAUS3506-13|United States|Texas|582[0n]|BOLD:ACI6921
Neumoegenia poetica|[7733]|NAMUM092-08|United States|Arizona|656[0n]|BOLD:AAF2638
Neumoegenia poetica|[7734]|RDNMD809-07|United States|Arizona|658[0n]|BOLD:AAF2638
Neumoegenia poetica|[7735]|RDNMD828-07|United States|Arizona|658[0n]|BOLD:AAF2638
Neumoegenia poetica|[7736]|CMAZA466-10|United States|Arizona|658[0n]|BOLD:AAF2638
Neumoegenia poetica|[7737]|AWCL009-09|United States|Arizona|658[0n]|BOLD:AAF2638
Neumoegenia poetica|[7738]|CMAZA465-10|United States|Arizona|658[0n]|BOLD:AAF2638
Neumoegenia poetica|[7739]|CMAZA960-12|United States|Arizona|658[0n]|BOLD:AAF2638
Chalcopasta acema|[7740]|RDNME879-08|United States|Arizona|658[0n]|BOLD:AAH4908
Chalcopasta acema|[7741]|AWCL010-09|United States|Arizona|658[0n]|BOLD:AAH4908
Chalcopasta territans|[7742]|CMAZA976-12|United States|Arizona|658[0n]|BOLD:ABW2952
Chalcopasta territans|[7743]|CMAZA990-12|United States|Arizona|658[0n]|BOLD:ABW2952
Plagiomimicus aureolum|[7744]|CMAZA1211-12|United States|Arizona|658[0n]|BOLD:ABW6338
Cirrhophanus pretiosa|[7745]|NOCNA047-14|United States|Texas|658[0n]|BOLD:ACN5518
Cirrhophanus pretiosa|[7746]|NOCNA048-14|United States|Oklahoma|658[0n]|BOLD:ACN5518
Plagiomimicus ochoa|[7747]|CNCLB195-14|United States|Texas|658[0n]|BOLD:ACM4213
Cirrhophanus dyari|[7748]|CMAZA988-12|United States|Arizona|658[0n]|BOLD:ABW2950
Cirrhophanus dyari|[7749]|CMAZA1215-12|United States|Arizona|658[0n]|BOLD:ABW2950
Cirrhophanus dyari|[7750]|CMAZA980-12|United States|Arizona|658[0n]|BOLD:ABW2950
Cirrhophanus dyari|[7751]|CMAZA1038-12|United States|Arizona|658[0n]|BOLD:ABW2950
Cirrhophanus dyari|[7752]|RDNMK903-13|Mexico|Sonora|658[0n]|BOLD:ABW2950
Angulostiria chryseochilus|[7753]|CNCLB1696-14|United States|Texas|552[0n]|BOLD:ACP3625
Xanthothrix ranunculi|[7754]|CNCLB953-14|United States|California|658[0n]|BOLD:ACM4354
Xanthothrix ranunculi|[7755]|CNCLB954-14|United States|California|658[0n]|BOLD:ACM4354
Chrysoecia scira|[7756]|CMAZA578-10|United States|Arizona|658[0n]|BOLD:AAJ4874
Chrysoecia scira|[7757]|RDNMG113-08|United States|Arizona|658[0n]|BOLD:AAJ4874
Chrysoecia scira|[7758]|CMAZA1134-12|United States|Arizona|658[0n]|BOLD:AAJ4874

[illegible]
Chrysoecia scira|[7756]|CMAZA578-10|United States|Arizona|658[0n]|BOLD:AAJ4874
Chrysoecia scira|[7757]|RDNMG113-08|United States|Arizona|658[0n]|BOLD:AAJ4874
Chrysoecia scira|[7758]|CMAZA1134-12|United States|Arizona|658[0n]|BOLD:AAJ4874
Chrysoecia gladiola|[7759]|RDNME878-08|United States|Arizona|658[0n]|BOLD:AAJ0189
Chrysoecia gladiola|[7760]|JBAZ119-09|United States|Arizona|634[0n]|BOLD:AAJ0189
Chrysoecia atrolinea|[7761]|JBAZ115-09|United States|Arizona|658[0n]|BOLD:AAI4062
Chrysoecia atrolinea|[7762]|JBAZ116-09|United States|Arizona|658[0n]|BOLD:AAI4062
Chrysoecia atrolinea|[7763]|CMAZA1216-12|United States|Arizona|658[0n]|BOLD:AAI4062
Chrysoecia atrolinea|[7764]|RDNMK931-13|Mexico|Sonora|658[0n]|BOLD:AAI4062
Eulithosia composita|[7765]|RDNMK171-11|United States|Arizona|658[0n]|BOLD:AAY8979
Eulithosia composita|[7766]|CNCLB198-14|United States|Arizona|658[0n]|BOLD:AAY8979
Eulithosia discistriga|[7767]|USLEP069-10|United States|Arizona|658[0n]|BOLD:AAM8592
Eulithosia plesioglauca|[7768]|RDNMD807-07|United States|Arizona|658[0n]|BOLD:AAY3455
Eulithosia papago|[7769]|CNCLB196-14|United States|Colorado|658[0n]|BOLD:ACM4313
Eulithosia papago|[7770]|CNCLB1069-14|United States|Colorado|658[0n]|BOLD:ACM4313
Eulithosia papago|[7771]|CNCLB1070-14|United States|Colorado|658[0n]|BOLD:ACM4313
Xanthothrix neumoegeni|[7772]|CNCLB951-14|United States|California|658[0n]|BOLD:ACM4355
Xanthothrix neumoegeni|[7773]|CNCLB952-14|United States|California|658[0n]|BOLD:ACM4355
Lineostriastiria hutsoni|[7774]|LOCBF339-13|United States|California|542[0n]|BOLD:AAH4958
Lineostriastiria hutsoni|[7775]|IAWL965-09|United States|Arizona|618[1n]|BOLD:AAH4958
Lineostriastiria hutsoni|[7776]|LOCBF1106-13|United States|California|658[0n]|BOLD:AAH4958
Lineostriastiria hutsoni|[7777]|LOCBF2103-13|United States|California|603[0n]|BOLD:AAH4958
Lineostriastiria hutsoni|[7778]|LOCBF340-13|United States|California|593[0n]|BOLD:AAH4958
Lineostriastiria hutsoni|[7779]|RDNMC749-06|United States|California|658[0n]|BOLD:AAH4958
Lineostriastiria hutsoni|[7780]|IAWL966-09|United States|Arizona|640[0n]|BOLD:AAH4958
Lineostriastiria hutsoni|[7781]|LOCBF322-13|United States|California|658[0n]|BOLD:AAH4958
Lineostriastiria hutsoni|[7782]|LOCBF2104-13|United States|California|606[0n]|BOLD:AAH4958
Lineostriastiria hutsoni|[7783]|LOCBF2136-13|United States|California|607[0n]|BOLD:AAH4958
Lineostriastiria hutsoni|[7784]|LOCBF2150-13|United States|California|607[0n]|BOLD:AAH4958
Lineostriastiria hutsoni|[7785]|LOCBF2151-13|United States|California|606[0n]|BOLD:AAH4958
Lineostriastiria hutsoni|[7786]|LOCBF2269-13|United States|California|606[0n]|BOLD:AAH4958
Lineostriastiria olivalis|[7787]|TML151-14|United States||599[0n]|BOLD:ACO1701
Lineostriastiria sexseriata|[7788]|CMAZA1129-12|United States|Arizona|658[0n]|BOLD:ABW6378
Lineostriastiria sexseriata|[7789]|CMAZA1218-12|United States|Arizona|658[0n]|BOLD:ABW6378
Lineostriastiria hachita|[7790]|CMAZA770-10|United States|Arizona|658[0n]|BOLD:AAH9282
Lineostriastiria hachita|[7791]|QUNOD124-10|United States|Texas|658[0n]|BOLD:AAH9282
Lineostriastiria hachita|[7792]|NAMUM102-08|United States|Texas|658[0n]|BOLD:AAH9282
Lineostriastiria hachita|[7793]|QUNOD123-10|United States|Texas|611[0n]|BOLD:AAH9282
Lineostriastiria hachita|[7794]|RDNMK008-11|United States|Arizona|658[0n]|BOLD:AAH9282
Lineostriastiria hachita|[7795]|RDNMK162-11|United States|Arizona|658[0n]|BOLD:AAH9282
Lineostriastiria hachita|[7796]|RDNMK164-11|United States|Arizona|658[0n]|BOLD:AAH9282
Lineostriastiria hachita|[7797]|CMAZA1081-12|United States|Arizona|658[0n]|BOLD:AAH9282
Lineostriastiria hachita|[7798]|CMAZA1094-12|United States|Arizona|658[0n]|BOLD:AAH9282
Lineostriastiria biundulalis|[7799]|CNCLB1697-14|United States|Texas|658[0n]|BOLD:ACP4416
Plagiomimicus olvello|[7800]|NAMUM104-08|United States|Texas|658[0n]|BOLD:ABY8220
Plagiomimicus manti|[7801]|NAMUM121-08|United States|Texas|658[0n]|BOLD:ABY8926
Plagiomimicus manti|[7802]|RDNMG290-08|United States|Arizona|658[0n]|BOLD:ABY8926
Plagiomimicus manti|[7803]|CMAZA977-12|United States|Arizona|658[0n]|BOLD:ABY8926
Plagiomimicus dimidiata|[7804]|NAMUM101-08|United States|Texas|658[0n]|BOLD:ABZ1816
Plagiomimicus dimidiata|[7805]|CMAZA1050-12|United States|Arizona|658[0n]|BOLD:ABZ1816
Plagiomimicus spumosum|[7806]|RDNMF652-08|United States|Missouri|658[2n]|BOLD:ABX4958
Plagiomimicus spumosum|[7807]|RDNMF463-08|United States|Arizona|609[0n]|BOLD:ABX4958
Plagiomimicus spumosum|[7808]|NAMUM055-08|United States|Arizona|657[0n]|BOLD:ABX4958
Plagiomimicus spumosum|[7809]|RDNMF462-08|United States|Arizona|658[0n]|BOLD:ABX4958
Plagiomimicus spumosum|[7810]|RDNMF461-08|United States|California|658[0n]|BOLD:ABX4958
Plagiomimicus spumosum|[7811]|BBLOD893-11|United States|California|658[0n]|BOLD:ABX4958
Plagiomimicus spumosum|[7812]|BBLOD944-11|United States|Texas|658[0n]|BOLD:ABX4958
Plagiomimicus spumosum|[7813]|BBLOD945-11|United States|Texas|658[0n]|BOLD:ABX4958
Plagiomimicus spumosum|[7814]|BBLOD948-11|United States|California|658[0n]|BOLD:ABX4958
Plagiomimicus spumosum|[7815]|BBLOD957-11|United States|Texas|658[0n]|BOLD:ABX4958
Plagiomimicus spumosum|[7816]|BBLOD958-11|United States|Texas|658[0n]|BOLD:ABX4958
Plagiomimicus caesium|[7817]|CNCLB955-14|United States|Texas|658[0n]|BOLD:ABX4958
Plagiomimicus caesium|[7818]|CNCLB956-14|United States|Texas|658[0n]|BOLD:ABX4958
Plagiomimicus curiosum|[7819]|CNCLB957-14|United States|Arizona|658[0n]|BOLD:ACM3986
Plagiomimicus curiosum|[7820]|CNCLB958-14|United States|Arizona|658[0n]|BOLD:ACM3986
Plagiomimicus astigmatosum|[7821]|CNCLB2449-14|United States|Texas|658[0n]|BOLD:ACR9293
Plagiomimicus heitzmani|[7822]|LPOKA408-09|United States|Oklahoma|658[0n]|BOLD:ABU7138
Plagiomimicus triplagiatus|[7823]|RDNMG551-08|United States|Colorado|658[0n]|BOLD:AAD0097
Plagiomimicus triplagiatus|[7824]|RDNMG552-08|United States|Colorado|658[0n]|BOLD:AAD0097
Plagiomimicus pityochromus|[7825]|LPOKA444-09|United States|Oklahoma|658[0n]|BOLD:AAD0097
Plagiomimicus pityochromus|[7826]|NAMUM117-08|United States|Texas|634[0n]|BOLD:AAD0097
Plagiomimicus pityochromus|[7827]|LPOKD225-09|United States|Oklahoma|657[0n]|BOLD:AAD0097
Plagiomimicus pityochromus|[7828]|LPOKA472-09|United States|Oklahoma|658[0n]|BOLD:AAD0097
Plagiomimicus pityochromus|[7829]|HKONB430-09|United States|Indiana|658[0n]|BOLD:AAD0097
Plagiomimicus pityochromus|[7830]|HKONB429-09|United States|Indiana|658[0n]|BOLD:AAD0097
Plagiomimicus pityochromus|[7831]|LPOKA232-08|United States|Oklahoma|658[0n]|BOLD:AAD0097
Plagiomimicus pityochromus|[7832]|NAMUM118-08|United States|Texas|658[0n]|BOLD:AAD0097
Plagiomimicus pityochromus|[7833]|LGSMG464-07|United States|North Carolina|658[0n]|BOLD:AAD0097
Plagiomimicus pityochromus|[7834]|NAMUM116-08|United States|Texas|658[0n]|BOLD:AAD0097
Plagiomimicus pityochromus|[7835]|LPOKD231-09|United States|Oklahoma|654[0n]|BOLD:AAD0097
Plagiomimicus pityochromus|[7836]|CNCLB2904-14|United States|North Carolina|658[0n]|BOLD:AAD0097
Plagiomimicus expallidus|[7837]|RDNMF465-08|United States|Kansas|658[0n]|BOLD:ABZ4305
Plagiomimicus expallidus|[7838]|RDMAB948-09|Canada|Alberta|586[0n]|BOLD:ABZ4305
Plagiomimicus hilli|[7839]|NAMUM176-08|United States|California|658[0n]|BOLD:ABZ4304
Plagiomimicus hilli|[7840]|NAMUM177-08|United States|California|658[0n]|BOLD:ABZ4304
Plagiomimicus hilli|[7841]|NAMUM178-08|United States|California|658[0n]|BOLD:ABZ4304
Plagiomimicus kathyae|[7842]|CNCLB2150-14|United States|Utah|407[0n]|
Plagiomimicus kathyae|[7843]|CNCLB3095-15|United States|Arizona|658[0n]|BOLD:ACW8572
Plagiomimicus tepperi|[7844]|CNCLB795-14|United States|Washington|658[0n]|BOLD:ACR9301
Plagiomimicus tepperi|[7845]|NAMUM100-08|United States|Texas|656[0n]|BOLD:AAF2198
Plagiomimicus tepperi|[7846]|NAMUM107-08|United States|Texas|655[0n]|BOLD:AAF2198
Plagiomimicus tepperi|[7847]|CMAZA1079-12|United States|Arizona|658[0n]|BOLD:ACR9303
Plagiomimicus tepperi|[7848]|CMAZA1119-12|United States|Arizona|658[0n]|BOLD:ACR9303
Plagiomimicus mimica|[7849]|RDNME981-08|United States|New Mexico|658[0n]|BOLD:ABZ5142
Plagiomimicus mimica|[7850]|CMAZA1140-12|United States|Arizona|658[0n]|BOLD:ACR9302
Plagiomimicus mimica|[7851]|CMAZA1082-12|United States|Arizona|658[0n]|BOLD:ACR9302
Plagiomimicus mimica|[7852]|CMAZA1158-12|United States|Arizona|658[0n]|BOLD:ACR9302
Plagiomimicus mimica|[7853]|NAMUM133-08|United States|California|657[0n]|BOLD:ABZ5142
Plagiomimicus mimica|[7854]|CNCLB2468-14|United States|California|658[0n]|BOLD:ABZ5142
Plagiomimicus sp.|[7855]|CNCLB2466-14|United States|California|636[0n]|BOLD:ACR9431
Plagiomimicus sp.|[7856]|CNCLB2467-14|United States|California|658[0n]|BOLD:ACR9431
Plagiomimicus sp.|[7857]|CNCLB2469-14|United States|California|658[0n]|BOLD:ACR9431

Plagiomimicus sp.|[7855]|CNCLB2466-14|United States|California|636[0n]|BOLD:ACR9431
Plagiomimicus sp.|[7856]|CNCLB2467-14|United States|California|658[0n]|BOLD:ACR9431
Plagiomimicus sp.|[7857]|CNCLB2469-14|United States|California|658[0n]|BOLD:ACR9431
Plagiomimicus sp.|[7858]|CNCLB2470-14|United States|California|658[0n]|BOLD:ACR9431
Argentostiria koebelei|[7859]|RDNMC696-06|United States|California|568[0n]|BOLD:AAF5400
Argentostiria koebelei|[7860]|NAMUM148-08|United States|California|658[0n]|BOLD:AAF5400
Argentostiria koebelei|[7861]|JBAZ073-09|United States|California|658[0n]|BOLD:AAF5400
Argentostiria koebelei|[7862]|CGLCA148-10|United States|California|658[0n]|BOLD:AAF5400
Plagiomimicus pyralina|[7863]|RDNMI118-10|United States|Arizona|658[0n]|BOLD:AAX5101
Plagiomimicus pyralina|[7864]|PSAT153-10|Mexico||658[0n]|BOLD:AAX5101
Fala ptychophora|[7865]|CGLCA031-10|United States|California|658[0n]|BOLD:AAM0919
Bistica noela|[7866]|CNCLB3204-15|Mexico|Chihuahua|658[0n]|BOLD:ACW9219
Agriopodes tybo|[7867]|RDNMH832-09|United States|Arizona|658[2n]|BOLD:AAF8092
Agriopodes tybo|[7868]|AWCL054-09|United States|Arizona|658[0n]|BOLD:AAF8092
Agriopodes tybo|[7869]|RDNMC362-05|United States|Arizona|553[0n]|BOLD:AAF8092
Agriopodes tybo|[7870]|NAMUM081-08|United States|Arizona|657[0n]|BOLD:AAF8092
Agriopodes tybo|[7871]|RDNMJ522-11|United States|Arizona|658[0n]|BOLD:AAF8092
Agriopodes tybo|[7872]|RDNMJ850-11|United States|Arizona|658[0n]|BOLD:AAF8092
Agriopodes tybo|[7873]|CMAZA1030-12|United States|Arizona|658[0n]|BOLD:AAF8092
Acronicta perblanda|[7874]|RDNMK826-12|United States|Georgia|658[0n]|BOLD:ABW0255
Acronicta perblanda|[7875]|RDNMK828-12|United States|Georgia|658[0n]|BOLD:ABW0255
Acronicta sp.|[7876]|RDNML039-13|United States|Louisiana|658[0n]|BOLD:AAS0521
Acronicta sp.|[7877]|QUNO839-08|United States|Louisiana|658[0n]|BOLD:AAS0521
Acronicta sp.|[7878]|RDNML040-13|United States|Louisiana|658[0n]|BOLD:AAS0521
Acronicta sp.|[7879]|RDNML041-13|United States|Louisiana|658[0n]|BOLD:AAS0521
Acronicta sp.|[7880]|RDNMJ052-10|United States|Arizona|658[0n]|BOLD:AAY8751
Acronicta sp.|[7881]|RDNMJ054-10|United States|Arizona|658[0n]|BOLD:AAY8751
Acronicta sp.|[7882]|RDNMK121-11|Mexico|Sonora|658[0n]|BOLD:AAY8751
Acronicta sp.|[7883]|RDNMJ053-10|Mexico|Sonora|658[0n]|BOLD:AAY8751
Acronicta sp.|[7884]|RDNMK679-11|Mexico|Sonora|658[0n]|BOLD:AAY8751
Acronicta beameri|[7885]|RDNMI007-10|United States|Arizona|658[0n]|BOLD:AAJ1595
Acronicta beameri|[7886]|RDNMJ626-11|United States|Arizona|658[0n]|BOLD:AAJ1595
Acronicta beameri|[7887]|RDNME857-08|United States|Arizona|658[0n]|BOLD:AAJ1595
Acronicta beameri|[7888]|RDNMJ627-11|United States|Arizona|658[0n]|BOLD:AAJ1595
Acronicta atristrigatus|[7889]|CMAZA327-10|United States|Arizona|658[0n]|BOLD:AAJ1596
Acronicta atristrigatus|[7890]|RDNMJ576-11|United States|Texas|571[1n]|BOLD:AAJ1596
Acronicta atristrigatus|[7891]|RDNMJ684-11|United States|Arizona|658[0n]|BOLD:AAJ1596
Acronicta atristrigatus|[7892]|IAWLB530-11|United States|Arizona|658[0n]|BOLD:AAJ1596
Acronicta atristrigatus|[7893]|JBAZ131-09|United States|Arizona|658[0n]|BOLD:AAJ1596
Acronicta atristrigatus|[7894]|RDNMJ612-11|United States|Arizona|658[0n]|BOLD:AAJ1596
Acronicta atristrigatus|[7895]|RDNMJ614-11|United States|Arizona|658[0n]|BOLD:AAJ1596
Acronicta atristrigatus|[7896]|RDNMI011-10|United States|Arizona|649[0n]|BOLD:AAJ1596
Acronicta atristrigatus|[7897]|RDNMI015-10|United States|Arizona|658[0n]|BOLD:AAJ1596
Acronicta atristrigatus|[7898]|RDNMJ613-11|United States|Arizona|658[0n]|BOLD:AAJ1596
Acronicta atristrigatus|[7899]|CMAZA911-12|United States|Arizona|658[0n]|BOLD:AAJ1596
Acronicta sp.|[7900]|RDNMK690-11|United States|Arizona|658[0n]|BOLD:ABA2128
Acronicta sp.|[7901]|RDNMK691-11|United States|Arizona|658[0n]|BOLD:ABA2128
Acronicta sp.|[7902]|RDNMK692-11|United States|Arizona|658[0n]|BOLD:ABA2128
Acronicta nr. brumosa|[7903]|PSAT143-10|United States|New Mexico|658[0n]|BOLD:AAC7219
Acronicta nr. brumosa|[7904]|RDNMG274-08|United States|Texas|658[0n]|BOLD:AAC7219
Acronicta nr. brumosa|[7905]|RDNMJ281-11|United States|New Mexico|658[0n]|BOLD:AAC7219
Acronicta nr. brumosa|[7906]|RDNMJ585-11|United States|New Mexico|658[0n]|BOLD:AAC7219
Acronicta nr. brumosa|[7907]|RDNMJ282-11|United States|New Mexico|658[0n]|BOLD:AAC7219
Acronicta nr. brumosa|[7908]|RDNMK615-11|United States|New Mexico|658[0n]|BOLD:AAC7219
Acronicta nr. brumosa|[7909]|CMAZA501-10|United States|Arizona|658[0n]|BOLD:AAC7219
Acronicta nr. brumosa|[7910]|CMAZA566-10|United States|Arizona|658[0n]|BOLD:AAC7219
Acronicta nr. brumosa|[7911]|RDNMJ377-11|United States|Arizona|658[0n]|BOLD:AAC7219
Acronicta nr. brumosa|[7912]|IAWLB529-11|United States|Arizona|658[0n]|BOLD:AAC7219
Acronicta nr. brumosa|[7913]|RDNMD940-07|United States|Arizona|655[0n]|BOLD:AAC7219
Acronicta nr. brumosa|[7914]|RDNMG587-08|United States|Arizona|658[0n]|BOLD:AAC7219
Acronicta nr. brumosa|[7915]|CMAZA934-12|United States|Arizona|658[0n]|BOLD:AAC7219
Acronicta nr. brumosa|[7916]|RDNMJ073-10|United States|Texas|658[0n]|BOLD:AAC7219
Acronicta nr. brumosa|[7917]|RDNMJ074-10|United States|Texas|658[0n]|BOLD:AAC7219
Acronicta nr. brumosa|[7918]|RDNML285-13|United States|Texas|658[0n]|BOLD:AAC7219
Acronicta brumosa|[7919]|RDNMC302-05|United States|Florida|561[0n]|BOLD:AAB3594
Acronicta brumosa|[7920]|RDNMD286-06|United States|Florida|658[0n]|BOLD:AAB3594
Acronicta brumosa|[7921]|LOFLB815-06|United States|Florida|656[0n]|BOLD:AAB3594
Acronicta brumosa|[7922]|LSEU091-06|United States|Georgia|595[0n]|BOLD:AAB3594
Acronicta brumosa|[7923]|RDNMC308-05|United States|Florida|592[0n]|BOLD:AAB3594
Acronicta brumosa|[7924]|LNCB840-09|United States|Alabama|658[0n]|BOLD:AAB3594
Acronicta brumosa|[7925]|LSEU090-06|United States|Georgia|605[0n]|BOLD:AAB3594
Acronicta brumosa|[7926]|RDNMD287-06|United States|Florida|658[0n]|BOLD:AAB3594
Acronicta brumosa|[7927]|LOFLC470-06|United States|Florida|658[0n]|BOLD:AAB3594
Acronicta brumosa|[7928]|LNCC155-10|United States|North Carolina|658[0n]|BOLD:AAB3594
Acronicta brumosa|[7929]|RDNML286-13|United States|Texas|658[0n]|BOLD:AAB3594
Acronicta marmorata|[7930]|LMH009-06|Canada|British Columbia|658[0n]|BOLD:AAD4321
Acronicta marmorata|[7931]|RDNMF119-08|Canada|British Columbia|658[0n]|BOLD:AAD4321
Acronicta marmorata|[7932]|RDNMF121-08|Canada|British Columbia|658[0n]|BOLD:AAD4321
Acronicta marmorata|[7933]|RDNMF122-08|Canada|British Columbia|658[0n]|BOLD:AAD4321
Acronicta marmorata|[7934]|CGLCA024-10|United States|California|658[0n]|BOLD:AAD4321
Acronicta marmorata|[7935]|NAMUM127-08|United States|California|658[0n]|BOLD:AAD4321
Acronicta marmorata|[7936]|RDNMF120-08|United States|California|658[0n]|BOLD:AAD4321
Acronicta marmorata|[7937]|RDNMF123-08|United States|California|658[0n]|BOLD:AAD4321
Acronicta marmorata|[7938]|CGLCA051-10|United States|California|658[0n]|BOLD:AAD4321
Acronicta marmorata|[7939]|IAWLB437-11|United States|California|658[0n]|BOLD:AAD4321
Acronicta marmorata|[7940]|GMLC1069-12|United States|California|658[0n]|BOLD:AAD4321
Acronicta afflicta|[7941]|PSAT163-10|United States|Florida|610[0n]|BOLD:AAB4198
Acronicta afflicta|[7942]|LPOKB384-09|United States|Oklahoma|630[0n]|BOLD:AAB4198
Acronicta afflicta|[7943]|RDNMC292-05|United States|Florida|589[0n]|BOLD:AAB4198
Acronicta afflicta|[7944]|RDNMC293-05|United States|Florida|593[1n]|BOLD:AAB4198
Acronicta afflicta|[7945]|LOT303-04|United States|Tennessee|658[0n]|BOLD:AAB4198
Acronicta afflicta|[7946]|LNCB070-06|United States|North Carolina|656[0n]|BOLD:AAB4198
Acronicta afflicta|[7947]|LGSMG960-10|United States|North Carolina|658[0n]|BOLD:AAB4198
Acronicta afflicta|[7948]|LPOKC776-09|United States|Oklahoma|658[0n]|BOLD:AAB4198
Acronicta afflicta|[7949]|NAMUM414-09|United States|West Virginia|658[0n]|BOLD:AAB4198
Acronicta afflicta|[7950]|LNCB510-07|United States|North Carolina|658[0n]|BOLD:AAB4198
Acronicta afflicta|[7951]|LSEU328-06|United States|Georgia|658[0n]|BOLD:AAB4198
Acronicta afflicta|[7952]|LSEU327-06|United States|Georgia|658[0n]|BOLD:AAB4198
Acronicta afflicta|[7953]|LOFLA927-06|United States|Florida|658[0n]|BOLD:AAB4198
Acronicta afflicta|[7954]|LOFLA522-06|United States|Florida|658[0n]|BOLD:AAB4198
Acronicta afflicta|[7955]|LOFLA215-06|United States|Florida|658[0n]|BOLD:AAB4198
Acronicta afflicta|[7956]|RDNMD285-06|United States|Florida|658[0n]|BOLD:AAB4198
Acronicta afflicta|[7957]|RDNMD284-06|United States|Florida|658[0n]|BOLD:AAB4198

[illegible]
Acronicta afflicta|[7955]|LOFLA215-06|United States|Florida|658[0n]|BOLD:AAB4198
Acronicta afflicta|[7956]|RDNMD285-06|United States|Florida|658[0n]|BOLD:AAB4198
Acronicta afflicta|[7957]|RDNMD284-06|United States|Florida|658[0n]|BOLD:AAB4198
Acronicta afflicta|[7958]|RDNMD283-06|United States|Florida|658[0n]|BOLD:AAB4198
Acronicta afflicta|[7959]|LNC755-06|United States|North Carolina|658[0n]|BOLD:AAB4198
Acronicta afflicta|[7960]|LOCT059-05|United States|Connecticut|658[0n]|BOLD:AAB4198
Acronicta afflicta|[7961]|LOTB330-05|United States|Tennessee|658[0n]|BOLD:AAB4198
Acronicta afflicta|[7962]|LOTB086-05|United States|Tennessee|658[0n]|BOLD:AAB4198
Acronicta afflicta|[7963]|LGSMC879-05|United States|Tennessee|658[0n]|BOLD:AAB4198
Acronicta afflicta|[7964]|PHMNB452-04|Canada|New Brunswick|629[0n]|BOLD:AAB4198
Acronicta afflicta|[7965]|LPOKC756-09|United States|Oklahoma|623[0n]|BOLD:AAB4198
Acronicta afflicta|[7966]|LNCC1062-11|United States|North Carolina|658[0n]|BOLD:AAB4198
Acronicta afflicta|[7967]|CNKJP1300-14|Canada|Nova Scotia|582[0n]|BOLD:AAB4198
Acronicta tristis|[7968]|PHMNB227-04|Canada|New Brunswick|609[0n]|BOLD:ACE9904
Acronicta tristis|[7969]|PHMNB217-04|Canada|New Brunswick|609[0n]|BOLD:ACE9904
Acronicta tristis|[7970]|PHMNB591-04|Canada|New Brunswick|658[0n]|BOLD:ACE9904
Acronicta tristis|[7971]|RDLQ132-05|Canada|Quebec|658[0n]|BOLD:ACE9904
Acronicta tristis|[7972]|MNBB615-05|Canada|New Brunswick|658[0n]|BOLD:ACE9904
Acronicta tristis|[7973]|RDNMI084-10|Canada|Ontario|658[0n]|BOLD:ACE9904
Acronicta tristis|[7974]|BBLCU207-09|United States|Michigan|658[0n]|BOLD:ACE9904
Acronicta tristis|[7975]|TMNBB162-06|Canada|New Brunswick|658[0n]|BOLD:ACE9904
Acronicta tristis|[7976]|TMNBB161-06|Canada|New Brunswick|658[0n]|BOLD:ACE9904
Acronicta tristis|[7977]|TTMNB326-06|Canada|New Brunswick|658[0n]|BOLD:ACE9904
Acronicta tristis|[7978]|TTMNB325-06|Canada|New Brunswick|658[0n]|BOLD:ACE9904
Acronicta tristis|[7979]|TTMNB004-06|Canada|New Brunswick|657[0n]|BOLD:ACE9904
Acronicta tristis|[7980]|TTMNB003-06|Canada|New Brunswick|657[0n]|BOLD:ACE9904
Acronicta tristis|[7981]|RDLQB553-05|Canada|Quebec|658[0n]|BOLD:ACE9904
Acronicta tristis|[7982]|MNBB506-05|Canada|New Brunswick|658[0n]|BOLD:ACE9904
Acronicta tristis|[7983]|MNBB505-05|Canada|New Brunswick|658[0n]|BOLD:ACE9904
Acronicta tristis|[7984]|MNBB395-05|Canada|New Brunswick|658[0n]|BOLD:ACE9904
Acronicta tristis|[7985]|MNBB394-05|Canada|New Brunswick|658[0n]|BOLD:ACE9904
Acronicta tristis|[7986]|MNBB393-05|Canada|New Brunswick|658[0n]|BOLD:ACE9904
Acronicta tristis|[7987]|MNBB392-05|Canada|New Brunswick|658[0n]|BOLD:ACE9904
Acronicta tristis|[7988]|MNBB195-05|Canada|New Brunswick|658[0n]|BOLD:ACE9904
Acronicta tristis|[7989]|RDLQ128-05|Canada|Quebec|658[0n]|BOLD:ACE9904
Acronicta tristis|[7990]|PHMNB750-05|Canada|New Brunswick|658[0n]|BOLD:ACE9904
Acronicta tristis|[7991]|PHMNB725-05|Canada|New Brunswick|658[0n]|BOLD:ACE9904
Acronicta tristis|[7992]|PHMNB589-04|Canada|New Brunswick|658[0n]|BOLD:ACE9904
Acronicta tristis|[7993]|PHMNB454-04|Canada|New Brunswick|658[0n]|BOLD:ACE9904
Acronicta tristis|[7994]|LOT310-04|United States|Tennessee|658[0n]|BOLD:ACE9904
Acronicta tristis|[7995]|LOT309-04|United States|Tennessee|658[0n]|BOLD:ACE9904
Acronicta tristis|[7996]|TMNBB160-06|Canada|New Brunswick|658[2n]|BOLD:ACE9904
Acronicta tristis|[7997]|PHMNB159-04|Canada|New Brunswick|658[0n]|BOLD:ACE9904
Acronicta tristis|[7998]|PHMNB038-03|Canada|New Brunswick|639[0n]|BOLD:ACE9904
Acronicta tristis|[7999]|PHMNB252-04|Canada|New Brunswick|616[0n]|BOLD:ACE9904
Acronicta tristis|[8000]|RDNMI086-10|Canada|Ontario|620[0n]|BOLD:ACE9904
Acronicta tristis|[8001]|RDNMI087-10|Canada|New Brunswick|620[0n]|BOLD:ACE9904
Acronicta tristis|[8002]|RDNMI088-10|Canada|New Brunswick|658[0n]|BOLD:ACE9904
Acronicta tristis|[8003]|RDNMI090-10|Canada|New Brunswick|658[0n]|BOLD:ACE9904
Acronicta tristis|[8004]|RDNMI093-10|Canada|New Brunswick|658[0n]|BOLD:ACE9904
Acronicta tristis|[8005]|RDNMJ642-11|United States|Kentucky|658[0n]|BOLD:ACE9904
Acronicta tristis|[8006]|MNBB616-05|Canada|New Brunswick|658[0n]|BOLD:ACE9904
Acronicta tristis|[8007]|LOT308-04|United States|Tennessee|658[0n]|BOLD:ACE9904
Acronicta tristis|[8008]|LGSMG955-10|United States|Tennessee|658[0n]|BOLD:ACE9904
Acronicta tristis|[8009]|LNCC1183-11|United States|North Carolina|658[0n]|BOLD:ACE9904
Acronicta ovata|[8010]|LGSMG944-10|United States|Tennessee|658[0n]|BOLD:ACF1195
Acronicta ovata|[8011]|LGSMG945-10|United States|North Carolina|658[0n]|BOLD:ACF1195
Acronicta ovata|[8012]|LGSMG946-10|United States|Tennessee|658[0n]|BOLD:ACF1195
Acronicta ovata|[8013]|RDNMI092-10|Canada|Ontario|658[0n]|BOLD:ACF1195
Acronicta ovata|[8014]|LOT486-04|United States|Tennessee|658[0n]|BOLD:ACF1195
Acronicta ovata|[8015]|LOT564-04|United States|Tennessee|598[0n]|BOLD:ACF1195
Acronicta ovata|[8016]|LNCC1048-11|United States|North Carolina|634[0n]|BOLD:ACF1195
Acronicta ovata|[8017]|BBLCU118-09|United States|Michigan|658[0n]|BOLD:ACF1195
Acronicta ovata|[8018]|NAMUM423-09|United States||658[0n]|BOLD:ACF1195
Acronicta ovata|[8019]|QUNOB544-09|United States|Kentucky|658[0n]|BOLD:ACF1195
Acronicta ovata|[8020]|QUNOB543-09|United States|Kentucky|658[0n]|BOLD:ACF1195
Acronicta ovata|[8021]|LOT292-04|United States|Tennessee|658[0n]|BOLD:ACF1195
Acronicta ovata|[8022]|LOT517-04|United States|Tennessee|658[0n]|BOLD:ACF1195
Acronicta ovata|[8023]|LSEU679-06|United States|Georgia|658[0n]|BOLD:ACF1195
Acronicta ovata|[8024]|LOT320-04|United States|Tennessee|658[0n]|BOLD:ACF1195
Acronicta ovata|[8025]|LGSM520-04|United States|Tennessee|658[0n]|BOLD:ACF1195
Acronicta ovata|[8026]|LGSM519-04|United States|North Carolina|658[0n]|BOLD:ACF1195
Acronicta ovata|[8027]|BBLCU172-09|United States|Michigan|599[0n]|BOLD:ACF1195
Acronicta ovata|[8028]|RDNMI091-10|Canada|Ontario|658[0n]|BOLD:ACF1195
Acronicta ovata|[8029]|RDNMI190-10|Canada||658[0n]|BOLD:ACF1195
Acronicta ovata|[8030]|LNCC1046-11|United States|North Carolina|658[0n]|BOLD:ACF1195
Acronicta ovata|[8031]|LNCC1047-11|United States|North Carolina|658[0n]|BOLD:ACF1195
Acronicta ovata|[8032]|LNCC1852-13|United States|North Carolina|658[0n]|BOLD:ACF1195
Acronicta sp. 1|[8033]|LOT323-04|United States|Tennessee|658[0n]|BOLD:ACF1194
Acronicta sp. 1|[8034]|LOTB483-05|United States|Tennessee|612[0n]|BOLD:ACF1194
Acronicta n sp.|[8035]|BBLCU024-09|United States|Michigan|621[0n]|BOLD:ACE9903
Acronicta n sp.|[8036]|BBLCU025-09|United States|Michigan|658[0n]|BOLD:ACE9903
Acronicta n sp.|[8037]|BBLCU026-09|United States|Michigan|658[0n]|BOLD:ACE9903
Acronicta n sp.|[8038]|BBLCU028-09|United States|Michigan|658[0n]|BOLD:ACE9903
Acronicta n sp.|[8039]|RDNMJ072-10|Canada|Ontario|658[0n]|BOLD:ACE9903
Acronicta n sp.|[8040]|RDNMJ629-11|Canada|Ontario|658[0n]|BOLD:ACE9903
Acronicta haesitata|[8041]|LOTB478-05|United States|Tennessee|610[0n]|BOLD:ACF1195
Acronicta haesitata|[8042]|RDLQ126-05|Canada|Quebec|658[0n]|BOLD:ACF1195
Acronicta haesitata|[8043]|LOTB292-05|United States|Tennessee|658[0n]|BOLD:ACF1195
Acronicta haesitata|[8044]|BBLCU185-09|United States|Michigan|658[0n]|BOLD:ACF1195
Acronicta haesitata|[8045]|LNCC100-10|United States|North Carolina|658[0n]|BOLD:ACF1195
Acronicta haesitata|[8046]|LNC245-05|United States|North Carolina|658[0n]|BOLD:ACF1195
Acronicta haesitata|[8047]|LNCC101-10|United States|North Carolina|658[0n]|BOLD:ACF1195
Acronicta haesitata|[8048]|RDNMI071-10|Canada|Ontario|621[0n]|BOLD:ACF1195
Acronicta haesitata|[8049]|LNCC1406-11|United States|North Carolina|658[0n]|BOLD:ACF1195
Acronicta haesitata|[8050]|LNCC1407-11|United States|North Carolina|658[0n]|BOLD:ACF1195
Acronicta haesitata|[8051]|RDNMI063-10|Canada|Ontario|658[0n]|BOLD:ACF1195
Acronicta haesitata|[8052]|LNC246-05|United States|North Carolina|658[0n]|BOLD:ACF1195
Acronicta haesitata|[8053]|RDNMI065-10|Canada|Ontario|612[0n]|BOLD:ACF1195
Acronicta albarufa|[8054]|RDNMG216-08|United States|New Jersey|658[0n]|BOLD:ACF1195
Acronicta exempta|[8055]|RDNMJ617-11|United States|New Mexico|658[0n]|BOLD:ACF1195
Acronicta exempta|[8056]|RDNMJ618-11|United States|New Mexico|658[0n]|BOLD:ACF1195

Acronicta albarufa|[8054]|RDNMG216-08|United States|New Jersey|658[0n]|BOLD:ACF1195
Acronicta exempta|[8055]|RDNMJ617-11|United States|New Mexico|658[0n]|BOLD:ACF1195
Acronicta exempta|[8056]|RDNMJ618-11|United States|New Mexico|658[0n]|BOLD:ACF1195
Acronicta haesitata|[8057]|RDNMI077-10|United States|Mississippi|658[0n]|BOLD:ACF1195
Acronicta haesitata|[8058]|LGSMC872-05|United States|Tennessee|591[0n]|BOLD:ACF1195
Acronicta haesitata|[8059]|LGSMG950-10|United States|Tennessee|658[0n]|BOLD:ACF1195
Acronicta haesitata|[8060]|LGSMG951-10|United States|Tennessee|658[0n]|BOLD:ACF1195
Acronicta haesitata|[8061]|LNCB960-10|United States|North Carolina|658[0n]|BOLD:ACF1195
Acronicta haesitata|[8062]|LNCC099-10|United States|North Carolina|658[0n]|BOLD:ACF1195
Acronicta haesitata|[8063]|QUNOB546-09|United States|Kentucky|658[0n]|BOLD:ACF1195
Acronicta haesitata|[8064]|BBLCU318-09|United States|Michigan|658[0n]|BOLD:ACF1195
Acronicta haesitata|[8065]|LSEU330-06|United States|Georgia|658[0n]|BOLD:ACF1195
Acronicta haesitata|[8066]|LSEU329-06|United States|Georgia|658[0n]|BOLD:ACF1195
Acronicta haesitata|[8067]|RDLQB564-05|Canada|Quebec|658[0n]|BOLD:ACF1195
Acronicta haesitata|[8068]|RDNM101-05|Canada|Ontario|658[0n]|BOLD:ACF1195
Acronicta haesitata|[8069]|LOTB480-05|United States|Tennessee|658[0n]|BOLD:ACF1195
Acronicta haesitata|[8070]|LOTB479-05|United States|Tennessee|658[0n]|BOLD:ACF1195
Acronicta haesitata|[8071]|LOTB477-05|United States|Tennessee|658[0n]|BOLD:ACF1195
Acronicta haesitata|[8072]|LOTB476-05|United States|Tennessee|658[0n]|BOLD:ACF1195
Acronicta haesitata|[8073]|LOTB475-05|United States|Tennessee|658[0n]|BOLD:ACF1195
Acronicta haesitata|[8074]|LOTB314-05|United States|Tennessee|658[0n]|BOLD:ACF1195
Acronicta haesitata|[8075]|LGSMC873-05|United States|Tennessee|658[0n]|BOLD:ACF1195
Acronicta haesitata|[8076]|LGSMC871-05|United States|Tennessee|658[0n]|BOLD:ACF1195
Acronicta haesitata|[8077]|LGSMC874-05|United States|Tennessee|615[0n]|BOLD:ACF1195
Acronicta haesitata|[8078]|LSEU389-06|United States|Georgia|612[0n]|BOLD:ACF1195
Acronicta haesitata|[8079]|QUNOB545-09|United States|Kentucky|658[0n]|BOLD:ACF1195
Acronicta haesitata|[8080]|RDNMI060-10|Canada|Ontario|658[0n]|BOLD:ACF1195
Acronicta haesitata|[8081]|RDNMI066-10|Canada|Ontario|658[0n]|BOLD:ACF1195
Acronicta haesitata|[8082]|RDNMJ078-10|United States|Texas|658[0n]|BOLD:ACF1195
Acronicta haesitata|[8083]|LNCC1855-13|United States|North Carolina|658[0n]|BOLD:ACF1195
Acronicta modica|[8084]|LOTB355-05|United States|Tennessee|614[0n]|BOLD:ACF1195
Acronicta modica|[8085]|BBLCU255-09|United States|Michigan|658[0n]|BOLD:ACF1195
Acronicta modica|[8086]|LOT293-04|United States|Tennessee|658[0n]|BOLD:ACF1195
Acronicta modica|[8087]|LOT315-04|United States|Tennessee|658[1n]|BOLD:ACF1195
Acronicta modica|[8088]|LOT294-04|United States|Tennessee|658[0n]|BOLD:ACF1195
Acronicta modica|[8089]|LOT314-04|United States|Tennessee|658[0n]|BOLD:ACF1195
Acronicta modica|[8090]|LGSMG952-10|United States|Tennessee|658[0n]|BOLD:ACF1195
Acronicta modica|[8091]|LNCC098-10|United States|North Carolina|658[0n]|BOLD:ACF1195
Acronicta modica|[8092]|RDNMI069-10|Canada|Ontario|658[0n]|BOLD:ACF1195
Acronicta modica|[8093]|RDNMK247-11|Canada|Ontario|658[0n]|BOLD:ACF1195
Acronicta modica|[8094]|RDNMK248-11|Canada|Ontario|658[0n]|BOLD:ACF1195
Acronicta modica|[8095]|LNCC1291-11|United States|North Carolina|658[0n]|BOLD:ACF1195
Acronicta modica|[8096]|LGSM522-04|United States|Tennessee|658[0n]|BOLD:ACF1195
Acronicta modica|[8097]|LOT316-04|United States|Tennessee|658[0n]|BOLD:ACF1195
Acronicta modica|[8098]|BBLCU173-09|United States|Michigan|658[0n]|BOLD:ACF1195
Acronicta modica|[8099]|LOT313-04|United States|Tennessee|658[0n]|BOLD:ACF1195
Acronicta modica|[8100]|BBLCU121-09|United States|Michigan|658[0n]|BOLD:ACF1195
Acronicta modica|[8101]|LGSMG947-10|United States|North Carolina|658[0n]|BOLD:ACF1195
Acronicta modica|[8102]|QUNOB547-09|United States|Kentucky|658[0n]|BOLD:ACF1195
Acronicta modica|[8103]|LGSMG948-10|United States|Tennessee|658[0n]|BOLD:ACF1195
Acronicta modica|[8104]|LSUSA156-06|United States|Kentucky|656[0n]|BOLD:ACF1195
Acronicta modica|[8105]|LGSM521-04|United States|Tennessee|658[0n]|BOLD:ACF1195
Acronicta modica|[8106]|LOT312-04|United States|Tennessee|658[0n]|BOLD:ACF1195
Acronicta modica|[8107]|LGSMC412-05|United States|Tennessee|658[0n]|BOLD:ACF1195
Acronicta modica|[8108]|BBLCU171-09|United States|Michigan|658[0n]|BOLD:ACF1195
Acronicta modica|[8109]|BBLCU317-09|United States|Michigan|658[0n]|BOLD:ACF1195
Acronicta modica|[8110]|LGSMG949-10|United States|North Carolina|658[0n]|BOLD:ACF1195
Acronicta modica|[8111]|QUNOB548-09|United States|Kentucky|658[0n]|BOLD:ACF1195
Acronicta modica|[8112]|BBLCU192-09|United States|Michigan|658[0n]|BOLD:ACF1195
Acronicta modica|[8113]|BBLCU200-09|United States|Michigan|658[0n]|BOLD:ACF1195
Acronicta modica|[8114]|RDNMI067-10|United States|North Carolina|658[0n]|BOLD:ACF1195
Acronicta modica|[8115]|BBLCU205-09|United States|Michigan|658[0n]|BOLD:ACF1195
Acronicta modica|[8116]|RDNMI068-10|United States|Mississippi|658[0n]|BOLD:ACF1195
Acronicta modica|[8117]|RDNMI070-10|United States|Mississippi|658[0n]|BOLD:ACF1195
Acronicta modica|[8118]|LNCC1856-13|United States|North Carolina|658[0n]|BOLD:ACF1195
Acronicta increta|[8119]|RDNMJ636-11|United States|Louisiana|525[0n]|BOLD:AAA7126
Acronicta increta|[8120]|RDNML034-13|United States|Louisiana|658[0n]|BOLD:AAA7126
Acronicta increta|[8121]|LPOKC629-09|United States|Oklahoma|658[0n]|BOLD:AAA7126
Acronicta increta|[8122]|LPOKD194-09|United States|Oklahoma|658[0n]|BOLD:AAA7126
Acronicta increta|[8123]|RDNMI073-10|United States|Mississippi|658[0n]|BOLD:AAA7126
Acronicta increta|[8124]|RDNMJ076-10|United States|Texas|658[0n]|BOLD:AAA7126
Acronicta increta|[8125]|RDNMI075-10|United States|Mississippi|658[0n]|BOLD:AAA7126
Acronicta increta|[8126]|RDNMI074-10|United States|Mississippi|658[0n]|BOLD:AAA7126
Acronicta increta|[8127]|LNCC104-10|United States|North Carolina|658[0n]|BOLD:AAA7126
Acronicta increta|[8128]|LNCC103-10|United States|North Carolina|658[0n]|BOLD:AAA7126
Acronicta increta|[8129]|LNCC102-10|United States|North Carolina|658[0n]|BOLD:AAA7126
Acronicta increta|[8130]|LNCB948-10|United States|North Carolina|658[0n]|BOLD:AAA7126
Acronicta increta|[8131]|LPOKC785-09|United States|Oklahoma|658[0n]|BOLD:AAA7126
Acronicta increta|[8132]|LPOKC773-09|United States|Oklahoma|658[0n]|BOLD:AAA7126
Acronicta increta|[8133]|LPOKB309-09|United States|Oklahoma|658[0n]|BOLD:AAA7126
Acronicta increta|[8134]|LPOKB279-09|United States|Oklahoma|658[0n]|BOLD:AAA7126
Acronicta increta|[8135]|LPOKB192-09|United States|Oklahoma|658[0n]|BOLD:AAA7126
Acronicta increta|[8136]|LPOKA403-09|United States|Oklahoma|658[0n]|BOLD:AAA7126
Acronicta increta|[8137]|LPOKA024-08|United States|Oklahoma|658[0n]|BOLD:AAA7126
Acronicta increta|[8138]|LSEU332-06|United States|Georgia|658[0n]|BOLD:AAA7126
Acronicta increta|[8139]|LSEU331-06|United States|Georgia|658[0n]|BOLD:AAA7126
Acronicta increta|[8140]|LOTB281-05|United States|Tennessee|658[0n]|BOLD:AAA7126
Acronicta increta|[8141]|LOTB274-05|United States|Tennessee|658[0n]|BOLD:AAA7126
Acronicta increta|[8142]|LGSMC862-05|United States|Tennessee|658[0n]|BOLD:AAA7126
Acronicta increta|[8143]|LOT523-04|United States|Tennessee|658[0n]|BOLD:AAA7126
Acronicta increta|[8144]|LOT324-04|United States|Tennessee|658[0n]|BOLD:AAA7126
Acronicta increta|[8145]|LOT306-04|United States|Tennessee|658[0n]|BOLD:AAA7126
Acronicta increta|[8146]|LGSM525-04|United States|Tennessee|658[0n]|BOLD:AAA7126
Acronicta increta|[8147]|RDNMJ077-10|United States|Texas|658[0n]|BOLD:AAA7126
Acronicta increta|[8148]|RDNMJ587-11|United States|Mississippi|658[0n]|BOLD:AAA7126
Acronicta increta|[8149]|LNCC676-11|United States|North Carolina|658[0n]|BOLD:AAA7126
Acronicta increta|[8150]|LNCC820-11|United States|North Carolina|658[0n]|BOLD:AAA7126
Acronicta increta|[8151]|LNCC1408-11|United States|North Carolina|658[0n]|BOLD:AAA7126
Acronicta increta|[8152]|RDNML030-13|United States|Louisiana|658[0n]|BOLD:AAA7126
Acronicta increta|[8153]|LNCC1060-11|United States|North Carolina|658[0n]|BOLD:AAA7126
Acronicta increta|[8154]|RDNMJ630-11|Canada|Ontario|658[0n]|BOLD:AAA7126
Acronicta increta|[8155]|LGSMC865-05|United States|Tennessee|658[0n]|BOLD:AAA7126
Acronicta increta|[8156]|LOTB282-05|United States|Tennessee|658[0n]|BOLD:AAA7126

Acronicta increta|[8153]|LNCC1060-11|United States|North Carolina|658[0n]|BOLD:AAA7126
Acronicta increta|[8154]|RDNMJ630-11|Canada|Ontario|658[0n]|BOLD:AAA7126
Acronicta increta|[8155]|LGSMC865-05|United States|Tennessee|658[0n]|BOLD:AAA7126
Acronicta increta|[8156]|LOTB282-05|United States|Tennessee|658[0n]|BOLD:AAA7126
Acronicta increta|[8157]|RDLQB552-05|Canada|Quebec|658[0n]|BOLD:AAA7126
Acronicta increta|[8158]|LGSMG953-10|United States|Tennessee|658[0n]|BOLD:AAA7126
Acronicta increta|[8159]|LGSMG956-10|United States|Tennessee|658[0n]|BOLD:AAA7126
Acronicta increta|[8160]|PSAT157-10|Canada|Ontario|658[0n]|BOLD:AAA7126
Acronicta increta|[8161]|LNCC1049-11|United States|North Carolina|658[0n]|BOLD:AAA7126
Acronicta increta|[8162]|LNCC1055-11|United States|North Carolina|658[0n]|BOLD:AAA7126
Acronicta increta|[8163]|LNCC1057-11|United States|North Carolina|658[0n]|BOLD:AAA7126
Acronicta increta|[8164]|LNCC1059-11|United States|North Carolina|658[0n]|BOLD:AAA7126
Acronicta increta|[8165]|LOT305-04|United States|Tennessee|658[0n]|BOLD:AAA7126
Acronicta increta|[8166]|LGSMC864-05|United States|Tennessee|658[0n]|BOLD:AAA7126
Acronicta increta|[8167]|LNCC1185-11|United States|North Carolina|658[0n]|BOLD:AAA7126
Acronicta increta|[8168]|XAE560-04|Canada|Ontario|568[0n]|BOLD:AAA7126
Acronicta increta|[8169]|MILEP366-10|United States|Alabama|658[0n]|BOLD:AAA7126
Acronicta increta|[8170]|PHMNB593-04|Canada|New Brunswick|658[0n]|BOLD:AAA7126
Acronicta increta|[8171]|LOTB279-05|United States|Tennessee|658[0n]|BOLD:AAA7126
Acronicta increta|[8172]|BBLCU027-09|United States|Michigan|658[0n]|BOLD:AAA7126
Acronicta increta|[8173]|RDNMI078-10|United States|Maryland|658[0n]|BOLD:AAA7126
Acronicta increta|[8174]|RDLQ133-05|Canada|Quebec|594[0n]|BOLD:AAA7126
Acronicta increta|[8175]|BBLPE262-09|Canada|Nova Scotia|658[0n]|BOLD:AAA7126
Acronicta increta|[8176]|LNCC1051-11|United States|North Carolina|658[0n]|BOLD:AAA7126
Acronicta increta|[8177]|LNCC1050-11|United States|North Carolina|658[0n]|BOLD:AAA7126
Acronicta increta|[8178]|RDNMK246-11|Canada|Ontario|658[0n]|BOLD:AAA7126
Acronicta increta|[8179]|RDNMK245-11|Canada|Ontario|658[0n]|BOLD:AAA7126
Acronicta increta|[8180]|RDNMJ659-11|Canada|Ontario|658[0n]|BOLD:AAA7126
Acronicta increta|[8181]|RDNMJ658-11|Canada|Ontario|658[0n]|BOLD:AAA7126
Acronicta increta|[8182]|RDNMJ336-11|Canada|Ontario|658[0n]|BOLD:AAA7126
Acronicta increta|[8183]|LNCC272-10|United States|North Carolina|658[0n]|BOLD:AAA7126
Acronicta increta|[8184]|LNCC271-10|United States|North Carolina|658[0n]|BOLD:AAA7126
Acronicta increta|[8185]|RDNMI189-10|Canada||658[0n]|BOLD:AAA7126
Acronicta increta|[8186]|RDNMI089-10|Canada|New Brunswick|658[0n]|BOLD:AAA7126
Acronicta increta|[8187]|RDNMI083-10|Canada|Ontario|658[0n]|BOLD:AAA7126
Acronicta increta|[8188]|RDNMI082-10|Canada|Ontario|658[0n]|BOLD:AAA7126
Acronicta increta|[8189]|RDNMI081-10|Canada|Ontario|658[0n]|BOLD:AAA7126
Acronicta increta|[8190]|RDNMI080-10|Canada|Ontario|658[0n]|BOLD:AAA7126
Acronicta increta|[8191]|RDNMI079-10|United States|Maryland|658[0n]|BOLD:AAA7126
Acronicta increta|[8192]|RDNMI064-10|Canada|New Brunswick|658[0n]|BOLD:AAA7126
Acronicta increta|[8193]|RDNMI062-10|Canada|Ontario|658[0n]|BOLD:AAA7126
Acronicta increta|[8194]|MILEP368-10|United States|Alabama|658[0n]|BOLD:AAA7126
Acronicta increta|[8195]|MILEP367-10|United States|Alabama|658[0n]|BOLD:AAA7126
Acronicta increta|[8196]|MILEP365-10|United States|Alabama|658[0n]|BOLD:AAA7126
Acronicta increta|[8197]|LNCC051-10|United States|North Carolina|658[0n]|BOLD:AAA7126
Acronicta increta|[8198]|LNCC050-10|United States|North Carolina|658[0n]|BOLD:AAA7126
Acronicta increta|[8199]|LNCC049-10|United States|North Carolina|658[0n]|BOLD:AAA7126
Acronicta increta|[8200]|LNCC048-10|United States|North Carolina|658[0n]|BOLD:AAA7126
Acronicta increta|[8201]|LNCB947-10|United States|North Carolina|658[0n]|BOLD:AAA7126
Acronicta increta|[8202]|LGSMG958-10|United States|Tennessee|658[0n]|BOLD:AAA7126
Acronicta increta|[8203]|LGSMG957-10|United States|Tennessee|658[0n]|BOLD:AAA7126
Acronicta increta|[8204]|LGSMG954-10|United States|Tennessee|658[0n]|BOLD:AAA7126
Acronicta increta|[8205]|BBLCU319-09|United States|Michigan|658[0n]|BOLD:AAA7126
Acronicta increta|[8206]|LPMNB322-09|Canada|Manitoba|658[0n]|BOLD:AAA7126
Acronicta increta|[8207]|NAMUM415-09|United States|Maryland|658[0n]|BOLD:AAA7126
Acronicta increta|[8208]|QUNOB549-09|United States|Kentucky|658[0n]|BOLD:AAA7126
Acronicta increta|[8209]|RDLQG038-06|Canada|Quebec|658[0n]|BOLD:AAA7126
Acronicta increta|[8210]|LSEU333-06|United States|Georgia|658[0n]|BOLD:AAA7126
Acronicta increta|[8211]|RDLQB554-05|Canada|Quebec|658[0n]|BOLD:AAA7126
Acronicta increta|[8212]|RDNM102-05|Canada|Ontario|658[0n]|BOLD:AAA7126
Acronicta increta|[8213]|XAF738-05|Canada|Ontario|658[0n]|BOLD:AAA7126
Acronicta increta|[8214]|LOTB481-05|United States|Tennessee|658[0n]|BOLD:AAA7126
Acronicta increta|[8215]|LGSMC870-05|United States|Tennessee|658[0n]|BOLD:AAA7126
Acronicta increta|[8216]|LGSMC411-05|United States|Tennessee|658[0n]|BOLD:AAA7126
Acronicta increta|[8217]|RDLQ140-05|Canada|Quebec|658[0n]|BOLD:AAA7126
Acronicta increta|[8218]|RDLQ139-05|Canada|Quebec|658[0n]|BOLD:AAA7126
Acronicta increta|[8219]|RDLQ138-05|Canada|Quebec|658[0n]|BOLD:AAA7126
Acronicta increta|[8220]|RDLQ136-05|Canada|Quebec|658[0n]|BOLD:AAA7126
Acronicta increta|[8221]|RDLQ135-05|Canada|Quebec|658[0n]|BOLD:AAA7126
Acronicta increta|[8222]|RDLQ134-05|Canada|Quebec|658[0n]|BOLD:AAA7126
Acronicta increta|[8223]|RDLQ131-05|Canada|Quebec|658[0n]|BOLD:AAA7126
Acronicta increta|[8224]|RDLQ130-05|Canada|Quebec|658[0n]|BOLD:AAA7126
Acronicta increta|[8225]|RDLQ129-05|Canada|Quebec|658[0n]|BOLD:AAA7126
Acronicta increta|[8226]|XAC664-04|Canada|Ontario|658[0n]|BOLD:AAA7126
Acronicta increta|[8227]|XAC663-04|Canada|Ontario|658[0n]|BOLD:AAA7126
Acronicta increta|[8228]|LOT318-04|United States|Tennessee|658[0n]|BOLD:AAA7126
Acronicta increta|[8229]|LOT307-04|United States|Tennessee|658[0n]|BOLD:AAA7126
Acronicta increta|[8230]|LGSM526-04|United States|Tennessee|658[0n]|BOLD:AAA7126
Acronicta increta|[8231]|LGSM524-04|United States|Tennessee|658[0n]|BOLD:AAA7126
Acronicta increta|[8232]|LGSM523-04|United States|North Carolina|658[0n]|BOLD:AAA7126
Acronicta increta|[8233]|LPOKB247-09|United States|Oklahoma|621[0n]|BOLD:AAA7126
Acronicta increta|[8234]|RDNMI085-10|Canada|New Brunswick|658[0n]|BOLD:AAA7126
Acronicta increta|[8235]|LGSMC863-05|United States|Tennessee|617[0n]|BOLD:AAA7126
Acronicta increta|[8236]|LNCC383-10|United States|North Carolina|624[0n]|BOLD:AAA7126
Acronicta increta|[8237]|TTMNB327-06|Canada|New Brunswick|649[3n]|BOLD:AAA7126
Acronicta increta|[8238]|RDNMI061-10|Canada|Manitoba|541[0n]|BOLD:AAA7126
Acronicta increta|[8239]|LNCC1056-11|United States|North Carolina|621[0n]|BOLD:AAA7126
Acronicta increta|[8240]|LNCC1058-11|United States|North Carolina|658[0n]|BOLD:AAA7126
Acronicta increta|[8241]|LNCC1063-11|United States|North Carolina|658[0n]|BOLD:AAA7126
Acronicta increta|[8242]|LNCC1064-11|United States|North Carolina|658[0n]|BOLD:AAA7126
Acronicta increta|[8243]|LNCC1065-11|United States|North Carolina|658[0n]|BOLD:AAA7126
Acronicta increta|[8244]|LNCC1184-11|United States|North Carolina|658[0n]|BOLD:AAA7126
Acronicta increta|[8245]|LNCC1186-11|United States|North Carolina|658[0n]|BOLD:AAA7126
Acronicta increta|[8246]|LNCC1368-11|United States|North Carolina|658[0n]|BOLD:AAA7126
Acronicta increta|[8247]|LNCC1409-11|United States|North Carolina|658[0n]|BOLD:AAA7126
Acronicta increta|[8248]|RDNML031-13|United States|Louisiana|658[0n]|BOLD:AAA7126
Acronicta increta|[8249]|RDNML036-13|United States|Louisiana|658[0n]|BOLD:AAA7126
Acronicta increta|[8250]|LNCC1853-13|United States|North Carolina|658[0n]|BOLD:AAA7126
Acronicta increta|[8251]|LNCC1854-13|United States|North Carolina|658[0n]|BOLD:AAA7126
Acronicta increta|[8252]|LNCC1857-13|United States|North Carolina|658[0n]|BOLD:AAA7126
Acronicta increta|[8253]|LNCC1858-13|United States|North Carolina|658[0n]|BOLD:AAA7126
Acronicta increta|[8254]|CNCLB2583-14|United States|North Carolina|658[0n]|BOLD:AAA7126
Acronicta exilis|[8255]|RDNMI072-10|United States|Mississippi|658[0n]|BOLD:AAA7126

Acronicta increta|[8253]|LNCC1858-13|United States|North Carolina|658[0n]|BOLD:AAA7126
Acronicta increta|[8254]|CNCLB2583-14|United States|North Carolina|658[0n]|BOLD:AAA7126
Acronicta exilis|[8255]|RDNMI072-10|United States|Mississippi|658[0n]|BOLD:AAA7126
Acronicta exilis|[8256]|BBLOC1192-11|United States|Texas|634[0n]|BOLD:AAA7126
Acronicta exilis|[8257]|LNCC384-10|United States|North Carolina|658[0n]|BOLD:AAA7126
Acronicta exilis|[8258]|LGSMG943-10|United States|Tennessee|658[0n]|BOLD:AAA7126
Acronicta exilis|[8259]|RDNMJ664-11|United States|Maryland|658[0n]|BOLD:AAA7126
Acronicta exilis|[8260]|LPOKC306-09|United States|Oklahoma|658[0n]|BOLD:AAA7126
Acronicta exilis|[8261]|RDNMJ075-10|United States|Texas|658[0n]|BOLD:AAA7126
Acronicta exilis|[8262]|LPOKB724-09|United States|Oklahoma|658[0n]|BOLD:AAA7126
Acronicta exilis|[8263]|LPOKB692-09|United States|Oklahoma|658[0n]|BOLD:AAA7126
Acronicta exilis|[8264]|LPOKB274-09|United States|Oklahoma|658[0n]|BOLD:AAA7126
Acronicta exilis|[8265]|LPOKA172-08|United States|Oklahoma|658[0n]|BOLD:AAA7126
Acronicta exilis|[8266]|LGSM562-04|United States|Tennessee|658[0n]|BOLD:AAA7126
Acronicta exilis|[8267]|RDNMJ644-11|United States|Florida|632[0n]|BOLD:AAA7126
Acronicta exilis|[8268]|QUNOD738-11|United States|Texas|658[0n]|BOLD:AAA7126
Acronicta exilis|[8269]|LILLA366-11|United States|Illinois|658[0n]|BOLD:AAA7126
Acronicta exilis|[8270]|CNCLB2603-14|United States|North Carolina|658[0n]|BOLD:AAA7126
Acronicta impressa|[8271]|LBCC003-05|Canada|British Columbia|658[0n]|BOLD:ACF2279
Acronicta impressa|[8272]|LBCC762-05|Canada|British Columbia|658[0n]|BOLD:ACF2279
Acronicta impressa|[8273]|RDNMK838-12|Canada|British Columbia|658[0n]|BOLD:ACF2279
Acronicta impressa|[8274]|LBCC291-05|Canada|British Columbia|658[0n]|BOLD:ACF2279
Acronicta impressa|[8275]|RWWA257-09|United States|Washington|658[0n]|BOLD:ACF2279
Acronicta impressa|[8276]|RWWA750-09|United States|Washington|658[0n]|BOLD:ACF2279
Acronicta impressa|[8277]|RWWB601-10|United States|Washington|658[0n]|BOLD:ACF2279
Acronicta impressa|[8278]|RWWC797-11|United States|Washington|658[0n]|BOLD:ACF2279
Acronicta impressa|[8279]|RWWB669-10|United States|Washington|658[0n]|BOLD:ACF2279
Acronicta impressa|[8280]|RDNMK684-11|United States|California|658[0n]|BOLD:ACF2279
Acronicta impressa|[8281]|RWWB640-10|United States|Washington|658[0n]|BOLD:ACF2279
Acronicta impressa|[8282]|RWWB614-10|United States|Washington|658[0n]|BOLD:ACF2279
Acronicta impressa|[8283]|RWWB609-10|United States|Washington|658[0n]|BOLD:ACF2279
Acronicta impressa|[8284]|RWWB602-10|United States|Washington|658[0n]|BOLD:ACF2279
Acronicta impressa|[8285]|RWWB005-09|United States|Washington|658[0n]|BOLD:ACF2279
Acronicta impressa|[8286]|RWWA818-09|United States|Washington|658[0n]|BOLD:ACF2279
Acronicta impressa|[8287]|RDNMG968-08|United States|California|658[0n]|BOLD:ACF2279
Acronicta impressa|[8288]|RDNMJ420-11|United States|California|658[0n]|BOLD:ACF2279
Acronicta impressa|[8289]|RWWC189-11|United States|Washington|651[0n]|BOLD:ACF2279
Acronicta impressa|[8290]|RDNMK767-12|Canada|Alberta|658[0n]|BOLD:ACF2279
Acronicta impressa|[8291]|RDNMK685-11|United States|Nevada|658[0n]|BOLD:ACF2279
Acronicta impressa|[8292]|RWWC1055-12|United States|Washington|658[0n]|BOLD:ACF2279
Acronicta impressa|[8293]|RDLQ192-05|Canada|Quebec|553[0n]|BOLD:AAA7955
Acronicta impressa|[8294]|RDLQB524-05|Canada|Quebec|658[0n]|BOLD:AAA7955
Acronicta impressa|[8295]|XAG707-05|Canada|Ontario|658[0n]|BOLD:AAA7955
Acronicta impressa|[8296]|RDLQ305-05|Canada|Quebec|656[0n]|BOLD:AAA7955
Acronicta impressa|[8297]|RDLQ291-05|Canada|Quebec|658[0n]|BOLD:AAA7955
Acronicta impressa|[8298]|PMG083-03|Canada|Ontario|617[0n]|BOLD:AAA7955
Acronicta impressa|[8299]|RDNMB609-05|Canada|Quebec|535[0n]|BOLD:AAA7955
Acronicta impressa|[8300]|XAJ136-06|Canada|Ontario|634[0n]|BOLD:AAA7955
Acronicta impressa|[8301]|XAF423-05|Canada|Ontario|658[0n]|BOLD:AAA7955
Acronicta impressa|[8302]|RDLQ405-05|Canada|Quebec|658[0n]|BOLD:AAA7955
Acronicta impressa|[8303]|RDNMB607-05|Canada|Quebec|658[0n]|BOLD:AAA7955
Acronicta impressa|[8304]|RDLQB736-05|Canada|Quebec|658[0n]|BOLD:AAA7955
Acronicta impressa|[8305]|XAK284-06|Canada|Ontario|658[0n]|BOLD:AAA7955
Acronicta impressa|[8306]|RDNMK687-11|Canada|Alberta|658[0n]|BOLD:AAA7955
Acronicta impressa|[8307]|RDNMK763-12|Canada|Alberta|658[0n]|BOLD:AAA7955
Acronicta impressa|[8308]|RDNMK824-12|United States|North Carolina|658[0n]|BOLD:AAA7955
Acronicta impressa|[8309]|RDNMK686-11|United States|Colorado|658[0n]|BOLD:AAA7955
Acronicta impressa|[8310]|RDNMK839-12|United States|New Mexico|658[0n]|BOLD:AAA7955
Acronicta impressa|[8311]|RDNMK765-12|Canada|Alberta|658[0n]|BOLD:AAA7955
Acronicta impressa|[8312]|LMIS024-05|Canada|Ontario|658[0n]|BOLD:AAA7955
Acronicta impressa|[8313]|RDLQ143-05|Canada|Quebec|658[0n]|BOLD:AAA7955
Acronicta impressa|[8314]|XAF406-05|Canada|Ontario|658[0n]|BOLD:AAA7955
Acronicta impressa|[8315]|TTMNB007-06|Canada|New Brunswick|656[0n]|BOLD:AAA7955
Acronicta impressa|[8316]|TTMNB005-06|Canada|New Brunswick|656[0n]|BOLD:AAA7955
Acronicta impressa|[8317]|TTMNB009-06|Canada|New Brunswick|657[0n]|BOLD:AAA7955
Acronicta impressa|[8318]|RDLQB166-05|Canada|Quebec|658[0n]|BOLD:AAA7955
Acronicta impressa|[8319]|RDNMB610-05|Canada|Quebec|658[0n]|BOLD:AAA7955
Acronicta impressa|[8320]|RDNMB608-05|Canada|Quebec|658[0n]|BOLD:AAA7955
Acronicta impressa|[8321]|RDLQB167-05|Canada|Quebec|658[0n]|BOLD:AAA7955
Acronicta impressa|[8322]|TMG114-03|Canada|Ontario|639[0n]|BOLD:AAA7955
Acronicta impressa|[8323]|RDLQ299-05|Canada|Quebec|593[0n]|BOLD:AAA7955
Acronicta impressa|[8324]|RDLQF690-06|Canada|Quebec|637[0n]|BOLD:AAA7955
Acronicta impressa|[8325]|XAD665-05|Canada|Ontario|658[0n]|BOLD:AAA7955
Acronicta impressa|[8326]|TTMNB008-06|Canada|New Brunswick|656[0n]|BOLD:AAA7955
Acronicta impressa|[8327]|RDNMK768-12|Canada|Alberta|658[0n]|BOLD:AAA7955
Acronicta impressa|[8328]|RDNMK766-12|Canada|Alberta|658[0n]|BOLD:AAA7955
Acronicta impressa|[8329]|RDNMK764-12|Canada|Alberta|658[0n]|BOLD:AAA7955
Acronicta impressa|[8330]|RDNMK689-11|Canada|Alberta|658[0n]|BOLD:AAA7955
Acronicta impressa|[8331]|RDNMK688-11|United States|Minnesota|658[0n]|BOLD:AAA7955
Acronicta impressa|[8332]|BBLPC511-09|Canada|New Brunswick|658[0n]|BOLD:AAA7955
Acronicta impressa|[8333]|BBLPC486-09|Canada|New Brunswick|658[0n]|BOLD:AAA7955
Acronicta impressa|[8334]|BBLPC381-09|Canada|New Brunswick|658[0n]|BOLD:AAA7955
Acronicta impressa|[8335]|CHLEP082-09|Canada|Manitoba|658[0n]|BOLD:AAA7955
Acronicta impressa|[8336]|LPSOD524-09|Canada|Ontario|658[0n]|BOLD:AAA7955
Acronicta impressa|[8337]|XAK011-06|Canada|Ontario|658[0n]|BOLD:AAA7955
Acronicta impressa|[8338]|MNBB449-05|Canada|New Brunswick|658[0n]|BOLD:AAA7955
Acronicta impressa|[8339]|RDLQ404-05|Canada|Quebec|658[0n]|BOLD:AAA7955
Acronicta impressa|[8340]|RDLQ453-07|Canada|Quebec|613[0n]|BOLD:AAA7955
Acronicta menyanthidis|[8341]|UAMIC479-13|United States|Alaska|635[0n]|BOLD:AAA7955
Acronicta menyanthidis|[8342]|RDNML284-13|United States|Alaska|658[0n]|BOLD:AAA7955
Acronicta sinescripta|[8343]|ABNCC461-07|United States|Florida|642[0n]|BOLD:AAS0516
Acronicta lanceolaria|[8344]|RDNMB892-05|Canada|Alberta|658[0n]|BOLD:AAJ1615
Acronicta lanceolaria|[8345]|RDNMG539-08|Canada|Alberta|658[0n]|BOLD:AAJ1615
Acronicta lanceolaria|[8346]|CNCLA553-13|United States|Florida|658[0n]|BOLD:AAJ1615
Acronicta extricata|[8347]|RDNMK804-12|United States|Oklahoma|658[0n]|BOLD:AAV9947
Acronicta extricata|[8348]|RDNMK801-12|United States|Texas|658[0n]|BOLD:AAV9947
Acronicta extricata|[8349]|RDNMK805-12|United States|Texas|658[0n]|BOLD:AAV9947
Acronicta extricata|[8350]|RDNMK806-12|United States|Texas|658[0n]|BOLD:AAV9947
Acronicta extricata|[8351]|RDNMK807-12|United States|Texas|658[0n]|BOLD:AAV9947
Acronicta edolata|[8352]|RDNMJ092-10|United States|Texas|658[2n]|BOLD:AAV9947
Acronicta edolata|[8353]|IAWLB613-11|United States|Arizona|658[0n]|BOLD:AAV9947
Acronicta edolata|[8354]|IAWLB614-11|United States|Arizona|658[0n]|BOLD:AAV9947
[illegible]

Acronicta edolata|[8352]|RDNMJ092-10|United States|Texas|658[2n]|BOLD:AAV9947
Acronicta edolata|[8353]|IAWLB613-11|United States|Arizona|658[0n]|BOLD:AAV9947
Acronicta edolata|[8354]|IAWLB614-11|United States|Arizona|658[0n]|BOLD:AAV9947
Acronicta edolata|[8355]|RDNMJ091-10|United States|Texas|658[0n]|BOLD:AAV9947
Acronicta edolata|[8356]|RDNMI008-10|United States|Arizona|658[0n]|BOLD:AAV9947
Acronicta edolata|[8357]|RDNMJ625-11|United States|Oklahoma|658[0n]|BOLD:AAV9947
Acronicta edolata|[8358]|RDNMK802-12|United States|New Mexico|658[0n]|BOLD:AAV9947
Acronicta edolata|[8359]|RDNMK803-12|United States|Wyoming|658[0n]|BOLD:AAV9947
Acronicta sagittata|[8360]|RDNML028-13|United States|Utah|658[0n]|BOLD:AAV9947
Acronicta insularis|[8361]|LNC517-06|United States|North Carolina|522[7n]|BOLD:AAA7111
Acronicta insularis|[8362]|BBLOD962-11|United States|Texas|658[0n]|BOLD:AAA7111
Acronicta insularis|[8363]|XAC154-04|Canada|Ontario|658[0n]|BOLD:AAA7111
Acronicta insularis|[8364]|BBLOD960-11|United States|Texas|658[0n]|BOLD:AAA7111
Acronicta insularis|[8365]|HKONB387-09|United States|Indiana|658[0n]|BOLD:AAA7111
Acronicta insularis|[8366]|LOFLB748-06|United States|Florida|636[0n]|BOLD:AAA7111
Acronicta insularis|[8367]|LOFLB485-06|United States|Florida|635[0n]|BOLD:AAA7111
Acronicta insularis|[8368]|LOFLB496-06|United States|Florida|658[0n]|BOLD:AAA7111
Acronicta insularis|[8369]|LNCB325-06|United States|North Carolina|658[0n]|BOLD:AAA7111
Acronicta insularis|[8370]|LPOKA148-08|United States|Oklahoma|658[0n]|BOLD:AAA7111
Acronicta insularis|[8371]|LOFLC407-06|United States|Florida|658[0n]|BOLD:AAA7111
Acronicta insularis|[8372]|LOFLC370-06|United States|Florida|658[0n]|BOLD:AAA7111
Acronicta insularis|[8373]|LOFLB720-06|United States|Florida|658[0n]|BOLD:AAA7111
Acronicta insularis|[8374]|LOFLB525-06|United States|Florida|658[0n]|BOLD:AAA7111
Acronicta insularis|[8375]|LOFLB517-06|United States|Florida|658[0n]|BOLD:AAA7111
Acronicta insularis|[8376]|LOFLB401-06|United States|Florida|658[0n]|BOLD:AAA7111
Acronicta insularis|[8377]|LOFLB390-06|United States|Florida|658[0n]|BOLD:AAA7111
Acronicta insularis|[8378]|LOFLB210-06|United States|Florida|658[0n]|BOLD:AAA7111
Acronicta insularis|[8379]|LOFLB140-06|United States|Florida|658[0n]|BOLD:AAA7111
Acronicta insularis|[8380]|LOFLB135-06|United States|Florida|658[0n]|BOLD:AAA7111
Acronicta insularis|[8381]|LOFLB123-06|United States|Florida|658[0n]|BOLD:AAA7111
Acronicta insularis|[8382]|LOFLB113-06|United States|Florida|658[0n]|BOLD:AAA7111
Acronicta insularis|[8383]|LOFLB264-06|United States|Florida|626[0n]|BOLD:AAA7111
Acronicta insularis|[8384]|LOFLB446-06|United States|Florida|627[0n]|BOLD:AAA7111
Acronicta insularis|[8385]|LNCB166-06|United States|North Carolina|658[0n]|BOLD:AAA7111
Acronicta insularis|[8386]|LOFLB473-06|United States|Florida|658[0n]|BOLD:AAA7111
Acronicta insularis|[8387]|LNCB841-09|United States|Alabama|658[0n]|BOLD:AAA7111
Acronicta insularis|[8388]|BBLOD943-11|United States|West Virginia|658[0n]|BOLD:AAA7111
Acronicta insularis|[8389]|BBLOD963-11|United States|Texas|658[0n]|BOLD:AAA7111
Acronicta insularis|[8390]|BBLOD961-11|United States|Texas|658[0n]|BOLD:AAA7111
Acronicta insularis|[8391]|RDLQB922-05|Canada|Quebec|580[3n]|BOLD:AAA7111
Acronicta insularis|[8392]|XAK532-07|Canada|Ontario|658[0n]|BOLD:AAA7111
Acronicta insularis|[8393]|XAK531-07|Canada|Ontario|658[0n]|BOLD:AAA7111
Acronicta insularis|[8394]|RDLQH082-06|Canada|Quebec|658[0n]|BOLD:AAA7111
Acronicta insularis|[8395]|RDLQG222-06|Canada|Quebec|658[0n]|BOLD:AAA7111
Acronicta insularis|[8396]|RDLQG221-06|Canada|Quebec|658[0n]|BOLD:AAA7111
Acronicta insularis|[8397]|XAK260-06|Canada|Ontario|658[0n]|BOLD:AAA7111
Acronicta insularis|[8398]|RDLQB921-05|Canada|Quebec|658[0n]|BOLD:AAA7111
Acronicta insularis|[8399]|RDLQB706-05|Canada|Quebec|658[0n]|BOLD:AAA7111
Acronicta insularis|[8400]|RDLQB499-05|Canada|Quebec|658[0n]|BOLD:AAA7111
Acronicta insularis|[8401]|XAG014-05|Canada|Ontario|658[0n]|BOLD:AAA7111
Acronicta insularis|[8402]|XAD660-05|Canada|Ontario|658[0n]|BOLD:AAA7111
Acronicta insularis|[8403]|XAC181-04|Canada|Ontario|658[0n]|BOLD:AAA7111
Acronicta insularis|[8404]|XAC153-04|Canada|Ontario|658[0n]|BOLD:AAA7111
Acronicta insularis|[8405]|PMG161-03|Canada|Ontario|617[0n]|BOLD:AAA7111
Acronicta insularis|[8406]|XAK600-07|Canada|Ontario|592[0n]|BOLD:AAA7111
Acronicta insularis|[8407]|BLTIB057-08|Canada|Ontario|658[0n]|BOLD:AAA7111
Acronicta insularis|[8408]|TMG115-03|Canada|Ontario|639[0n]|BOLD:AAA7111
Acronicta insularis|[8409]|RDMAB709-06|Canada|Alberta|566[0n]|BOLD:AAA7111
Acronicta insularis|[8410]|LOCBC662-06|United States|California|658[0n]|BOLD:AAA7111
Acronicta insularis|[8411]|RDMAB718-06|Canada|Alberta|658[0n]|BOLD:AAA7111
Acronicta insularis|[8412]|RDNME568-08|United States|California|658[0n]|BOLD:AAA7111
Acronicta insularis|[8413]|LPMN297-08|Canada|Manitoba|634[1n]|BOLD:AAA7111
Acronicta insularis|[8414]|IAWLB646-11|United States|Arizona|658[0n]|BOLD:AAA7111
Acronicta insularis|[8415]|BBLOD951-11|United States|Texas|658[0n]|BOLD:AAA7111
Acronicta insularis|[8416]|CMAZA906-12|United States|Arizona|658[0n]|BOLD:AAA7111
Acronicta insularis|[8417]|CMAZA937-12|United States|Arizona|658[0n]|BOLD:AAA7111
Acronicta insularis|[8418]|RDMAB710-06|Canada|Alberta|636[0n]|BOLD:AAA7111
Acronicta insularis|[8419]|XAE638-04|Canada|Ontario|658[0n]|BOLD:AAA7111
Acronicta insularis|[8420]|XAG013-05|Canada|Ontario|658[0n]|BOLD:AAA7111
Acronicta insularis|[8421]|RDLQB429-05|Canada|Quebec|658[0n]|BOLD:AAA7111
Acronicta insularis|[8422]|XAJ453-06|Canada|Ontario|658[0n]|BOLD:AAA7111
Acronicta insularis|[8423]|HKONB474-09|United States|Florida|658[0n]|BOLD:AAA7111
Acronicta insularis|[8424]|LPOKC777-09|United States|Oklahoma|658[0n]|BOLD:AAA7111
Acronicta insularis|[8425]|LPOKE284-11|United States|Oklahoma|658[0n]|BOLD:AAA7111
Acronicta insularis|[8426]|NCCH103-11|Canada|Ontario|658[0n]|BOLD:AAA7111
Acronicta insularis|[8427]|CNPPE1049-12|Canada|Ontario|638[0n]|BOLD:AAA7111
Acronicta insularis|[8428]|LOCBF3630-14|United States|California|588[0n]|BOLD:AAA7111
Acronicta oblinita|[8429]|LOCT237-05|United States|Connecticut|614[0n]|BOLD:AAB1100
Acronicta oblinita|[8430]|RWWC403-11|United States|Washington|658[0n]|BOLD:AAB1100
Acronicta oblinita|[8431]|XAE309-04|Canada|Ontario|658[0n]|BOLD:AAB1100
Acronicta oblinita|[8432]|RDLQB163-05|Canada|Quebec|658[0n]|BOLD:AAB1100
Acronicta oblinita|[8433]|RDLQB804-05|Canada|Quebec|615[0n]|BOLD:AAB1100
Acronicta oblinita|[8434]|XAF755-05|Canada|Ontario|587[4n]|BOLD:AAB1100
Acronicta oblinita|[8435]|XAI059-05|Canada|Ontario|658[0n]|BOLD:AAB1100
Acronicta oblinita|[8436]|RDLQB801-05|Canada|Quebec|658[0n]|BOLD:AAB1100
Acronicta oblinita|[8437]|RDLQB803-05|Canada|Quebec|658[0n]|BOLD:AAB1100
Acronicta oblinita|[8438]|BBLPE033-09|Canada|Nova Scotia|658[0n]|BOLD:AAB1100
Acronicta oblinita|[8439]|RWWA530-09|United States|Washington|658[0n]|BOLD:AAB1100
Acronicta oblinita|[8440]|RWWC996-12|United States|Washington|658[0n]|BOLD:AAB1100
Acronicta oblinita|[8441]|LPSOB600-08|Canada|Ontario|658[0n]|BOLD:AAB1100
Acronicta oblinita|[8442]|RDLQB802-05|Canada|Quebec|563[1n]|BOLD:AAB1100
Acronicta oblinita|[8443]|XAJ594-06|Canada|Ontario|656[0n]|BOLD:AAB1100
Acronicta oblinita|[8444]|XAJ400-06|Canada|Ontario|658[0n]|BOLD:AAB1100
Acronicta oblinita|[8445]|PHMNB211-04|Canada|New Brunswick|589[3n]|BOLD:AAB1100
Acronicta oblinita|[8446]|RDLQ290-05|Canada|Quebec|597[0n]|BOLD:AAB1100
Acronicta oblinita|[8447]|TTMNB030-06|Canada|New Brunswick|658[0n]|BOLD:AAB1100
Acronicta oblinita|[8448]|LOFLC377-06|United States|Florida|658[0n]|BOLD:AAB1100
Acronicta oblinita|[8449]|USLEP634-10|United States|Florida|658[0n]|BOLD:AAB1100
Acronicta oblinita|[8450]|LPSO544-08|Canada|Ontario|658[0n]|BOLD:AAB1100
Acronicta oblinita|[8451]|LPSO847-08|Canada|Ontario|658[0n]|BOLD:AAB1100
Acronicta oblinita|[8452]|LPSO525-08|Canada|Ontario|656[0n]|BOLD:AAB1100
Acronicta oblinita|[8453]|XAC625-04|Canada|Ontario|615[2n]|BOLD:AAB1100
Acronicta oblinita|[8454]|XAE206-04|Canada|Ontario|658[0n]|BOLD:AAB1100

Acronicta oblinita|[8452]|LPSO525-08|Canada|Ontario|656[0n]|BOLD:AAB1100
Acronicta oblinita|[8453]|XAC625-04|Canada|Ontario|615[2n]|BOLD:AAB1100
Acronicta oblinita|[8454]|XAE206-04|Canada|Ontario|658[0n]|BOLD:AAB1100
Acronicta oblinita|[8455]|LSEU507-06|United States|Georgia|658[0n]|BOLD:AAB1100
Acronicta oblinita|[8456]|LPOKA975-09|United States|Oklahoma|658[0n]|BOLD:AAB1100
Acronicta oblinita|[8457]|LPOKA1002-09|United States|Oklahoma|658[0n]|BOLD:AAB1100
Acronicta oblinita|[8458]|LPOKB120-09|United States|Oklahoma|658[0n]|BOLD:AAB1100
Acronicta oblinita|[8459]|LPOKB187-09|United States|Oklahoma|658[0n]|BOLD:AAB1100
Acronicta oblinita|[8460]|LPOKB260-09|United States|Oklahoma|658[0n]|BOLD:AAB1100
Acronicta oblinita|[8461]|LPOKB1005-09|United States|Oklahoma|658[0n]|BOLD:AAB1100
Acronicta oblinita|[8462]|LPOKB1006-09|United States|Oklahoma|658[0n]|BOLD:AAB1100
Acronicta oblinita|[8463]|LPOKD717-10|United States|Oklahoma|658[0n]|BOLD:AAB1100
Acronicta oblinita|[8464]|LPOKD726-10|United States|Oklahoma|658[0n]|BOLD:AAB1100
Acronicta oblinita|[8465]|RDNMJ090-10|United States|Texas|658[0n]|BOLD:AAB1100
Acronicta oblinita|[8466]|LPOKE279-11|United States|Oklahoma|658[0n]|BOLD:AAB1100
Acronicta oblinita|[8467]|LPOKE280-11|United States|Oklahoma|658[0n]|BOLD:AAB1100
Acronicta oblinita|[8468]|LNCC1259-11|United States|North Carolina|658[0n]|BOLD:AAB1100
Acronicta oblinita|[8469]|LNCC1521-13|United States|North Carolina|658[0n]|BOLD:AAB1100
Acronicta dolli|[8470]|NAMUM420-09|United States|West Virginia|658[0n]|BOLD:AAD5475
Acronicta dolli|[8471]|NAMUM421-09|United States|West Virginia|658[0n]|BOLD:AAD5475
Acronicta dolli|[8472]|RDNMG192-08|United States|New York|658[0n]|BOLD:AAD5475
Acronicta dolli|[8473]|RDNMK827-12|United States|Georgia|658[0n]|BOLD:AAD5475
Acronicta dolli|[8474]|RDNMK829-12|United States|Georgia|658[0n]|BOLD:AAD5475
Acronicta|[8475]|UAMIC550-13|United States|Alaska|649[0n]|BOLD:AAC4122
Acronicta lupini|[8476]|RDNMB455-05|Canada|British Columbia|658[0n]|BOLD:AAC4122
Acronicta lupini|[8477]|JMMMB582-13|United States|California|603[0n]|BOLD:AAC4122
Acronicta australis|[8478]|RDNMK123-11|United States|California|658[0n]|BOLD:AAC4122
Acronicta lupini|[8479]|LBCB937-05|Canada|British Columbia|624[1n]|BOLD:AAC4122
Acronicta lupini|[8480]|RDNMD281-06|Canada|British Columbia|608[0n]|BOLD:AAC4122
Acronicta lupini|[8481]|RDNMD282-06|Canada|British Columbia|658[0n]|BOLD:AAC4122
Acronicta lupini|[8482]|GWNC612-07|Canada|Yukon Territory|658[0n]|BOLD:AAC4122
Acronicta lupini|[8483]|LPMN928-08|Canada|Alberta|658[0n]|BOLD:AAC4122
Acronicta lupini|[8484]|LPABB404-08|Canada|Alberta|658[0n]|BOLD:AAC4122
Acronicta lupini|[8485]|RDNMJ628-11|United States|Utah|658[0n]|BOLD:AAC4122
Acronicta lupini|[8486]|RDLQF112-06|Canada|Quebec|656[0n]|BOLD:AAC4122
Acronicta lupini|[8487]|GWNC611-07|Canada|Yukon Territory|658[0n]|BOLD:AAC4122
Acronicta lupini|[8488]|RDLQ455-07|Canada|Newfoundland and Labrador|658[0n]|BOLD:AAC4122
Acronicta lupini|[8489]|CHLEP068-09|Canada|Manitoba|627[0n]|BOLD:AAC4122
Acronicta australis|[8490]|RDNMK120-11|United States|California|658[0n]|BOLD:AAC4122
Acronicta spinea|[8491]|CNCLB287-14|United States|California|658[0n]|BOLD:AAC4122
Acronicta spinea|[8492]|CNCLB286-14|United States|California|658[0n]|BOLD:AAC4122
Acronicta spinea|[8493]|CNCLB473-14|United States|California|658[0n]|BOLD:AAC4122
Acronicta spinea|[8494]|CNCLB475-14|United States|California|550[0n]|BOLD:AAC4122
Acronicta menyanthidis|[8495]|RDNML283-13|United States|Alaska|658[0n]|BOLD:AAE0830
Acronicta impleta|[8496]|RDNMB886-05|United States|California|658[0n]|BOLD:AAB0826
Acronicta impleta|[8497]|LGSM452-04|United States|Tennessee|658[0n]|BOLD:AAB0826
Acronicta impleta|[8498]|LPOKA587-09|United States|Oklahoma|628[0n]|BOLD:AAB0826
Acronicta impleta|[8499]|PHMNB017-03|Canada|New Brunswick|639[0n]|BOLD:AAB0826
Acronicta impleta|[8500]|RDLQ448-07|Canada|Quebec|658[0n]|BOLD:AAB0826
Acronicta impleta|[8501]|RDNMD290-06|United States|Florida|658[0n]|BOLD:AAB0826
Acronicta impleta|[8502]|RDNMB885-05|Canada|Ontario|658[0n]|BOLD:AAB0826
Acronicta impleta|[8503]|LPSOB376-08|Canada|Ontario|657[0n]|BOLD:AAB0826
Acronicta impleta|[8504]|RDLQ446-07|Canada|Quebec|645[0n]|BOLD:AAB0826
Acronicta impleta|[8505]|RDLQ447-07|Canada|Quebec|645[0n]|BOLD:AAB0826
Acronicta impleta|[8506]|LPSOD238-09|Canada|Ontario|658[1n]|BOLD:AAB0826
Acronicta impleta|[8507]|NAMUM409-09|United States|California|658[0n]|BOLD:AAB0826
Acronicta impleta|[8508]|LOTB473-05|United States|Tennessee|658[0n]|BOLD:AAB0826
Acronicta impleta|[8509]|LSEU325-06|United States|Georgia|658[0n]|BOLD:AAB0826
Acronicta impleta|[8510]|LSEU506-06|United States|Georgia|658[0n]|BOLD:AAB0826
Acronicta impleta|[8511]|LNCC211-10|United States|North Carolina|658[0n]|BOLD:AAB0826
Acronicta impleta|[8512]|RDNMD291-06|United States|Colorado|658[0n]|BOLD:AAB0826
Acronicta impleta|[8513]|RDNMD292-06|United States|Colorado|658[0n]|BOLD:AAB0826
Acronicta impleta|[8514]|BBLSX313-09|United States|Oklahoma|658[0n]|BOLD:AAB0826
Acronicta impleta|[8515]|LOTB474-05|United States|Tennessee|658[0n]|BOLD:AAB0826
Acronicta impleta|[8516]|LOTB270-05|United States|Tennessee|658[0n]|BOLD:AAB0826
Acronicta impleta|[8517]|LGSM453-04|United States|Tennessee|658[0n]|BOLD:AAB0826
Acronicta impleta|[8518]|RDNMD289-06|United States|Florida|658[0n]|BOLD:AAB0826
Acronicta impleta|[8519]|LSEU326-06|United States|Georgia|658[0n]|BOLD:AAB0826
Acronicta impleta|[8520]|LPVIA110-08|Canada|British Columbia|658[0n]|BOLD:AAB0826
Acronicta impleta|[8521]|LPOKA502-09|United States|Oklahoma|658[0n]|BOLD:AAB0826
Acronicta impleta|[8522]|UDLEP037-09|United States|Maryland|658[0n]|BOLD:AAB0826
Acronicta impleta|[8523]|LNCC210-10|United States|North Carolina|658[0n]|BOLD:AAB0826
Acronicta impleta|[8524]|PSAT135-10|Canada|British Columbia|658[0n]|BOLD:AAB0826
Acronicta impleta|[8525]|PSAT136-10|United States|Washington|658[0n]|BOLD:AAB0826
Acronicta impleta|[8526]|RDNMB884-05|Canada|Ontario|658[0n]|BOLD:AAB0826
Acronicta impleta|[8527]|RDMAB123-05|Canada|Alberta|658[0n]|BOLD:AAB0826
Acronicta impleta|[8528]|LPSOB590-08|Canada|Ontario|658[0n]|BOLD:AAB0826
Acronicta impleta|[8529]|BBLPB650-10|Canada|Ontario|658[0n]|BOLD:AAB0826
Acronicta impleta|[8530]|LSUSA110-06|United States|Kentucky|542[0n]|BOLD:AAB0826
Acronicta impleta|[8531]|LOTB348-05|United States|Tennessee|658[0n]|BOLD:AAB0826
Acronicta impleta|[8532]|LOTB329-05|United States|Tennessee|658[0n]|BOLD:AAB0826
Acronicta impleta|[8533]|LOTB472-05|United States|Tennessee|658[0n]|BOLD:AAB0826
Acronicta impleta|[8534]|LSUSA007-06|United States|Kentucky|658[0n]|BOLD:AAB0826
Acronicta impleta|[8535]|LPOKD159-09|United States|Oklahoma|658[0n]|BOLD:AAB0826
Acronicta impleta|[8536]|LGSMG961-10|United States|North Carolina|658[0n]|BOLD:AAB0826
Acronicta impleta|[8537]|LPOKE273-11|United States|Oklahoma|658[0n]|BOLD:AAB0826
Acronicta impleta|[8538]|CNEIF2209-12|Canada|Alberta|624[0n]|BOLD:AAB0826
Acronicta sperata|[8539]|RDNMB450-05|Canada|Ontario|658[0n]|BOLD:AAD7893
Acronicta sperata|[8540]|RDNMB451-05|Canada|Ontario|658[0n]|BOLD:AAD7893
Acronicta sperata|[8541]|RDNMB452-05|Canada|Ontario|658[0n]|BOLD:AAD7893
Acronicta sperata|[8542]|RDNMB453-05|Canada|Alberta|658[0n]|BOLD:AAD7893
Acronicta sperata|[8543]|RDNMB454-05|Canada|Alberta|658[0n]|BOLD:AAD7893
Acronicta sperata|[8544]|KPOEC142-08|Canada|Ontario|658[0n]|BOLD:AAD7893
Acronicta perdita|[8545]|RDNMF117-08|United States|California|625[3n]|BOLD:ABZ6712
Acronicta perdita|[8546]|RDNMF115-08|United States|Washington|636[0n]|BOLD:ABZ6712
Acronicta perdita|[8547]|LPVIB875-08|Canada|British Columbia|630[0n]|BOLD:ABZ6712
Acronicta perdita|[8548]|NAMUM256-08|United States|California|658[1n]|BOLD:ABZ6712
Acronicta perdita|[8549]|RDNMF118-08|United States|California|633[0n]|BOLD:ABZ6712
Acronicta perdita|[8550]|RDNMF114-08|Canada|British Columbia|647[0n]|BOLD:ABZ6712
Acronicta perdita|[8551]|RDNMF116-08|Canada|British Columbia|658[0n]|BOLD:ABZ6712
Acronicta perdita|[8552]|JMMMB399-11|United States|California|658[0n]|BOLD:ABZ6712
Acronicta barnesii|[8553]|RDNME571-08|United States|Colorado|658[0n]|BOLD:AAD5474
[illegible]

Acronicta perdita|[8551]|RDNMF116-08|Canada|British Columbia|658[0n]|BOLD:ABZ6712
Acronicta perdita|[8552]|JMMMB399-11|United States|California|658[0n]|BOLD:ABZ6712
Acronicta barnesii|[8553]|RDNME571-08|United States|Colorado|658[0n]|BOLD:AAD5474
Acronicta barnesii|[8554]|RDNMI013-10|United States|Colorado|658[0n]|BOLD:AAD5474
Acronicta barnesii|[8555]|RDNMI014-10|United States|Colorado|658[0n]|BOLD:AAD5474
Acronicta barnesii|[8556]|RDNMJ588-11|United States|Colorado|658[0n]|BOLD:AAD5474
Acronicta barnesii|[8557]|RDNMJ589-11|United States|Colorado|658[0n]|BOLD:AAD5474
Acronicta barnesii|[8558]|RDNMJ590-11|United States|Nevada|658[0n]|BOLD:AAD5474
Acronicta barnesii|[8559]|RDNMJ591-11|United States|Nevada|658[0n]|BOLD:AAD5474
Acronicta othello|[8560]|RDNMI018-10|United States|California|658[0n]|BOLD:AAD5473
Acronicta othello|[8561]|RDNMJ583-11|United States|California|658[0n]|BOLD:AAD5473
Acronicta othello|[8562]|LOCBE111-06|United States|California|658[0n]|BOLD:AAD5473
Acronicta othello|[8563]|RDNMJ578-11|United States|California|658[0n]|BOLD:AAD5473
Acronicta othello|[8564]|RDNMJ577-11|United States|California|658[0n]|BOLD:AAD5473
Acronicta othello|[8565]|RDNMJ584-11|United States|California|658[0n]|BOLD:AAD5473
Acronicta othello|[8566]|GMLC931-12|United States|California|658[0n]|BOLD:AAD5473
Acronicta longa|[8567]|RDNMK483-11|United States|Connecticut|658[0n]|BOLD:AAD2547
Acronicta longa|[8568]|RDNMJ051-10|Canada|Alberta|658[0n]|BOLD:AAD2547
Acronicta longa|[8569]|RDNMK683-11|Canada|Alberta|658[0n]|BOLD:AAD2547
Acronicta longa|[8570]|RDLQ289-05|Canada|Quebec|656[0n]|BOLD:AAD2547
Acronicta longa|[8571]|TTMNB035-06|Canada|New Brunswick|656[0n]|BOLD:AAD2547
Acronicta longa|[8572]|LILLB004-11|United States|Illinois|658[0n]|BOLD:AAD2547
Acronicta longa|[8573]|RDNMK052-11|United States|Florida|658[0n]|BOLD:AAD2547
Acronicta longa|[8574]|RDNMK800-12|United States|Texas|630[0n]|BOLD:AAD2547
Acronicta longa|[8575]|LPOKB149-09|United States|Oklahoma|658[0n]|BOLD:AAD2547
Acronicta longa|[8576]|LNC091-05|United States|North Carolina|658[0n]|BOLD:AAD2547
Acronicta longa|[8577]|LNC937-06|United States|North Carolina|658[0n]|BOLD:AAD2547
Acronicta longa|[8578]|LNC938-06|United States|North Carolina|658[0n]|BOLD:AAD2547
Acronicta longa|[8579]|LNC939-06|United States|North Carolina|658[0n]|BOLD:AAD2547
Acronicta longa|[8580]|LPOKB358-09|United States|Oklahoma|658[0n]|BOLD:AAD2547
Acronicta longa|[8581]|LPOKB362-09|United States|Oklahoma|658[0n]|BOLD:AAD2547
Acronicta longa|[8582]|LPOKD728-10|United States|Oklahoma|658[0n]|BOLD:AAD2547
Acronicta longa|[8583]|LNCC156-10|United States|North Carolina|658[0n]|BOLD:AAD2547
Acronicta longa|[8584]|RDNMJ611-11|United States|Oklahoma|658[0n]|BOLD:AAD2547
Acronicta longa|[8585]|RDNMK482-11|United States|Mississippi|658[0n]|BOLD:AAD2547
Acronicta longa|[8586]|RDNMK813-12|United States|Texas|658[0n]|BOLD:AAD2547
Acronicta longa|[8587]|RDNMK814-12|United States|Texas|658[0n]|BOLD:AAD2547
Acronicta noctivaga|[8588]|PHMNB251-04|Canada|New Brunswick|567[0n]|BOLD:AAC0015
Acronicta noctivaga|[8589]|RDLQ141-05|Canada|Quebec|614[0n]|BOLD:AAC0015
Acronicta noctivaga|[8590]|LGSM433-04|United States|Tennessee|609[0n]|BOLD:AAC0015
Acronicta noctivaga|[8591]|RDLQB165-05|Canada|Quebec|658[0n]|BOLD:AAC0015
Acronicta noctivaga|[8592]|TMNBB158-06|Canada|New Brunswick|658[0n]|BOLD:AAC0015
Acronicta noctivaga|[8593]|TMNBB159-06|Canada|New Brunswick|658[0n]|BOLD:AAC0015
Acronicta noctivaga|[8594]|XAJ352-06|Canada|Ontario|658[0n]|BOLD:AAC0015
Acronicta noctivaga|[8595]|RDLQF516-06|Canada|Quebec|658[0n]|BOLD:AAC0015
Acronicta noctivaga|[8596]|LGSM432-04|United States|Tennessee|658[0n]|BOLD:AAC0015
Acronicta noctivaga|[8597]|RDLQB164-05|Canada|Quebec|658[0n]|BOLD:AAC0015
Acronicta noctivaga|[8598]|RDLQB795-05|Canada|Quebec|658[0n]|BOLD:AAC0015
Acronicta noctivaga|[8599]|KPOEC131-08|Canada|Ontario|658[0n]|BOLD:AAC0015
Acronicta noctivaga|[8600]|LPSOD244-09|Canada|Ontario|658[0n]|BOLD:AAC0015
Acronicta noctivaga|[8601]|LPSOD874-09|Canada|Ontario|658[0n]|BOLD:AAC0015
Acronicta noctivaga|[8602]|RDNMJ089-10|United States|Texas|658[0n]|BOLD:AAC0015
Acronicta noctivaga|[8603]|HPPPD1638-13|Canada|Nova Scotia|580[0n]|BOLD:AAC0015
Acronicta noctivaga|[8604]|LOCT050-05|United States|Connecticut|658[0n]|BOLD:AAC0015
Acronicta noctivaga|[8605]|CNCLB1428-14|United States|North Carolina|658[0n]|BOLD:AAC0015
Acronicta noctivaga|[8606]|CNCLB1429-14|United States|North Carolina|658[0n]|BOLD:AAC0015
Acronicta cyanescens|[8607]|NAMUM410-09|United States|California|658[0n]|BOLD:AAC7207
Acronicta lepusculina|[8608]|RDNMJ603-11|United States|Colorado|658[0n]|BOLD:AAB0891
Acronicta cyanescens|[8609]|RDNMJ601-11|United States|Colorado|658[0n]|BOLD:AAB0891
Acronicta cyanescens|[8610]|RDNMJ602-11|United States|Colorado|658[0n]|BOLD:AAB0891
Acronicta cyanescens|[8611]|RDNMJ598-11|Canada|British Columbia|658[0n]|BOLD:AAB0891
Acronicta cyanescens|[8612]|RDNMJ599-11|Canada|British Columbia|658[0n]|BOLD:AAB0891
Acronicta cyanescens|[8613]|RDNMJ594-11|United States|Colorado|658[0n]|BOLD:AAB0891
Acronicta cyanescens|[8614]|RDNMJ593-11|United States|Colorado|658[0n]|BOLD:AAB0891
Acronicta cyanescens|[8615]|RDNMJ592-11|United States|Colorado|658[0n]|BOLD:AAB0891
Acronicta lepusculina|[8616]|LPOKD061-09|United States|Oklahoma|658[0n]|BOLD:AAB0891
Acronicta cyanescens|[8617]|LPABC976-09|Canada|Alberta|658[0n]|BOLD:AAB0891
Acronicta cyanescens|[8618]|LPABC812-09|Canada|Alberta|658[0n]|BOLD:AAB0891
Acronicta cyanescens|[8619]|LPABB609-08|Canada|Alberta|658[0n]|BOLD:AAB0891
Acronicta cyanescens|[8620]|RDNME193-07|Canada|Yukon Territory|658[0n]|BOLD:AAB0891
Acronicta lepusculina|[8621]|RDMAB614-06|Canada|Alberta|658[0n]|BOLD:AAB0891
Acronicta lepusculina|[8622]|RDMAB605-06|Canada|Alberta|658[0n]|BOLD:AAB0891
Acronicta cyanescens|[8623]|RDMAB572-06|Canada|Alberta|658[0n]|BOLD:AAB0891
Acronicta cyanescens|[8624]|RDNMB874-05|Canada|British Columbia|658[0n]|BOLD:AAB0891
Acronicta lepusculina|[8625]|LPOKB201-09|United States|Oklahoma|658[0n]|BOLD:AAB0891
Acronicta lepusculina|[8626]|RDMAB612-06|Canada|Alberta|658[0n]|BOLD:AAB0891
Acronicta lepusculina|[8627]|RDMAB615-06|Canada|Alberta|658[0n]|BOLD:AAB0891
Acronicta cyanescens|[8628]|RDNMB873-05|Canada|British Columbia|550[0n]|BOLD:AAB0891
Acronicta lepusculina|[8629]|RDNMJ604-11|United States|Colorado|658[0n]|BOLD:AAB0891
Acronicta cyanescens|[8630]|RDMAB573-06|Canada|Alberta|629[0n]|BOLD:AAB0891
Acronicta cyanescens|[8631]|LPABB608-08|Canada|Alberta|658[0n]|BOLD:AAB0891
Acronicta cyanescens|[8632]|RDNMJ595-11|Canada|British Columbia|658[0n]|BOLD:AAB0891
Acronicta cyanescens|[8633]|RDNMJ596-11|United States|Idaho|658[0n]|BOLD:AAB0891
Acronicta cyanescens|[8634]|RDNMJ623-11|Canada|Alberta|658[0n]|BOLD:AAB0891
Acronicta lepusculina|[8635]|RDMAB613-06|Canada|Alberta|658[0n]|BOLD:AAB0891
Acronicta lepusculina|[8636]|RDNME565-08|United States|Utah|658[1n]|BOLD:AAB0891
Acronicta lepusculina|[8637]|XAJ597-06|Canada|Ontario|656[0n]|BOLD:AAB0891
Acronicta lepusculina|[8638]|RDNMJ597-11|Canada|Alberta|658[0n]|BOLD:AAB0891
Acronicta lepusculina|[8639]|RDNMJ605-11|Canada|New Brunswick|658[0n]|BOLD:AAB0891
Acronicta lepusculina|[8640]|KPOEC129-08|Canada|Ontario|658[0n]|BOLD:AAB0891
Acronicta lepusculina|[8641]|KPOEC166-08|Canada|Ontario|658[0n]|BOLD:AAB0891
Acronicta lepusculina|[8642]|XAK537-07|Canada|Ontario|658[0n]|BOLD:AAB0891
Acronicta lepusculina|[8643]|LPSOB322-08|Canada|Ontario|658[0n]|BOLD:AAB0891
Acronicta lepusculina|[8644]|RDNMJ607-11|Canada|Alberta|658[0n]|BOLD:AAB0891
Acronicta lepusculina|[8645]|XAK509-07|Canada|Ontario|658[0n]|BOLD:AAB0891
Acronicta lepusculina|[8646]|RDLQ443-07|Canada|Quebec|658[0n]|BOLD:AAB0891
Acronicta lepusculina|[8647]|XAK159-06|Canada|Ontario|658[0n]|BOLD:AAB0891
Acronicta lepusculina|[8648]|RDNMD273-06|Canada|New Brunswick|658[0n]|BOLD:AAB0891
Acronicta lepusculina|[8649]|RDNMD272-06|Canada|New Brunswick|658[0n]|BOLD:AAB0891
Acronicta lepusculina|[8650]|RDNMD271-06|Canada|New Brunswick|658[0n]|BOLD:AAB0891
Acronicta lepusculina|[8651]|RDNMD270-06|Canada|New Brunswick|658[0n]|BOLD:AAB0891
Acronicta lepusculina|[8652]|RDMAB606-06|Canada|Alberta|658[0n]|BOLD:AAB0891
Acronicta lepusculina|[8653]|RDLQB157-05|Canada|Quebec|658[0n]|BOLD:AAB0891

Acronicta lepusculina|[8651]|RDNMD270-06|Canada|New Brunswick|658[0n]|BOLD:AAB0891
Acronicta lepusculina|[8652]|RDMAB606-06|Canada|Alberta|658[0n]|BOLD:AAB0891
Acronicta lepusculina|[8653]|RDLQB157-05|Canada|Quebec|658[0n]|BOLD:AAB0891
Acronicta lepusculina|[8654]|RDLQB156-05|Canada|Quebec|658[0n]|BOLD:AAB0891
Acronicta lepusculina|[8655]|RDLQB155-05|Canada|Quebec|658[0n]|BOLD:AAB0891
Acronicta lepusculina|[8656]|RDLQB154-05|Canada|Quebec|658[0n]|BOLD:AAB0891
Acronicta lepusculina|[8657]|RDLQB153-05|Canada|Quebec|658[0n]|BOLD:AAB0891
Acronicta lepusculina|[8658]|RDNMB878-05|Canada|New Brunswick|658[0n]|BOLD:AAB0891
Acronicta lepusculina|[8659]|XAF793-05|Canada|Ontario|658[0n]|BOLD:AAB0891
Acronicta lepusculina|[8660]|RDNMB879-05|Canada|New Brunswick|638[0n]|BOLD:AAB0891
Acronicta lepusculina|[8661]|PMG084-03|Canada|Ontario|617[0n]|BOLD:AAB0891
Acronicta lepusculina|[8662]|RDNMB875-05|United States|California|541[0n]|BOLD:AAB0891
Acronicta lepusculina|[8663]|RDNMB876-05|United States|California|506[1n]|BOLD:AAB0891
Acronicta lepusculina|[8664]|RDNMJ608-11|United States|California|538[0n]|BOLD:AAB0891
Acronicta lepusculina|[8665]|RDNMJ609-11|United States|California|580[0n]|BOLD:AAB0891
Acronicta lepusculina|[8666]|BBLOC1374-11|United States|California|658[0n]|BOLD:AAB0891
Acronicta vulpina|[8667]|RDNMD365-06|Canada|New Brunswick|656[0n]|BOLD:AAB3897
Acronicta vulpina|[8668]|XAF454-05|Canada|Ontario|658[0n]|BOLD:AAB3897
Acronicta vulpina|[8669]|XAJ530-06|Canada|Ontario|658[0n]|BOLD:AAB3897
Acronicta vulpina|[8670]|LPABB330-08|Canada|Alberta|658[0n]|BOLD:AAB3897
Acronicta vulpina|[8671]|BBLEC398-09|Canada|Newfoundland and Labrador|658[0n]|BOLD:AAB3897
Acronicta vulpina|[8672]|LPMN924-08|Canada|Alberta|648[0n]|BOLD:AAB3897
Acronicta vulpina|[8673]|RDLQ444-07|Canada|Quebec|650[0n]|BOLD:AAB3897
Acronicta vulpina|[8674]|LBCA576-05|Canada|British Columbia|658[0n]|BOLD:AAB3897
Acronicta vulpina|[8675]|PHMNB583-04|Canada|New Brunswick|658[0n]|BOLD:AAB3897
Acronicta vulpina|[8676]|XAB216-04|Canada|Ontario|658[0n]|BOLD:AAB3897
Acronicta vulpina|[8677]|LBCD045-05|Canada|British Columbia|596[1n]|BOLD:AAB3897
Acronicta vulpina|[8678]|RDNMD267-06|Canada|New Brunswick|658[0n]|BOLD:AAB3897
Acronicta vulpina|[8679]|RDNMD268-06|Canada|New Brunswick|658[0n]|BOLD:AAB3897
Acronicta vulpina|[8680]|RDNMD269-06|Canada|New Brunswick|658[0n]|BOLD:AAB3897
Acronicta vulpina|[8681]|RDNMD364-06|Canada|New Brunswick|658[0n]|BOLD:AAB3897
Acronicta vulpina|[8682]|RDLQ445-07|Canada|Quebec|658[0n]|BOLD:AAB3897
Acronicta vulpina|[8683]|LPSOB436-08|Canada|Ontario|658[0n]|BOLD:AAB3897
Acronicta vulpina|[8684]|LPMN172-08|Canada|Manitoba|658[0n]|BOLD:AAB3897
Acronicta vulpina|[8685]|LPMN308-08|Canada|Manitoba|658[0n]|BOLD:AAB3897
Acronicta vulpina|[8686]|LPMN312-08|Canada|Manitoba|658[0n]|BOLD:AAB3897
Acronicta vulpina|[8687]|LPMN320-08|Canada|Manitoba|658[0n]|BOLD:AAB3897
Acronicta vulpina|[8688]|LPMN321-08|Canada|Manitoba|658[0n]|BOLD:AAB3897
Acronicta vulpina|[8689]|LPMN563-08|Canada|Manitoba|658[0n]|BOLD:AAB3897
Acronicta vulpina|[8690]|LPMN916-08|Canada|Alberta|658[0n]|BOLD:AAB3897
Acronicta vulpina|[8691]|BBLPA381-10|Canada|Ontario|658[0n]|BOLD:AAB3897
Acronicta vulpina|[8692]|RDNMK126-11|United States|Colorado|658[0n]|BOLD:AAB3897
Acronicta vulpina|[8693]|CNCLB664-14|Japan||550[0n]|BOLD:AAB3897
Acronicta rubricoma|[8694]|LPOKB356-09|United States|Oklahoma|658[0n]|BOLD:AAB6117
Acronicta rubricoma|[8695]|LPOKB394-09|United States|Oklahoma|603[0n]|BOLD:AAB6117
Acronicta rubricoma|[8696]|LPOKA061-08|United States|Oklahoma|658[0n]|BOLD:AAB6117
Acronicta rubricoma|[8697]|LPOKC619-09|United States|Oklahoma|658[0n]|BOLD:AAB6117
Acronicta rubricoma|[8698]|LPOKD090-09|United States|Oklahoma|658[0n]|BOLD:AAB6117
Acronicta rubricoma|[8699]|RDNMF006-08|United States|Florida|658[0n]|BOLD:AAB6117
Acronicta rubricoma|[8700]|UDLEP145-09|United States|Pennsylvania|658[0n]|BOLD:AAB6117
Acronicta rubricoma|[8701]|LPSO870-08|Canada|Ontario|611[2n]|BOLD:AAB6117
Acronicta rubricoma|[8702]|LPSO843-08|Canada|Ontario|609[1n]|BOLD:AAB6117
Acronicta rubricoma|[8703]|LPSO649-08|Canada|Ontario|645[0n]|BOLD:AAB6117
Acronicta rubricoma|[8704]|LPOKB367-09|United States|Oklahoma|658[0n]|BOLD:AAB6117
Acronicta rubricoma|[8705]|LPOKB991-09|United States|Oklahoma|658[0n]|BOLD:AAB6117
Acronicta rubricoma|[8706]|LPOKE270-11|United States|Oklahoma|658[0n]|BOLD:AAB6117
Acronicta rubricoma|[8707]|LPSO139-08|Canada|Ontario|658[0n]|BOLD:AAB6117
Acronicta rubricoma|[8708]|LPSO342-08|Canada|Ontario|658[0n]|BOLD:AAB6117
Acronicta rubricoma|[8709]|LPSO349-08|Canada|Ontario|658[0n]|BOLD:AAB6117
Acronicta rubricoma|[8710]|LPSO363-08|Canada|Ontario|658[0n]|BOLD:AAB6117
Acronicta rubricoma|[8711]|LPSO450-08|Canada|Ontario|658[0n]|BOLD:AAB6117
Acronicta rubricoma|[8712]|LPSO738-08|Canada|Ontario|658[0n]|BOLD:AAB6117
Acronicta rubricoma|[8713]|LPSO739-08|Canada|Ontario|658[0n]|BOLD:AAB6117
Acronicta rubricoma|[8714]|LPSO845-08|Canada|Ontario|658[0n]|BOLD:AAB6117
Acronicta rubricoma|[8715]|LPSO869-08|Canada|Ontario|658[0n]|BOLD:AAB6117
Acronicta rubricoma|[8716]|LPSO954-08|Canada|Ontario|658[0n]|BOLD:AAB6117
Acronicta rubricoma|[8717]|LNCC1244-11|United States|North Carolina|658[0n]|BOLD:AAB6117
Acronicta americana|[8718]|LGSM441-04|United States|Tennessee|609[0n]|BOLD:AAA5139
Acronicta americana|[8719]|LGSMC847-05|United States|Tennessee|658[0n]|BOLD:AAA5139
Acronicta americana|[8720]|LOT299-04|United States|Tennessee|658[0n]|BOLD:AAA5139
Acronicta americana|[8721]|LGSMC848-05|United States|Tennessee|580[0n]|BOLD:AAA5139
Acronicta americana|[8722]|LOT302-04|United States|Tennessee|658[0n]|BOLD:AAA5139
Acronicta americana|[8723]|LGSMG644-07|United States|Tennessee|656[0n]|BOLD:AAA5139
Acronicta americana|[8724]|LOT300-04|United States|Tennessee|658[0n]|BOLD:AAA5139
Acronicta americana|[8725]|LOT298-04|United States|Tennessee|658[0n]|BOLD:AAA5139
Acronicta americana|[8726]|LOT297-04|United States|Tennessee|658[0n]|BOLD:AAA5139
Acronicta americana|[8727]|LOT296-04|United States|Tennessee|658[0n]|BOLD:AAA5139
Acronicta americana|[8728]|LOT295-04|United States|Tennessee|658[0n]|BOLD:AAA5139
Acronicta americana|[8729]|LGSM440-04|United States|Tennessee|658[0n]|BOLD:AAA5139
Acronicta americana|[8730]|LOT301-04|United States|Tennessee|658[0n]|BOLD:AAA5139
Acronicta americana|[8731]|LOTB271-05|United States|Tennessee|658[0n]|BOLD:AAA5139
Acronicta americana|[8732]|LOTB273-05|United States|Tennessee|658[0n]|BOLD:AAA5139
Acronicta americana|[8733]|LNC859-06|United States|North Carolina|658[0n]|BOLD:AAA5139
Acronicta americana|[8734]|LGSMG645-07|United States|North Carolina|658[0n]|BOLD:AAA5139
Acronicta americana|[8735]|LPOKA184-08|United States|Oklahoma|658[0n]|BOLD:AAA5139
Acronicta americana|[8736]|LPOKB975-09|United States|Oklahoma|658[0n]|BOLD:AAA5139
Acronicta americana|[8737]|RDNMJ633-11|United States|Arizona|604[0n]|BOLD:AAA5139
Acronicta americana|[8738]|XAK152-06|Canada|Ontario|658[0n]|BOLD:AAA5139
Acronicta americana|[8739]|MNBB149-05|Canada|New Brunswick|658[0n]|BOLD:AAA5139
Acronicta americana|[8740]|MNBB150-05|Canada|New Brunswick|658[0n]|BOLD:AAA5139
Acronicta americana|[8741]|MNBB553-05|Canada|New Brunswick|658[0n]|BOLD:AAA5139
Acronicta americana|[8742]|BLTIB1062-08|Canada|Ontario|658[0n]|BOLD:AAA5139
Acronicta americana|[8743]|MNBB320-05|Canada|New Brunswick|658[0n]|BOLD:AAA5139
Acronicta americana|[8744]|BBLPE314-09|Canada|Newfoundland and Labrador|658[0n]|BOLD:AAA5139
Acronicta americana|[8745]|LPSOC283-08|Canada|Ontario|658[0n]|BOLD:AAA5139
Acronicta americana|[8746]|MNBB266-05|Canada|New Brunswick|658[0n]|BOLD:AAA5139
Acronicta americana|[8747]|MNBB147-05|Canada|New Brunswick|658[0n]|BOLD:AAA5139
Acronicta americana|[8748]|XAC618-04|Canada|Ontario|658[0n]|BOLD:AAA5139
Acronicta americana|[8749]|XAE547-04|Canada|Ontario|617[1n]|BOLD:AAA5139
Acronicta americana|[8750]|PHMNB076-03|Canada|New Brunswick|639[0n]|BOLD:AAA5139
Acronicta americana|[8751]|PHMO390-03|Canada|Ontario|639[0n]|BOLD:AAA5139
Acronicta americana|[8752]|BBLPE482-09|Canada|Newfoundland and Labrador|632[0n]|BOLD:AAA5139
[illegible]

Acronicta americana|[8750]|PHMNB076-03|Canada|New Brunswick|639[0n]|BOLD:AAA5139
Acronicta americana|[8751]|PHMO390-03|Canada|Ontario|639[0n]|BOLD:AAA5139
Acronicta americana|[8752]|BBLPE482-09|Canada|Newfoundland and Labrador|632[0n]|BOLD:AAA5139
Acronicta americana|[8753]|BBLPE520-09|Canada|Newfoundland and Labrador|658[0n]|BOLD:AAA5139
Acronicta americana|[8754]|BBLPE521-09|Canada|Newfoundland and Labrador|658[0n]|BOLD:AAA5139
Acronicta americana|[8755]|BBLPE522-09|Canada|Newfoundland and Labrador|658[0n]|BOLD:AAA5139
Acronicta americana|[8756]|BBLPE523-09|Canada|Newfoundland and Labrador|658[0n]|BOLD:AAA5139
Acronicta americana|[8757]|BBLPE531-09|Canada|Newfoundland and Labrador|658[0n]|BOLD:AAA5139
Acronicta americana|[8758]|XAB120-04|Canada|Ontario|658[0n]|BOLD:AAA5139
Acronicta americana|[8759]|MNBB420-05|Canada|New Brunswick|658[0n]|BOLD:AAA5139
Acronicta americana|[8760]|XAE550-04|Canada|Ontario|625[0n]|BOLD:AAA5139
Acronicta americana|[8761]|PHMNB351-04|Canada|New Brunswick|654[0n]|BOLD:AAA5139
Acronicta americana|[8762]|BBLPE299-09|Canada|Newfoundland and Labrador|658[0n]|BOLD:AAA5139
Acronicta americana|[8763]|BBLEC042-09|Canada|New Brunswick|658[0n]|BOLD:AAA5139
Acronicta americana|[8764]|RDLQ442-07|Canada|Quebec|658[0n]|BOLD:AAA5139
Acronicta americana|[8765]|RDLQ441-07|Canada|Quebec|658[0n]|BOLD:AAA5139
Acronicta americana|[8766]|MNBB589-05|Canada|New Brunswick|658[0n]|BOLD:AAA5139
Acronicta americana|[8767]|MNBB588-05|Canada|New Brunswick|658[0n]|BOLD:AAA5139
Acronicta americana|[8768]|MNBB375-05|Canada|New Brunswick|658[0n]|BOLD:AAA5139
Acronicta americana|[8769]|MNBB354-05|Canada|New Brunswick|658[0n]|BOLD:AAA5139
Acronicta americana|[8770]|MNBB270-05|Canada|New Brunswick|658[0n]|BOLD:AAA5139
Acronicta americana|[8771]|MNBB269-05|Canada|New Brunswick|658[0n]|BOLD:AAA5139
Acronicta americana|[8772]|MNBB224-05|Canada|New Brunswick|658[0n]|BOLD:AAA5139
Acronicta americana|[8773]|MNBB223-05|Canada|New Brunswick|658[0n]|BOLD:AAA5139
Acronicta americana|[8774]|MNBB222-05|Canada|New Brunswick|658[0n]|BOLD:AAA5139
Acronicta americana|[8775]|MNBB221-05|Canada|New Brunswick|658[0n]|BOLD:AAA5139
Acronicta americana|[8776]|MNBB219-05|Canada|New Brunswick|658[0n]|BOLD:AAA5139
Acronicta americana|[8777]|MNBB218-05|Canada|New Brunswick|658[0n]|BOLD:AAA5139
Acronicta americana|[8778]|MNBB178-05|Canada|New Brunswick|658[0n]|BOLD:AAA5139
Acronicta americana|[8779]|MNBB155-05|Canada|New Brunswick|658[0n]|BOLD:AAA5139
Acronicta americana|[8780]|MNBB151-05|Canada|New Brunswick|658[0n]|BOLD:AAA5139
Acronicta americana|[8781]|MNBB092-05|Canada|New Brunswick|658[0n]|BOLD:AAA5139
Acronicta americana|[8782]|LOCT049-05|United States|Connecticut|657[0n]|BOLD:AAA5139
Acronicta americana|[8783]|PHMNB741-05|Canada|New Brunswick|658[0n]|BOLD:AAA5139
Acronicta americana|[8784]|PHMNB586-04|Canada|New Brunswick|658[0n]|BOLD:AAA5139
Acronicta americana|[8785]|XAB179-04|Canada|Ontario|658[0n]|BOLD:AAA5139
Acronicta americana|[8786]|XAC571-04|Canada|Ontario|658[0n]|BOLD:AAA5139
Acronicta americana|[8787]|BBLPE300-09|Canada|Newfoundland and Labrador|648[0n]|BOLD:AAA5139
Acronicta americana|[8788]|BBLPE371-09|Canada|Newfoundland and Labrador|658[0n]|BOLD:AAA5139
Acronicta americana|[8789]|BBLPE483-09|Canada|Newfoundland and Labrador|658[0n]|BOLD:AAA5139
Acronicta americana|[8790]|BBLPE532-09|Canada|Newfoundland and Labrador|658[0n]|BOLD:AAA5139
Acronicta americana|[8791]|BBLEC409-09|Canada|Newfoundland and Labrador|658[0n]|BOLD:AAA5139
Acronicta americana|[8792]|LILLA800-11|United States|Illinois|658[0n]|BOLD:AAA5139
Acronicta dactylina|[8793]|NAMUM260-08|United States|California|658[0n]|BOLD:AAA2802
Acronicta dactylina|[8794]|RDNMG431-08|United States|California|658[0n]|BOLD:AAA2802
Acronicta dactylina|[8795]|MNBB226-05|Canada|New Brunswick|658[0n]|BOLD:AAA2802
Acronicta dactylina|[8796]|XAC177-04|Canada|Ontario|656[0n]|BOLD:AAA2802
Acronicta dactylina|[8797]|MNBB268-05|Canada|New Brunswick|658[0n]|BOLD:AAA2802
Acronicta dactylina|[8798]|MNBB148-05|Canada|New Brunswick|658[0n]|BOLD:AAA2802
Acronicta dactylina|[8799]|MNBB513-05|Canada|New Brunswick|658[0n]|BOLD:AAA2802
Acronicta dactylina|[8800]|RDLQB151-05|Canada|Quebec|658[0n]|BOLD:AAA2802
Acronicta dactylina|[8801]|LHLEP367-06|Canada|British Columbia|657[0n]|BOLD:AAA2802
Acronicta dactylina|[8802]|PHMNB317-04|Canada|New Brunswick|658[0n]|BOLD:AAA2802
Acronicta dactylina|[8803]|MNBB353-05|Canada|New Brunswick|658[0n]|BOLD:AAA2802
Acronicta dactylina|[8804]|LBCS641-07|Canada|British Columbia|658[0n]|BOLD:AAA2802
Acronicta dactylina|[8805]|RWWA894-09|United States|Washington|658[0n]|BOLD:AAA2802
Acronicta dactylina|[8806]|RWWA955-09|United States|Washington|658[0n]|BOLD:AAA2802
Acronicta dactylina|[8807]|RWWB240-09|United States|Washington|658[0n]|BOLD:AAA2802
Acronicta dactylina|[8808]|RWWB886-10|United States|Washington|658[0n]|BOLD:AAA2802
Acronicta dactylina|[8809]|RWWA276-09|United States|Washington|658[0n]|BOLD:AAA2802
Acronicta dactylina|[8810]|RWWA310-09|United States|Washington|658[0n]|BOLD:AAA2802
Acronicta dactylina|[8811]|RWWC399-11|United States|Washington|658[0n]|BOLD:AAA2802
Acronicta dactylina|[8812]|RWWC461-11|United States|Washington|658[0n]|BOLD:AAA2802
Acronicta dactylina|[8813]|RWWA098-09|United States|Washington|658[0n]|BOLD:AAA2802
Acronicta dactylina|[8814]|RWWA196-09|United States|Washington|658[0n]|BOLD:AAA2802
Acronicta dactylina|[8815]|RWWC533-11|United States|Washington|658[0n]|BOLD:AAA2802
Acronicta dactylina|[8816]|LBCS640-07|Canada|British Columbia|658[0n]|BOLD:AAA2802
Acronicta dactylina|[8817]|MNBB158-05|Canada|New Brunswick|658[0n]|BOLD:AAA2802
Acronicta dactylina|[8818]|TMNBB152-06|Canada|New Brunswick|656[0n]|BOLD:AAA2802
Acronicta dactylina|[8819]|LBCA467-05|Canada|British Columbia|627[0n]|BOLD:AAA2802
Acronicta dactylina|[8820]|LBCA352-05|Canada|British Columbia|632[0n]|BOLD:AAA2802
Acronicta dactylina|[8821]|LBCA483-05|Canada|British Columbia|632[0n]|BOLD:AAA2802
Acronicta dactylina|[8822]|LOWCB855-05|Canada|British Columbia|658[0n]|BOLD:AAA2802
Acronicta dactylina|[8823]|LALPA909-11|Canada|British Columbia|658[0n]|BOLD:AAA2802
Acronicta dactylina|[8824]|LALPA1011-11|Canada|British Columbia|658[0n]|BOLD:AAA2802
Acronicta dactylina|[8825]|RWWC457-11|United States|Washington|658[0n]|BOLD:AAA2802
Acronicta dactylina|[8826]|LBCA466-05|Canada|British Columbia|651[0n]|BOLD:AAA2802
Acronicta dactylina|[8827]|LBCA746-05|Canada|British Columbia|631[0n]|BOLD:AAA2802
Acronicta dactylina|[8828]|MNBB661-05|Canada|New Brunswick|633[0n]|BOLD:AAA2802
Acronicta dactylina|[8829]|LBCB177-05|Canada|British Columbia|658[0n]|BOLD:AAA2802
Acronicta dactylina|[8830]|BBLPB667-10|Canada|British Columbia|658[0n]|BOLD:AAA2802
Acronicta dactylina|[8831]|RDNMK661-11|Canada|British Columbia|658[0n]|BOLD:AAA2802
Acronicta dactylina|[8832]|TTMNB320-06|Canada|New Brunswick|658[3n]|BOLD:AAA2802
Acronicta dactylina|[8833]|LALPA666-10|Canada|British Columbia|658[0n]|BOLD:AAA2802
Acronicta dactylina|[8834]|MNBB473-05|Canada|New Brunswick|658[0n]|BOLD:AAA2802
Acronicta dactylina|[8835]|RWWA193-09|United States|Washington|658[0n]|BOLD:AAA2802
Acronicta dactylina|[8836]|RWWA248-09|United States|Washington|658[0n]|BOLD:AAA2802
Acronicta dactylina|[8837]|LHLEP370-06|Canada|British Columbia|657[0n]|BOLD:AAA2802
Acronicta dactylina|[8838]|XAI057-05|Canada|Ontario|658[0n]|BOLD:AAA2802
Acronicta dactylina|[8839]|MNBB555-05|Canada|New Brunswick|658[0n]|BOLD:AAA2802
Acronicta dactylina|[8840]|MNBB273-05|Canada|New Brunswick|658[0n]|BOLD:AAA2802
Acronicta dactylina|[8841]|MNBB160-05|Canada|New Brunswick|658[0n]|BOLD:AAA2802
Acronicta dactylina|[8842]|MNBB154-05|Canada|New Brunswick|658[0n]|BOLD:AAA2802
Acronicta dactylina|[8843]|PHMNB742-05|Canada|New Brunswick|658[0n]|BOLD:AAA2802
Acronicta dactylina|[8844]|MNBB228-05|Canada|New Brunswick|543[0n]|BOLD:AAA2802
Acronicta dactylina|[8845]|RDLQB797-05|Canada|Quebec|658[0n]|BOLD:AAA2802
Acronicta dactylina|[8846]|TMNBB154-06|Canada|New Brunswick|658[0n]|BOLD:AAA2802
Acronicta dactylina|[8847]|LMH010-06|Canada|British Columbia|658[1n]|BOLD:AAA2802
Acronicta dactylina|[8848]|XAE367-04|Canada|Ontario|658[0n]|BOLD:AAA2802
Acronicta dactylina|[8849]|RWWC287-11|United States|Washington|658[0n]|BOLD:AAA2802
Acronicta dactylina|[8850]|RDLQG137-06|Canada|Quebec|658[4n]|BOLD:AAA2802
Acronicta dactylina|[8851]|RDLQB152-05|Canada|Quebec|658[0n]|BOLD:AAA2802
Acronicta dactylina|[8852]|PHMNB266-04|Canada|New Brunswick|609[0n]|BOLD:AAA2802

[illegible]
Acronicta dactylina|[8850]|RDLQG137-06|Canada|Quebec|658[4n]|BOLD:AAA2802
Acronicta dactylina|[8851]|RDLQB152-05|Canada|Quebec|658[0n]|BOLD:AAA2802
Acronicta dactylina|[8852]|PHMNB266-04|Canada|New Brunswick|609[0n]|BOLD:AAA2802
Acronicta dactylina|[8853]|MNBB602-05|Canada|New Brunswick|658[0n]|BOLD:AAA2802
Acronicta dactylina|[8854]|RDLQB150-05|Canada|Quebec|658[0n]|BOLD:AAA2802
Acronicta dactylina|[8855]|RWWA914-09|United States|Washington|658[0n]|BOLD:AAA2802
Acronicta dactylina|[8856]|RWWA834-09|United States|Washington|658[0n]|BOLD:AAA2802
Acronicta dactylina|[8857]|RWWA778-09|United States|Washington|658[0n]|BOLD:AAA2802
Acronicta dactylina|[8858]|RWWA770-09|United States|Washington|658[0n]|BOLD:AAA2802
Acronicta dactylina|[8859]|RWWA665-09|United States|Washington|658[0n]|BOLD:AAA2802
Acronicta dactylina|[8860]|RWWA274-09|United States|Washington|658[0n]|BOLD:AAA2802
Acronicta dactylina|[8861]|LPVIA508-08|Canada|British Columbia|658[0n]|BOLD:AAA2802
Acronicta dactylina|[8862]|LPMN667-08|Canada|Manitoba|658[0n]|BOLD:AAA2802
Acronicta dactylina|[8863]|LPMN666-08|Canada|Manitoba|658[0n]|BOLD:AAA2802
Acronicta dactylina|[8864]|LPMN565-08|Canada|Manitoba|658[0n]|BOLD:AAA2802
Acronicta dactylina|[8865]|LPSOC082-08|Canada|Ontario|658[0n]|BOLD:AAA2802
Acronicta dactylina|[8866]|LPSOC081-08|Canada|Ontario|658[0n]|BOLD:AAA2802
Acronicta dactylina|[8867]|LBCS642-07|Canada|British Columbia|658[0n]|BOLD:AAA2802
Acronicta dactylina|[8868]|LHLEP368-06|Canada|British Columbia|657[0n]|BOLD:AAA2802
Acronicta dactylina|[8869]|LHLEP366-06|Canada|British Columbia|657[0n]|BOLD:AAA2802
Acronicta dactylina|[8870]|LHLEP365-06|Canada|British Columbia|657[0n]|BOLD:AAA2802
Acronicta dactylina|[8871]|LHLEP364-06|Canada|British Columbia|657[0n]|BOLD:AAA2802
Acronicta dactylina|[8872]|LHLEP363-06|Canada|British Columbia|657[0n]|BOLD:AAA2802
Acronicta dactylina|[8873]|LHLEP362-06|Canada|British Columbia|657[0n]|BOLD:AAA2802
Acronicta dactylina|[8874]|LHLEP360-06|Canada|British Columbia|657[0n]|BOLD:AAA2802
Acronicta dactylina|[8875]|LHLEP358-06|Canada|British Columbia|657[0n]|BOLD:AAA2802
Acronicta dactylina|[8876]|LHLEP308-06|Canada|British Columbia|657[0n]|BOLD:AAA2802
Acronicta dactylina|[8877]|XAJ580-06|Canada|Ontario|658[0n]|BOLD:AAA2802
Acronicta dactylina|[8878]|TMNBB153-06|Canada|New Brunswick|658[0n]|BOLD:AAA2802
Acronicta dactylina|[8879]|TTMNB321-06|Canada|New Brunswick|658[0n]|BOLD:AAA2802
Acronicta dactylina|[8880]|TTMNB319-06|Canada|New Brunswick|658[0n]|BOLD:AAA2802
Acronicta dactylina|[8881]|TTMNB318-06|Canada|New Brunswick|658[0n]|BOLD:AAA2802
Acronicta dactylina|[8882]|RDLQB793-05|Canada|Quebec|658[0n]|BOLD:AAA2802
Acronicta dactylina|[8883]|RDNMB877-05|Canada|New Brunswick|658[0n]|BOLD:AAA2802
Acronicta dactylina|[8884]|MNBB662-05|Canada|New Brunswick|658[0n]|BOLD:AAA2802
Acronicta dactylina|[8885]|MNBB660-05|Canada|New Brunswick|658[0n]|BOLD:AAA2802
Acronicta dactylina|[8886]|MNBB647-05|Canada|New Brunswick|658[0n]|BOLD:AAA2802
Acronicta dactylina|[8887]|MNBB587-05|Canada|New Brunswick|658[0n]|BOLD:AAA2802
Acronicta dactylina|[8888]|MNBB554-05|Canada|New Brunswick|658[0n]|BOLD:AAA2802
Acronicta dactylina|[8889]|MNBB512-05|Canada|New Brunswick|658[0n]|BOLD:AAA2802
Acronicta dactylina|[8890]|MNBB511-05|Canada|New Brunswick|658[0n]|BOLD:AAA2802
Acronicta dactylina|[8891]|MNBB376-05|Canada|New Brunswick|658[0n]|BOLD:AAA2802
Acronicta dactylina|[8892]|MNBB322-05|Canada|New Brunswick|658[0n]|BOLD:AAA2802
Acronicta dactylina|[8893]|MNBB296-05|Canada|New Brunswick|658[0n]|BOLD:AAA2802
Acronicta dactylina|[8894]|MNBB272-05|Canada|New Brunswick|658[0n]|BOLD:AAA2802
Acronicta dactylina|[8895]|MNBB271-05|Canada|New Brunswick|658[0n]|BOLD:AAA2802
Acronicta dactylina|[8896]|MNBB267-05|Canada|New Brunswick|658[0n]|BOLD:AAA2802
Acronicta dactylina|[8897]|MNBB217-05|Canada|New Brunswick|658[0n]|BOLD:AAA2802
Acronicta dactylina|[8898]|MNBB157-05|Canada|New Brunswick|658[0n]|BOLD:AAA2802
Acronicta dactylina|[8899]|MNBB153-05|Canada|New Brunswick|658[0n]|BOLD:AAA2802
Acronicta dactylina|[8900]|MNBB093-05|Canada|New Brunswick|658[0n]|BOLD:AAA2802
Acronicta dactylina|[8901]|XAF572-05|Canada|Ontario|658[0n]|BOLD:AAA2802
Acronicta dactylina|[8902]|XAF455-05|Canada|Ontario|658[0n]|BOLD:AAA2802
Acronicta dactylina|[8903]|MNBB071-05|Canada|New Brunswick|658[0n]|BOLD:AAA2802
Acronicta dactylina|[8904]|RDLQ122-05|Canada|Quebec|658[0n]|BOLD:AAA2802
Acronicta dactylina|[8905]|PHMNB768-05|Canada|New Brunswick|658[0n]|BOLD:AAA2802
Acronicta dactylina|[8906]|PHMNB763-05|Canada|New Brunswick|658[0n]|BOLD:AAA2802
Acronicta dactylina|[8907]|PHMNB587-04|Canada|New Brunswick|658[0n]|BOLD:AAA2802
Acronicta dactylina|[8908]|PHMNB584-04|Canada|New Brunswick|658[0n]|BOLD:AAA2802
Acronicta dactylina|[8909]|PHMNB438-04|Canada|New Brunswick|658[0n]|BOLD:AAA2802
Acronicta dactylina|[8910]|XAC654-04|Canada|Ontario|658[0n]|BOLD:AAA2802
Acronicta dactylina|[8911]|XAC617-04|Canada|Ontario|658[0n]|BOLD:AAA2802
Acronicta dactylina|[8912]|MNBB227-05|Canada|New Brunswick|658[0n]|BOLD:AAA2802
Acronicta dactylina|[8913]|XAB476-04|Canada|Ontario|658[0n]|BOLD:AAA2802
Acronicta dactylina|[8914]|MNBB377-05|Canada|New Brunswick|622[0n]|BOLD:AAA2802
Acronicta dactylina|[8915]|PHMNB316-04|Canada|New Brunswick|658[0n]|BOLD:AAA2802
Acronicta dactylina|[8916]|RDLQ120-05|Canada|Quebec|658[0n]|BOLD:AAA2802
Acronicta dactylina|[8917]|LHLEP372-06|Canada|British Columbia|631[0n]|BOLD:AAA2802
Acronicta dactylina|[8918]|PHMNB762-05|Canada|New Brunswick|608[0n]|BOLD:AAA2802
Acronicta dactylina|[8919]|RDLQ121-05|Canada|Quebec|589[1n]|BOLD:AAA2802
Acronicta dactylina|[8920]|MNBB159-05|Canada|New Brunswick|658[0n]|BOLD:AAA2802
Acronicta dactylina|[8921]|LPVIA838-08|Canada|British Columbia|658[0n]|BOLD:AAA2802
Acronicta dactylina|[8922]|LHLEP371-06|Canada|British Columbia|648[0n]|BOLD:AAA2802
Acronicta dactylina|[8923]|LPVIB874-08|Canada|British Columbia|647[0n]|BOLD:AAA2802
Acronicta dactylina|[8924]|RDLQ123-05|Canada|Quebec|648[0n]|BOLD:AAA2802
Acronicta dactylina|[8925]|RWWA273-09|United States|Washington|615[0n]|BOLD:AAA2802
Acronicta dactylina|[8926]|LPMN313-08|Canada|Manitoba|636[0n]|BOLD:AAA2802
Acronicta dactylina|[8927]|LHLEP359-06|Canada|British Columbia|623[0n]|BOLD:AAA2802
Acronicta dactylina|[8928]|PHMNB013-03|Canada|New Brunswick|639[0n]|BOLD:AAA2802
Acronicta dactylina|[8929]|LPVIA629-08|Canada|British Columbia|634[0n]|BOLD:AAA2802
Acronicta dactylina|[8930]|MNBB220-05|Canada|New Brunswick|539[0n]|BOLD:AAA2802
Acronicta dactylina|[8931]|MNBB321-05|Canada|New Brunswick|576[2n]|BOLD:AAA2802
Acronicta dactylina|[8932]|LPVIA967-08|Canada|British Columbia|627[0n]|BOLD:AAA2802
Acronicta dactylina|[8933]|RWWA577-09|United States|Washington|642[0n]|BOLD:AAA2802
Acronicta dactylina|[8934]|LHLEP361-06|Canada|British Columbia|657[0n]|BOLD:AAA2802
Acronicta dactylina|[8935]|PHMNB701-04|Canada|New Brunswick|658[0n]|BOLD:AAA2802
Acronicta dactylina|[8936]|MNBB225-05|Canada|New Brunswick|585[0n]|BOLD:AAA2802
Acronicta dactylina|[8937]|LHLEP369-06|Canada|British Columbia|649[0n]|BOLD:AAA2802
Acronicta dactylina|[8938]|BBLCU112-09|United States|Michigan|658[0n]|BOLD:AAA2802
Acronicta dactylina|[8939]|BBLEC356-09|Canada|Newfoundland and Labrador|658[0n]|BOLD:AAA2802
Acronicta dactylina|[8940]|BBLEC839-09|Canada|Newfoundland and Labrador|658[0n]|BOLD:AAA2802
Acronicta dactylina|[8941]|BBLPC632-09|Canada|Newfoundland and Labrador|658[0n]|BOLD:AAA2802
Acronicta dactylina|[8942]|BBLPC639-09|Canada|Newfoundland and Labrador|658[0n]|BOLD:AAA2802
Acronicta dactylina|[8943]|BBLPC670-09|Canada|Newfoundland and Labrador|658[0n]|BOLD:AAA2802
Acronicta dactylina|[8944]|RDNMJ474-11|Canada|Ontario|658[0n]|BOLD:AAA2802
Acronicta dactylina|[8945]|LALPA1015-11|Canada|British Columbia|658[0n]|BOLD:AAA2802
Acronicta dactylina|[8946]|LALPA1121-11|Canada|British Columbia|658[0n]|BOLD:AAA2802
Acronicta dactylina|[8947]|RWWC450-11|United States|Washington|658[0n]|BOLD:AAA2802
Acronicta dactylina|[8948]|RWWC451-11|United States|Washington|658[0n]|BOLD:AAA2802
Acronicta dactylina|[8949]|RWWC650-11|United States|Washington|658[0n]|BOLD:AAA2802
Acronicta dactylina|[8950]|LALPA1199-11|Canada|British Columbia|658[0n]|BOLD:AAA2802
Acronicta dactylina|[8951]|RWWC951-12|United States|Washington|658[0n]|BOLD:AAA2802

Acronicta dactylina|[8949]|RWWC650-11|United States|Washington|658[0n]|BOLD:AAA2802
Acronicta dactylina|[8950]|LALPA1199-11|Canada|British Columbia|658[0n]|BOLD:AAA2802
Acronicta dactylina|[8951]|RWWC951-12|United States|Washington|658[0n]|BOLD:AAA2802
Acronicta dactylina|[8952]|PMG081-03|Canada|Ontario|617[0n]|BOLD:AAJ1616
Acronicta dactylina|[8953]|PHMO124-03|Canada|Ontario|639[0n]|BOLD:AAJ1616
Acronicta dactylina|[8954]|RDNMJ473-11|Canada|Ontario|658[0n]|BOLD:AAJ1616
Acronicta dactylina|[8955]|RDNMK507-11|Canada|Ontario|658[0n]|BOLD:AAJ1616
Acronicta dactylina|[8956]|RDNMK663-11|Canada|Ontario|658[0n]|BOLD:AAJ1616
Acronicta hastulifera|[8957]|LNCC1643-13|United States|North Carolina|658[0n]|BOLD:AAJ1616
Acronicta hastulifera|[8958]|RDNMK506-11|United States|Connecticut|658[0n]|BOLD:AAJ1616
Acronicta hastulifera|[8959]|RDNMK508-11|United States|Massachusetts|658[0n]|BOLD:AAJ1616
Acronicta hastulifera|[8960]|LNCC046-10|United States|North Carolina|658[0n]|BOLD:AAJ1616
Acronicta hastulifera|[8961]|LNCC047-10|United States|North Carolina|658[0n]|BOLD:AAJ1616
Acronicta hastulifera|[8962]|CNCLB2586-14|United States|North Carolina|658[0n]|BOLD:AAJ1616
Acronicta tota|[8963]|RDNMJ084-10|United States|Texas|658[0n]|BOLD:AAU4309
Acronicta tota|[8964]|RDNMJ573-11|United States|New Mexico|652[0n]|BOLD:AAU4309
Acronicta tota|[8965]|RDNMJ574-11|United States|New Mexico|658[0n]|BOLD:AAU4309
Acronicta retardata|[8966]|BBLCU016-09|United States|Michigan|658[0n]|BOLD:AAB2242
Acronicta retardata|[8967]|LPOKA001-08|United States|Oklahoma|658[0n]|BOLD:AAB2242
Acronicta retardata|[8968]|LPOKA025-08|United States|Oklahoma|658[0n]|BOLD:AAB2242
Acronicta retardata|[8969]|LPOKA139-08|United States|Oklahoma|657[0n]|BOLD:AAB2242
Acronicta retardata|[8970]|LGSMC880-05|United States|Tennessee|658[0n]|BOLD:AAB2242
Acronicta retardata|[8971]|LOT317-04|United States|Tennessee|658[0n]|BOLD:AAB2242
Acronicta retardata|[8972]|CNCLB2598-14|United States|North Carolina|636[1n]|BOLD:AAB2242
Acronicta retardata|[8973]|LSEU335-06|United States|Georgia|658[0n]|BOLD:AAB2242
Acronicta retardata|[8974]|LGSMG959-10|United States|Tennessee|658[0n]|BOLD:AAB2242
Acronicta retardata|[8975]|LSEU336-06|United States|Georgia|658[0n]|BOLD:AAB2242
Acronicta retardata|[8976]|RDNMJ639-11|United States|Kentucky|658[0n]|BOLD:AAB2242
Acronicta retardata|[8977]|LNCC1052-11|United States|North Carolina|658[0n]|BOLD:AAB2242
Acronicta retardata|[8978]|LNCC1053-11|United States|North Carolina|658[0n]|BOLD:AAB2242
Acronicta retardata|[8979]|LNCC1054-11|United States|North Carolina|658[0n]|BOLD:AAB2242
Acronicta retardata|[8980]|LOT319-04|United States|Tennessee|658[0n]|BOLD:AAB2242
Acronicta retardata|[8981]|RDNMJ638-11|United States|Kentucky|658[0n]|BOLD:AAB2242
Acronicta retardata|[8982]|RDNMJ079-10|United States|Texas|658[0n]|BOLD:AAB2242
Acronicta retardata|[8983]|LOTB280-05|United States|Tennessee|658[0n]|BOLD:AAB2242
Acronicta retardata|[8984]|LOT321-04|United States|Tennessee|658[0n]|BOLD:AAB2242
Acronicta retardata|[8985]|LGSM770-04|United States|Tennessee|586[0n]|BOLD:AAB2242
Acronicta retardata|[8986]|LOT322-04|United States|Tennessee|658[0n]|BOLD:AAB2242
Acronicta retardata|[8987]|LSUSA188-06|United States|Kentucky|658[0n]|BOLD:AAB2242
Acronicta retardata|[8988]|RDNMJ080-10|United States|Texas|658[0n]|BOLD:AAB2242
Acronicta retardata|[8989]|CNCLB2600-14|United States|North Carolina|658[0n]|BOLD:AAB2242
Acronicta retardata|[8990]|PSAT158-10|United States|Florida|658[0n]|BOLD:ACF2975
Acronicta retardata|[8991]|PSAT159-10|United States|Florida|636[0n]|BOLD:ACF2975
Acronicta retardata|[8992]|PSAT160-10|United States|Florida|636[0n]|BOLD:ACF2975
Acronicta retardata|[8993]|LNC942-06|United States|North Carolina|658[0n]|BOLD:ACF1696
Acronicta retardata|[8994]|LNCC660-11|United States|North Carolina|658[0n]|BOLD:ACF1696
Acronicta retardata|[8995]|LNCC549-11|United States|North Carolina|658[0n]|BOLD:ACF1696
Acronicta retardata|[8996]|CNCLB2601-14|United States|North Carolina|658[0n]|BOLD:ACF1696
Acronicta retardata|[8997]|MNBB138-05|Canada|New Brunswick|658[0n]|BOLD:ACF1696
Acronicta retardata|[8998]|RDLQ449-07|Canada|Quebec|658[0n]|BOLD:ACF1696
Acronicta retardata|[8999]|LOCT094-05|United States|Connecticut|658[0n]|BOLD:ACF1696
Acronicta retardata|[9000]|BBLEC112-09|Canada|Nova Scotia|632[0n]|BOLD:ACF1696
Acronicta retardata|[9001]|PSAT156-10|Canada|Ontario|658[0n]|BOLD:ACF1696
Acronicta retardata|[9002]|BBLPE146-09|Canada|Nova Scotia|658[0n]|BOLD:ACF1696
Acronicta retardata|[9003]|BBLPC264-09|Canada|Nova Scotia|658[0n]|BOLD:ACF1696
Acronicta retardata|[9004]|BBLEC575-09|Canada|Nova Scotia|658[0n]|BOLD:ACF1696
Acronicta retardata|[9005]|RDNM104-05|Canada|Ontario|658[0n]|BOLD:ACF1696
Acronicta retardata|[9006]|RDNM103-05|Canada|Ontario|658[0n]|BOLD:ACF1696
Acronicta retardata|[9007]|LOCT095-05|United States|Connecticut|658[0n]|BOLD:ACF1696
Acronicta retardata|[9008]|MNBB009-05|Canada|New Brunswick|658[0n]|BOLD:ACF1696
Acronicta retardata|[9009]|XAE352-04|Canada|Ontario|658[0n]|BOLD:ACF1696
Acronicta retardata|[9010]|XAE351-04|Canada|Ontario|658[0n]|BOLD:ACF1696
Acronicta retardata|[9011]|BBLPC238-09|Canada|Nova Scotia|658[0n]|BOLD:ACF1696
Acronicta retardata|[9012]|RDLQ317-05|Canada|Quebec|566[0n]|BOLD:ACF1696
Acronicta retardata|[9013]|LNCC381-10|United States|North Carolina|658[0n]|BOLD:ACF1696
Acronicta retardata|[9014]|LNCC382-10|United States|North Carolina|658[0n]|BOLD:ACF1696
Acronicta retardata|[9015]|LNCC661-11|United States|North Carolina|658[0n]|BOLD:ACF1696
Acronicta retardata|[9016]|CNCLB2605-14|United States|North Carolina|658[0n]|BOLD:ACF1696
Acronicta fragilis|[9017]|PHMNB221-04|Canada|New Brunswick|609[0n]|BOLD:ACF0048
Acronicta fragilis|[9018]|MNBB490-05|Canada|New Brunswick|658[0n]|BOLD:ACF0048
Acronicta fragilis|[9019]|RDLQ303-05|Canada|Quebec|656[0n]|BOLD:ACF0048
Acronicta fragilis|[9020]|PHMNB329-04|Canada|New Brunswick|658[0n]|BOLD:ACF0048
Acronicta fragilis|[9021]|TTMNB324-06|Canada|New Brunswick|658[0n]|BOLD:ACF0048
Acronicta fragilis|[9022]|BBLPB207-10|Canada|Ontario|658[0n]|BOLD:ACF0048
Acronicta fragilis|[9023]|CNPES012-14|Canada|Prince Edward Island|569[0n]|BOLD:ACF0048
Acronicta heitzmani|[9024]|RDNMK825-12|United States|Georgia|658[1n]|BOLD:ACF4251
Acronicta heitzmani|[9025]|RDNMK810-12|United States|Kentucky|658[0n]|BOLD:ACF4251
Acronicta heitzmani|[9026]|RDNMK841-12|Canada|Ontario|658[0n]|BOLD:ACF4251
Acronicta heitzmani|[9027]|RDNMK808-12|United States|Kentucky|658[0n]|BOLD:ACF4251
Acronicta heitzmani|[9028]|RDNMK809-12|United States|Kentucky|658[0n]|BOLD:ACF4251
Acronicta heitzmani|[9029]|RDNMK842-12|Canada|Ontario|658[0n]|BOLD:ACF4251
Acronicta fragilis|[9030]|PHMNB261-04|Canada|New Brunswick|597[0n]|BOLD:AAA9541
Acronicta fragilis|[9031]|LSEU089-06|United States|North Carolina|598[1n]|BOLD:AAA9541
Acronicta fragilis|[9032]|LGSM466-04|United States|North Carolina|658[0n]|BOLD:AAA9541
Acronicta fragilis|[9033]|LGSM467-04|United States|North Carolina|658[0n]|BOLD:AAA9541
Acronicta fragilis|[9034]|LSEU680-06|United States|Georgia|658[0n]|BOLD:AAA9541
Acronicta fragilis|[9035]|LSEU681-06|United States|Georgia|658[0n]|BOLD:AAA9541
Acronicta fragilis|[9036]|LSEU698-06|United States|Georgia|658[0n]|BOLD:AAA9541
Acronicta fragilis|[9037]|MMNA102-08|United States|North Carolina|658[0n]|BOLD:AAA9541
Acronicta fragilis|[9038]|LNCC1735-13|United States|North Carolina|658[0n]|BOLD:AAA9541
Acronicta fragilis|[9039]|BBLPC798-09|Canada|Newfoundland and Labrador|658[0n]|BOLD:AAA9541
Acronicta fragilis|[9040]|LBCG2546-09|Canada|British Columbia|658[0n]|BOLD:AAA9541
Acronicta fragilis|[9041]|LBCG3221-09|Canada|British Columbia|658[0n]|BOLD:AAA9541
Acronicta fragilis|[9042]|BBLPE400-09|Canada|Newfoundland and Labrador|658[0n]|BOLD:AAA9541
Acronicta fragilis|[9043]|CNPAC673-13|Canada|Saskatchewan|633[0n]|BOLD:AAA9541
Acronicta fragilis|[9044]|LBCH3031-10|Canada|British Columbia|658[0n]|BOLD:AAA9541
Acronicta fragilis|[9045]|BBLPB523-10|Canada|Ontario|658[0n]|BOLD:AAA9541
Acronicta fragilis|[9046]|BBLPE530-09|Canada|Newfoundland and Labrador|658[0n]|BOLD:AAA9541
Acronicta fragilis|[9047]|LBCH228-10|Canada|British Columbia|658[0n]|BOLD:AAA9541
Acronicta fragilis|[9048]|RDNMH339-09|Canada|New Brunswick|658[0n]|BOLD:AAA9541
Acronicta fragilis|[9049]|BBLEC336-09|Canada|Nova Scotia|658[0n]|BOLD:AAA9541
Acronicta fragilis|[9050]|LALPA1008-11|Canada|British Columbia|658[0n]|BOLD:AAA9541
Acronicta fragilis|[9051]|RDNMH338-09|Canada|New Brunswick|658[0n]|BOLD:AAA9541

[illegible]
Acronicta fragilis|[9049]|BBLEC336-09|Canada|Nova Scotia|658[0n]|BOLD:AAA9541
Acronicta fragilis|[9050]|LALPA1008-11|Canada|British Columbia|658[0n]|BOLD:AAA9541
Acronicta fragilis|[9051]|RDNMH338-09|Canada|New Brunswick|658[0n]|BOLD:AAA9541
Acronicta fragilis|[9052]|LBCG2545-09|Canada|British Columbia|658[0n]|BOLD:AAA9541
Acronicta fragilis|[9053]|LBCG2544-09|Canada|British Columbia|658[0n]|BOLD:AAA9541
Acronicta fragilis|[9054]|LBCG2543-09|Canada|British Columbia|658[0n]|BOLD:AAA9541
Acronicta fragilis|[9055]|LPMN397-08|Canada|Manitoba|658[0n]|BOLD:AAA9541
Acronicta fragilis|[9056]|LBCG177-08|Canada|British Columbia|658[0n]|BOLD:AAA9541
Acronicta fragilis|[9057]|RDLQG322-06|Canada|Quebec|658[0n]|BOLD:AAA9541
Acronicta fragilis|[9058]|RDLQF513-06|Canada|Quebec|658[0n]|BOLD:AAA9541
Acronicta fragilis|[9059]|TMNBB157-06|Canada|New Brunswick|658[0n]|BOLD:AAA9541
Acronicta fragilis|[9060]|TTMNB323-06|Canada|New Brunswick|658[0n]|BOLD:AAA9541
Acronicta fragilis|[9061]|LBCC442-05|Canada|British Columbia|658[0n]|BOLD:AAA9541
Acronicta fragilis|[9062]|LBCA830-05|Canada|British Columbia|658[0n]|BOLD:AAA9541
Acronicta fragilis|[9063]|LBCA505-05|Canada|British Columbia|658[0n]|BOLD:AAA9541
Acronicta fragilis|[9064]|BBLEC819-09|Canada|Newfoundland and Labrador|658[0n]|BOLD:AAA9541
Acronicta fragilis|[9065]|LALPA156-10|Canada|British Columbia|658[0n]|BOLD:AAA9541
Acronicta fragilis|[9066]|RDLQF514-06|Canada|Quebec|658[0n]|BOLD:AAA9541
Acronicta fragilis|[9067]|LALPA921-11|Canada|British Columbia|634[0n]|BOLD:AAA9541
Acronicta fragilis|[9068]|LALPA988-11|Canada|British Columbia|658[0n]|BOLD:AAA9541
Acronicta fragilis|[9069]|RWWC415-11|United States|Washington|658[0n]|BOLD:AAA9541
Acronicta fragilis|[9070]|CNTNC2657-14|Canada|Newfoundland and Labrador|593[0n]|BOLD:AAA9541
Acronicta lobeliae|[9071]|LOTB484-05|United States|Tennessee|658[0n]|BOLD:AAC7189
Acronicta lobeliae|[9072]|RDLQ316-05|Canada|Quebec|658[0n]|BOLD:AAC7189
Acronicta lobeliae|[9073]|LPOKC606-09|United States|Oklahoma|658[0n]|BOLD:AAC7189
Acronicta lobeliae|[9074]|LSEU324-06|United States|Georgia|658[0n]|BOLD:AAC7189
Acronicta lobeliae|[9075]|LSEU323-06|United States|Georgia|658[0n]|BOLD:AAC7189
Acronicta lobeliae|[9076]|LPOKD732-10|United States|Oklahoma|634[0n]|BOLD:AAC7189
Acronicta lobeliae|[9077]|LOTB219-05|United States|Tennessee|658[1n]|BOLD:AAC7189
Acronicta lobeliae|[9078]|LTOLB128-08|United States|Maryland|658[0n]|BOLD:AAC7189
Acronicta lobeliae|[9079]|RDLQ406-05|Canada|Quebec|658[0n]|BOLD:AAC7189
Acronicta lobeliae|[9080]|LOCT269-05|United States|Connecticut|658[0n]|BOLD:AAC7189
Acronicta lobeliae|[9081]|LPSO005-08|Canada|Ontario|658[0n]|BOLD:AAC7189
Acronicta lobeliae|[9082]|LGSMG938-10|United States|North Carolina|658[0n]|BOLD:AAC7189
Acronicta lobeliae|[9083]|LNCC056-10|United States|North Carolina|658[0n]|BOLD:AAC7189
Acronicta lobeliae|[9084]|LNCC219-10|United States|North Carolina|658[0n]|BOLD:AAC7189
Acronicta lobeliae|[9085]|LNCC220-10|United States|North Carolina|658[0n]|BOLD:AAC7189
Acronicta lobeliae|[9086]|LNCC1261-11|United States|North Carolina|658[0n]|BOLD:AAC7189
Acronicta sp.|[9087]|RDNMI012-10|United States|Texas|658[0n]|BOLD:AAY4986
Acronicta sp.|[9088]|RDNMK778-12|United States|Texas|658[0n]|BOLD:AAY4986
Acronicta sp.|[9089]|RDNMK779-12|United States|Texas|658[0n]|BOLD:AAY4986
Acronicta sp.|[9090]|RDNMI016-10|United States|Texas|658[0n]|BOLD:AAY4986
Acronicta sp.|[9091]|RDNMK816-12|United States|Texas|658[0n]|BOLD:AAY4986
Acronicta sp.|[9092]|RDNMK817-12|United States|Texas|658[0n]|BOLD:AAY4986
Acronicta sp.|[9093]|RDNMK821-12|United States|Texas|658[3n]|BOLD:AAY4986
Acronicta morula|[9094]|LPOKB119-09|United States|Oklahoma|658[0n]|BOLD:AAB3303
Acronicta morula|[9095]|LGSMC993-05|United States|Tennessee|603[0n]|BOLD:AAB3303
Acronicta morula|[9096]|RDLQ224-05|Canada|Quebec|658[0n]|BOLD:AAB3303
Acronicta morula|[9097]|PHMNB352-04|Canada|New Brunswick|658[0n]|BOLD:AAB3303
Acronicta morula|[9098]|XAC729-04|Canada|Ontario|658[0n]|BOLD:AAB3303
Acronicta morula|[9099]|XAB161-04|Canada|Ontario|658[0n]|BOLD:AAB3303
Acronicta morula|[9100]|XAB110-04|Canada|Ontario|658[0n]|BOLD:AAB3303
Acronicta morula|[9101]|PMG085-03|Canada|Ontario|617[0n]|BOLD:AAB3303
Acronicta morula|[9102]|TMG113-03|Canada|Ontario|639[0n]|BOLD:AAB3303
Acronicta morula|[9103]|PHMNB029-03|Canada|New Brunswick|639[0n]|BOLD:AAB3303
Acronicta morula|[9104]|PHMNB056-03|Canada|New Brunswick|639[0n]|BOLD:AAB3303
Acronicta morula|[9105]|XAC572-04|Canada|Ontario|594[0n]|BOLD:AAB3303
Acronicta morula|[9106]|XAE549-04|Canada|Ontario|582[0n]|BOLD:AAB3303
Acronicta morula|[9107]|XAF545-05|Canada|Ontario|658[0n]|BOLD:AAB3303
Acronicta morula|[9108]|XAF649-05|Canada|Ontario|658[0n]|BOLD:AAB3303
Acronicta morula|[9109]|MNBB561-05|Canada|New Brunswick|658[0n]|BOLD:AAB3303
Acronicta morula|[9110]|MNBB562-05|Canada|New Brunswick|658[0n]|BOLD:AAB3303
Acronicta morula|[9111]|MNBB601-05|Canada|New Brunswick|658[0n]|BOLD:AAB3303
Acronicta morula|[9112]|MNBB603-05|Canada|New Brunswick|658[0n]|BOLD:AAB3303
Acronicta morula|[9113]|BLTIB174-08|Canada|Ontario|658[0n]|BOLD:AAB3303
Acronicta morula|[9114]|PHMNB231-04|Canada|New Brunswick|609[0n]|BOLD:AAB3303
Acronicta morula|[9115]|LOT304-04|United States|Tennessee|658[0n]|BOLD:AAB3303
Acronicta morula|[9116]|LGSMC440-05|United States|Tennessee|658[0n]|BOLD:AAB3303
Acronicta morula|[9117]|XAJ401-06|Canada|Ontario|658[0n]|BOLD:AAB3303
Acronicta morula|[9118]|XAJ616-06|Canada|Ontario|658[0n]|BOLD:AAB3303
Acronicta morula|[9119]|LGSMG937-10|United States|North Carolina|658[0n]|BOLD:AAB3303
Acronicta morula|[9120]|LMDH182-11|United States|Minnesota|658[0n]|BOLD:AAB3303
Acronicta morula|[9121]|LNCC1044-11|United States|North Carolina|658[0n]|BOLD:AAB3303
Acronicta morula|[9122]|LNCC1045-11|United States|North Carolina|658[0n]|BOLD:AAB3303
Acronicta superans|[9123]|PHMNB444-04|Canada|New Brunswick|658[3n]|BOLD:AAB7444
Acronicta superans|[9124]|PHMNB223-04|Canada|New Brunswick|572[0n]|BOLD:AAB7444
Acronicta superans|[9125]|XAE396-04|Canada|Ontario|658[0n]|BOLD:AAB7444
Acronicta superans|[9126]|LPSOC365-08|Canada|Ontario|655[0n]|BOLD:AAB7444
Acronicta superans|[9127]|CNWLB641-12|Canada|Alberta|614[0n]|BOLD:AAB7444
Acronicta superans|[9128]|LGSM633-04|United States|Tennessee|609[0n]|BOLD:AAB7444
Acronicta superans|[9129]|LGSMG935-10|United States|North Carolina|658[0n]|BOLD:AAB7444
Acronicta superans|[9130]|MNBB515-05|Canada|New Brunswick|658[0n]|BOLD:AAB7444
Acronicta superans|[9131]|LPSOC312-08|Canada|Ontario|658[0n]|BOLD:AAB7444
Acronicta superans|[9132]|LGSMG652-07|United States|North Carolina|658[0n]|BOLD:AAB7444
Acronicta superans|[9133]|XAK156-06|Canada|Ontario|658[0n]|BOLD:AAB7444
Acronicta superans|[9134]|LMIS030-05|Canada|Ontario|658[0n]|BOLD:AAB7444
Acronicta superans|[9135]|MNBB282-05|Canada|New Brunswick|658[0n]|BOLD:AAB7444
Acronicta superans|[9136]|XAF327-05|Canada|Ontario|658[0n]|BOLD:AAB7444
Acronicta superans|[9137]|XAC730-04|Canada|Ontario|658[0n]|BOLD:AAB7444
Acronicta superans|[9138]|LOT518-04|United States|Tennessee|658[0n]|BOLD:AAB7444
Acronicta superans|[9139]|LOTB278-05|United States|Tennessee|658[0n]|BOLD:AAB7444
Acronicta superans|[9140]|LGSM561-04|United States|Tennessee|658[0n]|BOLD:AAB7444
Acronicta superans|[9141]|RDLQ454-07|Canada|Quebec|617[1n]|BOLD:AAB7444
Acronicta superans|[9142]|LPSOD337-09|Canada|Ontario|658[0n]|BOLD:AAB7444
Acronicta superans|[9143]|LPSOD366-09|Canada|Ontario|658[0n]|BOLD:AAB7444
Acronicta superans|[9144]|LNCC331-10|United States|North Carolina|658[0n]|BOLD:AAB7444
Acronicta superans|[9145]|BBLPB648-10|Canada|Ontario|658[0n]|BOLD:AAB7444
Acronicta superans|[9146]|PHMNB236-04|Canada|New Brunswick|526[0n]|BOLD:AAB7444
Acronicta superans|[9147]|CNRME1807-12|Canada|Manitoba|632[0n]|BOLD:AAB7444
Acronicta rapidan|[9148]|RDNMJ081-10|United States|Texas|618[0n]|BOLD:ABY7448
Acronicta rapidan|[9149]|RDNMJ082-10|United States|Texas|658[0n]|BOLD:ABY7448
Acronicta rapidan|[9150]|RDNMK815-12|United States|Texas|658[0n]|BOLD:ABY7448

Acronicta rapidan|[9148]|RDNMJ081-10|United States|Texas|618[0n]|BOLD:ABY7448
Acronicta rapidan|[9149]|RDNMJ082-10|United States|Texas|658[0n]|BOLD:ABY7448
Acronicta rapidan|[9150]|RDNMK815-12|United States|Texas|658[0n]|BOLD:ABY7448
Acronicta connecta|[9151]|LPOKD695-10|United States|Oklahoma|658[0n]|BOLD:AAF0028
Acronicta connecta|[9152]|LPOKB1028-09|United States|Oklahoma|658[0n]|BOLD:AAF0028
Acronicta connecta|[9153]|PHMO161-03|Canada|Ontario|639[0n]|BOLD:AAF0028
Acronicta connecta|[9154]|XAJ595-06|Canada|Ontario|656[0n]|BOLD:AAF0028
Acronicta connecta|[9155]|BLTIB281-08|Canada|Ontario|658[0n]|BOLD:AAF0028
Acronicta connecta|[9156]|RDNMI002-10|United States|Texas|621[0n]|BOLD:AAF0028
Acronicta connecta|[9157]|PSAT162-10|United States|Florida|636[0n]|BOLD:AAF0028
Acronicta connecta|[9158]|RDNMK819-12|United States|Florida|658[0n]|BOLD:AAF0028
Acronicta interrupta|[9159]|XAJ505-06|Canada|Ontario|644[0n]|BOLD:AAB7005
Acronicta interrupta|[9160]|MNBB240-05|Canada|New Brunswick|524[0n]|BOLD:AAB7005
Acronicta interrupta|[9161]|PHMNB585-04|Canada|New Brunswick|658[0n]|BOLD:AAB7005
Acronicta interrupta|[9162]|MNBB190-05|Canada|New Brunswick|658[0n]|BOLD:AAB7005
Acronicta interrupta|[9163]|MNBB480-05|Canada|New Brunswick|658[0n]|BOLD:AAB7005
Acronicta interrupta|[9164]|MNBB649-05|Canada|New Brunswick|658[0n]|BOLD:AAB7005
Acronicta interrupta|[9165]|XAJ455-06|Canada|Ontario|658[0n]|BOLD:AAB7005
Acronicta interrupta|[9166]|XAB303-04|Canada|Ontario|658[0n]|BOLD:AAB7005
Acronicta interrupta|[9167]|LOT291-04|United States|Tennessee|658[0n]|BOLD:AAB7005
Acronicta interrupta|[9168]|LGSM629-04|United States|North Carolina|658[0n]|BOLD:AAB7005
Acronicta interrupta|[9169]|LGSM630-04|United States|North Carolina|658[0n]|BOLD:AAB7005
Acronicta interrupta|[9170]|RDLQ264-05|Canada|Quebec|658[0n]|BOLD:AAB7005
Acronicta interrupta|[9171]|LOTB482-05|United States|Tennessee|658[1n]|BOLD:AAB7005
Acronicta interrupta|[9172]|ABNCC561-07|United States||600[0n]|BOLD:AAB7005
Acronicta interrupta|[9173]|ABNCC562-07|United States||619[0n]|BOLD:AAB7005
Acronicta interrupta|[9174]|LPSOB805-08|Canada|Ontario|658[0n]|BOLD:AAB7005
Acronicta interrupta|[9175]|UDLEP103-09|United States|Maryland|658[0n]|BOLD:AAB7005
Acronicta interrupta|[9176]|UDLEP133-09|United States|Pennsylvania|658[0n]|BOLD:AAB7005
Acronicta interrupta|[9177]|LPSOD276-09|Canada|Ontario|658[0n]|BOLD:AAB7005
Acronicta interrupta|[9178]|LPOKB1007-09|United States|Oklahoma|658[0n]|BOLD:AAB7005
Acronicta interrupta|[9179]|LPOKC623-09|United States|Oklahoma|658[0n]|BOLD:AAB7005
Acronicta interrupta|[9180]|LNCC337-10|United States|North Carolina|658[0n]|BOLD:AAB7005
Acronicta interrupta|[9181]|LNCC338-10|United States|North Carolina|658[0n]|BOLD:AAB7005
Acronicta quadrata|[9182]|RDNMB880-05|Canada|Saskatchewan|511[0n]|BOLD:AAF6773
Acronicta quadrata|[9183]|RDLQB161-05|Canada|Quebec|658[0n]|BOLD:AAF6773
Acronicta quadrata|[9184]|RDNMB881-05|Canada|Manitoba|658[0n]|BOLD:AAF6773
Acronicta quadrata|[9185]|BBLPB251-10|Canada|Ontario|658[0n]|BOLD:AAF6773
Acronicta quadrata|[9186]|BBLPB705-10|Canada|Ontario|658[0n]|BOLD:AAF6773
Acronicta radcliffei|[9187]|RDNMK677-11|United States||658[0n]|BOLD:AAC4642
Acronicta radcliffei|[9188]|CNRMC1485-12|Canada|Manitoba|629[0n]|BOLD:AAC4642
Acronicta radcliffei|[9189]|CNRME046-12|Canada|Manitoba|637[0n]|BOLD:AAC4642
Acronicta radcliffei|[9190]|DUNLP148-08|Canada|British Columbia|650[0n]|BOLD:AAC4642
Acronicta radcliffei|[9191]|LGSMC889-05|United States|Tennessee|658[0n]|BOLD:AAC4642
Acronicta radcliffei|[9192]|RDNMB487-05|United States|California|658[0n]|BOLD:AAC4642
Acronicta radcliffei|[9193]|LPABB502-08|Canada|Alberta|658[0n]|BOLD:AAC4642
Acronicta radcliffei|[9194]|LPVIA405-08|Canada|British Columbia|658[0n]|BOLD:AAC4642
Acronicta radcliffei|[9195]|LPVIA230-08|Canada|British Columbia|658[0n]|BOLD:AAC4642
Acronicta radcliffei|[9196]|LPVIA109-08|Canada|British Columbia|658[0n]|BOLD:AAC4642
Acronicta radcliffei|[9197]|RDLQB177-05|Canada|Quebec|658[0n]|BOLD:AAC4642
Acronicta radcliffei|[9198]|RDNMB889-05|Canada|British Columbia|658[0n]|BOLD:AAC4642
Acronicta radcliffei|[9199]|RDNMB488-05|United States|Georgia|658[0n]|BOLD:AAC4642
Acronicta radcliffei|[9200]|RDNMB486-05|Canada|British Columbia|658[0n]|BOLD:AAC4642
Acronicta radcliffei|[9201]|RDNMB485-05|Canada|British Columbia|658[0n]|BOLD:AAC4642
Acronicta radcliffei|[9202]|LBCA598-05|Canada|British Columbia|658[0n]|BOLD:AAC4642
Acronicta radcliffei|[9203]|LSEU390-06|United States|Georgia|622[0n]|BOLD:AAC4642
Acronicta radcliffei|[9204]|LALPA967-11|Canada|British Columbia|634[0n]|BOLD:AAC4642
Acronicta radcliffei|[9205]|LNCC1066-11|United States|North Carolina|658[0n]|BOLD:AAC4642
Acronicta radcliffei|[9206]|CNCLB1390-14|United States|North Carolina|658[0n]|BOLD:AAC4642
Acronicta laetifica|[9207]|LOT289-04|United States|Tennessee|658[0n]|BOLD:AAB3866
Acronicta laetifica|[9208]|RDLQ412-05|Canada|Quebec|601[0n]|BOLD:AAB3866
Acronicta laetifica|[9209]|LNC756-06|United States|North Carolina|658[0n]|BOLD:AAB3866
Acronicta laetifica|[9210]|LNC757-06|United States|North Carolina|658[0n]|BOLD:AAB3866
Acronicta laetifica|[9211]|LNC225-05|United States|North Carolina|658[0n]|BOLD:AAB3866
Acronicta laetifica|[9212]|LOTB277-05|United States|Tennessee|658[0n]|BOLD:AAB3866
Acronicta laetifica|[9213]|LGSMC855-05|United States|Tennessee|658[0n]|BOLD:AAB3866
Acronicta laetifica|[9214]|LOT522-04|United States|Tennessee|658[0n]|BOLD:AAB3866
Acronicta laetifica|[9215]|LOT290-04|United States|Tennessee|658[0n]|BOLD:AAB3866
Acronicta laetifica|[9216]|LGSM496-04|United States|Tennessee|658[0n]|BOLD:AAB3866
Acronicta laetifica|[9217]|LGSM495-04|United States|Tennessee|658[0n]|BOLD:AAB3866
Acronicta laetifica|[9218]|LSUSA157-06|United States|Kentucky|658[0n]|BOLD:AAB3866
Acronicta laetifica|[9219]|RDNMD277-06|Canada|New Brunswick|658[0n]|BOLD:AAB3866
Acronicta laetifica|[9220]|XAB207-04|Canada|Ontario|658[0n]|BOLD:AAB3866
Acronicta laetifica|[9221]|RDNM105-05|Canada|Ontario|658[0n]|BOLD:AAB3866
Acronicta laetifica|[9222]|RDLQ125-05|Canada|Quebec|658[0n]|BOLD:AAB3866
Acronicta laetifica|[9223]|RDLQ124-05|Canada|Quebec|658[0n]|BOLD:AAB3866
Acronicta laetifica|[9224]|LOT288-04|United States|Tennessee|658[0n]|BOLD:AAB3866
Acronicta laetifica|[9225]|RDNMB478-05|Canada|New Brunswick|614[0n]|BOLD:AAB3866
Acronicta laetifica|[9226]|RDNMB479-05|Canada|Ontario|658[0n]|BOLD:AAB3866
Acronicta laetifica|[9227]|RDLQB708-05|Canada|Quebec|658[0n]|BOLD:AAB3866
Acronicta laetifica|[9228]|RDNMD274-06|Canada|New Brunswick|658[0n]|BOLD:AAB3866
Acronicta laetifica|[9229]|RDNMD276-06|Canada|New Brunswick|658[0n]|BOLD:AAB3866
Acronicta laetifica|[9230]|RDLQ450-07|Canada|Quebec|658[0n]|BOLD:AAB3866
Acronicta laetifica|[9231]|BBLCU012-09|United States|Michigan|658[0n]|BOLD:AAB3866
Acronicta spinigera|[9232]|LPSOC080-08|Canada|Ontario|658[0n]|BOLD:ABY8980
Acronicta spinigera|[9233]|RDNMB882-05|Canada|Ontario|658[0n]|BOLD:ABY8980
Acronicta spinigera|[9234]|RDNMB883-05|Canada|Ontario|658[0n]|BOLD:ABY8980
Acronicta spinigera|[9235]|XAJ618-06|Canada|Ontario|633[0n]|BOLD:ABY8980
Acronicta spinigera|[9236]|LPSOC085-08|Canada|Ontario|658[0n]|BOLD:ABY8980
Acronicta spinigera|[9237]|RDLQ302-05|Canada|Quebec|621[0n]|BOLD:ABY8980
Acronicta spinigera|[9238]|IAWLB381-11|United States|Virginia|658[0n]|BOLD:ABY8980
Acronicta spinigera|[9239]|LGSMC888-05|United States|Tennessee|658[0n]|BOLD:ABY8980
Acronicta spinigera|[9240]|LGSMG939-10|United States|Tennessee|658[0n]|BOLD:ABY8980
Acronicta spinigera|[9241]|LGSMG940-10|United States|Tennessee|658[0n]|BOLD:ABY8980
Acronicta spinigera|[9242]|LGSMG941-10|United States|North Carolina|658[0n]|BOLD:ABY8980
Acronicta spinigera|[9243]|LGSMG942-10|United States|Tennessee|658[0n]|BOLD:ABY8980
Acronicta spinigera|[9244]|LGSMC887-05|United States|Tennessee|658[0n]|BOLD:ABY8980
Acronicta spinigera|[9245]|LGSM672-04|United States|Tennessee|658[0n]|BOLD:ABY8980
Acronicta spinigera|[9246]|LGSM671-04|United States|Tennessee|658[0n]|BOLD:ABY8980
Acronicta spinigera|[9247]|LGSMC886-05|United States|Tennessee|616[0n]|BOLD:ABY8980
Acronicta spinigera|[9248]|CNCLB1435-14|United States|North Carolina|658[0n]|BOLD:ABY8980
Acronicta spinigera|[9249]|CNCLB1436-14|United States|North Carolina|658[0n]|BOLD:ABY8980
Acronicta spinigera|[9250]|CNCLB2588-14|United States|North Carolina|658[0n]|BOLD:ABY8980

Acronicta spinigera|[9247]|LGSM[illegible]-05|United States|Tennessee|[illegible]|BOLD:ABY8980
Acronicta spinigera|[9248]|CNCLB1435-14|United States|North Carolina|658[0n]|BOLD:ABY8980
Acronicta spinigera|[9249]|CNCLB1436-14|United States|North Carolina|658[0n]|BOLD:ABY8980
Acronicta spinigera|[9250]|CNCLB2588-14|United States|North Carolina|658[0n]|BOLD:ABY8980
Acronicta hasta|[9251]|LGSM644-04|United States|Tennessee|609[0n]|BOLD:AAA6038
Acronicta hasta|[9252]|LPSO362-08|Canada|Ontario|658[0n]|BOLD:AAA6038
Acronicta hasta|[9253]|RDNMB481-05|Canada|Ontario|658[0n]|BOLD:AAA6038
Acronicta hasta|[9254]|LPSOB440-08|Canada|Ontario|658[0n]|BOLD:AAA6038
Acronicta hasta|[9255]|RDNM106-05|Canada|Ontario|658[0n]|BOLD:AAA6038
Acronicta hasta|[9256]|XAD099-04|Canada|Ontario|658[0n]|BOLD:AAA6038
Acronicta hasta|[9257]|XAE473-04|Canada|Ontario|658[6n]|BOLD:AAA6038
Acronicta hasta|[9258]|LGSMC853-05|United States|Tennessee|658[0n]|BOLD:AAA6038
Acronicta hasta|[9259]|RDNMB474-05|Canada|British Columbia|658[0n]|BOLD:AAA6038
Acronicta hasta|[9260]|RDNMB475-05|Canada|British Columbia|528[0n]|BOLD:AAA6038
Acronicta hasta|[9261]|LNCB517-07|United States|North Carolina|658[0n]|BOLD:AAA6038
Acronicta hasta|[9262]|UDLEP042-09|United States|Delaware|573[0n]|BOLD:AAA6038
Acronicta hasta|[9263]|UDLEP079-09|United States|Maryland|658[0n]|BOLD:AAA6038
Acronicta hasta|[9264]|XAJ488-06|Canada|Ontario|656[0n]|BOLD:AAA6038
Acronicta hasta|[9265]|RDLQ301-05|Canada|Quebec|608[0n]|BOLD:AAA6038
Acronicta hasta|[9266]|RDNMB477-05|Canada|Ontario|658[0n]|BOLD:AAA6038
Acronicta hasta|[9267]|BLTIB293-08|Canada|Ontario|658[0n]|BOLD:AAA6038
Acronicta hasta|[9268]|LNCC221-10|United States|North Carolina|658[0n]|BOLD:AAA6038
Acronicta hasta|[9269]|BBLPB644-10|Canada|Saskatchewan|658[0n]|BOLD:AAA6038
Acronicta hasta|[9270]|BBLPE263-09|Canada|Nova Scotia|658[0n]|BOLD:AAA6038
Acronicta hasta|[9271]|LGSMG936-10|United States|North Carolina|658[0n]|BOLD:AAA6038
Acronicta hasta|[9272]|LPSOC088-08|Canada|Ontario|658[0n]|BOLD:AAA6038
Acronicta hasta|[9273]|LPSOC289-08|Canada|Ontario|658[0n]|BOLD:AAA6038
Acronicta hasta|[9274]|LPSOB789-08|Canada|Ontario|658[0n]|BOLD:AAA6038
Acronicta hasta|[9275]|UDLEP268-09|United States|Delaware|658[0n]|BOLD:AAA6038
Acronicta hasta|[9276]|RDNMD280-06|Canada|New Brunswick|658[0n]|BOLD:AAA6038
Acronicta hasta|[9277]|XAJ289-06|Canada|Ontario|658[0n]|BOLD:AAA6038
Acronicta hasta|[9278]|XAJ399-06|Canada|Ontario|658[0n]|BOLD:AAA6038
Acronicta hasta|[9279]|XAJ409-06|Canada|Ontario|658[0n]|BOLD:AAA6038
Acronicta hasta|[9280]|XAJ454-06|Canada|Ontario|658[0n]|BOLD:AAA6038
Acronicta hasta|[9281]|XAJ582-06|Canada|Ontario|658[0n]|BOLD:AAA6038
Acronicta hasta|[9282]|LSUSA160-06|United States|Kentucky|658[0n]|BOLD:AAA6038
Acronicta hasta|[9283]|LSUSA167-06|United States|Kentucky|658[0n]|BOLD:AAA6038
Acronicta hasta|[9284]|RDNMB480-05|Canada|Alberta|658[0n]|BOLD:AAA6038
Acronicta hasta|[9285]|RDNMB482-05|Canada|New Brunswick|658[0n]|BOLD:AAA6038
Acronicta hasta|[9286]|XAG405-05|Canada|Ontario|658[0n]|BOLD:AAA6038
Acronicta hasta|[9287]|RDNMB476-05|Canada|Ontario|658[0n]|BOLD:AAA6038
Acronicta hasta|[9288]|RDNMB483-05|Canada|Alberta|658[0n]|BOLD:AAA6038
Acronicta hasta|[9289]|RDLQB160-05|Canada|Quebec|658[0n]|BOLD:AAA6038
Acronicta hasta|[9290]|XAJ979-06|Canada|Ontario|658[0n]|BOLD:AAA6038
Acronicta hasta|[9291]|LSEU676-06|United States|Georgia|658[0n]|BOLD:AAA6038
Acronicta hasta|[9292]|RDLQ451-07|Canada|Quebec|658[0n]|BOLD:AAA6038
Acronicta hasta|[9293]|RDLQ452-07|Canada|Quebec|658[0n]|BOLD:AAA6038
Acronicta hasta|[9294]|LPSOC030-08|Canada|Ontario|658[0n]|BOLD:AAA6038
Acronicta hasta|[9295]|LPSOC087-08|Canada|Ontario|658[0n]|BOLD:AAA6038
Acronicta hasta|[9296]|LSUSA094-06|United States|Kentucky|658[0n]|BOLD:AAA6038
Acronicta hasta|[9297]|RDNMD275-06|Canada|New Brunswick|658[0n]|BOLD:AAA6038
Acronicta hasta|[9298]|RDNMD278-06|Canada|New Brunswick|658[0n]|BOLD:AAA6038
Acronicta hasta|[9299]|RDNMD279-06|Canada|New Brunswick|658[0n]|BOLD:AAA6038
Acronicta hasta|[9300]|RDLQ269-05|Canada|Quebec|658[0n]|BOLD:AAA6038
Acronicta hasta|[9301]|RDLQ256-05|Canada|Quebec|658[0n]|BOLD:AAA6038
Acronicta hasta|[9302]|XAF643-05|Canada|Ontario|658[0n]|BOLD:AAA6038
Acronicta hasta|[9303]|XAF567-05|Canada|Ontario|658[0n]|BOLD:AAA6038
Acronicta hasta|[9304]|XAF543-05|Canada|Ontario|658[0n]|BOLD:AAA6038
Acronicta hasta|[9305]|LGSMC854-05|United States|Tennessee|658[0n]|BOLD:AAA6038
Acronicta hasta|[9306]|LGSMC432-05|United States|Tennessee|658[0n]|BOLD:AAA6038
Acronicta hasta|[9307]|PHMNB588-04|Canada|New Brunswick|658[0n]|BOLD:AAA6038
Acronicta hasta|[9308]|XAE621-04|Canada|Ontario|657[0n]|BOLD:AAA6038
Acronicta hasta|[9309]|XAE618-04|Canada|Ontario|658[0n]|BOLD:AAA6038
Acronicta hasta|[9310]|XAE583-04|Canada|Ontario|658[0n]|BOLD:AAA6038
Acronicta hasta|[9311]|XAE314-04|Canada|Ontario|658[0n]|BOLD:AAA6038
Acronicta hasta|[9312]|XAB164-04|Canada|Ontario|658[0n]|BOLD:AAA6038
Acronicta hasta|[9313]|LOT311-04|United States|Tennessee|658[0n]|BOLD:AAA6038
Acronicta hasta|[9314]|LOTB272-05|United States|Tennessee|658[0n]|BOLD:AAA6038
Acronicta hasta|[9315]|UDLEP194-09|United States|Delaware|658[0n]|BOLD:AAA6038
Acronicta hasta|[9316]|TMG112-03|Canada|Ontario|639[0n]|BOLD:AAA6038
Acronicta hasta|[9317]|PMG082-03|Canada|Ontario|617[0n]|BOLD:AAA6038
Acronicta hasta|[9318]|XAC626-04|Canada|Ontario|615[0n]|BOLD:AAA6038
Acronicta hasta|[9319]|LPMN572-08|Canada|Manitoba|651[0n]|BOLD:AAA6038
Acronicta hasta|[9320]|LNCC1061-11|United States|North Carolina|658[0n]|BOLD:AAA6038
Acronicta hasta|[9321]|CNCLB1431-14|United States|North Carolina|658[0n]|BOLD:AAA6038
Acronicta betulae|[9322]|LSUSA206-06|United States|Kentucky|658[0n]|BOLD:AAF0376
Acronicta betulae|[9323]|LNCB570-09|United States|North Carolina|646[0n]|BOLD:AAF0376
Acronicta betulae|[9324]|LNCB571-09|United States|North Carolina|658[0n]|BOLD:AAF0376
Acronicta betulae|[9325]|LPOKD242-09|United States|Oklahoma|657[0n]|BOLD:AAF0376
Acronicta betulae|[9326]|LSEU639-06|United States|Georgia|658[0n]|BOLD:AAF0376
Acronicta betulae|[9327]|LNCC385-10|United States|North Carolina|658[0n]|BOLD:AAF0376
Acronicta betulae|[9328]|LNCC1283-11|United States|North Carolina|658[0n]|BOLD:AAF0376
Acronicta betulae|[9329]|LNCC1284-11|United States|North Carolina|658[0n]|BOLD:AAF0376
Acronicta innotata|[9330]|XAB253-04|Canada|Ontario|658[2n]|BOLD:AAA3813
Acronicta innotata|[9331]|LOWCC350-05|Canada|British Columbia|658[0n]|BOLD:AAA3813
Acronicta innotata|[9332]|LOWCC352-05|Canada|British Columbia|658[0n]|BOLD:AAA3813
Acronicta innotata|[9333]|LOWCC351-05|Canada|British Columbia|658[0n]|BOLD:AAA3813
Acronicta innotata|[9334]|LBCD422-05|Canada|British Columbia|658[0n]|BOLD:AAA3813
Acronicta innotata|[9335]|LBCC410-05|Canada|British Columbia|658[0n]|BOLD:AAA3813
Acronicta innotata|[9336]|LBCC041-05|Canada|British Columbia|658[0n]|BOLD:AAA3813
Acronicta innotata|[9337]|LBCB629-05|Canada|British Columbia|658[0n]|BOLD:AAA3813
Acronicta innotata|[9338]|LOWCC353-05|Canada|British Columbia|658[0n]|BOLD:AAA3813
Acronicta innotata|[9339]|LBCB147-05|Canada|British Columbia|658[0n]|BOLD:AAA3813
Acronicta innotata|[9340]|LOWCC349-05|Canada|British Columbia|582[0n]|BOLD:AAA3813
Acronicta innotata|[9341]|LOWCC354-05|Canada|British Columbia|529[0n]|BOLD:AAA3813
Acronicta innotata|[9342]|LOWCC862-05|Canada|British Columbia|575[1n]|BOLD:AAA3813
Acronicta innotata|[9343]|LOWCE196-06|Canada|British Columbia|658[0n]|BOLD:AAA3813
Acronicta innotata|[9344]|LOWCE808-06|Canada|British Columbia|658[0n]|BOLD:AAA3813
Acronicta innotata|[9345]|LHLEP373-06|Canada|British Columbia|657[0n]|BOLD:AAA3813
Acronicta innotata|[9346]|LPABB392-08|Canada|Alberta|658[0n]|BOLD:AAA3813
Acronicta innotata|[9347]|BBLPA378-10|Canada|British Columbia|658[0n]|BOLD:AAA3813
Acronicta innotata|[9348]|BBLPA379-10|Canada|British Columbia|658[0n]|BOLD:AAA3813
Acronicta innotata|[9349]|LALPA872-11|Canada|British Columbia|658[0n]|BOLD:AAA3813

Acronicta innotata|[9347]|BBLPA378-10|Canada|British Columbia|658[0n]|BOLD:AAA3813
Acronicta innotata|[9348]|BBLPA379-10|Canada|British Columbia|658[0n]|BOLD:AAA3813
Acronicta innotata|[9349]|LALPA872-11|Canada|British Columbia|658[0n]|BOLD:AAA3813
Acronicta innotata|[9350]|LALPA875-11|Canada|British Columbia|658[0n]|BOLD:AAA3813
Acronicta innotata|[9351]|RDNMK654-11|Canada|British Columbia|658[0n]|BOLD:AAA3813
Acronicta innotata|[9352]|RDNMK658-11|Canada|British Columbia|658[0n]|BOLD:AAA3813
Acronicta innotata|[9353]|PHMNB590-04|Canada|New Brunswick|658[0n]|BOLD:AAA3813
Acronicta innotata|[9354]|BBLPC671-09|Canada|Newfoundland and Labrador|658[1n]|BOLD:AAA3813
Acronicta innotata|[9355]|MNBB280-05|Canada|New Brunswick|658[0n]|BOLD:AAA3813
Acronicta innotata|[9356]|MNBB483-05|Canada|New Brunswick|658[1n]|BOLD:AAA3813
Acronicta innotata|[9357]|MNBB192-05|Canada|New Brunswick|658[1n]|BOLD:AAA3813
Acronicta innotata|[9358]|BBLEC005-09|Canada|New Brunswick|632[0n]|BOLD:AAA3813
Acronicta innotata|[9359]|LGSM046-04|United States|North Carolina|658[0n]|BOLD:AAA3813
Acronicta innotata|[9360]|BBLPE175-09|Canada|Nova Scotia|639[0n]|BOLD:AAA3813
Acronicta innotata|[9361]|MNBB278-05|Canada|New Brunswick|658[0n]|BOLD:AAA3813
Acronicta innotata|[9362]|BBLEC075-09|Canada|New Brunswick|656[0n]|BOLD:AAA3813
Acronicta innotata|[9363]|LGSMG647-07|United States|Tennessee|655[0n]|BOLD:AAA3813
Acronicta innotata|[9364]|MNBB388-05|Canada|New Brunswick|658[1n]|BOLD:AAA3813
Acronicta innotata|[9365]|LGSMG648-07|United States|North Carolina|658[0n]|BOLD:AAA3813
Acronicta innotata|[9366]|LGSMG649-07|United States|North Carolina|658[0n]|BOLD:AAA3813
Acronicta innotata|[9367]|MNBB448-05|Canada|New Brunswick|658[0n]|BOLD:AAA3813
Acronicta innotata|[9368]|MNBB484-05|Canada|New Brunswick|658[0n]|BOLD:AAA3813
Acronicta innotata|[9369]|MNBB391-05|Canada|New Brunswick|658[0n]|BOLD:AAA3813
Acronicta innotata|[9370]|MNBB430-05|Canada|New Brunswick|658[0n]|BOLD:AAA3813
Acronicta innotata|[9371]|MNBB330-05|Canada|New Brunswick|658[0n]|BOLD:AAA3813
Acronicta innotata|[9372]|MNBB390-05|Canada|New Brunswick|658[0n]|BOLD:AAA3813
Acronicta innotata|[9373]|MNBB275-05|Canada|New Brunswick|658[0n]|BOLD:AAA3813
Acronicta innotata|[9374]|MNBB276-05|Canada|New Brunswick|658[0n]|BOLD:AAA3813
Acronicta innotata|[9375]|RDLQB162-05|Canada|Quebec|658[0n]|BOLD:AAA3813
Acronicta innotata|[9376]|XAJ407-06|Canada|Ontario|658[0n]|BOLD:AAA3813
Acronicta innotata|[9377]|XAK050-06|Canada|Ontario|658[0n]|BOLD:AAA3813
Acronicta innotata|[9378]|XAK053-06|Canada|Ontario|658[0n]|BOLD:AAA3813
Acronicta innotata|[9379]|LSEU677-06|United States|Georgia|658[0n]|BOLD:AAA3813
Acronicta innotata|[9380]|LGSMG646-07|United States|Tennessee|658[0n]|BOLD:AAA3813
Acronicta innotata|[9381]|MMNA083-08|United States|North Carolina|658[0n]|BOLD:AAA3813
Acronicta innotata|[9382]|LPSOB383-08|Canada|Ontario|658[0n]|BOLD:AAA3813
Acronicta innotata|[9383]|LPMN267-08|Canada|Manitoba|658[0n]|BOLD:AAA3813
Acronicta innotata|[9384]|BBLEC359-09|Canada|Newfoundland and Labrador|658[0n]|BOLD:AAA3813
Acronicta innotata|[9385]|BBLEC366-09|Canada|Newfoundland and Labrador|658[0n]|BOLD:AAA3813
Acronicta innotata|[9386]|BBLEC388-09|Canada|Newfoundland and Labrador|658[0n]|BOLD:AAA3813
Acronicta innotata|[9387]|BBLEC783-09|Canada|Newfoundland and Labrador|658[0n]|BOLD:AAA3813
Acronicta innotata|[9388]|BBLEC882-09|Canada|Newfoundland and Labrador|658[0n]|BOLD:AAA3813
Acronicta innotata|[9389]|BBLPC571-09|Canada|Nova Scotia|658[0n]|BOLD:AAA3813
Acronicta innotata|[9390]|BBLPC576-09|Canada|Nova Scotia|658[0n]|BOLD:AAA3813
Acronicta innotata|[9391]|BBLPC851-09|Canada|Newfoundland and Labrador|658[0n]|BOLD:AAA3813
Acronicta innotata|[9392]|BBLPC950-09|Canada|Newfoundland and Labrador|658[0n]|BOLD:AAA3813
Acronicta innotata|[9393]|BBLPE035-09|Canada|Nova Scotia|658[0n]|BOLD:AAA3813
Acronicta innotata|[9394]|BBLPE301-09|Canada|Newfoundland and Labrador|658[0n]|BOLD:AAA3813
Acronicta innotata|[9395]|BBLPE409-09|Canada|Newfoundland and Labrador|658[0n]|BOLD:AAA3813
Acronicta innotata|[9396]|BBLPE484-09|Canada|Newfoundland and Labrador|658[0n]|BOLD:AAA3813
Acronicta innotata|[9397]|BBLPE487-09|Canada|Newfoundland and Labrador|658[0n]|BOLD:AAA3813
Acronicta innotata|[9398]|BBLPE488-09|Canada|Newfoundland and Labrador|658[0n]|BOLD:AAA3813
Acronicta innotata|[9399]|BBLPE490-09|Canada|Newfoundland and Labrador|658[0n]|BOLD:AAA3813
Acronicta innotata|[9400]|BBLPE514-09|Canada|Newfoundland and Labrador|658[0n]|BOLD:AAA3813
Acronicta innotata|[9401]|BBLPA377-10|Canada|Ontario|658[0n]|BOLD:AAA3813
Acronicta innotata|[9402]|BBLPA380-10|Canada|Ontario|658[0n]|BOLD:AAA3813
Acronicta innotata|[9403]|MNBB504-05|Canada|New Brunswick|658[0n]|BOLD:AAA3813
Acronicta innotata|[9404]|MNBB604-05|Canada|New Brunswick|658[0n]|BOLD:AAA3813
Acronicta innotata|[9405]|MNBB485-05|Canada|New Brunswick|658[0n]|BOLD:AAA3813
Acronicta innotata|[9406]|MNBB503-05|Canada|New Brunswick|658[0n]|BOLD:AAA3813
Acronicta innotata|[9407]|MNBB194-05|Canada|New Brunswick|658[0n]|BOLD:AAA3813
Acronicta innotata|[9408]|MNBB241-05|Canada|New Brunswick|658[0n]|BOLD:AAA3813
Acronicta innotata|[9409]|BBLPA382-10|Canada|Ontario|658[0n]|BOLD:AAA3813
Acronicta innotata|[9410]|CNCLB2590-14|United States|North Carolina|658[0n]|BOLD:AAA3813
Acronicta innotata|[9411]|MNBB277-05|Canada|New Brunswick|658[0n]|BOLD:AAA3813
Acronicta innotata|[9412]|MNBB279-05|Canada|New Brunswick|658[0n]|BOLD:AAA3813
Acronicta innotata|[9413]|MNBB191-05|Canada|New Brunswick|658[0n]|BOLD:AAA3813
Acronicta innotata|[9414]|MNBB094-05|Canada|New Brunswick|658[0n]|BOLD:AAA3813
Acronicta innotata|[9415]|XAF517-05|Canada|Ontario|658[0n]|BOLD:AAA3813
Acronicta innotata|[9416]|MNBB045-05|Canada|New Brunswick|658[0n]|BOLD:AAA3813
Acronicta innotata|[9417]|MNBB043-05|Canada|New Brunswick|658[0n]|BOLD:AAA3813
Acronicta innotata|[9418]|PHMNB360-04|Canada|New Brunswick|658[0n]|BOLD:AAA3813
Acronicta innotata|[9419]|XAE461-04|Canada|Ontario|658[0n]|BOLD:AAA3813
Acronicta innotata|[9420]|XAE358-04|Canada|Ontario|658[0n]|BOLD:AAA3813
Acronicta innotata|[9421]|LOT504-04|United States|Tennessee|658[0n]|BOLD:AAA3813
Acronicta innotata|[9422]|LGSM461-04|United States|Tennessee|658[0n]|BOLD:AAA3813
Acronicta innotata|[9423]|LGSM460-04|United States|North Carolina|658[0n]|BOLD:AAA3813
Acronicta innotata|[9424]|XAE284-04|Canada|Ontario|658[0n]|BOLD:AAA3813
Acronicta innotata|[9425]|XAE465-04|Canada|Ontario|613[0n]|BOLD:AAA3813
Acronicta innotata|[9426]|MNBB389-05|Canada|New Brunswick|658[0n]|BOLD:AAA3813
Acronicta innotata|[9427]|MNBB648-05|Canada|New Brunswick|658[1n]|BOLD:AAA3813
Acronicta innotata|[9428]|LSEU678-06|United States|Georgia|658[0n]|BOLD:AAA3813
Acronicta innotata|[9429]|BBLEC382-09|Canada|Newfoundland and Labrador|648[0n]|BOLD:AAA3813
Acronicta innotata|[9430]|BBLEC346-09|Canada|Newfoundland and Labrador|633[0n]|BOLD:AAA3813
Acronicta innotata|[9431]|XAE470-04|Canada|Ontario|615[0n]|BOLD:AAA3813
Acronicta innotata|[9432]|LGSMC992-05|United States|Tennessee|584[0n]|BOLD:AAA3813
Acronicta innotata|[9433]|PHMO215-03|Canada|Ontario|639[0n]|BOLD:AAA3813
Acronicta innotata|[9434]|MNBB331-05|Canada|New Brunswick|570[0n]|BOLD:AAA3813
Acronicta innotata|[9435]|TTMNB322-06|Canada|New Brunswick|608[0n]|BOLD:AAA3813
Acronicta innotata|[9436]|XAK479-07|Canada|Ontario|594[0n]|BOLD:AAA3813
Acronicta innotata|[9437]|BBLPE541-09|Canada|Newfoundland and Labrador|627[0n]|BOLD:AAA3813
Acronicta innotata|[9438]|CNCLB2591-14|United States|North Carolina|658[0n]|BOLD:AAA3813
Agriopodes fallax|[9439]|BBLCU061-09|United States|Michigan|658[0n]|BOLD:AAB6889
Agriopodes fallax|[9440]|BBLCU124-09|United States|Michigan|658[0n]|BOLD:AAB6889
Agriopodes fallax|[9441]|BBLCU204-09|United States|Michigan|658[0n]|BOLD:AAB6889
Agriopodes fallax|[9442]|LSEU393-06|United States|Georgia|658[0n]|BOLD:AAB6889
Agriopodes fallax|[9443]|LSUSA001-06|United States|Kentucky|658[0n]|BOLD:AAB6889
Agriopodes fallax|[9444]|XAF497-05|Canada|Ontario|658[0n]|BOLD:AAB6889
Agriopodes fallax|[9445]|XAE561-04|Canada|Ontario|658[0n]|BOLD:AAB6889
Agriopodes fallax|[9446]|XAB606-04|Canada|Ontario|658[0n]|BOLD:AAB6889
Agriopodes fallax|[9447]|XAF754-05|Canada|Ontario|578[0n]|BOLD:AAB6889
Agriopodes fallax|[9448]|PHMO147-03|Canada|Ontario|639[0n]|BOLD:AAB6889
Agriopodes fallax|[9449]|PHMO[illegible]-03|Canada|Ontario|639[0n]|BOLD:AAB6889

Agriopodes fallax|[9446]|XAB606-04|Canada|Ontario|658[0n]|BOLD:AAB6889
Agriopodes fallax|[9447]|XAF754-05|Canada|Ontario|578[0n]|BOLD:AAB6889
Agriopodes fallax|[9448]|PHMO147-03|Canada|Ontario|639[0n]|BOLD:AAB6889
Agriopodes fallax|[9449]|PHMO133-03|Canada|Ontario|639[0n]|BOLD:AAB6889
Agriopodes fallax|[9450]|XAC797-04|Canada|Ontario|615[0n]|BOLD:AAB6889
Agriopodes fallax|[9451]|RDNMF449-08|Canada|Manitoba|641[1n]|BOLD:AAB6889
Agriopodes fallax|[9452]|UDLEP273-09|United States|Delaware|658[0n]|BOLD:AAB6889
Agriopodes fallax|[9453]|XAI039-05|Canada|Ontario|658[0n]|BOLD:AAB6889
Agriopodes fallax|[9454]|LPSO355-08|Canada|Ontario|658[0n]|BOLD:AAB6889
Agriopodes fallax|[9455]|LPSOC352-08|Canada|Ontario|658[0n]|BOLD:AAB6889
Agriopodes fallax|[9456]|LPOKB126-09|United States|Oklahoma|658[0n]|BOLD:AAB6889
Agriopodes fallax|[9457]|BBLCU123-09|United States|Michigan|658[0n]|BOLD:AAB6889
Agriopodes fallax|[9458]|USLEP664-10|United States|Florida|658[0n]|BOLD:AAB6889
Agriopodes fallax|[9459]|RDLQB064-05|Canada|Quebec|658[0n]|BOLD:AAB6889
Agriopodes fallax|[9460]|RDLQB065-05|Canada|Quebec|553[0n]|BOLD:AAB6889
Agriopodes fallax|[9461]|BBLPE029-09|Canada|Nova Scotia|658[0n]|BOLD:AAB6889
Agriopodes fallax|[9462]|BBLPE272-09|Canada|Nova Scotia|658[0n]|BOLD:AAB6889
Agriopodes fallax|[9463]|LOFLB816-06|United States|Florida|658[0n]|BOLD:AAB6889
Agriopodes fallax|[9464]|PHMNB596-04|Canada|New Brunswick|658[0n]|BOLD:AAB6889
Agriopodes fallax|[9465]|LNCC042-10|United States|North Carolina|658[0n]|BOLD:AAB6889
Agriopodes fallax|[9466]|LNCC043-10|United States|North Carolina|658[0n]|BOLD:AAB6889
Acronicta valliscola|[9467]|RDNMJ088-10|United States|Texas|658[0n]|BOLD:ABV4471
Acronicta valliscola|[9468]|RDNMK780-12|United States|Texas|658[0n]|BOLD:ABV4471
Acronicta valliscola|[9469]|RDNMK781-12|United States|Texas|658[0n]|BOLD:ABV4471
Acronicta valliscola|[9470]|RDNMK812-12|United States|Texas|658[0n]|BOLD:ABV4471
Acronicta clarescens|[9471]|UDLEP074-09|United States|Delaware|658[0n]|BOLD:AAH4715
Acronicta clarescens|[9472]|LNC941-06|United States|North Carolina|658[0n]|BOLD:AAH4715
Acronicta clarescens|[9473]|LNC940-06|United States|North Carolina|658[0n]|BOLD:AAH4715
Acronicta clarescens|[9474]|RDNMJ586-11|United States|Texas|619[0n]|BOLD:AAH4715
Acronicta thoracica|[9475]|RDNMG273-08|United States|Texas|658[0n]|BOLD:AAJ1619
Acronicta thoracica|[9476]|RDNMD937-07|United States|Arizona|653[0n]|BOLD:AAJ1619
Acronicta thoracica|[9477]|CMAZA940-12|United States|Arizona|658[0n]|BOLD:AAJ1619
Acronicta strigulata|[9478]|RDNMB887-05|Canada|British Columbia|658[0n]|BOLD:AAF5220
Acronicta strigulata|[9479]|RDNMB888-05|United States|Oregon|658[0n]|BOLD:AAF5220
Acronicta strigulata|[9480]|LOWCB176-05|Canada|British Columbia|658[0n]|BOLD:AAF5220
Acronicta strigulata|[9481]|LBCH5609-10|Canada|British Columbia|658[0n]|BOLD:AAF5220
Acronicta strigulata|[9482]|RDNMI010-10|United States|Colorado|658[0n]|BOLD:AAF5220
Acronicta strigulata|[9483]|RDNMJ619-11|United States|Utah|658[0n]|BOLD:AAF5220
Acronicta strigulata|[9484]|RDNMJ620-11|United States|Colorado|658[0n]|BOLD:AAF5220
Acronicta strigulata|[9485]|BBLOC205-11|United States|Texas|658[0n]|BOLD:AAF5220
Acronicta strigulata|[9486]|RDNMK822-12|United States|Colorado|658[0n]|BOLD:AAF5220
Acronicta browni|[9487]|RDNMI009-10|United States|California|658[0n]|BOLD:AAO0432
Acronicta tritona|[9488]|LGSMG651-07|United States|Tennessee|658[0n]|BOLD:AAC1000
Acronicta tritona|[9489]|LNC030-05|United States|North Carolina|658[0n]|BOLD:AAC1000
Acronicta tritona|[9490]|MMNA003-05|Canada|New Brunswick|658[0n]|BOLD:AAC1000
Acronicta tritona|[9491]|LSEU391-06|United States|Georgia|658[0n]|BOLD:AAC1000
Acronicta tritona|[9492]|LOFLC437-06|United States|Florida|658[0n]|BOLD:AAC1000
Acronicta tritona|[9493]|LOFLA226-06|United States|Florida|658[0n]|BOLD:AAC1000
Acronicta tritona|[9494]|LNC031-05|United States|North Carolina|658[0n]|BOLD:AAC1000
Acronicta tritona|[9495]|LGSMC388-05|United States|Tennessee|658[0n]|BOLD:AAC1000
Acronicta tritona|[9496]|LCH263-04|Canada|Manitoba|658[0n]|BOLD:AAC1000
Acronicta tritona|[9497]|LOFLB752-06|United States|Florida|658[0n]|BOLD:AAC1000
Acronicta tritona|[9498]|LOFLC449-06|United States|Florida|653[0n]|BOLD:AAC1000
Acronicta tritona|[9499]|LOFLC401-06|United States|Florida|630[0n]|BOLD:AAC1000
Acronicta tritona|[9500]|RDLQF711-06|Canada|Quebec|637[0n]|BOLD:AAC1000
Acronicta tritona|[9501]|LGSMG650-07|United States|Tennessee|658[0n]|BOLD:AAC1000
Acronicta tritona|[9502]|BBLOB1697-11|United States|Florida|658[0n]|BOLD:AAC1000
Acronicta grisea|[9503]|LALPA541-10|Canada|British Columbia|658[0n]|BOLD:AAA7688
Acronicta grisea|[9504]|RDLQB168-05|Canada|Quebec|513[0n]|BOLD:AAA7688
Acronicta grisea|[9505]|PHMNB592-04|Canada|New Brunswick|658[0n]|BOLD:AAA7688
Acronicta grisea|[9506]|LPSOD336-09|Canada|Ontario|658[0n]|BOLD:AAA7688
Acronicta grisea|[9507]|LBCA362-05|Canada|British Columbia|658[0n]|BOLD:AAA7688
Acronicta grisea|[9508]|PHMNB363-04|Canada|New Brunswick|658[0n]|BOLD:AAA7688
Acronicta grisea|[9509]|RDLQ292-05|Canada|Quebec|600[0n]|BOLD:AAA7688
Acronicta grisea|[9510]|BBLPB645-10|Canada|Alberta|658[0n]|BOLD:AAA7688
Acronicta grisea|[9511]|LOPN058-06|United States|Oregon|658[0n]|BOLD:AAA7688
Acronicta grisea|[9512]|LPVIA229-08|Canada|British Columbia|658[0n]|BOLD:AAA7688
Acronicta grisea|[9513]|PHMNB250-04|Canada|New Brunswick|609[0n]|BOLD:AAA7688
Acronicta grisea|[9514]|BBLPB778-10|Canada|Ontario|658[0n]|BOLD:AAA7688
Acronicta grisea|[9515]|LOWCE803-06|Canada|British Columbia|658[1n]|BOLD:AAA7688
Acronicta grisea|[9516]|NAMUM411-09|United States|California|658[0n]|BOLD:AAA7688
Acronicta grisea|[9517]|LBCA363-05|Canada|British Columbia|636[0n]|BOLD:AAA7688
Acronicta grisea|[9518]|RDLQB175-05|Canada|Quebec|658[0n]|BOLD:AAA7688
Acronicta grisea|[9519]|LPMN635-08|Canada|Manitoba|658[0n]|BOLD:AAA7688
Acronicta grisea|[9520]|LPABB371-08|Canada|Alberta|658[0n]|BOLD:AAA7688
Acronicta grisea|[9521]|BBLPC746-09|Canada|Newfoundland and Labrador|658[0n]|BOLD:AAA7688
Acronicta grisea|[9522]|BBLPE275-09|Canada|Nova Scotia|658[0n]|BOLD:AAA7688
Acronicta grisea|[9523]|BBLPE544-09|Canada|Newfoundland and Labrador|658[0n]|BOLD:AAA7688
Acronicta grisea|[9524]|RWWB864-10|United States|Washington|658[0n]|BOLD:AAA7688
Acronicta grisea|[9525]|BBLPB646-10|Canada|British Columbia|658[0n]|BOLD:AAA7688
Acronicta grisea|[9526]|LALPA920-11|Canada|British Columbia|658[0n]|BOLD:AAA7688
Acronicta grisea|[9527]|LPABC974-09|Canada|Alberta|658[0n]|BOLD:AAA7688
Acronicta grisea|[9528]|LPSOD586-09|Canada|Ontario|658[0n]|BOLD:AAA7688
Acronicta grisea|[9529]|LCHP361-07|Canada|Manitoba|658[0n]|BOLD:AAA7688
Acronicta grisea|[9530]|LPMN573-08|Canada|Manitoba|658[0n]|BOLD:AAA7688
Acronicta grisea|[9531]|LOWCE809-06|Canada|British Columbia|658[0n]|BOLD:AAA7688
Acronicta grisea|[9532]|RDLQF515-06|Canada|Quebec|658[0n]|BOLD:AAA7688
Acronicta grisea|[9533]|RDLQB174-05|Canada|Quebec|658[0n]|BOLD:AAA7688
Acronicta grisea|[9534]|RDLQB176-05|Canada|Quebec|658[0n]|BOLD:AAA7688
Acronicta grisea|[9535]|RDLQB172-05|Canada|Quebec|658[0n]|BOLD:AAA7688
Acronicta grisea|[9536]|RDLQB173-05|Canada|Quebec|658[0n]|BOLD:AAA7688
Acronicta grisea|[9537]|RDLQB171-05|Canada|Quebec|658[0n]|BOLD:AAA7688
Acronicta grisea|[9538]|RDLQB170-05|Canada|Quebec|658[0n]|BOLD:AAA7688
Acronicta grisea|[9539]|RDLQB169-05|Canada|Quebec|658[0n]|BOLD:AAA7688
Acronicta grisea|[9540]|LOWCC914-05|Canada|British Columbia|658[0n]|BOLD:AAA7688
Acronicta grisea|[9541]|RDNMB891-05|Canada|British Columbia|658[0n]|BOLD:AAA7688
Acronicta grisea|[9542]|RDNMB890-05|Canada|New Brunswick|658[0n]|BOLD:AAA7688
Acronicta grisea|[9543]|LBCA497-05|Canada|British Columbia|658[0n]|BOLD:AAA7688
Acronicta grisea|[9544]|PHMNB680-04|Canada|New Brunswick|658[0n]|BOLD:AAA7688
Acronicta grisea|[9545]|PHMNB678-04|Canada|New Brunswick|658[0n]|BOLD:AAA7688
Acronicta grisea|[9546]|PHMNB535-04|Canada|New Brunswick|658[0n]|BOLD:AAA7688
Acronicta grisea|[9547]|PHMNB527-04|Canada|New Brunswick|658[0n]|BOLD:AAA7688
Acronicta grisea|[9548]|LOWCB854-05|Canada|British Columbia|547[1n]|BOLD:AAA7688

Acronicta grisea|[9546]|PHMNB535-04|Canada|New Brunswick|658[0n]|BOLD:AAA7688
Acronicta grisea|[9547]|PHMNB527-04|Canada|New Brunswick|658[0n]|BOLD:AAA7688
Acronicta grisea|[9548]|LOWCB854-05|Canada|British Columbia|547[1n]|BOLD:AAA7688
Acronicta grisea|[9549]|LBCA391-05|Canada|British Columbia|658[1n]|BOLD:AAA7688
Acronicta grisea|[9550]|RDLQB800-05|Canada|Quebec|617[0n]|BOLD:AAA7688
Acronicta grisea|[9551]|LPVIA999-08|Canada|British Columbia|635[0n]|BOLD:AAA7688
Acronicta grisea|[9552]|LALPA943-11|Canada|British Columbia|658[0n]|BOLD:AAA7688
Acronicta grisea|[9553]|LOPN059-06|United States|Oregon|513[2n]|BOLD:AAA7688
Acronicta grisea|[9554]|LPMN322-08|Canada|Manitoba|658[0n]|BOLD:AAA7688
Acronicta grisea|[9555]|PHMNB027-03|Canada|New Brunswick|639[0n]|BOLD:AAA7688
Acronicta grisea|[9556]|LPMN347-08|Canada|Manitoba|658[0n]|BOLD:AAA7688
Acronicta grisea|[9557]|LPABB488-08|Canada|Alberta|658[0n]|BOLD:AAA7688
Acronicta grisea|[9558]|LBCD142-05|Canada|British Columbia|658[0n]|BOLD:AAA7688
Acronicta grisea|[9559]|LPAB012-08|Canada|Alberta|658[0n]|BOLD:AAA7688
Acronicta grisea|[9560]|LPABB448-08|Canada|Alberta|658[0n]|BOLD:AAA7688
Acronicta grisea|[9561]|RDNMK655-11|Canada|British Columbia|658[0n]|BOLD:AAA7688
Acronicta falcula|[9562]|LNAUS2783-13|United States|Connecticut|658[0n]|BOLD:ACI4248
Acronicta hamamelis|[9563]|QUNOC037-09|United States|Louisiana|658[0n]|BOLD:AAJ1606
Acronicta hamamelis|[9564]|LNCB069-06|United States|North Carolina|658[0n]|BOLD:AAJ1606
Acronicta hamamelis|[9565]|MILEQ293-11|United States|Alabama|658[0n]|BOLD:AAJ1606
Acronicta hamamelis|[9566]|CNCLB2585-14|United States|North Carolina|658[0n]|BOLD:AAJ1606
Acronicta lithospila|[9567]|CNCLB2593-14|United States|North Carolina|658[0n]|BOLD:AAF0152
Acronicta lithospila|[9568]|LSEU334-06|United States|Georgia|658[0n]|BOLD:AAF0152
Acronicta lithospila|[9569]|LOTB298-05|United States|Tennessee|658[0n]|BOLD:AAF0152
Acronicta lithospila|[9570]|LSEU392-06|United States|Georgia|658[0n]|BOLD:AAF0152
Acronicta lithospila|[9571]|LPOKB1035-09|United States|Oklahoma|658[0n]|BOLD:AAF0152
Acronicta lithospila|[9572]|LNCC657-11|United States|North Carolina|658[0n]|BOLD:AAF0152
Acronicta lithospila|[9573]|CNCLB2595-14|United States|North Carolina|658[0n]|BOLD:AAF0152
Acronicta lithospila|[9574]|CNCLB2596-14|United States|North Carolina|658[0n]|BOLD:AAF0152
Acronicta mansueta|[9575]|RDNMB459-05|United States|California|658[0n]|BOLD:AAD2021
Acronicta mansueta|[9576]|RDNMB463-05|United States|California|658[0n]|BOLD:AAD2021
Acronicta mansueta|[9577]|RDNMB464-05|United States|California|658[0n]|BOLD:AAD2021
Acronicta mansueta|[9578]|RDNMB465-05|United States|Colorado|658[0n]|BOLD:AAD2021
Acronicta mansueta|[9579]|RDNMB466-05|Canada|Alberta|658[0n]|BOLD:AAD2021
Acronicta mansueta|[9580]|PSAT148-10|United States|Colorado|658[0n]|BOLD:AAD2021
Acronicta mansueta|[9581]|RDNMB467-05|Canada|Alberta|583[1n]|BOLD:AAD2021
Acronicta mansueta|[9582]|RDNMB461-05|Canada|Alberta|658[0n]|BOLD:AAD2021
Acronicta mansueta|[9583]|RDNMB462-05|Canada|British Columbia|658[0n]|BOLD:AAD2021
Acronicta mansueta|[9584]|RDMAB118-05|Canada|Alberta|658[0n]|BOLD:AAD2021
Acronicta mansueta|[9585]|LBCG502-08|Canada|British Columbia|658[0n]|BOLD:AAD2021
Acronicta mansueta|[9586]|LPABC904-09|Canada|Alberta|658[0n]|BOLD:AAD2021
Acronicta mansueta|[9587]|LBCH5210-10|Canada|British Columbia|658[0n]|BOLD:AAD2021
Acronicta mansueta|[9588]|PSAT147-10|United States|Colorado|658[0n]|BOLD:AAD2021
Acronicta mansueta|[9589]|RDNMK145-11|United States|Utah|658[0n]|BOLD:AAD2021
Acronicta mansueta|[9590]|RDNMK147-11|United States|Utah|658[0n]|BOLD:AAD2021
Acronicta mansueta|[9591]|RDNMB468-05|United States|Colorado|530[1n]|BOLD:AAE9946
Acronicta mansueta|[9592]|RDNMJ615-11|United States|Oklahoma|658[0n]|BOLD:AAE9946
Acronicta parallela|[9593]|RDNMJ085-10|United States|Texas|658[0n]|BOLD:AAE9946
Acronicta parallela|[9594]|RDNMK820-12|United States|Oklahoma|658[0n]|BOLD:AAE9946
Acronicta parallela|[9595]|RDNMK823-12|United States|Kansas|658[0n]|BOLD:AAE9946
Acronicta sp.|[9596]|PSAT161-10|United States|Florida|620[0n]|BOLD:AAC1018
Acronicta vinnula|[9597]|LNCC1898-13|United States|North Carolina|658[0n]|BOLD:AAC1018
Acronicta vinnula|[9598]|RDLQH215-07|Canada|Quebec|644[0n]|BOLD:AAC1018
Acronicta vinnula|[9599]|XAJ553-06|Canada|Ontario|658[0n]|BOLD:AAC1018
Acronicta vinnula|[9600]|XAK319-06|Canada|Ontario|658[0n]|BOLD:AAC1018
Acronicta vinnula|[9601]|XAK332-06|Canada|Ontario|658[0n]|BOLD:AAC1018
Acronicta vinnula|[9602]|XAK333-06|Canada|Ontario|658[0n]|BOLD:AAC1018
Acronicta vinnula|[9603]|LPSOC357-08|Canada|Ontario|658[0n]|BOLD:AAC1018
Acronicta vinnula|[9604]|LPSOB825-08|Canada|Ontario|658[0n]|BOLD:AAC1018
Acronicta vinnula|[9605]|LGSM541-04|United States|Tennessee|658[0n]|BOLD:AAC1018
Acronicta vinnula|[9606]|LGSM542-04|United States|Tennessee|593[0n]|BOLD:AAC1018
Acronicta vinnula|[9607]|LSUSA162-06|United States|Kentucky|658[0n]|BOLD:AAC1018
Acronicta vinnula|[9608]|LILLA049-11|United States|Illinois|658[0n]|BOLD:AAC1018
Acronicta lepetita|[9609]|RDNMJ086-10|United States|Texas|658[0n]|BOLD:AAC1018
Acronicta paupercula|[9610]|RDNMI006-10|United States|Texas|605[0n]|BOLD:AAC1018
Acronicta vinnula|[9611]|BBLSW853-09|United States|Texas|658[0n]|BOLD:AAC1018
Acronicta vinnula|[9612]|LPOKB429-09|United States|Oklahoma|644[0n]|BOLD:AAC1018
Acronicta lepetita|[9613]|RDNMI003-10|United States|Texas|658[0n]|BOLD:AAC1018
Acronicta paupercula|[9614]|USLEP609-10|United States|Texas|658[0n]|BOLD:AAC1018
Acronicta lepetita|[9615]|RDNMI004-10|United States|Texas|658[0n]|BOLD:AAC1018
Acronicta vinnula|[9616]|RDNMJ579-11|United States|Texas|658[0n]|BOLD:AAC1018
Acronicta paupercula|[9617]|LPOKD344-09|United States|Oklahoma|658[0n]|BOLD:AAC1018
Acronicta vinnula|[9618]|LPOKC766-09|United States|Oklahoma|658[0n]|BOLD:AAC1018
Acronicta paupercula|[9619]|RDNMI005-10|United States|Mississippi|589[0n]|BOLD:AAC1018
Acronicta vinnula|[9620]|RDNMJ580-11|United States|Texas|658[0n]|BOLD:AAC1018
Acronicta vinnula|[9621]|RDNMJ581-11|United States|Texas|658[0n]|BOLD:AAC1018
Acronicta vinnula|[9622]|RDNMJ582-11|United States|Texas|658[0n]|BOLD:AAC1018
Acronicta lepetita|[9623]|RDNMJ610-11|United States|Texas|658[0n]|BOLD:AAC1018
Acronicta vinnula|[9624]|LNC034-05|United States|North Carolina|627[0n]|BOLD:AAC1018
Acronicta vinnula|[9625]|LNCC1681-13|United States|North Carolina|658[0n]|BOLD:AAC1018
Acronicta vinnula|[9626]|LNCC1891-13|United States|North Carolina|658[0n]|BOLD:AAC1018
Acronicta vinnula|[9627]|LNCC1899-13|United States|North Carolina|658[0n]|BOLD:AAC1018
Acronicta vinnula|[9628]|LNCC1900-13|United States|North Carolina|658[0n]|BOLD:AAC1018
Acronicta vinnula|[9629]|LNC035-05|United States|North Carolina|658[0n]|BOLD:AAC1018
Acronicta vinnula|[9630]|LNCC1680-13|United States|North Carolina|658[0n]|BOLD:AAC1018
Acronicta vinnula|[9631]|CNCLB2608-14|United States|North Carolina|658[0n]|BOLD:AAC1018
Comachara cadburyi|[9632]|LGSMC461-05|United States|Tennessee|658[0n]|BOLD:AAC8110
Comachara cadburyi|[9633]|LOCT226-05|United States|Connecticut|658[0n]|BOLD:AAC8110
Comachara cadburyi|[9634]|LOCT228-05|United States|Connecticut|658[0n]|BOLD:AAC8110
Comachara cadburyi|[9635]|LOCT227-05|United States|Connecticut|658[0n]|BOLD:AAC8110
Comachara cadburyi|[9636]|LOCT293-05|United States|Connecticut|619[0n]|BOLD:AAC8110
Comachara cadburyi|[9637]|HKONS309-08|United States|Florida|658[0n]|BOLD:AAC8110
Comachara cadburyi|[9638]|LOCT229-05|United States|Connecticut|658[0n]|BOLD:AAC8110
Comachara cadburyi|[9639]|LNC931-06|United States|North Carolina|658[0n]|BOLD:AAC8110
Comachara cadburyi|[9640]|LSEU339-06|United States|Georgia|658[0n]|BOLD:AAC8110
Comachara cadburyi|[9641]|LNCC662-11|United States|North Carolina|658[0n]|BOLD:AAC8110
Polygrammate hebraeicum|[9642]|LGSMC348-05|United States|Tennessee|658[0n]|BOLD:AAA8249
Polygrammate hebraeicum|[9643]|LOTB183-05|United States|Tennessee|616[0n]|BOLD:AAA8249
Polygrammate hebraeicum|[9644]|LOT209-04|United States|Tennessee|609[0n]|BOLD:AAA8249
Polygrammate hebraeicum|[9645]|LOT208-04|United States|Tennessee|609[0n]|BOLD:AAA8249
Polygrammate hebraeicum|[9646]|LOT207-04|United States|Tennessee|609[0n]|BOLD:AAA8249
Polygrammate hebraeicum|[9647]|LOT206-04|United States|Tennessee|609[0n]|BOLD:AAA8249
[illegible]

Polygrammate hebraeicum|[9645]|LOT208-04|United States|Tennessee|609[0n]|BOLD:AAA8249
Polygrammate hebraeicum|[9646]|LOT207-04|United States|Tennessee|609[0n]|BOLD:AAA8249
Polygrammate hebraeicum|[9647]|LOT206-04|United States|Tennessee|609[0n]|BOLD:AAA8249
Polygrammate hebraeicum|[9648]|LOT205-04|United States|Tennessee|609[0n]|BOLD:AAA8249
Polygrammate hebraeicum|[9649]|LOT204-04|United States|Tennessee|609[0n]|BOLD:AAA8249
Polygrammate hebraeicum|[9650]|LGSMD249-05|United States|Tennessee|658[0n]|BOLD:AAA8249
Polygrammate hebraeicum|[9651]|LILLA584-11|United States|Illinois|658[0n]|BOLD:AAA8249
Polygrammate hebraeicum|[9652]|LNCC1548-13|United States|North Carolina|658[0n]|BOLD:AAA8249
Polygrammate hebraeicum|[9653]|LSEU338-06|United States|Georgia|658[0n]|BOLD:AAA8249
Polygrammate hebraeicum|[9654]|LGSMG962-10|United States|Tennessee|658[0n]|BOLD:AAA8249
Polygrammate hebraeicum|[9655]|LSEU337-06|United States|Georgia|658[0n]|BOLD:AAA8249
Polygrammate hebraeicum|[9656]|LOCT067-05|United States|Connecticut|658[0n]|BOLD:AAA8249
Polygrammate hebraeicum|[9657]|LOTB184-05|United States|Tennessee|658[0n]|BOLD:AAA8249
Polygrammate hebraeicum|[9658]|LOTB181-05|United States|Tennessee|658[0n]|BOLD:AAA8249
Polygrammate hebraeicum|[9659]|LOTB180-05|United States|Tennessee|658[0n]|BOLD:AAA8249
Polygrammate hebraeicum|[9660]|LOTB179-05|United States|Tennessee|658[0n]|BOLD:AAA8249
Polygrammate hebraeicum|[9661]|LOTB178-05|United States|Tennessee|658[0n]|BOLD:AAA8249
Polygrammate hebraeicum|[9662]|LOTB177-05|United States|Tennessee|658[0n]|BOLD:AAA8249
Polygrammate hebraeicum|[9663]|LOTB176-05|United States|Tennessee|658[0n]|BOLD:AAA8249
Polygrammate hebraeicum|[9664]|LGSMC351-05|United States|Tennessee|658[0n]|BOLD:AAA8249
Polygrammate hebraeicum|[9665]|LGSMC350-05|United States|Tennessee|658[0n]|BOLD:AAA8249
Polygrammate hebraeicum|[9666]|LGSMC349-05|United States|Tennessee|658[0n]|BOLD:AAA8249
Polygrammate hebraeicum|[9667]|LGSM421-04|United States|Tennessee|658[0n]|BOLD:AAA8249
Polygrammate hebraeicum|[9668]|LGSM420-04|United States|Tennessee|658[0n]|BOLD:AAA8249
Polygrammate hebraeicum|[9669]|LOTB182-05|United States|Tennessee|617[0n]|BOLD:AAA8249
Polygrammate hebraeicum|[9670]|PSAT119-10|Canada|Ontario|658[0n]|BOLD:AAA8249
Polygrammate hebraeicum|[9671]|LNCC1549-13|United States|North Carolina|658[0n]|BOLD:AAA8249
Polygrammate hebraeicum|[9672]|LNCC1595-13|United States|North Carolina|658[0n]|BOLD:AAA8249
Polygrammate hebraeicum|[9673]|LNCC1596-13|United States|North Carolina|658[0n]|BOLD:AAA8249
Polygrammate hebraeicum|[9674]|LNCC1597-13|United States|North Carolina|658[0n]|BOLD:AAA8249
Polygrammate hebraeicum|[9675]|CNCLB2742-14|United States|North Carolina|658[1n]|BOLD:ACF4101
Polygrammate hebraeicum|[9676]|LOFLA203-06|United States|Florida|658[0n]|BOLD:ACF4101
Polygrammate hebraeicum|[9677]|LSUSA161-06|United States|Kentucky|658[0n]|BOLD:ACF4101
Polygrammate hebraeicum|[9678]|MILEP370-10|United States|Alabama|658[0n]|BOLD:ACF4101
Polygrammate hebraeicum|[9679]|LNC040-05|United States|North Carolina|626[0n]|BOLD:ACF4101
Polygrammate hebraeicum|[9680]|LOFLD266-07|United States|Florida|606[1n]|BOLD:ACF4101
Polygrammate hebraeicum|[9681]|MILEP372-10|United States|Alabama|658[0n]|BOLD:ACF4101
Polygrammate hebraeicum|[9682]|LOFLA050-06|United States|Florida|658[0n]|BOLD:ACF4101
Polygrammate hebraeicum|[9683]|LOFLA049-06|United States|Florida|658[0n]|BOLD:ACF4101
Polygrammate hebraeicum|[9684]|LNC041-05|United States|North Carolina|658[0n]|BOLD:ACF4101
Polygrammate hebraeicum|[9685]|LOFLA016-06|United States|Florida|656[0n]|BOLD:ACF4101
Polygrammate hebraeicum|[9686]|LOFLA051-06|United States|Florida|656[0n]|BOLD:ACF4101
Polygrammate hebraeicum|[9687]|LOFLA052-06|United States|Florida|658[0n]|BOLD:ACF4101
Polygrammate hebraeicum|[9688]|LOFLA262-06|United States|Florida|658[0n]|BOLD:ACF4101
Polygrammate hebraeicum|[9689]|LOFLA293-06|United States|Florida|658[0n]|BOLD:ACF4101
Polygrammate hebraeicum|[9690]|LOFLA342-06|United States|Florida|658[0n]|BOLD:ACF4101
Polygrammate hebraeicum|[9691]|LOFLA583-06|United States|Florida|658[0n]|BOLD:ACF4101
Polygrammate hebraeicum|[9692]|LOFLA585-06|United States|Florida|658[0n]|BOLD:ACF4101
Polygrammate hebraeicum|[9693]|LOFLA738-06|United States|Florida|658[0n]|BOLD:ACF4101
Polygrammate hebraeicum|[9694]|LOFLA843-06|United States|Florida|658[0n]|BOLD:ACF4101
Polygrammate hebraeicum|[9695]|LOFLD372-07|United States|Florida|658[0n]|BOLD:ACF4101
Polygrammate hebraeicum|[9696]|LNCC096-10|United States|North Carolina|658[0n]|BOLD:ACF4101
Polygrammate hebraeicum|[9697]|MILEP371-10|United States|Alabama|658[0n]|BOLD:ACF4101
Polygrammate hebraeicum|[9698]|RDNMK631-11|United States|Florida|658[0n]|BOLD:ACF4101
Polygrammate hebraeicum|[9699]|BBLOB917-11|United States|Florida|658[0n]|BOLD:ACF4101
Polygrammate hebraeicum|[9700]|BBLOB1446-11|United States|Florida|658[0n]|BOLD:ACF4101
Polygrammate hebraeicum|[9701]|CNCLB2738-14|United States|North Carolina|658[0n]|BOLD:ACF4101
Polygrammate hebraeicum|[9702]|CNCLB2746-14|United States|North Carolina|658[0n]|BOLD:ACF4101
Polygrammate hebraeicum|[9703]|CNCLB2750-14|United States|North Carolina|658[0n]|BOLD:ACF4101
Polygrammate hebraeicum|[9704]|CNCLB2758-14|United States|North Carolina|658[0n]|BOLD:ACF4101
Polygrammate hebraeicum|[9705]|CNCLB2763-14|United States|North Carolina|658[1n]|BOLD:ACF4101
Cerma cerintha|[9706]|LPOKA133-08|United States|Oklahoma|658[0n]|BOLD:AAB5015
Cerma cerintha|[9707]|LPOKB722-09|United States|Oklahoma|658[0n]|BOLD:AAB5015
Cerma cerintha|[9708]|LPOKC336-09|United States|Oklahoma|658[0n]|BOLD:AAB5015
Cerma cerintha|[9709]|LPOKC740-09|United States|Oklahoma|658[0n]|BOLD:AAB5015
Cerma cerintha|[9710]|LILLA301-11|United States|Illinois|658[0n]|BOLD:AAB5015
Cerma sirius|[9711]|RDNMH036-09|United States|Texas|658[0n]|BOLD:AAB5015
Cerma cerintha|[9712]|PMG100-03|Canada|Ontario|617[0n]|BOLD:AAB5015
Cerma cerintha|[9713]|LPSOC153-08|Canada|Ontario|621[0n]|BOLD:AAB5015
Cerma cerintha|[9714]|XAK178-06|Canada|Ontario|658[0n]|BOLD:AAB5015
Cerma cerintha|[9715]|XAK063-06|Canada|Ontario|658[0n]|BOLD:AAB5015
Cerma cerintha|[9716]|XAE333-04|Canada|Ontario|658[0n]|BOLD:AAB5015
Cerma cerintha|[9717]|XAB635-04|Canada|Ontario|658[0n]|BOLD:AAB5015
Cerma cerintha|[9718]|TMG101-03|Canada|Ontario|639[0n]|BOLD:AAB5015
Cerma cerintha|[9719]|BLTIB614-08|Canada|Ontario|649[0n]|BOLD:AAB5015
Cerma cerintha|[9720]|LGSM666-04|United States|Tennessee|658[0n]|BOLD:AAB5015
Cerma cerintha|[9721]|LOT353-04|United States|Tennessee|658[0n]|BOLD:AAB5015
Cerma cerintha|[9722]|LOT354-04|United States|Tennessee|658[0n]|BOLD:AAB5015
Cerma cerintha|[9723]|LOT514-04|United States|Tennessee|658[0n]|BOLD:AAB5015
Cerma cerintha|[9724]|XAF568-05|Canada|Ontario|658[0n]|BOLD:AAB5015
Cerma cerintha|[9725]|LSEU617-06|United States|Georgia|658[0n]|BOLD:AAB5015
Cerma cerintha|[9726]|RDLQF934-06|Canada|Quebec|658[0n]|BOLD:AAB5015
Cerma cerintha|[9727]|LGSMG637-07|United States|Tennessee|658[0n]|BOLD:AAB5015
Cerma cerintha|[9728]|LPSOC152-08|Canada|Ontario|658[0n]|BOLD:AAB5015
Cerma cerintha|[9729]|BLTIB503-08|Canada|Ontario|658[0n]|BOLD:AAB5015
Cerma cerintha|[9730]|UDLEP279-09|United States|Delaware|658[0n]|BOLD:AAB5015
Cerma cerintha|[9731]|UDLEP294-09|United States|Pennsylvania|658[0n]|BOLD:AAB5015
Cerma cerintha|[9732]|LNCC1068-11|United States|North Carolina|658[0n]|BOLD:AAB5015
Cerma cora|[9733]|RDLQF843-06|Canada|Quebec|658[0n]|BOLD:AAD3483
Cerma cora|[9734]|RDLQF844-06|Canada|Quebec|658[0n]|BOLD:AAD3483
Cerma cora|[9735]|RDNMK830-12|United States|Georgia|658[0n]|BOLD:AAD3483
Cerma cora|[9736]|RDNMK831-12|United States|Georgia|658[0n]|BOLD:AAD3483
Cerma cora|[9737]|PHMNB069-03|Canada|New Brunswick|639[0n]|BOLD:AAD3483
Cerma cora|[9738]|PHMNB481-04|Canada|New Brunswick|617[0n]|BOLD:AAD3483
Cerma cora|[9739]|CNCLB2778-14|United States|North Carolina|658[0n]|BOLD:AAD3483
Cerma cora|[9740]|MNAC056-07|Canada|Ontario|658[0n]|BOLD:AAD3483
Cerma cora|[9741]|RDLQF845-06|Canada|Quebec|658[0n]|BOLD:AAD3483
Cerma cora|[9742]|RDLQF452-06|Canada|Quebec|658[0n]|BOLD:AAD3483
Cerma cora|[9743]|CNCLB2780-14|United States|North Carolina|586[0n]|BOLD:AAD3483
Catabena sagittata|[9744]|RDNMB358-05|United States|California|604[0n]|BOLD:AAE9426
Catabena sagittata|[9745]|RDNMB357-05|United States|California|529[1n]|BOLD:AAE9426
Catabena sagittata|[9746]|RDNMD448-06|United States|California|658[0n]|BOLD:AAE9426
Catabena sagittata|[9747]|RDNME635-08|United States|California|658[0n]|BOLD:AAE9426

Catabena sagittata|[9745]|RDNMB357-05|United States|California|529[1n]|BOLD:AAE9426
Catabena sagittata|[9746]|RDNMD448-06|United States|California|658[0n]|BOLD:AAE9426
Catabena sagittata|[9747]|RDNME635-08|United States|California|658[0n]|BOLD:AAE9426
Pleromelloida bonuscula|[9748]|RDNMF025-08|Canada|British Columbia|658[0n]|BOLD:AAH9194
Pleromelloida bonuscula|[9749]|RDNMG782-08|Canada|British Columbia|643[0n]|BOLD:AAH9194
Pleromelloida bonuscula|[9750]|LBCH4975-10|Canada|British Columbia|658[0n]|BOLD:AAH9194
Pleromelloida bonuscula|[9751]|LBCH4988-10|Canada|British Columbia|658[0n]|BOLD:AAH9194
Pleromelloida bonuscula|[9752]|JMMMB406-11|United States|California|658[0n]|BOLD:AAH9194
Pleromelloida conserta|[9753]|CGLCA006-10|United States|California|658[0n]|BOLD:ABX4893
Pleromelloida conserta|[9754]|CGLCA007-10|United States|California|658[0n]|BOLD:ABX4893
Pleromelloida conserta|[9755]|CGLCA015-10|United States|California|658[0n]|BOLD:ABX4893
Pleromelloida conserta|[9756]|GMLC176-11|United States|California|658[0n]|BOLD:ABX4893
Pleromelloida conserta|[9757]|GMLC1330-12|United States|California|605[0n]|BOLD:ABX4893
Pleromelloida conserta|[9758]|GMLC1354-12|United States|California|613[0n]|BOLD:ABX4893
Pleromelloida conserta|[9759]|RDNME311-07|Canada|Alberta|658[0n]|BOLD:AAE2130
Pleromelloida conserta|[9760]|RDNME310-07|Canada|Alberta|657[0n]|BOLD:AAE2130
Pleromelloida conserta|[9761]|RDNME312-07|Canada|British Columbia|656[0n]|BOLD:AAE2130
Pleromelloida conserta|[9762]|LBCG306-08|Canada|British Columbia|658[0n]|BOLD:AAE2130
Pleromelloida conserta|[9763]|LALPA025-10|Canada|British Columbia|658[0n]|BOLD:AAE2130
Pleromelloida conserta|[9764]|LALPA056-10|Canada|British Columbia|658[0n]|BOLD:AAE2130
Pleromelloida conserta|[9765]|JMMMB326-11|United States|California|658[0n]|BOLD:AAE2130
Pleromelloida conserta|[9766]|LALPA024-10|Canada|British Columbia|658[0n]|BOLD:AAE2130
Pleromelloida conserta|[9767]|LALPA046-10|Canada|British Columbia|658[0n]|BOLD:AAE2130
Pleromelloida conserta|[9768]|LALPA1357-12|Canada|British Columbia|658[0n]|BOLD:AAE2130
Pleromelloida conserta|[9769]|RDNMG144-08|United States|Colorado|658[0n]|BOLD:ABX4893
Pleromelloida sp.|[9770]|LBCH7083-10|Canada|British Columbia|658[0n]|BOLD:ACE3622
Pleromelloida sp.|[9771]|LBCH7121-10|Canada|British Columbia|658[0n]|BOLD:ACE3622
Pleromelloida sp.|[9772]|LBCH7182-10|Canada|British Columbia|658[0n]|BOLD:ACE3622
Pleromelloida sp.|[9773]|JMMMB331-11|United States|California|658[0n]|BOLD:ACE3622
Pleromelloida sp.|[9774]|GMLC1269-12|United States|California|658[0n]|BOLD:ACE3622
Pleromelloida cinerea|[9775]|LALPA743-10|Canada|British Columbia|658[0n]|BOLD:AAD4314
Pleromelloida cinerea|[9776]|LALPA769-10|Canada|British Columbia|658[0n]|BOLD:AAD4314
Pleromelloida cinerea|[9777]|LOWCB491-05|Canada|British Columbia|621[2n]|BOLD:AAD4314
Pleromelloida cinerea|[9778]|LOWCB492-05|Canada|British Columbia|611[1n]|BOLD:AAD4314
Pleromelloida cinerea|[9779]|LOWCB493-05|Canada|British Columbia|607[6n]|
Pleromelloida cinerea|[9780]|LOWCB490-05|Canada|British Columbia|616[2n]|BOLD:AAD4314
Pleromelloida cinerea|[9781]|LOWCB487-05|Canada|British Columbia|658[0n]|BOLD:AAD4314
Pleromelloida cinerea|[9782]|LOWCB489-05|Canada|British Columbia|658[0n]|BOLD:AAD4314
Pleromelloida cinerea|[9783]|LOWCC193-05|Canada|British Columbia|658[0n]|BOLD:AAD4314
Pleromelloida cinerea|[9784]|JMMMB344-11|United States|California|645[0n]|BOLD:AAD4314
Pleromelloida cinerea|[9785]|LALPA1337-12|Canada|British Columbia|621[0n]|BOLD:AAD4314
Pleromelloida sp.|[9786]|RDNMK770-12|United States|Colorado|658[0n]|BOLD:ABW0041
Pleromelloida arizonata|[9787]|LNAUS5018-13|United States|California|658[0n]|BOLD:ACK3481
Pleromelloida arizonata|[9788]|LNAUS5019-13|United States|California|658[0n]|BOLD:ACK3481
Pleromelloida arizonata|[9789]|LNAUS5020-13|United States|California|658[0n]|BOLD:ACK3481
Pleromelloida arizonata|[9790]|LNAUS5021-13|United States|California|658[0n]|BOLD:ACK3481
Behrensia conchiformis|[9791]|RWWC182-11|United States|Washington|633[0n]|BOLD:AAC9584
Behrensia conchiformis|[9792]|RWWC196-11|United States|Washington|658[0n]|BOLD:AAC9584
Behrensia conchiformis|[9793]|RWWC184-11|United States|Washington|658[0n]|BOLD:AAC9584
Behrensia conchiformis|[9794]|LALPA1074-11|Canada|British Columbia|658[0n]|BOLD:AAC9584
Behrensia conchiformis|[9795]|LALPA1073-11|Canada|British Columbia|658[0n]|BOLD:AAC9584
Behrensia conchiformis|[9796]|LALPA1076-11|Canada|British Columbia|627[0n]|BOLD:AAC9584
Behrensia conchiformis|[9797]|GMLC1316-12|United States|California|618[0n]|BOLD:AAC9584
Behrensia conchiformis|[9798]|GMLC1371-12|United States|California|629[0n]|BOLD:AAC9584
Behrensia conchiformis|[9799]|NAMUM244-08|United States|California|656[0n]|BOLD:AAC9584
Behrensia conchiformis|[9800]|GMLC1374-12|United States|California|638[0n]|BOLD:AAC9584
Behrensia conchiformis|[9801]|GMLC1393-12|United States|California|601[0n]|BOLD:AAC9584
Behrensia conchiformis|[9802]|LOCB709-06|United States|California|658[0n]|BOLD:AAC9584
Behrensia conchiformis|[9803]|LOCBB200-06|United States|California|622[0n]|BOLD:AAC9584
Behrensia conchiformis|[9804]|LOCBB201-06|United States|California|626[0n]|BOLD:AAC9584
Behrensia conchiformis|[9805]|LOCB922-06|United States|California|605[0n]|BOLD:AAC9584
Behrensia conchiformis|[9806]|LOCB923-06|United States|California|605[0n]|BOLD:AAC9584
Behrensia conchiformis|[9807]|LOCBB202-06|United States|California|626[0n]|BOLD:AAC9584
Behrensia conchiformis|[9808]|LOCBB203-06|United States|California|649[0n]|BOLD:AAC9584
Behrensia conchiformis|[9809]|LOCBB204-06|United States|California|657[0n]|BOLD:AAC9584
Behrensia conchiformis|[9810]|CGLCA017-10|United States|California|658[0n]|BOLD:AAC9584
Behrensia conchiformis|[9811]|RDNMD451-06|United States|California|658[0n]|BOLD:AAC9584
Behrensia conchiformis|[9812]|LOCB708-06|United States|California|658[0n]|BOLD:AAC9584
Behrensia conchiformis|[9813]|RDNMD452-06|United States|California|658[0n]|BOLD:AAC9584
Behrensia conchiformis suffusa|[9814]|NAMUM204-08|United States|California|651[0n]|BOLD:AAC9584
Behrensia conchiformis suffusa|[9815]|NAMUM205-08|United States|California|644[0n]|BOLD:AAC9584
Behrensia conchiformis|[9816]|LOCBF3263-14|United States|California|561[0n]|BOLD:AAC9584
Behrensia bicolor|[9817]|LNAUS4394-13|United States|California|460[0n]|BOLD:ACM4259
Behrensia bicolor|[9818]|CNCLB189-14|United States|California|658[0n]|BOLD:ACM4259
Behrensia bicolor|[9819]|CNCLB551-14|United States|California|657[0n]|BOLD:ACM4259
Behrensia bicolor|[9820]|CNCLB190-14|United States|California|658[0n]|BOLD:ACM4259
Behrensia bicolor|[9821]|CNCLB552-14|United States|California|658[0n]|BOLD:ACM4259
Behrensia bicolor|[9822]|CNCLB553-14|United States|California|658[0n]|BOLD:ACM4259
Behrensia bicolor|[9823]|CNCLB554-14|United States|California|658[0n]|BOLD:ACM4259
Behrensia bicolor|[9824]|CNCLB555-14|United States|California|658[0n]|BOLD:ACM4259
Neogalea|[9825]|CNCLA5286-13|United States|Florida|658[0n]|BOLD:AAC5921
Neogalea sunia|[9826]|MHAUB213-05|Costa Rica|Guanacaste|526[0n]|BOLD:AAC5921
Neogalea sunia|[9827]|MHAUB215-05|Costa Rica|Guanacaste|527[0n]|BOLD:AAC5921
Neogalea sunia|[9828]|BLPCA461-08|Costa Rica|Guanacaste|658[0n]|BOLD:AAC5921
Neogalea sunia|[9829]|MHAUB212-05|Costa Rica|Guanacaste|544[0n]|BOLD:AAC5921
Neogalea sunia|[9830]|HKONB223-09|United States|Texas|658[0n]|BOLD:AAC5921
Neogalea sunia|[9831]|BLPEE719-12|Costa Rica|Guanacaste|658[0n]|BOLD:AAC5921
Neogalea sunia|[9832]|BLPBG821-07|Costa Rica|Guanacaste|655[0n]|BOLD:AAC5921
Neogalea sunia|[9833]|BLPCA458-08|Costa Rica|Guanacaste|658[0n]|BOLD:AAC5921
Neogalea sunia|[9834]|BLPCA844-08|Costa Rica|Guanacaste|658[0n]|BOLD:AAC5921
Neogalea sunia|[9835]|BLPEE562-12|Costa Rica|Guanacaste|658[0n]|BOLD:AAC5921
Neogalea sunia|[9836]|BLPEE564-12|Costa Rica|Guanacaste|658[0n]|BOLD:AAC5921
Neogalea sunia|[9837]|BLPEE561-12|Costa Rica|Guanacaste|658[0n]|BOLD:AAC5921
Neogalea sunia|[9838]|BLPED025-11|Costa Rica|Guanacaste|658[0n]|BOLD:AAC5921
Neogalea sunia|[9839]|MHMXZ915-09|Costa Rica||658[0n]|BOLD:AAC5921
Neogalea sunia|[9840]|HKONB222-09|United States|Texas|658[0n]|BOLD:AAC5921
Neogalea sunia|[9841]|BLPCA460-08|Costa Rica|Guanacaste|658[0n]|BOLD:AAC5921
Neogalea sunia|[9842]|BLPCA141-08|Costa Rica|Guanacaste|658[0n]|BOLD:AAC5921
Neogalea sunia|[9843]|BLPAE772-06|Costa Rica|Guanacaste|658[0n]|BOLD:AAC5921
Neogalea sunia|[9844]|BLPEE720-12|Costa Rica|Guanacaste|658[0n]|BOLD:AAC5921
Neogalea sunia|[9845]|BLPEE1064-12|Costa Rica|Guanacaste|658[0n]|BOLD:AAC5921
Neogalea sunia|[9846]|BLPEF4965-13|Costa Rica|Guanacaste|658[0n]|BOLD:AAC5921

Neogalea sunia|[9844]|BLPEE720-12|Costa Rica|Guanacaste|658[0n]|BOLD:AAC5921
Neogalea sunia|[9845]|BLPEE1064-12|Costa Rica|Guanacaste|658[0n]|BOLD:AAC5921
Neogalea sunia|[9846]|BLPEF4965-13|Costa Rica|Guanacaste|658[0n]|BOLD:AAC5921
Neogalea sunia|[9847]|BLPEE563-12|Costa Rica|Guanacaste|658[0n]|BOLD:AAC5921
Neogalea sunia|[9848]|BLPEF5676-13|Costa Rica||658[0n]|BOLD:AAC5921
Leucocnemis nivalis|[9849]|LSEU064-06|United States|Texas|421[0n]|
Leucocnemis nivalis|[9850]|CNCLB222-14|United States|Texas|307[0n]|
Leucocnemis nivalis|[9851]|CNCLB1693-14|United States|Texas|658[0n]|BOLD:ACP3572
Leucocnemis nivalis|[9852]|CNCLB1743-14|United States|Texas|658[0n]|BOLD:ACP3572
Leucocnemis perfundis|[9853]|CNCLB1691-14|United States|Texas|658[0n]|BOLD:ABW8311
Leucocnemis perfundis|[9854]|CNCLB1692-14|United States|Texas|658[0n]|BOLD:ABW8311
Leucocnemis perfundis|[9855]|CNCLB1813-14|United States|New Mexico|658[0n]|BOLD:ACP7061
Leucocnemis perfundis|[9856]|CNCLB1814-14|United States|New Mexico|658[0n]|BOLD:ACP7061
Supralathosea pronuba|[9857]|CNCLB358-14|United States|Arizona|658[0n]|BOLD:ACM7023
Supralathosea pronuba|[9858]|CNCLB359-14|United States|Arizona|658[0n]|BOLD:ACM7023
Supralathosea pronuba|[9859]|CNCLB360-14|United States|Arizona|658[0n]|BOLD:ACM7023
Supralathosea pronuba|[9860]|CNCLB361-14|United States|Arizona|658[0n]|BOLD:ACM7023
Supralathosea pronuba|[9861]|CNCLB362-14|United States|Arizona|658[0n]|BOLD:ACM7023
Supralathosea baboquivariensis|[9862]|LNAUS5028-13|United States|Texas|657[0n]|BOLD:AAH5315
Supralathosea baboquivariensis|[9863]|CNCLB1699-14|United States|Texas|658[0n]|BOLD:AAH5315
Supralathosea baboquivariensis|[9864]|BBLSW756-09|United States|Texas|658[0n]|BOLD:AAH5315
Supralathosea baboquivariensis|[9865]|BBLSW730-09|United States|Texas|658[0n]|BOLD:AAH5315
Supralathosea baboquivariensis|[9866]|LNAUS5030-13|United States|Texas|617[0n]|BOLD:AAH5315
Supralathosea baboquivariensis|[9867]|CNCLB906-14|United States|Texas|658[0n]|BOLD:AAH5315
Supralathosea baboquivariensis|[9868]|CNCLB1783-14|United States|Texas|657[0n]|BOLD:AAH5315
Supralathosea baboquivariensis|[9869]|CNCLB1698-14|United States|Texas|658[0n]|BOLD:AAH5315
Supralathosea baboquivariensis|[9870]|CNCLB2370-14|United States|Arizona|658[0n]|BOLD:AAH5315
Supralathosea baboquivariensis|[9871]|CNCLB2371-14|United States|Arizona|658[0n]|BOLD:AAH5315
Supralathosea baboquivariensis|[9872]|CNCLB2372-14|United States|Arizona|658[0n]|BOLD:AAH5315
Sympistis franclemonti|[9873]|LNAUS3220-13|United States|Texas|529[0n]|BOLD:ACH5478
Sympistis franclemonti|[9874]|LNAUS3221-13|United States|Texas|566[0n]|BOLD:ACH5477
Pseudacontia crustaria|[9875]|RDNMF785-08|Canada|Alberta|658[0n]|BOLD:AAX8202
Pseudacontia crustaria|[9876]|CNCLB1690-14|United States|Texas|658[0n]|BOLD:AAX8202
Sympistis corusca|[9877]|RDNMB122-05|United States|California|548[0n]|BOLD:AAX0343
Sympistis terminalis|[9878]|RDNMC184-05|United States|New Mexico|658[0n]|BOLD:AAI1720
Sympistis terminalis|[9879]|RDNMC185-05|United States|New Mexico|658[0n]|BOLD:AAI1720
Sympistis sorapis|[9880]|CMAZA998-12|United States|Arizona|658[0n]|BOLD:ABW3026
Sympistis sorapis|[9881]|CNCLB1673-14|United States|New Mexico|658[0n]|BOLD:ABW3026
Sympistis behrensi|[9882]|CNCLB144-14|United States|California|658[0n]|BOLD:ACM4246
Sympistis behrensi|[9883]|CNCLB145-14|United States|California|658[0n]|BOLD:ACM4246
Sympistis behrensi|[9884]|TML213-14|United States||658[0n]|BOLD:ACM4246
Sympistis melalutea|[9885]|NAMUM103-08|United States|Texas|655[0n]|BOLD:ABY9814
Sympistis laticosta|[9886]|RDNME983-08|United States|New Mexico|658[0n]|BOLD:ABY9813
Sympistis coprocolor|[9887]|RDNM080-05|Canada|British Columbia|622[0n]|BOLD:ACE9017
Sympistis coprocolor|[9888]|RDNM081-05|United States|Washington|615[0n]|BOLD:ACE9017
Sympistis coprocolor|[9889]|RDNM082-05|United States|Oregon|658[0n]|BOLD:ACE9017
Sympistis coprocolor|[9890]|RDNM083-05|United States|Oregon|658[0n]|BOLD:ACE9017
Sympistis coprocolor|[9891]|RDNMG813-08|Canada|British Columbia|658[0n]|BOLD:ACE9017
Sympistis coprocolor|[9892]|RDNMG814-08|United States|Oregon|658[0n]|BOLD:ACE9017
Sympistis perscripta|[9893]|CNCLB242-14|United States|Connecticut|658[0n]|BOLD:ACM4382
Sympistis perscripta|[9894]|CNCLB243-14|United States|Connecticut|658[0n]|BOLD:ACM4382
Sympistis perscripta|[9895]|LOCB669-06|United States|California|658[0n]|BOLD:AAB2958
Sympistis perscripta|[9896]|LOCBB171-06|United States|California|658[0n]|BOLD:AAB2958
Sympistis perscripta|[9897]|RDNMD449-06|United States|California|658[0n]|BOLD:AAB2958
Sympistis perscripta|[9898]|LOCBF283-13|United States|California|611[0n]|BOLD:AAB2958
Sympistis perscripta|[9899]|LOCB739-06|United States|California|658[0n]|BOLD:AAB2958
Sympistis perscripta|[9900]|LOCBB368-06|United States|California|658[0n]|BOLD:AAB2958
Sympistis perscripta|[9901]|LOCB624-06|United States|California|658[0n]|BOLD:AAB2958
Sympistis perscripta|[9902]|LOCB921-06|United States|California|608[0n]|BOLD:AAB2958
Sympistis perscripta|[9903]|LOCB674-06|United States|California|658[0n]|BOLD:AAB2958
Sympistis perscripta|[9904]|IAWLB651-11|United States|Arizona|622[0n]|BOLD:AAB2958
Sympistis perscripta|[9905]|LOCBB168-06|United States|California|658[0n]|BOLD:AAB2958
Sympistis perscripta|[9906]|LOCBB367-06|United States|California|658[0n]|BOLD:AAB2958
Sympistis perscripta|[9907]|LOCBB173-06|United States|California|658[0n]|BOLD:AAB2958
Sympistis perscripta|[9908]|LOCBB170-06|United States|California|658[0n]|BOLD:AAB2958
Sympistis perscripta|[9909]|LOCB740-06|United States|California|658[0n]|BOLD:AAB2958
Sympistis perscripta|[9910]|LOCB738-06|United States|California|658[0n]|BOLD:AAB2958
Sympistis perscripta|[9911]|LOCB676-06|United States|California|658[0n]|BOLD:AAB2958
Sympistis perscripta|[9912]|LOCB675-06|United States|California|658[0n]|BOLD:AAB2958
Sympistis perscripta|[9913]|LOCB667-06|United States|California|658[0n]|BOLD:AAB2958
Sympistis perscripta|[9914]|LOCB920-06|United States|California|617[0n]|BOLD:AAB2958
Sympistis perscripta|[9915]|LOCB919-06|United States|California|609[0n]|BOLD:AAB2958
Sympistis perscripta|[9916]|CNCLB241-14|United States|Arizona|658[0n]|BOLD:AAB2958
Sympistis perscripta|[9917]|LOCBB172-06|United States|California|658[0n]|BOLD:AAB2958
Sympistis perscripta|[9918]|LOCB777-06|United States|California|658[0n]|BOLD:AAB2958
Sympistis perscripta|[9919]|LOCB668-06|United States|California|658[0n]|BOLD:AAB2958
Sympistis perscripta|[9920]|LOCBB612-06|United States|California|658[0n]|BOLD:AAB2958
Sympistis perscripta|[9921]|LOCB741-06|United States|California|658[0n]|BOLD:AAB2958
Sympistis perscripta|[9922]|LOCBB169-06|United States|California|658[0n]|BOLD:AAB2958
Sympistis perscripta|[9923]|RDNMD450-06|United States|California|658[0n]|BOLD:AAB2958
Sympistis perscripta|[9924]|TML117-14|United States||658[0n]|BOLD:AAB2958
Sympistis shait|[9925]|CNCLB2071-14|United States|Arizona|307[0n]|
Sympistis shait|[9926]|CNCLB3177-15|United States|Arizona|658[0n]|BOLD:AAB2958
Sympistis kelsoensis|[9927]|CNCLB168-14|United States|California|658[0n]|BOLD:ACM4003
Sympistis intruda|[9928]|LNAUT2062-14|United States|New Mexico|658[0n]|BOLD:ACR1128
Sympistis intruda|[9929]|CNCLB1687-14|United States|Arizona|658[0n]|BOLD:ACP4144
Sympistis wilsonensis|[9930]|RDNM593-05|United States|California|589[0n]|BOLD:AAF4190
Sympistis wilsonensis|[9931]|RDNM594-05|United States|California|658[0n]|BOLD:AAF4190
Sympistis wilsonensis|[9932]|NAMUM168-08|United States|California|621[0n]|BOLD:AAF4191
Sympistis poliafascies|[9933]|RDNM592-05|United States|Texas|485[0n]|
Sympistis poliafascies|[9934]|RDNM591-05|Mexico|Chihuahua|658[0n]|BOLD:AAX0327
Sympistis poliafascies|[9935]|CNCLB1285-14|United States|Arizona|658[0n]|BOLD:AAX0327
Sympistis poliafascies|[9936]|CNCLB1286-14|United States|Arizona|658[0n]|BOLD:AAX0327
Sympistis toddi|[9937]|CNCLB1686-14|United States|Texas|658[0n]|BOLD:ACP4202
Oxycnemis acuna|[9938]|RDNMK181-11|United States|Arizona|658[0n]|BOLD:AAY9494
Leucocnemis variabilis|[9939]|NAMUM135-08|United States|California|658[0n]|BOLD:AAX4969
Oxycnemis gracillinea|[9940]|BBLOB132-11|United States|Arizona|658[0n]|BOLD:AAH5280
Oxycnemis gracillinea|[9941]|RDNMJ692-11|United States|Arizona|658[0n]|BOLD:AAH5280
Oxycnemis gracillinea|[9942]|USLEP1020-10|United States|New Mexico|658[0n]|BOLD:AAH5280
Oxycnemis gracillinea|[9943]|USLEP1019-10|United States|New Mexico|658[0n]|BOLD:AAH5280
Oxycnemis gracillinea|[9944]|BBLSY746-09|United States|Arizona|658[0n]|BOLD:AAH5280
Oxycnemis gracillinea|[9945]|BBLSY080-09|United States|Arizona|658[0n]|BOLD:AAH5280
Oxycnemis gracillinea|[9946]|BBLSY986-09|United States|Arizona|658[0n]|BOLD:AAH5280

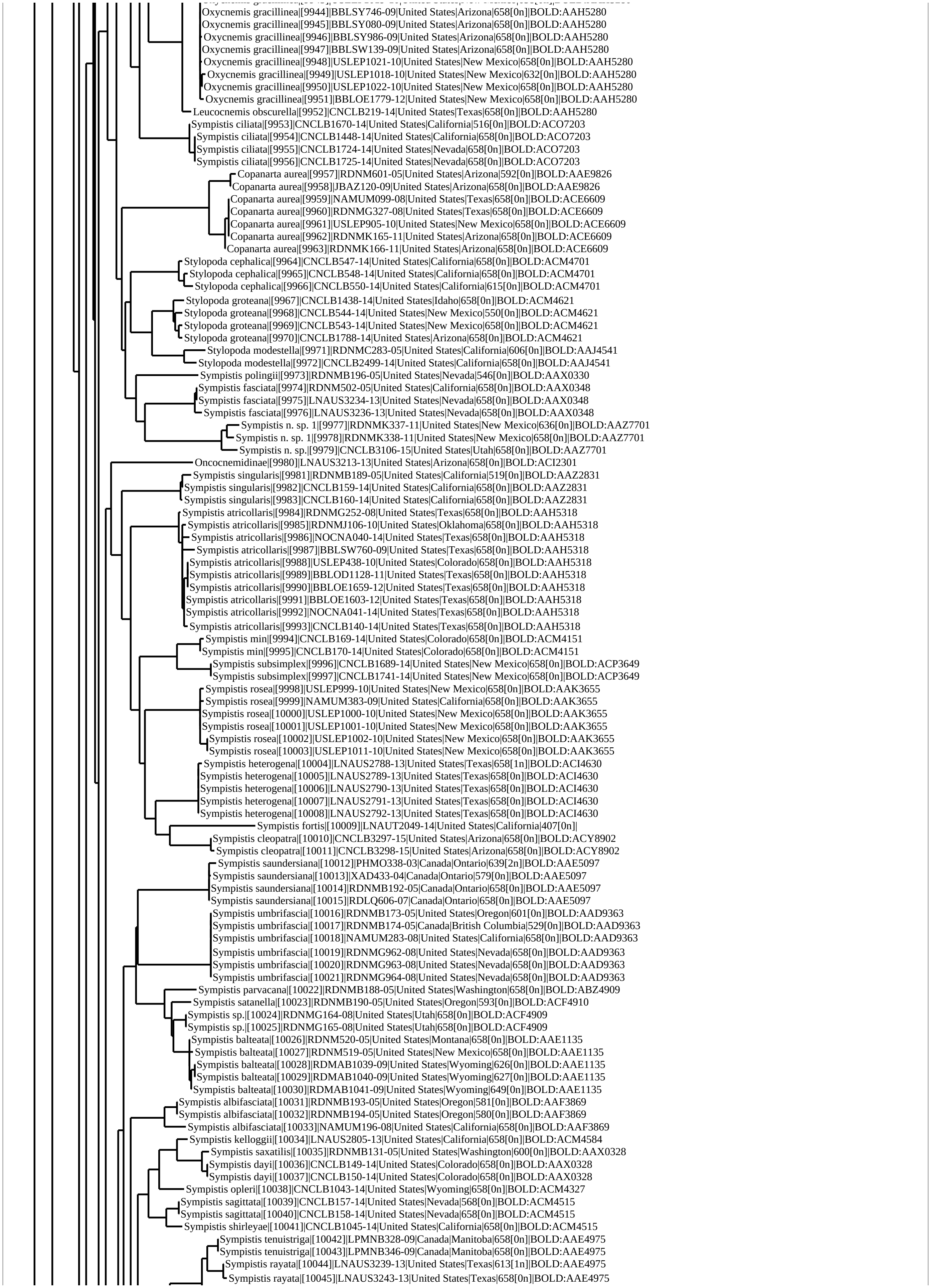
Oxycnemis gracillinea|[9944]|BBLSY746-09|United States|Arizona|658[0n]|BOLD:AAH5280
Oxycnemis gracillinea|[9945]|BBLSY080-09|United States|Arizona|658[0n]|BOLD:AAH5280
Oxycnemis gracillinea|[9946]|BBLSY986-09|United States|Arizona|658[0n]|BOLD:AAH5280
Oxycnemis gracillinea|[9947]|BBLSW139-09|United States|Arizona|658[0n]|BOLD:AAH5280
Oxycnemis gracillinea|[9948]|USLEP1021-10|United States|New Mexico|658[0n]|BOLD:AAH5280
Oxycnemis gracillinea|[9949]|USLEP1018-10|United States|New Mexico|632[0n]|BOLD:AAH5280
Oxycnemis gracillinea|[9950]|USLEP1022-10|United States|New Mexico|658[0n]|BOLD:AAH5280
Oxycnemis gracillinea|[9951]|BBLOE1779-12|United States|New Mexico|658[0n]|BOLD:AAH5280
Leucocnemis obscurella|[9952]|CNCLB219-14|United States|Texas|658[0n]|BOLD:AAH5280
Sympistis ciliata|[9953]|CNCLB1670-14|United States|California|516[0n]|BOLD:ACO7203
Sympistis ciliata|[9954]|CNCLB1448-14|United States|California|658[0n]|BOLD:ACO7203
Sympistis ciliata|[9955]|CNCLB1724-14|United States|Nevada|658[0n]|BOLD:ACO7203
Sympistis ciliata|[9956]|CNCLB1725-14|United States|Nevada|658[0n]|BOLD:ACO7203
Copanarta aurea|[9957]|RDNM601-05|United States|Arizona|592[0n]|BOLD:AAE9826
Copanarta aurea|[9958]|JBAZ120-09|United States|Arizona|658[0n]|BOLD:AAE9826
Copanarta aurea|[9959]|NAMUM099-08|United States|Texas|658[0n]|BOLD:ACE6609
Copanarta aurea|[9960]|RDNMG327-08|United States|Texas|658[0n]|BOLD:ACE6609
Copanarta aurea|[9961]|USLEP905-10|United States|New Mexico|658[0n]|BOLD:ACE6609
Copanarta aurea|[9962]|RDNMK165-11|United States|Arizona|658[0n]|BOLD:ACE6609
Copanarta aurea|[9963]|RDNMK166-11|United States|Arizona|658[0n]|BOLD:ACE6609
Stylopoda cephalica|[9964]|CNCLB547-14|United States|California|658[0n]|BOLD:ACM4701
Stylopoda cephalica|[9965]|CNCLB548-14|United States|California|658[0n]|BOLD:ACM4701
Stylopoda cephalica|[9966]|CNCLB550-14|United States|California|615[0n]|BOLD:ACM4701
Stylopoda groteana|[9967]|CNCLB1438-14|United States|Idaho|658[0n]|BOLD:ACM4621
Stylopoda groteana|[9968]|CNCLB544-14|United States|New Mexico|550[0n]|BOLD:ACM4621
Stylopoda groteana|[9969]|CNCLB543-14|United States|New Mexico|658[0n]|BOLD:ACM4621
Stylopoda groteana|[9970]|CNCLB1788-14|United States|Arizona|658[0n]|BOLD:ACM4621
Stylopoda modestella|[9971]|RDNMC283-05|United States|California|606[0n]|BOLD:AAJ4541
Stylopoda modestella|[9972]|CNCLB2499-14|United States|California|658[0n]|BOLD:AAJ4541
Sympistis polingii|[9973]|RDNMB196-05|United States|Nevada|546[0n]|BOLD:AAX0330
Sympistis fasciata|[9974]|RDNM502-05|United States|California|658[0n]|BOLD:AAX0348
Sympistis fasciata|[9975]|LNAUS3234-13|United States|Nevada|658[0n]|BOLD:AAX0348
Sympistis fasciata|[9976]|LNAUS3236-13|United States|Nevada|658[0n]|BOLD:AAX0348
Sympistis n. sp. 1|[9977]|RDNMK337-11|United States|New Mexico|636[0n]|BOLD:AAZ7701
Sympistis n. sp. 1|[9978]|RDNMK338-11|United States|New Mexico|658[0n]|BOLD:AAZ7701
Sympistis n. sp.|[9979]|CNCLB3106-15|United States|Utah|658[0n]|BOLD:AAZ7701
Oncocnemidinae|[9980]|LNAUS3213-13|United States|Arizona|658[0n]|BOLD:ACI2301
Sympistis singularis|[9981]|RDNMB189-05|United States|California|519[0n]|BOLD:AAZ2831
Sympistis singularis|[9982]|CNCLB159-14|United States|California|658[0n]|BOLD:AAZ2831
Sympistis singularis|[9983]|CNCLB160-14|United States|California|658[0n]|BOLD:AAZ2831
Sympistis atricollaris|[9984]|RDNMG252-08|United States|Texas|658[0n]|BOLD:AAH5318
Sympistis atricollaris|[9985]|RDNMJ106-10|United States|Oklahoma|658[0n]|BOLD:AAH5318
Sympistis atricollaris|[9986]|NOCNA040-14|United States|Texas|658[0n]|BOLD:AAH5318
Sympistis atricollaris|[9987]|BBLSW760-09|United States|Texas|658[0n]|BOLD:AAH5318
Sympistis atricollaris|[9988]|USLEP438-10|United States|Colorado|658[0n]|BOLD:AAH5318
Sympistis atricollaris|[9989]|BBLOD1128-11|United States|Texas|658[0n]|BOLD:AAH5318
Sympistis atricollaris|[9990]|BBLOE1659-12|United States|Texas|658[0n]|BOLD:AAH5318
Sympistis atricollaris|[9991]|BBLOE1603-12|United States|Texas|658[0n]|BOLD:AAH5318
Sympistis atricollaris|[9992]|NOCNA041-14|United States|Texas|658[0n]|BOLD:AAH5318
Sympistis atricollaris|[9993]|CNCLB140-14|United States|Texas|658[0n]|BOLD:AAH5318
Sympistis min|[9994]|CNCLB169-14|United States|Colorado|658[0n]|BOLD:ACM4151
Sympistis min|[9995]|CNCLB170-14|United States|Colorado|658[0n]|BOLD:ACM4151
Sympistis subsimplex|[9996]|CNCLB1689-14|United States|New Mexico|658[0n]|BOLD:ACP3649
Sympistis subsimplex|[9997]|CNCLB1741-14|United States|New Mexico|658[0n]|BOLD:ACP3649
Sympistis rosea|[9998]|USLEP999-10|United States|New Mexico|658[0n]|BOLD:AAK3655
Sympistis rosea|[9999]|NAMUM383-09|United States|California|658[0n]|BOLD:AAK3655
Sympistis rosea|[10000]|USLEP1000-10|United States|New Mexico|658[0n]|BOLD:AAK3655
Sympistis rosea|[10001]|USLEP1001-10|United States|New Mexico|658[0n]|BOLD:AAK3655
Sympistis rosea|[10002]|USLEP1002-10|United States|New Mexico|658[0n]|BOLD:AAK3655
Sympistis rosea|[10003]|USLEP1011-10|United States|New Mexico|658[0n]|BOLD:AAK3655
Sympistis heterogena|[10004]|LNAUS2788-13|United States|Texas|658[1n]|BOLD:ACI4630
Sympistis heterogena|[10005]|LNAUS2789-13|United States|Texas|658[0n]|BOLD:ACI4630
Sympistis heterogena|[10006]|LNAUS2790-13|United States|Texas|658[0n]|BOLD:ACI4630
Sympistis heterogena|[10007]|LNAUS2791-13|United States|Texas|658[0n]|BOLD:ACI4630
Sympistis heterogena|[10008]|LNAUS2792-13|United States|Texas|658[0n]|BOLD:ACI4630
Sympistis fortis|[10009]|LNAUT2049-14|United States|California|407[0n]|
Sympistis cleopatra|[10010]|CNCLB3297-15|United States|Arizona|658[0n]|BOLD:ACY8902
Sympistis cleopatra|[10011]|CNCLB3298-15|United States|Arizona|658[0n]|BOLD:ACY8902
Sympistis saundersiana|[10012]|PHMO338-03|Canada|Ontario|639[2n]|BOLD:AAE5097
Sympistis saundersiana|[10013]|XAD433-04|Canada|Ontario|579[0n]|BOLD:AAE5097
Sympistis saundersiana|[10014]|RDNMB192-05|Canada|Ontario|658[0n]|BOLD:AAE5097
Sympistis saundersiana|[10015]|RDLQ606-07|Canada|Ontario|658[0n]|BOLD:AAE5097
Sympistis umbrifascia|[10016]|RDNMB173-05|United States|Oregon|601[0n]|BOLD:AAD9363
Sympistis umbrifascia|[10017]|RDNMB174-05|Canada|British Columbia|529[0n]|BOLD:AAD9363
Sympistis umbrifascia|[10018]|NAMUM283-08|United States|California|658[0n]|BOLD:AAD9363
Sympistis umbrifascia|[10019]|RDNMG962-08|United States|Nevada|658[0n]|BOLD:AAD9363
Sympistis umbrifascia|[10020]|RDNMG963-08|United States|Nevada|658[0n]|BOLD:AAD9363
Sympistis umbrifascia|[10021]|RDNMG964-08|United States|Nevada|658[0n]|BOLD:AAD9363
Sympistis parvacana|[10022]|RDNMB188-05|United States|Washington|658[0n]|BOLD:ABZ4909
Sympistis satanella|[10023]|RDNMB190-05|United States|Oregon|593[0n]|BOLD:ACF4910
Sympistis sp.|[10024]|RDNMG164-08|United States|Utah|658[0n]|BOLD:ACF4909
Sympistis sp.|[10025]|RDNMG165-08|United States|Utah|658[0n]|BOLD:ACF4909
Sympistis balteata|[10026]|RDNM520-05|United States|Montana|658[0n]|BOLD:AAE1135
Sympistis balteata|[10027]|RDNM519-05|United States|New Mexico|658[0n]|BOLD:AAE1135
Sympistis balteata|[10028]|RDMAB1039-09|United States|Wyoming|626[0n]|BOLD:AAE1135
Sympistis balteata|[10029]|RDMAB1040-09|United States|Wyoming|627[0n]|BOLD:AAE1135
Sympistis balteata|[10030]|RDMAB1041-09|United States|Wyoming|649[0n]|BOLD:AAE1135
Sympistis albifasciata|[10031]|RDNMB193-05|United States|Oregon|581[0n]|BOLD:AAF3869
Sympistis albifasciata|[10032]|RDNMB194-05|United States|Oregon|580[0n]|BOLD:AAF3869
Sympistis albifasciata|[10033]|NAMUM196-08|United States|California|658[0n]|BOLD:AAF3869
Sympistis kelloggii|[10034]|LNAUS2805-13|United States|California|658[0n]|BOLD:ACM4584
Sympistis saxatilis|[10035]|RDNMB131-05|United States|Washington|600[0n]|BOLD:AAX0328
Sympistis dayi|[10036]|CNCLB149-14|United States|Colorado|658[0n]|BOLD:AAX0328
Sympistis dayi|[10037]|CNCLB150-14|United States|Colorado|658[0n]|BOLD:AAX0328
Sympistis opleri|[10038]|CNCLB1043-14|United States|Wyoming|658[0n]|BOLD:ACM4327
Sympistis sagittata|[10039]|CNCLB157-14|United States|Nevada|568[0n]|BOLD:ACM4515
Sympistis sagittata|[10040]|CNCLB158-14|United States|Nevada|658[0n]|BOLD:ACM4515
Sympistis shirleyae|[10041]|CNCLB1045-14|United States|California|658[0n]|BOLD:ACM4515
Sympistis tenuistriga|[10042]|LPMNB328-09|Canada|Manitoba|658[0n]|BOLD:AAE4975
Sympistis tenuistriga|[10043]|LPMNB346-09|Canada|Manitoba|658[0n]|BOLD:AAE4975
Sympistis rayata|[10044]|LNAUS3239-13|United States|Texas|613[1n]|BOLD:AAE4975
Sympistis rayata|[10045]|LNAUS3243-13|United States|Texas|658[0n]|BOLD:AAE4975

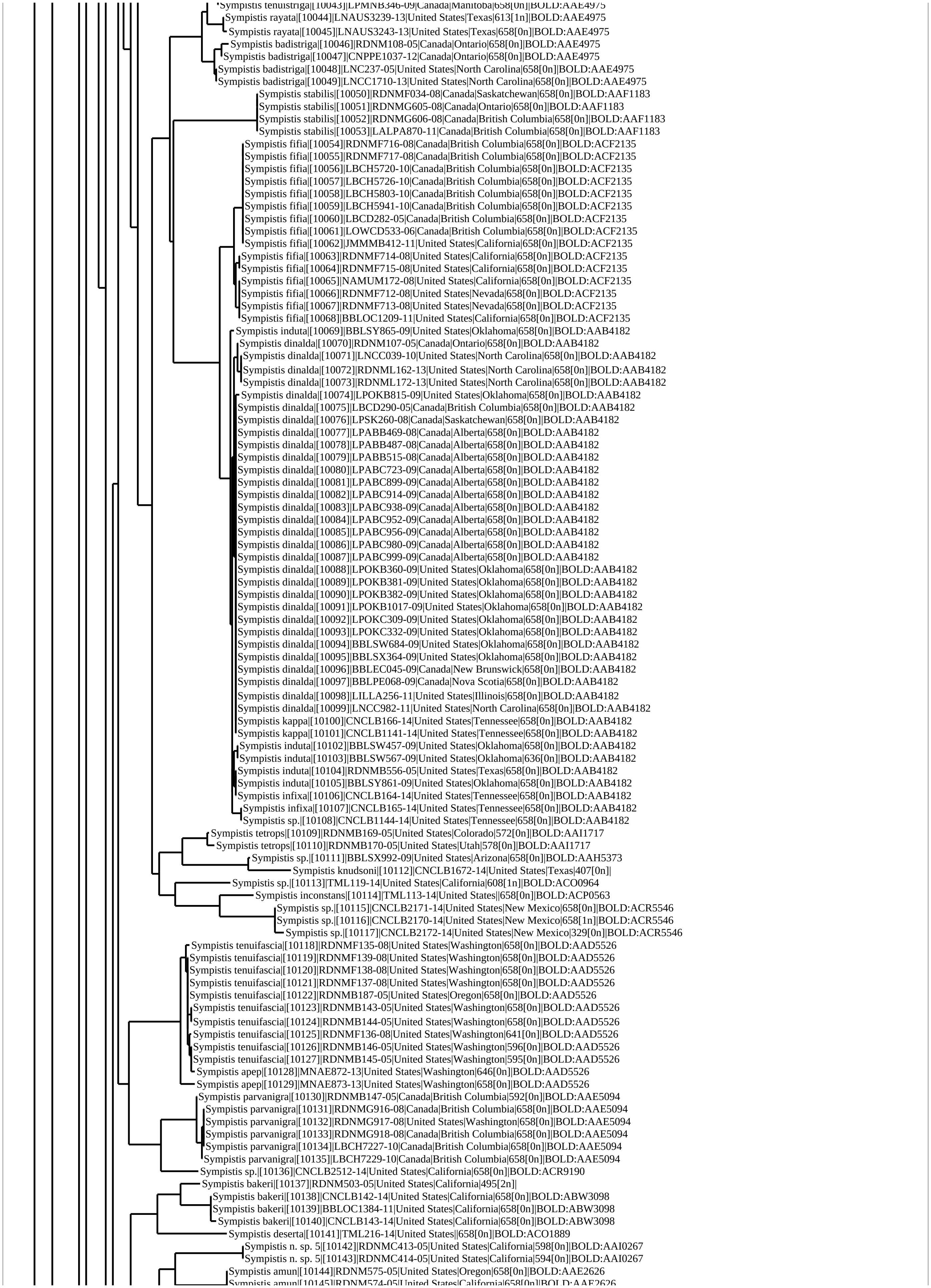
Sympistis tenuistriga|[10043]|LPMNB346-09|Canada|Manitoba|658[0n]|BOLD:AAE4975
Sympistis rayata|[10044]|LNAUS3239-13|United States|Texas|613[1n]|BOLD:AAE4975
Sympistis rayata|[10045]|LNAUS3243-13|United States|Texas|658[0n]|BOLD:AAE4975
Sympistis badistriga|[10046]|RDNM108-05|Canada|Ontario|658[0n]|BOLD:AAE4975
Sympistis badistriga|[10047]|CNPPE1037-12|Canada|Ontario|658[0n]|BOLD:AAE4975
Sympistis badistriga|[10048]|LNC237-05|United States|North Carolina|658[0n]|BOLD:AAE4975
Sympistis badistriga|[10049]|LNCC1710-13|United States|North Carolina|658[0n]|BOLD:AAE4975
Sympistis stabilis|[10050]|RDNMF034-08|Canada|Saskatchewan|658[0n]|BOLD:AAF1183
Sympistis stabilis|[10051]|RDNMG605-08|Canada|Ontario|658[0n]|BOLD:AAF1183
Sympistis stabilis|[10052]|RDNMG606-08|Canada|British Columbia|658[0n]|BOLD:AAF1183
Sympistis stabilis|[10053]|LALPA870-11|Canada|British Columbia|658[0n]|BOLD:AAF1183
Sympistis fifia|[10054]|RDNMF716-08|Canada|British Columbia|658[0n]|BOLD:ACF2135
Sympistis fifia|[10055]|RDNMF717-08|Canada|British Columbia|658[0n]|BOLD:ACF2135
Sympistis fifia|[10056]|LBCH5720-10|Canada|British Columbia|658[0n]|BOLD:ACF2135
Sympistis fifia|[10057]|LBCH5726-10|Canada|British Columbia|658[0n]|BOLD:ACF2135
Sympistis fifia|[10058]|LBCH5803-10|Canada|British Columbia|658[0n]|BOLD:ACF2135
Sympistis fifia|[10059]|LBCH5941-10|Canada|British Columbia|658[0n]|BOLD:ACF2135
Sympistis fifia|[10060]|LBCD282-05|Canada|British Columbia|658[0n]|BOLD:ACF2135
Sympistis fifia|[10061]|LOWCD533-06|Canada|British Columbia|658[0n]|BOLD:ACF2135
Sympistis fifia|[10062]|JMMMB412-11|United States|California|658[0n]|BOLD:ACF2135
Sympistis fifia|[10063]|RDNMF714-08|United States|California|658[0n]|BOLD:ACF2135
Sympistis fifia|[10064]|RDNMF715-08|United States|California|658[0n]|BOLD:ACF2135
Sympistis fifia|[10065]|NAMUM172-08|United States|California|658[0n]|BOLD:ACF2135
Sympistis fifia|[10066]|RDNMF712-08|United States|Nevada|658[0n]|BOLD:ACF2135
Sympistis fifia|[10067]|RDNMF713-08|United States|Nevada|658[0n]|BOLD:ACF2135
Sympistis fifia|[10068]|BBLOC1209-11|United States|California|658[0n]|BOLD:ACF2135
Sympistis induta|[10069]|BBLSY865-09|United States|Oklahoma|658[0n]|BOLD:AAB4182
Sympistis dinalda|[10070]|RDNM107-05|Canada|Ontario|658[0n]|BOLD:AAB4182
Sympistis dinalda|[10071]|LNCC039-10|United States|North Carolina|658[0n]|BOLD:AAB4182
Sympistis dinalda|[10072]|RDNML162-13|United States|North Carolina|658[0n]|BOLD:AAB4182
Sympistis dinalda|[10073]|RDNML172-13|United States|North Carolina|658[0n]|BOLD:AAB4182
Sympistis dinalda|[10074]|LPOKB815-09|United States|Oklahoma|658[0n]|BOLD:AAB4182
Sympistis dinalda|[10075]|LBCD290-05|Canada|British Columbia|658[0n]|BOLD:AAB4182
Sympistis dinalda|[10076]|LPSK260-08|Canada|Saskatchewan|658[0n]|BOLD:AAB4182
Sympistis dinalda|[10077]|LPABB469-08|Canada|Alberta|658[0n]|BOLD:AAB4182
Sympistis dinalda|[10078]|LPABB487-08|Canada|Alberta|658[0n]|BOLD:AAB4182
Sympistis dinalda|[10079]|LPABB515-08|Canada|Alberta|658[0n]|BOLD:AAB4182
Sympistis dinalda|[10080]|LPABC723-09|Canada|Alberta|658[0n]|BOLD:AAB4182
Sympistis dinalda|[10081]|LPABC899-09|Canada|Alberta|658[0n]|BOLD:AAB4182
Sympistis dinalda|[10082]|LPABC914-09|Canada|Alberta|658[0n]|BOLD:AAB4182
Sympistis dinalda|[10083]|LPABC938-09|Canada|Alberta|658[0n]|BOLD:AAB4182
Sympistis dinalda|[10084]|LPABC952-09|Canada|Alberta|658[0n]|BOLD:AAB4182
Sympistis dinalda|[10085]|LPABC956-09|Canada|Alberta|658[0n]|BOLD:AAB4182
Sympistis dinalda|[10086]|LPABC980-09|Canada|Alberta|658[0n]|BOLD:AAB4182
Sympistis dinalda|[10087]|LPABC999-09|Canada|Alberta|658[0n]|BOLD:AAB4182
Sympistis dinalda|[10088]|LPOKB360-09|United States|Oklahoma|658[0n]|BOLD:AAB4182
Sympistis dinalda|[10089]|LPOKB381-09|United States|Oklahoma|658[0n]|BOLD:AAB4182
Sympistis dinalda|[10090]|LPOKB382-09|United States|Oklahoma|658[0n]|BOLD:AAB4182
Sympistis dinalda|[10091]|LPOKB1017-09|United States|Oklahoma|658[0n]|BOLD:AAB4182
Sympistis dinalda|[10092]|LPOKC309-09|United States|Oklahoma|658[0n]|BOLD:AAB4182
Sympistis dinalda|[10093]|LPOKC332-09|United States|Oklahoma|658[0n]|BOLD:AAB4182
Sympistis dinalda|[10094]|BBLSW684-09|United States|Oklahoma|658[0n]|BOLD:AAB4182
Sympistis dinalda|[10095]|BBLSX364-09|United States|Oklahoma|658[0n]|BOLD:AAB4182
Sympistis dinalda|[10096]|BBLEC045-09|Canada|New Brunswick|658[0n]|BOLD:AAB4182
Sympistis dinalda|[10097]|BBLPE068-09|Canada|Nova Scotia|658[0n]|BOLD:AAB4182
Sympistis dinalda|[10098]|LILLA256-11|United States|Illinois|658[0n]|BOLD:AAB4182
Sympistis dinalda|[10099]|LNCC982-11|United States|North Carolina|658[0n]|BOLD:AAB4182
Sympistis kappa|[10100]|CNCLB166-14|United States|Tennessee|658[0n]|BOLD:AAB4182
Sympistis kappa|[10101]|CNCLB1141-14|United States|Tennessee|658[0n]|BOLD:AAB4182
Sympistis induta|[10102]|BBLSW457-09|United States|Oklahoma|658[0n]|BOLD:AAB4182
Sympistis induta|[10103]|BBLSW567-09|United States|Oklahoma|636[0n]|BOLD:AAB4182
Sympistis induta|[10104]|RDNMB556-05|United States|Texas|658[0n]|BOLD:AAB4182
Sympistis induta|[10105]|BBLSY861-09|United States|Oklahoma|658[0n]|BOLD:AAB4182
Sympistis infixa|[10106]|CNCLB164-14|United States|Tennessee|658[0n]|BOLD:AAB4182
Sympistis infixa|[10107]|CNCLB165-14|United States|Tennessee|658[0n]|BOLD:AAB4182
Sympistis sp.|[10108]|CNCLB1144-14|United States|Tennessee|658[0n]|BOLD:AAB4182
Sympistis tetrops|[10109]|RDNMB169-05|United States|Colorado|572[0n]|BOLD:AAI1717
Sympistis tetrops|[10110]|RDNMB170-05|United States|Utah|578[0n]|BOLD:AAI1717
Sympistis sp.|[10111]|BBLSX992-09|United States|Arizona|658[0n]|BOLD:AAH5373
Sympistis knudsoni|[10112]|CNCLB1672-14|United States|Texas|407[0n]|
Sympistis sp.|[10113]|TML119-14|United States|California|608[1n]|BOLD:ACO0964
Sympistis inconstans|[10114]|TML113-14|United States||658[0n]|BOLD:ACP0563
Sympistis sp.|[10115]|CNCLB2171-14|United States|New Mexico|658[0n]|BOLD:ACR5546
Sympistis sp.|[10116]|CNCLB2170-14|United States|New Mexico|658[1n]|BOLD:ACR5546
Sympistis sp.|[10117]|CNCLB2172-14|United States|New Mexico|329[0n]|BOLD:ACR5546
Sympistis tenuifascia|[10118]|RDNMF135-08|United States|Washington|658[0n]|BOLD:AAD5526
Sympistis tenuifascia|[10119]|RDNMF139-08|United States|Washington|658[0n]|BOLD:AAD5526
Sympistis tenuifascia|[10120]|RDNMF138-08|United States|Washington|658[0n]|BOLD:AAD5526
Sympistis tenuifascia|[10121]|RDNMF137-08|United States|Washington|658[0n]|BOLD:AAD5526
Sympistis tenuifascia|[10122]|RDNMB187-05|United States|Oregon|658[0n]|BOLD:AAD5526
Sympistis tenuifascia|[10123]|RDNMB143-05|United States|Washington|658[0n]|BOLD:AAD5526
Sympistis tenuifascia|[10124]|RDNMB144-05|United States|Washington|658[0n]|BOLD:AAD5526
Sympistis tenuifascia|[10125]|RDNMF136-08|United States|Washington|641[0n]|BOLD:AAD5526
Sympistis tenuifascia|[10126]|RDNMB146-05|United States|Washington|596[0n]|BOLD:AAD5526
Sympistis tenuifascia|[10127]|RDNMB145-05|United States|Washington|595[0n]|BOLD:AAD5526
Sympistis apep|[10128]|MNAE872-13|United States|Washington|646[0n]|BOLD:AAD5526
Sympistis apep|[10129]|MNAE873-13|United States|Washington|658[0n]|BOLD:AAD5526
Sympistis parvanigra|[10130]|RDNMB147-05|Canada|British Columbia|592[0n]|BOLD:AAE5094
Sympistis parvanigra|[10131]|RDNMG916-08|Canada|British Columbia|658[0n]|BOLD:AAE5094
Sympistis parvanigra|[10132]|RDNMG917-08|United States|Washington|658[0n]|BOLD:AAE5094
Sympistis parvanigra|[10133]|RDNMG918-08|Canada|British Columbia|658[0n]|BOLD:AAE5094
Sympistis parvanigra|[10134]|LBCH7227-10|Canada|British Columbia|658[0n]|BOLD:AAE5094
Sympistis parvanigra|[10135]|LBCH7229-10|Canada|British Columbia|658[0n]|BOLD:AAE5094
Sympistis sp.|[10136]|CNCLB2512-14|United States|California|658[0n]|BOLD:ACR9190
Sympistis bakeri|[10137]|RDNM503-05|United States|California|495[2n]|
Sympistis bakeri|[10138]|CNCLB142-14|United States|California|658[0n]|BOLD:ABW3098
Sympistis bakeri|[10139]|BBLOC1384-11|United States|California|658[0n]|BOLD:ABW3098
Sympistis bakeri|[10140]|CNCLB143-14|United States|California|658[0n]|BOLD:ABW3098
Sympistis deserta|[10141]|TML216-14|United States||658[0n]|BOLD:ACO1889
Sympistis n. sp. 5|[10142]|RDNMC413-05|United States|California|598[0n]|BOLD:AAI0267
Sympistis n. sp. 5|[10143]|RDNMC414-05|United States|California|594[0n]|BOLD:AAI0267
Sympistis amun|[10144]|RDNM575-05|United States|Oregon|658[0n]|BOLD:AAE2626
Sympistis amun|[10145]|RDNM574-05|United States|California|658[0n]|BOLD:AAE2626

[illegible]
Sympistis n. sp. 5|[10143]|RDNMC414-05|United States|California|594[0n]|BOLD:AAI0267
Sympistis amun|[10144]|RDNM575-05|United States|Oregon|658[0n]|BOLD:AAE2626
Sympistis amun|[10145]|RDNM574-05|United States|California|658[0n]|BOLD:AAE2626
Sympistis amun|[10146]|RDNM566-05|United States|Oregon|658[0n]|BOLD:AAE2626
Sympistis amun|[10147]|RDNM585-05|Canada|British Columbia|658[0n]|BOLD:AAE2626
Sympistis amun|[10148]|LOWCB731-05|Canada|British Columbia|658[0n]|BOLD:AAE2626
Sympistis amun|[10149]|LBCH5861-10|Canada|British Columbia|658[0n]|BOLD:AAE2626
Sympistis major|[10150]|RDNM560-05|United States|California|658[0n]|BOLD:ABZ1047
Sympistis major|[10151]|RDNM561-05|United States|California|658[0n]|BOLD:ABZ1047
Sympistis major|[10152]|RDNM562-05|United States|California|658[0n]|BOLD:ABZ1047
Sympistis major|[10153]|RDNMC415-05|United States|California|556[0n]|BOLD:ABZ1047
Sympistis major|[10154]|RDNMC833-06|United States|Utah|519[0n]|BOLD:ABZ1047
Sympistis major|[10155]|RDNM567-05|United States|Utah|512[0n]|BOLD:ABZ1047
Sympistis major|[10156]|RDNM635-05|United States|Colorado|658[0n]|BOLD:ABZ1047
Sympistis major|[10157]|RDNMC832-06|United States|Utah|568[0n]|BOLD:ABZ1047
Sympistis major|[10158]|RDNMD445-06|United States|California|658[0n]|BOLD:ABZ1047
Sympistis major|[10159]|RDNMD446-06|United States|California|658[0n]|BOLD:ABZ1047
Sympistis nenun|[10160]|RDNM636-05|United States|Utah|515[0n]|BOLD:ACF3395
Sympistis nenun|[10161]|RDNM637-05|United States|Utah|579[0n]|BOLD:ACF3395
Sympistis nenun|[10162]|RDNM638-05|United States|Utah|545[0n]|BOLD:ACF3395
Sympistis n. sp. 6|[10163]|NAMUM152-08|United States|California|658[0n]|BOLD:ABZ1046
Sympistis n. sp. 6|[10164]|BBLOC939-11|United States|Texas|658[0n]|BOLD:ABZ1046
Sympistis n. sp. 6|[10165]|BBLOC944-11|United States|Texas|658[0n]|BOLD:ABZ1046
Sympistis n. sp. 6|[10166]|BBLOC1002-11|United States|California|658[0n]|BOLD:ABZ1046
Sympistis n. sp. 6|[10167]|BBLOC1260-11|United States|California|658[0n]|BOLD:ABZ1046
Sympistis n. sp. 6|[10168]|BBLOC1279-11|United States|California|658[0n]|BOLD:ABZ1046
Sympistis n. sp. 6|[10169]|BBLOC1346-11|United States|California|658[0n]|BOLD:ABZ1046
Sympistis n. sp. 6|[10170]|BBLOC1349-11|United States|California|658[0n]|BOLD:ABZ1046
Sympistis n. sp. 6|[10171]|BBLOC1382-11|United States|California|658[0n]|BOLD:ABZ1046
Sympistis riparia|[10172]|RDNM569-05|Canada|Saskatchewan|548[0n]|BOLD:ACF5261
Sympistis riparia|[10173]|RDNM568-05|Canada|Saskatchewan|658[0n]|BOLD:ACF5261
Sympistis riparia|[10174]|RDMAB490-06|Canada|Alberta|658[0n]|BOLD:ACF5261
Sympistis riparia|[10175]|RDMAB452-05|Canada|Alberta|568[3n]|BOLD:ACF5261
Sympistis riparia|[10176]|RDNM571-05|Canada|Alberta|552[0n]|BOLD:ACF5261
Sympistis riparia|[10177]|RDNM570-05|Canada|Alberta|658[0n]|BOLD:ACF5261
Sympistis riparia|[10178]|RDNM572-05|Canada|Ontario|658[0n]|BOLD:ACF5261
Sympistis riparia|[10179]|RDNM573-05|Canada|Ontario|658[0n]|BOLD:ACF5261
Sympistis riparia|[10180]|QUNOE400-12|United States|Michigan|658[0n]|BOLD:ACF5261
Sympistis mut|[10181]|RDNM564-05|United States|California|524[0n]|BOLD:AAZ2689
Sympistis chons|[10182]|RDNMC411-05|United States|Arizona|548[0n]|BOLD:AAB4199
Sympistis chons|[10183]|RDNMC412-05|United States|Arizona|545[0n]|BOLD:AAB4199
Sympistis chons|[10184]|LBCH5709-10|Canada|British Columbia|658[0n]|BOLD:AAB4199
Sympistis chons|[10185]|LBCH5862-10|Canada|British Columbia|658[0n]|BOLD:AAB4199
Sympistis chons|[10186]|RDNM576-05|Canada|British Columbia|578[0n]|BOLD:AAB4199
Sympistis chons|[10187]|RDNM577-05|Canada|British Columbia|645[0n]|BOLD:AAB4199
Sympistis chons|[10188]|RDNM578-05|Canada|British Columbia|658[0n]|BOLD:AAB4199
Sympistis chons|[10189]|LOWCB729-05|Canada|British Columbia|515[0n]|BOLD:AAB4199
Sympistis chons|[10190]|LBCH5940-10|Canada|British Columbia|658[0n]|BOLD:AAB4199
Sympistis chons|[10191]|RDMAB453-05|Canada|Alberta|617[0n]|BOLD:AAB4199
Sympistis chons|[10192]|LOWCB730-05|Canada|British Columbia|658[1n]|BOLD:AAB4199
Sympistis chons|[10193]|LBCH5804-10|Canada|British Columbia|658[0n]|BOLD:AAB4199
Sympistis chons|[10194]|LBCH5670-10|Canada|British Columbia|658[0n]|BOLD:AAB4199
Sympistis chons|[10195]|LBCH5607-10|Canada|British Columbia|658[0n]|BOLD:AAB4199
Sympistis chons|[10196]|LBCH5573-10|Canada|British Columbia|658[0n]|BOLD:AAB4199
Sympistis chons|[10197]|LBCH5428-10|Canada|British Columbia|658[0n]|BOLD:AAB4199
Sympistis chons|[10198]|LPABB303-08|Canada|Alberta|658[0n]|BOLD:AAB4199
Sympistis chons|[10199]|RDMAB582-06|Canada|Alberta|658[0n]|BOLD:AAB4199
Sympistis chons|[10200]|RDMAB473-06|Canada|Alberta|658[0n]|BOLD:AAB4199
Sympistis chons|[10201]|LOWCB738-05|Canada|British Columbia|658[0n]|BOLD:AAB4199
Sympistis chons|[10202]|LOWCB734-05|Canada|British Columbia|658[0n]|BOLD:AAB4199
Sympistis chons|[10203]|LOWCB733-05|Canada|British Columbia|658[0n]|BOLD:AAB4199
Sympistis chons|[10204]|LOWCB732-05|Canada|British Columbia|658[0n]|BOLD:AAB4199
Sympistis chons|[10205]|RDNM583-05|Canada|Alberta|658[0n]|BOLD:AAB4199
Sympistis chons|[10206]|RDNM582-05|Canada|Alberta|658[0n]|BOLD:AAB4199
Sympistis chons|[10207]|RDNM581-05|Canada|British Columbia|658[0n]|BOLD:AAB4199
Sympistis chons|[10208]|RDNM580-05|Canada|British Columbia|658[0n]|BOLD:AAB4199
Sympistis chons|[10209]|RDNM559-05|United States|Nevada|658[0n]|BOLD:AAB4199
Sympistis chons|[10210]|RDNM558-05|United States|Nevada|658[0n]|BOLD:AAB4199
Sympistis chons|[10211]|RDNM557-05|United States|Nevada|658[0n]|BOLD:AAB4199
Sympistis chons|[10212]|LOWCB737-05|Canada|British Columbia|658[0n]|BOLD:AAB4199
Sympistis chons|[10213]|RDNM579-05|Canada|British Columbia|658[0n]|BOLD:AAB4199
Sympistis chons|[10214]|RDNM584-05|Canada|British Columbia|658[0n]|BOLD:AAB4199
Sympistis chons|[10215]|LOWCB736-05|Canada|British Columbia|559[0n]|BOLD:AAB4199
Sympistis chons|[10216]|LOWCB735-05|Canada|British Columbia|585[1n]|BOLD:AAB4199
Sympistis chons|[10217]|LBCH5913-10|Canada|British Columbia|644[0n]|BOLD:AAB4199
Sympistis chons|[10218]|LBCH6024-10|Canada|British Columbia|658[0n]|BOLD:AAB4199
Sympistis chons|[10219]|IAWLB441-11|United States|California|658[0n]|BOLD:AAB4199
Sympistis chons|[10220]|IAWLB442-11|United States|California|658[0n]|BOLD:AAB4199
Sympistis richersi|[10221]|CNCLB172-14|United States|California|658[0n]|BOLD:ACM4371
Sympistis richersi|[10222]|CNCLB171-14|United States|California|658[0n]|BOLD:ACM4371
Sympistis richersi|[10223]|LNAUT1117-14|United States|California|658[0n]|BOLD:ACM4371
Sympistis richersi|[10224]|LNAUT1118-14|United States|California|658[0n]|BOLD:ACM4371
Sympistis richersi|[10225]|LNAUT1119-14|United States|California|658[0n]|BOLD:ACM4371
Sympistis richersi|[10226]|LNAUT1120-14|United States|California|658[0n]|BOLD:ACM4371
Sympistis aqualis|[10227]|CNCLB1564-14|United States|Nevada|658[0n]|BOLD:ACO7690
Sympistis babi|[10228]|RDNM643-05|United States|Colorado|658[0n]|BOLD:AAK1079
Sympistis astrigata|[10229]|RDNMB171-05|United States|Nevada|597[0n]|BOLD:AAD7673
Sympistis astrigata|[10230]|RDNMB172-05|United States|California|567[0n]|BOLD:AAD7673
Sympistis astrigata|[10231]|LOCBE314-06|United States|California|658[0n]|BOLD:AAD7673
Sympistis astrigata|[10232]|LOCBE313-06|United States|California|658[0n]|BOLD:AAD7673
Sympistis astrigata|[10233]|LOCBD563-06|United States|California|658[0n]|BOLD:AAD7673
Sympistis astrigata|[10234]|NAMUM164-08|United States|California|645[0n]|BOLD:AAD7673
Sympistis minor|[10235]|RDNMB911-05|United States|Colorado|658[0n]|BOLD:AAX0324
Sympistis nita|[10236]|RDNMB139-05|United States|California|605[0n]|BOLD:AAI1670
Sympistis nita|[10237]|RDNMB140-05|United States|California|595[0n]|BOLD:AAI1670
Sympistis deceptiva|[10238]|CNCLB151-14|United States|Nevada|658[0n]|BOLD:ACM4456
Sympistis deceptiva|[10239]|TML215-14|United States||658[0n]|BOLD:ACM4456
Sympistis youngi|[10240]|RDNMB179-05|United States|Oregon|577[1n]|BOLD:AAE1045
Sympistis youngi|[10241]|RDNMB180-05|United States|Washington|654[0n]|BOLD:AAE1045
Sympistis youngi|[10242]|RDNMG921-08|United States|Oregon|658[0n]|BOLD:AAE1045
Sympistis youngi|[10243]|RDNMG922-08|United States|Washington|658[0n]|BOLD:AAE1045
Sympistis youngi|[10244]|RDNMG923-08|Canada|British Columbia|658[0n]|BOLD:AAE1045

Sympistis youngi|[10242]|RDNMG921-08|United States|Oregon|658[0n]|BOLD:AAE1045
Sympistis youngi|[10243]|RDNMG922-08|United States|Washington|658[0n]|BOLD:AAE1045
Sympistis youngi|[10244]|RDNMG923-08|Canada|British Columbia|658[0n]|BOLD:AAE1045
Sympistis cherti|[10245]|RDNM463-05|United States|Washington|658[0n]|BOLD:AAE2632
Sympistis cherti|[10246]|RDNM465-05|United States|Washington|658[0n]|BOLD:AAE2632
Sympistis cherti|[10247]|RDNM464-05|United States|Washington|658[0n]|BOLD:AAE2632
Sympistis cherti|[10248]|RDNM473-05|United States|Oregon|658[0n]|BOLD:AAE2632
Sympistis helena|[10249]|TML112-14|United States|California|658[0n]|BOLD:AAE2632
Sympistis cherti|[10250]|RDNM468-05|United States|Nevada|658[0n]|BOLD:AAE2632
Sympistis helena|[10251]|CNCLB1669-14|United States|California|658[0n]|BOLD:AAE2632
Sympistis columbia|[10252]|LOPN156-06|United States|Oregon|510[0n]|BOLD:AAE2638
Sympistis pallida|[10253]|RDNM630-05|United States|Colorado|584[1n]|BOLD:AAE2638
Sympistis pallida|[10254]|RDNM631-05|United States|Colorado|584[1n]|BOLD:AAE2638
Sympistis columbia|[10255]|RDNM481-05|United States|Washington|658[0n]|BOLD:AAE2638
Sympistis columbia|[10256]|RDNMB182-05|United States|Washington|547[1n]|BOLD:AAE2638
Sympistis columbia|[10257]|RDNM482-05|United States|Washington|658[0n]|BOLD:AAE2638
Sympistis pallida|[10258]|RDNM478-05|United States|Nevada|658[0n]|BOLD:AAE2638
Sympistis pallida|[10259]|RDNM477-05|United States|Nevada|658[0n]|BOLD:AAE2638
Sympistis pallida|[10260]|RDNMB181-05|United States|Nevada|608[0n]|BOLD:AAE2638
Sympistis columbia|[10261]|RDNMB183-05|Canada|British Columbia|585[0n]|BOLD:AAE2638
Sympistis columbia|[10262]|LOPN155-06|United States|Oregon|604[1n]|BOLD:AAE2638
Sympistis columbia|[10263]|LOPN157-06|United States|Oregon|586[0n]|BOLD:AAE2638
Sympistis pallida|[10264]|RDNMG157-08|United States|California|658[0n]|BOLD:AAE2638
Sympistis pallida|[10265]|RDNMG158-08|United States|Nevada|658[0n]|BOLD:AAE2638
Sympistis sobek|[10266]|CNCLB173-14|United States|Oregon|658[0n]|BOLD:AAI0269
Sympistis hathor|[10267]|RDNM466-05|United States|Oregon|658[0n]|BOLD:AAI0269
Sympistis hathor|[10268]|RDNM467-05|United States|Oregon|658[0n]|BOLD:AAI0269
Sympistis sobek|[10269]|CNCLB174-14|United States|California|658[0n]|BOLD:AAI0269
Sympistis septu|[10270]|RDNM639-05|United States|Colorado|658[0n]|BOLD:ACF2437
Sympistis septu|[10271]|RDNM640-05|United States|Colorado|658[0n]|BOLD:ACF2437
Sympistis ra|[10272]|RDNMB197-05|United States|California|316[0n]|
Sympistis ra|[10273]|CNCLB2176-14|United States|California|379[0n]|
Sympistis arizonensis|[10274]|RDNM474-05|United States|Colorado|658[0n]|BOLD:AAE2637
Sympistis arizonensis|[10275]|RDNM475-05|United States|Colorado|658[0n]|BOLD:AAE2637
Sympistis arizonensis|[10276]|RDNM476-05|United States|Colorado|658[0n]|BOLD:AAE2637
Sympistis arizonensis|[10277]|RDNMB553-05|United States|Colorado|658[0n]|BOLD:AAE2637
Sympistis arizonensis|[10278]|IAWLB548-11|United States|Arizona|658[0n]|BOLD:AAE2637
Sympistis arizonensis|[10279]|IAWLB549-11|United States|Arizona|658[0n]|BOLD:AAE2637
Sympistis arizonensis|[10280]|IAWLB550-11|United States|Arizona|658[0n]|BOLD:AAE2637
Sympistis arizonensis|[10281]|RDNMK565-11|United States|Arizona|658[0n]|BOLD:AAE2637
Sympistis homogena|[10282]|RDNM650-05|United States|Colorado|504[1n]|BOLD:AAZ2832
Sympistis homogena|[10283]|CNCLB2178-14|United States|Utah|540[0n]|BOLD:AAZ2832
Sympistis orbicularis|[10284]|RDNM589-05|United States|Oregon|658[0n]|BOLD:ACE5893
Sympistis orbicularis|[10285]|RDNM588-05|United States|Oregon|658[0n]|BOLD:ACE5893
Sympistis orbicularis|[10286]|RDNMC756-06|United States|California|658[0n]|BOLD:ACE5893
Sympistis orbicularis|[10287]|RDNMC754-06|United States|California|550[0n]|BOLD:ACE5893
Sympistis orbicularis|[10288]|RDNMC755-06|United States|California|577[1n]|BOLD:ACE5893
Sympistis orbicularis|[10289]|RDNMC753-06|United States|California|599[1n]|BOLD:ACE5893
Sympistis orbicularis|[10290]|RDNMC757-06|United States|California|590[1n]|BOLD:ACE5893
Sympistis sp.|[10291]|RDNMC759-06|United States|Washington|583[0n]|BOLD:AAC0956
Sympistis sp.|[10292]|RDNMC758-06|United States|Washington|658[0n]|BOLD:AAC0956
Sympistis sp.|[10293]|RDNM586-05|United States|Washington|658[0n]|BOLD:AAC0956
Sympistis sp.|[10294]|RDNMC760-06|United States|Washington|633[0n]|BOLD:AAC0956
Sympistis sp.|[10295]|RDNMC761-06|United States|Oregon|658[0n]|BOLD:AAC0956
Sympistis meadiana|[10296]|RDNM587-05|United States|Nevada|595[0n]|BOLD:ABZ4554
Sympistis meadiana|[10297]|RDNMC763-06|United States|Nevada|658[0n]|BOLD:ABZ4554
Sympistis meadiana|[10298]|RDNMC764-06|United States|Nevada|658[0n]|BOLD:ABZ4554
Sympistis meadiana|[10299]|RDNMC762-06|United States|Oregon|658[0n]|BOLD:ABZ4554
Sympistis meadiana|[10300]|RDNMC765-06|United States|Washington|612[0n]|BOLD:ABZ4554
Sympistis meadiana|[10301]|RDNMC766-06|United States|Washington|608[0n]|BOLD:ABZ4554
Sympistis meadiana|[10302]|RDNMC767-06|United States|Washington|612[0n]|BOLD:ABZ4554
Sympistis meadiana|[10303]|RDNMC768-06|United States|Colorado|599[0n]|BOLD:ABZ4554
Sympistis meadiana|[10304]|RDNMC769-06|United States|Colorado|574[1n]|BOLD:ABZ4554
Sympistis meadiana|[10305]|RDNMC770-06|United States|Colorado|596[0n]|BOLD:ABZ4554
Sympistis dunbari|[10306]|LOPN153-06|United States|Oregon|527[0n]|BOLD:AAE1138
Sympistis dunbari|[10307]|RDNM618-05|United States|Washington|658[0n]|BOLD:AAE1138
Sympistis dunbari|[10308]|RDNM619-05|United States|Washington|658[0n]|BOLD:AAE1138
Sympistis dunbari|[10309]|LOPN152-06|United States|Oregon|657[1n]|BOLD:AAE1138
Sympistis dunbari|[10310]|LOPN154-06|United States|Oregon|589[1n]|BOLD:AAE1138
Sympistis n. sp. 7|[10311]|RDNMJ840-11|United States|Arizona|658[0n]|BOLD:AAU2696
Sympistis n. sp. 7|[10312]|RDNMJ841-11|United States|Arizona|658[0n]|BOLD:AAU2696
Sympistis definita|[10313]|RDNM620-05|United States|Nevada|581[0n]|BOLD:AAE5103
Sympistis definita|[10314]|RDNM621-05|United States|Nevada|658[0n]|BOLD:AAE5103
Sympistis definita|[10315]|RDNME528-08|United States|Colorado|658[0n]|BOLD:AAE5103
Sympistis definita|[10316]|RDNME529-08|United States|Colorado|658[0n]|BOLD:AAE5103
Sympistis sp.|[10317]|IAWLB523-11|United States|Arizona|658[0n]|BOLD:AAZ0785
Sympistis wilsoni|[10318]|RDNMF453-08|Canada|British Columbia|641[0n]|BOLD:AAC8395
Sympistis heliophila|[10319]|RDNMB922-05|Canada|Yukon Territory|570[0n]|BOLD:AAC8395
Sympistis heliophila|[10320]|RDMAB213-05|Canada|Alberta|658[0n]|BOLD:AAC8395
Sympistis heliophila|[10321]|RDMAB214-05|Canada|Alberta|658[0n]|BOLD:AAC8395
Sympistis heliophila|[10322]|RDNME515-08|Canada|Yukon Territory|658[0n]|BOLD:AAC8395
Sympistis heliophila|[10323]|RDNME524-08|Canada|Yukon Territory|658[0n]|BOLD:AAC8395
Sympistis wilsoni|[10324]|RDNMF454-08|Canada|British Columbia|658[0n]|BOLD:AAC8395
Sympistis zetterstedtii|[10325]|LCHP336-07|Canada|Manitoba|658[1n]|BOLD:AAA7102
Sympistis zetterstedtii|[10326]|LCHP335-07|Canada|Manitoba|656[0n]|BOLD:AAA7102
Sympistis zetterstedtii|[10327]|RDNMB917-05|Canada|Manitoba|562[0n]|BOLD:AAA7102
Sympistis zetterstedtii|[10328]|RDNMB916-05|Canada|Manitoba|573[2n]|BOLD:AAA7102
Sympistis zetterstedtii|[10329]|RDNMB918-05|Canada|Manitoba|517[0n]|BOLD:AAA7102
Sympistis zetterstedtii|[10330]|LCHP023-07|Canada|Manitoba|658[0n]|BOLD:AAA7102
Sympistis zetterstedtii|[10331]|LCHP024-07|Canada|Manitoba|658[0n]|BOLD:AAA7102
Sympistis zetterstedtii|[10332]|LCHP052-07|Canada|Manitoba|657[0n]|BOLD:AAA7102
Sympistis zetterstedtii|[10333]|LCHP091-07|Canada|Manitoba|655[0n]|BOLD:AAA7102
Sympistis zetterstedtii|[10334]|MHLEP027-07|Canada|Manitoba|658[0n]|BOLD:AAA7102
Sympistis zetterstedtii|[10335]|LCHP921-07|Canada|Manitoba|658[0n]|BOLD:AAA7102
Sympistis zetterstedtii|[10336]|LCHQ925-08|Canada|Manitoba|658[0n]|BOLD:AAA7102
Sympistis zetterstedtii|[10337]|MHCOL104-07|Canada|Manitoba|620[0n]|BOLD:AAA7102
Sympistis zetterstedtii|[10338]|MHCOL161-07|Canada|Manitoba|654[0n]|BOLD:AAA7102
Sympistis zetterstedtii|[10339]|LCHP093-07|Canada|Manitoba|653[0n]|BOLD:AAA7102
Sympistis zetterstedtii|[10340]|LCHP025-07|Canada|Manitoba|656[0n]|BOLD:AAA7102
Sympistis zetterstedtii|[10341]|CHLEP278-09|Canada|Manitoba|658[0n]|BOLD:AAA7102
Sympistis zetterstedtii|[10342]|LCHQ924-08|Canada|Manitoba|658[0n]|BOLD:AAA7102
Sympistis zetterstedtii|[10343]|LCHQ914-08|Canada|Manitoba|658[0n]|BOLD:AAA7102
Sympistis zetterstedtii|[10344]|LCHQ906-08|Canada|Manitoba|658[0n]|BOLD:AAA7102

Sympistis zetterstedtii|[10341]|CHLEP278-09|Canada|Manitoba|658[0n]|BOLD:AAA7102
Sympistis zetterstedtii|[10342]|LCHQ924-08|Canada|Manitoba|658[0n]|BOLD:AAA7102
Sympistis zetterstedtii|[10343]|LCHQ914-08|Canada|Manitoba|658[0n]|BOLD:AAA7102
Sympistis zetterstedtii|[10344]|LCHQ906-08|Canada|Manitoba|658[0n]|BOLD:AAA7102
Sympistis zetterstedtii|[10345]|LCHP922-07|Canada|Manitoba|658[0n]|BOLD:AAA7102
Sympistis zetterstedtii|[10346]|LCHP584-07|Canada|Manitoba|658[0n]|BOLD:AAA7102
Sympistis zetterstedtii|[10347]|LCHP451-07|Canada|Manitoba|658[0n]|BOLD:AAA7102
Sympistis zetterstedtii|[10348]|LCHP448-07|Canada|Manitoba|658[0n]|BOLD:AAA7102
Sympistis zetterstedtii|[10349]|LCHP429-07|Canada|Manitoba|658[0n]|BOLD:AAA7102
Sympistis zetterstedtii|[10350]|LCHP427-07|Canada|Manitoba|658[0n]|BOLD:AAA7102
Sympistis zetterstedtii|[10351]|LCHP426-07|Canada|Manitoba|658[0n]|BOLD:AAA7102
Sympistis zetterstedtii|[10352]|LCHP425-07|Canada|Manitoba|658[0n]|BOLD:AAA7102
Sympistis zetterstedtii|[10353]|MHCOL163-07|Canada|Manitoba|658[0n]|BOLD:AAA7102
Sympistis zetterstedtii|[10354]|MHCOL159-07|Canada|Manitoba|658[0n]|BOLD:AAA7102
Sympistis zetterstedtii|[10355]|MHCOL158-07|Canada|Manitoba|658[0n]|BOLD:AAA7102
Sympistis zetterstedtii|[10356]|LCHP334-07|Canada|Manitoba|658[0n]|BOLD:AAA7102
Sympistis zetterstedtii|[10357]|LCHP115-07|Canada|Manitoba|658[0n]|BOLD:AAA7102
Sympistis zetterstedtii|[10358]|LCHP114-07|Canada|Manitoba|658[0n]|BOLD:AAA7102
Sympistis zetterstedtii|[10359]|LCHP113-07|Canada|Manitoba|658[0n]|BOLD:AAA7102
Sympistis zetterstedtii|[10360]|LCHP022-07|Canada|Manitoba|658[0n]|BOLD:AAA7102
Sympistis zetterstedtii|[10361]|LCH326-04|Canada|Manitoba|566[0n]|BOLD:AAA7102
Sympistis zetterstedtii|[10362]|MHCOL166-07|Canada|Manitoba|645[0n]|BOLD:AAA7102
Sympistis zetterstedtii|[10363]|CHLEP223-09|Canada|Manitoba|640[0n]|BOLD:AAA7102
Sympistis zetterstedtii|[10364]|CHLEP279-09|Canada|Manitoba|636[0n]|BOLD:AAA7102
Sympistis zetterstedtii|[10365]|RDNMB923-05|United States|Colorado|586[2n]|BOLD:AAA7102
Sympistis zetterstedtii|[10366]|RDNMB924-05|Canada|British Columbia|614[0n]|BOLD:AAA7102
Sympistis zetterstedtii|[10367]|RDNMB926-05|Canada|British Columbia|551[0n]|BOLD:AAA7102
Sympistis zetterstedtii|[10368]|RDNME514-08|Canada|Yukon Territory|658[0n]|BOLD:AAA7102
Sympistis zetterstedtii|[10369]|RDNME522-08|Canada|Yukon Territory|658[0n]|BOLD:AAA7102
Sympistis zetterstedtii|[10370]|RDNME523-08|Canada|Yukon Territory|658[0n]|BOLD:AAA7102
Sympistis zetterstedtii|[10371]|RDNME534-08|Canada|Yukon Territory|658[0n]|BOLD:AAA7102
Sympistis zetterstedtii|[10372]|RDNME533-08|Canada|Yukon Territory|658[0n]|BOLD:AAA7102
Sympistis zetterstedtii|[10373]|RDNME531-08|Canada|Yukon Territory|646[0n]|BOLD:AAA7102
Sympistis zetterstedtii|[10374]|RDNME532-08|Canada|Yukon Territory|658[0n]|BOLD:AAA7102
Sympistis zetterstedtii|[10375]|RDNME535-08|Canada|Yukon Territory|658[0n]|BOLD:AAA7102
Sympistis zetterstedtii|[10376]|RDNMI114-10|Canada|Yukon Territory|658[0n]|BOLD:AAA7102
Sympistis lapponica|[10377]|RDNMG783-08|Canada|Yukon Territory|590[0n]|BOLD:AAC8398
Sympistis lapponica|[10378]|LEFII107-10|Finland|Lapland|658[0n]|BOLD:AAC8398
Sympistis lapponica|[10379]|RDNMG785-08|Canada|Yukon Territory|658[0n]|BOLD:AAC8398
Sympistis lapponica|[10380]|RDNMG784-08|Canada|Northwest Territories|658[0n]|BOLD:AAC8398
Sympistis lapponica|[10381]|RDNME513-08|Canada|Yukon Territory|658[0n]|BOLD:AAC8398
Sympistis lapponica|[10382]|RDNMB921-05|Canada|Nunavut|588[0n]|BOLD:AAC8398
Sympistis lapponica|[10383]|RDNMB919-05|Norway|Finnmark|539[0n]|BOLD:AAC8398
Sympistis lapponica|[10384]|RDNMB920-05|Norway|Finnmark|556[0n]|BOLD:AAC8398
Sympistis lapponica|[10385]|LEFIF286-10|Finland||658[0n]|BOLD:AAC8398
Sympistis lapponica|[10386]|LEFIG492-10|Finland||658[0n]|BOLD:AAC8398
Sympistis lapponica|[10387]|LEFII108-10|Finland|Lapland|658[0n]|BOLD:AAC8398
Sympistis barnesii|[10388]|RDNMB184-05|United States|Washington|598[0n]|BOLD:ABZ4087
Sympistis barnesii|[10389]|RDNMB185-05|United States|Washington|541[0n]|BOLD:ABZ4087
Sympistis piffardi|[10390]|RDNM483-05|Canada|New Brunswick|658[0n]|BOLD:AAE1059
Sympistis piffardi|[10391]|RDNMG801-08|Canada|New Brunswick|658[0n]|BOLD:AAE1059
Sympistis piffardi|[10392]|RDNMG807-08|Canada|New Brunswick|658[0n]|BOLD:AAE1059
Sympistis piffardi|[10393]|RDLQF241-06|Canada|Quebec|636[0n]|BOLD:AAE1059
Sympistis piffardi|[10394]|RDNMG808-08|Canada|Alberta|658[0n]|BOLD:AAE1059
Sympistis chalybdis|[10395]|RDNM484-05|Canada|British Columbia|658[0n]|BOLD:AAE1059
Sympistis chalybdis|[10396]|RDNMG876-08|United States|Oregon|658[0n]|BOLD:AAE1059
Sympistis chalybdis|[10397]|RDNMG877-08|United States|Washington|658[0n]|BOLD:AAE1059
Sympistis chalybdis|[10398]|RDNMG878-08|Canada|British Columbia|658[0n]|BOLD:AAE1059
Sympistis funebris|[10399]|RDNMF455-08|Canada|Alberta|618[0n]|BOLD:AAD4608
Sympistis funebris|[10400]|RDNMF456-08|Canada|Yukon Territory|658[0n]|BOLD:AAD4608
Sympistis funebris|[10401]|RDNMF457-08|Canada|Yukon Territory|634[0n]|BOLD:AAD4608
Sympistis funebris|[10402]|RDNMF458-08|Canada|Yukon Territory|658[0n]|BOLD:AAD4608
Sympistis funebris|[10403]|CHLEP255-09|Canada|Manitoba|658[0n]|BOLD:AAD4608
Sympistis forbesi|[10404]|RDNMK490-11|United States|Iowa|573[0n]|BOLD:AAD7859
Sympistis forbesi|[10405]|RDNMK491-11|United States|Iowa|618[0n]|BOLD:AAD7859
Sympistis chionanthi|[10406]|LNCC1475-13|United States|North Carolina|658[0n]|BOLD:AAD7859
Sympistis chionanthi|[10407]|RDNMB214-05|Canada|Alberta|654[0n]|BOLD:AAD7859
Sympistis chionanthi|[10408]|RDNMK771-12|Canada|Alberta|658[0n]|BOLD:AAD7859
Sympistis chionanthi|[10409]|RDNMK772-12|Canada|Alberta|658[0n]|BOLD:AAD7859
Sympistis chionanthi|[10410]|RDNMB213-05|Canada|Ontario|658[0n]|BOLD:AAD7859
Sympistis chionanthi|[10411]|XAH314-05|Canada|Ontario|658[0n]|BOLD:AAD7859
Sympistis chionanthi|[10412]|XAH345-05|Canada|Ontario|658[0n]|BOLD:AAD7859
Sympistis chionanthi|[10413]|RDLQ604-07|Canada|Quebec|658[0n]|BOLD:AAD7859
Sympistis chionanthi|[10414]|RDLQ605-07|Canada|Quebec|658[0n]|BOLD:AAD7859
Sympistis chionanthi|[10415]|RDNMK488-11|United States|Connecticut|658[0n]|BOLD:AAD7859
Sympistis chionanthi|[10416]|CNCLB2461-14|Canada|Ontario|658[0n]|BOLD:AAD7859
Sympistis chionanthi|[10417]|CNCLB2462-14|Canada|Ontario|658[0n]|BOLD:AAD7859
Sympistis nigrocaput|[10418]|RDNMB195-05|United States|California|528[0n]|BOLD:AAI1672
Sympistis nigrocaput|[10419]|RDNMD447-06|United States|California|658[0n]|BOLD:AAI1672
Sympistis californiae|[10420]|RDNMB536-05|Canada|British Columbia|630[0n]|BOLD:AAF7139
Sympistis californiae|[10421]|RDNMB537-05|United States|California|603[0n]|BOLD:AAF7139
Sympistis californiae|[10422]|RDNMB538-05|United States|Oregon|658[0n]|BOLD:AAF7139
Sympistis anweileri|[10423]|RDMAB455-05|Canada|Alberta|506[0n]|BOLD:AAD4613
Sympistis anweileri|[10424]|RDNMB533-05|Canada|Alberta|549[0n]|BOLD:AAD4613
Sympistis anweileri|[10425]|RDNMB531-05|Canada|Alberta|658[0n]|BOLD:AAD4613
Sympistis anweileri|[10426]|RDNMB530-05|Canada|Alberta|658[0n]|BOLD:AAD4613
Sympistis anweileri|[10427]|RDNMB534-05|Canada|British Columbia|600[0n]|BOLD:AAD4613
Sympistis anweileri|[10428]|RDNMB535-05|Canada|British Columbia|658[0n]|BOLD:AAD4613
Sympistis anweileri|[10429]|RDMAB454-05|Canada|Alberta|658[0n]|BOLD:AAD4613
Sympistis anweileri|[10430]|LBCH4053-10|Canada|British Columbia|658[0n]|BOLD:AAD4613
Sympistis dentata|[10431]|RDLQB609-05|Canada|Quebec|571[2n]|BOLD:AAB6888
Sympistis dentata|[10432]|RDLQB607-05|Canada|Quebec|567[1n]|BOLD:AAB6888
Sympistis dentata|[10433]|RDNMB532-05|Canada|New Brunswick|565[1n]|BOLD:AAB6888
Sympistis dentata|[10434]|RDLQB616-05|Canada|Quebec|578[2n]|BOLD:AAB6888
Sympistis dentata|[10435]|LCHIP138-07|Canada|Manitoba|650[0n]|BOLD:AAB6888
Sympistis dentata|[10436]|RDLQB084-05|Canada|Quebec|658[0n]|BOLD:AAB6888
Sympistis dentata|[10437]|RDLQB613-05|Canada|Quebec|552[0n]|BOLD:AAB6888
Sympistis dentata|[10438]|RDLQB611-05|Canada|Quebec|569[0n]|BOLD:AAB6888
Sympistis dentata|[10439]|RDLQB612-05|Canada|Quebec|602[0n]|BOLD:AAB6888
Sympistis dentata|[10440]|RDLQB610-05|Canada|Quebec|596[1n]|BOLD:AAB6888
Sympistis dentata|[10441]|RDLQB614-05|Canada|Quebec|581[1n]|BOLD:AAB6888
Sympistis dentata|[10442]|RDLQB615-05|Canada|Quebec|596[0n]|BOLD:AAB6888
Sympistis dentata|[10443]|TTMNB086-06|Canada|New Brunswick|658[0n]|BOLD:AAB6888

Sympistis dentata|[10441]|RDLQB614-05|Canada|Quebec|581[1n]|BOLD:AAB6888
Sympistis dentata|[10442]|RDLQB615-05|Canada|Quebec|596[0n]|BOLD:AAB6888
Sympistis dentata|[10443]|TTMNB086-06|Canada|New Brunswick|658[0n]|BOLD:AAB6888
Sympistis dentata|[10444]|TTMNB087-06|Canada|New Brunswick|658[0n]|BOLD:AAB6888
Sympistis dentata|[10445]|BBLEC433-09|Canada|New Brunswick|658[0n]|BOLD:AAB6888
Sympistis dentata|[10446]|BBLPE129-09|Canada|Nova Scotia|658[0n]|BOLD:AAB6888
Sympistis dentata|[10447]|RDLQF364-06|Canada|Quebec|658[0n]|BOLD:AAB6888
Sympistis dentata|[10448]|RDLQB608-05|Canada|Quebec|567[0n]|BOLD:AAB6888
Sympistis dentata|[10449]|BBLPC096-09|Canada|New Brunswick|658[0n]|BOLD:AAB6888
Sympistis dentata|[10450]|LBCH6741-10|Canada|British Columbia|658[0n]|BOLD:AAB6888
Sympistis goedeni|[10451]|RDNMB121-05|United States|California|599[0n]|BOLD:AAX0345
Sympistis buchis|[10452]|MNAE870-13|United States|Colorado|658[0n]|BOLD:ACD7895
Sympistis buchis|[10453]|MNAE871-13|United States|Colorado|658[0n]|BOLD:ACD7895
Sympistis apis|[10454]|CNCLB2162-14|United States|Texas|307[0n]|
Sympistis apis|[10455]|CNCLB2159-14|United States|Texas|658[0n]|BOLD:ACD7895
Sympistis apis|[10456]|CNCLB2160-14|United States|Texas|658[0n]|BOLD:ACD7895
Sympistis apis|[10457]|CNCLB2161-14|United States|Texas|658[0n]|BOLD:ACD7895
Sympistis apis|[10458]|CNCLB2163-14|United States|Texas|658[0n]|BOLD:ACD7895
Sympistis pernotata|[10459]|CNCLB3439-15|United States|Arizona|627[0n]|BOLD:ACD7895
Sympistis sectiloides|[10460]|HKONB215-09|United States|Texas|658[0n]|BOLD:AAI1674
Sympistis sectilis|[10461]|RDNMG268-08|United States|Texas|658[0n]|BOLD:AAI1674
Sympistis sectiloides|[10462]|HKONB216-09|United States|Texas|658[0n]|BOLD:AAI1674
Sympistis griseicollis|[10463]|CNCLB153-14|United States|Arizona|658[0n]|BOLD:ABW8306
Sympistis griseicollis|[10464]|CNCLB154-14|United States|Arizona|658[0n]|BOLD:ABW8306
Sympistis griseicollis|[10465]|TML111-14|United States||658[0n]|BOLD:ABW8306
Sympistis occata|[10466]|LOCBF002-13|United States|California|629[0n]|BOLD:AAI1668
Sympistis occata|[10467]|LOCBF1030-13|United States|California|613[0n]|BOLD:AAI1668
Sympistis occata|[10468]|LOCBF345-13|United States|California|534[0n]|BOLD:AAI1668
Sympistis occata|[10469]|LOCBF1131-13|United States|California|558[0n]|BOLD:AAI1668
Sympistis occata|[10470]|LOCBF1054-13|United States|California|573[0n]|BOLD:AAI1668
Sympistis occata|[10471]|AWCLB538-11|United States|Arizona|658[0n]|BOLD:AAI1668
Sympistis occata|[10472]|LOCBF1018-13|United States|California|603[0n]|BOLD:AAI1668
Sympistis occata|[10473]|LOCBF1066-13|United States|California|561[0n]|BOLD:AAI1668
Sympistis occata|[10474]|LOCBF1132-13|United States|California|567[0n]|BOLD:AAI1668
Sympistis occata|[10475]|LOCBF3747-14|United States|California|564[0n]|BOLD:AAI1668
Sympistis occata|[10476]|AWCLB464-10|United States|Arizona|658[0n]|BOLD:AAI1668
Sympistis occata|[10477]|LOCBF344-13|United States|California|592[0n]|BOLD:AAI1668
Sympistis occata|[10478]|AWCLB664-11|United States|Arizona|658[0n]|BOLD:AAI1668
Sympistis occata|[10479]|AWCLB158-10|United States|Arizona|658[0n]|BOLD:AAI1668
Sympistis occata|[10480]|AWCLB124-10|United States|Arizona|658[0n]|BOLD:AAI1668
Sympistis occata|[10481]|AWCLB122-10|United States|Arizona|658[0n]|BOLD:AAI1668
Sympistis occata|[10482]|RDNMB201-05|United States|Washington|545[0n]|BOLD:AAI1668
Sympistis occata|[10483]|AWCLB110-10|United States|Arizona|658[0n]|BOLD:AAI1668
Sympistis occata|[10484]|RDNMB869-05|Canada|Alberta|658[0n]|BOLD:AAI1668
Sympistis occata|[10485]|AWCLB463-10|United States|Arizona|658[0n]|BOLD:AAI1668
Sympistis occata|[10486]|LOCBF1042-13|United States|California|588[0n]|BOLD:AAI1668
Sympistis occata|[10487]|CNCLB1666-14|United States|Nevada|658[0n]|BOLD:AAI1668
Sympistis semicollaris|[10488]|LOWCD347-06|Canada|British Columbia|590[0n]|BOLD:AAB6384
Sympistis semicollaris|[10489]|LOWCD348-06|Canada|British Columbia|587[0n]|BOLD:AAB6384
Sympistis semicollaris|[10490]|LOWCD352-06|Canada|British Columbia|658[0n]|BOLD:AAB6384
Sympistis semicollaris|[10491]|RDNM488-05|Canada|British Columbia|658[0n]|BOLD:AAB6384
Sympistis semicollaris|[10492]|RDNM487-05|United States|Oregon|658[0n]|BOLD:AAB6384
Sympistis semicollaris|[10493]|LOWCD359-06|Canada|British Columbia|574[0n]|BOLD:AAB6384
Sympistis semicollaris|[10494]|LOWCD353-06|Canada|British Columbia|582[0n]|BOLD:AAB6384
Sympistis semicollaris|[10495]|LOWCD356-06|Canada|British Columbia|563[0n]|BOLD:AAB6384
Sympistis semicollaris|[10496]|LOWCD355-06|Canada|British Columbia|565[0n]|BOLD:AAB6384
Sympistis semicollaris|[10497]|LOWCD354-06|Canada|British Columbia|616[0n]|BOLD:AAB6384
Sympistis semicollaris|[10498]|LOWCD349-06|Canada|British Columbia|600[0n]|BOLD:AAB6384
Sympistis semicollaris|[10499]|LOWCD345-06|Canada|British Columbia|615[0n]|BOLD:AAB6384
Sympistis semicollaris|[10500]|LOWCD360-06|Canada|British Columbia|592[0n]|BOLD:AAB6384
Sympistis semicollaris|[10501]|LOWCD367-06|Canada|British Columbia|581[0n]|BOLD:AAB6384
Sympistis semicollaris|[10502]|LOWCD357-06|Canada|British Columbia|556[0n]|BOLD:AAB6384
Sympistis semicollaris|[10503]|LOWCD363-06|Canada|British Columbia|612[0n]|BOLD:AAB6384
Sympistis semicollaris|[10504]|LOWCD350-06|Canada|British Columbia|604[0n]|BOLD:AAB6384
Sympistis semicollaris|[10505]|LOWCD366-06|Canada|British Columbia|585[0n]|BOLD:AAB6384
Sympistis semicollaris|[10506]|LOWCD368-06|Canada|British Columbia|600[0n]|BOLD:AAB6384
Sympistis semicollaris|[10507]|LBCH5610-10|Canada|British Columbia|658[0n]|BOLD:AAB6384
Sympistis semicollaris|[10508]|LBCH5705-10|Canada|British Columbia|658[0n]|BOLD:AAB6384
Sympistis semicollaris|[10509]|LBCH5815-10|Canada|British Columbia|658[0n]|BOLD:AAB6384
Sympistis semicollaris|[10510]|LBCH5816-10|Canada|British Columbia|658[0n]|BOLD:AAB6384
Sympistis semicollaris|[10511]|LBCH5817-10|Canada|British Columbia|658[0n]|BOLD:AAB6384
Sympistis semicollaris|[10512]|LALPA918-11|Canada|British Columbia|658[0n]|BOLD:AAB6384
Sympistis collaris|[10513]|RDNM501-05|United States|Texas|525[0n]|BOLD:ABX5588
Sympistis collaris|[10514]|CNCLB146-14|United States|Texas|658[0n]|BOLD:ABX5588
Sympistis ragani|[10515]|RDNM489-05|United States|Oregon|658[0n]|BOLD:AAB2084
Sympistis ragani|[10516]|LOCBF908-13|United States|California|576[0n]|BOLD:AAB2084
Sympistis ragani|[10517]|RDNM490-05|United States|California|538[0n]|BOLD:AAB2084
Sympistis ragani|[10518]|LOCBC047-06|United States|California|658[0n]|BOLD:AAB2084
Sympistis ragani|[10519]|LOCBB129-06|United States|California|658[0n]|BOLD:AAB2084
Sympistis ragani|[10520]|LOCBB127-06|United States|California|658[0n]|BOLD:AAB2084
Sympistis ragani|[10521]|LOCBB613-06|United States|California|656[0n]|BOLD:AAB2084
Sympistis ragani|[10522]|LOCBC199-06|United States|California|658[0n]|BOLD:AAB2084
Sympistis ragani|[10523]|LOCBC192-06|United States|California|658[0n]|BOLD:AAB2084
Sympistis ragani|[10524]|LOCBB611-06|United States|California|658[0n]|BOLD:AAB2084
Sympistis ragani|[10525]|LOCBB610-06|United States|California|658[0n]|BOLD:AAB2084
Sympistis ragani|[10526]|LOCBB128-06|United States|California|658[0n]|BOLD:AAB2084
Sympistis ragani|[10527]|LOCB489-06|United States|California|658[0n]|BOLD:AAB2084
Sympistis ragani|[10528]|LOCBE388-06|United States|California|594[0n]|BOLD:AAB2084
Sympistis ragani|[10529]|LOCBE389-06|United States|California|593[0n]|BOLD:AAB2084
Sympistis ragani|[10530]|LOCBD743-06|United States|California|663[0n]|BOLD:AAB2084
Sympistis ragani|[10531]|LOCB443-06|United States|California|658[0n]|BOLD:AAB2084
Sympistis ragani|[10532]|LOCB446-06|United States|California|658[0n]|BOLD:AAB2084
Sympistis ragani|[10533]|LOCB488-06|United States|California|658[0n]|BOLD:AAB2084
Sympistis ragani|[10534]|LOCBB130-06|United States|California|658[0n]|BOLD:AAB2084
Sympistis ragani|[10535]|LOCBB609-06|United States|California|658[0n]|BOLD:AAB2084
Sympistis ragani|[10536]|LOCBC193-06|United States|California|658[0n]|BOLD:AAB2084
Sympistis ragani|[10537]|LOCBC200-06|United States|California|658[0n]|BOLD:AAB2084
Sympistis ragani|[10538]|LOCBC233-06|United States|California|658[0n]|BOLD:AAB2084
Sympistis ragani|[10539]|LOCBD205-06|United States|California|658[0n]|BOLD:AAB2084
Sympistis ragani|[10540]|LOCBD207-06|United States|California|658[0n]|BOLD:AAB2084
Sympistis ragani|[10541]|LOCBD219-06|United States|California|658[0n]|BOLD:AAB2084
Sympistis ragani|[10542]|LOCBE083-06|United States|California|658[0n]|BOLD:AAB2084
[illegible]

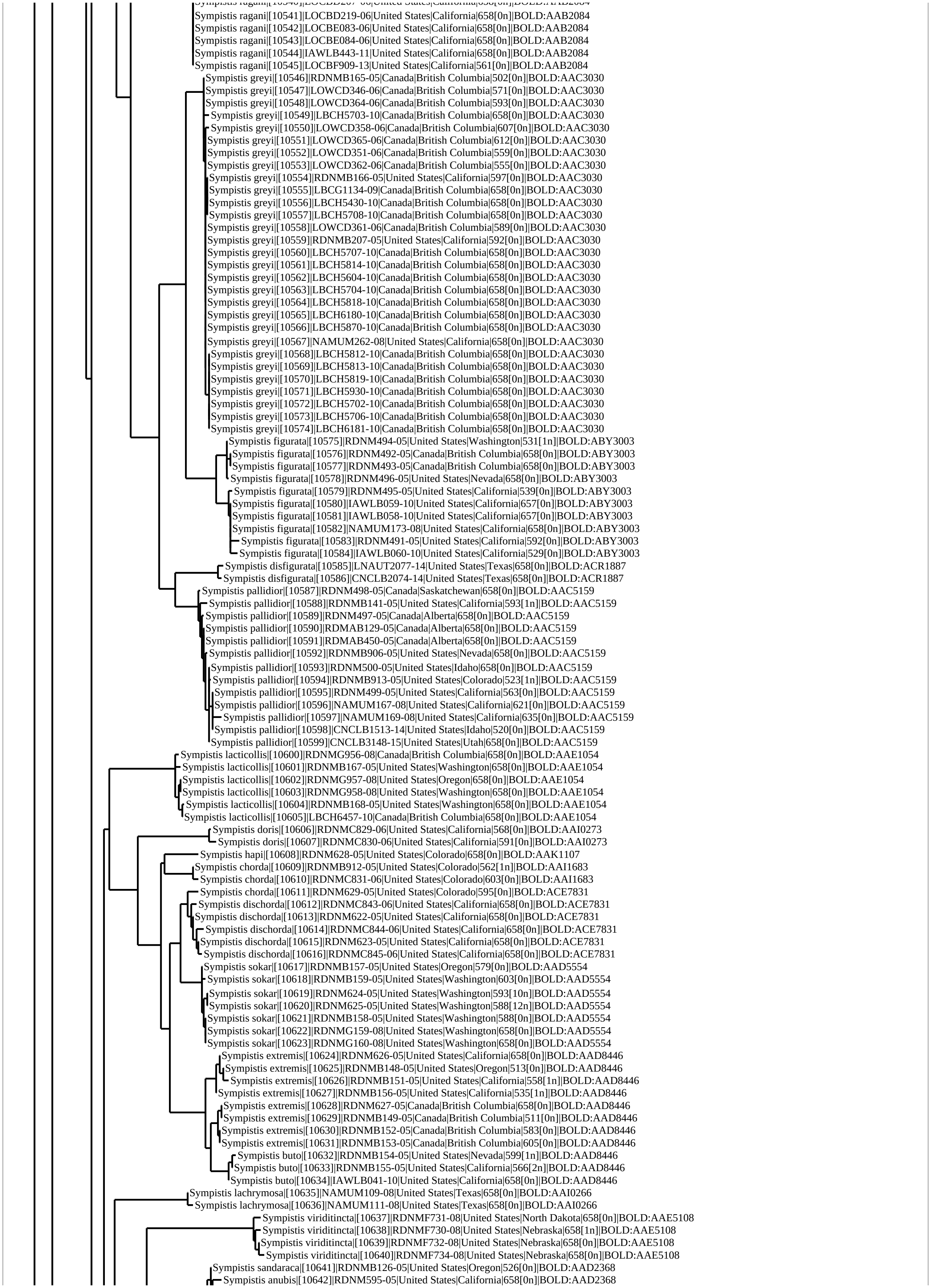
Sympistis ragani|[10540]|LOCBD207-06|United States|California|658[0n]|BOLD:AAB2084
Sympistis ragani|[10541]|LOCBD219-06|United States|California|658[0n]|BOLD:AAB2084
Sympistis ragani|[10542]|LOCBE083-06|United States|California|658[0n]|BOLD:AAB2084
Sympistis ragani|[10543]|LOCBE084-06|United States|California|658[0n]|BOLD:AAB2084
Sympistis ragani|[10544]|IAWLB443-11|United States|California|658[0n]|BOLD:AAB2084
Sympistis ragani|[10545]|LOCBF909-13|United States|California|561[0n]|BOLD:AAB2084
Sympistis greyi|[10546]|RDNMB165-05|Canada|British Columbia|502[0n]|BOLD:AAC3030
Sympistis greyi|[10547]|LOWCD346-06|Canada|British Columbia|571[0n]|BOLD:AAC3030
Sympistis greyi|[10548]|LOWCD364-06|Canada|British Columbia|593[0n]|BOLD:AAC3030
Sympistis greyi|[10549]|LBCH5703-10|Canada|British Columbia|658[0n]|BOLD:AAC3030
Sympistis greyi|[10550]|LOWCD358-06|Canada|British Columbia|607[0n]|BOLD:AAC3030
Sympistis greyi|[10551]|LOWCD365-06|Canada|British Columbia|612[0n]|BOLD:AAC3030
Sympistis greyi|[10552]|LOWCD351-06|Canada|British Columbia|559[0n]|BOLD:AAC3030
Sympistis greyi|[10553]|LOWCD362-06|Canada|British Columbia|555[0n]|BOLD:AAC3030
Sympistis greyi|[10554]|RDNMB166-05|United States|California|597[0n]|BOLD:AAC3030
Sympistis greyi|[10555]|LBCG1134-09|Canada|British Columbia|658[0n]|BOLD:AAC3030
Sympistis greyi|[10556]|LBCH5430-10|Canada|British Columbia|658[0n]|BOLD:AAC3030
Sympistis greyi|[10557]|LBCH5708-10|Canada|British Columbia|658[0n]|BOLD:AAC3030
Sympistis greyi|[10558]|LOWCD361-06|Canada|British Columbia|589[0n]|BOLD:AAC3030
Sympistis greyi|[10559]|RDNMB207-05|United States|California|592[0n]|BOLD:AAC3030
Sympistis greyi|[10560]|LBCH5707-10|Canada|British Columbia|658[0n]|BOLD:AAC3030
Sympistis greyi|[10561]|LBCH5814-10|Canada|British Columbia|658[0n]|BOLD:AAC3030
Sympistis greyi|[10562]|LBCH5604-10|Canada|British Columbia|658[0n]|BOLD:AAC3030
Sympistis greyi|[10563]|LBCH5704-10|Canada|British Columbia|658[0n]|BOLD:AAC3030
Sympistis greyi|[10564]|LBCH5818-10|Canada|British Columbia|658[0n]|BOLD:AAC3030
Sympistis greyi|[10565]|LBCH6180-10|Canada|British Columbia|658[0n]|BOLD:AAC3030
Sympistis greyi|[10566]|LBCH5870-10|Canada|British Columbia|658[0n]|BOLD:AAC3030
Sympistis greyi|[10567]|NAMUM262-08|United States|California|658[0n]|BOLD:AAC3030
Sympistis greyi|[10568]|LBCH5812-10|Canada|British Columbia|658[0n]|BOLD:AAC3030
Sympistis greyi|[10569]|LBCH5813-10|Canada|British Columbia|658[0n]|BOLD:AAC3030
Sympistis greyi|[10570]|LBCH5819-10|Canada|British Columbia|658[0n]|BOLD:AAC3030
Sympistis greyi|[10571]|LBCH5930-10|Canada|British Columbia|658[0n]|BOLD:AAC3030
Sympistis greyi|[10572]|LBCH5702-10|Canada|British Columbia|658[0n]|BOLD:AAC3030
Sympistis greyi|[10573]|LBCH5706-10|Canada|British Columbia|658[0n]|BOLD:AAC3030
Sympistis greyi|[10574]|LBCH6181-10|Canada|British Columbia|658[0n]|BOLD:AAC3030
Sympistis figurata|[10575]|RDNM494-05|United States|Washington|531[1n]|BOLD:ABY3003
Sympistis figurata|[10576]|RDNM492-05|Canada|British Columbia|658[0n]|BOLD:ABY3003
Sympistis figurata|[10577]|RDNM493-05|Canada|British Columbia|658[0n]|BOLD:ABY3003
Sympistis figurata|[10578]|RDNM496-05|United States|Nevada|658[0n]|BOLD:ABY3003
Sympistis figurata|[10579]|RDNM495-05|United States|California|539[0n]|BOLD:ABY3003
Sympistis figurata|[10580]|IAWLB059-10|United States|California|657[0n]|BOLD:ABY3003
Sympistis figurata|[10581]|IAWLB058-10|United States|California|657[0n]|BOLD:ABY3003
Sympistis figurata|[10582]|NAMUM173-08|United States|California|658[0n]|BOLD:ABY3003
Sympistis figurata|[10583]|RDNM491-05|United States|California|592[0n]|BOLD:ABY3003
Sympistis figurata|[10584]|IAWLB060-10|United States|California|529[0n]|BOLD:ABY3003
Sympistis disfigurata|[10585]|LNAUT2077-14|United States|Texas|658[0n]|BOLD:ACR1887
Sympistis disfigurata|[10586]|CNCLB2074-14|United States|Texas|658[0n]|BOLD:ACR1887
Sympistis pallidior|[10587]|RDNM498-05|Canada|Saskatchewan|658[0n]|BOLD:AAC5159
Sympistis pallidior|[10588]|RDNMB141-05|United States|California|593[1n]|BOLD:AAC5159
Sympistis pallidior|[10589]|RDNM497-05|Canada|Alberta|658[0n]|BOLD:AAC5159
Sympistis pallidior|[10590]|RDMAB129-05|Canada|Alberta|658[0n]|BOLD:AAC5159
Sympistis pallidior|[10591]|RDMAB450-05|Canada|Alberta|658[0n]|BOLD:AAC5159
Sympistis pallidior|[10592]|RDNMB906-05|United States|Nevada|658[0n]|BOLD:AAC5159
Sympistis pallidior|[10593]|RDNM500-05|United States|Idaho|658[0n]|BOLD:AAC5159
Sympistis pallidior|[10594]|RDNMB913-05|United States|Colorado|523[1n]|BOLD:AAC5159
Sympistis pallidior|[10595]|RDNM499-05|United States|California|563[0n]|BOLD:AAC5159
Sympistis pallidior|[10596]|NAMUM167-08|United States|California|621[0n]|BOLD:AAC5159
Sympistis pallidior|[10597]|NAMUM169-08|United States|California|635[0n]|BOLD:AAC5159
Sympistis pallidior|[10598]|CNCLB1513-14|United States|Idaho|520[0n]|BOLD:AAC5159
Sympistis pallidior|[10599]|CNCLB3148-15|United States|Utah|658[0n]|BOLD:AAC5159
Sympistis lacticollis|[10600]|RDNMG956-08|Canada|British Columbia|658[0n]|BOLD:AAE1054
Sympistis lacticollis|[10601]|RDNMB167-05|United States|Washington|658[0n]|BOLD:AAE1054
Sympistis lacticollis|[10602]|RDNMG957-08|United States|Oregon|658[0n]|BOLD:AAE1054
Sympistis lacticollis|[10603]|RDNMG958-08|United States|Washington|658[0n]|BOLD:AAE1054
Sympistis lacticollis|[10604]|RDNMB168-05|United States|Washington|658[0n]|BOLD:AAE1054
Sympistis lacticollis|[10605]|LBCH6457-10|Canada|British Columbia|658[0n]|BOLD:AAE1054
Sympistis doris|[10606]|RDNMC829-06|United States|California|568[0n]|BOLD:AAI0273
Sympistis doris|[10607]|RDNMC830-06|United States|California|591[0n]|BOLD:AAI0273
Sympistis hapi|[10608]|RDNM628-05|United States|Colorado|658[0n]|BOLD:AAK1107
Sympistis chorda|[10609]|RDNMB912-05|United States|Colorado|562[1n]|BOLD:AAI1683
Sympistis chorda|[10610]|RDNMC831-06|United States|Colorado|603[0n]|BOLD:AAI1683
Sympistis chorda|[10611]|RDNM629-05|United States|Colorado|595[0n]|BOLD:ACE7831
Sympistis dischorda|[10612]|RDNMC843-06|United States|California|658[0n]|BOLD:ACE7831
Sympistis dischorda|[10613]|RDNM622-05|United States|California|658[0n]|BOLD:ACE7831
Sympistis dischorda|[10614]|RDNMC844-06|United States|California|658[0n]|BOLD:ACE7831
Sympistis dischorda|[10615]|RDNM623-05|United States|California|658[0n]|BOLD:ACE7831
Sympistis dischorda|[10616]|RDNMC845-06|United States|California|658[0n]|BOLD:ACE7831
Sympistis sokar|[10617]|RDNMB157-05|United States|Oregon|579[0n]|BOLD:AAD5554
Sympistis sokar|[10618]|RDNMB159-05|United States|Washington|603[0n]|BOLD:AAD5554
Sympistis sokar|[10619]|RDNM624-05|United States|Washington|593[10n]|BOLD:AAD5554
Sympistis sokar|[10620]|RDNM625-05|United States|Washington|588[12n]|BOLD:AAD5554
Sympistis sokar|[10621]|RDNMB158-05|United States|Washington|588[0n]|BOLD:AAD5554
Sympistis sokar|[10622]|RDNMG159-08|United States|Washington|658[0n]|BOLD:AAD5554
Sympistis sokar|[10623]|RDNMG160-08|United States|Washington|658[0n]|BOLD:AAD5554
Sympistis extremis|[10624]|RDNM626-05|United States|California|658[0n]|BOLD:AAD8446
Sympistis extremis|[10625]|RDNMB148-05|United States|Oregon|513[0n]|BOLD:AAD8446
Sympistis extremis|[10626]|RDNMB151-05|United States|California|558[1n]|BOLD:AAD8446
Sympistis extremis|[10627]|RDNMB156-05|United States|California|535[1n]|BOLD:AAD8446
Sympistis extremis|[10628]|RDNM627-05|Canada|British Columbia|658[0n]|BOLD:AAD8446
Sympistis extremis|[10629]|RDNMB149-05|Canada|British Columbia|511[0n]|BOLD:AAD8446
Sympistis extremis|[10630]|RDNMB152-05|Canada|British Columbia|583[0n]|BOLD:AAD8446
Sympistis extremis|[10631]|RDNMB153-05|Canada|British Columbia|605[0n]|BOLD:AAD8446
Sympistis buto|[10632]|RDNMB154-05|United States|Nevada|599[1n]|BOLD:AAD8446
Sympistis buto|[10633]|RDNMB155-05|United States|California|566[2n]|BOLD:AAD8446
Sympistis buto|[10634]|IAWLB041-10|United States|California|658[0n]|BOLD:AAD8446
Sympistis lachrymosa|[10635]|NAMUM109-08|United States|Texas|658[0n]|BOLD:AAI0266
Sympistis lachrymosa|[10636]|NAMUM111-08|United States|Texas|658[0n]|BOLD:AAI0266
Sympistis viriditincta|[10637]|RDNMF731-08|United States|North Dakota|658[0n]|BOLD:AAE5108
Sympistis viriditincta|[10638]|RDNMF730-08|United States|Nebraska|658[1n]|BOLD:AAE5108
Sympistis viriditincta|[10639]|RDNMF732-08|United States|Nebraska|658[0n]|BOLD:AAE5108
Sympistis viriditincta|[10640]|RDNMF734-08|United States|Nebraska|658[0n]|BOLD:AAE5108
Sympistis sandaraca|[10641]|RDNMB126-05|United States|Oregon|526[0n]|BOLD:AAD2368
Sympistis anubis|[10642]|RDNM595-05|United States|California|658[0n]|BOLD:AAD2368

Sympistis viriditincta|[10640]|RDNMF734-08|United States|Nebraska|658[0n]|BOLD:AAE5108
Sympistis sandaraca|[10641]|RDNMB126-05|United States|Oregon|526[0n]|BOLD:AAD2368
Sympistis anubis|[10642]|RDNM595-05|United States|California|658[0n]|BOLD:AAD2368
Sympistis sandaraca|[10643]|RDNM597-05|United States|Oregon|658[0n]|BOLD:AAD2368
Sympistis sandaraca|[10644]|RDNMB893-05|United States|Washington|658[0n]|BOLD:AAD2368
Sympistis sandaraca|[10645]|RDNMB125-05|United States|Oregon|573[0n]|BOLD:AAD2368
Sympistis sandaraca|[10646]|RDNM596-05|United States|Washington|586[0n]|BOLD:AAD2368
Sympistis sandaraca|[10647]|RDNMB127-05|United States|California|658[0n]|BOLD:AAD2368
Sympistis sandaraca|[10648]|RDNM598-05|Canada|British Columbia|639[0n]|BOLD:AAD2368
Sympistis sandaraca|[10649]|RDNM600-05|United States|Washington|658[0n]|BOLD:AAD2368
Sympistis sandaraca|[10650]|RDNM599-05|United States|Washington|658[0n]|BOLD:AAD2368
Sympistis sandaraca|[10651]|RDNMB129-05|United States|Washington|593[0n]|BOLD:AAD2368
Sympistis sandaraca|[10652]|RDNMB128-05|United States|Washington|599[0n]|BOLD:AAD2368
Sympistis sandaraca|[10653]|RDNMB130-05|United States|California|582[0n]|BOLD:AAD2368
Sympistis sandaraca|[10654]|RDNMB915-05|United States|Washington|658[0n]|BOLD:AAD2368
Sympistis hayesi|[10655]|RDNMB132-05|United States|Colorado|549[0n]|BOLD:ACE7771
Sympistis hayesi|[10656]|RDNM518-05|United States|Utah|658[0n]|BOLD:ACE7771
Sympistis hayesi|[10657]|RDNMB894-05|United States|Colorado|658[0n]|BOLD:ACE7771
Sympistis melantho|[10658]|RDNMB123-05|United States|California|549[0n]|BOLD:ACE7771
Sympistis pudorata|[10659]|RDNMB124-05|Canada|British Columbia|570[1n]|BOLD:ACE7771
Sympistis pudorata|[10660]|RDMAB1036-09|Canada|British Columbia|658[0n]|BOLD:ACE7771
Sympistis pudorata|[10661]|RDMAB1037-09|Canada|British Columbia|658[0n]|BOLD:ACE7771
Sympistis pudorata|[10662]|RDMAB1038-09|Canada|British Columbia|658[0n]|BOLD:ACE7771
Sympistis sesmu|[10663]|RDNMB163-05|United States|Washington|597[0n]|BOLD:AAI0283
Sympistis sesmu|[10664]|RDNMB164-05|United States|Washington|604[0n]|BOLD:AAI0283
Sympistis sanina|[10665]|RDNM634-05|United States|Colorado|525[0n]|BOLD:AAD2911
Sympistis sanina|[10666]|RDNM647-05|United States|Colorado|515[1n]|BOLD:AAD2911
Sympistis sanina|[10667]|RDNMB558-05|United States|Colorado|506[1n]|BOLD:AAD2911
Sympistis sanina|[10668]|RDNM533-05|United States|New Mexico|658[0n]|BOLD:AAD2911
Sympistis sanina|[10669]|RDNM532-05|United States|New Mexico|658[0n]|BOLD:AAD2911
Sympistis insanina|[10670]|RDNM615-05|Canada|Alberta|658[0n]|BOLD:AAD2911
Sympistis insanina|[10671]|RDNM616-05|Canada|Alberta|626[0n]|BOLD:AAD2911
Sympistis insanina|[10672]|RDNM617-05|Canada|Saskatchewan|658[0n]|BOLD:AAD2911
Sympistis sanina|[10673]|RDNMB559-05|United States|New Mexico|658[0n]|BOLD:AAD2911
Sympistis sanina|[10674]|RDNM648-05|United States|New Mexico|658[0n]|BOLD:AAD2911
Sympistis sanina|[10675]|RDNMB560-05|United States|New Mexico|658[0n]|BOLD:AAD2911
Sympistis sanina|[10676]|RDNMB561-05|United States|New Mexico|658[0n]|BOLD:AAD2911
Sympistis sanina|[10677]|RDNMB176-05|United States|Wyoming|658[0n]|BOLD:AAD2911
Sympistis sanina|[10678]|RDNMB177-05|United States|Wyoming|592[0n]|BOLD:AAD2911
Sympistis sanina|[10679]|RDNM610-05|United States|Wyoming|658[0n]|BOLD:AAD2911
Sympistis sanina|[10680]|RDNMB178-05|United States|Wyoming|599[0n]|BOLD:AAD2911
Sympistis sanina|[10681]|RDNMG163-08|United States|Utah|658[0n]|BOLD:AAD2911
Sympistis augustus|[10682]|RDNMB137-05|United States|Washington|548[0n]|BOLD:AAE1134
Sympistis augustus|[10683]|RDNMB138-05|United States|Washington|581[0n]|BOLD:AAE1134
Sympistis augustus|[10684]|RDNMB632-05|Canada|Alberta|658[0n]|BOLD:AAE1134
Sympistis augustus|[10685]|RDNMB868-05|Canada|Alberta|658[0n]|BOLD:AAE1134
Sympistis augustus|[10686]|IAWLB503-11|United States|Arizona|658[0n]|BOLD:AAE1134
Sympistis augustus|[10687]|RDNMB557-05|United States|Texas|557[0n]|BOLD:AAE1134
Sympistis augustus|[10688]|AWCLB481-11|United States|Arizona|658[0n]|BOLD:AAE1134
Sympistis augustus|[10689]|IAWLB504-11|United States|Arizona|658[0n]|BOLD:AAE1134
Sympistis cocytus|[10690]|RDNMC805-06|United States|Washington|538[2n]|BOLD:ABZ6408
Sympistis cocytus|[10691]|RDNMC787-06|United States|Washington|620[0n]|BOLD:ABZ6408
Sympistis cocytus|[10692]|RDNMC788-06|United States|Washington|604[0n]|BOLD:ABZ6408
Sympistis cocytus|[10693]|RDNMC803-06|United States|Oregon|604[0n]|BOLD:ABZ6408
Sympistis cocytus|[10694]|RDNMC813-06|Canada|British Columbia|557[2n]|BOLD:ABZ6408
Sympistis cocytus|[10695]|RDNMC811-06|Canada|British Columbia|563[0n]|BOLD:ABZ6408
Sympistis cocytus|[10696]|RDNMC812-06|Canada|British Columbia|564[0n]|BOLD:ABZ6408
Sympistis cocytus|[10697]|RDNMB206-05|Canada|British Columbia|569[0n]|BOLD:ABZ6408
Sympistis cocytus|[10698]|RDNMC090-05|United States|Oregon|549[2n]|BOLD:ABZ6408
Sympistis cocytus|[10699]|RDNMC810-06|Canada|British Columbia|574[0n]|BOLD:ABZ6408
Sympistis cocytus|[10700]|RDNMC814-06|Canada|British Columbia|621[0n]|BOLD:ABZ6408
Sympistis cocytus|[10701]|RDNMC821-06|United States|Washington|597[0n]|BOLD:ABZ6408
Sympistis cocytus|[10702]|LBCH7217-10|Canada|British Columbia|658[0n]|BOLD:ABZ6408
Sympistis acheron|[10703]|RDNMC822-06|United States|California|542[0n]|BOLD:AAB6137
Sympistis acheron|[10704]|RDNMC794-06|United States|Oregon|532[0n]|BOLD:AAB6137
Sympistis acheron|[10705]|RDNMC816-06|United States|Washington|595[1n]|BOLD:AAB6137
Sympistis acheron|[10706]|RDNMC802-06|United States|Oregon|604[0n]|BOLD:AAB6137
Sympistis acheron|[10707]|RDNMC801-06|United States|Oregon|600[0n]|BOLD:AAB6137
Sympistis acheron|[10708]|RDNMC809-06|Canada|British Columbia|604[1n]|BOLD:AAB6137
Sympistis acheron|[10709]|RDNMC820-06|United States|Washington|600[1n]|BOLD:AAB6137
Sympistis acheron|[10710]|RDNMC823-06|United States|California|578[0n]|BOLD:AAB6137
Sympistis acheron|[10711]|RDNMC818-06|United States|Washington|568[0n]|BOLD:AAB6137
Sympistis acheron|[10712]|RDNMC798-06|United States|California|598[1n]|BOLD:AAB6137
Sympistis acheron|[10713]|RDNMC796-06|United States|California|572[0n]|BOLD:AAB6137
Sympistis acheron|[10714]|RDNMB205-05|United States|Washington|600[0n]|BOLD:AAB6137
Sympistis acheron|[10715]|RDNMC817-06|United States|Washington|597[2n]|BOLD:AAB6137
Sympistis acheron|[10716]|RDNMC799-06|United States|California|604[0n]|BOLD:AAB6137
Sympistis acheron|[10717]|RDNMC815-06|United States|Washington|604[1n]|BOLD:AAB6137
Sympistis acheron|[10718]|RDNMC800-06|United States|California|609[0n]|BOLD:AAB6137
Sympistis acheron|[10719]|RDNMB203-05|United States|California|592[0n]|BOLD:AAB6137
Sympistis acheron|[10720]|RDNMC795-06|United States|Oregon|616[0n]|BOLD:AAB6137
Sympistis acheron|[10721]|RDNMC797-06|United States|California|620[0n]|BOLD:AAB6137
Sympistis acheron|[10722]|RDNMC819-06|United States|Washington|592[0n]|BOLD:AAB6137
Sympistis acheron|[10723]|IAWLB100-10|United States|California|658[0n]|BOLD:AAB6137
Sympistis acheron|[10724]|IAWLB101-10|United States|California|658[0n]|BOLD:AAB6137
Sympistis jocelynae|[10725]|CNCLB161-14|United States|Utah|658[0n]|BOLD:ACM3911
Sympistis incubus|[10726]|RDNM606-05|United States|Washington|658[0n]|BOLD:ABZ0891
Sympistis incubus|[10727]|RDNM607-05|United States|Washington|658[0n]|BOLD:ABZ0891
Sympistis levis|[10728]|RDNM609-05|United States|Wyoming|658[0n]|BOLD:AAE1053
Sympistis levis|[10729]|RDNM646-05|United States|Colorado|593[0n]|BOLD:AAE1053
Sympistis levis|[10730]|RDNM608-05|United States|Wyoming|658[0n]|BOLD:AAE1053
Sympistis levis|[10731]|RDNM645-05|United States|Wyoming|658[0n]|BOLD:AAE1053
Sympistis levis|[10732]|RDNMB870-05|Canada|Alberta|658[0n]|BOLD:AAE1053
Sympistis levis|[10733]|RDNMG161-08|United States|Utah|658[0n]|BOLD:AAE1053
Sympistis seth|[10734]|RDNM612-05|United States|Oregon|658[0n]|BOLD:ACF4579
Sympistis seth|[10735]|RDNM613-05|United States|Oregon|658[0n]|BOLD:ACF4579
Sympistis sp.|[10736]|RDNMG162-08|United States|Utah|658[0n]|BOLD:ACF4579
Sympistis duplex|[10737]|RDNM604-05|United States|California|497[0n]|
Sympistis duplex|[10738]|LNAUT1123-14|United States|California|658[0n]|BOLD:ACF4579
Sympistis duplex|[10739]|RDNM603-05|United States|California|581[0n]|BOLD:ACF4579
Sympistis duplex|[10740]|LNAUT1121-14|United States|California|658[0n]|BOLD:ACF4579
Sympistis duplex|[10741]|LNAUT1122-14|United States|California|658[0n]|BOLD:ACF4579
[illegible]

Sympistis duplex|[10739]|RDNM603-05|United States|California|581[0n]|BOLD:ACF4579
Sympistis duplex|[10740]|LNAUT1121-14|United States|California|658[0n]|BOLD:ACF4579
Sympistis duplex|[10741]|LNAUT1122-14|United States|California|658[0n]|BOLD:ACF4579
Sympistis duplex|[10742]|LNAUT1124-14|United States|California|658[0n]|BOLD:ACF4579
Sympistis simplex|[10743]|RDNM605-05|United States|Washington|658[0n]|BOLD:AAI0281
Sympistis simplex|[10744]|LNAUS2810-13|United States|Washington|658[0n]|BOLD:AAI0281
Sympistis simplex|[10745]|LNAUS2811-13|United States|Washington|658[0n]|BOLD:AAI0281
Sympistis simplex|[10746]|LNAUS2812-13|United States|Washington|658[0n]|BOLD:AAI0281
Sympistis simplex|[10747]|LNAUS3193-13|United States|Washington|658[0n]|BOLD:AAI0281
Sympistis simplex|[10748]|LNAUS3194-13|United States|Washington|658[0n]|BOLD:AAI0281
Sympistis benjamini|[10749]|NAMUM175-08|United States|California|647[0n]|BOLD:AAX0340
Sympistis linda|[10750]|TML116-14|United States||658[0n]|BOLD:ACP1354
Sympistis linda|[10751]|CNCLB1684-14|United States|Arizona|658[0n]|BOLD:ACP1354
Sympistis iricolor|[10752]|RDNM512-05|United States|Arizona|658[0n]|BOLD:AAE5105
Sympistis iricolor|[10753]|RDNM511-05|United States|Montana|658[0n]|BOLD:AAE5105
Sympistis iricolor|[10754]|RDNM513-05|United States|Montana|658[0n]|BOLD:AAE5105
Sympistis iricolor|[10755]|RDNM649-05|United States|Oregon|658[0n]|BOLD:AAE5105
Sympistis iricolor|[10756]|RDNMB142-05|United States|Oregon|658[0n]|BOLD:AAE5105
Sympistis glennyi|[10757]|RDNMC804-06|United States|California|501[0n]|
Sympistis glennyi|[10758]|RDNMC826-06|Canada|British Columbia|501[0n]|
Sympistis glennyi|[10759]|RDNMC793-06|United States|Washington|533[0n]|BOLD:AAD1770
Sympistis glennyi|[10760]|RDNMB204-05|United States|Washington|658[0n]|BOLD:AAD1770
Sympistis glennyi|[10761]|RDNMC790-06|United States|Washington|564[0n]|BOLD:AAD1770
Sympistis glennyi|[10762]|RDNMC827-06|Canada|British Columbia|598[0n]|BOLD:AAD1770
Sympistis glennyi|[10763]|RDNMC807-06|Canada|British Columbia|598[0n]|BOLD:AAD1770
Sympistis glennyi|[10764]|RDNMC791-06|United States|Washington|598[0n]|BOLD:AAD1770
Sympistis glennyi|[10765]|RDNMC806-06|Canada|British Columbia|608[0n]|BOLD:AAD1770
Sympistis glennyi|[10766]|RDNMC792-06|United States|Washington|563[0n]|BOLD:AAD1770
Sympistis glennyi|[10767]|RDNMC824-06|Canada|British Columbia|560[1n]|BOLD:AAD1770
Sympistis glennyi|[10768]|RDNMC808-06|Canada|British Columbia|610[0n]|BOLD:AAD1770
Sympistis glennyi|[10769]|RDNMC825-06|Canada|British Columbia|560[0n]|BOLD:AAD1770
Sympistis glennyi|[10770]|RDNMC828-06|Canada|British Columbia|608[0n]|BOLD:AAD1770
Sympistis lepipoloides|[10771]|RDNMG914-08|Canada|Saskatchewan|588[1n]|BOLD:ACF5739
Sympistis lepipoloides|[10772]|RDNMF735-08|Canada|Saskatchewan|658[0n]|BOLD:ACF5739
Sympistis lepipoloides|[10773]|RDNMG915-08|Canada|Saskatchewan|587[0n]|BOLD:ACF5739
Sympistis sakhmet|[10774]|RDNM517-05|United States|New Mexico|658[0n]|BOLD:AAF3060
Sympistis sakhmet|[10775]|RDNM516-05|United States|New Mexico|658[0n]|BOLD:AAF3060
Sympistis sakhmet|[10776]|RDNM515-05|United States|New Mexico|658[0n]|BOLD:AAF3060
Sympistis sakhmet|[10777]|RDNM514-05|United States|New Mexico|658[0n]|BOLD:AAF3060
Sympistis ptah|[10778]|CNCLB155-14|United States|New Mexico|658[0n]|BOLD:AAF3060
Sympistis baloghi|[10779]|RDNM534-05|United States|New Mexico|658[0n]|BOLD:AAK1077
Sympistis tartarea|[10780]|RDNM614-05|United States|Oregon|658[0n]|BOLD:AAK1077
Sympistis osiris|[10781]|CNCLB156-14|United States|Utah|658[0n]|BOLD:ACM4031
Sympistis isis|[10782]|CNCLB162-14|United States|Utah|658[0n]|BOLD:ACM4031
Sympistis isis|[10783]|CNCLB163-14|United States|Utah|658[0n]|BOLD:ACM4031
Sympistis osiris|[10784]|RDNM505-05|United States|Texas|490[0n]|
Sympistis horus|[10785]|CNCLB1042-14|United States|New Mexico|658[0n]|BOLD:ACM4186
Sympistis jenniferae|[10786]|CNCLB1044-14|United States|New Mexico|658[0n]|BOLD:ACM4032
Sympistis basifugens|[10787]|RDNME849-08|||658[0n]|BOLD:AAH5682
Sympistis basifugens|[10788]|CMAZA285-09|United States|Arizona|658[0n]|BOLD:AAH5682
Sympistis curvicollis|[10789]|CNCLB147-14|United States|New Mexico|658[0n]|BOLD:ACM4083
Sympistis curvicollis|[10790]|CNCLB148-14|United States|New Mexico|658[0n]|BOLD:ACM4083
Sympistis poliochroa|[10791]|LOWCC198-05|Canada|British Columbia|591[1n]|BOLD:AAE1057
Sympistis poliochroa|[10792]|RDNMG961-08|United States|California|658[0n]|BOLD:AAE1057
Sympistis poliochroa|[10793]|RDNMB191-05|United States|Washington|595[0n]|BOLD:AAE1057
Sympistis poliochroa|[10794]|RDNMG960-08|Canada|British Columbia|658[0n]|BOLD:AAE1057
Sympistis poliochroa|[10795]|LBCH7224-10|Canada|British Columbia|658[0n]|BOLD:AAE1057
Sympistis poliochroa|[10796]|RDNMG959-08|United States|California|642[0n]|BOLD:ACE3576
Sympistis poliochroa|[10797]|LBCH7675-10|Canada|British Columbia|658[0n]|BOLD:ACE3576
Sympistis mirificalis|[10798]|RDNM506-05|United States|Washington|658[0n]|BOLD:ABY4268
Sympistis mirificalis|[10799]|RDNM507-05|United States|Washington|658[0n]|BOLD:ABY4268
Sympistis euta|[10800]|RDNM508-05|United States|California|658[0n]|BOLD:ABY4269
Sympistis euta|[10801]|RDNM509-05|United States|California|658[0n]|BOLD:ABY4269
Sympistis regina|[10802]|RDNMB133-05|United States|California|551[0n]|BOLD:ACE3022
Sympistis regina|[10803]|RDNMB134-05|United States|Oregon|590[0n]|BOLD:ACE3022
Sympistis regina|[10804]|RDNMB135-05|United States|Oregon|590[0n]|BOLD:ACE3022
Sympistis regina|[10805]|RDNM642-05|United States|Colorado|574[1n]|BOLD:ACF4288
Sympistis regina|[10806]|RDNMB136-05|United States|Colorado|581[0n]|BOLD:ACF4288
Sympistis regina|[10807]|RDNM641-05|United States|Colorado|549[0n]|BOLD:ACE3022
Sympistis regina|[10808]|RDNMB871-05|Canada|Alberta|658[0n]|BOLD:ACE3022
Sympistis regina|[10809]|RDNMB872-05|Canada|Alberta|658[0n]|BOLD:ACE3022
Sympistis cibalis|[10810]|RDNMB160-05|Canada|British Columbia|583[0n]|BOLD:AAC6246
Sympistis cibalis|[10811]|LOWCB485-05|Canada|British Columbia|589[0n]|BOLD:AAC6246
Sympistis cibalis|[10812]|LOWCC194-05|Canada|British Columbia|591[0n]|BOLD:AAC6246
Sympistis cibalis|[10813]|LOWCB488-05|Canada|British Columbia|658[0n]|BOLD:AAC6246
Sympistis cibalis|[10814]|RDNMB161-05|Canada|Alberta|598[0n]|BOLD:AAC6246
Sympistis cibalis|[10815]|LOWCB486-05|Canada|British Columbia|658[0n]|BOLD:AAC6246
Sympistis cibalis|[10816]|LOWCB484-05|Canada|British Columbia|615[0n]|BOLD:AAC6246
Sympistis cibalis|[10817]|LOWCB494-05|Canada|British Columbia|617[0n]|BOLD:AAC6246
Sympistis cibalis|[10818]|RDMAB451-05|Canada|Alberta|658[0n]|BOLD:AAC6246
Sympistis amenthes|[10819]|RDNMB162-05|United States|Washington|595[0n]|BOLD:ABY4270
Sympistis amenthes|[10820]|RDNMK769-12|United States|Colorado|658[0n]|BOLD:ABY4270
Sympistis amenthes|[10821]|MNAE875-13|United States|Utah|658[0n]|BOLD:ABY4270
Sympistis amenthes|[10822]|MNAE876-13|United States|Utah|658[0n]|BOLD:ABY4270
Sympistis utahensis|[10823]|RDNM510-05|United States|Colorado|507[0n]|BOLD:ACE9113
Sympistis mackiei|[10824]|RDNMB199-05|Canada|Alberta|590[1n]|BOLD:AAF3800
Sympistis mackiei|[10825]|RDNMB198-05|Canada|Alberta|598[0n]|BOLD:AAF3800
Sympistis mackiei|[10826]|RDNMB200-05|Canada|Alberta|597[1n]|BOLD:AAF3800
Sympistis chandleri|[10827]|RDNM632-05|United States|Colorado|562[0n]|BOLD:ACE9112
Sympistis chandleri|[10828]|RDNM633-05|United States|Colorado|516[0n]|BOLD:ACE9112
Sympistis extranea|[10829]|CNCLB1514-14|United States|Idaho|617[0n]|BOLD:ACM4693
Sympistis extranea|[10830]|CNCLB152-14|United States|Colorado|658[0n]|BOLD:ACM4693
Sympistis extranea|[10831]|CNCLB1685-14|United States|Utah|658[0n]|BOLD:ACM4693
Sympistis ibapahensis|[10832]|CNCLB1688-14|United States|Utah|658[0n]|BOLD:ACP4387
Sympistis pachet|[10833]|NAMUM139-08|United States|California|656[0n]|BOLD:AAI1669
Sympistis pachet|[10834]|CNCLB1674-14|United States|Utah|658[0n]|BOLD:AAI1669
Sympistis pachet|[10835]|LNAUT2603-14|United States||658[0n]|BOLD:AAI1669
Sympistis obscurata|[10836]|CNCLB3449-15|United States|New Mexico|658[0n]|BOLD:ACY5897
Sympistis obscurata|[10837]|CNCLB3450-15|United States|New Mexico|658[0n]|BOLD:ACY5897
Ozarba catilina|[10838]|CNCLB2430-14|United States|Texas|658[0n]|BOLD:AAK2161
Litoprosopus bahamensis|[10839]|CNCLB995-14|United States|Florida|658[0n]|BOLD:ACM4287
Litoprosopus bahamensis|[10840]|CNCLB822-14|United States|Florida|658[0n]|BOLD:ACM4287
Litoprosopus bahamensis|[10841]|CNCLB823-14|United States|Florida|658[0n]|BOLD:ACM4287

Litoprosopus bahamensis|[10839]|CNCLB995-14|United States|Florida|658[0n]|BOLD:ACM4287
Litoprosopus bahamensis|[10840]|CNCLB822-14|United States|Florida|658[0n]|BOLD:ACM4287
Litoprosopus bahamensis|[10841]|CNCLB823-14|United States|Florida|658[0n]|BOLD:ACM4287
Litoprosopus bahamensis|[10842]|CNCLB824-14|United States|Florida|658[0n]|BOLD:ACM4287
Litoprosopus bahamensis|[10843]|CNCLB825-14|United States|Florida|658[0n]|BOLD:ACM4287
Litoprosopus bahamensis|[10844]|CNCLB996-14|United States|Florida|658[0n]|BOLD:ACM4287
Litoprosopus configens|[10845]|BLPCD663-08|Costa Rica|Guanacaste|658[0n]|BOLD:AAE0783
Litoprosopus configens|[10846]|BLPDE117-09|Costa Rica|Guanacaste|658[0n]|BOLD:AAE0783
Litoprosopus configens|[10847]|RDNMJ662-11|Guatemala|Jalapa|658[0n]|BOLD:AAE0783
Litoprosopus configens|[10848]|QUNOD163-10|United States|Texas|658[0n]|BOLD:AAE0783
Litoprosopus configens|[10849]|BLPCD346-08|Costa Rica|Guanacaste|658[0n]|BOLD:AAE0783
Litoprosopus configens|[10850]|LOCRA555-07|Costa Rica|Alajuela|658[0n]|BOLD:AAE0783
Litoprosopus configens|[10851]|LOCRA554-07|Costa Rica|Alajuela|658[0n]|BOLD:AAE0783
Litoprosopus configens|[10852]|BLPCD662-08|Costa Rica|Guanacaste|658[0n]|BOLD:AAE0783
Litoprosopus configens|[10853]|MOTAR085-12|Argentina|Formosa|646[0n]|BOLD:AAE0783
Litoprosopus coachella|[10854]|IAWL010-09|United States|Arizona|658[0n]|BOLD:AAI1076
Litoprosopus coachella|[10855]|QUNOD169-10|United States|Arizona|658[0n]|BOLD:AAI1076
Litoprosopus coachella|[10856]|BBLOC1639-11|United States|California|658[0n]|BOLD:AAI1076
Litoprosopus futilis|[10857]|HKONB017-08|United States|Texas|658[0n]|BOLD:AAE0785
Litoprosopus futilis|[10858]|RDNME376-07|United States|Florida|587[0n]|BOLD:AAE0785
Litoprosopus futilis|[10859]|LSEU033-06|United States|Florida|526[0n]|BOLD:AAE0785
Litoprosopus futilis|[10860]|HKONS037-07|United States|Florida|658[0n]|BOLD:AAE0785
Litoprosopus futilis|[10861]|USLEP569-10|United States|Florida|658[0n]|BOLD:AAE0785
Litoprosopus futilis|[10862]|CNCLB1613-14|United States|Florida|658[0n]|BOLD:AAE0785
Litoprosopus sp.|[10863]|CNCLB826-14|United States|Florida|658[0n]|BOLD:ACM4126
Litoprosopus sp.|[10864]|CNCLB1753-14|United States|Florida|658[0n]|BOLD:ACM4126
Hemigrotella argenteostriata|[10865]|CNCLB943-14|United States|California|658[0n]|BOLD:ACK7012
Crambodes talidiformis|[10866]|BLTIB064-08|Canada|Ontario|606[6n]|
Crambodes talidiformis|[10867]|BBLSY169-09|United States|Texas|658[0n]|BOLD:AAD0878
Crambodes talidiformis|[10868]|LSUSA207-06|United States|Kentucky|656[0n]|BOLD:AAD0878
Crambodes talidiformis|[10869]|BBLSX257-09|United States|Texas|658[0n]|BOLD:AAD0878
Crambodes talidiformis|[10870]|BBLSX757-09|United States|Oklahoma|633[0n]|BOLD:AAD0878
Crambodes talidiformis|[10871]|BBLCU095-09|United States|Kansas|658[0n]|BOLD:AAD0878
Crambodes talidiformis|[10872]|BBLSX216-09|United States|Texas|658[0n]|BOLD:AAD0878
Crambodes talidiformis|[10873]|LPOKD320-09|United States|Oklahoma|658[0n]|BOLD:AAD0878
Crambodes talidiformis|[10874]|RDNMJ545-11|United States|Arizona|658[0n]|BOLD:AAD0878
Crambodes talidiformis|[10875]|RDNMJ839-11|United States|Arizona|658[0n]|BOLD:AAD0878
Crambodes talidiformis|[10876]|HKONS536-08|United States|Florida|609[0n]|BOLD:AAD0878
Crambodes talidiformis|[10877]|XAC702-04|Canada|Ontario|658[0n]|BOLD:AAD0878
Crambodes talidiformis|[10878]|LSEU774-06|United States|Missouri|658[0n]|BOLD:AAD0878
Crambodes talidiformis|[10879]|LPOKE086-10|United States|Oklahoma|658[0n]|BOLD:AAD0878
Crambodes talidiformis|[10880]|XAC158-04|Canada|Ontario|658[0n]|BOLD:AAD0878
Crambodes talidiformis|[10881]|RDLQF886-06|Canada|Quebec|611[0n]|BOLD:AAD0878
Crambodes talidiformis|[10882]|LSEU775-06|United States|Missouri|658[0n]|BOLD:AAD0878
Crambodes talidiformis|[10883]|UDLEP160-09|United States|Pennsylvania|658[0n]|BOLD:AAD0878
Crambodes talidiformis|[10884]|CNCLB2811-14|United States|North Carolina|658[0n]|BOLD:AAD0878
Crambodes talidiformis|[10885]|CNCLB2814-14|United States|North Carolina|658[0n]|BOLD:AAD0878
Podagra crassipes|[10886]|CNCLB1064-14|United States|California|658[0n]|BOLD:ACM6653
Podagra crassipes|[10887]|CNCLB1068-14|United States|California|658[0n]|BOLD:ACM6653
Podagra crassipes|[10888]|TML150-14|United States||658[0n]|BOLD:ACM6653
Grotella stretchi|[10889]|LNAUS4113-13|United States|California|658[0n]|BOLD:ACJ8871
Grotella stretchi|[10890]|LNAUS4114-13|United States|California|607[0n]|BOLD:ACJ8871
Grotella vagans|[10891]|CNCLB3102-15|United States|Arizona|658[0n]|BOLD:ACJ8871
Grotellaforma lactea|[10892]|RDNMC746-06|United States|California|658[0n]|BOLD:AAH4891
Grotellaforma lactea|[10893]|IAWL880-09|United States|Arizona|658[0n]|BOLD:AAH4891
Grotellaforma lactea|[10894]|BBLSW210-09|United States|Arizona|658[0n]|BOLD:AAH4891
Grotella blanchardi|[10895]|LNAUT812-14|United States|New Mexico|658[0n]|BOLD:AAL3475
Grotella blanchardi|[10896]|LNAUT813-14|United States|New Mexico|658[0n]|BOLD:AAL3475
Grotella blanchardi|[10897]|LNAUT814-14|United States|New Mexico|658[0n]|BOLD:AAL3475
Grotella blanchardi|[10898]|QUNOD118-10|United States|Texas|658[0n]|BOLD:AAL3475
Grotella blanchardi|[10899]|LNAUT811-14|United States|New Mexico|658[0n]|BOLD:AAL3475
Grotella blanchardi|[10900]|LNAUT815-14|United States|New Mexico|658[0n]|BOLD:AAL3475
Grotella nr. grisescens|[10901]|RDNMK188-11|United States|Arizona|658[0n]|BOLD:AAY9130
Grotella nr. grisescens|[10902]|RDNMK187-11|United States|Arizona|658[0n]|BOLD:AAY9130
Grotella grisescens|[10903]|CNCLB935-14|United States|Texas|658[0n]|BOLD:AAY9130
Grotella olivacea|[10904]|RDNML198-13|United States|Arizona|486[0n]|
Grotella citronella|[10905]|CNCLB2138-14|United States|California|658[0n]|BOLD:ACR4824
Grotella binda|[10906]|RDNMK013-11|United States|Arizona|658[0n]|BOLD:AAY8870
Grotella binda|[10907]|RDNMK014-11|United States|Arizona|658[0n]|BOLD:AAY8870
Grotella binda|[10908]|RDNMK163-11|United States|Arizona|658[0n]|BOLD:AAY8870
Grotella binda|[10909]|BBLOC692-11|United States|Arizona|658[0n]|BOLD:AAY8870
Grotella tricolor|[10910]|RDNME042-07|United States|Arizona|658[0n]|BOLD:AAL3477
Grotella tricolor|[10911]|CMAZA766-10|United States|Arizona|658[0n]|BOLD:AAL3477
Grotella tricolor|[10912]|CMAZA1120-12|United States|Arizona|658[0n]|BOLD:AAL3477
Grotella tricolor|[10913]|CMAZA1121-12|United States|Arizona|658[0n]|BOLD:AAL3477
Grotella margueritaria|[10914]|CNCLB2441-14|United States|Texas|407[0n]|
Grotella vauriae|[10915]|CNCLB939-14|United States|Texas|553[0n]|BOLD:ACM4663
Grotella vauriae|[10916]|CNCLB941-14|United States|Texas|603[0n]|BOLD:ACM4663
Grotella blanca|[10917]|RDNMJ260-11|United States|Arizona|649[0n]|BOLD:AAV6941
Grotella blanca|[10918]|RDNMJ304-11|United States|Arizona|658[0n]|BOLD:AAV6941
Grotella sp.|[10919]|LNAUS4094-13|United States|New Mexico|658[0n]|BOLD:ACJ9298
Grotella dis|[10920]|LNAUS4093-13|United States|New Mexico|658[0n]|BOLD:ACJ9297
Grotella dis|[10921]|CNCLB930-14|United States|New Mexico|658[0n]|BOLD:ACJ9297
Grotella dis|[10922]|CNCLB931-14|United States|New Mexico|658[0n]|BOLD:ACJ9297
Grotella parvipuncta|[10923]|LNAUS4098-13|United States|New Mexico|658[0n]|BOLD:ACJ9197
Grotella parvipuncta|[10924]|LNAUS4099-13|United States|New Mexico|658[0n]|BOLD:ACJ9197
Grotella parvipuncta|[10925]|LNAUS4100-13|United States|New Mexico|658[0n]|BOLD:ACJ9197
Grotella sp.|[10926]|CNCLB3099-15|United States|Arizona|658[0n]|BOLD:ACM4645
Grotella vagans|[10927]|CNCLB933-14|United States|Nevada|658[0n]|BOLD:ACM4645
Grotella sp.|[10928]|CNCLB3100-15|United States|Arizona|658[0n]|BOLD:ACM4645
Grotella sampita|[10929]|CMAZA605-10|United States|Arizona|658[0n]|BOLD:AAP6004
Grotella sampita|[10930]|CMAZA634-10|United States|Arizona|658[0n]|BOLD:AAP6004
Grotella sampita|[10931]|RDNMJ297-11|United States|Arizona|658[0n]|BOLD:AAP6004
Grotella sampita|[10932]|CMAZA1163-12|United States|Arizona|658[0n]|BOLD:AAP6004
Grotella sampita|[10933]|RDNMK929-13|Mexico|Sonora|658[0n]|BOLD:AAP6004
Grotella sampita|[10934]|RDNMK930-13|Mexico|Sonora|658[0n]|BOLD:AAP6004
Grotella septempunctata|[10935]|CNCLB927-14|United States|Kansas|658[0n]|BOLD:ACM4059
Grotella septempunctata|[10936]|CNCLB1519-14|United States|Kansas|658[0n]|BOLD:ACM4059
Grotella harveyi|[10937]|CNCLB929-14|United States|Kansas|610[0n]|BOLD:ACM4059
Grotella harveyi|[10938]|CNCLB1149-14|United States|Kansas|658[0n]|BOLD:ACM4059
Grotella harveyi|[10939]|CNCLB1521-14|United States|Kansas|658[0n]|BOLD:ACM4059
Grotella septempunctata|[10940]|CNCLB928-14|United States|Kansas|658[0n]|BOLD:ACM4059

Grotella harveyi|[10938]|CNCLB1149-14|United States|Kansas|658[0n]|BOLD:ACM4059
Grotella harveyi|[10939]|CNCLB1521-14|United States|Kansas|658[0n]|BOLD:ACM4059
Grotella septempunctata|[10940]|CNCLB928-14|United States|Kansas|658[0n]|BOLD:ACM4059
Grotella septempunctata|[10941]|CNCLB1148-14|United States|Kansas|658[0n]|BOLD:ACM4059
Grotella septempunctata|[10942]|CNCLB1520-14|United States|Kansas|658[0n]|BOLD:ACM4059
Grotella harveyi|[10943]|CNCLB3101-15|United States|New Mexico|658[0n]|BOLD:ACM4059
Neogrotella confusa|[10944]|RDNME897-08|United States|New Mexico|658[0n]|BOLD:AAH4887
Neogrotella confusa|[10945]|NOCNA051-14|United States|Texas|658[0n]|BOLD:AAH4887
Neogrotella spaldingi|[10946]|CNCLB938-14|United States|Nevada|658[0n]|BOLD:AAH4887
Neogrotella macdunnoughi|[10947]|CNCLB946-14|United States|California|658[0n]|BOLD:AAH4887
Neogrotella macdunnoughi|[10948]|IAWL850-09|United States|Arizona|658[0n]|BOLD:AAH4887
Neogrotella macdunnoughi|[10949]|CNCLB945-14|United States|California|658[0n]|BOLD:AAH4887
Neogrotella macdunnoughi|[10950]|CNCLB3103-15|United States|California|620[0n]|BOLD:AAH4887
Bagisara albicosta|[10951]|LNAUS3511-13|Costa Rica|Alajuela|658[0n]|BOLD:AAB3503
Bagisara albicosta|[10952]|LNAUS3512-13|Costa Rica|Alajuela|658[0n]|BOLD:AAB3503
Bagisara albicosta|[10953]|LNAUS3513-13|Costa Rica|Alajuela|658[0n]|BOLD:AAB3503
Bagisara albicosta|[10954]|LNAUS3514-13|Costa Rica|Alajuela|658[0n]|BOLD:AAB3503
Bagisara albicosta|[10955]|MXBLP168-11|Mexico|Jalisco|658[0n]|BOLD:AAB3503
Bagisara albicosta|[10956]|LNAUS3510-13|Costa Rica|Alajuela|658[0n]|BOLD:AAB3503
Bagisara albicosta|[10957]|CNCLB877-14|United States|Arizona|658[0n]|BOLD:AAB3503
Bagisara repanda|[10958]|LOFLB185-06|United States|Florida|658[0n]|BOLD:AAB2073
Bagisara repanda|[10959]|LOFLB193-06|United States|Florida|658[0n]|BOLD:AAB2073
Bagisara repanda|[10960]|LOFLC218-06|United States|Florida|658[0n]|BOLD:AAB2073
Bagisara repanda|[10961]|LOFLC253-06|United States|Florida|658[0n]|BOLD:AAB2073
Bagisara repanda|[10962]|HKONB221-09|United States|Texas|658[0n]|BOLD:AAB2073
Bagisara pacifica|[10963]|MHAUB094-05|Costa Rica|Guanacaste|515[0n]|BOLD:AAB2731
Bagisara pacifica|[10964]|MHMXO458-08|Costa Rica|Guanacaste|609[0n]|BOLD:AAB2731
Bagisara pacifica|[10965]|MHAUB095-05|Costa Rica|Guanacaste|658[0n]|BOLD:AAB2731
Bagisara pacifica|[10966]|MHMXF655-07|Costa Rica|Guanacaste|655[0n]|BOLD:AAB2731
Bagisara pacifica|[10967]|MHMXE318-06|Costa Rica|Guanacaste|657[0n]|BOLD:AAB2731
Bagisara pacifica|[10968]|MHMXF656-07|Costa Rica|Guanacaste|658[0n]|BOLD:AAB2731
Bagisara pacifica|[10969]|BLPCO815-08|Costa Rica|Guanacaste|658[0n]|BOLD:AAB2731
Bagisara pacifica|[10970]|MHMXZ855-09|Costa Rica||658[0n]|BOLD:AAB2731
Bagisara pacifica|[10971]|MHAUB089-05|Costa Rica|Guanacaste|658[0n]|BOLD:AAB2731
Bagisara pacifica|[10972]|MHMXI413-07|Costa Rica|Guanacaste|658[0n]|BOLD:AAB2731
Bagisara pacifica|[10973]|MHMXM278-07|Costa Rica|Guanacaste|658[0n]|BOLD:AAB2731
Bagisara pacifica|[10974]|MHMXZ857-09|Costa Rica||658[0n]|BOLD:AAB2731
Bagisara pacifica|[10975]|MHMXZ858-09|Costa Rica||658[0n]|BOLD:AAB2731
Bagisara pacifica|[10976]|BLPAG028-07|Costa Rica|Guanacaste|658[0n]|BOLD:AAB2731
Bagisara pacifica|[10977]|MHAUG111-07|Costa Rica|Guanacaste|658[0n]|BOLD:AAB2731
Bagisara pacifica|[10978]|MHMXZ859-09|Costa Rica||658[0n]|BOLD:AAB2731
Bagisara pacifica|[10979]|LPYPB277-08|Mexico|Quintana Roo|658[0n]|BOLD:AAB2731
Bagisara pacifica|[10980]|HKONB213-09|United States|Texas|658[0n]|BOLD:AAB2731
Bagisara pacifica|[10981]|MHMXO459-08|Costa Rica|Guanacaste|636[0n]|BOLD:AAB2731
Bagisara pacifica|[10982]|MHMXZ856-09|Costa Rica||658[0n]|BOLD:AAB2731
Bagisara pacifica|[10983]|HKONB212-09|United States|Texas|658[0n]|BOLD:AAB2731
Bagisara pacifica|[10984]|HKONB214-09|United States|Texas|658[0n]|BOLD:AAB2731
Bagisara pacifica|[10985]|MHAUB090-05|Costa Rica|Guanacaste|631[0n]|BOLD:AAB2731
Bagisara pacifica|[10986]|BLPCA462-08|Costa Rica|Guanacaste|658[0n]|BOLD:AAB2731
Bagisara pacifica|[10987]|MHAUB091-05|Costa Rica|Guanacaste|658[0n]|BOLD:AAB2731
Bagisara pacifica|[10988]|LYPAP567-09|Mexico|Quintana Roo|658[0n]|BOLD:AAB2731
Bagisara brouana|[10989]|HKONB325-09|United States|Florida|658[0n]|BOLD:ACE6881
Bagisara brouana|[10990]|HKONS065-07|United States|Florida|658[1n]|BOLD:ACE6881
Bagisara brouana|[10991]|MILEQ202-11|United States|Georgia|658[0n]|BOLD:ACE6881
Bagisara rectifascia|[10992]|LNCC1669-13|United States|North Carolina|658[0n]|BOLD:AAB2732
Bagisara rectifascia|[10993]|RDNMF525-08|United States|Maryland|609[0n]|BOLD:AAB2732
Bagisara rectifascia|[10994]|RDNMK481-11|United States|Massachusetts|658[0n]|BOLD:AAB2732
Bagisara rectifascia|[10995]|QUNOE467-12|United States|Florida|658[0n]|BOLD:AAB2732
Bagisara rectifascia|[10996]|RDNMF527-08|United States|Texas|609[0n]|BOLD:AAB2732
Bagisara rectifascia|[10997]|CNCLB881-14|United States|Texas|658[0n]|BOLD:AAB2732
Bagisara rectifascia|[10998]|CNCLB882-14|United States|Texas|658[0n]|BOLD:AAB2732
Bagisara rectifascia|[10999]|HKONB417-09|United States|Indiana|658[0n]|BOLD:AAB2732
Bagisara rectifascia|[11000]|RDNMF526-08|United States|Texas|658[0n]|BOLD:AAB2732
Bagisara rectifascia|[11001]|RDNMK480-11|United States|Connecticut|602[0n]|BOLD:AAB2732
Bagisara rectifascia|[11002]|MNAE869-13|United States|Texas|622[0n]|BOLD:AAB2732
Bagisara rectifascia|[11003]|CNCLB889-14|United States|Louisiana|658[0n]|BOLD:AAB2732
Bagisara tristicta|[11004]|RDNMH586-09|United States|Arizona|658[0n]|BOLD:AAC2622
Bagisara tristicta|[11005]|MHAUB459-05|Costa Rica|Guanacaste|658[0n]|BOLD:AAC2622
Bagisara tristicta|[11006]|MHMXF632-07|Costa Rica|Guanacaste|658[0n]|BOLD:AAC2622
Bagisara tristicta|[11007]|MHMXE883-07|Costa Rica|Guanacaste|658[0n]|BOLD:AAC2622
Bagisara tristicta|[11008]|MHAUB460-05|Costa Rica|Guanacaste|658[0n]|BOLD:AAC2622
Bagisara tristicta|[11009]|MHAUB458-05|Costa Rica|Guanacaste|658[0n]|BOLD:AAC2622
Bagisara tristicta|[11010]|MHAUG727-07|Costa Rica|Guanacaste|620[0n]|BOLD:AAC2622
Bagisara tristicta|[11011]|BLPAE685-06|Costa Rica|Guanacaste|658[0n]|BOLD:AAC2622
Bagisara tristicta|[11012]|MHAUB457-05|Costa Rica|Guanacaste|658[0n]|BOLD:AAC2622
Bagisara tristicta|[11013]|BLPAE686-06|Costa Rica|Guanacaste|658[0n]|BOLD:AAC2622
Bagisara tristicta|[11014]|MHMXO295-08|Costa Rica|Guanacaste|658[0n]|BOLD:AAC2622
Bagisara tristicta|[11015]|MHMXO457-08|Costa Rica|Guanacaste|642[0n]|BOLD:AAC2622
Bagisara tristicta|[11016]|BLPDK662-09|Costa Rica|Guanacaste|658[0n]|BOLD:AAC2622
Bagisara laverna|[11017]|BLPCA198-08|Costa Rica|Guanacaste|658[0n]|BOLD:AAB2474
Bagisara laverna|[11018]|BLPDW226-11|Costa Rica|Guanacaste|658[0n]|BOLD:AAB2474
Bagisara laverna|[11019]|BLPDU829-11|Costa Rica|Guanacaste|658[0n]|BOLD:AAB2474
Bagisara laverna|[11020]|BLPDU827-11|Costa Rica|Guanacaste|658[0n]|BOLD:AAB2474
Bagisara laverna|[11021]|MHMYC2220-09|Costa Rica||658[0n]|BOLD:AAB2474
Bagisara laverna|[11022]|BLPCL471-08|Costa Rica|Guanacaste|658[0n]|BOLD:AAB2474
Bagisara laverna|[11023]|BLPCI118-08|Costa Rica|Guanacaste|658[0n]|BOLD:AAB2474
Bagisara laverna|[11024]|BLPCH592-08|Costa Rica|Guanacaste|658[0n]|BOLD:AAB2474
Bagisara laverna|[11025]|BLPCD413-08|Costa Rica|Guanacaste|658[0n]|BOLD:AAB2474
Bagisara laverna|[11026]|BLPCD412-08|Costa Rica|Guanacaste|658[0n]|BOLD:AAB2474
Bagisara laverna|[11027]|BLPCC490-08|Costa Rica|Guanacaste|658[0n]|BOLD:AAB2474
Bagisara laverna|[11028]|LOCRB237-08|Costa Rica|Alajuela|658[0n]|BOLD:AAB2474
Bagisara laverna|[11029]|MHMXO294-08|Costa Rica|Guanacaste|658[0n]|BOLD:AAB2474
Bagisara laverna|[11030]|BLPBH445-07|Costa Rica|Guanacaste|658[0n]|BOLD:AAB2474
Bagisara laverna|[11031]|BLPBA209-07|Costa Rica|Guanacaste|658[0n]|BOLD:AAB2474
Bagisara laverna|[11032]|BLPBA208-07|Costa Rica|Guanacaste|658[0n]|BOLD:AAB2474
Bagisara laverna|[11033]|BLPAH304-07|Costa Rica|Guanacaste|658[0n]|BOLD:AAB2474
Bagisara laverna|[11034]|BLPAH302-07|Costa Rica|Guanacaste|658[0n]|BOLD:AAB2474
Bagisara laverna|[11035]|BLPAH299-07|Costa Rica|Guanacaste|658[0n]|BOLD:AAB2474
Bagisara laverna|[11036]|BLPAH298-07|Costa Rica|Guanacaste|658[0n]|BOLD:AAB2474
Bagisara laverna|[11037]|BLPAF394-07|Costa Rica|Guanacaste|658[0n]|BOLD:AAB2474
Bagisara laverna|[11038]|BLPAH303-07|Costa Rica|Guanacaste|658[1n]|BOLD:AAB2474
Bagisara laverna|[11039]|MHAUB087-05|Costa Rica|Guanacaste|571[0n]|BOLD:AAB2474
Bagisara laverna|[11040]|MHMXF655-07|Costa Rica|Alajuela|621[0n]|BOLD:AAB2474

[illegible]
Bagisara laverna|[11038]|BLPAH303-07|Costa Rica|Guanacaste|658[1n]|BOLD:AAB2474
Bagisara laverna|[11039]|MHAUB087-05|Costa Rica|Guanacaste|571[0n]|BOLD:AAB2474
Bagisara laverna|[11040]|MHMXE655-07|Costa Rica|Alajuela|621[0n]|BOLD:AAB2474
Bagisara laverna|[11041]|BLPAH300-07|Costa Rica|Guanacaste|658[0n]|BOLD:AAB2474
Bagisara laverna|[11042]|BLPDU828-11|Costa Rica|Guanacaste|658[0n]|BOLD:AAB2474
Bagisara laverna|[11043]|LNAUS3515-13|Guatemala|Suchitepequez|658[0n]|BOLD:AAB2474
Bagisara laverna|[11044]|BLPDK1310-09|Costa Rica|Guanacaste|658[1n]|BOLD:AAB2474
Bagisara laverna|[11045]|MHAUB088-05|Costa Rica|Guanacaste|570[0n]|BOLD:AAB2474
Bagisara laverna|[11046]|BLPAG033-07|Costa Rica|Guanacaste|658[0n]|BOLD:AAB2474
Bagisara laverna|[11047]|BLPAH301-07|Costa Rica|Guanacaste|658[0n]|BOLD:AAB2474
Bagisara laverna|[11048]|BLPCL470-08|Costa Rica|Guanacaste|658[0n]|BOLD:AAB2474
Bagisara laverna|[11049]|LNAUS3516-13|Guatemala|Suchitepequez|658[0n]|BOLD:AAB2474
Bagisara laverna|[11050]|CNCLB878-14|United States|Arizona|658[0n]|BOLD:AAB2474
Bagisara praecelsa|[11051]|HKONB326-09|United States|Texas|658[0n]|BOLD:AAW3006
Bagisara praecelsa|[11052]|CNCLB891-14|United States|Texas|658[0n]|BOLD:AAW3006
Bagisara gulnare|[11053]|NOCNA094-14|United States|Wisconsin|658[0n]|BOLD:ACN3193
Bagisara gulnare|[11054]|CNCLB892-14|United States|Ohio|658[0n]|BOLD:ACN3193
Bagisara oula|[11055]|RDNMH770-09|United States|Arizona|658[0n]|BOLD:AAD2139
Bagisara oula|[11056]|RDNMF829-08|United States|Arizona|658[0n]|BOLD:AAD2139
Bagisara oula|[11057]|RDNMG432-08|United States|Texas|640[0n]|BOLD:AAD2139
Bagisara oula|[11058]|RDNMF973-08|United States|Texas|609[0n]|BOLD:AAD2139
Bagisara oula|[11059]|RDNMF983-08|United States|Arizona|658[0n]|BOLD:AAD2139
Bagisara oula|[11060]|HKONB217-09|United States|Texas|658[0n]|BOLD:AAD2139
Bagisara oula|[11061]|RDNMF974-08|United States|Texas|658[0n]|BOLD:AAD2139
Bagisara oula|[11062]|HKONB218-09|United States|Texas|658[0n]|BOLD:AAD2139
Bagisara oula|[11063]|CNCLB879-14|United States|Texas|658[0n]|BOLD:AAD2139
Bagisara demura|[11064]|CNCLB890-14|United States|Arizona|658[0n]|BOLD:ACM4107
Bagisara buxea|[11065]|CMAZA793-10|United States|Arizona|658[0n]|BOLD:AAF2676
Bagisara buxea|[11066]|RDNMF843-08|United States|Arizona|658[1n]|BOLD:AAF2676
Bagisara buxea|[11067]|BBLSY944-09|United States|Texas|658[0n]|BOLD:AAF2676
Bagisara buxea|[11068]|USLEP071-10|United States|Texas|658[0n]|BOLD:AAF2676
Bagisara buxea|[11069]|CMAZA390-10|United States|Arizona|658[0n]|BOLD:AAF2676
Bagisara buxea|[11070]|BBLSY943-09|United States|Texas|658[0n]|BOLD:AAF2676
Bagisara buxea|[11071]|BBLSX795-09|United States|Texas|658[0n]|BOLD:AAF2676
Bagisara buxea|[11072]|HKONB327-09|United States|Texas|658[0n]|BOLD:AAF2676
Bagisara buxea|[11073]|RDNMH587-09|United States|Arizona|658[0n]|BOLD:AAF2676
Bagisara buxea|[11074]|BBLOD1113-11|United States|Texas|658[0n]|BOLD:AAF2676
Bagisara buxea|[11075]|CMAZA985-12|United States|Arizona|658[0n]|BOLD:AAF2676
Bagisara buxea|[11076]|CNCLB880-14|United States|Texas|583[0n]|BOLD:AAF2676
Bagisara buxea|[11077]|CNCLB1523-14|United States|Texas|658[0n]|BOLD:AAF2676
Metaponpneumata rogenhoferi|[11078]|BBLOD718-11|United States|Texas|636[0n]|BOLD:AAA0716
Metaponpneumata rogenhoferi|[11079]|BBLOD1112-11|United States|Texas|612[0n]|BOLD:AAA0716
Metaponpneumata rogenhoferi|[11080]|CMAZA469-10|United States|Arizona|658[0n]|BOLD:AAA0716
Metaponpneumata rogenhoferi|[11081]|BBLOE1289-12|United States|Texas|658[0n]|BOLD:AAA0716
Metaponpneumata rogenhoferi|[11082]|CMAZA742-10|United States|Arizona|658[0n]|BOLD:AAA0716
Metaponpneumata rogenhoferi|[11083]|BBLOC499-11|United States|Arizona|658[0n]|BOLD:AAA0716
Metaponpneumata rogenhoferi|[11084]|BBLOE1596-12|United States|Texas|658[0n]|BOLD:AAA0716
Metaponpneumata rogenhoferi|[11085]|BBLOC501-11|United States|Arizona|631[0n]|BOLD:AAA0716
Metaponpneumata rogenhoferi|[11086]|BBLSW085-09|United States|Texas|636[0n]|BOLD:AAA0716
Metaponpneumata rogenhoferi|[11087]|BBLOD1739-11|United States|Texas|627[0n]|BOLD:AAA0716
Metaponpneumata rogenhoferi|[11088]|BBLOD1504-11|United States|Texas|622[0n]|BOLD:AAA0716
Metaponpneumata rogenhoferi|[11089]|BBLOD1741-11|United States|Texas|636[0n]|BOLD:AAA0716
Metaponpneumata rogenhoferi|[11090]|BBLOD1161-11|United States|Arizona|658[0n]|BOLD:AAA0716
Metaponpneumata rogenhoferi|[11091]|BBLOE1813-12|United States|Arizona|658[0n]|BOLD:AAA0716
Metaponpneumata rogenhoferi|[11092]|BBLOC477-11|United States|Arizona|658[0n]|BOLD:AAA0716
Metaponpneumata rogenhoferi|[11093]|BBLOE1615-12|United States|Texas|658[0n]|BOLD:AAA0716
Metaponpneumata rogenhoferi|[11094]|BBLOE1847-12|United States|Texas|658[0n]|BOLD:AAA0716
Metaponpneumata rogenhoferi|[11095]|BBLOB714-11|United States|Texas|658[1n]|BOLD:AAA0716
Metaponpneumata rogenhoferi|[11096]|BBLOD1512-11|United States|Texas|658[0n]|BOLD:AAA0716
Metaponpneumata rogenhoferi|[11097]|BBLOE1614-12|United States|Texas|658[0n]|BOLD:AAA0716
Metaponpneumata rogenhoferi|[11098]|BBLOD721-11|United States|Texas|658[0n]|BOLD:AAA0716
Metaponpneumata rogenhoferi|[11099]|BBLSW076-09|United States|Texas|658[0n]|BOLD:AAA0716
Metaponpneumata rogenhoferi|[11100]|BBLOD1110-11|United States|Texas|658[0n]|BOLD:AAA0716
Metaponpneumata rogenhoferi|[11101]|BBLOC1770-11|United States|Texas|658[0n]|BOLD:AAA0716
Metaponpneumata rogenhoferi|[11102]|BBLOD728-11|United States|Texas|658[0n]|BOLD:AAA0716
Metaponpneumata rogenhoferi|[11103]|CMAZA743-10|United States|Arizona|658[0n]|BOLD:AAA0716
Metaponpneumata rogenhoferi|[11104]|BBLOB624-11|United States|Texas|658[0n]|BOLD:AAA0716
Metaponpneumata rogenhoferi|[11105]|BBLOC1758-11|United States|Texas|658[0n]|BOLD:AAA0716
Metaponpneumata rogenhoferi|[11106]|BBLOC1782-11|United States|Texas|658[0n]|BOLD:AAA0716
Metaponpneumata rogenhoferi|[11107]|BBLOD1025-11|United States|Texas|658[0n]|BOLD:AAA0716
Metaponpneumata rogenhoferi|[11108]|BBLOD973-11|United States|Texas|658[0n]|BOLD:AAA0716
Metaponpneumata rogenhoferi|[11109]|BBLOC518-11|United States|Arizona|658[0n]|BOLD:AAA0716
Metaponpneumata rogenhoferi|[11110]|BBLOB698-11|United States|Texas|658[0n]|BOLD:AAA0716
Metaponpneumata rogenhoferi|[11111]|BBLOC1791-11|United States|Texas|658[0n]|BOLD:AAA0716
Metaponpneumata rogenhoferi|[11112]|BBLOC813-11|United States|Arizona|658[0n]|BOLD:AAA0716
Metaponpneumata rogenhoferi|[11113]|BBLOC530-11|United States|Arizona|658[0n]|BOLD:AAA0716
Metaponpneumata rogenhoferi|[11114]|AWCLB490-11|United States|Arizona|658[0n]|BOLD:AAA0716
Metaponpneumata rogenhoferi|[11115]|BBLSW087-09|United States|Texas|658[0n]|BOLD:AAA0716
Metaponpneumata rogenhoferi|[11116]|BBLSW047-09|United States|Texas|658[0n]|BOLD:AAA0716
Metaponpneumata rogenhoferi|[11117]|BBLOC1785-11|United States|Texas|622[0n]|BOLD:AAA0716
Metaponpneumata rogenhoferi|[11118]|BBLOD720-11|United States|Texas|658[0n]|BOLD:AAA0716
Metaponpneumata rogenhoferi|[11119]|BBLOD1505-11|United States|Texas|658[0n]|BOLD:AAA0716
Metaponpneumata rogenhoferi|[11120]|BBLOE1595-12|United States|Texas|658[0n]|BOLD:AAA0716
Metaponpneumata rogenhoferi|[11121]|BBLOE1600-12|United States|Texas|658[0n]|BOLD:AAA0716
Metaponpneumata rogenhoferi|[11122]|BBLOD1019-11|United States|Texas|658[0n]|BOLD:AAA0716
Metaponpneumata rogenhoferi|[11123]|BBLOD1619-11|United States|Texas|658[0n]|BOLD:AAA0716
Metaponpneumata rogenhoferi|[11124]|BBLOE1393-12|United States|Texas|658[0n]|BOLD:AAA0716
Metaponpneumata rogenhoferi|[11125]|BBLOE1622-12|United States|Texas|658[0n]|BOLD:AAA0716
Metaponpneumata rogenhoferi|[11126]|BBLOC1792-11|United States|Texas|658[0n]|BOLD:AAA0716
Metaponpneumata rogenhoferi|[11127]|BBLSY173-09|United States|Texas|658[0n]|BOLD:AAA0716
Metaponpneumata rogenhoferi|[11128]|BBLOE1623-12|United States|Texas|658[0n]|BOLD:AAA0716
Metaponpneumata rogenhoferi|[11129]|BBLOC1778-11|United States|Texas|658[2n]|BOLD:AAA0716
Metaponpneumata rogenhoferi|[11130]|BBLOE1817-12|United States|Arizona|658[0n]|BOLD:AAA0716
Metaponpneumata rogenhoferi|[11131]|BBLOD1123-11|United States|Texas|658[0n]|BOLD:AAA0716
Metaponpneumata rogenhoferi|[11132]|BBLOD1107-11|United States|Texas|658[0n]|BOLD:AAA0716
Metaponpneumata rogenhoferi|[11133]|BBLSX816-09|United States|Texas|658[0n]|BOLD:AAA0716
Metaponpneumata rogenhoferi|[11134]|BBLOE1621-12|United States|Texas|658[0n]|BOLD:AAA0716
Metaponpneumata rogenhoferi|[11135]|BBLOE1822-12|United States|Texas|658[0n]|BOLD:AAA0716
Metaponpneumata rogenhoferi|[11136]|BBLOE1811-12|United States|Arizona|658[0n]|BOLD:AAA0716
Metaponpneumata rogenhoferi|[11137]|BBLOE1809-12|United States|Arizona|658[0n]|BOLD:AAA0716
Metaponpneumata rogenhoferi|[11138]|BBLOE1628-12|United States|Texas|658[0n]|BOLD:AAA0716
Metaponpneumata rogenhoferi|[11139]|BBLOE1626-12|United States|Texas|658[0n]|BOLD:AAA0716
[illegible]

Metaponpneumata rogenhoferi|[11137]|BBLOE1809-12|United States|Arizona|658[0n]|BOLD:AAA0716
Metaponpneumata rogenhoferi|[11138]|BBLOE1628-12|United States|Texas|658[0n]|BOLD:AAA0716
Metaponpneumata rogenhoferi|[11139]|BBLOE1626-12|United States|Texas|658[0n]|BOLD:AAA0716
Metaponpneumata rogenhoferi|[11140]|BBLOE1625-12|United States|Texas|658[0n]|BOLD:AAA0716
Metaponpneumata rogenhoferi|[11141]|BBLOE1616-12|United States|Texas|658[0n]|BOLD:AAA0716
Metaponpneumata rogenhoferi|[11142]|BBLOE1606-12|United States|Texas|658[0n]|BOLD:AAA0716
Metaponpneumata rogenhoferi|[11143]|BBLOE1604-12|United States|Texas|658[0n]|BOLD:AAA0716
Metaponpneumata rogenhoferi|[11144]|BBLOE1593-12|United States|Texas|658[0n]|BOLD:AAA0716
Metaponpneumata rogenhoferi|[11145]|BBLOE1518-12|United States|Texas|658[0n]|BOLD:AAA0716
Metaponpneumata rogenhoferi|[11146]|BBLOE1499-12|United States|Texas|658[0n]|BOLD:AAA0716
Metaponpneumata rogenhoferi|[11147]|BBLOE1395-12|United States|Texas|658[0n]|BOLD:AAA0716
Metaponpneumata rogenhoferi|[11148]|BBLOE1286-12|United States|Texas|658[0n]|BOLD:AAA0716
Metaponpneumata rogenhoferi|[11149]|BBLOE1285-12|United States|Texas|658[0n]|BOLD:AAA0716
Metaponpneumata rogenhoferi|[11150]|BBLOE1282-12|United States|Texas|658[0n]|BOLD:AAA0716
Metaponpneumata rogenhoferi|[11151]|BBLOE1278-12|United States|Texas|658[0n]|BOLD:AAA0716
Metaponpneumata rogenhoferi|[11152]|BBLOE1276-12|United States|Texas|658[0n]|BOLD:AAA0716
Metaponpneumata rogenhoferi|[11153]|BBLOE1175-12|United States|Texas|658[0n]|BOLD:AAA0716
Metaponpneumata rogenhoferi|[11154]|BBLOD1735-11|United States|Texas|658[0n]|BOLD:AAA0716
Metaponpneumata rogenhoferi|[11155]|BBLOD1734-11|United States|Texas|658[0n]|BOLD:AAA0716
Metaponpneumata rogenhoferi|[11156]|BBLOD1509-11|United States|Texas|658[0n]|BOLD:AAA0716
Metaponpneumata rogenhoferi|[11157]|BBLOD1507-11|United States|Texas|658[0n]|BOLD:AAA0716
Metaponpneumata rogenhoferi|[11158]|BBLOD1132-11|United States|Texas|658[0n]|BOLD:AAA0716
Metaponpneumata rogenhoferi|[11159]|BBLOD1121-11|United States|Texas|658[0n]|BOLD:AAA0716
Metaponpneumata rogenhoferi|[11160]|BBLOD1119-11|United States|Texas|658[0n]|BOLD:AAA0716
Metaponpneumata rogenhoferi|[11161]|BBLOD1115-11|United States|Texas|658[0n]|BOLD:AAA0716
Metaponpneumata rogenhoferi|[11162]|BBLOD1109-11|United States|Texas|658[0n]|BOLD:AAA0716
Metaponpneumata rogenhoferi|[11163]|BBLOD1065-11|United States|Texas|658[0n]|BOLD:AAA0716
Metaponpneumata rogenhoferi|[11164]|BBLOD1022-11|United States|Texas|658[0n]|BOLD:AAA0716
Metaponpneumata rogenhoferi|[11165]|BBLOD1017-11|United States|Texas|658[0n]|BOLD:AAA0716
Metaponpneumata rogenhoferi|[11166]|BBLOD1015-11|United States|Texas|658[0n]|BOLD:AAA0716
Metaponpneumata rogenhoferi|[11167]|BBLOD1014-11|United States|Texas|658[0n]|BOLD:AAA0716
Metaponpneumata rogenhoferi|[11168]|BBLOD1010-11|United States|Texas|658[0n]|BOLD:AAA0716
Metaponpneumata rogenhoferi|[11169]|BBLOD1009-11|United States|Texas|658[0n]|BOLD:AAA0716
Metaponpneumata rogenhoferi|[11170]|BBLOD1002-11|United States|Texas|658[0n]|BOLD:AAA0716
Metaponpneumata rogenhoferi|[11171]|BBLOD727-11|United States|Texas|658[0n]|BOLD:AAA0716
Metaponpneumata rogenhoferi|[11172]|BBLOD725-11|United States|Texas|658[0n]|BOLD:AAA0716
Metaponpneumata rogenhoferi|[11173]|BBLOD724-11|United States|Texas|658[0n]|BOLD:AAA0716
Metaponpneumata rogenhoferi|[11174]|BBLOD723-11|United States|Texas|658[0n]|BOLD:AAA0716
Metaponpneumata rogenhoferi|[11175]|BBLOD361-11|United States|Texas|658[0n]|BOLD:AAA0716
Metaponpneumata rogenhoferi|[11176]|BBLOD005-11|United States|Texas|658[0n]|BOLD:AAA0716
Metaponpneumata rogenhoferi|[11177]|BBLOC1793-11|United States|Texas|658[0n]|BOLD:AAA0716
Metaponpneumata rogenhoferi|[11178]|BBLOC1790-11|United States|Texas|658[0n]|BOLD:AAA0716
Metaponpneumata rogenhoferi|[11179]|BBLOC1786-11|United States|Texas|658[0n]|BOLD:AAA0716
Metaponpneumata rogenhoferi|[11180]|BBLOC1781-11|United States|Texas|658[0n]|BOLD:AAA0716
Metaponpneumata rogenhoferi|[11181]|BBLOC1773-11|United States|Texas|658[0n]|BOLD:AAA0716
Metaponpneumata rogenhoferi|[11182]|BBLOC1767-11|United States|Texas|658[0n]|BOLD:AAA0716
Metaponpneumata rogenhoferi|[11183]|BBLOC1760-11|United States|Texas|658[0n]|BOLD:AAA0716
Metaponpneumata rogenhoferi|[11184]|BBLOC1481-11|United States|Texas|658[0n]|BOLD:AAA0716
Metaponpneumata rogenhoferi|[11185]|BBLOC784-11|United States|Arizona|658[0n]|BOLD:AAA0716
Metaponpneumata rogenhoferi|[11186]|BBLOC440-11|United States|Arizona|658[0n]|BOLD:AAA0716
Metaponpneumata rogenhoferi|[11187]|BBLOC439-11|United States|Arizona|658[0n]|BOLD:AAA0716
Metaponpneumata rogenhoferi|[11188]|BBLOC422-11|United States|Arizona|658[0n]|BOLD:AAA0716
Metaponpneumata rogenhoferi|[11189]|BBLOC412-11|United States|Arizona|658[0n]|BOLD:AAA0716
Metaponpneumata rogenhoferi|[11190]|BBLOC356-11|United States|Arizona|658[0n]|BOLD:AAA0716
Metaponpneumata rogenhoferi|[11191]|BBLOC088-11|United States|Arizona|658[0n]|BOLD:AAA0716
Metaponpneumata rogenhoferi|[11192]|BBLOB1990-11|United States|Arizona|658[0n]|BOLD:AAA0716
Metaponpneumata rogenhoferi|[11193]|BBLOB707-11|United States|Texas|658[0n]|BOLD:AAA0716
Metaponpneumata rogenhoferi|[11194]|BBLOB706-11|United States|Texas|658[0n]|BOLD:AAA0716
Metaponpneumata rogenhoferi|[11195]|BBLOB704-11|United States|Texas|658[0n]|BOLD:AAA0716
Metaponpneumata rogenhoferi|[11196]|BBLOB702-11|United States|Texas|658[0n]|BOLD:AAA0716
Metaponpneumata rogenhoferi|[11197]|BBLOB625-11|United States|Texas|658[0n]|BOLD:AAA0716
Metaponpneumata rogenhoferi|[11198]|BBLOB616-11|United States|Texas|658[0n]|BOLD:AAA0716
Metaponpneumata rogenhoferi|[11199]|RDNMK183-11|United States|Arizona|658[0n]|BOLD:AAA0716
Metaponpneumata rogenhoferi|[11200]|RDNMK182-11|United States|Arizona|658[0n]|BOLD:AAA0716
Metaponpneumata rogenhoferi|[11201]|RDNMJ726-11|United States|Arizona|658[0n]|BOLD:AAA0716
Metaponpneumata rogenhoferi|[11202]|CMAZA754-10|United States|Arizona|658[0n]|BOLD:AAA0716
Metaponpneumata rogenhoferi|[11203]|BBLSX799-09|United States|Texas|658[0n]|BOLD:AAA0716
Metaponpneumata rogenhoferi|[11204]|BBLSX241-09|United States|Texas|658[0n]|BOLD:AAA0716
Metaponpneumata rogenhoferi|[11205]|BBLSX240-09|United States|Texas|658[0n]|BOLD:AAA0716
Metaponpneumata rogenhoferi|[11206]|BBLSX218-09|United States|Texas|658[0n]|BOLD:AAA0716
Metaponpneumata rogenhoferi|[11207]|BBLSX215-09|United States|Texas|658[0n]|BOLD:AAA0716
Metaponpneumata rogenhoferi|[11208]|BBLSX204-09|United States|Texas|658[0n]|BOLD:AAA0716
Metaponpneumata rogenhoferi|[11209]|BBLSX203-09|United States|Texas|658[0n]|BOLD:AAA0716
Metaponpneumata rogenhoferi|[11210]|BBLSW732-09|United States|Texas|658[0n]|BOLD:AAA0716
Metaponpneumata rogenhoferi|[11211]|BBLSW089-09|United States|Texas|658[0n]|BOLD:AAA0716
Metaponpneumata rogenhoferi|[11212]|BBLOC1783-11|United States|Texas|658[0n]|BOLD:AAA0716
Metaponpneumata rogenhoferi|[11213]|BBLSW079-09|United States|Texas|658[0n]|BOLD:AAA0716
Metaponpneumata rogenhoferi|[11214]|PSAT125-10|United States|Arizona|658[0n]|BOLD:AAA0716
Metaponpneumata rogenhoferi|[11215]|BBLSW088-09|United States|Texas|658[0n]|BOLD:AAA0716
Metaponpneumata rogenhoferi|[11216]|BBLOC434-11|United States|Arizona|658[0n]|BOLD:AAA0716
Metaponpneumata rogenhoferi|[11217]|BBLOC1795-11|United States|Texas|658[0n]|BOLD:AAA0716
Metaponpneumata rogenhoferi|[11218]|BBLOD857-11|United States|Texas|658[0n]|BOLD:AAA0716
Metaponpneumata rogenhoferi|[11219]|IAWL566-09|United States|Arizona|658[0n]|BOLD:AAA0716
Metaponpneumata rogenhoferi|[11220]|BBLOD1013-11|United States|Texas|658[0n]|BOLD:AAA0716
Metaponpneumata rogenhoferi|[11221]|RDNMD816-07|United States|Arizona|658[0n]|BOLD:AAA0716
Metaponpneumata rogenhoferi|[11222]|BBLOC1776-11|United States|Texas|658[0n]|BOLD:AAA0716
Metaponpneumata rogenhoferi|[11223]|BBLOB696-11|United States|Texas|658[0n]|BOLD:AAA0716
Metaponpneumata rogenhoferi|[11224]|BBLOC1789-11|United States|Texas|658[0n]|BOLD:AAA0716
Metaponpneumata rogenhoferi|[11225]|BBLSW077-09|United States|Texas|658[0n]|BOLD:AAA0716
Metaponpneumata rogenhoferi|[11226]|BBLOB695-11|United States|Texas|658[0n]|BOLD:AAA0716
Metaponpneumata rogenhoferi|[11227]|BBLOE1605-12|United States|Texas|658[0n]|BOLD:AAA0716
Metaponpneumata rogenhoferi|[11228]|BBLOD1511-11|United States|Texas|658[0n]|BOLD:AAA0716
Metaponpneumata rogenhoferi|[11229]|BBLOD1124-11|United States|Texas|658[1n]|BOLD:AAA0716
Metaponpneumata rogenhoferi|[11230]|BBLOC1765-11|United States|Texas|658[0n]|BOLD:AAA0716
Metaponpneumata rogenhoferi|[11231]|BBLOC1798-11|United States|Texas|621[0n]|BOLD:AAA0716
Metaponpneumata rogenhoferi|[11232]|BBLOD1000-11|United States|Texas|658[0n]|BOLD:AAA0716
Metaponpneumata rogenhoferi|[11233]|BBLOD1021-11|United States|Texas|658[0n]|BOLD:AAA0716
Metaponpneumata rogenhoferi|[11234]|BBLOD1130-11|United States|Texas|658[0n]|BOLD:AAA0716
Metaponpneumata rogenhoferi|[11235]|BBLOD1902-11|United States|Arizona|658[0n]|BOLD:AAA0716
Metaponpneumata rogenhoferi|[11236]|BBLSW078-09|United States|Texas|636[0n]|BOLD:AAA0716
Metaponpneumata rogenhoferi|[11237]|BBLOC1772-11|United States|Texas|622[0n]|BOLD:AAA0716
Metaponpneumata rogenhoferi|[11238]|BBLOC1761-11|United States|Texas|622[0n]|BOLD:AAA0716
Metaponpneumata rogenhoferi|[11239]|BBLOB699-11|United States|Texas|623[0n]|BOLD:AAA0716

[illegible]
Metaponpneumata rogenhoferi|[11237]|BBLOC1772-11|United States|Texas|622[0n]|BOLD:AAA0716
Metaponpneumata rogenhoferi|[11238]|BBLOC1761-11|United States|Texas|622[0n]|BOLD:AAA0716
Metaponpneumata rogenhoferi|[11239]|BBLOB699-11|United States|Texas|623[0n]|BOLD:AAA0716
Metaponpneumata rogenhoferi|[11240]|BBLOC1766-11|United States|Texas|622[0n]|BOLD:AAA0716
Metaponpneumata rogenhoferi|[11241]|BBLOC1780-11|United States|Texas|622[0n]|BOLD:AAA0716
Metaponpneumata rogenhoferi|[11242]|BBLOC1784-11|United States|Texas|622[0n]|BOLD:AAA0716
Metaponpneumata rogenhoferi|[11243]|BBLOE1824-12|United States|Texas|633[0n]|BOLD:AAA0716
Metaponpneumata rogenhoferi|[11244]|BBLOE1828-12|United States|Texas|658[0n]|BOLD:AAA0716
Metaponpneumata rogenhoferi|[11245]|BBLOE1838-12|United States|Texas|658[0n]|BOLD:AAA0716
Metaponpneumata rogenhoferi|[11246]|BBLOE1843-12|United States|Texas|658[0n]|BOLD:AAA0716
Metaponpneumata rogenhoferi|[11247]|BBLOB717-11|United States|Texas|658[0n]|BOLD:AAA0716
Metaponpneumata rogenhoferi|[11248]|BBLOD748-11|United States|Arizona|658[0n]|BOLD:AAA0716
Metaponpneumata rogenhoferi|[11249]|BBLOD1122-11|United States|Texas|658[0n]|BOLD:AAA0716
Metaponpneumata rogenhoferi|[11250]|BBLOE1845-12|United States|Texas|658[0n]|BOLD:AAA0716
Metaponpneumata rogenhoferi|[11251]|CMAZA1123-12|United States|Arizona|658[0n]|BOLD:AAA0716
Metaponpneumata rogenhoferi|[11252]|BBLSX812-09|United States|Texas|658[0n]|BOLD:AAA0716
Metaponpneumata rogenhoferi|[11253]|CMAZA1164-12|United States|Arizona|658[0n]|BOLD:AAA0716
Axenus arvalis|[11254]|NAMUM300-08|United States|California|658[0n]|BOLD:AAF4020
Axenus arvalis|[11255]|NAMUM375-09|United States|California|658[0n]|BOLD:AAF4020
Axenus arvalis|[11256]|NAMUM376-09|United States|California|658[0n]|BOLD:AAF4020
Axenus|[11257]|LNAUT500-14|United States|Oregon|572[0n]|BOLD:ACN8932
Axenus|[11258]|LNAUT501-14|United States|Oregon|658[0n]|BOLD:ACN8932
Axenus|[11259]|LNAUT502-14|United States|Oregon|658[0n]|BOLD:ACN8932
Cydosia nobilitella|[11260]|CNCLB1092-14|Jamaica||658[0n]|BOLD:AAM8442
Cydosia nobilitella|[11261]|CNCLB1093-14|Trinidad and Tobago||307[0n]|
Cydosia majuscula|[11262]|LMEM087-09|United States|Alabama|654[4n]|BOLD:ACE5918
Cydosia majuscula|[11263]|LMEM092-09|United States|Alabama|611[1n]|BOLD:ACE5918
Cydosia majuscula|[11264]|HKONB426-09|United States|Indiana|658[0n]|BOLD:ACE5918
Cydosia majuscula|[11265]|LMEM094-09|United States|Alabama|658[1n]|BOLD:ACE5918
Cydosia majuscula|[11266]|LMEM088-09|United States|Alabama|658[0n]|BOLD:ACE5918
Cydosia majuscula|[11267]|LMEM089-09|United States|Alabama|504[0n]|BOLD:ACE5918
Cydosia majuscula|[11268]|LMEM093-09|United States|Alabama|550[0n]|BOLD:ACE5918
Cydosia majuscula|[11269]|LMEM095-09|United States|Alabama|658[0n]|BOLD:ACE5918
Cydosia majuscula|[11270]|MNAO022-11|United States|Arkansas|658[0n]|BOLD:ACE5918
Cydosia aurivitta|[11271]|HKONB421-09|United States|Texas|658[0n]|BOLD:AAC9718
Cydosia aurivitta|[11272]|BBLSX845-09|United States|Texas|658[0n]|BOLD:AAC9718
Cydosia aurivitta|[11273]|USLEP498-10|United States|Texas|658[0n]|BOLD:AAC9718
Cydosia aurivitta|[11274]|RDNMK485-11|United States|Texas|658[0n]|BOLD:AAC9718
Cydosia aurivitta|[11275]|BBLSY933-09|United States|Texas|658[0n]|BOLD:AAC9718
Cydosia aurivitta|[11276]|BBLSX844-09|United States|Texas|658[0n]|BOLD:AAC9718
Cydosia aurivitta|[11277]|BBLSY255-09|United States|Texas|658[0n]|BOLD:AAC9718
Cydosia aurivitta|[11278]|BBLSX843-09|United States|Texas|639[0n]|BOLD:AAC9718
Cydosia aurivitta|[11279]|HKONB422-09|United States|Texas|635[0n]|BOLD:AAC9718
Cydosia aurivitta|[11280]|BBLSW874-09|United States|Texas|634[0n]|BOLD:AAC9718
Cydosia aurivitta|[11281]|USLEP499-10|United States|Texas|658[0n]|BOLD:AAC9718
Cydosia aurivitta|[11282]|BBLOC1566-11|United States|Texas|620[0n]|BOLD:AAC9718
Cydosia aurivitta|[11283]|BBLSX842-09|United States|Texas|658[0n]|BOLD:AAC9718
Cydosia aurivitta|[11284]|USLEP665-10|United States|Texas|658[0n]|BOLD:AAC9718
Cydosia aurivitta|[11285]|BBLOD1190-11|United States|Texas|658[0n]|BOLD:AAC9718
Cydosia curvinella|[11286]|BLPDB495-09|Costa Rica|Alajuela|658[0n]|BOLD:AAA6291
Cydosia curvinella|[11287]|BLPDC453-09|Costa Rica|Alajuela|618[0n]|BOLD:AAA6291
Cydosia curvinella|[11288]|MHMXQ556-08|Costa Rica|Guanacaste|658[0n]|BOLD:AAA6291
Cydosia curvinella|[11289]|BLPCA794-08|Costa Rica|Guanacaste|658[0n]|BOLD:AAA6291
Cydosia curvinella|[11290]|BLPAE706-06|Costa Rica|Guanacaste|658[0n]|BOLD:AAA6291
Cydosia curvinella|[11291]|BLPAE708-06|Costa Rica|Guanacaste|658[0n]|BOLD:AAA6291
Cydosia curvinella|[11292]|BLPAF783-07|Costa Rica|Guanacaste|658[0n]|BOLD:AAA6291
Cydosia curvinella|[11293]|BLPCJ345-08|Costa Rica|Guanacaste|658[0n]|BOLD:AAA6291
Cydosia curvinella|[11294]|BLPCO808-08|Costa Rica|Guanacaste|658[0n]|BOLD:AAA6291
Cydosia curvinella|[11295]|BLPDM327-10|Costa Rica|Guanacaste|658[0n]|BOLD:AAA6291
Cydosia curvinella|[11296]|MHMXZ881-09|Costa Rica||658[0n]|BOLD:AAA6291
Cydosia curvinella|[11297]|BLPDE276-09|Costa Rica|Guanacaste|658[0n]|BOLD:AAA6291
Cydosia curvinella|[11298]|BLPDC623-09|Costa Rica|Alajuela|658[0n]|BOLD:AAA6291
Cydosia curvinella|[11299]|BLPDB496-09|Costa Rica|Alajuela|658[0n]|BOLD:AAA6291
Cydosia curvinella|[11300]|BLPDA852-09|Costa Rica|Guanacaste|658[0n]|BOLD:AAA6291
Cydosia curvinella|[11301]|BLPCO810-08|Costa Rica|Guanacaste|658[0n]|BOLD:AAA6291
Cydosia curvinella|[11302]|BLPCO809-08|Costa Rica|Guanacaste|658[0n]|BOLD:AAA6291
Cydosia curvinella|[11303]|BLPCK250-08|Costa Rica|Alajuela|658[0n]|BOLD:AAA6291
Cydosia curvinella|[11304]|MHMXQ557-08|Costa Rica|Guanacaste|658[0n]|BOLD:AAA6291
Cydosia curvinella|[11305]|MHMXQ555-08|Costa Rica|Guanacaste|658[0n]|BOLD:AAA6291
Cydosia curvinella|[11306]|BLPCA040-08|Costa Rica|Guanacaste|658[0n]|BOLD:AAA6291
Cydosia curvinella|[11307]|BLPCA039-08|Costa Rica|Guanacaste|658[0n]|BOLD:AAA6291
Cydosia curvinella|[11308]|BLPBB042-07|Costa Rica|Guanacaste|658[0n]|BOLD:AAA6291
Cydosia curvinella|[11309]|BLPBB041-07|Costa Rica|Guanacaste|658[0n]|BOLD:AAA6291
Cydosia curvinella|[11310]|BLPBB040-07|Costa Rica|Guanacaste|658[0n]|BOLD:AAA6291
Cydosia curvinella|[11311]|BLPBB039-07|Costa Rica|Guanacaste|658[0n]|BOLD:AAA6291
Cydosia curvinella|[11312]|BLPBB038-07|Costa Rica|Guanacaste|658[0n]|BOLD:AAA6291
Cydosia curvinella|[11313]|BLPAH400-07|Costa Rica|Guanacaste|658[0n]|BOLD:AAA6291
Cydosia curvinella|[11314]|BLPAF787-07|Costa Rica|Guanacaste|658[0n]|BOLD:AAA6291
Cydosia curvinella|[11315]|BLPAF782-07|Costa Rica|Guanacaste|658[0n]|BOLD:AAA6291
Cydosia curvinella|[11316]|BLPAF781-07|Costa Rica|Guanacaste|658[0n]|BOLD:AAA6291
Cydosia curvinella|[11317]|BLPAF780-07|Costa Rica|Guanacaste|658[0n]|BOLD:AAA6291
Cydosia curvinella|[11318]|BLPAE707-06|Costa Rica|Guanacaste|658[0n]|BOLD:AAA6291
Cydosia curvinella|[11319]|BLPAE705-06|Costa Rica|Guanacaste|658[0n]|BOLD:AAA6291
Cydosia curvinella|[11320]|MHAUF444-06|Costa Rica|Guanacaste|658[0n]|BOLD:AAA6291
Cydosia curvinella|[11321]|MHAUF442-06|Costa Rica|Guanacaste|658[0n]|BOLD:AAA6291
Cydosia curvinella|[11322]|MHAUF441-06|Costa Rica|Guanacaste|658[0n]|BOLD:AAA6291
Cydosia curvinella|[11323]|MHAUB450-05|Costa Rica|Guanacaste|658[0n]|BOLD:AAA6291
Cydosia curvinella|[11324]|MHAUB449-05|Costa Rica|Guanacaste|658[0n]|BOLD:AAA6291
Cydosia curvinella|[11325]|BLPDE277-09|Costa Rica|Guanacaste|658[0n]|BOLD:AAA6291
Cydosia curvinella|[11326]|LOCRC516-09|Costa Rica|Guanacaste|658[0n]|BOLD:AAA6291
Cydosia curvinella|[11327]|LOCRC517-09|Costa Rica|Guanacaste|658[0n]|BOLD:AAA6291
Cydosia curvinella|[11328]|BLPDH796-09|Costa Rica|Alajuela|658[0n]|BOLD:AAA6291
Cydosia curvinella|[11329]|BLPDI773-09|Costa Rica|Guanacaste|658[0n]|BOLD:AAA6291
Cydosia curvinella|[11330]|BLPDI881-09|Costa Rica|Guanacaste|658[0n]|BOLD:AAA6291
Cydosia curvinella|[11331]|BLPDK666-09|Costa Rica|Guanacaste|658[0n]|BOLD:AAA6291
Cydosia curvinella|[11332]|BLPDL1683-10|Costa Rica|Guanacaste|658[0n]|BOLD:AAA6291
Cydosia curvinella|[11333]|BLPED2143-12|Costa Rica|Guanacaste|658[0n]|BOLD:AAA6291
Cydosia curvinella|[11334]|LYPIE282-09|Mexico|Yucatan|658[0n]|BOLD:AAA6291
Cydosia curvinella|[11335]|MHAUF443-06|Costa Rica|Guanacaste|658[0n]|BOLD:AAA6291
Cydosia curvinella|[11336]|BLPDA853-09|Costa Rica|Guanacaste|658[0n]|BOLD:AAA6291
Cydosia curvinella|[11337]|BLPCO685-08|Costa Rica|Guanacaste|658[0n]|BOLD:AAA6291
Cydosia curvinella|[11338]|BLPCK873-08|Costa Rica|Alajuela|658[0n]|BOLD:AAA6291

Cydosia curvinella|[11336]|BLPDA853-09|Costa Rica|Guanacaste|658[0n]|BOLD:AAA6291
Cydosia curvinella|[11337]|BLPCO685-08|Costa Rica|Guanacaste|658[0n]|BOLD:AAA6291
Cydosia curvinella|[11338]|BLPCK873-08|Costa Rica|Alajuela|658[0n]|BOLD:AAA6291
Cydosia curvinella|[11339]|LPYPC075-08|Mexico|Yucatan|658[0n]|BOLD:AAA6291
Cydosia curvinella|[11340]|LPYPB278-08|Mexico|Quintana Roo|658[0n]|BOLD:AAA6291
Cydosia curvinella|[11341]|BLPCD765-08|Costa Rica|Guanacaste|658[0n]|BOLD:AAA6291
Cydosia curvinella|[11342]|BLPAF788-07|Costa Rica|Guanacaste|658[0n]|BOLD:AAA6291
Cydosia curvinella|[11343]|BLPAF785-07|Costa Rica|Guanacaste|658[0n]|BOLD:AAA6291
Cydosia curvinella|[11344]|BLPAF784-07|Costa Rica|Guanacaste|658[0n]|BOLD:AAA6291
Cydosia curvinella|[11345]|LYHES267-09|Mexico|Quintana Roo|605[0n]|BOLD:AAA6291
Cydosia curvinella|[11346]|LYHES539-09|Mexico|Quintana Roo|656[0n]|BOLD:AAA6291
Cydosia curvinella|[11347]|BLPDK199-09|Costa Rica|Guanacaste|658[0n]|BOLD:AAA6291
Cydosia curvinella|[11348]|CNCLB1462-14|Ecuador|Napo|658[0n]|BOLD:AAA6291
PsaphidiniRZ new sp.|[11349]|RDNMG244-08|United States|Texas|658[0n]|BOLD:AAG1047
PsaphidiniRZ new sp.|[11350]|RDNMH037-09|United States|Texas|658[0n]|BOLD:AAG1047
PsaphidiniRZ new sp.|[11351]|HKONB542-09|United States|Texas|658[0n]|BOLD:AAG1047
PsaphidiniRZ new sp.|[11352]|RDNMK344-11|United States|New Mexico|658[0n]|BOLD:ACY8727
Paramiana canoa|[11353]|RDNMG008-08|United States|Arizona|658[0n]|BOLD:AAI6363
Paramiana canoa|[11354]|RDNMJ394-11|United States|Arizona|658[0n]|BOLD:AAI6363
Nacopa melanderi|[11355]|NAMUM119-08|United States|Texas|658[0n]|BOLD:AAF7411
Nacopa melanderi|[11356]|NAMUM120-08|United States|Texas|658[0n]|BOLD:AAF7411
Nacopa melanderi|[11357]|RDNME961-08|United States|New Mexico|658[0n]|BOLD:AAF7411
Nacopa bistrigata|[11358]|CNCLB894-14|United States|Arizona|658[0n]|BOLD:ACM4470
Nacopa bistrigata|[11359]|CNCLB895-14|United States|Arizona|658[0n]|BOLD:ACM4470
Cryphia albipuncta|[11360]|CNCLB226-14|United States|California|658[0n]|BOLD:ACM4455
Cryphia albipuncta|[11361]|CNCLB227-14|United States|California|658[0n]|BOLD:ACM4455
Cryphia flavipuncta|[11362]|CNCLB1077-14|United States|California|307[0n]|
Plagiomimicus navia|[11363]|BBLSX214-09|United States|Texas|658[0n]|BOLD:AAH5273
Plagiomimicus navia|[11364]|BBLSX254-09|United States|Texas|632[0n]|BOLD:AAH5273
Plagiomimicus navia|[11365]|BBLSY489-09|United States|New Mexico|658[0n]|BOLD:AAH5273
Plagiomimicus navia|[11366]|BBLSW098-09|United States|Texas|658[0n]|BOLD:AAH5273
Plagiomimicus navia|[11367]|BBLSY526-09|United States|New Mexico|658[0n]|BOLD:AAH5273
Airamia albiocula|[11368]|LNAUS4364-13|United States|Nevada|658[0n]|BOLD:ACJ7028
Airamia albiocula|[11369]|CNCLB896-14|United States|Nevada|658[0n]|BOLD:ACJ7028
Neotarache deserticola|[11370]|TML161-14|United States||625[0n]|BOLD:ACO4436
Aleptina junctimacula|[11371]|LNAUS3272-13|United States|Texas|658[0n]|BOLD:ACH8654
Aleptina junctimacula|[11372]|LNAUS3274-13|United States|Texas|625[0n]|BOLD:ACH8654
Aleptina semiatra|[11373]|BBLOC1494-11|United States|California|658[0n]|BOLD:AAU5834
Aleptina semiatra|[11374]|LNAUS3284-13|United States|California|658[0n]|BOLD:AAU5834
Aleptina semiatra|[11375]|RDNMJ134-10|United States|Arizona|658[0n]|BOLD:AAU5834
Aleptina semiatra|[11376]|LNAUS3281-13|United States|California|658[0n]|BOLD:AAU5834
Aleptina semiatra|[11377]|RDNMJ135-10|United States|Arizona|658[0n]|BOLD:AAU5834
Aleptina semiatra|[11378]|LOCBF1130-13|United States|California|570[0n]|BOLD:AAU5834
Aleptina semiatra|[11379]|LNAUS3283-13|United States|California|658[0n]|BOLD:AAU5834
Aleptina semiatra|[11380]|LNAUS3282-13|United States|California|658[0n]|BOLD:AAU5834
Aleptina semiatra|[11381]|LOCBF2147-13|United States|California|618[0n]|BOLD:AAU5834
Aleptina arenaria|[11382]|RDNMK327-11|United States|New Mexico|658[0n]|BOLD:AAZ9513
Aleptina clinopetes|[11383]|LNAUS3276-13|Mexico|Sinaloa|407[0n]|
Aleptina clinopetes|[11384]|CNCLB2125-14|United States|Texas|658[0n]|BOLD:ACR4684
Aleptina clinopetes|[11385]|CNCLB2428-14|United States|Texas|658[0n]|BOLD:ACR4684
Aleptina inca|[11386]|BBLSW082-09|United States|Texas|658[0n]|BOLD:AAH5263
Aleptina inca|[11387]|BBLSW081-09|United States|Texas|658[0n]|BOLD:AAH5263
Aleptina inca|[11388]|BBLOE1620-12|United States|Texas|658[0n]|BOLD:AAH5263
Aleptina inca|[11389]|BBLSX837-09|United States|Texas|658[0n]|BOLD:AAH5263
Aleptina inca|[11390]|BBLOE928-12|United States|Texas|658[0n]|BOLD:AAH5263
Aleptina inca|[11391]|BBLOD1803-11|United States|Arizona|624[0n]|BOLD:AAH5263
Aleptina inca|[11392]|BBLSX232-09|United States|Texas|658[0n]|BOLD:AAH5263
Aleptina inca|[11393]|BBLOD1795-11|United States|Arizona|658[0n]|BOLD:AAH5263
Aleptina inca|[11394]|BBLSX263-09|United States|Texas|658[0n]|BOLD:AAH5263
Aleptina inca|[11395]|BBLSW048-09|United States|Texas|658[0n]|BOLD:AAH5263
Aleptina inca|[11396]|BBLOD1525-11|United States|Arizona|658[0n]|BOLD:AAH5263
Aleptina inca|[11397]|BBLOD1538-11|United States|Arizona|658[0n]|BOLD:AAH5263
Aleptina inca|[11398]|BBLOD1888-11|United States|Arizona|658[0n]|BOLD:AAH5263
Aleptina inca|[11399]|BBLOD1918-11|United States|Arizona|658[0n]|BOLD:AAH5263
Aleptina inca|[11400]|BBLOE1832-12|United States|Texas|658[0n]|BOLD:AAH5263
Aleptina inca|[11401]|LNAUS3286-13|United States|New Mexico|658[0n]|BOLD:AAH5263
Aleptina inca|[11402]|BBLSW050-09|United States|Texas|658[0n]|BOLD:AAH5263
Aleptina inca|[11403]|BBLSW052-09|United States|Texas|658[0n]|BOLD:AAH5263
Aleptina inca|[11404]|BBLSW688-09|United States|New Mexico|658[0n]|BOLD:AAH5263
Aleptina inca|[11405]|BBLOD1068-11|United States|Texas|658[0n]|BOLD:AAH5263
Aleptina inca|[11406]|BBLOE1834-12|United States|Texas|658[0n]|BOLD:AAH5263
Aleptina inca|[11407]|BBLSX180-09|United States|Texas|658[0n]|BOLD:AAH5263
Aleptina inca|[11408]|BBLOD1528-11|United States|Arizona|658[0n]|BOLD:AAH5263
Aleptina inca|[11409]|USLEP417-10|United States|Colorado|658[0n]|BOLD:AAH5263
Aleptina inca|[11410]|BBLSX243-09|United States|Texas|658[0n]|BOLD:AAH5263
Aleptina inca|[11411]|BBLSW049-09|United States|Texas|658[0n]|BOLD:AAH5263
Aleptina inca|[11412]|BBLSW051-09|United States|Texas|658[0n]|BOLD:AAH5263
Aleptina inca|[11413]|BBLSW053-09|United States|Texas|658[0n]|BOLD:AAH5263
Aleptina inca|[11414]|BBLSW083-09|United States|Texas|658[0n]|BOLD:AAH5263
Aleptina inca|[11415]|BBLSX242-09|United States|Texas|658[0n]|BOLD:AAH5263
Aleptina inca|[11416]|RDNMK347-11|United States|New Mexico|658[0n]|BOLD:AAH5263
Aleptina inca|[11417]|RDNMK346-11|United States|New Mexico|658[0n]|BOLD:AAH5263
Aleptina inca|[11418]|BBLSX184-09|United States|Texas|658[0n]|BOLD:AAH5263
Aleptina inca|[11419]|RDNMK345-11|United States|New Mexico|658[0n]|BOLD:AAH5263
Aleptina inca|[11420]|BBLOE1516-12|United States|Texas|658[0n]|BOLD:AAH5263
Aleptina inca|[11421]|BBLOE1842-12|United States|Texas|658[0n]|BOLD:AAH5263
Aleptina inca|[11422]|BBLOE1848-12|United States|Texas|658[0n]|BOLD:AAH5263
Aleptina inca|[11423]|BBLSX238-09|United States|Texas|658[0n]|BOLD:AAH5263
Aleptina inca|[11424]|BBLSX211-09|United States|Texas|658[0n]|BOLD:AAH5263
Aleptina inca|[11425]|BBLSX209-09|United States|Texas|658[0n]|BOLD:AAH5263
Aleptina inca|[11426]|CNCLA5284-13|United States|Arizona|658[0n]|BOLD:AAH5263
Aleptina aleptivoides|[11427]|CNCLB3089-15|United States|California|658[0n]|BOLD:ACW8160
Catabenoides terminellus|[11428]|BBLOE1627-12|United States|Texas|658[0n]|BOLD:ABW8287
Catabenoides terminellus|[11429]|RDNML218-13|United States|Arizona|658[0n]|BOLD:ABW8287
Catabenoides terminellus|[11430]|RDNML219-13|United States|Arizona|658[0n]|BOLD:ABW8287
Catabenoides vitrina|[11431]|CNCLA5287-13|United States|Florida|658[0n]|BOLD:ACI9518
Amphipyra glabella|[11432]|BLTIB1002-08|Canada|Ontario|658[0n]|BOLD:AAE2518
Amphipyra glabella|[11433]|BLTIB1043-08|Canada|Ontario|658[0n]|BOLD:AAE2518
Amphipyra glabella|[11434]|RDLQB838-05|Canada|Quebec|623[1n]|BOLD:AAE2518
Amphipyra glabella|[11435]|RDMAB987-09|Canada|Alberta|634[0n]|BOLD:AAE2518
Amphipyra glabella|[11436]|RDMAB988-09|Canada|Manitoba|634[0n]|BOLD:AAE2518
Amphipyra glabella|[11437]|RDMAB989-09|Canada|Alberta|658[0n]|BOLD:AAE2518
Amphipyra brunneoatra|[11438]|LOCBF409-06|United States|California|597[0n]|BOLD:AAH4526

Amphipyra glabella|[11435]|RDMAB987-09|Canada|Alberta|634[0n]|BOLD:AAE2518
Amphipyra glabella|[11436]|RDMAB988-09|Canada|Manitoba|634[0n]|BOLD:AAE2518
Amphipyra glabella|[11437]|RDMAB989-09|Canada|Alberta|658[0n]|BOLD:AAE2518
Amphipyra brunneoatra|[11438]|LOCBE409-06|United States|California|597[0n]|BOLD:AAH4526
Amphipyra brunneoatra|[11439]|LOCBE417-06|United States|California|596[0n]|BOLD:AAH4526
Amphipyra brunneoatra|[11440]|LOCBE415-06|United States|California|585[0n]|BOLD:AAH4526
Amphipyra brunneoatra|[11441]|RDNMG678-08|United States|California|658[0n]|BOLD:AAH4526
Amphipyra brunneoatra|[11442]|BBLSX708-09|United States|Arizona|658[0n]|BOLD:AAH4526
Tyta luctuosa|[11443]|LEFIL011-10|Russia|Orenburg|658[0n]|BOLD:AAD5088
Tyta luctuosa|[11444]|IBLAO344-12|Spain|Andalusia|658[0n]|BOLD:AAD5088
Tyta luctuosa|[11445]|IBLAO345-12|Spain|Andalusia|658[0n]|BOLD:AAD5088
Tyta luctuosa|[11446]|RDNME664-08|United States|Utah|658[0n]|BOLD:AAD5088
Tyta luctuosa|[11447]|GWOTI360-12|Georgia||658[0n]|BOLD:AAD5088
Tyta luctuosa|[11448]|GWOTI361-12|Georgia||658[0n]|BOLD:AAD5088
Tyta luctuosa|[11449]|LEFIL012-10|Russia|Orenburg|658[0n]|BOLD:AAD5088
Tyta luctuosa|[11450]|LEFIB035-10|Greece|North Aegean|658[0n]|BOLD:AAD5088
Tyta luctuosa|[11451]|GSCMW1389-10|Austria|Upper Austria|658[0n]|BOLD:AAD5088
Tyta luctuosa|[11452]|FBLMV202-09|Germany|Bavaria|658[0n]|BOLD:AAD5088
Tyta luctuosa|[11453]|FBLMT407-09|Germany|Bavaria|658[0n]|BOLD:AAD5088
Tyta luctuosa|[11454]|GWORK239-09|Germany|Bavaria|658[0n]|BOLD:AAD5088
Tyta luctuosa|[11455]|QUNO365-08|France||658[0n]|BOLD:AAD5088
Tyta luctuosa|[11456]|LENOA043-11|France|Haute Normandie|407[0n]|
Tyta luctuosa|[11457]|LEFIJ1213-11|Finland||658[0n]|BOLD:AAD5088
Tyta luctuosa|[11458]|LEFIJ1214-11|Finland||658[0n]|BOLD:AAD5088
Tyta luctuosa|[11459]|GBLGC334-12|Germany|Bavaria|658[0n]|BOLD:AAD5088
Tyta luctuosa|[11460]|GBLAB297-13|Germany|Saarland|658[0n]|BOLD:AAD5088
Tyta luctuosa|[11461]|TRLEP105-13|Turkey|Mersin|658[0n]|BOLD:AAD5088
Grotella soror|[11462]|CNCLB937-14|United States|Arizona|307[0n]|
Grotella soror|[11463]|CNCLB2145-14|United States|Arizona|658[0n]|BOLD:ACR4987
Tristyla alboplagiata|[11464]|NAMUM151-08|United States|California|658[0n]|BOLD:AAW7928
Sexserrata hampsoni|[11465]|CNCLB3096-15|United States|California|658[0n]|BOLD:ACW8664
Condica parista|[11466]|RDNME659-08|United States|Texas|658[0n]|BOLD:AAD3843
Condica parista|[11467]|LOCRC559-09|Costa Rica|Heredia|658[0n]|BOLD:AAD3843
Harrisimemna trisignata|[11468]|RDLQB067-05|Canada|Quebec|658[0n]|BOLD:AAB3137
Harrisimemna trisignata|[11469]|XAE274-04|Canada|Ontario|658[0n]|BOLD:AAB3137
Harrisimemna trisignata|[11470]|PHMNB727-05|Canada|New Brunswick|658[0n]|BOLD:AAB3137
Harrisimemna trisignata|[11471]|RDLQB066-05|Canada|Quebec|581[0n]|BOLD:AAB3137
Harrisimemna trisignata|[11472]|RDLQB555-05|Canada|Quebec|658[0n]|BOLD:AAB3137
Harrisimemna trisignata|[11473]|RDLQB744-05|Canada|Quebec|658[0n]|BOLD:AAB3137
Harrisimemna trisignata|[11474]|RDLQB745-05|Canada|Quebec|658[0n]|BOLD:AAB3137
Harrisimemna trisignata|[11475]|MECD348-06|Canada|Quebec|658[0n]|BOLD:AAB3137
Harrisimemna trisignata|[11476]|TTMNB002-06|Canada|New Brunswick|657[0n]|BOLD:AAB3137
Harrisimemna trisignata|[11477]|XAJ927-06|Canada|Ontario|658[0n]|BOLD:AAB3137
Harrisimemna trisignata|[11478]|XAK062-06|Canada|Ontario|658[0n]|BOLD:AAB3137
Harrisimemna trisignata|[11479]|LPSOD1064-09|Canada|Ontario|658[0n]|BOLD:AAB3137
Harrisimemna trisignata|[11480]|BBLSW826-09|United States|Texas|658[0n]|BOLD:AAB3137
Harrisimemna trisignata|[11481]|LPMN714-08|Canada|Manitoba|658[0n]|BOLD:AAB3137
Harrisimemna trisignata|[11482]|LPOKA212-08|United States|Oklahoma|658[0n]|BOLD:AAB3137
Harrisimemna trisignata|[11483]|BBLSW844-09|United States|Texas|655[0n]|BOLD:AAB3137
Harrisimemna trisignata|[11484]|LPOKC760-09|United States|Oklahoma|658[0n]|BOLD:AAB3137
Harrisimemna trisignata|[11485]|LOFLA918-06|United States|Florida|658[0n]|BOLD:AAB3137
Harrisimemna trisignata|[11486]|LNC018-05|United States|North Carolina|658[0n]|BOLD:AAB3137
Harrisimemna trisignata|[11487]|TTMNB001-06|Canada|New Brunswick|658[0n]|BOLD:AAB3137
Harrisimemna trisignata|[11488]|LNC943-06|United States|North Carolina|658[0n]|BOLD:AAB3137
Harrisimemna trisignata|[11489]|MMNA070-08|United States|Georgia|658[0n]|BOLD:AAB3137
Harrisimemna trisignata|[11490]|MNBB288-05|Canada|New Brunswick|658[0n]|BOLD:AAB3137
Harrisimemna trisignata|[11491]|TTMNB029-06|Canada|New Brunswick|658[0n]|BOLD:AAB3137
Harrisimemna trisignata|[11492]|BBLPC156-09|Canada|Nova Scotia|658[0n]|BOLD:AAB3137
Harrisimemna trisignata|[11493]|BBLPC163-09|Canada|Nova Scotia|658[0n]|BOLD:AAB3137
Harrisimemna trisignata|[11494]|BBLPC901-09|Canada|Newfoundland and Labrador|658[0n]|BOLD:AAB3137
Homolagoa grotelliformis|[11495]|LMEMB444-09|United States|Arizona|658[2n]|BOLD:AAN9561
Homolagoa grotelliformis|[11496]|LMEMB445-09|United States|Arizona|658[0n]|BOLD:AAN9561
Homolagoa grotelliformis|[11497]|CMAZA594-10|United States|Arizona|658[0n]|BOLD:AAN9561
Homolagoa grotelliformis|[11498]|CMAZA1162-12|United States|Arizona|658[0n]|BOLD:AAN9561
Condica punctifera|[11499]|RDNML322-13|United States|Florida|658[0n]|BOLD:ACD8995
Condica punctifera|[11500]|RDNML323-13|United States|Florida|658[0n]|BOLD:ACD8995
Condica mobilis|[11501]|BLPBC606-07|Costa Rica|Guanacaste|632[0n]|BOLD:AAB3357
Condica mobilis|[11502]|LYHES389-09|Mexico|Quintana Roo|614[0n]|BOLD:AAB3357
Condica mobilis|[11503]|BLPCA456-08|Costa Rica|Guanacaste|658[2n]|BOLD:AAB3357
Condica mobilis|[11504]|LYPAP461-09|Mexico|Quintana Roo|625[0n]|BOLD:AAB3357
Condica mobilis|[11505]|BLPDK1706-09|Costa Rica|Guanacaste|658[0n]|BOLD:AAB3357
Condica mobilis|[11506]|LOFLA272-06|United States|Florida|657[0n]|BOLD:AAB3357
Condica mobilis|[11507]|GWOSU321-11|Argentina||658[0n]|BOLD:AAB3357
Condica mobilis|[11508]|GWOSU322-11|Argentina||658[0n]|BOLD:AAB3357
Condica mobilis|[11509]|GWOSZ809-11|Peru|Huanuco|658[0n]|BOLD:AAB3357
Condica mobilis|[11510]|MOTAR201-12|Argentina|Misiones|655[0n]|BOLD:AAB3357
Condica mobilis|[11511]|MOTAR202-12|Argentina|Misiones|658[0n]|BOLD:AAB3357
Condica mobilis|[11512]|RDNMH089-09|United States|Arizona|658[0n]|BOLD:AAB3357
Condica mobilis|[11513]|USLEP1225-10|United States|Florida|658[0n]|BOLD:AAB3357
Condica mobilis|[11514]|BLPDK1705-09|Costa Rica|Guanacaste|658[0n]|BOLD:AAB3357
Condica mobilis|[11515]|BLPCO350-08|Costa Rica|Guanacaste|658[0n]|BOLD:AAB3357
Condica mobilis|[11516]|BLPCK982-08|Costa Rica|Alajuela|658[0n]|BOLD:AAB3357
Condica mobilis|[11517]|BLPCK265-08|Costa Rica|Alajuela|658[0n]|BOLD:AAB3357
Condica mobilis|[11518]|BLPCJ492-08|Costa Rica|Guanacaste|658[0n]|BOLD:AAB3357
Condica mobilis|[11519]|BLPCJ056-08|Costa Rica|Guanacaste|658[0n]|BOLD:AAB3357
Condica mobilis|[11520]|BLPCA457-08|Costa Rica|Guanacaste|658[0n]|BOLD:AAB3357
Condica mobilis|[11521]|BLPAH363-07|Costa Rica|Guanacaste|658[0n]|BOLD:AAB3357
Condica mobilis|[11522]|BLPAH359-07|Costa Rica|Guanacaste|658[0n]|BOLD:AAB3357
Condica mobilis|[11523]|BLPAG253-07|Costa Rica|Guanacaste|658[0n]|BOLD:AAB3357
Condica mobilis|[11524]|RDNMD496-06|United States|Florida|658[0n]|BOLD:AAB3357
Condica mobilis|[11525]|RDNMD479-06|United States|Florida|658[0n]|BOLD:AAB3357
Condica mobilis|[11526]|BLPAD068-06|Costa Rica|Guanacaste|658[0n]|BOLD:AAB3357
Condica mobilis|[11527]|LOFLC214-06|United States|Florida|658[0n]|BOLD:AAB3357
Condica mobilis|[11528]|LYPIE242-09|Mexico|Quintana Roo|658[0n]|BOLD:AAB3357
Condica mobilis|[11529]|BLPDA773-09|Costa Rica|Guanacaste|658[0n]|BOLD:AAB3357
Condica mobilis|[11530]|RDNMG221-08|Dominican Republic||648[0n]|BOLD:AAB3357
Condica mobilis|[11531]|BLPAD057-06|Costa Rica|Guanacaste|634[1n]|BOLD:AAB3357
Condica mobilis|[11532]|BLPDI659-09|Costa Rica|Guanacaste|623[0n]|BOLD:AAB3357
Condica mobilis|[11533]|USLEP1226-10|United States|Florida|658[0n]|BOLD:AAB3357
Condica mobilis|[11534]|RDNMJ014-10|United States|Florida|658[0n]|BOLD:AAB3357
Condica mobilis|[11535]|RDNMJ015-10|United States|Florida|658[0n]|BOLD:AAB3357
Condica mobilis|[11536]|RDNMJ020-10|United States|Florida|658[0n]|BOLD:AAB3357
Condica mobilis|[11537]|RDNMJ022-10|United States|Florida|658[0n]|BOLD:AAB3357

Condica mobilis|[11535]|RDNMJ015-10|United States|Florida|658[0n]|BOLD:AAB3357
Condica mobilis|[11536]|RDNMJ020-10|United States|Florida|658[0n]|BOLD:AAB3357
Condica mobilis|[11537]|RDNMJ022-10|United States|Florida|658[0n]|BOLD:AAB3357
Condica mobilis|[11538]|RDNMJ024-10|United States|Florida|658[0n]|BOLD:AAB3357
Condica mobilis|[11539]|RDNMJ032-10|United States|Florida|658[0n]|BOLD:AAB3357
Condica mobilis|[11540]|RDNMJ033-10|United States|Florida|658[0n]|BOLD:AAB3357
Condica mobilis|[11541]|BLPDU208-11|Costa Rica|Guanacaste|658[0n]|BOLD:AAB3357
Condica mobilis|[11542]|MHMYS2781-13|Costa Rica|Guanacaste|658[0n]|BOLD:AAB3357
Condica mobilis|[11543]|MHMYS2910-13|Costa Rica|Guanacaste|658[0n]|BOLD:AAB3357
Condica mobilis|[11544]|BLPEE5336-14|Costa Rica|Guanacaste|658[0n]|BOLD:AAB3357
Condica albigera|[11545]|MHAUF134-06|Costa Rica|Guanacaste|512[1n]|BOLD:AAD8636
Condica albigera|[11546]|LYPIE314-09|Mexico|Yucatan|658[0n]|BOLD:AAD8636
Condica albigera|[11547]|HKONB189-08|United States|Texas|658[0n]|BOLD:AAD8636
Condica albigera|[11548]|LPYPB915-08|Mexico|Campeche|658[0n]|BOLD:AAD8636
Condica albigera|[11549]|BLPCC369-08|Costa Rica|Guanacaste|658[0n]|BOLD:AAD8636
Condica albigera|[11550]|BLPDB629-09|Costa Rica|Alajuela|639[1n]|BOLD:AAD8636
Condica albigera|[11551]|LYRIO466-10|Mexico|Yucatan|658[0n]|BOLD:AAD8636
Condica sutor|[11552]|RDNMH483-09|United States|Louisiana|658[0n]|BOLD:AAA5860
Condica sutor|[11553]|LNC511-06|United States|North Carolina|621[3n]|BOLD:AAA5860
Condica sutor|[11554]|LNCNW055-06|United States|North Carolina|658[0n]|BOLD:AAA5860
Condica sutor|[11555]|RDNMJ013-10|United States|Florida|658[0n]|BOLD:AAA5860
Condica sutor|[11556]|RDNMH487-09|United States|Louisiana|658[0n]|BOLD:AAA5860
Condica sutor|[11557]|USLEP299-10|United States|Florida|658[0n]|BOLD:AAA5860
Condica sutor|[11558]|RDNMJ016-10|United States|Florida|658[0n]|BOLD:AAA5860
Condica sutor|[11559]|RDNMJ018-10|United States|Florida|658[0n]|BOLD:AAA5860
Condica sutor|[11560]|BBLOC968-11|United States|Texas|658[0n]|BOLD:AAA5860
Condica sutor|[11561]|RDNMH497-09|United States|Louisiana|658[0n]|BOLD:AAA5860
Condica sutor|[11562]|BBLOC1434-11|United States|Texas|658[0n]|BOLD:AAA5860
Condica sutor|[11563]|RDNMH485-09|United States|Louisiana|658[0n]|BOLD:AAA5860
Condica sutor|[11564]|RDNMH486-09|United States|Louisiana|658[0n]|BOLD:AAA5860
Condica sutor|[11565]|RDNMH488-09|United States|Louisiana|658[0n]|BOLD:AAA5860
Condica sutor|[11566]|RDNMH500-09|United States|Louisiana|658[0n]|BOLD:AAA5860
Condica sutor|[11567]|RDNMH511-09|United States|Louisiana|658[0n]|BOLD:AAA5860
Condica sutor|[11568]|LGSMG1022-10|United States|North Carolina|658[0n]|BOLD:AAA5860
Condica sutor|[11569]|RDNMJ017-10|United States|Florida|658[0n]|BOLD:AAA5860
Condica sutor|[11570]|RDNMJ023-10|United States|Florida|658[0n]|BOLD:AAA5860
Condica sutor|[11571]|BBLOB1236-11|United States|Florida|658[0n]|BOLD:AAA5860
Condica sutor|[11572]|BBLOC123-11|United States|Florida|658[0n]|BOLD:AAA5860
Condica sutor|[11573]|BBLOC377-11|United States|Texas|658[0n]|BOLD:AAA5860
Condica sutor|[11574]|BBLOC1671-11|United States|Texas|658[0n]|BOLD:AAA5860
Condica sutor|[11575]|RDNMJ021-10|United States|Florida|658[0n]|BOLD:AAA5860
Condica sutor|[11576]|RDNMH499-09|United States|Louisiana|658[0n]|BOLD:AAA5860
Condica sutor|[11577]|RDNMH498-09|United States|Louisiana|658[0n]|BOLD:AAA5860
Condica sutor|[11578]|RDNMH496-09|United States|Louisiana|658[0n]|BOLD:AAA5860
Condica sutor|[11579]|RDNMH484-09|United States|Louisiana|658[0n]|BOLD:AAA5860
Condica sutor|[11580]|LNCNW054-06|United States|North Carolina|658[0n]|BOLD:AAA5860
Condica sutor|[11581]|RDNMH501-09|United States|Louisiana|658[0n]|BOLD:AAA5860
Condica sutor|[11582]|BBLOC1047-11|United States|Arkansas|658[0n]|BOLD:AAA5860
Condica sutor|[11583]|BBLOD1601-11|United States|Texas|658[0n]|BOLD:AAA5860
Condica concisa|[11584]|GWOSU327-11|Brazil||555[0n]|BOLD:AAD8631
Condica concisa|[11585]|MHAUC481-06|Costa Rica|Guanacaste|658[0n]|BOLD:AAD8631
Condica concisa|[11586]|LOFLB707-06|United States|Florida|658[0n]|BOLD:AAD8631
Condica concisa|[11587]|RDNMJ019-10|United States|Florida|658[0n]|BOLD:AAD8631
Condica concisa|[11588]|MOTAR049-12|Argentina|Entre Rios|658[0n]|BOLD:AAD8631
Condica concisa|[11589]|RDNML010-13|United States|Florida|658[0n]|BOLD:AAD8631
Condica concisa|[11590]|LYPIE239-09|Mexico|Quintana Roo|658[0n]|BOLD:AAD8631
Condica concisa|[11591]|MHMXD754-06|Costa Rica|Guanacaste|658[0n]|BOLD:AAD8631
Condica concisa|[11592]|BLPCM328-08|Costa Rica|Guanacaste|658[0n]|BOLD:AAD8631
Condica concisa|[11593]|LYPIE224-09|Mexico|Quintana Roo|658[0n]|BOLD:AAD8631
Condica concisa|[11594]|MOTAR351-12|Argentina|Misiones|658[0n]|BOLD:AAD8631
Condica concisa|[11595]|BLPEF4944-13|Costa Rica|Guanacaste|658[0n]|BOLD:AAD8631
Condica orta|[11596]|RDNMG193-08|United States|Texas|658[0n]|BOLD:AAF0911
Condica orta|[11597]|RDNME944-08|United States|Arizona|658[0n]|BOLD:AAF0911
Condica orta|[11598]|RDNME945-08|United States|Arizona|658[0n]|BOLD:AAF0911
Condica vecors|[11599]|LGSMG1020-10|United States|North Carolina|658[0n]|BOLD:AAB5995
Condica vecors|[11600]|LNC273-05|United States|North Carolina|658[0n]|BOLD:AAB5995
Condica vecors|[11601]|LSUSA002-06|United States|Kentucky|658[0n]|BOLD:AAB5995
Condica vecors|[11602]|LEFIA1061-10|United States|West Virginia|670[0n]|BOLD:AAB5995
Condica vecors|[11603]|LPOKB292-09|United States|Oklahoma|658[0n]|BOLD:AAB5995
Condica vecors|[11604]|LPOKA1030-09|United States|Oklahoma|658[0n]|BOLD:AAB5995
Condica vecors|[11605]|LPOKA436-09|United States|Oklahoma|658[0n]|BOLD:AAB5995
Condica vecors|[11606]|LTOLB127-08|United States|West Virginia|658[0n]|BOLD:AAB5995
Condica vecors|[11607]|LPSOC367-08|Canada|Ontario|655[0n]|BOLD:AAB5995
Condica vecors|[11608]|LSEU748-06|United States|Georgia|658[0n]|BOLD:AAB5995
Condica vecors|[11609]|LSEU358-06|United States|Georgia|658[0n]|BOLD:AAB5995
Condica vecors|[11610]|LNC848-06|United States|North Carolina|658[0n]|BOLD:AAB5995
Condica vecors|[11611]|LGSMC661-05|United States|Tennessee|658[0n]|BOLD:AAB5995
Condica vecors|[11612]|RDLQ155-05|Canada|Quebec|658[0n]|BOLD:AAB5995
Condica vecors|[11613]|LOT515-04|United States|Tennessee|658[0n]|BOLD:AAB5995
Condica vecors|[11614]|LGSM750-04|United States|Tennessee|658[0n]|BOLD:AAB5995
Condica vecors|[11615]|LOCT122-05|United States|Connecticut|658[1n]|BOLD:AAB5995
Condica vecors|[11616]|RDLQF653-06|Canada|Quebec|637[0n]|BOLD:AAB5995
Condica vecors|[11617]|RDLQF810-06|Canada|Quebec|591[1n]|BOLD:AAB5995
Condica vecors|[11618]|RDLQG275-06|Canada|Quebec|631[0n]|BOLD:AAB5995
Condica vecors|[11619]|LPOKB304-09|United States|Oklahoma|632[0n]|BOLD:AAB5995
Condica vecors|[11620]|LPOKB1025-09|United States|Oklahoma|658[0n]|BOLD:AAB5995
Condica vecors|[11621]|BBLSX576-09|United States|Oklahoma|658[0n]|BOLD:AAB5995
Condica vecors|[11622]|LGSMG1021-10|United States|Tennessee|658[0n]|BOLD:AAB5995
Condica vecors|[11623]|LILLB036-11|United States|Illinois|658[0n]|BOLD:AAB5995
Condica vecors|[11624]|LPOKE285-11|United States|Oklahoma|658[0n]|BOLD:AAB5995
Condica luxuriosa|[11625]|JBAZ124-09|United States|Arizona|636[0n]|BOLD:AAE1797
Condica luxuriosa|[11626]|RDNMJ668-11|United States|Arizona|658[0n]|BOLD:AAE1797
Condica luxuriosa|[11627]|RDNMK541-11|United States|Arizona|658[0n]|BOLD:AAE1797
Condica albolabes|[11628]|RDNMD938-07|United States|New Mexico|636[0n]|BOLD:AAE1796
Condica albolabes|[11629]|CMAZA986-12|United States|Arizona|658[0n]|BOLD:AAE1796
Condica albolabes|[11630]|RDNMJ540-11|United States|Arizona|658[0n]|BOLD:AAE1796
Condica albolabes|[11631]|RDNMG217-08|United States|Arizona|658[0n]|BOLD:AAE1796
Condica albolabes|[11632]|NAMUM091-08|United States|Arizona|658[0n]|BOLD:AAE1796
Condica albolabes|[11633]|RDNME1004-08|United States|Arizona|658[0n]|BOLD:AAE1796
Condica albolabes|[11634]|CMAZA1062-12|United States|Arizona|658[0n]|BOLD:AAE1796
Perigea bahamica|[11635]|RDNML006-13|United States|Florida|658[0n]|BOLD:ACD3453
Condica vacillans|[11636]|RDNMF977-08|United States|Texas|658[0n]|BOLD:AAD3844
Condica vacillans|[11637]|GNCLB229-14|United States|Texas|658[0n]|BOLD:AAD3844

[illegible]
Perigea bahamica|[11635]|RDNML006-13|United States|Florida|658[0n]|BOLD:ACD3453
Condica vacillans|[11636]|RDNMF977-08|United States|Texas|658[0n]|BOLD:AAD3844
Condica vacillans|[11637]|CNCLB230-14|United States|Texas|658[0n]|BOLD:AAD3844
Condica confederata|[11638]|LSEU100-06|United States|Florida|517[0n]|BOLD:AAE9644
Condica confederata|[11639]|RDNMH491-09|United States|Louisiana|658[0n]|BOLD:AAE9644
Condica confederata|[11640]|RDNMH492-09|United States|Louisiana|658[0n]|BOLD:AAE9644
Condica confederata|[11641]|RDNMH493-09|United States|Louisiana|658[0n]|BOLD:AAE9644
Condica confederata|[11642]|LNCC454-10|United States|North Carolina|658[0n]|BOLD:AAE9644
Condica confederata|[11643]|LNCC455-10|United States|North Carolina|658[0n]|BOLD:AAE9644
Condica cupentia|[11644]|MHAUF126-06|Costa Rica|Guanacaste|541[0n]|BOLD:AAA7985
Condica cupentia|[11645]|GMARM119-14|Argentina|Misiones|591[0n]|BOLD:AAA7985
Condica cupentia|[11646]|GMARH002-14|Argentina|Misiones|531[0n]|
Condica cupentia|[11647]|MHAUF128-06|Costa Rica|Guanacaste|555[0n]|BOLD:AAA7985
Condica cupentia|[11648]|MHAUF127-06|Costa Rica|Guanacaste|569[1n]|BOLD:AAA7985
Condica cupentia|[11649]|RDNMF619-08|United States|Florida|615[0n]|BOLD:AAA7985
Condica cupentia|[11650]|RDNMH494-09|United States|Louisiana|658[0n]|BOLD:AAA7985
Condica cupentia|[11651]|RDNMH495-09|United States|Louisiana|658[0n]|BOLD:AAA7985
Condica cupentia|[11652]|RDNMJ025-10|United States|Florida|658[0n]|BOLD:AAA7985
Condica cupentia|[11653]|RDNMJ026-10|United States|Florida|658[0n]|BOLD:AAA7985
Condica cupentia|[11654]|RDNMK088-11|United States|Florida|658[0n]|BOLD:AAA7985
Condica cupentia|[11655]|BBLOB477-11|United States|Florida|627[0n]|BOLD:AAA7985
Condica cupentia|[11656]|BLPDW637-11|Costa Rica|Guanacaste|658[0n]|BOLD:AAA7985
Condica cupentia|[11657]|BLPDC462-09|Costa Rica|Alajuela|598[0n]|BOLD:AAA7985
Condica cupentia|[11658]|MHAUF125-06|Costa Rica|Guanacaste|594[0n]|BOLD:AAA7985
Condica cupentia|[11659]|MHAUF124-06|Costa Rica|Guanacaste|592[0n]|BOLD:AAA7985
Condica cupentia|[11660]|BLPBA016-07|Costa Rica|Guanacaste|658[0n]|BOLD:AAA7985
Condica cupentia|[11661]|BLPDS508-10|Costa Rica|Alajuela|658[0n]|BOLD:AAA7985
Condica cupentia|[11662]|MHMXE647-07|Costa Rica|Guanacaste|644[0n]|BOLD:AAA7985
Condica cupentia|[11663]|MOTAR1044-12|Argentina|Misiones|658[0n]|BOLD:AAA7985
Condica cupentia|[11664]|MOTAR238-12|Argentina|Misiones|658[0n]|BOLD:AAA7985
Condica cupentia|[11665]|MOTAR235-12|Argentina|Misiones|658[0n]|BOLD:AAA7985
Condica cupentia|[11666]|MOTAR220-12|Argentina|Misiones|658[0n]|BOLD:AAA7985
Condica cupentia|[11667]|ARMOT227-12|Argentina|Misiones|657[0n]|BOLD:AAA7985
Condica cupentia|[11668]|ARMOT224-12|Argentina|Misiones|657[0n]|BOLD:AAA7985
Condica cupentia|[11669]|ARMOT220-12|Argentina|Formosa|657[0n]|BOLD:AAA7985
Condica cupentia|[11670]|ARMOT201-12|Argentina|Misiones|657[0n]|BOLD:AAA7985
Condica cupentia|[11671]|ARMOT197-12|Argentina|Misiones|657[0n]|BOLD:AAA7985
Condica cupentia|[11672]|MHMYO244-11|Costa Rica||658[0n]|BOLD:AAA7985
Condica cupentia|[11673]|BLPDW638-11|Costa Rica|Guanacaste|658[0n]|BOLD:AAA7985
Condica cupentia|[11674]|BLPDV006-11|Costa Rica|Guanacaste|658[0n]|BOLD:AAA7985
Condica cupentia|[11675]|BLPDP296-10|Costa Rica|Alajuela|658[0n]|BOLD:AAA7985
Condica cupentia|[11676]|MHMYF789-10|Costa Rica|Alajuela|658[0n]|BOLD:AAA7985
Condica cupentia|[11677]|BLPDL1459-10|Costa Rica|Alajuela|658[0n]|BOLD:AAA7985
Condica cupentia|[11678]|BLPDD563-09|Costa Rica|Guanacaste|658[0n]|BOLD:AAA7985
Condica cupentia|[11679]|BLPDD562-09|Costa Rica|Guanacaste|658[0n]|BOLD:AAA7985
Condica cupentia|[11680]|BLPDD457-09|Costa Rica|Guanacaste|658[0n]|BOLD:AAA7985
Condica cupentia|[11681]|BLPDD452-09|Costa Rica|Guanacaste|658[0n]|BOLD:AAA7985
Condica cupentia|[11682]|BLPDD350-09|Costa Rica|Alajuela|658[0n]|BOLD:AAA7985
Condica cupentia|[11683]|BLPDC906-09|Costa Rica|Alajuela|658[0n]|BOLD:AAA7985
Condica cupentia|[11684]|BLPDC262-09|Costa Rica|Guanacaste|658[0n]|BOLD:AAA7985
Condica cupentia|[11685]|BLPDA465-09|Costa Rica|Alajuela|658[0n]|BOLD:AAA7985
Condica cupentia|[11686]|BLPDA464-09|Costa Rica|Alajuela|658[0n]|BOLD:AAA7985
Condica cupentia|[11687]|BLPDA333-09|Costa Rica|Guanacaste|658[0n]|BOLD:AAA7985
Condica cupentia|[11688]|BLPCM340-08|Costa Rica|Guanacaste|658[0n]|BOLD:AAA7985
Condica cupentia|[11689]|RDNMG220-08|Costa Rica||658[0n]|BOLD:AAA7985
Condica cupentia|[11690]|BLPCK669-08|Costa Rica|Alajuela|658[0n]|BOLD:AAA7985
Condica cupentia|[11691]|BLPCG779-08|Costa Rica|Guanacaste|658[0n]|BOLD:AAA7985
Condica cupentia|[11692]|BLPCG190-08|Costa Rica|Alajuela|658[0n]|BOLD:AAA7985
Condica cupentia|[11693]|BLPCD642-08|Costa Rica|Guanacaste|658[0n]|BOLD:AAA7985
Condica cupentia|[11694]|BLPCD405-08|Costa Rica|Guanacaste|658[0n]|BOLD:AAA7985
Condica cupentia|[11695]|BLPCD404-08|Costa Rica|Guanacaste|658[0n]|BOLD:AAA7985
Condica cupentia|[11696]|BLPCC454-08|Costa Rica|Guanacaste|658[0n]|BOLD:AAA7985
Condica cupentia|[11697]|BLPCC451-08|Costa Rica|Guanacaste|658[0n]|BOLD:AAA7985
Condica cupentia|[11698]|BLPCB425-08|Costa Rica|Alajuela|658[0n]|BOLD:AAA7985
Condica cupentia|[11699]|BLPBA206-07|Costa Rica|Guanacaste|658[0n]|BOLD:AAA7985
Condica cupentia|[11700]|BLPAE881-07|Costa Rica|Guanacaste|658[0n]|BOLD:AAA7985
Condica cupentia|[11701]|MHMXE646-07|Costa Rica|Guanacaste|658[0n]|BOLD:AAA7985
Condica cupentia|[11702]|MHMXE225-06|Costa Rica|Guanacaste|658[0n]|BOLD:AAA7985
Condica cupentia|[11703]|BLPAE141-06|Costa Rica|Alajuela|658[0n]|BOLD:AAA7985
Condica cupentia|[11704]|BLPAD086-06|Costa Rica|Guanacaste|658[0n]|BOLD:AAA7985
Condica cupentia|[11705]|BLPAD085-06|Costa Rica|Guanacaste|658[0n]|BOLD:AAA7985
Condica cupentia|[11706]|BLPAC359-06|Costa Rica|Guanacaste|658[0n]|BOLD:AAA7985
Condica cupentia|[11707]|BLPAA845-06|Costa Rica|Alajuela|658[0n]|BOLD:AAA7985
Condica cupentia|[11708]|MHAUF360-06|Costa Rica|Guanacaste|658[0n]|BOLD:AAA7985
Condica cupentia|[11709]|MHAUF130-06|Costa Rica|Alajuela|658[0n]|BOLD:AAA7985
Condica cupentia|[11710]|MOTAR230-12|Argentina|Misiones|643[0n]|BOLD:AAA7985
Condica cupentia|[11711]|LEPPA673-13|Argentina|Formosa|619[0n]|BOLD:AAA7985
Condica cupentia|[11712]|LPYPB512-08|Mexico|Quintana Roo|658[0n]|BOLD:AAA7985
Condica cupentia|[11713]|GMARN160-14|Argentina|Misiones|658[0n]|BOLD:AAA7985
Condica mersa|[11714]|CNCLB228-14|United States|Oregon|658[0n]|BOLD:ACM4632
Condica mersa|[11715]|CNCLB229-14|United States|Oregon|658[0n]|BOLD:ACM4632
Condica mersa|[11716]|CNCLB1567-14|United States|Oregon|658[0n]|BOLD:ACM4632
Condica sp. 1 nr. discistriga|[11717]|LBCH5671-10|Canada|British Columbia|658[0n]|BOLD:AAC3223
Condica sp. 1 nr. discistriga|[11718]|LBCH5722-10|Canada|British Columbia|658[0n]|BOLD:AAC3223
Condica sp. 1 nr. discistriga|[11719]|LBCH5085-10|Canada|British Columbia|658[0n]|BOLD:AAC3223
Condica sp. 1 nr. discistriga|[11720]|LBCH5606-10|Canada|British Columbia|658[0n]|BOLD:AAC3223
Condica sp. 1 nr. discistriga|[11721]|LBCH5806-10|Canada|British Columbia|658[0n]|BOLD:AAC3223
Condica sp. 1 nr. discistriga|[11722]|LOWCB869-05|Canada|British Columbia|648[0n]|BOLD:AAC3223
Condica sp. 1 nr. discistriga|[11723]|LBCH5526-10|Canada|British Columbia|658[0n]|BOLD:AAC3223
Condica sp. 1 nr. discistriga|[11724]|LBCH5549-10|Canada|British Columbia|658[0n]|BOLD:AAC3223
Condica sp. 1 nr. discistriga|[11725]|LBCH5557-10|Canada|British Columbia|658[0n]|BOLD:AAC3223
Condica sp. 1 nr. discistriga|[11726]|LBCH5574-10|Canada|British Columbia|658[0n]|BOLD:AAC3223
Condica sp. 1 nr. discistriga|[11727]|LBCH5996-10|Canada|British Columbia|658[0n]|BOLD:AAC3223
Condica sp. 1 nr. discistriga|[11728]|LBCH5997-10|Canada|British Columbia|658[0n]|BOLD:AAC3223
Condica sp. 1 nr. discistriga|[11729]|LBCH5992-10|Canada|British Columbia|658[0n]|BOLD:AAC3223
Condica sp. 1 nr. discistriga|[11730]|LBCH5993-10|Canada|British Columbia|658[0n]|BOLD:AAC3223
Condica sp. 1 nr. discistriga|[11731]|LBCH5994-10|Canada|British Columbia|658[0n]|BOLD:AAC3223
Condica sp. 1 nr. discistriga|[11732]|LBCH5995-10|Canada|British Columbia|658[0n]|BOLD:AAC3223
Condica sp. 1 nr. discistriga|[11733]|LBCH5998-10|Canada|British Columbia|658[0n]|BOLD:AAC3223
Condica sp. 1 nr. discistriga|[11734]|LBCH5999-10|Canada|British Columbia|658[0n]|BOLD:AAC3223
Condica sp. 1 nr. discistriga|[11735]|LBCH6048-10|Canada|British Columbia|658[0n]|BOLD:AAC3223
Condica sp. 1 nr. discistriga|[11736]|LBCH6049-10|Canada|British Columbia|658[0n]|BOLD:AAC3223

Condica sp. 1 nr. discistriga|[11734]|LBCH5999-10|Canada|British Columbia|658[0n]|BOLD:AAC3223
Condica sp. 1 nr. discistriga|[11735]|LBCH6048-10|Canada|British Columbia|658[0n]|BOLD:AAC3223
Condica sp. 1 nr. discistriga|[11736]|LBCH6049-10|Canada|British Columbia|658[0n]|BOLD:AAC3223
Condica sp. 1 nr. discistriga|[11737]|LBCH6050-10|Canada|British Columbia|658[0n]|BOLD:AAC3223
Condica sp. 1 nr. discistriga|[11738]|LBCH6051-10|Canada|British Columbia|658[0n]|BOLD:AAC3223
Condica sp. 1 nr. discistriga|[11739]|LBCH6052-10|Canada|British Columbia|658[0n]|BOLD:AAC3223
Condica sp. 1 nr. discistriga|[11740]|LBCH6053-10|Canada|British Columbia|658[0n]|BOLD:AAC3223
Condica sp. 1 nr. discistriga|[11741]|LBCH5524-10|Canada|British Columbia|658[0n]|BOLD:AAC3223
Condica sp. 1 nr. discistriga|[11742]|LBCH5525-10|Canada|British Columbia|658[0n]|BOLD:AAC3223
Condica sp. 1 nr. discistriga|[11743]|LBCH6046-10|Canada|British Columbia|658[0n]|BOLD:AAC3223
Condica sp. 1 nr. discistriga|[11744]|LBCH6047-10|Canada|British Columbia|658[0n]|BOLD:AAC3223
Condica sp. 1 nr. discistriga|[11745]|LBCH5522-10|Canada|British Columbia|658[0n]|BOLD:AAC3223
Condica sp. 1 nr. discistriga|[11746]|LBCH5523-10|Canada|British Columbia|658[0n]|BOLD:AAC3223
Condica sp. 1 nr. discistriga|[11747]|LBCH5519-10|Canada|British Columbia|658[0n]|BOLD:AAC3223
Condica sp. 1 nr. discistriga|[11748]|LBCH5520-10|Canada|British Columbia|658[0n]|BOLD:AAC3223
Condica sp. 1 nr. discistriga|[11749]|LBCH5495-10|Canada|British Columbia|658[0n]|BOLD:AAC3223
Condica sp. 1 nr. discistriga|[11750]|LBCH5496-10|Canada|British Columbia|658[0n]|BOLD:AAC3223
Condica sp. 1 nr. discistriga|[11751]|LOWCB874-05|Canada|British Columbia|658[0n]|BOLD:AAC3223
Condica sp. 1 nr. discistriga|[11752]|LOWCB873-05|Canada|British Columbia|658[0n]|BOLD:AAC3223
Condica sp. 1 nr. discistriga|[11753]|LOWCB872-05|Canada|British Columbia|658[0n]|BOLD:AAC3223
Condica sp. 1 nr. discistriga|[11754]|LOWCB871-05|Canada|British Columbia|658[0n]|BOLD:AAC3223
Condica sp. 1 nr. discistriga|[11755]|LOWCB870-05|Canada|British Columbia|658[0n]|BOLD:AAC3223
Condica sp. 1 nr. discistriga|[11756]|LOWCB868-05|Canada|British Columbia|658[0n]|BOLD:AAC3223
Condica sp. 1 nr. discistriga|[11757]|LOWCB864-05|Canada|British Columbia|658[0n]|BOLD:AAC3223
Condica sp. 1 nr. discistriga|[11758]|LOWCB866-05|Canada|British Columbia|568[0n]|BOLD:AAC3223
Condica sp. 1 nr. discistriga|[11759]|LOWCB867-05|Canada|British Columbia|565[0n]|BOLD:AAC3223
Condica sp. 1 nr. discistriga|[11760]|LOWCB865-05|Canada|British Columbia|587[0n]|BOLD:AAC3223
Condica sp. 1 nr. discistriga|[11761]|LOWCD117-06|Canada|British Columbia|597[0n]|BOLD:AAC3223
Condica sp. 1 nr. discistriga|[11762]|LBCH5039-10|Canada|British Columbia|658[0n]|BOLD:AAC3223
Condica sp. 1 nr. discistriga|[11763]|LBCH5254-10|Canada|British Columbia|658[0n]|BOLD:AAC3223
Condica sp. 1 nr. discistriga|[11764]|LBCH5347-10|Canada|British Columbia|658[0n]|BOLD:AAC3223
Condica sp. 1 nr. discistriga|[11765]|LBCH5481-10|Canada|British Columbia|658[0n]|BOLD:AAC3223
Condica sp. 1 nr. discistriga|[11766]|LBCH5521-10|Canada|British Columbia|658[0n]|BOLD:AAC3223
Condica sp. 1 nr. discistriga|[11767]|LBCH6093-10|Canada|British Columbia|658[0n]|BOLD:AAC3223
Condica sp. 1 nr. discistriga|[11768]|LBCH6453-10|Canada|British Columbia|658[0n]|BOLD:AAC3223
Condica videns|[11769]|PHMO070-03|Canada|Ontario|639[0n]|BOLD:AAA9166
Condica videns|[11770]|LOFLB204-06|United States|Florida|658[0n]|BOLD:AAA9166
Condica videns|[11771]|KPOEC072-08|Canada|Ontario|656[0n]|BOLD:AAA9166
Condica videns|[11772]|LOFLB279-06|United States|Florida|658[0n]|BOLD:AAA9166
Condica videns|[11773]|XAJ301-06|Canada|Ontario|658[0n]|BOLD:AAA9166
Condica videns|[11774]|RDLQB673-05|Canada|Quebec|658[0n]|BOLD:AAA9166
Condica videns|[11775]|XAH458-05|Canada|Ontario|658[0n]|BOLD:AAA9166
Condica videns|[11776]|XAH319-05|Canada|Ontario|658[0n]|BOLD:AAA9166
Condica videns|[11777]|LNC037-05|United States|North Carolina|657[0n]|BOLD:AAA9166
Condica videns|[11778]|LNC036-05|United States|North Carolina|658[0n]|BOLD:AAA9166
Condica videns|[11779]|XAB292-04|Canada|Ontario|658[0n]|BOLD:AAA9166
Condica videns|[11780]|PMG103-03|Canada|Ontario|617[0n]|BOLD:AAA9166
Condica videns|[11781]|LNCNW041-06|United States|North Carolina|658[0n]|BOLD:AAA9166
Condica videns|[11782]|XAK305-06|Canada|Ontario|658[0n]|BOLD:AAA9166
Condica videns|[11783]|RDLQF368-06|Canada|Quebec|658[0n]|BOLD:AAA9166
Condica videns|[11784]|RDLQF811-06|Canada|Quebec|658[0n]|BOLD:AAA9166
Condica videns|[11785]|LPSO267-08|Canada|Ontario|658[0n]|BOLD:AAA9166
Condica videns|[11786]|KPOEC181-08|Canada|Ontario|658[0n]|BOLD:AAA9166
Condica videns|[11787]|RDNMH506-09|United States|Louisiana|658[0n]|BOLD:AAA9166
Condica videns|[11788]|BBLSX897-09|United States|Oklahoma|658[0n]|BOLD:AAA9166
Condica videns|[11789]|XAH696-05|Canada|Ontario|658[0n]|BOLD:AAA9166
Condica videns|[11790]|XAD628-05|Canada|Ontario|658[0n]|BOLD:AAA9166
Condica videns|[11791]|XAG240-05|Canada|Ontario|658[0n]|BOLD:AAA9166
Condica videns|[11792]|LPOKA316-08|United States|Oklahoma|658[0n]|BOLD:AAA9166
Condica videns|[11793]|LPOKA553-09|United States|Oklahoma|658[0n]|BOLD:AAA9166
Condica videns|[11794]|RDNMH503-09|United States|Louisiana|658[0n]|BOLD:AAA9166
Condica videns|[11795]|LPOKD285-09|United States|Oklahoma|657[0n]|BOLD:AAA9166
Condica videns|[11796]|XAD629-05|Canada|Ontario|658[0n]|BOLD:AAA9166
Condica videns|[11797]|XAB271-04|Canada|Ontario|658[0n]|BOLD:AAA9166
Condica videns|[11798]|PHMO071-03|Canada|Ontario|639[0n]|BOLD:AAA9166
Condica videns|[11799]|PHMO235-03|Canada|Ontario|639[0n]|BOLD:AAA9166
Condica videns|[11800]|LOFLB402-06|United States|Florida|658[0n]|BOLD:AAA9166
Condica videns|[11801]|XAK297-06|Canada|Ontario|658[0n]|BOLD:AAA9166
Condica videns|[11802]|LPSO687-08|Canada|Ontario|658[0n]|BOLD:AAA9166
Condica videns|[11803]|LPOKA999-09|United States|Oklahoma|658[0n]|BOLD:AAA9166
Condica videns|[11804]|RDNMH502-09|United States|Louisiana|658[0n]|BOLD:AAA9166
Condica videns|[11805]|RDNMH504-09|United States|Louisiana|658[0n]|BOLD:AAA9166
Condica videns|[11806]|RDNMH505-09|United States|Louisiana|658[0n]|BOLD:AAA9166
Condica videns|[11807]|RDNMH507-09|United States|Louisiana|658[0n]|BOLD:AAA9166
Condica videns|[11808]|RDNMH508-09|United States|Louisiana|658[0n]|BOLD:AAA9166
Condica videns|[11809]|RDNMH509-09|United States|Louisiana|658[0n]|BOLD:AAA9166
Condica videns|[11810]|LILLA888-11|United States|Illinois|658[0n]|BOLD:AAA9166
Condica videns|[11811]|BBLOB1314-11|United States|Florida|658[0n]|BOLD:AAA9166
Condica videns|[11812]|LPOKE468-12|United States|Oklahoma|627[0n]|BOLD:AAA9166
Condica videns|[11813]|RBINA5359-13|Canada|Ontario|576[0n]|BOLD:AAA9166
Condica temecula|[11814]|RDNMK102-11|United States|Arizona|576[0n]|BOLD:ACF1097
Condica temecula|[11815]|NAMUM088-08|United States|Arizona|655[0n]|BOLD:ACF1097
Condica temecula|[11816]|RDNMH844-09|United States|Arizona|658[0n]|BOLD:ACF1097
Condica temecula|[11817]|RDNMG269-08|United States|Texas|658[0n]|BOLD:ACF1097
Condica temecula|[11818]|LNAUT809-14|United States|New Mexico|658[0n]|BOLD:ACF1097
Condica sp. 2 nr. discistriga|[11819]|RDNMD437-06|United States|California|658[0n]|BOLD:ACE6394
Condica sp. 2 nr. discistriga|[11820]|RDNME075-07|United States|California|658[0n]|BOLD:ACE6394
Condica sp. 2 nr. discistriga|[11821]|RDNME076-07|United States|California|658[0n]|BOLD:ACE6394
Condica leucorena|[11822]|RDNME969-08|United States|New Mexico|658[0n]|BOLD:ACE6393
Condica leucorena|[11823]|RDNMJ837-11|United States|New Mexico|658[0n]|BOLD:ACE6393
Condica leucorena|[11824]|AWCLB502-11|United States|Arizona|658[0n]|BOLD:ACE6393
Condica leucorena|[11825]|AWCLB580-11|United States|Arizona|658[0n]|BOLD:ACE6393
Condica leucorena|[11826]|AWCLB193-10|United States|Arizona|658[0n]|BOLD:ACE6393
Condica leucorena|[11827]|RDNMJ838-11|United States|New Mexico|658[0n]|BOLD:ACE6393
Condica leucorena|[11828]|IAWLB653-11|United States|Arizona|658[0n]|BOLD:ACE6393
Condica discistriga|[11829]|RDNML205-13|United States|Wyoming|627[0n]|BOLD:ACE6393
Condica discistriga|[11830]|RDNML013-13|United States|Colorado|658[0n]|BOLD:ACE6393
Condica discistriga|[11831]|CNCLB1568-14|United States|Idaho|658[0n]|BOLD:ACE6393
Condica claufacta|[11832]|RDNMH512-09|United States|Louisiana|658[0n]|BOLD:AAA9267
Condica claufacta|[11833]|RDNMJ030-10|United States|Florida|658[0n]|BOLD:AAA9267
Condica claufacta|[11834]|RDNMJ029-10|United States|Florida|658[0n]|BOLD:AAA9267
Condica claufacta|[11835]|RDNMJ028-10|United States|Florida|658[0n]|BOLD:AAA9267
[illegible]

Condica claufacta|[11833]|RDNMJ030-10|United States|Florida|658[0n]|BOLD:AAA9267
Condica claufacta|[11834]|RDNMJ029-10|United States|Florida|658[0n]|BOLD:AAA9267
Condica claufacta|[11835]|RDNMJ028-10|United States|Florida|658[0n]|BOLD:AAA9267
Condica claufacta|[11836]|RDNMJ027-10|United States|Florida|658[0n]|BOLD:AAA9267
Condica claufacta|[11837]|PSAT138-10|United States|Florida|658[0n]|BOLD:AAA9267
Condica claufacta|[11838]|USLEP1218-10|United States|Florida|658[0n]|BOLD:AAA9267
Condica claufacta|[11839]|RDNMH513-09|United States|Louisiana|658[0n]|BOLD:AAA9267
Condica claufacta|[11840]|LOFLB274-06|United States|Florida|658[0n]|BOLD:AAA9267
Condica claufacta|[11841]|BBLOB509-11|United States|Florida|576[1n]|BOLD:AAA9267
Condica sp. nr. claufacta|[11842]|CMAZA1001-12|United States|Arizona|658[0n]|BOLD:AAA9267
Condica morsa|[11843]|NOCNA039-14|United States|Texas|658[0n]|BOLD:AAA9267
Condica andrena|[11844]|TML154-14|United States||550[0n]|BOLD:ACO2340
Condica andrena|[11845]|CNCLB1661-14|United States|California|407[0n]|BOLD:ACO2340
Condica charada|[11846]|CMAZA103-09|United States|Arizona|658[0n]|BOLD:AAH5647
Condica charada|[11847]|CMAZA102-09|United States|Arizona|658[0n]|BOLD:AAH5647
Condica charada|[11848]|CMAZA387-10|United States|Arizona|658[0n]|BOLD:AAH5647
Condica charada|[11849]|CMAZA888-12|United States|Arizona|658[0n]|BOLD:AAH5647
Condica charada|[11850]|TXLEP188-15|United States|Texas|588[0n]|BOLD:AAH5647
Condica charada|[11851]|TXLEP189-15|United States|Texas|594[0n]|BOLD:AAH5647
Diastema sp.|[11852]|RDNME614-08|United States|Texas|609[0n]|BOLD:AAI9433
Diastema sp.|[11853]|RDNME657-08|United States|Texas|590[0n]|BOLD:AAI9433
Diastema cnossia|[11854]|HKONB169-08|United States|Texas|658[0n]|BOLD:AAB8830
Diastema cnossia|[11855]|HKONB170-08|United States|Texas|658[0n]|BOLD:AAB8830
Diastema tigris|[11856]|HKONB190-09|United States|Texas|658[0n]|BOLD:AAA8374
Diastema tigris|[11857]|HKONB191-09|United States|Texas|658[0n]|BOLD:AAA8374
Diastema sp.|[11858]|BBLOB1285-11|United States|Florida|658[0n]|BOLD:ABA1169
Leuconycta lepidula|[11859]|LSEU753-06|United States|Georgia|658[0n]|BOLD:AAB1221
Leuconycta lepidula|[11860]|LPSO427-08|Canada|Ontario|658[0n]|BOLD:AAB1221
Leuconycta lepidula|[11861]|TTMNB312-06|Canada|New Brunswick|658[0n]|BOLD:AAB1221
Leuconycta lepidula|[11862]|PHMNB387-04|Canada|New Brunswick|658[0n]|BOLD:AAB1221
Leuconycta lepidula|[11863]|PHMNB425-04|Canada|New Brunswick|658[0n]|BOLD:AAB1221
Leuconycta lepidula|[11864]|XAK325-06|Canada|Ontario|658[0n]|BOLD:AAB1221
Leuconycta lepidula|[11865]|LGSMG639-07|United States|Tennessee|658[0n]|BOLD:AAB1221
Leuconycta lepidula|[11866]|LGSM478-04|United States|Tennessee|609[0n]|BOLD:AAB1221
Leuconycta lepidula|[11867]|XAE270-04|Canada|Ontario|658[2n]|BOLD:AAB1221
Leuconycta lepidula|[11868]|BBLSX425-09|United States|Oklahoma|658[0n]|BOLD:AAB1221
Leuconycta lepidula|[11869]|XAJ718-06|Canada|Ontario|658[0n]|BOLD:AAB1221
Leuconycta lepidula|[11870]|TMNBB120-06|Canada|New Brunswick|658[0n]|BOLD:AAB1221
Leuconycta lepidula|[11871]|RDMAB534-06|Canada|Alberta|658[0n]|BOLD:AAB1221
Leuconycta lepidula|[11872]|XAB593-04|Canada|Ontario|658[0n]|BOLD:AAB1221
Leuconycta lepidula|[11873]|XAK299-06|Canada|Ontario|658[0n]|BOLD:AAB1221
Leuconycta lepidula|[11874]|XAB578-04|Canada|Ontario|658[0n]|BOLD:AAB1221
Leuconycta lepidula|[11875]|LGSM479-04|United States|Tennessee|658[0n]|BOLD:AAB1221
Leuconycta lepidula|[11876]|PHMO146-03|Canada|Ontario|639[0n]|BOLD:AAB1221
Leuconycta lepidula|[11877]|PHMNB154-04|Canada|New Brunswick|522[5n]|BOLD:AAB1221
Leuconycta lepidula|[11878]|RDMAB038-05|Canada|Alberta|652[0n]|BOLD:AAB1221
Leuconycta lepidula|[11879]|PHMO173-03|Canada|Ontario|639[0n]|BOLD:AAB1221
Leuconycta lepidula|[11880]|PHMO164-03|Canada|Ontario|639[0n]|BOLD:AAB1221
Leuconycta lepidula|[11881]|XAI038-05|Canada|Ontario|643[0n]|BOLD:AAB1221
Leuconycta lepidula|[11882]|RDLQF651-06|Canada|Quebec|643[0n]|BOLD:AAB1221
Leuconycta lepidula|[11883]|LPSO059-08|Canada|Ontario|658[0n]|BOLD:AAB1221
Leuconycta lepidula|[11884]|LPSO174-08|Canada|Ontario|658[0n]|BOLD:AAB1221
Leuconycta lepidula|[11885]|HKONB453-09|United States|Indiana|658[0n]|BOLD:AAB1221
Leuconycta lepidula|[11886]|HKONB454-09|United States|Texas|658[0n]|BOLD:AAB1221
Leuconycta lepidula|[11887]|HKONB455-09|United States|Louisiana|658[0n]|BOLD:AAB1221
Leuconycta lepidula|[11888]|BBLSW486-09|United States|Oklahoma|658[0n]|BOLD:AAB1221
Leuconycta lepidula|[11889]|BBLSX283-09|United States|Oklahoma|658[0n]|BOLD:AAB1221
Leuconycta lepidula|[11890]|IAWLB390-11|United States|Virginia|658[0n]|BOLD:AAB1221
Leuconycta lepidula|[11891]|PHMNB699-04|Canada|New Brunswick|658[0n]|BOLD:AAB1221
Leuconycta lepidula|[11892]|PHMNB703-04|Canada|New Brunswick|658[0n]|BOLD:AAB1221
Leuconycta lepidula|[11893]|HPPPE1554-13|Canada|Nova Scotia|551[1n]|BOLD:AAB1221
Leuconycta diphteroides|[11894]|LPSO668-08|Canada|Ontario|577[0n]|BOLD:AAA6913
Leuconycta diphteroides|[11895]|TMG102-03|Canada|Ontario|617[0n]|BOLD:AAA6913
Leuconycta diphteroides|[11896]|TMG120-03|Canada|Ontario|617[0n]|BOLD:AAA6913
Leuconycta diphteroides|[11897]|XAB116-04|Canada|Ontario|658[0n]|BOLD:AAA6913
Leuconycta diphteroides|[11898]|XAB172-04|Canada|Ontario|658[0n]|BOLD:AAA6913
Leuconycta diphteroides|[11899]|KPOEC087-08|Canada|Ontario|658[0n]|BOLD:AAA6913
Leuconycta diphteroides|[11900]|PHMNB214-04|Canada|New Brunswick|609[0n]|BOLD:AAA6913
Leuconycta diphteroides|[11901]|PHMNB463-04|Canada|New Brunswick|658[0n]|BOLD:AAA6913
Leuconycta diphteroides|[11902]|TTMNB310-06|Canada|New Brunswick|658[1n]|BOLD:AAA6913
Leuconycta diphteroides|[11903]|XAB245-04|Canada|Ontario|658[0n]|BOLD:AAA6913
Leuconycta diphteroides|[11904]|LPSOB813-08|Canada|Ontario|657[0n]|BOLD:AAA6913
Leuconycta diphteroides|[11905]|PHMNB176-04|Canada|New Brunswick|609[0n]|BOLD:AAA6913
Leuconycta diphteroides|[11906]|PHMNB520-04|Canada|New Brunswick|658[0n]|BOLD:AAA6913
Leuconycta diphteroides|[11907]|PHMNB739-05|Canada|New Brunswick|658[0n]|BOLD:AAA6913
Leuconycta diphteroides|[11908]|BLTIB710-08|Canada|Ontario|658[0n]|BOLD:AAA6913
Leuconycta diphteroides|[11909]|BLTIB675-08|Canada|Ontario|658[0n]|BOLD:AAA6913
Leuconycta diphteroides|[11910]|BLTIB596-08|Canada|Ontario|658[0n]|BOLD:AAA6913
Leuconycta diphteroides|[11911]|BLTIB533-08|Canada|Ontario|658[0n]|
Leuconycta diphteroides|[11912]|BLTIB521-08|Canada|Ontario|658[0n]|BOLD:AAA6913
Leuconycta diphteroides|[11913]|BLTIB278-08|Canada|Ontario|658[0n]|BOLD:AAA6913
Leuconycta diphteroides|[11914]|BLTIB259-08|Canada|Ontario|658[0n]|BOLD:AAA6913
Leuconycta diphteroides|[11915]|LPSOC356-08|Canada|Ontario|655[0n]|BOLD:AAA6913
Leuconycta diphteroides|[11916]|LPSO562-08|Canada|Ontario|658[0n]|BOLD:AAA6913
Leuconycta diphteroides|[11917]|RDLQG277-06|Canada|Quebec|658[0n]|BOLD:AAA6913
Leuconycta diphteroides|[11918]|RDLQF428-06|Canada|Quebec|658[0n]|BOLD:AAA6913
Leuconycta diphteroides|[11919]|XAJ537-06|Canada|Ontario|658[0n]|BOLD:AAA6913
Leuconycta diphteroides|[11920]|XAJ343-06|Canada|Ontario|658[0n]|BOLD:AAA6913
Leuconycta diphteroides|[11921]|XAH280-05|Canada|Ontario|658[0n]|BOLD:AAA6913
Leuconycta diphteroides|[11922]|XAF505-05|Canada|Ontario|658[0n]|BOLD:AAA6913
Leuconycta diphteroides|[11923]|LOCT083-05|United States|Connecticut|658[0n]|BOLD:AAA6913
Leuconycta diphteroides|[11924]|PHMNB625-04|Canada|New Brunswick|658[0n]|BOLD:AAA6913
Leuconycta diphteroides|[11925]|PHMNB149-04|Canada|New Brunswick|658[0n]|BOLD:AAA6913
Leuconycta diphteroides|[11926]|LPSOB814-08|Canada|Ontario|648[0n]|BOLD:AAA6913
Leuconycta diphteroides|[11927]|TTMNB311-06|Canada|New Brunswick|583[0n]|BOLD:AAA6913
Leuconycta diphteroides|[11928]|PMG127-03|Canada|Ontario|617[0n]|BOLD:AAA6913
Leuconycta diphteroides|[11929]|PHMO139-03|Canada|Ontario|639[0n]|BOLD:AAA6913
Leuconycta diphteroides|[11930]|PHMNB026-03|Canada|New Brunswick|639[0n]|BOLD:AAA6913
Leuconycta diphteroides|[11931]|BLTIB660-08|Canada|Ontario|634[0n]|BOLD:AAA6913
Leuconycta diphteroides|[11932]|BLTIB479-08|Canada|Ontario|658[0n]|BOLD:AAA6913
Leuconycta diphteroides|[11933]|LPSOD250-09|Canada|Ontario|658[0n]|BOLD:AAA6913
Leuconycta diphteroides|[11934]|BBLPE079-09|Canada|Nova Scotia|658[0n]|BOLD:AAA6913
Leuconycta diphteroides|[11935]|BBLCU125-09|United States|Michigan|658[0n]|BOLD:AAA6913

Leuconycta diphteroides|[11933]|LPSOD250-09|Canada|Ontario|658[0n]|BOLD:AAA6913
Leuconycta diphteroides|[11934]|BBLPE079-09|Canada|Nova Scotia|658[0n]|BOLD:AAA6913
Leuconycta diphteroides|[11935]|BBLCU125-09|United States|Michigan|658[0n]|BOLD:AAA6913
Leuconycta diphteroides|[11936]|XAF610-05|Canada|Ontario|658[0n]|BOLD:AAA6913
Leuconycta diphteroides|[11937]|LPSO417-08|Canada|Ontario|658[0n]|BOLD:AAA6913
Leuconycta diphteroides|[11938]|LPSO553-08|Canada|Ontario|658[0n]|BOLD:AAA6913
Leuconycta diphteroides|[11939]|LNCB938-10|United States|North Carolina|658[0n]|BOLD:AAA6913
Leuconycta diphteroides|[11940]|LNCC349-10|United States|North Carolina|658[0n]|BOLD:AAA6913
Leuconycta diphteroides|[11941]|BBLCU030-09|United States|Michigan|658[0n]|BOLD:AAA6913
Leuconycta diphteroides|[11942]|BBLCU208-09|United States|Michigan|658[0n]|BOLD:AAA6913
Leuconycta diphteroides|[11943]|LPSOB808-08|Canada|Ontario|658[0n]|BOLD:AAA6913
Leuconycta diphteroides|[11944]|LPSOC353-08|Canada|Ontario|657[0n]|BOLD:AAA6913
Leuconycta diphteroides|[11945]|LPSOC035-08|Canada|Ontario|658[0n]|BOLD:AAA6913
Leuconycta diphteroides|[11946]|LPSO667-08|Canada|Ontario|658[0n]|BOLD:AAA6913
Leuconycta diphteroides|[11947]|LPSO549-08|Canada|Ontario|658[0n]|BOLD:AAA6913
Leuconycta diphteroides|[11948]|LPSO428-08|Canada|Ontario|658[0n]|BOLD:AAA6913
Leuconycta diphteroides|[11949]|LGSMG638-07|United States|Tennessee|658[0n]|BOLD:AAA6913
Leuconycta diphteroides|[11950]|LOCT061-05|United States|Connecticut|658[0n]|BOLD:AAA6913
Leuconycta diphteroides|[11951]|XAB320-04|Canada|Ontario|658[0n]|BOLD:AAA6913
Leuconycta diphteroides|[11952]|LPSOC355-08|Canada|Ontario|656[1n]|BOLD:AAA6913
Leuconycta diphteroides|[11953]|LPSOC354-08|Canada|Ontario|647[0n]|BOLD:AAA6913
Leuconycta diphteroides|[11954]|LGSM570-04|United States|Tennessee|605[0n]|BOLD:AAA6913
Leuconycta diphteroides|[11955]|UDLEP308-09|United States|Pennsylvania|634[0n]|BOLD:AAA6913
Leuconycta diphteroides|[11956]|LSUSA125-06|United States|Kentucky|658[0n]|BOLD:AAA6913
Leuconycta diphteroides|[11957]|LILLA733-11|United States|Illinois|658[0n]|BOLD:AAA6913
Leuconycta diphteroides|[11958]|LILLA617-11|United States|Illinois|658[0n]|BOLD:AAA6913
Leuconycta diphteroides|[11959]|LNCC1189-11|United States|North Carolina|658[0n]|BOLD:AAA6913
Leuconycta diphteroides|[11960]|RBINA2466-13|Canada|Ontario|582[0n]|BOLD:AAA6913
Alypiodes bimaculata|[11961]|JBAZ128-09|United States|Arizona|658[0n]|BOLD:AAJ5319
Alypiodes bimaculata|[11962]|DMAZ153-10|United States|Arizona|658[0n]|BOLD:AAJ5319
Alypiodes bimaculata|[11963]|DMAZ155-10|United States|Arizona|658[0n]|BOLD:AAJ5319
Alypiodes bimaculata|[11964]|AWCLB034-10|United States|Arizona|658[0n]|BOLD:AAJ5319
Alypiodes bimaculata|[11965]|AWCLB035-10|United States|Arizona|576[0n]|BOLD:AAJ5319
Euscirrhopterus cosyra|[11966]|BBLSW029-09|United States|Texas|658[0n]|BOLD:AAH5258
Euscirrhopterus cosyra|[11967]|BBLSW765-09|United States|Texas|658[0n]|BOLD:AAH5258
Euscirrhopterus cosyra|[11968]|AWCLB036-10|United States|Arizona|658[0n]|BOLD:AAH5258
Euscirrhopterus cosyra|[11969]|RDNMD928-07|United States|Arizona|655[0n]|BOLD:AAH5258
Euscirrhopterus cosyra|[11970]|BBLOC631-11|United States|Arizona|658[0n]|BOLD:AAH5258
Euscirrhopterus poeyi|[11971]|GWOSU378-11|Cuba|Holguin|658[0n]|BOLD:ABA3355
Euscirrhopterus poeyi|[11972]|GWOSU379-11|Cuba|Holguin|658[0n]|BOLD:ABA3355
Euscirrhopterus poeyi|[11973]|GWOSU380-11|Cuba|Holguin|658[0n]|BOLD:ABA3355
Euscirrhopterus gloveri|[11974]|CMAZA504-10|United States|Arizona|658[0n]|BOLD:AAL4952
Euscirrhopterus gloveri|[11975]|NAMUM067-08|United States|Arizona|657[0n]|BOLD:AAL4952
Euscirrhopterus gloveri|[11976]|AWCLB040-10|United States|Arizona|658[0n]|BOLD:AAL4952
Euscirrhopterus gloveri|[11977]|CMAZA814-10|United States|Arizona|658[0n]|BOLD:AAL4952
Euscirrhopterus gloveri|[11978]|CMAZA875-12|United States|Arizona|658[0n]|BOLD:AAL4952
Psychomorpha epimenis|[11979]|LSUSA060-06|United States|Kentucky|504[0n]|
Psychomorpha epimenis|[11980]|LOCT002-05|United States|Connecticut|658[0n]|BOLD:AAE0982
Psychomorpha epimenis|[11981]|LOCT004-05|United States|Connecticut|658[0n]|BOLD:AAE0982
Psychomorpha epimenis|[11982]|LOCT003-05|United States|Connecticut|658[0n]|BOLD:AAE0982
Psychomorpha epimenis|[11983]|QUNOE469-12|United States|Florida|658[0n]|BOLD:AAE0982
Psychomorpha epimenis|[11984]|CNCLB1522-14|United States|Florida|658[0n]|BOLD:AAE0982
Gerrodes minatea|[11985]|NAMUM094-08|United States|Arizona|658[0n]|BOLD:AAL2563
Gerrodes minatea|[11986]|CMAZA559-10|United States|Arizona|658[0n]|BOLD:AAL2563
Xerociris wilsonii|[11987]|HKONB143-08|United States|Texas|658[0n]|BOLD:AAH5314
Xerociris wilsonii|[11988]|BBLSW728-09|United States|Texas|658[0n]|BOLD:AAH5314
Xerociris wilsonii|[11989]|USLEP494-10|United States|Texas|658[0n]|BOLD:AAH5314
Xerociris wilsonii|[11990]|BBLOE1454-12|United States|Texas|658[0n]|BOLD:AAH5314
Xerociris wilsonii|[11991]|USLEP495-10|United States|Texas|658[0n]|BOLD:AAH5314
Xerociris wilsonii|[11992]|HKONB142-08|United States|Texas|658[0n]|BOLD:AAH5314
Xerociris wilsonii|[11993]|USLEP493-10|United States|Texas|658[0n]|BOLD:AAH5314
Xerociris wilsonii|[11994]|BBLOE1458-12|United States|Texas|658[0n]|BOLD:AAH5314
Eudryas grata|[11995]|LGSM504-04|United States|Tennessee|608[3n]|BOLD:AAB0562
Eudryas grata|[11996]|LPOKC757-09|United States|Oklahoma|658[0n]|BOLD:AAB0562
Eudryas grata|[11997]|LTOLB144-08|United States|Maryland|658[0n]|BOLD:AAB0562
Eudryas grata|[11998]|LPOKB1020-09|United States|Oklahoma|658[0n]|BOLD:AAB0562
Eudryas grata|[11999]|LOT275-04|United States|Tennessee|658[0n]|BOLD:AAB0562
Eudryas grata|[12000]|XAH054-05|Canada|Ontario|658[0n]|BOLD:AAB0562
Eudryas grata|[12001]|LOT277-04|United States|Tennessee|658[0n]|BOLD:AAB0562
Eudryas grata|[12002]|XAK059-06|Canada|Ontario|656[0n]|BOLD:AAB0562
Eudryas grata|[12003]|XAK258-06|Canada|Ontario|658[0n]|BOLD:AAB0562
Eudryas grata|[12004]|XAK060-06|Canada|Ontario|658[0n]|BOLD:AAB0562
Eudryas grata|[12005]|XAJ986-06|Canada|Ontario|658[0n]|BOLD:AAB0562
Eudryas grata|[12006]|XAJ985-06|Canada|Ontario|658[0n]|BOLD:AAB0562
Eudryas grata|[12007]|XAI043-05|Canada|Ontario|658[0n]|BOLD:AAB0562
Eudryas grata|[12008]|XAI042-05|Canada|Ontario|657[0n]|BOLD:AAB0562
Eudryas grata|[12009]|XAI040-05|Canada|Ontario|658[0n]|BOLD:AAB0562
Eudryas grata|[12010]|MNBB039-05|Canada|New Brunswick|658[0n]|BOLD:AAB0562
Eudryas grata|[12011]|XAE598-04|Canada|Ontario|658[0n]|BOLD:AAB0562
Eudryas grata|[12012]|XAC620-04|Canada|Ontario|658[0n]|BOLD:AAB0562
Eudryas grata|[12013]|XAC567-04|Canada|Ontario|658[0n]|BOLD:AAB0562
Eudryas grata|[12014]|LOT361-04|United States|Tennessee|658[0n]|BOLD:AAB0562
Eudryas grata|[12015]|LOT276-04|United States|Tennessee|658[0n]|BOLD:AAB0562
Eudryas grata|[12016]|PMG110-03|Canada|Ontario|617[0n]|BOLD:AAB0562
Eudryas grata|[12017]|LOT566-04|United States|Tennessee|658[0n]|BOLD:AAB0562
Eudryas grata|[12018]|XAC623-04|Canada|Ontario|596[2n]|BOLD:AAB0562
Eudryas grata|[12019]|RDLQ457-07|Canada|Quebec|658[0n]|BOLD:AAB0562
Eudryas grata|[12020]|LPSOD205-09|Canada|Ontario|658[0n]|BOLD:AAB0562
Eudryas grata|[12021]|BBLPA600-10|Canada|Ontario|658[0n]|BOLD:AAB0562
Eudryas grata|[12022]|BBLPA601-10|Canada|Ontario|658[0n]|BOLD:AAB0562
Eudryas grata|[12023]|LGSM503-04|United States|Tennessee|658[0n]|BOLD:AAB0562
Eudryas grata|[12024]|LOT362-04|United States|Tennessee|658[0n]|BOLD:AAB0562
Eudryas grata|[12025]|LOT278-04|United States|Tennessee|658[0n]|BOLD:AAB0562
Eudryas grata|[12026]|LOT490-04|United States|Tennessee|658[0n]|BOLD:AAB0562
Eudryas grata|[12027]|PHMO106-03|Canada|Ontario|639[0n]|BOLD:AAB0562
Eudryas grata|[12028]|LNCB442-07|United States|North Carolina|639[0n]|BOLD:AAB0562
Eudryas grata|[12029]|LNCC054-10|United States|North Carolina|658[0n]|BOLD:AAB0562
Eudryas grata|[12030]|LILLA995-11|United States|Illinois|658[0n]|BOLD:AAB0562
Eudryas unio|[12031]|LPSO434-08|Canada|Ontario|658[0n]|BOLD:ACF1723
Eudryas unio|[12032]|LPSO053-08|Canada|Ontario|658[0n]|BOLD:ACF1723
Eudryas unio|[12033]|LPSO054-08|Canada|Ontario|646[0n]|BOLD:ACF1723
Eudryas unio|[12034]|LPSO432-08|Canada|Ontario|658[0n]|BOLD:ACF1723

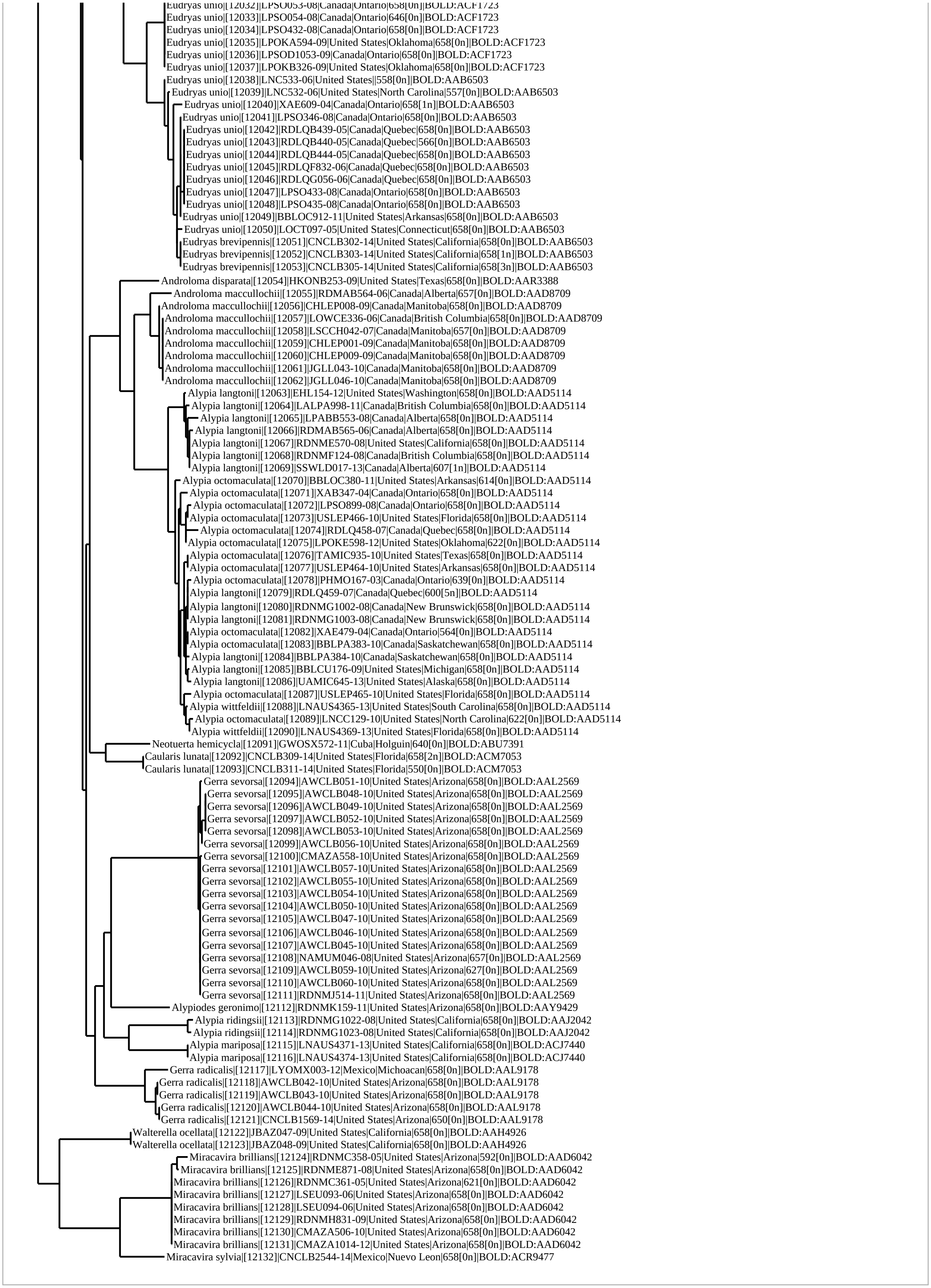
Eudryas unio|[12032]|LPSO053-08|Canada|Ontario|658[0n]|BOLD:ACF1723
Eudryas unio|[12033]|LPSO054-08|Canada|Ontario|646[0n]|BOLD:ACF1723
Eudryas unio|[12034]|LPSO432-08|Canada|Ontario|658[0n]|BOLD:ACF1723
Eudryas unio|[12035]|LPOKA594-09|United States|Oklahoma|658[0n]|BOLD:ACF1723
Eudryas unio|[12036]|LPSOD1053-09|Canada|Ontario|658[0n]|BOLD:ACF1723
Eudryas unio|[12037]|LPOKB326-09|United States|Oklahoma|658[0n]|BOLD:ACF1723
Eudryas unio|[12038]|LNC533-06|United States||558[0n]|BOLD:AAB6503
Eudryas unio|[12039]|LNC532-06|United States|North Carolina|557[0n]|BOLD:AAB6503
Eudryas unio|[12040]|XAE609-04|Canada|Ontario|658[1n]|BOLD:AAB6503
Eudryas unio|[12041]|LPSO346-08|Canada|Ontario|658[0n]|BOLD:AAB6503
Eudryas unio|[12042]|RDLQB439-05|Canada|Quebec|658[0n]|BOLD:AAB6503
Eudryas unio|[12043]|RDLQB440-05|Canada|Quebec|566[0n]|BOLD:AAB6503
Eudryas unio|[12044]|RDLQB444-05|Canada|Quebec|658[0n]|BOLD:AAB6503
Eudryas unio|[12045]|RDLQF832-06|Canada|Quebec|658[0n]|BOLD:AAB6503
Eudryas unio|[12046]|RDLQG056-06|Canada|Quebec|658[0n]|BOLD:AAB6503
Eudryas unio|[12047]|LPSO433-08|Canada|Ontario|658[0n]|BOLD:AAB6503
Eudryas unio|[12048]|LPSO435-08|Canada|Ontario|658[0n]|BOLD:AAB6503
Eudryas unio|[12049]|BBLOC912-11|United States|Arkansas|658[0n]|BOLD:AAB6503
Eudryas unio|[12050]|LOCT097-05|United States|Connecticut|658[0n]|BOLD:AAB6503
Eudryas brevipennis|[12051]|CNCLB302-14|United States|California|658[0n]|BOLD:AAB6503
Eudryas brevipennis|[12052]|CNCLB303-14|United States|California|658[1n]|BOLD:AAB6503
Eudryas brevipennis|[12053]|CNCLB305-14|United States|California|658[3n]|BOLD:AAB6503
Androloma disparata|[12054]|HKONB253-09|United States|Texas|658[0n]|BOLD:AAR3388
Androloma maccullochii|[12055]|RDMAB564-06|Canada|Alberta|657[0n]|BOLD:AAD8709
Androloma maccullochii|[12056]|CHLEP008-09|Canada|Manitoba|658[0n]|BOLD:AAD8709
Androloma maccullochii|[12057]|LOWCE336-06|Canada|British Columbia|658[0n]|BOLD:AAD8709
Androloma maccullochii|[12058]|LSCCH042-07|Canada|Manitoba|657[0n]|BOLD:AAD8709
Androloma maccullochii|[12059]|CHLEP001-09|Canada|Manitoba|658[0n]|BOLD:AAD8709
Androloma maccullochii|[12060]|CHLEP009-09|Canada|Manitoba|658[0n]|BOLD:AAD8709
Androloma maccullochii|[12061]|JGLL043-10|Canada|Manitoba|658[0n]|BOLD:AAD8709
Androloma maccullochii|[12062]|JGLL046-10|Canada|Manitoba|658[0n]|BOLD:AAD8709
Alypia langtoni|[12063]|EHL154-12|United States|Washington|658[0n]|BOLD:AAD5114
Alypia langtoni|[12064]|LALPA998-11|Canada|British Columbia|658[0n]|BOLD:AAD5114
Alypia langtoni|[12065]|LPABB553-08|Canada|Alberta|658[0n]|BOLD:AAD5114
Alypia langtoni|[12066]|RDMAB565-06|Canada|Alberta|658[0n]|BOLD:AAD5114
Alypia langtoni|[12067]|RDNME570-08|United States|California|658[0n]|BOLD:AAD5114
Alypia langtoni|[12068]|RDNMF124-08|Canada|British Columbia|658[0n]|BOLD:AAD5114
Alypia langtoni|[12069]|SSWLD017-13|Canada|Alberta|607[1n]|BOLD:AAD5114
Alypia octomaculata|[12070]|BBLOC380-11|United States|Arkansas|614[0n]|BOLD:AAD5114
Alypia octomaculata|[12071]|XAB347-04|Canada|Ontario|658[0n]|BOLD:AAD5114
Alypia octomaculata|[12072]|LPSO899-08|Canada|Ontario|658[0n]|BOLD:AAD5114
Alypia octomaculata|[12073]|USLEP466-10|United States|Florida|658[0n]|BOLD:AAD5114
Alypia octomaculata|[12074]|RDLQ458-07|Canada|Quebec|658[0n]|BOLD:AAD5114
Alypia octomaculata|[12075]|LPOKE598-12|United States|Oklahoma|622[0n]|BOLD:AAD5114
Alypia octomaculata|[12076]|TAMIC935-10|United States|Texas|658[0n]|BOLD:AAD5114
Alypia octomaculata|[12077]|USLEP464-10|United States|Arkansas|658[0n]|BOLD:AAD5114
Alypia octomaculata|[12078]|PHMO167-03|Canada|Ontario|639[0n]|BOLD:AAD5114
Alypia langtoni|[12079]|RDLQ459-07|Canada|Quebec|600[5n]|BOLD:AAD5114
Alypia langtoni|[12080]|RDNMG1002-08|Canada|New Brunswick|658[0n]|BOLD:AAD5114
Alypia langtoni|[12081]|RDNMG1003-08|Canada|New Brunswick|658[0n]|BOLD:AAD5114
Alypia octomaculata|[12082]|XAE479-04|Canada|Ontario|564[0n]|BOLD:AAD5114
Alypia octomaculata|[12083]|BBLPA383-10|Canada|Saskatchewan|658[0n]|BOLD:AAD5114
Alypia langtoni|[12084]|BBLPA384-10|Canada|Saskatchewan|658[0n]|BOLD:AAD5114
Alypia langtoni|[12085]|BBLCU176-09|United States|Michigan|658[0n]|BOLD:AAD5114
Alypia langtoni|[12086]|UAMIC645-13|United States|Alaska|658[0n]|BOLD:AAD5114
Alypia octomaculata|[12087]|USLEP465-10|United States|Florida|658[0n]|BOLD:AAD5114
Alypia wittfeldii|[12088]|LNAUS4365-13|United States|South Carolina|658[0n]|BOLD:AAD5114
Alypia octomaculata|[12089]|LNCC129-10|United States|North Carolina|622[0n]|BOLD:AAD5114
Alypia wittfeldii|[12090]|LNAUS4369-13|United States|Florida|658[0n]|BOLD:AAD5114
Neotuerta hemicycla|[12091]|GWOSX572-11|Cuba|Holguin|640[0n]|BOLD:ABU7391
Caularis lunata|[12092]|CNCLB309-14|United States|Florida|658[2n]|BOLD:ACM7053
Caularis lunata|[12093]|CNCLB311-14|United States|Florida|550[0n]|BOLD:ACM7053
Gerra sevorsa|[12094]|AWCLB051-10|United States|Arizona|658[0n]|BOLD:AAL2569
Gerra sevorsa|[12095]|AWCLB048-10|United States|Arizona|658[0n]|BOLD:AAL2569
Gerra sevorsa|[12096]|AWCLB049-10|United States|Arizona|658[0n]|BOLD:AAL2569
Gerra sevorsa|[12097]|AWCLB052-10|United States|Arizona|658[0n]|BOLD:AAL2569
Gerra sevorsa|[12098]|AWCLB053-10|United States|Arizona|658[0n]|BOLD:AAL2569
Gerra sevorsa|[12099]|AWCLB056-10|United States|Arizona|658[0n]|BOLD:AAL2569
Gerra sevorsa|[12100]|CMAZA558-10|United States|Arizona|658[0n]|BOLD:AAL2569
Gerra sevorsa|[12101]|AWCLB057-10|United States|Arizona|658[0n]|BOLD:AAL2569
Gerra sevorsa|[12102]|AWCLB055-10|United States|Arizona|658[0n]|BOLD:AAL2569
Gerra sevorsa|[12103]|AWCLB054-10|United States|Arizona|658[0n]|BOLD:AAL2569
Gerra sevorsa|[12104]|AWCLB050-10|United States|Arizona|658[0n]|BOLD:AAL2569
Gerra sevorsa|[12105]|AWCLB047-10|United States|Arizona|658[0n]|BOLD:AAL2569
Gerra sevorsa|[12106]|AWCLB046-10|United States|Arizona|658[0n]|BOLD:AAL2569
Gerra sevorsa|[12107]|AWCLB045-10|United States|Arizona|658[0n]|BOLD:AAL2569
Gerra sevorsa|[12108]|NAMUM046-08|United States|Arizona|657[0n]|BOLD:AAL2569
Gerra sevorsa|[12109]|AWCLB059-10|United States|Arizona|627[0n]|BOLD:AAL2569
Gerra sevorsa|[12110]|AWCLB060-10|United States|Arizona|658[0n]|BOLD:AAL2569
Gerra sevorsa|[12111]|RDNMJ514-11|United States|Arizona|658[0n]|BOLD:AAL2569
Alypiodes geronimo|[12112]|RDNMK159-11|United States|Arizona|658[0n]|BOLD:AAY9429
Alypia ridingsii|[12113]|RDNMG1022-08|United States|California|658[0n]|BOLD:AAJ2042
Alypia ridingsii|[12114]|RDNMG1023-08|United States|California|658[0n]|BOLD:AAJ2042
Alypia mariposa|[12115]|LNAUS4371-13|United States|California|658[0n]|BOLD:ACJ7440
Alypia mariposa|[12116]|LNAUS4374-13|United States|California|658[0n]|BOLD:ACJ7440
Gerra radicalis|[12117]|LYOMX003-12|Mexico|Michoacan|658[0n]|BOLD:AAL9178
Gerra radicalis|[12118]|AWCLB042-10|United States|Arizona|658[0n]|BOLD:AAL9178
Gerra radicalis|[12119]|AWCLB043-10|United States|Arizona|658[0n]|BOLD:AAL9178
Gerra radicalis|[12120]|AWCLB044-10|United States|Arizona|658[0n]|BOLD:AAL9178
Gerra radicalis|[12121]|CNCLB1569-14|United States|Arizona|650[0n]|BOLD:AAL9178
Walterella ocellata|[12122]|JBAZ047-09|United States|California|658[0n]|BOLD:AAH4926
Walterella ocellata|[12123]|JBAZ048-09|United States|California|658[0n]|BOLD:AAH4926
Miracavira brillians|[12124]|RDNMC358-05|United States|Arizona|592[0n]|BOLD:AAD6042
Miracavira brillians|[12125]|RDNME871-08|United States|Arizona|658[0n]|BOLD:AAD6042
Miracavira brillians|[12126]|RDNMC361-05|United States|Arizona|621[0n]|BOLD:AAD6042
Miracavira brillians|[12127]|LSEU093-06|United States|Arizona|658[0n]|BOLD:AAD6042
Miracavira brillians|[12128]|LSEU094-06|United States|Arizona|658[0n]|BOLD:AAD6042
Miracavira brillians|[12129]|RDNMH831-09|United States|Arizona|658[0n]|BOLD:AAD6042
Miracavira brillians|[12130]|CMAZA506-10|United States|Arizona|658[0n]|BOLD:AAD6042
Miracavira brillians|[12131]|CMAZA1014-12|United States|Arizona|658[0n]|BOLD:AAD6042
Miracavira sylvia|[12132]|CNCLB2544-14|Mexico|Nuevo Leon|658[0n]|BOLD:ACR9477
