# Supplementary material for: Probing planetary biodiversity with DNA barcodes: The Noctuoidea of North America
Source: PLoS One. 2017 Jun 1;12(6):e0178548. doi: 10.1371/journal.pone.0178548 (PMC5453547; doi:10.1371/journal.pone.0178548)
Supplement: S7 Tree — NJ tree based on sequence variation in the barcode region of the cytochrome c oxidase I gene for North American species in the family Noctuidae-2. (PDF) [file pone.0178548.s020.pdf]

BOLD TaxonID Tree

Title	:	Tree Result - Search (19787 records)
Date	:	21-April-2016
Data Type	:	Nucleotide
Distance Model	:	Kimura 2 Parameter
Marker	:	COI-5P

Label	:	Process ID
Label	:	Taxon
Label	:	Country
Label	:	Province/State
Label	:	Sequence Length
Label	:	Barcode Cluster (BIN)

Sequence Count	:	19787
Species count	:	1029
Genus count	:	185
Family count	:	1
Unidentified	:	0

BIN Count	:	1000

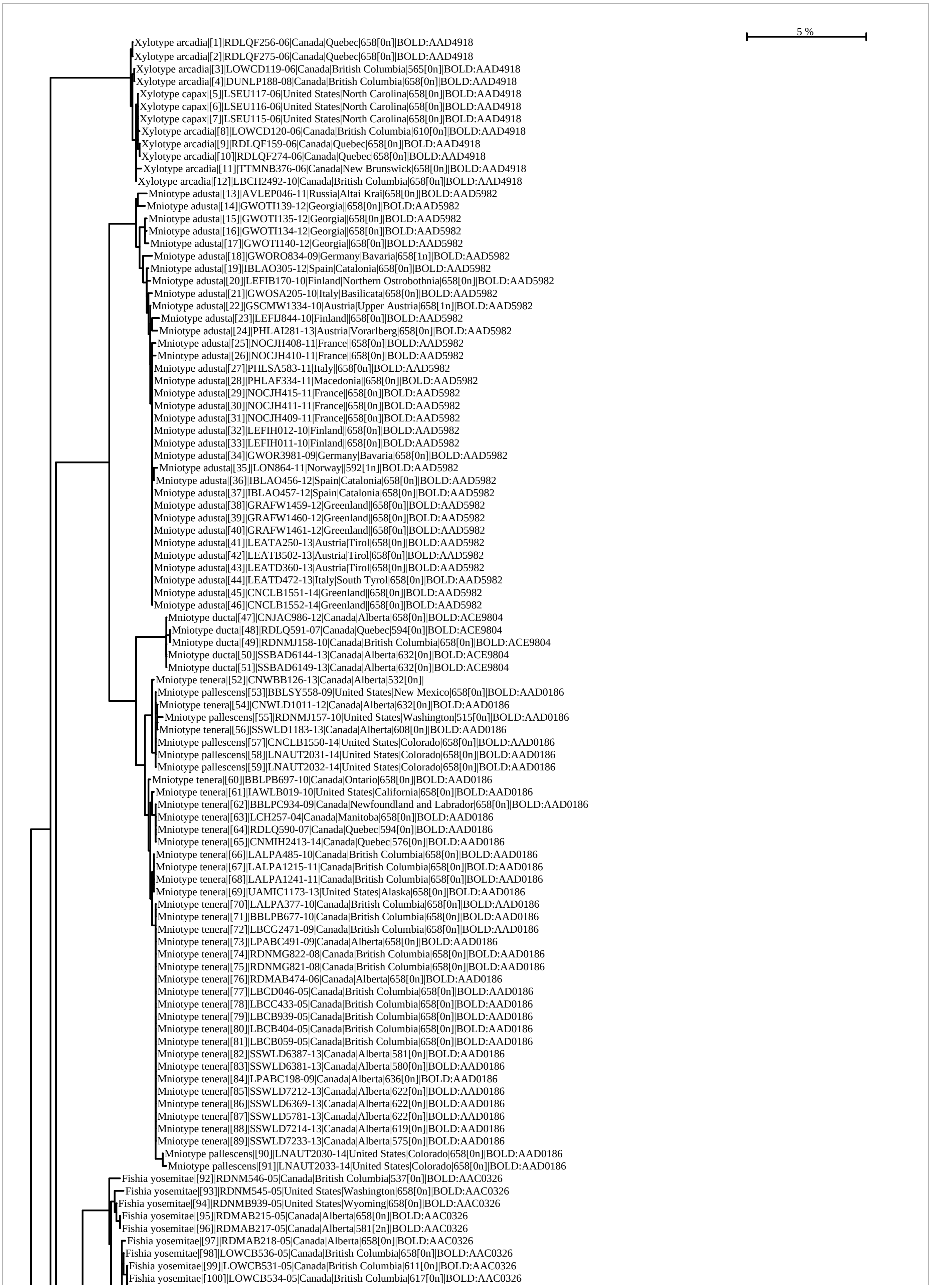
5 %
Xylotype arcadia|[1]|RDLQF256-06|Canada|Quebec|658[0n]|BOLD:AAD4918
Xylotype arcadia|[2]|RDLQF275-06|Canada|Quebec|658[0n]|BOLD:AAD4918
Xylotype arcadia|[3]|LOWCD119-06|Canada|British Columbia|565[0n]|BOLD:AAD4918
Xylotype arcadia|[4]|DUNLP188-08|Canada|British Columbia|658[0n]|BOLD:AAD4918
Xylotype capax|[5]|LSEU117-06|United States|North Carolina|658[0n]|BOLD:AAD4918
Xylotype capax|[6]|LSEU116-06|United States|North Carolina|658[0n]|BOLD:AAD4918
Xylotype capax|[7]|LSEU115-06|United States|North Carolina|658[0n]|BOLD:AAD4918
Xylotype arcadia|[8]|LOWCD120-06|Canada|British Columbia|610[0n]|BOLD:AAD4918
Xylotype arcadia|[9]|RDLQF159-06|Canada|Quebec|658[0n]|BOLD:AAD4918
Xylotype arcadia|[10]|RDLQF274-06|Canada|Quebec|658[0n]|BOLD:AAD4918
Xylotype arcadia|[11]|TTMNB376-06|Canada|New Brunswick|658[0n]|BOLD:AAD4918
Xylotype arcadia|[12]|LBCH2492-10|Canada|British Columbia|658[0n]|BOLD:AAD4918
Mniotype adusta|[13]|AVLEP046-11|Russia|Altai Krai|658[0n]|BOLD:AAD5982
Mniotype adusta|[14]|GWOTI139-12|Georgia||658[0n]|BOLD:AAD5982
Mniotype adusta|[15]|GWOTI135-12|Georgia||658[0n]|BOLD:AAD5982
Mniotype adusta|[16]|GWOTI134-12|Georgia||658[0n]|BOLD:AAD5982
Mniotype adusta|[17]|GWOTI140-12|Georgia||658[0n]|BOLD:AAD5982
Mniotype adusta|[18]|GWORO834-09|Germany|Bavaria|658[1n]|BOLD:AAD5982
Mniotype adusta|[19]|IBLAO305-12|Spain|Catalonia|658[0n]|BOLD:AAD5982
Mniotype adusta|[20]|LEFIB170-10|Finland|Northern Ostrobothnia|658[0n]|BOLD:AAD5982
Mniotype adusta|[21]|GWOSA205-10|Italy|Basilicata|658[0n]|BOLD:AAD5982
Mniotype adusta|[22]|GSCMW1334-10|Austria|Upper Austria|658[1n]|BOLD:AAD5982
Mniotype adusta|[23]|LEFIJ844-10|Finland||658[0n]|BOLD:AAD5982
Mniotype adusta|[24]|PHLAI281-13|Austria|Vorarlberg|658[0n]|BOLD:AAD5982
Mniotype adusta|[25]|NOCJH408-11|France||658[0n]|BOLD:AAD5982
Mniotype adusta|[26]|NOCJH410-11|France||658[0n]|BOLD:AAD5982
Mniotype adusta|[27]|PHLSA583-11|Italy||658[0n]|BOLD:AAD5982
Mniotype adusta|[28]|PHLAF334-11|Macedonia||658[0n]|BOLD:AAD5982
Mniotype adusta|[29]|NOCJH415-11|France||658[0n]|BOLD:AAD5982
Mniotype adusta|[30]|NOCJH411-11|France||658[0n]|BOLD:AAD5982
Mniotype adusta|[31]|NOCJH409-11|France||658[0n]|BOLD:AAD5982
Mniotype adusta|[32]|LEFIH012-10|Finland||658[0n]|BOLD:AAD5982
Mniotype adusta|[33]|LEFIH011-10|Finland||658[0n]|BOLD:AAD5982
Mniotype adusta|[34]|GWOR3981-09|Germany|Bavaria|658[0n]|BOLD:AAD5982
Mniotype adusta|[35]|LON864-11|Norway||592[1n]|BOLD:AAD5982
Mniotype adusta|[36]|IBLAO456-12|Spain|Catalonia|658[0n]|BOLD:AAD5982
Mniotype adusta|[37]|IBLAO457-12|Spain|Catalonia|658[0n]|BOLD:AAD5982
Mniotype adusta|[38]|GRAFW1459-12|Greenland||658[0n]|BOLD:AAD5982
Mniotype adusta|[39]|GRAFW1460-12|Greenland||658[0n]|BOLD:AAD5982
Mniotype adusta|[40]|GRAFW1461-12|Greenland||658[0n]|BOLD:AAD5982
Mniotype adusta|[41]|LEATA250-13|Austria|Tirol|658[0n]|BOLD:AAD5982
Mniotype adusta|[42]|LEATB502-13|Austria|Tirol|658[0n]|BOLD:AAD5982
Mniotype adusta|[43]|LEATD360-13|Austria|Tirol|658[0n]|BOLD:AAD5982
Mniotype adusta|[44]|LEATD472-13|Italy|South Tyrol|658[0n]|BOLD:AAD5982
Mniotype adusta|[45]|CNCLB1551-14|Greenland||658[0n]|BOLD:AAD5982
Mniotype adusta|[46]|CNCLB1552-14|Greenland||658[0n]|BOLD:AAD5982
Mniotype ducta|[47]|CNJAC986-12|Canada|Alberta|658[0n]|BOLD:ACE9804
Mniotype ducta|[48]|RDLQ591-07|Canada|Quebec|594[0n]|BOLD:ACE9804
Mniotype ducta|[49]|RDNMJ158-10|Canada|British Columbia|658[0n]|BOLD:ACE9804
Mniotype ducta|[50]|SSBAD6144-13|Canada|Alberta|632[0n]|BOLD:ACE9804
Mniotype ducta|[51]|SSBAD6149-13|Canada|Alberta|632[0n]|BOLD:ACE9804
Mniotype tenera|[52]|CNWBB126-13|Canada|Alberta|532[0n]|
Mniotype pallescens|[53]|BBLSY558-09|United States|New Mexico|658[0n]|BOLD:AAD0186
Mniotype tenera|[54]|CNWLD1011-12|Canada|Alberta|632[0n]|BOLD:AAD0186
Mniotype pallescens|[55]|RDNMJ157-10|United States|Washington|515[0n]|BOLD:AAD0186
Mniotype tenera|[56]|SSWLD1183-13|Canada|Alberta|608[0n]|BOLD:AAD0186
Mniotype pallescens|[57]|CNCLB1550-14|United States|Colorado|658[0n]|BOLD:AAD0186
Mniotype pallescens|[58]|LNAUT2031-14|United States|Colorado|658[0n]|BOLD:AAD0186
Mniotype pallescens|[59]|LNAUT2032-14|United States|Colorado|658[0n]|BOLD:AAD0186
Mniotype tenera|[60]|BBLPB697-10|Canada|Ontario|658[0n]|BOLD:AAD0186
Mniotype tenera|[61]|IAWLB019-10|United States|California|658[0n]|BOLD:AAD0186
Mniotype tenera|[62]|BBLPC934-09|Canada|Newfoundland and Labrador|658[0n]|BOLD:AAD0186
Mniotype tenera|[63]|LCH257-04|Canada|Manitoba|658[0n]|BOLD:AAD0186
Mniotype tenera|[64]|RDLQ590-07|Canada|Quebec|594[0n]|BOLD:AAD0186
Mniotype tenera|[65]|CNMIH2413-14|Canada|Quebec|576[0n]|BOLD:AAD0186
Mniotype tenera|[66]|LALPA485-10|Canada|British Columbia|658[0n]|BOLD:AAD0186
Mniotype tenera|[67]|LALPA1215-11|Canada|British Columbia|658[0n]|BOLD:AAD0186
Mniotype tenera|[68]|LALPA1241-11|Canada|British Columbia|658[0n]|BOLD:AAD0186
Mniotype tenera|[69]|UAMIC1173-13|United States|Alaska|658[0n]|BOLD:AAD0186
Mniotype tenera|[70]|LALPA377-10|Canada|British Columbia|658[0n]|BOLD:AAD0186
Mniotype tenera|[71]|BBLPB677-10|Canada|British Columbia|658[0n]|BOLD:AAD0186
Mniotype tenera|[72]|LBCG2471-09|Canada|British Columbia|658[0n]|BOLD:AAD0186
Mniotype tenera|[73]|LPABC491-09|Canada|Alberta|658[0n]|BOLD:AAD0186
Mniotype tenera|[74]|RDNMG822-08|Canada|British Columbia|658[0n]|BOLD:AAD0186
Mniotype tenera|[75]|RDNMG821-08|Canada|British Columbia|658[0n]|BOLD:AAD0186
Mniotype tenera|[76]|RDMAB474-06|Canada|Alberta|658[0n]|BOLD:AAD0186
Mniotype tenera|[77]|LBCD046-05|Canada|British Columbia|658[0n]|BOLD:AAD0186
Mniotype tenera|[78]|LBCC433-05|Canada|British Columbia|658[0n]|BOLD:AAD0186
Mniotype tenera|[79]|LBCB939-05|Canada|British Columbia|658[0n]|BOLD:AAD0186
Mniotype tenera|[80]|LBCB404-05|Canada|British Columbia|658[0n]|BOLD:AAD0186
Mniotype tenera|[81]|LBCB059-05|Canada|British Columbia|658[0n]|BOLD:AAD0186
Mniotype tenera|[82]|SSWLD6387-13|Canada|Alberta|581[0n]|BOLD:AAD0186
Mniotype tenera|[83]|SSWLD6381-13|Canada|Alberta|580[0n]|BOLD:AAD0186
Mniotype tenera|[84]|LPABC198-09|Canada|Alberta|636[0n]|BOLD:AAD0186
Mniotype tenera|[85]|SSWLD7212-13|Canada|Alberta|622[0n]|BOLD:AAD0186
Mniotype tenera|[86]|SSWLD6369-13|Canada|Alberta|622[0n]|BOLD:AAD0186
Mniotype tenera|[87]|SSWLD5781-13|Canada|Alberta|622[0n]|BOLD:AAD0186
Mniotype tenera|[88]|SSWLD7214-13|Canada|Alberta|619[0n]|BOLD:AAD0186
Mniotype tenera|[89]|SSWLD7233-13|Canada|Alberta|575[0n]|BOLD:AAD0186
Mniotype pallescens|[90]|LNAUT2030-14|United States|Colorado|658[0n]|BOLD:AAD0186
Mniotype pallescens|[91]|LNAUT2033-14|United States|Colorado|658[0n]|BOLD:AAD0186
Fishia yosemitae|[92]|RDNM546-05|Canada|British Columbia|537[0n]|BOLD:AAC0326
Fishia yosemitae|[93]|RDNM545-05|United States|Washington|658[0n]|BOLD:AAC0326
Fishia yosemitae|[94]|RDNMB939-05|United States|Wyoming|658[0n]|BOLD:AAC0326
Fishia yosemitae|[95]|RDMAB215-05|Canada|Alberta|658[0n]|BOLD:AAC0326
Fishia yosemitae|[96]|RDMAB217-05|Canada|Alberta|581[2n]|BOLD:AAC0326
Fishia yosemitae|[97]|RDMAB218-05|Canada|Alberta|658[0n]|BOLD:AAC0326
Fishia yosemitae|[98]|LOWCB536-05|Canada|British Columbia|658[0n]|BOLD:AAC0326
Fishia yosemitae|[99]|LOWCB531-05|Canada|British Columbia|611[0n]|BOLD:AAC0326
Fishia yosemitae|[100]|LOWCB534-05|Canada|British Columbia|617[0n]|BOLD:AAC0326

Fishia yosemitae|[98]|LOWCB536-05|Canada|British Columbia|658[0n]|BOLD:AAC0326
Fishia yosemitae|[99]|LOWCB531-05|Canada|British Columbia|611[0n]|BOLD:AAC0326
Fishia yosemitae|[100]|LOWCB534-05|Canada|British Columbia|617[0n]|BOLD:AAC0326
Fishia yosemitae|[101]|LOWCB538-05|Canada|British Columbia|629[0n]|BOLD:AAC0326
Fishia yosemitae|[102]|RDNM547-05|Canada|Ontario|658[0n]|BOLD:AAC0326
Fishia yosemitae|[103]|RDNM548-05|Canada|Ontario|658[0n]|BOLD:AAC0326
Fishia yosemitae|[104]|LOWCB532-05|Canada|British Columbia|658[0n]|BOLD:AAC0326
Fishia yosemitae|[105]|LOWCB533-05|Canada|British Columbia|658[0n]|BOLD:AAC0326
Fishia yosemitae|[106]|LOWCB535-05|Canada|British Columbia|658[0n]|BOLD:AAC0326
Fishia yosemitae|[107]|LOWCB537-05|Canada|British Columbia|658[0n]|BOLD:AAC0326
Fishia yosemitae|[108]|LOWCB539-05|Canada|British Columbia|658[0n]|BOLD:AAC0326
Fishia yosemitae|[109]|LOWCB540-05|Canada|British Columbia|658[0n]|BOLD:AAC0326
Fishia yosemitae|[110]|RDMAB216-05|Canada|Alberta|658[0n]|BOLD:AAC0326
Fishia yosemitae|[111]|RDNMF750-08|Canada|Ontario|658[0n]|BOLD:AAC0326
Fishia discors|[112]|LOWCD933-06|Canada|British Columbia|584[0n]|BOLD:AAB3715
Fishia discors|[113]|LOWCD931-06|Canada|British Columbia|658[0n]|BOLD:AAB3715
Fishia discors|[114]|LOWCD932-06|Canada|British Columbia|658[0n]|BOLD:AAB3715
Fishia discors|[115]|LOWCD929-06|Canada|British Columbia|658[0n]|BOLD:AAB3715
Fishia discors|[116]|LOWCD930-06|Canada|British Columbia|658[0n]|BOLD:AAB3715
Fishia discors|[117]|LOWCC038-05|Canada|British Columbia|658[0n]|BOLD:AAB3715
Fishia discors|[118]|LOWCC039-05|Canada|British Columbia|658[0n]|BOLD:AAB3715
Fishia discors|[119]|LOWCC037-05|Canada|British Columbia|658[0n]|BOLD:AAB3715
Fishia discors|[120]|LOWCC036-05|Canada|British Columbia|658[0n]|BOLD:AAB3715
Fishia discors|[121]|LOWCC035-05|Canada|British Columbia|658[0n]|BOLD:AAB3715
Fishia discors|[122]|LOWCC034-05|Canada|British Columbia|658[0n]|BOLD:AAB3715
Fishia discors|[123]|LOWCC033-05|Canada|British Columbia|658[0n]|BOLD:AAB3715
Fishia discors|[124]|LOWCC032-05|Canada|British Columbia|658[0n]|BOLD:AAB3715
Fishia discors|[125]|LOWCC031-05|Canada|British Columbia|658[0n]|BOLD:AAB3715
Fishia discors|[126]|LOWCC030-05|Canada|British Columbia|658[0n]|BOLD:AAB3715
Fishia discors|[127]|LOWCC029-05|Canada|British Columbia|658[0n]|BOLD:AAB3715
Fishia discors|[128]|LOWCC028-05|Canada|British Columbia|658[0n]|BOLD:AAB3715
Fishia discors|[129]|LOWCC027-05|Canada|British Columbia|658[0n]|BOLD:AAB3715
Fishia discors|[130]|LOWCC026-05|Canada|British Columbia|658[0n]|BOLD:AAB3715
Fishia discors|[131]|LOWCC025-05|Canada|British Columbia|658[0n]|BOLD:AAB3715
Fishia discors|[132]|LOWCD935-06|Canada|British Columbia|596[0n]|BOLD:AAB3715
Fishia discors|[133]|LOWCD934-06|Canada|British Columbia|658[0n]|BOLD:AAB3715
Fishia discors|[134]|LOWCD936-06|Canada|British Columbia|658[0n]|BOLD:AAB3715
Fishia discors|[135]|RDMAB578-06|Canada|Alberta|658[0n]|BOLD:AAB3715
Fishia discors|[136]|LALPA791-10|Canada|British Columbia|658[0n]|BOLD:AAB3715
Fishia discors|[137]|LALPA810-10|Canada|British Columbia|658[0n]|BOLD:AAB3715
Fishia illocata|[138]|TMNBB184-06|Canada|New Brunswick|658[0n]|BOLD:AAA9136
Fishia illocata|[139]|TMNBB185-06|Canada|New Brunswick|658[0n]|BOLD:AAA9136
Fishia illocata|[140]|PHMO334-03|Canada|Ontario|637[5n]|BOLD:AAA9136
Fishia illocata|[141]|LOWCD700-06|Canada|British Columbia|589[0n]|BOLD:AAA9136
Fishia illocata|[142]|LOWCD703-06|Canada|British Columbia|591[0n]|BOLD:AAA9136
Fishia illocata|[143]|LOWCB459-05|Canada|British Columbia|658[0n]|BOLD:AAA9136
Fishia illocata|[144]|LOWCD155-06|Canada|British Columbia|658[0n]|BOLD:AAA9136
Fishia illocata|[145]|LOWCD704-06|Canada|British Columbia|658[0n]|BOLD:AAA9136
Fishia illocata|[146]|LOWCD122-06|Canada|British Columbia|658[0n]|BOLD:AAA9136
Fishia illocata|[147]|LSEU195-06|United States|North Carolina|658[0n]|BOLD:AAA9136
Fishia illocata|[148]|LSEU194-06|United States|North Carolina|658[0n]|BOLD:AAA9136
Fishia illocata|[149]|RDLQB518-05|Canada|Quebec|658[0n]|BOLD:AAA9136
Fishia illocata|[150]|LOWCB461-05|Canada|British Columbia|658[0n]|BOLD:AAA9136
Fishia illocata|[151]|LOWCB460-05|Canada|British Columbia|658[0n]|BOLD:AAA9136
Fishia illocata|[152]|LOWCB455-05|Canada|British Columbia|658[0n]|BOLD:AAA9136
Fishia illocata|[153]|LOWCB453-05|Canada|British Columbia|658[0n]|BOLD:AAA9136
Fishia illocata|[154]|LOWCB452-05|Canada|British Columbia|658[0n]|BOLD:AAA9136
Fishia illocata|[155]|XAH565-05|Canada|Ontario|658[0n]|BOLD:AAA9136
Fishia illocata|[156]|RDNMC272-05|Canada|New Brunswick|617[0n]|BOLD:AAA9136
Fishia illocata|[157]|XAH312-05|Canada|Ontario|639[0n]|BOLD:AAA9136
Fishia illocata|[158]|RDNMC271-05|Canada|New Brunswick|591[0n]|BOLD:AAA9136
Fishia illocata|[159]|LOWCD702-06|Canada|British Columbia|588[0n]|BOLD:AAA9136
Fishia illocata|[160]|LOWCD699-06|Canada|British Columbia|588[0n]|BOLD:AAA9136
Fishia illocata|[161]|TTMNB353-06|Canada|New Brunswick|613[0n]|BOLD:AAA9136
Fishia illocata|[162]|RDLQF277-06|Canada|Quebec|658[0n]|BOLD:AAA9136
Fishia illocata|[163]|RDLQF278-06|Canada|Quebec|658[0n]|BOLD:AAA9136
Fishia illocata|[164]|LBCH2704-10|Canada|British Columbia|658[0n]|BOLD:AAA9136
Fishia illocata|[165]|LBCH2789-10|Canada|British Columbia|658[0n]|BOLD:AAA9136
Fishia illocata|[166]|LBCH2861-10|Canada|British Columbia|658[0n]|BOLD:AAA9136
Fishia illocata|[167]|RDLQB517-05|Canada|Quebec|658[0n]|BOLD:AAA9136
Fishia illocata|[168]|MJMSL046-10|United States|Massachusetts|658[0n]|BOLD:AAA9136
Fishia illocata|[169]|LOWCD701-06|Canada|British Columbia|658[0n]|BOLD:AAA9136
Fishia illocata|[170]|LSEU193-06|United States|North Carolina|658[0n]|BOLD:AAA9136
Fishia illocata|[171]|LOWCB454-05|Canada|British Columbia|658[0n]|BOLD:AAA9136
Fishia illocata|[172]|LPVIB633-08|Canada|British Columbia|634[0n]|BOLD:AAA9136
Fishia illocata|[173]|RWWB245-09|United States|Washington|658[0n]|BOLD:AAA9136
Fishia illocata|[174]|RWWB294-09|United States|Washington|658[0n]|BOLD:AAA9136
Fishia illocata|[175]|RWWB379-09|United States|Washington|658[0n]|BOLD:AAA9136
Fishia illocata|[176]|LBCH2607-10|Canada|British Columbia|658[0n]|BOLD:AAA9136
Fishia illocata|[177]|MJMSL005-10|United States|Massachusetts|658[0n]|BOLD:AAA9136
Fishia illocata|[178]|LALPA748-10|Canada|British Columbia|658[0n]|BOLD:AAA9136
Fishia illocata|[179]|LALPA771-10|Canada|British Columbia|658[0n]|BOLD:AAA9136
Fishia illocata|[180]|LALPA795-10|Canada|British Columbia|658[0n]|BOLD:AAA9136
Fishia illocata|[181]|RWWB362-09|United States|Washington|658[0n]|BOLD:AAA9136
Fishia illocata|[182]|RWWB344-09|United States|Washington|658[0n]|BOLD:AAA9136
Fishia illocata|[183]|LALPA747-10|Canada|British Columbia|644[0n]|BOLD:AAA9136
Fishia illocata|[184]|LALPA757-10|Canada|British Columbia|658[0n]|BOLD:AAA9136
Fishia illocata|[185]|LALPA779-10|Canada|British Columbia|658[0n]|BOLD:AAA9136
Fishia illocata|[186]|LALPA1300-11|Canada|British Columbia|658[0n]|BOLD:AAA9136
Fishia illocata|[187]|RWWC830-12|United States|Washington|658[0n]|BOLD:AAA9136
Platypolia mactata|[188]|LNCC428-10|United States|North Carolina|658[0n]|BOLD:AAC7837
Platypolia mactata|[189]|LBCH2535-10|Canada|British Columbia|658[0n]|BOLD:AAC7837
Platypolia mactata|[190]|LOWCE651-06|Canada|British Columbia|658[0n]|BOLD:AAC7837
Platypolia mactata|[191]|LOWCE650-06|Canada|British Columbia|658[0n]|BOLD:AAC7837
Platypolia mactata|[192]|LOWCD537-06|Canada|British Columbia|658[0n]|BOLD:AAC7837
Platypolia mactata|[193]|LOWCD535-06|Canada|British Columbia|658[0n]|BOLD:AAC7837
Platypolia mactata|[194]|LOWCD161-06|Canada|British Columbia|658[0n]|BOLD:AAC7837
Platypolia mactata|[195]|RDNMC274-05|Canada|Alberta|597[0n]|BOLD:AAC7837
Platypolia mactata|[196]|RDNMC273-05|Canada|New Brunswick|589[0n]|BOLD:AAC7837
Platypolia mactata|[197]|LOWCD191-06|Canada|British Columbia|616[0n]|BOLD:AAC7837
Platypolia mactata|[198]|RDLQ463-07|Canada|Quebec|585[1n]|BOLD:AAC7837
Platypolia mactata|[199]|RDLQ464-07|Canada|Quebec|615[0n]|BOLD:AAC7837
Platypolia mactata|[200]|CNWLH466-12|Canada|Alberta|632[0n]|BOLD:AAC7837

Platypolia mactata|[198]|RDLQ463-07|Canada|Quebec|585[1n]|BOLD:AAC7837
Platypolia mactata|[199]|RDLQ464-07|Canada|Quebec|615[0n]|BOLD:AAC7837
Platypolia mactata|[200]|CNWLH466-12|Canada|Alberta|632[0n]|BOLD:AAC7837
Platypolia mactata|[201]|CNWLH465-12|Canada|Alberta|632[0n]|BOLD:AAC7837
Platypolia mactata|[202]|CNRMG778-12|Canada|Manitoba|630[0n]|BOLD:AAC7837
Platypolia mactata|[203]|CNWLH468-12|Canada|Alberta|630[0n]|BOLD:AAC7837
Platypolia mactata|[204]|CNWLI318-12|Canada|Alberta|630[0n]|BOLD:AAC7837
Platypolia mactata|[205]|CNWLI321-12|Canada|Alberta|632[0n]|BOLD:AAC7837
Platypolia mactata|[206]|LNCC1482-13|United States|North Carolina|658[0n]|BOLD:AAC7837
Platypolia mactata|[207]|LNCC1483-13|United States|North Carolina|658[0n]|BOLD:AAC7837
Platypolia mactata|[208]|CNWLO771-13|Canada|Alberta|598[0n]|BOLD:AAC7837
Fishia connecta|[209]|LNAUS5377-13|United States|Arizona|610[0n]|BOLD:ACK3425
Fishia connecta|[210]|CNCLB096-14|United States|Colorado|658[0n]|BOLD:ACK3425
Dryotype opina|[211]|RDNMF795-08|Canada|British Columbia|658[0n]|BOLD:AAF6858
Dryotype opina|[212]|RDNMF796-08|Canada|British Columbia|658[0n]|BOLD:AAF6858
Dryotype opina|[213]|RDNMF797-08|Canada|British Columbia|658[0n]|BOLD:AAF6858
Dryotype opina|[214]|LALPA772-10|Canada|British Columbia|658[0n]|BOLD:AAF6858
Sutyna privata|[215]|TMNBB278-06|Canada|New Brunswick|658[0n]|BOLD:AAB3593
Sutyna privata|[216]|TTMNB379-06|Canada|New Brunswick|658[0n]|BOLD:AAB3593
Sutyna privata|[217]|TTMNB378-06|Canada|New Brunswick|658[1n]|BOLD:AAB3593
Sutyna privata|[218]|TMNBB276-06|Canada|New Brunswick|658[0n]|BOLD:AAB3593
Sutyna privata|[219]|TMNBB275-06|Canada|New Brunswick|658[0n]|BOLD:AAB3593
Sutyna privata|[220]|TMNBB273-06|Canada|New Brunswick|658[0n]|BOLD:AAB3593
Sutyna privata|[221]|TTMNB384-06|Canada|New Brunswick|658[0n]|BOLD:AAB3593
Sutyna privata|[222]|TTMNB383-06|Canada|New Brunswick|658[0n]|BOLD:AAB3593
Sutyna privata|[223]|TTMNB382-06|Canada|New Brunswick|658[0n]|BOLD:AAB3593
Sutyna privata|[224]|TTMNB380-06|Canada|New Brunswick|658[0n]|BOLD:AAB3593
Sutyna privata|[225]|TMNBB272-06|Canada|New Brunswick|656[0n]|BOLD:AAB3593
Sutyna privata|[226]|TMNBB274-06|Canada|New Brunswick|656[0n]|BOLD:AAB3593
Sutyna privata|[227]|TMNBB279-06|Canada|New Brunswick|658[0n]|BOLD:AAB3593
Sutyna privata|[228]|TMNBB281-06|Canada|New Brunswick|658[0n]|BOLD:AAB3593
Sutyna privata|[229]|TTMNB377-06|Canada|New Brunswick|658[0n]|BOLD:AAB3593
Sutyna privata|[230]|TMNBB277-06|Canada|New Brunswick|656[0n]|BOLD:AAB3593
Sutyna privata|[231]|LOWCB175-05|Canada|British Columbia|658[0n]|BOLD:AAB3593
Sutyna privata|[232]|RDMAB952-09|Canada|Alberta|602[0n]|BOLD:AAB3593
Sutyna privata|[233]|RDMAB953-09|Canada|Alberta|620[0n]|BOLD:AAB3593
Sutyna privata|[234]|RDNMH130-09|United States|Florida|658[0n]|BOLD:AAB3593
Sutyna privata|[235]|RDLQ585-07|Canada|Quebec|588[1n]|BOLD:AAB3593
Sutyna privata|[236]|RDLQ584-07|Canada|Quebec|584[1n]|BOLD:AAB3593
Sutyna privata|[237]|RDLQ586-07|Canada|Quebec|595[1n]|BOLD:AAB3593
Sutyna privata|[238]|RDLQ587-07|Canada|Quebec|595[1n]|BOLD:AAB3593
Sutyna privata|[239]|RDLQ589-07|Canada|Quebec|590[0n]|BOLD:AAB3593
Sutyna privata|[240]|PSAT133-10|United States|Florida|589[0n]|BOLD:AAB3593
Sutyna privata|[241]|TMNBB280-06|Canada|New Brunswick|658[0n]|BOLD:AAB3593
Sutyna privata|[242]|TTMNB381-06|Canada|New Brunswick|658[0n]|BOLD:AAB3593
Sutyna privata|[243]|RDLQ588-07|Canada|Quebec|587[1n]|BOLD:AAB3593
Sutyna privata|[244]|RDMAB954-09|Canada|Alberta|609[0n]|BOLD:AAB3593
Sutyna privata|[245]|GWOTA066-12|United States|Massachusetts|658[0n]|BOLD:AAB3593
Fishia nigrescens|[246]|NAMUM185-08|United States|California|658[0n]|BOLD:AAE6644
Fishia nigrescens|[247]|RDNMF746-08|United States|Nevada|658[0n]|BOLD:AAE6644
Fishia nigrescens|[248]|RDNMF747-08|United States|California|658[0n]|BOLD:AAE6644
Fishia nigrescens|[249]|RDNMF748-08|United States|California|658[0n]|BOLD:AAE6644
Fishia dispar|[250]|RDNME856-08|United States|Arizona|658[0n]|BOLD:AAE6644
Fishia dispar|[251]|RDNMG020-08|United States|Arizona|658[0n]|BOLD:AAE6644
Fishia dispar|[252]|CNCLB097-14|United States|Colorado|658[0n]|BOLD:AAE6644
Fishia dispar|[253]|CNCLB098-14|United States|Colorado|658[0n]|BOLD:AAE6644
Pachypolia atricornis|[254]|LNCC424-10|United States|North Carolina|658[0n]|BOLD:AAQ1857
Pachypolia atricornis|[255]|LNCC425-10|United States|North Carolina|658[0n]|BOLD:AAQ1857
Pachypolia atricornis|[256]|LNCC426-10|United States|North Carolina|658[0n]|BOLD:AAQ1857
Pachypolia atricornis|[257]|LNCC427-10|United States|North Carolina|658[0n]|BOLD:AAQ1857
Pachypolia atricornis|[258]|LNAUT2654-14|United States|Illinois|658[0n]|BOLD:AAQ1857
Platypolia contadina|[259]|RDNM732-05|United States|Oregon|505[0n]|BOLD:ABY9730
Platypolia contadina|[260]|RDMAB609-06|Canada|Alberta|658[0n]|BOLD:AAD4229
Platypolia contadina|[261]|RDMAB610-06|Canada|Alberta|658[0n]|BOLD:AAD4229
Platypolia contadina|[262]|RDNM733-05|Canada|British Columbia|658[0n]|BOLD:AAD4229
Platypolia contadina|[263]|RDNM734-05|United States|Washington|658[0n]|BOLD:AAD4229
Platypolia contadina|[264]|RDMAB611-06|Canada|Alberta|658[0n]|BOLD:AAD4229
Platypolia contadina|[265]|LOWCE596-06|Canada|British Columbia|656[0n]|BOLD:AAD4229
Platypolia loda|[266]|LOWCE582-06|Canada|British Columbia|658[0n]|BOLD:AAD1063
Platypolia loda|[267]|LOWCE584-06|Canada|British Columbia|658[0n]|BOLD:AAD1063
Platypolia loda|[268]|LOWCE586-06|Canada|British Columbia|658[0n]|BOLD:AAD1063
Platypolia loda|[269]|LOWCE587-06|Canada|British Columbia|658[0n]|BOLD:AAD1063
Platypolia loda|[270]|LOWCE583-06|Canada|British Columbia|658[0n]|BOLD:AAD1063
Platypolia loda|[271]|LOWCE594-06|Canada|British Columbia|658[0n]|BOLD:AAD1063
Platypolia loda|[272]|LOWCE585-06|Canada|British Columbia|658[0n]|BOLD:AAD1063
Platypolia loda|[273]|LOWCE595-06|Canada|British Columbia|658[0n]|BOLD:AAD1063
Platypolia loda|[274]|JMMMB583-13|United States|California|540[0n]|
Platypolia anceps|[275]|LBCH2608-10|Canada|British Columbia|658[0n]|BOLD:AAD1074
Platypolia anceps|[276]|LBCH2591-10|Canada|British Columbia|658[0n]|BOLD:AAD1074
Platypolia anceps|[277]|LBCH2505-10|Canada|British Columbia|658[0n]|BOLD:AAD1074
Platypolia anceps|[278]|LOWCB529-05|Canada|British Columbia|658[0n]|BOLD:AAD1074
Platypolia anceps|[279]|LOWCB528-05|Canada|British Columbia|658[0n]|BOLD:AAD1074
Platypolia anceps|[280]|LOWCB527-05|Canada|British Columbia|658[0n]|BOLD:AAD1074
Platypolia anceps|[281]|LOWCB526-05|Canada|British Columbia|658[0n]|BOLD:AAD1074
Platypolia anceps|[282]|LOWCB525-05|Canada|British Columbia|658[0n]|BOLD:AAD1074
Platypolia anceps|[283]|LOWCB524-05|Canada|British Columbia|658[0n]|BOLD:AAD1074
Platypolia anceps|[284]|LOWCB530-05|Canada|British Columbia|597[0n]|BOLD:AAD1074
Platypolia anceps|[285]|LOWCC836-05|Canada|British Columbia|558[0n]|BOLD:AAD1074
Platypolia anceps|[286]|CNCLB2990-14|United States|North Carolina|658[0n]|BOLD:AAD1074
Platypolia anceps|[287]|CNCLB2991-14|United States|North Carolina|658[0n]|BOLD:AAD1074
Platypolia anceps|[288]|CNCLB2993-14|United States|North Carolina|658[0n]|BOLD:AAD1074
Platypolia anceps|[289]|CNCLB2994-14|United States|North Carolina|658[0n]|BOLD:AAD1074
Magusa orbifera|[290]|LNAUS4492-13|British Virgin Islands|Guana Island|658[0n]|BOLD:ACD9027
Magusa orbifera|[291]|LNAUS4493-13|British Virgin Islands|Guana Island|658[0n]|BOLD:ACD9027
Magusa orbifera|[292]|RDNML321-13|United States|Florida|658[0n]|BOLD:ACD9027
Magusa orbifera|[293]|LNAUS4491-13|British Virgin Islands|Guana Island|658[0n]|BOLD:ACD9027
Magusa orbifera|[294]|LNAUS4494-13|British Virgin Islands|Guana Island|658[0n]|BOLD:ACD9027
Magusa orbifera|[295]|LNAUS4495-13|British Virgin Islands|Guana Island|658[0n]|BOLD:ACD9027
Magusa divaricata|[296]|BLPBG815-07|Costa Rica|Guanacaste|636[0n]|BOLD:AAA4337
Magusa divaricata|[297]|MHAUB064-05|Costa Rica|Guanacaste|617[0n]|BOLD:AAA4337
Magusa divaricata|[298]|BLPCO030-08|Costa Rica|Guanacaste|658[0n]|BOLD:AAA4337
Magusa divaricata|[299]|BLPCD391-08|Costa Rica|Guanacaste|658[0n]|BOLD:AAA4337
Magusa divaricata|[300]|BLPDU247-11|Costa Rica|Guanacaste|658[0n]|BOLD:AAA4337

Magusa divaricata|[298]|BLPCO030-08|Costa Rica|Guanacaste|658[0n]|BOLD:AAA4337
Magusa divaricata|[299]|BLPCD391-08|Costa Rica|Guanacaste|658[0n]|BOLD:AAA4337
Magusa divaricata|[300]|BLPDU247-11|Costa Rica|Guanacaste|658[0n]|BOLD:AAA4337
Magusa divaricata|[301]|BLPDW247-11|Costa Rica|Guanacaste|658[0n]|BOLD:AAA4337
Magusa divaricata|[302]|LOCRG467-11|Costa Rica|Cartago|658[1n]|BOLD:AAA4337
Magusa divaricata|[303]|BLPDK1898-09|Costa Rica|Guanacaste|639[0n]|BOLD:AAA4337
Magusa divaricata|[304]|BLPDU245-11|Costa Rica|Guanacaste|658[0n]|BOLD:AAA4337
Magusa divaricata|[305]|BLPDU249-11|Costa Rica|Guanacaste|658[0n]|BOLD:AAA4337
Magusa divaricata|[306]|BLPDV402-11|Costa Rica|Guanacaste|658[0n]|BOLD:AAA4337
Magusa divaricata|[307]|BLPDX147-11|Costa Rica|Guanacaste|658[0n]|BOLD:AAA4337
Magusa divaricata|[308]|BLPDX167-11|Costa Rica|Guanacaste|658[0n]|BOLD:AAA4337
Magusa divaricata|[309]|BLPDW244-11|Costa Rica|Guanacaste|658[0n]|BOLD:AAA4337
Magusa divaricata|[310]|BLPDW248-11|Costa Rica|Guanacaste|658[0n]|BOLD:AAA4337
Magusa divaricata|[311]|BLPDW251-11|Costa Rica|Guanacaste|658[0n]|BOLD:AAA4337
Magusa divaricata|[312]|LEMMZ158-10|Brazil|Parana|658[0n]|BOLD:AAA4337
Magusa divaricata|[313]|GWOSH601-10|Brazil|Rio Grande do Sul|658[0n]|BOLD:AAA4337
Magusa divaricata|[314]|LEMMZ026-10|Brazil|Parana|658[0n]|BOLD:AAA4337
Magusa divaricata|[315]|LEMMZ028-10|Brazil|Parana|658[0n]|BOLD:AAA4337
Magusa divaricata|[316]|BLPDK1900-09|Costa Rica|Guanacaste|658[0n]|BOLD:AAA4337
Magusa orbifera|[317]|INCTA997-10|Brazil|Maranhao|658[0n]|BOLD:AAA4337
Magusa divaricata|[318]|BLPDK1297-09|Costa Rica|Guanacaste|658[0n]|BOLD:AAA4337
Magusa divaricata|[319]|BLPDK1298-09|Costa Rica|Guanacaste|658[0n]|BOLD:AAA4337
Magusa divaricata|[320]|BLPCO132-08|Costa Rica|Guanacaste|658[0n]|BOLD:AAA4337
Magusa divaricata|[321]|BLPDI684-09|Costa Rica|Guanacaste|658[0n]|BOLD:AAA4337
Magusa divaricata|[322]|BLPCO034-08|Costa Rica|Guanacaste|658[0n]|BOLD:AAA4337
Magusa divaricata|[323]|HKONB016-08|United States|Texas|658[0n]|BOLD:AAA4337
Magusa divaricata|[324]|BLPCN107-08|Costa Rica|Guanacaste|658[0n]|BOLD:AAA4337
Magusa divaricata|[325]|BLPCD388-08|Costa Rica|Guanacaste|658[0n]|BOLD:AAA4337
Magusa divaricata|[326]|BLPCC505-08|Costa Rica|Guanacaste|658[0n]|BOLD:AAA4337
Magusa divaricata|[327]|BLPCA791-08|Costa Rica|Guanacaste|658[0n]|BOLD:AAA4337
Magusa divaricata|[328]|BLPBH778-07|Costa Rica|Guanacaste|658[0n]|BOLD:AAA4337
Magusa divaricata|[329]|BLPBH777-07|Costa Rica|Guanacaste|658[0n]|BOLD:AAA4337
Magusa divaricata|[330]|RDNME051-07|United States|Arizona|658[0n]|BOLD:AAA4337
Magusa divaricata|[331]|MHAUB056-05|Costa Rica|Guanacaste|658[0n]|BOLD:AAA4337
Magusa divaricata|[332]|BLPAA383-06|Costa Rica|Guanacaste|658[0n]|BOLD:AAA4337
Magusa divaricata|[333]|BLPDV058-11|Costa Rica|Guanacaste|658[0n]|BOLD:AAA4337
Magusa divaricata|[334]|BLPBG491-07|Costa Rica|Guanacaste|645[0n]|BOLD:AAA4337
Magusa divaricata|[335]|BLPDE166-09|Costa Rica|Guanacaste|658[0n]|BOLD:AAA4337
Magusa divaricata|[336]|BLPBG838-07|Costa Rica|Guanacaste|621[0n]|BOLD:AAA4337
Magusa divaricata|[337]|BLPCO306-08|Costa Rica|Guanacaste|622[2n]|BOLD:AAA4337
Magusa divaricata|[338]|MHMYS2788-13|Costa Rica|Guanacaste|634[0n]|BOLD:AAA4337
Magusa divaricata|[339]|BLPEF4966-13|Costa Rica|Guanacaste|658[0n]|BOLD:AAA4337
Magusa divaricata|[340]|MHMYS3015-13|Costa Rica|Guanacaste|658[0n]|BOLD:AAA4337
Magusa divaricata|[341]|BLPAA704-06|Costa Rica|Guanacaste|658[0n]|BOLD:AAA4337
Magusa divaricata|[342]|XAH035-05|Canada|Ontario|658[0n]|BOLD:AAA4337
Magusa divaricata|[343]|RDNMD850-07|United States|Arizona|655[0n]|BOLD:AAA4337
Magusa divaricata|[344]|BLPDK1295-09|Costa Rica|Guanacaste|658[0n]|BOLD:AAA4337
Magusa divaricata|[345]|BLPDK1292-09|Costa Rica|Guanacaste|658[0n]|BOLD:AAA4337
Magusa divaricata|[346]|MHMYS546-12|Costa Rica||658[0n]|BOLD:AAA4337
Magusa divaricata|[347]|BLPDK1293-09|Costa Rica|Guanacaste|658[0n]|BOLD:AAA4337
Magusa divaricata|[348]|BLPDW654-11|Costa Rica|Guanacaste|658[0n]|BOLD:AAA4337
Magusa divaricata|[349]|LNOUB710-10|French Guiana||658[0n]|BOLD:AAA4337
Magusa divaricata|[350]|LPOKA358-08|United States|Oklahoma|658[0n]|BOLD:AAA4337
Magusa divaricata|[351]|BLPCO029-08|Costa Rica|Guanacaste|658[0n]|BOLD:AAA4337
Magusa divaricata|[352]|MHMYL3453-11|Costa Rica||658[0n]|BOLD:AAA4337
Magusa divaricata|[353]|CMAZA904-12|United States|Arizona|658[0n]|BOLD:AAA4337
Magusa divaricata|[354]|BLPEF1432-12|Costa Rica|Guanacaste|658[0n]|BOLD:AAA4337
Magusa divaricata|[355]|BLPCK993-08|Costa Rica|Alajuela|658[0n]|BOLD:AAA4337
Magusa divaricata|[356]|LEMMZ159-10|Brazil|Parana|658[0n]|BOLD:AAA4337
Magusa divaricata|[357]|BLPCB929-08|Costa Rica|Alajuela|658[1n]|BOLD:AAA4337
Magusa divaricata|[358]|BLPCA553-08|Costa Rica|Guanacaste|632[0n]|BOLD:AAA4337
Magusa divaricata|[359]|BLPDV399-11|Costa Rica|Guanacaste|658[0n]|BOLD:AAA4337
Magusa divaricata|[360]|BLPDK1896-09|Costa Rica|Guanacaste|658[0n]|BOLD:AAA4337
Magusa divaricata|[361]|BLPDK1300-09|Costa Rica|Guanacaste|658[1n]|BOLD:AAA4337
Magusa divaricata|[362]|MHMYS3116-13|Costa Rica|Guanacaste|658[0n]|BOLD:AAA4337
Magusa divaricata|[363]|BLPDK1895-09|Costa Rica|Guanacaste|658[0n]|BOLD:AAA4337
Magusa divaricata|[364]|BLPDK1899-09|Costa Rica|Guanacaste|658[0n]|BOLD:AAA4337
Magusa divaricata|[365]|BLPDT1691-10|Costa Rica|Guanacaste|658[0n]|BOLD:AAA4337
Magusa divaricata|[366]|LEMMZ157-10|Brazil|Parana|658[0n]|BOLD:AAA4337
Magusa divaricata|[367]|BLPDU225-11|Costa Rica|Guanacaste|658[0n]|BOLD:AAA4337
Magusa divaricata|[368]|BLPDU246-11|Costa Rica|Guanacaste|658[0n]|BOLD:AAA4337
Magusa divaricata|[369]|BLPDU251-11|Costa Rica|Guanacaste|658[0n]|BOLD:AAA4337
Magusa divaricata|[370]|BLPDU254-11|Costa Rica|Guanacaste|658[0n]|BOLD:AAA4337
Magusa divaricata|[371]|BLPDU255-11|Costa Rica|Guanacaste|658[0n]|BOLD:AAA4337
Magusa divaricata|[372]|BLPDU863-11|Costa Rica|Guanacaste|658[0n]|BOLD:AAA4337
Magusa divaricata|[373]|BLPDU1181-11|Costa Rica|Guanacaste|658[0n]|BOLD:AAA4337
Magusa divaricata|[374]|BLPDV400-11|Costa Rica|Guanacaste|658[0n]|BOLD:AAA4337
Magusa divaricata|[375]|BLPDW243-11|Costa Rica|Guanacaste|658[0n]|BOLD:AAA4337
Magusa divaricata|[376]|BLPDW245-11|Costa Rica|Guanacaste|658[0n]|BOLD:AAA4337
Magusa divaricata|[377]|MHMYS3014-13|Costa Rica|Guanacaste|658[0n]|BOLD:AAA4337
Magusa divaricata|[378]|MHMYS3117-13|Costa Rica|Guanacaste|658[0n]|BOLD:AAA4337
Magusa divaricata|[379]|BLPDK1289-09|Costa Rica|Guanacaste|658[0n]|BOLD:AAA4337
Magusa divaricata|[380]|BLPDK1296-09|Costa Rica|Guanacaste|658[0n]|BOLD:AAA4337
Magusa divaricata|[381]|BLPDW253-11|Costa Rica|Guanacaste|658[0n]|BOLD:AAA4337
Magusa divaricata|[382]|BLPDW255-11|Costa Rica|Guanacaste|658[0n]|BOLD:AAA4337
Magusa divaricata|[383]|BLPDW256-11|Costa Rica|Guanacaste|658[0n]|BOLD:AAA4337
Magusa divaricata|[384]|BLPDW651-11|Costa Rica|Guanacaste|658[0n]|BOLD:AAA4337
Magusa divaricata|[385]|MILEQ351-11|Ecuador|Orellana|658[0n]|BOLD:AAA4337
Magusa divaricata|[386]|CMAZA918-12|United States|Arizona|658[0n]|BOLD:AAA4337
Magusa divaricata|[387]|BLPEE721-12|Costa Rica|Guanacaste|658[0n]|BOLD:AAA4337
Magusa divaricata|[388]|BLPEF849-12|Costa Rica|Guanacaste|658[0n]|BOLD:AAA4337
Magusa divaricata|[389]|BLPDC915-09|Costa Rica|Alajuela|658[0n]|BOLD:AAA4337
Magusa divaricata|[390]|BLPDI662-09|Costa Rica|Guanacaste|658[0n]|BOLD:AAA4337
Magusa divaricata|[391]|GWORH237-09|Brazil|Rio Grande do Sul|658[0n]|BOLD:AAA4337
Magusa divaricata|[392]|BLPDA733-09|Costa Rica|Guanacaste|658[0n]|BOLD:AAA4337
Magusa divaricata|[393]|BLPCO032-08|Costa Rica|Guanacaste|658[0n]|BOLD:AAA4337
Magusa divaricata|[394]|BLPCO131-08|Costa Rica|Guanacaste|658[0n]|BOLD:AAA4337
Magusa divaricata|[395]|BLPCO028-08|Costa Rica|Guanacaste|658[0n]|BOLD:AAA4337
Magusa divaricata|[396]|BLPCO031-08|Costa Rica|Guanacaste|658[0n]|BOLD:AAA4337
Magusa divaricata|[397]|HKONB015-08|United States|Texas|658[0n]|BOLD:AAA4337
Magusa divaricata|[398]|BLPBH776-07|Costa Rica|Guanacaste|658[0n]|BOLD:AAA4337
Magusa divaricata|[399]|BLPBG890-07|Costa Rica|Guanacaste|658[0n]|BOLD:AAA4337
Magusa divaricata|[400]|RDNMD830-07|United States|Arizona|658[0n]|BOLD:AAA4337

Magusa divaricata|[398]|BLPBH776-07|Costa Rica|Guanacaste|658[0n]|BOLD:AAA4337
Magusa divaricata|[399]|BLPBG890-07|Costa Rica|Guanacaste|658[0n]|BOLD:AAA4337
Magusa divaricata|[400]|RDNMD830-07|United States|Arizona|658[0n]|BOLD:AAA4337
Magusa divaricata|[401]|LSUSA061-06|United States|Kentucky|658[0n]|BOLD:AAA4337
Magusa divaricata|[402]|MHAUB063-05|Costa Rica|Guanacaste|658[0n]|BOLD:AAA4337
Magusa divaricata|[403]|MHAUB061-05|Costa Rica|Guanacaste|658[0n]|BOLD:AAA4337
Magusa divaricata|[404]|MHAUB059-05|Costa Rica|Guanacaste|658[0n]|BOLD:AAA4337
Magusa divaricata|[405]|MHAUB057-05|Costa Rica|Guanacaste|658[0n]|BOLD:AAA4337
Magusa divaricata|[406]|BLPDK1460-09|Costa Rica|Guanacaste|658[0n]|BOLD:AAA4337
Magusa divaricata|[407]|BLPDK1897-09|Costa Rica|Guanacaste|658[0n]|BOLD:AAA4337
Magusa divaricata|[408]|BLPDW652-11|Costa Rica|Guanacaste|658[0n]|BOLD:AAA4337
Magusa divaricata|[409]|BLPCK992-08|Costa Rica|Alajuela|658[0n]|BOLD:AAA4337
Magusa divaricata|[410]|BLPBF912-07|Costa Rica|Guanacaste|655[0n]|BOLD:AAA4337
Magusa divaricata|[411]|BLPBF644-07|Costa Rica|Guanacaste|645[0n]|BOLD:AAA4337
Magusa divaricata|[412]|LPOKA632-09|United States|Oklahoma|614[0n]|BOLD:AAA4337
Magusa divaricata|[413]|MHAUB062-05|Costa Rica|Guanacaste|566[0n]|BOLD:AAA4337
Magusa divaricata|[414]|BLPDV057-11|Costa Rica|Guanacaste|635[0n]|BOLD:AAA4337
Magusa divaricata|[415]|MILEP895-11|Ecuador|Napo|548[0n]|BOLD:AAA4337
Magusa divaricata|[416]|MHMYS3119-13|Costa Rica|Guanacaste|658[0n]|BOLD:AAA4337
Magusa divaricata|[417]|BLPEF2328-13|Costa Rica|Guanacaste|620[0n]|BOLD:AAA4337
Magusa divaricata|[418]|BLPDF744-09|Costa Rica|Guanacaste|658[0n]|BOLD:AAA4337
Magusa divaricata|[419]|BLPDW653-11|Costa Rica|Guanacaste|658[0n]|BOLD:AAA4337
Magusa divaricata|[420]|BLPDW254-11|Costa Rica|Guanacaste|658[0n]|BOLD:AAA4337
Magusa divaricata|[421]|BLPDW252-11|Costa Rica|Guanacaste|658[0n]|BOLD:AAA4337
Magusa divaricata|[422]|BLPDW250-11|Costa Rica|Guanacaste|658[0n]|BOLD:AAA4337
Magusa divaricata|[423]|BLPDW249-11|Costa Rica|Guanacaste|658[0n]|BOLD:AAA4337
Magusa divaricata|[424]|BLPDW246-11|Costa Rica|Guanacaste|658[0n]|BOLD:AAA4337
Magusa divaricata|[425]|BLPDV401-11|Costa Rica|Guanacaste|658[0n]|BOLD:AAA4337
Magusa divaricata|[426]|BLPDU864-11|Costa Rica|Guanacaste|658[0n]|BOLD:AAA4337
Magusa divaricata|[427]|BLPDU256-11|Costa Rica|Guanacaste|658[0n]|BOLD:AAA4337
Magusa divaricata|[428]|BLPDU253-11|Costa Rica|Guanacaste|658[0n]|BOLD:AAA4337
Magusa divaricata|[429]|BLPDU252-11|Costa Rica|Guanacaste|658[0n]|BOLD:AAA4337
Magusa divaricata|[430]|BLPDU250-11|Costa Rica|Guanacaste|658[0n]|BOLD:AAA4337
Magusa divaricata|[431]|BLPDU248-11|Costa Rica|Guanacaste|658[0n]|BOLD:AAA4337
Magusa divaricata|[432]|LEMMZ156-10|Brazil|Parana|658[0n]|BOLD:AAA4337
Magusa divaricata|[433]|BLPDK1299-09|Costa Rica|Guanacaste|658[0n]|BOLD:AAA4337
Magusa divaricata|[434]|BLPDK1294-09|Costa Rica|Guanacaste|658[0n]|BOLD:AAA4337
Magusa divaricata|[435]|BLPDK1291-09|Costa Rica|Guanacaste|658[0n]|BOLD:AAA4337
Magusa divaricata|[436]|BLPDK1290-09|Costa Rica|Guanacaste|658[0n]|BOLD:AAA4337
Magusa divaricata|[437]|LPOKA602-09|United States|Oklahoma|658[0n]|BOLD:AAA4337
Magusa divaricata|[438]|BLPCO305-08|Costa Rica|Guanacaste|658[0n]|BOLD:AAA4337
Magusa divaricata|[439]|BLPCO133-08|Costa Rica|Guanacaste|658[0n]|BOLD:AAA4337
Magusa divaricata|[440]|BLPCO033-08|Costa Rica|Guanacaste|658[0n]|BOLD:AAA4337
Magusa divaricata|[441]|BLPCN105-08|Costa Rica|Guanacaste|658[0n]|BOLD:AAA4337
Magusa divaricata|[442]|BLPCB395-08|Costa Rica|Alajuela|658[0n]|BOLD:AAA4337
Magusa divaricata|[443]|BLPBH779-07|Costa Rica|Guanacaste|658[0n]|BOLD:AAA4337
Magusa divaricata|[444]|RDLQB627-05|Canada|Quebec|658[0n]|BOLD:AAA4337
Magusa divaricata|[445]|MHAUB060-05|Costa Rica|Guanacaste|658[0n]|BOLD:AAA4337
Magusa divaricata|[446]|BLPBG500-07|Costa Rica|Guanacaste|658[0n]|BOLD:AAA4337
Magusa divaricata|[447]|MHAUB058-05|Costa Rica|Guanacaste|658[0n]|BOLD:AAA4337
Magusa divaricata|[448]|BLPDK1719-09|Costa Rica|Guanacaste|658[0n]|BOLD:AAA4337
Magusa divaricata|[449]|BLPBG502-07|Costa Rica|Guanacaste|650[0n]|BOLD:AAA4337
Magusa divaricata|[450]|BLPBG493-07|Costa Rica|Guanacaste|652[0n]|BOLD:AAA4337
Magusa divaricata|[451]|BLPBG492-07|Costa Rica|Guanacaste|641[0n]|BOLD:AAA4337
Magusa divaricata|[452]|BLPDV056-11|Costa Rica|Guanacaste|636[0n]|BOLD:AAA4337
Magusa divaricata|[453]|BBLOD519-11|United States|Texas|658[0n]|BOLD:AAA4337
Magusa divaricata|[454]|BLPEF976-12|Costa Rica|Alajuela|658[0n]|BOLD:AAA4337
Magusa divaricata|[455]|BLPEF2508-13|Costa Rica|Guanacaste|658[0n]|BOLD:AAA4337
Magusa divaricata|[456]|MHMYS2913-13|Costa Rica|Guanacaste|658[0n]|BOLD:AAA4337
Magusa divaricata|[457]|MHMYS3016-13|Costa Rica|Guanacaste|658[0n]|BOLD:AAA4337
Magusa divaricata|[458]|MHMYS3118-13|Costa Rica|Guanacaste|658[0n]|BOLD:AAA4337
Magusa divaricata|[459]|BLPEE6869-14|Costa Rica||658[0n]|BOLD:AAA4337
Magusa divaricata|[460]|BLPEE6870-14|Costa Rica||658[0n]|BOLD:AAA4337
Magusa divaricata|[461]|BLPEE6871-14|Costa Rica||658[0n]|BOLD:AAA4337
Magusa divaricata|[462]|BLPEE6872-14|Costa Rica||658[0n]|BOLD:AAA4337
Magusa divaricata|[463]|BLPEE6873-14|Costa Rica||658[0n]|BOLD:AAA4337
Neophaenis boucheri|[464]|MHAUB217-05|Costa Rica|Guanacaste|658[0n]|BOLD:AAC2394
Neophaenis boucheri|[465]|BLPAE417-06|Costa Rica|Guanacaste|658[0n]|BOLD:AAC2394
Neophaenis boucheri|[466]|BLPCD486-08|Costa Rica|Guanacaste|658[0n]|BOLD:AAC2394
Neophaenis boucheri|[467]|BLPCM572-08|Costa Rica|Guanacaste|658[1n]|BOLD:AAC2394
Neophaenis boucheri|[468]|BLPDW257-11|Costa Rica|Guanacaste|658[0n]|BOLD:AAC2394
Neophaenis boucheri|[469]|BLPDM586-10|Costa Rica|Guanacaste|658[0n]|BOLD:AAC2394
Neophaenis boucheri|[470]|BLPDU517-11|Costa Rica|Guanacaste|658[0n]|BOLD:AAC2394
Neophaenis boucheri|[471]|MHMYS3415-13|Costa Rica|Guanacaste|658[0n]|BOLD:AAC2394
Neophaenis boucheri|[472]|CNCLB1957-14|Guatemala||658[0n]|BOLD:AAC2394
Neophaenis boucheri|[473]|MHMXM100-07|Costa Rica|Guanacaste|599[0n]|BOLD:AAC2394
Neophaenis boucheri|[474]|BLPDU516-11|Costa Rica|Guanacaste|658[0n]|BOLD:AAC2394
Neophaenis boucheri|[475]|BLPAE697-06|Costa Rica|Guanacaste|658[0n]|BOLD:AAC2394
Neophaenis boucheri|[476]|BLPAA096-06|Costa Rica|Guanacaste|658[1n]|BOLD:AAC2394
Neophaenis boucheri|[477]|BLPCD483-08|Costa Rica|Guanacaste|658[0n]|BOLD:AAC2394
Neophaenis boucheri|[478]|MHMYS3115-13|Costa Rica|Guanacaste|658[0n]|BOLD:AAC2394
Neophaenis boucheri|[479]|MHMYS3210-13|Costa Rica|Guanacaste|658[0n]|BOLD:AAC2394
Neophaenis boucheri|[480]|BLPCD485-08|Costa Rica|Guanacaste|658[0n]|BOLD:AAC2394
Neophaenis boucheri|[481]|BLPCD484-08|Costa Rica|Guanacaste|658[0n]|BOLD:AAC2394
Neophaenis boucheri|[482]|BLPCI623-08|Costa Rica|Guanacaste|658[0n]|BOLD:AAC2394
Neophaenis boucheri|[483]|BLPDK1459-09|Costa Rica|Guanacaste|658[2n]|BOLD:AAC2394
Neophaenis boucheri|[484]|CNCLB1958-14|Guatemala||658[0n]|BOLD:AAC2394
Neophaenis nr. boucheri|[485]|CNCLB2434-14|United States|Texas|407[0n]|
Trachea delicata|[486]|RDLQB695-05|Canada|Quebec|658[0n]|BOLD:AAE8397
Trachea delicata|[487]|RDMAB103-05|Canada|Alberta|658[0n]|BOLD:AAE8397
Trachea delicata|[488]|LOCT046-05|United States|Connecticut|658[0n]|BOLD:AAE8397
Trachea delicata|[489]|RDMAB533-06|Canada|Alberta|658[0n]|BOLD:AAE8397
Pseudomarimatha flava|[490]|RDNME933-08|United States|Arizona|658[0n]|BOLD:AAX7120
Elaphria exesa|[491]|LOFLA035-06|United States|Florida|653[0n]|BOLD:AAB3726
Elaphria exesa|[492]|RDNMD535-06|United States|Florida|653[0n]|BOLD:AAB3726
Elaphria exesa|[493]|LOFLA038-06|United States|Florida|653[0n]|BOLD:AAB3726
Elaphria exesa|[494]|LOFLB778-06|United States|Florida|674[0n]|BOLD:AAB3726
Elaphria exesa|[495]|LOFLB788-06|United States|Florida|658[0n]|BOLD:AAB3726
Elaphria exesa|[496]|LOFLC405-06|United States|Florida|658[0n]|BOLD:AAB3726
Elaphria exesa|[497]|LOFLB776-06|United States|Florida|658[0n]|BOLD:AAB3726
Elaphria exesa|[498]|LOFLB780-06|United States|Florida|658[0n]|BOLD:AAB3726
Elaphria exesa|[499]|LOFLA019-06|United States|Florida|658[0n]|BOLD:AAB3726
Elaphria exesa|[500]|LOFLA394-06|United States|Florida|658[0n]|BOLD:AAB3726

Elaphria exesa|[498]|LOFLB780-06|United States|Florida|658[0n]|BOLD:AAB3726
Elaphria exesa|[499]|LOFLA019-06|United States|Florida|658[0n]|BOLD:AAB3726
Elaphria exesa|[500]|LOFLA394-06|United States|Florida|658[0n]|BOLD:AAB3726
Elaphria exesa|[501]|LOFLA598-06|United States|Florida|658[0n]|BOLD:AAB3726
Elaphria exesa|[502]|LOFLA697-06|United States|Florida|658[0n]|BOLD:AAB3726
Elaphria exesa|[503]|LOFLA699-06|United States|Florida|658[0n]|BOLD:AAB3726
Elaphria exesa|[504]|LOFLB272-06|United States|Florida|658[0n]|BOLD:AAB3726
Elaphria exesa|[505]|LOFLB713-06|United States|Florida|658[0n]|BOLD:AAB3726
Elaphria exesa|[506]|LOFLB760-06|United States|Florida|658[0n]|BOLD:AAB3726
Elaphria exesa|[507]|LOFLC440-06|United States|Florida|658[0n]|BOLD:AAB3726
Elaphria exesa|[508]|MILEP304-10|United States|Georgia|658[0n]|BOLD:AAB3726
Elaphria exesa|[509]|LSEU187-06|United States|Georgia|658[0n]|BOLD:AAB3726
Elaphria exesa|[510]|LSEU188-06|United States|Georgia|658[0n]|BOLD:AAB3726
Elaphria exesa|[511]|BBLOB644-11|United States|Florida|658[0n]|BOLD:AAB3726
Elaphria trolia|[512]|HKONB237-09|United States|Texas|658[0n]|BOLD:AAA4745
Elaphria trolia|[513]|HKONB234-09|United States|Texas|658[0n]|BOLD:AAA4745
Elaphria trolia|[514]|HKONB235-09|United States|Texas|658[0n]|BOLD:AAA4745
Elaphria trolia|[515]|HKONB236-09|United States|Texas|658[0n]|BOLD:AAA4745
Elaphria trolia|[516]|HKONB238-09|United States|Texas|658[0n]|BOLD:AAA4745
Elaphria chalcedonia|[517]|LNC501-06|United States|North Carolina|619[1n]|BOLD:AAB6018
Elaphria chalcedonia|[518]|LSUSA067-06|United States|Kentucky|606[0n]|BOLD:AAB6018
Elaphria chalcedonia|[519]|LPOKA596-09|United States|Oklahoma|636[0n]|BOLD:AAB6018
Elaphria chalcedonia|[520]|LPOKA331-08|United States|Oklahoma|658[0n]|BOLD:AAB6018
Elaphria chalcedonia|[521]|LOFLB464-06|United States|Florida|657[0n]|BOLD:AAB6018
Elaphria chalcedonia|[522]|LOFLB462-06|United States|Florida|657[0n]|BOLD:AAB6018
Elaphria chalcedonia|[523]|LOFLB405-06|United States|Florida|657[0n]|BOLD:AAB6018
Elaphria chalcedonia|[524]|LOFLB275-06|United States|Florida|657[0n]|BOLD:AAB6018
Elaphria chalcedonia|[525]|LOFLB192-06|United States|Florida|657[0n]|BOLD:AAB6018
Elaphria chalcedonia|[526]|LOFLA708-06|United States|Florida|657[0n]|BOLD:AAB6018
Elaphria chalcedonia|[527]|LOFLA597-06|United States|Florida|657[0n]|BOLD:AAB6018
Elaphria chalcedonia|[528]|LOFLA560-06|United States|Florida|657[0n]|BOLD:AAB6018
Elaphria chalcedonia|[529]|LOFLA200-06|United States|Florida|657[0n]|BOLD:AAB6018
Elaphria chalcedonia|[530]|LNC789-06|United States|North Carolina|657[0n]|BOLD:AAB6018
Elaphria chalcedonia|[531]|LNC437-05|United States|North Carolina|657[0n]|BOLD:AAB6018
Elaphria chalcedonia|[532]|PHFLO075-10|United States|Florida|658[0n]|BOLD:AAB6018
Elaphria chalcedonia|[533]|LPOKA296-08|United States|Oklahoma|658[0n]|BOLD:AAB6018
Elaphria chalcedonia|[534]|LPOKA342-08|United States|Oklahoma|658[0n]|BOLD:AAB6018
Elaphria chalcedonia|[535]|LPOKA649-09|United States|Oklahoma|658[0n]|BOLD:AAB6018
Elaphria chalcedonia|[536]|LPOKB623-09|United States|Oklahoma|658[0n]|BOLD:AAB6018
Elaphria chalcedonia|[537]|LPOKC737-09|United States|Oklahoma|658[0n]|BOLD:AAB6018
Elaphria chalcedonia|[538]|LGSMG1010-10|United States|North Carolina|658[0n]|BOLD:AAB6018
Elaphria chalcedonia|[539]|LGSMG1011-10|United States|North Carolina|658[0n]|BOLD:AAB6018
Elaphria chalcedonia|[540]|USLEP633-10|United States|Texas|658[0n]|BOLD:AAB6018
Elaphria chalcedonia|[541]|LILLA831-11|United States|Illinois|658[0n]|BOLD:AAB6018
Elaphria chalcedonia|[542]|LILLA994-11|United States|Illinois|658[0n]|BOLD:AAB6018
Elaphria chalcedonia|[543]|LILLB023-11|United States|Illinois|658[0n]|BOLD:AAB6018
Elaphria chalcedonia|[544]|LILLB040-11|United States|Illinois|658[0n]|BOLD:AAB6018
Elaphria chalcedonia|[545]|BBLOC1050-11|United States|Arkansas|658[0n]|BOLD:AAB6018
Elaphria chalcedonia|[546]|BBLOC1629-11|United States|Texas|658[0n]|BOLD:AAB6018
Elaphria agrotina|[547]|LPYPA260-08|Mexico|Quintana Roo|658[0n]|BOLD:ABZ4872
Elaphria agrotina|[548]|LPYPC123-08|Mexico|Quintana Roo|549[0n]|BOLD:ABZ4872
Elaphria agrotina|[549]|HKONB229-09|United States|Texas|655[0n]|BOLD:ABZ4872
Elaphria agrotina|[550]|BLPDK1332-09|Costa Rica|Guanacaste|658[0n]|BOLD:ABZ4872
Elaphria agrotina|[551]|LOCRC428-08|Costa Rica|Guanacaste|658[0n]|BOLD:AAA4470
Elaphria agrotina|[552]|BLPCA242-08|Costa Rica|Guanacaste|658[0n]|BOLD:AAA4470
Elaphria agrotina|[553]|BLPDM2146-10|Costa Rica|Guanacaste|621[0n]|BOLD:AAA4470
Elaphria agrotina|[554]|BLPAA145-06|Costa Rica|Guanacaste|658[0n]|BOLD:AAA4470
Elaphria agrotina|[555]|BLPCA299-08|Costa Rica|Guanacaste|658[0n]|BOLD:AAA4470
Elaphria agrotina|[556]|BLPCA780-08|Costa Rica|Guanacaste|658[0n]|BOLD:AAA4470
Elaphria agrotina|[557]|BLPDJ076-09|Costa Rica|Guanacaste|658[0n]|BOLD:AAA4470
Elaphria agrotina|[558]|BLPDK1910-09|Costa Rica|Guanacaste|658[0n]|BOLD:AAA4470
Elaphria agrotina|[559]|LOCRI448-11|Costa Rica|Limon|658[0n]|BOLD:AAA4470
Elaphria agrotina|[560]|BLPED006-11|Costa Rica|Guanacaste|658[0n]|BOLD:AAA4470
Elaphria agrotina|[561]|BLPED668-11|Costa Rica|Guanacaste|658[0n]|BOLD:AAA4470
Elaphria agrotina|[562]|BLPEF845-12|Costa Rica|Guanacaste|658[0n]|BOLD:AAA4470
Elaphria agrotina|[563]|BLPDK1331-09|Costa Rica|Guanacaste|658[0n]|BOLD:AAA4470
Elaphria agrotina|[564]|BLPCA786-08|Costa Rica|Guanacaste|658[0n]|BOLD:AAA4470
Elaphria agrotina|[565]|BLPEF408-12|Costa Rica|Guanacaste|658[0n]|BOLD:AAA4470
Elaphria agrotina|[566]|BLPCA779-08|Costa Rica|Guanacaste|658[0n]|BOLD:AAA4470
Elaphria agrotina|[567]|BLPDK1342-09|Costa Rica|Guanacaste|658[0n]|BOLD:AAA4470
Elaphria agrotina|[568]|BLPED516-11|Costa Rica|Guanacaste|658[0n]|BOLD:AAA4470
Elaphria agrotina|[569]|BLPCA297-08|Costa Rica|Guanacaste|658[0n]|BOLD:AAA4470
Elaphria agrotina|[570]|BLPCA785-08|Costa Rica|Guanacaste|658[0n]|BOLD:AAA4470
Elaphria agrotina|[571]|BLPED008-11|Costa Rica|Guanacaste|658[0n]|BOLD:AAA4470
Elaphria agrotina|[572]|BLPEF2163-13|Costa Rica|Guanacaste|658[0n]|BOLD:AAA4470
Elaphria agrotina|[573]|BLPEF2164-13|Costa Rica|Guanacaste|658[0n]|BOLD:AAA4470
Elaphria agrotina|[574]|BLPEF5695-13|Costa Rica||658[0n]|BOLD:AAA4470
Elaphria agrotina|[575]|BLPEF4928-13|Costa Rica|Guanacaste|658[0n]|BOLD:AAA4470
Elaphria agrotina|[576]|BLPDK499-09|Costa Rica|Guanacaste|658[1n]|BOLD:AAA4470
Elaphria agrotina|[577]|BLPDJ428-09|Costa Rica|Guanacaste|658[0n]|BOLD:AAA4470
Elaphria agrotina|[578]|BLPEF040-12|Costa Rica|Alajuela|658[0n]|BOLD:AAA4470
Elaphria agrotina|[579]|BLPDK838-09|Costa Rica|Guanacaste|658[0n]|BOLD:AAA4470
Elaphria agrotina|[580]|BLPDK759-09|Costa Rica|Guanacaste|658[0n]|BOLD:AAA4470
Elaphria agrotina|[581]|BLPDK486-09|Costa Rica|Guanacaste|658[0n]|BOLD:AAA4470
Elaphria agrotina|[582]|BLPDJ427-09|Costa Rica|Guanacaste|658[0n]|BOLD:AAA4470
Elaphria agrotina|[583]|HKONB230-09|United States|Texas|658[0n]|BOLD:AAA4470
Elaphria agrotina|[584]|LOCRC430-08|Costa Rica|Guanacaste|658[0n]|BOLD:AAA4470
Elaphria agrotina|[585]|LOCRC429-08|Costa Rica|Guanacaste|658[0n]|BOLD:AAA4470
Elaphria agrotina|[586]|BLPCJ462-08|Costa Rica|Guanacaste|658[0n]|BOLD:AAA4470
Elaphria agrotina|[587]|BLPCA784-08|Costa Rica|Guanacaste|658[0n]|BOLD:AAA4470
Elaphria agrotina|[588]|BLPCA778-08|Costa Rica|Guanacaste|658[0n]|BOLD:AAA4470
Elaphria agrotina|[589]|BLPCA298-08|Costa Rica|Guanacaste|658[0n]|BOLD:AAA4470
Elaphria agrotina|[590]|BLPAG211-07|Costa Rica|Guanacaste|658[0n]|BOLD:AAA4470
Elaphria agrotina|[591]|BLPAG078-07|Costa Rica|Guanacaste|658[0n]|BOLD:AAA4470
Elaphria agrotina|[592]|BLPAF597-07|Costa Rica|Guanacaste|658[0n]|BOLD:AAA4470
Elaphria agrotina|[593]|BLPAC496-06|Costa Rica|Guanacaste|658[0n]|BOLD:AAA4470
Elaphria agrotina|[594]|BLPAA446-06|Costa Rica|Guanacaste|658[0n]|BOLD:AAA4470
Elaphria agrotina|[595]|BLPAA275-06|Costa Rica|Guanacaste|658[0n]|BOLD:AAA4470
Elaphria agrotina|[596]|BLPAA269-06|Costa Rica|Guanacaste|658[0n]|BOLD:AAA4470
Elaphria agrotina|[597]|BLPDJ097-09|Costa Rica|Guanacaste|623[0n]|BOLD:AAA4470
Elaphria agrotina|[598]|BLPDM1716-10|Costa Rica|Guanacaste|629[0n]|BOLD:AAA4470
Elaphria agrotina|[599]|BLPED005-11|Costa Rica|Guanacaste|658[0n]|BOLD:AAA4470
Elaphria agrotina|[600]|BLPED007-11|Costa Rica|Guanacaste|658[0n]|BOLD:AAA4470

Elaphria agrotina|[598]|BLPDM1716-10|Costa Rica|Guanacaste|629[0n]|BOLD:AAA4470
Elaphria agrotina|[599]|BLPED005-11|Costa Rica|Guanacaste|658[0n]|BOLD:AAA4470
Elaphria agrotina|[600]|BLPED007-11|Costa Rica|Guanacaste|658[0n]|BOLD:AAA4470
Elaphria agrotina|[601]|BLPED670-11|Costa Rica|Guanacaste|658[0n]|BOLD:AAA4470
Elaphria agrotina|[602]|BLPEE1978-12|Costa Rica|Guanacaste|658[0n]|BOLD:AAA4470
Elaphria agrotina|[603]|BLPEF962-12|Costa Rica|Alajuela|658[0n]|BOLD:AAA4470
Elaphria agrotina|[604]|BLPEF2324-13|Costa Rica|Guanacaste|658[0n]|BOLD:AAA4470
Elaphria agrotina|[605]|BLPDJ098-09|Costa Rica|Guanacaste|615[0n]|BOLD:AAA4470
Elaphria agrotina|[606]|BLPCA296-08|Costa Rica|Guanacaste|658[0n]|BOLD:AAA4470
Elaphria agrotina|[607]|BLPCO054-08|Costa Rica|Guanacaste|658[0n]|BOLD:AAA4470
Elaphria agrotina|[608]|MHMYS3201-13|Costa Rica|Guanacaste|658[0n]|BOLD:AAA4470
Elaphria agrotina|[609]|BLPEF1135-12|Costa Rica|Guanacaste|658[0n]|BOLD:AAA4470
Elaphria agrotina|[610]|BLPAG092-07|Costa Rica|Guanacaste|658[0n]|BOLD:AAA4470
Elaphria agrotina|[611]|MHMYS3103-13|Costa Rica|Guanacaste|658[0n]|BOLD:AAA4470
Elaphria agrotina|[612]|MHMYS2996-13|Costa Rica|Guanacaste|658[0n]|BOLD:AAA4470
Elaphria agrotina|[613]|BLPEF2422-13|Costa Rica|Guanacaste|658[0n]|BOLD:AAA4470
Elaphria agrotina|[614]|BLPAF598-07|Costa Rica|Guanacaste|658[0n]|BOLD:AAA4470
Elaphria agrotina|[615]|BLPDK839-09|Costa Rica|Guanacaste|638[0n]|BOLD:AAA4470
Elaphria agrotina|[616]|MHMYS2777-13|Costa Rica|Guanacaste|636[0n]|BOLD:AAA4470
Elaphria agrotina|[617]|MHMYS3200-13|Costa Rica|Guanacaste|658[0n]|BOLD:AAA4470
Elaphria agrotina|[618]|BLPED515-11|Costa Rica|Guanacaste|658[0n]|BOLD:AAA4470
Elaphria agrotina|[619]|BLPED1742-12|Costa Rica|Guanacaste|658[0n]|BOLD:AAA4470
Elaphria agrotina|[620]|LPYPB813-08|Mexico|Quintana Roo|658[0n]|BOLD:AAA4470
Elaphria agrotina|[621]|LOCRC427-08|Costa Rica|Guanacaste|658[0n]|BOLD:AAA4470
Elaphria agrotina|[622]|BLPDK658-09|Costa Rica|Guanacaste|658[0n]|BOLD:AAA4470
Elaphria agrotina|[623]|BLPDK764-09|Costa Rica|Guanacaste|658[0n]|BOLD:AAA4470
Elaphria agrotina|[624]|BLPEF2160-13|Costa Rica|Guanacaste|658[0n]|BOLD:AAA4470
Elaphria agrotina|[625]|BLPCB026-08|Costa Rica|Guanacaste|658[0n]|BOLD:AAA4470
Elaphria agrotina|[626]|BLPCA788-08|Costa Rica|Guanacaste|658[3n]|BOLD:AAA4470
Elaphria agrotina|[627]|BLPEF4927-13|Costa Rica|Guanacaste|658[0n]|BOLD:AAA4470
Elaphria agrotina|[628]|BLPDK484-09|Costa Rica|Guanacaste|658[0n]|BOLD:AAA4470
Elaphria agrotina|[629]|BLPEE1443-12|Costa Rica|Guanacaste|658[0n]|BOLD:AAA4470
Elaphria agrotina|[630]|BLPAF591-07|Costa Rica|Guanacaste|658[0n]|BOLD:AAA4470
Elaphria agrotina|[631]|BLPDK1646-09|Costa Rica|Guanacaste|656[0n]|BOLD:AAA4470
Elaphria agrotina|[632]|BLPAF050-07|Costa Rica|Guanacaste|658[0n]|BOLD:AAA4470
Elaphria agrotina|[633]|BLPBD643-07|Costa Rica|Guanacaste|658[0n]|BOLD:AAA4470
Elaphria agrotina|[634]|BLPDK841-09|Costa Rica|Guanacaste|645[0n]|BOLD:AAA4470
Elaphria agrotina|[635]|BLPAC475-06|Costa Rica|Guanacaste|658[0n]|BOLD:AAA4470
Elaphria agrotina|[636]|BLPAA440-06|Costa Rica|Guanacaste|658[0n]|BOLD:AAA4470
Elaphria agrotina|[637]|BLPCA789-08|Costa Rica|Guanacaste|632[0n]|BOLD:AAA4470
Elaphria agrotina|[638]|BLPED1743-12|Costa Rica|Guanacaste|658[0n]|BOLD:AAA4470
Elaphria agrotina|[639]|BLPDK1576-09|Costa Rica|Guanacaste|658[0n]|BOLD:AAA4470
Elaphria agrotina|[640]|BLPCA781-08|Costa Rica|Guanacaste|658[0n]|BOLD:AAA4470
Elaphria agrotina|[641]|BLPDK842-09|Costa Rica|Guanacaste|658[0n]|BOLD:AAA4470
Elaphria agrotina|[642]|BLPDK191-09|Costa Rica|Guanacaste|658[0n]|BOLD:AAA4470
Elaphria agrotina|[643]|BLPCB025-08|Costa Rica|Guanacaste|658[0n]|BOLD:AAA4470
Elaphria agrotina|[644]|BLPCN064-08|Costa Rica|Guanacaste|658[0n]|BOLD:AAA4470
Elaphria agrotina|[645]|BLPCN712-08|Costa Rica|Guanacaste|658[0n]|BOLD:AAA4470
Elaphria agrotina|[646]|HKONB231-09|United States|Texas|658[0n]|BOLD:AAA4470
Elaphria agrotina|[647]|BLPDD416-09|Costa Rica|Guanacaste|658[0n]|BOLD:AAA4470
Elaphria agrotina|[648]|MHMYC2184-09|Costa Rica|Guanacaste|658[0n]|BOLD:AAA4470
Elaphria agrotina|[649]|BLPDK410-09|Costa Rica|Guanacaste|658[0n]|BOLD:AAA4470
Elaphria agrotina|[650]|BLPDK485-09|Costa Rica|Guanacaste|658[0n]|BOLD:AAA4470
Elaphria agrotina|[651]|BLPDK840-09|Costa Rica|Guanacaste|658[0n]|BOLD:AAA4470
Elaphria agrotina|[652]|BLPDK1152-09|Costa Rica|Guanacaste|658[0n]|BOLD:AAA4470
Elaphria agrotina|[653]|BLPDK1165-09|Costa Rica|Guanacaste|658[0n]|BOLD:AAA4470
Elaphria agrotina|[654]|BLPDK1575-09|Costa Rica|Guanacaste|658[0n]|BOLD:AAA4470
Elaphria agrotina|[655]|BLPDK1605-09|Costa Rica|Guanacaste|658[0n]|BOLD:AAA4470
Elaphria agrotina|[656]|BLPDL554-10|Costa Rica|Guanacaste|658[0n]|BOLD:AAA4470
Elaphria agrotina|[657]|LOCRE580-10|Costa Rica|San Jose|658[0n]|BOLD:AAA4470
Elaphria agrotina|[658]|BLPED669-11|Costa Rica|Guanacaste|658[0n]|BOLD:AAA4470
Elaphria agrotina|[659]|BLPED1741-12|Costa Rica|Guanacaste|658[0n]|BOLD:AAA4470
Elaphria agrotina|[660]|BLPEF662-12|Costa Rica|Guanacaste|658[0n]|BOLD:AAA4470
Elaphria agrotina|[661]|BLPEF2325-13|Costa Rica|Guanacaste|658[0n]|BOLD:AAA4470
Elaphria agrotina|[662]|BLPEF4915-13|Costa Rica|Guanacaste|658[0n]|BOLD:AAA4470
Elaphria agrotina|[663]|BLPEF4919-13|Costa Rica|Guanacaste|658[0n]|BOLD:AAA4470
Elaphria agrotina|[664]|BLPEF4930-13|Costa Rica|Guanacaste|658[0n]|BOLD:AAA4470
Elaphria agrotina|[665]|MHMYS2995-13|Costa Rica|Guanacaste|658[0n]|BOLD:AAA4470
Elaphria agrotina|[666]|MHMYS3104-13|Costa Rica|Guanacaste|658[0n]|BOLD:AAA4470
Elaphria agrotina|[667]|MHMYS3105-13|Costa Rica|Guanacaste|658[0n]|BOLD:AAA4470
Elaphria agrotina|[668]|BLPCA790-08|Costa Rica|Guanacaste|658[0n]|BOLD:AAA4470
Elaphria agrotina|[669]|BLPCA787-08|Costa Rica|Guanacaste|658[0n]|BOLD:AAA4470
Elaphria agrotina|[670]|BLPCA783-08|Costa Rica|Guanacaste|658[0n]|BOLD:AAA4470
Elaphria agrotina|[671]|BLPAG212-07|Costa Rica|Guanacaste|658[0n]|BOLD:AAA4470
Elaphria agrotina|[672]|BLPAG210-07|Costa Rica|Guanacaste|658[0n]|BOLD:AAA4470
Elaphria agrotina|[673]|BLPAG077-07|Costa Rica|Guanacaste|658[0n]|BOLD:AAA4470
Elaphria agrotina|[674]|BLPAG076-07|Costa Rica|Guanacaste|658[0n]|BOLD:AAA4470
Elaphria agrotina|[675]|BLPAC888-06|Costa Rica|Guanacaste|658[0n]|BOLD:AAA4470
Elaphria agrotina|[676]|BLPCA777-08|Costa Rica|Guanacaste|658[0n]|BOLD:AAA4470
Elaphria agrotina|[677]|LPYPA261-08|Mexico|Quintana Roo|581[0n]|BOLD:AAA4470
Elaphria agrotina|[678]|BLPAA594-06|Costa Rica|Guanacaste|658[0n]|BOLD:AAA4470
Elaphria agrotina|[679]|BLPCA292-08|Costa Rica|Guanacaste|658[0n]|BOLD:AAA4470
Elaphria agrotina|[680]|BLPDJ191-09|Costa Rica|Guanacaste|658[0n]|BOLD:AAA4470
Elaphria agrotina|[681]|HKONB232-09|United States|Texas|640[0n]|BOLD:AAA4470
Elaphria agrotina|[682]|BLPAE845-06|Costa Rica|Guanacaste|630[0n]|BOLD:AAA4470
Elaphria agrotina|[683]|BLPAG087-07|Costa Rica|Guanacaste|616[4n]|BOLD:AAA4470
Elaphria agrotina|[684]|LYPIE206-09|Mexico|Quintana Roo|658[0n]|BOLD:AAA4470
Elaphria agrotina|[685]|BLPED514-11|Costa Rica|Guanacaste|658[0n]|BOLD:AAA4470
Elaphria agrotina|[686]|BLPCJ463-08|Costa Rica|Guanacaste|658[0n]|BOLD:AAA4470
Elaphria agrotina|[687]|BLPEF2036-13|Costa Rica|Guanacaste|658[0n]|BOLD:AAA4470
Elaphria agrotina|[688]|BLPEF2102-13|Costa Rica|Guanacaste|658[0n]|BOLD:AAA4470
Elaphria agrotina|[689]|MHMYS3411-13|Costa Rica|Guanacaste|658[0n]|BOLD:AAA4470
Elaphria grata|[690]|LGSMG730-07|United States|Tennessee|658[0n]|BOLD:AAB4093
Elaphria grata|[691]|BBLSW443-09|United States|Oklahoma|658[0n]|BOLD:AAB4093
Elaphria grata|[692]|LPOKB271-09|United States|Oklahoma|658[0n]|BOLD:AAB4093
Elaphria grata|[693]|LPOKB008-09|United States|Oklahoma|658[0n]|BOLD:AAB4093
Elaphria grata|[694]|LPOKA1019-09|United States|Oklahoma|658[0n]|BOLD:AAB4093
Elaphria grata|[695]|LPOKA1010-09|United States|Oklahoma|658[0n]|BOLD:AAB4093
Elaphria grata|[696]|LPOKA645-09|United States|Oklahoma|658[0n]|BOLD:AAB4093
Elaphria grata|[697]|LPOKA554-09|United States|Oklahoma|658[0n]|BOLD:AAB4093
Elaphria grata|[698]|HKONB463-09|United States|Florida|658[0n]|BOLD:AAB4093
Elaphria grata|[699]|LPSO945-08|Canada|Ontario|658[0n]|BOLD:AAB4093
Elaphria grata|[700]|LPSO758-08|Canada|Ontario|656[0n]|BOLD:AAB4093

Elaphria grata|[698]|HKONB463-09|United States|Florida|658[0n]|BOLD:AAB4093
Elaphria grata|[699]|LPSO945-08|Canada|Ontario|658[0n]|BOLD:AAB4093
Elaphria grata|[700]|LPSO758-08|Canada|Ontario|656[0n]|BOLD:AAB4093
Elaphria grata|[701]|LPSO485-08|Canada|Ontario|658[0n]|BOLD:AAB4093
Elaphria grata|[702]|LPSO195-08|Canada|Ontario|658[0n]|BOLD:AAB4093
Elaphria grata|[703]|LPSO106-08|Canada|Ontario|658[0n]|BOLD:AAB4093
Elaphria grata|[704]|LPSO096-08|Canada|Ontario|658[0n]|BOLD:AAB4093
Elaphria grata|[705]|LNCNW064-06|United States|North Carolina|658[0n]|BOLD:AAB4093
Elaphria grata|[706]|LOCT253-05|United States|Connecticut|658[0n]|BOLD:AAB4093
Elaphria grata|[707]|LGSM735-04|United States|Tennessee|658[0n]|BOLD:AAB4093
Elaphria grata|[708]|LNCNW065-06|United States|North Carolina|658[0n]|BOLD:AAB4093
Elaphria grata|[709]|LGSM560-04|United States|North Carolina|658[0n]|BOLD:AAB4093
Elaphria grata|[710]|LPSO505-08|Canada|Ontario|633[0n]|BOLD:AAB4093
Elaphria grata|[711]|LPOKA641-09|United States|Oklahoma|639[0n]|BOLD:AAB4093
Elaphria grata|[712]|LPOKA633-09|United States|Oklahoma|639[0n]|BOLD:AAB4093
Elaphria grata|[713]|BBLSW569-09|United States|Oklahoma|636[0n]|BOLD:AAB4093
Elaphria grata|[714]|BBLSW651-09|United States|Oklahoma|658[0n]|BOLD:AAB4093
Elaphria grata|[715]|BBLSY029-09|United States|Oklahoma|658[0n]|BOLD:AAB4093
Elaphria grata|[716]|BBLSX370-09|United States|Oklahoma|658[0n]|BOLD:AAB4093
Elaphria grata|[717]|BBLSX437-09|United States|Oklahoma|658[0n]|BOLD:AAB4093
Elaphria grata|[718]|BBLSX497-09|United States|Oklahoma|658[0n]|BOLD:AAB4093
Elaphria grata|[719]|BBLSX896-09|United States|Oklahoma|658[0n]|BOLD:AAB4093
Elaphria grata|[720]|BBLSX900-09|United States|Oklahoma|658[0n]|BOLD:AAB4093
Elaphria grata|[721]|BBLSU118-09|United States|Mississippi|658[0n]|BOLD:AAB4093
Elaphria grata|[722]|LGSMG1014-10|United States|Tennessee|658[0n]|BOLD:AAB4093
Elaphria grata|[723]|LGSMG1015-10|United States|North Carolina|658[0n]|BOLD:AAB4093
Elaphria grata|[724]|LILLA034-11|United States|Illinois|658[0n]|BOLD:AAB4093
Elaphria grata|[725]|LILLA375-11|United States|Illinois|658[0n]|BOLD:AAB4093
Elaphria grata|[726]|LILLB050-11|United States|Illinois|658[0n]|BOLD:AAB4093
Elaphria fuscimacula group|[727]|BBLSX070-09|United States|Texas|658[0n]|BOLD:ABZ6467
Elaphria fuscimacula group|[728]|LNCB787-09|United States|North Carolina|658[0n]|BOLD:ABZ6467
Elaphria hypophaea|[729]|RDNMC739-06|United States|Texas|525[2n]|BOLD:AAA6881
Elaphria hypophaea|[730]|HKONB233-09|United States|Texas|658[0n]|BOLD:AAA6881
Elaphria hypophaea|[731]|RDNMF976-08|United States|Texas|658[0n]|BOLD:AAA6881
Elaphria hypophaea|[732]|PSAT154-10|United States|Texas|658[0n]|BOLD:AAA6881
Elaphria fuscimacula|[733]|BBLOB1241-11|United States|Florida|658[0n]|BOLD:ACF0733
Elaphria fuscimacula|[734]|BBLOB1286-11|United States|Florida|658[0n]|BOLD:ACF0733
Elaphria fuscimacula group|[735]|USLEP616-10|United States|Florida|658[0n]|BOLD:ABZ6468
Elaphria fuscimacula group|[736]|BBLOB1612-11|United States|Florida|658[0n]|BOLD:ABZ6468
Elaphria subobliqua|[737]|LNAUT2078-14|Dominican Republic|La Vega|658[0n]|BOLD:ACR1647
Elaphria subobliqua complex|[738]|CNCLB2214-14|Puerto Rico|Mayaguez|658[0n]|BOLD:ACD9415
Elaphria subobliqua|[739]|BLPDT1432-10|Costa Rica|Guanacaste|658[0n]|BOLD:AAB0340
Elaphria subobliqua|[740]|BLPDE864-09|Costa Rica||658[0n]|BOLD:AAB0340
Elaphria subobliqua|[741]|BLPCK951-08|Costa Rica|Alajuela|658[0n]|BOLD:AAB0340
Elaphria subobliqua|[742]|BLPDD428-09|Costa Rica|Guanacaste|658[0n]|BOLD:AAB0340
Elaphria subobliqua|[743]|BLPDD429-09|Costa Rica|Guanacaste|658[0n]|BOLD:AAB0340
Elaphria subobliqua|[744]|BLPAC032-06|Costa Rica|Guanacaste|658[0n]|BOLD:AAB0340
Elaphria subobliqua|[745]|BLPDD619-09|Costa Rica|Guanacaste|658[0n]|BOLD:AAB0340
Elaphria subobliqua|[746]|BLPDD620-09|Costa Rica|Guanacaste|658[0n]|BOLD:AAB0340
Elaphria subobliqua|[747]|BLPDK487-09|Costa Rica|Guanacaste|658[0n]|BOLD:AAB0340
Elaphria subobliqua|[748]|BLPAC004-06|Costa Rica|Guanacaste|658[0n]|BOLD:AAB0340
Elaphria subobliquaCR1|[749]|LOCRI444-11|Costa Rica|Limon|658[0n]|BOLD:AAB0340
Elaphria subobliqua|[750]|BLPEF1424-12|Costa Rica|Guanacaste|658[0n]|BOLD:AAB0340
Elaphria subobliquaDHJ02|[751]|LOCRE578-10|Costa Rica|San Jose|658[0n]|BOLD:AAB0340
Elaphria subobliqua|[752]|BLPDW606-11|Costa Rica|Guanacaste|658[0n]|BOLD:AAB0340
Elaphria subobliqua|[753]|BLPDT1664-10|Costa Rica|Guanacaste|658[0n]|BOLD:AAB0340
Elaphria subobliqua|[754]|BLPDT1665-10|Costa Rica|Guanacaste|658[0n]|BOLD:AAB0340
Elaphria subobliqua|[755]|BLPCG931-08|Costa Rica|Guanacaste|658[0n]|BOLD:AAB0340
Elaphria subobliqua|[756]|BLPDD427-09|Costa Rica|Guanacaste|658[0n]|BOLD:AAB0340
Elaphria subobliqua|[757]|BLPDD621-09|Costa Rica|Guanacaste|658[0n]|BOLD:AAB0340
Elaphria subobliqua|[758]|BLPDK488-09|Costa Rica|Guanacaste|658[0n]|BOLD:AAB0340
Elaphria subobliqua|[759]|BLPDD430-09|Costa Rica|Guanacaste|658[0n]|BOLD:AAB0340
Elaphria subobliqua|[760]|BLPDD431-09|Costa Rica|Guanacaste|658[0n]|BOLD:AAB0340
Elaphria subobliqua|[761]|BLPCG930-08|Costa Rica|Guanacaste|658[0n]|BOLD:AAB0340
Elaphria subobliqua|[762]|BLPCH556-08|Costa Rica|Guanacaste|658[0n]|BOLD:AAB0340
Elaphria subobliqua|[763]|BLPBD308-07|Costa Rica|Guanacaste|658[0n]|BOLD:AAB0340
Elaphria subobliqua|[764]|BLPBA240-07|Costa Rica|Guanacaste|658[0n]|BOLD:AAB0340
Elaphria subobliqua|[765]|BLPAH373-07|Costa Rica|Guanacaste|658[0n]|BOLD:AAB0340
Elaphria subobliqua|[766]|BLPAD021-06|Costa Rica|Guanacaste|658[0n]|BOLD:AAB0340
Elaphria subobliqua|[767]|BLPAC502-06|Costa Rica|Guanacaste|658[0n]|BOLD:AAB0340
Elaphria subobliqua|[768]|BLPAC499-06|Costa Rica|Guanacaste|658[0n]|BOLD:AAB0340
Elaphria subobliqua|[769]|BLPAC001-06|Costa Rica|Guanacaste|658[0n]|BOLD:AAB0340
Elaphria subobliqua|[770]|BLPAB909-06|Costa Rica|Guanacaste|658[0n]|BOLD:AAB0340
Elaphria subobliqua|[771]|BLPBD307-07|Costa Rica|Guanacaste|656[0n]|BOLD:AAB0340
Elaphria subobliqua|[772]|BLPDG227-09|Costa Rica|Guanacaste|621[0n]|BOLD:AAB0340
Elaphria subobliqua|[773]|GMCRA1692-13|Costa Rica|Guanacaste|618[0n]|BOLD:AAB0340
Elaphria subobliqua|[774]|BLPAH372-07|Costa Rica|Guanacaste|658[0n]|BOLD:AAB0340
Elaphria subobliqua|[775]|BLPDT1663-10|Costa Rica|Guanacaste|628[0n]|BOLD:AAB0340
Elaphria subobliqua|[776]|BLPEF3917-13|Costa Rica|Guanacaste|630[0n]|BOLD:AAB0340
Elaphria subobliqua|[777]|BLPEF3916-13|Costa Rica|Guanacaste|658[0n]|BOLD:AAB0340
Elaphria subobliqua|[778]|CNCLB2283-14|Guatemala|San Marcos|658[0n]|BOLD:AAB0340
Elaphria georgei|[779]|LSEU192-06|United States|Georgia|658[1n]|BOLD:AAC1403
Elaphria georgei|[780]|LSEU191-06|United States|Georgia|550[1n]|BOLD:AAC1403
Elaphria georgei|[781]|LNC934-06|United States|North Carolina|658[0n]|BOLD:AAC1403
Elaphria georgei|[782]|RDLQG281-06|Canada|Quebec|658[0n]|BOLD:AAC1403
Elaphria georgei|[783]|LPSOD356-09|Canada|Ontario|658[0n]|BOLD:AAC1403
Elaphria georgei|[784]|LPSOD242-09|Canada|Ontario|658[0n]|BOLD:AAC1403
Elaphria georgei|[785]|RDLQ476-07|Canada|Quebec|616[0n]|BOLD:AAC1403
Elaphria georgei|[786]|LPSOD357-09|Canada|Ontario|658[0n]|BOLD:AAC1403
Elaphria georgei|[787]|RDLQG504-06|Canada|Quebec|621[3n]|BOLD:AAC1403
Elaphria georgei|[788]|RDLQ475-07|Canada|Quebec|616[0n]|BOLD:AAC1403
Elaphria georgei|[789]|LPSOD287-09|Canada|Ontario|658[0n]|BOLD:AAC1403
Elaphria georgei|[790]|LPSOD297-09|Canada|Ontario|658[0n]|BOLD:AAC1403
Elaphria georgei|[791]|LPSOD292-09|Canada|Ontario|658[0n]|BOLD:AAC1403
Elaphria georgei|[792]|LPSOD286-09|Canada|Ontario|658[0n]|BOLD:AAC1403
Elaphria georgei|[793]|RDLQG503-06|Canada|Quebec|658[0n]|BOLD:AAC1403
Elaphria georgei|[794]|LPSOD304-09|Canada|Ontario|658[0n]|BOLD:AAC1403
Elaphria georgei|[795]|RDNMK087-11|United States|Florida|658[0n]|BOLD:AAC1403
Elaphria sp. nr. alapallida|[796]|USLEP436-10|United States|Texas|658[0n]|BOLD:ACE4740
Elaphria sp. nr. alapallida|[797]|USLEP959-10|United States|Texas|658[0n]|BOLD:ACE4740
Elaphria sp. nr. alapallida|[798]|BBLSX526-09|United States|Oklahoma|658[0n]|BOLD:ACE4740
Elaphria sp. nr. alapallida|[799]|BBLSW790-09|United States|Oklahoma|658[0n]|BOLD:ACE4740
Elaphria sp. nr. alapallida|[800]|LPOKD342-09|United States|Oklahoma|658[0n]|BOLD:ACE4740

[illegible]
Elaphria sp. nr. alapallida|[798]|BBLSX526-09|United States|Oklahoma|658[0n]|BOLD:ACE4740
Elaphria sp. nr. alapallida|[799]|BBLSW790-09|United States|Oklahoma|658[0n]|BOLD:ACE4740
Elaphria sp. nr. alapallida|[800]|LPOKD342-09|United States|Oklahoma|658[0n]|BOLD:ACE4740
Elaphria sp. nr. alapallida|[801]|USLEP766-10|United States|Texas|658[0n]|BOLD:ACE4740
Elaphria sp. nr. alapallida|[802]|LPOKB278-09|United States|Oklahoma|658[0n]|BOLD:ACE4740
Elaphria sp. nr. alapallida|[803]|BBLSW485-09|United States|Oklahoma|658[0n]|BOLD:ACE4740
Elaphria sp. nr. alapallida|[804]|LPOKA221-08|United States|Oklahoma|658[0n]|BOLD:ACE4740
Elaphria sp. nr. alapallida|[805]|BBLSX590-09|United States|Oklahoma|658[0n]|BOLD:ACE4740
Elaphria sp. nr. alapallida|[806]|LPOKD297-09|United States|Oklahoma|658[0n]|BOLD:ACE4740
Elaphria sp. nr. alapallida|[807]|BBLOD1224-11|United States|Oklahoma|658[0n]|BOLD:ACE4740
Elaphria sp. nr. alapallida|[808]|BBLSX533-09|United States|Oklahoma|658[0n]|BOLD:ACE4740
Elaphria sp. nr. alapallida|[809]|BBLSW475-09|United States|Oklahoma|658[0n]|BOLD:ACE4740
Elaphria sp. nr. alapallida|[810]|BBLSW794-09|United States|Oklahoma|658[0n]|BOLD:ACE4740
Elaphria sp. nr. alapallida|[811]|BBLSX549-09|United States|Oklahoma|658[0n]|BOLD:ACE4740
Elaphria sp. nr. alapallida|[812]|BBLOD1232-11|United States|Oklahoma|658[0n]|BOLD:ACE4740
Elaphria sp. nr. alapallida|[813]|USLEP958-10|United States|Texas|614[0n]|BOLD:ACE4740
Elaphria sp. nr. alapallida|[814]|BBLSW496-09|United States|Oklahoma|658[0n]|BOLD:ACE4740
Elaphria sp. nr. alapallida|[815]|BBLSX964-09|United States|Oklahoma|658[0n]|BOLD:ACE4740
Elaphria sp. nr. alapallida|[816]|BBLSW511-09|United States|Oklahoma|655[0n]|BOLD:ACE4740
Elaphria sp. nr. alapallida|[817]|BBLSW509-09|United States|Oklahoma|658[0n]|BOLD:ACE4740
Elaphria sp. nr. alapallida|[818]|BBLSW492-09|United States|Oklahoma|632[0n]|BOLD:ACE4740
Elaphria sp. nr. alapallida|[819]|BBLSW796-09|United States|Oklahoma|650[0n]|BOLD:ACE4740
Elaphria sp. nr. alapallida|[820]|BBLSX084-09|United States|Oklahoma|658[0n]|BOLD:ACE4740
Elaphria sp. nr. alapallida|[821]|USLEP778-10|United States|Texas|658[0n]|BOLD:ACE4740
Elaphria sp. nr. alapallida|[822]|USLEP960-10|United States|Texas|658[0n]|BOLD:ACE4740
Elaphria sp. nr. alapallida|[823]|USLEP1067-10|United States|Texas|658[0n]|BOLD:ACE4740
Elaphria sp. nr. alapallida|[824]|BBLOE1642-12|United States|Oklahoma|658[0n]|BOLD:ACE4740
Elaphria festivoides|[825]|LNC801-06|United States|North Carolina|658[0n]|BOLD:ACE4741
Elaphria festivoides|[826]|LNCB709-09|United States|North Carolina|658[0n]|BOLD:ACE4741
Elaphria festivoides|[827]|RDNMD512-06|United States|Florida|658[0n]|BOLD:ACE4741
Elaphria festivoides|[828]|RDNMD536-06|United States|Florida|658[0n]|BOLD:ACE4741
Elaphria festivoides|[829]|RDNMD513-06|United States|Florida|658[0n]|BOLD:ACE4741
Elaphria festivoides|[830]|LNCB946-10|United States|North Carolina|658[1n]|BOLD:ACE4741
Elaphria festivoides|[831]|RDNMF978-08|United States|Colorado|658[0n]|BOLD:ACE4741
Elaphria festivoides|[832]|LNC800-06|United States|North Carolina|658[0n]|BOLD:ACE4741
Elaphria festivoides|[833]|LNCB708-09|United States|North Carolina|658[0n]|BOLD:ACE4741
Elaphria festivoides|[834]|USLEP615-10|United States|Florida|658[0n]|BOLD:ACE4741
Elaphria alapallida|[835]|LPSO319-08|Canada|Ontario|658[0n]|BOLD:AAA7700
Elaphria alapallida|[836]|LPSOD295-09|Canada|Ontario|658[0n]|BOLD:AAA7700
Elaphria alapallida|[837]|BBLCU044-09|United States|Michigan|658[0n]|BOLD:AAA7700
Elaphria alapallida|[838]|CNWBA498-13|Canada|Alberta|559[0n]|BOLD:AAA7700
Elaphria alapallida|[839]|TMNBB221-06|Canada|New Brunswick|658[0n]|BOLD:AAA7700
Elaphria alapallida|[840]|LOWCB518-05|Canada|British Columbia|658[0n]|BOLD:AAA7700
Elaphria alapallida|[841]|RDLQG826-06|Canada|Quebec|658[0n]|BOLD:AAA7700
Elaphria alapallida|[842]|RDLQG731-06|Canada|Quebec|658[0n]|BOLD:AAA7700
Elaphria alapallida|[843]|RDLQG723-06|Canada|Quebec|658[0n]|BOLD:AAA7700
Elaphria alapallida|[844]|RDLQG521-06|Canada|Quebec|658[0n]|BOLD:AAA7700
Elaphria alapallida|[845]|LGSMC333-05|United States|Tennessee|658[0n]|BOLD:AAA7700
Elaphria alapallida|[846]|RDLQG502-06|Canada|Quebec|658[0n]|BOLD:AAA7700
Elaphria alapallida|[847]|MEC704-04|Canada|Quebec|658[0n]|BOLD:AAA7700
Elaphria alapallida|[848]|RDLQH212-07|Canada|Quebec|621[0n]|BOLD:AAA7700
Elaphria alapallida|[849]|LPSOD372-09|Canada|Ontario|658[0n]|BOLD:AAA7700
Elaphria alapallida|[850]|LPSOD469-09|Canada|Ontario|658[0n]|BOLD:AAA7700
Elaphria alapallida|[851]|BBLCU049-09|United States|Michigan|658[0n]|BOLD:AAA7700
Elaphria alapallida|[852]|BBLCU183-09|United States|Michigan|658[0n]|BOLD:AAA7700
Elaphria alapallida|[853]|RDMAB130-05|Canada|Alberta|621[1n]|BOLD:AAA7700
Elaphria alapallida|[854]|RDLQG535-06|Canada|Quebec|658[0n]|BOLD:AAA7700
Elaphria alapallida|[855]|TMNBB216-06|Canada|New Brunswick|658[0n]|BOLD:AAA7700
Elaphria alapallida|[856]|LSEU516-06|United States|Georgia|658[0n]|BOLD:AAA7700
Elaphria alapallida|[857]|BBLCU226-09|United States|Michigan|658[0n]|BOLD:AAA7700
Elaphria alapallida|[858]|LPSOD373-09|Canada|Ontario|658[0n]|BOLD:AAA7700
Elaphria alapallida|[859]|BBLPB741-10|Canada|Ontario|658[0n]|BOLD:AAA7700
Elaphria alapallida|[860]|LPSOD294-09|Canada|Ontario|658[0n]|BOLD:AAA7700
Elaphria alapallida|[861]|BBLCU235-09|United States|Michigan|658[0n]|BOLD:AAA7700
Elaphria alapallida|[862]|BBLCU142-09|United States|Michigan|658[0n]|BOLD:AAA7700
Elaphria alapallida|[863]|BBLCU080-09|United States|Michigan|658[0n]|BOLD:AAA7700
Elaphria alapallida|[864]|BBLCU055-09|United States|Michigan|658[0n]|BOLD:AAA7700
Elaphria alapallida|[865]|BBLCU018-09|United States|Michigan|658[0n]|BOLD:AAA7700
Elaphria alapallida|[866]|LPSOD655-09|Canada|Ontario|658[0n]|BOLD:AAA7700
Elaphria alapallida|[867]|LPSOD377-09|Canada|Ontario|658[0n]|BOLD:AAA7700
Elaphria alapallida|[868]|LPSOD374-09|Canada|Ontario|658[0n]|BOLD:AAA7700
Elaphria alapallida|[869]|LPSOD359-09|Canada|Ontario|658[0n]|BOLD:AAA7700
Elaphria alapallida|[870]|LPSOD342-09|Canada|Ontario|658[0n]|BOLD:AAA7700
Elaphria alapallida|[871]|LPSOD309-09|Canada|Ontario|658[0n]|BOLD:AAA7700
Elaphria alapallida|[872]|LPSOD285-09|Canada|Ontario|658[0n]|BOLD:AAA7700
Elaphria alapallida|[873]|LPSOD284-09|Canada|Ontario|658[0n]|BOLD:AAA7700
Elaphria alapallida|[874]|LPMN795-08|Canada|Manitoba|658[0n]|BOLD:AAA7700
Elaphria alapallida|[875]|LPMN090-08|Canada|Manitoba|658[0n]|BOLD:AAA7700
Elaphria alapallida|[876]|LPSOB488-08|Canada|Ontario|658[0n]|BOLD:AAA7700
Elaphria alapallida|[877]|RDLQG821-06|Canada|Quebec|658[0n]|BOLD:AAA7700
Elaphria alapallida|[878]|RDLQG500-06|Canada|Quebec|658[0n]|BOLD:AAA7700
Elaphria alapallida|[879]|RDLQG282-06|Canada|Quebec|658[0n]|BOLD:AAA7700
Elaphria alapallida|[880]|TMNBB220-06|Canada|New Brunswick|658[0n]|BOLD:AAA7700
Elaphria alapallida|[881]|TMNBB219-06|Canada|New Brunswick|658[0n]|BOLD:AAA7700
Elaphria alapallida|[882]|TMNBB217-06|Canada|New Brunswick|658[0n]|BOLD:AAA7700
Elaphria alapallida|[883]|TMNBB214-06|Canada|New Brunswick|658[0n]|BOLD:AAA7700
Elaphria alapallida|[884]|TTMNB058-06|Canada|New Brunswick|658[0n]|BOLD:AAA7700
Elaphria alapallida|[885]|RDLQB271-05|Canada|Quebec|658[0n]|BOLD:AAA7700
Elaphria alapallida|[886]|LOWCC481-05|Canada|British Columbia|658[0n]|BOLD:AAA7700
Elaphria alapallida|[887]|LOWCC480-05|Canada|British Columbia|658[0n]|BOLD:AAA7700
Elaphria alapallida|[888]|PHMNB641-04|Canada|New Brunswick|658[0n]|BOLD:AAA7700
Elaphria alapallida|[889]|TMNBB215-06|Canada|New Brunswick|658[0n]|BOLD:AAA7700
Elaphria alapallida|[890]|BBLCU048-09|United States|Michigan|658[0n]|BOLD:AAA7700
Elaphria alapallida|[891]|LPSOD367-09|Canada|Ontario|658[0n]|BOLD:AAA7700
Elaphria alapallida|[892]|XAE417-04|Canada|Ontario|571[0n]|BOLD:AAA7700
Elaphria alapallida|[893]|RDLQG501-06|Canada|Quebec|647[0n]|BOLD:AAA7700
Elaphria alapallida|[894]|LGSMC910-05|United States|Tennessee|617[0n]|BOLD:AAA7700
Elaphria alapallida|[895]|LOWCB516-05|Canada|British Columbia|591[0n]|BOLD:AAA7700
Elaphria alapallida|[896]|RDLQB776-05|Canada|Quebec|617[0n]|BOLD:AAA7700
Elaphria alapallida|[897]|RDLQG536-06|Canada|Quebec|658[0n]|BOLD:AAA7700
Elaphria alapallida|[898]|LPSOD262-09|Canada|Ontario|582[0n]|BOLD:AAA7700
Elaphria alapallida|[899]|BBLPC809-09|Canada|Newfoundland and Labrador|658[0n]|BOLD:AAA7700
Elaphria alapallida|[900]|BBLPC844-09|Canada|Newfoundland and Labrador|658[0n]|BOLD:AAA7700

[illegible]
Elaphria alapallida|[898]|LPSOD262-09|Canada|Ontario|582[0n]|BOLD:AAA7700
Elaphria alapallida|[899]|BBLPC809-09|Canada|Newfoundland and Labrador|658[0n]|BOLD:AAA7700
Elaphria alapallida|[900]|BBLPC844-09|Canada|Newfoundland and Labrador|658[0n]|BOLD:AAA7700
Elaphria alapallida|[901]|LALPA289-10|Canada|British Columbia|658[0n]|BOLD:AAA7700
Elaphria alapallida|[902]|RDNMK229-11|Canada|Ontario|658[0n]|BOLD:AAA7700
Elaphria alapallida|[903]|RDNMK230-11|Canada|Ontario|658[0n]|BOLD:AAA7700
Elaphria alapallida|[904]|RDNMK231-11|Canada|Ontario|658[0n]|BOLD:AAA7700
Elaphria alapallida|[905]|RDLQB924-05|Canada|Quebec|560[0n]|BOLD:AAA7700
Elaphria alapallida|[906]|CNWBE388-13|Canada|Alberta|558[0n]|BOLD:AAA7700
Elaphria cornutinus|[907]|LGSM559-04|United States|North Carolina|609[0n]|BOLD:ACE7923
Elaphria cornutinus|[908]|LGSMC875-05|United States|Tennessee|658[0n]|BOLD:ACE7923
Elaphria cornutinus|[909]|LGSMC876-05|United States|Tennessee|658[0n]|BOLD:ACE7923
Elaphria cornutinus|[910]|LOTB122-05|United States|Tennessee|658[0n]|BOLD:ACE7923
Elaphria cornutinus|[911]|LSEU354-06|United States|Georgia|658[0n]|BOLD:ACE7923
Elaphria cornutinus|[912]|LSEU614-06|United States|Georgia|658[0n]|BOLD:ACE7923
Elaphria cornutinus|[913]|LNCB651-09|United States|North Carolina|658[0n]|BOLD:ACE7923
Elaphria cornutinus|[914]|LGSMG1012-10|United States|Tennessee|658[0n]|BOLD:ACE7923
Elaphria cornutinus|[915]|LGSMG1013-10|United States|Tennessee|658[0n]|BOLD:ACE7923
Elaphria cornutinus|[916]|LNCC550-11|United States|North Carolina|658[0n]|BOLD:ACE7923
Elaphria cornutinus|[917]|LNCC679-11|United States|North Carolina|658[0n]|BOLD:ACE7923
Elaphria cornutinus|[918]|LNCC861-11|United States|North Carolina|658[0n]|BOLD:ACE7923
Elaphria cornutinus|[919]|LGSM410-04|United States|Tennessee|658[0n]|BOLD:ACE7923
Elaphria cornutinus|[920]|LNCC862-11|United States|North Carolina|658[0n]|BOLD:ACE7923
Elaphria cornutinus|[921]|LNCC108-10|United States|North Carolina|658[0n]|BOLD:ACE7923
Elaphria cornutinus|[922]|LNCC109-10|United States|North Carolina|658[0n]|BOLD:ACE7923
Elaphria cornutinus|[923]|LSEU355-06|United States|Georgia|658[0n]|BOLD:ACE7923
Elaphria cornutinus|[924]|LSEU615-06|United States|Georgia|658[0n]|BOLD:ACE7923
Elaphria cornutinus|[925]|LNCC1552-13|United States|North Carolina|658[0n]|BOLD:ACE7923
Elaphria cornutinus|[926]|LOT340-04|United States|Tennessee|658[0n]|BOLD:ACE7923
Elaphria cornutinus|[927]|LOT339-04|United States|Tennessee|658[0n]|BOLD:ACE7923
Elaphria cornutinus|[928]|LGSM411-04|United States|Tennessee|658[0n]|BOLD:ACE7923
Elaphria cornutinus|[929]|LOT548-04|United States|Tennessee|597[0n]|BOLD:ACE7923
Elaphria cornutinus|[930]|LSEU613-06|United States|Georgia|658[0n]|BOLD:ACE7923
Elaphria cornutinus|[931]|LNCC107-10|United States|North Carolina|658[0n]|BOLD:ACE7923
Elaphria festivoides|[932]|CNCLB2452-14|United States|Maryland|658[0n]|BOLD:ACE7923
Spodoptera hipparis|[933]|RDNMD637-06|United States|New Mexico|658[0n]|BOLD:AAJ2429
Spodoptera hipparis|[934]|RDNMD638-06|United States|New Mexico|658[0n]|BOLD:AAJ2429
Spodoptera hipparis|[935]|USLEP846-10|United States|Arizona|658[0n]|BOLD:AAJ2429
Spodoptera hipparis|[936]|RDNMJ682-11|United States|Arizona|658[0n]|BOLD:AAJ2429
Spodoptera hipparis|[937]|IAWLB524-11|United States|Arizona|658[0n]|BOLD:AAJ2429
Spodoptera hipparis|[938]|IAWLB525-11|United States|Arizona|658[0n]|BOLD:AAJ2429
Spodoptera exigua|[939]|AWCLB162-10|United States|Arizona|571[0n]|BOLD:AAA6644
Spodoptera exigua|[940]|LOCBF012-13|United States|California|616[0n]|BOLD:AAA6644
Spodoptera exigua|[941]|LOCBF016-13|United States|California|602[0n]|BOLD:AAA6644
Spodoptera exigua|[942]|LOCBF011-13|United States|California|608[0n]|BOLD:AAA6644
Spodoptera exigua|[943]|GMLC086-09|United States|California|658[0n]|BOLD:AAA6644
Spodoptera exigua|[944]|LOCBB360-06|United States|California|656[0n]|BOLD:AAA6644
Spodoptera exigua|[945]|AWCLB115-10|United States|Arizona|622[0n]|BOLD:AAA6644
Spodoptera exigua|[946]|BBLOD698-11|United States|California|658[0n]|BOLD:AAA6644
Spodoptera exigua|[947]|BBLOD706-11|United States|California|658[0n]|BOLD:AAA6644
Spodoptera exigua|[948]|GMLC1153-12|United States|California|658[0n]|BOLD:AAA6644
Spodoptera exigua|[949]|GBMIN38621-13|Spain||641[0n]|BOLD:AAA6644
Spodoptera exigua|[950]|LOCBF465-13|United States|California|618[0n]|BOLD:AAA6644
Spodoptera exigua|[951]|BBLOC426-11|United States|Arizona|614[0n]|BOLD:AAA6644
Spodoptera exigua|[952]|BBLOC511-11|United States|Arizona|658[0n]|BOLD:AAA6644
Spodoptera exigua|[953]|LEFIJ339-10|Finland||658[0n]|BOLD:AAA6644
Spodoptera exigua|[954]|GBMIN38631-13|Thailand||641[0n]|BOLD:AAA6644
Spodoptera exigua|[955]|GWORR384-10|Italy|Calabria|630[0n]|BOLD:AAA6644
Spodoptera exigua|[956]|GBMIN12119-13|Japan||573[0n]|BOLD:AAA6644
Spodoptera exigua|[957]|GBMIN12118-13|Japan||573[0n]|BOLD:AAA6644
Spodoptera exigua|[958]|MAMOT651-10|Pakistan|Punjab|658[0n]|BOLD:AAA6644
Spodoptera exigua|[959]|GBMIN12111-13|Japan||573[0n]|BOLD:AAA6644
Spodoptera exigua|[960]|MAMOT470-10|Pakistan|Punjab|658[1n]|BOLD:AAA6644
Spodoptera exigua|[961]|GWOTF353-12|Italy|Basilicata|658[0n]|BOLD:AAA6644
Spodoptera exigua|[962]|IPI840-14|India|Maharashtra|658[0n]|BOLD:AAA6644
Spodoptera exigua|[963]|IPI864-14|India|Maharashtra|658[0n]|BOLD:AAA6644
Spodoptera exigua|[964]|MAMOT3830-13|Pakistan|Khyber Pakhtunkhwa|658[0n]|BOLD:AAA6644
Spodoptera exigua|[965]|LEPIN046-13|India|Punjab|658[0n]|BOLD:AAA6644
Spodoptera exigua|[966]|MAMOT3484-13|Pakistan|Punjab|658[0n]|BOLD:AAA6644
Spodoptera exigua|[967]|MAMOT3591-13|Pakistan|Khyber Pakhtunkhwa|658[0n]|BOLD:AAA6644
Spodoptera exigua|[968]|IBLAO585-12|Spain|Murcia|658[0n]|BOLD:AAA6644
Spodoptera exigua|[969]|MAMOT1474-12|Pakistan|Sind|658[0n]|BOLD:AAA6644
Spodoptera exigua|[970]|PHLSA535-11|Spain|Comunidad Valenciana|658[0n]|BOLD:AAA6644
Spodoptera exigua|[971]|IBLAO061-11|Spain|Murcia|658[0n]|BOLD:AAA6644
Spodoptera exigua|[972]|MAMOT751-10|Pakistan|Punjab|658[0n]|BOLD:AAA6644
Spodoptera exigua|[973]|MAMOT663-10|Pakistan|Punjab|658[0n]|BOLD:AAA6644
Spodoptera exigua|[974]|MAMOT652-10|Pakistan|Punjab|658[0n]|BOLD:AAA6644
Spodoptera exigua|[975]|MAMOT247-10|Pakistan|Punjab|658[0n]|BOLD:AAA6644
Spodoptera exigua|[976]|MAMOT243-10|Pakistan|Punjab|658[0n]|BOLD:AAA6644
Spodoptera exigua|[977]|GWORZ535-10|Germany|Bavaria|658[0n]|BOLD:AAA6644
Spodoptera exigua|[978]|PMANL404-09|Namibia|Kunene|658[0n]|BOLD:AAA6644
Spodoptera exigua|[979]|PMANL191-09|Kenya|Laikipia County|658[0n]|BOLD:AAA6644
Spodoptera exigua|[980]|CGUKC200-09|United Kingdom|England|658[0n]|BOLD:AAA6644
Spodoptera exigua|[981]|FBLMV381-09|Germany|Bavaria|596[1n]|BOLD:AAA6644
Spodoptera exigua|[982]|IPI848-14|India|Maharashtra|658[0n]|BOLD:AAA6644
Spodoptera exigua|[983]|IPI1042-14|India|Maharashtra|658[0n]|BOLD:AAA6644
Spodoptera exigua|[984]|GBMIN38597-13|||641[0n]|BOLD:AAA6644
Spodoptera exigua|[985]|GBMIN38646-13|||641[0n]|BOLD:AAA6644
Spodoptera exigua|[986]|GBMIN38596-13|||641[0n]|BOLD:AAA6644
Spodoptera exigua|[987]|BBLOC532-11|United States|Arizona|621[0n]|BOLD:AAA6644
Spodoptera exigua|[988]|BBLOC524-11|United States|Arizona|621[0n]|BOLD:AAA6644
Spodoptera exigua|[989]|LOCBF472-13|United States|California|621[0n]|BOLD:AAA6644
Spodoptera exigua|[990]|BBLOC476-11|United States|Arizona|621[0n]|BOLD:AAA6644
Spodoptera exigua|[991]|BBLOC474-11|United States|Arizona|621[0n]|BOLD:AAA6644
Spodoptera exigua|[992]|GMLC1351-12|United States|California|632[0n]|BOLD:AAA6644
Spodoptera exigua|[993]|LOFLA396-06|United States|Florida|658[0n]|BOLD:AAA6644
Spodoptera exigua|[994]|LOCBB601-06|United States|California|658[0n]|BOLD:AAA6644
Spodoptera exigua|[995]|BBLSW315-09|United States|Arizona|658[0n]|BOLD:AAA6644
Spodoptera exigua|[996]|BBLSW328-09|United States|Arizona|658[0n]|BOLD:AAA6644
Spodoptera exigua|[997]|CMAZA251-09|United States|Arizona|658[0n]|BOLD:AAA6644
Spodoptera exigua|[998]|AWCLB643-11|United States|Arizona|658[0n]|BOLD:AAA6644
Spodoptera exigua|[999]|AWCLB661-11|United States|Arizona|658[0n]|BOLD:AAA6644
Spodoptera exigua|[1000]|BBLOC507-11|United States|Arizona|658[0n]|BOLD:AAA6644

Spodoptera exigua|[997]|CMAZA251-09|United States|Arizona|658[0n]|BOLD:AAA6644
Spodoptera exigua|[998]|AWCLB643-11|United States|Arizona|658[0n]|BOLD:AAA6644
Spodoptera exigua|[999]|AWCLB661-11|United States|Arizona|658[0n]|BOLD:AAA6644
Spodoptera exigua|[1000]|BBLOC507-11|United States|Arizona|658[0n]|BOLD:AAA6644
Spodoptera exigua|[1001]|BBLOD705-11|United States|California|658[0n]|BOLD:AAA6644
Spodoptera exigua|[1002]|GMLC1161-12|United States|California|658[0n]|BOLD:AAA6644
Spodoptera exigua|[1003]|BBLOC451-11|United States|Arizona|658[0n]|BOLD:AAA6644
Spodoptera exigua|[1004]|GMLC1290-12|United States|California|658[0n]|BOLD:AAA6644
Spodoptera exigua|[1005]|GMLC1282-12|United States|California|658[0n]|BOLD:AAA6644
Spodoptera exigua|[1006]|GMLC1277-12|United States|California|658[0n]|BOLD:AAA6644
Spodoptera exigua|[1007]|GMLC1267-12|United States|California|658[0n]|BOLD:AAA6644
Spodoptera exigua|[1008]|GMLC1241-12|United States|California|658[0n]|BOLD:AAA6644
Spodoptera exigua|[1009]|GMLC1197-12|United States|California|658[0n]|BOLD:AAA6644
Spodoptera exigua|[1010]|GMLC1190-12|United States|California|658[0n]|BOLD:AAA6644
Spodoptera exigua|[1011]|GMLC1150-12|United States|California|658[0n]|BOLD:AAA6644
Spodoptera exigua|[1012]|GMLC1038-12|United States|California|658[0n]|BOLD:AAA6644
Spodoptera exigua|[1013]|CMAZA1107-12|United States|Arizona|658[0n]|BOLD:AAA6644
Spodoptera exigua|[1014]|BBLOE1966-12|United States|California|658[0n]|BOLD:AAA6644
Spodoptera exigua|[1015]|BBLOE1808-12|United States|Arizona|658[0n]|BOLD:AAA6644
Spodoptera exigua|[1016]|BBLOE1806-12|United States|Arizona|658[0n]|BOLD:AAA6644
Spodoptera exigua|[1017]|BBLOE1756-12|United States|Texas|658[0n]|BOLD:AAA6644
Spodoptera exigua|[1018]|BBLOE1694-12|United States|Oklahoma|658[0n]|BOLD:AAA6644
Spodoptera exigua|[1019]|BBLOD1880-11|United States|Arizona|658[0n]|BOLD:AAA6644
Spodoptera exigua|[1020]|BBLOD1573-11|United States|Oklahoma|658[0n]|BOLD:AAA6644
Spodoptera exigua|[1021]|BBLOD942-11|United States|Texas|658[0n]|BOLD:AAA6644
Spodoptera exigua|[1022]|BBLOD850-11|United States|Arizona|658[0n]|BOLD:AAA6644
Spodoptera exigua|[1023]|BBLOD848-11|United States|Arizona|658[0n]|BOLD:AAA6644
Spodoptera exigua|[1024]|BBLOD846-11|United States|Arizona|658[0n]|BOLD:AAA6644
Spodoptera exigua|[1025]|BBLOD802-11|United States|California|658[0n]|BOLD:AAA6644
Spodoptera exigua|[1026]|BBLOD707-11|United States|California|658[0n]|BOLD:AAA6644
Spodoptera exigua|[1027]|BBLOD697-11|United States|California|658[0n]|BOLD:AAA6644
Spodoptera exigua|[1028]|BBLOD588-11|United States|California|658[0n]|BOLD:AAA6644
Spodoptera exigua|[1029]|BBLOD131-11|United States|California|658[0n]|BOLD:AAA6644
Spodoptera exigua|[1030]|BBLOD098-11|United States|Texas|658[0n]|BOLD:AAA6644
Spodoptera exigua|[1031]|BBLOC1673-11|United States|Arkansas|658[0n]|BOLD:AAA6644
Spodoptera exigua|[1032]|BBLOC1661-11|United States|Arizona|658[0n]|BOLD:AAA6644
Spodoptera exigua|[1033]|BBLOC1660-11|United States|Arizona|658[0n]|BOLD:AAA6644
Spodoptera exigua|[1034]|BBLOC1648-11|United States|Arizona|658[0n]|BOLD:AAA6644
Spodoptera exigua|[1035]|BBLOC1640-11|United States|Arizona|658[0n]|BOLD:AAA6644
Spodoptera exigua|[1036]|BBLOC1620-11|United States|Arizona|658[0n]|BOLD:AAA6644
Spodoptera exigua|[1037]|BBLOC1613-11|United States|Arizona|658[0n]|BOLD:AAA6644
Spodoptera exigua|[1038]|BBLOC1582-11|United States|California|658[0n]|BOLD:AAA6644
Spodoptera exigua|[1039]|BBLOC1577-11|United States|California|658[0n]|BOLD:AAA6644
Spodoptera exigua|[1040]|BBLOC1576-11|United States|California|658[0n]|BOLD:AAA6644
Spodoptera exigua|[1041]|BBLOC1551-11|United States|California|658[0n]|BOLD:AAA6644
Spodoptera exigua|[1042]|LNCC1328-11|United States|North Carolina|658[0n]|BOLD:AAA6644
Spodoptera exigua|[1043]|LNCC1327-11|United States|North Carolina|658[0n]|BOLD:AAA6644
Spodoptera exigua|[1044]|BBLOC894-11|United States|Arizona|658[0n]|BOLD:AAA6644
Spodoptera exigua|[1045]|BBLOC796-11|United States|Arizona|658[0n]|BOLD:AAA6644
Spodoptera exigua|[1046]|BBLOC551-11|United States|Arizona|658[0n]|BOLD:AAA6644
Spodoptera exigua|[1047]|BBLOC547-11|United States|Arizona|658[0n]|BOLD:AAA6644
Spodoptera exigua|[1048]|BBLOC542-11|United States|Arizona|658[0n]|BOLD:AAA6644
Spodoptera exigua|[1049]|BBLOC533-11|United States|Arizona|658[0n]|BOLD:AAA6644
Spodoptera exigua|[1050]|BBLOC527-11|United States|Arizona|658[0n]|BOLD:AAA6644
Spodoptera exigua|[1051]|BBLOC526-11|United States|Arizona|658[0n]|BOLD:AAA6644
Spodoptera exigua|[1052]|BBLOC525-11|United States|Arizona|658[0n]|BOLD:AAA6644
Spodoptera exigua|[1053]|BBLOC522-11|United States|Arizona|658[0n]|BOLD:AAA6644
Spodoptera exigua|[1054]|BBLOC521-11|United States|Arizona|658[0n]|BOLD:AAA6644
Spodoptera exigua|[1055]|BBLOC519-11|United States|Arizona|658[0n]|BOLD:AAA6644
Spodoptera exigua|[1056]|BBLOC513-11|United States|Arizona|658[0n]|BOLD:AAA6644
Spodoptera exigua|[1057]|BBLOC512-11|United States|Arizona|658[0n]|BOLD:AAA6644
Spodoptera exigua|[1058]|BBLOC510-11|United States|Arizona|658[0n]|BOLD:AAA6644
Spodoptera exigua|[1059]|BBLOC508-11|United States|Arizona|658[0n]|BOLD:AAA6644
Spodoptera exigua|[1060]|BBLOC506-11|United States|Arizona|658[0n]|BOLD:AAA6644
Spodoptera exigua|[1061]|BBLOC503-11|United States|Arizona|658[0n]|BOLD:AAA6644
Spodoptera exigua|[1062]|BBLOC500-11|United States|Arizona|658[0n]|BOLD:AAA6644
Spodoptera exigua|[1063]|BBLOC498-11|United States|Arizona|658[0n]|BOLD:AAA6644
Spodoptera exigua|[1064]|BBLOC495-11|United States|Arizona|658[0n]|BOLD:AAA6644
Spodoptera exigua|[1065]|BBLOC486-11|United States|Arizona|658[0n]|BOLD:AAA6644
Spodoptera exigua|[1066]|BBLOC485-11|United States|Arizona|658[0n]|BOLD:AAA6644
Spodoptera exigua|[1067]|BBLOC472-11|United States|Arizona|658[0n]|BOLD:AAA6644
Spodoptera exigua|[1068]|BBLOC464-11|United States|Arizona|658[0n]|BOLD:AAA6644
Spodoptera exigua|[1069]|BBLOC462-11|United States|Arizona|658[0n]|BOLD:AAA6644
Spodoptera exigua|[1070]|BBLOC438-11|United States|Arizona|658[0n]|BOLD:AAA6644
Spodoptera exigua|[1071]|BBLOC432-11|United States|Arizona|658[0n]|BOLD:AAA6644
Spodoptera exigua|[1072]|BBLOC431-11|United States|Arizona|658[0n]|BOLD:AAA6644
Spodoptera exigua|[1073]|BBLOC430-11|United States|Arizona|658[0n]|BOLD:AAA6644
Spodoptera exigua|[1074]|BBLOC428-11|United States|Arizona|658[0n]|BOLD:AAA6644
Spodoptera exigua|[1075]|BBLOC425-11|United States|Arizona|658[0n]|BOLD:AAA6644
Spodoptera exigua|[1076]|BBLOC423-11|United States|Arizona|658[0n]|BOLD:AAA6644
Spodoptera exigua|[1077]|BBLOC399-11|United States|Arizona|658[0n]|BOLD:AAA6644
Spodoptera exigua|[1078]|BBLOC396-11|United States|Arizona|658[0n]|BOLD:AAA6644
Spodoptera exigua|[1079]|BBLOC278-11|United States|Arizona|658[0n]|BOLD:AAA6644
Spodoptera exigua|[1080]|BBLOC237-11|United States|Arizona|658[0n]|BOLD:AAA6644
Spodoptera exigua|[1081]|BBLOC161-11|United States|Arizona|658[0n]|BOLD:AAA6644
Spodoptera exigua|[1082]|BBLOC151-11|United States|Arizona|658[0n]|BOLD:AAA6644
Spodoptera exigua|[1083]|BBLOC082-11|United States|Arizona|658[0n]|BOLD:AAA6644
Spodoptera exigua|[1084]|BBLOB1951-11|United States|Arizona|658[0n]|BOLD:AAA6644
Spodoptera exigua|[1085]|AWCLB662-11|United States|Arizona|658[0n]|BOLD:AAA6644
Spodoptera exigua|[1086]|AWCLB647-11|United States|Arizona|658[0n]|BOLD:AAA6644
Spodoptera exigua|[1087]|AWCLB644-11|United States|Arizona|658[0n]|BOLD:AAA6644
Spodoptera exigua|[1088]|AWCLB642-11|United States|Arizona|658[0n]|BOLD:AAA6644
Spodoptera exigua|[1089]|AWCLB641-11|United States|Arizona|658[0n]|BOLD:AAA6644
Spodoptera exigua|[1090]|AWCLB640-11|United States|Arizona|658[0n]|BOLD:AAA6644
Spodoptera exigua|[1091]|AWCLB527-11|United States|Arizona|658[0n]|BOLD:AAA6644
Spodoptera exigua|[1092]|AWCLB526-11|United States|Arizona|658[0n]|BOLD:AAA6644
Spodoptera exigua|[1093]|AWCLB520-11|United States|Arizona|658[0n]|BOLD:AAA6644
Spodoptera exigua|[1094]|AWCLB519-11|United States|Arizona|658[0n]|BOLD:AAA6644
Spodoptera exigua|[1095]|AWCLB503-11|United States|Arizona|658[0n]|BOLD:AAA6644
Spodoptera exigua|[1096]|AWCLB501-11|United States|Arizona|658[0n]|BOLD:AAA6644
Spodoptera exigua|[1097]|AWCLB500-11|United States|Arizona|658[0n]|BOLD:AAA6644
Spodoptera exigua|[1098]|AWCLB494-11|United States|Arizona|658[0n]|BOLD:AAA6644
Spodoptera exigua|[1099]|CMAZA261-09|United States|Arizona|658[0n]|BOLD:AAA6644
Spodoptera exigua|[1100]|BBLSY433-09|United States|Arizona|658[0n]|BOLD:AAA6644

Spodoptera exigua|[1097]|AWCLB500-11|United States|Arizona|658[0n]|BOLD:AAA6644
Spodoptera exigua|[1098]|AWCLB494-11|United States|Arizona|658[0n]|BOLD:AAA6644
Spodoptera exigua|[1099]|CMAZA261-09|United States|Arizona|658[0n]|BOLD:AAA6644
Spodoptera exigua|[1100]|BBLSY433-09|United States|Arizona|658[0n]|BOLD:AAA6644
Spodoptera exigua|[1101]|BBLSW352-09|United States|Arizona|658[0n]|BOLD:AAA6644
Spodoptera exigua|[1102]|BBLSW327-09|United States|Arizona|658[0n]|BOLD:AAA6644
Spodoptera exigua|[1103]|BBLSW326-09|United States|Arizona|658[0n]|BOLD:AAA6644
Spodoptera exigua|[1104]|BBLSW311-09|United States|Arizona|658[0n]|BOLD:AAA6644
Spodoptera exigua|[1105]|LPOKA1042-09|United States|Oklahoma|658[0n]|BOLD:AAA6644
Spodoptera exigua|[1106]|LPOKA1038-09|United States|Oklahoma|658[0n]|BOLD:AAA6644
Spodoptera exigua|[1107]|LPOKA913-09|United States|Oklahoma|658[0n]|BOLD:AAA6644
Spodoptera exigua|[1108]|LPOKA887-09|United States|Oklahoma|658[0n]|BOLD:AAA6644
Spodoptera exigua|[1109]|GMLC106-09|United States|California|658[0n]|BOLD:AAA6644
Spodoptera exigua|[1110]|GMLC079-09|United States|California|658[0n]|BOLD:AAA6644
Spodoptera exigua|[1111]|GMLC041-09|United States|California|658[0n]|BOLD:AAA6644
Spodoptera exigua|[1112]|LPOKA631-09|United States|Oklahoma|658[0n]|BOLD:AAA6644
Spodoptera exigua|[1113]|LPOKA611-09|United States|Oklahoma|658[0n]|BOLD:AAA6644
Spodoptera exigua|[1114]|LPOKA352-08|United States|Oklahoma|658[0n]|BOLD:AAA6644
Spodoptera exigua|[1115]|RDNMD853-07|United States|New Mexico|655[0n]|BOLD:AAA6644
Spodoptera exigua|[1116]|LOCBE251-06|United States|California|658[0n]|BOLD:AAA6644
Spodoptera exigua|[1117]|LOCBE157-06|United States|California|658[0n]|BOLD:AAA6644
Spodoptera exigua|[1118]|LOCBE147-06|United States|California|658[0n]|BOLD:AAA6644
Spodoptera exigua|[1119]|LOCBE088-06|United States|California|658[0n]|BOLD:AAA6644
Spodoptera exigua|[1120]|LOCBE087-06|United States|California|658[0n]|BOLD:AAA6644
Spodoptera exigua|[1121]|LOCBD963-06|United States|California|658[0n]|BOLD:AAA6644
Spodoptera exigua|[1122]|LOCBD898-06|United States|California|658[0n]|BOLD:AAA6644
Spodoptera exigua|[1123]|LOCBD543-06|United States|California|658[0n]|BOLD:AAA6644
Spodoptera exigua|[1124]|RDNMD436-06|United States|California|658[0n]|BOLD:AAA6644
Spodoptera exigua|[1125]|LOCBB606-06|United States|California|658[0n]|BOLD:AAA6644
Spodoptera exigua|[1126]|LOCBB603-06|United States|California|658[0n]|BOLD:AAA6644
Spodoptera exigua|[1127]|LOFLC242-06|United States|Florida|658[0n]|BOLD:AAA6644
Spodoptera exigua|[1128]|LOFLC241-06|United States|Florida|658[0n]|BOLD:AAA6644
Spodoptera exigua|[1129]|LOFLB270-06|United States|Florida|658[0n]|BOLD:AAA6644
Spodoptera exigua|[1130]|LOCBB363-06|United States|California|658[0n]|BOLD:AAA6644
Spodoptera exigua|[1131]|LOCBB362-06|United States|California|658[0n]|BOLD:AAA6644
Spodoptera exigua|[1132]|LOCBB361-06|United States|California|658[0n]|BOLD:AAA6644
Spodoptera exigua|[1133]|BBLOE1807-12|United States|Arizona|658[0n]|BOLD:AAA6644
Spodoptera exigua|[1134]|LOCBB771-06|United States|California|617[0n]|BOLD:AAA6644
Spodoptera exigua|[1135]|BBLOC509-11|United States|Arizona|658[0n]|BOLD:AAA6644
Spodoptera exigua|[1136]|JMMMB350-11|United States|California|658[2n]|BOLD:AAA6644
Spodoptera exigua|[1137]|BBLOC482-11|United States|Arizona|648[0n]|BOLD:AAA6644
Spodoptera exigua|[1138]|LOCBB602-06|United States|California|658[0n]|BOLD:AAA6644
Spodoptera exigua|[1139]|LOCBB741-06|United States|California|604[0n]|BOLD:AAA6644
Spodoptera exigua|[1140]|BBLOC1579-11|United States|California|658[0n]|BOLD:AAA6644
Spodoptera exigua|[1141]|BBLOD1117-11|United States|Texas|658[0n]|BOLD:AAA6644
Spodoptera exigua|[1142]|AWCLB504-11|United States|Arizona|658[0n]|BOLD:AAA6644
Spodoptera exigua|[1143]|BBLOC454-11|United States|Arizona|658[0n]|BOLD:AAA6644
Spodoptera exigua|[1144]|CMAZA1171-12|United States|Arizona|658[0n]|BOLD:AAA6644
Spodoptera exigua|[1145]|LPOKA644-09|United States|Oklahoma|609[0n]|BOLD:AAA6644
Spodoptera exigua|[1146]|PHMO362-03|Canada|Ontario|639[0n]|BOLD:AAA6644
Spodoptera exigua|[1147]|BBLOB018-11|United States|Arizona|602[0n]|BOLD:AAA6644
Spodoptera exigua|[1148]|BBLOC1615-11|United States|Arizona|616[0n]|BOLD:AAA6644
Spodoptera exigua|[1149]|MAMOT1678-12|Pakistan|Punjab|616[0n]|BOLD:AAA6644
Spodoptera exigua|[1150]|LOCBF2614-13|United States|California|658[0n]|BOLD:AAA6644
Spodoptera exigua|[1151]|BCMI283-11|Israel||658[0n]|BOLD:AAA6644
Spodoptera exigua|[1152]|LEFIJ1186-11|Finland||627[0n]|BOLD:AAA6644
Spodoptera exigua|[1153]|LEPIN012-12|India|Punjab|658[0n]|BOLD:AAA6644
Spodoptera exigua|[1154]|MAMOT734-10|Pakistan|Punjab|658[0n]|BOLD:AAA6644
Spodoptera exigua|[1155]|MAMOT3077-13|Pakistan|Punjab|658[0n]|BOLD:AAA6644
Spodoptera exigua|[1156]|IPI723-13|India|Maharashtra|658[0n]|BOLD:AAA6644
Spodoptera exigua|[1157]|IPI862-14|India|Maharashtra|658[0n]|BOLD:AAA6644
Spodoptera exigua|[1158]|GWORN403-09|Mali||658[0n]|BOLD:AAA6644
Spodoptera exigua|[1159]|GWOSA298-10|Italy|Basilicata|658[0n]|BOLD:AAA6644
Spodoptera exigua|[1160]|GWORO707-09|Germany|Bavaria|658[0n]|BOLD:AAA6644
Spodoptera exigua|[1161]|MAMOT402-10|Pakistan|Punjab|658[0n]|BOLD:AAA6644
Spodoptera exigua|[1162]|MAMOT468-10|Pakistan|Punjab|658[0n]|BOLD:AAA6644
Spodoptera exigua|[1163]|MAMOT469-10|Pakistan|Punjab|658[0n]|BOLD:AAA6644
Spodoptera exigua|[1164]|MAMOT657-10|Pakistan|Punjab|658[0n]|BOLD:AAA6644
Spodoptera exigua|[1165]|MAMOT659-10|Pakistan|Sind|658[0n]|BOLD:AAA6644
Spodoptera exigua|[1166]|LEFIJ340-10|Finland||658[0n]|BOLD:AAA6644
Spodoptera exigua|[1167]|BCMI003-11|Israel||658[0n]|BOLD:AAA6644
Spodoptera exigua|[1168]|BCMI432-11|Israel||658[0n]|BOLD:AAA6644
Spodoptera exigua|[1169]|PHLSA742-11|Austria|Vorarlberg|658[0n]|BOLD:AAA6644
Spodoptera exigua|[1170]|MAMOT1473-12|Pakistan|Sind|655[0n]|BOLD:AAA6644
Spodoptera exigua|[1171]|MAMOT3078-13|Pakistan|Punjab|658[0n]|BOLD:AAA6644
Spodoptera exigua|[1172]|TRLEP084-13|Turkey|Mersin|658[0n]|BOLD:AAA6644
Spodoptera exigua|[1173]|IPI751-13|India|Maharashtra|658[0n]|BOLD:AAA6644
Spodoptera exigua|[1174]|GBLAC762-13|Germany|Bavaria|658[0n]|BOLD:AAA6644
Spodoptera exigua|[1175]|LEPIN067-14|India|Punjab|658[0n]|BOLD:AAA6644
Spodoptera albula|[1176]|JAT127-10|Mexico|Chiapas|658[0n]|BOLD:AAD0740
Spodoptera albula|[1177]|BLPCA843-08|Costa Rica|Guanacaste|658[0n]|BOLD:AAD0740
Spodoptera albula|[1178]|MHMXM176-07|Costa Rica|Alajuela|652[0n]|BOLD:AAD0740
Spodoptera albula|[1179]|MHMXI542-07|Costa Rica|Alajuela|658[0n]|BOLD:AAD0740
Spodoptera albula|[1180]|GBGL12590-13|||1000[1n]|
Spodoptera albula|[1181]|GBGL12589-13|||1000[1n]|
Spodoptera albula|[1182]|GBGL12591-13|||1000[1n]|
Spodoptera albula|[1183]|GBGL12588-13|||1000[1n]|
Spodoptera albula|[1184]|GBGL12592-13|||1000[1n]|
Spodoptera albula|[1185]|HKONB226-09|United States|Texas|658[0n]|BOLD:AAD0740
Spodoptera albula|[1186]|MHMXI511-07|Costa Rica|Alajuela|658[0n]|BOLD:AAD0740
Spodoptera albula|[1187]|MHMXI512-07|Costa Rica|Alajuela|658[0n]|BOLD:AAD0740
Spodoptera albula|[1188]|HKONB225-09|United States|Texas|658[0n]|BOLD:AAD0740
Spodoptera albula|[1189]|MHMYS2786-13|Costa Rica|Guanacaste|658[0n]|BOLD:AAD0740
Spodoptera albula|[1190]|LYHES391-09|Mexico|Quintana Roo|658[0n]|BOLD:AAD0740
Spodoptera albula|[1191]|LOCRE458-10|Costa Rica|San Jose|658[0n]|BOLD:AAD0740
Spodoptera albula|[1192]|LOFLB202-06|United States|Florida|658[0n]|BOLD:AAD0740
Spodoptera albula|[1193]|LOFLB437-06|United States|Florida|658[0n]|BOLD:AAD0740
Spodoptera albula|[1194]|BBLOB1596-11|United States|Florida|658[0n]|BOLD:AAD0740
Spodoptera albula|[1195]|MHMXI510-07|Costa Rica|Alajuela|658[0n]|BOLD:AAD0740
Spodoptera albula|[1196]|BLPED1867-12|Costa Rica|Guanacaste|658[0n]|BOLD:AAD0740
Spodoptera albula|[1197]|MHMXI544-07|Costa Rica|Alajuela|658[0n]|BOLD:AAD0740
Spodoptera albula|[1198]|LOCRE491-10|Costa Rica|San Jose|658[0n]|BOLD:AAD0740
Spodoptera albula|[1199]|MHMYS2787-13|Costa Rica|Guanacaste|658[0n]|BOLD:AAD0740
Spodoptera albula|[1200]|BLPEF1672-12|Costa Rica|Guanacaste|658[0n]|BOLD:AAD0740

Spodoptera albula|[1197]|MHMXI544-07|Costa Rica|Alajuela|658[0n]|BOLD:AAD0740
Spodoptera albula|[1198]|LOCRE491-10|Costa Rica|San Jose|658[0n]|BOLD:AAD0740
Spodoptera albula|[1199]|MHMYS2787-13|Costa Rica|Guanacaste|658[0n]|BOLD:AAD0740
Spodoptera albula|[1200]|BLPEE1672-12|Costa Rica|Guanacaste|658[0n]|BOLD:AAD0740
Spodoptera albula|[1201]|GBGL12647-13|Brazil||658[0n]|BOLD:AAD0740
Spodoptera albula|[1202]|GBGL12648-13|Brazil||658[0n]|BOLD:AAD0740
Spodoptera albula|[1203]|GBGL12649-13|Brazil||658[0n]|BOLD:AAD0740
Spodoptera albula|[1204]|GBGL12650-13|Brazil||658[0n]|BOLD:AAD0740
Spodoptera albula|[1205]|GBGL12651-13|Brazil||658[0n]|BOLD:AAD0740
Spodoptera albula|[1206]|GBGL12652-13|Brazil||658[0n]|BOLD:AAD0740
Spodoptera albula|[1207]|GBGL12653-13|Brazil||658[0n]|BOLD:AAD0740
Spodoptera albula|[1208]|GBGL12654-13|Brazil||658[0n]|BOLD:AAD0740
Spodoptera albula|[1209]|GBGL12655-13|Brazil||658[0n]|BOLD:AAD0740
Spodoptera albula|[1210]|GBGL12656-13|Brazil||658[0n]|BOLD:AAD0740
Spodoptera albula|[1211]|GBGL12657-13|Brazil||658[0n]|BOLD:AAD0740
Spodoptera albula|[1212]|GBGL12658-13|Brazil||658[0n]|BOLD:AAD0740
Spodoptera albula|[1213]|GBGL12659-13|Brazil||658[0n]|BOLD:AAD0740
Spodoptera albula|[1214]|GBGL12660-13|Brazil||658[0n]|BOLD:AAD0740
Spodoptera albula|[1215]|GBGL12661-13|Brazil||658[0n]|BOLD:AAD0740
Spodoptera albula|[1216]|GBGL12662-13|Brazil||658[0n]|BOLD:AAD0740
Spodoptera albula|[1217]|GBGL12663-13|Brazil||658[0n]|BOLD:AAD0740
Spodoptera albula|[1218]|GBGL12664-13|Brazil||658[0n]|BOLD:AAD0740
Spodoptera albula|[1219]|BLPCA212-08|Costa Rica|Guanacaste|658[0n]|BOLD:AAD0740
Spodoptera albula|[1220]|MHMYS3110-13|Costa Rica|Guanacaste|658[0n]|BOLD:AAD0740
Spodoptera eridania|[1221]|MXBLP243-11|Mexico|Jalisco|523[1n]|BOLD:AAA6521
Spodoptera eridania|[1222]|BLPDC450-09|Costa Rica|Alajuela|614[0n]|BOLD:AAA6521
Spodoptera eridania|[1223]|GBGL10121-12|||771[0n]|BOLD:AAA6521
Spodoptera eridania|[1224]|GBGL10122-12|||771[0n]|BOLD:AAA6521
Spodoptera eridania|[1225]|MHMXI513-07|Costa Rica|Alajuela|658[0n]|BOLD:AAA6521
Spodoptera eridania|[1226]|MHMXI526-07|Costa Rica|Alajuela|658[0n]|BOLD:AAA6521
Spodoptera eridania|[1227]|MHMXI529-07|Costa Rica|Alajuela|658[0n]|BOLD:AAA6521
Spodoptera eridania|[1228]|MHMXI530-07|Costa Rica|Alajuela|658[0n]|BOLD:AAA6521
Spodoptera eridania|[1229]|MHMXI534-07|Costa Rica|Alajuela|658[0n]|BOLD:AAA6521
Spodoptera eridania|[1230]|MHMXI537-07|Costa Rica|Alajuela|658[0n]|BOLD:AAA6521
Spodoptera eridania|[1231]|MHMXI514-07|Costa Rica|Alajuela|658[0n]|BOLD:AAA6521
Spodoptera eridania|[1232]|MHMXI528-07|Costa Rica|Alajuela|658[0n]|BOLD:AAA6521
Spodoptera eridania|[1233]|MHMXI546-07|Costa Rica|Alajuela|658[0n]|BOLD:AAA6521
Spodoptera eridania|[1234]|GBGL10123-12|||771[0n]|BOLD:AAA6521
Spodoptera eridania|[1235]|GBGL10119-12|||771[0n]|BOLD:AAA6521
Spodoptera eridania|[1236]|LPOKA535-09|United States|Oklahoma|658[0n]|BOLD:AAA6521
Spodoptera eridania|[1237]|MHMYH830-10|Costa Rica||658[0n]|BOLD:AAA6521
Spodoptera eridania|[1238]|MHMYH831-10|Costa Rica||658[0n]|BOLD:AAA6521
Spodoptera eridania|[1239]|GBGL12635-13|Brazil|Goias|658[0n]|BOLD:AAA6521
Spodoptera eridania|[1240]|GBGL12636-13|Brazil|Goias|658[0n]|BOLD:AAA6521
Spodoptera eridania|[1241]|GBGL12637-13|Brazil|Goias|658[0n]|BOLD:AAA6521
Spodoptera eridania|[1242]|GBGL12638-13|Brazil|Goias|658[0n]|BOLD:AAA6521
Spodoptera eridania|[1243]|MOTAR137-12|Argentina|Formosa|658[0n]|BOLD:AAA6521
Spodoptera eridania|[1244]|GBGL12630-13|Brazil|Goias|658[0n]|BOLD:AAA6521
Spodoptera eridania|[1245]|MHMYH827-10|Costa Rica||658[0n]|BOLD:AAA6521
Spodoptera eridania|[1246]|MHMYH828-10|Costa Rica||658[0n]|BOLD:AAA6521
Spodoptera eridania|[1247]|GBGL12633-13|Brazil|Goias|658[0n]|BOLD:AAA6521
Spodoptera eridania|[1248]|GBGL12634-13|Brazil|Goias|658[0n]|BOLD:AAA6521
Spodoptera eridania|[1249]|GBGL12631-13|Brazil|Goias|658[0n]|BOLD:AAA6521
Spodoptera eridania|[1250]|GBGL12632-13|Brazil|Goias|658[0n]|BOLD:AAA6521
Spodoptera eridania|[1251]|GBGL12639-13|Brazil|Goias|658[0n]|BOLD:AAA6521
Spodoptera eridania|[1252]|GBGL10120-12|||771[0n]|BOLD:AAA6521
Spodoptera eridania|[1253]|MHAUB899-05|Costa Rica|Guanacaste|593[0n]|BOLD:AAA6521
Spodoptera eridania|[1254]|LEMMZ129-10|Brazil|Parana|658[0n]|BOLD:AAA6521
Spodoptera eridania|[1255]|MHAUB895-05|Costa Rica|Guanacaste|592[0n]|BOLD:AAA6521
Spodoptera eridania|[1256]|MHAUB898-05|Costa Rica|Guanacaste|596[0n]|BOLD:AAA6521
Spodoptera eridania|[1257]|ECPD091-14|Argentina|Buenos Aires|658[0n]|BOLD:AAA6521
Spodoptera eridania|[1258]|LNAUT1244-14|United States|South Carolina|658[0n]|BOLD:AAA6521
Spodoptera eridania|[1259]|ECPD078-14|Argentina|Buenos Aires|658[0n]|BOLD:AAA6521
Spodoptera eridania|[1260]|ECPD089-14|Argentina|Buenos Aires|658[0n]|BOLD:AAA6521
Spodoptera eridania|[1261]|GBGL12646-13|Brazil|Goias|658[0n]|BOLD:AAA6521
Spodoptera eridania|[1262]|ECPD076-14|Argentina|Buenos Aires|658[0n]|BOLD:AAA6521
Spodoptera eridania|[1263]|GBGL12645-13|Brazil|Goias|658[0n]|BOLD:AAA6521
Spodoptera eridania|[1264]|GBGL12640-13|Brazil|Goias|658[0n]|BOLD:AAA6521
Spodoptera eridania|[1265]|CSEB672-13|Ecuador||658[0n]|BOLD:AAA6521
Spodoptera eridania|[1266]|MHMYT328-13|Costa Rica||658[0n]|BOLD:AAA6521
Spodoptera eridania|[1267]|MOTAR153-12|Argentina|Misiones|658[0n]|BOLD:AAA6521
Spodoptera eridania|[1268]|MOTAR151-12|Argentina|Formosa|658[0n]|BOLD:AAA6521
Spodoptera eridania|[1269]|MHMYH829-10|Costa Rica||658[0n]|BOLD:AAA6521
Spodoptera eridania|[1270]|MHMYA1159-09|Costa Rica||658[0n]|BOLD:AAA6521
Spodoptera eridania|[1271]|BLPDA469-09|Costa Rica|Alajuela|658[0n]|BOLD:AAA6521
Spodoptera eridania|[1272]|BLPCK258-08|Costa Rica|Alajuela|658[0n]|BOLD:AAA6521
Spodoptera eridania|[1273]|MHMXM118-07|Costa Rica|Alajuela|658[0n]|BOLD:AAA6521
Spodoptera eridania|[1274]|MHMXI541-07|Costa Rica|Alajuela|658[0n]|BOLD:AAA6521
Spodoptera eridania|[1275]|BLPEC679-11|Costa Rica|Alajuela|658[0n]|BOLD:AAA6521
Spodoptera eridania|[1276]|GBGL12552-13|||1000[1n]|
Spodoptera eridania|[1277]|GBGL12550-13|||1000[1n]|
Spodoptera eridania|[1278]|GBGL12551-13|||925[1n]|
Spodoptera eridania|[1279]|GBGL12556-13|||455[0n]|
Spodoptera eridania|[1280]|GBGL12557-13|||1000[1n]|
Spodoptera eridania|[1281]|GBGL12549-13|||959[1n]|
Spodoptera eridania|[1282]|GBGL12555-13|||1000[1n]|
Spodoptera eridania|[1283]|GBGL12553-13|||1000[1n]|
Spodoptera eridania|[1284]|GBGL12554-13|||1000[1n]|
Spodoptera eridania|[1285]|GBGL12558-13|||1000[1n]|
Spodoptera eridania|[1286]|LNAUT1259-14|United States|Mississippi|658[0n]|BOLD:AAA6521
Spodoptera eridania|[1287]|LNAUT1248-14|United States|Texas|658[0n]|BOLD:AAA6521
Spodoptera eridania|[1288]|LNAUT1261-14|United States|Mississippi|658[0n]|BOLD:AAA6521
Spodoptera eridania|[1289]|MHAUB897-05|Costa Rica|Guanacaste|593[0n]|BOLD:AAA6521
Spodoptera eridania|[1290]|GWORH245-09|Brazil|Rio Grande do Sul|658[0n]|BOLD:AAA6521
Spodoptera eridania|[1291]|MHAUB896-05|Costa Rica|Guanacaste|593[0n]|BOLD:AAA6521
Spodoptera eridania|[1292]|MHMXI536-07|Costa Rica|Alajuela|658[0n]|BOLD:AAA6521
Spodoptera eridania|[1293]|MHMXI532-07|Costa Rica|Alajuela|658[0n]|BOLD:AAA6521
Spodoptera eridania|[1294]|MHMXI523-07|Costa Rica|Alajuela|658[0n]|BOLD:AAA6521
Spodoptera eridania|[1295]|MHMXI522-07|Costa Rica|Alajuela|658[0n]|BOLD:AAA6521
Spodoptera eridania|[1296]|MHMXI518-07|Costa Rica|Alajuela|658[0n]|BOLD:AAA6521
Spodoptera eridania|[1297]|MHMXI517-07|Costa Rica|Alajuela|658[0n]|BOLD:AAA6521
Spodoptera eridania|[1298]|MHMXI516-07|Costa Rica|Alajuela|658[0n]|BOLD:AAA6521
Spodoptera eridania|[1299]|MHMXI515-07|Costa Rica|Alajuela|658[0n]|BOLD:AAA6521
Spodoptera eridania|[1300]|MHMXI538-07|Costa Rica|Alajuela|655[0n]|BOLD:AAA6521

Spodoptera eridania|[1297]|MHMXI517-07|Costa Rica|Alajuela|658[0n]|BOLD:AAA6521
Spodoptera eridania|[1298]|MHMXI516-07|Costa Rica|Alajuela|658[0n]|BOLD:AAA6521
Spodoptera eridania|[1299]|MHMXI515-07|Costa Rica|Alajuela|658[0n]|BOLD:AAA6521
Spodoptera eridania|[1300]|MHMXI538-07|Costa Rica|Alajuela|655[0n]|BOLD:AAA6521
Spodoptera eridania|[1301]|MHMXI545-07|Costa Rica|Alajuela|658[0n]|BOLD:AAA6521
Spodoptera eridania|[1302]|MHMXI549-07|Costa Rica|Alajuela|658[0n]|BOLD:AAA6521
Spodoptera eridania|[1303]|MOTAR177-12|Argentina|Misiones|632[0n]|BOLD:AAA6521
Spodoptera eridania|[1304]|MHMXI219-07|Costa Rica|Guanacaste|659[0n]|BOLD:AAA6521
Spodoptera eridania|[1305]|MHMXI539-07|Costa Rica|Alajuela|658[0n]|BOLD:AAA6521
Spodoptera eridania|[1306]|MHMXI543-07|Costa Rica|Alajuela|658[0n]|BOLD:AAA6521
Spodoptera eridania|[1307]|MHMXI547-07|Costa Rica|Alajuela|658[0n]|BOLD:AAA6521
Spodoptera eridania|[1308]|MHMXI548-07|Costa Rica|Alajuela|658[0n]|BOLD:AAA6521
Spodoptera eridania|[1309]|MHMXM102-07|Costa Rica|Alajuela|658[0n]|BOLD:AAA6521
Spodoptera eridania|[1310]|MHMYA1158-09|Costa Rica||658[0n]|BOLD:AAA6521
Spodoptera eridania|[1311]|MHMYA1160-09|Costa Rica||658[0n]|BOLD:AAA6521
Spodoptera eridania|[1312]|GWOSU375-11|Peru|Huanuco|658[0n]|BOLD:AAA6521
Spodoptera eridania|[1313]|GBGL12641-13|Brazil|Goias|658[0n]|BOLD:AAA6521
Spodoptera eridania|[1314]|GBGL12642-13|Brazil|Goias|658[0n]|BOLD:AAA6521
Spodoptera eridania|[1315]|GBGL12643-13|Brazil|Goias|658[0n]|BOLD:AAA6521
Spodoptera eridania|[1316]|GBGL12644-13|Brazil|Goias|658[0n]|BOLD:AAA6521
Spodoptera eridania|[1317]|LNAUT1246-14|United States|Virginia|658[0n]|BOLD:AAA6521
Spodoptera eridania|[1318]|LNAUT1260-14|United States|Mississippi|658[0n]|BOLD:AAA6521
Spodoptera eridania|[1319]|MHMXI533-07|Costa Rica|Alajuela|658[0n]|BOLD:AAA6521
Spodoptera eridania|[1320]|MHMXI535-07|Costa Rica|Alajuela|658[0n]|BOLD:AAA6521
Spodoptera eridania|[1321]|MHMXI527-07|Costa Rica|Alajuela|658[0n]|BOLD:AAA6521
Spodoptera eridania|[1322]|MHMXI531-07|Costa Rica|Alajuela|658[0n]|BOLD:AAA6521
Spodoptera eridania|[1323]|MHMXI525-07|Costa Rica|Alajuela|658[0n]|BOLD:AAA6521
Spodoptera eridania|[1324]|MHMXI524-07|Costa Rica|Alajuela|658[0n]|BOLD:AAA6521
Spodoptera eridania|[1325]|MHMXI521-07|Costa Rica|Alajuela|658[0n]|BOLD:AAA6521
Spodoptera eridania|[1326]|MHMXI520-07|Costa Rica|Alajuela|658[0n]|BOLD:AAA6521
Spodoptera eridania|[1327]|MHMXI519-07|Costa Rica|Alajuela|658[0n]|BOLD:AAA6521
Spodoptera eridania|[1328]|MHMXI509-07|Costa Rica|Guanacaste|658[0n]|BOLD:AAA6521
Spodoptera eridania|[1329]|MHMXI508-07|Costa Rica|Guanacaste|658[0n]|BOLD:AAA6521
Spodoptera eridania|[1330]|MHMXI507-07|Costa Rica|Guanacaste|658[0n]|BOLD:AAA6521
Spodoptera eridania|[1331]|MHMXI506-07|Costa Rica|Guanacaste|658[0n]|BOLD:AAA6521
Spodoptera eridania|[1332]|MHMXI505-07|Costa Rica|Guanacaste|658[0n]|BOLD:AAA6521
Spodoptera eridania|[1333]|MHMXF638-07|Costa Rica|Guanacaste|658[0n]|BOLD:AAA6521
Spodoptera eridania|[1334]|MHMXI540-07|Costa Rica|Alajuela|656[0n]|BOLD:AAA6521
Spodoptera eridania|[1335]|LNAUT1262-14|United States|Mississippi|658[0n]|BOLD:AAA6521
Spodoptera eridania|[1336]|LNAUT1263-14|United States|Mississippi|658[0n]|BOLD:AAA6521
Spodoptera frugiperda sp. 1|[1337]|BBLOD526-11|United States|Texas|632[0n]|BOLD:ACE4783
Spodoptera frugiperda sp. 1|[1338]|MJMSL047-10|United States|Massachusetts|642[0n]|BOLD:ACE4783
Spodoptera frugiperda sp. 1|[1339]|LEMMZ132-10|Brazil|Parana|658[0n]|BOLD:ACE4783
Spodoptera frugiperda sp. 1|[1340]|LEMMZ133-10|Brazil|Parana|658[0n]|BOLD:ACE4783
Spodoptera frugiperda sp. 1|[1341]|LPOKA648-09|United States|Oklahoma|637[0n]|BOLD:ACE4783
Spodoptera frugiperda sp. 1|[1342]|RDNMD540-06|United States|Florida|658[1n]|BOLD:ACE4783
Spodoptera frugiperda sp. 1|[1343]|LTOL082-06|United States|Maryland|658[0n]|BOLD:ACE4783
Spodoptera frugiperda sp. 1|[1344]|LPOKA402-09|United States|Oklahoma|608[0n]|BOLD:ACE4783
Spodoptera frugiperda sp. 1|[1345]|MOTAR370-12|Argentina|Entre Rios|658[0n]|BOLD:ACE4783
Spodoptera frugiperda sp. 1|[1346]|MOTAR030-12|Argentina|Entre Rios|658[0n]|BOLD:ACE4783
Spodoptera frugiperda sp. 1|[1347]|BBLOD514-11|United States|Texas|658[0n]|BOLD:ACE4783
Spodoptera frugiperda sp. 1|[1348]|BBLOD185-11|United States|Texas|658[0n]|BOLD:ACE4783
Spodoptera frugiperda sp. 1|[1349]|BBLOD088-11|United States|Texas|658[0n]|BOLD:ACE4783
Spodoptera frugiperda sp. 1|[1350]|BBLOC1564-11|United States|Texas|658[0n]|BOLD:ACE4783
Spodoptera frugiperda sp. 1|[1351]|BBLOC1435-11|United States|Texas|658[0n]|BOLD:ACE4783
Spodoptera frugiperda sp. 1|[1352]|BBLOC905-11|United States|Arkansas|658[0n]|BOLD:ACE4783
Spodoptera frugiperda sp. 1|[1353]|GWOST420-11|Dominican Republic||658[0n]|BOLD:ACE4783
Spodoptera frugiperda sp. 1|[1354]|GWOST419-11|Dominican Republic||658[0n]|BOLD:ACE4783
Spodoptera frugiperda sp. 1|[1355]|GWOST418-11|Argentina||658[0n]|BOLD:ACE4783
Spodoptera frugiperda sp. 1|[1356]|LILLA993-11|United States|Illinois|658[0n]|BOLD:ACE4783
Spodoptera frugiperda sp. 1|[1357]|LILLA892-11|United States|Illinois|658[0n]|BOLD:ACE4783
Spodoptera frugiperda sp. 1|[1358]|LILLA880-11|United States|Illinois|658[0n]|BOLD:ACE4783
Spodoptera frugiperda sp. 1|[1359]|MILEP713-11|French Guiana||658[0n]|BOLD:ACE4783
Spodoptera frugiperda sp. 1|[1360]|LPOKE209-10|United States|Oklahoma|658[0n]|BOLD:ACE4783
Spodoptera frugiperda sp. 1|[1361]|LEMMZ131-10|Brazil|Parana|658[0n]|BOLD:ACE4783
Spodoptera frugiperda sp. 1|[1362]|LEMMZ130-10|Brazil|Parana|658[0n]|BOLD:ACE4783
Spodoptera frugiperda sp. 1|[1363]|LEMMZ128-10|Brazil|Parana|658[0n]|BOLD:ACE4783
Spodoptera frugiperda sp. 1|[1364]|LEMMZ127-10|Brazil|Parana|658[0n]|BOLD:ACE4783
Spodoptera frugiperda sp. 1|[1365]|MJMSL151-10|United States|Massachusetts|658[0n]|BOLD:ACE4783
Spodoptera frugiperda sp. 1|[1366]|MJMSL150-10|United States|Massachusetts|658[0n]|BOLD:ACE4783
Spodoptera frugiperda sp. 1|[1367]|MJMSL149-10|United States|Massachusetts|658[0n]|BOLD:ACE4783
Spodoptera frugiperda sp. 1|[1368]|MJMSL099-10|United States|Massachusetts|658[0n]|BOLD:ACE4783
Spodoptera frugiperda sp. 1|[1369]|MJMSL098-10|United States|Massachusetts|658[0n]|BOLD:ACE4783
Spodoptera frugiperda sp. 1|[1370]|MJMSL097-10|United States|Massachusetts|658[0n]|BOLD:ACE4783
Spodoptera frugiperda sp. 1|[1371]|MJMSL096-10|United States|Massachusetts|658[0n]|BOLD:ACE4783
Spodoptera frugiperda sp. 1|[1372]|MJMSL095-10|United States|Massachusetts|658[0n]|BOLD:ACE4783
Spodoptera frugiperda sp. 1|[1373]|MJMSL048-10|United States|Massachusetts|658[0n]|BOLD:ACE4783
Spodoptera frugiperda sp. 1|[1374]|MJMSL003-10|United States|Massachusetts|658[0n]|BOLD:ACE4783
Spodoptera frugiperda sp. 1|[1375]|MJMSL002-10|United States|Massachusetts|658[0n]|BOLD:ACE4783
Spodoptera frugiperda sp. 1|[1376]|MJMSL001-10|United States|Massachusetts|658[0n]|BOLD:ACE4783
Spodoptera frugiperda sp. 1|[1377]|LGSMG1002-10|United States|North Carolina|658[0n]|BOLD:ACE4783
Spodoptera frugiperda sp. 1|[1378]|LPOKD100-09|United States|Oklahoma|658[0n]|BOLD:ACE4783
Spodoptera frugiperda sp. 1|[1379]|BBLSW809-09|United States|Oklahoma|658[0n]|BOLD:ACE4783
Spodoptera frugiperda sp. 1|[1380]|LPOKA1041-09|United States|Oklahoma|658[0n]|BOLD:ACE4783
Spodoptera frugiperda sp. 1|[1381]|LPOKA662-09|United States|Oklahoma|658[0n]|BOLD:ACE4783
Spodoptera frugiperda sp. 1|[1382]|LPOKA530-09|United States|Oklahoma|658[0n]|BOLD:ACE4783
Spodoptera frugiperda sp. 1|[1383]|LPOKA523-09|United States|Oklahoma|658[0n]|BOLD:ACE4783
Spodoptera frugiperda sp. 1|[1384]|LPOKA520-09|United States|Oklahoma|658[0n]|BOLD:ACE4783
Spodoptera frugiperda sp. 1|[1385]|LPOKA505-09|United States|Oklahoma|658[0n]|BOLD:ACE4783
Spodoptera frugiperda sp. 1|[1386]|LPOKA491-09|United States|Oklahoma|658[0n]|BOLD:ACE4783
Spodoptera frugiperda sp. 1|[1387]|LPOKA470-09|United States|Oklahoma|658[0n]|BOLD:ACE4783
Spodoptera frugiperda sp. 1|[1388]|LPOKA406-09|United States|Oklahoma|658[0n]|BOLD:ACE4783
Spodoptera frugiperda sp. 1|[1389]|LPOKA393-09|United States|Oklahoma|658[0n]|BOLD:ACE4783
Spodoptera frugiperda sp. 1|[1390]|LPOKA357-08|United States|Oklahoma|658[0n]|BOLD:ACE4783
Spodoptera frugiperda sp. 1|[1391]|LPOKA353-08|United States|Oklahoma|658[0n]|BOLD:ACE4783
Spodoptera frugiperda sp. 1|[1392]|LPOKA259-08|United States|Oklahoma|658[0n]|BOLD:ACE4783
Spodoptera frugiperda sp. 1|[1393]|LPOKA103-08|United States|Oklahoma|658[0n]|BOLD:ACE4783
Spodoptera frugiperda sp. 1|[1394]|RDNMD541-06|United States|Florida|658[0n]|BOLD:ACE4783
Spodoptera frugiperda sp. 1|[1395]|LNC851-06|United States|North Carolina|658[0n]|BOLD:ACE4783
Spodoptera frugiperda sp. 1|[1396]|MNBB563-05|Canada|New Brunswick|658[0n]|BOLD:ACE4783
Spodoptera frugiperda sp. 1|[1397]|XAD239-04|Canada|Ontario|658[0n]|BOLD:ACE4783
Spodoptera frugiperda sp. 1|[1398]|LILLA677-11|United States|Illinois|658[0n]|BOLD:ACE4783
Spodoptera frugiperda sp. 1|[1399]|BBLOB697-11|United States|Texas|655[0n]|BOLD:ACE4783
Spodoptera frugiperda sp. 1|[1400]|GBGL3730-06||684[0n]|BOLD:ACE4783

Spodoptera frugiperda sp. 1|[1397]|XAD239-04|Canada|Ontario|658[0n]|BOLD:ACE4783
Spodoptera frugiperda sp. 1|[1398]|LILLA677-11|United States|Illinois|658[0n]|BOLD:ACE4783
Spodoptera frugiperda sp. 1|[1399]|BBLOB697-11|United States|Texas|655[0n]|BOLD:ACE4783
Spodoptera frugiperda sp. 1|[1400]|GBGL3730-06|||684[0n]|BOLD:ACE4783
Spodoptera frugiperda sp. 1|[1401]|XAD265-04|Canada|Ontario|594[0n]|BOLD:ACE4783
Spodoptera frugiperda sp. 1|[1402]|PHMO358-03|Canada|Ontario|638[5n]|BOLD:ACE4783
Spodoptera frugiperda sp. 1|[1403]|RDLQ494-07|Canada|Quebec|594[0n]|BOLD:ACE4783
Spodoptera frugiperda sp. 1|[1404]|GBGL10077-12|||938[0n]|BOLD:ACE4783
Spodoptera frugiperda sp. 1|[1405]|LPOKD615-09|United States|Oklahoma|639[0n]|BOLD:ACE4783
Spodoptera frugiperda sp. 1|[1406]|LPOKA1039-09|United States|Oklahoma|630[0n]|BOLD:ACE4783
Spodoptera frugiperda sp. 1|[1407]|LILLA910-11|United States|Illinois|622[0n]|BOLD:ACE4783
Spodoptera frugiperda sp. 1|[1408]|BBLOD182-11|United States|Texas|629[0n]|BOLD:ACE4783
Spodoptera frugiperda sp. 1|[1409]|GBGL10076-12|||938[0n]|BOLD:ACE4783
Spodoptera frugiperda sp. 1|[1410]|GBGL10079-12|||938[0n]|BOLD:ACE4783
Spodoptera frugiperda sp. 1|[1411]|GBGL10080-12|||938[0n]|BOLD:ACE4783
Spodoptera frugiperda sp. 1|[1412]|GBGL10081-12|||938[0n]|BOLD:ACE4783
Spodoptera frugiperda sp. 2|[1413]|XAD490-04|Canada|Ontario|557[0n]|BOLD:AAA4532
Spodoptera frugiperda sp. 2|[1414]|GBGL10071-12|||938[0n]|BOLD:AAA4532
Spodoptera frugiperda sp. 2|[1415]|LPOKA533-09|United States|Oklahoma|621[0n]|BOLD:AAA4532
Spodoptera frugiperda sp. 2|[1416]|GBGL3728-06|||684[0n]|BOLD:AAA4532
Spodoptera frugiperda sp. 2|[1417]|LPOKA531-09|United States|Oklahoma|636[0n]|BOLD:AAA4532
Spodoptera frugiperda sp. 2|[1418]|RDLQB738-05|Canada|Quebec|593[0n]|BOLD:AAA4532
Spodoptera frugiperda sp. 2|[1419]|GBGL3729-06|||684[0n]|BOLD:AAA4532
Spodoptera frugiperda sp. 2|[1420]|GBGL5879-09|||696[0n]|BOLD:AAA4532
Spodoptera frugiperda sp. 2|[1421]|CMAZA278-09|United States|Arizona|658[0n]|BOLD:AAA4532
Spodoptera frugiperda sp. 2|[1422]|MJMSL196-10|United States|Massachusetts|658[0n]|BOLD:AAA4532
Spodoptera frugiperda sp. 2|[1423]|LPOKA153-08|United States|Oklahoma|658[0n]|BOLD:AAA4532
Spodoptera frugiperda sp. 2|[1424]|LPOKA287-08|United States|Oklahoma|658[0n]|BOLD:AAA4532
Spodoptera frugiperda sp. 2|[1425]|LYHES537-09|Mexico|Quintana Roo|658[0n]|BOLD:AAA4532
Spodoptera frugiperda sp. 2|[1426]|LYRIO470-10|Mexico|Yucatan|658[0n]|BOLD:AAA4532
Spodoptera frugiperda sp. 2|[1427]|GBGL10074-12|||938[0n]|BOLD:AAA4532
Spodoptera frugiperda sp. 2|[1428]|GBGL10075-12|||938[0n]|BOLD:AAA4532
Spodoptera frugiperda sp. 2|[1429]|GBGL10069-12|||938[0n]|BOLD:AAA4532
Spodoptera frugiperda sp. 2|[1430]|GBGL10070-12|||938[0n]|BOLD:AAA4532
Spodoptera frugiperda sp. 2|[1431]|LGSMG1005-10|United States|North Carolina|658[0n]|BOLD:AAA4532
Spodoptera frugiperda sp. 2|[1432]|GBGL10072-12|||938[0n]|BOLD:AAA4532
Spodoptera frugiperda sp. 2|[1433]|LPOKA579-09|United States|Oklahoma|610[0n]|BOLD:AAA4532
Spodoptera frugiperda sp. 2|[1434]|GBGL10073-12|||938[0n]|BOLD:AAA4532
Spodoptera frugiperda sp. 2|[1435]|LPOKA564-09|United States|Oklahoma|637[0n]|BOLD:AAA4532
Spodoptera frugiperda sp. 2|[1436]|CMAZA277-09|United States|Arizona|658[0n]|BOLD:AAA4532
Spodoptera frugiperda sp. 2|[1437]|GWOSZ763-11|Ecuador||658[0n]|BOLD:AAA4532
Spodoptera frugiperda sp. 2|[1438]|BBLOE1983-12|United States|Texas|658[0n]|BOLD:AAA4532
Spodoptera frugiperda sp. 2|[1439]|LGSMG1003-10|United States|North Carolina|658[1n]|BOLD:AAA4532
Spodoptera frugiperda sp. 2|[1440]|AWCLB505-11|United States|Arizona|658[0n]|BOLD:AAA4532
Spodoptera frugiperda sp. 2|[1441]|LEMMZ134-10|Brazil|Parana|658[0n]|BOLD:AAA4532
Spodoptera frugiperda sp. 2|[1442]|USLEP1223-10|United States|Arizona|658[0n]|BOLD:AAA4532
Spodoptera frugiperda sp. 2|[1443]|LPOKD179-09|United States|Oklahoma|658[0n]|BOLD:AAA4532
Spodoptera frugiperda sp. 2|[1444]|LYNYM309-09|Mexico|Quintana Roo|658[0n]|BOLD:AAA4532
Spodoptera frugiperda sp. 2|[1445]|LPOKA1036-09|United States|Oklahoma|658[0n]|BOLD:AAA4532
Spodoptera frugiperda sp. 2|[1446]|LPOKA432-09|United States|Oklahoma|658[0n]|BOLD:AAA4532
Spodoptera frugiperda sp. 2|[1447]|LPOKA320-08|United States|Oklahoma|658[0n]|BOLD:AAA4532
Spodoptera frugiperda sp. 2|[1448]|LPOKA299-08|United States|Oklahoma|658[0n]|BOLD:AAA4532
Spodoptera frugiperda sp. 2|[1449]|LPOKA283-08|United States|Oklahoma|658[0n]|BOLD:AAA4532
Spodoptera frugiperda sp. 2|[1450]|TZBCA211-06|Canada|Ontario|658[0n]|BOLD:AAA4532
Spodoptera frugiperda sp. 2|[1451]|TZBCA210-06|Canada|Ontario|658[0n]|BOLD:AAA4532
Spodoptera frugiperda sp. 2|[1452]|LOCBD958-06|United States|California|658[0n]|BOLD:AAA4532
Spodoptera frugiperda sp. 2|[1453]|RDLQB505-05|Canada|Quebec|658[0n]|BOLD:AAA4532
Spodoptera frugiperda sp. 2|[1454]|XAH550-05|Canada|Ontario|658[0n]|BOLD:AAA4532
Spodoptera frugiperda sp. 2|[1455]|XAH548-05|Canada|Ontario|658[0n]|BOLD:AAA4532
Spodoptera frugiperda sp. 2|[1456]|XAD236-04|Canada|Ontario|658[0n]|BOLD:AAA4532
Spodoptera frugiperda sp. 2|[1457]|NAGEO074-09|United States|Maryland|658[0n]|
Spodoptera frugiperda sp. 2|[1458]|LYRIO468-10|Mexico|Yucatan|658[0n]|BOLD:AAA4532
Spodoptera frugiperda sp. 2|[1459]|CMAZA061-09|United States|Arizona|650[0n]|BOLD:AAA4532
Spodoptera frugiperda sp. 2|[1460]|GBGL3727-06|||684[0n]|BOLD:AAA4532
Spodoptera frugiperda sp. 2|[1461]|PHMO299-03|Canada|Ontario|639[0n]|BOLD:AAA4532
Spodoptera frugiperda sp. 2|[1462]|PHMO305-03|Canada|Ontario|639[0n]|BOLD:AAA4532
Spodoptera frugiperda sp. 2|[1463]|XAD509-04|Canada|Ontario|617[0n]|BOLD:AAA4532
Spodoptera frugiperda sp. 2|[1464]|PHJUL020-11|Canada|Ontario|616[0n]|BOLD:AAA4532
Spodoptera frugiperda sp. 2|[1465]|PHJUL079-11|Canada|Ontario|607[0n]|BOLD:AAA4532
Spodoptera frugiperda sp. 2|[1466]|IAWLB193-11|United States|Arizona|658[0n]|BOLD:AAA4532
Spodoptera frugiperda sp. 2|[1467]|IAWLB282-11|United States|Arizona|658[0n]|BOLD:AAA4532
Spodoptera frugiperda sp. 2|[1468]|BBLOD130-11|United States|California|658[0n]|BOLD:AAA4532
Spodoptera frugiperda sp. 2|[1469]|BBLOD188-11|United States|Texas|658[0n]|BOLD:AAA4532
Spodoptera frugiperda sp. 2|[1470]|BBLOD509-11|United States|Texas|658[0n]|BOLD:AAA4532
Spodoptera frugiperda sp. 2|[1471]|MOTAR347-12|Argentina|Entre Rios|658[0n]|BOLD:AAA4532
Spodoptera frugiperda sp. 2|[1472]|ITLP234-13|United States||658[0n]|BOLD:AAA4532
Spodoptera ornithogalli|[1473]|LOCBF3740-14|United States|California|603[1n]|BOLD:ABY9250
Spodoptera ornithogalli|[1474]|LPOKB359-09|United States|Oklahoma|658[0n]|BOLD:ABY9250
Spodoptera ornithogalli|[1475]|GWOST424-11|Dominican Republic||658[1n]|BOLD:ABY9250
Spodoptera ornithogalli|[1476]|LOT326-04|United States|Tennessee|658[0n]|BOLD:ABY9250
Spodoptera ornithogalli|[1477]|CNPPB2269-12|Canada|Ontario|632[0n]|BOLD:ABY9250
Spodoptera ornithogalli|[1478]|BBLWU305-09|United States|Oklahoma|641[0n]|BOLD:ABY9250
Spodoptera ornithogalli|[1479]|LGSMG1007-10|United States|North Carolina|658[0n]|BOLD:ABY9250
Spodoptera ornithogalli|[1480]|XAB643-04|Canada|Ontario|658[4n]|BOLD:ABY9250
Spodoptera ornithogalli|[1481]|RDLQ498-07|Canada|Quebec|592[0n]|BOLD:ABY9250
Spodoptera ornithogalli|[1482]|UDLEP088-09|United States|Maryland|658[0n]|BOLD:ABY9250
Spodoptera ornithogalli|[1483]|LPOKA562-09|United States|Oklahoma|658[0n]|BOLD:ABY9250
Spodoptera ornithogalli|[1484]|LPOKA556-09|United States|Oklahoma|658[0n]|BOLD:ABY9250
Spodoptera ornithogalli|[1485]|LPOKA433-09|United States|Oklahoma|658[0n]|BOLD:ABY9250
Spodoptera ornithogalli|[1486]|LPOKA329-08|United States|Oklahoma|658[0n]|BOLD:ABY9250
Spodoptera ornithogalli|[1487]|LGSME168-06|United States|Tennessee|658[0n]|BOLD:ABY9250
Spodoptera ornithogalli|[1488]|LGSMG1008-10|United States|North Carolina|585[0n]|BOLD:ABY9250
Spodoptera ornithogalli|[1489]|LPOKA220-08|United States|Oklahoma|658[0n]|BOLD:ABY9250
Spodoptera ornithogalli|[1490]|LOT327-04|United States|Tennessee|658[0n]|BOLD:ABY9250
Spodoptera ornithogalli|[1491]|LGSM429-04|United States|Tennessee|658[0n]|BOLD:ABY9250
Spodoptera ornithogalli|[1492]|LGSM428-04|United States|Tennessee|658[0n]|BOLD:ABY9250
Spodoptera ornithogalli|[1493]|BBLOC1412-11|United States|Texas|658[0n]|BOLD:ABY9250
Spodoptera ornithogalli|[1494]|MOTAR117-12|Argentina|Formosa|658[0n]|BOLD:ABY9250
Spodoptera ornithogalli|[1495]|LPOKA907-09|United States|Oklahoma|658[0n]|BOLD:ABY9250
Spodoptera ornithogalli|[1496]|LPOKA926-09|United States|Oklahoma|658[0n]|BOLD:ABY9250
Spodoptera ornithogalli|[1497]|BBLSX117-09|United States|Oklahoma|658[0n]|BOLD:ABY9250
Spodoptera ornithogalli|[1498]|BBLSX697-09|United States|Texas|658[0n]|BOLD:ABY9250
Spodoptera ornithogalli|[1499]|LGSMG1004-10|United States|North Carolina|658[0n]|BOLD:ABY9250
[illegible]

Spodoptera ornithogalli|[1497]|BBLSX117-09|United States|Oklahoma|658[0n]|BOLD:ABY9250
Spodoptera ornithogalli|[1498]|BBLSX697-09|United States|Texas|658[0n]|BOLD:ABY9250
Spodoptera ornithogalli|[1499]|LGSMG1004-10|United States|North Carolina|658[0n]|BOLD:ABY9250
Spodoptera ornithogalli|[1500]|LGSMG1006-10|United States|North Carolina|658[0n]|BOLD:ABY9250
Spodoptera ornithogalli|[1501]|MJMSL004-10|United States|Massachusetts|658[0n]|BOLD:ABY9250
Spodoptera ornithogalli|[1502]|LILLA135-11|United States|Illinois|658[0n]|BOLD:ABY9250
Spodoptera ornithogalli|[1503]|LILLA601-11|United States|Illinois|658[0n]|BOLD:ABY9250
Spodoptera ornithogalli|[1504]|BBLOC911-11|United States|Arkansas|658[0n]|BOLD:ABY9250
Spodoptera ornithogalli|[1505]|BBLOD1282-11|United States|Texas|658[0n]|BOLD:ABY9250
Spodoptera ornithogalli|[1506]|CMAZA1033-12|United States|Arizona|658[0n]|BOLD:ABY9250
Spodoptera ornithogalli|[1507]|CMAZA1046-12|United States|Arizona|658[0n]|BOLD:ABY9250
Spodoptera ornithogalli|[1508]|CNCLB1266-14|United States|Maryland|658[0n]|BOLD:ABY9250
Spodoptera praefica|[1509]|RDNMF643-08|United States|California|609[0n]|BOLD:ACE4784
Spodoptera praefica|[1510]|BBLOD964-11|United States|Texas|658[0n]|BOLD:ACE4784
Spodoptera praefica|[1511]|BBLOC1216-11|United States|California|658[0n]|BOLD:ACE4784
Spodoptera praefica|[1512]|RDNMF642-08|United States|California|658[0n]|BOLD:ACE4784
Spodoptera praefica|[1513]|NAMUM208-08|United States|California|646[0n]|BOLD:ACE4784
Spodoptera praefica|[1514]|RDNMF644-08|Canada|Alberta|658[1n]|BOLD:ACE4784
Spodoptera praefica|[1515]|RDNMF645-08|United States|California|658[1n]|BOLD:ACE4784
Spodoptera praefica|[1516]|LBCH5807-10|Canada|British Columbia|658[0n]|BOLD:ACE4784
Spodoptera praefica|[1517]|JMMMB318-11|United States|California|658[0n]|BOLD:ACE4784
Spodoptera praefica|[1518]|GMLC613-11|United States|California|658[0n]|BOLD:ACE4784
Spodoptera praefica|[1519]|GMLC627-11|United States|California|658[0n]|BOLD:ACE4784
Spodoptera praefica|[1520]|GMLC642-11|United States|California|658[0n]|BOLD:ACE4784
Spodoptera praefica|[1521]|GMLC651-11|United States|California|658[0n]|BOLD:ACE4784
Spodoptera praefica|[1522]|BBLOD946-11|United States|Texas|658[0n]|BOLD:ACE4784
Spodoptera praefica|[1523]|BBLOD947-11|United States|Texas|658[0n]|BOLD:ACE4784
Spodoptera praefica|[1524]|BBLOD954-11|United States|Texas|658[0n]|BOLD:ACE4784
Spodoptera praefica|[1525]|GMLC893-12|United States|California|658[0n]|BOLD:ACE4784
Spodoptera praefica|[1526]|GMLC897-12|United States|California|658[0n]|BOLD:ACE4784
Spodoptera praefica|[1527]|GMLC973-12|United States|California|658[0n]|BOLD:ACE4784
Spodoptera praefica|[1528]|GMLC1328-12|United States|California|614[0n]|BOLD:ACE4784
Spodoptera dolichos|[1529]|GWOST421-11|Cuba|Holguin|658[0n]|BOLD:ABY5226
Spodoptera dolichos|[1530]|BLPBB293-07|Costa Rica|Guanacaste|510[0n]|BOLD:ABY5226
Spodoptera dolichos|[1531]|BLPDV1041-11|Costa Rica|Guanacaste|658[0n]|BOLD:ABY5226
Spodoptera dolichos|[1532]|GBGL10124-12|||771[0n]|BOLD:ABY5226
Spodoptera dolichos|[1533]|GBGL10125-12|||771[0n]|BOLD:ABY5226
Spodoptera dolichos|[1534]|BLPAH330-07|Costa Rica|Guanacaste|658[0n]|BOLD:ABY5226
Spodoptera dolichos|[1535]|BLPAB239-06|Costa Rica|Alajuela|658[0n]|BOLD:ABY5226
Spodoptera dolichos|[1536]|BLPAA926-06|Costa Rica|Alajuela|658[0n]|BOLD:ABY5226
Spodoptera dolichos|[1537]|MHAUB915-05|Costa Rica|Guanacaste|594[0n]|BOLD:ABY5226
Spodoptera dolichos|[1538]|BLPBH662-07|Costa Rica|Guanacaste|634[0n]|BOLD:ABY5226
Spodoptera dolichos|[1539]|BLPCD472-08|Costa Rica|Guanacaste|658[0n]|BOLD:ABY5226
Spodoptera dolichos|[1540]|LEMMZ124-10|Brazil|Parana|658[0n]|BOLD:ABY5226
Spodoptera dolichos|[1541]|MHMYO321-11|Costa Rica||658[0n]|BOLD:ABY5226
Spodoptera dolichos|[1542]|GBGL10126-12|||771[0n]|BOLD:ABY5226
Spodoptera dolichos|[1543]|GWOST422-11|Dominican Republic||630[0n]|BOLD:ABY5226
Spodoptera dolichos|[1544]|MHMXO341-08|Costa Rica|Guanacaste|629[0n]|BOLD:ABY5226
Spodoptera dolichos|[1545]|BLPAF454-07|Costa Rica|Guanacaste|658[0n]|BOLD:ABY5226
Spodoptera dolichos|[1546]|MHAUB913-05|Costa Rica|Alajuela|594[0n]|BOLD:ABY5226
Spodoptera dolichos|[1547]|LGSME061-06|United States|Tennessee|658[0n]|BOLD:ABY5226
Spodoptera dolichos|[1548]|LGSME062-06|United States|Tennessee|658[0n]|BOLD:ABY5226
Spodoptera dolichos|[1549]|BLPBG173-07|Costa Rica|Guanacaste|658[0n]|BOLD:ABY5226
Spodoptera dolichos|[1550]|BLPDV1039-11|Costa Rica|Guanacaste|658[0n]|BOLD:ABY5226
Spodoptera dolichos|[1551]|BLPAF456-07|Costa Rica|Guanacaste|658[0n]|BOLD:ABY5226
Spodoptera dolichos|[1552]|BLPAB037-06|Costa Rica|Alajuela|658[0n]|BOLD:ABY5226
Spodoptera dolichos|[1553]|LPOKA388-09|United States|Oklahoma|578[0n]|BOLD:ABY5226
Spodoptera dolichos|[1554]|GBGL10127-12|||771[0n]|BOLD:ABY5226
Spodoptera dolichos|[1555]|BLPAH331-07|Costa Rica|Guanacaste|658[0n]|BOLD:ABY5226
Spodoptera dolichos|[1556]|BLPAG030-07|Costa Rica|Guanacaste|658[0n]|BOLD:ABY5226
Spodoptera dolichos|[1557]|BLPAF455-07|Costa Rica|Guanacaste|658[0n]|BOLD:ABY5226
Spodoptera dolichos|[1558]|BLPAD217-06|Costa Rica|Guanacaste|658[0n]|BOLD:ABY5226
Spodoptera dolichos|[1559]|BLPAC328-06|Costa Rica|Guanacaste|658[0n]|BOLD:ABY5226
Spodoptera dolichos|[1560]|BLPAB276-06|Costa Rica|Alajuela|658[0n]|BOLD:ABY5226
Spodoptera dolichos|[1561]|BLPAB275-06|Costa Rica|Alajuela|658[0n]|BOLD:ABY5226
Spodoptera dolichos|[1562]|BLPAB238-06|Costa Rica|Alajuela|658[0n]|BOLD:ABY5226
Spodoptera dolichos|[1563]|BLPAA927-06|Costa Rica|Alajuela|658[0n]|BOLD:ABY5226
Spodoptera dolichos|[1564]|BLPAA820-06|Costa Rica|Alajuela|658[0n]|BOLD:ABY5226
Spodoptera dolichos|[1565]|MHMXA733-06|Costa Rica|Alajuela|658[0n]|BOLD:ABY5226
Spodoptera dolichos|[1566]|MHAUF369-06|Costa Rica|Guanacaste|658[0n]|BOLD:ABY5226
Spodoptera dolichos|[1567]|BLPAG055-07|Costa Rica|Guanacaste|658[0n]|BOLD:ABY5226
Spodoptera dolichos|[1568]|MHAUC210-06|Costa Rica|Alajuela|658[0n]|BOLD:ABY5226
Spodoptera dolichos|[1569]|MHAUB910-05|Costa Rica|Guanacaste|589[0n]|BOLD:ABY5226
Spodoptera dolichos|[1570]|MHAUB911-05|Costa Rica|Guanacaste|599[0n]|BOLD:ABY5226
Spodoptera dolichos|[1571]|MHAUB914-05|Costa Rica|Alajuela|603[0n]|BOLD:ABY5226
Spodoptera dolichos|[1572]|MHAUB912-05|Costa Rica|Alajuela|603[0n]|BOLD:ABY5226
Spodoptera dolichos|[1573]|BLPBH664-07|Costa Rica|Guanacaste|634[0n]|BOLD:ABY5226
Spodoptera dolichos|[1574]|MHMXM111-07|Costa Rica|Alajuela|631[0n]|BOLD:ABY5226
Spodoptera dolichos|[1575]|MHMXO338-08|Costa Rica|Guanacaste|658[0n]|BOLD:ABY5226
Spodoptera dolichos|[1576]|MHMXO340-08|Costa Rica|Guanacaste|655[0n]|BOLD:ABY5226
Spodoptera dolichos|[1577]|MHMXO342-08|Costa Rica|Guanacaste|658[0n]|BOLD:ABY5226
Spodoptera dolichos|[1578]|MHMXO344-08|Costa Rica|Guanacaste|658[0n]|BOLD:ABY5226
Spodoptera dolichos|[1579]|MHMXQ639-08|Costa Rica|Guanacaste|658[0n]|BOLD:ABY5226
Spodoptera dolichos|[1580]|BLPCD470-08|Costa Rica|Guanacaste|658[0n]|BOLD:ABY5226
Spodoptera dolichos|[1581]|BLPCD473-08|Costa Rica|Guanacaste|658[0n]|BOLD:ABY5226
Spodoptera dolichos|[1582]|BLPCD476-08|Costa Rica|Guanacaste|658[0n]|BOLD:ABY5226
Spodoptera dolichos|[1583]|BLPCD478-08|Costa Rica|Guanacaste|658[0n]|BOLD:ABY5226
Spodoptera dolichos|[1584]|BLPCH049-08|Costa Rica|Guanacaste|658[0n]|BOLD:ABY5226
Spodoptera dolichos|[1585]|BLPCJ098-08|Costa Rica|Guanacaste|658[0n]|BOLD:ABY5226
Spodoptera dolichos|[1586]|BLPCI619-08|Costa Rica|Guanacaste|658[0n]|BOLD:ABY5226
Spodoptera dolichos|[1587]|BLPCK133-08|Costa Rica|Alajuela|658[0n]|BOLD:ABY5226
Spodoptera dolichos|[1588]|BLPCO135-08|Costa Rica|Guanacaste|658[0n]|BOLD:ABY5226
Spodoptera dolichos|[1589]|BLPCP113-08|Costa Rica|Guanacaste|658[0n]|BOLD:ABY5226
Spodoptera dolichos|[1590]|BLPDB841-09|Costa Rica|Guanacaste|658[0n]|BOLD:ABY5226
Spodoptera dolichos|[1591]|BLPDC205-09|Costa Rica|Guanacaste|658[0n]|BOLD:ABY5226
Spodoptera dolichos|[1592]|BLPDH180-09|Costa Rica|Guanacaste|658[0n]|BOLD:ABY5226
Spodoptera dolichos|[1593]|MHMXZ666-09|Costa Rica||658[0n]|BOLD:ABY5226
Spodoptera dolichos|[1594]|MHMXZ667-09|Costa Rica||658[0n]|BOLD:ABY5226
Spodoptera dolichos|[1595]|MHMYC2259-09|Costa Rica|Alajuela|658[0n]|BOLD:ABY5226
Spodoptera dolichos|[1596]|BLPDK1928-09|Costa Rica|Guanacaste|658[0n]|BOLD:ABY5226
Spodoptera dolichos|[1597]|INCTA724-10|Brazil|Para|658[0n]|BOLD:ABY5226
Spodoptera dolichos|[1598]|BLPDT1400-10|Costa Rica|Guanacaste|658[0n]|BOLD:ABY5226
Spodoptera dolichos|[1599]|BLPDT1684-10|Costa Rica|Guanacaste|658[0n]|BOLD:ABY5226
[illegible]

Spodoptera dolichos|[1597]|INCTA724-10|Brazil|Para|658[0n]|BOLD:ABY5226
Spodoptera dolichos|[1598]|BLPDT1400-10|Costa Rica|Guanacaste|658[0n]|BOLD:ABY5226
Spodoptera dolichos|[1599]|BLPDT1684-10|Costa Rica|Guanacaste|658[0n]|BOLD:ABY5226
Spodoptera dolichos|[1600]|MHMYL3604-11|Costa Rica||658[0n]|BOLD:ABY5226
Spodoptera dolichos|[1601]|MHMYL3693-11|Costa Rica||658[0n]|BOLD:ABY5226
Spodoptera dolichos|[1602]|BLPED2105-12|Costa Rica|Guanacaste|658[0n]|BOLD:ABY5226
Spodoptera dolichos|[1603]|MOTAR157-12|Argentina|Misiones|658[0n]|BOLD:ABY5226
Spodoptera dolichos|[1604]|LNAUT1254-14|United States|Mississippi|658[0n]|BOLD:ABY5226
Spodoptera dolichos|[1605]|LNAUT1255-14|United States|Mississippi|658[0n]|BOLD:ABY5226
Spodoptera dolichos|[1606]|LNAUT1256-14|United States|Mississippi|658[0n]|BOLD:ABY5226
Spodoptera dolichos|[1607]|BLPBA273-07|Costa Rica|Guanacaste|658[0n]|BOLD:ABY5226
Spodoptera dolichos|[1608]|LNAUT1258-14|United States|Mississippi|658[0n]|BOLD:ABY5226
Spodoptera latifascia|[1609]|BLPCA522-08|Costa Rica|Guanacaste|632[0n]|BOLD:AAA5443
Spodoptera latifascia|[1610]|BLPCA523-08|Costa Rica|Guanacaste|632[0n]|BOLD:AAA5443
Spodoptera latifascia|[1611]|MHAUB918-05|Costa Rica|Guanacaste|594[0n]|BOLD:AAA5443
Spodoptera latifascia|[1612]|MHAUB916-05|Costa Rica|Guanacaste|658[0n]|BOLD:AAA5443
Spodoptera latifascia|[1613]|MHAUB919-05|Costa Rica|Guanacaste|658[0n]|BOLD:AAA5443
Spodoptera latifascia|[1614]|MHAUB920-05|Costa Rica|Guanacaste|594[0n]|BOLD:AAA5443
Spodoptera latifascia|[1615]|BLPAB893-06|Costa Rica|Guanacaste|658[0n]|BOLD:AAA5443
Spodoptera latifascia|[1616]|LPYPB078-08|Mexico|Campeche|658[0n]|BOLD:AAA5443
Spodoptera latifascia|[1617]|BLPAA394-06|Costa Rica|Guanacaste|658[0n]|BOLD:AAA5443
Spodoptera latifascia|[1618]|BLPAA922-06|Costa Rica|Alajuela|658[0n]|BOLD:AAA5443
Spodoptera latifascia|[1619]|BLPDY798-11|Costa Rica|Guanacaste|658[0n]|BOLD:AAA5443
Spodoptera latifascia|[1620]|BLPCA525-08|Costa Rica|Guanacaste|627[1n]|BOLD:AAA5443
Spodoptera latifascia|[1621]|BLPCA521-08|Costa Rica|Guanacaste|632[0n]|BOLD:AAA5443
Spodoptera latifascia|[1622]|BLPCA519-08|Costa Rica|Guanacaste|632[0n]|BOLD:AAA5443
Spodoptera latifascia|[1623]|BLPCA520-08|Costa Rica|Guanacaste|658[0n]|BOLD:AAA5443
Spodoptera latifascia|[1624]|BLPAA730-06|Costa Rica|Alajuela|658[0n]|BOLD:AAA5443
Spodoptera latifascia|[1625]|BLPCA208-08|Costa Rica|Guanacaste|658[0n]|BOLD:AAA5443
Spodoptera latifascia|[1626]|MHAUB924-05|Costa Rica|Guanacaste|594[0n]|BOLD:AAA5443
Spodoptera latifascia|[1627]|MHAUB921-05|Costa Rica|Guanacaste|594[0n]|BOLD:AAA5443
Spodoptera latifascia|[1628]|LOCRI1884-13|Costa Rica|San Jose|658[0n]|BOLD:AAA5443
Spodoptera latifascia|[1629]|MHAUB917-05|Costa Rica|Alajuela|658[3n]|BOLD:AAA5443
Spodoptera latifascia|[1630]|BLPBH663-07|Costa Rica|Guanacaste|658[0n]|BOLD:AAA5443
Spodoptera latifascia|[1631]|BLPCK290-08|Costa Rica|Alajuela|658[0n]|BOLD:AAA5443
Spodoptera latifascia|[1632]|BLPCL008-08|Costa Rica|Alajuela|658[0n]|BOLD:AAA5443
Spodoptera latifascia|[1633]|BLPDE114-09|Costa Rica|Guanacaste|658[0n]|BOLD:AAA5443
Spodoptera latifascia|[1634]|BLPDE115-09|Costa Rica|Guanacaste|658[0n]|BOLD:AAA5443
Spodoptera latifascia|[1635]|LYPAP083-09|Mexico|Quintana Roo|658[0n]|BOLD:AAA5443
Spodoptera latifascia|[1636]|MHMYC2258-09|Costa Rica|Alajuela|658[0n]|BOLD:AAA5443
Spodoptera latifascia|[1637]|BLPDT1683-10|Costa Rica|Guanacaste|658[0n]|BOLD:AAA5443
Spodoptera latifascia|[1638]|BLPDU282-11|Costa Rica|Guanacaste|658[0n]|BOLD:AAA5443
Spodoptera latifascia|[1639]|BLPDU284-11|Costa Rica|Guanacaste|658[0n]|BOLD:AAA5443
Spodoptera latifascia|[1640]|MHMYS2797-13|Costa Rica|Guanacaste|658[0n]|BOLD:AAA5443
Spodoptera latifascia|[1641]|BLPCK289-08|Costa Rica|Alajuela|658[0n]|BOLD:AAA5443
Spodoptera latifascia|[1642]|LPYPC069-08|Mexico|Yucatan|658[0n]|BOLD:AAA5443
Spodoptera latifascia|[1643]|BLPCH656-08|Costa Rica|Guanacaste|658[0n]|BOLD:AAA5443
Spodoptera latifascia|[1644]|BLPCD479-08|Costa Rica|Guanacaste|658[0n]|BOLD:AAA5443
Spodoptera latifascia|[1645]|BLPCD477-08|Costa Rica|Guanacaste|658[0n]|BOLD:AAA5443
Spodoptera latifascia|[1646]|BLPCA159-08|Costa Rica|Guanacaste|658[0n]|BOLD:AAA5443
Spodoptera latifascia|[1647]|MHMXM116-07|Costa Rica|Alajuela|658[0n]|BOLD:AAA5443
Spodoptera latifascia|[1648]|BLPBF915-07|Costa Rica|Guanacaste|658[0n]|BOLD:AAA5443
Spodoptera latifascia|[1649]|MHAUG697-07|Costa Rica|Alajuela|658[0n]|BOLD:AAA5443
Spodoptera latifascia|[1650]|BLPBA005-07|Costa Rica|Guanacaste|658[0n]|BOLD:AAA5443
Spodoptera latifascia|[1651]|BLPAG029-07|Costa Rica|Guanacaste|658[0n]|BOLD:AAA5443
Spodoptera latifascia|[1652]|BLPAF457-07|Costa Rica|Guanacaste|658[0n]|BOLD:AAA5443
Spodoptera latifascia|[1653]|BLPAD014-06|Costa Rica|Guanacaste|658[0n]|BOLD:AAA5443
Spodoptera latifascia|[1654]|MHMXA735-06|Costa Rica|Guanacaste|658[0n]|BOLD:AAA5443
Spodoptera latifascia|[1655]|BLPAA463-06|Costa Rica|Guanacaste|658[0n]|BOLD:AAA5443
Spodoptera latifascia|[1656]|MHAUC211-06|Costa Rica|Guanacaste|658[0n]|BOLD:AAA5443
Spodoptera latifascia|[1657]|JAT126-10|Mexico|Chiapas|656[0n]|BOLD:AAA5443
Spodoptera latifascia|[1658]|MHMXM110-07|Costa Rica|Guanacaste|649[0n]|BOLD:AAA5443
Spodoptera latifascia|[1659]|LYPAP059-09|Mexico|Quintana Roo|607[0n]|BOLD:AAA5443
Spodoptera latifascia|[1660]|MHMXA728-06|Costa Rica|Guanacaste|627[0n]|BOLD:AAA5443
Spodoptera latifascia|[1661]|MHMXI195-07|Costa Rica|Guanacaste|501[0n]|
Spodoptera latifascia|[1662]|GBGL12517-13|||937[2n]|
Spodoptera latifascia|[1663]|LNAUT1249-14|United States|Mississippi|658[0n]|BOLD:AAA5443
Spodoptera latifascia|[1664]|LNAUT1250-14|United States|Mississippi|658[0n]|BOLD:AAA5443
Spodoptera latifascia|[1665]|LNAUT1253-14|United States|Florida|658[0n]|BOLD:AAA5443
Spodoptera latifascia|[1666]|MHMXM115-07|Costa Rica|Guanacaste|658[0n]|BOLD:AAA5443
Spodoptera latifascia|[1667]|MHAUB922-05|Costa Rica|Guanacaste|594[0n]|BOLD:AAA5443
Spodoptera latifascia|[1668]|BLPAA386-06|Costa Rica|Guanacaste|658[0n]|BOLD:AAA5443
Spodoptera latifascia|[1669]|MHAUB925-05|Costa Rica|Guanacaste|594[0n]|BOLD:AAA5443
Spodoptera latifascia|[1670]|BLPCI616-08|Costa Rica|Guanacaste|658[0n]|BOLD:AAA5443
Spodoptera latifascia|[1671]|LPYPC063-08|Mexico|Yucatan|658[0n]|BOLD:AAA5443
Spodoptera latifascia|[1672]|BLPAG031-07|Costa Rica|Guanacaste|658[0n]|BOLD:AAA5443
Spodoptera latifascia|[1673]|BLPCD480-08|Costa Rica|Guanacaste|658[0n]|BOLD:AAA5443
Spodoptera latifascia|[1674]|BLPDV1050-11|Costa Rica|Guanacaste|658[0n]|BOLD:AAA5443
Spodoptera latifascia|[1675]|LYPIE245-09|Mexico|Quintana Roo|658[0n]|BOLD:AAA5443
Spodoptera latifascia|[1676]|BLPCI617-08|Costa Rica|Guanacaste|658[0n]|BOLD:AAA5443
Spodoptera latifascia|[1677]|MHMXU488-08|||658[0n]|BOLD:AAA5443
Spodoptera latifascia|[1678]|BLPCJ553-08|Costa Rica|Guanacaste|658[0n]|BOLD:AAA5443
Spodoptera latifascia|[1679]|BLPCJ554-08|Costa Rica|Guanacaste|658[0n]|BOLD:AAA5443
Spodoptera latifascia|[1680]|BLPCA207-08|Costa Rica|Guanacaste|658[0n]|BOLD:AAA5443
Spodoptera latifascia|[1681]|BLPCI137-08|Costa Rica|Guanacaste|658[0n]|BOLD:AAA5443
Spodoptera latifascia|[1682]|MHMXM112-07|Costa Rica|Alajuela|658[0n]|BOLD:AAA5443
Spodoptera latifascia|[1683]|BLPAC725-06|Costa Rica|Guanacaste|658[0n]|BOLD:AAA5443
Spodoptera latifascia|[1684]|BLPAC723-06|Costa Rica|Guanacaste|658[0n]|BOLD:AAA5443
Spodoptera latifascia|[1685]|BLPAC247-06|Costa Rica|Guanacaste|658[0n]|BOLD:AAA5443
Spodoptera latifascia|[1686]|BLPAC244-06|Costa Rica|Guanacaste|658[0n]|BOLD:AAA5443
Spodoptera latifascia|[1687]|BLPAA707-06|Costa Rica|Guanacaste|658[0n]|BOLD:AAA5443
Spodoptera latifascia|[1688]|MHMXA734-06|Costa Rica|Guanacaste|658[0n]|BOLD:AAA5443
Spodoptera latifascia|[1689]|BLPAA382-06|Costa Rica|Guanacaste|658[0n]|BOLD:AAA5443
Spodoptera latifascia|[1690]|MHAUB923-05|Costa Rica|Guanacaste|658[0n]|BOLD:AAA5443
Spodoptera latifascia|[1691]|MHMXM114-07|Costa Rica|Guanacaste|658[1n]|BOLD:AAA5443
Spodoptera latifascia|[1692]|BLPCD474-08|Costa Rica|Guanacaste|658[0n]|BOLD:AAA5443
Spodoptera latifascia|[1693]|BLPDB355-09|Costa Rica|Alajuela|650[0n]|BOLD:AAA5443
Spodoptera latifascia|[1694]|BLPDE113-09|Costa Rica|Guanacaste|658[0n]|BOLD:AAA5443
Spodoptera latifascia|[1695]|BLPDU281-11|Costa Rica|Guanacaste|658[0n]|BOLD:AAA5443
Spodoptera latifascia|[1696]|BLPDU283-11|Costa Rica|Guanacaste|658[0n]|BOLD:AAA5443
Spodoptera latifascia|[1697]|BLPDU884-11|Costa Rica|Guanacaste|658[0n]|BOLD:AAA5443
Spodoptera latifascia|[1698]|BLPDV1051-11|Costa Rica|Guanacaste|658[0n]|BOLD:AAA5443
Spodoptera latifascia|[1699]|BLPDV1056-11|Costa Rica|Guanacaste|658[0n]|BOLD:AAA5443
[illegible]

Spodoptera latifascia|[1697]|BLPDU884-11|Costa Rica|Guanacaste|658[0n]|BOLD:AAA5443
Spodoptera latifascia|[1698]|BLPDV1051-11|Costa Rica|Guanacaste|658[0n]|BOLD:AAA5443
Spodoptera latifascia|[1699]|BLPDV1056-11|Costa Rica|Guanacaste|658[0n]|BOLD:AAA5443
Spodoptera latifascia|[1700]|BBLOB1534-11|United States|Florida|658[0n]|BOLD:AAA5443
Spodoptera latifascia|[1701]|GWOST423-11|Cuba|Holguin|658[0n]|BOLD:AAA5443
Spodoptera latifascia|[1702]|BLPEF2317-13|Costa Rica|Guanacaste|658[0n]|BOLD:AAA5443
Spodoptera latifascia|[1703]|BLPEG1337-14|Costa Rica|Guanacaste|658[0n]|BOLD:AAA5443
Spodoptera androgea|[1704]|BLPDA718-09|Costa Rica|Guanacaste|632[0n]|BOLD:ABZ1417
Spodoptera androgea|[1705]|MHAUB909-05|Costa Rica|Guanacaste|603[0n]|BOLD:ABZ1417
Spodoptera androgea|[1706]|MHAUB908-05|Costa Rica|Guanacaste|603[0n]|BOLD:ABZ1417
Spodoptera androgea|[1707]|MHAUF370-06|Costa Rica|Guanacaste|588[0n]|BOLD:ABZ1417
Spodoptera androgea|[1708]|MHAUB902-05|Costa Rica|Alajuela|588[0n]|BOLD:ABZ1417
Spodoptera androgea|[1709]|MHAUB906-05|Costa Rica|Guanacaste|658[0n]|BOLD:ABZ1417
Spodoptera androgea|[1710]|BLPAA925-06|Costa Rica|Alajuela|658[0n]|BOLD:ABZ1417
Spodoptera androgea|[1711]|BLPAC246-06|Costa Rica|Guanacaste|658[0n]|BOLD:ABZ1417
Spodoptera androgea|[1712]|BLPAC327-06|Costa Rica|Guanacaste|658[0n]|BOLD:ABZ1417
Spodoptera androgea|[1713]|MHMXD719-06|Costa Rica|Alajuela|658[0n]|BOLD:ABZ1417
Spodoptera androgea|[1714]|BLPDR506-10|Costa Rica|Alajuela|658[0n]|BOLD:ABZ1417
Spodoptera androgea|[1715]|MHAUB901-05|Costa Rica|Guanacaste|594[0n]|BOLD:ABZ1417
Spodoptera androgea|[1716]|MHAUB900-05|Costa Rica|Alajuela|594[0n]|BOLD:ABZ1417
Spodoptera androgea|[1717]|BLPAC245-06|Costa Rica|Guanacaste|658[0n]|BOLD:ABZ1417
Spodoptera androgea|[1718]|BLPCC488-08|Costa Rica|Guanacaste|658[0n]|BOLD:ABZ1417
Spodoptera androgea|[1719]|BLPCD475-08|Costa Rica|Guanacaste|658[0n]|BOLD:ABZ1417
Spodoptera androgea|[1720]|MHMXZ754-09|Costa Rica||658[0n]|BOLD:ABZ1417
Spodoptera androgea|[1721]|BLPDT1399-10|Costa Rica|Guanacaste|658[0n]|BOLD:ABZ1417
Spodoptera androgea|[1722]|BLPDW646-11|Costa Rica|Guanacaste|658[0n]|BOLD:ABZ1417
Spodoptera androgea|[1723]|MHAUB903-05|Costa Rica|Alajuela|595[0n]|BOLD:ABZ1417
Spodoptera androgea|[1724]|BLPCF456-08|Costa Rica|Alajuela|658[0n]|BOLD:ABZ1417
Spodoptera androgea|[1725]|MHMXM109-07|Costa Rica|Alajuela|658[0n]|BOLD:ABZ1417
Spodoptera androgea|[1726]|MHAUB904-05|Costa Rica|Guanacaste|658[0n]|BOLD:ABZ1417
Spodoptera androgea|[1727]|GWOSZ845-11|Peru|Huanuco|658[0n]|BOLD:ABZ1417
Spodoptera androgea|[1728]|MILEQ376-11|Ecuador|Pichincha|658[0n]|BOLD:ABZ1417
Spodoptera androgea|[1729]|GWOST417-11|Peru|Huanuco|658[0n]|BOLD:ABZ1417
Spodoptera androgea|[1730]|GWOSS501-11|Honduras|Atlantida|658[0n]|BOLD:ABZ1417
Spodoptera androgea|[1731]|BLPDU883-11|Costa Rica|Guanacaste|658[0n]|BOLD:ABZ1417
Spodoptera androgea|[1732]|BLPDU882-11|Costa Rica|Guanacaste|658[0n]|BOLD:ABZ1417
Spodoptera androgea|[1733]|LNOUB495-10|French Guiana||658[0n]|BOLD:ABZ1417
Spodoptera androgea|[1734]|GWORQ095-10|Ecuador|Pichincha|658[0n]|BOLD:ABZ1417
Spodoptera androgea|[1735]|MHMYF655-10|Costa Rica|Alajuela|658[0n]|BOLD:ABZ1417
Spodoptera androgea|[1736]|BLPDK1305-09|Costa Rica|Guanacaste|658[0n]|BOLD:ABZ1417
Spodoptera androgea|[1737]|MHMYC2261-09|Costa Rica|Alajuela|658[0n]|BOLD:ABZ1417
Spodoptera androgea|[1738]|MHMYC2260-09|Costa Rica|Alajuela|658[0n]|BOLD:ABZ1417
Spodoptera androgea|[1739]|MHMYC2257-09|Costa Rica|Alajuela|658[0n]|BOLD:ABZ1417
Spodoptera androgea|[1740]|MHMXZ753-09|Costa Rica||657[0n]|BOLD:ABZ1417
Spodoptera androgea|[1741]|MHMXZ668-09|Costa Rica||658[0n]|BOLD:ABZ1417
Spodoptera androgea|[1742]|BLPDD686-09|Costa Rica|Guanacaste|658[0n]|BOLD:ABZ1417
Spodoptera androgea|[1743]|BLPDD685-09|Costa Rica|Guanacaste|658[0n]|BOLD:ABZ1417
Spodoptera androgea|[1744]|BLPDD478-09|Costa Rica|Guanacaste|658[0n]|BOLD:ABZ1417
Spodoptera androgea|[1745]|BLPDD477-09|Costa Rica|Guanacaste|658[0n]|BOLD:ABZ1417
Spodoptera androgea|[1746]|BLPDC921-09|Costa Rica|Alajuela|658[0n]|BOLD:ABZ1417
Spodoptera androgea|[1747]|BLPDA435-09|Costa Rica|Alajuela|658[0n]|BOLD:ABZ1417
Spodoptera androgea|[1748]|MHMXV046-08|Costa Rica||658[0n]|BOLD:ABZ1417
Spodoptera androgea|[1749]|BLPCO066-08|Costa Rica|Guanacaste|658[0n]|BOLD:ABZ1417
Spodoptera androgea|[1750]|BLPCL010-08|Costa Rica|Alajuela|658[0n]|BOLD:ABZ1417
Spodoptera androgea|[1751]|BLPCL009-08|Costa Rica|Alajuela|658[0n]|BOLD:ABZ1417
Spodoptera androgea|[1752]|BLPCI618-08|Costa Rica|Guanacaste|658[0n]|BOLD:ABZ1417
Spodoptera androgea|[1753]|BLPCJ205-08|Costa Rica|Guanacaste|658[0n]|BOLD:ABZ1417
Spodoptera androgea|[1754]|BLPCF455-08|Costa Rica|Alajuela|658[0n]|BOLD:ABZ1417
Spodoptera androgea|[1755]|BLPCF454-08|Costa Rica|Alajuela|658[0n]|BOLD:ABZ1417
Spodoptera androgea|[1756]|BLPCD471-08|Costa Rica|Guanacaste|658[0n]|BOLD:ABZ1417
Spodoptera androgea|[1757]|BLPCC004-08|Costa Rica|Alajuela|658[0n]|BOLD:ABZ1417
Spodoptera androgea|[1758]|BLPCC003-08|Costa Rica|Alajuela|658[0n]|BOLD:ABZ1417
Spodoptera androgea|[1759]|MHMXO345-08|Costa Rica|Guanacaste|658[0n]|BOLD:ABZ1417
Spodoptera androgea|[1760]|MHMXO343-08|Costa Rica|Guanacaste|658[0n]|BOLD:ABZ1417
Spodoptera androgea|[1761]|BLPBF049-07|Costa Rica|Guanacaste|658[0n]|BOLD:ABZ1417
Spodoptera androgea|[1762]|BLPBF047-07|Costa Rica|Guanacaste|658[0n]|BOLD:ABZ1417
Spodoptera androgea|[1763]|BLPBD224-07|Costa Rica|Guanacaste|658[0n]|BOLD:ABZ1417
Spodoptera androgea|[1764]|MHAUG093-07|Costa Rica|Alajuela|655[0n]|BOLD:ABZ1417
Spodoptera androgea|[1765]|BLPBB638-07|Costa Rica|Guanacaste|658[0n]|BOLD:ABZ1417
Spodoptera androgea|[1766]|BLPAG449-07|Costa Rica|Guanacaste|658[0n]|BOLD:ABZ1417
Spodoptera androgea|[1767]|BLPAE061-06|Costa Rica|Alajuela|658[0n]|BOLD:ABZ1417
Spodoptera androgea|[1768]|BLPAE060-06|Costa Rica|Alajuela|658[0n]|BOLD:ABZ1417
Spodoptera androgea|[1769]|BLPAC726-06|Costa Rica|Guanacaste|658[0n]|BOLD:ABZ1417
Spodoptera androgea|[1770]|BLPAB894-06|Costa Rica|Guanacaste|658[0n]|BOLD:ABZ1417
Spodoptera androgea|[1771]|BLPAB812-06|Costa Rica|Guanacaste|658[0n]|BOLD:ABZ1417
Spodoptera androgea|[1772]|BLPAA821-06|Costa Rica|Alajuela|658[0n]|BOLD:ABZ1417
Spodoptera androgea|[1773]|MHMXA730-06|Costa Rica|Alajuela|658[0n]|BOLD:ABZ1417
Spodoptera androgea|[1774]|MHMXA727-06|Costa Rica|Guanacaste|658[0n]|BOLD:ABZ1417
Spodoptera androgea|[1775]|MHAUB907-05|Costa Rica|Alajuela|658[0n]|BOLD:ABZ1417
Spodoptera androgea|[1776]|MHAUB905-05|Costa Rica|Alajuela|658[0n]|BOLD:ABZ1417
Spodoptera androgea|[1777]|BLPBF048-07|Costa Rica|Guanacaste|658[1n]|BOLD:ABZ1417
Spodoptera androgea|[1778]|BLPBF046-07|Costa Rica|Guanacaste|644[1n]|BOLD:ABZ1417
Spodoptera androgea|[1779]|MHMXA729-06|Costa Rica|Guanacaste|621[0n]|BOLD:ABZ1417
Spodoptera androgea|[1780]|GBGL12587-13|||1000[1n]|
Spodoptera androgea|[1781]|BLPBC143-07|Costa Rica|Guanacaste|629[0n]|BOLD:ABZ1417
Spodoptera androgea|[1782]|LNAUT1251-14|United States|Florida|658[0n]|BOLD:ABZ1417
Spodoptera androgea|[1783]|RDNML258-13|Puerto Rico||658[0n]|BOLD:ABZ1417
Spodoptera androgea|[1784]|RDNML351-13|United States|Florida|658[0n]|BOLD:ABZ1417
Spodoptera androgea|[1785]|RDNML352-13|United States|Florida|658[0n]|BOLD:ABZ1417
Spodoptera androgea|[1786]|LNAUT1252-14|United States|Florida|658[0n]|BOLD:ABZ1417
Spodoptera pulchella|[1787]|LNAUS3498-13|Jamaica||658[2n]|BOLD:ACB5436
Spodoptera pulchella|[1788]|GBGL10114-12|||771[0n]|BOLD:ACB5436
Spodoptera pulchella|[1789]|LNAUS3499-13|Dominican Republic|La Vega|658[0n]|BOLD:ACB5436
Spodoptera pulchella|[1790]|GBGL10113-12|||771[0n]|BOLD:ACB5436
Spodoptera pulchella|[1791]|LNAUS3496-13|Puerto Rico||658[0n]|BOLD:ACB5436
Spodoptera pulchella|[1792]|CNCLB1761-14|United States|Florida|658[0n]|BOLD:ACB5436
Spodoptera pulchella|[1793]|CNCLB1762-14|United States|Florida|658[0n]|BOLD:ACB5436
Phosphila miselioides|[1794]|LGSMC905-05|United States|Tennessee|658[0n]|BOLD:AAB2662
Phosphila miselioides|[1795]|LPSO343-08|Canada|Ontario|658[0n]|BOLD:AAB2662
Phosphila miselioides|[1796]|LPSO745-08|Canada|Ontario|658[0n]|BOLD:AAB2662
Phosphila miselioides|[1797]|BBLSW792-09|United States|Oklahoma|634[0n]|BOLD:AAB2662
Phosphila miselioides|[1798]|LPSOB081-08|Canada|Ontario|658[0n]|BOLD:AAB2662
Phosphila miselioides|[1799]|LOFLB711-06|United States|Florida|658[0n]|BOLD:AAB2662

Phosphila miselioides|[1797]|BBLSW792-09|United States|Oklahoma|634[0n]|BOLD:AAB2662
Phosphila miselioides|[1798]|LPSOB081-08|Canada|Ontario|658[0n]|BOLD:AAB2662
Phosphila miselioides|[1799]|LOFLB711-06|United States|Florida|658[0n]|BOLD:AAB2662
Phosphila miselioides|[1800]|LGSMG999-10|United States|Tennessee|658[0n]|BOLD:AAB2662
Phosphila miselioides|[1801]|TAMIC936-10|United States|Texas|658[0n]|BOLD:AAB2662
Phosphila miselioides|[1802]|LPSO883-08|Canada|Ontario|658[0n]|BOLD:AAB2662
Phosphila miselioides|[1803]|LSEU356-06|United States|Georgia|658[0n]|BOLD:AAB2662
Phosphila miselioides|[1804]|LOFLC381-06|United States|Florida|658[0n]|BOLD:AAB2662
Phosphila miselioides|[1805]|LOFLB406-06|United States|Florida|658[0n]|BOLD:AAB2662
Phosphila miselioides|[1806]|MECD362-06|United States|Maryland|658[0n]|BOLD:AAB2662
Phosphila miselioides|[1807]|LNC039-05|United States|North Carolina|658[0n]|BOLD:AAB2662
Phosphila miselioides|[1808]|LNC038-05|United States|North Carolina|658[0n]|BOLD:AAB2662
Phosphila miselioides|[1809]|LOCT222-05|United States|Connecticut|658[0n]|BOLD:AAB2662
Phosphila miselioides|[1810]|LGSM510-04|United States|Tennessee|658[0n]|BOLD:AAB2662
Phosphila miselioides|[1811]|LSEU357-06|United States|Georgia|618[0n]|BOLD:AAB2662
Phosphila miselioides|[1812]|LSEU616-06|United States|Georgia|638[0n]|BOLD:AAB2662
Phosphila miselioides|[1813]|LPOKA527-09|United States|Oklahoma|638[0n]|BOLD:AAB2662
Phosphila miselioides|[1814]|BBLSW922-09|United States|Texas|658[0n]|BOLD:AAB2662
Phosphila miselioides|[1815]|BBLSX574-09|United States|Oklahoma|658[0n]|BOLD:AAB2662
Phosphila miselioides|[1816]|USLEP078-10|United States|Texas|658[0n]|BOLD:AAB2662
Phosphila miselioides|[1817]|LOT198-04|United States|Tennessee|609[0n]|BOLD:AAB2662
Phosphila miselioides|[1818]|LGSM509-04|United States|North Carolina|609[0n]|BOLD:AAB2662
Phosphila miselioides|[1819]|USLEP883-10|United States|Texas|658[0n]|BOLD:AAB2662
Phosphila miselioides|[1820]|CNCLB511-14|United States|Texas|658[2n]|BOLD:AAB2662
Phosphila miselioides|[1821]|USLEP079-10|United States|Texas|658[0n]|BOLD:AAB2662
Phosphila miselioides|[1822]|BBLSX048-09|United States|Texas|658[0n]|BOLD:AAB2662
Phosphila miselioides|[1823]|HKONB396-09|United States|Texas|658[0n]|BOLD:AAB2662
Phosphila miselioides|[1824]|BBLSW561-09|United States|Oklahoma|638[0n]|BOLD:AAB2662
Phosphila miselioides|[1825]|BBLSW923-09|United States|Texas|638[0n]|BOLD:AAB2662
Phosphila miselioides|[1826]|USLEP080-10|United States|Texas|658[0n]|BOLD:AAB2662
Phosphila miselioides|[1827]|USLEP571-10|United States|Texas|658[0n]|BOLD:AAB2662
Phosphila miselioides|[1828]|CNCLB512-14|United States|Texas|658[0n]|BOLD:AAB2662
Phosphila miselioides|[1829]|LSEU236-06|United States|North Carolina|658[1n]|BOLD:AAB2662
Phosphila miselioides|[1830]|USLEP882-10|United States|Florida|658[0n]|BOLD:AAB2662
Phosphila miselioides|[1831]|USLEP884-10|United States|Florida|658[0n]|BOLD:AAB2662
Phosphila miselioides|[1832]|USLEP885-10|United States|Florida|658[0n]|BOLD:AAB2662
Phosphila miselioides|[1833]|USLEP886-10|United States|Florida|658[0n]|BOLD:AAB2662
Phosphila miselioides|[1834]|BBLSX955-09|United States|Oklahoma|658[0n]|BOLD:AAB2662
Phosphila miselioides|[1835]|LPOKC765-09|United States|Oklahoma|658[0n]|BOLD:AAB2662
Phosphila miselioides|[1836]|BBLSX913-09|United States|Arizona|658[0n]|BOLD:AAB2662
Phosphila miselioides|[1837]|BBLSX901-09|United States|Oklahoma|658[0n]|BOLD:AAB2662
Phosphila miselioides|[1838]|BBLSX499-09|United States|Oklahoma|658[0n]|BOLD:AAB2662
Phosphila miselioides|[1839]|LPOKB295-09|United States|Oklahoma|658[0n]|BOLD:AAB2662
Phosphila miselioides|[1840]|RDNMD785-07|United States|North Carolina|658[0n]|BOLD:AAB2662
Phosphila miselioides|[1841]|LSUSA211-06|United States|Kentucky|658[0n]|BOLD:AAB2662
Phosphila miselioides|[1842]|LSUSA197-06|United States|Kentucky|658[0n]|BOLD:AAB2662
Phosphila miselioides|[1843]|LOFLA010-06|United States|Florida|658[0n]|BOLD:AAB2662
Phosphila miselioides|[1844]|LGSMC904-05|United States|Tennessee|658[0n]|BOLD:AAB2662
Phosphila miselioides|[1845]|BBLSU033-09|United States|Arkansas|658[0n]|BOLD:AAB2662
Phosphila miselioides|[1846]|CNCLB513-14|United States|Texas|658[0n]|BOLD:AAB2662
Phosphila fernae|[1847]|LNAUT418-14|Mexico|Tamaulipas|658[0n]|BOLD:ACN8553
Phosphila fernae|[1848]|LNAUT419-14|Mexico|Tamaulipas|658[0n]|BOLD:ACN8553
Galgula partita PS1|[1849]|RDNMK914-13|Mexico|Sonora|658[0n]|BOLD:AAH5650
Galgula partita PS1|[1850]|LNAUT408-14|United States|Arizona|658[0n]|BOLD:AAH5650
Galgula partita PS1|[1851]|LNAUT409-14|United States|Arizona|658[0n]|BOLD:AAH5650
Galgula partita PS1|[1852]|LNAUT410-14|United States|Arizona|658[0n]|BOLD:AAH5650
Galgula partita PS1|[1853]|CMAZA117-09|United States|Arizona|658[0n]|BOLD:AAH5650
Galgula partita PS1|[1854]|CMAZA1083-12|United States|Arizona|658[0n]|BOLD:AAH5650
Galgula partita PS1|[1855]|LNAUT411-14|United States|Arizona|658[0n]|BOLD:AAH5650
Galgula partita|[1856]|LPOKA251-08|United States|Oklahoma|658[0n]|BOLD:AAA6919
Galgula partita|[1857]|USLEP534-10|United States|Florida|658[0n]|BOLD:AAA6919
Galgula partita|[1858]|PHFLO069-10|United States|Florida|658[0n]|BOLD:AAA6919
Galgula partita|[1859]|LOFLD285-07|United States|Florida|658[0n]|BOLD:AAA6919
Galgula partita|[1860]|LOFLD044-07|United States|Florida|658[0n]|BOLD:AAA6919
Galgula partita|[1861]|LOFLB465-06|United States|Florida|658[0n]|BOLD:AAA6919
Galgula partita|[1862]|LOFLB347-06|United States|Florida|658[0n]|BOLD:AAA6919
Galgula partita|[1863]|RDNMD344-06|United States|Florida|658[0n]|BOLD:AAA6919
Galgula partita|[1864]|PHFLO074-10|United States|Florida|634[0n]|BOLD:AAA6919
Galgula partita|[1865]|RWWB306-09|United States|Washington|614[0n]|BOLD:AAA6919
Galgula partita|[1866]|RWWB417-09|United States|Washington|658[0n]|BOLD:AAA6919
Galgula partita|[1867]|RWWB359-09|United States|Washington|658[0n]|BOLD:AAA6919
Galgula partita|[1868]|RWWB361-09|United States|Washington|658[0n]|BOLD:AAA6919
Galgula partita|[1869]|RWWA712-09|United States|Washington|649[0n]|BOLD:AAA6919
Galgula partita|[1870]|RWWB274-09|United States|Washington|658[0n]|BOLD:AAA6919
Galgula partita|[1871]|RWWB281-09|United States|Washington|658[0n]|BOLD:AAA6919
Galgula partita|[1872]|RWWB284-09|United States|Washington|658[0n]|BOLD:AAA6919
Galgula partita|[1873]|RWWB285-09|United States|Washington|658[0n]|BOLD:AAA6919
Galgula partita|[1874]|RWWB290-09|United States|Washington|658[0n]|BOLD:AAA6919
Galgula partita|[1875]|RWWB292-09|United States|Washington|658[0n]|BOLD:AAA6919
Galgula partita|[1876]|RWWB297-09|United States|Washington|658[0n]|BOLD:AAA6919
Galgula partita|[1877]|RWWB312-09|United States|Washington|658[0n]|BOLD:AAA6919
Galgula partita|[1878]|RWWB328-09|United States|Washington|658[0n]|BOLD:AAA6919
Galgula partita|[1879]|RWWB329-09|United States|Washington|658[0n]|BOLD:AAA6919
Galgula partita|[1880]|RWWB346-09|United States|Washington|658[0n]|BOLD:AAA6919
Galgula partita|[1881]|RWWC802-11|United States|Washington|658[0n]|BOLD:AAA6919
Galgula partita|[1882]|RWWC833-12|United States|Washington|658[0n]|BOLD:AAA6919
Galgula partita|[1883]|CNPPA3973-12|Canada|Ontario|599[0n]|BOLD:AAA6919
Galgula partita|[1884]|GMLC1437-12|United States|California|614[0n]|BOLD:AAA6919
Galgula partita|[1885]|GMLC1426-12|United States|California|614[0n]|BOLD:AAA6919
Galgula partita|[1886]|USLEP1129-10|United States|Texas|658[0n]|BOLD:AAA6919
Galgula partita|[1887]|BBLOE1381-12|United States|Arkansas|658[0n]|BOLD:AAA6919
Galgula partita|[1888]|LPOKD657-10|United States|Oklahoma|658[0n]|BOLD:AAA6919
Galgula partita|[1889]|NHMNO003-11|Portugal|Azores|658[0n]|BOLD:AAA6919
Galgula partita|[1890]|NHMNO005-11|Portugal|Madeira|658[0n]|BOLD:AAA6919
Galgula partita|[1891]|BBLSX927-09|United States|Arizona|658[0n]|BOLD:AAA6919
Galgula partita|[1892]|USLEP763-10|United States|Texas|658[0n]|BOLD:AAA6919
Galgula partita|[1893]|BBLSX891-09|United States|Oklahoma|658[0n]|BOLD:AAA6919
Galgula partita|[1894]|LPOKA957-09|United States|Oklahoma|658[0n]|BOLD:AAA6919
Galgula partita|[1895]|LILLA075-11|United States|Illinois|658[0n]|BOLD:AAA6919
Galgula partita|[1896]|MECD346-06|United States|Maryland|658[0n]|BOLD:AAA6919
Galgula partita|[1897]|LPOKA959-09|United States|Oklahoma|658[0n]|BOLD:AAA6919
Galgula partita|[1898]|LPOKB770-09|United States|Oklahoma|658[0n]|BOLD:AAA6919
Galgula partita|[1899]|LPOKC314-09|United States|Oklahoma|658[0n]|BOLD:AAA6919

Galgula partita|[1897]|LPOKA959-09|United States|Oklahoma|658[0n]|BOLD:AAA6919
Galgula partita|[1898]|LPOKB770-09|United States|Oklahoma|658[0n]|BOLD:AAA6919
Galgula partita|[1899]|LPOKC314-09|United States|Oklahoma|658[0n]|BOLD:AAA6919
Galgula partita|[1900]|LPOKC553-09|United States|Oklahoma|658[0n]|BOLD:AAA6919
Galgula partita|[1901]|BBLSW006-09|United States|Oklahoma|658[0n]|BOLD:AAA6919
Galgula partita|[1902]|BBLSW812-09|United States|Oklahoma|658[0n]|BOLD:AAA6919
Galgula partita|[1903]|BBLSW964-09|United States|Texas|658[0n]|BOLD:AAA6919
Galgula partita|[1904]|BBLSX407-09|United States|Oklahoma|658[0n]|BOLD:AAA6919
Galgula partita|[1905]|BBLSX408-09|United States|Oklahoma|658[0n]|BOLD:AAA6919
Galgula partita|[1906]|BBLSX654-09|United States|Oklahoma|658[0n]|BOLD:AAA6919
Galgula partita|[1907]|BBLSX952-09|United States|Oklahoma|658[0n]|BOLD:AAA6919
Galgula partita|[1908]|BBLSX962-09|United States|Oklahoma|658[0n]|BOLD:AAA6919
Galgula partita|[1909]|BBLSX963-09|United States|Oklahoma|658[0n]|BOLD:AAA6919
Galgula partita|[1910]|BBLSX967-09|United States|Oklahoma|658[0n]|BOLD:AAA6919
Galgula partita|[1911]|BBLSX968-09|United States|Oklahoma|658[0n]|BOLD:AAA6919
Galgula partita|[1912]|LPOKD287-09|United States|Oklahoma|657[0n]|BOLD:AAA6919
Galgula partita|[1913]|LPOKD670-10|United States|Oklahoma|658[0n]|BOLD:AAA6919
Galgula partita|[1914]|LGSMG1016-10|United States|Tennessee|658[0n]|BOLD:AAA6919
Galgula partita|[1915]|LGSMG1017-10|United States|North Carolina|658[0n]|BOLD:AAA6919
Galgula partita|[1916]|USLEP640-10|United States|Florida|658[0n]|BOLD:AAA6919
Galgula partita|[1917]|USLEP764-10|United States|Texas|658[0n]|BOLD:AAA6919
Galgula partita|[1918]|USLEP765-10|United States|Arkansas|658[0n]|BOLD:AAA6919
Galgula partita|[1919]|USLEP918-10|United States|Florida|658[0n]|BOLD:AAA6919
Galgula partita|[1920]|USLEP1128-10|United States|Texas|658[0n]|BOLD:AAA6919
Galgula partita|[1921]|MJMSL045-10|United States|Massachusetts|658[0n]|BOLD:AAA6919
Galgula partita|[1922]|NHMNO002-11|United Kingdom|England|658[0n]|BOLD:AAA6919
Galgula partita|[1923]|NHMNO004-11|Spain|Canary Islands|658[0n]|BOLD:AAA6919
Galgula partita|[1924]|LILLA523-11|United States|Illinois|658[0n]|BOLD:AAA6919
Galgula partita|[1925]|LILLA997-11|United States|Illinois|658[0n]|BOLD:AAA6919
Galgula partita|[1926]|BBLOC838-11|United States|Arkansas|658[0n]|BOLD:AAA6919
Galgula partita|[1927]|BBLOE1375-12|United States|Arkansas|658[0n]|BOLD:AAA6919
Galgula partita|[1928]|PHFLO077-10|United States|Florida|658[0n]|BOLD:AAA6919
Galgula partita|[1929]|BBLPB883-10|Canada|Ontario|658[0n]|BOLD:AAA6919
Galgula partita|[1930]|BBLPD449-10|Canada|Ontario|658[0n]|BOLD:AAA6919
Galgula partita|[1931]|RDNMJ706-11|United States|Arizona|658[0n]|BOLD:AAA6919
Galgula partita|[1932]|BBLOE1384-12|United States|Arkansas|658[0n]|BOLD:AAA6919
Galgula partita|[1933]|BBLOE1928-12|United States|Oklahoma|658[0n]|BOLD:AAA6919
Galgula partita|[1934]|GMLC748-12|United States|California|658[0n]|BOLD:AAA6919
Galgula partita|[1935]|GMLC1154-12|United States|California|658[0n]|BOLD:AAA6919
Galgula partita|[1936]|LPOKA227-08|United States|Oklahoma|658[0n]|BOLD:AAA6919
Galgula partita|[1937]|NAMUM419-09|United States|Texas|658[0n]|BOLD:AAA6919
Galgula partita|[1938]|BBLSX972-09|United States|Oklahoma|658[0n]|BOLD:AAA6919
Galgula partita|[1939]|BBLSU063-09|United States|Arkansas|658[0n]|BOLD:AAA6919
Galgula partita|[1940]|RDLQG810-06|Canada|Quebec|658[0n]|BOLD:AAA6919
Galgula partita|[1941]|LNCB525-07|United States|North Carolina|658[0n]|BOLD:AAA6919
Galgula partita|[1942]|LOCBE137-06|United States|California|658[0n]|BOLD:AAA6919
Galgula partita|[1943]|RDLQG800-06|Canada|Quebec|658[0n]|BOLD:AAA6919
Galgula partita|[1944]|XAJ775-06|Canada|Ontario|658[0n]|BOLD:AAA6919
Galgula partita|[1945]|XAK306-06|Canada|Ontario|658[0n]|BOLD:AAA6919
Galgula partita|[1946]|LNCNW094-06|United States|North Carolina|658[0n]|BOLD:AAA6919
Galgula partita|[1947]|LSEU365-06|United States|Georgia|658[0n]|BOLD:AAA6919
Galgula partita|[1948]|LOFLB543-06|United States|Florida|658[0n]|BOLD:AAA6919
Galgula partita|[1949]|LOFLA759-06|United States|Florida|658[0n]|BOLD:AAA6919
Galgula partita|[1950]|LOFLA053-06|United States|Florida|658[0n]|BOLD:AAA6919
Galgula partita|[1951]|LGSMD678-05|United States|North Carolina|658[0n]|BOLD:AAA6919
Galgula partita|[1952]|LGSMC450-05|United States|Tennessee|658[0n]|BOLD:AAA6919
Galgula partita|[1953]|XAD568-04|Canada|Ontario|658[0n]|BOLD:AAA6919
Galgula partita|[1954]|BBLPB882-10|Canada|Ontario|658[0n]|BOLD:AAA6919
Galgula partita|[1955]|LGSM720-04|United States|North Carolina|658[0n]|BOLD:AAA6919
Galgula partita|[1956]|LGSM719-04|United States|North Carolina|658[0n]|BOLD:AAA6919
Galgula partita|[1957]|LNC254-05|United States|North Carolina|658[0n]|BOLD:AAA6919
Galgula partita|[1958]|MECD347-06|United States|Maryland|656[0n]|BOLD:AAA6919
Galgula partita|[1959]|TMG119-03|Canada|Ontario|639[0n]|BOLD:AAA6919
Galgula partita|[1960]|PMG117-03|Canada|Ontario|617[0n]|BOLD:AAA6919
Galgula partita|[1961]|RDLQB778-05|Canada|Quebec|617[0n]|BOLD:AAA6919
Galgula partita|[1962]|XAD010-04|Canada|Ontario|602[0n]|BOLD:AAA6919
Galgula partita|[1963]|TMNBB697-06|Canada|New Brunswick|643[0n]|BOLD:AAA6919
Galgula partita|[1964]|GBGL5941-09|||1392[0n]|BOLD:AAA6919
Galgula partita|[1965]|RDNMJ711-11|United States|Arizona|632[0n]|BOLD:AAA6919
Galgula partita|[1966]|CNCLB1812-14|United States|New Mexico|658[0n]|BOLD:AAA6919
Elaphria cyanympha|[1967]|LSEU081-06|United States|Florida|533[0n]|BOLD:AAD6810
Elaphria cyanympha|[1968]|LOFLA497-06|United States|Florida|658[0n]|BOLD:AAD6810
Elaphria cyanympha|[1969]|RDNMD351-06|United States|Florida|658[0n]|BOLD:AAD6810
Elaphria cyanympha|[1970]|MNAC303-07|United States|Florida|658[0n]|BOLD:AAD6810
Elaphria cyanympha|[1971]|HKONB317-09|United States|Florida|658[0n]|BOLD:AAD6810
Elaphria cyanympha|[1972]|LNCB683-09|United States|North Carolina|521[0n]|BOLD:AAD6811
Bryolymnia ensina|[1973]|RDNME989-08|United States|New Mexico|658[0n]|BOLD:AAW7335
Bryolymnia viridimedia|[1974]|CNCLB563-14|Mexico||611[0n]|BOLD:ACM3969
Bryolymnia mixta|[1975]|RDNME882-08|United States|Arizona|658[0n]|BOLD:AAE8300
Bryolymnia semifascia|[1976]|RDNMD655-06|United States|New Mexico|658[0n]|BOLD:AAI8506
Bryolymnia semifascia|[1977]|RDNMD656-06|United States|New Mexico|658[0n]|BOLD:AAI8506
Bryolymnia semifascia|[1978]|CMAZA403-10|United States|Arizona|658[0n]|BOLD:ACE4343
Bryolymnia semifascia|[1979]|CMAZA1155-12|United States|Arizona|658[0n]|BOLD:ACE4343
Bryolymnia anthracitaria|[1980]|RDNMD848-07|United States|Arizona|655[0n]|BOLD:AAW7331
Bryolymnia marti|[1981]|RDNME853-08|United States|Arizona|658[0n]|BOLD:AAE8299
Bryolymnia marti|[1982]|RDNME852-08|United States|New Mexico|658[0n]|BOLD:AAE8299
Bryolymnia marti|[1983]|RDNME854-08|United States|New Mexico|658[0n]|BOLD:AAE8299
Bryolymnia viridimedia|[1984]|IAWLB379-11|United States|Arizona|658[0n]|BOLD:AAZ0700
Bryolymnia viridimedia|[1985]|CNCLB558-14|United States|Arizona|658[0n]|BOLD:AAZ0700
Bryolymnia viridimedia|[1986]|CNCLB560-14|Mexico||469[1n]|
Bryolymnia viridimedia|[1987]|CNCLB561-14|Mexico||550[0n]|BOLD:ACQ8304
Bryolymnia viridimedia|[1988]|CNCLB559-14|Mexico||550[0n]|BOLD:ACQ8304
Bryolymnia viridimedia|[1989]|CNCLB562-14|Mexico||550[0n]|BOLD:ACQ8304
Bryolymnia biformata|[1990]|RDNMH1030-09|United States|Arizona|658[0n]|BOLD:AAN6784
Bryolymnia biformata|[1991]|LNAUT851-14|United States|Arizona|658[0n]|BOLD:AAN6784
Bryolymnia biformata|[1992]|LNAUT852-14|United States|Arizona|658[0n]|BOLD:ACN5580
Bryolymnia biformata|[1993]|LNAUT853-14|United States|Arizona|658[0n]|BOLD:ACN5580
Elaphria nucicolora|[1994]|LOFLB476-06|United States|Florida|658[0n]|BOLD:AAB8482
Elaphria nucicolora|[1995]|LOFLB843-06|United States|Florida|658[0n]|BOLD:AAB8482
Elaphria nucicolora|[1996]|RDNMF975-08|United States|Texas|658[0n]|BOLD:AAB8482
Elaphria nucicolora|[1997]|LSEU190-06|United States|Florida|658[0n]|BOLD:AAB8482
Elaphria nucicolora|[1998]|LOFLA444-06|United States|Florida|620[0n]|BOLD:AAB8482
Elaphria nucicolora|[1999]|LSEU189-06|United States|Florida|658[0n]|BOLD:AAB8482

Elaphria nucicolora|[1997]|LSEU190-06|United States|Florida|658[0n]|BOLD:AAB8482
Elaphria nucicolora|[1998]|LOFLA444-06|United States|Florida|620[0n]|BOLD:AAB8482
Elaphria nucicolora|[1999]|LSEU189-06|United States|Florida|658[0n]|BOLD:AAB8482
Elaphria nucicolora|[2000]|LOFLA261-06|United States|Florida|658[0n]|BOLD:AAB8482
Elaphria nucicolora|[2001]|LOFLA449-06|United States|Florida|617[0n]|BOLD:AAB8482
Elaphria nucicolora|[2002]|LOFLA706-06|United States|Florida|658[0n]|BOLD:AAB8482
Elaphria nucicolora|[2003]|LOFLA586-06|United States|Florida|658[0n]|BOLD:AAB8482
Elaphria nucicolora|[2004]|LOFLC255-06|United States|Florida|658[0n]|BOLD:AAB8482
Elaphria nucicolora|[2005]|LOFLA427-06|United States|Florida|658[0n]|BOLD:AAB8482
Elaphria nucicolora|[2006]|LOFLB531-06|United States|Florida|658[0n]|BOLD:AAB8482
Elaphria nucicolora|[2007]|LOFLC229-06|United States|Florida|658[0n]|BOLD:AAB8482
Elaphria nucicolora|[2008]|LOFLB276-06|United States|Florida|658[0n]|BOLD:AAB8482
Elaphria nucicolora|[2009]|LOFLA601-06|United States|Florida|658[0n]|BOLD:AAB8482
Elaphria nucicolora|[2010]|LOFLB847-06|United States|Florida|658[0n]|BOLD:AAB8482
Elaphria nucicolora|[2011]|USLEP996-10|United States|Florida|653[0n]|BOLD:AAB8482
Elaphria nucicolora|[2012]|RDNMJ004-10|United States|Florida|658[0n]|BOLD:AAB8482
Elaphria deltoides|[2013]|RDNMJ133-10|United States|Florida|658[0n]|BOLD:AAU0963
Elaphria deltoides|[2014]|RDNMJ132-10|United States|Florida|658[0n]|BOLD:AAU0963
Elaphria deltoides|[2015]|RDNMK176-11|United States|Florida|658[0n]|BOLD:AAU0963
Elaphria deltoides|[2016]|RDNMK177-11|United States|Florida|658[0n]|BOLD:AAU0963
Elaphria deltoides|[2017]|RDNMK178-11|United States|Florida|658[0n]|BOLD:AAU0963
Bryolymnia viridata|[2018]|CGLCA014-10|United States|California|658[0n]|BOLD:AAG5836
Bryolymnia viridata|[2019]|CGLCA018-10|United States|California|658[0n]|BOLD:AAG5836
Bryolymnia viridata|[2020]|CGLCA019-10|United States|California|658[0n]|BOLD:AAG5836
Bryolymnia viridata|[2021]|GMLC882-12|United States|California|658[0n]|BOLD:AAG5836
Bryolymnia viridata|[2022]|GMLC612-11|United States|California|658[0n]|BOLD:AAG5836
Bryolymnia viridata|[2023]|GMLC245-11|United States|California|658[0n]|BOLD:AAG5836
Bryolymnia viridata|[2024]|GMLC667-11|United States|California|658[0n]|BOLD:AAG5836
Bryolymnia viridata|[2025]|GMLC904-12|United States|California|658[0n]|BOLD:AAG5836
Bryolymnia viridata|[2026]|GMLC988-12|United States|California|658[0n]|BOLD:AAG5836
Bryolymnia viridata|[2027]|GMLC099-09|United States|California|658[0n]|BOLD:AAG5836
Bryolymnia viridata|[2028]|GMLC288-11|United States|California|658[0n]|BOLD:AAG5836
Bryolymnia viridata|[2029]|GMLC1016-12|United States|California|658[0n]|BOLD:AAG5836
Bryolymnia viridata|[2030]|LOCBC010-06|United States|California|658[0n]|BOLD:AAG5836
Bryolymnia viridata|[2031]|LOCBB232-06|United States|California|658[0n]|BOLD:AAG5836
Bryolymnia viridata|[2032]|LOCBB231-06|United States|California|658[0n]|BOLD:AAG5836
Bryolymnia viridata|[2033]|LOCB661-06|United States|California|658[0n]|BOLD:AAG5836
Bryolymnia viridata|[2034]|LOCB395-06|United States|California|658[0n]|BOLD:AAG5836
Bryolymnia viridata|[2035]|LOCBC247-06|United States|California|656[0n]|BOLD:AAG5836
Bryolymnia viridata|[2036]|LOCBC248-06|United States|California|658[0n]|BOLD:AAG5836
Bryolymnia viridata|[2037]|LOCBD031-06|United States|California|658[0n]|BOLD:AAG5836
Bryolymnia viridata|[2038]|BBLOD564-11|United States|California|658[0n]|BOLD:AAG5836
Bryolymnia viridata|[2039]|LOCBB233-06|United States|California|658[0n]|BOLD:AAG5836
Bryolymnia viridata|[2040]|LOCBC673-06|United States|California|658[0n]|BOLD:AAG5836
Bryolymnia viridata|[2041]|LOCBB229-06|United States|California|658[0n]|BOLD:AAG5836
Bryolymnia viridata|[2042]|LOCBB230-06|United States|California|658[0n]|BOLD:AAG5836
Bryolymnia viridata|[2043]|LOCBB234-06|United States|California|658[0n]|BOLD:AAG5836
Bryolymnia viridata|[2044]|LOCBB616-06|United States|California|658[0n]|BOLD:AAG5836
Bryolymnia viridata|[2045]|LOCBC674-06|United States|California|658[0n]|BOLD:AAG5836
Bryolymnia viridata|[2046]|LOCBD032-06|United States|California|658[0n]|BOLD:AAG5836
Bryolymnia viridata|[2047]|LOCBD033-06|United States|California|658[0n]|BOLD:AAG5836
Bryolymnia viridata|[2048]|LOCBE146-06|United States|California|658[0n]|BOLD:AAG5836
Bryolymnia viridata|[2049]|RDNMH320-09|United States|California|658[0n]|BOLD:AAG5836
Bryolymnia viridata|[2050]|LOCBF2634-13|United States|California|658[0n]|BOLD:AAG5836
Elaphria versicolor|[2051]|LNCNW066-06|United States|North Carolina|658[0n]|BOLD:AAA4393
Elaphria versicolor|[2052]|LOTB123-05|United States|Tennessee|658[0n]|BOLD:AAA4393
Elaphria versicolor|[2053]|LGSMD246-05|United States|Tennessee|658[0n]|BOLD:AAA4393
Elaphria versicolor|[2054]|LNC280-05|United States|North Carolina|658[0n]|BOLD:AAA4393
Elaphria versicolor|[2055]|LNCB377-06|United States|North Carolina|658[0n]|BOLD:AAA4393
Elaphria versicolor|[2056]|LSUSA068-06|United States|Kentucky|506[0n]|BOLD:AAA4393
Elaphria versicolor|[2057]|LOT338-04|United States|Tennessee|658[0n]|BOLD:AAA4393
Elaphria versicolor|[2058]|LPOKA605-09|United States|Oklahoma|639[0n]|BOLD:AAA4393
Elaphria versicolor|[2059]|LPOKA1001-09|United States|Oklahoma|658[0n]|BOLD:AAA4393
Elaphria versicolor|[2060]|LPOKA991-09|United States|Oklahoma|658[0n]|BOLD:AAA4393
Elaphria versicolor|[2061]|LPOKA963-09|United States|Oklahoma|658[0n]|BOLD:AAA4393
Elaphria versicolor|[2062]|LPOKA140-08|United States|Oklahoma|658[0n]|BOLD:AAA4393
Elaphria versicolor|[2063]|LPSO691-08|Canada|Ontario|658[0n]|BOLD:AAA4393
Elaphria versicolor|[2064]|LOFLA614-06|United States|Florida|658[0n]|BOLD:AAA4393
Elaphria versicolor|[2065]|LOFLA605-06|United States|Florida|658[0n]|BOLD:AAA4393
Elaphria versicolor|[2066]|LNC258-05|United States|North Carolina|658[0n]|BOLD:AAA4393
Elaphria versicolor|[2067]|LGSMD247-05|United States|Tennessee|658[0n]|BOLD:AAA4393
Elaphria versicolor|[2068]|LOCT284-05|United States|Connecticut|658[0n]|BOLD:AAA4393
Elaphria versicolor|[2069]|LOCT118-05|United States|Connecticut|658[0n]|BOLD:AAA4393
Elaphria versicolor|[2070]|LOTB353-05|United States|Tennessee|658[0n]|BOLD:AAA4393
Elaphria versicolor|[2071]|LOTB126-05|United States|Tennessee|658[0n]|BOLD:AAA4393
Elaphria versicolor|[2072]|LOTB124-05|United States|Tennessee|658[0n]|BOLD:AAA4393
Elaphria versicolor|[2073]|LGSMC867-05|United States|Tennessee|658[0n]|BOLD:AAA4393
Elaphria versicolor|[2074]|LOT337-04|United States|Tennessee|658[0n]|BOLD:AAA4393
Elaphria versicolor|[2075]|LOT335-04|United States|Tennessee|658[0n]|BOLD:AAA4393
Elaphria versicolor|[2076]|LOT336-04|United States|Tennessee|658[1n]|BOLD:AAA4393
Elaphria versicolor|[2077]|LOCT361-05|United States|Connecticut|655[0n]|BOLD:AAA4393
Elaphria versicolor|[2078]|LPOKB242-09|United States|Oklahoma|624[1n]|BOLD:AAA4393
Elaphria versicolor|[2079]|LGSMD245-05|United States|Tennessee|627[0n]|BOLD:AAA4393
Elaphria versicolor|[2080]|LSUSA069-06|United States|Kentucky|609[0n]|BOLD:AAA4393
Elaphria versicolor|[2081]|LPOKB258-09|United States|Oklahoma|622[1n]|BOLD:AAA4393
Elaphria versicolor|[2082]|LPOKC800-09|United States|Oklahoma|658[0n]|BOLD:AAA4393
Elaphria versicolor|[2083]|LGSMG1009-10|United States|Tennessee|658[0n]|BOLD:AAA4393
Elaphria versicolor|[2084]|LGSM753-04|United States|Tennessee|658[0n]|BOLD:AAA4393
Elaphria versicolor|[2085]|BBLPC171-09|Canada|Nova Scotia|658[0n]|BOLD:AAA4393
Elaphria versicolor|[2086]|CNKOE2199-14|Canada|New Brunswick|537[0n]|BOLD:AAA4393
Elaphria versicolor|[2087]|CNKJD175-14|Canada|Nova Scotia|531[0n]|
Elaphria versicolor|[2088]|ALLEP097-13|Canada|Ontario|658[0n]|BOLD:AAA4393
Elaphria versicolor|[2089]|ALLEP062-13|Canada|Ontario|658[0n]|BOLD:AAA4393
Elaphria versicolor|[2090]|BBLPE273-09|Canada|Nova Scotia|658[0n]|BOLD:AAA4393
Elaphria versicolor|[2091]|BBLPC148-09|Canada|Nova Scotia|658[0n]|BOLD:AAA4393
Elaphria versicolor|[2092]|BBLEC587-09|Canada|Nova Scotia|658[0n]|BOLD:AAA4393
Elaphria versicolor|[2093]|LPSOD782-09|Canada|Ontario|658[0n]|BOLD:AAA4393
Elaphria versicolor|[2094]|LPSOC179-08|Canada|Ontario|658[0n]|BOLD:AAA4393
Elaphria versicolor|[2095]|MNBB142-05|Canada|New Brunswick|658[0n]|BOLD:AAA4393
Elaphria versicolor|[2096]|LGSMC332-05|United States|Tennessee|658[0n]|BOLD:AAA4393
Elaphria versicolor|[2097]|LOT334-04|United States|Tennessee|658[0n]|BOLD:AAA4393
Elaphria versicolor|[2098]|LGSMC866-05|United States|Tennessee|658[0n]|BOLD:AAA4393
Elaphria versicolor|[2099]|LOTB125-05|United States|Tennessee|658[0n]|BOLD:AAA4393

Elaphria versicolor|[2097]|LOT334-04|United States|Tennessee|658[0n]|BOLD:AAA4393
Elaphria versicolor|[2098]|LGSMC866-05|United States|Tennessee|658[0n]|BOLD:AAA4393
Elaphria versicolor|[2099]|LOTB125-05|United States|Tennessee|658[0n]|BOLD:AAA4393
Elaphria versicolor|[2100]|XAB309-04|Canada|Ontario|658[0n]|BOLD:AAA4393
Elaphria versicolor|[2101]|RDLQG534-06|Canada|Quebec|621[4n]|BOLD:AAA4393
Elaphria versicolor|[2102]|BBLEC667-09|Canada|Nova Scotia|643[0n]|BOLD:AAA4393
Elaphria versicolor|[2103]|CNKJO840-14|Canada|Nova Scotia|600[0n]|BOLD:AAA4393
Elaphria versicolor|[2104]|CNPEE2056-14|Canada|Prince Edward Island|605[0n]|BOLD:AAA4393
Elaphria versicolor|[2105]|CNSLD232-12|Canada|Ontario|632[0n]|BOLD:AAA4393
Elaphria versicolor|[2106]|RDLQG699-06|Canada|Quebec|632[0n]|BOLD:AAA4393
Elaphria versicolor|[2107]|XAJ382-06|Canada|Ontario|632[0n]|BOLD:AAA4393
Elaphria versicolor|[2108]|CNKOI002-14|Canada|New Brunswick|594[0n]|BOLD:AAA4393
Elaphria versicolor|[2109]|CNKOH2030-14|Canada|New Brunswick|576[0n]|BOLD:AAA4393
Elaphria versicolor|[2110]|RDLQG507-06|Canada|Quebec|620[0n]|BOLD:AAA4393
Elaphria versicolor|[2111]|CNPED1511-14|Canada|Prince Edward Island|614[0n]|BOLD:AAA4393
Elaphria versicolor|[2112]|CNBPD282-12|Canada|Ontario|618[0n]|BOLD:AAA4393
Elaphria versicolor|[2113]|CNBPD281-12|Canada|Ontario|618[0n]|BOLD:AAA4393
Elaphria versicolor|[2114]|PHMO189-03|Canada|Ontario|639[0n]|BOLD:AAA4393
Elaphria versicolor|[2115]|CNKOH023-14|Canada|New Brunswick|612[2n]|BOLD:AAA4393
Elaphria versicolor|[2116]|RDLQG506-06|Canada|Quebec|634[0n]|BOLD:AAA4393
Elaphria versicolor|[2117]|CNKOO180-14|Canada|New Brunswick|588[0n]|BOLD:AAA4393
Elaphria versicolor|[2118]|CNKOO179-14|Canada|New Brunswick|588[0n]|BOLD:AAA4393
Elaphria versicolor|[2119]|CNFDE1639-14|Canada|New Brunswick|589[0n]|BOLD:AAA4393
Elaphria versicolor|[2120]|CNSLO024-13|Canada|Ontario|585[0n]|BOLD:AAA4393
Elaphria versicolor|[2121]|CNBPR292-13|Canada|Ontario|567[0n]|BOLD:AAA4393
Elaphria versicolor|[2122]|CNKOE2208-14|Canada|New Brunswick|579[0n]|BOLD:AAA4393
Elaphria versicolor|[2123]|CNKOG1108-14|Canada|New Brunswick|552[0n]|BOLD:AAA4393
Elaphria versicolor|[2124]|CNKOQ429-14|Canada|New Brunswick|567[0n]|BOLD:AAA4393
Elaphria versicolor|[2125]|CNKJP1288-14|Canada|Nova Scotia|591[0n]|BOLD:AAA4393
Elaphria versicolor|[2126]|RDLQG739-06|Canada|Quebec|658[0n]|BOLD:AAA4393
Elaphria versicolor|[2127]|TMNBB218-06|Canada|New Brunswick|658[0n]|BOLD:AAA4393
Elaphria versicolor|[2128]|RDLQG537-06|Canada|Quebec|658[0n]|BOLD:AAA4393
Elaphria versicolor|[2129]|CNSLE006-12|Canada|Ontario|632[0n]|BOLD:AAA4393
Elaphria versicolor|[2130]|LPSOB865-08|Canada|Ontario|658[0n]|BOLD:AAA4393
Elaphria versicolor|[2131]|ALLEP077-13|Canada|Ontario|658[0n]|BOLD:AAA4393
Elaphria versicolor|[2132]|ALLEP171-13|Canada|Ontario|658[0n]|BOLD:AAA4393
Elaphria versicolor|[2133]|ALLEP204-13|Canada|Ontario|658[0n]|BOLD:AAA4393
Elaphria versicolor|[2134]|RDLQG838-06|Canada|Quebec|658[0n]|BOLD:AAA4393
Elaphria versicolor|[2135]|RDLQG839-06|Canada|Quebec|658[0n]|BOLD:AAA4393
Elaphria versicolor|[2136]|RDLQG813-06|Canada|Quebec|658[0n]|BOLD:AAA4393
Elaphria versicolor|[2137]|RDLQG816-06|Canada|Quebec|655[0n]|BOLD:AAA4393
Elaphria versicolor|[2138]|XAJ648-06|Canada|Ontario|658[0n]|BOLD:AAA4393
Elaphria versicolor|[2139]|RDLQG533-06|Canada|Quebec|658[0n]|BOLD:AAA4393
Elaphria versicolor|[2140]|XAJ383-06|Canada|Ontario|658[0n]|BOLD:AAA4393
Elaphria versicolor|[2141]|PHMNB649-04|Canada|New Brunswick|658[0n]|BOLD:AAA4393
Elaphria versicolor|[2142]|PHMNB643-04|Canada|New Brunswick|658[0n]|BOLD:AAA4393
Elaphria versicolor|[2143]|PHMNB635-04|Canada|New Brunswick|658[0n]|BOLD:AAA4393
Elaphria versicolor|[2144]|PHMNB546-04|Canada|New Brunswick|658[0n]|BOLD:AAA4393
Elaphria versicolor|[2145]|PHMNB497-04|Canada|New Brunswick|658[0n]|BOLD:AAA4393
Elaphria versicolor|[2146]|PHMNB495-04|Canada|New Brunswick|658[0n]|BOLD:AAA4393
Elaphria versicolor|[2147]|PHMNB493-04|Canada|New Brunswick|658[0n]|BOLD:AAA4393
Elaphria versicolor|[2148]|PHMNB386-04|Canada|New Brunswick|658[0n]|BOLD:AAA4393
Elaphria versicolor|[2149]|XAC325-04|Canada|Ontario|658[0n]|BOLD:AAA4393
Elaphria versicolor|[2150]|XAB370-04|Canada|Ontario|658[0n]|BOLD:AAA4393
Elaphria versicolor|[2151]|RDLQB923-05|Canada|Quebec|658[0n]|BOLD:AAA4393
Elaphria versicolor|[2152]|PHMTV428-10|Canada|Ontario|658[0n]|BOLD:AAA4393
Elaphria versicolor|[2153]|RDLQB925-05|Canada|Quebec|658[0n]|BOLD:AAA4393
Elaphria versicolor|[2154]|CNBPD741-13|Canada|Ontario|614[0n]|BOLD:AAA4393
Elaphria versicolor|[2155]|CNSLP1129-13|Canada|Ontario|608[0n]|BOLD:AAA4393
Elaphria versicolor|[2156]|MEC706-04|Canada|Quebec|658[0n]|BOLD:AAA4393
Elaphria versicolor|[2157]|CNPEE2053-14|Canada|Prince Edward Island|604[0n]|BOLD:AAA4393
Elaphria versicolor|[2158]|CNLMN302-14|Canada|Quebec|600[0n]|BOLD:AAA4393
Elaphria versicolor|[2159]|XAI025-05|Canada|Ontario|565[0n]|BOLD:AAA4393
Elaphria versicolor|[2160]|CNKOE2204-14|Canada|New Brunswick|600[0n]|BOLD:AAA4393
Elaphria versicolor|[2161]|BBLPC566-09|Canada|Nova Scotia|638[0n]|BOLD:AAA4393
Elaphria versicolor|[2162]|RDLQG510-06|Canada|Quebec|646[0n]|BOLD:AAA4393
Elaphria versicolor|[2163]|CNKOE2201-14|Canada|New Brunswick|597[0n]|BOLD:AAA4393
Elaphria versicolor|[2164]|CNPKG1461-14|Canada|Ontario|601[0n]|BOLD:AAA4393
Elaphria versicolor|[2165]|CNBPQ438-13|Canada|Ontario|603[3n]|BOLD:AAA4393
Elaphria versicolor|[2166]|CNFDG382-14|Canada|New Brunswick|621[0n]|BOLD:AAA4393
Elaphria versicolor|[2167]|CNBPE746-13|Canada|Ontario|614[0n]|BOLD:AAA4393
Elaphria versicolor|[2168]|CNBPD745-13|Canada|Ontario|614[0n]|BOLD:AAA4393
Elaphria versicolor|[2169]|CNKOH024-14|Canada|New Brunswick|591[0n]|BOLD:AAA4393
Elaphria versicolor|[2170]|CNBPD740-13|Canada|Ontario|611[0n]|BOLD:AAA4393
Elaphria versicolor|[2171]|CNKOR1761-14|Canada|New Brunswick|516[0n]|
Elaphria versicolor|[2172]|CNKOE2203-14|Canada|New Brunswick|588[0n]|BOLD:AAA4393
Elaphria versicolor|[2173]|BBLPE278-09|Canada|Nova Scotia|616[0n]|BOLD:AAA4393
Elaphria versicolor|[2174]|CNFNF2491-14|Canada|Quebec|571[0n]|BOLD:AAA4393
Elaphria versicolor|[2175]|CNPED152-14|Canada|Prince Edward Island|563[0n]|BOLD:AAA4393
Elaphria versicolor|[2176]|CNSLO020-13|Canada|Ontario|555[0n]|BOLD:AAA4393
Elaphria versicolor|[2177]|XAB297-04|Canada|Ontario|614[0n]|BOLD:AAA4393
Elaphria versicolor|[2178]|XAE499-04|Canada|Ontario|574[2n]|BOLD:AAA4393
Elaphria versicolor|[2179]|TTMNB370-06|Canada|New Brunswick|519[0n]|BOLD:AAA4393
Elaphria versicolor|[2180]|RDLQG505-06|Canada|Quebec|605[0n]|BOLD:AAA4393
Elaphria versicolor|[2181]|RDLQG508-06|Canada|Quebec|605[0n]|BOLD:AAA4393
Elaphria versicolor|[2182]|RDLQG509-06|Canada|Quebec|605[0n]|BOLD:AAA4393
Elaphria versicolor|[2183]|CNBPE744-13|Canada|Ontario|563[0n]|BOLD:AAA4393
Elaphria versicolor|[2184]|CNKOE408-14|Canada|New Brunswick|568[0n]|BOLD:AAA4393
Elaphria versicolor|[2185]|CNKOR1758-14|Canada|New Brunswick|576[0n]|BOLD:AAA4393
Elaphria versicolor|[2186]|CNLMO2451-14|Canada|Quebec|564[0n]|BOLD:AAA4393
Elaphria devara|[2187]|MOTAR226-12|Argentina|Formosa|647[0n]|BOLD:AAB2202
Elaphria devara|[2188]|BLPDG267-09|Costa Rica|Guanacaste|658[0n]|BOLD:AAB2202
Elaphria devara|[2189]|BLPDZ687-11|Costa Rica|Alajuela|658[0n]|BOLD:AAB2202
Elaphria devara|[2190]|BLPEE5044-14|Costa Rica|Guanacaste|658[0n]|BOLD:AAB2202
Elaphria devara|[2191]|BLPCA302-08|Costa Rica|Guanacaste|658[0n]|BOLD:AAB2202
Elaphria devara|[2192]|LPYPB498-08|Mexico|Quintana Roo|658[0n]|BOLD:AAB2202
Elaphria devara|[2193]|BLPDD609-09|Costa Rica|Guanacaste|658[0n]|BOLD:AAB2202
Elaphria devara|[2194]|BLPDM1470-10|Costa Rica|Guanacaste|658[0n]|BOLD:AAB2202
Elaphria devara|[2195]|BLPDD605-09|Costa Rica|Guanacaste|658[0n]|BOLD:AAB2202
Elaphria devara|[2196]|LPYPB429-08|Mexico|Campeche|652[0n]|BOLD:AAB2202
Elaphria devara|[2197]|BLPDD607-09|Costa Rica|Guanacaste|658[0n]|BOLD:AAB2202
Elaphria devara|[2198]|MHMXA812-06|Costa Rica|Guanacaste|658[0n]|BOLD:AAB2202
Elaphria devara|[2199]|LPMX249-07|Mexico|Campeche|658[0n]|BOLD:AAB2202

Elaphria devara|[2197]|BLPDD607-09|Costa Rica|Guanacaste|658[0n]|BOLD:AAB2202
Elaphria devara|[2198]|MHMXA812-06|Costa Rica|Guanacaste|658[0n]|BOLD:AAB2202
Elaphria devara|[2199]|LPMX249-07|Mexico|Campeche|658[0n]|BOLD:AAB2202
Elaphria devara|[2200]|BLPBE416-07|Costa Rica|Guanacaste|658[0n]|BOLD:AAB2202
Elaphria devara|[2201]|BLPCG886-08|Costa Rica|Guanacaste|658[0n]|BOLD:AAB2202
Elaphria devara|[2202]|BLPCI729-08|Costa Rica|Guanacaste|658[0n]|BOLD:AAB2202
Elaphria devara|[2203]|BLPDE161-09|Costa Rica|Guanacaste|658[0n]|BOLD:AAB2202
Elaphria devara|[2204]|BLPDE233-09|Costa Rica|Guanacaste|658[0n]|BOLD:AAB2202
Elaphria devara|[2205]|BLPED1773-12|Costa Rica|Guanacaste|658[0n]|BOLD:AAB2202
Elaphria devara|[2206]|BLPEE4389-14|Costa Rica|Guanacaste|658[0n]|BOLD:AAB2202
Elaphria devara|[2207]|BLPEE5328-14|Costa Rica|Guanacaste|658[0n]|BOLD:AAB2202
Elaphria devara|[2208]|LYPIE208-09|Mexico|Quintana Roo|658[0n]|BOLD:AAB2202
Elaphria devara|[2209]|LPMX257-07|Mexico|Campeche|646[0n]|BOLD:AAB2202
Elaphria devara|[2210]|BLPEE5042-14|Costa Rica|Guanacaste|658[0n]|BOLD:AAB2202
Elaphria devara|[2211]|BLPDD413-09|Costa Rica|Guanacaste|658[0n]|BOLD:AAB2202
Elaphria devara|[2212]|BLPDD411-09|Costa Rica|Guanacaste|658[0n]|BOLD:AAB2202
Elaphria devara|[2213]|BLPDD410-09|Costa Rica|Guanacaste|658[0n]|BOLD:AAB2202
Elaphria devara|[2214]|BLPCI728-08|Costa Rica|Guanacaste|658[0n]|BOLD:AAB2202
Elaphria devara|[2215]|BLPCH605-08|Costa Rica|Guanacaste|658[0n]|BOLD:AAB2202
Elaphria devara|[2216]|LPMX443-07|Mexico|Campeche|658[0n]|BOLD:AAB2202
Elaphria devara|[2217]|LPMX250-07|Mexico|Campeche|658[0n]|BOLD:AAB2202
Elaphria devara|[2218]|LPMX201-07|Mexico|Campeche|658[0n]|BOLD:AAB2202
Elaphria devara|[2219]|BLPAB264-06|Costa Rica|Alajuela|658[0n]|BOLD:AAB2202
Elaphria devara|[2220]|BLPCJ468-08|Costa Rica|Guanacaste|658[0n]|BOLD:AAB2202
Elaphria devara|[2221]|BLPBC862-07|Costa Rica|Alajuela|630[1n]|BOLD:AAB2202
Elaphria devara|[2222]|LPMX235-07|Mexico|Campeche|623[0n]|BOLD:AAB2202
Elaphria devara|[2223]|MXBLP208-11|Mexico|Jalisco|633[0n]|BOLD:AAB2202
Elaphria devara|[2224]|MXBLP261-11|Mexico|Jalisco|625[0n]|BOLD:AAB2202
Elaphria devara|[2225]|BLPDE178-09|Costa Rica|Guanacaste|658[0n]|BOLD:AAB2202
Elaphria devara|[2226]|BLPDZ815-11|Costa Rica|Alajuela|658[0n]|BOLD:AAB2202
Elaphria devara|[2227]|BLPED2504-12|Costa Rica|Guanacaste|658[0n]|BOLD:AAB2202
Elaphria devara|[2228]|BLPEE2283-14|Costa Rica|Guanacaste|613[0n]|BOLD:AAB2202
Elaphria devara|[2229]|BLPEE4831-14|Costa Rica|Guanacaste|658[0n]|BOLD:AAB2202
Elaphria devara|[2230]|BLPEE5037-14|Costa Rica|Guanacaste|658[0n]|BOLD:AAB2202
Elaphria devara|[2231]|BLPEE5327-14|Costa Rica|Guanacaste|658[0n]|BOLD:AAB2202
Elaphria devara|[2232]|BLPEE5394-14|Costa Rica|Guanacaste|658[0n]|BOLD:AAB2202
Elaphria devara|[2233]|BLPED2137-12|Costa Rica|Guanacaste|658[0n]|BOLD:AAB2202
Elaphria devara|[2234]|CQR493-13|Mexico|Quintana Roo|658[0n]|BOLD:AAB2202
Elaphria devara|[2235]|BLPEE5395-14|Costa Rica|Guanacaste|658[0n]|BOLD:AAB2202
Elaphria devara|[2236]|LNAUT413-14|Mexico|Veracruz|658[0n]|BOLD:AAB2202
Elaphria devara|[2237]|LNAUT415-14|Mexico|Veracruz|658[0n]|BOLD:AAB2202
Elaphria devara|[2238]|BLPEG1073-14|Costa Rica|Guanacaste|658[0n]|BOLD:AAB2202
Elaphria devara|[2239]|BLPEE5115-14|Costa Rica|Guanacaste|658[0n]|BOLD:AAB2202
Elaphria devara|[2240]|BLPEE5326-14|Costa Rica|Guanacaste|658[0n]|BOLD:AAB2202
Elaphria devara|[2241]|BLPEF3765-13|Costa Rica|Guanacaste|658[0n]|BOLD:AAB2202
Elaphria devara|[2242]|BLPEE4661-14|Costa Rica|Guanacaste|658[0n]|BOLD:AAB2202
Elaphria devara|[2243]|BLPEE4869-14|Costa Rica|Guanacaste|658[0n]|BOLD:AAB2202
Elaphria devara|[2244]|BLPEE5035-14|Costa Rica|Guanacaste|658[0n]|BOLD:AAB2202
Elaphria devara|[2245]|BLPEE5038-14|Costa Rica|Guanacaste|658[0n]|BOLD:AAB2202
Elaphria devara|[2246]|BLPEE5043-14|Costa Rica|Guanacaste|658[0n]|BOLD:AAB2202
Elaphria devara|[2247]|BLPED667-11|Costa Rica|Guanacaste|658[0n]|BOLD:AAB2202
Elaphria devara|[2248]|BLPED1534-12|Costa Rica|Guanacaste|658[0n]|BOLD:AAB2202
Elaphria devara|[2249]|BLPDE331-09|Costa Rica|Guanacaste|658[0n]|BOLD:AAB2202
Elaphria devara|[2250]|BLPDX102-11|Costa Rica|Guanacaste|658[0n]|BOLD:AAB2202
Elaphria devara|[2251]|BLPDY012-11|Costa Rica|Guanacaste|658[0n]|BOLD:AAB2202
Elaphria devara|[2252]|MHMYL3481-11|Costa Rica||658[0n]|BOLD:AAB2202
Elaphria devara|[2253]|BLPDE169-09|Costa Rica|Guanacaste|658[0n]|BOLD:AAB2202
Elaphria devara|[2254]|BLPDE177-09|Costa Rica|Guanacaste|658[0n]|BOLD:AAB2202
Elaphria devara|[2255]|BLPDE179-09|Costa Rica|Guanacaste|658[0n]|BOLD:AAB2202
Elaphria devara|[2256]|BLPDE180-09|Costa Rica|Guanacaste|658[0n]|BOLD:AAB2202
Elaphria devara|[2257]|BLPDD606-09|Costa Rica|Guanacaste|658[0n]|BOLD:AAB2202
Elaphria devara|[2258]|BLPDD608-09|Costa Rica|Guanacaste|658[0n]|BOLD:AAB2202
Elaphria devara|[2259]|CNCLB2415-14|United States|Texas|658[0n]|BOLD:AAB2202
Dypterygia patina|[2260]|NAMUM069-08|United States|Arizona|657[0n]|BOLD:AAK4105
Dypterygia patina|[2261]|RDNMJ112-10|United States|Arizona|658[0n]|BOLD:AAK4105
Dypterygia patina|[2262]|RDNMJ369-11|United States|Arizona|600[0n]|BOLD:AAK4105
Dypterygia patina|[2263]|USLEP719-10|United States|Texas|658[0n]|BOLD:AAK4105
Dypterygia patina|[2264]|CMAZA1042-12|United States|Arizona|658[0n]|BOLD:AAK4105
Gonodes liquida|[2265]|BLPBC369-07|Costa Rica|Alajuela|610[0n]|BOLD:AAA2909
Gonodes liquida|[2266]|LOCRA881-08|Costa Rica|Alajuela|658[0n]|BOLD:AAA2909
Gonodes liquida|[2267]|BLPBD280-07|Costa Rica|Guanacaste|656[0n]|BOLD:AAA2909
Gonodes liquida|[2268]|BLPDC889-09|Costa Rica|Alajuela|658[0n]|BOLD:AAA2909
Gonodes liquida|[2269]|BLPDD624-09|Costa Rica|Guanacaste|658[0n]|BOLD:AAA2909
Gonodes liquida|[2270]|BLPDM754-10|Costa Rica|Guanacaste|658[0n]|BOLD:AAA2909
Gonodes liquida|[2271]|BLPDV940-11|Costa Rica|Guanacaste|658[0n]|BOLD:AAA2909
Gonodes liquida|[2272]|BLPDY769-11|Costa Rica|Guanacaste|658[0n]|BOLD:AAA2909
Gonodes liquida|[2273]|BLPEF3587-13|Costa Rica|Guanacaste|658[0n]|BOLD:AAA2909
Gonodes liquida|[2274]|BLPCG858-08|Costa Rica|Guanacaste|658[1n]|BOLD:AAA2909
Gonodes liquida|[2275]|BLPEF965-12|Costa Rica|Alajuela|658[0n]|BOLD:AAA2909
Gonodes liquida|[2276]|BBLOB511-11|United States|Florida|658[0n]|BOLD:AAA2909
Gonodes liquida|[2277]|BLPDW1156-11|Costa Rica|Guanacaste|658[0n]|BOLD:AAA2909
Gonodes liquida|[2278]|BLPDW200-11|Costa Rica|Guanacaste|658[0n]|BOLD:AAA2909
Gonodes liquida|[2279]|BLPDX139-11|Costa Rica|Guanacaste|658[0n]|BOLD:AAA2909
Gonodes liquida|[2280]|BLPDN181-10|Costa Rica|Alajuela|658[0n]|BOLD:AAA2909
Gonodes liquida|[2281]|BLPDM028-10|Costa Rica|Guanacaste|658[0n]|BOLD:AAA2909
Gonodes liquida|[2282]|BLPDF532-09|Costa Rica|Alajuela|658[0n]|BOLD:AAA2909
Gonodes liquida|[2283]|BLPDD625-09|Costa Rica|Guanacaste|658[0n]|BOLD:AAA2909
Gonodes liquida|[2284]|BLPDD623-09|Costa Rica|Guanacaste|658[0n]|BOLD:AAA2909
Gonodes liquida|[2285]|BLPDD423-09|Costa Rica|Guanacaste|658[0n]|BOLD:AAA2909
Gonodes liquida|[2286]|BLPDC890-09|Costa Rica|Alajuela|658[0n]|BOLD:AAA2909
Gonodes liquida|[2287]|BLPDC620-09|Costa Rica|Alajuela|658[0n]|BOLD:AAA2909
Gonodes liquida|[2288]|BLPDB901-09|Costa Rica|Guanacaste|658[0n]|BOLD:AAA2909
Gonodes liquida|[2289]|BLPCK112-08|Costa Rica|Alajuela|658[0n]|BOLD:AAA2909
Gonodes liquida|[2290]|BLPCI709-08|Costa Rica|Guanacaste|658[0n]|BOLD:AAA2909
Gonodes liquida|[2291]|BLPCG860-08|Costa Rica|Guanacaste|658[0n]|BOLD:AAA2909
Gonodes liquida|[2292]|BLPCG859-08|Costa Rica|Guanacaste|658[0n]|BOLD:AAA2909
Gonodes liquida|[2293]|BLPCG801-08|Costa Rica|Guanacaste|658[0n]|BOLD:AAA2909
Gonodes liquida|[2294]|BLPCH586-08|Costa Rica|Guanacaste|658[0n]|BOLD:AAA2909
Gonodes liquida|[2295]|BLPCF142-08|Costa Rica|Alajuela|658[0n]|BOLD:AAA2909
Gonodes liquida|[2296]|BLPCC552-08|Costa Rica|Guanacaste|658[0n]|BOLD:AAA2909
Gonodes liquida|[2297]|BLPCC403-08|Costa Rica|Guanacaste|658[0n]|BOLD:AAA2909
Gonodes liquida|[2298]|BLPBC866-07|Costa Rica|Alajuela|658[0n]|BOLD:AAA2909
Gonodes liquida|[2299]|BLPBA707-07|Costa Rica|Guanacaste|658[0n]|BOLD:AAA2909

Gonodes liquida|[2297]|BLPCC403-08|Costa Rica|Guanacaste|658[0n]|BOLD:AAA2909
Gonodes liquida|[2298]|BLPBC866-07|Costa Rica|Alajuela|658[0n]|BOLD:AAA2909
Gonodes liquida|[2299]|BLPBA707-07|Costa Rica|Guanacaste|658[0n]|BOLD:AAA2909
Gonodes liquida|[2300]|LPMX151-07|Mexico|Campeche|658[0n]|BOLD:AAA2909
Gonodes liquida|[2301]|BLPAH367-07|Costa Rica|Guanacaste|658[0n]|BOLD:AAA2909
Gonodes liquida|[2302]|BLPAH123-07|Costa Rica|Guanacaste|658[0n]|BOLD:AAA2909
Gonodes liquida|[2303]|BLPAC506-06|Costa Rica|Guanacaste|658[0n]|BOLD:AAA2909
Gonodes liquida|[2304]|BLPAA937-06|Costa Rica|Alajuela|658[0n]|BOLD:AAA2909
Gonodes liquida|[2305]|BLPBB789-07|Costa Rica|Guanacaste|630[0n]|BOLD:AAA2909
Gonodes liquida|[2306]|BLPBC746-07|Costa Rica|Guanacaste|630[0n]|BOLD:AAA2909
Gonodes liquida|[2307]|BLPAH366-07|Costa Rica|Guanacaste|642[0n]|BOLD:AAA2909
Gonodes liquida|[2308]|BLPDW115-11|Costa Rica|Guanacaste|611[1n]|BOLD:AAA2909
Gonodes liquida|[2309]|BBLOB486-11|United States|Florida|658[0n]|BOLD:AAA2909
Gonodes liquida|[2310]|BLPBC867-07|Costa Rica|Alajuela|622[1n]|BOLD:AAA2909
Gonodes liquida|[2311]|BLPEF966-12|Costa Rica|Alajuela|658[0n]|BOLD:AAA2909
Gonodes liquida|[2312]|BLPDW201-11|Costa Rica|Guanacaste|658[0n]|BOLD:AAA2909
Gonodes liquida|[2313]|BLPDX479-11|Costa Rica|Guanacaste|658[0n]|BOLD:AAA2909
Gonodes liquida|[2314]|BLPBA193-07|Costa Rica|Guanacaste|658[0n]|BOLD:AAA2909
Gonodes liquida|[2315]|BLPAB006-06|Costa Rica|Alajuela|658[0n]|BOLD:AAA2909
Gonodes liquida|[2316]|BLPEF3772-13|Costa Rica|Guanacaste|566[0n]|BOLD:AAA2909
Gonodes liquida|[2317]|BLPBD316-07|Costa Rica|Guanacaste|603[0n]|BOLD:AAA2909
Gonodes liquida|[2318]|BLPCG857-08|Costa Rica|Guanacaste|658[2n]|BOLD:AAA2909
Gonodes liquida|[2319]|BLPCK263-08|Costa Rica|Alajuela|658[0n]|BOLD:AAA2909
Gonodes liquida|[2320]|BLPCO802-08|Costa Rica|Guanacaste|658[0n]|BOLD:AAA2909
Gonodes liquida|[2321]|BLPDD622-09|Costa Rica|Guanacaste|658[0n]|BOLD:AAA2909
Gonodes liquida|[2322]|BLPDH759-09|Costa Rica|Alajuela|658[0n]|BOLD:AAA2909
Gonodes liquida|[2323]|BLPEF1297-12|Costa Rica|Guanacaste|658[0n]|BOLD:AAA2909
Gonodes liquida|[2324]|BLPEF4036-13|Costa Rica|Guanacaste|658[0n]|BOLD:AAA2909
Chytonix palliatricula|[2325]|LGSMC390-05|United States|Tennessee|658[0n]|BOLD:AAA6619
Chytonix palliatricula|[2326]|LSUSA242-06|United States|Kentucky|658[0n]|BOLD:AAA6619
Chytonix palliatricula|[2327]|LOT355-04|United States|Tennessee|657[0n]|BOLD:AAA6619
Chytonix palliatricula|[2328]|LGSMC391-05|United States|Tennessee|658[0n]|BOLD:AAA6619
Chytonix palliatricula|[2329]|LOTB207-05|United States|Tennessee|658[0n]|BOLD:AAA6619
Chytonix palliatricula|[2330]|BBLSW484-09|United States|Oklahoma|644[0n]|BOLD:AAA6619
Chytonix palliatricula|[2331]|LGSM507-04|United States|Tennessee|658[0n]|BOLD:AAA6619
Chytonix palliatricula|[2332]|LOT547-04|United States|Tennessee|595[0n]|BOLD:AAA6619
Chytonix palliatricula|[2333]|LOT505-04|United States|Tennessee|658[0n]|BOLD:AAA6619
Chytonix palliatricula|[2334]|LNC100-05|United States|North Carolina|658[0n]|BOLD:AAA6619
Chytonix palliatricula|[2335]|BBLSU046-09|United States|Arkansas|658[0n]|BOLD:AAA6619
Chytonix palliatricula|[2336]|CNSLE370-12|Canada|Ontario|633[0n]|BOLD:AAA6619
Chytonix palliatricula|[2337]|CNSLD272-12|Canada|Ontario|632[0n]|BOLD:AAA6619
Chytonix palliatricula|[2338]|XAC596-04|Canada|Ontario|617[0n]|BOLD:AAA6619
Chytonix palliatricula|[2339]|CNSLD556-12|Canada|Ontario|632[3n]|BOLD:AAA6619
Chytonix palliatricula|[2340]|LGSMG995-10|United States|Tennessee|658[0n]|BOLD:AAA6619
Chytonix palliatricula|[2341]|LOT191-04|United States|Tennessee|609[0n]|BOLD:AAA6619
Chytonix palliatricula|[2342]|LOT358-04|United States|Tennessee|657[0n]|BOLD:AAA6619
Chytonix palliatricula|[2343]|LOT359-04|United States|Tennessee|658[0n]|BOLD:AAA6619
Chytonix palliatricula|[2344]|LOT520-04|United States|Tennessee|658[0n]|BOLD:AAA6619
Chytonix palliatricula|[2345]|LNC101-05|United States|North Carolina|657[0n]|BOLD:AAA6619
Chytonix palliatricula|[2346]|XAI045-05|Canada|Ontario|658[0n]|BOLD:AAA6619
Chytonix palliatricula|[2347]|XAJ693-06|Canada|Ontario|657[0n]|BOLD:AAA6619
Chytonix palliatricula|[2348]|LSEU623-06|United States|Georgia|657[0n]|BOLD:AAA6619
Chytonix palliatricula|[2349]|LILLA134-11|United States|Illinois|658[0n]|BOLD:AAA6619
Chytonix palliatricula|[2350]|RDNMK219-11|Canada|Ontario|658[0n]|BOLD:AAA6619
Chytonix palliatricula|[2351]|RDNMK220-11|Canada|Ontario|658[0n]|BOLD:AAA6619
Chytonix palliatricula|[2352]|LOT562-04|United States|Tennessee|589[0n]|BOLD:AAA6619
Chytonix palliatricula|[2353]|CNBPM451-13|Canada|Ontario|593[0n]|BOLD:AAA6619
Chytonix palliatricula|[2354]|CNSLP067-13|Canada|Ontario|552[0n]|BOLD:AAA6619
Chytonix palliatricula|[2355]|RDNMD503-06|United States|Florida|658[0n]|BOLD:AAA6619
Chytonix palliatricula|[2356]|LPSOD325-09|Canada|Ontario|614[0n]|BOLD:AAA6619
Chytonix palliatricula|[2357]|LPSOB599-08|Canada|Ontario|658[0n]|BOLD:AAA6619
Chytonix palliatricula|[2358]|LPSOD321-09|Canada|Ontario|658[0n]|BOLD:AAA6619
Chytonix palliatricula|[2359]|LPSOD347-09|Canada|Ontario|658[0n]|BOLD:AAA6619
Chytonix palliatricula|[2360]|PHMNB470-04|Canada|New Brunswick|658[0n]|BOLD:AAA6619
Chytonix palliatricula|[2361]|RDNMD299-06|Canada|New Brunswick|653[0n]|BOLD:AAA6619
Chytonix palliatricula|[2362]|LOT506-04|United States|Tennessee|658[0n]|BOLD:AAA6619
Chytonix palliatricula|[2363]|RDNMD298-06|Canada|New Brunswick|658[0n]|BOLD:AAA6619
Chytonix palliatricula|[2364]|RDNMD301-06|Canada|New Brunswick|658[0n]|BOLD:AAA6619
Chytonix palliatricula|[2365]|BBLEC579-09|Canada|Nova Scotia|658[1n]|BOLD:AAA6619
Chytonix palliatricula|[2366]|RDNMD300-06|Canada|New Brunswick|653[0n]|BOLD:AAA6619
Chytonix palliatricula|[2367]|LPMN010-08|Canada|Manitoba|658[0n]|BOLD:AAA6619
Chytonix palliatricula|[2368]|LPOKA529-09|United States|Oklahoma|658[0n]|BOLD:AAA6619
Chytonix palliatricula|[2369]|LPOKB225-09|United States|Oklahoma|658[0n]|BOLD:AAA6619
Chytonix palliatricula|[2370]|LPOKB248-09|United States|Oklahoma|658[0n]|BOLD:AAA6619
Chytonix palliatricula|[2371]|LPOKB322-09|United States|Oklahoma|658[0n]|BOLD:AAA6619
Chytonix palliatricula|[2372]|BBLSX588-09|United States|Oklahoma|658[0n]|BOLD:AAA6619
Chytonix palliatricula|[2373]|BBLSZ126-09|United States|Oklahoma|658[0n]|BOLD:AAA6619
Chytonix palliatricula|[2374]|LPOKB270-09|United States|Oklahoma|658[0n]|BOLD:AAA6619
Chytonix palliatricula|[2375]|LPOKB307-09|United States|Oklahoma|658[0n]|BOLD:AAA6619
Chytonix palliatricula|[2376]|LPSOB468-08|Canada|Ontario|658[0n]|BOLD:AAA6619
Chytonix palliatricula|[2377]|PHMO231-03|Canada|Ontario|639[0n]|BOLD:AAA6619
Chytonix palliatricula|[2378]|LOTB261-05|United States|Tennessee|658[0n]|BOLD:AAA6619
Chytonix palliatricula|[2379]|LGSM508-04|United States|North Carolina|658[0n]|BOLD:AAA6619
Chytonix palliatricula|[2380]|LSEU353-06|United States|Georgia|632[0n]|BOLD:AAA6619
Chytonix palliatricula|[2381]|LSEU622-06|United States|Georgia|658[0n]|BOLD:AAA6619
Chytonix palliatricula|[2382]|CNRMD2060-12|Canada|Manitoba|635[0n]|BOLD:AAA6619
Chytonix palliatricula|[2383]|CNSLE374-12|Canada|Ontario|638[0n]|BOLD:AAA6619
Chytonix palliatricula|[2384]|CNSLP059-13|Canada|Ontario|603[0n]|BOLD:AAA6619
Chytonix palliatricula|[2385]|LOCT066-05|United States|Connecticut|658[0n]|BOLD:AAA6619
Chytonix palliatricula|[2386]|LOTB260-05|United States|Tennessee|658[0n]|BOLD:AAA6619
Chytonix palliatricula|[2387]|LGSMC688-05|United States|Tennessee|658[0n]|BOLD:AAA6619
Chytonix palliatricula|[2388]|LGSMC389-05|United States|Tennessee|658[0n]|BOLD:AAA6619
Chytonix palliatricula|[2389]|LGSM747-04|United States|Tennessee|658[0n]|BOLD:AAA6619
Chytonix palliatricula|[2390]|TMNBB193-06|Canada|New Brunswick|657[0n]|BOLD:AAA6619
Chytonix palliatricula|[2391]|TMNBB194-06|Canada|New Brunswick|658[0n]|BOLD:AAA6619
Chytonix palliatricula|[2392]|TMNBB195-06|Canada|New Brunswick|657[0n]|BOLD:AAA6619
Chytonix palliatricula|[2393]|XAK110-06|Canada|Ontario|658[0n]|BOLD:AAA6619
Chytonix palliatricula|[2394]|LPSOC138-08|Canada|Ontario|658[0n]|BOLD:AAA6619
Chytonix palliatricula|[2395]|LPOKB748-09|United States|Oklahoma|658[0n]|BOLD:AAA6619
Chytonix palliatricula|[2396]|BBLSX073-09|United States|Oklahoma|658[0n]|BOLD:AAA6619
Chytonix palliatricula|[2397]|BBLCU131-09|United States|Michigan|658[0n]|BOLD:AAA6619
Chytonix palliatricula|[2398]|RDMAB077-05|Canada|Alberta|571[0n]|BOLD:AAA6619
Chytonix palliatricula|[2399]|BBLCU227-09|United States|Michigan|658[0n]|BOLD:AAA6619

Chytonix palliatricula|[2397]|BBLCU131-09|United States|Michigan|658[0n]|BOLD:AAA6619
Chytonix palliatricula|[2398]|RDMAB077-05|Canada|Alberta|571[0n]|BOLD:AAA6619
Chytonix palliatricula|[2399]|BBLCU227-09|United States|Michigan|658[0n]|BOLD:AAA6619
Chytonix palliatricula|[2400]|BBLCU033-09|United States|Michigan|658[0n]|BOLD:AAA6619
Chytonix palliatricula|[2401]|LOCT065-05|United States|Connecticut|616[0n]|BOLD:AAA6619
Chytonix palliatricula|[2402]|SSEIA3359-13|Canada|Alberta|627[0n]|BOLD:AAA6619
Chytonix palliatricula|[2403]|CNRME1814-12|Canada|Manitoba|612[0n]|BOLD:AAA6619
Chytonix palliatricula|[2404]|BBLPB409-10|Canada|Ontario|658[0n]|BOLD:AAA6619
Chytonix palliatricula|[2405]|RDLQB268-05|Canada|Quebec|658[0n]|BOLD:AAA6619
Chytonix palliatricula|[2406]|CNSLF925-12|Canada|Ontario|567[0n]|BOLD:AAA6619
Chytonix palliatricula|[2407]|CNSLP057-13|Canada|Ontario|564[4n]|BOLD:AAA6619
Chytonix palliatricula|[2408]|CNSLP1135-13|Canada|Ontario|611[0n]|BOLD:AAA6619
Chytonix palliatricula|[2409]|CNSLP1140-13|Canada|Ontario|614[0n]|BOLD:AAA6619
Chytonix sp.|[2410]|RDNMD296-06|United States|Florida|658[0n]|BOLD:ABA8707
Chytonix sp.|[2411]|RDNMD297-06|United States|Florida|656[0n]|BOLD:ABA8707
Chytonix sp.|[2412]|PSAT114-10|United States|Florida|632[1n]|BOLD:ABA8707
Chytonix sensilis|[2413]|RDNMK060-11|United States|Florida|658[0n]|BOLD:AAD8379
Chytonix sensilis|[2414]|RDLQF374-06|Canada|Quebec|658[0n]|BOLD:AAD8379
Chytonix sensilis|[2415]|RDLQF375-06|Canada|Quebec|658[0n]|BOLD:AAD8379
Chytonix sensilis|[2416]|PSAT117-10|Canada|Ontario|658[0n]|BOLD:AAD8379
Chytonix sensilis|[2417]|RDNMK062-11|United States|Florida|658[0n]|BOLD:AAD8379
Chytonix sensilis|[2418]|LSEU185-06|United States|Georgia|658[0n]|BOLD:AAD8379
Chytonix sensilis|[2419]|LSEU186-06|United States|Georgia|658[0n]|BOLD:AAD8379
Chytonix sensilis|[2420]|CNCLB2786-14|United States|North Carolina|658[0n]|BOLD:AAD8379
Marilopteryx carancahua|[2421]|CNCLB246-14|United States|Texas|658[0n]|BOLD:ACM4103
Marilopteryx lutina|[2422]|LNAUT474-14|United States|Florida|658[0n]|BOLD:ACM4271
Marilopteryx lutina|[2423]|CNCLB247-14|United States|Florida|658[0n]|BOLD:ACM4271
Marilopteryx lutina|[2424]|LNAUT855-14|United States|Florida|658[0n]|BOLD:ACM4271
Marilopteryx lutina|[2425]|CNCLB248-14|United States|Florida|658[0n]|BOLD:ACM4271
Marilopteryx lutina|[2426]|LNAUT472-14|United States|Florida|658[0n]|BOLD:ACM4271
Marilopteryx lutina|[2427]|LNAUT473-14|United States|Florida|658[0n]|BOLD:ACM4271
Marilopteryx lutina|[2428]|LNAUT856-14|United States|Florida|658[0n]|BOLD:ACM4271
Alastria chico|[2429]|RDNMD354-06|Canada|British Columbia|658[0n]|BOLD:AAF0646
Alastria chico|[2430]|RWWB078-09|United States|Washington|658[0n]|BOLD:AAF0646
Alastria chico|[2431]|RWWB159-09|United States|Washington|658[0n]|BOLD:AAF0646
Alastria chico|[2432]|RWWC1089-12|United States|Washington|658[0n]|BOLD:AAF0646
Iodopepla u-album|[2433]|LSEU512-06|United States|Georgia|658[0n]|BOLD:AAC1012
Iodopepla u-album|[2434]|USLEP618-10|United States|Florida|658[0n]|BOLD:AAC1012
Iodopepla u-album|[2435]|LNCNW110-06|United States|North Carolina|658[0n]|BOLD:AAC1012
Iodopepla u-album|[2436]|LNC738-06|United States|North Carolina|658[0n]|BOLD:AAC1012
Iodopepla u-album|[2437]|RDNMG167-08|Canada|Ontario|658[0n]|BOLD:AAC1012
Iodopepla u-album|[2438]|LSEU058-06|United States|Georgia|591[0n]|BOLD:AAC1012
Iodopepla u-album|[2439]|LSEU059-06|United States|Florida|597[0n]|BOLD:AAC1012
Iodopepla u-album|[2440]|LSEU060-06|United States|Georgia|602[0n]|BOLD:AAC1012
Iodopepla u-album|[2441]|LNC739-06|United States|North Carolina|658[0n]|BOLD:AAC1012
Iodopepla u-album|[2442]|LNC740-06|United States|North Carolina|658[0n]|BOLD:AAC1012
Iodopepla u-album|[2443]|LOFLB434-06|United States|Florida|658[0n]|BOLD:AAC1012
Iodopepla u-album|[2444]|LOFLB730-06|United States|Florida|658[0n]|BOLD:AAC1012
Iodopepla u-album|[2445]|LOFLC301-06|United States|Florida|658[0n]|BOLD:AAC1012
Iodopepla u-album|[2446]|NAMUM112-08|United States|Texas|658[0n]|BOLD:AAC1012
Iodopepla u-album|[2447]|RDNMG166-08|United States|Louisiana|658[0n]|BOLD:AAC1012
Iodopepla u-album|[2448]|BBLOB1667-11|United States|Florida|658[0n]|BOLD:AAC1012
Nedra ramosula|[2449]|XAC803-04|Canada|Ontario|658[0n]|BOLD:AAB3074
Nedra ramosula|[2450]|XAK538-07|Canada|Ontario|658[5n]|BOLD:AAB3074
Nedra ramosula|[2451]|XAH201-05|Canada|Ontario|658[0n]|BOLD:AAB3074
Nedra ramosula|[2452]|XAD659-05|Canada|Ontario|658[0n]|BOLD:AAB3074
Nedra ramosula|[2453]|PHMNB685-04|Canada|New Brunswick|658[0n]|BOLD:AAB3074
Nedra ramosula|[2454]|XAE300-04|Canada|Ontario|658[0n]|BOLD:AAB3074
Nedra ramosula|[2455]|PMG137-03|Canada|Ontario|617[0n]|BOLD:AAB3074
Nedra ramosula|[2456]|XAH265-05|Canada|Ontario|523[2n]|BOLD:AAB3074
Nedra ramosula|[2457]|XAD272-04|Canada|Ontario|605[0n]|BOLD:AAB3074
Nedra ramosula|[2458]|XAH274-05|Canada|Ontario|495[0n]|BOLD:AAB3074
Nedra ramosula|[2459]|XAH375-05|Canada|Ontario|658[0n]|BOLD:AAB3074
Nedra ramosula|[2460]|TTMNB548-06|Canada|New Brunswick|658[0n]|BOLD:AAB3074
Nedra ramosula|[2461]|XAJ276-06|Canada|Ontario|658[0n]|BOLD:AAB3074
Nedra ramosula|[2462]|XAJ510-06|Canada|Ontario|658[0n]|BOLD:AAB3074
Nedra ramosula|[2463]|XAJ556-06|Canada|Ontario|658[0n]|BOLD:AAB3074
Nedra ramosula|[2464]|RDLQG278-06|Canada|Quebec|658[0n]|BOLD:AAB3074
Nedra ramosula|[2465]|RDLQG320-06|Canada|Quebec|658[0n]|BOLD:AAB3074
Nedra ramosula|[2466]|RDLQH083-06|Canada|Quebec|658[0n]|BOLD:AAB3074
Nedra ramosula|[2467]|LILLA068-11|United States|Illinois|658[0n]|BOLD:AAB3074
Nedra ramosula|[2468]|GBGL5897-09|||722[0n]|BOLD:AAB3074
Nedra ramosula|[2469]|XAF345-05|Canada|Ontario|559[0n]|BOLD:AAB3074
Nedra ramosula|[2470]|TMG117-03|Canada|Ontario|639[0n]|BOLD:AAB3074
Nedra ramosula|[2471]|XAD599-05|Canada|Ontario|658[0n]|BOLD:AAB3074
Nedra ramosula|[2472]|XAH200-05|Canada|Ontario|658[0n]|BOLD:AAB3074
Nedra ramosula|[2473]|XAH133-05|Canada|Ontario|553[0n]|BOLD:AAB3074
Nedra ramosula|[2474]|XAH503-05|Canada|Ontario|658[0n]|BOLD:AAB3074
Nedra ramosula|[2475]|LSEU223-06|United States|North Carolina|658[0n]|BOLD:AAB3074
Nedra ramosula|[2476]|XAJ227-06|Canada|Ontario|658[0n]|BOLD:AAB3074
Nedra ramosula|[2477]|LILLA893-11|United States|Illinois|658[0n]|BOLD:AAB3074
Nedra ramosula|[2478]|LPOKE470-12|United States|Oklahoma|632[0n]|BOLD:AAB3074
Nedra stewarti|[2479]|NAMUM263-08|United States|California|658[0n]|BOLD:AAG3683
Nedra stewarti|[2480]|RDNMG154-08|United States|Oregon|641[0n]|BOLD:AAG3683
Nedra stewarti|[2481]|CNCLB1545-14|United States|California|658[0n]|BOLD:AAG3683
Nedra stewarti|[2482]|CNCLB1491-14|United States|California|658[0n]|BOLD:AAG3683
Nedra hoeffleri|[2483]|CNCLB1439-14|United States|Washington|658[0n]|BOLD:AAG3683
Nedra stewarti|[2484]|LNAUT1243-14|United States|California|658[0n]|BOLD:AAG3683
Nedra stewarti|[2485]|LNAUT1239-14|United States|Washington|658[0n]|BOLD:AAG3683
Nedra stewarti|[2486]|LNAUT1240-14|United States|Washington|614[0n]|BOLD:AAG3683
Nedra stewarti|[2487]|CNCLB1544-14|United States|California|568[0n]|BOLD:AAG3683
Nedra stewarti|[2488]|RDNMG156-08|United States|Oregon|609[0n]|BOLD:AAG3683
Nedra stewarti|[2489]|RDNMG155-08|United States|Oregon|558[3n]|BOLD:AAG3683
Nedra stewarti|[2490]|LNAUT2611-14|United States||390[0n]|BOLD:AAG3683
Fagitana littera|[2491]|RDLQ491-07|Canada|Quebec|615[0n]|BOLD:AAD1237
Fagitana littera|[2492]|LNCB303-06|United States|North Carolina|658[0n]|BOLD:AAD1237
Fagitana littera|[2493]|LNC084-05|United States|North Carolina|658[0n]|BOLD:AAD1237
Fagitana littera|[2494]|LNC085-05|United States|North Carolina|658[0n]|BOLD:AAD1237
Fagitana littera|[2495]|LOFLA340-06|United States|Florida|658[0n]|BOLD:AAD1237
Fagitana littera|[2496]|LNCB304-06|United States|North Carolina|658[0n]|BOLD:AAD1237
Fagitana littera|[2497]|LNCB305-06|United States|North Carolina|658[0n]|BOLD:AAD1237
Fagitana littera|[2498]|HKONS238-08|United States|Florida|658[0n]|BOLD:AAD1237
Fagitana littera|[2499]|MILEP338-10|United States|Alabama|658[0n]|BOLD:AAD1237

[illegible]
Fagitana littera|[2497]|LNCB305-06|United States|North Carolina|658[0n]|BOLD:AAD1237
Fagitana littera|[2498]|HKONS238-08|United States|Florida|658[0n]|BOLD:AAD1237
Fagitana littera|[2499]|MILEP338-10|United States|Alabama|658[0n]|BOLD:AAD1237
Dypterygia dolens|[2500]|BLPAF922-07|Costa Rica|Guanacaste|658[0n]|BOLD:AAB6030
Dypterygia dolens|[2501]|BLPDM035-10|Costa Rica|Guanacaste|658[0n]|BOLD:AAB6030
Dypterygia dolens|[2502]|MHAUA855-05|Costa Rica|Guanacaste|579[0n]|BOLD:AAB6030
Dypterygia dolens|[2503]|MHAUA853-05|Costa Rica|Guanacaste|581[0n]|BOLD:AAB6030
Dypterygia dolens|[2504]|MHAUA852-05|Costa Rica|Guanacaste|566[0n]|BOLD:AAB6030
Dypterygia dolens|[2505]|MHAUA851-05|Costa Rica|Guanacaste|658[0n]|BOLD:AAB6030
Dypterygia dolens|[2506]|MHAUA854-05|Costa Rica|Guanacaste|531[0n]|BOLD:AAB6030
Dypterygia dolens|[2507]|MHAUA856-05|Costa Rica|Guanacaste|555[0n]|BOLD:AAB6030
Dypterygia dolens|[2508]|BLPCI592-08|Costa Rica|Guanacaste|658[0n]|BOLD:AAB6030
Dypterygia dolens|[2509]|BLPCA556-08|Costa Rica|Guanacaste|658[0n]|BOLD:AAB6030
Dypterygia dolens|[2510]|BLPCG849-08|Costa Rica|Guanacaste|658[0n]|BOLD:AAB6030
Dypterygia dolens|[2511]|BLPCI591-08|Costa Rica|Guanacaste|658[0n]|BOLD:AAB6030
Dypterygia dolens|[2512]|BLPDC504-09|Costa Rica|Guanacaste|658[0n]|BOLD:AAB6030
Dypterygia dolens|[2513]|BLPDD882-09|Costa Rica|Guanacaste|658[0n]|BOLD:AAB6030
Dypterygia dolens|[2514]|BLPDV077-11|Costa Rica|Guanacaste|658[0n]|BOLD:AAB6030
Dypterygia dolens|[2515]|MHAUA850-05|Costa Rica|Guanacaste|658[0n]|BOLD:AAB6030
Dypterygia dolens|[2516]|BLPBB223-07|Costa Rica|Guanacaste|658[0n]|BOLD:AAB6030
Dypterygia dolens|[2517]|BLPED2151-12|Costa Rica|Guanacaste|658[0n]|BOLD:AAB6030
Dypterygia dolens|[2518]|LYRIO175-09|Mexico|Yucatan|630[0n]|BOLD:AAB6030
Dypterygia dolens|[2519]|LYRIO177-09|Mexico|Yucatan|621[0n]|BOLD:AAB6030
Dypterygia dolens|[2520]|LYHES024-09|Mexico|Yucatan|574[0n]|BOLD:AAB6030
Dypterygia dolens|[2521]|QUNOD148-10|United States|Texas|658[0n]|BOLD:AAB6030
Dypterygia dolens|[2522]|LNAUT854-14|United States|Texas|658[0n]|BOLD:AAB6030
Dypterygia dolens|[2523]|LNAUT1235-14|United States|Texas|658[0n]|BOLD:AAB6030
Dypterygia dolens|[2524]|LNAUT1236-14|United States|Texas|658[0n]|BOLD:AAB6030
Dypterygia dolens|[2525]|LNAUT1238-14|United States|Texas|658[0n]|BOLD:AAB6030
Orthosia tenuimacula|[2526]|CNCLB486-14|United States|Texas|569[0n]|BOLD:AAW9526
Orthosia mediomacula|[2527]|LNAUT899-14|United States|Texas|658[0n]|BOLD:AAW9526
Orthosia nongenerica|[2528]|RDNME864-08|United States|New Mexico|658[0n]|BOLD:AAW9526
Orthosia mediomacula|[2529]|LNAUT901-14|United States|Texas|658[0n]|BOLD:AAW9526
Orthosia mediomacula|[2530]|LNAUT903-14|United States|Texas|658[0n]|BOLD:AAW9526
Pseudeustrotia carneola|[2531]|LOCT281-05|United States|Connecticut|658[0n]|BOLD:AAA6268
Pseudeustrotia carneola|[2532]|LNCNW095-06|United States|North Carolina|658[0n]|BOLD:AAA6268
Pseudeustrotia carneola|[2533]|BLTIB302-08|Canada|Ontario|658[0n]|BOLD:AAA6268
Pseudeustrotia carneola|[2534]|LPSOD218-09|Canada|Ontario|636[0n]|BOLD:AAA6268
Pseudeustrotia carneola|[2535]|PMG156-03|Canada|Ontario|617[0n]|BOLD:AAA6268
Pseudeustrotia carneola|[2536]|RDLQG538-06|Canada|Quebec|658[0n]|BOLD:AAA6268
Pseudeustrotia carneola|[2537]|XAG304-05|Canada|Ontario|658[0n]|BOLD:AAA6268
Pseudeustrotia carneola|[2538]|XAG487-05|Canada|Ontario|518[3n]|BOLD:AAA6268
Pseudeustrotia carneola|[2539]|RDLQE356-06|Canada|Quebec|658[0n]|BOLD:AAA6268
Pseudeustrotia carneola|[2540]|TTMNB306-06|Canada|New Brunswick|658[0n]|BOLD:AAA6268
Pseudeustrotia carneola|[2541]|TMG99-03|Canada|Ontario|639[0n]|BOLD:AAA6268
Pseudeustrotia carneola|[2542]|PHMNB558-04|Canada|New Brunswick|658[0n]|BOLD:AAA6268
Pseudeustrotia carneola|[2543]|LSUSA238-06|United States|Kentucky|658[0n]|BOLD:AAA6268
Pseudeustrotia carneola|[2544]|XAH159-05|Canada|Ontario|658[1n]|BOLD:AAA6268
Pseudeustrotia carneola|[2545]|LGSM676-04|United States|North Carolina|658[0n]|BOLD:AAA6268
Pseudeustrotia carneola|[2546]|LGSMG635-07|United States|Tennessee|658[0n]|BOLD:AAA6268
Pseudeustrotia carneola|[2547]|MNBB210-05|Canada|New Brunswick|539[0n]|BOLD:AAA6268
Pseudeustrotia carneola|[2548]|MNBB471-05|Canada|New Brunswick|658[0n]|BOLD:AAA6268
Pseudeustrotia carneola|[2549]|PHMNB030-03|Canada|New Brunswick|639[0n]|BOLD:AAA6268
Pseudeustrotia carneola|[2550]|TMG100-03|Canada|Ontario|639[0n]|BOLD:AAA6268
Pseudeustrotia carneola|[2551]|RDLQG703-06|Canada|Quebec|598[0n]|BOLD:AAA6268
Pseudeustrotia carneola|[2552]|LOCT399-05|United States|Connecticut|601[0n]|BOLD:AAA6268
Pseudeustrotia carneola|[2553]|LPSOB1000-08|Canada|Ontario|658[0n]|BOLD:AAA6268
Pseudeustrotia carneola|[2554]|LPMN094-08|Canada|Manitoba|658[0n]|BOLD:AAA6268
Pseudeustrotia carneola|[2555]|LPMN132-08|Canada|Manitoba|658[0n]|BOLD:AAA6268
Pseudeustrotia carneola|[2556]|LTOLB146-08|United States|Maryland|658[0n]|BOLD:AAA6268
Pseudeustrotia carneola|[2557]|BLGSM003-09|Canada|Ontario|658[0n]|BOLD:AAA6268
Pseudeustrotia carneola|[2558]|LPOKB282-09|United States|Oklahoma|658[0n]|BOLD:AAA6268
Pseudeustrotia carneola|[2559]|LPOKB420-09|United States|Oklahoma|658[0n]|BOLD:AAA6268
Pseudeustrotia carneola|[2560]|BBLCU062-09|United States|Michigan|658[0n]|BOLD:AAA6268
Pseudeustrotia carneola|[2561]|BBLCU063-09|United States|Michigan|658[0n]|BOLD:AAA6268
Pseudeustrotia carneola|[2562]|BBLPE008-09|Canada|Nova Scotia|658[0n]|BOLD:AAA6268
Pseudeustrotia carneola|[2563]|BBLPA654-10|Canada|Ontario|658[0n]|BOLD:AAA6268
Pseudeustrotia carneola|[2564]|LILLA108-11|United States|Illinois|658[0n]|BOLD:AAA6268
Pseudeustrotia carneola|[2565]|RDLQG775-06|Canada|Quebec|658[0n]|BOLD:AAA6268
Pseudeustrotia carneola|[2566]|RDLQG895-06|Canada|Quebec|658[0n]|BOLD:AAA6268
Pseudeustrotia carneola|[2567]|LGSMG636-07|United States|North Carolina|658[0n]|BOLD:AAA6268
Pseudeustrotia carneola|[2568]|LPSO468-08|Canada|Ontario|658[0n]|BOLD:AAA6268
Pseudeustrotia carneola|[2569]|LPSOB035-08|Canada|Ontario|658[0n]|BOLD:AAA6268
Pseudeustrotia carneola|[2570]|LPSOC406-08|Canada|Ontario|658[0n]|BOLD:AAA6268
Pseudeustrotia carneola|[2571]|XAJ783-06|Canada|Ontario|658[0n]|BOLD:AAA6268
Pseudeustrotia carneola|[2572]|RDLQG714-06|Canada|Quebec|658[0n]|BOLD:AAA6268
Pseudeustrotia carneola|[2573]|TMNBB119-06|Canada|New Brunswick|658[0n]|BOLD:AAA6268
Pseudeustrotia carneola|[2574]|XAJ529-06|Canada|Ontario|658[0n]|BOLD:AAA6268
Pseudeustrotia carneola|[2575]|LPSOC428-08|Canada|Ontario|658[0n]|BOLD:AAA6268
Pseudeustrotia carneola|[2576]|LPSOC429-08|Canada|Ontario|658[0n]|BOLD:AAA6268
Pseudeustrotia carneola|[2577]|TTMNB308-06|Canada|New Brunswick|658[0n]|BOLD:AAA6268
Pseudeustrotia carneola|[2578]|TMNBB118-06|Canada|New Brunswick|658[0n]|BOLD:AAA6268
Pseudeustrotia carneola|[2579]|TTMNB307-06|Canada|New Brunswick|658[0n]|BOLD:AAA6268
Pseudeustrotia carneola|[2580]|XAH470-05|Canada|Ontario|658[0n]|BOLD:AAA6268
Pseudeustrotia carneola|[2581]|XAH253-05|Canada|Ontario|658[0n]|BOLD:AAA6268
Pseudeustrotia carneola|[2582]|XAG868-05|Canada|Ontario|658[0n]|BOLD:AAA6268
Pseudeustrotia carneola|[2583]|XAG402-05|Canada|Ontario|658[0n]|BOLD:AAA6268
Pseudeustrotia carneola|[2584]|LOCT310-05|United States|Connecticut|658[0n]|BOLD:AAA6268
Pseudeustrotia carneola|[2585]|LOCT280-05|United States|Connecticut|658[0n]|BOLD:AAA6268
Pseudeustrotia carneola|[2586]|XAE249-04|Canada|Ontario|658[0n]|BOLD:AAA6268
Pseudeustrotia carneola|[2587]|XAB616-04|Canada|Ontario|658[0n]|BOLD:AAA6268
Pseudeustrotia carneola|[2588]|MEC342-04|Canada|Quebec|658[0n]|BOLD:AAA6268
Pseudeustrotia carneola|[2589]|BBLEC711-09|Canada|Nova Scotia|658[0n]|BOLD:AAA6268
Pseudeustrotia carneola|[2590]|BBLPC462-09|Canada|New Brunswick|658[0n]|BOLD:AAA6268
Pseudeustrotia carneola|[2591]|XAH328-05|Canada|Ontario|658[0n]|BOLD:AAA6268
Pseudeustrotia carneola|[2592]|RDMAB070-05|Canada|Alberta|636[0n]|BOLD:AAA6268
Pseudeustrotia carneola|[2593]|RDMAB009-05|Canada|Alberta|637[0n]|BOLD:AAA6268
Pseudeustrotia carneola|[2594]|RDLQG480-06|Canada|Quebec|635[0n]|BOLD:AAA6268
Pseudeustrotia carneola|[2595]|BBLPE109-09|Canada|Nova Scotia|635[0n]|BOLD:AAA6268
Pseudeustrotia carneola|[2596]|LILLA394-11|United States|Illinois|658[0n]|BOLD:AAA6268
Pseudeustrotia indeterminata|[2597]|CNCLB1259-14|United States|Louisiana|658[0n]|BOLD:ABW0894
Pseudeustrotia indeterminata|[2598]|BBLOE1382-12|United States|Arkansas|658[0n]|BOLD:ABW0894
Pseudeustrotia indeterminata|[2599]|BBLOE1177-12|United States|Texas|658[0n]|BOLD:ABW0894

[illegible]
Pseudeustrotia indeterminata|[2597]|CNCLB1259-14|United States|Louisiana|658[0n]|BOLD:ABW0894
Pseudeustrotia indeterminata|[2598]|BBLOE1382-12|United States|Arkansas|658[0n]|BOLD:ABW0894
Pseudeustrotia indeterminata|[2599]|BBLOE1177-12|United States|Texas|658[0n]|BOLD:ABW0894
Pseudeustrotia indeterminata|[2600]|CNCLB506-14|United States|Texas|658[0n]|BOLD:ABW0894
Pseudeustrotia indeterminata|[2601]|CNCLB507-14|United States|Texas|658[0n]|BOLD:ABW0894
Pseudeustrotia indeterminata|[2602]|CNCLB510-14|United States|Texas|658[0n]|BOLD:ABW0894
Pseudeustrotia indeterminata|[2603]|BBLOE1179-12|United States|Texas|658[0n]|BOLD:ABW0894
Pseudeustrotia indeterminata|[2604]|CNCLB1260-14|United States|Louisiana|658[0n]|BOLD:ABW0894
Cerapteryx graminis|[2605]|GMNWJ3468-14|Norway|Sor-Trondelag|522[1n]|BOLD:AAB4284
Cerapteryx graminis|[2606]|GMNWJ3481-14|Norway|Sor-Trondelag|519[0n]|BOLD:AAB4284
Cerapteryx graminis|[2607]|PHLAE302-11|Austria|Vorarlberg|634[0n]|BOLD:AAB4284
Cerapteryx graminis|[2608]|LEATB539-13|Italy|South Tyrol|658[0n]|BOLD:AAB4284
Cerapteryx graminis|[2609]|GWOR4035-09|Germany|Bavaria|658[0n]|BOLD:AAB4284
Cerapteryx graminis|[2610]|GWORK481-09|Germany|Bavaria|658[0n]|BOLD:AAB4284
Cerapteryx graminis|[2611]|PHLAC593-10|Italy|South Tyrol|658[0n]|BOLD:AAB4284
Cerapteryx graminis|[2612]|LEATG312-14|Austria|Tirol|658[0n]|BOLD:AAB4284
Cerapteryx graminis|[2613]|LEATG313-14|Austria|Tirol|658[0n]|BOLD:AAB4284
Cerapteryx graminis|[2614]|GMNWK4215-14|Norway|Sor-Trondelag|591[0n]|BOLD:AAB4284
Cerapteryx graminis|[2615]|GMNWK4214-14|Norway|Sor-Trondelag|591[0n]|BOLD:AAB4284
Cerapteryx graminis|[2616]|LEFIA529-10|Finland|South Karelia|637[0n]|BOLD:AAB4284
Cerapteryx graminis|[2617]|GMNWK4213-14|Norway|Sor-Trondelag|603[0n]|BOLD:AAB4284
Cerapteryx graminis|[2618]|GMNWK4201-14|Norway|Sor-Trondelag|603[0n]|BOLD:AAB4284
Cerapteryx graminis|[2619]|GMNWJ3472-14|Norway|Sor-Trondelag|567[0n]|BOLD:AAB4284
Cerapteryx graminis|[2620]|GBLAF282-14|Germany|Brandenburg|658[0n]|BOLD:AAB4284
Cerapteryx graminis|[2621]|GWOTL094-13|Germany|Saarland|658[0n]|BOLD:AAB4284
Cerapteryx graminis|[2622]|LON863-11|Norway||658[0n]|BOLD:AAB4284
Cerapteryx graminis|[2623]|LEFIE021-10|Finland||658[0n]|BOLD:AAB4284
Cerapteryx graminis|[2624]|LEFIA528-10|Finland|South Karelia|658[0n]|BOLD:AAB4284
Cerapteryx graminis|[2625]|RDNMF441-08|Finland||658[0n]|BOLD:AAB4284
Cerapteryx graminis|[2626]|RDNMF440-08|Finland|Uusimaa|658[0n]|BOLD:AAB4284
Cerapteryx graminis|[2627]|RDNMF439-08|Finland||658[0n]|BOLD:AAB4284
Cerapteryx graminis|[2628]|RDNMF438-08|Finland||658[0n]|BOLD:AAB4284
Cerapteryx graminis|[2629]|NORIN043-13|Norway|Akershus|656[0n]|BOLD:AAB4284
Cerapteryx graminis|[2630]|RDNMD740-06|Denmark||645[0n]|BOLD:AAB4284
Cerapteryx graminis|[2631]|RDNMG508-08|Finland||640[0n]|BOLD:AAB4284
Cerapteryx graminis|[2632]|LEFIB299-10|Finland|Northern Ostrobothnia|636[0n]|BOLD:AAB4284
Cerapteryx graminis|[2633]|GMNWJ3471-14|Norway|Sor-Trondelag|516[1n]|BOLD:AAB4284
Cerapteryx graminis|[2634]|GMNWJ3486-14|Norway|Sor-Trondelag|579[0n]|BOLD:AAB4284
Cerapteryx graminis|[2635]|GMNWL3212-14|Norway|Sor-Trondelag|564[0n]|BOLD:AAB4284
Cerapteryx graminis|[2636]|GMNWL3218-14|Norway|Sor-Trondelag|576[1n]|BOLD:AAB4284
Cerapteryx graminis|[2637]|GMNWJ3476-14|Norway|Sor-Trondelag|576[0n]|BOLD:AAB4284
Cerapteryx graminis|[2638]|GMNWK4220-14|Norway|Sor-Trondelag|606[0n]|BOLD:AAB4284
Cerapteryx graminis|[2639]|GMNWK4196-14|Norway|Sor-Trondelag|606[0n]|BOLD:AAB4284
Cerapteryx graminis|[2640]|GMNWL3225-14|Norway|Sor-Trondelag|588[0n]|BOLD:AAB4284
Cerapteryx graminis|[2641]|GMNWK4202-14|Norway|Sor-Trondelag|603[0n]|BOLD:AAB4284
Cerapteryx graminis|[2642]|GMNWK4169-14|Norway|Sor-Trondelag|576[0n]|BOLD:AAB4284
Cerapteryx graminis|[2643]|GMNWL3209-14|Norway|Sor-Trondelag|555[1n]|BOLD:AAB4284
Cerapteryx graminis|[2644]|GMNWL3226-14|Norway|Sor-Trondelag|579[0n]|BOLD:AAB4284
Cerapteryx graminis|[2645]|GMNWL3210-14|Norway|Sor-Trondelag|609[0n]|BOLD:AAB4284
Cerapteryx graminis|[2646]|GMNWL3211-14|Norway|Sor-Trondelag|609[1n]|BOLD:AAB4284
Cerapteryx graminis|[2647]|GMNWL3217-14|Norway|Sor-Trondelag|576[0n]|BOLD:AAB4284
Cerapteryx graminis|[2648]|GMNWL3205-14|Norway|Sor-Trondelag|576[0n]|BOLD:AAB4284
Cerapteryx graminis|[2649]|GMNWK4218-14|Norway|Sor-Trondelag|591[0n]|BOLD:AAB4284
Cerapteryx graminis|[2650]|GMNWJ3473-14|Norway|Sor-Trondelag|577[0n]|BOLD:AAB4284
Cerapteryx graminis|[2651]|GMNWJ3469-14|Norway|Sor-Trondelag|576[0n]|BOLD:AAB4284
Cerapteryx graminis|[2652]|GMNWK4226-14|Norway|Sor-Trondelag|606[0n]|BOLD:AAB4284
Cerapteryx graminis|[2653]|GMNWK4210-14|Norway|Sor-Trondelag|607[0n]|BOLD:AAB4284
Cerapteryx graminis|[2654]|GMNWK4208-14|Norway|Sor-Trondelag|606[0n]|BOLD:AAB4284
Cerapteryx graminis|[2655]|GMNWK4199-14|Norway|Sor-Trondelag|606[0n]|BOLD:AAB4284
Cerapteryx graminis|[2656]|GMNWK4198-14|Norway|Sor-Trondelag|606[0n]|BOLD:AAB4284
Cerapteryx graminis|[2657]|GMNWK4192-14|Norway|Sor-Trondelag|606[0n]|BOLD:AAB4284
Cerapteryx graminis|[2658]|GMNWL3229-14|Norway|Sor-Trondelag|588[0n]|BOLD:AAB4284
Cerapteryx graminis|[2659]|GMNWL3222-14|Norway|Sor-Trondelag|588[0n]|BOLD:AAB4284
Cerapteryx graminis|[2660]|GMNWK4219-14|Norway|Sor-Trondelag|603[0n]|BOLD:AAB4284
Cerapteryx graminis|[2661]|GMNWK4216-14|Norway|Sor-Trondelag|603[0n]|BOLD:AAB4284
Cerapteryx graminis|[2662]|GMNWK4212-14|Norway|Sor-Trondelag|603[0n]|BOLD:AAB4284
Cerapteryx graminis|[2663]|GMNWK4190-14|Norway|Sor-Trondelag|603[0n]|BOLD:AAB4284
Cerapteryx graminis|[2664]|RDNMF759-08|Canada|Newfoundland and Labrador|658[0n]|BOLD:AAB4284
Cerapteryx graminis|[2665]|RDNMF760-08|Canada|Newfoundland and Labrador|658[0n]|BOLD:AAB4284
Cerapteryx graminis|[2666]|RDNMF761-08|Canada|Newfoundland and Labrador|658[0n]|BOLD:AAB4284
Cerapteryx graminis|[2667]|RDNMF762-08|Canada|Newfoundland and Labrador|658[0n]|BOLD:AAB4284
Cerapteryx graminis|[2668]|GMNWK4178-14|Norway|Sor-Trondelag|603[0n]|BOLD:AAB4284
Cerapteryx graminis|[2669]|GMNWK4174-14|Norway|Sor-Trondelag|604[0n]|BOLD:AAB4284
Cerapteryx graminis|[2670]|GMNWK4173-14|Norway|Sor-Trondelag|603[0n]|BOLD:AAB4284
Cerapteryx graminis|[2671]|GMNWM145-14|Norway|Sor-Trondelag|564[0n]|BOLD:AAB4284
Cerapteryx graminis|[2672]|GMNWL3203-14|Norway|Sor-Trondelag|564[0n]|BOLD:AAB4284
Cerapteryx graminis|[2673]|GMNWK4221-14|Norway|Sor-Trondelag|576[0n]|BOLD:AAB4284
Cerapteryx graminis|[2674]|GMNWK4209-14|Norway|Sor-Trondelag|576[0n]|BOLD:AAB4284
Cerapteryx graminis|[2675]|GMNWK4206-14|Norway|Sor-Trondelag|576[0n]|BOLD:AAB4284
Cerapteryx graminis|[2676]|GMNWK4191-14|Norway|Sor-Trondelag|576[0n]|BOLD:AAB4284
Cerapteryx graminis|[2677]|GMNWK4170-14|Norway|Sor-Trondelag|576[0n]|BOLD:AAB4284
Cerapteryx graminis|[2678]|GMNWL3219-14|Norway|Sor-Trondelag|567[0n]|BOLD:AAB4284
Cerapteryx graminis|[2679]|GMNWK4171-14|Norway|Sor-Trondelag|582[0n]|BOLD:AAB4284
Cerapteryx graminis|[2680]|GMNWJ3466-14|Norway|Sor-Trondelag|567[0n]|BOLD:AAB4284
Cerapteryx graminis|[2681]|GMNWK4224-14|Norway|Sor-Trondelag|600[0n]|BOLD:AAB4284
Cerapteryx graminis|[2682]|GMNWK4172-14|Norway|Sor-Trondelag|573[0n]|BOLD:AAB4284
Cerapteryx graminis|[2683]|GMNWJ3467-14|Norway|Sor-Trondelag|561[0n]|BOLD:AAB4284
Cerapteryx graminis|[2684]|GMNWK4211-14|Norway|Sor-Trondelag|597[0n]|BOLD:AAB4284
Cerapteryx graminis|[2685]|GMNWK4205-14|Norway|Sor-Trondelag|597[0n]|BOLD:AAB4284
Cerapteryx graminis|[2686]|CGUKB261-09|United Kingdom|England|595[0n]|BOLD:AAB4284
Cerapteryx graminis|[2687]|GBLAA1002-14|Germany|Rhineland-Palatinate|658[0n]|BOLD:AAB4284
Cerapteryx graminis|[2688]|GBLAD130-14|Germany|Saxony|658[0n]|BOLD:AAB4284
Cerapteryx graminis|[2689]|GWOSC413-10|Germany|Bavaria|658[0n]|BOLD:AAB4284
Cerapteryx graminis|[2690]|FBLMV074-09|Germany|Bavaria|658[0n]|BOLD:AAB4284
Cerapteryx graminis|[2691]|RDNMG509-08|Germany||658[0n]|BOLD:AAB4284
Cerapteryx graminis|[2692]|RDNMD607-06|Denmark||658[0n]|BOLD:AAB4284
Cerapteryx graminis|[2693]|RDNMD606-06|Denmark||658[0n]|BOLD:AAB4284
Cerapteryx graminis|[2694]|GMNWK4217-14|Norway|Sor-Trondelag|576[0n]|BOLD:AAB4284
Cerapteryx graminis|[2695]|GMNWL3206-14|Norway|Sor-Trondelag|579[0n]|BOLD:AAB4284
Cerapteryx graminis|[2696]|CGUKD457-09|United Kingdom|England|594[0n]|BOLD:AAB4284
Cerapteryx graminis|[2697]|GMNWK4197-14|Norway|Sor-Trondelag|567[0n]|BOLD:AAB4284
Cerapteryx graminis|[2698]|GMNWJ3477-14|Norway|Sor-Trondelag|555[0n]|BOLD:AAB4284
Cerapteryx graminis|[2699]|GMNWK4225-14|Norway|Sor-Trondelag|546[0n]|BOLD:AAB4284

[illegible]
Cerapteryx graminis|[2697]|GMNWK4197-14|Norway|Sor-Trondelag|567[0n]|BOLD:AAB4284
Cerapteryx graminis|[2698]|GMNWJ3477-14|Norway|Sor-Trondelag|555[0n]|BOLD:AAB4284
Cerapteryx graminis|[2699]|GMNWK4225-14|Norway|Sor-Trondelag|546[0n]|BOLD:AAB4284
Cerapteryx graminis|[2700]|GMNWJ3482-14|Norway|Sor-Trondelag|552[1n]|BOLD:AAB4284
Cerapteryx graminis|[2701]|CGUKA866-09|United Kingdom|England|636[0n]|BOLD:AAB4284
Cerapteryx graminis|[2702]|GMNWJ3479-14|Norway|Sor-Trondelag|582[0n]|BOLD:AAB4284
Cerapteryx graminis|[2703]|GMNWJ3480-14|Norway|Sor-Trondelag|534[0n]|BOLD:AAB4284
Cerapteryx graminis|[2704]|GMNWL3204-14|Norway|Sor-Trondelag|573[1n]|BOLD:AAB4284
Cerapteryx graminis|[2705]|GMNWL3208-14|Norway|Sor-Trondelag|525[0n]|BOLD:AAB4284
Cerapteryx graminis|[2706]|GMNWM146-14|Norway|Sor-Trondelag|555[0n]|BOLD:AAB4284
Nephelodes carminata|[2707]|CNCLB521-14|United States|Colorado|658[0n]|BOLD:ACM4411
Nephelodes carminata|[2708]|CNCLB522-14|United States|Colorado|658[0n]|BOLD:ACM4411
Nephelodes carminata|[2709]|CNCLB523-14|United States|Arizona|658[0n]|BOLD:ACM4264
Nephelodes carminata|[2710]|CNCLB524-14|United States|Arizona|658[0n]|BOLD:ACM4264
Nephelodes carminata|[2711]|CNCLB525-14|United States|Arizona|550[0n]|BOLD:ACM4264
Tholera americana|[2712]|LBCH7529-10|Canada|British Columbia|636[0n]|BOLD:AAB9282
Tholera americana|[2713]|LOWC076-05|Canada|British Columbia|658[0n]|BOLD:AAB9282
Tholera americana|[2714]|LBCH6878-10|Canada|British Columbia|635[0n]|BOLD:AAB9282
Tholera americana|[2715]|LBCH6875-10|Canada|British Columbia|636[0n]|BOLD:AAB9282
Tholera americana|[2716]|LBCH6944-10|Canada|British Columbia|658[0n]|BOLD:AAB9282
Tholera americana|[2717]|LBCH6986-10|Canada|British Columbia|640[0n]|BOLD:AAB9282
Tholera americana|[2718]|LBCH7912-10|Canada|British Columbia|658[0n]|BOLD:AAB9282
Tholera americana|[2719]|LBCH7913-10|Canada|British Columbia|658[0n]|BOLD:AAB9282
Tholera americana|[2720]|LBCH7910-10|Canada|British Columbia|658[0n]|BOLD:AAB9282
Tholera americana|[2721]|LBCH7911-10|Canada|British Columbia|658[0n]|BOLD:AAB9282
Tholera americana|[2722]|LBCH6991-10|Canada|British Columbia|658[0n]|BOLD:AAB9282
Tholera americana|[2723]|LBCH7745-10|Canada|British Columbia|658[0n]|BOLD:AAB9282
Tholera americana|[2724]|LBCH6984-10|Canada|British Columbia|658[0n]|BOLD:AAB9282
Tholera americana|[2725]|LBCH6989-10|Canada|British Columbia|658[0n]|BOLD:AAB9282
Tholera americana|[2726]|LBCH6951-10|Canada|British Columbia|658[0n]|BOLD:AAB9282
Tholera americana|[2727]|LBCH6950-10|Canada|British Columbia|658[0n]|BOLD:AAB9282
Tholera americana|[2728]|LBCH6949-10|Canada|British Columbia|658[0n]|BOLD:AAB9282
Tholera americana|[2729]|LBCH6877-10|Canada|British Columbia|658[0n]|BOLD:AAB9282
Tholera americana|[2730]|LBCH6873-10|Canada|British Columbia|658[0n]|BOLD:AAB9282
Tholera americana|[2731]|LBCH6800-10|Canada|British Columbia|658[0n]|BOLD:AAB9282
Tholera americana|[2732]|LBCH6799-10|Canada|British Columbia|658[0n]|BOLD:AAB9282
Tholera americana|[2733]|LBCH6797-10|Canada|British Columbia|658[0n]|BOLD:AAB9282
Tholera americana|[2734]|LBCH6796-10|Canada|British Columbia|658[0n]|BOLD:AAB9282
Tholera americana|[2735]|LBCH6762-10|Canada|British Columbia|658[0n]|BOLD:AAB9282
Tholera americana|[2736]|LBCH6933-10|Canada|British Columbia|658[0n]|BOLD:AAB9282
Tholera americana|[2737]|LBCH6988-10|Canada|British Columbia|658[0n]|BOLD:AAB9282
Tholera americana|[2738]|LBCH6795-10|Canada|British Columbia|644[0n]|BOLD:AAB9282
Tholera americana|[2739]|LBCH6876-10|Canada|British Columbia|634[0n]|BOLD:AAB9282
Tholera americana|[2740]|LBCH7528-10|Canada|British Columbia|639[0n]|BOLD:AAB9282
Tholera americana|[2741]|LBCH7914-10|Canada|British Columbia|658[0n]|BOLD:AAB9282
Tholera americana|[2742]|LOWCD636-06|Canada|British Columbia|658[0n]|BOLD:AAB9282
Tholera americana|[2743]|LOWC077-05|Canada|British Columbia|658[0n]|BOLD:AAB9282
Tholera americana|[2744]|LBCH7368-10|Canada|British Columbia|658[0n]|BOLD:AAB9282
Tholera americana|[2745]|LBCH7522-10|Canada|British Columbia|658[0n]|BOLD:AAB9282
Tholera americana|[2746]|LBCH7074-10|Canada|British Columbia|658[0n]|BOLD:AAB9282
Tholera americana|[2747]|LBCH7225-10|Canada|British Columbia|658[0n]|BOLD:AAB9282
Tholera americana|[2748]|LBCH7071-10|Canada|British Columbia|658[0n]|BOLD:AAB9282
Tholera americana|[2749]|LBCH7072-10|Canada|British Columbia|658[0n]|BOLD:AAB9282
Tholera americana|[2750]|LBCH6952-10|Canada|British Columbia|658[0n]|BOLD:AAB9282
Tholera americana|[2751]|LBCH7069-10|Canada|British Columbia|658[0n]|BOLD:AAB9282
Tholera americana|[2752]|LBCH6872-10|Canada|British Columbia|658[0n]|BOLD:AAB9282
Tholera americana|[2753]|LBCH6874-10|Canada|British Columbia|658[0n]|BOLD:AAB9282
Tholera americana|[2754]|LOWCD640-06|Canada|British Columbia|657[0n]|BOLD:AAB9282
Tholera americana|[2755]|LOWCD639-06|Canada|British Columbia|658[0n]|BOLD:AAB9282
Tholera americana|[2756]|LOWCD638-06|Canada|British Columbia|658[0n]|BOLD:AAB9282
Tholera americana|[2757]|LOWCD637-06|Canada|British Columbia|658[0n]|BOLD:AAB9282
Tholera americana|[2758]|LOWCD635-06|Canada|British Columbia|658[0n]|BOLD:AAB9282
Tholera americana|[2759]|LOWC075-05|Canada|British Columbia|658[0n]|BOLD:AAB9282
Tholera americana|[2760]|LOWC074-05|Canada|British Columbia|658[0n]|BOLD:AAB9282
Tholera americana|[2761]|LOWC073-05|Canada|British Columbia|658[0n]|BOLD:AAB9282
Tholera americana|[2762]|LOWC072-05|Canada|British Columbia|658[0n]|BOLD:AAB9282
Tholera americana|[2763]|LOWC071-05|Canada|British Columbia|658[0n]|BOLD:AAB9282
Tholera americana|[2764]|LBCH7067-10|Canada|British Columbia|658[0n]|BOLD:AAB9282
Tholera americana|[2765]|LBCH7068-10|Canada|British Columbia|658[0n]|BOLD:AAB9282
Tholera americana|[2766]|LBCH7070-10|Canada|British Columbia|658[0n]|BOLD:AAB9282
Tholera americana|[2767]|LBCH7073-10|Canada|British Columbia|658[0n]|BOLD:AAB9282
Tholera americana|[2768]|LOWCD642-06|Canada|British Columbia|600[0n]|BOLD:AAB9282
Tholera americana|[2769]|LBCH7168-10|Canada|British Columbia|658[0n]|BOLD:AAB9282
Tholera americana|[2770]|LBCH6917-10|Canada|British Columbia|614[0n]|BOLD:AAB9282
Tholera americana|[2771]|RDMAB685-06|Canada|Alberta|605[0n]|BOLD:AAB9282
Tholera americana|[2772]|LOWCD641-06|Canada|British Columbia|601[0n]|BOLD:AAB9282
Tholera americana|[2773]|LBCH7526-10|Canada|British Columbia|634[0n]|BOLD:AAB9282
Tholera americana|[2774]|LBCH7523-10|Canada|British Columbia|658[0n]|BOLD:AAB9282
Tholera americana|[2775]|LBCH7691-10|Canada|British Columbia|658[0n]|BOLD:AAB9282
Tholera americana|[2776]|LBCH7915-10|Canada|British Columbia|658[0n]|BOLD:AAB9282
Tholera americana|[2777]|LBCH7916-10|Canada|British Columbia|658[0n]|BOLD:AAB9282
Tholera americana|[2778]|LBCH6930-10|Canada|British Columbia|658[0n]|BOLD:AAB9282
Tholera americana|[2779]|LBCH6985-10|Canada|British Columbia|658[0n]|BOLD:AAB9282
Tholera americana|[2780]|LBCH6879-10|Canada|British Columbia|658[0n]|BOLD:AAB9282
Tholera americana|[2781]|LBCH6948-10|Canada|British Columbia|658[0n]|BOLD:AAB9282
Tholera americana|[2782]|LBCH7524-10|Canada|British Columbia|658[0n]|BOLD:AAB9282
Tholera americana|[2783]|LBCH6801-10|Canada|British Columbia|658[0n]|BOLD:AAB9282
Tholera americana|[2784]|LBCH6945-10|Canada|British Columbia|658[0n]|BOLD:AAB9282
Tholera americana|[2785]|LBCH6987-10|Canada|British Columbia|658[0n]|BOLD:AAB9282
Tholera americana|[2786]|LBCH6990-10|Canada|British Columbia|658[0n]|BOLD:AAB9282
Tholera americana|[2787]|LBCH7525-10|Canada|British Columbia|658[0n]|BOLD:AAB9282
Tholera americana|[2788]|LBCH7527-10|Canada|British Columbia|658[0n]|BOLD:AAB9282
Tholera americana|[2789]|LBCH6794-10|Canada|British Columbia|658[0n]|BOLD:AAB9282
Tholera americana|[2790]|LBCH6798-10|Canada|British Columbia|658[0n]|BOLD:AAB9282
Tholera americana|[2791]|LBCH7917-10|Canada|British Columbia|658[0n]|BOLD:AAB9282
Nephelodes minians|[2792]|LOWC085-05|Canada|British Columbia|538[0n]|BOLD:AAA5081
Nephelodes minians|[2793]|BBLEC062-09|Canada|New Brunswick|658[0n]|BOLD:AAA5081
Nephelodes minians|[2794]|BBLEC438-09|Canada|New Brunswick|658[0n]|BOLD:AAA5081
Nephelodes minians|[2795]|BBLPC348-09|Canada|New Brunswick|658[0n]|BOLD:AAA5081
Nephelodes minians|[2796]|BBLPC368-09|Canada|New Brunswick|658[0n]|BOLD:AAA5081
Nephelodes minians|[2797]|LPOKD612-09|United States|Oklahoma|658[0n]|BOLD:AAA5081
Nephelodes demaculata|[2798]|JMMMB592-13|United States|California|573[0n]|BOLD:AAA5081
Nephelodes demaculata|[2799]|JMMMB320-11|United States|California|658[0n]|BOLD:AAA5081

Nephelodes minians|[27[illegible]]|[illegible]|Canada|New Brunswick|658[0n]|BOLD:AAA5081
Nephelodes minians|[2797]|LPOKD612-09|United States|Oklahoma|658[0n]|BOLD:AAA5081
Nephelodes demaculata|[2798]|JMMMB592-13|United States|California|573[0n]|BOLD:AAA5081
Nephelodes demaculata|[2799]|JMMMB320-11|United States|California|658[0n]|BOLD:AAA5081
Nephelodes demaculata|[2800]|CNCLB179-14|United States|California|658[0n]|BOLD:AAA5081
Nephelodes minians|[2801]|LPOKA369-08|United States|Oklahoma|658[0n]|BOLD:AAA5081
Nephelodes minians|[2802]|BBLOC998-11|United States|California|658[0n]|BOLD:AAA5081
Nephelodes minians|[2803]|BBLOC1220-11|United States|California|658[0n]|BOLD:AAA5081
Nephelodes minians|[2804]|BBLEC488-09|Canada|New Brunswick|658[0n]|BOLD:AAA5081
Nephelodes minians|[2805]|RDLQB930-05|Canada|Quebec|530[0n]|BOLD:AAA5081
Nephelodes minians|[2806]|BBLPC365-09|Canada|New Brunswick|658[0n]|BOLD:AAA5081
Nephelodes minians|[2807]|BBLPC367-09|Canada|New Brunswick|658[0n]|BOLD:AAA5081
Nephelodes minians|[2808]|XAB415-04|Canada|Ontario|658[0n]|BOLD:AAA5081
Nephelodes minians|[2809]|RDLQB929-05|Canada|Quebec|658[0n]|BOLD:AAA5081
Nephelodes minians|[2810]|BBLPC370-09|Canada|New Brunswick|658[0n]|BOLD:AAA5081
Nephelodes minians|[2811]|ALLEP455-13|Canada|Ontario|658[0n]|BOLD:AAA5081
Nephelodes minians|[2812]|LPOKA589-09|United States|Oklahoma|617[0n]|BOLD:AAA5081
Nephelodes minians|[2813]|RDNMC296-05|United States|Colorado|598[0n]|BOLD:AAA5081
Nephelodes minians|[2814]|BBLPB589-10|Canada|Saskatchewan|658[0n]|BOLD:AAA5081
Nephelodes minians|[2815]|CNCLB176-14|United States|New Mexico|658[0n]|BOLD:AAA5081
Nephelodes minians|[2816]|HESEP1375-12|Canada|Ontario|658[0n]|BOLD:AAA5081
Nephelodes minians|[2817]|BBLPC027-09|Canada|New Brunswick|658[0n]|BOLD:AAA5081
Nephelodes minians|[2818]|RDLQB884-05|Canada|Quebec|658[0n]|BOLD:AAA5081
Nephelodes minians|[2819]|BBLPC371-09|Canada|New Brunswick|658[0n]|BOLD:AAA5081
Nephelodes minians|[2820]|XAH207-05|Canada|Ontario|658[0n]|BOLD:AAA5081
Nephelodes minians|[2821]|BBLEC508-09|Canada|New Brunswick|658[0n]|BOLD:AAA5081
Nephelodes minians|[2822]|LNCNW023-06|United States|North Carolina|612[0n]|BOLD:AAA5081
Nephelodes minians|[2823]|LNCNW024-06|United States|North Carolina|658[0n]|BOLD:AAA5081
Nephelodes minians|[2824]|BBLEC443-09|Canada|New Brunswick|658[0n]|BOLD:AAA5081
Nephelodes minians|[2825]|BBLPC355-09|Canada|New Brunswick|658[0n]|BOLD:AAA5081
Nephelodes minians|[2826]|CNCLB2912-14|United States|North Carolina|658[0n]|BOLD:AAA5081
Nephelodes minians|[2827]|LOWC078-05|Canada|British Columbia|658[0n]|BOLD:AAA5081
Nephelodes minians|[2828]|XAB414-04|Canada|Ontario|611[0n]|BOLD:AAA5081
Nephelodes minians|[2829]|XAB426-04|Canada|Ontario|658[0n]|BOLD:AAA5081
Nephelodes minians|[2830]|XAH241-05|Canada|Ontario|658[0n]|BOLD:AAA5081
Nephelodes minians|[2831]|BBLPC431-09|Canada|New Brunswick|658[0n]|BOLD:AAA5081
Nephelodes minians|[2832]|BBLPB598-10|Canada|Saskatchewan|658[0n]|BOLD:AAA5081
Nephelodes minians|[2833]|BBLPB591-10|Canada|Alberta|658[1n]|BOLD:AAA5081
Nephelodes minians|[2834]|BBLPB588-10|Canada|Alberta|658[0n]|BOLD:AAA5081
Nephelodes minians|[2835]|BBLPB597-10|Canada|Saskatchewan|658[0n]|BOLD:AAA5081
Nephelodes minians|[2836]|BBLPB498-10|Canada|Saskatchewan|658[0n]|BOLD:AAA5081
Nephelodes minians|[2837]|LPMNB229-09|Canada|Manitoba|658[0n]|BOLD:AAA5081
Nephelodes minians|[2838]|BBLPB600-10|Canada|Saskatchewan|658[0n]|BOLD:AAA5081
Nephelodes minians|[2839]|BBLPB603-10|Canada|Saskatchewan|658[0n]|BOLD:AAA5081
Nephelodes minians|[2840]|RDLQB625-05|Canada|Quebec|515[1n]|BOLD:AAA5081
Nephelodes minians|[2841]|RDLQB626-05|Canada|Quebec|607[0n]|BOLD:AAA5081
Nephelodes minians|[2842]|XAD349-04|Canada|Ontario|592[0n]|BOLD:AAA5081
Nephelodes minians|[2843]|XAH266-05|Canada|Ontario|658[0n]|BOLD:AAA5081
Nephelodes minians|[2844]|XAH204-05|Canada|Ontario|658[0n]|BOLD:AAA5081
Nephelodes minians|[2845]|XAH239-05|Canada|Ontario|658[0n]|BOLD:AAA5081
Nephelodes minians|[2846]|XAB436-04|Canada|Ontario|657[0n]|BOLD:AAA5081
Nephelodes minians|[2847]|XAD352-04|Canada|Ontario|658[0n]|BOLD:AAA5081
Nephelodes minians|[2848]|XAH294-05|Canada|Ontario|658[0n]|BOLD:AAA5081
Nephelodes minians|[2849]|TTMNB412-06|Canada|New Brunswick|658[0n]|BOLD:AAA5081
Nephelodes minians|[2850]|XAH392-05|Canada|Ontario|658[0n]|BOLD:AAA5081
Nephelodes minians|[2851]|XAD370-04|Canada|Ontario|658[0n]|BOLD:AAA5081
Nephelodes minians|[2852]|XAD245-04|Canada|Ontario|658[0n]|BOLD:AAA5081
Nephelodes minians|[2853]|XAB457-04|Canada|Ontario|658[0n]|BOLD:AAA5081
Nephelodes minians|[2854]|TMNBB362-06|Canada|New Brunswick|636[0n]|BOLD:AAA5081
Nephelodes minians|[2855]|TMNBB363-06|Canada|New Brunswick|658[0n]|BOLD:AAA5081
Nephelodes minians|[2856]|LPSOD965-09|Canada|Ontario|658[0n]|BOLD:AAA5081
Nephelodes minians|[2857]|BBLEC037-09|Canada|New Brunswick|658[0n]|BOLD:AAA5081
Nephelodes minians|[2858]|BBLEC038-09|Canada|New Brunswick|658[0n]|BOLD:AAA5081
Nephelodes minians|[2859]|BBLEC462-09|Canada|New Brunswick|658[0n]|BOLD:AAA5081
Nephelodes minians|[2860]|BBLPC358-09|Canada|New Brunswick|658[0n]|BOLD:AAA5081
Nephelodes minians|[2861]|BBLPC372-09|Canada|New Brunswick|658[0n]|BOLD:AAA5081
Nephelodes minians|[2862]|BBLPB599-10|Canada|Saskatchewan|658[0n]|BOLD:AAA5081
Nephelodes minians|[2863]|JSSEP1088-11|Canada|Ontario|658[0n]|BOLD:AAA5081
Nephelodes minians|[2864]|RDNMC301-05|United States|Colorado|614[0n]|BOLD:AAA5081
Nephelodes minians|[2865]|CNCLB177-14|United States|New Mexico|658[0n]|BOLD:AAA5081
Nephelodes minians|[2866]|CNCLB1145-14|United States|New Mexico|658[0n]|BOLD:AAA5081
Nephelodes minians|[2867]|XAH310-05|Canada|Ontario|606[0n]|BOLD:AAA5081
Nephelodes minians|[2868]|LOWC079-05|Canada|British Columbia|658[0n]|BOLD:AAA5081
Nephelodes minians|[2869]|LOWC080-05|Canada|British Columbia|658[0n]|BOLD:AAA5081
Nephelodes minians|[2870]|LOWC081-05|Canada|British Columbia|658[0n]|BOLD:AAA5081
Nephelodes minians|[2871]|LOWC082-05|Canada|British Columbia|658[0n]|BOLD:AAA5081
Nephelodes minians|[2872]|LOWC083-05|Canada|British Columbia|658[0n]|BOLD:AAA5081
Nephelodes minians|[2873]|LOWC084-05|Canada|British Columbia|658[0n]|BOLD:AAA5081
Nephelodes minians|[2874]|LOWC086-05|Canada|British Columbia|658[0n]|BOLD:AAA5081
Nephelodes minians|[2875]|LOWCD182-06|Canada|British Columbia|658[0n]|BOLD:AAA5081
Nephelodes minians|[2876]|LOWCD643-06|Canada|British Columbia|658[0n]|BOLD:AAA5081
Nephelodes minians|[2877]|LOWCD644-06|Canada|British Columbia|657[0n]|BOLD:AAA5081
Nephelodes minians|[2878]|LOWCD645-06|Canada|British Columbia|658[0n]|BOLD:AAA5081
Nephelodes minians|[2879]|LOWCD646-06|Canada|British Columbia|658[0n]|BOLD:AAA5081
Nephelodes minians|[2880]|LOWCD647-06|Canada|British Columbia|658[0n]|BOLD:AAA5081
Nephelodes minians|[2881]|LOWCD648-06|Canada|British Columbia|658[0n]|BOLD:AAA5081
Nephelodes minians|[2882]|BBLPB590-10|Canada|Alberta|658[0n]|BOLD:AAA5081
Nephelodes minians|[2883]|BBLPB604-10|Canada|Saskatchewan|658[0n]|BOLD:AAA5081
Nephelodes minians|[2884]|BBLPB605-10|Canada|Saskatchewan|658[0n]|BOLD:AAA5081
Nephelodes minians|[2885]|CNCLB178-14|United States|Oregon|658[0n]|BOLD:AAA5081
Nephelodes minians|[2886]|CNCLB519-14|United States|Nevada|658[0n]|BOLD:AAA5081
Nephelodes minians|[2887]|CNCLB520-14|United States|Nevada|658[0n]|BOLD:AAA5081
Nephelodes minians|[2888]|CNCLB1143-14|United States|Nevada|658[0n]|BOLD:AAA5081
Nephelodes demaculata|[2889]|CNCLB1146-14|United States|Oregon|658[0n]|BOLD:AAA5081
Nephelodes minians|[2890]|XAD358-04|Canada|Ontario|559[0n]|BOLD:AAA5081
Nephelodes minians|[2891]|LSUSA013-06|United States|Kentucky|658[0n]|BOLD:AAA5081
Nephelodes minians|[2892]|LPOKA642-09|United States|Oklahoma|650[0n]|BOLD:AAA5081
Nephelodes minians|[2893]|XAB434-04|Canada|Ontario|658[0n]|BOLD:AAA5081
Nephelodes minians|[2894]|LPOKA367-08|United States|Oklahoma|658[0n]|BOLD:AAA5081
Nephelodes minians|[2895]|LPSOD984-09|Canada|Ontario|658[0n]|BOLD:AAA5081
Nephelodes minians|[2896]|LPOKD457-09|United States|Oklahoma|658[0n]|BOLD:AAA5081
Nephelodes minians|[2897]|LMDH004-11|United States|Minnesota|658[0n]|BOLD:AAA5081
Nephelodes minians|[2898]|NCCH062-11|Canada|Ontario|673[0n]|BOLD:AAA5081
Nephelodes minians|[2899]|LNCC1320-11|United States|North Carolina|658[0n]|BOLD:AAA5081

[illegible]
Nephelodes minians|[2897]|LMDH004-11|United States|Minnesota|658[0n]|BOLD:AAA5081
Nephelodes minians|[2898]|NCCH062-11|Canada|Ontario|673[0n]|BOLD:AAA5081
Nephelodes minians|[2899]|LNCC1320-11|United States|North Carolina|658[0n]|BOLD:AAA5081
Nephelodes minians|[2900]|CNCLB2969-14|United States|North Carolina|658[0n]|BOLD:AAA5081
Nephelodes minians|[2901]|LNCC456-10|United States|North Carolina|658[0n]|BOLD:AAA5081
Nephelodes minians|[2902]|CNCLB2970-14|United States|North Carolina|658[0n]|BOLD:AAA5081
Acerra normalis|[2903]|LALPA005-10|Canada|British Columbia|658[0n]|BOLD:AAD6503
Acerra normalis|[2904]|LALPA006-10|Canada|British Columbia|658[0n]|BOLD:AAD6503
Acerra normalis|[2905]|GMLC309-11|United States|California|658[0n]|BOLD:AAD6503
Acerra normalis|[2906]|CGLCA136-10|United States|California|658[0n]|BOLD:AAD6503
Acerra normalis|[2907]|CGLCA012-10|United States|California|658[0n]|BOLD:AAD6503
Acerra normalis|[2908]|CGLCA011-10|United States|California|658[0n]|BOLD:AAD6503
Acerra normalis|[2909]|NAMUM245-08|United States|California|658[0n]|BOLD:AAD6503
Acerra normalis|[2910]|RDNMF245-08|Canada|British Columbia|658[0n]|BOLD:AAD6503
Acerra normalis|[2911]|RDNMF244-08|Canada|British Columbia|658[0n]|BOLD:AAD6503
Acerra normalis|[2912]|RDNMF242-08|Canada|British Columbia|609[1n]|BOLD:AAD6503
Acerra normalis|[2913]|RDNMF243-08|Canada|British Columbia|640[0n]|BOLD:AAD6503
Acerra normalis|[2914]|RDNMF246-08|Canada|British Columbia|609[0n]|BOLD:AAD6503
Acerra normalis|[2915]|GMLC1364-12|United States|California|634[0n]|BOLD:AAD6503
Acerra normalis|[2916]|GMLC1380-12|United States|California|627[0n]|BOLD:AAD6503
Acerra normalis|[2917]|GMLC1389-12|United States|California|614[0n]|BOLD:AAD6503
Orthosia segregata|[2918]|LOWC068-05|Canada|British Columbia|530[0n]|BOLD:AAB8772
Orthosia segregata|[2919]|LOWC067-05|Canada|British Columbia|525[0n]|BOLD:AAB8772
Orthosia segregata|[2920]|LOWC066-05|Canada|British Columbia|574[0n]|BOLD:AAB8772
Orthosia segregata|[2921]|LOWCE239-06|Canada|British Columbia|658[0n]|BOLD:AAB8772
Orthosia segregata|[2922]|LOWCE407-06|Canada|British Columbia|599[0n]|BOLD:AAB8772
Orthosia segregata|[2923]|LOWCD626-06|Canada|British Columbia|657[0n]|BOLD:AAB8772
Orthosia segregata|[2924]|LOWCE024-06|Canada|British Columbia|658[0n]|BOLD:AAB8772
Orthosia segregata|[2925]|RDLQ671-07|Canada|Ontario|658[0n]|BOLD:AAB8772
Orthosia segregata|[2926]|LBCG470-08|Canada|British Columbia|658[0n]|BOLD:AAB8772
Orthosia segregata|[2927]|LBCH5157-10|Canada|British Columbia|658[0n]|BOLD:AAB8772
Orthosia segregata|[2928]|LBCH5209-10|Canada|British Columbia|658[0n]|BOLD:AAB8772
Orthosia segregata|[2929]|LBCH5255-10|Canada|British Columbia|658[0n]|BOLD:AAB8772
Orthosia segregata|[2930]|LOWCD627-06|Canada|British Columbia|658[0n]|BOLD:AAB8772
Orthosia segregata|[2931]|LOWCD628-06|Canada|British Columbia|658[0n]|BOLD:AAB8772
Orthosia segregata|[2932]|LBCH5343-10|Canada|British Columbia|658[0n]|BOLD:AAB8772
Orthosia segregata|[2933]|CNWBE852-13|Canada|Alberta|579[0n]|BOLD:AAB8772
Orthosia segregata|[2934]|CNWBE854-13|Canada|Alberta|585[0n]|BOLD:AAB8772
Orthosia segregata|[2935]|RDNMH391-09|Canada|Alberta|658[0n]|BOLD:AAB8772
Orthosia segregata|[2936]|LOWCE233-06|Canada|British Columbia|658[0n]|BOLD:AAB8772
Orthosia segregata|[2937]|LOWCD624-06|Canada|British Columbia|658[0n]|BOLD:AAB8772
Orthosia segregata|[2938]|LOWC069-05|Canada|British Columbia|658[0n]|BOLD:AAB8772
Orthosia segregata|[2939]|LOWCD756-06|Canada|British Columbia|656[0n]|BOLD:AAB8772
Orthosia segregata|[2940]|CNWBE856-13|Canada|Alberta|604[0n]|BOLD:AAB8772
Morrisonia triangula|[2941]|LNC107-05|United States|North Carolina|657[0n]|BOLD:AAE4501
Morrisonia triangula|[2942]|LNC108-05|United States|North Carolina|658[0n]|BOLD:AAE4501
Morrisonia triangula|[2943]|HKONS513-08|United States|Florida|658[0n]|BOLD:AAE4501
Morrisonia triangula|[2944]|HKONS514-08|United States|Florida|658[0n]|BOLD:AAE4501
Orthosia pacifica|[2945]|RDNMC464-05|Canada|British Columbia|584[1n]|BOLD:AAE4149
Orthosia pacifica|[2946]|RDMAB1043-09|Canada|British Columbia|635[0n]|BOLD:AAE4149
Orthosia pacifica|[2947]|RDNMG518-08|Canada|British Columbia|652[0n]|BOLD:AAE4149
Orthosia pacifica|[2948]|RDMAB1042-09|Canada|British Columbia|658[0n]|BOLD:AAE4149
Orthosia pacifica|[2949]|IAWLB201-11|United States|California|658[0n]|BOLD:AAE4149
Orthosia pacifica|[2950]|IAWLB202-11|United States|California|658[0n]|BOLD:AAE4149
Orthosia pacifica|[2951]|GMLC1356-12|United States|California|613[0n]|BOLD:AAE4149
Orthosia pacifica|[2952]|CGLCA153-10|United States|California|658[0n]|BOLD:AAE4149
Orthosia pacifica|[2953]|CGLCA154-10|United States|California|658[0n]|BOLD:AAE4149
Orthosia pacifica|[2954]|GMLC202-11|United States|California|658[0n]|BOLD:AAE4149
Orthosia pacifica|[2955]|GMLC216-11|United States|California|658[0n]|BOLD:AAE4149
Orthosia pacifica|[2956]|RDNMG517-08|Canada|British Columbia|658[0n]|BOLD:AAE4149
Orthosia pacifica|[2957]|CGLCA013-10|United States|California|658[0n]|BOLD:AAE4149
Orthosia pacifica|[2958]|GMLC226-11|United States|California|658[0n]|BOLD:AAE4149
Orthosia pacifica|[2959]|GMLC1360-12|United States|California|623[0n]|BOLD:AAE4149
Orthosia sp.|[2960]|RDNMC738-06|United States|Texas|658[0n]|BOLD:AAI3496
Orthosia alurina|[2961]|RDNMC466-05|Canada|Ontario|658[0n]|BOLD:ACF2855
Orthosia alurina|[2962]|MEC135-04|Canada|Quebec|658[0n]|BOLD:ACF2855
Orthosia alurina|[2963]|MEC118-04|Canada|Quebec|658[0n]|BOLD:ACF2855
Orthosia alurina|[2964]|PMG142-03|Canada|Ontario|617[0n]|BOLD:ACF2855
Orthosia alurina|[2965]|MEC127-04|Canada|Quebec|593[2n]|BOLD:ACF2855
Orthosia alurina|[2966]|RDLQ669-07|Canada|Quebec|646[0n]|BOLD:ACF2855
Orthosia alurina|[2967]|LPOKA892-09|United States|Oklahoma|658[0n]|BOLD:ABX5265
Orthosia alurina|[2968]|LPOKA903-09|United States|Oklahoma|658[0n]|BOLD:ABX5265
Orthosia alurina|[2969]|LPOKA904-09|United States|Oklahoma|658[0n]|BOLD:ABX5265
Orthosia alurina|[2970]|LPOKA920-09|United States|Oklahoma|658[0n]|BOLD:ABX5265
Orthosia alurina|[2971]|LPOKA922-09|United States|Oklahoma|658[0n]|BOLD:ABX5265
Orthosia alurina|[2972]|LPOKA947-09|United States|Oklahoma|658[0n]|BOLD:ABX5265
Orthosia alurina|[2973]|LPOKA974-09|United States|Oklahoma|658[0n]|BOLD:ABX5265
Orthosia alurina|[2974]|LNCC523-11|United States|North Carolina|658[0n]|BOLD:ABX5265
Orthosia alurina|[2975]|LNCC524-11|United States|North Carolina|658[0n]|BOLD:ABX5265
Orthosia hibisci|[2976]|LOCBB197-06|United States|California|658[1n]|BOLD:ACF3074
Orthosia hibisci|[2977]|RWWB552-10|United States|Washington|658[0n]|BOLD:ACF3074
Orthosia hibisci|[2978]|LALPA088-10|Canada|British Columbia|658[0n]|BOLD:ACF3074
Orthosia hibisci|[2979]|LALPA055-10|Canada|British Columbia|658[0n]|BOLD:ACF3074
Orthosia hibisci|[2980]|LALPA051-10|Canada|British Columbia|658[0n]|BOLD:ACF3074
Orthosia hibisci|[2981]|LALPA050-10|Canada|British Columbia|658[0n]|BOLD:ACF3074
Orthosia hibisci|[2982]|LALPA049-10|Canada|British Columbia|658[0n]|BOLD:ACF3074
Orthosia hibisci|[2983]|RWWB610-10|United States|Washington|658[0n]|BOLD:ACF3074
Orthosia hibisci|[2984]|RWWB599-10|United States|Washington|658[0n]|BOLD:ACF3074
Orthosia hibisci|[2985]|RWWB586-10|United States|Washington|658[0n]|BOLD:ACF3074
Orthosia hibisci|[2986]|RWWB584-10|United States|Washington|658[0n]|BOLD:ACF3074
Orthosia hibisci|[2987]|RWWB547-10|United States|Washington|658[0n]|BOLD:ACF3074
Orthosia hibisci|[2988]|RWWB542-10|United States|Washington|658[0n]|BOLD:ACF3074
Orthosia hibisci|[2989]|RWWB538-10|United States|Washington|658[0n]|BOLD:ACF3074
Orthosia hibisci|[2990]|RWWB535-10|United States|Washington|658[0n]|BOLD:ACF3074
Orthosia hibisci|[2991]|RWWB533-10|United States|Washington|658[0n]|BOLD:ACF3074
Orthosia hibisci|[2992]|RWWB524-10|United States|Washington|658[0n]|BOLD:ACF3074
Orthosia hibisci|[2993]|RWWB517-10|United States|Washington|658[0n]|BOLD:ACF3074
Orthosia hibisci|[2994]|RWWB505-10|United States|Washington|658[0n]|BOLD:ACF3074
Orthosia hibisci|[2995]|RWWB501-10|United States|Washington|658[0n]|BOLD:ACF3074
Orthosia hibisci|[2996]|LOCBB195-06|United States|California|658[0n]|BOLD:ACF3074
Orthosia hibisci|[2997]|LOCBB198-06|United States|California|654[0n]|BOLD:ACF3074
Orthosia hibisci|[2998]|LOCBB196-06|United States|California|652[1n]|BOLD:ACF3074
[illegible]

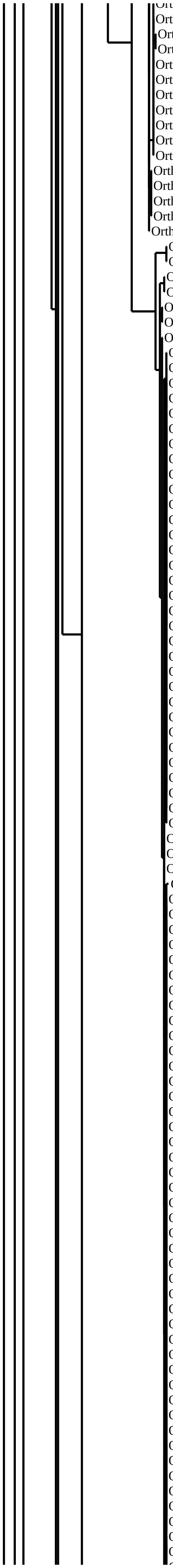

Orthosia hibisci|[2996]|LOCBB195-06|United States|California|658[0n]|BOLD:ACF3074
Orthosia hibisci|[2997]|LOCBB198-06|United States|California|654[0n]|BOLD:ACF3074
Orthosia hibisci|[2998]|LOCBB196-06|United States|California|652[1n]|BOLD:ACF3074
Orthosia hibisci|[2999]|LOCBB199-06|United States|California|617[0n]|BOLD:ACF3074
Orthosia hibisci|[3000]|RDNMD396-06|United States|California|658[0n]|BOLD:ACF3074
Orthosia hibisci|[3001]|RDNMD397-06|United States|California|658[0n]|BOLD:ACF3074
Orthosia hibisci|[3002]|RDNMD398-06|United States|California|658[0n]|BOLD:ACF3074
Orthosia hibisci|[3003]|RDNMD399-06|United States|California|658[0n]|BOLD:ACF3074
Orthosia hibisci|[3004]|IAWLB415-11|United States|California|658[0n]|BOLD:ACF3074
Orthosia hibisci|[3005]|IAWLB416-11|United States|California|658[0n]|BOLD:ACF3074
Orthosia hibisci|[3006]|IAWLB417-11|United States|California|658[0n]|BOLD:ACF3074
Orthosia hibisci|[3007]|RWWB500-10|United States|Washington|658[0n]|BOLD:ACF3074
Orthosia hibisci|[3008]|LOPN141-06|United States|Oregon|545[1n]|BOLD:ACF3074
Orthosia hibisci|[3009]|LOPN142-06|United States|Oregon|578[0n]|BOLD:ACF3074
Orthosia hibisci|[3010]|RWWC187-11|United States|Washington|651[0n]|BOLD:ACF3074
Orthosia hibisci|[3011]|RWWC906-12|United States|Washington|658[0n]|BOLD:ACF3074
Orthosia hibisci|[3012]|RDNMH591-09|United States|Colorado|658[0n]|BOLD:AAA4128
Orthosia hibisci|[3013]|RDNMH592-09|United States|Colorado|658[0n]|BOLD:AAA4128
Orthosia hibisci|[3014]|XAE007-04|Canada|Ontario|658[0n]|BOLD:AAA4128
Orthosia hibisci|[3015]|LBCH2427-10|Canada|British Columbia|658[0n]|BOLD:AAA4128
Orthosia hibisci|[3016]|XAF308-05|Canada|Ontario|658[0n]|BOLD:AAA4128
Orthosia hibisci|[3017]|LOWCD623-06|Canada|British Columbia|658[0n]|BOLD:AAA4128
Orthosia hibisci|[3018]|LOWCE362-06|Canada|British Columbia|586[6n]|BOLD:AAA4128
Orthosia hibisci|[3019]|LNC270-05|United States|North Carolina|658[0n]|BOLD:AAA4128
Orthosia hibisci|[3020]|MEC129-04|Canada|Quebec|658[0n]|BOLD:AAA4128
Orthosia hibisci|[3021]|CNPED058-14|Canada|Prince Edward Island|534[0n]|BOLD:AAA4128
Orthosia hibisci|[3022]|RDLQ666-07|Canada|Quebec|647[0n]|BOLD:AAA4128
Orthosia hibisci|[3023]|CNPEM3409-14|Canada|Prince Edward Island|592[0n]|BOLD:AAA4128
Orthosia hibisci|[3024]|SMTPB4562-13|Canada|Ontario|576[0n]|BOLD:AAA4128
Orthosia hibisci|[3025]|PHMO039-03|Canada|Ontario|639[0n]|BOLD:AAA4128
Orthosia hibisci|[3026]|PHMO035-03|Canada|Ontario|639[0n]|BOLD:AAA4128
Orthosia hibisci|[3027]|PHMO034-03|Canada|Ontario|639[0n]|BOLD:AAA4128
Orthosia hibisci|[3028]|PHMO027-03|Canada|Ontario|639[0n]|BOLD:AAA4128
Orthosia hibisci|[3029]|PHMO026-03|Canada|Ontario|639[0n]|BOLD:AAA4128
Orthosia hibisci|[3030]|PHMO021-03|Canada|Ontario|639[0n]|BOLD:AAA4128
Orthosia hibisci|[3031]|PHMO011-03|Canada|Ontario|639[0n]|BOLD:AAA4128
Orthosia hibisci|[3032]|PHMO007-03|Canada|Ontario|639[0n]|BOLD:AAA4128
Orthosia hibisci|[3033]|TMG133-03|Canada|Ontario|639[0n]|BOLD:AAA4128
Orthosia hibisci|[3034]|RDLQH106-06|Canada|Quebec|621[0n]|BOLD:AAA4128
Orthosia hibisci|[3035]|LOWCE365-06|Canada|British Columbia|578[0n]|BOLD:AAA4128
Orthosia hibisci|[3036]|LOWCE455-06|Canada|British Columbia|617[0n]|BOLD:AAA4128
Orthosia hibisci|[3037]|RDLQ667-07|Canada|Quebec|580[0n]|BOLD:AAA4128
Orthosia hibisci|[3038]|RDLQ668-07|Canada|Quebec|583[0n]|BOLD:AAA4128
Orthosia hibisci|[3039]|GMGSB635-12|United States|Tennessee|614[0n]|BOLD:AAA4128
Orthosia hibisci|[3040]|HEAPR3743-12|Canada|Ontario|613[0n]|BOLD:AAA4128
Orthosia hibisci|[3041]|CNPEB1909-14|Canada|Prince Edward Island|574[1n]|BOLD:AAA4128
Orthosia hibisci|[3042]|HEMAY1064-12|Canada|Ontario|583[0n]|BOLD:AAA4128
Orthosia hibisci|[3043]|HEAPR3776-12|Canada|Ontario|605[0n]|BOLD:AAA4128
Orthosia hibisci|[3044]|HEAPR3774-12|Canada|Ontario|605[0n]|BOLD:AAA4128
Orthosia hibisci|[3045]|HEAPR3773-12|Canada|Ontario|605[0n]|BOLD:AAA4128
Orthosia hibisci|[3046]|CNROE001-13|Canada|Ontario|594[0n]|BOLD:AAA4128
Orthosia hibisci|[3047]|CNROD001-13|Canada|Ontario|591[0n]|BOLD:AAA4128
Orthosia hibisci|[3048]|CNPEB1911-14|Canada|Prince Edward Island|592[0n]|BOLD:AAA4128
Orthosia hibisci|[3049]|CNPEM3413-14|Canada|Prince Edward Island|576[0n]|BOLD:AAA4128
Orthosia hibisci|[3050]|CNBRN327-14|Canada|Nova Scotia|621[1n]|BOLD:AAA4128
Orthosia hibisci|[3051]|LSEU226-06|United States|North Carolina|616[0n]|BOLD:AAA4128
Orthosia hibisci|[3052]|PMG143-03|Canada|Ontario|617[0n]|BOLD:AAA4128
Orthosia hibisci|[3053]|DUNLP178-08|Canada|British Columbia|604[0n]|BOLD:AAA4128
Orthosia hibisci|[3054]|LNC645-06|United States|North Carolina|658[0n]|BOLD:AAA4128
Orthosia hibisci|[3055]|XAF194-05|Canada|Ontario|658[0n]|BOLD:AAA4128
Orthosia hibisci|[3056]|XAF195-05|Canada|Ontario|658[0n]|BOLD:AAA4128
Orthosia hibisci|[3057]|XAF196-05|Canada|Ontario|658[0n]|BOLD:AAA4128
Orthosia hibisci|[3058]|XAF257-05|Canada|Ontario|658[0n]|BOLD:AAA4128
Orthosia hibisci|[3059]|XAF259-05|Canada|Ontario|658[0n]|BOLD:AAA4128
Orthosia hibisci|[3060]|XAF261-05|Canada|Ontario|658[0n]|BOLD:AAA4128
Orthosia hibisci|[3061]|XAF285-05|Canada|Ontario|658[0n]|BOLD:AAA4128
Orthosia hibisci|[3062]|XAF291-05|Canada|Ontario|658[0n]|BOLD:AAA4128
Orthosia hibisci|[3063]|XAF297-05|Canada|Ontario|658[0n]|BOLD:AAA4128
Orthosia hibisci|[3064]|XAF307-05|Canada|Ontario|658[0n]|BOLD:AAA4128
Orthosia hibisci|[3065]|XAF433-05|Canada|Ontario|658[0n]|BOLD:AAA4128
Orthosia hibisci|[3066]|LNC269-05|United States|North Carolina|658[0n]|BOLD:AAA4128
Orthosia hibisci|[3067]|LMIS032-05|Canada|Ontario|658[0n]|BOLD:AAA4128
Orthosia hibisci|[3068]|LSEU227-06|United States|North Carolina|658[0n]|BOLD:AAA4128
Orthosia hibisci|[3069]|LSEU228-06|United States|North Carolina|658[0n]|BOLD:AAA4128
Orthosia hibisci|[3070]|LOWCD621-06|Canada|British Columbia|658[0n]|BOLD:AAA4128
Orthosia hibisci|[3071]|LOWCD622-06|Canada|British Columbia|658[0n]|BOLD:AAA4128
Orthosia hibisci|[3072]|LOWCE010-06|Canada|British Columbia|658[0n]|BOLD:AAA4128
Orthosia hibisci|[3073]|LOWCE011-06|Canada|British Columbia|658[0n]|BOLD:AAA4128
Orthosia hibisci|[3074]|LOWCE012-06|Canada|British Columbia|658[0n]|BOLD:AAA4128
Orthosia hibisci|[3075]|LOWCE013-06|Canada|British Columbia|658[0n]|BOLD:AAA4128
Orthosia hibisci|[3076]|LOWCE014-06|Canada|British Columbia|658[0n]|BOLD:AAA4128
Orthosia hibisci|[3077]|LOWCE017-06|Canada|British Columbia|658[0n]|BOLD:AAA4128
Orthosia hibisci|[3078]|LOWCE018-06|Canada|British Columbia|658[0n]|BOLD:AAA4128
Orthosia hibisci|[3079]|LOWCE019-06|Canada|British Columbia|658[0n]|BOLD:AAA4128
Orthosia hibisci|[3080]|LOWCE210-06|Canada|British Columbia|658[0n]|BOLD:AAA4128
Orthosia hibisci|[3081]|LOWCE269-06|Canada|British Columbia|658[0n]|BOLD:AAA4128
Orthosia hibisci|[3082]|LOWCE409-06|Canada|British Columbia|658[0n]|BOLD:AAA4128
Orthosia hibisci|[3083]|LOWCE457-06|Canada|British Columbia|658[0n]|BOLD:AAA4128
Orthosia hibisci|[3084]|XAJ061-06|Canada|Ontario|658[0n]|BOLD:AAA4128
Orthosia hibisci|[3085]|XAJ062-06|Canada|Ontario|658[0n]|BOLD:AAA4128
Orthosia hibisci|[3086]|XAJ063-06|Canada|Ontario|658[0n]|BOLD:AAA4128
Orthosia hibisci|[3087]|XAJ087-06|Canada|Ontario|658[0n]|BOLD:AAA4128
Orthosia hibisci|[3088]|XAJ101-06|Canada|Ontario|658[0n]|BOLD:AAA4128
Orthosia hibisci|[3089]|XAJ125-06|Canada|Ontario|658[0n]|BOLD:AAA4128
Orthosia hibisci|[3090]|XAJ189-06|Canada|Ontario|658[0n]|BOLD:AAA4128
Orthosia hibisci|[3091]|XAJ336-06|Canada|Ontario|658[0n]|BOLD:AAA4128
Orthosia hibisci|[3092]|LPOKA977-09|United States|Oklahoma|658[0n]|BOLD:AAA4128
Orthosia hibisci|[3093]|LBCH5001-10|Canada|British Columbia|658[0n]|BOLD:AAA4128
Orthosia hibisci|[3094]|LNCC525-11|United States|North Carolina|658[0n]|BOLD:AAA4128
Orthosia hibisci|[3095]|LNCC526-11|United States|North Carolina|658[0n]|BOLD:AAA4128
Orthosia hibisci|[3096]|LNCC609-11|United States|North Carolina|658[0n]|BOLD:AAA4128
Orthosia hibisci|[3097]|XAE150-04|Canada|Ontario|658[0n]|BOLD:AAA4128
Orthosia hibisci|[3098]|XAF028-05|Canada|Ontario|658[0n]|BOLD:AAA4128
Orthosia hibisci|[3099]|LOWCE220-06|Canada|British Columbia|658[0n]|BOLD:AAA4128

Orthosia hibisci|[3096]|LNCC609-11|United States|North Carolina|658[0n]|BOLD:AAA4128
Orthosia hibisci|[3097]|XAE150-04|Canada|Ontario|658[0n]|BOLD:AAA4128
Orthosia hibisci|[3098]|XAF028-05|Canada|Ontario|658[0n]|BOLD:AAA4128
Orthosia hibisci|[3099]|LOWCE220-06|Canada|British Columbia|658[0n]|BOLD:AAA4128
Orthosia hibisci|[3100]|LOWCE268-06|Canada|British Columbia|658[0n]|BOLD:AAA4128
Orthosia hibisci|[3101]|LOWCE015-06|Canada|British Columbia|658[0n]|BOLD:AAA4128
Orthosia hibisci|[3102]|LOWCE016-06|Canada|British Columbia|658[0n]|BOLD:AAA4128
Orthosia hibisci|[3103]|XAE132-04|Canada|Ontario|658[0n]|BOLD:AAA4128
Orthosia hibisci|[3104]|XAE042-04|Canada|Ontario|658[0n]|BOLD:AAA4128
Orthosia hibisci|[3105]|XAE041-04|Canada|Ontario|658[0n]|BOLD:AAA4128
Orthosia hibisci|[3106]|XAC096-04|Canada|Ontario|658[0n]|BOLD:AAA4128
Orthosia hibisci|[3107]|MEC185-04|Canada|Quebec|658[0n]|BOLD:AAA4128
Orthosia hibisci|[3108]|MEC119-04|Canada|Quebec|658[0n]|BOLD:AAA4128
Orthosia hibisci|[3109]|MEC117-04|Canada|Quebec|658[0n]|BOLD:AAA4128
Orthosia hibisci|[3110]|MEC116-04|Canada|Quebec|658[0n]|BOLD:AAA4128
Orthosia hibisci|[3111]|LPOKA918-09|United States|Oklahoma|658[0n]|BOLD:AAA4128
Orthosia hibisci|[3112]|LNCC610-11|United States|North Carolina|658[0n]|BOLD:AAA4128
Orthosia hibisci|[3113]|CNPEN004-14|Canada|Prince Edward Island|552[1n]|BOLD:AAA4128
Engelhardtia ursina|[3114]|CNCLB1704-14|United States|Colorado|658[0n]|BOLD:ACP3573
Orthosia praeses|[3115]|RWWB506-10|United States|Washington|658[0n]|BOLD:AAD6082
Orthosia praeses|[3116]|LALPA012-10|Canada|British Columbia|658[0n]|BOLD:AAD6082
Orthosia praeses|[3117]|IAWLB238-11|United States|California|658[0n]|BOLD:AAD6082
Orthosia praeses|[3118]|LALPA1032-11|Canada|British Columbia|658[0n]|BOLD:AAD6082
Orthosia praeses|[3119]|LALPA011-10|Canada|British Columbia|658[0n]|BOLD:AAD6082
Orthosia praeses|[3120]|GMLC094-09|United States|California|658[0n]|BOLD:AAD6082
Orthosia praeses|[3121]|LALPA1031-11|Canada|British Columbia|658[0n]|BOLD:AAD6082
Orthosia praeses|[3122]|LALPA1042-11|Canada|British Columbia|658[0n]|BOLD:AAD6082
Orthosia praeses|[3123]|RWWB541-10|United States|Washington|658[0n]|BOLD:AAD6082
Orthosia praeses|[3124]|RWWB555-10|United States|Washington|658[0n]|BOLD:AAD6082
Orthosia praeses|[3125]|RWWC899-12|United States|Washington|658[0n]|BOLD:AAD6082
Orthosia praeses|[3126]|RWWB543-10|United States|Washington|658[0n]|BOLD:AAD6082
Orthosia praeses|[3127]|RWWB546-10|United States|Washington|658[0n]|BOLD:AAD6082
Orthosia praeses|[3128]|RWWB512-10|United States|Washington|658[0n]|BOLD:AAD6082
Orthosia praeses|[3129]|RWWB515-10|United States|Washington|658[0n]|BOLD:AAD6082
Orthosia praeses|[3130]|RWWC917-12|United States|Washington|658[0n]|BOLD:AAD6082
Orthosia praeses|[3131]|RWWB511-10|United States|Washington|658[0n]|BOLD:AAD6082
Orthosia praeses|[3132]|RWWB498-10|United States|Washington|658[0n]|BOLD:AAD6082
Orthosia praeses|[3133]|RWWB492-10|United States|Washington|658[0n]|BOLD:AAD6082
Orthosia praeses|[3134]|RWWB489-10|United States|Washington|658[0n]|BOLD:AAD6082
Orthosia praeses|[3135]|RDNMG520-08|Canada|British Columbia|658[0n]|BOLD:AAD6082
Orthosia praeses|[3136]|RDNMG519-08|Canada|British Columbia|658[0n]|BOLD:AAD6082
Orthosia praeses|[3137]|NAMUM293-08|United States|California|658[0n]|BOLD:AAD6082
Orthosia praeses|[3138]|RWWB551-10|United States|Washington|658[0n]|BOLD:AAD6082
Orthosia praeses|[3139]|GMLC1309-12|United States|California|635[0n]|BOLD:AAD6082
Orthosia praeses|[3140]|RWWB553-10|United States|Washington|658[0n]|BOLD:AAD6082
Orthosia praeses|[3141]|RWWB508-10|United States|Washington|658[0n]|BOLD:AAD6082
Orthosia praeses|[3142]|RWWB572-10|United States|Washington|658[0n]|BOLD:AAD6082
Orthosia praeses|[3143]|RWWB528-10|United States|Washington|658[0n]|BOLD:AAD6082
Orthosia praeses|[3144]|RWWB525-10|United States|Washington|658[0n]|BOLD:AAD6082
Orthosia praeses|[3145]|RWWB510-10|United States|Washington|658[0n]|BOLD:AAD6082
Orthosia praeses|[3146]|RDNMD433-06|United States|California|658[0n]|BOLD:AAD6082
Orthosia praeses|[3147]|LOCB915-06|United States|California|658[0n]|BOLD:AAD6082
Orthosia praeses|[3148]|GMLC1275-12|United States|California|658[0n]|BOLD:AAD6082
Orthosia praeses|[3149]|GMLC310-11|United States|California|658[0n]|BOLD:AAD6082
Orthosia praeses|[3150]|GMLC181-11|United States|California|658[0n]|BOLD:AAD6082
Orthosia praeses|[3151]|GMLC1361-12|United States|California|629[0n]|BOLD:AAD6082
Orthosia praeses|[3152]|RWWB523-10|United States|Washington|658[0n]|BOLD:AAD6082
Orthosia praeses|[3153]|RWWB507-10|United States|Washington|658[0n]|BOLD:AAD6082
Orthosia praeses|[3154]|CGLCA157-10|United States|California|658[0n]|BOLD:AAD6082
Orthosia praeses|[3155]|CGLCA156-10|United States|California|658[0n]|BOLD:AAD6082
Orthosia praeses|[3156]|CGLCA141-10|United States|California|658[0n]|BOLD:AAD6082
Orthosia praeses|[3157]|CGLCA139-10|United States|California|658[0n]|BOLD:AAD6082
Orthosia praeses|[3158]|CGLCA057-10|United States|California|658[0n]|BOLD:AAD6082
Orthosia praeses|[3159]|CGLCA056-10|United States|California|658[0n]|BOLD:AAD6082
Orthosia praeses|[3160]|CGLCA055-10|United States|California|658[0n]|BOLD:AAD6082
Orthosia praeses|[3161]|GMLC1317-12|United States|California|632[0n]|BOLD:AAD6082
Orthosia praeses|[3162]|GMLC1391-12|United States|California|632[0n]|BOLD:AAD6082
Egira variabilis|[3163]|RDNMB361-05|United States|California|601[1n]|BOLD:AAD0722
Egira variabilis|[3164]|RDNMD913-07|United States|Wyoming|655[0n]|BOLD:ACM4081
Egira variabilis|[3165]|RDNMG993-08|United States|Wyoming|658[0n]|BOLD:ACM4081
Egira variabilis|[3166]|RDNMG992-08|United States|Wyoming|658[0n]|BOLD:ACM4081
Egira variabilis|[3167]|RDNMD912-07|United States|Wyoming|655[0n]|BOLD:ACM4081
Egira variabilis|[3168]|NAMUM386-09|United States|California|658[0n]|BOLD:ACM4081
Egira variabilis|[3169]|NAMUM385-09|United States|California|658[0n]|BOLD:ACM4081
Egira variabilis|[3170]|NAMUM394-09|United States|California|658[0n]|BOLD:ACM4081
Egira baueri|[3171]|CNCLB490-14|United States|California|658[0n]|BOLD:ACM4548
Egira baueri|[3172]|CNCLB491-14|United States|California|658[0n]|BOLD:ACM4548
Egira baueri|[3173]|CNCLB492-14|United States|California|658[0n]|BOLD:ACM4548
Crocigrapha normani|[3174]|LOCT084-05|United States|Connecticut|658[0n]|BOLD:AAA6924
Crocigrapha normani|[3175]|KPOEC138-08|Canada|Ontario|658[0n]|BOLD:AAA6924
Crocigrapha normani|[3176]|PMG105-03|Canada|Ontario|617[0n]|BOLD:AAA6924
Crocigrapha normani|[3177]|BLTIB091-08|Canada|Ontario|658[1n]|BOLD:AAA6924
Crocigrapha normani|[3178]|TMNBB337-06|Canada|New Brunswick|658[0n]|BOLD:AAA6924
Crocigrapha normani|[3179]|TMNBB339-06|Canada|New Brunswick|658[0n]|BOLD:AAA6924
Crocigrapha normani|[3180]|XAJ216-06|Canada|Ontario|658[0n]|BOLD:AAA6924
Crocigrapha normani|[3181]|XAJ410-06|Canada|Ontario|658[0n]|BOLD:AAA6924
Crocigrapha normani|[3182]|XAJ456-06|Canada|Ontario|658[0n]|BOLD:AAA6924
Crocigrapha normani|[3183]|XAJ508-06|Canada|Ontario|658[0n]|BOLD:AAA6924
Crocigrapha normani|[3184]|RDLQH070-06|Canada|Quebec|658[0n]|BOLD:AAA6924
Crocigrapha normani|[3185]|RDLQ665-07|Canada|Quebec|658[0n]|BOLD:AAA6924
Crocigrapha normani|[3186]|KPOEC041-08|Canada|Ontario|658[0n]|BOLD:AAA6924
Crocigrapha normani|[3187]|KPOEC070-08|Canada|Ontario|658[0n]|BOLD:AAA6924
Crocigrapha normani|[3188]|LPSO258-08|Canada|Ontario|658[0n]|BOLD:AAA6924
Crocigrapha normani|[3189]|KPOEC114-08|Canada|Ontario|658[0n]|BOLD:AAA6924
Crocigrapha normani|[3190]|KPOEC141-08|Canada|Ontario|658[0n]|BOLD:AAA6924
Crocigrapha normani|[3191]|KPOEC151-08|Canada|Ontario|658[0n]|BOLD:AAA6924
Crocigrapha normani|[3192]|KPOEC154-08|Canada|Ontario|658[0n]|BOLD:AAA6924
Crocigrapha normani|[3193]|KPOEC175-08|Canada|Ontario|658[0n]|BOLD:AAA6924
Crocigrapha normani|[3194]|TMNBB336-06|Canada|New Brunswick|658[0n]|BOLD:AAA6924
Crocigrapha normani|[3195]|TMNBB335-06|Canada|New Brunswick|658[0n]|BOLD:AAA6924
Crocigrapha normani|[3196]|TTMNB579-06|Canada|New Brunswick|658[0n]|BOLD:AAA6924
Crocigrapha normani|[3197]|TTMNB577-06|Canada|New Brunswick|658[0n]|BOLD:AAA6924
Crocigrapha normani|[3198]|TTMNB576-06|Canada|New Brunswick|658[0n]|BOLD:AAA6924
Crocigrapha normani|[3199]|TTMNB091-06|Canada|New Brunswick|657[0n]|BOLD:AAA6924

Crocigrapha normani|[3196]|TTMNB579-06|Canada|New Brunswick|658[0n]|BOLD:AAA6924
Crocigrapha normani|[3197]|TTMNB577-06|Canada|New Brunswick|658[0n]|BOLD:AAA6924
Crocigrapha normani|[3198]|TTMNB576-06|Canada|New Brunswick|658[0n]|BOLD:AAA6924
Crocigrapha normani|[3199]|TTMNB091-06|Canada|New Brunswick|657[0n]|BOLD:AAA6924
Crocigrapha normani|[3200]|TTMNB049-06|Canada|New Brunswick|658[0n]|BOLD:AAA6924
Crocigrapha normani|[3201]|LOCT320-05|United States|Connecticut|658[0n]|BOLD:AAA6924
Crocigrapha normani|[3202]|LOCT106-05|United States|Connecticut|658[0n]|BOLD:AAA6924
Crocigrapha normani|[3203]|LOCT098-05|United States|Connecticut|658[0n]|BOLD:AAA6924
Crocigrapha normani|[3204]|XAF407-05|Canada|Ontario|658[0n]|BOLD:AAA6924
Crocigrapha normani|[3205]|KPOEC133-08|Canada|Ontario|658[0n]|BOLD:AAA6924
Crocigrapha normani|[3206]|TMG134-03|Canada|Ontario|639[0n]|BOLD:AAA6924
Crocigrapha normani|[3207]|TMG135-03|Canada|Ontario|639[0n]|BOLD:AAA6924
Crocigrapha normani|[3208]|TMG136-03|Canada|Ontario|639[0n]|BOLD:AAA6924
Crocigrapha normani|[3209]|KPOEC082-08|Canada|Ontario|646[0n]|BOLD:AAA6924
Crocigrapha normani|[3210]|KPOEC183-08|Canada|Ontario|655[0n]|BOLD:AAA6924
Crocigrapha normani|[3211]|BLTIB033-08|Canada|Ontario|658[0n]|BOLD:AAA6924
Crocigrapha normani|[3212]|LPSOD275-09|Canada|Ontario|658[0n]|BOLD:AAA6924
Crocigrapha normani|[3213]|XAF333-05|Canada|Ontario|658[0n]|BOLD:AAA6924
Crocigrapha normani|[3214]|LMIS020-05|Canada|Ontario|658[0n]|BOLD:AAA6924
Crocigrapha normani|[3215]|KPOEC124-08|Canada|Ontario|658[0n]|BOLD:AAA6924
Crocigrapha normani|[3216]|KPOEC125-08|Canada|Ontario|658[0n]|BOLD:AAA6924
Crocigrapha normani|[3217]|TTMNB411-06|Canada|New Brunswick|658[0n]|BOLD:AAA6924
Crocigrapha normani|[3218]|LOCT317-05|United States|Connecticut|658[0n]|BOLD:AAA6924
Crocigrapha normani|[3219]|XAF434-05|Canada|Ontario|658[0n]|BOLD:AAA6924
Crocigrapha normani|[3220]|TMNBB338-06|Canada|New Brunswick|656[0n]|BOLD:AAA6924
Crocigrapha normani|[3221]|RDLQG463-06|Canada|Quebec|658[0n]|BOLD:AAA6924
Crocigrapha normani|[3222]|KPOEC042-08|Canada|Ontario|658[0n]|BOLD:AAA6924
Crocigrapha normani|[3223]|KPOEC045-08|Canada|Ontario|658[0n]|BOLD:AAA6924
Crocigrapha normani|[3224]|LPSO550-08|Canada|Ontario|658[0n]|BOLD:AAA6924
Crocigrapha normani|[3225]|USLEP566-10|United States|Colorado|658[0n]|BOLD:AAA6924
Crocigrapha normani|[3226]|LNCB953-10|United States|North Carolina|658[0n]|BOLD:ABZ2369
Crocigrapha normani|[3227]|LNCC673-11|United States|North Carolina|658[0n]|BOLD:ABZ2369
Crocigrapha normani|[3228]|LSUSA101-06|United States|Kentucky|603[0n]|BOLD:ABZ2369
Crocigrapha normani|[3229]|LNCC674-11|United States|North Carolina|658[0n]|BOLD:ABZ2369
Egira dolosa|[3230]|XAF335-05|Canada|Ontario|658[0n]|BOLD:AAA5658
Egira dolosa|[3231]|LOWCD631-06|Canada|British Columbia|658[0n]|BOLD:AAA5658
Egira dolosa|[3232]|LOWCE245-06|Canada|British Columbia|658[0n]|BOLD:AAA5658
Egira dolosa|[3233]|XAF278-05|Canada|Ontario|658[0n]|BOLD:AAA5658
Egira dolosa|[3234]|PHMO031-03|Canada|Ontario|639[0n]|BOLD:AAA5658
Egira dolosa|[3235]|XAJ186-06|Canada|Ontario|607[0n]|BOLD:AAA5658
Egira dolosa|[3236]|PMG109-03|Canada|Ontario|617[0n]|BOLD:AAA5658
Egira dolosa|[3237]|XAJ187-06|Canada|Ontario|658[0n]|BOLD:AAA5658
Egira dolosa|[3238]|XAJ185-06|Canada|Ontario|658[0n]|BOLD:AAA5658
Egira dolosa|[3239]|XAJ140-06|Canada|Ontario|658[0n]|BOLD:AAA5658
Egira dolosa|[3240]|XAJ138-06|Canada|Ontario|658[0n]|BOLD:AAA5658
Egira dolosa|[3241]|XAJ134-06|Canada|Ontario|658[0n]|BOLD:AAA5658
Egira dolosa|[3242]|XAJ132-06|Canada|Ontario|658[0n]|BOLD:AAA5658
Egira dolosa|[3243]|XAJ131-06|Canada|Ontario|658[0n]|BOLD:AAA5658
Egira dolosa|[3244]|XAJ129-06|Canada|Ontario|658[0n]|BOLD:AAA5658
Egira dolosa|[3245]|XAJ128-06|Canada|Ontario|658[0n]|BOLD:AAA5658
Egira dolosa|[3246]|XAJ122-06|Canada|Ontario|658[0n]|BOLD:AAA5658
Egira dolosa|[3247]|LOWCE240-06|Canada|British Columbia|658[0n]|BOLD:AAA5658
Egira dolosa|[3248]|LOWCD634-06|Canada|British Columbia|658[0n]|BOLD:AAA5658
Egira dolosa|[3249]|LOWCD633-06|Canada|British Columbia|658[0n]|BOLD:AAA5658
Egira dolosa|[3250]|LOWCD632-06|Canada|British Columbia|658[0n]|BOLD:AAA5658
Egira dolosa|[3251]|LOWCD630-06|Canada|British Columbia|658[0n]|BOLD:AAA5658
Egira dolosa|[3252]|LOWCD629-06|Canada|British Columbia|658[0n]|BOLD:AAA5658
Egira dolosa|[3253]|XAD666-05|Canada|Ontario|658[0n]|BOLD:AAA5658
Egira dolosa|[3254]|XAD648-05|Canada|Ontario|658[0n]|BOLD:AAA5658
Egira dolosa|[3255]|XAD644-05|Canada|Ontario|658[0n]|BOLD:AAA5658
Egira dolosa|[3256]|XAF431-05|Canada|Ontario|658[0n]|BOLD:AAA5658
Egira dolosa|[3257]|XAF418-05|Canada|Ontario|658[0n]|BOLD:AAA5658
Egira dolosa|[3258]|XAF415-05|Canada|Ontario|658[0n]|BOLD:AAA5658
Egira dolosa|[3259]|XAF413-05|Canada|Ontario|658[0n]|BOLD:AAA5658
Egira dolosa|[3260]|XAF382-05|Canada|Ontario|658[0n]|BOLD:AAA5658
Egira dolosa|[3261]|XAF347-05|Canada|Ontario|658[0n]|BOLD:AAA5658
Egira dolosa|[3262]|XAF342-05|Canada|Ontario|658[0n]|BOLD:AAA5658
Egira dolosa|[3263]|XAF341-05|Canada|Ontario|658[0n]|BOLD:AAA5658
Egira dolosa|[3264]|XAF292-05|Canada|Ontario|658[0n]|BOLD:AAA5658
Egira dolosa|[3265]|XAB055-04|Canada|Ontario|658[0n]|BOLD:AAA5658
Egira dolosa|[3266]|XAF414-05|Canada|Ontario|650[0n]|BOLD:AAA5658
Egira dolosa|[3267]|XAC126-04|Canada|Ontario|525[0n]|BOLD:AAA5658
Egira dolosa|[3268]|XAF417-05|Canada|Ontario|647[0n]|BOLD:AAA5658
Egira dolosa|[3269]|XAD614-05|Canada|Ontario|524[0n]|BOLD:AAA5658
Egira dolosa|[3270]|XAF430-05|Canada|Ontario|588[0n]|BOLD:AAA5658
Egira dolosa|[3271]|XAJ198-06|Canada|Ontario|608[0n]|BOLD:AAA5658
Egira dolosa|[3272]|XAF429-05|Canada|Ontario|612[0n]|BOLD:AAA5658
Egira dolosa|[3273]|PHMO043-03|Canada|Ontario|639[0n]|BOLD:AAA5658
Egira dolosa|[3274]|PHMO042-03|Canada|Ontario|639[0n]|BOLD:AAA5658
Egira dolosa|[3275]|PHMO041-03|Canada|Ontario|639[0n]|BOLD:AAA5658
Egira dolosa|[3276]|PHMO040-03|Canada|Ontario|639[0n]|BOLD:AAA5658
Egira dolosa|[3277]|TMG139-03|Canada|Ontario|639[0n]|BOLD:AAA5658
Egira dolosa|[3278]|TMG138-03|Canada|Ontario|639[0n]|BOLD:AAA5658
Egira dolosa|[3279]|TMG137-03|Canada|Ontario|639[0n]|BOLD:AAA5658
Egira dolosa|[3280]|XAJ199-06|Canada|Ontario|607[0n]|BOLD:AAA5658
Egira dolosa|[3281]|XAJ090-06|Canada|Ontario|618[0n]|BOLD:AAA5658
Egira dolosa|[3282]|XAF416-05|Canada|Ontario|598[0n]|BOLD:AAA5658
Egira dolosa|[3283]|LOWCE368-06|Canada|British Columbia|577[0n]|BOLD:AAA5658
Egira dolosa|[3284]|XAJ201-06|Canada|Ontario|600[0n]|BOLD:AAA5658
Egira dolosa|[3285]|XAJ206-06|Canada|Ontario|658[0n]|BOLD:AAA5658
Egira dolosa|[3286]|XAJ213-06|Canada|Ontario|658[0n]|BOLD:AAA5658
Egira dolosa|[3287]|XAJ215-06|Canada|Ontario|658[0n]|BOLD:AAA5658
Egira dolosa|[3288]|XAJ225-06|Canada|Ontario|658[0n]|BOLD:AAA5658
Egira dolosa|[3289]|XAJ234-06|Canada|Ontario|658[0n]|BOLD:AAA5658
Egira dolosa|[3290]|XAJ235-06|Canada|Ontario|658[0n]|BOLD:AAA5658
Egira dolosa|[3291]|XAJ269-06|Canada|Ontario|658[0n]|BOLD:AAA5658
Egira dolosa|[3292]|XAJ393-06|Canada|Ontario|658[0n]|BOLD:AAA5658
Egira dolosa|[3293]|XAJ443-06|Canada|Ontario|658[0n]|BOLD:AAA5658
Egira dolosa|[3294]|RDLQH104-06|Canada|Quebec|658[0n]|BOLD:AAA5658
Egira dolosa|[3295]|LMDH026-11|United States|Minnesota|658[0n]|BOLD:AAA5658
Egira dolosa|[3296]|LMDH077-11|United States|Minnesota|658[0n]|BOLD:AAA5658
Orthosia revicta|[3297]|RDNMH620-09|United States|Colorado|658[0n]|BOLD:AAA9607
Orthosia revicta|[3298]|KPOEC160-08|Canada|Ontario|658[0n]|BOLD:AAA9607
[illegible]

Egira dolosa|[3296]|LMDH077-11|United States|Minnesota|658[0n]|BOLD:AAA5658
Orthosia revicta|[3297]|RDNMH620-09|United States|Colorado|658[0n]|BOLD:AAA9607
Orthosia revicta|[3298]|KPOEC160-08|Canada|Ontario|658[0n]|BOLD:AAA9607
Orthosia revicta|[3299]|LSUSA027-06|United States|Kentucky|658[0n]|BOLD:AAA9607
Orthosia revicta|[3300]|LBCH5346-10|Canada|British Columbia|658[0n]|BOLD:AAA9607
Orthosia revicta|[3301]|LOWCE243-06|Canada|British Columbia|658[0n]|BOLD:AAA9607
Orthosia revicta|[3302]|LOWCE248-06|Canada|British Columbia|658[0n]|BOLD:AAA9607
Orthosia revicta|[3303]|XAE068-04|Canada|Ontario|575[0n]|BOLD:AAA9607
Orthosia revicta|[3304]|TTMNB050-06|Canada|New Brunswick|658[0n]|BOLD:AAA9607
Orthosia revicta|[3305]|LNCC151-10|United States|North Carolina|658[0n]|BOLD:AAA9607
Orthosia revicta|[3306]|LNCC152-10|United States|North Carolina|658[0n]|BOLD:AAA9607
Orthosia revicta|[3307]|KPOEC076-08|Canada|Ontario|658[0n]|BOLD:AAA9607
Orthosia revicta|[3308]|KPOEC127-08|Canada|Ontario|658[0n]|BOLD:AAA9607
Orthosia revicta|[3309]|LBCH5038-10|Canada|British Columbia|658[0n]|BOLD:AAA9607
Orthosia revicta|[3310]|LALPA079-10|Canada|British Columbia|658[0n]|BOLD:AAA9607
Orthosia revicta|[3311]|LNCC149-10|United States|North Carolina|658[0n]|BOLD:AAA9607
Orthosia revicta|[3312]|LNCC150-10|United States|North Carolina|658[0n]|BOLD:AAA9607
Orthosia revicta|[3313]|LALPA096-10|Canada|British Columbia|658[0n]|BOLD:AAA9607
Orthosia revicta|[3314]|LNCC148-10|United States|North Carolina|658[0n]|BOLD:AAA9607
Orthosia revicta|[3315]|LOWCE267-06|Canada|British Columbia|658[0n]|BOLD:AAA9607
Orthosia revicta|[3316]|TMNBB333-06|Canada|New Brunswick|658[0n]|BOLD:AAA9607
Orthosia revicta|[3317]|LOWCE251-06|Canada|British Columbia|658[0n]|BOLD:AAA9607
Orthosia revicta|[3318]|LOWCE252-06|Canada|British Columbia|658[0n]|BOLD:AAA9607
Orthosia revicta|[3319]|TMNBB334-06|Canada|New Brunswick|658[0n]|BOLD:AAA9607
Orthosia revicta|[3320]|LOWCE249-06|Canada|British Columbia|658[0n]|BOLD:AAA9607
Orthosia revicta|[3321]|LOWCE232-06|Canada|British Columbia|658[0n]|BOLD:AAA9607
Orthosia revicta|[3322]|LOWCE231-06|Canada|British Columbia|658[0n]|BOLD:AAA9607
Orthosia revicta|[3323]|TTMNB582-06|Canada|New Brunswick|658[0n]|BOLD:AAA9607
Orthosia revicta|[3324]|TTMNB094-06|Canada|New Brunswick|658[0n]|BOLD:AAA9607
Orthosia revicta|[3325]|LOWCD620-06|Canada|British Columbia|658[0n]|BOLD:AAA9607
Orthosia revicta|[3326]|LSEU233-06|United States|North Carolina|658[0n]|BOLD:AAA9607
Orthosia revicta|[3327]|LSEU232-06|United States|North Carolina|658[0n]|BOLD:AAA9607
Orthosia revicta|[3328]|LSEU231-06|United States|North Carolina|658[0n]|BOLD:AAA9607
Orthosia revicta|[3329]|RDNMC468-05|Canada|British Columbia|658[0n]|BOLD:AAA9607
Orthosia revicta|[3330]|RDNMC467-05|Canada|Quebec|658[0n]|BOLD:AAA9607
Orthosia revicta|[3331]|PMG144-03|Canada|Ontario|617[0n]|BOLD:AAA9607
Orthosia revicta|[3332]|RDLQG465-06|Canada|Quebec|656[0n]|BOLD:AAA9607
Orthosia revicta|[3333]|TTMNB583-06|Canada|New Brunswick|656[0n]|BOLD:AAA9607
Orthosia revicta|[3334]|TTMNB093-06|Canada|New Brunswick|656[0n]|BOLD:AAA9607
Orthosia revicta|[3335]|LOWCE371-06|Canada|British Columbia|622[0n]|BOLD:AAA9607
Orthosia revicta|[3336]|LOWCE266-06|Canada|British Columbia|526[0n]|BOLD:AAA9607
Orthosia revicta|[3337]|LOWCE462-06|Canada|British Columbia|616[0n]|BOLD:AAA9607
Orthosia revicta|[3338]|LBCH5280-10|Canada|British Columbia|658[0n]|BOLD:AAA9607
Orthosia revicta|[3339]|LMDH078-11|United States|Minnesota|658[0n]|BOLD:AAA9607
Orthosia revicta|[3340]|LOWC070-05|Canada|British Columbia|600[0n]|BOLD:AAA9607
Orthosia revicta|[3341]|LOWCE370-06|Canada|British Columbia|586[0n]|BOLD:AAA9607
Orthosia revicta|[3342]|LOWCE456-06|Canada|British Columbia|658[0n]|BOLD:AAA9607
Orthosia revicta|[3343]|SSWLA4429-13|Canada|Alberta|555[1n]|BOLD:AAA9607
Orthosia revicta|[3344]|LOWCD215-06|Canada|British Columbia|658[0n]|BOLD:AAA9607
Orthosia revicta|[3345]|SSEIA7708-13|Canada|Alberta|608[0n]|BOLD:AAA9607
Orthosia revicta|[3346]|CNPKH116-14|Canada|Ontario|603[0n]|BOLD:AAA9607
Stretchia sp.|[3347]|RDNMD409-06|United States|California|658[0n]|BOLD:AAB2916
Stretchia pacifica|[3348]|CGLCA027-10|United States|California|658[0n]|BOLD:AAB2916
Stretchia pacifica|[3349]|CNCLB503-14|United States|California|550[0n]|BOLD:AAB2916
Stretchia pacifica|[3350]|CNCLB505-14|United States|California|550[0n]|BOLD:AAB2916
Stretchia nr. pacifica|[3351]|CGLCA009-10|United States|California|658[0n]|BOLD:AAB2916
Stretchia nr. pacifica|[3352]|LOCBF3254-14|United States|California|576[0n]|BOLD:AAB2916
Stretchia nr. pacifica|[3353]|RDNMD408-06|United States|California|658[0n]|BOLD:AAB2916
Stretchia nr. pacifica|[3354]|LNAUS4836-13|United States|California|658[0n]|BOLD:AAB2916
Stretchia nr. pacifica|[3355]|LOCBB178-06|United States|California|658[0n]|BOLD:AAB2916
Stretchia nr. pacifica|[3356]|LOCBB345-06|United States|California|658[0n]|BOLD:AAB2916
Stretchia nr. pacifica|[3357]|LOCBB174-06|United States|California|658[0n]|BOLD:AAB2916
Stretchia nr. pacifica|[3358]|LOCBB175-06|United States|California|658[0n]|BOLD:AAB2916
Stretchia nr. pacifica|[3359]|LOCBB176-06|United States|California|658[0n]|BOLD:AAB2916
Stretchia nr. pacifica|[3360]|LOCBB177-06|United States|California|658[0n]|BOLD:AAB2916
Stretchia nr. pacifica|[3361]|LOCBB346-06|United States|California|658[0n]|BOLD:AAB2916
Stretchia nr. pacifica|[3362]|RDNMD407-06|United States|California|658[0n]|BOLD:AAB2916
Stretchia nr. pacifica|[3363]|LNAUS4837-13|United States|California|658[0n]|BOLD:AAB2916
Stretchia nr. pacifica|[3364]|LOCBF4254-14|United States|California|658[0n]|BOLD:AAB2916
Stretchia nr. pacifica|[3365]|LOCBF4258-14|United States|California|658[0n]|BOLD:AAB2916
Stretchia nr. pacifica|[3366]|LOCBF4259-14|United States|California|658[0n]|BOLD:AAB2916
Stretchia nr. pacifica|[3367]|LOCBF4260-14|United States|California|658[0n]|BOLD:AAB2916
Stretchia nr. pacifica|[3368]|LOCBF4261-14|United States|California|658[0n]|BOLD:AAB2916
Stretchia nr. pacifica|[3369]|LOCBF4263-14|United States|California|658[0n]|BOLD:AAB2916
Stretchia nr. pacifica|[3370]|LOCBF4264-14|United States|California|658[0n]|BOLD:AAB2916
Stretchia nr. pacifica|[3371]|LOCBF4265-14|United States|California|658[0n]|BOLD:AAB2916
Stretchia nr. pacifica|[3372]|LOCBF4266-14|United States|California|658[0n]|BOLD:AAB2916
Stretchia nr. pacifica|[3373]|LOCBF4267-14|United States|California|658[0n]|BOLD:AAB2916
Stretchia nr. pacifica|[3374]|LOCBF4268-14|United States|California|658[0n]|BOLD:AAB2916
Stretchia nr. pacifica|[3375]|LOCBF4269-14|United States|California|658[0n]|BOLD:AAB2916
Stretchia nr. pacifica|[3376]|LOCBF4270-14|United States|California|658[0n]|BOLD:AAB2916
Stretchia nr. pacifica|[3377]|LOCB714-06|United States|California|658[0n]|BOLD:AAB2916
Stretchia nr. pacifica|[3378]|LOCB715-06|United States|California|658[0n]|BOLD:AAB2916
Stretchia nr. pacifica|[3379]|LOCB712-06|United States|California|658[0n]|BOLD:AAB2916
Stretchia nr. pacifica|[3380]|LOCB713-06|United States|California|658[0n]|BOLD:AAB2916
Stretchia nr. pacifica|[3381]|LOCB710-06|United States|California|658[0n]|BOLD:AAB2916
Stretchia nr. pacifica|[3382]|LOCB711-06|United States|California|658[0n]|BOLD:AAB2916
Stretchia nr. pacifica|[3383]|LOCBF4271-14|United States|California|658[0n]|BOLD:AAB2916
Stretchia nr. pacifica|[3384]|LOCB685-06|United States|California|658[0n]|BOLD:AAB2916
Stretchia nr. pacifica|[3385]|LOCB684-06|United States|California|658[0n]|BOLD:AAB2916
Stretchia nr. pacifica|[3386]|LOCB683-06|United States|California|658[0n]|BOLD:AAB2916
Stretchia nr. pacifica|[3387]|LOCB672-06|United States|California|658[0n]|BOLD:AAB2916
Stretchia nr. pacifica|[3388]|LOCB640-06|United States|California|658[0n]|BOLD:AAB2916
Stretchia nr. pacifica|[3389]|LOCB638-06|United States|California|658[0n]|BOLD:AAB2916
Stretchia nr. pacifica|[3390]|LOCB631-06|United States|California|658[0n]|BOLD:AAB2916
Stretchia nr. pacifica|[3391]|LOCB630-06|United States|California|658[0n]|BOLD:AAB2916
Stretchia nr. pacifica|[3392]|LOCB629-06|United States|California|658[0n]|BOLD:AAB2916
Stretchia nr. pacifica|[3393]|LOCB628-06|United States|California|658[0n]|BOLD:AAB2916
Stretchia nr. pacifica|[3394]|LOCB625-06|United States|California|658[0n]|BOLD:AAB2916
Stretchia nr. pacifica|[3395]|LOCBF3258-14|United States|California|564[0n]|BOLD:AAB2916
Stretchia nr. pacifica|[3396]|LOCBF4262-14|United States|California|627[0n]|BOLD:AAB2916
Stretchia nr. pacifica|[3397]|LOCBF3255-14|United States|California|561[0n]|BOLD:AAB2916
Stretchia nr. pacifica|[3398]|LNAUS4835-13|United States|California|615[0n]|BOLD:AAB2916
[illegible]

Stretchia nr. pacifica|[3396]|LOCBF4262-14|United States|California|627[0n]|BOLD:AAB2916
Stretchia nr. pacifica|[3397]|LOCBF3255-14|United States|California|561[0n]|BOLD:AAB2916
Stretchia nr. pacifica|[3398]|LNAUS4835-13|United States|California|615[0n]|BOLD:AAB2916
Stretchia nr. pacifica|[3399]|LOCBF3753-14|United States|California|588[0n]|BOLD:AAB2916
Stretchia nr. pacifica|[3400]|LOCBF3256-14|United States|California|546[0n]|BOLD:AAB2916
Stretchia nr. pacifica|[3401]|LOCBF3257-14|United States|California|549[0n]|BOLD:AAB2916
Stretchia nr. pacifica|[3402]|LOCBF3624-14|United States|California|582[0n]|BOLD:AAB2916
Stretchia nr. pacifica|[3403]|LOCBF4272-14|United States|California|631[0n]|BOLD:AAB2916
Stretchia muricina|[3404]|RDNMG344-08|Canada|Alberta|658[0n]|BOLD:AAB2916
Stretchia muricina|[3405]|RDNMG347-08|Canada|Alberta|658[0n]|BOLD:AAB2916
Stretchia muricina|[3406]|RDNMH018-09|United States|Colorado|658[0n]|BOLD:AAB2916
Stretchia muricina|[3407]|IAWLB421-11|United States|California|658[0n]|BOLD:AAB2916
Stretchia muricina|[3408]|IAWLB422-11|United States|California|658[0n]|BOLD:AAB2916
Stretchia muricina|[3409]|LALPA1052-11|Canada|British Columbia|658[0n]|BOLD:AAB2916
Stretchia muricina|[3410]|RDNMG067-08|United States|Colorado|658[0n]|BOLD:AAB2916
Stretchia muricina|[3411]|RDNMG343-08|Canada|Alberta|658[0n]|BOLD:AAB2916
Stretchia muricina|[3412]|RDNMG345-08|Canada|Alberta|599[1n]|BOLD:AAB2916
Stretchia muricina|[3413]|RDNMG346-08|Canada|Alberta|543[0n]|BOLD:AAB2916
Stretchia muricina|[3414]|RWWB619-10|United States|Washington|658[1n]|BOLD:AAB2916
Stretchia muricina|[3415]|RWWA041-09|United States|Washington|658[0n]|BOLD:AAB2916
Stretchia muricina|[3416]|RWWB595-10|United States|Washington|658[0n]|BOLD:AAB2916
Stretchia muricina|[3417]|RWWC922-12|United States|Washington|658[0n]|BOLD:AAB2916
Stretchia muricina|[3418]|RDNMF266-08|Canada|British Columbia|658[0n]|BOLD:AAB2916
Stretchia muricina|[3419]|RDNMF267-08|Canada|British Columbia|658[0n]|BOLD:AAB2916
Stretchia muricina|[3420]|RDNMF268-08|Canada|British Columbia|658[0n]|BOLD:AAB2916
Stretchia muricina|[3421]|CNCLB1703-14|United States|Washington|658[0n]|BOLD:AAB2916
Stretchia plusiaeformis|[3422]|RDNMF293-08|Canada|Quebec|640[0n]|BOLD:ACE6587
Stretchia plusiaeformis|[3423]|RDNMG348-08|Canada|Alberta|658[0n]|BOLD:ACE6587
Stretchia plusiaeformis|[3424]|RDNMG349-08|Canada|Alberta|658[0n]|BOLD:ACE6587
Stretchia plusiaeformis|[3425]|RDNMG350-08|Canada|Alberta|658[0n]|BOLD:ACE6587
Stretchia plusiaeformis|[3426]|RDNMG351-08|Canada|Alberta|658[0n]|BOLD:ACE6587
Stretchia plusiaeformis|[3427]|RDNMG066-08|United States|Colorado|658[0n]|BOLD:ACE6587
Stretchia plusiaeformis|[3428]|RDNMG068-08|United States|Colorado|658[0n]|BOLD:ACE6587
Stretchia plusiaeformis|[3429]|RDNMG354-08|United States|Wyoming|658[0n]|BOLD:ACE6587
Stretchia|[3430]|CNCLB1796-14|United States|New Mexico|658[0n]|BOLD:ACE6587
Stretchia plusiaeformis|[3431]|RDNMG352-08|United States|Wyoming|658[0n]|BOLD:AAB6980
Stretchia plusiaeformis|[3432]|RDNMF262-08|United States|Wyoming|658[0n]|BOLD:AAB6980
Stretchia plusiaeformis|[3433]|RDNMG353-08|United States|Wyoming|658[0n]|BOLD:AAB6980
Stretchia plusiaeformis|[3434]|RDNMF263-08|United States|Wyoming|658[0n]|BOLD:AAB6980
Stretchia sp.|[3435]|RDNMH449-09|United States|Colorado|658[0n]|BOLD:AAB6980
Stretchia pictipennis|[3436]|RDNMG356-08|United States|California|658[0n]|BOLD:AAB6980
Stretchia pictipennis|[3437]|RDNMH019-09|United States|California|658[0n]|BOLD:AAB6980
Stretchia pictipennis|[3438]|CNCLB500-14|United States|California|658[0n]|BOLD:AAB6980
Stretchia pictipennis|[3439]|CNCLB502-14|United States|California|550[0n]|BOLD:AAB6980
Stretchia pictipennis|[3440]|RDNMG357-08|United States|California|658[0n]|BOLD:AAB6980
Stretchia pictipennis|[3441]|CNCLB501-14|United States|California|658[0n]|BOLD:AAB6980
Stretchia plusiaeformis|[3442]|RDNMF260-08|Canada|British Columbia|658[0n]|BOLD:AAB6980
Stretchia plusiaeformis|[3443]|RDNMF261-08|Canada|British Columbia|658[0n]|BOLD:AAB6980
Stretchia plusiaeformis|[3444]|RDNMF264-08|Canada|British Columbia|658[0n]|BOLD:AAB6980
Stretchia prima|[3445]|CNCLB1702-14|United States|Oregon|614[1n]|BOLD:AAB6980
Stretchia plusiaeformis|[3446]|RDNMG355-08|United States|Wyoming|658[0n]|BOLD:AAB6980
Stretchia sp.|[3447]|RDNMH448-09|United States|Colorado|658[0n]|BOLD:AAB6980
Stretchia plusiaeformis|[3448]|NAMUM198-08|United States|California|658[0n]|BOLD:AAB6980
Stretchia prima|[3449]|CNCLB2108-14|United States|California|658[0n]|BOLD:AAB6980
Stretchia prima|[3450]|CNCLB2109-14|United States|California|658[0n]|BOLD:AAB6980
Stretchia prima|[3451]|CNCLB2110-14|United States|California|658[0n]|BOLD:AAB6980
Orthosia behrensiana|[3452]|LOCBB190-06|United States|California|658[1n]|BOLD:AAG7397
Orthosia behrensiana|[3453]|GMLC298-11|United States|California|658[0n]|BOLD:AAG7397
Orthosia behrensiana|[3454]|CGLCA140-10|United States|California|658[0n]|BOLD:AAG7397
Orthosia behrensiana|[3455]|CGLCA138-10|United States|California|658[0n]|BOLD:AAG7397
Orthosia behrensiana|[3456]|CGLCA008-10|United States|California|658[0n]|BOLD:AAG7397
Orthosia behrensiana|[3457]|GMLC207-11|United States|California|652[0n]|BOLD:AAG7397
Orthosia behrensiana|[3458]|LOCBB191-06|United States|California|658[0n]|BOLD:AAG7397
Orthosia behrensiana|[3459]|LOCBB194-06|United States|California|630[0n]|BOLD:AAG7397
Orthosia behrensiana|[3460]|GMLC206-11|United States|California|647[0n]|BOLD:AAG7397
Orthosia behrensiana|[3461]|GMLC1359-12|United States|California|633[0n]|BOLD:AAG7397
Orthosia behrensiana|[3462]|GMLC1272-12|United States|California|658[0n]|BOLD:AAG7397
Orthosia behrensiana|[3463]|LOCBB192-06|United States|California|626[0n]|BOLD:AAG7397
Orthosia behrensiana|[3464]|GMLC1362-12|United States|California|623[0n]|BOLD:AAG7397
Orthosia behrensiana|[3465]|GMLC1280-12|United States|California|658[0n]|BOLD:AAG7397
Orthosia behrensiana|[3466]|GMLC1276-12|United States|California|658[0n]|BOLD:AAG7397
Orthosia behrensiana|[3467]|GMLC1273-12|United States|California|658[0n]|BOLD:AAG7397
Orthosia behrensiana|[3468]|GMLC219-11|United States|California|658[0n]|BOLD:AAG7397
Orthosia behrensiana|[3469]|GMLC215-11|United States|California|658[0n]|BOLD:AAG7397
Orthosia behrensiana|[3470]|GMLC139-09|United States|California|658[0n]|BOLD:AAG7397
Orthosia behrensiana|[3471]|LOCBB193-06|United States|California|658[0n]|BOLD:AAG7397
Orthosia behrensiana|[3472]|GMLC1305-12|United States|California|613[0n]|BOLD:AAG7397
Orthosia behrensiana|[3473]|GMLC1367-12|United States|California|622[0n]|BOLD:AAG7397
Orthosia behrensiana|[3474]|GMLC134-09|United States|California|658[0n]|BOLD:AAG7397
Orthosia behrensiana|[3475]|GMLC1368-12|United States|California|614[0n]|BOLD:AAG7397
Perigonica pectinata|[3476]|RDNMI043-10|United States|California|658[0n]|BOLD:AAY5140
Perigonica nr. pectinata|[3477]|LNAUS4820-13|United States|Arizona|658[0n]|BOLD:AAY5140
Perigonica nr. pectinata|[3478]|LNAUS4822-13|United States|Arizona|658[0n]|BOLD:AAY5140
Perigonica nr. pectinata|[3479]|LNAUS4823-13|United States|Arizona|658[0n]|BOLD:AAY5140
Perigonica nr. pectinata|[3480]|LNAUS4824-13|United States|Arizona|658[0n]|BOLD:AAY5140
Orthosia annulimacula|[3481]|RDNML326-13|United States|New Mexico|658[0n]|BOLD:ACD9083
Orthosia annulimacula|[3482]|RDNML327-13|United States|New Mexico|658[0n]|BOLD:ACD9083
Orthosia annulimacula|[3483]|LNAUS4841-13|United States|Arizona|658[0n]|BOLD:ACD9083
Orthosia annulimacula|[3484]|CNCLB2102-14|United States|Arizona|658[0n]|BOLD:ACD9083
Orthosia annulimacula|[3485]|LNAUS4842-13|United States|Arizona|587[0n]|BOLD:ACD9083
Orthosia annulimacula|[3486]|LNAUS4845-13|United States|Arizona|658[0n]|BOLD:ACD9083
Orthosia annulimacula|[3487]|LNAUS4844-13|United States|Arizona|658[0n]|BOLD:ACD9083
Orthosia annulimacula|[3488]|CNCLB489-14|United States|Texas|540[0n]|BOLD:ACD9083
Orthosia annulimacula|[3489]|CNCLB2103-14|United States|Arizona|658[0n]|BOLD:ACD9083
Orthosia ferrigera|[3490]|LOCBB187-06|United States|California|658[0n]|BOLD:AAC8451
Orthosia ferrigera|[3491]|LOCBB188-06|United States|California|658[0n]|BOLD:AAC8451
Orthosia ferrigera|[3492]|LOCBB186-06|United States|California|658[0n]|BOLD:AAC8451
Orthosia ferrigera|[3493]|LOCBB185-06|United States|California|658[0n]|BOLD:AAC8451
Orthosia ferrigera|[3494]|LOCBB189-06|United States|California|626[0n]|BOLD:AAC8451
Orthosia ferrigera|[3495]|RDNMF257-08|United States|Oregon|658[0n]|BOLD:AAC8451
Orthosia ferrigera|[3496]|RDNMF258-08|United States|Oregon|658[0n]|BOLD:AAC8451
Orthosia ferrigera|[3497]|RDNMF259-08|United States|Oregon|658[0n]|BOLD:AAC8451
Orthosia ferrigera|[3498]|NAMUM248-08|United States|California|658[0n]|BOLD:AAC8451

Orthosia ferrigera|[3496]|RDNMF258-08|United States|Oregon|658[0n]|BOLD:AAC8451
Orthosia ferrigera|[3497]|RDNMF259-08|United States|Oregon|658[0n]|BOLD:AAC8451
Orthosia ferrigera|[3498]|NAMUM248-08|United States|California|658[0n]|BOLD:AAC8451
Orthosia terminata|[3499]|LNAUS2766-13|United States|California|658[0n]|BOLD:ACI4148
Orthosia sp.|[3500]|CNCLB2105-14|United States|New Mexico|658[0n]|BOLD:ACR4910
Orthosia sp.|[3501]|CNCLB2496-14|United States|Arizona|658[0n]|BOLD:ACR4910
Egira hiemalis|[3502]|RWWA040-09|United States|Washington|658[0n]|BOLD:ACE7810
Egira hiemalis|[3503]|DUNLP360-08|Canada|British Columbia|632[0n]|BOLD:ACE7810
Egira hiemalis|[3504]|RDNMF253-08|Canada|British Columbia|658[0n]|BOLD:ACE7810
Egira hiemalis|[3505]|RDNMF254-08|Canada|British Columbia|658[0n]|BOLD:ACE7810
Egira hiemalis|[3506]|RDNMF255-08|Canada|British Columbia|658[0n]|BOLD:ACE7810
Egira hiemalis|[3507]|LALPA1037-11|Canada|British Columbia|658[0n]|BOLD:ACE7810
Egira hiemalis|[3508]|GMLC1302-12|United States|California|629[0n]|BOLD:ACE7810
Egira hiemalis|[3509]|GMLC177-11|United States|California|658[0n]|BOLD:AAC1822
Egira hiemalis|[3510]|GMLC208-11|United States|California|658[0n]|BOLD:AAC1822
Egira hiemalis|[3511]|RWWB497-10|United States|Washington|658[0n]|BOLD:AAC1822
Egira hiemalis|[3512]|RDNMG1012-08|United States|California|658[0n]|BOLD:AAC1822
Egira hiemalis|[3513]|GMLC1271-12|United States|California|658[0n]|BOLD:AAC1822
Egira hiemalis|[3514]|GMLC157-09|United States|California|658[0n]|BOLD:AAC1822
Egira hiemalis|[3515]|GMLC156-09|United States|California|658[0n]|BOLD:AAC1822
Egira hiemalis|[3516]|GMLC158-09|United States|California|658[0n]|BOLD:AAC1822
Egira hiemalis|[3517]|GMLC1286-12|United States|California|658[0n]|BOLD:AAC1822
Egira hiemalis|[3518]|RDNMF252-08|United States|California|658[1n]|BOLD:AAC1822
Egira hiemalis|[3519]|RDNMF256-08|United States|California|658[0n]|BOLD:AAC1822
Egira hiemalis|[3520]|RDNMG1011-08|United States|California|658[0n]|BOLD:AAC1822
Egira hiemalis|[3521]|GMLC155-09|United States|California|658[0n]|BOLD:AAC1822
Egira hiemalis|[3522]|CGLCA053-10|United States|California|658[0n]|BOLD:AAC1822
Egira hiemalis|[3523]|GMLC227-11|United States|California|632[0n]|BOLD:AAC1822
Egira hiemalis|[3524]|GMLC1303-12|United States|California|615[0n]|BOLD:AAC1822
Egira hiemalis|[3525]|GMLC1301-12|United States|California|622[0n]|BOLD:AAC1822
Egira hiemalis|[3526]|GMLC1310-12|United States|California|607[0n]|BOLD:AAC1822
Egira hiemalis|[3527]|GMLC175-11|United States|California|658[0n]|BOLD:AAC1822
Egira hiemalis|[3528]|GMLC1292-12|United States|California|634[0n]|BOLD:AAC1822
Egira hiemalis|[3529]|GMLC1388-12|United States|California|614[0n]|BOLD:AAC1822
Himella fidelis|[3530]|LSEU235-06|United States|Georgia|640[1n]|BOLD:AAC5638
Himella fidelis|[3531]|RDNMF705-08|United States|South Carolina|658[0n]|BOLD:AAC5638
Himella fidelis|[3532]|RDNMF706-08|United States|Massachusetts|658[0n]|BOLD:AAC5638
Himella fidelis|[3533]|LPOKB220-09|United States|Oklahoma|658[0n]|BOLD:AAC5638
Himella fidelis|[3534]|LPOKA937-09|United States|Oklahoma|658[0n]|BOLD:AAC5638
Himella fidelis|[3535]|NAMUM357-08|United States|Maryland|658[0n]|BOLD:AAC5638
Himella fidelis|[3536]|NAMUM356-08|United States|Maryland|658[0n]|BOLD:AAC5638
Himella fidelis|[3537]|RDNMF703-08|United States|Louisiana|658[0n]|BOLD:AAC5638
Himella fidelis|[3538]|LPSO677-08|Canada|Ontario|658[0n]|BOLD:AAC5638
Himella fidelis|[3539]|LPSO047-08|Canada|Ontario|658[0n]|BOLD:AAC5638
Himella fidelis|[3540]|RDNMF704-08|United States|South Carolina|658[0n]|BOLD:AAC5638
Himella fidelis|[3541]|GMGSK011-12|United States|Tennessee|614[0n]|BOLD:AAC5638
Egira simplex|[3542]|RDNMG473-08|United States|Oregon|623[9n]|
Egira simplex|[3543]|RWWB589-10|United States|Washington|658[0n]|BOLD:ACE7118
Egira simplex|[3544]|RWWB490-10|United States|Washington|658[0n]|BOLD:ACE7118
Egira simplex|[3545]|RWWB596-10|United States|Washington|658[0n]|BOLD:ACE7118
Egira simplex|[3546]|RWWA075-09|United States|Washington|658[0n]|BOLD:ACE7118
Egira simplex|[3547]|RWWA014-09|United States|Washington|658[0n]|BOLD:ACE7118
Egira simplex|[3548]|RWWA010-09|United States|Washington|658[0n]|BOLD:ACE7118
Egira simplex|[3549]|RWWB607-10|United States|Washington|658[1n]|BOLD:ACE7118
Egira simplex|[3550]|RWWB648-10|United States|Washington|658[0n]|BOLD:ACE7118
Egira simplex|[3551]|DUNLP154-08|Canada|British Columbia|658[0n]|BOLD:ACE7118
Egira simplex|[3552]|LALPA064-10|Canada|British Columbia|658[0n]|BOLD:ACE7118
Egira simplex|[3553]|DUNLP153-08|Canada|British Columbia|658[0n]|BOLD:ACE7118
Egira simplex|[3554]|RWWA030-09|United States|Washington|658[0n]|BOLD:ACE7118
Egira simplex|[3555]|LALPA063-10|Canada|British Columbia|658[0n]|BOLD:ACE7118
Egira simplex|[3556]|LALPA065-10|Canada|British Columbia|658[0n]|BOLD:ACE7118
Egira simplex|[3557]|LALPA066-10|Canada|British Columbia|658[0n]|BOLD:ACE7118
Egira simplex|[3558]|RWWB699-10|United States|Washington|658[0n]|BOLD:ACE7118
Egira simplex|[3559]|LBCH4974-10|Canada|British Columbia|658[0n]|BOLD:ACE7118
Egira simplex|[3560]|RDNMG573-08|Canada|British Columbia|658[0n]|BOLD:ACE7118
Egira simplex|[3561]|RDNMG472-08|Canada|British Columbia|658[0n]|BOLD:ACE7118
Egira simplex|[3562]|RWWA023-09|United States|Washington|658[0n]|BOLD:ACE7118
Egira simplex|[3563]|LBCH4978-10|Canada|British Columbia|658[0n]|BOLD:ACE7118
Egira simplex|[3564]|RWWB577-10|United States|Washington|658[0n]|BOLD:ACE7118
Egira simplex|[3565]|RWWC214-11|United States|Washington|658[0n]|BOLD:ACE7118
Egira simplex|[3566]|USLEP1156-10|United States|Colorado|658[0n]|BOLD:AAC1794
Egira simplex|[3567]|USLEP1157-10|United States|Colorado|658[0n]|BOLD:AAC1794
Egira simplex|[3568]|RDNMF033-08|Canada|British Columbia|658[0n]|BOLD:AAC1794
Egira simplex|[3569]|USLEP1158-10|United States|Colorado|658[0n]|BOLD:AAC1794
Egira crucialis|[3570]|GMLC344-11|United States|California|658[0n]|BOLD:AAC1794
Egira crucialis|[3571]|GMLC506-11|United States|California|658[0n]|BOLD:AAC1794
Egira crucialis|[3572]|GMLC559-11|United States|California|658[0n]|BOLD:AAC1794
Egira crucialis|[3573]|RWWB513-10|United States|Washington|658[0n]|BOLD:AAC1794
Egira crucialis|[3574]|GMLC531-11|United States|California|658[0n]|BOLD:AAC1794
Egira crucialis|[3575]|CGLCA020-10|United States|California|658[0n]|BOLD:AAC1794
Egira crucialis|[3576]|CGLCA021-10|United States|California|658[0n]|BOLD:AAC1794
Egira crucialis|[3577]|JMMMB373-11|United States|California|658[0n]|BOLD:AAC1794
Egira crucialis|[3578]|GMLC402-11|United States|California|658[0n]|BOLD:AAC1794
Egira crucialis|[3579]|LOCBB167-06|United States|California|658[0n]|BOLD:AAC1794
Egira crucialis|[3580]|NAMUM174-08|United States|California|632[0n]|BOLD:AAC1794
Egira crucialis|[3581]|RWWC914-12|United States|Washington|658[0n]|BOLD:AAC1794
Egira crucialis|[3582]|RWWC911-12|United States|Washington|658[0n]|BOLD:AAC1794
Egira crucialis|[3583]|LALPA1049-11|Canada|British Columbia|658[0n]|BOLD:AAC1794
Egira crucialis|[3584]|LALPA009-10|Canada|British Columbia|658[0n]|BOLD:AAC1794
Egira crucialis|[3585]|LALPA008-10|Canada|British Columbia|658[0n]|BOLD:AAC1794
Egira crucialis|[3586]|LALPA007-10|Canada|British Columbia|658[0n]|BOLD:AAC1794
Egira crucialis|[3587]|RWWB580-10|United States|Washington|658[0n]|BOLD:AAC1794
Egira crucialis|[3588]|RWWB573-10|United States|Washington|658[0n]|BOLD:AAC1794
Egira crucialis|[3589]|RWWB567-10|United States|Washington|658[0n]|BOLD:AAC1794
Egira crucialis|[3590]|RWWB566-10|United States|Washington|658[0n]|BOLD:AAC1794
Egira crucialis|[3591]|RWWB558-10|United States|Washington|658[0n]|BOLD:AAC1794
Egira crucialis|[3592]|RWWB520-10|United States|Washington|658[0n]|BOLD:AAC1794
Egira crucialis|[3593]|RWWB516-10|United States|Washington|658[0n]|BOLD:AAC1794
Egira crucialis|[3594]|LBCH5284-10|Canada|British Columbia|658[0n]|BOLD:AAC1794
Egira crucialis|[3595]|RWWA275-09|United States|Washington|658[0n]|BOLD:AAC1794
Egira crucialis|[3596]|RWWA060-09|United States|Washington|658[0n]|BOLD:AAC1794
Egira crucialis|[3597]|RWWA045-09|United States|Washington|658[0n]|BOLD:AAC1794
Egira crucialis|[3598]|RWWA043-09|United States|Washington|658[0n]|BOLD:AAC1794

Egira crucialis|[3596]|RWWA060-09|United States|Washington|658[0n]|BOLD:AAC1794
Egira crucialis|[3597]|RWWA045-09|United States|Washington|658[0n]|BOLD:AAC1794
Egira crucialis|[3598]|RWWA043-09|United States|Washington|658[0n]|BOLD:AAC1794
Egira crucialis|[3599]|RWWA015-09|United States|Washington|658[0n]|BOLD:AAC1794
Egira crucialis|[3600]|RDNMF026-08|Canada|British Columbia|658[0n]|BOLD:AAC1794
Egira crucialis|[3601]|LBCF005-07|Canada|British Columbia|658[0n]|BOLD:AAC1794
Egira crucialis|[3602]|LBCF004-07|Canada|British Columbia|658[0n]|BOLD:AAC1794
Egira crucialis|[3603]|LOPN072-06|United States|Oregon|658[0n]|BOLD:AAC1794
Egira crucialis|[3604]|LOPN071-06|United States|Oregon|658[0n]|BOLD:AAC1794
Egira crucialis|[3605]|LOPN073-06|United States|Oregon|521[1n]|BOLD:AAC1794
Egira crucialis|[3606]|CNGIE024-12|Canada|British Columbia|601[0n]|BOLD:AAC1794
Egira crucialis|[3607]|GMLC225-11|United States|California|658[0n]|BOLD:AAC1794
Egira crucialis|[3608]|GMLC360-11|United States|California|658[0n]|BOLD:AAC1794
Egira crucialis|[3609]|GMLC1382-12|United States|California|618[0n]|BOLD:AAC1794
Egira vanduzeei|[3610]|CNCLB494-14|United States|California|658[0n]|BOLD:ACM4251
Egira vanduzeei|[3611]|LNAUT909-14|United States|California|658[0n]|BOLD:ACM4251
Orthosia transparens|[3612]|LOCB717-06|United States|California|658[0n]|BOLD:AAB8764
Orthosia transparens|[3613]|LOCB745-06|United States|California|658[0n]|BOLD:AAB8764
Orthosia transparens|[3614]|LOCB647-06|United States|California|601[0n]|BOLD:AAB8764
Orthosia transparens|[3615]|LOCB748-06|United States|California|656[2n]|BOLD:AAB8764
Orthosia transparens|[3616]|LOCB916-06|United States|California|658[0n]|BOLD:AAB8764
Orthosia transparens|[3617]|LOCB747-06|United States|California|658[0n]|BOLD:AAB8764
Orthosia transparens|[3618]|LOCB746-06|United States|California|658[0n]|BOLD:AAB8764
Orthosia transparens|[3619]|LOCB635-06|United States|California|658[0n]|BOLD:AAB8764
Orthosia transparens|[3620]|LOCB917-06|United States|California|603[0n]|BOLD:AAB8764
Orthosia transparens|[3621]|LOCB924-06|United States|California|606[0n]|BOLD:AAB8764
Orthosia transparens|[3622]|LOCB932-06|United States|California|618[0n]|BOLD:AAB8764
Orthosia transparens|[3623]|RDNMD400-06|United States|California|658[0n]|BOLD:AAB8764
Orthosia transparens|[3624]|RDNMD401-06|United States|California|658[0n]|BOLD:AAB8764
Orthosia transparens|[3625]|RDNMD402-06|United States|California|658[0n]|BOLD:AAB8764
Orthosia transparens|[3626]|JBAZ046-09|United States|California|658[0n]|BOLD:AAB8764
Orthosia transparens|[3627]|RWWB608-10|United States|Washington|658[0n]|BOLD:AAB8764
Orthosia transparens|[3628]|GMLC209-11|United States|California|627[0n]|BOLD:AAB8764
Orthosia transparens|[3629]|GMLC359-11|United States|California|658[0n]|BOLD:AAB8764
Orthosia transparens|[3630]|GMLC385-11|United States|California|658[0n]|BOLD:AAB8764
Orthosia transparens|[3631]|RWWB532-10|United States|Washington|658[0n]|BOLD:AAB8764
Orthosia transparens|[3632]|GMLC335-11|United States|California|658[0n]|BOLD:AAB8764
Orthosia transparens|[3633]|GMLC522-11|United States|California|658[0n]|BOLD:AAB8764
Orthosia transparens|[3634]|RWWB590-10|United States|Washington|658[0n]|BOLD:AAB8764
Orthosia transparens|[3635]|LALPA023-10|Canada|British Columbia|658[0n]|BOLD:AAB8764
Orthosia transparens|[3636]|LBCF006-07|Canada|British Columbia|658[0n]|BOLD:AAB8764
Orthosia transparens|[3637]|RWWB540-10|United States|Washington|658[0n]|BOLD:AAB8764
Orthosia transparens|[3638]|LALPA048-10|Canada|British Columbia|658[0n]|BOLD:AAB8764
Orthosia transparens|[3639]|RWWB626-10|United States|Washington|658[0n]|BOLD:AAB8764
Orthosia transparens|[3640]|RWWC185-11|United States|Washington|658[0n]|BOLD:AAB8764
Orthosia transparens|[3641]|LALPA1065-11|Canada|British Columbia|658[0n]|BOLD:AAB8764
Orthosia transparens|[3642]|LALPA1103-11|Canada|British Columbia|658[0n]|BOLD:AAB8764
Orthosia erythrolita|[3643]|LOCB928-06|United States|California|607[0n]|BOLD:AAB2229
Orthosia erythrolita|[3644]|GMLC617-11|United States|California|658[0n]|BOLD:AAB2229
Orthosia erythrolita|[3645]|LOCB447-06|United States|California|658[0n]|BOLD:AAB2229
Orthosia erythrolita|[3646]|LOCB690-06|United States|California|658[0n]|BOLD:AAB2229
Orthosia erythrolita|[3647]|CGLCA010-10|United States|California|658[0n]|BOLD:AAB2229
Orthosia erythrolita|[3648]|LOCB930-06|United States|California|627[0n]|BOLD:AAB2229
Orthosia erythrolita|[3649]|LOCB440-06|United States|California|658[0n]|BOLD:AAB2229
Orthosia erythrolita|[3650]|LOCB646-06|United States|California|617[0n]|BOLD:AAB2229
Orthosia erythrolita|[3651]|LOCB918-06|United States|California|658[0n]|BOLD:AAB2229
Orthosia erythrolita|[3652]|LOCB925-06|United States|California|596[0n]|BOLD:AAB2229
Orthosia erythrolita|[3653]|LOCB445-06|United States|California|658[0n]|BOLD:AAB2229
Orthosia erythrolita|[3654]|LOCB751-06|United States|California|658[0n]|BOLD:AAB2229
Orthosia erythrolita|[3655]|RDNMD395-06|United States|California|658[0n]|BOLD:AAB2229
Orthosia erythrolita|[3656]|RDNMD394-06|United States|California|658[0n]|BOLD:AAB2229
Orthosia erythrolita|[3657]|LOCBB647-06|United States|California|658[0n]|BOLD:AAB2229
Orthosia erythrolita|[3658]|LOCB926-06|United States|California|658[0n]|BOLD:AAB2229
Orthosia erythrolita|[3659]|LOCB752-06|United States|California|658[0n]|BOLD:AAB2229
Orthosia erythrolita|[3660]|LOCB750-06|United States|California|658[0n]|BOLD:AAB2229
Orthosia erythrolita|[3661]|LOCB749-06|United States|California|658[0n]|BOLD:AAB2229
Orthosia erythrolita|[3662]|LOCB722-06|United States|California|658[0n]|BOLD:AAB2229
Orthosia erythrolita|[3663]|LOCB705-06|United States|California|658[0n]|BOLD:AAB2229
Orthosia erythrolita|[3664]|LOCB450-06|United States|California|658[0n]|BOLD:AAB2229
Orthosia erythrolita|[3665]|CGLCA181-10|United States|California|658[0n]|BOLD:AAB2229
Orthosia erythrolita|[3666]|LOCB448-06|United States|California|658[0n]|BOLD:AAB2229
Orthosia erythrolita|[3667]|LOCB633-06|United States|California|658[0n]|BOLD:AAB2229
Orthosia erythrolita|[3668]|RDNMD393-06|United States|California|658[0n]|BOLD:AAB2229
Orthosia erythrolita|[3669]|LOCB618-06|United States|California|619[0n]|BOLD:AAB2229
Orthosia erythrolita|[3670]|LOCB931-06|United States|California|604[0n]|BOLD:AAB2229
Orthosia erythrolita|[3671]|LOCB636-06|United States|California|610[0n]|BOLD:AAB2229
Orthosia erythrolita|[3672]|GMLC1320-12|United States|California|634[0n]|BOLD:AAB2229
Orthosia erythrolita|[3673]|LOCB929-06|United States|California|618[0n]|BOLD:AAB2229
Orthosia erythrolita|[3674]|LOCB927-06|United States|California|618[0n]|BOLD:AAB2229
Orthosia erythrolita|[3675]|GMLC1321-12|United States|California|620[0n]|BOLD:AAB2229
Orthosia erythrolita|[3676]|GMLC1268-12|United States|California|658[0n]|BOLD:AAB2229
Orthosia erythrolita|[3677]|GMLC377-11|United States|California|658[0n]|BOLD:AAB2229
Orthosia erythrolita|[3678]|GMLC1342-12|United States|California|631[0n]|BOLD:AAB2229
Orthosia erythrolita|[3679]|GMLC1307-12|United States|California|630[0n]|BOLD:AAB2229
Orthosia erythrolita|[3680]|GMLC1343-12|United States|California|615[0n]|BOLD:AAB2229
Orthosia mys|[3681]|CMAZA265-09|United States|Arizona|658[0n]|BOLD:ABZ3062
Orthosia mys|[3682]|CMAZA268-09|United States|Arizona|658[0n]|BOLD:ABZ3062
Orthosia mys|[3683]|CMAZA270-09|United States|Arizona|658[0n]|BOLD:ABZ3062
Orthosia mys|[3684]|CGLCA069-10|United States|California|658[0n]|BOLD:AAD3143
Orthosia mys|[3685]|JBAZ041-09|United States|California|658[0n]|BOLD:AAD3143
Orthosia mys|[3686]|GMLC073-09|United States|California|658[0n]|BOLD:AAD3143
Orthosia mys|[3687]|GMLC1230-12|United States|California|658[0n]|BOLD:AAD3143
Orthosia mys|[3688]|GMLC1261-12|United States|California|658[0n]|BOLD:AAD3143
Orthosia mys|[3689]|GMLC1262-12|United States|California|658[0n]|BOLD:AAD3143
Orthosia mys|[3690]|LOPN093-06|United States|Oregon|587[1n]|BOLD:AAD3143
Orthosia mys|[3691]|LOPN094-06|United States|Oregon|508[0n]|BOLD:AAD3143
Orthosia mys|[3692]|GMLC1263-12|United States|California|658[0n]|BOLD:AAD3143
Orthosia mys|[3693]|GMLC1264-12|United States|California|658[0n]|BOLD:AAD3143
Orthosia mys|[3694]|RDNMC469-05|Canada|British Columbia|597[0n]|BOLD:AAD3143
Orthosia mys|[3695]|RDNMC470-05|Canada|British Columbia|581[0n]|BOLD:AAD3143
Orthosia mys|[3696]|LOPN091-06|United States|Oregon|514[0n]|BOLD:AAD3143
Orthosia mys|[3697]|LALPA1349-12|Canada|British Columbia|629[0n]|BOLD:AAD3143
Egira alternans|[3698]|LNC086-05|United States|North Carolina|658[0n]|BOLD:AAB6897

Orthosia mys|[3696]|LOPN091-06|United States|Oregon|514[0n]|BOLD:AAD3143
Orthosia mys|[3697]|LALPA1349-12|Canada|British Columbia|629[0n]|BOLD:AAD3143
Egira alternans|[3698]|LNC086-05|United States|North Carolina|658[0n]|BOLD:AAB6897
Egira alternans|[3699]|LNC087-05|United States|North Carolina|658[0n]|BOLD:AAB6897
Egira alternans|[3700]|LGSMC687-05|United States|Tennessee|658[0n]|BOLD:AAB6897
Egira alternans|[3701]|LSEU518-06|United States|Georgia|658[0n]|BOLD:AAB6897
Egira alternans|[3702]|LGSMC381-05|United States|Tennessee|585[0n]|BOLD:AAB6897
Egira alternans|[3703]|LGSMC382-05|United States|Tennessee|658[0n]|BOLD:AAB6897
Egira alternans|[3704]|TTMNB406-06|Canada|New Brunswick|658[0n]|BOLD:AAB6897
Egira alternans|[3705]|TTMNB407-06|Canada|New Brunswick|658[0n]|BOLD:AAB6897
Egira alternans|[3706]|TTMNB409-06|Canada|New Brunswick|658[0n]|BOLD:AAB6897
Egira alternans|[3707]|TMNBB342-06|Canada|New Brunswick|658[0n]|BOLD:AAB6897
Egira alternans|[3708]|TMNBB344-06|Canada|New Brunswick|658[0n]|BOLD:AAB6897
Egira alternans|[3709]|TTMNB405-06|Canada|New Brunswick|658[0n]|BOLD:AAB6897
Egira alternans|[3710]|TTMNB404-06|Canada|New Brunswick|658[0n]|BOLD:AAB6897
Egira alternans|[3711]|TMNBB343-06|Canada|New Brunswick|652[0n]|BOLD:AAB6897
Egira alternans|[3712]|TMNBB340-06|Canada|New Brunswick|658[0n]|BOLD:AAB6897
Egira alternans|[3713]|LGSMC686-05|United States|Tennessee|658[0n]|BOLD:AAB6897
Egira alternans|[3714]|TTMNB408-06|Canada|New Brunswick|636[0n]|BOLD:AAB6897
Egira alternans|[3715]|TMNBB341-06|Canada|New Brunswick|658[0n]|BOLD:AAB6897
Egira alternans|[3716]|RDLQG319-06|Canada|Quebec|658[0n]|BOLD:AAB6897
Achatia distincta|[3717]|LSUSA104-06|United States|Kentucky|583[0n]|BOLD:AAB7392
Achatia distincta|[3718]|XAJ408-06|Canada|Ontario|658[0n]|BOLD:AAB7392
Achatia distincta|[3719]|LPOKE289-11|United States|Oklahoma|658[0n]|BOLD:AAB7392
Achatia distincta|[3720]|KPOEC180-08|Canada|Ontario|658[0n]|BOLD:AAB7392
Achatia distincta|[3721]|RDLQH107-06|Canada|Quebec|654[0n]|BOLD:AAB7392
Achatia distincta|[3722]|XAJ130-06|Canada|Ontario|658[0n]|BOLD:AAB7392
Achatia distincta|[3723]|LNC795-06|United States|Tennessee|658[0n]|BOLD:AAB7392
Achatia distincta|[3724]|LNCC588-11|United States|North Carolina|658[0n]|BOLD:AAB7392
Achatia distincta|[3725]|LNCC658-11|United States|North Carolina|658[0n]|BOLD:AAB7392
Achatia distincta|[3726]|AHLEP095-10|United States|Pennsylvania|658[0n]|BOLD:AAB7392
Achatia distincta|[3727]|LPOKD697-10|United States|Oklahoma|658[0n]|BOLD:AAB7392
Achatia distincta|[3728]|AHLEP050-10|United States|Pennsylvania|658[0n]|BOLD:AAB7392
Achatia distincta|[3729]|AHLEP051-10|United States|Pennsylvania|658[0n]|BOLD:AAB7392
Achatia distincta|[3730]|RDNMH616-09|Canada|Quebec|658[0n]|BOLD:AAB7392
Achatia distincta|[3731]|RDNMH617-09|Canada|Ontario|658[0n]|BOLD:AAB7392
Achatia distincta|[3732]|KPOEC123-08|Canada|Ontario|658[0n]|BOLD:AAB7392
Achatia distincta|[3733]|KPOEC043-08|Canada|Ontario|658[0n]|BOLD:AAB7392
Achatia distincta|[3734]|XAJ337-06|Canada|Ontario|658[0n]|BOLD:AAB7392
Achatia distincta|[3735]|XAJ230-06|Canada|Ontario|658[0n]|BOLD:AAB7392
Achatia distincta|[3736]|LSUSA038-06|United States|Kentucky|658[0n]|BOLD:AAB7392
Achatia distincta|[3737]|LOCT283-05|United States|Connecticut|658[0n]|BOLD:AAB7392
Achatia distincta|[3738]|KPOEC073-08|Canada|Ontario|650[0n]|BOLD:AAB7392
Achatia distincta|[3739]|KPOEC126-08|Canada|Ontario|658[0n]|BOLD:AAB7392
Achatia distincta|[3740]|KPOEC177-08|Canada|Ontario|658[0n]|BOLD:AAB7392
Achatia distincta|[3741]|KPOEC178-08|Canada|Ontario|658[0n]|BOLD:AAB7392
Achatia distincta|[3742]|UDLEP003-09|United States|Delaware|658[0n]|BOLD:AAB7392
Achatia distincta|[3743]|LNCC675-11|United States|North Carolina|658[0n]|BOLD:AAB7392
Achatia distincta|[3744]|GMGSK035-12|United States|Tennessee|615[0n]|BOLD:AAB7392
Egira curialis|[3745]|LALPA1097-11|Canada|British Columbia|658[0n]|BOLD:AAC7972
Egira curialis|[3746]|LBCH5013-10|Canada|British Columbia|658[0n]|BOLD:AAC7972
Egira curialis|[3747]|LBCH4987-10|Canada|British Columbia|658[0n]|BOLD:AAC7972
Egira curialis|[3748]|LBCH5109-10|Canada|British Columbia|658[0n]|BOLD:AAC7972
Egira curialis|[3749]|LBCH5158-10|Canada|British Columbia|658[0n]|BOLD:AAC7972
Egira curialis|[3750]|NAMUM384-09|United States|California|658[0n]|BOLD:AAC7972
Egira curialis|[3751]|IAWLB213-11|United States|Arizona|658[0n]|BOLD:AAC7972
Egira curialis|[3752]|RDNMG479-08|Canada|British Columbia|658[0n]|BOLD:AAC7972
Egira curialis|[3753]|LBCH4973-10|Canada|British Columbia|658[0n]|BOLD:AAC7972
Egira curialis|[3754]|LBCH5161-10|Canada|British Columbia|658[0n]|BOLD:AAC7972
Egira curialis|[3755]|GMLC445-11|United States|California|658[0n]|BOLD:AAC7972
Egira curialis|[3756]|NAMUM297-08|United States|California|658[0n]|BOLD:AAC7972
Egira curialis|[3757]|RDNMG478-08|United States|California|649[0n]|BOLD:AAC7972
Egira curialis|[3758]|GMLC1358-12|United States|California|618[0n]|BOLD:AAC7972
Egira curialis|[3759]|GMLC1418-12|United States|California|607[0n]|BOLD:AAC7972
Egira curialis|[3760]|NAMUM296-08|United States|California|658[0n]|BOLD:AAC7972
Egira curialis|[3761]|GMLC371-11|United States|California|658[0n]|BOLD:AAC7972
Egira curialis|[3762]|LBCH5345-10|Canada|British Columbia|658[0n]|BOLD:AAC7972
Egira curialis|[3763]|LBCH5287-10|Canada|British Columbia|658[0n]|BOLD:AAC7972
Egira curialis|[3764]|LBCH5251-10|Canada|British Columbia|658[0n]|BOLD:AAC7972
Egira curialis|[3765]|LBCH5002-10|Canada|British Columbia|658[0n]|BOLD:AAC7972
Egira curialis|[3766]|LBCH4994-10|Canada|British Columbia|658[0n]|BOLD:AAC7972
Egira curialis|[3767]|CGLCA058-10|United States|California|658[0n]|BOLD:AAC7972
Egira curialis|[3768]|RDNMG477-08|United States|California|658[0n]|BOLD:AAC7972
Egira curialis|[3769]|LBCG469-08|Canada|British Columbia|658[0n]|BOLD:AAC7972
Egira curialis|[3770]|LBCG468-08|Canada|British Columbia|658[0n]|BOLD:AAC7972
Egira curialis|[3771]|LBCG465-08|Canada|British Columbia|658[0n]|BOLD:AAC7972
Egira curialis|[3772]|LOCB734-06|United States|California|658[0n]|BOLD:AAC7972
Egira curialis|[3773]|GMLC460-11|United States|California|658[0n]|BOLD:AAC7972
Egira curialis|[3774]|GMLC1365-12|United States|California|634[0n]|BOLD:AAC7972
Egira curialis|[3775]|GMLC1366-12|United States|California|634[0n]|BOLD:AAC7972
Egira curialis|[3776]|LBCH5014-10|Canada|British Columbia|631[0n]|BOLD:AAC7972
Egira curialis|[3777]|GMLC1420-12|United States|California|629[0n]|BOLD:AAC7972
Egira nr. rubrica|[3778]|USLEP1149-10|United States|Arizona|658[0n]|BOLD:AAM8638
Egira nr. rubrica|[3779]|USLEP1150-10|United States|Arizona|658[0n]|BOLD:AAM8638
Egira rubrica|[3780]|LOCB664-06|United States|California|658[0n]|BOLD:AAC4246
Egira rubrica|[3781]|LOCB830-06|United States|California|658[0n]|BOLD:AAC4246
Egira rubrica|[3782]|LOCB660-06|United States|California|658[0n]|BOLD:AAC4246
Egira rubrica|[3783]|LOCB662-06|United States|California|658[0n]|BOLD:AAC4246
Egira rubrica|[3784]|LOCB698-06|United States|California|658[0n]|BOLD:AAC4246
Egira rubrica|[3785]|LOCB700-06|United States|California|658[0n]|BOLD:AAC4246
Egira rubrica|[3786]|LOCB703-06|United States|California|658[0n]|BOLD:AAC4246
Egira rubrica|[3787]|RDNMD405-06|United States|California|658[0n]|BOLD:AAC4246
Egira rubrica|[3788]|RDNMD406-06|United States|California|658[0n]|BOLD:AAC4246
Egira rubrica|[3789]|CGLCA016-10|United States|California|658[0n]|BOLD:AAC4246
Egira rubrica|[3790]|GMLC295-11|United States|California|658[0n]|BOLD:ABY7940
Egira rubrica|[3791]|GMLC376-11|United States|California|658[0n]|BOLD:ABY7940
Egira rubrica|[3792]|RWWB641-10|United States|Washington|658[1n]|BOLD:ABY7939
Egira rubrica|[3793]|RWWA081-09|United States|Washington|658[0n]|BOLD:ABY7939
Egira rubrica|[3794]|LBCH5000-10|Canada|British Columbia|658[0n]|BOLD:ABY7939
Egira rubrica|[3795]|JMMMB371-11|United States|California|658[0n]|BOLD:ABY7939
Egira rubrica|[3796]|NAMUM295-08|United States|California|658[0n]|BOLD:ABY7939
Egira rubrica|[3797]|RWWA120-09|United States|Washington|658[0n]|BOLD:ABY7939
Egira rubrica|[3798]|CGLCA023-10|United States|California|658[0n]|BOLD:ABY7939

Egira rubrica|[3796]|NAMUM295-08|United States|California|658[0n]|BOLD:ABY7939
Egira rubrica|[3797]|RWWA120-09|United States|Washington|658[0n]|BOLD:ABY7939
Egira rubrica|[3798]|CGLCA023-10|United States|California|658[0n]|BOLD:ABY7939
Egira rubrica|[3799]|LALPA068-10|Canada|British Columbia|658[0n]|BOLD:ABY7939
Egira rubrica|[3800]|RWWC195-11|United States|Washington|658[0n]|BOLD:ABY7939
Egira rubrica|[3801]|LALPA069-10|Canada|British Columbia|658[0n]|BOLD:ABY7939
Egira rubrica|[3802]|RWWB612-10|United States|Washington|658[0n]|BOLD:ABY7939
Egira rubrica|[3803]|RWWB654-10|United States|Washington|658[1n]|BOLD:ABY7939
Egira rubrica|[3804]|LALPA067-10|Canada|British Columbia|658[0n]|BOLD:ABY7939
Egira rubrica|[3805]|RWWB650-10|United States|Washington|658[0n]|BOLD:ABY7939
Egira rubrica|[3806]|RWWC915-12|United States|Washington|658[0n]|BOLD:ABY7939
Egira perlubens|[3807]|GMLC441-11|United States|California|658[0n]|BOLD:AAC9111
Egira perlubens|[3808]|RDNMF251-08|Canada|British Columbia|658[0n]|BOLD:AAC9111
Egira perlubens|[3809]|GMLC596-11|United States|California|658[0n]|BOLD:AAC9111
Egira perlubens|[3810]|GMLC410-11|United States|California|658[0n]|BOLD:AAC9111
Egira perlubens|[3811]|GMLC401-11|United States|California|658[0n]|BOLD:AAC9111
Egira perlubens|[3812]|GMLC372-11|United States|California|658[0n]|BOLD:AAC9111
Egira perlubens|[3813]|GMLC341-11|United States|California|658[0n]|BOLD:AAC9111
Egira perlubens|[3814]|GMLC338-11|United States|California|658[0n]|BOLD:AAC9111
Egira perlubens|[3815]|JMMMB369-11|United States|California|658[0n]|BOLD:AAC9111
Egira perlubens|[3816]|LALPA072-10|Canada|British Columbia|658[0n]|BOLD:AAC9111
Egira perlubens|[3817]|LALPA071-10|Canada|British Columbia|658[0n]|BOLD:AAC9111
Egira perlubens|[3818]|LALPA070-10|Canada|British Columbia|658[0n]|BOLD:AAC9111
Egira perlubens|[3819]|USLEP1160-10|United States|Colorado|658[0n]|BOLD:AAC9111
Egira perlubens|[3820]|LBCH5155-10|Canada|British Columbia|658[0n]|BOLD:AAC9111
Egira perlubens|[3821]|LBCH5096-10|Canada|British Columbia|658[0n]|BOLD:AAC9111
Egira perlubens|[3822]|LBCH4990-10|Canada|British Columbia|658[0n]|BOLD:AAC9111
Egira perlubens|[3823]|LBCH4983-10|Canada|British Columbia|658[0n]|BOLD:AAC9111
Egira perlubens|[3824]|CGLCA135-10|United States|California|658[0n]|BOLD:AAC9111
Egira perlubens|[3825]|CGLCA134-10|United States|California|658[0n]|BOLD:AAC9111
Egira perlubens|[3826]|JBAZ025-09|United States|California|658[0n]|BOLD:AAC9111
Egira perlubens|[3827]|LPVIC123-08|Canada|British Columbia|658[0n]|BOLD:AAC9111
Egira perlubens|[3828]|DUNLP152-08|Canada|British Columbia|658[0n]|BOLD:AAC9111
Egira perlubens|[3829]|DUNLP151-08|Canada|British Columbia|658[0n]|BOLD:AAC9111
Egira perlubens|[3830]|RDNMF250-08|United States|Oregon|658[0n]|BOLD:AAC9111
Egira perlubens|[3831]|RDNMF249-08|Canada|British Columbia|658[0n]|BOLD:AAC9111
Egira perlubens|[3832]|RDNMF248-08|Canada|British Columbia|658[0n]|BOLD:AAC9111
Egira perlubens|[3833]|RDNMF247-08|Canada|British Columbia|658[0n]|BOLD:AAC9111
Egira perlubens|[3834]|USLEP1159-10|United States|Colorado|658[0n]|BOLD:AAC9111
Egira perlubens|[3835]|GMLC1379-12|United States|California|634[0n]|BOLD:AAC9111
Egira perlubens|[3836]|GMLC1378-12|United States|California|636[0n]|BOLD:AAC9111
Egira perlubens|[3837]|NAMUM162-08|United States|California|632[0n]|BOLD:AAC9111
Egira perlubens|[3838]|GMLC1404-12|United States|California|632[0n]|BOLD:AAC9111
Egira purpurea|[3839]|LNAUT913-14|United States|Arizona|628[0n]|BOLD:ACN8805
Egira purpurea|[3840]|LNAUT914-14|United States|Arizona|658[0n]|BOLD:ACN8805
Orthosia garmani|[3841]|RDNMF824-08|Canada|Ontario|658[0n]|BOLD:AAD0700
Orthosia garmani|[3842]|LSEU234-06|United States|Georgia|579[1n]|BOLD:AAD0700
Orthosia garmani|[3843]|LPOKA1005-09|United States|Oklahoma|658[0n]|BOLD:AAD0700
Orthosia garmani|[3844]|LPOKA1007-09|United States|Oklahoma|658[0n]|BOLD:AAD0700
Orthosia garmani|[3845]|RDNMF823-08|||658[0n]|BOLD:AAD0700
Orthosia garmani|[3846]|LPOKA951-09|United States|Oklahoma|658[0n]|BOLD:AAD0700
Orthosia garmani|[3847]|LPOKA967-09|United States|Oklahoma|658[0n]|BOLD:AAD0700
Orthosia garmani|[3848]|LPOKA984-09|United States|Oklahoma|658[0n]|BOLD:AAD0700
Orthosia garmani|[3849]|LNCB975-10|United States|North Carolina|658[0n]|BOLD:AAD0700
Orthosia garmani|[3850]|LNCC577-11|United States|North Carolina|658[0n]|BOLD:AAD0700
Morrisonia mucens|[3851]|USLEP597-10|United States|Florida|658[0n]|BOLD:AAK6533
Morrisonia mucens|[3852]|USLEP620-10|United States|Florida|658[0n]|BOLD:AAK6533
Morrisonia mucens|[3853]|USLEP621-10|United States|Florida|658[0n]|BOLD:AAK6533
Morrisonia mucens|[3854]|USLEP1219-10|United States|Florida|658[0n]|BOLD:AAK6533
Morrisonia mucens|[3855]|LSEU510-06|United States|Georgia|658[0n]|BOLD:AAK6533
Morrisonia mucens|[3856]|USLEP598-10|United States|Florida|658[0n]|BOLD:AAK6533
Morrisonia mucens|[3857]|USLEP1220-10|United States|Florida|658[0n]|BOLD:AAK6533
Morrisonia mucens|[3858]|LNCC154-10|United States|North Carolina|658[0n]|BOLD:AAK6533
Egira cognata|[3859]|LOCBF3637-14|United States|California|567[0n]|BOLD:AAD4439
Egira cognata|[3860]|LOCBF014-13|United States|California|604[0n]|BOLD:AAD4439
Egira cognata|[3861]|RDNMG475-08|United States|California|658[6n]|BOLD:AAD4439
Egira cognata|[3862]|RDNMG474-08|United States|California|658[0n]|BOLD:AAD4439
Egira cognata|[3863]|RDNMD404-06|United States|California|658[0n]|BOLD:AAD4439
Egira cognata|[3864]|RDNMD403-06|United States|California|658[0n]|BOLD:AAD4439
Egira cognata|[3865]|RDNMG476-08|United States|California|658[0n]|BOLD:AAD4439
Egira cognata|[3866]|RDNMG1014-08|United States|California|658[0n]|BOLD:AAD4439
Egira cognata|[3867]|LOCBF3650-14|United States|California|594[0n]|BOLD:AAD4439
Egira cognata|[3868]|GMLC153-09|United States|California|658[0n]|BOLD:AAD4439
Egira cognata|[3869]|RWWB496-10|United States|Washington|658[0n]|BOLD:AAD4439
Egira cognata|[3870]|RWWB504-10|United States|Washington|633[0n]|BOLD:AAD4439
Egira cognata|[3871]|RWWB514-10|United States|Washington|658[0n]|BOLD:AAD4439
Egira cognata|[3872]|RWWB522-10|United States|Washington|658[0n]|BOLD:AAD4439
Egira cognata|[3873]|RWWB526-10|United States|Washington|658[0n]|BOLD:AAD4439
Egira cognata|[3874]|RWWB527-10|United States|Washington|658[0n]|BOLD:AAD4439
Egira cognata|[3875]|RWWB550-10|United States|Washington|658[0n]|BOLD:AAD4439
Egira cognata|[3876]|RWWB594-10|United States|Washington|658[0n]|BOLD:AAD4439
Egira cognata|[3877]|RWWC172-11|United States|Washington|658[0n]|BOLD:AAD4439
Egira cognata|[3878]|RWWC898-12|United States|Washington|658[0n]|BOLD:AAD4439
Egira februalis|[3879]|LNAUT904-14|United States|California|658[0n]|BOLD:AAD4439
Egira februalis|[3880]|LNAUT908-14|United States|California|658[0n]|BOLD:AAD4439
Egira februalis|[3881]|LNAUT905-14|United States|California|658[0n]|BOLD:AAD4439
Egira februalis|[3882]|LNAUT907-14|United States|California|658[0n]|BOLD:AAD4439
Egira februalis|[3883]|CNCLB499-14|United States|California|658[0n]|BOLD:AAD4439
Egira februalis|[3884]|LNAUS4849-13|United States|Oregon|658[0n]|BOLD:AAD4439
Egira februalis|[3885]|LNAUT906-14|United States|California|658[0n]|BOLD:AAD4439
Egira februalis|[3886]|TML199-14|United States||621[0n]|BOLD:AAD4439
Orthosia rubescens|[3887]|PMG145-03|Canada|Ontario|617[0n]|BOLD:AAC0946
Orthosia rubescens|[3888]|PHMO032-03|Canada|Ontario|639[0n]|BOLD:AAC0946
Orthosia rubescens|[3889]|TMNBB330-06|Canada|New Brunswick|658[0n]|BOLD:AAC0946
Orthosia rubescens|[3890]|TMNBB332-06|Canada|New Brunswick|658[0n]|BOLD:AAC0946
Orthosia rubescens|[3891]|RDLQ670-07|Canada|Quebec|655[0n]|BOLD:AAC0946
Orthosia rubescens|[3892]|LGSMG691-07|United States|Tennessee|658[0n]|BOLD:AAC0946
Orthosia rubescens|[3893]|AHLEP069-10|United States|Pennsylvania|658[0n]|BOLD:AAC0946
Orthosia rubescens|[3894]|AHLEP070-10|United States|Pennsylvania|658[0n]|BOLD:AAC0946
Orthosia rubescens|[3895]|AHLEP097-10|United States|Pennsylvania|658[0n]|BOLD:AAC0946
Orthosia rubescens|[3896]|LPOKE272-11|United States|Oklahoma|658[0n]|BOLD:AAC0946
Orthosia rubescens|[3897]|TMNBB331-06|Canada|New Brunswick|658[0n]|BOLD:AAC0946
Orthosia rubescens|[3898]|XAE170-04|Canada|Ontario|658[0n]|BOLD:AAC0946

Orthosia rubescens|[3896]|LPOKE272-11|United States|Oklahoma|658[0n]|BOLD:AAC0946
Orthosia rubescens|[3897]|TMNBB331-06|Canada|New Brunswick|658[0n]|BOLD:AAC0946
Orthosia rubescens|[3898]|XAE170-04|Canada|Ontario|658[0n]|BOLD:AAC0946
Orthosia rubescens|[3899]|AHLEP045-10|United States|Pennsylvania|593[0n]|BOLD:AAC0946
Orthosia rubescens|[3900]|ALLEP443-13|Canada|Ontario|658[0n]|BOLD:AAC0946
Orthosia rubescens|[3901]|CNPEM3412-14|Canada|Prince Edward Island|549[0n]|BOLD:AAC0946
Orthosia rubescens|[3902]|LGSMC882-05|United States|North Carolina|658[0n]|BOLD:AAC0946
Orthosia rubescens|[3903]|CNBRB452-14|Canada|Nova Scotia|592[0n]|BOLD:AAC0946
Orthosia rubescens|[3904]|CNBRD1066-14|Canada|Nova Scotia|630[0n]|BOLD:AAC0946
Orthosia rubescens|[3905]|RDNMC465-05|Canada|Ontario|614[0n]|BOLD:AAC0946
Orthosia rubescens|[3906]|LSEU229-06|United States|North Carolina|658[0n]|BOLD:AAC0946
Orthosia rubescens|[3907]|LSEU230-06|United States|North Carolina|658[0n]|BOLD:AAC0946
Orthosia rubescens|[3908]|LGSMG690-07|United States|Tennessee|658[0n]|BOLD:AAC0946
Orthosia rubescens|[3909]|KPOEC173-08|Canada|Ontario|655[0n]|BOLD:AAC0946
Orthosia rubescens|[3910]|LMDH027-11|United States|Minnesota|658[0n]|BOLD:AAC0946
Orthosia rubescens|[3911]|AHLEP777-12|United States|Pennsylvania|658[0n]|BOLD:AAC0946
Orthosia rubescens|[3912]|CNBRM848-14|Canada|Nova Scotia|622[0n]|BOLD:AAC0946
Perigonica nr. angulata|[3913]|RDNMJ830-11|United States|Arizona|658[1n]|BOLD:ABZ7149
Perigonica nr. angulata|[3914]|RDNMJ831-11|United States|Arizona|658[4n]|BOLD:ABZ7149
Perigonica angulata|[3915]|LOCBB120-06|United States|California|658[0n]|BOLD:AAD1622
Perigonica angulata|[3916]|LOCBB119-06|United States|California|656[0n]|BOLD:AAD1622
Perigonica angulata|[3917]|JMMMB363-11|United States|California|658[0n]|BOLD:AAD1622
Perigonica angulata|[3918]|NAMUM243-08|United States|California|658[0n]|BOLD:AAD1622
Perigonica angulata|[3919]|RDNMD411-06|United States|California|658[0n]|BOLD:AAD1622
Perigonica angulata|[3920]|RDNMD410-06|United States|California|658[0n]|BOLD:AAD1622
Perigonica angulata|[3921]|LOCBB121-06|United States|California|658[0n]|BOLD:AAD1622
Perigonica angulata|[3922]|LOCBB117-06|United States|California|658[0n]|BOLD:AAD1622
Perigonica angulata|[3923]|LOCBB116-06|United States|California|658[0n]|BOLD:AAD1622
Perigonica angulata|[3924]|LOCBF2308-13|United States|California|594[0n]|BOLD:AAD1622
Perigonica fulminans|[3925]|LOCBB118-06|United States|California|656[0n]|BOLD:AAI2376
Perigonica eldana|[3926]|CNCLB2062-14|United States|Arizona|307[0n]|
Perigonica fulminans|[3927]|USLEP557-10|United States|Colorado|589[1n]|BOLD:AAI2376
Perigonica fulminans|[3928]|USLEP559-10|United States|Colorado|658[0n]|BOLD:AAI2376
Perigonica fulminans|[3929]|USLEP556-10|United States|Colorado|658[0n]|BOLD:AAI2376
Perigonica fulminans|[3930]|USLEP558-10|United States|Colorado|658[0n]|BOLD:AAI2376
Perigonica fulminans|[3931]|USLEP560-10|United States|Colorado|658[0n]|BOLD:AAI2376
Perigonica tertia|[3932]|RDNMI041-10|United States|California|658[0n]|BOLD:AAI2376
Perigonica tertia|[3933]|RDNMI044-10|United States|California|658[0n]|BOLD:AAI2376
Perigonica tertia|[3934]|RDNMI188-10|United States|California|658[0n]|BOLD:AAI2376
Perigonica tertia|[3935]|RDNMI042-10|United States|California|658[0n]|BOLD:AAI2376
Perigonica tertia|[3936]|LOCBF2305-13|United States|California|594[0n]|BOLD:AAI2376
Perigonica tertia|[3937]|LOCBF2304-13|United States|California|558[0n]|BOLD:AAI2376
Perigonica tertia|[3938]|LOCBF2307-13|United States|California|564[0n]|BOLD:AAI2376
Perigonica tertia|[3939]|LOCBF2306-13|United States|California|561[0n]|BOLD:AAI2376
Perigonica tertia|[3940]|JBAZ022-09|United States|California|658[0n]|BOLD:AAI2376
Perigonica tertia|[3941]|LOCBF3999-14|United States|California|603[0n]|BOLD:AAI2376
Perigonica tertia|[3942]|LOCBF4003-14|United States|California|588[0n]|BOLD:AAI2376
Perigonica tertia|[3943]|LOCBF4004-14|United States|California|588[0n]|BOLD:AAI2376
Perigonica fulminans|[3944]|RDNMJ854-11|United States|Utah|658[0n]|BOLD:AAI2376
Perigonica eldana|[3945]|CNCLB2059-14|United States|Arizona|625[0n]|BOLD:AAI2376
Perigonica fulminans|[3946]|RDNMJ853-11|United States|Utah|658[0n]|BOLD:AAI2376
Perigonica eldana|[3947]|CNCLB2060-14|United States|Arizona|658[0n]|BOLD:AAI2376
Perigonica eldana|[3948]|CNCLB2058-14|United States|Arizona|658[0n]|BOLD:AAI2376
Perigonica eldana|[3949]|CNCLB2057-14|United States|Arizona|658[0n]|BOLD:AAI2376
Perigonica eldana|[3950]|CNCLB2061-14|United States|Arizona|658[0n]|BOLD:AAI2376
Perigonica eldana|[3951]|CNCLB2063-14|United States|Arizona|658[0n]|BOLD:AAI2376
Morrisonia evicta|[3952]|LSEU057-06|United States|North Carolina|658[0n]|BOLD:AAB1504
Morrisonia evicta|[3953]|LSEU056-06|United States|North Carolina|595[1n]|BOLD:AAB1504
Morrisonia evicta|[3954]|TMNBB347-06|Canada|New Brunswick|658[0n]|BOLD:AAB1504
Morrisonia evicta|[3955]|TMNBB345-06|Canada|New Brunswick|658[0n]|BOLD:AAB1504
Morrisonia evicta|[3956]|XAF323-05|Canada|Ontario|658[0n]|BOLD:AAB1504
Morrisonia evicta|[3957]|RDLQG467-06|Canada|Quebec|658[0n]|BOLD:AAB1504
Morrisonia evicta|[3958]|TMNBB346-06|Canada|New Brunswick|658[0n]|BOLD:AAB1504
Morrisonia evicta|[3959]|TTMNB587-06|Canada|New Brunswick|658[0n]|BOLD:AAB1504
Morrisonia evicta|[3960]|LSUSA029-06|United States|Kentucky|658[0n]|BOLD:AAB1504
Morrisonia evicta|[3961]|LOCT075-05|United States|Connecticut|658[0n]|BOLD:AAB1504
Morrisonia evicta|[3962]|XAF337-05|Canada|Ontario|658[0n]|BOLD:AAB1504
Morrisonia evicta|[3963]|PMG136-03|Canada|Ontario|617[0n]|BOLD:AAB1504
Morrisonia evicta|[3964]|TTMNB056-06|Canada|New Brunswick|613[0n]|BOLD:AAB1504
Morrisonia evicta|[3965]|TMG140-03|Canada|Ontario|639[0n]|BOLD:AAB1504
Morrisonia evicta|[3966]|LOCT074-05|United States|Connecticut|639[0n]|BOLD:AAB1504
Morrisonia evicta|[3967]|RDLQG464-06|Canada|Quebec|645[0n]|BOLD:AAB1504
Morrisonia evicta|[3968]|XAJ229-06|Canada|Ontario|658[0n]|BOLD:AAB1504
Morrisonia evicta|[3969]|LPSO150-08|Canada|Ontario|658[0n]|BOLD:AAB1504
Morrisonia evicta|[3970]|LPSO155-08|Canada|Ontario|658[0n]|BOLD:AAB1504
Morrisonia evicta|[3971]|LPSO251-08|Canada|Ontario|658[0n]|BOLD:AAB1504
Morrisonia evicta|[3972]|LPSO253-08|Canada|Ontario|657[0n]|BOLD:AAB1504
Morrisonia evicta|[3973]|BLTIB054-08|Canada|Ontario|658[0n]|BOLD:AAB1504
Morrisonia evicta|[3974]|XAJ226-06|Canada|Ontario|658[0n]|BOLD:AAB1504
Morrisonia evicta|[3975]|LMDH064-11|United States|Minnesota|658[0n]|BOLD:AAB1504
Morrisonia evicta|[3976]|XAE154-04|Canada|Ontario|658[0n]|BOLD:AAB1504
Morrisonia evicta|[3977]|LOTB107-05|United States|Tennessee|658[0n]|BOLD:AAB1504
Morrisonia evicta|[3978]|XAJ111-06|Canada|Ontario|658[0n]|BOLD:AAB1504
Morrisonia evicta|[3979]|XAJ243-06|Canada|Ontario|658[0n]|BOLD:AAB1504
Morrisonia evicta|[3980]|XAJ267-06|Canada|Ontario|658[0n]|BOLD:AAB1504
Morrisonia evicta|[3981]|LGSMC912-05|United States|Tennessee|658[0n]|BOLD:AAB1504
Morrisonia evicta|[3982]|LSEU509-06|United States|Georgia|658[0n]|BOLD:AAB1504
Morrisonia evicta|[3983]|SMTPB12941-13|Canada|Ontario|591[0n]|BOLD:AAB1504
Morrisonia confusa|[3984]|XAE281-04|Canada|Ontario|658[0n]|BOLD:AAA6652
Morrisonia confusa|[3985]|CNSLF295-12|Canada|Ontario|616[0n]|BOLD:AAA6652
Morrisonia confusa|[3986]|LILLA058-11|United States|Illinois|658[0n]|BOLD:AAA6652
Morrisonia confusa|[3987]|BBLSX519-09|United States|Oklahoma|658[0n]|BOLD:AAA6652
Morrisonia confusa|[3988]|LPOKB232-09|United States|Oklahoma|658[0n]|BOLD:AAA6652
Morrisonia confusa|[3989]|LPSO955-08|Canada|Ontario|658[0n]|BOLD:AAA6652
Morrisonia confusa|[3990]|LPSO374-08|Canada|Ontario|658[0n]|BOLD:AAA6652
Morrisonia confusa|[3991]|LPSO372-08|Canada|Ontario|658[0n]|BOLD:AAA6652
Morrisonia confusa|[3992]|LPSO236-08|Canada|Ontario|658[0n]|BOLD:AAA6652
Morrisonia confusa|[3993]|LPSO049-08|Canada|Ontario|658[0n]|BOLD:AAA6652
Morrisonia confusa|[3994]|LPSO038-08|Canada|Ontario|658[0n]|BOLD:AAA6652
Morrisonia confusa|[3995]|LSEU322-06|United States|Georgia|657[0n]|BOLD:AAA6652
Morrisonia confusa|[3996]|XAJ404-06|Canada|Ontario|658[0n]|BOLD:AAA6652
Morrisonia confusa|[3997]|XAJ355-06|Canada|Ontario|658[0n]|BOLD:AAA6652
Morrisonia confusa|[3998]|LNC762-06|United States|North Carolina|658[0n]|BOLD:AAA6652

Morrisonia confusa|[3996]|XAJ404-06|Canada|Ontario|658[0n]|BOLD:AAA6652
Morrisonia confusa|[3997]|XAJ355-06|Canada|Ontario|658[0n]|BOLD:AAA6652
Morrisonia confusa|[3998]|LNC762-06|United States|North Carolina|658[0n]|BOLD:AAA6652
Morrisonia confusa|[3999]|LSUSA033-06|United States|Kentucky|658[0n]|BOLD:AAA6652
Morrisonia confusa|[4000]|LNC090-05|United States|North Carolina|658[0n]|BOLD:AAA6652
Morrisonia confusa|[4001]|LOTB240-05|United States|Tennessee|658[0n]|BOLD:AAA6652
Morrisonia confusa|[4002]|LOTB153-05|United States|Tennessee|658[0n]|BOLD:AAA6652
Morrisonia confusa|[4003]|LGSMC484-05|United States|Tennessee|658[0n]|BOLD:AAA6652
Morrisonia confusa|[4004]|XAC157-04|Canada|Ontario|658[0n]|BOLD:AAA6652
Morrisonia confusa|[4005]|XAB277-04|Canada|Ontario|658[0n]|BOLD:AAA6652
Morrisonia confusa|[4006]|LGSMC479-05|United States|Tennessee|600[0n]|BOLD:AAA6652
Morrisonia confusa|[4007]|GMGSA073-12|United States|Tennessee|632[0n]|BOLD:AAA6652
Morrisonia confusa|[4008]|CNROD531-13|Canada|Ontario|606[0n]|BOLD:AAA6652
Morrisonia confusa|[4009]|CNROE1157-13|Canada|Ontario|546[0n]|BOLD:AAA6652
Morrisonia confusa|[4010]|NOCNA080-14|United States|Florida|658[0n]|BOLD:AAA6652
Morrisonia confusa|[4011]|XAD669-05|Canada|Ontario|658[0n]|BOLD:AAA6652
Morrisonia confusa|[4012]|RDLQ684-07|Canada|Quebec|592[0n]|BOLD:AAA6652
Morrisonia confusa|[4013]|AHLEP1075-12|United States|Pennsylvania|658[0n]|BOLD:AAA6652
Morrisonia confusa|[4014]|PMG135-03|Canada|Ontario|617[0n]|BOLD:AAA6652
Morrisonia confusa|[4015]|LGSMC482-05|United States|Tennessee|584[0n]|BOLD:AAA6652
Morrisonia confusa|[4016]|CNSLP001-13|Canada|Ontario|581[0n]|BOLD:AAA6652
Morrisonia confusa|[4017]|GMGSB177-12|United States|Tennessee|632[0n]|BOLD:AAA6652
Morrisonia confusa|[4018]|CNSLC438-12|Canada|Ontario|614[0n]|BOLD:AAA6652
Morrisonia confusa|[4019]|GMGSD645-12|United States|Tennessee|614[0n]|BOLD:AAA6652
Morrisonia confusa|[4020]|CNSLO872-13|Canada|Ontario|620[0n]|BOLD:AAA6652
Morrisonia confusa|[4021]|CNROM1171-13|Canada|Ontario|576[0n]|BOLD:AAA6652
Morrisonia confusa|[4022]|CNSLO871-13|Canada|Ontario|602[0n]|BOLD:AAA6652
Morrisonia confusa|[4023]|CNSLD281-12|Canada|Ontario|630[0n]|BOLD:AAA6652
Morrisonia confusa|[4024]|GMGSF384-12|United States|Tennessee|629[0n]|BOLD:AAA6652
Morrisonia confusa|[4025]|BBLCU122-09|United States|Michigan|658[0n]|BOLD:AAA6652
Morrisonia confusa|[4026]|AHLEP660-12|United States|Pennsylvania|658[0n]|BOLD:AAA6652
Morrisonia confusa|[4027]|RDLQ683-07|Canada|Quebec|658[0n]|BOLD:AAA6652
Morrisonia confusa|[4028]|BLTIB032-08|Canada|Ontario|658[0n]|BOLD:AAA6652
Morrisonia confusa|[4029]|UDLEP155-09|United States|Pennsylvania|658[0n]|BOLD:AAA6652
Morrisonia confusa|[4030]|UDLEP311-09|United States|Pennsylvania|658[0n]|BOLD:AAA6652
Morrisonia confusa|[4031]|TMNBB355-06|Canada|New Brunswick|658[0n]|BOLD:AAA6652
Morrisonia confusa|[4032]|TMNBB356-06|Canada|New Brunswick|658[0n]|BOLD:AAA6652
Morrisonia confusa|[4033]|TMNBB357-06|Canada|New Brunswick|658[0n]|BOLD:AAA6652
Morrisonia confusa|[4034]|TMNBB359-06|Canada|New Brunswick|658[0n]|BOLD:AAA6652
Morrisonia confusa|[4035]|TMNBB361-06|Canada|New Brunswick|658[0n]|BOLD:AAA6652
Morrisonia confusa|[4036]|LSEU340-06|United States|Georgia|658[0n]|BOLD:AAA6652
Morrisonia confusa|[4037]|TMNBB353-06|Canada|New Brunswick|658[0n]|BOLD:AAA6652
Morrisonia confusa|[4038]|TMNBB354-06|Canada|New Brunswick|658[0n]|BOLD:AAA6652
Morrisonia confusa|[4039]|TMNBB351-06|Canada|New Brunswick|658[0n]|BOLD:AAA6652
Morrisonia confusa|[4040]|TMNBB352-06|Canada|New Brunswick|658[0n]|BOLD:AAA6652
Morrisonia confusa|[4041]|TMNBB350-06|Canada|New Brunswick|658[0n]|BOLD:AAA6652
Morrisonia confusa|[4042]|TMNBB349-06|Canada|New Brunswick|658[0n]|BOLD:AAA6652
Morrisonia confusa|[4043]|TMNBB348-06|Canada|New Brunswick|658[0n]|BOLD:AAA6652
Morrisonia confusa|[4044]|LOCT316-05|United States|Connecticut|658[0n]|BOLD:AAA6652
Morrisonia confusa|[4045]|LOCT079-05|United States|Connecticut|658[0n]|BOLD:AAA6652
Morrisonia confusa|[4046]|LOCT078-05|United States|Connecticut|658[0n]|BOLD:AAA6652
Morrisonia confusa|[4047]|LOTB239-05|United States|Tennessee|658[0n]|BOLD:AAA6652
Morrisonia confusa|[4048]|LOTB156-05|United States|Tennessee|658[0n]|BOLD:AAA6652
Morrisonia confusa|[4049]|LOTB155-05|United States|Tennessee|658[0n]|BOLD:AAA6652
Morrisonia confusa|[4050]|LOTB154-05|United States|Tennessee|658[0n]|BOLD:AAA6652
Morrisonia confusa|[4051]|LGSMC480-05|United States|Tennessee|658[0n]|BOLD:AAA6652
Morrisonia confusa|[4052]|MEC177-04|Canada|Quebec|658[0n]|BOLD:AAA6652
Morrisonia confusa|[4053]|CNSLF332-12|Canada|Ontario|616[0n]|BOLD:AAA6652
Morrisonia confusa|[4054]|GMGSK036-12|United States|Tennessee|625[0n]|BOLD:AAA6652
Morrisonia confusa|[4055]|LGSMC483-05|United States|Tennessee|607[0n]|BOLD:AAA6652
Morrisonia confusa|[4056]|XAJ411-06|Canada|Ontario|656[0n]|BOLD:AAA6652
Morrisonia confusa|[4057]|TMNBB360-06|Canada|New Brunswick|656[0n]|BOLD:AAA6652
Morrisonia confusa|[4058]|LMIS015-05|Canada|Ontario|658[0n]|BOLD:AAA6652
Morrisonia confusa|[4059]|CNSLE404-12|Canada|Ontario|632[0n]|BOLD:AAA6652
Morrisonia confusa|[4060]|TMNBB358-06|Canada|New Brunswick|656[0n]|BOLD:AAA6652
Morrisonia confusa|[4061]|TMG141-03|Canada|Ontario|639[0n]|BOLD:AAA6652
Morrisonia confusa|[4062]|LGSMC481-05|United States|Tennessee|600[0n]|BOLD:AAA6652
Morrisonia confusa|[4063]|CNSLH030-12|Canada|Ontario|634[0n]|BOLD:AAA6652
Morrisonia confusa|[4064]|CNSLG080-12|Canada|Ontario|631[0n]|BOLD:AAA6652
Morrisonia confusa|[4065]|CNSLE001-12|Canada|Ontario|631[0n]|BOLD:AAA6652
Morrisonia confusa|[4066]|CNSLD294-12|Canada|Ontario|632[0n]|BOLD:AAA6652
Morrisonia confusa|[4067]|CNSLD282-12|Canada|Ontario|631[0n]|BOLD:AAA6652
Morrisonia confusa|[4068]|CNSLC439-12|Canada|Ontario|633[0n]|BOLD:AAA6652
Morrisonia confusa|[4069]|CNSLC435-12|Canada|Ontario|631[0n]|BOLD:AAA6652
Morrisonia confusa|[4070]|CNSLN446-13|Canada|Ontario|588[0n]|BOLD:AAA6652
Morrisonia confusa|[4071]|CNSLN447-13|Canada|Ontario|588[0n]|BOLD:AAA6652
Morrisonia confusa|[4072]|CNROR024-13|Canada|Ontario|606[0n]|BOLD:AAA6652
Morrisonia confusa|[4073]|AHLEP1141-13|United States|Pennsylvania|658[0n]|BOLD:AAA6652
Morrisonia confusa|[4074]|CNCLB2977-14|United States|North Carolina|658[0n]|BOLD:AAA6652
Anorthodes indigena|[4075]|RDNMJ816-11|United States|Arizona|658[0n]|BOLD:AAP5984
Anorthodes indigena|[4076]|CMAZA360-10|United States|Arizona|658[0n]|BOLD:AAP5984
Anorthodes indigena|[4077]|RDNMJ817-11|United States|Arizona|658[0n]|BOLD:AAP5984
Anorthodes triquetra|[4078]|RDNMG280-08|United States|Texas|658[0n]|BOLD:AAJ2734
Anorthodes triquetra|[4079]|RDNMG903-08|United States|Arizona|658[0n]|BOLD:AAJ2734
Anorthodes triquetra|[4080]|CMAZA784-10|United States|Arizona|658[0n]|BOLD:AAJ2734
Anorthodes triquetra|[4081]|CMAZA1154-12|United States|Arizona|658[0n]|BOLD:AAJ2734
Proxenus mendosa|[4082]|BBLPB451-10|Canada|Alberta|658[0n]|BOLD:AAC9107
Proxenus mendosa|[4083]|RDMAB333-05|Canada|Alberta|561[1n]|BOLD:AAC9107
Proxenus mendosa|[4084]|RDMAB501-06|Canada|Alberta|658[0n]|BOLD:AAC9107
Proxenus mendosa|[4085]|RDNMC735-06|Canada|Quebec|658[0n]|BOLD:AAC9107
Proxenus mendosa|[4086]|LPABB051-08|Canada|Alberta|658[0n]|BOLD:AAC9107
Proxenus mendosa|[4087]|LPABB412-08|Canada|Alberta|658[0n]|BOLD:AAC9107
Proxenus mendosa|[4088]|LPABB434-08|Canada|Alberta|658[0n]|BOLD:AAC9107
Proxenus mendosa|[4089]|LBCC285-05|Canada|British Columbia|658[0n]|BOLD:AAC9107
Proxenus mendosa|[4090]|LBCC284-05|Canada|British Columbia|658[0n]|BOLD:AAC9107
Proxenus mendosa|[4091]|RDMAB331-05|Canada|Alberta|505[0n]|BOLD:AAC9107
Proxenus mendosa|[4092]|BBLPF009-10|Canada|Alberta|658[0n]|BOLD:AAC9107
Proxenus mendosa|[4093]|BBLPF010-10|Canada|Alberta|658[0n]|BOLD:AAC9107
Proxenus miranda|[4094]|RDLQB072-05|Canada|Quebec|658[0n]|BOLD:AAA5599
Proxenus miranda|[4095]|LPSO763-08|Canada|Ontario|658[0n]|BOLD:AAA5599
Proxenus miranda|[4096]|LPSO999-08|Canada|Ontario|658[0n]|BOLD:AAA5599
Proxenus miranda|[4097]|RDLQG557-06|Canada|Quebec|658[0n]|BOLD:AAA5599
Proxenus miranda|[4098]|RDLQG314-06|Canada|Quebec|658[0n]|BOLD:AAA5599

Proxenus miranda|[4096]|LPSO999-08|Canada|Ontario|658[0n]|BOLD:AAA5599
Proxenus miranda|[4097]|RDLQG557-06|Canada|Quebec|658[0n]|BOLD:AAA5599
Proxenus miranda|[4098]|RDLQG314-06|Canada|Quebec|658[0n]|BOLD:AAA5599
Proxenus miranda|[4099]|KPOEC048-08|Canada|Ontario|658[0n]|BOLD:AAA5599
Proxenus miranda|[4100]|LPSO770-08|Canada|Ontario|658[0n]|BOLD:AAA5599
Proxenus miranda|[4101]|LPSO753-08|Canada|Ontario|658[0n]|BOLD:AAA5599
Proxenus miranda|[4102]|LPSO690-08|Canada|Ontario|658[0n]|BOLD:AAA5599
Proxenus miranda|[4103]|LPSO303-08|Canada|Ontario|658[0n]|BOLD:AAA5599
Proxenus miranda|[4104]|LGSM656-04|United States|North Carolina|658[0n]|BOLD:AAA5599
Proxenus miranda|[4105]|PMG153-03|Canada|Ontario|617[0n]|BOLD:AAA5599
Proxenus miranda|[4106]|XAK274-06|Canada|Ontario|658[0n]|BOLD:AAA5599
Proxenus miranda|[4107]|LPSO302-08|Canada|Ontario|658[0n]|BOLD:AAA5599
Proxenus miranda|[4108]|LPSO833-08|Canada|Ontario|609[0n]|BOLD:AAA5599
Proxenus miranda|[4109]|LPSO921-08|Canada|Ontario|658[0n]|BOLD:AAA5599
Proxenus miranda|[4110]|LPSOB211-08|Canada|Ontario|658[0n]|BOLD:AAA5599
Proxenus miranda|[4111]|BBLCU093-09|United States|Kansas|658[0n]|BOLD:AAA5599
Proxenus miranda|[4112]|BBLCU379-09|United States|Kansas|658[0n]|BOLD:AAA5599
Proxenus miranda|[4113]|LNCB945-10|United States|North Carolina|658[0n]|BOLD:AAA5599
Proxenus miranda|[4114]|BBLOE1671-12|United States|Texas|658[0n]|BOLD:AAA5599
Proxenus miranda|[4115]|LPSK468-08|Canada|Saskatchewan|658[0n]|BOLD:AAA5599
Proxenus miranda|[4116]|LILLA493-11|United States|Illinois|658[0n]|BOLD:AAA5599
Proxenus miranda|[4117]|KPOEC144-08|Canada|Ontario|658[0n]|BOLD:AAA5599
Proxenus miranda|[4118]|XAG079-05|Canada|Ontario|658[0n]|BOLD:AAA5599
Proxenus miranda|[4119]|LPOKC296-09|United States|Oklahoma|658[0n]|BOLD:AAA5599
Proxenus miranda|[4120]|XAD282-04|Canada|Ontario|593[0n]|BOLD:AAA5599
Proxenus miranda|[4121]|XAK540-07|Canada|Ontario|594[0n]|BOLD:AAA5599
Proxenus miranda|[4122]|CNGRJ009-13|Canada|Saskatchewan|566[0n]|BOLD:AAA5599
Proxenus miranda|[4123]|RDMAB345-05|Canada|Alberta|658[0n]|BOLD:AAA5599
Proxenus miranda|[4124]|LOWCB912-05|Canada|British Columbia|658[0n]|BOLD:AAA5599
Proxenus miranda|[4125]|LPSO755-08|Canada|Ontario|658[0n]|BOLD:AAA5599
Proxenus miranda|[4126]|KPOEC118-08|Canada|Ontario|621[2n]|BOLD:AAA5599
Proxenus miranda|[4127]|LPMN949-08|Canada|Alberta|658[0n]|BOLD:AAA5599
Proxenus miranda|[4128]|LPAB105-08|Canada|Alberta|658[1n]|BOLD:AAA5599
Proxenus miranda|[4129]|LBCG3059-09|Canada|British Columbia|658[0n]|BOLD:AAA5599
Proxenus miranda|[4130]|LBCG2470-09|Canada|British Columbia|658[0n]|BOLD:AAA5599
Proxenus miranda|[4131]|LBCG1881-09|Canada|British Columbia|658[0n]|BOLD:AAA5599
Proxenus miranda|[4132]|LPABC880-09|Canada|Alberta|658[0n]|BOLD:AAA5599
Proxenus miranda|[4133]|LPABC757-09|Canada|Alberta|658[0n]|BOLD:AAA5599
Proxenus miranda|[4134]|LPABB536-08|Canada|Alberta|658[0n]|BOLD:AAA5599
Proxenus miranda|[4135]|LPAB095-08|Canada|Alberta|658[0n]|BOLD:AAA5599
Proxenus miranda|[4136]|LPMN944-08|Canada|Alberta|658[0n]|BOLD:AAA5599
Proxenus miranda|[4137]|LPMN393-08|Canada|Manitoba|658[0n]|BOLD:AAA5599
Proxenus miranda|[4138]|LPMN335-08|Canada|Manitoba|658[0n]|BOLD:AAA5599
Proxenus miranda|[4139]|LBCG393-08|Canada|British Columbia|658[0n]|BOLD:AAA5599
Proxenus miranda|[4140]|LBCG391-08|Canada|British Columbia|658[0n]|BOLD:AAA5599
Proxenus miranda|[4141]|LPSO459-08|Canada|Ontario|658[0n]|BOLD:AAA5599
Proxenus miranda|[4142]|LPSO301-08|Canada|Ontario|658[0n]|BOLD:AAA5599
Proxenus miranda|[4143]|RDLQG556-06|Canada|Quebec|658[0n]|BOLD:AAA5599
Proxenus miranda|[4144]|RDLQG313-06|Canada|Quebec|658[0n]|BOLD:AAA5599
Proxenus miranda|[4145]|MECC102-06|Canada|Ontario|658[0n]|BOLD:AAA5599
Proxenus miranda|[4146]|RDMAB326-05|Canada|Alberta|658[0n]|BOLD:AAA5599
Proxenus miranda|[4147]|RDMAB115-05|Canada|Alberta|658[0n]|BOLD:AAA5599
Proxenus miranda|[4148]|RDLQB071-05|Canada|Quebec|658[0n]|BOLD:AAA5599
Proxenus miranda|[4149]|LOWCB915-05|Canada|British Columbia|658[0n]|BOLD:AAA5599
Proxenus miranda|[4150]|LOWCB911-05|Canada|British Columbia|658[0n]|BOLD:AAA5599
Proxenus miranda|[4151]|LOWCB910-05|Canada|British Columbia|658[0n]|BOLD:AAA5599
Proxenus miranda|[4152]|LOWCB909-05|Canada|British Columbia|658[0n]|BOLD:AAA5599
Proxenus miranda|[4153]|LOWCB906-05|Canada|British Columbia|658[0n]|BOLD:AAA5599
Proxenus miranda|[4154]|XAH557-05|Canada|Ontario|658[0n]|BOLD:AAA5599
Proxenus miranda|[4155]|XAG699-05|Canada|Ontario|658[0n]|BOLD:AAA5599
Proxenus miranda|[4156]|KPOEC095-08|Canada|Ontario|658[0n]|BOLD:AAA5599
Proxenus miranda|[4157]|BBLPB442-10|Canada|Ontario|658[0n]|BOLD:AAA5599
Proxenus miranda|[4158]|LOWCD300-06|Canada|British Columbia|654[0n]|BOLD:AAA5599
Proxenus miranda|[4159]|LOWCB913-05|Canada|British Columbia|585[0n]|BOLD:AAA5599
Proxenus miranda|[4160]|LOWCB914-05|Canada|British Columbia|585[0n]|BOLD:AAA5599
Proxenus miranda|[4161]|CNWBB132-13|Canada|Alberta|591[0n]|BOLD:AAA5599
Proxenus miranda|[4162]|CNWBB130-13|Canada|Alberta|583[0n]|BOLD:AAA5599
Proxenus miranda|[4163]|CNWBB129-13|Canada|Alberta|585[0n]|BOLD:AAA5599
Proxenus miranda|[4164]|RDLQB070-05|Canada|Quebec|571[0n]|BOLD:AAA5599
Proxenus miranda|[4165]|XAK564-07|Canada|Ontario|584[0n]|BOLD:AAA5599
Proxenus miranda|[4166]|RDMAB330-05|Canada|Alberta|558[0n]|BOLD:AAA5599
Proxenus miranda|[4167]|CNGRI312-13|Canada|Saskatchewan|552[0n]|BOLD:AAA5599
Proxenus miranda|[4168]|LOWCB907-05|Canada|British Columbia|589[0n]|BOLD:AAA5599
Proxenus miranda|[4169]|RDMAB320-05|Canada|Alberta|517[0n]|BOLD:AAA5599
Proxenus miranda|[4170]|CNGRI313-13|Canada|Saskatchewan|543[0n]|BOLD:AAA5599
Proxenus mindara|[4171]|LOCBB255-06|United States|California|658[0n]|BOLD:AAB9054
Proxenus mindara|[4172]|LOCBB257-06|United States|California|658[0n]|BOLD:AAB9054
Proxenus mindara|[4173]|LOCBB256-06|United States|California|658[0n]|BOLD:AAB9054
Proxenus mindara|[4174]|LOCBB254-06|United States|California|658[0n]|BOLD:AAB9054
Proxenus mindara|[4175]|LOCBB253-06|United States|California|658[0n]|BOLD:AAB9054
Proxenus mindara|[4176]|RDMAB325-05|United States|Washington|517[1n]|BOLD:AAB9054
Proxenus mindara|[4177]|RDNMC750-06|United States|California|658[0n]|BOLD:AAB9054
Proxenus mindara|[4178]|LOCBC195-06|United States|California|656[0n]|BOLD:AAB9054
Proxenus mindara|[4179]|LOCBC580-06|United States|California|658[0n]|BOLD:AAB9054
Proxenus mindara|[4180]|LOCBC671-06|United States|California|658[0n]|BOLD:AAB9054
Proxenus mindara|[4181]|LOCBD926-06|United States|California|658[0n]|BOLD:AAB9054
Proxenus mindara|[4182]|LOCBF2615-13|United States|California|658[0n]|BOLD:AAB9054
Proxenus mindara|[4183]|RWWC324-11|United States|Washington|658[0n]|BOLD:AAB9054
Proxenus mindara|[4184]|RWWC456-11|United States|Washington|658[0n]|BOLD:AAB9054
Proxenus mindara|[4185]|RWWB935-10|United States|Washington|658[0n]|BOLD:AAB9054
Proxenus mindara|[4186]|RWWA938-09|United States|Washington|658[0n]|BOLD:AAB9054
Proxenus mindara|[4187]|RWWA633-09|United States|Washington|658[0n]|BOLD:AAB9054
Proxenus mindara|[4188]|RWWA613-09|United States|Washington|658[0n]|BOLD:AAB9054
Proxenus mindara|[4189]|RWWA605-09|United States|Washington|658[0n]|BOLD:AAB9054
Proxenus mindara|[4190]|RWWA578-09|United States|Washington|658[0n]|BOLD:AAB9054
Proxenus mindara|[4191]|RWWB856-10|United States|Washington|632[0n]|BOLD:AAB9054
Proxenus mindara|[4192]|RWWC1142-13|United States|Washington|601[0n]|BOLD:AAB9054
Athetis tarda|[4193]|LGSMC339-05|United States|Tennessee|658[0n]|BOLD:AAA8313
Athetis tarda|[4194]|BBLSX522-09|United States|Oklahoma|658[0n]|BOLD:AAA8313
Athetis tarda|[4195]|BBLSX074-09|United States|Oklahoma|658[0n]|BOLD:AAA8313
Athetis tarda|[4196]|LOTB143-05|United States|Tennessee|616[0n]|BOLD:AAA8313
Athetis tarda|[4197]|LOTB374-05|United States|Tennessee|658[0n]|BOLD:AAA8313
Athetis tarda|[4198]|BBLSX303-09|United States|Oklahoma|658[0n]|BOLD:AAA8313

Athetis tarda|[4196]|LOTB143-05|United States|Tennessee|616[0n]|BOLD:AAA8313
Athetis tarda|[4197]|LOTB374-05|United States|Tennessee|658[0n]|BOLD:AAA8313
Athetis tarda|[4198]|BBLSX303-09|United States|Oklahoma|658[0n]|BOLD:AAA8313
Athetis tarda|[4199]|LOTB244-05|United States|Tennessee|658[0n]|BOLD:AAA8313
Athetis tarda|[4200]|LOTB238-05|United States|Tennessee|658[0n]|BOLD:AAA8313
Athetis tarda|[4201]|BBLSX525-09|United States|Oklahoma|658[0n]|BOLD:AAA8313
Athetis tarda|[4202]|LPOKA387-09|United States|Oklahoma|658[0n]|BOLD:AAA8313
Athetis tarda|[4203]|BBLSW487-09|United States|Oklahoma|623[0n]|BOLD:AAA8313
Athetis tarda|[4204]|LGSMC342-05|United States|Tennessee|658[0n]|BOLD:AAA8313
Athetis tarda|[4205]|LNC438-05|United States|North Carolina|614[0n]|BOLD:AAA8313
Athetis tarda|[4206]|BBLSX080-09|United States|Oklahoma|623[0n]|BOLD:AAA8313
Athetis tarda|[4207]|BBLSW503-09|United States|Oklahoma|658[0n]|BOLD:AAA8313
Athetis tarda|[4208]|BBLSX530-09|United States|Oklahoma|658[0n]|BOLD:AAA8313
Athetis tarda|[4209]|LGSMC340-05|United States|Tennessee|658[0n]|BOLD:AAA8313
Athetis tarda|[4210]|LGSMC338-05|United States|Tennessee|658[0n]|BOLD:AAA8313
Athetis tarda|[4211]|LGSMC337-05|United States|Tennessee|658[0n]|BOLD:AAA8313
Athetis tarda|[4212]|LGSMC341-05|United States|Tennessee|658[0n]|BOLD:AAA8313
Athetis tarda|[4213]|LOTB189-05|United States|Tennessee|658[0n]|BOLD:AAA8313
Athetis tarda|[4214]|LOTB190-05|United States|Tennessee|658[0n]|BOLD:AAA8313
Athetis tarda|[4215]|LOTB191-05|United States|Tennessee|658[0n]|BOLD:AAA8313
Athetis tarda|[4216]|LOTB237-05|United States|Tennessee|658[0n]|BOLD:AAA8313
Athetis tarda|[4217]|LOTB242-05|United States|Tennessee|658[0n]|BOLD:AAA8313
Athetis tarda|[4218]|LOTB245-05|United States|Tennessee|658[0n]|BOLD:AAA8313
Athetis tarda|[4219]|LOTB246-05|United States|Tennessee|658[0n]|BOLD:AAA8313
Athetis tarda|[4220]|LOTB352-05|United States|Tennessee|658[0n]|BOLD:AAA8313
Athetis tarda|[4221]|LOCT277-05|United States|Connecticut|658[0n]|BOLD:AAA8313
Athetis tarda|[4222]|LOCT279-05|United States|Connecticut|658[0n]|BOLD:AAA8313
Athetis tarda|[4223]|LPSO684-08|Canada|Ontario|658[0n]|BOLD:AAA8313
Athetis tarda|[4224]|LPSO749-08|Canada|Ontario|658[0n]|BOLD:AAA8313
Athetis tarda|[4225]|LPSO751-08|Canada|Ontario|658[0n]|BOLD:AAA8313
Athetis tarda|[4226]|BBLSW505-09|United States|Oklahoma|658[0n]|BOLD:AAA8313
Athetis tarda|[4227]|LPSO437-08|Canada|Ontario|658[0n]|BOLD:AAA8313
Athetis tarda|[4228]|LPSO551-08|Canada|Ontario|658[0n]|BOLD:AAA8313
Athetis tarda|[4229]|LNC082-05|United States|North Carolina|658[0n]|BOLD:AAA8313
Athetis tarda|[4230]|LNC767-06|United States|North Carolina|658[0n]|BOLD:AAA8313
Athetis tarda|[4231]|LSEU348-06|United States|Georgia|658[0n]|BOLD:AAA8313
Athetis tarda|[4232]|LSEU349-06|United States|Georgia|658[0n]|BOLD:AAA8313
Athetis tarda|[4233]|LSUSA203-06|United States|Kentucky|658[0n]|BOLD:AAA8313
Athetis tarda|[4234]|LPSO275-08|Canada|Ontario|658[0n]|BOLD:AAA8313
Athetis tarda|[4235]|BBLSX081-09|United States|Oklahoma|658[0n]|BOLD:AAA8313
Athetis tarda|[4236]|BBLSX289-09|United States|Oklahoma|658[0n]|BOLD:AAA8313
Athetis tarda|[4237]|BBLSX291-09|United States|Oklahoma|658[0n]|BOLD:AAA8313
Athetis tarda|[4238]|BBLSX532-09|United States|Oklahoma|658[0n]|BOLD:AAA8313
Athetis tarda|[4239]|BBLSX596-09|United States|Oklahoma|658[0n]|BOLD:AAA8313
Athetis tarda|[4240]|BBLSX960-09|United States|Oklahoma|658[0n]|BOLD:AAA8313
Athetis tarda|[4241]|LPOKB285-09|United States|Oklahoma|658[0n]|BOLD:AAA8313
Athetis tarda|[4242]|BBLSX292-09|United States|Oklahoma|658[0n]|BOLD:AAA8313
Athetis tarda|[4243]|BBLSW498-09|United States|Oklahoma|658[0n]|BOLD:AAA8313
Athetis tarda|[4244]|LOTB236-05|United States|Tennessee|658[0n]|BOLD:AAA8313
Athetis tarda|[4245]|BBLSW499-09|United States|Oklahoma|658[0n]|BOLD:AAA8313
Athetis tarda|[4246]|LILLA037-11|United States|Illinois|658[0n]|BOLD:AAA8313
Athetis tarda|[4247]|LPSO671-08|Canada|Ontario|658[0n]|BOLD:AAA8313
Athetis tarda|[4248]|BBLSX287-09|United States|Oklahoma|658[0n]|BOLD:AAA8313
Athetis tarda|[4249]|LNCNW061-06|United States|North Carolina|658[0n]|BOLD:AAA8313
Athetis tarda|[4250]|LOTB243-05|United States|Tennessee|658[0n]|BOLD:AAA8313
Athetis tarda|[4251]|LOCT278-05|United States|Connecticut|658[0n]|BOLD:AAA8313
Athetis tarda|[4252]|LPOKA394-09|United States|Oklahoma|658[0n]|BOLD:AAA8313
Athetis tarda|[4253]|LPOKB236-09|United States|Oklahoma|658[0n]|BOLD:AAA8313
Athetis tarda|[4254]|LPOKB321-09|United States|Oklahoma|658[0n]|BOLD:AAA8313
Athetis tarda|[4255]|BBLSW490-09|United States|Oklahoma|658[0n]|BOLD:AAA8313
Athetis tarda|[4256]|BBLSX077-09|United States|Oklahoma|658[0n]|BOLD:AAA8313
Athetis tarda|[4257]|BBLSX579-09|United States|Oklahoma|658[0n]|BOLD:AAA8313
Athetis tarda|[4258]|BBLSU049-09|United States|Arkansas|658[0n]|BOLD:AAA8313
Athetis tarda|[4259]|LNCC1687-13|United States|North Carolina|658[0n]|BOLD:AAA8313
Caradrina clavipalpis|[4260]|GWOTF298-12|Italy|Calabria|658[0n]|BOLD:AAB6999
Caradrina clavipalpis|[4261]|NOCJH319-10|France||658[0n]|BOLD:AAB6999
Caradrina clavipalpis|[4262]|GWORR899-10|Spain|Murcia|658[0n]|BOLD:AAB6999
Caradrina clavipalpis|[4263]|GWORR898-10|Spain|Murcia|658[0n]|BOLD:AAB6999
Caradrina clavipalpis|[4264]|NOCJH318-10|France||658[0n]|BOLD:AAB6999
Caradrina clavipalpis|[4265]|LEFID808-10|Finland|Aland Islands|658[0n]|BOLD:AAB6999
Caradrina clavipalpis|[4266]|CGUKC268-09|United Kingdom|England|658[0n]|BOLD:AAB6999
Caradrina clavipalpis|[4267]|CGUKB884-09|United Kingdom|Wales|658[0n]|BOLD:AAB6999
Caradrina clavipalpis|[4268]|CGUKA916-09|United Kingdom|England|658[0n]|BOLD:AAB6999
Caradrina clavipalpis|[4269]|CGUKA701-09|United Kingdom||658[0n]|BOLD:AAB6999
Caradrina clavipalpis|[4270]|CGUKA409-09|United Kingdom|England|658[0n]|BOLD:AAB6999
Caradrina clavipalpis|[4271]|CGUKA197-09|United Kingdom|England|658[0n]|BOLD:AAB6999
Caradrina clavipalpis|[4272]|CGUKA142-09|United Kingdom|England|658[0n]|BOLD:AAB6999
Caradrina clavipalpis|[4273]|LEFIC454-10|Finland|Uusimaa|658[0n]|BOLD:AAB6999
Caradrina clavipalpis|[4274]|CGUKA550-09|United Kingdom|England|621[0n]|BOLD:AAB6999
Caradrina clavipalpis|[4275]|NOCJH314-10|France||577[1n]|BOLD:AAB6999
Caradrina clavipalpis|[4276]|NOCJH321-10|France||658[0n]|BOLD:AAB6999
Caradrina clavipalpis|[4277]|FBLMX241-11|Germany|Bavaria|658[0n]|BOLD:AAB6999
Caradrina clavipalpis|[4278]|LEFIC682-10|Finland|Finland Proper|658[0n]|BOLD:AAB6999
Caradrina clavipalpis|[4279]|RDNMH1006-09|United States|New York|658[0n]|BOLD:AAB6999
Caradrina clavipalpis|[4280]|GWOSZ130-11|Italy|Trentino-Alto Adige|658[0n]|BOLD:AAB6999
Caradrina clavipalpis|[4281]|LEFIK474-10|Finland||658[0n]|BOLD:AAB6999
Caradrina clavipalpis|[4282]|LEFID260-10|Finland||658[0n]|BOLD:AAB6999
Caradrina clavipalpis|[4283]|FBLMT913-09|Germany|Bavaria|658[0n]|BOLD:AAB6999
Caradrina clavipalpis|[4284]|CGUKD292-09|United Kingdom|England|658[0n]|BOLD:AAB6999
Caradrina clavipalpis|[4285]|CGUKC247-09|United Kingdom|England|658[0n]|BOLD:AAB6999
Caradrina clavipalpis|[4286]|CGUKB111-09|United Kingdom|England|658[0n]|BOLD:AAB6999
Caradrina clavipalpis|[4287]|CGUKB948-09|United Kingdom|Wales|658[0n]|BOLD:AAB6999
Caradrina clavipalpis|[4288]|CGUKA421-09|United Kingdom|England|658[0n]|BOLD:AAB6999
Caradrina clavipalpis|[4289]|RDNMD675-06|Denmark||658[0n]|BOLD:AAB6999
Caradrina clavipalpis|[4290]|CGUKB805-09|United Kingdom|England|638[0n]|BOLD:AAB6999
Caradrina clavipalpis|[4291]|CGUKB305-09|United Kingdom|England|623[0n]|BOLD:AAB6999
Caradrina clavipalpis|[4292]|GWORZ538-10|Germany|Bavaria|623[0n]|BOLD:AAB6999
Caradrina clavipalpis|[4293]|NOCJH322-10|France||611[0n]|BOLD:AAB6999
Caradrina clavipalpis|[4294]|GWOTI301-12|Germany|Saxony|658[0n]|BOLD:AAB6999
Caradrina clavipalpis|[4295]|GWOTL007-13|Germany|Saarland|658[0n]|BOLD:AAB6999
Caradrina multifera|[4296]|TMNBB213-06|Canada|New Brunswick|658[0n]|BOLD:AAA8639
Caradrina multifera|[4297]|TMNBB211-06|Canada|New Brunswick|658[0n]|BOLD:AAA8639
Caradrina multifera|[4298]|TMNBB212-06|Canada|New Brunswick|658[0n]|BOLD:AAA8639

[illegible]
Caradrina multifera|[4296]|TMNBB213-06|Canada|New Brunswick|658[0n]|BOLD:AAA8639
Caradrina multifera|[4297]|TMNBB211-06|Canada|New Brunswick|658[0n]|BOLD:AAA8639
Caradrina multifera|[4298]|TMNBB212-06|Canada|New Brunswick|658[0n]|BOLD:AAA8639
Caradrina multifera|[4299]|RDNMB216-05|Canada|Ontario|588[0n]|BOLD:AAA8639
Caradrina multifera|[4300]|RDLQ492-07|Canada|Quebec|604[1n]|BOLD:AAA8639
Caradrina multifera|[4301]|RDLQ493-07|Canada|Quebec|608[0n]|BOLD:AAA8639
Caradrina montana|[4302]|LALPA505-10|Canada|British Columbia|658[0n]|BOLD:AAA8639
Caradrina montana|[4303]|LOWCB884-05|Canada|British Columbia|658[0n]|BOLD:AAA8639
Caradrina montana|[4304]|LOWCB881-05|Canada|British Columbia|658[0n]|BOLD:AAA8639
Caradrina montana|[4305]|LOWCB891-05|Canada|British Columbia|656[0n]|BOLD:AAA8639
Caradrina montana|[4306]|LOWCB886-05|Canada|British Columbia|588[0n]|BOLD:AAA8639
Caradrina montana|[4307]|LOWCB880-05|Canada|British Columbia|573[0n]|BOLD:AAA8639
Caradrina montana|[4308]|LOWCB878-05|Canada|British Columbia|564[0n]|BOLD:AAA8639
Caradrina montana|[4309]|LOWCB877-05|Canada|British Columbia|566[0n]|BOLD:AAA8639
Caradrina montana|[4310]|LOWCB894-05|Canada|British Columbia|565[0n]|BOLD:AAA8639
Caradrina montana|[4311]|LBCG501-08|Canada|British Columbia|658[0n]|BOLD:AAA8639
Caradrina montana|[4312]|LPVIB763-08|Canada|British Columbia|658[0n]|BOLD:AAA8639
Caradrina montana|[4313]|LBCG2582-09|Canada|British Columbia|658[0n]|BOLD:AAA8639
Caradrina montana|[4314]|LBCH6643-10|Canada|British Columbia|658[0n]|BOLD:AAA8639
Caradrina montana|[4315]|LBCH6644-10|Canada|British Columbia|658[0n]|BOLD:AAA8639
Caradrina montana|[4316]|LBCH7377-10|Canada|British Columbia|658[0n]|BOLD:AAA8639
Caradrina montana|[4317]|LBCH7617-10|Canada|British Columbia|658[0n]|BOLD:AAA8639
Caradrina montana|[4318]|LALPA625-10|Canada|British Columbia|658[0n]|BOLD:AAA8639
Caradrina montana|[4319]|LALPA953-11|Canada|British Columbia|658[0n]|BOLD:AAA8639
Caradrina montana|[4320]|LALPA1239-11|Canada|British Columbia|658[0n]|BOLD:AAA8639
Caradrina sp.|[4321]|LOCBD545-06|United States|California|658[0n]|BOLD:AAA8639
Caradrina sp.|[4322]|LOCBD547-06|United States|California|658[0n]|BOLD:AAA8639
Caradrina sp.|[4323]|LOCBC173-06|United States|California|658[0n]|BOLD:AAA8639
Caradrina sp.|[4324]|LOCBD254-06|United States|California|658[0n]|BOLD:AAA8639
Caradrina sp.|[4325]|LOCBB879-06|United States|California|658[0n]|BOLD:AAA8639
Caradrina sp.|[4326]|LOCBB353-06|United States|California|658[0n]|BOLD:AAA8639
Caradrina sp.|[4327]|LOCBB352-06|United States|California|658[0n]|BOLD:AAA8639
Caradrina sp.|[4328]|LOCBB351-06|United States|California|658[0n]|BOLD:AAA8639
Caradrina sp.|[4329]|LOCBB349-06|United States|California|658[0n]|BOLD:AAA8639
Caradrina sp.|[4330]|LOCBB348-06|United States|California|658[0n]|BOLD:AAA8639
Caradrina sp.|[4331]|LOCB778-06|United States|California|658[0n]|BOLD:AAA8639
Caradrina sp.|[4332]|LOCB490-06|United States|California|658[0n]|BOLD:AAA8639
Caradrina sp.|[4333]|LOCBC394-06|United States|California|658[0n]|BOLD:AAA8639
Caradrina sp.|[4334]|LOCBD549-06|United States|California|656[0n]|BOLD:AAA8639
Caradrina sp.|[4335]|GMLC816-12|United States|California|615[0n]|BOLD:AAA8639
Caradrina montana|[4336]|LCHQ718-08|Canada|Manitoba|657[0n]|BOLD:AAA8639
Caradrina montana|[4337]|LCHQ760-08|Canada|Manitoba|658[0n]|BOLD:AAA8639
Caradrina montana|[4338]|LBCC838-05|Canada|British Columbia|658[0n]|BOLD:AAA8639
Caradrina montana|[4339]|LBCH7797-10|Canada|British Columbia|658[0n]|BOLD:AAA8639
Caradrina montana|[4340]|BBLSW380-09|United States|Arizona|658[0n]|BOLD:AAA8639
Caradrina montana|[4341]|BBLPB427-10|Canada|Alberta|658[0n]|BOLD:AAA8639
Caradrina montana|[4342]|BBLPB439-10|Canada|British Columbia|658[0n]|BOLD:AAA8639
Caradrina montana|[4343]|BBLPB424-10|Canada|Alberta|658[0n]|BOLD:AAA8639
Caradrina montana|[4344]|BBLPB425-10|Canada|Alberta|658[0n]|BOLD:AAA8639
Caradrina montana|[4345]|LBCH7856-10|Canada|British Columbia|658[0n]|BOLD:AAA8639
Caradrina montana|[4346]|LBCH7857-10|Canada|British Columbia|658[0n]|BOLD:AAA8639
Caradrina montana|[4347]|LBCH7858-10|Canada|British Columbia|658[0n]|BOLD:AAA8639
Caradrina montana|[4348]|LBCH7862-10|Canada|British Columbia|658[0n]|BOLD:AAA8639
Caradrina montana|[4349]|LALPA815-10|Canada|British Columbia|658[0n]|BOLD:AAA8639
Caradrina montana|[4350]|BBLPB423-10|Canada|Alberta|658[0n]|BOLD:AAA8639
Caradrina montana|[4351]|LBCH7801-10|Canada|British Columbia|658[0n]|BOLD:AAA8639
Caradrina montana|[4352]|LBCH7855-10|Canada|British Columbia|658[0n]|BOLD:AAA8639
Caradrina montana|[4353]|BBLCU094-09|United States|Kansas|658[0n]|BOLD:AAA8639
Caradrina montana|[4354]|BBLCU380-09|United States|Kansas|658[0n]|BOLD:AAA8639
Caradrina montana|[4355]|BBLWU065-09|United States|Colorado|658[0n]|BOLD:AAA8639
Caradrina montana|[4356]|BBLWU092-09|United States|Colorado|658[0n]|BOLD:AAA8639
Caradrina montana|[4357]|BBLWU106-09|United States|Colorado|658[0n]|BOLD:AAA8639
Caradrina montana|[4358]|BBLCU092-09|United States|Kansas|658[0n]|BOLD:AAA8639
Caradrina montana|[4359]|BBLSW422-09|United States|New Mexico|658[0n]|BOLD:AAA8639
Caradrina montana|[4360]|BBLWU046-09|United States|Colorado|658[0n]|BOLD:AAA8639
Caradrina montana|[4361]|BBLWU058-09|United States|Colorado|658[0n]|BOLD:AAA8639
Caradrina montana|[4362]|BBLWU063-09|United States|Colorado|658[0n]|BOLD:AAA8639
Caradrina montana|[4363]|LBCH6128-10|Canada|British Columbia|658[0n]|BOLD:AAA8639
Caradrina montana|[4364]|LBCH6459-10|Canada|British Columbia|658[0n]|BOLD:AAA8639
Caradrina montana|[4365]|LBCH6546-10|Canada|British Columbia|658[0n]|BOLD:AAA8639
Caradrina montana|[4366]|LBCH6645-10|Canada|British Columbia|658[0n]|BOLD:AAA8639
Caradrina montana|[4367]|LBCH6646-10|Canada|British Columbia|658[0n]|BOLD:AAA8639
Caradrina montana|[4368]|LBCH6648-10|Canada|British Columbia|658[0n]|BOLD:AAA8639
Caradrina montana|[4369]|LBCH6983-10|Canada|British Columbia|658[0n]|BOLD:AAA8639
Caradrina montana|[4370]|LBCH7796-10|Canada|British Columbia|658[0n]|BOLD:AAA8639
Caradrina montana|[4371]|LBCH5431-10|Canada|British Columbia|658[0n]|BOLD:AAA8639
Caradrina montana|[4372]|LBCH6001-10|Canada|British Columbia|658[0n]|BOLD:AAA8639
Caradrina montana|[4373]|LBCG2107-09|Canada|British Columbia|658[0n]|BOLD:AAA8639
Caradrina montana|[4374]|BBLSW366-09|United States|Arizona|658[0n]|BOLD:AAA8639
Caradrina montana|[4375]|LPSK155-08|Canada|Saskatchewan|658[0n]|BOLD:AAA8639
Caradrina montana|[4376]|LPSK386-08|Canada|Saskatchewan|658[0n]|BOLD:AAA8639
Caradrina montana|[4377]|LPSK152-08|Canada|Saskatchewan|658[0n]|BOLD:AAA8639
Caradrina montana|[4378]|LPSK154-08|Canada|Saskatchewan|658[0n]|BOLD:AAA8639
Caradrina montana|[4379]|LOWCE847-06|Canada|British Columbia|658[0n]|BOLD:AAA8639
Caradrina montana|[4380]|LPSK147-08|Canada|Saskatchewan|658[0n]|BOLD:AAA8639
Caradrina montana|[4381]|RDMAB136-05|Canada|Alberta|658[0n]|BOLD:AAA8639
Caradrina montana|[4382]|LOWCB896-05|Canada|British Columbia|658[0n]|BOLD:AAA8639
Caradrina montana|[4383]|LOWCB895-05|Canada|British Columbia|658[0n]|BOLD:AAA8639
Caradrina montana|[4384]|LOWCB892-05|Canada|British Columbia|658[0n]|BOLD:AAA8639
Caradrina montana|[4385]|LOWCB889-05|Canada|British Columbia|658[0n]|BOLD:AAA8639
Caradrina montana|[4386]|LOWCB888-05|Canada|British Columbia|658[0n]|BOLD:AAA8639
Caradrina montana|[4387]|LOWCB885-05|Canada|British Columbia|658[0n]|BOLD:AAA8639
Caradrina montana|[4388]|LOWCB883-05|Canada|British Columbia|658[0n]|BOLD:AAA8639
Caradrina montana|[4389]|LOWCB879-05|Canada|British Columbia|658[0n]|BOLD:AAA8639
Caradrina montana|[4390]|LPSK571-08|Canada|Saskatchewan|658[0n]|BOLD:AAA8639
Caradrina montana|[4391]|LOWCB875-05|Canada|British Columbia|658[0n]|BOLD:AAA8639
Caradrina montana|[4392]|LCHQ313-08|Canada|Manitoba|656[0n]|BOLD:AAA8639
Caradrina montana|[4393]|LPSK123-08|Canada|Saskatchewan|658[0n]|BOLD:AAA8639
Caradrina montana|[4394]|LBCH7860-10|Canada|British Columbia|642[0n]|BOLD:AAA8639
Caradrina montana|[4395]|RDMAB040-05|Canada|Alberta|631[0n]|BOLD:AAA8639
Caradrina montana|[4396]|LOWCB897-05|Canada|British Columbia|589[0n]|BOLD:AAA8639
Caradrina montana|[4397]|LOWCB882-05|Canada|British Columbia|595[0n]|BOLD:AAA8639
Caradrina montana|[4398]|LOWCB887-05|Canada|British Columbia|501[0n]|

[illegible]
Caradrina montana|[4396]|LOWCB897-05|Canada|British Columbia|589[0n]|BOLD:AAA8639
Caradrina montana|[4397]|LOWCB882-05|Canada|British Columbia|595[0n]|BOLD:AAA8639
Caradrina montana|[4398]|LOWCB887-05|Canada|British Columbia|501[0n]|
Caradrina montana|[4399]|LOWCB890-05|Canada|British Columbia|563[0n]|BOLD:AAA8639
Caradrina montana|[4400]|LOWCB876-05|Canada|British Columbia|582[0n]|BOLD:AAA8639
Caradrina montana|[4401]|LOWCB893-05|Canada|British Columbia|566[0n]|BOLD:AAA8639
Caradrina montana|[4402]|LOWCB898-05|Canada|British Columbia|503[0n]|BOLD:AAA8639
Caradrina montana|[4403]|RDNMI196-12|Canada|Ontario|614[0n]|BOLD:AAA8639
Protoperigea n. sp.|[4404]|RDNME893-08|United States|Arizona|658[0n]|BOLD:AAX8569
Protoperigea calientensis|[4405]|CNCLB1142-14|United States|California|307[0n]|
Protoperigea calientensis|[4406]|TML167-14|United States||621[0n]|BOLD:ACO6109
Protoperigea subterminata|[4407]|RDNME892-08|United States|Arizona|658[0n]|BOLD:AAX8570
Protoperigea anotha|[4408]|RDNMF602-08|Canada|Alberta|658[0n]|BOLD:AAF6358
Protoperigea anotha|[4409]|RDNMF601-08|Canada|Alberta|658[0n]|BOLD:AAF6358
Protoperigea anotha|[4410]|RDNMF603-08|Canada|Alberta|652[0n]|BOLD:AAF6358
Protoperigea anotha|[4411]|BBLPB421-10|Canada|Alberta|658[0n]|BOLD:AAF6358
Protoperigea umbricata|[4412]|RDNMD825-07|United States|California|658[0n]|BOLD:AAX8571
Protoperigea umbricata|[4413]|GMLC1156-12|United States|California|658[0n]|BOLD:AAX8571
Protoperigea umbricata|[4414]|GMLC1219-12|United States|California|658[0n]|BOLD:AAX8571
Protoperigea posticata|[4415]|RDNMC583-06|Canada|Alberta|658[0n]|BOLD:AAF6359
Protoperigea posticata|[4416]|RDNMJ848-11|United States|New Mexico|658[0n]|BOLD:AAF6359
Protoperigea posticata|[4417]|RDMAB591-06|Canada|Alberta|615[0n]|BOLD:AAF6359
Protoperigea posticata|[4418]|RDNMC582-06|United States|Oregon|658[0n]|BOLD:AAF6359
Protoperigea posticata|[4419]|RDNMJ379-11|United States|New Mexico|658[0n]|BOLD:AAF6359
Protoperigea posticata|[4420]|RDNMJ849-11|United States|New Mexico|658[0n]|BOLD:AAF6359
Protoperigea posticata|[4421]|CMAZA1043-12|United States|Arizona|658[0n]|BOLD:AAF6359
Protoperigea parvulata|[4422]|CNCLB923-14|United States|California|658[0n]|BOLD:AAF6359
Protoperigea parvulata|[4423]|CNCLB924-14|United States|California|658[0n]|BOLD:AAF6359
Protoperigea parvulata|[4424]|CNCLB925-14|United States|California|658[0n]|BOLD:AAF6359
Caradrina meralis|[4425]|LTOL930-08|United States|California|655[0n]|BOLD:AAB9848
Caradrina mona|[4426]|RDNMK110-11|United States|California|658[0n]|BOLD:AAB9848
Caradrina mona|[4427]|RDNMK113-11|United States|California|658[0n]|BOLD:AAB9848
Caradrina meralis|[4428]|LBCG2874-09|Canada|British Columbia|658[0n]|BOLD:AAB9848
Caradrina meralis|[4429]|LBCH6174-10|Canada|British Columbia|658[0n]|BOLD:AAB9848
Caradrina meralis|[4430]|LBCH6292-10|Canada|British Columbia|658[0n]|BOLD:AAB9848
Caradrina meralis|[4431]|LBCH6297-10|Canada|British Columbia|658[0n]|BOLD:AAB9848
Caradrina meralis|[4432]|LBCH6650-10|Canada|British Columbia|658[0n]|BOLD:AAB9848
Caradrina meralis|[4433]|LBCH7190-10|Canada|British Columbia|658[0n]|BOLD:AAB9848
Caradrina meralis|[4434]|LBCH7473-10|Canada|British Columbia|658[0n]|BOLD:AAB9848
Caradrina meralis|[4435]|LBCH7501-10|Canada|British Columbia|658[0n]|BOLD:AAB9848
Caradrina meralis|[4436]|LBCH6169-10|Canada|British Columbia|658[0n]|BOLD:AAB9848
Caradrina meralis|[4437]|LBCH6307-10|Canada|British Columbia|658[0n]|BOLD:AAB9848
Caradrina meralis|[4438]|LBCG498-08|Canada|British Columbia|658[0n]|BOLD:AAB9848
Caradrina meralis|[4439]|LOWCD860-06|Canada|British Columbia|658[0n]|BOLD:AAB9848
Caradrina meralis|[4440]|LBCG249-08|Canada|British Columbia|658[0n]|BOLD:AAB9848
Caradrina meralis|[4441]|LBCG2115-09|Canada|British Columbia|658[0n]|BOLD:AAB9848
Caradrina meralis|[4442]|LBCG2866-09|Canada|British Columbia|658[0n]|BOLD:AAB9848
Caradrina meralis|[4443]|LBCH6304-10|Canada|British Columbia|658[0n]|BOLD:AAB9848
Caradrina meralis|[4444]|LBCH7398-10|Canada|British Columbia|658[0n]|BOLD:AAB9848
Caradrina meralis|[4445]|LBCH7401-10|Canada|British Columbia|658[0n]|BOLD:AAB9848
Caradrina meralis|[4446]|LBCH7798-10|Canada|British Columbia|658[0n]|BOLD:AAB9848
Caradrina meralis|[4447]|LBCH7802-10|Canada|British Columbia|658[0n]|BOLD:AAB9848
Caradrina meralis|[4448]|LBCH7859-10|Canada|British Columbia|658[0n]|BOLD:AAB9848
Caradrina meralis|[4449]|LBCH7105-10|Canada|British Columbia|658[0n]|BOLD:AAB9848
Caradrina meralis|[4450]|LBCH7096-10|Canada|British Columbia|658[0n]|BOLD:AAB9848
Caradrina meralis|[4451]|LBCH6649-10|Canada|British Columbia|658[0n]|BOLD:AAB9848
Caradrina meralis|[4452]|LBCH6310-10|Canada|British Columbia|658[0n]|BOLD:AAB9848
Caradrina meralis|[4453]|LBCH6308-10|Canada|British Columbia|658[0n]|BOLD:AAB9848
Caradrina meralis|[4454]|LBCH6305-10|Canada|British Columbia|658[0n]|BOLD:AAB9848
Caradrina meralis|[4455]|LBCH6303-10|Canada|British Columbia|658[0n]|BOLD:AAB9848
Caradrina meralis|[4456]|LBCH6302-10|Canada|British Columbia|658[0n]|BOLD:AAB9848
Caradrina meralis|[4457]|LBCH6300-10|Canada|British Columbia|658[0n]|BOLD:AAB9848
Caradrina meralis|[4458]|LBCH6240-10|Canada|British Columbia|658[0n]|BOLD:AAB9848
Caradrina meralis|[4459]|LBCH6235-10|Canada|British Columbia|658[0n]|BOLD:AAB9848
Caradrina meralis|[4460]|LBCH6175-10|Canada|British Columbia|658[0n]|BOLD:AAB9848
Caradrina meralis|[4461]|LBCH6173-10|Canada|British Columbia|658[0n]|BOLD:AAB9848
Caradrina meralis|[4462]|LBCH6172-10|Canada|British Columbia|658[0n]|BOLD:AAB9848
Caradrina meralis|[4463]|LBCH6171-10|Canada|British Columbia|658[0n]|BOLD:AAB9848
Caradrina meralis|[4464]|LBCH6170-10|Canada|British Columbia|658[0n]|BOLD:AAB9848
Caradrina meralis|[4465]|LBCH6168-10|Canada|British Columbia|658[0n]|BOLD:AAB9848
Caradrina meralis|[4466]|LBCH6127-10|Canada|British Columbia|658[0n]|BOLD:AAB9848
Caradrina meralis|[4467]|LBCH6126-10|Canada|British Columbia|658[0n]|BOLD:AAB9848
Caradrina meralis|[4468]|LBCG2864-09|Canada|British Columbia|658[0n]|BOLD:AAB9848
Caradrina meralis|[4469]|LBCG2114-09|Canada|British Columbia|658[0n]|BOLD:AAB9848
Caradrina meralis|[4470]|LPVIB270-08|Canada|British Columbia|658[0n]|BOLD:AAB9848
Caradrina meralis|[4471]|LBCG504-08|Canada|British Columbia|658[0n]|BOLD:AAB9848
Caradrina meralis|[4472]|LBCG492-08|Canada|British Columbia|658[0n]|BOLD:AAB9848
Caradrina meralis|[4473]|LOWCD861-06|Canada|British Columbia|658[0n]|BOLD:AAB9848
Caradrina meralis|[4474]|LBCH6301-10|Canada|British Columbia|658[0n]|BOLD:AAB9848
Caradrina meralis|[4475]|LBCG2113-09|Canada|British Columbia|658[0n]|BOLD:AAB9848
Caradrina meralis|[4476]|LPVIB671-08|Canada|British Columbia|633[0n]|BOLD:AAB9848
Caradrina meralis|[4477]|LOWCD863-06|Canada|British Columbia|658[0n]|BOLD:AAB9848
Caradrina meralis|[4478]|LPVIB269-08|Canada|British Columbia|646[0n]|BOLD:AAB9848
Caradrina meralis|[4479]|LOWCD862-06|Canada|British Columbia|596[0n]|BOLD:AAB9848
Caradrina meralis|[4480]|LBCH7124-10|Canada|British Columbia|636[0n]|BOLD:AAB9848
Caradrina meralis|[4481]|LBCH7219-10|Canada|British Columbia|658[0n]|BOLD:AAB9848
Caradrina meralis|[4482]|LBCH7339-10|Canada|British Columbia|658[0n]|BOLD:AAB9848
Caradrina meralis|[4483]|LBCH7396-10|Canada|British Columbia|658[0n]|BOLD:AAB9848
Caradrina meralis|[4484]|LBCH7399-10|Canada|British Columbia|658[0n]|BOLD:AAB9848
Caradrina meralis|[4485]|LBCH7400-10|Canada|British Columbia|658[0n]|BOLD:AAB9848
Caradrina meralis|[4486]|LBCH7402-10|Canada|British Columbia|658[0n]|BOLD:AAB9848
Caradrina meralis|[4487]|LBCH7403-10|Canada|British Columbia|658[0n]|BOLD:AAB9848
Caradrina meralis|[4488]|LBCH7404-10|Canada|British Columbia|658[0n]|BOLD:AAB9848
Caradrina meralis|[4489]|LBCH7405-10|Canada|British Columbia|658[0n]|BOLD:AAB9848
Caradrina meralis|[4490]|LBCH7432-10|Canada|British Columbia|658[0n]|BOLD:AAB9848
Caradrina meralis|[4491]|LBCH7795-10|Canada|British Columbia|658[0n]|BOLD:AAB9848
Caradrina meralis|[4492]|LBCH7799-10|Canada|British Columbia|658[0n]|BOLD:AAB9848
Caradrina meralis|[4493]|LBCH7800-10|Canada|British Columbia|658[0n]|BOLD:AAB9848
Caradrina meralis|[4494]|LBCH7821-10|Canada|British Columbia|658[0n]|BOLD:AAB9848
Caradrina meralis|[4495]|LBCH7861-10|Canada|British Columbia|658[0n]|BOLD:AAB9848
Caradrina meralis|[4496]|RDNMG826-08|United States|Oregon|658[0n]|BOLD:AAB9848
Caradrina meralis|[4497]|JMMMB152-11|United States|California|658[0n]|BOLD:AAB9848
Caradrina sp.|[4498]|LNAUS4439-13|United States|California|658[0n]|BOLD:AAB9848

Caradrina meralis|[4495]|[illegible]|Canada|British Columbia|658[0n]|BOLD:AAB9848
Caradrina meralis|[4496]|RDNMG826-08|United States|Oregon|658[0n]|BOLD:AAB9848
Caradrina meralis|[4497]|JMMMB152-11|United States|California|658[0n]|BOLD:AAB9848
Caradrina sp.|[4498]|LNAUS4439-13|United States|California|658[0n]|BOLD:AAB9848
Caradrina sp.|[4499]|LNAUS4440-13|United States|California|658[0n]|BOLD:AAB9848
Caradrina sp.|[4500]|LNAUS4441-13|United States|California|658[0n]|BOLD:AAB9848
Caradrina sp.|[4501]|LNAUS4442-13|United States|Colorado|658[0n]|BOLD:AAB9848
Caradrina sp.|[4502]|RDNMK112-11|United States|Colorado|658[0n]|BOLD:AAY8710
Caradrina sp.|[4503]|LNAUS4443-13|United States|Texas|658[0n]|BOLD:AAY8710
Caradrina distinctoides|[4504]|RDNMC687-06|United States|California|658[0n]|BOLD:ACF3775
Caradrina distinctoides|[4505]|RDNMC688-06|United States|California|658[0n]|BOLD:ACF3775
Caradrina meralis|[4506]|RDNMK111-11|United States|Utah|658[0n]|BOLD:ACM2685
Caradrina sp.|[4507]|RDNMJ360-11|United States|New Mexico|658[0n]|BOLD:AAF7866
Caradrina beta|[4508]|RDNMC708-06|United States|Arizona|617[2n]|BOLD:AAF7866
Caradrina beta|[4509]|CMAZA098-09|United States|Arizona|658[0n]|BOLD:AAF7866
Caradrina beta|[4510]|CMAZA114-09|United States|Arizona|658[0n]|BOLD:AAF7866
Caradrina beta|[4511]|RDNMD854-07|United States|Arizona|655[0n]|BOLD:AAF7866
Caradrina beta|[4512]|RDNMD855-07|United States|Arizona|655[0n]|BOLD:AAF7866
Caradrina beta|[4513]|CMAZA162-09|United States|Arizona|658[0n]|BOLD:AAF7866
Caradrina beta|[4514]|CMAZA165-09|United States|Arizona|658[0n]|BOLD:AAF7866
Caradrina beta|[4515]|CMAZA247-09|United States|Arizona|658[0n]|BOLD:AAF7866
Caradrina beta|[4516]|CMAZA996-12|United States|Arizona|658[0n]|BOLD:AAF7866
Caradrina camina|[4517]|NAMUM238-08|United States|California|658[0n]|BOLD:ACE8449
Caradrina camina|[4518]|RDNMK074-11|United States|Colorado|658[0n]|BOLD:ACE8449
Caradrina camina|[4519]|NGNAQ1468-14|Canada|British Columbia|577[0n]|BOLD:ABY7042
Caradrina camina|[4520]|NGNAQ1461-14|Canada|British Columbia|577[0n]|BOLD:ABY7042
Caradrina camina|[4521]|RDNMC707-06|Canada|British Columbia|591[0n]|BOLD:ABY7042
Caradrina camina|[4522]|LBCH7908-10|Canada|British Columbia|658[0n]|BOLD:ABY7042
Caradrina camina|[4523]|LBCH7906-10|Canada|British Columbia|658[0n]|BOLD:ABY7042
Caradrina camina|[4524]|LBCH7909-10|Canada|British Columbia|658[0n]|BOLD:ABY7042
Caradrina camina|[4525]|LBCH7905-10|Canada|British Columbia|658[0n]|BOLD:ABY7042
Caradrina camina|[4526]|LBCH7904-10|Canada|British Columbia|658[0n]|BOLD:ABY7042
Caradrina camina|[4527]|LBCH7903-10|Canada|British Columbia|658[0n]|BOLD:ABY7042
Caradrina camina|[4528]|LBCH7902-10|Canada|British Columbia|658[0n]|BOLD:ABY7042
Caradrina camina|[4529]|LBCH7822-10|Canada|British Columbia|658[0n]|BOLD:ABY7042
Caradrina camina|[4530]|LBCH7561-10|Canada|British Columbia|658[0n]|BOLD:ABY7042
Caradrina camina|[4531]|LBCH7065-10|Canada|British Columbia|658[0n]|BOLD:ABY7042
Caradrina camina|[4532]|LBCH6926-10|Canada|British Columbia|658[0n]|BOLD:ABY7042
Caradrina camina|[4533]|LBCH6533-10|Canada|British Columbia|658[0n]|BOLD:ABY7042
Caradrina camina|[4534]|LBCH7907-10|Canada|British Columbia|658[0n]|BOLD:ABY7042
Caradrina camina|[4535]|LBCH7973-10|Canada|British Columbia|658[0n]|BOLD:ABY7042
Caradrina camina|[4536]|RDNMG827-08|United States|Washington|658[0n]|BOLD:ABY7042
Caradrina camina|[4537]|CNCLB1581-14|United States|Idaho|658[0n]|BOLD:ABY7042
Caradrina morpheus|[4538]|PHLAF249-11|Macedonia||658[0n]|BOLD:AAA6694
Caradrina morpheus|[4539]|LEATC636-13|Italy|South Tyrol|658[0n]|BOLD:AAA6694
Caradrina morpheus|[4540]|ABOLA083-14|Austria|Carinthia|658[0n]|BOLD:AAA6694
Caradrina morpheus|[4541]|RDNMD665-06|Denmark||658[0n]|BOLD:AAA6694
Caradrina morpheus|[4542]|CGUKA918-09|United Kingdom|England|658[0n]|BOLD:AAA6694
Caradrina morpheus|[4543]|CGUKB112-09|United Kingdom|England|658[0n]|BOLD:AAA6694
Caradrina morpheus|[4544]|CGUKB733-09|United Kingdom|England|658[0n]|BOLD:AAA6694
Caradrina morpheus|[4545]|CGUKB932-09|United Kingdom|Wales|658[0n]|BOLD:AAA6694
Caradrina morpheus|[4546]|LBCG1905-09|Canada|British Columbia|658[0n]|BOLD:AAA6694
Caradrina morpheus|[4547]|GWORL444-09|Germany|Bavaria|658[0n]|BOLD:AAA6694
Caradrina morpheus|[4548]|RWWA195-09|United States|Washington|656[0n]|BOLD:AAA6694
Caradrina morpheus|[4549]|RWWA212-09|United States|Washington|658[0n]|BOLD:AAA6694
Caradrina morpheus|[4550]|RWWA225-09|United States|Washington|658[0n]|BOLD:AAA6694
Caradrina morpheus|[4551]|RWWA271-09|United States|Washington|658[0n]|BOLD:AAA6694
Caradrina morpheus|[4552]|RWWA300-09|United States|Washington|658[0n]|BOLD:AAA6694
Caradrina morpheus|[4553]|RWWA329-09|United States|Washington|658[0n]|BOLD:AAA6694
Caradrina morpheus|[4554]|RWWA338-09|United States|Washington|658[0n]|BOLD:AAA6694
Caradrina morpheus|[4555]|RWWA405-09|United States|Washington|658[0n]|BOLD:AAA6694
Caradrina morpheus|[4556]|RWWA433-09|United States|Washington|658[0n]|BOLD:AAA6694
Caradrina morpheus|[4557]|RWWA439-09|United States|Washington|658[0n]|BOLD:AAA6694
Caradrina morpheus|[4558]|RWWA521-09|United States|Washington|658[0n]|BOLD:AAA6694
Caradrina morpheus|[4559]|RWWA594-09|United States|Washington|658[0n]|BOLD:AAA6694
Caradrina morpheus|[4560]|CGUKD1003-09|United Kingdom|England|658[0n]|BOLD:AAA6694
Caradrina morpheus|[4561]|FBLMV126-09|Germany|Bavaria|658[0n]|BOLD:AAA6694
Caradrina morpheus|[4562]|BBLPC623-09|Canada|Newfoundland and Labrador|658[0n]|BOLD:AAA6694
Caradrina morpheus|[4563]|BBLPE416-09|Canada|Newfoundland and Labrador|658[0n]|BOLD:AAA6694
Caradrina morpheus|[4564]|LEFIA556-10|Finland|South Karelia|658[0n]|BOLD:AAA6694
Caradrina morpheus|[4565]|LBCH2252-10|Canada|British Columbia|658[0n]|BOLD:AAA6694
Caradrina morpheus|[4566]|LBCH5614-10|Canada|British Columbia|658[0n]|BOLD:AAA6694
Caradrina morpheus|[4567]|LEFID577-10|Finland||658[0n]|BOLD:AAA6694
Caradrina morpheus|[4568]|RWWB711-10|United States|Washington|658[0n]|BOLD:AAA6694
Caradrina morpheus|[4569]|NOCJH323-10|France||658[0n]|BOLD:AAA6694
Caradrina morpheus|[4570]|NOCJH324-10|France||658[0n]|BOLD:AAA6694
Caradrina morpheus|[4571]|NOCJH325-10|France||658[0n]|BOLD:AAA6694
Caradrina morpheus|[4572]|NOCJH326-10|France||658[0n]|BOLD:AAA6694
Caradrina morpheus|[4573]|LALPA234-10|Canada|British Columbia|658[0n]|BOLD:AAA6694
Caradrina morpheus|[4574]|LALPA359-10|Canada|British Columbia|658[0n]|BOLD:AAA6694
Caradrina morpheus|[4575]|LALPA393-10|Canada|British Columbia|658[0n]|BOLD:AAA6694
Caradrina morpheus|[4576]|LALPA420-10|Canada|British Columbia|658[0n]|BOLD:AAA6694
Caradrina morpheus|[4577]|BBLPB389-10|Canada|British Columbia|658[0n]|BOLD:AAA6694
Caradrina morpheus|[4578]|BBLPB392-10|Canada|British Columbia|658[0n]|BOLD:AAA6694
Caradrina morpheus|[4579]|BBLPB393-10|Canada|British Columbia|658[0n]|BOLD:AAA6694
Caradrina morpheus|[4580]|LPVIB841-08|Canada|British Columbia|658[0n]|BOLD:AAA6694
Caradrina morpheus|[4581]|CGUKA071-09|United Kingdom|England|658[0n]|BOLD:AAA6694
Caradrina morpheus|[4582]|CGUKA722-09|United Kingdom||658[0n]|BOLD:AAA6694
Caradrina morpheus|[4583]|CGUKA917-09|United Kingdom|England|658[0n]|BOLD:AAA6694
Caradrina morpheus|[4584]|LHLEP253-06|Canada|British Columbia|658[0n]|BOLD:AAA6694
Caradrina morpheus|[4585]|LHLEP254-06|Canada|British Columbia|658[0n]|BOLD:AAA6694
Caradrina morpheus|[4586]|LHLEP255-06|Canada|British Columbia|658[0n]|BOLD:AAA6694
Caradrina morpheus|[4587]|LBCS485-07|Canada|British Columbia|658[0n]|BOLD:AAA6694
Caradrina morpheus|[4588]|LBCG008-08|Canada|British Columbia|658[0n]|BOLD:AAA6694
Caradrina morpheus|[4589]|LPVIA409-08|Canada|British Columbia|658[0n]|BOLD:AAA6694
Caradrina morpheus|[4590]|LPVIA613-08|Canada|British Columbia|658[0n]|BOLD:AAA6694
Caradrina morpheus|[4591]|LPVIB007-08|Canada|British Columbia|658[0n]|BOLD:AAA6694
Caradrina morpheus|[4592]|IBLAO258-11|Spain|Castilla-La Mancha|658[0n]|BOLD:AAA6694
Caradrina morpheus|[4593]|LENOA616-11|France|Haute Normandie|658[0n]|BOLD:AAA6694
Caradrina morpheus|[4594]|RWWC339-11|United States|Washington|658[0n]|BOLD:AAA6694
Caradrina morpheus|[4595]|RWWC355-11|United States|Washington|658[0n]|BOLD:AAA6694
Caradrina morpheus|[4596]|NLLEA214-12|Netherlands|South Holland|658[0n]|BOLD:AAA6694
Caradrina morpheus|[4597]|NLLEA526-12|Netherlands|South Holland|658[0n]|BOLD:AAA6694
Caradrina morpheus|[4598]|CWOTC934-12|Russia|Irkutsk|658[0n]|BOLD:AAA6694

Caradrina morpheus|[4595]|RWWC353-11|United States|Washington|658[0n]|BOLD:AAA6694
Caradrina morpheus|[4596]|NLLEA214-12|Netherlands|South Holland|658[0n]|BOLD:AAA6694
Caradrina morpheus|[4597]|NLLEA526-12|Netherlands|South Holland|658[0n]|BOLD:AAA6694
Caradrina morpheus|[4598]|GWOTG934-12|Russia|Irkutsk|658[0n]|BOLD:AAA6694
Caradrina morpheus|[4599]|IBLAO1041-14|Spain||658[0n]|BOLD:AAA6694
Caradrina morpheus|[4600]|BBLPB747-10|Canada|British Columbia|658[0n]|BOLD:AAA6694
Caradrina morpheus|[4601]|BBLPB748-10|Canada|British Columbia|658[0n]|BOLD:AAA6694
Caradrina morpheus|[4602]|BBLPB390-10|Canada|British Columbia|658[0n]|BOLD:AAA6694
Caradrina morpheus|[4603]|BBLPB391-10|Canada|British Columbia|658[0n]|BOLD:AAA6694
Caradrina morpheus|[4604]|LHLEP251-06|Canada|British Columbia|658[0n]|BOLD:AAA6694
Caradrina morpheus|[4605]|LHLEP252-06|Canada|British Columbia|658[0n]|BOLD:AAA6694
Caradrina morpheus|[4606]|LHLEP078-06|Canada|British Columbia|658[0n]|BOLD:AAA6694
Caradrina morpheus|[4607]|LHLEP250-06|Canada|British Columbia|658[0n]|BOLD:AAA6694
Caradrina morpheus|[4608]|GBLAD714-14|Germany|Bavaria|658[0n]|BOLD:AAA6694
Caradrina morpheus|[4609]|LHLEP077-06|Canada|British Columbia|658[0n]|BOLD:AAA6694
Caradrina morpheus|[4610]|LHLEP076-06|Canada|British Columbia|658[0n]|BOLD:AAA6694
Caradrina morpheus|[4611]|RDNMD664-06|Denmark||658[0n]|BOLD:AAA6694
Caradrina morpheus|[4612]|RDNMD663-06|Denmark||658[0n]|BOLD:AAA6694
Caradrina morpheus|[4613]|LOWCB904-05|Canada|British Columbia|658[0n]|BOLD:AAA6694
Caradrina morpheus|[4614]|LOWCB903-05|Canada|British Columbia|658[0n]|BOLD:AAA6694
Caradrina morpheus|[4615]|LOWCB901-05|Canada|British Columbia|658[0n]|BOLD:AAA6694
Caradrina morpheus|[4616]|LBCA083-05|Canada|British Columbia|658[0n]|BOLD:AAA6694
Caradrina morpheus|[4617]|PHMNB180-04|Canada|New Brunswick|658[0n]|BOLD:AAA6694
Caradrina morpheus|[4618]|RWWC1363-14|United States|Washington|591[0n]|BOLD:AAA6694
Caradrina morpheus|[4619]|LPVIB949-08|Canada|British Columbia|647[0n]|BOLD:AAA6694
Caradrina morpheus|[4620]|LEFIA557-10|Finland|South Karelia|641[0n]|BOLD:AAA6694
Caradrina morpheus|[4621]|LPVIB915-08|Canada|British Columbia|634[0n]|BOLD:AAA6694
Caradrina morpheus|[4622]|LPVIB880-08|Canada|British Columbia|634[0n]|BOLD:AAA6694
Caradrina morpheus|[4623]|LPVIB873-08|Canada|British Columbia|634[0n]|BOLD:AAA6694
Caradrina morpheus|[4624]|LOWCB900-05|Canada|British Columbia|606[0n]|BOLD:AAA6694
Caradrina morpheus|[4625]|RDMAB015-05|Canada|Alberta|622[0n]|BOLD:AAA6694
Caradrina morpheus|[4626]|RDMAB014-05|Canada|Alberta|567[0n]|BOLD:AAA6694
Caradrina morpheus|[4627]|RDLQ495-07|Canada|Quebec|601[2n]|BOLD:AAA6694
Caradrina morpheus|[4628]|LOWCB902-05|Canada|British Columbia|587[0n]|BOLD:AAA6694
Caradrina morpheus|[4629]|LOWCB905-05|Canada|British Columbia|580[0n]|BOLD:AAA6694
Caradrina morpheus|[4630]|RDLQ496-07|Canada|Quebec|607[0n]|BOLD:AAA6694
Caradrina morpheus|[4631]|RWWC437-11|United States|Washington|631[0n]|BOLD:AAA6694
Caradrina morpheus|[4632]|RWWC1364-14|United States|Washington|603[0n]|BOLD:AAA6694
Homorthodes NEW|[4633]|CMAZA294-09|United States|Arizona|658[0n]|BOLD:AAH5684
Homorthodes sp.|[4634]|RDNMH782-09|United States|Texas|658[0n]|BOLD:AAB4380
Homorthodes sp.|[4635]|RDNMH777-09|United States|Texas|658[0n]|BOLD:AAB4380
Homorthodes sp.|[4636]|RDNMH778-09|United States|Texas|658[0n]|BOLD:AAB4380
Homorthodes sp.|[4637]|RDNMH779-09|United States|Texas|658[0n]|BOLD:AAB4380
Homorthodes sp.|[4638]|RDNMJ142-10|United States|Texas|658[0n]|BOLD:AAB4380
Homorthodes perturba|[4639]|RDNMJ778-11|United States|Arizona|658[0n]|BOLD:AAT9246
Mythimna sequax|[4640]|RDNMH141-09|United States|Florida|658[0n]|BOLD:AAB1969
Mythimna sequax|[4641]|GWOSU300-11|Ecuador|Pichincha|658[0n]|BOLD:AAB1969
Mythimna sequax|[4642]|ARMOT127-12|Argentina|Formosa|658[0n]|BOLD:AAB1969
Mythimna unipuncta|[4643]|CNPPA3920-12|Canada|Ontario|589[0n]|BOLD:AAA2482
Mythimna unipuncta|[4644]|SMTPB22047-13|Canada|Ontario|549[0n]|BOLD:AAA2482
Mythimna unipuncta|[4645]|SMTPB3165-13|Canada|Ontario|549[0n]|BOLD:AAA2482
Mythimna unipuncta|[4646]|SMTPB13112-13|Canada|Ontario|537[0n]|BOLD:AAA2482
Mythimna unipuncta|[4647]|SMTPB9117-13|Canada|Ontario|537[0n]|BOLD:AAA2482
Mythimna unipuncta|[4648]|SSPAG300-13|Canada|Saskatchewan|559[0n]|BOLD:AAA2482
Mythimna unipuncta|[4649]|LOCBB865-06|United States|California|658[2n]|BOLD:AAA2482
Mythimna unipuncta|[4650]|LPSOD448-09|Canada|Ontario|658[0n]|BOLD:AAA2482
Mythimna unipuncta|[4651]|GMLC1100-12|United States|California|658[0n]|BOLD:AAA2482
Mythimna unipuncta|[4652]|SMTPB12940-13|Canada|Ontario|532[0n]|
Mythimna unipuncta|[4653]|SMTPB9028-13|Canada|Ontario|532[0n]|
Mythimna unipuncta|[4654]|SMTPB5749-13|Canada|Ontario|561[1n]|BOLD:AAA2482
Mythimna unipuncta|[4655]|SMTPB1145-13|Canada|Ontario|531[0n]|BOLD:AAA2482
Mythimna unipuncta|[4656]|LOCBB745-06|United States|California|617[0n]|BOLD:AAA2482
Mythimna unipuncta|[4657]|LPSOB146-08|Canada|Ontario|658[0n]|BOLD:AAA2482
Mythimna unipuncta|[4658]|LPSOD424-09|Canada|Ontario|658[0n]|BOLD:AAA2482
Mythimna unipuncta|[4659]|SMTPB19513-13|Canada|Ontario|576[0n]|BOLD:AAA2482
Mythimna unipuncta|[4660]|SMTPB3163-13|Canada|Ontario|552[0n]|BOLD:AAA2482
Mythimna unipuncta|[4661]|SMTPB19945-13|Canada|Ontario|591[0n]|BOLD:AAA2482
Mythimna unipuncta|[4662]|SMTPB16092-13|Canada|Ontario|591[0n]|BOLD:AAA2482
Mythimna unipuncta|[4663]|SMTPB16090-13|Canada|Ontario|591[0n]|BOLD:AAA2482
Mythimna unipuncta|[4664]|RWWB192-09|United States|Washington|658[0n]|BOLD:AAA2482
Mythimna unipuncta|[4665]|AWCLB590-11|United States|Arizona|658[0n]|BOLD:AAA2482
Mythimna unipuncta|[4666]|SMTPB19946-13|Canada|Ontario|582[0n]|BOLD:AAA2482
Mythimna unipuncta|[4667]|SMTPB5714-13|Canada|Ontario|589[0n]|BOLD:AAA2482
Mythimna unipuncta|[4668]|LOCBB572-06|United States|California|616[0n]|BOLD:AAA2482
Mythimna unipuncta|[4669]|LBCS134-07|Canada|British Columbia|658[0n]|BOLD:AAA2482
Mythimna unipuncta|[4670]|BBLPA828-10|Canada|Saskatchewan|658[0n]|BOLD:AAA2482
Mythimna unipuncta|[4671]|GMLC408-11|United States|California|658[0n]|BOLD:AAA2482
Mythimna unipuncta|[4672]|SMTPB13111-13|Canada|Ontario|516[0n]|
Mythimna unipuncta|[4673]|SMTPB19514-13|Canada|Ontario|588[0n]|BOLD:AAA2482
Mythimna unipuncta|[4674]|SMTPB13012-13|Canada|Ontario|588[0n]|BOLD:AAA2482
Mythimna unipuncta|[4675]|XAB623-04|Canada|Ontario|658[0n]|BOLD:AAA2482
Mythimna unipuncta|[4676]|SMTPB19947-13|Canada|Ontario|555[0n]|BOLD:AAA2482
Mythimna unipuncta|[4677]|SMTPB19515-13|Canada|Ontario|556[0n]|BOLD:AAA2482
Mythimna unipuncta|[4678]|SMTPB13013-13|Canada|Ontario|555[0n]|BOLD:AAA2482
Mythimna unipuncta|[4679]|SMTPB3225-13|Canada|Ontario|555[0n]|BOLD:AAA2482
Mythimna unipuncta|[4680]|LOCBF062-13|United States|California|611[0n]|BOLD:AAA2482
Mythimna unipuncta|[4681]|SMTPB19949-13|Canada|Ontario|564[0n]|BOLD:AAA2482
Mythimna unipuncta|[4682]|SMTPB19948-13|Canada|Ontario|564[0n]|BOLD:AAA2482
Mythimna unipuncta|[4683]|SMTPB13003-13|Canada|Ontario|564[0n]|BOLD:AAA2482
Mythimna unipuncta|[4684]|SMTPB10851-13|Canada|Ontario|564[0n]|BOLD:AAA2482
Mythimna unipuncta|[4685]|SMTPB6733-13|Canada|Ontario|565[0n]|BOLD:AAA2482
Mythimna unipuncta|[4686]|SMTPB3719-13|Canada|Ontario|565[0n]|BOLD:AAA2482
Mythimna unipuncta|[4687]|SMTPB2111-13|Canada|Ontario|564[0n]|BOLD:AAA2482
Mythimna unipuncta|[4688]|SMTPB16091-13|Canada|Ontario|582[0n]|BOLD:AAA2482
Mythimna unipuncta|[4689]|SMTPB13014-13|Canada|Ontario|582[0n]|BOLD:AAA2482
Mythimna unipuncta|[4690]|SMTPB12963-13|Canada|Ontario|582[0n]|BOLD:AAA2482
Mythimna unipuncta|[4691]|BBLSX284-09|United States|Oklahoma|658[0n]|BOLD:AAA2482
Mythimna unipuncta|[4692]|SMTPB22049-13|Canada|Ontario|561[0n]|BOLD:AAA2482
Mythimna unipuncta|[4693]|SMTPB10081-13|Canada|Ontario|583[0n]|BOLD:AAA2482
Mythimna unipuncta|[4694]|SMTPB8684-13|Canada|Ontario|582[0n]|BOLD:AAA2482
Mythimna unipuncta|[4695]|SMTPB6732-13|Canada|Ontario|582[0n]|BOLD:AAA2482
Mythimna unipuncta|[4696]|SMTPB5701-13|Canada|Ontario|582[0n]|BOLD:AAA2482
Mythimna unipuncta|[4697]|SMTPB22048-13|Canada|Ontario|561[0n]|BOLD:AAA2482
Mythimna unipuncta|[4698]|PHMNB350-04|Canada|New Brunswick|658[0n]|BOLD:AAA2482

[illegible]
Mythimna unipuncta|[4696]|SMTPB5701-13|Canada|Ontario|582[0n]|BOLD:AAA2482
Mythimna unipuncta|[4697]|SMTPB22048-13|Canada|Ontario|561[0n]|BOLD:AAA2482
Mythimna unipuncta|[4698]|PHMNB350-04|Canada|New Brunswick|658[0n]|BOLD:AAA2482
Mythimna unipuncta|[4699]|LPSOD449-09|Canada|Ontario|658[0n]|BOLD:AAA2482
Mythimna unipuncta|[4700]|LPSOD519-09|Canada|Ontario|658[0n]|BOLD:AAA2482
Mythimna unipuncta|[4701]|BBLCU263-09|United States|Illinois|658[0n]|BOLD:AAA2482
Mythimna unipuncta|[4702]|SMTPB12939-13|Canada|Ontario|561[0n]|BOLD:AAA2482
Mythimna unipuncta|[4703]|CNBPK388-13|Canada|Ontario|582[0n]|BOLD:AAA2482
Mythimna unipuncta|[4704]|SMTPB6150-13|Canada|Ontario|546[0n]|BOLD:AAA2482
Mythimna unipuncta|[4705]|LGSM412-04|United States|Tennessee|658[0n]|BOLD:AAA2482
Mythimna unipuncta|[4706]|BBLCU120-09|United States|Michigan|658[0n]|BOLD:AAA2482
Mythimna unipuncta|[4707]|SMTPB17406-13|Canada|Ontario|573[0n]|BOLD:AAA2482
Mythimna unipuncta|[4708]|XAH004-05|Canada|Ontario|658[0n]|BOLD:AAA2482
Mythimna unipuncta|[4709]|LPSOD331-09|Canada|Ontario|658[0n]|BOLD:AAA2482
Mythimna unipuncta|[4710]|LPSOD570-09|Canada|Ontario|658[0n]|BOLD:AAA2482
Mythimna unipuncta|[4711]|RWWB537-10|United States|Washington|658[0n]|BOLD:AAA2482
Mythimna unipuncta|[4712]|BBLSX954-09|United States|Oklahoma|658[0n]|BOLD:AAA2482
Mythimna unipuncta|[4713]|LPSOC321-08|Canada|Ontario|658[0n]|BOLD:AAA2482
Mythimna unipuncta|[4714]|LPSOD640-09|Canada|Ontario|658[0n]|BOLD:AAA2482
Mythimna unipuncta|[4715]|LPSOD733-09|Canada|Ontario|658[0n]|BOLD:AAA2482
Mythimna unipuncta|[4716]|USLEP587-10|United States|Texas|658[0n]|BOLD:AAA2482
Mythimna unipuncta|[4717]|USLEP631-10|United States|Texas|658[0n]|BOLD:AAA2482
Mythimna unipuncta|[4718]|MJMSL017-10|United States|Massachusetts|658[0n]|BOLD:AAA2482
Mythimna unipuncta|[4719]|RWWC118-10|United States|Washington|658[0n]|BOLD:AAA2482
Mythimna unipuncta|[4720]|LILLA011-11|United States|Illinois|658[0n]|BOLD:AAA2482
Mythimna unipuncta|[4721]|LILLA447-11|United States|Illinois|658[0n]|BOLD:AAA2482
Mythimna unipuncta|[4722]|LILLA767-11|United States|Illinois|658[0n]|BOLD:AAA2482
Mythimna unipuncta|[4723]|LILLA982-11|United States|Illinois|658[0n]|BOLD:AAA2482
Mythimna unipuncta|[4724]|BBLPA825-10|Canada|Ontario|658[0n]|BOLD:AAA2482
Mythimna unipuncta|[4725]|BBLPA826-10|Canada|Ontario|658[0n]|BOLD:AAA2482
Mythimna unipuncta|[4726]|BBLPA827-10|Canada|Ontario|658[0n]|BOLD:AAA2482
Mythimna unipuncta|[4727]|JMMMB387-11|United States|California|658[0n]|BOLD:AAA2482
Mythimna unipuncta|[4728]|LPSOD569-09|Canada|Ontario|658[0n]|BOLD:AAA2482
Mythimna unipuncta|[4729]|LPSOD571-09|Canada|Ontario|658[0n]|BOLD:AAA2482
Mythimna unipuncta|[4730]|LPSOD567-09|Canada|Ontario|658[0n]|BOLD:AAA2482
Mythimna unipuncta|[4731]|LPSOD568-09|Canada|Ontario|658[0n]|BOLD:AAA2482
Mythimna unipuncta|[4732]|BLTIB081-08|Canada|Ontario|658[0n]|BOLD:AAA2482
Mythimna unipuncta|[4733]|BLTIB109-08|Canada|Ontario|658[0n]|BOLD:AAA2482
Mythimna unipuncta|[4734]|LPSOB153-08|Canada|Ontario|658[0n]|BOLD:AAA2482
Mythimna unipuncta|[4735]|LPSOC098-08|Canada|Ontario|658[0n]|BOLD:AAA2482
Mythimna unipuncta|[4736]|LPSOB110-08|Canada|Ontario|658[0n]|BOLD:AAA2482
Mythimna unipuncta|[4737]|LPSOB111-08|Canada|Ontario|658[0n]|BOLD:AAA2482
Mythimna unipuncta|[4738]|LPSO935-08|Canada|Ontario|658[0n]|BOLD:AAA2482
Mythimna unipuncta|[4739]|LPSOB074-08|Canada|Ontario|658[0n]|BOLD:AAA2482
Mythimna unipuncta|[4740]|LPSO144-08|Canada|Ontario|658[0n]|BOLD:AAA2482
Mythimna unipuncta|[4741]|LPSO328-08|Canada|Ontario|658[0n]|BOLD:AAA2482
Mythimna unipuncta|[4742]|LPSO028-08|Canada|Ontario|658[0n]|BOLD:AAA2482
Mythimna unipuncta|[4743]|LPSO029-08|Canada|Ontario|658[0n]|BOLD:AAA2482
Mythimna unipuncta|[4744]|LTOL928-08|United States|Maryland|658[0n]|BOLD:AAA2482
Mythimna unipuncta|[4745]|LPSO027-08|Canada|Ontario|658[0n]|BOLD:AAA2482
Mythimna unipuncta|[4746]|RDLQF871-06|Canada|Quebec|658[0n]|BOLD:AAA2482
Mythimna unipuncta|[4747]|MHCOL287-07|Canada|Manitoba|658[0n]|BOLD:AAA2482
Mythimna unipuncta|[4748]|LOCBC166-06|United States|California|658[0n]|BOLD:AAA2482
Mythimna unipuncta|[4749]|RDNMD391-06|United States|California|658[0n]|BOLD:AAA2482
Mythimna unipuncta|[4750]|LOCBB296-06|United States|California|658[0n]|BOLD:AAA2482
Mythimna unipuncta|[4751]|LOCBB297-06|United States|California|658[0n]|BOLD:AAA2482
Mythimna unipuncta|[4752]|LPSO334-08|Canada|Ontario|658[0n]|BOLD:AAA2482
Mythimna unipuncta|[4753]|LPSO886-08|Canada|Ontario|658[0n]|BOLD:AAA2482
Mythimna unipuncta|[4754]|LPSOC205-08|Canada|Ontario|658[0n]|BOLD:AAA2482
Mythimna unipuncta|[4755]|LPSOC288-08|Canada|Ontario|658[0n]|BOLD:AAA2482
Mythimna unipuncta|[4756]|LPSOC318-08|Canada|Ontario|658[0n]|BOLD:AAA2482
Mythimna unipuncta|[4757]|LPSOC320-08|Canada|Ontario|658[0n]|BOLD:AAA2482
Mythimna unipuncta|[4758]|LPSOC322-08|Canada|Ontario|658[0n]|BOLD:AAA2482
Mythimna unipuncta|[4759]|LPSOB213-08|Canada|Ontario|658[0n]|BOLD:AAA2482
Mythimna unipuncta|[4760]|LPSOB215-08|Canada|Ontario|658[0n]|BOLD:AAA2482
Mythimna unipuncta|[4761]|LPSOB291-08|Canada|Ontario|658[0n]|BOLD:AAA2482
Mythimna unipuncta|[4762]|LPSOB459-08|Canada|Ontario|658[0n]|BOLD:AAA2482
Mythimna unipuncta|[4763]|LPSOB587-08|Canada|Ontario|658[0n]|BOLD:AAA2482
Mythimna unipuncta|[4764]|LPSOB697-08|Canada|Ontario|655[0n]|BOLD:AAA2482
Mythimna unipuncta|[4765]|LPSOB703-08|Canada|Ontario|657[0n]|BOLD:AAA2482
Mythimna unipuncta|[4766]|KPOEC139-08|Canada|Ontario|658[0n]|BOLD:AAA2482
Mythimna unipuncta|[4767]|KPOEC153-08|Canada|Ontario|658[0n]|BOLD:AAA2482
Mythimna unipuncta|[4768]|DES031-08|United States|New York|658[0n]|
Mythimna unipuncta|[4769]|DES032-08|United States|New York|658[0n]|
Mythimna unipuncta|[4770]|BLTIB214-08|Canada|Ontario|658[0n]|BOLD:AAA2482
Mythimna unipuncta|[4771]|BLTIB505-08|Canada|Ontario|658[0n]|BOLD:AAA2482
Mythimna unipuncta|[4772]|BLTIB509-08|Canada|Ontario|658[0n]|BOLD:AAA2482
Mythimna unipuncta|[4773]|BLTIB510-08|Canada|Ontario|658[0n]|BOLD:AAA2482
Mythimna unipuncta|[4774]|BLTIB548-08|Canada|Ontario|658[0n]|BOLD:AAA2482
Mythimna unipuncta|[4775]|BLTIB735-08|Canada|Ontario|658[0n]|BOLD:AAA2482
Mythimna unipuncta|[4776]|BLTIB912-08|Canada|Ontario|658[0n]|BOLD:AAA2482
Mythimna unipuncta|[4777]|BLTIB972-08|Canada|Ontario|658[0n]|BOLD:AAA2482
Mythimna unipuncta|[4778]|BLTIB975-08|Canada|Ontario|658[0n]|BOLD:AAA2482
Mythimna unipuncta|[4779]|LPOKA361-08|United States|Oklahoma|658[0n]|BOLD:AAA2482
Mythimna unipuncta|[4780]|BLGSM027-09|Canada|Ontario|658[0n]|BOLD:AAA2482
Mythimna unipuncta|[4781]|UDLEP001-09|United States|Delaware|658[0n]|BOLD:AAA2482
Mythimna unipuncta|[4782]|UDLEP330-09|United States|Pennsylvania|658[0n]|BOLD:AAA2482
Mythimna unipuncta|[4783]|UDLEP332-09|United States|Pennsylvania|658[0n]|BOLD:AAA2482
Mythimna unipuncta|[4784]|LPSOD273-09|Canada|Ontario|658[0n]|BOLD:AAA2482
Mythimna unipuncta|[4785]|LPSOD315-09|Canada|Ontario|658[0n]|BOLD:AAA2482
Mythimna unipuncta|[4786]|LPSOD324-09|Canada|Ontario|658[0n]|BOLD:AAA2482
Mythimna unipuncta|[4787]|LPSOD338-09|Canada|Ontario|658[0n]|BOLD:AAA2482
Mythimna unipuncta|[4788]|LPSOD425-09|Canada|Ontario|658[0n]|BOLD:AAA2482
Mythimna unipuncta|[4789]|LPSOD487-09|Canada|Ontario|658[0n]|BOLD:AAA2482
Mythimna unipuncta|[4790]|LPSOD509-09|Canada|Ontario|658[0n]|BOLD:AAA2482
Mythimna unipuncta|[4791]|LPSOD517-09|Canada|Ontario|658[0n]|BOLD:AAA2482
Mythimna unipuncta|[4792]|LPSOD518-09|Canada|Ontario|658[0n]|BOLD:AAA2482
Mythimna unipuncta|[4793]|LPSOD566-09|Canada|Ontario|658[0n]|BOLD:AAA2482
Mythimna unipuncta|[4794]|LPSOD743-09|Canada|Ontario|658[0n]|BOLD:AAA2482
Mythimna unipuncta|[4795]|LPSOD799-09|Canada|Ontario|658[0n]|BOLD:AAA2482
Mythimna unipuncta|[4796]|RWWA171-09|United States|Washington|658[0n]|BOLD:AAA2482
Mythimna unipuncta|[4797]|BBLSW340-09|United States|Arizona|658[0n]|BOLD:AAA2482
[illegible]

Mythimna unipuncta|[4795]|LPSOD799-09|Canada|Ontario|658[0n]|BOLD:AAA2482
Mythimna unipuncta|[4796]|RWWA171-09|United States|Washington|658[0n]|BOLD:AAA2482
Mythimna unipuncta|[4797]|BBLSW340-09|United States|Arizona|658[0n]|BOLD:AAA2482
Mythimna unipuncta|[4798]|BBLSY415-09|United States|Arizona|658[0n]|BOLD:AAA2482
Mythimna unipuncta|[4799]|BBLWU139-09|United States|Colorado|658[0n]|BOLD:AAA2482
Mythimna unipuncta|[4800]|BBLCU090-09|United States|Kansas|658[0n]|BOLD:AAA2482
Mythimna unipuncta|[4801]|BBLCU262-09|United States|Illinois|658[0n]|BOLD:AAA2482
Mythimna unipuncta|[4802]|RWWB345-09|United States|Washington|658[0n]|BOLD:AAA2482
Mythimna unipuncta|[4803]|RWWB401-09|United States|Washington|658[0n]|BOLD:AAA2482
Mythimna unipuncta|[4804]|BBLPC056-09|Canada|New Brunswick|658[0n]|BOLD:AAA2482
Mythimna unipuncta|[4805]|BBLPC065-09|Canada|New Brunswick|658[0n]|BOLD:AAA2482
Mythimna unipuncta|[4806]|LGSMG861-10|United States|North Carolina|658[0n]|BOLD:AAA2482
Mythimna unipuncta|[4807]|RWWB443-10|United States|Washington|658[0n]|BOLD:AAA2482
Mythimna unipuncta|[4808]|BBLSX888-09|United States|Oklahoma|658[0n]|BOLD:AAA2482
Mythimna unipuncta|[4809]|BBLSX910-09|United States|Arizona|658[0n]|BOLD:AAA2482
Mythimna unipuncta|[4810]|BBLSX743-09|United States|Arizona|658[0n]|BOLD:AAA2482
Mythimna unipuncta|[4811]|BBLSX884-09|United States|Oklahoma|658[0n]|BOLD:AAA2482
Mythimna unipuncta|[4812]|BBLSW804-09|United States|Oklahoma|658[0n]|BOLD:AAA2482
Mythimna unipuncta|[4813]|BBLSX586-09|United States|Oklahoma|658[0n]|BOLD:AAA2482
Mythimna unipuncta|[4814]|LPOKB143-09|United States|Oklahoma|658[0n]|BOLD:AAA2482
Mythimna unipuncta|[4815]|LPOKB997-09|United States|Oklahoma|658[0n]|BOLD:AAA2482
Mythimna unipuncta|[4816]|LPOKA667-09|United States|Oklahoma|658[0n]|BOLD:AAA2482
Mythimna unipuncta|[4817]|LPOKA1009-09|United States|Oklahoma|658[0n]|BOLD:AAA2482
Mythimna unipuncta|[4818]|LPSOD866-09|Canada|Ontario|658[0n]|BOLD:AAA2482
Mythimna unipuncta|[4819]|LPSOD1050-09|Canada|Ontario|658[0n]|BOLD:AAA2482
Mythimna unipuncta|[4820]|LPSOD802-09|Canada|Ontario|658[0n]|BOLD:AAA2482
Mythimna unipuncta|[4821]|LPSOD806-09|Canada|Ontario|658[0n]|BOLD:AAA2482
Mythimna unipuncta|[4822]|BBLOC650-11|United States|Arkansas|658[0n]|BOLD:AAA2482
Mythimna unipuncta|[4823]|RWWC735-11|United States|Washington|658[0n]|BOLD:AAA2482
Mythimna unipuncta|[4824]|BBLOD144-11|United States|Texas|658[0n]|BOLD:AAA2482
Mythimna unipuncta|[4825]|BBLOD965-11|United States|Texas|658[0n]|BOLD:AAA2482
Mythimna unipuncta|[4826]|BBLOE2019-12|United States|Texas|658[0n]|BOLD:AAA2482
Mythimna unipuncta|[4827]|CMAZA1055-12|United States|Arizona|658[0n]|BOLD:AAA2482
Mythimna unipuncta|[4828]|GMLC881-12|United States|California|658[0n]|BOLD:AAA2482
Mythimna unipuncta|[4829]|GMLC888-12|United States|California|658[0n]|BOLD:AAA2482
Mythimna unipuncta|[4830]|GMLC1002-12|United States|California|658[0n]|BOLD:AAA2482
Mythimna unipuncta|[4831]|GMLC1099-12|United States|California|658[0n]|BOLD:AAA2482
Mythimna unipuncta|[4832]|GMLC1102-12|United States|California|658[0n]|BOLD:AAA2482
Mythimna unipuncta|[4833]|GMLC1106-12|United States|California|658[0n]|BOLD:AAA2482
Mythimna unipuncta|[4834]|GMLC1118-12|United States|California|658[0n]|BOLD:AAA2482
Mythimna unipuncta|[4835]|GMLC1209-12|United States|California|658[0n]|BOLD:AAA2482
Mythimna unipuncta|[4836]|LNC448-05|United States|North Carolina|658[0n]|BOLD:AAA2482
Mythimna unipuncta|[4837]|LOCBB295-06|United States|California|658[0n]|BOLD:AAA2482
Mythimna unipuncta|[4838]|RDNMD392-06|United States|California|658[0n]|BOLD:AAA2482
Mythimna unipuncta|[4839]|LOCBC458-06|United States|California|658[0n]|BOLD:AAA2482
Mythimna unipuncta|[4840]|LOCBF2598-13|United States|California|658[0n]|BOLD:AAA2482
Mythimna unipuncta|[4841]|XAH651-05|Canada|Ontario|658[0n]|BOLD:AAA2482
Mythimna unipuncta|[4842]|LNC236-05|United States|North Carolina|658[0n]|BOLD:AAA2482
Mythimna unipuncta|[4843]|MNBB445-05|Canada|New Brunswick|658[0n]|BOLD:AAA2482
Mythimna unipuncta|[4844]|LOTB487-05|United States|Tennessee|658[0n]|BOLD:AAA2482
Mythimna unipuncta|[4845]|PHMNB355-04|Canada|New Brunswick|658[0n]|BOLD:AAA2482
Mythimna unipuncta|[4846]|JSJUL2390-11|Canada|Ontario|658[0n]|BOLD:AAA2482
Mythimna unipuncta|[4847]|LOCT105-05|United States|Connecticut|658[0n]|BOLD:AAA2482
Mythimna unipuncta|[4848]|PHMNB315-04|Canada|New Brunswick|658[0n]|BOLD:AAA2482
Mythimna unipuncta|[4849]|PHMNB308-04|Canada|New Brunswick|658[0n]|BOLD:AAA2482
Mythimna unipuncta|[4850]|XAD438-04|Canada|Ontario|658[0n]|BOLD:AAA2482
Mythimna unipuncta|[4851]|LOT507-04|United States|Tennessee|658[0n]|BOLD:AAA2482
Mythimna unipuncta|[4852]|BLTIB734-08|Canada|Ontario|658[0n]|BOLD:AAA2482
Mythimna unipuncta|[4853]|LOT345-04|United States|Tennessee|658[0n]|BOLD:AAA2482
Mythimna unipuncta|[4854]|LOT344-04|United States|Tennessee|658[0n]|BOLD:AAA2482
Mythimna unipuncta|[4855]|RDNMH140-09|United States|Florida|658[0n]|BOLD:AAA2482
Mythimna unipuncta|[4856]|LGSM413-04|United States|Tennessee|658[0n]|BOLD:AAA2482
Mythimna unipuncta|[4857]|LOCT062-05|United States|Connecticut|658[0n]|BOLD:AAA2482
Mythimna unipuncta|[4858]|BLTIB875-08|Canada|Ontario|658[0n]|BOLD:AAA2482
Mythimna unipuncta|[4859]|BLTIB689-08|Canada|Ontario|658[1n]|BOLD:AAA2482
Mythimna unipuncta|[4860]|LPSO324-08|Canada|Ontario|658[0n]|BOLD:AAA2482
Mythimna unipuncta|[4861]|BBLSX909-09|United States|Arizona|658[0n]|BOLD:AAA2482
Mythimna unipuncta|[4862]|XAH176-05|Canada|Ontario|658[1n]|BOLD:AAA2482
Mythimna unipuncta|[4863]|BLTIB291-08|Canada|Ontario|649[1n]|BOLD:AAA2482
Mythimna unipuncta|[4864]|LPSOB694-08|Canada|Ontario|645[0n]|BOLD:AAA2482
Mythimna unipuncta|[4865]|LTOL936-08|United States|California|645[0n]|BOLD:AAA2482
Mythimna unipuncta|[4866]|XAG895-05|Canada|Ontario|643[0n]|BOLD:AAA2482
Mythimna unipuncta|[4867]|LOCT319-05|United States|Connecticut|643[0n]|BOLD:AAA2482
Mythimna unipuncta|[4868]|LPSOB711-08|Canada|Ontario|653[0n]|BOLD:AAA2482
Mythimna unipuncta|[4869]|LPSOB698-08|Canada|Ontario|653[0n]|BOLD:AAA2482
Mythimna unipuncta|[4870]|BLTIB478-08|Canada|Ontario|656[0n]|BOLD:AAA2482
Mythimna unipuncta|[4871]|LPSOC093-08|Canada|Ontario|658[0n]|BOLD:AAA2482
Mythimna unipuncta|[4872]|LPSOB619-08|Canada|Ontario|650[0n]|BOLD:AAA2482
Mythimna unipuncta|[4873]|MJMSL049-10|United States|Massachusetts|640[0n]|BOLD:AAA2482
Mythimna unipuncta|[4874]|LOCB537-06|United States|California|619[0n]|BOLD:AAA2482
Mythimna unipuncta|[4875]|LTOL935-08|United States|California|650[0n]|BOLD:AAA2482
Mythimna unipuncta|[4876]|LPSOB706-08|Canada|Ontario|648[0n]|BOLD:AAA2482
Mythimna unipuncta|[4877]|LPSOB670-08|Canada|Ontario|646[0n]|BOLD:AAA2482
Mythimna unipuncta|[4878]|LPSOB663-08|Canada|Ontario|646[0n]|BOLD:AAA2482
Mythimna unipuncta|[4879]|PMG154-03|Canada|Ontario|617[0n]|BOLD:AAA2482
Mythimna unipuncta|[4880]|CNPPH804-12|Canada|Ontario|637[0n]|BOLD:AAA2482
Mythimna unipuncta|[4881]|CNPPE1032-12|Canada|Ontario|635[0n]|BOLD:AAA2482
Mythimna unipuncta|[4882]|CNPPE1031-12|Canada|Ontario|635[0n]|BOLD:AAA2482
Mythimna unipuncta|[4883]|LOCBB294-06|United States|California|632[0n]|BOLD:AAA2482
Mythimna unipuncta|[4884]|BLTIB613-08|Canada|Ontario|643[1n]|BOLD:AAA2482
Mythimna unipuncta|[4885]|GMLC1392-12|United States|California|614[0n]|BOLD:AAA2482
Mythimna unipuncta|[4886]|BBLPE191-09|Canada|Newfoundland and Labrador|614[0n]|BOLD:AAA2482
Mythimna unipuncta|[4887]|XAH003-05|Canada|Ontario|621[0n]|BOLD:AAA2482
Mythimna unipuncta|[4888]|LOCBC167-06|United States|California|617[0n]|BOLD:AAA2482
Mythimna unipuncta|[4889]|BLTIB586-08|Canada|Ontario|638[0n]|BOLD:AAA2482
Mythimna unipuncta|[4890]|LOCBC086-06|United States|California|626[0n]|BOLD:AAA2482
Mythimna unipuncta|[4891]|TMG132-03|Canada|Ontario|639[0n]|BOLD:AAA2482
Mythimna unipuncta|[4892]|XAD480-04|Canada|Ontario|583[0n]|BOLD:AAA2482
Mythimna unipuncta|[4893]|LOCBC168-06|United States|California|617[0n]|BOLD:AAA2482
Mythimna unipuncta|[4894]|LPSOD651-09|Canada|Ontario|582[0n]|BOLD:AAA2482
Mythimna unipuncta|[4895]|RWWB459-10|United States|Washington|635[0n]|BOLD:AAA2482
Mythimna unipuncta|[4896]|BLTIB108-08|Canada|Ontario|519[0n]|BOLD:AAA2482
Mythimna unipuncta|[4897]|SMTPB1146-13|Canada|Ontario|629[0n]|BOLD:AAA2482
Mythimna unipuncta|[4898]|CNPPA[illegible]-12|Canada|Ontario|606[0n]|BOLD:AAA2482

Mythimna unipuncta|[4895]|RWWB459-10|United States|Washington|633[0n]|BOLD:AAA2482
Mythimna unipuncta|[4896]|BLTIB108-08|Canada|Ontario|519[0n]|BOLD:AAA2482
Mythimna unipuncta|[4897]|SMTPB1146-13|Canada|Ontario|629[0n]|BOLD:AAA2482
Mythimna unipuncta|[4898]|CNPPA3923-12|Canada|Ontario|606[0n]|BOLD:AAA2482
Mythimna unipuncta|[4899]|CNPPA3922-12|Canada|Ontario|606[0n]|BOLD:AAA2482
Mythimna unipuncta|[4900]|CNPPA3925-12|Canada|Ontario|602[0n]|BOLD:AAA2482
Mythimna unipuncta|[4901]|CNPPA3921-12|Canada|Ontario|601[0n]|BOLD:AAA2482
Mythimna unipuncta|[4902]|LOCBF2630-13|United States|California|658[0n]|BOLD:AAA2482
Mythimna unipuncta|[4903]|SMTPB3164-13|Canada|Ontario|552[0n]|BOLD:AAA2482
Mythimna unipuncta|[4904]|XAD347-04|Canada|Ontario|658[0n]|BOLD:AAA2482
Mythimna unipuncta|[4905]|SMTPB3720-13|Canada|Ontario|552[0n]|BOLD:AAA2482
Mythimna unipuncta|[4906]|RBINA884-13|Canada|Ontario|564[0n]|BOLD:AAA2482
Mythimna unipuncta|[4907]|SMTPD3726-13|Canada|Ontario|603[0n]|BOLD:AAA2482
Leucania insueta|[4908]|RDLQB909-05|Canada|Quebec|504[0n]|BOLD:AAA3150
Leucania insueta|[4909]|XAE556-04|Canada|Ontario|658[3n]|BOLD:AAA3150
Leucania insueta|[4910]|RDLQB910-05|Canada|Quebec|658[0n]|BOLD:AAA3150
Leucania insueta|[4911]|XAE428-04|Canada|Ontario|658[0n]|BOLD:AAA3150
Leucania insueta|[4912]|LOWCD233-06|Canada|British Columbia|569[0n]|BOLD:AAA3150
Leucania insueta|[4913]|LOWCD231-06|Canada|British Columbia|563[0n]|BOLD:AAA3150
Leucania insueta|[4914]|LOWC059-05|Canada|British Columbia|513[0n]|BOLD:AAA3150
Leucania insueta|[4915]|LOWCD244-06|Canada|British Columbia|567[0n]|BOLD:AAA3150
Leucania insueta|[4916]|RDMAB125-05|Canada|Alberta|658[0n]|BOLD:AAA3150
Leucania insueta|[4917]|RDNMJ129-10|United States|Arizona|658[0n]|BOLD:AAA3150
Leucania insueta|[4918]|BBLPE306-09|Canada|Newfoundland and Labrador|658[0n]|BOLD:AAA3150
Leucania insueta|[4919]|BBLPC718-09|Canada|Newfoundland and Labrador|658[0n]|BOLD:AAA3150
Leucania insueta|[4920]|BBLPC698-09|Canada|Newfoundland and Labrador|658[0n]|BOLD:AAA3150
Leucania insueta|[4921]|LPSK447-08|Canada|Saskatchewan|658[0n]|BOLD:AAA3150
Leucania insueta|[4922]|RDLQF842-06|Canada|Quebec|658[0n]|BOLD:AAA3150
Leucania insueta|[4923]|RDLQF841-06|Canada|Quebec|658[0n]|BOLD:AAA3150
Leucania insueta|[4924]|RDLQF840-06|Canada|Quebec|658[0n]|BOLD:AAA3150
Leucania insueta|[4925]|RDLQF839-06|Canada|Quebec|658[0n]|BOLD:AAA3150
Leucania insueta|[4926]|XAJ836-06|Canada|Ontario|658[0n]|BOLD:AAA3150
Leucania insueta|[4927]|RDLQF026-06|Canada|Quebec|658[0n]|BOLD:AAA3150
Leucania insueta|[4928]|LOWCD243-06|Canada|British Columbia|658[0n]|BOLD:AAA3150
Leucania insueta|[4929]|LOWCD242-06|Canada|British Columbia|658[0n]|BOLD:AAA3150
Leucania insueta|[4930]|LOWCD241-06|Canada|British Columbia|658[0n]|BOLD:AAA3150
Leucania insueta|[4931]|LOWCD239-06|Canada|British Columbia|658[0n]|BOLD:AAA3150
Leucania insueta|[4932]|LOWCD238-06|Canada|British Columbia|658[0n]|BOLD:AAA3150
Leucania insueta|[4933]|RDLQB911-05|Canada|Quebec|658[0n]|BOLD:AAA3150
Leucania insueta|[4934]|LOWC065-05|Canada|British Columbia|658[0n]|BOLD:AAA3150
Leucania insueta|[4935]|LOWC064-05|Canada|British Columbia|658[0n]|BOLD:AAA3150
Leucania insueta|[4936]|LOWC063-05|Canada|British Columbia|658[0n]|BOLD:AAA3150
Leucania insueta|[4937]|LOWC060-05|Canada|British Columbia|658[0n]|BOLD:AAA3150
Leucania insueta|[4938]|LOWC057-05|Canada|British Columbia|658[0n]|BOLD:AAA3150
Leucania insueta|[4939]|LOWC052-05|Canada|British Columbia|658[0n]|BOLD:AAA3150
Leucania insueta|[4940]|LOWC025-05|Canada|British Columbia|658[0n]|BOLD:AAA3150
Leucania insueta|[4941]|RDNMB429-05|Canada|Alberta|658[0n]|BOLD:AAA3150
Leucania insueta|[4942]|PHMNB610-04|Canada|New Brunswick|658[0n]|BOLD:AAA3150
Leucania insueta|[4943]|PHMNB328-04|Canada|New Brunswick|658[0n]|BOLD:AAA3150
Leucania insueta|[4944]|LPSK599-08|Canada|Saskatchewan|658[0n]|BOLD:AAA3150
Leucania insueta|[4945]|LPSK560-08|Canada|Saskatchewan|658[0n]|BOLD:AAA3150
Leucania insueta|[4946]|RDLQF838-06|Canada|Quebec|658[0n]|BOLD:AAA3150
Leucania insueta|[4947]|RDNMD563-06|United States|Colorado|658[1n]|BOLD:AAA3150
Leucania insueta|[4948]|RDNMB428-05|Canada|British Columbia|541[1n]|BOLD:AAA3150
Leucania insueta|[4949]|RDNMD564-06|United States|Colorado|658[0n]|BOLD:AAA3150
Leucania insueta|[4950]|LOWCD240-06|Canada|British Columbia|596[0n]|BOLD:AAA3150
Leucania insueta|[4951]|RDNME291-07|United States|Colorado|593[1n]|BOLD:AAA3150
Leucania insueta|[4952]|LOWC058-05|Canada|British Columbia|658[0n]|BOLD:AAA3150
Leucania dia|[4953]|CNGRB517-12|Canada|Saskatchewan|633[0n]|BOLD:AAA3150
Leucania insueta|[4954]|CNGRB520-12|Canada|Saskatchewan|636[0n]|BOLD:AAA3150
Leucania insueta|[4955]|HPPPE1552-13|Canada|Nova Scotia|558[0n]|BOLD:AAA3150
Leucania dia|[4956]|LHLEP098-06|Canada|British Columbia|658[0n]|BOLD:AAA3150
Leucania dia|[4957]|LBCG376-08|Canada|British Columbia|658[0n]|BOLD:AAA3150
Leucania dia|[4958]|RDLQF025-06|Canada|Quebec|658[0n]|BOLD:AAA3150
Leucania dia|[4959]|LBCH5420-10|Canada|British Columbia|658[0n]|BOLD:AAA3150
Leucania dia|[4960]|LBCH5484-10|Canada|British Columbia|658[0n]|BOLD:AAA3150
Leucania dia|[4961]|LBCH5088-10|Canada|British Columbia|658[0n]|BOLD:AAA3150
Leucania dia|[4962]|LBCH5090-10|Canada|British Columbia|658[0n]|BOLD:AAA3150
Leucania dia|[4963]|LBCH5092-10|Canada|British Columbia|658[0n]|BOLD:AAA3150
Leucania dia|[4964]|LBCH5093-10|Canada|British Columbia|658[0n]|BOLD:AAA3150
Leucania dia|[4965]|LBCH5162-10|Canada|British Columbia|658[0n]|BOLD:AAA3150
Leucania dia|[4966]|LBCH5163-10|Canada|British Columbia|658[0n]|BOLD:AAA3150
Leucania dia|[4967]|LBCH5164-10|Canada|British Columbia|658[0n]|BOLD:AAA3150
Leucania dia|[4968]|LBCH5167-10|Canada|British Columbia|658[0n]|BOLD:AAA3150
Leucania dia|[4969]|LBCH5168-10|Canada|British Columbia|658[0n]|BOLD:AAA3150
Leucania dia|[4970]|LBCH5359-10|Canada|British Columbia|658[0n]|BOLD:AAA3150
Leucania dia|[4971]|LBCH5364-10|Canada|British Columbia|658[0n]|BOLD:AAA3150
Leucania dia|[4972]|LBCH5419-10|Canada|British Columbia|658[0n]|BOLD:AAA3150
Leucania dia|[4973]|LBCH5009-10|Canada|British Columbia|658[0n]|BOLD:AAA3150
Leucania dia|[4974]|LBCH5010-10|Canada|British Columbia|658[0n]|BOLD:AAA3150
Leucania dia|[4975]|LBCH5052-10|Canada|British Columbia|658[0n]|BOLD:AAA3150
Leucania dia|[4976]|LBCH5087-10|Canada|British Columbia|658[0n]|BOLD:AAA3150
Leucania dia|[4977]|LBCH5004-10|Canada|British Columbia|658[0n]|BOLD:AAA3150
Leucania dia|[4978]|LBCH5005-10|Canada|British Columbia|658[0n]|BOLD:AAA3150
Leucania dia|[4979]|LBCG426-08|Canada|British Columbia|658[0n]|BOLD:AAA3150
Leucania dia|[4980]|LBCH5003-10|Canada|British Columbia|658[0n]|BOLD:AAA3150
Leucania dia|[4981]|LBCG392-08|Canada|British Columbia|658[0n]|BOLD:AAA3150
Leucania dia|[4982]|LBCG416-08|Canada|British Columbia|658[0n]|BOLD:AAA3150
Leucania dia|[4983]|LBCG375-08|Canada|British Columbia|658[0n]|BOLD:AAA3150
Leucania dia|[4984]|LBCG379-08|Canada|British Columbia|658[0n]|BOLD:AAA3150
Leucania dia|[4985]|LBCG370-08|Canada|British Columbia|658[0n]|BOLD:AAA3150
Leucania dia|[4986]|LBCG372-08|Canada|British Columbia|658[0n]|BOLD:AAA3150
Leucania dia|[4987]|LBCG363-08|Canada|British Columbia|658[0n]|BOLD:AAA3150
Leucania dia|[4988]|LBCG369-08|Canada|British Columbia|658[0n]|BOLD:AAA3150
Leucania dia|[4989]|LBCG127-08|Canada|British Columbia|658[0n]|BOLD:AAA3150
Leucania dia|[4990]|LBCG345-08|Canada|British Columbia|658[0n]|BOLD:AAA3150
Leucania dia|[4991]|LBCG117-08|Canada|British Columbia|658[0n]|BOLD:AAA3150
Leucania dia|[4992]|LBCG122-08|Canada|British Columbia|658[0n]|BOLD:AAA3150
Leucania dia|[4993]|LBCG110-08|Canada|British Columbia|658[0n]|BOLD:AAA3150
Leucania dia|[4994]|LBCG114-08|Canada|British Columbia|658[0n]|BOLD:AAA3150
Leucania dia|[4995]|LBCH5572-10|Canada|British Columbia|658[0n]|BOLD:AAA3150
Leucania dia|[4996]|LBCG105-08|Canada|British Columbia|658[0n]|BOLD:AAA3150
Leucania dia|[4997]|RDNMF575-08|Canada|British Columbia|658[0n]|BOLD:AAA3150
[illegible]

Leucania dia|[4995]|LBCH5572-10|Canada|British Columbia|658[0n]|BOLD:AAA3150
Leucania dia|[4996]|LBCG105-08|Canada|British Columbia|658[0n]|BOLD:AAA3150
Leucania dia|[4997]|RDNMF575-08|Canada|British Columbia|658[0n]|BOLD:AAA3150
Leucania dia|[4998]|RDNMF572-08|Canada|British Columbia|658[0n]|BOLD:AAA3150
Leucania dia|[4999]|RDNME362-07|Canada|Yukon Territory|658[0n]|BOLD:AAA3150
Leucania dia|[5000]|LOWCE111-06|Canada|British Columbia|658[0n]|BOLD:AAA3150
Leucania dia|[5001]|LOWCD236-06|Canada|British Columbia|658[0n]|BOLD:AAA3150
Leucania dia|[5002]|LOWCD235-06|Canada|British Columbia|658[0n]|BOLD:AAA3150
Leucania dia|[5003]|LOWCD234-06|Canada|British Columbia|658[0n]|BOLD:AAA3150
Leucania dia|[5004]|LBCG373-08|Canada|British Columbia|658[0n]|BOLD:AAA3150
Leucania dia|[5005]|LBCG358-08|Canada|British Columbia|658[0n]|BOLD:AAA3150
Leucania dia|[5006]|RDNMF574-08|Canada|British Columbia|652[0n]|BOLD:AAA3150
Leucania dia|[5007]|LBCG382-08|Canada|British Columbia|658[0n]|BOLD:AAA3150
Leucania dia|[5008]|LOWCD232-06|Canada|British Columbia|591[0n]|BOLD:AAA3150
Leucania dia|[5009]|RDNME364-07|Canada|Yukon Territory|608[0n]|BOLD:AAA3150
Leucania dia|[5010]|LBCG1352-09|Canada|British Columbia|636[0n]|BOLD:AAA3150
Leucania dia|[5011]|UAMIC522-13|United States|Alaska|648[0n]|BOLD:AAA3150
Leucania dia|[5012]|SSWLB052-13|Canada|Alberta|562[0n]|BOLD:AAA3150
Leucania dia|[5013]|RDLQB912-05|Canada|Quebec|658[0n]|BOLD:AAA3150
Leucania dia|[5014]|LOWCD237-06|Canada|British Columbia|614[0n]|BOLD:AAA3150
Leucania dia|[5015]|LPABB151-08|Canada|Alberta|658[0n]|BOLD:AAA3150
Leucania dia|[5016]|BBLPA831-10|Canada|Alberta|658[0n]|BOLD:AAA3150
Leucania dia|[5017]|BBLPA832-10|Canada|Alberta|658[0n]|BOLD:AAA3150
Leucania dia|[5018]|BBLPA834-10|Canada|Alberta|658[0n]|BOLD:AAA3150
Leucania dia|[5019]|LBCG106-08|Canada|British Columbia|658[0n]|BOLD:AAA3150
Leucania dia|[5020]|LHLEP099-06|Canada|British Columbia|658[0n]|BOLD:AAA3150
Leucania dia|[5021]|LHLEP095-06|Canada|British Columbia|658[0n]|BOLD:AAA3150
Leucania dia|[5022]|LPVIB013-08|Canada|British Columbia|641[0n]|BOLD:AAA3150
Leucania dia|[5023]|LPABB137-08|Canada|Alberta|638[0n]|BOLD:AAA3150
Leucania dia|[5024]|LPABC016-09|Canada|Alberta|633[0n]|BOLD:AAA3150
Leucania dia|[5025]|SSJAB013-13|Canada|Alberta|554[0n]|BOLD:AAA3150
Leucania dia|[5026]|BBLSY556-09|United States|New Mexico|658[0n]|BOLD:AAA3150
Leucania dia|[5027]|BBLSY583-09|United States|New Mexico|578[0n]|BOLD:AAA3150
Leucania dia|[5028]|LBCH5165-10|Canada|British Columbia|658[0n]|BOLD:AAA3150
Leucania dia|[5029]|LBCH5805-10|Canada|British Columbia|658[0n]|BOLD:AAA3150
Leucania dia|[5030]|RDNME363-07|Canada|Yukon Territory|656[0n]|BOLD:AAA3150
Leucania dia|[5031]|BBLPA833-10|Canada|Alberta|658[0n]|BOLD:AAA3150
Leucania dia|[5032]|BBLPA830-10|Canada|Alberta|658[0n]|BOLD:AAA3150
Leucania dia|[5033]|CNJAC987-12|Canada|Alberta|638[0n]|BOLD:AAA3150
Leucania dia|[5034]|SSJAB014-13|Canada|Alberta|598[0n]|BOLD:AAA3150
Leucania dia|[5035]|LBCH5166-10|Canada|British Columbia|658[0n]|BOLD:AAA3150
Leucania dia|[5036]|LBCH5608-10|Canada|British Columbia|658[0n]|BOLD:AAA3150
Leucania dia|[5037]|LBCH5558-10|Canada|British Columbia|658[0n]|BOLD:AAA3150
Leucania dia|[5038]|LBCH5518-10|Canada|British Columbia|658[0n]|BOLD:AAA3150
Leucania dia|[5039]|LBCH5485-10|Canada|British Columbia|658[0n]|BOLD:AAA3150
Leucania dia|[5040]|LBCH5469-10|Canada|British Columbia|658[0n]|BOLD:AAA3150
Leucania dia|[5041]|LBCH5363-10|Canada|British Columbia|658[0n]|BOLD:AAA3150
Leucania dia|[5042]|LBCH5362-10|Canada|British Columbia|658[0n]|BOLD:AAA3150
Leucania dia|[5043]|LBCH5361-10|Canada|British Columbia|658[0n]|BOLD:AAA3150
Leucania dia|[5044]|LBCH5360-10|Canada|British Columbia|658[0n]|BOLD:AAA3150
Leucania dia|[5045]|LBCH5358-10|Canada|British Columbia|658[0n]|BOLD:AAA3150
Leucania dia|[5046]|LBCH5285-10|Canada|British Columbia|658[0n]|BOLD:AAA3150
Leucania dia|[5047]|LBCH5264-10|Canada|British Columbia|658[0n]|BOLD:AAA3150
Leucania dia|[5048]|LBCH5169-10|Canada|British Columbia|658[0n]|BOLD:AAA3150
Leucania dia|[5049]|LBCH5091-10|Canada|British Columbia|658[0n]|BOLD:AAA3150
Leucania dia|[5050]|LBCH5089-10|Canada|British Columbia|658[0n]|BOLD:AAA3150
Leucania dia|[5051]|LBCH5086-10|Canada|British Columbia|658[0n]|BOLD:AAA3150
Leucania dia|[5052]|LBCH5008-10|Canada|British Columbia|658[0n]|BOLD:AAA3150
Leucania dia|[5053]|LBCH5007-10|Canada|British Columbia|658[0n]|BOLD:AAA3150
Leucania dia|[5054]|LBCH5006-10|Canada|British Columbia|658[0n]|BOLD:AAA3150
Leucania dia|[5055]|LBCH4984-10|Canada|British Columbia|658[0n]|BOLD:AAA3150
Leucania dia|[5056]|LBCG466-08|Canada|British Columbia|658[0n]|BOLD:AAA3150
Leucania dia|[5057]|LBCG464-08|Canada|British Columbia|658[0n]|BOLD:AAA3150
Leucania dia|[5058]|LBCG415-08|Canada|British Columbia|658[0n]|BOLD:AAA3150
Leucania dia|[5059]|LBCG377-08|Canada|British Columbia|658[0n]|BOLD:AAA3150
Leucania dia|[5060]|LBCG374-08|Canada|British Columbia|658[0n]|BOLD:AAA3150
Leucania dia|[5061]|LBCG360-08|Canada|British Columbia|658[0n]|BOLD:AAA3150
Leucania dia|[5062]|LBCG356-08|Canada|British Columbia|658[0n]|BOLD:AAA3150
Leucania dia|[5063]|LBCG354-08|Canada|British Columbia|658[0n]|BOLD:AAA3150
Leucania dia|[5064]|LBCG346-08|Canada|British Columbia|658[0n]|BOLD:AAA3150
Leucania dia|[5065]|LBCG344-08|Canada|British Columbia|658[0n]|BOLD:AAA3150
Leucania dia|[5066]|LBCG341-08|Canada|British Columbia|658[0n]|BOLD:AAA3150
Leucania dia|[5067]|LBCG338-08|Canada|British Columbia|658[0n]|BOLD:AAA3150
Leucania dia|[5068]|LBCG336-08|Canada|British Columbia|658[0n]|BOLD:AAA3150
Leucania dia|[5069]|LBCG334-08|Canada|British Columbia|658[0n]|BOLD:AAA3150
Leucania dia|[5070]|LBCG126-08|Canada|British Columbia|658[0n]|BOLD:AAA3150
Leucania dia|[5071]|LBCG120-08|Canada|British Columbia|658[0n]|BOLD:AAA3150
Leucania dia|[5072]|LBCG115-08|Canada|British Columbia|658[0n]|BOLD:AAA3150
Leucania dia|[5073]|LBCG103-08|Canada|British Columbia|658[0n]|BOLD:AAA3150
Leucania dia|[5074]|LBCG043-08|Canada|British Columbia|658[0n]|BOLD:AAA3150
Leucania dia|[5075]|LBCG042-08|Canada|British Columbia|657[0n]|BOLD:AAA3150
Leucania dia|[5076]|RDNME688-08|United States|California|658[0n]|BOLD:AAA3150
Leucania dia|[5077]|RDNME562-08|United States|California|658[0n]|BOLD:AAA3150
Leucania dia|[5078]|RDNMF573-08|Canada|British Columbia|640[0n]|BOLD:AAA3150
Leucania dia|[5079]|LBCH5357-10|Canada|British Columbia|644[0n]|BOLD:AAA3150
Leucania dia|[5080]|LALPA403-10|Canada|British Columbia|658[0n]|BOLD:AAA3150
Leucania dia|[5081]|JMMMB374-11|United States|California|658[0n]|BOLD:AAA3150
Leucania dia|[5082]|LALPA213-10|Canada|British Columbia|658[0n]|BOLD:AAA3150
Leucania dia|[5083]|LALPA228-10|Canada|British Columbia|658[0n]|BOLD:AAA3150
Leucania dia|[5084]|LPVIB852-08|Canada|British Columbia|658[0n]|BOLD:AAA3150
Leucania dia|[5085]|LPVIA298-08|Canada|British Columbia|658[0n]|BOLD:AAA3150
Leucania dia|[5086]|LBCG365-08|Canada|British Columbia|658[0n]|BOLD:AAA3150
Leucania dia|[5087]|RDNME600-08|United States|California|658[0n]|BOLD:AAA3150
Leucania dia|[5088]|RDNME599-08|United States|California|658[0n]|BOLD:AAA3150
Leucania dia|[5089]|LHLEP100-06|Canada|British Columbia|658[0n]|BOLD:AAA3150
Leucania dia|[5090]|LHLEP097-06|Canada|British Columbia|658[0n]|BOLD:AAA3150
Leucania dia|[5091]|LHLEP096-06|Canada|British Columbia|658[0n]|BOLD:AAA3150
Leucania dia|[5092]|RDNMD561-06|United States|Colorado|658[5n]|BOLD:AAA3150
Leucania dia|[5093]|RDNME288-07|United States|Colorado|658[0n]|BOLD:AAA3150
Leucania dia|[5094]|RDNMD562-06|United States|Colorado|658[1n]|BOLD:AAA3150
Leucania dia|[5095]|RDNME289-07|United States|Colorado|565[1n]|BOLD:AAA3150
Leucania dia|[5096]|RDNME290-07|United States|Colorado|607[0n]|BOLD:AAA3150
Leucania dia|[5097]|LPVIB881-08|Canada|British Columbia|639[0n]|BOLD:AAA3150
[illegible]

Leucania dia|[5095]|RDNME289-07|United States|Colorado|565[1n]|BOLD:AAA3150
Leucania dia|[5096]|RDNME290-07|United States|Colorado|607[0n]|BOLD:AAA3150
Leucania dia|[5097]|LPVIB881-08|Canada|British Columbia|639[0n]|BOLD:AAA3150
Leucania dia|[5098]|LPVIB907-08|Canada|British Columbia|638[0n]|BOLD:AAA3150
Leucania dia|[5099]|LPVIC099-08|Canada|British Columbia|637[0n]|BOLD:AAA3150
Leucania dia|[5100]|LALPA103-10|Canada|British Columbia|658[0n]|BOLD:AAA3150
Leucania dia|[5101]|LALPA192-10|Canada|British Columbia|658[0n]|BOLD:AAA3150
Leucania dia|[5102]|LALPA1278-11|Canada|British Columbia|658[0n]|BOLD:AAA3150
Leucania dia|[5103]|CNCLB2475-14|United States|California|658[0n]|BOLD:AAA3150
Mythimna oxygala|[5104]|BLTIB237-08|Canada|Ontario|658[0n]|BOLD:AAA2815
Mythimna oxygala|[5105]|LNAUP159-13|United States|Maryland|652[0n]|BOLD:AAA2815
Mythimna oxygala|[5106]|RDNMC032-05|United States|Wyoming|658[0n]|BOLD:AAA2815
Mythimna oxygala|[5107]|LSEU785-06|United States|Colorado|658[0n]|BOLD:AAA2815
Mythimna oxygala|[5108]|BBLPC057-09|Canada|New Brunswick|658[0n]|BOLD:AAA2815
Mythimna oxygala|[5109]|BBLPE352-09|Canada|Newfoundland and Labrador|658[0n]|BOLD:AAA2815
Mythimna oxygala|[5110]|LNAUP157-13|United States|New Mexico|658[0n]|BOLD:AAA2815
Mythimna oxygala|[5111]|LNAUP156-13|United States|Colorado|655[0n]|BOLD:AAA2815
Mythimna oxygala|[5112]|LNAUP155-13|United States|Colorado|658[0n]|BOLD:AAA2815
Mythimna oxygala|[5113]|LNAUP158-13|United States|New Mexico|658[0n]|BOLD:AAA2815
Mythimna oxygala|[5114]|LPSOC336-08|Canada|Ontario|658[0n]|BOLD:AAA2815
Mythimna oxygala|[5115]|XAD719-05|Canada|Ontario|658[0n]|BOLD:AAA2815
Mythimna oxygala|[5116]|RDLQF884-06|Canada|Quebec|658[0n]|BOLD:AAA2815
Mythimna oxygala|[5117]|XAB366-04|Canada|Ontario|658[0n]|BOLD:AAA2815
Mythimna oxygala|[5118]|BLTIB268-08|Canada|Ontario|658[0n]|BOLD:AAA2815
Mythimna oxygala|[5119]|LPMNB225-09|Canada|Manitoba|658[0n]|BOLD:AAA2815
Mythimna oxygala|[5120]|LPABC367-09|Canada|Alberta|638[0n]|BOLD:AAA2815
Mythimna oxygala|[5121]|BBLPA847-10|Canada|Alberta|658[0n]|BOLD:AAA2815
Mythimna oxygala|[5122]|BBLPA846-10|Canada|Alberta|658[0n]|BOLD:AAA2815
Mythimna oxygala|[5123]|BBLPA839-10|Canada|Saskatchewan|658[0n]|BOLD:AAA2815
Mythimna oxygala|[5124]|LPABC246-09|Canada|Alberta|658[0n]|BOLD:AAA2815
Mythimna oxygala|[5125]|BLTIB273-08|Canada|Ontario|658[0n]|BOLD:AAA2815
Mythimna oxygala|[5126]|LPSOC340-08|Canada|Ontario|658[0n]|BOLD:AAA2815
Mythimna oxygala|[5127]|LPSOC133-08|Canada|Ontario|658[0n]|BOLD:AAA2815
Mythimna oxygala|[5128]|LCH557-04|Canada|Manitoba|658[0n]|BOLD:AAA2815
Mythimna oxygala|[5129]|RDMAB062-05|Canada|Alberta|645[1n]|BOLD:AAA2815
Mythimna oxygala|[5130]|XAH171-05|Canada|Ontario|658[1n]|BOLD:AAA2815
Mythimna oxygala|[5131]|LPABC315-09|Canada|Alberta|633[0n]|BOLD:AAA2815
Mythimna oxygala|[5132]|BLGSM028-09|Canada|Ontario|646[0n]|BOLD:AAA2815
Mythimna oxygala|[5133]|PHMO117-03|Canada|Ontario|639[0n]|BOLD:AAA2815
Mythimna oxygala|[5134]|LPSOC338-08|Canada|Ontario|609[0n]|BOLD:AAA2815
Mythimna oxygala|[5135]|XAD366-04|Canada|Ontario|587[0n]|BOLD:AAA2815
Mythimna oxygala|[5136]|XAD365-04|Canada|Ontario|572[0n]|BOLD:AAA2815
Mythimna oxygala|[5137]|RDMAB063-05|Canada|Alberta|573[0n]|BOLD:AAA2815
Mythimna oxygala|[5138]|RDNMC193-05|Canada|Quebec|585[0n]|BOLD:AAA2815
Mythimna oxygala|[5139]|RDLQ658-07|Canada|Quebec|552[0n]|BOLD:AAA2815
Mythimna oxygala|[5140]|CNWBC136-13|Canada|Alberta|594[0n]|BOLD:AAA2815
Mythimna oxygala|[5141]|XAD717-05|Canada|Ontario|650[0n]|BOLD:AAA2815
Mythimna oxygala|[5142]|BLGSM065-09|Canada|Ontario|658[0n]|BOLD:AAA2815
Mythimna oxygala|[5143]|RDNMC035-05|Canada|Alberta|658[0n]|BOLD:AAA2815
Mythimna oxygala|[5144]|LPABC337-09|Canada|Alberta|658[0n]|BOLD:AAA2815
Mythimna oxygala|[5145]|BLGSM094-09|Canada|Ontario|658[0n]|BOLD:AAA2815
Mythimna oxygala|[5146]|BLGSM076-09|Canada|Ontario|658[0n]|BOLD:AAA2815
Mythimna oxygala|[5147]|BLGSM067-09|Canada|Ontario|658[0n]|BOLD:AAA2815
Mythimna oxygala|[5148]|BLTIB790-08|Canada|Ontario|658[0n]|BOLD:AAA2815
Mythimna oxygala|[5149]|BLTIB047-08|Canada|Ontario|658[0n]|BOLD:AAA2815
Mythimna oxygala|[5150]|LPSOC342-08|Canada|Ontario|658[0n]|BOLD:AAA2815
Mythimna oxygala|[5151]|LPSOC339-08|Canada|Ontario|658[0n]|BOLD:AAA2815
Mythimna oxygala|[5152]|LPSOC337-08|Canada|Ontario|657[0n]|BOLD:AAA2815
Mythimna oxygala|[5153]|LPSOC328-08|Canada|Ontario|658[0n]|BOLD:AAA2815
Mythimna oxygala|[5154]|LPSOC229-08|Canada|Ontario|658[0n]|BOLD:AAA2815
Mythimna yukonensis|[5155]|ABKWR093-07|United States|Alaska|658[0n]|BOLD:AAA2815
Mythimna yukonensis|[5156]|ABKWR055-07|United States|Alaska|658[0n]|BOLD:AAA2815
Mythimna oxygala|[5157]|XAJ628-06|Canada|Ontario|658[0n]|BOLD:AAA2815
Mythimna oxygala|[5158]|XAJ452-06|Canada|Ontario|658[0n]|BOLD:AAA2815
Mythimna oxygala|[5159]|XAJ316-06|Canada|Ontario|658[0n]|BOLD:AAA2815
Mythimna oxygala|[5160]|XAH454-05|Canada|Ontario|658[0n]|BOLD:AAA2815
Mythimna oxygala|[5161]|XAH380-05|Canada|Ontario|658[0n]|BOLD:AAA2815
Mythimna oxygala|[5162]|LBCC028-05|Canada|British Columbia|658[0n]|BOLD:AAA2815
Mythimna oxygala|[5163]|XAD718-05|Canada|Ontario|658[0n]|BOLD:AAA2815
Mythimna oxygala|[5164]|XAJ668-06|Canada|Ontario|658[0n]|BOLD:AAA2815
Mythimna oxygala|[5165]|XAF825-05|Canada|Ontario|658[0n]|BOLD:AAA2815
Mythimna oxygala|[5166]|XAD361-04|Canada|Ontario|658[0n]|BOLD:AAA2815
Mythimna oxygala|[5167]|XAB612-04|Canada|Ontario|658[0n]|BOLD:AAA2815
Mythimna oxygala|[5168]|XAK166-06|Canada|Ontario|658[0n]|BOLD:AAA2815
Mythimna oxygala|[5169]|BLTIB678-08|Canada|Ontario|658[1n]|BOLD:AAA2815
Mythimna oxygala|[5170]|LPSOB618-08|Canada|Ontario|646[0n]|BOLD:AAA2815
Mythimna oxygala|[5171]|BLTIB511-08|Canada|Ontario|606[0n]|BOLD:AAA2815
Mythimna oxygala|[5172]|LPSOC341-08|Canada|Ontario|646[0n]|BOLD:AAA2815
Mythimna oxygala|[5173]|PHMO065-03|Canada|Ontario|639[0n]|BOLD:AAA2815
Mythimna oxygala|[5174]|LPSOB724-08|Canada|Ontario|603[0n]|BOLD:AAA2815
Mythimna oxygala|[5175]|LPABC477-09|Canada|Alberta|609[0n]|BOLD:AAA2815
Mythimna oxygala|[5176]|BBLEC277-09|Canada|Nova Scotia|658[0n]|BOLD:AAA2815
Mythimna oxygala|[5177]|BBLPA841-10|Canada|British Columbia|658[0n]|BOLD:AAA2815
Mythimna oxygala|[5178]|BBLPA844-10|Canada|Alberta|658[0n]|BOLD:AAA2815
Mythimna oxygala|[5179]|BBLPA845-10|Canada|Alberta|658[0n]|BOLD:AAA2815
Mythimna oxygala|[5180]|LNAUP160-13|United States|Maryland|658[0n]|BOLD:AAA2815
Mythimna oxygala|[5181]|XAB516-04|Canada|Ontario|658[0n]|BOLD:AAA2815
Mythimna oxygala|[5182]|BLTIB338-08|Canada|Ontario|658[0n]|BOLD:AAA2815
Mythimna yukonensis|[5183]|UAMIC523-13|United States|Alaska|599[5n]|BOLD:AAA2815
Mythimna oxygala|[5184]|RWWA570-09|United States|Washington|658[0n]|BOLD:AAA2815
Mythimna oxygala|[5185]|LHLEP134-06|Canada|British Columbia|658[0n]|BOLD:AAA2815
Mythimna oxygala|[5186]|RWWA989-09|United States|Washington|658[0n]|BOLD:AAA2815
Mythimna oxygala|[5187]|LHLEP144-06|Canada|British Columbia|658[0n]|BOLD:AAA2815
Mythimna oxygala|[5188]|LHLEP145-06|Canada|British Columbia|658[0n]|BOLD:AAA2815
Mythimna oxygala|[5189]|LHLEP133-06|Canada|British Columbia|658[0n]|BOLD:AAA2815
Mythimna oxygala|[5190]|LHLEP141-06|Canada|British Columbia|658[0n]|BOLD:AAA2815
Mythimna oxygala|[5191]|LPSK462-08|Canada|Saskatchewan|658[0n]|BOLD:AAA2815
Mythimna oxygala|[5192]|LPABB382-08|Canada|Alberta|658[0n]|BOLD:AAA2815
Mythimna oxygala|[5193]|LBCC830-05|Canada|British Columbia|658[0n]|BOLD:AAA2815
Mythimna oxygala|[5194]|LOWCD142-06|Canada|British Columbia|658[0n]|BOLD:AAA2815
Mythimna oxygala|[5195]|BBLPA838-10|Canada|Alberta|658[0n]|BOLD:AAA2815
Mythimna oxygala|[5196]|LOWC045-05|Canada|British Columbia|658[0n]|BOLD:AAA2815
Mythimna oxygala|[5197]|LBCA206-05|Canada|British Columbia|658[0n]|BOLD:AAA2815

Mythimna oxygala|[5195]|BBLPA838-10|Canada|Alberta|658[0n]|BOLD:AAA2815
Mythimna oxygala|[5196]|LOWC045-05|Canada|British Columbia|658[0n]|BOLD:AAA2815
Mythimna oxygala|[5197]|LBCA206-05|Canada|British Columbia|658[0n]|BOLD:AAA2815
Mythimna oxygala|[5198]|LOWC036-05|Canada|British Columbia|658[0n]|BOLD:AAA2815
Mythimna oxygala|[5199]|LOWC040-05|Canada|British Columbia|575[0n]|BOLD:AAA2815
Mythimna oxygala|[5200]|LOWC041-05|Canada|British Columbia|658[0n]|BOLD:AAA2815
Mythimna oxygala|[5201]|LPABC454-09|Canada|Alberta|658[0n]|BOLD:AAA2815
Mythimna oxygala|[5202]|LBCC831-05|Canada|British Columbia|658[0n]|BOLD:AAA2815
Mythimna oxygala|[5203]|LBCD319-05|Canada|British Columbia|643[0n]|BOLD:AAA2815
Mythimna oxygala|[5204]|LPGVA609-08|Canada|British Columbia|636[0n]|BOLD:AAA2815
Mythimna oxygala|[5205]|LHLEP080-06|Canada|British Columbia|624[0n]|BOLD:AAA2815
Mythimna oxygala|[5206]|LPGVA637-08|Canada|British Columbia|622[0n]|BOLD:AAA2815
Mythimna oxygala|[5207]|RWWA199-09|United States|Washington|658[0n]|BOLD:AAA2815
Mythimna oxygala|[5208]|RWWA282-09|United States|Washington|658[0n]|BOLD:AAA2815
Mythimna oxygala|[5209]|LMH041-06|Canada|British Columbia|658[0n]|BOLD:AAA2815
Mythimna oxygala|[5210]|LHLEP079-06|Canada|British Columbia|658[0n]|BOLD:AAA2815
Mythimna oxygala|[5211]|LOWCD778-06|Canada|British Columbia|658[0n]|BOLD:AAA2815
Mythimna oxygala|[5212]|LOWCD779-06|Canada|British Columbia|658[0n]|BOLD:AAA2815
Mythimna oxygala|[5213]|LHLEP137-06|Canada|British Columbia|658[0n]|BOLD:AAA2815
Mythimna oxygala|[5214]|LHLEP142-06|Canada|British Columbia|658[0n]|BOLD:AAA2815
Mythimna oxygala|[5215]|LHLEP143-06|Canada|British Columbia|658[0n]|BOLD:AAA2815
Mythimna oxygala|[5216]|LHLEP146-06|Canada|British Columbia|658[0n]|BOLD:AAA2815
Mythimna oxygala|[5217]|LHLEP147-06|Canada|British Columbia|658[0n]|BOLD:AAA2815
Mythimna oxygala|[5218]|LHLEP148-06|Canada|British Columbia|658[0n]|BOLD:AAA2815
Mythimna oxygala|[5219]|RWWA456-09|United States|Washington|658[0n]|BOLD:AAA2815
Mythimna oxygala|[5220]|RWWA689-09|United States|Washington|658[0n]|BOLD:AAA2815
Mythimna oxygala|[5221]|RWWA973-09|United States|Washington|658[0n]|BOLD:AAA2815
Mythimna oxygala|[5222]|RWWB301-09|United States|Washington|658[0n]|BOLD:AAA2815
Mythimna oxygala|[5223]|RWWB309-09|United States|Washington|658[0n]|BOLD:AAA2815
Mythimna oxygala|[5224]|RWWB326-09|United States|Washington|658[0n]|BOLD:AAA2815
Mythimna oxygala|[5225]|RWWC133-10|United States|Washington|658[0n]|BOLD:AAA2815
Mythimna oxygala|[5226]|BBLPA837-10|Canada|Alberta|658[0n]|BOLD:AAA2815
Mythimna oxygala|[5227]|BBLPA842-10|Canada|British Columbia|658[0n]|BOLD:AAA2815
Mythimna oxygala|[5228]|RWWC240-11|United States|Washington|658[0n]|BOLD:AAA2815
Mythimna oxygala|[5229]|LPABC420-09|Canada|Alberta|658[0n]|BOLD:AAA2815
Mythimna oxygala|[5230]|RWWA084-09|United States|Washington|658[0n]|BOLD:AAA2815
Mythimna oxygala|[5231]|RWWA101-09|United States|Washington|658[0n]|BOLD:AAA2815
Mythimna oxygala|[5232]|RWWA102-09|United States|Washington|658[0n]|BOLD:AAA2815
Mythimna oxygala|[5233]|LPSK254-08|Canada|Saskatchewan|658[0n]|BOLD:AAA2815
Mythimna oxygala|[5234]|LPGVA611-08|Canada|British Columbia|658[0n]|BOLD:AAA2815
Mythimna oxygala|[5235]|LPABC363-09|Canada|Alberta|658[0n]|BOLD:AAA2815
Mythimna oxygala|[5236]|LPABC378-09|Canada|Alberta|658[0n]|BOLD:AAA2815
Mythimna oxygala|[5237]|LHLEP132-06|Canada|British Columbia|658[0n]|BOLD:AAA2815
Mythimna oxygala|[5238]|LHLEP135-06|Canada|British Columbia|658[0n]|BOLD:AAA2815
Mythimna oxygala|[5239]|LOWCD162-06|Canada|British Columbia|658[0n]|BOLD:AAA2815
Mythimna oxygala|[5240]|LOWCD188-06|Canada|British Columbia|658[0n]|BOLD:AAA2815
Mythimna oxygala|[5241]|LHLEP130-06|Canada|British Columbia|658[0n]|BOLD:AAA2815
Mythimna oxygala|[5242]|LHLEP131-06|Canada|British Columbia|658[0n]|BOLD:AAA2815
Mythimna oxygala|[5243]|LBCH350-10|Canada|British Columbia|658[0n]|BOLD:AAA2815
Mythimna oxygala|[5244]|LBCH1316-10|Canada|British Columbia|658[0n]|BOLD:AAA2815
Mythimna oxygala|[5245]|LOWC046-05|Canada|British Columbia|658[0n]|BOLD:AAA2815
Mythimna oxygala|[5246]|LOWC044-05|Canada|British Columbia|658[0n]|BOLD:AAA2815
Mythimna oxygala|[5247]|LOWC043-05|Canada|British Columbia|658[0n]|BOLD:AAA2815
Mythimna oxygala|[5248]|LOWC042-05|Canada|British Columbia|658[0n]|BOLD:AAA2815
Mythimna oxygala|[5249]|LOWC039-05|Canada|British Columbia|658[0n]|BOLD:AAA2815
Mythimna oxygala|[5250]|LOWC038-05|Canada|British Columbia|658[0n]|BOLD:AAA2815
Mythimna oxygala|[5251]|LOWC037-05|Canada|British Columbia|658[0n]|BOLD:AAA2815
Mythimna oxygala|[5252]|RDNMC034-05|Canada|British Columbia|657[0n]|BOLD:AAA2815
Mythimna oxygala|[5253]|RDNMC033-05|Canada|British Columbia|658[0n]|BOLD:AAA2815
Mythimna oxygala|[5254]|LBCC832-05|Canada|British Columbia|658[0n]|BOLD:AAA2815
Mythimna oxygala|[5255]|LBCA207-05|Canada|British Columbia|653[0n]|BOLD:AAA2815
Mythimna oxygala|[5256]|LHLEP140-06|Canada|British Columbia|652[0n]|BOLD:AAA2815
Mythimna oxygala|[5257]|LHLEP136-06|Canada|British Columbia|646[0n]|BOLD:AAA2815
Mythimna oxygala|[5258]|RWWB371-09|United States|Washington|633[0n]|BOLD:AAA2815
Mythimna oxygala|[5259]|LHLEP138-06|Canada|British Columbia|624[0n]|BOLD:AAA2815
Mythimna oxygala|[5260]|LPABC171-09|Canada|Alberta|613[0n]|BOLD:AAA2815
Mythimna oxygala|[5261]|LBCA374-05|Canada|British Columbia|622[0n]|BOLD:AAA2815
Mythimna oxygala|[5262]|LOWCC899-05|Canada|British Columbia|583[1n]|BOLD:AAA2815
Mythimna oxygala|[5263]|LOWCD770-06|Canada|British Columbia|591[0n]|BOLD:AAA2815
Mythimna oxygala|[5264]|LHLEP139-06|Canada|British Columbia|612[0n]|BOLD:AAA2815
Mythimna oxygala|[5265]|RWWB725-10|United States|Washington|602[0n]|BOLD:AAA2815
Mythimna oxygala|[5266]|RWWB860-10|United States|Washington|642[0n]|BOLD:AAA2815
Mythimna oxygala|[5267]|LALPA1204-11|Canada|British Columbia|658[0n]|BOLD:AAA2815
Mythimna oxygala|[5268]|LPVIB062-08|Canada|British Columbia|658[0n]|BOLD:AAA2815
Mythimna oxygala|[5269]|LPVIB006-08|Canada|British Columbia|658[0n]|BOLD:AAA2815
Mythimna oxygala|[5270]|LPGVA610-08|Canada|British Columbia|618[0n]|BOLD:AAA2815
Mythimna oxygala|[5271]|LPGVA636-08|Canada|British Columbia|632[0n]|BOLD:AAA2815
Mythimna oxygala|[5272]|JMMMB534-13|United States|California|597[0n]|BOLD:AAA2815
Leucania pilipalpis|[5273]|RDNMI045-10|United States|Florida|550[0n]|BOLD:AAM8639
Leucania pilipalpis|[5274]|USLEP1161-10|United States|Florida|658[0n]|BOLD:AAM8639
Leucania pilipalpis|[5275]|RDNMK116-11|United States|Florida|658[0n]|BOLD:AAM8639
Leucania phragmitidicola|[5276]|XAD288-04|Canada|Ontario|591[0n]|BOLD:ABX6101
Leucania phragmitidicola|[5277]|XAD578-04|Canada|Ontario|588[3n]|BOLD:ABX6101
Leucania phragmitidicola|[5278]|XAD720-05|Canada|Ontario|658[0n]|BOLD:ABX6101
Leucania phragmitidicola|[5279]|XAH134-05|Canada|Ontario|658[1n]|BOLD:ABX6101
Leucania phragmitidicola|[5280]|XAB257-04|Canada|Ontario|658[13n]|BOLD:ABX6101
Leucania phragmitidicola|[5281]|XAB595-04|Canada|Ontario|590[0n]|BOLD:ABX6101
Leucania phragmitidicola|[5282]|RDNMD646-06|United States|New Mexico|658[0n]|BOLD:ABX6101
Leucania phragmitidicola|[5283]|LPSOB212-08|Canada|Ontario|658[0n]|BOLD:ABX6101
Leucania phragmitidicola|[5284]|LPSOB227-08|Canada|Ontario|658[0n]|BOLD:ABX6101
Leucania phragmitidicola|[5285]|LPSOB385-08|Canada|Ontario|658[0n]|BOLD:ABX6101
Leucania phragmitidicola|[5286]|LPOKA377-08|United States|Oklahoma|658[0n]|BOLD:ABX6101
Leucania phragmitidicola|[5287]|LPOKB1032-09|United States|Oklahoma|658[0n]|BOLD:ABX6101
Leucania phragmitidicola|[5288]|BBLSW805-09|United States|Oklahoma|658[0n]|BOLD:ABX6101
Leucania phragmitidicola|[5289]|BBLCU091-09|United States|Kansas|658[0n]|BOLD:ABX6101
Leucania phragmitidicola|[5290]|USLEP1047-10|United States|Colorado|658[0n]|BOLD:ABX6101
Leucania phragmitidicola|[5291]|LNCC1229-11|United States|North Carolina|658[0n]|BOLD:ABX6101
Leucania phragmitidicola|[5292]|BBLOD887-11|United States|California|658[0n]|BOLD:ABX6101
Leucania phragmitidicola|[5293]|BBLOE1461-12|United States|Texas|658[0n]|BOLD:ABX6101
Leucania phragmitidicola|[5294]|BBLOE1711-12|United States|Texas|658[0n]|BOLD:ABX6101
Leucania commoides|[5295]|XAC613-04|Canada|Ontario|658[0n]|BOLD:AAA8386
Leucania commoides|[5296]|XAJ841-06|Canada|Ontario|658[0n]|BOLD:AAA8386
Leucania commoides|[5297]|XAJ917-06|Canada|Ontario|658[0n]|BOLD:AAA8386

Leucania commoides|[5295]|XAC613-04|Canada|Ontario|658[0n]|BOLD:AAA8386
Leucania commoides|[5296]|XAJ841-06|Canada|Ontario|658[0n]|BOLD:AAA8386
Leucania commoides|[5297]|XAJ917-06|Canada|Ontario|658[0n]|BOLD:AAA8386
Leucania commoides|[5298]|XAJ844-06|Canada|Ontario|658[0n]|BOLD:AAA8386
Leucania commoides|[5299]|XAB496-04|Canada|Ontario|658[0n]|BOLD:AAA8386
Leucania commoides|[5300]|RDLQB913-05|Canada|Quebec|549[0n]|BOLD:AAA8386
Leucania commoides|[5301]|XAJ826-06|Canada|Ontario|596[0n]|BOLD:AAA8386
Leucania commoides|[5302]|RDLQG129-06|Canada|Quebec|607[2n]|BOLD:AAA8386
Leucania commoides|[5303]|LPMN085-08|Canada|Manitoba|658[0n]|BOLD:AAA8386
Leucania commoides|[5304]|BLTIB786-08|Canada|Ontario|658[0n]|BOLD:AAA8386
Leucania commoides|[5305]|XAC461-04|Canada|Ontario|613[0n]|BOLD:AAA8386
Leucania commoides|[5306]|MNBB442-05|Canada|New Brunswick|658[0n]|BOLD:AAA8386
Leucania commoides|[5307]|MNBB444-05|Canada|New Brunswick|658[0n]|BOLD:AAA8386
Leucania commoides|[5308]|MNBB650-05|Canada|New Brunswick|658[0n]|BOLD:AAA8386
Leucania commoides|[5309]|TTMNB413-06|Canada|New Brunswick|658[0n]|BOLD:AAA8386
Leucania commoides|[5310]|RDNME583-08|United States|Colorado|658[0n]|BOLD:AAA8386
Leucania commoides|[5311]|LPSK007-08|Canada|Saskatchewan|658[0n]|BOLD:AAA8386
Leucania commoides|[5312]|LPMNB418-09|Canada|Manitoba|658[0n]|BOLD:AAA8386
Leucania commoides|[5313]|BBLEC696-09|Canada|Nova Scotia|658[0n]|BOLD:AAA8386
Leucania commoides|[5314]|MNBB162-05|Canada|New Brunswick|658[0n]|BOLD:AAA8386
Leucania commoides|[5315]|MNBB327-05|Canada|New Brunswick|658[0n]|BOLD:AAA8386
Leucania commoides|[5316]|MNBB609-05|Canada|New Brunswick|658[0n]|BOLD:AAA8386
Leucania commoides|[5317]|MNBB113-05|Canada|New Brunswick|658[0n]|BOLD:AAA8386
Leucania commoides|[5318]|MNBB112-05|Canada|New Brunswick|658[0n]|BOLD:AAA8386
Leucania commoides|[5319]|MNBB111-05|Canada|New Brunswick|658[0n]|BOLD:AAA8386
Leucania commoides|[5320]|MNBB110-05|Canada|New Brunswick|658[0n]|BOLD:AAA8386
Leucania commoides|[5321]|MNBB109-05|Canada|New Brunswick|658[0n]|BOLD:AAA8386
Leucania commoides|[5322]|MNBB108-05|Canada|New Brunswick|658[0n]|BOLD:AAA8386
Leucania commoides|[5323]|PHMNB748-05|Canada|New Brunswick|658[0n]|BOLD:AAA8386
Leucania commoides|[5324]|PHMNB096-04|Canada|New Brunswick|658[0n]|BOLD:AAA8386
Leucania commoides|[5325]|XAC714-04|Canada|Ontario|658[0n]|BOLD:AAA8386
Leucania commoides|[5326]|XAB175-04|Canada|Ontario|658[0n]|BOLD:AAA8386
Leucania commoides|[5327]|MNBB610-05|Canada|New Brunswick|658[0n]|BOLD:AAA8386
Leucania commoides|[5328]|MNBB164-05|Canada|New Brunswick|534[2n]|BOLD:AAA8386
Leucania commoides|[5329]|XAC715-04|Canada|Ontario|658[0n]|BOLD:AAA8386
Leucania commoides|[5330]|MNBB106-05|Canada|New Brunswick|658[0n]|BOLD:AAA8386
Leucania commoides|[5331]|MNBB239-05|Canada|New Brunswick|538[0n]|BOLD:AAA8386
Leucania commoides|[5332]|MNBB107-05|Canada|New Brunswick|548[0n]|BOLD:AAA8386
Leucania commoides|[5333]|XAC073-04|Canada|Ontario|582[0n]|BOLD:AAA8386
Leucania commoides|[5334]|MNBB163-05|Canada|New Brunswick|547[2n]|BOLD:AAA8386
Leucania commoides|[5335]|RDLQG128-06|Canada|Quebec|603[0n]|BOLD:AAA8386
Leucania commoides|[5336]|BBLEC708-09|Canada|Nova Scotia|656[0n]|BOLD:AAA8386
Leucania linda|[5337]|LILLB065-11|United States|Illinois|658[0n]|BOLD:ABY6338
Leucania linda|[5338]|LPOKA679-09|United States|Oklahoma|658[0n]|BOLD:ABY6338
Leucania linda|[5339]|BBLSY089-09|United States|Oklahoma|658[0n]|BOLD:ABY6338
Leucania linda|[5340]|LPOKD673-10|United States|Oklahoma|658[0n]|BOLD:ABY6338
Leucania linda|[5341]|LNCC1249-11|United States|North Carolina|658[0n]|BOLD:ABY6338
Leucania linda|[5342]|LSUSA194-06|United States|Kentucky|658[0n]|BOLD:ABY6338
Leucania linda|[5343]|LPSO992-08|Canada|Ontario|658[0n]|BOLD:ABY6338
Leucania linda|[5344]|LOT487-04|United States|Tennessee|658[0n]|BOLD:ABY6338
Leucania linda|[5345]|LGSMC684-05|United States|Tennessee|658[0n]|BOLD:ABY6338
Leucania linda|[5346]|LPOKA546-09|United States|Oklahoma|658[0n]|BOLD:ABY6338
Leucania linda|[5347]|LPOKA1037-09|United States|Oklahoma|658[0n]|BOLD:ABY6338
Leucania linda|[5348]|BBLSY859-09|United States|Oklahoma|658[0n]|BOLD:ABY6338
Leucania linda|[5349]|BBLSZ130-09|United States|Oklahoma|658[0n]|BOLD:ABY6338
Leucania linda|[5350]|BBLPA582-10|Canada|Ontario|658[0n]|BOLD:ABY6338
Leucania linda|[5351]|LILLA288-11|United States|Illinois|658[0n]|BOLD:ABY6338
Leucania linda|[5352]|LNCNW059-06|United States|North Carolina|658[0n]|BOLD:ABY6338
Leucania linda|[5353]|LILLA315-11|United States|Illinois|658[0n]|BOLD:ABY6338
Leucania linda|[5354]|LNCC1317-11|United States|North Carolina|658[0n]|BOLD:ABY6338
Leucania linda|[5355]|LNCC1226-11|United States|North Carolina|658[0n]|BOLD:ABY6338
Leucania linda|[5356]|LPOKE099-10|United States|Oklahoma|658[0n]|BOLD:ABY6338
Leucania linda|[5357]|LGSMG863-10|United States|North Carolina|658[0n]|BOLD:ABY6338
Leucania linda|[5358]|LPOKA561-09|United States|Oklahoma|658[0n]|BOLD:ABY6338
Leucania linda|[5359]|LPSOB970-08|Canada|Ontario|658[0n]|BOLD:ABY6338
Leucania linda|[5360]|XAH504-05|Canada|Ontario|658[0n]|BOLD:ABY6338
Leucania linda|[5361]|LGSMC683-05|United States|Tennessee|658[0n]|BOLD:ABY6338
Leucania linda|[5362]|LOT488-04|United States|Tennessee|658[0n]|BOLD:ABY6338
Leucania linda|[5363]|LNCNW046-06|United States|North Carolina|654[0n]|BOLD:ABY6338
Leucania linda|[5364]|CNPPI012-12|Canada|Ontario|627[0n]|BOLD:ABY6338
Leucania incognita|[5365]|LOFLC357-06|United States|Florida|658[0n]|BOLD:AAB1342
Leucania incognita|[5366]|BBLOB480-11|United States|Florida|658[0n]|BOLD:AAB1342
Leucania incognita|[5367]|LOFLC466-06|United States|Florida|658[0n]|BOLD:AAB1342
Leucania incognita|[5368]|LOFLC400-06|United States|Florida|658[0n]|BOLD:AAB1342
Leucania incognita|[5369]|LOFLC341-06|United States|Florida|658[0n]|BOLD:AAB1342
Leucania incognita|[5370]|LOFLC339-06|United States|Florida|658[0n]|BOLD:AAB1342
Leucania incognita|[5371]|LOFLC327-06|United States|Florida|658[0n]|BOLD:AAB1342
Leucania incognita|[5372]|LOFLC310-06|United States|Florida|658[0n]|BOLD:AAB1342
Leucania incognita|[5373]|LOFLB400-06|United States|Florida|658[0n]|BOLD:AAB1342
Leucania incognita|[5374]|LOFLB220-06|United States|Florida|658[0n]|BOLD:AAB1342
Leucania incognita|[5375]|BBLOB507-11|United States|Florida|618[0n]|BOLD:AAB1342
Leucania incognita|[5376]|LOCRE429-10|Costa Rica|San Jose|658[0n]|BOLD:AAB1342
Leucania incognita|[5377]|LOCRE427-10|Costa Rica|San Jose|658[0n]|BOLD:AAB1342
Leucania incognita|[5378]|BLPCA488-08|Costa Rica|Guanacaste|632[0n]|BOLD:AAB1342
Leucania incognita|[5379]|BLPAF771-07|Costa Rica|Guanacaste|658[0n]|BOLD:AAB1342
Leucania incognita|[5380]|RDNMD567-06|United States|Florida|658[0n]|BOLD:AAB1342
Leucania incognita|[5381]|LOFLB700-06|United States|Florida|658[0n]|BOLD:AAB1342
Leucania incognita|[5382]|BLPCA487-08|Costa Rica|Guanacaste|646[0n]|BOLD:AAB1342
Leucania incognita|[5383]|LOCRE428-10|Costa Rica|San Jose|658[0n]|BOLD:AAB1342
Leucania incognita|[5384]|BLPCK757-08|Costa Rica|Alajuela|658[0n]|BOLD:AAB1342
Leucania incognita|[5385]|RDNMD568-06|United States|Florida|658[0n]|BOLD:AAB1342
Leucania incognita|[5386]|BLPAA457-06|Costa Rica|Guanacaste|613[0n]|BOLD:AAB1342
Leucania incognita|[5387]|BLPDA475-09|Costa Rica|Alajuela|649[0n]|BOLD:AAB1342
Leucania incognita|[5388]|BBLSX699-09|United States|Texas|658[0n]|BOLD:AAB1342
Leucania incognita|[5389]|LOCRE432-10|Costa Rica|San Jose|658[0n]|BOLD:AAB1342
Leucania incognita|[5390]|LOCRE446-10|Costa Rica|San Jose|658[0n]|BOLD:AAB1342
Leucania incognita|[5391]|LOCRE435-10|Costa Rica|San Jose|658[0n]|BOLD:AAB1342
Leucania incognita|[5392]|BLPAA527-06|Costa Rica|Guanacaste|658[0n]|BOLD:AAB1342
Leucania incognita|[5393]|BLPAG412-07|Costa Rica|Guanacaste|658[0n]|BOLD:AAB1342
Leucania incognita|[5394]|BLPDE134-09|Costa Rica|Guanacaste|658[0n]|BOLD:AAB1342
Leucania incognita|[5395]|BBLSW845-09|United States|Texas|658[0n]|BOLD:AAB1342
Leucania incognita|[5396]|LYPAP464-09|Mexico|Quintana Roo|658[0n]|BOLD:AAB1342
Leucania incognita|[5397]|LYHES251-09|Mexico|Quintana Roo|658[0n]|BOLD:AAB1342

Leucania incognita|[5395]|BBLSW845-09|United States|Texas|658[0n]|BOLD:AAB1342
Leucania incognita|[5396]|LYPAP464-09|Mexico|Quintana Roo|658[0n]|BOLD:AAB1342
Leucania incognita|[5397]|LYHES251-09|Mexico|Quintana Roo|658[0n]|BOLD:AAB1342
Leucania incognita|[5398]|BBLSX696-09|United States|Texas|658[0n]|BOLD:AAB1342
Leucania incognita|[5399]|BBLOC1561-11|United States|Texas|658[0n]|BOLD:AAB1342
Leucania incognita|[5400]|BLPEE938-12|Costa Rica|Guanacaste|658[0n]|BOLD:AAB1342
Leucania incognita|[5401]|BLPDA738-09|Costa Rica|Guanacaste|658[0n]|BOLD:AAB1342
Leucania incognita|[5402]|LOCRE426-10|Costa Rica|San Jose|658[0n]|BOLD:AAB1342
Leucania incognita|[5403]|LOCRE430-10|Costa Rica|San Jose|658[0n]|BOLD:AAB1342
Leucania incognita|[5404]|BLPAA184-06|Costa Rica|Guanacaste|658[1n]|BOLD:AAB1342
Leucania incognita|[5405]|BLPBG130-07|Costa Rica|Guanacaste|654[0n]|BOLD:AAB1342
Leucania incognita|[5406]|IBOLG152-08|Costa Rica|Alajuela|657[0n]|BOLD:AAB1342
Leucania incognita|[5407]|IBOLG148-08|Costa Rica|Alajuela|657[0n]|BOLD:AAB1342
Leucania incognita|[5408]|BLPAH355-07|Costa Rica|Guanacaste|658[0n]|BOLD:AAB1342
Leucania incognita|[5409]|BLPDA782-09|Costa Rica|Guanacaste|658[0n]|BOLD:AAB1342
Leucania incognita|[5410]|LOCRE434-10|Costa Rica|San Jose|658[0n]|BOLD:AAB1342
Leucania incognita|[5411]|LOCRE440-10|Costa Rica|San Jose|658[0n]|BOLD:AAB1342
Leucania incognita|[5412]|LOCRE449-10|Costa Rica|San Jose|658[0n]|BOLD:AAB1342
Leucania incognita|[5413]|BLPEE1334-12|Costa Rica|Guanacaste|658[0n]|BOLD:AAB1342
Leucania extincta|[5414]|LOFLC308-06|United States|Florida|658[0n]|BOLD:AAD1190
Leucania extincta|[5415]|RDNMD569-06|United States|Florida|658[4n]|BOLD:AAD1190
Leucania extincta|[5416]|LOFLB818-06|United States|Florida|658[0n]|BOLD:AAD1190
Leucania extincta|[5417]|LSEU083-06|United States|Georgia|591[0n]|BOLD:AAD1190
Leucania extincta|[5418]|LOFLB538-06|United States|Florida|658[0n]|BOLD:AAD1190
Leucania extincta|[5419]|LOFLC442-06|United States|Florida|658[0n]|BOLD:AAD1190
Leucania extincta|[5420]|LNC088-05|United States|North Carolina|658[0n]|BOLD:AAD1190
Leucania extincta|[5421]|RDNMD570-06|United States|Florida|658[0n]|BOLD:AAD1190
Leucania extincta|[5422]|CNCLB1782-14|United States|Indiana|658[0n]|BOLD:AAD1190
Leucania adjuta|[5423]|LGSM529-04|United States|Tennessee|657[2n]|BOLD:AAC0179
Leucania adjuta|[5424]|LGSMC682-05|United States|Tennessee|658[0n]|BOLD:AAC0179
Leucania adjuta|[5425]|LNC430-05|United States|North Carolina|616[1n]|BOLD:AAC0179
Leucania adjuta|[5426]|LGSMC685-05|United States|Tennessee|658[1n]|BOLD:AAC0179
Leucania adjuta|[5427]|LGSMD680-05|United States|North Carolina|658[1n]|BOLD:AAC0179
Leucania adjuta|[5428]|LOFLA274-06|United States|Florida|657[0n]|BOLD:AAC0179
Leucania adjuta|[5429]|JRLAA011-09|United States|Alabama|658[0n]|BOLD:AAC0179
Leucania adjuta|[5430]|LGSM530-04|United States|Tennessee|658[0n]|BOLD:AAC0179
Leucania adjuta|[5431]|LGSMC380-05|United States|Tennessee|658[0n]|BOLD:AAC0179
Leucania adjuta|[5432]|LNC640-06|United States|North Carolina|657[0n]|BOLD:AAC0179
Leucania adjuta|[5433]|LOFLA170-06|United States|Florida|657[0n]|BOLD:AAC0179
Leucania adjuta|[5434]|RDNMD565-06|United States|Florida|657[0n]|BOLD:AAC0179
Leucania adjuta|[5435]|RDNMD571-06|United States|Florida|657[0n]|BOLD:AAC0179
Leucania adjuta|[5436]|RDNMD572-06|United States|Florida|657[0n]|BOLD:AAC0179
Leucania adjuta|[5437]|LPOKA284-08|United States|Oklahoma|658[0n]|BOLD:AAC0179
Leucania adjuta|[5438]|BBLSW298-09|United States|Oklahoma|658[0n]|BOLD:AAC0179
Leucania adjuta|[5439]|BBLSW454-09|United States|Oklahoma|658[0n]|BOLD:AAC0179
Leucania adjuta|[5440]|BBLSU088-09|United States|Mississippi|658[0n]|BOLD:AAC0179
Leucania adjuta|[5441]|LPOKD610-09|United States|Oklahoma|658[0n]|BOLD:AAC0179
Leucania adjuta|[5442]|LGSMG862-10|United States|North Carolina|658[0n]|BOLD:AAC0179
Leucania adjuta|[5443]|USLEP1193-10|United States|Florida|658[0n]|BOLD:AAC0179
Leucania adjuta|[5444]|BBLSX285-09|United States|Oklahoma|657[0n]|BOLD:AAC0179
Leucania adjuta|[5445]|LPOKC782-09|United States|Oklahoma|658[0n]|BOLD:AAC0179
Leucania adjuta|[5446]|LILLA948-11|United States|Illinois|658[0n]|BOLD:AAC0179
Leucania dorsalis|[5447]|BLPDB645-09|Costa Rica|Alajuela|608[0n]|BOLD:AAC7012
Leucania dorsalis|[5448]|BLPEF5673-13|Costa Rica||658[0n]|BOLD:AAC7012
Leucania dorsalis|[5449]|RDNML255-13|Puerto Rico||658[0n]|BOLD:AAC7012
Leucania dorsalis|[5450]|LOCRF650-11|Costa Rica|Cartago|658[0n]|BOLD:AAC7012
Leucania dorsalis|[5451]|LOCRE438-10|Costa Rica|San Jose|658[0n]|BOLD:AAC7012
Leucania dorsalis|[5452]|BLPDQ993-10|Costa Rica|Alajuela|658[0n]|BOLD:AAC7012
Leucania dorsalis|[5453]|USLEP1050-10|United States|Florida|658[0n]|BOLD:AAC7012
Leucania dorsalis|[5454]|USLEP784-10|United States|Florida|658[0n]|BOLD:AAC7012
Leucania dorsalis|[5455]|BLPAC899-06|Costa Rica|Guanacaste|658[0n]|BOLD:AAC7012
Leucania dorsalis|[5456]|BLPAB084-06|Costa Rica|Alajuela|658[0n]|BOLD:AAC7012
Leucania dorsalis|[5457]|MHMXE642-07|Costa Rica|Guanacaste|654[0n]|BOLD:AAC7012
Leucania dorsalis|[5458]|BLPEF5675-13|Costa Rica||658[0n]|BOLD:AAC7012
Leucania subpunctata|[5459]|RDNMD573-06|United States|Florida|657[0n]|BOLD:ACF4644
Leucania subpunctata|[5460]|RDNMK053-11|United States|Florida|658[0n]|BOLD:ACF4644
Leucania subpunctata|[5461]|GWOTA074-12|United States|Texas|658[0n]|BOLD:ACF4644
Leucania infatuans|[5462]|IAWLB497-11|United States|Arizona|658[0n]|BOLD:AAE3308
Leucania infatuans|[5463]|IAWLB496-11|United States|Arizona|658[0n]|BOLD:AAE3308
Leucania infatuans|[5464]|BLPCK128-08|Costa Rica|Alajuela|658[0n]|BOLD:AAE3308
Leucania infatuans|[5465]|BLPCK255-08|Costa Rica|Alajuela|658[0n]|BOLD:AAE3308
Leucania infatuans|[5466]|LOCRE441-10|Costa Rica|San Jose|658[0n]|BOLD:AAE3308
Leucania infatuans|[5467]|LYHES545-09|Mexico|Quintana Roo|658[0n]|BOLD:AAE3308
Leucania infatuans|[5468]|LOCRE451-10|Costa Rica|San Jose|658[0n]|BOLD:AAE3308
Leucania infatuans|[5469]|LYPAP423-09|Mexico|Quintana Roo|658[0n]|BOLD:AAE3308
Leucania infatuans|[5470]|CNCLA5281-13|United States|Florida|658[0n]|BOLD:AAE3308
Leucania infatuans|[5471]|LOCRE437-10|Costa Rica|San Jose|658[0n]|BOLD:AAE3308
Leucania infatuans|[5472]|LOCRE439-10|Costa Rica|San Jose|658[0n]|BOLD:AAE3308
Leucania infatuans|[5473]|USLEP1048-10|United States|Florida|658[0n]|BOLD:AAE3308
Leucania infatuans|[5474]|USLEP1049-10|United States|Florida|658[0n]|BOLD:AAE3308
Leucania infatuans|[5475]|RDNML324-13|United States|Florida|658[0n]|BOLD:AAE3308
Leucania infatuans|[5476]|RDNML325-13|United States|Florida|658[0n]|BOLD:AAE3308
Leucania infatuans|[5477]|CNCLB2115-14|United States|Florida|658[0n]|BOLD:AAE3308
Leucania oaxacana|[5478]|BBLOD1700-11|United States|California|658[0n]|BOLD:AAH5359
Leucania oaxacana|[5479]|CMAZA104-09|United States|Arizona|636[0n]|BOLD:AAH5359
Leucania oaxacana|[5480]|BBLOD1701-11|United States|California|658[0n]|BOLD:AAH5359
Leucania oaxacana|[5481]|BBLOD1699-11|United States|California|658[0n]|BOLD:AAH5359
Leucania oaxacana|[5482]|IAWLB192-11|United States|Arizona|658[0n]|BOLD:AAH5359
Leucania oaxacana|[5483]|RDNMJ127-10|United States|Arizona|658[0n]|BOLD:AAH5359
Leucania oaxacana|[5484]|CMAZA289-09|United States|Arizona|658[0n]|BOLD:AAH5359
Leucania oaxacana|[5485]|BBLSX721-09|United States|Arizona|658[0n]|BOLD:AAH5359
Leucania oaxacana|[5486]|BBLOD1702-11|United States|California|658[0n]|BOLD:AAH5359
Leucania oaxacana|[5487]|BBLOD1709-11|United States|California|658[0n]|BOLD:AAH5359
Leucania oaxacana|[5488]|BBLOD1710-11|United States|California|658[0n]|BOLD:AAH5359
Leucania imperfecta|[5489]|RDNMD898-07|United States|New Mexico|655[0n]|BOLD:ACE8617
Leucania imperfecta|[5490]|RDNMJ535-11|United States|Arizona|658[0n]|BOLD:ACE8617
Leucania imperfecta|[5491]|RDNMJ560-11|United States|Arizona|657[0n]|BOLD:ACE8617
Leucania imperfecta|[5492]|RDNMD636-06|United States|Wyoming|658[0n]|BOLD:ACE8617
Leucania imperfecta|[5493]|BBLSY379-09|United States|Arizona|658[0n]|BOLD:ACE8617
Leucania imperfecta|[5494]|IAWLB560-11|United States|Arizona|658[0n]|BOLD:ACE8617
Leucania imperfecta|[5495]|IAWLB562-11|United States|Arizona|658[0n]|BOLD:ACE8617
Leucania imperfecta|[5496]|RDNMD897-07|United States|New Mexico|655[0n]|BOLD:ACE8618
Leucania imperfecta|[5497]|RDNME049-07|United States|New Mexico|658[0n]|BOLD:ACE8618

Leucania imperfecta|[5495]|IAWLB562-11|United States|Arizona|658[0n]|BOLD:ACE8617
Leucania imperfecta|[5496]|RDNMD897-07|United States|New Mexico|655[0n]|BOLD:ACE8618
Leucania imperfecta|[5497]|RDNME049-07|United States|New Mexico|658[0n]|BOLD:ACE8618
Leucania imperfecta|[5498]|RDNMJ126-10|United States|Arizona|658[0n]|BOLD:ACE8618
Leucania imperfecta|[5499]|RDNMG258-08|United States|Texas|658[0n]|BOLD:ACE8618
Leucania imperfecta|[5500]|IAWLB561-11|United States|Arizona|658[0n]|BOLD:ACE8618
Leucania imperfecta|[5501]|LYOMX119-12|Mexico|Michoacan|658[0n]|BOLD:ACE8618
Leucania oregona|[5502]|RDNMF577-08|Canada|British Columbia|609[0n]|BOLD:AAC7175
Leucania oregona|[5503]|LOCBE092-06|United States|California|658[0n]|BOLD:AAC7175
Leucania oregona|[5504]|GMLC405-11|United States|California|658[0n]|BOLD:AAC7175
Leucania oregona|[5505]|RDNMF576-08|Canada|British Columbia|609[0n]|BOLD:AAC7175
Leucania oregona|[5506]|BBLOC934-11|United States|California|658[0n]|BOLD:AAC7175
Leucania oregona|[5507]|BBLOC932-11|United States|California|658[0n]|BOLD:AAC7175
Leucania oregona|[5508]|GMLC686-11|United States|California|658[0n]|BOLD:AAC7175
Leucania oregona|[5509]|GMLC387-11|United States|California|658[0n]|BOLD:AAC7175
Leucania oregona|[5510]|GMLC763-12|United States|California|651[0n]|BOLD:AAC7175
Leucania oregona|[5511]|GMLC854-12|United States|California|658[0n]|BOLD:AAC7175
Leucania oregona|[5512]|GMLC1233-12|United States|California|658[0n]|BOLD:AAC7175
Leucania senescens|[5513]|BLPAE671-06|Costa Rica|Guanacaste|655[0n]|BOLD:AAC6983
Leucania senescens|[5514]|LOCRE433-10|Costa Rica|San Jose|658[0n]|BOLD:AAC6983
Leucania senescens|[5515]|BLPDB382-09|Costa Rica|Alajuela|578[0n]|BOLD:AAC6983
Leucania senescens|[5516]|LNOUB684-10|French Guiana||658[0n]|BOLD:AAC6983
Leucania senescens|[5517]|MILEP717-11|French Guiana||658[0n]|BOLD:AAC6983
Leucania senescens|[5518]|BLPDB891-09|Costa Rica|Guanacaste|658[0n]|BOLD:AAC6983
Leucania senescens|[5519]|MHAUC460-06|Costa Rica|Guanacaste|658[0n]|BOLD:AAC6983
Leucania senescens|[5520]|BLPDB646-09|Costa Rica|Alajuela|658[0n]|BOLD:AAC6983
Leucania senescens|[5521]|BLPDD448-09|Costa Rica|Guanacaste|658[0n]|BOLD:AAC6983
Leucania senescens|[5522]|BLPAA686-06|Costa Rica|Guanacaste|658[0n]|BOLD:AAC6983
Leucania senescens|[5523]|BLPBG131-07|Costa Rica|Guanacaste|658[0n]|BOLD:AAC6983
Leucania senescens|[5524]|IBOLG002-08|Costa Rica|Alajuela|657[0n]|BOLD:AAC6983
Leucania senescens|[5525]|BLPCK956-08|Costa Rica|Alajuela|658[0n]|BOLD:AAC6983
Leucania senescens|[5526]|RDNMJ009-10|United States|Florida|658[0n]|BOLD:AAC6983
Leucania senescens|[5527]|BLPEE1459-12|Costa Rica|Guanacaste|658[0n]|BOLD:AAC6983
Leucania scirpicola|[5528]|LOFLB743-06|United States|Florida|658[0n]|BOLD:AAD1128
Leucania scirpicola|[5529]|LNC788-06|United States|North Carolina|658[0n]|BOLD:AAD1128
Leucania scirpicola|[5530]|LNC489-06|United States|North Carolina|658[0n]|BOLD:AAD1128
Leucania scirpicola|[5531]|LOFLB741-06|United States|Florida|658[0n]|BOLD:AAD1128
Leucania scirpicola|[5532]|LNC431-05|United States|North Carolina|616[0n]|BOLD:AAD1128
Leucania scirpicola|[5533]|LOFLC290-06|United States|Florida|621[0n]|BOLD:AAD1128
Leucania scirpicola|[5534]|LOFLC439-06|United States|Florida|658[0n]|BOLD:AAD1128
Leucania scirpicola|[5535]|RDNMD574-06|United States|Florida|658[0n]|BOLD:AAD1128
Leucania scirpicola|[5536]|RDNME292-07|United States|Florida|658[0n]|BOLD:AAD1128
Leucania scirpicola|[5537]|RDNML361-13|United States|Florida|658[0n]|BOLD:AAD1128
Leucania inconspicua|[5538]|BLPDA238-09|Costa Rica|Guanacaste|658[0n]|BOLD:AAA9170
Leucania inconspicua|[5539]|BLPCO387-08|Costa Rica|Guanacaste|658[0n]|BOLD:AAA9170
Leucania inconspicua|[5540]|BLPAC033-06|Costa Rica|Guanacaste|658[0n]|BOLD:AAA9170
Leucania inconspicua|[5541]|BLPAE672-06|Costa Rica|Guanacaste|658[0n]|BOLD:AAA9170
Leucania inconspicua|[5542]|BLPDD449-09|Costa Rica|Guanacaste|658[0n]|BOLD:AAA9170
Leucania inconspicua|[5543]|BLPAA584-06|Costa Rica|Guanacaste|658[0n]|BOLD:AAA9170
Leucania inconspicua|[5544]|BLPAA797-06|Costa Rica|Alajuela|658[0n]|BOLD:AAA9170
Leucania inconspicua|[5545]|BLPBG133-07|Costa Rica|Guanacaste|658[0n]|BOLD:AAA9170
Leucania inconspicua|[5546]|BLPEE4968-14|Costa Rica|Guanacaste|658[0n]|BOLD:AAA9170
Leucania inconspicua|[5547]|BLPAA868-06|Costa Rica|Alajuela|658[0n]|BOLD:AAA9170
Leucania inconspicua|[5548]|BLPAB068-06|Costa Rica|Alajuela|658[0n]|BOLD:AAA9170
Leucania inconspicua|[5549]|BLPCG765-08|Costa Rica|Guanacaste|658[0n]|BOLD:AAA9170
Leucania inconspicua|[5550]|BLPCE574-08|Costa Rica|Guanacaste|658[0n]|BOLD:AAA9170
Leucania inconspicua|[5551]|BLPCK762-08|Costa Rica|Alajuela|658[0n]|BOLD:AAA9170
Leucania inconspicua|[5552]|BLPDG519-09|Costa Rica|Guanacaste|658[0n]|BOLD:AAA9170
Leucania inconspicua|[5553]|LOCRF651-11|Costa Rica|Cartago|658[0n]|BOLD:AAA9170
Leucania inconspicua|[5554]|LOCRI606-11|Costa Rica|Cartago|658[0n]|BOLD:AAA9170
Leucania inconspicua|[5555]|MHMYS3013-13|Costa Rica|Guanacaste|658[0n]|BOLD:AAA9170
Leucania inconspicua|[5556]|BLPBD263-07|Costa Rica|Guanacaste|658[0n]|BOLD:AAA9170
Leucania inconspicua|[5557]|BLPCA255-08|Costa Rica|Guanacaste|658[0n]|BOLD:AAA9170
Leucania inconspicua|[5558]|BLPBA236-07|Costa Rica|Guanacaste|658[0n]|BOLD:AAA9170
Leucania inconspicua|[5559]|BLPBA710-07|Costa Rica|Guanacaste|658[0n]|BOLD:AAA9170
Leucania inconspicua|[5560]|BLPAC897-06|Costa Rica|Guanacaste|658[0n]|BOLD:AAA9170
Leucania inconspicua|[5561]|BLPAG420-07|Costa Rica|Guanacaste|658[0n]|BOLD:AAA9170
Leucania inconspicua|[5562]|BLPAC895-06|Costa Rica|Guanacaste|658[0n]|BOLD:AAA9170
Leucania inconspicua|[5563]|BLPAC758-06|Costa Rica|Guanacaste|658[0n]|BOLD:AAA9170
Leucania inconspicua|[5564]|BLPAC757-06|Costa Rica|Guanacaste|658[0n]|BOLD:AAA9170
Leucania inconspicua|[5565]|BLPAC347-06|Costa Rica|Guanacaste|658[0n]|BOLD:AAA9170
Leucania inconspicua|[5566]|BLPAC346-06|Costa Rica|Guanacaste|658[0n]|BOLD:AAA9170
Leucania inconspicua|[5567]|BLPAB018-06|Costa Rica|Alajuela|658[0n]|BOLD:AAA9170
Leucania inconspicua|[5568]|BLPAA860-06|Costa Rica|Alajuela|658[0n]|BOLD:AAA9170
Leucania inconspicua|[5569]|BLPAA799-06|Costa Rica|Alajuela|658[0n]|BOLD:AAA9170
Leucania inconspicua|[5570]|BLPAA696-06|Costa Rica|Guanacaste|658[0n]|BOLD:AAA9170
Leucania inconspicua|[5571]|BLPAA412-06|Costa Rica|Guanacaste|658[0n]|BOLD:AAA9170
Leucania inconspicua|[5572]|BLPAH356-07|Costa Rica|Guanacaste|658[0n]|BOLD:AAA9170
Leucania inconspicua|[5573]|BLPAB014-06|Costa Rica|Alajuela|658[0n]|BOLD:AAA9170
Leucania inconspicua|[5574]|MHAUF162-06|Costa Rica|Guanacaste|658[0n]|BOLD:AAA9170
Leucania inconspicua|[5575]|BLPAC896-06|Costa Rica|Guanacaste|658[0n]|BOLD:AAA9170
Leucania inconspicua|[5576]|CNCLA5279-13|United States|Florida|454[0n]|
Leucania inconspicua|[5577]|LNAUS5064-13|Cuba|Cienfuegos|624[0n]|BOLD:AAA9170
Leucania inconspicua|[5578]|CNCLB1700-14|United States|Florida|658[0n]|BOLD:AAA9170
Leucania linita|[5579]|HKONB406-09|United States|Texas|658[0n]|BOLD:ACF5143
Leucania linita|[5580]|HKONB397-09|United States|Texas|658[0n]|BOLD:ACF5143
Leucania linita|[5581]|HKONB398-09|United States|Texas|658[0n]|BOLD:ACF5143
Leucania linita|[5582]|HKONB407-09|United States|Texas|658[0n]|BOLD:ACF5143
Leucania linita|[5583]|MILEP295-10|United States|Georgia|658[0n]|BOLD:ACF5143
Leucania linita|[5584]|LNCB226-06|United States|North Carolina|658[0n]|BOLD:ACF5143
Leucania linita|[5585]|LNCB227-06|United States|North Carolina|658[0n]|BOLD:ACF5143
Leucania linita|[5586]|LNCB228-06|United States|North Carolina|658[0n]|BOLD:ACF5143
Leucania linita|[5587]|MILEP296-10|United States|Georgia|658[0n]|BOLD:ACF5143
Leucania linita|[5588]|MILEP297-10|United States|Georgia|658[0n]|BOLD:ACF5143
Leucania linita|[5589]|CNCLB1547-14|United States|Maryland|658[0n]|BOLD:ACF5143
Leucania amygdalina|[5590]|LPSO840-08|Canada|Ontario|658[1n]|BOLD:AAC3156
Leucania amygdalina|[5591]|HKONB408-09|United States|Indiana|658[0n]|BOLD:AAC3156
Leucania amygdalina|[5592]|LOCT236-05|United States|Connecticut|658[0n]|BOLD:AAC3156
Leucania amygdalina|[5593]|CNCLB1780-14|United States|Indiana|658[0n]|BOLD:AAC3156
Leucania amygdalina|[5594]|PHMO171-03|Canada|Ontario|639[0n]|BOLD:AAC3156
Leucania amygdalina|[5595]|RDLQ646-07|Canada|Quebec|621[0n]|BOLD:AAC3156
Leucania amygdalina|[5596]|RDLQ647-07|Canada|Quebec|646[0n]|BOLD:AAC3156
Leucania amygdalina|[5597]|LPMN796-08|Canada|Manitoba|658[0n]|BOLD:AAC3156

Leucania amygdalina|[5595]|RDLQ646-07|Canada|Quebec|621[0n]|BOLD:AAC3156
Leucania amygdalina|[5596]|RDLQ647-07|Canada|Quebec|646[0n]|BOLD:AAC3156
Leucania amygdalina|[5597]|LPMN796-08|Canada|Manitoba|658[0n]|BOLD:AAC3156
Leucania amygdalina|[5598]|LPMN797-08|Canada|Manitoba|658[0n]|BOLD:AAC3156
Leucania amygdalina|[5599]|LPMN804-08|Canada|Manitoba|658[0n]|BOLD:AAC3156
Leucania amygdalina|[5600]|LPSO572-08|Canada|Ontario|658[0n]|BOLD:AAC3156
Leucania amygdalina|[5601]|LILLA536-11|United States|Illinois|658[0n]|BOLD:AAC3156
Leucania amygdalina|[5602]|CNCLB1781-14|United States|Indiana|658[0n]|BOLD:AAC3156
Leucania lobrega|[5603]|MHMXZ927-09|Costa Rica||658[1n]|BOLD:AAB6105
Leucania lobrega|[5604]|RDNMI050-10|United States|Florida|658[0n]|BOLD:AAB6105
Leucania lobrega|[5605]|BLPCK745-08|Costa Rica|Alajuela|658[0n]|BOLD:AAB6105
Leucania lobrega|[5606]|MHMXZ936-09|Costa Rica||658[1n]|BOLD:AAB6105
Leucania lobrega|[5607]|LYPAP586-09|Mexico|Quintana Roo|658[0n]|BOLD:AAB6105
Leucania lobrega|[5608]|MHMXZ904-09|Costa Rica||658[0n]|BOLD:AAB6105
Leucania lobrega|[5609]|BLPCK763-08|Costa Rica|Alajuela|658[0n]|BOLD:AAB6105
Leucania lobrega|[5610]|LYNYM311-09|Mexico|Quintana Roo|625[0n]|BOLD:AAB6105
Leucania lobrega|[5611]|BLPCK958-08|Costa Rica|Alajuela|658[0n]|BOLD:AAB6105
Leucania lobrega|[5612]|BLPAB087-06|Costa Rica|Alajuela|658[0n]|BOLD:AAB6105
Leucania lobrega|[5613]|BLPAC034-06|Costa Rica|Guanacaste|658[0n]|BOLD:AAB6105
Leucania lobrega|[5614]|BLPAB122-06|Costa Rica|Alajuela|658[0n]|BOLD:AAB6105
Leucania lobrega|[5615]|BLPCF859-08|Costa Rica|Alajuela|658[0n]|BOLD:AAB6105
Leucania lobrega|[5616]|MHMXZ1128-09|Costa Rica||658[1n]|BOLD:AAB6105
Leucania lobrega|[5617]|LYHES535-09|Mexico|Quintana Roo|655[0n]|BOLD:AAB6105
Leucania lobrega|[5618]|BLPEF4963-13|Costa Rica|Guanacaste|658[0n]|BOLD:AAB6105
Leucania chejela|[5619]|CNCLB1660-14|United States|Florida|658[0n]|BOLD:AAX5629
Leucania calidior|[5620]|LNC754-06|United States|North Carolina|658[0n]|BOLD:ABZ4145
Leucania calidior|[5621]|RDNML177-13|United States|North Carolina|658[0n]|BOLD:ABZ4145
Leucania calidior|[5622]|CNCLB264-14|United States|Missouri|658[0n]|BOLD:ABZ4145
Leucania calidior|[5623]|LGSMG866-10|United States|Tennessee|658[0n]|BOLD:ABZ4145
Leucania calidior|[5624]|LNAUT1134-14|United States|Georgia|626[0n]|BOLD:ABZ4145
Leucania ursula|[5625]|LILLA062-11|United States|Illinois|658[0n]|BOLD:ACE7380
Leucania ursula|[5626]|LPSO656-08|Canada|Ontario|658[0n]|BOLD:ACE7380
Leucania ursula|[5627]|LPSO561-08|Canada|Ontario|658[0n]|BOLD:ACE7380
Leucania ursula|[5628]|LPSO257-08|Canada|Ontario|658[0n]|BOLD:ACE7380
Leucania ursula|[5629]|LNCNW048-06|United States|North Carolina|657[0n]|BOLD:ACE7380
Leucania ursula|[5630]|LNCNW047-06|United States|North Carolina|657[0n]|BOLD:ACE7380
Leucania ursula|[5631]|LOCT318-05|United States|Connecticut|658[0n]|BOLD:ACE7380
Leucania ursula|[5632]|LOCT282-05|United States|Connecticut|658[0n]|BOLD:ACE7380
Leucania ursula|[5633]|LOCT073-05|United States|Connecticut|658[0n]|BOLD:ACE7380
Leucania ursula|[5634]|LOCT053-05|United States|Connecticut|658[0n]|BOLD:ACE7380
Leucania ursula|[5635]|LOTB138-05|United States|Tennessee|658[0n]|BOLD:ACE7380
Leucania ursula|[5636]|LGSMC858-05|United States|Tennessee|658[0n]|BOLD:ACE7380
Leucania ursula|[5637]|LGSMC696-05|United States|Tennessee|658[0n]|BOLD:ACE7380
Leucania ursula|[5638]|LPSO429-08|Canada|Ontario|658[0n]|BOLD:ACE7380
Leucania ursula|[5639]|LPSO238-08|Canada|Ontario|656[0n]|BOLD:ACE7380
Leucania ursula|[5640]|LGSM675-04|United States|Tennessee|586[0n]|BOLD:ACE7380
Leucania ursula|[5641]|LPSO430-08|Canada|Ontario|638[0n]|BOLD:ACE7380
Leucania ursula|[5642]|LPSO741-08|Canada|Ontario|658[0n]|BOLD:ACE7380
Leucania ursula|[5643]|LGSMG864-10|United States|North Carolina|658[0n]|BOLD:ACE7380
Leucania ursula|[5644]|LPSO919-08|Canada|Ontario|658[0n]|BOLD:ACE7380
Leucania ursula|[5645]|LPSO920-08|Canada|Ontario|658[0n]|BOLD:ACE7380
Leucania ursula|[5646]|LGSMG865-10|United States|North Carolina|658[0n]|BOLD:ACE7380
Leucania ursula|[5647]|LILLA095-11|United States|Illinois|658[0n]|BOLD:ACE7380
Leucania ursula|[5648]|LPOKE278-11|United States|Oklahoma|658[0n]|BOLD:ACE7380
Leucania ursula|[5649]|LNCC1528-13|United States|North Carolina|658[0n]|BOLD:ACE7380
Leucania nr. pseudargyria|[5650]|LSEU735-06|United States|Georgia|657[0n]|BOLD:ACE4427
Leucania pseudargyria|[5651]|RDNME530-08|United States|Virginia|658[3n]|BOLD:AAB6246
Leucania pseudargyria|[5652]|LPMN988-09|Canada|Manitoba|658[0n]|BOLD:AAB6246
Leucania pseudargyria|[5653]|MILEP051-09|United States|North Carolina|658[0n]|BOLD:AAB6246
Leucania pseudargyria|[5654]|LGSM722-04|United States|North Carolina|658[0n]|BOLD:AAB6246
Leucania pseudargyria|[5655]|MNBB116-05|Canada|New Brunswick|658[0n]|BOLD:AAB6246
Leucania pseudargyria|[5656]|TTMNB414-06|Canada|New Brunswick|657[0n]|BOLD:AAB6246
Leucania pseudargyria|[5657]|XAK052-06|Canada|Ontario|657[0n]|BOLD:AAB6246
Leucania pseudargyria|[5658]|PHJUN3995-11|Canada|Ontario|658[0n]|BOLD:AAB6246
Leucania pseudargyria|[5659]|PMG126-03|Canada|Ontario|617[0n]|BOLD:AAB6246
Leucania pseudargyria|[5660]|XAE524-04|Canada|Ontario|592[0n]|BOLD:AAB6246
Leucania pseudargyria|[5661]|MNBB021-05|Canada|New Brunswick|658[0n]|BOLD:AAB6246
Leucania pseudargyria|[5662]|MNBB166-05|Canada|New Brunswick|658[0n]|BOLD:AAB6246
Leucania pseudargyria|[5663]|BLTIB504-08|Canada|Ontario|657[0n]|BOLD:AAB6246
Leucania pseudargyria|[5664]|PHJUN3994-11|Canada|Ontario|658[0n]|BOLD:AAB6246
Leucania pseudargyria|[5665]|PHJUN3998-11|Canada|Ontario|658[0n]|BOLD:AAB6246
Leucania pseudargyria|[5666]|RDLQG039-06|Canada|Quebec|658[0n]|BOLD:AAB6246
Leucania pseudargyria|[5667]|LPMN996-09|Canada|Manitoba|658[0n]|BOLD:AAB6246
Leucania pseudargyria|[5668]|LPMN987-09|Canada|Manitoba|658[0n]|BOLD:AAB6246
Leucania pseudargyria|[5669]|XAJ828-06|Canada|Ontario|657[0n]|BOLD:AAB6246
Leucania pseudargyria|[5670]|LPMNB319-09|Canada|Manitoba|658[0n]|BOLD:AAB6246
Leucania pseudargyria|[5671]|BBLEC559-09|Canada|Nova Scotia|643[0n]|BOLD:AAB6246
Leucania pseudargyria|[5672]|BBLEC561-09|Canada|Nova Scotia|658[0n]|BOLD:AAB6246
Leucania pseudargyria|[5673]|CNRMF524-12|Canada|Manitoba|614[0n]|BOLD:AAB6246
Leucania pseudargyria|[5674]|MNBB165-05|Canada|New Brunswick|658[0n]|BOLD:AAB6246
Leucania pseudargyria|[5675]|HPPPE1544-13|Canada|Nova Scotia|586[0n]|BOLD:AAB6246
Leucania pseudargyria|[5676]|HPPPE1545-13|Canada|Nova Scotia|578[0n]|BOLD:AAB6246
Leucania pseudargyria|[5677]|HPPPE1561-13|Canada|Nova Scotia|555[0n]|BOLD:AAB6246
Leucania inermis|[5678]|RDLQ659-07|Canada|Quebec|611[1n]|BOLD:ACE5714
Leucania inermis|[5679]|RDLQF935-06|Canada|Quebec|658[0n]|BOLD:ACE5714
Leucania inermis|[5680]|LNC095-05|United States|North Carolina|658[0n]|BOLD:ACE5714
Leucania inermis|[5681]|PHMO056-03|Canada|Ontario|639[0n]|BOLD:ACE5714
Leucania inermis|[5682]|LNC096-05|United States|North Carolina|581[0n]|BOLD:ACE5714
Leucania inermis|[5683]|RDLQ660-07|Canada|Quebec|644[0n]|BOLD:ACE5714
Leucania inermis|[5684]|LPMN072-08|Canada|Manitoba|658[0n]|BOLD:ACE5714
Leucania inermis|[5685]|CNCLB1467-14|United States|Louisiana|658[0n]|BOLD:ACE5714
Leucania inermis|[5686]|CNCLB1468-14|United States|Louisiana|583[0n]|BOLD:ACE5714
Leucania februalis|[5687]|LNAUT2021-14|United States|California|658[0n]|BOLD:ABW8345
Leucania februalis|[5688]|BBLOD1712-11|United States|California|658[0n]|BOLD:ABW8345
Leucania februalis|[5689]|LNAUT2019-14|United States|California|649[0n]|BOLD:ABW8345
Leucania februalis|[5690]|LNAUT2022-14|United States|California|658[0n]|BOLD:ABW8345
Leucania n. sp. 2 nr. humidicola|[5691]|CNCLA5283-13|United States|Florida|658[0n]|BOLD:ACP6589
Leucania n. sp. 2 nr. humidicola|[5692]|CNCLB1732-14|United States|Florida|658[0n]|BOLD:ACP6589
Leucania n. sp. 2 nr. humidicola|[5693]|CNCLB1733-14|United States|Texas|658[0n]|BOLD:ACP6589
Leucania n. sp. 2 nr. humidicola|[5694]|CNCLB1734-14|United States|Texas|658[0n]|BOLD:ACP6589
Leucania humidicola|[5695]|CNCLB2484-14|Venezuela||658[0n]|BOLD:AAD6861
Leucania humidicola|[5696]|CNCLB2485-14|Venezuela||459[0n]|
Leucania solita|[5697]|BLPAF776-07|Costa Rica|Guanacaste|658[0n]|BOLD:AAD6861

Leucania humidicola|[5695]|CNCLB2484-14|Venezuela||658[0n]|BOLD:AAD6861
Leucania humidicola|[5696]|CNCLB2485-14|Venezuela||459[0n]|
Leucania solita|[5697]|BLPAF776-07|Costa Rica|Guanacaste|658[0n]|BOLD:AAD6861
Leucania solita|[5698]|BLPAF778-07|Costa Rica|Guanacaste|658[0n]|BOLD:AAD6861
Leucania solita|[5699]|BLPBG132-07|Costa Rica|Guanacaste|658[0n]|BOLD:AAD6861
Leucania solita|[5700]|BLPCA841-08|Costa Rica|Guanacaste|658[0n]|BOLD:AAD6861
Leucania solita|[5701]|LOCRE444-10|Costa Rica|San Jose|658[0n]|BOLD:AAD6861
Leucania solita|[5702]|BLPAF777-07|Costa Rica|Guanacaste|658[0n]|BOLD:AAD6861
Leucania solita|[5703]|BLPBH528-07|Costa Rica|Guanacaste|655[0n]|BOLD:AAD6861
Leucania solita|[5704]|BLPEE1061-12|Costa Rica|Guanacaste|658[0n]|BOLD:AAD6861
Leucania solita|[5705]|TML179-14|United States|California|658[0n]|BOLD:AAD6861
Leucania solita|[5706]|CNCLB2486-14|United States|California|658[0n]|BOLD:AAD6861
Leucania multilinea|[5707]|LHLEP114-06|Canada|British Columbia|658[0n]|BOLD:ACF1540
Leucania multilinea|[5708]|LHLEP115-06|Canada|British Columbia|658[0n]|BOLD:ACF1540
Leucania multilinea|[5709]|LHLEP116-06|Canada|British Columbia|658[0n]|BOLD:ACF1540
Leucania multilinea|[5710]|LPSK444-08|Canada|Saskatchewan|658[0n]|BOLD:ACF1540
Leucania multilinea|[5711]|RDLQ650-07|Canada|Quebec|608[1n]|BOLD:ACF1540
Leucania multilinea|[5712]|LOWC055-05|Canada|British Columbia|658[0n]|BOLD:ACF1540
Leucania multilinea|[5713]|LPABC158-09|Canada|Alberta|658[0n]|BOLD:ACF1540
Leucania multilinea|[5714]|LPSK508-08|Canada|Saskatchewan|658[0n]|BOLD:ACF1540
Leucania multilinea|[5715]|LPSK443-08|Canada|Saskatchewan|658[0n]|BOLD:ACF1540
Leucania multilinea|[5716]|LPMN819-08|Canada|Manitoba|658[0n]|BOLD:ACF1540
Leucania multilinea|[5717]|LPSK253-08|Canada|Saskatchewan|658[0n]|BOLD:ACF1540
Leucania multilinea|[5718]|LPSK010-08|Canada|Saskatchewan|658[0n]|BOLD:ACF1540
Leucania multilinea|[5719]|LBCC027-05|Canada|British Columbia|658[0n]|BOLD:ACF1540
Leucania multilinea|[5720]|LPABC157-09|Canada|Alberta|645[1n]|BOLD:ACF1540
Leucania multilinea|[5721]|BBLPA824-10|Canada|British Columbia|658[0n]|BOLD:ACF1540
Leucania multilinea|[5722]|LNCC1157-11|United States|North Carolina|633[0n]|BOLD:ACF1540
Leucania multilinea|[5723]|LNCC1158-11|United States|North Carolina|658[0n]|BOLD:ACF1540
Leucania multilinea|[5724]|LBCH6366-10|Canada|British Columbia|658[0n]|BOLD:ACF1540
Leucania multilinea|[5725]|LHLEP119-06|Canada|British Columbia|658[0n]|BOLD:ACF1540
Leucania multilinea|[5726]|XAB107-04|Canada|Ontario|658[0n]|BOLD:ACF1540
Leucania multilinea|[5727]|LPSK252-08|Canada|Saskatchewan|658[0n]|BOLD:ACF1540
Leucania multilinea|[5728]|LNCC1156-11|United States|North Carolina|658[0n]|BOLD:ACF1540
Leucania multilinea|[5729]|BBLPE343-09|Canada|Newfoundland and Labrador|658[0n]|BOLD:ACF1540
Leucania multilinea|[5730]|BBLPE141-09|Canada|Nova Scotia|658[0n]|BOLD:ACF1540
Leucania multilinea|[5731]|BBLPC601-09|Canada|Nova Scotia|658[0n]|BOLD:ACF1540
Leucania multilinea|[5732]|XAB156-04|Canada|Ontario|658[1n]|BOLD:ACF1540
Leucania multilinea|[5733]|XAK051-06|Canada|Ontario|658[0n]|BOLD:ACF1540
Leucania multilinea|[5734]|BBLPC943-09|Canada|Newfoundland and Labrador|658[0n]|BOLD:ACF1540
Leucania multilinea|[5735]|BBLPC699-09|Canada|Newfoundland and Labrador|658[0n]|BOLD:ACF1540
Leucania multilinea|[5736]|BBLPC655-09|Canada|Newfoundland and Labrador|658[0n]|BOLD:ACF1540
Leucania multilinea|[5737]|BBLEC414-09|Canada|Newfoundland and Labrador|658[0n]|BOLD:ACF1540
Leucania multilinea|[5738]|BBLEC347-09|Canada|Newfoundland and Labrador|658[0n]|BOLD:ACF1540
Leucania multilinea|[5739]|BBLEC268-09|Canada|Nova Scotia|658[0n]|BOLD:ACF1540
Leucania multilinea|[5740]|BLTIB393-08|Canada|Ontario|658[0n]|BOLD:ACF1540
Leucania multilinea|[5741]|RDLQ652-07|Canada|Quebec|658[0n]|BOLD:ACF1540
Leucania multilinea|[5742]|RDLQ651-07|Canada|Quebec|658[0n]|BOLD:ACF1540
Leucania multilinea|[5743]|RDLQ649-07|Canada|Ontario|658[0n]|BOLD:ACF1540
Leucania multilinea|[5744]|MNBB443-05|Canada|New Brunswick|658[0n]|BOLD:ACF1540
Leucania multilinea|[5745]|MNBB023-05|Canada|New Brunswick|658[0n]|BOLD:ACF1540
Leucania multilinea|[5746]|XAC713-04|Canada|Ontario|658[0n]|BOLD:ACF1540
Leucania multilinea|[5747]|RDLQ654-07|Canada|Quebec|623[0n]|BOLD:ACF1540
Leucania multilinea|[5748]|LOWCE827-06|Canada|British Columbia|609[2n]|BOLD:ACF1540
Leucania multilinea|[5749]|RDLQ648-07|Canada|Ontario|624[0n]|BOLD:ACF1540
Leucania multilinea|[5750]|RDLQ657-07|Canada|Quebec|601[0n]|BOLD:ACF1540
Leucania multilinea|[5751]|BBLPE122-09|Canada|Nova Scotia|636[0n]|BOLD:ACF1540
Leucania multilinea|[5752]|BBLPE320-09|Canada|Newfoundland and Labrador|658[0n]|BOLD:ACF1540
Leucania multilinea|[5753]|BBLPE333-09|Canada|Newfoundland and Labrador|658[0n]|BOLD:ACF1540
Leucania multilinea|[5754]|BBLPE338-09|Canada|Newfoundland and Labrador|658[0n]|BOLD:ACF1540
Leucania multilinea|[5755]|BBLPE339-09|Canada|Newfoundland and Labrador|658[0n]|BOLD:ACF1540
Leucania multilinea|[5756]|BBLPE340-09|Canada|Newfoundland and Labrador|658[0n]|BOLD:ACF1540
Leucania multilinea|[5757]|BBLPE360-09|Canada|Newfoundland and Labrador|658[0n]|BOLD:ACF1540
Leucania multilinea|[5758]|BLTIB789-08|Canada|Ontario|658[0n]|BOLD:ACF1540
Leucania multilinea|[5759]|LPSK446-08|Canada|Saskatchewan|658[0n]|BOLD:ACF1540
Leucania multilinea|[5760]|LPSK445-08|Canada|Saskatchewan|658[0n]|BOLD:ACF1540
Leucania multilinea|[5761]|LHLEP118-06|Canada|British Columbia|658[0n]|BOLD:ACF1540
Leucania multilinea|[5762]|LHLEP117-06|Canada|British Columbia|658[0n]|BOLD:ACF1540
Leucania multilinea|[5763]|LOWCD225-06|Canada|British Columbia|658[0n]|BOLD:ACF1540
Leucania multilinea|[5764]|XAC668-04|Canada|Ontario|658[0n]|BOLD:ACF1540
Leucania multilinea|[5765]|PHMO156-03|Canada|Ontario|639[0n]|BOLD:ACF1540
Leucania multilinea|[5766]|XAB195-04|Canada|Ontario|658[0n]|BOLD:ACF1540
Leucania multilinea|[5767]|LNCC1159-11|United States|North Carolina|658[0n]|BOLD:ACF1540
Leucania multilinea|[5768]|CNGRJ062-13|Canada|Saskatchewan|591[0n]|BOLD:ACF1540
Leucania multilinea|[5769]|PHMNB761-05|Canada|New Brunswick|658[0n]|BOLD:ACF1540
Leucania multilinea|[5770]|LPSK017-08|Canada|Saskatchewan|658[0n]|BOLD:ACF1540
Leucania multilinea|[5771]|LPSK005-08|Canada|Saskatchewan|658[0n]|BOLD:ACF1540
Leucania multilinea|[5772]|LPSK589-08|Canada|Saskatchewan|658[0n]|BOLD:ACF1540
Leucania multilinea|[5773]|LPSK600-08|Canada|Saskatchewan|658[0n]|BOLD:ACF1540
Leucania multilinea|[5774]|LPMNB563-09|Canada|Manitoba|658[0n]|BOLD:ACF1540
Leucania multilinea|[5775]|LPMNB567-09|Canada|Manitoba|658[0n]|BOLD:ACF1540
Leucania multilinea|[5776]|LPSK548-08|Canada|Saskatchewan|658[0n]|BOLD:ACF1540
Leucania multilinea|[5777]|LPSK568-08|Canada|Saskatchewan|658[0n]|BOLD:ACF1540
Leucania multilinea|[5778]|LPSK530-08|Canada|Saskatchewan|658[0n]|BOLD:ACF1540
Leucania multilinea|[5779]|LPSK531-08|Canada|Saskatchewan|658[0n]|BOLD:ACF1540
Leucania multilinea|[5780]|LPSK509-08|Canada|Saskatchewan|658[0n]|BOLD:ACF1540
Leucania multilinea|[5781]|LPSK510-08|Canada|Saskatchewan|658[0n]|BOLD:ACF1540
Leucania multilinea|[5782]|BBLPA835-10|Canada|Alberta|658[0n]|BOLD:ACF1540
Leucania multilinea|[5783]|LPSK495-08|Canada|Saskatchewan|658[0n]|BOLD:ACF1540
Leucania multilinea|[5784]|LPSK486-08|Canada|Saskatchewan|658[0n]|BOLD:ACF1540
Leucania multilinea|[5785]|LPSK471-08|Canada|Saskatchewan|658[0n]|BOLD:ACF1540
Leucania multilinea|[5786]|LPSK440-08|Canada|Saskatchewan|658[0n]|BOLD:ACF1540
Leucania multilinea|[5787]|LPSK120-08|Canada|Saskatchewan|658[0n]|BOLD:ACF1540
Leucania multilinea|[5788]|LPSK049-08|Canada|Saskatchewan|658[0n]|BOLD:ACF1540
Leucania multilinea|[5789]|LPSK046-08|Canada|Saskatchewan|658[0n]|BOLD:ACF1540
Leucania multilinea|[5790]|LPSK032-08|Canada|Saskatchewan|658[0n]|BOLD:ACF1540
Leucania multilinea|[5791]|LPSK025-08|Canada|Saskatchewan|658[0n]|BOLD:ACF1540
Leucania multilinea|[5792]|LPSK006-08|Canada|Saskatchewan|658[0n]|BOLD:ACF1540
Leucania multilinea|[5793]|LPSK558-08|Canada|Saskatchewan|658[0n]|BOLD:ACF1540
Leucania multilinea|[5794]|LPSK598-08|Canada|Saskatchewan|658[0n]|BOLD:ACF1540
Leucania multilinea|[5795]|CNGRK438-13|Canada|Saskatchewan|637[0n]|BOLD:ACF1540
Leucania multilinea|[5796]|CNGRJ085-13|Canada|Saskatchewan|585[0n]|BOLD:ACF1540
Leucania farcta fractal|[5797]|RDNMD652-06|United States|New Mexico|658[0n]|BOLD:ABZ5867

Leucania multilinea|[5795]|CNGRK438-13|Canada|Saskatchewan|637[0n]|BOLD:ACF1540
Leucania multilinea|[5796]|CNGRJ085-13|Canada|Saskatchewan|585[0n]|BOLD:ACF1540
Leucania farcta fracta|[5797]|RDNMD652-06|United States|New Mexico|658[0n]|BOLD:ABZ5867
Leucania farcta fracta|[5798]|LOCBC351-06|United States|California|658[0n]|BOLD:ABZ5867
Leucania farcta fracta|[5799]|LOCBC405-06|United States|California|658[0n]|BOLD:ABZ5867
Leucania farcta fracta|[5800]|LOCBC403-06|United States|California|658[0n]|BOLD:ABZ5867
Leucania farcta fracta|[5801]|JMMMB536-13|United States|California|576[0n]|BOLD:ABZ5867
Leucania farcta fracta|[5802]|BBLOC1370-11|United States|California|614[0n]|BOLD:ABZ5867
Leucania farcta fracta|[5803]|BBLOC212-11|United States|Texas|540[0n]|BOLD:ABZ5867
Leucania farcta fracta|[5804]|BBLOD949-11|United States|Arizona|658[0n]|BOLD:ABZ5867
Leucania farcta fracta|[5805]|LOCBC348-06|United States|California|658[0n]|BOLD:ABZ5867
Leucania farcta fracta|[5806]|BBLOC941-11|United States|Texas|658[0n]|BOLD:ABZ5867
Leucania farcta fracta|[5807]|RDNMD654-06|United States|New Mexico|658[0n]|BOLD:ABZ5867
Leucania farcta fracta|[5808]|RDNMD653-06|United States|New Mexico|658[0n]|BOLD:ABZ5867
Leucania farcta fracta|[5809]|RDNMD560-06|United States|Colorado|658[0n]|BOLD:ABZ5867
Leucania farcta fracta|[5810]|LOCBD660-06|United States|California|658[0n]|BOLD:ABZ5867
Leucania farcta fracta|[5811]|LOCBD417-06|United States|California|658[0n]|BOLD:ABZ5867
Leucania farcta fracta|[5812]|LOCBC408-06|United States|California|658[0n]|BOLD:ABZ5867
Leucania farcta fracta|[5813]|LOCBC407-06|United States|California|658[0n]|BOLD:ABZ5867
Leucania farcta fracta|[5814]|LOCBC404-06|United States|California|658[0n]|BOLD:ABZ5867
Leucania farcta fracta|[5815]|LOCBC402-06|United States|California|658[0n]|BOLD:ABZ5867
Leucania farcta fracta|[5816]|LOCBC349-06|United States|California|658[0n]|BOLD:ABZ5867
Leucania farcta fracta|[5817]|LOCBC347-06|United States|California|658[0n]|BOLD:ABZ5867
Leucania farcta fracta|[5818]|LOCBC346-06|United States|California|658[0n]|BOLD:ABZ5867
Leucania farcta fracta|[5819]|LOCBC345-06|United States|California|658[0n]|BOLD:ABZ5867
Leucania farcta fracta|[5820]|LOCBC344-06|United States|California|658[0n]|BOLD:ABZ5867
Leucania farcta fracta|[5821]|LOCBC343-06|United States|California|658[0n]|BOLD:ABZ5867
Leucania farcta fracta|[5822]|LOCBC342-06|United States|California|658[0n]|BOLD:ABZ5867
Leucania farcta fracta|[5823]|LOCBC341-06|United States|California|658[0n]|BOLD:ABZ5867
Leucania farcta fracta|[5824]|LOCBC350-06|United States|California|656[0n]|BOLD:ABZ5867
Leucania farcta fracta|[5825]|LOCBE359-06|United States|California|605[0n]|BOLD:ABZ5867
Leucania farcta fracta|[5826]|BBLOC1205-11|United States|California|639[0n]|BOLD:ABZ5867
Leucania farcta fracta|[5827]|BBLOC1262-11|United States|California|658[0n]|BOLD:ABZ5867
Leucania farcta fracta|[5828]|BBLOC1275-11|United States|California|658[0n]|BOLD:ABZ5867
Leucania farcta fracta|[5829]|GMLC1087-12|United States|California|658[0n]|BOLD:ABZ5867
Leucania farcta fracta|[5830]|CNCLA5282-13|United States|New Mexico|658[0n]|BOLD:ABZ5867
Leucania anteroclara|[5831]|LOWC048-05|Canada|British Columbia|658[1n]|BOLD:ACE5750
Leucania anteroclara|[5832]|LOWC061-05|Canada|British Columbia|658[2n]|BOLD:ACE5750
Leucania anteroclara|[5833]|LOWC047-05|Canada|British Columbia|658[0n]|BOLD:ACE5750
Leucania anteroclara|[5834]|LOWC054-05|Canada|British Columbia|658[0n]|BOLD:ACE5750
Leucania anteroclara|[5835]|LPABB304-08|Canada|Alberta|658[0n]|BOLD:ACE5750
Leucania anteroclara|[5836]|LPABB393-08|Canada|Alberta|658[0n]|BOLD:ACE5750
Leucania anteroclara|[5837]|LPABC004-09|Canada|Alberta|658[1n]|BOLD:ACE5750
Leucania anteroclara|[5838]|LPABB118-08|Canada|Alberta|647[0n]|BOLD:ACE5750
Leucania anteroclara|[5839]|LPABC431-09|Canada|Alberta|658[0n]|BOLD:ACE5750
Leucania anteroclara|[5840]|LPABC005-09|Canada|Alberta|658[2n]|BOLD:ACE5750
Leucania anteroclara|[5841]|LOWC051-05|Canada|British Columbia|658[0n]|BOLD:ACE5750
Leucania anteroclara|[5842]|LOWC026-05|Canada|British Columbia|658[0n]|BOLD:ACE5750
Leucania anteroclara|[5843]|LOWCD230-06|Canada|British Columbia|583[0n]|BOLD:ACE5750
Leucania anteroclara|[5844]|LPABC824-09|Canada|Alberta|658[0n]|BOLD:ACE5750
Leucania anteroclara|[5845]|LPABC853-09|Canada|Alberta|658[0n]|BOLD:ACE5750
Leucania anteroclara|[5846]|LPABC946-09|Canada|Alberta|658[0n]|BOLD:ACE5750
Leucania anteroclara|[5847]|LBCH5559-10|Canada|British Columbia|658[0n]|BOLD:ACE5750
Leucania anteroclara|[5848]|LPABC065-09|Canada|Alberta|658[0n]|BOLD:ACE5750
Leucania anteroclara|[5849]|LPABC321-09|Canada|Alberta|658[0n]|BOLD:ACE5750
Leucania anteroclara|[5850]|LPABC411-09|Canada|Alberta|658[0n]|BOLD:ACE5750
Leucania anteroclara|[5851]|LPABC466-09|Canada|Alberta|658[0n]|BOLD:ACE5750
Leucania anteroclara|[5852]|LPABB193-08|Canada|Alberta|658[0n]|BOLD:ACE5750
Leucania anteroclara|[5853]|LPABB194-08|Canada|Alberta|658[0n]|BOLD:ACE5750
Leucania anteroclara|[5854]|LPABB402-08|Canada|Alberta|658[0n]|BOLD:ACE5750
Leucania anteroclara|[5855]|LPABB450-08|Canada|Alberta|658[0n]|BOLD:ACE5750
Leucania anteroclara|[5856]|LPABB843-09|Canada|Alberta|658[0n]|BOLD:ACE5750
Leucania anteroclara|[5857]|LPABB848-09|Canada|Alberta|658[0n]|BOLD:ACE5750
Leucania anteroclara|[5858]|LPABB573-08|Canada|Alberta|658[0n]|BOLD:ACE5750
Leucania anteroclara|[5859]|LPABB579-08|Canada|Alberta|658[0n]|BOLD:ACE5750
Leucania anteroclara|[5860]|LPABB630-08|Canada|Alberta|658[0n]|BOLD:ACE5750
Leucania anteroclara|[5861]|LPABB723-08|Canada|Alberta|658[0n]|BOLD:ACE5750
Leucania anteroclara|[5862]|LPABB868-09|Canada|Alberta|658[0n]|BOLD:ACE5750
Leucania anteroclara|[5863]|LBCH6523-10|Canada|British Columbia|658[0n]|BOLD:ACE5750
Leucania anteroclara|[5864]|LBCH6627-10|Canada|British Columbia|658[0n]|BOLD:ACE5750
Leucania anteroclara|[5865]|LBCH6044-10|Canada|British Columbia|658[0n]|BOLD:ACE5750
Leucania anteroclara|[5866]|LBCH6094-10|Canada|British Columbia|658[0n]|BOLD:ACE5750
Leucania anteroclara|[5867]|LBCH6441-10|Canada|British Columbia|658[0n]|BOLD:ACE5750
Leucania anteroclara|[5868]|BBLPB453-10|Canada|Alberta|658[0n]|BOLD:ACE5750
Leucania anteroclara|[5869]|LPABB145-08|Canada|Alberta|658[0n]|BOLD:ACE5750
Leucania anteroclara|[5870]|LPAB239-08|Canada|Alberta|658[0n]|BOLD:ACE5750
Leucania anteroclara|[5871]|LPMN898-08|Canada|Alberta|658[0n]|BOLD:ACE5750
Leucania anteroclara|[5872]|LPSK585-08|Canada|Saskatchewan|658[0n]|BOLD:ACE5750
Leucania anteroclara|[5873]|LPSK452-08|Canada|Saskatchewan|658[0n]|BOLD:ACE5750
Leucania anteroclara|[5874]|LPSK040-08|Canada|Saskatchewan|658[0n]|BOLD:ACE5750
Leucania anteroclara|[5875]|RDNMD556-06|United States|Colorado|658[0n]|BOLD:ACE5750
Leucania anteroclara|[5876]|LOWCD777-06|Canada|British Columbia|657[0n]|BOLD:ACE5750
Leucania anteroclara|[5877]|LOWCD775-06|Canada|British Columbia|657[0n]|BOLD:ACE5750
Leucania anteroclara|[5878]|LOWCD773-06|Canada|British Columbia|657[0n]|BOLD:ACE5750
Leucania anteroclara|[5879]|LOWCD771-06|Canada|British Columbia|657[0n]|BOLD:ACE5750
Leucania anteroclara|[5880]|RDMAB128-05|Canada|Alberta|658[0n]|BOLD:ACE5750
Leucania anteroclara|[5881]|LOWC062-05|Canada|British Columbia|658[0n]|BOLD:ACE5750
Leucania anteroclara|[5882]|LOWC049-05|Canada|British Columbia|658[0n]|BOLD:ACE5750
Leucania anteroclara|[5883]|LOWCD228-06|Canada|British Columbia|658[0n]|BOLD:ACE5750
Leucania anteroclara|[5884]|LPABB849-09|Canada|Alberta|658[0n]|BOLD:ACE5750
Leucania anteroclara|[5885]|LOWC056-05|Canada|British Columbia|658[0n]|BOLD:ACE5750
Leucania anteroclara|[5886]|LOWCD229-06|Canada|British Columbia|605[0n]|BOLD:ACE5750
Leucania anteroclara|[5887]|LOWCD772-06|Canada|British Columbia|656[0n]|BOLD:ACE5750
Leucania anteroclara|[5888]|LPABB365-08|Canada|Alberta|638[0n]|BOLD:ACE5750
Leucania anteroclara|[5889]|LPABB359-08|Canada|Alberta|639[0n]|BOLD:ACE5750
Leucania anteroclara|[5890]|LOWCD227-06|Canada|British Columbia|610[0n]|BOLD:ACE5750
Leucania anteroclara|[5891]|LOWCD226-06|Canada|British Columbia|607[0n]|BOLD:ACE5750
Leucania anteroclara|[5892]|LOWCD769-06|Canada|British Columbia|609[0n]|BOLD:ACE5750
Leucania anteroclara|[5893]|LOWC050-05|Canada|British Columbia|583[0n]|BOLD:ACE5750
Leucania anteroclara|[5894]|LOWCD774-06|Canada|British Columbia|604[1n]|BOLD:ACE5750
Leucania anteroclara|[5895]|RDNMD557-06|United States|Colorado|614[3n]|BOLD:ACE5750
Leucania anteroclara|[5896]|LBCH5953-10|Canada|British Columbia|641[0n]|BOLD:ACE5750
Leucania anteroclara|[5897]|SSWLE1805-13|Canada|Alberta|599[0n]|BOLD:ACE5750

Leucania anteroclara|[5895]|RDNMD557-06|United States|Colorado|614[3n]|BOLD:ACE5750
Leucania anteroclara|[5896]|LBCH5953-10|Canada|British Columbia|641[0n]|BOLD:ACE5750
Leucania anteroclara|[5897]|SSWLE1805-13|Canada|Alberta|599[0n]|BOLD:ACE5750
Leucania farcta roseola|[5898]|RWWA458-09|United States|Washington|658[0n]|BOLD:AAA2483
Leucania farcta roseola|[5899]|LHLEP105-06|Canada|British Columbia|658[2n]|BOLD:AAA2483
Leucania farcta roseola|[5900]|RWWA224-09|United States|Washington|658[0n]|BOLD:AAA2483
Leucania farcta roseola|[5901]|LALPA339-10|Canada|British Columbia|658[0n]|BOLD:AAA2483
Leucania farcta roseola|[5902]|LALPA496-10|Canada|British Columbia|658[0n]|BOLD:AAA2483
Leucania farcta roseola|[5903]|LPVIA303-08|Canada|British Columbia|658[0n]|BOLD:AAA2483
Leucania farcta roseola|[5904]|LPGVA713-08|Canada|British Columbia|658[0n]|BOLD:AAA2483
Leucania farcta roseola|[5905]|LHLEP113-06|Canada|British Columbia|658[0n]|BOLD:AAA2483
Leucania farcta roseola|[5906]|LHLEP112-06|Canada|British Columbia|658[0n]|BOLD:AAA2483
Leucania farcta roseola|[5907]|LHLEP109-06|Canada|British Columbia|658[0n]|BOLD:AAA2483
Leucania farcta roseola|[5908]|LHLEP106-06|Canada|British Columbia|658[0n]|BOLD:AAA2483
Leucania farcta roseola|[5909]|LHLEP103-06|Canada|British Columbia|658[0n]|BOLD:AAA2483
Leucania farcta roseola|[5910]|LHLEP102-06|Canada|British Columbia|658[0n]|BOLD:AAA2483
Leucania farcta roseola|[5911]|LHLEP101-06|Canada|British Columbia|658[0n]|BOLD:AAA2483
Leucania farcta roseola|[5912]|LPGVA714-08|Canada|British Columbia|638[0n]|BOLD:AAA2483
Leucania farcta roseola|[5913]|LPVIA410-08|Canada|British Columbia|658[0n]|BOLD:AAA2483
Leucania farcta roseola|[5914]|LPVIB061-08|Canada|British Columbia|658[0n]|BOLD:AAA2483
Leucania farcta roseola|[5915]|LPVIB980-08|Canada|British Columbia|658[0n]|BOLD:AAA2483
Leucania farcta roseola|[5916]|RWWA247-09|United States|Washington|658[0n]|BOLD:AAA2483
Leucania farcta roseola|[5917]|LALPA552-10|Canada|British Columbia|658[0n]|BOLD:AAA2483
Leucania farcta roseola|[5918]|RWWB052-09|United States|Washington|658[0n]|BOLD:AAA2483
Leucania farcta roseola|[5919]|LOWCE813-06|Canada|British Columbia|657[0n]|BOLD:AAA2483
Leucania farcta roseola|[5920]|LOWCE832-06|Canada|British Columbia|657[0n]|BOLD:AAA2483
Leucania farcta roseola|[5921]|LALPA888-11|Canada|British Columbia|658[0n]|BOLD:AAA2483
Leucania farcta roseola|[5922]|LBCA208-05|Canada|British Columbia|658[0n]|BOLD:AAA2483
Leucania farcta roseola|[5923]|LPVIC031-08|Canada|British Columbia|658[1n]|BOLD:AAA2483
Leucania farcta roseola|[5924]|RWWA479-09|United States|Washington|658[0n]|BOLD:AAA2483
Leucania farcta roseola|[5925]|LHLEP104-06|Canada|British Columbia|658[2n]|BOLD:AAA2483
Leucania farcta roseola|[5926]|LBCD320-05|Canada|British Columbia|658[0n]|BOLD:AAA2483
Leucania farcta roseola|[5927]|LBCD474-05|Canada|British Columbia|646[0n]|BOLD:AAA2483
Leucania farcta roseola|[5928]|LHLEP110-06|Canada|British Columbia|658[0n]|BOLD:AAA2483
Leucania farcta roseola|[5929]|LHLEP107-06|Canada|British Columbia|658[0n]|BOLD:AAA2483
Leucania farcta roseola|[5930]|LBCD476-05|Canada|British Columbia|658[0n]|BOLD:AAA2483
Leucania farcta roseola|[5931]|LBCD475-05|Canada|British Columbia|658[0n]|BOLD:AAA2483
Leucania farcta roseola|[5932]|LBCD428-05|Canada|British Columbia|658[0n]|BOLD:AAA2483
Leucania farcta roseola|[5933]|LBCD321-05|Canada|British Columbia|658[0n]|BOLD:AAA2483
Leucania farcta roseola|[5934]|LBCC301-05|Canada|British Columbia|658[0n]|BOLD:AAA2483
Leucania farcta roseola|[5935]|LPVIB520-08|Canada|British Columbia|658[0n]|BOLD:AAA2483
Leucania farcta roseola|[5936]|RWWA370-09|United States|Washington|658[0n]|BOLD:AAA2483
Leucania farcta roseola|[5937]|RWWA438-09|United States|Washington|658[0n]|BOLD:AAA2483
Leucania farcta roseola|[5938]|RWWA453-09|United States|Washington|658[0n]|BOLD:AAA2483
Leucania farcta roseola|[5939]|RWWA481-09|United States|Washington|658[0n]|BOLD:AAA2483
Leucania farcta roseola|[5940]|RWWA566-09|United States|Washington|658[0n]|BOLD:AAA2483
Leucania farcta roseola|[5941]|RWWA579-09|United States|Washington|658[0n]|BOLD:AAA2483
Leucania farcta roseola|[5942]|LBCH5605-10|Canada|British Columbia|658[0n]|BOLD:AAA2483
Leucania farcta roseola|[5943]|LBCH7880-10|Canada|British Columbia|658[0n]|BOLD:AAA2483
Leucania farcta roseola|[5944]|LALPA397-10|Canada|British Columbia|658[0n]|BOLD:AAA2483
Leucania farcta roseola|[5945]|LALPA494-10|Canada|British Columbia|658[0n]|BOLD:AAA2483
Leucania farcta roseola|[5946]|LALPA498-10|Canada|British Columbia|658[0n]|BOLD:AAA2483
Leucania farcta roseola|[5947]|BBLPA816-10|Canada|British Columbia|658[0n]|BOLD:AAA2483
Leucania farcta roseola|[5948]|BBLPA817-10|Canada|British Columbia|658[0n]|BOLD:AAA2483
Leucania farcta roseola|[5949]|BBLPA818-10|Canada|British Columbia|658[0n]|BOLD:AAA2483
Leucania farcta roseola|[5950]|BBLPA820-10|Canada|British Columbia|658[0n]|BOLD:AAA2483
Leucania farcta roseola|[5951]|BBLPA821-10|Canada|British Columbia|658[0n]|BOLD:AAA2483
Leucania farcta roseola|[5952]|BBLPA823-10|Canada|British Columbia|658[0n]|BOLD:AAA2483
Leucania farcta roseola|[5953]|LPVIA839-08|Canada|British Columbia|658[1n]|BOLD:AAA2483
Leucania farcta roseola|[5954]|RWWA345-09|United States|Washington|658[0n]|BOLD:AAA2483
Leucania farcta roseola|[5955]|RWWA510-09|United States|Washington|658[0n]|BOLD:AAA2483
Leucania farcta roseola|[5956]|BBLPA819-10|Canada|British Columbia|658[0n]|BOLD:AAA2483
Leucania farcta roseola|[5957]|RWWC275-11|United States|Washington|658[0n]|BOLD:AAA2483
Leucania farcta roseola|[5958]|LALPA895-11|Canada|British Columbia|658[0n]|BOLD:AAA2483
Leucania farcta roseola|[5959]|RDNMB439-05|Canada|British Columbia|608[1n]|BOLD:AAA2483
Leucania farcta roseola|[5960]|LOWCE825-06|Canada|British Columbia|625[0n]|BOLD:AAA2483
Leucania farcta roseola|[5961]|LPVIA627-08|Canada|British Columbia|623[0n]|BOLD:AAA2483
Leucania farcta roseola|[5962]|RWWB145-09|United States|Washington|646[0n]|BOLD:AAA2483
Leucania farcta roseola|[5963]|LMH040-06|Canada|British Columbia|658[0n]|BOLD:AAA2483
Leucania farcta roseola|[5964]|LHLEP111-06|Canada|British Columbia|658[0n]|BOLD:AAA2483
Leucania farcta roseola|[5965]|LALPA368-10|Canada|British Columbia|658[0n]|BOLD:AAA2483
Leucania farcta roseola|[5966]|LALPA474-10|Canada|British Columbia|658[0n]|BOLD:AAA2483
Leucania farcta roseola|[5967]|LHLEP108-06|Canada|British Columbia|658[0n]|BOLD:AAA2483
Leucania farcta roseola|[5968]|BBLPA822-10|Canada|British Columbia|658[0n]|BOLD:AAA2483
Leucania farcta roseola|[5969]|LALPA1172-11|Canada|British Columbia|658[0n]|BOLD:AAA2483
Leucania farcta roseola|[5970]|RWWC1171-13|United States|Washington|592[0n]|BOLD:AAA2483
Leucania stolata|[5971]|BBLSW426-09|United States|New Mexico|658[0n]|BOLD:AAH5307
Leucania stolata|[5972]|BBLSW428-09|United States|New Mexico|658[0n]|BOLD:AAH5307
Leucania stolata|[5973]|BBLSW432-09|United States|New Mexico|658[0n]|BOLD:AAH5307
Leucania stolata|[5974]|BBLSW433-09|United States|New Mexico|644[0n]|BOLD:AAH5307
Leucania stolata|[5975]|BBLSW430-09|United States|New Mexico|658[0n]|BOLD:AAH5307
Leucania stolata|[5976]|BBLSW434-09|United States|New Mexico|658[0n]|BOLD:AAH5307
Leucania stolata|[5977]|BBLSW435-09|United States|New Mexico|658[0n]|BOLD:AAH5307
Leucania stolata|[5978]|RDNMJ559-11|United States|Arizona|658[0n]|BOLD:AAH5307
Leucania stolata|[5979]|RDNMG247-08|United States|Texas|658[0n]|BOLD:AAH5307
Leucania stolata|[5980]|BBLSW429-09|United States|New Mexico|640[0n]|BOLD:AAH5307
Leucania stolata|[5981]|BBLSW431-09|United States|New Mexico|658[0n]|BOLD:AAH5307
Leucania stolata|[5982]|BBLWU060-09|United States|Colorado|658[0n]|BOLD:AAH5307
Leucania stolata|[5983]|BBLSW687-09|United States|New Mexico|658[0n]|BOLD:AAH5307
Leucania stolata|[5984]|BBLWU160-09|United States|Colorado|658[0n]|BOLD:AAH5307
Leucania stolata|[5985]|CMAZA391-10|United States|Arizona|658[0n]|BOLD:AAH5307
Leucania stolata|[5986]|RDNMJ104-10|United States|New Mexico|658[0n]|BOLD:AAH5307
Leucania stolata|[5987]|RDNMJ128-10|United States|Arizona|658[0n]|BOLD:AAH5307
Leucania stolata|[5988]|CNCLA5280-13|United States|New Mexico|658[0n]|BOLD:AAH5307
Leucania lapidaria|[5989]|XAG976-05|Canada|Ontario|585[0n]|BOLD:AAB8995
Leucania lapidaria|[5990]|RDLQ655-07|Canada|Quebec|623[0n]|BOLD:AAB8995
Leucania lapidaria|[5991]|RDLQ653-07|Canada|Quebec|658[0n]|BOLD:AAB8995
Leucania lapidaria|[5992]|LOCT259-05|United States|Connecticut|658[0n]|BOLD:AAB8995
Leucania lapidaria|[5993]|LOCT258-05|United States|Connecticut|658[0n]|BOLD:AAB8995
Leucania lapidaria|[5994]|XAJ689-06|Canada|Ontario|656[0n]|BOLD:AAB8995
Leucania lapidaria|[5995]|LNCNW049-06|United States|North Carolina|657[0n]|BOLD:AAB8995
Leucania lapidaria|[5996]|XAJ690-06|Canada|Ontario|656[0n]|BOLD:AAB8995
Leucania lapidaria|[5997]|XAD302-04|Canada|Ontario|600[1n]|BOLD:AAB8995

[illegible]
Leucania lapidaria|[5995]|LNCNW049-06|United States|North Carolina|657[0n]|BOLD:AAB8995
Leucania lapidaria|[5996]|XAJ690-06|Canada|Ontario|656[0n]|BOLD:AAB8995
Leucania lapidaria|[5997]|XAD302-04|Canada|Ontario|600[1n]|BOLD:AAB8995
Leucania lapidaria|[5998]|RDLQ656-07|Canada|Quebec|625[0n]|BOLD:AAB8995
Leucania lapidaria|[5999]|LNCC292-10|United States|North Carolina|658[0n]|BOLD:AAB8995
Leucania lapidaria|[6000]|XAJ451-06|Canada|Ontario|657[1n]|BOLD:AAB8995
Leucania lapidaria|[6001]|XAJ449-06|Canada|Ontario|657[1n]|BOLD:AAB8995
Leucania lapidaria|[6002]|LOCT261-05|United States|Connecticut|632[0n]|BOLD:AAB8995
Leucania lapidaria|[6003]|LOCT260-05|United States|Connecticut|632[0n]|BOLD:AAB8995
Leucania lapidaria|[6004]|XAB260-04|Canada|Ontario|658[0n]|BOLD:AAB8995
Leucania lapidaria|[6005]|XAK164-06|Canada|Ontario|657[0n]|BOLD:AAB8995
Leucania lapidaria|[6006]|BLGSM066-09|Canada|Ontario|658[0n]|BOLD:AAB8995
Leucania lapidaria|[6007]|CNCLB2880-14|United States|North Carolina|658[0n]|BOLD:AAB8995
Properigea loculosa|[6008]|RDNMD933-07|United States|Arizona|655[0n]|BOLD:AAH7706
Properigea loculosa|[6009]|RDNMG266-08|United States|Texas|658[0n]|BOLD:AAH7706
Properigea loculosa|[6010]|CMAZA1206-12|United States|Arizona|658[0n]|BOLD:AAH7706
Phlogophora periculosa|[6011]|XAD087-04|Canada|Ontario|576[0n]|BOLD:AAA7228
Phlogophora periculosa|[6012]|BBLEC010-09|Canada|New Brunswick|658[0n]|BOLD:AAA7228
Phlogophora periculosa|[6013]|LGSMG994-10|United States|North Carolina|658[0n]|BOLD:AAA7228
Phlogophora periculosa|[6014]|BBLPB633-10|Canada|Alberta|658[0n]|BOLD:AAA7228
Phlogophora periculosa|[6015]|BBLEC009-09|Canada|New Brunswick|658[0n]|BOLD:AAA7228
Phlogophora periculosa|[6016]|LPSOD1001-09|Canada|Ontario|658[0n]|BOLD:AAA7228
Phlogophora periculosa|[6017]|LNCNW021-06|United States|North Carolina|658[0n]|BOLD:AAA7228
Phlogophora periculosa|[6018]|LGSMG993-10|United States|North Carolina|623[0n]|BOLD:AAA7228
Phlogophora periculosa|[6019]|LNCC1258-11|United States|North Carolina|658[0n]|BOLD:AAA7228
Phlogophora periculosa|[6020]|LNCNW022-06|United States|North Carolina|605[0n]|BOLD:AAA7228
Phlogophora periculosa|[6021]|XAD305-04|Canada|Ontario|592[0n]|BOLD:AAA7228
Phlogophora periculosa|[6022]|XAD351-04|Canada|Ontario|576[1n]|BOLD:AAA7228
Phlogophora periculosa|[6023]|DUNLP181-08|Canada|British Columbia|658[0n]|BOLD:AAA7228
Phlogophora periculosa|[6024]|RWWC682-11|United States|Washington|658[0n]|BOLD:AAA7228
Phlogophora periculosa|[6025]|MJMSL087-10|United States|Massachusetts|658[0n]|BOLD:AAA7228
Phlogophora periculosa|[6026]|MJMSL086-10|United States|Massachusetts|658[0n]|BOLD:AAA7228
Phlogophora periculosa|[6027]|LALPA577-10|Canada|British Columbia|658[0n]|BOLD:AAA7228
Phlogophora periculosa|[6028]|LALPA572-10|Canada|British Columbia|658[0n]|BOLD:AAA7228
Phlogophora periculosa|[6029]|LALPA555-10|Canada|British Columbia|658[0n]|BOLD:AAA7228
Phlogophora periculosa|[6030]|LBCH4402-10|Canada|British Columbia|658[0n]|BOLD:AAA7228
Phlogophora periculosa|[6031]|LBCH4401-10|Canada|British Columbia|658[0n]|BOLD:AAA7228
Phlogophora periculosa|[6032]|LBCH4400-10|Canada|British Columbia|658[0n]|BOLD:AAA7228
Phlogophora periculosa|[6033]|LBCH4399-10|Canada|British Columbia|658[0n]|BOLD:AAA7228
Phlogophora periculosa|[6034]|LBCH4398-10|Canada|British Columbia|658[0n]|BOLD:AAA7228
Phlogophora periculosa|[6035]|LBCH4396-10|Canada|British Columbia|658[0n]|BOLD:AAA7228
Phlogophora periculosa|[6036]|LBCH4395-10|Canada|British Columbia|658[0n]|BOLD:AAA7228
Phlogophora periculosa|[6037]|LBCH4121-10|Canada|British Columbia|658[0n]|BOLD:AAA7228
Phlogophora periculosa|[6038]|LBCH4120-10|Canada|British Columbia|658[0n]|BOLD:AAA7228
Phlogophora periculosa|[6039]|LBCH3775-10|Canada|British Columbia|658[0n]|BOLD:AAA7228
Phlogophora periculosa|[6040]|LBCH3774-10|Canada|British Columbia|658[0n]|BOLD:AAA7228
Phlogophora periculosa|[6041]|LBCH3773-10|Canada|British Columbia|658[0n]|BOLD:AAA7228
Phlogophora periculosa|[6042]|LBCH3772-10|Canada|British Columbia|658[0n]|BOLD:AAA7228
Phlogophora periculosa|[6043]|LBCH3771-10|Canada|British Columbia|658[0n]|BOLD:AAA7228
Phlogophora periculosa|[6044]|LBCH3770-10|Canada|British Columbia|658[0n]|BOLD:AAA7228
Phlogophora periculosa|[6045]|LBCH3769-10|Canada|British Columbia|658[0n]|BOLD:AAA7228
Phlogophora periculosa|[6046]|LBCH3768-10|Canada|British Columbia|658[0n]|BOLD:AAA7228
Phlogophora periculosa|[6047]|LBCH3767-10|Canada|British Columbia|658[0n]|BOLD:AAA7228
Phlogophora periculosa|[6048]|LBCH3479-10|Canada|British Columbia|658[0n]|BOLD:AAA7228
Phlogophora periculosa|[6049]|LBCH3478-10|Canada|British Columbia|658[0n]|BOLD:AAA7228
Phlogophora periculosa|[6050]|LBCH3477-10|Canada|British Columbia|658[0n]|BOLD:AAA7228
Phlogophora periculosa|[6051]|LBCH3476-10|Canada|British Columbia|658[0n]|BOLD:AAA7228
Phlogophora periculosa|[6052]|LBCH3475-10|Canada|British Columbia|658[0n]|BOLD:AAA7228
Phlogophora periculosa|[6053]|LBCH3474-10|Canada|British Columbia|658[0n]|BOLD:AAA7228
Phlogophora periculosa|[6054]|LBCH3473-10|Canada|British Columbia|658[0n]|BOLD:AAA7228
Phlogophora periculosa|[6055]|LBCH3472-10|Canada|British Columbia|658[0n]|BOLD:AAA7228
Phlogophora periculosa|[6056]|LBCH3329-10|Canada|British Columbia|658[0n]|BOLD:AAA7228
Phlogophora periculosa|[6057]|LBCH3067-10|Canada|British Columbia|658[0n]|BOLD:AAA7228
Phlogophora periculosa|[6058]|LBCH2084-10|Canada|British Columbia|658[0n]|BOLD:AAA7228
Phlogophora periculosa|[6059]|LBCH1500-10|Canada|British Columbia|658[0n]|BOLD:AAA7228
Phlogophora periculosa|[6060]|LBCH945-10|Canada|British Columbia|658[0n]|BOLD:AAA7228
Phlogophora periculosa|[6061]|BBLPC123-09|Canada|New Brunswick|658[0n]|BOLD:AAA7228
Phlogophora periculosa|[6062]|BBLPC107-09|Canada|New Brunswick|658[0n]|BOLD:AAA7228
Phlogophora periculosa|[6063]|BBLEC528-09|Canada|New Brunswick|658[0n]|BOLD:AAA7228
Phlogophora periculosa|[6064]|BBLEC043-09|Canada|New Brunswick|658[0n]|BOLD:AAA7228
Phlogophora periculosa|[6065]|RWWB019-09|United States|Washington|658[0n]|BOLD:AAA7228
Phlogophora periculosa|[6066]|RWWA957-09|United States|Washington|658[0n]|BOLD:AAA7228
Phlogophora periculosa|[6067]|RWWA935-09|United States|Washington|658[0n]|BOLD:AAA7228
Phlogophora periculosa|[6068]|LPMNB531-09|Canada|Manitoba|658[0n]|BOLD:AAA7228
Phlogophora periculosa|[6069]|LPMNB248-09|Canada|Manitoba|658[0n]|BOLD:AAA7228
Phlogophora periculosa|[6070]|LHLEP264-06|Canada|British Columbia|658[0n]|BOLD:AAA7228
Phlogophora periculosa|[6071]|LHLEP263-06|Canada|British Columbia|658[0n]|BOLD:AAA7228
Phlogophora periculosa|[6072]|LHLEP262-06|Canada|British Columbia|658[0n]|BOLD:AAA7228
Phlogophora periculosa|[6073]|LHLEP261-06|Canada|British Columbia|658[0n]|BOLD:AAA7228
Phlogophora periculosa|[6074]|LHLEP260-06|Canada|British Columbia|658[0n]|BOLD:AAA7228
Phlogophora periculosa|[6075]|LHLEP259-06|Canada|British Columbia|658[0n]|BOLD:AAA7228
Phlogophora periculosa|[6076]|LHLEP258-06|Canada|British Columbia|658[0n]|BOLD:AAA7228
Phlogophora periculosa|[6077]|LHLEP257-06|Canada|British Columbia|658[0n]|BOLD:AAA7228
Phlogophora periculosa|[6078]|TMNBB191-06|Canada|New Brunswick|658[0n]|BOLD:AAA7228
Phlogophora periculosa|[6079]|LOWCD156-06|Canada|British Columbia|658[0n]|BOLD:AAA7228
Phlogophora periculosa|[6080]|RDLQB483-05|Canada|Quebec|658[0n]|BOLD:AAA7228
Phlogophora periculosa|[6081]|LOWCC017-05|Canada|British Columbia|658[0n]|BOLD:AAA7228
Phlogophora periculosa|[6082]|LOWCC015-05|Canada|British Columbia|658[0n]|BOLD:AAA7228
Phlogophora periculosa|[6083]|LOWCC008-05|Canada|British Columbia|620[0n]|BOLD:AAA7228
Phlogophora periculosa|[6084]|TMNBB190-06|Canada|New Brunswick|658[0n]|BOLD:AAA7228
Phlogophora periculosa|[6085]|LOWCC014-05|Canada|British Columbia|658[0n]|BOLD:AAA7228
Phlogophora periculosa|[6086]|LOWCC013-05|Canada|British Columbia|658[0n]|BOLD:AAA7228
Phlogophora periculosa|[6087]|LOWCC012-05|Canada|British Columbia|658[0n]|BOLD:AAA7228
Phlogophora periculosa|[6088]|LOWCC011-05|Canada|British Columbia|658[0n]|BOLD:AAA7228
Phlogophora periculosa|[6089]|LOWCC010-05|Canada|British Columbia|658[0n]|BOLD:AAA7228
Phlogophora periculosa|[6090]|LOWCC009-05|Canada|British Columbia|658[0n]|BOLD:AAA7228
Phlogophora periculosa|[6091]|LOWCC007-05|Canada|British Columbia|658[0n]|BOLD:AAA7228
Phlogophora periculosa|[6092]|LBCC443-05|Canada|British Columbia|658[0n]|BOLD:AAA7228
Phlogophora periculosa|[6093]|XAG942-05|Canada|Ontario|658[0n]|BOLD:AAA7228
Phlogophora periculosa|[6094]|MJMSL085-10|United States|Massachusetts|658[0n]|BOLD:AAA7228
Phlogophora periculosa|[6095]|TTMNB363-06|Canada|New Brunswick|658[3n]|BOLD:AAA7228
Phlogophora periculosa|[6096]|XAH001-05|Canada|Ontario|614[0n]|BOLD:AAA7228
Phlogophora periculosa|[6097]|BBLPC106-09|Canada|New Brunswick|633[0n]|BOLD:AAA7228

Phlogophora periculosa|[illegible]
Phlogophora periculosa|[6095]|TTMNB363-06|Canada|New Brunswick|658[3n]|BOLD:AAA7228
Phlogophora periculosa|[6096]|XAH001-05|Canada|Ontario|614[0n]|BOLD:AAA7228
Phlogophora periculosa|[6097]|BBLPC106-09|Canada|New Brunswick|633[0n]|BOLD:AAA7228
Phlogophora periculosa|[6098]|BBLPC103-09|Canada|New Brunswick|631[0n]|BOLD:AAA7228
Phlogophora periculosa|[6099]|LBCH944-10|Canada|British Columbia|658[0n]|BOLD:AAA7228
Phlogophora periculosa|[6100]|LBCH4397-10|Canada|British Columbia|658[0n]|BOLD:AAA7228
Phlogophora periculosa|[6101]|LOWCC016-05|Canada|British Columbia|653[0n]|BOLD:AAA7228
Phlogophora periculosa|[6102]|PHAUG1779-11|Canada|Ontario|658[0n]|BOLD:AAA7228
Phlogophora iris|[6103]|PHMNB151-04|Canada|New Brunswick|565[5n]|BOLD:AAA9019
Phlogophora iris|[6104]|BBLEC364-09|Canada|Newfoundland and Labrador|658[0n]|BOLD:AAA9019
Phlogophora iris|[6105]|TMNBB188-06|Canada|New Brunswick|658[1n]|BOLD:AAA9019
Phlogophora iris|[6106]|PHMNB318-04|Canada|New Brunswick|658[0n]|BOLD:AAA9019
Phlogophora iris|[6107]|PHMNB563-04|Canada|New Brunswick|658[1n]|BOLD:AAA9019
Phlogophora iris|[6108]|BBLEC358-09|Canada|Newfoundland and Labrador|658[0n]|BOLD:AAA9019
Phlogophora iris|[6109]|BBLPC321-09|Canada|Newfoundland and Labrador|658[0n]|BOLD:AAA9019
Phlogophora iris|[6110]|BBLPE492-09|Canada|Newfoundland and Labrador|658[0n]|BOLD:AAA9019
Phlogophora iris|[6111]|BBLPE494-09|Canada|Newfoundland and Labrador|658[0n]|BOLD:AAA9019
Phlogophora iris|[6112]|BBLPE501-09|Canada|Newfoundland and Labrador|658[0n]|BOLD:AAA9019
Phlogophora iris|[6113]|BBLPB516-10|Canada|Ontario|658[0n]|BOLD:AAA9019
Phlogophora iris|[6114]|BBLPB517-10|Canada|Ontario|658[0n]|BOLD:AAA9019
Phlogophora iris|[6115]|BBLPB518-10|Canada|Ontario|658[0n]|BOLD:AAA9019
Phlogophora iris|[6116]|BBLPB519-10|Canada|Ontario|658[0n]|BOLD:AAA9019
Phlogophora iris|[6117]|BBLPB520-10|Canada|Ontario|658[0n]|BOLD:AAA9019
Phlogophora iris|[6118]|BBLCU299-09|United States|Michigan|658[0n]|BOLD:AAA9019
Phlogophora iris|[6119]|BBLEC348-09|Canada|Newfoundland and Labrador|658[0n]|BOLD:AAA9019
Phlogophora iris|[6120]|LPMN340-08|Canada|Manitoba|658[0n]|BOLD:AAA9019
Phlogophora iris|[6121]|LPMN814-08|Canada|Manitoba|658[0n]|BOLD:AAA9019
Phlogophora iris|[6122]|LPSOB784-08|Canada|Ontario|658[0n]|BOLD:AAA9019
Phlogophora iris|[6123]|LPSOB785-08|Canada|Ontario|658[0n]|BOLD:AAA9019
Phlogophora iris|[6124]|LPSOC311-08|Canada|Ontario|658[0n]|BOLD:AAA9019
Phlogophora iris|[6125]|LPSOB313-08|Canada|Ontario|658[0n]|BOLD:AAA9019
Phlogophora iris|[6126]|TMNBB189-06|Canada|New Brunswick|658[0n]|BOLD:AAA9019
Phlogophora iris|[6127]|LPSOB008-08|Canada|Ontario|658[0n]|BOLD:AAA9019
Phlogophora iris|[6128]|TMNBB187-06|Canada|New Brunswick|658[0n]|BOLD:AAA9019
Phlogophora iris|[6129]|TTMNB362-06|Canada|New Brunswick|658[0n]|BOLD:AAA9019
Phlogophora iris|[6130]|PHMNB691-04|Canada|New Brunswick|658[0n]|BOLD:AAA9019
Phlogophora iris|[6131]|PHMNB469-04|Canada|New Brunswick|658[0n]|BOLD:AAA9019
Phlogophora iris|[6132]|PHMNB464-04|Canada|New Brunswick|658[0n]|BOLD:AAA9019
Phlogophora iris|[6133]|PHMNB461-04|Canada|New Brunswick|658[0n]|BOLD:AAA9019
Phlogophora iris|[6134]|PHMNB319-04|Canada|New Brunswick|658[0n]|BOLD:AAA9019
Phlogophora iris|[6135]|XAE455-04|Canada|Ontario|658[0n]|BOLD:AAA9019
Phlogophora iris|[6136]|XAE276-04|Canada|Ontario|658[0n]|BOLD:AAA9019
Phlogophora iris|[6137]|XAB551-04|Canada|Ontario|658[0n]|BOLD:AAA9019
Phlogophora iris|[6138]|PHMNB380-04|Canada|New Brunswick|658[0n]|BOLD:AAA9019
Phlogophora iris|[6139]|CNRMD2054-12|Canada|Manitoba|637[0n]|BOLD:AAA9019
Phlogophora iris|[6140]|CNRMD2077-12|Canada|Manitoba|637[0n]|BOLD:AAA9019
Phlogophora iris|[6141]|XAB397-04|Canada|Ontario|658[0n]|BOLD:AAA9019
Phlogophora iris|[6142]|PHMNB235-04|Canada|New Brunswick|568[3n]|BOLD:AAA9019
Phlogophora iris|[6143]|PHMNB720-05|Canada|New Brunswick|616[0n]|BOLD:AAA9019
Phlogophora iris|[6144]|PHMNB679-04|Canada|New Brunswick|616[0n]|BOLD:AAA9019
Phlogophora iris|[6145]|PHMNB066-03|Canada|New Brunswick|639[0n]|BOLD:AAA9019
Phlogophora iris|[6146]|PHMO084-03|Canada|Ontario|639[0n]|BOLD:AAA9019
Phlogophora iris|[6147]|PHMO083-03|Canada|Ontario|639[0n]|BOLD:AAA9019
Phlogophora iris|[6148]|PHMNB188-04|Canada|New Brunswick|609[0n]|BOLD:AAA9019
Phlogophora iris|[6149]|RDLQ486-07|Canada|Quebec|599[0n]|BOLD:AAA9019
Phlogophora iris|[6150]|SSPAA6091-13|Canada|Saskatchewan|560[0n]|BOLD:AAA9019
Phlogophora iris|[6151]|HPPPC1200-13|Canada|Nova Scotia|587[0n]|BOLD:AAA9019
Conservula anodonta|[6152]|RDLQB674-05|Canada|Quebec|658[0n]|BOLD:AAF1231
Conservula anodonta|[6153]|RDLQG132-06|Canada|Quebec|574[0n]|BOLD:AAF1231
Conservula anodonta|[6154]|RDLQF545-06|Canada|Quebec|658[0n]|BOLD:AAF1231
Conservula anodonta|[6155]|LMDH172-11|United States|Minnesota|658[0n]|BOLD:AAF1231
Euplexia triplaga|[6156]|LOCRA805-07|Costa Rica|Alajuela|594[0n]|BOLD:ABZ7420
Euplexia triplaga|[6157]|RDNME891-08|United States|Arizona|658[0n]|BOLD:ABZ7420
Euplexia triplaga|[6158]|LOCRF664-11|Costa Rica|Cartago|658[0n]|BOLD:ABZ7420
Euplexia triplaga|[6159]|LOCRA804-07|Costa Rica|Alajuela|615[0n]|BOLD:ABZ7420
Euplexia triplaga|[6160]|LOCRF663-11|Costa Rica|Cartago|658[0n]|BOLD:ABZ7420
Euplexia triplaga|[6161]|LOCRF665-11|Costa Rica|Cartago|658[0n]|BOLD:ABZ7420
Euplexia benesimilis|[6162]|GMGSH414-12|United States|Tennessee|638[0n]|BOLD:AAA4098
Euplexia benesimilis|[6163]|LSEU658-06|United States|Georgia|658[0n]|BOLD:AAA4098
Euplexia benesimilis|[6164]|LGSM489-04|United States|North Carolina|658[0n]|BOLD:AAA4098
Euplexia benesimilis|[6165]|LOCT093-05|United States|Connecticut|632[0n]|BOLD:AAA4098
Euplexia benesimilis|[6166]|LSEU078-06|United States|North Carolina|658[0n]|BOLD:AAA4098
Euplexia benesimilis|[6167]|LNCNW063-06|United States|North Carolina|658[0n]|BOLD:AAA4098
Euplexia benesimilis|[6168]|LSEU659-06|United States|Georgia|658[0n]|BOLD:AAA4098
Euplexia benesimilis|[6169]|LSEU660-06|United States|Georgia|658[0n]|BOLD:AAA4098
Euplexia benesimilis|[6170]|LPSO842-08|Canada|Ontario|658[0n]|BOLD:AAA4098
Euplexia benesimilis|[6171]|LNCC279-10|United States|North Carolina|658[0n]|BOLD:AAA4098
Euplexia benesimilis|[6172]|LNCC1729-13|United States|North Carolina|658[0n]|BOLD:AAA4098
Euplexia benesimilis|[6173]|HPPPE1553-13|Canada|Nova Scotia|658[0n]|BOLD:ABZ7421
Euplexia benesimilis|[6174]|HPPPC1206-13|Canada|Nova Scotia|661[0n]|BOLD:ABZ7421
Euplexia benesimilis|[6175]|LNCC1109-11|United States|North Carolina|658[0n]|BOLD:ABZ7421
Euplexia benesimilis|[6176]|CNKJK1591-14|Canada|Nova Scotia|591[0n]|BOLD:ABZ7421
Euplexia benesimilis|[6177]|LPSO384-08|Canada|Ontario|658[0n]|BOLD:AAA4097
Euplexia benesimilis|[6178]|CNPEP084-14|Canada|Prince Edward Island|534[0n]|BOLD:AAA4097
Euplexia benesimilis|[6179]|LPSO359-08|Canada|Ontario|658[0n]|BOLD:AAA4097
Euplexia benesimilis|[6180]|XAC799-04|Canada|Ontario|658[0n]|BOLD:AAA4097
Euplexia benesimilis|[6181]|BLTIB113-08|Canada|Ontario|609[1n]|BOLD:AAA4097
Euplexia benesimilis|[6182]|LPSO364-08|Canada|Ontario|658[0n]|BOLD:AAA4097
Euplexia benesimilis|[6183]|LPSO495-08|Canada|Ontario|658[0n]|BOLD:AAA4097
Euplexia benesimilis|[6184]|LPSOB816-08|Canada|Ontario|658[0n]|BOLD:AAA4097
Euplexia benesimilis|[6185]|LNCC1523-13|United States|North Carolina|658[0n]|BOLD:AAA4097
Euplexia benesimilis|[6186]|LNCC1728-13|United States|North Carolina|658[0n]|BOLD:AAA4097
Euplexia benesimilis|[6187]|LNCC1730-13|United States|North Carolina|658[0n]|BOLD:AAA4097
Euplexia benesimilis|[6188]|CNROI023-13|Canada|Ontario|614[0n]|BOLD:AAA4097
Euplexia benesimilis|[6189]|LGSMC658-05|United States|Tennessee|616[0n]|BOLD:AAA4097
Euplexia benesimilis|[6190]|LGSM490-04|United States|North Carolina|609[0n]|BOLD:AAA4097
Euplexia benesimilis|[6191]|RDLQG202-06|Canada|Quebec|658[0n]|BOLD:AAA4097
Euplexia benesimilis|[6192]|BLTIB459-08|Canada|Ontario|658[0n]|BOLD:AAA4097
Euplexia benesimilis|[6193]|LGSMG992-10|United States|North Carolina|658[0n]|BOLD:AAA4097
Euplexia benesimilis|[6194]|BLTIB145-08|Canada|Ontario|658[0n]|BOLD:AAA4097
Euplexia benesimilis|[6195]|BLTIB090-08|Canada|Ontario|658[0n]|BOLD:AAA4097
Euplexia benesimilis|[6196]|LPSOB818-08|Canada|Ontario|658[0n]|BOLD:AAA4097
Euplexia benesimilis|[6197]|LPSOC111-08|Canada|Ontario|658[0n]|BOLD:AAA4097

Euplexia benesimilis|[6194]|BLTIB145-08|Canada|Ontario|658[0n]|BOLD:AAA4097
Euplexia benesimilis|[6195]|BLTIB090-08|Canada|Ontario|658[0n]|BOLD:AAA4097
Euplexia benesimilis|[6196]|LPSOB818-08|Canada|Ontario|658[0n]|BOLD:AAA4097
Euplexia benesimilis|[6197]|LPSOC111-08|Canada|Ontario|658[0n]|BOLD:AAA4097
Euplexia benesimilis|[6198]|LPSOB093-08|Canada|Ontario|658[0n]|BOLD:AAA4097
Euplexia benesimilis|[6199]|LPSO496-08|Canada|Ontario|658[0n]|BOLD:AAA4097
Euplexia benesimilis|[6200]|RDLQF566-06|Canada|Quebec|658[0n]|BOLD:AAA4097
Euplexia benesimilis|[6201]|TTMNB361-06|Canada|New Brunswick|658[0n]|BOLD:AAA4097
Euplexia benesimilis|[6202]|LOCT089-05|United States|Connecticut|658[0n]|BOLD:AAA4097
Euplexia benesimilis|[6203]|LGSMC659-05|United States|Tennessee|658[0n]|BOLD:AAA4097
Euplexia benesimilis|[6204]|XAC601-04|Canada|Ontario|658[0n]|BOLD:AAA4097
Euplexia benesimilis|[6205]|PHMO049-03|Canada|Ontario|639[0n]|BOLD:AAA4097
Euplexia benesimilis|[6206]|BLGSM059-09|Canada|Ontario|621[0n]|BOLD:AAA4097
Euplexia benesimilis|[6207]|CNKJB129-14|Canada|Nova Scotia|549[0n]|BOLD:AAA4097
Euplexia benesimilis|[6208]|CNKJK1901-14|Canada|Nova Scotia|564[0n]|BOLD:AAA4097
Euplexia benesimilis|[6209]|CNPAC666-13|Canada|Saskatchewan|637[0n]|BOLD:AAA4097
Euplexia benesimilis|[6210]|LPMN724-08|Canada|Manitoba|658[0n]|BOLD:AAA4097
Euplexia benesimilis|[6211]|SSWLD5780-13|Canada|Alberta|622[0n]|BOLD:AAA4097
Euplexia benesimilis|[6212]|LOWCB437-05|Canada|British Columbia|590[0n]|BOLD:AAA4097
Euplexia benesimilis|[6213]|LOWCB438-05|Canada|British Columbia|626[1n]|BOLD:AAA4097
Euplexia benesimilis|[6214]|LOWCB458-05|Canada|British Columbia|617[0n]|BOLD:AAA4097
Euplexia benesimilis|[6215]|RWWC372-11|United States|Washington|658[0n]|BOLD:AAA4097
Euplexia benesimilis|[6216]|LALPA1122-11|Canada|British Columbia|658[0n]|BOLD:AAA4097
Euplexia benesimilis|[6217]|RWWC332-11|United States|Washington|658[0n]|BOLD:AAA4097
Euplexia benesimilis|[6218]|RWWC225-11|United States|Washington|658[0n]|BOLD:AAA4097
Euplexia benesimilis|[6219]|BBLPB852-10|Canada|British Columbia|658[0n]|BOLD:AAA4097
Euplexia benesimilis|[6220]|BBLPA815-10|Canada|British Columbia|658[0n]|BOLD:AAA4097
Euplexia benesimilis|[6221]|BBLPA814-10|Canada|British Columbia|658[0n]|BOLD:AAA4097
Euplexia benesimilis|[6222]|BBLPA813-10|Canada|British Columbia|658[0n]|BOLD:AAA4097
Euplexia benesimilis|[6223]|BBLPA812-10|Canada|British Columbia|658[0n]|BOLD:AAA4097
Euplexia benesimilis|[6224]|LALPA601-10|Canada|British Columbia|658[0n]|BOLD:AAA4097
Euplexia benesimilis|[6225]|LBCH2983-10|Canada|British Columbia|658[0n]|BOLD:AAA4097
Euplexia benesimilis|[6226]|LBCH2958-10|Canada|British Columbia|658[0n]|BOLD:AAA4097
Euplexia benesimilis|[6227]|RWWA719-09|United States|Washington|658[0n]|BOLD:AAA4097
Euplexia benesimilis|[6228]|RWWA554-09|United States|Washington|658[0n]|BOLD:AAA4097
Euplexia benesimilis|[6229]|RWWA512-09|United States|Washington|658[0n]|BOLD:AAA4097
Euplexia benesimilis|[6230]|RWWA160-09|United States|Washington|658[0n]|BOLD:AAA4097
Euplexia benesimilis|[6231]|RWWA157-09|United States|Washington|655[0n]|BOLD:AAA4097
Euplexia benesimilis|[6232]|LBCG2542-09|Canada|British Columbia|658[0n]|BOLD:AAA4097
Euplexia benesimilis|[6233]|LPVIA236-08|Canada|British Columbia|658[0n]|BOLD:AAA4097
Euplexia benesimilis|[6234]|LBCS749-07|Canada|British Columbia|658[0n]|BOLD:AAA4097
Euplexia benesimilis|[6235]|LHLEP428-06|Canada|British Columbia|658[0n]|BOLD:AAA4097
Euplexia benesimilis|[6236]|LOWCB457-05|Canada|British Columbia|658[0n]|BOLD:AAA4097
Euplexia benesimilis|[6237]|LOWCB456-05|Canada|British Columbia|658[0n]|BOLD:AAA4097
Euplexia benesimilis|[6238]|LBCD103-05|Canada|British Columbia|658[0n]|BOLD:AAA4097
Euplexia benesimilis|[6239]|LBCD056-05|Canada|British Columbia|658[0n]|BOLD:AAA4097
Euplexia benesimilis|[6240]|LBCD052-05|Canada|British Columbia|658[0n]|BOLD:AAA4097
Euplexia benesimilis|[6241]|LBCC440-05|Canada|British Columbia|658[0n]|BOLD:AAA4097
Euplexia benesimilis|[6242]|LBCC335-05|Canada|British Columbia|658[0n]|BOLD:AAA4097
Euplexia benesimilis|[6243]|LBCC334-05|Canada|British Columbia|658[0n]|BOLD:AAA4097
Euplexia benesimilis|[6244]|LBCC303-05|Canada|British Columbia|658[0n]|BOLD:AAA4097
Euplexia benesimilis|[6245]|LBCC296-05|Canada|British Columbia|658[0n]|BOLD:AAA4097
Euplexia benesimilis|[6246]|LBCC042-05|Canada|British Columbia|658[0n]|BOLD:AAA4097
Euplexia benesimilis|[6247]|LBCC008-05|Canada|British Columbia|658[0n]|BOLD:AAA4097
Euplexia benesimilis|[6248]|LBCC007-05|Canada|British Columbia|658[0n]|BOLD:AAA4097
Euplexia benesimilis|[6249]|LBCC006-05|Canada|British Columbia|658[0n]|BOLD:AAA4097
Euplexia benesimilis|[6250]|LBCB622-05|Canada|British Columbia|658[0n]|BOLD:AAA4097
Euplexia benesimilis|[6251]|LBCB552-05|Canada|British Columbia|658[0n]|BOLD:AAA4097
Euplexia benesimilis|[6252]|LBCB407-05|Canada|British Columbia|658[0n]|BOLD:AAA4097
Euplexia benesimilis|[6253]|LBCB206-05|Canada|British Columbia|658[0n]|BOLD:AAA4097
Euplexia benesimilis|[6254]|LBCB075-05|Canada|British Columbia|658[0n]|BOLD:AAA4097
Euplexia benesimilis|[6255]|LBCB073-05|Canada|British Columbia|658[0n]|BOLD:AAA4097
Euplexia benesimilis|[6256]|LALPA141-10|Canada|British Columbia|658[0n]|BOLD:AAA4097
Euplexia benesimilis|[6257]|LBCD489-05|Canada|British Columbia|654[0n]|BOLD:AAA4097
Euplexia benesimilis|[6258]|CNRMC1486-12|Canada|Manitoba|615[0n]|BOLD:AAA4097
Euplexia benesimilis|[6259]|CNRMD2090-12|Canada|Manitoba|633[0n]|BOLD:AAA4097
Euplexia benesimilis|[6260]|CNRMD2059-12|Canada|Manitoba|632[0n]|BOLD:AAA4097
Euplexia benesimilis|[6261]|BBLPC222-09|Canada|Nova Scotia|633[0n]|BOLD:AAA4097
Euplexia benesimilis|[6262]|LBCD460-05|Canada|British Columbia|645[0n]|BOLD:AAA4097
Euplexia benesimilis|[6263]|CNEIC3024-12|Canada|Alberta|633[0n]|BOLD:AAA4097
Euplexia benesimilis|[6264]|CNEIB1522-12|Canada|Alberta|625[0n]|BOLD:AAA4097
Euplexia benesimilis|[6265]|CNRMD2089-12|Canada|Manitoba|638[0n]|BOLD:AAA4097
Euplexia benesimilis|[6266]|CNRMD2080-12|Canada|Manitoba|637[0n]|BOLD:AAA4097
Euplexia benesimilis|[6267]|CNRMD2058-12|Canada|Manitoba|637[0n]|BOLD:AAA4097
Euplexia benesimilis|[6268]|CNRMD2055-12|Canada|Manitoba|638[0n]|BOLD:AAA4097
Euplexia benesimilis|[6269]|CNRMD2051-12|Canada|Manitoba|637[0n]|BOLD:AAA4097
Euplexia benesimilis|[6270]|CNPAC674-13|Canada|Saskatchewan|635[0n]|BOLD:AAA4097
Euplexia benesimilis|[6271]|CNPAC672-13|Canada|Saskatchewan|634[0n]|BOLD:AAA4097
Euplexia benesimilis|[6272]|CNRMD2079-12|Canada|Manitoba|635[0n]|BOLD:AAA4097
Euplexia benesimilis|[6273]|CNEIC3031-12|Canada|Alberta|634[0n]|BOLD:AAA4097
Euplexia benesimilis|[6274]|LBCD104-05|Canada|British Columbia|636[0n]|BOLD:AAA4097
Euplexia benesimilis|[6275]|CNPAL1082-13|Canada|Saskatchewan|614[0n]|BOLD:AAA4097
Euplexia benesimilis|[6276]|LCH556-04|Canada|Manitoba|585[0n]|BOLD:AAA4097
Euplexia benesimilis|[6277]|RWWA516-09|United States|Washington|632[0n]|BOLD:AAA4097
Euplexia benesimilis|[6278]|CNEIC2822-12|Canada|Alberta|564[0n]|BOLD:AAA4097
Euplexia benesimilis|[6279]|SSPAB028-13|Canada|Saskatchewan|592[0n]|BOLD:AAA4097
Euplexia benesimilis|[6280]|CNEIC2824-12|Canada|Alberta|563[0n]|BOLD:AAA4097
Euplexia benesimilis|[6281]|CNRMC1487-12|Canada|Manitoba|637[0n]|BOLD:AAA4097
Euplexia benesimilis|[6282]|RDMAB075-05|Canada|Alberta|611[0n]|BOLD:AAA4097
Euplexia benesimilis|[6283]|SSEIC7299-13|Canada|Alberta|570[0n]|BOLD:AAA4097
Euplexia benesimilis|[6284]|BBLPB542-10|Canada|Ontario|658[0n]|BOLD:AAA4097
Euplexia benesimilis|[6285]|LPSO743-08|Canada|Ontario|658[0n]|BOLD:AAA4097
Euplexia benesimilis|[6286]|BBLPC973-09|Canada|Newfoundland and Labrador|658[0n]|BOLD:AAA4097
Euplexia benesimilis|[6287]|BBLPC629-09|Canada|Newfoundland and Labrador|658[0n]|BOLD:AAA4097
Euplexia benesimilis|[6288]|BBLPB749-10|Canada|Ontario|658[0n]|BOLD:AAA4097
Euplexia benesimilis|[6289]|BBLPA811-10|Canada|Ontario|658[0n]|BOLD:AAA4097
Euplexia benesimilis|[6290]|BBLPA810-10|Canada|Ontario|658[0n]|BOLD:AAA4097
Euplexia benesimilis|[6291]|BBLPA809-10|Canada|Ontario|658[0n]|BOLD:AAA4097
Euplexia benesimilis|[6292]|BBLPA808-10|Canada|Ontario|658[0n]|BOLD:AAA4097
Euplexia benesimilis|[6293]|BBLPE357-09|Canada|Newfoundland and Labrador|658[0n]|BOLD:AAA4097
Euplexia benesimilis|[6294]|BBLPE347-09|Canada|Newfoundland and Labrador|658[0n]|BOLD:AAA4097
Euplexia benesimilis|[6295]|BBLPE266-09|Canada|Nova Scotia|658[0n]|BOLD:AAA4097
Euplexia benesimilis|[6296]|BBLPE128-09|Canada|Nova Scotia|658[0n]|BOLD:AAA4097
Euplexia benesimilis|[6297]|BBLPE126-09|Canada|Nova Scotia|658[0n]|BOLD:AAA4097

Euplexia benesimilis|[6294]|BBLPE347-09|Canada|Newfoundland and Labrador|658[0n]|BOLD:AAA4097
Euplexia benesimilis|[6295]|BBLPE266-09|Canada|Nova Scotia|658[0n]|BOLD:AAA4097
Euplexia benesimilis|[6296]|BBLPE128-09|Canada|Nova Scotia|658[0n]|BOLD:AAA4097
Euplexia benesimilis|[6297]|BBLPE126-09|Canada|Nova Scotia|658[0n]|BOLD:AAA4097
Euplexia benesimilis|[6298]|BBLPE100-09|Canada|Nova Scotia|658[0n]|BOLD:AAA4097
Euplexia benesimilis|[6299]|BBLPE014-09|Canada|Nova Scotia|658[0n]|BOLD:AAA4097
Euplexia benesimilis|[6300]|BBLPC266-09|Canada|Nova Scotia|658[0n]|BOLD:AAA4097
Euplexia benesimilis|[6301]|BBLEC804-09|Canada|Newfoundland and Labrador|658[0n]|BOLD:AAA4097
Euplexia benesimilis|[6302]|BBLEC383-09|Canada|Newfoundland and Labrador|658[0n]|BOLD:AAA4097
Euplexia benesimilis|[6303]|BBLEC357-09|Canada|Newfoundland and Labrador|658[0n]|BOLD:AAA4097
Euplexia benesimilis|[6304]|TTMNB034-06|Canada|New Brunswick|658[0n]|BOLD:AAA4097
Euplexia benesimilis|[6305]|LBCC766-05|Canada|British Columbia|658[0n]|BOLD:AAA4097
Euplexia benesimilis|[6306]|CNEIB1520-12|Canada|Alberta|613[0n]|BOLD:AAA4097
Euplexia benesimilis|[6307]|CNBRN829-14|Canada|Nova Scotia|561[0n]|BOLD:AAA4097
Euplexia benesimilis|[6308]|CNPKF2672-14|Canada|Ontario|617[0n]|BOLD:AAA4097
Euplexia benesimilis|[6309]|CNFNF2497-14|Canada|Quebec|540[0n]|BOLD:AAA4097
Euplexia benesimilis|[6310]|CNKOO178-14|Canada|New Brunswick|588[0n]|BOLD:AAA4097
Euplexia benesimilis|[6311]|CNFNR4007-14|Canada|Quebec|558[0n]|BOLD:AAA4097
Euplexia benesimilis|[6312]|CNFNS1487-14|Canada|Quebec|564[0n]|BOLD:AAA4097
Euplexia benesimilis|[6313]|SSPAB029-13|Canada|Saskatchewan|571[0n]|BOLD:AAA4097
Euplexia benesimilis|[6314]|CNTNC2655-14|Canada|Newfoundland and Labrador|581[0n]|BOLD:AAA4097
Euplexia benesimilis|[6315]|BBLPE099-09|Canada|Nova Scotia|658[0n]|BOLD:AAA4097
Euplexia benesimilis|[6316]|LOCT325-05|United States|Connecticut|631[0n]|BOLD:AAA4097
Euplexia benesimilis|[6317]|LPVIA237-08|Canada|British Columbia|658[0n]|BOLD:AAA4097
Euplexia benesimilis|[6318]|LPABB581-08|Canada|Alberta|658[0n]|BOLD:AAA4097
Euplexia benesimilis|[6319]|LGSMC478-05|United States|Tennessee|617[0n]|BOLD:AAA4097
Euplexia benesimilis|[6320]|CNFNR4024-14|Canada|Quebec|591[0n]|BOLD:AAA4097
Euplexia benesimilis|[6321]|CNFNR4006-14|Canada|Quebec|591[0n]|BOLD:AAA4097
Euplexia benesimilis|[6322]|CNPAL1079-13|Canada|Saskatchewan|603[0n]|BOLD:AAA4097
Euplexia benesimilis|[6323]|CNTNC028-14|Canada|Newfoundland and Labrador|567[0n]|BOLD:AAA4097
Euplexia benesimilis|[6324]|CNTNC2656-14|Canada|Newfoundland and Labrador|543[0n]|BOLD:AAA4097
Euplexia benesimilis|[6325]|HPPPD1644-13|Canada|Nova Scotia|560[0n]|BOLD:AAA4097
Euplexia benesimilis|[6326]|PHMNB058-03|Canada|New Brunswick|639[0n]|BOLD:AAA4097
Euplexia benesimilis|[6327]|CNPEE143-14|Canada|Prince Edward Island|593[0n]|BOLD:AAA4097
Euplexia benesimilis|[6328]|HPPPI1423-13|Canada|Nova Scotia|598[0n]|BOLD:AAA4097
Euplexia benesimilis|[6329]|CNTNE1283-14|Canada|Newfoundland and Labrador|588[0n]|BOLD:AAA4097
Dypterygia rozmani|[6330]|RDLQF563-06|Canada|Quebec|658[0n]|BOLD:AAE8028
Dypterygia rozmani|[6331]|RDLQB561-05|Canada|Quebec|658[0n]|BOLD:AAE8028
Dypterygia rozmani|[6332]|LSEU766-06|United States|Missouri|658[0n]|BOLD:AAE8028
Dypterygia rozmani|[6333]|RDLQG058-06|Canada|Quebec|658[0n]|BOLD:AAE8028
Dypterygia rozmani|[6334]|LPOKE241-10|United States|Oklahoma|658[0n]|BOLD:AAE8028
Dypterygia rozmani|[6335]|LNCC1838-13|United States|North Carolina|658[0n]|BOLD:AAE8028
Dypterygia rozmani|[6336]|CNCLB2871-14|United States|North Carolina|658[0n]|BOLD:AAE8028
Hexorthodes senatoria|[6337]|RDNME975-08|United States|New Mexico|658[0n]|BOLD:AAE5369
Hexorthodes senatoria|[6338]|RDNMG296-08|United States|Arizona|658[0n]|BOLD:AAE5369
Hexorthodes senatoria|[6339]|RDNME631-08|United States|New Mexico|658[0n]|BOLD:AAE5369
Hexorthodes senatoria|[6340]|NAMUM059-08|United States|Arizona|657[0n]|BOLD:AAE5369
Hexorthodes senatoria|[6341]|RDNMJ517-11|United States|Arizona|658[0n]|BOLD:AAE5369
Hexorthodes senatoria|[6342]|IAWLB566-11|United States|Arizona|658[0n]|BOLD:AAE5369
Hexorthodes senatoria|[6343]|IAWLB567-11|United States|Arizona|658[0n]|BOLD:AAE5369
Hexorthodes inconspicua|[6344]|CNCLB265-14|United States|Arizona|658[0n]|BOLD:ACM4163
Anhimella perbrunnea|[6345]|RWWB150-09|United States|Washington|658[0n]|BOLD:ACE4177
Anhimella perbrunnea|[6346]|RWWC008-10|United States|Washington|634[0n]|BOLD:ACE4177
Anhimella perbrunnea|[6347]|RWWC625-11|United States|Washington|658[0n]|BOLD:ACE4177
Anhimella perbrunnea|[6348]|GMLC1223-12|United States|California|658[0n]|BOLD:AAF1328
Anhimella perbrunnea|[6349]|RDNMF586-08|United States|Washington|658[2n]|BOLD:AAF1328
Anhimella perbrunnea|[6350]|RDNMF585-08|United States|Oregon|658[0n]|BOLD:AAF1328
Anhimella perbrunnea|[6351]|GMLC1185-12|United States|California|658[0n]|BOLD:AAF1328
Anhimella perbrunnea|[6352]|GMLC1447-12|United States|California|658[0n]|BOLD:AAF1328
Anhimella pacifica|[6353]|RDNMG944-08|Canada|British Columbia|658[0n]|BOLD:AAE5018
Anhimella pacifica|[6354]|RDNMG973-08|United States|California|658[0n]|BOLD:AAJ2705
Anhimella pacifica|[6355]|AWCLB271-10|United States|Arizona|658[0n]|BOLD:AAJ2705
Anhimella pacifica|[6356]|AWCLB272-10|United States|Arizona|658[0n]|BOLD:AAJ2705
Anhimella contrahens|[6357]|RDNMG945-08|Canada|Ontario|658[0n]|BOLD:AAE5019
Anhimella contrahens|[6358]|XAG420-05|Canada|Ontario|658[0n]|BOLD:AAE5017
Anhimella contrahens|[6359]|RDNMG946-08|Canada|Alberta|658[0n]|BOLD:AAE5017
Anhimella contrahens|[6360]|RDNMF587-08|United States|California|658[0n]|BOLD:AAJ2704
Anhimella contrahens|[6361]|JMMMB516-13|United States|California|658[0n]|BOLD:AAJ2704
Anhimella contrahens|[6362]|SDRVA048-13|United States|California|664[0n]|BOLD:AAJ2704
Hexorthodes tuana|[6363]|LNAUT873-14|United States|Texas|658[0n]|BOLD:AAB4381
Hexorthodes tuana|[6364]|LNAUT874-14|United States|Texas|658[0n]|BOLD:AAB4381
Hexorthodes tuana|[6365]|RDNMH780-09|United States|Texas|658[0n]|BOLD:AAB4381
Hexorthodes tuana|[6366]|LNAUT875-14|United States|Texas|658[0n]|BOLD:AAB4381
Hyssia degenerans|[6367]|LNAUT469-14|United States|Texas|550[0n]|BOLD:AAB4382
Hyssia degenerans|[6368]|RDNMH781-09|United States|Texas|658[0n]|BOLD:AAB4382
Hyssia degenerans|[6369]|LNAUT470-14|United States|Texas|658[0n]|BOLD:AAB4382
Hexorthodes nipana|[6370]|LOCBF400-13|United States|California|641[0n]|BOLD:AAH5406
Hexorthodes nipana|[6371]|LOCBF401-13|United States|California|628[0n]|BOLD:AAH5406
Hexorthodes nipana|[6372]|LOCBF2367-13|United States|California|603[0n]|BOLD:AAH5406
Hexorthodes nipana|[6373]|SDRAW002-14|United States|California|582[0n]|BOLD:AAH5406
Hexorthodes nipana|[6374]|SDRAU009-14|United States|California|582[0n]|BOLD:AAH5406
Hexorthodes nipana|[6375]|SDRAO035-14|United States|California|582[0n]|BOLD:AAH5406
Hexorthodes nipana|[6376]|LOCBF3518-14|United States|California|555[0n]|BOLD:AAH5406
Hexorthodes nipana|[6377]|LOCBF3498-14|United States|California|555[0n]|BOLD:AAH5406
Hexorthodes nipana|[6378]|LOCBF471-13|United States|California|603[0n]|BOLD:AAH5406
Hexorthodes nipana|[6379]|LOCBF473-13|United States|California|595[0n]|BOLD:AAH5406
Hexorthodes nipana|[6380]|LOCBF398-13|United States|California|611[0n]|BOLD:AAH5406
Hexorthodes nipana|[6381]|LOCBF397-13|United States|California|610[0n]|BOLD:AAH5406
Hexorthodes nipana|[6382]|LOCBF2366-13|United States|California|615[0n]|BOLD:AAH5406
Hexorthodes nipana|[6383]|RDNMH067-09|United States|Arizona|658[0n]|BOLD:AAH5406
Hexorthodes nipana|[6384]|BBLSY713-09|United States|Arizona|658[0n]|BOLD:AAH5406
Hexorthodes nipana|[6385]|RDNMJ791-11|United States|Arizona|658[0n]|BOLD:AAH5406
Hexorthodes nipana|[6386]|RDNMJ803-11|United States|Arizona|658[0n]|BOLD:AAH5406
Hexorthodes nipana|[6387]|LOCBF399-13|United States|California|629[0n]|BOLD:AAH5406
Hexorthodes nipana|[6388]|LNAUS2837-13|United States|California|658[0n]|BOLD:AAH5406
Hexorthodes nipana|[6389]|LNAUS2838-13|United States|California|658[0n]|BOLD:AAH5406
Hexorthodes nipana|[6390]|LNAUS2834-13|United States|California|658[0n]|BOLD:AAH5406
Hexorthodes nipana|[6391]|LNAUS2836-13|United States|California|658[0n]|BOLD:AAH5406
Hexorthodes nipana|[6392]|LNAUT2944-14|United States|California|658[0n]|BOLD:AAH5406
Morrisonia latex|[6393]|UDLEP027-09|United States|Maryland|658[0n]|BOLD:AAB0781
Morrisonia latex|[6394]|UDLEP028-09|United States|Maryland|658[0n]|BOLD:AAB0781
Morrisonia latex|[6395]|RDLQG270-06|Canada|Quebec|658[0n]|BOLD:AAB0781
Morrisonia latex|[6396]|NAMUM342-08|United States|West Virginia|658[0n]|BOLD:AAB0781
Morrisonia latex|[6397]|LOTB498-05|United States|Tennessee|658[2n]|BOLD:AAB0781

Morrisonia latex|[6394]|UDLEP028-09|United States|Maryland|658[0n]|BOLD:AAB0781
Morrisonia latex|[6395]|RDLQG270-06|Canada|Quebec|658[0n]|BOLD:AAB0781
Morrisonia latex|[6396]|NAMUM342-08|United States|West Virginia|658[0n]|BOLD:AAB0781
Morrisonia latex|[6397]|LOTB498-05|United States|Tennessee|658[2n]|BOLD:AAB0781
Morrisonia latex|[6398]|CNSLF294-12|Canada|Ontario|613[0n]|BOLD:AAB0781
Morrisonia latex|[6399]|LOTB150-05|United States|Tennessee|619[0n]|BOLD:AAB0781
Morrisonia latex|[6400]|XAC151-04|Canada|Ontario|619[0n]|BOLD:AAB0781
Morrisonia latex|[6401]|XAB617-04|Canada|Ontario|619[0n]|BOLD:AAB0781
Morrisonia latex|[6402]|PHJUL926-11|Canada|Ontario|658[0n]|BOLD:AAB0781
Morrisonia latex|[6403]|LGSMG850-10|United States|North Carolina|658[0n]|BOLD:AAB0781
Morrisonia latex|[6404]|LPSOD693-09|Canada|Ontario|658[0n]|BOLD:AAB0781
Morrisonia latex|[6405]|LPSOD504-09|Canada|Ontario|658[0n]|BOLD:AAB0781
Morrisonia latex|[6406]|LPSOD322-09|Canada|Ontario|658[0n]|BOLD:AAB0781
Morrisonia latex|[6407]|LPSOD255-09|Canada|Ontario|658[0n]|BOLD:AAB0781
Morrisonia latex|[6408]|UDLEP256-09|United States|Pennsylvania|658[0n]|BOLD:AAB0781
Morrisonia latex|[6409]|LPSOB968-08|Canada|Ontario|658[0n]|BOLD:AAB0781
Morrisonia latex|[6410]|LPSOC317-08|Canada|Ontario|658[0n]|BOLD:AAB0781
Morrisonia latex|[6411]|MMNA106-08|United States|North Carolina|658[0n]|BOLD:AAB0781
Morrisonia latex|[6412]|LSEU662-06|United States|Georgia|658[0n]|BOLD:AAB0781
Morrisonia latex|[6413]|XAK161-06|Canada|Ontario|658[0n]|BOLD:AAB0781
Morrisonia latex|[6414]|XAJ632-06|Canada|Ontario|658[0n]|BOLD:AAB0781
Morrisonia latex|[6415]|TTMNB410-06|Canada|New Brunswick|658[0n]|BOLD:AAB0781
Morrisonia latex|[6416]|XAI066-05|Canada|Ontario|658[0n]|BOLD:AAB0781
Morrisonia latex|[6417]|XAI063-05|Canada|Ontario|658[0n]|BOLD:AAB0781
Morrisonia latex|[6418]|LOCT107-05|United States|Connecticut|658[0n]|BOLD:AAB0781
Morrisonia latex|[6419]|LOTB499-05|United States|Tennessee|658[0n]|BOLD:AAB0781
Morrisonia latex|[6420]|LGSMC355-05|United States|Tennessee|658[0n]|BOLD:AAB0781
Morrisonia latex|[6421]|LGSMC354-05|United States|Tennessee|658[0n]|BOLD:AAB0781
Morrisonia latex|[6422]|LGSMC353-05|United States|Tennessee|658[0n]|BOLD:AAB0781
Morrisonia latex|[6423]|LGSMC352-05|United States|Tennessee|658[0n]|BOLD:AAB0781
Morrisonia latex|[6424]|PHMNB453-04|Canada|New Brunswick|658[0n]|BOLD:AAB0781
Morrisonia latex|[6425]|XAE491-04|Canada|Ontario|568[0n]|BOLD:AAB0781
Morrisonia latex|[6426]|LGSM474-04|United States|North Carolina|631[0n]|BOLD:AAB0781
Morrisonia latex|[6427]|CNSLO878-13|Canada|Ontario|600[0n]|BOLD:AAB0781
Morrisonia latex|[6428]|XAE345-04|Canada|Ontario|658[0n]|BOLD:AAB0781
Morrisonia latex|[6429]|LGSM475-04|United States|North Carolina|658[0n]|BOLD:AAB0781
Morrisonia latex|[6430]|LSEU661-06|United States|Georgia|658[0n]|BOLD:AAB0781
Morrisonia latex|[6431]|LPSOC306-08|Canada|Ontario|654[0n]|BOLD:AAB0781
Morrisonia latex|[6432]|CNSLF712-12|Canada|Ontario|631[0n]|BOLD:AAB0781
Morrisonia latex|[6433]|UDLEP017-09|United States|Maryland|601[0n]|BOLD:AAB0781
Morrisonia latex|[6434]|CNSLE023-12|Canada|Ontario|636[0n]|BOLD:AAB0781
Morrisonia latex|[6435]|CNSLO880-13|Canada|Ontario|586[0n]|BOLD:AAB0781
Pseudorthodes sp.|[6436]|NAMUM237-08|United States|California|658[0n]|BOLD:AAB0357
Pseudorthodes puerilis|[6437]|GMLC870-12|United States|California|658[0n]|BOLD:AAB0357
Pseudorthodes puerilis|[6438]|CGLCA144-10|United States|California|658[0n]|BOLD:AAB0357
Pseudorthodes puerilis|[6439]|CGLCA143-10|United States|California|634[0n]|BOLD:AAB0357
Pseudorthodes puerilis|[6440]|CGLCA145-10|United States|California|658[0n]|BOLD:AAB0357
Pseudorthodes puerilis|[6441]|GMLC738-12|United States|California|658[0n]|BOLD:AAB0357
Pseudorthodes puerilis|[6442]|GMLC874-12|United States|California|658[0n]|BOLD:AAB0357
Pseudorthodes puerilis|[6443]|GMLC985-12|United States|California|658[0n]|BOLD:AAB0357
Pseudorthodes puerilis|[6444]|GMLC740-12|United States|California|658[0n]|BOLD:AAB0357
Pseudorthodes puerilis|[6445]|GMLC675-11|United States|California|658[0n]|BOLD:AAB0357
Pseudorthodes puerilis|[6446]|GMLC1008-12|United States|California|658[0n]|BOLD:AAB0357
Pseudorthodes puerilis|[6447]|GMLC1026-12|United States|California|658[0n]|BOLD:AAB0357
Pseudorthodes puerilis|[6448]|GMLC1051-12|United States|California|658[0n]|BOLD:AAB0357
Pseudorthodes puerilis|[6449]|GMLC767-12|United States|California|658[0n]|BOLD:AAB0357
Pseudorthodes puerilis|[6450]|GMLC095-09|United States|California|632[0n]|BOLD:AAB0357
Pseudorthodes puerilis|[6451]|GMLC429-11|United States|California|658[0n]|BOLD:AAB0357
Pseudorthodes puerilis|[6452]|GMLC483-11|United States|California|658[0n]|BOLD:AAB0357
Pseudorthodes puerilis|[6453]|GMLC557-11|United States|California|658[0n]|BOLD:AAB0357
Pseudorthodes puerilis|[6454]|GMLC585-11|United States|California|658[0n]|BOLD:AAB0357
Pseudorthodes puerilis|[6455]|GMLC588-11|United States|California|658[0n]|BOLD:AAB0357
Pseudorthodes puerilis|[6456]|GMLC692-11|United States|California|658[0n]|BOLD:AAB0357
Pseudorthodes puerilis|[6457]|GMLC861-12|United States|California|658[0n]|BOLD:AAB0357
Pseudorthodes puerilis|[6458]|GMLC875-12|United States|California|658[0n]|BOLD:AAB0357
Pseudorthodes puerilis|[6459]|GMLC899-12|United States|California|658[0n]|BOLD:AAB0357
Pseudorthodes puerilis|[6460]|GMLC1194-12|United States|California|636[0n]|BOLD:AAB0357
Pseudorthodes puerilis|[6461]|GMLC689-11|United States|California|658[0n]|BOLD:AAB0357
Pseudorthodes puerilis|[6462]|GMLC524-11|United States|California|658[0n]|BOLD:AAB0357
Pseudorthodes puerilis|[6463]|GMLC543-11|United States|California|658[0n]|BOLD:AAB0357
Pseudorthodes puerilis|[6464]|GMLC720-11|United States|California|658[0n]|BOLD:AAB0357
Pseudorthodes puerilis|[6465]|GMLC739-12|United States|California|658[0n]|BOLD:AAB0357
Pseudorthodes puerilis|[6466]|GMLC992-12|United States|California|658[0n]|BOLD:AAB0357
Pseudorthodes puerilis|[6467]|CNCLA5034-13|United States|Arizona|658[0n]|BOLD:AAB0357
Pseudorthodes iole|[6468]|LOCRA192-06|Costa Rica|San Jose|658[0n]|BOLD:AAH7972
Pseudorthodes iole|[6469]|LOCRE057-10|Costa Rica|Cartago|658[2n]|BOLD:AAH7972
Pseudorthodes iole|[6470]|LOCRA191-06|Costa Rica|San Jose|658[0n]|BOLD:AAH7972
Pseudorthodes iole|[6471]|LOCRE058-10|Costa Rica|Cartago|658[0n]|BOLD:AAH7972
Pseudorthodes keela|[6472]|RDNMH727-09|United States|New Mexico|658[0n]|BOLD:AAL1608
Hexorthodes agrotiformis|[6473]|RDNMD925-07|United States|Arizona|653[0n]|BOLD:AAE4087
Hexorthodes agrotiformis|[6474]|RDNMD923-07|United States|New Mexico|655[0n]|BOLD:AAE4087
Hexorthodes agrotiformis|[6475]|RDNMD921-07|United States|Wyoming|655[0n]|BOLD:AAE4087
Hexorthodes agrotiformis|[6476]|RDNMD922-07|United States|Wyoming|651[0n]|BOLD:AAE4087
Hexorthodes agrotiformis|[6477]|RDNMD924-07|United States|Arizona|655[0n]|BOLD:AAE4087
Hexorthodes agrotiformis|[6478]|RDNMJ479-11|United States|Arizona|658[0n]|BOLD:AAE4087
Hexorthodes agrotiformis|[6479]|RDNMJ524-11|United States|Arizona|648[0n]|BOLD:AAE4087
Pseudorthodes irrorata|[6480]|LBCG2249-09|Canada|British Columbia|658[0n]|BOLD:ABZ1590
Pseudorthodes irrorata|[6481]|LBCS486-07|Canada|British Columbia|658[0n]|BOLD:ABZ1590
Pseudorthodes irrorata|[6482]|LBCS487-07|Canada|British Columbia|658[0n]|BOLD:ABZ1590
Pseudorthodes irrorata|[6483]|LBCS675-07|Canada|British Columbia|658[0n]|BOLD:ABZ1590
Pseudorthodes irrorata|[6484]|LBCS676-07|Canada|British Columbia|658[0n]|BOLD:ABZ1590
Pseudorthodes irrorata|[6485]|LBCS677-07|Canada|British Columbia|658[0n]|BOLD:ABZ1590
Pseudorthodes irrorata|[6486]|LBCS678-07|Canada|British Columbia|658[0n]|BOLD:ABZ1590
Pseudorthodes irrorata|[6487]|LBCS679-07|Canada|British Columbia|658[0n]|BOLD:ABZ1590
Pseudorthodes irrorata|[6488]|LBCH4016-10|Canada|British Columbia|658[0n]|BOLD:ABZ1590
Pseudorthodes irrorata|[6489]|LBCH4473-10|Canada|British Columbia|658[0n]|BOLD:ABZ1590
Pseudorthodes irrorata|[6490]|LBCH4739-10|Canada|British Columbia|658[0n]|BOLD:ABZ1590
Pseudorthodes irrorata|[6491]|LHLEP431-06|Canada|British Columbia|658[0n]|BOLD:ABZ1590
Pseudorthodes irrorata|[6492]|LBCS137-07|Canada|British Columbia|658[0n]|BOLD:ABZ1590
Pseudorthodes irrorata|[6493]|LALPA932-11|Canada|British Columbia|658[0n]|BOLD:ABZ1590
Pseudorthodes irrorata|[6494]|LPVIA844-08|Canada|British Columbia|658[0n]|BOLD:ABZ1590
Pseudorthodes irrorata|[6495]|RWWA596-09|United States|Washington|658[0n]|BOLD:ABZ1590
Pseudorthodes irrorata|[6496]|RWWB882-10|United States|Washington|658[0n]|BOLD:ABZ1590
Pseudorthodes irrorata|[6497]|RWWB879-10|United States|Washington|658[0n]|BOLD:ABZ1590

Pseudorthodes irrorata|[6494]|LPVIA844-08|Canada|British Columbia|658[0n]|BOLD:ABZ1590
Pseudorthodes irrorata|[6495]|RWWA596-09|United States|Washington|658[0n]|BOLD:ABZ1590
Pseudorthodes irrorata|[6496]|RWWB882-10|United States|Washington|658[0n]|BOLD:ABZ1590
Pseudorthodes irrorata|[6497]|RWWB970-10|United States|Washington|658[0n]|BOLD:ABZ1590
Pseudorthodes irrorata|[6498]|RWWB977-10|United States|Washington|658[0n]|BOLD:ABZ1590
Pseudorthodes irrorata|[6499]|RWWA975-09|United States|Washington|658[0n]|BOLD:ABZ1590
Pseudorthodes irrorata|[6500]|RWWA631-09|United States|Washington|658[0n]|BOLD:ABZ1590
Pseudorthodes irrorata|[6501]|RWWB867-10|United States|Washington|658[0n]|BOLD:ABZ1590
Pseudorthodes irrorata|[6502]|RWWA619-09|United States|Washington|658[0n]|BOLD:ABZ1590
Pseudorthodes irrorata|[6503]|RWWA630-09|United States|Washington|658[0n]|BOLD:ABZ1590
Pseudorthodes irrorata|[6504]|RWWA597-09|United States|Washington|658[0n]|BOLD:ABZ1590
Pseudorthodes irrorata|[6505]|RWWA616-09|United States|Washington|658[0n]|BOLD:ABZ1590
Pseudorthodes irrorata|[6506]|RWWA581-09|United States|Washington|658[0n]|BOLD:ABZ1590
Pseudorthodes irrorata|[6507]|RWWA568-09|United States|Washington|658[0n]|BOLD:ABZ1590
Pseudorthodes irrorata|[6508]|RWWA553-09|United States|Washington|658[0n]|BOLD:ABZ1590
Pseudorthodes irrorata|[6509]|RWWA551-09|United States|Washington|658[0n]|BOLD:ABZ1590
Pseudorthodes irrorata|[6510]|RWWA493-09|United States|Washington|658[0n]|BOLD:ABZ1590
Pseudorthodes irrorata|[6511]|RWWA434-09|United States|Washington|658[0n]|BOLD:ABZ1590
Pseudorthodes irrorata|[6512]|RWWA430-09|United States|Washington|658[0n]|BOLD:ABZ1590
Pseudorthodes irrorata|[6513]|RWWA414-09|United States|Washington|658[0n]|BOLD:ABZ1590
Pseudorthodes irrorata|[6514]|RWWA361-09|United States|Washington|658[0n]|BOLD:ABZ1590
Pseudorthodes irrorata|[6515]|RWWA608-09|United States|Washington|654[0n]|BOLD:ABZ1590
Pseudorthodes irrorata|[6516]|RWWA590-09|United States|Washington|654[0n]|BOLD:ABZ1590
Pseudorthodes irrorata|[6517]|DUNLP189-08|Canada|British Columbia|650[0n]|BOLD:ABZ1590
Pseudorthodes irrorata|[6518]|RWWA749-09|United States|Washington|633[0n]|BOLD:ABZ1590
Pseudorthodes irrorata|[6519]|RWWC388-11|United States|Washington|638[0n]|BOLD:ABZ1590
Pseudorthodes irrorata|[6520]|RWWC376-11|United States|Washington|658[0n]|BOLD:ABZ1590
Pseudorthodes irrorata|[6521]|RWWC434-11|United States|Washington|658[0n]|BOLD:ABZ1590
Pseudorthodes sp.|[6522]|RDNMK545-11|United States|Colorado|658[0n]|BOLD:ABZ1591
Pseudorthodes sp.|[6523]|NAMUM282-08|United States|California|658[0n]|BOLD:ACE4572
Pseudorthodes sp.|[6524]|IAWLB079-10|United States|California|658[0n]|BOLD:ACE4572
Pseudorthodes virgula|[6525]|RDNMK542-11|United States|Wyoming|611[0n]|BOLD:ABZ1589
Pseudorthodes virgula|[6526]|RDNMJ721-11|United States|Arizona|658[0n]|BOLD:ABZ1589
Pseudorthodes virgula|[6527]|RDNMK543-11|United States|Arizona|658[0n]|BOLD:ABZ1589
Pseudorthodes virgula|[6528]|RDNMK544-11|United States|Arizona|658[0n]|BOLD:ABZ1589
Pseudorthodes virgula|[6529]|RDNMK546-11|United States|Arizona|568[0n]|BOLD:ABZ1589
Pseudorthodes virgula|[6530]|RDNMK547-11|United States|Arizona|658[0n]|BOLD:ABZ1589
Pseudorthodes vecors|[6531]|LGSM039-04|United States|North Carolina|658[0n]|BOLD:AAA8885
Pseudorthodes vecors|[6532]|LGSMC475-05|United States|Tennessee|658[0n]|BOLD:AAA8885
Pseudorthodes vecors|[6533]|LGSM044-04|United States|North Carolina|658[0n]|BOLD:AAA8885
Pseudorthodes vecors|[6534]|LNCC1199-11|United States|North Carolina|658[0n]|BOLD:AAA8885
Pseudorthodes vecors|[6535]|LSEU652-06|United States|Georgia|658[0n]|BOLD:AAA8885
Pseudorthodes vecors|[6536]|LNCC1197-11|United States|North Carolina|658[0n]|BOLD:AAA8885
Pseudorthodes vecors|[6537]|LGSMC474-05|United States|Tennessee|616[0n]|BOLD:AAA8885
Pseudorthodes vecors|[6538]|LGSM456-04|United States|North Carolina|658[0n]|BOLD:AAA8885
Pseudorthodes vecors|[6539]|LGSMC471-05|United States|Tennessee|618[0n]|BOLD:AAA8885
Pseudorthodes vecors|[6540]|LOCT271-05|United States|Connecticut|658[0n]|BOLD:AAA8885
Pseudorthodes vecors|[6541]|RDLQ306-05|Canada|Quebec|658[0n]|BOLD:AAA8885
Pseudorthodes vecors|[6542]|RDLQB069-05|Canada|Quebec|627[0n]|BOLD:AAA8885
Pseudorthodes vecors|[6543]|LGSMC470-05|United States|Tennessee|658[1n]|BOLD:AAA8885
Pseudorthodes vecors|[6544]|LNCC291-10|United States|North Carolina|658[0n]|BOLD:AAA8885
Pseudorthodes vecors|[6545]|LGSMG870-10|United States|North Carolina|658[0n]|BOLD:AAA8885
Pseudorthodes vecors|[6546]|RDLQ674-07|Canada|Quebec|658[0n]|BOLD:AAA8885
Pseudorthodes vecors|[6547]|LGSMC473-05|United States|Tennessee|658[0n]|BOLD:AAA8885
Pseudorthodes vecors|[6548]|LNCC1198-11|United States|North Carolina|658[0n]|BOLD:AAA8885
Pseudorthodes vecors|[6549]|LGSM771-04|United States|Tennessee|658[0n]|BOLD:AAA8885
Pseudorthodes vecors|[6550]|LNCC218-10|United States|North Carolina|658[0n]|BOLD:AAA8885
Pseudorthodes vecors|[6551]|LOCT272-05|United States|Connecticut|658[0n]|BOLD:AAA8885
Pseudorthodes vecors|[6552]|LOCT270-05|United States|Connecticut|658[0n]|BOLD:AAA8885
Pseudorthodes vecors|[6553]|LOCT313-05|United States|Connecticut|654[0n]|BOLD:AAA8885
Pseudorthodes vecors|[6554]|PHJUN4007-11|Canada|Ontario|658[0n]|BOLD:AAA8885
Pseudorthodes vecors|[6555]|LGSM457-04|United States|North Carolina|615[0n]|BOLD:AAA8885
Pseudorthodes vecors|[6556]|PHMNB681-04|Canada|New Brunswick|658[0n]|BOLD:AAA8885
Pseudorthodes vecors|[6557]|LGSMG869-10|United States|North Carolina|658[0n]|BOLD:AAA8885
Pseudorthodes vecors|[6558]|PMG155-03|Canada|Ontario|617[1n]|BOLD:AAA8885
Pseudorthodes vecors|[6559]|LGSMC472-05|United States|Tennessee|574[3n]|BOLD:AAA8885
Pseudorthodes vecors|[6560]|LGSMC476-05|United States|Tennessee|594[0n]|BOLD:AAA8885
Pseudorthodes vecors|[6561]|RDLQF035-06|Canada|Quebec|658[0n]|BOLD:AAA8885
Pseudorthodes vecors|[6562]|PHJUN4015-11|Canada|Ontario|658[0n]|BOLD:AAA8885
Pseudorthodes vecors|[6563]|LGSMG871-10|United States|Tennessee|658[0n]|BOLD:ACF5054
Pseudorthodes vecors|[6564]|HPPPE1563-13|Canada|Nova Scotia|541[0n]|BOLD:ACF5054
Pseudorthodes vecors|[6565]|HPPPE1548-13|Canada|Nova Scotia|547[1n]|BOLD:ACF5054
Pseudorthodes vecors|[6566]|RDLQ675-07|Canada|Quebec|658[0n]|BOLD:ACF5054
Pseudorthodes vecors|[6567]|RDLQG382-06|Canada|Quebec|658[0n]|BOLD:ACF5054
Pseudorthodes vecors|[6568]|RDLQG381-06|Canada|Quebec|658[0n]|BOLD:ACF5054
Pseudorthodes vecors|[6569]|RDLQG365-06|Canada|Quebec|658[0n]|BOLD:ACF5054
Pseudorthodes vecors|[6570]|RDLQF455-06|Canada|Quebec|658[0n]|BOLD:ACF5054
Pseudorthodes vecors|[6571]|XAJ843-06|Canada|Ontario|658[0n]|BOLD:ACF5054
Pseudorthodes vecors|[6572]|RDLQF034-06|Canada|Quebec|658[0n]|BOLD:ACF5054
Pseudorthodes vecors|[6573]|LSEU653-06|United States|Georgia|658[0n]|BOLD:ACF5054
Pseudorthodes vecors|[6574]|RDLQ672-07|Canada|Quebec|608[0n]|BOLD:ACF5054
Pseudorthodes vecors|[6575]|RDLQ304-05|Canada|Quebec|608[0n]|BOLD:ACF5054
Pseudorthodes vecors|[6576]|PHMO185-03|Canada|Ontario|639[0n]|BOLD:ACF5054
Pseudorthodes vecors|[6577]|RDLQ673-07|Canada|Quebec|617[0n]|BOLD:ACF5054
Pseudorthodes vecors|[6578]|RDLQ676-07|Canada|Quebec|632[0n]|BOLD:ACF5054
Pseudorthodes vecors|[6579]|RDLQ677-07|Canada|Quebec|658[0n]|BOLD:ACF5054
Pseudorthodes vecors|[6580]|RDLQ678-07|Canada|Quebec|625[0n]|BOLD:ACF5054
Pseudorthodes vecors|[6581]|RDLQ679-07|Canada|Quebec|658[0n]|BOLD:ACF5054
Pseudorthodes vecors|[6582]|HPPPI1422-13|Canada|Nova Scotia|522[0n]|BOLD:ACF5054
Pseudorthodes vecors|[6583]|HPPPJ1072-13|Canada|Nova Scotia|560[0n]|BOLD:ACF5054
Homorthodes communis|[6584]|BBLOE1238-12|United States|California|658[0n]|BOLD:AAX1186
Homorthodes communis|[6585]|GMLC010-09|United States|California|658[0n]|BOLD:AAX1186
Homorthodes communis|[6586]|GMLC272-11|United States|California|658[0n]|BOLD:AAX1186
Homorthodes communis|[6587]|GMLC1125-12|United States|California|658[0n]|BOLD:AAX1186
Homorthodes communis|[6588]|RDNMG433-08|United States|California|658[0n]|BOLD:AAC1982
Homorthodes communis|[6589]|RDNMG434-08|United States|California|658[0n]|BOLD:AAC1982
Homorthodes communis|[6590]|LPVIB427-08|Canada|British Columbia|658[0n]|BOLD:AAC1982
Homorthodes communis|[6591]|RDNMG908-08|Canada|British Columbia|658[0n]|BOLD:AAC1982
Homorthodes communis|[6592]|LPVIB666-08|Canada|British Columbia|575[0n]|BOLD:AAC1982
Homorthodes communis|[6593]|LPVIB665-08|Canada|British Columbia|634[0n]|BOLD:AAC1982
Homorthodes communis|[6594]|LPVIB664-08|Canada|British Columbia|601[0n]|BOLD:AAC1982
Homorthodes communis|[6595]|LPVIB673-08|Canada|British Columbia|606[0n]|BOLD:AAC1982
Homorthodes communis|[6596]|LPVIB765-08|Canada|British Columbia|658[0n]|BOLD:AAC1982
Homorthodes communis|[6597]|NAMUM134-08|United States|California|658[0n]|BOLD:AAC1982

Homorthodes communis|[6594]|LPVIB664-08|Canada|British Columbia|601[0n]|BOLD:AAC1982
Homorthodes communis|[6595]|LPVIB673-08|Canada|British Columbia|606[0n]|BOLD:AAC1982
Homorthodes communis|[6596]|LPVIB765-08|Canada|British Columbia|658[0n]|BOLD:AAC1982
Homorthodes communis|[6597]|NAMUM124-08|United States|California|658[0n]|BOLD:AAC1982
Homorthodes communis|[6598]|RDNMG815-08|United States|California|658[0n]|BOLD:AAC1982
Homorthodes communis|[6599]|RDNMG907-08|Canada|British Columbia|658[0n]|BOLD:AAC1982
Homorthodes communis|[6600]|GMLC1216-12|United States|California|658[0n]|BOLD:AAC1982
Homorthodes communis|[6601]|LBCW058-08|Canada|British Columbia|658[0n]|BOLD:AAC1982
Homorthodes communis|[6602]|LPVIB667-08|Canada|British Columbia|631[0n]|BOLD:AAC1982
Homorthodes communis|[6603]|LPVIB679-08|Canada|British Columbia|637[1n]|BOLD:AAC1982
Homorthodes communis|[6604]|LPVIB273-08|Canada|British Columbia|658[0n]|BOLD:AAC1982
Homorthodes communis|[6605]|LPVIB663-08|Canada|British Columbia|634[0n]|BOLD:AAC1982
Homorthodes communis|[6606]|LPVIB495-08|Canada|British Columbia|658[0n]|BOLD:AAC1982
Homorthodes communis|[6607]|LPVIB426-08|Canada|British Columbia|658[0n]|BOLD:AAC1982
Homorthodes communis|[6608]|LPVIB315-08|Canada|British Columbia|658[0n]|BOLD:AAC1982
Homorthodes communis|[6609]|LPVIB272-08|Canada|British Columbia|658[0n]|BOLD:AAC1982
Homorthodes communis|[6610]|RDNMG909-08|United States|Oregon|658[0n]|BOLD:AAC1982
Homorthodes communis|[6611]|LPVIB672-08|Canada|British Columbia|639[0n]|BOLD:AAC1982
Homorthodes communis|[6612]|LPVIB764-08|Canada|British Columbia|658[0n]|BOLD:AAC1982
Homorthodes communis|[6613]|RWWA949-09|United States|Washington|658[0n]|BOLD:AAC1982
Homorthodes communis|[6614]|RWWB032-09|United States|Washington|658[0n]|BOLD:AAC1982
Homorthodes communis|[6615]|RWWB033-09|United States|Washington|658[0n]|BOLD:AAC1982
Homorthodes communis|[6616]|RWWB137-09|United States|Washington|658[0n]|BOLD:AAC1982
Homorthodes communis|[6617]|RWWB167-09|United States|Washington|658[0n]|BOLD:AAC1982
Homorthodes communis|[6618]|RWWC047-10|United States|Washington|658[0n]|BOLD:AAC1982
Homorthodes communis|[6619]|RWWC551-11|United States|Washington|658[0n]|BOLD:AAC1982
Homorthodes communis|[6620]|LALPA1201-11|Canada|British Columbia|658[0n]|BOLD:AAC1982
Homorthodes communis|[6621]|RWWC1066-12|United States|Washington|658[0n]|BOLD:AAC1982
Homorthodes fractura|[6622]|RDNMH064-09|United States|New Mexico|658[0n]|BOLD:AAB4377
Homorthodes fractura|[6623]|RDNMH073-09|United States|New Mexico|658[0n]|BOLD:AAB4377
Homorthodes fractura|[6624]|RDNMH074-09|United States|Arizona|658[0n]|BOLD:AAB4377
Homorthodes fractura|[6625]|RDNMJ836-11|United States|Arizona|658[0n]|BOLD:AAB4377
Homorthodes fractura|[6626]|LALPA287-10|Canada|British Columbia|658[0n]|BOLD:AAB4377
Homorthodes fractura|[6627]|LALPA504-10|Canada|British Columbia|658[0n]|BOLD:AAB4377
Homorthodes fractura|[6628]|BBLPB447-10|Canada|British Columbia|658[0n]|BOLD:AAB4377
Homorthodes fractura|[6629]|LALPA934-11|Canada|British Columbia|658[0n]|BOLD:AAB4377
Homorthodes fractura|[6630]|LALPA979-11|Canada|British Columbia|658[0n]|BOLD:AAB4377
Homorthodes fractura|[6631]|LALPA1017-11|Canada|British Columbia|658[0n]|BOLD:AAB4377
Homorthodes fractura|[6632]|BBLOC1344-11|United States|California|658[0n]|BOLD:AAB4377
Homorthodes fractura|[6633]|GMLC921-12|United States|California|658[0n]|BOLD:AAB4377
Homorthodes fractura|[6634]|RWWA638-09|United States|Washington|658[0n]|BOLD:AAB4377
Homorthodes fractura|[6635]|RWWA781-09|United States|Washington|658[0n]|BOLD:AAB4377
Homorthodes fractura|[6636]|RWWB965-10|United States|Washington|658[0n]|BOLD:AAB4377
Homorthodes fractura|[6637]|RWWC288-11|United States|Washington|658[0n]|BOLD:AAB4377
Homorthodes fractura|[6638]|RWWA239-09|United States|Washington|658[0n]|BOLD:AAB4377
Homorthodes fractura|[6639]|RWWA617-09|United States|Washington|658[0n]|BOLD:AAB4377
Homorthodes fractura|[6640]|RWWC350-11|United States|Washington|658[0n]|BOLD:AAB4377
Homorthodes fractura|[6641]|LOCB774-06|United States|California|658[0n]|BOLD:AAB4377
Homorthodes fractura|[6642]|LOCB775-06|United States|California|658[0n]|BOLD:AAB4377
Homorthodes fractura|[6643]|LOCB776-06|United States|California|658[0n]|BOLD:AAB4377
Homorthodes fractura|[6644]|RDNMD428-06|United States|California|658[0n]|BOLD:AAB4377
Homorthodes fractura|[6645]|RDNMF409-08|United States|California|658[0n]|BOLD:AAB4377
Homorthodes fractura|[6646]|RDNMG886-08|United States|California|658[0n]|BOLD:AAB4377
Homorthodes fractura|[6647]|BBLOD331-11|United States|California|658[0n]|BOLD:AAB4377
Homorthodes fractura|[6648]|BBLOD1706-11|United States|California|658[0n]|BOLD:AAB4377
Homorthodes fractura|[6649]|BBLOD1708-11|United States|California|658[0n]|BOLD:AAB4377
Homorthodes fractura|[6650]|BBLOE1304-12|United States|California|658[0n]|BOLD:AAB4377
Homorthodes fractura|[6651]|GMLC1107-12|United States|California|658[0n]|BOLD:AAB4377
Homorthodes fractura|[6652]|RWWC560-11|United States|Washington|658[0n]|BOLD:AAB4377
Homorthodes fractura|[6653]|GMLC721-11|United States|California|658[0n]|BOLD:AAB4377
Homorthodes fractura|[6654]|LALPA540-10|Canada|British Columbia|658[0n]|BOLD:AAB4377
Homorthodes fractura|[6655]|LALPA539-10|Canada|British Columbia|658[0n]|BOLD:AAB4377
Homorthodes fractura|[6656]|LALPA538-10|Canada|British Columbia|658[0n]|BOLD:AAB4377
Homorthodes fractura|[6657]|LALPA183-10|Canada|British Columbia|658[0n]|BOLD:AAB4377
Homorthodes fractura|[6658]|RWWA738-09|United States|Washington|658[0n]|BOLD:AAB4377
Homorthodes fractura|[6659]|RWWA629-09|United States|Washington|658[0n]|BOLD:AAB4377
Homorthodes fractura|[6660]|RWWA560-09|United States|Washington|658[0n]|BOLD:AAB4377
Homorthodes fractura|[6661]|RWWA518-09|United States|Washington|658[0n]|BOLD:AAB4377
Homorthodes fractura|[6662]|RWWA389-09|United States|Washington|658[0n]|BOLD:AAB4377
Homorthodes fractura|[6663]|RWWA297-09|United States|Washington|658[0n]|BOLD:AAB4377
Homorthodes fractura|[6664]|LPVIB058-08|Canada|British Columbia|658[0n]|BOLD:AAB4377
Homorthodes fractura|[6665]|LHLEP430-06|Canada|British Columbia|658[0n]|BOLD:AAB4377
Homorthodes fractura|[6666]|LHLEP429-06|Canada|British Columbia|658[0n]|BOLD:AAB4377
Homorthodes fractura|[6667]|LHLEP075-06|Canada|British Columbia|658[0n]|BOLD:AAB4377
Homorthodes fractura|[6668]|RWWA377-09|United States|Washington|658[0n]|BOLD:AAB4377
Homorthodes fractura|[6669]|RDNMF408-08|Canada|British Columbia|652[0n]|BOLD:AAB4377
Homorthodes fractura|[6670]|LPVIA626-08|Canada|British Columbia|638[0n]|BOLD:AAB4377
Homorthodes fractura|[6671]|GMLC1005-12|United States|California|658[0n]|BOLD:AAB4377
Homorthodes fractura|[6672]|GMLC1004-12|United States|California|658[0n]|BOLD:AAB4377
Homorthodes fractura|[6673]|GMLC1428-12|United States|California|629[0n]|BOLD:AAB4377
Homorthodes fractura|[6674]|GMLC1440-12|United States|California|630[0n]|BOLD:AAB4377
Homorthodes sp.|[6675]|RDNMG334-08|United States|Arizona|658[0n]|BOLD:AAB4373
Homorthodes sp.|[6676]|RDNMH057-09|United States|Arizona|658[0n]|BOLD:AAB4373
Homorthodes sp.|[6677]|RDNMH058-09|United States|Arizona|658[0n]|BOLD:AAB4373
Homorthodes gigantoides|[6678]|RDNMG867-08|United States|Colorado|658[0n]|BOLD:AAX1188
Homorthodes gigantoides|[6679]|LNAUT896-14|United States|New Mexico|658[0n]|BOLD:AAX1188
Homorthodes rubritincta|[6680]|RDNMF407-08|United States|Arizona|658[0n]|BOLD:AAX1187
Homorthodes discreta|[6681]|LBCH6634-10|Canada|British Columbia|658[0n]|BOLD:AAD9145
Homorthodes discreta|[6682]|LBCH7341-10|Canada|British Columbia|658[0n]|BOLD:AAD9145
Homorthodes discreta|[6683]|LBCH6296-10|Canada|British Columbia|658[0n]|BOLD:AAD9145
Homorthodes discreta|[6684]|LBCH6295-10|Canada|British Columbia|658[0n]|BOLD:AAD9145
Homorthodes discreta|[6685]|LBCH6239-10|Canada|British Columbia|658[0n]|BOLD:AAD9145
Homorthodes discreta|[6686]|LBCH6238-10|Canada|British Columbia|658[0n]|BOLD:AAD9145
Homorthodes discreta|[6687]|LBCH6166-10|Canada|British Columbia|658[0n]|BOLD:AAD9145
Homorthodes discreta|[6688]|LBCG1102-09|Canada|British Columbia|658[0n]|BOLD:AAD9145
Homorthodes discreta|[6689]|RDNMG911-08|United States|Oregon|658[0n]|BOLD:AAD9145
Homorthodes discreta|[6690]|RDNMG851-08|United States|Oregon|658[0n]|BOLD:AAD9145
Homorthodes discreta|[6691]|LOWCB899-05|Canada|British Columbia|658[0n]|BOLD:AAD9145
Homorthodes discreta|[6692]|RDNMF588-08|United States|Oregon|612[0n]|BOLD:AAD9145
Homorthodes discreta|[6693]|RDNMF590-08|United States|Oregon|640[0n]|BOLD:AAD9145
Homorthodes discreta|[6694]|LBCH7376-10|Canada|British Columbia|636[0n]|BOLD:AAD9145
Homorthodes discreta|[6695]|LBCH7474-10|Canada|British Columbia|658[0n]|BOLD:AAD9145
Homorthodes discreta|[6696]|RDNMJ140-10|United States|Washington|658[0n]|BOLD:AAD9145
[illegible]

Homorthodes discreta|[6694]|LBCH7376-10|Canada|British Columbia|636[0n]|BOLD:AAD9145
Homorthodes discreta|[6695]|LBCH7474-10|Canada|British Columbia|658[0n]|BOLD:AAD9145
Homorthodes discreta|[6696]|RDNMJ140-10|United States|Washington|658[0n]|BOLD:AAD9145
Homorthodes discreta|[6697]|RDNMJ144-10|United States|Washington|658[0n]|BOLD:AAD9145
Homorthodes sp. A|[6698]|RDNMH766-09|United States|Texas|658[0n]|BOLD:AAB4379
Homorthodes sp. A|[6699]|LNAUS4430-13|United States|Texas|658[0n]|BOLD:AAK5427
Homorthodes sp. A|[6700]|LNAUS4428-13|United States|Arizona|658[0n]|BOLD:AAK5427
Homorthodes sp. A|[6701]|LNAUS4429-13|United States|Arizona|658[0n]|BOLD:AAK5427
Homorthodes sp. A|[6702]|RDNMC606-06|United States|Arizona|658[0n]|BOLD:AAK5427
Homorthodes sp. A|[6703]|RDNMJ776-11|United States|Arizona|658[0n]|BOLD:AAK5427
Homorthodes sp. A|[6704]|LNAUS4431-13|United States|Arizona|658[0n]|BOLD:AAK5427
Homorthodes reliqua|[6705]|RDNMG953-08|United States|Wyoming|658[0n]|BOLD:AAF1163
Homorthodes reliqua|[6706]|RDNMG954-08|United States|Wyoming|658[0n]|BOLD:AAF1163
Homorthodes reliqua|[6707]|RDNMG955-08|United States|Wyoming|658[0n]|BOLD:AAF1163
Homorthodes reliqua|[6708]|RDNMJ712-11|United States|Arizona|658[0n]|BOLD:AAF1163
Homorthodes sp.|[6709]|RDNMJ772-11|United States|Arizona|658[0n]|BOLD:AAT9245
Homorthodes sp.|[6710]|RDNMJ773-11|United States|Arizona|658[0n]|BOLD:AAT9245
Homorthodes sp.|[6711]|BBLSY435-09|United States|Arizona|658[0n]|BOLD:AAB4375
Homorthodes sp.|[6712]|RDNMH062-09|United States|Arizona|658[0n]|BOLD:AAB4375
Homorthodes sp.|[6713]|RDNMH061-09|United States|New Mexico|658[0n]|BOLD:AAB4375
Homorthodes sp.|[6714]|BBLSY799-09|United States|New Mexico|641[0n]|BOLD:AAB4375
Homorthodes sp.|[6715]|USLEP718-10|United States|Colorado|658[0n]|BOLD:AAB4375
Homorthodes sp.|[6716]|RDNMJ790-11|United States|New Mexico|658[0n]|BOLD:AAB4375
Homorthodes sp.|[6717]|RDNMJ806-11|United States|New Mexico|658[0n]|BOLD:AAB4375
Homorthodes sp.|[6718]|IAWLB563-11|United States|Arizona|658[0n]|BOLD:ACF2106
Homorthodes sp.|[6719]|IAWLB564-11|United States|Arizona|658[0n]|BOLD:ACF2106
Homorthodes sp.|[6720]|RDNMJ143-10|United States|Arizona|658[0n]|BOLD:AAU5168
Homorthodes sp.|[6721]|IAWLB565-11|United States|Arizona|658[0n]|BOLD:AAU5168
Homorthodes mania|[6722]|RDNMJ767-11|United States|New Mexico|658[0n]|BOLD:ACE7787
Homorthodes mania|[6723]|RDNMG414-08|United States|Colorado|658[0n]|BOLD:ACE7787
Homorthodes mania|[6724]|RDNMG415-08|United States|New Mexico|658[0n]|BOLD:ACE7787
Homorthodes mania|[6725]|LNAUT897-14|United States|Colorado|658[0n]|BOLD:ACE7787
Homorthodes mania|[6726]|RDNMG413-08|United States|Colorado|658[0n]|BOLD:AAF1164
Homorthodes mania|[6727]|RDNMI036-10|United States|Colorado|658[0n]|BOLD:AAF1164
Homorthodes mania|[6728]|LNAUT898-14|United States|Colorado|658[0n]|BOLD:AAF1164
Homorthodes carneola|[6729]|RDNMG017-08|United States|Arizona|658[0n]|BOLD:AAF1154
Homorthodes carneola|[6730]|RDNMG466-08|United States|Colorado|658[0n]|BOLD:AAF1154
Homorthodes carneola|[6731]|RDNMG467-08|United States|Colorado|658[0n]|BOLD:AAF1154
Homorthodes carneola|[6732]|CNCLB1763-14|Canada|British Columbia|658[0n]|BOLD:AAF1154
Homorthodes euxoiformis|[6733]|RDNMG335-08|United States|Arizona|658[0n]|BOLD:AAB4376
Homorthodes euxoiformis|[6734]|RDNMH063-09|United States|Arizona|658[0n]|BOLD:AAB4376
Homorthodes rectiflava|[6735]|RDNMC607-06|United States|New Mexico|658[0n]|BOLD:AAF1165
Homorthodes rectiflava|[6736]|RDNMD908-07|United States|Arizona|655[0n]|BOLD:AAF1165
Homorthodes rectiflava|[6737]|RDNMD909-07|United States|Arizona|655[0n]|BOLD:AAF1165
Homorthodes rectiflava|[6738]|RDNMJ774-11|United States|Arizona|658[0n]|BOLD:AAF1165
Orthosia arthrolita|[6739]|GMLC045-09|United States|California|658[0n]|BOLD:AAE4007
Orthosia arthrolita|[6740]|GMLC050-09|United States|California|658[0n]|BOLD:AAE4007
Orthosia arthrolita|[6741]|GMLC122-09|United States|California|658[0n]|BOLD:AAE4007
Orthosia arthrolita|[6742]|GMLC126-09|United States|California|658[0n]|BOLD:AAE4007
Orthosia arthrolita|[6743]|GMLC129-09|United States|California|658[0n]|BOLD:AAE4007
Orthosia arthrolita|[6744]|GMLC1246-12|United States|California|658[0n]|BOLD:AAE4007
Orthosia arthrolita|[6745]|GMLC1249-12|United States|California|658[0n]|BOLD:AAE4007
Orthosia arthrolita|[6746]|GMLC1256-12|United States|California|658[0n]|BOLD:AAE4007
Orthosia pulchella|[6747]|RDNMC463-05|Canada|British Columbia|591[0n]|BOLD:AAF0758
Orthosia pulchella|[6748]|LOWCD781-06|Canada|British Columbia|657[0n]|BOLD:AAF0758
Orthosia pulchella|[6749]|RDNMC462-05|United States|Oregon|539[0n]|BOLD:AAF0758
Orthosia pulchella|[6750]|JMMMB336-11|United States|California|658[0n]|BOLD:AAF0758
Orthosia pulchella|[6751]|JMMMB337-11|United States|California|658[0n]|BOLD:AAF0758
Orthosia pulchella|[6752]|JMMMB338-11|United States|California|658[0n]|BOLD:AAF0758
Orthosia pulchella|[6753]|JMMMB380-11|United States|California|658[0n]|BOLD:AAF0758
Homorthodes sp. B|[6754]|RDNMJ807-11|United States|Arizona|658[0n]|BOLD:AAE1667
Homorthodes sp. B|[6755]|RDNMJ145-10|United States|Arizona|658[0n]|BOLD:AAE1667
Homorthodes sp. B|[6756]|RDNMJ368-11|United States|Arizona|658[0n]|BOLD:AAE1667
Homorthodes sp. B|[6757]|RDNMJ783-11|United States|Arizona|658[0n]|BOLD:AAE1667
Homorthodes sp. B|[6758]|RDNMD906-07|United States|Arizona|655[0n]|BOLD:AAE1667
Homorthodes sp. B|[6759]|RDNMD907-07|United States|Arizona|655[0n]|BOLD:AAE1667
Homorthodes sp. B|[6760]|RDNMF410-08|United States|Arizona|658[0n]|BOLD:AAE1667
Homorthodes sp. B|[6761]|RDNMF411-08|United States|Arizona|658[0n]|BOLD:AAE1667
Homorthodes sp. B|[6762]|RDNMG471-08|United States|California|658[0n]|BOLD:AAE1667
Homorthodes sp. B|[6763]|RDNMJ653-11|United States|Arizona|658[0n]|BOLD:AAE1667
Homorthodes sp. B|[6764]|RDNMJ760-11|United States|Arizona|658[0n]|BOLD:AAE1667
Homorthodes sp. B|[6765]|RDNMJ763-11|United States|Arizona|658[0n]|BOLD:AAE1667
Homorthodes sp. B|[6766]|RDNMJ770-11|United States|Arizona|658[0n]|BOLD:AAE1667
Homorthodes sp. B|[6767]|RDNMJ782-11|United States|Arizona|658[0n]|BOLD:AAE1667
Homorthodes sp. B|[6768]|RDNMJ784-11|United States|Arizona|658[0n]|BOLD:AAE1667
Homorthodes sp. B|[6769]|RDNMJ789-11|United States|Arizona|658[0n]|BOLD:AAE1667
Homorthodes sp. B|[6770]|RDNMK566-11|United States|Arizona|658[2n]|BOLD:AAE1667
Homorthodes sp.|[6771]|RDNMJ768-11|United States|Arizona|658[0n]|BOLD:ABX5429
Homorthodes sp.|[6772]|RDNMJ769-11|United States|Arizona|658[0n]|BOLD:ABX5429
Homorthodes sp.|[6773]|RDNMJ483-11|United States|Arizona|658[0n]|BOLD:ABZ5531
Homorthodes sp.|[6774]|RDNMJ484-11|United States|Arizona|658[0n]|BOLD:ABZ5531
Homorthodes sp.|[6775]|RDNMJ526-11|United States|Arizona|658[0n]|BOLD:ABZ5531
Homorthodes sp.|[6776]|RDNMJ527-11|United States|Arizona|658[0n]|BOLD:ABZ5531
Homorthodes sp.|[6777]|RDNMJ528-11|United States|Arizona|658[0n]|BOLD:ABZ5531
Homorthodes sp.|[6778]|RDNMJ529-11|United States|Arizona|658[0n]|BOLD:ABZ5531
Homorthodes sp.|[6779]|RDNMJ530-11|United States|Arizona|658[0n]|BOLD:ABZ5531
Homorthodes sp.|[6780]|RDNMJ713-11|United States|Arizona|658[0n]|BOLD:ABZ5531
Homorthodes new sp.|[6781]|RDNMD904-07|United States|New Mexico|610[0n]|BOLD:AAF1152
Homorthodes new sp.|[6782]|RDNMC593-06|United States|New Mexico|657[0n]|BOLD:AAF1152
Homorthodes new sp.|[6783]|RDNMD905-07|United States|New Mexico|655[0n]|BOLD:AAF1152
Homorthodes new sp.|[6784]|RDNMG550-08|United States|New Mexico|658[0n]|BOLD:AAF1152
Homorthodes new sp.|[6785]|RDNMJ141-10|United States|New Mexico|658[0n]|BOLD:AAF1152
Homorthodes new sp.|[6786]|RDNMJ182-10|United States|New Mexico|658[0n]|BOLD:AAF1152
Homorthodes new sp.|[6787]|RDNMJ785-11|United States|New Mexico|658[0n]|BOLD:AAF1152
Homorthodes sp.|[6788]|RDNMJ771-11|United States|New Mexico|658[0n]|BOLD:ACF5962
Homorthodes sp.|[6789]|RDNMJ808-11|United States|New Mexico|658[0n]|BOLD:ACF5962
Homorthodes sp.|[6790]|IAWLB254-11|United States|Arizona|658[0n]|BOLD:ACF5960
Homorthodes sp.|[6791]|RDNMJ655-11|United States|Arizona|658[0n]|BOLD:ABZ0662
Homorthodes sp.|[6792]|RDNMJ786-11|United States|Arizona|658[0n]|BOLD:ABZ0662
Homorthodes dubia|[6793]|RDNMH076-09|United States|Arizona|658[0n]|BOLD:ABZ0661
Homorthodes dubia|[6794]|CMAZA296-09|United States|Arizona|658[0n]|BOLD:ABZ0661
Homorthodes dubia|[6795]|CMAZA297-09|United States|Arizona|658[0n]|BOLD:ABZ0661
Homorthodes dubia|[6796]|RDNMJ761-11|United States|Arizona|658[0n]|BOLD:ABZ0661

Homorthodes dubia|[6794]|CMAZA296-09|United States|Arizona|658[0n]|BOLD:ABZ0661
Homorthodes dubia|[6795]|CMAZA297-09|United States|Arizona|658[0n]|BOLD:ABZ0661
Homorthodes dubia|[6796]|RDNMJ761-11|United States|Arizona|658[0n]|BOLD:ABZ0661
Homorthodes dubia|[6797]|RDNMJ762-11|United States|Arizona|658[0n]|BOLD:ABZ0661
Homorthodes dubia|[6798]|RDNMJ764-11|United States|Arizona|658[0n]|BOLD:ABZ0661
Homorthodes dubia|[6799]|RDNMJ775-11|United States|Arizona|658[0n]|BOLD:ABZ0661
Homorthodes dubia|[6800]|RDNMJ777-11|United States|Arizona|658[0n]|BOLD:ABZ0661
Homorthodes dubia|[6801]|RDNMJ787-11|United States|Arizona|658[0n]|BOLD:ABZ0661
Homorthodes dubia|[6802]|RDNMJ788-11|United States|Arizona|658[0n]|BOLD:ABZ0661
Homorthodes dubia|[6803]|RDNMG1015-08|United States|Arizona|658[0n]|BOLD:ABZ0661
Homorthodes dubia|[6804]|RDNMH075-09|United States|Arizona|658[0n]|BOLD:ABZ0661
Homorthodes dubia|[6805]|LNAUS4432-13|United States|Arizona|658[0n]|BOLD:ABZ0661
Homorthodes new sp.|[6806]|RDNME1013-08|United States|New Mexico|658[0n]|BOLD:AAB4378
Homorthodes new sp.|[6807]|RDNMH068-09|United States|Arizona|658[0n]|BOLD:AAB4378
Homorthodes new sp.|[6808]|RDNMH069-09|United States|Arizona|658[0n]|BOLD:AAB4378
Homorthodes new sp.|[6809]|RDNMH776-09|United States|Arizona|658[0n]|BOLD:AAB4378
Homorthodes hanhami|[6810]|RDNMF405-08|United States|Oregon|658[0n]|BOLD:AAC8664
Homorthodes hanhami|[6811]|RDNMF406-08|United States|Oregon|658[0n]|BOLD:AAC8664
Homorthodes hanhami|[6812]|RDNMG430-08|United States|California|658[0n]|BOLD:AAC8664
Homorthodes hanhami|[6813]|LPVIA973-08|Canada|British Columbia|613[0n]|BOLD:AAC8664
Homorthodes hanhami|[6814]|LPVIB043-08|Canada|British Columbia|619[0n]|BOLD:AAC8664
Homorthodes hanhami|[6815]|LPVIA842-08|Canada|British Columbia|658[0n]|BOLD:AAC8664
Homorthodes hanhami|[6816]|LPVIA606-08|Canada|British Columbia|658[0n]|BOLD:AAC8664
Homorthodes hanhami|[6817]|LPVIA605-08|Canada|British Columbia|658[0n]|BOLD:AAC8664
Homorthodes hanhami|[6818]|LPVIA571-08|Canada|British Columbia|658[0n]|BOLD:AAC8664
Homorthodes hanhami|[6819]|LPVIA277-08|Canada|British Columbia|658[0n]|BOLD:AAC8664
Homorthodes hanhami|[6820]|LPVIA275-08|Canada|British Columbia|658[0n]|BOLD:AAC8664
Homorthodes hanhami|[6821]|LPVIA274-08|Canada|British Columbia|658[0n]|BOLD:AAC8664
Homorthodes hanhami|[6822]|LPVIA272-08|Canada|British Columbia|658[0n]|BOLD:AAC8664
Homorthodes hanhami|[6823]|LPVIA268-08|Canada|British Columbia|658[0n]|BOLD:AAC8664
Homorthodes hanhami|[6824]|LPVIA101-08|Canada|British Columbia|658[0n]|BOLD:AAC8664
Homorthodes hanhami|[6825]|LPVIA276-08|Canada|British Columbia|658[2n]|BOLD:AAC8664
Homorthodes hanhami|[6826]|LPVIA969-08|Canada|British Columbia|636[0n]|BOLD:AAC8664
Homorthodes hanhami|[6827]|LPVIA971-08|Canada|British Columbia|636[0n]|BOLD:AAC8664
Homorthodes hanhami|[6828]|LPVIB044-08|Canada|British Columbia|658[0n]|BOLD:AAC8664
Homorthodes hanhami|[6829]|LPVIB056-08|Canada|British Columbia|658[0n]|BOLD:AAC8664
Homorthodes hanhami|[6830]|LPVIA234-08|Canada|British Columbia|658[0n]|BOLD:AAC8664
Homorthodes hanhami|[6831]|LPVIA269-08|Canada|British Columbia|658[0n]|BOLD:AAC8664
Homorthodes hanhami|[6832]|LPVIA102-08|Canada|British Columbia|658[0n]|BOLD:AAC8664
Homorthodes hanhami|[6833]|LPVIA273-08|Canada|British Columbia|644[0n]|BOLD:AAC8664
Homorthodes hanhami|[6834]|LPVIA287-08|Canada|British Columbia|658[0n]|BOLD:AAC8664
Homorthodes hanhami|[6835]|LPVIB042-08|Canada|British Columbia|658[0n]|BOLD:AAC8664
Homorthodes hanhami|[6836]|LALPA218-10|Canada|British Columbia|658[0n]|BOLD:AAC8664
Homorthodes hanhami|[6837]|LALPA223-10|Canada|British Columbia|658[0n]|BOLD:AAC8664
Homorthodes hanhami|[6838]|LALPA240-10|Canada|British Columbia|658[0n]|BOLD:AAC8664
Homorthodes hanhami|[6839]|LALPA245-10|Canada|British Columbia|658[0n]|BOLD:AAC8664
Homorthodes hanhami|[6840]|RDNMG865-08|United States|California|658[0n]|BOLD:AAC8664
Homorthodes hanhami|[6841]|RDNMG864-08|United States|California|658[0n]|BOLD:AAC8664
Homorthodes hanhami|[6842]|LOCBC237-06|United States|California|658[0n]|BOLD:AAC8664
Homorthodes hanhami|[6843]|LOCBC014-06|United States|California|658[0n]|BOLD:AAC8664
Homorthodes hanhami|[6844]|LOCBC670-06|United States|California|648[0n]|BOLD:AAC8664
Homorthodes hanhami|[6845]|RDNMG866-08|United States|California|658[0n]|BOLD:AAC8664
Homorthodes hanhami|[6846]|CGLCA161-10|United States|California|658[0n]|BOLD:AAC8664
Homorthodes hanhami|[6847]|IAWLB458-11|United States|California|658[0n]|BOLD:AAC8664
Homorthodes hanhami|[6848]|IAWLB459-11|United States|California|658[0n]|BOLD:AAC8664
Homorthodes hanhami|[6849]|IAWLB460-11|United States|California|658[0n]|BOLD:AAC8664
Homorthodes furfurata|[6850]|IAWLB075-10|United States|California|658[0n]|BOLD:AAA3454
Homorthodes furfurata|[6851]|BBLOC067-11|United States|Texas|658[0n]|BOLD:AAA3454
Homorthodes furfurata|[6852]|BBLOC1004-11|United States|California|658[0n]|BOLD:AAA3454
Homorthodes furfurata|[6853]|IAWLB078-10|United States|California|658[0n]|BOLD:AAA3454
Homorthodes furfurata|[6854]|IAWLB076-10|United States|California|658[0n]|BOLD:AAA3454
Homorthodes furfurata|[6855]|JMMMB609-13|United States|California|561[0n]|BOLD:AAA3454
Homorthodes furfurata|[6856]|RDNME1009-08|United States|New Mexico|658[0n]|BOLD:AAA3454
Homorthodes furfurata|[6857]|RDNME1011-08|United States|New Mexico|658[0n]|BOLD:AAA3454
Homorthodes furfurata|[6858]|RDNMG416-08|United States|New Mexico|658[0n]|BOLD:AAA3454
Homorthodes furfurata|[6859]|RDNMH071-09|United States|Arizona|658[0n]|BOLD:AAA3454
Homorthodes furfurata|[6860]|RDNMJ766-11|United States|Arizona|658[0n]|BOLD:AAA3454
Homorthodes furfurata|[6861]|RDNMJ809-11|United States|Arizona|658[0n]|BOLD:AAA3454
Homorthodes furfurata|[6862]|RDLQ681-07|Canada|Quebec|594[0n]|BOLD:AAA3454
Homorthodes furfurata|[6863]|RDLQ680-07|Canada|Quebec|591[0n]|BOLD:AAA3454
Homorthodes furfurata|[6864]|RDLQB309-05|Canada|Quebec|658[0n]|BOLD:AAA3454
Homorthodes furfurata|[6865]|LSEU097-06|United States|North Carolina|658[0n]|BOLD:AAA3454
Homorthodes furfurata|[6866]|LSEU098-06|United States|North Carolina|658[0n]|BOLD:AAA3454
Homorthodes furfurata|[6867]|LSEU099-06|United States|North Carolina|658[0n]|BOLD:AAA3454
Homorthodes furfurata|[6868]|MMNA096-08|United States|North Carolina|658[0n]|BOLD:AAA3454
Homorthodes furfurata|[6869]|LNCC1071-11|United States|North Carolina|658[0n]|BOLD:AAA3454
Homorthodes furfurata|[6870]|LNCC1070-11|United States|North Carolina|658[0n]|BOLD:AAA3454
Homorthodes furfurata|[6871]|LNCC1072-11|United States|North Carolina|658[0n]|BOLD:AAA3454
Homorthodes furfurata|[6872]|LBCH5959-10|Canada|British Columbia|658[0n]|BOLD:AAA3454
Homorthodes furfurata|[6873]|LBCH6100-10|Canada|British Columbia|658[0n]|BOLD:AAA3454
Homorthodes furfurata|[6874]|LOWCD554-06|Canada|British Columbia|658[0n]|BOLD:AAA3454
Homorthodes furfurata|[6875]|LOWC102-05|Canada|British Columbia|610[1n]|BOLD:AAA3454
Homorthodes furfurata|[6876]|LOWC100-05|Canada|British Columbia|604[0n]|BOLD:AAA3454
Homorthodes furfurata|[6877]|LBCH5961-10|Canada|British Columbia|658[0n]|BOLD:AAA3454
Homorthodes furfurata|[6878]|LBCG1360-09|Canada|British Columbia|658[0n]|BOLD:AAA3454
Homorthodes furfurata|[6879]|LBCH5963-10|Canada|British Columbia|644[0n]|BOLD:AAA3454
Homorthodes furfurata|[6880]|LOWCD650-06|Canada|British Columbia|600[1n]|BOLD:AAA3454
Homorthodes furfurata|[6881]|LBCB119-05|Canada|British Columbia|658[0n]|BOLD:AAA3454
Homorthodes furfurata|[6882]|BBLPB358-10|Canada|British Columbia|658[0n]|BOLD:AAA3454
Homorthodes furfurata|[6883]|BBLPB357-10|Canada|British Columbia|658[0n]|BOLD:AAA3454
Homorthodes furfurata|[6884]|LBCH6754-10|Canada|British Columbia|658[0n]|BOLD:AAA3454
Homorthodes furfurata|[6885]|LBCH6298-10|Canada|British Columbia|658[0n]|BOLD:AAA3454
Homorthodes furfurata|[6886]|LBCH6294-10|Canada|British Columbia|658[0n]|BOLD:AAA3454
Homorthodes furfurata|[6887]|LBCH6291-10|Canada|British Columbia|658[0n]|BOLD:AAA3454
Homorthodes furfurata|[6888]|LBCH6167-10|Canada|British Columbia|658[0n]|BOLD:AAA3454
Homorthodes furfurata|[6889]|LBCH6099-10|Canada|British Columbia|658[0n]|BOLD:AAA3454
Homorthodes furfurata|[6890]|LBCH6025-10|Canada|British Columbia|658[0n]|BOLD:AAA3454
Homorthodes furfurata|[6891]|LBCH5969-10|Canada|British Columbia|658[0n]|BOLD:AAA3454
Homorthodes furfurata|[6892]|LBCH5968-10|Canada|British Columbia|658[0n]|BOLD:AAA3454
Homorthodes furfurata|[6893]|LBCH5967-10|Canada|British Columbia|658[0n]|BOLD:AAA3454
Homorthodes furfurata|[6894]|LBCH5966-10|Canada|British Columbia|658[0n]|BOLD:AAA3454
Homorthodes furfurata|[6895]|LBCH5965-10|Canada|British Columbia|658[0n]|BOLD:AAA3454
Homorthodes furfurata|[6896]|LBCH5962-10|Canada|British Columbia|658[0n]|BOLD:AAA3454
[illegible]

Homorthodes furfurata|[6894]|LBCH5966-10|Canada|British Columbia|658[0n]|BOLD:AAA3454
Homorthodes furfurata|[6895]|LBCH5965-10|Canada|British Columbia|658[0n]|BOLD:AAA3454
Homorthodes furfurata|[6896]|LBCH5962-10|Canada|British Columbia|658[0n]|BOLD:AAA3454
Homorthodes furfurata|[6897]|LBCH5960-10|Canada|British Columbia|658[0n]|BOLD:AAA3454
Homorthodes furfurata|[6898]|LBCH5875-10|Canada|British Columbia|658[0n]|BOLD:AAA3454
Homorthodes furfurata|[6899]|LBCH5874-10|Canada|British Columbia|658[0n]|BOLD:AAA3454
Homorthodes furfurata|[6900]|LBCH5873-10|Canada|British Columbia|658[0n]|BOLD:AAA3454
Homorthodes furfurata|[6901]|LBCH5795-10|Canada|British Columbia|658[0n]|BOLD:AAA3454
Homorthodes furfurata|[6902]|LBCH5613-10|Canada|British Columbia|658[0n]|BOLD:AAA3454
Homorthodes furfurata|[6903]|LBCH5560-10|Canada|British Columbia|658[0n]|BOLD:AAA3454
Homorthodes furfurata|[6904]|LBCH5550-10|Canada|British Columbia|658[0n]|BOLD:AAA3454
Homorthodes furfurata|[6905]|LBCG2865-09|Canada|British Columbia|658[0n]|BOLD:AAA3454
Homorthodes furfurata|[6906]|LBCG2256-09|Canada|British Columbia|658[0n]|BOLD:AAA3454
Homorthodes furfurata|[6907]|LBCG1368-09|Canada|British Columbia|658[0n]|BOLD:AAA3454
Homorthodes furfurata|[6908]|LBCG1367-09|Canada|British Columbia|658[0n]|BOLD:AAA3454
Homorthodes furfurata|[6909]|LBCG1366-09|Canada|British Columbia|658[0n]|BOLD:AAA3454
Homorthodes furfurata|[6910]|LBCG1365-09|Canada|British Columbia|658[0n]|BOLD:AAA3454
Homorthodes furfurata|[6911]|LBCG1364-09|Canada|British Columbia|658[0n]|BOLD:AAA3454
Homorthodes furfurata|[6912]|LBCG1363-09|Canada|British Columbia|658[0n]|BOLD:AAA3454
Homorthodes furfurata|[6913]|LBCG1362-09|Canada|British Columbia|658[0n]|BOLD:AAA3454
Homorthodes furfurata|[6914]|LBCG1361-09|Canada|British Columbia|658[0n]|BOLD:AAA3454
Homorthodes furfurata|[6915]|LBCG1359-09|Canada|British Columbia|658[0n]|BOLD:AAA3454
Homorthodes furfurata|[6916]|LBCG1358-09|Canada|British Columbia|658[0n]|BOLD:AAA3454
Homorthodes furfurata|[6917]|LBCG1357-09|Canada|British Columbia|658[0n]|BOLD:AAA3454
Homorthodes furfurata|[6918]|LBCG1354-09|Canada|British Columbia|658[0n]|BOLD:AAA3454
Homorthodes furfurata|[6919]|LBCG425-08|Canada|British Columbia|658[0n]|BOLD:AAA3454
Homorthodes furfurata|[6920]|LOWCD656-06|Canada|British Columbia|658[0n]|BOLD:AAA3454
Homorthodes furfurata|[6921]|LOWCD655-06|Canada|British Columbia|658[0n]|BOLD:AAA3454
Homorthodes furfurata|[6922]|LOWCD654-06|Canada|British Columbia|658[0n]|BOLD:AAA3454
Homorthodes furfurata|[6923]|LOWCD653-06|Canada|British Columbia|658[0n]|BOLD:AAA3454
Homorthodes furfurata|[6924]|LOWCD652-06|Canada|British Columbia|658[0n]|BOLD:AAA3454
Homorthodes furfurata|[6925]|LOWCD651-06|Canada|British Columbia|658[0n]|BOLD:AAA3454
Homorthodes furfurata|[6926]|LOWCD555-06|Canada|British Columbia|658[0n]|BOLD:AAA3454
Homorthodes furfurata|[6927]|LOWCD553-06|Canada|British Columbia|658[0n]|BOLD:AAA3454
Homorthodes furfurata|[6928]|LOWCD552-06|Canada|British Columbia|658[0n]|BOLD:AAA3454
Homorthodes furfurata|[6929]|LOWCD551-06|Canada|British Columbia|658[0n]|BOLD:AAA3454
Homorthodes furfurata|[6930]|LOWCD550-06|Canada|British Columbia|658[0n]|BOLD:AAA3454
Homorthodes furfurata|[6931]|LOWCD549-06|Canada|British Columbia|658[0n]|BOLD:AAA3454
Homorthodes furfurata|[6932]|LOWCD548-06|Canada|British Columbia|658[0n]|BOLD:AAA3454
Homorthodes furfurata|[6933]|LOWCD546-06|Canada|British Columbia|658[0n]|BOLD:AAA3454
Homorthodes furfurata|[6934]|LOWCD545-06|Canada|British Columbia|658[0n]|BOLD:AAA3454
Homorthodes furfurata|[6935]|LOWCD544-06|Canada|British Columbia|658[0n]|BOLD:AAA3454
Homorthodes furfurata|[6936]|LOWCD543-06|Canada|British Columbia|658[0n]|BOLD:AAA3454
Homorthodes furfurata|[6937]|LOWCD542-06|Canada|British Columbia|658[0n]|BOLD:AAA3454
Homorthodes furfurata|[6938]|LOWCD540-06|Canada|British Columbia|658[0n]|BOLD:AAA3454
Homorthodes furfurata|[6939]|LOWCD539-06|Canada|British Columbia|658[0n]|BOLD:AAA3454
Homorthodes furfurata|[6940]|LOWC101-05|Canada|British Columbia|658[0n]|BOLD:AAA3454
Homorthodes furfurata|[6941]|LOWC099-05|Canada|British Columbia|658[0n]|BOLD:AAA3454
Homorthodes furfurata|[6942]|LOWC098-05|Canada|British Columbia|658[0n]|BOLD:AAA3454
Homorthodes furfurata|[6943]|LOWC096-05|Canada|British Columbia|658[0n]|BOLD:AAA3454
Homorthodes furfurata|[6944]|LOWC094-05|Canada|British Columbia|658[0n]|BOLD:AAA3454
Homorthodes furfurata|[6945]|LOWC093-05|Canada|British Columbia|658[0n]|BOLD:AAA3454
Homorthodes furfurata|[6946]|LOWC092-05|Canada|British Columbia|658[0n]|BOLD:AAA3454
Homorthodes furfurata|[6947]|LOWC091-05|Canada|British Columbia|658[0n]|BOLD:AAA3454
Homorthodes furfurata|[6948]|LOWC089-05|Canada|British Columbia|658[0n]|BOLD:AAA3454
Homorthodes furfurata|[6949]|LOWC088-05|Canada|British Columbia|658[0n]|BOLD:AAA3454
Homorthodes furfurata|[6950]|LOWC087-05|Canada|British Columbia|658[0n]|BOLD:AAA3454
Homorthodes furfurata|[6951]|LBCC646-05|Canada|British Columbia|658[0n]|BOLD:AAA3454
Homorthodes furfurata|[6952]|LBCC458-05|Canada|British Columbia|658[0n]|BOLD:AAA3454
Homorthodes furfurata|[6953]|LBCC457-05|Canada|British Columbia|658[0n]|BOLD:AAA3454
Homorthodes furfurata|[6954]|LBCC455-05|Canada|British Columbia|658[0n]|BOLD:AAA3454
Homorthodes furfurata|[6955]|LBCC454-05|Canada|British Columbia|658[0n]|BOLD:AAA3454
Homorthodes furfurata|[6956]|LBCC351-05|Canada|British Columbia|658[0n]|BOLD:AAA3454
Homorthodes furfurata|[6957]|LBCC350-05|Canada|British Columbia|658[0n]|BOLD:AAA3454
Homorthodes furfurata|[6958]|LBCC349-05|Canada|British Columbia|658[0n]|BOLD:AAA3454
Homorthodes furfurata|[6959]|LBCC348-05|Canada|British Columbia|658[0n]|BOLD:AAA3454
Homorthodes furfurata|[6960]|LBCC347-05|Canada|British Columbia|658[0n]|BOLD:AAA3454
Homorthodes furfurata|[6961]|LBCC346-05|Canada|British Columbia|658[0n]|BOLD:AAA3454
Homorthodes furfurata|[6962]|LBCC345-05|Canada|British Columbia|658[0n]|BOLD:AAA3454
Homorthodes furfurata|[6963]|LBCB627-05|Canada|British Columbia|658[0n]|BOLD:AAA3454
Homorthodes furfurata|[6964]|LBCB626-05|Canada|British Columbia|658[0n]|BOLD:AAA3454
Homorthodes furfurata|[6965]|LBCB354-05|Canada|British Columbia|658[0n]|BOLD:AAA3454
Homorthodes furfurata|[6966]|LBCB116-05|Canada|British Columbia|658[0n]|BOLD:AAA3454
Homorthodes furfurata|[6967]|LBCB114-05|Canada|British Columbia|658[0n]|BOLD:AAA3454
Homorthodes furfurata|[6968]|LBCB080-05|Canada|British Columbia|658[0n]|BOLD:AAA3454
Homorthodes furfurata|[6969]|LBCB079-05|Canada|British Columbia|658[0n]|BOLD:AAA3454
Homorthodes furfurata|[6970]|LBCA827-05|Canada|British Columbia|658[0n]|BOLD:AAA3454
Homorthodes furfurata|[6971]|LOWC090-05|Canada|British Columbia|658[0n]|BOLD:AAA3454
Homorthodes furfurata|[6972]|LOWCD547-06|Canada|British Columbia|658[0n]|BOLD:AAA3454
Homorthodes furfurata|[6973]|LOWCD657-06|Canada|British Columbia|658[0n]|BOLD:AAA3454
Homorthodes furfurata|[6974]|LBCG1356-09|Canada|British Columbia|641[0n]|BOLD:AAA3454
Homorthodes furfurata|[6975]|LOWC097-05|Canada|British Columbia|658[0n]|BOLD:AAA3454
Homorthodes furfurata|[6976]|LOWC095-05|Canada|British Columbia|617[0n]|BOLD:AAA3454
Homorthodes furfurata|[6977]|LBCG1355-09|Canada|British Columbia|643[0n]|BOLD:AAA3454
Homorthodes furfurata|[6978]|LBCH5964-10|Canada|British Columbia|644[0n]|BOLD:AAA3454
Homorthodes furfurata|[6979]|LBCH6059-10|Canada|British Columbia|646[0n]|BOLD:AAA3454
Homorthodes furfurata|[6980]|LBCH6060-10|Canada|British Columbia|646[0n]|BOLD:AAA3454
Homorthodes furfurata|[6981]|LALPA887-11|Canada|British Columbia|640[0n]|BOLD:AAA3454
Homorthodes furfurata|[6982]|LBCA615-05|Canada|British Columbia|658[0n]|BOLD:AAA3454
Homorthodes furfurata|[6983]|LOWCD649-06|Canada|British Columbia|658[0n]|BOLD:AAA3454
Homorthodes furfurata|[6984]|LBCC456-05|Canada|British Columbia|658[0n]|BOLD:AAA3454
Homorthodes furfurata|[6985]|RDNMH070-09|United States|Wyoming|658[0n]|BOLD:AAA3454
Homorthodes furfurata|[6986]|LPABB529-08|Canada|Alberta|658[0n]|BOLD:AAA3454
Homorthodes furfurata|[6987]|LBCB625-05|Canada|British Columbia|658[0n]|BOLD:AAA3454
Homorthodes furfurata|[6988]|LBCB624-05|Canada|British Columbia|658[0n]|BOLD:AAA3454
Homorthodes furfurata|[6989]|LBCA701-05|Canada|British Columbia|658[0n]|BOLD:AAA3454
Homorthodes furfurata|[6990]|LBCA699-05|Canada|British Columbia|658[0n]|BOLD:AAA3454
Homorthodes furfurata|[6991]|LBCA698-05|Canada|British Columbia|658[0n]|BOLD:AAA3454
Homorthodes furfurata|[6992]|LBCA697-05|Canada|British Columbia|658[0n]|BOLD:AAA3454
Homorthodes furfurata|[6993]|LBCA696-05|Canada|British Columbia|658[0n]|BOLD:AAA3454
Homorthodes furfurata|[6994]|LBCA613-05|Canada|British Columbia|653[0n]|BOLD:AAA3454
Homorthodes furfurata|[6995]|LBCA618-05|Canada|British Columbia|633[0n]|BOLD:AAA3454
Homorthodes furfurata|[6996]|CNWLF2000-12|Canada|Alberta|636[0n]|BOLD:AAA3454

Homorthodes furfurata|[6994]|LBCA613-05|Canada|British Columbia|653[0n]|BOLD:AAA3454
Homorthodes furfurata|[6995]|LBCA618-05|Canada|British Columbia|633[0n]|BOLD:AAA3454
Homorthodes furfurata|[6996]|CNWLF2000-12|Canada|Alberta|636[0n]|BOLD:AAA3454
Homorthodes furfurata|[6997]|LBCA617-05|Canada|British Columbia|634[0n]|BOLD:AAA3454
Homorthodes furfurata|[6998]|LBCA616-05|Canada|British Columbia|634[0n]|BOLD:AAA3454
Homorthodes furfurata|[6999]|LBCA700-05|Canada|British Columbia|647[0n]|BOLD:AAA3454
Homorthodes furfurata|[7000]|LBCA614-05|Canada|British Columbia|634[0n]|BOLD:AAA3454
Homorthodes furfurata|[7001]|RDMAB292-05|Canada|Alberta|614[1n]|BOLD:AAA3454
Homorthodes furfurata|[7002]|RDNME245-07|Canada|Alberta|616[0n]|BOLD:AAA3454
Homorthodes furfurata|[7003]|CNWLM034-13|Canada|Alberta|576[0n]|BOLD:AAA3454
Homorthodes furfurata|[7004]|CNWLM042-13|Canada|Alberta|540[0n]|BOLD:AAA3454
Homorthodes sp.|[7005]|RDNMG328-08|United States|Texas|658[0n]|BOLD:ACE3898
Homorthodes sp.|[7006]|RDNMH784-09|United States|Texas|626[0n]|BOLD:ACE3898
Homorthodes lindseyi|[7007]|LGSMC401-05|United States|Tennessee|658[0n]|BOLD:ACE3897
Homorthodes lindseyi|[7008]|LGSMC400-05|United States|Tennessee|658[0n]|BOLD:ACE3897
Homorthodes lindseyi|[7009]|LSEU096-06|United States|Georgia|658[0n]|BOLD:ACE3897
Homorthodes lindseyi|[7010]|LSEU095-06|United States|Georgia|658[0n]|BOLD:ACE3897
Homorthodes lindseyi|[7011]|LOCT070-05|United States|Connecticut|658[0n]|BOLD:ACE3897
Homorthodes lindseyi|[7012]|LOTB210-05|United States|Tennessee|658[0n]|BOLD:ACE3897
Homorthodes lindseyi|[7013]|LGSMC402-05|United States|Tennessee|658[0n]|BOLD:ACE3897
Homorthodes lindseyi|[7014]|LGSMC399-05|United States|Tennessee|658[0n]|BOLD:ACE3897
Homorthodes lindseyi|[7015]|RDNMD903-07|United States|Massachusetts|653[0n]|BOLD:ACE3897
Homorthodes lindseyi|[7016]|LGSMG867-10|United States|Tennessee|658[0n]|BOLD:ACE3897
Homorthodes lindseyi|[7017]|LGSMG868-10|United States|Tennessee|658[0n]|BOLD:ACE3897
Homorthodes lindseyi|[7018]|LNCC057-10|United States|North Carolina|658[0n]|BOLD:ACE3897
Homorthodes lindseyi|[7019]|LNCC677-11|United States|North Carolina|658[0n]|BOLD:ACE3897
Homorthodes lindseyi|[7020]|CNCLB2529-14|United States|Maryland|658[0n]|BOLD:ACE3897
Anhypotrix tristis|[7021]|RDNME875-08|United States|New Mexico|658[0n]|BOLD:AAH8070
Anhypotrix tristis|[7022]|RDNMG294-08|United States|Arizona|658[0n]|BOLD:AAH8070
Anhypotrix tristis|[7023]|RDNMI106-10|United States|Arizona|658[0n]|BOLD:AAH8070
Orthodes cynica|[7024]|NAMUM340-08|United States|Maryland|658[0n]|BOLD:AAA2411
Orthodes cynica|[7025]|LOCT324-05|United States|Connecticut|658[0n]|BOLD:AAA2411
Orthodes cynica|[7026]|LPSOB983-08|Canada|Ontario|658[0n]|BOLD:AAA2411
Orthodes cynica|[7027]|LPSOB994-08|Canada|Ontario|658[0n]|BOLD:AAA2411
Orthodes cynica|[7028]|XAB592-04|Canada|Ontario|658[0n]|BOLD:AAA2411
Orthodes cynica|[7029]|LGSMC409-05|United States|North Carolina|619[0n]|BOLD:AAA2411
Orthodes cynica|[7030]|PMG141-03|Canada|Ontario|617[0n]|BOLD:AAA2411
Orthodes cynica|[7031]|XAE299-04|Canada|Ontario|617[0n]|BOLD:AAA2411
Orthodes cynica|[7032]|RDLQB812-05|Canada|Quebec|617[0n]|BOLD:AAA2411
Orthodes cynica|[7033]|LPSO263-08|Canada|Ontario|657[0n]|BOLD:AAA2411
Orthodes cynica|[7034]|XAB078-04|Canada|Ontario|569[0n]|BOLD:AAA2411
Orthodes cynica|[7035]|MEC587-04|Canada|Quebec|583[0n]|BOLD:AAA2411
Orthodes cynica|[7036]|LGSMC407-05|United States|Tennessee|658[0n]|BOLD:AAA2411
Orthodes cynica|[7037]|RDLQ312-05|Canada|Quebec|582[0n]|BOLD:AAA2411
Orthodes cynica|[7038]|XAE251-04|Canada|Ontario|616[1n]|BOLD:AAA2411
Orthodes cynica|[7039]|RDLQB050-05|Canada|Quebec|658[0n]|BOLD:AAA2411
Orthodes cynica|[7040]|RDLQ313-05|Canada|Quebec|578[0n]|BOLD:AAA2411
Orthodes cynica|[7041]|RDLQ308-05|Canada|Quebec|591[0n]|BOLD:AAA2411
Orthodes cynica|[7042]|RDLQ307-05|Canada|Quebec|557[0n]|BOLD:AAA2411
Orthodes cynica|[7043]|RDLQ309-05|Canada|Quebec|571[1n]|BOLD:AAA2411
Orthodes cynica|[7044]|XAJ554-06|Canada|Ontario|658[0n]|BOLD:AAA2411
Orthodes cynica|[7045]|TTMNB557-06|Canada|New Brunswick|658[1n]|BOLD:AAA2411
Orthodes cynica|[7046]|RDLQ310-05|Canada|Quebec|600[1n]|BOLD:AAA2411
Orthodes cynica|[7047]|LPSOC369-08|Canada|Ontario|643[0n]|BOLD:AAA2411
Orthodes cynica|[7048]|LPSO282-08|Canada|Ontario|643[0n]|BOLD:AAA2411
Orthodes cynica|[7049]|LPSOD694-09|Canada|Ontario|658[0n]|BOLD:AAA2411
Orthodes cynica|[7050]|LPSOD572-09|Canada|Ontario|658[0n]|BOLD:AAA2411
Orthodes cynica|[7051]|LPSOD521-09|Canada|Ontario|658[0n]|BOLD:AAA2411
Orthodes cynica|[7052]|LPSOD440-09|Canada|Ontario|658[0n]|BOLD:AAA2411
Orthodes cynica|[7053]|LPSOD434-09|Canada|Ontario|658[0n]|BOLD:AAA2411
Orthodes cynica|[7054]|BLTIB254-08|Canada|Ontario|658[0n]|BOLD:AAA2411
Orthodes cynica|[7055]|BLTIB252-08|Canada|Ontario|658[0n]|BOLD:AAA2411
Orthodes cynica|[7056]|BLTIB184-08|Canada|Ontario|658[0n]|BOLD:AAA2411
Orthodes cynica|[7057]|LPMN578-08|Canada|Manitoba|658[0n]|BOLD:AAA2411
Orthodes cynica|[7058]|LPMN528-08|Canada|Manitoba|658[0n]|BOLD:AAA2411
Orthodes cynica|[7059]|LPMN333-08|Canada|Manitoba|658[0n]|BOLD:AAA2411
Orthodes cynica|[7060]|KPOEC176-08|Canada|Ontario|658[0n]|BOLD:AAA2411
Orthodes cynica|[7061]|KPOEC162-08|Canada|Ontario|658[0n]|BOLD:AAA2411
Orthodes cynica|[7062]|LPSOB999-08|Canada|Ontario|658[0n]|BOLD:AAA2411
Orthodes cynica|[7063]|LPSOB998-08|Canada|Ontario|658[0n]|BOLD:AAA2411
Orthodes cynica|[7064]|LPSOB997-08|Canada|Ontario|658[0n]|BOLD:AAA2411
Orthodes cynica|[7065]|LPSOB995-08|Canada|Ontario|658[0n]|BOLD:AAA2411
Orthodes cynica|[7066]|LPSOB993-08|Canada|Ontario|658[0n]|BOLD:AAA2411
Orthodes cynica|[7067]|LPSOB991-08|Canada|Ontario|658[0n]|BOLD:AAA2411
Orthodes cynica|[7068]|LPSOB987-08|Canada|Ontario|658[0n]|BOLD:AAA2411
Orthodes cynica|[7069]|LPSOB829-08|Canada|Ontario|658[0n]|BOLD:AAA2411
Orthodes cynica|[7070]|LPSOB819-08|Canada|Ontario|658[0n]|BOLD:AAA2411
Orthodes cynica|[7071]|LPSOB809-08|Canada|Ontario|658[0n]|BOLD:AAA2411
Orthodes cynica|[7072]|LPSOB804-08|Canada|Ontario|658[0n]|BOLD:AAA2411
Orthodes cynica|[7073]|LPSOB803-08|Canada|Ontario|658[0n]|BOLD:AAA2411
Orthodes cynica|[7074]|LPSOB720-08|Canada|Ontario|658[0n]|BOLD:AAA2411
Orthodes cynica|[7075]|LPSOB232-08|Canada|Ontario|658[0n]|BOLD:AAA2411
Orthodes cynica|[7076]|LPSOC385-08|Canada|Ontario|658[0n]|BOLD:AAA2411
Orthodes cynica|[7077]|LPSOC371-08|Canada|Ontario|655[0n]|BOLD:AAA2411
Orthodes cynica|[7078]|LPSOC106-08|Canada|Ontario|658[0n]|BOLD:AAA2411
Orthodes cynica|[7079]|LPSOC097-08|Canada|Ontario|658[0n]|BOLD:AAA2411
Orthodes cynica|[7080]|LPSOC044-08|Canada|Ontario|658[0n]|BOLD:AAA2411
Orthodes cynica|[7081]|LPSOB120-08|Canada|Ontario|658[0n]|BOLD:AAA2411
Orthodes cynica|[7082]|LPSOB119-08|Canada|Ontario|658[0n]|BOLD:AAA2411
Orthodes cynica|[7083]|LPSOB094-08|Canada|Ontario|658[0n]|BOLD:AAA2411
Orthodes cynica|[7084]|LPSO926-08|Canada|Ontario|658[0n]|BOLD:AAA2411
Orthodes cynica|[7085]|LPSO889-08|Canada|Ontario|658[0n]|BOLD:AAA2411
Orthodes cynica|[7086]|LPSO747-08|Canada|Ontario|658[0n]|BOLD:AAA2411
Orthodes cynica|[7087]|LPSO742-08|Canada|Ontario|658[0n]|BOLD:AAA2411
Orthodes cynica|[7088]|LPSO740-08|Canada|Ontario|658[0n]|BOLD:AAA2411
Orthodes cynica|[7089]|LPSO669-08|Canada|Ontario|658[0n]|BOLD:AAA2411
Orthodes cynica|[7090]|LPSO645-08|Canada|Ontario|658[0n]|BOLD:AAA2411
Orthodes cynica|[7091]|LPSO547-08|Canada|Ontario|658[0n]|BOLD:AAA2411
Orthodes cynica|[7092]|LPSO543-08|Canada|Ontario|657[0n]|BOLD:AAA2411
Orthodes cynica|[7093]|LPSO502-08|Canada|Ontario|658[0n]|BOLD:AAA2411
Orthodes cynica|[7094]|LPSO499-08|Canada|Ontario|658[0n]|BOLD:AAA2411
Orthodes cynica|[7095]|LPSO493-08|Canada|Ontario|658[0n]|BOLD:AAA2411
Orthodes cynica|[7096]|LPSO492-08|Canada|Ontario|658[0n]|BOLD:AAA2411

Orthodes cynica|[7094]|LPSO499-08|Canada|Ontario|658[0n]|BOLD:AAA2411
Orthodes cynica|[7095]|LPSO493-08|Canada|Ontario|658[0n]|BOLD:AAA2411
Orthodes cynica|[7096]|LPSO492-08|Canada|Ontario|658[0n]|BOLD:AAA2411
Orthodes cynica|[7097]|LPSO409-08|Canada|Ontario|658[0n]|BOLD:AAA2411
Orthodes cynica|[7098]|LPSO360-08|Canada|Ontario|658[0n]|BOLD:AAA2411
Orthodes cynica|[7099]|LPSO348-08|Canada|Ontario|658[0n]|BOLD:AAA2411
Orthodes cynica|[7100]|LPSO277-08|Canada|Ontario|658[0n]|BOLD:AAA2411
Orthodes cynica|[7101]|LPSO266-08|Canada|Ontario|658[0n]|BOLD:AAA2411
Orthodes cynica|[7102]|LPSO254-08|Canada|Ontario|658[0n]|BOLD:AAA2411
Orthodes cynica|[7103]|LPSO250-08|Canada|Ontario|658[0n]|BOLD:AAA2411
Orthodes cynica|[7104]|LPSO249-08|Canada|Ontario|658[0n]|BOLD:AAA2411
Orthodes cynica|[7105]|LPSO055-08|Canada|Ontario|658[0n]|BOLD:AAA2411
Orthodes cynica|[7106]|LPSO051-08|Canada|Ontario|658[0n]|BOLD:AAA2411
Orthodes cynica|[7107]|LPSO050-08|Canada|Ontario|658[0n]|BOLD:AAA2411
Orthodes cynica|[7108]|LPSO037-08|Canada|Ontario|658[0n]|BOLD:AAA2411
Orthodes cynica|[7109]|LPSO036-08|Canada|Ontario|658[0n]|BOLD:AAA2411
Orthodes cynica|[7110]|KPOEC084-08|Canada|Ontario|658[0n]|BOLD:AAA2411
Orthodes cynica|[7111]|RDLQG397-06|Canada|Quebec|658[0n]|BOLD:AAA2411
Orthodes cynica|[7112]|RDLQG396-06|Canada|Quebec|658[0n]|BOLD:AAA2411
Orthodes cynica|[7113]|RDLQG392-06|Canada|Quebec|658[0n]|BOLD:AAA2411
Orthodes cynica|[7114]|RDLQG391-06|Canada|Quebec|658[0n]|BOLD:AAA2411
Orthodes cynica|[7115]|RDLQG390-06|Canada|Quebec|658[0n]|BOLD:AAA2411
Orthodes cynica|[7116]|RDLQG389-06|Canada|Quebec|658[0n]|BOLD:AAA2411
Orthodes cynica|[7117]|RDLQG388-06|Canada|Quebec|658[0n]|BOLD:AAA2411
Orthodes cynica|[7118]|RDLQG367-06|Canada|Quebec|658[0n]|BOLD:AAA2411
Orthodes cynica|[7119]|RDLQG366-06|Canada|Quebec|658[0n]|BOLD:AAA2411
Orthodes cynica|[7120]|RDLQG274-06|Canada|Quebec|658[0n]|BOLD:AAA2411
Orthodes cynica|[7121]|RDLQG255-06|Canada|Quebec|658[0n]|BOLD:AAA2411
Orthodes cynica|[7122]|RDLQF936-06|Canada|Quebec|658[0n]|BOLD:AAA2411
Orthodes cynica|[7123]|RDLQF846-06|Canada|Quebec|658[0n]|BOLD:AAA2411
Orthodes cynica|[7124]|XAK180-06|Canada|Ontario|658[0n]|BOLD:AAA2411
Orthodes cynica|[7125]|LSEU517-06|United States|Georgia|658[0n]|BOLD:AAA2411
Orthodes cynica|[7126]|XAJ713-06|Canada|Ontario|658[0n]|BOLD:AAA2411
Orthodes cynica|[7127]|XAJ712-06|Canada|Ontario|658[0n]|BOLD:AAA2411
Orthodes cynica|[7128]|XAJ653-06|Canada|Ontario|658[0n]|BOLD:AAA2411
Orthodes cynica|[7129]|XAJ615-06|Canada|Ontario|658[0n]|BOLD:AAA2411
Orthodes cynica|[7130]|TTMNB561-06|Canada|New Brunswick|658[0n]|BOLD:AAA2411
Orthodes cynica|[7131]|TTMNB559-06|Canada|New Brunswick|658[0n]|BOLD:AAA2411
Orthodes cynica|[7132]|TTMNB558-06|Canada|New Brunswick|658[0n]|BOLD:AAA2411
Orthodes cynica|[7133]|TTMNB556-06|Canada|New Brunswick|658[0n]|BOLD:AAA2411
Orthodes cynica|[7134]|RDLQB068-05|Canada|Quebec|658[0n]|BOLD:AAA2411
Orthodes cynica|[7135]|RDLQ261-05|Canada|Quebec|658[0n]|BOLD:AAA2411
Orthodes cynica|[7136]|XAF821-05|Canada|Ontario|658[0n]|BOLD:AAA2411
Orthodes cynica|[7137]|XAF586-05|Canada|Ontario|658[0n]|BOLD:AAA2411
Orthodes cynica|[7138]|XAF470-05|Canada|Ontario|658[0n]|BOLD:AAA2411
Orthodes cynica|[7139]|LOCT322-05|United States|Connecticut|658[0n]|BOLD:AAA2411
Orthodes cynica|[7140]|LOCT321-05|United States|Connecticut|658[0n]|BOLD:AAA2411
Orthodes cynica|[7141]|LOCT252-05|United States|Connecticut|658[0n]|BOLD:AAA2411
Orthodes cynica|[7142]|LOCT251-05|United States|Connecticut|658[0n]|BOLD:AAA2411
Orthodes cynica|[7143]|LOCT250-05|United States|Connecticut|658[0n]|BOLD:AAA2411
Orthodes cynica|[7144]|LOCT249-05|United States|Connecticut|658[0n]|BOLD:AAA2411
Orthodes cynica|[7145]|LOTB485-05|United States|Tennessee|658[0n]|BOLD:AAA2411
Orthodes cynica|[7146]|LGSMC410-05|United States|Tennessee|658[0n]|BOLD:AAA2411
Orthodes cynica|[7147]|LGSMC408-05|United States|Tennessee|658[0n]|BOLD:AAA2411
Orthodes cynica|[7148]|LGSMC406-05|United States|Tennessee|658[0n]|BOLD:AAA2411
Orthodes cynica|[7149]|LGSMC405-05|United States|Tennessee|658[0n]|BOLD:AAA2411
Orthodes cynica|[7150]|LGSMC404-05|United States|North Carolina|658[0n]|BOLD:AAA2411
Orthodes cynica|[7151]|LGSMC403-05|United States|Tennessee|658[0n]|BOLD:AAA2411
Orthodes cynica|[7152]|PHMNB413-04|Canada|New Brunswick|658[0n]|BOLD:AAA2411
Orthodes cynica|[7153]|PHMNB382-04|Canada|New Brunswick|658[0n]|BOLD:AAA2411
Orthodes cynica|[7154]|XAC597-04|Canada|Ontario|658[0n]|BOLD:AAA2411
Orthodes cynica|[7155]|XAC296-04|Canada|Ontario|658[0n]|BOLD:AAA2411
Orthodes cynica|[7156]|XAC292-04|Canada|Ontario|658[0n]|BOLD:AAA2411
Orthodes cynica|[7157]|XAC180-04|Canada|Ontario|658[0n]|BOLD:AAA2411
Orthodes cynica|[7158]|XAB266-04|Canada|Ontario|658[0n]|BOLD:AAA2411
Orthodes cynica|[7159]|XAB259-04|Canada|Ontario|658[0n]|BOLD:AAA2411
Orthodes cynica|[7160]|XAB251-04|Canada|Ontario|658[0n]|BOLD:AAA2411
Orthodes cynica|[7161]|LOTB247-05|United States|Tennessee|658[0n]|BOLD:AAA2411
Orthodes cynica|[7162]|XAB217-04|Canada|Ontario|658[0n]|BOLD:AAA2411
Orthodes cynica|[7163]|LPSO035-08|Canada|Ontario|658[0n]|BOLD:AAA2411
Orthodes cynica|[7164]|RDLQ409-05|Canada|Quebec|658[1n]|BOLD:AAA2411
Orthodes cynica|[7165]|LPSOB158-08|Canada|Ontario|656[0n]|BOLD:AAA2411
Orthodes cynica|[7166]|TTMNB560-06|Canada|New Brunswick|656[0n]|BOLD:AAA2411
Orthodes cynica|[7167]|RDLQ287-05|Canada|Quebec|656[0n]|BOLD:AAA2411
Orthodes cynica|[7168]|TMG144-03|Canada|Ontario|639[0n]|BOLD:AAA2411
Orthodes cynica|[7169]|TMG143-03|Canada|Ontario|639[0n]|BOLD:AAA2411
Orthodes cynica|[7170]|PHMO059-03|Canada|Ontario|639[0n]|BOLD:AAA2411
Orthodes cynica|[7171]|PHMO145-03|Canada|Ontario|639[0n]|BOLD:AAA2411
Orthodes cynica|[7172]|LOCT323-05|United States|Connecticut|634[0n]|BOLD:AAA2411
Orthodes cynica|[7173]|LPSOB664-08|Canada|Ontario|646[0n]|BOLD:AAA2411
Orthodes cynica|[7174]|LPSOB692-08|Canada|Ontario|646[0n]|BOLD:AAA2411
Orthodes cynica|[7175]|LPSOB992-08|Canada|Ontario|646[0n]|BOLD:AAA2411
Orthodes cynica|[7176]|LPSOD707-09|Canada|Ontario|658[0n]|BOLD:AAA2411
Orthodes cynica|[7177]|LPSOD771-09|Canada|Ontario|658[0n]|BOLD:AAA2411
Orthodes cynica|[7178]|LPSOD885-09|Canada|Ontario|658[0n]|BOLD:AAA2411
Orthodes cynica|[7179]|LPSOD886-09|Canada|Ontario|658[0n]|BOLD:AAA2411
Orthodes cynica|[7180]|LPSOD894-09|Canada|Ontario|658[0n]|BOLD:AAA2411
Orthodes cynica|[7181]|BBLCU119-09|United States|Michigan|658[0n]|BOLD:AAA2411
Orthodes cynica|[7182]|BBLCU130-09|United States|Michigan|658[0n]|BOLD:AAA2411
Orthodes cynica|[7183]|PHMTV426-10|Canada|Ontario|658[0n]|BOLD:AAA2411
Orthodes cynica|[7184]|BBLPB454-10|Canada|Ontario|658[0n]|BOLD:AAA2411
Orthodes cynica|[7185]|BBLPB585-10|Canada|Ontario|658[0n]|BOLD:AAA2411
Orthodes cynica|[7186]|HPPPC1207-13|Canada|Nova Scotia|564[3n]|BOLD:AAA2411
Orthodes adiastola|[7187]|RDNME980-08|United States|New Mexico|658[0n]|BOLD:AAI2218
Orthodes adiastola|[7188]|RDNME988-08|United States|New Mexico|658[0n]|BOLD:AAI2218
Orthodes adiastola|[7189]|RDNMJ810-11|United States|New Mexico|658[0n]|BOLD:AAI2218
Orthodes adiastola|[7190]|RDNMJ811-11|United States|Arizona|658[0n]|BOLD:AAI2218
Synorthodes typhedana|[7191]|RDNMJ699-11|United States|Arizona|658[0n]|BOLD:AAH9681
Synorthodes typhedana|[7192]|RDNMJ683-11|United States|Arizona|658[0n]|BOLD:AAH9681
Synorthodes typhedana|[7193]|RDNME901-08|United States|Arizona|658[0n]|BOLD:AAH9681
Synorthodes typhedana|[7194]|RDNME605-08|United States|Arizona|658[0n]|BOLD:AAH9681
Synorthodes typhedana|[7195]|RDNMJ813-11|United States|Arizona|658[0n]|BOLD:AAH9681
Synorthodes typhedana|[7196]|RDNMJ814-11|United States|New Mexico|658[0n]|BOLD:AAH9681

Synorthodes typhedana|[7194]|RDNME605-08|United States|Arizona|658[0n]|BOLD:AAH9681
Synorthodes typhedana|[7195]|RDNMJ813-11|United States|Arizona|658[0n]|BOLD:AAH9681
Synorthodes typhedana|[7196]|RDNMJ814-11|United States|New Mexico|658[0n]|BOLD:AAH9681
Orthodes majuscula|[7197]|BBLOC1040-11|United States|Arkansas|658[0n]|BOLD:AAB4054
Orthodes majuscula|[7198]|LPOKD727-10|United States|Oklahoma|658[0n]|BOLD:AAB4054
Orthodes majuscula|[7199]|BBLSU056-09|United States|Arkansas|658[0n]|BOLD:AAB4054
Orthodes majuscula|[7200]|BBLSX883-09|United States|Oklahoma|658[0n]|BOLD:AAB4054
Orthodes majuscula|[7201]|BBLSW666-09|United States|Oklahoma|658[0n]|BOLD:AAB4054
Orthodes majuscula|[7202]|LPOKB226-09|United States|Oklahoma|658[0n]|BOLD:AAB4054
Orthodes majuscula|[7203]|LPOKA373-08|United States|Oklahoma|658[0n]|BOLD:AAB4054
Orthodes majuscula|[7204]|LPOKA285-08|United States|Oklahoma|658[0n]|BOLD:AAB4054
Orthodes majuscula|[7205]|RDLQG188-06|Canada|Quebec|655[0n]|BOLD:AAB4054
Orthodes majuscula|[7206]|LPMNB335-09|Canada|Manitoba|640[0n]|BOLD:AAB4054
Orthodes majuscula|[7207]|CNPPG799-12|Canada|Ontario|636[0n]|BOLD:AAB4054
Orthodes majuscula|[7208]|BBLSY860-09|United States|Oklahoma|658[0n]|BOLD:AAB4054
Orthodes majuscula|[7209]|LGSMC491-05|United States|Tennessee|612[0n]|BOLD:AAB4054
Orthodes majuscula|[7210]|LPSO487-08|Canada|Ontario|658[0n]|BOLD:AAB4054
Orthodes majuscula|[7211]|LNCB519-07|United States|North Carolina|658[6n]|BOLD:AAB4054
Orthodes majuscula|[7212]|BBLSX890-09|United States|Oklahoma|658[0n]|BOLD:AAB4054
Orthodes majuscula|[7213]|BBLSX316-09|United States|Oklahoma|658[0n]|BOLD:AAB4054
Orthodes majuscula|[7214]|BBLSY085-09|United States|Oklahoma|658[0n]|BOLD:AAB4054
Orthodes majuscula|[7215]|BBLSW570-09|United States|Oklahoma|658[0n]|BOLD:AAB4054
Orthodes majuscula|[7216]|LPOKB288-09|United States|Oklahoma|658[0n]|BOLD:AAB4054
Orthodes majuscula|[7217]|NAMUM358-08|United States|Maryland|658[0n]|BOLD:AAB4054
Orthodes majuscula|[7218]|LPSOB082-08|Canada|Ontario|658[0n]|BOLD:AAB4054
Orthodes majuscula|[7219]|LPSO500-08|Canada|Ontario|658[1n]|BOLD:AAB4054
Orthodes majuscula|[7220]|RDLQG135-06|Canada|Quebec|658[0n]|BOLD:AAB4054
Orthodes majuscula|[7221]|RDLQF544-06|Canada|Quebec|658[0n]|BOLD:AAB4054
Orthodes majuscula|[7222]|LNCB327-06|United States|North Carolina|658[0n]|BOLD:AAB4054
Orthodes majuscula|[7223]|LSUSA178-06|United States|Kentucky|658[0n]|BOLD:AAB4054
Orthodes majuscula|[7224]|TTMNB037-06|Canada|New Brunswick|658[0n]|BOLD:AAB4054
Orthodes majuscula|[7225]|LOCT235-05|United States|Connecticut|658[0n]|BOLD:AAB4054
Orthodes majuscula|[7226]|LGSMC656-05|United States|Tennessee|658[0n]|BOLD:AAB4054
Orthodes majuscula|[7227]|LGSMC490-05|United States|Tennessee|658[0n]|BOLD:AAB4054
Orthodes majuscula|[7228]|LGSM728-04|United States|Tennessee|658[0n]|BOLD:AAB4054
Orthodes majuscula|[7229]|RDNMC288-05|United States|Florida|594[0n]|BOLD:AAB4054
Orthodes majuscula|[7230]|LPOKD261-09|United States|Oklahoma|657[0n]|BOLD:AAB4054
Orthodes majuscula|[7231]|LGSMG872-10|United States|North Carolina|658[0n]|BOLD:AAB4054
Orthodes majuscula|[7232]|LGSMG873-10|United States|Tennessee|658[0n]|BOLD:AAB4054
Orthodes majuscula|[7233]|CNPPH805-12|Canada|Ontario|643[0n]|BOLD:AAB4054
Orthodes sp.|[7234]|RDNME954-08|United States|New Mexico|658[0n]|BOLD:AAD8937
Orthodes sp.|[7235]|RDNML380-13|United States|Arizona|658[0n]|BOLD:AAD8937
Orthodes furtiva|[7236]|LNAUS4873-13|United States|Texas|658[0n]|BOLD:ACK4025
Orthodes furtiva|[7237]|LNAUS4874-13|United States|Texas|658[0n]|BOLD:ACK4025
Orthodes furtiva|[7238]|LNAUS4876-13|United States|Texas|566[0n]|BOLD:ACK4025
Orthodes furtiva|[7239]|CNCLB690-14|United States|Texas|658[0n]|BOLD:ACK4025
Orthodes furtiva|[7240]|CNCLB689-14|United States|Texas|658[0n]|BOLD:ACK4025
Orthodes furtiva|[7241]|CNCLB691-14|United States|Texas|602[2n]|BOLD:ACK4025
Orthodes furtiva|[7242]|LNAUT453-14|United States|Texas|658[0n]|BOLD:ACK4025
Lacinipolia mimula|[7243]|RDNMD375-06|United States|Arizona|658[0n]|BOLD:AAX0569
Hypotrix parallela|[7244]|RDNME634-08|United States|Arizona|658[0n]|BOLD:AAF5377
Hypotrix parallela|[7245]|NAMUM062-08|United States|Arizona|657[0n]|BOLD:AAF5377
Hypotrix parallela|[7246]|RDNMG1006-08|United States|Arizona|658[0n]|BOLD:AAF5377
Hypotrix rubra|[7247]|RDNME967-08|United States|New Mexico|658[0n]|BOLD:AAH8075
Hypotrix rubra|[7248]|RDNMG910-08|United States|New Mexico|658[0n]|BOLD:AAH8075
Hypotrix rubra|[7249]|RDNMF833-08|United States|Arizona|658[0n]|BOLD:AAH8075
Hypotrix diplogramma|[7250]|RDNMF831-08|United States|Arizona|658[0n]|BOLD:AAH8074
Hypotrix diplogramma|[7251]|RDNMF832-08|United States|Arizona|658[0n]|BOLD:AAH8074
Hypotrix ferricola|[7252]|NAMUM060-08|United States|Arizona|657[0n]|BOLD:AAF5374
Hypotrix ferricola|[7253]|RDNME970-08|United States|Arizona|658[0n]|BOLD:AAF5374
Hypotrix ferricola|[7254]|RDNMF830-08|United States|Arizona|658[0n]|BOLD:AAF5374
Hypotrix ferricola|[7255]|RDNMJ724-11|United States|Arizona|658[0n]|BOLD:AAF5374
Hypotrix ocularis|[7256]|RDNME1001-08|United States|Arizona|447[0n]||
Hypotrix trifascia|[7257]|RDNMJ832-11|United States|New Mexico|658[0n]|BOLD:AAW9460
Hypotrix trifascia|[7258]|LNAUT464-14|United States|New Mexico|658[0n]|BOLD:AAW9460
Hypotrix trifascia|[7259]|LNAUT465-14|United States|New Mexico|658[0n]|BOLD:AAW9460
Hypotrix trifascia|[7260]|LNAUT466-14|United States|New Mexico|658[0n]|BOLD:AAW9460
Hypotrix trifascia|[7261]|LNAUT467-14|United States|New Mexico|658[0n]|BOLD:AAW9460
Hypotrix trifascia|[7262]|LNAUT468-14|United States|New Mexico|658[0n]|BOLD:AAW9460
Eriopyga new sp.|[7263]|IAWLB520-11|United States|Arizona|658[0n]|BOLD:AAZ0784
Eriopyga new sp.|[7264]|IAWLB522-11|United States|Arizona|658[0n]|BOLD:AAZ0784
Eriopyga new sp.|[7265]|IAWLB521-11|United States|Arizona|658[0n]|BOLD:AAZ0784
Eriopyga new sp.|[7266]|RDNMK568-11|United States|Arizona|658[0n]|BOLD:AAZ0784
Eriopyga new sp.|[7267]|RDNML369-13|United States|Arizona|658[0n]|BOLD:AAZ0784
Hypotrix lunata|[7268]|CMAZA397-10|United States|Arizona|658[0n]|BOLD:AAH5619
Hypotrix lunata|[7269]|CMAZA036-09|United States|Arizona|658[0n]|BOLD:AAH5619
Hypotrix lunata|[7270]|CMAZA545-10|United States|Arizona|658[0n]|BOLD:AAH5619
Hypotrix lunata|[7271]|NAMUM074-08|United States|Arizona|657[0n]|BOLD:AAH5619
Hypotrix lunata|[7272]|RDNMH731-09|United States|Arizona|658[0n]|BOLD:AAH5619
Hypotrix lunata|[7273]|RDNMJ503-11|United States|Arizona|658[0n]|BOLD:AAH5619
Hypotrix lunata|[7274]|CMAZA995-12|United States|Arizona|658[0n]|BOLD:AAH5619
Hypotrix spinosa|[7275]|NAMUM050-08|United States|Arizona|657[0n]|BOLD:AAJ8654
Hypotrix basistriga|[7276]|RDNMG336-08|United States|Arizona|658[0n]|BOLD:AAW9382
Hypotrix naglei|[7277]|RDNMC742-06|United States|Arizona|658[0n]|BOLD:AAF8679
Hypotrix naglei|[7278]|RDNME371-07|United States|Arizona|655[1n]|BOLD:AAF8679
Hypotrix naglei|[7279]|BBLSY565-09|United States|New Mexico|658[0n]|BOLD:AAF8679
Hypotrix optima|[7280]|RDNMG298-08|United States|Arizona|658[0n]|BOLD:AAX0812
Hypotrix optima|[7281]|LNAUT881-14|United States|Arizona|658[0n]|BOLD:AAX0812
Hypotrix alamosa|[7282]|RDNMG295-08|United States|Arizona|658[0n]|BOLD:AAX0826
Hypotrix hueco|[7283]|JBAZ125-09|United States|Arizona|658[0n]|BOLD:AAI8440
Hypotrix hueco|[7284]|RDNMH817-09|United States|Arizona|658[0n]|BOLD:AAI8440
Hypotrix n sp.|[7285]|CNCLB1560-14|United States|Arizona|658[0n]|BOLD:ACO7143
Hypotrix n sp.|[7286]|CNCLB1561-14|United States|Arizona|643[0n]|BOLD:ACO7143
Agrotisia evelinae|[7287]|RDNME855-08|United States|Arizona|658[0n]|BOLD:AAJ1415
Agrotisia evelinae|[7288]|RDNMG326-08|United States|Texas|658[0n]|BOLD:AAJ1415
Lasionycta conjugata|[7289]|RDNM269-05|United States|Colorado|658[0n]|BOLD:ACE5772
Lasionycta conjugata|[7290]|RDNM268-05|United States|Wyoming|658[0n]|BOLD:ACE5772
Lasionycta conjugata|[7291]|RDNM267-05|United States|Wyoming|658[0n]|BOLD:ACE5772
Lasionycta conjugata|[7292]|RDNM270-05|United States|Colorado|514[0n]|BOLD:ACE5772
Lasionycta subdita|[7293]|RDLQ664-07|Canada|Newfoundland and Labrador|611[0n]|BOLD:AAC3208
Lasionycta subdita|[7294]|LCHP380-07|Canada|Manitoba|645[0n]|BOLD:AAC3208
Lasionycta subdita|[7295]|LCHP377-07|Canada|Manitoba|641[0n]|BOLD:AAC3208
Lasionycta subdita|[7296]|LCHP010-07|Canada|Manitoba|658[0n]|BOLD:AAC3208

Lasionycta subdita|[7294]|LCHP380-07|Canada|Manitoba|645[0n]|BOLD:AAC3208
Lasionycta subdita|[7295]|LCHP377-07|Canada|Manitoba|641[0n]|BOLD:AAC3208
Lasionycta subdita|[7296]|LCHP010-07|Canada|Manitoba|658[0n]|BOLD:AAC3208
Lasionycta subdita|[7297]|RDNMB712-05|Canada|Manitoba|658[0n]|BOLD:AAC3208
Lasionycta subdita|[7298]|RDNMB711-05|Canada|Manitoba|658[0n]|BOLD:AAC3208
Lasionycta subdita|[7299]|LCHP150-07|Canada|Manitoba|656[0n]|BOLD:AAC3208
Lasionycta subdita|[7300]|LCHP151-07|Canada|Manitoba|656[0n]|BOLD:AAC3208
Lasionycta subdita|[7301]|LCHP242-07|Canada|Manitoba|658[0n]|BOLD:AAC3208
Lasionycta subdita|[7302]|LCHP418-07|Canada|Manitoba|658[0n]|BOLD:AAC3208
Lasionycta subdita|[7303]|LCHP796-07|Canada|Manitoba|658[0n]|BOLD:AAC3208
Lasionycta subdita|[7304]|CHLEP214-09|Canada|Manitoba|658[0n]|BOLD:AAC3208
Lasionycta fergusoni|[7305]|RDNM264-05|Canada|Alberta|658[0n]|BOLD:ACF5159
Lasionycta fergusoni|[7306]|RDNM265-05|Canada|British Columbia|513[0n]|BOLD:ACF5159
Lasionycta fergusoni|[7307]|LBCG3045-09|Canada|British Columbia|658[0n]|BOLD:ACF5159
Lasionycta fergusoni|[7308]|RDNM266-05|Canada|British Columbia|658[0n]|BOLD:ACF5159
Lasionycta fergusoni|[7309]|LBCG2486-09|Canada|British Columbia|621[0n]|BOLD:ACF5159
Lasionycta fergusoni|[7310]|SSWLC3100-13|Canada|Alberta|565[0n]|BOLD:ACF5159
Lasionycta haida|[7311]|RDNM258-05|Canada|British Columbia|658[0n]|BOLD:ACE5758
Lasionycta haida|[7312]|RDNM257-05|Canada|British Columbia|658[0n]|BOLD:ACE5758
Lasionycta haida|[7313]|RDNM256-05|Canada|British Columbia|658[0n]|BOLD:ACE5758
Lasionycta haida|[7314]|RDNM255-05|Canada|British Columbia|570[0n]|BOLD:ACE5758
Lasionycta haida|[7315]|RDNM259-05|Canada|British Columbia|540[0n]|BOLD:ACE5758
Lasionycta haida|[7316]|RDNM260-05|Canada|British Columbia|565[0n]|BOLD:ACE5758
Lasionycta mutilata|[7317]|RDNM263-05|Canada|British Columbia|658[0n]|BOLD:AAA5423
Lasionycta mutilata|[7318]|RDMAB459-05|Canada|Alberta|658[0n]|BOLD:AAA5423
Lasionycta mutilata|[7319]|RDNM261-05|Canada|British Columbia|658[0n]|BOLD:AAA5423
Lasionycta mutilata|[7320]|RDNM262-05|Canada|British Columbia|658[0n]|BOLD:AAA5423
Lasionycta mutilata|[7321]|LPAB075-08|Canada|Alberta|658[0n]|BOLD:AAA5423
Lasionycta mutilata|[7322]|LBCH2192-10|Canada|British Columbia|658[0n]|BOLD:AAA5423
Lasionycta mutilata|[7323]|LBCH2191-10|Canada|British Columbia|658[0n]|BOLD:AAA5423
Lasionycta mutilata|[7324]|LBCH2190-10|Canada|British Columbia|658[0n]|BOLD:AAA5423
Lasionycta mutilata|[7325]|LBCH2189-10|Canada|British Columbia|658[0n]|BOLD:AAA5423
Lasionycta mutilata|[7326]|LBCH2188-10|Canada|British Columbia|658[0n]|BOLD:AAA5423
Lasionycta mutilata|[7327]|LBCH2127-10|Canada|British Columbia|658[0n]|BOLD:AAA5423
Lasionycta mutilata|[7328]|LBCH2126-10|Canada|British Columbia|658[0n]|BOLD:AAA5423
Lasionycta mutilata|[7329]|LBCH2125-10|Canada|British Columbia|658[0n]|BOLD:AAA5423
Lasionycta mutilata|[7330]|LBCH2124-10|Canada|British Columbia|658[0n]|BOLD:AAA5423
Lasionycta mutilata|[7331]|LBCH2123-10|Canada|British Columbia|658[0n]|BOLD:AAA5423
Lasionycta mutilata|[7332]|LBCH2122-10|Canada|British Columbia|658[0n]|BOLD:AAA5423
Lasionycta mutilata|[7333]|LBCH2121-10|Canada|British Columbia|658[0n]|BOLD:AAA5423
Lasionycta mutilata|[7334]|LBCH2120-10|Canada|British Columbia|658[0n]|BOLD:AAA5423
Lasionycta mutilata|[7335]|LBCH2089-10|Canada|British Columbia|658[0n]|BOLD:AAA5423
Lasionycta mutilata|[7336]|LBCH2028-10|Canada|British Columbia|658[0n]|BOLD:AAA5423
Lasionycta mutilata|[7337]|LBCH1934-10|Canada|British Columbia|658[0n]|BOLD:AAA5423
Lasionycta mutilata|[7338]|LBCH1920-10|Canada|British Columbia|658[0n]|BOLD:AAA5423
Lasionycta mutilata|[7339]|LBCH1828-10|Canada|British Columbia|658[0n]|BOLD:AAA5423
Lasionycta mutilata|[7340]|LBCH1773-10|Canada|British Columbia|658[0n]|BOLD:AAA5423
Lasionycta mutilata|[7341]|LBCH1760-10|Canada|British Columbia|658[0n]|BOLD:AAA5423
Lasionycta mutilata|[7342]|LBCH1749-10|Canada|British Columbia|658[0n]|BOLD:AAA5423
Lasionycta mutilata|[7343]|LBCH1716-10|Canada|British Columbia|658[0n]|BOLD:AAA5423
Lasionycta mutilata|[7344]|LBCH1678-10|Canada|British Columbia|658[0n]|BOLD:AAA5423
Lasionycta mutilata|[7345]|LBCH1579-10|Canada|British Columbia|658[0n]|BOLD:AAA5423
Lasionycta mutilata|[7346]|LBCH1577-10|Canada|British Columbia|658[0n]|BOLD:AAA5423
Lasionycta mutilata|[7347]|LBCH1539-10|Canada|British Columbia|658[0n]|BOLD:AAA5423
Lasionycta mutilata|[7348]|LBCH1538-10|Canada|British Columbia|658[0n]|BOLD:AAA5423
Lasionycta mutilata|[7349]|LBCH1506-10|Canada|British Columbia|658[0n]|BOLD:AAA5423
Lasionycta mutilata|[7350]|LBCH1168-10|Canada|British Columbia|658[0n]|BOLD:AAA5423
Lasionycta mutilata|[7351]|LBCH1115-10|Canada|British Columbia|658[0n]|BOLD:AAA5423
Lasionycta mutilata|[7352]|LBCH1114-10|Canada|British Columbia|658[0n]|BOLD:AAA5423
Lasionycta mutilata|[7353]|LBCH1113-10|Canada|British Columbia|658[0n]|BOLD:AAA5423
Lasionycta mutilata|[7354]|LBCH1112-10|Canada|British Columbia|658[0n]|BOLD:AAA5423
Lasionycta mutilata|[7355]|LBCH1111-10|Canada|British Columbia|658[0n]|BOLD:AAA5423
Lasionycta mutilata|[7356]|LBCH1110-10|Canada|British Columbia|658[0n]|BOLD:AAA5423
Lasionycta mutilata|[7357]|LBCH1109-10|Canada|British Columbia|658[0n]|BOLD:AAA5423
Lasionycta mutilata|[7358]|LBCG3051-09|Canada|British Columbia|658[0n]|BOLD:AAA5423
Lasionycta mutilata|[7359]|LBCG3050-09|Canada|British Columbia|658[0n]|BOLD:AAA5423
Lasionycta mutilata|[7360]|LBCG3049-09|Canada|British Columbia|658[0n]|BOLD:AAA5423
Lasionycta mutilata|[7361]|LBCG3047-09|Canada|British Columbia|658[0n]|BOLD:AAA5423
Lasionycta mutilata|[7362]|LBCG3046-09|Canada|British Columbia|658[0n]|BOLD:AAA5423
Lasionycta mutilata|[7363]|LBCG3044-09|Canada|British Columbia|658[0n]|BOLD:AAA5423
Lasionycta mutilata|[7364]|LBCG3042-09|Canada|British Columbia|658[0n]|BOLD:AAA5423
Lasionycta mutilata|[7365]|LBCG3041-09|Canada|British Columbia|658[0n]|BOLD:AAA5423
Lasionycta mutilata|[7366]|LBCG3040-09|Canada|British Columbia|658[0n]|BOLD:AAA5423
Lasionycta mutilata|[7367]|LBCG3039-09|Canada|British Columbia|658[0n]|BOLD:AAA5423
Lasionycta mutilata|[7368]|LBCG3038-09|Canada|British Columbia|658[0n]|BOLD:AAA5423
Lasionycta mutilata|[7369]|LBCG3037-09|Canada|British Columbia|658[0n]|BOLD:AAA5423
Lasionycta mutilata|[7370]|LBCG3035-09|Canada|British Columbia|658[0n]|BOLD:AAA5423
Lasionycta mutilata|[7371]|LBCG3034-09|Canada|British Columbia|658[0n]|BOLD:AAA5423
Lasionycta mutilata|[7372]|LBCG2832-09|Canada|British Columbia|658[0n]|BOLD:AAA5423
Lasionycta mutilata|[7373]|LBCG2830-09|Canada|British Columbia|658[0n]|BOLD:AAA5423
Lasionycta mutilata|[7374]|LBCG2829-09|Canada|British Columbia|658[0n]|BOLD:AAA5423
Lasionycta mutilata|[7375]|LBCG2770-09|Canada|British Columbia|658[0n]|BOLD:AAA5423
Lasionycta mutilata|[7376]|LBCG2769-09|Canada|British Columbia|658[0n]|BOLD:AAA5423
Lasionycta mutilata|[7377]|LBCG2768-09|Canada|British Columbia|658[0n]|BOLD:AAA5423
Lasionycta mutilata|[7378]|LBCG2485-09|Canada|British Columbia|658[0n]|BOLD:AAA5423
Lasionycta mutilata|[7379]|LBCG2484-09|Canada|British Columbia|658[0n]|BOLD:AAA5423
Lasionycta mutilata|[7380]|LBCG2482-09|Canada|British Columbia|658[0n]|BOLD:AAA5423
Lasionycta mutilata|[7381]|LBCG2481-09|Canada|British Columbia|658[0n]|BOLD:AAA5423
Lasionycta mutilata|[7382]|LBCG2480-09|Canada|British Columbia|658[0n]|BOLD:AAA5423
Lasionycta mutilata|[7383]|LBCG2479-09|Canada|British Columbia|658[0n]|BOLD:AAA5423
Lasionycta mutilata|[7384]|LBCG2478-09|Canada|British Columbia|658[0n]|BOLD:AAA5423
Lasionycta mutilata|[7385]|LBCG2477-09|Canada|British Columbia|658[0n]|BOLD:AAA5423
Lasionycta mutilata|[7386]|LBCG2476-09|Canada|British Columbia|658[0n]|BOLD:AAA5423
Lasionycta mutilata|[7387]|LBCG1899-09|Canada|British Columbia|658[0n]|BOLD:AAA5423
Lasionycta mutilata|[7388]|LBCG1898-09|Canada|British Columbia|658[0n]|BOLD:AAA5423
Lasionycta mutilata|[7389]|LBCG1897-09|Canada|British Columbia|658[0n]|BOLD:AAA5423
Lasionycta mutilata|[7390]|LBCG1896-09|Canada|British Columbia|658[0n]|BOLD:AAA5423
Lasionycta mutilata|[7391]|LBCG1895-09|Canada|British Columbia|658[0n]|BOLD:AAA5423
Lasionycta mutilata|[7392]|LBCG1894-09|Canada|British Columbia|658[0n]|BOLD:AAA5423
Lasionycta mutilata|[7393]|LBCG1893-09|Canada|British Columbia|658[0n]|BOLD:AAA5423
Lasionycta mutilata|[7394]|LBCG1891-09|Canada|British Columbia|658[0n]|BOLD:AAA5423
Lasionycta mutilata|[7395]|LBCG1890-09|Canada|British Columbia|658[0n]|BOLD:AAA5423
Lasionycta mutilata|[7396]|LBCG1889-09|Canada|British Columbia|658[0n]|BOLD:AAA5423

Lasionycta mutilata|[7394]|LBCG1891-09|Canada|British Columbia|658[0n]|BOLD:AAA5423
Lasionycta mutilata|[7395]|LBCG1890-09|Canada|British Columbia|658[0n]|BOLD:AAA5423
Lasionycta mutilata|[7396]|LBCG1889-09|Canada|British Columbia|658[0n]|BOLD:AAA5423
Lasionycta mutilata|[7397]|LBCG1888-09|Canada|British Columbia|658[0n]|BOLD:AAA5423
Lasionycta mutilata|[7398]|LBCG1887-09|Canada|British Columbia|658[0n]|BOLD:AAA5423
Lasionycta mutilata|[7399]|LBCG2475-09|Canada|British Columbia|631[1n]|BOLD:AAA5423
Lasionycta mutilata|[7400]|LBCG3052-09|Canada|British Columbia|631[0n]|BOLD:AAA5423
Lasionycta mutilata|[7401]|LBCH1852-10|Canada|British Columbia|641[0n]|BOLD:AAA5423
Lasionycta mutilata|[7402]|LBCH1868-10|Canada|British Columbia|642[0n]|BOLD:AAA5423
Lasionycta mutilata|[7403]|LBCH2193-10|Canada|British Columbia|647[0n]|BOLD:AAA5423
Lasionycta mutilata|[7404]|LBCH2194-10|Canada|British Columbia|658[0n]|BOLD:AAA5423
Lasionycta mutilata|[7405]|LBCH2195-10|Canada|British Columbia|658[0n]|BOLD:AAA5423
Lasionycta mutilata|[7406]|LBCH2257-10|Canada|British Columbia|658[0n]|BOLD:AAA5423
Lasionycta mutilata|[7407]|LBCH2258-10|Canada|British Columbia|658[0n]|BOLD:AAA5423
Lasionycta mutilata|[7408]|LBCH2259-10|Canada|British Columbia|658[0n]|BOLD:AAA5423
Lasionycta mutilata|[7409]|LBCH2260-10|Canada|British Columbia|658[0n]|BOLD:AAA5423
Lasionycta mutilata|[7410]|LBCH2261-10|Canada|British Columbia|658[0n]|BOLD:AAA5423
Lasionycta mutilata|[7411]|LBCH2262-10|Canada|British Columbia|658[0n]|BOLD:AAA5423
Lasionycta mutilata|[7412]|LBCH2263-10|Canada|British Columbia|658[0n]|BOLD:AAA5423
Lasionycta mutilata|[7413]|LBCH2264-10|Canada|British Columbia|658[0n]|BOLD:AAA5423
Lasionycta taigata|[7414]|RDLQB232-05|Canada|Quebec|658[0n]|BOLD:AAB7616
Lasionycta taigata|[7415]|RDLQB245-05|Canada|Quebec|658[0n]|BOLD:AAB7616
Lasionycta taigata|[7416]|RDNMF580-08|Canada|Quebec|658[0n]|BOLD:AAB7616
Lasionycta taigata|[7417]|RDNMB713-05|Canada|Quebec|658[0n]|BOLD:AAB7616
Lasionycta taigata|[7418]|RDLQB247-05|Canada|Quebec|658[0n]|BOLD:AAB7616
Lasionycta taigata|[7419]|RDNMF582-08|Canada|Quebec|658[0n]|BOLD:AAB7616
Lasionycta taigata|[7420]|RDNMB714-05|Canada|Manitoba|658[0n]|BOLD:AAB7616
Lasionycta taigata|[7421]|LCHP609-07|Canada|Manitoba|658[0n]|BOLD:AAB7616
Lasionycta taigata|[7422]|LCHP171-07|Canada|Manitoba|658[0n]|BOLD:AAB7616
Lasionycta taigata|[7423]|LCHP363-07|Canada|Manitoba|632[0n]|BOLD:AAB7616
Lasionycta taigata|[7424]|LCHP375-07|Canada|Manitoba|654[0n]|BOLD:AAB7616
Lasionycta taigata|[7425]|LCHP378-07|Canada|Manitoba|655[0n]|BOLD:AAB7616
Lasionycta taigata|[7426]|LCHP483-07|Canada|Manitoba|658[0n]|BOLD:AAB7616
Lasionycta taigata|[7427]|RDNMF581-08|Canada|Alberta|658[0n]|BOLD:AAB7616
Lasionycta taigata|[7428]|LPMN713-08|Canada|Manitoba|658[0n]|BOLD:AAB7616
Lasionycta skraelingia|[7429]|RDNMF579-08|Canada|Yukon Territory|658[0n]|BOLD:AAB7616
Lasionycta skraelingia|[7430]|RDNME679-08|Canada|Yukon Territory|658[0n]|BOLD:AAB7616
Lasionycta taigata|[7431]|RDLQB246-05|Canada|Quebec|658[0n]|BOLD:AAB7616
Lasionycta taigata|[7432]|RDLQB233-05|Canada|Quebec|658[0n]|BOLD:AAB7616
Lasionycta taigata|[7433]|RDNMH187-09|Canada|Newfoundland and Labrador|637[0n]|BOLD:AAB7616
Orthodes detracta|[7434]|TMNBB309-06|Canada|New Brunswick|658[0n]|BOLD:AAA6122
Orthodes detracta|[7435]|RDLQB927-05|Canada|Quebec|658[0n]|BOLD:AAA6122
Orthodes detracta|[7436]|RDLQB926-05|Canada|Quebec|658[0n]|BOLD:AAA6122
Orthodes detracta|[7437]|RDLQB057-05|Canada|Quebec|658[0n]|BOLD:AAA6122
Orthodes detracta|[7438]|RDLQB056-05|Canada|Quebec|658[0n]|BOLD:AAA6122
Orthodes detracta|[7439]|RDLQB054-05|Canada|Quebec|658[0n]|BOLD:AAA6122
Orthodes detracta|[7440]|RDLQB053-05|Canada|Quebec|658[0n]|BOLD:AAA6122
Orthodes detracta|[7441]|RDLQB051-05|Canada|Quebec|658[0n]|BOLD:AAA6122
Orthodes detracta|[7442]|RDLQB049-05|Canada|Quebec|658[0n]|BOLD:AAA6122
Orthodes detracta|[7443]|RDLQB047-05|Canada|Quebec|658[0n]|BOLD:AAA6122
Orthodes detracta|[7444]|RDLQB045-05|Canada|Quebec|658[0n]|BOLD:AAA6122
Orthodes detracta|[7445]|RDLQB055-05|Canada|Quebec|602[0n]|BOLD:AAA6122
Orthodes detracta|[7446]|TMNBB310-06|Canada|New Brunswick|656[0n]|BOLD:AAA6122
Orthodes detracta|[7447]|TMNBB412-06|Canada|New Brunswick|658[0n]|BOLD:AAA6122
Orthodes detracta|[7448]|RDLQG191-06|Canada|Quebec|658[0n]|BOLD:AAA6122
Orthodes detracta|[7449]|BBLPA649-10|Canada|Ontario|658[0n]|BOLD:AAA6122
Orthodes detracta|[7450]|LGSM663-04|United States|North Carolina|658[0n]|BOLD:AAA6122
Orthodes detracta|[7451]|IAWLB387-11|United States|Virginia|658[0n]|BOLD:AAA6122
Orthodes detracta|[7452]|LBCH354-10|Canada|British Columbia|658[0n]|BOLD:AAA6122
Orthodes detracta|[7453]|BBLCU045-09|United States|Michigan|658[0n]|BOLD:AAA6122
Orthodes detracta|[7454]|NAMUM343-08|United States|West Virginia|658[0n]|BOLD:AAA6122
Orthodes detracta|[7455]|LPMN250-08|Canada|Manitoba|658[0n]|BOLD:AAA6122
Orthodes detracta|[7456]|LPMN089-08|Canada|Manitoba|658[0n]|BOLD:AAA6122
Orthodes detracta|[7457]|XAJ850-06|Canada|Ontario|658[0n]|BOLD:AAA6122
Orthodes detracta|[7458]|LOWCD748-06|Canada|British Columbia|658[0n]|BOLD:AAA6122
Orthodes detracta|[7459]|LOWCD747-06|Canada|British Columbia|657[0n]|BOLD:AAA6122
Orthodes detracta|[7460]|LOWCD744-06|Canada|British Columbia|658[0n]|BOLD:AAA6122
Orthodes detracta|[7461]|LOWCD743-06|Canada|British Columbia|658[0n]|BOLD:AAA6122
Orthodes detracta|[7462]|LOWCD742-06|Canada|British Columbia|658[0n]|BOLD:AAA6122
Orthodes detracta|[7463]|LOWCD741-06|Canada|British Columbia|658[0n]|BOLD:AAA6122
Orthodes detracta|[7464]|LOWCD738-06|Canada|British Columbia|658[0n]|BOLD:AAA6122
Orthodes detracta|[7465]|RDLQB058-05|Canada|Quebec|657[0n]|BOLD:AAA6122
Orthodes detracta|[7466]|RDLQB052-05|Canada|Quebec|658[0n]|BOLD:AAA6122
Orthodes detracta|[7467]|RDLQB048-05|Canada|Quebec|658[0n]|BOLD:AAA6122
Orthodes detracta|[7468]|LOWCB315-05|Canada|British Columbia|658[0n]|BOLD:AAA6122
Orthodes detracta|[7469]|LNC072-05|United States|North Carolina|658[0n]|BOLD:AAA6122
Orthodes detracta|[7470]|LNC071-05|United States|North Carolina|658[0n]|BOLD:AAA6122
Orthodes detracta|[7471]|LPMN343-08|Canada|Manitoba|658[0n]|BOLD:AAA6122
Orthodes detracta|[7472]|LGSM657-04|United States|North Carolina|658[0n]|BOLD:AAA6122
Orthodes detracta|[7473]|MMNA084-08|United States|North Carolina|636[0n]|BOLD:AAA6122
Orthodes detracta|[7474]|LBCA601-05|Canada|British Columbia|638[0n]|BOLD:AAA6122
Orthodes detracta|[7475]|LOWCB319-05|Canada|British Columbia|574[0n]|BOLD:AAA6122
Orthodes detracta|[7476]|LOWCD745-06|Canada|British Columbia|610[0n]|BOLD:AAA6122
Orthodes detracta|[7477]|LOWCD740-06|Canada|British Columbia|610[0n]|BOLD:AAA6122
Orthodes detracta|[7478]|LOWCD746-06|Canada|British Columbia|582[0n]|BOLD:AAA6122
Orthodes detracta|[7479]|LOWCD736-06|Canada|British Columbia|595[0n]|BOLD:AAA6122
Orthodes detracta|[7480]|LOWCC091-05|Canada|British Columbia|587[0n]|BOLD:AAA6122
Orthodes detracta|[7481]|RDNMB251-05|Canada|Quebec|588[0n]|BOLD:AAA6122
Orthodes detracta|[7482]|LOWCB321-05|Canada|British Columbia|606[0n]|BOLD:AAA6122
Orthodes detracta|[7483]|LOWCB318-05|Canada|British Columbia|606[0n]|BOLD:AAA6122
Orthodes detracta|[7484]|LOWCB312-05|Canada|British Columbia|606[0n]|BOLD:AAA6122
Orthodes detracta|[7485]|RDLQB046-05|Canada|Quebec|524[1n]|BOLD:AAA6122
Orthodes detracta|[7486]|LOWCB311-05|Canada|British Columbia|606[0n]|BOLD:AAA6122
Orthodes detracta|[7487]|RDNMB249-05|Canada|British Columbia|554[1n]|BOLD:AAA6122
Orthodes detracta|[7488]|LOWCD735-06|Canada|British Columbia|576[0n]|BOLD:AAA6122
Orthodes detracta|[7489]|LOWCB320-05|Canada|British Columbia|577[1n]|BOLD:AAA6122
Orthodes detracta|[7490]|LOWCB310-05|Canada|British Columbia|591[0n]|BOLD:AAA6122
Orthodes detracta|[7491]|LOWCB309-05|Canada|British Columbia|579[0n]|BOLD:AAA6122
Orthodes detracta|[7492]|LOWCB313-05|Canada|British Columbia|595[0n]|BOLD:AAA6122
Orthodes detracta|[7493]|LOWCC867-05|Canada|British Columbia|599[0n]|BOLD:AAA6122
Orthodes detracta|[7494]|LOWCD739-06|Canada|British Columbia|564[0n]|BOLD:AAA6122
Orthodes detracta|[7495]|LOWCE275-06|Canada|British Columbia|605[0n]|BOLD:AAA6122
Orthodes detracta|[7496]|RDNME246-07|Canada|Alberta|620[1n]|BOLD:AAA6122

Orthodes detracta|[7494]|LOWCD739-06|Canada|British Columbia|564[0n]|BOLD:AAA6122
Orthodes detracta|[7495]|LOWCE275-06|Canada|British Columbia|605[0n]|BOLD:AAA6122
Orthodes detracta|[7496]|RDNME246-07|Canada|Alberta|620[1n]|BOLD:AAA6122
Orthodes detracta|[7497]|CNRME052-12|Canada|Manitoba|634[0n]|BOLD:AAA6122
Orthodes detracta|[7498]|CNSLE371-12|Canada|Ontario|638[0n]|BOLD:AAA6122
Lasionycta luteola|[7499]|RDNMB499-05|Canada|British Columbia|534[0n]|BOLD:AAE5146
Lasionycta luteola|[7500]|RDNM403-05|Canada|British Columbia|658[0n]|BOLD:AAE5146
Lasionycta luteola|[7501]|RDNM402-05|Canada|British Columbia|581[0n]|BOLD:AAE5146
Lasionycta luteola|[7502]|LPABB810-09|Canada|Alberta|658[0n]|BOLD:AAE5146
Lasionycta phaea|[7503]|RDNMB710-05|Canada|Nunavut|594[0n]|BOLD:AAX0117
Lasionycta secedens|[7504]|RDLQ643-07|Canada|Quebec|514[8n]|
Lasionycta secedens|[7505]|RDLQ644-07|Canada|Quebec|632[0n]|BOLD:AAB0756
Lasionycta secedens|[7506]|LOWCD266-06|Canada|British Columbia|658[0n]|BOLD:AAB0756
Lasionycta secedens|[7507]|LCHQ134-07|Canada|Manitoba|658[4n]|BOLD:AAB0756
Lasionycta secedens|[7508]|LOWCC102-05|Canada|British Columbia|596[0n]|BOLD:AAB0756
Lasionycta secedens|[7509]|RDNMB729-05|Canada|Quebec|658[0n]|BOLD:AAB0756
Lasionycta secedens|[7510]|RDNMB730-05|Canada|Quebec|658[0n]|BOLD:AAB0756
Lasionycta secedens|[7511]|LOWCC098-05|Canada|British Columbia|658[0n]|BOLD:AAB0756
Lasionycta secedens|[7512]|LOWCC099-05|Canada|British Columbia|658[0n]|BOLD:AAB0756
Lasionycta secedens|[7513]|LOWCC100-05|Canada|British Columbia|658[0n]|BOLD:AAB0756
Lasionycta secedens|[7514]|LOWCC101-05|Canada|British Columbia|658[0n]|BOLD:AAB0756
Lasionycta secedens|[7515]|LOWCC103-05|Canada|British Columbia|658[0n]|BOLD:AAB0756
Lasionycta secedens|[7516]|LOWCC104-05|Canada|British Columbia|658[0n]|BOLD:AAB0756
Lasionycta secedens|[7517]|LOWCC105-05|Canada|British Columbia|658[0n]|BOLD:AAB0756
Lasionycta secedens|[7518]|LOWCC106-05|Canada|British Columbia|658[0n]|BOLD:AAB0756
Lasionycta secedens|[7519]|LOWCC107-05|Canada|British Columbia|658[0n]|BOLD:AAB0756
Lasionycta secedens|[7520]|LOWCD265-06|Canada|British Columbia|658[0n]|BOLD:AAB0756
Lasionycta secedens|[7521]|LOWCD267-06|Canada|British Columbia|658[0n]|BOLD:AAB0756
Lasionycta secedens|[7522]|RDNME516-08|Canada|Yukon Territory|658[1n]|BOLD:AAB0756
Lasionycta secedens|[7523]|LCHP307-07|Canada|Manitoba|658[0n]|BOLD:AAB0756
Lasionycta secedens|[7524]|LOWCD268-06|Canada|British Columbia|658[0n]|BOLD:AAB0756
Lasionycta secedens|[7525]|LCHP306-07|Canada|Manitoba|656[0n]|BOLD:AAB0756
Lasionycta secedens|[7526]|LCH317-04|Canada|Manitoba|658[0n]|BOLD:AAB0756
Lasionycta secedens|[7527]|LCHQ037-07|Canada|Manitoba|658[0n]|BOLD:AAB0756
Lasionycta secedens|[7528]|LCHQ033-07|Canada|Manitoba|658[0n]|BOLD:AAB0756
Lasionycta secedens|[7529]|LOWCD269-06|Canada|British Columbia|658[0n]|BOLD:AAB0756
Lasionycta secedens|[7530]|LOWCC159-05|Canada|British Columbia|658[0n]|BOLD:AAB0756
Lasionycta secedens|[7531]|LOWCC158-05|Canada|British Columbia|658[0n]|BOLD:AAB0756
Lasionycta secedens|[7532]|LCHP412-07|Canada|Manitoba|658[1n]|BOLD:AAB0756
Lasionycta secedens|[7533]|LCHP376-07|Canada|Manitoba|622[0n]|BOLD:AAB0756
Lasionycta secedens|[7534]|LPMN760-08|Canada|Manitoba|567[0n]|BOLD:AAB0756
Lasionycta leucocycla|[7535]|RDNMB753-05|Canada|Ontario|568[1n]|BOLD:ACF3546
Lasionycta leucocycla|[7536]|RDNMB683-05|Canada|Manitoba|537[2n]|BOLD:ACF3546
Lasionycta leucocycla|[7537]|RDNMB758-05|Canada|Nunavut|572[1n]|BOLD:ACF3546
Lasionycta leucocycla|[7538]|RDNMB696-05|Canada|Nunavut|540[1n]|BOLD:ACF3546
Lasionycta leucocycla|[7539]|RDNMB698-05|Canada|Yukon Territory|530[0n]|BOLD:ACF3546
Lasionycta leucocycla|[7540]|RDNM444-05|Canada|Northwest Territories|658[0n]|BOLD:ACF3546
Lasionycta leucocycla|[7541]|RDNMB699-05|Canada|Yukon Territory|557[0n]|BOLD:ACF3546
Lasionycta leucocycla|[7542]|RDNMB700-05|Canada|Yukon Territory|557[0n]|BOLD:ACF3546
Lasionycta leucocycla|[7543]|RDNMB697-05|Canada|Yukon Territory|544[0n]|BOLD:ACF3546
Lasionycta frigida|[7544]|RDNM426-05|Canada|Alberta|658[1n]|BOLD:ACF3546
Lasionycta frigida|[7545]|RDNM445-05|Canada|Alberta|658[0n]|BOLD:ACF3546
Lasionycta leucocycla|[7546]|RDNMB682-05|Canada|Yukon Territory|658[0n]|BOLD:ACF3546
Lasionycta leucocycla|[7547]|RDNMB681-05|Canada|Yukon Territory|658[0n]|BOLD:ACF3546
Lasionycta leucocycla|[7548]|RDNMB494-05|Canada|British Columbia|658[0n]|BOLD:ACF3546
Lasionycta leucocycla|[7549]|RDNMB493-05|Canada|British Columbia|658[0n]|BOLD:ACF3546
Lasionycta leucocycla|[7550]|RDNMB491-05|Canada|British Columbia|658[0n]|BOLD:ACF3546
Lasionycta leucocycla|[7551]|RDNM405-05|Canada|British Columbia|572[0n]|BOLD:ACF3546
Lasionycta leucocycla|[7552]|RDNM404-05|Canada|Yukon Territory|564[0n]|BOLD:ACF3546
Lasionycta leucocycla|[7553]|RDNMB692-05|Canada|Nunavut|573[0n]|BOLD:ACF3546
Lasionycta leucocycla|[7554]|RDNMB703-05|Canada|British Columbia|603[0n]|BOLD:ACF3546
Lasionycta leucocycla|[7555]|RDNMB695-05|Canada|Nunavut|544[0n]|BOLD:ACF3546
Lasionycta leucocycla|[7556]|RDNMB694-05|Canada|Nunavut|570[0n]|BOLD:ACF3546
Lasionycta leucocycla|[7557]|RDNMB756-05|Canada|Nunavut|573[0n]|BOLD:ACF3546
Lasionycta leucocycla|[7558]|RDNMB701-05|Canada|Yukon Territory|577[0n]|BOLD:ACF3546
Lasionycta leucocycla|[7559]|RDNMB508-05|Canada|Manitoba|558[0n]|BOLD:ACF3546
Lasionycta leucocycla|[7560]|RDNMB509-05|Canada|Manitoba|596[0n]|BOLD:ACF3546
Lasionycta anthracina|[7561]|RDNMB506-05|Canada|Alberta|568[1n]|BOLD:ACF3546
Lasionycta leucocycla|[7562]|RDNMB705-05|Canada|British Columbia|567[2n]|BOLD:ACF3546
Lasionycta leucocycla|[7563]|RDNMB693-05|Canada|Nunavut|576[0n]|BOLD:ACF3546
Lasionycta leucocycla|[7564]|RDNM451-05|Canada|Manitoba|658[0n]|BOLD:ACF3546
Lasionycta leucocycla|[7565]|RDNMB688-05|Canada|Manitoba|614[0n]|BOLD:ACF3546
Lasionycta leucocycla|[7566]|RDNMB691-05|Canada|Manitoba|658[0n]|BOLD:ACF3546
Lasionycta leucocycla|[7567]|RDNMB687-05|Canada|Manitoba|655[0n]|BOLD:ACF3546
Lasionycta leucocycla|[7568]|RDNMB689-05|Canada|Manitoba|657[0n]|BOLD:ACF3546
Lasionycta leucocycla|[7569]|RDNMB690-05|Canada|Manitoba|658[0n]|BOLD:ACF3546
Lasionycta leucocycla|[7570]|RDNMB492-05|Canada|British Columbia|658[0n]|BOLD:ACF3546
Lasionycta leucocycla|[7571]|RDNMB490-05|Canada|British Columbia|658[0n]|BOLD:ACF3546
Lasionycta leucocycla|[7572]|RDNMB489-05|Canada|Yukon Territory|658[0n]|BOLD:ACF3546
Lasionycta leucocycla|[7573]|RDNMB704-05|Canada|British Columbia|605[0n]|BOLD:ACF3546
Lasionycta leucocycla|[7574]|RDNM406-05|Canada|British Columbia|593[0n]|BOLD:ACF3546
Lasionycta leucocycla|[7575]|RDNMB702-05|Canada|British Columbia|615[2n]|BOLD:ACF3546
Lasionycta leucocycla|[7576]|RDNMB757-05|Canada|Nunavut|605[0n]|BOLD:ACF3546
Lasionycta leucocycla|[7577]|MNAG254-08|Canada|Nunavut|658[0n]|BOLD:ACF3546
Lasionycta leucocycla|[7578]|LARC047-10|Canada|Nunavut|658[0n]|BOLD:ACF3546
Lasionycta leucocycla|[7579]|RDNMC351-05|Canada|Quebec|564[0n]|BOLD:ACF3546
Lasionycta anthracina|[7580]|RDNM272-05|Canada|Quebec|658[0n]|BOLD:ACF3546
Lasionycta anthracina|[7581]|RDLQB075-05|Canada|Quebec|658[0n]|BOLD:ACF3546
Lasionycta anthracina|[7582]|RDLQB076-05|Canada|Quebec|658[0n]|BOLD:ACF3546
Lasionycta flanda|[7583]|RDNMH188-09|Canada|Newfoundland and Labrador|637[0n]|BOLD:ACF3546
Lasionycta leucocycla|[7584]|RDNM407-05|Canada|Nunavut|535[0n]|BOLD:ACF3546
Lasionycta coracina|[7585]|RDNMB752-05|Canada|Yukon Territory|546[1n]|BOLD:ACF3546
Lasionycta leucocycla|[7586]|RDNMB686-05|Canada|Manitoba|591[1n]|BOLD:ACF3546
Lasionycta coracina|[7587]|RDNMB510-05|Canada|Yukon Territory|613[4n]|BOLD:ACF3546
Lasionycta leucocycla|[7588]|RDNMB507-05|Canada|Manitoba|576[0n]|BOLD:ACF3546
Lasionycta leucocycla|[7589]|RDNMB685-05|Canada|Manitoba|616[1n]|BOLD:ACF3546
Lasionycta leucocycla|[7590]|RDNM450-05|Canada|Manitoba|658[0n]|BOLD:ACF3546
Lasionycta leucocycla|[7591]|LCHP069-07|Canada|Manitoba|658[0n]|BOLD:ACF3546
Lasionycta staudingeri|[7592]|LEFIK438-10|Finland||658[0n]|BOLD:ACF3546
Lasionycta staudingeri|[7593]|LEFIK439-10|Finland||658[0n]|BOLD:ACF3546
Lasionycta staudingeri|[7594]|LEFIK440-10|Finland||658[0n]|BOLD:ACF3546
Lasionycta subfumosa|[7595]|LEFII274-11|Canada||658[0n]|BOLD:ACF3546
Lasionycta subfumosa|[7596]|LEFII275-11|Canada||658[0n]|BOLD:ACF3546

Lasionycta staudingeri|[7594]|LEFIK440-10|Finland||658[0n]|BOLD:ACF3546
Lasionycta subfumosa|[7595]|LEFII274-11|Canada||658[0n]|BOLD:ACF3546
Lasionycta subfumosa|[7596]|LEFII275-11|Canada||658[0n]|BOLD:ACF3546
Lasionycta staudingeri|[7597]|RDNM420-05|Canada|Nunavut|546[0n]|BOLD:ACF3546
Lasionycta staudingeri|[7598]|RDNM419-05|Canada|Nunavut|544[0n]|BOLD:ACF3546
Lasionycta staudingeri|[7599]|RDNMB754-05|Canada|Yukon Territory|657[1n]|BOLD:ACF3546
Lasionycta staudingeri|[7600]|RDNMB755-05|Canada|Yukon Territory|658[0n]|BOLD:ACF3546
Lasionycta staudingeri|[7601]|RDNMB751-05|United States|Alaska|570[0n]|BOLD:ACF3546
Lasionycta staudingeri|[7602]|RDNMK712-11|Canada|Northwest Territories|658[0n]|BOLD:ACF3546
Lasionycta subalpina|[7603]|RDNMF958-08|United States|Wyoming|658[0n]|BOLD:ACF4310
Lasionycta subalpina|[7604]|RDNMF959-08|United States|Wyoming|658[0n]|BOLD:ACF4310
Lasionycta subalpina|[7605]|RDNMF960-08|United States|Texas|658[0n]|BOLD:ACF4310
Lasionycta subalpina|[7606]|RDNMC584-06|United States|Wyoming|591[0n]|BOLD:ACF4310
Lasionycta subalpina|[7607]|RDNMC585-06|United States|Wyoming|569[3n]|BOLD:ACF4310
Lasionycta subalpina|[7608]|RDNMG105-08|United States|Wyoming|658[0n]|BOLD:ACF4310
Lasionycta subalpina|[7609]|RDNMG106-08|United States|Wyoming|658[0n]|BOLD:ACF4310
Lasionycta subalpina|[7610]|RDNMH048-09|United States|Wyoming|658[0n]|BOLD:ACF4310
Lasionycta benjamini|[7611]|RDNM424-05|United States|Colorado|658[0n]|BOLD:ACF3554
Lasionycta benjamini|[7612]|RDNMB715-05|United States|Colorado|615[0n]|BOLD:ACF3554
Lasionycta benjamini|[7613]|RDNMB716-05|United States|Colorado|615[0n]|BOLD:ACF3554
Lasionycta benjamini|[7614]|RDNMB745-05|United States|California|588[0n]|BOLD:ACE3544
Lasionycta benjamini|[7615]|RDNM425-05|United States|California|575[0n]|BOLD:ACE3544
Lasionycta benjamini|[7616]|RDNMB746-05|United States|California|571[0n]|BOLD:ACE3544
Lasionycta benjamini|[7617]|RDNMB747-05|United States|California|574[0n]|BOLD:ACE3544
Lasionycta silacea|[7618]|RDNM176-05|Canada|British Columbia|658[0n]|BOLD:AAA8091
Lasionycta silacea|[7619]|RDNM178-05|Canada|British Columbia|658[0n]|BOLD:AAA8091
Lasionycta silacea|[7620]|RDNM183-05|United States|Washington|570[0n]|BOLD:AAA8091
Lasionycta silacea|[7621]|RDNM436-05|Canada|British Columbia|658[0n]|BOLD:AAA8091
Lasionycta silacea|[7622]|RDMAB290-05|Canada|Alberta|596[0n]|BOLD:AAA8091
Lasionycta promulsa|[7623]|RDNME680-08|United States|Utah|658[0n]|BOLD:AAA8091
Lasionycta pulverea|[7624]|RDNMB749-05|Canada|Alberta|658[0n]|BOLD:AAA8091
Lasionycta sierra|[7625]|RDNM384-05|United States|California|567[0n]|BOLD:AAA8091
Lasionycta sierra|[7626]|RDNM383-05|United States|California|553[0n]|BOLD:AAA8091
Lasionycta sierra|[7627]|RDNM385-05|United States|California|556[0n]|BOLD:AAA8091
Lasionycta pulverea|[7628]|RDNM452-05|Canada|Alberta|601[0n]|BOLD:AAA8091
Lasionycta pulverea|[7629]|RDNMB750-05|Canada|Alberta|554[0n]|BOLD:AAA8091
Lasionycta pulverea|[7630]|RDNMB748-05|Canada|Alberta|560[0n]|BOLD:AAA8091
Lasionycta pulverea|[7631]|RDNM453-05|Canada|Alberta|542[0n]|BOLD:AAA8091
Lasionycta pulverea|[7632]|RDMAB460-05|Canada|Alberta|596[0n]|BOLD:AAA8091
Lasionycta promulsa|[7633]|RDNMB742-05|Canada|Alberta|658[0n]|BOLD:AAA8091
Lasionycta promulsa|[7634]|RDNMB740-05|Canada|Alberta|658[0n]|BOLD:AAA8091
Lasionycta promulsa|[7635]|RDNMB738-05|Canada|Alberta|658[0n]|BOLD:AAA8091
Lasionycta promulsa|[7636]|RDNMB737-05|Canada|Alberta|658[0n]|BOLD:AAA8091
Lasionycta promulsa|[7637]|RDNM427-05|Canada|Alberta|658[0n]|BOLD:AAA8091
Lasionycta promulsa|[7638]|RDMAB672-06|Canada|Alberta|562[0n]|BOLD:AAA8091
Lasionycta promulsa|[7639]|RDMAB674-06|Canada|Alberta|561[0n]|BOLD:AAA8091
Lasionycta promulsa|[7640]|RDNMB743-05|Canada|Alberta|658[0n]|BOLD:AAA8091
Lasionycta promulsa|[7641]|BBLPB380-10|Canada|British Columbia|658[0n]|BOLD:AAA8091
Lasionycta promulsa|[7642]|RDNMB739-05|Canada|Alberta|658[0n]|BOLD:AAA8091
Lasionycta promulsa|[7643]|RDNMB741-05|Canada|Alberta|658[0n]|BOLD:AAA8091
Lasionycta promulsa|[7644]|RDNMB736-05|Canada|Alberta|658[0n]|BOLD:AAA8091
Lasionycta promulsa|[7645]|RDNMB735-05|Canada|Alberta|658[0n]|BOLD:AAA8091
Lasionycta promulsa|[7646]|RDNMB733-05|Canada|British Columbia|658[0n]|BOLD:AAA8091
Lasionycta promulsa|[7647]|RDNM435-05|Canada|British Columbia|658[0n]|BOLD:AAA8091
Lasionycta promulsa|[7648]|RDNM434-05|Canada|British Columbia|658[0n]|BOLD:AAA8091
Lasionycta promulsa|[7649]|RDNM433-05|Canada|British Columbia|658[0n]|BOLD:AAA8091
Lasionycta promulsa|[7650]|RDNM431-05|Canada|British Columbia|658[0n]|BOLD:AAA8091
Lasionycta promulsa|[7651]|RDNM430-05|Canada|British Columbia|658[0n]|BOLD:AAA8091
Lasionycta promulsa|[7652]|RDNM429-05|Canada|British Columbia|658[0n]|BOLD:AAA8091
Lasionycta promulsa|[7653]|RDNMB744-05|Canada|Alberta|615[0n]|BOLD:AAA8091
Lasionycta promulsa|[7654]|RDNM428-05|United States|Colorado|579[0n]|BOLD:AAA8091
Lasionycta promulsa|[7655]|RDMAB675-06|Canada|Alberta|658[0n]|BOLD:AAA8091
Lasionycta promulsa|[7656]|RDNMB734-05|Canada|Alberta|598[0n]|BOLD:AAA8091
Lasionycta promulsa|[7657]|RDNM432-05|Canada|British Columbia|581[0n]|BOLD:AAA8091
Lasionycta promulsa|[7658]|RDNMB732-05|Canada|British Columbia|570[0n]|BOLD:AAA8091
Lasionycta promulsa|[7659]|RDMAB673-06|Canada|Alberta|621[1n]|BOLD:AAA8091
Lasionycta promulsa|[7660]|RDNM454-05|Canada|Alberta|524[0n]|BOLD:AAA8091
Lasionycta promulsa|[7661]|RDNME681-08|United States|Utah|658[0n]|BOLD:AAA8091
Lasionycta promulsa|[7662]|RDNME682-08|United States|Utah|658[0n]|BOLD:AAA8091
Lasionycta promulsa|[7663]|BBLPB464-10|Canada|British Columbia|658[0n]|BOLD:AAA8091
Lasionycta impingens|[7664]|RDNM416-05|United States|Colorado|582[0n]|BOLD:AAA8091
Lasionycta impingens|[7665]|LSEU799-06|United States|Colorado|658[0n]|BOLD:AAA8091
Lasionycta impingens|[7666]|LSEU800-06|United States|Colorado|657[0n]|BOLD:AAA8091
Lasionycta impingens|[7667]|LOWCD114-06|Canada|British Columbia|658[0n]|BOLD:AAA8091
Lasionycta impingens|[7668]|RDNMB717-05|Canada|British Columbia|658[0n]|BOLD:AAA8091
Lasionycta impingens|[7669]|RDNM415-05|Canada|British Columbia|592[0n]|BOLD:AAA8091
Lasionycta impingens|[7670]|RDNMB718-05|Canada|Alberta|563[2n]|BOLD:AAA8091
Lasionycta impingens|[7671]|RDNMH054-09|United States|Wyoming|658[0n]|BOLD:AAA8091
Lasionycta impingens|[7672]|RDNMH055-09|United States|Wyoming|658[0n]|BOLD:AAA8091
Lasionycta impingens|[7673]|BBLPB466-10|Canada|Alberta|658[0n]|BOLD:AAA8091
Lasionycta brunnea|[7674]|RDNM374-05|Canada|British Columbia|658[0n]|BOLD:AAA8091
Lasionycta brunnea|[7675]|RDNM369-05|Canada|British Columbia|658[0n]|BOLD:AAA8091
Lasionycta brunnea|[7676]|RDNM180-05|Canada|British Columbia|658[0n]|BOLD:AAA8091
Lasionycta brunnea|[7677]|RDNM377-05|Canada|British Columbia|601[0n]|BOLD:AAA8091
Lasionycta brunnea|[7678]|RDNM179-05|Canada|British Columbia|534[0n]|BOLD:AAA8091
Lasionycta brunnea|[7679]|RDNM181-05|Canada|British Columbia|567[0n]|BOLD:AAA8091
Lasionycta brunnea|[7680]|RDNM380-05|Canada|British Columbia|568[0n]|BOLD:AAA8091
Lasionycta brunnea|[7681]|RDNMB542-05|Canada|British Columbia|658[0n]|BOLD:AAA8091
Lasionycta brunnea|[7682]|RDNME684-08|United States|Washington|658[0n]|BOLD:AAA8091
Lasionycta brunnea|[7683]|RDNME686-08|United States|Washington|658[0n]|BOLD:AAA8091
Lasionycta brunnea|[7684]|LPABB349-08|Canada|Alberta|658[0n]|BOLD:AAA8091
Lasionycta dolosa|[7685]|RDNM417-05|United States|Colorado|558[0n]|BOLD:ACE6976
Lasionycta dolosa|[7686]|RDNM418-05|United States|Colorado|555[2n]|BOLD:ACE6976
Lasionycta discolor|[7687]|RDNM440-05|United States|Colorado|658[0n]|BOLD:AAA8091
Lasionycta discolor|[7688]|RDNM439-05|United States|Colorado|593[0n]|BOLD:AAA8091
Lasionycta discolor|[7689]|RDNM441-05|United States|Colorado|587[0n]|BOLD:AAA8091
Lasionycta discolor|[7690]|RDNM443-05|United States|Colorado|658[0n]|BOLD:AAA8091
Lasionycta discolor|[7691]|RDNM460-05|United States|Colorado|658[0n]|BOLD:AAA8091
Lasionycta discolor|[7692]|RDNMH056-09|United States|Wyoming|658[0n]|BOLD:AAA8091
Lasionycta uniformis|[7693]|RDNMB708-05|United States|California|658[0n]|BOLD:AAA8091
Lasionycta uniformis|[7694]|RDNMB500-05|Canada|British Columbia|658[0n]|BOLD:AAA8091
Lasionycta uniformis|[7695]|RDNM458-05|Canada|British Columbia|658[0n]|BOLD:AAA8091
Lasionycta uniformis|[7696]|RDNM449-05|Canada|British Columbia|658[0n]|BOLD:AAA8091

[illegible]
Lasionycta uniformis|[7694]|RDNMB500-05|Canada|British Columbia|658[0n]|BOLD:AAA8091
Lasionycta uniformis|[7695]|RDNM458-05|Canada|British Columbia|658[0n]|BOLD:AAA8091
Lasionycta uniformis|[7696]|RDNM449-05|Canada|British Columbia|658[0n]|BOLD:AAA8091
Lasionycta uniformis|[7697]|RDNM455-05|Canada|British Columbia|564[0n]|BOLD:AAA8091
Lasionycta uniformis|[7698]|RDNM456-05|Canada|British Columbia|558[0n]|BOLD:AAA8091
Lasionycta uniformis|[7699]|RDNMB504-05|Canada|British Columbia|559[0n]|BOLD:AAA8091
Lasionycta uniformis|[7700]|RDNMB511-05|Canada|British Columbia|658[0n]|BOLD:AAA8091
Lasionycta uniformis|[7701]|RDNM387-05|Canada|British Columbia|658[0n]|BOLD:AAA8091
Lasionycta uniformis|[7702]|RDNM388-05|Canada|British Columbia|658[0n]|BOLD:AAA8091
Lasionycta uniformis|[7703]|RDNM457-05|United States|Washington|658[0n]|BOLD:AAA8091
Lasionycta uniformis|[7704]|RDNM459-05|Canada|British Columbia|658[0n]|BOLD:AAA8091
Lasionycta uniformis|[7705]|RDNM462-05|Canada|British Columbia|658[0n]|BOLD:AAA8091
Lasionycta uniformis|[7706]|RDNMB502-05|Canada|British Columbia|658[0n]|BOLD:AAA8091
Lasionycta uniformis|[7707]|RDNMB588-05|United States|Washington|658[0n]|BOLD:AAA8091
Lasionycta uniformis|[7708]|RDNMB709-05|Canada|British Columbia|658[0n]|BOLD:AAA8091
Lasionycta uniformis|[7709]|RDNM382-05|United States|Colorado|569[1n]|BOLD:AAA8091
Lasionycta uniformis|[7710]|RDNM437-05|United States|Colorado|658[0n]|BOLD:AAA8091
Lasionycta uniformis|[7711]|RDNM381-05|United States|Colorado|528[1n]|BOLD:AAA8091
Lasionycta uniformis|[7712]|RDNM438-05|United States|Colorado|658[0n]|BOLD:AAA8091
Lasionycta uniformis|[7713]|RDNM401-05|United States|Wyoming|658[0n]|BOLD:AAA8091
Lasionycta uniformis|[7714]|RDNM399-05|United States|Wyoming|658[0n]|BOLD:AAA8091
Lasionycta uniformis|[7715]|RDNM370-05|Canada|British Columbia|567[0n]|BOLD:AAA8091
Lasionycta uniformis|[7716]|RDNM400-05|United States|Wyoming|612[0n]|BOLD:AAA8091
Lasionycta uniformis|[7717]|RDNMB539-05|Canada|Alberta|581[1n]|BOLD:AAA8091
Lasionycta uniformis|[7718]|RDNM375-05|Canada|British Columbia|590[0n]|BOLD:AAA8091
Lasionycta uniformis|[7719]|RDNM371-05|Canada|British Columbia|612[0n]|BOLD:AAA8091
Lasionycta uniformis|[7720]|RDNM379-05|Canada|British Columbia|567[0n]|BOLD:AAA8091
Lasionycta uniformis|[7721]|RDNM378-05|Canada|British Columbia|567[0n]|BOLD:AAA8091
Lasionycta uniformis|[7722]|RDNM372-05|Canada|British Columbia|563[0n]|BOLD:AAA8091
Lasionycta uniformis|[7723]|RDNMB540-05|Canada|Alberta|559[0n]|BOLD:AAA8091
Lasionycta uniformis|[7724]|RDNM376-05|Canada|British Columbia|658[0n]|BOLD:AAA8091
Lasionycta uniformis|[7725]|RDNM373-05|Canada|British Columbia|658[0n]|BOLD:AAA8091
Lasionycta uniformis|[7726]|RDNMB541-05|Canada|Alberta|658[0n]|BOLD:AAA8091
Lasionycta uniformis|[7727]|LSEU796-06|United States|Colorado|658[0n]|BOLD:AAA8091
Lasionycta uniformis|[7728]|RDNM386-05|Canada|British Columbia|658[0n]|BOLD:AAA8091
Lasionycta uniformis|[7729]|RDNMB498-05|Canada|British Columbia|658[0n]|BOLD:AAA8091
Lasionycta uniformis|[7730]|RDNMB497-05|Canada|British Columbia|613[0n]|BOLD:AAA8091
Lasionycta uniformis|[7731]|RDNMB503-05|Canada|British Columbia|614[0n]|BOLD:AAA8091
Lasionycta uniformis|[7732]|RDNMB505-05|Canada|British Columbia|658[0n]|BOLD:AAA8091
Lasionycta uniformis|[7733]|RDNMB587-05|Canada|British Columbia|658[0n]|BOLD:AAA8091
Lasionycta phoca|[7734]|RDNMB770-05|Canada|Quebec|534[0n]|BOLD:AAA8091
Lasionycta phoca|[7735]|LCHP235-07|Canada|Manitoba|656[0n]|BOLD:AAA8091
Lasionycta phoca|[7736]|RDLQ663-07|Canada|Newfoundland and Labrador|648[0n]|BOLD:AAA8091
Lasionycta phoca|[7737]|LCHP385-07|Canada|Manitoba|632[0n]|BOLD:AAA8091
Lasionycta phoca|[7738]|LCHP393-07|Canada|Manitoba|658[0n]|BOLD:AAA8091
Lasionycta phoca|[7739]|LCHP308-07|Canada|Manitoba|658[0n]|BOLD:AAA8091
Lasionycta phoca|[7740]|LCHP219-07|Canada|Manitoba|658[0n]|BOLD:AAA8091
Lasionycta phoca|[7741]|RDNM271-05|Canada|Manitoba|658[0n]|BOLD:AAA8091
Lasionycta phoca|[7742]|RDNM185-05|Canada|Manitoba|658[0n]|BOLD:AAA8091
Lasionycta phoca|[7743]|RDNM184-05|Canada|Manitoba|658[0n]|BOLD:AAA8091
Lasionycta phoca|[7744]|LCHP218-07|Canada|Manitoba|636[0n]|BOLD:AAA8091
Lasionycta phoca|[7745]|LCHP823-07|Canada|Manitoba|631[0n]|BOLD:AAA8091
Lasionycta uniformis|[7746]|RDNM446-05|Canada|British Columbia|658[0n]|BOLD:AAA8091
Lasionycta uniformis|[7747]|RDNM447-05|Canada|British Columbia|658[0n]|BOLD:AAA8091
Lasionycta uniformis|[7748]|RDNM448-05|Canada|British Columbia|571[0n]|BOLD:AAA8091
Lasionycta uniformis|[7749]|RDNME683-08|United States|Utah|658[0n]|BOLD:AAA8091
Lasionycta gelida|[7750]|RDNMG095-08|Canada|British Columbia|609[0n]|BOLD:AAA8091
Lasionycta gelida|[7751]|RDNMG096-08|Canada|British Columbia|658[0n]|BOLD:AAA8091
Lasionycta lagganata|[7752]|RDNM442-05|Canada|British Columbia|605[0n]|BOLD:AAA8091
Lasionycta lagganata|[7753]|RDNMG843-08|Canada|British Columbia|595[0n]|BOLD:AAA8091
Lasionycta lagganata|[7754]|RDNMG842-08|Canada|British Columbia|643[1n]|BOLD:AAA8091
Lasionycta lagganata|[7755]|RDNMG844-08|Canada|British Columbia|643[0n]|BOLD:AAA8091
Lasionycta carolynae|[7756]|RDNMH920-09|Canada||658[0n]|BOLD:AAA8091
Lasionycta carolynae|[7757]|RDNMH921-09|Canada||658[0n]|BOLD:AAA8091
Lasionycta carolynae|[7758]|RDNMH922-09|Canada||658[0n]|BOLD:AAA8091
Lasionycta quadrilunata|[7759]|RDNMI115-10|Canada|Yukon Territory|658[0n]|BOLD:AAA8091
Lasionycta quadrilunata|[7760]|RDNM461-05|Canada|Alberta|658[0n]|BOLD:AAA8091
Lasionycta quadrilunata|[7761]|RDNM422-05|United States|Colorado|542[0n]|BOLD:AAA8091
Lasionycta quadrilunata|[7762]|RDNMG845-08|United States|Colorado|658[0n]|BOLD:AAA8091
Lasionycta quadrilunata|[7763]|RDNMK517-11|Canada|Yukon Territory|658[0n]|BOLD:AAA8091
Lasionycta quadrilunata|[7764]|RDNMK518-11|Canada|Yukon Territory|658[0n]|BOLD:AAA8091
Lasionycta quadrilunata|[7765]|RDNMK519-11|Canada|Yukon Territory|658[0n]|BOLD:AAA8091
Lasionycta quadrilunata|[7766]|RDNMK520-11|Canada|Yukon Territory|658[0n]|BOLD:AAA8091
Lasionycta quadrilunata|[7767]|RDNMK521-11|Canada|Yukon Territory|658[0n]|BOLD:AAA8091
Lasionycta subfuscula|[7768]|RDNMB769-05|United States|Colorado|615[0n]|BOLD:ACE5913
Lasionycta subfuscula|[7769]|RDNMB768-05|United States|Colorado|614[0n]|BOLD:ACE5913
Lasionycta subfuscula|[7770]|RDNMB763-05|United States|Colorado|658[0n]|BOLD:ACE5913
Lasionycta subfuscula|[7771]|RDNMB772-05|United States|Colorado|658[0n]|BOLD:ACE5913
Lasionycta subfuscula|[7772]|LSEU795-06|United States|Colorado|658[0n]|BOLD:ACE5913
Lasionycta caesia|[7773]|RDNM239-05|Canada|British Columbia|562[0n]|BOLD:AAA8091
Lasionycta caesia|[7774]|RDNM389-05|Canada|British Columbia|579[0n]|BOLD:AAA8091
Lasionycta caesia|[7775]|RDNM390-05|Canada|British Columbia|658[0n]|BOLD:AAA8091
Lasionycta subfuscula|[7776]|RDNMB519-05|Canada|British Columbia|539[2n]|BOLD:ACF3546
Lasionycta subfuscula|[7777]|RDNMB766-05|United States|Washington|575[0n]|BOLD:ACF3546
Lasionycta subfuscula|[7778]|RDNMB764-05|United States|Oregon|576[0n]|BOLD:ACF3546
Lasionycta subfuscula|[7779]|RDNMB518-05|Canada|British Columbia|576[0n]|BOLD:ACF3546
Lasionycta subfuscula|[7780]|RDNMB762-05|Canada|British Columbia|572[0n]|BOLD:ACF3546
Lasionycta subfuscula|[7781]|RDNMB760-05|Canada|British Columbia|571[0n]|BOLD:ACF3546
Lasionycta subfuscula|[7782]|RDNMB767-05|United States|Washington|606[1n]|BOLD:ACF3546
Lasionycta subfuscula|[7783]|RDNMB516-05|Canada|British Columbia|658[0n]|BOLD:ACF3546
Lasionycta subfuscula|[7784]|RDNMB514-05|Canada|British Columbia|658[0n]|BOLD:ACF3546
Lasionycta subfuscula|[7785]|RDNMB512-05|United States|Oregon|658[0n]|BOLD:ACF3546
Lasionycta subfuscula|[7786]|RDNM397-05|Canada|British Columbia|658[0n]|BOLD:ACF3546
Lasionycta subfuscula|[7787]|RDNMB765-05|United States|Oregon|615[0n]|BOLD:ACF3546
Lasionycta subfuscula|[7788]|RDNMB771-05|Canada|British Columbia|658[0n]|BOLD:ACF3546
Lasionycta subfuscula|[7789]|RDNMJ475-11|United States|Washington|658[0n]|BOLD:ACF3546
Lasionycta subfuscula|[7790]|RDNMG099-08|United States|Oregon|643[0n]|BOLD:ACF3546
Lasionycta subfuscula|[7791]|RDNMH050-09|United States|Utah|658[0n]|BOLD:ACF3546
Lasionycta subfuscula|[7792]|RDNMC038-05|United States|Utah|658[0n]|BOLD:ACF3546
Lasionycta subfuscula|[7793]|RDNMH053-09|United States|Utah|658[0n]|BOLD:ACF3546
Lasionycta subfuscula|[7794]|RDNMB761-05|Canada|British Columbia|579[0n]|BOLD:ACF3546
Lasionycta subfuscula|[7795]|RDNMB517-05|Canada|British Columbia|658[0n]|BOLD:ACF3546
Lasionycta subfuscula|[7796]|RDNMB513-05|Canada|British Columbia|658[0n]|BOLD:ACF3546

[illegible]
Lasionycta subfuscula|[7794]|RDNMB761-05|Canada|British Columbia|579[0n]|BOLD:ACF3546
Lasionycta subfuscula|[7795]|RDNMB517-05|Canada|British Columbia|658[0n]|BOLD:ACF3546
Lasionycta subfuscula|[7796]|RDNMB513-05|Canada|British Columbia|658[0n]|BOLD:ACF3546
Lasionycta subfuscula|[7797]|RDNM398-05|Canada|British Columbia|658[0n]|BOLD:ACF3546
Lasionycta subfuscula|[7798]|RDNMB759-05|Canada|British Columbia|617[0n]|BOLD:ACF3546
Lasionycta subfuscula|[7799]|RDNMB773-05|Canada|British Columbia|658[0n]|BOLD:ACF3546
Lasionycta subfuscula|[7800]|RDNMG097-08|Canada|British Columbia|658[0n]|BOLD:ACF3546
Lasionycta subfuscula|[7801]|RDNMG101-08|Canada|British Columbia|658[0n]|BOLD:ACF3546
Lasionycta subfuscula|[7802]|LPABB116-08|Canada|Alberta|658[0n]|BOLD:ACF3546
Lasionycta subfuscula|[7803]|BBLPB758-10|Canada|Alberta|634[0n]|BOLD:ACF3546
Lasionycta subfuscula|[7804]|SSBAD6368-13|Canada|Alberta|562[0n]|BOLD:ACF3546
Lasionycta perplexella|[7805]|RDNMB724-05|Canada|British Columbia|658[1n]|BOLD:ACF3557
Lasionycta perplexella|[7806]|RDNM392-05|Canada|British Columbia|597[0n]|BOLD:ACF3557
Lasionycta perplexella|[7807]|RDNMB520-05|Canada|British Columbia|658[0n]|BOLD:ACF3557
Lasionycta perplexella|[7808]|RDNMB521-05|United States|Washington|658[0n]|BOLD:ACF3557
Lasionycta perplexella|[7809]|RDNMB725-05|Canada|British Columbia|658[0n]|BOLD:ACF3557
Lasionycta perplexella|[7810]|RDNMB726-05|Canada|British Columbia|658[0n]|BOLD:ACF3557
Lasionycta perplexella|[7811]|RDNMB728-05|Canada|British Columbia|563[0n]|BOLD:ACF3557
Lasionycta perplexa|[7812]|RDNMB515-05|United States|Washington|546[3n]|BOLD:ACF3546
Lasionycta perplexa|[7813]|RDNM393-05|United States|Washington|561[1n]|BOLD:ACF3546
Lasionycta perplexa|[7814]|RDNM394-05|United States|Oregon|658[0n]|BOLD:ACF3546
Lasionycta perplexa|[7815]|RDNM396-05|Canada|British Columbia|658[0n]|BOLD:ACF3546
Lasionycta perplexa|[7816]|RDMAB464-05|Canada|Alberta|585[4n]|BOLD:ACF3546
Lasionycta perplexa|[7817]|LPABB610-08|Canada|Alberta|658[0n]|BOLD:ACF3546
Lasionycta perplexa|[7818]|LPABB627-08|Canada|Alberta|658[0n]|BOLD:ACF3546
Lasionycta perplexa|[7819]|LPABB586-08|Canada|Alberta|658[0n]|BOLD:ACF3546
Lasionycta perplexa|[7820]|LPABB492-08|Canada|Alberta|658[0n]|BOLD:ACF3546
Lasionycta perplexa|[7821]|LPABB485-08|Canada|Alberta|658[0n]|BOLD:ACF3546
Lasionycta perplexa|[7822]|LPABB400-08|Canada|Alberta|658[0n]|BOLD:ACF3546
Lasionycta perplexa|[7823]|LPABB340-08|Canada|Alberta|658[0n]|BOLD:ACF3546
Lasionycta perplexa|[7824]|LPABB268-08|Canada|Alberta|658[0n]|BOLD:ACF3546
Lasionycta perplexa|[7825]|LPABB121-08|Canada|Alberta|658[0n]|BOLD:ACF3546
Lasionycta perplexa|[7826]|LPABB016-08|Canada|Alberta|658[0n]|BOLD:ACF3546
Lasionycta perplexa|[7827]|LSEU798-06|United States|Colorado|658[0n]|BOLD:ACF3546
Lasionycta perplexa|[7828]|RDNMC586-06|United States|Wyoming|658[0n]|BOLD:ACF3546
Lasionycta perplexa|[7829]|RDNM391-05|United States|Nevada|658[0n]|BOLD:ACF3546
Lasionycta perplexa|[7830]|RDNMB707-05|United States|Nevada|617[0n]|BOLD:ACF3546
Lasionycta perplexa|[7831]|LPAB228-08|Canada|Alberta|656[1n]|BOLD:ACF3546
Lasionycta perplexa|[7832]|LPABB391-08|Canada|Alberta|658[0n]|BOLD:ACF3546
Lasionycta perplexa|[7833]|LPAB064-08|Canada|Alberta|658[0n]|BOLD:ACF3546
Lasionycta perplexa|[7834]|LPAB021-08|Canada|Alberta|658[0n]|BOLD:ACF3546
Lasionycta perplexa|[7835]|LPAB008-08|Canada|Alberta|658[0n]|BOLD:ACF3546
Lasionycta perplexa|[7836]|RDMAB468-05|Canada|Alberta|658[0n]|BOLD:ACF3546
Lasionycta perplexa|[7837]|RDMAB467-05|Canada|Yukon Territory|658[0n]|BOLD:ACF3546
Lasionycta perplexa|[7838]|RDMAB462-05|Canada|Alberta|658[0n]|BOLD:ACF3546
Lasionycta perplexa|[7839]|RDMAB461-05|Canada|Alberta|658[0n]|BOLD:ACF3546
Lasionycta perplexa|[7840]|RDMAB250-05|Canada|Alberta|658[0n]|BOLD:ACF3546
Lasionycta perplexa|[7841]|RDMAB249-05|Canada|Alberta|658[0n]|BOLD:ACF3546
Lasionycta perplexa|[7842]|RDNMB723-05|Canada|British Columbia|658[0n]|BOLD:ACF3546
Lasionycta perplexa|[7843]|RDNM395-05|Canada|British Columbia|658[0n]|BOLD:ACF3546
Lasionycta perplexa|[7844]|RDMAB463-05|Canada|Alberta|658[0n]|BOLD:ACF3546
Lasionycta perplexa|[7845]|RDNMB727-05|Canada|Alberta|571[0n]|BOLD:ACF3546
Lasionycta perplexa|[7846]|LOWCB314-05|Canada|British Columbia|609[0n]|BOLD:ACF3546
Lasionycta perplexa|[7847]|RDMAB465-05|Canada|Alberta|604[0n]|BOLD:ACF3546
Lasionycta perplexa|[7848]|RDMAB466-05|Canada|Alberta|604[1n]|BOLD:ACF3546
Lasionycta perplexa|[7849]|LOWCD737-06|Canada|British Columbia|608[0n]|BOLD:ACF3546
Lasionycta perplexa|[7850]|LPMN954-08|Canada|Alberta|631[0n]|BOLD:ACF3546
Lasionycta perplexa|[7851]|LPABB120-08|Canada|Alberta|635[0n]|BOLD:ACF3546
Lasionycta perplexa|[7852]|LPABB296-08|Canada|Alberta|658[0n]|BOLD:ACF3546
Lasionycta perplexa|[7853]|LPABB338-08|Canada|Alberta|658[0n]|BOLD:ACF3546
Lasionycta perplexa|[7854]|LPABB339-08|Canada|Alberta|658[0n]|BOLD:ACF3546
Lasionycta perplexa|[7855]|LPABB341-08|Canada|Alberta|658[0n]|BOLD:ACF3546
Lasionycta perplexa|[7856]|LPABB354-08|Canada|Alberta|658[0n]|BOLD:ACF3546
Lasionycta perplexa|[7857]|LPABB374-08|Canada|Alberta|658[0n]|BOLD:ACF3546
Lasionycta perplexa|[7858]|LPABB379-08|Canada|Alberta|658[0n]|BOLD:ACF3546
Lasionycta perplexa|[7859]|LPABB387-08|Canada|Alberta|658[0n]|BOLD:ACF3546
Lasionycta perplexa|[7860]|LPABB407-08|Canada|Alberta|658[0n]|BOLD:ACF3546
Lasionycta perplexa|[7861]|LPABB408-08|Canada|Alberta|658[0n]|BOLD:ACF3546
Lasionycta perplexa|[7862]|LPABB414-08|Canada|Alberta|658[0n]|BOLD:ACF3546
Lasionycta perplexa|[7863]|LPABB441-08|Canada|Alberta|658[0n]|BOLD:ACF3546
Lasionycta perplexa|[7864]|LPABB453-08|Canada|Alberta|658[0n]|BOLD:ACF3546
Lasionycta perplexa|[7865]|LPABB455-08|Canada|Alberta|658[0n]|BOLD:ACF3546
Lasionycta perplexa|[7866]|LPABB484-08|Canada|Alberta|658[0n]|BOLD:ACF3546
Lasionycta perplexa|[7867]|BBLPB333-10|Canada|British Columbia|658[0n]|BOLD:ACF3546
Lasionycta perplexa|[7868]|BBLPB339-10|Canada|British Columbia|658[0n]|BOLD:ACF3546
Lasionycta perplexa|[7869]|BBLPB757-10|Canada|Alberta|658[0n]|BOLD:ACF3546
Lasionycta perplexa|[7870]|BBLPB764-10|Canada|British Columbia|658[0n]|BOLD:ACF3546
Lasionycta perplexa|[7871]|LALPA993-11|Canada|British Columbia|658[0n]|BOLD:ACF3546
Lasionycta coloradensis|[7872]|RDNM412-05|United States|Wyoming|598[0n]|BOLD:ACF3546
Lasionycta coloradensis|[7873]|RDNM413-05|United States|Wyoming|658[0n]|BOLD:ACF3546
Lasionycta coloradensis|[7874]|RDNM414-05|United States|Wyoming|605[0n]|BOLD:ACF3546
Lasionycta sasquatch|[7875]|RDNM410-05|United States|Washington|533[1n]|BOLD:ACF3546
Lasionycta poca|[7876]|RDNM408-05|Canada|British Columbia|658[0n]|BOLD:ACF3546
Lasionycta poca|[7877]|RDNM187-05|Canada|British Columbia|658[0n]|BOLD:ACF3546
Lasionycta poca|[7878]|RDNM186-05|Canada|British Columbia|658[0n]|BOLD:ACF3546
Lasionycta poca|[7879]|RDNM188-05|Canada|Alberta|564[0n]|BOLD:ACF3546
Lasionycta sasquatch|[7880]|RDNM409-05|United States|Washington|568[1n]|BOLD:ACF3546
Lasionycta sasquatch|[7881]|RDNM411-05|United States|Washington|571[1n]|BOLD:ACF3546
Lasionycta poca|[7882]|RDNMB484-05|Canada|Yukon Territory|658[0n]|BOLD:ACF3546
Lasionycta illima|[7883]|CNCLB680-14|Canada|Yukon Territory|658[0n]|BOLD:ACF3546
Anterastria teratophora|[7884]|XAB600-04|Canada|Ontario|552[0n]|BOLD:AAE5228
Anterastria teratophora|[7885]|XAC696-04|Canada|Ontario|658[0n]|BOLD:AAE5228
Anterastria teratophora|[7886]|XAE244-04|Canada|Ontario|658[0n]|BOLD:AAE5228
Anterastria teratophora|[7887]|RDNM098-05|Canada|Ontario|658[0n]|BOLD:AAE5228
Anterastria teratophora|[7888]|LNCC343-10|United States|North Carolina|658[0n]|BOLD:AAE5228
Anterastria teratophora|[7889]|LNCC344-10|United States|North Carolina|658[0n]|BOLD:AAE5228
Anterastria teratophora|[7890]|LILLB008-11|United States|Illinois|658[0n]|BOLD:AAE5228
Phosphila turbulenta|[7891]|LPSO888-08|Canada|Ontario|657[0n]|BOLD:AAD5305
Phosphila turbulenta|[7892]|LSEU238-06|United States|Georgia|658[0n]|BOLD:AAD5305
Phosphila turbulenta|[7893]|LNCC173-10|United States|North Carolina|658[0n]|BOLD:AAD5305
Phosphila turbulenta|[7894]|LOT343-04|United States|Tennessee|658[0n]|BOLD:AAD5305
Phosphila turbulenta|[7895]|LSEU237-06|United States|Georgia|658[0n]|BOLD:AAD5305
Phosphila turbulenta|[7896]|RDNMD480-06|United States|Florida|658[1n]|BOLD:AAD5305

[illegible]
Phosphila turbulenta|[7894]|LOT343-04|United States|Tennessee|658[0n]|BOLD:AAD5305
Phosphila turbulenta|[7895]|LSEU237-06|United States|Georgia|658[0n]|BOLD:AAD5305
Phosphila turbulenta|[7896]|RDNMD480-06|United States|Florida|658[1n]|BOLD:AAD5305
Phosphila turbulenta|[7897]|LNCC172-10|United States|North Carolina|658[0n]|BOLD:AAD5305
Phosphila turbulenta|[7898]|MMNA097-08|United States|North Carolina|658[0n]|BOLD:AAD5305
Phosphila turbulenta|[7899]|LSEU654-06|United States|Georgia|611[0n]|BOLD:AAD5305
Phosphila turbulenta|[7900]|LNCC170-10|United States|North Carolina|658[0n]|BOLD:AAD5305
Phosphila turbulenta|[7901]|LNCC171-10|United States|North Carolina|658[0n]|BOLD:AAD5305
Phosphila turbulenta|[7902]|LNCC174-10|United States|North Carolina|658[0n]|BOLD:AAD5305
Phosphila turbulenta|[7903]|LNCC1255-11|United States|North Carolina|658[0n]|BOLD:AAD5305
Acherdoa ferraria|[7904]|BBLOB1456-11|United States|Florida|658[0n]|BOLD:ABA1176
Acherdoa ferraria|[7905]|MNAE854-13|United States|Florida|657[0n]|BOLD:ABA1176
Acherdoa ferraria|[7906]|RDNML319-13|United States|Florida|658[0n]|BOLD:ABA1176
Acherdoa ferraria|[7907]|RDNML320-13|United States|Florida|658[0n]|BOLD:ABA1176
Hexorthodes catalina|[7908]|NAMUM061-08|United States|Arizona|657[0n]|BOLD:AAI8439
Hexorthodes catalina|[7909]|RDNME602-08|United States|Arizona|658[0n]|BOLD:AAI8439
Hexorthodes catalina|[7910]|RDNMJ513-11|United States|Arizona|658[0n]|BOLD:AAI8439
Dargida quadrannulata|[7911]|CNCLB723-14|United States|Florida|615[0n]|BOLD:ACM4041
Dargida procinctus|[7912]|LBCS129-07|Canada|British Columbia|658[0n]|BOLD:AAB3336
Dargida procinctus|[7913]|LBCS126-07|Canada|British Columbia|658[0n]|BOLD:AAB3336
Dargida procinctus|[7914]|RWWB453-10|United States|Washington|614[0n]|BOLD:AAB3336
Dargida procinctus|[7915]|LOCBB243-06|United States|California|658[0n]|BOLD:AAB3336
Dargida procinctus|[7916]|GMLC1297-12|United States|California|629[0n]|BOLD:AAB3336
Dargida procinctus|[7917]|LALPA649-10|Canada|British Columbia|658[0n]|BOLD:AAB3336
Dargida procinctus|[7918]|RWWC105-10|United States|Washington|658[0n]|BOLD:AAB3336
Dargida procinctus|[7919]|LBCS650-07|Canada|British Columbia|658[0n]|BOLD:AAB3336
Dargida procinctus|[7920]|LBCG821-09|Canada|British Columbia|658[0n]|BOLD:AAB3336
Dargida procinctus|[7921]|LBCS648-07|Canada|British Columbia|658[0n]|BOLD:AAB3336
Dargida procinctus|[7922]|LBCS649-07|Canada|British Columbia|658[0n]|BOLD:AAB3336
Dargida procinctus|[7923]|LBCS128-07|Canada|British Columbia|658[0n]|BOLD:AAB3336
Dargida procinctus|[7924]|LBCS251-07|Canada|British Columbia|658[0n]|BOLD:AAB3336
Dargida procinctus|[7925]|LBCS022-07|Canada|British Columbia|658[0n]|BOLD:AAB3336
Dargida procinctus|[7926]|LBCS127-07|Canada|British Columbia|658[0n]|BOLD:AAB3336
Dargida procinctus|[7927]|LBCS021-07|Canada|British Columbia|658[0n]|BOLD:AAB3336
Dargida procinctus|[7928]|RDNMD419-06|United States|California|658[0n]|BOLD:AAB3336
Dargida procinctus|[7929]|RDNMD418-06|United States|California|658[0n]|BOLD:AAB3336
Dargida procinctus|[7930]|LOCBB246-06|United States|California|658[0n]|BOLD:AAB3336
Dargida procinctus|[7931]|LOCBB245-06|United States|California|658[0n]|BOLD:AAB3336
Dargida procinctus|[7932]|LOCBB244-06|United States|California|658[0n]|BOLD:AAB3336
Dargida procinctus|[7933]|LOCB820-06|United States|California|658[0n]|BOLD:AAB3336
Dargida procinctus|[7934]|LOWCE206-06|Canada|British Columbia|658[0n]|BOLD:AAB3336
Dargida procinctus|[7935]|LBCA276-05|Canada|British Columbia|658[0n]|BOLD:AAB3336
Dargida procinctus|[7936]|RWWA034-09|United States|Washington|618[0n]|BOLD:AAB3336
Dargida procinctus|[7937]|LBCA277-05|Canada|British Columbia|601[0n]|BOLD:AAB3336
Dargida procinctus|[7938]|RWWA085-09|United States|Washington|572[0n]|BOLD:AAB3336
Dargida procinctus|[7939]|RWWC924-12|United States|Washington|658[0n]|BOLD:AAB3336
Dargida diffusa|[7940]|RDNMF562-08|United States|Oregon|609[0n]|BOLD:AAB3769
Dargida diffusa|[7941]|RDNMF563-08|Canada|British Columbia|609[0n]|BOLD:AAB3769
Dargida diffusa|[7942]|RDNMF564-08|United States|Oregon|658[0n]|BOLD:AAB3769
Dargida diffusa|[7943]|RDNMF565-08|United States|Oregon|658[0n]|BOLD:AAB3769
Dargida diffusa|[7944]|RDNMF566-08|United States|Oregon|658[0n]|BOLD:AAB3769
Dargida diffusa|[7945]|LNCC230-10|United States|North Carolina|658[0n]|BOLD:AAB3769
Dargida diffusa|[7946]|BBLPA836-10|Canada|Alberta|658[0n]|BOLD:AAB3769
Dargida diffusa|[7947]|BBLSX966-09|United States|Oklahoma|658[0n]|BOLD:AAB3769
Dargida diffusa|[7948]|LPMN064-08|Canada|Manitoba|658[0n]|BOLD:AAB3769
Dargida diffusa|[7949]|LPOKA932-09|United States|Oklahoma|658[0n]|BOLD:AAB3769
Dargida diffusa|[7950]|LPSOB226-08|Canada|Ontario|658[0n]|BOLD:AAB3769
Dargida diffusa|[7951]|LPMN042-08|Canada|Manitoba|658[0n]|BOLD:AAB3769
Dargida diffusa|[7952]|BBLSY128-09|United States|Texas|658[0n]|BOLD:AAB3769
Dargida diffusa|[7953]|LNCC231-10|United States|North Carolina|658[0n]|BOLD:AAB3769
Dargida diffusa|[7954]|BBLPA840-10|Canada|Saskatchewan|658[0n]|BOLD:AAB3769
Dargida diffusa|[7955]|LPSOB225-08|Canada|Ontario|658[0n]|BOLD:AAB3769
Dargida diffusa|[7956]|LPSOB223-08|Canada|Ontario|658[0n]|BOLD:AAB3769
Dargida diffusa|[7957]|RDLQG317-06|Canada|Quebec|658[0n]|BOLD:AAB3769
Dargida diffusa|[7958]|RDLQG266-06|Canada|Quebec|658[0n]|BOLD:AAB3769
Dargida diffusa|[7959]|RDLQB823-05|Canada|Quebec|658[0n]|BOLD:AAB3769
Dargida diffusa|[7960]|RDLQB755-05|Canada|Quebec|658[0n]|BOLD:AAB3769
Dargida diffusa|[7961]|RDLQB743-05|Canada|Quebec|658[0n]|BOLD:AAB3769
Dargida diffusa|[7962]|RDLQB728-05|Canada|Quebec|658[0n]|BOLD:AAB3769
Dargida diffusa|[7963]|XAI033-05|Canada|Ontario|658[0n]|BOLD:AAB3769
Dargida diffusa|[7964]|XAD344-04|Canada|Ontario|658[0n]|BOLD:AAB3769
Dargida diffusa|[7965]|LPSOB224-08|Canada|Ontario|658[0n]|BOLD:AAB3769
Dargida diffusa|[7966]|XAE478-04|Canada|Ontario|658[1n]|BOLD:AAB3769
Dargida diffusa|[7967]|PMG112-03|Canada|Ontario|617[0n]|BOLD:AAB3769
Dargida diffusa|[7968]|XAG762-05|Canada|Ontario|651[0n]|BOLD:AAB3769
Dargida diffusa|[7969]|RDLQB826-05|Canada|Quebec|622[0n]|BOLD:AAB3769
Dargida diffusa|[7970]|XAK601-07|Canada|Ontario|609[1n]|BOLD:AAB3769
Dargida diffusa|[7971]|PHMNB609-04|Canada|New Brunswick|658[0n]|BOLD:AAB3769
Dargida diffusa|[7972]|RDLQB928-05|Canada|Quebec|658[0n]|BOLD:AAB3769
Dargida diffusa|[7973]|NCCH100-11|Canada|Ontario|658[0n]|BOLD:AAB3769
Dargida tetera|[7974]|CMAZA534-10|United States|Arizona|658[0n]|BOLD:ACN3644
Dargida tetera|[7975]|DMAZ174-10|United States|Arizona|658[0n]|BOLD:ACN6780
Dargida tetera|[7976]|RDNMJ557-11|United States|Arizona|658[0n]|BOLD:ACN6780
Dargida tetera|[7977]|CNCLB268-14|United States|Arizona|658[0n]|BOLD:ACN6780
Dargida nr. aleada|[7978]|CNCLB724-14|United States|North Carolina|658[0n]|BOLD:ACM4338
Dargida aleada|[7979]|CNCLB727-14|United States|Texas|658[0n]|BOLD:ACM3912
Dargida aleada|[7980]|CNCLB728-14|United States|Texas|406[0n]|
Dargida rubripennis|[7981]|LPOKC761-09|United States|Oklahoma|658[0n]|BOLD:AAD2899
Dargida rubripennis|[7982]|RDNMF571-08|United States|Texas|658[0n]|BOLD:AAD2899
Dargida rubripennis|[7983]|RDNMF568-08|United States|New Jersey|658[0n]|BOLD:AAD2899
Dargida rubripennis|[7984]|RDNMF569-08|United States|New Jersey|609[0n]|BOLD:AAD2899
Dargida rubripennis|[7985]|RDNMF567-08|United States|Texas|609[0n]|BOLD:AAD2899
Dargida rubripennis|[7986]|RDNMF570-08|United States|New Jersey|646[0n]|BOLD:AAD2899
Dargida tetera|[7987]|CNCLB1626-14|United States|New Mexico|658[0n]|BOLD:AAD2899
Dargida sp.|[7988]|LNAUS4815-13|United States|Arizona|658[0n]|BOLD:AAZ0786
Dargida sp.|[7989]|IAWLB535-11|United States|Arizona|658[0n]|BOLD:AAZ0786
Dargida sp.|[7990]|LNAUS4816-13|United States|Arizona|632[0n]|BOLD:AAZ0786
Dargida sp.|[7991]|LNAUS4817-13|United States|Arizona|658[1n]|BOLD:AAZ0786
Dargida grammivora|[7992]|CNCLB1820-14|Mexico||658[0n]|BOLD:AAI9612
Dargida grammivora|[7993]|CNCLB1817-14|Guatemala||658[0n]|BOLD:AAI9612
Dargida grammivora|[7994]|CNCLB1818-14|Mexico||658[0n]|BOLD:AAI9612
Dargida grammivora|[7995]|CNCLB1819-14|Mexico||658[0n]|BOLD:AAI9612
Dargida grammivora|[7996]|CNCLB1821-14|Mexico||658[0n]|BOLD:AAI9612

Dargida grammivora|[7993]|CNCLB1817-14|Guatemala||658[0n]|BOLD:AAI9612
Dargida grammivora|[7994]|CNCLB1818-14|Mexico||658[0n]|BOLD:AAI9612
Dargida grammivora|[7995]|CNCLB1819-14|Mexico||658[0n]|BOLD:AAI9612
Dargida grammivora|[7996]|CNCLB1821-14|Mexico||658[0n]|BOLD:AAI9612
Lacinipolia lorea|[7997]|LOWC001-05|Canada|British Columbia|658[0n]|BOLD:AAA5449
Lacinipolia lorea|[7998]|LOWC006-05|Canada|British Columbia|658[0n]|BOLD:AAA5449
Lacinipolia lorea|[7999]|LOWC012-05|Canada|British Columbia|658[0n]|BOLD:AAA5449
Lacinipolia lorea|[8000]|LOWCD216-06|Canada|British Columbia|656[1n]|BOLD:AAA5449
Lacinipolia lorea|[8001]|PHMNB434-04|Canada|New Brunswick|658[0n]|BOLD:AAA5449
Lacinipolia lorea|[8002]|PHMNB682-04|Canada|New Brunswick|658[0n]|BOLD:AAA5449
Lacinipolia lorea|[8003]|RDMAB013-05|Canada|Alberta|631[0n]|BOLD:AAA5449
Lacinipolia lorea|[8004]|BBLPB623-10|Canada|Ontario|658[0n]|BOLD:AAA5449
Lacinipolia lorea|[8005]|LOWCC843-05|Canada|British Columbia|658[0n]|BOLD:AAA5449
Lacinipolia lorea|[8006]|LPSOB611-08|Canada|Ontario|658[0n]|BOLD:AAA5449
Lacinipolia lorea|[8007]|LOWCD223-06|Canada|British Columbia|658[0n]|BOLD:AAA5449
Lacinipolia lorea|[8008]|LOWCD224-06|Canada|British Columbia|658[0n]|BOLD:AAA5449
Lacinipolia lorea|[8009]|LOWCE799-06|Canada|British Columbia|658[0n]|BOLD:AAA5449
Lacinipolia lorea|[8010]|TMNBB329-06|Canada|New Brunswick|658[0n]|BOLD:AAA5449
Lacinipolia lorea|[8011]|LPSOB974-08|Canada|Ontario|658[0n]|BOLD:AAA5449
Lacinipolia lorea|[8012]|LPMN332-08|Canada|Manitoba|658[0n]|BOLD:AAA5449
Lacinipolia lorea|[8013]|LPMN357-08|Canada|Manitoba|658[0n]|BOLD:AAA5449
Lacinipolia lorea|[8014]|LPMN606-08|Canada|Manitoba|658[0n]|BOLD:AAA5449
Lacinipolia lorea|[8015]|LPMN634-08|Canada|Manitoba|658[0n]|BOLD:AAA5449
Lacinipolia lorea|[8016]|LPMN658-08|Canada|Manitoba|658[0n]|BOLD:AAA5449
Lacinipolia lorea|[8017]|LPMN730-08|Canada|Manitoba|658[0n]|BOLD:AAA5449
Lacinipolia lorea|[8018]|LPMN934-08|Canada|Alberta|658[0n]|BOLD:AAA5449
Lacinipolia lorea|[8019]|LPMN940-08|Canada|Alberta|658[0n]|BOLD:AAA5449
Lacinipolia lorea|[8020]|LPABB012-08|Canada|Alberta|658[0n]|BOLD:AAA5449
Lacinipolia lorea|[8021]|LPABB014-08|Canada|Alberta|658[0n]|BOLD:AAA5449
Lacinipolia lorea|[8022]|LPABB053-08|Canada|Alberta|658[0n]|BOLD:AAA5449
Lacinipolia lorea|[8023]|LPABB183-08|Canada|Alberta|658[0n]|BOLD:AAA5449
Lacinipolia lorea|[8024]|LPABB209-08|Canada|Alberta|658[0n]|BOLD:AAA5449
Lacinipolia lorea|[8025]|LPABB226-08|Canada|Alberta|658[0n]|BOLD:AAA5449
Lacinipolia lorea|[8026]|LPABB494-08|Canada|Alberta|658[0n]|BOLD:AAA5449
Lacinipolia lorea|[8027]|LPABB495-08|Canada|Alberta|658[0n]|BOLD:AAA5449
Lacinipolia lorea|[8028]|LPABB569-08|Canada|Alberta|658[0n]|BOLD:AAA5449
Lacinipolia lorea|[8029]|LPABB595-08|Canada|Alberta|658[0n]|BOLD:AAA5449
Lacinipolia lorea|[8030]|LPSOD260-09|Canada|Ontario|658[0n]|BOLD:AAA5449
Lacinipolia lorea|[8031]|LPSOD307-09|Canada|Ontario|658[0n]|BOLD:AAA5449
Lacinipolia lorea|[8032]|LPSOD343-09|Canada|Ontario|658[0n]|BOLD:AAA5449
Lacinipolia lorea|[8033]|LPSOD376-09|Canada|Ontario|658[0n]|BOLD:AAA5449
Lacinipolia lorea|[8034]|BBLPB361-10|Canada|Saskatchewan|658[0n]|BOLD:AAA5449
Lacinipolia lorea|[8035]|BBLPB363-10|Canada|British Columbia|658[0n]|BOLD:AAA5449
Lacinipolia lorea|[8036]|BBLPB364-10|Canada|British Columbia|658[0n]|BOLD:AAA5449
Lacinipolia lorea|[8037]|BBLPB621-10|Canada|Ontario|658[0n]|BOLD:AAA5449
Lacinipolia lorea|[8038]|LMDH158-11|United States|Minnesota|658[0n]|BOLD:AAA5449
Lacinipolia lorea|[8039]|LOWCD220-06|Canada|British Columbia|658[0n]|BOLD:AAA5449
Lacinipolia lorea|[8040]|LOWCD222-06|Canada|British Columbia|658[0n]|BOLD:AAA5449
Lacinipolia lorea|[8041]|LOWCD217-06|Canada|British Columbia|658[0n]|BOLD:AAA5449
Lacinipolia lorea|[8042]|LOWCD219-06|Canada|British Columbia|658[0n]|BOLD:AAA5449
Lacinipolia lorea|[8043]|LOWCC887-05|Canada|British Columbia|658[0n]|BOLD:AAA5449
Lacinipolia lorea|[8044]|RDLQB265-05|Canada|Quebec|658[0n]|BOLD:AAA5449
Lacinipolia lorea|[8045]|LOWC013-05|Canada|British Columbia|658[0n]|BOLD:AAA5449
Lacinipolia lorea|[8046]|LOWCC886-05|Canada|British Columbia|658[0n]|BOLD:AAA5449
Lacinipolia lorea|[8047]|LOWC011-05|Canada|British Columbia|658[0n]|BOLD:AAA5449
Lacinipolia lorea|[8048]|LOWC010-05|Canada|British Columbia|658[0n]|BOLD:AAA5449
Lacinipolia lorea|[8049]|LOWC009-05|Canada|British Columbia|658[0n]|BOLD:AAA5449
Lacinipolia lorea|[8050]|LOWC007-05|Canada|British Columbia|658[0n]|BOLD:AAA5449
Lacinipolia lorea|[8051]|LOWC004-05|Canada|British Columbia|658[0n]|BOLD:AAA5449
Lacinipolia lorea|[8052]|LOWC003-05|Canada|British Columbia|658[0n]|BOLD:AAA5449
Lacinipolia lorea|[8053]|LOWC002-05|Canada|British Columbia|658[0n]|BOLD:AAA5449
Lacinipolia lorea|[8054]|RDMAB012-05|Canada|Alberta|658[0n]|BOLD:AAA5449
Lacinipolia lorea|[8055]|PHMNB347-04|Canada|New Brunswick|658[0n]|BOLD:AAA5449
Lacinipolia lorea|[8056]|BBLEC574-09|Canada|Nova Scotia|658[0n]|BOLD:AAA5449
Lacinipolia lorea|[8057]|BBLPB362-10|Canada|British Columbia|658[0n]|BOLD:AAA5449
Lacinipolia lorea|[8058]|BBLPB622-10|Canada|Ontario|658[0n]|BOLD:AAA5449
Lacinipolia lorea|[8059]|LOWC008-05|Canada|British Columbia|534[0n]|BOLD:AAA5449
Lacinipolia lorea|[8060]|LOWCD221-06|Canada|British Columbia|624[0n]|BOLD:AAA5449
Lacinipolia lorea|[8061]|PHMNB772-05|Canada|New Brunswick|615[0n]|BOLD:AAA5449
Lacinipolia lorea|[8062]|PMG123-03|Canada|Ontario|617[0n]|BOLD:AAA5449
Lacinipolia lorea|[8063]|LOWCC907-05|Canada|British Columbia|557[1n]|BOLD:AAA5449
Lacinipolia lorea|[8064]|LOWCC909-05|Canada|British Columbia|550[1n]|BOLD:AAA5449
Lacinipolia lorea|[8065]|RDNMC402-05|Canada|Ontario|595[0n]|BOLD:AAA5449
Lacinipolia lorea|[8066]|SSWLB053-13|Canada|Alberta|574[0n]|BOLD:AAA5449
Lacinipolia lorea|[8067]|LGSMC892-05|United States|Tennessee|658[0n]|BOLD:AAA5449
Lacinipolia lorea|[8068]|LGSMC486-05|United States|Tennessee|658[0n]|BOLD:AAA5449
Lacinipolia lorea|[8069]|LGSMC894-05|United States|Tennessee|658[0n]|BOLD:AAA5449
Lacinipolia lorea|[8070]|XAC660-04|Canada|Ontario|658[0n]|BOLD:AAA5449
Lacinipolia lorea|[8071]|XAC703-04|Canada|Ontario|658[0n]|BOLD:AAA5449
Lacinipolia lorea|[8072]|RDNMC282-05|United States|North Carolina|658[0n]|BOLD:AAA5449
Lacinipolia lorea|[8073]|LPSOB975-08|Canada|Ontario|658[0n]|BOLD:AAA5449
Lacinipolia lorea|[8074]|LSEU624-06|United States|Georgia|658[0n]|BOLD:AAA5449
Lacinipolia lorea|[8075]|XAI060-05|Canada|Ontario|658[0n]|BOLD:AAA5449
Lacinipolia lorea|[8076]|LGSMC893-05|United States|Tennessee|658[0n]|BOLD:AAA5449
Lacinipolia lorea|[8077]|CNCLB2873-14|United States|North Carolina|658[0n]|BOLD:AAA5449
Lacinipolia lorea|[8078]|CNCLB2874-14|United States|North Carolina|658[0n]|BOLD:AAA5449
Tricholita palmillo|[8079]|RDNME937-08|United States|Arizona|658[0n]|BOLD:AAJ6711
Hydroeciodes auripurpura|[8080]|LNAUS5574-13|United States|Texas|658[0n]|BOLD:ACK9589
Hydroeciodes auripurpura|[8081]|CNCLB567-14|United States|Texas|656[0n]|BOLD:ACK9589
Hydroeciodes juvenilis|[8082]|LNAUT436-14|United States|New Mexico|658[0n]|BOLD:AAX2672
Hydroeciodes juvenilis|[8083]|LNAUT437-14|United States|New Mexico|658[0n]|BOLD:AAX2672
Hydroeciodes juvenilis|[8084]|RDNMD368-06|United States|Arizona|658[0n]|BOLD:AAX2672
Hydroeciodes juvenilis|[8085]|LNAUT435-14|United States|New Mexico|658[0n]|BOLD:AAX2672
Hydroeciodes juvenilis|[8086]|LNAUT438-14|United States|New Mexico|658[0n]|BOLD:AAX2672
Hydroeciodes serrata|[8087]|RDNMG764-08|United States|Colorado|658[0n]|BOLD:AAE5159
Hydroeciodes serrata|[8088]|LPABC693-09|Canada|Alberta|658[0n]|BOLD:AAE5159
Hydroeciodes serrata|[8089]|RDNMF442-08|Canada|Alberta|658[0n]|BOLD:AAE5159
Hydroeciodes serrata|[8090]|RDNME625-08|United States|Colorado|658[0n]|BOLD:AAE5159
Hydroeciodes ochrimacula|[8091]|CNCLB565-14|United States|Arizona|658[1n]|BOLD:AAE5159
Hydroeciodes ochrimacula|[8092]|CNCLB566-14|United States|Arizona|658[0n]|BOLD:AAE5159
Hydroeciodes repleta|[8093]|CNCLB569-14|United States|Arizona|658[0n]|BOLD:AAE5159
Hydroeciodes repleta|[8094]|CNCLB570-14|United States|Arizona|658[0n]|BOLD:AAE5159
Hydroeciodes ochrimacula|[8095]|LNAUT423-14|United States|Arizona|658[0n]|BOLD:AAE5159
Hydroeciodes repleta|[8096]|LNAUT430-14|United States|Arizona|658[0n]|BOLD:AAE5159

[illegible]
Hydroeciodes repleta|[8094]|CNCLB570-14|United States|Arizona|658[0n]|BOLD:AAE5159
Hydroeciodes ochrimacula|[8095]|LNAUT423-14|United States|Arizona|658[0n]|BOLD:AAE5159
Hydroeciodes repleta|[8096]|LNAUT430-14|United States|Arizona|658[0n]|BOLD:AAE5159
Hydroeciodes repleta|[8097]|TML171-14|United States||658[0n]|BOLD:AAE5159
Tricholita signata|[8098]|LPMNB227-09|Canada|Manitoba|658[0n]|BOLD:AAA6002
Tricholita signata|[8099]|XAG550-05|Canada|Ontario|597[1n]|BOLD:AAA6002
Tricholita signata|[8100]|XAG547-05|Canada|Ontario|604[1n]|BOLD:AAA6002
Tricholita signata|[8101]|XAG103-05|Canada|Ontario|658[0n]|BOLD:AAA6002
Tricholita signata|[8102]|XAD582-04|Canada|Ontario|658[0n]|BOLD:AAA6002
Tricholita signata|[8103]|XAG471-05|Canada|Ontario|546[2n]|BOLD:AAA6002
Tricholita signata|[8104]|XAG473-05|Canada|Ontario|580[1n]|BOLD:AAA6002
Tricholita signata|[8105]|XAK257-06|Canada|Ontario|658[0n]|BOLD:AAA6002
Tricholita signata|[8106]|LGSMG877-10|United States|North Carolina|658[0n]|BOLD:AAA6002
Tricholita signata|[8107]|LGSMG879-10|United States|North Carolina|658[0n]|BOLD:AAA6002
Tricholita signata|[8108]|XAG862-05|Canada|Ontario|658[0n]|BOLD:AAA6002
Tricholita signata|[8109]|XAG649-05|Canada|Ontario|658[0n]|BOLD:AAA6002
Tricholita signata|[8110]|LNC480-06|United States|North Carolina|658[0n]|BOLD:AAA6002
Tricholita signata|[8111]|RDNME623-08|United States|Colorado|658[0n]|BOLD:AAA6002
Tricholita signata|[8112]|LGSM038-04|United States|North Carolina|658[0n]|BOLD:AAA6002
Tricholita signata|[8113]|LGSM764-04|United States|Tennessee|658[0n]|BOLD:AAA6002
Tricholita signata|[8114]|XAG853-05|Canada|Ontario|658[0n]|BOLD:AAA6002
Tricholita signata|[8115]|XAG764-05|Canada|Ontario|658[0n]|BOLD:AAA6002
Tricholita signata|[8116]|XAG528-05|Canada|Ontario|658[0n]|BOLD:AAA6002
Tricholita signata|[8117]|XAG345-05|Canada|Ontario|658[0n]|BOLD:AAA6002
Tricholita signata|[8118]|XAG107-05|Canada|Ontario|658[0n]|BOLD:AAA6002
Tricholita signata|[8119]|XAC834-04|Canada|Ontario|658[0n]|BOLD:AAA6002
Tricholita signata|[8120]|XAC723-04|Canada|Ontario|658[0n]|BOLD:AAA6002
Tricholita signata|[8121]|XAC462-04|Canada|Ontario|658[0n]|BOLD:AAA6002
Tricholita signata|[8122]|PMG164-03|Canada|Ontario|617[0n]|BOLD:AAA6002
Tricholita signata|[8123]|XAG654-05|Canada|Ontario|657[1n]|BOLD:AAA6002
Tricholita signata|[8124]|XAG672-05|Canada|Ontario|658[1n]|BOLD:AAA6002
Tricholita signata|[8125]|XAG670-05|Canada|Ontario|658[0n]|BOLD:AAA6002
Tricholita signata|[8126]|TMG145-03|Canada|Ontario|639[0n]|BOLD:AAA6002
Tricholita signata|[8127]|XAC850-04|Canada|Ontario|592[0n]|BOLD:AAA6002
Tricholita signata|[8128]|XAD101-04|Canada|Ontario|593[0n]|BOLD:AAA6002
Tricholita signata|[8129]|XAD585-04|Canada|Ontario|557[0n]|BOLD:AAA6002
Tricholita signata|[8130]|XAG673-05|Canada|Ontario|614[0n]|BOLD:AAA6002
Tricholita signata|[8131]|XAG885-05|Canada|Ontario|632[0n]|BOLD:AAA6002
Tricholita signata|[8132]|XAI047-05|Canada|Ontario|658[0n]|BOLD:AAA6002
Tricholita signata|[8133]|XAI048-05|Canada|Ontario|658[0n]|BOLD:AAA6002
Tricholita signata|[8134]|XAK605-07|Canada|Ontario|658[0n]|BOLD:AAA6002
Tricholita signata|[8135]|BLTIB862-08|Canada|Ontario|658[0n]|BOLD:AAA6002
Tricholita signata|[8136]|BLTIB914-08|Canada|Ontario|658[0n]|BOLD:AAA6002
Tricholita signata|[8137]|BLTIB1031-08|Canada|Ontario|658[0n]|BOLD:AAA6002
Tricholita signata|[8138]|LNCNW018-06|United States|North Carolina|658[0n]|BOLD:AAA6002
Tricholita signata|[8139]|LNC424-05|United States|North Carolina|658[0n]|BOLD:AAA6002
Tricholita signata|[8140]|BBLPC543-09|Canada|New Brunswick|639[0n]|BOLD:AAA6002
Tricholita signata|[8141]|BBLPE560-09|Canada|Nova Scotia|658[0n]|BOLD:AAA6002
Tricholita signata|[8142]|LGSMG876-10|United States|North Carolina|658[0n]|BOLD:AAA6002
Tricholita signata|[8143]|LGSMG875-10|United States|North Carolina|658[0n]|BOLD:AAA6002
Tricholita signata|[8144]|XAI049-05|Canada|Ontario|658[0n]|BOLD:AAA6002
Tricholita signata|[8145]|XAK604-07|Canada|Ontario|658[0n]|BOLD:AAA6002
Tricholita signata|[8146]|XAK416-06|Canada|Ontario|656[0n]|BOLD:AAA6002
Tricholita signata|[8147]|BLTIB845-08|Canada|Ontario|658[0n]|BOLD:AAA6002
Tricholita signata|[8148]|BBLPC095-09|Canada|New Brunswick|658[0n]|BOLD:AAA6002
Tricholita signata|[8149]|BLTIB848-08|Canada|Ontario|658[0n]|BOLD:AAA6002
Tricholita signata|[8150]|BLTIB989-08|Canada|Ontario|658[0n]|BOLD:AAA6002
Tricholita signata|[8151]|BLTIB863-08|Canada|Ontario|658[0n]|BOLD:AAA6002
Tricholita signata|[8152]|BLTIB799-08|Canada|Ontario|658[0n]|BOLD:AAA6002
Tricholita signata|[8153]|BLTIB773-08|Canada|Ontario|658[0n]|BOLD:AAA6002
Tricholita signata|[8154]|RDLQF373-06|Canada|Quebec|658[0n]|BOLD:AAA6002
Tricholita signata|[8155]|XAK228-06|Canada|Ontario|658[0n]|BOLD:AAA6002
Tricholita signata|[8156]|XAB013-04|Canada|Ontario|658[0n]|BOLD:AAA6002
Tricholita signata|[8157]|LNCNW017-06|United States|North Carolina|658[0n]|BOLD:AAA6002
Tricholita signata|[8158]|XAG650-05|Canada|Ontario|658[0n]|BOLD:AAA6002
Tricholita signata|[8159]|XAG472-05|Canada|Ontario|658[0n]|BOLD:AAA6002
Tricholita signata|[8160]|XAC837-04|Canada|Ontario|658[0n]|BOLD:AAA6002
Tricholita signata|[8161]|XAC042-04|Canada|Ontario|592[0n]|BOLD:AAA6002
Tricholita signata|[8162]|RDLQB649-05|Canada|Quebec|569[0n]|BOLD:AAA6002
Tricholita signata|[8163]|BLTIB1019-08|Canada|Ontario|658[0n]|BOLD:AAA6002
Tricholita signata|[8164]|LGSMG878-10|United States|North Carolina|658[0n]|BOLD:AAA6002
Tricholita signata|[8165]|LGSMG880-10|United States|North Carolina|658[0n]|BOLD:AAA6002
Tricholita sp.|[8166]|RDNMJ697-11|United States|Arizona|658[0n]|BOLD:AAV7891
Tricholita knudsoni|[8167]|RDNME611-08|United States|Texas|649[2n]|BOLD:AAG0357
Tricholita bisulca|[8168]|RDNMJ815-11|United States|New Mexico|658[0n]|BOLD:AAV8593
Tricholita bisulca|[8169]|RDNML206-13|United States|New Mexico|658[0n]|BOLD:AAV8593
Tricholita elsinora|[8170]|LNAUS5571-13|United States|Arizona|658[0n]|BOLD:ACK9356
Tricholita elsinora|[8171]|CNCLB697-14|United States|Arizona|597[1n]|BOLD:ACK9356
Tricholita elsinora|[8172]|CNCLB694-14|Mexico||658[0n]|BOLD:ACM4300
Tricholita elsinora|[8173]|CNCLB695-14|Mexico||658[0n]|BOLD:ACM4300
Tricholita elsinora|[8174]|CNCLB698-14|Mexico||658[0n]|BOLD:ACM4300
Tricholita ferrisi|[8175]|RDNMG098-08|United States|Arizona|658[0n]|BOLD:AAK7887
Tricholita ferrisi|[8176]|RDNMJ558-11|United States|Arizona|658[0n]|BOLD:AAK7887
Tricholita ferrisi|[8177]|IAWLB568-11|United States|Arizona|658[0n]|BOLD:AAK7887
Tricholita notata|[8178]|RDNMF738-08|United States|Oklahoma|640[0n]|BOLD:AAJ8475
Tricholita n. sp.|[8179]|LOCBB289-06|United States|California|658[0n]|BOLD:AAG0356
Tricholita n. sp.|[8180]|LOCBB290-06|United States|California|658[0n]|BOLD:AAG0356
Tricholita n. sp.|[8181]|LOCBB291-06|United States|California|658[0n]|BOLD:AAG0356
Tricholita n. sp.|[8182]|LOCBB292-06|United States|California|658[0n]|BOLD:AAG0356
Tricholita n. sp.|[8183]|LOCBB293-06|United States|California|658[0n]|BOLD:AAG0356
Tricholita chipeta|[8184]|NAMUM136-08|United States|California|658[0n]|BOLD:AAJ8473
Tricholita sp.|[8185]|QUNOD033-10|United States|Texas|658[0n]|BOLD:AAJ8473
Tricholita fistula|[8186]|NAMUM214-08|United States|California|658[0n]|BOLD:AAH8100
Tricholita fistula|[8187]|NAMUM215-08|United States|California|658[0n]|BOLD:AAH8100
Tricholita chipeta|[8188]|LNAUT884-14|United States|California|658[0n]|BOLD:ACN7614
Tricholita chipeta|[8189]|LNAUT885-14|United States|California|658[0n]|BOLD:ACN7614
Tricholita chipeta|[8190]|LNAUT882-14|United States|California|658[0n]|BOLD:ACN7614
Tricholita chipeta|[8191]|LNAUT883-14|United States|California|658[0n]|BOLD:ACN7614
Tricholita chipeta|[8192]|LNAUT886-14|United States|California|658[0n]|BOLD:ACN7614
Tricholita chipeta|[8193]|LNAUT1125-14|United States|California|658[0n]|BOLD:ACN7614
Tricholita baranca|[8194]|CNCLB267-14|United States|Texas|658[0n]|BOLD:ACM4700
Lophoceramica artega|[8195]|NAMUM080-08|United States|Arizona|644[0n]|BOLD:AAK6879
[illegible]

Tricholita chipeta|[8193]|LNAUT1125-14|United States|California|658[0n]|BOLD:ACN7614
Tricholita baranca|[8194]|CNCLB267-14|United States|Texas|658[0n]|BOLD:ACM4700
Lophoceramica artega|[8195]|NAMUM080-08|United States|Arizona|644[0n]|BOLD:AAK6879
Lophoceramica artega|[8196]|RDNMJ539-11|United States|Arizona|658[0n]|BOLD:AAK6879
Lophoceramica artega|[8197]|IAWLB300-11|United States|Arizona|658[0n]|BOLD:AAK6879
Lophoceramica artega|[8198]|CNCLB1565-14|United States|Arizona|658[0n]|BOLD:AAK6879
Hemibryomima chryselectra|[8199]|RDNMD931-07|United States|Arizona|655[0n]|BOLD:AAI6711
Hemibryomima chryselectra|[8200]|RDNMD932-07|United States|Arizona|613[0n]|BOLD:AAI6711
Hemibryomima chryselectra|[8201]|CMAZA527-10|United States|Arizona|658[0n]|BOLD:AAI6711
Hemibryomima chryselectra|[8202]|RDNMK106-11|United States|Arizona|658[0n]|BOLD:AAI6711
Hemibryomima chryselectra|[8203]|RDNMJ729-11|United States|Arizona|658[0n]|BOLD:AAI6711
Hemibryomima chryselectra|[8204]|CMAZA1092-12|United States|Arizona|658[0n]|BOLD:AAI6711
Synorthodes auriginea|[8205]|RDNMK938-13|Mexico|Sonora|658[0n]|BOLD:ACC9154
Xanthopastis regnatrix|[8206]|RDNMK090-11|United States|Louisiana|658[0n]|BOLD:AAD8033
Xanthopastis regnatrix|[8207]|HKONS062-07|United States|Florida|658[1n]|BOLD:AAD8033
Xanthopastis regnatrix|[8208]|NAMUM261-08|United States|Florida|658[0n]|BOLD:AAD8033
Xanthopastis regnatrix|[8209]|NAMUM292-08|United States|Florida|658[0n]|BOLD:AAD8033
Xanthopastis regnatrix|[8210]|DMAZ129-09|United States|Florida|658[0n]|BOLD:AAD8033
Xanthopastis regnatrix|[8211]|USLEP661-10|United States|Florida|658[0n]|BOLD:AAD8033
Xanthopastis regnatrix|[8212]|LNAUT447-14|United States|Florida|658[0n]|BOLD:AAD8033
Xanthopastis regnatrix|[8213]|LNAUT448-14|United States|Florida|658[0n]|BOLD:AAD8033
Xanthopastis regnatrix|[8214]|LNAUT449-14|United States|Florida|658[0n]|BOLD:AAD8033
Xanthopastis regnatrix|[8215]|LNAUT450-14|United States|Florida|658[0n]|BOLD:AAD8033
Xanthopastis moctezuma|[8216]|RDNMK859-13|Mexico|Sonora|658[0n]|BOLD:ACC8932
Xanthopastis moctezuma|[8217]|CNCLB1722-14|Mexico|Chiapas|658[0n]|BOLD:ACC8932
Xanthopastis moctezuma|[8218]|CNCLB1952-14|Guatemala||658[0n]|BOLD:ACC8932
Xanthopastis moctezuma|[8219]|CNCLB2007-14|Mexico||658[0n]|BOLD:ACC8932
Xanthopastis moctezuma|[8220]|CNCLB2008-14|Mexico||658[0n]|BOLD:ACC8932
Xanthopastis moctezuma|[8221]|CNCLB2009-14|Mexico||658[0n]|BOLD:ACC8932
Xanthopastis moctezuma|[8222]|CNCLB2195-14|Mexico||658[0n]|BOLD:ACC8932
Xanthopastis moctezuma|[8223]|CNCLB2196-14|Mexico||658[0n]|BOLD:ACC8932
Mimobarathra antonito|[8224]|RDNMD896-07|United States|New Mexico|617[0n]|BOLD:AAF6347
Mimobarathra antonito|[8225]|RDNMD895-07|United States|New Mexico|655[0n]|BOLD:AAF6347
Mimobarathra antonito|[8226]|RDNME627-08|United States|New Mexico|658[0n]|BOLD:AAF6347
Dypterygia ligata|[8227]|RDNML349-13|United States|Florida|658[0n]|BOLD:AAK3945
Dypterygia ligata|[8228]|RDNML350-13|United States|Florida|658[0n]|BOLD:AAK3945
Dypterygia ordinarius|[8229]|LMEMB715-09|Puerto Rico||656[0n]|BOLD:AAA5250
Dypterygia ordinarius|[8230]|BLPAE005-06|Costa Rica|Alajuela|658[0n]|BOLD:AAA5250
Dypterygia ordinarius|[8231]|MHMYN116-11|Costa Rica||658[0n]|BOLD:AAA5250
Dypterygia ordinarius|[8232]|MHMYN118-11|Costa Rica||658[0n]|BOLD:AAA5250
Dypterygia ordinarius|[8233]|MHMYQ1776-12|Costa Rica||658[0n]|BOLD:AAA5250
Dypterygia ordinarius|[8234]|MHMYT215-13|Costa Rica||658[0n]|BOLD:AAA5250
Dypterygia ordinarius|[8235]|LNAUS4488-13|Costa Rica|Alajuela|658[0n]|BOLD:AAA5250
Dypterygia ordinarius|[8236]|MHAUA864-05|Costa Rica|Guanacaste|658[0n]|BOLD:AAA5250
Dypterygia ordinarius|[8237]|BLPAE504-06|Costa Rica|Guanacaste|658[0n]|BOLD:AAA5250
Dypterygia ordinarius|[8238]|BLPAF665-07|Costa Rica|Guanacaste|658[0n]|BOLD:AAA5250
Dypterygia ordinarius|[8239]|BLPAG044-07|Costa Rica|Guanacaste|658[0n]|BOLD:AAA5250
Dypterygia ordinarius|[8240]|BLPCA314-08|Costa Rica|Guanacaste|658[0n]|BOLD:AAA5250
Dypterygia ordinarius|[8241]|BLPCN097-08|Costa Rica|Guanacaste|658[0n]|BOLD:AAA5250
Dypterygia ordinarius|[8242]|BLPDK1303-09|Costa Rica|Guanacaste|658[0n]|BOLD:AAA5250
Dypterygia ordinarius|[8243]|BLPDK1883-09|Costa Rica|Guanacaste|658[0n]|BOLD:AAA5250
Dypterygia ordinarius|[8244]|MHMYH735-10|Costa Rica||658[0n]|BOLD:AAA5250
Dypterygia ordinarius|[8245]|LNOUB791-10|French Guiana||658[0n]|BOLD:AAA5250
Dypterygia ordinarius|[8246]|BLPDT1428-10|Costa Rica|Guanacaste|658[0n]|BOLD:AAA5250
Dypterygia ordinarius|[8247]|BLPDU243-11|Costa Rica|Guanacaste|658[0n]|BOLD:AAA5250
Dypterygia ordinarius|[8248]|BLPDV345-11|Costa Rica|Guanacaste|658[0n]|BOLD:AAA5250
Dypterygia ordinarius|[8249]|BLPDW624-11|Costa Rica|Guanacaste|658[0n]|BOLD:AAA5250
Dypterygia ordinarius|[8250]|BLPEF810-12|Costa Rica|Guanacaste|658[0n]|BOLD:AAA5250
Dypterygia ordinarius|[8251]|BLPEF981-12|Costa Rica|Alajuela|658[0n]|BOLD:AAA5250
Dypterygia ordinarius|[8252]|LNAUS4486-13|Costa Rica|Alajuela|658[0n]|BOLD:AAA5250
Dypterygia ordinarius|[8253]|BLPEF4952-13|Costa Rica|Guanacaste|658[0n]|BOLD:AAA5250
Dypterygia ordinarius|[8254]|BLPCA862-08|Costa Rica|Guanacaste|658[0n]|BOLD:AAA5250
Dypterygia ordinarius|[8255]|CNCLB2001-14|Guatemala||658[0n]|BOLD:AAA5250
Dypterygia ordinarius|[8256]|BLPDK671-09|Costa Rica|Guanacaste|658[0n]|BOLD:AAA5250
Dypterygia ordinarius|[8257]|BLPDT1427-10|Costa Rica|Guanacaste|658[0n]|BOLD:AAA5250
Dypterygia ordinarius|[8258]|BLPDU241-11|Costa Rica|Guanacaste|658[0n]|BOLD:AAA5250
Dypterygia ordinarius|[8259]|BLPDU825-11|Costa Rica|Guanacaste|658[2n]|BOLD:AAA5250
Dypterygia ordinarius|[8260]|MHMYL3222-11|Costa Rica||658[0n]|BOLD:AAA5250
Dypterygia ordinarius|[8261]|BLPDV347-11|Costa Rica|Guanacaste|658[0n]|BOLD:AAA5250
Dypterygia ordinarius|[8262]|BLPDU824-11|Costa Rica|Guanacaste|658[0n]|BOLD:AAA5250
Dypterygia ordinarius|[8263]|BLPDU242-11|Costa Rica|Guanacaste|658[0n]|BOLD:AAA5250
Dypterygia ordinarius|[8264]|BLPDU240-11|Costa Rica|Guanacaste|658[0n]|BOLD:AAA5250
Dypterygia ordinarius|[8265]|BLPDK1882-09|Costa Rica|Guanacaste|658[0n]|BOLD:AAA5250
Dypterygia ordinarius|[8266]|BLPDK1708-09|Costa Rica|Guanacaste|658[0n]|BOLD:AAA5250
Dypterygia ordinarius|[8267]|BLPDK207-09|Costa Rica|Guanacaste|658[0n]|BOLD:AAA5250
Dypterygia ordinarius|[8268]|BLPDB895-09|Costa Rica|Guanacaste|658[0n]|BOLD:AAA5250
Dypterygia ordinarius|[8269]|BLPCB895-08|Costa Rica|Alajuela|658[0n]|BOLD:AAA5250
Dypterygia ordinarius|[8270]|BLPCA307-08|Costa Rica|Guanacaste|658[0n]|BOLD:AAA5250
Dypterygia ordinarius|[8271]|MHMXM231-07|Costa Rica|Guanacaste|658[0n]|BOLD:AAA5250
Dypterygia ordinarius|[8272]|BLPBH419-07|Costa Rica|Guanacaste|658[0n]|BOLD:AAA5250
Dypterygia ordinarius|[8273]|BLPAE682-06|Costa Rica|Guanacaste|658[0n]|BOLD:AAA5250
Dypterygia ordinarius|[8274]|MHAUA867-05|Costa Rica|Guanacaste|658[0n]|BOLD:AAA5250
Dypterygia ordinarius|[8275]|BLPBH420-07|Costa Rica|Guanacaste|658[0n]|BOLD:AAA5250
Dypterygia ordinarius|[8276]|BLPDK206-09|Costa Rica|Guanacaste|658[0n]|BOLD:AAA5250
Dypterygia ordinarius|[8277]|MHMYO240-11|Costa Rica||658[0n]|BOLD:AAA5250
Dypterygia ordinarius|[8278]|MHMYQ027-12|Costa Rica||658[0n]|BOLD:AAA5250
Dypterygia ordinarius|[8279]|MHMYQ028-12|Costa Rica||658[0n]|BOLD:AAA5250
Dypterygia ordinarius|[8280]|LNAUS4489-13|Costa Rica|Alajuela|658[0n]|BOLD:AAA5250
Dypterygia ordinarius|[8281]|BLPEF4955-13|Costa Rica|Guanacaste|658[0n]|BOLD:AAA5250
Dypterygia ordinarius|[8282]|MHMYS2789-13|Costa Rica|Guanacaste|658[0n]|BOLD:AAA5250
Dypterygia ordinarius|[8283]|BLPEE8246-14|Costa Rica||658[0n]|BOLD:AAA5250
Dypterygia ordinarius|[8284]|MHAUA871-05|Costa Rica|Guanacaste|531[0n]|BOLD:AAA5250
Dypterygia ordinarius|[8285]|BLPDV973-11|Costa Rica|Guanacaste|658[0n]|BOLD:AAA5250
Dypterygia ordinarius|[8286]|BLPCC724-08|Costa Rica|Guanacaste|658[0n]|BOLD:AAA5250
Dypterygia ordinarius|[8287]|BLPBH424-07|Costa Rica|Guanacaste|658[0n]|BOLD:AAA5250
Dypterygia ordinarius|[8288]|GMCRA1705-13|Costa Rica|Guanacaste|631[0n]|BOLD:AAA5250
Dypterygia ordinarius|[8289]|MHMYO239-11|Costa Rica||658[0n]|BOLD:AAA5250
Dypterygia ordinarius|[8290]|MHMYO238-11|Costa Rica||658[0n]|BOLD:AAA5250
Dypterygia ordinarius|[8291]|MHMYL3387-11|Costa Rica||658[0n]|BOLD:AAA5250
Dypterygia ordinarius|[8292]|BLPDU244-11|Costa Rica|Guanacaste|658[0n]|BOLD:AAA5250
Dypterygia ordinarius|[8293]|BLPDT1648-10|Costa Rica|Guanacaste|658[0n]|BOLD:AAA5250
Dypterygia ordinarius|[8294]|BLPDT1429-10|Costa Rica|Guanacaste|658[0n]|BOLD:AAA5250
Dypterygia ordinarius|[8295]|BLPDK1707-09|Costa Rica|Guanacaste|658[0n]|BOLD:AAA5250
[illegible]

Dypterygia ordinarius|[8293]|BLPDT1648-10|Costa Rica|Guanacaste|658[0n]|BOLD:AAA5250
Dypterygia ordinarius|[8294]|BLPDT1429-10|Costa Rica|Guanacaste|658[0n]|BOLD:AAA5250
Dypterygia ordinarius|[8295]|BLPDK1707-09|Costa Rica|Guanacaste|658[0n]|BOLD:AAA5250
Dypterygia ordinarius|[8296]|BLPDK1302-09|Costa Rica|Guanacaste|658[0n]|BOLD:AAA5250
Dypterygia ordinarius|[8297]|BLPDK515-09|Costa Rica|Guanacaste|658[0n]|BOLD:AAA5250
Dypterygia ordinarius|[8298]|BLPCO355-08|Costa Rica|Guanacaste|658[0n]|BOLD:AAA5250
Dypterygia ordinarius|[8299]|BLPCO354-08|Costa Rica|Guanacaste|658[0n]|BOLD:AAA5250
Dypterygia ordinarius|[8300]|BLPCO353-08|Costa Rica|Guanacaste|658[0n]|BOLD:AAA5250
Dypterygia ordinarius|[8301]|BLPCO352-08|Costa Rica|Guanacaste|658[0n]|BOLD:AAA5250
Dypterygia ordinarius|[8302]|BLPCN034-08|Costa Rica|Guanacaste|658[0n]|BOLD:AAA5250
Dypterygia ordinarius|[8303]|BLPCD616-08|Costa Rica|Guanacaste|658[0n]|BOLD:AAA5250
Dypterygia ordinarius|[8304]|BLPCB466-08|Costa Rica|Alajuela|658[0n]|BOLD:AAA5250
Dypterygia ordinarius|[8305]|BLPCA867-08|Costa Rica|Guanacaste|658[0n]|BOLD:AAA5250
Dypterygia ordinarius|[8306]|BLPBH782-07|Costa Rica|Guanacaste|658[0n]|BOLD:AAA5250
Dypterygia ordinarius|[8307]|BLPBH423-07|Costa Rica|Guanacaste|658[0n]|BOLD:AAA5250
Dypterygia ordinarius|[8308]|BLPBH416-07|Costa Rica|Guanacaste|658[0n]|BOLD:AAA5250
Dypterygia ordinarius|[8309]|BLPBG813-07|Costa Rica|Guanacaste|658[0n]|BOLD:AAA5250
Dypterygia ordinarius|[8310]|BLPAF562-07|Costa Rica|Guanacaste|658[0n]|BOLD:AAA5250
Dypterygia ordinarius|[8311]|BLPAF061-07|Costa Rica|Guanacaste|658[0n]|BOLD:AAA5250
Dypterygia ordinarius|[8312]|MHAUA870-05|Costa Rica|Guanacaste|553[0n]|BOLD:AAA5250
Dypterygia ordinarius|[8313]|BLPDJ124-09|Costa Rica|Guanacaste|614[0n]|BOLD:AAA5250
Dypterygia ordinarius|[8314]|BLPDT1649-10|Costa Rica|Guanacaste|635[0n]|BOLD:AAA5250
Dypterygia ordinarius|[8315]|MHAUA862-05|Costa Rica|Guanacaste|558[0n]|BOLD:AAA5250
Dypterygia ordinarius|[8316]|MHAUA863-05|Costa Rica|Guanacaste|555[0n]|BOLD:AAA5250
Dypterygia ordinarius|[8317]|MHMYL3388-11|Costa Rica||621[0n]|BOLD:AAA5250
Dypterygia ordinarius|[8318]|MHMYO241-11|Costa Rica||658[0n]|BOLD:AAA5250
Dypterygia ordinarius|[8319]|MHMYT214-13|Costa Rica||658[0n]|BOLD:AAA5250
Dypterygia ordinarius|[8320]|BLPEF4953-13|Costa Rica|Guanacaste|658[0n]|BOLD:AAA5250
Dypterygia ordinarius|[8321]|BLPEF4954-13|Costa Rica|Guanacaste|658[0n]|BOLD:AAA5250
Dypterygia ordinarius|[8322]|CNCLB2002-14|Mexico||658[0n]|BOLD:AAA5250
Dypterygia ordinarius|[8323]|BLPDA461-09|Costa Rica|Alajuela|658[0n]|BOLD:AAA5250
Dypterygia ordinarius|[8324]|BLPDJ223-09|Costa Rica|Guanacaste|658[0n]|BOLD:AAA5250
Dypterygia ordinarius|[8325]|BLPAE678-06|Costa Rica|Guanacaste|658[0n]|BOLD:AAA5250
Dypterygia ordinarius|[8326]|BLPAF563-07|Costa Rica|Guanacaste|658[0n]|BOLD:AAA5250
Dypterygia ordinarius|[8327]|MHAUA865-05|Costa Rica|Guanacaste|658[0n]|BOLD:AAA5250
Dypterygia ordinarius|[8328]|MHAUA866-05|Costa Rica|Guanacaste|658[0n]|BOLD:AAA5250
Dypterygia ordinarius|[8329]|BLPAE679-06|Costa Rica|Guanacaste|658[0n]|BOLD:AAA5250
Dypterygia ordinarius|[8330]|BLPAE680-06|Costa Rica|Guanacaste|658[0n]|BOLD:AAA5250
Dypterygia ordinarius|[8331]|BLPAE681-06|Costa Rica|Guanacaste|658[0n]|BOLD:AAA5250
Dypterygia ordinarius|[8332]|BLPBH418-07|Costa Rica|Guanacaste|658[0n]|BOLD:AAA5250
Dypterygia ordinarius|[8333]|BLPBH421-07|Costa Rica|Guanacaste|658[0n]|BOLD:AAA5250
Dypterygia ordinarius|[8334]|BLPBH422-07|Costa Rica|Guanacaste|658[0n]|BOLD:AAA5250
Dypterygia ordinarius|[8335]|BLPCA262-08|Costa Rica|Guanacaste|658[0n]|BOLD:AAA5250
Dypterygia ordinarius|[8336]|BLPCN025-08|Costa Rica|Guanacaste|658[0n]|BOLD:AAA5250
Dypterygia ordinarius|[8337]|BLPCN866-08|Costa Rica|Guanacaste|658[0n]|BOLD:AAA5250
Dypterygia ordinarius|[8338]|BLPDT1650-10|Costa Rica|Guanacaste|658[0n]|BOLD:AAA5250
Dypterygia ordinarius|[8339]|BLPDT1660-10|Costa Rica|Guanacaste|658[0n]|BOLD:AAA5250
Dypterygia ordinarius|[8340]|MHMYQ026-12|Costa Rica||658[0n]|BOLD:AAA5250
Dypterygia ordinarius|[8341]|LNAUS4487-13|Costa Rica|Alajuela|658[0n]|BOLD:AAA5250
Dypterygia ordinarius|[8342]|BLPBH425-07|Costa Rica|Guanacaste|658[0n]|BOLD:AAA5250
Dypterygia ordinarius|[8343]|BLPCN098-08|Costa Rica|Guanacaste|658[0n]|BOLD:AAA5250
Dypterygia ordinarius|[8344]|BLPDK1709-09|Costa Rica|Guanacaste|658[0n]|BOLD:AAA5250
Dypterygia ordinarius|[8345]|BLPDU833-11|Costa Rica|Guanacaste|658[0n]|BOLD:AAA5250
Dypterygia ordinarius|[8346]|BLPEC610-11|Costa Rica|Alajuela|658[0n]|BOLD:AAA5250
Dypterygia ordinarius|[8347]|MHMYO236-11|Costa Rica||658[0n]|BOLD:AAA5250
Dypterygia ordinarius|[8348]|MHMYQ1777-12|Costa Rica||658[0n]|BOLD:AAA5250
Dypterygia ordinarius|[8349]|MHMYQ1778-12|Costa Rica||658[0n]|BOLD:AAA5250
Dypterygia ordinarius|[8350]|BLPAF693-07|Costa Rica|Guanacaste|658[0n]|BOLD:AAA5250
Dypterygia ordinarius|[8351]|BLPBH417-07|Costa Rica|Guanacaste|658[0n]|BOLD:AAA5250
Dypterygia ordinarius|[8352]|MHAUA869-05|Costa Rica|Alajuela|658[0n]|BOLD:AAA5250
Dypterygia ordinarius|[8353]|BLPAD906-06|Costa Rica|Alajuela|658[0n]|BOLD:AAA5250
Dypterygia ordinarius|[8354]|LNAUS4490-13|Costa Rica|Alajuela|658[0n]|BOLD:AAA5250
Dypterygia ordinarius|[8355]|BLPDJ222-09|Costa Rica|Guanacaste|658[0n]|BOLD:AAA5250
Dypterygia ordinarius|[8356]|BLPDT1651-10|Costa Rica|Guanacaste|658[0n]|BOLD:AAA5250
Dypterygia ordinarius|[8357]|BLPDV349-11|Costa Rica|Guanacaste|658[0n]|BOLD:AAA5250
Dypterygia ordinarius|[8358]|BLPBG814-07|Costa Rica|Guanacaste|655[0n]|BOLD:AAA5250
Dypterygia ordinarius|[8359]|CNCLB2003-14|Mexico||658[0n]|BOLD:AAA5250
Dypterygia ordinarius|[8360]|CNCLB2274-14|Puerto Rico|Mayaguez|658[0n]|BOLD:AAA5250
Orthodes bolteri|[8361]|RDNMD911-07|United States|Arizona|613[0n]|BOLD:AAI2219
Orthodes bolteri|[8362]|RDNMD910-07|United States|Arizona|655[0n]|BOLD:AAI2219
Orthodes bolteri|[8363]|RDNMG837-08|United States|Arizona|658[0n]|BOLD:AAI2219
Orthodes bolteri|[8364]|RDNMJ708-11|United States|Arizona|658[0n]|BOLD:AAI2219
Hexorthodes accurata|[8365]|CMAZA508-10|United States|Arizona|658[0n]|BOLD:AAD0730
Hexorthodes accurata|[8366]|RDNMG297-08|United States|Arizona|658[0n]|BOLD:AAD0730
Hexorthodes accurata|[8367]|AWCLB087-10|United States|Arizona|658[0n]|BOLD:AAD0730
Hexorthodes accurata|[8368]|CMAZA771-10|United States|Arizona|658[0n]|BOLD:AAD0730
Hexorthodes accurata|[8369]|JBAZ130-09|United States|Arizona|658[0n]|BOLD:AAD0730
Hexorthodes accurata|[8370]|CMAZA389-10|United States|Arizona|658[0n]|BOLD:AAD0730
Hexorthodes accurata|[8371]|CMAZA607-10|United States|Arizona|658[0n]|BOLD:AAD0730
Hexorthodes accurata|[8372]|AWCLB285-10|United States|Arizona|658[0n]|BOLD:AAD0730
Hexorthodes accurata|[8373]|RDNMJ737-11|United States|Arizona|658[0n]|BOLD:AAD0730
Hexorthodes accurata|[8374]|CMAZA979-12|United States|Arizona|658[0n]|BOLD:AAD0730
Hexorthodes emendata|[8375]|RDNMG262-08|United States|Texas|658[0n]|BOLD:AAD0730
Hexorthodes emendata|[8376]|LNAUT869-14|United States|Texas|658[0n]|BOLD:AAD0730
Hexorthodes emendata|[8377]|LNAUT870-14|United States|Texas|485[0n]|
Hexorthodes emendata|[8378]|LNAUT872-14|United States|Texas|658[1n]|BOLD:AAD0730
Hexorthodes citeria|[8379]|NAMUM085-08|United States|Arizona|658[0n]|BOLD:AAD0730
Hexorthodes citeria|[8380]|TML170-14|United States||658[0n]|BOLD:AAD0730
Hexorthodes citeria|[8381]|LNAUT2083-14|United States|Texas|658[0n]|BOLD:AAD0730
Hexorthodes citeria|[8382]|CNCLB2402-14|United States|Texas|658[0n]|BOLD:AAD0730
Pseudanarta flavidens|[8383]|RDNMD935-07|United States|Arizona|655[0n]|BOLD:AAH8187
Pseudanarta flavidens|[8384]|NAMUM064-08|United States|Arizona|657[0n]|BOLD:AAH8187
Pseudanarta flavidens|[8385]|RDNMK096-11|United States|New Mexico|658[0n]|BOLD:AAH8187
Protorthodes alfkenii|[8386]|BBLSY657-09|United States|Arizona|564[1n]|
Protorthodes alfkenii|[8387]|USLEP770-10|United States|Arizona|658[0n]|BOLD:AAC9621
Protorthodes alfkenii|[8388]|BBLSY042-09|United States|Arizona|658[0n]|BOLD:AAC9621
Protorthodes alfkenii|[8389]|BBLOE1342-12|United States|Arizona|658[0n]|BOLD:AAC9621
Protorthodes alfkenii|[8390]|BBLOB356-11|United States|Arizona|658[0n]|BOLD:AAC9621
Protorthodes alfkenii|[8391]|BBLSW783-09|United States|Arizona|654[0n]|BOLD:AAC9621
Protorthodes alfkenii|[8392]|BBLSX712-09|United States|Arizona|658[0n]|BOLD:AAC9621
Protorthodes alfkenii|[8393]|BBLSY387-09|United States|Arizona|658[0n]|BOLD:AAC9621
Protorthodes alfkenii|[8394]|BBLSX736-09|United States|Arizona|658[0n]|BOLD:AAC9621
Protorthodes alfkenii|[8395]|BBLSY388-09|United States|Arizona|658[0n]|BOLD:AAC9621
Protorthodes alfkenii|[8396]|BBLSY734-09|United States|Arizona|658[0n]|BOLD:AAC9621

Protorthodes alfkenii|[8393]|BBLSY387-09|United States|Arizona|658[0n]|BOLD:AAC9621
Protorthodes alfkenii|[8394]|BBLSX736-09|United States|Arizona|658[0n]|BOLD:AAC9621
Protorthodes alfkenii|[8395]|BBLSY388-09|United States|Arizona|658[0n]|BOLD:AAC9621
Protorthodes alfkenii|[8396]|BBLSY734-09|United States|Arizona|658[0n]|BOLD:AAC9621
Protorthodes alfkenii|[8397]|CMAZA146-09|United States|Arizona|658[0n]|BOLD:AAC9621
Protorthodes alfkenii|[8398]|LOCBD554-06|United States|California|658[0n]|BOLD:AAC9621
Protorthodes alfkenii|[8399]|BBLSY301-09|United States|Arizona|658[1n]|BOLD:AAC9621
Protorthodes alfkenii|[8400]|BBLSY305-09|United States|Arizona|658[0n]|BOLD:AAC9621
Protorthodes alfkenii|[8401]|BBLOE1358-12|United States|Arizona|658[0n]|BOLD:AAC9621
Protorthodes alfkenii|[8402]|BBLOE1336-12|United States|Arizona|658[0n]|BOLD:AAC9621
Protorthodes alfkenii|[8403]|BBLSY596-09|United States|Arizona|589[0n]|BOLD:AAC9621
Protorthodes alfkenii|[8404]|BBLSY619-09|United States|Arizona|589[0n]|BOLD:AAC9621
Protorthodes alfkenii|[8405]|BBLSY302-09|United States|Arizona|658[0n]|BOLD:AAC9621
Protorthodes alfkenii|[8406]|RDNMC597-06|United States|Wyoming|658[0n]|BOLD:AAC9621
Protorthodes alfkenii|[8407]|BBLSW424-09|United States|New Mexico|658[0n]|BOLD:AAC9621
Protorthodes alfkenii|[8408]|IAWLB279-11|United States|Arizona|658[0n]|BOLD:AAC9621
Protorthodes alfkenii|[8409]|BBLOE1330-12|United States|Arizona|658[0n]|BOLD:AAC9621
Protorthodes alfkenii|[8410]|BBLSW416-09|United States|New Mexico|658[0n]|BOLD:AAC9621
Protorthodes alfkenii|[8411]|BBLSX996-09|United States|Arizona|658[0n]|BOLD:AAC9621
Protorthodes alfkenii|[8412]|BBLSX751-09|United States|Arizona|658[0n]|BOLD:AAC9621
Protorthodes alfkenii|[8413]|BBLSX711-09|United States|Arizona|658[0n]|BOLD:AAC9621
Protorthodes alfkenii|[8414]|BBLSX619-09|United States|Arizona|658[0n]|BOLD:AAC9621
Protorthodes alfkenii|[8415]|BBLSW423-09|United States|New Mexico|658[0n]|BOLD:AAC9621
Protorthodes alfkenii|[8416]|RDNME978-08|United States|New Mexico|658[0n]|BOLD:AAC9621
Protorthodes alfkenii|[8417]|RDNME977-08|United States|New Mexico|658[0n]|BOLD:AAC9621
Protorthodes alfkenii|[8418]|RDNMG276-08|United States|Texas|658[0n]|BOLD:AAC9621
Protorthodes alfkenii|[8419]|RDNMC596-06|United States|New Mexico|658[0n]|BOLD:AAC9621
Protorthodes alfkenii|[8420]|BBLSX718-09|United States|Arizona|658[0n]|BOLD:AAC9621
Protorthodes alfkenii|[8421]|BBLSX998-09|United States|Arizona|658[0n]|BOLD:AAC9621
Protorthodes alfkenii|[8422]|BBLSY307-09|United States|Arizona|658[0n]|BOLD:AAC9621
Protorthodes alfkenii|[8423]|BBLSY386-09|United States|Arizona|658[0n]|BOLD:AAC9621
Protorthodes alfkenii|[8424]|BBLSY418-09|United States|Arizona|658[0n]|BOLD:AAC9621
Protorthodes alfkenii|[8425]|BBLSY681-09|United States|Arizona|658[0n]|BOLD:AAC9621
Protorthodes alfkenii|[8426]|BBLSY685-09|United States|Arizona|658[0n]|BOLD:AAC9621
Protorthodes alfkenii|[8427]|BBLSY733-09|United States|Arizona|658[0n]|BOLD:AAC9621
Protorthodes alfkenii|[8428]|BBLSY833-09|United States|Arizona|658[0n]|BOLD:AAC9621
Protorthodes alfkenii|[8429]|CMAZA154-09|United States|Arizona|658[0n]|BOLD:AAC9621
Protorthodes alfkenii|[8430]|IAWLB277-11|United States|Arizona|658[0n]|BOLD:AAC9621
Protorthodes alfkenii|[8431]|BBLOB556-11|United States|Arizona|658[0n]|BOLD:AAC9621
Protorthodes alfkenii|[8432]|BBLOE1340-12|United States|Arizona|658[0n]|BOLD:AAC9621
Protorthodes alfkenii|[8433]|BBLOE1345-12|United States|Arizona|658[0n]|BOLD:AAC9621
Protorthodes alfkenii|[8434]|BBLOE1357-12|United States|Arizona|658[0n]|BOLD:AAC9621
Protorthodes alfkenii|[8435]|CMAZA1045-12|United States|Arizona|658[0n]|BOLD:AAC9621
Protorthodes alfkenii|[8436]|CMAZA1049-12|United States|Arizona|658[0n]|BOLD:AAC9621
Protorthodes alfkenii|[8437]|LOCBF1243-13|United States|California|613[0n]|BOLD:AAC9621
Protorthodes alfkenii|[8438]|BBLOE1705-12|United States|Arizona|658[0n]|BOLD:AAC9621
Protorthodes alfkenii|[8439]|BBLOE1297-12|United States|Arizona|658[0n]|BOLD:AAC9621
Protorthodes alfkenii|[8440]|BBLOE1298-12|United States|Arizona|658[0n]|BOLD:AAC9621
Protorthodes alfkenii|[8441]|BBLSY390-09|United States|Arizona|658[0n]|BOLD:AAC9621
Protorthodes alfkenii|[8442]|BBLSW415-09|United States|New Mexico|658[0n]|BOLD:AAC9621
Protorthodes alfkenii|[8443]|LOCBF1213-13|United States|California|614[0n]|BOLD:AAC9621
Protorthodes alfkenii|[8444]|BBLOE1296-12|United States|Arizona|658[0n]|BOLD:AAC9621
Protorthodes alfkenii|[8445]|BBLOE1349-12|United States|Arizona|658[0n]|BOLD:AAC9621
Protorthodes alfkenii|[8446]|RDNMJ780-11|United States|Arizona|658[0n]|BOLD:AAC9621
Protorthodes alfkenii|[8447]|IAWLB195-11|United States|Arizona|658[0n]|BOLD:AAC9621
Protorthodes alfkenii|[8448]|BBLOB548-11|United States|Arizona|658[0n]|BOLD:AAC9621
Protorthodes alfkenii|[8449]|BBLSZ077-09|United States|Arizona|658[0n]|BOLD:AAC9621
Protorthodes alfkenii|[8450]|BBLSY738-09|United States|Arizona|658[0n]|BOLD:AAC9621
Protorthodes alfkenii|[8451]|BBLSY667-09|United States|Arizona|658[0n]|BOLD:AAC9621
Protorthodes alfkenii|[8452]|BBLSY391-09|United States|Arizona|658[0n]|BOLD:AAC9621
Protorthodes alfkenii|[8453]|BBLSX749-09|United States|Arizona|658[0n]|BOLD:AAC9621
Protorthodes alfkenii|[8454]|BBLSY019-09|United States|Arizona|658[0n]|BOLD:AAC9621
Protorthodes alfkenii|[8455]|BBLSY013-09|United States|Arizona|658[0n]|BOLD:AAC9621
Protorthodes alfkenii|[8456]|BBLSW436-09|United States|New Mexico|658[0n]|BOLD:AAC9621
Protorthodes alfkenii|[8457]|BBLSW425-09|United States|New Mexico|658[0n]|BOLD:AAC9621
Protorthodes alfkenii|[8458]|BBLSW126-09|United States|Arizona|658[0n]|BOLD:AAC9621
Protorthodes alfkenii|[8459]|BBLSW125-09|United States|Arizona|658[0n]|BOLD:AAC9621
Protorthodes alfkenii|[8460]|BBLOB551-11|United States|Arizona|658[0n]|BOLD:AAC9621
Protorthodes alfkenii|[8461]|RDNMH066-09|United States|Arizona|658[0n]|BOLD:AAC9621
Protorthodes alfkenii|[8462]|BBLSY605-09|United States|Arizona|589[0n]|BOLD:AAC9621
Protorthodes alfkenii|[8463]|BBLSY599-09|United States|Arizona|589[0n]|BOLD:AAC9621
Protorthodes alfkenii|[8464]|BBLSY293-09|United States|Arizona|639[0n]|BOLD:AAC9621
Protorthodes alfkenii|[8465]|BBLSY303-09|United States|Arizona|639[0n]|BOLD:AAC9621
Protorthodes alfkenii|[8466]|BBLSY304-09|United States|Arizona|622[0n]|BOLD:AAC9621
Protorthodes alfkenii|[8467]|BBLSY593-09|United States|Arizona|589[0n]|BOLD:AAC9621
Protorthodes alfkenii|[8468]|BBLSY594-09|United States|Arizona|589[0n]|BOLD:AAC9621
Protorthodes alfkenii|[8469]|LOCBB373-06|United States|California|658[0n]|BOLD:AAC9621
Protorthodes alfkenii|[8470]|BBLSY012-09|United States|Arizona|658[0n]|BOLD:AAC9621
Protorthodes alfkenii|[8471]|BBLSY389-09|United States|Arizona|658[0n]|BOLD:AAC9621
Protorthodes alfkenii|[8472]|BBLSY704-09|United States|Arizona|658[0n]|BOLD:AAC9621
Protorthodes alfkenii|[8473]|BBLOC1696-11|United States|California|658[0n]|BOLD:AAC9621
Protorthodes alfkenii|[8474]|BBLOD562-11|United States|California|658[0n]|BOLD:AAC9621
Protorthodes alfkenii|[8475]|BBLOE1339-12|United States|Arizona|658[0n]|BOLD:AAC9621
Protorthodes alfkenii|[8476]|BBLOE1355-12|United States|Arizona|658[0n]|BOLD:AAC9621
Protorthodes alfkenii|[8477]|BBLOE1874-12|United States|Arizona|523[0n]|BOLD:AAC9621
Protorthodes alfkenii|[8478]|BBLSX999-09|United States|Arizona|658[0n]|BOLD:AAC9621
Protorthodes alfkenii|[8479]|BBLSY737-09|United States|Arizona|658[0n]|BOLD:AAC9621
Protorthodes alfkenii|[8480]|BBLOE1300-12|United States|Arizona|658[0n]|BOLD:AAC9621
Protorthodes alfkenii|[8481]|BBLSY309-09|United States|Arizona|622[0n]|BOLD:AAC9621
Protorthodes alfkenii|[8482]|BBLSW129-09|United States|Arizona|658[0n]|BOLD:AAC9621
Protorthodes alfkenii|[8483]|BBLSY306-09|United States|Arizona|658[0n]|BOLD:AAC9621
Protorthodes alfkenii|[8484]|BBLSZ070-09|United States|Arizona|658[0n]|BOLD:AAC9621
Protorthodes alfkenii|[8485]|IAWLB194-11|United States|Arizona|658[0n]|BOLD:AAC9621
Protorthodes alfkenii|[8486]|BBLSY621-09|United States|Arizona|637[0n]|BOLD:AAC9621
Protorthodes alfkenii|[8487]|BBLSY624-09|United States|Arizona|589[0n]|BOLD:AAC9621
Protorthodes alfkenii|[8488]|BBLOE2017-12|United States|Arizona|658[0n]|BOLD:AAC9621
Protorthodes alfkenii|[8489]|SDRVF1666-14|United States|California|606[0n]|BOLD:AAC9621
Protorthodes alfkenii|[8490]|LOCBD421-06|United States|California|658[0n]|BOLD:AAC9621
Protorthodes alfkenii|[8491]|LOCBF3466-14|United States|California|549[0n]|BOLD:AAC9621
Protorthodes alfkenii|[8492]|BBLOD556-11|United States|California|658[0n]|BOLD:AAC9621
Protorthodes alfkenii|[8493]|LOCBF2593-13|United States|California|658[0n]|BOLD:AAC9621
Protorthodes alfkenii|[8494]|BBLOC1697-11|United States|California|658[0n]|BOLD:AAC9621
Protorthodes alfkenii|[8495]|BBLOD329-11|United States|California|658[0n]|BOLD:AAC9621
[illegible]

Protorthodes alfkenii|[8493]|LOCBF2593-13|United States|California|658[0n]|BOLD:AAC9621
Protorthodes alfkenii|[8494]|BBLOC1697-11|United States|California|658[0n]|BOLD:AAC9621
Protorthodes alfkenii|[8495]|BBLOD329-11|United States|California|658[0n]|BOLD:AAC9621
Protorthodes alfkenii|[8496]|BBLOD332-11|United States|California|658[0n]|BOLD:AAC9621
Protorthodes alfkenii|[8497]|BBLOD568-11|United States|California|658[0n]|BOLD:AAC9621
Protorthodes alfkenii|[8498]|BBLOE1262-12|United States|California|658[0n]|BOLD:AAC9621
Protorthodes alfkenii|[8499]|GMLC1056-12|United States|California|658[0n]|BOLD:AAC9621
Protorthodes alfkenii|[8500]|LOCBF2594-13|United States|California|658[0n]|BOLD:AAC9621
Protorthodes alfkenii|[8501]|BBLOC1700-11|United States|California|658[0n]|BOLD:AAC9621
Protorthodes alfkenii|[8502]|BBLOD565-11|United States|California|658[0n]|BOLD:AAC9621
Protorthodes alfkenii|[8503]|LOCBF2597-13|United States|California|658[0n]|BOLD:AAC9621
Protorthodes alfkenii|[8504]|BBLOC1704-11|United States|California|658[0n]|BOLD:AAC9621
Protorthodes alfkenii|[8505]|BBLOC1705-11|United States|California|658[0n]|BOLD:AAC9621
Protorthodes alfkenii|[8506]|BBLOD495-11|United States|California|658[0n]|BOLD:AAC9621
Protorthodes alfkenii|[8507]|LOCBF2606-13|United States|California|658[0n]|BOLD:AAC9621
Protorthodes alfkenii|[8508]|LOCBF2662-13|United States|California|658[0n]|BOLD:AAC9621
Protorthodes alfkenii|[8509]|IAWLB278-11|United States|Arizona|658[0n]|BOLD:AAC9621
Protorthodes alfkenii|[8510]|BBLOD1707-11|United States|California|658[0n]|BOLD:AAC9621
Protorthodes alfkenii|[8511]|GMLC1191-12|United States|California|658[0n]|BOLD:AAC9621
Protorthodes alfkenii|[8512]|LOCBF3472-14|United States|California|591[0n]|BOLD:AAC9621
Hyperepia jugifera|[8513]|CNCLB281-14|United States|Texas|658[0n]|BOLD:ACM4005
Hyperepia jugifera|[8514]|CNCLB282-14|United States|Texas|658[0n]|BOLD:ACM4005
Protorthodes antennata|[8515]|RDNMJ781-11|United States|Arizona|658[0n]|BOLD:AAV3077
Protorthodes antennata|[8516]|BBLOB550-11|United States|Arizona|658[0n]|BOLD:AAV3077
Protorthodes antennata|[8517]|BBLOD1846-11|United States|Arizona|658[0n]|BOLD:AAV3077
Protorthodes antennata|[8518]|BBLOE1343-12|United States|Arizona|658[0n]|BOLD:AAV3077
Protorthodes antennata|[8519]|RDNMJ654-11|United States|Arizona|658[0n]|BOLD:AAV3077
Protorthodes antennata|[8520]|BBLOE1338-12|United States|Arizona|658[0n]|BOLD:AAV3077
Protorthodes antennata|[8521]|BBLOE1744-12|United States|Arizona|658[0n]|BOLD:AAV3077
Protorthodes antennata|[8522]|CMAZA953-12|United States|Arizona|658[0n]|BOLD:AAV3077
Protorthodes perforata|[8523]|RDNMG820-08|United States|California|658[0n]|BOLD:AAH7638
Protorthodes perforata|[8524]|RDNMH085-09|United States|New Mexico|658[0n]|BOLD:AAH7638
Protorthodes rufula|[8525]|LOCB686-06|United States|California|658[0n]|BOLD:AAB0747
Protorthodes rufula|[8526]|LOCBC012-06|United States|California|658[0n]|BOLD:AAB0747
Protorthodes rufula|[8527]|RDNMC592-06|United States|Oregon|519[0n]|BOLD:AAB0747
Protorthodes rufula|[8528]|GMLC1018-12|United States|California|658[0n]|BOLD:AAB0747
Protorthodes rufula|[8529]|GMLC1204-12|United States|California|658[0n]|BOLD:AAB0747
Protorthodes rufula|[8530]|LOCB688-06|United States|California|658[0n]|BOLD:AAB0747
Protorthodes rufula|[8531]|LOCB699-06|United States|California|658[0n]|BOLD:AAB0747
Protorthodes rufula|[8532]|RDNMG859-08|United States|California|658[0n]|BOLD:AAB0747
Protorthodes rufula|[8533]|LOCBB302-06|United States|California|627[0n]|BOLD:AAB0747
Protorthodes rufula|[8534]|LOCBB298-06|United States|California|658[0n]|BOLD:AAB0747
Protorthodes rufula|[8535]|LOCBB651-06|United States|California|658[0n]|BOLD:AAB0747
Protorthodes rufula|[8536]|LOCB707-06|United States|California|658[0n]|BOLD:AAB0747
Protorthodes rufula|[8537]|LOCB831-06|United States|California|658[0n]|BOLD:AAB0747
Protorthodes rufula|[8538]|RDNMD426-06|United States|California|658[0n]|BOLD:AAB0747
Protorthodes rufula|[8539]|LOCB494-06|United States|California|658[0n]|BOLD:AAB0747
Protorthodes rufula|[8540]|LOCB634-06|United States|California|658[0n]|BOLD:AAB0747
Protorthodes rufula|[8541]|LOCB687-06|United States|California|658[0n]|BOLD:AAB0747
Protorthodes rufula|[8542]|LOCB689-06|United States|California|658[0n]|BOLD:AAB0747
Protorthodes rufula|[8543]|LOCB701-06|United States|California|658[0n]|BOLD:AAB0747
Protorthodes rufula|[8544]|LOCB702-06|United States|California|658[0n]|BOLD:AAB0747
Protorthodes rufula|[8545]|LOCB704-06|United States|California|658[0n]|BOLD:AAB0747
Protorthodes rufula|[8546]|LOCB706-06|United States|California|658[0n]|BOLD:AAB0747
Protorthodes rufula|[8547]|LOCB716-06|United States|California|658[0n]|BOLD:AAB0747
Protorthodes rufula|[8548]|LOCB773-06|United States|California|658[0n]|BOLD:AAB0747
Protorthodes rufula|[8549]|LOCBB299-06|United States|California|658[0n]|BOLD:AAB0747
Protorthodes rufula|[8550]|LOCBB300-06|United States|California|658[0n]|BOLD:AAB0747
Protorthodes rufula|[8551]|LOCBB301-06|United States|California|658[0n]|BOLD:AAB0747
Protorthodes rufula|[8552]|RDNMD425-06|United States|California|658[0n]|BOLD:AAB0747
Protorthodes rufula|[8553]|RDNMD427-06|United States|California|658[0n]|BOLD:AAB0747
Protorthodes rufula|[8554]|RDNMG860-08|United States|California|658[0n]|BOLD:AAB0747
Protorthodes rufula|[8555]|RWWB635-10|United States|Washington|639[0n]|BOLD:AAB0747
Protorthodes rufula|[8556]|GMLC477-11|United States|California|658[0n]|BOLD:AAB0747
Protorthodes rufula|[8557]|CGLCA155-10|United States|California|658[0n]|BOLD:AAB0747
Protorthodes rufula|[8558]|GMLC229-11|United States|California|658[0n]|BOLD:AAB0747
Protorthodes rufula|[8559]|GMLC352-11|United States|California|658[0n]|BOLD:AAB0747
Protorthodes rufula|[8560]|GMLC368-11|United States|California|658[0n]|BOLD:AAB0747
Protorthodes rufula|[8561]|GMLC369-11|United States|California|658[0n]|BOLD:AAB0747
Protorthodes rufula|[8562]|GMLC424-11|United States|California|658[0n]|BOLD:AAB0747
Protorthodes rufula|[8563]|GMLC448-11|United States|California|658[0n]|BOLD:AAB0747
Protorthodes rufula|[8564]|GMLC450-11|United States|California|658[0n]|BOLD:AAB0747
Protorthodes rufula|[8565]|GMLC452-11|United States|California|658[0n]|BOLD:AAB0747
Protorthodes rufula|[8566]|GMLC453-11|United States|California|658[0n]|BOLD:AAB0747
Protorthodes rufula|[8567]|GMLC462-11|United States|California|658[0n]|BOLD:AAB0747
Protorthodes rufula|[8568]|GMLC465-11|United States|California|658[0n]|BOLD:AAB0747
Protorthodes rufula|[8569]|GMLC500-11|United States|California|658[0n]|BOLD:AAB0747
Protorthodes rufula|[8570]|GMLC514-11|United States|California|658[0n]|BOLD:AAB0747
Protorthodes rufula|[8571]|GMLC1017-12|United States|California|658[0n]|BOLD:AAB0747
Protorthodes rufula|[8572]|GMLC1095-12|United States|California|658[0n]|BOLD:AAB0747
Protorthodes rufula|[8573]|GMLC510-11|United States|California|658[0n]|BOLD:AAB0747
Protorthodes rufula|[8574]|RWWA977-09|United States|Washington|658[0n]|BOLD:AAB0747
Protorthodes rufula|[8575]|RDNMD423-06|United States|California|658[0n]|BOLD:AAB0747
Protorthodes rufula|[8576]|RWWB073-09|United States|Washington|658[0n]|BOLD:AAB0747
Protorthodes rufula|[8577]|GMLC386-11|United States|California|658[0n]|BOLD:AAB0747
Protorthodes rufula|[8578]|GMLC451-11|United States|California|658[0n]|BOLD:AAB0747
Protorthodes rufula|[8579]|GMLC511-11|United States|California|658[0n]|BOLD:AAB0747
Protorthodes rufula|[8580]|GMLC1020-12|United States|California|658[0n]|BOLD:AAB0747
Protorthodes rufula|[8581]|GMLC1126-12|United States|California|658[0n]|BOLD:AAB0747
Protorthodes rufula|[8582]|GMLC1175-12|United States|California|658[0n]|BOLD:AAB0747
Protorthodes rufula|[8583]|GMLC1449-12|United States|California|600[0n]|BOLD:AAB0747
Protorthodes rufula|[8584]|GMLC1450-12|United States|California|600[0n]|BOLD:AAB0747
Protorthodes rufula|[8585]|LOCBF204-13|United States|California|611[0n]|BOLD:AAB0747
Protorthodes argentoppida|[8586]|RDNMC745-06|United States|New Mexico|658[0n]|BOLD:AAH7634
Protorthodes argentoppida|[8587]|RDNMG871-08|United States|New Mexico|592[0n]|BOLD:AAH7634
Trichopolia dentatella|[8588]|RDNME979-08|United States|New Mexico|658[0n]|BOLD:AAD9929
Trichopolia sp.|[8589]|RDNMJ359-11|United States|New Mexico|658[0n]|BOLD:AAD9929
Trichopolia dentatella|[8590]|NAMUM134-08|United States|California|658[0n]|BOLD:AAD9928
Trichopolia dentatella|[8591]|NAMUM144-08|United States|California|658[0n]|BOLD:AAD9928
Trichopolia dentatella|[8592]|IAWLB274-11|United States|Arizona|658[0n]|BOLD:AAD9928
Trichopolia dentatella|[8593]|RDNME991-08|United States|New Mexico|658[0n]|BOLD:AAD9928
Trichopolia dentatella|[8594]|NAMUM110-08|United States|Texas|656[0n]|BOLD:AAD9928
Trichopolia dentatella|[8595]|IAWLB275-11|United States|Arizona|658[0n]|BOLD:AAD9928
[illegible]

Trichopolia dentatella|[8593]|RDNME991-08|United States|New Mexico|658[0n]|BOLD:AAD9928
Trichopolia dentatella|[8594]|NAMUM110-08|United States|Texas|656[0n]|BOLD:AAD9928
Trichopolia dentatella|[8595]|IAWLB275-11|United States|Arizona|658[0n]|BOLD:AAD9928
Trichopolia dentatella|[8596]|IAWLB276-11|United States|Arizona|658[0n]|BOLD:AAD9928
Protorthodes ustulata|[8597]|RDNMG566-08|United States|Colorado|658[0n]|BOLD:AAF6263
Protorthodes ustulata|[8598]|RDNMH083-09|United States|Arizona|658[0n]|BOLD:AAF6263
Protorthodes ustulata|[8599]|USLEP563-10|United States|Colorado|658[0n]|BOLD:AAF6263
Protorthodes ustulata|[8600]|RDNMH078-09|United States|Arizona|658[0n]|BOLD:AAF6263
Protorthodes ustulata|[8601]|USLEP564-10|United States|Colorado|658[0n]|BOLD:AAF6263
Protorthodes ustulata|[8602]|USLEP565-10|United States|Colorado|658[0n]|BOLD:AAF6263
Protorthodes ustulata|[8603]|RDNME1007-08|United States|New Mexico|658[0n]|BOLD:AAF6263
Protorthodes ustulata|[8604]|RDNME1008-08|United States|New Mexico|658[0n]|BOLD:AAF6263
Protorthodes ustulata|[8605]|RDNMG887-08|United States|New Mexico|658[0n]|BOLD:AAF6263
Protorthodes ustulata|[8606]|RDNMG990-08|United States|Colorado|658[0n]|BOLD:AAF6263
Protorthodes ustulata|[8607]|USLEP561-10|United States|Colorado|658[0n]|BOLD:AAF6263
Protorthodes ustulata|[8608]|USLEP562-10|United States|Colorado|658[0n]|BOLD:AAF6263
Protorthodes ustulata|[8609]|USLEP568-10|United States|Colorado|658[0n]|BOLD:AAF6263
Protorthodes ustulata|[8610]|IAWLB585-11|United States|Arizona|658[0n]|BOLD:AAF6263
Protorthodes incincta|[8611]|RDNMB284-05|Canada|Manitoba|658[0n]|BOLD:AAB5164
Protorthodes incincta|[8612]|RDNMF758-08|Canada|Alberta|658[0n]|BOLD:AAB5164
Protorthodes incincta|[8613]|RDNMB282-05|Canada|Ontario|658[0n]|BOLD:AAB5164
Protorthodes incincta|[8614]|RDNMB283-05|Canada|Ontario|598[0n]|BOLD:AAB5164
Protorthodes incincta|[8615]|RDMAB711-06|Canada|Alberta|576[1n]|BOLD:AAB5164
Protorthodes incincta|[8616]|RDMAB712-06|Canada|Alberta|658[0n]|BOLD:AAB5164
Protorthodes incincta|[8617]|RDNMF764-08|Canada|Alberta|658[0n]|BOLD:AAB5164
Protorthodes incincta|[8618]|RDNMF763-08|Canada|Alberta|658[0n]|BOLD:AAB5164
Protorthodes incincta|[8619]|RDNMG408-08|United States|Colorado|658[0n]|BOLD:AAB5164
Protorthodes incincta|[8620]|RDNMG407-08|United States|Colorado|658[0n]|BOLD:AAB5164
Protorthodes incincta|[8621]|RDNMF591-08|Canada|Saskatchewan|658[0n]|BOLD:AAB5164
Protorthodes incincta|[8622]|RDNMB280-05|Canada|Alberta|658[0n]|BOLD:AAB5164
Protorthodes incincta|[8623]|RDNMB277-05|Canada|Alberta|658[0n]|BOLD:AAB5164
Protorthodes incincta|[8624]|RDNMB278-05|Canada|Alberta|573[0n]|BOLD:AAB5164
Protorthodes incincta|[8625]|RDNMB279-05|Canada|Alberta|658[0n]|BOLD:AAB5164
Protorthodes incincta|[8626]|RDNMB281-05|Canada|Alberta|658[0n]|BOLD:AAB5164
Protorthodes incincta|[8627]|RDNMF765-08|Canada|Alberta|658[0n]|BOLD:AAB5164
Protorthodes incincta|[8628]|RDNMF766-08|Canada|Alberta|658[0n]|BOLD:AAB5164
Protorthodes incincta|[8629]|RDNMF767-08|Canada|Alberta|658[0n]|BOLD:AAB5164
Protorthodes incincta|[8630]|RDNMG881-08|United States|Colorado|658[0n]|BOLD:AAB5164
Protorthodes incincta|[8631]|RDNMG862-08|United States|Wyoming|658[0n]|BOLD:AAB5164
Protorthodes incincta|[8632]|RDNMG869-08|United States|Wyoming|658[0n]|BOLD:AAB5164
Protorthodes incincta|[8633]|RDNMG870-08|United States|Wyoming|658[0n]|BOLD:AAB5164
Protorthodes incincta|[8634]|RDNMH080-09|United States|Wyoming|658[0n]|BOLD:AAB5164
Protorthodes incincta|[8635]|RDNMG883-08|United States|Colorado|658[0n]|BOLD:AAB5164
Protorthodes incincta|[8636]|RDNMJ793-11|United States|Utah|658[0n]|BOLD:AAB5164
Protorthodes incincta|[8637]|CMAZA170-09|United States|Arizona|658[0n]|BOLD:AAB5164
Protorthodes incincta|[8638]|CMAZA147-09|United States|Arizona|658[0n]|BOLD:AAB5164
Protorthodes incincta|[8639]|RDNMH079-09|United States|New Mexico|658[0n]|BOLD:AAB5164
Protorthodes incincta|[8640]|RDNMG882-08|United States|New Mexico|658[0n]|BOLD:AAB5164
Protorthodes incincta|[8641]|BBLSY383-09|United States|Arizona|658[0n]|BOLD:AAB5164
Protorthodes incincta|[8642]|CMAZA119-09|United States|Arizona|622[0n]|BOLD:AAB5164
Protorthodes incincta|[8643]|RDNME1014-08|United States|New Mexico|658[0n]|BOLD:AAB5164
Protorthodes incincta|[8644]|RDNMH077-09|United States|New Mexico|658[0n]|BOLD:AAB5164
Protorthodes incincta|[8645]|RDNME1010-08|United States|New Mexico|658[0n]|BOLD:AAB5164
Protorthodes incincta|[8646]|RDNME1012-08|United States|New Mexico|658[0n]|BOLD:AAB5164
Protorthodes incincta|[8647]|CMAZA116-09|United States|Arizona|658[0n]|BOLD:AAB5164
Protorthodes incincta|[8648]|CMAZA956-12|United States|Arizona|658[0n]|BOLD:AAB5164
Protorthodes eureka|[8649]|RDMAB713-06|Canada|Alberta|607[0n]|BOLD:AAD9514
Protorthodes eureka|[8650]|RDMAB714-06|Canada|Alberta|658[0n]|BOLD:AAD9514
Protorthodes eureka|[8651]|RDNMB285-05|United States|Montana|571[1n]|BOLD:AAD9514
Protorthodes eureka|[8652]|RDNMG868-08|United States|Wyoming|658[0n]|BOLD:AAD9514
Protorthodes eureka|[8653]|RDNMH081-09|United States|Wyoming|658[0n]|BOLD:AAD9514
Protorthodes curtica|[8654]|RDNMG817-08|United States|California|592[0n]|BOLD:ABZ7207
Protorthodes curtica|[8655]|JMMMB590-13|United States|California|603[0n]|BOLD:ABZ7207
Protorthodes curtica|[8656]|RDNMG818-08|United States|California|609[1n]|BOLD:ABZ7207
Protorthodes curtica|[8657]|LBCH7185-10|Canada|British Columbia|658[0n]|BOLD:ABZ7207
Protorthodes curtica|[8658]|LBCH7258-10|Canada|British Columbia|626[0n]|BOLD:ABZ7207
Protorthodes curtica|[8659]|LBCH7340-10|Canada|British Columbia|658[0n]|BOLD:ABZ7207
Protorthodes curtica|[8660]|LBCH7336-10|Canada|British Columbia|636[0n]|BOLD:ABZ7207
Protorthodes curtica|[8661]|LBCH7812-10|Canada|British Columbia|658[0n]|BOLD:ABZ7207
Protorthodes curtica|[8662]|LBCH7794-10|Canada|British Columbia|658[0n]|BOLD:ABZ7207
Protorthodes curtica|[8663]|LBCH7679-10|Canada|British Columbia|658[0n]|BOLD:ABZ7207
Protorthodes curtica|[8664]|LBCH7616-10|Canada|British Columbia|658[0n]|BOLD:ABZ7207
Protorthodes curtica|[8665]|LBCH7615-10|Canada|British Columbia|658[0n]|BOLD:ABZ7207
Protorthodes curtica|[8666]|LBCH7614-10|Canada|British Columbia|658[0n]|BOLD:ABZ7207
Protorthodes curtica|[8667]|LBCH7613-10|Canada|British Columbia|658[0n]|BOLD:ABZ7207
Protorthodes curtica|[8668]|LBCH7610-10|Canada|British Columbia|658[0n]|BOLD:ABZ7207
Protorthodes curtica|[8669]|LBCH7609-10|Canada|British Columbia|658[0n]|BOLD:ABZ7207
Protorthodes curtica|[8670]|LBCH7556-10|Canada|British Columbia|658[0n]|BOLD:ABZ7207
Protorthodes curtica|[8671]|LBCH7477-10|Canada|British Columbia|658[0n]|BOLD:ABZ7207
Protorthodes curtica|[8672]|LBCH7430-10|Canada|British Columbia|658[0n]|BOLD:ABZ7207
Protorthodes curtica|[8673]|LBCH7335-10|Canada|British Columbia|658[0n]|BOLD:ABZ7207
Protorthodes curtica|[8674]|LBCH7295-10|Canada|British Columbia|658[0n]|BOLD:ABZ7207
Protorthodes curtica|[8675]|LBCH7294-10|Canada|British Columbia|658[0n]|BOLD:ABZ7207
Protorthodes curtica|[8676]|LBCH7293-10|Canada|British Columbia|658[0n]|BOLD:ABZ7207
Protorthodes curtica|[8677]|LBCH7291-10|Canada|British Columbia|658[0n]|BOLD:ABZ7207
Protorthodes curtica|[8678]|LBCH7290-10|Canada|British Columbia|658[0n]|BOLD:ABZ7207
Protorthodes curtica|[8679]|LBCH7289-10|Canada|British Columbia|658[0n]|BOLD:ABZ7207
Protorthodes curtica|[8680]|LBCH7288-10|Canada|British Columbia|658[0n]|BOLD:ABZ7207
Protorthodes curtica|[8681]|LBCH7260-10|Canada|British Columbia|658[0n]|BOLD:ABZ7207
Protorthodes curtica|[8682]|LBCH7254-10|Canada|British Columbia|658[0n]|BOLD:ABZ7207
Protorthodes curtica|[8683]|LBCH7253-10|Canada|British Columbia|658[0n]|BOLD:ABZ7207
Protorthodes curtica|[8684]|LBCH7191-10|Canada|British Columbia|658[0n]|BOLD:ABZ7207
Protorthodes curtica|[8685]|LBCH7189-10|Canada|British Columbia|658[0n]|BOLD:ABZ7207
Protorthodes curtica|[8686]|LBCH7188-10|Canada|British Columbia|658[0n]|BOLD:ABZ7207
Protorthodes curtica|[8687]|LBCH7186-10|Canada|British Columbia|658[0n]|BOLD:ABZ7207
Protorthodes curtica|[8688]|LBCH7184-10|Canada|British Columbia|658[0n]|BOLD:ABZ7207
Protorthodes curtica|[8689]|LBCH7183-10|Canada|British Columbia|658[0n]|BOLD:ABZ7207
Protorthodes curtica|[8690]|LBCH7113-10|Canada|British Columbia|658[0n]|BOLD:ABZ7207
Protorthodes curtica|[8691]|LBCH7112-10|Canada|British Columbia|658[0n]|BOLD:ABZ7207
Protorthodes curtica|[8692]|LBCH7111-10|Canada|British Columbia|658[0n]|BOLD:ABZ7207
Protorthodes curtica|[8693]|LBCH7110-10|Canada|British Columbia|658[0n]|BOLD:ABZ7207
Protorthodes curtica|[8694]|LBCH7109-10|Canada|British Columbia|658[0n]|BOLD:ABZ7207
Protorthodes curtica|[8695]|LBCH7108-10|Canada|British Columbia|658[0n]|BOLD:ABZ7207

Protorthodes curtica|[8693]|LBCH7110-10|Canada|British Columbia|658[0n]|BOLD:ABZ7207
Protorthodes curtica|[8694]|LBCH7109-10|Canada|British Columbia|658[0n]|BOLD:ABZ7207
Protorthodes curtica|[8695]|LBCH7108-10|Canada|British Columbia|658[0n]|BOLD:ABZ7207
Protorthodes curtica|[8696]|LBCH7107-10|Canada|British Columbia|658[0n]|BOLD:ABZ7207
Protorthodes curtica|[8697]|LBCH7106-10|Canada|British Columbia|658[0n]|BOLD:ABZ7207
Protorthodes curtica|[8698]|LBCH7104-10|Canada|British Columbia|658[0n]|BOLD:ABZ7207
Protorthodes curtica|[8699]|LBCH6999-10|Canada|British Columbia|658[0n]|BOLD:ABZ7207
Protorthodes curtica|[8700]|LBCH6998-10|Canada|British Columbia|658[0n]|BOLD:ABZ7207
Protorthodes curtica|[8701]|LBCH6997-10|Canada|British Columbia|658[0n]|BOLD:ABZ7207
Protorthodes curtica|[8702]|LBCH6996-10|Canada|British Columbia|658[0n]|BOLD:ABZ7207
Protorthodes curtica|[8703]|LBCH6995-10|Canada|British Columbia|658[0n]|BOLD:ABZ7207
Protorthodes curtica|[8704]|LBCH6994-10|Canada|British Columbia|658[0n]|BOLD:ABZ7207
Protorthodes curtica|[8705]|LBCH6993-10|Canada|British Columbia|658[0n]|BOLD:ABZ7207
Protorthodes curtica|[8706]|LBCH6992-10|Canada|British Columbia|658[0n]|BOLD:ABZ7207
Protorthodes curtica|[8707]|LBCH6934-10|Canada|British Columbia|658[0n]|BOLD:ABZ7207
Protorthodes curtica|[8708]|LBCH6928-10|Canada|British Columbia|658[0n]|BOLD:ABZ7207
Protorthodes curtica|[8709]|LBCH6831-10|Canada|British Columbia|658[0n]|BOLD:ABZ7207
Protorthodes curtica|[8710]|LBCH6830-10|Canada|British Columbia|658[0n]|BOLD:ABZ7207
Protorthodes curtica|[8711]|LBCH6792-10|Canada|British Columbia|658[0n]|BOLD:ABZ7207
Protorthodes curtica|[8712]|LBCH6791-10|Canada|British Columbia|658[0n]|BOLD:ABZ7207
Protorthodes curtica|[8713]|LBCH6764-10|Canada|British Columbia|658[0n]|BOLD:ABZ7207
Protorthodes curtica|[8714]|LBCH6547-10|Canada|British Columbia|658[0n]|BOLD:ABZ7207
Protorthodes curtica|[8715]|RDNMG816-08|United States|California|658[0n]|BOLD:ABZ7207
Protorthodes curtica|[8716]|LBCH7612-10|Canada|British Columbia|644[0n]|BOLD:ABZ7207
Protorthodes curtica|[8717]|LBCH7611-10|Canada|British Columbia|642[0n]|BOLD:ABZ7207
Protorthodes curtica|[8718]|LBCH6916-10|Canada|British Columbia|629[0n]|BOLD:ABZ7207
Protorthodes curtica|[8719]|LBCH6929-10|Canada|British Columbia|634[0n]|BOLD:ABZ7207
Protorthodes curtica|[8720]|LBCH7256-10|Canada|British Columbia|641[0n]|BOLD:ABZ7207
Protorthodes curtica|[8721]|LBCH7257-10|Canada|British Columbia|622[0n]|BOLD:ABZ7207
Protorthodes curtica|[8722]|LBCH7259-10|Canada|British Columbia|626[0n]|BOLD:ABZ7207
Protorthodes curtica|[8723]|LBCH7292-10|Canada|British Columbia|626[0n]|BOLD:ABZ7207
Protorthodes curtica|[8724]|LBCH7883-10|Canada|British Columbia|658[0n]|BOLD:ABZ7207
Protorthodes curtica|[8725]|LBCH7886-10|Canada|British Columbia|658[0n]|BOLD:ABZ7207
Protorthodes curtica|[8726]|LBCH7888-10|Canada|British Columbia|658[0n]|BOLD:ABZ7207
Protorthodes curtica|[8727]|LBCH7889-10|Canada|British Columbia|658[0n]|BOLD:ABZ7207
Protorthodes curtica|[8728]|LBCH7890-10|Canada|British Columbia|658[0n]|BOLD:ABZ7207
Protorthodes curtica|[8729]|LBCH7891-10|Canada|British Columbia|658[0n]|BOLD:ABZ7207
Protorthodes curtica|[8730]|LBCH7892-10|Canada|British Columbia|658[0n]|BOLD:ABZ7207
Protorthodes curtica|[8731]|LBCH7893-10|Canada|British Columbia|658[0n]|BOLD:ABZ7207
Protorthodes curtica|[8732]|JMMMB354-11|United States|California|658[0n]|BOLD:ABZ7207
Protorthodes curtica|[8733]|JMMMB403-11|United States|California|658[0n]|BOLD:ABZ7207
Protorthodes curtica|[8734]|JMMMB408-11|United States|California|658[0n]|BOLD:ABZ7207
Protorthodes curtica|[8735]|RDNMB274-05|Canada|British Columbia|538[0n]|BOLD:ABZ7207
Protorthodes curtica|[8736]|LBCH7255-10|Canada|British Columbia|658[0n]|BOLD:ABZ7207
Protorthodes curtica|[8737]|JMMMB604-13|United States|California|600[0n]|BOLD:ABZ7207
Protorthodes texicana|[8738]|RDNMG872-08|United States|Texas|642[0n]|BOLD:AAH7637
Protorthodes texicana|[8739]|RDNMG873-08|United States|Texas|658[0n]|BOLD:AAH7637
Protorthodes texicana|[8740]|USLEP1066-10|United States|Texas|658[0n]|BOLD:AAH7637
Protorthodes mulina|[8741]|RDNMG762-08|United States|Arizona|658[0n]|BOLD:AAF6275
Protorthodes mulina|[8742]|RDNMG896-08|United States|Arizona|658[0n]|BOLD:AAF6275
Protorthodes mulina|[8743]|BBLSY382-09|United States|Arizona|658[0n]|BOLD:AAF6275
Protorthodes mulina|[8744]|CMAZA003-09|United States|Arizona|658[0n]|BOLD:AAF6275
Protorthodes mulina|[8745]|CMAZA292-09|United States|Arizona|658[0n]|BOLD:AAF6275
Protorthodes mulina|[8746]|RDNMG763-08|United States|Arizona|658[0n]|BOLD:AAF6275
Protorthodes mulina|[8747]|CMAZA983-12|United States|Arizona|658[0n]|BOLD:AAF6275
Protorthodes mulina|[8748]|CMAZA121-09|United States|Arizona|658[0n]|BOLD:AAF6275
Protorthodes mulina|[8749]|IAWLB248-11|United States|Arizona|658[0n]|BOLD:AAF6275
Protorthodes mulina|[8750]|BBLSY384-09|United States|Arizona|658[0n]|BOLD:AAF6275
Protorthodes mulina|[8751]|BBLSY385-09|United States|Arizona|658[0n]|BOLD:AAF6275
Protorthodes mulina|[8752]|LYOMX041-12|Mexico|Michoacan|658[0n]|BOLD:AAF6275
Protorthodes mulina|[8753]|LYOMX146-12|Mexico|Michoacan|658[0n]|BOLD:AAF6275
Protorthodes oviduca|[8754]|LOWCE755-06|Canada|British Columbia|658[0n]|BOLD:AAB0331
Protorthodes oviduca|[8755]|LOWCE756-06|Canada|British Columbia|658[0n]|BOLD:AAB0331
Protorthodes oviduca|[8756]|LOWCD541-06|Canada|British Columbia|658[0n]|BOLD:AAB0331
Protorthodes oviduca|[8757]|LOWCE754-06|Canada|British Columbia|658[0n]|BOLD:AAB0331
Protorthodes oviduca|[8758]|RDNMH082-09|United States|Wyoming|658[0n]|BOLD:AAB0331
Protorthodes oviduca|[8759]|BBLSX741-09|United States|New Mexico|658[0n]|BOLD:AAB0331
Protorthodes oviduca|[8760]|BBLSX742-09|United States|New Mexico|658[0n]|BOLD:AAB0331
Protorthodes oviduca|[8761]|BBLSY548-09|United States|New Mexico|658[0n]|BOLD:AAB0331
Protorthodes oviduca|[8762]|LNC600-06|United States|North Carolina|658[0n]|BOLD:AAB0331
Protorthodes oviduca|[8763]|RDNMJ812-11|United States|Utah|658[0n]|BOLD:AAB0331
Protorthodes oviduca|[8764]|XAD647-05|Canada|Ontario|639[0n]|BOLD:AAB0331
Protorthodes oviduca|[8765]|XAC152-04|Canada|Ontario|658[0n]|BOLD:AAB0331
Protorthodes oviduca|[8766]|PMG152-03|Canada|Ontario|617[0n]|BOLD:AAB0331
Protorthodes oviduca|[8767]|PHMO079-03|Canada|Ontario|639[0n]|BOLD:AAB0331
Protorthodes oviduca|[8768]|XAF556-05|Canada|Ontario|658[0n]|BOLD:AAB0331
Protorthodes oviduca|[8769]|RDNMG379-08|Canada|Newfoundland and Labrador|658[0n]|BOLD:AAB0331
Protorthodes oviduca|[8770]|LPSOD834-09|Canada|Ontario|658[0n]|BOLD:AAB0331
Protorthodes oviduca|[8771]|LNCB993-10|United States|North Carolina|658[0n]|BOLD:AAB0331
Protorthodes oviduca|[8772]|BBLPB366-10|Canada|Ontario|658[0n]|BOLD:AAB0331
Protorthodes oviduca|[8773]|LPSOD725-09|Canada|Ontario|658[0n]|BOLD:AAB0331
Protorthodes oviduca|[8774]|LPSOD887-09|Canada|Ontario|658[0n]|BOLD:AAB0331
Protorthodes oviduca|[8775]|RDLQG265-06|Canada|Quebec|658[0n]|BOLD:AAB0331
Protorthodes oviduca|[8776]|RDLQF507-06|Canada|Quebec|658[0n]|BOLD:AAB0331
Protorthodes oviduca|[8777]|XAJ392-06|Canada|Ontario|658[0n]|BOLD:AAB0331
Protorthodes oviduca|[8778]|XAF581-05|Canada|Ontario|658[0n]|BOLD:AAB0331
Protorthodes oviduca|[8779]|XAC123-04|Canada|Ontario|658[0n]|BOLD:AAB0331
Protorthodes oviduca|[8780]|XAB353-04|Canada|Ontario|658[0n]|BOLD:AAB0331
Protorthodes oviduca|[8781]|TMG142-03|Canada|Ontario|639[0n]|BOLD:AAB0331
Protorthodes oviduca|[8782]|PHMO073-03|Canada|Ontario|639[0n]|BOLD:AAB0331
Protorthodes oviduca|[8783]|RDLQ288-05|Canada|Quebec|656[0n]|BOLD:AAB0331
Protorthodes oviduca|[8784]|RDLQG383-06|Canada|Quebec|658[0n]|BOLD:AAB0331
Protorthodes oviduca|[8785]|RDLQG384-06|Canada|Quebec|658[0n]|BOLD:AAB0331
Protorthodes oviduca|[8786]|RDLQG385-06|Canada|Quebec|658[0n]|BOLD:AAB0331
Protorthodes oviduca|[8787]|RDLQG386-06|Canada|Quebec|658[0n]|BOLD:AAB0331
Protorthodes oviduca|[8788]|RDLQG387-06|Canada|Quebec|658[0n]|BOLD:AAB0331
Protorthodes oviduca|[8789]|LPSOB242-08|Canada|Ontario|658[0n]|BOLD:AAB0331
Protorthodes oviduca|[8790]|LPSOD256-09|Canada|Ontario|658[0n]|BOLD:AAB0331
Protorthodes oviduca|[8791]|LPSOD395-09|Canada|Ontario|658[0n]|BOLD:AAB0331
Protorthodes oviduca|[8792]|LPSOD624-09|Canada|Ontario|658[0n]|BOLD:AAB0331
Protorthodes oviduca|[8793]|LPSOD652-09|Canada|Ontario|658[0n]|BOLD:AAB0331
Protorthodes oviduca|[8794]|BBLPB367-10|Canada|Saskatchewan|658[0n]|BOLD:AAB0331
Protorthodes oviduca|[8795]|BBLPB368-10|Canada|Saskatchewan|658[0n]|BOLD:AAB0331

Protorthodes oviduca|[8793]|LPSOD652-09|Canada|Ontario|658[0n]|BOLD:AAB0331
Protorthodes oviduca|[8794]|BBLPB367-10|Canada|Saskatchewan|658[0n]|BOLD:AAB0331
Protorthodes oviduca|[8795]|BBLPB368-10|Canada|Saskatchewan|658[0n]|BOLD:AAB0331
Protorthodes oviduca|[8796]|BBLPB440-10|Canada|Saskatchewan|658[0n]|BOLD:AAB0331
Protorthodes oviduca|[8797]|BBLPB445-10|Canada|Saskatchewan|658[0n]|BOLD:AAB0331
Protorthodes oviduca|[8798]|RDNMG380-08|Canada|Newfoundland and Labrador|658[0n]|BOLD:AAB0331
Protorthodes oviduca|[8799]|CNPKF2681-14|Canada|Ontario|564[0n]|BOLD:AAB0331
Protorthodes orobia|[8800]|LNAUT887-14|United States|Texas|658[0n]|BOLD:ACN8729
Protorthodes orobia|[8801]|LNAUT889-14|United States|Texas|658[0n]|BOLD:ACN8729
Protorthodes orobia|[8802]|LNAUT888-14|United States|Texas|658[0n]|BOLD:ACN8729
Protorthodes orobia|[8803]|LNAUT890-14|United States|Texas|658[0n]|BOLD:ACN8729
Protorthodes melanopis|[8804]|GMRMA239-14|United States|California|551[0n]|BOLD:AAD0713
Protorthodes melanopis|[8805]|RDNMG839-08|United States|New Mexico|658[0n]|BOLD:AAD0713
Protorthodes melanopis|[8806]|LOCB619-06|United States|California|658[0n]|BOLD:AAD0713
Protorthodes melanopis|[8807]|IAWLB655-11|United States|Arizona|658[0n]|BOLD:AAD0713
Protorthodes melanopis|[8808]|IAWLB656-11|United States|Arizona|658[0n]|BOLD:AAD0713
Protorthodes melanopis|[8809]|IAWLB657-11|United States|Arizona|658[0n]|BOLD:AAD0713
Protorthodes melanopis|[8810]|IAWLB652-11|United States|Arizona|658[0n]|BOLD:AAD0713
Protorthodes melanopis|[8811]|RDNMH084-09|United States|New Mexico|658[0n]|BOLD:AAD0713
Protorthodes melanopis|[8812]|RDNMG838-08|United States|New Mexico|658[0n]|BOLD:AAD0713
Protorthodes melanopis|[8813]|LOCBC194-06|United States|California|658[2n]|BOLD:AAD0713
Protorthodes melanopis|[8814]|GMRMC005-14|United States|California|592[0n]|BOLD:AAD0713
Protorthodes melanopis|[8815]|LOCBF324-13|United States|California|571[0n]|BOLD:AAD0713
Protorthodes melanopis|[8816]|LOCBF3456-14|United States|California|576[0n]|BOLD:AAD0713
Protorthodes melanopis|[8817]|RDNMG840-08|United States|New Mexico|658[0n]|BOLD:AAD0713
Protorthodes melanopis|[8818]|LOCBB579-06|United States|California|658[0n]|BOLD:AAD0713
Protorthodes melanopis|[8819]|RDNMG874-08|United States|California|658[1n]|BOLD:AAD0713
Protorthodes melanopis|[8820]|GMRMK200-14|United States|California|591[0n]|BOLD:AAD0713
Lacinipolia aileenae|[8821]|RDNMH726-09|United States|New Mexico|658[0n]|BOLD:AAJ3208
Lacinipolia aileenae|[8822]|RDNME876-08|United States|Arizona|658[0n]|BOLD:AAJ3208
Lacinipolia aileenae|[8823]|CMAZA1027-12|United States|Arizona|658[0n]|BOLD:AAJ3208
Nudorthodes molino|[8824]|RDNMC705-06|United States|Arizona|658[0n]|BOLD:AAF9071
Nudorthodes molino|[8825]|RDNMC706-06|United States|Arizona|658[0n]|BOLD:AAF9071
Nudorthodes molino|[8826]|RDNME370-07|United States|Arizona|658[0n]|BOLD:AAF9071
Nudorthodes texana|[8827]|RDNMF893-08|United States|Oregon|658[0n]|BOLD:AAD8014
Nudorthodes texana|[8828]|RDNMG863-08|United States|Nevada|658[0n]|BOLD:AAD8014
Nudorthodes texana|[8829]|RDNMG861-08|United States|Oregon|658[0n]|BOLD:AAD8014
Nudorthodes texana|[8830]|RDNMF894-08|United States|Oregon|658[0n]|BOLD:AAD8014
Nudorthodes texana|[8831]|RDNMG452-08|United States|Utah|658[0n]|BOLD:AAD8014
Nudorthodes texana|[8832]|AWCLB634-11|United States|Arizona|658[0n]|BOLD:AAD8014
Nudorthodes texana|[8833]|RDNMG451-08|United States|Utah|658[0n]|BOLD:AAD8014
Nudorthodes texana|[8834]|AWCLB638-11|United States|Arizona|658[0n]|BOLD:AAD8014
Nudorthodes texana|[8835]|AWCLB636-11|United States|Arizona|658[0n]|BOLD:AAD8014
Nudorthodes texana|[8836]|AWCLB663-11|United States|Arizona|658[0n]|BOLD:AAD8014
Nudorthodes texana|[8837]|AWCLB650-11|United States|Arizona|658[0n]|BOLD:AAD8014
Nudorthodes texana|[8838]|AWCLB639-11|United States|Arizona|658[0n]|BOLD:AAD8014
Nudorthodes texana|[8839]|AWCLB523-11|United States|Arizona|658[0n]|BOLD:AAD8014
Nudorthodes texana|[8840]|AWCLB637-11|United States|Arizona|647[0n]|BOLD:AAD8014
Nudorthodes texana|[8841]|AWCLB632-11|United States|Arizona|658[0n]|BOLD:AAD8014
Nudorthodes texana|[8842]|AWCLB665-11|United States|Arizona|658[0n]|BOLD:AAD8014
Nudorthodes texana|[8843]|IAWLB509-11|United States|Arizona|658[0n]|BOLD:AAD8014
Nudorthodes texana|[8844]|IAWLB510-11|United States|Arizona|658[0n]|BOLD:AAD8014
Nudorthodes texana|[8845]|AWCLB649-11|United States|Arizona|658[0n]|BOLD:AAU7968
Nudorthodes texana|[8846]|AWCLB531-11|United States|Arizona|658[0n]|BOLD:AAU7968
Nudorthodes texana|[8847]|AWCLB633-11|United States|Arizona|658[0n]|BOLD:AAU7968
Nudorthodes texana|[8848]|AWCLB635-11|United States|Arizona|658[0n]|BOLD:AAU7968
Nudorthodes texana|[8849]|IAWLB508-11|United States|Arizona|658[0n]|BOLD:AAU7968
Nudorthodes variabilis|[8850]|LOCBE149-06|United States|California|658[0n]|BOLD:AAG5847
Nudorthodes variabilis|[8851]|RDNMH086-09|United States|California|658[0n]|BOLD:AAG5847
Nudorthodes variabilis|[8852]|LOCBF3444-14|United States|California|588[0n]|BOLD:AAG5847
Nudorthodes variabilis|[8853]|LOCBF3445-14|United States|California|576[0n]|BOLD:AAG5847
Nudorthodes variabilis|[8854]|LOCBF3446-14|United States|California|576[0n]|BOLD:AAG5847
Nudorthodes variabilis|[8855]|LOCBF3453-14|United States|California|549[0n]|BOLD:AAG5847
Pseudanarta basivirida|[8856]|LNAUS4735-13|United States|Arizona|658[2n]|BOLD:ACJ6778
Homoanarta peralta|[8857]|LNAUS4713-13|United States|Arizona|658[0n]|BOLD:ACJ6968
Homoanarta peralta|[8858]|LNAUS4714-13|United States|Arizona|658[0n]|BOLD:ACJ6968
Homoanarta nr. falcata|[8859]|NAMUM106-08|United States|Texas|658[0n]|BOLD:AAK9754
Homoanarta nr. falcata|[8860]|QUNOD020-10|United States|Texas|658[0n]|BOLD:AAK9754
Homoanarta falcata|[8861]|CMAZA1132-12|United States|Arizona|658[0n]|BOLD:ABW6369
Homoanarta falcata|[8862]|RDNMK917-13|Mexico|Sonora|658[0n]|BOLD:ABW6369
Pseudanthoecia tumida|[8863]|LNAUS4519-13|United States|New Mexico|658[0n]|BOLD:ACJ8904
Pseudanthoecia tumida|[8864]|LNAUS4518-13|United States|New Mexico|658[0n]|BOLD:ACJ8904
Pseudanthoecia tumida|[8865]|CNCLB175-14|United States|New Mexico|621[0n]|BOLD:ACJ8904
Pseudanarta crocea|[8866]|GWOSN433-11|United States|California|658[0n]|BOLD:AAD4581
Pseudanarta crocea|[8867]|LBCH7635-10|Canada|British Columbia|658[0n]|BOLD:AAD4581
Pseudanarta crocea|[8868]|LBCH7642-10|Canada|British Columbia|658[1n]|BOLD:AAD4581
Pseudanarta crocea|[8869]|LBCH7637-10|Canada|British Columbia|658[0n]|BOLD:AAD4581
Pseudanarta crocea|[8870]|LBCH7638-10|Canada|British Columbia|658[0n]|BOLD:AAD4581
Pseudanarta crocea|[8871]|LBCH7558-10|Canada|British Columbia|658[0n]|BOLD:AAD4581
Pseudanarta crocea|[8872]|LBCH7636-10|Canada|British Columbia|658[0n]|BOLD:AAD4581
Pseudanarta crocea|[8873]|LBCH7639-10|Canada|British Columbia|658[0n]|BOLD:AAD4581
Pseudanarta crocea|[8874]|LBCH7640-10|Canada|British Columbia|658[0n]|BOLD:AAD4581
Pseudanarta crocea|[8875]|LBCH7641-10|Canada|British Columbia|658[0n]|BOLD:AAD4581
Pseudanarta crocea|[8876]|LBCH6972-10|Canada|British Columbia|658[0n]|BOLD:AAD4581
Pseudanarta crocea|[8877]|LBCH6971-10|Canada|British Columbia|658[0n]|BOLD:AAD4581
Pseudanarta crocea|[8878]|LBCH6970-10|Canada|British Columbia|658[0n]|BOLD:AAD4581
Pseudanarta crocea|[8879]|LBCH6969-10|Canada|British Columbia|658[0n]|BOLD:AAD4581
Pseudanarta crocea|[8880]|LBCH6968-10|Canada|British Columbia|658[0n]|BOLD:AAD4581
Pseudanarta crocea|[8881]|LBCH6967-10|Canada|British Columbia|658[0n]|BOLD:AAD4581
Pseudanarta crocea|[8882]|LBCH6966-10|Canada|British Columbia|658[0n]|BOLD:AAD4581
Pseudanarta crocea|[8883]|LBCH6965-10|Canada|British Columbia|658[0n]|BOLD:AAD4581
Pseudanarta crocea|[8884]|LBCH6386-10|Canada|British Columbia|658[0n]|BOLD:AAD4581
Pseudanarta crocea|[8885]|RDNMF157-08|Canada|British Columbia|658[0n]|BOLD:AAD4581
Pseudanarta crocea|[8886]|RDNMF154-08|Canada|British Columbia|658[0n]|BOLD:AAD4581
Pseudanarta crocea|[8887]|RDNMF153-08|Canada|British Columbia|658[0n]|BOLD:AAD4581
Pseudanarta crocea|[8888]|NGNAQ1467-14|Canada|British Columbia|580[0n]|BOLD:AAD4581
Pseudanarta crocea|[8889]|NGAAB063-14|Canada|British Columbia|555[0n]|BOLD:AAD4581
Pseudanarta crocea|[8890]|RDNMF156-08|United States|Washington|637[1n]|BOLD:AAD4581
Pseudanarta crocea|[8891]|NAMUM108-08|United States|Texas|657[0n]|BOLD:AAD4581
Pseudanarta crocea|[8892]|CMAZA1186-12|United States|Arizona|658[0n]|BOLD:AAD4581
Pseudanarta crocea|[8893]|LNAUT478-14|United States|New Mexico|658[0n]|BOLD:AAD4581
Pseudanarta crocea|[8894]|RDNMF155-08|United States|Nevada|646[0n]|BOLD:AAD4581
Pseudanarta crocea|[8895]|NAMUM137-08|United States|California|656[0n]|BOLD:AAD4581

Pseudanarta crocea|[8893]|LNAUT478-14|United States|New Mexico|658[0n]|BOLD:AAD4581
Pseudanarta crocea|[8894]|RDNMF155-08|United States|Nevada|646[0n]|BOLD:AAD4581
Pseudanarta crocea|[8895]|NAMUM137-08|United States|California|656[0n]|BOLD:AAD4581
Pseudanarta crocea|[8896]|LBCH7098-10|Canada|British Columbia|658[0n]|BOLD:AAD4581
Pseudanarta crocea|[8897]|LBCH7232-10|Canada|British Columbia|658[0n]|BOLD:AAD4581
Pseudanarta crocea|[8898]|LBCH7352-10|Canada|British Columbia|658[0n]|BOLD:AAD4581
Pseudanarta crocea|[8899]|LNAUT479-14|United States|New Mexico|658[0n]|BOLD:AAD4581
Pseudanarta crocea|[8900]|LNAUT480-14|United States|New Mexico|658[0n]|BOLD:AAD4581
Pseudanarta singula|[8901]|RDNME617-08|United States|Colorado|658[0n]|BOLD:AAH5608
Pseudanarta singula|[8902]|CMAZA006-09|United States|Arizona|658[0n]|BOLD:AAH5608
Pseudanarta singula|[8903]|GWOSN432-11|United States|Arizona|658[0n]|BOLD:AAH5608
Pseudanarta singula|[8904]|CMAZA1080-12|United States|Arizona|658[0n]|BOLD:AAH5608
Pseudanarta singula|[8905]|CMAZA1213-12|United States|Arizona|658[0n]|BOLD:AAH5608
Pseudanarta caeca|[8906]|LNAUT476-14|United States|New Mexico|658[0n]|BOLD:AAX8341
Pseudanarta caeca|[8907]|NAMUM186-08|United States|California|650[0n]|BOLD:AAX8341
Pseudanarta caeca|[8908]|RDNMK105-11|United States|New Mexico|658[0n]|BOLD:AAX8341
Pseudanarta caeca|[8909]|LNAUT477-14|United States|New Mexico|658[0n]|BOLD:AAX8341
Homoanarta carneola|[8910]|LNAUS4718-13|United States|New Mexico|658[0n]|BOLD:ACJ7401
Homoanarta carneola|[8911]|LNAUS4719-13|United States|New Mexico|658[0n]|BOLD:ACJ7401
Homoanarta carneola|[8912]|LNAUS4720-13|United States|Arizona|607[0n]|BOLD:ACJ7401
Homoanarta carneola|[8913]|LNAUS4722-13|United States|Arizona|601[0n]|BOLD:ACJ7401
Pseudanarta pulverulenta|[8914]|CMAZA1209-12|United States|Arizona|658[0n]|BOLD:ACF3029
Pseudanarta pulverulenta|[8915]|RDNMK097-11|United States|New Mexico|658[0n]|BOLD:ACF3029
Pseudanarta pulverulenta|[8916]|RDNMK100-11|United States|Arizona|658[0n]|BOLD:ACF3029
Pseudanarta pulverulenta|[8917]|LNAUS4729-13|United States|Arizona|658[0n]|BOLD:ACF3029
Pseudanarta pulverulenta|[8918]|CNCLB1758-14|United States|Arizona|658[0n]|BOLD:ACF3029
Pseudanarta pulverulenta|[8919]|CNCLB1759-14|United States|Arizona|658[0n]|BOLD:ACF3029
Pseudanarta pulverulenta|[8920]|CNCLB1774-14|United States|Arizona|658[0n]|BOLD:ACF3029
Pseudanarta pulverulenta|[8921]|CNCLB1806-14|United States|Arizona|658[0n]|BOLD:ACF3029
Pseudanarta sp.|[8922]|CNCLB1779-14|United States|Arizona|658[0n]|BOLD:ABZ1959
Pseudanarta vexata|[8923]|RDNMH065-09|United States|Arizona|658[0n]|BOLD:ABZ1959
Pseudanarta vexata|[8924]|RDNMJ802-11|United States|New Mexico|658[0n]|BOLD:ABZ1959
Pseudanarta exasperata|[8925]|RDNME618-08|United States|Colorado|658[0n]|BOLD:ABZ1959
Pseudanarta exasperata|[8926]|RDNMJ846-11|United States|New Mexico|658[0n]|BOLD:ABZ1959
Pseudanarta exasperata|[8927]|RDNMK098-11|United States|New Mexico|658[0n]|BOLD:ABZ1959
Pseudanarta exasperata|[8928]|RDNMK099-11|United States|Arizona|658[0n]|BOLD:ABZ1959
Pseudanarta damnata|[8929]|CNCLB1751-14|United States|Arizona|658[0n]|BOLD:ABZ1959
Pseudanarta damnata|[8930]|CNCLB1757-14|United States|Arizona|658[0n]|BOLD:ABZ1959
Pseudanarta damnata|[8931]|CNCLB1778-14|United States|Arizona|658[0n]|BOLD:ABZ1959
Pseudanarta damnata|[8932]|CNCLB2069-14|United States||658[0n]|BOLD:ABZ1959
Pseudanarta damnata|[8933]|CNCLB2070-14|United States||632[0n]|BOLD:ABZ1959
Pseudanarta perplexa|[8934]|RDNMD832-07|United States|Arizona|658[0n]|BOLD:AAH8186
Pseudanarta perplexa|[8935]|RDNME621-08|United States|Colorado|658[0n]|BOLD:AAH8186
Pseudanarta perplexa|[8936]|RDNMD497-06|United States|Colorado|658[0n]|BOLD:AAH8186
Pseudanarta perplexa|[8937]|LNAUS4728-13|United States|Arizona|658[0n]|BOLD:AAH8186
Pseudanarta daemonalis|[8938]|CNCLB2410-14|United States|Texas|658[0n]|BOLD:ACR8363
Pseudanarta flava|[8939]|RDNMD936-07|United States|Utah|655[0n]|BOLD:AAE0933
Pseudanarta flava|[8940]|RDNMD498-06|United States|Colorado|658[0n]|BOLD:AAE0933
Pseudanarta flava|[8941]|NAMUM189-08|United States|California|645[0n]|BOLD:AAE0933
Pseudanarta flava|[8942]|RDNMG498-08|United States|Washington|640[0n]|BOLD:AAE0933
Pseudanarta flava|[8943]|CGLCA063-10|United States|California|658[0n]|BOLD:AAE0933
Pseudanarta flava|[8944]|LALPA818-10|Canada|British Columbia|658[0n]|BOLD:AAE0933
Pseudanarta flava|[8945]|NGAAB064-14|Canada|British Columbia|546[3n]|BOLD:AAE0933
Pseudanarta flava|[8946]|LBCH6973-10|Canada|British Columbia|658[0n]|BOLD:AAE0933
Pseudanarta flava|[8947]|CGLCA062-10|United States|California|658[0n]|BOLD:AAE0933
Pseudanarta flava|[8948]|RDNMG525-08|United States|Washington|640[0n]|BOLD:AAE0933
Pseudanarta flava|[8949]|NGNAQ1459-14|Canada|British Columbia|592[0n]|BOLD:AAE0933
Pseudanarta flava|[8950]|NGNAR106-14|Canada|British Columbia|592[0n]|BOLD:AAE0933
Pseudanarta flava|[8951]|NGAAA1900-14|Canada|British Columbia|588[0n]|BOLD:AAE0933
Pseudanarta flava|[8952]|NGNAW112-14|Canada|British Columbia|588[0n]|BOLD:AAE0933
Pseudanarta flava|[8953]|NGNAQ1466-14|Canada|British Columbia|591[0n]|BOLD:AAE0933
Pseudanarta flava|[8954]|NGNAR1051-14|Canada|British Columbia|589[0n]|BOLD:AAE0933
Pseudanarta flava|[8955]|NGNAR1050-14|Canada|British Columbia|589[0n]|BOLD:AAE0933
Pseudanarta flava|[8956]|NGNAR107-14|Canada|British Columbia|589[0n]|BOLD:AAE0933
Pseudanarta flava|[8957]|NGNAR104-14|Canada|British Columbia|591[0n]|BOLD:AAE0933
Pseudanarta flava|[8958]|NGNAR103-14|Canada|British Columbia|591[0n]|BOLD:AAE0933
Pseudanarta flava|[8959]|NGAAB065-14|Canada|British Columbia|564[0n]|BOLD:AAE0933
Pseudanarta flava|[8960]|NGNAW110-14|Canada|British Columbia|564[0n]|BOLD:AAE0933
Pseudanarta flava|[8961]|NGNAQ343-14|Canada|British Columbia|581[0n]|BOLD:AAE0933
Pseudanarta flava|[8962]|NGNAR102-14|Canada|British Columbia|571[0n]|BOLD:AAE0933
Pseudanarta flava|[8963]|NGNAQ1463-14|Canada|British Columbia|580[0n]|BOLD:AAE0933
Pseudanarta flava|[8964]|NGNAQ1465-14|Canada|British Columbia|566[0n]|BOLD:AAE0933
Pseudanarta flava|[8965]|NGAAB062-14|Canada|British Columbia|552[0n]|BOLD:AAE0933
Pseudanarta flava|[8966]|NGAAB067-14|Canada|British Columbia|555[0n]|BOLD:AAE0933
Pseudanarta actura|[8967]|CNCLB2067-14|United States|Arizona|658[0n]|BOLD:ACN8047
Pseudanarta actura|[8968]|LNAUT475-14|United States|Arizona|658[0n]|BOLD:ACN8047
Pseudanarta actura|[8969]|LNAUT947-14|United States|Texas|658[0n]|BOLD:ACN8047
Pseudanarta actura|[8970]|CNCLB2064-14|United States|Arizona|658[0n]|BOLD:ACN8047
Pseudanarta actura|[8971]|CNCLB2068-14|United States|New Mexico|658[0n]|BOLD:ACN8047
Pseudanarta actura|[8972]|CNCLB2066-14|United States|Arizona|658[0n]|BOLD:ACN8047
Pseudanarta actura|[8973]|CNCLB2065-14|United States|Arizona|658[0n]|BOLD:ACN8047
Pseudanarta actura|[8974]|LNAUT2915-14|United States|Texas|370[0n]|
Pseudanarta actura|[8975]|LNAUT2916-14|United States|Texas|370[0n]|
Lacinipolia parvula|[8976]|RDNMJ005-10|United States|Florida|658[0n]|BOLD:AAV6673
Lacinipolia parvula|[8977]|RDNMJ006-10|United States|Florida|658[0n]|BOLD:AAV6673
Elaphria perigeanaDHJ02|[8978]|BBLOD180-11|United States|Texas|623[0n]|BOLD:AAA9267
Elaphria perigeanaDHJ02|[8979]|BBLOD354-11|United States|Texas|658[0n]|BOLD:AAA9267
Lacinipolia|[8980]|RDNMC421-05|United States|Washington|529[13n]|
Lacinipolia palilis|[8981]|CNCLB670-14|United States|Texas|658[0n]|BOLD:ACM3926
Lacinipolia vittula|[8982]|LNAUS2822-13|United States|New Mexico|658[0n]|BOLD:AAF2596
Lacinipolia stenotis|[8983]|NAMUM132-08|United States|California|658[0n]|BOLD:AAF2596
Lacinipolia stenotis|[8984]|RDNMC400-05|United States|Oregon|579[0n]|BOLD:AAF2596
Lacinipolia stenotis|[8985]|NAMUM143-08|United States|California|656[0n]|BOLD:AAF2596
Lacinipolia stenotis|[8986]|CMAZA1210-12|United States|Arizona|658[0n]|BOLD:AAF2596
Lacinipolia vittula|[8987]|LNAUS2819-13|United States|New Mexico|658[0n]|BOLD:AAF2596
Lacinipolia vittula|[8988]|LNAUS2820-13|United States|New Mexico|658[1n]|BOLD:AAF2596
Lacinipolia vittula|[8989]|LNAUS2821-13|United States|New Mexico|658[0n]|BOLD:AAF2596
Lacinipolia vittula|[8990]|LNAUS2823-13|United States|New Mexico|658[0n]|BOLD:AAF2596
Lacinipolia sp. A|[8991]|RDNMH588-09|United States|Colorado|658[0n]|BOLD:AAX0568
Lacinipolia uliginosa|[8992]|LNAUS2830-13|United States|Texas|407[2n]|
Lacinipolia uliginosa|[8993]|LNAUS2832-13|United States|Texas|658[0n]|BOLD:AAX0568
Lacinipolia umbrosa|[8994]|CNCLB668-14|United States|New Mexico|658[0n]|BOLD:ACM4262
Lacinipolia umbrosa|[8995]|CNCLB666-14|United States|Arizona|658[0n]|BOLD:ACM4262

Lacinipolia uliginosa|[8993]|LNAUS2832-13|United States|Texas|658[0n]|BOLD:AAX0568
Lacinipolia umbrosa|[8994]|CNCLB668-14|United States|New Mexico|658[0n]|BOLD:ACM4262
Lacinipolia umbrosa|[8995]|CNCLB666-14|United States|Arizona|658[0n]|BOLD:ACM4262
Lacinipolia umbrosa|[8996]|CNCLB669-14|United States|Arizona|658[0n]|BOLD:ACM4262
Lacinipolia umbrosa|[8997]|CNCLB1140-14|United States|New Mexico|658[0n]|BOLD:ACM4262
Lacinipolia longiclava|[8998]|RDMAB107-05|Canada|Alberta|658[1n]|BOLD:ACE5163
Lacinipolia longiclava|[8999]|USLEP713-10|United States|Colorado|658[0n]|BOLD:ACE5163
Lacinipolia longiclava|[9000]|USLEP712-10|United States|Colorado|658[0n]|BOLD:ACE5163
Lacinipolia longiclava|[9001]|USLEP711-10|United States|Colorado|658[0n]|BOLD:ACE5163
Lacinipolia longiclava|[9002]|RDMAB310-05|Canada|Alberta|658[0n]|BOLD:ACE5163
Lacinipolia longiclava|[9003]|RDMAB308-05|Canada|Alberta|658[0n]|BOLD:ACE5163
Lacinipolia longiclava|[9004]|RDMAB307-05|Canada|Alberta|658[0n]|BOLD:ACE5163
Lacinipolia longiclava|[9005]|USLEP714-10|United States|Colorado|636[0n]|BOLD:ACE5163
Lacinipolia longiclava|[9006]|USLEP715-10|United States|Colorado|658[0n]|BOLD:ACE5163
Lacinipolia longiclava|[9007]|USLEP975-10|United States|Colorado|658[0n]|BOLD:ACE5163
Lacinipolia incurva|[9008]|RDNMG306-08|United States|Arizona|658[0n]|BOLD:ACE5163
Lacinipolia incurva|[9009]|RDNMG001-08|United States|Arizona|609[0n]|BOLD:ACE5163
Lacinipolia incurva|[9010]|IAWLB621-11|United States|Arizona|581[1n]|BOLD:ACE5163
Lacinipolia anguina|[9011]|LOTB327-05|United States|Tennessee|658[0n]|BOLD:AAB8456
Lacinipolia anguina|[9012]|TMNBB324-06|Canada|New Brunswick|658[0n]|BOLD:AAB8456
Lacinipolia anguina|[9013]|RDLQB074-05|Canada|Quebec|658[0n]|BOLD:AAB8456
Lacinipolia anguina|[9014]|RDLQB073-05|Canada|Quebec|658[0n]|BOLD:AAB8456
Lacinipolia anguina|[9015]|LGSMC920-05|United States|Tennessee|658[0n]|BOLD:AAB8456
Lacinipolia anguina|[9016]|TTMNB555-06|Canada|New Brunswick|656[0n]|BOLD:AAB8456
Lacinipolia anguina|[9017]|TMNBB325-06|Canada|New Brunswick|656[0n]|BOLD:AAB8456
Lacinipolia anguina|[9018]|LPSOD902-09|Canada|Ontario|658[0n]|BOLD:AAB8456
Lacinipolia anguina|[9019]|BBLPB335-10|Canada|Ontario|658[0n]|BOLD:AAB8456
Lacinipolia anguina|[9020]|BBLPB477-10|Canada|British Columbia|658[0n]|BOLD:AAB8456
Lacinipolia anguina|[9021]|LBCH5515-10|Canada|British Columbia|658[0n]|BOLD:AAB8456
Lacinipolia anguina|[9022]|LBCH5339-10|Canada|British Columbia|658[0n]|BOLD:AAB8456
Lacinipolia anguina|[9023]|BBLSY405-09|United States|Arizona|658[0n]|BOLD:AAB8456
Lacinipolia anguina|[9024]|BBLSW368-09|United States|Arizona|658[0n]|BOLD:AAB8456
Lacinipolia anguina|[9025]|LBCG045-08|Canada|British Columbia|658[0n]|BOLD:AAB8456
Lacinipolia anguina|[9026]|RDMAB640-06|Canada|Alberta|658[0n]|BOLD:AAB8456
Lacinipolia anguina|[9027]|RDMAB309-05|Canada|Alberta|658[0n]|BOLD:AAB8456
Lacinipolia anguina|[9028]|LOWCC083-05|Canada|British Columbia|658[0n]|BOLD:AAB8456
Lacinipolia anguina|[9029]|BBLWU169-09|United States|Colorado|640[0n]|BOLD:AAB8456
Lacinipolia anguina|[9030]|RDMAB369-05|Canada|Alberta|617[0n]|BOLD:AAB8456
Lacinipolia anguina|[9031]|LOWCE553-06|Canada|British Columbia|627[0n]|BOLD:AAB8456
Lacinipolia anguina|[9032]|LOWCE549-06|Canada|British Columbia|616[0n]|BOLD:AAB8456
Lacinipolia anguina|[9033]|LOWCE550-06|Canada|British Columbia|607[0n]|BOLD:AAB8456
Lacinipolia anguina|[9034]|RDMAB261-05|Canada|Alberta|570[0n]|BOLD:AAB8456
Lacinipolia anguina|[9035]|BBLPB479-10|Canada|Alberta|522[1n]|BOLD:AAB8456
Lacinipolia anguina|[9036]|BBLPB481-10|Canada|Alberta|658[0n]|BOLD:AAB8456
Lacinipolia anguina|[9037]|BBLPB482-10|Canada|Alberta|658[0n]|BOLD:AAB8456
Lacinipolia anguina|[9038]|BBLPB483-10|Canada|Alberta|658[0n]|BOLD:AAB8456
Lacinipolia anguina|[9039]|BBLPB491-10|Canada|Alberta|658[0n]|BOLD:AAB8456
Lacinipolia anguina|[9040]|BBLPB492-10|Canada|Alberta|658[0n]|BOLD:AAB8456
Lacinipolia anguina|[9041]|BBLPB493-10|Canada|Alberta|658[0n]|BOLD:AAB8456
Lacinipolia anguina|[9042]|CNCLB2520-14|Canada|Ontario|658[0n]|BOLD:AAB8456
Lacinipolia fordi|[9043]|RDNMG311-08|United States|Arizona|658[0n]|BOLD:AAJ2867
Lacinipolia fordi|[9044]|RDNME1021-08|United States|Arizona|658[0n]|BOLD:AAJ2867
Lacinipolia stricta|[9045]|LOWCC076-05|Canada|British Columbia|658[0n]|BOLD:AAB2880
Lacinipolia stricta|[9046]|LOWCC077-05|Canada|British Columbia|658[0n]|BOLD:AAB2880
Lacinipolia stricta|[9047]|LOWCC079-05|Canada|British Columbia|658[0n]|BOLD:AAB2880
Lacinipolia stricta|[9048]|LOWCE529-06|Canada|British Columbia|658[0n]|BOLD:AAB2880
Lacinipolia stricta|[9049]|LBCH6536-10|Canada|British Columbia|658[0n]|BOLD:AAB2880
Lacinipolia stricta|[9050]|LBCH7475-10|Canada|British Columbia|658[0n]|BOLD:AAB2880
Lacinipolia stricta|[9051]|LBCH7123-10|Canada|British Columbia|658[0n]|BOLD:AAB2880
Lacinipolia stricta|[9052]|LBCH7354-10|Canada|British Columbia|658[0n]|BOLD:AAB2880
Lacinipolia stricta|[9053]|LOWCE531-06|Canada|British Columbia|658[0n]|BOLD:AAB2880
Lacinipolia stricta|[9054]|LOWCD190-06|Canada|British Columbia|658[0n]|BOLD:AAB2880
Lacinipolia stricta|[9055]|LOWCC082-05|Canada|British Columbia|658[0n]|BOLD:AAB2880
Lacinipolia stricta|[9056]|LOWCC081-05|Canada|British Columbia|658[0n]|BOLD:AAB2880
Lacinipolia stricta|[9057]|LOWCC080-05|Canada|British Columbia|658[0n]|BOLD:AAB2880
Lacinipolia stricta|[9058]|LOWCC078-05|Canada|British Columbia|658[0n]|BOLD:AAB2880
Lacinipolia stricta|[9059]|LOWCC075-05|Canada|British Columbia|658[0n]|BOLD:AAB2880
Lacinipolia stricta|[9060]|LOWCC074-05|Canada|British Columbia|658[0n]|BOLD:AAB2880
Lacinipolia stricta|[9061]|LOWCC073-05|Canada|British Columbia|658[0n]|BOLD:AAB2880
Lacinipolia stricta|[9062]|LOWCC071-05|Canada|British Columbia|658[0n]|BOLD:AAB2880
Lacinipolia stricta|[9063]|LOWCC072-05|Canada|British Columbia|658[0n]|BOLD:AAB2880
Lacinipolia stricta|[9064]|LOWC005-05|Canada|British Columbia|658[0n]|BOLD:AAB2880
Lacinipolia stricta|[9065]|RDNMB220-05|Canada|British Columbia|599[0n]|BOLD:AAB2880
Lacinipolia stricta|[9066]|LOWCE530-06|Canada|British Columbia|614[0n]|BOLD:AAB2880
Lacinipolia stricta|[9067]|LBCH7222-10|Canada|British Columbia|642[0n]|BOLD:AAB2880
Lacinipolia stricta|[9068]|LALPA745-10|Canada|British Columbia|658[0n]|BOLD:AAB2880
Lacinipolia sp.|[9069]|RDMAB314-05|Canada|Alberta|658[0n]|BOLD:AAB2883
Lacinipolia sp.|[9070]|RDNMC405-05|United States|Oregon|568[0n]|BOLD:AAB2883
Lacinipolia sp.|[9071]|RDNMC407-05|United States|California|594[0n]|BOLD:ABZ8179
Lacinipolia sp.|[9072]|LOCBB371-06|United States|California|658[0n]|BOLD:ABZ8179
Lacinipolia circumcincta|[9073]|JMMMB325-11|United States|California|658[0n]|BOLD:AAB2881
Lacinipolia sp.|[9074]|RDNMC408-05|United States|Oregon|550[0n]|BOLD:ABZ8180
Lacinipolia circumcincta|[9075]|RDNMC403-05|United States|Oregon|579[0n]|BOLD:AAB2881
Lacinipolia circumcincta|[9076]|RDNMC406-05|United States|Oregon|602[0n]|BOLD:AAB2881
Lacinipolia circumcincta|[9077]|RDNMB221-05|United States|California|599[0n]|BOLD:AAB2881
Lacinipolia circumcincta|[9078]|JMMMB381-11|United States|California|658[0n]|BOLD:AAB2881
Lacinipolia circumcincta|[9079]|GMLC022-09|United States|California|658[0n]|BOLD:ABZ8181
Lacinipolia circumcincta|[9080]|GMLC1226-12|United States|California|658[0n]|BOLD:ABZ8181
Lacinipolia sp.|[9081]|CNCLB1131-14|Mexico||658[0n]|BOLD:ACM6980
Lacinipolia spiculosa|[9082]|IAWLB250-11|United States|Arizona|658[0n]|BOLD:AAZ0778
Lacinipolia spiculosa|[9083]|BBLOB774-11|United States|Arizona|658[0n]|BOLD:AAZ0778
Lacinipolia spiculosa|[9084]|CNCLB1128-14|United States|Arizona|658[0n]|BOLD:AAZ0778
Lacinipolia spiculosa|[9085]|BBLOB776-11|United States|Arizona|658[0n]|BOLD:AAZ0778
Lacinipolia spiculosa|[9086]|CNCLB1129-14|United States|Arizona|658[0n]|BOLD:AAZ0778
Lacinipolia spiculosa|[9087]|CNCLB1132-14|United States|Arizona|658[0n]|BOLD:AAZ0778
Lacinipolia spiculosa|[9088]|CNCLB1133-14|United States|Arizona|550[0n]|BOLD:AAZ0778
Lacinipolia renigera|[9089]|LPSOB237-08|Canada|Ontario|632[1n]|BOLD:AAA2636
Lacinipolia renigera|[9090]|XAK283-06|Canada|Ontario|658[0n]|BOLD:AAA2636
Lacinipolia renigera|[9091]|LPSOB245-08|Canada|Ontario|658[0n]|BOLD:AAA2636
Lacinipolia renigera|[9092]|BBLCU233-09|United States|Michigan|658[0n]|BOLD:AAA2636
Lacinipolia renigera|[9093]|BBLPC391-09|Canada|New Brunswick|658[0n]|BOLD:AAA2636
Lacinipolia renigera|[9094]|BLTIB829-08|Canada|Ontario|658[0n]|BOLD:ACE7009
Lacinipolia renigera|[9095]|BBLPB468-10|Canada|Alberta|658[0n]|BOLD:ACE7009

Lacinipolia renigera|[9093]|BBLPC391-09|Canada|New Brunswick|658[0n]|BOLD:AAA2636
Lacinipolia renigera|[9094]|BLTIB829-08|Canada|Ontario|658[0n]|BOLD:ACE7009
Lacinipolia renigera|[9095]|BBLPB468-10|Canada|Alberta|658[0n]|BOLD:ACE7009
Lacinipolia renigera|[9096]|BLTIB711-08|Canada|Ontario|658[0n]|BOLD:ACE7009
Lacinipolia renigera|[9097]|BBLPC507-09|Canada|New Brunswick|614[0n]|BOLD:ACE7009
Lacinipolia renigera|[9098]|LPABC137-09|Canada|Alberta|658[2n]|BOLD:ACE7009
Lacinipolia renigera|[9099]|BBLEC742-09|Canada|Nova Scotia|633[0n]|BOLD:ACE7009
Lacinipolia renigera|[9100]|RDLQF370-06|Canada|Quebec|658[0n]|BOLD:ACE7009
Lacinipolia renigera|[9101]|LPSK245-08|Canada|Saskatchewan|658[0n]|BOLD:ACE7009
Lacinipolia renigera|[9102]|LPMN800-08|Canada|Manitoba|658[0n]|BOLD:ACE7009
Lacinipolia renigera|[9103]|BLTIB1102-08|Canada|Ontario|658[0n]|BOLD:ACE7009
Lacinipolia renigera|[9104]|LPMNB347-09|Canada|Manitoba|658[0n]|BOLD:ACE7009
Lacinipolia renigera|[9105]|LPMNB509-09|Canada|Manitoba|658[0n]|BOLD:ACE7009
Lacinipolia renigera|[9106]|BBLSZ107-09|United States|Oklahoma|658[0n]|BOLD:ACE7009
Lacinipolia renigera|[9107]|BBLSZ127-09|United States|Oklahoma|658[0n]|BOLD:ACE7009
Lacinipolia renigera|[9108]|BBLEC706-09|Canada|Nova Scotia|658[0n]|BOLD:ACE7009
Lacinipolia renigera|[9109]|BBLEC707-09|Canada|Nova Scotia|658[0n]|BOLD:ACE7009
Lacinipolia renigera|[9110]|BBLEC767-09|Canada|Nova Scotia|658[0n]|BOLD:ACE7009
Lacinipolia renigera|[9111]|BBLEC968-09|Canada|Nova Scotia|658[0n]|BOLD:ACE7009
Lacinipolia renigera|[9112]|BBLPC387-09|Canada|New Brunswick|658[0n]|BOLD:ACE7009
Lacinipolia renigera|[9113]|BBLPC420-09|Canada|New Brunswick|658[0n]|BOLD:ACE7009
Lacinipolia renigera|[9114]|LNCC041-10|United States|North Carolina|658[0n]|BOLD:ACE7009
Lacinipolia renigera|[9115]|LNCC1307-11|United States|North Carolina|658[0n]|BOLD:ACE7009
Lacinipolia renigera|[9116]|BBLEC545-09|Canada|Nova Scotia|658[0n]|BOLD:ACE7009
Lacinipolia renigera|[9117]|BBLEC688-09|Canada|Nova Scotia|658[0n]|BOLD:ACE7009
Lacinipolia renigera|[9118]|LPABB206-08|Canada|Alberta|658[0n]|BOLD:ACE7009
Lacinipolia renigera|[9119]|LPMNB246-09|Canada|Manitoba|658[0n]|BOLD:ACE7009
Lacinipolia renigera|[9120]|RDLQF208-06|Canada|Quebec|658[0n]|BOLD:ACE7009
Lacinipolia renigera|[9121]|LNCNW080-06|United States|North Carolina|658[0n]|BOLD:ACE7009
Lacinipolia renigera|[9122]|RDLQF073-06|Canada|Quebec|658[0n]|BOLD:ACE7009
Lacinipolia renigera|[9123]|RDLQF070-06|Canada|Quebec|658[0n]|BOLD:ACE7009
Lacinipolia renigera|[9124]|RDMAB648-06|Canada|Alberta|658[0n]|BOLD:ACE7009
Lacinipolia renigera|[9125]|RDMAB499-06|Canada|Alberta|658[0n]|BOLD:ACE7009
Lacinipolia renigera|[9126]|RDLQB538-05|Canada|Quebec|658[0n]|BOLD:ACE7009
Lacinipolia renigera|[9127]|RDMAB010-05|Canada|Alberta|658[0n]|BOLD:ACE7009
Lacinipolia renigera|[9128]|LOTB257-05|United States|Tennessee|658[0n]|BOLD:ACE7009
Lacinipolia renigera|[9129]|LGSMC443-05|United States|Tennessee|658[0n]|BOLD:ACE7009
Lacinipolia renigera|[9130]|PHMNB749-05|Canada|New Brunswick|658[0n]|BOLD:ACE7009
Lacinipolia renigera|[9131]|BBLEC710-09|Canada|Nova Scotia|655[0n]|BOLD:ACE7009
Lacinipolia renigera|[9132]|BBLEC253-09|Canada|Nova Scotia|635[0n]|BOLD:ACE7009
Lacinipolia renigera|[9133]|BBLEC716-09|Canada|Nova Scotia|641[0n]|BOLD:ACE7009
Lacinipolia renigera|[9134]|BBLEC768-09|Canada|Nova Scotia|650[0n]|BOLD:ACE7009
Lacinipolia renigera|[9135]|LNCC1676-13|United States|North Carolina|658[0n]|BOLD:ACE7009
Lacinipolia renigera|[9136]|LPSOB177-08|Canada|Ontario|658[0n]|BOLD:AAA2636
Lacinipolia renigera|[9137]|LPSOB209-08|Canada|Ontario|658[0n]|BOLD:AAA2636
Lacinipolia renigera|[9138]|BBLEC252-09|Canada|Nova Scotia|658[0n]|BOLD:AAA2636
Lacinipolia renigera|[9139]|LPSK246-08|Canada|Saskatchewan|658[0n]|BOLD:AAA2636
Lacinipolia renigera|[9140]|RDNMD759-07|United States|North Carolina|658[0n]|BOLD:AAA2636
Lacinipolia renigera|[9141]|PHMNB379-04|Canada|New Brunswick|658[0n]|BOLD:AAA2636
Lacinipolia renigera|[9142]|MNBB127-05|Canada|New Brunswick|584[20n]|
Lacinipolia renigera|[9143]|UDLEP069-09|United States|Delaware|608[0n]|BOLD:AAA2636
Lacinipolia renigera|[9144]|BBLEC180-09|Canada|Nova Scotia|647[0n]|BOLD:AAA2636
Lacinipolia renigera|[9145]|BBLEC547-09|Canada|Nova Scotia|654[0n]|BOLD:AAA2636
Lacinipolia renigera|[9146]|BBLEC550-09|Canada|Nova Scotia|658[0n]|BOLD:AAA2636
Lacinipolia renigera|[9147]|BBLPE572-09|Canada|Nova Scotia|658[0n]|BOLD:AAA2636
Lacinipolia renigera|[9148]|LNCC664-11|United States|North Carolina|658[0n]|BOLD:AAA2636
Lacinipolia renigera|[9149]|LNCC682-11|United States|North Carolina|658[0n]|BOLD:AAA2636
Lacinipolia renigera|[9150]|LNCC1308-11|United States|North Carolina|658[0n]|BOLD:AAA2636
Lacinipolia renigera|[9151]|LNCC1309-11|United States|North Carolina|658[0n]|BOLD:AAA2636
Lacinipolia renigera|[9152]|LNCC1330-11|United States|North Carolina|658[0n]|BOLD:AAA2636
Lacinipolia renigera|[9153]|XAH037-05|Canada|Ontario|658[1n]|BOLD:AAA2636
Lacinipolia renigera|[9154]|XAC801-04|Canada|Ontario|658[0n]|BOLD:AAA2636
Lacinipolia renigera|[9155]|XAB641-04|Canada|Ontario|658[0n]|BOLD:AAA2636
Lacinipolia renigera|[9156]|XAB456-04|Canada|Ontario|658[0n]|BOLD:AAA2636
Lacinipolia renigera|[9157]|XAB446-04|Canada|Ontario|658[0n]|BOLD:AAA2636
Lacinipolia renigera|[9158]|XAB383-04|Canada|Ontario|658[0n]|BOLD:AAA2636
Lacinipolia renigera|[9159]|XAB354-04|Canada|Ontario|658[0n]|BOLD:AAA2636
Lacinipolia renigera|[9160]|XAB124-04|Canada|Ontario|658[0n]|BOLD:AAA2636
Lacinipolia renigera|[9161]|XAB117-04|Canada|Ontario|658[0n]|BOLD:AAA2636
Lacinipolia renigera|[9162]|XAG261-05|Canada|Ontario|655[0n]|BOLD:AAA2636
Lacinipolia renigera|[9163]|XAH613-05|Canada|Ontario|658[0n]|BOLD:AAA2636
Lacinipolia renigera|[9164]|XAH665-05|Canada|Ontario|658[0n]|BOLD:AAA2636
Lacinipolia renigera|[9165]|XAI029-05|Canada|Ontario|658[0n]|BOLD:AAA2636
Lacinipolia renigera|[9166]|RDLQF067-06|Canada|Quebec|658[0n]|BOLD:AAA2636
Lacinipolia renigera|[9167]|RDLQF069-06|Canada|Quebec|658[0n]|BOLD:AAA2636
Lacinipolia renigera|[9168]|TMNBB326-06|Canada|New Brunswick|658[0n]|BOLD:AAA2636
Lacinipolia renigera|[9169]|XAJ846-06|Canada|Ontario|658[0n]|BOLD:AAA2636
Lacinipolia renigera|[9170]|XAJ858-06|Canada|Ontario|658[0n]|BOLD:AAA2636
Lacinipolia renigera|[9171]|XAK109-06|Canada|Ontario|658[0n]|BOLD:AAA2636
Lacinipolia renigera|[9172]|XAK145-06|Canada|Ontario|658[0n]|BOLD:AAA2636
Lacinipolia renigera|[9173]|XAK184-06|Canada|Ontario|658[0n]|BOLD:AAA2636
Lacinipolia renigera|[9174]|BLTIB570-08|Canada|Ontario|658[0n]|BOLD:AAA2636
Lacinipolia renigera|[9175]|BLTIB712-08|Canada|Ontario|658[0n]|BOLD:AAA2636
Lacinipolia renigera|[9176]|BLTIB897-08|Canada|Ontario|658[0n]|BOLD:AAA2636
Lacinipolia renigera|[9177]|BLTIB1132-08|Canada|Ontario|658[0n]|BOLD:AAA2636
Lacinipolia renigera|[9178]|BBLPC404-09|Canada|New Brunswick|658[0n]|BOLD:AAA2636
Lacinipolia renigera|[9179]|XAC839-04|Canada|Ontario|658[0n]|BOLD:AAA2636
Lacinipolia renigera|[9180]|XAH198-05|Canada|Ontario|658[0n]|BOLD:AAA2636
Lacinipolia renigera|[9181]|LGSMG853-10|United States|North Carolina|658[0n]|BOLD:AAA2636
Lacinipolia renigera|[9182]|LPMN742-08|Canada|Manitoba|621[0n]|BOLD:AAA2636
Lacinipolia renigera|[9183]|BBLPB740-10|Canada|Alberta|658[0n]|BOLD:AAA2636
Lacinipolia renigera|[9184]|BBLPB429-10|Canada|Saskatchewan|658[0n]|BOLD:AAA2636
Lacinipolia renigera|[9185]|LPSOD1000-09|Canada|Ontario|658[0n]|BOLD:AAA2636
Lacinipolia renigera|[9186]|LPMNB391-09|Canada|Manitoba|658[0n]|BOLD:AAA2636
Lacinipolia renigera|[9187]|LPMNB334-09|Canada|Manitoba|658[0n]|BOLD:AAA2636
Lacinipolia renigera|[9188]|LPMN403-08|Canada|Manitoba|658[0n]|BOLD:AAA2636
Lacinipolia renigera|[9189]|LPMN334-08|Canada|Manitoba|658[0n]|BOLD:AAA2636
Lacinipolia renigera|[9190]|XAH377-05|Canada|Ontario|643[0n]|BOLD:AAA2636
Lacinipolia renigera|[9191]|CNGBG1122-14|Canada|Ontario|551[0n]|BOLD:AAA2636
Lacinipolia renigera|[9192]|BLTIB896-08|Canada|Ontario|638[0n]|BOLD:AAA2636
Lacinipolia renigera|[9193]|TMG131-03|Canada|Ontario|639[0n]|BOLD:AAA2636
Lacinipolia renigera|[9194]|XAK486-07|Canada|Ontario|583[1n]|BOLD:AAA2636
Lacinipolia renigera|[9195]|GBGL5899-09|||1392[0n]|BOLD:AAA2636

[illegible]
Lacinipolia renigera|[9193]|TMG131-03|Canada|Ontario|639[0n]|BOLD:AAA2636
Lacinipolia renigera|[9194]|XAK486-07|Canada|Ontario|583[1n]|BOLD:AAA2636
Lacinipolia renigera|[9195]|GBGL5899-09|||1392[0n]|BOLD:AAA2636
Lacinipolia renigera|[9196]|PMG124-03|Canada|Ontario|617[0n]|BOLD:AAA2636
Lacinipolia renigera|[9197]|CNGBN1753-14|Canada|Ontario|546[0n]|BOLD:AAA2636
Lacinipolia renigera|[9198]|RDMAB011-05|Canada|Alberta|627[0n]|BOLD:ACE7010
Lacinipolia renigera|[9199]|RDNMC409-05|Canada|Alberta|658[0n]|BOLD:ACE7010
Lacinipolia renigera|[9200]|RDMAB649-06|Canada|Alberta|658[0n]|BOLD:ACE7010
Lacinipolia renigera|[9201]|LPABB499-08|Canada|Alberta|636[0n]|BOLD:ACE7010
Lacinipolia renigera|[9202]|BBLPB426-10|Canada|Alberta|658[0n]|BOLD:ACE7010
Lacinipolia renigera|[9203]|BBLPB428-10|Canada|Alberta|658[0n]|BOLD:ACE7010
Lacinipolia renigera|[9204]|BBLPB739-10|Canada|Alberta|658[0n]|BOLD:ACE7010
Lacinipolia renigera|[9205]|XAJ692-06|Canada|Ontario|658[0n]|BOLD:ACE7010
Lacinipolia renigera|[9206]|LNCC336-10|United States|North Carolina|658[0n]|BOLD:ACE7010
Lacinipolia renigera|[9207]|BBLSY868-09|United States|Oklahoma|658[0n]|BOLD:ACE7010
Lacinipolia renigera|[9208]|RDLQB920-05|Canada|Quebec|658[0n]|BOLD:ACE7010
Lacinipolia renigera|[9209]|RDLQB919-05|Canada|Quebec|658[0n]|BOLD:ACE7010
Lacinipolia renigera|[9210]|RDLQB918-05|Canada|Quebec|658[0n]|BOLD:ACE7010
Lacinipolia renigera|[9211]|BBLPC265-09|Canada|Nova Scotia|658[0n]|BOLD:ACE7010
Lacinipolia renigera|[9212]|BBLEC265-09|Canada|Nova Scotia|658[0n]|BOLD:ACE7010
Lacinipolia renigera|[9213]|BBLPE271-09|Canada|Nova Scotia|622[0n]|BOLD:ACE7010
Lacinipolia renigera|[9214]|BBLPE309-09|Canada|Newfoundland and Labrador|658[0n]|BOLD:ACE7010
Lacinipolia renigera|[9215]|LNCC335-10|United States|North Carolina|658[0n]|BOLD:ACE7010
Lacinipolia renigera|[9216]|LNCC333-10|United States|North Carolina|658[0n]|BOLD:ACE7010
Lacinipolia renigera|[9217]|BBLPC421-09|Canada|New Brunswick|658[0n]|BOLD:ACE7010
Lacinipolia renigera|[9218]|BBLPC398-09|Canada|New Brunswick|658[0n]|BOLD:ACE7010
Lacinipolia renigera|[9219]|XAK301-06|Canada|Ontario|658[0n]|BOLD:ACE7010
Lacinipolia renigera|[9220]|TTMNB401-06|Canada|New Brunswick|658[0n]|BOLD:ACE7010
Lacinipolia renigera|[9221]|RDLQF072-06|Canada|Quebec|658[0n]|BOLD:ACE7010
Lacinipolia renigera|[9222]|RDLQF071-06|Canada|Quebec|658[0n]|BOLD:ACE7010
Lacinipolia renigera|[9223]|XAD716-05|Canada|Ontario|658[0n]|BOLD:ACE7010
Lacinipolia renigera|[9224]|XAD478-04|Canada|Ontario|658[0n]|BOLD:ACE7010
Lacinipolia renigera|[9225]|RDMAB266-05|Canada|Alberta|658[0n]|BOLD:ACE7010
Lacinipolia renigera|[9226]|XAD091-04|Canada|Ontario|580[0n]|BOLD:ACE7010
Lacinipolia renigera|[9227]|XAD481-04|Canada|Ontario|600[0n]|BOLD:ACE7010
Lacinipolia renigera|[9228]|RDLQF699-06|Canada|Quebec|637[0n]|BOLD:ACE7010
Lacinipolia renigera|[9229]|SMTPI2743-14|Canada|Ontario|534[0n]|BOLD:ACE7010
Lacinipolia renigera|[9230]|MJMSL018-10|United States|Massachusetts|658[0n]|BOLD:ABZ4680
Lacinipolia renigera|[9231]|BBLSX314-09|United States|Oklahoma|658[0n]|BOLD:ABZ4680
Lacinipolia renigera|[9232]|BBLSZ129-09|United States|Oklahoma|658[0n]|BOLD:ABZ4680
Lacinipolia renigera|[9233]|BBLSX320-09|United States|Oklahoma|658[0n]|BOLD:ABZ4680
Lacinipolia renigera|[9234]|BBLSY864-09|United States|Oklahoma|658[0n]|BOLD:ABZ4680
Lacinipolia renigera|[9235]|BBLSY873-09|United States|Oklahoma|658[0n]|BOLD:ABZ4680
Lacinipolia renigera|[9236]|BBLSX319-09|United States|Oklahoma|658[0n]|BOLD:ABZ4680
Lacinipolia renigera|[9237]|LGSMC445-05|United States|Tennessee|576[0n]|BOLD:ABZ4680
Lacinipolia renigera|[9238]|BBLSY872-09|United States|Oklahoma|658[0n]|BOLD:ABZ4680
Lacinipolia renigera|[9239]|LSEU352-06|United States|Georgia|658[0n]|BOLD:ABZ4680
Lacinipolia renigera|[9240]|LPOKB298-09|United States|Oklahoma|658[0n]|BOLD:ABZ4680
Lacinipolia renigera|[9241]|LPOKB327-09|United States|Oklahoma|658[0n]|BOLD:ABZ4680
Lacinipolia renigera|[9242]|BBLSY875-09|United States|Oklahoma|658[0n]|BOLD:ABZ4680
Lacinipolia renigera|[9243]|BBLSZ099-09|United States|Oklahoma|658[0n]|BOLD:ABZ4680
Lacinipolia renigera|[9244]|BBLSZ118-09|United States|Oklahoma|658[0n]|BOLD:ABZ4680
Lacinipolia renigera|[9245]|BBLSZ128-09|United States|Oklahoma|658[0n]|BOLD:ABZ4680
Lacinipolia renigera|[9246]|BBLSZ132-09|United States|Oklahoma|658[0n]|BOLD:ABZ4680
Lacinipolia renigera|[9247]|BBLSZ137-09|United States|Oklahoma|658[0n]|BOLD:ABZ4680
Lacinipolia renigera|[9248]|BBLSU003-09|United States|Arkansas|658[0n]|BOLD:ABZ4680
Lacinipolia renigera|[9249]|BBLSU034-09|United States|Arkansas|658[0n]|BOLD:ABZ4680
Lacinipolia renigera|[9250]|BBLSY863-09|United States|Oklahoma|658[0n]|BOLD:ABZ4680
Lacinipolia renigera|[9251]|BBLSY874-09|United States|Oklahoma|658[0n]|BOLD:ABZ4680
Lacinipolia renigera|[9252]|BBLSX315-09|United States|Oklahoma|658[0n]|BOLD:ABZ4680
Lacinipolia renigera|[9253]|BBLSX317-09|United States|Oklahoma|658[0n]|BOLD:ABZ4680
Lacinipolia renigera|[9254]|LPOKB340-09|United States|Oklahoma|658[0n]|BOLD:ABZ4680
Lacinipolia renigera|[9255]|LPOKB600-09|United States|Oklahoma|658[0n]|BOLD:ABZ4680
Lacinipolia renigera|[9256]|BBLSY854-09|United States|Oklahoma|658[0n]|BOLD:ABZ4680
Lacinipolia renigera|[9257]|BBLSY862-09|United States|Oklahoma|658[0n]|BOLD:ABZ4680
Lacinipolia renigera|[9258]|LPOKB206-09|United States|Oklahoma|658[0n]|BOLD:ABZ4680
Lacinipolia renigera|[9259]|LPSOB246-08|Canada|Ontario|658[0n]|BOLD:ABZ4680
Lacinipolia renigera|[9260]|LNCNW081-06|United States|North Carolina|658[0n]|BOLD:ABZ4680
Lacinipolia renigera|[9261]|LSUSA252-06|United States|Kentucky|658[0n]|BOLD:ABZ4680
Lacinipolia renigera|[9262]|LOTB255-05|United States|Tennessee|658[0n]|BOLD:ABZ4680
Lacinipolia renigera|[9263]|LOTB253-05|United States|Tennessee|658[0n]|BOLD:ABZ4680
Lacinipolia renigera|[9264]|LOTB217-05|United States|Tennessee|658[0n]|BOLD:ABZ4680
Lacinipolia renigera|[9265]|BBLSZ109-09|United States|Oklahoma|658[0n]|BOLD:ABZ4680
Lacinipolia renigera|[9266]|LGSMC442-05|United States|Tennessee|658[0n]|BOLD:ABZ4680
Lacinipolia renigera|[9267]|LGSMC441-05|United States|Tennessee|658[0n]|BOLD:ABZ4680
Lacinipolia renigera|[9268]|BBLSY870-09|United States|Oklahoma|658[0n]|BOLD:ABZ4680
Lacinipolia renigera|[9269]|LPSO835-08|Canada|Ontario|658[0n]|BOLD:ABZ4680
Lacinipolia renigera|[9270]|BBLSX318-09|United States|Oklahoma|658[0n]|BOLD:ABZ4680
Lacinipolia renigera|[9271]|BBLSZ133-09|United States|Oklahoma|658[0n]|BOLD:ABZ4680
Lacinipolia renigera|[9272]|BBLSZ135-09|United States|Oklahoma|658[0n]|BOLD:ABZ4680
Lacinipolia renigera|[9273]|LPOKB331-09|United States|Oklahoma|658[0n]|BOLD:ABZ4680
Lacinipolia renigera|[9274]|LPOKA630-09|United States|Oklahoma|616[0n]|BOLD:ABZ4680
Lacinipolia renigera|[9275]|BBLCU341-09|United States|Michigan|623[0n]|BOLD:ABZ4680
Lacinipolia renigera|[9276]|LILLA222-11|United States|Illinois|658[0n]|BOLD:ABZ4680
Lacinipolia renigera|[9277]|LILLA258-11|United States|Illinois|658[0n]|BOLD:ABZ4680
Lacinipolia renigera|[9278]|BBLSY857-09|United States|Oklahoma|658[0n]|BOLD:ABZ4680
Lacinipolia renigera|[9279]|LGSMC444-05|United States|Tennessee|658[0n]|BOLD:ABZ4680
Lacinipolia renigera|[9280]|BBLSZ125-09|United States|Oklahoma|658[0n]|BOLD:ABZ4680
Lacinipolia renigera|[9281]|SMTPI5899-14|Canada|Ontario|624[0n]|BOLD:ABZ4680
Lacinipolia renigera|[9282]|LNCC1675-13|United States|North Carolina|658[0n]|BOLD:ABZ4680
Lacinipolia renigera|[9283]|LNCC1311-11|United States|North Carolina|658[0n]|BOLD:ABZ4680
Lacinipolia renigera|[9284]|LNCC1107-11|United States|North Carolina|658[0n]|BOLD:ABZ4680
Lacinipolia renigera|[9285]|LPSOB236-08|Canada|Ontario|658[0n]|BOLD:ABZ4680
Lacinipolia renigera|[9286]|LPSOB235-08|Canada|Ontario|658[0n]|BOLD:ABZ4680
Lacinipolia renigera|[9287]|LNCNW079-06|United States|North Carolina|658[0n]|BOLD:ABZ4680
Lacinipolia renigera|[9288]|LOTB256-05|United States|Tennessee|658[0n]|BOLD:ABZ4680
Lacinipolia renigera|[9289]|BBLCU137-09|United States|Michigan|658[0n]|BOLD:ABZ4680
Lacinipolia renigera|[9290]|LPSO685-08|Canada|Ontario|658[1n]|BOLD:ABZ4680
Lacinipolia renigera|[9291]|LOTB254-05|United States|Tennessee|612[1n]|BOLD:ABZ4680
Lacinipolia renigera|[9292]|SMTPI5895-14|Canada|Ontario|579[0n]|BOLD:ABZ4680
Lacinipolia renigera|[9293]|LOCT092-05|United States|Connecticut|658[0n]|BOLD:ABZ4680
Lacinipolia renigera|[9294]|LOTB252-05|United States|Tennessee|658[0n]|BOLD:ABZ4680
Lacinipolia renigera|[9295]|BBLSZ124-09|United States|Oklahoma|658[0n]|BOLD:ABZ4680

Lacinipolia renigera|[9293]|LOCT092-05|United States|Connecticut|658[0n]|BOLD:ABZ4680
Lacinipolia renigera|[9294]|LOTB252-05|United States|Tennessee|658[0n]|BOLD:ABZ4680
Lacinipolia renigera|[9295]|BBLSZ124-09|United States|Oklahoma|658[0n]|BOLD:ABZ4680
Lacinipolia renigera|[9296]|BBLSZ134-09|United States|Oklahoma|658[0n]|BOLD:ABZ4680
Lacinipolia renigera|[9297]|LNCC1310-11|United States|North Carolina|658[0n]|BOLD:ABZ4680
Lacinipolia renigera|[9298]|LPSOB222-08|Canada|Ontario|658[0n]|BOLD:ABZ4680
Lacinipolia renigera|[9299]|LNCB962-10|United States|North Carolina|658[0n]|BOLD:ABZ4680
Lacinipolia renigera|[9300]|LNCB995-10|United States|North Carolina|658[0n]|BOLD:ABZ4680
Lacinipolia renigera|[9301]|BBLPA650-10|Canada|Ontario|658[0n]|BOLD:ABZ4680
Lacinipolia renigera|[9302]|LNCC1329-11|United States|North Carolina|658[0n]|BOLD:ABZ4680
Lacinipolia renigera|[9303]|LPSOB238-08|Canada|Ontario|658[0n]|BOLD:ABZ4680
Lacinipolia renigera|[9304]|RBINA2150-13|Canada|Ontario|570[0n]|BOLD:ABZ4680
Lacinipolia renigera|[9305]|SMTPJ4029-14|Canada|Ontario|606[0n]|BOLD:ABZ4680
Psammopolia arietis|[9306]|NAMUM232-08|United States|California|655[0n]|BOLD:ACE5307
Psammopolia arietis|[9307]|NAMUM233-08|United States|California|658[0n]|BOLD:ACE5307
Psammopolia arietis|[9308]|RWWC574-11|United States|Washington|658[0n]|BOLD:AAC7832
Psammopolia arietis|[9309]|RWWB104-09|United States|Washington|658[0n]|BOLD:AAC7832
Psammopolia arietis|[9310]|RWWB223-09|United States|Washington|658[0n]|BOLD:AAC7832
Psammopolia arietis|[9311]|RWWA858-09|United States|Washington|658[0n]|BOLD:AAC7832
Psammopolia arietis|[9312]|RDNMB721-05|United States|Oregon|572[1n]|BOLD:AAC7832
Psammopolia arietis|[9313]|RDNMB722-05|United States|Washington|651[0n]|BOLD:AAC7832
Psammopolia arietis|[9314]|RWWB185-09|United States|Washington|658[0n]|BOLD:AAC7832
Psammopolia arietis|[9315]|RWWB243-09|United States|Washington|658[0n]|BOLD:AAC7832
Psammopolia arietis|[9316]|RWWB266-09|United States|Washington|658[0n]|BOLD:AAC7832
Psammopolia arietis|[9317]|RWWC636-11|United States|Washington|658[0n]|BOLD:AAC7832
Psammopolia arietis|[9318]|RWWC767-11|United States|Washington|658[0n]|BOLD:AAC7832
Psammopolia arietis|[9319]|RWWC773-11|United States|Washington|658[0n]|BOLD:AAC7832
Psammopolia arietis|[9320]|RWWC1106-12|United States|Washington|658[0n]|BOLD:AAC7832
Psammopolia sp.|[9321]|BBLPB769-10|Canada|British Columbia|658[0n]|BOLD:AAP8033
Psammopolia sp.|[9322]|UAMIC1160-13|United States|Alaska|658[0n]|BOLD:AAP8033
Psammopolia sala|[9323]|CGLCA159-10|United States|California|658[0n]|BOLD:AAL7125
Psammopolia sala|[9324]|CNCLB270-14|United States|California|658[0n]|BOLD:AAL7125
Psammopolia insolens|[9325]|CNCLB684-14|United States|California|658[0n]|BOLD:ACM4665
Psammopolia wyatti|[9326]|RDNMB719-05|Canada|British Columbia|538[1n]|BOLD:AAB0902
Psammopolia wyatti|[9327]|RWWA727-09|United States|Washington|658[0n]|BOLD:AAB0902
Psammopolia wyatti|[9328]|RWWA839-09|United States|Washington|658[0n]|BOLD:AAB0902
Psammopolia wyatti|[9329]|RWWB049-09|United States|Washington|658[0n]|BOLD:AAB0902
Psammopolia wyatti|[9330]|RWWA780-09|United States|Washington|658[0n]|BOLD:AAB0902
Psammopolia wyatti|[9331]|RWWA873-09|United States|Washington|658[0n]|BOLD:AAB0902
Psammopolia wyatti|[9332]|RWWA760-09|United States|Washington|658[0n]|BOLD:AAB0902
Psammopolia wyatti|[9333]|RWWB047-09|United States|Washington|658[0n]|BOLD:AAB0902
Psammopolia wyatti|[9334]|RWWB154-09|United States|Washington|658[0n]|BOLD:AAB0902
Psammopolia wyatti|[9335]|RWWB156-09|United States|Washington|658[0n]|BOLD:AAB0902
Psammopolia wyatti|[9336]|RWWB113-09|United States|Washington|658[0n]|BOLD:AAB0902
Psammopolia wyatti|[9337]|RWWB117-09|United States|Washington|658[0n]|BOLD:AAB0902
Psammopolia wyatti|[9338]|RWWB083-09|United States|Washington|658[0n]|BOLD:AAB0902
Psammopolia wyatti|[9339]|RWWB106-09|United States|Washington|658[0n]|BOLD:AAB0902
Psammopolia wyatti|[9340]|RWWB196-09|United States|Washington|658[0n]|BOLD:AAB0902
Psammopolia wyatti|[9341]|RWWB034-09|United States|Washington|658[0n]|BOLD:AAB0902
Psammopolia wyatti|[9342]|RWWA950-09|United States|Washington|658[0n]|BOLD:AAB0902
Psammopolia wyatti|[9343]|RWWA947-09|United States|Washington|658[0n]|BOLD:AAB0902
Psammopolia wyatti|[9344]|RWWA921-09|United States|Washington|658[0n]|BOLD:AAB0902
Psammopolia wyatti|[9345]|RWWA872-09|United States|Washington|658[0n]|BOLD:AAB0902
Psammopolia wyatti|[9346]|RWWA833-09|United States|Washington|658[0n]|BOLD:AAB0902
Psammopolia wyatti|[9347]|RWWA790-09|United States|Washington|658[0n]|BOLD:AAB0902
Psammopolia wyatti|[9348]|RWWA777-09|United States|Washington|658[0n]|BOLD:AAB0902
Psammopolia wyatti|[9349]|RWWA753-09|United States|Washington|658[0n]|BOLD:AAB0902
Psammopolia wyatti|[9350]|RWWA688-09|United States|Washington|658[0n]|BOLD:AAB0902
Psammopolia wyatti|[9351]|RWWA680-09|United States|Washington|658[0n]|BOLD:AAB0902
Psammopolia wyatti|[9352]|RWWB065-09|United States|Washington|658[0n]|BOLD:AAB0902
Psammopolia wyatti|[9353]|RDNMB720-05|Canada|British Columbia|562[1n]|BOLD:AAB0902
Psammopolia wyatti|[9354]|RWWB217-09|United States|Washington|638[0n]|BOLD:AAB0902
Psammopolia wyatti|[9355]|RWWB224-09|United States|Washington|658[0n]|BOLD:AAB0902
Psammopolia wyatti|[9356]|RWWB242-09|United States|Washington|658[0n]|BOLD:AAB0902
Psammopolia wyatti|[9357]|RWWB820-10|United States|Washington|658[0n]|BOLD:AAB0902
Psammopolia ochracea|[9358]|CNCLB685-14|United States|California|658[0n]|BOLD:ACM4664
Trichocerapoda oceanis|[9359]|LNAUT486-14|United States|California|658[0n]|BOLD:ACN7388
Trichocerapoda oceanis|[9360]|LNAUT487-14|United States|California|658[0n]|BOLD:ACN7388
Trichocerapoda oceanis|[9361]|LNAUT488-14|United States|California|658[0n]|BOLD:ACN7388
Trichocerapoda oceanis|[9362]|LNAUT489-14|United States|California|658[0n]|BOLD:ACN7388
Trichocerapoda oblita|[9363]|NAMUM187-08|United States|California|658[0n]|BOLD:AAF4923
Trichocerapoda oblita|[9364]|RDNMC404-05|Canada|Saskatchewan|595[0n]|BOLD:AAF4923
Trichocerapoda oblita|[9365]|NAMUM188-08|United States|California|658[0n]|BOLD:AAF4923
Trichocerapoda oblita|[9366]|CNCLB1570-14|United States|Oregon|658[0n]|BOLD:AAF4923
Trichocerapoda comstocki|[9367]|IAWLB507-11|United States|Arizona|658[0n]|BOLD:AAU7967
Trichocerapoda comstocki|[9368]|IAWLB505-11|United States|Arizona|658[0n]|BOLD:AAU7967
Trichocerapoda comstocki|[9369]|AWCLB530-11|United States|Arizona|658[0n]|BOLD:AAU7967
Trichocerapoda comstocki|[9370]|IAWLB506-11|United States|Arizona|658[0n]|BOLD:AAU7967
Trichocerapoda comstocki|[9371]|LNAUS4723-13|United States|California|658[0n]|BOLD:AAU7967
Trichocerapoda comstocki|[9372]|LNAUS4725-13|United States|California|658[0n]|BOLD:AAU7967
Lacinipolia leucogramma|[9373]|NAMUM213-08|United States|California|658[0n]|BOLD:AAJ3034
Lacinipolia leucogramma|[9374]|CNCLB1557-14|United States|California|658[0n]|BOLD:AAJ3034
Lacinipolia leucogramma|[9375]|NAMUM212-08|United States|California|658[0n]|BOLD:AAJ3034
Lacinipolia leucogramma|[9376]|CNCLB250-14|United States|California|658[0n]|BOLD:AAJ3034
Lacinipolia leucogramma|[9377]|CNCLB1558-14|United States|California|658[0n]|BOLD:AAJ3034
Trichocerapoda strigata|[9378]|RDNMC692-06|United States|California|658[0n]|BOLD:AAJ8683
Trichocerapoda strigata|[9379]|LNAUT481-14|United States|Texas|658[0n]|BOLD:AAJ8683
Trichocerapoda strigata|[9380]|LNAUT482-14|United States|Texas|658[0n]|BOLD:AAJ8683
Trichocerapoda strigata|[9381]|CNCLB1580-14|United States|Oregon|573[0n]|BOLD:AAJ8683
Lacinipolia rectilinea|[9382]|RDNMF583-08|United States|Washington|609[1n]|BOLD:AAJ2964
Lacinipolia rectilinea|[9383]|RDNMF584-08|United States|Washington|658[0n]|BOLD:AAJ2964
Lacinipolia davena|[9384]|LHLEP567-06|Canada|British Columbia|658[0n]|BOLD:AAB3076
Lacinipolia davena|[9385]|LOWCE279-06|Canada|British Columbia|658[1n]|BOLD:AAB3076
Lacinipolia davena|[9386]|RDMAB251-05|Canada|Alberta|658[0n]|BOLD:AAB3076
Lacinipolia davena|[9387]|LBCD146-05|Canada|British Columbia|658[0n]|BOLD:AAB3076
Lacinipolia davena|[9388]|LBCD145-05|Canada|British Columbia|658[0n]|BOLD:AAB3076
Lacinipolia davena|[9389]|LBCA371-05|Canada|British Columbia|610[1n]|BOLD:AAB3076
Lacinipolia davena|[9390]|LBCA506-05|Canada|British Columbia|658[0n]|BOLD:AAB3076
Lacinipolia davena|[9391]|LBCA370-05|Canada|British Columbia|658[0n]|BOLD:AAB3076
Lacinipolia davena|[9392]|LBCA369-05|Canada|British Columbia|658[0n]|BOLD:AAB3076
Lacinipolia davena|[9393]|RDMAB253-05|Canada|Alberta|655[0n]|BOLD:AAB3076
Lacinipolia davena|[9394]|LBCD147-05|Canada|British Columbia|596[0n]|BOLD:AAB3076
Lacinipolia davena|[9395]|RDNMC390-05|United States|Oregon|551[0n]|BOLD:AAB3076

Lacinipolia davena|[9393]|RDMAB253-05|Canada|Alberta|655[0n]|BOLD:AAB3076
Lacinipolia davena|[9394]|LBCD147-05|Canada|British Columbia|596[0n]|BOLD:AAB3076
Lacinipolia davena|[9395]|RDNMC390-05|United States|Oregon|551[0n]|BOLD:AAB3076
Lacinipolia davena|[9396]|LOWCE278-06|Canada|British Columbia|658[0n]|BOLD:AAB3076
Lacinipolia davena|[9397]|LOWCE280-06|Canada|British Columbia|658[0n]|BOLD:AAB3076
Lacinipolia davena|[9398]|LHLEP568-06|Canada|British Columbia|658[0n]|BOLD:AAB3076
Lacinipolia davena|[9399]|LPAB020-08|Canada|Alberta|658[0n]|BOLD:AAB3076
Lacinipolia davena|[9400]|LPABB122-08|Canada|Alberta|658[0n]|BOLD:AAB3076
Lacinipolia davena|[9401]|LPABB123-08|Canada|Alberta|658[0n]|BOLD:AAB3076
Lacinipolia davena|[9402]|LPABB267-08|Canada|Alberta|658[0n]|BOLD:AAB3076
Lacinipolia davena|[9403]|LPABB376-08|Canada|Alberta|658[0n]|BOLD:AAB3076
Lacinipolia davena|[9404]|LALPA610-10|Canada|British Columbia|658[0n]|BOLD:AAB3076
Lacinipolia davena|[9405]|RWWC400-11|United States|Washington|658[0n]|BOLD:AAB3076
Lacinipolia davena|[9406]|LALPA253-10|Canada|British Columbia|658[0n]|BOLD:AAB3076
Lacinipolia davena|[9407]|LALPA341-10|Canada|British Columbia|658[0n]|BOLD:AAB3076
Lacinipolia davena|[9408]|LALPA216-10|Canada|British Columbia|658[0n]|BOLD:AAB3076
Lacinipolia davena|[9409]|LALPA237-10|Canada|British Columbia|658[0n]|BOLD:AAB3076
Lacinipolia davena|[9410]|RWWA476-09|United States|Washington|658[0n]|BOLD:AAB3076
Lacinipolia davena|[9411]|RWWA912-09|United States|Washington|658[0n]|BOLD:AAB3076
Lacinipolia davena|[9412]|LPABB394-08|Canada|Alberta|658[0n]|BOLD:AAB3076
Lacinipolia davena|[9413]|LPABB411-08|Canada|Alberta|658[0n]|BOLD:AAB3076
Lacinipolia davena|[9414]|LPABB631-08|Canada|Alberta|658[0n]|BOLD:AAB3076
Lacinipolia davena|[9415]|LPABC905-09|Canada|Alberta|658[0n]|BOLD:AAB3076
Lacinipolia davena|[9416]|RDNMK659-11|Canada|British Columbia|658[0n]|BOLD:AAB3076
Lacinipolia davena|[9417]|RDNMK660-11|Canada|British Columbia|658[0n]|BOLD:AAB3076
Lacinipolia comis|[9418]|LBCH4050-10|Canada|British Columbia|658[0n]|BOLD:AAC2984
Lacinipolia comis|[9419]|LBCH4438-10|Canada|British Columbia|658[0n]|BOLD:AAC2984
Lacinipolia comis|[9420]|LBCH938-10|Canada|British Columbia|658[0n]|BOLD:AAC2984
Lacinipolia comis|[9421]|LBCH3785-10|Canada|British Columbia|658[0n]|BOLD:AAC2984
Lacinipolia comis|[9422]|LBCH4440-10|Canada|British Columbia|658[0n]|BOLD:AAC2984
Lacinipolia comis|[9423]|RWWC1093-12|United States|Washington|658[0n]|BOLD:AAC2984
Lacinipolia comis|[9424]|GMLC1165-12|United States|California|658[0n]|BOLD:AAC2984
Lacinipolia comis|[9425]|LALPA1314-11|Canada|British Columbia|658[0n]|BOLD:AAC2984
Lacinipolia comis|[9426]|LALPA982-11|Canada|British Columbia|658[0n]|BOLD:AAC2984
Lacinipolia comis|[9427]|LALPA942-11|Canada|British Columbia|658[0n]|BOLD:AAC2984
Lacinipolia comis|[9428]|BBLPB470-10|Canada|British Columbia|658[0n]|BOLD:AAC2984
Lacinipolia comis|[9429]|LALPA617-10|Canada|British Columbia|658[0n]|BOLD:AAC2984
Lacinipolia comis|[9430]|LBCH6739-10|Canada|British Columbia|658[0n]|BOLD:AAC2984
Lacinipolia comis|[9431]|LBCH4052-10|Canada|British Columbia|658[0n]|BOLD:AAC2984
Lacinipolia comis|[9432]|LBCH2086-10|Canada|British Columbia|658[0n]|BOLD:AAC2984
Lacinipolia comis|[9433]|LBCH1952-10|Canada|British Columbia|658[0n]|BOLD:AAC2984
Lacinipolia comis|[9434]|LBCH355-10|Canada|British Columbia|658[0n]|BOLD:AAC2984
Lacinipolia comis|[9435]|RWWB189-09|United States|Washington|658[0n]|BOLD:AAC2984
Lacinipolia comis|[9436]|RWWB122-09|United States|Washington|658[0n]|BOLD:AAC2984
Lacinipolia comis|[9437]|RWWA970-09|United States|Washington|658[0n]|BOLD:AAC2984
Lacinipolia comis|[9438]|RWWA765-09|United States|Washington|658[0n]|BOLD:AAC2984
Lacinipolia comis|[9439]|LPABC958-09|Canada|Alberta|658[0n]|BOLD:AAC2984
Lacinipolia comis|[9440]|LBCD464-05|Canada|British Columbia|658[0n]|BOLD:AAC2984
Lacinipolia comis|[9441]|LBCB074-05|Canada|British Columbia|658[0n]|BOLD:AAC2984
Lacinipolia comis|[9442]|LBCH1973-10|Canada|British Columbia|642[0n]|BOLD:AAC2984
Lacinipolia comis|[9443]|RDNMC396-05|United States|Oregon|598[0n]|BOLD:AAC2984
Lacinipolia comis|[9444]|RDNMC395-05|United States|Oregon|589[0n]|BOLD:AAC2984
Lacinipolia comis|[9445]|RDNMC397-05|United States|Oregon|587[0n]|BOLD:AAC2984
Lacinipolia comis|[9446]|JMMMB595-13|United States|California|603[0n]|BOLD:AAC2984
Lacinipolia sharonae|[9447]|RDNME1015-08|United States|Arizona|658[0n]|BOLD:ABX5834
Lacinipolia sharonae|[9448]|RDNME1017-08|United States|Arizona|658[0n]|BOLD:ABX5834
Lacinipolia sharonae|[9449]|BBLSY587-09|United States|New Mexico|589[0n]|BOLD:ABX5834
Lacinipolia olivacea|[9450]|XAG798-05|Canada|Ontario|658[0n]|BOLD:AAA2596
Lacinipolia olivacea|[9451]|XAK322-06|Canada|Ontario|658[0n]|BOLD:AAA2596
Lacinipolia olivacea|[9452]|LGSMG857-10|United States|North Carolina|658[0n]|BOLD:AAA2596
Lacinipolia olivacea|[9453]|LGSMG855-10|United States|North Carolina|658[0n]|BOLD:AAA2596
Lacinipolia olivacea|[9454]|LGSMG858-10|United States|North Carolina|658[0n]|BOLD:AAA2596
Lacinipolia olivacea|[9455]|LGSMG854-10|United States|North Carolina|658[0n]|BOLD:AAA2596
Lacinipolia olivacea|[9456]|BBLPC520-09|Canada|New Brunswick|658[0n]|BOLD:AAA2596
Lacinipolia olivacea|[9457]|BBLEC573-09|Canada|Nova Scotia|658[0n]|BOLD:AAA2596
Lacinipolia olivacea|[9458]|BBLPE334-09|Canada|Newfoundland and Labrador|658[0n]|BOLD:AAA2596
Lacinipolia olivacea|[9459]|XAK334-06|Canada|Ontario|658[0n]|BOLD:AAA2596
Lacinipolia olivacea|[9460]|MNBB670-05|Canada|New Brunswick|658[0n]|BOLD:AAA2596
Lacinipolia olivacea|[9461]|RDLQB638-05|Canada|Quebec|614[1n]|BOLD:AAA2596
Lacinipolia olivacea|[9462]|BBLPC070-09|Canada|New Brunswick|658[0n]|BOLD:AAA2596
Lacinipolia olivacea|[9463]|BBLPC529-09|Canada|New Brunswick|658[0n]|BOLD:AAA2596
Lacinipolia olivacea|[9464]|BBLPC109-09|Canada|New Brunswick|658[0n]|BOLD:AAA2596
Lacinipolia olivacea|[9465]|BBLPC388-09|Canada|New Brunswick|658[0n]|BOLD:AAA2596
Lacinipolia olivacea|[9466]|BBLPC389-09|Canada|New Brunswick|658[0n]|BOLD:AAA2596
Lacinipolia olivacea|[9467]|BBLPC401-09|Canada|New Brunswick|658[0n]|BOLD:AAA2596
Lacinipolia olivacea|[9468]|BBLPC040-09|Canada|New Brunswick|658[0n]|BOLD:AAA2596
Lacinipolia olivacea|[9469]|BBLEC745-09|Canada|Nova Scotia|658[0n]|BOLD:AAA2596
Lacinipolia olivacea|[9470]|BBLEC470-09|Canada|New Brunswick|658[0n]|BOLD:AAA2596
Lacinipolia olivacea|[9471]|BBLEC431-09|Canada|New Brunswick|658[0n]|BOLD:AAA2596
Lacinipolia olivacea|[9472]|BBLEC031-09|Canada|New Brunswick|658[0n]|BOLD:AAA2596
Lacinipolia olivacea|[9473]|TMNBB328-06|Canada|New Brunswick|658[0n]|BOLD:AAA2596
Lacinipolia olivacea|[9474]|TMNBB327-06|Canada|New Brunswick|658[0n]|BOLD:AAA2596
Lacinipolia olivacea|[9475]|TTMNB402-06|Canada|New Brunswick|658[0n]|BOLD:AAA2596
Lacinipolia olivacea|[9476]|MNBB671-05|Canada|New Brunswick|658[0n]|BOLD:AAA2596
Lacinipolia olivacea|[9477]|MNBB622-05|Canada|New Brunswick|658[0n]|BOLD:AAA2596
Lacinipolia olivacea|[9478]|MNBB619-05|Canada|New Brunswick|658[0n]|BOLD:AAA2596
Lacinipolia olivacea|[9479]|BBLPC043-09|Canada|New Brunswick|656[0n]|BOLD:AAA2596
Lacinipolia olivacea|[9480]|PHMNB006-03|Canada|New Brunswick|639[0n]|BOLD:AAA2596
Lacinipolia olivacea|[9481]|BBLEC748-09|Canada|Nova Scotia|632[0n]|BOLD:AAA2596
Lacinipolia olivacea|[9482]|BBLPC422-09|Canada|New Brunswick|632[0n]|BOLD:AAA2596
Lacinipolia olivacea|[9483]|BBLPC410-09|Canada|New Brunswick|658[0n]|BOLD:AAA2596
Lacinipolia olivacea|[9484]|BBLPC424-09|Canada|New Brunswick|658[0n]|BOLD:AAA2596
Lacinipolia olivacea|[9485]|BBLPC488-09|Canada|New Brunswick|658[0n]|BOLD:AAA2596
Lacinipolia olivacea|[9486]|BBLPC555-09|Canada|New Brunswick|658[0n]|BOLD:AAA2596
Lacinipolia olivacea|[9487]|MNBB672-05|Canada|New Brunswick|658[0n]|BOLD:AAA2596
Lacinipolia olivacea|[9488]|BBLPC067-09|Canada|New Brunswick|658[0n]|BOLD:AAA2596
Lacinipolia olivacea|[9489]|XAK323-06|Canada|Ontario|658[0n]|BOLD:AAA2596
Lacinipolia olivacea|[9490]|BLTIB800-08|Canada|Ontario|658[0n]|BOLD:AAA2596
Lacinipolia olivacea|[9491]|BLTIB839-08|Canada|Ontario|658[0n]|BOLD:AAA2596
Lacinipolia olivacea|[9492]|BLTIB944-08|Canada|Ontario|658[0n]|BOLD:AAA2596
Lacinipolia olivacea|[9493]|BLTIB1063-08|Canada|Ontario|658[0n]|BOLD:AAA2596
Lacinipolia olivacea|[9494]|BBLPC059-09|Canada|New Brunswick|658[0n]|BOLD:AAA2596
Lacinipolia olivacea|[9495]|TTMNB403-06|Canada|New Brunswick|658[0n]|BOLD:AAA2596

[illegible]
Lacinipolia olivacea|[9493]|BLTIB1063-08|Canada|Ontario|658[0n]|BOLD:AAA2596
Lacinipolia olivacea|[9494]|BBLPC059-09|Canada|New Brunswick|658[0n]|BOLD:AAA2596
Lacinipolia olivacea|[9495]|TTMNB403-06|Canada|New Brunswick|658[0n]|BOLD:AAA2596
Lacinipolia olivacea|[9496]|XAK321-06|Canada|Ontario|658[0n]|BOLD:AAA2596
Lacinipolia olivacea|[9497]|RDLQB632-05|Canada|Quebec|658[0n]|BOLD:AAA2596
Lacinipolia olivacea|[9498]|RDLQB747-05|Canada|Quebec|658[0n]|BOLD:AAA2596
Lacinipolia olivacea|[9499]|XAI037-05|Canada|Ontario|658[0n]|BOLD:AAA2596
Lacinipolia olivacea|[9500]|XAI036-05|Canada|Ontario|658[0n]|BOLD:AAA2596
Lacinipolia olivacea|[9501]|XAI035-05|Canada|Ontario|658[0n]|BOLD:AAA2596
Lacinipolia olivacea|[9502]|XAI034-05|Canada|Ontario|658[0n]|BOLD:AAA2596
Lacinipolia olivacea|[9503]|XAG711-05|Canada|Ontario|658[0n]|BOLD:AAA2596
Lacinipolia olivacea|[9504]|LGSM743-04|United States|North Carolina|658[0n]|BOLD:AAA2596
Lacinipolia olivacea|[9505]|MNBB621-05|Canada|New Brunswick|658[0n]|BOLD:AAA2596
Lacinipolia olivacea|[9506]|MNBB620-05|Canada|New Brunswick|658[0n]|BOLD:AAA2596
Lacinipolia olivacea|[9507]|MNBB452-05|Canada|New Brunswick|658[0n]|BOLD:AAA2596
Lacinipolia olivacea|[9508]|XAG210-05|Canada|Ontario|658[0n]|BOLD:AAA2596
Lacinipolia olivacea|[9509]|LGSM744-04|United States|North Carolina|658[0n]|BOLD:AAA2596
Lacinipolia olivacea|[9510]|BBLPC064-09|Canada|New Brunswick|658[0n]|BOLD:AAA2596
Lacinipolia olivacea|[9511]|XAG815-05|Canada|Ontario|658[1n]|BOLD:AAA2596
Lacinipolia olivacea|[9512]|XAC854-04|Canada|Ontario|581[0n]|BOLD:AAA2596
Lacinipolia olivacea|[9513]|XAD249-04|Canada|Ontario|593[0n]|BOLD:AAA2596
Lacinipolia olivacea|[9514]|PHMO222-03|Canada|Ontario|639[0n]|BOLD:AAA2596
Lacinipolia olivacea|[9515]|XAK485-07|Canada|Ontario|598[0n]|BOLD:AAA2596
Lacinipolia olivacea|[9516]|XAD008-04|Canada|Ontario|599[0n]|BOLD:AAA2596
Lacinipolia olivacea|[9517]|XAK560-07|Canada|Ontario|595[0n]|BOLD:AAA2596
Lacinipolia olivacea|[9518]|XAK561-07|Canada|Ontario|604[0n]|BOLD:AAA2596
Lacinipolia olivacea|[9519]|BBLPC412-09|Canada|New Brunswick|632[0n]|BOLD:AAA2596
Lacinipolia olivacea|[9520]|LGSMG859-10|United States|North Carolina|658[0n]|BOLD:AAA2596
Lacinipolia olivacea|[9521]|BBLEC576-09|Canada|Nova Scotia|658[0n]|BOLD:AAA2596
Lacinipolia olivacea|[9522]|LGSMG856-10|United States|North Carolina|658[0n]|BOLD:AAA2596
Lacinipolia olivacea|[9523]|LGSMG860-10|United States|North Carolina|658[0n]|BOLD:AAA2596
Lacinipolia olivacea|[9524]|LNCC215-10|United States|North Carolina|658[0n]|BOLD:AAA2596
Lacinipolia olivacea|[9525]|LNCC216-10|United States|North Carolina|658[0n]|BOLD:AAA2596
Lacinipolia olivacea|[9526]|LNCC233-10|United States|North Carolina|658[0n]|BOLD:AAA2596
Lacinipolia olivacea|[9527]|LPMNB359-09|Canada|Manitoba|658[0n]|BOLD:ACF0280
Lacinipolia olivacea|[9528]|LPMNB456-09|Canada|Manitoba|658[0n]|BOLD:ACF0280
Lacinipolia olivacea|[9529]|RDLQG064-06|Canada|Quebec|656[0n]|BOLD:ACF0280
Lacinipolia olivacea|[9530]|LPMNB411-09|Canada|Manitoba|658[0n]|BOLD:ACF0280
Lacinipolia olivacea|[9531]|LPMNB380-09|Canada|Manitoba|658[0n]|BOLD:ACF0280
Lacinipolia olivacea|[9532]|LPMNB354-09|Canada|Manitoba|658[0n]|BOLD:ACF0280
Lacinipolia olivacea|[9533]|LPMNB330-09|Canada|Manitoba|658[0n]|BOLD:ACF0280
Lacinipolia olivacea|[9534]|LPMNB245-09|Canada|Manitoba|658[0n]|BOLD:ACF0280
Lacinipolia olivacea|[9535]|LPMNB417-09|Canada|Manitoba|658[0n]|BOLD:ACF0280
Lacinipolia olivacea|[9536]|LPMNB445-09|Canada|Manitoba|658[0n]|BOLD:ACF0280
Lacinipolia olivacea|[9537]|LPMNB448-09|Canada|Manitoba|658[0n]|BOLD:ACF0280
Lacinipolia olivacea|[9538]|LPMNB474-09|Canada|Manitoba|658[0n]|BOLD:ACF0280
Lacinipolia olivacea|[9539]|RDNME1018-08|United States|Arizona|658[0n]|BOLD:ACF0282
Lacinipolia olivacea|[9540]|LPAB251-08|Canada|Alberta|658[0n]|BOLD:ACE9063
Lacinipolia olivacea|[9541]|LPABC821-09|Canada|Alberta|658[0n]|BOLD:ACE9063
Lacinipolia olivacea|[9542]|CNWBG3075-13|Canada|Alberta|573[0n]|BOLD:ACE9063
Lacinipolia olivacea|[9543]|CNWBG3072-13|Canada|Alberta|602[0n]|BOLD:ACE9063
Lacinipolia olivacea|[9544]|LOWCE541-06|Canada|British Columbia|598[0n]|BOLD:ACE9063
Lacinipolia olivacea|[9545]|LPABB632-08|Canada|Alberta|658[0n]|BOLD:ACE9063
Lacinipolia olivacea|[9546]|LPABC132-09|Canada|Alberta|658[0n]|BOLD:ACE9063
Lacinipolia olivacea|[9547]|LPABB276-08|Canada|Alberta|658[0n]|BOLD:ACE9063
Lacinipolia olivacea|[9548]|LPABB504-08|Canada|Alberta|658[0n]|BOLD:ACE9063
Lacinipolia olivacea|[9549]|LPABC136-09|Canada|Alberta|658[0n]|BOLD:ACE9063
Lacinipolia olivacea|[9550]|LPABC138-09|Canada|Alberta|658[0n]|BOLD:ACE9063
Lacinipolia olivacea|[9551]|LPABC654-09|Canada|Alberta|658[0n]|BOLD:ACE9063
Lacinipolia olivacea|[9552]|LPABC655-09|Canada|Alberta|658[0n]|BOLD:ACE9063
Lacinipolia olivacea|[9553]|LPABC769-09|Canada|Alberta|658[0n]|BOLD:ACE9063
Lacinipolia olivacea|[9554]|LPABC770-09|Canada|Alberta|658[0n]|BOLD:ACE9063
Lacinipolia olivacea|[9555]|LPSOD935-09|Canada|Ontario|658[0n]|BOLD:ACE9063
Lacinipolia olivacea|[9556]|BBLPC906-09|Canada|Newfoundland and Labrador|658[0n]|BOLD:ACE9063
Lacinipolia olivacea|[9557]|LBCH2206-10|Canada|British Columbia|658[0n]|BOLD:ACE9063
Lacinipolia olivacea|[9558]|BBLPB472-10|Canada|British Columbia|658[0n]|BOLD:ACE9063
Lacinipolia olivacea|[9559]|BBLPB473-10|Canada|British Columbia|658[0n]|BOLD:ACE9063
Lacinipolia olivacea|[9560]|BBLPB474-10|Canada|British Columbia|658[0n]|BOLD:ACE9063
Lacinipolia olivacea|[9561]|BBLPB475-10|Canada|British Columbia|658[0n]|BOLD:ACE9063
Lacinipolia olivacea|[9562]|BBLPB476-10|Canada|British Columbia|658[0n]|BOLD:ACE9063
Lacinipolia olivacea|[9563]|BBLPB484-10|Canada|Alberta|658[0n]|BOLD:ACE9063
Lacinipolia olivacea|[9564]|BBLPB485-10|Canada|Alberta|658[0n]|BOLD:ACE9063
Lacinipolia olivacea|[9565]|BBLPB487-10|Canada|Saskatchewan|658[0n]|BOLD:ACE9063
Lacinipolia olivacea|[9566]|BBLPB488-10|Canada|Saskatchewan|658[0n]|BOLD:ACE9063
Lacinipolia olivacea|[9567]|BBLPB494-10|Canada|Alberta|658[0n]|BOLD:ACE9063
Lacinipolia olivacea|[9568]|BBLPB854-10|Canada|Alberta|658[0n]|BOLD:ACE9063
Lacinipolia olivacea|[9569]|LPABB528-08|Canada|Alberta|658[0n]|BOLD:ACE9063
Lacinipolia olivacea|[9570]|LPABB618-08|Canada|Alberta|658[0n]|BOLD:ACE9063
Lacinipolia olivacea|[9571]|LALPA860-11|Canada|British Columbia|658[0n]|BOLD:ACE9063
Lacinipolia olivacea|[9572]|LALPA869-11|Canada|British Columbia|658[0n]|BOLD:ACE9063
Lacinipolia olivacea|[9573]|LPABB248-08|Canada|Alberta|658[0n]|BOLD:ACE9063
Lacinipolia olivacea|[9574]|LPABB249-08|Canada|Alberta|658[0n]|BOLD:ACE9063
Lacinipolia olivacea|[9575]|RDMAB647-06|Canada|Alberta|658[0n]|BOLD:ACE9063
Lacinipolia olivacea|[9576]|LOWCD179-06|Canada|British Columbia|658[0n]|BOLD:ACE9063
Lacinipolia olivacea|[9577]|BBLPB489-10|Canada|Alberta|658[0n]|BOLD:ACE9063
Lacinipolia olivacea|[9578]|BBLPB490-10|Canada|Alberta|658[0n]|BOLD:ACE9063
Lacinipolia olivacea|[9579]|LPABB175-08|Canada|Alberta|658[0n]|BOLD:ACE9063
Lacinipolia olivacea|[9580]|LPABB222-08|Canada|Alberta|658[0n]|BOLD:ACE9063
Lacinipolia olivacea|[9581]|LOWCC180-05|Canada|British Columbia|658[0n]|BOLD:ACE9063
Lacinipolia olivacea|[9582]|RDMAB646-06|Canada|Alberta|658[0n]|BOLD:ACE9063
Lacinipolia olivacea|[9583]|LPABC820-09|Canada|Alberta|658[0n]|BOLD:ACE9063
Lacinipolia olivacea|[9584]|LPABC935-09|Canada|Alberta|658[0n]|BOLD:ACE9063
Lacinipolia olivacea|[9585]|LPAB236-08|Canada|Alberta|658[0n]|BOLD:ACE9063
Lacinipolia olivacea|[9586]|LPABB127-08|Canada|Alberta|658[0n]|BOLD:ACE9063
Lacinipolia olivacea|[9587]|LOWC023-05|Canada|British Columbia|658[0n]|BOLD:ACE9063
Lacinipolia olivacea|[9588]|LOWC024-05|Canada|British Columbia|658[0n]|BOLD:ACE9063
Lacinipolia olivacea|[9589]|LALPA873-11|Canada|British Columbia|658[0n]|BOLD:ACE9063
Lacinipolia olivacea|[9590]|LOWC022-05|Canada|British Columbia|658[0n]|BOLD:ACE9063
Lacinipolia olivacea|[9591]|LOWC021-05|Canada|British Columbia|658[0n]|BOLD:ACE9063
Lacinipolia olivacea|[9592]|LOWC020-05|Canada|British Columbia|658[0n]|BOLD:ACE9063
Lacinipolia olivacea|[9593]|LOWC019-05|Canada|British Columbia|658[0n]|BOLD:ACE9063
Lacinipolia olivacea|[9594]|LOWC018-05|Canada|British Columbia|658[0n]|BOLD:ACE9063
Lacinipolia olivacea|[9595]|LOWC017-05|Canada|British Columbia|658[0n]|BOLD:ACE9063

[illegible]
Lacinipolia olivacea|[9593]|LOWC019-05|Canada|British Columbia|658[0n]|BOLD:ACE9063
Lacinipolia olivacea|[9594]|LOWC018-05|Canada|British Columbia|658[0n]|BOLD:ACE9063
Lacinipolia olivacea|[9595]|LOWC017-05|Canada|British Columbia|658[0n]|BOLD:ACE9063
Lacinipolia olivacea|[9596]|LOWC016-05|Canada|British Columbia|658[0n]|BOLD:ACE9063
Lacinipolia olivacea|[9597]|CNWBG3148-13|Canada|Alberta|595[0n]|BOLD:ACE9063
Lacinipolia olivacea|[9598]|LOWC014-05|Canada|British Columbia|658[0n]|BOLD:ACE9063
Lacinipolia olivacea|[9599]|LBCC302-05|Canada|British Columbia|658[0n]|BOLD:ACE9063
Lacinipolia olivacea|[9600]|LBCB553-05|Canada|British Columbia|658[0n]|BOLD:ACE9063
Lacinipolia olivacea|[9601]|BBLPB486-10|Canada|Alberta|658[0n]|BOLD:ACE9063
Lacinipolia olivacea|[9602]|LPABC133-09|Canada|Alberta|654[0n]|BOLD:ACE9063
Lacinipolia olivacea|[9603]|LPABC134-09|Canada|Alberta|633[0n]|BOLD:ACE9063
Lacinipolia olivacea|[9604]|LPABC276-09|Canada|Alberta|630[1n]|BOLD:ACE9063
Lacinipolia olivacea|[9605]|LOWCE543-06|Canada|British Columbia|617[1n]|BOLD:ACE9063
Lacinipolia olivacea|[9606]|LOWCE535-06|Canada|British Columbia|627[0n]|BOLD:ACE9063
Lacinipolia olivacea|[9607]|LOWCE540-06|Canada|British Columbia|610[0n]|BOLD:ACE9063
Lacinipolia olivacea|[9608]|LOWCE538-06|Canada|British Columbia|610[0n]|BOLD:ACE9063
Lacinipolia olivacea|[9609]|LOWCE534-06|Canada|British Columbia|610[0n]|BOLD:ACE9063
Lacinipolia olivacea|[9610]|LOWCE533-06|Canada|British Columbia|610[0n]|BOLD:ACE9063
Lacinipolia olivacea|[9611]|RDNMC391-05|Canada|Alberta|610[0n]|BOLD:ACE9063
Lacinipolia olivacea|[9612]|LPABC129-09|Canada|Alberta|623[2n]|BOLD:ACE9063
Lacinipolia olivacea|[9613]|LOWCE539-06|Canada|British Columbia|617[0n]|BOLD:ACE9063
Lacinipolia olivacea|[9614]|LOWCE536-06|Canada|British Columbia|617[0n]|BOLD:ACE9063
Lacinipolia olivacea|[9615]|LOWCE537-06|Canada|British Columbia|608[0n]|BOLD:ACE9063
Lacinipolia olivacea|[9616]|LOWCE542-06|Canada|British Columbia|606[0n]|BOLD:ACE9063
Lacinipolia olivacea|[9617]|LPABC135-09|Canada|Alberta|568[0n]|BOLD:ACE9063
Lacinipolia olivacea|[9618]|BBLPB478-10|Canada|British Columbia|609[0n]|BOLD:ACE9063
Lacinipolia olivacea|[9619]|SSWLD7936-13|Canada|Alberta|628[0n]|BOLD:ACE9063
Lacinipolia petita|[9620]|JMMMB315-11|United States|California|582[0n]|BOLD:ACE9063
Lacinipolia petita|[9621]|BBLOC1249-11|United States|California|658[0n]|BOLD:ACE9063
Lacinipolia petita|[9622]|BBLOC1251-11|United States|California|658[0n]|BOLD:ACE9063
Lacinipolia petita|[9623]|BBLOC1255-11|United States|California|658[0n]|BOLD:ACE9063
Lacinipolia petita|[9624]|BBLOC1256-11|United States|California|658[0n]|BOLD:ACE9063
Lacinipolia petita|[9625]|RWWA797-09|United States|Washington|658[0n]|BOLD:ACE9063
Lacinipolia petita|[9626]|RWWA827-09|United States|Washington|658[0n]|BOLD:ACE9063
Lacinipolia petita|[9627]|RWWC369-11|United States|Washington|658[0n]|BOLD:ACE9063
Lacinipolia petita|[9628]|RWWA825-09|United States|Washington|658[0n]|BOLD:ACE9063
Lacinipolia petita|[9629]|LALPA511-10|Canada|British Columbia|658[0n]|BOLD:ACE9063
Lacinipolia petita|[9630]|RWWB206-09|United States|Washington|658[0n]|BOLD:ACE9063
Lacinipolia petita|[9631]|RWWB102-09|United States|Washington|658[0n]|BOLD:ACE9063
Lacinipolia petita|[9632]|RWWB028-09|United States|Washington|658[0n]|BOLD:ACE9063
Lacinipolia petita|[9633]|RWWB011-09|United States|Washington|658[0n]|BOLD:ACE9063
Lacinipolia petita|[9634]|RWWA735-09|United States|Washington|658[0n]|BOLD:ACE9063
Lacinipolia petita|[9635]|CNCLB673-14|United States|Oregon|586[3n]|BOLD:ACE9063
Lacinipolia erecta|[9636]|USLEP088-10|United States|Texas|658[0n]|BOLD:AAH5321
Lacinipolia erecta|[9637]|USLEP772-10|United States|Texas|658[0n]|BOLD:AAH5321
Lacinipolia erecta|[9638]|BBLSX887-09|United States|Oklahoma|658[0n]|BOLD:AAH5321
Lacinipolia erecta|[9639]|BBLSW793-09|United States|Oklahoma|658[0n]|BOLD:AAH5321
Lacinipolia erecta|[9640]|LPOKE295-11|United States|Oklahoma|658[0n]|BOLD:AAH5321
Lacinipolia triplehorni|[9641]|CMAZA468-10|United States|Arizona|658[0n]|BOLD:AAH5676
Lacinipolia triplehorni|[9642]|CMAZA239-09|United States|Arizona|658[0n]|BOLD:AAH5676
Lacinipolia triplehorni|[9643]|CMAZA1051-12|United States|Arizona|658[0n]|BOLD:AAH5676
Lacinipolia triplehorni|[9644]|CMAZA1073-12|United States|Arizona|658[0n]|BOLD:AAH5676
Lacinipolia triplehorni|[9645]|CNCLB257-14|United States|Arizona|658[0n]|BOLD:AAH5676
Lacinipolia triplehorni|[9646]|CNCLB258-14|United States|Arizona|658[0n]|BOLD:AAH5676
Lacinipolia triplehorni|[9647]|CNCLB2446-14|United States|Texas|658[0n]|BOLD:AAH5676
Lacinipolia rodora|[9648]|RDNMG257-08|United States|Texas|658[0n]|BOLD:AAH5662
Lacinipolia rodora|[9649]|RDNMG310-08|United States|Arizona|658[0n]|BOLD:AAH5662
Lacinipolia rodora|[9650]|CMAZA169-09|United States|Arizona|658[0n]|BOLD:AAH5662
Lacinipolia rodora|[9651]|CMAZA370-10|United States|Arizona|658[0n]|BOLD:AAH5662
Lacinipolia sp.|[9652]|BBLSX753-09|United States|Oklahoma|658[0n]|BOLD:AAB4695
Lacinipolia sp.|[9653]|LGSMC906-05|United States|Tennessee|658[0n]|BOLD:AAB4695
Lacinipolia sp.|[9654]|BBLSW799-09|United States|Oklahoma|658[0n]|BOLD:AAB4695
Lacinipolia sp.|[9655]|BBLSW648-09|United States|Oklahoma|658[0n]|BOLD:AAB4695
Lacinipolia sp.|[9656]|BBLSX755-09|United States|Oklahoma|658[0n]|BOLD:AAB4695
Lacinipolia sp.|[9657]|BBLSX754-09|United States|Oklahoma|658[0n]|BOLD:AAB4695
Lacinipolia sp.|[9658]|BBLSX295-09|United States|Oklahoma|658[0n]|BOLD:AAB4695
Lacinipolia sp.|[9659]|BBLSY090-09|United States|Oklahoma|658[0n]|BOLD:AAB4695
Lacinipolia sp.|[9660]|BBLSW447-09|United States|Oklahoma|658[0n]|BOLD:AAB4695
Lacinipolia sp.|[9661]|BBLSW302-09|United States|Oklahoma|658[0n]|BOLD:AAB4695
Lacinipolia sp.|[9662]|BBLSW301-09|United States|Oklahoma|658[0n]|BOLD:AAB4695
Lacinipolia sp.|[9663]|BBLSX396-09|United States|Oklahoma|640[0n]|BOLD:AAB4695
Lacinipolia sp.|[9664]|USLEP1152-10|United States|Texas|641[0n]|BOLD:AAB4695
Lacinipolia laudabilis|[9665]|GMCRK2655-13|Costa Rica|Guanacaste|591[0n]|BOLD:AAB4696
Lacinipolia strigicollis|[9666]|RDNMC281-05|Canada|British Columbia|602[0n]|BOLD:AAB7768
Lacinipolia strigicollis|[9667]|LBCG1106-09|Canada|British Columbia|658[0n]|BOLD:AAB7768
Lacinipolia strigicollis|[9668]|LBCH5947-10|Canada|British Columbia|658[0n]|BOLD:AAB7768
Lacinipolia strigicollis|[9669]|LBCH5948-10|Canada|British Columbia|658[0n]|BOLD:AAB7768
Lacinipolia strigicollis|[9670]|LBCH6129-10|Canada|British Columbia|658[0n]|BOLD:AAB7768
Lacinipolia strigicollis|[9671]|LBCH6130-10|Canada|British Columbia|658[0n]|BOLD:AAB7768
Lacinipolia strigicollis|[9672]|LBCH6157-10|Canada|British Columbia|658[0n]|BOLD:AAB7768
Lacinipolia strigicollis|[9673]|LBCH6159-10|Canada|British Columbia|658[0n]|BOLD:AAB7768
Lacinipolia strigicollis|[9674]|LBCH6160-10|Canada|British Columbia|658[0n]|BOLD:AAB7768
Lacinipolia strigicollis|[9675]|LBCH6161-10|Canada|British Columbia|658[0n]|BOLD:AAB7768
Lacinipolia strigicollis|[9676]|LBCH6162-10|Canada|British Columbia|658[0n]|BOLD:AAB7768
Lacinipolia strigicollis|[9677]|LBCH6163-10|Canada|British Columbia|658[0n]|BOLD:AAB7768
Lacinipolia strigicollis|[9678]|LBCH6225-10|Canada|British Columbia|658[0n]|BOLD:AAB7768
Lacinipolia strigicollis|[9679]|LBCH6227-10|Canada|British Columbia|658[0n]|BOLD:AAB7768
Lacinipolia strigicollis|[9680]|LBCH6228-10|Canada|British Columbia|658[0n]|BOLD:AAB7768
Lacinipolia strigicollis|[9681]|LBCH6281-10|Canada|British Columbia|658[0n]|BOLD:AAB7768
Lacinipolia strigicollis|[9682]|LBCH6282-10|Canada|British Columbia|658[0n]|BOLD:AAB7768
Lacinipolia strigicollis|[9683]|LBCH6382-10|Canada|British Columbia|658[0n]|BOLD:AAB7768
Lacinipolia strigicollis|[9684]|LBCH6056-10|Canada|British Columbia|658[0n]|BOLD:AAB7768
Lacinipolia strigicollis|[9685]|LBCG2855-09|Canada|British Columbia|658[0n]|BOLD:AAB7768
Lacinipolia strigicollis|[9686]|LBCG2852-09|Canada|British Columbia|658[0n]|BOLD:AAB7768
Lacinipolia strigicollis|[9687]|LBCG2851-09|Canada|British Columbia|658[0n]|BOLD:AAB7768
Lacinipolia strigicollis|[9688]|LBCG2845-09|Canada|British Columbia|658[0n]|BOLD:AAB7768
Lacinipolia strigicollis|[9689]|LBCG2843-09|Canada|British Columbia|658[0n]|BOLD:AAB7768
Lacinipolia strigicollis|[9690]|LBCH6603-10|Canada|British Columbia|658[0n]|BOLD:AAB7768
Lacinipolia strigicollis|[9691]|LBCH6607-10|Canada|British Columbia|658[0n]|BOLD:AAB7768
Lacinipolia strigicollis|[9692]|LBCH6609-10|Canada|British Columbia|658[0n]|BOLD:AAB7768
Lacinipolia strigicollis|[9693]|LBCH6610-10|Canada|British Columbia|658[0n]|BOLD:AAB7768
Lacinipolia strigicollis|[9694]|LBCH7767-10|Canada|British Columbia|658[0n]|BOLD:AAB7768
Lacinipolia strigicollis|[9695]|LBCH7781-10|Canada|British Columbia|658[0n]|BOLD:AAB7768

Lacinipolia strigicollis|[9692]|LBCH6609-10|Canada|British Columbia|658[0n]|BOLD:AAB7768
Lacinipolia strigicollis|[9693]|LBCH6610-10|Canada|British Columbia|658[0n]|BOLD:AAB7768
Lacinipolia strigicollis|[9694]|LBCH7767-10|Canada|British Columbia|658[0n]|BOLD:AAB7768
Lacinipolia strigicollis|[9695]|LBCH7781-10|Canada|British Columbia|658[0n]|BOLD:AAB7768
Lacinipolia strigicollis|[9696]|RDNMC392-05|Canada|British Columbia|591[0n]|BOLD:AAB7768
Lacinipolia strigicollis|[9697]|LALPA889-11|Canada|British Columbia|658[0n]|BOLD:AAB7768
Lacinipolia strigicollis|[9698]|BBLOD561-11|United States|California|658[0n]|BOLD:AAB7768
Lacinipolia strigicollis|[9699]|BBLOC1710-11|United States|California|658[0n]|BOLD:AAB7768
Lacinipolia strigicollis|[9700]|BBLOC1698-11|United States|California|658[0n]|BOLD:AAB7768
Lacinipolia strigicollis|[9701]|BBLOC1328-11|United States|California|658[0n]|BOLD:AAB7768
Lacinipolia strigicollis|[9702]|BBLOC864-11|United States|California|658[0n]|BOLD:AAB7768
Lacinipolia strigicollis|[9703]|BBLOC858-11|United States|California|658[0n]|BOLD:AAB7768
Lacinipolia strigicollis|[9704]|BBLOD443-11|United States|California|658[1n]|BOLD:AAB7768
Lacinipolia strigicollis|[9705]|BBLOD454-11|United States|California|658[0n]|BOLD:AAB7768
Lacinipolia strigicollis|[9706]|BBLOD566-11|United States|California|658[0n]|BOLD:AAB7768
Lacinipolia strigicollis|[9707]|BBLOD687-11|United States|California|658[0n]|BOLD:AAB7768
Lacinipolia strigicollis|[9708]|BBLOD688-11|United States|California|658[0n]|BOLD:AAB7768
Lacinipolia strigicollis|[9709]|BBLOD835-11|United States|California|658[0n]|BOLD:AAB7768
Lacinipolia strigicollis|[9710]|BBLOD1705-11|United States|California|658[0n]|BOLD:AAB7768
Lacinipolia strigicollis|[9711]|BBLOD1726-11|United States|California|658[0n]|BOLD:AAB7768
Lacinipolia strigicollis|[9712]|BBLOE1259-12|United States|California|658[0n]|BOLD:AAB7768
Lacinipolia strigicollis|[9713]|GMLC1050-12|United States|California|658[0n]|BOLD:AAB7768
Lacinipolia strigicollis|[9714]|GMLC1134-12|United States|California|658[0n]|BOLD:AAB7768
Lacinipolia strigicollis|[9715]|GMLC1213-12|United States|California|658[0n]|BOLD:AAB7768
Lacinipolia buscki|[9716]|RDNMG254-08|United States|Texas|658[0n]|BOLD:ACF0239
Lacinipolia buscki|[9717]|LBCG2850-09|Canada|British Columbia|658[0n]|BOLD:ACF0239
Lacinipolia buscki|[9718]|LOWCE532-06|Canada|British Columbia|658[0n]|BOLD:ACF0239
Lacinipolia buscki|[9719]|LBCG250-08|Canada|British Columbia|658[0n]|BOLD:ACF0239
Lacinipolia buscki|[9720]|LBCG2854-09|Canada|British Columbia|658[0n]|BOLD:ACF0239
Lacinipolia buscki|[9721]|LBCH6384-10|Canada|British Columbia|658[0n]|BOLD:ACF0239
Lacinipolia buscki|[9722]|LBCH6606-10|Canada|British Columbia|658[0n]|BOLD:ACF0239
Lacinipolia buscki|[9723]|LBCG248-08|Canada|British Columbia|658[0n]|BOLD:ACF0239
Lacinipolia buscki|[9724]|LBCG2867-09|Canada|British Columbia|658[0n]|BOLD:ACF0239
Lacinipolia buscki|[9725]|LBCG3246-09|Canada|British Columbia|630[0n]|BOLD:ACF0239
Lacinipolia buscki|[9726]|LBCH6461-10|Canada|British Columbia|658[0n]|BOLD:ACF0239
Lacinipolia buscki|[9727]|LBCH6605-10|Canada|British Columbia|658[0n]|BOLD:ACF0239
Lacinipolia buscki|[9728]|LBCH6608-10|Canada|British Columbia|658[0n]|BOLD:ACF0239
Lacinipolia buscki|[9729]|LBCH7342-10|Canada|British Columbia|658[0n]|BOLD:ACF0239
Lacinipolia buscki|[9730]|LBCH7562-10|Canada|British Columbia|658[0n]|BOLD:ACF0239
Lacinipolia buscki|[9731]|LBCH6226-10|Canada|British Columbia|658[0n]|BOLD:ACF0239
Lacinipolia buscki|[9732]|LBCH6557-10|Canada|British Columbia|658[0n]|BOLD:ACF0239
Lacinipolia buscki|[9733]|LBCG2848-09|Canada|British Columbia|658[0n]|BOLD:ACF0239
Lacinipolia buscki|[9734]|LBCG2849-09|Canada|British Columbia|658[0n]|BOLD:ACF0239
Lacinipolia buscki|[9735]|LBCG2853-09|Canada|British Columbia|658[0n]|BOLD:ACF0239
Lacinipolia buscki|[9736]|LBCH6158-10|Canada|British Columbia|658[0n]|BOLD:ACF0239
Lacinipolia buscki|[9737]|LBCG2846-09|Canada|British Columbia|658[0n]|BOLD:ACF0239
Lacinipolia buscki|[9738]|LBCG2847-09|Canada|British Columbia|658[0n]|BOLD:ACF0239
Lacinipolia buscki|[9739]|LBCG500-08|Canada|British Columbia|658[0n]|BOLD:ACF0239
Lacinipolia buscki|[9740]|LBCG2844-09|Canada|British Columbia|658[0n]|BOLD:ACF0239
Lacinipolia buscki|[9741]|LBCG483-08|Canada|British Columbia|658[0n]|BOLD:ACF0239
Lacinipolia buscki|[9742]|LBCG495-08|Canada|British Columbia|658[0n]|BOLD:ACF0239
Lacinipolia buscki|[9743]|LOWC035-05|Canada|British Columbia|658[0n]|BOLD:ACF0239
Lacinipolia buscki|[9744]|LOWC034-05|Canada|British Columbia|658[0n]|BOLD:ACF0239
Lacinipolia buscki|[9745]|LOWC032-05|Canada|British Columbia|658[0n]|BOLD:ACF0239
Lacinipolia buscki|[9746]|LOWC031-05|Canada|British Columbia|658[0n]|BOLD:ACF0239
Lacinipolia buscki|[9747]|LOWC030-05|Canada|British Columbia|658[0n]|BOLD:ACF0239
Lacinipolia buscki|[9748]|LOWC029-05|Canada|British Columbia|658[0n]|BOLD:ACF0239
Lacinipolia buscki|[9749]|LBCH6604-10|Canada|British Columbia|658[0n]|BOLD:ACF0239
Lacinipolia buscki|[9750]|LBCH6632-10|Canada|British Columbia|658[0n]|BOLD:ACF0239
Lacinipolia buscki|[9751]|LOWC028-05|Canada|British Columbia|658[0n]|BOLD:ACF0239
Lacinipolia buscki|[9752]|LBCH6164-10|Canada|British Columbia|658[0n]|BOLD:ACF0239
Lacinipolia buscki|[9753]|LOWC027-05|Canada|British Columbia|658[0n]|BOLD:ACF0239
Lacinipolia buscki|[9754]|LBCH7372-10|Canada|British Columbia|658[0n]|BOLD:ACF0239
Lacinipolia buscki|[9755]|LBCH7769-10|Canada|British Columbia|658[0n]|BOLD:ACF0239
Lacinipolia buscki|[9756]|AWCLB041-10|United States|Idaho|658[0n]|BOLD:ACF0239
Lacinipolia buscki|[9757]|CGLCA046-10|United States|California|658[0n]|BOLD:ACF0239
Lacinipolia buscki|[9758]|BBLOD438-11|United States|California|658[0n]|BOLD:ACF0239
Lacinipolia buscki|[9759]|BBLOD1715-11|United States|California|658[0n]|BOLD:ACF0239
Lacinipolia buscki|[9760]|BBLOC863-11|United States|California|658[0n]|BOLD:ACF0239
Lacinipolia buscki|[9761]|RDNMB225-05|United States|California|589[0n]|BOLD:ACF0239
Lacinipolia buscki|[9762]|RDNMC394-05|United States|California|593[0n]|BOLD:ACF0239
Lacinipolia buscki|[9763]|CGLCA045-10|United States|California|658[0n]|BOLD:ACF0239
Lacinipolia buscki|[9764]|BBLOD439-11|United States|California|658[0n]|BOLD:ACF0239
Lacinipolia buscki|[9765]|BBLOE1240-12|United States|California|658[0n]|BOLD:ACF0239
Lacinipolia buscki|[9766]|LOCBE259-06|United States|California|592[0n]|BOLD:ACF0239
Lacinipolia buscki|[9767]|LOCBD730-06|United States|California|623[0n]|BOLD:ACF0239
Lacinipolia buscki|[9768]|LOCBE365-06|United States|California|592[0n]|BOLD:ACF0239
Lacinipolia buscki|[9769]|LOCBE366-06|United States|California|605[0n]|BOLD:ACF0239
Lacinipolia buscki|[9770]|LOCBD296-06|United States|California|658[0n]|BOLD:ACF0239
Lacinipolia buscki|[9771]|LOCBD696-06|United States|California|644[0n]|BOLD:ACF0239
Lacinipolia buscki|[9772]|RDNME1023-08|United States|California|587[0n]|BOLD:ACF0239
Lacinipolia buscki|[9773]|JBAZ049-09|United States|California|517[0n]|
Lacinipolia buscki|[9774]|LOCBD877-06|United States|California|658[0n]|BOLD:ACF0239
Lacinipolia buscki|[9775]|LOCBD879-06|United States|California|658[0n]|BOLD:ACF0239
Lacinipolia buscki|[9776]|LOCBE143-06|United States|California|658[0n]|BOLD:ACF0239
Lacinipolia buscki|[9777]|BBLOE1263-12|United States|California|658[0n]|BOLD:ACF0239
Lacinipolia buscki|[9778]|LOCBD574-06|United States|California|658[0n]|BOLD:ACF0239
Lacinipolia buscki|[9779]|LOCBE274-06|United States|California|592[0n]|BOLD:ACF0239
Lacinipolia buscki|[9780]|LOWC033-05|Canada|British Columbia|570[0n]|BOLD:ACF0239
Lacinipolia buscki|[9781]|BBLOD774-11|United States|California|658[0n]|BOLD:ACF0239
Lacinipolia buscki|[9782]|LBCG3245-09|Canada|British Columbia|632[0n]|BOLD:ACF0239
Lacinipolia buscki|[9783]|BBLOD1750-11|United States|California|658[0n]|BOLD:ACF0239
Lacinipolia buscki|[9784]|LOCBE363-06|United States|California|658[0n]|BOLD:ACF0239
Lacinipolia buscki|[9785]|BBLOC1701-11|United States|California|622[0n]|BOLD:ACF0239
Lacinipolia buscki|[9786]|BBLOD1747-11|United States|California|658[0n]|BOLD:ACF0239
Lacinipolia buscki|[9787]|LOCBF2609-13|United States|California|658[0n]|BOLD:ACF0239
Lacinipolia buscki|[9788]|LOCBC468-06|United States|California|658[0n]|BOLD:ACF0239
Lacinipolia buscki|[9789]|LOCBC576-06|United States|California|658[0n]|BOLD:ACF0239
Lacinipolia buscki|[9790]|LOCBF2653-13|United States|California|658[0n]|BOLD:ACF0239
Lacinipolia buscki|[9791]|LOCBE368-06|United States|California|658[0n]|BOLD:ACF0239
Lacinipolia buscki|[9792]|LOCBD881-06|United States|California|658[0n]|BOLD:ACF0239
Lacinipolia buscki|[9793]|LOCBD035-06|United States|California|658[0n]|BOLD:ACF0239
Lacinipolia buscki|[9794]|LOCBF2600-13|United States|California|658[0n]|BOLD:ACF0239
Lacinipolia buscki|[9795]|LOCBC469-06|United States|California|658[0n]|BOLD:ACF0239

Lacinipolia buscki|[9792]|LOCBD881-06|United States|California|658[0n]|BOLD:ACF0239
Lacinipolia buscki|[9793]|LOCBD035-06|United States|California|658[0n]|BOLD:ACF0239
Lacinipolia buscki|[9794]|LOCBF2600-13|United States|California|658[0n]|BOLD:ACF0239
Lacinipolia buscki|[9795]|LOCBC469-06|United States|California|658[0n]|BOLD:ACF0239
Lacinipolia buscki|[9796]|LOCBE275-06|United States|California|592[0n]|BOLD:ACF0239
Lacinipolia buscki|[9797]|LOCBD167-06|United States|California|658[0n]|BOLD:ACF0239
Lacinipolia buscki|[9798]|LOCBC672-06|United States|California|658[0n]|BOLD:ACF0239
Lacinipolia buscki|[9799]|LOCBD036-06|United States|California|658[0n]|BOLD:ACF0239
Lacinipolia buscki|[9800]|LOCBD729-06|United States|California|623[0n]|BOLD:ACF0239
Lacinipolia buscki|[9801]|LOCBF2610-13|United States|California|658[0n]|BOLD:ACF0239
Lacinipolia buscki|[9802]|LOCBD738-06|United States|California|663[0n]|BOLD:ACF0239
Lacinipolia buscki|[9803]|LOCBE266-06|United States|California|593[1n]|BOLD:ACF0239
Lacinipolia buscki|[9804]|LOCBE268-06|United States|California|581[0n]|BOLD:ACF0239
Lacinipolia buscki|[9805]|BBLOE1261-12|United States|California|658[0n]|BOLD:ACF0239
Lacinipolia buscki|[9806]|LOCBE141-06|United States|California|658[0n]|BOLD:ACF0239
Lacinipolia buscki|[9807]|RDNMC393-05|United States|California|565[0n]|BOLD:ACF0239
Lacinipolia buscki|[9808]|LOCBD733-06|United States|California|623[0n]|BOLD:ACF0239
Lacinipolia buscki|[9809]|LOCBD731-06|United States|California|623[0n]|BOLD:ACF0239
Lacinipolia buscki|[9810]|LOCBE142-06|United States|California|658[0n]|BOLD:ACF0239
Lacinipolia buscki|[9811]|LOCBD736-06|United States|California|647[0n]|BOLD:ACF0239
Lacinipolia buscki|[9812]|LOCBE267-06|United States|California|603[0n]|BOLD:ACF0239
Lacinipolia buscki|[9813]|LOCBE271-06|United States|California|577[0n]|BOLD:ACF0239
Lacinipolia buscki|[9814]|LOCBE145-06|United States|California|629[0n]|BOLD:ACF0239
Lacinipolia buscki|[9815]|LOCBE269-06|United States|California|593[0n]|BOLD:ACF0239
Lacinipolia buscki|[9816]|LOCBE272-06|United States|California|593[0n]|BOLD:ACF0239
Lacinipolia buscki|[9817]|LOCBE273-06|United States|California|593[0n]|BOLD:ACF0239
Lacinipolia buscki|[9818]|LOCBD732-06|United States|California|623[0n]|BOLD:ACF0239
Lacinipolia buscki|[9819]|LOCBE364-06|United States|California|593[0n]|BOLD:ACF0239
Lacinipolia buscki|[9820]|BBLOC1699-11|United States|California|658[0n]|BOLD:ACF0239
Lacinipolia buscki|[9821]|LOCBF2612-13|United States|California|658[0n]|BOLD:ACF0239
Lacinipolia buscki|[9822]|LOCBF2607-13|United States|California|658[0n]|BOLD:ACF0239
Lacinipolia buscki|[9823]|LOCBF2644-13|United States|California|658[0n]|BOLD:ACF0239
Lacinipolia buscki|[9824]|LOCBD925-06|United States|California|658[0n]|BOLD:ACF0239
Lacinipolia buscki|[9825]|BBLOE1305-12|United States|California|658[0n]|BOLD:ACF0239
Lacinipolia buscki|[9826]|LOCBF2654-13|United States|California|658[0n]|BOLD:ACF0239
Lacinipolia buscki|[9827]|LOCBD878-06|United States|California|658[0n]|BOLD:ACF0239
Lacinipolia buscki|[9828]|LOCBD876-06|United States|California|658[0n]|BOLD:ACF0239
Lacinipolia buscki|[9829]|LOCBD695-06|United States|California|658[0n]|BOLD:ACF0239
Lacinipolia buscki|[9830]|LOCBD295-06|United States|California|658[0n]|BOLD:ACF0239
Lacinipolia buscki|[9831]|LOCBD294-06|United States|California|658[0n]|BOLD:ACF0239
Lacinipolia buscki|[9832]|LOCBD168-06|United States|California|658[0n]|BOLD:ACF0239
Lacinipolia buscki|[9833]|LOCBD880-06|United States|California|658[0n]|BOLD:ACF0239
Lacinipolia buscki|[9834]|LOCBD166-06|United States|California|658[0n]|BOLD:ACF0239
Lacinipolia buscki|[9835]|LOCBC844-06|United States|California|658[0n]|BOLD:ACF0239
Lacinipolia buscki|[9836]|RDNMD432-06|United States|California|658[0n]|BOLD:ACF0239
Lacinipolia buscki|[9837]|LOCBC440-06|United States|California|658[0n]|BOLD:ACF0239
Lacinipolia buscki|[9838]|LOCBD734-06|United States|California|663[0n]|BOLD:ACF0239
Lacinipolia buscki|[9839]|LOCBD740-06|United States|California|663[0n]|BOLD:ACF0239
Lacinipolia buscki|[9840]|LOCBD918-06|United States|California|658[0n]|BOLD:ACF0239
Lacinipolia buscki|[9841]|LOCBD034-06|United States|California|658[0n]|BOLD:ACF0239
Lacinipolia buscki|[9842]|LOCBC441-06|United States|California|658[0n]|BOLD:ACF0239
Lacinipolia buscki|[9843]|LOCBE270-06|United States|California|593[0n]|BOLD:ACF0239
Lacinipolia buscki|[9844]|LOCBD735-06|United States|California|663[0n]|BOLD:ACF0239
Lacinipolia buscki|[9845]|LOCBD737-06|United States|California|663[0n]|BOLD:ACF0239
Lacinipolia buscki|[9846]|LOCBD739-06|United States|California|663[0n]|BOLD:ACF0239
Lacinipolia buscki|[9847]|LOCBD742-06|United States|California|663[0n]|BOLD:ACF0239
Lacinipolia buscki|[9848]|LOCBE367-06|United States|California|654[0n]|BOLD:ACF0239
Lacinipolia buscki|[9849]|BBLOE1260-12|United States|California|658[0n]|BOLD:ACF0239
Lacinipolia buscki|[9850]|LOCBF2667-13|United States|California|658[0n]|BOLD:ACF0239
Lacinipolia buscki|[9851]|BBLOE1350-12|United States|Arizona|658[0n]|BOLD:ACF0239
Lacinipolia buscki|[9852]|BBLOB090-11|United States|Arizona|658[0n]|BOLD:ACF0239
Lacinipolia buscki|[9853]|BBLOB142-11|United States|Arizona|658[0n]|BOLD:ACF0239
Lacinipolia buscki|[9854]|BBLOE1353-12|United States|Arizona|658[0n]|BOLD:ACF0239
Lacinipolia buscki|[9855]|RDNMG302-08|United States|Arizona|658[0n]|BOLD:AAA5200
Lacinipolia buscki|[9856]|RDNME1026-08|United States|New Mexico|658[0n]|BOLD:AAA5200
Lacinipolia buscki|[9857]|BBLSY966-09|United States|Arizona|658[0n]|BOLD:AAA5200
Lacinipolia buscki|[9858]|RDNMG303-08|United States|Arizona|658[1n]|BOLD:AAA5200
Lacinipolia buscki|[9859]|AWCLB061-10|United States|Arizona|658[0n]|BOLD:AAA5200
Lacinipolia buscki|[9860]|BBLOE1702-12|United States|Arizona|658[0n]|BOLD:AAA5200
Lacinipolia buscki|[9861]|BBLSW119-09|United States|Arizona|658[0n]|BOLD:AAA5200
Lacinipolia buscki|[9862]|CMAZA1131-12|United States|Arizona|658[0n]|BOLD:AAA5200
Lacinipolia buscki|[9863]|RDNMH072-09|United States|Arizona|658[0n]|BOLD:AAA5200
Lacinipolia buscki|[9864]|BBLOB113-11|United States|Arizona|658[0n]|BOLD:AAA5200
Lacinipolia buscki|[9865]|BBLOE1356-12|United States|Arizona|658[0n]|BOLD:AAA5200
Lacinipolia buscki|[9866]|BBLOE1995-12|United States|Arizona|658[0n]|BOLD:AAA5200
Lacinipolia buscki|[9867]|BBLOE1703-12|United States|Arizona|658[0n]|BOLD:AAA5200
Lacinipolia buscki|[9868]|BBLSZ019-09|United States|Arizona|658[0n]|BOLD:AAA5200
Lacinipolia buscki|[9869]|CMAZA1084-12|United States|Arizona|658[0n]|BOLD:AAA5200
Lacinipolia buscki|[9870]|BBLSY825-09|United States|Arizona|615[0n]|BOLD:AAA5200
Lacinipolia buscki|[9871]|BBLOE1348-12|United States|Arizona|658[0n]|BOLD:AAA5200
Lacinipolia buscki|[9872]|BBLSZ084-09|United States|Arizona|658[0n]|BOLD:AAA5200
Lacinipolia buscki|[9873]|BBLSY675-09|United States|Arizona|658[0n]|BOLD:AAA5200
Lacinipolia buscki|[9874]|BBLSW195-09|United States|Arizona|658[0n]|BOLD:AAA5200
Lacinipolia buscki|[9875]|CMAZA774-10|United States|Arizona|648[0n]|BOLD:AAA5200
Lacinipolia buscki|[9876]|BBLOB111-11|United States|Arizona|658[0n]|BOLD:AAA5200
Lacinipolia buscki|[9877]|BBLOB116-11|United States|Arizona|658[0n]|BOLD:AAA5200
Lacinipolia buscki|[9878]|BBLOB591-11|United States|Arizona|658[0n]|BOLD:AAA5200
Lacinipolia buscki|[9879]|BBLOE1321-12|United States|Arizona|658[0n]|BOLD:AAA5200
Lacinipolia buscki|[9880]|BBLOE1875-12|United States|Arizona|658[0n]|BOLD:AAA5200
Lacinipolia buscki|[9881]|CMAZA1142-12|United States|Arizona|658[0n]|BOLD:AAA5200
Lacinipolia buscki|[9882]|CMAZA1208-12|United States|Arizona|658[0n]|BOLD:AAA5200
Lacinipolia buscki|[9883]|CNCLB251-14|United States|Arizona|658[0n]|BOLD:AAA5200
Lacinipolia tricornuta|[9884]|RDNME1025-08|United States|Arizona|658[1n]|BOLD:AAA5202
Lacinipolia tricornuta|[9885]|RDNMK323-11|United States|New Mexico|637[0n]|BOLD:AAA5202
Lacinipolia tricornuta|[9886]|TAMIC931-10|United States|Texas|658[0n]|BOLD:ABZ1329
Lacinipolia tricornuta|[9887]|TAMIC932-10|United States|Texas|658[0n]|BOLD:ABZ1329
Lacinipolia tricornuta|[9888]|CNCLB252-14|United States|Texas|658[0n]|BOLD:ABZ1329
Lacinipolia marinitincta|[9889]|RDNMC740-06|United States|Texas|658[0n]|BOLD:ABZ7134
Lacinipolia marinitincta|[9890]|RDNME1002-08|United States|New Mexico|658[0n]|BOLD:ABZ7134
Lacinipolia implicata|[9891]|RDLQ662-07|Canada|Quebec|658[0n]|BOLD:AAE5535
Lacinipolia implicata|[9892]|LNCNW026-06|United States|North Carolina|617[1n]|BOLD:AAE5535
Lacinipolia implicata|[9893]|LNCNW025-06|United States|North Carolina|658[0n]|BOLD:AAE5535
Lacinipolia implicata|[9894]|LNCC1247-11|United States|North Carolina|658[0n]|BOLD:AAE5535
Lacinipolia implicata|[9895]|LNCC1306-11|United States|North Carolina|658[0n]|BOLD:AAE5535

Lacinipolia implicata|[9892]|LNCNW026-06|United States|North Carolina|617[1n]|BOLD:AAE5535
Lacinipolia implicata|[9893]|LNCNW025-06|United States|North Carolina|658[0n]|BOLD:AAE5535
Lacinipolia implicata|[9894]|LNCC1247-11|United States|North Carolina|658[0n]|BOLD:AAE5535
Lacinipolia implicata|[9895]|LNCC1306-11|United States|North Carolina|658[0n]|BOLD:AAE5535
Lacinipolia implicata|[9896]|LNCC1305-11|United States|North Carolina|658[0n]|BOLD:AAE5535
Lacinipolia implicata|[9897]|RDLQ661-07|Canada|Quebec|644[0n]|BOLD:AAE5535
Lacinipolia implicata|[9898]|LNCC1246-11|United States|North Carolina|658[0n]|BOLD:AAE5535
Lacinipolia implicata|[9899]|GMGSV005-13|United States|Tennessee|589[0n]|BOLD:AAE5535
Lacinipolia quadrilineata|[9900]|GMLC1407-12|United States|California|601[0n]|BOLD:ACF0721
Lacinipolia quadrilineata|[9901]|GMLC1408-12|United States|California|621[0n]|BOLD:ACF0721
Lacinipolia quadrilineata|[9902]|GMLC976-12|United States|California|658[0n]|BOLD:ACF0721
Lacinipolia quadrilineata|[9903]|RDNMB246-05|United States|Oregon|570[0n]|BOLD:ACF0721
Lacinipolia quadrilineata|[9904]|GMLC438-11|United States|California|658[0n]|BOLD:ACF0721
Lacinipolia quadrilineata|[9905]|GMLC393-11|United States|California|658[0n]|BOLD:ACF0721
Lacinipolia quadrilineata|[9906]|GMLC390-11|United States|California|658[0n]|BOLD:ACF0721
Lacinipolia quadrilineata|[9907]|GMLC389-11|United States|California|658[0n]|BOLD:ACF0721
Lacinipolia quadrilineata|[9908]|GMLC113-09|United States|California|658[0n]|BOLD:ACF0721
Lacinipolia quadrilineata|[9909]|NAMUM231-08|United States|California|658[0n]|BOLD:ACF0721
Lacinipolia quadrilineata|[9910]|LOCBB356-06|United States|California|658[0n]|BOLD:ACF0721
Lacinipolia quadrilineata|[9911]|LOCB840-06|United States|California|658[0n]|BOLD:ACF0721
Lacinipolia quadrilineata|[9912]|LOCB483-06|United States|California|658[0n]|BOLD:ACF0721
Lacinipolia quadrilineata|[9913]|GMLC378-11|United States|California|658[0n]|BOLD:ACF0721
Lacinipolia quadrilineata|[9914]|GMLC440-11|United States|California|658[0n]|BOLD:ACF0721
Lacinipolia quadrilineata|[9915]|GMLC447-11|United States|California|658[0n]|BOLD:ACF0721
Lacinipolia quadrilineata|[9916]|GMLC459-11|United States|California|658[0n]|BOLD:ACF0721
Lacinipolia quadrilineata|[9917]|GMLC478-11|United States|California|658[0n]|BOLD:ACF0721
Lacinipolia quadrilineata|[9918]|GMLC484-11|United States|California|658[0n]|BOLD:ACF0721
Lacinipolia quadrilineata|[9919]|GMLC499-11|United States|California|658[0n]|BOLD:ACF0721
Lacinipolia quadrilineata|[9920]|GMLC621-11|United States|California|658[0n]|BOLD:ACF0721
Lacinipolia quadrilineata|[9921]|GMLC1411-12|United States|California|632[0n]|BOLD:ACF0721
Lacinipolia quadrilineata|[9922]|GMLC1419-12|United States|California|632[0n]|BOLD:ACF0721
Lacinipolia martini|[9923]|LOCB836-06|United States|California|658[0n]|BOLD:AAC2836
Lacinipolia martini|[9924]|LOCBB742-06|United States|California|658[0n]|BOLD:AAC2836
Lacinipolia martini|[9925]|CMAZA1207-12|United States|Arizona|658[0n]|BOLD:AAC2836
Lacinipolia martini|[9926]|IAWLB280-11|United States|Arizona|658[0n]|BOLD:AAC2836
Lacinipolia martini|[9927]|RDNMC594-06|United States|Arizona|658[0n]|BOLD:AAC2836
Lacinipolia martini|[9928]|RDNME1027-08|United States|New Mexico|658[0n]|BOLD:AAC2836
Lacinipolia martini|[9929]|CMAZA149-09|United States|Arizona|658[0n]|BOLD:AAC2836
Lacinipolia martini|[9930]|RDNMK326-11|United States|New Mexico|658[0n]|BOLD:AAC2836
Lacinipolia martini|[9931]|BBLOB378-11|United States|Arizona|658[0n]|BOLD:AAC2836
Lacinipolia martini|[9932]|BBLOB1499-11|United States|Arizona|658[0n]|BOLD:AAC2836
Lacinipolia martini|[9933]|BBLOB1510-11|United States|Arizona|658[0n]|BOLD:AAC2836
Lacinipolia martini|[9934]|CMAZA954-12|United States|Arizona|658[0n]|BOLD:AAC2836
Lacinipolia martini|[9935]|CMAZA1201-12|United States|Arizona|658[0n]|BOLD:AAC2836
Lacinipolia martini|[9936]|CMAZA1204-12|United States|Arizona|658[0n]|BOLD:AAC2836
Lacinipolia martini|[9937]|BBLSW227-09|United States|Arizona|658[0n]|BOLD:AAC2836
Lacinipolia martini|[9938]|BBLSX734-09|United States|Arizona|658[0n]|BOLD:AAC2836
Lacinipolia martini|[9939]|LOCBC011-06|United States|California|658[0n]|BOLD:AAC2836
Lacinipolia martini|[9940]|LOCBB358-06|United States|California|658[0n]|BOLD:AAC2836
Lacinipolia martini|[9941]|LOCBB355-06|United States|California|658[0n]|BOLD:AAC2836
Lacinipolia martini|[9942]|LOCBB354-06|United States|California|658[0n]|BOLD:AAC2836
Lacinipolia martini|[9943]|LOCB900-06|United States|California|658[0n]|BOLD:AAC2836
Lacinipolia martini|[9944]|LOCB837-06|United States|California|658[0n]|BOLD:AAC2836
Lacinipolia martini|[9945]|LOCB627-06|United States|California|658[0n]|BOLD:AAC2836
Lacinipolia martini|[9946]|LOCB527-06|United States|California|658[0n]|BOLD:AAC2836
Lacinipolia martini|[9947]|LOCBC092-06|United States|California|598[0n]|BOLD:AAC2836
Lacinipolia martini|[9948]|LOCBF474-13|United States|California|598[0n]|BOLD:AAC2836
Lacinipolia martini|[9949]|LOCBF475-13|United States|California|575[0n]|BOLD:AAC2836
Lacinipolia explicata|[9950]|LPOKA398-09|United States|Oklahoma|658[0n]|BOLD:AAB5863
Lacinipolia explicata|[9951]|BBLSX756-09|United States|Oklahoma|658[0n]|BOLD:AAB5863
Lacinipolia explicata|[9952]|LPOKC286-09|United States|Oklahoma|658[0n]|BOLD:AAB5863
Lacinipolia explicata|[9953]|LOTB136-05|United States|Tennessee|658[0n]|BOLD:AAB5863
Lacinipolia explicata|[9954]|LOTB206-05|United States|Tennessee|658[0n]|BOLD:AAB5863
Lacinipolia explicata|[9955]|LOTB134-05|United States|Tennessee|615[0n]|BOLD:AAB5863
Lacinipolia explicata|[9956]|LPOKA627-09|United States|Oklahoma|639[0n]|BOLD:AAB5863
Lacinipolia explicata|[9957]|LPOKB269-09|United States|Oklahoma|658[0n]|BOLD:AAB5863
Lacinipolia explicata|[9958]|LNCC040-10|United States|North Carolina|658[0n]|BOLD:AAB5863
Lacinipolia explicata|[9959]|LPOKA458-09|United States|Oklahoma|658[0n]|BOLD:AAB5863
Lacinipolia explicata|[9960]|LPOKA427-09|United States|Oklahoma|658[0n]|BOLD:AAB5863
Lacinipolia explicata|[9961]|LPOKB135-09|United States|Oklahoma|658[0n]|BOLD:AAB5863
Lacinipolia explicata|[9962]|BBLSY028-09|United States|Oklahoma|658[0n]|BOLD:AAB5863
Lacinipolia explicata|[9963]|BBLSX583-09|United States|Oklahoma|658[0n]|BOLD:AAB5863
Lacinipolia explicata|[9964]|BBLSW788-09|United States|Oklahoma|658[0n]|BOLD:AAB5863
Lacinipolia explicata|[9965]|LPOKB231-09|United States|Oklahoma|621[0n]|BOLD:AAB5863
Lacinipolia explicata|[9966]|BBLSY027-09|United States|Oklahoma|658[0n]|BOLD:AAB5863
Lacinipolia explicata|[9967]|LOTB137-05|United States|Tennessee|658[0n]|BOLD:AAB5863
Lacinipolia explicata|[9968]|LOTB135-05|United States|Tennessee|614[0n]|BOLD:AAB5863
Lacinipolia explicata|[9969]|LSUSA075-06|United States|Kentucky|553[0n]|BOLD:AAB5863
Lacinipolia explicata|[9970]|LPOKB233-09|United States|Oklahoma|658[0n]|BOLD:AAB5863
Lacinipolia explicata|[9971]|LPOKA275-08|United States|Oklahoma|658[0n]|BOLD:AAB5863
Lacinipolia explicata|[9972]|LPOKA392-09|United States|Oklahoma|658[0n]|BOLD:AAB5863
Lacinipolia explicata|[9973]|LPOKA410-09|United States|Oklahoma|658[0n]|BOLD:AAB5863
Lacinipolia explicata|[9974]|LPOKB312-09|United States|Oklahoma|658[0n]|BOLD:AAB5863
Lacinipolia explicata|[9975]|BBLSX294-09|United States|Oklahoma|658[0n]|BOLD:AAB5863
Lacinipolia explicata|[9976]|LNCC656-11|United States|North Carolina|658[0n]|BOLD:AAB5863
Lacinipolia explicata|[9977]|LPOKA581-09|United States|Oklahoma|658[0n]|BOLD:AAB5863
Lacinipolia explicata|[9978]|CNCLB2876-14|United States|North Carolina|658[0n]|BOLD:AAB5863
Lacinipolia gnata|[9979]|RDNME1019-08|United States|Arizona|658[0n]|BOLD:AAF2822
Lacinipolia gnata|[9980]|RDNMG309-08|United States|Arizona|658[0n]|BOLD:AAF2822
Lacinipolia gnata|[9981]|RDNME1028-08|United States|New Mexico|658[0n]|BOLD:AAF2822
Lacinipolia meditata|[9982]|LGSMG852-10|United States|North Carolina|658[0n]|BOLD:AAA8562
Lacinipolia meditata|[9983]|XAD253-04|Canada|Ontario|646[0n]|BOLD:AAA8562
Lacinipolia meditata|[9984]|XAD256-04|Canada|Ontario|599[0n]|BOLD:AAA8562
Lacinipolia meditata|[9985]|XAD275-04|Canada|Ontario|589[0n]|BOLD:AAA8562
Lacinipolia meditata|[9986]|XAG845-05|Canada|Ontario|658[0n]|BOLD:AAA8562
Lacinipolia meditata|[9987]|XAD242-04|Canada|Ontario|658[0n]|BOLD:AAA8562
Lacinipolia meditata|[9988]|RDNMG585-08|United States|Colorado|658[0n]|BOLD:AAA8562
Lacinipolia meditata|[9989]|RDNMG586-08|United States|Colorado|658[0n]|BOLD:AAA8562
Lacinipolia meditata|[9990]|RDNMB248-05|Canada|Alberta|658[0n]|BOLD:AAA8562
Lacinipolia meditata|[9991]|LPMN902-08|Canada|Alberta|658[0n]|BOLD:AAA8562
Lacinipolia meditata|[9992]|LPABB184-08|Canada|Alberta|658[0n]|BOLD:AAA8562
Lacinipolia meditata|[9993]|LPABC942-09|Canada|Alberta|658[0n]|BOLD:AAA8562
Lacinipolia meditata|[9994]|XAD364-04|Canada|Ontario|569[1n]|BOLD:AAA8562
Lacinipolia meditata|[9995]|XAG646-05|Canada|Ontario|658[0n]|BOLD:AAA8562

Lacinipolia meditata|[9992]|LPABB184-08|Canada|Alberta|658[0n]|BOLD:AAA8562
Lacinipolia meditata|[9993]|LPABC942-09|Canada|Alberta|658[0n]|BOLD:AAA8562
Lacinipolia meditata|[9994]|XAD364-04|Canada|Ontario|569[1n]|BOLD:AAA8562
Lacinipolia meditata|[9995]|XAG646-05|Canada|Ontario|658[0n]|BOLD:AAA8562
Lacinipolia meditata|[9996]|XAH208-05|Canada|Ontario|658[0n]|BOLD:AAA8562
Lacinipolia meditata|[9997]|RDLQF209-06|Canada|Quebec|658[0n]|BOLD:AAA8562
Lacinipolia meditata|[9998]|LNCC1303-11|United States|North Carolina|658[0n]|BOLD:AAA8562
Lacinipolia meditata|[9999]|LSEU101-06|United States|North Carolina|658[0n]|BOLD:AAA8562
Lacinipolia meditata|[10000]|LNCC332-10|United States|North Carolina|658[0n]|BOLD:AAA8562
Lacinipolia meditata|[10001]|LNCC1301-11|United States|North Carolina|658[0n]|BOLD:AAA8562
Lacinipolia meditata|[10002]|XAD346-04|Canada|Ontario|658[0n]|BOLD:AAA8562
Lacinipolia meditata|[10003]|PHMO392-03|Canada|Ontario|639[0n]|BOLD:AAA8562
Lacinipolia meditata|[10004]|XAG944-05|Canada|Ontario|658[0n]|BOLD:AAA8562
Lacinipolia meditata|[10005]|XAG995-05|Canada|Ontario|658[0n]|BOLD:AAA8562
Lacinipolia meditata|[10006]|RDNMB247-05|Canada|Ontario|658[0n]|BOLD:AAA8562
Lacinipolia meditata|[10007]|XAG978-05|Canada|Ontario|633[0n]|BOLD:AAA8562
Lacinipolia meditata|[10008]|XAG671-05|Canada|Ontario|658[1n]|BOLD:AAA8562
Lacinipolia meditata|[10009]|XAH127-05|Canada|Ontario|658[0n]|BOLD:AAA8562
Lacinipolia meditata|[10010]|XAH028-05|Canada|Ontario|658[0n]|BOLD:AAA8562
Lacinipolia meditata|[10011]|XAG914-05|Canada|Ontario|658[0n]|BOLD:AAA8562
Lacinipolia meditata|[10012]|XAD003-04|Canada|Ontario|580[0n]|BOLD:AAA8562
Lacinipolia meditata|[10013]|XAD273-04|Canada|Ontario|604[0n]|BOLD:AAA8562
Lacinipolia meditata|[10014]|XAH151-05|Canada|Ontario|658[1n]|BOLD:AAA8562
Lacinipolia meditata|[10015]|XAH152-05|Canada|Ontario|658[0n]|BOLD:AAA8562
Lacinipolia meditata|[10016]|XAH153-05|Canada|Ontario|658[0n]|BOLD:AAA8562
Lacinipolia meditata|[10017]|XAH270-05|Canada|Ontario|658[0n]|BOLD:AAA8562
Lacinipolia meditata|[10018]|XAH281-05|Canada|Ontario|658[0n]|BOLD:AAA8562
Lacinipolia meditata|[10019]|XAD254-04|Canada|Ontario|607[0n]|BOLD:AAA8562
Lacinipolia meditata|[10020]|XAG852-05|Canada|Ontario|658[0n]|BOLD:AAA8562
Lacinipolia meditata|[10021]|XAH036-05|Canada|Ontario|658[0n]|BOLD:AAA8562
Lacinipolia meditata|[10022]|XAK245-06|Canada|Ontario|658[0n]|BOLD:AAA8562
Lacinipolia meditata|[10023]|XAK271-06|Canada|Ontario|658[0n]|BOLD:AAA8562
Lacinipolia meditata|[10024]|XAK291-06|Canada|Ontario|658[0n]|BOLD:AAA8562
Lacinipolia meditata|[10025]|LNCC452-10|United States|North Carolina|658[0n]|BOLD:AAA8562
Lacinipolia meditata|[10026]|LNCC1302-11|United States|North Carolina|658[0n]|BOLD:AAA8562
Lacinipolia meditata|[10027]|XAD362-04|Canada|Ontario|658[0n]|BOLD:AAA8562
Lacinipolia meditata|[10028]|XAG733-05|Canada|Ontario|658[0n]|BOLD:AAA8562
Lacinipolia meditata|[10029]|LNCC1304-11|United States|North Carolina|658[0n]|BOLD:AAA8562
Lacinipolia delongi|[10030]|RDNME880-08|United States|Arizona|658[0n]|BOLD:ABZ4814
Lacinipolia delongi|[10031]|RDNMG305-08|United States|Arizona|658[0n]|BOLD:ABZ4814
Lacinipolia naevia|[10032]|RDMAB374-05|Canada|Alberta|575[1n]|BOLD:AAH5399
Lacinipolia naevia|[10033]|BBLSY557-09|United States|New Mexico|658[0n]|BOLD:AAH5399
Lacinipolia lustralis|[10034]|RDMAB034-05|Canada|Alberta|628[0n]|BOLD:AAA7130
Lacinipolia lustralis|[10035]|RDMAB368-05|Canada|Alberta|587[0n]|BOLD:AAA7130
Lacinipolia lustralis|[10036]|RDMAB522-06|Canada|Alberta|658[0n]|BOLD:AAA7130
Lacinipolia lustralis|[10037]|LPMN013-08|Canada|Manitoba|658[0n]|BOLD:AAA7130
Lacinipolia lustralis|[10038]|LOWCC092-05|Canada|British Columbia|542[0n]|BOLD:AAA7130
Lacinipolia lustralis|[10039]|LPMN633-08|Canada|Manitoba|658[0n]|BOLD:AAA7130
Lacinipolia lustralis|[10040]|LOWCE568-06|Canada|British Columbia|658[0n]|BOLD:AAA7130
Lacinipolia lustralis|[10041]|RDMAB111-05|Canada|Alberta|658[0n]|BOLD:AAA7130
Lacinipolia lustralis|[10042]|LOWCE277-06|Canada|British Columbia|563[1n]|BOLD:AAA7130
Lacinipolia lustralis|[10043]|LPMN264-08|Canada|Manitoba|658[0n]|BOLD:AAA7130
Lacinipolia lustralis|[10044]|LOWCE575-06|Canada|British Columbia|658[0n]|BOLD:AAA7130
Lacinipolia lustralis|[10045]|LOWCE577-06|Canada|British Columbia|658[0n]|BOLD:AAA7130
Lacinipolia lustralis|[10046]|LOWCE578-06|Canada|British Columbia|658[0n]|BOLD:AAA7130
Lacinipolia lustralis|[10047]|LOWCE579-06|Canada|British Columbia|658[0n]|BOLD:AAA7130
Lacinipolia lustralis|[10048]|LOWCE580-06|Canada|British Columbia|658[0n]|BOLD:AAA7130
Lacinipolia lustralis|[10049]|LOWCE581-06|Canada|British Columbia|658[0n]|BOLD:AAA7130
Lacinipolia lustralis|[10050]|LOWCE569-06|Canada|British Columbia|658[0n]|BOLD:AAA7130
Lacinipolia lustralis|[10051]|LOWCE570-06|Canada|British Columbia|658[0n]|BOLD:AAA7130
Lacinipolia lustralis|[10052]|LOWCE566-06|Canada|British Columbia|658[0n]|BOLD:AAA7130
Lacinipolia lustralis|[10053]|LOWCE567-06|Canada|British Columbia|658[0n]|BOLD:AAA7130
Lacinipolia lustralis|[10054]|LOWCE565-06|Canada|British Columbia|658[0n]|BOLD:AAA7130
Lacinipolia lustralis|[10055]|LOWCE564-06|Canada|British Columbia|658[0n]|BOLD:AAA7130
Lacinipolia lustralis|[10056]|LOWCE563-06|Canada|British Columbia|658[0n]|BOLD:AAA7130
Lacinipolia lustralis|[10057]|RDMAB576-06|Canada|Alberta|658[0n]|BOLD:AAA7130
Lacinipolia lustralis|[10058]|RDMAB575-06|Canada|Alberta|658[0n]|BOLD:AAA7130
Lacinipolia lustralis|[10059]|RDMAB099-05|Canada|Alberta|658[0n]|BOLD:AAA7130
Lacinipolia lustralis|[10060]|LOWCC097-05|Canada|British Columbia|658[0n]|BOLD:AAA7130
Lacinipolia lustralis|[10061]|LOWCC096-05|Canada|British Columbia|658[0n]|BOLD:AAA7130
Lacinipolia lustralis|[10062]|LOWCC095-05|Canada|British Columbia|658[0n]|BOLD:AAA7130
Lacinipolia lustralis|[10063]|LOWCC094-05|Canada|British Columbia|658[0n]|BOLD:AAA7130
Lacinipolia lustralis|[10064]|LOWCC093-05|Canada|British Columbia|658[0n]|BOLD:AAA7130
Lacinipolia lustralis|[10065]|LOWCC090-05|Canada|British Columbia|658[0n]|BOLD:AAA7130
Lacinipolia lustralis|[10066]|LOWCE561-06|Canada|British Columbia|658[0n]|BOLD:AAA7130
Lacinipolia lustralis|[10067]|LOWCE562-06|Canada|British Columbia|654[0n]|BOLD:AAA7130
Lacinipolia lustralis|[10068]|LOWCE576-06|Canada|British Columbia|649[0n]|BOLD:AAA7130
Lacinipolia lustralis|[10069]|RDMAB289-05|Canada|Alberta|612[1n]|BOLD:AAA7130
Lacinipolia lustralis|[10070]|RDNMC387-05|Canada|Alberta|578[0n]|BOLD:AAA7130
Lacinipolia lustralis|[10071]|LOWCD759-06|Canada|British Columbia|583[1n]|BOLD:AAA7130
Lacinipolia lustralis|[10072]|RDMAB312-05|Canada|Alberta|611[0n]|BOLD:AAA7130
Lacinipolia lustralis|[10073]|RDMAB313-05|Canada|Alberta|612[0n]|BOLD:AAA7130
Lacinipolia lustralis|[10074]|LOWCE862-06|Canada|British Columbia|617[0n]|BOLD:AAA7130
Lacinipolia lustralis|[10075]|LPMN165-08|Canada|Manitoba|658[0n]|BOLD:AAA7130
Lacinipolia lustralis|[10076]|LOWCE571-06|Canada|British Columbia|658[0n]|BOLD:AAA7130
Lacinipolia lustralis|[10077]|LOWCE573-06|Canada|British Columbia|658[0n]|BOLD:AAA7130
Lacinipolia lustralis|[10078]|LPSK251-08|Canada|Saskatchewan|658[0n]|BOLD:AAA7130
Lacinipolia lustralis|[10079]|LPMN323-08|Canada|Manitoba|658[0n]|BOLD:AAA7130
Lacinipolia lustralis|[10080]|LPMN527-08|Canada|Manitoba|658[0n]|BOLD:AAA7130
Lacinipolia lustralis|[10081]|LPMN643-08|Canada|Manitoba|658[0n]|BOLD:AAA7130
Lacinipolia lustralis|[10082]|LPSK442-08|Canada|Saskatchewan|658[0n]|BOLD:AAA7130
Lacinipolia lustralis|[10083]|LPSK466-08|Canada|Saskatchewan|658[0n]|BOLD:AAA7130
Lacinipolia lustralis|[10084]|RDMAB258-05|Canada|Alberta|658[0n]|BOLD:AAA7130
Lacinipolia lustralis|[10085]|PHMNB138-04|Canada|New Brunswick|658[0n]|BOLD:AAA7130
Lacinipolia lustralis|[10086]|PHMNB483-04|Canada|New Brunswick|658[0n]|BOLD:AAA7130
Lacinipolia lustralis|[10087]|RDLQB062-05|Canada|Quebec|658[0n]|BOLD:AAA7130
Lacinipolia lustralis|[10088]|RDLQB061-05|Canada|Quebec|658[0n]|BOLD:AAA7130
Lacinipolia lustralis|[10089]|RDLQB060-05|Canada|Quebec|658[0n]|BOLD:AAA7130
Lacinipolia lustralis|[10090]|XAC621-04|Canada|Ontario|617[0n]|BOLD:AAA7130
Lacinipolia lustralis|[10091]|RDNMC401-05|Canada|Ontario|613[0n]|BOLD:AAA7130
Lacinipolia lustralis|[10092]|PHMNB477-04|Canada|New Brunswick|658[0n]|BOLD:AAA7130
Lacinipolia lustralis|[10093]|RDLQB063-05|Canada|Quebec|658[0n]|BOLD:AAA7130
Lacinipolia lustralis|[10094]|LPSOD252-09|Canada|Ontario|658[0n]|BOLD:AAA7130
[illegible]

Lacinipolia lustralis|[10092]|PHMNB477-04|Canada|New Brunswick|658[0n]|BOLD:AAA7130
Lacinipolia lustralis|[10093]|RDLQB063-05|Canada|Quebec|658[0n]|BOLD:AAA7130
Lacinipolia lustralis|[10094]|LPSOD252-09|Canada|Ontario|658[0n]|BOLD:AAA7130
Lacinipolia sp.|[10095]|RDNMG188-08|United States|Arizona|630[0n]|BOLD:AAE5526
Lacinipolia agnata|[10096]|RDNMG304-08|United States|Arizona|658[0n]|BOLD:AAE5527
Lacinipolia agnata|[10097]|RDNME1016-08|United States|Arizona|643[0n]|BOLD:AAE5527
Lacinipolia agnata|[10098]|RDNME1020-08|United States|Arizona|658[0n]|BOLD:AAE5527
Lacinipolia lepidula|[10099]|BBLSW358-09|United States|Arizona|658[0n]|BOLD:AAH5298
Lacinipolia lepidula|[10100]|RDNMG301-08|United States|Arizona|658[0n]|BOLD:AAH5298
Lacinipolia lepidula|[10101]|BBLSW359-09|United States|Arizona|658[0n]|BOLD:AAH5298
Lacinipolia sp.|[10102]|RDNMJ851-11|United States|New Mexico|658[0n]|BOLD:AAT8999
Lacinipolia franclemonti|[10103]|IAWLB547-11|United States|Arizona|658[0n]|BOLD:AAT8999
Lacinipolia franclemonti|[10104]|IAWLB559-11|United States|Arizona|658[0n]|BOLD:AAT8999
Lacinipolia prognata|[10105]|CNCLB1135-14|United States|Arizona|550[0n]|BOLD:ACQ0345
Lacinipolia prognata|[10106]|CNCLB1136-14|United States|Arizona|550[0n]|BOLD:ACQ0345
Lacinipolia basiplaga|[10107]|RDNMG300-08|United States|Arizona|658[0n]|BOLD:AAX0582
Lacinipolia basiplaga|[10108]|RDNMK943-13|Mexico|Sonora|658[0n]|BOLD:AAX0582
Lacinipolia consimilis|[10109]|IAWLB569-11|United States|Arizona|658[0n]|BOLD:AAZ0787
Lacinipolia consimilis|[10110]|RDNMG307-08|United States|Arizona|407[0n]|
Lacinipolia consimilis|[10111]|CNCLB255-14|United States|Arizona|658[0n]|BOLD:AAZ0787
Lacinipolia consimilis|[10112]|CNCLB256-14|United States|Arizona|658[0n]|BOLD:AAZ0787
Lacinipolia viridifera|[10113]|RDNMG299-08|United States|Arizona|658[0n]|BOLD:AAJ3059
Lacinipolia viridifera|[10114]|RDNMG308-08|United States|Arizona|658[0n]|BOLD:AAJ3059
Lacinipolia viridifera|[10115]|CMAZA1151-12|United States|Arizona|658[0n]|BOLD:AAJ3059
Lacinipolia viridifera|[10116]|CMAZA1141-12|United States|Arizona|658[0n]|BOLD:AAJ3059
Lacinipolia viridifera|[10117]|CMAZA1156-12|United States|Arizona|658[0n]|BOLD:AAJ3059
Lacinipolia runica|[10118]|CNCLB677-14|United States|Arizona|658[0n]|BOLD:ACM3927
Lacinipolia runica|[10119]|CNCLB679-14|United States|Arizona|407[0n]|BOLD:ACM3927
Lacinipolia pensilis|[10120]|LOWCE525-06|Canada|British Columbia|570[1n]|BOLD:ABZ1332
Lacinipolia pensilis|[10121]|LOWCE526-06|Canada|British Columbia|548[2n]|BOLD:ABZ1332
Lacinipolia pensilis|[10122]|RDNMB231-05|United States|Washington|595[0n]|BOLD:AAA5195
Lacinipolia pensilis|[10123]|RDNMB230-05|United States|Washington|505[0n]|BOLD:AAA5195
Lacinipolia pensilis|[10124]|LOWCE557-06|Canada|British Columbia|617[0n]|BOLD:AAA5195
Lacinipolia pensilis|[10125]|LOWCE544-06|Canada|British Columbia|605[0n]|BOLD:AAA5195
Lacinipolia pensilis|[10126]|RDNMC081-05|Canada|Alberta|658[0n]|BOLD:AAA5195
Lacinipolia pensilis|[10127]|LBCH6740-10|Canada|British Columbia|658[0n]|BOLD:AAA5195
Lacinipolia pensilis|[10128]|RDNMB233-05|Canada|British Columbia|592[0n]|BOLD:AAA5195
Lacinipolia pensilis|[10129]|LBCH7394-10|Canada|British Columbia|658[0n]|BOLD:AAA5195
Lacinipolia pensilis|[10130]|LBCG2863-09|Canada|British Columbia|658[0n]|BOLD:AAA5195
Lacinipolia pensilis|[10131]|LBCG3273-09|Canada|British Columbia|641[0n]|BOLD:AAA5195
Lacinipolia pensilis|[10132]|LPVIB253-08|Canada|British Columbia|658[0n]|BOLD:AAA5195
Lacinipolia pensilis|[10133]|LPVIB634-08|Canada|British Columbia|597[0n]|BOLD:AAA5195
Lacinipolia pensilis|[10134]|LBCH7231-10|Canada|British Columbia|643[0n]|BOLD:AAA5195
Lacinipolia pensilis|[10135]|LBCG3247-09|Canada|British Columbia|643[0n]|BOLD:AAA5195
Lacinipolia pensilis|[10136]|RDMAB623-06|Canada|Alberta|658[0n]|BOLD:AAA5195
Lacinipolia pensilis|[10137]|LPVIB637-08|Canada|British Columbia|634[0n]|BOLD:AAA5195
Lacinipolia pensilis|[10138]|LOWCC086-05|Canada|British Columbia|658[0n]|BOLD:AAA5195
Lacinipolia pensilis|[10139]|LPVIB258-08|Canada|British Columbia|658[0n]|BOLD:AAA5195
Lacinipolia pensilis|[10140]|LBCG2862-09|Canada|British Columbia|658[0n]|BOLD:AAA5195
Lacinipolia pensilis|[10141]|LOWCE556-06|Canada|British Columbia|617[0n]|BOLD:AAA5195
Lacinipolia pensilis|[10142]|LOWCE551-06|Canada|British Columbia|617[0n]|BOLD:AAA5195
Lacinipolia pensilis|[10143]|LPABC387-09|Canada|Alberta|658[0n]|BOLD:AAA5195
Lacinipolia pensilis|[10144]|LBCG3257-09|Canada|British Columbia|642[0n]|BOLD:AAA5195
Lacinipolia pensilis|[10145]|LBCH6497-10|Canada|British Columbia|658[0n]|BOLD:AAA5195
Lacinipolia pensilis|[10146]|LBCG2873-09|Canada|British Columbia|658[0n]|BOLD:AAA5195
Lacinipolia pensilis|[10147]|LBCH6540-10|Canada|British Columbia|658[0n]|BOLD:AAA5195
Lacinipolia pensilis|[10148]|LBCG2868-09|Canada|British Columbia|658[0n]|BOLD:AAA5195
Lacinipolia pensilis|[10149]|LBCG2871-09|Canada|British Columbia|658[0n]|BOLD:AAA5195
Lacinipolia pensilis|[10150]|LBCG1346-09|Canada|British Columbia|658[0n]|BOLD:AAA5195
Lacinipolia pensilis|[10151]|LBCG2108-09|Canada|British Columbia|658[0n]|BOLD:AAA5195
Lacinipolia pensilis|[10152]|LPABC389-09|Canada|Alberta|658[0n]|BOLD:AAA5195
Lacinipolia pensilis|[10153]|LBCG1344-09|Canada|British Columbia|658[0n]|BOLD:AAA5195
Lacinipolia pensilis|[10154]|LBCH7476-10|Canada|British Columbia|658[0n]|BOLD:AAA5195
Lacinipolia pensilis|[10155]|LPVIB636-08|Canada|British Columbia|658[0n]|BOLD:AAA5195
Lacinipolia pensilis|[10156]|LPABB011-08|Canada|Alberta|658[0n]|BOLD:AAA5195
Lacinipolia pensilis|[10157]|LBCG480-08|Canada|British Columbia|658[0n]|BOLD:AAA5195
Lacinipolia pensilis|[10158]|LBCG252-08|Canada|British Columbia|658[0n]|BOLD:AAA5195
Lacinipolia pensilis|[10159]|LOWCE574-06|Canada|British Columbia|658[0n]|BOLD:AAA5195
Lacinipolia pensilis|[10160]|LOWCE559-06|Canada|British Columbia|658[0n]|BOLD:AAA5195
Lacinipolia pensilis|[10161]|RDMAB622-06|Canada|Alberta|658[0n]|BOLD:AAA5195
Lacinipolia pensilis|[10162]|LOWCC088-05|Canada|British Columbia|658[0n]|BOLD:AAA5195
Lacinipolia pensilis|[10163]|LOWCC087-05|Canada|British Columbia|658[0n]|BOLD:AAA5195
Lacinipolia pensilis|[10164]|LBCD461-05|Canada|British Columbia|658[0n]|BOLD:AAA5195
Lacinipolia pensilis|[10165]|LBCD293-05|Canada|British Columbia|633[0n]|BOLD:AAA5195
Lacinipolia pensilis|[10166]|LBCG3251-09|Canada|British Columbia|638[0n]|BOLD:AAA5195
Lacinipolia pensilis|[10167]|LALPA1276-11|Canada|British Columbia|658[0n]|BOLD:AAA5195
Lacinipolia pensilis|[10168]|SSBAA5642-12|Canada|Alberta|634[0n]|BOLD:AAA5195
Lacinipolia sareta|[10169]|RDMAB621-06|Canada|Alberta|658[0n]|BOLD:AAD4273
Lacinipolia sareta|[10170]|RDMAB303-05|Canada|Alberta|658[0n]|BOLD:AAD4273
Lacinipolia sareta|[10171]|RDMAB683-06|Canada|Alberta|574[0n]|BOLD:AAD4273
Lacinipolia dimocki|[10172]|RDNMB226-05|United States|California|580[0n]|BOLD:ACF5304
Lacinipolia dimocki|[10173]|RDNMB222-05|United States|California|579[0n]|BOLD:ACF5304
Lacinipolia dimocki|[10174]|RDNMB223-05|United States|California|583[0n]|BOLD:ACF5304
Lacinipolia dimocki|[10175]|RDNMB227-05|United States|California|582[0n]|BOLD:ACF5304
Lacinipolia dimocki|[10176]|LOCBC310-06|United States|California|658[0n]|BOLD:ABZ7491
Lacinipolia dimocki|[10177]|LOCBC307-06|United States|California|654[0n]|BOLD:ABZ7491
Lacinipolia dimocki|[10178]|RDNMD431-06|United States|California|656[0n]|BOLD:ABZ7491
Lacinipolia dimocki|[10179]|LOCBE391-06|United States|California|566[0n]|BOLD:ABZ7491
Lacinipolia dimocki|[10180]|LOCBD220-06|United States|California|658[0n]|BOLD:ABZ7491
Lacinipolia dimocki|[10181]|LOCBD466-06|United States|California|657[0n]|BOLD:ABZ7491
Lacinipolia dimocki|[10182]|RDNMG1009-08|United States|California|658[0n]|BOLD:ABZ7491
Lacinipolia dimocki|[10183]|LOCBC311-06|United States|California|658[0n]|BOLD:ABZ7491
Lacinipolia dimocki|[10184]|LOCBC413-06|United States|California|658[0n]|BOLD:ABZ7491
Lacinipolia dimocki|[10185]|LOCBE390-06|United States|California|585[0n]|BOLD:ABZ7491
Lacinipolia dimocki|[10186]|RDNMG1010-08|United States|California|658[0n]|BOLD:ABZ7491
Lacinipolia sareta|[10187]|BBLOC133-11|United States|Texas|658[0n]|BOLD:ACF5303
Lacinipolia sareta|[10188]|BBLOC1201-11|United States|California|658[0n]|BOLD:ACF5303
Lacinipolia sareta|[10189]|BBLOC1204-11|United States|California|658[0n]|BOLD:ACF5303
Lacinipolia sareta|[10190]|BBLOC1206-11|United States|California|658[0n]|BOLD:ACF5303
Lacinipolia sareta|[10191]|IAWLB074-10|United States|California|658[0n]|BOLD:ACF5303
Lacinipolia sareta|[10192]|BBLOC131-11|United States|Texas|658[0n]|BOLD:ACF5303
Lacinipolia sareta|[10193]|RDNMB229-05|United States|California|658[0n]|BOLD:ACF5303
Lacinipolia sareta|[10194]|IAWLB073-10|United States|California|658[0n]|BOLD:ACF5303
[illegible]

Lacinipolia sareta|[10192]|BBLOC131-11|United States|Texas|658[0n]|BOLD:ACF5303
Lacinipolia sareta|[10193]|RDNMB229-05|United States|California|658[0n]|BOLD:ACF5303
Lacinipolia sareta|[10194]|IAWLB073-10|United States|California|658[0n]|BOLD:ACF5303
Lacinipolia sareta|[10195]|BBLOC1211-11|United States|California|658[0n]|BOLD:ACF5303
Lacinipolia sareta|[10196]|RDNMD650-06|United States|New Mexico|658[0n]|BOLD:ABY4469
Lacinipolia sareta|[10197]|BBLSW367-09|United States|Arizona|658[0n]|BOLD:ABY4469
Lacinipolia sareta|[10198]|BBLSY491-09|United States|New Mexico|658[0n]|BOLD:ABY4469
Lacinipolia sareta|[10199]|RDNMD649-06|United States|New Mexico|656[0n]|BOLD:ABY4469
Lacinipolia sareta|[10200]|CMAZA317-10|United States|Arizona|658[0n]|BOLD:ABY4469
Lacinipolia sareta|[10201]|CMAZA163-09|United States|Arizona|658[0n]|BOLD:ABY4469
Lacinipolia sareta|[10202]|CMAZA284-09|United States|Arizona|658[0n]|BOLD:ABY4469
Lacinipolia sareta|[10203]|RDNME624-08|United States|New Mexico|658[0n]|BOLD:ABY4469
Lacinipolia sareta|[10204]|BBLSY011-09|United States|Arizona|658[0n]|BOLD:ABY4469
Lacinipolia sareta|[10205]|IAWLB249-11|United States|Arizona|658[0n]|BOLD:ABY4469
Lacinipolia sareta|[10206]|IAWLB632-11|United States|Arizona|658[0n]|BOLD:ABY4469
Lacinipolia sareta|[10207]|CMAZA952-12|United States|Arizona|658[0n]|BOLD:ABY4469
Lacinipolia sareta|[10208]|CMAZA955-12|United States|Arizona|658[0n]|BOLD:ABY4469
Lacinipolia sareta|[10209]|LOWCC089-05|Canada|British Columbia|658[0n]|BOLD:AAA6056
Lacinipolia sareta|[10210]|BBLPB469-10|Canada|Ontario|658[0n]|BOLD:AAA6056
Lacinipolia sareta|[10211]|LBCH5820-10|Canada|British Columbia|658[0n]|BOLD:AAA6056
Lacinipolia sareta|[10212]|BBLPB467-10|Canada|British Columbia|658[0n]|BOLD:AAA6056
Lacinipolia sareta|[10213]|LBCG132-08|Canada|British Columbia|658[0n]|BOLD:AAA6056
Lacinipolia sareta|[10214]|LOWCD763-06|Canada|British Columbia|656[0n]|BOLD:AAA6056
Lacinipolia sareta|[10215]|LOWCD765-06|Canada|British Columbia|657[1n]|BOLD:AAA6056
Lacinipolia sareta|[10216]|LOWCD767-06|Canada|British Columbia|658[0n]|BOLD:AAA6056
Lacinipolia sareta|[10217]|LOWCD762-06|Canada|British Columbia|657[0n]|BOLD:AAA6056
Lacinipolia sareta|[10218]|LOWCC085-05|Canada|British Columbia|658[0n]|BOLD:AAA6056
Lacinipolia sareta|[10219]|LOWCD764-06|Canada|British Columbia|658[0n]|BOLD:AAA6056
Lacinipolia sareta|[10220]|LOWCE545-06|Canada|British Columbia|626[0n]|BOLD:AAA6056
Lacinipolia sareta|[10221]|RDNMB237-05|Canada|British Columbia|588[0n]|BOLD:AAA6056
Lacinipolia sareta|[10222]|LOWCE546-06|Canada|British Columbia|598[0n]|BOLD:AAA6056
Lacinipolia sareta|[10223]|LOWCE547-06|Canada|British Columbia|615[0n]|BOLD:AAA6056
Lacinipolia sareta|[10224]|LOWCE548-06|Canada|British Columbia|626[0n]|BOLD:AAA6056
Lacinipolia sareta|[10225]|LOWCE558-06|Canada|British Columbia|658[0n]|BOLD:AAA6056
Lacinipolia sareta|[10226]|LBCG119-08|Canada|British Columbia|658[0n]|BOLD:AAA6056
Lacinipolia sareta|[10227]|LBCG128-08|Canada|British Columbia|658[0n]|BOLD:AAA6056
Lacinipolia sareta|[10228]|LBCH5514-10|Canada|British Columbia|658[0n]|BOLD:AAA6056
Lacinipolia sareta|[10229]|LBCH5548-10|Canada|British Columbia|658[0n]|BOLD:AAA6056
Lacinipolia sareta|[10230]|LBCH5575-10|Canada|British Columbia|658[0n]|BOLD:AAA6056
Lacinipolia sareta|[10231]|LBCH6098-10|Canada|British Columbia|658[0n]|BOLD:AAA6056
Lacinipolia sareta|[10232]|LBCH5717-10|Canada|British Columbia|658[0n]|BOLD:AAA6056
Lacinipolia sareta|[10233]|BBLPB336-10|Canada|British Columbia|658[0n]|BOLD:AAA6056
Lacinipolia sareta|[10234]|LBCH5602-10|Canada|British Columbia|658[0n]|BOLD:AAA6056
Lacinipolia sareta|[10235]|LBCH5429-10|Canada|British Columbia|658[0n]|BOLD:AAA6056
Lacinipolia sareta|[10236]|LBCH5715-10|Canada|British Columbia|658[0n]|BOLD:AAA6056
Lacinipolia sareta|[10237]|LBCH5821-10|Canada|British Columbia|658[0n]|BOLD:AAA6056
Lacinipolia sareta|[10238]|LBCH5946-10|Canada|British Columbia|658[0n]|BOLD:AAA6056
Lacinipolia sareta|[10239]|BBLPB569-10|Canada|British Columbia|658[0n]|BOLD:AAA6056
Lacinipolia sareta|[10240]|RDMAB638-06|Canada|Alberta|658[0n]|BOLD:AAA6056
Lacinipolia sareta|[10241]|RDMAB639-06|Canada|Alberta|658[0n]|BOLD:AAA6056
Lacinipolia sareta|[10242]|LPABB431-08|Canada|Alberta|658[0n]|BOLD:AAA6056
Lacinipolia sareta|[10243]|LPABC139-09|Canada|Alberta|658[0n]|BOLD:AAA6056
Lacinipolia sareta|[10244]|LPABC986-09|Canada|Alberta|658[0n]|BOLD:AAA6056
Lacinipolia sareta|[10245]|LPABB582-08|Canada|Alberta|658[0n]|BOLD:AAA6056
Lacinipolia sareta|[10246]|LPABB440-08|Canada|Alberta|658[0n]|BOLD:AAA6056
Lacinipolia sareta|[10247]|LPABB418-08|Canada|Alberta|658[0n]|BOLD:AAA6056
Lacinipolia sareta|[10248]|LPABB353-08|Canada|Alberta|658[0n]|BOLD:AAA6056
Lacinipolia sareta|[10249]|LPABB068-08|Canada|Alberta|658[0n]|BOLD:AAA6056
Lacinipolia sareta|[10250]|LPABB031-08|Canada|Alberta|658[0n]|BOLD:AAA6056
Lacinipolia sareta|[10251]|LPAB249-08|Canada|Alberta|658[0n]|BOLD:AAA6056
Lacinipolia sareta|[10252]|LPMN885-08|Canada|Alberta|658[0n]|BOLD:AAA6056
Lacinipolia sareta|[10253]|RDMAB635-06|Canada|Alberta|658[0n]|BOLD:AAA6056
Lacinipolia sareta|[10254]|RDMAB632-06|Canada|Alberta|658[0n]|BOLD:AAA6056
Lacinipolia sareta|[10255]|RDMAB620-06|Canada|Alberta|658[0n]|BOLD:AAA6056
Lacinipolia sareta|[10256]|RDMAB619-06|Canada|Alberta|658[0n]|BOLD:AAA6056
Lacinipolia sareta|[10257]|RDMAB263-05|Canada|Alberta|658[0n]|BOLD:AAA6056
Lacinipolia sareta|[10258]|RDMAB254-05|Canada|Alberta|658[0n]|BOLD:AAA6056
Lacinipolia sareta|[10259]|LPABB015-08|Canada|Alberta|633[0n]|BOLD:AAA6056
Lacinipolia sareta|[10260]|RDMAB288-05|Canada|Alberta|634[0n]|BOLD:AAA6056
Lacinipolia sareta|[10261]|CNWLE2517-12|Canada|Alberta|640[0n]|BOLD:AAA6056
Lacinipolia sareta|[10262]|LPABC370-09|Canada|Alberta|658[0n]|BOLD:AAA6056
Lacinipolia sareta|[10263]|BBLPB480-10|Canada|Alberta|658[0n]|BOLD:AAA6056
Lacinipolia sareta|[10264]|CNCLB2522-14|Canada|Yukon Territory|658[0n]|BOLD:AAA6056
Lacinipolia sareta|[10265]|RDLQB448-05|Canada|Quebec|658[0n]|BOLD:AAA6057
Lacinipolia sareta|[10266]|CNCLB2519-14|Canada|Quebec|658[0n]|BOLD:AAA6057
Lacinipolia sareta|[10267]|CNCLB2541-14|Canada|Ontario|658[0n]|BOLD:AAA6057
Lacinipolia sareta|[10268]|RDMAB271-05|Canada|Saskatchewan|658[0n]|BOLD:AAA6058
Lacinipolia sareta|[10269]|RDMAB634-06|Canada|Alberta|658[0n]|BOLD:AAA6058
Lacinipolia sareta|[10270]|LPSK492-08|Canada|Saskatchewan|658[0n]|BOLD:AAA6058
Lacinipolia sareta|[10271]|LPSK529-08|Canada|Saskatchewan|658[0n]|BOLD:AAA6058
Lacinipolia sareta|[10272]|CNGRJ075-13|Canada|Saskatchewan|529[5n]|BOLD:AAA6058
Lacinipolia sareta|[10273]|RDMAB643-06|Canada|Alberta|658[0n]|BOLD:AAA6058
Lacinipolia sareta|[10274]|LPSK553-08|Canada|Saskatchewan|658[0n]|BOLD:AAA6058
Lacinipolia sareta|[10275]|LPSK593-08|Canada|Saskatchewan|658[0n]|BOLD:AAA6058
Lacinipolia sareta|[10276]|RDMAB277-05|Canada|Alberta|587[0n]|BOLD:AAA6058
Lacinipolia sareta|[10277]|RDMAB636-06|Canada|Alberta|658[0n]|BOLD:AAA6058
Lacinipolia sareta|[10278]|LPSK473-08|Canada|Saskatchewan|658[0n]|BOLD:AAA6058
Lacinipolia sareta|[10279]|LPSK475-08|Canada|Saskatchewan|658[0n]|BOLD:AAA6058
Lacinipolia sareta|[10280]|LPSK491-08|Canada|Saskatchewan|658[0n]|BOLD:AAA6058
Lacinipolia sareta|[10281]|LPSK496-08|Canada|Saskatchewan|658[0n]|BOLD:AAA6058
Lacinipolia sareta|[10282]|LPSK501-08|Canada|Saskatchewan|658[0n]|BOLD:AAA6058
Lacinipolia sareta|[10283]|LPSK503-08|Canada|Saskatchewan|658[0n]|BOLD:AAA6058
Lacinipolia sareta|[10284]|LPSK602-08|Canada|Saskatchewan|658[0n]|BOLD:AAA6058
Lacinipolia sareta|[10285]|TTCFW629-08|Canada|Alberta|658[0n]|
Lacinipolia sareta|[10286]|LPMN337-08|Canada|Manitoba|658[0n]|BOLD:AAA6058
Lacinipolia sareta|[10287]|LPSK454-08|Canada|Saskatchewan|658[0n]|BOLD:AAA6058
Lacinipolia sareta|[10288]|LPSK464-08|Canada|Saskatchewan|658[0n]|BOLD:AAA6058
Lacinipolia sareta|[10289]|LPSK469-08|Canada|Saskatchewan|658[0n]|BOLD:AAA6058
Lacinipolia sareta|[10290]|LPSK044-08|Canada|Saskatchewan|658[0n]|BOLD:AAA6058
Lacinipolia sareta|[10291]|LPSK247-08|Canada|Saskatchewan|658[0n]|BOLD:AAA6058
Lacinipolia sareta|[10292]|LPSK587-08|Canada|Saskatchewan|658[0n]|BOLD:AAA6058
Lacinipolia sareta|[10293]|LPSK588-08|Canada|Saskatchewan|658[0n]|BOLD:AAA6058
Lacinipolia sareta|[10294]|LPSK566-08|Canada|Saskatchewan|658[0n]|BOLD:AAA6058
[illegible]

Lacinipolia sareta|[10292]|LPSK587-08|Canada|Saskatchewan|658[0n]|BOLD:AAA6058
Lacinipolia sareta|[10293]|LPSK588-08|Canada|Saskatchewan|658[0n]|BOLD:AAA6058
Lacinipolia sareta|[10294]|LPSK566-08|Canada|Saskatchewan|658[0n]|BOLD:AAA6058
Lacinipolia sareta|[10295]|LPSK577-08|Canada|Saskatchewan|658[0n]|BOLD:AAA6058
Lacinipolia sareta|[10296]|LPSK540-08|Canada|Saskatchewan|658[0n]|BOLD:AAA6058
Lacinipolia sareta|[10297]|LPSK561-08|Canada|Saskatchewan|658[0n]|BOLD:AAA6058
Lacinipolia sareta|[10298]|LPSK515-08|Canada|Saskatchewan|658[0n]|BOLD:AAA6058
Lacinipolia sareta|[10299]|LPSK539-08|Canada|Saskatchewan|658[0n]|BOLD:AAA6058
Lacinipolia sareta|[10300]|LPSK041-08|Canada|Saskatchewan|658[0n]|BOLD:AAA6058
Lacinipolia sareta|[10301]|LPSK042-08|Canada|Saskatchewan|658[0n]|BOLD:AAA6058
Lacinipolia sareta|[10302]|RDMAB645-06|Canada|Alberta|658[0n]|BOLD:AAA6058
Lacinipolia sareta|[10303]|RDMAB644-06|Canada|Alberta|658[0n]|BOLD:AAA6058
Lacinipolia sareta|[10304]|RDMAB642-06|Canada|Alberta|658[0n]|BOLD:AAA6058
Lacinipolia sareta|[10305]|RDMAB637-06|Canada|Alberta|658[0n]|BOLD:AAA6058
Lacinipolia sareta|[10306]|RDMAB633-06|Canada|Alberta|658[0n]|BOLD:AAA6058
Lacinipolia sareta|[10307]|RDMAB521-06|Canada|Alberta|658[0n]|BOLD:AAA6058
Lacinipolia sareta|[10308]|RDMAB497-06|Canada|Alberta|658[0n]|BOLD:AAA6058
Lacinipolia sareta|[10309]|RDMAB366-05|Canada|Alberta|658[0n]|BOLD:AAA6058
Lacinipolia sareta|[10310]|RDMAB276-05|Canada|Alberta|658[0n]|BOLD:AAA6058
Lacinipolia sareta|[10311]|LPSK573-08|Canada|Saskatchewan|658[0n]|BOLD:AAA6058
Lacinipolia sareta|[10312]|RDMAB270-05|Canada|Saskatchewan|658[0n]|BOLD:AAA6058
Lacinipolia sareta|[10313]|RDMAB274-05|Canada|Alberta|658[0n]|BOLD:AAA6058
Lacinipolia sareta|[10314]|RDNMB236-05|Canada|Alberta|658[0n]|BOLD:AAA6058
Lacinipolia sareta|[10315]|CNGRD1024-12|Canada|Saskatchewan|612[0n]|BOLD:AAA6058
Lacinipolia sareta|[10316]|CNGRD1030-12|Canada|Saskatchewan|638[0n]|BOLD:AAA6058
Lacinipolia sareta|[10317]|CNGRD1023-12|Canada|Saskatchewan|638[0n]|BOLD:AAA6058
Lacinipolia sareta|[10318]|RDMAB367-05|Canada|Alberta|603[0n]|BOLD:AAA6058
Lacinipolia sareta|[10319]|RDNMB238-05|Canada|Alberta|602[0n]|BOLD:AAA6058
Lacinipolia sareta|[10320]|RDMAB311-05|Canada|Alberta|605[0n]|BOLD:AAA6058
Lacinipolia sareta|[10321]|CNGRJ074-13|Canada|Saskatchewan|588[0n]|BOLD:AAA6058
Lacinipolia sareta|[10322]|CNGRK550-13|Canada|Saskatchewan|564[0n]|BOLD:AAA6058
Lacinipolia sareta|[10323]|LPSK043-08|Canada|Saskatchewan|658[0n]|BOLD:AAA6058
Lacinipolia sareta|[10324]|CNGRL485-13|Canada|Saskatchewan|552[0n]|BOLD:AAA6058
Lacinipolia sareta|[10325]|LPSK249-08|Canada|Saskatchewan|658[0n]|BOLD:AAA6058
Lacinipolia sareta|[10326]|CNGRJ066-13|Canada|Saskatchewan|576[0n]|BOLD:AAA6058
Lacinipolia sareta|[10327]|RDMAB273-05|Canada|Saskatchewan|658[0n]|BOLD:AAA6058
Lacinipolia sareta|[10328]|LPSK525-08|Canada|Saskatchewan|658[0n]|BOLD:AAA6058
Lacinipolia sareta|[10329]|RDMAB631-06|Canada|Alberta|658[0n]|BOLD:AAA6058
Lacinipolia sareta|[10330]|RDMAB630-06|Canada|Alberta|658[0n]|BOLD:AAA6058
Lacinipolia sareta|[10331]|RDMAB491-06|Canada|Alberta|658[0n]|BOLD:AAA6058
Lacinipolia sareta|[10332]|RDMAB275-05|Canada|Alberta|658[0n]|BOLD:AAA6058
Lacinipolia sareta|[10333]|RDMAB272-05|Canada|Alberta|658[0n]|BOLD:AAA6058
Lacinipolia sareta|[10334]|CNGRD1031-12|Canada|Saskatchewan|638[0n]|BOLD:AAA6058
Lacinipolia sareta|[10335]|RDMAB641-06|Canada|Alberta|658[0n]|BOLD:AAA6058
Lacinipolia sareta|[10336]|CNGRL486-13|Canada|Saskatchewan|597[0n]|BOLD:AAA6058
Lacinipolia sareta|[10337]|RDMAB110-05|Canada|Alberta|658[0n]|BOLD:AAA6058
Lacinipolia sareta|[10338]|LPSK563-08|Canada|Saskatchewan|658[0n]|BOLD:AAA6058
Lacinipolia sareta|[10339]|CNCLB249-14|Canada|Ontario|658[0n]|BOLD:AAA6058
Lacinipolia imbuna|[10340]|CNCLB2388-14|Canada|Ontario|658[0n]|BOLD:AAA6058
Lacinipolia sareta|[10341]|CNCLB2521-14|Canada|Ontario|658[0n]|BOLD:AAA6058
Lacinipolia sareta|[10342]|CNCLB2542-14|Canada|Ontario|658[0n]|BOLD:AAA6058
Lacinipolia acutipennis|[10343]|JMMMB379-11|United States|California|658[0n]|BOLD:ACQ7349
Lacinipolia acutipennis|[10344]|JMMMB430-11|United States|California|658[0n]|BOLD:ACQ7349
Lacinipolia acutipennis|[10345]|JMMMB602-13|United States|California|658[0n]|BOLD:ACQ7349
Lacinipolia acutipennis|[10346]|RDNMB235-05|United States|Oregon|597[0n]|BOLD:ACJ0862
Lacinipolia acutipennis|[10347]|RDMAB601-06|Canada|Alberta|658[0n]|BOLD:ABZ7094
Lacinipolia acutipennis|[10348]|RDMAB304-05|Canada|Alberta|658[0n]|BOLD:ABZ7094
Lacinipolia acutipennis|[10349]|RDMAB305-05|Canada|Alberta|658[0n]|BOLD:ABZ7094
Lacinipolia acutipennis|[10350]|RDMAB602-06|Canada|Alberta|658[0n]|BOLD:ABZ7094
Lacinipolia acutipennis|[10351]|RDNMB234-05|United States|Washington|545[0n]|BOLD:ABZ7094
Lacinipolia acutipennis|[10352]|LOWCD766-06|Canada|British Columbia|657[0n]|BOLD:ABZ7094
Lacinipolia acutipennis|[10353]|RDNMB232-05|Canada|British Columbia|615[0n]|BOLD:ABZ7094
Lacinipolia acutipennis|[10354]|RDNMB224-05|Canada|British Columbia|594[0n]|BOLD:ABZ7094
Lacinipolia acutipennis|[10355]|LOWCD768-06|Canada|British Columbia|591[0n]|BOLD:ABZ7094
Lacinipolia acutipennis|[10356]|LOWCE554-06|Canada|British Columbia|610[0n]|BOLD:ABZ7094
Lacinipolia acutipennis|[10357]|LOWCE555-06|Canada|British Columbia|658[0n]|BOLD:ABZ7094
Lacinipolia acutipennis|[10358]|LBCH7671-10|Canada|British Columbia|658[0n]|BOLD:ABZ7094
Lacinipolia acutipennis|[10359]|LBCH7673-10|Canada|British Columbia|658[0n]|BOLD:ABZ7094
Lacinipolia acutipennis|[10360]|LBCH7672-10|Canada|British Columbia|636[0n]|BOLD:ABZ7094
Lacinipolia acutipennis|[10361]|LBCH7435-10|Canada|British Columbia|658[0n]|BOLD:ABZ7094
Lacinipolia acutipennis|[10362]|LBCH7670-10|Canada|British Columbia|658[0n]|BOLD:ABZ7094
Lacinipolia acutipennis|[10363]|LBCH7669-10|Canada|British Columbia|658[0n]|BOLD:ABZ7094
Lacinipolia acutipennis|[10364]|LBCH7668-10|Canada|British Columbia|658[0n]|BOLD:ABZ7094
Lacinipolia acutipennis|[10365]|LBCH7667-10|Canada|British Columbia|658[0n]|BOLD:ABZ7094
Lacinipolia acutipennis|[10366]|LBCH7122-10|Canada|British Columbia|658[0n]|BOLD:ABZ7094
Lacinipolia acutipennis|[10367]|LBCH7001-10|Canada|British Columbia|658[0n]|BOLD:ABZ7094
Lacinipolia acutipennis|[10368]|LBCH6456-10|Canada|British Columbia|658[0n]|BOLD:ABZ7094
Lacinipolia acutipennis|[10369]|LBCH6381-10|Canada|British Columbia|658[0n]|BOLD:ABZ7094
Lacinipolia acutipennis|[10370]|LBCG3324-09|Canada|British Columbia|658[0n]|BOLD:ABZ7094
Lacinipolia acutipennis|[10371]|LOWCE552-06|Canada|British Columbia|610[0n]|BOLD:ABZ7094
Lacinipolia acutipennis|[10372]|LBCH6724-10|Canada|British Columbia|640[0n]|BOLD:ABZ7094
Lacinipolia acutipennis|[10373]|LBCH7674-10|Canada|British Columbia|658[0n]|BOLD:ABZ7094
Lacinipolia acutipennis|[10374]|LBCH7810-10|Canada|British Columbia|658[0n]|BOLD:ABZ7094
Lacinipolia acutipennis|[10375]|LBCH7884-10|Canada|British Columbia|658[0n]|BOLD:ABZ7094
Lacinipolia acutipennis|[10376]|CNCLB253-14|United States|California|658[0n]|BOLD:ACM4566
Lacinipolia acutipennis|[10377]|CNCLB254-14|United States|California|658[0n]|BOLD:ACM4566
Lacinipolia vicina|[10378]|CNCLB2500-14|United States|North Carolina|658[0n]|BOLD:ACL6183
Lacinipolia vicina|[10379]|CNCLB2954-14|United States|North Carolina|658[0n]|BOLD:ACL6183
Ulolonche culea|[10380]|BBLCU193-09|United States|Michigan|658[0n]|BOLD:AAE0713
Ulolonche culea|[10381]|BBLCU243-09|United States|Michigan|658[0n]|BOLD:AAE0713
Ulolonche culea|[10382]|BBLCU214-09|United States|Michigan|658[0n]|BOLD:AAE0713
Ulolonche culea|[10383]|BBLCU126-09|United States|Michigan|658[0n]|BOLD:AAE0713
Ulolonche culea|[10384]|LOCT051-05|United States|Connecticut|633[0n]|BOLD:AAE0713
Ulolonche culea|[10385]|BBLSX953-09|United States|Oklahoma|644[0n]|BOLD:AAE0713
Ulolonche culea|[10386]|BBLCU246-09|United States|Michigan|614[0n]|BOLD:AAE0713
Ulolonche culea|[10387]|LGSMC419-05|United States|Tennessee|658[0n]|BOLD:AAE0713
Ulolonche culea|[10388]|LOCT052-05|United States|Connecticut|658[0n]|BOLD:AAE0713
Ulolonche culea|[10389]|LSEU347-06|United States|Georgia|658[0n]|BOLD:AAE0713
Ulolonche culea|[10390]|LNCB951-10|United States|North Carolina|658[0n]|BOLD:AAE0713
Ulolonche culea|[10391]|LSEU346-06|United States|Georgia|658[0n]|BOLD:AAE0713
Ulolonche culea|[10392]|LNCC161-10|United States|North Carolina|658[0n]|BOLD:AAE0713
Ulolonche consopita|[10393]|RDNMG263-08|United States|Texas|658[0n]|BOLD:AAH7583
Ulolonche consopita|[10394]|USLEP567-10|United States|Colorado|658[0n]|BOLD:AAH7583

Ulolonche culea|[10392]|LNCC161-10|United States|North Carolina|658[0n]|BOLD:AAE0713
Ulolonche consopita|[10393]|RDNMG263-08|United States|Texas|658[0n]|BOLD:AAH7583
Ulolonche consopita|[10394]|USLEP567-10|United States|Colorado|658[0n]|BOLD:AAH7583
Ulolonche consopita|[10395]|RDNMJ167-10|United States|Colorado|658[0n]|BOLD:ABY8831
Ulolonche consopita|[10396]|RDNMJ168-10|United States|Colorado|658[0n]|BOLD:ABY8831
Ulolonche consopita|[10397]|RDNME962-08|United States|New Mexico|658[0n]|BOLD:ABY8831
Ulolonche consopita|[10398]|IAWLB246-11|United States|Arizona|658[0n]|BOLD:ABY8831
Ulolonche consopita|[10399]|IAWLB247-11|United States|Arizona|658[0n]|BOLD:ABY8831
Ulolonche consopita|[10400]|CMAZA1018-12|United States|Arizona|658[0n]|BOLD:ABY8831
Ulolonche modesta|[10401]|LSEU224-06|United States|Georgia|658[0n]|BOLD:ACE3249
Ulolonche modesta|[10402]|LSEU225-06|United States|Georgia|658[0n]|BOLD:ACE3249
Ulolonche modesta|[10403]|LOCT091-05|United States|Connecticut|658[0n]|BOLD:AAD9680
Ulolonche modesta|[10404]|RDLQ682-07|Canada|Quebec|631[0n]|BOLD:AAD9680
Ulolonche modesta|[10405]|LPSO552-08|Canada|Ontario|657[0n]|BOLD:AAD9680
Ulolonche modesta|[10406]|LNCC144-10|United States|North Carolina|658[0n]|BOLD:AAD9680
Ulolonche modesta|[10407]|LNCC145-10|United States|North Carolina|658[0n]|BOLD:AAD9680
Ulolonche modesta|[10408]|LNCC143-10|United States|North Carolina|658[0n]|BOLD:AAD9680
Ulolonche modesta|[10409]|LNCC142-10|United States|North Carolina|658[0n]|BOLD:AAD9680
Ulolonche modesta|[10410]|LNCC146-10|United States|North Carolina|658[0n]|BOLD:AAD9680
Ulolonche modesta|[10411]|LNCC147-10|United States|North Carolina|658[0n]|BOLD:AAD9680
Ulolonche orbiculata|[10412]|RDNME966-08|United States|New Mexico|658[0n]|BOLD:AAD7571
Ulolonche orbiculata|[10413]|RDNMF369-08|Canada|Alberta|658[0n]|BOLD:AAD7571
Ulolonche orbiculata|[10414]|RDNMF371-08|Canada|Alberta|658[0n]|BOLD:AAD7571
Ulolonche orbiculata|[10415]|RDNMF372-08|Canada|Alberta|658[0n]|BOLD:AAD7571
Ulolonche orbiculata|[10416]|RDNMF373-08|Canada|Alberta|658[0n]|BOLD:AAD7571
Ulolonche orbiculata|[10417]|RDNMF370-08|Canada|Alberta|609[0n]|BOLD:AAD7571
Ulolonche orbiculata|[10418]|BBLSY471-09|United States|Arizona|658[0n]|BOLD:AAD7571
Ulolonche orbiculata|[10419]|BBLSW385-09|United States|Arizona|658[0n]|BOLD:AAD7571
Ulolonche orbiculata|[10420]|BBLSY469-09|United States|Arizona|658[0n]|BOLD:AAD7571
Ulolonche orbiculata|[10421]|BBLWU010-09|United States|Colorado|658[0n]|BOLD:AAD7571
Ulolonche orbiculata|[10422]|RDNMJ169-10|United States|New Mexico|658[0n]|BOLD:AAD7571
Ulolonche orbiculata|[10423]|RDNMJ170-10|United States|New Mexico|658[0n]|BOLD:AAD7571
Ulolonche orbiculata|[10424]|CMAZA969-12|United States|Arizona|658[0n]|BOLD:AAD7571
Ulolonche niveiguttata|[10425]|GMLC247-11|United States|California|658[4n]|BOLD:ABY4723
Ulolonche niveiguttata|[10426]|CGLCA146-10|United States|California|658[0n]|BOLD:ABY5071
Ulolonche niveiguttata|[10427]|CGLCA158-10|United States|California|658[0n]|BOLD:ABY5071
Ulolonche niveiguttata|[10428]|LOCBB764-06|United States|California|658[11n]|
Ulolonche niveiguttata|[10429]|LOCBB760-06|United States|California|658[0n]|BOLD:AAF0383
Ulolonche niveiguttata|[10430]|LOCB449-06|United States|California|658[0n]|BOLD:AAF0383
Ulolonche niveiguttata|[10431]|LOCBB765-06|United States|California|613[0n]|BOLD:AAF0383
Ulolonche niveiguttata|[10432]|RDNML208-13|United States|California|658[0n]|BOLD:AAF0383
Ulolonche fasciata|[10433]|RDNME1006-08|United States|New Mexico|658[0n]|BOLD:AAJ7861
Ulolonche fasciata|[10434]|RDNMJ159-10|United States|Kansas|658[0n]|BOLD:AAJ7861
Ulolonche fasciata|[10435]|RDNMJ160-10|United States|Kansas|658[0n]|BOLD:AAJ7861
Ulolonche fasciata|[10436]|RDNMJ165-10|United States|Utah|658[0n]|BOLD:AAJ7861
Ulolonche fasciata|[10437]|RDNMJ166-10|United States|New Mexico|658[0n]|BOLD:AAJ7861
Ulolonche dilecta|[10438]|LOCBC319-06|United States|California|658[0n]|BOLD:AAC5238
Ulolonche dilecta|[10439]|LOCBD263-06|United States|California|658[0n]|BOLD:AAC5238
Ulolonche dilecta|[10440]|LOCBD212-06|United States|California|658[0n]|BOLD:AAC5238
Ulolonche dilecta|[10441]|LOCBC920-06|United States|California|658[0n]|BOLD:AAC5238
Ulolonche dilecta|[10442]|RDNMD429-06|United States|California|658[0n]|BOLD:AAC5238
Ulolonche dilecta|[10443]|LOCBC321-06|United States|California|658[0n]|BOLD:AAC5238
Ulolonche dilecta|[10444]|LOCBC320-06|United States|California|658[0n]|BOLD:AAC5238
Ulolonche dilecta|[10445]|LOCBC318-06|United States|California|658[0n]|BOLD:AAC5238
Ulolonche dilecta|[10446]|LOCBD278-06|United States|California|658[0n]|BOLD:AAC5238
Ulolonche dilecta|[10447]|LOCBD406-06|United States|California|658[0n]|BOLD:AAC5238
Ulolonche dilecta|[10448]|LOCBD422-06|United States|California|658[0n]|BOLD:AAC5238
Ulolonche dilecta|[10449]|LOCBD544-06|United States|California|658[0n]|BOLD:AAC5238
Ulolonche dilecta|[10450]|RDNMD829-07|United States|California|658[0n]|BOLD:AAC5238
Ulolonche dilecta|[10451]|RDNMD915-07|United States|California|655[0n]|BOLD:AAC5238
Ulolonche dilecta|[10452]|RDNMD916-07|United States|California|598[0n]|BOLD:AAC5238
Ulolonche sp.|[10453]|RDNMJ161-10|United States|Utah|658[0n]|BOLD:ABZ0723
Ulolonche sp.|[10454]|RDNMD917-07|United States|Arizona|655[0n]|BOLD:ABZ0723
Ulolonche sp.|[10455]|RDNMD644-06|United States|New Mexico|658[0n]|BOLD:ABZ0723
Ulolonche sp.|[10456]|RDNMD643-06|United States|New Mexico|658[0n]|BOLD:ABZ0723
Ulolonche sp.|[10457]|RDNMD642-06|United States|New Mexico|658[0n]|BOLD:ABZ0723
Ulolonche sp.|[10458]|RDNMD920-07|United States|Arizona|613[0n]|BOLD:ABZ0723
Ulolonche sp.|[10459]|CMAZA338-10|United States|Arizona|658[0n]|BOLD:ABZ0723
Ulolonche sp.|[10460]|RDNMJ162-10|United States|Utah|658[0n]|BOLD:ABZ0723
Ulolonche sp.|[10461]|IAWLB251-11|United States|Arizona|658[0n]|BOLD:ABZ0723
Ulolonche sp.|[10462]|CMAZA1048-12|United States|Arizona|658[0n]|BOLD:ABZ0723
Ulolonche sp.|[10463]|CMAZA1052-12|United States|Arizona|658[0n]|BOLD:ABZ0723
Ulolonche sp.|[10464]|RDNMJ163-10|United States|New Mexico|658[0n]|BOLD:ABZ0725
Ulolonche sp.|[10465]|RDNMJ164-10|United States|New Mexico|658[0n]|BOLD:ABZ0725
Ulolonche disticha|[10466]|CMAZA1028-12|United States|Arizona|658[0n]|BOLD:ABZ0724
Ulolonche disticha|[10467]|RDNMD919-07|United States|Wyoming|655[0n]|BOLD:ABZ0724
Ulolonche disticha|[10468]|BBLWU153-09|United States|Colorado|658[0n]|BOLD:ABZ0724
Ulolonche disticha|[10469]|RDNMD914-07|Canada|Alberta|655[0n]|BOLD:ABZ0724
Ulolonche disticha|[10470]|RDNMD918-07|United States|Wyoming|655[0n]|BOLD:ABZ0724
Ulolonche disticha|[10471]|BBLWU195-09|United States|Colorado|658[0n]|BOLD:ABZ0724
Ulolonche disticha|[10472]|BBLWU196-09|United States|Colorado|658[0n]|BOLD:ABZ0724
Ulolonche disticha|[10473]|BBLWU197-09|United States|Colorado|658[0n]|BOLD:ABZ0724
Ulolonche disticha|[10474]|CMAZA797-10|United States|Arizona|658[0n]|BOLD:ABZ0724
Ulolonche disticha|[10475]|RDNMD639-06|United States|New Mexico|658[0n]|BOLD:ABZ0724
Ulolonche disticha|[10476]|RDNMD640-06|United States|New Mexico|632[0n]|BOLD:ABZ0724
Ulolonche disticha|[10477]|RDNMD641-06|United States|New Mexico|658[0n]|BOLD:ABZ0724
Ulolonche disticha|[10478]|IAWLB252-11|United States|Arizona|658[0n]|BOLD:ABZ0724
Ulolonche disticha|[10479]|RDNMJ818-11|United States|Arizona|658[0n]|BOLD:ABZ0724
Ulolonche disticha|[10480]|CNCLB2492-14|United States|Arizona|658[0n]|BOLD:ABZ0724
Homorthodes flosca|[10481]|RDNMH059-09|United States|Arizona|658[0n]|BOLD:AAB4374
Homorthodes flosca|[10482]|RDNMH060-09|United States|Arizona|658[0n]|BOLD:AAB4374
Homorthodes flosca|[10483]|RDNMJ652-11|United States|Arizona|658[0n]|BOLD:AAB4374
Homorthodes flosca|[10484]|RDNMJ779-11|United States|Arizona|658[0n]|BOLD:AAB4374
Trichofeltia circumdata|[10485]|RDNMD893-07|United States|Arizona|651[0n]|BOLD:AAH8083
Trichofeltia circumdata|[10486]|RDNMD894-07|United States|Arizona|655[0n]|BOLD:AAH8083
Trichofeltia circumdata|[10487]|IAWLB545-11|United States|Arizona|658[0n]|BOLD:AAH8083
Neleucania patricia|[10488]|NAMUM051-08|United States|Arizona|657[0n]|BOLD:AAX6069
Neleucania patricia|[10489]|LNAUT857-14|United States|New Mexico|658[0n]|BOLD:AAX6069
Neleucania patricia|[10490]|LNAUT859-14|United States|New Mexico|658[0n]|BOLD:AAX6069
Neleucania patricia|[10491]|LNAUT858-14|United States|New Mexico|658[0n]|BOLD:AAX6069
Neleucania patricia|[10492]|LNAUT860-14|United States|New Mexico|658[0n]|BOLD:AAX6069
Neleucania patricia|[10493]|LNAUT861-14|United States|New Mexico|658[0n]|BOLD:AAX6069
Neleucania praegracilis|[10494]|RDNMF229-08|United States|Wyoming|658[0n]|BOLD:AAE6946

Neleucania patricia|[10492]|LNAUT860-14|United States|New Mexico|658[0n]|BOLD:AAX6069
Neleucania patricia|[10493]|LNAUT861-14|United States|New Mexico|658[0n]|BOLD:AAX6069
Neleucania praegracilis|[10494]|RDNMF229-08|United States|Wyoming|658[0n]|BOLD:AAE6946
Neleucania praegracilis|[10495]|RDNMF230-08|United States|Wyoming|658[0n]|BOLD:AAE6946
Neleucania praegracilis|[10496]|RDNMF231-08|United States|Wyoming|658[0n]|BOLD:AAE6946
Neleucania praegracilis|[10497]|RDNMG265-08|United States|Texas|658[0n]|BOLD:AAE6946
Zosteropoda hirtipes|[10498]|LPABB506-08|Canada|Alberta|658[0n]|BOLD:AAB4289
Zosteropoda hirtipes|[10499]|LALPA286-10|Canada|British Columbia|658[0n]|BOLD:AAB4289
Zosteropoda hirtipes|[10500]|RWWA127-09|United States|Washington|658[0n]|BOLD:AAB4289
Zosteropoda hirtipes|[10501]|RWWA322-09|United States|Washington|658[0n]|BOLD:AAB4289
Zosteropoda hirtipes|[10502]|RWWA893-09|United States|Washington|658[0n]|BOLD:AAB4289
Zosteropoda hirtipes|[10503]|LBCH5516-10|Canada|British Columbia|658[0n]|BOLD:AAB4289
Zosteropoda hirtipes|[10504]|LBCH5576-10|Canada|British Columbia|658[0n]|BOLD:AAB4289
Zosteropoda hirtipes|[10505]|RWWB719-10|United States|Washington|658[0n]|BOLD:AAB4289
Zosteropoda hirtipes|[10506]|RWWB831-10|United States|Washington|658[0n]|BOLD:AAB4289
Zosteropoda hirtipes|[10507]|LALPA272-10|Canada|British Columbia|658[0n]|BOLD:AAB4289
Zosteropoda hirtipes|[10508]|LALPA315-10|Canada|British Columbia|658[0n]|BOLD:AAB4289
Zosteropoda hirtipes|[10509]|CGLCA050-10|United States|California|658[0n]|BOLD:ABX6400
Zosteropoda hirtipes|[10510]|GMLC231-11|United States|California|622[0n]|BOLD:ABX6400
Zosteropoda hirtipes|[10511]|LOCBB650-06|United States|California|658[0n]|BOLD:ABX6400
Zosteropoda hirtipes|[10512]|LOCBB866-06|United States|California|658[0n]|BOLD:ABX6400
Zosteropoda hirtipes|[10513]|NAMUM219-08|United States|California|657[0n]|BOLD:ABX6400
Zosteropoda hirtipes|[10514]|NAMUM230-08|United States|California|658[0n]|BOLD:ABX6400
Zosteropoda hirtipes|[10515]|GMLC294-11|United States|California|658[0n]|BOLD:ABX6400
Zosteropoda hirtipes|[10516]|GMLC454-11|United States|California|658[0n]|BOLD:ABX6400
Zosteropoda hirtipes|[10517]|GMLC577-11|United States|California|658[0n]|BOLD:ABX6400
Zosteropoda hirtipes|[10518]|GMLC578-11|United States|California|658[0n]|BOLD:ABX6400
Zosteropoda hirtipes|[10519]|BBLOD146-11|United States|California|658[0n]|BOLD:ABX6400
Zosteropoda hirtipes|[10520]|BBLOD312-11|United States|California|658[0n]|BOLD:ABX6400
Zosteropoda hirtipes|[10521]|BBLOD575-11|United States|California|658[0n]|BOLD:ABX6400
Zosteropoda hirtipes|[10522]|BBLOD933-11|United States|Arizona|658[0n]|BOLD:ABX6400
Zosteropoda hirtipes|[10523]|GMLC857-12|United States|California|658[0n]|BOLD:ABX6400
Zosteropoda hirtipes|[10524]|RDNML204-13|United States|California|658[0n]|BOLD:ABX6400
Zosteropoda hirtipes|[10525]|LOCBB868-06|United States|California|658[0n]|BOLD:ABX6400
Zosteropoda hirtipes|[10526]|RDNMD430-06|United States|California|658[0n]|BOLD:ABX6400
Zosteropoda hirtipes|[10527]|LOCBB646-06|United States|California|658[0n]|BOLD:ABX6400
Zosteropoda hirtipes|[10528]|LOCBB581-06|United States|California|658[0n]|BOLD:ABX6400
Zosteropoda hirtipes|[10529]|LOCBB580-06|United States|California|658[0n]|BOLD:ABX6400
Zosteropoda hirtipes|[10530]|LOCBB272-06|United States|California|658[0n]|BOLD:ABX6400
Zosteropoda hirtipes|[10531]|LOCBB271-06|United States|California|658[0n]|BOLD:ABX6400
Zosteropoda hirtipes|[10532]|LOCBB270-06|United States|California|658[0n]|BOLD:ABX6400
Zosteropoda hirtipes|[10533]|LOCBB269-06|United States|California|658[0n]|BOLD:ABX6400
Zosteropoda hirtipes|[10534]|LOCB526-06|United States|California|658[0n]|BOLD:ABX6400
Zosteropoda hirtipes|[10535]|LOCB487-06|United States|California|658[0n]|BOLD:ABX6400
Zosteropoda hirtipes|[10536]|LOCB486-06|United States|California|658[0n]|BOLD:ABX6400
Zosteropoda hirtipes|[10537]|LOCB452-06|United States|California|658[0n]|BOLD:ABX6400
Zosteropoda hirtipes|[10538]|LOCB396-06|United States|California|658[0n]|BOLD:ABX6400
Zosteropoda hirtipes|[10539]|LOCBB762-06|United States|California|611[0n]|BOLD:ABX6400
Zosteropoda hirtipes|[10540]|GMLC234-11|United States|California|643[0n]|BOLD:ABX6400
Zosteropoda hirtipes|[10541]|BBLOD310-11|United States|California|658[0n]|BOLD:ABX6400
Zosteropoda hirtipes|[10542]|LOCBF2622-13|United States|California|658[0n]|BOLD:ABX6400
Zosteropoda clementei|[10543]|LNAUS4879-13|United States|California|658[0n]|BOLD:ABX6400
Zosteropoda clementei|[10544]|CNCLB1656-14|United States|California|658[0n]|BOLD:ABX6400
Lacinipolia cuneata|[10545]|LPVIA098-08|Canada|British Columbia|658[0n]|BOLD:AAB1084
Lacinipolia cuneata|[10546]|LPVIA293-08|Canada|British Columbia|658[0n]|BOLD:AAB1084
Lacinipolia cuneata|[10547]|LPVIA292-08|Canada|British Columbia|658[0n]|BOLD:AAB1084
Lacinipolia cuneata|[10548]|LPVIA100-08|Canada|British Columbia|658[0n]|BOLD:AAB1084
Lacinipolia cuneata|[10549]|LPVIA235-08|Canada|British Columbia|637[0n]|BOLD:AAB1084
Lacinipolia cuneata|[10550]|LPVIB929-08|Canada|British Columbia|648[0n]|BOLD:AAB1084
Lacinipolia cuneata|[10551]|LALPA186-10|Canada|British Columbia|658[0n]|BOLD:AAB1084
Lacinipolia cuneata|[10552]|LALPA227-10|Canada|British Columbia|658[0n]|BOLD:AAB1084
Lacinipolia cuneata|[10553]|LALPA290-10|Canada|British Columbia|658[0n]|BOLD:AAB1084
Lacinipolia cuneata|[10554]|LALPA293-10|Canada|British Columbia|658[0n]|BOLD:AAB1084
Lacinipolia cuneata|[10555]|LALPA321-10|Canada|British Columbia|658[0n]|BOLD:AAB1084
Lacinipolia cuneata|[10556]|LALPA423-10|Canada|British Columbia|658[0n]|BOLD:AAB1084
Lacinipolia cuneata|[10557]|LALPA1188-11|Canada|British Columbia|658[0n]|BOLD:AAB1084
Lacinipolia cuneata|[10558]|LOCBB761-06|United States|California|577[8n]|BOLD:AAB1084
Lacinipolia cuneata|[10559]|LOCBB648-06|United States|California|658[0n]|BOLD:AAB1084
Lacinipolia cuneata|[10560]|LOCB481-06|United States|California|658[0n]|BOLD:AAB1084
Lacinipolia cuneata|[10561]|LOCB772-06|United States|California|658[0n]|BOLD:AAB1084
Lacinipolia cuneata|[10562]|LOCB484-06|United States|California|658[0n]|BOLD:AAB1084
Lacinipolia cuneata|[10563]|LOCBB653-06|United States|California|658[0n]|BOLD:AAB1084
Lacinipolia cuneata|[10564]|LOCBB578-06|United States|California|658[0n]|BOLD:AAB1084
Lacinipolia cuneata|[10565]|LOCBB649-06|United States|California|658[0n]|BOLD:AAB1084
Lacinipolia cuneata|[10566]|LOCB901-06|United States|California|658[0n]|BOLD:AAB1084
Lacinipolia cuneata|[10567]|LOCBB763-06|United States|California|626[0n]|BOLD:AAB1084
Lacinipolia cuneata|[10568]|RWWB672-10|United States|Washington|658[0n]|BOLD:AAB1084
Lacinipolia cuneata|[10569]|RWWB769-10|United States|Washington|658[0n]|BOLD:AAB1084
Lacinipolia cuneata|[10570]|RWWB690-10|United States|Washington|658[0n]|BOLD:AAB1084
Lacinipolia cuneata|[10571]|RWWB664-10|United States|Washington|658[0n]|BOLD:AAB1084
Lacinipolia cuneata|[10572]|RWWA497-09|United States|Washington|658[0n]|BOLD:AAB1084
Lacinipolia cuneata|[10573]|RWWA474-09|United States|Washington|658[0n]|BOLD:AAB1084
Lacinipolia cuneata|[10574]|RWWA469-09|United States|Washington|658[0n]|BOLD:AAB1084
Lacinipolia cuneata|[10575]|RWWA423-09|United States|Washington|658[0n]|BOLD:AAB1084
Lacinipolia cuneata|[10576]|RWWA380-09|United States|Washington|658[0n]|BOLD:AAB1084
Lacinipolia cuneata|[10577]|RWWA360-09|United States|Washington|658[0n]|BOLD:AAB1084
Lacinipolia cuneata|[10578]|RWWA356-09|United States|Washington|658[0n]|BOLD:AAB1084
Lacinipolia cuneata|[10579]|RWWA331-09|United States|Washington|658[0n]|BOLD:AAB1084
Lacinipolia cuneata|[10580]|RWWA321-09|United States|Washington|658[0n]|BOLD:AAB1084
Lacinipolia cuneata|[10581]|RWWA319-09|United States|Washington|658[0n]|BOLD:AAB1084
Lacinipolia cuneata|[10582]|RWWA317-09|United States|Washington|658[0n]|BOLD:AAB1084
Lacinipolia cuneata|[10583]|RWWA308-09|United States|Washington|658[0n]|BOLD:AAB1084
Lacinipolia cuneata|[10584]|RWWA279-09|United States|Washington|658[0n]|BOLD:AAB1084
Lacinipolia cuneata|[10585]|RWWA259-09|United States|Washington|658[0n]|BOLD:AAB1084
Lacinipolia cuneata|[10586]|RWWA240-09|United States|Washington|658[0n]|BOLD:AAB1084
Lacinipolia cuneata|[10587]|RWWA229-09|United States|Washington|658[0n]|BOLD:AAB1084
Lacinipolia cuneata|[10588]|RWWA174-09|United States|Washington|658[0n]|BOLD:AAB1084
Lacinipolia cuneata|[10589]|RWWA097-09|United States|Washington|658[0n]|BOLD:AAB1084
Lacinipolia cuneata|[10590]|RDNMC399-05|United States|Oregon|658[0n]|BOLD:AAB1084
Lacinipolia cuneata|[10591]|RWWA639-09|United States|Washington|658[0n]|BOLD:AAB1084
Lacinipolia cuneata|[10592]|RWWB816-10|United States|Washington|636[0n]|BOLD:AAB1084
Lacinipolia cuneata|[10593]|RDNMC398-05|United States|Oregon|595[0n]|BOLD:AAB1084
Lacinipolia cuneata|[10594]|RWWB846-10|United States|Washington|635[0n]|BOLD:AAB1084

Lacinipolia cuneata|[10592]|RWWB816-10|United States|Washington|636[0n]|BOLD:AAB1084
Lacinipolia cuneata|[10593]|RDNMC398-05|United States|Oregon|595[0n]|BOLD:AAB1084
Lacinipolia cuneata|[10594]|RWWB846-10|United States|Washington|635[0n]|BOLD:AAB1084
Lacinipolia cuneata|[10595]|LALPA320-10|Canada|British Columbia|658[0n]|BOLD:AAB1084
Lacinipolia cuneata|[10596]|LALPA479-10|Canada|British Columbia|658[0n]|BOLD:AAB1084
Lacinipolia cuneata|[10597]|GMLC572-11|United States|California|658[0n]|BOLD:AAB1084
Lacinipolia cuneata|[10598]|RWWC244-11|United States|Washington|655[0n]|BOLD:AAB1084
Lacinipolia cuneata|[10599]|RWWC274-11|United States|Washington|658[0n]|BOLD:AAB1084
Lacinipolia cuneata|[10600]|LALPA1104-11|Canada|British Columbia|658[0n]|BOLD:AAB1084
Lacinipolia cuneata|[10601]|RWWC377-11|United States|Washington|658[0n]|BOLD:AAB1084
Lacinipolia patalis|[10602]|RWWA264-09|United States|Washington|658[0n]|BOLD:AAB1769
Lacinipolia patalis|[10603]|RWWA268-09|United States|Washington|658[0n]|BOLD:AAB1769
Lacinipolia patalis|[10604]|RWWA358-09|United States|Washington|658[0n]|BOLD:AAB1769
Lacinipolia patalis|[10605]|RWWB668-10|United States|Washington|658[0n]|BOLD:AAB1769
Lacinipolia patalis|[10606]|RWWA139-09|United States|Washington|658[0n]|BOLD:AAB1769
Lacinipolia patalis|[10607]|RWWA211-09|United States|Washington|658[0n]|BOLD:AAB1769
Lacinipolia patalis|[10608]|RWWB673-10|United States|Washington|658[0n]|BOLD:AAB1769
Lacinipolia patalis|[10609]|LHLEP267-06|Canada|British Columbia|658[0n]|BOLD:AAB1769
Lacinipolia patalis|[10610]|LOCBC171-06|United States|California|658[0n]|BOLD:AAB1769
Lacinipolia patalis|[10611]|GMLC820-12|United States|California|658[0n]|BOLD:AAB1769
Lacinipolia patalis|[10612]|RWWA161-09|United States|Washington|658[0n]|BOLD:AAB1769
Lacinipolia patalis|[10613]|RWWA175-09|United States|Washington|658[0n]|BOLD:AAB1769
Lacinipolia patalis|[10614]|GMLC232-11|United States|California|658[0n]|BOLD:AAB1769
Lacinipolia patalis|[10615]|GMLC890-12|United States|California|622[0n]|BOLD:AAB1769
Lacinipolia patalis|[10616]|GMLC475-11|United States|California|658[0n]|BOLD:AAB1769
Lacinipolia patalis|[10617]|GMLC513-11|United States|California|658[0n]|BOLD:AAB1769
Lacinipolia patalis|[10618]|GMLC562-11|United States|California|658[0n]|BOLD:AAB1769
Lacinipolia patalis|[10619]|GMLC650-11|United States|California|658[0n]|BOLD:AAB1769
Lacinipolia patalis|[10620]|GMLC652-11|United States|California|658[0n]|BOLD:AAB1769
Lacinipolia patalis|[10621]|GMLC684-11|United States|California|658[0n]|BOLD:AAB1769
Lacinipolia patalis|[10622]|GMLC707-11|United States|California|658[0n]|BOLD:AAB1769
Lacinipolia patalis|[10623]|GMLC715-11|United States|California|658[0n]|BOLD:AAB1769
Lacinipolia patalis|[10624]|LALPA117-10|Canada|British Columbia|658[0n]|BOLD:AAB1769
Lacinipolia patalis|[10625]|RWWB757-10|United States|Washington|658[0n]|BOLD:AAB1769
Lacinipolia patalis|[10626]|RWWB776-10|United States|Washington|658[0n]|BOLD:AAB1769
Lacinipolia patalis|[10627]|RWWB808-10|United States|Washington|658[0n]|BOLD:AAB1769
Lacinipolia patalis|[10628]|LALPA204-10|Canada|British Columbia|658[0n]|BOLD:AAB1769
Lacinipolia patalis|[10629]|LALPA230-10|Canada|British Columbia|658[0n]|BOLD:AAB1769
Lacinipolia patalis|[10630]|RWWC209-11|United States|Washington|658[0n]|BOLD:AAB1769
Lacinipolia patalis|[10631]|RWWC231-11|United States|Washington|658[0n]|BOLD:AAB1769
Lacinipolia patalis|[10632]|BBLPB407-10|Canada|British Columbia|658[0n]|BOLD:AAB1769
Lacinipolia patalis|[10633]|BBLPB465-10|Canada|British Columbia|658[0n]|BOLD:AAB1769
Lacinipolia patalis|[10634]|RWWC306-11|United States|Washington|658[0n]|BOLD:AAB1769
Lacinipolia patalis|[10635]|GMLC725-12|United States|California|658[0n]|BOLD:AAB1769
Lacinipolia patalis|[10636]|GMLC880-12|United States|California|658[0n]|BOLD:AAB1769
Lacinipolia patalis|[10637]|GMLC895-12|United States|California|658[0n]|BOLD:AAB1769
Lacinipolia patalis|[10638]|GMLC918-12|United States|California|658[0n]|BOLD:AAB1769
Lacinipolia patalis|[10639]|GMLC942-12|United States|California|658[0n]|BOLD:AAB1769
Lacinipolia patalis|[10640]|GMLC956-12|United States|California|658[0n]|BOLD:AAB1769
Lacinipolia patalis|[10641]|GMLC994-12|United States|California|658[0n]|BOLD:AAB1769
Lacinipolia patalis|[10642]|GMLC734-12|United States|California|658[0n]|BOLD:AAB1769
Lacinipolia patalis|[10643]|GMLC879-12|United States|California|658[0n]|BOLD:AAB1769
Lacinipolia patalis|[10644]|RWWA634-09|United States|Washington|658[0n]|BOLD:AAB1769
Lacinipolia patalis|[10645]|CGLCA149-10|United States|California|658[0n]|BOLD:AAB1769
Lacinipolia patalis|[10646]|RWWA215-09|United States|Washington|658[0n]|BOLD:AAB1769
Lacinipolia patalis|[10647]|RWWA270-09|United States|Washington|658[0n]|BOLD:AAB1769
Lacinipolia patalis|[10648]|LPVIA294-08|Canada|British Columbia|658[0n]|BOLD:AAB1769
Lacinipolia patalis|[10649]|RWWA092-09|United States|Washington|658[0n]|BOLD:AAB1769
Lacinipolia patalis|[10650]|LPVIA104-08|Canada|British Columbia|658[0n]|BOLD:AAB1769
Lacinipolia patalis|[10651]|LPVIA238-08|Canada|British Columbia|658[0n]|BOLD:AAB1769
Lacinipolia patalis|[10652]|LBCS103-07|Canada|British Columbia|658[0n]|BOLD:AAB1769
Lacinipolia patalis|[10653]|LPVIA103-08|Canada|British Columbia|658[0n]|BOLD:AAB1769
Lacinipolia patalis|[10654]|LHLEP268-06|Canada|British Columbia|658[0n]|BOLD:AAB1769
Lacinipolia patalis|[10655]|LMH008-06|Canada|British Columbia|658[0n]|BOLD:AAB1769
Lacinipolia patalis|[10656]|LOCBC249-06|United States|California|658[0n]|BOLD:AAB1769
Lacinipolia patalis|[10657]|LOCBC236-06|United States|California|658[0n]|BOLD:AAB1769
Lacinipolia patalis|[10658]|LOCBC235-06|United States|California|658[0n]|BOLD:AAB1769
Lacinipolia patalis|[10659]|LOCBC234-06|United States|California|658[0n]|BOLD:AAB1769
Lacinipolia patalis|[10660]|LOCBC172-06|United States|California|658[0n]|BOLD:AAB1769
Lacinipolia patalis|[10661]|LOCBB754-06|United States|California|656[0n]|BOLD:AAB1769
Lacinipolia patalis|[10662]|GMLC705-11|United States|California|658[0n]|BOLD:AAB1769
Lacinipolia patalis|[10663]|LOCBB753-06|United States|California|658[0n]|BOLD:AAB1769
Lacinipolia patalis|[10664]|LOCB663-06|United States|California|658[0n]|BOLD:AAB1769
Lacinipolia patalis|[10665]|LOCB528-06|United States|California|658[0n]|BOLD:AAB1769
Lacinipolia patalis|[10666]|GMLC233-11|United States|California|636[0n]|BOLD:AAB1769
Lacinipolia patalis|[10667]|LPVIA267-08|Canada|British Columbia|644[0n]|BOLD:AAB1769
Lacinipolia patalis|[10668]|RWWB735-10|United States|Washington|621[1n]|BOLD:AAB1769
Lacinipolia patalis|[10669]|RWWA062-09|United States|Washington|614[0n]|BOLD:AAB1769
Lacinipolia patalis|[10670]|GMLC244-11|United States|California|618[0n]|BOLD:AAB1769
Lacinipolia patalis|[10671]|RDNMC388-05|Canada|British Columbia|579[0n]|BOLD:AAB1769
Lacinipolia patalis|[10672]|LOCBB772-06|United States|California|577[0n]|BOLD:AAB1769
Lacinipolia patalis|[10673]|RDNMC280-05|Canada|British Columbia|602[0n]|BOLD:AAB1769
Lacinipolia patalis|[10674]|GMLC1425-12|United States|California|601[0n]|BOLD:AAB1769
Lacinipolia patalis|[10675]|GMLC1429-12|United States|California|601[0n]|BOLD:AAB1769
Hexorthodes jocosa|[10676]|RDNMG181-08|United States|New Mexico|658[0n]|BOLD:ACW8852
Hexorthodes jocosa|[10677]|RDNMG182-08|United States|New Mexico|658[0n]|BOLD:ACW8852
Hexorthodes jocosa|[10678]|RDNME628-08|United States|New Mexico|658[0n]|BOLD:AAF0579
Hexorthodes jocosa|[10679]|CNCLB266-14|United States|Arizona|658[0n]|BOLD:AAF0579
Hexorthodes serrata|[10680]|CNCLB3145-15|United States|Texas|658[0n]|BOLD:ACW9811
Hexorthodes serrata|[10681]|CNCLB3146-15|United States|Texas|658[0n]|BOLD:ACW9811
Hexorthodes serrata|[10682]|CNCLB3147-15|United States|Texas|658[0n]|BOLD:ACW9811
Miodera stigmata|[10683]|LOCB691-06|United States|California|658[0n]|BOLD:AAC7041
Miodera stigmata|[10684]|LOCB673-06|United States|California|632[0n]|BOLD:AAC7041
Miodera stigmata|[10685]|LOCB620-06|United States|California|658[0n]|BOLD:AAC7041
Miodera stigmata|[10686]|LOCB639-06|United States|California|658[0n]|BOLD:AAC7041
Miodera stigmata|[10687]|RDNMD415-06|United States|California|658[0n]|BOLD:AAC7041
Miodera stigmata|[10688]|RDNMD416-06|United States|California|658[0n]|BOLD:AAC7041
Miodera stigmata|[10689]|LOCB626-06|United States|California|658[0n]|BOLD:AAC7041
Miodera stigmata|[10690]|LOCB670-06|United States|California|658[0n]|BOLD:AAC7041
Miodera stigmata|[10691]|LOCB692-06|United States|California|658[0n]|BOLD:AAC7041
Miodera stigmata|[10692]|RDNMD417-06|United States|California|658[0n]|BOLD:AAC7041
Miodera stigmata|[10693]|LOCBF015-13|United States|California|583[0n]|BOLD:AAC7041
Polia richardsoni|[10694]|RDNME517-08|Canada|Yukon Territory|658[0n]|BOLD:AAA9583

Miodera stigmata|[10692]|RDNMD417-06|United States|California|658[0n]|BOLD:AAC7041
Miodera stigmata|[10693]|LOCBF015-13|United States|California|583[0n]|BOLD:AAC7041
Polia richardsoni|[10694]|RDNME517-08|Canada|Yukon Territory|658[0n]|BOLD:AAA9583
Polia richardsoni|[10695]|RDNME525-08|Canada|Yukon Territory|658[0n]|BOLD:AAA9583
Polia richardsoni|[10696]|RDNMF051-08|Canada|Yukon Territory|658[0n]|BOLD:AAA9583
Polia rogenhoferi|[10697]|LCHP828-07|Canada|Manitoba|658[0n]|BOLD:AAA9583
Polia rogenhoferi|[10698]|LCHP201-07|Canada|Manitoba|658[1n]|BOLD:AAA9583
Polia rogenhoferi|[10699]|LCHQ140-07|Canada|Manitoba|658[0n]|BOLD:AAA9583
Polia rogenhoferi|[10700]|LCHP202-07|Canada|Manitoba|658[0n]|BOLD:AAA9583
Polia rogenhoferi|[10701]|LCHP272-07|Canada|Manitoba|657[0n]|BOLD:AAA9583
Polia rogenhoferi|[10702]|LCHP623-07|Canada|Manitoba|658[0n]|BOLD:AAA9583
Polia rogenhoferi|[10703]|LCHQ139-07|Canada|Manitoba|658[0n]|BOLD:AAA9583
Polia rogenhoferi|[10704]|GWNC610-07|Canada|Alberta|658[0n]|BOLD:AAA9583
Polia rogenhoferi|[10705]|LCHQ888-08|Canada|Manitoba|658[0n]|BOLD:AAA9583
Polia rogenhoferi|[10706]|LCHQ889-08|Canada|Manitoba|658[0n]|BOLD:AAA9583
Polia rogenhoferi|[10707]|LCHQ044-07|Canada|Manitoba|658[0n]|BOLD:AAA9583
Polia rogenhoferi|[10708]|LCHQ045-07|Canada|Manitoba|658[0n]|BOLD:AAA9583
Polia rogenhoferi|[10709]|LCHP811-07|Canada|Manitoba|658[0n]|BOLD:AAA9583
Polia rogenhoferi|[10710]|LCHP930-07|Canada|Manitoba|658[0n]|BOLD:AAA9583
Polia rogenhoferi|[10711]|LCHP637-07|Canada|Manitoba|658[0n]|BOLD:AAA9583
Polia rogenhoferi|[10712]|LCHP705-07|Canada|Manitoba|658[0n]|BOLD:AAA9583
Polia rogenhoferi|[10713]|LCHP156-07|Canada|Manitoba|658[0n]|BOLD:AAA9583
Polia rogenhoferi|[10714]|LCHP155-07|Canada|Manitoba|658[0n]|BOLD:AAA9583
Polia rogenhoferi|[10715]|LCHP154-07|Canada|Manitoba|658[0n]|BOLD:AAA9583
Polia rogenhoferi|[10716]|LCHP153-07|Canada|Manitoba|658[0n]|BOLD:AAA9583
Polia rogenhoferi|[10717]|LCHP152-07|Canada|Manitoba|656[0n]|BOLD:AAA9583
Polia rogenhoferi|[10718]|LCHP198-07|Canada|Manitoba|632[0n]|BOLD:AAA9583
Polia rogenhoferi|[10719]|LCHP199-07|Canada|Manitoba|658[0n]|BOLD:AAA9583
Polia rogenhoferi|[10720]|LCHP200-07|Canada|Manitoba|658[0n]|BOLD:AAA9583
Polia rogenhoferi|[10721]|LCHP203-07|Canada|Manitoba|658[0n]|BOLD:AAA9583
Polia rogenhoferi|[10722]|LCHP204-07|Canada|Manitoba|658[0n]|BOLD:AAA9583
Polia rogenhoferi|[10723]|LCHP205-07|Canada|Manitoba|658[0n]|BOLD:AAA9583
Polia rogenhoferi|[10724]|LCHP232-07|Canada|Manitoba|658[0n]|BOLD:AAA9583
Polia rogenhoferi|[10725]|LCHP233-07|Canada|Manitoba|658[0n]|BOLD:AAA9583
Polia rogenhoferi|[10726]|CHLEP154-09|Canada|Manitoba|658[0n]|BOLD:AAA9583
Polia rogenhoferi|[10727]|LSEU801-06|United States|Colorado|658[0n]|BOLD:AAA9583
Polia rogenhoferi|[10728]|RDLQF037-06|Canada|Quebec|658[0n]|BOLD:AAA9583
Polia rogenhoferi|[10729]|RDLQF036-06|Canada|Quebec|658[0n]|BOLD:AAA9583
Polia rogenhoferi|[10730]|RDLQF038-06|Canada|Quebec|573[1n]|BOLD:AAA9583
Polia rogenhoferi|[10731]|GWNC608-07|Canada|Alberta|658[0n]|BOLD:AAA9583
Polia rogenhoferi|[10732]|RDNME649-08|Canada|British Columbia|658[0n]|BOLD:AAA9583
Polia rogenhoferi|[10733]|LBCG797-09|Canada|British Columbia|658[0n]|BOLD:AAA9583
Polia rogenhoferi|[10734]|RDLQB219-05|Canada|Quebec|658[0n]|BOLD:AAA9583
Polia rogenhoferi|[10735]|RDLQB218-05|Canada|Quebec|658[0n]|BOLD:AAA9583
Polia rogenhoferi|[10736]|RDLQB221-05|Canada|Quebec|658[1n]|BOLD:AAA9583
Polia rogenhoferi|[10737]|BBLPB525-10|Canada|British Columbia|658[0n]|BOLD:AAA9583
Polia propodea|[10738]|LCHP520-07|Canada|Manitoba|658[0n]|BOLD:AAA9949
Polia propodea|[10739]|LCH239-04|Canada|Manitoba|658[0n]|BOLD:AAA9949
Polia propodea|[10740]|LCH240-04|Canada|Manitoba|658[0n]|BOLD:AAA9949
Polia propodea|[10741]|GWNC609-07|Canada|Alberta|658[0n]|BOLD:AAA9949
Polia propodea|[10742]|RDNME646-08|Canada|Alberta|658[0n]|BOLD:AAA9949
Polia propodea|[10743]|RDNME672-08|Canada|Quebec|658[0n]|BOLD:AAA9949
Polia propodea|[10744]|RDNME711-08|Canada|Quebec|658[0n]|BOLD:AAA9949
Polia propodea|[10745]|LOWCC834-05|Canada|British Columbia|658[0n]|BOLD:AAA9949
Polia propodea|[10746]|LBCG952-09|Canada|British Columbia|658[2n]|BOLD:AAA9949
Polia propodea|[10747]|LOWCD247-06|Canada|British Columbia|658[2n]|BOLD:AAA9949
Polia propodea|[10748]|LOWCD260-06|Canada|British Columbia|658[0n]|BOLD:AAA9949
Polia propodea|[10749]|LBCH1432-10|Canada|British Columbia|658[0n]|BOLD:AAA9949
Polia propodea|[10750]|LBCH1496-10|Canada|British Columbia|658[0n]|BOLD:AAA9949
Polia propodea|[10751]|LBCH1164-10|Canada|British Columbia|658[0n]|BOLD:AAA9949
Polia propodea|[10752]|LBCH1237-10|Canada|British Columbia|658[0n]|BOLD:AAA9949
Polia propodea|[10753]|LBCG1866-09|Canada|British Columbia|658[0n]|BOLD:AAA9949
Polia propodea|[10754]|LBCG948-09|Canada|British Columbia|658[0n]|BOLD:AAA9949
Polia propodea|[10755]|RDNME647-08|Canada|Alberta|658[0n]|BOLD:AAA9949
Polia propodea|[10756]|GWNC615-07|Canada|Alberta|658[0n]|BOLD:AAA9949
Polia propodea|[10757]|LOWCD256-06|Canada|British Columbia|658[0n]|BOLD:AAA9949
Polia propodea|[10758]|LOWCD254-06|Canada|British Columbia|658[0n]|BOLD:AAA9949
Polia propodea|[10759]|LOWCD248-06|Canada|British Columbia|658[0n]|BOLD:AAA9949
Polia propodea|[10760]|LOWCC833-05|Canada|British Columbia|658[0n]|BOLD:AAA9949
Polia propodea|[10761]|LOWCC831-05|Canada|British Columbia|658[0n]|BOLD:AAA9949
Polia propodea|[10762]|LOWCC829-05|Canada|British Columbia|658[0n]|BOLD:AAA9949
Polia propodea|[10763]|LBCG707-09|Canada|British Columbia|658[0n]|BOLD:AAA9949
Polia propodea|[10764]|LOWCD253-06|Canada|British Columbia|654[0n]|BOLD:AAA9949
Polia propodea|[10765]|LOWCD249-06|Canada|British Columbia|658[0n]|BOLD:AAA9949
Polia propodea|[10766]|LOWCD257-06|Canada|British Columbia|658[0n]|BOLD:AAA9949
Polia propodea|[10767]|LOWCD245-06|Canada|British Columbia|546[0n]|BOLD:AAA9949
Polia propodea|[10768]|LOWCC830-05|Canada|British Columbia|593[0n]|BOLD:AAA9949
Polia propodea|[10769]|LOWCD251-06|Canada|British Columbia|608[0n]|BOLD:AAA9949
Polia propodea|[10770]|LOWCD246-06|Canada|British Columbia|599[0n]|BOLD:AAA9949
Polia propodea|[10771]|LOWCD258-06|Canada|British Columbia|591[0n]|BOLD:AAA9949
Polia propodea|[10772]|LBCG1865-09|Canada|British Columbia|621[0n]|BOLD:AAA9949
Polia propodea|[10773]|LBCH2100-10|Canada|British Columbia|642[0n]|BOLD:AAA9949
Polia propodea|[10774]|LBCH2057-10|Canada|British Columbia|658[0n]|BOLD:AAA9949
Polia propodea|[10775]|LBCH2102-10|Canada|British Columbia|658[0n]|BOLD:AAA9949
Polia propodea|[10776]|RDNMK047-11|United States|Colorado|658[0n]|BOLD:AAA9949
Polia propodea|[10777]|RDNMK046-11|United States|Colorado|658[0n]|BOLD:AAA9949
Polia propodea|[10778]|RDNMK045-11|United States|Colorado|658[0n]|BOLD:AAA9949
Polia propodea|[10779]|RDNMK044-11|United States|Colorado|658[0n]|BOLD:AAA9949
Polia propodea|[10780]|RDNMK048-11|United States|Colorado|631[0n]|BOLD:AAA9949
Polia purpurissata|[10781]|LOWCE679-06|Canada|British Columbia|631[0n]|BOLD:AAA6697
Polia nugatis|[10782]|LBCH6932-10|Canada|British Columbia|658[0n]|BOLD:AAD3359
Polia nugatis|[10783]|LBCH6881-10|Canada|British Columbia|658[0n]|BOLD:AAD3359
Polia nugatis|[10784]|LBCH6884-10|Canada|British Columbia|658[0n]|BOLD:AAD3359
Polia nugatis|[10785]|LBCH7849-10|Canada|British Columbia|658[0n]|BOLD:AAD3359
Polia nugatis|[10786]|LBCH7850-10|Canada|British Columbia|658[0n]|BOLD:AAD3359
Polia nugatis|[10787]|LBCH7852-10|Canada|British Columbia|658[0n]|BOLD:AAD3359
Polia nugatis|[10788]|LBCH6882-10|Canada|British Columbia|658[0n]|BOLD:AAD3359
Polia nugatis|[10789]|LBCH6526-10|Canada|British Columbia|658[0n]|BOLD:AAD3359
Polia nugatis|[10790]|LBCH6542-10|Canada|British Columbia|658[0n]|BOLD:AAD3359
Polia nugatis|[10791]|LBCH6885-10|Canada|British Columbia|658[0n]|BOLD:AAD3359
Polia nugatis|[10792]|LBCH6887-10|Canada|British Columbia|658[0n]|BOLD:AAD3359
Polia nugatis|[10793]|LBCH7853-10|Canada|British Columbia|658[0n]|BOLD:AAD3359
Polia nugatis|[10794]|LBCH7599-10|Canada|British Columbia|658[0n]|BOLD:AAD3359

Polia nugatis|[10792]|LBCH6887-10|Canada|British Columbia|658[0n]|BOLD:AAD3359
Polia nugatis|[10793]|LBCH7853-10|Canada|British Columbia|658[0n]|BOLD:AAD3359
Polia nugatis|[10794]|LBCH7599-10|Canada|British Columbia|658[0n]|BOLD:AAD3359
Polia nugatis|[10795]|LBCH6962-10|Canada|British Columbia|658[0n]|BOLD:AAD3359
Polia nugatis|[10796]|LBCH6907-10|Canada|British Columbia|658[0n]|BOLD:AAD3359
Polia nugatis|[10797]|LBCH6888-10|Canada|British Columbia|658[0n]|BOLD:AAD3359
Polia nugatis|[10798]|LBCH6886-10|Canada|British Columbia|658[0n]|BOLD:AAD3359
Polia nugatis|[10799]|LBCH6883-10|Canada|British Columbia|658[0n]|BOLD:AAD3359
Polia nugatis|[10800]|LBCH6880-10|Canada|British Columbia|658[0n]|BOLD:AAD3359
Polia nugatis|[10801]|LBCH6525-10|Canada|British Columbia|658[0n]|BOLD:AAD3359
Polia nugatis|[10802]|LBCH6368-10|Canada|British Columbia|658[0n]|BOLD:AAD3359
Polia nugatis|[10803]|LOWCB326-05|Canada|British Columbia|658[0n]|BOLD:AAD3359
Polia nugatis|[10804]|LOWCB323-05|Canada|British Columbia|658[0n]|BOLD:AAD3359
Polia nugatis|[10805]|LBCH6922-10|Canada|British Columbia|624[0n]|BOLD:AAD3359
Polia nugatis|[10806]|LBCH7747-10|Canada|British Columbia|646[0n]|BOLD:AAD3359
Polia nugatis|[10807]|LBCH7748-10|Canada|British Columbia|658[0n]|BOLD:AAD3359
Polia nugatis|[10808]|LBCH7750-10|Canada|British Columbia|658[0n]|BOLD:AAD3359
Polia nugatis|[10809]|LBCH7751-10|Canada|British Columbia|658[0n]|BOLD:AAD3359
Polia nugatis|[10810]|LBCH7752-10|Canada|British Columbia|658[0n]|BOLD:AAD3359
Polia nugatis|[10811]|LBCH7753-10|Canada|British Columbia|658[0n]|BOLD:AAD3359
Polia nugatis|[10812]|LBCH7754-10|Canada|British Columbia|658[0n]|BOLD:AAD3359
Polia nugatis|[10813]|LBCH7847-10|Canada|British Columbia|658[0n]|BOLD:AAD3359
Polia nugatis|[10814]|LBCH7848-10|Canada|British Columbia|658[0n]|BOLD:AAD3359
Polia nugatis|[10815]|LBCH7851-10|Canada|British Columbia|658[0n]|BOLD:AAD3359
Polia nugatis|[10816]|LBCH7854-10|Canada|British Columbia|658[0n]|BOLD:AAD3359
Polia nugatis|[10817]|IAWLB141-10|United States|California|658[0n]|BOLD:AAD3359
Polia nugatis|[10818]|RDNMB243-05|United States|Oregon|658[0n]|BOLD:AAD3359
Polia nugatis|[10819]|IAWLB142-10|United States|California|559[0n]|BOLD:AAD3359
Polia purpurissata|[10820]|LOWCE811-06|Canada|British Columbia|658[0n]|BOLD:AAA6697
Polia purpurissata|[10821]|TMNBB307-06|Canada|New Brunswick|658[0n]|BOLD:AAA6697
Polia purpurissata|[10822]|LPABC467-09|Canada|Alberta|658[0n]|BOLD:AAA6697
Polia purpurissata|[10823]|LBCG3320-09|Canada|British Columbia|658[0n]|BOLD:AAA6697
Polia purpurissata|[10824]|LOWCE684-06|Canada|British Columbia|658[0n]|BOLD:AAA6697
Polia purpurissata|[10825]|LOWCE677-06|Canada|British Columbia|658[0n]|BOLD:AAA6697
Polia purpurissata|[10826]|LOWCE675-06|Canada|British Columbia|658[0n]|BOLD:AAA6697
Polia purpurissata|[10827]|LOWCC172-05|Canada|British Columbia|658[0n]|BOLD:AAA6697
Polia purpurissata|[10828]|LOWCB328-05|Canada|British Columbia|515[0n]|BOLD:AAA6697
Polia purpurissata|[10829]|LOWCB334-05|Canada|British Columbia|558[0n]|BOLD:AAA6697
Polia purpurissata|[10830]|LOWCE674-06|Canada|British Columbia|616[0n]|BOLD:AAA6697
Polia purpurissata|[10831]|LOWCE676-06|Canada|British Columbia|596[0n]|BOLD:AAA6697
Polia purpurissata|[10832]|LBCH5952-10|Canada|British Columbia|634[0n]|BOLD:AAA6697
Polia purpurissata|[10833]|LBCH6623-10|Canada|British Columbia|658[0n]|BOLD:AAA6697
Polia purpurissata|[10834]|BBLPB665-10|Canada|British Columbia|658[0n]|BOLD:AAA6697
Polia purpurissata|[10835]|BBLPB666-10|Canada|British Columbia|658[0n]|BOLD:AAA6697
Polia purpurissata|[10836]|XAG311-05|Canada|Ontario|658[1n]|BOLD:AAA6697
Polia purpurissata|[10837]|XAG801-05|Canada|Ontario|658[0n]|BOLD:AAA6697
Polia purpurissata|[10838]|TTMNB507-06|Canada|New Brunswick|658[0n]|BOLD:AAA6697
Polia purpurissata|[10839]|RDLQ623-07|Canada|Quebec|632[0n]|BOLD:AAA6697
Polia purpurissata|[10840]|LPSK231-08|Canada|Saskatchewan|658[0n]|BOLD:AAA6697
Polia purpurissata|[10841]|LPSK448-08|Canada|Saskatchewan|658[0n]|BOLD:AAA6697
Polia purpurissata|[10842]|LPABC027-09|Canada|Alberta|658[0n]|BOLD:AAA6697
Polia purpurissata|[10843]|TMNBB308-06|Canada|New Brunswick|658[0n]|BOLD:AAA6697
Polia purpurissata|[10844]|LPMNB436-09|Canada|Manitoba|658[0n]|BOLD:AAA6697
Polia purpurissata|[10845]|LPABC114-09|Canada|Alberta|658[1n]|BOLD:AAA6697
Polia purpurissata|[10846]|LBCC828-05|Canada|British Columbia|658[0n]|BOLD:AAA6697
Polia purpurissata|[10847]|BBLPB319-10|Canada|British Columbia|658[0n]|BOLD:AAA6697
Polia purpurissata|[10848]|BBLPB320-10|Canada|British Columbia|658[0n]|BOLD:AAA6697
Polia purpurissata|[10849]|BBLPB671-10|Canada|British Columbia|658[0n]|BOLD:AAA6697
Polia nugatis|[10850]|LMDH204-11|United States|Minnesota|658[0n]|BOLD:AAA6697
Polia purpurissata|[10851]|LALPA878-11|Canada|British Columbia|658[0n]|BOLD:AAA6697
Polia purpurissata|[10852]|LALPA884-11|Canada|British Columbia|658[0n]|BOLD:AAA6697
Polia purpurissata|[10853]|LPABC927-09|Canada|Alberta|658[0n]|BOLD:AAA6697
Polia purpurissata|[10854]|LBCH4666-10|Canada|British Columbia|658[0n]|BOLD:AAA6697
Polia purpurissata|[10855]|LPABC336-09|Canada|Alberta|658[0n]|BOLD:AAA6697
Polia purpurissata|[10856]|LPABC397-09|Canada|Alberta|658[0n]|BOLD:AAA6697
Polia purpurissata|[10857]|LPABB189-08|Canada|Alberta|658[0n]|BOLD:AAA6697
Polia purpurissata|[10858]|LPABC094-09|Canada|Alberta|658[0n]|BOLD:AAA6697
Polia purpurissata|[10859]|LHLEP256-06|Canada|British Columbia|658[0n]|BOLD:AAA6697
Polia purpurissata|[10860]|LPABB040-08|Canada|Alberta|658[0n]|BOLD:AAA6697
Polia purpurissata|[10861]|LOWCE683-06|Canada|British Columbia|658[0n]|BOLD:AAA6697
Polia purpurissata|[10862]|LOWCE682-06|Canada|British Columbia|658[0n]|BOLD:AAA6697
Polia purpurissata|[10863]|LOWCE678-06|Canada|British Columbia|658[0n]|BOLD:AAA6697
Polia purpurissata|[10864]|TTMNB510-06|Canada|New Brunswick|658[0n]|BOLD:AAA6697
Polia purpurissata|[10865]|TTMNB508-06|Canada|New Brunswick|658[0n]|BOLD:AAA6697
Polia purpurissata|[10866]|TTMNB506-06|Canada|New Brunswick|658[0n]|BOLD:AAA6697
Polia purpurissata|[10867]|LOWCB331-05|Canada|British Columbia|658[0n]|BOLD:AAA6697
Polia purpurissata|[10868]|LOWCB325-05|Canada|British Columbia|658[0n]|BOLD:AAA6697
Polia purpurissata|[10869]|XAD354-04|Canada|Ontario|658[0n]|BOLD:AAA6697
Polia purpurissata|[10870]|LBCD221-05|Canada|British Columbia|657[0n]|BOLD:AAA6697
Polia nugatis|[10871]|RDMAB546-06|Canada|Alberta|622[2n]|BOLD:AAA6697
Polia nugatis|[10872]|RDNMB242-05|Canada|Alberta|594[0n]|BOLD:AAA6697
Polia purpurissata|[10873]|TTMNB509-06|Canada|New Brunswick|658[0n]|BOLD:AAA6697
Polia purpurissata|[10874]|XAH051-05|Canada|Ontario|645[0n]|BOLD:AAA6697
Polia purpurissata|[10875]|BBLPB700-10|Canada|British Columbia|632[0n]|BOLD:AAA6697
Polia purpurissata|[10876]|LBCA594-05|Canada|British Columbia|632[0n]|BOLD:AAA6697
Polia nugatis|[10877]|RDMAB545-06|Canada|Alberta|629[0n]|BOLD:AAA6697
Polia purpurissata|[10878]|LBCA592-05|Canada|British Columbia|617[0n]|BOLD:AAA6697
Polia purpurissata|[10879]|PMG151-03|Canada|Ontario|617[0n]|BOLD:AAA6697
Polia purpurissata|[10880]|LOWCB322-05|Canada|British Columbia|596[1n]|BOLD:AAA6697
Polia purpurissata|[10881]|XAD577-04|Canada|Ontario|589[0n]|BOLD:AAA6697
Polia purpurissata|[10882]|LOWCB329-05|Canada|British Columbia|557[0n]|BOLD:AAA6697
Polia purpurissata|[10883]|LOWCB332-05|Canada|British Columbia|557[0n]|BOLD:AAA6697
Polia purpurissata|[10884]|LOWCB324-05|Canada|British Columbia|594[0n]|BOLD:AAA6697
Polia purpurissata|[10885]|LOWCB327-05|Canada|British Columbia|591[0n]|BOLD:AAA6697
Polia purpurissata|[10886]|LOWCB330-05|Canada|British Columbia|582[0n]|BOLD:AAA6697
Polia purpurissata|[10887]|LOWCB333-05|Canada|British Columbia|594[0n]|BOLD:AAA6697
Polia purpurissata|[10888]|RDLQ624-07|Canada|Quebec|624[0n]|BOLD:AAA6697
Polia purpurissata|[10889]|CNWBG3135-13|Canada|Alberta|589[0n]|BOLD:AAA6697
Polia discalis|[10890]|RDNMG541-08|United States|Wyoming|658[0n]|BOLD:ACE7737
Polia discalis|[10891]|RDNMG540-08|United States|Wyoming|658[0n]|BOLD:ACE7737
Polia discalis|[10892]|RDMAB019-05|Canada|Alberta|617[0n]|BOLD:ACE7737
Polia discalis|[10893]|RDNMB245-05|Canada|Saskatchewan|599[0n]|BOLD:ACE7737
Polia discalis|[10894]|LPABC102-09|Canada|Alberta|658[0n]|BOLD:ACE7737

Polia discalis|[10892]|RDMAB019-05|Canada|Alberta|617[0n]|BOLD:ACE7737
Polia discalis|[10893]|RDNMB245-05|Canada|Saskatchewan|599[0n]|BOLD:ACE7737
Polia discalis|[10894]|LPABC102-09|Canada|Alberta|658[0n]|BOLD:ACE7737
Polia piniae|[10895]|LPABB836-09|Canada|Alberta|658[0n]|BOLD:AAA6561
Polia piniae|[10896]|BBLPB321-10|Canada|Alberta|658[0n]|BOLD:AAA6561
Polia piniae|[10897]|BBLPB323-10|Canada|Alberta|658[0n]|BOLD:AAA6561
Polia piniae|[10898]|BBLPB670-10|Canada|Alberta|658[0n]|BOLD:AAA6561
Polia piniae|[10899]|LOWCD848-06|Canada|British Columbia|576[0n]|BOLD:AAA6561
Polia piniae|[10900]|LOWCD840-06|Canada|British Columbia|658[0n]|BOLD:AAA6561
Polia piniae|[10901]|LOWCD842-06|Canada|British Columbia|577[7n]|
Polia piniae|[10902]|LPABB325-08|Canada|Alberta|658[0n]|BOLD:AAA6561
Polia piniae|[10903]|LOWCD844-06|Canada|British Columbia|658[0n]|BOLD:AAA6561
Polia piniae|[10904]|LPABB065-08|Canada|Alberta|615[0n]|BOLD:AAA6561
Polia piniae|[10905]|LPABB082-08|Canada|Alberta|658[0n]|BOLD:AAA6561
Polia piniae|[10906]|LBCD229-05|Canada|British Columbia|658[0n]|BOLD:AAA6561
Polia piniae|[10907]|LPABB084-08|Canada|Alberta|641[0n]|BOLD:AAA6561
Polia piniae|[10908]|LPABB501-08|Canada|Alberta|658[0n]|BOLD:AAA6561
Polia piniae|[10909]|LBCH5797-10|Canada|British Columbia|658[0n]|BOLD:AAA6561
Polia piniae|[10910]|LBCH6624-10|Canada|British Columbia|658[0n]|BOLD:AAA6561
Polia piniae|[10911]|LBCD235-05|Canada|British Columbia|658[0n]|BOLD:AAA6561
Polia piniae|[10912]|LBCH1931-10|Canada|British Columbia|658[0n]|BOLD:AAA6561
Polia piniae|[10913]|LBCH2085-10|Canada|British Columbia|658[0n]|BOLD:AAA6561
Polia piniae|[10914]|LBCG2841-09|Canada|British Columbia|658[0n]|BOLD:AAA6561
Polia piniae|[10915]|LBCH1520-10|Canada|British Columbia|658[0n]|BOLD:AAA6561
Polia piniae|[10916]|LBCG817-09|Canada|British Columbia|658[0n]|BOLD:AAA6561
Polia piniae|[10917]|LPABC831-09|Canada|Alberta|658[0n]|BOLD:AAA6561
Polia piniae|[10918]|LPABB039-08|Canada|Alberta|658[0n]|BOLD:AAA6561
Polia piniae|[10919]|LPABB200-08|Canada|Alberta|658[0n]|BOLD:AAA6561
Polia piniae|[10920]|LPABB324-08|Canada|Alberta|658[0n]|BOLD:AAA6561
Polia piniae|[10921]|LPABB519-08|Canada|Alberta|658[0n]|BOLD:AAA6561
Polia piniae|[10922]|LBCH2244-10|Canada|British Columbia|658[0n]|BOLD:AAA6561
Polia piniae|[10923]|LBCH5417-10|Canada|British Columbia|658[0n]|BOLD:AAA6561
Polia piniae|[10924]|LBCH5691-10|Canada|British Columbia|658[0n]|BOLD:AAA6561
Polia piniae|[10925]|LBCH5798-10|Canada|British Columbia|658[0n]|BOLD:AAA6561
Polia piniae|[10926]|LBCH7365-10|Canada|British Columbia|658[0n]|BOLD:AAA6561
Polia piniae|[10927]|LALPA578-10|Canada|British Columbia|658[0n]|BOLD:AAA6561
Polia piniae|[10928]|LALPA592-10|Canada|British Columbia|658[0n]|BOLD:AAA6561
Polia piniae|[10929]|LALPA665-10|Canada|British Columbia|658[0n]|BOLD:AAA6561
Polia piniae|[10930]|LPABB025-08|Canada|Alberta|658[0n]|BOLD:AAA6561
Polia piniae|[10931]|LPABB038-08|Canada|Alberta|658[0n]|BOLD:AAA6561
Polia piniae|[10932]|LOWCD843-06|Canada|British Columbia|658[0n]|BOLD:AAA6561
Polia piniae|[10933]|RDNMG438-08|United States|California|658[0n]|BOLD:AAA6561
Polia piniae|[10934]|RDNMG439-08|United States|California|658[0n]|BOLD:AAA6561
Polia piniae|[10935]|LPABB024-08|Canada|Alberta|658[0n]|BOLD:AAA6561
Polia piniae|[10936]|LOWCD841-06|Canada|British Columbia|658[0n]|BOLD:AAA6561
Polia piniae|[10937]|LOWCD839-06|Canada|British Columbia|658[0n]|BOLD:AAA6561
Polia piniae|[10938]|RDMAB472-06|Canada|Alberta|658[0n]|BOLD:AAA6561
Polia piniae|[10939]|LBCD236-05|Canada|British Columbia|657[0n]|BOLD:AAA6561
Polia piniae|[10940]|LBCD230-05|Canada|British Columbia|658[0n]|BOLD:AAA6561
Polia piniae|[10941]|LBCD228-05|Canada|British Columbia|658[0n]|BOLD:AAA6561
Polia piniae|[10942]|LBCD227-05|Canada|British Columbia|655[0n]|BOLD:AAA6561
Polia piniae|[10943]|LBCD225-05|Canada|British Columbia|658[0n]|BOLD:AAA6561
Polia piniae|[10944]|LBCD224-05|Canada|British Columbia|657[0n]|BOLD:AAA6561
Polia piniae|[10945]|LBCA585-05|Canada|British Columbia|638[0n]|BOLD:AAA6561
Polia piniae|[10946]|LBCD226-05|Canada|British Columbia|651[0n]|BOLD:AAA6561
Polia piniae|[10947]|LBCH1564-10|Canada|British Columbia|658[0n]|BOLD:AAA6561
Polia piniae|[10948]|LBCD220-05|Canada|British Columbia|658[0n]|BOLD:AAA6561
Polia piniae|[10949]|LOWCE833-06|Canada|British Columbia|656[0n]|BOLD:AAA6561
Polia piniae|[10950]|LBCB889-05|Canada|British Columbia|615[1n]|BOLD:AAA6561
Polia piniae|[10951]|LBCA593-05|Canada|British Columbia|650[0n]|BOLD:AAA6561
Polia piniae|[10952]|LPABB187-08|Canada|Alberta|648[0n]|BOLD:AAA6561
Polia piniae|[10953]|LBCD231-05|Canada|British Columbia|638[0n]|BOLD:AAA6561
Polia piniae|[10954]|LBCC643-05|Canada|British Columbia|632[0n]|BOLD:AAA6561
Polia piniae|[10955]|LOWCB716-05|Canada|British Columbia|587[0n]|BOLD:AAA6561
Polia piniae|[10956]|LOWCB717-05|Canada|British Columbia|587[0n]|BOLD:AAA6561
Polia piniae|[10957]|LOWCD847-06|Canada|British Columbia|583[0n]|BOLD:AAA6561
Polia piniae|[10958]|LPABB085-08|Canada|Alberta|636[0n]|BOLD:AAA6561
Polia piniae|[10959]|LOWCB714-05|Canada|British Columbia|586[0n]|BOLD:AAA6561
Polia piniae|[10960]|LOWCD838-06|Canada|British Columbia|600[0n]|BOLD:AAA6561
Polia piniae|[10961]|RDNMB244-05|Canada|British Columbia|595[0n]|BOLD:AAA6561
Polia piniae|[10962]|LOWCD846-06|Canada|British Columbia|596[0n]|BOLD:AAA6561
Polia piniae|[10963]|LOWCD849-06|Canada|British Columbia|596[0n]|BOLD:AAA6561
Polia piniae|[10964]|LPABC408-09|Canada|Alberta|634[0n]|BOLD:AAA6561
Polia piniae|[10965]|LPABC959-09|Canada|Alberta|560[0n]|BOLD:AAA6561
Polia piniae|[10966]|IAWLB139-10|United States|California|624[0n]|BOLD:AAA6561
Polia piniae|[10967]|BBLPB672-10|Canada|British Columbia|658[0n]|BOLD:AAA6561
Polia nimbosa|[10968]|LGSM032-04|United States|North Carolina|609[0n]|BOLD:AAA4058
Polia nimbosa|[10969]|LGSM033-04|United States|North Carolina|658[0n]|BOLD:AAA4058
Polia nimbosa|[10970]|LSEU199-06|United States|North Carolina|658[0n]|BOLD:AAA4058
Polia nimbosa|[10971]|LSEU200-06|United States|North Carolina|658[0n]|BOLD:AAA4058
Polia nimbosa|[10972]|LSEU201-06|United States|North Carolina|658[0n]|BOLD:AAA4058
Polia nimbosa|[10973]|LSEU751-06|United States|Georgia|658[0n]|BOLD:AAA4058
Polia nimbosa|[10974]|LSEU752-06|United States|Georgia|658[0n]|BOLD:AAA4058
Polia nimbosa|[10975]|LGSMG842-10|United States|Tennessee|658[0n]|BOLD:AAA4058
Polia nimbosa|[10976]|LGSMG843-10|United States|Tennessee|658[0n]|BOLD:AAA4058
Polia nimbosa|[10977]|LGSMG844-10|United States|Tennessee|658[0n]|BOLD:AAA4058
Polia nimbosa|[10978]|PHMNB267-04|Canada|New Brunswick|658[0n]|BOLD:AAA4058
Polia nimbosa|[10979]|LBCA351-05|Canada|British Columbia|658[0n]|BOLD:AAA4058
Polia nimbosa|[10980]|LOWCC873-05|Canada|British Columbia|561[0n]|BOLD:AAA4058
Polia nimbosa|[10981]|LBCA356-05|Canada|British Columbia|646[0n]|BOLD:AAA4058
Polia nimbosa|[10982]|RDLQ622-07|Canada|Quebec|645[0n]|BOLD:AAA4058
Polia nimbosa|[10983]|LBCH504-10|Canada|British Columbia|658[0n]|BOLD:AAA4058
Polia nimbosa|[10984]|LBCH346-10|Canada|British Columbia|658[0n]|BOLD:AAA4058
Polia nimbosa|[10985]|LBCH219-10|Canada|British Columbia|658[0n]|BOLD:AAA4058
Polia nimbosa|[10986]|LBCH111-10|Canada|British Columbia|658[0n]|BOLD:AAA4058
Polia nimbosa|[10987]|LBCH108-10|Canada|British Columbia|658[0n]|BOLD:AAA4058
Polia nimbosa|[10988]|LBCH020-10|Canada|British Columbia|658[0n]|BOLD:AAA4058
Polia nimbosa|[10989]|LBCH019-10|Canada|British Columbia|658[0n]|BOLD:AAA4058
Polia nimbosa|[10990]|BBLPC999-09|Canada|Nova Scotia|658[0n]|BOLD:AAA4058
Polia nimbosa|[10991]|BBLPC998-09|Canada|Nova Scotia|658[0n]|BOLD:AAA4058
Polia nimbosa|[10992]|BBLPC987-09|Canada|Nova Scotia|658[0n]|BOLD:AAA4058
Polia nimbosa|[10993]|BBLPC986-09|Canada|Nova Scotia|658[0n]|BOLD:AAA4058
Polia nimbosa|[10994]|BBLEC093-09|Canada|Nova Scotia|658[0n]|BOLD:AAA4058

Polia nimbosa|[10992]|BBLPC987-09|Canada|Nova Scotia|658[0n]|BOLD:AAA4058
Polia nimbosa|[10993]|BBLPC986-09|Canada|Nova Scotia|658[0n]|BOLD:AAA4058
Polia nimbosa|[10994]|BBLEC093-09|Canada|Nova Scotia|658[0n]|BOLD:AAA4058
Polia nimbosa|[10995]|RWWA802-09|United States|Washington|658[0n]|BOLD:AAA4058
Polia nimbosa|[10996]|RWWA792-09|United States|Washington|658[0n]|BOLD:AAA4058
Polia nimbosa|[10997]|RWWA744-09|United States|Washington|658[0n]|BOLD:AAA4058
Polia nimbosa|[10998]|RWWA726-09|United States|Washington|658[0n]|BOLD:AAA4058
Polia nimbosa|[10999]|RWWA539-09|United States|Washington|658[0n]|BOLD:AAA4058
Polia nimbosa|[11000]|LPABB362-08|Canada|Alberta|658[0n]|BOLD:AAA4058
Polia nimbosa|[11001]|LPABB355-08|Canada|Alberta|658[0n]|BOLD:AAA4058
Polia nimbosa|[11002]|LPMN331-08|Canada|Manitoba|658[0n]|BOLD:AAA4058
Polia nimbosa|[11003]|LPMN123-08|Canada|Manitoba|658[0n]|BOLD:AAA4058
Polia nimbosa|[11004]|LHLEP249-06|Canada|British Columbia|658[0n]|BOLD:AAA4058
Polia nimbosa|[11005]|LOWCE852-06|Canada|British Columbia|658[0n]|BOLD:AAA4058
Polia nimbosa|[11006]|LOWCE814-06|Canada|British Columbia|658[0n]|BOLD:AAA4058
Polia nimbosa|[11007]|LOWCD255-06|Canada|British Columbia|658[0n]|BOLD:AAA4058
Polia nimbosa|[11008]|LOWCD250-06|Canada|British Columbia|658[0n]|BOLD:AAA4058
Polia nimbosa|[11009]|LOWCD180-06|Canada|British Columbia|658[0n]|BOLD:AAA4058
Polia nimbosa|[11010]|LOWCC197-05|Canada|British Columbia|658[0n]|BOLD:AAA4058
Polia nimbosa|[11011]|LOWCC196-05|Canada|British Columbia|658[0n]|BOLD:AAA4058
Polia nimbosa|[11012]|LOWCB335-05|Canada|British Columbia|658[0n]|BOLD:AAA4058
Polia nimbosa|[11013]|MNBB421-05|Canada|New Brunswick|658[0n]|BOLD:AAA4058
Polia nimbosa|[11014]|MNBB156-05|Canada|New Brunswick|658[0n]|BOLD:AAA4058
Polia nimbosa|[11015]|MNBB152-05|Canada|New Brunswick|658[0n]|BOLD:AAA4058
Polia nimbosa|[11016]|LBCA577-05|Canada|British Columbia|655[0n]|BOLD:AAA4058
Polia nimbosa|[11017]|LBCA490-05|Canada|British Columbia|658[0n]|BOLD:AAA4058
Polia nimbosa|[11018]|LBCA489-05|Canada|British Columbia|658[0n]|BOLD:AAA4058
Polia nimbosa|[11019]|LBCA488-05|Canada|British Columbia|658[0n]|BOLD:AAA4058
Polia nimbosa|[11020]|LBCA486-05|Canada|British Columbia|658[0n]|BOLD:AAA4058
Polia nimbosa|[11021]|LBCA485-05|Canada|British Columbia|658[0n]|BOLD:AAA4058
Polia nimbosa|[11022]|LBCA484-05|Canada|British Columbia|658[0n]|BOLD:AAA4058
Polia nimbosa|[11023]|LBCA482-05|Canada|British Columbia|658[0n]|BOLD:AAA4058
Polia nimbosa|[11024]|LBCA481-05|Canada|British Columbia|658[0n]|BOLD:AAA4058
Polia nimbosa|[11025]|LBCA479-05|Canada|British Columbia|658[0n]|BOLD:AAA4058
Polia nimbosa|[11026]|LBCA478-05|Canada|British Columbia|658[0n]|BOLD:AAA4058
Polia nimbosa|[11027]|LBCA477-05|Canada|British Columbia|658[0n]|BOLD:AAA4058
Polia nimbosa|[11028]|LBCA476-05|Canada|British Columbia|658[0n]|BOLD:AAA4058
Polia nimbosa|[11029]|LBCA475-05|Canada|British Columbia|658[0n]|BOLD:AAA4058
Polia nimbosa|[11030]|LBCA474-05|Canada|British Columbia|658[0n]|BOLD:AAA4058
Polia nimbosa|[11031]|LBCA473-05|Canada|British Columbia|658[0n]|BOLD:AAA4058
Polia nimbosa|[11032]|LBCA472-05|Canada|British Columbia|658[0n]|BOLD:AAA4058
Polia nimbosa|[11033]|LBCA471-05|Canada|British Columbia|658[0n]|BOLD:AAA4058
Polia nimbosa|[11034]|LBCA470-05|Canada|British Columbia|658[0n]|BOLD:AAA4058
Polia nimbosa|[11035]|LBCA469-05|Canada|British Columbia|658[0n]|BOLD:AAA4058
Polia nimbosa|[11036]|LBCA360-05|Canada|British Columbia|658[0n]|BOLD:AAA4058
Polia nimbosa|[11037]|LBCA353-05|Canada|British Columbia|658[0n]|BOLD:AAA4058
Polia nimbosa|[11038]|LBCA347-05|Canada|British Columbia|658[0n]|BOLD:AAA4058
Polia nimbosa|[11039]|LBCH649-10|Canada|British Columbia|658[0n]|BOLD:AAA4058
Polia nimbosa|[11040]|LBCH814-10|Canada|British Columbia|658[0n]|BOLD:AAA4058
Polia nimbosa|[11041]|LBCH2377-10|Canada|British Columbia|658[0n]|BOLD:AAA4058
Polia nimbosa|[11042]|LBCH3071-10|Canada|British Columbia|658[0n]|BOLD:AAA4058
Polia nimbosa|[11043]|RWWB969-10|United States|Washington|658[0n]|BOLD:AAA4058
Polia nimbosa|[11044]|BBLPB673-10|Canada|British Columbia|658[0n]|BOLD:AAA4058
Polia nimbosa|[11045]|BBLPB674-10|Canada|British Columbia|658[0n]|BOLD:AAA4058
Polia nimbosa|[11046]|LALPA913-11|Canada|British Columbia|658[0n]|BOLD:AAA4058
Polia nimbosa|[11047]|RWWC446-11|United States|Washington|658[0n]|BOLD:AAA4058
Polia nimbosa|[11048]|LBCA349-05|Canada|British Columbia|640[0n]|BOLD:AAA4058
Polia nimbosa|[11049]|RDMAB017-05|Canada|Alberta|641[0n]|BOLD:AAA4058
Polia nimbosa|[11050]|LBCA359-05|Canada|British Columbia|636[0n]|BOLD:AAA4058
Polia nimbosa|[11051]|RWWA716-09|United States|Washington|658[0n]|BOLD:AAA4058
Polia nimbosa|[11052]|RWWA756-09|United States|Washington|658[0n]|BOLD:AAA4058
Polia nimbosa|[11053]|RDLQ621-07|Canada|Quebec|594[0n]|BOLD:AAA4058
Polia nimbosa|[11054]|LBCA361-05|Canada|British Columbia|633[0n]|BOLD:AAA4058
Polia nimbosa|[11055]|LBCA354-05|Canada|British Columbia|658[0n]|BOLD:AAA4058
Polia nimbosa|[11056]|LOWCE829-06|Canada|British Columbia|603[0n]|BOLD:AAA4058
Polia nimbosa|[11057]|BBLPC252-09|Canada|Nova Scotia|614[0n]|BOLD:AAA4058
Polia nimbosa|[11058]|PHMO230-03|Canada|Ontario|639[0n]|BOLD:AAA4058
Polia nimbosa|[11059]|LBCA348-05|Canada|British Columbia|628[0n]|BOLD:AAA4058
Polia nimbosa|[11060]|LOWCC195-05|Canada|British Columbia|579[0n]|BOLD:AAA4058
Polia nimbosa|[11061]|LBCA350-05|Canada|British Columbia|615[0n]|BOLD:AAA4058
Polia nimbosa|[11062]|LBCA355-05|Canada|British Columbia|610[0n]|BOLD:AAA4058
Polia nimbosa|[11063]|LBCA358-05|Canada|British Columbia|608[0n]|BOLD:AAA4058
Polia nimbosa|[11064]|LBCA480-05|Canada|British Columbia|658[0n]|BOLD:AAA4058
Polia nimbosa|[11065]|LBCA487-05|Canada|British Columbia|658[0n]|BOLD:AAA4058
Polia nimbosa|[11066]|PHMNB228-04|Canada|New Brunswick|586[0n]|BOLD:AAA4058
Polia nimbosa|[11067]|LOWCC853-05|Canada|British Columbia|586[1n]|BOLD:AAA4058
Polia nimbosa|[11068]|LBCA357-05|Canada|British Columbia|614[0n]|BOLD:AAA4058
Polia nimbosa|[11069]|RDMAB016-05|Canada|Alberta|566[0n]|BOLD:AAA4058
Polia nimbosa|[11070]|LOWCD252-06|Canada|British Columbia|582[0n]|BOLD:AAA4058
Polia nimbosa|[11071]|CNRME5004-12|Canada|Manitoba|621[0n]|BOLD:AAA4058
Polia imbrifera|[11072]|XAK460-06|Canada|Ontario|658[0n]|BOLD:AAB3022
Polia imbrifera|[11073]|XAK457-06|Canada|Ontario|658[0n]|BOLD:AAB3022
Polia imbrifera|[11074]|RDLQ627-07|Canada|Quebec|631[0n]|BOLD:AAB3022
Polia imbrifera|[11075]|RDLQ625-07|Canada|Quebec|658[0n]|BOLD:AAB3022
Polia imbrifera|[11076]|LSEU203-06|United States|North Carolina|658[0n]|BOLD:AAB3022
Polia imbrifera|[11077]|LSEU202-06|United States|North Carolina|658[0n]|BOLD:AAB3022
Polia imbrifera|[11078]|LGSM034-04|United States|North Carolina|658[0n]|BOLD:AAB3022
Polia imbrifera|[11079]|LGSM035-04|United States|North Carolina|658[0n]|BOLD:AAB3022
Polia imbrifera|[11080]|BBLPE217-09|Canada|Newfoundland and Labrador|634[0n]|BOLD:AAB3022
Polia imbrifera|[11081]|RDLQB220-05|Canada|Quebec|658[0n]|BOLD:AAB3022
Polia imbrifera|[11082]|BLTIB567-08|Canada|Ontario|658[0n]|BOLD:AAB3022
Polia imbrifera|[11083]|BBLEC091-09|Canada|Nova Scotia|658[0n]|BOLD:AAB3022
Polia imbrifera|[11084]|BBLPC992-09|Canada|Nova Scotia|658[0n]|BOLD:AAB3022
Polia imbrifera|[11085]|LPMN915-08|Canada|Alberta|658[0n]|BOLD:AAB3022
Polia imbrifera|[11086]|RDLQ626-07|Canada|Quebec|658[0n]|BOLD:AAB3022
Polia imbrifera|[11087]|RDLQG126-06|Canada|Quebec|658[0n]|BOLD:AAB3022
Polia imbrifera|[11088]|XAK459-06|Canada|Ontario|658[0n]|BOLD:AAB3022
Polia imbrifera|[11089]|XAK458-06|Canada|Ontario|658[0n]|BOLD:AAB3022
Polia imbrifera|[11090]|XAK455-06|Canada|Ontario|658[0n]|BOLD:AAB3022
Polia imbrifera|[11091]|XAK040-06|Canada|Ontario|658[0n]|BOLD:AAB3022
Polia imbrifera|[11092]|TMNBB306-06|Canada|New Brunswick|658[0n]|BOLD:AAB3022
Polia imbrifera|[11093]|TTMNB393-06|Canada|New Brunswick|658[0n]|BOLD:AAB3022
Polia imbrifera|[11094]|RDLQB217-05|Canada|Quebec|658[0n]|BOLD:AAB3022

Polia imbrifera|[11092]|TMNBB306-06|Canada|New Brunswick|658[0n]|BOLD:AAB3022
Polia imbrifera|[11093]|TTMNB393-06|Canada|New Brunswick|658[0n]|BOLD:AAB3022
Polia imbrifera|[11094]|RDLQB217-05|Canada|Quebec|658[0n]|BOLD:AAB3022
Polia imbrifera|[11095]|LBCA582-05|Canada|British Columbia|655[0n]|BOLD:AAB3022
Polia imbrifera|[11096]|XAC709-04|Canada|Ontario|658[0n]|BOLD:AAB3022
Polia imbrifera|[11097]|CNEID3343-12|Canada|Alberta|641[0n]|BOLD:AAB3022
Polia imbrifera|[11098]|CNEID3345-12|Canada|Alberta|638[0n]|BOLD:AAB3022
Polia imbrifera|[11099]|CNEID3342-12|Canada|Alberta|638[0n]|BOLD:AAB3022
Polia imbrifera|[11100]|CNEID3334-12|Canada|Alberta|638[0n]|BOLD:AAB3022
Polia imbrifera|[11101]|CNEID3332-12|Canada|Alberta|637[0n]|BOLD:AAB3022
Polia imbrifera|[11102]|CNEID3328-12|Canada|Alberta|638[0n]|BOLD:AAB3022
Polia imbrifera|[11103]|CNEID3317-12|Canada|Alberta|638[0n]|BOLD:AAB3022
Polia imbrifera|[11104]|CNEID3315-12|Canada|Alberta|638[0n]|BOLD:AAB3022
Polia imbrifera|[11105]|CNEID3316-12|Canada|Alberta|634[0n]|BOLD:AAB3022
Polia imbrifera|[11106]|CNEII009-13|Canada|Alberta|632[0n]|BOLD:AAB3022
Polia imbrifera|[11107]|CNEII008-13|Canada|Alberta|632[0n]|BOLD:AAB3022
Polia imbrifera|[11108]|CNEII007-13|Canada|Alberta|632[0n]|BOLD:AAB3022
Polia imbrifera|[11109]|CNEIH041-13|Canada|Alberta|632[0n]|BOLD:AAB3022
Polia imbrifera|[11110]|CNEIE1803-12|Canada|Alberta|627[0n]|BOLD:AAB3022
Polia imbrifera|[11111]|PMG150-03|Canada|Ontario|617[0n]|BOLD:AAB3022
Polia imbrifera|[11112]|SSEIB12889-13|Canada|Alberta|556[0n]|BOLD:AAB3022
Polia imbrifera|[11113]|CNEII642-13|Canada|Alberta|548[0n]|BOLD:AAB3022
Polia imbrifera|[11114]|HPPPE1543-13|Canada|Nova Scotia|552[0n]|BOLD:AAB3022
Polia imbrifera|[11115]|SSPAC3387-13|Canada|Saskatchewan|583[0n]|BOLD:AAB3022
Properigea seitzi|[11116]|LNAUS4744-13|United States|Texas|658[0n]|BOLD:ACJ7063
Properigea seitzi|[11117]|LNAUS4746-13|United States|Texas|658[0n]|BOLD:ACJ7063
Properigea seitzi|[11118]|LNAUS4745-13|United States|Texas|658[0n]|BOLD:ACJ7063
Properigea seitzi|[11119]|LNAUS4747-13|United States|Texas|658[0n]|BOLD:ACJ7063
Properigea nr. continens|[11120]|RDNME040-07|United States|Arizona|658[0n]|BOLD:AAF6873
Properigea nr. continens|[11121]|CMAZA529-10|United States|Arizona|658[0n]|BOLD:AAF6873
Properigea nr. continens|[11122]|CMAZA582-10|United States|Arizona|658[0n]|BOLD:AAF6873
Properigea nr. continens|[11123]|CMAZA587-10|United States|Arizona|658[0n]|BOLD:AAF6873
Properigea nr. continens|[11124]|CMAZA773-10|United States|Arizona|658[0n]|BOLD:AAF6873
Properigea nr. continens|[11125]|CMAZA775-10|United States|Arizona|658[0n]|BOLD:AAF6873
Properigea tapeta|[11126]|LOFLA741-06|United States|Florida|658[0n]|BOLD:AAF6874
Properigea|[11127]|RDNMG281-08|United States|Texas|658[0n]|BOLD:AAF6874
Properigea mephisto|[11128]|LNAUT490-14|United States|New Mexico|658[0n]|BOLD:ACN8675
Properigea continens|[11129]|RDNMK023-11|United States|Arizona|658[0n]|BOLD:AAF6872
Properigea continens|[11130]|LNAUT491-14|United States|New Mexico|658[0n]|BOLD:AAF6872
Properigea continens|[11131]|RDNMD934-07|United States|Arizona|655[0n]|BOLD:AAF6872
Properigea continens|[11132]|RDNMJ365-11|United States|Arizona|658[0n]|BOLD:AAF6872
Properigea continens|[11133]|LNAUT492-14|United States|New Mexico|658[0n]|BOLD:AAF6872
Properigea continens|[11134]|LNAUT493-14|United States|New Mexico|658[0n]|BOLD:AAF6872
Pseudobryomima n. sp. nr. distans|[11135]|NAMUM159-08|United States|California|658[0n]|BOLD:AAD7262
Pseudobryomima muscosa|[11136]|RDNMF158-08|United States|Oregon|658[0n]|BOLD:AAD7262
Pseudobryomima muscosa|[11137]|RDNMF160-08|United States|California|658[0n]|BOLD:AAD7262
Pseudobryomima muscosa|[11138]|RDNMF159-08|Canada|British Columbia|658[0n]|BOLD:AAD7262
Pseudobryomima muscosa|[11139]|RDNMF161-08|United States|California|632[0n]|BOLD:AAD7262
Pseudobryomima muscosa|[11140]|RDNMF162-08|United States|Oregon|658[0n]|BOLD:AAD7262
Pseudobryomima muscosa|[11141]|LALPA813-10|Canada|British Columbia|658[0n]|BOLD:AAD7262
Pseudobryomima distans|[11142]|RDNME909-08|United States|Arizona|658[0n]|BOLD:AAD7263
Pseudobryomima fallax|[11143]|NAMUM257-08|United States|California|658[1n]|BOLD:AAD7263
Pseudobryomima fallax|[11144]|NAMUM299-08|United States|California|658[0n]|BOLD:AAD7263
Pseudobryomima fallax|[11145]|IAWLB228-11|United States|California|658[0n]|BOLD:AAD7263
Pseudobryomima fallax|[11146]|IAWLB227-11|United States|California|658[0n]|BOLD:AAD7263
Pseudobryomima fallax|[11147]|LOCBF019-13|United States|California|601[0n]|BOLD:AAD7263
Pseudobryomima fallax|[11148]|LNAUT497-14|United States|California|614[0n]|BOLD:AAD7263
Pseudobryomima fallax|[11149]|LNAUT498-14|United States|California|658[0n]|BOLD:AAD7263
Pseudobryomima fallax|[11150]|NAMUM199-08|United States|California|646[0n]|BOLD:AAD7263
Pseudobryomima fallax|[11151]|SDRVO001-14|United States|California|588[0n]|BOLD:AAD7263
Properigea costa|[11152]|CMAZA1068-12|United States|Arizona|658[0n]|BOLD:ABW6337
Properigea costa|[11153]|RDNMF528-08|United States|Arizona|658[0n]|BOLD:AAH7705
Properigea costa|[11154]|RDNMF529-08|United States|Arizona|609[0n]|BOLD:AAH7705
Properigea sp.|[11155]|CNCLA5276-13|Canada|Ontario|609[0n]|BOLD:ACI9597
Properigea sp.|[11156]|CNCLA5277-13|Canada|Ontario|658[0n]|BOLD:ACI9597
Properigea sp.|[11157]|CNCLA7370-13|Canada|Ontario|658[0n]|BOLD:ACI9597
Properigea sp.|[11158]|LNCC1678-13|United States|North Carolina|658[0n]|BOLD:ACI9597
Properigea sp.|[11159]|LNCC1679-13|United States|North Carolina|658[0n]|BOLD:ACI9597
Properigea albimacula|[11160]|BBLOC1315-11|United States|California|614[0n]|BOLD:AAC7834
Properigea albimacula|[11161]|LBCH6018-10|Canada|British Columbia|658[0n]|BOLD:AAC7834
Properigea albimacula|[11162]|RDNMF403-08|United States|Oregon|658[0n]|BOLD:AAC7834
Properigea albimacula|[11163]|RDNMF404-08|United States|Oregon|658[0n]|BOLD:AAC7834
Properigea albimacula|[11164]|LPVIA572-08|Canada|British Columbia|658[0n]|BOLD:AAC7834
Properigea albimacula|[11165]|LBCH5932-10|Canada|British Columbia|658[0n]|BOLD:AAC7834
Properigea albimacula|[11166]|LBCH5933-10|Canada|British Columbia|658[0n]|BOLD:AAC7834
Properigea albimacula|[11167]|LBCH5934-10|Canada|British Columbia|658[0n]|BOLD:AAC7834
Properigea albimacula|[11168]|LBCH5935-10|Canada|British Columbia|658[0n]|BOLD:AAC7834
Properigea albimacula|[11169]|LBCH5936-10|Canada|British Columbia|658[0n]|BOLD:AAC7834
Properigea albimacula|[11170]|LBCH5937-10|Canada|British Columbia|658[0n]|BOLD:AAC7834
Properigea albimacula|[11171]|LBCH5938-10|Canada|British Columbia|658[0n]|BOLD:AAC7834
Properigea albimacula|[11172]|LBCH6233-10|Canada|British Columbia|658[0n]|BOLD:AAC7834
Properigea albimacula|[11173]|BBLOE1246-12|United States|California|658[0n]|BOLD:AAC7834
Properigea albimacula|[11174]|LOCBC455-06|United States|California|658[0n]|BOLD:AAC7834
Properigea albimacula|[11175]|LOCBB145-06|United States|California|658[0n]|BOLD:AAC7834
Properigea albimacula|[11176]|LOCBB144-06|United States|California|658[0n]|BOLD:AAC7834
Properigea albimacula|[11177]|LOCBB141-06|United States|California|658[0n]|BOLD:AAC7834
Properigea albimacula|[11178]|LOCBB143-06|United States|California|658[0n]|BOLD:AAC7834
Properigea albimacula|[11179]|LOCBB142-06|United States|California|623[0n]|BOLD:AAC7834
Properigea albimacula|[11180]|LOCBC057-06|United States|California|608[0n]|BOLD:AAC7834
Properigea albimacula|[11181]|LOCBC058-06|United States|California|632[0n]|BOLD:AAC7834
Properigea albimacula|[11182]|BBLOE1258-12|United States|California|648[0n]|BOLD:AAC7834
Properigea albimacula|[11183]|GMLC1042-12|United States|California|658[0n]|BOLD:AAC7834
Properigea albimacula|[11184]|GMLC1078-12|United States|California|658[0n]|BOLD:AAC7834
Properigea albimacula|[11185]|GMLC1124-12|United States|California|658[0n]|BOLD:AAC7834
Properigea albimacula|[11186]|GMLC1138-12|United States|California|658[0n]|BOLD:AAC7834
Properigea albimacula|[11187]|GMLC1176-12|United States|California|658[0n]|BOLD:AAC7834
Properigea albimacula|[11188]|GMLC1179-12|United States|California|658[0n]|BOLD:AAC7834
Properigea albimacula|[11189]|GMLC1186-12|United States|California|658[0n]|BOLD:AAC7834
Properigea albimacula|[11190]|GMLC1214-12|United States|California|658[0n]|BOLD:AAC7834
Properigea albimacula|[11191]|GMLC1215-12|United States|California|658[0n]|BOLD:AAC7834
Properigea albimacula|[11192]|LOCBF3703-14|United States|California|588[0n]|BOLD:AAC7834
Properigea albimacula|[11193]|LOCBF3709-14|United States|California|588[0n]|BOLD:AAC7834
Properigea niveirena|[11194]|RDNMD824-07|United States|Arizona|658[0n]|BOLD:AAF0135

[illegible]
Properigea albimacula|[11192]|LOCBF3703-14|United States|California|588[0n]|BOLD:AAC7834
Properigea albimacula|[11193]|LOCBF3709-14|United States|California|588[0n]|BOLD:AAC7834
Properigea niveirena|[11194]|RDNMD824-07|United States|Arizona|658[0n]|BOLD:AAF0135
Properigea niveirena|[11195]|RDNMG548-08|United States|Oregon|658[0n]|BOLD:AAF0135
Properigea niveirena|[11196]|RDNMG549-08|United States|Oregon|658[0n]|BOLD:AAF0135
Properigea niveirena|[11197]|LALPA911-11|Canada|British Columbia|658[0n]|BOLD:AAF0135
Properigea sp.|[11198]|BBLSW130-09|United States|Arizona|658[0n]|BOLD:ABZ2463
Properigea sp.|[11199]|BBLOE1484-12|United States|Arizona|658[0n]|BOLD:ABZ2463
Properigea perolivalis|[11200]|LOCBD259-06|United States|California|658[0n]|BOLD:AAE0191
Properigea perolivalis|[11201]|LOCBD068-06|United States|California|658[0n]|BOLD:AAE0191
Properigea perolivalis|[11202]|LOCBC323-06|United States|California|658[0n]|BOLD:AAE0191
Properigea perolivalis|[11203]|LOCBC325-06|United States|California|658[0n]|BOLD:AAE0191
Properigea perolivalis|[11204]|LOCBC614-06|United States|California|658[0n]|BOLD:AAE0191
Properigea perolivalis|[11205]|LOCBC430-06|United States|California|658[0n]|BOLD:AAE0191
Properigea perolivalis|[11206]|LOCBD311-06|United States|California|628[0n]|BOLD:AAE0191
Properigea perolivalis|[11207]|LOCBD412-06|United States|California|658[0n]|BOLD:AAE0191
Properigea perolivalis|[11208]|IAWLB445-11|United States|California|658[0n]|BOLD:AAE0191
Properigea suffusa|[11209]|RDNMD831-07|United States|California|658[0n]|BOLD:AAH7695
Properigea suffusa|[11210]|RDNMK109-11|United States|Arizona|658[0n]|BOLD:ACP9154
Properigea suffusa|[11211]|LNAUS4743-13|United States|Arizona|658[0n]|BOLD:ACP9154
Properigea suffusa|[11212]|NAMUM128-08|United States|California|646[0n]|BOLD:ACP9153
Properigea suffusa|[11213]|SDRVH001-14|United States|California|579[0n]|BOLD:ACP9152
Properigea suffusa|[11214]|SDRVZ008-14|United States|California|564[0n]|BOLD:ACP9152
Melanchra adjuncta|[11215]|LGSMG851-10|United States|North Carolina|658[0n]|BOLD:ACF4823
Melanchra adjuncta|[11216]|LGSMC649-05|United States|Tennessee|658[0n]|BOLD:ACF4823
Melanchra adjuncta|[11217]|LOTB309-05|United States|Tennessee|658[0n]|BOLD:ACF4823
Melanchra adjuncta|[11218]|PMG134-03|Canada|Ontario|617[0n]|BOLD:ACF4823
Melanchra adjuncta|[11219]|XAE562-04|Canada|Ontario|530[0n]|BOLD:ACF4823
Melanchra adjuncta|[11220]|PHMNB446-04|Canada|New Brunswick|658[0n]|BOLD:ACF4823
Melanchra adjuncta|[11221]|XAF548-05|Canada|Ontario|658[0n]|BOLD:ACF4823
Melanchra adjuncta|[11222]|LNCC342-10|United States|North Carolina|658[0n]|BOLD:ACF4823
Melanchra adjuncta|[11223]|BBLPC903-09|Canada|Newfoundland and Labrador|658[0n]|BOLD:AAA4742
Melanchra adjuncta|[11224]|TMG128-03|Canada|Ontario|639[0n]|BOLD:AAA4742
Melanchra adjuncta|[11225]|XAD343-04|Canada|Ontario|658[0n]|BOLD:AAA4742
Melanchra adjuncta|[11226]|XAF469-05|Canada|Ontario|654[0n]|BOLD:AAA4742
Melanchra adjuncta|[11227]|LBCA808-05|Canada|British Columbia|658[0n]|BOLD:AAA4742
Melanchra adjuncta|[11228]|XAI030-05|Canada|Ontario|658[0n]|BOLD:AAA4742
Melanchra adjuncta|[11229]|LBCB616-05|Canada|British Columbia|658[0n]|BOLD:AAA4742
Melanchra adjuncta|[11230]|LPVIA227-08|Canada|British Columbia|658[0n]|BOLD:AAA4742
Melanchra adjuncta|[11231]|LPVIA112-08|Canada|British Columbia|658[0n]|BOLD:AAA4742
Melanchra adjuncta|[11232]|LPSOC313-08|Canada|Ontario|658[0n]|BOLD:AAA4742
Melanchra adjuncta|[11233]|LPSOC122-08|Canada|Ontario|658[0n]|BOLD:AAA4742
Melanchra adjuncta|[11234]|LPSOC101-08|Canada|Ontario|658[0n]|BOLD:AAA4742
Melanchra adjuncta|[11235]|LPSOC090-08|Canada|Ontario|658[0n]|BOLD:AAA4742
Melanchra adjuncta|[11236]|KPOEC037-08|Canada|Ontario|658[0n]|BOLD:AAA4742
Melanchra adjuncta|[11237]|RDLQ630-07|Canada|Quebec|658[0n]|BOLD:AAA4742
Melanchra adjuncta|[11238]|LHLEP383-06|Canada|British Columbia|658[0n]|BOLD:AAA4742
Melanchra adjuncta|[11239]|XAJ676-06|Canada|Ontario|658[0n]|BOLD:AAA4742
Melanchra adjuncta|[11240]|XAJ675-06|Canada|Ontario|658[0n]|BOLD:AAA4742
Melanchra adjuncta|[11241]|XAJ584-06|Canada|Ontario|658[0n]|BOLD:AAA4742
Melanchra adjuncta|[11242]|XAJ350-06|Canada|Ontario|658[0n]|BOLD:AAA4742
Melanchra adjuncta|[11243]|TMNBB312-06|Canada|New Brunswick|658[0n]|BOLD:AAA4742
Melanchra adjuncta|[11244]|TMNBB311-06|Canada|New Brunswick|658[0n]|BOLD:AAA4742
Melanchra adjuncta|[11245]|LOWCE838-06|Canada|British Columbia|658[0n]|BOLD:AAA4742
Melanchra adjuncta|[11246]|TTMNB396-06|Canada|New Brunswick|658[0n]|BOLD:AAA4742
Melanchra adjuncta|[11247]|TTMNB395-06|Canada|New Brunswick|658[0n]|BOLD:AAA4742
Melanchra adjuncta|[11248]|TTMNB394-06|Canada|New Brunswick|658[0n]|BOLD:AAA4742
Melanchra adjuncta|[11249]|TTMNB085-06|Canada|New Brunswick|658[0n]|BOLD:AAA4742
Melanchra adjuncta|[11250]|XAI032-05|Canada|Ontario|658[0n]|BOLD:AAA4742
Melanchra adjuncta|[11251]|XAI031-05|Canada|Ontario|658[0n]|BOLD:AAA4742
Melanchra adjuncta|[11252]|LBCC763-05|Canada|British Columbia|658[0n]|BOLD:AAA4742
Melanchra adjuncta|[11253]|LBCC436-05|Canada|British Columbia|658[0n]|BOLD:AAA4742
Melanchra adjuncta|[11254]|LBCC435-05|Canada|British Columbia|658[0n]|BOLD:AAA4742
Melanchra adjuncta|[11255]|LBCC434-05|Canada|British Columbia|658[0n]|BOLD:AAA4742
Melanchra adjuncta|[11256]|LBCC290-05|Canada|British Columbia|658[0n]|BOLD:AAA4742
Melanchra adjuncta|[11257]|LBCB066-05|Canada|British Columbia|658[0n]|BOLD:AAA4742
Melanchra adjuncta|[11258]|LBCB065-05|Canada|British Columbia|658[0n]|BOLD:AAA4742
Melanchra adjuncta|[11259]|LBCB064-05|Canada|British Columbia|658[0n]|BOLD:AAA4742
Melanchra adjuncta|[11260]|LBCB063-05|Canada|British Columbia|658[0n]|BOLD:AAA4742
Melanchra adjuncta|[11261]|XAG970-05|Canada|Ontario|658[0n]|BOLD:AAA4742
Melanchra adjuncta|[11262]|LBCA790-05|Canada|British Columbia|658[0n]|BOLD:AAA4742
Melanchra adjuncta|[11263]|XAF803-05|Canada|Ontario|658[0n]|BOLD:AAA4742
Melanchra adjuncta|[11264]|XAF779-05|Canada|Ontario|658[0n]|BOLD:AAA4742
Melanchra adjuncta|[11265]|XAF739-05|Canada|Ontario|658[0n]|BOLD:AAA4742
Melanchra adjuncta|[11266]|PHMNB600-04|Canada|New Brunswick|658[0n]|BOLD:AAA4742
Melanchra adjuncta|[11267]|PHMNB599-04|Canada|New Brunswick|658[0n]|BOLD:AAA4742
Melanchra adjuncta|[11268]|PHMNB381-04|Canada|New Brunswick|658[0n]|BOLD:AAA4742
Melanchra adjuncta|[11269]|PHMNB322-04|Canada|New Brunswick|658[0n]|BOLD:AAA4742
Melanchra adjuncta|[11270]|XAE095-04|Canada|Ontario|658[0n]|BOLD:AAA4742
Melanchra adjuncta|[11271]|XAE094-04|Canada|Ontario|658[0n]|BOLD:AAA4742
Melanchra adjuncta|[11272]|XAC179-04|Canada|Ontario|658[0n]|BOLD:AAA4742
Melanchra adjuncta|[11273]|XAC161-04|Canada|Ontario|658[0n]|BOLD:AAA4742
Melanchra adjuncta|[11274]|XAC160-04|Canada|Ontario|658[0n]|BOLD:AAA4742
Melanchra adjuncta|[11275]|XAB604-04|Canada|Ontario|658[0n]|BOLD:AAA4742
Melanchra adjuncta|[11276]|RDLQ629-07|Canada|Quebec|644[0n]|BOLD:AAA4742
Melanchra adjuncta|[11277]|PHMNB061-03|Canada|New Brunswick|639[0n]|BOLD:AAA4742
Melanchra adjuncta|[11278]|PHMNB090-03|Canada|New Brunswick|639[0n]|BOLD:AAA4742
Melanchra adjuncta|[11279]|TMG129-03|Canada|Ontario|639[0n]|BOLD:AAA4742
Melanchra adjuncta|[11280]|XAC189-04|Canada|Ontario|658[0n]|BOLD:AAA4742
Melanchra adjuncta|[11281]|PHMNB206-04|Canada|New Brunswick|609[0n]|BOLD:AAA4742
Melanchra adjuncta|[11282]|PHMNB243-04|Canada|New Brunswick|609[0n]|BOLD:AAA4742
Melanchra adjuncta|[11283]|LBCB940-05|Canada|British Columbia|615[0n]|BOLD:AAA4742
Melanchra adjuncta|[11284]|KPOEC174-08|Canada|Ontario|647[0n]|BOLD:AAA4742
Melanchra adjuncta|[11285]|XAJ442-06|Canada|Ontario|658[0n]|BOLD:AAA4742
Melanchra adjuncta|[11286]|RDLQ628-07|Canada|Quebec|602[1n]|BOLD:AAA4742
Melanchra adjuncta|[11287]|MEC586-04|Canada|Quebec|618[0n]|BOLD:AAA4742
Melanchra adjuncta|[11288]|PHMNB538-04|Canada|New Brunswick|616[0n]|BOLD:AAA4742
Melanchra adjuncta|[11289]|PMG133-03|Canada|Ontario|617[0n]|BOLD:AAA4742
Melanchra adjuncta|[11290]|LOWCD275-06|Canada|British Columbia|593[0n]|BOLD:AAA4742
Melanchra adjuncta|[11291]|TMG130-03|Canada|Ontario|639[0n]|BOLD:AAA4742
Melanchra adjuncta|[11292]|TMG127-03|Canada|Ontario|639[0n]|BOLD:AAA4742
Melanchra adjuncta|[11293]|BLTIB001-08|Canada|Ontario|579[0n]|BOLD:AAA4742
Melanchra adjuncta|[11294]|LPABB073-08|Canada|Alberta|658[0n]|BOLD:AAA4742

[illegible]
Melanchra adjuncta|[11292]|TMG127-03|Canada|Ontario|639[0n]|BOLD:AAA4742
Melanchra adjuncta|[11293]|BLTIB001-08|Canada|Ontario|579[0n]|BOLD:AAA4742
Melanchra adjuncta|[11294]|LPABB073-08|Canada|Alberta|658[0n]|BOLD:AAA4742
Melanchra adjuncta|[11295]|LPVIA228-08|Canada|British Columbia|636[0n]|BOLD:AAA4742
Melanchra adjuncta|[11296]|RWWA363-09|United States|Washington|658[0n]|BOLD:AAA4742
Melanchra adjuncta|[11297]|RWWA422-09|United States|Washington|658[0n]|BOLD:AAA4742
Melanchra adjuncta|[11298]|RWWA664-09|United States|Washington|658[0n]|BOLD:AAA4742
Melanchra adjuncta|[11299]|RWWA667-09|United States|Washington|658[0n]|BOLD:AAA4742
Melanchra adjuncta|[11300]|RWWA718-09|United States|Washington|658[0n]|BOLD:AAA4742
Melanchra adjuncta|[11301]|RWWA789-09|United States|Washington|658[0n]|BOLD:AAA4742
Melanchra adjuncta|[11302]|RWWA915-09|United States|Washington|658[0n]|BOLD:AAA4742
Melanchra adjuncta|[11303]|BBLPE119-09|Canada|Nova Scotia|658[0n]|BOLD:AAA4742
Melanchra adjuncta|[11304]|BBLPE318-09|Canada|Newfoundland and Labrador|658[0n]|BOLD:AAA4742
Melanchra adjuncta|[11305]|RWWB947-10|United States|Washington|658[0n]|BOLD:AAA4742
Melanchra adjuncta|[11306]|BBLPB642-10|Canada|Alberta|658[0n]|BOLD:AAA4742
Melanchra adjuncta|[11307]|BBLPB643-10|Canada|Alberta|658[0n]|BOLD:AAA4742
Melanchra adjuncta|[11308]|RWWC470-11|United States|Washington|658[0n]|BOLD:AAA4742
Melanchra adjuncta|[11309]|JSAUG1677-11|Canada|Ontario|658[0n]|BOLD:AAA4742
Admetovis oxymorus|[11310]|RDNMF548-08|United States|Oregon|609[0n]|BOLD:AAD7455
Admetovis oxymorus|[11311]|IAWLB099-10|United States|California|658[0n]|BOLD:AAD7455
Admetovis oxymorus|[11312]|IAWLB098-10|United States|California|658[0n]|BOLD:AAD7455
Admetovis oxymorus|[11313]|IAWLB097-10|United States|California|658[0n]|BOLD:AAD7455
Admetovis oxymorus|[11314]|LBCH6013-10|Canada|British Columbia|658[0n]|BOLD:AAD7455
Admetovis oxymorus|[11315]|LBCH5696-10|Canada|British Columbia|658[0n]|BOLD:AAD7455
Admetovis oxymorus|[11316]|LBCH5440-10|Canada|British Columbia|658[0n]|BOLD:AAD7455
Admetovis oxymorus|[11317]|RDNMF542-08|United States|Oregon|658[0n]|BOLD:AAD7455
Admetovis oxymorus|[11318]|RDNMF544-08|United States|Oregon|658[0n]|BOLD:AAD7455
Admetovis oxymorus|[11319]|RDNMF540-08|Canada|British Columbia|658[0n]|BOLD:AAD7455
Admetovis oxymorus|[11320]|IAWLB096-10|United States|California|593[0n]|BOLD:AAD7455
Admetovis oxymorus|[11321]|IAWLB123-10|United States|California|658[0n]|BOLD:AAD7455
Admetovis sp.|[11322]|RDNMF541-08|United States|Washington|658[0n]|BOLD:AAD7456
Admetovis sp.|[11323]|RDNMF543-08|United States|Utah|658[0n]|BOLD:AAD7456
Admetovis similaris|[11324]|RDNMF545-08|United States|Washington|658[3n]|BOLD:AAB7673
Admetovis similaris|[11325]|RDNMF546-08|United States|Washington|658[1n]|BOLD:AAB7673
Admetovis similaris|[11326]|NAMUM206-08|United States|California|645[0n]|BOLD:AAB7673
Admetovis similaris|[11327]|RDNMF549-08|United States|Washington|609[0n]|BOLD:AAB7673
Admetovis similaris|[11328]|LBCH5083-10|Canada|British Columbia|658[0n]|BOLD:AAB7673
Admetovis similaris|[11329]|IAWLB647-11|United States|Arizona|658[0n]|BOLD:AAB7673
Admetovis similaris|[11330]|LOCBE244-06|United States|California|658[0n]|BOLD:AAB7673
Admetovis similaris|[11331]|LOCBD657-06|United States|California|658[0n]|BOLD:AAB7673
Admetovis similaris|[11332]|LOCBD561-06|United States|California|658[0n]|BOLD:AAB7673
Admetovis similaris|[11333]|LOCBD560-06|United States|California|658[0n]|BOLD:AAB7673
Admetovis similaris|[11334]|LOCBD173-06|United States|California|658[0n]|BOLD:AAB7673
Admetovis similaris|[11335]|LOCBC354-06|United States|California|658[0n]|BOLD:AAB7673
Admetovis similaris|[11336]|LOCBC352-06|United States|California|658[0n]|BOLD:AAB7673
Admetovis similaris|[11337]|LOCBC328-06|United States|California|658[0n]|BOLD:AAB7673
Admetovis similaris|[11338]|LOCBC327-06|United States|California|658[0n]|BOLD:AAB7673
Admetovis similaris|[11339]|LOCBC326-06|United States|California|658[0n]|BOLD:AAB7673
Admetovis similaris|[11340]|LOCBC353-06|United States|California|630[0n]|BOLD:AAB7673
Admetovis similaris|[11341]|LOCBE243-06|United States|California|646[1n]|BOLD:AAB7673
Admetovis similaris|[11342]|RDNMF547-08|United States|California|658[0n]|BOLD:AAB7673
Admetovis similaris|[11343]|JBAZ026-09|United States|California|658[0n]|BOLD:AAB7673
Admetovis similaris|[11344]|CGLCA030-10|United States|California|658[0n]|BOLD:AAB7673
Admetovis similaris|[11345]|CGLCA061-10|United States|California|658[0n]|BOLD:AAB7673
Admetovis similaris|[11346]|USLEP637-10|United States|Arizona|658[0n]|BOLD:AAB7673
Admetovis similaris|[11347]|GMLC948-12|United States|California|658[0n]|BOLD:AAB7673
Hecatera dysodea|[11348]|GWORO998-09|Italy|Basilicata|658[0n]|BOLD:AAD0557
Hecatera dysodea|[11349]|GWORL409-09|Germany|Bavaria|632[3n]|BOLD:AAD0557
Hecatera dysodea|[11350]|GWORZ236-10|Italy|Basilicata|658[0n]|BOLD:AAD0557
Hecatera dysodea|[11351]|CGUKA513-09|United Kingdom|England|634[0n]|BOLD:AAD0557
Hecatera dysodea|[11352]|LNAUT1128-14|United States|Oregon|658[0n]|BOLD:AAD0557
Hecatera dysodea|[11353]|LNAUT1129-14|United States|Oregon|658[0n]|BOLD:AAD0557
Hecatera dysodea|[11354]|FBLMX176-11|Germany||658[0n]|BOLD:AAD0557
Hecatera dysodea|[11355]|PHLAC556-10|Italy|South Tyrol|658[0n]|BOLD:AAD0557
Hecatera dysodea|[11356]|GWORL398-09|Germany|Bavaria|658[0n]|BOLD:AAD0557
Hecatera dysodea|[11357]|GWORK445-09|Germany|Bavaria|658[0n]|BOLD:AAD0557
Hecatera dysodea|[11358]|GWOR4012-09|Germany|Bavaria|658[0n]|BOLD:AAD0557
Hecatera dysodea|[11359]|CGUKC067-09|United Kingdom|England|638[1n]|BOLD:AAD0557
Hecatera dysodea|[11360]|CGUKC384-09|United Kingdom|England|613[0n]|BOLD:AAD0557
Hecatera dysodea|[11361]|GWOSA437-10|Germany|Bavaria|600[0n]|BOLD:AAD0557
Hecatera dysodea|[11362]|LENOA227-11|France|Haute Normandie|605[0n]|BOLD:AAD0557
Hecatera dysodea|[11363]|IBLAO366-12|Spain|Murcia|658[0n]|BOLD:AAD0557
Hecatera dysodea|[11364]|IBLAO723-12|Spain|Andalusia|658[0n]|BOLD:AAD0557
Hecatera dysodea|[11365]|GBLAA131-14|Germany|Baden-Wuerttemberg|658[0n]|BOLD:AAD0557
Hecatera dysodea|[11366]|GBLAA239-14|Germany|Schleswig-Holstein|658[0n]|BOLD:AAD0557
Hecatera dysodea|[11367]|LNAUT1126-14|United States|Washington|658[0n]|BOLD:AAD0557
Hecatera dysodea|[11368]|LNAUT1127-14|United States|Washington|658[0n]|BOLD:AAD0557
Hecatera dysodea|[11369]|LNAUT1130-14|United States|Oregon|658[0n]|BOLD:AAD0557
Hada sutrina|[11370]|RDMAB363-05|Canada|Alberta|616[0n]|BOLD:AAC2435
Hada sutrina|[11371]|RDLQ636-07|Canada|Quebec|633[0n]|BOLD:AAC2435
Hada sutrina|[11372]|RDLQ635-07|Canada|Newfoundland and Labrador|633[0n]|BOLD:AAC2435
Hada sutrina|[11373]|LSEU797-06|United States|Colorado|657[0n]|BOLD:AAC2435
Hada sutrina|[11374]|LBCA195-05|Canada|British Columbia|658[0n]|BOLD:AAC2435
Hada sutrina|[11375]|BBLPB761-10|Canada|British Columbia|658[0n]|BOLD:AAC2435
Hada sutrina|[11376]|RDNME521-08|Canada|Yukon Territory|658[0n]|BOLD:AAC2435
Hada sutrina|[11377]|BBLPB772-10|Canada|British Columbia|658[0n]|BOLD:AAC2435
Hada sutrina|[11378]|LBCH2196-10|Canada|British Columbia|658[0n]|BOLD:AAC2435
Hada sutrina|[11379]|LBCH1921-10|Canada|British Columbia|658[0n]|BOLD:AAC2435
Hada sutrina|[11380]|LBCH1717-10|Canada|British Columbia|658[0n]|BOLD:AAC2435
Hada sutrina|[11381]|LBCG3043-09|Canada|British Columbia|658[0n]|BOLD:AAC2435
Hada sutrina|[11382]|LBCG2831-09|Canada|British Columbia|658[0n]|BOLD:AAC2435
Hada sutrina|[11383]|LBCG2483-09|Canada|British Columbia|658[0n]|BOLD:AAC2435
Hada sutrina|[11384]|LBCG2395-09|Canada|British Columbia|658[0n]|BOLD:AAC2435
Hada sutrina|[11385]|LBCG1345-09|Canada|British Columbia|658[0n]|BOLD:AAC2435
Hada sutrina|[11386]|LBCC336-05|Canada|British Columbia|658[0n]|BOLD:AAC2435
Hada sutrina|[11387]|LBCH5490-10|Canada|British Columbia|658[0n]|BOLD:AAC2435
Hada sutrina|[11388]|LALPA834-11|Canada|British Columbia|658[0n]|BOLD:AAC2435
Hada sutrina|[11389]|LALPA1088-11|Canada|British Columbia|658[0n]|BOLD:AAC2435
Hada sutrina|[11390]|LALPA1139-11|Canada|British Columbia|658[0n]|BOLD:AAC2435
Hadena capsularis|[11391]|RDNMG987-08|United States|Colorado|658[0n]|BOLD:AAD5556
Hadena capsularis|[11392]|RDNMG988-08|United States|Colorado|658[0n]|BOLD:AAD5556
Hadena capsularis|[11393]|RDNMB240-05|United States|Oregon|552[0n]|BOLD:AAD5556
Hadena capsularis|[11394]|RDNMB239-05|Canada|Ontario|603[0n]|BOLD:AAD5556

Hadena capsularis|[11391]|RDNMG987-08|United States|Colorado|658[0n]|BOLD:AAD5556
Hadena capsularis|[11392]|RDNMG988-08|United States|Colorado|658[0n]|BOLD:AAD5556
Hadena capsularis|[11393]|RDNMB240-05|United States|Oregon|552[0n]|BOLD:AAD5556
Hadena capsularis|[11394]|RDNMB239-05|Canada|Ontario|603[0n]|BOLD:AAD5556
Hadena capsularis|[11395]|RDLQ645-07|Canada|Quebec|657[0n]|BOLD:AAD5556
Hadena capsularis|[11396]|RDNMG989-08|United States|Colorado|658[0n]|BOLD:AAD5556
Hadena capsularis|[11397]|RDNMF886-08|Canada|Ontario|658[0n]|BOLD:AAD5556
Hadena capsularis|[11398]|BBLWU194-09|United States|Colorado|658[0n]|BOLD:AAD5556
Hadena lafontainei|[11399]|NAMUM096-08|United States|Arizona|657[0n]|BOLD:ABZ4581
Hadena lafontainei|[11400]|LNAUT1111-14|United States|Arizona|658[0n]|BOLD:ABZ4581
Hadena lafontainei|[11401]|CNCLB262-14|United States|New Mexico|658[0n]|BOLD:ABZ4581
Hadena lafontainei|[11402]|CNCLB263-14|United States|New Mexico|658[0n]|BOLD:ABZ4581
Hadena lafontainei|[11403]|LNAUT1108-14|United States|Colorado|658[0n]|BOLD:ABZ4581
Hadena lafontainei|[11404]|LNAUT1109-14|United States|Colorado|658[0n]|BOLD:ABZ4581
Hadena lafontainei|[11405]|LNAUT1110-14|United States|Arizona|658[0n]|BOLD:ABZ4581
Hadena lafontainei|[11406]|LNAUT1112-14|United States|Colorado|658[0n]|BOLD:ABZ4581
Hadena lafontainei|[11407]|LNAUT1113-14|United States|New Mexico|658[0n]|BOLD:ABZ4581
Hadena lafontainei|[11408]|LNAUT1114-14|United States|New Mexico|658[0n]|BOLD:ABZ4581
Hadena lafontainei|[11409]|LNAUT1115-14|United States|New Mexico|658[0n]|BOLD:ABZ4581
Hadena variolata|[11410]|RDNMG662-08|United States|Washington|658[0n]|BOLD:AAE6874
Hadena variolata|[11411]|RDMAB526-06|Canada|Alberta|658[0n]|BOLD:AAE6874
Hadena variolata|[11412]|RDNMG663-08|United States|Washington|642[0n]|BOLD:AAE6874
Hadena variolata|[11413]|RDNMG664-08|United States|Washington|658[0n]|BOLD:AAE6874
Hadena variolata|[11414]|LBCH5611-10|Canada|British Columbia|658[0n]|BOLD:AAE6874
Hadena variolata|[11415]|LBCH5802-10|Canada|British Columbia|658[0n]|BOLD:AAE6874
Hadena ectypa|[11416]|LGSM566-04|United States|Tennessee|658[0n]|BOLD:AAI6514
Hadena ectypa|[11417]|LGSM567-04|United States|North Carolina|658[0n]|BOLD:AAI6514
Hadena minorata|[11418]|CNCLB259-14|United States|California|658[0n]|BOLD:ACM4471
Hadena caelestis|[11419]|RDNMF240-08|Canada|British Columbia|658[0n]|BOLD:ACE5504
Hadena caelestis|[11420]|RDNMF239-08|Canada|British Columbia|658[0n]|BOLD:ACE5504
Hadena caelestis|[11421]|RDNMF241-08|Canada|British Columbia|658[0n]|BOLD:ACE5504
Hadena caelestis|[11422]|RDNMF237-08|Canada|British Columbia|658[0n]|BOLD:ACE5504
Hadena caelestis|[11423]|RDNMF238-08|Canada|British Columbia|658[0n]|BOLD:ACE5504
Hadena caelestis|[11424]|NAMUM287-08|United States|California|658[0n]|BOLD:ACE5504
Hadena amabilis|[11425]|RDNMD420-06|United States|California|658[0n]|BOLD:ACE5504
Hadena amabilis|[11426]|RDNMD421-06|United States|California|658[0n]|BOLD:ACE5504
Hadena circumvadis|[11427]|RDNMJ485-11|United States|Arizona|658[0n]|BOLD:ACE5504
Hadena circumvadis|[11428]|RDNMF235-08|Canada|Saskatchewan|658[0n]|BOLD:ACE5504
Hadena circumvadis|[11429]|LPMN946-08|Canada|Alberta|658[0n]|BOLD:ACE5504
Hadena circumvadis|[11430]|RDNMF232-08|Canada|Alberta|658[0n]|BOLD:ACE5504
Hadena circumvadis|[11431]|RDNMF233-08|Canada|Saskatchewan|658[0n]|BOLD:ACE5504
Hadena circumvadis|[11432]|RDNMF234-08|United States|Wyoming|658[0n]|BOLD:ACE5504
Hadena circumvadis|[11433]|RDNMF236-08|United States|Montana|658[0n]|BOLD:ACE5504
Hadena glaciata|[11434]|RDNML377-13|United States|Arizona|658[0n]|BOLD:ACE5504
Hadena glaciata|[11435]|CNCLA546-13|United States|Arizona|658[0n]|BOLD:ACE5504
Hadena paulula|[11436]|CNCLB730-14|United States|Arizona|658[0n]|BOLD:AAB7648
Hadena paulula|[11437]|CNCLB731-14|United States|Arizona|658[0n]|BOLD:AAB7648
Hadena ectrapela|[11438]|RDNM090-05|United States|Oregon|591[0n]|BOLD:AAB7648
Hadena ectrapela|[11439]|RDNM837-05|United States|California|616[0n]|BOLD:AAB7648
Hadena ectrapela|[11440]|RDNMB544-05|United States|Oregon|658[0n]|BOLD:AAB7648
Hadena ectrapela|[11441]|RDNMB548-05|United States|Oregon|658[0n]|BOLD:AAB7648
Hadena maccabei|[11442]|RDNM836-05|United States|California|583[0n]|BOLD:AAB7648
Hadena maccabei|[11443]|RDNM834-05|United States|California|658[0n]|BOLD:AAB7648
Hadena maccabei|[11444]|RDNM835-05|United States|California|658[0n]|BOLD:AAB7648
Hadena ectrapela|[11445]|RDNM838-05|United States|Oregon|616[0n]|BOLD:AAB7648
Hadena ectrapela|[11446]|RDNM091-05|United States|Oregon|591[0n]|BOLD:AAB7648
Hadena ectrapela|[11447]|RDNM089-05|United States|Oregon|591[0n]|BOLD:AAB7648
Hadena ectrapela|[11448]|RDNMB543-05|United States|Oregon|613[0n]|BOLD:AAB7648
Hadena ectrapela|[11449]|RDNMB549-05|United States|Oregon|658[0n]|BOLD:AAB7648
Hadena ectrapela|[11450]|RDNMB547-05|United States|Oregon|658[0n]|BOLD:AAB7648
Hadena ectrapela|[11451]|RDNMB546-05|United States|Oregon|658[0n]|BOLD:AAB7648
Hadena ectrapela|[11452]|RDNMB550-05|United States|Oregon|580[0n]|BOLD:AAB7648
Hadena ectrapela|[11453]|RDNMB545-05|United States|Oregon|613[1n]|BOLD:AAB7648
Hadena ectrapela|[11454]|RDNM001-05|United States|Washington|615[0n]|BOLD:AAB7648
Hadena ectrapela|[11455]|RDNM833-05|United States|Washington|616[0n]|BOLD:AAB7648
Hadena ectrapela|[11456]|RDMAB574-06|Canada|Alberta|658[0n]|BOLD:AAB7648
Hadena ectrapela|[11457]|LBCH1578-10|Canada|British Columbia|658[0n]|BOLD:AAB7648
Hadena siskiyou|[11458]|RDNM093-05|United States|Oregon|596[0n]|BOLD:AAB7648
Hadena siskiyou|[11459]|RDNM094-05|United States|Oregon|658[0n]|BOLD:AAB7648
Hadena siskiyou|[11460]|RDNM092-05|United States|Oregon|658[0n]|BOLD:AAB7648
Hadena gabrieli|[11461]|RDNMB241-05|United States|California|658[0n]|BOLD:AAB7648
Hadena plumasata|[11462]|JMMMB340-11|United States|California|658[0n]|BOLD:AAB7648
Hadena plumasata|[11463]|CNCLB260-14|United States|California|658[0n]|BOLD:AAB7648
Hadena plumasata|[11464]|CNCLB261-14|United States|California|658[0n]|BOLD:AAB7648
Hadena plumasata|[11465]|LNAUT1116-14|United States|California|658[0n]|BOLD:AAB7648
Coranarta macrostigma|[11466]|RDMAB090-05|Canada|Alberta|652[0n]|BOLD:AAD9178
Coranarta macrostigma|[11467]|RDMAB260-05|Canada|Alberta|658[0n]|BOLD:AAD9178
Coranarta macrostigma|[11468]|RDMAB432-05|Canada|Alberta|658[0n]|BOLD:AAD9178
Coranarta macrostigma|[11469]|RDNMG824-08|Canada|Alberta|658[0n]|BOLD:AAD9178
Coranarta macrostigma|[11470]|RDNMG825-08|Canada|Alberta|658[0n]|BOLD:AAD9178
Coranarta macrostigma|[11471]|RDMAB431-05|Canada|Alberta|658[0n]|BOLD:AAD9178
Coranarta macrostigma|[11472]|NOCNA091-14|United States|Michigan|658[0n]|BOLD:AAD9178
Coranarta macrostigma|[11473]|NOCNA092-14|United States|Michigan|658[0n]|BOLD:AAD9178
Coranarta luteola|[11474]|CNWBA499-13|Canada|Alberta|658[0n]|BOLD:ACO9759
Coranarta luteola|[11475]|RDMAB430-05|Canada|Alberta|599[0n]|BOLD:ABY9453
Coranarta luteola|[11476]|RDNMG765-08|Canada|Yukon Territory|590[0n]|BOLD:ABY9453
Coranarta luteola|[11477]|RDLQ634-07|Canada|Quebec|592[2n]|BOLD:ABY9453
Coranarta luteola|[11478]|RDNMG766-08|Canada|Manitoba|658[0n]|BOLD:ABY9453
Coranarta luteola|[11479]|RDNMG767-08|Canada|Quebec|658[0n]|BOLD:ABY9453
Coranarta luteola|[11480]|RDNMG768-08|Canada|Yukon Territory|658[0n]|BOLD:ABY9453
Coranarta luteola|[11481]|BBLPB316-10|Canada|British Columbia|658[0n]|BOLD:ABY9453
Coranarta luteola|[11482]|CNCLB3152-15|Canada|Yukon Territory|658[0n]|BOLD:ABY9453
Coranarta luteola|[11483]|CNCLB3153-15|United States|Alaska|658[0n]|BOLD:ABY9453
Mamestra configurata|[11484]|LBCC755-05|Canada|British Columbia|658[0n]|BOLD:ABY4746
Mamestra configurata|[11485]|LOWCD325-06|Canada|British Columbia|614[0n]|BOLD:ABY4746
Mamestra configurata|[11486]|LHLEP066-06|Canada|British Columbia|658[0n]|BOLD:ABY4746
Mamestra configurata|[11487]|LBCS263-07|Canada|British Columbia|658[0n]|BOLD:ABY4746
Mamestra configurata|[11488]|LPVIC124-08|Canada|British Columbia|658[0n]|BOLD:ABY4746
Mamestra configurata|[11489]|RWWA803-09|United States|Washington|658[0n]|BOLD:ABY4746
Mamestra configurata|[11490]|LALPA534-10|Canada|British Columbia|658[0n]|BOLD:ABY4746
Mamestra configurata|[11491]|RWWC969-12|United States|Washington|658[0n]|BOLD:ABY4746
Mamestra curialis|[11492]|LOWCD274-06|Canada|British Columbia|598[0n]|BOLD:ABZ3096
Mamestra curialis|[11493]|LPMN816-08|Canada|Manitoba|658[0n]|BOLD:ABZ3096
Mamestra curialis|[11494]|RDLQE453-06|Canada|Quebec|658[0n]|BOLD:ABZ3096

Mamestra configurata|[11491]|RWWC909-12|United States|Washington|658[0n]|BOLD:[illegible]
Mamestra curialis|[11492]|LOWCD274-06|Canada|British Columbia|598[0n]|BOLD:ABZ3096
Mamestra curialis|[11493]|LPMN816-08|Canada|Manitoba|658[0n]|BOLD:ABZ3096
Mamestra curialis|[11494]|RDLQF453-06|Canada|Quebec|658[0n]|BOLD:ABZ3096
Mamestra curialis|[11495]|LBCB361-05|Canada|British Columbia|658[0n]|BOLD:ABZ3096
Mamestra curialis|[11496]|RDMAB547-06|Canada|Alberta|606[0n]|BOLD:ABZ3096
Mamestra curialis|[11497]|SSPAC13761-13|Canada|Saskatchewan|595[0n]|BOLD:ABZ3096
Mamestra curialis|[11498]|SSPAC13762-13|Canada|Saskatchewan|595[0n]|BOLD:ABZ3096
Spiramater lutra|[11499]|LOWCD146-06|Canada|British Columbia|525[0n]|BOLD:AAA7346
Spiramater lutra|[11500]|LOWCC816-05|Canada|British Columbia|658[1n]|BOLD:AAA7346
Spiramater lutra|[11501]|LBCA215-05|Canada|British Columbia|616[0n]|BOLD:AAA7346
Spiramater lutra|[11502]|LPVIA233-08|Canada|British Columbia|658[0n]|BOLD:AAA7346
Spiramater lutra|[11503]|LBCG034-08|Canada|British Columbia|658[0n]|BOLD:AAA7346
Spiramater lutra|[11504]|LPSOD249-09|Canada|Ontario|658[0n]|BOLD:AAA7346
Spiramater lutra|[11505]|LPSOD340-09|Canada|Ontario|658[0n]|BOLD:AAA7346
Spiramater lutra|[11506]|RWWA582-09|United States|Washington|658[0n]|BOLD:AAA7346
Spiramater lutra|[11507]|BBLPC644-09|Canada|Newfoundland and Labrador|658[0n]|BOLD:AAA7346
Spiramater lutra|[11508]|BBLPC666-09|Canada|Newfoundland and Labrador|658[0n]|BOLD:AAA7346
Spiramater lutra|[11509]|BBLPC684-09|Canada|Newfoundland and Labrador|658[0n]|BOLD:AAA7346
Spiramater lutra|[11510]|BBLPC774-09|Canada|Newfoundland and Labrador|658[0n]|BOLD:AAA7346
Spiramater lutra|[11511]|TTMNB554-06|Canada|New Brunswick|658[0n]|BOLD:AAA7346
Spiramater lutra|[11512]|LOWCE816-06|Canada|British Columbia|658[0n]|BOLD:AAA7346
Spiramater lutra|[11513]|TMNBB322-06|Canada|New Brunswick|658[0n]|BOLD:AAA7346
Spiramater lutra|[11514]|TMNBB323-06|Canada|New Brunswick|658[0n]|BOLD:AAA7346
Spiramater lutra|[11515]|BBLPC778-09|Canada|Newfoundland and Labrador|658[0n]|BOLD:AAA7346
Spiramater lutra|[11516]|BBLPC855-09|Canada|Newfoundland and Labrador|658[0n]|BOLD:AAA7346
Spiramater lutra|[11517]|BBLPE307-09|Canada|Newfoundland and Labrador|658[0n]|BOLD:AAA7346
Spiramater lutra|[11518]|BBLPE310-09|Canada|Newfoundland and Labrador|658[0n]|BOLD:AAA7346
Spiramater lutra|[11519]|BBLPE349-09|Canada|Newfoundland and Labrador|658[0n]|BOLD:AAA7346
Spiramater lutra|[11520]|BBLPE358-09|Canada|Newfoundland and Labrador|658[0n]|BOLD:AAA7346
Spiramater lutra|[11521]|BBLPE391-09|Canada|Newfoundland and Labrador|658[0n]|BOLD:AAA7346
Spiramater lutra|[11522]|LALPA313-10|Canada|British Columbia|658[0n]|BOLD:AAA7346
Spiramater lutra|[11523]|RWWC269-11|United States|Washington|658[0n]|BOLD:AAA7346
Spiramater lutra|[11524]|RWWC294-11|United States|Washington|658[0n]|BOLD:AAA7346
Spiramater lutra|[11525]|LALPA892-11|Canada|British Columbia|658[0n]|BOLD:AAA7346
Spiramater lutra|[11526]|LALPA1167-11|Canada|British Columbia|658[0n]|BOLD:AAA7346
Spiramater lutra|[11527]|LALPA1184-11|Canada|British Columbia|658[0n]|BOLD:AAA7346
Spiramater lutra|[11528]|RWWC428-11|United States|Washington|658[0n]|BOLD:AAA7346
Spiramater lutra|[11529]|TTMNB551-06|Canada|New Brunswick|658[0n]|BOLD:AAA7346
Spiramater lutra|[11530]|TTMNB553-06|Canada|New Brunswick|658[0n]|BOLD:AAA7346
Spiramater lutra|[11531]|LOWCD273-06|Canada|British Columbia|658[0n]|BOLD:AAA7346
Spiramater lutra|[11532]|TTMNB398-06|Canada|New Brunswick|658[0n]|BOLD:AAA7346
Spiramater lutra|[11533]|LOWCD272-06|Canada|British Columbia|658[0n]|BOLD:AAA7346
Spiramater lutra|[11534]|LOWCD271-06|Canada|British Columbia|658[0n]|BOLD:AAA7346
Spiramater lutra|[11535]|RDLQB281-05|Canada|Quebec|658[0n]|BOLD:AAA7346
Spiramater lutra|[11536]|RDLQB280-05|Canada|Quebec|658[0n]|BOLD:AAA7346
Spiramater lutra|[11537]|RDLQB279-05|Canada|Quebec|658[0n]|BOLD:AAA7346
Spiramater lutra|[11538]|LOWCC814-05|Canada|British Columbia|658[0n]|BOLD:AAA7346
Spiramater lutra|[11539]|LOWCC817-05|Canada|British Columbia|658[0n]|BOLD:AAA7346
Spiramater lutra|[11540]|LOWCD270-06|Canada|British Columbia|658[0n]|BOLD:AAA7346
Spiramater lutra|[11541]|LBCB060-05|Canada|British Columbia|658[0n]|BOLD:AAA7346
Spiramater lutra|[11542]|LBCA800-05|Canada|British Columbia|658[0n]|BOLD:AAA7346
Spiramater lutra|[11543]|BBLPC667-09|Canada|Newfoundland and Labrador|658[0n]|BOLD:AAA7346
Spiramater lutra|[11544]|LPSOD245-09|Canada|Ontario|658[0n]|BOLD:AAA7346
Spiramater lutra|[11545]|LBCA605-05|Canada|British Columbia|651[0n]|BOLD:AAA7346
Spiramater lutra|[11546]|LOWCD131-06|Canada|British Columbia|579[0n]|BOLD:AAA7346
Spiramater lutra|[11547]|LPVIC119-08|Canada|British Columbia|658[0n]|BOLD:AAA7346
Spiramater lutra|[11548]|LOWCC820-05|Canada|British Columbia|615[0n]|BOLD:AAA7346
Spiramater lutra|[11549]|LOWCC815-05|Canada|British Columbia|585[1n]|BOLD:AAA7346
Spiramater lutra|[11550]|TTMNB552-06|Canada|New Brunswick|656[0n]|BOLD:AAA7346
Spiramater lutra|[11551]|LBCA604-05|Canada|British Columbia|628[0n]|BOLD:AAA7346
Spiramater lutra|[11552]|LOWCC818-05|Canada|British Columbia|639[0n]|BOLD:AAA7346
Spiramater lutra|[11553]|LOWCC819-05|Canada|British Columbia|639[1n]|BOLD:AAA7346
Spiramater lutra|[11554]|BBLPE372-09|Canada|Newfoundland and Labrador|638[0n]|BOLD:AAA7346
Spiramater lutra|[11555]|BBLPE420-09|Canada|Newfoundland and Labrador|635[0n]|BOLD:AAA7346
Spiramater lutra|[11556]|RWWB877-10|United States|Washington|636[0n]|BOLD:AAA7346
Spiramater lutra|[11557]|RWWC495-11|United States|Washington|658[0n]|BOLD:AAA7346
Spiramater lutra|[11558]|LOCT060-05|United States|Connecticut|658[0n]|BOLD:AAA7346
Spiramater lutra|[11559]|LSEU139-06|United States|North Carolina|658[0n]|BOLD:AAA7346
Spiramater lutra|[11560]|BBLPC963-09|Canada|Newfoundland and Labrador|658[0n]|BOLD:AAA7346
Spiramater lutra|[11561]|GWOTA072-12|United States|Massachusetts|658[0n]|BOLD:AAA7346
Melanchra picta|[11562]|PHMO302-03|Canada|Ontario|639[0n]|BOLD:AAC8574
Melanchra picta|[11563]|XAC156-04|Canada|Ontario|639[0n]|BOLD:AAC8574
Melanchra picta|[11564]|XAC159-04|Canada|Ontario|658[0n]|BOLD:AAC8574
Melanchra picta|[11565]|LHLEP414-06|Canada|British Columbia|658[0n]|BOLD:AAC8574
Melanchra picta|[11566]|RDLQ631-07|Canada|Ontario|658[0n]|BOLD:AAC8574
Melanchra picta|[11567]|RWWA162-09|United States|Washington|658[0n]|BOLD:AAC8574
Melanchra picta|[11568]|RWWA180-09|United States|Washington|658[0n]|BOLD:AAC8574
Melanchra picta|[11569]|RWWA337-09|United States|Washington|658[0n]|BOLD:AAC8574
Melanchra picta|[11570]|RWWA410-09|United States|Washington|658[0n]|BOLD:AAC8574
Melanchra picta|[11571]|RWWB787-10|United States|Washington|658[0n]|BOLD:AAC8574
Melanchra picta|[11572]|RWWB794-10|United States|Washington|658[0n]|BOLD:AAC8574
Melanchra picta|[11573]|RWWB896-10|United States|Washington|658[0n]|BOLD:AAC8574
Melanchra picta|[11574]|RWWC283-11|United States|Washington|658[0n]|BOLD:AAC8574
Melanchra assimilis|[11575]|RDNMB367-05|Canada|Ontario|658[0n]|BOLD:ABZ8175
Melanchra assimilis|[11576]|RDNMB366-05|Canada|Ontario|658[0n]|BOLD:ABZ8175
Melanchra assimilis|[11577]|RDLQB091-05|Canada|Quebec|658[0n]|BOLD:ABZ8175
Melanchra assimilis|[11578]|TMNBB315-06|Canada|New Brunswick|658[0n]|BOLD:ABZ8175
Melanchra assimilis|[11579]|RDLQG361-06|Canada|Quebec|658[0n]|BOLD:ABZ8175
Melanchra assimilis|[11580]|BBLPC196-09|Canada|Nova Scotia|658[0n]|BOLD:ABZ8175
Melanchra assimilis|[11581]|BBLPE135-09|Canada|Nova Scotia|658[0n]|BOLD:ABZ8175
Melanchra pulverulenta|[11582]|SSJAE5040-13|Canada|Alberta|538[0n]|BOLD:AAB0758
Melanchra pulverulenta|[11583]|SSPAA10032-13|Canada|Saskatchewan|526[0n]|BOLD:AAB0758
Melanchra pulverulenta|[11584]|SSPAA10027-13|Canada|Saskatchewan|539[0n]|BOLD:AAB0758
Melanchra pulverulenta|[11585]|LCH248-04|Canada|Manitoba|658[0n]|BOLD:AAB0758
Melanchra pulverulenta|[11586]|SSJAE6811-13|Canada|Alberta|546[1n]|BOLD:AAB0758
Melanchra pulverulenta|[11587]|TMNBB313-06|Canada|New Brunswick|658[0n]|BOLD:AAB0758
Melanchra pulverulenta|[11588]|RDNMB364-05|United States|Oregon|609[0n]|BOLD:AAB0758
Melanchra pulverulenta|[11589]|SSWLD6373-13|Canada|Alberta|604[0n]|BOLD:AAB0758
Melanchra pulverulenta|[11590]|LBCA447-05|Canada|British Columbia|658[1n]|BOLD:AAB0758
Melanchra pulverulenta|[11591]|RDNMB363-05|Canada|Quebec|658[0n]|BOLD:AAB0758
Melanchra pulverulenta|[11592]|RDNMB365-05|Canada|British Columbia|658[0n]|BOLD:AAB0758
Melanchra pulverulenta|[11593]|LOWCC084-05|Canada|British Columbia|658[0n]|BOLD:AAB0758
Melanchra pulverulenta|[11594]|RDLQB089-05|Canada|Quebec|658[0n]|BOLD:AAB0758

Melanchra pulverulenta|[11591]|RDNMB363-05|Canada|Quebec|658[0n]|BOLD:AAB0758
Melanchra pulverulenta|[11592]|RDNMB365-05|Canada|British Columbia|658[0n]|BOLD:AAB0758
Melanchra pulverulenta|[11593]|LOWCC084-05|Canada|British Columbia|658[0n]|BOLD:AAB0758
Melanchra pulverulenta|[11594]|RDLQB089-05|Canada|Quebec|658[0n]|BOLD:AAB0758
Melanchra pulverulenta|[11595]|RDLQB090-05|Canada|Quebec|658[0n]|BOLD:AAB0758
Melanchra pulverulenta|[11596]|TMNBB314-06|Canada|New Brunswick|658[0n]|BOLD:AAB0758
Melanchra pulverulenta|[11597]|LCHP822-07|Canada|Manitoba|658[0n]|BOLD:AAB0758
Melanchra pulverulenta|[11598]|LBCG2468-09|Canada|British Columbia|658[0n]|BOLD:AAB0758
Melanchra pulverulenta|[11599]|BBLPE220-09|Canada|Newfoundland and Labrador|658[0n]|BOLD:AAB0758
Melanchra pulverulenta|[11600]|BBLPE242-09|Canada|Newfoundland and Labrador|658[0n]|BOLD:AAB0758
Melanchra pulverulenta|[11601]|LBCH2197-10|Canada|British Columbia|658[0n]|BOLD:AAB0758
Melanchra pulverulenta|[11602]|LBCH2253-10|Canada|British Columbia|658[0n]|BOLD:AAB0758
Melanchra pulverulenta|[11603]|LBCH2254-10|Canada|British Columbia|658[0n]|BOLD:AAB0758
Melanchra pulverulenta|[11604]|PHLCH306-10|Canada|Manitoba|658[0n]|BOLD:AAB0758
Melanchra pulverulenta|[11605]|BBLPC745-09|Canada|Newfoundland and Labrador|658[0n]|BOLD:AAB0758
Melanchra pulverulenta|[11606]|BBLPC808-09|Canada|Newfoundland and Labrador|658[0n]|BOLD:AAB0758
Melanchra pulverulenta|[11607]|LBCD099-05|Canada|British Columbia|658[0n]|BOLD:AAB0758
Melanchra pulverulenta|[11608]|LBCD610-05|Canada|British Columbia|658[0n]|BOLD:AAB0758
Melanchra pulverulenta|[11609]|LBCD097-05|Canada|British Columbia|658[0n]|BOLD:AAB0758
Melanchra pulverulenta|[11610]|LBCD098-05|Canada|British Columbia|658[0n]|BOLD:AAB0758
Melanchra pulverulenta|[11611]|BBLPB338-10|Canada|British Columbia|658[0n]|BOLD:AAB0758
Melanchra pulverulenta|[11612]|LBCC557-05|Canada|British Columbia|658[0n]|BOLD:AAB0758
Melanchra pulverulenta|[11613]|LBCC295-05|Canada|British Columbia|658[0n]|BOLD:AAB0758
Melanchra pulverulenta|[11614]|LBCC011-05|Canada|British Columbia|658[0n]|BOLD:AAB0758
Melanchra pulverulenta|[11615]|LBCC001-05|Canada|British Columbia|658[0n]|BOLD:AAB0758
Melanchra pulverulenta|[11616]|BBLPE218-09|Canada|Newfoundland and Labrador|658[0n]|BOLD:AAB0758
Melanchra pulverulenta|[11617]|LBCB187-05|Canada|British Columbia|658[0n]|BOLD:AAB0758
Melanchra pulverulenta|[11618]|LBCB186-05|Canada|British Columbia|658[0n]|BOLD:AAB0758
Melanchra pulverulenta|[11619]|LBCB185-05|Canada|British Columbia|658[0n]|BOLD:AAB0758
Melanchra pulverulenta|[11620]|LBCB067-05|Canada|British Columbia|658[0n]|BOLD:AAB0758
Melanchra pulverulenta|[11621]|LBCA932-05|Canada|British Columbia|658[0n]|BOLD:AAB0758
Melanchra pulverulenta|[11622]|LCH249-04|Canada|Manitoba|658[0n]|BOLD:AAB0758
Melanchra pulverulenta|[11623]|SSBAF7414-13|Canada|Alberta|620[0n]|BOLD:AAB0758
Melanchra pulverulenta|[11624]|LBCD096-05|Canada|British Columbia|624[0n]|BOLD:AAB0758
Melanchra pulverulenta|[11625]|RDNMB362-05|Canada|British Columbia|580[0n]|BOLD:AAB0758
Melanchra pulverulenta|[11626]|LOWCD159-06|Canada|British Columbia|616[0n]|BOLD:AAB0758
Melanchra pulverulenta|[11627]|SSPAA9985-13|Canada|Saskatchewan|607[1n]|BOLD:AAB0758
Melanchra pulverulenta|[11628]|SSBAA5712-12|Canada|Alberta|631[0n]|BOLD:AAB0758
Melanchra pulverulenta|[11629]|SSWLD7230-13|Canada|Alberta|591[0n]|BOLD:AAB0758
Melanchra pulverulenta|[11630]|SSWLD6370-13|Canada|Alberta|619[0n]|BOLD:AAB0758
Melanchra pulverulenta|[11631]|SSWLD5787-13|Canada|Alberta|619[0n]|BOLD:AAB0758
Melanchra pulverulenta|[11632]|SSWLD6367-13|Canada|Alberta|617[0n]|BOLD:AAB0758
Melanchra pulverulenta|[11633]|SSPAA9988-13|Canada|Saskatchewan|591[0n]|BOLD:AAB0758
Melanchra pulverulenta|[11634]|SSBAF7753-13|Canada|Alberta|613[0n]|BOLD:AAB0758
Melanchra pulverulenta|[11635]|SSJAE9455-13|Canada|Alberta|601[0n]|BOLD:AAB0758
Lacanobia grandis|[11636]|RDLQB274-05|Canada|Quebec|658[0n]|BOLD:AAB6987
Lacanobia grandis|[11637]|RDLQB275-05|Canada|Quebec|658[0n]|BOLD:AAB6987
Lacanobia grandis|[11638]|RDMAB117-05|Canada|Alberta|658[0n]|BOLD:AAB6987
Lacanobia grandis|[11639]|TTMNB550-06|Canada|New Brunswick|658[0n]|BOLD:AAB6987
Lacanobia grandis|[11640]|PHMNB537-04|Canada|New Brunswick|658[0n]|BOLD:AAB6987
Lacanobia grandis|[11641]|RDLQB273-05|Canada|Quebec|658[0n]|BOLD:AAB6987
Lacanobia grandis|[11642]|XAB242-04|Canada|Ontario|658[0n]|BOLD:AAB6987
Lacanobia grandis|[11643]|XAC294-04|Canada|Ontario|658[0n]|BOLD:AAB6987
Lacanobia grandis|[11644]|PHMNB445-04|Canada|New Brunswick|658[0n]|BOLD:AAB6987
Lacanobia grandis|[11645]|PHMNB459-04|Canada|New Brunswick|658[0n]|BOLD:AAB6987
Lacanobia grandis|[11646]|RDLQB276-05|Canada|Quebec|658[0n]|BOLD:AAB6987
Lacanobia grandis|[11647]|RDLQB277-05|Canada|Quebec|658[0n]|BOLD:AAB6987
Lacanobia grandis|[11648]|LPSOB316-08|Canada|Ontario|658[0n]|BOLD:AAB6987
Lacanobia grandis|[11649]|LPSOB379-08|Canada|Ontario|658[0n]|BOLD:AAB6987
Lacanobia grandis|[11650]|LPVIC118-08|Canada|British Columbia|617[0n]|BOLD:AAB6987
Lacanobia grandis|[11651]|RDLQB278-05|Canada|Quebec|658[0n]|BOLD:AAB6987
Lacanobia grandis|[11652]|RDLQB272-05|Canada|Quebec|658[0n]|BOLD:AAB6987
Lacanobia grandis|[11653]|RDMAB537-06|Canada|Alberta|658[0n]|BOLD:AAB6987
Lacanobia grandis|[11654]|LPMN815-08|Canada|Manitoba|658[0n]|BOLD:AAB6987
Lacanobia grandis|[11655]|LPSOD430-09|Canada|Ontario|658[0n]|BOLD:AAB6987
Papestra brenda|[11656]|RDNMF560-08|Canada|British Columbia|609[0n]|BOLD:ABY8229
Papestra brenda|[11657]|RDMAB1058-09|Canada|British Columbia|600[0n]|BOLD:ABY8229
Papestra brenda|[11658]|RDMAB1059-09|Canada|British Columbia|658[0n]|BOLD:ABY8229
Papestra brenda|[11659]|LBCH5712-10|Canada|British Columbia|658[0n]|BOLD:ABY8229
Papestra brenda|[11660]|IAWLB024-10|United States|California|658[0n]|BOLD:ABY8229
Papestra invalida|[11661]|RDNMF559-08|Canada|British Columbia|658[0n]|BOLD:ACF1818
Papestra invalida|[11662]|RDNMF557-08|United States|California|658[0n]|BOLD:ACF1818
Papestra invalida|[11663]|RDNMF556-08|United States|California|658[0n]|BOLD:ACF1818
Papestra invalida|[11664]|RDNMF558-08|United States|California|658[0n]|BOLD:ACF1818
Papestra invalida|[11665]|LBCH5145-10|Canada|British Columbia|658[0n]|BOLD:ACF1818
Papestra invalida|[11666]|JMMMB419-11|United States|California|658[0n]|BOLD:ACF1818
Papestra cristifera|[11667]|RWWC224-11|United States|Washington|658[0n]|BOLD:AAB4212
Papestra cristifera|[11668]|SSWLD7228-13|Canada|Alberta|560[0n]|BOLD:AAB4212
Papestra cristifera|[11669]|SSWLD7216-13|Canada|Alberta|591[0n]|BOLD:AAB4212
Papestra cristifera|[11670]|SSWLD6375-13|Canada|Alberta|591[0n]|BOLD:AAB4212
Papestra cristifera|[11671]|SSWLD5783-13|Canada|Alberta|602[0n]|BOLD:AAB4212
Papestra cristifera|[11672]|SSWLD6402-13|Canada|Alberta|558[0n]|BOLD:AAB4212
Papestra cristifera|[11673]|SSWLD5786-13|Canada|Alberta|584[0n]|BOLD:AAB4212
Papestra cristifera|[11674]|LPABC081-09|Canada|Alberta|658[1n]|BOLD:AAB4212
Papestra cristifera|[11675]|LALPA1098-11|Canada|British Columbia|658[0n]|BOLD:AAB4212
Papestra cristifera|[11676]|SSWLD6407-13|Canada|Alberta|520[4n]|BOLD:AAB4212
Papestra cristifera|[11677]|SSWLD6374-13|Canada|Alberta|622[0n]|BOLD:AAB4212
Papestra cristifera|[11678]|SSWLD6371-13|Canada|Alberta|622[0n]|BOLD:AAB4212
Papestra cristifera|[11679]|SSWLD6368-13|Canada|Alberta|622[0n]|BOLD:AAB4212
Papestra cristifera|[11680]|SSWLD5788-13|Canada|Alberta|623[0n]|BOLD:AAB4212
Papestra cristifera|[11681]|LBCD047-05|Canada|British Columbia|658[0n]|BOLD:AAB4212
Papestra cristifera|[11682]|LPABC323-09|Canada|Alberta|658[0n]|BOLD:AAB4212
Papestra cristifera|[11683]|LPSOD888-09|Canada|Ontario|658[0n]|BOLD:AAB4212
Papestra cristifera|[11684]|BBLPB762-10|Canada|British Columbia|658[0n]|BOLD:AAB4212
Papestra cristifera|[11685]|SSWLD5785-13|Canada|Alberta|622[0n]|BOLD:AAB4212
Papestra cristifera|[11686]|SSWLD5782-13|Canada|Alberta|622[0n]|BOLD:AAB4212
Papestra cristifera|[11687]|SSWLD5779-13|Canada|Alberta|622[0n]|BOLD:AAB4212
Papestra cristifera|[11688]|LPABC475-09|Canada|Alberta|658[0n]|BOLD:AAB4212
Papestra cristifera|[11689]|SSWLD6366-13|Canada|Alberta|621[0n]|BOLD:AAB4212
Papestra cristifera|[11690]|SSWLD5796-13|Canada|Alberta|591[1n]|BOLD:AAB4212
Papestra cristifera|[11691]|LBCA603-05|Canada|British Columbia|629[0n]|BOLD:AAB4212
Papestra cristifera|[11692]|SSWLD6372-13|Canada|Alberta|615[0n]|BOLD:AAB4212
Papestra cristifera|[11693]|LBCA738-05|Canada|British Columbia|646[0n]|BOLD:AAB4212
Papestra cristifera|[11694]|SSWLD5781-13|Canada|Alberta|613[0n]|BOLD:AAB4212

Papestra cristifera|[11691]|LBCA603-05|Canada|British Columbia|629[0n]|BOLD:AAB4212
Papestra cristifera|[11692]|SSWLD6372-13|Canada|Alberta|615[0n]|BOLD:AAB4212
Papestra cristifera|[11693]|LBCA738-05|Canada|British Columbia|646[0n]|BOLD:AAB4212
Papestra cristifera|[11694]|SSWLD5791-13|Canada|Alberta|613[0n]|BOLD:AAB4212
Papestra cristifera|[11695]|SSWLD5784-13|Canada|Alberta|606[0n]|BOLD:AAB4212
Papestra cristifera|[11696]|LBCD292-05|Canada|British Columbia|627[0n]|BOLD:AAB4212
Papestra cristifera|[11697]|RDLQ642-07|Canada|Newfoundland and Labrador|616[0n]|BOLD:AAB4212
Papestra cristifera|[11698]|SSWLD7227-13|Canada|Alberta|577[0n]|BOLD:AAB4212
Papestra cristifera|[11699]|SSJAD6428-13|Canada|Alberta|595[0n]|BOLD:AAB4212
Papestra quadrata|[11700]|LOWCC157-05|Canada|British Columbia|533[0n]|BOLD:AAA9849
Papestra quadrata|[11701]|LOWCD755-06|Canada|British Columbia|656[0n]|BOLD:AAA9849
Papestra quadrata|[11702]|LOWCD757-06|Canada|British Columbia|656[0n]|BOLD:AAA9849
Papestra quadrata|[11703]|LOWCD758-06|Canada|British Columbia|658[0n]|BOLD:AAA9849
Papestra quadrata|[11704]|RDLQ638-07|Canada|Newfoundland and Labrador|620[0n]|BOLD:AAA9849
Papestra quadrata|[11705]|LCHP381-07|Canada|Manitoba|658[0n]|BOLD:AAA9849
Papestra quadrata|[11706]|CHLEP105-09|Canada|Manitoba|658[0n]|BOLD:AAA9849
Papestra quadrata|[11707]|LBCG2474-09|Canada|British Columbia|621[0n]|BOLD:AAA9849
Papestra quadrata|[11708]|LBCH2255-10|Canada|British Columbia|658[0n]|BOLD:AAA9849
Papestra quadrata|[11709]|LBCH2198-10|Canada|British Columbia|658[0n]|BOLD:AAA9849
Papestra quadrata|[11710]|LBCH1853-10|Canada|British Columbia|658[0n]|BOLD:AAA9849
Papestra quadrata|[11711]|LBCH1750-10|Canada|British Columbia|658[0n]|BOLD:AAA9849
Papestra quadrata|[11712]|LBCH1733-10|Canada|British Columbia|658[0n]|BOLD:AAA9849
Papestra quadrata|[11713]|LBCG3033-09|Canada|British Columbia|658[0n]|BOLD:AAA9849
Papestra quadrata|[11714]|LBCG3032-09|Canada|British Columbia|658[0n]|BOLD:AAA9849
Papestra quadrata|[11715]|LBCG2775-09|Canada|British Columbia|658[0n]|BOLD:AAA9849
Papestra quadrata|[11716]|LBCG2774-09|Canada|British Columbia|658[0n]|BOLD:AAA9849
Papestra quadrata|[11717]|LBCG2773-09|Canada|British Columbia|658[0n]|BOLD:AAA9849
Papestra quadrata|[11718]|LBCG2488-09|Canada|British Columbia|658[0n]|BOLD:AAA9849
Papestra quadrata|[11719]|LBCG2487-09|Canada|British Columbia|658[0n]|BOLD:AAA9849
Papestra quadrata|[11720]|LBCH2265-10|Canada|British Columbia|658[0n]|BOLD:AAA9849
Papestra quadrata|[11721]|BBLPB873-10|Canada|British Columbia|658[0n]|BOLD:AAA9849
Papestra quadrata|[11722]|RDNMD554-06|United States|Colorado|658[6n]|BOLD:AAA9849
Papestra quadrata|[11723]|RDNMD555-06|United States|Colorado|622[8n]|
Papestra quadrata|[11724]|IAWLB046-10|United States|California|658[0n]|BOLD:AAA9849
Papestra quadrata|[11725]|IAWLB045-10|United States|California|658[0n]|BOLD:AAA9849
Papestra quadrata|[11726]|CHLEP215-09|Canada|Manitoba|658[0n]|BOLD:AAA9849
Papestra quadrata|[11727]|CHLEP157-09|Canada|Manitoba|658[0n]|BOLD:AAA9849
Papestra quadrata|[11728]|LCHP794-07|Canada|Manitoba|658[0n]|BOLD:AAA9849
Papestra quadrata|[11729]|LCHP243-07|Canada|Manitoba|658[0n]|BOLD:AAA9849
Papestra quadrata|[11730]|LBCG2834-09|Canada|British Columbia|658[0n]|BOLD:AAA9849
Papestra quadrata|[11731]|RDMAB608-06|Canada|Alberta|658[0n]|BOLD:AAA9849
Papestra quadrata|[11732]|CNWBE858-13|Canada|Alberta|592[0n]|BOLD:AAA9849
Papestra quadrata|[11733]|LCHP251-07|Canada|Manitoba|656[0n]|BOLD:AAA9849
Papestra quadrata|[11734]|IAWLB048-10|United States|California|650[0n]|BOLD:AAA9849
Papestra quadrata|[11735]|CHLEP218-09|Canada|Manitoba|643[0n]|BOLD:AAA9849
Papestra quadrata|[11736]|RDMAB607-06|Canada|Alberta|624[0n]|BOLD:AAA9849
Papestra quadrata|[11737]|IAWLB049-10|United States|California|605[0n]|BOLD:AAA9849
Papestra quadrata|[11738]|RDLQ637-07|Canada|Quebec|618[0n]|BOLD:AAA9849
Papestra quadrata|[11739]|CNWBE859-13|Canada|Alberta|588[0n]|BOLD:AAA9849
Papestra quadrata|[11740]|LCHP465-07|Canada|Manitoba|632[0n]|BOLD:AAA9849
Papestra quadrata|[11741]|LCH259-04|Canada|Manitoba|658[0n]|BOLD:AAA9849
Papestra quadrata|[11742]|LCH260-04|Canada|Manitoba|658[0n]|BOLD:AAA9849
Papestra quadrata|[11743]|LCHP009-07|Canada|Manitoba|658[0n]|BOLD:AAA9849
Papestra quadrata|[11744]|LCHP240-07|Canada|Manitoba|658[0n]|BOLD:AAA9849
Papestra quadrata|[11745]|MHCOL149-07|Canada|Manitoba|658[0n]|BOLD:AAA9849
Papestra quadrata|[11746]|CHLEP217-09|Canada|Manitoba|658[0n]|BOLD:AAA9849
Papestra quadrata|[11747]|CNWBE862-13|Canada|Alberta|606[0n]|BOLD:AAA9849
Papestra biren|[11748]|KSPBL172-10|Austria|Styria|614[0n]|BOLD:AAA9849
Papestra biren|[11749]|PHLAE337-11|Austria|Vorarlberg|658[0n]|BOLD:AAA9849
Papestra biren|[11750]|LEATD036-13|Austria|Tirol|658[0n]|BOLD:AAA9849
Papestra biren|[11751]|RDNMD113-06|Italy||658[0n]|BOLD:AAA9849
Papestra biren|[11752]|KSPBL173-10|Austria|Styria|658[0n]|BOLD:AAA9849
Papestra biren|[11753]|LEATD362-13|Austria|Tirol|658[0n]|BOLD:AAA9849
Papestra biren|[11754]|RDNMD484-06|United States|Colorado|658[0n]|BOLD:AAA9849
Papestra biren|[11755]|RDNMD485-06|United States|Colorado|658[0n]|BOLD:AAA9849
Papestra biren|[11756]|RDLQ641-07|Canada|Newfoundland and Labrador|658[0n]|BOLD:AAA9849
Papestra biren|[11757]|IBLAO415-12|Spain|Catalonia|658[0n]|BOLD:AAA9849
Papestra biren|[11758]|KSPBL171-10|Czech Republic|Southern Bohemia|658[0n]|BOLD:AAA9849
Papestra biren|[11759]|KSPBL170-10|Czech Republic|Southern Bohemia|658[0n]|BOLD:AAA9849
Papestra biren|[11760]|KSPBL169-10|Czech Republic|Southern Bohemia|658[0n]|BOLD:AAA9849
Papestra biren|[11761]|KSPBL168-10|Czech Republic|Southern Bohemia|658[0n]|BOLD:AAA9849
Papestra biren|[11762]|KSPBL167-10|Czech Republic|Southern Bohemia|658[0n]|BOLD:AAA9849
Papestra biren|[11763]|KSPBL166-10|Czech Republic|Southern Bohemia|658[0n]|BOLD:AAA9849
Papestra biren|[11764]|LEFIG616-10|Finland||658[0n]|BOLD:AAA9849
Papestra biren|[11765]|LEFIF629-10|Finland||658[0n]|BOLD:AAA9849
Papestra biren|[11766]|FBLMV063-09|Germany|Bavaria|658[0n]|BOLD:AAA9849
Papestra biren|[11767]|GWORK556-09|Germany|Bavaria|658[0n]|BOLD:AAA9849
Papestra biren|[11768]|RDNMF561-08|Canada|British Columbia|658[0n]|BOLD:AAA9849
Papestra biren|[11769]|TMNBB321-06|Canada|New Brunswick|658[0n]|BOLD:AAA9849
Papestra biren|[11770]|TMNBB320-06|Canada|New Brunswick|658[0n]|BOLD:AAA9849
Papestra biren|[11771]|TMNBB319-06|Canada|New Brunswick|658[0n]|BOLD:AAA9849
Papestra biren|[11772]|TMNBB317-06|Canada|New Brunswick|658[0n]|BOLD:AAA9849
Papestra biren|[11773]|TMNBB318-06|Canada|New Brunswick|658[0n]|BOLD:AAA9849
Papestra biren|[11774]|TMNBB316-06|Canada|New Brunswick|658[0n]|BOLD:AAA9849
Papestra biren|[11775]|RDNMD112-06|Russia|Magadan|658[0n]|BOLD:AAA9849
Papestra biren|[11776]|GWORO835-09|Germany|Bavaria|658[0n]|BOLD:AAA9849
Papestra biren|[11777]|CGUKD868-09|United Kingdom|England|624[0n]|BOLD:AAA9849
Papestra biren|[11778]|CHLEP310-09|Canada|Manitoba|639[0n]|BOLD:AAA9849
Papestra biren|[11779]|LEFIA747-10|Finland|Lapland|632[0n]|BOLD:AAA9849
Papestra biren|[11780]|KSPBL165-10|Czech Republic|Southern Bohemia|632[0n]|BOLD:AAA9849
Papestra biren|[11781]|GWORL329-09|Germany|Bavaria|658[0n]|BOLD:AAA9849
Papestra biren|[11782]|GWOSZ117-11|Italy|Trentino-Alto Adige|658[0n]|BOLD:AAA9849
Papestra biren|[11783]|IBLAO394-12|Spain|Catalonia|658[0n]|BOLD:AAA9849
Papestra biren|[11784]|GWOTG904-12|Russia|Irkutsk|658[0n]|BOLD:AAA9849
Papestra biren|[11785]|LEATD035-13|Austria|Tirol|658[0n]|BOLD:AAA9849
Papestra biren|[11786]|GBLAF549-14|Germany|Brandenburg|658[0n]|BOLD:AAA9849
Lacanobia radix|[11787]|SSWLA3858-13|Canada|Alberta|577[0n]|BOLD:AAB6755
Lacanobia radix|[11788]|LOWCC189-05|Canada|British Columbia|658[0n]|BOLD:AAB6755
Lacanobia radix|[11789]|LBCA600-05|Canada|British Columbia|650[0n]|BOLD:AAB6755
Lacanobia radix|[11790]|LOWCC201-05|Canada|British Columbia|603[0n]|BOLD:AAB6755
Lacanobia radix|[11791]|LOWCC202-05|Canada|British Columbia|599[0n]|BOLD:AAB6755
Lacanobia radix|[11792]|LALPA412-10|Canada|British Columbia|658[0n]|BOLD:AAB6755
Lacanobia radix|[11793]|CNRMC1483-12|Canada|Manitoba|615[0n]|BOLD:AAB6755
Lacanobia radix|[11794]|LOWCF376-06|Canada|British Columbia|[illegible]|BOLD:AAB6755

Lacanobia radix|[11791]|LOWCC202-05|Canada|British Columbia|599[0n]|BOLD:AAB6755
Lacanobia radix|[11792]|LALPA412-10|Canada|British Columbia|658[0n]|BOLD:AAB6755
Lacanobia radix|[11793]|CNRMC1483-12|Canada|Manitoba|615[0n]|BOLD:AAB6755
Lacanobia radix|[11794]|LOWCE276-06|Canada|British Columbia|590[0n]|BOLD:AAB6755
Lacanobia radix|[11795]|BBLSY562-09|United States|New Mexico|658[0n]|BOLD:AAB6755
Lacanobia radix|[11796]|BBLPB348-10|Canada|British Columbia|658[0n]|BOLD:AAB6755
Lacanobia radix|[11797]|LPABB878-09|Canada|Alberta|658[0n]|BOLD:AAB6755
Lacanobia radix|[11798]|LPMN712-08|Canada|Manitoba|658[0n]|BOLD:AAB6755
Lacanobia radix|[11799]|LCHP965-07|Canada|Manitoba|658[0n]|BOLD:AAB6755
Lacanobia radix|[11800]|LCHP835-07|Canada|Manitoba|658[0n]|BOLD:AAB6755
Lacanobia radix|[11801]|LCHP543-07|Canada|Manitoba|658[0n]|BOLD:AAB6755
Lacanobia radix|[11802]|RDLQG371-06|Canada|Quebec|658[0n]|BOLD:AAB6755
Lacanobia radix|[11803]|RDLQB807-05|Canada|Quebec|658[0n]|BOLD:AAB6755
Lacanobia radix|[11804]|LOWCC200-05|Canada|British Columbia|658[0n]|BOLD:AAB6755
Lacanobia radix|[11805]|LBCA547-05|Canada|British Columbia|658[0n]|BOLD:AAB6755
Lacanobia radix|[11806]|LCH586-04|Canada|Manitoba|658[0n]|BOLD:AAB6755
Lacanobia radix|[11807]|LCH247-04|Canada|Manitoba|658[0n]|BOLD:AAB6755
Lacanobia radix|[11808]|LHLEP566-06|Canada|British Columbia|653[1n]|BOLD:AAB6755
Lacanobia radix|[11809]|LPABC251-09|Canada|Alberta|625[1n]|BOLD:AAB6755
Lacanobia radix|[11810]|LPABC396-09|Canada|Alberta|658[0n]|BOLD:AAB6755
Lacanobia radix|[11811]|BBLSY560-09|United States|New Mexico|658[0n]|BOLD:AAB6755
Lacanobia radix|[11812]|LALPA956-11|Canada|British Columbia|658[0n]|BOLD:AAB6755
Lacanobia radix|[11813]|SSWLA3859-13|Canada|Alberta|604[0n]|BOLD:AAB6755
Lacanobia radix|[11814]|CNWBF029-13|Canada|Alberta|587[0n]|BOLD:AAB6755
Lacanobia radix|[11815]|CNWLL015-13|Canada|Alberta|555[0n]|BOLD:AAB6755
Lacanobia atlantica|[11816]|TTMNB397-06|Canada|New Brunswick|658[0n]|BOLD:AAB7145
Lacanobia atlantica|[11817]|RDNM088-05|Canada|Ontario|658[0n]|BOLD:AAB7145
Lacanobia atlantica|[11818]|LPSOB230-08|Canada|Ontario|646[0n]|BOLD:AAB7145
Lacanobia atlantica|[11819]|LPSOB243-08|Canada|Ontario|658[0n]|BOLD:AAB7145
Lacanobia atlantica|[11820]|LPSK124-08|Canada|Saskatchewan|658[0n]|BOLD:AAB7145
Lacanobia atlantica|[11821]|RDNM084-05|Canada|British Columbia|573[1n]|BOLD:AAB7145
Lacanobia atlantica|[11822]|RDNM085-05|Canada|British Columbia|566[0n]|BOLD:AAB7145
Lacanobia atlantica|[11823]|LPMN802-08|Canada|Manitoba|658[0n]|BOLD:AAB7145
Lacanobia atlantica|[11824]|BBLPB800-10|Canada|British Columbia|658[0n]|BOLD:AAB7145
Lacanobia atlantica|[11825]|RDNM086-05|Canada|British Columbia|500[0n]|
Lacanobia atlantica|[11826]|RDNM192-05|Canada|British Columbia|658[0n]|BOLD:AAB7145
Lacanobia atlantica|[11827]|RDNM193-05|Canada|British Columbia|658[0n]|BOLD:AAB7145
Lacanobia atlantica|[11828]|RDNM087-05|Canada|Ontario|658[0n]|BOLD:AAB7145
Lacanobia atlantica|[11829]|RDNM189-05|Canada|Alberta|658[0n]|BOLD:AAB7145
Lacanobia atlantica|[11830]|RDNM190-05|Canada|Alberta|658[0n]|BOLD:AAB7145
Lacanobia atlantica|[11831]|RDNM191-05|Canada|British Columbia|658[0n]|BOLD:AAB7145
Lacanobia atlantica|[11832]|RDLQ632-07|Canada|Quebec|658[0n]|BOLD:AAB7145
Lacanobia atlantica|[11833]|RDLQ633-07|Canada|Quebec|658[0n]|BOLD:AAB7145
Lacanobia atlantica|[11834]|LPMN338-08|Canada|Manitoba|658[0n]|BOLD:AAB7145
Lacanobia atlantica|[11835]|BBLPB805-10|Canada|Saskatchewan|658[0n]|BOLD:AAB7145
Lacanobia atlantica|[11836]|BBLPB806-10|Canada|Ontario|658[0n]|BOLD:AAB7145
Lacanobia atlantica|[11837]|BBLPB857-10|Canada|Ontario|658[0n]|BOLD:AAB7145
Lacanobia nevadae|[11838]|BBLPE419-09|Canada|Newfoundland and Labrador|658[0n]|BOLD:ABY4611
Lacanobia nevadae|[11839]|CHLEP280-09|Canada|Manitoba|658[0n]|BOLD:ABY4611
Lacanobia nevadae|[11840]|LPABC010-09|Canada|Alberta|658[0n]|BOLD:ABY4611
Lacanobia nevadae|[11841]|LPABB078-08|Canada|Alberta|658[0n]|BOLD:ABY4611
Lacanobia nevadae|[11842]|LPAB235-08|Canada|Alberta|658[0n]|BOLD:ABY4611
Lacanobia nevadae|[11843]|RDLQF457-06|Canada|Quebec|658[0n]|BOLD:ABY4611
Lacanobia nevadae|[11844]|LOWCD761-06|Canada|British Columbia|657[0n]|BOLD:ABY4611
Lacanobia nevadae|[11845]|LBCD454-05|Canada|British Columbia|658[0n]|BOLD:ABY4611
Lacanobia nevadae|[11846]|LBCD105-05|Canada|British Columbia|658[0n]|BOLD:ABY4611
Lacanobia nevadae|[11847]|LBCB825-05|Canada|British Columbia|658[0n]|BOLD:ABY4611
Lacanobia nevadae|[11848]|SSPAA10030-13|Canada|Saskatchewan|542[0n]|BOLD:ABY4611
Lacanobia nevadae|[11849]|LPABC479-09|Canada|Alberta|623[0n]|BOLD:ABY4611
Lacanobia nevadae|[11850]|BBLPC714-09|Canada|Newfoundland and Labrador|547[0n]|BOLD:ABY4611
Lacanobia nevadae|[11851]|SSPAA10031-13|Canada|Saskatchewan|565[0n]|BOLD:ABY4611
Lacanobia nevadae|[11852]|SSPAA10033-13|Canada|Saskatchewan|602[0n]|BOLD:ABY4611
Lacanobia subjuncta|[11853]|RDNMJ370-11|United States|Arizona|658[0n]|BOLD:ACF0664
Lacanobia subjuncta|[11854]|IAWLB531-11|United States|Arizona|658[0n]|BOLD:ACF0664
Lacanobia subjuncta|[11855]|LBCH5697-10|Canada|British Columbia|658[0n]|BOLD:ACF0859
Lacanobia subjuncta|[11856]|LBCH5673-10|Canada|British Columbia|658[0n]|BOLD:ACF0859
Lacanobia subjuncta|[11857]|LBCH5912-10|Canada|British Columbia|658[0n]|BOLD:ACF0859
Lacanobia subjuncta|[11858]|IAWLB118-10|United States|California|658[0n]|BOLD:ACF0859
Lacanobia subjuncta|[11859]|LALPA924-11|Canada|British Columbia|658[0n]|BOLD:ACF0859
Lacanobia subjuncta|[11860]|LPVIA231-08|Canada|British Columbia|658[0n]|BOLD:AAB0925
Lacanobia subjuncta|[11861]|RWWA924-09|United States|Washington|658[0n]|BOLD:AAB0925
Lacanobia subjuncta|[11862]|LALPA1116-11|Canada|British Columbia|658[0n]|BOLD:AAB0925
Lacanobia subjuncta|[11863]|XAB563-04|Canada|Ontario|658[0n]|BOLD:AAB0925
Lacanobia subjuncta|[11864]|XAB103-04|Canada|Ontario|658[0n]|BOLD:AAB0925
Lacanobia subjuncta|[11865]|PHMO342-03|Canada|Ontario|639[0n]|BOLD:AAB0925
Lacanobia subjuncta|[11866]|XAH009-05|Canada|Ontario|647[0n]|BOLD:AAB0925
Lacanobia subjuncta|[11867]|RDLQB508-05|Canada|Quebec|658[0n]|BOLD:AAB0925
Lacanobia subjuncta|[11868]|RDMAB124-05|Canada|Alberta|658[0n]|BOLD:AAB0925
Lacanobia subjuncta|[11869]|RDLQF271-06|Canada|Quebec|658[0n]|BOLD:AAB0925
Lacanobia subjuncta|[11870]|LPSOC315-08|Canada|Ontario|658[0n]|BOLD:AAB0925
Lacanobia subjuncta|[11871]|UDLEP254-09|United States|Pennsylvania|658[0n]|BOLD:AAB0925
Lacanobia subjuncta|[11872]|LALPA257-10|Canada|British Columbia|658[0n]|BOLD:AAB0925
Lacanobia subjuncta|[11873]|LALPA338-10|Canada|British Columbia|658[0n]|BOLD:AAB0925
Lacanobia subjuncta|[11874]|LALPA838-11|Canada|British Columbia|658[0n]|BOLD:AAB0925
Lacanobia subjuncta|[11875]|LBCA583-05|Canada|British Columbia|658[0n]|BOLD:AAB0925
Lacanobia subjuncta|[11876]|LOWCC823-05|Canada|British Columbia|588[0n]|BOLD:AAB0925
Lacanobia subjuncta|[11877]|LOWCC825-05|Canada|British Columbia|658[0n]|BOLD:AAB0925
Lacanobia subjuncta|[11878]|LOWCC824-05|Canada|British Columbia|594[0n]|BOLD:AAB0925
Lacanobia subjuncta|[11879]|LOWCD753-06|Canada|British Columbia|594[0n]|BOLD:AAB0925
Lacanobia subjuncta|[11880]|XAH428-05|Canada|Ontario|658[0n]|BOLD:AAB0925
Lacanobia subjuncta|[11881]|LBCA581-05|Canada|British Columbia|658[0n]|BOLD:AAB0925
Lacanobia subjuncta|[11882]|LBCB149-05|Canada|British Columbia|658[0n]|BOLD:AAB0925
Lacanobia subjuncta|[11883]|LBCB184-05|Canada|British Columbia|658[0n]|BOLD:AAB0925
Lacanobia subjuncta|[11884]|LBCA584-05|Canada|British Columbia|652[0n]|BOLD:AAB0925
Lacanobia subjuncta|[11885]|PHMO257-03|Canada|Ontario|639[0n]|BOLD:AAB0925
Lacanobia subjuncta|[11886]|XAD287-04|Canada|Ontario|598[0n]|BOLD:AAB0925
Lacanobia subjuncta|[11887]|LOWCC821-05|Canada|British Columbia|606[0n]|BOLD:AAB0925
Lacanobia subjuncta|[11888]|LOWCC822-05|Canada|British Columbia|658[0n]|BOLD:AAB0925
Lacanobia subjuncta|[11889]|LOWCC826-05|Canada|British Columbia|658[0n]|BOLD:AAB0925
Lacanobia subjuncta|[11890]|LOWCC827-05|Canada|British Columbia|658[0n]|BOLD:AAB0925
Lacanobia subjuncta|[11891]|LOWCC828-05|Canada|British Columbia|658[0n]|BOLD:AAB0925
Lacanobia subjuncta|[11892]|LOWCD749-06|Canada|British Columbia|658[0n]|BOLD:AAB0925
Lacanobia subjuncta|[11893]|LOWCD750-06|Canada|British Columbia|658[0n]|BOLD:AAB0925
[illegible]

Lacanobia subjuncta|[11891]|LOWCC828-05|Canada|British Columbia|658[0n]|BOLD:AAB0925
Lacanobia subjuncta|[11892]|LOWCD749-06|Canada|British Columbia|658[0n]|BOLD:AAB0925
Lacanobia subjuncta|[11893]|LOWCD750-06|Canada|British Columbia|658[0n]|BOLD:AAB0925
Lacanobia subjuncta|[11894]|LOWCD751-06|Canada|British Columbia|658[0n]|BOLD:AAB0925
Lacanobia subjuncta|[11895]|LOWCD752-06|Canada|British Columbia|658[0n]|BOLD:AAB0925
Lacanobia subjuncta|[11896]|LOWCD754-06|Canada|British Columbia|657[0n]|BOLD:AAB0925
Lacanobia subjuncta|[11897]|CNCLB2862-14|United States|North Carolina|658[0n]|BOLD:AAB0925
Trichordestra prodeniformis|[11898]|NAMUM072-08|United States|Arizona|657[0n]|BOLD:AAF5386
Trichordestra prodeniformis|[11899]|CMAZA569-10|United States|Arizona|658[0n]|BOLD:AAF5386
Trichordestra prodeniformis|[11900]|RDNMJ735-11|United States|Arizona|658[0n]|BOLD:AAF5386
Trichordestra lilacina|[11901]|BTWC018-09|Canada|Ontario|553[2n]|
Trichordestra lilacina|[11902]|LOWCD263-06|Canada|British Columbia|656[1n]|BOLD:AAB1044
Trichordestra lilacina|[11903]|RWWB812-10|United States|Washington|658[0n]|BOLD:AAB1044
Trichordestra lilacina|[11904]|LALPA318-10|Canada|British Columbia|658[0n]|BOLD:AAB1044
Trichordestra lilacina|[11905]|RWWC411-11|United States|Washington|658[0n]|BOLD:AAB1044
Trichordestra lilacina|[11906]|RWWB698-10|United States|Washington|658[0n]|BOLD:AAB1044
Trichordestra lilacina|[11907]|RWWA473-09|United States|Washington|658[0n]|BOLD:AAB1044
Trichordestra lilacina|[11908]|RWWA350-09|United States|Washington|658[0n]|BOLD:AAB1044
Trichordestra lilacina|[11909]|RWWA333-09|United States|Washington|658[0n]|BOLD:AAB1044
Trichordestra lilacina|[11910]|LHLEP565-06|Canada|British Columbia|658[0n]|BOLD:AAB1044
Trichordestra lilacina|[11911]|LHLEP270-06|Canada|British Columbia|658[0n]|BOLD:AAB1044
Trichordestra lilacina|[11912]|LHLEP269-06|Canada|British Columbia|658[0n]|BOLD:AAB1044
Trichordestra lilacina|[11913]|LHLEP068-06|Canada|British Columbia|658[0n]|BOLD:AAB1044
Trichordestra lilacina|[11914]|LBCA501-05|Canada|British Columbia|658[0n]|BOLD:AAB1044
Trichordestra lilacina|[11915]|LBCA504-05|Canada|British Columbia|658[0n]|BOLD:AAB1044
Trichordestra lilacina|[11916]|LBCA498-05|Canada|British Columbia|658[0n]|BOLD:AAB1044
Trichordestra lilacina|[11917]|LOWCC125-05|Canada|British Columbia|546[5n]|BOLD:AAB1044
Trichordestra lilacina|[11918]|LOWCC117-05|Canada|British Columbia|537[0n]|BOLD:AAB1044
Trichordestra lilacina|[11919]|LOWCD262-06|Canada|British Columbia|605[1n]|BOLD:AAB1044
Trichordestra lilacina|[11920]|RDLQG049-06|Canada|Quebec|643[0n]|BOLD:AAB1044
Trichordestra lilacina|[11921]|RWWC1147-13|United States|Washington|579[0n]|BOLD:AAB1044
Trichordestra lilacina|[11922]|CNGRK426-13|Canada|Saskatchewan|615[0n]|BOLD:AAB1044
Trichordestra lilacina|[11923]|SSBAD6150-13|Canada|Alberta|613[0n]|BOLD:AAB1044
Trichordestra lilacina|[11924]|RWWA172-09|United States|Washington|629[0n]|BOLD:AAB1044
Trichordestra lilacina|[11925]|BTWC057-09|Canada|Ontario|550[0n]|
Trichordestra lilacina|[11926]|RWWC348-11|United States|Washington|658[0n]|BOLD:AAB1044
Trichordestra lilacina|[11927]|RWWB931-10|United States|Washington|658[0n]|BOLD:AAB1044
Trichordestra lilacina|[11928]|RWWB922-10|United States|Washington|658[0n]|BOLD:AAB1044
Trichordestra lilacina|[11929]|RWWB647-10|United States|Washington|658[0n]|BOLD:AAB1044
Trichordestra lilacina|[11930]|RWWA536-09|United States|Washington|658[0n]|BOLD:AAB1044
Trichordestra lilacina|[11931]|RWWA197-09|United States|Washington|658[0n]|BOLD:AAB1044
Trichordestra lilacina|[11932]|RWWA106-09|United States|Washington|658[0n]|BOLD:AAB1044
Trichordestra lilacina|[11933]|LPSK554-08|Canada|Saskatchewan|658[0n]|BOLD:AAB1044
Trichordestra lilacina|[11934]|RDLQ639-07|Canada|Quebec|658[0n]|BOLD:AAB1044
Trichordestra lilacina|[11935]|RDLQG051-06|Canada|Quebec|658[0n]|BOLD:AAB1044
Trichordestra lilacina|[11936]|RDLQB437-05|Canada|Quebec|658[0n]|BOLD:AAB1044
Trichordestra lilacina|[11937]|LBCA500-05|Canada|British Columbia|658[0n]|BOLD:AAB1044
Trichordestra lilacina|[11938]|LBCA499-05|Canada|British Columbia|658[0n]|BOLD:AAB1044
Trichordestra lilacina|[11939]|PHMNB755-05|Canada|New Brunswick|658[0n]|BOLD:AAB1044
Trichordestra lilacina|[11940]|PHMNB371-04|Canada|New Brunswick|658[0n]|BOLD:AAB1044
Trichordestra lilacina|[11941]|PHMNB370-04|Canada|New Brunswick|658[0n]|BOLD:AAB1044
Trichordestra lilacina|[11942]|RDNMF553-08|Canada|Ontario|658[1n]|BOLD:AAB1044
Trichordestra lilacina|[11943]|RDLQ640-07|Canada|Quebec|631[0n]|BOLD:AAB1044
Trichordestra lilacina|[11944]|LOWCC123-05|Canada|British Columbia|587[1n]|BOLD:AAB1044
Trichordestra lilacina|[11945]|LOWCC113-05|Canada|British Columbia|579[1n]|BOLD:AAB1044
Trichordestra lilacina|[11946]|RWWA591-09|United States|Washington|621[0n]|BOLD:AAB1044
Trichordestra lilacina|[11947]|NGNAX941-14|Canada|British Columbia|588[0n]|BOLD:AAB1044
Trichordestra tacoma|[11948]|CDIBC001-06|Canada|British Columbia|658[0n]|BOLD:ABY8753
Trichordestra tacoma|[11949]|JSBIC003-08|Canada|Ontario|648[0n]|BOLD:ABY8753
Trichordestra tacoma|[11950]|JSBIC004-08|Canada|New Brunswick|658[0n]|BOLD:ABY8753
Trichordestra tacoma|[11951]|LPMN245-08|Canada|Manitoba|658[0n]|BOLD:ABY8753
Trichordestra tacoma|[11952]|LPAB234-08|Canada|Alberta|658[0n]|BOLD:ABY8753
Trichordestra tacoma|[11953]|BBLPB868-10|Canada|British Columbia|658[0n]|BOLD:ABY8753
Trichordestra liquida|[11954]|LBCA028-05|Canada|British Columbia|658[0n]|BOLD:AAA8701
Trichordestra liquida|[11955]|LBCA306-05|Canada|British Columbia|606[92n]|
Trichordestra liquida|[11956]|RWWB723-10|United States|Washington|658[0n]|BOLD:AAA8701
Trichordestra liquida|[11957]|LBCA040-05|Canada|British Columbia|658[2n]|BOLD:AAA8701
Trichordestra liquida|[11958]|LBCA039-05|Canada|British Columbia|658[3n]|BOLD:AAA8701
Trichordestra liquida|[11959]|RWWA618-09|United States|Washington|658[0n]|BOLD:AAA8701
Trichordestra liquida|[11960]|RWWA309-09|United States|Washington|658[0n]|BOLD:AAA8701
Trichordestra liquida|[11961]|RWWA205-09|United States|Washington|656[0n]|BOLD:AAA8701
Trichordestra liquida|[11962]|RWWA125-09|United States|Washington|658[0n]|BOLD:AAA8701
Trichordestra liquida|[11963]|LPMN917-08|Canada|Alberta|658[0n]|BOLD:AAA8701
Trichordestra liquida|[11964]|LOWCC121-05|Canada|British Columbia|646[0n]|BOLD:AAA8701
Trichordestra liquida|[11965]|LOWCD261-06|Canada|British Columbia|611[2n]|BOLD:AAA8701
Trichordestra liquida|[11966]|LOWCC114-05|Canada|British Columbia|621[0n]|BOLD:AAA8701
Trichordestra liquida|[11967]|LOWCC110-05|Canada|British Columbia|548[1n]|BOLD:AAA8701
Trichordestra liquida|[11968]|LOWCC108-05|Canada|British Columbia|554[0n]|BOLD:AAA8701
Trichordestra liquida|[11969]|LOWCC115-05|Canada|British Columbia|549[0n]|BOLD:AAA8701
Trichordestra liquida|[11970]|LOWCC120-05|Canada|British Columbia|567[0n]|BOLD:AAA8701
Trichordestra liquida|[11971]|LPVIC120-08|Canada|British Columbia|608[0n]|BOLD:AAA8701
Trichordestra liquida|[11972]|LALPA193-10|Canada|British Columbia|658[0n]|BOLD:AAA8701
Trichordestra liquida|[11973]|JMMMB384-11|United States|California|658[0n]|BOLD:AAA8701
Trichordestra liquida|[11974]|LBCA502-05|Canada|British Columbia|658[0n]|BOLD:AAA8701
Trichordestra liquida|[11975]|LPVIC098-08|Canada|British Columbia|616[0n]|BOLD:AAA8701
Trichordestra liquida|[11976]|RWWA318-09|United States|Washington|658[0n]|BOLD:AAA8701
Trichordestra liquida|[11977]|RWWA472-09|United States|Washington|658[0n]|BOLD:AAA8701
Trichordestra liquida|[11978]|RWWA721-09|United States|Washington|658[0n]|BOLD:AAA8701
Trichordestra liquida|[11979]|RWWA923-09|United States|Washington|658[0n]|BOLD:AAA8701
Trichordestra liquida|[11980]|RWWC208-11|United States|Washington|658[0n]|BOLD:AAA8701
Trichordestra liquida|[11981]|LBCA246-05|Canada|British Columbia|658[0n]|BOLD:AAA8701
Trichordestra liquida|[11982]|LBCA244-05|Canada|British Columbia|658[0n]|BOLD:AAA8701
Trichordestra liquida|[11983]|LBCA196-05|Canada|British Columbia|658[0n]|BOLD:AAA8701
Trichordestra liquida|[11984]|LBCA248-05|Canada|British Columbia|643[0n]|BOLD:AAA8701
Trichordestra liquida|[11985]|LBCA449-05|Canada|British Columbia|632[0n]|BOLD:AAA8701
Trichordestra liquida|[11986]|LBCA930-05|Canada|British Columbia|604[0n]|BOLD:AAA8701
Trichordestra liquida|[11987]|LBCB483-05|Canada|British Columbia|643[0n]|BOLD:AAA8701
Trichordestra liquida|[11988]|BBLPB708-10|Canada|British Columbia|658[0n]|BOLD:AAA8701
Trichordestra liquida|[11989]|BBLPB709-10|Canada|British Columbia|658[0n]|BOLD:AAA8701
Trichordestra liquida|[11990]|LBCB484-05|Canada|British Columbia|649[2n]|BOLD:AAA8701
Trichordestra liquida|[11991]|LBCA198-05|Canada|British Columbia|658[0n]|BOLD:AAA8701
Trichordestra liquida|[11992]|LBCA197-05|Canada|British Columbia|658[0n]|BOLD:AAA8701
Trichordestra liquida|[11993]|LBCA307-05|Canada|British Columbia|645[0n]|BOLD:AAA8701
[illegible]

Trichordestra liquida|[11991]|LBCA198-05|Canada|British Columbia|658[0n]|BOLD:AAA8701
Trichordestra liquida|[11992]|LBCA197-05|Canada|British Columbia|658[0n]|BOLD:AAA8701
Trichordestra liquida|[11993]|LBCA307-05|Canada|British Columbia|645[0n]|BOLD:AAA8701
Trichordestra liquida|[11994]|LBCA432-05|Canada|British Columbia|658[0n]|BOLD:AAA8701
Trichordestra liquida|[11995]|LOWCC109-05|Canada|British Columbia|658[0n]|BOLD:AAA8701
Trichordestra liquida|[11996]|LOWCC111-05|Canada|British Columbia|658[0n]|BOLD:AAA8701
Trichordestra liquida|[11997]|LOWCC112-05|Canada|British Columbia|658[0n]|BOLD:AAA8701
Trichordestra liquida|[11998]|LOWCC118-05|Canada|British Columbia|658[0n]|BOLD:AAA8701
Trichordestra liquida|[11999]|LOWCC119-05|Canada|British Columbia|658[0n]|BOLD:AAA8701
Trichordestra liquida|[12000]|LOWCC122-05|Canada|British Columbia|658[0n]|BOLD:AAA8701
Trichordestra liquida|[12001]|LPVIA105-08|Canada|British Columbia|658[0n]|BOLD:AAA8701
Trichordestra liquida|[12002]|LALPA116-10|Canada|British Columbia|658[0n]|BOLD:AAA8701
Trichordestra liquida|[12003]|LALPA136-10|Canada|British Columbia|658[0n]|BOLD:AAA8701
Trichordestra liquida|[12004]|LALPA317-10|Canada|British Columbia|658[0n]|BOLD:AAA8701
Trichordestra liquida|[12005]|BBLPB710-10|Canada|British Columbia|658[0n]|BOLD:AAA8701
Trichordestra liquida|[12006]|LALPA1126-11|Canada|British Columbia|658[0n]|BOLD:AAA8701
Trichordestra beanii|[12007]|CNCLB737-14|United States|Arkansas|658[0n]|BOLD:ACK4362
Trichordestra beanii|[12008]|CNCLB738-14|United States|North Carolina|658[0n]|BOLD:ACK4362
Trichordestra beanii|[12009]|CNCLB739-14|United States|Arkansas|658[0n]|BOLD:ACK4362
Trichordestra beanii|[12010]|CNCLB740-14|United States|Arkansas|658[0n]|BOLD:ACK4362
Trichordestra beanii|[12011]|LNAUS4811-13|United States|South Carolina|658[0n]|BOLD:ACK4362
Trichordestra beanii|[12012]|LNAUS4813-13|United States|Texas|658[0n]|BOLD:ACK4362
Trichordestra beanii|[12013]|CNCLB1261-14|United States|Louisiana|658[0n]|BOLD:ACK4362
Trichordestra rugosa|[12014]|RDLQG372-06|Canada|Quebec|658[0n]|BOLD:ACE8278
Trichordestra rugosa|[12015]|RDLQB081-05|Canada|Quebec|658[0n]|BOLD:ACE8278
Trichordestra rugosa|[12016]|RDNMG772-08|Canada|Quebec|658[0n]|BOLD:ACE8278
Trichordestra rugosa|[12017]|RDNMG773-08|Canada|Quebec|658[0n]|BOLD:ACE8278
Trichordestra dodii|[12018]|RDNMF554-08|Canada|British Columbia|609[0n]|BOLD:AAB6376
Trichordestra dodii|[12019]|BBLSY566-09|United States|New Mexico|658[0n]|BOLD:AAB6376
Trichordestra legitima|[12020]|BBLEC784-09|Canada|Newfoundland and Labrador|658[0n]|BOLD:AAB6376
Trichordestra legitima|[12021]|XAC795-04|Canada|Ontario|658[0n]|BOLD:AAB6376
Trichordestra legitima|[12022]|XAE572-04|Canada|Ontario|658[0n]|BOLD:AAB6376
Trichordestra dodii|[12023]|RDNMF555-08|Canada|Alberta|658[0n]|BOLD:AAB6376
Trichordestra legitima|[12024]|BBLPC638-09|Canada|Newfoundland and Labrador|658[0n]|BOLD:AAB6376
Trichordestra legitima|[12025]|BBLPE311-09|Canada|Newfoundland and Labrador|658[0n]|BOLD:AAB6376
Trichordestra legitima|[12026]|BBLPC636-09|Canada|Newfoundland and Labrador|658[0n]|BOLD:AAB6376
Trichordestra legitima|[12027]|BBLPC654-09|Canada|Newfoundland and Labrador|658[0n]|BOLD:AAB6376
Trichordestra legitima|[12028]|BBLEC597-09|Canada|Nova Scotia|658[0n]|BOLD:AAB6376
Trichordestra legitima|[12029]|BBLEC823-09|Canada|Newfoundland and Labrador|658[0n]|BOLD:AAB6376
Trichordestra legitima|[12030]|BBLEC565-09|Canada|Nova Scotia|658[0n]|BOLD:AAB6376
Trichordestra legitima|[12031]|LPOKD293-09|United States|Oklahoma|658[0n]|BOLD:AAB6376
Trichordestra legitima|[12032]|LPOKD216-09|United States|Oklahoma|657[0n]|BOLD:AAB6376
Trichordestra legitima|[12033]|NAMUM324-08|United States|Maryland|658[0n]|BOLD:AAB6376
Trichordestra legitima|[12034]|RDLQG050-06|Canada|Quebec|658[0n]|BOLD:AAB6376
Trichordestra legitima|[12035]|TTMNB400-06|Canada|New Brunswick|658[0n]|BOLD:AAB6376
Trichordestra legitima|[12036]|TTMNB399-06|Canada|New Brunswick|658[0n]|BOLD:AAB6376
Trichordestra legitima|[12037]|MNBB183-05|Canada|New Brunswick|658[0n]|BOLD:AAB6376
Trichordestra legitima|[12038]|PHMNB348-04|Canada|New Brunswick|658[0n]|BOLD:AAB6376
Trichordestra legitima|[12039]|XAC599-04|Canada|Ontario|658[0n]|BOLD:AAB6376
Trichordestra legitima|[12040]|XAB199-04|Canada|Ontario|614[1n]|BOLD:AAB6376
Trichordestra legitima|[12041]|PHMO216-03|Canada|Ontario|639[0n]|BOLD:AAB6376
Trichordestra legitima|[12042]|XAE569-04|Canada|Ontario|551[0n]|BOLD:AAB6376
Trichordestra legitima|[12043]|RDLQF685-06|Canada|Quebec|611[0n]|BOLD:AAB6376
Trichordestra legitima|[12044]|LPOKD249-09|United States|Oklahoma|635[0n]|BOLD:AAB6376
Trichordestra legitima|[12045]|RBINA4166-13|Canada|Ontario|591[0n]|BOLD:AAB6376
Trichordestra legitima|[12046]|CNCLB2866-14|United States|North Carolina|658[0n]|BOLD:AAB6376
Trichordestra legitima|[12047]|CNCLB2868-14|United States|North Carolina|658[0n]|BOLD:AAB6376
Sideridis rosea|[12048]|LPSK439-08|Canada|Saskatchewan|658[0n]|BOLD:AAC8783
Sideridis rosea|[12049]|RDNMG835-08|Canada|New Brunswick|658[0n]|BOLD:AAC8783
Sideridis rosea|[12050]|RDNMG834-08|United States|Washington|658[0n]|BOLD:AAC8783
Sideridis rosea|[12051]|RDNMG833-08|Canada|New Brunswick|658[0n]|BOLD:AAC8783
Sideridis rosea|[12052]|KPOEC140-08|Canada|Ontario|658[0n]|BOLD:AAC8783
Sideridis rosea|[12053]|PHMO052-03|Canada|Ontario|639[0n]|BOLD:AAC8783
Sideridis rosea|[12054]|PHMO048-03|Canada|Ontario|639[0n]|BOLD:AAC8783
Sideridis rosea|[12055]|RDLQ619-07|Canada|Quebec|573[0n]|BOLD:AAC8783
Sideridis rosea|[12056]|LPVIC125-08|Canada|British Columbia|658[0n]|BOLD:AAC8783
Sideridis rosea|[12057]|LBCH5037-10|Canada|British Columbia|658[0n]|BOLD:AAC8783
Sideridis rosea|[12058]|LBCH5084-10|Canada|British Columbia|658[0n]|BOLD:AAC8783
Sideridis rosea|[12059]|LBCH5171-10|Canada|British Columbia|658[0n]|BOLD:AAC8783
Sideridis rosea|[12060]|LBCH5340-10|Canada|British Columbia|658[0n]|BOLD:AAC8783
Sideridis rosea|[12061]|LBCH5416-10|Canada|British Columbia|658[0n]|BOLD:AAC8783
Sideridis rosea|[12062]|RWWA406-09|United States|Washington|658[0n]|BOLD:AAC8783
Sideridis rosea|[12063]|RWWC230-11|United States|Washington|658[0n]|BOLD:AAC8783
Sideridis ruisa|[12064]|CNCLB277-14|United States|Texas|658[0n]|BOLD:ACM4385
Sideridis ruisa|[12065]|CNCLB278-14|United States|Texas|658[0n]|BOLD:ACM4385
Sideridis vindemialis|[12066]|CNCLB279-14|United States|Florida|658[0n]|BOLD:AAM8601
Sideridis vindemialis|[12067]|USLEP612-10|United States|Florida|658[0n]|BOLD:AAM8601
Sideridis vindemialis|[12068]|CNCLB280-14|United States|Florida|658[0n]|BOLD:AAM8601
Sideridis fuscolutea|[12069]|RDNMF286-08|United States|Nevada|658[0n]|BOLD:AAF4049
Sideridis fuscolutea|[12070]|RDNMF287-08|United States|Oregon|658[0n]|BOLD:AAF4049
Sideridis fuscolutea|[12071]|RDNMF288-08|United States|Oregon|658[0n]|BOLD:AAF4049
Sideridis mojave|[12072]|CNCLB734-14|United States|California|658[0n]|BOLD:ACM3954
Sideridis artesta|[12073]|RDMAB538-06|Canada|Alberta|658[0n]|BOLD:ABX6687
Sideridis artesta|[12074]|RDNMG579-08|Canada|Alberta|658[0n]|BOLD:ABX6687
Sideridis artesta|[12075]|RDNMG847-08|Canada|Alberta|658[0n]|BOLD:ABX6687
Sideridis uscripta|[12076]|RDNMD083-06|United States||658[0n]|BOLD:AAB1581
Sideridis uscripta|[12077]|RDNMG447-08|United States|Wyoming|658[0n]|BOLD:ACE5146
Sideridis uscripta|[12078]|RDNMG448-08|United States|Wyoming|658[0n]|BOLD:ACE5146
Sideridis uscripta|[12079]|RDNMG446-08|United States|Wyoming|658[0n]|BOLD:ACE5146
Sideridis uscripta|[12080]|NAMUM259-08|United States|California|658[0n]|BOLD:ACE5146
Sideridis uscripta|[12081]|IAWLB125-10|United States|California|658[0n]|BOLD:ACE5146
Sideridis uscripta|[12082]|JMMMB584-13|United States|California|606[0n]|BOLD:ACE5146
Sideridis maryx|[12083]|LOWCC203-05|Canada|British Columbia|585[0n]|BOLD:AAB1581
Sideridis maryx|[12084]|LOWCC204-05|Canada|British Columbia|658[0n]|BOLD:AAB1581
Sideridis maryx|[12085]|LPMN588-08|Canada|Manitoba|658[0n]|BOLD:AAB1581
Sideridis maryx|[12086]|LALPA471-10|Canada|British Columbia|658[0n]|BOLD:AAB1581
Sideridis maryx|[12087]|LALPA841-11|Canada|British Columbia|658[0n]|BOLD:AAB1581
Sideridis maryx|[12088]|RDLQB225-05|Canada|Quebec|617[0n]|BOLD:AAB1581
Sideridis maryx|[12089]|RDLQB229-05|Canada|Quebec|658[0n]|BOLD:AAB1581
Sideridis maryx|[12090]|TMNBB304-06|Canada|New Brunswick|658[0n]|BOLD:AAB1581
Sideridis maryx|[12091]|TMNBB305-06|Canada|New Brunswick|658[0n]|BOLD:AAB1581
Sideridis maryx|[12092]|BBLPC816-09|Canada|Newfoundland and Labrador|658[0n]|BOLD:AAB1581
Sideridis maryx|[12093]|BBLPB789-10|Canada|Ontario|658[0n]|BOLD:AAB1581
[illegible]

Sideridis marxy|[12091]|TMNBB305-06|Canada|New Brunswick|658[0n]|BOLD:AAB1581
Sideridis marxy|[12092]|BBLPC816-09|Canada|Newfoundland and Labrador|658[0n]|BOLD:AAB1581
Sideridis marxy|[12093]|BBLPB789-10|Canada|Ontario|658[0n]|BOLD:AAB1581
Sideridis marxy|[12094]|TMNBB299-06|Canada|New Brunswick|658[0n]|BOLD:AAB1581
Sideridis marxy|[12095]|TMNBB303-06|Canada|New Brunswick|658[0n]|BOLD:AAB1581
Sideridis marxy|[12096]|TMNBB297-06|Canada|New Brunswick|658[0n]|BOLD:AAB1581
Sideridis marxy|[12097]|TMNBB298-06|Canada|New Brunswick|658[0n]|BOLD:AAB1581
Sideridis marxy|[12098]|TMNBB294-06|Canada|New Brunswick|658[0n]|BOLD:AAB1581
Sideridis marxy|[12099]|TMNBB295-06|Canada|New Brunswick|658[0n]|BOLD:AAB1581
Sideridis marxy|[12100]|RDLQB805-05|Canada|Quebec|658[0n]|BOLD:AAB1581
Sideridis marxy|[12101]|TMNBB293-06|Canada|New Brunswick|658[0n]|BOLD:AAB1581
Sideridis marxy|[12102]|RDLQB231-05|Canada|Quebec|658[0n]|BOLD:AAB1581
Sideridis marxy|[12103]|RDLQB228-05|Canada|Quebec|658[0n]|BOLD:AAB1581
Sideridis marxy|[12104]|RDLQB227-05|Canada|Quebec|658[0n]|BOLD:AAB1581
Sideridis marxy|[12105]|RDLQB226-05|Canada|Quebec|658[0n]|BOLD:AAB1581
Sideridis marxy|[12106]|RDLQB224-05|Canada|Quebec|658[0n]|BOLD:AAB1581
Sideridis marxy|[12107]|RDLQB223-05|Canada|Quebec|658[0n]|BOLD:AAB1581
Sideridis marxy|[12108]|RDLQB222-05|Canada|Quebec|658[0n]|BOLD:AAB1581
Sideridis marxy|[12109]|TTMNB549-06|Canada|New Brunswick|658[0n]|BOLD:AAB1581
Sideridis marxy|[12110]|TMNBB292-06|Canada|New Brunswick|658[0n]|BOLD:AAB1581
Sideridis marxy|[12111]|TMNBB296-06|Canada|New Brunswick|658[0n]|BOLD:AAB1581
Sideridis marxy|[12112]|TMNBB302-06|Canada|New Brunswick|656[0n]|BOLD:AAB1581
Sideridis marxy|[12113]|TMNBB301-06|Canada|New Brunswick|656[0n]|BOLD:AAB1581
Sideridis marxy|[12114]|TMNBB300-06|Canada|New Brunswick|656[0n]|BOLD:AAB1581
Sideridis marxy|[12115]|RDLQB230-05|Canada|Quebec|658[1n]|BOLD:AAB1581
Sideridis marxy|[12116]|CNPKG1473-14|Canada|Ontario|594[0n]|BOLD:AAB1581
Sideridis congermana|[12117]|RDNMG846-08|Canada|Ontario|658[0n]|BOLD:AAF4037
Sideridis congermana|[12118]|LPSOB219-08|Canada|Ontario|658[0n]|BOLD:AAF4037
Sideridis congermana|[12119]|RDLQ620-07|Canada|Quebec|658[0n]|BOLD:AAF4037
Sideridis congermana|[12120]|LNCC1460-13|United States|North Carolina|658[1n]|BOLD:AAF4037
Sideridis congermana|[12121]|LNCC1461-13|United States|North Carolina|658[0n]|BOLD:AAF4037
Sideridis congermana|[12122]|LNCC1462-13|United States|North Carolina|658[0n]|BOLD:AAF4037
Sideridis congermana|[12123]|CNCLB2878-14|United States|North Carolina|658[0n]|BOLD:AAF4037
Tridepia nova|[12124]|AWCLB262-10|United States|Arizona|658[0n]|BOLD:AAD9766
Tridepia nova|[12125]|AWCLB274-10|United States|Arizona|658[1n]|BOLD:AAD9766
Tridepia nova|[12126]|AWCLB506-11|United States|Arizona|658[0n]|BOLD:AAD9766
Tridepia nova|[12127]|AWCLB419-10|United States|Arizona|658[0n]|BOLD:AAD9766
Tridepia nova|[12128]|RDNMD821-07|United States|Arizona|658[0n]|BOLD:AAD9766
Tridepia nova|[12129]|NAMUM150-08|United States|California|658[0n]|BOLD:AAD9766
Tridepia nova|[12130]|NAMUM170-08|United States|California|658[0n]|BOLD:AAD9766
Tridepia nova|[12131]|NAMUM392-09|United States|California|658[0n]|BOLD:AAD9766
Tridepia nova|[12132]|AWCLB450-10|United States|Arizona|658[0n]|BOLD:AAD9766
Tridepia nova|[12133]|AWCLB473-10|United States|Arizona|658[0n]|BOLD:AAD9766
Tridepia nova|[12134]|BBLSW321-09|United States|Arizona|658[0n]|BOLD:AAD9766
Tridepia nova|[12135]|AWCLB410-10|United States|Arizona|658[0n]|BOLD:AAD9766
Tridepia nova|[12136]|IAWLB488-11|United States|Arizona|658[0n]|BOLD:AAD9766
Tridepia nova|[12137]|AWCLB449-10|United States|Arizona|658[0n]|BOLD:AAD9766
Tridepia nova|[12138]|AWCLB420-10|United States|Arizona|658[0n]|BOLD:AAD9766
Tridepia nova|[12139]|LOCBB744-06|United States|California|590[0n]|BOLD:AAD9766
Tridepia nova|[12140]|IAWLB489-11|United States|Arizona|658[0n]|BOLD:AAD9766
Tridepia nova|[12141]|IAWLB490-11|United States|Arizona|658[0n]|BOLD:AAD9766
Anarta antica|[12142]|RDNME971-08|United States|New Mexico|658[0n]|BOLD:AAF3901
Anarta antica|[12143]|AWCLB117-10|United States|Arizona|658[0n]|BOLD:AAF3901
Anarta antica|[12144]|RDNME976-08|United States|New Mexico|658[0n]|BOLD:AAF3901
Anarta antica|[12145]|AWCLB374-10|United States|Arizona|658[0n]|BOLD:AAF3901
Anarta antica|[12146]|RDMAB688-06|Canada|Alberta|632[0n]|BOLD:AAF3901
Anarta antica|[12147]|IAWLB491-11|United States|Arizona|658[0n]|BOLD:AAF3901
Anarta antica|[12148]|RDNMK259-11|United States|New Mexico|658[0n]|BOLD:AAF3901
Anarta antica|[12149]|RDNMK260-11|United States|Texas|658[0n]|BOLD:AAF3901
Anarta decepta|[12150]|IAWLB499-11|United States|Arizona|658[0n]|BOLD:AAE1460
Anarta decepta|[12151]|AWCLB101-10|United States|Arizona|658[0n]|BOLD:AAE1460
Anarta decepta|[12152]|AWCLB451-10|United States|Arizona|658[0n]|BOLD:AAE1460
Anarta decepta|[12153]|AWCLB532-11|United States|Arizona|658[0n]|BOLD:AAE1460
Anarta decepta|[12154]|LOCBF038-13|United States|California|628[0n]|BOLD:AAE1460
Anarta decepta|[12155]|RDNME973-08|United States|New Mexico|658[0n]|BOLD:AAE1460
Anarta decepta|[12156]|RDNMF345-08|Canada|British Columbia|658[0n]|BOLD:AAE1460
Anarta decepta|[12157]|LBCH5097-10|Canada|British Columbia|658[0n]|BOLD:AAE1460
Anarta decepta|[12158]|LBCH5170-10|Canada|British Columbia|658[0n]|BOLD:AAE1460
Anarta decepta|[12159]|LBCH5871-10|Canada|British Columbia|658[0n]|BOLD:AAE1460
Anarta decepta|[12160]|LBCH6154-10|Canada|British Columbia|658[0n]|BOLD:AAE1460
Anarta decepta|[12161]|LBCH7094-10|Canada|British Columbia|658[0n]|BOLD:AAE1460
Anarta decepta|[12162]|RDNMG632-08|United States|New Mexico|658[0n]|BOLD:AAE1460
Anarta decepta|[12163]|JMMMB401-11|United States|California|658[0n]|BOLD:AAE1460
Anarta decepta|[12164]|RDNMK256-11|United States|Utah|658[0n]|BOLD:AAE1460
Anarta decepta|[12165]|RDNME972-08|United States|New Mexico|569[0n]|BOLD:AAE1460
Anarta decepta|[12166]|RDNMG633-08|United States|New Mexico|658[0n]|BOLD:AAE1460
Anarta decepta|[12167]|CNCLB720-14|United States|Texas|658[0n]|BOLD:AAE1460
Anarta decepta|[12168]|CNCLB721-14|United States|Texas|658[0n]|BOLD:AAE1460
Anarta florida|[12169]|CNCLB722-14|United States|Florida|658[0n]|BOLD:AAE1460
Trichocosmia inornata|[12170]|AWCLB645-11|United States|Arizona|658[0n]|BOLD:AAJ8684
Trichocosmia inornata|[12171]|AWCLB421-10|United States|Arizona|658[0n]|BOLD:AAJ8684
Trichocosmia inornata|[12172]|AWCLB646-11|United States|Arizona|658[0n]|BOLD:AAJ8684
Trichocosmia inornata|[12173]|IAWLB281-11|United States|Arizona|658[0n]|BOLD:AAJ8684
Trichocosmia inornata|[12174]|RDNME896-08|United States|Arizona|658[0n]|BOLD:AAJ8684
Trichocosmia inornata|[12175]|BBLOE1841-12|United States|Texas|658[0n]|BOLD:AAJ8684
Trichocosmia inornata|[12176]|BBLOD1535-11|United States|Arizona|658[0n]|BOLD:AAJ8684
Trichocosmia inornata|[12177]|BBLOD730-11|United States|Arizona|658[0n]|BOLD:AAJ8684
Trichocosmia inornata|[12178]|BBLOD1916-11|United States|Arizona|658[0n]|BOLD:AAJ8684
Trichocosmia inornata|[12179]|USLEP1110-10|United States|Arizona|614[0n]|BOLD:AAJ8684
Trichocosmia inornata|[12180]|BBLOE1793-12|United States|Arizona|658[0n]|BOLD:AAJ8684
Trichocosmia inornata|[12181]|BBLOD1870-11|United States|Arizona|658[0n]|BOLD:AAJ8684
Trichocosmia inornata|[12182]|BBLOD732-11|United States|Arizona|658[0n]|BOLD:AAJ8684
Trichocosmia inornata|[12183]|BBLOB868-11|United States|Arizona|658[0n]|BOLD:AAJ8684
Trichocosmia inornata|[12184]|BBLOE1802-12|United States|Arizona|633[0n]|BOLD:AAJ8684
Trichocosmia inornata|[12185]|CMAZA1160-12|United States|Arizona|658[0n]|BOLD:AAJ8684
Anarta edwardsii|[12186]|LOCBF025-13|United States|California|628[0n]|BOLD:AAF3996
Anarta edwardsii|[12187]|LOCBF061-13|United States|California|628[0n]|BOLD:AAF3996
Anarta edwardsii|[12188]|LOCBF2596-13|United States|California|658[0n]|BOLD:AAF3996
Anarta edwardsii|[12189]|RDNMF342-08|United States|Washington|658[0n]|BOLD:AAF3996
Anarta edwardsii|[12190]|RDNMF344-08|Canada|British Columbia|658[0n]|BOLD:AAF3996
Anarta edwardsii|[12191]|RDNMF343-08|Canada|British Columbia|658[0n]|BOLD:AAF3996
Anarta edwardsii|[12192]|LOCBF2663-13|United States|California|658[0n]|BOLD:AAF3996
Scotogramma gatei|[12193]|AWCLB116-10|United States|Arizona|658[0n]|BOLD:AAE9852

Anarta edwardsii|[12191]|RDNMF343-08|Canada|British Columbia|658[0n]|BOLD:AAF3996
Anarta edwardsii|[12192]|LOCBF2663-13|United States|California|658[0n]|BOLD:AAF3996
Scotogramma gatei|[12193]|AWCLB116-10|United States|Arizona|658[0n]|BOLD:AAE9852
Scotogramma fieldi|[12194]|LOCBF3640-14|United States|California|555[0n]|BOLD:AAE9852
Scotogramma fieldi|[12195]|LOCBF196-13|United States|California|598[0n]|BOLD:AAE9852
Scotogramma fieldi|[12196]|LOCBF3485-14|United States|California|549[0n]|BOLD:AAE9852
Scotogramma fieldi|[12197]|LOCBF065-13|United States|California|601[0n]|BOLD:AAE9852
Scotogramma fieldi|[12198]|LOCBF075-13|United States|California|602[0n]|BOLD:AAE9852
Scotogramma fieldi|[12199]|LOCBF074-13|United States|California|589[0n]|BOLD:AAE9852
Scotogramma fieldi|[12200]|LOCBB607-06|United States|California|658[0n]|BOLD:AAE9852
Scotogramma fieldi|[12201]|LOCBB766-06|United States|California|658[1n]|BOLD:AAE9852
Scotogramma fieldi|[12202]|LOCBF064-13|United States|California|628[0n]|BOLD:AAE9852
Scotogramma fieldi|[12203]|LOCBF073-13|United States|California|628[0n]|BOLD:AAE9852
Scotogramma fieldi|[12204]|LOCBF197-13|United States|California|630[0n]|BOLD:AAE9852
Scotogramma fieldi|[12205]|LOCBF203-13|United States|California|630[0n]|BOLD:AAE9852
Scotogramma fieldi|[12206]|LOCBF3646-14|United States|California|588[0n]|BOLD:AAE9852
Scotogramma fieldi|[12207]|LOCBF3648-14|United States|California|588[0n]|BOLD:AAE9852
Scotogramma fieldi|[12208]|BBLOD1719-11|United States|California|658[0n]|BOLD:AAE9852
Scotogramma fieldi|[12209]|LOCB397-06|United States|California|658[0n]|BOLD:AAE9852
Scotogramma fieldi|[12210]|LOCBB370-06|United States|California|658[0n]|BOLD:AAE9852
Scotogramma fieldi|[12211]|LOCBF3655-14|United States|California|588[0n]|BOLD:AAE9852
Scotogramma sp.|[12212]|RDNMJ341-11|United States|New Mexico|658[0n]|BOLD:AAV7632
Scotogramma sp.|[12213]|RDNMJ342-11|United States|New Mexico|658[0n]|BOLD:AAV7632
Scotogramma orida|[12214]|NAMUM190-08|United States|California|658[1n]|BOLD:ACE3947
Scotogramma gatei|[12215]|AWCLB097-10|United States|Arizona|658[1n]|BOLD:ACE3947
Scotogramma gatei|[12216]|AWCLB098-10|United States|Arizona|658[0n]|BOLD:ACE3947
Scotogramma gatei|[12217]|RDNME974-08|United States|New Mexico|658[0n]|BOLD:ACE3947
Scotogramma gatei|[12218]|AWCLB105-10|United States|Arizona|635[0n]|BOLD:ACE3947
Scotogramma gatei|[12219]|AWCLB106-10|United States|Arizona|555[0n]|BOLD:ACE3947
Scotogramma gatei|[12220]|AWCLB482-11|United States|Arizona|658[0n]|BOLD:ACE3947
Scotogramma gatei|[12221]|AWCLB533-11|United States|Arizona|658[0n]|BOLD:ACE3947
Scotogramma gatei|[12222]|IAWLB485-11|United States|Arizona|658[0n]|BOLD:ACE3947
Scotogramma gatei|[12223]|IAWLB486-11|United States|Arizona|658[0n]|BOLD:ACE3947
Scotogramma gatei|[12224]|AWCLB111-10|United States|Arizona|621[0n]|BOLD:ACE3947
Scotogramma gatei|[12225]|AWCLB107-10|United States|Arizona|658[0n]|BOLD:ACE3947
Scotogramma gatei|[12226]|AWCLB113-10|United States|Arizona|658[0n]|BOLD:ACE3947
Scotogramma gatei|[12227]|AWCLB112-10|United States|Arizona|658[0n]|BOLD:ACE3947
Scotogramma gatei|[12228]|AWCLB108-10|United States|Arizona|658[0n]|BOLD:ACE3947
Scotogramma gatei|[12229]|AWCLB102-10|United States|Arizona|647[0n]|BOLD:ACE3947
Scotogramma gatei|[12230]|AWCLB114-10|United States|Arizona|572[0n]|BOLD:ACE3947
Scotogramma gatei|[12231]|AWCLB475-10|United States|Arizona|658[0n]|BOLD:ACE3947
Scotogramma gatei|[12232]|IAWLB487-11|United States|Arizona|658[0n]|BOLD:ACE3947
Scotogramma sp.|[12233]|GMLC914-12|United States|California|658[0n]|BOLD:ABY4673
Scotogramma yakima|[12234]|CNCLB272-14|United States|Nevada|658[0n]|BOLD:ACM4592
Scotogramma yakima|[12235]|CNCLB273-14|United States|Oregon|658[0n]|BOLD:ACM4592
Sparkia immacula|[12236]|RDNMH092-09|United States|New Mexico|658[0n]|BOLD:AAG1049
Sparkia immacula|[12237]|RDNMH629-09|United States|New Mexico|658[0n]|BOLD:AAG1049
Scotogramma densa|[12238]|RDNMC452-05|United States|Nevada|500[0n]|
Scotogramma densa|[12239]|RDNMC450-05|United States|Oregon|595[0n]|BOLD:AAE9851
Scotogramma densa|[12240]|RDNMC453-05|United States|Oregon|658[0n]|BOLD:AAE9851
Scotogramma densa|[12241]|RDNME563-08|United States|California|658[0n]|BOLD:AAE9851
Scotogramma submarina|[12242]|RDMAB106-05|Canada|Alberta|658[0n]|BOLD:AAE9851
Scotogramma submarina|[12243]|RDNMC454-05|Canada|Alberta|657[0n]|BOLD:AAE9851
Scotogramma submarina|[12244]|RDNMC451-05|United States|Utah|536[0n]|BOLD:AAE9851
Scotogramma submarina|[12245]|RDNMK255-11|United States|Arizona|658[0n]|BOLD:AAE9851
Scotogramma harnardi|[12246]|RDNMJ364-11|United States|Colorado|658[0n]|BOLD:ABY6488
Scotogramma harnardi|[12247]|RDNMC326-05|United States|Oregon|587[0n]|BOLD:ACF4639
Scotogramma harnardi|[12248]|RDNMJ363-11|United States|New Mexico|658[0n]|BOLD:ACF4638
Scotogramma harnardi|[12249]|RDNMK253-11|United States|New Mexico|658[0n]|BOLD:ACF4638
Scotogramma harnardi|[12250]|RDNMJ833-11|United States|Utah|658[0n]|BOLD:AAH9699
Scotogramma harnardi|[12251]|RDMAB963-09|United States|Utah|643[0n]|BOLD:AAH9699
Scotogramma harnardi|[12252]|RDNMK252-11|United States|Arizona|658[0n]|BOLD:AAH9699
Scotogramma harnardi|[12253]|CNCLB1553-14|United States|Nevada|658[0n]|BOLD:AAH9699
Scotogramma harnardi|[12254]|CNCLB1554-14|United States|Utah|658[0n]|BOLD:AAH9699
Scotogramma stretchii|[12255]|RDNMC455-05|United States|Washington|550[0n]|BOLD:AAH9701
Scotogramma stretchii|[12256]|CNCLB1562-14|United States|Nevada|658[0n]|BOLD:AAH9701
Scotogramma stretchii|[12257]|CNCLB1563-14|United States|Nevada|658[0n]|BOLD:AAH9701
Hadenella pergentilis|[12258]|RDNMF532-08|United States|Colorado|658[0n]|BOLD:AAD7037
Hadenella pergentilis|[12259]|NAMUM146-08|United States|California|657[0n]|BOLD:AAD7036
Hadenella pergentilis|[12260]|RDNMF534-08|United States|California|658[0n]|BOLD:AAD7036
Hadenella pergentilis|[12261]|RDNMF533-08|United States|Oregon|658[0n]|BOLD:ACE6084
Hadenella pergentilis|[12262]|RDNMF531-08|United States|Oregon|658[0n]|BOLD:ACE6084
Hadenella pergentilis|[12263]|RDNMF535-08|United States|Oregon|658[1n]|BOLD:ACE6084
Afotella cylindrica|[12264]|RDMAB551-06|Canada|Alberta|658[0n]|BOLD:AAE6413
Afotella cylindrica|[12265]|RDNMG554-08|Canada|Alberta|658[0n]|BOLD:AAE6413
Afotella cylindrica|[12266]|RDNMG555-08|Canada|Alberta|658[0n]|BOLD:AAE6413
Afotella cylindrica|[12267]|RDNMG556-08|United States|Oregon|640[0n]|BOLD:AAE6413
Scotogramma fervida|[12268]|RDNMB960-05|United States|Wyoming|658[0n]|BOLD:AAC4952
Scotogramma fervida|[12269]|RDNMB961-05|Canada|Alberta|658[0n]|BOLD:AAC4952
Scotogramma fervida|[12270]|LPSK465-08|Canada|Saskatchewan|658[0n]|BOLD:AAC4952
Scotogramma fervida|[12271]|LPSK522-08|Canada|Saskatchewan|658[0n]|BOLD:AAC4952
Scotogramma fervida|[12272]|LPSK537-08|Canada|Saskatchewan|658[0n]|BOLD:AAC4952
Scotogramma fervida|[12273]|RDNMF899-08|United States|Oregon|595[0n]|BOLD:AAC4952
Scotogramma fervida|[12274]|RDNMB958-05|United States|Nevada|658[0n]|BOLD:AAC4952
Scotogramma fervida|[12275]|RDNMB959-05|United States|Oregon|658[0n]|BOLD:AAC4952
Scotogramma fervida|[12276]|RDNMC574-06|United States|Nevada|658[0n]|BOLD:AAC4952
Scotogramma fervida|[12277]|NAMUM276-08|United States|California|658[1n]|BOLD:AAC4952
Scotogramma fervida|[12278]|RDNMC573-06|United States|California|658[0n]|BOLD:AAC4952
Scotogramma fervida|[12279]|RDNMC577-06|United States|California|658[0n]|BOLD:AAC4952
Scotogramma fervida|[12280]|CNCLB275-14|United States|California|658[0n]|BOLD:AAC4952
Anarta chartaria|[12281]|LOCBF2595-13|United States|California|658[0n]|BOLD:AAB9765
Anarta chartaria|[12282]|LOCBB278-06|United States|California|658[0n]|BOLD:AAB9765
Anarta chartaria|[12283]|LOCBB280-06|United States|California|658[0n]|BOLD:AAB9765
Anarta chartaria|[12284]|LOCBE082-06|United States|California|621[0n]|BOLD:AAB9765
Anarta chartaria|[12285]|LOCBF3439-14|United States|California|576[0n]|BOLD:AAB9765
Anarta chartaria|[12286]|LOCBF3441-14|United States|California|576[0n]|BOLD:AAB9765
Anarta chartaria|[12287]|LOCBF3442-14|United States|California|579[0n]|BOLD:AAB9765
Anarta chartaria|[12288]|LOCBF3494-14|United States|California|549[0n]|BOLD:AAB9765
Anarta chartaria|[12289]|RDNMD820-07|United States|California|658[0n]|BOLD:AAB9765
Anarta chartaria|[12290]|LOCBC133-06|United States|California|658[0n]|BOLD:AAB9765
Anarta chartaria|[12291]|LOCBB283-06|United States|California|658[0n]|BOLD:AAB9765
Anarta chartaria|[12292]|LOCBB282-06|United States|California|658[0n]|BOLD:AAB9765
Anarta chartaria|[12293]|LOCBC135-06|United States|California|605[0n]|BOLD:AAB9765

Anarta chartaria|[12291]|LOCBB283-06|United States|California|658[0n]|BOLD:AAB9765
Anarta chartaria|[12292]|LOCBB282-06|United States|California|658[0n]|BOLD:AAB9765
Anarta chartaria|[12293]|LOCBC135-06|United States|California|605[0n]|BOLD:AAB9765
Anarta chartaria|[12294]|LOCBF3440-14|United States|California|582[0n]|BOLD:AAB9765
Anarta chartaria|[12295]|LOCBF3512-14|United States|California|588[0n]|BOLD:AAB9765
Anarta chartaria|[12296]|LOCBF3669-14|United States|California|588[0n]|BOLD:AAB9765
Anarta chartaria|[12297]|LOCBB279-06|United States|California|658[0n]|BOLD:AAB9765
Anarta chartaria|[12298]|LOCBC136-06|United States|California|658[0n]|BOLD:AAB9765
Anarta chartaria|[12299]|LOCBC137-06|United States|California|658[0n]|BOLD:AAB9765
Anarta chartaria|[12300]|LOCBE080-06|United States|California|658[0n]|BOLD:AAB9765
Anarta chartaria|[12301]|LOCBE081-06|United States|California|658[0n]|BOLD:AAB9765
Anarta chartaria|[12302]|LOCBF2611-13|United States|California|658[0n]|BOLD:AAB9765
Anarta chartaria|[12303]|LOCBC134-06|United States|California|658[0n]|BOLD:AAB9765
Anarta chartaria|[12304]|LOCBB281-06|United States|California|658[0n]|BOLD:AAB9765
Anarta chartaria|[12305]|LOCBF3744-14|United States|California|588[0n]|BOLD:AAB9765
Anarta mutata|[12306]|IAWLB645-11|United States|Arizona|658[0n]|BOLD:AAD7628
Anarta mutata|[12307]|CMAZA802-10|United States|Arizona|658[0n]|BOLD:AAD7628
Anarta mutata|[12308]|BBLOD1691-11|United States|Arizona|658[0n]|BOLD:AAD7628
Anarta mutata|[12309]|BBLOC265-11|United States|Arizona|658[0n]|BOLD:AAD7628
Anarta mutata|[12310]|CMAZA341-10|United States|Arizona|658[0n]|BOLD:AAD7628
Anarta mutata|[12311]|CMAZA1065-12|United States|Arizona|658[0n]|BOLD:AAD7628
Anarta mutata|[12312]|LPSK499-08|Canada|Saskatchewan|658[0n]|BOLD:AAD7628
Anarta mutata|[12313]|BBLSW372-09|United States|Arizona|658[0n]|BOLD:AAD7628
Anarta mutata|[12314]|LPSK562-08|Canada|Saskatchewan|658[0n]|BOLD:AAD7628
Anarta mutata|[12315]|LPSK511-08|Canada|Saskatchewan|658[0n]|BOLD:AAD7628
Anarta mutata|[12316]|RDNME056-07|United States|Arizona|658[0n]|BOLD:AAD7628
Anarta mutata|[12317]|RDNME055-07|United States|Arizona|658[0n]|BOLD:AAD7628
Anarta mutata|[12318]|BBLSW344-09|United States|Arizona|658[0n]|BOLD:AAD7628
Anarta mutata|[12319]|RDNMC303-05|United States|Colorado|577[0n]|BOLD:AAD7628
Anarta mutata|[12320]|LBCH6451-10|Canada|British Columbia|642[0n]|BOLD:AAD7628
Anarta mutata|[12321]|RDNMJ482-11|United States|Arizona|658[0n]|BOLD:AAD7628
Anarta mutata|[12322]|BBLOD1267-11|United States|Arizona|658[0n]|BOLD:AAD7628
Anarta mutata|[12323]|CMAZA922-12|United States|Arizona|658[0n]|BOLD:AAD7628
Anarta mutata|[12324]|CMAZA1032-12|United States|Arizona|658[0n]|BOLD:AAD7628
Anarta mutata|[12325]|CMAZA1058-12|United States|Arizona|658[0n]|BOLD:AAD7628
Anarta mutata|[12326]|CNCLB1451-14|United States|Louisiana|658[0n]|BOLD:AAD7628
Scotogramma ptilodonta|[12327]|RDNME959-08|United States|New Mexico|658[0n]|BOLD:AAH5414
Scotogramma ptilodonta|[12328]|RDNMK020-11|United States|Arizona|658[0n]|BOLD:AAH5414
Scotogramma ptilodonta|[12329]|RDNMK258-11|United States|Arizona|658[0n]|BOLD:AAH5414
Scotogramma ptilodonta|[12330]|RDNMK302-11|United States|Arizona|658[0n]|BOLD:AAH5414
Scotogramma ptilodonta|[12331]|BBLSY822-09|United States|Arizona|634[0n]|BOLD:AAH5414
Scotogramma ptilodonta|[12332]|RDNML210-13|United States|Arizona|658[0n]|BOLD:AAH5414
Escaria clauda|[12333]|LNAUS4363-13|United States|California|658[0n]|BOLD:AAE2691
Escaria homogena|[12334]|RDNMG1033-08|Canada|Alberta|658[0n]|BOLD:AAE2691
Escaria homogena|[12335]|RDMAB530-06|Canada|Alberta|658[0n]|BOLD:AAE2691
Escaria homogena|[12336]|RDMAB990-09|Canada|Alberta|649[0n]|BOLD:AAE2691
Escaria homogena|[12337]|RDMAB991-09|Canada|Alberta|635[0n]|BOLD:AAE2691
Escaria homogena|[12338]|RDMAB992-09|Canada|Alberta|634[0n]|BOLD:AAE2691
Escaria clauda|[12339]|LNAUS4362-13|United States|Nevada|658[0n]|BOLD:AAE2691
Escaria clauda|[12340]|LNAUS4508-13|United States|Nevada|658[0n]|BOLD:AAE2691
Escaria clauda|[12341]|AWCLB606-11|United States|Arizona|658[0n]|BOLD:AAE2691
Escaria clauda|[12342]|LNAUS4509-13|United States|California|658[0n]|BOLD:AAE2691
Escaria clauda|[12343]|LNAUS4510-13|United States|California|658[0n]|BOLD:AAE2691
Escaria clauda|[12344]|LNAUS4511-13|United States|California|658[0n]|BOLD:AAE2691
Escaria clauda|[12345]|LNAUS4512-13|United States|California|658[0n]|BOLD:AAE2691
Anarta fulgora|[12346]|RDNMC575-06|United States|California|658[0n]|BOLD:AAJ2341
Anarta fulgora|[12347]|RDNMC576-06|United States|California|658[0n]|BOLD:AAJ2341
Anarta fulgora|[12348]|CNCLB276-14|United States|Nevada|658[0n]|BOLD:AAJ2341
Anarta fulgora|[12349]|CNCLB1701-14|United States|Utah|658[0n]|BOLD:AAJ2341
Anarta farnhami|[12350]|IAWLB042-10|United States|California|658[0n]|BOLD:AAA8739
Anarta farnhami|[12351]|IAWLB043-10|United States|California|658[0n]|BOLD:AAA8739
Anarta farnhami|[12352]|CHLEP096-09|Canada|Manitoba|658[0n]|BOLD:AAA8739
Anarta farnhami|[12353]|CHLEP097-09|Canada|Manitoba|658[0n]|BOLD:AAA8739
Anarta farnhami|[12354]|CHLEP098-09|Canada|Manitoba|658[0n]|BOLD:AAA8739
Anarta farnhami|[12355]|CHLEP099-09|Canada|Manitoba|658[0n]|BOLD:AAA8739
Anarta farnhami|[12356]|CHLEP100-09|Canada|Manitoba|658[0n]|BOLD:AAA8739
Anarta farnhami|[12357]|CHLEP101-09|Canada|Manitoba|658[0n]|BOLD:AAA8739
Anarta farnhami|[12358]|CHLEP090-09|Canada|Manitoba|658[0n]|BOLD:AAA8739
Anarta farnhami|[12359]|CHLEP091-09|Canada|Manitoba|658[0n]|BOLD:AAA8739
Anarta farnhami|[12360]|CHLEP204-09|Canada|Manitoba|658[0n]|BOLD:AAA8739
Anarta farnhami|[12361]|CHLEP206-09|Canada|Manitoba|658[0n]|BOLD:AAA8739
Anarta farnhami|[12362]|CHLEP092-09|Canada|Manitoba|658[0n]|BOLD:AAA8739
Anarta farnhami|[12363]|CHLEP093-09|Canada|Manitoba|658[0n]|BOLD:AAA8739
Anarta farnhami|[12364]|CHLEP207-09|Canada|Manitoba|658[0n]|BOLD:AAA8739
Anarta farnhami|[12365]|CHLEP220-09|Canada|Manitoba|658[0n]|BOLD:AAA8739
Anarta farnhami|[12366]|CHLEP221-09|Canada|Manitoba|658[0n]|BOLD:AAA8739
Anarta farnhami|[12367]|CHLEP291-09|Canada|Manitoba|658[0n]|BOLD:AAA8739
Anarta farnhami|[12368]|CHLEP292-09|Canada|Manitoba|658[0n]|BOLD:AAA8739
Anarta farnhami|[12369]|CHLEP294-09|Canada|Manitoba|658[0n]|BOLD:AAA8739
Anarta farnhami|[12370]|IAWLB044-10|United States|California|658[0n]|BOLD:AAA8739
Anarta farnhami|[12371]|PHLCH830-11|Canada|Manitoba|658[0n]|BOLD:AAA8739
Anarta farnhami|[12372]|CHLEP094-09|Canada|Manitoba|658[0n]|BOLD:AAA8739
Anarta farnhami|[12373]|CHLEP095-09|Canada|Manitoba|658[0n]|BOLD:AAA8739
Anarta farnhami|[12374]|CHLEP156-09|Canada|Manitoba|658[0n]|BOLD:AAA8739
Anarta farnhami|[12375]|CHLEP188-09|Canada|Manitoba|658[0n]|BOLD:AAA8739
Anarta farnhami|[12376]|CHLEP102-09|Canada|Manitoba|658[0n]|BOLD:AAA8739
Anarta farnhami|[12377]|CHLEP103-09|Canada|Manitoba|658[0n]|BOLD:AAA8739
Anarta farnhami|[12378]|LCHP839-07|Canada|Manitoba|658[0n]|BOLD:AAA8739
Anarta farnhami|[12379]|LCHQ123-07|Canada|Manitoba|658[0n]|BOLD:AAA8739
Anarta farnhami|[12380]|LCHP829-07|Canada|Manitoba|658[0n]|BOLD:AAA8739
Anarta farnhami|[12381]|LCHP787-07|Canada|Manitoba|658[0n]|BOLD:AAA8739
Anarta farnhami|[12382]|LCHP391-07|Canada|Manitoba|658[0n]|BOLD:AAA8739
Anarta farnhami|[12383]|MHLEP086-07|Canada|Manitoba|658[0n]|BOLD:AAA8739
Anarta farnhami|[12384]|MHLEP055-07|Canada|Manitoba|658[0n]|
Anarta farnhami|[12385]|MHLEP029-07|Canada|Manitoba|658[0n]|BOLD:AAA8739
Anarta farnhami|[12386]|HMCOL071-07|Canada|Manitoba|658[0n]|BOLD:AAA8739
Anarta farnhami|[12387]|LCHP221-07|Canada|Manitoba|658[0n]|BOLD:AAA8739
Anarta farnhami|[12388]|LCHP220-07|Canada|Manitoba|658[0n]|BOLD:AAA8739
Anarta farnhami|[12389]|LCH238-04|Canada|Manitoba|658[0n]|BOLD:AAA8739
Anarta farnhami|[12390]|LCH237-04|Canada|Manitoba|658[0n]|BOLD:AAA8739
Anarta farnhami|[12391]|MHCOL362-07|Canada|Manitoba|645[0n]|BOLD:AAA8739
Anarta farnhami|[12392]|CHLEP205-09|Canada|Manitoba|634[0n]|BOLD:AAA8739
Anarta farnhami|[12393]|CHLEP212-09|Canada|Manitoba|626[0n]|BOLD:AAA8739

Anarta farnhami|[12391]|MHCOL362-07|Canada|Manitoba|645[0n]|BOLD:AAA8739
Anarta farnhami|[12392]|CHLEP205-09|Canada|Manitoba|634[0n]|BOLD:AAA8739
Anarta farnhami|[12393]|CHLEP212-09|Canada|Manitoba|626[0n]|BOLD:AAA8739
Anarta farnhami|[12394]|PHLCH831-11|Canada|Manitoba|658[0n]|BOLD:AAA8739
Anarta hamata|[12395]|RDNM873-05|United States|California|658[0n]|BOLD:ABZ1428
Anarta hamata|[12396]|RDNM874-05|United States|California|575[1n]|BOLD:ABZ1428
Anarta hamata|[12397]|RDNM876-05|United States|Washington|658[0n]|BOLD:ABZ1428
Anarta hamata|[12398]|RDNM875-05|United States|Wyoming|658[0n]|BOLD:ABZ1428
Anarta hamata|[12399]|RDNM877-05|United States|Washington|575[2n]|BOLD:ABZ1428
Anarta alta|[12400]|RDNM891-05|Canada|Alberta|512[0n]|BOLD:ACE5827
Anarta alta|[12401]|RDNM881-05|Canada|Alberta|658[0n]|BOLD:ACE5827
Anarta alta|[12402]|RDNM882-05|Canada|Alberta|562[4n]|BOLD:ACE5827
Anarta alta|[12403]|LPMN929-08|Canada|Alberta|658[0n]|BOLD:ACE5827
Anarta alta|[12404]|LPAB022-08|Canada|Alberta|658[0n]|BOLD:ACE5827
Anarta alta|[12405]|LPABB396-08|Canada|Alberta|658[0n]|BOLD:ACE5827
Anarta alta|[12406]|LPABB399-08|Canada|Alberta|658[0n]|BOLD:ACE5827
Anarta alta|[12407]|LPABB416-08|Canada|Alberta|658[0n]|BOLD:ACE5827
Anarta alta|[12408]|LPABB634-08|Canada|Alberta|658[0n]|BOLD:ACE5827
Anarta oregonica|[12409]|RDNM883-05|Canada|British Columbia|658[0n]|BOLD:ACE5827
Anarta oregonica|[12410]|RDNM893-05|Canada|British Columbia|570[1n]|BOLD:ACE5827
Anarta oregonica|[12411]|RDNM894-05|Canada|British Columbia|505[0n]|BOLD:ACE5827
Anarta oregonica|[12412]|RDNM897-05|United States|Oregon|658[0n]|BOLD:ACE5827
Anarta oregonica|[12413]|LBCD148-05|Canada|British Columbia|658[0n]|BOLD:ACE5827
Anarta oregonica|[12414]|LBCG2367-09|Canada|British Columbia|658[0n]|BOLD:ACE5827
Anarta oregonica|[12415]|RDNM892-05|Canada|British Columbia|658[0n]|BOLD:ACE5827
Anarta oregonica|[12416]|LBCC331-05|Canada|British Columbia|658[0n]|BOLD:ACE5827
Anarta oregonica|[12417]|LBCG2473-09|Canada|British Columbia|658[0n]|BOLD:ACE5827
Anarta oregonica|[12418]|RDNM896-05|United States|California|504[0n]|BOLD:ACE5827
Anarta oregonica|[12419]|RDNM895-05|United States|California|564[2n]|BOLD:ACE5827
Anarta oregonica|[12420]|NAMUM258-08|United States|California|658[0n]|BOLD:ACE5827
Anarta oregonica|[12421]|IAWLB021-10|United States|California|658[0n]|BOLD:ACE5827
Anarta oregonica|[12422]|IAWLB047-10|United States|California|658[0n]|BOLD:ACE5827
Anarta oregonica|[12423]|IAWLB051-10|United States|California|658[0n]|BOLD:ACE5827
Anarta inconcinna|[12424]|LBCG3319-09|Canada|British Columbia|658[0n]|BOLD:ABZ1962
Anarta inconcinna|[12425]|RDNM880-05|Canada|British Columbia|658[0n]|BOLD:ABZ1962
Anarta inconcinna|[12426]|RDNM879-05|Canada|Alberta|658[0n]|BOLD:ABZ1962
Anarta inconcinna|[12427]|RDNM878-05|Canada|Alberta|658[0n]|BOLD:ABZ1962
Anarta inconcinna|[12428]|LOWCD720-06|Canada|British Columbia|658[0n]|BOLD:ABZ1962
Anarta inconcinna|[12429]|LOWCD717-06|Canada|British Columbia|658[0n]|BOLD:ABZ1962
Anarta inconcinna|[12430]|LOWCC192-05|Canada|British Columbia|658[0n]|BOLD:ABZ1962
Anarta inconcinna|[12431]|LOWCB723-05|Canada|British Columbia|658[0n]|BOLD:ABZ1962
Anarta inconcinna|[12432]|LOWCB721-05|Canada|British Columbia|658[0n]|BOLD:ABZ1962
Anarta inconcinna|[12433]|LOWCD731-06|Canada|British Columbia|568[0n]|BOLD:ABZ1962
Anarta inconcinna|[12434]|LOWCD733-06|Canada|British Columbia|658[0n]|BOLD:ABZ1962
Anarta inconcinna|[12435]|LBCG2878-09|Canada|British Columbia|658[0n]|BOLD:ABZ1962
Anarta inconcinna|[12436]|LBCH5051-10|Canada|British Columbia|658[0n]|BOLD:ABZ1962
Anarta inconcinna|[12437]|LBCH5492-10|Canada|British Columbia|658[0n]|BOLD:ABZ1962
Anarta inconcinna|[12438]|LBCH5493-10|Canada|British Columbia|658[0n]|BOLD:ABZ1962
Anarta inconcinna|[12439]|RDNMJ105-10|United States|New Mexico|658[0n]|BOLD:ABZ1962
Anarta obesula|[12440]|RDNMF537-08|United States|Colorado|609[0n]|BOLD:AAA9985
Anarta obesula|[12441]|RDNMG829-08|United States|Wyoming|592[0n]|BOLD:AAA9985
Anarta obesula|[12442]|RDNMF536-08|United States|Wyoming|609[0n]|BOLD:AAA9985
Anarta obesula|[12443]|RDNMG830-08|United States|Wyoming|609[0n]|BOLD:AAA9985
Anarta columbica|[12444]|LOWCD729-06|Canada|British Columbia|506[0n]|
Anarta columbica|[12445]|LOWCD719-06|Canada|British Columbia|558[0n]|BOLD:AAA9985
Anarta columbica|[12446]|LOWCD716-06|Canada|British Columbia|657[2n]|BOLD:AAA9985
Anarta columbica|[12447]|LOWCB724-05|Canada|British Columbia|658[0n]|BOLD:AAA9985
Anarta columbica|[12448]|LBCH5491-10|Canada|British Columbia|658[0n]|BOLD:AAA9985
Anarta columbica|[12449]|RDNM886-05|United States|Washington|658[0n]|BOLD:AAA9985
Anarta columbica|[12450]|LBCG116-08|Canada|British Columbia|658[0n]|BOLD:AAA9985
Anarta columbica|[12451]|LBCH5447-10|Canada|British Columbia|658[0n]|BOLD:AAA9985
Anarta columbica|[12452]|LBCH5094-10|Canada|British Columbia|658[0n]|BOLD:AAA9985
Anarta columbica|[12453]|LBCH5827-10|Canada|British Columbia|658[0n]|BOLD:AAA9985
Anarta columbica|[12454]|LBCH5444-10|Canada|British Columbia|658[0n]|BOLD:AAA9985
Anarta columbica|[12455]|LBCH5830-10|Canada|British Columbia|658[0n]|BOLD:AAA9985
Anarta columbica|[12456]|LBCH5826-10|Canada|British Columbia|658[0n]|BOLD:AAA9985
Anarta columbica|[12457]|LBCH5825-10|Canada|British Columbia|658[0n]|BOLD:AAA9985
Anarta columbica|[12458]|LBCH5809-10|Canada|British Columbia|658[0n]|BOLD:AAA9985
Anarta columbica|[12459]|LBCH5728-10|Canada|British Columbia|658[0n]|BOLD:AAA9985
Anarta columbica|[12460]|LBCH5601-10|Canada|British Columbia|658[0n]|BOLD:AAA9985
Anarta columbica|[12461]|LBCH5494-10|Canada|British Columbia|658[0n]|BOLD:AAA9985
Anarta columbica|[12462]|LBCH5486-10|Canada|British Columbia|658[0n]|BOLD:AAA9985
Anarta columbica|[12463]|LBCH5482-10|Canada|British Columbia|658[0n]|BOLD:AAA9985
Anarta columbica|[12464]|LBCH5449-10|Canada|British Columbia|658[0n]|BOLD:AAA9985
Anarta columbica|[12465]|LBCH5448-10|Canada|British Columbia|658[0n]|BOLD:AAA9985
Anarta columbica|[12466]|LBCH5446-10|Canada|British Columbia|658[0n]|BOLD:AAA9985
Anarta columbica|[12467]|LBCH5445-10|Canada|British Columbia|658[0n]|BOLD:AAA9985
Anarta columbica|[12468]|LBCH5443-10|Canada|British Columbia|658[0n]|BOLD:AAA9985
Anarta columbica|[12469]|LBCH5427-10|Canada|British Columbia|658[0n]|BOLD:AAA9985
Anarta columbica|[12470]|LBCH5426-10|Canada|British Columbia|658[0n]|BOLD:AAA9985
Anarta columbica|[12471]|LBCH5424-10|Canada|British Columbia|658[0n]|BOLD:AAA9985
Anarta columbica|[12472]|LBCH5423-10|Canada|British Columbia|658[0n]|BOLD:AAA9985
Anarta columbica|[12473]|LBCH5344-10|Canada|British Columbia|658[0n]|BOLD:AAA9985
Anarta columbica|[12474]|LBCH5286-10|Canada|British Columbia|658[0n]|BOLD:AAA9985
Anarta columbica|[12475]|LBCH5252-10|Canada|British Columbia|658[0n]|BOLD:AAA9985
Anarta columbica|[12476]|LBCH5213-10|Canada|British Columbia|658[0n]|BOLD:AAA9985
Anarta columbica|[12477]|LBCH5212-10|Canada|British Columbia|658[0n]|BOLD:AAA9985
Anarta columbica|[12478]|LBCH5160-10|Canada|British Columbia|658[0n]|BOLD:AAA9985
Anarta columbica|[12479]|LBCH5095-10|Canada|British Columbia|658[0n]|BOLD:AAA9985
Anarta columbica|[12480]|LBCG371-08|Canada|British Columbia|658[0n]|BOLD:AAA9985
Anarta columbica|[12481]|LBCG343-08|Canada|British Columbia|658[0n]|BOLD:AAA9985
Anarta columbica|[12482]|LBCG335-08|Canada|British Columbia|658[0n]|BOLD:AAA9985
Anarta columbica|[12483]|LBCG121-08|Canada|British Columbia|658[0n]|BOLD:AAA9985
Anarta columbica|[12484]|LBCG118-08|Canada|British Columbia|658[0n]|BOLD:AAA9985
Anarta columbica|[12485]|LBCG101-08|Canada|British Columbia|658[0n]|BOLD:AAA9985
Anarta columbica|[12486]|LOWCD732-06|Canada|British Columbia|658[0n]|BOLD:AAA9985
Anarta columbica|[12487]|LOWCD726-06|Canada|British Columbia|658[0n]|BOLD:AAA9985
Anarta columbica|[12488]|LOWCD725-06|Canada|British Columbia|658[0n]|BOLD:AAA9985
Anarta columbica|[12489]|LOWCD724-06|Canada|British Columbia|657[0n]|BOLD:AAA9985
Anarta columbica|[12490]|LOWCD718-06|Canada|British Columbia|658[0n]|BOLD:AAA9985
Anarta columbica|[12491]|LOWCD714-06|Canada|British Columbia|658[0n]|BOLD:AAA9985
Anarta columbica|[12492]|LOWCB727-05|Canada|British Columbia|658[0n]|BOLD:AAA9985
Anarta columbica|[12493]|LOWCB726-05|Canada|British Columbia|658[0n]|BOLD:AAA9985

Anarta columbica|[12491]|LOWCD714-06|Canada|British Columbia|658[0n]|BOLD:AAA9985
Anarta columbica|[12492]|LOWCB727-05|Canada|British Columbia|658[0n]|BOLD:AAA9985
Anarta columbica|[12493]|LOWCB726-05|Canada|British Columbia|658[0n]|BOLD:AAA9985
Anarta columbica|[12494]|LOWCB725-05|Canada|British Columbia|658[0n]|BOLD:AAA9985
Anarta columbica|[12495]|LOWCB720-05|Canada|British Columbia|658[0n]|BOLD:AAA9985
Anarta columbica|[12496]|LOWCB719-05|Canada|British Columbia|658[0n]|BOLD:AAA9985
Anarta columbica|[12497]|RDNM885-05|United States|Oregon|658[0n]|BOLD:AAA9985
Anarta columbica|[12498]|RDNM884-05|Canada|British Columbia|658[0n]|BOLD:AAA9985
Anarta columbica|[12499]|RDNM872-05|Canada|British Columbia|658[0n]|BOLD:AAA9985
Anarta columbica|[12500]|RDNM871-05|Canada|British Columbia|658[0n]|BOLD:AAA9985
Anarta columbica|[12501]|LOWCD723-06|Canada|British Columbia|641[0n]|BOLD:AAA9985
Anarta columbica|[12502]|LOWCD727-06|Canada|British Columbia|587[0n]|BOLD:AAA9985
Anarta columbica|[12503]|LBCG124-08|Canada|British Columbia|640[0n]|BOLD:AAA9985
Anarta columbica|[12504]|LOWCB316-05|Canada|British Columbia|581[0n]|BOLD:AAA9985
Anarta columbica|[12505]|LOWCD728-06|Canada|British Columbia|574[0n]|BOLD:AAA9985
Anarta columbica|[12506]|LOWCD721-06|Canada|British Columbia|657[0n]|BOLD:AAA9985
Anarta columbica|[12507]|LOWCD715-06|Canada|British Columbia|594[0n]|BOLD:AAA9985
Anarta columbica|[12508]|LBCH5159-10|Canada|British Columbia|623[0n]|BOLD:AAA9985
Anarta columbica|[12509]|LBCH5600-10|Canada|British Columbia|644[0n]|BOLD:AAA9985
Anarta columbica|[12510]|RDNMJ380-11|United States|California|658[0n]|BOLD:AAA9985
Anarta trifolii|[12511]|BBLWU004-09|United States|Colorado|658[0n]|BOLD:ABZ7601
Anarta trifolii|[12512]|BBLWU008-09|United States|Colorado|658[0n]|BOLD:ABZ7601
Anarta trifolii|[12513]|BBLWU057-09|United States|Colorado|658[0n]|BOLD:ABZ7601
Anarta trifolii|[12514]|BBLWU140-09|United States|Colorado|658[0n]|BOLD:ABZ7601
Anarta trifolii|[12515]|BBLWU168-09|United States|Colorado|658[0n]|BOLD:ABZ7601
Anarta trifolii|[12516]|RDNMK261-11|United States|North Dakota|658[0n]|BOLD:ABZ7601
Anarta trifolii|[12517]|BBLCU381-09|United States|Kansas|658[0n]|BOLD:ABZ7601
Anarta trifolii|[12518]|RDNMB963-05|Canada|Alberta|658[0n]|BOLD:ABZ7601
Anarta trifolii|[12519]|XAH268-05|Canada|Ontario|658[0n]|BOLD:ABZ7601
Anarta trifolii|[12520]|RDNMC304-05|United States|Colorado|581[0n]|BOLD:ABZ7601
Anarta trifolii|[12521]|BBLWU002-09|United States|Colorado|658[0n]|BOLD:ABZ7601
Anarta trifolii|[12522]|BBLWU007-09|United States|Colorado|658[0n]|BOLD:ABZ7601
Anarta trifolii|[12523]|BBLWU107-09|United States|Colorado|658[0n]|BOLD:ABZ7601
Anarta trifolii|[12524]|BBLWU167-09|United States|Colorado|658[0n]|BOLD:ABZ7601
Anarta trifolii|[12525]|RDNMJ109-10|United States|New Mexico|658[0n]|BOLD:ABZ7601
Anarta trifolii|[12526]|CMAZA921-12|United States|Arizona|658[0n]|BOLD:ABZ7601
Anarta trifolii|[12527]|CMAZA928-12|United States|Arizona|658[0n]|BOLD:ABZ7601
Anarta trifolii|[12528]|CMAZA1067-12|United States|Arizona|638[0n]|BOLD:ABZ7601
Anarta trifolii|[12529]|RDNMB962-05|United States|Washington|658[0n]|BOLD:ABZ7601
Anarta trifolii|[12530]|RDNMB964-05|Canada|British Columbia|658[0n]|BOLD:ABZ7601
Anarta trifolii|[12531]|RWWC270-11|United States|Washington|658[0n]|BOLD:ABZ7601
Anarta trifolii|[12532]|ABCNA889-08|United States|Wisconsin|609[0n]|BOLD:ABZ7601
Anarta trifolii|[12533]|GWOSK878-11|Cyprus||658[0n]|BOLD:ABZ7601
Anarta trifolii|[12534]|PHLSA539-11|Spain|Comunidad Valenciana|658[0n]|BOLD:ABZ7601
Anarta trifolii|[12535]|GWOTI161-12|Germany|Saxony|658[0n]|BOLD:ABZ7601
Anarta trifolii|[12536]|GWOTI162-12|Georgia||658[0n]|BOLD:ABZ7601
Anarta trifolii|[12537]|LEFIF621-10|Finland||658[0n]|BOLD:ABZ7601
Anarta trifolii|[12538]|LEFIF685-10|Finland||658[0n]|BOLD:ABZ7601
Anarta trifolii|[12539]|GWOR4010-09|Germany|Bavaria|658[0n]|BOLD:ABZ7601
Anarta trifolii|[12540]|LEFID051-10|Finland|Ostrobothnia|658[0n]|BOLD:ABZ7601
Anarta trifolii|[12541]|ABCNA890-08|United States|Wisconsin|658[0n]|BOLD:ABZ7601
Anarta trifolii|[12542]|CGUKB696-09|United Kingdom|England|658[0n]|BOLD:ABZ7601
Anarta trifolii|[12543]|LCHQ118-07|Canada|Manitoba|658[0n]|BOLD:ABZ7601
Anarta trifolii|[12544]|LCHP407-07|Canada|Manitoba|658[0n]|BOLD:ABZ7601
Anarta trifolii|[12545]|RDNMD737-06|Denmark||658[0n]|BOLD:ABZ7601
Anarta trifolii|[12546]|RDLQG057-06|Canada|Quebec|658[0n]|BOLD:ABZ7601
Anarta trifolii|[12547]|RDLQG024-06|Canada|Quebec|658[0n]|BOLD:ABZ7601
Anarta trifolii|[12548]|RDLQF229-06|Canada|Quebec|658[0n]|BOLD:ABZ7601
Anarta trifolii|[12549]|RDNMC203-05|Canada|New Brunswick|658[0n]|BOLD:ABZ7601
Anarta trifolii|[12550]|GWOR3902-09|Germany|Bavaria|658[0n]|BOLD:ABZ7601
Anarta trifolii|[12551]|RDLQB741-05|Canada|Quebec|658[0n]|BOLD:ABZ7601
Anarta trifolii|[12552]|RDNMB057-05|Denmark||658[0n]|BOLD:ABZ7601
Anarta trifolii|[12553]|GWORZ226-10|Italy|Basilicata|658[0n]|BOLD:ABZ7601
Anarta trifolii|[12554]|RDLQB765-05|Canada|Quebec|617[0n]|BOLD:ABZ7601
Anarta trifolii|[12555]|RDNMC204-05|Canada|New Brunswick|587[1n]|BOLD:ABZ7601
Anarta trifolii|[12556]|ABCNA888-08|United States|Wisconsin|646[0n]|BOLD:ABZ7601
Anarta trifolii|[12557]|BLTIB673-08|Canada|Ontario|658[1n]|BOLD:ABZ7601
Anarta trifolii|[12558]|CGUKD538-09|United Kingdom|England|633[0n]|BOLD:ABZ7601
Anarta trifolii|[12559]|MAMOT1500-12|Pakistan|Punjab|654[0n]|BOLD:ABZ7601
Anarta trifolii|[12560]|GRAFW1455-12|Greenland||658[0n]|BOLD:ABZ7601
Anarta trifolii|[12561]|NGNAI442-13|Canada|British Columbia|592[0n]|BOLD:ABZ7601
Anarta trifolii|[12562]|LON374-08|Norway|Ostfold|657[0n]|BOLD:ABZ7601
Anarta trifolii|[12563]|CGUKA304-09|United Kingdom|England|658[0n]|BOLD:ABZ7601
Anarta trifolii|[12564]|CGUKA314-09|United Kingdom|England|658[0n]|BOLD:ABZ7601
Anarta trifolii|[12565]|CGUKA761-09|United Kingdom||658[0n]|BOLD:ABZ7601
Anarta trifolii|[12566]|GWOR3895-09|Germany|Bavaria|658[0n]|BOLD:ABZ7601
Anarta trifolii|[12567]|CGUKD258-09|United Kingdom|England|658[0n]|BOLD:ABZ7601
Anarta trifolii|[12568]|LENOA219-11|France|Haute Normandie|658[0n]|BOLD:ABZ7601
Anarta trifolii|[12569]|RDNMB056-05|Denmark||658[0n]|BOLD:ABZ7601
Anarta trifolii|[12570]|GWOSA421-10|Germany|Bavaria|658[0n]|BOLD:ABZ7601
Anarta trifolii|[12571]|FBLMW379-10|Germany|Bavaria|658[0n]|BOLD:ABZ7601
Anarta trifolii|[12572]|LENOA218-11|France|Haute Normandie|658[0n]|BOLD:ABZ7601
Anarta trifolii|[12573]|GWOTL077-13|Germany|Saarland|658[0n]|BOLD:ABZ7601
Anarta trifolii|[12574]|LEATB745-13|Italy|South Tyrol|658[0n]|BOLD:ABZ7601
Anarta trifolii|[12575]|LEATB746-13|Italy|South Tyrol|658[0n]|BOLD:ABZ7601
Anarta trifolii|[12576]|LEATG114-14|Austria|Tirol|658[0n]|BOLD:ABZ7601
Anarta trifolii|[12577]|LEATG174-14|Austria|Tirol|658[0n]|BOLD:ABZ7601
Anarta nigrolunata|[12578]|RDNMC352-05|Canada|Yukon Territory|658[0n]|BOLD:AAD2342
Anarta nigrolunata|[12579]|RDNME419-08|Canada|Yukon Territory|658[0n]|BOLD:AAD2342
Anarta nigrolunata|[12580]|CHIP153-12|Canada|Manitoba|658[0n]|BOLD:AAD2342
Anarta nigrolunata|[12581]|CHLEP123-09|Canada|Manitoba|658[0n]|BOLD:AAD2342
Anarta nigrolunata|[12582]|LPABB809-09|Canada|Alberta|658[0n]|BOLD:AAD2342
Anarta nigrolunata|[12583]|LPABB806-09|Canada|Alberta|658[0n]|BOLD:AAD2342
Anarta nigrolunata|[12584]|LPABB805-09|Canada|Alberta|658[0n]|BOLD:AAD2342
Anarta nigrolunata|[12585]|LCHQ867-08|Canada|Manitoba|658[0n]|BOLD:AAD2342
Anarta nigrolunata|[12586]|LOWCD112-06|Canada|British Columbia|658[0n]|BOLD:AAD2342
Anarta nigrolunata|[12587]|CNCLB1499-14|Canada|Yukon Territory|604[0n]|BOLD:AAD2342
Anarta nigrolunata|[12588]|CNCLB1500-14|Canada|Yukon Territory|593[0n]|BOLD:AAD2342
Anarta nigrolunata|[12589]|CNCLB1794-14|Canada|Yukon Territory|658[0n]|BOLD:AAD2342
Anarta sierrae|[12590]|RDNMC733-06|United States|California|658[0n]|BOLD:ABZ3207
Anarta melanopa|[12591]|LEFIG500-10|Finland||658[0n]|BOLD:AAF8108
Anarta melanopa|[12592]|LEFIA755-10|Finland|Lapland|658[0n]|BOLD:AAF8108
Anarta melanopa|[12593]|LEFIA749-10|Finland|Lapland|658[0n]|BOLD:AAF8108

Anarta melanopa|[12591]|LEFIG500-10|Finland||658[0n]|BOLD:AAF8108
Anarta melanopa|[12592]|LEFIA755-10|Finland|Lapland|658[0n]|BOLD:AAF8108
Anarta melanopa|[12593]|LEFIA749-10|Finland|Lapland|658[0n]|BOLD:AAF8108
Anarta melanopa|[12594]|LON166-08|Norway|Finnmark|657[0n]|BOLD:AAF8108
Anarta melanopa|[12595]|LEFIK422-10|Finland||658[0n]|BOLD:AAF8108
Anarta melanopa|[12596]|LEATC092-13|Italy|Trentino-Alto Adige|658[0n]|BOLD:AAF8108
Anarta melanopa|[12597]|UAMIC1144-13|United States|Alaska|658[0n]|BOLD:AAF8108
Anarta melanopa|[12598]|PHLAC668-10|Switzerland|Graubunden|658[0n]|BOLD:AAF8108
Anarta melanopa|[12599]|PHLAB1138-10|Austria|Tirol|658[0n]|BOLD:AAF8108
Anarta melanopa|[12600]|GWOSA803-10|Germany|Bavaria|624[0n]|BOLD:AAF8108
Anarta melanopa|[12601]|PHLAI866-13|Austria|Carinthia|658[0n]|BOLD:AAF8108
Anarta melanopa|[12602]|LEATD132-13|Switzerland|Graubunden|658[0n]|BOLD:AAF8108
Anarta melanopa|[12603]|CNCLB1498-14|United States|Alaska|596[0n]|BOLD:AAF8108
Anarta melanopa|[12604]|CNCLB1793-14|Canada|Yukon Territory|658[0n]|BOLD:AAF8108
Anarta melanopa|[12605]|CNCLB1795-14|Canada|Yukon Territory|658[0n]|BOLD:AAF8108
Anarta crotchii|[12606]|RDNMH867-09|United States|California|658[0n]|BOLD:AAB9779
Anarta crotchii|[12607]|RDNMC786-06|United States|Wyoming|549[1n]|BOLD:AAB9779
Anarta crotchii|[12608]|NAMUM382-09|United States|California|658[0n]|BOLD:AAB9779
Anarta crotchii|[12609]|NAMUM390-09|United States|California|658[0n]|BOLD:AAB9779
Anarta crotchii|[12610]|NAMUM393-09|United States|California|658[0n]|BOLD:AAB9779
Anarta crotchii|[12611]|RDNMC785-06|United States|Wyoming|574[0n]|BOLD:AAB9779
Anarta crotchii|[12612]|RDNM888-05|Canada|British Columbia|577[3n]|BOLD:AAB9779
Anarta crotchii|[12613]|RDNMC346-05|Canada|British Columbia|559[0n]|BOLD:AAB9779
Anarta crotchii|[12614]|RDNMC784-06|Canada|British Columbia|549[2n]|BOLD:AAB9779
Anarta crotchii|[12615]|LBCH4977-10|Canada|British Columbia|658[0n]|BOLD:AAB9779
Anarta crotchii|[12616]|LBCH4981-10|Canada|British Columbia|658[0n]|BOLD:AAB9779
Anarta crotchii|[12617]|RDNMC182-05|Canada|British Columbia|658[0n]|BOLD:AAB9779
Anarta crotchii|[12618]|RDNMC772-06|United States|Oregon|565[0n]|BOLD:AAB9779
Anarta crotchii|[12619]|RDNMC349-05|Canada|British Columbia|579[0n]|BOLD:AAB9779
Anarta crotchii|[12620]|RDNMC348-05|United States|Oregon|586[0n]|BOLD:AAB9779
Anarta crotchii|[12621]|RDNMC347-05|United States|Oregon|576[0n]|BOLD:AAB9779
Anarta crotchii|[12622]|RDNMC344-05|Canada|British Columbia|582[3n]|BOLD:AAB9779
Anarta crotchii|[12623]|RDNMC774-06|United States|Oregon|549[0n]|BOLD:AAB9779
Anarta crotchii|[12624]|RDNMC775-06|United States|Oregon|572[0n]|BOLD:AAB9779
Anarta crotchii|[12625]|RDNMC777-06|Canada|British Columbia|574[0n]|BOLD:AAB9779
Anarta crotchii|[12626]|LBCH4993-10|Canada|British Columbia|658[0n]|BOLD:AAB9779
Anarta crotchii|[12627]|LBCH4999-10|Canada|British Columbia|658[0n]|BOLD:AAB9779
Anarta crotchii|[12628]|LBCH5011-10|Canada|British Columbia|658[0n]|BOLD:AAB9779
Anarta crotchii|[12629]|IAWLB419-11|United States|California|658[0n]|BOLD:AAB9779
Anarta crotchii|[12630]|RDNMC778-06|Canada|British Columbia|588[1n]|BOLD:AAB9779
Anarta crotchii|[12631]|RDNMK249-11|United States|Utah|658[0n]|BOLD:AAB9779
Anarta crotchii|[12632]|RDNMK251-11|United States|Utah|658[0n]|BOLD:AAB9779
Anarta crotchii|[12633]|RDNMK300-11|United States|Utah|658[0n]|BOLD:AAB9779
Anarta oaklandiae|[12634]|NAMUM203-08|United States|California|615[0n]|BOLD:AAB9779
Anarta oaklandiae|[12635]|CNCLB1516-14|United States|California|658[0n]|BOLD:AAB9779
Anarta oaklandiae|[12636]|NAMUM202-08|United States|California|658[1n]|BOLD:AAB9779
Anarta oaklandiae|[12637]|CNCLB1517-14|United States|California|544[0n]|BOLD:AAB9779
Anarta oaklandiae|[12638]|CNCLB1518-14|United States|California|528[0n]|BOLD:AAB9779
Anarta crotchii|[12639]|RDNMC782-06|United States|California|585[0n]|BOLD:AAB8611
Anarta crotchii|[12640]|RDNM889-05|Canada|British Columbia|612[1n]|BOLD:AAB8611
Anarta crotchii|[12641]|RDMAB471-06|Canada|Alberta|658[0n]|BOLD:AAB8611
Anarta crotchii|[12642]|RDNMB965-05|Canada|Alberta|658[0n]|BOLD:AAB8611
Anarta crotchii|[12643]|RDNMB116-05|Canada|Alberta|658[0n]|BOLD:AAB8611
Anarta crotchii|[12644]|RDNM890-05|Canada|British Columbia|658[0n]|BOLD:AAB8611
Anarta crotchii|[12645]|RDNMC779-06|Canada|British Columbia|615[0n]|BOLD:AAB8611
Anarta crotchii|[12646]|RDNMC350-05|Canada|British Columbia|585[0n]|BOLD:AAB8611
Anarta crotchii|[12647]|RDNMC345-05|Canada|British Columbia|608[0n]|BOLD:AAB8611
Anarta crotchii|[12648]|RDNMC780-06|Canada|British Columbia|604[0n]|BOLD:AAB8611
Anarta crotchii|[12649]|RDNMC773-06|United States|Oregon|565[0n]|BOLD:AAB8611
Anarta crotchii|[12650]|RDNMC181-05|United States|Oregon|658[0n]|BOLD:AAB8611
Anarta crotchii|[12651]|RDNMC771-06|United States|Wyoming|614[0n]|BOLD:AAB8611
Anarta crotchii|[12652]|RDNM066-05|United States|Oregon|616[0n]|BOLD:AAB8611
Anarta crotchii|[12653]|RDNMC776-06|United States|Oregon|608[0n]|BOLD:AAB8611
Anarta crotchii|[12654]|RDNMC781-06|United States|California|604[0n]|BOLD:AAB8611
Anarta crotchii|[12655]|RDNMF538-08|United States|California|658[0n]|BOLD:AAB8611
Anarta crotchii|[12656]|RDNMF539-08|United States|California|658[0n]|BOLD:AAB8611
Anarta crotchii|[12657]|IAWLB420-11|United States|California|658[0n]|BOLD:AAB8611
Anarta crotchii|[12658]|JMMMB588-13|United States|California|603[0n]|BOLD:AAB8611
Trudestra hadeniformis|[12659]|CNCLB3237-15|United States|Nevada|658[0n]|BOLD:ACW8873
Apamea devastator|[12660]|CNGRF001-12|Canada|Saskatchewan|618[4n]|BOLD:ABY5257
Apamea devastator|[12661]|RWWB094-09|United States|Washington|577[0n]|BOLD:ABY5257
Apamea devastator|[12662]|CNGRF026-12|Canada|Saskatchewan|618[0n]|BOLD:ABY5257
Apamea devastator|[12663]|CNGRE016-12|Canada|Saskatchewan|618[0n]|BOLD:ABY5257
Apamea devastator|[12664]|CNGRL422-13|Canada|Saskatchewan|582[0n]|BOLD:ABY5257
Apamea devastator|[12665]|LBCB621-05|Canada|British Columbia|597[0n]|BOLD:ABY5257
Apamea devastator|[12666]|CNGRL419-13|Canada|Saskatchewan|576[0n]|BOLD:ABY5257
Apamea devastator|[12667]|CNGRL405-13|Canada|Saskatchewan|594[0n]|BOLD:ABY5257
Apamea devastator|[12668]|CNGRK432-13|Canada|Saskatchewan|592[0n]|BOLD:ABY5257
Apamea devastator|[12669]|CNWBG3104-13|Canada|Alberta|607[0n]|BOLD:ABY5257
Apamea devastator|[12670]|TTMNB336-06|Canada|New Brunswick|658[1n]|BOLD:ABY5257
Apamea devastator|[12671]|UAMIC548-13|United States|Alaska|633[0n]|BOLD:ABY5257
Apamea devastator|[12672]|CNGRK441-13|Canada|Saskatchewan|629[0n]|BOLD:ABY5257
Apamea devastator|[12673]|CNWLF2008-12|Canada|Alberta|630[0n]|BOLD:ABY5257
Apamea devastator|[12674]|CNGRF014-12|Canada|Saskatchewan|629[0n]|BOLD:ABY5257
Apamea devastator|[12675]|CNGRF005-12|Canada|Saskatchewan|629[0n]|BOLD:ABY5257
Apamea devastator|[12676]|CNGRF004-12|Canada|Saskatchewan|629[0n]|BOLD:ABY5257
Apamea devastator|[12677]|CNGRE026-12|Canada|Saskatchewan|629[0n]|BOLD:ABY5257
Apamea devastator|[12678]|CNGRE024-12|Canada|Saskatchewan|629[0n]|BOLD:ABY5257
Apamea devastator|[12679]|CNGRE022-12|Canada|Saskatchewan|629[0n]|BOLD:ABY5257
Apamea devastator|[12680]|CNGRE020-12|Canada|Saskatchewan|629[0n]|BOLD:ABY5257
Apamea devastator|[12681]|CNGRE011-12|Canada|Saskatchewan|629[0n]|BOLD:ABY5257
Apamea devastator|[12682]|CNGRE002-12|Canada|Saskatchewan|629[0n]|BOLD:ABY5257
Apamea devastator|[12683]|PHMNB240-04|Canada|New Brunswick|609[0n]|BOLD:ABY5257
Apamea devastator|[12684]|CNGRL431-13|Canada|Saskatchewan|579[0n]|BOLD:ABY5257
Apamea devastator|[12685]|GMLC1159-12|United States|California|658[0n]|BOLD:ABY5257
Apamea devastator|[12686]|RWWC718-11|United States|Washington|658[0n]|BOLD:ABY5257
Apamea devastator|[12687]|RWWC690-11|United States|Washington|658[0n]|BOLD:ABY5257
Apamea devastator|[12688]|RWWC536-11|United States|Washington|658[0n]|BOLD:ABY5257
Apamea devastator|[12689]|RWWC404-11|United States|Washington|658[0n]|BOLD:ABY5257
Apamea devastator|[12690]|BBLPB802-10|Canada|British Columbia|658[0n]|BOLD:ABY5257
Apamea devastator|[12691]|BBLPB360-10|Canada|Alberta|658[0n]|BOLD:ABY5257
Apamea devastator|[12692]|RWWC058-10|United States|Washington|658[0n]|BOLD:ABY5257
Apamea devastator|[12693]|RWWC003-10|United States|Washington|658[0n]|BOLD:ABY5257

Apamea devastator|[12691]|BBLPB360-10|Canada|Alberta|658[0n]|BOLD:ABY5257
Apamea devastator|[12692]|RWWC058-10|United States|Washington|658[0n]|BOLD:ABY5257
Apamea devastator|[12693]|RWWC003-10|United States|Washington|658[0n]|BOLD:ABY5257
Apamea devastator|[12694]|RWWC002-10|United States|Washington|658[0n]|BOLD:ABY5257
Apamea devastator|[12695]|LNCC294-10|United States|North Carolina|658[0n]|BOLD:ABY5257
Apamea devastator|[12696]|LNCC293-10|United States|North Carolina|658[0n]|BOLD:ABY5257
Apamea devastator|[12697]|LBCH7684-10|Canada|British Columbia|658[0n]|BOLD:ABY5257
Apamea devastator|[12698]|LBCH7328-10|Canada|British Columbia|658[0n]|BOLD:ABY5257
Apamea devastator|[12699]|LBCH7120-10|Canada|British Columbia|658[0n]|BOLD:ABY5257
Apamea devastator|[12700]|LBCH6038-10|Canada|British Columbia|658[0n]|BOLD:ABY5257
Apamea devastator|[12701]|LBCH6026-10|Canada|British Columbia|658[0n]|BOLD:ABY5257
Apamea devastator|[12702]|LBCH2095-10|Canada|British Columbia|658[0n]|BOLD:ABY5257
Apamea devastator|[12703]|LBCH1970-10|Canada|British Columbia|658[0n]|BOLD:ABY5257
Apamea devastator|[12704]|LBCH1866-10|Canada|British Columbia|658[0n]|BOLD:ABY5257
Apamea devastator|[12705]|LBCH1167-10|Canada|British Columbia|658[0n]|BOLD:ABY5257
Apamea devastator|[12706]|RWWB197-09|United States|Washington|658[0n]|BOLD:ABY5257
Apamea devastator|[12707]|RWWB180-09|United States|Washington|658[0n]|BOLD:ABY5257
Apamea devastator|[12708]|RWWB178-09|United States|Washington|658[0n]|BOLD:ABY5257
Apamea devastator|[12709]|RWWB121-09|United States|Washington|658[0n]|BOLD:ABY5257
Apamea devastator|[12710]|RWWB088-09|United States|Washington|658[0n]|BOLD:ABY5257
Apamea devastator|[12711]|RWWB062-09|United States|Washington|658[0n]|BOLD:ABY5257
Apamea devastator|[12712]|RWWB061-09|United States|Washington|658[0n]|BOLD:ABY5257
Apamea devastator|[12713]|RWWB043-09|United States|Washington|658[0n]|BOLD:ABY5257
Apamea devastator|[12714]|RWWB036-09|United States|Washington|658[0n]|BOLD:ABY5257
Apamea devastator|[12715]|RWWB025-09|United States|Washington|658[0n]|BOLD:ABY5257
Apamea devastator|[12716]|RWWA998-09|United States|Washington|658[0n]|BOLD:ABY5257
Apamea devastator|[12717]|RWWA995-09|United States|Washington|658[0n]|BOLD:ABY5257
Apamea devastator|[12718]|RWWA937-09|United States|Washington|658[0n]|BOLD:ABY5257
Apamea devastator|[12719]|RWWA804-09|United States|Washington|658[0n]|BOLD:ABY5257
Apamea devastator|[12720]|LBCG3323-09|Canada|British Columbia|658[0n]|BOLD:ABY5257
Apamea devastator|[12721]|LBCG3282-09|Canada|British Columbia|658[0n]|BOLD:ABY5257
Apamea devastator|[12722]|LBCG3280-09|Canada|British Columbia|658[0n]|BOLD:ABY5257
Apamea devastator|[12723]|LBCG2890-09|Canada|British Columbia|658[0n]|BOLD:ABY5257
Apamea devastator|[12724]|LBCG2580-09|Canada|British Columbia|658[0n]|BOLD:ABY5257
Apamea devastator|[12725]|LPABC414-09|Canada|Alberta|658[0n]|BOLD:ABY5257
Apamea devastator|[12726]|LPABB727-08|Canada|Alberta|658[0n]|BOLD:ABY5257
Apamea devastator|[12727]|LPABB636-08|Canada|Alberta|658[0n]|BOLD:ABY5257
Apamea devastator|[12728]|LPABB390-08|Canada|Alberta|658[0n]|BOLD:ABY5257
Apamea devastator|[12729]|LPVIC028-08|Canada|British Columbia|658[0n]|BOLD:ABY5257
Apamea devastator|[12730]|LPVIB570-08|Canada|British Columbia|658[0n]|BOLD:ABY5257
Apamea devastator|[12731]|LPVIB238-08|Canada|British Columbia|658[0n]|BOLD:ABY5257
Apamea devastator|[12732]|LPVIB069-08|Canada|British Columbia|658[0n]|BOLD:ABY5257
Apamea devastator|[12733]|BLTIB864-08|Canada|Ontario|658[0n]|BOLD:ABY5257
Apamea devastator|[12734]|BLTIB569-08|Canada|Ontario|658[0n]|BOLD:ABY5257
Apamea devastator|[12735]|LPSK590-08|Canada|Saskatchewan|658[0n]|BOLD:ABY5257
Apamea devastator|[12736]|LPSK550-08|Canada|Saskatchewan|658[0n]|BOLD:ABY5257
Apamea devastator|[12737]|LPSK536-08|Canada|Saskatchewan|658[0n]|BOLD:ABY5257
Apamea devastator|[12738]|LPSK514-08|Canada|Saskatchewan|658[0n]|BOLD:ABY5257
Apamea devastator|[12739]|LPSK500-08|Canada|Saskatchewan|658[0n]|BOLD:ABY5257
Apamea devastator|[12740]|LPSK483-08|Canada|Saskatchewan|658[0n]|BOLD:ABY5257
Apamea devastator|[12741]|LPSK461-08|Canada|Saskatchewan|658[0n]|BOLD:ABY5257
Apamea devastator|[12742]|LPSK450-08|Canada|Saskatchewan|658[0n]|BOLD:ABY5257
Apamea devastator|[12743]|LPSK449-08|Canada|Saskatchewan|658[0n]|BOLD:ABY5257
Apamea devastator|[12744]|LPSK435-08|Canada|Saskatchewan|658[0n]|BOLD:ABY5257
Apamea devastator|[12745]|LPSK427-08|Canada|Saskatchewan|658[0n]|BOLD:ABY5257
Apamea devastator|[12746]|LPSK426-08|Canada|Saskatchewan|658[0n]|BOLD:ABY5257
Apamea devastator|[12747]|LPSK115-08|Canada|Saskatchewan|658[0n]|BOLD:ABY5257
Apamea devastator|[12748]|LPSK020-08|Canada|Saskatchewan|658[0n]|BOLD:ABY5257
Apamea devastator|[12749]|LBCG471-08|Canada|British Columbia|658[0n]|BOLD:ABY5257
Apamea devastator|[12750]|LHLEP418-06|Canada|British Columbia|658[0n]|BOLD:ABY5257
Apamea devastator|[12751]|XAJ980-06|Canada|Ontario|658[0n]|BOLD:ABY5257
Apamea devastator|[12752]|TMNBB164-06|Canada|New Brunswick|658[0n]|BOLD:ABY5257
Apamea devastator|[12753]|LOWCE831-06|Canada|British Columbia|658[0n]|BOLD:ABY5257
Apamea devastator|[12754]|LOWCE705-06|Canada|British Columbia|658[0n]|BOLD:ABY5257
Apamea devastator|[12755]|LOWCE703-06|Canada|British Columbia|658[0n]|BOLD:ABY5257
Apamea devastator|[12756]|LOWCE702-06|Canada|British Columbia|658[0n]|BOLD:ABY5257
Apamea devastator|[12757]|LOWCE701-06|Canada|British Columbia|658[0n]|BOLD:ABY5257
Apamea devastator|[12758]|LOWCE700-06|Canada|British Columbia|658[0n]|BOLD:ABY5257
Apamea devastator|[12759]|LOWCE698-06|Canada|British Columbia|658[0n]|BOLD:ABY5257
Apamea devastator|[12760]|LOWCE694-06|Canada|British Columbia|658[0n]|BOLD:ABY5257
Apamea devastator|[12761]|LOWCE692-06|Canada|British Columbia|658[0n]|BOLD:ABY5257
Apamea devastator|[12762]|LOWCE691-06|Canada|British Columbia|658[0n]|BOLD:ABY5257
Apamea devastator|[12763]|LOWCE690-06|Canada|British Columbia|658[0n]|BOLD:ABY5257
Apamea devastator|[12764]|TTMNB569-06|Canada|New Brunswick|658[0n]|BOLD:ABY5257
Apamea devastator|[12765]|TTMNB567-06|Canada|New Brunswick|658[0n]|BOLD:ABY5257
Apamea devastator|[12766]|TTMNB335-06|Canada|New Brunswick|658[0n]|BOLD:ABY5257
Apamea devastator|[12767]|RDNMD136-06|United States|Colorado|658[0n]|BOLD:ABY5257
Apamea devastator|[12768]|RDNMD135-06|United States|Colorado|658[0n]|BOLD:ABY5257
Apamea devastator|[12769]|RDLQB825-05|Canada|Quebec|658[0n]|BOLD:ABY5257
Apamea devastator|[12770]|LOWCC440-05|Canada|British Columbia|658[0n]|BOLD:ABY5257
Apamea devastator|[12771]|LOWCB480-05|Canada|British Columbia|658[0n]|BOLD:ABY5257
Apamea devastator|[12772]|LOWCB172-05|Canada|British Columbia|658[0n]|BOLD:ABY5257
Apamea devastator|[12773]|LOWCB170-05|Canada|British Columbia|658[0n]|BOLD:ABY5257
Apamea devastator|[12774]|LOWCB168-05|Canada|British Columbia|658[0n]|BOLD:ABY5257
Apamea devastator|[12775]|LBCC299-05|Canada|British Columbia|658[0n]|BOLD:ABY5257
Apamea devastator|[12776]|XAG016-05|Canada|Ontario|658[0n]|BOLD:ABY5257
Apamea devastator|[12777]|CNGRL408-13|Canada|Saskatchewan|558[0n]|BOLD:ABY5257
Apamea devastator|[12778]|XAD014-04|Canada|Ontario|658[0n]|BOLD:ABY5257
Apamea devastator|[12779]|XAE626-04|Canada|Ontario|658[0n]|BOLD:ABY5257
Apamea devastator|[12780]|CNGRL429-13|Canada|Saskatchewan|555[0n]|BOLD:ABY5257
Apamea devastator|[12781]|CNGRL406-13|Canada|Saskatchewan|564[0n]|BOLD:ABY5257
Apamea devastator|[12782]|CNGRF009-12|Canada|Saskatchewan|638[0n]|BOLD:ABY5257
Apamea devastator|[12783]|CNGRF011-12|Canada|Saskatchewan|639[0n]|BOLD:ABY5257
Apamea devastator|[12784]|CNGRE018-12|Canada|Saskatchewan|632[0n]|BOLD:ABY5257
Apamea devastator|[12785]|CNGRF012-12|Canada|Saskatchewan|633[0n]|BOLD:ABY5257
Apamea devastator|[12786]|CNGRF016-12|Canada|Saskatchewan|636[0n]|BOLD:ABY5257
Apamea devastator|[12787]|CNGRF017-12|Canada|Saskatchewan|636[0n]|BOLD:ABY5257
Apamea devastator|[12788]|CNGRF022-12|Canada|Saskatchewan|636[0n]|BOLD:ABY5257
Apamea devastator|[12789]|CNGRF024-12|Canada|Saskatchewan|636[0n]|BOLD:ABY5257
Apamea devastator|[12790]|CNGRE023-12|Canada|Saskatchewan|636[0n]|BOLD:ABY5257
Apamea devastator|[12791]|CNGRF006-12|Canada|Saskatchewan|636[0n]|BOLD:ABY5257
Apamea devastator|[12792]|CNGRE006-12|Canada|Saskatchewan|636[0n]|BOLD:ABY5257
Apamea devastator|[12793]|CNGRE007-12|Canada|Saskatchewan|636[0n]|BOLD:ABY5257

Apamea devastator|[12791]|CNGRF006-12|Canada|Saskatchewan|636[0n]|BOLD:ABY5257
Apamea devastator|[12792]|CNGRE006-12|Canada|Saskatchewan|636[0n]|BOLD:ABY5257
Apamea devastator|[12793]|CNGRE007-12|Canada|Saskatchewan|636[0n]|BOLD:ABY5257
Apamea devastator|[12794]|CNGRD1020-12|Canada|Saskatchewan|636[0n]|BOLD:ABY5257
Apamea devastator|[12795]|CNGRE003-12|Canada|Saskatchewan|636[0n]|BOLD:ABY5257
Apamea devastator|[12796]|CNGRE013-12|Canada|Saskatchewan|637[0n]|BOLD:ABY5257
Apamea devastator|[12797]|CNGRK440-13|Canada|Saskatchewan|634[0n]|BOLD:ABY5257
Apamea devastator|[12798]|CNGRF027-12|Canada|Saskatchewan|637[0n]|BOLD:ABY5257
Apamea devastator|[12799]|CNGRF028-12|Canada|Saskatchewan|638[0n]|BOLD:ABY5257
Apamea devastator|[12800]|CNGRE001-12|Canada|Saskatchewan|638[0n]|BOLD:ABY5257
Apamea devastator|[12801]|CNGRE017-12|Canada|Saskatchewan|637[0n]|BOLD:ABY5257
Apamea devastator|[12802]|CNGRD1027-12|Canada|Saskatchewan|638[0n]|BOLD:ABY5257
Apamea devastator|[12803]|CNGRD1028-12|Canada|Saskatchewan|639[0n]|BOLD:ABY5257
Apamea devastator|[12804]|XAG732-05|Canada|Ontario|638[0n]|BOLD:ABY5257
Apamea devastator|[12805]|LPABC068-09|Canada|Alberta|637[0n]|BOLD:ABY5257
Apamea devastator|[12806]|LOWCE697-06|Canada|British Columbia|605[0n]|BOLD:ABY5257
Apamea devastator|[12807]|LOWCE696-06|Canada|British Columbia|605[0n]|BOLD:ABY5257
Apamea devastator|[12808]|LOWCE695-06|Canada|British Columbia|608[0n]|BOLD:ABY5257
Apamea devastator|[12809]|LOWCE693-06|Canada|British Columbia|608[0n]|BOLD:ABY5257
Apamea devastator|[12810]|RDLQB758-05|Canada|Quebec|631[0n]|BOLD:ABY5257
Apamea devastator|[12811]|PMG091-03|Canada|Ontario|617[0n]|BOLD:ABY5257
Apamea devastator|[12812]|LPVIC029-08|Canada|British Columbia|592[0n]|BOLD:ABY5257
Apamea devastator|[12813]|RDNMC489-06|United States|Wyoming|591[1n]|BOLD:ABY5257
Apamea devastator|[12814]|TTMNB332-06|Canada|New Brunswick|611[0n]|BOLD:ABY5257
Apamea devastator|[12815]|BBLPB663-10|Canada|British Columbia|658[0n]|BOLD:ABY5257
Apamea devastator|[12816]|BBLPB678-10|Canada|Alberta|658[0n]|BOLD:ABY5257
Apamea devastator|[12817]|CNGRK424-13|Canada|Saskatchewan|540[2n]|BOLD:ABY5257
Apamea devastator|[12818]|RDLQ144-05|Canada|Quebec|531[0n]|BOLD:ABY5257
Apamea devastator|[12819]|CNGRM228-13|Canada|Saskatchewan|525[0n]|BOLD:ABY5257
Apamea devastator|[12820]|CNGRL420-13|Canada|Saskatchewan|558[0n]|BOLD:ABY5257
Apamea devastator|[12821]|CNGRE025-12|Canada|Saskatchewan|637[0n]|BOLD:ABY5257
Apamea devastator|[12822]|CNGRE027-12|Canada|Saskatchewan|637[0n]|BOLD:ABY5257
Apamea devastator|[12823]|CNGRF023-12|Canada|Saskatchewan|633[0n]|BOLD:ABY5257
Apamea devastator|[12824]|CNGRK434-13|Canada|Saskatchewan|631[0n]|BOLD:ABY5257
Apamea devastator|[12825]|CNGRL401-13|Canada|Saskatchewan|591[0n]|BOLD:ABY5257
Apamea devastator|[12826]|CNGRL407-13|Canada|Saskatchewan|591[0n]|BOLD:ABY5257
Apamea devastator|[12827]|CNGRK429-13|Canada|Saskatchewan|588[0n]|BOLD:ABY5257
Apamea devastator|[12828]|CNGRM227-13|Canada|Saskatchewan|588[0n]|BOLD:ABY5257
Apamea devastator|[12829]|CNWLG952-12|Canada|Alberta|639[0n]|BOLD:ABY5257
Apamea devastator|[12830]|CNGRL403-13|Canada|Saskatchewan|588[0n]|BOLD:ABY5257
Apamea devastator|[12831]|CNGRL411-13|Canada|Saskatchewan|579[0n]|BOLD:ABY5257
Apamea devastator|[12832]|CNGRL432-13|Canada|Saskatchewan|579[0n]|BOLD:ABY5257
Apamea devastator|[12833]|CNGRL430-13|Canada|Saskatchewan|591[0n]|BOLD:ABY5257
Apamea devastator|[12834]|CNGRJ087-13|Canada|Saskatchewan|591[0n]|BOLD:ABY5257
Apamea devastator|[12835]|CNGRL409-13|Canada|Saskatchewan|591[0n]|BOLD:ABY5257
Apamea devastator|[12836]|CNGRL424-13|Canada|Saskatchewan|591[0n]|BOLD:ABY5257
Apamea devastator|[12837]|CNGRM229-13|Canada|Saskatchewan|588[0n]|BOLD:ABY5257
Apamea devastator|[12838]|CNGRL400-13|Canada|Saskatchewan|591[0n]|BOLD:ABY5257
Apamea devastator|[12839]|CNGRK443-13|Canada|Saskatchewan|576[0n]|BOLD:ABY5257
Apamea devastator|[12840]|CNWLN1522-13|Canada|Alberta|600[0n]|BOLD:ABY5257
Apamea devastator|[12841]|CNGRK435-13|Canada|Saskatchewan|585[0n]|BOLD:ABY5257
Apamea devastator|[12842]|CNWLN1523-13|Canada|Alberta|583[0n]|BOLD:ABY5257
Apamea devastator|[12843]|CNGRL428-13|Canada|Saskatchewan|567[0n]|BOLD:ABY5257
Apamea devastator|[12844]|CNGRJ078-13|Canada|Saskatchewan|567[0n]|BOLD:ABY5257
Apamea devastator|[12845]|CNGRL417-13|Canada|Saskatchewan|567[0n]|BOLD:ABY5257
Apamea devastator|[12846]|CNGRL423-13|Canada|Saskatchewan|567[0n]|BOLD:ABY5257
Apamea devastator|[12847]|CNGRL402-13|Canada|Saskatchewan|567[0n]|BOLD:ABY5257
Apamea devastator|[12848]|CNGRL399-13|Canada|Saskatchewan|567[0n]|BOLD:ABY5257
Apamea devastator|[12849]|CNGRL398-13|Canada|Saskatchewan|567[0n]|BOLD:ABY5257
Apamea devastator|[12850]|CNGRK428-13|Canada|Saskatchewan|564[0n]|BOLD:ABY5257
Apamea devastator|[12851]|CNWBG3093-13|Canada|Alberta|587[0n]|BOLD:ABY5257
Apamea devastator|[12852]|BBLPB664-10|Canada|British Columbia|636[0n]|BOLD:ABY5257
Apamea devastator|[12853]|CNWBG3143-13|Canada|Alberta|603[0n]|BOLD:ABY5257
Apamea devastator|[12854]|CNGRM230-13|Canada|Saskatchewan|582[2n]|BOLD:ABY5257
Apamea devastator|[12855]|CNGRK427-13|Canada|Saskatchewan|564[0n]|BOLD:ABY5257
Apamea devastator|[12856]|CNGRE008-12|Canada|Saskatchewan|613[0n]|BOLD:ABY5257
Apamea devastator|[12857]|XAG848-05|Canada|Ontario|658[0n]|BOLD:ABY5257
Apamea devastator|[12858]|CNGRK425-13|Canada|Saskatchewan|612[0n]|BOLD:ABY5257
Apamea devastator|[12859]|XAC036-04|Canada|Ontario|583[0n]|BOLD:ABY5257
Apamea devastator|[12860]|LPVIB507-08|Canada|British Columbia|658[0n]|BOLD:ABY5257
Apamea devastator|[12861]|SSJAE1893-13|Canada|Alberta|614[0n]|BOLD:ABY5257
Apamea devastator|[12862]|XAC804-04|Canada|Ontario|658[0n]|BOLD:ABY5257
Apamea devastator|[12863]|XAC039-04|Canada|Ontario|658[0n]|BOLD:ABY5257
Apamea devastator|[12864]|XAB162-04|Canada|Ontario|658[0n]|BOLD:ABY5257
Apamea devastator|[12865]|TTMNB334-06|Canada|New Brunswick|658[1n]|BOLD:ABY5257
Apamea devastator|[12866]|TTMNB331-06|Canada|New Brunswick|658[1n]|BOLD:ABY5257
Apamea devastator|[12867]|LBCH2098-10|Canada|British Columbia|658[0n]|BOLD:ABY5257
Apamea devastator|[12868]|LOWCB477-05|Canada|British Columbia|658[1n]|BOLD:ABY5257
Apamea devastator|[12869]|LOWCB478-05|Canada|British Columbia|658[0n]|BOLD:ABY5257
Apamea devastator|[12870]|LSEU781-06|United States|Colorado|658[0n]|BOLD:ABY5257
Apamea devastator|[12871]|LPABB833-09|Canada|Alberta|658[0n]|BOLD:ABY5257
Apamea devastator|[12872]|JMMMB398-11|United States|California|658[0n]|BOLD:ABY5257
Apamea devastator|[12873]|RWWB234-09|United States|Washington|658[0n]|BOLD:ABY5257
Apamea devastator|[12874]|RWWB112-09|United States|Washington|658[0n]|BOLD:ABY5257
Apamea devastator|[12875]|LBCG2886-09|Canada|British Columbia|658[0n]|BOLD:ABY5257
Apamea devastator|[12876]|CNGRE015-12|Canada|Saskatchewan|637[0n]|BOLD:ABY5257
Apamea devastator|[12877]|CNWLN1067-13|Canada|Alberta|613[0n]|BOLD:ABY5257
Apamea devastator|[12878]|LPABB614-08|Canada|Alberta|658[0n]|BOLD:ABY5257
Apamea devastator|[12879]|LPABC099-09|Canada|Alberta|658[0n]|BOLD:ABY5257
Apamea devastator|[12880]|LPSK597-08|Canada|Saskatchewan|658[0n]|BOLD:ABY5257
Apamea devastator|[12881]|LPABB119-08|Canada|Alberta|658[0n]|BOLD:ABY5257
Apamea devastator|[12882]|LPSK458-08|Canada|Saskatchewan|658[0n]|BOLD:ABY5257
Apamea devastator|[12883]|LPSK552-08|Canada|Saskatchewan|658[0n]|BOLD:ABY5257
Apamea devastator|[12884]|LPSK428-08|Canada|Saskatchewan|658[0n]|BOLD:ABY5257
Apamea devastator|[12885]|LPSK457-08|Canada|Saskatchewan|658[0n]|BOLD:ABY5257
Apamea devastator|[12886]|CNGRF007-12|Canada|Saskatchewan|618[0n]|BOLD:ABY5257
Apamea devastator|[12887]|RDNMJ657-11|Canada|Alberta|658[0n]|BOLD:ABY5257
Apamea devastator|[12888]|CNGRE010-12|Canada|Saskatchewan|636[0n]|BOLD:ABY5257
Apamea devastator|[12889]|LSEU780-06|United States|Colorado|658[0n]|BOLD:ABY5257
Apamea devastator|[12890]|CNGRF021-12|Canada|Saskatchewan|638[0n]|BOLD:ABY5257
Apamea devastator|[12891]|TTMNB333-06|Canada|New Brunswick|658[0n]|BOLD:ABY5257
Apamea devastator|[12892]|RDNMC683-06|United States|California|658[0n]|BOLD:ABY5257
Apamea devastator|[12893]|LOWCB482-05|Canada|British Columbia|658[0n]|BOLD:ABY5257

[illegible]
Apamea devastator|[12891]|TTMNB333-06|Canada|New Brunswick|658[0n]|BOLD:ABY5257
Apamea devastator|[12892]|RDNMC683-06|United States|California|658[0n]|BOLD:ABY5257
Apamea devastator|[12893]|LOWCB482-05|Canada|British Columbia|658[0n]|BOLD:ABY5257
Apamea devastator|[12894]|BBLPB767-10|Canada|Alberta|658[0n]|BOLD:ABY5257
Apamea devastator|[12895]|LOWCB481-05|Canada|British Columbia|658[0n]|BOLD:ABY5257
Apamea devastator|[12896]|LOWCB171-05|Canada|British Columbia|658[0n]|BOLD:ABY5257
Apamea devastator|[12897]|LOWCB169-05|Canada|British Columbia|658[0n]|BOLD:ABY5257
Apamea devastator|[12898]|CNGRF008-12|Canada|Saskatchewan|638[3n]|BOLD:ABY5257
Apamea devastator|[12899]|CNGRE009-12|Canada|Saskatchewan|636[0n]|BOLD:ABY5257
Apamea devastator|[12900]|CNGRF029-12|Canada|Saskatchewan|637[0n]|BOLD:ABY5257
Apamea devastator|[12901]|CNGRF003-12|Canada|Saskatchewan|638[0n]|BOLD:ABY5257
Apamea devastator|[12902]|CNGRE004-12|Canada|Saskatchewan|638[0n]|BOLD:ABY5257
Apamea devastator|[12903]|CNGRF025-12|Canada|Saskatchewan|629[0n]|BOLD:ABY5257
Apamea devastator|[12904]|CNGRE021-12|Canada|Saskatchewan|629[0n]|BOLD:ABY5257
Apamea devastator|[12905]|CNGRD1016-12|Canada|Saskatchewan|629[0n]|BOLD:ABY5257
Apamea devastator|[12906]|CNGRE014-12|Canada|Saskatchewan|632[0n]|BOLD:ABY5257
Apamea devastator|[12907]|CNGRL415-13|Canada|Saskatchewan|582[0n]|BOLD:ABY5257
Apamea devastator|[12908]|RDNMC488-06|United States|Wyoming|577[1n]|BOLD:ABY5257
Apamea devastator|[12909]|CNGRK436-13|Canada|Saskatchewan|585[0n]|BOLD:ABY5257
Apamea devastator|[12910]|JMMMB367-11|United States|California|658[0n]|BOLD:ABY5257
Apamea devastator|[12911]|BBLPB676-10|Canada|Alberta|658[0n]|BOLD:ABY5257
Apamea devastator|[12912]|LBCH1106-10|Canada|British Columbia|658[0n]|BOLD:ABY5257
Apamea devastator|[12913]|LPABC704-09|Canada|Alberta|658[0n]|BOLD:ABY5257
Apamea devastator|[12914]|LPABC042-09|Canada|Alberta|658[0n]|BOLD:ABY5257
Apamea devastator|[12915]|LPABB845-09|Canada|Alberta|658[0n]|BOLD:ABY5257
Apamea devastator|[12916]|LPABB196-08|Canada|Alberta|658[0n]|BOLD:ABY5257
Apamea devastator|[12917]|LPABB117-08|Canada|Alberta|658[0n]|BOLD:ABY5257
Apamea devastator|[12918]|LOWCE699-06|Canada|British Columbia|658[0n]|BOLD:ABY5257
Apamea devastator|[12919]|LOWCB479-05|Canada|British Columbia|658[0n]|BOLD:ABY5257
Apamea devastator|[12920]|LBCD441-05|Canada|British Columbia|658[0n]|BOLD:ABY5257
Apamea devastator|[12921]|LOWCB483-05|Canada|British Columbia|611[0n]|BOLD:ABY5257
Apamea devastator|[12922]|BBLPB711-10|Canada|Alberta|612[0n]|BOLD:ABY5257
Apamea devastator|[12923]|CNGRF002-12|Canada|Saskatchewan|637[0n]|BOLD:ABY5257
Apamea devastator|[12924]|CNGRK433-13|Canada|Saskatchewan|564[0n]|BOLD:ABY5257
Apamea devastator|[12925]|CNGRL404-13|Canada|Saskatchewan|564[0n]|BOLD:ABY5257
Apamea devastator|[12926]|CNGRL425-13|Canada|Saskatchewan|555[0n]|BOLD:ABY5257
Apamea devastator|[12927]|CNGRL427-13|Canada|Saskatchewan|582[0n]|BOLD:ABY5257
Apamea devastator|[12928]|CNWLN1057-13|Canada|Alberta|613[0n]|BOLD:ABY5257
Apamea devastator|[12929]|CNWLN1066-13|Canada|Alberta|613[0n]|BOLD:ABY5257
Apamea devastator|[12930]|CNGRF010-12|Canada|Saskatchewan|613[0n]|BOLD:ABY5257
Apamea devastator|[12931]|CNGRE005-12|Canada|Saskatchewan|614[0n]|BOLD:ABY5257
Apamea devastator|[12932]|CNGRD1013-12|Canada|Saskatchewan|614[0n]|BOLD:ABY5257
Apamea devastator|[12933]|LPSK564-08|Canada|Saskatchewan|658[0n]|BOLD:ABY5257
Apamea devastator|[12934]|LPABC069-09|Canada|Alberta|658[0n]|BOLD:ABY5257
Apamea devastator|[12935]|BBLPB702-10|Canada|Alberta|658[0n]|BOLD:ABY5257
Apamea devastator|[12936]|CNGRF013-12|Canada|Saskatchewan|629[0n]|BOLD:ABY5257
Apamea lintneri|[12937]|RDNMC266-05|Canada|Quebec|658[0n]|BOLD:AAE5928
Apamea lintneri|[12938]|RDNMF742-08|Canada|Quebec|614[0n]|BOLD:AAE5928
Apamea lintneri|[12939]|RDNMF741-08|Canada|Quebec|658[0n]|BOLD:AAE5928
Apamea lintneri|[12940]|RDNMG924-08|Canada|Quebec|643[0n]|BOLD:AAE5928
Mesapamea arbora|[12941]|RDNMD795-07|United States|Arizona|658[0n]|BOLD:ABZ5995
Mesapamea arbora|[12942]|RDNMD799-07|United States|California|658[0n]|BOLD:ABZ5995
Mesapamea arbora|[12943]|RDNME894-08|United States|Arizona|658[0n]|BOLD:ABZ5995
Mesapamea sp.|[12944]|CMAZA1063-12|United States|Arizona|658[0n]|BOLD:ABZ5996
Mesapamea fractilinea|[12945]|XAD297-04|Canada|Ontario|579[0n]|BOLD:AAC5476
Mesapamea fractilinea|[12946]|RDNME266-07|United States|Texas|658[0n]|BOLD:AAC5476
Mesapamea fractilinea|[12947]|RDLQ156-05|Canada|Quebec|609[0n]|BOLD:AAC5476
Mesapamea fractilinea|[12948]|RDNME265-07|United States|Texas|658[0n]|BOLD:AAC5476
Mesapamea fractilinea|[12949]|PHSEP346-11|Canada|Ontario|652[0n]|BOLD:AAC5476
Mesapamea fractilinea|[12950]|PHAUG1513-11|Canada|Ontario|658[0n]|BOLD:AAC5476
Mesapamea fractilinea|[12951]|HEAUG1529-12|Canada|Ontario|618[0n]|BOLD:AAC5476
Mesapamea fractilinea|[12952]|LNCC398-10|United States|North Carolina|658[0n]|BOLD:AAC5476
Mesapamea fractilinea|[12953]|XAD342-04|Canada|Ontario|658[0n]|BOLD:AAC5476
Mesapamea fractilinea|[12954]|RDNMC294-05|United States|Florida|592[0n]|BOLD:AAC5476
Mesapamea fractilinea|[12955]|RDLQ462-07|Canada|Quebec|608[0n]|BOLD:AAC5476
Mesapamea fractilinea|[12956]|RDLQB813-05|Canada|Quebec|658[0n]|BOLD:AAC5476
Mesapamea fractilinea|[12957]|LPOKE130-10|United States|Oklahoma|658[0n]|BOLD:AAC5476
Mesapamea fractilinea|[12958]|RDLQB734-05|Canada|Quebec|658[0n]|BOLD:AAC5476
Mesapamea fractilinea|[12959]|CNCLA7374-13|Canada|Ontario|658[0n]|BOLD:AAC5476
Apamea longula|[12960]|RDNMC295-05|United States|Colorado|563[0n]|BOLD:ABZ5701
Apamea longula|[12961]|RDNMD159-06|United States|Colorado|658[0n]|BOLD:ABZ5701
Apamea longula|[12962]|RDNMD160-06|United States|Colorado|658[0n]|BOLD:ABZ5701
Apamea longula|[12963]|RDNMC014-05|United States|Oregon|658[0n]|BOLD:ABZ5701
Apamea longula|[12964]|RDNMC298-05|United States|Colorado|627[0n]|BOLD:ABZ5701
Apamea longula|[12965]|RDNMC017-05|Canada|Alberta|658[0n]|BOLD:ABZ5701
Apamea longula|[12966]|RDNM969-05|United States|Oregon|658[0n]|BOLD:ABZ5701
Apamea longula|[12967]|RDNMC012-05|Canada|Alberta|583[1n]|BOLD:ABZ5701
Apamea longula|[12968]|RDNMC019-05|United States|Oregon|576[0n]|BOLD:ABZ5701
Apamea longula|[12969]|RDNMC297-05|United States|Colorado|605[0n]|BOLD:ABZ5701
Apamea longula|[12970]|RDNMD156-06|United States|Colorado|658[0n]|BOLD:ABZ5701
Apamea longula|[12971]|RDNMC016-05|United States|Wyoming|658[0n]|BOLD:ABZ5701
Apamea longula|[12972]|RDNMD157-06|United States|Colorado|613[0n]|BOLD:ABZ5701
Apamea longula|[12973]|RDNMG972-08|United States|Colorado|658[0n]|BOLD:ABZ5701
Apamea bernardino|[12974]|RDNMC679-06|United States|California|658[0n]|BOLD:ABZ5701
Apamea longula|[12975]|RDMAB441-05|Canada|Alberta|578[0n]|BOLD:ABZ5701
Apamea longula|[12976]|RDNMC018-05|United States|Oregon|559[3n]|BOLD:ABZ5701
Apamea longula|[12977]|IAWLB103-10|United States|California|658[0n]|BOLD:ABZ5701
Apamea longula|[12978]|LBCG2885-09|Canada|British Columbia|638[0n]|BOLD:ABZ5701
Apamea longula|[12979]|LBCH7757-10|Canada|British Columbia|658[0n]|BOLD:ABZ5701
Apamea longula|[12980]|LBCH7468-10|Canada|British Columbia|658[0n]|BOLD:ABZ5701
Apamea longula|[12981]|LBCH6757-10|Canada|British Columbia|658[0n]|BOLD:ABZ5701
Apamea longula|[12982]|LOWCE689-06|Canada|British Columbia|658[0n]|BOLD:ABZ5701
Apamea longula|[12983]|LOWCE688-06|Canada|British Columbia|658[0n]|BOLD:ABZ5701
Apamea longula|[12984]|LOWCE685-06|Canada|British Columbia|658[0n]|BOLD:ABZ5701
Apamea longula|[12985]|RDNMD158-06|United States|Colorado|658[0n]|BOLD:ABZ5701
Apamea longula|[12986]|RDMAB442-05|Canada|Alberta|658[0n]|BOLD:ABZ5701
Apamea longula|[12987]|LOWCB476-05|Canada|British Columbia|658[0n]|BOLD:ABZ5701
Apamea longula|[12988]|LOWCB475-05|Canada|British Columbia|658[0n]|BOLD:ABZ5701
Apamea longula|[12989]|LOWCB474-05|Canada|British Columbia|658[0n]|BOLD:ABZ5701
Apamea longula|[12990]|LOWCB473-05|Canada|British Columbia|658[0n]|BOLD:ABZ5701
Apamea longula|[12991]|LOWCB471-05|Canada|British Columbia|658[0n]|BOLD:ABZ5701
Apamea longula|[12992]|LOWCB470-05|Canada|British Columbia|658[0n]|BOLD:ABZ5701
Apamea longula|[12993]|LOWCB469-05|Canada|British Columbia|658[0n]|BOLD:ABZ5701

[illegible]
Apamea longula|[12991]|LOWCB471-05|Canada|British Columbia|658[0n]|BOLD:ABZ5701
Apamea longula|[12992]|LOWCB470-05|Canada|British Columbia|658[0n]|BOLD:ABZ5701
Apamea longula|[12993]|LOWCB469-05|Canada|British Columbia|658[0n]|BOLD:ABZ5701
Apamea longula|[12994]|LOWCB468-05|Canada|British Columbia|658[0n]|BOLD:ABZ5701
Apamea longula|[12995]|LOWCB467-05|Canada|British Columbia|658[0n]|BOLD:ABZ5701
Apamea longula|[12996]|LOWCB466-05|Canada|British Columbia|658[0n]|BOLD:ABZ5701
Apamea longula|[12997]|LOWCB465-05|Canada|British Columbia|658[0n]|BOLD:ABZ5701
Apamea longula|[12998]|LOWCB464-05|Canada|British Columbia|658[0n]|BOLD:ABZ5701
Apamea longula|[12999]|LOWCB463-05|Canada|British Columbia|658[0n]|BOLD:ABZ5701
Apamea longula|[13000]|RDNM553-05|United States|Washington|658[0n]|BOLD:ABZ5701
Apamea longula|[13001]|RDNMC020-05|United States|Oregon|658[0n]|BOLD:ABZ5701
Apamea longula|[13002]|RDNM970-05|Canada|British Columbia|590[0n]|BOLD:ABZ5701
Apamea longula|[13003]|RDNMC215-05|United States|Oregon|578[0n]|BOLD:ABZ5701
Apamea longula|[13004]|RDNMC373-05|Canada|British Columbia|617[0n]|BOLD:ABZ5701
Apamea longula|[13005]|RDNMC015-05|United States|Washington|578[1n]|BOLD:ABZ5701
Apamea longula|[13006]|LOWCB472-05|Canada|British Columbia|615[0n]|BOLD:ABZ5701
Apamea longula|[13007]|LOWCE687-06|Canada|British Columbia|605[1n]|BOLD:ABZ5701
Apamea longula|[13008]|LOWCE686-06|Canada|British Columbia|605[1n]|BOLD:ABZ5701
Apamea longula|[13009]|RDNMC376-05|Canada|British Columbia|571[0n]|BOLD:ABZ5701
Apamea longula|[13010]|RDMAB440-05|Canada|Yukon Territory|658[0n]|BOLD:ABZ5701
Apamea longula|[13011]|RDMAB439-05|Canada|Yukon Territory|658[0n]|BOLD:ABZ5701
Apamea longula|[13012]|RDNM075-05|United States|Nevada|658[0n]|BOLD:ABZ5701
Apamea longula|[13013]|RDNMC370-05|Canada|British Columbia|598[0n]|BOLD:ABZ5701
Apamea longula|[13014]|RDNMC371-05|Canada|British Columbia|598[1n]|BOLD:ABZ5701
Apamea longula|[13015]|RDNMC013-05|United States|Washington|557[0n]|BOLD:ABZ5701
Apamea longula|[13016]|RDNMC372-05|Canada|British Columbia|552[2n]|BOLD:ABZ5701
Apamea longula|[13017]|RDNMC374-05|Canada|British Columbia|597[0n]|BOLD:ABZ5701
Apamea longula|[13018]|IAWLB102-10|United States|California|658[0n]|BOLD:ABZ5701
Apamea longula|[13019]|IAWLB131-10|United States|California|658[0n]|BOLD:ABZ5701
Apamea cuculliformis|[13020]|GMLC1119-12|United States|California|658[0n]|BOLD:AAF1821
Apamea cuculliformis|[13021]|GMLC649-11|United States|California|658[0n]|BOLD:AAF1821
Apamea cuculliformis|[13022]|NAMUM153-08|United States|California|658[0n]|BOLD:AAF1821
Apamea cuculliformis|[13023]|LOCBC287-06|United States|California|658[0n]|BOLD:AAF1821
Apamea cuculliformis|[13024]|RDNMC677-06|United States|California|658[0n]|BOLD:AAF1821
Apamea cuculliformis|[13025]|GMLC1081-12|United States|California|658[0n]|BOLD:AAF1821
Apamea cuculliformis|[13026]|GMLC1423-12|United States|California|614[0n]|BOLD:AAF1821
Apamea cuculliformis|[13027]|GMLC1427-12|United States|California|614[0n]|BOLD:AAF1821
Apamea lutosa|[13028]|RDNMG809-08|Canada|Ontario|658[0n]|BOLD:AAE3213
Apamea lutosa|[13029]|RDNM203-05|Canada|Ontario|658[0n]|BOLD:AAE3213
Apamea lutosa|[13030]|RDNM204-05|Canada|Ontario|653[0n]|BOLD:AAE3213
Apamea lutosa|[13031]|RDNMG810-08|Canada|Ontario|658[0n]|BOLD:AAE3213
Apamea lutosa|[13032]|RDNMG811-08|Canada|Ontario|658[0n]|BOLD:AAE3213
Apamea quinteri|[13033]|RDNMC528-06|United States|Mississippi|511[0n]|BOLD:AAF1794
Apamea quinteri|[13034]|RDNMC255-05|United States|Missouri|577[1n]|BOLD:AAF1794
Apamea quinteri|[13035]|RDNMC714-06|United States|North Carolina|658[0n]|BOLD:AAF1794
Apamea quinteri|[13036]|LPOKE276-11|United States|Oklahoma|658[0n]|BOLD:AAF1794
Apamea quinteri|[13037]|CNCLB2646-14|United States|North Carolina|658[0n]|BOLD:AAF1794
Apamea cariosa|[13038]|RDNM202-05|Canada|Ontario|658[0n]|BOLD:AAE3183
Apamea cariosa|[13039]|RDNM201-05|Canada|Ontario|658[0n]|BOLD:AAE3183
Apamea cariosa|[13040]|RDNM200-05|Canada|Ontario|658[0n]|BOLD:AAE3183
Apamea cariosa|[13041]|XAE254-04|Canada|Ontario|658[0n]|BOLD:AAE3183
Apamea cariosa|[13042]|RDNMD152-06|United States|Colorado|576[0n]|BOLD:AAE3183
Apamea cariosa|[13043]|BBLSW455-09|United States|Oklahoma|658[0n]|BOLD:AAE3183
Apamea cariosa|[13044]|BBLSW456-09|United States|Oklahoma|658[0n]|BOLD:AAE3183
Apamea cariosa|[13045]|LNCC1674-13|United States|North Carolina|658[0n]|BOLD:AAE3183
Apamea cariosa|[13046]|LNCC1860-13|United States|North Carolina|658[0n]|BOLD:AAE3183
Apamea wikeri|[13047]|RDNMC529-06|United States|Mississippi|569[2n]|BOLD:AAJ1432
Apamea wikeri|[13048]|RDNMC715-06|United States|Missouri|558[1n]|BOLD:AAJ1432
Apamea verbascoides|[13049]|RDLQF543-06|Canada|Quebec|658[0n]|BOLD:AAD6273
Apamea verbascoides|[13050]|RDLQF047-06|Canada|Quebec|658[0n]|BOLD:AAD6273
Apamea verbascoides|[13051]|RDNM199-05|Canada|Ontario|658[0n]|BOLD:AAD6273
Apamea verbascoides|[13052]|RDLQF045-06|Canada|Quebec|658[0n]|BOLD:AAD6273
Apamea verbascoides|[13053]|RDLQG127-06|Canada|Quebec|643[0n]|BOLD:AAD6273
Apamea verbascoides|[13054]|BBLPC683-09|Canada|Newfoundland and Labrador|658[0n]|BOLD:AAD6273
Apamea verbascoides|[13055]|PHMTV424-10|Canada|Ontario|658[0n]|BOLD:AAD6273
Apamea verbascoides|[13056]|LNCC989-11|United States|North Carolina|658[0n]|BOLD:AAD6273
Apamea verbascoides|[13057]|LNCC1160-11|United States|North Carolina|658[0n]|BOLD:AAD6273
Apamea verbascoides|[13058]|LNCC1161-11|United States|North Carolina|658[0n]|BOLD:AAD6273
Apamea verbascoides|[13059]|LNCC1162-11|United States|North Carolina|658[0n]|BOLD:AAD6273
Apamea cristata|[13060]|RDLQF041-06|Canada|Quebec|658[0n]|BOLD:AAF1717
Apamea cristata|[13061]|RDLQF042-06|Canada|Quebec|658[0n]|BOLD:AAF1717
Apamea cristata|[13062]|RDLQF043-06|Canada|Quebec|658[0n]|BOLD:AAF1717
Apamea inebriata|[13063]|RDNMG209-08|United States|New Jersey|609[0n]|BOLD:AAW5920
Apamea inebriata|[13064]|LNAUT2655-14|United States|Massachusetts|658[0n]|BOLD:AAW5920
Apamea nigrior|[13065]|PHMNB582-04|Canada|New Brunswick|658[0n]|BOLD:AAE7006
Apamea nigrior|[13066]|PHMNB321-04|Canada|New Brunswick|658[0n]|BOLD:AAE7006
Apamea nigrior|[13067]|RDNM197-05|Canada|Ontario|638[0n]|BOLD:AAE7006
Apamea nigrior|[13068]|RDNM198-05|Canada|Ontario|658[0n]|BOLD:AAE7006
Apamea nigrior|[13069]|CNCLB2641-14|United States|North Carolina|658[0n]|BOLD:AAE7006
Apamea nigrior|[13070]|CNCLB2643-14|United States|North Carolina|658[0n]|BOLD:AAE7006
Apamea vulgaris|[13071]|RDNMD120-06|United States|Alabama|605[0n]|BOLD:AAB7575
Apamea vulgaris|[13072]|RDNMG210-08|United States|Maryland|658[0n]|BOLD:AAB7575
Apamea vulgaris|[13073]|MMNA099-08|United States|North Carolina|658[0n]|BOLD:AAB7575
Apamea vulgaris|[13074]|RDNMD118-06|United States|North Carolina|605[0n]|BOLD:AAB7575
Apamea vulgaris|[13075]|RDNMD119-06|United States|North Carolina|585[0n]|BOLD:AAB7575
Apamea vulgaris|[13076]|LPSO641-08|Canada|Ontario|624[0n]|BOLD:AAB7575
Apamea vulgaris|[13077]|RDNMG211-08|United States|Maryland|649[0n]|BOLD:AAB7575
Apamea vulgaris|[13078]|RDNMG855-08|Canada|Ontario|658[0n]|BOLD:AAB7575
Apamea vulgaris|[13079]|CNCLB1614-14|Canada|Ontario|658[0n]|BOLD:AAB7575
Apamea vulgaris|[13080]|CNCLB2650-14|United States|North Carolina|658[0n]|BOLD:AAB7575
Apamea vulgaris|[13081]|CNCLB2651-14|United States|North Carolina|658[0n]|BOLD:AAB7575
Apamea vulgaris|[13082]|CNCLB2653-14|United States|North Carolina|658[0n]|BOLD:AAB7575
Apamea cogitata|[13083]|RDNMC256-05|United States|Colorado|537[1n]|BOLD:AAA2865
Apamea cogitata|[13084]|RDNMC521-06|United States|Colorado|658[0n]|BOLD:AAA2865
Apamea cogitata|[13085]|IAWLB156-10|United States|California|658[0n]|BOLD:AAA2865
Apamea cogitata|[13086]|JMMMB348-11|United States|California|658[0n]|BOLD:AAA2865
Apamea cogitata|[13087]|LBCH1304-10|Canada|British Columbia|633[0n]|BOLD:AAA2865
Apamea cogitata|[13088]|LPABB726-08|Canada|Alberta|658[0n]|BOLD:AAA2865
Apamea cogitata|[13089]|LPABC208-09|Canada|Alberta|658[0n]|BOLD:AAA2865
Apamea cogitata|[13090]|LPABB716-08|Canada|Alberta|658[0n]|BOLD:AAA2865
Apamea cogitata|[13091]|LPABB715-08|Canada|Alberta|658[0n]|BOLD:AAA2865
Apamea cogitata|[13092]|RDLQF064-06|Canada|Quebec|658[0n]|BOLD:AAA2865
Apamea cogitata|[13093]|RDLQF062-06|Canada|Quebec|658[0n]|BOLD:AAA2865

[illegible]
Apamea cogitata|[13091]|LPABB715-08|Canada|Alberta|658[0n]|BOLD:AAA2865
Apamea cogitata|[13092]|RDLQF064-06|Canada|Quebec|658[0n]|BOLD:AAA2865
Apamea cogitata|[13093]|RDLQF062-06|Canada|Quebec|658[0n]|BOLD:AAA2865
Apamea cogitata|[13094]|LCH627-05|Canada|Manitoba|658[0n]|BOLD:AAA2865
Apamea cogitata|[13095]|RDLQF063-06|Canada|Quebec|658[0n]|BOLD:AAA2865
Apamea cogitata|[13096]|LPABC254-09|Canada|Alberta|633[1n]|BOLD:AAA2865
Apamea cogitata|[13097]|LPABC342-09|Canada|Alberta|638[0n]|BOLD:AAA2865
Apamea cogitata|[13098]|LPABC399-09|Canada|Alberta|658[0n]|BOLD:AAA2865
Apamea cogitata|[13099]|LPABC460-09|Canada|Alberta|658[0n]|BOLD:AAA2865
Apamea cogitata|[13100]|BBLPB787-10|Canada|Alberta|658[0n]|BOLD:AAA2865
Apamea cogitata|[13101]|BBLPB788-10|Canada|Alberta|658[0n]|BOLD:AAA2865
Apamea cogitata|[13102]|LPABC202-09|Canada|Alberta|658[0n]|BOLD:AAA2865
Apamea cogitata|[13103]|RWWA764-09|United States|Washington|658[0n]|BOLD:AAA2865
Apamea cogitata|[13104]|RWWB936-10|United States|Washington|658[0n]|BOLD:AAA2865
Apamea cogitata|[13105]|RWWB945-10|United States|Washington|658[0n]|BOLD:AAA2865
Apamea cogitata|[13106]|RWWA848-09|United States|Washington|658[0n]|BOLD:AAA2865
Apamea cogitata|[13107]|RWWA913-09|United States|Washington|658[0n]|BOLD:AAA2865
Apamea cogitata|[13108]|RWWA767-09|United States|Washington|658[0n]|BOLD:AAA2865
Apamea cogitata|[13109]|RWWA769-09|United States|Washington|658[0n]|BOLD:AAA2865
Apamea cogitata|[13110]|RWWB973-10|United States|Washington|658[0n]|BOLD:AAA2865
Apamea cogitata|[13111]|RWWC448-11|United States|Washington|658[0n]|BOLD:AAA2865
Apamea cogitata|[13112]|LBCH2096-10|Canada|British Columbia|658[0n]|BOLD:AAA2865
Apamea cogitata|[13113]|RWWB865-10|United States|Washington|658[0n]|BOLD:AAA2865
Apamea cogitata|[13114]|RWWA538-09|United States|Washington|658[0n]|BOLD:AAA2865
Apamea cogitata|[13115]|LHLEP417-06|Canada|British Columbia|658[0n]|BOLD:AAA2865
Apamea cogitata|[13116]|RWWA672-09|United States|Washington|626[0n]|BOLD:AAA2865
Apamea cogitata|[13117]|RWWA722-09|United States|Washington|658[0n]|BOLD:AAA2865
Apamea cogitata|[13118]|RWWA761-09|United States|Washington|658[0n]|BOLD:AAA2865
Apamea cogitata|[13119]|RWWC609-11|United States|Washington|658[0n]|BOLD:AAA2865
Apamea cogitata|[13120]|UAMIC557-13|United States|Alaska|634[0n]|BOLD:AAA2865
Apamea cogitata|[13121]|LOWCD309-06|Canada|British Columbia|610[0n]|BOLD:AAA2865
Apamea cogitata|[13122]|LOWCD308-06|Canada|British Columbia|608[0n]|BOLD:AAA2865
Apamea cogitata|[13123]|TTMNB328-06|Canada|New Brunswick|626[1n]|BOLD:AAA2865
Apamea cogitata|[13124]|CNWLN1056-13|Canada|Alberta|588[0n]|BOLD:AAA2865
Apamea cogitata|[13125]|LBCH1583-10|Canada|British Columbia|658[0n]|BOLD:AAA2865
Apamea cogitata|[13126]|LBCH1862-10|Canada|British Columbia|658[0n]|BOLD:AAA2865
Apamea cogitata|[13127]|LBCH1497-10|Canada|British Columbia|658[0n]|BOLD:AAA2865
Apamea cogitata|[13128]|LBCH1582-10|Canada|British Columbia|658[0n]|BOLD:AAA2865
Apamea cogitata|[13129]|LBCH796-10|Canada|British Columbia|658[0n]|BOLD:AAA2865
Apamea cogitata|[13130]|LBCH1169-10|Canada|British Columbia|658[0n]|BOLD:AAA2865
Apamea cogitata|[13131]|LBCH223-10|Canada|British Columbia|658[0n]|BOLD:AAA2865
Apamea cogitata|[13132]|LBCH694-10|Canada|British Columbia|658[0n]|BOLD:AAA2865
Apamea cogitata|[13133]|LBCG3322-09|Canada|British Columbia|658[0n]|BOLD:AAA2865
Apamea cogitata|[13134]|LBCH113-10|Canada|British Columbia|658[0n]|BOLD:AAA2865
Apamea cogitata|[13135]|BBLPB790-10|Canada|British Columbia|658[0n]|BOLD:AAA2865
Apamea cogitata|[13136]|RWWC547-11|United States|Washington|658[0n]|BOLD:AAA2865
Apamea cogitata|[13137]|LBCG2776-09|Canada|British Columbia|658[0n]|BOLD:AAA2865
Apamea cogitata|[13138]|LBCG3281-09|Canada|British Columbia|658[0n]|BOLD:AAA2865
Apamea cogitata|[13139]|LBCH3344-10|Canada|British Columbia|658[0n]|BOLD:AAA2865
Apamea cogitata|[13140]|LBCH4113-10|Canada|British Columbia|658[0n]|BOLD:AAA2865
Apamea cogitata|[13141]|LBCG2364-09|Canada|British Columbia|658[0n]|BOLD:AAA2865
Apamea cogitata|[13142]|LBCG2365-09|Canada|British Columbia|658[0n]|BOLD:AAA2865
Apamea cogitata|[13143]|LPABC908-09|Canada|Alberta|658[0n]|BOLD:AAA2865
Apamea cogitata|[13144]|LBCG2066-09|Canada|British Columbia|658[0n]|BOLD:AAA2865
Apamea cogitata|[13145]|LBCH2241-10|Canada|British Columbia|658[0n]|BOLD:AAA2865
Apamea cogitata|[13146]|LBCH3070-10|Canada|British Columbia|658[0n]|BOLD:AAA2865
Apamea cogitata|[13147]|LBCH2097-10|Canada|British Columbia|658[0n]|BOLD:AAA2865
Apamea cogitata|[13148]|LBCH2240-10|Canada|British Columbia|658[0n]|BOLD:AAA2865
Apamea cogitata|[13149]|LBCH1935-10|Canada|British Columbia|658[0n]|BOLD:AAA2865
Apamea cogitata|[13150]|LBCH1997-10|Canada|British Columbia|658[0n]|BOLD:AAA2865
Apamea cogitata|[13151]|LPABC255-09|Canada|Alberta|658[0n]|BOLD:AAA2865
Apamea cogitata|[13152]|LPABC436-09|Canada|Alberta|658[0n]|BOLD:AAA2865
Apamea cogitata|[13153]|LPABC207-09|Canada|Alberta|658[0n]|BOLD:AAA2865
Apamea cogitata|[13154]|LPABC212-09|Canada|Alberta|658[0n]|BOLD:AAA2865
Apamea cogitata|[13155]|LPABB873-09|Canada|Alberta|658[0n]|BOLD:AAA2865
Apamea cogitata|[13156]|LPABB882-09|Canada|Alberta|658[0n]|BOLD:AAA2865
Apamea cogitata|[13157]|LPSK453-08|Canada|Saskatchewan|658[0n]|BOLD:AAA2865
Apamea cogitata|[13158]|LPSK459-08|Canada|Saskatchewan|658[0n]|BOLD:AAA2865
Apamea cogitata|[13159]|LPABB629-08|Canada|Alberta|658[0n]|BOLD:AAA2865
Apamea cogitata|[13160]|LPABB713-08|Canada|Alberta|658[0n]|BOLD:AAA2865
Apamea cogitata|[13161]|LPABB604-08|Canada|Alberta|658[0n]|BOLD:AAA2865
Apamea cogitata|[13162]|LPABB615-08|Canada|Alberta|658[0n]|BOLD:AAA2865
Apamea cogitata|[13163]|LPSK460-08|Canada|Saskatchewan|658[0n]|BOLD:AAA2865
Apamea cogitata|[13164]|LPABB577-08|Canada|Alberta|658[0n]|BOLD:AAA2865
Apamea cogitata|[13165]|LPSK434-08|Canada|Saskatchewan|658[0n]|BOLD:AAA2865
Apamea cogitata|[13166]|LPSK437-08|Canada|Saskatchewan|658[0n]|BOLD:AAA2865
Apamea cogitata|[13167]|LPSK432-08|Canada|Saskatchewan|658[0n]|BOLD:AAA2865
Apamea cogitata|[13168]|LPSK433-08|Canada|Saskatchewan|658[0n]|BOLD:AAA2865
Apamea cogitata|[13169]|LPSK429-08|Canada|Saskatchewan|658[0n]|BOLD:AAA2865
Apamea cogitata|[13170]|LPSK431-08|Canada|Saskatchewan|658[0n]|BOLD:AAA2865
Apamea cogitata|[13171]|LBCS187-07|Canada|British Columbia|658[0n]|BOLD:AAA2865
Apamea cogitata|[13172]|LBCG472-08|Canada|British Columbia|658[0n]|BOLD:AAA2865
Apamea cogitata|[13173]|ABKWR116-07|United States|Alaska|658[0n]|BOLD:AAA2865
Apamea cogitata|[13174]|ABKWR119-07|United States|Alaska|658[0n]|BOLD:AAA2865
Apamea cogitata|[13175]|LHLEP442-06|Canada|British Columbia|658[0n]|BOLD:AAA2865
Apamea cogitata|[13176]|ABKWR110-07|United States|Alaska|658[0n]|BOLD:AAA2865
Apamea cogitata|[13177]|LHLEP063-06|Canada|British Columbia|658[0n]|BOLD:AAA2865
Apamea cogitata|[13178]|LHLEP416-06|Canada|British Columbia|658[0n]|BOLD:AAA2865
Apamea cogitata|[13179]|LOWCD304-06|Canada|British Columbia|658[0n]|BOLD:AAA2865
Apamea cogitata|[13180]|LOWCD307-06|Canada|British Columbia|658[0n]|BOLD:AAA2865
Apamea cogitata|[13181]|LOWCC436-05|Canada|British Columbia|658[0n]|BOLD:AAA2865
Apamea cogitata|[13182]|LOWCC437-05|Canada|British Columbia|658[0n]|BOLD:AAA2865
Apamea cogitata|[13183]|LOWCC432-05|Canada|British Columbia|658[0n]|BOLD:AAA2865
Apamea cogitata|[13184]|LOWCC434-05|Canada|British Columbia|658[0n]|BOLD:AAA2865
Apamea cogitata|[13185]|LOWCC418-05|Canada|British Columbia|658[0n]|BOLD:AAA2865
Apamea cogitata|[13186]|LOWCC419-05|Canada|British Columbia|658[0n]|BOLD:AAA2865
Apamea cogitata|[13187]|LOWCC416-05|Canada|British Columbia|658[0n]|BOLD:AAA2865
Apamea cogitata|[13188]|LOWCC417-05|Canada|British Columbia|658[0n]|BOLD:AAA2865
Apamea cogitata|[13189]|LOWCC420-05|Canada|British Columbia|658[0n]|BOLD:AAA2865
Apamea cogitata|[13190]|LOWCC422-05|Canada|British Columbia|658[0n]|BOLD:AAA2865
Apamea cogitata|[13191]|LBCD449-05|Canada|British Columbia|658[0n]|BOLD:AAA2865
Apamea cogitata|[13192]|LBCD450-05|Canada|British Columbia|658[0n]|BOLD:AAA2865
Apamea cogitata|[13193]|LBCD442-05|Canada|British Columbia|658[0n]|BOLD:AAA2865

Apamea cogitata|[13190]|LOWCC422-05|Canada|British Columbia|658[0n]|BOLD:AAA2865
Apamea cogitata|[13191]|LBCD449-05|Canada|British Columbia|658[0n]|BOLD:AAA2865
Apamea cogitata|[13192]|LBCD450-05|Canada|British Columbia|658[0n]|BOLD:AAA2865
Apamea cogitata|[13193]|LBCD442-05|Canada|British Columbia|658[0n]|BOLD:AAA2865
Apamea cogitata|[13194]|LBCD443-05|Canada|British Columbia|658[0n]|BOLD:AAA2865
Apamea cogitata|[13195]|LBCD273-05|Canada|British Columbia|658[0n]|BOLD:AAA2865
Apamea cogitata|[13196]|LBCD310-05|Canada|British Columbia|656[0n]|BOLD:AAA2865
Apamea cogitata|[13197]|LBCC869-05|Canada|British Columbia|658[0n]|BOLD:AAA2865
Apamea cogitata|[13198]|LBCD048-05|Canada|British Columbia|658[0n]|BOLD:AAA2865
Apamea cogitata|[13199]|LBCC556-05|Canada|British Columbia|658[0n]|BOLD:AAA2865
Apamea cogitata|[13200]|LBCC824-05|Canada|British Columbia|658[0n]|BOLD:AAA2865
Apamea cogitata|[13201]|BBLPB785-10|Canada|British Columbia|658[0n]|BOLD:AAA2865
Apamea cogitata|[13202]|BBLPB786-10|Canada|British Columbia|658[0n]|BOLD:AAA2865
Apamea cogitata|[13203]|LBCH4434-10|Canada|British Columbia|658[0n]|BOLD:AAA2865
Apamea cogitata|[13204]|LBCH4694-10|Canada|British Columbia|658[0n]|BOLD:AAA2865
Apamea cogitata|[13205]|LBCB802-05|Canada|British Columbia|658[0n]|BOLD:AAA2865
Apamea cogitata|[13206]|LBCC338-05|Canada|British Columbia|658[0n]|BOLD:AAA2865
Apamea cogitata|[13207]|UAMIC558-13|United States|Alaska|658[0n]|BOLD:AAA2865
Apamea cogitata|[13208]|LBCB753-05|Canada|British Columbia|658[0n]|BOLD:AAA2865
Apamea cogitata|[13209]|LBCB752-05|Canada|British Columbia|658[0n]|BOLD:AAA2865
Apamea cogitata|[13210]|LBCB581-05|Canada|British Columbia|658[0n]|BOLD:AAA2865
Apamea cogitata|[13211]|LBCB070-05|Canada|British Columbia|658[0n]|BOLD:AAA2865
Apamea cogitata|[13212]|LPGVA631-08|Canada|British Columbia|658[0n]|BOLD:AAA2865
Apamea cogitata|[13213]|LBCG582-09|Canada|British Columbia|658[0n]|BOLD:AAA2865
Apamea cogitata|[13214]|LBCG2366-09|Canada|British Columbia|658[0n]|BOLD:AAA2865
Apamea cogitata|[13215]|LOWCD305-06|Canada|British Columbia|658[0n]|BOLD:AAA2865
Apamea cogitata|[13216]|LOWCD310-06|Canada|British Columbia|658[0n]|BOLD:AAA2865
Apamea cogitata|[13217]|LOWCC935-05|Canada|British Columbia|658[0n]|BOLD:AAA2865
Apamea cogitata|[13218]|LOWCC872-05|Canada|British Columbia|658[0n]|BOLD:AAA2865
Apamea cogitata|[13219]|LOWCC435-05|Canada|British Columbia|658[0n]|BOLD:AAA2865
Apamea cogitata|[13220]|LOWCC415-05|Canada|British Columbia|658[0n]|BOLD:AAA2865
Apamea cogitata|[13221]|LBCB069-05|Canada|British Columbia|658[0n]|BOLD:AAA2865
Apamea cogitata|[13222]|LHLEP064-06|Canada|British Columbia|646[0n]|BOLD:AAA2865
Apamea cogitata|[13223]|LBCG2884-09|Canada|British Columbia|658[0n]|BOLD:AAA2865
Apamea cogitata|[13224]|LBCH017-10|Canada|British Columbia|658[0n]|BOLD:AAA2865
Apamea cogitata|[13225]|LBCH917-10|Canada|British Columbia|658[0n]|BOLD:AAA2865
Apamea cogitata|[13226]|LBCH1581-10|Canada|British Columbia|658[0n]|BOLD:AAA2865
Apamea cogitata|[13227]|LBCH2203-10|Canada|British Columbia|658[0n]|BOLD:AAA2865
Apamea cogitata|[13228]|LBCH4695-10|Canada|British Columbia|658[0n]|BOLD:AAA2865
Apamea cogitata|[13229]|RDMAB073-05|Canada|Alberta|633[0n]|BOLD:AAA2865
Apamea cogitata|[13230]|LPABB725-08|Canada|Alberta|658[0n]|BOLD:AAA2865
Apamea cogitata|[13231]|BBLPB784-10|Canada|Alberta|658[0n]|BOLD:AAA2865
Apamea cogitata|[13232]|BBLPB791-10|Canada|Alberta|658[0n]|BOLD:AAA2865
Apamea cogitata|[13233]|LBCD326-05|Canada|British Columbia|650[0n]|BOLD:AAA2865
Apamea cogitata|[13234]|LBCD444-05|Canada|British Columbia|658[0n]|BOLD:AAA2865
Apamea cogitata|[13235]|CNWLF2009-12|Canada|Alberta|638[0n]|BOLD:AAA2865
Apamea cogitata|[13236]|ABKWR095-07|United States|Alaska|647[0n]|BOLD:AAA2865
Apamea cogitata|[13237]|LPABB816-09|Canada|Alberta|644[0n]|BOLD:AAA2865
Apamea cogitata|[13238]|LPSK455-08|Canada|Saskatchewan|643[0n]|BOLD:AAA2865
Apamea cogitata|[13239]|CNWLE2505-12|Canada|Alberta|640[0n]|BOLD:AAA2865
Apamea cogitata|[13240]|CNWLF2012-12|Canada|Alberta|639[0n]|BOLD:AAA2865
Apamea cogitata|[13241]|ABKWR106-07|United States|Alaska|635[0n]|BOLD:AAA2865
Apamea cogitata|[13242]|CNWLF2015-12|Canada|Alberta|633[0n]|BOLD:AAA2865
Apamea cogitata|[13243]|CNWLE2502-12|Canada|Alberta|631[0n]|BOLD:AAA2865
Apamea cogitata|[13244]|CNWLE2397-12|Canada|Alberta|632[0n]|BOLD:AAA2865
Apamea cogitata|[13245]|LOWCD306-06|Canada|British Columbia|593[0n]|BOLD:AAA2865
Apamea cogitata|[13246]|LPABB719-08|Canada|Alberta|635[0n]|BOLD:AAA2865
Apamea cogitata|[13247]|CNWLF2018-12|Canada|Alberta|629[0n]|BOLD:AAA2865
Apamea cogitata|[13248]|CNWLE2389-12|Canada|Alberta|632[0n]|BOLD:AAA2865
Apamea cogitata|[13249]|RDMAB498-06|Canada|Alberta|608[0n]|BOLD:AAA2865
Apamea cogitata|[13250]|LOWCC421-05|Canada|British Columbia|604[0n]|BOLD:AAA2865
Apamea cogitata|[13251]|CNWLF2002-12|Canada|Alberta|618[0n]|BOLD:AAA2865
Apamea cogitata|[13252]|LPSK430-08|Canada|Saskatchewan|609[0n]|BOLD:AAA2865
Apamea cogitata|[13253]|LOWCC433-05|Canada|British Columbia|589[0n]|BOLD:AAA2865
Apamea cogitata|[13254]|LOWCD303-06|Canada|British Columbia|585[0n]|BOLD:AAA2865
Apamea cogitata|[13255]|CNWLM2434-13|Canada|Alberta|582[0n]|BOLD:AAA2865
Apamea cogitata|[13256]|CNWLM2399-13|Canada|Alberta|613[0n]|BOLD:AAA2865
Apamea cogitata|[13257]|CNWLM041-13|Canada|Alberta|552[0n]|BOLD:AAA2865
Apamea cogitata|[13258]|CNWLM030-13|Canada|Alberta|579[0n]|BOLD:AAA2865
Apamea cogitata|[13259]|CNWLM049-13|Canada|Alberta|579[0n]|BOLD:AAA2865
Apamea cogitata|[13260]|CNWLM057-13|Canada|Alberta|579[0n]|BOLD:AAA2865
Apamea cogitata|[13261]|CNWLM2410-13|Canada|Alberta|610[0n]|BOLD:AAA2865
Apamea cogitata|[13262]|CNWLM2441-13|Canada|Alberta|613[0n]|BOLD:AAA2865
Apamea cogitata|[13263]|LBCH7755-10|Canada|British Columbia|658[0n]|BOLD:AAA2865
Apamea cogitata|[13264]|LBCH1681-10|Canada|British Columbia|658[0n]|BOLD:AAA2865
Apamea cogitata|[13265]|CNWLF2033-12|Canada|Alberta|637[0n]|BOLD:AAA2865
Apamea cogitata|[13266]|CNWLE2503-12|Canada|Alberta|636[0n]|BOLD:AAA2865
Apamea cogitata|[13267]|LPABC204-09|Canada|Alberta|634[0n]|BOLD:AAA2865
Apamea cogitata|[13268]|CNWLF2017-12|Canada|Alberta|613[0n]|BOLD:AAA2865
Apamea cogitata|[13269]|CNWLN1073-13|Canada|Alberta|601[0n]|BOLD:AAA2865
Apamea dubitans|[13270]|PHMO298-03|Canada|Ontario|639[0n]|BOLD:ACF4111
Apamea dubitans|[13271]|LNCNW057-06|United States|North Carolina|658[0n]|BOLD:ACF4111
Apamea dubitans|[13272]|BBLEC473-09|Canada|New Brunswick|638[0n]|BOLD:ACF4111
Apamea dubitans|[13273]|RDLQF066-06|Canada|Quebec|658[0n]|BOLD:ACF4111
Apamea dubitans|[13274]|RDLQF065-06|Canada|Quebec|658[0n]|BOLD:ACF4111
Apamea dubitans|[13275]|LGSM563-04|United States|North Carolina|658[0n]|BOLD:ACF4111
Apamea dubitans|[13276]|XAH170-05|Canada|Ontario|579[4n]|BOLD:ACF4111
Apamea dubitans|[13277]|RDNMD121-06|United States|North Carolina|617[0n]|BOLD:ACF4111
Apamea dubitans|[13278]|LNCNW020-06|United States|North Carolina|658[0n]|BOLD:ACF4111
Apamea dubitans|[13279]|LNCNW058-06|United States|North Carolina|658[0n]|BOLD:ACF4111
Apamea dubitans|[13280]|RDLQF552-06|Canada|Quebec|658[0n]|BOLD:ACF4111
Apamea dubitans|[13281]|BLTIB955-08|Canada|Ontario|658[0n]|BOLD:ACF4111
Apamea dubitans|[13282]|LPMNB240-09|Canada|Manitoba|658[0n]|BOLD:ACF4111
Apamea dubitans|[13283]|LPSOD973-09|Canada|Ontario|658[0n]|BOLD:ACF4111
Apamea dubitans|[13284]|LGSMG966-10|United States|North Carolina|658[0n]|BOLD:ACF4111
Apamea dubitans|[13285]|LGSMG967-10|United States|North Carolina|658[0n]|BOLD:ACF4111
Apamea dubitans|[13286]|LGSMG968-10|United States|North Carolina|658[0n]|BOLD:ACF4111
Apamea dubitans|[13287]|LGSMG969-10|United States|North Carolina|658[0n]|BOLD:ACF4111
Apamea dubitans|[13288]|CNCLB2638-14|United States|North Carolina|658[0n]|BOLD:ACF4111
Apamea geminimacula|[13289]|RDNMC275-05|United States|Colorado|573[0n]|BOLD:AAF1836
Apamea geminimacula|[13290]|RDNMD134-06|United States|Colorado|658[0n]|BOLD:AAF1836
Apamea geminimacula|[13291]|RDNMD849-07|United States|New Mexico|655[0n]|BOLD:AAF1836
Apamea helva|[13292]|RDNM972-05|Canada|Ontario|658[0n]|BOLD:AAC5412
Apamea helva|[13293]|LNCNW037-06|United States|North Carolina|658[0n]|BOLD:AAC5412

Apamea geminimacula|[13290]|RDNMD154-06|United States|Colorado|658[0n]|BOLD:AAF1836
Apamea geminimacula|[13291]|RDNMD849-07|United States|New Mexico|655[0n]|BOLD:AAF1836
Apamea helva|[13292]|RDNM972-05|Canada|Ontario|658[0n]|BOLD:AAC5412
Apamea helva|[13293]|LNCNW037-06|United States|North Carolina|658[0n]|BOLD:AAC5412
Apamea helva|[13294]|RDLQF061-06|Canada|Quebec|658[0n]|BOLD:AAC5412
Apamea helva|[13295]|LSEU210-06|United States|North Carolina|658[0n]|BOLD:AAC5412
Apamea helva|[13296]|LNCNW038-06|United States|North Carolina|658[0n]|BOLD:AAC5412
Apamea helva|[13297]|RDLQF553-06|Canada|Quebec|658[0n]|BOLD:AAC5412
Apamea helva|[13298]|LPOKA416-09|United States|Oklahoma|630[0n]|BOLD:AAC5412
Apamea helva|[13299]|RDLQF060-06|Canada|Quebec|658[0n]|BOLD:AAC5412
Apamea helva|[13300]|RDLQF059-06|Canada|Quebec|658[0n]|BOLD:AAC5412
Apamea helva|[13301]|RDNMD155-06|United States|Colorado|605[0n]|BOLD:AAC5412
Apamea helva|[13302]|LPOKA426-09|United States|Oklahoma|658[0n]|BOLD:AAC5412
Loscopia roblei|[13303]|RDNMC712-06|United States|North Carolina|658[0n]|BOLD:AAW5921
Apamea scoparia gabrieli|[13304]|RDNMC684-06|United States|California|658[0n]|BOLD:AAA8064
Apamea scoparia|[13305]|CNGRJ063-13|Canada|Saskatchewan|613[0n]|BOLD:AAA8064
Apamea scoparia|[13306]|CNGRD1015-12|Canada|Saskatchewan|614[0n]|BOLD:AAA8064
Apamea scoparia|[13307]|CNWLM047-13|Canada|Alberta|582[0n]|BOLD:AAA8064
Apamea scoparia|[13308]|LPSK438-08|Canada|Saskatchewan|609[0n]|BOLD:AAA8064
Apamea scoparia|[13309]|CNWLM033-13|Canada|Alberta|576[0n]|BOLD:AAA8064
Apamea scoparia|[13310]|CNWLE2388-12|Canada|Alberta|603[0n]|BOLD:AAA8064
Apamea scoparia|[13311]|CNGRD1014-12|Canada|Saskatchewan|633[0n]|BOLD:AAA8064
Apamea scoparia|[13312]|CNGRD1022-12|Canada|Saskatchewan|633[0n]|BOLD:AAA8064
Apamea scoparia|[13313]|LOWCC426-05|Canada|British Columbia|584[0n]|BOLD:AAA8064
Apamea scoparia|[13314]|CNWLM036-13|Canada|Alberta|558[0n]|BOLD:AAA8064
Apamea scoparia|[13315]|CNWLN1071-13|Canada|Alberta|613[0n]|BOLD:AAA8064
Apamea scoparia|[13316]|CNGRK431-13|Canada|Saskatchewan|592[0n]|BOLD:AAA8064
Apamea scoparia|[13317]|CNGLF020-13|Canada|British Columbia|579[0n]|BOLD:AAA8064
Apamea scoparia|[13318]|CNGRJ083-13|Canada|Saskatchewan|558[0n]|BOLD:AAA8064
Apamea scoparia|[13319]|LOWCC214-05|Canada|British Columbia|608[0n]|BOLD:AAA8064
Apamea scoparia|[13320]|CNGRJ086-13|Canada|Saskatchewan|591[0n]|BOLD:AAA8064
Apamea scoparia|[13321]|CNGRJ077-13|Canada|Saskatchewan|591[0n]|BOLD:AAA8064
Apamea scoparia|[13322]|CNGRD1012-12|Canada|Saskatchewan|635[0n]|BOLD:AAA8064
Apamea scoparia|[13323]|LPSK519-08|Canada|Saskatchewan|658[0n]|BOLD:AAA8064
Apamea scoparia|[13324]|LPSK521-08|Canada|Saskatchewan|658[0n]|BOLD:AAA8064
Apamea scoparia|[13325]|LPSK485-08|Canada|Saskatchewan|658[0n]|BOLD:AAA8064
Apamea scoparia|[13326]|LPSK493-08|Canada|Saskatchewan|658[0n]|BOLD:AAA8064
Apamea scoparia|[13327]|LPSK111-08|Canada|Saskatchewan|658[0n]|BOLD:AAA8064
Apamea scoparia|[13328]|LPSK474-08|Canada|Saskatchewan|658[0n]|BOLD:AAA8064
Apamea scoparia|[13329]|RDNMD126-06|United States|Colorado|658[0n]|BOLD:AAA8064
Apamea scoparia|[13330]|DSCNI039-07|Canada|Manitoba|658[0n]|BOLD:AAA8064
Apamea scoparia|[13331]|LPSK527-08|Canada|Saskatchewan|658[0n]|BOLD:AAA8064
Apamea scoparia|[13332]|LPSK528-08|Canada|Saskatchewan|658[0n]|BOLD:AAA8064
Apamea scoparia|[13333]|LOWCD282-06|Canada|British Columbia|657[0n]|BOLD:AAA8064
Apamea scoparia|[13334]|RDNMC483-06|Canada|Quebec|658[0n]|BOLD:AAA8064
Apamea scoparia|[13335]|LOWCC423-05|Canada|British Columbia|658[0n]|BOLD:AAA8064
Apamea scoparia|[13336]|LBCB406-05|Canada|British Columbia|658[0n]|BOLD:AAA8064
Apamea scoparia|[13337]|RDNMD127-06|United States|Colorado|658[0n]|BOLD:AAA8064
Apamea scoparia|[13338]|LBCB620-05|Canada|British Columbia|658[0n]|BOLD:AAA8064
Apamea scoparia|[13339]|LPABB029-08|Canada|Alberta|658[0n]|BOLD:AAA8064
Apamea scoparia|[13340]|LPSK579-08|Canada|Saskatchewan|658[0n]|BOLD:AAA8064
Apamea scoparia|[13341]|LPABB722-08|Canada|Alberta|658[0n]|BOLD:AAA8064
Apamea scoparia|[13342]|LPABC072-09|Canada|Alberta|658[0n]|BOLD:AAA8064
Apamea scoparia|[13343]|LALPA1216-11|Canada|British Columbia|658[0n]|BOLD:AAA8064
Apamea scoparia|[13344]|LOWCC424-05|Canada|British Columbia|658[0n]|BOLD:AAA8064
Apamea scoparia|[13345]|LOWCC425-05|Canada|British Columbia|658[0n]|BOLD:AAA8064
Apamea scoparia|[13346]|LHLEP062-06|Canada|British Columbia|658[0n]|BOLD:AAA8064
Apamea scoparia|[13347]|LCHQ517-08|Canada|Manitoba|658[0n]|BOLD:AAA8064
Apamea scoparia|[13348]|LPSK108-08|Canada|Saskatchewan|658[0n]|BOLD:AAA8064
Apamea scoparia|[13349]|LPSK213-08|Canada|Saskatchewan|658[0n]|BOLD:AAA8064
Apamea scoparia|[13350]|LPSK220-08|Canada|Saskatchewan|658[0n]|BOLD:AAA8064
Apamea scoparia|[13351]|LPSK451-08|Canada|Saskatchewan|658[0n]|BOLD:AAA8064
Apamea scoparia|[13352]|LPSK518-08|Canada|Saskatchewan|658[0n]|BOLD:AAA8064
Apamea scoparia|[13353]|LPSK549-08|Canada|Saskatchewan|658[0n]|BOLD:AAA8064
Apamea scoparia|[13354]|LPSK551-08|Canada|Saskatchewan|658[0n]|BOLD:AAA8064
Apamea scoparia|[13355]|LPSK580-08|Canada|Saskatchewan|658[0n]|BOLD:AAA8064
Apamea scoparia|[13356]|LPSK582-08|Canada|Saskatchewan|658[0n]|BOLD:AAA8064
Apamea scoparia|[13357]|LPSK583-08|Canada|Saskatchewan|658[0n]|BOLD:AAA8064
Apamea scoparia|[13358]|LPSK586-08|Canada|Saskatchewan|658[0n]|BOLD:AAA8064
Apamea scoparia|[13359]|LPSK592-08|Canada|Saskatchewan|658[0n]|BOLD:AAA8064
Apamea scoparia|[13360]|LPSK596-08|Canada|Saskatchewan|658[0n]|BOLD:AAA8064
Apamea scoparia|[13361]|LPABB336-08|Canada|Alberta|658[0n]|BOLD:AAA8064
Apamea scoparia|[13362]|LPABB721-08|Canada|Alberta|658[0n]|BOLD:AAA8064
Apamea scoparia|[13363]|LPABB728-08|Canada|Alberta|658[0n]|BOLD:AAA8064
Apamea scoparia|[13364]|BBLPB793-10|Canada|British Columbia|658[0n]|BOLD:AAA8064
Apamea scoparia|[13365]|JMMMB347-11|United States|California|658[0n]|BOLD:AAA8064
Apamea scoparia|[13366]|LALPA864-11|Canada|British Columbia|658[0n]|BOLD:AAA8064
Apamea scoparia|[13367]|LALPA886-11|Canada|British Columbia|658[0n]|BOLD:AAA8064
Apamea scoparia|[13368]|CNGRJ061-13|Canada|Saskatchewan|564[0n]|BOLD:AAA8064
Apamea scoparia|[13369]|CNWLM029-13|Canada|Alberta|565[0n]|BOLD:AAA8064
Apamea scoparia|[13370]|LCHQ524-08|Canada|Manitoba|643[1n]|BOLD:AAA8064
Apamea scoparia|[13371]|CNWLM050-13|Canada|Alberta|564[0n]|BOLD:AAA8064
Apamea scoparia|[13372]|CNWLM052-13|Canada|Alberta|564[0n]|BOLD:AAA8064
Apamea scoparia|[13373]|CNWLN1079-13|Canada|Alberta|577[0n]|BOLD:AAA8064
Apamea scoparia|[13374]|CNWLN1529-13|Canada|Alberta|601[0n]|BOLD:AAA8064
Apamea inficita|[13375]|RDNMD128-06|United States|Colorado|594[0n]|BOLD:ACF0441
Apamea inficita|[13376]|RDNMC299-05|United States|Colorado|594[0n]|BOLD:ACF0441
Apamea inficita|[13377]|RDNMC300-05|United States|Colorado|587[0n]|BOLD:ACF0441
Apamea inficita|[13378]|RDNMD129-06|United States|Colorado|658[0n]|BOLD:ACF0441
Apamea inficita|[13379]|RDNMD131-06|United States|Colorado|658[0n]|BOLD:ACF0441
Apamea inficita|[13380]|RDNMD133-06|United States|Colorado|628[0n]|BOLD:ACF0441
Apamea inficita|[13381]|RDNMD132-06|United States|Colorado|657[0n]|BOLD:ABZ5959
Apamea inficita|[13382]|LOWCE706-06|Canada|British Columbia|576[1n]|BOLD:ABZ5959
Apamea inficita|[13383]|LOWCE711-06|Canada|British Columbia|584[1n]|BOLD:ABZ5959
Apamea inficita|[13384]|LOWCB861-05|Canada|British Columbia|557[0n]|BOLD:ABZ5959
Apamea inficita|[13385]|LOWCB857-05|Canada|British Columbia|581[0n]|BOLD:ABZ5959
Apamea inficita|[13386]|LOWCB860-05|Canada|British Columbia|658[0n]|BOLD:ABZ5959
Apamea inficita|[13387]|LOWCE708-06|Canada|British Columbia|658[0n]|BOLD:ABZ5959
Apamea inficita|[13388]|LOWCE710-06|Canada|British Columbia|658[0n]|BOLD:ABZ5959
Apamea inficita|[13389]|LBCG188-08|Canada|British Columbia|658[0n]|BOLD:ABZ5959
Apamea inficita|[13390]|LBCG3311-09|Canada|British Columbia|658[0n]|BOLD:ABZ5959
Apamea inficita|[13391]|LOWCB856-05|Canada|British Columbia|573[0n]|BOLD:ABZ5959
Apamea inficita|[13392]|RDNMC363-05|United States|Colorado|577[0n]|BOLD:ABZ5959
Apamea inficita|[13393]|LPABC836-09|Canada|Alberta|658[0n]|BOLD:ABZ5959

Apamea inficita|[13390]|LBCG3311-09|Canada|British Columbia|658[0n]|BOLD:ABZ5959
Apamea inficita|[13391]|LOWCB856-05|Canada|British Columbia|573[0n]|BOLD:ABZ5959
Apamea inficita|[13392]|RDNMC363-05|United States|Colorado|577[0n]|BOLD:ABZ5959
Apamea inficita|[13393]|LPABC826-09|Canada|Alberta|658[0n]|BOLD:ABZ5959
Apamea inficita|[13394]|RDNMD130-06|United States|Colorado|658[0n]|BOLD:ABZ5959
Apamea inficita|[13395]|RDNM968-05|Canada|Alberta|534[0n]|BOLD:ABZ5959
Apamea inficita|[13396]|LOWCB858-05|Canada|British Columbia|593[0n]|BOLD:ABZ5959
Apamea inficita|[13397]|LPABC206-09|Canada|Alberta|658[0n]|BOLD:ABZ5959
Apamea inficita|[13398]|LPABC827-09|Canada|Alberta|658[0n]|BOLD:ABZ5959
Apamea inficita|[13399]|LPABC224-09|Canada|Alberta|658[0n]|BOLD:ABZ5959
Apamea inficita|[13400]|LPABB860-09|Canada|Alberta|658[0n]|BOLD:ABZ5959
Apamea inficita|[13401]|LPABB858-09|Canada|Alberta|658[0n]|BOLD:ABZ5959
Apamea inficita|[13402]|LPABB847-09|Canada|Alberta|658[0n]|BOLD:ABZ5959
Apamea inficita|[13403]|LPABB724-08|Canada|Alberta|658[0n]|BOLD:ABZ5959
Apamea inficita|[13404]|LPABB710-08|Canada|Alberta|658[0n]|BOLD:ABZ5959
Apamea inficita|[13405]|LPABB017-08|Canada|Alberta|658[0n]|BOLD:ABZ5959
Apamea inficita|[13406]|LPAB247-08|Canada|Alberta|658[0n]|BOLD:ABZ5959
Apamea inficita|[13407]|LBCG187-08|Canada|British Columbia|658[0n]|BOLD:ABZ5959
Apamea inficita|[13408]|LOWCE707-06|Canada|British Columbia|658[0n]|BOLD:ABZ5959
Apamea inficita|[13409]|RDMAB457-05|Canada|Alberta|658[0n]|BOLD:ABZ5959
Apamea inficita|[13410]|RDMAB456-05|Canada|Alberta|658[0n]|BOLD:ABZ5959
Apamea inficita|[13411]|LOWC053-05|Canada|British Columbia|658[0n]|BOLD:ABZ5959
Apamea inficita|[13412]|LPABB714-08|Canada|Alberta|631[0n]|BOLD:ABZ5959
Apamea inficita|[13413]|RDNM967-05|Canada|British Columbia|658[0n]|BOLD:ABZ5959
Apamea inficita|[13414]|LPABB711-08|Canada|Alberta|637[0n]|BOLD:ABZ5959
Apamea inficita|[13415]|LPABB880-09|Canada|Alberta|636[0n]|BOLD:ABZ5959
Apamea inficita|[13416]|LPABB712-08|Canada|Alberta|658[0n]|BOLD:ABZ5959
Apamea inficita|[13417]|LPABC811-09|Canada|Alberta|658[0n]|BOLD:ABZ5959
Apamea inficita|[13418]|CNWLF2029-12|Canada|Alberta|634[0n]|BOLD:ABZ5959
Apamea inficita|[13419]|BBLEC464-09|Canada|New Brunswick|658[0n]|BOLD:ABZ5959
Apamea inficita|[13420]|LOWCE713-06|Canada|British Columbia|658[0n]|BOLD:ABZ5959
Apamea inficita|[13421]|BBLPB438-10|Canada|Alberta|658[0n]|BOLD:ABZ5959
Apamea inficita|[13422]|RDNMC355-05|United States|Alaska|523[0n]|BOLD:ABZ5959
Apamea inficita|[13423]|RDMAB458-05|Canada|Yukon Territory|658[0n]|BOLD:ABZ5959
Apamea inficita|[13424]|RDMAB448-05|Canada|Yukon Territory|658[0n]|BOLD:ABZ5959
Apamea inficita|[13425]|UAMIC546-13|United States|Alaska|632[0n]|BOLD:ABZ5959
Apamea commoda|[13426]|LPABC463-09|Canada|Alberta|658[0n]|BOLD:AAA6405
Apamea commoda|[13427]|RDMAB443-05|Canada|Alberta|658[0n]|BOLD:AAA6405
Apamea commoda|[13428]|LOWCC441-05|Canada|British Columbia|658[1n]|BOLD:AAA6405
Apamea commoda|[13429]|RDMAB445-05|Canada|Yukon Territory|658[0n]|BOLD:AAA6405
Apamea commoda|[13430]|LOWCC445-05|Canada|British Columbia|658[0n]|BOLD:AAA6405
Apamea commoda|[13431]|LOWCC442-05|Canada|British Columbia|658[0n]|BOLD:AAA6405
Apamea commoda|[13432]|RDNMC356-05|Canada|Newfoundland and Labrador|565[0n]|BOLD:AAA6405
Apamea commoda|[13433]|RDNMC200-05|Canada|Quebec|557[0n]|BOLD:AAA6405
Apamea commoda|[13434]|RDNMC201-05|Canada|Quebec|603[1n]|BOLD:AAA6405
Apamea commoda|[13435]|RDNM060-05|Canada|Alberta|600[0n]|BOLD:AAA6405
Apamea commoda|[13436]|RDLQ456-07|Canada|Quebec|594[0n]|BOLD:AAA6405
Apamea commoda|[13437]|LPAB212-08|Canada|Alberta|658[0n]|BOLD:AAA6405
Apamea commoda|[13438]|RDNM933-05|Canada|British Columbia|616[0n]|BOLD:AAA6405
Apamea commoda|[13439]|BBLPB792-10|Canada|Alberta|658[0n]|BOLD:AAA6405
Apamea commoda|[13440]|BBLPB752-10|Canada|Alberta|658[0n]|BOLD:AAA6405
Apamea commoda|[13441]|LPABC320-09|Canada|Alberta|658[0n]|BOLD:AAA6405
Apamea commoda|[13442]|LPABC274-09|Canada|Alberta|658[0n]|BOLD:AAA6405
Apamea commoda|[13443]|LPABC273-09|Canada|Alberta|658[0n]|BOLD:AAA6405
Apamea commoda|[13444]|LPABC180-09|Canada|Alberta|658[0n]|BOLD:AAA6405
Apamea commoda|[13445]|LPABB871-09|Canada|Alberta|658[0n]|BOLD:AAA6405
Apamea commoda|[13446]|LPABB859-09|Canada|Alberta|658[0n]|BOLD:AAA6405
Apamea commoda|[13447]|LPABB857-09|Canada|Alberta|658[0n]|BOLD:AAA6405
Apamea commoda|[13448]|LPABB846-09|Canada|Alberta|658[0n]|BOLD:AAA6405
Apamea commoda|[13449]|LPABB329-08|Canada|Alberta|658[0n]|BOLD:AAA6405
Apamea commoda|[13450]|LPAB211-08|Canada|Alberta|658[0n]|BOLD:AAA6405
Apamea commoda|[13451]|LOWCE807-06|Canada|British Columbia|658[0n]|BOLD:AAA6405
Apamea commoda parcata|[13452]|RDNMD161-06|United States|Colorado|658[0n]|BOLD:AAA6405
Apamea commoda parcata|[13453]|RDNMD124-06|United States|South Dakota|658[0n]|BOLD:AAA6405
Apamea commoda|[13454]|RDMAB481-06|Canada|Alberta|658[0n]|BOLD:AAA6405
Apamea commoda|[13455]|RDMAB480-06|Canada|Alberta|658[0n]|BOLD:AAA6405
Apamea commoda|[13456]|RDMAB479-06|Canada|Alberta|658[0n]|BOLD:AAA6405
Apamea commoda|[13457]|RDMAB475-06|Canada|Alberta|658[0n]|BOLD:AAA6405
Apamea commoda|[13458]|RDMAB444-05|Canada|Alberta|658[0n]|BOLD:AAA6405
Apamea commoda|[13459]|RDMAB436-05|Canada|Yukon Territory|658[0n]|BOLD:AAA6405
Apamea commoda|[13460]|LOWCC443-05|Canada|British Columbia|658[0n]|BOLD:AAA6405
Apamea commoda|[13461]|LOWCC427-05|Canada|British Columbia|658[0n]|BOLD:AAA6405
Apamea commoda|[13462]|LOWCC206-05|Canada|British Columbia|658[0n]|BOLD:AAA6405
Apamea commoda parcata|[13463]|RDNMC066-05|United States|Wyoming|658[0n]|BOLD:AAA6405
Apamea commoda|[13464]|RDNMC062-05|Canada|Alberta|658[0n]|BOLD:AAA6405
Apamea commoda|[13465]|RDNM943-05|Canada|Alberta|658[0n]|BOLD:AAA6405
Apamea commoda|[13466]|RDNM147-05|Canada|Alberta|658[0n]|BOLD:AAA6405
Apamea commoda|[13467]|BBLPE355-09|Canada|Newfoundland and Labrador|643[0n]|BOLD:AAA6405
Apamea commoda|[13468]|LPABB530-08|Canada|Alberta|658[0n]|BOLD:AAA6405
Apamea commoda|[13469]|CNWLE2405-12|Canada|Alberta|632[0n]|BOLD:AAA6405
Apamea commoda|[13470]|RDMAB483-06|Canada|Alberta|638[0n]|BOLD:AAA6405
Apamea commoda|[13471]|RDNM062-05|Canada|Alberta|577[0n]|BOLD:AAA6405
Apamea commoda|[13472]|RDNM061-05|Canada|British Columbia|614[0n]|BOLD:AAA6405
Apamea commoda|[13473]|LOWCB523-05|Canada|British Columbia|608[0n]|BOLD:AAA6405
Apamea commoda|[13474]|RDMAB346-05|Canada|Alberta|604[0n]|BOLD:AAA6405
Apamea commoda|[13475]|RDMAB332-05|Canada|Alberta|603[0n]|BOLD:AAA6405
Apamea commoda|[13476]|RDNMC365-05|Canada|British Columbia|601[0n]|BOLD:AAA6405
Apamea commoda|[13477]|RDNMC366-05|Canada|British Columbia|599[0n]|BOLD:AAA6405
Apamea commoda parcata|[13478]|RDNMC367-05|United States|Wyoming|576[0n]|BOLD:AAA6405
Apamea commoda|[13479]|RDMAB482-06|Canada|Alberta|627[0n]|BOLD:AAA6405
Apamea commoda|[13480]|LPABB854-09|Canada|Alberta|634[0n]|BOLD:AAA6405
Apamea commoda|[13481]|CNWLE2511-12|Canada|Alberta|642[0n]|BOLD:AAA6405
Apamea commoda|[13482]|CNWBG3079-13|Canada|Alberta|594[0n]|BOLD:AAA6405
Apamea commoda|[13483]|LPSK556-08|Canada|Saskatchewan|658[0n]|BOLD:AAA6405
Apamea commoda|[13484]|RDMAB121-05|Canada|Alberta|658[0n]|BOLD:AAA6405
Apamea commoda|[13485]|RDNMC064-05|Canada|Alberta|658[0n]|BOLD:AAA6405
Apamea commoda|[13486]|RDNM944-05|Canada|Saskatchewan|658[0n]|BOLD:AAA6405
Apamea commoda|[13487]|RDNM941-05|Canada|Alberta|658[0n]|BOLD:AAA6405
Apamea commoda|[13488]|CNGRD1026-12|Canada|Saskatchewan|638[0n]|BOLD:AAA6405
Apamea commoda|[13489]|RDNM069-05|Canada|Alberta|622[0n]|BOLD:AAA6405
Apamea commoda|[13490]|RDMAB327-05|Canada|Alberta|606[0n]|BOLD:AAA6405
Apamea commoda|[13491]|CNGRJ071-13|Canada|Saskatchewan|522[5n]|BOLD:AAA6405
Apamea commoda|[13492]|RDNM938-05|Canada|British Columbia|658[0n]|BOLD:ACE3024
[illegible]

Apamea commoda|[13490]|RDMAB327-05|Canada|Alberta|606[0n]|BOLD:AAA6405
Apamea commoda|[13491]|CNGRJ071-13|Canada|Saskatchewan|522[5n]|BOLD:AAA6405
Apamea commoda|[13492]|RDNM938-05|Canada|British Columbia|658[0n]|BOLD:ACE3024
Apamea commoda|[13493]|RDNM937-05|Canada|British Columbia|658[0n]|BOLD:ACE3024
Apamea commoda|[13494]|RDNMD247-06|Canada|British Columbia|617[0n]|BOLD:ACE3024
Apamea commoda|[13495]|RDNMC368-05|Canada|British Columbia|616[0n]|BOLD:ACE3024
Apamea centralis|[13496]|RDNMD243-06|United States|Oregon|604[0n]|BOLD:ACE3024
Apamea commoda|[13497]|RDNM939-05|Canada|British Columbia|605[0n]|BOLD:ACE3024
Apamea commoda|[13498]|RDNMD248-06|Canada|British Columbia|603[0n]|BOLD:ACE3024
Apamea centralis|[13499]|LOWCB462-05|Canada|British Columbia|658[0n]|BOLD:ACE3024
Apamea centralis|[13500]|LOWCC431-05|Canada|British Columbia|658[0n]|BOLD:ACE3024
Apamea centralis|[13501]|LOWCE749-06|Canada|British Columbia|658[0n]|BOLD:ACE3024
Apamea centralis|[13502]|RDNMD244-06|Canada|British Columbia|658[0n]|BOLD:ACE3024
Apamea centralis|[13503]|RDNMD242-06|Canada|British Columbia|658[0n]|BOLD:ACE3024
Apamea centralis|[13504]|LOWCD298-06|Canada|British Columbia|658[0n]|BOLD:ACE3024
Apamea centralis|[13505]|RDMAB476-06|Canada|Alberta|658[0n]|BOLD:ACE3024
Apamea centralis|[13506]|RDNMC681-06|United States|California|658[0n]|BOLD:ACE3024
Apamea centralis|[13507]|RDMAB447-05|Canada|Alberta|658[0n]|BOLD:ACE3024
Apamea centralis|[13508]|LOWCC429-05|Canada|British Columbia|658[0n]|BOLD:ACE3024
Apamea centralis|[13509]|LOWCC428-05|Canada|British Columbia|658[0n]|BOLD:ACE3024
Apamea centralis|[13510]|RDNMC027-05|United States|Washington|658[0n]|BOLD:ACE3024
Apamea centralis|[13511]|RDNMC024-05|United States|Oregon|658[0n]|BOLD:ACE3024
Apamea centralis|[13512]|RDNMC023-05|United States|Oregon|658[0n]|BOLD:ACE3024
Apamea centralis|[13513]|RDNMD246-06|Canada|British Columbia|596[0n]|BOLD:ACE3024
Apamea centralis|[13514]|RDNMC026-05|United States|Oregon|591[0n]|BOLD:ACE3024
Apamea centralis|[13515]|RDNMC025-05|Canada|British Columbia|541[1n]|BOLD:ACE3024
Apamea centralis|[13516]|RDNMD245-06|United States|Oregon|604[0n]|BOLD:ACE3024
Apamea centralis|[13517]|IAWLB032-10|United States|California|543[0n]|BOLD:ACE3024
Apamea centralis|[13518]|IAWLB033-10|United States|California|658[0n]|BOLD:ACE3024
Apamea centralis|[13519]|IAWLB034-10|United States|California|658[0n]|BOLD:ACE3024
Apamea centralis|[13520]|IAWLB035-10|United States|California|658[0n]|BOLD:ACE3024
Apamea centralis|[13521]|IAWLB036-10|United States|California|658[0n]|BOLD:ACE3024
Apamea commoda|[13522]|BBLPB770-10|Canada|British Columbia|658[0n]|BOLD:ACE3024
Apamea commoda|[13523]|LPSK142-08|Canada|Saskatchewan|658[0n]|BOLD:ACE3024
Apamea commoda|[13524]|LPSK128-08|Canada|Saskatchewan|658[0n]|BOLD:ACE3024
Apamea commoda|[13525]|RDMAB097-05|Canada|Alberta|658[0n]|BOLD:ACE3024
Apamea commoda|[13526]|RDNMC065-05|Canada|Alberta|658[0n]|BOLD:ACE3024
Apamea commoda|[13527]|RDNMC063-05|Canada|Saskatchewan|658[0n]|BOLD:ACE3024
Apamea commoda|[13528]|RDNM945-05|Canada|Alberta|658[0n]|BOLD:ACE3024
Apamea commoda|[13529]|RDNM942-05|Canada|Alberta|658[0n]|BOLD:ACE3024
Apamea commoda parcata|[13530]|RDNM940-05|United States|Wyoming|658[0n]|BOLD:ACE3024
Apamea commoda parcata|[13531]|RDNM934-05|United States|Washington|658[0n]|BOLD:ACE3024
Apamea commoda|[13532]|RDNM146-05|Canada|Saskatchewan|658[0n]|BOLD:ACE3024
Apamea commoda|[13533]|RDNM064-05|Canada|Alberta|658[0n]|BOLD:ACE3024
Apamea commoda|[13534]|RDNM063-05|Canada|Alberta|658[0n]|BOLD:ACE3024
Apamea commoda|[13535]|BBLPB699-10|Canada|British Columbia|634[0n]|BOLD:ACE3024
Apamea commoda parcata|[13536]|RDNM065-05|United States|Oregon|602[1n]|BOLD:ACE3024
Apamea commoda|[13537]|RDNM946-05|Canada|Saskatchewan|567[0n]|BOLD:ACE3024
Apamea commoda|[13538]|CNGRJ064-13|Canada|Saskatchewan|579[0n]|BOLD:ACE3024
Apamea commoda|[13539]|CNGRJ079-13|Canada|Saskatchewan|591[0n]|BOLD:ACE3024
Apamea siskiyou|[13540]|RDNM013-05|United States|Oregon|658[0n]|BOLD:ABX6485
Apamea siskiyou|[13541]|RDNM014-05|United States|Oregon|658[0n]|BOLD:ABX6485
Apamea siskiyou|[13542]|RDNM015-05|United States|Oregon|658[0n]|BOLD:ABX6485
Apamea siskiyou|[13543]|RDNME687-08|United States|Washington|658[0n]|BOLD:ABX6485
Apamea antennata|[13544]|RDNM959-05|United States|Idaho|658[0n]|BOLD:AAB3665
Apamea antennata|[13545]|RDNMC678-06|United States|California|658[0n]|BOLD:AAB3665
Apamea antennata|[13546]|IAWLB435-11|United States|California|658[0n]|BOLD:AAB3665
Apamea antennata|[13547]|RDMAB375-05|Canada|Alberta|631[0n]|BOLD:AAB3665
Apamea antennata|[13548]|LOWCC397-05|Canada|British Columbia|658[0n]|BOLD:AAB3665
Apamea antennata|[13549]|LOWCC401-05|Canada|British Columbia|658[0n]|BOLD:AAB3665
Apamea antennata|[13550]|JMMMB422-11|United States|California|658[0n]|BOLD:AAB3665
Apamea antennata|[13551]|LALPA957-11|Canada|British Columbia|640[0n]|BOLD:AAB3665
Apamea antennata|[13552]|LOWCC394-05|Canada|British Columbia|658[0n]|BOLD:AAB3665
Apamea antennata|[13553]|LBCH5853-10|Canada|British Columbia|658[0n]|BOLD:AAB3665
Apamea antennata|[13554]|LBCH5854-10|Canada|British Columbia|658[0n]|BOLD:AAB3665
Apamea antennata|[13555]|LBCH5710-10|Canada|British Columbia|658[0n]|BOLD:AAB3665
Apamea antennata|[13556]|LBCH5711-10|Canada|British Columbia|658[0n]|BOLD:AAB3665
Apamea antennata|[13557]|LBCH5569-10|Canada|British Columbia|658[0n]|BOLD:AAB3665
Apamea antennata|[13558]|LBCH5612-10|Canada|British Columbia|658[0n]|BOLD:AAB3665
Apamea antennata|[13559]|LBCG1074-09|Canada|British Columbia|658[0n]|BOLD:AAB3665
Apamea antennata|[13560]|LBCG1340-09|Canada|British Columbia|658[0n]|BOLD:AAB3665
Apamea antennata|[13561]|LPABB619-08|Canada|Alberta|658[0n]|BOLD:AAB3665
Apamea antennata|[13562]|LBCG1073-09|Canada|British Columbia|658[0n]|BOLD:AAB3665
Apamea antennata|[13563]|LPABB473-08|Canada|Alberta|658[0n]|BOLD:AAB3665
Apamea antennata|[13564]|LPABB572-08|Canada|Alberta|658[0n]|BOLD:AAB3665
Apamea antennata|[13565]|LPABB033-08|Canada|Alberta|658[0n]|BOLD:AAB3665
Apamea antennata|[13566]|LPABB045-08|Canada|Alberta|658[0n]|BOLD:AAB3665
Apamea antennata|[13567]|LOWCC399-05|Canada|British Columbia|658[0n]|BOLD:AAB3665
Apamea antennata|[13568]|LOWCC400-05|Canada|British Columbia|658[0n]|BOLD:AAB3665
Apamea antennata|[13569]|LOWCC398-05|Canada|British Columbia|658[0n]|BOLD:AAB3665
Apamea antennata|[13570]|LOWCC396-05|Canada|British Columbia|658[0n]|BOLD:AAB3665
Apamea antennata|[13571]|LOWCC395-05|Canada|British Columbia|658[0n]|BOLD:AAB3665
Apamea antennata|[13572]|LBCA580-05|Canada|British Columbia|658[0n]|BOLD:AAB3665
Apamea antennata|[13573]|RDNM958-05|Canada|British Columbia|658[0n]|BOLD:AAB3665
Apamea antennata|[13574]|RDNM957-05|Canada|British Columbia|658[0n]|BOLD:AAB3665
Apamea antennata|[13575]|RDNM956-05|Canada|British Columbia|658[0n]|BOLD:AAB3665
Apamea antennata|[13576]|LBCH5796-10|Canada|British Columbia|649[0n]|BOLD:AAB3665
Apamea antennata|[13577]|LBCH6015-10|Canada|British Columbia|658[0n]|BOLD:AAB3665
Apamea antennata|[13578]|LBCH6090-10|Canada|British Columbia|658[0n]|BOLD:AAB3665
Apamea antennata|[13579]|LALPA355-10|Canada|British Columbia|658[0n]|BOLD:AAB3665
Apamea antennata|[13580]|LALPA999-11|Canada|British Columbia|658[0n]|BOLD:AAB3665
Apamea lignicolora|[13581]|RDNMD125-06|United States|South Dakota|658[0n]|BOLD:AAB0880
Apamea lignicolora|[13582]|LPSK488-08|Canada|Saskatchewan|658[0n]|BOLD:AAB0880
Apamea lignicolora|[13583]|LPSK489-08|Canada|Saskatchewan|658[0n]|BOLD:AAB0880
Apamea lignicolora|[13584]|LPSK479-08|Canada|Saskatchewan|658[0n]|BOLD:AAB0880
Apamea lignicolora|[13585]|LPSK487-08|Canada|Saskatchewan|658[0n]|BOLD:AAB0880
Apamea lignicolora|[13586]|LPSK121-08|Canada|Saskatchewan|658[0n]|BOLD:AAB0880
Apamea lignicolora|[13587]|LPSK477-08|Canada|Saskatchewan|658[0n]|BOLD:AAB0880
Apamea lignicolora|[13588]|LPSK019-08|Canada|Saskatchewan|658[0n]|BOLD:AAB0880
Apamea lignicolora|[13589]|LPSK022-08|Canada|Saskatchewan|658[0n]|BOLD:AAB0880
Apamea lignicolora|[13590]|TMNBB163-06|Canada|New Brunswick|658[0n]|BOLD:AAB0880
Apamea lignicolora|[13591]|XAI171-06|Canada|Ontario|658[0n]|BOLD:AAB0880
Apamea lignicolora|[13592]|RDLQF054-06|Canada|Quebec|658[0n]|BOLD:AAB0880
Apamea lignicolora|[13593]|TTMNB56[illegible]-06|Canada|New Brunswick|658[0n]|BOLD:AAB0880

Apamea lignicolora|[13590]|TTMNBB163-06|Canada|New Brunswick|658[0n]|BOLD:AAB0880
Apamea lignicolora|[13591]|XAI171-06|Canada|Ontario|658[0n]|BOLD:AAB0880
Apamea lignicolora|[13592]|RDLQF054-06|Canada|Quebec|658[0n]|BOLD:AAB0880
Apamea lignicolora|[13593]|TTMNB568-06|Canada|New Brunswick|658[0n]|BOLD:AAB0880
Apamea lignicolora|[13594]|RDLQF052-06|Canada|Quebec|658[0n]|BOLD:AAB0880
Apamea lignicolora|[13595]|RDLQF051-06|Canada|Quebec|658[0n]|BOLD:AAB0880
Apamea lignicolora|[13596]|PHMNB451-04|Canada|New Brunswick|658[0n]|BOLD:AAB0880
Apamea lignicolora|[13597]|XAC072-04|Canada|Ontario|658[0n]|BOLD:AAB0880
Apamea lignicolora|[13598]|LGSM662-04|United States|Tennessee|658[0n]|BOLD:AAB0880
Apamea lignicolora|[13599]|PMG092-03|Canada|Ontario|617[0n]|BOLD:AAB0880
Apamea lignicolora|[13600]|PHMO176-03|Canada|Ontario|639[0n]|BOLD:AAB0880
Apamea lignicolora|[13601]|PHMO194-03|Canada|Ontario|639[0n]|BOLD:AAB0880
Apamea lignicolora|[13602]|PHMNB088-03|Canada|New Brunswick|639[0n]|BOLD:AAB0880
Apamea lignicolora|[13603]|LPSK478-08|Canada|Saskatchewan|609[0n]|BOLD:AAB0880
Apamea lignicolora|[13604]|RDMAB478-06|Canada|Alberta|658[0n]|BOLD:AAB0880
Apamea lignicolora|[13605]|XAC669-04|Canada|Ontario|658[0n]|BOLD:AAB0880
Apamea lignicolora|[13606]|LPSK490-08|Canada|Saskatchewan|658[0n]|BOLD:AAB0880
Apamea lignicolora|[13607]|LPSK546-08|Canada|Saskatchewan|658[0n]|BOLD:AAB0880
Apamea lignicolora|[13608]|LPSK524-08|Canada|Saskatchewan|658[0n]|BOLD:AAB0880
Apamea lignicolora|[13609]|LPSK547-08|Canada|Saskatchewan|658[0n]|BOLD:AAB0880
Apamea lignicolora|[13610]|LPSK595-08|Canada|Saskatchewan|658[0n]|BOLD:AAB0880
Apamea lignicolora|[13611]|PHJUN3999-11|Canada|Ontario|658[0n]|BOLD:AAB0880
Apamea atriclava|[13612]|RDNM955-05|Canada|British Columbia|658[0n]|BOLD:AAD5818
Apamea atriclava|[13613]|RDNM018-05|United States|Oregon|658[0n]|BOLD:AAD5818
Apamea atriclava|[13614]|RDNM017-05|United States|Oregon|658[0n]|BOLD:AAD5818
Apamea atriclava|[13615]|RDNM016-05|United States|Oregon|658[0n]|BOLD:AAD5818
Apamea atriclava|[13616]|RDNMD263-06|Canada|British Columbia|608[0n]|BOLD:AAD5818
Apamea atriclava|[13617]|RDNMD264-06|United States|Oregon|658[0n]|BOLD:AAD5818
Apamea atriclava|[13618]|LPVIA270-08|Canada|British Columbia|658[0n]|BOLD:AAD5818
Apamea atriclava|[13619]|RWWB946-10|United States|Washington|658[0n]|BOLD:AAD5818
Apamea auranticolor|[13620]|RDNMB585-05|United States|Colorado|658[0n]|BOLD:ACF2002
Apamea auranticolor|[13621]|RDNMB584-05|United States|Nevada|658[0n]|BOLD:ACF2002
Apamea auranticolor|[13622]|RDNMB586-05|United States|Nevada|658[0n]|BOLD:ACF2002
Apamea auranticolor|[13623]|RDNMC250-05|United States|Nevada|658[0n]|BOLD:ACF2002
Apamea auranticolor|[13624]|RDNMB583-05|United States|Colorado|658[0n]|BOLD:ACF2002
Apamea auranticolor|[13625]|RDNMC526-06|United States|Colorado|658[0n]|BOLD:ACF2002
Apamea auranticolor|[13626]|RDNMC525-06|United States|Wyoming|575[4n]|BOLD:ACF2002
Apamea auranticolor|[13627]|RDNMD145-06|United States|Colorado|608[0n]|BOLD:ACF2002
Apamea auranticolor|[13628]|RDNMD146-06|United States|Colorado|658[0n]|BOLD:ACF2002
Apamea tahoeensis|[13629]|IAWLB040-10|United States|California|658[0n]|BOLD:ACF2002
Apamea tahoeensis|[13630]|IAWLB039-10|United States|California|658[0n]|BOLD:ACF2002
Apamea tahoeensis|[13631]|IAWLB038-10|United States|California|658[0n]|BOLD:ACF2002
Apamea tahoeensis|[13632]|IAWLB037-10|United States|California|658[0n]|BOLD:ACF2002
Apamea tahoeensis|[13633]|NAMUM406-09|United States|California|658[0n]|BOLD:ACF2002
Apamea tahoeensis|[13634]|RDNMC524-06|United States|California|658[0n]|BOLD:ACF2002
Apamea tahoeensis|[13635]|RDNMC523-06|United States|California|658[0n]|BOLD:ACF2002
Apamea tahoeensis|[13636]|RDNMC522-06|United States|California|658[0n]|BOLD:ACF2002
Apamea tahoeensis|[13637]|RDNM954-05|United States|California|658[0n]|BOLD:ACF2002
Apamea tahoeensis|[13638]|NAMUM171-08|United States|California|646[0n]|BOLD:ACF2002
Apamea tahoeensis|[13639]|RDNM022-05|United States|Oregon|615[0n]|BOLD:ACF2002
Apamea tahoeensis|[13640]|RDNM019-05|United States|Oregon|608[0n]|BOLD:ACF2002
Apamea tahoeensis|[13641]|RDNM020-05|United States|Oregon|622[0n]|BOLD:ACF2002
Apamea tahoeensis|[13642]|RDNM953-05|United States|California|554[1n]|BOLD:ACF2002
Apamea tahoeensis|[13643]|JMMMB586-13|United States|California|591[3n]|BOLD:ACF2002
Apamea sora|[13644]|LOWCC404-05|Canada|British Columbia|658[1n]|BOLD:AAA8200
Apamea sora|[13645]|BBLPB707-10|Canada|British Columbia|658[0n]|BOLD:AAA8200
Apamea sora|[13646]|BBLPB689-10|Canada|British Columbia|658[0n]|BOLD:AAA8200
Apamea sora|[13647]|BBLPB634-10|Canada|Alberta|658[0n]|BOLD:AAA8200
Apamea sora|[13648]|LPABC895-09|Canada|Alberta|658[0n]|BOLD:AAA8200
Apamea sora|[13649]|LPABC458-09|Canada|Alberta|658[0n]|BOLD:AAA8200
Apamea sora|[13650]|LPABC419-09|Canada|Alberta|658[0n]|BOLD:AAA8200
Apamea sora|[13651]|LPABC201-09|Canada|Alberta|658[0n]|BOLD:AAA8200
Apamea sora|[13652]|LPABC183-09|Canada|Alberta|658[0n]|BOLD:AAA8200
Apamea sora|[13653]|LPABC070-09|Canada|Alberta|658[0n]|BOLD:AAA8200
Apamea sora|[13654]|LPABB814-09|Canada|Alberta|658[0n]|BOLD:AAA8200
Apamea sora|[13655]|LPABB730-08|Canada|Alberta|658[0n]|BOLD:AAA8200
Apamea sora|[13656]|LPABB717-08|Canada|Alberta|658[0n]|BOLD:AAA8200
Apamea sora|[13657]|LPABB642-08|Canada|Alberta|658[0n]|BOLD:AAA8200
Apamea sora|[13658]|LPABB191-08|Canada|Alberta|658[0n]|BOLD:AAA8200
Apamea sora|[13659]|LPAB202-08|Canada|Alberta|658[0n]|BOLD:AAA8200
Apamea sora|[13660]|RDMAB485-06|Canada|Alberta|658[0n]|BOLD:AAA8200
Apamea sora|[13661]|RDMAB477-06|Canada|Alberta|658[0n]|BOLD:AAA8200
Apamea sora|[13662]|RDMAB449-05|Canada|Yukon Territory|658[0n]|BOLD:AAA8200
Apamea sora|[13663]|LOWCC430-05|Canada|British Columbia|658[0n]|BOLD:AAA8200
Apamea sora|[13664]|LOWCC413-05|Canada|British Columbia|658[0n]|BOLD:AAA8200
Apamea sora|[13665]|LOWCC412-05|Canada|British Columbia|658[0n]|BOLD:AAA8200
Apamea sora|[13666]|LOWCC411-05|Canada|British Columbia|658[0n]|BOLD:AAA8200
Apamea sora|[13667]|LOWCC410-05|Canada|British Columbia|658[0n]|BOLD:AAA8200
Apamea sora|[13668]|LOWCC409-05|Canada|British Columbia|658[0n]|BOLD:AAA8200
Apamea sora|[13669]|LOWCC408-05|Canada|British Columbia|658[0n]|BOLD:AAA8200
Apamea sora|[13670]|LOWCC407-05|Canada|British Columbia|658[0n]|BOLD:AAA8200
Apamea sora|[13671]|LOWCC406-05|Canada|British Columbia|658[0n]|BOLD:AAA8200
Apamea sora|[13672]|LOWCC405-05|Canada|British Columbia|658[0n]|BOLD:AAA8200
Apamea sora|[13673]|LOWCC403-05|Canada|British Columbia|658[0n]|BOLD:AAA8200
Apamea sora|[13674]|LOWCC402-05|Canada|British Columbia|658[0n]|BOLD:AAA8200
Apamea sora|[13675]|LOWCB183-05|Canada|British Columbia|658[0n]|BOLD:AAA8200
Apamea sora|[13676]|RDNMC078-05|Canada|British Columbia|658[0n]|BOLD:AAA8200
Apamea sora|[13677]|LBCD327-05|Canada|British Columbia|658[0n]|BOLD:AAA8200
Apamea sora|[13678]|RDNM936-05|Canada|British Columbia|658[0n]|BOLD:AAA8200
Apamea sora|[13679]|RDNM077-05|Canada|British Columbia|658[0n]|BOLD:AAA8200
Apamea sora|[13680]|RDNM076-05|Canada|British Columbia|658[0n]|BOLD:AAA8200
Apamea sora|[13681]|LPABC030-09|Canada|Alberta|658[1n]|BOLD:AAA8200
Apamea sora|[13682]|RDMAB486-06|Canada|Alberta|646[0n]|BOLD:AAA8200
Apamea sora|[13683]|RDNM024-05|Canada|British Columbia|598[0n]|BOLD:AAA8200
Apamea sora|[13684]|RDNM078-05|Canada|Alberta|623[0n]|BOLD:AAA8200
Apamea sora|[13685]|RDNM079-05|Canada|British Columbia|622[0n]|BOLD:AAA8200
Apamea sora|[13686]|RDMAB484-06|Canada|Alberta|615[0n]|BOLD:AAA8200
Apamea sora|[13687]|LPAB207-08|Canada|Alberta|655[0n]|BOLD:AAA8200
Apamea sora|[13688]|BBLPB696-10|Canada|British Columbia|658[0n]|BOLD:AAA8200
Apamea sora|[13689]|LPABB815-09|Canada|Alberta|637[0n]|BOLD:AAA8200
Apamea sora|[13690]|CNWLE2403-12|Canada|Alberta|632[0n]|BOLD:AAA8200
Apamea sora|[13691]|LPABC200-09|Canada|Alberta|632[0n]|BOLD:AAA8200
Apamea sora|[13692]|SSBAD6393-13|Canada|Alberta|591[0n]|BOLD:AAA8200
[illegible]

Apamea sora|[13690]|CNWLE2403-12|Canada|Alberta|632[0n]|BOLD:AAA8200
Apamea sora|[13691]|LPABC200-09|Canada|Alberta|632[0n]|BOLD:AAA8200
Apamea sora|[13692]|SSBAD6393-13|Canada|Alberta|591[0n]|BOLD:AAA8200
Apamea sora|[13693]|SSBAD6363-13|Canada|Alberta|577[0n]|BOLD:AAA8200
Apamea sora|[13694]|CNWLM056-13|Canada|Alberta|549[0n]|BOLD:AAA8200
Apamea sora|[13695]|CNWLN1077-13|Canada|Alberta|601[0n]|BOLD:AAA8200
Apamea amputatrix|[13696]|LBCS119-07|Canada|British Columbia|658[0n]|BOLD:AAA3867
Apamea amputatrix|[13697]|RWWC968-12|United States|Washington|658[0n]|BOLD:AAA3867
Apamea amputatrix|[13698]|LPMN935-08|Canada|Alberta|658[0n]|BOLD:AAA3867
Apamea amputatrix|[13699]|RDMAB126-05|Canada|Alberta|658[0n]|BOLD:AAA3867
Apamea amputatrix|[13700]|LPABB188-08|Canada|Alberta|658[0n]|BOLD:AAA3867
Apamea amputatrix|[13701]|LBCS652-07|Canada|British Columbia|658[0n]|BOLD:AAA3867
Apamea amputatrix|[13702]|XAC852-04|Canada|Ontario|590[1n]|BOLD:AAA3867
Apamea amputatrix|[13703]|LPVIA507-08|Canada|British Columbia|658[0n]|BOLD:AAA3867
Apamea amputatrix|[13704]|XAI044-05|Canada|Ontario|658[0n]|BOLD:AAA3867
Apamea amputatrix|[13705]|JMMMB581-13|United States|California|570[0n]|BOLD:AAA3867
Apamea amputatrix|[13706]|BBLPC607-09|Canada|Nova Scotia|658[0n]|BOLD:AAA3867
Apamea amputatrix|[13707]|LGSMG963-10|United States|North Carolina|658[0n]|BOLD:AAA3867
Apamea amputatrix|[13708]|JMMMB356-11|United States|California|658[0n]|BOLD:AAA3867
Apamea amputatrix|[13709]|RWWC436-11|United States|Washington|658[0n]|BOLD:AAA3867
Apamea amputatrix|[13710]|BBLEC353-09|Canada|Newfoundland and Labrador|658[0n]|BOLD:AAA3867
Apamea amputatrix|[13711]|BBLEC483-09|Canada|New Brunswick|658[0n]|BOLD:AAA3867
Apamea amputatrix|[13712]|BBLEC690-09|Canada|Nova Scotia|658[0n]|BOLD:AAA3867
Apamea amputatrix|[13713]|BBLPC165-09|Canada|Nova Scotia|658[0n]|BOLD:AAA3867
Apamea amputatrix|[13714]|LNCC296-10|United States|North Carolina|658[0n]|BOLD:AAA3867
Apamea amputatrix|[13715]|RWWC079-10|United States|Washington|658[0n]|BOLD:AAA3867
Apamea amputatrix|[13716]|LBCS653-07|Canada|British Columbia|658[0n]|BOLD:AAA3867
Apamea amputatrix|[13717]|LPSK578-08|Canada|Saskatchewan|658[0n]|BOLD:AAA3867
Apamea amputatrix|[13718]|LPSK581-08|Canada|Saskatchewan|658[0n]|BOLD:AAA3867
Apamea amputatrix|[13719]|LPMN914-08|Canada|Alberta|658[0n]|BOLD:AAA3867
Apamea amputatrix|[13720]|RWWB009-09|United States|Washington|658[0n]|BOLD:AAA3867
Apamea amputatrix|[13721]|RWWB187-09|United States|Washington|658[0n]|BOLD:AAA3867
Apamea amputatrix|[13722]|LPABC829-09|Canada|Alberta|658[0n]|BOLD:AAA3867
Apamea amputatrix|[13723]|LPABC845-09|Canada|Alberta|658[0n]|BOLD:AAA3867
Apamea amputatrix|[13724]|RWWA697-09|United States|Washington|658[0n]|BOLD:AAA3867
Apamea amputatrix|[13725]|RWWA754-09|United States|Washington|658[0n]|BOLD:AAA3867
Apamea amputatrix|[13726]|LHLEP422-06|Canada|British Columbia|658[0n]|BOLD:AAA3867
Apamea amputatrix|[13727]|LHLEP423-06|Canada|British Columbia|658[0n]|BOLD:AAA3867
Apamea amputatrix|[13728]|LHLEP573-06|Canada|British Columbia|658[0n]|BOLD:AAA3867
Apamea amputatrix|[13729]|LBCS249-07|Canada|British Columbia|658[0n]|BOLD:AAA3867
Apamea amputatrix|[13730]|LPAB001-08|Canada|Alberta|658[0n]|BOLD:AAA3867
Apamea amputatrix|[13731]|BLTIB349-08|Canada|Ontario|658[0n]|BOLD:AAA3867
Apamea amputatrix|[13732]|BLTIB439-08|Canada|Ontario|658[0n]|BOLD:AAA3867
Apamea amputatrix|[13733]|BLTIB494-08|Canada|Ontario|658[0n]|BOLD:AAA3867
Apamea amputatrix|[13734]|LPVIA414-08|Canada|British Columbia|658[0n]|BOLD:AAA3867
Apamea amputatrix|[13735]|LPVIA573-08|Canada|British Columbia|658[0n]|BOLD:AAA3867
Apamea amputatrix|[13736]|LPVIB052-08|Canada|British Columbia|658[0n]|BOLD:AAA3867
Apamea amputatrix|[13737]|LPABB621-08|Canada|Alberta|658[0n]|BOLD:AAA3867
Apamea amputatrix|[13738]|LALPA522-10|Canada|British Columbia|658[0n]|BOLD:AAA3867
Apamea amputatrix|[13739]|LALPA533-10|Canada|British Columbia|658[0n]|BOLD:AAA3867
Apamea amputatrix|[13740]|LALPA581-10|Canada|British Columbia|658[0n]|BOLD:AAA3867
Apamea amputatrix|[13741]|LNCC295-10|United States|North Carolina|658[0n]|BOLD:AAA3867
Apamea amputatrix|[13742]|RWWB880-10|United States|Washington|658[0n]|BOLD:AAA3867
Apamea amputatrix|[13743]|LALPA487-10|Canada|British Columbia|658[0n]|BOLD:AAA3867
Apamea amputatrix|[13744]|RWWC468-11|United States|Washington|658[0n]|BOLD:AAA3867
Apamea amputatrix|[13745]|RWWC469-11|United States|Washington|658[0n]|BOLD:AAA3867
Apamea amputatrix|[13746]|RWWC501-11|United States|Washington|658[0n]|BOLD:AAA3867
Apamea amputatrix|[13747]|RWWC542-11|United States|Washington|658[0n]|BOLD:AAA3867
Apamea amputatrix|[13748]|LNCC1028-11|United States|North Carolina|658[0n]|BOLD:AAA3867
Apamea amputatrix|[13749]|RWWC554-11|United States|Washington|658[0n]|BOLD:AAA3867
Apamea amputatrix|[13750]|RWWC571-11|United States|Washington|658[0n]|BOLD:AAA3867
Apamea amputatrix|[13751]|RWWC626-11|United States|Washington|658[0n]|BOLD:AAA3867
Apamea amputatrix|[13752]|GMLC859-12|United States|California|658[0n]|BOLD:AAA3867
Apamea amputatrix|[13753]|GMLC964-12|United States|California|658[0n]|BOLD:AAA3867
Apamea amputatrix|[13754]|GMLC1142-12|United States|California|658[0n]|BOLD:AAA3867
Apamea amputatrix|[13755]|GMLC1193-12|United States|California|658[0n]|BOLD:AAA3867
Apamea amputatrix|[13756]|GMLC1201-12|United States|California|658[0n]|BOLD:AAA3867
Apamea amputatrix|[13757]|RWWC989-12|United States|Washington|658[0n]|BOLD:AAA3867
Apamea amputatrix|[13758]|LMH044-06|Canada|British Columbia|658[0n]|BOLD:AAA3867
Apamea amputatrix|[13759]|LHLEP421-06|Canada|British Columbia|658[0n]|BOLD:AAA3867
Apamea amputatrix|[13760]|LMH043-06|Canada|British Columbia|656[0n]|BOLD:AAA3867
Apamea amputatrix|[13761]|LMH042-06|Canada|British Columbia|658[0n]|BOLD:AAA3867
Apamea amputatrix|[13762]|RDLQF040-06|Canada|Quebec|658[0n]|BOLD:AAA3867
Apamea amputatrix|[13763]|RDLQF039-06|Canada|Quebec|658[0n]|BOLD:AAA3867
Apamea amputatrix|[13764]|MNBB117-05|Canada|New Brunswick|658[0n]|BOLD:AAA3867
Apamea amputatrix|[13765]|RDNM951-05|United States|Oregon|658[0n]|BOLD:AAA3867
Apamea amputatrix|[13766]|RDNM949-05|Canada|Ontario|658[0n]|BOLD:AAA3867
Apamea amputatrix|[13767]|PHMNB756-05|Canada|New Brunswick|658[0n]|BOLD:AAA3867
Apamea amputatrix|[13768]|XAC835-04|Canada|Ontario|658[0n]|BOLD:AAA3867
Apamea amputatrix|[13769]|XAC832-04|Canada|Ontario|658[0n]|BOLD:AAA3867
Apamea amputatrix|[13770]|XAC037-04|Canada|Ontario|658[0n]|BOLD:AAA3867
Apamea amputatrix|[13771]|LGSM455-04|United States|North Carolina|658[0n]|BOLD:AAA3867
Apamea amputatrix|[13772]|LGSM454-04|United States|North Carolina|658[0n]|BOLD:AAA3867
Apamea amputatrix|[13773]|LPABC098-09|Canada|Alberta|658[3n]|BOLD:AAA3867
Apamea amputatrix|[13774]|BLTIB242-08|Canada|Ontario|658[0n]|BOLD:AAA3867
Apamea amputatrix|[13775]|LPABC118-09|Canada|Alberta|641[1n]|BOLD:AAA3867
Apamea amputatrix|[13776]|LPVIB909-08|Canada|British Columbia|647[0n]|BOLD:AAA3867
Apamea amputatrix|[13777]|LPVIB885-08|Canada|British Columbia|639[0n]|BOLD:AAA3867
Apamea amputatrix|[13778]|BLTIB663-08|Canada|Ontario|648[0n]|BOLD:AAA3867
Apamea amputatrix|[13779]|LOWCD278-06|Canada|British Columbia|599[0n]|BOLD:AAA3867
Apamea amputatrix|[13780]|LPGVA601-08|Canada|British Columbia|623[0n]|BOLD:AAA3867
Apamea amputatrix|[13781]|LPGVA602-08|Canada|British Columbia|627[0n]|BOLD:AAA3867
Apamea amputatrix|[13782]|LPGVA600-08|Canada|British Columbia|632[0n]|BOLD:AAA3867
Apamea amputatrix|[13783]|LPVIA966-08|Canada|British Columbia|631[0n]|BOLD:AAA3867
Apamea amputatrix|[13784]|PHMNB234-04|Canada|New Brunswick|573[2n]|BOLD:AAA3867
Apamea amputatrix|[13785]|RDNM950-05|Canada|British Columbia|540[1n]|BOLD:AAA3867
Apamea amputatrix|[13786]|LBCC431-05|Canada|British Columbia|658[0n]|BOLD:AAA3867
Apamea amputatrix|[13787]|RWWC1173-13|United States|Washington|587[0n]|BOLD:AAA3867
Apamea amputatrix|[13788]|RWWC1194-13|United States|Washington|593[0n]|BOLD:AAA3867
Apamea amputatrix|[13789]|BTWC005-09|Canada|Ontario|658[229n]|
Apamea amputatrix|[13790]|RWWA849-09|United States|Washington|658[0n]|BOLD:AAA3867
Apamea amputatrix|[13791]|LPABC117-09|Canada|Alberta|636[0n]|BOLD:AAA3867
Apamea amputatrix|[13792]|CNGRJ080-13|Canada|Saskatchewan|591[0n]|BOLD:AAA3867
[illegible]

Apamea amputatrix|[13790]|RWWA849-09|United States|Washington|658[0n]|BOLD:AAA3867
Apamea amputatrix|[13791]|LPABC117-09|Canada|Alberta|636[0n]|BOLD:AAA3867
Apamea amputatrix|[13792]|CNGRJ080-13|Canada|Saskatchewan|591[0n]|BOLD:AAA3867
Apamea amputatrix|[13793]|CNGRJ084-13|Canada|Saskatchewan|591[0n]|BOLD:AAA3867
Apamea amputatrix|[13794]|BBLPB293-10|Canada|Alberta|658[0n]|BOLD:AAA3867
Apamea amputatrix|[13795]|MNBB429-05|Canada|New Brunswick|658[0n]|BOLD:AAA3867
Apamea amputatrix|[13796]|BLTIB241-08|Canada|Ontario|658[0n]|BOLD:AAA3867
Apamea amputatrix|[13797]|LOWCC414-05|Canada|British Columbia|658[0n]|BOLD:AAA3867
Apamea amputatrix|[13798]|LBCS248-07|Canada|British Columbia|658[0n]|BOLD:AAA3867
Apamea amputatrix|[13799]|LALPA542-10|Canada|British Columbia|658[0n]|BOLD:AAA3867
Apamea amputatrix|[13800]|BBLPB291-10|Canada|Alberta|658[0n]|BOLD:AAA3867
Apamea amputatrix|[13801]|BBLPB292-10|Canada|Alberta|658[0n]|BOLD:AAA3867
Apamea amputatrix|[13802]|RWWC727-11|United States|Washington|658[0n]|BOLD:AAA3867
Apamea amputatrix|[13803]|GMLC954-12|United States|California|658[0n]|BOLD:AAA3867
Apamea amputatrix|[13804]|GMLC970-12|United States|California|658[0n]|BOLD:AAA3867
Apamea amputatrix|[13805]|BBLEC440-09|Canada|New Brunswick|658[0n]|BOLD:AAA3867
Apamea amputatrix|[13806]|LGSMG964-10|United States|North Carolina|658[0n]|BOLD:AAA3867
Apamea amputatrix|[13807]|LGSMG965-10|United States|North Carolina|658[0n]|BOLD:AAA3867
Apamea amputatrix|[13808]|RWWB972-10|United States|Washington|658[0n]|BOLD:AAA3867
Apamea amputatrix|[13809]|LPABB335-08|Canada|Alberta|658[0n]|BOLD:AAA3867
Apamea amputatrix|[13810]|LPABB344-08|Canada|Alberta|658[0n]|BOLD:AAA3867
Apamea amputatrix|[13811]|LPABC846-09|Canada|Alberta|658[0n]|BOLD:AAA3867
Apamea amputatrix|[13812]|LPABC847-09|Canada|Alberta|658[0n]|BOLD:AAA3867
Apamea amputatrix|[13813]|LPABC121-09|Canada|Alberta|658[0n]|BOLD:AAA3867
Apamea amputatrix|[13814]|LPABC842-09|Canada|Alberta|658[0n]|BOLD:AAA3867
Apamea amputatrix|[13815]|LPABB481-08|Canada|Alberta|658[0n]|BOLD:AAA3867
Apamea amputatrix|[13816]|LPABB568-08|Canada|Alberta|658[0n]|BOLD:AAA3867
Apamea amputatrix|[13817]|LPMN936-08|Canada|Alberta|658[0n]|BOLD:AAA3867
Apamea amputatrix|[13818]|LPABB028-08|Canada|Alberta|658[0n]|BOLD:AAA3867
Apamea amputatrix|[13819]|LPMN901-08|Canada|Alberta|658[0n]|BOLD:AAA3867
Apamea amputatrix|[13820]|LPMN913-08|Canada|Alberta|658[0n]|BOLD:AAA3867
Apamea amputatrix|[13821]|LBCS651-07|Canada|British Columbia|658[0n]|BOLD:AAA3867
Apamea amputatrix|[13822]|LPSK004-08|Canada|Saskatchewan|658[0n]|BOLD:AAA3867
Apamea amputatrix|[13823]|LBCS120-07|Canada|British Columbia|658[0n]|BOLD:AAA3867
Apamea amputatrix|[13824]|LBCS185-07|Canada|British Columbia|658[0n]|BOLD:AAA3867
Apamea amputatrix|[13825]|LHLEP405-06|Canada|British Columbia|658[0n]|BOLD:AAA3867
Apamea amputatrix|[13826]|LHLEP572-06|Canada|British Columbia|658[0n]|BOLD:AAA3867
Apamea amputatrix|[13827]|RDNMD163-06|United States|Colorado|658[0n]|BOLD:AAA3867
Apamea amputatrix|[13828]|LBCD477-05|Canada|British Columbia|658[0n]|BOLD:AAA3867
Apamea amputatrix|[13829]|LBCC432-05|Canada|British Columbia|658[0n]|BOLD:AAA3867
Apamea amputatrix|[13830]|MNBB652-05|Canada|New Brunswick|658[0n]|BOLD:AAA3867
Apamea amputatrix|[13831]|MNBB285-05|Canada|New Brunswick|658[0n]|BOLD:AAA3867
Apamea amputatrix|[13832]|RDNM952-05|United States|California|658[0n]|BOLD:AAA3867
Apamea amputatrix|[13833]|LPABC182-09|Canada|Alberta|603[0n]|BOLD:AAA3867
Apamea amputatrix|[13834]|MNBB216-05|Canada|New Brunswick|658[0n]|BOLD:AAA3867
Apamea amputatrix|[13835]|PHMNB150-04|Canada|New Brunswick|658[0n]|BOLD:AAA3867
Apamea amputatrix|[13836]|XAD581-04|Canada|Ontario|658[0n]|BOLD:AAA3867
Apamea amputatrix|[13837]|GMLC1012-12|United States|California|658[0n]|BOLD:AAA3867
Apamea amputatrix|[13838]|LPABC127-09|Canada|Alberta|612[0n]|BOLD:AAA3867
Apamea amputatrix|[13839]|LPABC119-09|Canada|Alberta|608[0n]|BOLD:AAA3867
Apamea amputatrix|[13840]|PHMNB230-04|Canada|New Brunswick|609[0n]|BOLD:AAA3867
Apamea amputatrix|[13841]|LPABC128-09|Canada|Alberta|620[2n]|BOLD:AAA3867
Apamea amputatrix|[13842]|LPABC498-09|Canada|Alberta|611[0n]|BOLD:AAA3867
Apamea amputatrix|[13843]|LPABC097-09|Canada|Alberta|636[1n]|BOLD:AAA3867
Apamea amputatrix|[13844]|PHMNB077-03|Canada|New Brunswick|639[0n]|BOLD:AAA3867
Apamea amputatrix|[13845]|LPABC124-09|Canada|Alberta|616[0n]|BOLD:AAA3867
Apamea amputatrix|[13846]|CNWLE2398-12|Canada|Alberta|627[0n]|BOLD:AAA3867
Apamea amputatrix|[13847]|RWWC1153-13|United States|Washington|593[0n]|BOLD:AAA3867
Apamea amputatrix|[13848]|LPVIC030-08|Canada|British Columbia|658[0n]|BOLD:AAA3867
Apamea amputatrix|[13849]|CNWLM2450-13|Canada|Alberta|583[0n]|BOLD:AAA3867
Apamea amputatrix|[13850]|LPVIB015-08|Canada|British Columbia|658[0n]|BOLD:AAA3867
Apamea amputatrix|[13851]|RWWC1210-13|United States|Washington|536[0n]|BOLD:AAA3867
Apamea atrosuffusa|[13852]|RDNMC243-05|United States|Wyoming|658[0n]|BOLD:AAB3666
Apamea atrosuffusa|[13853]|RDNMC244-05|United States|Wyoming|658[0n]|BOLD:AAB3666
Apamea atrosuffusa|[13854]|RDNMD114-06|United States|Colorado|658[0n]|BOLD:AAB3666
Apamea atrosuffusa|[13855]|RDNMD115-06|United States|South Dakota|658[0n]|BOLD:AAB3666
Apamea atrosuffusa|[13856]|RDNMD116-06|United States|Arizona|658[0n]|BOLD:AAB3666
Apamea atrosuffusa|[13857]|RDNMD117-06|United States|Colorado|658[0n]|BOLD:AAB3666
Apamea atrosuffusa|[13858]|NAMUM398-09|United States|Arizona|658[0n]|BOLD:AAB3666
Apamea atrosuffusa|[13859]|IAWLB517-11|United States|Arizona|658[0n]|BOLD:AAB3666
Apamea atrosuffusa|[13860]|IAWLB532-11|United States|Arizona|658[0n]|BOLD:AAB3666
Apamea relicina|[13861]|RDNMC254-05|United States|New Jersey|567[2n]|BOLD:AAF4056
Apamea relicina|[13862]|RDNMD123-06|United States|New Jersey|604[0n]|BOLD:AAF4056
Apamea relicina|[13863]|RDNMD467-06|United States|Indiana|658[0n]|BOLD:AAF4056
Apamea burgessi|[13864]|RDNMD250-06|United States|Oregon|617[0n]|BOLD:AAC5458
Apamea burgessi|[13865]|RDNMD251-06|United States|Oregon|617[0n]|BOLD:AAC5458
Apamea burgessi|[13866]|RDNMD464-06|United States|Colorado|597[0n]|BOLD:AAC5458
Apamea burgessi|[13867]|RDNMD154-06|United States|Colorado|605[0n]|BOLD:AAC5458
Apamea burgessi|[13868]|RDNMD465-06|United States|Colorado|658[0n]|BOLD:AAC5458
Apamea burgessi|[13869]|RDNMD153-06|United States|Colorado|658[0n]|BOLD:AAC5457
Apamea burgessi|[13870]|RDNMD249-06|United States|Montana|617[0n]|BOLD:AAC5459
Apamea burgessi|[13871]|RDNMD252-06|United States|Arizona|591[0n]|BOLD:AAC5459
Apamea burgessi|[13872]|RDNMD253-06|United States|Wyoming|617[0n]|BOLD:AAC5459
Apamea burgessi|[13873]|RDNMG899-08|United States|Colorado|658[0n]|BOLD:AAC5459
Apamea burgessi|[13874]|RDNMD336-06|United States|Massachusetts|658[0n]|BOLD:AAC5459
Apamea burgessi|[13875]|GWOTA073-12|United States|Massachusetts|658[0n]|BOLD:AAC5459
Apamea acera|[13876]|RDNMG461-08|United States|Oregon|658[0n]|BOLD:AAE6994
Apamea acera|[13877]|RDNMF296-08|United States|Oregon|658[0n]|BOLD:AAE6994
Apamea acera|[13878]|RDNMF003-08|Canada|British Columbia|658[0n]|BOLD:AAE6994
Apamea acera|[13879]|RDNMG462-08|United States|Washington|640[0n]|BOLD:AAE6994
Apamea acera|[13880]|LBCH6217-10|Canada|British Columbia|658[0n]|BOLD:AAE6994
Apamea niveivenosa|[13881]|LPABC111-09|Canada|Alberta|658[0n]|BOLD:AAB8174
Apamea niveivenosa|[13882]|RDNMD148-06|United States|Colorado|605[0n]|BOLD:AAB8174
Apamea niveivenosa|[13883]|RDNMD149-06|United States|Colorado|658[0n]|BOLD:AAB8174
Apamea niveivenosa|[13884]|RDNM057-05|Canada|Alberta|611[0n]|BOLD:AAB8174
Apamea niveivenosa|[13885]|RDNM055-05|United States|Oregon|612[0n]|BOLD:AAB8174
Apamea niveivenosa|[13886]|RDNM056-05|United States|Oregon|609[2n]|BOLD:AAB8174
Apamea niveivenosa|[13887]|RDNMC324-05|United States|Nevada|600[0n]|BOLD:AAB8174
Apamea niveivenosa|[13888]|RDNM058-05|Canada|Alberta|600[2n]|BOLD:AAB8174
Apamea niveivenosa|[13889]|RDNM059-05|Canada|Alberta|601[0n]|BOLD:AAB8174
Apamea niveivenosa|[13890]|RDNM054-05|United States|California|584[0n]|BOLD:AAB8174
Apamea niveivenosa|[13891]|LPABC112-09|Canada|Alberta|632[2n]|BOLD:AAB8174
Apamea niveivenosa|[13892]|BBLOC1368-11|United States|California|658[0n]|BOLD:AAB8174

Apamea niveivenosa|[13890]|RDNM054-05|United States|California|584[0n]|BOLD:AAB8174
Apamea niveivenosa|[13891]|LPABC112-09|Canada|Alberta|632[2n]|BOLD:AAB8174
Apamea niveivenosa|[13892]|BBLOC1368-11|United States|California|658[0n]|BOLD:AAB8174
Apamea niveivenosa|[13893]|BBLOC1361-11|United States|California|658[0n]|BOLD:AAB8174
Apamea niveivenosa|[13894]|BBLOC1265-11|United States|California|658[0n]|BOLD:AAB8174
Apamea niveivenosa|[13895]|LPMNB471-09|Canada|Manitoba|658[0n]|BOLD:AAB8174
Apamea niveivenosa|[13896]|LPABB647-08|Canada|Alberta|658[0n]|BOLD:AAB8174
Apamea niveivenosa|[13897]|LPABB384-08|Canada|Alberta|658[0n]|BOLD:AAB8174
Apamea niveivenosa|[13898]|LPABB077-08|Canada|Alberta|658[0n]|BOLD:AAB8174
Apamea niveivenosa|[13899]|RDNMD053-06|United States||658[0n]|BOLD:AAB8174
Apamea niveivenosa|[13900]|RDNM053-05|United States|Oregon|658[0n]|BOLD:AAB8174
Apamea niveivenosa|[13901]|RDNM052-05|United States|Oregon|658[0n]|BOLD:AAB8174
Apamea niveivenosa|[13902]|JMMMB593-13|United States|California|571[0n]|BOLD:AAB8174
Apamea zeta|[13903]|RDNMB605-05|Canada|Quebec|658[0n]|BOLD:ACE6001
Apamea zeta|[13904]|LCH236-04|Canada|Manitoba|658[0n]|BOLD:ACE6001
Apamea zeta|[13905]|RDNMB606-05|Canada|Quebec|658[0n]|BOLD:ACE6001
Apamea contradicta|[13906]|RDLQB891-05|Canada|Newfoundland and Labrador|658[0n]|BOLD:ACE6002
Apamea contradicta|[13907]|LPMN716-08|Canada|Manitoba|658[0n]|BOLD:ACE6002
Apamea contradicta|[13908]|LPMN636-08|Canada|Manitoba|658[0n]|BOLD:ACE6002
Apamea contradicta|[13909]|RDNMD162-06|United States|Colorado|605[0n]|BOLD:ACE6002
Apamea contradicta|[13910]|LOWCD302-06|Canada|British Columbia|585[0n]|BOLD:ACE6002
Apamea contradicta|[13911]|RDNMC211-05|Canada|Quebec|576[0n]|BOLD:ACE6002
Apamea contradicta|[13912]|RDNMC209-05|Canada|Quebec|658[0n]|BOLD:ACE6002
Apamea contradicta|[13913]|RDNMC369-05|Canada|British Columbia|600[0n]|BOLD:ACE6002
Apamea contradicta|[13914]|LPABB850-09|Canada|Alberta|658[0n]|BOLD:ACE6002
Apamea zeta nichollae|[13915]|RDNMD847-07|United States|Colorado|655[0n]|BOLD:ACE6000
Apamea alticola|[13916]|RDNM522-05|United States|Colorado|658[0n]|BOLD:ACE6000
Apamea alticola|[13917]|RDNM521-05|United States|Colorado|658[0n]|BOLD:ACE6000
Apamea alticola|[13918]|RDNME285-07|United States|Colorado|638[0n]|BOLD:ACE6000
Apamea alticola|[13919]|RDNME286-07|United States|Colorado|658[0n]|BOLD:ACE6000
Apamea zeta|[13920]|RDNM148-05|Canada|British Columbia|569[0n]|BOLD:AAA5797
Apamea zeta|[13921]|RDMAB438-05|Canada|Yukon Territory|658[0n]|BOLD:AAA5797
Apamea zeta|[13922]|RDNMD227-06|Canada|British Columbia|658[0n]|BOLD:AAA5797
Apamea zeta|[13923]|RDNM530-05|Canada|British Columbia|658[0n]|BOLD:AAA5797
Apamea zeta|[13924]|RDNM149-05|Canada|British Columbia|534[0n]|BOLD:AAA5797
Apamea zeta|[13925]|RDNMD231-06|United States|Washington|608[0n]|BOLD:AAA5797
Apamea zeta|[13926]|RDNMD233-06|Canada|British Columbia|619[0n]|BOLD:AAA5797
Apamea zeta|[13927]|RDNMD234-06|Canada|British Columbia|658[0n]|BOLD:AAA5797
Apamea zeta|[13928]|RDNMD235-06|Canada|British Columbia|596[0n]|BOLD:AAA5797
Apamea zeta|[13929]|RDNMB603-05|Canada|Nunavut|658[0n]|BOLD:AAA5797
Apamea zeta|[13930]|RDNMB604-05|Canada|Nunavut|658[0n]|BOLD:AAA5797
Apamea zeta|[13931]|RDLQB856-05|Canada|Quebec|516[0n]|BOLD:AAA5797
Apamea zeta|[13932]|RDNM531-05|Canada|British Columbia|583[0n]|BOLD:AAA5797
Apamea zeta|[13933]|RDNMD229-06|Canada|British Columbia|658[0n]|BOLD:AAA5797
Apamea zeta|[13934]|RDNMD230-06|Canada|British Columbia|658[0n]|BOLD:AAA5797
Apamea zeta|[13935]|RDNMD232-06|Canada|British Columbia|658[0n]|BOLD:AAA5797
Apamea zeta|[13936]|RDNM536-05|Canada|Manitoba|658[0n]|BOLD:AAA5797
Apamea zeta|[13937]|RDNMD236-06|Canada|Manitoba|591[0n]|BOLD:AAA5797
Apamea zeta|[13938]|LCHP579-07|Canada|Manitoba|658[0n]|BOLD:AAA5797
Apamea zeta|[13939]|LCHP627-07|Canada|Manitoba|658[0n]|BOLD:AAA5797
Apamea zeta|[13940]|LCHP783-07|Canada|Manitoba|658[0n]|BOLD:AAA5797
Apamea zeta|[13941]|LCHP784-07|Canada|Manitoba|658[0n]|BOLD:AAA5797
Apamea zeta|[13942]|LCHP816-07|Canada|Manitoba|658[0n]|BOLD:AAA5797
Apamea zeta|[13943]|LCHP821-07|Canada|Manitoba|658[0n]|BOLD:AAA5797
Apamea zeta|[13944]|LCHP860-07|Canada|Manitoba|658[0n]|BOLD:AAA5797
Apamea zeta|[13945]|LCHP898-07|Canada|Manitoba|658[0n]|BOLD:AAA5797
Apamea zeta|[13946]|LCHQ115-07|Canada|Manitoba|658[0n]|BOLD:AAA5797
Apamea zeta|[13947]|RDNM537-05|Canada|Manitoba|584[0n]|BOLD:AAA5797
Apamea zeta|[13948]|RDNM529-05|Canada|Manitoba|579[0n]|BOLD:AAA5797
Apamea zeta|[13949]|RDNM527-05|Canada|Manitoba|582[0n]|BOLD:AAA5797
Apamea zeta|[13950]|LCHP846-07|Canada|Manitoba|655[0n]|BOLD:AAA5797
Apamea zeta|[13951]|LCHP847-07|Canada|Manitoba|658[0n]|BOLD:AAA5797
Apamea zeta|[13952]|LCHP849-07|Canada|Manitoba|658[0n]|BOLD:AAA5797
Apamea zeta|[13953]|LCHP850-07|Canada|Manitoba|658[0n]|BOLD:AAA5797
Apamea zeta|[13954]|LCHP853-07|Canada|Manitoba|658[0n]|BOLD:AAA5797
Apamea zeta|[13955]|LCHP858-07|Canada|Manitoba|658[0n]|BOLD:AAA5797
Apamea zeta|[13956]|LCHP859-07|Canada|Manitoba|658[0n]|BOLD:AAA5797
Apamea zeta|[13957]|LCHP861-07|Canada|Manitoba|658[0n]|BOLD:AAA5797
Apamea zeta|[13958]|LCHP838-07|Canada|Manitoba|658[0n]|BOLD:AAA5797
Apamea zeta|[13959]|LCHP844-07|Canada|Manitoba|657[0n]|BOLD:AAA5797
Apamea zeta|[13960]|LCHP635-07|Canada|Manitoba|658[0n]|BOLD:AAA5797
Apamea zeta|[13961]|LCHP837-07|Canada|Manitoba|658[0n]|BOLD:AAA5797
Apamea zeta|[13962]|LCHP625-07|Canada|Manitoba|658[0n]|BOLD:AAA5797
Apamea zeta|[13963]|LCHP632-07|Canada|Manitoba|658[0n]|BOLD:AAA5797
Apamea zeta|[13964]|LCHP526-07|Canada|Manitoba|658[0n]|BOLD:AAA5797
Apamea zeta|[13965]|LCHP580-07|Canada|Manitoba|658[0n]|BOLD:AAA5797
Apamea zeta|[13966]|LCHP415-07|Canada|Manitoba|658[0n]|BOLD:AAA5797
Apamea zeta|[13967]|LCHP420-07|Canada|Manitoba|658[0n]|BOLD:AAA5797
Apamea zeta|[13968]|LCHP410-07|Canada|Manitoba|658[0n]|BOLD:AAA5797
Apamea zeta|[13969]|LCHP414-07|Canada|Manitoba|658[0n]|BOLD:AAA5797
Apamea zeta|[13970]|LCHP384-07|Canada|Manitoba|658[0n]|BOLD:AAA5797
Apamea zeta|[13971]|LCHP395-07|Canada|Manitoba|658[0n]|BOLD:AAA5797
Apamea zeta|[13972]|LCHP260-07|Canada|Manitoba|658[0n]|BOLD:AAA5797
Apamea zeta|[13973]|LCHP277-07|Canada|Manitoba|656[0n]|BOLD:AAA5797
Apamea zeta|[13974]|LCHP216-07|Canada|Manitoba|658[0n]|BOLD:AAA5797
Apamea zeta|[13975]|LCHP217-07|Canada|Manitoba|658[0n]|BOLD:AAA5797
Apamea zeta|[13976]|LCHP209-07|Canada|Manitoba|658[0n]|BOLD:AAA5797
Apamea zeta|[13977]|LCHP215-07|Canada|Manitoba|658[0n]|BOLD:AAA5797
Apamea zeta|[13978]|LCHP005-07|Canada|Manitoba|658[0n]|BOLD:AAA5797
Apamea zeta|[13979]|LCHP208-07|Canada|Manitoba|658[0n]|BOLD:AAA5797
Apamea zeta|[13980]|RDNMD226-06|Canada|Manitoba|658[0n]|BOLD:AAA5797
Apamea zeta|[13981]|RDNME084-07|United States|Utah|658[0n]|BOLD:AAA5797
Apamea zeta|[13982]|RDNM525-05|Canada|Manitoba|658[0n]|BOLD:AAA5797
Apamea zeta|[13983]|RDNM538-05|Canada|Manitoba|658[0n]|BOLD:AAA5797
Apamea zeta|[13984]|RDNM524-05|Canada|Manitoba|658[0n]|BOLD:AAA5797
Apamea zeta|[13985]|RDNM523-05|Canada|Nunavut|658[0n]|BOLD:AAA5797
Apamea zeta|[13986]|LCH235-04|Canada|Manitoba|658[0n]|BOLD:AAA5797
Apamea zeta|[13987]|LCH234-04|Canada|Manitoba|658[0n]|BOLD:AAA5797
Apamea zeta|[13988]|RDNMD228-06|Canada|Manitoba|603[0n]|BOLD:AAA5797
Apamea zeta|[13989]|LCHP004-07|Canada|Manitoba|655[0n]|BOLD:AAA5797
Apamea zeta|[13990]|RDNMD237-06|Canada|Manitoba|583[0n]|BOLD:AAA5797
Apamea zeta|[13991]|LCHP854-07|Canada|Manitoba|658[0n]|BOLD:AAA5797
Apamea zeta|[13992]|LCHP855-07|Canada|Manitoba|658[0n]|BOLD:AAA5797

Apamea zeta|[13990]|RDNMD237-06|Canada|Manitoba|583[0n]|BOLD:AAA5797
Apamea zeta|[13991]|LCHP854-07|Canada|Manitoba|658[0n]|BOLD:AAA5797
Apamea zeta|[13992]|LCHP855-07|Canada|Manitoba|658[0n]|BOLD:AAA5797
Apamea zeta|[13993]|LCHP857-07|Canada|Manitoba|634[0n]|BOLD:AAA5797
Apamea zeta|[13994]|LCHQ170-07|Canada|Manitoba|656[0n]|BOLD:AAA5797
Apamea zeta|[13995]|LCHP887-07|Canada|Manitoba|658[0n]|BOLD:AAA5797
Apamea zeta|[13996]|LCHQ117-07|Canada|Manitoba|658[0n]|BOLD:AAA5797
Apamea zeta|[13997]|LCHQ168-07|Canada|Manitoba|658[0n]|BOLD:AAA5797
Apamea zeta|[13998]|CHLEP158-09|Canada|Manitoba|658[0n]|BOLD:AAA5797
Apamea rubrirena|[13999]|GWORL330-09|Germany|Bavaria|658[0n]|BOLD:ACE5999
Apamea rubrirena|[14000]|PHLAA271-09|Austria|Vorarlberg|658[0n]|BOLD:ACE5999
Apamea rubrirena|[14001]|LEATB378-13|Austria|Tirol|658[0n]|BOLD:ACE5999
Apamea rubrirena|[14002]|LEATB452-13|Austria|Tirol|658[0n]|BOLD:ACE5999
Apamea rubrirena|[14003]|PHLAC438-10|Italy|South Tyrol|631[0n]|BOLD:ACE5999
Apamea rubrirena|[14004]|GWORO833-09|Germany|Bavaria|658[0n]|BOLD:ACE5999
Apamea rubrirena|[14005]|NOCJH064-09|France||658[0n]|BOLD:ACE5999
Apamea rubrirena|[14006]|LEFID212-10|Finland||658[0n]|BOLD:ACE5999
Apamea rubrirena|[14007]|LEFIF547-10|Finland||658[0n]|BOLD:ACE5999
Apamea rubrirena|[14008]|RDNMC499-06|Finland|Uusimaa|658[0n]|BOLD:ACE5999
Apamea rubrirena|[14009]|LEFIK457-10|Finland||658[0n]|BOLD:ACE5999
Apamea rubrirena|[14010]|NOCJH471-11|France||658[0n]|BOLD:ACE5999
Apamea rubrirena|[14011]|NOCJH480-11|France||658[0n]|BOLD:ACE5999
Apamea rubrirena|[14012]|GWOSP002-11|Italy|Lazio|658[0n]|BOLD:ACE5999
Apamea rubrirena|[14013]|GWOSP003-11|Italy|Lazio|658[0n]|BOLD:ACE5999
Apamea rubrirena|[14014]|PHLAE655-11|Italy||658[0n]|BOLD:ACE5999
Apamea rubrirena|[14015]|PHLAE657-11|Macedonia||658[0n]|BOLD:ACE5999
Apamea rubrirena|[14016]|PHLAF288-11|Macedonia||658[0n]|BOLD:ACE5999
Apamea rubrirena|[14017]|PHLSA432-11|Italy||658[0n]|BOLD:ACE5999
Apamea rubrirena|[14018]|LEATB578-13|Italy|South Tyrol|658[0n]|BOLD:ACE5999
Loscopia velata|[14019]|LSEU657-06|United States|Georgia|658[0n]|BOLD:AAB5969
Loscopia velata|[14020]|LGSM680-04|United States|North Carolina|658[0n]|BOLD:AAB5969
Loscopia velata|[14021]|LGSM776-04|United States|North Carolina|658[0n]|BOLD:AAB5969
Loscopia velata|[14022]|XAJ852-06|Canada|Ontario|658[0n]|BOLD:AAB5969
Loscopia velata|[14023]|LSEU656-06|United States|Georgia|658[0n]|BOLD:AAB5969
Loscopia velata|[14024]|RDNMD776-07|United States|North Carolina|658[0n]|BOLD:AAB5969
Loscopia velata|[14025]|PHSEP340-11|Canada|Ontario|657[0n]|BOLD:AAB5969
Loscopia velata|[14026]|RDNMD775-07|United States|North Carolina|658[0n]|BOLD:AAB5969
Loscopia velata|[14027]|MNBB526-05|Canada|New Brunswick|658[0n]|BOLD:AAB5969
Loscopia velata|[14028]|MNBB525-05|Canada|New Brunswick|658[0n]|BOLD:AAB5969
Loscopia velata|[14029]|MNBB076-05|Canada|New Brunswick|658[0n]|BOLD:AAB5969
Loscopia velata|[14030]|XAC849-04|Canada|Ontario|658[0n]|BOLD:AAB5969
Loscopia velata|[14031]|LGSM491-04|United States|North Carolina|658[0n]|BOLD:AAB5969
Loscopia velata|[14032]|RDLQB648-05|Canada|Quebec|601[0n]|BOLD:AAB5969
Loscopia velata|[14033]|RDLQ472-07|Canada|Quebec|585[0n]|BOLD:AAB5969
Loscopia velata|[14034]|BLTIB913-08|Canada|Ontario|658[0n]|BOLD:AAB5969
Loscopia velata|[14035]|BLTIB1125-08|Canada|Ontario|658[0n]|BOLD:AAB5969
Loscopia velata|[14036]|BBLEC544-09|Canada|Nova Scotia|658[0n]|BOLD:AAB5969
Loscopia velata|[14037]|BBLPC112-09|Canada|New Brunswick|658[0n]|BOLD:AAB5969
Loscopia velata|[14038]|BBLPB572-10|Canada|Saskatchewan|658[0n]|BOLD:AAB5969
Loscopia velata|[14039]|HEJUL2101-12|Canada|Ontario|658[0n]|BOLD:AAB5969
Loscopia velata|[14040]|RDLQ465-07|Canada|Quebec|585[0n]|BOLD:AAB5969
Loscopia velata|[14041]|BLTIB532-08|Canada|Ontario|658[0n]|BOLD:AAB5969
Loscopia velata|[14042]|SSPAG165-13|Canada|Saskatchewan|541[0n]|BOLD:AAB5969
Loscopia velata|[14043]|HPPPE1562-13|Canada|Nova Scotia|554[0n]|BOLD:AAB5969
Apamea fergusoni|[14044]|RDNMD142-06|United States|Colorado|658[0n]|BOLD:AAW5928
Apamea spaldingi|[14045]|RDNME939-08|United States|Oregon|609[0n]|BOLD:AAD3063
Apamea spaldingi|[14046]|LBCH5040-10|Canada|British Columbia|658[0n]|BOLD:AAD3063
Apamea spaldingi|[14047]|RDNM961-05|United States|Oregon|658[0n]|BOLD:AAD3063
Apamea spaldingi|[14048]|RDNM960-05|United States|Oregon|658[0n]|BOLD:AAD3063
Apamea spaldingi|[14049]|RDNM948-05|United States|Oregon|658[0n]|BOLD:AAD3063
Apamea spaldingi|[14050]|RDMAB487-06|Canada|Alberta|634[0n]|BOLD:AAD3063
Apamea spaldingi|[14051]|RDNMD150-06|United States|Colorado|654[0n]|BOLD:AAD3063
Apamea spaldingi|[14052]|RDNMD151-06|United States|Colorado|658[0n]|BOLD:AAD3063
Apamea spaldingi|[14053]|LBCH5041-10|Canada|British Columbia|658[0n]|BOLD:AAD3063
Apamea spaldingi|[14054]|LBCH5042-10|Canada|British Columbia|658[0n]|BOLD:AAD3063
Apamea spaldingi|[14055]|LBCH5043-10|Canada|British Columbia|658[0n]|BOLD:AAD3063
Apamea spaldingi|[14056]|LBCH5044-10|Canada|British Columbia|658[0n]|BOLD:AAD3063
Apamea spaldingi|[14057]|LBCH5045-10|Canada|British Columbia|658[0n]|BOLD:AAD3063
Apamea spaldingi|[14058]|LBCH5046-10|Canada|British Columbia|658[0n]|BOLD:AAD3063
Apamea spaldingi|[14059]|LBCH5047-10|Canada|British Columbia|658[0n]|BOLD:AAD3063
Apamea spaldingi|[14060]|LBCH5283-10|Canada|British Columbia|658[0n]|BOLD:AAD3063
Apamea spaldingi|[14061]|LBCH5348-10|Canada|British Columbia|658[0n]|BOLD:AAD3063
Apamea spaldingi|[14062]|LBCH5421-10|Canada|British Columbia|658[0n]|BOLD:AAD3063
Apamea spaldingi|[14063]|LBCH5517-10|Canada|British Columbia|658[0n]|BOLD:AAD3063
Apamea spaldingi|[14064]|JMMMB394-11|United States|California|658[0n]|BOLD:AAD3063
Apamea cinefacta|[14065]|GMLC409-11|United States|California|658[0n]|BOLD:AAD3412
Apamea cinefacta|[14066]|GMLC528-11|United States|California|658[0n]|BOLD:AAD3412
Apamea cinefacta|[14067]|IAWLB236-11|United States|California|658[0n]|BOLD:AAD3412
Apamea cinefacta|[14068]|GMLC411-11|United States|California|658[0n]|BOLD:AAD3412
Apamea cinefacta|[14069]|GMLC395-11|United States|California|658[0n]|BOLD:AAD3412
Apamea cinefacta|[14070]|GMLC392-11|United States|California|658[0n]|BOLD:AAD3412
Apamea cinefacta|[14071]|GMLC237-11|United States|California|658[0n]|BOLD:AAD3412
Apamea cinefacta|[14072]|LOCB641-06|United States|California|658[0n]|BOLD:AAD3412
Apamea cinefacta|[14073]|RDNMC680-06|United States|California|658[0n]|BOLD:AAD3412
Apamea cinefacta|[14074]|RDNM935-05|United States|Oregon|658[0n]|BOLD:AAD3412
Apamea cinefacta|[14075]|RDNM963-05|United States|California|658[0n]|BOLD:AAD3412
Apamea cinefacta|[14076]|GMLC384-11|United States|California|644[0n]|BOLD:AAD3412
Apamea cinefacta|[14077]|RDNM068-05|United States|California|614[0n]|BOLD:AAD3412
Apamea cinefacta|[14078]|RDNM067-05|United States|Oregon|624[0n]|BOLD:AAD3412
Apamea cinefacta|[14079]|RDNM962-05|Canada|British Columbia|558[0n]|BOLD:AAD3412
Apamea cinefacta|[14080]|GMLC431-11|United States|California|632[0n]|BOLD:AAD3412
Apamea cinefacta|[14081]|GMLC463-11|United States|California|658[0n]|BOLD:AAD3412
Apamea cinefacta|[14082]|IAWLB237-11|United States|California|658[0n]|BOLD:AAD3412
Apamea cinefacta|[14083]|GMLC1403-12|United States|California|601[0n]|BOLD:AAD3412
Apamea inordinata|[14084]|RDNMD141-06|United States|Colorado|597[0n]|BOLD:AAC7156
Apamea inordinata|[14085]|RDNME608-08|United States|Washington|658[0n]|BOLD:AAC7156
Apamea inordinata|[14086]|RDNME660-08|United States|Washington|658[0n]|BOLD:AAC7156
Apamea inordinata|[14087]|LBCH5257-10|Canada|British Columbia|658[0n]|BOLD:AAC7156
Apamea inordinata|[14088]|LBCH5256-10|Canada|British Columbia|658[0n]|BOLD:AAC7156
Apamea inordinata|[14089]|LBCH5050-10|Canada|British Columbia|658[0n]|BOLD:AAC7156
Apamea inordinata|[14090]|LBCH5049-10|Canada|British Columbia|658[0n]|BOLD:AAC7156
Apamea inordinata|[14091]|LBCH5048-10|Canada|British Columbia|658[0n]|BOLD:AAC7156
Apamea inordinata|[14092]|LBCG342-08|Canada|British Columbia|658[0n]|BOLD:AAC7156

Apamea inordinata|[14090]|LBCH5049-10|Canada|British Columbia|658[0n]|BOLD:AAC7156
Apamea inordinata|[14091]|LBCH5048-10|Canada|British Columbia|658[0n]|BOLD:AAC7156
Apamea inordinata|[14092]|LBCG342-08|Canada|British Columbia|658[0n]|BOLD:AAC7156
Apamea inordinata|[14093]|LBCG340-08|Canada|British Columbia|658[0n]|BOLD:AAC7156
Apamea inordinata|[14094]|RDNMD241-06|Canada|Ontario|658[0n]|BOLD:AAC7156
Apamea inordinata|[14095]|RDNMD240-06|Canada|Ontario|658[0n]|BOLD:AAC7156
Apamea inordinata|[14096]|RDNMD238-06|United States|Oregon|658[0n]|BOLD:AAC7156
Apamea inordinata|[14097]|RDMAB446-05|Canada|Alberta|658[0n]|BOLD:AAC7156
Apamea inordinata|[14098]|LBCH5258-10|Canada|British Columbia|642[0n]|BOLD:AAC7156
Apamea inordinata|[14099]|RDNMD239-06|United States|Washington|618[0n]|BOLD:AAC7156
Apamea inordinata|[14100]|LBCH5259-10|Canada|British Columbia|641[0n]|BOLD:AAC7156
Apamea inordinata|[14101]|LBCH5260-10|Canada|British Columbia|658[0n]|BOLD:AAC7156
Apamea inordinata|[14102]|LBCH5261-10|Canada|British Columbia|658[0n]|BOLD:AAC7156
Apamea inordinata|[14103]|LBCH5262-10|Canada|British Columbia|658[0n]|BOLD:AAC7156
Apamea inordinata|[14104]|LBCH5263-10|Canada|British Columbia|658[0n]|BOLD:AAC7156
Apamea inordinata|[14105]|LBCH5349-10|Canada|British Columbia|658[0n]|BOLD:AAC7156
Apamea inordinata|[14106]|LBCH5350-10|Canada|British Columbia|658[0n]|BOLD:AAC7156
Apamea inordinata|[14107]|LBCH5351-10|Canada|British Columbia|658[0n]|BOLD:AAC7156
Apamea inordinata|[14108]|LBCH5352-10|Canada|British Columbia|658[0n]|BOLD:AAC7156
Apamea inordinata|[14109]|LBCH5353-10|Canada|British Columbia|658[0n]|BOLD:AAC7156
Apamea inordinata|[14110]|LBCH5354-10|Canada|British Columbia|658[0n]|BOLD:AAC7156
Apamea inordinata|[14111]|LBCH5355-10|Canada|British Columbia|658[0n]|BOLD:AAC7156
Apamea inordinata|[14112]|LBCH5356-10|Canada|British Columbia|658[0n]|BOLD:AAC7156
Apamea inordinata|[14113]|LNAUT2644-14|United States|Massachusetts|658[1n]|BOLD:ACR7361
Apamea inordinata|[14114]|LNAUT2645-14|United States|Massachusetts|658[0n]|BOLD:ACR7361
Protapamea danieli|[14115]|HKONS519-08|United States|Florida|658[2n]|BOLD:AAD5877
Protapamea danieli|[14116]|HKONS749-08|United States|Florida|658[0n]|BOLD:AAD5877
Protapamea danieli|[14117]|RDNMD753-07|United States|Illinois|658[0n]|BOLD:AAD5877
Protapamea danieli|[14118]|RDNMD754-07|United States|Virginia|658[0n]|BOLD:AAD5877
Protapamea danieli|[14119]|HKONS520-08|United States|Florida|658[0n]|BOLD:AAD5877
Protapamea danieli|[14120]|HKONS750-08|United States|Florida|658[0n]|BOLD:AAD5877
Protapamea danieli|[14121]|MILEP095-09|United States|North Carolina|658[0n]|BOLD:AAD5877
Protapamea danieli|[14122]|LNCC097-10|United States|North Carolina|658[0n]|BOLD:AAD5877
Protapamea danieli|[14123]|LNCC138-10|United States|North Carolina|658[0n]|BOLD:AAD5877
Protapamea danieli|[14124]|LNCC743-11|United States|North Carolina|658[0n]|BOLD:AAD5877
Protapamea danieli|[14125]|LNCC744-11|United States|North Carolina|658[0n]|BOLD:AAD5877
Protapamea danieli|[14126]|LNCC745-11|United States|North Carolina|658[0n]|BOLD:AAD5877
Protapamea danieli|[14127]|LNCC926-11|United States|North Carolina|658[0n]|BOLD:AAD5877
Protapamea danieli|[14128]|LNCC927-11|United States|North Carolina|658[0n]|BOLD:AAD5877
Protapamea louisae|[14129]|RDNMD755-07|United States|Tennessee|658[0n]|BOLD:AAJ1410
Protapamea louisae|[14130]|RDNMD756-07|United States|Missouri|658[0n]|BOLD:AAJ1410
Protapamea louisae|[14131]|RDNML014-13|United States|Louisiana|658[0n]|BOLD:AAJ1410
Neoligia lancea|[14132]|RDNMF174-08|Canada|British Columbia|658[0n]|BOLD:AAD9222
Neoligia lancea|[14133]|RDNMF175-08|United States|Washington|658[0n]|BOLD:AAD9222
Neoligia lancea|[14134]|RDNMF178-08|United States|Washington|645[1n]|BOLD:AAD9222
Neoligia lancea|[14135]|RDNMF177-08|United States|Washington|646[0n]|BOLD:AAD9222
Neoligia lancea|[14136]|RDNMF176-08|United States|Washington|647[0n]|BOLD:AAD9222
Neoligia lancea|[14137]|RDNMF180-08|United States|Washington|658[0n]|BOLD:AAD9222
Neoligia lancea|[14138]|LBCH7353-10|Canada|British Columbia|658[0n]|BOLD:AAD9222
Neoligia albirena|[14139]|RDNMF163-08|Canada|British Columbia|658[0n]|BOLD:ABX4938
Neoligia albirena|[14140]|RDNMF165-08|Canada|British Columbia|658[0n]|BOLD:ABX4938
Neoligia albirena|[14141]|RDNMF166-08|Canada|British Columbia|647[0n]|BOLD:ABX4938
Neoligia albirena|[14142]|RDNMF167-08|United States|Nevada|657[0n]|BOLD:ABX4938
Neoligia albirena|[14143]|RDNMF164-08|United States|Nevada|658[0n]|BOLD:ABX4938
Neoligia albirena|[14144]|RDNMF185-08|United States|California|658[0n]|BOLD:ABX4938
Neoligia lillooet|[14145]|RDNMF179-08|Canada|British Columbia|645[0n]|BOLD:AAD8644
Neoligia lillooet|[14146]|LBCH7559-10|Canada|British Columbia|658[0n]|BOLD:AAD8644
Neoligia lillooet|[14147]|LOWCC626-05|Canada|British Columbia|658[0n]|BOLD:AAD8644
Neoligia lillooet|[14148]|LOWCC627-05|Canada|British Columbia|596[0n]|BOLD:AAD8644
Neoligia lillooet|[14149]|RDNMF366-08|Canada|Alberta|657[0n]|BOLD:AAD8644
Neoligia lillooet|[14150]|RDNMF367-08|Canada|Alberta|658[0n]|BOLD:AAD8644
Neoligia lillooet|[14151]|RDNMF368-08|Canada|Alberta|658[0n]|BOLD:AAD8644
Neoligia lillooet|[14152]|LBCH7953-10|Canada|British Columbia|658[0n]|BOLD:AAD8644
Neoligia elephas|[14153]|RDNMH584-09|United States|Arizona|658[0n]|BOLD:ACF5557
Neoligia elephas|[14154]|RDNMJ525-11|United States|Arizona|658[0n]|BOLD:ACF5557
Neoligia elephas|[14155]|RDNMK569-11|United States|Arizona|658[0n]|BOLD:ACF5557
Neoligia elephas|[14156]|RDNML194-13|United States|Arizona|658[0n]|BOLD:ACF5557
Neoligia invenusta|[14157]|LBCH7560-10|Canada|British Columbia|658[0n]|BOLD:AAD8668
Neoligia invenusta|[14158]|RDNMF172-08|United States|California|658[0n]|BOLD:AAD8668
Neoligia invenusta|[14159]|LBCG1388-09|Canada|British Columbia|658[0n]|BOLD:AAD8668
Neoligia invenusta|[14160]|LBCH6165-10|Canada|British Columbia|658[0n]|BOLD:AAD8668
Neoligia invenusta|[14161]|LBCH6383-10|Canada|British Columbia|658[0n]|BOLD:AAD8668
Neoligia invenusta|[14162]|LBCH6385-10|Canada|British Columbia|658[0n]|BOLD:AAD8668
Neoligia invenusta|[14163]|RDNMF171-08|Canada|British Columbia|658[0n]|BOLD:AAD8668
Neoligia invenusta|[14164]|RDNMF173-08|Canada|British Columbia|658[0n]|BOLD:AAD8668
Neoligia invenusta|[14165]|LBCH6725-10|Canada|British Columbia|658[0n]|BOLD:AAD8668
Neoligia invenusta|[14166]|LBCH7664-10|Canada|British Columbia|658[0n]|BOLD:AAD8668
Neoligia invenusta|[14167]|LALPA675-10|Canada|British Columbia|658[0n]|BOLD:AAD8668
Neoligia invenusta|[14168]|LALPA730-10|Canada|British Columbia|658[0n]|BOLD:AAD8668
Neoligia invenusta|[14169]|RDNMF169-08|Canada|British Columbia|658[0n]|BOLD:AAD8668
Neoligia invenusta|[14170]|RDNMF170-08|Canada|British Columbia|658[0n]|BOLD:AAD8668
Neoligia invenusta|[14171]|LALPA755-10|Canada|British Columbia|658[0n]|BOLD:AAD8668
Neoligia invenusta|[14172]|JMMMB515-13|United States|California|549[0n]|BOLD:AAD8668
Neoligia tonsa|[14173]|RDNMF183-08|Canada|British Columbia|658[0n]|BOLD:AAE1791
Neoligia tonsa|[14174]|LBCH7662-10|Canada|British Columbia|658[0n]|BOLD:AAE1791
Neoligia tonsa|[14175]|RDNMF182-08|Canada|British Columbia|658[0n]|BOLD:AAE1791
Neoligia tonsa|[14176]|LBCG1401-09|Canada|British Columbia|658[0n]|BOLD:AAE1791
Neoligia tonsa|[14177]|LBCH7660-10|Canada|British Columbia|658[0n]|BOLD:AAE1791
Neoligia tonsa|[14178]|LBCH6556-10|Canada|British Columbia|658[0n]|BOLD:AAE1791
Neoligia tonsa|[14179]|LBCH6727-10|Canada|British Columbia|658[0n]|BOLD:AAE1791
Neoligia tonsa|[14180]|LBCH7659-10|Canada|British Columbia|658[0n]|BOLD:AAE1791
Neoligia tonsa|[14181]|LBCH7663-10|Canada|British Columbia|658[0n]|BOLD:AAE1791
Neoligia tonsa|[14182]|LBCH7666-10|Canada|British Columbia|658[0n]|BOLD:AAE1791
Neoligia tonsa|[14183]|LBCH7823-10|Canada|British Columbia|658[0n]|BOLD:AAE1791
Neoligia tonsa|[14184]|LBCH6558-10|Canada|British Columbia|658[0n]|BOLD:AAE1791
Neoligia tonsa|[14185]|LBCH6387-10|Canada|British Columbia|658[0n]|BOLD:AAE1791
Neoligia tonsa|[14186]|RDNMF181-08|Canada|British Columbia|658[0n]|BOLD:AAE1791
Neoligia tonsa|[14187]|RDNMF184-08|Canada|British Columbia|632[0n]|BOLD:AAE1791
Neoligia tonsa|[14188]|LBCH7661-10|Canada|British Columbia|636[0n]|BOLD:AAE1791
Neoligia tonsa|[14189]|LBCH7665-10|Canada|British Columbia|658[0n]|BOLD:AAE1791
Neoligia tonsa|[14190]|LBCH7824-10|Canada|British Columbia|658[0n]|BOLD:AAE1791
Neoligia tonsa|[14191]|IAWLB081-10|United States|California|658[0n]|BOLD:AAE1791
Neoligia tonsa|[14192]|IAWLB172-10|United States|California|658[0n]|BOLD:AAE1791

Neoligia tonsa|[14190]|LBCH7824-10|Canada|British Columbia|658[0n]|BOLD:AAE1791
Neoligia tonsa|[14191]|IAWLB081-10|United States|California|658[0n]|BOLD:AAE1791
Neoligia tonsa|[14192]|IAWLB172-10|United States|California|658[0n]|BOLD:AAE1791
Neoligia tonsa|[14193]|LPOKE301-11|United States|Oklahoma|658[0n]|BOLD:AAE1791
Amphipoea pacifica|[14194]|RDNMK563-11|United States|Oregon|609[0n]|BOLD:ABU6210
Amphipoea pacifica|[14195]|GMLC1170-12|United States|California|658[0n]|BOLD:ABU6210
Amphipoea americana|[14196]|RDLQ469-07|Canada|Quebec|596[3n]|BOLD:AAC0644
Amphipoea americana|[14197]|LGSMG972-10|United States|North Carolina|658[0n]|BOLD:AAC0644
Amphipoea americana|[14198]|BLTIB1044-08|Canada|Ontario|658[0n]|BOLD:AAC0644
Amphipoea americana|[14199]|LGSMG976-10|United States|North Carolina|658[1n]|BOLD:AAC0644
Amphipoea americana|[14200]|BBLEC709-09|Canada|Nova Scotia|658[0n]|BOLD:AAC0644
Amphipoea americana|[14201]|TTMNB360-06|Canada|New Brunswick|658[1n]|BOLD:AAC0644
Amphipoea americana|[14202]|BBLPB630-10|Canada|Saskatchewan|658[0n]|BOLD:AAC0644
Amphipoea americana|[14203]|BBLPB629-10|Canada|Saskatchewan|658[0n]|BOLD:AAC0644
Amphipoea americana|[14204]|BBLPB628-10|Canada|Alberta|658[0n]|BOLD:AAC0644
Amphipoea americana|[14205]|BBLPB627-10|Canada|Alberta|658[0n]|BOLD:AAC0644
Amphipoea americana|[14206]|BBLPB626-10|Canada|Alberta|658[0n]|BOLD:AAC0644
Amphipoea americana|[14207]|BBLPB625-10|Canada|Alberta|658[0n]|BOLD:AAC0644
Amphipoea americana|[14208]|BBLPB624-10|Canada|Alberta|658[0n]|BOLD:AAC0644
Amphipoea americana|[14209]|LNCC348-10|United States|North Carolina|658[0n]|BOLD:AAC0644
Amphipoea americana|[14210]|LNCC330-10|United States|North Carolina|658[0n]|BOLD:AAC0644
Amphipoea americana|[14211]|LGSMG981-10|United States|North Carolina|658[0n]|BOLD:AAC0644
Amphipoea americana|[14212]|LGSMG980-10|United States|North Carolina|658[0n]|BOLD:AAC0644
Amphipoea americana|[14213]|LGSMG979-10|United States|North Carolina|658[0n]|BOLD:AAC0644
Amphipoea americana|[14214]|LGSMG978-10|United States|North Carolina|658[0n]|BOLD:AAC0644
Amphipoea americana|[14215]|LGSMG977-10|United States|North Carolina|658[0n]|BOLD:AAC0644
Amphipoea americana|[14216]|LGSMG975-10|United States|North Carolina|658[0n]|BOLD:AAC0644
Amphipoea americana|[14217]|LGSMG974-10|United States|North Carolina|658[0n]|BOLD:AAC0644
Amphipoea americana|[14218]|LGSMG973-10|United States|North Carolina|658[0n]|BOLD:AAC0644
Amphipoea americana|[14219]|BBLEC692-09|Canada|Nova Scotia|658[0n]|BOLD:AAC0644
Amphipoea americana|[14220]|XAK415-06|Canada|Ontario|658[0n]|BOLD:AAC0644
Amphipoea americana|[14221]|XAK339-06|Canada|Ontario|658[0n]|BOLD:AAC0644
Amphipoea americana|[14222]|LSEU750-06|United States|Georgia|658[0n]|BOLD:AAC0644
Amphipoea americana|[14223]|XAJ984-06|Canada|Ontario|657[0n]|BOLD:AAC0644
Amphipoea americana|[14224]|TMNBB186-06|Canada|New Brunswick|658[0n]|BOLD:AAC0644
Amphipoea americana|[14225]|TTMNB359-06|Canada|New Brunswick|658[0n]|BOLD:AAC0644
Amphipoea americana|[14226]|TTMNB356-06|Canada|New Brunswick|658[0n]|BOLD:AAC0644
Amphipoea americana|[14227]|LOWCD178-06|Canada|British Columbia|658[0n]|BOLD:AAC0644
Amphipoea americana|[14228]|RDLQB724-05|Canada|Quebec|658[0n]|BOLD:AAC0644
Amphipoea americana|[14229]|BBLPB632-10|Canada|Saskatchewan|658[0n]|BOLD:AAC0644
Amphipoea americana|[14230]|XAH323-05|Canada|Ontario|658[0n]|BOLD:AAC0644
Amphipoea americana|[14231]|MNBB664-05|Canada|New Brunswick|658[0n]|BOLD:AAC0644
Amphipoea americana|[14232]|BBLEC137-09|Canada|Nova Scotia|658[0n]|BOLD:AAC0644
Amphipoea americana|[14233]|BBLPB631-10|Canada|Saskatchewan|658[0n]|BOLD:AAC0644
Amphipoea americana|[14234]|TTMNB357-06|Canada|New Brunswick|599[0n]|BOLD:AAC0644
Amphipoea americana|[14235]|XAD304-04|Canada|Ontario|584[0n]|BOLD:AAC0644
Amphipoea americana|[14236]|RDLQ470-07|Canada|Quebec|608[0n]|BOLD:AAC0644
Amphipoea americana|[14237]|TTMNB358-06|Canada|New Brunswick|611[0n]|BOLD:AAC0644
Amphipoea americana|[14238]|PAJUL2050-12|Canada|Ontario|561[0n]|BOLD:AAC0644
Amphipoea americana|[14239]|LNAUT837-14|United States|Indiana|658[0n]|BOLD:AAC0644
Amphipoea americana|[14240]|LNAUT841-14|United States|Wisconsin|658[0n]|BOLD:AAC0644
Amphipoea cottlei|[14241]|NAMUM194-08|United States|California|647[0n]|BOLD:AAK1208
Amphipoea keiferi|[14242]|CNCLB1110-14|United States|Oregon|658[0n]|BOLD:AAK1208
Amphipoea keiferi|[14243]|CNCLB1111-14|United States|Oregon|658[0n]|BOLD:AAK1208
Amphipoea keiferi|[14244]|CNCLB1112-14|United States|Oregon|658[0n]|BOLD:AAK1208
Amphipoea keiferi|[14245]|CNCLB1114-14|United States|Oregon|658[0n]|BOLD:AAK1208
Amphipoea interoceanica|[14246]|RWWB168-09|United States|Washington|658[0n]|BOLD:ABZ0147
Amphipoea interoceanica|[14247]|RDNML375-13|Canada|Ontario|658[0n]|BOLD:ABZ0147
Amphipoea interoceanica|[14248]|TTMNB355-06|Canada|New Brunswick|658[0n]|BOLD:ABZ0147
Amphipoea interoceanica|[14249]|LSEU749-06|United States|Georgia|658[0n]|BOLD:ABZ0147
Amphipoea interoceanica|[14250]|BBLEC699-09|Canada|Nova Scotia|658[0n]|BOLD:ABZ0147
Amphipoea interoceanica|[14251]|RDNML376-13|Canada|Alberta|658[0n]|BOLD:ABZ0147
Amphipoea interoceanica|[14252]|CNCLA548-13|Canada|Ontario|658[0n]|BOLD:ABZ0147
Amphipoea interoceanica|[14253]|CNCLA549-13|Canada|Ontario|658[0n]|BOLD:ABZ0147
Amphipoea interoceanica|[14254]|CNCLA550-13|Canada|Ontario|658[0n]|BOLD:ABZ0147
Amphipoea interoceanica|[14255]|CNCLA551-13|Canada|Ontario|658[0n]|BOLD:ABZ0147
Amphipoea interoceanica|[14256]|CNCLA552-13|Canada|Ontario|658[0n]|BOLD:ABZ0147
Amphipoea interoceanica|[14257]|LNAUT838-14|United States|Indiana|658[0n]|BOLD:ABZ0147
Amphipoea interoceanica|[14258]|LNAUT839-14|United States|Indiana|658[0n]|BOLD:ABZ0147
Amphipoea interoceanica|[14259]|LNAUT840-14|United States|Indiana|658[0n]|BOLD:ABZ0147
Amphipoea senilis|[14260]|RDNMD491-06|United States|Colorado|658[0n]|BOLD:AAK1201
Amphipoea senilis|[14261]|LNAUT842-14|United States|New Mexico|658[0n]|BOLD:AAK1201
Amphipoea senilis|[14262]|LNAUT844-14|United States|New Mexico|658[0n]|BOLD:AAK1201
Amphipoea senilis|[14263]|LNAUT845-14|United States|New Mexico|658[0n]|BOLD:AAK1201
Amphipoea senilis|[14264]|LNAUT846-14|United States|New Mexico|658[0n]|BOLD:AAK1201
Neoligia semicana|[14265]|RDNMG948-08|Canada|Ontario|658[0n]|BOLD:AAG4393
Neoligia semicana|[14266]|RDNMG949-08|Canada|Ontario|658[0n]|BOLD:AAG4393
Neoligia canadensis|[14267]|RDNM097-05|Canada|Ontario|658[0n]|BOLD:AAE1829
Neoligia canadensis|[14268]|RDNM096-05|Canada|Ontario|658[0n]|BOLD:AAE1829
Neoligia canadensis|[14269]|RDLQB414-05|Canada|Quebec|658[0n]|BOLD:AAE1829
Neoligia canadensis|[14270]|RDNME639-08|Canada|Quebec|658[0n]|BOLD:AAE1829
Neoligia canadensis|[14271]|RDNMG950-08|Canada|Ontario|658[0n]|BOLD:AAE1829
Neoligia atlantica|[14272]|RDNMF168-08|Canada|Nova Scotia|632[0n]|BOLD:AAK7888
Neoligia atlantica|[14273]|BBLPC712-09|Canada|Newfoundland and Labrador|658[0n]|BOLD:AAK7888
Neoligia inermis|[14274]|RDNML193-13|United States|Arizona|658[0n]|BOLD:ACD9275
Neoligia inermis|[14275]|RDNML195-13|United States|Arizona|658[0n]|BOLD:ACD9275
Neoligia inermis|[14276]|RDNML196-13|United States|Arizona|658[0n]|BOLD:ACD9275
Neoligia subjuncta|[14277]|LBCH1335-10|Canada|British Columbia|658[0n]|BOLD:AAA8279
Neoligia subjuncta|[14278]|LOWCD530-06|Canada|British Columbia|658[0n]|BOLD:AAA8279
Neoligia subjuncta|[14279]|BLTIB542-08|Canada|Ontario|658[0n]|BOLD:AAA8279
Neoligia subjuncta|[14280]|BLTIB498-08|Canada|Ontario|658[0n]|BOLD:AAA8279
Neoligia subjuncta|[14281]|BLTIB455-08|Canada|Ontario|658[0n]|BOLD:AAA8279
Neoligia subjuncta|[14282]|LPSK262-08|Canada|Saskatchewan|658[0n]|BOLD:AAA8279
Neoligia subjuncta|[14283]|LPMN070-08|Canada|Manitoba|658[0n]|BOLD:AAA8279
Neoligia subjuncta|[14284]|RDLQG807-06|Canada|Quebec|658[0n]|BOLD:AAA8279
Neoligia subjuncta|[14285]|RDLQG795-06|Canada|Quebec|658[0n]|BOLD:AAA8279
Neoligia subjuncta|[14286]|RDLQG788-06|Canada|Quebec|658[0n]|BOLD:AAA8279
Neoligia subjuncta|[14287]|RDLQG773-06|Canada|Quebec|658[0n]|BOLD:AAA8279
Neoligia subjuncta|[14288]|RDLQB411-05|Canada|Quebec|658[0n]|BOLD:AAA8279
Neoligia subjuncta|[14289]|RDLQB398-05|Canada|Quebec|658[0n]|BOLD:AAA8279
Neoligia subjuncta|[14290]|RDLQB397-05|Canada|Quebec|658[0n]|BOLD:AAA8279
Neoligia subjuncta|[14291]|LOWCB521-05|Canada|British Columbia|658[0n]|BOLD:AAA8279
Neoligia subjuncta|[14292]|LBCC241-05|Canada|British Columbia|658[0n]|BOLD:AAA8279

Neoligia subjuncta|[14290]|RDLQB397-05|Canada|Quebec|658[0n]|BOLD:AAA8279
Neoligia subjuncta|[14291]|LOWCB521-05|Canada|British Columbia|658[0n]|BOLD:AAA8279
Neoligia subjuncta|[14292]|LBCC241-05|Canada|British Columbia|658[0n]|BOLD:AAA8279
Neoligia subjuncta|[14293]|MNBB083-05|Canada|New Brunswick|658[0n]|BOLD:AAA8279
Neoligia subjuncta|[14294]|XAC710-04|Canada|Ontario|658[0n]|BOLD:AAA8279
Neoligia subjuncta|[14295]|XAB131-04|Canada|Ontario|658[0n]|BOLD:AAA8279
Neoligia subjuncta|[14296]|XAB122-04|Canada|Ontario|658[0n]|BOLD:AAA8279
Neoligia subjuncta|[14297]|RDLQG806-06|Canada|Quebec|658[0n]|BOLD:AAA8279
Neoligia subjuncta|[14298]|RDLQB396-05|Canada|Quebec|658[0n]|BOLD:AAA8279
Neoligia subjuncta|[14299]|RDMAB078-05|Canada|Alberta|622[0n]|BOLD:AAA8279
Neoligia subjuncta|[14300]|LOWCB515-05|Canada|British Columbia|610[0n]|BOLD:AAA8279
Neoligia subjuncta|[14301]|LOWCB514-05|Canada|British Columbia|615[0n]|BOLD:AAA8279
Neoligia subjuncta|[14302]|PMG140-03|Canada|Ontario|617[0n]|BOLD:AAA8279
Neoligia subjuncta|[14303]|LOWCB513-05|Canada|British Columbia|605[0n]|BOLD:AAA8279
Neoligia subjuncta|[14304]|LOWCB517-05|Canada|British Columbia|608[0n]|BOLD:AAA8279
Neoligia subjuncta|[14305]|BLTIB615-08|Canada|Ontario|631[0n]|BOLD:AAA8279
Neoligia subjuncta|[14306]|LPMNB420-09|Canada|Manitoba|658[0n]|BOLD:AAA8279
Neoligia subjuncta|[14307]|LPABC359-09|Canada|Alberta|658[0n]|BOLD:AAA8279
Neoligia subjuncta|[14308]|LPABC361-09|Canada|Alberta|658[0n]|BOLD:AAA8279
Neoligia subjuncta|[14309]|LPABC435-09|Canada|Alberta|658[0n]|BOLD:AAA8279
Neoligia subjuncta|[14310]|LPABC469-09|Canada|Alberta|658[0n]|BOLD:AAA8279
Neoligia subjuncta|[14311]|BBLEC875-09|Canada|Newfoundland and Labrador|658[0n]|BOLD:AAA8279
Neoligia subjuncta|[14312]|BBLPC716-09|Canada|Newfoundland and Labrador|658[0n]|BOLD:AAA8279
Neoligia subjuncta|[14313]|BBLPE389-09|Canada|Newfoundland and Labrador|658[0n]|BOLD:AAA8279
Neoligia subjuncta|[14314]|BBLPB190-10|Canada|Alberta|658[0n]|BOLD:AAA8279
Neoligia subjuncta|[14315]|BBLPB471-10|Canada|British Columbia|658[0n]|BOLD:AAA8279
Neoligia subjuncta|[14316]|BBLPD546-10|Canada|British Columbia|658[0n]|BOLD:AAA8279
Neoligia subjuncta|[14317]|BBLPD856-10|Canada|British Columbia|658[0n]|BOLD:AAA8279
Neoligia subjuncta|[14318]|LALPA938-11|Canada|British Columbia|658[0n]|BOLD:AAA8279
Neoligia subjuncta|[14319]|LPABC155-09|Canada|Alberta|627[0n]|BOLD:AAA8279
Neoligia subjuncta|[14320]|LOWCB520-05|Canada|British Columbia|658[0n]|BOLD:AAA8279
Neoligia subjuncta|[14321]|LPABC896-09|Canada|Alberta|658[0n]|BOLD:AAA8279
Neoligia subjuncta|[14322]|LPABB537-08|Canada|Alberta|658[0n]|BOLD:AAA8279
Neoligia subjuncta|[14323]|LOWCD531-06|Canada|British Columbia|658[0n]|BOLD:AAA8279
Neoligia subjuncta|[14324]|LOWCB519-05|Canada|British Columbia|658[0n]|BOLD:AAA8279
Neoligia subjuncta|[14325]|LOWCB522-05|Canada|British Columbia|605[0n]|BOLD:AAA8279
Neoligia subjuncta|[14326]|CNWLM2402-13|Canada|Alberta|600[0n]|BOLD:AAA8279
Neoligia subjuncta|[14327]|CNWLM2443-13|Canada|Alberta|608[0n]|BOLD:AAA8279
Neoligia surdirena|[14328]|CNCLB1717-14|United States|New Mexico|658[0n]|BOLD:ACU1096
Neoligia hardwicki|[14329]|CNCLB1718-14|United States|Utah|658[0n]|BOLD:ACU1096
Neoligia crytora|[14330]|RDLQG749-06|Canada|Quebec|658[0n]|BOLD:AAF0862
Neoligia crytora|[14331]|RDLQG750-06|Canada|Quebec|658[0n]|BOLD:AAF0862
Neoligia crytora|[14332]|RDLQG751-06|Canada|Quebec|658[0n]|BOLD:AAF0862
Neoligia crytora|[14333]|LNCC1108-11|United States|North Carolina|658[0n]|BOLD:AAF0862
Neoligia crytora|[14334]|LNCC1150-11|United States|North Carolina|658[0n]|BOLD:AAF0862
Benjaminiola colorada|[14335]|LOCBB311-06|United States|California|658[0n]|BOLD:AAD2278
Benjaminiola colorada|[14336]|LOCBB309-06|United States|California|658[0n]|BOLD:AAD2278
Benjaminiola colorada|[14337]|LOCBB308-06|United States|California|658[0n]|BOLD:AAD2278
Benjaminiola colorada|[14338]|LOCBB310-06|United States|California|658[0n]|BOLD:AAD2278
Benjaminiola colorada|[14339]|LOCBB312-06|United States|California|658[0n]|BOLD:AAD2278
Benjaminiola colorada|[14340]|RDNMF284-08|United States|Oregon|658[0n]|BOLD:ACF2615
Benjaminiola colorada|[14341]|RDNMF283-08|Canada|British Columbia|658[0n]|BOLD:ACF2615
Benjaminiola colorada|[14342]|RDNMF285-08|United States|Oregon|658[0n]|BOLD:ACF2615
Photedes didonea|[14343]|RDMAB956-09|United States|Washington|642[0n]|BOLD:AAF1495
Photedes didonea|[14344]|RDNMD792-07|United States|Oregon|658[0n]|BOLD:AAF1495
Photedes didonea|[14345]|RDMAB955-09|United States|Oregon|621[0n]|BOLD:AAF1495
Photedes didonea|[14346]|RDMAB957-09|United States|Oregon|638[0n]|BOLD:AAF1495
Hypocoena sofiae|[14347]|RDNMC752-06|United States|California|596[1n]|BOLD:ABA1196
Hypocoena sofiae|[14348]|BBLOC128-11|United States|Texas|658[0n]|BOLD:ABA1196
Hypocoena inquinata|[14349]|RDNMG503-08|Canada|Manitoba|658[0n]|BOLD:AAD0213
Hypocoena inquinata|[14350]|RDNMG504-08|Canada|Ontario|658[0n]|BOLD:ACE4044
Hypocoena inquinata|[14351]|BBLPE618-09|Canada|Nova Scotia|632[0n]|BOLD:ACE4044
Hypocoena inquinata|[14352]|RDNMG939-08|Canada|Ontario|658[0n]|BOLD:AAD0213
Hypocoena inquinata|[14353]|RDNMG938-08|Canada|Alberta|658[0n]|BOLD:AAD0213
Hypocoena inquinata|[14354]|BBLPB388-10|Canada|Alberta|658[0n]|BOLD:AAD0213
Hypocoena inquinata|[14355]|LPABC290-09|Canada|Alberta|658[0n]|BOLD:AAD0213
Hypocoena inquinata|[14356]|BBLPA843-10|Canada|Alberta|658[0n]|BOLD:AAD0213
Hypocoena inquinata|[14357]|BBLPB387-10|Canada|Alberta|658[0n]|BOLD:AAD0213
Hypocoena inquinata|[14358]|RDNMG940-08|Canada|Alberta|658[0n]|BOLD:AAD0213
Hypocoena inquinata|[14359]|LPABC266-09|Canada|Alberta|658[0n]|BOLD:AAD0213
Hypocoena inquinata|[14360]|CNEID3323-12|Canada|Alberta|638[0n]|BOLD:AAD0213
Hypocoena inquinata|[14361]|CNEID3339-12|Canada|Alberta|637[0n]|BOLD:AAD0213
Hypocoena inquinata|[14362]|BBLPB385-10|Canada|Alberta|658[0n]|BOLD:AAD0213
Hypocoena inquinata|[14363]|BBLPB386-10|Canada|Alberta|658[0n]|BOLD:AAD0213
Hypocoena inquinata|[14364]|CNEID3340-12|Canada|Alberta|638[0n]|BOLD:AAD0213
Oligia minuscula|[14365]|TTMNB343-06|Canada|New Brunswick|526[1n]|BOLD:AAB2890
Oligia minuscula|[14366]|TTMNB346-06|Canada|New Brunswick|558[3n]|BOLD:AAB2890
Oligia minuscula|[14367]|TTMNB345-06|Canada|New Brunswick|575[0n]|BOLD:AAB2890
Oligia minuscula|[14368]|TTMNB341-06|Canada|New Brunswick|658[0n]|BOLD:AAB2890
Oligia minuscula|[14369]|TMNBB179-06|Canada|New Brunswick|658[0n]|BOLD:AAB2890
Oligia minuscula|[14370]|TMNBB183-06|Canada|New Brunswick|658[0n]|BOLD:AAB2890
Oligia minuscula|[14371]|TTMNB344-06|Canada|New Brunswick|579[0n]|BOLD:AAB2890
Oligia minuscula|[14372]|TTMNB352-06|Canada|New Brunswick|658[1n]|BOLD:AAB2890
Oligia minuscula|[14373]|TTMNB350-06|Canada|New Brunswick|612[1n]|BOLD:AAB2890
Oligia minuscula|[14374]|TTMNB348-06|Canada|New Brunswick|658[1n]|BOLD:AAB2890
Oligia minuscula|[14375]|TMNBB178-06|Canada|New Brunswick|658[0n]|BOLD:AAB2890
Oligia minuscula|[14376]|TTMNB349-06|Canada|New Brunswick|658[5n]|BOLD:AAB2890
Oligia minuscula|[14377]|TTMNB347-06|Canada|New Brunswick|658[6n]|BOLD:AAB2890
Oligia minuscula|[14378]|TMNBB181-06|Canada|New Brunswick|658[0n]|BOLD:AAB2890
Oligia minuscula|[14379]|RDLQB773-05|Canada|Quebec|618[0n]|BOLD:AAB2890
Oligia minuscula|[14380]|RDLQB769-05|Canada|Quebec|617[0n]|BOLD:AAB2890
Oligia minuscula|[14381]|TMNBB180-06|Canada|New Brunswick|658[0n]|BOLD:AAB2890
Oligia minuscula|[14382]|TMNBB182-06|Canada|New Brunswick|658[0n]|BOLD:AAB2890
Oligia minuscula|[14383]|TMNBB177-06|Canada|New Brunswick|658[0n]|BOLD:AAB2890
Oligia minuscula|[14384]|TTMNB351-06|Canada|New Brunswick|658[2n]|BOLD:AAB2890
Oligia minuscula|[14385]|TTMNB342-06|Canada|New Brunswick|658[1n]|BOLD:AAB2890
Oligia minuscula|[14386]|RDLQB516-05|Canada|Quebec|658[0n]|BOLD:AAB2890
Oligia minuscula|[14387]|RDLQB763-05|Canada|Quebec|658[0n]|BOLD:AAB2890
Oligia minuscula|[14388]|RDLQB774-05|Canada|Quebec|658[0n]|BOLD:AAB2890
Oligia minuscula|[14389]|RDLQB817-05|Canada|Quebec|658[0n]|BOLD:AAB2890
Oligia minuscula|[14390]|RDLQF312-06|Canada|Quebec|658[0n]|BOLD:AAB2890
Photedes panatela|[14391]|LPSOB827-08|Canada|Ontario|658[0n]|BOLD:AAC7821
Photedes panatela|[14392]|LPSOB852-08|Canada|Ontario|658[0n]|BOLD:AAC7821

Oligia minuscula|[14390]|RDLQF312-06|Canada|Quebec|658[0n]|BOLD:AAB2890
Photedes panatela|[14391]|LPSOB827-08|Canada|Ontario|658[0n]|BOLD:AAC7821
Photedes panatela|[14392]|LPSOB852-08|Canada|Ontario|658[0n]|BOLD:AAC7821
Photedes panatela|[14393]|TMNBB470-06|Canada|New Brunswick|658[0n]|BOLD:AAC7821
Photedes panatela|[14394]|RDNMF280-08|Canada|New Brunswick|609[0n]|BOLD:AAC7821
Photedes panatela|[14395]|LPSOB837-08|Canada|Ontario|658[0n]|BOLD:AAC7821
Photedes panatela|[14396]|LPSOC195-08|Canada|Ontario|658[0n]|BOLD:AAC7821
Photedes panatela|[14397]|RDNMF282-08|Canada|Ontario|658[0n]|BOLD:AAC7821
Photedes panatela|[14398]|RDNMF281-08|Canada|New Brunswick|658[0n]|BOLD:AAC7821
Photedes panatela|[14399]|RDLQB270-05|Canada|Quebec|658[0n]|BOLD:AAC7821
Photedes panatela|[14400]|BLGSM046-09|Canada|Ontario|658[0n]|BOLD:AAC7821
Photedes panatela|[14401]|LPSOD296-09|Canada|Ontario|658[0n]|BOLD:AAC7821
Agrochola lota|[14402]|NMNHL219-10|Bulgaria||658[0n]|BOLD:AAC0283
Agrochola lota|[14403]|LEFIB987-10|Finland|Aland Islands|658[0n]|BOLD:AAC0283
Agrochola lota|[14404]|FBLMV166-09|Germany|Bavaria|658[0n]|BOLD:AAC0283
Agrochola lota|[14405]|RDNMG507-08|Germany||658[0n]|BOLD:AAC0283
Agrochola lota|[14406]|NMNHL218-10|Bulgaria||658[0n]|BOLD:AAC0283
Agrochola lota|[14407]|RDNMG506-08|Germany||658[0n]|BOLD:AAC0283
Agrochola lota|[14408]|LEATF435-14|Austria|Tirol|658[0n]|BOLD:AAC0283
Agrochola lota|[14409]|GBLAA209-14|Germany|Schleswig-Holstein|658[0n]|BOLD:AAC0283
Agrochola lota|[14410]|LEFIB788-10|Finland|South Karelia|658[0n]|BOLD:AAC0283
Agrochola lota|[14411]|CGUKB277-09|United Kingdom|England|625[0n]|BOLD:AAC0283
Agrochola lota|[14412]|GWORK421-09|Germany|Bavaria|658[0n]|BOLD:AAC0283
Agrochola lota|[14413]|RDNMF486-08|||638[0n]|BOLD:AAC0283
Agrochola lota|[14414]|CGUKA1004-09|United Kingdom|England|657[0n]|BOLD:AAC0283
Agrochola lota|[14415]|NOCJH431-11|France||658[0n]|BOLD:AAC0283
Agrochola lota|[14416]|LEATD501-13|Austria|Tirol|637[0n]|BOLD:AAC0283
Agrochola lota|[14417]|GWOTL036-13|Germany|Saarland|658[0n]|BOLD:AAC0283
Agrochola lota|[14418]|NLLEA1120-12|Netherlands|South Holland|658[0n]|BOLD:AAC0283
Agrochola lota|[14419]|PHLSA688-11|Austria|Vorarlberg|658[0n]|BOLD:AAC0283
Agrochola lota|[14420]|LENOA364-11|France|Haute Normandie|658[0n]|BOLD:AAC0283
Agrochola lota|[14421]|IBLAO143-11|Spain|Castilla-La Mancha|658[0n]|BOLD:AAC0283
Agrochola lota|[14422]|NOCJH443-11|France||658[0n]|BOLD:AAC0283
Agrochola lota|[14423]|NOCJH442-11|France||658[0n]|BOLD:AAC0283
Agrochola lota|[14424]|LEFIK466-10|Finland||658[0n]|BOLD:AAC0283
Agrochola lota|[14425]|CGUKB663-09|United Kingdom|England|658[0n]|BOLD:AAC0283
Agrochola lota|[14426]|CGUKC033-09|United Kingdom|England|611[0n]|BOLD:AAC0283
Agrochola lota|[14427]|CGUKD543-09|United Kingdom|England|658[0n]|BOLD:AAC0283
Agrochola lota|[14428]|CGUKA905-09|United Kingdom|England|658[0n]|BOLD:AAC0283
Agrochola lota|[14429]|CGUKA904-09|United Kingdom|England|658[0n]|BOLD:AAC0283
Agrochola lota|[14430]|GBLAC972-13|Germany|Thuringia|658[0n]|BOLD:AAC0283
Agrochola lota|[14431]|GBLAC988-13|Germany||658[0n]|BOLD:AAC0283
Agrochola lota|[14432]|RDNMF487-08|Canada|Newfoundland and Labrador|609[0n]|BOLD:AAC0283
Agrochola lota|[14433]|IBLAO146-11|Spain|Castilla-La Mancha|601[0n]|BOLD:AAC0283
Agrochola lota|[14434]|NORIN248-14|Norway|Akershus|650[0n]|BOLD:AAC0283
Capsula laeta|[14435]|RDNMC031-05|Canada|Ontario|658[0n]|BOLD:ABY6766
Capsula laeta|[14436]|RDNMG905-08|Canada|Ontario|658[0n]|BOLD:ABY6766
Capsula laeta|[14437]|RDNMG906-08|Canada|Ontario|658[0n]|BOLD:ABY6766
Capsula oblonga|[14438]|RDNMC030-05|Canada|Ontario|658[0n]|BOLD:AAB3171
Capsula oblonga|[14439]|TTMNB354-06|Canada|New Brunswick|658[1n]|BOLD:AAB3171
Capsula oblonga|[14440]|RDLQB837-05|Canada|Quebec|658[0n]|BOLD:AAB3171
Capsula oblonga|[14441]|RDLQB840-05|Canada|Quebec|617[0n]|BOLD:AAB3171
Capsula oblonga|[14442]|RDLQF555-06|Canada|Quebec|658[0n]|BOLD:AAB3171
Capsula oblonga|[14443]|RDLQF556-06|Canada|Quebec|658[0n]|BOLD:AAB3171
Capsula oblonga|[14444]|RDLQF550-06|Canada|Quebec|658[0n]|BOLD:AAB3171
Capsula oblonga|[14445]|RDLQF557-06|Canada|Quebec|658[0n]|BOLD:AAB3171
Capsula oblonga|[14446]|BBLOD1666-11|United States|Arizona|658[0n]|BOLD:AAB3171
Capsula oblonga|[14447]|MILEP094-09|United States|North Carolina|658[0n]|BOLD:AAB3171
Capsula oblonga|[14448]|RWWB300-09|United States|Washington|658[0n]|BOLD:AAB3171
Capsula oblonga|[14449]|RWWC780-11|United States|Washington|658[0n]|BOLD:AAB3171
Capsula oblonga|[14450]|RWWC812-11|United States|Washington|658[0n]|BOLD:AAB3171
Capsula oblonga|[14451]|AWCLB435-10|United States|Arizona|658[0n]|BOLD:AAB3171
Capsula oblonga|[14452]|RWWC766-11|United States|Washington|658[0n]|BOLD:AAB3171
Capsula oblonga|[14453]|HKONB385-09|United States|Texas|658[0n]|BOLD:AAB3171
Capsula oblonga|[14454]|MILEP093-09|United States|North Carolina|658[0n]|BOLD:AAB3171
Capsula oblonga|[14455]|LOCBD960-06|United States|California|658[0n]|BOLD:AAB3171
Capsula oblonga|[14456]|LOCBE110-06|United States|California|658[0n]|BOLD:AAB3171
Capsula oblonga|[14457]|LPGVA632-08|Canada|British Columbia|658[0n]|BOLD:AAB3171
Capsula oblonga|[14458]|LPGVA633-08|Canada|British Columbia|658[0n]|BOLD:AAB3171
Capsula oblonga|[14459]|RDNME559-08|United States|California|658[0n]|BOLD:AAB3171
Capsula oblonga|[14460]|LBCW040-08|Canada|British Columbia|658[0n]|BOLD:AAB3171
Capsula oblonga|[14461]|LOFLB794-06|United States|Florida|658[0n]|BOLD:AAB3171
Capsula oblonga|[14462]|LOCBB221-06|United States|California|658[0n]|BOLD:AAB3171
Capsula oblonga|[14463]|LOCBB220-06|United States|California|658[0n]|BOLD:AAB3171
Capsula oblonga|[14464]|LOCBB219-06|United States|California|658[0n]|BOLD:AAB3171
Capsula oblonga|[14465]|LOCBB218-06|United States|California|658[0n]|BOLD:AAB3171
Capsula oblonga|[14466]|RDLQB500-05|Canada|Quebec|658[0n]|BOLD:AAB3171
Capsula oblonga|[14467]|LNC285-05|United States|North Carolina|658[0n]|BOLD:AAB3171
Capsula oblonga|[14468]|XAD356-04|Canada|Ontario|658[0n]|BOLD:AAB3171
Capsula oblonga|[14469]|XAD100-04|Canada|Ontario|579[0n]|BOLD:AAB3171
Capsula oblonga|[14470]|LBCW041-08|Canada|British Columbia|658[0n]|BOLD:AAB3171
Capsula oblonga|[14471]|RWWC838-12|United States|Washington|658[0n]|BOLD:AAB3171
Capsula oblonga|[14472]|CNCLB1815-14|United States|New Mexico|658[0n]|BOLD:AAB3171
Capsula oblonga|[14473]|CNCLB1816-14|United States|New Mexico|658[0n]|BOLD:AAB3171
Capsula subflava|[14474]|RDMAB558-06|Canada|Alberta|658[0n]|BOLD:AAC6808
Capsula subflava|[14475]|RDNMB023-05|Canada|Ontario|658[0n]|BOLD:AAC6807
Capsula subflava|[14476]|RDNME560-08|United States|California|658[0n]|BOLD:AAC6807
Capsula subflava|[14477]|RDNMC028-05|Canada|Ontario|658[0n]|BOLD:AAC6807
Capsula subflava|[14478]|RDNME566-08|United States|California|658[0n]|BOLD:AAC6807
Capsula subflava|[14479]|LBCH6522-10|Canada|British Columbia|658[0n]|BOLD:AAC6807
Capsula subflava|[14480]|RDMAB557-06|Canada|Alberta|658[0n]|BOLD:AAC6807
Capsula subflava|[14481]|RDNMC029-05|Canada|Ontario|658[0n]|BOLD:AAC6807
Capsula subflava|[14482]|RDNMB024-05|Canada|Ontario|658[0n]|BOLD:AAC6807
Capsula subflava|[14483]|RDNMB022-05|Canada|Ontario|658[0n]|BOLD:AAC6807
Capsula subflava|[14484]|RDNMB021-05|Canada|Ontario|658[0n]|BOLD:AAC6807
Capsula subflava|[14485]|CNWBD036-13|Canada|Alberta|605[0n]|BOLD:AAC6807
Capsula alameda|[14486]|BBLOD966-11|United States|Texas|658[0n]|BOLD:ACF2699
Capsula alameda|[14487]|RDNMH636-09|United States|California|658[0n]|BOLD:ACF2699
Capsula alameda|[14488]|CNCLB2471-14|United States|California|658[0n]|BOLD:ACF2699
Oligia chlorostigma|[14489]|HKONS477-08|United States|Florida|658[0n]|BOLD:AAE5953
Oligia chlorostigma|[14490]|RDNMF379-08|Canada|Quebec|658[0n]|BOLD:AAE5953
Oligia chlorostigma|[14491]|RDNMF380-08|Canada|Quebec|658[0n]|BOLD:AAE5953
Oligia chlorostigma|[14492]|RDNMG512-08|Canada|New Brunswick|658[0n]|BOLD:AAE5953

Oligia chlorostigma|[14490]|RDNMF379-08|Canada|Quebec|658[0n]|BOLD:AAE5953
Oligia chlorostigma|[14491]|RDNMF380-08|Canada|Quebec|658[0n]|BOLD:AAE5953
Oligia chlorostigma|[14492]|RDNMG512-08|Canada|New Brunswick|658[0n]|BOLD:AAE5953
Oligia chlorostigma|[14493]|LNCC748-11|United States|North Carolina|658[0n]|BOLD:AAE5953
Oligia chlorostigma|[14494]|LNCC749-11|United States|North Carolina|658[0n]|BOLD:AAE5953
Oligia chlorostigma|[14495]|LNCC750-11|United States|North Carolina|658[0n]|BOLD:AAE5953
Oligia chlorostigma|[14496]|RDNML148-13|United States|North Carolina|658[0n]|BOLD:AAE5953
Sericaglaea signata|[14497]|LPOKE603-12|United States|Oklahoma|620[0n]|BOLD:AAJ7714
Sericaglaea signata|[14498]|LNC029-05|United States|North Carolina|658[0n]|BOLD:AAJ7714
Sericaglaea signata|[14499]|LNCC521-11|United States|North Carolina|658[0n]|BOLD:AAJ7714
Sericaglaea signata|[14500]|RDNMK029-11|United States|Florida|658[0n]|BOLD:AAJ7714
Sericaglaea signata|[14501]|RDNMK030-11|United States|Florida|658[0n]|BOLD:AAJ7714
Sericaglaea signata|[14502]|RDNMK033-11|United States|Florida|658[0n]|BOLD:AAJ7714
Sericaglaea signata|[14503]|LNCC1471-13|United States|North Carolina|658[0n]|BOLD:AAJ7714
Sericaglaea signata|[14504]|CNCLB2555-14|United States|North Carolina|658[0n]|BOLD:AAJ7714
Sericaglaea signata|[14505]|RDNMK032-11|United States|Florida|658[0n]|BOLD:AAJ7714
Sericaglaea signata|[14506]|LNCC522-11|United States|North Carolina|658[0n]|BOLD:AAJ7714
Sericaglaea signata|[14507]|LNCB990-10|United States|North Carolina|658[0n]|BOLD:AAJ7714
Sericaglaea signata|[14508]|CNCLB2782-14|United States|North Carolina|658[0n]|BOLD:AAJ7714
Papaipema marginidens|[14509]|QUNOE404-12|United States|Wisconsin|658[0n]|BOLD:ABV5327
Papaipema marginidens|[14510]|RDNML144-13|United States|North Carolina|632[0n]|BOLD:ABV5327
Papaipema stenocelis|[14511]|LNCC819-11|United States|North Carolina|658[0n]|BOLD:ABU6938
Papaipema stenocelis|[14512]|LNCC1945-14|United States|North Carolina|658[0n]|BOLD:ABU6938
Papaipema stenocelis|[14513]|LNAUT786-14|United States|Florida|658[0n]|BOLD:ABU6938
Papaipema stenocelis|[14514]|LNAUT787-14|United States|Florida|550[0n]|BOLD:ABU6938
Papaipema sp.|[14515]|LSUSA019-06|United States|Kentucky|658[0n]|BOLD:AAI5207
Papaipema sp.|[14516]|LSUSA023-06|United States|Kentucky|658[0n]|BOLD:AAI5207
Papaipema cerussata|[14517]|LNCC396-10|United States|North Carolina|658[0n]|BOLD:AAE7004
Papaipema cerussata|[14518]|LSEU207-06|United States|Georgia|658[0n]|BOLD:AAE7004
Papaipema cerussata|[14519]|LSEU208-06|United States|Georgia|658[0n]|BOLD:AAE7004
Papaipema cerussata|[14520]|LNCC395-10|United States|North Carolina|658[0n]|BOLD:AAE7004
Papaipema cerussata|[14521]|LSEU209-06|United States|Georgia|658[0n]|BOLD:AAE7004
Papaipema cerussata|[14522]|LSUSA017-06|United States|Kentucky|658[0n]|BOLD:AAE7004
Papaipema cerussata|[14523]|LNCC394-10|United States|North Carolina|658[0n]|BOLD:AAE7004
Papaipema cerussata|[14524]|LNCC397-10|United States|North Carolina|658[0n]|BOLD:AAE7004
Papaipema unimoda|[14525]|XAH564-05|Canada|Ontario|658[0n]|BOLD:AAC2395
Papaipema unimoda|[14526]|RDNMC579-06|Canada|New Brunswick|658[0n]|BOLD:AAC2395
Papaipema unimoda|[14527]|RDLQ477-07|Canada|Quebec|622[0n]|BOLD:AAC2395
Papaipema unimoda|[14528]|RDLQ479-07|Canada|Quebec|627[1n]|BOLD:AAC2395
Papaipema unimoda|[14529]|RDLQ478-07|Canada|Quebec|613[0n]|BOLD:AAC2395
Papaipema unimoda|[14530]|RDLQ480-07|Canada|Quebec|623[0n]|BOLD:AAC2395
Papaipema unimoda|[14531]|RDLQ483-07|Canada|Quebec|630[0n]|BOLD:AAC2395
Papaipema unimoda|[14532]|RDNMC580-06|Canada|New Brunswick|658[0n]|BOLD:AAC2395
Papaipema unimoda|[14533]|RDLQ481-07|Canada|Quebec|622[0n]|BOLD:AAC2395
Papaipema unimoda|[14534]|RDLQ482-07|Canada|Quebec|627[0n]|BOLD:AAC2395
Papaipema unimoda|[14535]|RDLQ484-07|Canada|Quebec|619[0n]|BOLD:AAC2395
Papaipema unimoda|[14536]|RDNMC581-06|Canada|Alberta|658[0n]|BOLD:AAC2395
Papaipema unimoda|[14537]|LMDH015-11|United States|Minnesota|658[0n]|BOLD:AAC2395
Papaipema furcata|[14538]|LPOKE282-11|United States|Oklahoma|658[0n]|BOLD:AAF1779
Papaipema furcata|[14539]|RDNML139-13|United States|North Carolina|658[0n]|BOLD:AAF1779
Papaipema furcata|[14540]|RDNML140-13|United States|North Carolina|658[0n]|BOLD:AAF1779
Papaipema furcata|[14541]|RDNMG777-08|Canada|Ontario|658[0n]|BOLD:AAF1779
Papaipema furcata|[14542]|LSEU197-06|United States|Georgia|595[0n]|BOLD:AAF1779
Papaipema furcata|[14543]|LSEU198-06|United States|Georgia|658[0n]|BOLD:AAF1779
Papaipema furcata|[14544]|RDNML141-13|United States|North Carolina|658[0n]|BOLD:AAF1779
Papaipema leucostigma|[14545]|RDNM761-05|Canada|Ontario|658[0n]|BOLD:AAB8711
Papaipema leucostigma|[14546]|RDNM818-05|Canada|Ontario|658[0n]|BOLD:AAB8711
Papaipema leucostigma|[14547]|RDNM819-05|Canada|Ontario|658[0n]|BOLD:AAB8711
Papaipema leucostigma|[14548]|RDNM772-05|Canada|Ontario|658[0n]|BOLD:AAB8711
Papaipema leucostigma|[14549]|RDNM771-05|Canada|Ontario|658[0n]|BOLD:AAB8711
Papaipema leucostigma|[14550]|RDNM763-05|Canada|Ontario|658[0n]|BOLD:AAB8711
Papaipema leucostigma|[14551]|RDNM762-05|Canada|Ontario|658[0n]|BOLD:AAB8711
Papaipema leucostigma|[14552]|RDNM760-05|Canada|Ontario|658[0n]|BOLD:AAB8711
Papaipema leucostigma|[14553]|RDNM759-05|Canada|Ontario|658[0n]|BOLD:AAB8711
Papaipema leucostigma|[14554]|RDNM010-05|Canada|Ontario|658[0n]|BOLD:AAB8711
Papaipema leucostigma|[14555]|RDMAB1050-09|Canada|Ontario|649[0n]|BOLD:AAB8711
Papaipema leucostigma|[14556]|RDNM828-05|Canada|Ontario|658[0n]|BOLD:AAB8711
Papaipema leucostigma|[14557]|LMDH002-11|United States|Minnesota|658[0n]|BOLD:AAB8711
Papaipema pterisii|[14558]|RDLQ286-05|Canada|Quebec|576[0n]|BOLD:AAB8711
Papaipema pterisii|[14559]|RDNM002-05|Canada|Ontario|658[0n]|BOLD:AAB8711
Papaipema pterisii|[14560]|RDNM708-05|Canada|Ontario|601[0n]|BOLD:AAB8711
Papaipema pterisii|[14561]|RDNM829-05|Canada|Ontario|658[0n]|BOLD:AAB8711
Papaipema pterisii|[14562]|RDNM004-05|Canada|Ontario|658[0n]|BOLD:AAB8711
Papaipema pterisii|[14563]|RDNM765-05|Canada|Ontario|658[0n]|BOLD:AAB8711
Papaipema pterisii|[14564]|RDNM766-05|Canada|Ontario|658[0n]|BOLD:AAB8711
Papaipema pterisii|[14565]|RDNM767-05|Canada|Ontario|658[0n]|BOLD:AAB8711
Papaipema pterisii|[14566]|RDNM821-05|Canada|Ontario|658[0n]|BOLD:AAB8711
Papaipema pterisii|[14567]|RDNM822-05|Canada|Ontario|658[0n]|BOLD:AAB8711
Papaipema pterisii|[14568]|RDNM827-05|Canada|Ontario|658[0n]|BOLD:AAB8711
Papaipema pterisii|[14569]|RDNM830-05|Canada|Ontario|658[0n]|BOLD:AAB8711
Papaipema pterisii|[14570]|RDNMB590-05|Canada|Ontario|658[0n]|BOLD:AAB8711
Papaipema circumlucens|[14571]|RDNMF475-08|Canada|Saskatchewan|609[0n]|BOLD:AAB8711
Papaipema circumlucens|[14572]|QUNOD669-11|United States|Michigan|658[0n]|BOLD:AAB8711
Papaipema circumlucens|[14573]|QUNOE080-11|United States|Michigan|658[0n]|BOLD:AAB8711
Papaipema cataphracta|[14574]|RDNMG929-08|Canada|Ontario|658[0n]|BOLD:ABZ6984
Papaipema lysimachiae|[14575]|RDLQ147-05|Canada|Quebec|658[0n]|BOLD:ACF1518
Papaipema lysimachiae|[14576]|RDMAB1049-09|Canada|Ontario|656[0n]|BOLD:ACF1518
Papaipema sauzalitae|[14577]|NAMUM373-09|United States|California|658[0n]|BOLD:ABY6163
Papaipema angelica|[14578]|LNAUS4466-13|United States|California|540[0n]|BOLD:ACQ7575
Papaipema angelica|[14579]|LNAUS4469-13|United States|California|540[0n]|BOLD:ACQ7575
Papaipema araliae|[14580]|LNAUS4471-13|United States|North Carolina|658[0n]|BOLD:AAB8711
Papaipema araliae|[14581]|LNAUS4473-13|United States|North Carolina|658[0n]|BOLD:AAB8711
Papaipema araliae|[14582]|LNAUS4474-13|United States|North Carolina|658[0n]|BOLD:AAB8711
Papaipema araliae|[14583]|CNCLB1115-14|||658[0n]|BOLD:AAB8711
Papaipema araliae|[14584]|LNCC1488-13|United States|North Carolina|658[0n]|BOLD:AAB8711
Papaipema araliae|[14585]|LNAUS4470-13|United States|North Carolina|658[0n]|BOLD:AAB8711
Papaipema araliae|[14586]|LNAUT402-14|United States|North Carolina|658[0n]|BOLD:AAB8711
Papaipema araliae|[14587]|LNAUS4472-13|United States|North Carolina|658[0n]|BOLD:AAB8711
Papaipema araliae|[14588]|LNAUT403-14|United States|North Carolina|658[0n]|BOLD:AAB8711
Papaipema sp.|[14589]|RDLQ025-05|Canada|Quebec|658[0n]|BOLD:AAB8711
Papaipema sp.|[14590]|RDNMD846-07|Canada|Quebec|658[0n]|BOLD:AAB8711
Papaipema nr. pterisii|[14591]|RDNM007-05|Canada|Ontario|658[0n]|BOLD:AAB8711
Papaipema nr. pterisii|[14592]|RDNM008-05|Canada|New Brunswick|627[0n]|BOLD:AAB8711

Papaipema sp.|[14590]|RDNMD846-07|Canada|Quebec|658[0n]|BOLD:AAB8711
Papaipema nr. pterisii|[14591]|RDNM007-05|Canada|Ontario|658[0n]|BOLD:AAB8711
Papaipema nr. pterisii|[14592]|RDNM008-05|Canada|New Brunswick|627[0n]|BOLD:AAB8711
Papaipema nr. pterisii|[14593]|RDNM009-05|Canada|New Brunswick|625[0n]|BOLD:AAB8711
Papaipema nr. pterisii|[14594]|RDNM823-05|Canada|Ontario|658[0n]|BOLD:AAB8711
Papaipema nr. pterisii|[14595]|XAH453-05|Canada|Ontario|658[0n]|BOLD:AAB8711
Papaipema nr. pterisii|[14596]|RDNM820-05|Canada|Ontario|658[0n]|BOLD:AAB8711
Papaipema nr. pterisii|[14597]|XAH559-05|Canada|Ontario|658[0n]|BOLD:AAB8711
Papaipema nr. pterisii|[14598]|RDNM005-05|Canada|Ontario|658[0n]|BOLD:AAB8711
Papaipema nr. pterisii|[14599]|RDNM769-05|Canada|Ontario|658[0n]|BOLD:AAB8711
Papaipema nr. pterisii|[14600]|RDNM006-05|Canada|Ontario|658[0n]|BOLD:AAB8711
Papaipema nr. pterisii|[14601]|XAB453-04|Canada|Ontario|658[0n]|BOLD:AAB8711
Papaipema nr. pterisii|[14602]|RDLQ297-05|Canada|Quebec|607[0n]|BOLD:AAB8711
Papaipema nr. pterisii|[14603]|RDNM832-05|Canada|Ontario|617[0n]|BOLD:AAB8711
Papaipema nr. pterisii|[14604]|RDNM831-05|Canada|Ontario|617[0n]|BOLD:AAB8711
Papaipema nr. pterisii|[14605]|RDNM003-05|Canada|Ontario|658[0n]|BOLD:AAB8711
Papaipema nr. pterisii|[14606]|RDLQ314-05|Canada|Quebec|658[0n]|BOLD:AAB8711
Papaipema nr. pterisii|[14607]|RDNM770-05|Canada|New Brunswick|603[0n]|BOLD:AAB8711
Papaipema nr. pterisii|[14608]|RDNMB589-05|Canada|Ontario|617[0n]|BOLD:AAB8711
Papaipema nr. pterisii|[14609]|RDLQF266-06|Canada|Quebec|658[0n]|BOLD:AAB8711
Papaipema harrisii|[14610]|PHMNB300-04|Canada|New Brunswick|658[0n]|BOLD:AAB8711
Papaipema harrisii|[14611]|RDLQ390-05|Canada|New Brunswick|658[0n]|BOLD:AAB8711
Papaipema harrisii|[14612]|RDLQ391-05|Canada|New Brunswick|658[0n]|BOLD:AAB8711
Papaipema harrisii|[14613]|RDLQ392-05|Canada|New Brunswick|658[0n]|BOLD:AAB8711
Papaipema harrisii|[14614]|RDNMB119-05|Canada|Alberta|614[0n]|BOLD:AAB8711
Papaipema harrisii|[14615]|RDLQ389-05|Canada|New Brunswick|658[0n]|BOLD:AAB8711
Papaipema polymniae|[14616]|LSEU205-06|United States|Georgia|658[0n]|BOLD:AAB8711
Papaipema polymniae|[14617]|LSEU206-06|United States|Georgia|658[0n]|BOLD:AAB8711
Papaipema harrisii|[14618]|RDNMB120-05|Canada|Alberta|614[0n]|BOLD:AAB8711
Papaipema harrisii|[14619]|CNRME2835-12|Canada|Manitoba|603[0n]|BOLD:AAB8711
Papaipema sp.|[14620]|LSUSA015-06|United States|Kentucky|658[0n]|BOLD:AAB8711
Papaipema impecuniosa|[14621]|XAD270-04|Canada|Ontario|658[0n]|BOLD:AAB8711
Papaipema impecuniosa|[14622]|XAH560-05|Canada|Ontario|658[0n]|BOLD:AAB8711
Papaipema impecuniosa|[14623]|PHMO356-03|Canada|Ontario|639[0n]|BOLD:AAB8711
Papaipema impecuniosa|[14624]|LNCC415-10|United States|North Carolina|658[0n]|BOLD:AAB8711
Papaipema impecuniosa|[14625]|RDLQH038-06|Canada|Quebec|658[0n]|BOLD:AAB8711
Papaipema impecuniosa|[14626]|RDLQF268-06|Canada|Quebec|658[0n]|BOLD:AAB8711
Papaipema impecuniosa|[14627]|CNCLB2945-14|United States|North Carolina|658[0n]|BOLD:AAB8711
Papaipema impecuniosa|[14628]|CNCLB2959-14|United States|North Carolina|658[0n]|BOLD:AAB8711
Papaipema impecuniosa|[14629]|CNCLB2961-14|United States|North Carolina|658[0n]|BOLD:AAB8711
Papaipema silphii|[14630]|QUNOE401-12|United States|Wisconsin|658[0n]|BOLD:AAB8711
Papaipema duplicatus|[14631]|LNAUS2740-13|United States|Virginia|658[0n]|BOLD:AAB8711
Papaipema duplicatus|[14632]|LNAUS2741-13|United States|Virginia|658[0n]|BOLD:AAB8711
Papaipema duplicatus|[14633]|LNAUS2744-13|United States|Virginia|658[0n]|BOLD:AAB8711
Papaipema duplicatus|[14634]|LNAUS2742-13|United States|Virginia|658[0n]|BOLD:AAB8711
Papaipema duplicatus|[14635]|LNAUS2743-13|United States|Virginia|658[0n]|BOLD:AAB8711
Papaipema silphii|[14636]|LNAUT784-14|United States|Illinois|658[0n]|BOLD:AAB8711
Papaipema sp.|[14637]|RDNMH868-09|Canada|Nova Scotia|658[0n]|BOLD:AAB8711
Papaipema sp.|[14638]|RDNMH869-09|Canada|Nova Scotia|658[0n]|BOLD:AAB8711
Papaipema nelita|[14639]|LGSMG990-10|United States|North Carolina|658[0n]|BOLD:AAB8711
Papaipema nelita|[14640]|JSAUG1675-11|Canada|Ontario|658[0n]|BOLD:AAB8711
Papaipema nelita|[14641]|LGSMG989-10|United States|North Carolina|658[0n]|BOLD:AAB8711
Papaipema nelita|[14642]|LGSMG988-10|United States|North Carolina|658[0n]|BOLD:AAB8711
Papaipema nelita|[14643]|LGSMG987-10|United States|North Carolina|658[0n]|BOLD:AAB8711
Papaipema nelita|[14644]|LGSMG986-10|United States|North Carolina|658[0n]|BOLD:AAB8711
Papaipema nelita|[14645]|LGSMG985-10|United States|North Carolina|658[0n]|BOLD:AAB8711
Papaipema nelita|[14646]|LGSMG984-10|United States|North Carolina|658[0n]|BOLD:AAB8711
Papaipema nelita|[14647]|LGSMG983-10|United States|North Carolina|658[0n]|BOLD:AAB8711
Papaipema nelita|[14648]|LGSMG982-10|United States|North Carolina|658[0n]|BOLD:AAB8711
Papaipema sp.|[14649]|HKONB386-09|United States|Indiana|658[0n]|BOLD:AAB8711
Papaipema nelita|[14650]|RDNMD600-06|United States|Colorado|658[0n]|BOLD:AAB8711
Papaipema nelita|[14651]|RDLQB470-05|Canada|Quebec|658[0n]|BOLD:AAB8711
Papaipema nelita|[14652]|RDLQB464-05|Canada|Quebec|658[0n]|BOLD:AAB8711
Papaipema nelita|[14653]|RDLQ151-05|Canada|Quebec|658[0n]|BOLD:AAB8711
Papaipema nelita|[14654]|RDLQ148-05|Canada|Quebec|658[0n]|BOLD:AAB8711
Papaipema nelita|[14655]|XAB215-04|Canada|Ontario|658[0n]|BOLD:AAB8711
Papaipema nelita|[14656]|XAB213-04|Canada|Ontario|658[0n]|BOLD:AAB8711
Papaipema nelita|[14657]|LGSM661-04|United States|North Carolina|658[0n]|BOLD:AAB8711
Papaipema nelita|[14658]|RDLQ149-05|Canada|Quebec|597[0n]|BOLD:AAB8711
Papaipema nelita|[14659]|RDLQ150-05|Canada|Quebec|573[0n]|BOLD:AAB8711
Papaipema nelita|[14660]|RDLQ154-05|Canada|Quebec|569[0n]|BOLD:AAB8711
Papaipema nelita|[14661]|RDLQB846-05|Canada|Quebec|620[1n]|BOLD:AAB8711
Papaipema nelita|[14662]|RDLQ485-07|Canada|Quebec|619[0n]|BOLD:AAB8711
Papaipema nelita|[14663]|LNCC232-10|United States|North Carolina|658[0n]|BOLD:AAB8711
Papaipema nelita|[14664]|LNCC341-10|United States|North Carolina|658[0n]|BOLD:AAB8711
Papaipema nelita|[14665]|LNCC1943-14|United States|North Carolina|658[0n]|BOLD:AAB8711
Papaipema impecuniosa|[14666]|RDNML183-13|United States|North Carolina|658[0n]|BOLD:AAB8711
Papaipema impecuniosa|[14667]|CNCLB2942-14|United States|North Carolina|658[0n]|BOLD:AAB8711
Papaipema impecuniosa|[14668]|CNCLB2960-14|United States|North Carolina|658[0n]|BOLD:AAB8711
Papaipema impecuniosa|[14669]|CNCLB2962-14|United States|North Carolina|658[0n]|BOLD:AAB8711
Papaipema nebris|[14670]|RDNML179-13|United States|North Carolina|658[0n]|BOLD:ACF1624
Papaipema nebris|[14671]|RDNML180-13|United States|North Carolina|658[0n]|BOLD:ACF1624
Papaipema nebris|[14672]|RDNML186-13|United States|North Carolina|658[0n]|BOLD:ACF1624
Papaipema nebris|[14673]|LPOKA322-08|United States|Oklahoma|658[0n]|BOLD:ACF1624
Papaipema nebris|[14674]|LPOKD617-09|United States|Oklahoma|658[0n]|BOLD:ACF1624
Papaipema nebris|[14675]|CNCLB2390-14|Canada|Ontario|658[0n]|BOLD:ACF1624
Papaipema nebris|[14676]|LPOKD409-09|United States|Oklahoma|658[0n]|BOLD:ACF1624
Papaipema nebris|[14677]|CNCLB2963-14|United States|North Carolina|658[0n]|BOLD:ACF1624
Papaipema nebris|[14678]|LPOKA072-08|United States|Oklahoma|658[0n]|BOLD:ACF1624
Papaipema nebris|[14679]|LILLA862-11|United States|Illinois|658[0n]|BOLD:ACF1624
Papaipema nebris|[14680]|LPOKD603-09|United States|Oklahoma|658[0n]|BOLD:ACF1624
Papaipema nebris|[14681]|LPOKA366-08|United States|Oklahoma|658[0n]|BOLD:ACF1624
Papaipema nebris|[14682]|LSUSA074-06|United States|Kentucky|658[0n]|BOLD:ACF1624
Papaipema nebris|[14683]|LSUSA014-06|United States|Kentucky|658[0n]|BOLD:ACF1624
Papaipema nebris|[14684]|LSUSA016-06|United States|Kentucky|658[0n]|BOLD:ACF1624
Papaipema nebris|[14685]|LILLA943-11|United States|Illinois|658[0n]|BOLD:ACF1624
Papaipema nebris|[14686]|LPOKE287-11|United States|Oklahoma|658[0n]|BOLD:ACF1624
Papaipema nebris|[14687]|RDNML178-13|United States|North Carolina|658[0n]|BOLD:ACF1624
Papaipema nebris|[14688]|CNCLB2964-14|United States|North Carolina|658[0n]|BOLD:ACF1624
Papaipema maritima|[14689]|QUNOE403-12|United States|Wisconsin|658[0n]|BOLD:ACE4040
Papaipema eryngii|[14690]|LNAUS2746-13|United States|Illinois|658[0n]|BOLD:ACI4471
Papaipema eryngii|[14691]|LNAUS2747-13|United States|Illinois|658[0n]|BOLD:ACI4471
Papaipema eryngii|[14692]|LNAUS2748-13|United States|Illinois|658[0n]|BOLD:ACI4471

[illegible]
Papaipema eryngii|[14690]|LNAUS2746-13|United States|Illinois|658[0n]|BOLD:ACI4471
Papaipema eryngii|[14691]|LNAUS2747-13|United States|Illinois|658[0n]|BOLD:ACI4471
Papaipema eryngii|[14692]|LNAUS2748-13|United States|Illinois|658[0n]|BOLD:ACI4471
Papaipema sp.|[14693]|LNCB455-07|United States|North Carolina|658[0n]|BOLD:AAC7956
Papaipema sp.|[14694]|LNCB456-07|United States|North Carolina|658[0n]|BOLD:AAC7956
Papaipema sp.|[14695]|LNCB457-07|United States|North Carolina|658[0n]|BOLD:AAC7956
Papaipema sp.|[14696]|LNCB454-07|United States|North Carolina|658[0n]|BOLD:AAC7956
Papaipema sp.|[14697]|LNAUT790-14|United States|North Carolina|658[0n]|BOLD:AAC7956
Papaipema sp.|[14698]|LNAUT791-14|United States|North Carolina|658[0n]|BOLD:AAC7956
Papaipema sp.|[14699]|LNAUT792-14|United States|North Carolina|658[0n]|BOLD:AAC7956
Papaipema aweme|[14700]|RDNMD635-06|Canada|Ontario|658[1n]|BOLD:ABU6937
Papaipema aweme|[14701]|NOCNA088-14|United States|Michigan|658[0n]|BOLD:ABU6937
Papaipema cataphracta|[14702]|LSEU204-06|United States|Georgia|658[0n]|BOLD:ACF1229
Papaipema cataphracta|[14703]|LSUSA093-06|United States|Kentucky|658[0n]|BOLD:ACF1229
Papaipema cataphracta|[14704]|RDNMG930-08|United States|South Carolina|641[0n]|BOLD:ACF1229
Papaipema cataphracta|[14705]|RDNML145-13|United States|North Carolina|658[0n]|BOLD:ACF1229
Papaipema cataphracta|[14706]|RDNML146-13|United States|North Carolina|658[0n]|BOLD:ACF1229
Papaipema sulphurata|[14707]|CNCLB893-14|United States|Massachusetts|658[0n]|BOLD:ABZ0884
Papaipema sulphurata|[14708]|CNCLB1147-14|United States|Massachusetts|658[0n]|BOLD:ABZ0884
Papaipema insulidens|[14709]|RDNM707-05|United States|Oregon|598[0n]|BOLD:ABZ0884
Papaipema insulidens|[14710]|RWWC064-10|United States|Washington|307[0n]|
Papaipema insulidens|[14711]|LNCC340-10|United States|North Carolina|658[0n]|BOLD:ABZ0884
Papaipema insulidens|[14712]|RDNML181-13|United States|North Carolina|658[0n]|BOLD:ABZ0884
Papaipema insulidens|[14713]|XAB433-04|Canada|Ontario|658[0n]|BOLD:ABZ0884
Papaipema insulidens|[14714]|RDNML182-13|United States|North Carolina|658[0n]|BOLD:ABZ0884
Papaipema insulidens|[14715]|RDMAB1056-09|Canada|British Columbia|658[0n]|BOLD:ABZ0884
Papaipema insulidens|[14716]|CNCLB1151-14|Canada|British Columbia|658[0n]|BOLD:ABZ0884
Papaipema insulidens|[14717]|CNCLB283-14|Canada|British Columbia|658[0n]|BOLD:ABZ0884
Papaipema insulidens|[14718]|LNCC1485-13|United States|North Carolina|658[0n]|BOLD:ABZ0884
Papaipema insulidens|[14719]|LMDH006-11|United States|Minnesota|658[0n]|BOLD:ABZ0884
Papaipema insulidens|[14720]|RDMAB1057-09|Canada|British Columbia|658[0n]|BOLD:ABZ0884
Papaipema insulidens|[14721]|RDNMF474-08|Canada|British Columbia|658[0n]|BOLD:ABZ0884
Papaipema insulidens|[14722]|RDNME420-08|Canada|Ontario|658[0n]|BOLD:ABZ0884
Papaipema insulidens|[14723]|RDLQF264-06|Canada|Quebec|658[0n]|BOLD:ABZ0884
Papaipema insulidens|[14724]|RDMAB1052-09|United States|Oregon|651[0n]|BOLD:ABZ0884
Papaipema insulidens|[14725]|RDMAB1053-09|United States|Oregon|656[0n]|BOLD:ABZ0884
Papaipema insulidens|[14726]|RDMAB1054-09|United States|Oregon|630[0n]|BOLD:ABZ0884
Papaipema insulidens|[14727]|RDMAB1055-09|Canada|British Columbia|635[0n]|BOLD:ABZ0884
Papaipema insulidens|[14728]|LNAUT862-14|United States|Oregon|658[0n]|BOLD:ABZ0884
Papaipema insulidens|[14729]|LNAUT863-14|United States|Oregon|658[0n]|BOLD:ABZ0884
Papaipema insulidens|[14730]|LNAUT864-14|United States|Oregon|658[0n]|BOLD:ABZ0884
Papaipema insulidens|[14731]|LNAUT865-14|United States|Oregon|658[0n]|BOLD:ABZ0884
Papaipema insulidens|[14732]|RWWB120-09|United States|Washington|573[0n]|BOLD:ABZ0884
Papaipema insulidens|[14733]|RWWB199-09|United States|Washington|658[0n]|BOLD:ABZ0884
Papaipema insulidens|[14734]|RWWB310-09|United States|Washington|658[0n]|BOLD:ABZ0884
Papaipema insulidens|[14735]|RWWB391-09|United States|Washington|658[0n]|BOLD:ABZ0884
Papaipema insulidens|[14736]|RWWC080-10|United States|Washington|658[0n]|BOLD:ABZ0884
Papaipema insulidens|[14737]|RWWC092-10|United States|Washington|658[0n]|BOLD:ABZ0884
Papaipema insulidens|[14738]|RWWC093-10|United States|Washington|658[0n]|BOLD:ABZ0884
Papaipema insulidens|[14739]|RWWC095-10|United States|Washington|658[0n]|BOLD:ABZ0884
Papaipema insulidens|[14740]|RWWC098-10|United States|Washington|658[0n]|BOLD:ABZ0884
Papaipema insulidens|[14741]|RWWC099-10|United States|Washington|658[0n]|BOLD:ABZ0884
Papaipema insulidens|[14742]|RWWC101-10|United States|Washington|658[0n]|BOLD:ABZ0884
Papaipema insulidens|[14743]|RWWC117-10|United States|Washington|658[0n]|BOLD:ABZ0884
Papaipema insulidens|[14744]|RWWC123-10|United States|Washington|658[0n]|BOLD:ABZ0884
Papaipema insulidens|[14745]|RWWC760-11|United States|Washington|658[0n]|BOLD:ABZ0884
Papaipema insulidens|[14746]|RWWC804-11|United States|Washington|658[0n]|BOLD:ABZ0884
Papaipema insulidens|[14747]|LNAUT866-14|United States|Oregon|658[0n]|BOLD:ABZ0884
Papaipema insulidens|[14748]|TML166-14|United States||658[0n]|BOLD:ABZ0884
Papaipema necopina|[14749]|RDNMF476-08|Canada|Ontario|587[0n]|BOLD:ABZ0884
Papaipema necopina|[14750]|CNCLB2958-14|United States|North Carolina|658[0n]|BOLD:ABZ0884
Papaipema necopina|[14751]|LNCC1939-14|United States|North Carolina|658[0n]|BOLD:ABZ0884
Papaipema necopina|[14752]|CNCLB2988-14|United States|North Carolina|658[0n]|BOLD:ABZ0884
Papaipema limpida|[14753]|CNCLB284-14|United States|Texas|658[0n]|BOLD:ACM4135
Papaipema limpida|[14754]|CNCLB285-14|United States|Nebraska|658[0n]|BOLD:ACM4135
Papaipema rigida|[14755]|LNCC1474-13|United States|North Carolina|658[0n]|BOLD:ACF1228
Papaipema rigida|[14756]|RDLQ152-05|Canada|Quebec|658[0n]|BOLD:ACF1228
Papaipema rigida|[14757]|RDNMG802-08|Canada|New Brunswick|658[0n]|BOLD:ACF1228
Papaipema rigida|[14758]|RDNMG803-08|Canada|New Brunswick|658[0n]|BOLD:ACF1228
Papaipema rigida|[14759]|CNCLB2389-14|Canada|Ontario|658[0n]|BOLD:ACF1228
Papaipema rigida|[14760]|LNCC1473-13|United States|North Carolina|658[0n]|BOLD:ACF1228
Papaipema rigida|[14761]|CNCLB2946-14|United States|North Carolina|658[0n]|BOLD:ACF1228
Papaipema duovata|[14762]|LNC487-06|United States|North Carolina|658[0n]|BOLD:ABX6803
Papaipema duovata|[14763]|LNC488-06|United States|North Carolina|658[0n]|BOLD:ABX6803
Papaipema speciosissima|[14764]|LNCB458-07|United States|North Carolina|649[0n]|BOLD:AAF1797
Papaipema speciosissima|[14765]|LNCC408-10|United States|North Carolina|658[0n]|BOLD:AAF1797
Papaipema speciosissima|[14766]|LSUSA271-06|United States|Florida|658[0n]|BOLD:AAF1797
Papaipema speciosissima|[14767]|RDNMJ366-11|United States|Florida|658[0n]|BOLD:AAF1797
Papaipema speciosissima|[14768]|RDNMG774-08|Canada|Ontario|658[0n]|BOLD:AAF1797
Papaipema speciosissima|[14769]|LNCC409-10|United States|North Carolina|658[0n]|BOLD:AAF1797
Papaipema speciosissima|[14770]|LNCC1539-13|United States|North Carolina|658[0n]|BOLD:AAF1797
Papaipema speciosissima|[14771]|LNAUT2637-14|United States|Massachusetts|658[0n]|BOLD:AAF1797
Papaipema cerina|[14772]|RDNMF473-08|Canada|Ontario|658[0n]|BOLD:ABZ5369
Papaipema cerina|[14773]|LNAUT799-14|United States|Wisconsin|658[0n]|BOLD:ABZ5369
Papaipema cerina|[14774]|LNAUT800-14|United States|Illinois|658[0n]|BOLD:ABZ5369
Papaipema cerina|[14775]|CNCLB2984-14|United States|North Carolina|658[0n]|BOLD:ABZ5369
Papaipema inquaesita|[14776]|XAH707-05|Canada|Ontario|658[0n]|BOLD:ACF3648
Papaipema inquaesita|[14777]|XAH733-05|Canada|Ontario|658[0n]|BOLD:ACF3648
Papaipema inquaesita|[14778]|PHMO355-03|Canada|Ontario|639[0n]|BOLD:ACF3648
Papaipema inquaesita|[14779]|XAH581-05|Canada|Ontario|658[0n]|BOLD:ACF3648
Papaipema inquaesita|[14780]|XAH705-05|Canada|Ontario|658[0n]|BOLD:ACF3648
Papaipema inquaesita|[14781]|XAH695-05|Canada|Ontario|658[0n]|BOLD:ACF3648
Papaipema inquaesita|[14782]|XAH606-05|Canada|Ontario|658[0n]|BOLD:ACF3648
Papaipema inquaesita|[14783]|XAH558-05|Canada|Ontario|658[0n]|BOLD:ACF3648
Papaipema inquaesita|[14784]|XAH546-05|Canada|Ontario|658[0n]|BOLD:ACF3648
Papaipema inquaesita|[14785]|XAH706-05|Canada|Ontario|612[0n]|BOLD:ACF3648
Papaipema inquaesita|[14786]|RDLQF265-06|Canada|Quebec|658[0n]|BOLD:ACF3648
Papaipema inquaesita|[14787]|LNCC1319-11|United States|North Carolina|658[0n]|BOLD:ACF3648
Papaipema inquaesita|[14788]|RDNML184-13|United States|North Carolina|658[0n]|BOLD:ACF3648
Papaipema inquaesita|[14789]|PHMO327-03|Canada|Ontario|639[0n]|BOLD:ACF3648
Papaipema inquaesita|[14790]|XAD471-04|Canada|Ontario|566[0n]|BOLD:ACF3648
Papaipema inquaesita|[14791]|NAMUM403-09|United States|Maryland|658[0n]|BOLD:ACF3648
Papaipema inquaesita|[14792]|LNCC1944-14|United States|North Carolina|658[0n]|BOLD:ACF3648

[illegible]
Papaipema inquaesita|[14790]|XAD471-04|Canada|Ontario|566[0n]|BOLD:ACF3648
Papaipema inquaesita|[14791]|NAMUM403-09|United States|Maryland|658[0n]|BOLD:ACF3648
Papaipema inquaesita|[14792]|LNCC1944-14|United States|North Carolina|658[0n]|BOLD:ACF3648
Papaipema inquaesita|[14793]|CNCLB2957-14|United States|North Carolina|658[0n]|BOLD:ACF3648
Papaipema arctivorens|[14794]|XAH246-05|Canada|Ontario|658[0n]|BOLD:ACF1624
Papaipema arctivorens|[14795]|LNAUS2730-13|United States|Virginia|658[0n]|BOLD:ACF1624
Papaipema arctivorens|[14796]|LSUSA063-06|United States|Kentucky|589[0n]|BOLD:ACF1624
Papaipema arctivorens|[14797]|XAH150-05|Canada|Ontario|658[0n]|BOLD:ACF1624
Papaipema arctivorens|[14798]|XAH147-05|Canada|Ontario|658[0n]|BOLD:ACF1624
Papaipema arctivorens|[14799]|RDNM768-05|Canada|Ontario|658[0n]|BOLD:ACF1624
Papaipema arctivorens|[14800]|RDNM764-05|Canada|Ontario|658[0n]|BOLD:ACF1624
Papaipema arctivorens|[14801]|XAB411-04|Canada|Ontario|658[0n]|BOLD:ACF1624
Papaipema arctivorens|[14802]|PHMO301-03|Canada|Ontario|639[0n]|BOLD:ACF1624
Papaipema arctivorens|[14803]|PHMO330-03|Canada|Ontario|639[0n]|BOLD:ACF1624
Papaipema arctivorens|[14804]|RDLQB824-05|Canada|Quebec|617[0n]|BOLD:ACF1624
Papaipema arctivorens|[14805]|RDLQB844-05|Canada|Quebec|627[0n]|BOLD:ACF1624
Papaipema arctivorens|[14806]|XAI170-06|Canada|Ontario|656[0n]|BOLD:ACF1624
Papaipema arctivorens|[14807]|RDLQG060-06|Canada|Quebec|658[0n]|BOLD:ACF1624
Papaipema arctivorens|[14808]|RDNML185-13|United States|North Carolina|658[0n]|BOLD:ACF1624
Papaipema arctivorens|[14809]|LNAUS2731-13|United States|Virginia|658[0n]|BOLD:ACF1624
Papaipema arctivorens|[14810]|XAD237-04|Canada|Ontario|658[0n]|BOLD:ACF1624
Papaipema arctivorens|[14811]|SMTPJ537-14|Canada|Ontario|606[0n]|BOLD:ACF1624
Papaipema limata|[14812]|CNCLB1447-14|United States|Oregon|658[0n]|BOLD:ACF1624
Papaipema baptisiae|[14813]|PHMO248-03|Canada|Ontario|639[1n]|BOLD:ACF1624
Papaipema baptisiae|[14814]|CNCLB2853-14|United States|North Carolina|658[0n]|BOLD:ACF1624
Papaipema baptisiae|[14815]|RDNML142-13|United States|North Carolina|658[0n]|BOLD:ACF1624
Papaipema baptisiae|[14816]|SMTPD2018-13|Canada|Ontario|567[0n]|BOLD:ACF1624
Papaipema baptisiae|[14817]|LPOKD625-09|United States|Oklahoma|658[0n]|BOLD:ACF1624
Papaipema baptisiae|[14818]|RDNML143-13|United States|North Carolina|658[0n]|BOLD:ACF1624
Papaipema baptisiae|[14819]|CNCLB2856-14|United States|North Carolina|658[0n]|BOLD:ACF1624
Papaipema baptisiae|[14820]|LNCC1338-11|United States|North Carolina|658[0n]|BOLD:ACF1624
Papaipema baptisiae|[14821]|LPOKE261-10|United States|Oklahoma|658[0n]|BOLD:ACF1624
Papaipema baptisiae|[14822]|LPOKD607-09|United States|Oklahoma|658[0n]|BOLD:ACF1624
Papaipema rutila|[14823]|CNCLB2889-14|United States|North Carolina|658[0n]|BOLD:ACF1624
Papaipema baptisiae|[14824]|XAB439-04|Canada|Ontario|658[0n]|BOLD:ACF1624
Papaipema baptisiae|[14825]|LNAUS2732-13|United States|Virginia|658[0n]|BOLD:ACF1624
Papaipema baptisiae|[14826]|LNAUT401-14|United States|Maryland|658[0n]|BOLD:ACF1624
Papaipema baptisiae|[14827]|XAD467-04|Canada|Ontario|588[0n]|BOLD:ACF1624
Papaipema baptisiae|[14828]|RDLQ251-05|Canada|Quebec|614[0n]|BOLD:ACF1624
Papaipema baptisiae|[14829]|NAMUM341-08|United States|Maryland|658[0n]|BOLD:ACF1624
Papaipema baptisiae|[14830]|RDMAB1051-09|Canada|Ontario|653[0n]|BOLD:ACF1624
Papaipema baptisiae|[14831]|LPOKD425-09|United States|Oklahoma|658[0n]|BOLD:ACF1624
Papaipema baptisiae|[14832]|LPOKD441-09|United States|Oklahoma|658[0n]|BOLD:ACF1624
Papaipema baptisiae|[14833]|LPOKD447-09|United States|Oklahoma|658[0n]|BOLD:ACF1624
Papaipema baptisiae|[14834]|MJMSL006-10|United States|Massachusetts|658[0n]|BOLD:ACF1624
Papaipema baptisiae|[14835]|LNCC1318-11|United States|North Carolina|658[0n]|BOLD:ACF1624
Papaipema rutila|[14836]|LNAUS2753-13|United States|Maryland|658[0n]|BOLD:ACF1624
Papaipema rutila|[14837]|LNAUS2754-13|United States|Maryland|658[0n]|BOLD:ACF1624
Papaipema rutila|[14838]|LNAUS2755-13|United States|Maryland|658[0n]|BOLD:ACF1624
Papaipema baptisiae|[14839]|CNCLB2965-14|United States|North Carolina|658[0n]|BOLD:ACF1624
Papaipema rutila|[14840]|CNCLB2985-14|United States|North Carolina|658[0n]|BOLD:ACF1624
Papaipema baptisiae|[14841]|CNCLB2986-14|United States|North Carolina|658[0n]|BOLD:ACF1624
Papaipema beeriana|[14842]|LNAUS2736-13|United States|Illinois|658[0n]|BOLD:ACI4617
Papaipema beeriana|[14843]|NOCNA084-14|United States|Michigan|658[0n]|BOLD:ACI4617
Papaipema beeriana|[14844]|LNAUS2735-13|United States|Illinois|658[0n]|BOLD:ACI4617
Papaipema beeriana|[14845]|NOCNA085-14|United States|Michigan|658[0n]|BOLD:ACI4617
Papaipema beeriana|[14846]|NOCNA086-14|United States|Michigan|658[0n]|BOLD:ACI4617
Papaipema beeriana|[14847]|NOCNA087-14|United States|Michigan|658[0n]|BOLD:ACI4617
Papaipema nepheleptena|[14848]|RDNMG778-08|Canada|Ontario|658[0n]|BOLD:ACF3647
Papaipema sciata|[14849]|QUNOE402-12|United States|Wisconsin|658[0n]|BOLD:ACF3647
Papaipema sciata|[14850]|LNAUT797-14|United States|Illinois|658[0n]|BOLD:ACF3647
Papaipema eupatorii|[14851]|RDNMG793-08|Canada|New Brunswick|658[0n]|BOLD:ACF3647
Papaipema eupatorii|[14852]|RDNME421-08|Canada|Ontario|658[0n]|BOLD:ACF3647
Papaipema eupatorii|[14853]|RDLQF263-06|Canada|Quebec|658[0n]|BOLD:ACF3647
Papaipema eupatorii|[14854]|RDNMG794-08|Canada|New Brunswick|592[0n]|BOLD:ACF3647
Papaipema eupatorii|[14855]|RDNMG792-08|United States|Georgia|658[0n]|BOLD:ACF3647
Papaipema eupatorii|[14856]|CNCLB2989-14|United States|North Carolina|658[0n]|BOLD:ACF3647
Cherokeea attakullakulla|[14857]|RDNMD767-07|United States|North Carolina|658[4n]|BOLD:AAG0719
Cherokeea attakullakulla|[14858]|LNCC1704-13|United States|North Carolina|658[0n]|BOLD:AAG0719
Cherokeea attakullakulla|[14859]|LNCC1705-13|United States|North Carolina|658[0n]|BOLD:AAG0719
Cherokeea attakullakulla|[14860]|RDNMD766-07|United States|North Carolina|658[0n]|BOLD:AAG0719
Cherokeea attakullakulla|[14861]|LNCC1703-13|United States|North Carolina|658[0n]|BOLD:AAG0719
Cherokeea attakullakulla|[14862]|LNCC1706-13|United States|North Carolina|658[0n]|BOLD:AAG0719
Cherokeea attakullakulla|[14863]|LNCC1707-13|United States|North Carolina|658[3n]|BOLD:AAG0719
Resapamea trigona|[14864]|RDNMC717-06|United States|Arkansas|658[0n]|BOLD:AAG2802
Resapamea trigona|[14865]|RDNMD345-06|United States|Georgia|656[0n]|BOLD:AAG2802
Apameagenn sp. 4|[14866]|LNCC1700-13|United States|North Carolina|658[0n]|BOLD:ACJ4815
Apameagenn sp. 4|[14867]|LNCC1848-13|United States|North Carolina|658[0n]|BOLD:ACJ4815
Apameagenn sp. 4|[14868]|LNCC1701-13|United States|North Carolina|658[0n]|BOLD:ACJ4815
Apameagenn sp. 4|[14869]|LNCC1849-13|United States|North Carolina|658[0n]|BOLD:ACJ4815
Apameagenn sp. 1|[14870]|MILEP362-10|United States|Alabama|658[0n]|BOLD:AAP1020
Apameagenn sp. 3|[14871]|LNCC017-10|United States|North Carolina|658[0n]|BOLD:AAK1847
Apameagenn sp. 3|[14872]|LNCC018-10|United States|North Carolina|658[0n]|BOLD:AAK1847
Apameagenn sp. 3|[14873]|RDNMD761-07|United States|Virginia|658[0n]|BOLD:AAK1847
Apameagenn sp. 3|[14874]|LNCC016-10|United States|North Carolina|658[0n]|BOLD:AAK1847
Apameagenn sp. 3|[14875]|CNCLB2474-14|United States|Maryland|658[0n]|BOLD:AAK1847
Apameagenn sp. 2|[14876]|LNCC070-10|United States|North Carolina|658[0n]|BOLD:AAE2902
Apameagenn sp. 2|[14877]|HKONS480-08|United States|Florida|658[0n]|BOLD:AAE2902
Apameagenn sp. 2|[14878]|RDNMD760-07|United States|North Carolina|658[0n]|BOLD:AAE2902
Apameagenn sp. 2|[14879]|LNCB200-06|United States|North Carolina|658[0n]|BOLD:AAE2902
Apameagenn sp. 2|[14880]|LNCB199-06|United States|North Carolina|658[0n]|BOLD:AAE2902
Apameagenn sp. 2|[14881]|LNCC068-10|United States|North Carolina|658[0n]|BOLD:AAE2902
Apameagenn sp. 2|[14882]|LNCC069-10|United States|North Carolina|658[0n]|BOLD:AAE2902
Apameagenn sp. 2|[14883]|LNCC071-10|United States|North Carolina|658[0n]|BOLD:AAE2902
Apameagenn sp. 2|[14884]|CNCLB1457-14|United States|Louisiana|658[0n]|BOLD:AAE2902
Apameagenn sp. 2|[14885]|HKONS478-08|United States|Florida|658[0n]|BOLD:AAE2902
Apameagenn sp. 2|[14886]|HKONS479-08|United States|Florida|658[0n]|BOLD:AAE2902
Apameagenn sp. 2|[14887]|CNCLB1546-14|United States|Louisiana|658[0n]|BOLD:AAE2902
Apameagenn sp. 5|[14888]|HKONS374-08|United States|Florida|658[0n]|BOLD:AAC5372
Apameagenn sp. 5|[14889]|HKONB308-09|United States|Texas|658[0n]|BOLD:AAC5372
Apameagenn sp. 5|[14890]|HKONB309-09|United States|Texas|658[0n]|BOLD:AAC5372
Apameagenn sp. 5|[14891]|HKONS083-07|United States|Florida|658[2n]|BOLD:AAC5372
Apameagenn sp. 5|[14892]|HKONB306-09|United States|Texas|658[0n]|BOLD:AAC5372

Apameagenn sp. 5|[14889]|HKONB308-09|United States|Texas|658[0n]|BOLD:AAC5372
Apameagenn sp. 5|[14890]|HKONB309-09|United States|Texas|658[0n]|BOLD:AAC5372
Apameagenn sp. 5|[14891]|HKONS083-07|United States|Florida|658[2n]|BOLD:AAC5372
Apameagenn sp. 5|[14892]|HKONB306-09|United States|Texas|658[0n]|BOLD:AAC5372
Apameagenn sp. 5|[14893]|HKONB310-09|United States|Texas|658[0n]|BOLD:AAC5372
Apameagenn sp. 5|[14894]|HKONB307-09|United States|Texas|658[0n]|BOLD:AAC5372
Apameagenn sp. 5|[14895]|HKONS527-08|United States|Florida|591[0n]|BOLD:AAC5372
Apameagenn sp. 5|[14896]|HKONB311-09|United States|Texas|658[0n]|BOLD:AAC5372
Apameagenn sp. 5|[14897]|HKONB312-09|United States|Texas|658[0n]|BOLD:AAC5372
Apameagenn sp. 5|[14898]|HKONB313-09|United States|Texas|658[0n]|BOLD:AAC5372
Apameagenn sp. 5|[14899]|RDNMH489-09|United States|Louisiana|658[0n]|BOLD:AAC5372
Apamea alia|[14900]|LPVIA297-08|Canada|British Columbia|658[0n]|BOLD:AAB7612
Apamea alia|[14901]|LPVIB847-08|Canada|British Columbia|658[0n]|BOLD:AAB7612
Apamea alia|[14902]|LPVIB851-08|Canada|British Columbia|658[0n]|BOLD:AAB7612
Apamea alia|[14903]|LPVIB864-08|Canada|British Columbia|658[0n]|BOLD:AAB7612
Apamea alia|[14904]|LALPA200-10|Canada|British Columbia|658[0n]|BOLD:AAB7612
Apamea alia|[14905]|LALPA201-10|Canada|British Columbia|658[0n]|BOLD:AAB7612
Apamea alia|[14906]|LALPA369-10|Canada|British Columbia|658[0n]|BOLD:AAB7612
Apamea alia|[14907]|LALPA384-10|Canada|British Columbia|658[0n]|BOLD:AAB7612
Apamea alia|[14908]|LOWCC850-05|Canada|British Columbia|580[4n]|BOLD:AAB7612
Apamea alia|[14909]|RDLQF049-06|Canada|Quebec|658[2n]|BOLD:AAB7612
Apamea alia|[14910]|LPSK506-08|Canada|Saskatchewan|658[0n]|BOLD:AAB7612
Apamea alia|[14911]|XAC297-04|Canada|Ontario|658[0n]|BOLD:AAB7612
Apamea alia|[14912]|JMMMB346-11|United States|California|658[0n]|BOLD:AAB7612
Apamea alia|[14913]|LPMN793-08|Canada|Manitoba|658[0n]|BOLD:AAB7612
Apamea alia|[14914]|LPMN579-08|Canada|Manitoba|658[0n]|BOLD:AAB7612
Apamea alia|[14915]|LPSK034-08|Canada|Saskatchewan|658[0n]|BOLD:AAB7612
Apamea alia|[14916]|RDLQF048-06|Canada|Quebec|658[0n]|BOLD:AAB7612
Apamea alia|[14917]|XAB636-04|Canada|Ontario|658[0n]|BOLD:AAB7612
Apamea alia|[14918]|XAF583-05|Canada|Ontario|658[0n]|BOLD:AAB7612
Apamea alia|[14919]|PHMO112-03|Canada|Ontario|639[1n]|BOLD:AAB7612
Apamea alia|[14920]|LOWCD279-06|Canada|British Columbia|578[0n]|BOLD:AAB7612
Apamea alia|[14921]|PHMO210-03|Canada|Ontario|639[0n]|BOLD:AAB7612
Apamea alia|[14922]|CNEIC3028-12|Canada|Alberta|634[0n]|BOLD:AAB7612
Apamea alia|[14923]|CNWLF2004-12|Canada|Alberta|634[0n]|BOLD:AAB7612
Apamea alia|[14924]|BBLPB635-10|Canada|British Columbia|658[0n]|BOLD:AAB7612
Apamea alia|[14925]|RDLQB693-05|Canada|Quebec|658[0n]|BOLD:AAB7612
Apamea alia|[14926]|RDNMD147-06|United States|Colorado|604[0n]|BOLD:AAB7612
Apamea alia|[14927]|LPABC330-09|Canada|Alberta|650[0n]|BOLD:AAB7612
Apamea alia|[14928]|LOWCD280-06|Canada|British Columbia|658[0n]|BOLD:AAB7612
Apamea alia|[14929]|LBCA508-05|Canada|British Columbia|658[0n]|BOLD:AAB7612
Apamea alia|[14930]|CNWLE2414-12|Canada|Alberta|632[0n]|BOLD:AAB7612
Apamea alia|[14931]|CNWBG3149-13|Canada|Alberta|588[0n]|BOLD:AAB7612
Macronoctua onusta|[14932]|PHMO247-03|Canada|Ontario|639[0n]|BOLD:AAD1618
Macronoctua onusta|[14933]|XAD469-04|Canada|Ontario|658[0n]|BOLD:AAD1618
Macronoctua onusta|[14934]|RDLQF243-06|Canada|Quebec|658[0n]|BOLD:AAD1618
Macronoctua onusta|[14935]|RDNMD786-07|United States|Connecticut|658[0n]|BOLD:AAD1618
Macronoctua onusta|[14936]|XAH563-05|Canada|Ontario|580[0n]|BOLD:AAD1618
Macronoctua onusta|[14937]|RDLQF242-06|Canada|Quebec|658[0n]|BOLD:AAD1618
Macronoctua onusta|[14938]|XAH730-05|Canada|Ontario|658[0n]|BOLD:AAD1618
Macronoctua onusta|[14939]|PHMO335-03|Canada|Ontario|639[0n]|BOLD:AAD1618
Macronoctua onusta|[14940]|SMTPD4323-13|Canada|Ontario|591[0n]|BOLD:AAD1618
Lemmeria digitalis|[14941]|RDLQF259-06|Canada|Quebec|658[0n]|BOLD:AAF0679
Lemmeria digitalis|[14942]|RDLQF260-06|Canada|Quebec|658[0n]|BOLD:AAF0679
Lemmeria digitalis|[14943]|RDNMD791-07|Canada|Ontario|658[0n]|BOLD:AAF0679
Eremobina claudens|[14944]|LALPA1286-11|Canada|British Columbia|658[0n]|BOLD:AAC2614
Eremobina unicincta|[14945]|NAMUM285-08|United States|California|658[0n]|BOLD:AAC2614
Eremobina claudens|[14946]|RWWB184-09|United States|Washington|658[0n]|BOLD:AAC2614
Eremobina claudens|[14947]|RWWC1031-12|United States|Washington|658[0n]|BOLD:AAC2614
Eremobina claudens|[14948]|RWWC820-11|United States|Washington|658[0n]|BOLD:AAC2614
Eremobina claudens|[14949]|RWWC772-11|United States|Washington|658[0n]|BOLD:AAC2614
Eremobina claudens|[14950]|RWWC730-11|United States|Washington|658[0n]|BOLD:AAC2614
Eremobina claudens|[14951]|RWWC707-11|United States|Washington|658[0n]|BOLD:AAC2614
Eremobina claudens|[14952]|LBCH4433-10|Canada|British Columbia|658[0n]|BOLD:AAC2614
Eremobina claudens|[14953]|RWWB351-09|United States|Washington|658[0n]|BOLD:AAC2614
Eremobina claudens|[14954]|RWWB226-09|United States|Washington|658[0n]|BOLD:AAC2614
Eremobina claudens|[14955]|RDNMB469-05|Canada|Alberta|658[0n]|BOLD:AAC2614
Eremobina claudens|[14956]|RWWA979-09|United States|Washington|658[0n]|BOLD:AAC2614
Eremobina claudens|[14957]|RDLQ461-07|Canada|Quebec|608[0n]|BOLD:AAC2614
Eremobina claudens|[14958]|SSEIB11413-13|Canada|Alberta|558[0n]|BOLD:AAC2614
Eremobina leucoscelis|[14959]|RDNMB471-05|Canada|Alberta|658[0n]|BOLD:AAC2614
Eremobina leucoscelis|[14960]|RDNMB472-05|Canada|Alberta|658[0n]|BOLD:AAC2614
Eremobina leucoscelis|[14961]|BBLPB773-10|Canada|Alberta|658[0n]|BOLD:AAC2614
Eremobina claudens|[14962]|LBCH2058-10|Canada|British Columbia|658[0n]|BOLD:AAC2614
Eremobina claudens|[14963]|LBCH1553-10|Canada|British Columbia|658[0n]|BOLD:AAC2614
Eremobina leucoscelis|[14964]|XAD379-04|Canada|Ontario|658[0n]|BOLD:AAC2614
Eremobina leucoscelis|[14965]|RDLQB642-05|Canada|Quebec|578[0n]|BOLD:AAC2614
Eremobina leucoscelis|[14966]|RDLQ468-07|Canada|Quebec|601[0n]|BOLD:AAC2614
Eremobina leucoscelis|[14967]|RDNMI168-10|Canada|New Brunswick|658[0n]|BOLD:AAC2614
Eremobina leucoscelis|[14968]|BBLPB768-10|Canada|Alberta|658[0n]|BOLD:AAC2614
Eremobina claudens|[14969]|LBCH1239-10|Canada|British Columbia|658[0n]|BOLD:AAC2614
Eremobina unicincta|[14970]|JMMMB587-13|United States|California|592[0n]|BOLD:AAC2614
Photedes carterae|[14971]|LNAUT829-14|United States|North Carolina|658[0n]|BOLD:AAJ3892
Photedes carterae|[14972]|RDNMD789-07|United States|Georgia|656[0n]|BOLD:AAJ3892
Photedes carterae|[14973]|LNAUT830-14|United States|New Jersey|658[0n]|BOLD:AAJ3892
Photedes carterae|[14974]|LNAUT831-14|United States|New Jersey|658[0n]|BOLD:AAJ3892
Photedes carterae|[14975]|LNAUT832-14|United States|New Jersey|658[0n]|BOLD:AAJ3892
Neoligia rubirena|[14976]|RDNML192-13|United States|Arizona|658[0n]|BOLD:ACD9295
Xylomoia chagnoni|[14977]|RDNMD783-07|United States|Connecticut|658[0n]|BOLD:AAE4227
Xylomoia chagnoni|[14978]|RDNMG580-08|Canada|Ontario|658[0n]|BOLD:AAE4227
Xylomoia chagnoni|[14979]|RDNMG581-08|Canada|Ontario|658[0n]|BOLD:AAE4227
Xylomoia chagnoni|[14980]|RDNMG582-08|Canada|Ontario|658[0n]|BOLD:AAE4227
Xylomoia chagnoni|[14981]|BLTIB367-08|Canada|Ontario|642[0n]|BOLD:AAE4227
Xylomoia chagnoni|[14982]|LILLA473-11|United States|Illinois|658[0n]|BOLD:AAE4227
Xylomoia indirecta|[14983]|LALPA468-10|Canada|British Columbia|658[0n]|BOLD:AAB1776
Xylomoia indirecta|[14984]|LALPA418-10|Canada|British Columbia|658[0n]|BOLD:AAB1776
Xylomoia indirecta|[14985]|LPVIA407-08|Canada|British Columbia|658[0n]|BOLD:AAB1776
Xylomoia indirecta|[14986]|LALPA414-10|Canada|British Columbia|658[0n]|BOLD:AAB1776
Xylomoia indirecta|[14987]|LALPA480-10|Canada|British Columbia|658[0n]|BOLD:AAB1776
Xylomoia indirecta|[14988]|LPVIA517-08|Canada|British Columbia|658[0n]|BOLD:AAB1776
Xylomoia indirecta|[14989]|LALPA410-10|Canada|British Columbia|658[0n]|BOLD:AAB1776
Xylomoia indirecta|[14990]|LALPA532-10|Canada|British Columbia|658[0n]|BOLD:AAB1776
Xylomoia indirecta|[14991]|RWWA684-09|United States|Washington|658[0n]|BOLD:AAB1776
Xylomoia indirecta|[14992]|LHLEP388-06|Canada|British Columbia|658[0n]|BOLD:AAB1776

Xylomoia indirecta|[illegible]|LALPA410-10|Canada|British Columbia|658[0n]|BOLD:AAB1776
Xylomoia indirecta|[14990]|LALPA532-10|Canada|British Columbia|658[0n]|BOLD:AAB1776
Xylomoia indirecta|[14991]|RWWA684-09|United States|Washington|658[0n]|BOLD:AAB1776
Xylomoia indirecta|[14992]|LHLEP388-06|Canada|British Columbia|658[0n]|BOLD:AAB1776
Xylomoia indirecta|[14993]|LHLEP419-06|Canada|British Columbia|658[0n]|BOLD:AAB1776
Xylomoia indirecta|[14994]|RWWA733-09|United States|Washington|658[0n]|BOLD:AAB1776
Xylomoia indirecta|[14995]|RWWA808-09|United States|Washington|658[0n]|BOLD:AAB1776
Xylomoia indirecta|[14996]|RWWA768-09|United States|Washington|658[0n]|BOLD:AAB1776
Xylomoia indirecta|[14997]|RWWA656-09|United States|Washington|658[0n]|BOLD:AAB1776
Xylomoia indirecta|[14998]|RWWA646-09|United States|Washington|658[0n]|BOLD:AAB1776
Xylomoia indirecta|[14999]|LHLEP409-06|Canada|British Columbia|658[0n]|BOLD:AAB1776
Xylomoia indirecta|[15000]|LHLEP384-06|Canada|British Columbia|658[0n]|BOLD:AAB1776
Xylomoia indirecta|[15001]|RWWA557-09|United States|Washington|618[0n]|BOLD:AAB1776
Xylomoia indirecta|[15002]|RDNMB527-05|Canada|British Columbia|565[1n]|BOLD:AAB1776
Xylomoia indirecta|[15003]|LHLEP385-06|Canada|British Columbia|658[0n]|BOLD:AAB1776
Xylomoia indirecta|[15004]|RWWA676-09|United States|Washington|620[0n]|BOLD:AAB1776
Xylomoia indirecta|[15005]|RWWA829-09|United States|Washington|634[0n]|BOLD:AAB1776
Xylomoia indirecta|[15006]|RWWA931-09|United States|Washington|658[0n]|BOLD:AAB1776
Xylomoia indirecta|[15007]|RWWB928-10|United States|Washington|658[0n]|BOLD:AAB1776
Xylomoia indirecta|[15008]|JMMMB323-11|United States|California|658[0n]|BOLD:AAB1776
Xylomoia indirecta|[15009]|RDNMB526-05|United States|Oregon|658[0n]|BOLD:AAB1776
Xylomoia indirecta|[15010]|RWWB286-09|United States|Washington|658[0n]|BOLD:AAB1776
Xylomoia indirecta|[15011]|RWWB040-09|United States|Washington|658[0n]|BOLD:AAB1776
Xylomoia indirecta|[15012]|RWWA828-09|United States|Washington|658[0n]|BOLD:AAB1776
Xylomoia indirecta|[15013]|LPVIA609-08|Canada|British Columbia|658[0n]|BOLD:AAB1776
Xylomoia indirecta|[15014]|LBCS267-07|Canada|British Columbia|658[0n]|BOLD:AAB1776
Xylomoia indirecta|[15015]|LHLEP438-06|Canada|British Columbia|658[0n]|BOLD:AAB1776
Xylomoia indirecta|[15016]|LHLEP436-06|Canada|British Columbia|658[0n]|BOLD:AAB1776
Xylomoia indirecta|[15017]|LHLEP420-06|Canada|British Columbia|658[0n]|BOLD:AAB1776
Xylomoia indirecta|[15018]|LHLEP408-06|Canada|British Columbia|658[0n]|BOLD:AAB1776
Xylomoia indirecta|[15019]|LHLEP386-06|Canada|British Columbia|658[0n]|BOLD:AAB1776
Xylomoia indirecta|[15020]|RWWB953-10|United States|Washington|630[0n]|BOLD:AAB1776
Xylomoia indirecta|[15021]|LHLEP387-06|Canada|British Columbia|658[0n]|BOLD:AAB1776
Xylomoia indirecta|[15022]|RWWA693-09|United States|Washington|658[0n]|BOLD:AAB1776
Xylomoia indirecta|[15023]|RWWC482-11|United States|Washington|658[0n]|BOLD:AAB1776
Xylomoia indirecta|[15024]|LOWCD281-06|Canada|British Columbia|658[0n]|BOLD:AAB1776
Xylomoia indirecta|[15025]|RWWC555-11|United States|Washington|658[0n]|BOLD:AAB1776
Brachylomia populi|[15026]|RDNMF205-08|United States|Washington|658[0n]|BOLD:AAE0555
Brachylomia populi|[15027]|RDNMF206-08|United States|Washington|658[0n]|BOLD:AAE0555
Brachylomia populi|[15028]|RDNMF204-08|United States|Washington|658[0n]|BOLD:AAE0555
Brachylomia populi|[15029]|RDNMF207-08|United States|Washington|658[0n]|BOLD:AAE0555
Brachylomia populi|[15030]|RDNMF208-08|United States|Washington|658[0n]|BOLD:AAE0555
Rhizedra lutosa|[15031]|RDLQB761-05|Canada|Quebec|589[7n]|
Rhizedra lutosa|[15032]|NLLEA1119-12|Netherlands|South Holland|658[0n]|BOLD:AAB8633
Rhizedra lutosa|[15033]|LEFIC674-10|Finland|Finland Proper|658[0n]|BOLD:AAB8633
Rhizedra lutosa|[15034]|LEFIF504-10|Finland||658[0n]|BOLD:AAB8633
Rhizedra lutosa|[15035]|GBMIN22526-13|||666[1n]|BOLD:AAB8633
Rhizedra lutosa|[15036]|RDLQH037-06|Canada|Quebec|658[0n]|BOLD:AAB8633
Rhizedra lutosa|[15037]|LEFIH015-10|Finland||658[0n]|BOLD:AAB8633
Rhizedra lutosa|[15038]|LEFIH016-10|Finland||658[0n]|BOLD:AAB8633
Rhizedra lutosa|[15039]|RDLQB459-05|Canada|Quebec|658[0n]|BOLD:AAB8633
Rhizedra lutosa|[15040]|RDLQF882-06|Canada|Quebec|658[0n]|BOLD:AAB8633
Rhizedra lutosa|[15041]|RDLQG217-06|Canada|Quebec|658[0n]|BOLD:AAB8633
Rhizedra lutosa|[15042]|RDLQH035-06|Canada|Quebec|658[0n]|BOLD:AAB8633
Rhizedra lutosa|[15043]|RDLQH036-06|Canada|Quebec|658[0n]|BOLD:AAB8633
Rhizedra lutosa|[15044]|MJMSL083-10|United States|Massachusetts|648[0n]|BOLD:AAB8633
Rhizedra lutosa|[15045]|GWOR3875-09|Germany|Bavaria|658[0n]|BOLD:AAB8633
Rhizedra lutosa|[15046]|CGUKB657-09|United Kingdom|England|658[0n]|BOLD:AAB8633
Rhizedra lutosa|[15047]|CGUKA887-09|United Kingdom|England|642[0n]|BOLD:AAB8633
Rhizedra lutosa|[15048]|GWORK460-09|Germany|Bavaria|658[0n]|BOLD:AAB8633
Rhizedra lutosa|[15049]|CGUKA1000-09|United Kingdom|England|657[0n]|BOLD:AAB8633
Rhizedra lutosa|[15050]|GWORO988-09|Germany|Bavaria|658[0n]|BOLD:AAB8633
Rhizedra lutosa|[15051]|LENOA568-11|France|Haute Normandie|658[0n]|BOLD:AAB8633
Rhizedra lutosa|[15052]|LENOA569-11|France|Haute Normandie|658[0n]|BOLD:AAB8633
Rhizedra lutosa|[15053]|LENOA570-11|France|Haute Normandie|658[0n]|BOLD:AAB8633
Rhizedra lutosa|[15054]|GBLAF586-14|Germany|Brandenburg|658[0n]|BOLD:AAB8633
Rhizedra lutosa|[15055]|LASTS034-14|Austria||658[0n]|BOLD:AAB8633
Rhizedra lutosa|[15056]|LEATG276-14|Austria|Tirol|658[0n]|BOLD:AAB8633
Rhizedra lutosa|[15057]|LEATG275-14|Austria|Vorarlberg|658[0n]|BOLD:AAB8633
Rhizedra lutosa|[15058]|GWOR4001-09|Germany|Bavaria|658[0n]|BOLD:AAB8633
Rhizedra lutosa|[15059]|FBLMV120-09|Germany|Bavaria|658[0n]|BOLD:AAB8633
Rhizedra lutosa|[15060]|GWOSA396-10|Germany|Bavaria|658[0n]|BOLD:AAB8633
Rhizedra lutosa|[15061]|LEATH608-14|Italy|South Tyrol|658[0n]|BOLD:AAB8633
Meropleon diversicolor|[15062]|XAD399-04|Canada|Ontario|577[0n]|BOLD:AAF0922
Meropleon diversicolor|[15063]|RDLQ466-07|Canada|Quebec|608[0n]|BOLD:AAF0922
Meropleon diversicolor|[15064]|RDNMD777-07|United States|Connecticut|658[0n]|BOLD:AAF0922
Meropleon diversicolor|[15065]|RDNML160-13|United States|North Carolina|658[0n]|BOLD:AAF0922
Meropleon diversicolor|[15066]|RDNML161-13|United States|North Carolina|658[0n]|BOLD:AAF0922
Meropleon cinnamicolor|[15067]|LNAUS4450-13|United States|South Carolina|658[0n]|BOLD:ACJ9003
Meropleon cinnamicolor|[15068]|LNAUS4452-13|United States|South Carolina|658[0n]|BOLD:ACJ9003
Meropleon cinnamicolor|[15069]|LNAUS4449-13|United States|South Carolina|658[0n]|BOLD:ACJ9003
Meropleon cinnamicolor|[15070]|LNAUS4451-13|United States|South Carolina|658[0n]|BOLD:ACJ9003
Meropleon cinnamicolor|[15071]|LNAUS4453-13|United States|South Carolina|658[0n]|BOLD:ACJ9003
Meropleon cosmion|[15072]|RDNML318-13|United States|Florida|658[0n]|BOLD:AAX4662
Meropleon cosmion|[15073]|RDNMI183-10|United States|South Carolina|658[0n]|BOLD:AAX4662
Meropleon cosmion|[15074]|LNAUS4454-13|United States|Maryland|658[0n]|BOLD:AAX4662
Meropleon cosmion|[15075]|LNAUS4458-13|United States|Maryland|658[0n]|BOLD:ACJ8154
Meropleon cosmion|[15076]|LNAUS4455-13|United States|Maryland|658[0n]|BOLD:ACJ8154
Meropleon cosmion|[15077]|LNAUS4456-13|United States|Maryland|658[0n]|BOLD:ACJ8154
Meropleon cosmion|[15078]|LNAUS4457-13|United States|Maryland|658[0n]|BOLD:ACJ8154
Meropleon cosmion|[15079]|LNAUS4459-13|United States|Maryland|658[0n]|BOLD:ACJ8154
Meropleon ambifusca|[15080]|LPOKA407-09|United States|Oklahoma|658[0n]|BOLD:AAD8634
Meropleon ambifusca|[15081]|LSEU114-06|United States|Georgia|658[0n]|BOLD:AAD8634
Meropleon ambifusca|[15082]|RDNMC709-06|United States|Oklahoma|658[0n]|BOLD:AAD8634
Meropleon ambifusca|[15083]|LSEU777-06|United States|Missouri|658[0n]|BOLD:AAD8634
Meropleon ambifusca|[15084]|LSEU778-06|United States|Missouri|656[0n]|BOLD:AAD8634
Meropleon ambifusca|[15085]|LSEU776-06|United States|Missouri|658[0n]|BOLD:AAD8634
Meropleon ambifusca|[15086]|LILLA726-11|United States|Illinois|658[0n]|BOLD:AAD8634
Meropleon linae|[15087]|LNAUT405-14|United States|Kansas|658[0n]|BOLD:ACN8106
Meropleon linae|[15088]|LNAUT406-14|United States|Kansas|658[0n]|BOLD:ACN8106
Meropleon titan|[15089]|CNCLB2906-14|United States|North Carolina|658[0n]|BOLD:AAH5691
Meropleon titan|[15090]|LPOKD622-09|United States|Oklahoma|658[0n]|BOLD:AAH5691
Meropleon titan|[15091]|CNCLB207-14|United States|New Jersey|658[0n]|BOLD:AAH5691
[illegible]

Meropleon titan|[15089]|CNCLB2906-14|United States|North Carolina|658[0n]|BOLD:AAH5691
Meropleon titan|[15090]|LPOKD622-09|United States|Oklahoma|658[0n]|BOLD:AAH5691
Meropleon titan|[15091]|CNCLB207-14|United States|New Jersey|658[0n]|BOLD:AAH5691
Meropleon titan|[15092]|CNCLB2974-14|United States|North Carolina|658[0n]|BOLD:AAH5691
Meropleon titan|[15093]|CNCLB2978-14|United States|North Carolina|658[0n]|BOLD:AAH5691
Meropleon titan|[15094]|CNCLB2979-14|United States|North Carolina|658[0n]|BOLD:AAH5691
Meropleon titan|[15095]|LNCC399-10|United States|North Carolina|658[0n]|BOLD:AAH5691
Meropleon titan|[15096]|CNCLB2980-14|United States|North Carolina|658[0n]|BOLD:AAH5691
Epidemas cinerea|[15097]|LOWCB503-05|Canada|British Columbia|658[0n]|BOLD:ABY4336
Epidemas cinerea|[15098]|LOWCB508-05|Canada|British Columbia|658[0n]|BOLD:ABY4336
Epidemas cinerea|[15099]|LOWCB505-05|Canada|British Columbia|658[0n]|BOLD:ABY4336
Epidemas cinerea|[15100]|LOWCB498-05|Canada|British Columbia|658[0n]|BOLD:ABY4336
Epidemas cinerea|[15101]|LOWCB497-05|Canada|British Columbia|658[0n]|BOLD:ABY4336
Epidemas cinerea|[15102]|LOWCB500-05|Canada|British Columbia|615[1n]|BOLD:ABY4336
Epidemas cinerea|[15103]|LOWCB507-05|Canada|British Columbia|616[1n]|BOLD:ABY4336
Epidemas cinerea|[15104]|LOWCB506-05|Canada|British Columbia|658[0n]|BOLD:ABY4336
Epidemas cinerea|[15105]|LOWCB509-05|Canada|British Columbia|658[0n]|BOLD:ABY4336
Epidemas cinerea|[15106]|LOWCB502-05|Canada|British Columbia|615[0n]|BOLD:ABY4336
Epidemas cinerea|[15107]|LOWCB496-05|Canada|British Columbia|658[0n]|BOLD:ABY4336
Epidemas cinerea|[15108]|LOWCB499-05|Canada|British Columbia|598[2n]|BOLD:ABY4336
Epidemas cinerea|[15109]|LOWCB511-05|Canada|British Columbia|658[0n]|BOLD:ABY4336
Epidemas cinerea|[15110]|RDNM125-05|Canada|Alberta|658[0n]|BOLD:ACF0588
Epidemas cinerea|[15111]|RDNM126-05|Canada|Alberta|658[0n]|BOLD:ACF0588
Epidemas cinerea|[15112]|RDNM127-05|Canada|Alberta|658[0n]|BOLD:ACF0588
Epidemas cinerea|[15113]|RDNM133-05|United States|Wyoming|658[0n]|BOLD:ACF0588
Epidemas cinerea|[15114]|RDNM123-05|United States|Oregon|658[0n]|BOLD:ACF0588
Epidemas cinerea|[15115]|RDNM122-05|United States|Oregon|658[0n]|BOLD:ACF0588
Epidemas cinerea|[15116]|RDNM134-05|United States|Wyoming|658[0n]|BOLD:ACF0588
Epidemas cinerea|[15117]|RDNM135-05|United States|Wyoming|658[0n]|BOLD:ACF0588
Epidemas cinerea|[15118]|RDNM136-05|United States|Wyoming|658[0n]|BOLD:ACF0588
Epidemas cinerea|[15119]|RDNM137-05|United States|Wyoming|658[0n]|BOLD:ACF0588
Epidemas cinerea|[15120]|RDNMB208-05|United States|Washington|602[0n]|BOLD:AAB1675
Epidemas cinerea|[15121]|RDNMB212-05|United States|Washington|594[0n]|BOLD:AAB1675
Epidemas cinerea|[15122]|RDNMB210-05|United States|Washington|593[0n]|BOLD:AAB1675
Epidemas cinerea|[15123]|RDNMB209-05|United States|Washington|593[0n]|BOLD:AAB1675
Epidemas cinerea|[15124]|RDNMB211-05|United States|Washington|601[0n]|BOLD:AAB1675
Epidemas cinerea|[15125]|RDNM124-05|United States|Washington|658[0n]|BOLD:AAB1675
Epidemas cinerea|[15126]|LOWCB504-05|Canada|British Columbia|658[0n]|BOLD:AAB1675
Epidemas cinerea|[15127]|LOWCB510-05|Canada|British Columbia|658[0n]|BOLD:AAB1675
Epidemas cinerea|[15128]|LOWCB495-05|Canada|British Columbia|658[0n]|BOLD:AAB1675
Epidemas cinerea|[15129]|LOWCB501-05|Canada|British Columbia|658[0n]|BOLD:AAB1675
Epidemas cinerea|[15130]|LOWCB512-05|Canada|British Columbia|658[0n]|BOLD:AAB1675
Epidemas cinerea|[15131]|LOWCC191-05|Canada|British Columbia|605[0n]|BOLD:AAB1675
Epidemas obscurus|[15132]|RDNM138-05|United States|Oregon|658[0n]|BOLD:AAB1674
Epidemas obscurus|[15133]|RDNM140-05|United States|Oregon|658[0n]|BOLD:AAB1674
Epidemas obscurus|[15134]|RDNM141-05|United States|Oregon|658[0n]|BOLD:AAB1674
Epidemas obscurus|[15135]|RDNM128-05|United States|Washington|658[0n]|BOLD:AAB1674
Epidemas obscurus|[15136]|LBCH6849-10|Canada|British Columbia|658[0n]|BOLD:AAB1674
Epidemas obscurus|[15137]|LBCH6782-10|Canada|British Columbia|658[0n]|BOLD:AAB1674
Epidemas obscurus|[15138]|RDNM130-05|United States|Washington|658[0n]|BOLD:AAB1674
Epidemas obscurus|[15139]|RDNM139-05|United States|Oregon|658[0n]|BOLD:AAB1674
Epidemas obscurus|[15140]|RDNM142-05|United States|Oregon|658[0n]|BOLD:AAB1674
Epidemas obscurus|[15141]|RDNM144-05|United States|Oregon|581[0n]|BOLD:AAB1674
Epidemas obscurus|[15142]|RDNM143-05|United States|Oregon|658[0n]|BOLD:AAB1674
Epidemas obscurus|[15143]|RDNMF498-08|United States|Washington|658[0n]|BOLD:AAB1674
Epidemas obscurus|[15144]|RDNMF499-08|Canada|British Columbia|658[0n]|BOLD:AAB1674
Epidemas obscurus|[15145]|RDNMF500-08|Canada|British Columbia|609[0n]|BOLD:AAB1674
Epidemas obscurus|[15146]|RDNM129-05|United States|Washington|658[0n]|BOLD:AAB1674
Epidemas obscurus|[15147]|LBCH6927-10|Canada|British Columbia|658[0n]|BOLD:AAB1674
Epidemas obscurus|[15148]|LBCH6833-10|Canada|British Columbia|658[0n]|BOLD:AAB1674
Epidemas obscurus|[15149]|LBCH7051-10|Canada|British Columbia|658[0n]|BOLD:AAB1674
Epidemas obscurus|[15150]|LBCH7052-10|Canada|British Columbia|658[0n]|BOLD:AAB1674
Epidemas obscurus|[15151]|LBCH7230-10|Canada|British Columbia|658[0n]|BOLD:AAB1674
Photedes inops|[15152]|RDNMF277-08|Canada|Alberta|658[0n]|BOLD:AAD7226
Photedes inops|[15153]|RDNMF275-08|Canada|Alberta|658[0n]|BOLD:AAD7226
Photedes inops|[15154]|RDNMD790-07|Canada|Alberta|658[0n]|BOLD:AAD7226
Photedes inops|[15155]|RDNMF276-08|Canada|Alberta|658[0n]|BOLD:AAD7226
Photedes inops|[15156]|RDNMF278-08|Canada|Alberta|658[0n]|BOLD:AAD7226
Photedes inops|[15157]|RDNMF279-08|Canada|Alberta|658[0n]|BOLD:AAD7226
Hypocoena rufostrigata|[15158]|RDNMF270-08|Canada|Saskatchewan|658[0n]|BOLD:AAD8056
Hypocoena rufostrigata|[15159]|RDNMF273-08|Canada|Saskatchewan|658[0n]|BOLD:AAD8056
Hypocoena rufostrigata|[15160]|RDNMF274-08|Canada|Alberta|658[0n]|BOLD:AAD8056
Hypocoena rufostrigata|[15161]|RDNMF272-08|Canada|Saskatchewan|658[1n]|BOLD:AAD8056
Hypocoena rufostrigata|[15162]|RDNMF271-08|Canada|Saskatchewan|658[0n]|BOLD:AAD8056
Hypocoena rufostrigata|[15163]|LPMN733-08|Canada|Manitoba|658[0n]|BOLD:AAD8056
Hypocoena rufostrigata|[15164]|JMMMB328-11|United States|California|658[0n]|BOLD:AAD8056
Resapamea angelika|[15165]|RDNM036-05|United States|Nevada|658[0n]|BOLD:AAY0558
Resapamea diluvius|[15166]|RDNM027-05|United States|Washington|658[0n]|BOLD:AAF7494
Resapamea diluvius|[15167]|RDNM028-05|United States|Washington|658[0n]|BOLD:AAF7494
Resapamea diluvius|[15168]|RDNM029-05|United States|Washington|658[0n]|BOLD:AAF7494
Resapamea diluvius|[15169]|RWWA494-09|United States|Washington|658[0n]|BOLD:AAF7494
Resapamea diluvius|[15170]|RDNMJ151-10|United States|Washington|658[0n]|BOLD:AAF7494
Resapamea diluvius|[15171]|RDNMJ153-10|United States|Washington|658[0n]|BOLD:AAF7494
Resapamea enargia|[15172]|LNAUS4445-13|United States|California|658[0n]|BOLD:ACJ8064
Resapamea innota|[15173]|RDNME569-08|United States|California|658[0n]|BOLD:AAE7363
Resapamea innota|[15174]|RDNM543-05|United States|Oregon|574[0n]|BOLD:AAE7363
Resapamea innota|[15175]|RDNM030-05|United States|Oregon|623[0n]|BOLD:AAE7363
Resapamea innota|[15176]|RDNM544-05|United States|Oregon|579[0n]|BOLD:AAE7363
Resapamea innota|[15177]|RDNMJ156-10|United States|Oregon|658[0n]|BOLD:AAE7363
Resapamea mammuthus|[15178]|CNCLA568-13|Canada|Yukon Territory|658[0n]|BOLD:ACV2136
Resapamea passer|[15179]|RDNMJ155-10|United States|California|658[0n]|BOLD:AAB5260
Resapamea venosa|[15180]|RDMAB1045-09|United States|Washington|658[0n]|BOLD:AAB5260
Resapamea passer|[15181]|LBCA373-05|Canada|British Columbia|582[0n]|BOLD:AAB5260
Resapamea passer|[15182]|LPVIB840-08|Canada|British Columbia|658[0n]|BOLD:AAB5260
Resapamea passer|[15183]|LPSO877-08|Canada|Ontario|658[0n]|BOLD:AAB5260
Resapamea venosa|[15184]|RDNMF723-08|Canada|British Columbia|658[0n]|BOLD:AAB5260
Resapamea passer|[15185]|LCHQ171-07|Canada|Manitoba|658[0n]|BOLD:AAB5260
Resapamea passer|[15186]|RDNMD782-07|United States|Kentucky|658[0n]|BOLD:AAB5260
Resapamea passer|[15187]|LBCA038-05|Canada|British Columbia|658[0n]|BOLD:AAB5260
Resapamea passer|[15188]|LBCA032-05|Canada|British Columbia|658[0n]|BOLD:AAB5260
Resapamea passer|[15189]|LBCA031-05|Canada|British Columbia|658[0n]|BOLD:AAB5260
Resapamea passer|[15190]|RDNM973-05|Canada|Alberta|658[0n]|BOLD:AAB5260
Resapamea passer|[15191]|RDNM035-05|United States|Nevada|658[0n]|BOLD:AAB5260
[illegible]

Resapamea passer|[15189]|LBCA031-05|Canada|British Columbia|658[0n]|BOLD:AAB5260
Resapamea passer|[15190]|RDNM973-05|Canada|Alberta|658[0n]|BOLD:AAB5260
Resapamea passer|[15191]|RDNM035-05|United States|Nevada|658[0n]|BOLD:AAB5260
Resapamea passer|[15192]|RDNM034-05|Canada|Alberta|658[0n]|BOLD:AAB5260
Resapamea passer|[15193]|RDNM033-05|Canada|Alberta|658[0n]|BOLD:AAB5260
Resapamea passer|[15194]|RDNM032-05|Canada|Alberta|658[0n]|BOLD:AAB5260
Resapamea passer|[15195]|RDNM539-05|United States|Washington|561[0n]|BOLD:AAB5260
Resapamea passer|[15196]|RDNM541-05|Canada|British Columbia|581[0n]|BOLD:AAB5260
Resapamea passer|[15197]|RDNM540-05|United States|Washington|580[0n]|BOLD:AAB5260
Resapamea passer|[15198]|RDNM542-05|Canada|British Columbia|567[0n]|BOLD:AAB5260
Resapamea passer|[15199]|RDNM031-05|Canada|Alberta|624[0n]|BOLD:AAB5260
Resapamea passer|[15200]|LPVIA232-08|Canada|British Columbia|631[0n]|BOLD:AAB5260
Resapamea venosa|[15201]|RDMAB1044-09|United States|Washington|654[0n]|BOLD:AAB5260
Resapamea passer|[15202]|LALPA340-10|Canada|British Columbia|658[0n]|BOLD:AAB5260
Resapamea passer|[15203]|BBLPB870-10|Canada|Alberta|658[0n]|BOLD:AAB5260
Resapamea passer|[15204]|RDNM974-05|United States|Wyoming|658[0n]|BOLD:AAB5260
Resapamea passer|[15205]|LBCA037-05|Canada|British Columbia|658[0n]|BOLD:AAB5260
Resapamea passer|[15206]|LBCA033-05|Canada|British Columbia|658[0n]|BOLD:AAB5260
Resapamea passer|[15207]|LBCA041-05|Canada|British Columbia|658[0n]|BOLD:AAB5260
Resapamea passer|[15208]|LNCC1508-13|United States|North Carolina|658[0n]|BOLD:AAB5260
Resapamea passer|[15209]|CNCLB2875-14|United States|North Carolina|658[0n]|BOLD:AAB5260
Oligia latruncula|[15210]|FBLMV394-09|Germany|Bavaria|658[1n]|BOLD:AAB7661
Oligia latruncula|[15211]|GWOTL067-13|Germany|Saarland|658[0n]|BOLD:AAB7661
Oligia latruncula|[15212]|GWORO673-09|Germany|Bavaria|658[0n]|BOLD:AAB7661
Oligia latruncula|[15213]|GWOSA383-10|Germany|Bavaria|658[0n]|BOLD:AAB7661
Oligia latruncula|[15214]|ODOPE368-11|Germany|Bavaria|658[0n]|BOLD:AAB7661
Oligia latruncula|[15215]|LEFIA570-10|Finland|South Karelia|658[0n]|BOLD:AAB7661
Oligia latruncula|[15216]|LEFIA571-10|Finland|South Karelia|658[0n]|BOLD:AAB7661
Oligia latruncula|[15217]|PHLAC454-10|Italy|South Tyrol|658[0n]|BOLD:AAB7661
Oligia latruncula|[15218]|GWOTG947-12|Bulgaria|Sofiya|658[0n]|BOLD:AAB7661
Oligia latruncula|[15219]|PHLAF317-11|Macedonia||658[0n]|BOLD:AAB7661
Oligia latruncula|[15220]|PHLAB1207-10|Italy|Lazio|658[0n]|BOLD:AAB7661
Oligia latruncula|[15221]|LENOA558-11|France|Provence-Alpes-Cote d'Azur|658[0n]|BOLD:AAB7661
Oligia latruncula|[15222]|GWOSZ238-11|Italy|Trentino-Alto Adige|658[0n]|BOLD:AAB7661
Oligia latruncula|[15223]|PHLAF613-11|Italy|South Tyrol|658[0n]|BOLD:AAB7661
Oligia latruncula|[15224]|NOCJH179-09|France||658[0n]|BOLD:AAB7661
Oligia latruncula|[15225]|RDNMD707-06|Denmark||658[0n]|BOLD:AAB7661
Oligia latruncula|[15226]|GWOTI294-12|Georgia||658[0n]|BOLD:AAB7661
Oligia latruncula|[15227]|LEATD195-13|Austria|Tirol|658[0n]|BOLD:AAB7661
Oligia latruncula|[15228]|CNCLB2527-14|United States|New York|658[0n]|BOLD:AAB7661
Oligia latruncula|[15229]|NOCJH174-09|France||636[0n]|BOLD:AAB7661
Oligia latruncula|[15230]|NOCJH177-09|France||658[0n]|BOLD:AAB7661
Oligia latruncula|[15231]|CGUKB121-09|United Kingdom|England|658[0n]|BOLD:AAB7661
Oligia latruncula|[15232]|NLLEA368-12|Netherlands|South Holland|658[0n]|BOLD:AAB7661
Oligia latruncula|[15233]|CGUKA765-09|United Kingdom||658[0n]|BOLD:AAB7661
Oligia latruncula|[15234]|CGUKA444-09|United Kingdom|England|658[0n]|BOLD:AAB7661
Oligia latruncula|[15235]|CGUKA480-09|United Kingdom|England|638[0n]|BOLD:AAB7661
Oligia latruncula|[15236]|CGUKA597-09|United Kingdom|England|640[0n]|BOLD:AAB7661
Oligia latruncula|[15237]|CGUKA818-09|United Kingdom||658[0n]|BOLD:AAB7661
Oligia latruncula|[15238]|CGUKA928-09|United Kingdom|England|658[0n]|BOLD:AAB7661
Oligia latruncula|[15239]|CGUKB721-09|United Kingdom|England|658[0n]|BOLD:AAB7661
Oligia latruncula|[15240]|CGUKB770-09|Ireland||658[0n]|BOLD:AAB7661
Oligia latruncula|[15241]|CGUKD364-09|United Kingdom|England|658[0n]|BOLD:AAB7661
Oligia latruncula|[15242]|NOCJH048-09|France||658[0n]|BOLD:AAB7661
Oligia latruncula|[15243]|NOCJH138-09|France||658[0n]|BOLD:AAB7661
Oligia latruncula|[15244]|NOCJH150-09|France||658[0n]|BOLD:AAB7661
Oligia latruncula|[15245]|NOCJH152-09|France||658[0n]|BOLD:AAB7661
Oligia latruncula|[15246]|NOCJH163-09|France||658[0n]|BOLD:AAB7661
Oligia latruncula|[15247]|NOCJH165-09|France||658[0n]|BOLD:AAB7661
Oligia latruncula|[15248]|NOCJH166-09|France||658[0n]|BOLD:AAB7661
Oligia latruncula|[15249]|NOCJH167-09|France||658[0n]|BOLD:AAB7661
Oligia latruncula|[15250]|NOCJH171-09|France||658[0n]|BOLD:AAB7661
Oligia latruncula|[15251]|NOCJH175-09|France||658[0n]|BOLD:AAB7661
Oligia latruncula|[15252]|NOCJH178-09|France||658[0n]|BOLD:AAB7661
Oligia latruncula|[15253]|LEFID619-10|Finland||658[0n]|BOLD:AAB7661
Oligia latruncula|[15254]|LEFIF714-10|Finland||658[0n]|BOLD:AAB7661
Oligia latruncula|[15255]|LENOA557-11|France|Haute Normandie|658[0n]|BOLD:AAB7661
Oligia latruncula|[15256]|NLLEA337-12|Netherlands|South Holland|658[0n]|BOLD:AAB7661
Oligia latruncula|[15257]|NLLEA359-12|Netherlands|South Holland|658[0n]|BOLD:AAB7661
Oligia latruncula|[15258]|FBLMZ142-12|Germany|Bavaria|658[0n]|BOLD:AAB7661
Oligia latruncula|[15259]|GWOTL068-13|Germany|Saarland|658[0n]|BOLD:AAB7661
Oligia latruncula|[15260]|CNCLB2528-14|United States|New York|658[0n]|BOLD:AAB7661
Oligia strigilis|[15261]|PHLAF270-11|Macedonia||658[0n]|BOLD:AAB4833
Oligia strigilis|[15262]|TTNFS132-09|Serbia||658[2n]|BOLD:AAB4833
Oligia strigilis|[15263]|LENOA554-11|France|Haute Normandie|658[1n]|BOLD:AAB4833
Oligia strigilis|[15264]|IBLAO416-12|Spain|Catalonia|658[0n]|BOLD:AAB4833
Oligia strigilis|[15265]|GWOTL269-13|Germany|Saarland|658[1n]|BOLD:AAB4833
Oligia strigilis|[15266]|CGUKB965-09|United Kingdom|Wales|645[0n]|BOLD:AAB4833
Oligia strigilis|[15267]|LEATD218-13|Austria|Tirol|658[1n]|BOLD:AAB4833
Oligia strigilis|[15268]|GBLAA966-14|Germany|North Rhine-Westphalia|658[0n]|BOLD:AAB4833
Oligia strigilis|[15269]|GWOTL064-13|Germany|Saarland|626[1n]|BOLD:AAB4833
Oligia strigilis|[15270]|GWOSA279-10|Italy|Calabria|658[0n]|BOLD:AAB4833
Oligia strigilis|[15271]|PHLAC358-10|Italy|South Tyrol|658[0n]|BOLD:AAB4833
Oligia strigilis|[15272]|PHLAC419-10|Italy|South Tyrol|658[0n]|BOLD:AAB4833
Oligia strigilis|[15273]|GWOSZ237-11|Italy|Trentino-Alto Adige|658[0n]|BOLD:AAB4833
Oligia strigilis|[15274]|GWOSZ239-11|Italy|Trentino-Alto Adige|658[0n]|BOLD:AAB4833
Oligia strigilis|[15275]|NOCJH159-09|France||614[0n]|BOLD:AAB4833
Oligia strigilis|[15276]|NOCJH153-09|France||627[0n]|BOLD:AAB4833
Oligia strigilis|[15277]|CGUKD259-09|United Kingdom|England|643[0n]|BOLD:AAB4833
Oligia strigilis|[15278]|NOCJH142-09|France||658[0n]|BOLD:AAB4833
Oligia strigilis|[15279]|NOCJH168-09|France||658[0n]|BOLD:AAB4833
Oligia strigilis|[15280]|NOCJH180-09|France||658[0n]|BOLD:AAB4833
Oligia strigilis|[15281]|NOCJH183-09|France||658[0n]|BOLD:AAB4833
Oligia strigilis|[15282]|NOCJH185-09|France||658[0n]|BOLD:AAB4833
Oligia strigilis|[15283]|NOCJH187-09|France||658[0n]|BOLD:AAB4833
Oligia strigilis|[15284]|NOCJH148-09|France||658[0n]|BOLD:AAB4833
Oligia strigilis|[15285]|NOCJH147-09|France||658[0n]|BOLD:AAB4833
Oligia strigilis|[15286]|NOCJH146-09|France||658[0n]|BOLD:AAB4833
Oligia strigilis|[15287]|NOCJH141-09|France||658[0n]|BOLD:AAB4833
Oligia strigilis|[15288]|NOCJH140-09|France||658[0n]|BOLD:AAB4833
Oligia strigilis|[15289]|NOCJH139-09|France||658[0n]|BOLD:AAB4833
Oligia strigilis|[15290]|GWORK414-09|Germany|Bavaria|658[0n]|BOLD:AAB4833
Oligia strigilis|[15291]|CGUKD314-09|United Kingdom|England|658[0n]|BOLD:AAB4833
Oligia strigilis|[15292]|CGUKB729-09|United Kingdom|England|658[0n]|BOLD:AAB4833

Oligia strigilis|[15289]|NOCJH159-09|France||658[0n]|BOLD:AAB4833
Oligia strigilis|[15290]|GWORK414-09|Germany|Bavaria|658[0n]|BOLD:AAB4833
Oligia strigilis|[15291]|CGUKD314-09|United Kingdom|England|658[0n]|BOLD:AAB4833
Oligia strigilis|[15292]|CGUKB729-09|United Kingdom|England|658[0n]|BOLD:AAB4833
Oligia strigilis|[15293]|CGUKA927-09|United Kingdom|England|658[0n]|BOLD:AAB4833
Oligia strigilis|[15294]|CGUKA465-09|United Kingdom|England|658[0n]|BOLD:AAB4833
Oligia strigilis|[15295]|CGUKA458-09|United Kingdom|England|658[0n]|BOLD:AAB4833
Oligia strigilis|[15296]|CGUKA452-09|United Kingdom|England|658[0n]|BOLD:AAB4833
Oligia strigilis|[15297]|CGUKA043-09|United Kingdom|England|658[0n]|BOLD:AAB4833
Oligia strigilis|[15298]|CGUKA016-09|United Kingdom|England|658[0n]|BOLD:AAB4833
Oligia strigilis|[15299]|RDNMG511-08|Canada|Ontario|658[0n]|BOLD:AAB4833
Oligia strigilis|[15300]|RDNMD715-06|Denmark||658[0n]|BOLD:AAB4833
Oligia strigilis|[15301]|RDNMD706-06|Denmark||658[0n]|BOLD:AAB4833
Oligia strigilis|[15302]|GWOR3996-09|Germany|Bavaria|583[0n]|BOLD:AAB4833
Oligia strigilis|[15303]|CGUKC256-09|United Kingdom|England|638[0n]|BOLD:AAB4833
Oligia strigilis|[15304]|CGUKB293-09|United Kingdom|England|638[0n]|BOLD:AAB4833
Oligia strigilis|[15305]|CGUKA596-09|United Kingdom|England|638[0n]|BOLD:AAB4833
Oligia strigilis|[15306]|RDLQ467-07|Canada|Quebec|606[0n]|BOLD:AAB4833
Oligia strigilis|[15307]|NOCJH143-09|France||627[0n]|BOLD:AAB4833
Oligia strigilis|[15308]|NOCJH158-09|France||630[0n]|BOLD:AAB4833
Oligia strigilis|[15309]|NOCJH161-09|France||658[0n]|BOLD:AAB4833
Oligia strigilis|[15310]|NOCJH170-09|France||658[0n]|BOLD:AAB4833
Oligia strigilis|[15311]|NOCJH173-09|France||658[0n]|BOLD:AAB4833
Oligia strigilis|[15312]|NOCJH184-09|France||658[0n]|BOLD:AAB4833
Oligia strigilis|[15313]|NOCJH186-09|France||658[0n]|BOLD:AAB4833
Oligia strigilis|[15314]|NOCJH188-09|France||658[0n]|BOLD:AAB4833
Oligia strigilis|[15315]|LEFIA572-10|Finland|South Karelia|658[0n]|BOLD:AAB4833
Oligia strigilis|[15316]|LEFIA573-10|Finland|South Karelia|658[0n]|BOLD:AAB4833
Oligia strigilis|[15317]|LEFIE577-10|Finland|Aland Islands|658[0n]|BOLD:AAB4833
Oligia strigilis|[15318]|LEFIC251-10|Finland|Uusimaa|658[0n]|BOLD:AAB4833
Oligia strigilis|[15319]|GWOSA382-10|Germany|Bavaria|658[0n]|BOLD:AAB4833
Oligia strigilis|[15320]|LENOA553-11|France|Haute Normandie|658[0n]|BOLD:AAB4833
Oligia strigilis|[15321]|IBLAO301-12|Spain|Catalonia|658[0n]|BOLD:AAB4833
Oligia strigilis|[15322]|GWOTG928-12|Russia|Irkutsk|658[0n]|BOLD:AAB4833
Oligia strigilis|[15323]|LON1036-12|Norway|Aust-Agder|658[0n]|BOLD:AAB4833
Oligia strigilis|[15324]|PHLAV041-12|Austria|Vorarlberg|658[0n]|BOLD:AAB4833
Oligia strigilis|[15325]|PHLAV068-12|Austria|Vorarlberg|658[0n]|BOLD:AAB4833
Oligia strigilis|[15326]|GWOTL065-13|Germany|Saarland|658[0n]|BOLD:AAB4833
Oligia strigilis|[15327]|GWOTL066-13|Germany|Saarland|658[0n]|BOLD:AAB4833
Oligia strigilis|[15328]|LEATD281-13|Austria|Tirol|658[0n]|BOLD:AAB4833
Oligia strigilis|[15329]|GBLAC606-13|Germany|Bavaria|658[0n]|BOLD:AAB4833
Oligia strigilis|[15330]|GBLAC613-13|Germany|Bavaria|658[0n]|BOLD:AAB4833
Oligia strigilis|[15331]|GBLAC1069-13|Germany|Bavaria|658[0n]|BOLD:AAB4833
Oligia strigilis|[15332]|GBLAC1070-13|Germany|Bavaria|658[0n]|BOLD:AAB4833
Oligia strigilis|[15333]|NORIN152-13|Norway|Akershus|657[0n]|BOLD:AAB4833
Oligia strigilis|[15334]|GBLAA967-14|Germany|Rhineland-Palatinate|658[0n]|BOLD:AAB4833
Oligia strigilis|[15335]|GBLAD759-14|Germany|Saxony|658[0n]|BOLD:AAB4833
Oligia strigilis|[15336]|GBLAD806-14|Germany|Bavaria|658[0n]|BOLD:AAB4833
Oligia strigilis|[15337]|GBLAD812-14|Germany|Bavaria|658[0n]|BOLD:AAB4833
Oligia strigilis|[15338]|GBLAD813-14|Germany|Bavaria|658[0n]|BOLD:AAB4833
Oligia strigilis|[15339]|ABOLA485-14|Austria|Tirol|658[0n]|BOLD:AAB4833
Neoligia exhausta|[15340]|XAB144-04|Canada|Ontario|658[0n]|BOLD:AAB6095
Neoligia exhausta|[15341]|XAC733-04|Canada|Ontario|658[0n]|BOLD:AAB6095
Neoligia exhausta|[15342]|LMDH200-11|United States|Minnesota|658[0n]|BOLD:AAB6095
Neoligia exhausta|[15343]|BLTIB1122-08|Canada|Ontario|658[0n]|BOLD:AAB6095
Neoligia exhausta|[15344]|RDNME669-08|Canada|New Brunswick|658[0n]|BOLD:AAB6095
Neoligia exhausta|[15345]|RDLQG917-06|Canada|Quebec|658[0n]|BOLD:AAB6095
Neoligia exhausta|[15346]|RDLQG776-06|Canada|Quebec|658[0n]|BOLD:AAB6095
Neoligia exhausta|[15347]|RDLQG753-06|Canada|Quebec|658[0n]|BOLD:AAB6095
Neoligia exhausta|[15348]|RDLQG591-06|Canada|Quebec|658[0n]|BOLD:AAB6095
Neoligia exhausta|[15349]|XAJ865-06|Canada|Ontario|658[0n]|BOLD:AAB6095
Neoligia exhausta|[15350]|XAJ863-06|Canada|Ontario|658[0n]|BOLD:AAB6095
Neoligia exhausta|[15351]|RDLQB395-05|Canada|Quebec|658[0n]|BOLD:AAB6095
Neoligia exhausta|[15352]|RDNM095-05|Canada|Ontario|658[0n]|BOLD:AAB6095
Neoligia exhausta|[15353]|XAG058-05|Canada|Ontario|658[0n]|BOLD:AAB6095
Neoligia exhausta|[15354]|XAC602-04|Canada|Ontario|658[0n]|BOLD:AAB6095
Neoligia exhausta|[15355]|RDLQG805-06|Canada|Quebec|653[0n]|BOLD:AAB6095
Neoligia exhausta|[15356]|PHMO188-03|Canada|Ontario|639[2n]|BOLD:AAB6095
Neoligia exhausta|[15357]|BLTIB577-08|Canada|Ontario|619[0n]|BOLD:AAB6095
Neoligia exhausta|[15358]|XAJ802-06|Canada|Ontario|632[0n]|BOLD:AAB6095
Neoligia exhausta|[15359]|PHMO278-03|Canada|Ontario|638[5n]|BOLD:AAB6095
Neoligia exhausta|[15360]|BLTIB602-08|Canada|Ontario|634[0n]|BOLD:AAB6095
Neoligia exhausta|[15361]|HEJUL255-12|Canada|Ontario|604[0n]|BOLD:AAB6095
Oligia tusa|[15362]|RWWA990-09|United States|Washington|658[0n]|BOLD:AAB5984
Oligia tusa|[15363]|RDNMF724-08|Canada|British Columbia|658[0n]|BOLD:AAB5984
Oligia tusa|[15364]|RDNMF725-08|Canada|British Columbia|658[0n]|BOLD:AAB5984
Oligia tusa|[15365]|RDNMF726-08|Canada|British Columbia|658[0n]|BOLD:AAB5984
Oligia tusa|[15366]|RDNMF728-08|United States|Washington|658[0n]|BOLD:AAB5984
Oligia tusa|[15367]|GMLC982-12|United States|California|658[0n]|BOLD:AAB5984
Oligia tusa|[15368]|RWWA820-09|United States|Washington|658[0n]|BOLD:AAB5984
Oligia tusa|[15369]|RWWA800-09|United States|Washington|639[0n]|BOLD:AAB5984
Oligia tusa|[15370]|RWWB013-09|United States|Washington|633[0n]|BOLD:AAB5984
Oligia tusa|[15371]|RWWB023-09|United States|Washington|658[0n]|BOLD:AAB5984
Oligia tusa|[15372]|RWWB031-09|United States|Washington|658[0n]|BOLD:AAB5984
Oligia tusa|[15373]|RWWB069-09|United States|Washington|658[0n]|BOLD:AAB5984
Oligia tusa|[15374]|RWWB084-09|United States|Washington|658[0n]|BOLD:AAB5984
Oligia tusa|[15375]|RWWB085-09|United States|Washington|658[0n]|BOLD:AAB5984
Oligia tusa|[15376]|RWWB142-09|United States|Washington|658[0n]|BOLD:AAB5984
Oligia tusa|[15377]|RWWB158-09|United States|Washington|658[0n]|BOLD:AAB5984
Oligia tusa|[15378]|RWWB204-09|United States|Washington|658[0n]|BOLD:AAB5984
Oligia tusa|[15379]|RWWB271-09|United States|Washington|658[0n]|BOLD:AAB5984
Oligia tusa|[15380]|RWWC1068-12|United States|Washington|658[0n]|BOLD:AAB5984
Oligia tusa|[15381]|RWWC1110-12|United States|Washington|658[0n]|BOLD:AAB5984
Oligia tusa|[15382]|RWWB014-09|United States|Washington|658[0n]|BOLD:AAB5984
Oligia tusa|[15383]|RWWA809-09|United States|Washington|658[0n]|BOLD:AAB5984
Oligia tusa|[15384]|RDNMF727-08|United States|Washington|658[0n]|BOLD:AAB5984
Oligia tusa|[15385]|RWWC1192-13|United States|Washington|591[0n]|BOLD:AAB5984
Oligia n. sp.|[15386]|RDNMG272-08|United States|Arizona|658[0n]|BOLD:AAK3519
Oligia n. sp.|[15387]|RDNMJ116-10|United States|Arizona|658[0n]|BOLD:AAK3519
Oligia modica|[15388]|CNCLB2947-14|United States|North Carolina|658[0n]|BOLD:ABZ2241
Oligia modica|[15389]|LNCC1422-11|United States|North Carolina|658[0n]|BOLD:ABZ2241
Oligia modica|[15390]|CNCLB2953-14|United States|North Carolina|658[0n]|BOLD:ABZ2241
Oligia modica|[15391]|LNCB759-09|United States|North Carolina|658[0n]|BOLD:ABZ8003
[illegible]

Oligia modica|[15389]|LNCC1422-11|United States|North Carolina|658[0n]|BOLD:ABZ2241
Oligia modica|[15390]|CNCLB2953-14|United States|North Carolina|658[0n]|BOLD:ABZ2241
Oligia modica|[15391]|LNCB759-09|United States|North Carolina|658[0n]|BOLD:ABZ8003
Oligia modica|[15392]|LNCC1312-11|United States|North Carolina|658[0n]|BOLD:ABZ8003
Oligia modica|[15393]|CNCLB2716-14|United States|North Carolina|658[0n]|BOLD:ABZ8003
Oligia modica|[15394]|LGSMG970-10|United States|North Carolina|658[0n]|BOLD:ABZ8003
Oligia modica|[15395]|LGSMG971-10|United States|North Carolina|658[0n]|BOLD:ABZ8003
Oligia modica|[15396]|CNCLB2713-14|United States|North Carolina|658[0n]|BOLD:ABZ8003
Oligia modica|[15397]|CNCLB2715-14|United States|North Carolina|658[0n]|BOLD:ABZ8003
Oligia modica|[15398]|CNCLB2943-14|United States|North Carolina|658[0n]|BOLD:ABZ8003
Oligia modica|[15399]|CNCLB2950-14|United States|North Carolina|658[0n]|BOLD:ABZ8003
Oligia modica|[15400]|CNCLB2955-14|United States|North Carolina|658[0n]|BOLD:ABZ8003
Oligia modica|[15401]|RDLQB427-05|Canada|Quebec|658[0n]|BOLD:AAE1462
Oligia modica|[15402]|CNGBJ1971-14|Canada|Ontario|548[1n]|BOLD:AAE1462
Oligia modica|[15403]|RDNMD780-07|United States|Connecticut|658[0n]|BOLD:AAE1462
Oligia modica|[15404]|CNCLB2944-14|United States|North Carolina|658[0n]|BOLD:AAE1462
Oligia modica|[15405]|TTMNB330-06|Canada|New Brunswick|585[0n]|BOLD:ABZ2233
Oligia modica|[15406]|RDLQF835-06|Canada|Quebec|658[0n]|BOLD:ABZ2233
Oligia modica|[15407]|HPPPL632-13|Canada|Nova Scotia|658[0n]|BOLD:ABZ2233
Oligia modica|[15408]|CNCLB2956-14|United States|North Carolina|658[0n]|BOLD:ABZ2233
Lateroligia ophiogramma|[15409]|LPVIA299-08|Canada|British Columbia|658[0n]|BOLD:AAB0872
Lateroligia ophiogramma|[15410]|LENOA532-11|France|Haute Normandie|658[0n]|BOLD:AAB0872
Lateroligia ophiogramma|[15411]|XAG548-05|Canada|Ontario|605[1n]|BOLD:AAB0872
Lateroligia ophiogramma|[15412]|LPVIB977-08|Canada|British Columbia|658[0n]|BOLD:AAB0872
Lateroligia ophiogramma|[15413]|RDLQB436-05|Canada|Quebec|576[0n]|BOLD:AAB0872
Lateroligia ophiogramma|[15414]|RWWC980-12|United States|Washington|658[0n]|BOLD:AAB0872
Lateroligia ophiogramma|[15415]|FGMLD015-13|Germany|Bavaria|658[0n]|BOLD:AAB0872
Lateroligia ophiogramma|[15416]|LEATG136-14|Austria|Tirol|658[0n]|BOLD:AAB0872
Lateroligia ophiogramma|[15417]|LEATG137-14|Austria|Tirol|658[0n]|BOLD:AAB0872
Lateroligia ophiogramma|[15418]|RWWB921-10|United States|Washington|658[0n]|BOLD:AAB0872
Lateroligia ophiogramma|[15419]|LALPA312-10|Canada|British Columbia|658[0n]|BOLD:AAB0872
Lateroligia ophiogramma|[15420]|LALPA455-10|Canada|British Columbia|658[0n]|BOLD:AAB0872
Lateroligia ophiogramma|[15421]|LALPA575-10|Canada|British Columbia|658[0n]|BOLD:AAB0872
Lateroligia ophiogramma|[15422]|RWWA615-09|United States|Washington|658[0n]|BOLD:AAB0872
Lateroligia ophiogramma|[15423]|RWWA705-09|United States|Washington|658[0n]|BOLD:AAB0872
Lateroligia ophiogramma|[15424]|RWWA812-09|United States|Washington|658[0n]|BOLD:AAB0872
Lateroligia ophiogramma|[15425]|RWWA946-09|United States|Washington|658[0n]|BOLD:AAB0872
Lateroligia ophiogramma|[15426]|RWWB026-09|United States|Washington|658[0n]|BOLD:AAB0872
Lateroligia ophiogramma|[15427]|BBLPC068-09|Canada|New Brunswick|658[0n]|BOLD:AAB0872
Lateroligia ophiogramma|[15428]|BBLPC409-09|Canada|New Brunswick|658[0n]|BOLD:AAB0872
Lateroligia ophiogramma|[15429]|LEFIC756-10|Finland|Finland Proper|658[0n]|BOLD:AAB0872
Lateroligia ophiogramma|[15430]|LEFIF192-10|Finland||658[0n]|BOLD:AAB0872
Lateroligia ophiogramma|[15431]|LEFIF682-10|Finland||658[0n]|BOLD:AAB0872
Lateroligia ophiogramma|[15432]|LALPA683-10|Canada|British Columbia|658[0n]|BOLD:AAB0872
Lateroligia ophiogramma|[15433]|BBLPB346-10|Canada|Alberta|658[0n]|BOLD:AAB0872
Lateroligia ophiogramma|[15434]|NOCJH489-11|France||658[0n]|BOLD:AAB0872
Lateroligia ophiogramma|[15435]|LENOA531-11|France|Haute Normandie|658[0n]|BOLD:AAB0872
Lateroligia ophiogramma|[15436]|GWORK446-09|Germany|Bavaria|658[0n]|BOLD:AAB0872
Lateroligia ophiogramma|[15437]|RWWA610-09|United States|Washington|658[0n]|BOLD:AAB0872
Lateroligia ophiogramma|[15438]|CGUKC332-09|United Kingdom|England|658[0n]|BOLD:AAB0872
Lateroligia ophiogramma|[15439]|GWOR3995-09|Germany|Bavaria|658[0n]|BOLD:AAB0872
Lateroligia ophiogramma|[15440]|LPVIB978-08|Canada|British Columbia|658[0n]|BOLD:AAB0872
Lateroligia ophiogramma|[15441]|LPMN1002-09|Canada|Manitoba|658[0n]|BOLD:AAB0872
Lateroligia ophiogramma|[15442]|LPVIB075-08|Canada|British Columbia|658[0n]|BOLD:AAB0872
Lateroligia ophiogramma|[15443]|LPVIB087-08|Canada|British Columbia|658[0n]|BOLD:AAB0872
Lateroligia ophiogramma|[15444]|LPVIA302-08|Canada|British Columbia|658[0n]|BOLD:AAB0872
Lateroligia ophiogramma|[15445]|LPVIA610-08|Canada|British Columbia|658[0n]|BOLD:AAB0872
Lateroligia ophiogramma|[15446]|BLTIB507-08|Canada|Ontario|658[0n]|BOLD:AAB0872
Lateroligia ophiogramma|[15447]|BLTIB658-08|Canada|Ontario|658[0n]|BOLD:AAB0872
Lateroligia ophiogramma|[15448]|LPVIA300-08|Canada|British Columbia|658[0n]|BOLD:AAB0872
Lateroligia ophiogramma|[15449]|LPVIA301-08|Canada|British Columbia|658[0n]|BOLD:AAB0872
Lateroligia ophiogramma|[15450]|LON563-08|Norway|Ostfold|657[0n]|BOLD:AAB0872
Lateroligia ophiogramma|[15451]|LPABB067-08|Canada|Alberta|658[0n]|BOLD:AAB0872
Lateroligia ophiogramma|[15452]|LHLEP576-06|Canada|British Columbia|658[0n]|BOLD:AAB0872
Lateroligia ophiogramma|[15453]|LHLEP575-06|Canada|British Columbia|658[0n]|BOLD:AAB0872
Lateroligia ophiogramma|[15454]|LHLEP074-06|Canada|British Columbia|658[0n]|BOLD:AAB0872
Lateroligia ophiogramma|[15455]|RDLQG054-06|Canada|Quebec|658[0n]|BOLD:AAB0872
Lateroligia ophiogramma|[15456]|LMH038-06|Canada|British Columbia|658[0n]|BOLD:AAB0872
Lateroligia ophiogramma|[15457]|RDLQB729-05|Canada|Quebec|658[0n]|BOLD:AAB0872
Lateroligia ophiogramma|[15458]|RDLQB722-05|Canada|Quebec|658[0n]|BOLD:AAB0872
Lateroligia ophiogramma|[15459]|MNBB125-05|Canada|New Brunswick|658[0n]|BOLD:AAB0872
Lateroligia ophiogramma|[15460]|CGUKC427-09|United Kingdom|England|633[0n]|BOLD:AAB0872
Lateroligia ophiogramma|[15461]|PHLAV375-12|Austria|Vorarlberg|658[0n]|BOLD:AAB0872
Lateroligia ophiogramma|[15462]|LPVIB001-08|Canada|British Columbia|622[0n]|BOLD:AAB0872
Lateroligia ophiogramma|[15463]|LHLEP574-06|Canada|British Columbia|622[0n]|BOLD:AAB0872
Lateroligia ophiogramma|[15464]|LHLEP073-06|Canada|British Columbia|644[0n]|BOLD:AAB0872
Lateroligia ophiogramma|[15465]|PHMO264-03|Canada|Ontario|639[0n]|BOLD:AAB0872
Lateroligia ophiogramma|[15466]|LHLEP577-06|Canada|British Columbia|634[0n]|BOLD:AAB0872
Lateroligia ophiogramma|[15467]|LEATH702-14|Italy|South Tyrol|634[0n]|BOLD:AAB0872
Melanapamea mixta|[15468]|RDNM194-05|Canada|Ontario|658[0n]|BOLD:AAE7007
Melanapamea mixta|[15469]|RDNM025-05|United States|Oregon|588[0n]|BOLD:AAE7007
Melanapamea mixta|[15470]|RDNM026-05|United States|Oregon|543[0n]|BOLD:AAE7007
Melanapamea mixta|[15471]|RDNM195-05|United States|Oregon|658[0n]|BOLD:AAE7007
Photedes includens|[15472]|RDNMG848-08|Canada|Ontario|658[0n]|BOLD:AAE2566
Photedes includens|[15473]|RDLQB404-05|Canada|Quebec|658[0n]|BOLD:AAE2566
Photedes includens|[15474]|RDMAB941-09|Canada|Ontario|619[0n]|BOLD:AAE2566
Photedes includens|[15475]|BBLPB994-10|Canada|Alberta|658[0n]|BOLD:AAE2566
Photedes includens|[15476]|RDMAB940-09|Canada|Alberta|610[0n]|BOLD:AAE2566
Photedes includens|[15477]|RDMAB942-09|Canada|Alberta|585[0n]|BOLD:AAE2566
Photedes includens|[15478]|BBLPB405-10|Canada|Alberta|658[0n]|BOLD:AAE2566
Photedes includens|[15479]|BBLPB406-10|Canada|Alberta|658[0n]|BOLD:AAE2566
Photedes includens|[15480]|BBLPB995-10|Canada|Alberta|658[0n]|BOLD:AAE2566
Amphipoea erepta|[15481]|LNC234-05|United States|North Carolina|658[0n]|BOLD:AAF4308
Amphipoea erepta|[15482]|RDNMD769-07|United States|New York|658[0n]|BOLD:AAF4308
Amphipoea erepta|[15483]|RDNMD770-07|United States|New York|658[0n]|BOLD:AAF4308
Oligia obtusa|[15484]|RDNMG952-08|Canada|Ontario|658[0n]|BOLD:AAD7847
Oligia obtusa|[15485]|PHMO324-03|Canada|Ontario|639[1n]|BOLD:AAD7847
Oligia obtusa|[15486]|PHMO328-03|Canada|Ontario|639[0n]|BOLD:AAD7847
Oligia obtusa|[15487]|RDLQB822-05|Canada|Quebec|621[0n]|BOLD:AAD7847
Oligia obtusa|[15488]|RDNMG951-08|Canada|British Columbia|658[0n]|BOLD:AAD7847
Oligia obtusa|[15489]|HKONB395-09|United States|Indiana|658[0n]|BOLD:AAD7847
Mammifrontia sp.|[15490]|LOCBC875-06|United States|California|616[8n]|
Mammifrontia rileyi|[15491]|LOCBD899-06|United States|California|652[0n]|BOLD:AAB0456
[illegible]

Oligia obtusa|[15489]|HKONB395-09|United States|Indiana|658[0n]|BOLD:AAD7847
Mammifrontia sp.|[15490]|LOCBC875-06|United States|California|616[8n]|
Mammifrontia rileyi|[15491]|LOCBD899-06|United States|California|652[0n]|BOLD:AAB0456
Mammifrontia rileyi|[15492]|BBLOD1703-11|United States|California|658[0n]|BOLD:AAB0456
Mammifrontia rileyi|[15493]|BBLOC937-11|United States|California|658[0n]|BOLD:AAB0456
Mammifrontia rileyi|[15494]|BBLOD560-11|United States|California|658[0n]|BOLD:AAB0456
Mammifrontia rileyi|[15495]|BBLOE1405-12|United States|California|658[0n]|BOLD:AAB0456
Mammifrontia rileyi|[15496]|LOCBD002-06|United States|California|654[0n]|BOLD:AAB0456
Mammifrontia rileyi|[15497]|LOCBC049-06|United States|California|658[0n]|BOLD:AAB0456
Mammifrontia rileyi|[15498]|LOCBC665-06|United States|California|658[0n]|BOLD:AAB0456
Mammifrontia rileyi|[15499]|LOCBD004-06|United States|California|654[0n]|BOLD:AAB0456
Mammifrontia rileyi|[15500]|LOCBC821-06|United States|California|658[0n]|BOLD:AAB0456
Mammifrontia rileyi|[15501]|LOCBD903-06|United States|California|658[0n]|BOLD:AAB0456
Mammifrontia rileyi|[15502]|LOCBD912-06|United States|California|653[0n]|BOLD:AAB0456
Mammifrontia rileyi|[15503]|BBLOE1998-12|United States|California|658[1n]|BOLD:AAB0456
Mammifrontia rileyi|[15504]|BBLOE1997-12|United States|California|658[1n]|BOLD:AAB0456
Mammifrontia rileyi|[15505]|LOCBD001-06|United States|California|654[0n]|BOLD:AAB0456
Mammifrontia rileyi|[15506]|LOCBC940-06|United States|California|656[0n]|BOLD:AAB0456
Mammifrontia rileyi|[15507]|LOCBC669-06|United States|California|654[0n]|BOLD:AAB0456
Mammifrontia rileyi|[15508]|LOCBC663-06|United States|California|656[0n]|BOLD:AAB0456
Mammifrontia rileyi|[15509]|LOCBD908-06|United States|California|655[0n]|BOLD:AAB0456
Mammifrontia rileyi|[15510]|LOCBD132-06|United States|California|658[0n]|BOLD:AAB0456
Mammifrontia rileyi|[15511]|LOCBC502-06|United States|California|658[0n]|BOLD:AAB0456
Mammifrontia rileyi|[15512]|LOCBD907-06|United States|California|638[0n]|BOLD:AAB0456
Mammifrontia rileyi|[15513]|LOCBF3319-14|United States|California|546[0n]|BOLD:AAB0456
Mammifrontia rileyi|[15514]|LOCBF3320-14|United States|California|549[0n]|BOLD:AAB0456
Mammifrontia sp.|[15515]|LOCBF3321-14|United States|California|534[0n]|BOLD:AAB0456
Mammifrontia rileyi|[15516]|LOCBF3322-14|United States|California|546[0n]|BOLD:AAB0456
Mammifrontia rileyi|[15517]|LOCBF3323-14|United States|California|552[0n]|BOLD:AAB0456
Mammifrontia rileyi|[15518]|LOCBD906-06|United States|California|652[0n]|BOLD:AAB0456
Mammifrontia rileyi|[15519]|LOCBD904-06|United States|California|652[0n]|BOLD:AAB0456
Mammifrontia rileyi|[15520]|LOCBD902-06|United States|California|652[0n]|BOLD:AAB0456
Mammifrontia rileyi|[15521]|LOCBD901-06|United States|California|650[0n]|BOLD:AAB0456
Mammifrontia rileyi|[15522]|BBLOD1711-11|United States|California|658[0n]|BOLD:AAB0456
Mammifrontia rileyi|[15523]|BBLOD1713-11|United States|California|658[0n]|BOLD:AAB0456
Mammifrontia rileyi|[15524]|LOCBD911-06|United States|California|658[0n]|BOLD:AAB0456
Mammifrontia rileyi|[15525]|RDNMD819-07|United States|California|658[0n]|BOLD:AAB0456
Mammifrontia rileyi|[15526]|LOCBD909-06|United States|California|658[0n]|BOLD:AAB0456
Mammifrontia rileyi|[15527]|LOCBD910-06|United States|California|658[0n]|BOLD:AAB0456
Mammifrontia rileyi|[15528]|LOCBD003-06|United States|California|658[0n]|BOLD:AAB0456
Mammifrontia rileyi|[15529]|LOCBD905-06|United States|California|658[0n]|BOLD:AAB0456
Mammifrontia rileyi|[15530]|LOCBC876-06|United States|California|658[0n]|BOLD:AAB0456
Mammifrontia rileyi|[15531]|LOCBC668-06|United States|California|658[0n]|BOLD:AAB0456
Mammifrontia rileyi|[15532]|LOCBC667-06|United States|California|658[0n]|BOLD:AAB0456
Mammifrontia rileyi|[15533]|LOCBC666-06|United States|California|658[0n]|BOLD:AAB0456
Mammifrontia rileyi|[15534]|LOCBC664-06|United States|California|658[0n]|BOLD:AAB0456
Mammifrontia rileyi|[15535]|LOCBC457-06|United States|California|658[0n]|BOLD:AAB0456
Mammifrontia rileyi|[15536]|LOCBD900-06|United States|California|658[0n]|BOLD:AAB0456
Mammifrontia rileyi|[15537]|BBLOE1404-12|United States|California|658[0n]|BOLD:AAB0456
Mammifrontia sarae|[15538]|CNCLB1119-14|United States|California|658[0n]|BOLD:AAB0456
Mammifrontia sarae|[15539]|CNCLB1120-14|United States|California|658[0n]|BOLD:AAB0456
Mammifrontia rileyi|[15540]|TML165-14|United States||658[0n]|BOLD:AAB0456
Acrapex relicta|[15541]|HKONS500-08|United States|Florida|574[1n]|BOLD:AAD8753
Acrapex relicta|[15542]|LNCB285-06|United States|North Carolina|658[0n]|BOLD:AAD8753
Acrapex relicta|[15543]|MILEP011-09|United States|North Carolina|626[0n]|BOLD:AAD8753
Acrapex relicta|[15544]|MILEP036-09|United States|North Carolina|613[0n]|BOLD:AAD8753
Acrapex relicta|[15545]|LNCB283-06|United States|North Carolina|658[0n]|BOLD:AAD8753
Acrapex relicta|[15546]|LNCB284-06|United States|North Carolina|658[0n]|BOLD:AAD8753
Acrapex relicta|[15547]|LNCC702-11|United States|North Carolina|658[0n]|BOLD:AAD8753
Hypocoena basistriga|[15548]|DSCNI065-07|Canada|Manitoba|658[0n]|BOLD:AAE2019
Hypocoena basistriga|[15549]|DSCNI037-07|Canada|Manitoba|658[0n]|BOLD:AAE2019
Hypocoena basistriga|[15550]|DSCNI064-07|Canada|Manitoba|658[1n]|BOLD:AAE2019
Hypocoena basistriga|[15551]|DSCNI027-07|Canada|Manitoba|658[1n]|BOLD:AAE2019
Hypocoena basistriga|[15552]|LCHIP030-07|Canada|Manitoba|650[0n]|BOLD:AAE2019
Hypocoena basistriga|[15553]|CNBAF154-12|Canada|Alberta|632[0n]|BOLD:AAE2019
Hypocoena basistriga|[15554]|CNBAF155-12|Canada|Alberta|632[0n]|BOLD:AAE2019
Hypocoena basistriga|[15555]|CNBAE257-12|Canada|Alberta|632[0n]|BOLD:AAE2019
Hypocoena basistriga|[15556]|CNBAE258-12|Canada|Alberta|632[0n]|BOLD:AAE2019
Hypocoena basistriga|[15557]|CNBAF156-12|Canada|Alberta|632[0n]|BOLD:AAE2019
Helotropha reniformis|[15558]|AWCLB584-11|United States|Arizona|618[0n]|BOLD:ACE3287
Helotropha reniformis|[15559]|AWCLB587-11|United States|Arizona|540[0n]|BOLD:ACE3287
Helotropha reniformis|[15560]|RWWB038-09|United States|Washington|658[0n]|BOLD:AAB8703
Helotropha reniformis|[15561]|CNPKK2094-14|Canada|Ontario|591[0n]|BOLD:AAB8703
Helotropha reniformis|[15562]|CNEIG1486-13|Canada|Alberta|606[0n]|BOLD:AAB8703
Helotropha reniformis|[15563]|CNEIH1557-13|Canada|Alberta|591[0n]|BOLD:AAB8703
Helotropha reniformis|[15564]|CNWBD035-13|Canada|Alberta|576[0n]|BOLD:AAB8703
Helotropha reniformis|[15565]|CNEIH1558-13|Canada|Alberta|600[0n]|BOLD:AAB8703
Helotropha reniformis|[15566]|CNEIG1485-13|Canada|Alberta|600[0n]|BOLD:AAB8703
Helotropha reniformis|[15567]|BBLPB751-10|Canada|Alberta|638[0n]|BOLD:AAB8703
Helotropha reniformis|[15568]|RBINA4109-13|Canada|Ontario|582[0n]|BOLD:AAB8703
Helotropha reniformis|[15569]|RDLQB601-05|Canada|Quebec|658[0n]|BOLD:AAB8703
Helotropha reniformis|[15570]|BBLPB803-10|Canada|Alberta|658[0n]|BOLD:AAB8703
Helotropha reniformis|[15571]|RWWC122-10|United States|Washington|658[0n]|BOLD:AAB8703
Helotropha reniformis|[15572]|RWWC097-10|United States|Washington|658[0n]|BOLD:AAB8703
Helotropha reniformis|[15573]|RWWC090-10|United States|Washington|658[0n]|BOLD:AAB8703
Helotropha reniformis|[15574]|RWWC083-10|United States|Washington|658[0n]|BOLD:AAB8703
Helotropha reniformis|[15575]|LALPA786-10|Canada|British Columbia|658[0n]|BOLD:AAB8703
Helotropha reniformis|[15576]|LALPA690-10|Canada|British Columbia|658[0n]|BOLD:AAB8703
Helotropha reniformis|[15577]|RWWB202-09|United States|Washington|658[0n]|BOLD:AAB8703
Helotropha reniformis|[15578]|RWWB194-09|United States|Washington|658[0n]|BOLD:AAB8703
Helotropha reniformis|[15579]|RWWB174-09|United States|Washington|658[0n]|BOLD:AAB8703
Helotropha reniformis|[15580]|RWWB133-09|United States|Washington|658[0n]|BOLD:AAB8703
Helotropha reniformis|[15581]|RWWB125-09|United States|Washington|658[0n]|BOLD:AAB8703
Helotropha reniformis|[15582]|RWWB116-09|United States|Washington|658[0n]|BOLD:AAB8703
Helotropha reniformis|[15583]|RWWB067-09|United States|Washington|658[0n]|BOLD:AAB8703
Helotropha reniformis|[15584]|RWWB029-09|United States|Washington|658[0n]|BOLD:AAB8703
Helotropha reniformis|[15585]|RWWA954-09|United States|Washington|658[0n]|BOLD:AAB8703
Helotropha reniformis|[15586]|LHLEP407-06|Canada|British Columbia|658[0n]|BOLD:AAB8703
Helotropha reniformis|[15587]|RDNMD466-06|United States|Minnesota|658[0n]|BOLD:AAB8703
Helotropha reniformis|[15588]|RDNMB938-05|Canada|Alberta|655[0n]|BOLD:AAB8703
Helotropha reniformis|[15589]|XAH219-05|Canada|Ontario|658[0n]|BOLD:AAB8703
Helotropha reniformis|[15590]|LBCH4430-10|Canada|British Columbia|658[0n]|BOLD:AAB8703
Helotropha reniformis|[15591]|BBLPB804-10|Canada|Alberta|658[0n]|BOLD:AAB8703

Helotropha reniformis|[15589]|XAH219-05|Canada|Ontario|658[0n]|BOLD:AAB8703
Helotropha reniformis|[15590]|LBCH4430-10|Canada|British Columbia|658[0n]|BOLD:AAB8703
Helotropha reniformis|[15591]|BBLPB804-10|Canada|Alberta|658[0n]|BOLD:AAB8703
Helotropha reniformis|[15592]|BBLPB872-10|Canada|Alberta|658[0n]|BOLD:AAB8703
Helotropha reniformis|[15593]|RWWC683-11|United States|Washington|658[0n]|BOLD:AAB8703
Helotropha reniformis|[15594]|LALPA1348-12|Canada|British Columbia|658[0n]|BOLD:AAB8703
Helotropha reniformis|[15595]|CNEIE1809-12|Canada|Alberta|564[0n]|BOLD:AAB8703
Helotropha reniformis|[15596]|RDNMB937-05|Canada|British Columbia|658[0n]|BOLD:AAB8703
Helotropha reniformis|[15597]|RWWC1314-14|United States|Washington|576[0n]|BOLD:AAB8703
Helotropha reniformis|[15598]|RWWC1482-14|United States|Washington|561[1n]|BOLD:AAB8703
Viridiseptis marina|[15599]|NAMUM266-08|United States|California|658[0n]|BOLD:ABY6208
Aseptis marina|[15600]|CGLCA119-10|United States|California|630[0n]|BOLD:ABY6208
Viridiseptis marina|[15601]|GMLC665-11|United States|California|658[0n]|BOLD:ABY6208
Viridiseptis marina|[15602]|GMLC761-12|United States|California|658[0n]|BOLD:ABY6208
Viridiseptis marina|[15603]|GMLC1058-12|United States|California|658[0n]|BOLD:ABY6208
Viridiseptis marina|[15604]|NAMUM265-08|United States|California|658[0n]|BOLD:ABY6208
Viridiseptis marina|[15605]|GMLC243-11|United States|California|658[0n]|BOLD:ABY6208
Viridiseptis marina|[15606]|GMLC716-11|United States|California|658[0n]|BOLD:ABY6208
Viridiseptis marina|[15607]|GMLC746-12|United States|California|658[0n]|BOLD:ABY6208
Viridiseptis marina|[15608]|GMLC877-12|United States|California|658[0n]|BOLD:ABY6208
Viridiseptis marina|[15609]|GMLC1065-12|United States|California|658[0n]|BOLD:ABY6208
Viridiseptis marina|[15610]|CGLCA116-10|United States|California|630[0n]|BOLD:AAB4861
Viridiseptis marina|[15611]|CGLCA200-10|United States|California|658[0n]|BOLD:AAB4861
Viridiseptis marina|[15612]|CGLCA185-10|United States|California|658[0n]|BOLD:AAB4861
Viridiseptis marina|[15613]|LOCBC205-06|United States|California|658[0n]|BOLD:AAB4861
Viridiseptis marina|[15614]|LOCBC204-06|United States|California|658[0n]|BOLD:AAB4861
Viridiseptis marina|[15615]|LOCBC056-06|United States|California|658[0n]|BOLD:AAB4861
Viridiseptis marina|[15616]|LOCBC055-06|United States|California|658[0n]|BOLD:AAB4861
Viridiseptis marina|[15617]|LOCBC046-06|United States|California|658[0n]|BOLD:AAB4861
Viridiseptis marina|[15618]|LOCBB877-06|United States|California|658[0n]|BOLD:AAB4861
Viridiseptis marina|[15619]|LOCBB876-06|United States|California|658[0n]|BOLD:AAB4861
Viridiseptis marina|[15620]|LOCBB875-06|United States|California|658[0n]|BOLD:AAB4861
Viridiseptis marina|[15621]|LOCBB874-06|United States|California|658[0n]|BOLD:AAB4861
Viridiseptis marina|[15622]|LOCBB873-06|United States|California|658[0n]|BOLD:AAB4861
Viridiseptis marina|[15623]|LOCBB872-06|United States|California|658[0n]|BOLD:AAB4861
Viridiseptis marina|[15624]|LOCBB213-06|United States|California|658[0n]|BOLD:AAB4861
Viridiseptis marina|[15625]|LOCBB151-06|United States|California|658[0n]|BOLD:AAB4861
Viridiseptis marina|[15626]|LOCBB150-06|United States|California|658[0n]|BOLD:AAB4861
Viridiseptis marina|[15627]|LOCBB149-06|United States|California|658[0n]|BOLD:AAB4861
Viridiseptis marina|[15628]|LOCBB148-06|United States|California|658[0n]|BOLD:AAB4861
Viridiseptis marina|[15629]|LOCBB146-06|United States|California|658[0n]|BOLD:AAB4861
Viridiseptis marina|[15630]|RDNMC695-06|United States|California|658[0n]|BOLD:AAB4861
Viridiseptis marina|[15631]|GMLC647-11|United States|California|658[0n]|BOLD:AAB4861
Viridiseptis marina|[15632]|GMLC894-12|United States|California|658[0n]|BOLD:AAB4861
Viridiseptis marina|[15633]|LOCBB878-06|United States|California|656[0n]|BOLD:AAB4861
Viridiseptis marina|[15634]|LOCBB212-06|United States|California|658[0n]|BOLD:AAB4861
Viridiseptis marina|[15635]|LOCBC184-06|United States|California|590[0n]|BOLD:AAB4861
Viridiseptis marina|[15636]|GMLC950-12|United States|California|658[0n]|BOLD:AAB4861
Viridiseptis marina|[15637]|LOCBF2617-13|United States|California|658[0n]|BOLD:AAB4861
Aseptis torreyana|[15638]|RDNME009-07|United States|California|609[0n]|BOLD:AAA7137
Aseptis torreyana|[15639]|RDNME021-07|United States|California|609[0n]|BOLD:AAA7137
Aseptis torreyana|[15640]|RDNME032-07|United States|Colorado|609[0n]|BOLD:AAA7137
Paraseptis adnixa|[15641]|LHLEP411-06|Canada|British Columbia|658[0n]|BOLD:AAA7134
Paraseptis adnixa|[15642]|LBCS671-07|Canada|British Columbia|658[0n]|BOLD:AAA7134
Paraseptis adnixa|[15643]|LBCS673-07|Canada|British Columbia|658[0n]|BOLD:AAA7134
Paraseptis adnixa|[15644]|LMHRG037-06|Canada|British Columbia|658[0n]|BOLD:AAA7134
Paraseptis adnixa|[15645]|LBCS672-07|Canada|British Columbia|658[0n]|BOLD:AAA7134
Paraseptis adnixa|[15646]|LHLEP413-06|Canada|British Columbia|658[0n]|BOLD:AAA7134
Paraseptis adnixa|[15647]|RDNMC666-06|Canada|British Columbia|658[0n]|BOLD:AAA7134
Paraseptis adnixa|[15648]|LBCS670-07|Canada|British Columbia|658[0n]|BOLD:AAA7134
Paraseptis adnixa|[15649]|LBCS674-07|Canada|British Columbia|658[0n]|BOLD:AAA7134
Paraseptis adnixa|[15650]|RDNMC661-06|Canada|British Columbia|658[0n]|BOLD:AAA7134
Paraseptis adnixa|[15651]|LHLEP412-06|Canada|British Columbia|658[0n]|BOLD:AAA7134
Paraseptis adnixa|[15652]|LPVIA291-08|Canada|British Columbia|658[0n]|BOLD:AAA7134
Paraseptis adnixa|[15653]|BBLOC139-11|United States|Texas|658[0n]|BOLD:AAA7135
Paraseptis adnixa|[15654]|RDNMC662-06|United States|California|658[0n]|BOLD:AAA7135
Paraseptis adnixa|[15655]|RDNME027-07|United States|California|609[0n]|BOLD:AAA7135
Paraseptis adnixa|[15656]|BBLOC1213-11|United States|California|658[0n]|BOLD:AAA7135
Paraseptis adnixa|[15657]|BBLOC1264-11|United States|California|658[0n]|BOLD:AAA7135
Paraseptis adnixa|[15658]|BBLOC1367-11|United States|California|658[0n]|BOLD:AAA7135
Paraseptis adnixa|[15659]|LOCBC293-06|United States|California|658[0n]|BOLD:AAA7136
Paraseptis adnixa|[15660]|LOCBC294-06|United States|California|658[0n]|BOLD:AAA7136
Paraseptis adnixa|[15661]|RDNMC663-06|United States|California|658[0n]|BOLD:AAA7136
Paraseptis adnixa|[15662]|LOCBC306-06|United States|California|658[0n]|BOLD:AAA7136
Paraseptis adnixa|[15663]|LOCBC090-06|United States|California|658[3n]|BOLD:AAA7136
Paraseptis adnixa|[15664]|LOCB394-06|United States|California|658[0n]|BOLD:AAA7136
Paraseptis adnixa|[15665]|LOCB392-06|United States|California|658[0n]|BOLD:AAA7136
Paraseptis adnixa|[15666]|LOCB531-06|United States|California|601[0n]|BOLD:AAA7136
Paraseptis adnixa|[15667]|LOCB532-06|United States|California|658[0n]|BOLD:AAA7136
Paraseptis adnixa|[15668]|LOCBC170-06|United States|California|658[0n]|BOLD:AAA7136
Paraseptis adnixa|[15669]|LOCBC201-06|United States|California|658[0n]|BOLD:AAA7136
Paraseptis adnixa|[15670]|LOCBC211-06|United States|California|658[0n]|BOLD:AAA7136
Paraseptis adnixa|[15671]|LOCBC213-06|United States|California|658[0n]|BOLD:AAA7136
Paraseptis adnixa|[15672]|LOCBB574-06|United States|California|618[0n]|BOLD:AAA7136
Paraseptis adnixa|[15673]|LOCBC183-06|United States|California|623[0n]|BOLD:AAA7136
Paraseptis adnixa|[15674]|LOCBC209-06|United States|California|658[0n]|BOLD:AAA7136
Paraseptis adnixa|[15675]|LOCBC208-06|United States|California|658[0n]|BOLD:AAA7136
Paraseptis adnixa|[15676]|LOCBC206-06|United States|California|658[0n]|BOLD:AAA7136
Paraseptis adnixa|[15677]|LOCBC182-06|United States|California|658[0n]|BOLD:AAA7136
Paraseptis adnixa|[15678]|LOCBB573-06|United States|California|658[0n]|BOLD:AAA7136
Paraseptis adnixa|[15679]|LOCB480-06|United States|California|658[0n]|BOLD:AAA7136
Paraseptis adnixa|[15680]|LOCB478-06|United States|California|658[0n]|BOLD:AAA7136
Paraseptis adnixa|[15681]|LOCB477-06|United States|California|658[0n]|BOLD:AAA7136
Paraseptis adnixa|[15682]|LOCB393-06|United States|California|658[0n]|BOLD:AAA7136
Paraseptis adnixa|[15683]|RDNMC664-06|United States|California|658[0n]|BOLD:AAA7136
Paraseptis adnixa|[15684]|LOCBB678-06|United States|California|625[0n]|BOLD:AAA7136
Paraseptis adnixa|[15685]|LOCBB679-06|United States|California|617[0n]|BOLD:AAA7136
Paraseptis adnixa|[15686]|LOCBB676-06|United States|California|608[0n]|BOLD:AAA7136
Paraseptis adnixa|[15687]|LOCBC210-06|United States|California|658[0n]|BOLD:AAA7136
Paraseptis adnixa|[15688]|LOCBC212-06|United States|California|658[0n]|BOLD:AAA7136
Paraseptis adnixa|[15689]|LOCBC214-06|United States|California|658[0n]|BOLD:AAA7136
Paraseptis adnixa|[15690]|LOCBC215-06|United States|California|658[0n]|BOLD:AAA7136
Paraseptis adnixa|[15691]|LOCBC216-06|United States|California|658[0n]|BOLD:AAA7136

Paraseptis adnixa|[15689]|LOCBC214-06|United States|California|658[0n]|BOLD:AAA7136
Paraseptis adnixa|[15690]|LOCBC215-06|United States|California|658[0n]|BOLD:AAA7136
Paraseptis adnixa|[15691]|LOCBC216-06|United States|California|658[0n]|BOLD:AAA7136
Paraseptis adnixa|[15692]|LOCBC229-06|United States|California|658[0n]|BOLD:AAA7136
Paraseptis adnixa|[15693]|LOCBC305-06|United States|California|658[0n]|BOLD:AAA7136
Paraseptis adnixa|[15694]|CGLCA068-10|United States|California|658[0n]|BOLD:AAA7136
Paraseptis adnixa|[15695]|CGLCA151-10|United States|California|658[0n]|BOLD:ACE9575
Paraseptis adnixa|[15696]|RDNME607-08|United States|California|658[0n]|BOLD:ACE9575
Paraseptis adnixa|[15697]|JMMMB366-11|United States|California|658[0n]|BOLD:ACE9575
Paraseptis adnixa|[15698]|GMLC843-12|United States|California|658[0n]|BOLD:ACE9575
Litholomia napaea|[15699]|LOWCE480-06|Canada|British Columbia|588[0n]|BOLD:AAB0068
Litholomia napaea|[15700]|RWWA042-09|United States|Washington|658[0n]|BOLD:AAB0068
Litholomia napaea|[15701]|LOWCE030-06|Canada|British Columbia|596[2n]|BOLD:AAB0068
Litholomia napaea|[15702]|RWWC091-10|United States|Washington|658[0n]|BOLD:AAB0068
Litholomia napaea|[15703]|ABKWR125-07|United States|Alaska|648[0n]|BOLD:AAB0068
Litholomia napaea|[15704]|LOWCE032-06|Canada|British Columbia|658[0n]|BOLD:AAB0068
Litholomia napaea|[15705]|LBCG271-08|Canada|British Columbia|658[0n]|BOLD:AAB0068
Litholomia napaea|[15706]|JMMMB402-11|United States|California|658[0n]|BOLD:AAB0068
Litholomia napaea|[15707]|JMMMB418-11|United States|California|658[0n]|BOLD:AAB0068
Litholomia napaea|[15708]|RWWC103-10|United States|Washington|658[0n]|BOLD:AAB0068
Litholomia napaea|[15709]|LOWCE001-06|Canada|British Columbia|609[0n]|BOLD:AAB0068
Litholomia napaea|[15710]|RWWC192-11|United States|Washington|658[0n]|BOLD:AAB0068
Litholomia napaea|[15711]|LOWCE002-06|Canada|British Columbia|658[0n]|BOLD:AAB0068
Litholomia napaea|[15712]|LOWCE034-06|Canada|British Columbia|658[0n]|BOLD:AAB0068
Litholomia napaea|[15713]|LOWCE375-06|Canada|British Columbia|658[0n]|BOLD:AAB0068
Litholomia napaea|[15714]|LOWCE373-06|Canada|British Columbia|658[0n]|BOLD:AAB0068
Litholomia napaea|[15715]|LOWCE244-06|Canada|British Columbia|658[0n]|BOLD:AAB0068
Litholomia napaea|[15716]|LOWCE241-06|Canada|British Columbia|658[0n]|BOLD:AAB0068
Litholomia napaea|[15717]|LOWCE033-06|Canada|British Columbia|658[0n]|BOLD:AAB0068
Litholomia napaea|[15718]|LOWCE031-06|Canada|British Columbia|658[0n]|BOLD:AAB0068
Litholomia napaea|[15719]|LOWCE007-06|Canada|British Columbia|658[0n]|BOLD:AAB0068
Litholomia napaea|[15720]|LOWCE006-06|Canada|British Columbia|658[0n]|BOLD:AAB0068
Litholomia napaea|[15721]|LOWCE004-06|Canada|British Columbia|658[0n]|BOLD:AAB0068
Litholomia napaea|[15722]|LOWCE003-06|Canada|British Columbia|658[0n]|BOLD:AAB0068
Litholomia napaea|[15723]|LOWCD938-06|Canada|British Columbia|658[0n]|BOLD:AAB0068
Litholomia napaea|[15724]|LOWCD937-06|Canada|British Columbia|658[0n]|BOLD:AAB0068
Litholomia napaea|[15725]|LOWCB862-05|Canada|British Columbia|658[0n]|BOLD:AAB0068
Litholomia napaea|[15726]|LOWCE506-06|Canada|British Columbia|620[0n]|BOLD:AAB0068
Litholomia napaea|[15727]|LBCH1241-10|Canada|British Columbia|658[0n]|BOLD:AAB0068
Litholomia napaea|[15728]|LALPA013-10|Canada|British Columbia|658[0n]|BOLD:AAB0068
Litholomia napaea|[15729]|LALPA032-10|Canada|British Columbia|658[0n]|BOLD:AAB0068
Litholomia napaea|[15730]|LALPA076-10|Canada|British Columbia|658[0n]|BOLD:AAB0068
Litholomia napaea|[15731]|JMMMB322-11|United States|California|658[0n]|BOLD:AAB0068
Litholomia napaea|[15732]|JMMMB342-11|United States|California|658[0n]|BOLD:AAB0068
Litholomia napaea|[15733]|RWWC220-11|United States|Washington|658[0n]|BOLD:AAB0068
Litholomia napaea|[15734]|RWWC806-11|United States|Washington|658[0n]|BOLD:AAB0068
Litholomia napaea|[15735]|RWWC910-12|United States|Washington|658[0n]|BOLD:AAB0068
Litholomia napaea|[15736]|SSEIA7706-13|Canada|Alberta|543[0n]|BOLD:AAB0068
Litholomia napaea|[15737]|LOWCD939-06|Canada|British Columbia|658[0n]|BOLD:AAB0068
Litholomia napaea|[15738]|TMNBB234-06|Canada|New Brunswick|656[0n]|BOLD:AAB0068
Litholomia napaea|[15739]|TMNBB233-06|Canada|New Brunswick|631[0n]|BOLD:AAB0068
Litholomia napaea|[15740]|TMNBB230-06|Canada|New Brunswick|658[0n]|BOLD:AAB0068
Litholomia napaea|[15741]|TMNBB235-06|Canada|New Brunswick|658[0n]|BOLD:AAB0068
Litholomia napaea|[15742]|TMNBB237-06|Canada|New Brunswick|658[0n]|BOLD:AAB0068
Litholomia napaea|[15743]|TMNBB231-06|Canada|New Brunswick|658[0n]|BOLD:AAB0068
Litholomia napaea|[15744]|TMNBB236-06|Canada|New Brunswick|658[0n]|BOLD:AAB0068
Litholomia napaea|[15745]|TMNBB232-06|Canada|New Brunswick|658[0n]|BOLD:AAB0068
Litholomia napaea|[15746]|RDLQ505-07|Canada|Quebec|608[0n]|BOLD:AAB0068
Litholomia napaea|[15747]|SSEIA2171-13|Canada|Alberta|608[0n]|BOLD:AAB0068
Litholomia napaea|[15748]|SSEIA7707-13|Canada|Alberta|608[0n]|BOLD:AAB0068
Mesogona subcuprea|[15749]|RDNMF483-08|United States|Nevada|658[0n]|BOLD:AAC4482
Mesogona subcuprea|[15750]|LOPN130-06|United States|Oregon|567[0n]|BOLD:AAC4482
Mesogona subcuprea|[15751]|RDNMF484-08|United States|Oregon|658[0n]|BOLD:AAC4482
Mesogona subcuprea|[15752]|RDNMF481-08|United States|Washington|658[0n]|BOLD:AAC4482
Mesogona subcuprea|[15753]|RDNMF482-08|United States|Washington|658[0n]|BOLD:AAC4482
Mesogona subcuprea|[15754]|RDNMF485-08|United States|California|658[0n]|BOLD:AAC4482
Mesogona rubra|[15755]|LOPN126-06|United States|Oregon|591[1n]|BOLD:AAC4484
Mesogona rubra|[15756]|LOPN127-06|United States|Oregon|525[0n]|BOLD:AAC4484
Mesogona rubra|[15757]|NAMUM399-09|United States|California|658[0n]|BOLD:AAC4484
Mesogona olivata|[15758]|RDNMJ187-10|United States|Texas|658[0n]|BOLD:AAU1648
Mesogona olivata|[15759]|RDNMJ174-10|United States|Arizona|658[0n]|BOLD:AAC4483
Mesogona olivata|[15760]|LOCBB240-06|United States|California|658[0n]|BOLD:AAC4483
Mesogona olivata|[15761]|LOCBB239-06|United States|California|658[0n]|BOLD:AAC4483
Mesogona olivata|[15762]|LOCBB241-06|United States|California|632[0n]|BOLD:AAC4483
Mesogona olivata|[15763]|LOCBB242-06|United States|California|658[0n]|BOLD:AAC4483
Mesogona olivata|[15764]|RDNMD434-06|United States|California|658[0n]|BOLD:AAC4483
Mesogona olivata|[15765]|RDNMD435-06|United States|California|658[0n]|BOLD:AAC4483
Mesogona olivata|[15766]|CGLCA052-10|United States|California|658[0n]|BOLD:AAC4483
Mesogona olivata|[15767]|RDNMJ175-10|United States|Utah|658[0n]|BOLD:AAC4483
Mesogona olivata|[15768]|RDNMJ176-10|United States|New Mexico|658[0n]|BOLD:AAC4483
Mesogona olivata|[15769]|RDNMJ177-10|United States|New Mexico|658[0n]|BOLD:AAC4483
Mesogona olivata|[15770]|RDNMJ178-10|United States|New Mexico|658[0n]|BOLD:AAC4483
Mesogona olivata|[15771]|RDNMJ179-10|United States|Arizona|658[0n]|BOLD:AAC4483
Mesogona sp.|[15772]|RDNMJ186-10|United States|Wyoming|658[0n]|BOLD:AAC4483
Mesogona sp.|[15773]|RDNMJ188-10|United States|Wyoming|658[0n]|BOLD:AAC4483
Mesogona olivata|[15774]|LBCH7151-10|Canada|British Columbia|658[0n]|BOLD:ACE4866
Mesogona olivata|[15775]|LPVIB642-08|Canada|British Columbia|641[0n]|BOLD:ACE4866
Mesogona olivata|[15776]|LPVIB643-08|Canada|British Columbia|631[0n]|BOLD:ACE4866
Mesogona olivata|[15777]|LBCH7250-10|Canada|British Columbia|658[0n]|BOLD:ACE4866
Mesogona olivata|[15778]|LBCH7315-10|Canada|British Columbia|642[0n]|BOLD:ACE4866
Mesogona olivata|[15779]|LOPN132-06|United States|Oregon|658[0n]|BOLD:ACE4866
Mesogona olivata|[15780]|LOPN133-06|United States|Oregon|542[0n]|BOLD:ACE4866
Mesogona olivata|[15781]|LALPA1297-11|Canada|British Columbia|658[0n]|BOLD:ACE4866
Mesogona olivata|[15782]|NAMUM400-09|United States|California|658[0n]|BOLD:AAC4485
Mesogona olivata|[15783]|NAMUM401-09|United States|California|658[0n]|BOLD:AAC4485
Mesogona olivata|[15784]|GMLC032-09|United States|California|658[0n]|BOLD:AAC4485
Mesogona olivata|[15785]|GMLC1229-12|United States|California|658[0n]|BOLD:AAC4485
Mesogona olivata|[15786]|GMLC1231-12|United States|California|658[0n]|BOLD:AAC4485
Metaxaglaea australis|[15787]|RDNMK118-11|United States|Florida|658[0n]|BOLD:ACF1931
Metaxaglaea australis|[15788]|RDNMH137-09|United States|Florida|658[0n]|BOLD:ACF1931
Metaxaglaea australis|[15789]|RDNMK035-11|United States|Florida|658[0n]|BOLD:ACF1931
Metaxaglaea australis|[15790]|RDNMK037-11|United States|Florida|658[0n]|BOLD:ACF1931
Metaxaglaea australis|[15791]|LNC472-06|United States|North Carolina|658[0n]|BOLD:ACF1931

Metaxaglaea australis|[15789]|RDNMK035-11|United States|Florida|658[0n]|BOLD:ACF1931
Metaxaglaea australis|[15790]|RDNMK037-11|United States|Florida|658[0n]|BOLD:ACF1931
Metaxaglaea australis|[15791]|LNC472-06|United States|North Carolina|658[0n]|BOLD:ACF1931
Metaxaglaea australis|[15792]|RDNMI021-10|United States|Florida|658[0n]|BOLD:ACF1931
Metaxaglaea australis|[15793]|RDNMI026-10|United States|Florida|658[0n]|BOLD:ACF1931
Metaxaglaea semitaria|[15794]|GWOTA069-12|United States|Massachusetts|658[0n]|BOLD:ACF1931
Metaxaglaea semitaria|[15795]|LNC498-06|United States|North Carolina|602[3n]|BOLD:ACF1931
Metaxaglaea semitaria|[15796]|RDNML136-13|United States|New Jersey|658[0n]|BOLD:ACF1931
Metaxaglaea australis|[15797]|RDNMH136-09|United States|Florida|658[0n]|BOLD:ACF1931
Metaxaglaea australis|[15798]|RDNMI023-10|United States|Florida|658[0n]|BOLD:ACF1931
Metaxaglaea australis|[15799]|RDNMK036-11|United States|Florida|631[0n]|BOLD:ACF1931
Metaxaglaea semitaria|[15800]|GWOTA070-12|United States|Massachusetts|658[0n]|BOLD:ACF1931
Metaxaglaea australis|[15801]|LNC471-06|United States|North Carolina|658[0n]|BOLD:ACF1931
Metaxaglaea australis|[15802]|RDNMI020-10|United States|Florida|658[0n]|BOLD:ACF1931
Metaxaglaea semitaria|[15803]|RDNML137-13|United States|New Jersey|658[0n]|BOLD:ACF1931
Metaxaglaea australis|[15804]|RDNMK034-11|United States|Florida|658[0n]|BOLD:ACF1931
Metaxaglaea australis|[15805]|RDNMH138-09|United States|Florida|658[0n]|BOLD:ACF1931
Metaxaglaea australis|[15806]|RDNMK119-11|United States|Florida|658[0n]|BOLD:ACF1931
Metaxaglaea semitaria|[15807]|RDNML138-13|United States|New Jersey|658[0n]|BOLD:ACF1931
Metaxaglaea inulta|[15808]|PHMO360-03|Canada|Ontario|639[0n]|BOLD:AAC1629
Metaxaglaea inulta|[15809]|RDLQB504-05|Canada|Quebec|658[0n]|BOLD:AAC1629
Metaxaglaea inulta|[15810]|RDLQF235-06|Canada|Quebec|658[0n]|BOLD:AAC1629
Metaxaglaea inulta|[15811]|RDLQF238-06|Canada|Quebec|658[0n]|BOLD:AAC1629
Metaxaglaea inulta|[15812]|RDLQF239-06|Canada|Quebec|656[0n]|BOLD:AAC1629
Metaxaglaea inulta|[15813]|TMNBB253-06|Canada|New Brunswick|658[0n]|BOLD:AAC1629
Metaxaglaea inulta|[15814]|XAH700-05|Canada|Ontario|658[0n]|BOLD:AAC1629
Metaxaglaea inulta|[15815]|PHMO190-03|Canada|Ontario|639[1n]|BOLD:AAC1629
Metaxaglaea inulta|[15816]|PHMO329-03|Canada|Ontario|639[6n]|BOLD:AAC1629
Metaxaglaea inulta|[15817]|TMNBB252-06|Canada|New Brunswick|658[0n]|BOLD:AAC1629
Metaxaglaea inulta|[15818]|TTMNB373-06|Canada|New Brunswick|658[0n]|BOLD:AAC1629
Metaxaglaea inulta|[15819]|TMNBB254-06|Canada|New Brunswick|656[0n]|BOLD:AAC1629
Metaxaglaea inulta|[15820]|LNCC561-11|United States|North Carolina|658[0n]|BOLD:AAC1629
Metaxaglaea violacea|[15821]|LNCB201-06|United States|North Carolina|658[0n]|BOLD:AAX4238
Metaxaglaea violacea|[15822]|RDNML134-13|United States|New Jersey|658[0n]|BOLD:AAX4238
Metaxaglaea violacea|[15823]|RDNML135-13|United States|New Jersey|658[0n]|BOLD:AAX4238
Metaxaglaea viatica|[15824]|RDNML170-13|United States|North Carolina|658[0n]|BOLD:AAC1630
Metaxaglaea viatica|[15825]|RDNML132-13|United States|New Jersey|625[0n]|BOLD:AAC1630
Metaxaglaea viatica|[15826]|RDNML133-13|United States|New Jersey|658[0n]|BOLD:AAC1630
Metaxaglaea viatica|[15827]|LNCC423-10|United States|North Carolina|658[0n]|BOLD:AAC1630
Metaxaglaea viatica|[15828]|LPOKA666-09|United States|Oklahoma|658[0n]|BOLD:AAC1630
Metaxaglaea viatica|[15829]|RDNML167-13|United States|North Carolina|658[0n]|BOLD:AAC1630
Metaxaglaea viatica|[15830]|RDNML169-13|United States|North Carolina|658[0n]|BOLD:AAC1630
Metaxaglaea viatica|[15831]|LSUSA102-06|United States|Kentucky|658[0n]|BOLD:AAC1630
Metaxaglaea viatica|[15832]|RDNML168-13|United States|North Carolina|658[0n]|BOLD:AAC1630
Metaxaglaea viatica|[15833]|RDNML171-13|United States|North Carolina|658[0n]|BOLD:AAC1630
Pseudohadena vulnerea|[15834]|IAWLB493-11|United States|Arizona|658[0n]|BOLD:AAL1819
Pseudohadena vulnerea|[15835]|AWCLB322-10|United States|Arizona|658[0n]|BOLD:AAL1819
Pseudohadena vulnerea|[15836]|AWCLB245-10|United States|Arizona|658[0n]|BOLD:AAL1819
Pseudohadena vulnerea|[15837]|AWCLB254-10|United States|Arizona|559[3n]|BOLD:AAL1819
Pseudohadena vulnerea|[15838]|AWCLB244-10|United States|Arizona|658[0n]|BOLD:AAL1819
Pseudohadena vulnerea|[15839]|IAWLB492-11|United States|Arizona|658[0n]|BOLD:AAL1819
Pseudohadena vulnerea|[15840]|LOCBF2322-13|United States|California|606[0n]|BOLD:AAL1819
Pseudohadena vulnerea|[15841]|LOCBF2323-13|United States|California|603[0n]|BOLD:AAL1819
Pseudohadena vulnerea|[15842]|IAWLB494-11|United States|Arizona|658[0n]|BOLD:AAL1819
Pseudohadena vulnerea|[15843]|AWCLB417-10|United States|Arizona|658[0n]|BOLD:AAL1819
Pseudohadena vulnerea|[15844]|AWCLB263-10|United States|Arizona|658[0n]|BOLD:AAL1819
Pseudohadena vulnerea|[15845]|RDNMC609-06|United States|California|658[0n]|BOLD:AAL1819
Pseudohadena vulnerea|[15846]|LOCBF2320-13|United States|California|606[0n]|BOLD:AAL1819
Pseudohadena vulnerea|[15847]|AWCLB252-10|United States|Arizona|658[0n]|BOLD:AAL1819
Pseudohadena vulnerea|[15848]|LOCBF2321-13|United States|California|576[0n]|BOLD:AAL1819
Pseudohadena vulnerea|[15849]|LOCBF2324-13|United States|California|606[0n]|BOLD:AAL1819
Hillia maida|[15850]|RDNMF497-08|United States|Colorado|658[0n]|BOLD:AAI8474
Hillia maida|[15851]|NAMUM280-08|United States|California|542[0n]|BOLD:AAI8475
Hillia iris|[15852]|LCHQ823-08|Canada|Manitoba|658[0n]|BOLD:AAA1264
Hillia iris|[15853]|LCHQ717-08|Canada|Manitoba|658[0n]|BOLD:AAA1264
Hillia iris|[15854]|LCHIP092-07|Canada|Manitoba|650[0n]|BOLD:AAA1264
Hillia iris|[15855]|LCHIP123-07|Canada|Manitoba|650[0n]|BOLD:AAA1264
Hillia iris|[15856]|LCHIP212-07|Canada|Manitoba|650[0n]|BOLD:AAA1264
Hillia iris|[15857]|LCHQ744-08|Canada|Manitoba|656[0n]|BOLD:AAA1264
Hillia iris|[15858]|LCHIP198-07|Canada|Manitoba|650[0n]|BOLD:AAA1264
Hillia iris|[15859]|LCHIP085-07|Canada|Manitoba|650[0n]|BOLD:AAA1264
Hillia iris|[15860]|LCHQ294-08|Canada|Manitoba|658[0n]|BOLD:AAA1264
Hillia iris|[15861]|LCHQ806-08|Canada|Manitoba|657[0n]|BOLD:AAA1264
Hillia iris|[15862]|LCHIP003-07|Canada|Manitoba|650[0n]|BOLD:AAA1264
Hillia iris|[15863]|LCHQ231-08|Canada|Manitoba|656[0n]|BOLD:AAA1264
Hillia iris|[15864]|LCHQ362-08|Canada|Manitoba|658[0n]|BOLD:AAA1264
Hillia iris|[15865]|LCHQ794-08|Canada|Manitoba|658[0n]|BOLD:AAA1264
Hillia iris|[15866]|LCHQ824-08|Canada|Manitoba|658[0n]|BOLD:AAA1264
Hillia iris|[15867]|LCHQ784-08|Canada|Manitoba|658[0n]|BOLD:AAA1264
Hillia iris|[15868]|LCHQ791-08|Canada|Manitoba|658[0n]|BOLD:AAA1264
Hillia iris|[15869]|LCHQ779-08|Canada|Manitoba|656[0n]|BOLD:AAA1264
Hillia iris|[15870]|LCHQ780-08|Canada|Manitoba|655[0n]|BOLD:AAA1264
Hillia iris|[15871]|LCHQ762-08|Canada|Manitoba|658[0n]|BOLD:AAA1264
Hillia iris|[15872]|LCHQ772-08|Canada|Manitoba|658[0n]|BOLD:AAA1264
Hillia iris|[15873]|LCHQ757-08|Canada|Manitoba|658[0n]|BOLD:AAA1264
Hillia iris|[15874]|LCHQ761-08|Canada|Manitoba|658[0n]|BOLD:AAA1264
Hillia iris|[15875]|LCHQ750-08|Canada|Manitoba|658[0n]|BOLD:AAA1264
Hillia iris|[15876]|LCHQ754-08|Canada|Manitoba|658[0n]|BOLD:AAA1264
Hillia iris|[15877]|LCHQ739-08|Canada|Manitoba|658[0n]|BOLD:AAA1264
Hillia iris|[15878]|LCHQ740-08|Canada|Manitoba|658[0n]|BOLD:AAA1264
Hillia iris|[15879]|LCHQ736-08|Canada|Manitoba|658[0n]|BOLD:AAA1264
Hillia iris|[15880]|LCHQ606-08|Canada|Manitoba|658[0n]|BOLD:AAA1264
Hillia iris|[15881]|LCHQ400-08|Canada|Manitoba|658[0n]|BOLD:AAA1264
Hillia iris|[15882]|LCHQ373-08|Canada|Manitoba|658[0n]|BOLD:AAA1264
Hillia iris|[15883]|LCHQ360-08|Canada|Manitoba|658[0n]|BOLD:AAA1264
Hillia iris|[15884]|LCHQ298-08|Canada|Manitoba|657[0n]|BOLD:AAA1264
Hillia iris|[15885]|LCHQ297-08|Canada|Manitoba|657[0n]|BOLD:AAA1264
Hillia iris|[15886]|LCHQ277-08|Canada|Manitoba|658[0n]|BOLD:AAA1264
Hillia iris|[15887]|MHLEP128-07|Canada|Manitoba|658[0n]|BOLD:AAA1264
Hillia iris|[15888]|LCHQ743-08|Canada|Manitoba|655[0n]|BOLD:AAA1264
Hillia iris|[15889]|LCHQ729-08|Canada|Manitoba|656[0n]|BOLD:AAA1264
Hillia iris|[15890]|LCHQ357-08|Canada|Manitoba|656[0n]|BOLD:AAA1264
Hillia iris|[15891]|LCHQ283-08|Canada|Manitoba|655[0n]|BOLD:AAA1264

Hillia iris|[15889]|LCHQ729-08|Canada|Manitoba|656[0n]|BOLD:AAA1264
Hillia iris|[15890]|LCHQ357-08|Canada|Manitoba|656[0n]|BOLD:AAA1264
Hillia iris|[15891]|LCHQ283-08|Canada|Manitoba|655[0n]|BOLD:AAA1264
Hillia iris|[15892]|LCHIP280-07|Canada|Manitoba|650[0n]|BOLD:AAA1264
Hillia iris|[15893]|LCHIP266-07|Canada|Manitoba|650[0n]|BOLD:AAA1264
Hillia iris|[15894]|LCHIP217-07|Canada|Manitoba|650[0n]|BOLD:AAA1264
Hillia iris|[15895]|LCHIP151-07|Canada|Manitoba|650[0n]|BOLD:AAA1264
Hillia iris|[15896]|LCHIP135-07|Canada|Manitoba|650[0n]|BOLD:AAA1264
Hillia iris|[15897]|LCHIP108-07|Canada|Manitoba|650[0n]|BOLD:AAA1264
Hillia iris|[15898]|LCHIP106-07|Canada|Manitoba|650[0n]|BOLD:AAA1264
Hillia iris|[15899]|LCHIP019-07|Canada|Manitoba|650[0n]|BOLD:AAA1264
Hillia iris|[15900]|LCHIP016-07|Canada|Manitoba|650[0n]|BOLD:AAA1264
Hillia iris|[15901]|LCHQ407-08|Canada|Manitoba|633[0n]|BOLD:AAA1264
Hillia iris|[15902]|LCHQ774-08|Canada|Manitoba|646[0n]|BOLD:AAA1264
Hillia iris|[15903]|LCHQ832-08|Canada|Manitoba|657[0n]|BOLD:AAA1264
Hillia iris|[15904]|LCHQ842-08|Canada|Manitoba|658[0n]|BOLD:AAA1264
Hillia iris|[15905]|LCHQ849-08|Canada|Manitoba|658[0n]|BOLD:AAA1264
Hillia iris|[15906]|LCHQ856-08|Canada|Manitoba|658[0n]|BOLD:AAA1264
Hillia iris|[15907]|LCHIP039-07|Canada|Manitoba|650[1n]|BOLD:AAA1264
Hillia iris|[15908]|LCHQ228-08|Canada|Manitoba|658[0n]|BOLD:AAA1264
Hillia iris|[15909]|LCHQ272-08|Canada|Manitoba|656[0n]|BOLD:AAA1264
Hillia iris|[15910]|LCHQ308-08|Canada|Manitoba|657[0n]|BOLD:AAA1264
Hillia iris|[15911]|LCHQ838-08|Canada|Manitoba|658[0n]|BOLD:AAA1264
Hillia iris|[15912]|LCHIP254-07|Canada|Manitoba|650[0n]|BOLD:AAA1264
Hillia iris|[15913]|MHLEP122-07|Canada|Manitoba|658[0n]|BOLD:AAA1264
Hillia iris|[15914]|LCHQ296-08|Canada|Manitoba|658[0n]|BOLD:AAA1264
Hillia iris|[15915]|PHMNB284-04|Canada|New Brunswick|571[1n]|BOLD:AAA1264
Hillia iris|[15916]|RDLQ592-07|Canada|Quebec|596[0n]|BOLD:AAA1264
Hillia iris|[15917]|LCHQ507-08|Canada|Manitoba|622[0n]|BOLD:AAA1264
Hillia iris|[15918]|LCHQ732-08|Canada|Manitoba|658[0n]|BOLD:AAA1264
Hillia iris|[15919]|LCHIP113-07|Canada|Manitoba|650[0n]|BOLD:AAA1264
Hillia iris|[15920]|LCHQ285-08|Canada|Manitoba|658[0n]|BOLD:AAA1264
Hillia iris|[15921]|LCHIP048-07|Canada|Manitoba|650[0n]|BOLD:AAA1264
Hillia iris|[15922]|LCHIP063-07|Canada|Manitoba|650[0n]|BOLD:AAA1264
Hillia iris|[15923]|LCHIP276-07|Canada|Manitoba|650[0n]|BOLD:AAA1264
Hillia iris|[15924]|LCHQ580-08|Canada|Manitoba|656[0n]|BOLD:AAA1264
Hillia iris|[15925]|LCHQ725-08|Canada|Manitoba|658[0n]|BOLD:AAA1264
Hillia iris|[15926]|LCHQ752-08|Canada|Manitoba|658[0n]|BOLD:AAA1264
Hillia iris|[15927]|LCHIP034-07|Canada|Manitoba|650[0n]|BOLD:AAA1264
Hillia iris|[15928]|MHLEP120-07|Canada|Manitoba|658[0n]|BOLD:AAA1264
Hillia iris|[15929]|LCHQ374-08|Canada|Manitoba|658[0n]|BOLD:AAA1264
Hillia iris|[15930]|LCHIP090-07|Canada|Manitoba|650[0n]|BOLD:AAA1264
Hillia iris|[15931]|LCHQ443-08|Canada|Manitoba|658[0n]|BOLD:AAA1264
Hillia iris|[15932]|LCHQ438-08|Canada|Manitoba|658[0n]|BOLD:AAA1264
Hillia iris|[15933]|LCHQ410-08|Canada|Manitoba|658[0n]|BOLD:AAA1264
Hillia iris|[15934]|LCHQ398-08|Canada|Manitoba|657[0n]|BOLD:AAA1264
Hillia iris|[15935]|LCHQ275-08|Canada|Manitoba|658[0n]|BOLD:AAA1264
Hillia iris|[15936]|LCHIP213-07|Canada|Manitoba|650[0n]|BOLD:AAA1264
Hillia iris|[15937]|LCHIP027-07|Canada|Manitoba|650[0n]|BOLD:AAA1264
Hillia iris|[15938]|LCHQ764-08|Canada|Manitoba|621[0n]|BOLD:AAA1264
Hillia iris|[15939]|LCHQ787-08|Canada|Manitoba|656[0n]|BOLD:AAA1264
Hillia iris|[15940]|LCHQ728-08|Canada|Manitoba|658[0n]|BOLD:AAA1264
Hillia iris|[15941]|LCHQ836-08|Canada|Manitoba|658[0n]|BOLD:AAA1264
Hillia iris|[15942]|LCHIP121-07|Canada|Manitoba|650[0n]|BOLD:AAA1264
Hillia iris|[15943]|LCHQ379-08|Canada|Manitoba|658[0n]|BOLD:AAA1264
Hillia iris|[15944]|LCHQ821-08|Canada|Manitoba|658[0n]|BOLD:AAA1264
Hillia iris|[15945]|LCHQ710-08|Canada|Manitoba|621[0n]|BOLD:AAA1264
Hillia iris|[15946]|LCHIP235-07|Canada|Manitoba|650[0n]|BOLD:AAA1264
Hillia iris|[15947]|LCHQ594-08|Canada|Manitoba|658[0n]|BOLD:AAA1264
Hillia iris|[15948]|LCHQ711-08|Canada|Manitoba|658[0n]|BOLD:AAA1264
Hillia iris|[15949]|MHLEP125-07|Canada|Manitoba|658[0n]|BOLD:AAA1264
Hillia iris|[15950]|LCHIP258-07|Canada|Manitoba|650[0n]|BOLD:AAA1264
Hillia iris|[15951]|LCHQ286-08|Canada|Manitoba|658[0n]|BOLD:AAA1264
Hillia iris|[15952]|LCHIP037-07|Canada|Manitoba|650[0n]|BOLD:AAA1264
Hillia iris|[15953]|LCHQ748-08|Canada|Manitoba|632[0n]|BOLD:AAA1264
Hillia iris|[15954]|LCHQ281-08|Canada|Manitoba|658[0n]|BOLD:AAA1264
Hillia iris|[15955]|LCHQ723-08|Canada|Manitoba|658[0n]|BOLD:AAA1264
Hillia iris|[15956]|LCHIP020-07|Canada|Manitoba|650[0n]|BOLD:AAA1264
Hillia iris|[15957]|LCHQ301-08|Canada|Manitoba|658[0n]|BOLD:AAA1264
Hillia iris|[15958]|LCHQ284-08|Canada|Manitoba|658[0n]|BOLD:AAA1264
Hillia iris|[15959]|LCHIP047-07|Canada|Manitoba|650[0n]|BOLD:AAA1264
Hillia iris|[15960]|LCHQ405-08|Canada|Manitoba|658[1n]|BOLD:AAA1264
Hillia iris|[15961]|LCHQ721-08|Canada|Manitoba|658[0n]|BOLD:AAA1264
Hillia iris|[15962]|LCHQ822-08|Canada|Manitoba|658[0n]|BOLD:AAA1264
Hillia iris|[15963]|DSCNI062-07|Canada|Manitoba|658[0n]|BOLD:AAA1264
Hillia iris|[15964]|LCHQ609-08|Canada|Manitoba|657[0n]|BOLD:AAA1264
Hillia iris|[15965]|LCHQ227-08|Canada|Manitoba|657[0n]|BOLD:AAA1264
Hillia iris|[15966]|LCHQ229-08|Canada|Manitoba|658[0n]|BOLD:AAA1264
Hillia iris|[15967]|LCHQ292-08|Canada|Manitoba|657[0n]|BOLD:AAA1264
Hillia iris|[15968]|LCHQ295-08|Canada|Manitoba|658[0n]|BOLD:AAA1264
Hillia iris|[15969]|LCHQ290-08|Canada|Manitoba|658[0n]|BOLD:AAA1264
Hillia iris|[15970]|LCHQ291-08|Canada|Manitoba|658[0n]|BOLD:AAA1264
Hillia iris|[15971]|MHLEP129-07|Canada|Manitoba|658[0n]|BOLD:AAA1264
Hillia iris|[15972]|MHLEP130-07|Canada|Manitoba|658[0n]|BOLD:AAA1264
Hillia iris|[15973]|LCHQ288-08|Canada|Manitoba|658[0n]|BOLD:AAA1264
Hillia iris|[15974]|LCHQ289-08|Canada|Manitoba|658[0n]|BOLD:AAA1264
Hillia iris|[15975]|LCHQ282-08|Canada|Manitoba|658[0n]|BOLD:AAA1264
Hillia iris|[15976]|LCHQ287-08|Canada|Manitoba|658[0n]|BOLD:AAA1264
Hillia iris|[15977]|MHLEP126-07|Canada|Manitoba|658[0n]|BOLD:AAA1264
Hillia iris|[15978]|MHLEP127-07|Canada|Manitoba|658[0n]|BOLD:AAA1264
Hillia iris|[15979]|LCHIP234-07|Canada|Manitoba|650[0n]|BOLD:AAA1264
Hillia iris|[15980]|LCHIP261-07|Canada|Manitoba|650[0n]|BOLD:AAA1264
Hillia iris|[15981]|LCHQ274-08|Canada|Manitoba|658[0n]|BOLD:AAA1264
Hillia iris|[15982]|MHLEP123-07|Canada|Manitoba|658[0n]|BOLD:AAA1264
Hillia iris|[15983]|MHLEP121-07|Canada|Manitoba|658[0n]|BOLD:AAA1264
Hillia iris|[15984]|DSCNI036-07|Canada|Manitoba|658[0n]|BOLD:AAA1264
Hillia iris|[15985]|DSCNI029-07|Canada|Manitoba|658[0n]|BOLD:AAA1264
Hillia iris|[15986]|DSCNI028-07|Canada|Manitoba|658[0n]|BOLD:AAA1264
Hillia iris|[15987]|DSCNI026-07|Canada|Manitoba|658[0n]|BOLD:AAA1264
Hillia iris|[15988]|DSCNI025-07|Canada|Manitoba|658[0n]|BOLD:AAA1264
Hillia iris|[15989]|LOWCC837-05|Canada|British Columbia|658[0n]|BOLD:AAA1264
Hillia iris|[15990]|LCHQ406-08|Canada|Manitoba|639[1n]|BOLD:AAA1264
Hillia iris|[15991]|LCHQ434-08|Canada|Manitoba|656[0n]|BOLD:AAA1264

Hillia iris|[15989]|LOWCC837-05|Canada|British Columbia|658[0n]|BOLD:AAA1264
Hillia iris|[15990]|LCHQ406-08|Canada|Manitoba|639[1n]|BOLD:AAA1264
Hillia iris|[15991]|LCHQ434-08|Canada|Manitoba|656[0n]|BOLD:AAA1264
Hillia iris|[15992]|LCHQ844-08|Canada|Manitoba|658[0n]|BOLD:AAA1264
Hillia iris|[15993]|LCH583-04|Canada|Manitoba|658[0n]|BOLD:AAA1264
Hillia iris|[15994]|LCHQ833-08|Canada|Manitoba|658[0n]|BOLD:AAA1264
Hillia iris|[15995]|LCHQ828-08|Canada|Manitoba|658[0n]|BOLD:AAA1264
Hillia iris|[15996]|LCHQ741-08|Canada|Manitoba|658[0n]|BOLD:AAA1264
Hillia iris|[15997]|LCHQ596-08|Canada|Manitoba|657[0n]|BOLD:AAA1264
Hillia iris|[15998]|LCHQ402-08|Canada|Manitoba|657[0n]|BOLD:AAA1264
Hillia iris|[15999]|LCHQ271-08|Canada|Manitoba|658[0n]|BOLD:AAA1264
Hillia iris|[16000]|LCHQ232-08|Canada|Manitoba|658[0n]|BOLD:AAA1264
Hillia iris|[16001]|LCHIP149-07|Canada|Manitoba|650[1n]|BOLD:AAA1264
Hillia iris|[16002]|LCHIP012-07|Canada|Manitoba|650[0n]|BOLD:AAA1264
Hillia iris|[16003]|LCHIP064-07|Canada|Manitoba|650[0n]|BOLD:AAA1264
Hillia iris|[16004]|LCHIP066-07|Canada|Manitoba|650[0n]|BOLD:AAA1264
Hillia iris|[16005]|LCHIP059-07|Canada|Manitoba|650[0n]|BOLD:AAA1264
Hillia iris|[16006]|LCHIP107-07|Canada|Manitoba|650[0n]|BOLD:AAA1264
Hillia iris|[16007]|LCHQ835-08|Canada|Manitoba|656[0n]|BOLD:AAA1264
Hillia iris|[16008]|LCHQ439-08|Canada|Manitoba|658[0n]|BOLD:AAA1264
Hillia iris|[16009]|LCHQ477-08|Canada|Manitoba|658[0n]|BOLD:AAA1264
Hillia iris|[16010]|LCHIP111-07|Canada|Manitoba|650[1n]|BOLD:AAA1264
Hillia iris|[16011]|LCHIP221-07|Canada|Manitoba|650[0n]|BOLD:AAA1264
Hillia iris|[16012]|LCHQ709-08|Canada|Manitoba|656[0n]|BOLD:AAA1264
Hillia iris|[16013]|LCHQ795-08|Canada|Manitoba|658[0n]|BOLD:AAA1264
Hillia iris|[16014]|LCHQ839-08|Canada|Manitoba|656[0n]|BOLD:AAA1264
Hillia iris|[16015]|LCHQ846-08|Canada|Manitoba|656[0n]|BOLD:AAA1264
Hillia iris|[16016]|LCHQ827-08|Canada|Manitoba|655[0n]|BOLD:AAA1264
Hillia iris|[16017]|LCHQ830-08|Canada|Manitoba|655[0n]|BOLD:AAA1264
Hillia iris|[16018]|LCHQ847-08|Canada|Manitoba|656[0n]|BOLD:AAA1264
Hillia iris|[16019]|LCHQ735-08|Canada|Manitoba|656[0n]|BOLD:AAA1264
Hillia iris|[16020]|LCHQ727-08|Canada|Manitoba|656[0n]|BOLD:AAA1264
Hillia iris|[16021]|LCHQ712-08|Canada|Manitoba|655[0n]|BOLD:AAA1264
Hillia iris|[16022]|LCHQ604-08|Canada|Manitoba|656[0n]|BOLD:AAA1264
Hillia iris|[16023]|LCHQ525-08|Canada|Manitoba|655[0n]|BOLD:AAA1264
Hillia iris|[16024]|LCHQ404-08|Canada|Manitoba|656[0n]|BOLD:AAA1264
Hillia iris|[16025]|LCHQ380-08|Canada|Manitoba|656[0n]|BOLD:AAA1264
Hillia iris|[16026]|LCHQ280-08|Canada|Manitoba|656[0n]|BOLD:AAA1264
Hillia iris|[16027]|LCHQ279-08|Canada|Manitoba|656[0n]|BOLD:AAA1264
Hillia iris|[16028]|LCHQ273-08|Canada|Manitoba|656[0n]|BOLD:AAA1264
Hillia iris|[16029]|LCHIP002-07|Canada|Manitoba|628[0n]|BOLD:AAA1264
Hillia iris|[16030]|LCHQ412-08|Canada|Manitoba|657[0n]|BOLD:AAA1264
Hillia iris|[16031]|LCHIP263-07|Canada|Manitoba|650[0n]|BOLD:AAA1264
Hillia iris|[16032]|LCHIP156-07|Canada|Manitoba|650[0n]|BOLD:AAA1264
Hillia iris|[16033]|LCHIP160-07|Canada|Manitoba|650[0n]|BOLD:AAA1264
Hillia iris|[16034]|LCHIP146-07|Canada|Manitoba|650[0n]|BOLD:AAA1264
Hillia iris|[16035]|LCHIP155-07|Canada|Manitoba|650[0n]|BOLD:AAA1264
Hillia iris|[16036]|LCHIP143-07|Canada|Manitoba|650[0n]|BOLD:AAA1264
Hillia iris|[16037]|LCHIP144-07|Canada|Manitoba|650[0n]|BOLD:AAA1264
Hillia iris|[16038]|LCHIP140-07|Canada|Manitoba|650[0n]|BOLD:AAA1264
Hillia iris|[16039]|LCHIP142-07|Canada|Manitoba|650[0n]|BOLD:AAA1264
Hillia iris|[16040]|LCHIP136-07|Canada|Manitoba|650[0n]|BOLD:AAA1264
Hillia iris|[16041]|LCHIP139-07|Canada|Manitoba|650[0n]|BOLD:AAA1264
Hillia iris|[16042]|LCHIP130-07|Canada|Manitoba|650[0n]|BOLD:AAA1264
Hillia iris|[16043]|LCHIP131-07|Canada|Manitoba|650[0n]|BOLD:AAA1264
Hillia iris|[16044]|LCHIP101-07|Canada|Manitoba|650[0n]|BOLD:AAA1264
Hillia iris|[16045]|LCHIP102-07|Canada|Manitoba|650[0n]|BOLD:AAA1264
Hillia iris|[16046]|LCHIP098-07|Canada|Manitoba|650[0n]|BOLD:AAA1264
Hillia iris|[16047]|LCHIP099-07|Canada|Manitoba|650[0n]|BOLD:AAA1264
Hillia iris|[16048]|LCHIP093-07|Canada|Manitoba|650[0n]|BOLD:AAA1264
Hillia iris|[16049]|LCHIP094-07|Canada|Manitoba|650[0n]|BOLD:AAA1264
Hillia iris|[16050]|LCHIP086-07|Canada|Manitoba|650[0n]|BOLD:AAA1264
Hillia iris|[16051]|LCHIP089-07|Canada|Manitoba|650[0n]|BOLD:AAA1264
Hillia iris|[16052]|LCHIP080-07|Canada|Manitoba|650[0n]|BOLD:AAA1264
Hillia iris|[16053]|LCHIP081-07|Canada|Manitoba|650[0n]|BOLD:AAA1264
Hillia iris|[16054]|LCHIP072-07|Canada|Manitoba|650[0n]|BOLD:AAA1264
Hillia iris|[16055]|LCHIP079-07|Canada|Manitoba|650[0n]|BOLD:AAA1264
Hillia iris|[16056]|LCHIP070-07|Canada|Manitoba|650[0n]|BOLD:AAA1264
Hillia iris|[16057]|LCHIP071-07|Canada|Manitoba|650[0n]|BOLD:AAA1264
Hillia iris|[16058]|LCHIP068-07|Canada|Manitoba|650[0n]|BOLD:AAA1264
Hillia iris|[16059]|LCHIP069-07|Canada|Manitoba|650[0n]|BOLD:AAA1264
Hillia iris|[16060]|LCHIP061-07|Canada|Manitoba|650[0n]|BOLD:AAA1264
Hillia iris|[16061]|LCHIP067-07|Canada|Manitoba|650[0n]|BOLD:AAA1264
Hillia iris|[16062]|LCHIP056-07|Canada|Manitoba|650[0n]|BOLD:AAA1264
Hillia iris|[16063]|LCHIP057-07|Canada|Manitoba|650[0n]|BOLD:AAA1264
Hillia iris|[16064]|LCHIP038-07|Canada|Manitoba|650[0n]|BOLD:AAA1264
Hillia iris|[16065]|LCHIP040-07|Canada|Manitoba|650[0n]|BOLD:AAA1264
Hillia iris|[16066]|LCHIP035-07|Canada|Manitoba|650[0n]|BOLD:AAA1264
Hillia iris|[16067]|LCHIP036-07|Canada|Manitoba|650[0n]|BOLD:AAA1264
Hillia iris|[16068]|LCHIP029-07|Canada|Manitoba|650[0n]|BOLD:AAA1264
Hillia iris|[16069]|LCHIP033-07|Canada|Manitoba|650[0n]|BOLD:AAA1264
Hillia iris|[16070]|LCHIP211-07|Canada|Manitoba|650[0n]|BOLD:AAA1264
Hillia iris|[16071]|LCHIP214-07|Canada|Manitoba|650[0n]|BOLD:AAA1264
Hillia iris|[16072]|LCHIP218-07|Canada|Manitoba|650[0n]|BOLD:AAA1264
Hillia iris|[16073]|LCHIP219-07|Canada|Manitoba|650[0n]|BOLD:AAA1264
Hillia iris|[16074]|LCHIP224-07|Canada|Manitoba|650[0n]|BOLD:AAA1264
Hillia iris|[16075]|LCHIP225-07|Canada|Manitoba|650[0n]|BOLD:AAA1264
Hillia iris|[16076]|LCHIP226-07|Canada|Manitoba|650[0n]|BOLD:AAA1264
Hillia iris|[16077]|LCHIP228-07|Canada|Manitoba|650[0n]|BOLD:AAA1264
Hillia iris|[16078]|LCHIP230-07|Canada|Manitoba|650[0n]|BOLD:AAA1264
Hillia iris|[16079]|LCHIP232-07|Canada|Manitoba|650[0n]|BOLD:AAA1264
Hillia iris|[16080]|LCHIP233-07|Canada|Manitoba|650[0n]|BOLD:AAA1264
Hillia iris|[16081]|LCHIP247-07|Canada|Manitoba|650[0n]|BOLD:AAA1264
Hillia iris|[16082]|LCHIP248-07|Canada|Manitoba|650[0n]|BOLD:AAA1264
Hillia iris|[16083]|LCHIP249-07|Canada|Manitoba|650[0n]|BOLD:AAA1264
Hillia iris|[16084]|LCHIP250-07|Canada|Manitoba|650[0n]|BOLD:AAA1264
Hillia iris|[16085]|LCHIP252-07|Canada|Manitoba|650[0n]|BOLD:AAA1264
Hillia iris|[16086]|LCHIP253-07|Canada|Manitoba|650[0n]|BOLD:AAA1264
Hillia iris|[16087]|LCHIP255-07|Canada|Manitoba|650[0n]|BOLD:AAA1264
Hillia iris|[16088]|LCHIP256-07|Canada|Manitoba|650[0n]|BOLD:AAA1264
Hillia iris|[16089]|LCHIP260-07|Canada|Manitoba|650[0n]|BOLD:AAA1264
Hillia iris|[16090]|LCHIP262-07|Canada|Manitoba|650[0n]|BOLD:AAA1264
Hillia iris|[16091]|LCHIP264-07|Canada|Manitoba|650[0n]|BOLD:AAA1264

Hillia iris|[16089]|LCHIP260-07|Canada|Manitoba|650[0n]|BOLD:AAA1264
Hillia iris|[16090]|LCHIP262-07|Canada|Manitoba|650[0n]|BOLD:AAA1264
Hillia iris|[16091]|LCHIP264-07|Canada|Manitoba|650[0n]|BOLD:AAA1264
Hillia iris|[16092]|LCHIP265-07|Canada|Manitoba|650[0n]|BOLD:AAA1264
Hillia iris|[16093]|LCHIP267-07|Canada|Manitoba|650[0n]|BOLD:AAA1264
Hillia iris|[16094]|LCHIP045-07|Canada|Manitoba|650[0n]|BOLD:AAA1264
Hillia iris|[16095]|LCHIP046-07|Canada|Manitoba|650[0n]|BOLD:AAA1264
Hillia iris|[16096]|LCHIP273-07|Canada|Manitoba|650[0n]|BOLD:AAA1264
Hillia iris|[16097]|LCHIP274-07|Canada|Manitoba|650[0n]|BOLD:AAA1264
Hillia iris|[16098]|LCHIP049-07|Canada|Manitoba|650[0n]|BOLD:AAA1264
Hillia iris|[16099]|LCHIP050-07|Canada|Manitoba|650[0n]|BOLD:AAA1264
Hillia iris|[16100]|LCHIP275-07|Canada|Manitoba|650[0n]|BOLD:AAA1264
Hillia iris|[16101]|LCHIP277-07|Canada|Manitoba|650[0n]|BOLD:AAA1264
Hillia iris|[16102]|LCHIP183-07|Canada|Manitoba|650[0n]|BOLD:AAA1264
Hillia iris|[16103]|LCHIP210-07|Canada|Manitoba|650[0n]|BOLD:AAA1264
Hillia iris|[16104]|LCHIP026-07|Canada|Manitoba|650[0n]|BOLD:AAA1264
Hillia iris|[16105]|LCHIP028-07|Canada|Manitoba|650[0n]|BOLD:AAA1264
Hillia iris|[16106]|LCHIP124-07|Canada|Manitoba|650[0n]|BOLD:AAA1264
Hillia iris|[16107]|LCHIP125-07|Canada|Manitoba|650[0n]|BOLD:AAA1264
Hillia iris|[16108]|LCHIP127-07|Canada|Manitoba|650[0n]|BOLD:AAA1264
Hillia iris|[16109]|LCHIP129-07|Canada|Manitoba|650[0n]|BOLD:AAA1264
Hillia iris|[16110]|LCHIP018-07|Canada|Manitoba|650[0n]|BOLD:AAA1264
Hillia iris|[16111]|LCHIP025-07|Canada|Manitoba|650[0n]|BOLD:AAA1264
Hillia iris|[16112]|LCHIP115-07|Canada|Manitoba|650[0n]|BOLD:AAA1264
Hillia iris|[16113]|LCHIP119-07|Canada|Manitoba|650[0n]|BOLD:AAA1264
Hillia iris|[16114]|LCHIP103-07|Canada|Manitoba|650[0n]|BOLD:AAA1264
Hillia iris|[16115]|LCHIP109-07|Canada|Manitoba|650[0n]|BOLD:AAA1264
Hillia iris|[16116]|LCHIP053-07|Canada|Manitoba|650[0n]|BOLD:AAA1264
Hillia iris|[16117]|LCHIP054-07|Canada|Manitoba|650[0n]|BOLD:AAA1264
Hillia iris|[16118]|LCHIP279-07|Canada|Manitoba|650[0n]|BOLD:AAA1264
Hillia iris|[16119]|LCHIP281-07|Canada|Manitoba|650[0n]|BOLD:AAA1264
Hillia iris|[16120]|LCHIP268-07|Canada|Manitoba|650[0n]|BOLD:AAA1264
Hillia iris|[16121]|LCHIP269-07|Canada|Manitoba|650[0n]|BOLD:AAA1264
Hillia iris|[16122]|LCHIP043-07|Canada|Manitoba|650[0n]|BOLD:AAA1264
Hillia iris|[16123]|LCHIP044-07|Canada|Manitoba|650[0n]|BOLD:AAA1264
Hillia iris|[16124]|LCHIP015-07|Canada|Manitoba|650[0n]|BOLD:AAA1264
Hillia iris|[16125]|LCHIP017-07|Canada|Manitoba|650[0n]|BOLD:AAA1264
Hillia iris|[16126]|LCHIP220-07|Canada|Manitoba|650[0n]|BOLD:AAA1264
Hillia iris|[16127]|LCHIP222-07|Canada|Manitoba|650[0n]|BOLD:AAA1264
Hillia iris|[16128]|LCHQ408-08|Canada|Manitoba|652[0n]|BOLD:AAA1264
Hillia iris|[16129]|LCHIP014-07|Canada|Manitoba|650[0n]|BOLD:AAA1264
Hillia iris|[16130]|LCHIP011-07|Canada|Manitoba|650[0n]|BOLD:AAA1264
Hillia iris|[16131]|LCHIP010-07|Canada|Manitoba|650[0n]|BOLD:AAA1264
Hillia iris|[16132]|LCHIP009-07|Canada|Manitoba|650[0n]|BOLD:AAA1264
Hillia iris|[16133]|LCHIP008-07|Canada|Manitoba|650[0n]|BOLD:AAA1264
Hillia iris|[16134]|LCHIP007-07|Canada|Manitoba|650[0n]|BOLD:AAA1264
Hillia iris|[16135]|LCHIP006-07|Canada|Manitoba|650[0n]|BOLD:AAA1264
Hillia iris|[16136]|LCHIP004-07|Canada|Manitoba|650[0n]|BOLD:AAA1264
Hillia iris|[16137]|LCHIP001-07|Canada|Manitoba|650[0n]|BOLD:AAA1264
Hillia iris|[16138]|LCHIP259-07|Canada|Manitoba|650[1n]|BOLD:AAA1264
Hillia iris|[16139]|LCHIP278-07|Canada|Manitoba|650[0n]|BOLD:AAA1264
Hillia iris|[16140]|LCHIP227-07|Canada|Manitoba|650[1n]|BOLD:AAA1264
Hillia iris|[16141]|LCHQ293-08|Canada|Manitoba|646[0n]|BOLD:AAA1264
Hillia iris|[16142]|LCHQ793-08|Canada|Manitoba|646[0n]|BOLD:AAA1264
Hillia iris|[16143]|LCHQ399-08|Canada|Manitoba|637[0n]|BOLD:AAA1264
Hillia iris|[16144]|LCHIP005-07|Canada|Manitoba|650[0n]|BOLD:AAA1264
Hillia iris|[16145]|LCHQ449-08|Canada|Manitoba|634[0n]|BOLD:AAA1264
Hillia iris|[16146]|LCHIP032-07|Canada|Manitoba|621[0n]|BOLD:AAA1264
Hillia iris|[16147]|LCHIP041-07|Canada|Manitoba|619[0n]|BOLD:AAA1264
Hillia iris|[16148]|LCHIP013-07|Canada|Manitoba|618[0n]|BOLD:AAA1264
Hillia iris|[16149]|LCHIP065-07|Canada|Manitoba|650[0n]|BOLD:AAA1264
Hillia iris|[16150]|LCHIP073-07|Canada|Manitoba|650[0n]|BOLD:AAA1264
Hillia iris|[16151]|LCHIP100-07|Canada|Manitoba|650[0n]|BOLD:AAA1264
Hillia iris|[16152]|LCHIP208-07|Canada|Manitoba|650[0n]|BOLD:AAA1264
Hillia iris|[16153]|LCHIP209-07|Canada|Manitoba|650[0n]|BOLD:AAA1264
Hillia iris|[16154]|LCHIP251-07|Canada|Manitoba|650[1n]|BOLD:AAA1264
Hillia iris|[16155]|LCHQ733-08|Canada|Manitoba|632[0n]|BOLD:AAA1264
Hillia iris|[16156]|LCHQ746-08|Canada|Manitoba|632[0n]|BOLD:AAA1264
Hillia iris|[16157]|LCHQ749-08|Canada|Manitoba|632[0n]|BOLD:AAA1264
Hillia iris|[16158]|LCHQ818-08|Canada|Manitoba|631[0n]|BOLD:AAA1264
Hillia iris|[16159]|LCHQ851-08|Canada|Manitoba|632[0n]|BOLD:AAA1264
Hillia iris|[16160]|LCHQ843-08|Canada|Manitoba|658[0n]|BOLD:AAA1264
Hillia iris|[16161]|LCHQ845-08|Canada|Manitoba|658[0n]|BOLD:AAA1264
Hillia iris|[16162]|LCHQ848-08|Canada|Manitoba|658[0n]|BOLD:AAA1264
Hillia iris|[16163]|LCHQ850-08|Canada|Manitoba|658[0n]|BOLD:AAA1264
Hillia iris|[16164]|LCHQ276-08|Canada|Manitoba|658[0n]|BOLD:AAA1264
Hillia iris|[16165]|LCHQ278-08|Canada|Manitoba|658[0n]|BOLD:AAA1264
Hillia iris|[16166]|LCHQ299-08|Canada|Manitoba|658[0n]|BOLD:AAA1264
Hillia iris|[16167]|LCHQ300-08|Canada|Manitoba|658[0n]|BOLD:AAA1264
Hillia iris|[16168]|LCHQ302-08|Canada|Manitoba|657[0n]|BOLD:AAA1264
Hillia iris|[16169]|LCHQ305-08|Canada|Manitoba|658[0n]|BOLD:AAA1264
Hillia iris|[16170]|LCHQ314-08|Canada|Manitoba|658[0n]|BOLD:AAA1264
Hillia iris|[16171]|LCHQ356-08|Canada|Manitoba|658[0n]|BOLD:AAA1264
Hillia iris|[16172]|LCHQ358-08|Canada|Manitoba|657[0n]|BOLD:AAA1264
Hillia iris|[16173]|LCHQ359-08|Canada|Manitoba|658[0n]|BOLD:AAA1264
Hillia iris|[16174]|LCHQ361-08|Canada|Manitoba|658[0n]|BOLD:AAA1264
Hillia iris|[16175]|LCHQ363-08|Canada|Manitoba|658[0n]|BOLD:AAA1264
Hillia iris|[16176]|LCHQ364-08|Canada|Manitoba|658[0n]|BOLD:AAA1264
Hillia iris|[16177]|LCHQ365-08|Canada|Manitoba|658[0n]|BOLD:AAA1264
Hillia iris|[16178]|LCHQ366-08|Canada|Manitoba|658[0n]|BOLD:AAA1264
Hillia iris|[16179]|LCHQ367-08|Canada|Manitoba|658[0n]|BOLD:AAA1264
Hillia iris|[16180]|LCHQ368-08|Canada|Manitoba|658[0n]|BOLD:AAA1264
Hillia iris|[16181]|LCHQ369-08|Canada|Manitoba|658[0n]|BOLD:AAA1264
Hillia iris|[16182]|LCHQ370-08|Canada|Manitoba|658[0n]|BOLD:AAA1264
Hillia iris|[16183]|LCHQ371-08|Canada|Manitoba|658[0n]|BOLD:AAA1264
Hillia iris|[16184]|LCHQ372-08|Canada|Manitoba|658[0n]|BOLD:AAA1264
Hillia iris|[16185]|LCHQ375-08|Canada|Manitoba|657[0n]|BOLD:AAA1264
Hillia iris|[16186]|LCHQ376-08|Canada|Manitoba|658[0n]|BOLD:AAA1264
Hillia iris|[16187]|LCHQ377-08|Canada|Manitoba|658[0n]|BOLD:AAA1264
Hillia iris|[16188]|LCHQ397-08|Canada|Manitoba|658[0n]|BOLD:AAA1264
Hillia iris|[16189]|LCHQ401-08|Canada|Manitoba|658[0n]|BOLD:AAA1264
Hillia iris|[16190]|LCHQ435-08|Canada|Manitoba|658[0n]|BOLD:AAA1264
Hillia iris|[16191]|LCHQ436-08|Canada|Manitoba|657[0n]|BOLD:AAA1264

Hillia iris|[16189]|LCHQ401-08|Canada|Manitoba|658[0n]|BOLD:AAA1264
Hillia iris|[16190]|LCHQ435-08|Canada|Manitoba|658[0n]|BOLD:AAA1264
Hillia iris|[16191]|LCHQ436-08|Canada|Manitoba|657[0n]|BOLD:AAA1264
Hillia iris|[16192]|LCHQ230-08|Canada|Manitoba|658[0n]|BOLD:AAA1264
Hillia iris|[16193]|LCHQ233-08|Canada|Manitoba|658[0n]|BOLD:AAA1264
Hillia iris|[16194]|LCHQ378-08|Canada|Manitoba|658[0n]|BOLD:AAA1264
Hillia iris|[16195]|LCHQ396-08|Canada|Manitoba|658[0n]|BOLD:AAA1264
Hillia iris|[16196]|LCHQ523-08|Canada|Manitoba|658[0n]|BOLD:AAA1264
Hillia iris|[16197]|LCHQ591-08|Canada|Manitoba|658[0n]|BOLD:AAA1264
Hillia iris|[16198]|LCHQ745-08|Canada|Manitoba|658[0n]|BOLD:AAA1264
Hillia iris|[16199]|LCHQ747-08|Canada|Manitoba|657[0n]|BOLD:AAA1264
Hillia iris|[16200]|LCHQ796-08|Canada|Manitoba|658[0n]|BOLD:AAA1264
Hillia iris|[16201]|LCHQ797-08|Canada|Manitoba|658[0n]|BOLD:AAA1264
Hillia iris|[16202]|LCHQ738-08|Canada|Manitoba|658[0n]|BOLD:AAA1264
Hillia iris|[16203]|LCHQ742-08|Canada|Manitoba|658[0n]|BOLD:AAA1264
Hillia iris|[16204]|LCHQ781-08|Canada|Manitoba|658[0n]|BOLD:AAA1264
Hillia iris|[16205]|LCHQ782-08|Canada|Manitoba|657[0n]|BOLD:AAA1264
Hillia iris|[16206]|LCHQ785-08|Canada|Manitoba|658[0n]|BOLD:AAA1264
Hillia iris|[16207]|LCHQ786-08|Canada|Manitoba|658[0n]|BOLD:AAA1264
Hillia iris|[16208]|LCHQ734-08|Canada|Manitoba|658[0n]|BOLD:AAA1264
Hillia iris|[16209]|LCHQ737-08|Canada|Manitoba|658[0n]|BOLD:AAA1264
Hillia iris|[16210]|LCHQ759-08|Canada|Manitoba|658[0n]|BOLD:AAA1264
Hillia iris|[16211]|LCHQ771-08|Canada|Manitoba|658[0n]|BOLD:AAA1264
Hillia iris|[16212]|LCHQ484-08|Canada|Manitoba|657[0n]|BOLD:AAA1264
Hillia iris|[16213]|LCHQ505-08|Canada|Manitoba|658[0n]|BOLD:AAA1264
Hillia iris|[16214]|LCHQ506-08|Canada|Manitoba|658[0n]|BOLD:AAA1264
Hillia iris|[16215]|LCHQ508-08|Canada|Manitoba|658[0n]|BOLD:AAA1264
Hillia iris|[16216]|LCHQ403-08|Canada|Manitoba|658[0n]|BOLD:AAA1264
Hillia iris|[16217]|LCHQ409-08|Canada|Manitoba|658[0n]|BOLD:AAA1264
Hillia iris|[16218]|LCHQ411-08|Canada|Manitoba|658[0n]|BOLD:AAA1264
Hillia iris|[16219]|LCHQ433-08|Canada|Manitoba|658[0n]|BOLD:AAA1264
Hillia iris|[16220]|LCHQ447-08|Canada|Manitoba|657[0n]|BOLD:AAA1264
Hillia iris|[16221]|LCHQ448-08|Canada|Manitoba|658[0n]|BOLD:AAA1264
Hillia iris|[16222]|LCHQ442-08|Canada|Manitoba|657[0n]|BOLD:AAA1264
Hillia iris|[16223]|LCHQ444-08|Canada|Manitoba|657[0n]|BOLD:AAA1264
Hillia iris|[16224]|LCHQ445-08|Canada|Manitoba|657[0n]|BOLD:AAA1264
Hillia iris|[16225]|LCHQ446-08|Canada|Manitoba|658[0n]|BOLD:AAA1264
Hillia iris|[16226]|LCHQ437-08|Canada|Manitoba|658[0n]|BOLD:AAA1264
Hillia iris|[16227]|LCHQ441-08|Canada|Manitoba|658[0n]|BOLD:AAA1264
Hillia iris|[16228]|LCHQ610-08|Canada|Manitoba|658[0n]|BOLD:AAA1264
Hillia iris|[16229]|LCHQ611-08|Canada|Manitoba|658[0n]|BOLD:AAA1264
Hillia iris|[16230]|LCHQ707-08|Canada|Manitoba|658[0n]|BOLD:AAA1264
Hillia iris|[16231]|LCHQ730-08|Canada|Manitoba|658[0n]|BOLD:AAA1264
Hillia iris|[16232]|LCHQ756-08|Canada|Manitoba|658[0n]|BOLD:AAA1264
Hillia iris|[16233]|LCHQ758-08|Canada|Manitoba|658[0n]|BOLD:AAA1264
Hillia iris|[16234]|LCHQ603-08|Canada|Manitoba|658[0n]|BOLD:AAA1264
Hillia iris|[16235]|LCHQ605-08|Canada|Manitoba|658[0n]|BOLD:AAA1264
Hillia iris|[16236]|LCHQ607-08|Canada|Manitoba|658[0n]|BOLD:AAA1264
Hillia iris|[16237]|LCHQ608-08|Canada|Manitoba|658[0n]|BOLD:AAA1264
Hillia iris|[16238]|LCHQ753-08|Canada|Manitoba|658[0n]|BOLD:AAA1264
Hillia iris|[16239]|LCHQ755-08|Canada|Manitoba|658[0n]|BOLD:AAA1264
Hillia iris|[16240]|LCHQ825-08|Canada|Manitoba|658[0n]|BOLD:AAA1264
Hillia iris|[16241]|LCHQ826-08|Canada|Manitoba|658[0n]|BOLD:AAA1264
Hillia iris|[16242]|LCHQ829-08|Canada|Manitoba|658[0n]|BOLD:AAA1264
Hillia iris|[16243]|LCHQ831-08|Canada|Manitoba|658[0n]|BOLD:AAA1264
Hillia iris|[16244]|LCHQ834-08|Canada|Manitoba|657[0n]|BOLD:AAA1264
Hillia iris|[16245]|LCHQ837-08|Canada|Manitoba|658[0n]|BOLD:AAA1264
Hillia iris|[16246]|LCHQ840-08|Canada|Manitoba|658[0n]|BOLD:AAA1264
Hillia iris|[16247]|LCHQ841-08|Canada|Manitoba|658[0n]|BOLD:AAA1264
Hillia iris|[16248]|LCHQ852-08|Canada|Manitoba|658[0n]|BOLD:AAA1264
Hillia iris|[16249]|LCHQ853-08|Canada|Manitoba|658[0n]|BOLD:AAA1264
Hillia iris|[16250]|LCHQ854-08|Canada|Manitoba|658[0n]|BOLD:AAA1264
Hillia iris|[16251]|LCHQ855-08|Canada|Manitoba|658[0n]|BOLD:AAA1264
Hillia iris|[16252]|LCHQ788-08|Canada|Manitoba|658[0n]|BOLD:AAA1264
Hillia iris|[16253]|LCHQ790-08|Canada|Manitoba|658[0n]|BOLD:AAA1264
Hillia iris|[16254]|LCHQ857-08|Canada|Manitoba|658[0n]|BOLD:AAA1264
Hillia iris|[16255]|ENLCW002-09|Canada|Manitoba|658[0n]|BOLD:AAA1264
Hillia iris|[16256]|ENLCW008-09|Canada|Manitoba|658[0n]|BOLD:AAA1264
Hillia iris|[16257]|ENLCW083-09|Canada|Manitoba|658[0n]|BOLD:AAA1264
Hillia iris|[16258]|ENLCW091-09|Canada|Manitoba|658[0n]|BOLD:AAA1264
Hillia iris|[16259]|ENLCW093-09|Canada|Manitoba|658[0n]|BOLD:AAA1264
Epiglaea apiata|[16260]|TMNBB256-06|Canada|New Brunswick|658[0n]|BOLD:AAC2729
Epiglaea apiata|[16261]|TMNBB260-06|Canada|New Brunswick|658[0n]|BOLD:AAC2729
Epiglaea apiata|[16262]|RDLQF236-06|Canada|Quebec|658[0n]|BOLD:AAC2729
Epiglaea apiata|[16263]|RDLQF234-06|Canada|Quebec|658[0n]|BOLD:AAC2729
Epiglaea apiata|[16264]|RDLQF237-06|Canada|Quebec|658[0n]|BOLD:AAC2729
Epiglaea apiata|[16265]|PSAT165-10|United States|Florida|618[0n]|BOLD:AAC2729
Epiglaea apiata|[16266]|RDNMH133-09|United States|Florida|658[0n]|BOLD:AAC2729
Epiglaea apiata|[16267]|LNC492-06|United States|North Carolina|658[0n]|BOLD:AAC2729
Epiglaea apiata|[16268]|TMNBB257-06|Canada|New Brunswick|658[0n]|BOLD:AAC2729
Epiglaea apiata|[16269]|TMNBB259-06|Canada|New Brunswick|658[0n]|BOLD:AAC2729
Epiglaea apiata|[16270]|LNC493-06|United States|North Carolina|658[0n]|BOLD:AAC2729
Epiglaea apiata|[16271]|RDLQF272-06|Canada|Quebec|658[0n]|BOLD:AAC2729
Epiglaea apiata|[16272]|TMNBB255-06|Canada|New Brunswick|658[0n]|BOLD:AAC2729
Epiglaea apiata|[16273]|TMNBB258-06|Canada|New Brunswick|658[0n]|BOLD:AAC2729
Epiglaea apiata|[16274]|PSAT166-10|United States|Florida|617[0n]|BOLD:AAC2729
Parastichtis suspecta|[16275]|LCHIP257-07|Canada|Manitoba|650[0n]|BOLD:ABY7871
Parastichtis suspecta|[16276]|BBLPB459-10|Canada|Alberta|658[0n]|BOLD:ABY7871
Parastichtis suspecta|[16277]|BBLPB350-10|Canada|Alberta|658[0n]|BOLD:ABY7871
Parastichtis suspecta|[16278]|MHLEP132-07|Canada|Manitoba|658[0n]|BOLD:ABY7871
Parastichtis suspecta|[16279]|LOWCD534-06|Canada|British Columbia|658[0n]|BOLD:ABY7871
Parastichtis suspecta|[16280]|LOWCD299-06|Canada|British Columbia|658[0n]|BOLD:ABY7871
Parastichtis suspecta|[16281]|LOWCB129-05|Canada|British Columbia|658[0n]|BOLD:ABY7871
Parastichtis suspecta|[16282]|CNPAF975-13|Canada|Saskatchewan|638[0n]|BOLD:ABY7871
Parastichtis suspecta|[16283]|CNWBG3123-13|Canada|Alberta|608[0n]|BOLD:ABY7871
Parastichtis suspecta|[16284]|CNWBG3097-13|Canada|Alberta|607[0n]|BOLD:ABY7871
Parastichtis suspecta|[16285]|CNWBG3085-13|Canada|Alberta|607[0n]|BOLD:ABY7871
Parastichtis suspecta|[16286]|CNWBG3054-13|Canada|Alberta|587[0n]|BOLD:ABY7871
Parastichtis suspecta|[16287]|CNWBC131-13|Canada|Alberta|577[0n]|BOLD:ABY7871
Parastichtis suspecta|[16288]|CNWBG3073-13|Canada|Alberta|592[0n]|BOLD:ABY7871
Parastichtis suspecta|[16289]|CNWBG3129-13|Canada|Alberta|588[0n]|BOLD:ABY7871
Parastichtis suspecta|[16290]|CNWBG3131-13|Canada|Alberta|583[2n]|BOLD:ABY7871
Parastichtis suspecta|[16291]|RDNMC278-05|Canada|British Columbia|579[0n]|BOLD:ABY7871

Parastichtis suspecta|[16289]|CNWBG3129-13|Canada|Alberta|588[0n]|BOLD:ABY7871
Parastichtis suspecta|[16290]|CNWBG3131-13|Canada|Alberta|583[2n]|BOLD:ABY7871
Parastichtis suspecta|[16291]|RDNMC278-05|Canada|British Columbia|579[0n]|BOLD:ABY7871
Parastichtis suspecta|[16292]|LOWCB130-05|Canada|British Columbia|577[0n]|BOLD:ABY7871
Parastichtis suspecta|[16293]|BBLPB743-10|Canada|Alberta|658[0n]|BOLD:ABY7871
Parastichtis suspecta|[16294]|BBLPB570-10|Canada|British Columbia|658[0n]|BOLD:ABY7871
Parastichtis suspecta|[16295]|BBLPB422-10|Canada|Alberta|658[0n]|BOLD:ABY7871
Parastichtis suspecta|[16296]|BBLPB352-10|Canada|Alberta|658[0n]|BOLD:ABY7871
Parastichtis suspecta|[16297]|BBLPB351-10|Canada|Alberta|658[0n]|BOLD:ABY7871
Parastichtis suspecta|[16298]|RDNMB935-05|Canada|British Columbia|658[0n]|BOLD:ABY7871
Parastichtis suspecta|[16299]|CNWBG3142-13|Canada|Alberta|605[0n]|BOLD:ABY7871
Parastichtis suspecta|[16300]|CNWBG3050-13|Canada|Alberta|606[1n]|BOLD:ABY7871
Parastichtis suspecta|[16301]|CNWBG3098-13|Canada|Alberta|608[0n]|BOLD:ABY7871
Parastichtis suspecta|[16302]|LOWCB131-05|Canada|British Columbia|582[1n]|BOLD:ABY7871
Parastichtis suspecta|[16303]|CNWBG3080-13|Canada|Alberta|587[0n]|BOLD:ABY7871
Parastichtis suspecta|[16304]|CNWBG3083-13|Canada|Alberta|582[1n]|BOLD:ABY7871
Parastichtis suspecta|[16305]|CNWBE861-13|Canada|Alberta|575[0n]|BOLD:ABY7871
Parastichtis suspecta|[16306]|CNWBG3087-13|Canada|Alberta|575[1n]|BOLD:ABY7871
Parastichtis suspecta|[16307]|CNWBG3144-13|Canada|Alberta|589[0n]|BOLD:ABY7871
Parastichtis suspecta|[16308]|CNWBG3166-13|Canada|Alberta|588[0n]|BOLD:ABY7871
Parastichtis suspecta|[16309]|CNWBG3168-13|Canada|Alberta|588[0n]|BOLD:ABY7871
Parastichtis suspecta|[16310]|LASTS002-14|Italy|South Tyrol|610[0n]|BOLD:AAB4551
Parastichtis suspecta|[16311]|LBCD416-05|Canada|British Columbia|658[0n]|BOLD:AAB4551
Parastichtis suspecta|[16312]|LBCH3334-10|Canada|British Columbia|658[0n]|BOLD:AAB4551
Parastichtis suspecta|[16313]|RDNMB052-05|Denmark||550[0n]|BOLD:AAB4551
Parastichtis suspecta|[16314]|LEFIA604-10|Finland|South Karelia|632[2n]|BOLD:AAB4551
Parastichtis suspecta|[16315]|RDNMC277-05|Canada|Alberta|616[0n]|BOLD:AAB4551
Parastichtis suspecta|[16316]|CNWBG3163-13|Canada|Alberta|589[0n]|BOLD:AAB4551
Parastichtis suspecta|[16317]|CNWBG3159-13|Canada|Alberta|588[0n]|BOLD:AAB4551
Parastichtis suspecta|[16318]|CNEIF2112-12|Canada|Alberta|603[0n]|BOLD:AAB4551
Parastichtis suspecta|[16319]|CNWBG3158-13|Canada|Alberta|576[0n]|BOLD:AAB4551
Parastichtis suspecta|[16320]|CNWBG3048-13|Canada|Alberta|602[0n]|BOLD:AAB4551
Parastichtis suspecta|[16321]|LEFIA605-10|Finland|South Karelia|621[1n]|BOLD:AAB4551
Parastichtis suspecta|[16322]|CNWLF2037-12|Canada|Alberta|638[0n]|BOLD:AAB4551
Parastichtis suspecta|[16323]|CNEID3322-12|Canada|Alberta|637[0n]|BOLD:AAB4551
Parastichtis suspecta|[16324]|SSWLA1825-13|Canada|Alberta|632[0n]|BOLD:AAB4551
Parastichtis suspecta|[16325]|CNWLG955-12|Canada|Alberta|632[0n]|BOLD:AAB4551
Parastichtis suspecta|[16326]|CNWLF2023-12|Canada|Alberta|631[0n]|BOLD:AAB4551
Parastichtis suspecta|[16327]|CNWBC135-13|Canada|Alberta|564[0n]|BOLD:AAB4551
Parastichtis suspecta|[16328]|CNWBG3095-13|Canada|Alberta|595[0n]|BOLD:AAB4551
Parastichtis suspecta|[16329]|CNWBG3059-13|Canada|Alberta|595[0n]|BOLD:AAB4551
Parastichtis suspecta|[16330]|CNWBG3102-13|Canada|Alberta|559[0n]|BOLD:AAB4551
Parastichtis suspecta|[16331]|CNWBG3111-13|Canada|Alberta|596[3n]|BOLD:AAB4551
Parastichtis suspecta|[16332]|CNWBG3065-13|Canada|Alberta|590[0n]|BOLD:AAB4551
Parastichtis suspecta|[16333]|SSWLD7954-13|Canada|Alberta|576[0n]|BOLD:AAB4551
Parastichtis suspecta|[16334]|CNWLN1080-13|Canada|Alberta|612[0n]|BOLD:AAB4551
Parastichtis suspecta|[16335]|GBLAA231-14|Germany|Schleswig-Holstein|658[0n]|BOLD:AAB4551
Parastichtis suspecta|[16336]|LASTS001-14|Austria|Tirol|658[0n]|BOLD:AAB4551
Parastichtis suspecta|[16337]|NLLEA1273-14|Netherlands|South Holland|658[0n]|BOLD:AAB4551
Parastichtis suspecta|[16338]|GBLAD153-14|Germany|Saxony|658[0n]|BOLD:AAB4551
Parastichtis suspecta|[16339]|LEATD125-13|Austria|Tirol|658[0n]|BOLD:AAB4551
Parastichtis suspecta|[16340]|GBLAC799-13|Germany|Bavaria|658[0n]|BOLD:AAB4551
Parastichtis suspecta|[16341]|ODOPE266-11|Germany|Bavaria|658[0n]|BOLD:AAB4551
Parastichtis suspecta|[16342]|LEATA419-13|Austria|Vorarlberg|658[0n]|BOLD:AAB4551
Parastichtis suspecta|[16343]|BBLPB460-10|Canada|Alberta|658[0n]|BOLD:AAB4551
Parastichtis suspecta|[16344]|LENOA374-11|France|Haute Normandie|658[0n]|BOLD:AAB4551
Parastichtis suspecta|[16345]|BBLPB419-10|Canada|Alberta|658[0n]|BOLD:AAB4551
Parastichtis suspecta|[16346]|LEFIB824-10|Finland|Northern Ostrobothnia|658[0n]|BOLD:AAB4551
Parastichtis suspecta|[16347]|FBLMV167-09|Germany|Bavaria|658[0n]|BOLD:AAB4551
Parastichtis suspecta|[16348]|CGUKD414-09|United Kingdom|England|658[0n]|BOLD:AAB4551
Parastichtis suspecta|[16349]|CGUKB334-09|United Kingdom|England|658[0n]|BOLD:AAB4551
Parastichtis suspecta|[16350]|RDNMC279-05|Canada|British Columbia|658[0n]|BOLD:AAB4551
Parastichtis suspecta|[16351]|RDNMB936-05|Canada|Alberta|658[0n]|BOLD:AAB4551
Parastichtis suspecta|[16352]|RDNMB053-05|Denmark||658[0n]|BOLD:AAB4551
Parastichtis suspecta|[16353]|LEATC158-13|Austria|Tirol|658[1n]|BOLD:AAB4551
Parastichtis suspecta|[16354]|LASTS028-14|Austria|Tirol|658[0n]|BOLD:AAB4551
Chaetaglaea fergusoni|[16355]|RDNMH134-09|United States|Florida|658[0n]|BOLD:AAI7071
Chaetaglaea fergusoni|[16356]|RDNMH135-09|United States|Florida|658[0n]|BOLD:AAI7071
Chaetaglaea cerata|[16357]|RDNMF718-08|Canada|Ontario|658[0n]|BOLD:AAF4072
Chaetaglaea cerata|[16358]|RDNMG521-08|Canada|Ontario|658[0n]|BOLD:AAF4072
Chaetaglaea cerata|[16359]|RDNMG522-08|United States|Wyoming|658[0n]|BOLD:AAF4072
Chaetaglaea sericea|[16360]|LNC452-05|United States|North Carolina|658[0n]|BOLD:AAE1199
Chaetaglaea sericea|[16361]|LNC453-05|United States|North Carolina|658[0n]|BOLD:AAE1199
Chaetaglaea sericea|[16362]|RDNMG544-08|Canada|Ontario|658[0n]|BOLD:AAE1199
Chaetaglaea sericea|[16363]|RDNMG543-08|Canada|Ontario|658[0n]|BOLD:AAE1199
Chaetaglaea sericea|[16364]|RDNMG542-08|Canada|Ontario|658[0n]|BOLD:AAE1199
Chaetaglaea sericea|[16365]|RDNMI025-10|United States|Florida|658[0n]|BOLD:AAE1199
Chaetaglaea sericea|[16366]|RDNMI027-10|United States|Florida|658[0n]|BOLD:AAE1199
Chaetaglaea sericea|[16367]|RDNMK031-11|United States|Florida|658[0n]|BOLD:AAE1199
Chaetaglaea sericea|[16368]|RDNMK136-11|United States|Florida|658[0n]|BOLD:AAE1199
Chaetaglaea rhonda|[16369]|LNC513-06|United States|North Carolina|618[0n]|BOLD:AAE1201
Chaetaglaea rhonda|[16370]|LNC514-06|United States|North Carolina|638[0n]|BOLD:AAE1201
Chaetaglaea rhonda|[16371]|RDNMG1029-08|Canada|Ontario|658[0n]|BOLD:AAE1201
Chaetaglaea rhonda|[16372]|RDNMG1030-08|Canada|Ontario|658[0n]|BOLD:AAE1201
Chaetaglaea rhonda|[16373]|RDNMG1031-08|Canada|Ontario|658[0n]|BOLD:AAE1201
Chaetaglaea rhonda|[16374]|MJMSL012-10|United States|Massachusetts|658[0n]|BOLD:AAE1201
Chaetaglaea rhonda|[16375]|MJMSL010-10|United States|Massachusetts|658[0n]|BOLD:AAE1201
Chaetaglaea rhonda|[16376]|MJMSL011-10|United States|Massachusetts|658[0n]|BOLD:AAE1201
Chaetaglaea rhonda|[16377]|MJMSL013-10|United States|Massachusetts|658[0n]|BOLD:AAE1201
Chaetaglaea rhonda|[16378]|MJMSL014-10|United States|Massachusetts|658[0n]|BOLD:AAE1201
Chaetaglaea rhonda|[16379]|MJMSL015-10|United States|Massachusetts|658[0n]|BOLD:AAE1201
Chaetaglaea rhonda|[16380]|GWOTA067-12|United States|Massachusetts|658[0n]|BOLD:AAE1201
Chaetaglaea rhonda|[16381]|GWOTA068-12|United States|Massachusetts|658[0n]|BOLD:AAE1201
Chaetaglaea tremula|[16382]|CNCLA5256-13|United States|Mississippi|658[0n]|BOLD:ABZ6246
Chaetaglaea tremula|[16383]|CNCLA5254-13|United States|Mississippi|658[1n]|BOLD:ABZ6246
Chaetaglaea tremula|[16384]|CNCLA5255-13|United States|Mississippi|658[0n]|BOLD:ABZ6246
Chaetaglaea tremula|[16385]|RDNMI024-10|United States|Florida|658[0n]|BOLD:ABZ6246
Chaetaglaea tremula|[16386]|RDNMK137-11|United States|Florida|658[0n]|BOLD:ABZ6246
Chaetaglaea tremula|[16387]|RDNMH139-09|United States|Florida|658[0n]|BOLD:ABZ6246
Chaetaglaea tremula|[16388]|RDNMK141-11|United States|Florida|658[0n]|BOLD:ABZ6246
Chaetaglaea tremula|[16389]|RDNMK142-11|United States|Florida|658[0n]|BOLD:ABZ6246
Chaetaglaea tremula|[16390]|RDNMI028-10|United States|Florida|658[0n]|BOLD:ABZ6246
Chaetaglaea tremula|[16391]|RDNMK140-11|United States|Florida|658[0n]|BOLD:ABZ6246

[illegible]
Chaetaglaea tremula|[16389]|RDNMK142-11|United States|Florida|658[0n]|BOLD:ABZ6246
Chaetaglaea tremula|[16390]|RDNMI028-10|United States|Florida|658[0n]|BOLD:ABZ6246
Chaetaglaea tremula|[16391]|RDNMK140-11|United States|Florida|658[0n]|BOLD:ABZ6246
Chaetaglaea tremula|[16392]|RDNMK143-11|United States|Florida|658[0n]|BOLD:ABZ6246
Chaetaglaea tremula|[16393]|CNCLB1337-14|United States|Louisiana|658[0n]|BOLD:ABZ6246
Chaetaglaea tremula|[16394]|CNCLB1338-14|United States|Louisiana|658[0n]|BOLD:ABZ6246
Orthomoia bloomfieldi|[16395]|LOCBC013-06|United States|California|656[0n]|BOLD:AAG5839
Orthomoia bloomfieldi|[16396]|LNAUS4460-13|United States|California|658[0n]|BOLD:AAG5839
Eucirroedia pampina|[16397]|JRLAA016-09|United States|Georgia|637[0n]|BOLD:AAB8057
Eucirroedia pampina|[16398]|LOWCD115-06|Canada|British Columbia|614[0n]|BOLD:AAB8057
Eucirroedia pampina|[16399]|TMNBB269-06|Canada|New Brunswick|658[0n]|BOLD:AAB8057
Eucirroedia pampina|[16400]|TMNBB268-06|Canada|New Brunswick|658[0n]|BOLD:AAB8057
Eucirroedia pampina|[16401]|TMNBB267-06|Canada|New Brunswick|658[0n]|BOLD:AAB8057
Eucirroedia pampina|[16402]|TMNBB266-06|Canada|New Brunswick|658[0n]|BOLD:AAB8057
Eucirroedia pampina|[16403]|TMNBB265-06|Canada|New Brunswick|658[0n]|BOLD:AAB8057
Eucirroedia pampina|[16404]|TMNBB264-06|Canada|New Brunswick|658[0n]|BOLD:AAB8057
Eucirroedia pampina|[16405]|TMNBB263-06|Canada|New Brunswick|658[0n]|BOLD:AAB8057
Eucirroedia pampina|[16406]|TMNBB262-06|Canada|New Brunswick|658[0n]|BOLD:AAB8057
Eucirroedia pampina|[16407]|TTMNB374-06|Canada|New Brunswick|658[0n]|BOLD:AAB8057
Eucirroedia pampina|[16408]|TMNBB261-06|Canada|New Brunswick|656[0n]|BOLD:AAB8057
Eucirroedia pampina|[16409]|TMNBB270-06|Canada|New Brunswick|658[0n]|BOLD:AAB8057
Eucirroedia pampina|[16410]|TMNBB271-06|Canada|New Brunswick|658[0n]|BOLD:AAB8057
Eucirroedia pampina|[16411]|RDLQF262-06|Canada|Quebec|658[0n]|BOLD:AAB8057
Eucirroedia pampina|[16412]|MJMSL009-10|United States|Massachusetts|658[0n]|BOLD:AAB8057
Eucirroedia pampina|[16413]|LMDH007-11|United States|Minnesota|658[0n]|BOLD:AAB8057
Eucirroedia pampina|[16414]|LNAUS5002-13|United States|Virginia|658[0n]|BOLD:AAB8057
Orthodes obscura|[16415]|RDLQ618-07|Canada|Quebec|636[0n]|BOLD:AAD1496
Orthodes obscura|[16416]|RDNMB255-05|Canada|Ontario|658[0n]|BOLD:AAD1496
Orthodes obscura|[16417]|KPOEC044-08|Canada|Ontario|658[0n]|BOLD:AAD1496
Orthodes obscura|[16418]|LOWCD734-06|Canada|British Columbia|658[0n]|BOLD:AAD1496
Orthodes obscura|[16419]|LBCG355-08|Canada|British Columbia|658[0n]|BOLD:AAD1496
Orthodes obscura|[16420]|LBCG381-08|Canada|British Columbia|658[0n]|BOLD:AAD1496
Orthodes obscura|[16421]|LPABB419-08|Canada|Alberta|658[0n]|BOLD:AAD1496
Orthodes obscura|[16422]|LPABB425-08|Canada|Alberta|658[0n]|BOLD:AAD1496
Orthodes obscura|[16423]|BBLPB345-10|Canada|British Columbia|658[0n]|BOLD:AAD1496
Orthodes noverca|[16424]|RDNMG831-08|United States|Washington|658[0n]|BOLD:AAE1343
Orthodes noverca|[16425]|RDNMJ834-11|United States|Utah|658[0n]|BOLD:AAE1343
Orthodes noverca|[16426]|RDNMJ835-11|United States|Utah|658[0n]|BOLD:AAE1343
Orthodes noverca|[16427]|LOWCD625-06|Canada|British Columbia|658[0n]|BOLD:AAE1343
Orthodes noverca|[16428]|RDNMB250-05|Canada|British Columbia|565[0n]|BOLD:AAE1343
Orthodes noverca|[16429]|RDMAB949-09|United States|Oregon|578[0n]|BOLD:AAE1343
Orthodes noverca|[16430]|RDNMG634-08|United States|New Mexico|658[0n]|BOLD:AAE1343
Orthodes noverca|[16431]|RDNMG832-08|United States|New Mexico|658[0n]|BOLD:AAE1343
Orthodes noverca|[16432]|RDMAB951-09|Canada|British Columbia|568[1n]|BOLD:AAE1343
Orthodes noverca|[16433]|RDMAB950-09|Canada|British Columbia|621[0n]|BOLD:AAE1343
Orthodes noverca|[16434]|LBCH5108-10|Canada|British Columbia|658[0n]|BOLD:AAE1343
Orthodes noverca|[16435]|LBCH5422-10|Canada|British Columbia|658[0n]|BOLD:AAE1343
Orthodes noverca|[16436]|LBCH5450-10|Canada|British Columbia|658[0n]|BOLD:AAE1343
Orthodes noverca|[16437]|LBCH5451-10|Canada|British Columbia|658[0n]|BOLD:AAE1343
Orthodes noverca|[16438]|LBCH5452-10|Canada|British Columbia|658[0n]|BOLD:AAE1343
Orthodes noverca|[16439]|LBCH5594-10|Canada|British Columbia|658[0n]|BOLD:AAE1343
Orthodes noverca|[16440]|LBCH5596-10|Canada|British Columbia|658[0n]|BOLD:AAE1343
Orthodes noverca|[16441]|LBCH5823-10|Canada|British Columbia|658[0n]|BOLD:AAE1343
Orthodes noverca|[16442]|LBCH5828-10|Canada|British Columbia|658[0n]|BOLD:AAE1343
Orthodes noverca|[16443]|LBCH5829-10|Canada|British Columbia|658[0n]|BOLD:AAE1343
Orthodes noverca|[16444]|LBCH5939-10|Canada|British Columbia|658[0n]|BOLD:AAE1343
Orthodes noverca|[16445]|LBCH6097-10|Canada|British Columbia|658[0n]|BOLD:AAE1343
Orthodes noverca|[16446]|LBCH7370-10|Canada|British Columbia|658[0n]|BOLD:AAE1343
Orthodes noverca|[16447]|CGLCA152-10|United States|California|658[0n]|BOLD:AAE1343
Orthodes noverca|[16448]|RDNMK271-11|United States|California|658[0n]|BOLD:AAE1343
Orthodes noverca|[16449]|RDNMK301-11|United States|California|658[0n]|BOLD:AAE1343
Orthodes noverca|[16450]|BBLOE1237-12|United States|California|658[0n]|BOLD:AAE1343
Orthodes noverca|[16451]|BBLOE1406-12|United States|California|658[0n]|BOLD:AAE1343
Niphonyx segregata|[16452]|RDNME268-07|United States|Connecticut|609[0n]|BOLD:AAG5871
Niphonyx segregata|[16453]|GBGL15788-14|||625[0n]|BOLD:AAG5871
Niphonyx segregata|[16454]|LNCC2033-14|United States|North Carolina|658[0n]|BOLD:AAG5871
Niphonyx segregata|[16455]|LNCC2032-14|United States|North Carolina|658[0n]|BOLD:AAG5871
Niphonyx segregata|[16456]|GBMIN18112-13|South Korea|658[0n]|BOLD:AAG5871
Niphonyx segregata|[16457]|GBMIN17988-13|South Korea|658[0n]|BOLD:AAG5871
Niphonyx segregata|[16458]|LTOLB1038-11|Japan|Chubu|658[0n]|BOLD:AAG5871
Niphonyx segregata|[16459]|GBGL12725-13|||603[0n]|BOLD:AAG5871
Niphonyx segregata|[16460]|GBGL15789-14|||625[0n]|BOLD:AAG5871
Niphonyx segregata|[16461]|GBGL15787-14|||625[0n]|BOLD:AAG5871
Niphonyx segregata|[16462]|GBGL15786-14|||625[0n]|BOLD:AAG5871
Niphonyx segregata|[16463]|GBGL15785-14|||625[0n]|BOLD:AAG5871
Niphonyx segregata|[16464]|GBGL15784-14|||625[0n]|BOLD:AAG5871
Niphonyx segregata|[16465]|GBGL12724-13|||603[0n]|BOLD:AAG5871
Niphonyx segregata|[16466]|GBGL12723-13|||603[0n]|BOLD:AAG5871
Niphonyx segregata|[16467]|GBGL12722-13|||603[0n]|BOLD:AAG5871
Niphonyx segregata|[16468]|GBGL15783-14|||612[0n]|BOLD:AAG5871
Niphonyx segregata|[16469]|GBGL15782-14|||612[0n]|BOLD:AAG5871
Niphonyx segregata|[16470]|GBGL15781-14|||612[0n]|BOLD:AAG5871
Niphonyx segregata|[16471]|GBGL15790-14|||625[0n]|BOLD:AAG5871
Niphonyx segregata|[16472]|GBGL15791-14|||625[0n]|BOLD:AAG5871
Niphonyx segregata|[16473]|GBGL15792-14|||625[0n]|BOLD:AAG5871
Niphonyx segregata|[16474]|GBGL15795-14|||588[0n]|BOLD:AAG5871
Niphonyx segregata|[16475]|GBGL15796-14|||588[0n]|BOLD:AAG5871
Niphonyx segregata|[16476]|GBGL15797-14|||588[0n]|BOLD:AAG5871
Niphonyx segregata|[16477]|GBGL15798-14|||588[0n]|BOLD:AAG5871
Niphonyx segregata|[16478]|GBGL15799-14|||588[0n]|BOLD:AAG5871
Niphonyx segregata|[16479]|GBGL15800-14|||588[0n]|BOLD:AAG5871
Fergusonix januaris|[16480]|LOCB839-06|United States|California|658[0n]|BOLD:AAC0348
Fergusonix januaris|[16481]|LOCB771-06|United States|California|658[0n]|BOLD:AAC0348
Fergusonix januaris|[16482]|LOCB770-06|United States|California|658[0n]|BOLD:AAC0348
Fergusonix januaris|[16483]|LOCB769-06|United States|California|658[0n]|BOLD:AAC0348
Fergusonix januaris|[16484]|LOCB768-06|United States|California|658[0n]|BOLD:AAC0348
Fergusonix januaris|[16485]|LOCB718-06|United States|California|658[0n]|BOLD:AAC0348
Fergusonix januaris|[16486]|LOCB485-06|United States|California|658[0n]|BOLD:AAC0348
Fergusonix januaris|[16487]|LOCB482-06|United States|California|658[0n]|BOLD:AAC0348
Fergusonix januaris|[16488]|LOCB442-06|United States|California|658[0n]|BOLD:AAC0348
Fergusonix januaris|[16489]|LOCB479-06|United States|California|658[0n]|BOLD:AAC0348
Fergusonix januaris|[16490]|LOCB899-06|United States|California|658[0n]|BOLD:AAC0348
Fergusonix januaris|[16491]|RDNMD413-06|United States|California|658[0n]|BOLD:AAC0348

[illegible]
Fergusonix januaris|[16489]|LOCB479-06|United States|California|658[0n]|BOLD:AAC0348
Fergusonix januaris|[16490]|LOCB899-06|United States|California|658[0n]|BOLD:AAC0348
Fergusonix januaris|[16491]|RDNMD413-06|United States|California|658[0n]|BOLD:AAC0348
Fergusonix januaris|[16492]|LOCB832-06|United States|California|658[0n]|BOLD:AAC0348
Fergusonix januaris|[16493]|RDNMD412-06|United States|California|658[0n]|BOLD:AAC0348
Fergusonix januaris|[16494]|RDNMD414-06|United States|California|658[0n]|BOLD:AAC0348
Fergusonix januaris|[16495]|LOCBF085-13|United States|California|619[0n]|BOLD:ACN3261
Fergusonix januaris|[16496]|LOCBF086-13|United States|California|586[0n]|BOLD:ACN3261
Orthodes goodelli|[16497]|RDNMC291-05|United States|Florida|568[0n]|BOLD:AAB4023
Orthodes goodelli|[16498]|RDNME274-07|United States|Florida|658[0n]|BOLD:AAB4023
Orthodes goodelli|[16499]|RDLQB308-05|Canada|Quebec|559[0n]|BOLD:AAB4023
Orthodes goodelli|[16500]|LGSMG846-10|United States|North Carolina|658[0n]|BOLD:AAB4023
Orthodes goodelli|[16501]|LSEU764-06|United States|Georgia|658[0n]|BOLD:AAB4023
Orthodes goodelli|[16502]|LSEU638-06|United States|Georgia|658[0n]|BOLD:AAB4023
Orthodes goodelli|[16503]|LSEU637-06|United States|Georgia|658[0n]|BOLD:AAB4023
Orthodes goodelli|[16504]|LSEU636-06|United States|Georgia|658[0n]|BOLD:AAB4023
Orthodes goodelli|[16505]|LSEU220-06|United States|North Carolina|658[0n]|BOLD:AAB4023
Orthodes goodelli|[16506]|LGSM777-04|United States|North Carolina|658[0n]|BOLD:AAB4023
Orthodes goodelli|[16507]|LGSMG848-10|United States|North Carolina|658[0n]|BOLD:AAB4023
Orthodes goodelli|[16508]|LGSM749-04|United States|North Carolina|658[0n]|BOLD:AAB4023
Orthodes goodelli|[16509]|LGSMG849-10|United States|Tennessee|658[0n]|BOLD:AAB4023
Orthodes goodelli|[16510]|RDNMB252-05|Canada|Alberta|658[0n]|BOLD:AAB4023
Orthodes goodelli|[16511]|LPMN654-08|Canada|Manitoba|658[0n]|BOLD:AAB4023
Orthodes goodelli|[16512]|LPMN798-08|Canada|Manitoba|658[0n]|BOLD:AAB4023
Orthodes goodelli|[16513]|LGSMC495-05|United States|Tennessee|658[0n]|BOLD:AAB4023
Orthodes goodelli|[16514]|LGSMC493-05|United States|Tennessee|658[0n]|BOLD:AAB4023
Orthodes goodelli|[16515]|LGSMC901-05|United States|Tennessee|607[0n]|BOLD:AAB4023
Orthodes goodelli|[16516]|RDMAB046-05|Canada|Alberta|630[0n]|BOLD:AAB4023
Orthodes goodelli|[16517]|RDNMB253-05|Canada|Ontario|658[0n]|BOLD:AAB4023
Orthodes goodelli|[16518]|RDNMB254-05|Canada|Ontario|658[0n]|BOLD:AAB4023
Orthodes goodelli|[16519]|LPSOC107-08|Canada|Ontario|658[0n]|BOLD:AAB4023
Orthodes goodelli|[16520]|BLTIB400-08|Canada|Ontario|658[0n]|BOLD:AAB4023
Orthodes goodelli|[16521]|LNCC325-10|United States|North Carolina|658[0n]|BOLD:AAB4023
Orthodes goodelli|[16522]|LNCC326-10|United States|North Carolina|658[0n]|BOLD:AAB4023
Orthodes goodelli|[16523]|LGSMC494-05|United States|Tennessee|658[0n]|BOLD:AAB4023
Orthodes goodelli|[16524]|LGSMC492-05|United States|Tennessee|658[0n]|BOLD:AAB4023
Orthodes goodelli|[16525]|LGSM778-04|United States|North Carolina|658[0n]|BOLD:AAB4023
Orthodes goodelli|[16526]|GMGST028-13|United States|Tennessee|593[0n]|BOLD:AAB4023
Orthodes goodelli|[16527]|CNCLB2948-14|United States|North Carolina|658[0n]|BOLD:AAB4023
Cosmia sp.|[16528]|LTOLB1315-11|United States|West Virginia|658[0n]|BOLD:ABZ2712
Cosmia calami|[16529]|RDNMD851-07|United States|Arizona|655[0n]|BOLD:AAA4468
Cosmia calami|[16530]|JBAZ160-09|United States|Arizona|658[0n]|BOLD:AAA4468
Cosmia calami|[16531]|LSEU650-06|United States|Georgia|658[1n]|BOLD:AAA4468
Cosmia calami|[16532]|RDLQB526-05|Canada|Quebec|658[0n]|BOLD:AAA4468
Cosmia calami|[16533]|RDLQB742-05|Canada|Quebec|658[0n]|BOLD:AAA4468
Cosmia calami|[16534]|LSUSA126-06|United States|Kentucky|658[0n]|BOLD:AAA4468
Cosmia calami|[16535]|LSEU651-06|United States|Georgia|658[0n]|BOLD:AAA4468
Cosmia calami|[16536]|XAK298-06|Canada|Ontario|658[0n]|BOLD:AAA4468
Cosmia calami|[16537]|BBLEC140-09|Canada|Nova Scotia|658[0n]|BOLD:AAA4468
Cosmia calami|[16538]|BBLEC730-09|Canada|Nova Scotia|632[0n]|BOLD:AAA4468
Cosmia calami|[16539]|BBLEC221-09|Canada|Nova Scotia|636[0n]|BOLD:AAA4468
Cosmia calami|[16540]|BBLEC142-09|Canada|Nova Scotia|658[0n]|BOLD:AAA4468
Cosmia calami|[16541]|BBLEC155-09|Canada|Nova Scotia|658[0n]|BOLD:AAA4468
Cosmia calami|[16542]|BBLEC907-09|Canada|Nova Scotia|658[0n]|BOLD:AAA4468
Cosmia calami|[16543]|BBLEC908-09|Canada|Nova Scotia|658[0n]|BOLD:AAA4468
Cosmia calami|[16544]|BBLEC916-09|Canada|Nova Scotia|658[0n]|BOLD:AAA4468
Cosmia calami|[16545]|BBLEC934-09|Canada|Nova Scotia|658[0n]|BOLD:AAA4468
Cosmia calami|[16546]|BBLEC945-09|Canada|Nova Scotia|658[0n]|BOLD:AAA4468
Cosmia calami|[16547]|BBLPE562-09|Canada|Nova Scotia|658[0n]|BOLD:AAA4468
Cosmia calami|[16548]|BBLPE571-09|Canada|Nova Scotia|658[0n]|BOLD:AAA4468
Cosmia calami|[16549]|BBLPE586-09|Canada|Nova Scotia|658[0n]|BOLD:AAA4468
Cosmia calami|[16550]|BBLPE595-09|Canada|Nova Scotia|658[0n]|BOLD:AAA4468
Cosmia calami|[16551]|BBLPE600-09|Canada|Nova Scotia|658[0n]|BOLD:AAA4468
Cosmia calami|[16552]|BBLPE607-09|Canada|Nova Scotia|658[0n]|BOLD:AAA4468
Cosmia calami|[16553]|BBLPE615-09|Canada|Nova Scotia|658[0n]|BOLD:AAA4468
Cosmia calami|[16554]|BBLPE620-09|Canada|Nova Scotia|658[0n]|BOLD:AAA4468
Cosmia calami|[16555]|LPMNB373-09|Canada|Manitoba|658[0n]|BOLD:AAA4468
Cosmia calami|[16556]|LPMNB393-09|Canada|Manitoba|658[0n]|BOLD:AAA4468
Cosmia calami|[16557]|LPMNB388-09|Canada|Manitoba|658[0n]|BOLD:AAA4468
Cosmia calami|[16558]|LPMNB387-09|Canada|Manitoba|658[0n]|BOLD:AAA4468
Cosmia calami|[16559]|LPMNB385-09|Canada|Manitoba|658[0n]|BOLD:AAA4468
Cosmia calami|[16560]|LPMNB383-09|Canada|Manitoba|658[0n]|BOLD:AAA4468
Cosmia calami|[16561]|LPMNB381-09|Canada|Manitoba|658[0n]|BOLD:AAA4468
Cosmia calami|[16562]|LPMNB379-09|Canada|Manitoba|658[0n]|BOLD:AAA4468
Cosmia calami|[16563]|LPMNB371-09|Canada|Manitoba|658[0n]|BOLD:AAA4468
Cosmia calami|[16564]|LPMNB369-09|Canada|Manitoba|658[0n]|BOLD:AAA4468
Cosmia calami|[16565]|LPMNB367-09|Canada|Manitoba|658[0n]|BOLD:AAA4468
Cosmia calami|[16566]|LPMNB366-09|Canada|Manitoba|658[0n]|BOLD:AAA4468
Cosmia calami|[16567]|LPMNB365-09|Canada|Manitoba|658[0n]|BOLD:AAA4468
Cosmia calami|[16568]|LPMNB361-09|Canada|Manitoba|658[0n]|BOLD:AAA4468
Cosmia calami|[16569]|LPMNB357-09|Canada|Manitoba|658[0n]|BOLD:AAA4468
Cosmia calami|[16570]|LPMNB349-09|Canada|Manitoba|658[0n]|BOLD:AAA4468
Cosmia calami|[16571]|LPMNB345-09|Canada|Manitoba|658[0n]|BOLD:AAA4468
Cosmia calami|[16572]|LPMNB344-09|Canada|Manitoba|658[0n]|BOLD:AAA4468
Cosmia calami|[16573]|LPMNB333-09|Canada|Manitoba|658[0n]|BOLD:AAA4468
Cosmia calami|[16574]|LPMNB374-09|Canada|Manitoba|609[0n]|BOLD:AAA4468
Cosmia calami|[16575]|LPMNB390-09|Canada|Manitoba|637[0n]|BOLD:AAA4468
Cosmia calami|[16576]|LPMNB394-09|Canada|Manitoba|609[0n]|BOLD:AAA4468
Cosmia calami|[16577]|LPMNB396-09|Canada|Manitoba|658[0n]|BOLD:AAA4468
Cosmia calami|[16578]|LPMNB399-09|Canada|Manitoba|658[0n]|BOLD:AAA4468
Cosmia calami|[16579]|LPMNB401-09|Canada|Manitoba|658[0n]|BOLD:AAA4468
Cosmia calami|[16580]|LPMNB402-09|Canada|Manitoba|658[0n]|BOLD:AAA4468
Cosmia calami|[16581]|LPMNB407-09|Canada|Manitoba|658[0n]|BOLD:AAA4468
Cosmia calami|[16582]|LPMNB408-09|Canada|Manitoba|658[0n]|BOLD:AAA4468
Cosmia calami|[16583]|LPMNB409-09|Canada|Manitoba|658[0n]|BOLD:AAA4468
Cosmia calami|[16584]|LPMNB414-09|Canada|Manitoba|658[0n]|BOLD:AAA4468
Cosmia calami|[16585]|LPMNB475-09|Canada|Manitoba|658[0n]|BOLD:AAA4468
Cosmia calami|[16586]|LPMNB476-09|Canada|Manitoba|658[0n]|BOLD:AAA4468
Cosmia calami|[16587]|LPMNB480-09|Canada|Manitoba|658[0n]|BOLD:AAA4468
Cosmia calami|[16588]|LPMNB481-09|Canada|Manitoba|658[0n]|BOLD:AAA4468
Cosmia calami|[16589]|LPOKD841-10|United States|Oklahoma|658[0n]|BOLD:AAA4468
Cosmia calami|[16590]|LOCBC818-06|United States|California|658[0n]|BOLD:ACE9329
Cosmia calami|[16591]|LOCBF256-06|United States|California|658[0n]|BOLD:ACE9329

Cosmia calami|[16588]|LPMND401-09|Canada|Manitoba|658[0n]|BOLD:AAA4468
Cosmia calami|[16589]|LPOKD841-10|United States|Oklahoma|658[0n]|BOLD:AAA4468
Cosmia calami|[16590]|LOCBC818-06|United States|California|658[0n]|BOLD:ACE9329
Cosmia calami|[16591]|LOCBE256-06|United States|California|658[0n]|BOLD:ACE9329
Cosmia calami|[16592]|LOCBE254-06|United States|California|658[0n]|BOLD:ACE9329
Cosmia calami|[16593]|LOCBC931-06|United States|California|658[0n]|BOLD:ACE9329
Cosmia calami|[16594]|LOCBC923-06|United States|California|658[0n]|BOLD:ACE9329
Cosmia calami|[16595]|LOCBC897-06|United States|California|658[0n]|BOLD:ACE9329
Cosmia calami|[16596]|LOCBC820-06|United States|California|658[0n]|BOLD:ACE9329
Cosmia calami|[16597]|LOCBD717-06|United States|California|663[0n]|BOLD:ACE9329
Cosmia calami|[16598]|LOCBE376-06|United States|California|593[1n]|BOLD:ACE9329
Cosmia calami|[16599]|LOCBE378-06|United States|California|658[0n]|BOLD:ACE9329
Cosmia calami|[16600]|LOCBD066-06|United States|California|658[0n]|BOLD:ACE9329
Cosmia calami|[16601]|LOCBD077-06|United States|California|658[0n]|BOLD:ACE9329
Cosmia calami|[16602]|LOCBD079-06|United States|California|569[0n]|BOLD:ACE9329
Cosmia calami|[16603]|LOCBC896-06|United States|California|658[0n]|BOLD:ACE9329
Cosmia calami|[16604]|LOCBC432-06|United States|California|658[0n]|BOLD:ACE9329
Cosmia calami|[16605]|LOCBD716-06|United States|California|663[0n]|BOLD:ACE9329
Cosmia calami|[16606]|LOCBD160-06|United States|California|658[0n]|BOLD:ACE9329
Cosmia calami|[16607]|LOCBE371-06|United States|California|658[1n]|BOLD:ACE9329
Cosmia calami|[16608]|LOCBD164-06|United States|California|658[0n]|BOLD:ACE9329
Cosmia calami|[16609]|LOCBD175-06|United States|California|658[0n]|BOLD:ACE9329
Cosmia calami|[16610]|LOCBE250-06|United States|California|658[0n]|BOLD:ACE9329
Cosmia calami|[16611]|LOCBE252-06|United States|California|658[0n]|BOLD:ACE9329
Cosmia calami|[16612]|LOCBE261-06|United States|California|658[0n]|BOLD:ACE9329
Cosmia calami|[16613]|LOCBE369-06|United States|California|657[0n]|BOLD:ACE9329
Cosmia calami|[16614]|LOCBD162-06|United States|California|658[0n]|BOLD:ACE9329
Cosmia calami|[16615]|LOCBD163-06|United States|California|658[0n]|BOLD:ACE9329
Cosmia calami|[16616]|LOCBD159-06|United States|California|658[0n]|BOLD:ACE9329
Cosmia calami|[16617]|LOCBD161-06|United States|California|658[0n]|BOLD:ACE9329
Cosmia calami|[16618]|LOCBD070-06|United States|California|658[0n]|BOLD:ACE9329
Cosmia calami|[16619]|LOCBD076-06|United States|California|658[0n]|BOLD:ACE9329
Cosmia calami|[16620]|LOCBC935-06|United States|California|658[0n]|BOLD:ACE9329
Cosmia calami|[16621]|LOCBD069-06|United States|California|658[0n]|BOLD:ACE9329
Cosmia calami|[16622]|LOCBC933-06|United States|California|658[0n]|BOLD:ACE9329
Cosmia calami|[16623]|LOCBC934-06|United States|California|658[0n]|BOLD:ACE9329
Cosmia calami|[16624]|LOCBC930-06|United States|California|658[0n]|BOLD:ACE9329
Cosmia calami|[16625]|LOCBC932-06|United States|California|658[0n]|BOLD:ACE9329
Cosmia calami|[16626]|LOCBC928-06|United States|California|658[0n]|BOLD:ACE9329
Cosmia calami|[16627]|LOCBC927-06|United States|California|658[0n]|BOLD:ACE9329
Cosmia calami|[16628]|LOCBC926-06|United States|California|658[0n]|BOLD:ACE9329
Cosmia calami|[16629]|LOCBC925-06|United States|California|658[0n]|BOLD:ACE9329
Cosmia calami|[16630]|LOCBC924-06|United States|California|658[0n]|BOLD:ACE9329
Cosmia calami|[16631]|LOCBC922-06|United States|California|658[0n]|BOLD:ACE9329
Cosmia calami|[16632]|LOCBC921-06|United States|California|658[0n]|BOLD:ACE9329
Cosmia calami|[16633]|LOCBC895-06|United States|California|658[0n]|BOLD:ACE9329
Cosmia calami|[16634]|LOCBC877-06|United States|California|658[0n]|BOLD:ACE9329
Cosmia calami|[16635]|LOCBC819-06|United States|California|658[0n]|BOLD:ACE9329
Cosmia calami|[16636]|LOCBE257-06|United States|California|658[0n]|BOLD:ACE9329
Cosmia calami|[16637]|LOCBD714-06|United States|California|663[0n]|BOLD:ACE9329
Cosmia calami|[16638]|LOCBD713-06|United States|California|663[0n]|BOLD:ACE9329
Cosmia calami|[16639]|LOCBD724-06|United States|California|663[0n]|BOLD:ACE9329
Cosmia calami|[16640]|LOCBE255-06|United States|California|646[0n]|BOLD:ACE9329
Cosmia calami|[16641]|LOCBE383-06|United States|California|584[0n]|BOLD:ACE9329
Cosmia calami|[16642]|LOCBE386-06|United States|California|594[0n]|BOLD:ACE9329
Cosmia calami|[16643]|LOCBE379-06|United States|California|658[0n]|BOLD:ACE9329
Cosmia calami|[16644]|LOCBE387-06|United States|California|658[0n]|BOLD:ACE9329
Cosmia calami|[16645]|GMLC928-12|United States|California|658[0n]|BOLD:ACE9329
Cosmia calami|[16646]|GMLC1076-12|United States|California|633[0n]|BOLD:ACE9329
Cosmia calami|[16647]|GMLC1146-12|United States|California|658[0n]|BOLD:ACE9329
Cosmia calami|[16648]|GMLC1080-12|United States|California|658[0n]|BOLD:ACE9329
Cosmia calami|[16649]|GMLC1060-12|United States|California|658[0n]|BOLD:ACE9329
Cosmia calami|[16650]|GMLC1027-12|United States|California|658[0n]|BOLD:ACE9329
Cosmia calami|[16651]|GMLC1013-12|United States|California|658[0n]|BOLD:ACE9329
Cosmia calami|[16652]|GMLC1006-12|United States|California|658[0n]|BOLD:ACE9329
Cosmia calami|[16653]|GMLC1000-12|United States|California|658[0n]|BOLD:ACE9329
Cosmia calami|[16654]|GMLC935-12|United States|California|658[0n]|BOLD:ACE9329
Cosmia calami|[16655]|GMLC934-12|United States|California|658[0n]|BOLD:ACE9329
Cosmia calami|[16656]|GMLC924-12|United States|California|658[0n]|BOLD:ACE9329
Cosmia calami|[16657]|GMLC922-12|United States|California|658[0n]|BOLD:ACE9329
Cosmia calami|[16658]|GMLC909-12|United States|California|658[0n]|BOLD:ACE9329
Cosmia calami|[16659]|GMLC905-12|United States|California|658[0n]|BOLD:ACE9329
Cosmia calami|[16660]|GMLC901-12|United States|California|658[0n]|BOLD:ACE9329
Cosmia calami|[16661]|GMLC872-12|United States|California|658[0n]|BOLD:ACE9329
Cosmia calami|[16662]|GMLC868-12|United States|California|658[0n]|BOLD:ACE9329
Cosmia calami|[16663]|GMLC860-12|United States|California|658[0n]|BOLD:ACE9329
Cosmia calami|[16664]|GMLC847-12|United States|California|658[0n]|BOLD:ACE9329
Cosmia calami|[16665]|GMLC778-12|United States|California|658[0n]|BOLD:ACE9329
Cosmia calami|[16666]|GMLC259-11|United States|California|658[0n]|BOLD:ACE9329
Cosmia calami|[16667]|GMLC858-12|United States|California|624[0n]|BOLD:ACE9329
Cosmia calami|[16668]|GMLC1097-12|United States|California|632[1n]|BOLD:ACE9329
Cosmia calami|[16669]|GMLC1183-12|United States|California|634[0n]|BOLD:ACE9329
Cosmia calami|[16670]|GMLC1187-12|United States|California|658[0n]|BOLD:ACE9329
Cosmia elisae|[16671]|RDNMC710-06|United States|Arizona|658[0n]|BOLD:AAB0874
Cosmia elisae|[16672]|LBCH4049-10|Canada|British Columbia|620[0n]|BOLD:AAB0874
Cosmia elisae|[16673]|LBCH4437-10|Canada|British Columbia|658[0n]|BOLD:AAB0874
Cosmia elisae|[16674]|LBCH4669-10|Canada|British Columbia|658[0n]|BOLD:AAB0874
Cosmia elisae|[16675]|LBCD297-05|Canada|British Columbia|616[0n]|BOLD:AAB0874
Cosmia elisae|[16676]|LOWCC474-05|Canada|British Columbia|580[0n]|BOLD:AAB0874
Cosmia elisae|[16677]|LOWCC190-05|Canada|British Columbia|592[0n]|BOLD:AAB0874
Cosmia elisae|[16678]|LBCH6007-10|Canada|British Columbia|643[0n]|BOLD:AAB0874
Cosmia elisae|[16679]|LOWCC055-05|Canada|British Columbia|658[0n]|BOLD:AAB0874
Cosmia elisae|[16680]|LOWCC069-05|Canada|British Columbia|658[0n]|BOLD:AAB0874
Cosmia elisae|[16681]|LOWCC053-05|Canada|British Columbia|658[0n]|BOLD:AAB0874
Cosmia elisae|[16682]|LOWCC054-05|Canada|British Columbia|658[0n]|BOLD:AAB0874
Cosmia elisae|[16683]|LOWCC049-05|Canada|British Columbia|658[0n]|BOLD:AAB0874
Cosmia elisae|[16684]|LOWCC050-05|Canada|British Columbia|658[0n]|BOLD:AAB0874
Cosmia elisae|[16685]|LOWCC047-05|Canada|British Columbia|658[0n]|BOLD:AAB0874
Cosmia elisae|[16686]|LOWCC048-05|Canada|British Columbia|658[0n]|BOLD:AAB0874
Cosmia elisae|[16687]|LOWCC051-05|Canada|British Columbia|658[0n]|BOLD:AAB0874
Cosmia elisae|[16688]|LOWCC052-05|Canada|British Columbia|658[0n]|BOLD:AAB0874
Cosmia elisae|[16689]|LOWCC045-05|Canada|British Columbia|658[0n]|BOLD:AAB0874
Cosmia elisae|[16690]|LOWCC044-05|Canada|British Columbia|658[0n]|BOLD:AAB0874
Cosmia elisae|[16691]|LOWCC043-05|Canada|British Columbia|658[0n]|BOLD:AAB0874

Cosmia elisae|[16688]|LOWCC052-05|Canada|British Columbia|658[0n]|BOLD:AAB0874
Cosmia elisae|[16689]|LOWCC045-05|Canada|British Columbia|658[0n]|BOLD:AAB0874
Cosmia elisae|[16690]|LOWCC044-05|Canada|British Columbia|658[0n]|BOLD:AAB0874
Cosmia elisae|[16691]|LOWCC043-05|Canada|British Columbia|658[0n]|BOLD:AAB0874
Cosmia elisae|[16692]|LOWCC042-05|Canada|British Columbia|658[0n]|BOLD:AAB0874
Cosmia elisae|[16693]|LOWCC041-05|Canada|British Columbia|657[0n]|BOLD:AAB0874
Cosmia elisae|[16694]|LOWCB174-05|Canada|British Columbia|658[0n]|BOLD:AAB0874
Cosmia elisae|[16695]|LOWCB173-05|Canada|British Columbia|658[0n]|BOLD:AAB0874
Cosmia elisae|[16696]|LBCD275-05|Canada|British Columbia|658[0n]|BOLD:AAB0874
Cosmia elisae|[16697]|RDMAB347-05|Canada|Alberta|605[0n]|BOLD:AAB0874
Cosmia elisae|[16698]|LOWCD529-06|Canada|British Columbia|658[0n]|BOLD:AAB0874
Cosmia elisae|[16699]|LOWCD536-06|Canada|British Columbia|658[0n]|BOLD:AAB0874
Cosmia elisae|[16700]|DUNLP149-08|Canada|British Columbia|658[0n]|BOLD:AAB0874
Cosmia elisae|[16701]|LBCH1240-10|Canada|British Columbia|658[0n]|BOLD:AAB0874
Cosmia elisae|[16702]|LBCH6008-10|Canada|British Columbia|658[0n]|BOLD:AAB0874
Cosmia elisae|[16703]|LBCH6055-10|Canada|British Columbia|658[0n]|BOLD:AAB0874
Cosmia praeacuta|[16704]|LPVIA828-08|Canada|British Columbia|618[1n]|BOLD:AAB0874
Cosmia praeacuta|[16705]|IAWLB557-11|United States|Arizona|658[0n]|BOLD:AAB0874
Cosmia praeacuta|[16706]|RDNMJ477-11|United States|Arizona|658[0n]|BOLD:AAB0874
Cosmia praeacuta|[16707]|RDNMJ478-11|United States|Arizona|658[0n]|BOLD:AAB0874
Cosmia praeacuta|[16708]|RDNMD927-07|United States|New Mexico|655[0n]|BOLD:AAB0874
Cosmia praeacuta|[16709]|IAWLB555-11|United States|Arizona|658[0n]|BOLD:AAB0874
Cosmia praeacuta|[16710]|RDNMC711-06|United States|New Mexico|658[0n]|BOLD:AAB0874
Cosmia praeacuta|[16711]|RDNMD926-07|United States|New Mexico|655[0n]|BOLD:AAB0874
Cosmia praeacuta|[16712]|IAWLB556-11|United States|Arizona|658[0n]|BOLD:AAB0874
Cosmia praeacuta|[16713]|RDNMK570-11|United States|Arizona|658[0n]|BOLD:AAB0874
Cosmia praeacuta|[16714]|LOWCC046-05|Canada|British Columbia|658[0n]|BOLD:AAB0874
Cosmia praeacuta|[16715]|LOWCC058-05|Canada|British Columbia|658[0n]|BOLD:AAB0874
Cosmia praeacuta|[16716]|LOWCC059-05|Canada|British Columbia|658[0n]|BOLD:AAB0874
Cosmia praeacuta|[16717]|LOWCC060-05|Canada|British Columbia|658[0n]|BOLD:AAB0874
Cosmia praeacuta|[16718]|LOWCC061-05|Canada|British Columbia|658[0n]|BOLD:AAB0874
Cosmia praeacuta|[16719]|LOWCC062-05|Canada|British Columbia|658[0n]|BOLD:AAB0874
Cosmia praeacuta|[16720]|LOWCC063-05|Canada|British Columbia|658[0n]|BOLD:AAB0874
Cosmia praeacuta|[16721]|LOWCC064-05|Canada|British Columbia|658[0n]|BOLD:AAB0874
Cosmia praeacuta|[16722]|LOWCC065-05|Canada|British Columbia|658[0n]|BOLD:AAB0874
Cosmia praeacuta|[16723]|LOWCC066-05|Canada|British Columbia|658[0n]|BOLD:AAB0874
Cosmia praeacuta|[16724]|LOWCC067-05|Canada|British Columbia|658[0n]|BOLD:AAB0874
Cosmia praeacuta|[16725]|LOWCC068-05|Canada|British Columbia|658[0n]|BOLD:AAB0874
Cosmia praeacuta|[16726]|LOWCC070-05|Canada|British Columbia|658[0n]|BOLD:AAB0874
Cosmia praeacuta|[16727]|LOWCD295-06|Canada|British Columbia|658[0n]|BOLD:AAB0874
Cosmia praeacuta|[16728]|LPVIA565-08|Canada|British Columbia|658[0n]|BOLD:AAB0874
Cosmia praeacuta|[16729]|LPVIB169-08|Canada|British Columbia|658[0n]|BOLD:AAB0874
Cosmia praeacuta|[16730]|LOWCC056-05|Canada|British Columbia|658[0n]|BOLD:AAB0874
Cosmia praeacuta|[16731]|LOWCC057-05|Canada|British Columbia|658[0n]|BOLD:AAB0874
Cosmia praeacuta|[16732]|LPVIB493-08|Canada|British Columbia|658[0n]|BOLD:AAB0874
Cosmia praeacuta|[16733]|LPVIB250-08|Canada|British Columbia|658[0n]|BOLD:AAB0874
Cosmia praeacuta|[16734]|LPVIA831-08|Canada|British Columbia|633[0n]|BOLD:AAB0874
Cosmia praeacuta|[16735]|LPVIA830-08|Canada|British Columbia|633[0n]|BOLD:AAB0874
Cosmia praeacuta|[16736]|LPVIA406-08|Canada|British Columbia|658[0n]|BOLD:AAB0874
Cosmia praeacuta|[16737]|DUNLP150-08|Canada|British Columbia|643[0n]|BOLD:AAB0874
Cosmia praeacuta|[16738]|LALPA647-10|Canada|British Columbia|658[0n]|BOLD:AAB0874
Cosmia praeacuta|[16739]|LALPA599-10|Canada|British Columbia|658[0n]|BOLD:AAB0874
Cosmia praeacuta|[16740]|LPVIC027-08|Canada|British Columbia|658[0n]|BOLD:AAB0874
Cosmia praeacuta|[16741]|LPVIC026-08|Canada|British Columbia|658[0n]|BOLD:AAB0874
Cosmia praeacuta|[16742]|LPVIC025-08|Canada|British Columbia|658[0n]|BOLD:AAB0874
Cosmia praeacuta|[16743]|LPVIB255-08|Canada|British Columbia|658[0n]|BOLD:AAB0874
Cosmia praeacuta|[16744]|LPVIB254-08|Canada|British Columbia|658[0n]|BOLD:AAB0874
Cosmia praeacuta|[16745]|LPVIB252-08|Canada|British Columbia|658[0n]|BOLD:AAB0874
Cosmia praeacuta|[16746]|LPVIB240-08|Canada|British Columbia|658[0n]|BOLD:AAB0874
Cosmia praeacuta|[16747]|LPVIB236-08|Canada|British Columbia|658[0n]|BOLD:AAB0874
Cosmia praeacuta|[16748]|LPVIB235-08|Canada|British Columbia|658[0n]|BOLD:AAB0874
Cosmia praeacuta|[16749]|LPVIB170-08|Canada|British Columbia|658[0n]|BOLD:AAB0874
Cosmia praeacuta|[16750]|LPVIB168-08|Canada|British Columbia|658[0n]|BOLD:AAB0874
Cosmia praeacuta|[16751]|LPVIB167-08|Canada|British Columbia|658[0n]|BOLD:AAB0874
Cosmia praeacuta|[16752]|LPVIB076-08|Canada|British Columbia|658[0n]|BOLD:AAB0874
Cosmia praeacuta|[16753]|LPVIB071-08|Canada|British Columbia|658[0n]|BOLD:AAB0874
Cosmia praeacuta|[16754]|LPVIA829-08|Canada|British Columbia|658[0n]|BOLD:AAB0874
Cosmia praeacuta|[16755]|LPVIA827-08|Canada|British Columbia|658[0n]|BOLD:AAB0874
Cosmia praeacuta|[16756]|LPVIA570-08|Canada|British Columbia|658[0n]|BOLD:AAB0874
Cosmia praeacuta|[16757]|LPVIA569-08|Canada|British Columbia|658[0n]|BOLD:AAB0874
Cosmia praeacuta|[16758]|LPVIA568-08|Canada|British Columbia|658[0n]|BOLD:AAB0874
Cosmia praeacuta|[16759]|LPVIA567-08|Canada|British Columbia|658[0n]|BOLD:AAB0874
Cosmia praeacuta|[16760]|LPVIA566-08|Canada|British Columbia|658[0n]|BOLD:AAB0874
Cosmia praeacuta|[16761]|LPVIA564-08|Canada|British Columbia|658[0n]|BOLD:AAB0874
Cosmia praeacuta|[16762]|LPVIA560-08|Canada|British Columbia|658[0n]|BOLD:AAB0874
Cosmia praeacuta|[16763]|LPVIA408-08|Canada|British Columbia|658[0n]|BOLD:AAB0874
Cosmia praeacuta|[16764]|LBCS264-07|Canada|British Columbia|658[0n]|BOLD:AAB0874
Cosmia praeacuta|[16765]|LBCS192-07|Canada|British Columbia|658[0n]|BOLD:AAB0874
Cosmia praeacuta|[16766]|LHLEP571-06|Canada|British Columbia|658[0n]|BOLD:AAB0874
Cosmia praeacuta|[16767]|LHLEP439-06|Canada|British Columbia|658[0n]|BOLD:AAB0874
Cosmia praeacuta|[16768]|LHLEP374-06|Canada|British Columbia|657[0n]|BOLD:AAB0874
Cosmia praeacuta|[16769]|LHLEP211-06|Canada|British Columbia|658[0n]|BOLD:AAB0874
Cosmia praeacuta|[16770]|LHLEP210-06|Canada|British Columbia|658[0n]|BOLD:AAB0874
Cosmia praeacuta|[16771]|LHLEP209-06|Canada|British Columbia|658[0n]|BOLD:AAB0874
Cosmia praeacuta|[16772]|LPVIB249-08|Canada|British Columbia|658[0n]|BOLD:AAB0874
Cosmia praeacuta|[16773]|LHLEP208-06|Canada|British Columbia|658[0n]|BOLD:AAB0874
Cosmia praeacuta|[16774]|LHLEP375-06|Canada|British Columbia|656[0n]|BOLD:AAB0874
Cosmia praeacuta|[16775]|LHLEP376-06|Canada|British Columbia|656[0n]|BOLD:AAB0874
Cosmia praeacuta|[16776]|LALPA556-10|Canada|British Columbia|658[0n]|BOLD:AAB0874
Cosmia praeacuta|[16777]|LALPA645-10|Canada|British Columbia|658[0n]|BOLD:AAB0874
Cosmia praeacuta|[16778]|LALPA658-10|Canada|British Columbia|658[0n]|BOLD:AAB0874
Cosmia praeacuta|[16779]|LALPA659-10|Canada|British Columbia|658[0n]|BOLD:AAB0874
Cosmia praeacuta|[16780]|LALPA664-10|Canada|British Columbia|658[0n]|BOLD:AAB0874
Cosmia praeacuta|[16781]|LALPA1332-12|Canada|British Columbia|614[0n]|BOLD:AAB0874
Enargia infumata|[16782]|CNPAA043-13|Canada|Saskatchewan|559[0n]|BOLD:AAA7455
Enargia infumata|[16783]|CNWBG3124-13|Canada|Alberta|571[0n]|BOLD:AAA7455
Enargia infumata|[16784]|CNWBG3094-13|Canada|Alberta|579[1n]|BOLD:AAA7455
Enargia infumata|[16785]|CNWBG3105-13|Canada|Alberta|606[1n]|BOLD:AAA7455
Enargia infumata|[16786]|CNWBG3147-13|Canada|Alberta|576[0n]|BOLD:AAA7455
Enargia infumata|[16787]|LBCD301-05|Canada|British Columbia|618[0n]|BOLD:AAA7455
Enargia infumata|[16788]|RDMAB284-05|Canada|Alberta|509[0n]|BOLD:AAA7455
Enargia infumata|[16789]|CNWBG3107-13|Canada|Alberta|608[1n]|BOLD:AAA7455
Enargia infumata|[16790]|CNWBG3096-13|Canada|Alberta|608[1n]|BOLD:AAA7455
Enargia infumata|[16791]|CNWBG3154-13|Canada|Alberta|588[0n]|BOLD:AAA7455

Enargia infumata|[illegible]|RDMAB284-05|Canada|Alberta|[illegible]|BOLD:AAA7455
Enargia infumata|[16789]|CNWBG3107-13|Canada|Alberta|608[1n]|BOLD:AAA7455
Enargia infumata|[16790]|CNWBG3096-13|Canada|Alberta|608[1n]|BOLD:AAA7455
Enargia infumata|[16791]|CNWBG3154-13|Canada|Alberta|588[0n]|BOLD:AAA7455
Enargia infumata|[16792]|CNWBG3134-13|Canada|Alberta|588[0n]|BOLD:AAA7455
Enargia infumata|[16793]|XAB147-04|Canada|Ontario|658[0n]|BOLD:AAA7455
Enargia infumata|[16794]|RDMAB659-06|Canada|Alberta|547[0n]|BOLD:AAA7455
Enargia infumata|[16795]|CNWBG3052-13|Canada|Alberta|611[2n]|BOLD:AAA7455
Enargia infumata|[16796]|RDMAB662-06|Canada|Alberta|606[2n]|BOLD:AAA7455
Enargia infumata|[16797]|CNWBG3055-13|Canada|Alberta|607[0n]|BOLD:AAA7455
Enargia infumata|[16798]|LBCD305-05|Canada|British Columbia|618[0n]|BOLD:AAA7455
Enargia infumata|[16799]|LBCD304-05|Canada|British Columbia|618[0n]|BOLD:AAA7455
Enargia infumata|[16800]|LBCA595-05|Canada|British Columbia|633[0n]|BOLD:AAA7455
Enargia infumata|[16801]|RDNME190-07|Canada|Ontario|604[1n]|BOLD:AAA7455
Enargia infumata|[16802]|LBCD300-05|Canada|British Columbia|618[0n]|BOLD:AAA7455
Enargia infumata|[16803]|LBCD309-05|Canada|British Columbia|639[0n]|BOLD:AAA7455
Enargia infumata|[16804]|CNWBG3046-13|Canada|Alberta|596[0n]|BOLD:AAA7455
Enargia infumata|[16805]|CNWBG3066-13|Canada|Alberta|594[0n]|BOLD:AAA7455
Enargia infumata|[16806]|ABKWR135-07|United States|Alaska|658[3n]|BOLD:AAA7455
Enargia infumata|[16807]|RDNME186-07|Canada|New Brunswick|640[0n]|BOLD:AAA7455
Enargia infumata|[16808]|LBCA597-05|Canada|British Columbia|641[0n]|BOLD:AAA7455
Enargia infumata|[16809]|RDLQF541-06|Canada|Quebec|658[0n]|BOLD:AAA7455
Enargia infumata|[16810]|XAK054-06|Canada|Ontario|658[0n]|BOLD:AAA7455
Enargia infumata|[16811]|RDMAB660-06|Canada|Alberta|658[0n]|BOLD:AAA7455
Enargia infumata|[16812]|RDMAB658-06|Canada|Alberta|658[0n]|BOLD:AAA7455
Enargia infumata|[16813]|RDMAB657-06|Canada|Alberta|658[0n]|BOLD:AAA7455
Enargia infumata|[16814]|LBCD467-05|Canada|British Columbia|658[0n]|BOLD:AAA7455
Enargia infumata|[16815]|LBCD427-05|Canada|British Columbia|658[0n]|BOLD:AAA7455
Enargia infumata|[16816]|LBCD308-05|Canada|British Columbia|658[0n]|BOLD:AAA7455
Enargia infumata|[16817]|LBCD307-05|Canada|British Columbia|658[0n]|BOLD:AAA7455
Enargia infumata|[16818]|LBCB199-05|Canada|British Columbia|658[0n]|BOLD:AAA7455
Enargia infumata|[16819]|XAC594-04|Canada|Ontario|658[0n]|BOLD:AAA7455
Enargia infumata|[16820]|XAC566-04|Canada|Ontario|658[0n]|BOLD:AAA7455
Enargia infumata|[16821]|XAC038-04|Canada|Ontario|658[0n]|BOLD:AAA7455
Enargia infumata|[16822]|XAB493-04|Canada|Ontario|658[0n]|BOLD:AAA7455
Enargia infumata|[16823]|XAB194-04|Canada|Ontario|658[0n]|BOLD:AAA7455
Enargia infumata|[16824]|RDNME187-07|Canada|New Brunswick|641[0n]|BOLD:AAA7455
Enargia infumata|[16825]|LPVIB233-08|Canada|British Columbia|658[0n]|BOLD:AAA7455
Enargia infumata|[16826]|LPVIC003-08|Canada|British Columbia|658[0n]|BOLD:AAA7455
Enargia infumata|[16827]|RDNMH999-09|Canada|British Columbia|658[0n]|BOLD:AAA7455
Enargia infumata|[16828]|RDNMH1001-09|Canada|British Columbia|658[0n]|BOLD:AAA7455
Enargia infumata|[16829]|RDNMH1003-09|Canada|British Columbia|658[0n]|BOLD:AAA7455
Enargia infumata|[16830]|RDNMH1005-09|Canada|British Columbia|658[0n]|BOLD:AAA7455
Enargia infumata|[16831]|RDNMI147-10|United States|Colorado|658[0n]|BOLD:AAA7455
Enargia infumata|[16832]|RDNMI149-10|United States|Colorado|658[0n]|BOLD:AAA7455
Enargia infumata|[16833]|RDNMI155-10|United States|Washington|658[0n]|BOLD:AAA7455
Enargia infumata|[16834]|RDNMI157-10|United States|Wyoming|658[0n]|BOLD:AAA7455
Enargia infumata|[16835]|CNWLF2035-12|Canada|Alberta|636[0n]|BOLD:AAA7455
Enargia infumata|[16836]|LBCB036-05|Canada|British Columbia|658[0n]|BOLD:AAA7455
Enargia infumata|[16837]|CNWBG3090-13|Canada|Alberta|596[0n]|BOLD:AAA7455
Enargia infumata|[16838]|CNWBG3155-13|Canada|Alberta|576[0n]|BOLD:AAA7455
Enargia infumata|[16839]|CNWBG3156-13|Canada|Alberta|579[0n]|BOLD:AAA7455
Enargia infumata|[16840]|CNWBG3157-13|Canada|Alberta|588[0n]|BOLD:AAA7455
Enargia sp.|[16841]|RDNMI148-10|United States|Arizona|658[0n]|BOLD:AAA7455
Enargia sp.|[16842]|RDNMI159-10|United States|Colorado|658[0n]|BOLD:AAA7455
Enargia sp.|[16843]|RDNME050-07|United States|Utah|658[0n]|BOLD:AAA7455
Enargia sp.|[16844]|RDNMD856-07|United States|Utah|655[0n]|BOLD:AAA7455
Enargia sp.|[16845]|RDNMD857-07|United States|Utah|619[0n]|BOLD:AAA7455
Enargia sp.|[16846]|RDNMI158-10|United States|Colorado|658[0n]|BOLD:AAA7455
Enargia sp.|[16847]|RDNMI160-10|United States|Colorado|658[0n]|BOLD:AAA7455
Enargia fausta|[16848]|LPMNB339-09|Canada|Manitoba|658[0n]|BOLD:AAA7455
Enargia fausta|[16849]|BBLPB237-10|Canada|Saskatchewan|658[0n]|BOLD:AAA7455
Enargia fausta|[16850]|TMNBB192-06|Canada|New Brunswick|658[0n]|BOLD:AAA7455
Enargia fausta|[16851]|LPMNB430-09|Canada|Manitoba|658[0n]|BOLD:AAA7455
Enargia fausta|[16852]|RDNMH997-09|Canada|New Brunswick|658[0n]|BOLD:AAA7455
Enargia fausta|[16853]|BBLPB239-10|Canada|Saskatchewan|658[0n]|BOLD:AAA7455
Enargia fausta|[16854]|BBLPB247-10|Canada|Saskatchewan|658[0n]|BOLD:AAA7455
Enargia infumata|[16855]|RDMAB285-05|Canada|Alberta|502[1n]|
Enargia infumata|[16856]|LBCD302-05|Canada|British Columbia|618[0n]|BOLD:AAA7455
Enargia infumata|[16857]|CNWBG3089-13|Canada|Alberta|596[0n]|BOLD:AAA7455
Enargia infumata|[16858]|CNWBG3138-13|Canada|Alberta|588[0n]|BOLD:AAA7455
Enargia infumata|[16859]|LBCD303-05|Canada|British Columbia|618[0n]|BOLD:AAA7455
Enargia infumata|[16860]|LBCA596-05|Canada|British Columbia|647[0n]|BOLD:AAA7455
Enargia infumata|[16861]|RDMAB661-06|Canada|Alberta|601[0n]|BOLD:AAA7455
Enargia infumata|[16862]|RDNME185-07|Canada|New Brunswick|642[0n]|BOLD:AAA7455
Enargia infumata|[16863]|RDNME188-07|Canada|New Brunswick|642[0n]|BOLD:AAA7455
Enargia infumata|[16864]|RDNME189-07|Canada|Ontario|619[0n]|BOLD:AAA7455
Enargia infumata|[16865]|RDNMI156-10|United States|Washington|658[0n]|BOLD:AAA7455
Enargia infumata|[16866]|LPMN993-09|Canada|Manitoba|658[0n]|BOLD:AAA7455
Enargia infumata|[16867]|LBCD306-05|Canada|British Columbia|658[0n]|BOLD:AAA7455
Enargia infumata|[16868]|XAB198-04|Canada|Ontario|658[0n]|BOLD:AAA7455
Enargia infumata|[16869]|RDLQB598-05|Canada|Quebec|519[0n]|BOLD:AAA7455
Enargia infumata|[16870]|BBLPB238-10|Canada|Saskatchewan|658[0n]|BOLD:AAA7455
Enargia infumata|[16871]|BBLPB240-10|Canada|Saskatchewan|658[0n]|BOLD:AAA7455
Enargia infumata|[16872]|CNWLF2027-12|Canada|Alberta|637[0n]|BOLD:AAA7455
Enargia infumata|[16873]|LPMNB323-09|Canada|Manitoba|658[0n]|BOLD:AAA7455
Enargia infumata|[16874]|CNWBG3061-13|Canada|Alberta|596[0n]|BOLD:AAA7455
Enargia infumata|[16875]|CNWBG3091-13|Canada|Alberta|596[0n]|BOLD:AAA7455
Enargia infumata|[16876]|CNPAJ104-13|Canada|Saskatchewan|627[2n]|BOLD:AAA7455
Enargia decolor|[16877]|RDMAB287-05|Canada|Alberta|517[0n]|BOLD:AAA7455
Enargia decolor|[16878]|CNWBD039-13|Canada|Alberta|554[0n]|BOLD:AAA7455
Enargia decolor|[16879]|CNWBD037-13|Canada|Alberta|576[0n]|BOLD:AAA7455
Enargia decolor|[16880]|CNWBD033-13|Canada|Alberta|548[0n]|BOLD:AAA7455
Enargia decolor|[16881]|CNWBC129-13|Canada|Alberta|541[0n]|BOLD:AAA7455
Enargia decolor|[16882]|CNPAO673-13|Canada|Saskatchewan|586[0n]|BOLD:AAA7455
Enargia decolor|[16883]|CNPAB339-13|Canada|Saskatchewan|603[0n]|BOLD:AAA7455
Enargia decolor|[16884]|CNRMG749-12|Canada|Manitoba|620[0n]|BOLD:AAA7455
Enargia decolor|[16885]|CNWBD032-13|Canada|Alberta|599[0n]|BOLD:AAA7455
Enargia decolor|[16886]|CNWLH467-12|Canada|Alberta|632[0n]|BOLD:AAA7455
Enargia decolor|[16887]|CNRMH353-12|Canada|Manitoba|633[0n]|BOLD:AAA7455
Enargia decolor|[16888]|CNRMG755-12|Canada|Manitoba|633[0n]|BOLD:AAA7455
Enargia decolor|[16889]|XAG971-05|Canada|Ontario|629[0n]|BOLD:AAA7455
Enargia decolor|[16890]|XAG614-05|Canada|Ontario|658[0n]|BOLD:AAA7455
Enargia decolor|[16891]|XAG758-05|Canada|Ontario|658[0n]|BOLD:AAA7455

Enargia decolor|[16888]|CNRMG755-12|Canada|Manitoba|633[0n]|BOLD:AAA7455
Enargia decolor|[16889]|XAG971-05|Canada|Ontario|629[0n]|BOLD:AAA7455
Enargia decolor|[16890]|XAG614-05|Canada|Ontario|658[0n]|BOLD:AAA7455
Enargia decolor|[16891]|XAG758-05|Canada|Ontario|658[0n]|BOLD:AAA7455
Enargia decolor|[16892]|LOWCC006-05|Canada|British Columbia|658[0n]|BOLD:AAA7455
Enargia decolor|[16893]|BBLPB243-10|Canada|Saskatchewan|658[0n]|BOLD:AAA7455
Enargia decolor|[16894]|BBLPB245-10|Canada|Saskatchewan|658[0n]|BOLD:AAA7455
Enargia decolor|[16895]|CNPAG337-13|Canada|Saskatchewan|636[0n]|BOLD:AAA7455
Enargia decolor|[16896]|CNPAO674-13|Canada|Saskatchewan|600[0n]|BOLD:AAA7455
Enargia decolor|[16897]|CNWLO775-13|Canada|Alberta|599[0n]|BOLD:AAA7455
Enargia decolor|[16898]|CNWBD031-13|Canada|Alberta|566[0n]|BOLD:AAA7455
Enargia decolor|[16899]|CNWBD034-13|Canada|Alberta|576[0n]|BOLD:AAA7455
Enargia decolor|[16900]|RDMAB286-05|Canada|Alberta|548[0n]|BOLD:AAA7455
Enargia decolor|[16901]|RDLQB597-05|Canada|Quebec|537[0n]|BOLD:AAA7455
Enargia decolor|[16902]|XAD353-04|Canada|Ontario|549[0n]|BOLD:AAA7455
Enargia decolor|[16903]|CNRMG753-12|Canada|Manitoba|633[0n]|BOLD:AAA7455
Enargia decolor|[16904]|CNRMG751-12|Canada|Manitoba|632[0n]|BOLD:AAA7455
Enargia decolor|[16905]|XAD391-04|Canada|Ontario|658[0n]|BOLD:AAA7455
Enargia decolor|[16906]|XAH052-05|Canada|Ontario|658[0n]|BOLD:AAA7455
Enargia decolor|[16907]|RDNMH998-09|Canada|New Brunswick|658[0n]|BOLD:AAA7455
Enargia decolor|[16908]|CNRMA334-12|Canada|Manitoba|622[0n]|BOLD:AAA7455
Enargia decolor|[16909]|CNRMH351-12|Canada|Manitoba|633[0n]|BOLD:AAA7455
Enargia decolor|[16910]|CNWLQ395-13|Canada|Alberta|584[0n]|BOLD:AAA7455
Enargia decolor|[16911]|CNEIG1484-13|Canada|Alberta|606[0n]|BOLD:AAA7455
Enargia decolor|[16912]|LOWCC001-05|Canada|British Columbia|658[0n]|BOLD:AAA7455
Enargia decolor|[16913]|RDMAB663-06|Canada|Alberta|639[2n]|BOLD:AAA7455
Enargia decolor|[16914]|CNPAG341-13|Canada|Saskatchewan|637[0n]|BOLD:AAA7455
Enargia decolor|[16915]|CNWLP386-13|Canada|Alberta|599[0n]|BOLD:AAA7455
Enargia decolor|[16916]|CNWBD030-13|Canada|Alberta|604[0n]|BOLD:AAA7455
Enargia decolor|[16917]|CNRMG771-12|Canada|Manitoba|633[0n]|BOLD:AAA7455
Enargia decolor|[16918]|CNRMG009-12|Canada|Manitoba|635[0n]|BOLD:AAA7455
Enargia decolor|[16919]|CNPAC658-13|Canada|Saskatchewan|614[0n]|BOLD:AAA7455
Enargia decolor|[16920]|CNPAC657-13|Canada|Saskatchewan|614[0n]|BOLD:AAA7455
Enargia decolor|[16921]|CNPAB331-13|Canada|Saskatchewan|614[0n]|BOLD:AAA7455
Enargia decolor|[16922]|CNRMG743-12|Canada|Manitoba|614[0n]|BOLD:AAA7455
Enargia decolor|[16923]|CNRMG731-12|Canada|Manitoba|614[0n]|BOLD:AAA7455
Enargia decolor|[16924]|CNRMG729-12|Canada|Manitoba|614[0n]|BOLD:AAA7455
Enargia decolor|[16925]|CNRMG758-12|Canada|Manitoba|615[0n]|BOLD:AAA7455
Enargia decolor|[16926]|CNRMG760-12|Canada|Manitoba|614[0n]|BOLD:AAA7455
Enargia decolor|[16927]|CNPAJ103-13|Canada|Saskatchewan|621[4n]|BOLD:AAA7455
Enargia decolor|[16928]|CNRMG772-12|Canada|Manitoba|614[0n]|BOLD:AAA7455
Enargia decolor|[16929]|CNRMG730-12|Canada|Manitoba|615[0n]|BOLD:AAA7455
Enargia decolor|[16930]|CNBPI569-13|Canada|Ontario|614[0n]|BOLD:AAA7455
Enargia decolor|[16931]|CNRMG736-12|Canada|Manitoba|621[0n]|BOLD:AAA7455
Enargia decolor|[16932]|CNRMG735-12|Canada|Manitoba|621[0n]|BOLD:AAA7455
Enargia decolor|[16933]|CNRMG745-12|Canada|Manitoba|618[0n]|BOLD:AAA7455
Enargia decolor|[16934]|CNRMG741-12|Canada|Manitoba|618[0n]|BOLD:AAA7455
Enargia decolor|[16935]|CNRMH352-12|Canada|Manitoba|633[0n]|BOLD:AAA7455
Enargia decolor|[16936]|CNRMG006-12|Canada|Manitoba|634[0n]|BOLD:AAA7455
Enargia decolor|[16937]|CNRMG733-12|Canada|Manitoba|633[0n]|BOLD:AAA7455
Enargia decolor|[16938]|CNRMG747-12|Canada|Manitoba|632[0n]|BOLD:AAA7455
Enargia decolor|[16939]|CNRMG768-12|Canada|Manitoba|632[0n]|BOLD:AAA7455
Enargia decolor|[16940]|CNRMG770-12|Canada|Manitoba|632[0n]|BOLD:AAA7455
Enargia decolor|[16941]|CNRMG773-12|Canada|Manitoba|633[0n]|BOLD:AAA7455
Enargia decolor|[16942]|CNRMG774-12|Canada|Manitoba|632[0n]|BOLD:AAA7455
Enargia decolor|[16943]|CNRMG775-12|Canada|Manitoba|632[0n]|BOLD:AAA7455
Enargia decolor|[16944]|CNRMG776-12|Canada|Manitoba|631[0n]|BOLD:AAA7455
Enargia decolor|[16945]|CNRMG777-12|Canada|Manitoba|632[0n]|BOLD:AAA7455
Enargia decolor|[16946]|CNRMH348-12|Canada|Manitoba|633[0n]|BOLD:AAA7455
Enargia decolor|[16947]|CNWLH460-12|Canada|Alberta|632[0n]|BOLD:AAA7455
Enargia decolor|[16948]|CNWLH461-12|Canada|Alberta|632[0n]|BOLD:AAA7455
Enargia decolor|[16949]|CNWLI319-12|Canada|Alberta|632[0n]|BOLD:AAA7455
Enargia decolor|[16950]|CNPAA047-13|Canada|Saskatchewan|632[0n]|BOLD:AAA7455
Enargia decolor|[16951]|CNPAG338-13|Canada|Saskatchewan|632[0n]|BOLD:AAA7455
Enargia decolor|[16952]|CNRMG765-12|Canada|Manitoba|633[0n]|BOLD:AAA7455
Enargia decolor|[16953]|CNRMG769-12|Canada|Manitoba|632[0n]|BOLD:AAA7455
Enargia decolor|[16954]|CNRMG762-12|Canada|Manitoba|633[0n]|BOLD:AAA7455
Enargia decolor|[16955]|CNRMG763-12|Canada|Manitoba|632[0n]|BOLD:AAA7455
Enargia decolor|[16956]|CNRMG752-12|Canada|Manitoba|633[0n]|BOLD:AAA7455
Enargia decolor|[16957]|CNRMG756-12|Canada|Manitoba|632[0n]|BOLD:AAA7455
Enargia decolor|[16958]|CNRMG744-12|Canada|Manitoba|632[0n]|BOLD:AAA7455
Enargia decolor|[16959]|CNRMG748-12|Canada|Manitoba|632[0n]|BOLD:AAA7455
Enargia decolor|[16960]|CNRMG738-12|Canada|Manitoba|632[0n]|BOLD:AAA7455
Enargia decolor|[16961]|CNRMG734-12|Canada|Manitoba|633[0n]|BOLD:AAA7455
Enargia decolor|[16962]|CNRMG727-12|Canada|Manitoba|633[0n]|BOLD:AAA7455
Enargia decolor|[16963]|CNRMG016-12|Canada|Manitoba|633[0n]|BOLD:AAA7455
Enargia decolor|[16964]|CNRMG007-12|Canada|Manitoba|631[0n]|BOLD:AAA7455
Enargia decolor|[16965]|CNRMG728-12|Canada|Manitoba|632[0n]|BOLD:AAA7455
Enargia decolor|[16966]|CNRMG761-12|Canada|Manitoba|633[1n]|BOLD:AAA7455
Enargia decolor|[16967]|LMDH011-11|United States|Minnesota|658[0n]|BOLD:AAA7455
Enargia decolor|[16968]|CNRMG764-12|Canada|Manitoba|630[0n]|BOLD:AAA7455
Enargia decolor|[16969]|CNRMG754-12|Canada|Manitoba|629[0n]|BOLD:AAA7455
Enargia decolor|[16970]|CNRMG015-12|Canada|Manitoba|629[0n]|BOLD:AAA7455
Enargia decolor|[16971]|CNRMG750-12|Canada|Manitoba|635[0n]|BOLD:AAA7455
Enargia decolor|[16972]|CNRMG766-12|Canada|Manitoba|635[0n]|BOLD:AAA7455
Enargia decolor|[16973]|CNRMG737-12|Canada|Manitoba|634[0n]|BOLD:AAA7455
Enargia decolor|[16974]|CNRMG739-12|Canada|Manitoba|635[0n]|BOLD:AAA7455
Enargia decolor|[16975]|CNRMG726-12|Canada|Manitoba|636[0n]|BOLD:AAA7455
Enargia decolor|[16976]|CNRMG017-12|Canada|Manitoba|636[0n]|BOLD:AAA7455
Enargia decolor|[16977]|CNRMG011-12|Canada|Manitoba|635[0n]|BOLD:AAA7455
Enargia decolor|[16978]|CNRMG008-12|Canada|Manitoba|634[0n]|BOLD:AAA7455
Enargia decolor|[16979]|CNRMG005-12|Canada|Manitoba|634[0n]|BOLD:AAA7455
Enargia decolor|[16980]|CNRMA927-12|Canada|Manitoba|635[0n]|BOLD:AAA7455
Enargia decolor|[16981]|CNRMA910-12|Canada|Manitoba|636[0n]|BOLD:AAA7455
Enargia decolor|[16982]|XAG969-05|Canada|Ontario|636[0n]|BOLD:AAA7455
Enargia decolor|[16983]|CNRMA917-12|Canada|Manitoba|638[0n]|BOLD:AAA7455
Enargia decolor|[16984]|CNRMG013-12|Canada|Manitoba|634[2n]|BOLD:AAA7455
Enargia decolor|[16985]|PHMO337-03|Canada|Ontario|639[1n]|BOLD:AAA7455
Enargia decolor|[16986]|RDNMI150-10|United States|Washington|658[0n]|BOLD:AAA7455
Enargia decolor|[16987]|RDNMI154-10|United States|Washington|658[0n]|BOLD:AAA7455
Enargia decolor|[16988]|TTMNB364-06|Canada|New Brunswick|658[4n]|BOLD:AAA7455
Enargia decolor|[16989]|LBCH2784-10|Canada|British Columbia|658[0n]|BOLD:AAA7455
Enargia decolor|[16990]|TTMNB365-06|Canada|New Brunswick|658[0n]|BOLD:AAA7455
Enargia decolor|[16991]|LOWCD174-06|Canada|British Columbia|658[0n]|BOLD:AAA7455

Enargia decolor|[16988]|TTMNB364-06|Canada|New Brunswick|658[4n]|BOLD:AAA7455
Enargia decolor|[16989]|LBCH2784-10|Canada|British Columbia|658[0n]|BOLD:AAA7455
Enargia decolor|[16990]|TTMNB365-06|Canada|New Brunswick|658[0n]|BOLD:AAA7455
Enargia decolor|[16991]|LOWCD174-06|Canada|British Columbia|658[0n]|BOLD:AAA7455
Enargia decolor|[16992]|RDMAB664-06|Canada|Alberta|658[0n]|BOLD:AAA7455
Enargia decolor|[16993]|RDLQB596-05|Canada|Quebec|658[0n]|BOLD:AAA7455
Enargia decolor|[16994]|LOWCC004-05|Canada|British Columbia|658[0n]|BOLD:AAA7455
Enargia decolor|[16995]|LOWCC003-05|Canada|British Columbia|658[0n]|BOLD:AAA7455
Enargia decolor|[16996]|XAH467-05|Canada|Ontario|658[0n]|BOLD:AAA7455
Enargia decolor|[16997]|XAH173-05|Canada|Ontario|658[0n]|BOLD:AAA7455
Enargia decolor|[16998]|XAH172-05|Canada|Ontario|658[0n]|BOLD:AAA7455
Enargia decolor|[16999]|XAG943-05|Canada|Ontario|658[0n]|BOLD:AAA7455
Enargia decolor|[17000]|XAG849-05|Canada|Ontario|658[0n]|BOLD:AAA7455
Enargia decolor|[17001]|LOWCC005-05|Canada|British Columbia|658[0n]|BOLD:AAA7455
Enargia decolor|[17002]|RDMAB665-06|Canada|Alberta|658[0n]|BOLD:AAA7455
Enargia decolor|[17003]|PHMO350-03|Canada|Ontario|639[0n]|BOLD:AAA7455
Enargia decolor|[17004]|LOWCD125-06|Canada|British Columbia|614[0n]|BOLD:AAA7455
Enargia decolor|[17005]|DSCNI038-07|Canada|Manitoba|655[0n]|BOLD:AAA7455
Enargia decolor|[17006]|LPMN995-09|Canada|Manitoba|658[0n]|BOLD:AAA7455
Enargia decolor|[17007]|RDNMH996-09|Canada|New Brunswick|658[0n]|BOLD:AAA7455
Enargia decolor|[17008]|LBCH2507-10|Canada|British Columbia|658[0n]|BOLD:AAA7455
Enargia decolor|[17009]|LBCH2543-10|Canada|British Columbia|658[0n]|BOLD:AAA7455
Enargia decolor|[17010]|LBCH2610-10|Canada|British Columbia|658[0n]|BOLD:AAA7455
Enargia decolor|[17011]|LBCH2683-10|Canada|British Columbia|658[0n]|BOLD:AAA7455
Enargia decolor|[17012]|LBCH2781-10|Canada|British Columbia|658[0n]|BOLD:AAA7455
Enargia decolor|[17013]|LBCH2782-10|Canada|British Columbia|658[0n]|BOLD:AAA7455
Enargia decolor|[17014]|LBCH2783-10|Canada|British Columbia|658[0n]|BOLD:AAA7455
Enargia decolor|[17015]|LBCH2785-10|Canada|British Columbia|658[0n]|BOLD:AAA7455
Enargia decolor|[17016]|LBCH2786-10|Canada|British Columbia|658[0n]|BOLD:AAA7455
Enargia decolor|[17017]|LBCH2787-10|Canada|British Columbia|658[0n]|BOLD:AAA7455
Enargia decolor|[17018]|LBCH2788-10|Canada|British Columbia|658[0n]|BOLD:AAA7455
Enargia decolor|[17019]|LBCH2820-10|Canada|British Columbia|658[0n]|BOLD:AAA7455
Enargia decolor|[17020]|LBCH2837-10|Canada|British Columbia|658[0n]|BOLD:AAA7455
Enargia decolor|[17021]|BBLPB244-10|Canada|Saskatchewan|658[0n]|BOLD:AAA7455
Enargia decolor|[17022]|BBLPB246-10|Canada|Saskatchewan|658[0n]|BOLD:AAA7455
Enargia decolor|[17023]|BBLPB248-10|Canada|Saskatchewan|658[0n]|BOLD:AAA7455
Enargia decolor|[17024]|CNRMA368-12|Canada|Manitoba|637[0n]|BOLD:AAA7455
Enargia decolor|[17025]|CNRMH350-12|Canada|Manitoba|637[0n]|BOLD:AAA7455
Enargia decolor|[17026]|LPMN994-09|Canada|Manitoba|658[0n]|BOLD:AAA7455
Enargia decolor|[17027]|CNRMG757-12|Canada|Manitoba|622[0n]|BOLD:AAA7455
Enargia decolor|[17028]|CNRMH354-12|Canada|Manitoba|632[0n]|BOLD:AAA7455
Enargia decolor|[17029]|CNPAG339-13|Canada|Saskatchewan|633[0n]|BOLD:AAA7455
Enargia decolor|[17030]|CNWLI322-12|Canada|Alberta|632[0n]|BOLD:AAA7455
Enargia decolor|[17031]|CNWLI324-12|Canada|Alberta|632[0n]|BOLD:AAA7455
Enargia decolor|[17032]|CNPAG340-13|Canada|Saskatchewan|633[0n]|BOLD:AAA7455
Enargia decolor|[17033]|CNPAG342-13|Canada|Saskatchewan|633[0n]|BOLD:AAA7455
Enargia decolor|[17034]|CNRMG732-12|Canada|Manitoba|615[0n]|BOLD:AAA7455
Enargia decolor|[17035]|CNRMG746-12|Canada|Manitoba|614[0n]|BOLD:AAA7455
Enargia decolor|[17036]|CNRMG740-12|Canada|Manitoba|621[0n]|BOLD:AAA7455
Enargia decolor|[17037]|CNRMA921-12|Canada|Manitoba|630[0n]|BOLD:AAA7455
Enargia decolor|[17038]|CNRMG014-12|Canada|Manitoba|634[0n]|BOLD:AAA7455
Enargia decolor|[17039]|CNRMG012-12|Canada|Manitoba|634[0n]|BOLD:AAA7455
Enargia decolor|[17040]|CNRMG010-12|Canada|Manitoba|640[0n]|BOLD:AAA7455
Enargia decolor|[17041]|BBLPB241-10|Canada|Saskatchewan|658[0n]|BOLD:AAA7455
Enargia decolor|[17042]|CNRMG767-12|Canada|Manitoba|632[0n]|BOLD:AAA7455
Enargia decolor|[17043]|CNWBH1366-13|Canada|Alberta|619[0n]|BOLD:AAA7455
Enargia decolor|[17044]|LOWCC002-05|Canada|British Columbia|658[0n]|BOLD:AAA7455
Enargia decolor|[17045]|BBLPB242-10|Canada|Saskatchewan|658[0n]|BOLD:AAA7455
Enargia decolor|[17046]|CNRMG759-12|Canada|Manitoba|632[0n]|BOLD:AAA7455
Enargia decolor|[17047]|CNPAO662-13|Canada|Saskatchewan|594[0n]|BOLD:AAA7455
Enargia decolor|[17048]|LPMNB320-09|Canada|Manitoba|658[0n]|BOLD:AAA7455
Enargia decolor|[17049]|CNRMG742-12|Canada|Manitoba|632[0n]|BOLD:AAA7455
Enargia decolor|[17050]|CNWLP385-13|Canada|Alberta|602[0n]|BOLD:AAA7455
Enargia decolor|[17051]|CNWLQ396-13|Canada|Alberta|623[1n]|BOLD:AAA7455
Andropolia diversilineata|[17052]|RDNMF315-08|United States|Wyoming|658[0n]|BOLD:AAE1573
Andropolia diversilineata|[17053]|JMMMB362-11|United States|California|658[0n]|BOLD:AAE1573
Andropolia diversilineata|[17054]|RDNMF312-08|United States|Nevada|658[0n]|BOLD:AAE1573
Andropolia diversilineata|[17055]|RDNMF313-08|United States|Nevada|658[0n]|BOLD:AAE1573
Andropolia diversilineata|[17056]|BBLOC994-11|United States|California|658[0n]|BOLD:AAE1573
Andropolia diversilineata|[17057]|BBLOC1215-11|United States|California|658[0n]|BOLD:AAE1573
Andropolia diversilineata|[17058]|BBLOC1375-11|United States|California|658[0n]|BOLD:AAE1573
Andropolia diversilineata|[17059]|BBLOC1369-11|United States|California|658[0n]|BOLD:AAE1573
Andropolia diversilineata|[17060]|BBLOC1214-11|United States|California|658[0n]|BOLD:AAE1573
Andropolia diversilineata|[17061]|RDNMF314-08|United States|California|658[0n]|BOLD:AAE1573
Andropolia diversilineata|[17062]|NAMUM165-08|United States|California|658[0n]|BOLD:AAE1573
Andropolia diversilineata|[17063]|BBLOC1377-11|United States|California|628[0n]|BOLD:AAE1573
Andropolia diversilineata|[17064]|BBLOC1381-11|United States|California|658[0n]|BOLD:AAE1573
Rhizagrotis stylata|[17065]|RDNMF092-08|United States|Colorado|658[0n]|BOLD:AAE1530
Rhizagrotis stylata|[17066]|RDNMF004-08|United States|Colorado|658[0n]|BOLD:AAE1530
Rhizagrotis stylata|[17067]|RDNME601-08|United States|Colorado|658[0n]|BOLD:AAE1530
Rhizagrotis stylata|[17068]|NAMUM275-08|United States|California|658[0n]|BOLD:AAE1530
Rhizagrotis stylata|[17069]|RDNMG440-08|United States|Utah|658[0n]|BOLD:AAE1530
Rhizagrotis stylata|[17070]|RDNMG442-08|United States|Utah|636[0n]|BOLD:AAE1530
Rhizagrotis cloanthoides|[17071]|RDNMD647-06|United States|New Mexico|658[0n]|BOLD:AAE3645
Rhizagrotis cloanthoides|[17072]|RDNMD648-06|United States|New Mexico|658[0n]|BOLD:AAE3645
Rhizagrotis cloanthoides|[17073]|RDNMG468-08|United States|Oregon|658[0n]|BOLD:AAE3645
Rhizagrotis cloanthoides|[17074]|RDNMG469-08|United States|Oregon|658[0n]|BOLD:AAE3645
Rhizagrotis cloanthoides|[17075]|RDNMG470-08|United States|Oregon|658[0n]|BOLD:AAE3645
Rhizagrotis albalis|[17076]|RDNMG441-08|United States|Colorado|658[0n]|BOLD:AAF7150
Rhizagrotis albalis|[17077]|BBLSX617-09|United States|Arizona|658[0n]|BOLD:AAF7150
Rhizagrotis albalis|[17078]|RDNMD645-06|United States|New Mexico|658[0n]|BOLD:AAF7150
Rhizagrotis albalis|[17079]|BBLSW413-09|United States|New Mexico|658[0n]|BOLD:AAF7150
Rhizagrotis albalis|[17080]|BBLSX618-09|United States|Arizona|658[0n]|BOLD:AAF7150
Rhizagrotis albalis|[17081]|BBLOC146-11|United States|Texas|658[0n]|BOLD:AAF7150
Rhizagrotis modesta|[17082]|NAMUM191-08|United States|California|647[0n]|BOLD:AAF5146
Rhizagrotis modesta|[17083]|NAMUM192-08|United States|California|650[0n]|BOLD:AAF5146
Rhizagrotis modesta|[17084]|NAMUM193-08|United States|California|650[0n]|BOLD:AAF5146
Andropolia contacta|[17085]|LBCD248-05|Canada|British Columbia|616[1n]|BOLD:AAC6243
Andropolia contacta|[17086]|LBCD421-05|Canada|British Columbia|632[0n]|BOLD:AAC6243
Andropolia contacta|[17087]|RDLQ487-07|Canada|Quebec|618[0n]|BOLD:AAC6243
Andropolia contacta|[17088]|RDLQ488-07|Canada|Quebec|618[0n]|BOLD:AAC6243
Andropolia contacta|[17089]|RDLQ489-07|Canada|Quebec|594[0n]|BOLD:AAC6243
Andropolia contacta|[17090]|LCHIP104-07|Canada|Manitoba|649[0n]|BOLD:AAC6243
[illegible]

Andropolia contacta|[17088]|RDLQ488-07|Canada|Quebec|618[0n]|BOLD:AAC6243
Andropolia contacta|[17089]|RDLQ489-07|Canada|Quebec|594[0n]|BOLD:AAC6243
Andropolia contacta|[17090]|LCHIP104-07|Canada|Manitoba|649[0n]|BOLD:AAC6243
Andropolia contacta|[17091]|RDLQB574-05|Canada|Quebec|658[0n]|BOLD:AAC6243
Andropolia contacta|[17092]|RDMAB507-06|Canada|Alberta|658[0n]|BOLD:AAC6243
Andropolia contacta|[17093]|DSCNI034-07|Canada|Manitoba|658[0n]|BOLD:AAC6243
Andropolia contacta|[17094]|LPABC957-09|Canada|Alberta|658[0n]|BOLD:AAC6243
Andropolia contacta|[17095]|CNWLG951-12|Canada|Alberta|641[0n]|BOLD:AAC6243
Andropolia contacta|[17096]|UAMIC549-13|United States|Alaska|551[1n]|BOLD:AAC6243
Andropolia contacta|[17097]|UAMIC561-13|United States|Alaska|550[0n]|BOLD:AAC6243
Andropolia aedon|[17098]|LOPN135-06|United States|Oregon|529[0n]|BOLD:ADA4376
Andropolia olga|[17099]|RDNME938-08|United States|California|658[0n]|BOLD:ADA4376
Andropolia olga|[17100]|RDNMI126-10|United States|Nevada|658[0n]|BOLD:ADA4376
Andropolia aedon|[17101]|BBLOC202-11|United States|Texas|658[0n]|BOLD:ADA4376
Andropolia aedon|[17102]|BBLOC213-11|United States|Texas|658[0n]|BOLD:ADA4376
Andropolia aedon|[17103]|LOPN136-06|United States|Oregon|557[0n]|BOLD:ADA4376
Andropolia aedon|[17104]|LOPN134-06|United States|Oregon|658[0n]|BOLD:ADA4376
Andropolia aedon|[17105]|RDMAB506-06|Canada|Alberta|631[0n]|BOLD:ADA4376
Andropolia aedon|[17106]|LBCH7327-10|Canada|British Columbia|658[0n]|BOLD:ADA4376
Andropolia aedon|[17107]|LALPA618-10|Canada|British Columbia|658[0n]|BOLD:ADA4376
Andropolia aedon|[17108]|LALPA637-10|Canada|British Columbia|658[0n]|BOLD:ADA4376
Andropolia aedon|[17109]|LALPA1202-11|Canada|British Columbia|658[0n]|BOLD:ADA4376
Andropolia theodori|[17110]|RDNM552-05|United States|Washington|658[0n]|BOLD:ADA4376
Andropolia theodori|[17111]|LBCH7551-10|Canada|British Columbia|634[0n]|BOLD:ADA4376
Andropolia theodori|[17112]|LBCH7749-10|Canada|British Columbia|658[0n]|BOLD:ADA4376
Andropolia theodori|[17113]|LALPA667-10|Canada|British Columbia|658[0n]|BOLD:ADA4376
Andropolia theodori|[17114]|RDMAB505-06|Canada|Alberta|658[0n]|BOLD:ADA4376
Andropolia theodori|[17115]|RDNM551-05|United States|California|658[0n]|BOLD:ADA4376
Andropolia theodori|[17116]|RDNMI124-10|United States|California|658[0n]|BOLD:ADA4376
Andropolia theodori|[17117]|RDNMI125-10|United States|California|658[0n]|BOLD:ADA4376
Andropolia theodori|[17118]|RDNM549-05|United States|Oregon|658[0n]|BOLD:ADA4376
Andropolia theodori|[17119]|RDNM550-05|United States|Oregon|658[0n]|BOLD:ADA4376
Andropolia theodori|[17120]|RDNMI129-10|United States|Arizona|658[0n]|BOLD:ADA4376
Andropolia theodori|[17121]|RDNMK151-11|United States|Utah|549[0n]|BOLD:ADA4376
Andropolia olorina|[17122]|NAMUM155-08|United States|California|634[0n]|BOLD:ADA4376
Andropolia olorina|[17123]|RDNMI128-10|United States|California|658[0n]|BOLD:ADA4376
Andropolia olorina|[17124]|CNCLB1548-14|United States|California|658[0n]|BOLD:ADA4376
Andropolia olorina|[17125]|CGLCA060-10|United States|California|658[0n]|BOLD:ADA4376
Andropolia olorina|[17126]|RDNMI127-10|United States|California|658[0n]|BOLD:ADA4376
Andropolia olorina|[17127]|CNCLB1549-14|United States|California|658[0n]|BOLD:ADA4376
Homoglaea californica|[17128]|RDNMF210-08|United States|Oregon|609[1n]|BOLD:AAI8247
Homoglaea californica|[17129]|RDNMG890-08|Canada|British Columbia|658[0n]|BOLD:AAI8247
Xylena nupera|[17130]|RDLQH092-06|Canada|Quebec|658[0n]|BOLD:AAD3567
Xylena nupera|[17131]|RDLQH091-06|Canada|Quebec|658[0n]|BOLD:AAD3567
Xylena nupera|[17132]|RDLQH089-06|Canada|Quebec|658[0n]|BOLD:AAD3567
Xylena nupera|[17133]|LCHIP182-07|Canada|Manitoba|641[0n]|BOLD:AAD3567
Xylena nupera|[17134]|LOWCC040-05|Canada|British Columbia|658[0n]|BOLD:AAD3566
Xylena nupera|[17135]|LALPA062-10|Canada|British Columbia|658[0n]|BOLD:AAD3566
Xylena nupera|[17136]|LOWCC212-05|Canada|British Columbia|658[0n]|BOLD:AAD3566
Xylena nupera|[17137]|RWWA038-09|United States|Washington|658[0n]|BOLD:AAD3566
Xylena nupera|[17138]|LALPA1050-11|Canada|British Columbia|658[0n]|BOLD:AAD3566
Xylena brucei|[17139]|RDNMG410-08|United States|Wyoming|658[0n]|BOLD:AAE4681
Xylena brucei|[17140]|LOWCC213-05|Canada|British Columbia|658[0n]|BOLD:AAE4681
Xylena brucei|[17141]|LOWCD925-06|Canada|British Columbia|576[0n]|BOLD:AAE4681
Xylena brucei|[17142]|RDNMG411-08|United States|Wyoming|658[0n]|BOLD:AAE4681
Xylena brucei|[17143]|JMMMB358-11|United States|California|658[0n]|BOLD:AAE4681
Xylena curvimacula|[17144]|LMIS014-05|Canada|Ontario|658[0n]|BOLD:ABZ7519
Xylena curvimacula|[17145]|XAF293-05|Canada|Ontario|658[0n]|BOLD:ABZ7519
Xylena curvimacula|[17146]|LOWCC211-05|Canada|British Columbia|618[0n]|BOLD:ABZ7519
Xylena curvimacula|[17147]|LOWCE065-06|Canada|British Columbia|658[0n]|BOLD:ABZ7519
Xylena curvimacula|[17148]|LOWCE064-06|Canada|British Columbia|658[0n]|BOLD:ABZ7519
Xylena curvimacula|[17149]|LOWCE061-06|Canada|British Columbia|658[0n]|BOLD:ABZ7519
Xylena curvimacula|[17150]|LOWCE060-06|Canada|British Columbia|658[0n]|BOLD:ABZ7519
Xylena curvimacula|[17151]|LOWCD296-06|Canada|British Columbia|658[0n]|BOLD:ABZ7519
Xylena curvimacula|[17152]|LOWCC024-05|Canada|British Columbia|658[0n]|BOLD:ABZ7519
Xylena curvimacula|[17153]|LOWCC022-05|Canada|British Columbia|658[0n]|BOLD:ABZ7519
Xylena curvimacula|[17154]|LOWCC021-05|Canada|British Columbia|658[0n]|BOLD:ABZ7519
Xylena curvimacula|[17155]|RDLQH085-06|Canada|Quebec|621[0n]|BOLD:ABZ7519
Xylena curvimacula|[17156]|PMG165-03|Canada|Ontario|617[0n]|BOLD:ABZ7519
Xylena curvimacula|[17157]|RDLQ499-07|Canada|Quebec|621[0n]|BOLD:ABZ7519
Xylena curvimacula|[17158]|LOWCC020-05|Canada|British Columbia|555[0n]|BOLD:ABZ7519
Xylena curvimacula|[17159]|RDLQ500-07|Canada|Quebec|601[0n]|BOLD:ABZ7519
Xylena curvimacula|[17160]|LALPA031-10|Canada|British Columbia|658[0n]|BOLD:ABZ7519
Xylena curvimacula|[17161]|LMDH067-11|United States|Minnesota|658[0n]|BOLD:ABZ7519
Xylena curvimacula|[17162]|LALPA1024-11|Canada|British Columbia|658[0n]|BOLD:ABZ7519
Xylena curvimacula|[17163]|LALPA1043-11|Canada|British Columbia|658[0n]|BOLD:ABZ7519
Xylena cineritia|[17164]|LOWCD922-06|Canada|British Columbia|658[0n]|BOLD:ABZ7519
Xylena cineritia|[17165]|LOWCE412-06|Canada|British Columbia|587[1n]|BOLD:ABZ7519
Xylena cineritia|[17166]|LOWCE057-06|Canada|British Columbia|658[0n]|BOLD:ABZ7519
Xylena cineritia|[17167]|LOWCE207-06|Canada|British Columbia|632[0n]|BOLD:ABZ7519
Xylena cineritia|[17168]|LBCH4991-10|Canada|British Columbia|658[0n]|BOLD:ABZ7519
Xylena cineritia|[17169]|LOWCE209-06|Canada|British Columbia|658[0n]|BOLD:ABZ7519
Xylena cineritia|[17170]|LOWCE205-06|Canada|British Columbia|658[0n]|BOLD:ABZ7519
Xylena cineritia|[17171]|LOWCD923-06|Canada|British Columbia|658[0n]|BOLD:ABZ7519
Xylena cineritia|[17172]|LOWCD921-06|Canada|British Columbia|658[0n]|BOLD:ABZ7519
Xylena cineritia|[17173]|LOWCE356-06|Canada|British Columbia|577[0n]|BOLD:ABZ7519
Xylena cineritia|[17174]|LOWCE360-06|Canada|British Columbia|604[0n]|BOLD:ABZ7519
Xylena cineritia|[17175]|LOWCE411-06|Canada|British Columbia|608[0n]|BOLD:ABZ7519
Xylena cineritia|[17176]|JMMMB357-11|United States|California|658[0n]|BOLD:ABZ7519
Xylena cineritia|[17177]|LOWCE059-06|Canada|British Columbia|658[0n]|BOLD:ABZ7519
Xylena cineritia|[17178]|LOWCD924-06|Canada|British Columbia|658[0n]|BOLD:ABZ7519
Xylena cineritia|[17179]|LOWCB541-05|Canada|British Columbia|658[0n]|BOLD:ABZ7519
Xylena cineritia|[17180]|LOWCB542-05|Canada|British Columbia|658[0n]|BOLD:ABZ7519
Xylena cineritia|[17181]|LOWCC210-05|Canada|British Columbia|658[0n]|BOLD:ABZ7519
Xylena cineritia|[17182]|LOWCD920-06|Canada|British Columbia|658[0n]|BOLD:ABZ7519
Xylena cineritia|[17183]|LOWCE058-06|Canada|British Columbia|658[0n]|BOLD:ABZ7519
Xylena cineritia|[17184]|LOWCE465-06|Canada|British Columbia|658[0n]|BOLD:ABZ7519
Xylena|[17185]|SSJAB4203-13|Canada|Alberta|610[0n]|BOLD:ABZ7519
Xylena thoracica|[17186]|DSCNI032-07|Canada|Manitoba|658[0n]|BOLD:ACF2663
Xylena thoracica|[17187]|DSCNI033-07|Canada|Manitoba|658[0n]|BOLD:ACF2663
Xylena thoracica|[17188]|LOWCD926-06|Canada|British Columbia|658[0n]|BOLD:ACF2663
Xylena thoracica|[17189]|LOWCD708-06|Canada|British Columbia|658[0n]|BOLD:ACF2663
Xylena thoracica|[17190]|RDNME237-07|Canada|Alberta|618[0n]|BOLD:ACF2663
[illegible]

Xylena thoracica|[17188]|LOWCD926-06|Canada|British Columbia|658[0n]|BOLD:ACF2663
Xylena thoracica|[17189]|LOWCD708-06|Canada|British Columbia|658[0n]|BOLD:ACF2663
Xylena thoracica|[17190]|RDNME237-07|Canada|Alberta|618[0n]|BOLD:ACF2663
Xylena thoracica|[17191]|LBCG467-08|Canada|British Columbia|658[0n]|BOLD:ACF2663
Xylena thoracica|[17192]|RDNMG969-08|United States|California|658[0n]|BOLD:ACF2663
Xylena thoracica|[17193]|LBCH4992-10|Canada|British Columbia|658[0n]|BOLD:ACF2663
Xylena thoracica|[17194]|LBCH5342-10|Canada|British Columbia|658[0n]|BOLD:ACF2663
Xylena thoracica|[17195]|LALPA001-10|Canada|British Columbia|658[0n]|BOLD:ACF2663
Xylena thoracica|[17196]|LALPA030-10|Canada|British Columbia|658[0n]|BOLD:ACF2663
Xylena thoracica|[17197]|RDNME238-07|Canada|Alberta|658[0n]|BOLD:AAB5195
Xylena thoracica|[17198]|TMNBB224-06|Canada|New Brunswick|658[0n]|BOLD:AAB5195
Xylena thoracica|[17199]|TMNBB223-06|Canada|New Brunswick|658[0n]|BOLD:AAB5195
Xylena thoracica|[17200]|TTMNB581-06|Canada|New Brunswick|658[0n]|BOLD:AAB5195
Xylena thoracica|[17201]|LOWCD712-06|Canada|British Columbia|658[0n]|BOLD:AAB5195
Xylena thoracica|[17202]|LOWCD710-06|Canada|British Columbia|658[0n]|BOLD:AAB5195
Xylena thoracica|[17203]|LOWCD709-06|Canada|British Columbia|658[0n]|BOLD:AAB5195
Xylena thoracica|[17204]|LOWCD707-06|Canada|British Columbia|658[0n]|BOLD:AAB5195
Xylena thoracica|[17205]|LOWCD706-06|Canada|British Columbia|658[0n]|BOLD:AAB5195
Xylena thoracica|[17206]|LOWCD705-06|Canada|British Columbia|658[0n]|BOLD:AAB5195
Xylena thoracica|[17207]|TMNBB222-06|Canada|New Brunswick|658[0n]|BOLD:AAB5195
Xylena thoracica|[17208]|LOWCD711-06|Canada|British Columbia|654[0n]|BOLD:AAB5195
Xylena thoracica|[17209]|LOWCB543-05|Canada|British Columbia|511[1n]|BOLD:AAB5195
Xylena thoracica|[17210]|RDLQ501-07|Canada|Ontario|597[0n]|BOLD:AAB5195
Xylena thoracica|[17211]|CNBAB411-12|Canada|Alberta|624[0n]|BOLD:AAB5195
Xylena thoracica|[17212]|SSJAB4206-13|Canada|Alberta|608[0n]|BOLD:AAB5195
Aseptis serrula|[17213]|RDNME033-07|United States|California|572[0n]|BOLD:AAK0112
Aseptis perfumosa|[17214]|GMLC682-11|United States|California|658[0n]|BOLD:AAB1913
Aseptis perfumosa|[17215]|LOCBC265-06|United States|California|658[0n]|BOLD:AAB1913
Aseptis perfumosa|[17216]|LOCBC266-06|United States|California|658[0n]|BOLD:AAB1913
Aseptis perfumosa|[17217]|LOCBC399-06|United States|California|658[0n]|BOLD:AAB1913
Aseptis perfumosa|[17218]|RDNMC587-06|United States|California|658[0n]|BOLD:AAB1913
Aseptis perfumosa|[17219]|RDNMC588-06|United States|California|658[0n]|BOLD:AAB1913
Aseptis perfumosa|[17220]|RDNMC589-06|United States|California|658[0n]|BOLD:AAB1913
Aseptis perfumosa|[17221]|LOCBB869-06|United States|California|658[0n]|BOLD:AAB1913
Aseptis perfumosa|[17222]|LOCBB870-06|United States|California|658[0n]|BOLD:AAB1913
Aseptis perfumosa|[17223]|LOCBC232-06|United States|California|658[0n]|BOLD:AAB1913
Aseptis perfumosa|[17224]|LOCBC251-06|United States|California|658[0n]|BOLD:AAB1913
Aseptis perfumosa|[17225]|LOCBC252-06|United States|California|658[0n]|BOLD:AAB1913
Aseptis perfumosa|[17226]|LOCBC254-06|United States|California|658[0n]|BOLD:AAB1913
Aseptis perfumosa|[17227]|LOCBC256-06|United States|California|658[0n]|BOLD:AAB1913
Aseptis perfumosa|[17228]|LOCBC257-06|United States|California|658[0n]|BOLD:AAB1913
Aseptis perfumosa|[17229]|LOCBC258-06|United States|California|658[0n]|BOLD:AAB1913
Aseptis perfumosa|[17230]|LOCBC260-06|United States|California|658[0n]|BOLD:AAB1913
Aseptis perfumosa|[17231]|LOCBC262-06|United States|California|658[0n]|BOLD:AAB1913
Aseptis perfumosa|[17232]|LOCBC263-06|United States|California|658[0n]|BOLD:AAB1913
Aseptis perfumosa|[17233]|LOCBC264-06|United States|California|658[0n]|BOLD:AAB1913
Aseptis perfumosa|[17234]|GMLC550-11|United States|California|658[0n]|BOLD:AAB1913
Aseptis perfumosa|[17235]|GMLC595-11|United States|California|658[0n]|BOLD:AAB1913
Aseptis perfumosa|[17236]|IAWLB444-11|United States|California|658[0n]|BOLD:AAB1913
Aseptis perfumosa|[17237]|GMLC706-11|United States|California|658[0n]|BOLD:AAB1913
Aseptis perfumosa|[17238]|GMLC710-11|United States|California|658[0n]|BOLD:AAB1913
Aseptis perfumosa|[17239]|GMLC762-12|United States|California|658[0n]|BOLD:AAB1913
Aseptis perfumosa|[17240]|RDNME026-07|United States|California|609[0n]|BOLD:AAB1913
Aseptis perfumosa|[17241]|LOCBC253-06|United States|California|658[0n]|BOLD:AAB1913
Aseptis perfumosa|[17242]|LOCBC261-06|United States|California|658[0n]|BOLD:AAB1913
Aseptis perfumosa|[17243]|GMLC676-11|United States|California|658[0n]|BOLD:AAB1913
Aseptis perfumosa|[17244]|LOCBD210-06|United States|California|658[0n]|BOLD:AAB1913
Aseptis perfumosa|[17245]|LOCBC259-06|United States|California|658[0n]|BOLD:AAB1913
Aseptis perfumosa|[17246]|LOCBC255-06|United States|California|658[0n]|BOLD:AAB1913
Aseptis perfumosa|[17247]|LOCBC250-06|United States|California|658[0n]|BOLD:AAB1913
Aseptis perfumosa|[17248]|LOCBC231-06|United States|California|658[0n]|BOLD:AAB1913
Aseptis perfumosa|[17249]|LOCBB871-06|United States|California|658[0n]|BOLD:AAB1913
Aseptis perfumosa|[17250]|LOCBC089-06|United States|California|607[0n]|BOLD:AAB1913
Aseptis perfumosa|[17251]|CNCLB1535-14|United States|California|591[0n]|BOLD:AAB1913
Aseptis susquesa|[17252]|RDNME002-07|United States|California|603[0n]|BOLD:AAJ3115
Aseptis susquesa|[17253]|RDNME001-07|United States|California|609[0n]|BOLD:AAJ3115
Aseptis susquesa|[17254]|RDNME038-07|United States|California|609[0n]|BOLD:AAJ3115
Aseptis catalina|[17255]|RDNMC656-06|United States|California|658[0n]|BOLD:AAD2465
Aseptis catalina|[17256]|USLEP760-10|United States|Arizona|658[0n]|BOLD:AAD2465
Aseptis catalina|[17257]|USLEP131-10|United States|Arizona|658[0n]|BOLD:AAD2465
Aseptis catalina|[17258]|USLEP134-10|United States|Arizona|658[0n]|BOLD:AAD2465
Aseptis catalina|[17259]|USLEP135-10|United States|Arizona|658[0n]|BOLD:AAD2465
Aseptis catalina|[17260]|USLEP132-10|United States|Arizona|658[0n]|BOLD:AAD2465
Aseptis catalina|[17261]|RDNMC653-06|United States|California|658[0n]|BOLD:AAD2465
Aseptis catalina|[17262]|RDNMC657-06|United States|Arizona|658[0n]|BOLD:AAD2465
Aseptis catalina|[17263]|RDNMC655-06|United States|California|658[0n]|BOLD:AAD2465
Aseptis catalina|[17264]|RDNMC654-06|United States|California|658[0n]|BOLD:AAD2465
Aseptis catalina|[17265]|RDNMC652-06|United States|California|658[0n]|BOLD:AAD2465
Aseptis catalina|[17266]|RDNMC658-06|United States|Arizona|658[1n]|BOLD:AAD2465
Aseptis catalina|[17267]|RDNME037-07|United States|California|609[0n]|BOLD:AAD2465
Aseptis catalina|[17268]|BBLSW192-09|United States|Arizona|658[0n]|BOLD:AAD2465
Aseptis catalina|[17269]|USLEP133-10|United States|Arizona|658[0n]|BOLD:AAD2465
Aseptis catalina|[17270]|USLEP136-10|United States|Arizona|658[0n]|BOLD:AAD2465
Aseptis catalina|[17271]|USLEP137-10|United States|Arizona|658[0n]|BOLD:AAD2465
Aseptis catalina|[17272]|USLEP138-10|United States|Arizona|658[0n]|BOLD:AAD2465
Aseptis catalina|[17273]|USLEP139-10|United States|Arizona|658[0n]|BOLD:AAD2465
Aseptis catalina|[17274]|LOCBF1064-13|United States|California|599[0n]|BOLD:AAD2465
Aseptis pseudolichena|[17275]|RDNMC608-06|United States|California|658[0n]|BOLD:AAD8383
Aseptis pseudolichena|[17276]|RDNMC612-06|United States|California|658[1n]|BOLD:AAD8383
Aseptis pseudolichena|[17277]|LOCBD293-06|United States|California|640[0n]|BOLD:AAD8383
Aseptis pseudolichena|[17278]|LOCBC324-06|United States|California|658[0n]|BOLD:AAD8383
Aseptis pseudolichena|[17279]|RDNMC659-06|United States|California|626[0n]|BOLD:AAD8383
Aseptis pseudolichena|[17280]|LOCBD413-06|United States|California|658[0n]|BOLD:AAD8383
Aseptis lichena|[17281]|RDNME035-07|United States|California|609[0n]|BOLD:AAK0146
Aseptis fanatica|[17282]|RDNMD823-07|United States|California|658[0n]|BOLD:ACF2527
Aseptis fanatica|[17283]|RDNMC631-06|United States|California|589[0n]|BOLD:ACF2527
Aseptis fanatica|[17284]|RDNME017-07|United States|California|609[0n]|BOLD:ACF2527
Aseptis fanatica|[17285]|RDNME080-07|United States|California|658[0n]|BOLD:ACF2527
Aseptis fanatica|[17286]|RDNME081-07|United States|California|658[0n]|BOLD:ACF2527
Aseptis fanatica|[17287]|IAWLB434-11|United States|California|658[0n]|BOLD:ACF2527
Aseptis sp. nr. fanatica|[17288]|RDNME082-07|United States|California|658[11n]|
Aseptis sp. nr. fanatica|[17289]|RDNMC640-06|United States|Oregon|658[0n]|BOLD:AAB5240
Aseptis sp. nr. fanatica|[17290]|LOPN137-06|United States|Oregon|574[1n]|BOLD:AAB5240
[illegible]

Aseptis sp. nr. fanatica|[17288]|RDNME082-07|United States|California|658[11n]|
Aseptis sp. nr. fanatica|[17289]|RDNMC640-06|United States|Oregon|658[0n]|BOLD:AAB5240
Aseptis sp. nr. fanatica|[17290]|LOPN137-06|United States|Oregon|574[1n]|BOLD:AAB5240
Aseptis sp. nr. fanatica|[17291]|LOPN138-06|United States|Oregon|658[0n]|BOLD:AAB5240
Aseptis sp. nr. fanatica|[17292]|GMLC789-12|United States|California|658[0n]|BOLD:AAB5240
Aseptis sp. nr. fanatica|[17293]|GMLC898-12|United States|California|658[0n]|BOLD:AAB5240
Aseptis sp. nr. fanatica|[17294]|GMLC923-12|United States|California|658[0n]|BOLD:AAB5240
Aseptis sp. nr. fanatica|[17295]|GMLC966-12|United States|California|658[0n]|BOLD:AAB5240
Aseptis sp. nr. fanatica|[17296]|GMLC967-12|United States|California|658[0n]|BOLD:AAB5240
Aseptis sp. nr. fanatica|[17297]|GMLC979-12|United States|California|658[0n]|BOLD:AAB5240
Aseptis sp. nr. fanatica|[17298]|GMLC986-12|United States|California|658[0n]|BOLD:AAB5240
Aseptis sp. nr. fanatica|[17299]|GMLC989-12|United States|California|658[0n]|BOLD:AAB5240
Aseptis sp. nr. fanatica|[17300]|GMLC993-12|United States|California|658[0n]|BOLD:AAB5240
Aseptis sp. nr. fanatica|[17301]|GMLC1096-12|United States|California|658[0n]|BOLD:AAB5240
Aseptis sp. nr. fanatica|[17302]|GMLC1108-12|United States|California|658[0n]|BOLD:AAB5240
Aseptis sp. nr. fanatica|[17303]|GMLC1174-12|United States|California|658[0n]|BOLD:AAB5240
Aseptis sp. nr. fanatica|[17304]|CNCLB1526-14|United States|California|571[0n]|BOLD:AAB5240
Aseptis sp. nr. fanatica|[17305]|LOPN140-06|United States|Oregon|658[0n]|BOLD:AAB5240
Aseptis sp. nr. fanatica|[17306]|RDNMC641-06|United States|California|658[2n]|BOLD:AAB5240
Aseptis sp. nr. fanatica|[17307]|RDNMC639-06|United States|Oregon|584[0n]|BOLD:AAB5240
Aseptis sp. nr. fanatica|[17308]|RDNME011-07|United States|California|609[0n]|BOLD:AAB5240
Aseptis sp. nr. fanatica|[17309]|CNCLB1530-14|United States|California|599[0n]|BOLD:AAB5240
Aseptis sp. nr. fanatica|[17310]|CNCLB1532-14|United States|California|658[0n]|BOLD:AAB5240
Aseptis ferruginea|[17311]|LOCBD556-06|United States|California|658[0n]|BOLD:ABZ6812
Aseptis ferruginea|[17312]|LOCBD418-06|United States|California|658[0n]|BOLD:ABZ6812
Aseptis ferruginea|[17313]|RDNMC634-06|United States|California|658[0n]|BOLD:ABZ6812
Aseptis ferruginea|[17314]|RDNMC630-06|United States|California|658[0n]|BOLD:ABZ6812
Aseptis ferruginea|[17315]|LOCBC881-06|United States|California|629[0n]|BOLD:ABZ6812
Aseptis ferruginea|[17316]|LOCBD557-06|United States|California|656[0n]|BOLD:ABZ6812
Aseptis ferruginea|[17317]|LOCBD558-06|United States|California|656[0n]|BOLD:ABZ6812
Aseptis ferruginea|[17318]|LOCBD655-06|United States|California|658[0n]|BOLD:ABZ6812
Aseptis ferruginea|[17319]|LOCBE350-06|United States|California|658[0n]|BOLD:ABZ6812
Aseptis fumeola|[17320]|LOCBC611-06|United States|California|658[0n]|BOLD:ACF1264
Aseptis fumeola|[17321]|RDNMC636-06|United States|California|658[0n]|BOLD:ACF1264
Aseptis fumeola|[17322]|RDNMD822-07|United States|California|658[0n]|BOLD:ACF1264
Aseptis fumeola|[17323]|LOCBD063-06|United States|California|658[0n]|BOLD:ACF1264
Aseptis fumeola|[17324]|RDNMC638-06|United States|California|658[0n]|BOLD:ACF1264
Aseptis fumeola|[17325]|LOCBE235-06|United States|California|648[1n]|BOLD:ACF1264
Aseptis fumeola|[17326]|RDNMC637-06|United States|California|568[0n]|BOLD:ACF1264
Aseptis fumeola|[17327]|LOCBE236-06|United States|California|594[0n]|BOLD:ACF1264
Aseptis fumeola|[17328]|RDNME007-07|United States|California|609[0n]|BOLD:ACF1264
Aseptis fumeola|[17329]|LOCBE347-06|United States|California|658[0n]|BOLD:ACF1264
Aseptis fumeola|[17330]|RDNME083-07|United States|California|658[0n]|BOLD:ACF1264
Aseptis ethnica|[17331]|LOPN139-06|United States|Oregon|570[2n]|BOLD:ACF1265
Aseptis ethnica|[17332]|LOPN212-06|United States|Oregon|604[0n]|BOLD:ACF1265
Aseptis ethnica|[17333]|LOPN213-06|United States|Oregon|615[0n]|BOLD:ACF1265
Aseptis ethnica|[17334]|LOCBE311-06|United States|California|658[0n]|BOLD:ACF2525
Aseptis ethnica|[17335]|LOCBE349-06|United States|California|601[2n]|BOLD:ACF2525
Aseptis ethnica|[17336]|LOCBC802-06|United States|California|658[0n]|BOLD:ACF2525
Aseptis ethnica|[17337]|LOCBC609-06|United States|California|658[0n]|BOLD:ACF2525
Aseptis ethnica|[17338]|RDNME016-07|United States|California|592[0n]|BOLD:ACF2525
Aseptis ethnica|[17339]|CNCLB1527-14|United States|California|615[0n]|BOLD:ACF2525
Aseptis ethnica|[17340]|RDNMC635-06|United States|California|658[0n]|BOLD:ACF2525
Aseptis ethnica|[17341]|CNCLB1528-14|United States|California|572[0n]|BOLD:ACF2525
Aseptis ethnica|[17342]|RDNMC642-06|United States|Oregon|576[1n]|BOLD:ACF2525
Aseptis ethnica|[17343]|LOCBC608-06|United States|California|643[0n]|BOLD:ACF2525
Aseptis ethnica|[17344]|RDNME019-07|United States|Oregon|609[0n]|BOLD:ACF2525
Aseptis ethnica|[17345]|CNCLB1529-14|United States|California|572[0n]|BOLD:ACF2525
Aseptis murina|[17346]|RDNMC632-06|United States|California|500[2n]|
Aseptis murina|[17347]|RDNMC633-06|United States|California|612[0n]|BOLD:ACF1263
Aseptis fanatica|[17348]|CNCLB1531-14|United States|California|547[0n]|BOLD:ACF2527
Aseptis fanatica|[17349]|CNCLB1533-14|United States|California|569[0n]|BOLD:ACF2527
Aseptis binotata|[17350]|LOWCB445-05|Canada|British Columbia|508[1n]|
Aseptis binotata|[17351]|RDNMC676-06|United States|Colorado|658[0n]|BOLD:AAA4141
Aseptis binotata|[17352]|RDNMK254-11|United States|Utah|658[0n]|BOLD:AAA4141
Aseptis binotata|[17353]|RDNMC660-06|United States|Utah|658[0n]|BOLD:AAA4141
Aseptis binotata|[17354]|RDNME036-07|United States|Colorado|609[0n]|BOLD:AAA4141
Aseptis binotata|[17355]|LOWCB439-05|Canada|British Columbia|658[0n]|BOLD:AAA4141
Aseptis binotata|[17356]|RDNMC624-06|United States|Wyoming|658[0n]|BOLD:AAA4141
Aseptis binotata|[17357]|RDNME020-07|United States|Wyoming|609[0n]|BOLD:AAA4141
Aseptis binotata|[17358]|RDNME031-07|Canada|British Columbia|609[0n]|BOLD:AAA4141
Aseptis binotata|[17359]|RDNME029-07|United States|Wyoming|609[0n]|BOLD:AAA4141
Aseptis binotata|[17360]|RDNMC647-06|United States|Washington|571[1n]|BOLD:AAA4141
Aseptis binotata|[17361]|RDNME022-07|United States|Wyoming|609[1n]|BOLD:AAA4141
Aseptis binotata|[17362]|LBCC645-05|Canada|British Columbia|658[0n]|BOLD:AAA4141
Aseptis binotata|[17363]|LOWCB442-05|Canada|British Columbia|658[0n]|BOLD:AAA4141
Aseptis binotata|[17364]|RDNMC723-06|United States|Colorado|658[0n]|BOLD:AAA4141
Aseptis binotata|[17365]|LOPN114-06|United States|Oregon|658[0n]|BOLD:AAA4141
Aseptis binotata|[17366]|LOPN115-06|United States|Oregon|658[0n]|BOLD:AAA4141
Aseptis binotata|[17367]|LOWCE859-06|Canada|British Columbia|658[0n]|BOLD:AAA4141
Aseptis binotata|[17368]|LOWCB450-05|Canada|British Columbia|658[0n]|BOLD:AAA4141
Aseptis binotata|[17369]|LOWCB451-05|Canada|British Columbia|658[0n]|BOLD:AAA4141
Aseptis binotata|[17370]|LOWCC205-05|Canada|British Columbia|658[0n]|BOLD:AAA4141
Aseptis binotata|[17371]|RDNMC626-06|United States|Colorado|658[0n]|BOLD:AAA4141
Aseptis binotata|[17372]|LOWCB443-05|Canada|British Columbia|658[0n]|BOLD:AAA4141
Aseptis binotata|[17373]|LOWCB444-05|Canada|British Columbia|658[0n]|BOLD:AAA4141
Aseptis binotata|[17374]|LBCD296-05|Canada|British Columbia|658[0n]|BOLD:AAA4141
Aseptis binotata|[17375]|LBCD424-05|Canada|British Columbia|658[0n]|BOLD:AAA4141
Aseptis binotata|[17376]|LOWCB446-05|Canada|British Columbia|658[0n]|BOLD:AAA4141
Aseptis binotata|[17377]|LOWCB447-05|Canada|British Columbia|658[0n]|BOLD:AAA4141
Aseptis binotata|[17378]|LOWCB448-05|Canada|British Columbia|658[0n]|BOLD:AAA4141
Aseptis binotata|[17379]|LOWCB449-05|Canada|British Columbia|658[0n]|BOLD:AAA4141
Aseptis binotata|[17380]|LHLEP432-06|Canada|British Columbia|658[0n]|BOLD:AAA4141
Aseptis binotata|[17381]|RDNME034-07|United States|Wyoming|658[0n]|BOLD:AAA4141
Aseptis binotata|[17382]|LPMN950-08|Canada|Alberta|658[0n]|BOLD:AAA4141
Aseptis binotata|[17383]|LPAB085-08|Canada|Alberta|658[0n]|BOLD:AAA4141
Aseptis binotata|[17384]|LPAB086-08|Canada|Alberta|658[0n]|BOLD:AAA4141
Aseptis binotata|[17385]|LPABB628-08|Canada|Alberta|658[0n]|BOLD:AAA4141
Aseptis binotata|[17386]|LPABC830-09|Canada|Alberta|658[0n]|BOLD:AAA4141
Aseptis binotata|[17387]|LPABC851-09|Canada|Alberta|658[0n]|BOLD:AAA4141
Aseptis binotata|[17388]|LPABC924-09|Canada|Alberta|658[0n]|BOLD:AAA4141
Aseptis binotata|[17389]|LPABC992-09|Canada|Alberta|658[0n]|BOLD:AAA4141
Aseptis binotata|[17390]|LALPA514-10|Canada|British Columbia|658[0n]|BOLD:AAA4141

Aseptis binotata|[17388]|LPABC924-09|Canada|Alberta|658[0n]|BOLD:AAA4141
Aseptis binotata|[17389]|LPABC992-09|Canada|Alberta|658[0n]|BOLD:AAA4141
Aseptis binotata|[17390]|LALPA514-10|Canada|British Columbia|658[0n]|BOLD:AAA4141
Aseptis binotata|[17391]|LALPA1193-11|Canada|British Columbia|658[0n]|BOLD:AAA4141
Aseptis binotata|[17392]|LBCD286-05|Canada|British Columbia|658[0n]|BOLD:AAA4141
Aseptis binotata|[17393]|LBCD295-05|Canada|British Columbia|658[0n]|BOLD:AAA4141
Aseptis binotata|[17394]|LBCD278-05|Canada|British Columbia|658[0n]|BOLD:AAA4141
Aseptis binotata|[17395]|LBCD285-05|Canada|British Columbia|658[0n]|BOLD:AAA4141
Aseptis binotata|[17396]|LBCD274-05|Canada|British Columbia|658[0n]|BOLD:AAA4141
Aseptis binotata|[17397]|LBCD276-05|Canada|British Columbia|658[0n]|BOLD:AAA4141
Aseptis binotata|[17398]|LBCC441-05|Canada|British Columbia|658[0n]|BOLD:AAA4141
Aseptis binotata|[17399]|LBCC764-05|Canada|British Columbia|658[0n]|BOLD:AAA4141
Aseptis binotata|[17400]|LBCC438-05|Canada|British Columbia|658[0n]|BOLD:AAA4141
Aseptis binotata|[17401]|LBCC437-05|Canada|British Columbia|658[0n]|BOLD:AAA4141
Aseptis binotata|[17402]|LBCC337-05|Canada|British Columbia|658[0n]|BOLD:AAA4141
Aseptis binotata|[17403]|LBCC333-05|Canada|British Columbia|658[0n]|BOLD:AAA4141
Aseptis binotata|[17404]|LBCC332-05|Canada|British Columbia|658[0n]|BOLD:AAA4141
Aseptis binotata|[17405]|LBCC330-05|Canada|British Columbia|658[0n]|BOLD:AAA4141
Aseptis binotata|[17406]|LBCC329-05|Canada|British Columbia|658[0n]|BOLD:AAA4141
Aseptis binotata|[17407]|LBCB062-05|Canada|British Columbia|658[0n]|BOLD:AAA4141
Aseptis binotata|[17408]|LBCD294-05|Canada|British Columbia|617[0n]|BOLD:AAA4141
Aseptis binotata|[17409]|LBCD283-05|Canada|British Columbia|616[0n]|BOLD:AAA4141
Aseptis binotata|[17410]|LBCC328-05|Canada|British Columbia|658[0n]|BOLD:AAA4141
Aseptis binotata|[17411]|LBCB061-05|Canada|British Columbia|658[0n]|BOLD:AAA4141
Aseptis binotata|[17412]|RDNMC627-06|United States|Colorado|644[0n]|BOLD:AAA4141
Aseptis binotata|[17413]|LBCD277-05|Canada|British Columbia|658[1n]|BOLD:AAA4141
Aseptis binotata|[17414]|RDNMC651-06|United States|Washington|532[0n]|BOLD:AAA4141
Aseptis binotata|[17415]|LOWCB440-05|Canada|British Columbia|590[2n]|BOLD:AAA4141
Aseptis binotata|[17416]|LOWCB441-05|Canada|British Columbia|587[0n]|BOLD:AAA4141
Aseptis binotata|[17417]|LOWCD527-06|Canada|British Columbia|623[0n]|BOLD:AAA4141
Aseptis binotata|[17418]|LHLEP433-06|Canada|British Columbia|658[0n]|BOLD:AAA4141
Aseptis binotata|[17419]|LHLEP570-06|Canada|British Columbia|658[0n]|BOLD:AAA4141
Aseptis binotata|[17420]|TML205-14|United States||658[0n]|BOLD:AAA4141
Aseptis binotata|[17421]|RWWA823-09|United States|Washington|658[0n]|BOLD:AAA4141
Aseptis binotata|[17422]|RWWA880-09|United States|Washington|658[0n]|BOLD:AAA4141
Aseptis binotata|[17423]|RWWA876-09|United States|Washington|658[0n]|BOLD:AAA4141
Aseptis binotata|[17424]|RWWA850-09|United States|Washington|658[0n]|BOLD:AAA4141
Aseptis binotata|[17425]|RWWA888-09|United States|Washington|658[0n]|BOLD:AAA4141
Aseptis binotata|[17426]|RWWA932-09|United States|Washington|658[0n]|BOLD:AAA4141
Aseptis binotata|[17427]|RWWB173-09|United States|Washington|658[0n]|BOLD:AAA4141
Aseptis binotata|[17428]|RWWA838-09|United States|Washington|658[0n]|BOLD:AAA4141
Aseptis binotata|[17429]|RWWA879-09|United States|Washington|658[0n]|BOLD:AAA4141
Aseptis binotata|[17430]|LALPA512-10|Canada|British Columbia|658[0n]|BOLD:AAA4141
Aseptis binotata|[17431]|LPVIB073-08|Canada|British Columbia|658[0n]|BOLD:AAA4141
Aseptis binotata|[17432]|NAGEO261-09|Canada|British Columbia|658[0n]|
Aseptis binotata|[17433]|LPVIB232-08|Canada|British Columbia|658[0n]|BOLD:AAA4141
Aseptis binotata|[17434]|LALPA530-10|Canada|British Columbia|658[0n]|BOLD:AAA4141
Aseptis binotata|[17435]|RWWA857-09|United States|Washington|658[0n]|BOLD:AAA4141
Aseptis binotata|[17436]|RWWA845-09|United States|Washington|658[0n]|BOLD:AAA4141
Aseptis binotata|[17437]|RWWA819-09|United States|Washington|658[0n]|BOLD:AAA4141
Aseptis binotata|[17438]|LPVIB509-08|Canada|British Columbia|658[0n]|BOLD:AAA4141
Aseptis binotata|[17439]|LPVIB262-08|Canada|British Columbia|658[0n]|BOLD:AAA4141
Aseptis binotata|[17440]|LPVIB251-08|Canada|British Columbia|658[0n]|BOLD:AAA4141
Aseptis binotata|[17441]|LPVIA837-08|Canada|British Columbia|658[0n]|BOLD:AAA4141
Aseptis binotata|[17442]|LPVIA563-08|Canada|British Columbia|658[0n]|BOLD:AAA4141
Aseptis binotata|[17443]|LPVIA562-08|Canada|British Columbia|658[0n]|BOLD:AAA4141
Aseptis binotata|[17444]|LPVIA561-08|Canada|British Columbia|658[0n]|BOLD:AAA4141
Aseptis binotata|[17445]|LBCS266-07|Canada|British Columbia|658[0n]|BOLD:AAA4141
Aseptis binotata|[17446]|LPVIB074-08|Canada|British Columbia|658[0n]|BOLD:AAA4141
Aseptis binotata|[17447]|LHLEP437-06|Canada|British Columbia|658[0n]|BOLD:AAA4141
Aseptis binotata|[17448]|LALPA1010-11|Canada|British Columbia|658[0n]|BOLD:AAA4141
Aseptis binotata|[17449]|LPVIB263-08|Canada|British Columbia|658[0n]|BOLD:AAA4141
Aseptis binotata|[17450]|LPVIB016-08|Canada|British Columbia|658[0n]|BOLD:AAA4141
Aseptis binotata|[17451]|LOPN118-06|United States|Oregon|518[1n]|BOLD:AAA4141
Aseptis binotata|[17452]|RDNMC644-06|Canada|British Columbia|519[0n]|BOLD:AAA4141
Aseptis binotata|[17453]|RWWA874-09|United States|Washington|634[0n]|BOLD:AAA4141
Aseptis binotata|[17454]|LALPA528-10|Canada|British Columbia|633[0n]|BOLD:AAA4141
Aseptis binotata|[17455]|RWWC576-11|United States|Washington|634[0n]|BOLD:AAA4141
Aseptis binotata|[17456]|LALPA1196-11|Canada|British Columbia|658[0n]|BOLD:AAA4141
Aseptis binotata|[17457]|LALPA1313-11|Canada|British Columbia|658[0n]|BOLD:AAA4141
Aseptis binotata|[17458]|RDNMC646-06|United States|California|603[0n]|BOLD:AAA4141
Aseptis binotata|[17459]|RDNMC625-06|United States|Nevada|658[0n]|BOLD:AAA4141
Aseptis binotata|[17460]|RDNMC648-06|United States|California|658[0n]|BOLD:AAA4141
Aseptis binotata|[17461]|GMLC1021-12|United States|California|658[0n]|BOLD:AAA4141
Aseptis binotata|[17462]|GMLC732-12|United States|California|658[0n]|BOLD:AAA4141
Aseptis binotata|[17463]|GMLC1039-12|United States|California|658[0n]|BOLD:AAA4141
Aseptis binotata|[17464]|LOCBB147-06|United States|California|658[0n]|BOLD:AAA4141
Aseptis binotata|[17465]|LOPN116-06|United States|Oregon|567[0n]|BOLD:AAA4141
Aseptis binotata|[17466]|LOPN117-06|United States|Oregon|576[4n]|BOLD:AAA4141
Aseptis binotata|[17467]|LOCBF3436-14|United States|California|564[0n]|BOLD:AAA4141
Aseptis binotata|[17468]|LOCBF3434-14|United States|California|555[0n]|BOLD:AAA4141
Aseptis binotata|[17469]|LOCBC053-06|United States|California|619[0n]|BOLD:AAA4141
Aseptis binotata|[17470]|GMLC1022-12|United States|California|658[0n]|BOLD:AAA4141
Aseptis binotata|[17471]|LOCBF3435-14|United States|California|552[0n]|BOLD:AAA4141
Aseptis binotata|[17472]|LOCBF2628-13|United States|California|658[0n]|BOLD:AAA4141
Aseptis binotata|[17473]|GMLC1114-12|United States|California|658[0n]|BOLD:AAA4141
Aseptis binotata|[17474]|GMLC1023-12|United States|California|658[0n]|BOLD:AAA4141
Aseptis binotata|[17475]|GMLC981-12|United States|California|658[0n]|BOLD:AAA4141
Aseptis binotata|[17476]|GMLC818-12|United States|California|658[0n]|BOLD:AAA4141
Aseptis binotata|[17477]|LOCBC463-06|United States|California|658[0n]|BOLD:AAA4141
Aseptis binotata|[17478]|LOCBC461-06|United States|California|658[0n]|BOLD:AAA4141
Aseptis binotata|[17479]|LOCBC459-06|United States|California|658[0n]|BOLD:AAA4141
Aseptis binotata|[17480]|LOCBC453-06|United States|California|658[0n]|BOLD:AAA4141
Aseptis binotata|[17481]|LOCBC452-06|United States|California|658[0n]|BOLD:AAA4141
Aseptis binotata|[17482]|LOCBC207-06|United States|California|658[0n]|BOLD:AAA4141
Aseptis binotata|[17483]|LOCBC203-06|United States|California|658[0n]|BOLD:AAA4141
Aseptis binotata|[17484]|LOCBC202-06|United States|California|658[0n]|BOLD:AAA4141
Aseptis binotata|[17485]|LOCBC052-06|United States|California|658[0n]|BOLD:AAA4141
Aseptis binotata|[17486]|LOCBB677-06|United States|California|658[0n]|BOLD:AAA4141
Aseptis binotata|[17487]|RDNMC650-06|United States|California|658[0n]|BOLD:AAA4141
Aseptis binotata|[17488]|RDNME012-07|United States|California|549[0n]|BOLD:AAA4141
Aseptis binotata|[17489]|LOCBB211-06|United States|California|658[0n]|BOLD:AAA4141
Aseptis binotata|[17490]|GMLC769-12|United States|California|638[0n]|BOLD:AAA4141

Aseptis binotata|[17488]|RDNME012-07|United States|California|549[0n]|BOLD:AAA4141
Aseptis binotata|[17489]|LOCBB211-06|United States|California|658[0n]|BOLD:AAA4141
Aseptis binotata|[17490]|GMLC769-12|United States|California|638[0n]|BOLD:AAA4141
Aseptis binotata|[17491]|LOCBC050-06|United States|California|617[0n]|BOLD:AAA4141
Aseptis binotata|[17492]|LOCBC051-06|United States|California|616[0n]|BOLD:AAA4141
Aseptis binotata|[17493]|LOCBB210-06|United States|California|631[0n]|BOLD:AAA4141
Aseptis binotata|[17494]|LOCBB209-06|United States|California|643[0n]|BOLD:AAA4141
Aseptis binotata|[17495]|LOCBC054-06|United States|California|608[0n]|BOLD:AAA4141
Aseptis binotata|[17496]|CNCLB1534-14|United States|California|596[0n]|BOLD:AAA4141
Aseptis characta|[17497]|LOCBC805-06|United States|California|658[0n]|BOLD:AAA6683
Aseptis characta|[17498]|LOCBD201-06|United States|California|658[0n]|BOLD:AAA6683
Aseptis characta|[17499]|LOCBD198-06|United States|California|658[0n]|BOLD:AAA6683
Aseptis characta|[17500]|LOCBD216-06|United States|California|658[0n]|BOLD:AAA6683
Aseptis characta|[17501]|LOCBC298-06|United States|California|658[0n]|BOLD:AAA6683
Aseptis characta|[17502]|RDNMC667-06|United States|Washington|658[0n]|BOLD:AAA6683
Aseptis characta|[17503]|LOCBD214-06|United States|California|658[0n]|BOLD:AAA6683
Aseptis characta|[17504]|LOCBD215-06|United States|California|658[0n]|BOLD:AAA6683
Aseptis characta|[17505]|LOCBD206-06|United States|California|658[0n]|BOLD:AAA6683
Aseptis characta|[17506]|LOCBD208-06|United States|California|658[0n]|BOLD:AAA6683
Aseptis characta|[17507]|LOCBC807-06|United States|California|658[0n]|BOLD:AAA6683
Aseptis characta|[17508]|LOCBD203-06|United States|California|658[0n]|BOLD:AAA6683
Aseptis characta|[17509]|LOCBC288-06|United States|California|649[0n]|BOLD:AAA6683
Aseptis characta|[17510]|LOCBC289-06|United States|California|656[0n]|BOLD:AAA6683
Aseptis characta|[17511]|LOCBC290-06|United States|California|658[0n]|BOLD:AAA6683
Aseptis characta|[17512]|LOCBC292-06|United States|California|658[0n]|BOLD:AAA6683
Aseptis characta|[17513]|LOCBC295-06|United States|California|658[0n]|BOLD:AAA6683
Aseptis characta|[17514]|LOCBC296-06|United States|California|658[0n]|BOLD:AAA6683
Aseptis characta|[17515]|LOCBC300-06|United States|California|658[0n]|BOLD:AAA6683
Aseptis characta|[17516]|LOCBC302-06|United States|California|658[0n]|BOLD:AAA6683
Aseptis characta|[17517]|LOCBC303-06|United States|California|658[0n]|BOLD:AAA6683
Aseptis characta|[17518]|LOCBC308-06|United States|California|658[0n]|BOLD:AAA6683
Aseptis characta|[17519]|LOCBC309-06|United States|California|658[0n]|BOLD:AAA6683
Aseptis characta|[17520]|LOCBC804-06|United States|California|658[0n]|BOLD:AAA6683
Aseptis characta|[17521]|LOCBC806-06|United States|California|658[0n]|BOLD:AAA6683
Aseptis characta|[17522]|LOCBD218-06|United States|California|658[0n]|BOLD:AAA6683
Aseptis characta|[17523]|LOCBC304-06|United States|California|658[0n]|BOLD:AAA6683
Aseptis characta|[17524]|LOCBC393-06|United States|California|609[0n]|BOLD:AAA6683
Aseptis characta|[17525]|LOCBD200-06|United States|California|658[0n]|BOLD:AAA6683
Aseptis characta|[17526]|LOCBC297-06|United States|California|658[0n]|BOLD:AAA6683
Aseptis characta|[17527]|LOCBD204-06|United States|California|658[0n]|BOLD:AAA6683
Aseptis characta|[17528]|LOCBD590-06|United States|California|658[0n]|BOLD:AAA6683
Aseptis characta|[17529]|LOCBD202-06|United States|California|658[0n]|BOLD:AAA6683
Aseptis characta|[17530]|LOCBD199-06|United States|California|658[0n]|BOLD:AAA6683
Aseptis characta|[17531]|LOCBD197-06|United States|California|658[0n]|BOLD:AAA6683
Aseptis characta|[17532]|LOCBD196-06|United States|California|658[0n]|BOLD:AAA6683
Aseptis characta|[17533]|LOCBC810-06|United States|California|658[0n]|BOLD:AAA6683
Aseptis characta|[17534]|LOCBC809-06|United States|California|657[0n]|BOLD:AAA6683
Aseptis characta|[17535]|LOCBC301-06|United States|California|658[0n]|BOLD:AAA6683
Aseptis characta|[17536]|LOCBC299-06|United States|California|658[0n]|BOLD:AAA6683
Aseptis characta|[17537]|LOCBC291-06|United States|California|658[0n]|BOLD:AAA6683
Aseptis characta|[17538]|LOCBC808-06|United States|California|633[0n]|BOLD:AAA6683
Aseptis characta|[17539]|RDNME030-07|United States|California|609[0n]|BOLD:AAA6683
Aseptis characta|[17540]|RDNME077-07|United States|California|658[0n]|BOLD:AAA6683
Aseptis characta|[17541]|RDNME079-07|United States|California|658[0n]|BOLD:AAA6683
Aseptis characta|[17542]|RDNMC669-06|Canada|British Columbia|658[0n]|BOLD:AAA6683
Aseptis characta|[17543]|IAWLB054-10|United States|California|658[0n]|BOLD:AAA6683
Aseptis characta|[17544]|IAWLB056-10|United States|California|658[0n]|BOLD:AAA6683
Aseptis characta|[17545]|IAWLB053-10|United States|California|658[0n]|BOLD:AAA6683
Aseptis characta|[17546]|IAWLB055-10|United States|California|658[0n]|BOLD:AAA6683
Aseptis characta|[17547]|BBLOC1218-11|United States|California|658[0n]|BOLD:AAA6683
Aseptis characta|[17548]|BBLOC1266-11|United States|California|658[0n]|BOLD:AAA6683
Aseptis characta|[17549]|BBLOC1202-11|United States|California|658[0n]|BOLD:AAA6683
Aseptis characta|[17550]|RDMAB134-05|Canada|Alberta|658[0n]|BOLD:AAA6683
Aseptis characta|[17551]|RDNMC670-06|United States|Nevada|658[0n]|BOLD:AAA6683
Aseptis characta|[17552]|RDNMC674-06|United States|Wyoming|658[0n]|BOLD:AAA6683
Aseptis characta|[17553]|BBLOC1210-11|United States|California|658[0n]|BOLD:AAA6683
Aseptis characta|[17554]|RDNMC675-06|United States|Washington|549[0n]|BOLD:AAA6683
Aseptis characta|[17555]|RDNMC672-06|United States|Nevada|658[0n]|BOLD:AAA6683
Aseptis characta|[17556]|BBLOC940-11|United States|Texas|658[0n]|BOLD:AAA6683
Aseptis characta|[17557]|BBLOC1348-11|United States|California|658[0n]|BOLD:AAA6683
Aseptis characta|[17558]|NAMUM267-08|United States|California|658[0n]|BOLD:AAA6683
Aseptis characta|[17559]|IAWLB052-10|United States|California|658[0n]|BOLD:AAA6683
Aseptis characta|[17560]|RDNME078-07|United States|California|658[0n]|BOLD:AAA6683
Aseptis characta|[17561]|RDNMC673-06|United States|Wyoming|658[0n]|BOLD:AAA6683
Aseptis characta|[17562]|RDNMC617-06|Canada|British Columbia|658[0n]|BOLD:AAA6683
Aseptis characta|[17563]|RDNMC611-06|Canada|Alberta|658[0n]|BOLD:AAA6683
Aseptis characta|[17564]|CNGRJ069-13|Canada|Saskatchewan|633[0n]|BOLD:AAA6683
Aseptis characta|[17565]|RDMAB039-05|Canada|Alberta|558[0n]|BOLD:AAA6683
Aseptis characta|[17566]|JMMMB528-13|United States|California|588[0n]|BOLD:AAA6683
Aseptis fumosa|[17567]|LOPN158-06|United States|Oregon|581[0n]|BOLD:AAC3839
Aseptis fumosa|[17568]|LOPN160-06|United States|Oregon|526[0n]|BOLD:AAC3839
Aseptis fumosa|[17569]|LOCBD211-06|United States|California|658[0n]|BOLD:AAC3839
Aseptis fumosa|[17570]|RDNMC623-06|United States|California|658[0n]|BOLD:AAC3839
Aseptis fumosa|[17571]|RDNMC622-06|United States|Nevada|658[0n]|BOLD:AAC3839
Aseptis fumosa|[17572]|RDNMC621-06|United States|California|658[0n]|BOLD:AAC3839
Aseptis fumosa|[17573]|LOPN159-06|United States|Oregon|589[0n]|BOLD:AAC3839
Aseptis fumosa|[17574]|RDNMC619-06|United States|Nevada|593[1n]|BOLD:AAC3839
Aseptis fumosa|[17575]|LOPN161-06|United States|Oregon|592[2n]|BOLD:AAC3839
Aseptis fumosa|[17576]|RDNME004-07|United States|California|609[0n]|BOLD:AAC3839
Aseptis fumosa|[17577]|RDNME014-07|United States|California|609[0n]|BOLD:AAC3839
Aseptis fumosa|[17578]|RDNME025-07|United States|California|609[0n]|BOLD:AAC3839
Aseptis fumosa|[17579]|CGLCA186-10|United States|California|658[0n]|BOLD:AAC3839
Aseptis fumosa|[17580]|BBLOC207-11|United States|Texas|658[0n]|BOLD:AAC3839
Aseptis fumosa|[17581]|BBLOC215-11|United States|Texas|658[0n]|BOLD:AAC3839
Aseptis fumosa|[17582]|BBLOC942-11|United States|Texas|658[0n]|BOLD:AAC3839
Aseptis fumosa|[17583]|BBLOC1212-11|United States|California|658[0n]|BOLD:AAC3839
Aseptis fumosa|[17584]|BBLOC1269-11|United States|California|658[0n]|BOLD:AAC3839
Aseptis fumosa|[17585]|CNCLB1536-14|United States|California|571[0n]|BOLD:AAC3839
Xystopeplus rufago|[17586]|LSEU519-06|United States|Georgia|658[0n]|BOLD:AAH9397
Xystopeplus rufago|[17587]|RDNMH830-09|United States|Georgia|658[0n]|BOLD:AAH9397
Pyreferra sp.|[17588]|CNCLA5265-13|United States|Mississippi|658[0n]|BOLD:ACI9598
Pyreferra sp.|[17589]|CNCLB1375-14|United States|North Carolina|658[0n]|BOLD:ACI9598
Pyreferra ceromatica|[17590]|CNCLB613-14|||658[0n]|BOLD:ACM4432

Pyreferra sp.|[17588]|CNCLA5265-13|United States|Mississippi|658[0n]|BOLD:ACI9598
Pyreferra sp.|[17589]|CNCLB1375-14|United States|North Carolina|658[0n]|BOLD:ACI9598
Pyreferra ceromatica|[17590]|CNCLB613-14|||658[0n]|BOLD:ACM4432
Pyreferra citrombra|[17591]|MEC126-04|Canada|Quebec|658[0n]|BOLD:AAD2862
Pyreferra citrombra|[17592]|RDNML190-13|United States|North Carolina|658[0n]|BOLD:AAD2862
Pyreferra citrombra|[17593]|RDLQH113-06|Canada|Quebec|658[0n]|BOLD:AAD2862
Pyreferra citrombra|[17594]|RDNMJ285-11|United States|Connecticut|655[0n]|BOLD:AAD2862
Pyreferra citrombra|[17595]|LNCC607-11|United States|North Carolina|658[0n]|BOLD:AAD2862
Pyreferra citrombra|[17596]|RDLQH112-06|Canada|Quebec|621[0n]|BOLD:AAD2862
Pyreferra citrombra|[17597]|NAMUM359-08|United States|West Virginia|658[0n]|BOLD:AAD2862
Pyreferra citrombra|[17598]|RDNMH625-09|Canada|Ontario|658[0n]|BOLD:AAD2862
Pyreferra citrombra|[17599]|LNCC1481-13|United States|North Carolina|658[0n]|BOLD:AAD2862
Pyreferra sp.|[17600]|LSEU121-06|United States|North Carolina|658[0n]|BOLD:AAD2863
Pyreferra sp.|[17601]|LSEU122-06|United States|North Carolina|658[0n]|BOLD:AAD2863
Pyreferra sp.|[17602]|LNCC606-11|United States|North Carolina|658[0n]|BOLD:AAD2863
Pyreferra sp.|[17603]|LNAUS5001-13|United States|Virginia|658[0n]|BOLD:AAD2863
Pyreferra sp.|[17604]|RDNML189-13|United States|North Carolina|658[0n]|BOLD:AAD2863
Pyreferra sp.|[17605]|LSEU123-06|United States|North Carolina|658[0n]|BOLD:AAD2863
Pyreferra sp.|[17606]|CNCLA7373-13|Canada|Ontario|658[0n]|BOLD:AAD2863
Pyreferra sp.|[17607]|RDNMH866-09|Canada|Ontario|658[0n]|BOLD:AAD2863
Pyreferra sp.|[17608]|CNCLB1342-14|United States|North Carolina|658[0n]|BOLD:AAD2863
Pyreferra sp.|[17609]|CNCLB1343-14|United States|North Carolina|658[0n]|BOLD:AAD2863
Pyreferra pettiti|[17610]|XAF279-05|Canada|Ontario|658[0n]|BOLD:AAD2999
Pyreferra pettiti|[17611]|RDLQ573-07|Canada|Quebec|556[0n]|BOLD:AAD2999
Pyreferra pettiti|[17612]|XAF051-05|Canada|Ontario|658[0n]|BOLD:AAD2999
Pyreferra pettiti|[17613]|CNCLB1345-14|United States|North Carolina|658[0n]|BOLD:AAD2999
Pyreferra pettiti|[17614]|MEC186-04|Canada|Quebec|658[0n]|BOLD:AAD2999
Pyreferra pettiti|[17615]|RDLQ571-07|Canada|Quebec|566[0n]|BOLD:AAD2999
Pyreferra pettiti|[17616]|MECB881-05|Canada|Quebec|658[0n]|BOLD:AAD2999
Pyreferra pettiti|[17617]|MECB882-05|Canada|Quebec|658[0n]|BOLD:AAD2999
Pyreferra pettiti|[17618]|CNCLB1347-14|United States|North Carolina|658[0n]|BOLD:AAD2999
Pyreferra pettiti|[17619]|CNCLB1346-14|United States|North Carolina|658[0n]|BOLD:AAD2999
Pyreferra pettiti|[17620]|CNCLB1376-14|United States|North Carolina|658[0n]|BOLD:AAD2999
Pyreferra hesperidago|[17621]|LOTB205-05|United States|Tennessee|658[0n]|BOLD:AAF5736
Pyreferra hesperidago|[17622]|RDLQ570-07|Canada|Quebec|575[0n]|BOLD:AAF5736
Pyreferra hesperidago|[17623]|LNCC608-11|United States|North Carolina|658[0n]|BOLD:AAF5736
Pyreferra hesperidago|[17624]|LNCC1930-14|United States|North Carolina|658[0n]|BOLD:AAF5736
Pyreferra hesperidago|[17625]|CNCLB1344-14|United States|North Carolina|658[0n]|BOLD:AAF5736
Pyreferra hesperidago|[17626]|CNCLB1377-14|United States|North Carolina|658[0n]|BOLD:AAF5736
Homoglaea hircina|[17627]|LOWCE589-06|Canada|British Columbia|658[0n]|BOLD:AAE1607
Homoglaea hircina|[17628]|LOWCE588-06|Canada|British Columbia|656[0n]|BOLD:AAE1607
Homoglaea hircina|[17629]|XAE639-04|Canada|Ontario|610[0n]|BOLD:AAE1607
Homoglaea hircina|[17630]|TMNBB228-06|Canada|New Brunswick|658[0n]|BOLD:AAE1607
Homoglaea hircina|[17631]|TMNBB229-06|Canada|New Brunswick|658[0n]|BOLD:AAE1607
Homoglaea carbonaria|[17632]|LALPA1023-11|Canada|British Columbia|658[0n]|BOLD:AAE4974
Homoglaea carbonaria|[17633]|LOWCD927-06|Canada|British Columbia|658[0n]|BOLD:AAE4974
Homoglaea carbonaria|[17634]|LOWCD928-06|Canada|British Columbia|521[1n]|BOLD:AAE4974
Homoglaea carbonaria|[17635]|LOWCE590-06|Canada|British Columbia|658[0n]|BOLD:AAE4974
Homoglaea carbonaria|[17636]|LOWCE591-06|Canada|British Columbia|658[0n]|BOLD:AAE4974
Homoglaea carbonaria|[17637]|LOWCE592-06|Canada|British Columbia|658[0n]|BOLD:AAE4974
Homoglaea carbonaria|[17638]|LOWCE593-06|Canada|British Columbia|658[0n]|BOLD:AAE4974
Homoglaea carbonaria|[17639]|IAWLB080-10|United States|California|658[0n]|BOLD:AAE4974
Homoglaea carbonaria|[17640]|LALPA814-10|Canada|British Columbia|658[0n]|BOLD:AAE4974
Homoglaea carbonaria|[17641]|RDNMG553-08|United States|California|658[0n]|BOLD:AAE4974
Homoglaea carbonaria|[17642]|GMLC1265-12|United States|California|658[0n]|BOLD:AAE4974
Homoglaea dives|[17643]|RWWB484-10|United States|Washington|658[0n]|BOLD:AAM3001
Homoglaea dives|[17644]|RWWB491-10|United States|Washington|658[0n]|BOLD:AAM3001
Homoglaea dives|[17645]|RWWB529-10|United States|Washington|658[0n]|BOLD:AAM3001
Homoglaea dives|[17646]|RWWC111-10|United States|Washington|658[0n]|BOLD:AAM3001
Homoglaea dives|[17647]|GMLC1266-12|United States|California|658[0n]|BOLD:AAM3001
Epiglaea decliva|[17648]|RDLQ574-07|Canada|Quebec|658[0n]|BOLD:AAX7020
Epiglaea decliva|[17649]|SMTPD2877-13|Canada|Ontario|576[0n]|BOLD:AAX7020
Epiglaea decliva|[17650]|CNCLB1369-14|United States|North Carolina|654[0n]|BOLD:AAX7020
Xanthia tatago|[17651]|PHMNB296-04|Canada|New Brunswick|657[1n]|BOLD:AAE4535
Xanthia tatago|[17652]|LOWCD167-06|Canada|British Columbia|614[0n]|BOLD:AAE4535
Xanthia tatago|[17653]|PHMNB272-04|Canada|New Brunswick|658[0n]|BOLD:AAE4535
Xanthia tatago|[17654]|PHMNB283-04|Canada|New Brunswick|658[0n]|BOLD:AAE4535
Xanthia tatago|[17655]|LBCH2864-10|Canada|British Columbia|658[0n]|BOLD:AAE4535
Xanthia tatago|[17656]|LALPA809-10|Canada|British Columbia|658[0n]|BOLD:AAE4535
Anathix ralla|[17657]|LNCC451-10|United States|North Carolina|658[0n]|BOLD:AAC9569
Anathix ralla|[17658]|LNCC412-10|United States|North Carolina|658[0n]|BOLD:AAC9569
Anathix ralla|[17659]|LNCC269-10|United States|North Carolina|658[0n]|BOLD:AAC9569
Anathix ralla|[17660]|MNAC922-07|Canada|Quebec|657[0n]|BOLD:AAC9569
Anathix ralla|[17661]|LSEU126-06|United States|North Carolina|658[0n]|BOLD:AAC9569
Anathix ralla|[17662]|LSEU125-06|United States|North Carolina|658[0n]|BOLD:AAC9569
Anathix ralla|[17663]|LSEU124-06|United States|North Carolina|658[0n]|BOLD:AAC9569
Anathix ralla|[17664]|RDLQB502-05|Canada|Quebec|658[0n]|BOLD:AAC9569
Anathix ralla|[17665]|RDNMB956-05|Canada|Ontario|658[0n]|BOLD:AAC9569
Anathix ralla|[17666]|RDLQ575-07|Canada|Quebec|593[0n]|BOLD:AAC9569
Anathix ralla|[17667]|RDLQ576-07|Canada|Quebec|594[0n]|BOLD:AAC9569
Anathix ralla|[17668]|CNSLJ441-12|Canada|Ontario|603[0n]|BOLD:AAC9569
Anathix aggressa|[17669]|RDNMB950-05|United States|New Mexico|658[0n]|BOLD:ABY4570
Anathix aggressa|[17670]|RDNMB951-05|United States|New Mexico|658[0n]|BOLD:ABY4570
Anathix aggressa|[17671]|RDNM728-05|United States|Washington|649[0n]|BOLD:ABY4570
Anathix aggressa|[17672]|RDNM729-05|United States|Washington|579[0n]|BOLD:ABY4570
Anathix aggressa|[17673]|RDNMB954-05|Canada|Alberta|658[0n]|BOLD:ABY4570
Anathix aggressa|[17674]|CMAZA105-09|United States|Arizona|658[0n]|BOLD:ABY4570
Anathix aggressa|[17675]|RDNM730-05|Canada|Alberta|606[0n]|BOLD:ACF4918
Anathix aggressa|[17676]|RDNMB955-05|Canada|Alberta|658[0n]|BOLD:ACF4918
Anathix aggressa|[17677]|RDNMB953-05|United States|Oregon|658[0n]|BOLD:ACE8281
Anathix aggressa|[17678]|LMDH008-11|United States|Minnesota|658[0n]|BOLD:ACE8281
Anathix puta|[17679]|RDNMB957-05|Canada|Alberta|658[0n]|BOLD:AAD2871
Anathix puta|[17680]|RDLQB514-05|Canada|Quebec|658[0n]|BOLD:AAD2871
Anathix puta|[17681]|BBLPB432-10|Canada|Saskatchewan|658[0n]|BOLD:AAD2871
Anathix puta|[17682]|BBLPB365-10|Canada|Alberta|658[0n]|BOLD:AAD2871
Anathix puta|[17683]|SSWLA1827-13|Canada|Alberta|608[0n]|BOLD:AAD2871
Anathix puta|[17684]|RDLQ577-07|Canada|Quebec|586[0n]|BOLD:AAD2871
Anathix puta|[17685]|CNWLG946-12|Canada|Alberta|635[0n]|BOLD:AAD2871
Anathix puta|[17686]|JMMMB351-11|United States|California|658[0n]|BOLD:AAD2871
Anathix puta|[17687]|BBLPB441-10|Canada|Saskatchewan|658[0n]|BOLD:AAD2871
Anathix puta|[17688]|BBLPB436-10|Canada|Alberta|658[0n]|BOLD:AAD2871
Anathix puta|[17689]|BBLPB435-10|Canada|Alberta|658[0n]|BOLD:AAD2871
Anathix puta|[17690]|BBLPB433-10|Canada|Alberta|658[0n]|BOLD:AAD2871

Anathix puta|[17688]|BBLPB436-10|Canada|Alberta|658[0n]|BOLD:AAD2871
Anathix puta|[17689]|BBLPB435-10|Canada|Alberta|658[0n]|BOLD:AAD2871
Anathix puta|[17690]|BBLPB433-10|Canada|Alberta|658[0n]|BOLD:AAD2871
Anathix puta|[17691]|BBLPB430-10|Canada|Saskatchewan|658[0n]|BOLD:AAD2871
Anathix puta|[17692]|BBLPB400-10|Canada|Alberta|658[0n]|BOLD:AAD2871
Anathix puta|[17693]|BBLPB369-10|Canada|Saskatchewan|658[0n]|BOLD:AAD2871
Anathix puta|[17694]|RDLQB515-05|Canada|Quebec|658[0n]|BOLD:AAD2871
Anathix puta|[17695]|RDNMC089-05|United States|Washington|658[0n]|BOLD:AAD2871
Anathix puta|[17696]|RDNMB952-05|United States|Washington|658[0n]|BOLD:AAD2871
Anathix puta|[17697]|XAH434-05|Canada|Ontario|658[0n]|BOLD:AAD2871
Anathix puta|[17698]|BBLPB431-10|Canada|Saskatchewan|658[0n]|BOLD:AAD2871
Anathix puta|[17699]|RDLQF174-06|Canada|Quebec|658[0n]|BOLD:AAD2871
Anathix puta|[17700]|RDNM731-05|United States|Washington|606[0n]|BOLD:AAD2871
Anathix puta|[17701]|RDLQ578-07|Canada|Quebec|567[0n]|BOLD:AAD2871
Anathix puta|[17702]|CNWLG953-12|Canada|Alberta|632[0n]|BOLD:AAD2871
Anathix puta|[17703]|CNWLH464-12|Canada|Alberta|632[0n]|BOLD:AAD2871
Anathix puta|[17704]|CNPAF976-13|Canada|Saskatchewan|638[0n]|BOLD:AAD2871
Anathix puta|[17705]|CNWLO772-13|Canada|Alberta|600[0n]|BOLD:AAD2871
Sunira verberata|[17706]|RWWB441-10|United States|Washington|658[0n]|BOLD:AAD2187
Sunira verberata|[17707]|RWWC166-10|United States|Washington|632[0n]|BOLD:AAD2187
Sunira verberata|[17708]|RWWC877-12|United States|Washington|658[0n]|BOLD:AAD2187
Sunira verberata|[17709]|RWWC886-12|United States|Washington|658[0n]|BOLD:AAD2187
Sunira verberata|[17710]|LBCH2509-10|Canada|British Columbia|658[0n]|BOLD:AAD2187
Sunira verberata|[17711]|RWWB421-09|United States|Washington|658[0n]|BOLD:AAD2187
Sunira verberata|[17712]|RWWB413-09|United States|Washington|658[0n]|BOLD:AAD2187
Sunira verberata|[17713]|RWWB425-09|United States|Washington|658[0n]|BOLD:AAD2187
Sunira verberata|[17714]|RDNMC196-05|Canada|New Brunswick|658[0n]|BOLD:AAD2187
Sunira verberata|[17715]|RWWB412-09|United States|Washington|658[0n]|BOLD:AAD2187
Sunira verberata|[17716]|RWWB437-10|United States|Washington|658[0n]|BOLD:AAD2187
Sunira verberata|[17717]|RDNMC195-05|Canada|New Brunswick|594[1n]|BOLD:AAD2187
Sunira verberata|[17718]|LOWCE560-06|Canada|British Columbia|605[0n]|BOLD:AAD2187
Sunira verberata|[17719]|RWWC129-10|United States|Washington|658[0n]|BOLD:AAD2187
Sunira verberata|[17720]|LBCH2638-10|Canada|British Columbia|658[0n]|BOLD:AAD2187
Sunira verberata|[17721]|LBCH2536-10|Canada|British Columbia|658[0n]|BOLD:AAD2187
Sunira verberata|[17722]|LBCH2510-10|Canada|British Columbia|658[0n]|BOLD:AAD2187
Sunira verberata|[17723]|LBCH2508-10|Canada|British Columbia|658[0n]|BOLD:AAD2187
Sunira verberata|[17724]|RDMAB580-06|Canada|Alberta|658[0n]|BOLD:AAD2187
Sunira verberata|[17725]|RDNMC198-05|Canada|New Brunswick|658[0n]|BOLD:AAD2187
Sunira verberata|[17726]|RDNMC197-05|Canada|New Brunswick|658[0n]|BOLD:AAD2187
Sunira verberata|[17727]|ABKWR133-07|United States|Alaska|658[1n]|BOLD:AAD2187
Sunira verberata|[17728]|CNWLI323-12|Canada|Alberta|632[0n]|BOLD:AAD2187
Sunira verberata|[17729]|LBCH2571-10|Canada|British Columbia|632[0n]|BOLD:AAD2187
Sunira verberata|[17730]|RDNMC199-05|Canada|New Brunswick|593[0n]|BOLD:AAD2187
Sunira verberata|[17731]|UAMIC520-13|United States|Alaska|643[0n]|BOLD:AAD2187
Sunira verberata|[17732]|UAMIC521-13|United States|Alaska|649[0n]|BOLD:AAD2187
Sunira verberata|[17733]|UAMIC1161-13|United States|Alaska|658[0n]|BOLD:AAD2187
Sunira verberata|[17734]|JMMMB530-13|United States|California|570[0n]|BOLD:AAD2187
Sunira decipiens|[17735]|LALPA826-10|Canada|British Columbia|658[0n]|BOLD:AAH9895
Sunira decipiens|[17736]|RDNM743-05|Canada|British Columbia|529[1n]|BOLD:AAH9895
Sunira decipiens|[17737]|LALPA825-10|Canada|British Columbia|658[0n]|BOLD:AAH9895
Sunira decipiens|[17738]|RDNM742-05|Canada|British Columbia|565[3n]|BOLD:AAH9895
Sunira decipiens|[17739]|RDMAB943-09|Canada|British Columbia|634[0n]|BOLD:AAH9895
Sunira decipiens|[17740]|LALPA751-10|Canada|British Columbia|658[0n]|BOLD:AAH9895
Sunira decipiens|[17741]|LALPA777-10|Canada|British Columbia|658[0n]|BOLD:AAH9895
Sunira decipiens|[17742]|LALPA824-10|Canada|British Columbia|658[0n]|BOLD:AAH9895
Sunira decipiens|[17743]|RWWC887-12|United States|Washington|658[0n]|BOLD:AAH9895
Sunira bicolorago|[17744]|SMTPD2324-13|Canada|Ontario|543[0n]|
Sunira bicolorago|[17745]|SMTPJ1704-14|Canada|Ontario|591[0n]|BOLD:AAA4426
Sunira bicolorago|[17746]|SMTPD1837-13|Canada|Ontario|543[0n]|BOLD:AAA4426
Sunira bicolorago|[17747]|SMTPD637-13|Canada|Ontario|552[0n]|BOLD:AAA4426
Sunira bicolorago|[17748]|SMTPD1838-13|Canada|Ontario|549[0n]|BOLD:AAA4426
Sunira bicolorago|[17749]|SMTPI7502-14|Canada|Ontario|576[0n]|BOLD:AAA4426
Sunira bicolorago|[17750]|XAD497-04|Canada|Ontario|581[0n]|BOLD:AAA4426
Sunira bicolorago|[17751]|XAH824-05|Canada|Ontario|658[0n]|BOLD:AAA4426
Sunira bicolorago|[17752]|SMTPD2019-13|Canada|Ontario|561[0n]|BOLD:AAA4426
Sunira bicolorago|[17753]|XAB654-04|Canada|Ontario|658[0n]|BOLD:AAA4426
Sunira bicolorago|[17754]|XAB655-04|Canada|Ontario|658[0n]|BOLD:AAA4426
Sunira bicolorago|[17755]|XAD494-04|Canada|Ontario|588[0n]|BOLD:AAA4426
Sunira bicolorago|[17756]|SMTPD2325-13|Canada|Ontario|552[0n]|BOLD:AAA4426
Sunira bicolorago|[17757]|SMTPI10021-14|Canada|Ontario|573[0n]|BOLD:AAA4426
Sunira bicolorago|[17758]|ALLEP310-13|Canada|Ontario|658[0n]|BOLD:AAA4426
Sunira bicolorago|[17759]|SMTPD1184-13|Canada|Ontario|603[0n]|BOLD:AAA4426
Sunira bicolorago|[17760]|SMTPD2613-13|Canada|Ontario|552[0n]|BOLD:AAA4426
Sunira bicolorago|[17761]|PAOCT102-12|Canada|Ontario|606[0n]|BOLD:AAA4426
Sunira bicolorago|[17762]|SMTPJ1706-14|Canada|Ontario|606[0n]|BOLD:AAA4426
Sunira bicolorago|[17763]|PAOCT099-12|Canada|Ontario|588[0n]|BOLD:AAA4426
Sunira bicolorago|[17764]|PAOCT100-12|Canada|Ontario|600[0n]|BOLD:AAA4426
Sunira bicolorago|[17765]|LOCT005-05|United States|Connecticut|658[0n]|BOLD:AAA4426
Sunira bicolorago|[17766]|XAH851-05|Canada|Ontario|658[0n]|BOLD:AAA4426
Sunira bicolorago|[17767]|ALLEP519-14|Canada|Ontario|658[0n]|BOLD:AAA4426
Sunira bicolorago|[17768]|SMTPF8663-14|Canada|Ontario|550[0n]|BOLD:AAA4426
Sunira bicolorago|[17769]|PHSEP397-11|Canada|Ontario|658[0n]|BOLD:AAA4426
Sunira bicolorago|[17770]|SMTPD1567-13|Canada|Ontario|603[0n]|BOLD:AAA4426
Sunira bicolorago|[17771]|HEOCT1004-12|Canada|Ontario|658[0n]|BOLD:AAA4426
Sunira bicolorago|[17772]|XAH775-05|Canada|Ontario|658[0n]|BOLD:AAA4426
Sunira bicolorago|[17773]|XAH607-05|Canada|Ontario|658[0n]|BOLD:AAA4426
Sunira bicolorago|[17774]|SMTPD3728-13|Canada|Ontario|597[0n]|BOLD:AAA4426
Sunira bicolorago|[17775]|XAH627-05|Canada|Ontario|658[0n]|BOLD:AAA4426
Sunira bicolorago|[17776]|SMTPI2728-14|Canada|Ontario|588[0n]|BOLD:AAA4426
Sunira bicolorago|[17777]|LNC454-05|United States|North Carolina|658[0n]|BOLD:AAA4426
Sunira bicolorago|[17778]|LPOKA1034-09|United States|Oklahoma|624[0n]|BOLD:AAA4426
Sunira bicolorago|[17779]|LALPA1321-12|Canada|British Columbia|601[0n]|BOLD:AAA4426
Sunira bicolorago|[17780]|ALLEP405-13|Canada|Ontario|658[0n]|BOLD:AAA4426
Sunira bicolorago|[17781]|SMTPD5502-13|Canada|Ontario|603[0n]|BOLD:AAA4426
Sunira bicolorago|[17782]|PAOCT101-12|Canada|Ontario|585[0n]|BOLD:AAA4426
Sunira bicolorago|[17783]|SMTPI5891-14|Canada|Ontario|591[0n]|BOLD:AAA4426
Sunira bicolorago|[17784]|XAH786-05|Canada|Ontario|658[0n]|BOLD:AAA4426
Sunira bicolorago|[17785]|XAH689-05|Canada|Ontario|658[0n]|BOLD:AAA4426
Sunira bicolorago|[17786]|XAH582-05|Canada|Ontario|658[0n]|BOLD:AAA4426
Sunira bicolorago|[17787]|PAOCT109-12|Canada|Ontario|561[0n]|BOLD:AAA4426
Sunira bicolorago|[17788]|XAH661-05|Canada|Ontario|658[0n]|BOLD:AAA4426
Sunira bicolorago|[17789]|RDNM741-05|Canada|Ontario|658[0n]|BOLD:AAA4426
Sunira bicolorago|[17790]|PHMNB303-04|Canada|New Brunswick|658[2n]|BOLD:AAA4426

Sunira bicolorago|[17788]|XAH661-05|Canada|Ontario|658[0n]|BOLD:AAA4426
Sunira bicolorago|[17789]|RDNM741-05|Canada|Ontario|658[0n]|BOLD:AAA4426
Sunira bicolorago|[17790]|PHMNB303-04|Canada|New Brunswick|658[2n]|BOLD:AAA4426
Sunira bicolorago|[17791]|RDMAB944-09|Canada|British Columbia|620[0n]|BOLD:AAA4426
Sunira bicolorago|[17792]|PAOCT110-12|Canada|Ontario|585[0n]|BOLD:AAA4426
Sunira bicolorago|[17793]|SMTPI5893-14|Canada|Ontario|588[0n]|BOLD:AAA4426
Sunira bicolorago|[17794]|PASEP431-12|Canada|Ontario|579[0n]|BOLD:AAA4426
Sunira bicolorago|[17795]|SMTPD347-13|Canada|Ontario|561[0n]|BOLD:AAA4426
Sunira bicolorago|[17796]|XAB656-04|Canada|Ontario|658[0n]|BOLD:AAA4426
Sunira bicolorago|[17797]|PHSEP400-11|Canada|Ontario|658[0n]|BOLD:AAA4426
Sunira bicolorago|[17798]|ALLEP190-13|Canada|Ontario|658[0n]|BOLD:AAA4426
Sunira bicolorago|[17799]|SMTPD4324-13|Canada|Ontario|594[0n]|BOLD:AAA4426
Sunira bicolorago|[17800]|XAH633-05|Canada|Ontario|658[0n]|BOLD:AAA4426
Sunira bicolorago|[17801]|JSSEP1089-11|Canada|Ontario|658[0n]|BOLD:AAA4426
Sunira bicolorago|[17802]|PAOCT095-12|Canada|Ontario|519[0n]|BOLD:AAA4426
Sunira bicolorago|[17803]|LPOKA348-08|United States|Oklahoma|658[0n]|BOLD:AAA4426
Sunira bicolorago|[17804]|XAB658-04|Canada|Ontario|658[0n]|BOLD:AAA4426
Sunira bicolorago|[17805]|SMTPD1185-13|Canada|Ontario|576[0n]|BOLD:AAA4426
Sunira bicolorago|[17806]|XAH708-05|Canada|Ontario|650[0n]|BOLD:AAA4426
Sunira bicolorago|[17807]|SMTPD2882-13|Canada|Ontario|561[0n]|BOLD:AAA4426
Sunira bicolorago|[17808]|PHSEP391-11|Canada|Ontario|658[0n]|BOLD:AAA4426
Sunira bicolorago|[17809]|HEOCT1006-12|Canada|Ontario|658[0n]|BOLD:AAA4426
Sunira bicolorago|[17810]|HEOCT1005-12|Canada|Ontario|658[0n]|BOLD:AAA4426
Sunira bicolorago|[17811]|PHSEP403-11|Canada|Ontario|658[0n]|BOLD:AAA4426
Sunira bicolorago|[17812]|LNC456-05|United States|North Carolina|658[0n]|BOLD:AAA4426
Sunira bicolorago|[17813]|XAH698-05|Canada|Ontario|658[0n]|BOLD:AAA4426
Sunira bicolorago|[17814]|XAH664-05|Canada|Ontario|658[0n]|BOLD:AAA4426
Sunira bicolorago|[17815]|XAH637-05|Canada|Ontario|658[0n]|BOLD:AAA4426
Sunira bicolorago|[17816]|XAH635-05|Canada|Ontario|658[0n]|BOLD:AAA4426
Sunira bicolorago|[17817]|XAH844-05|Canada|Ontario|658[0n]|BOLD:AAA4426
Sunira bicolorago|[17818]|XAH803-05|Canada|Ontario|658[0n]|BOLD:AAA4426
Sunira bicolorago|[17819]|XAB673-04|Canada|Ontario|658[0n]|BOLD:AAA4426
Sunira bicolorago|[17820]|PAOCT103-12|Canada|Ontario|594[0n]|BOLD:AAA4426
Sunira bicolorago|[17821]|PAOCT104-12|Canada|Ontario|597[0n]|BOLD:AAA4426
Sunira bicolorago|[17822]|SMTPD112-13|Canada|Ontario|603[0n]|BOLD:AAA4426
Sunira bicolorago|[17823]|SMTPJ4572-14|Canada|Ontario|603[0n]|BOLD:AAA4426
Sunira bicolorago|[17824]|XAH636-05|Canada|Ontario|658[0n]|BOLD:AAA4426
Sunira bicolorago|[17825]|MJMSL088-10|United States|Massachusetts|658[0n]|BOLD:AAA4426
Sunira bicolorago|[17826]|XAH785-05|Canada|Ontario|658[0n]|BOLD:AAA4426
Sunira bicolorago|[17827]|SMTPI5892-14|Canada|Ontario|579[0n]|BOLD:AAA4426
Sunira bicolorago|[17828]|SMTPD1186-13|Canada|Ontario|576[0n]|BOLD:AAA4426
Sunira bicolorago|[17829]|SMTPI5008-14|Canada|Alberta|576[0n]|BOLD:AAA4426
Sunira bicolorago|[17830]|SMTPI2727-14|Canada|Ontario|576[0n]|BOLD:AAA4426
Sunira bicolorago|[17831]|MBION019-14|Canada|Ontario|578[0n]|BOLD:AAA4426
Sunira bicolorago|[17832]|SMTPD2614-13|Canada|Ontario|591[0n]|BOLD:AAA4426
Sunira bicolorago|[17833]|SMTPD2881-13|Canada|Ontario|570[0n]|BOLD:AAA4426
Sunira bicolorago|[17834]|XAH764-05|Canada|Ontario|658[0n]|BOLD:AAA4426
Sunira bicolorago|[17835]|SMTPD2323-13|Canada|Ontario|561[0n]|BOLD:AAA4426
Sunira bicolorago|[17836]|SMTPD3727-13|Canada|Ontario|561[0n]|BOLD:AAA4426
Sunira bicolorago|[17837]|SMTPD2611-13|Canada|Ontario|561[0n]|BOLD:AAA4426
Sunira bicolorago|[17838]|SMTPF10033-14|Canada|Ontario|537[1n]|BOLD:AAA4426
Sunira bicolorago|[17839]|XAH789-05|Canada|Ontario|658[0n]|BOLD:AAA4426
Sunira bicolorago|[17840]|XAH710-05|Canada|Ontario|645[1n]|BOLD:AAA4426
Sunira bicolorago|[17841]|XAH762-05|Canada|Ontario|658[0n]|BOLD:AAA4426
Sunira bicolorago|[17842]|XAB700-04|Canada|Ontario|658[0n]|BOLD:AAA4426
Sunira bicolorago|[17843]|XAB662-04|Canada|Ontario|658[0n]|BOLD:AAA4426
Sunira bicolorago|[17844]|RDLQ581-07|Canada|Quebec|592[0n]|BOLD:AAA4426
Sunira bicolorago|[17845]|LPOKA345-08|United States|Oklahoma|636[0n]|BOLD:AAA4426
Sunira bicolorago|[17846]|SMTPI10020-14|Canada|Ontario|606[0n]|BOLD:AAA4426
Sunira bicolorago|[17847]|LNC455-05|United States|North Carolina|616[0n]|BOLD:AAA4426
Sunira bicolorago|[17848]|SMTPI10022-14|Canada|Ontario|603[0n]|BOLD:AAA4426
Sunira bicolorago|[17849]|XAH748-05|Canada|Ontario|658[0n]|BOLD:AAA4426
Sunira bicolorago|[17850]|SMTPD111-13|Canada|Ontario|576[0n]|BOLD:AAA4426
Sunira bicolorago|[17851]|SMTPI990-14|Canada|Ontario|607[0n]|BOLD:AAA4426
Sunira bicolorago|[17852]|SMTPI989-14|Canada|Ontario|603[0n]|BOLD:AAA4426
Sunira bicolorago|[17853]|SMTPD5500-13|Canada|Ontario|603[0n]|BOLD:AAA4426
Sunira bicolorago|[17854]|XAH603-05|Canada|Ontario|658[0n]|BOLD:AAA4426
Sunira bicolorago|[17855]|PAOCT108-12|Canada|Ontario|600[0n]|BOLD:AAA4426
Sunira bicolorago|[17856]|CNPEU114-14|Canada|Prince Edward Island|569[0n]|BOLD:AAA4426
Sunira bicolorago|[17857]|PAOCT094-12|Canada|Ontario|600[0n]|BOLD:AAA4426
Sunira bicolorago|[17858]|XAD495-04|Canada|Ontario|527[0n]|BOLD:AAA4426
Sunira bicolorago|[17859]|XAD491-04|Canada|Ontario|583[0n]|BOLD:AAA4426
Sunira bicolorago|[17860]|XAB657-04|Canada|Ontario|658[0n]|BOLD:AAA4426
Sunira bicolorago|[17861]|XAD492-04|Canada|Ontario|589[0n]|BOLD:AAA4426
Sunira bicolorago|[17862]|XAH632-05|Canada|Ontario|658[0n]|BOLD:AAA4426
Sunira bicolorago|[17863]|SMTPI2742-14|Canada|Ontario|609[1n]|BOLD:AAA4426
Sunira bicolorago|[17864]|XAH612-05|Canada|Ontario|633[0n]|BOLD:AAA4426
Sunira bicolorago|[17865]|PHSEP407-11|Canada|Ontario|658[0n]|BOLD:AAA4426
Sunira bicolorago|[17866]|PAOCT096-12|Canada|Ontario|579[0n]|BOLD:AAA4426
Sunira bicolorago|[17867]|SMTPD630-13|Canada|Ontario|552[0n]|BOLD:AAA4426
Sunira bicolorago|[17868]|PAOCT107-12|Canada|Ontario|564[0n]|BOLD:AAA4426
Sunira bicolorago|[17869]|ALLEP521-14|Canada|Ontario|658[0n]|BOLD:AAA4426
Sunira bicolorago|[17870]|SMTPD5186-13|Canada|Ontario|549[0n]|BOLD:AAA4426
Sunira bicolorago|[17871]|SMTPJ1705-14|Canada|Ontario|606[0n]|BOLD:AAA4426
Sunira bicolorago|[17872]|SMTPD3162-13|Canada|Ontario|606[0n]|BOLD:AAA4426
Sunira bicolorago|[17873]|ALLEP266-13|Canada|Ontario|658[0n]|BOLD:AAA4426
Sunira bicolorago|[17874]|SMTPJ930-14|Canada|Ontario|603[0n]|BOLD:AAA4426
Sunira bicolorago|[17875]|SMTPI10024-14|Canada|Ontario|603[0n]|BOLD:AAA4426
Sunira bicolorago|[17876]|SMTPI10023-14|Canada|Ontario|603[0n]|BOLD:AAA4426
Sunira bicolorago|[17877]|SMTPI10018-14|Canada|Ontario|603[0n]|BOLD:AAA4426
Sunira bicolorago|[17878]|PAOCT106-12|Canada|Ontario|603[0n]|BOLD:AAA4426
Sunira bicolorago|[17879]|XAH561-05|Canada|Ontario|658[0n]|BOLD:AAA4426
Sunira bicolorago|[17880]|XAH535-05|Canada|Ontario|658[0n]|BOLD:AAA4426
Sunira bicolorago|[17881]|XAH477-05|Canada|Ontario|658[0n]|BOLD:AAA4426
Sunira bicolorago|[17882]|LOCT006-05|United States|Connecticut|658[0n]|BOLD:AAA4426
Sunira bicolorago|[17883]|XAB540-04|Canada|Ontario|658[0n]|BOLD:AAA4426
Sunira bicolorago|[17884]|XAB696-04|Canada|Ontario|658[0n]|BOLD:AAA4426
Sunira bicolorago|[17885]|XAB698-04|Canada|Ontario|658[0n]|BOLD:AAA4426
Sunira bicolorago|[17886]|PHMNB287-04|Canada|New Brunswick|658[0n]|BOLD:AAA4426
Sunira bicolorago|[17887]|XAB663-04|Canada|Ontario|658[0n]|BOLD:AAA4426
Sunira bicolorago|[17888]|XAH574-05|Canada|Ontario|658[0n]|BOLD:AAA4426
Sunira bicolorago|[17889]|XAH662-05|Canada|Ontario|658[0n]|BOLD:AAA4426
Sunira bicolorago|[17890]|XAH663-05|Canada|Ontario|658[0n]|BOLD:AAA4426

Sunira bicolorago|[17888]|XAH574-05|Canada|Ontario|658[0n]|BOLD:AAA4426
Sunira bicolorago|[17889]|XAH662-05|Canada|Ontario|658[0n]|BOLD:AAA4426
Sunira bicolorago|[17890]|XAH663-05|Canada|Ontario|658[0n]|BOLD:AAA4426
Sunira bicolorago|[17891]|XAH734-05|Canada|Ontario|658[0n]|BOLD:AAA4426
Sunira bicolorago|[17892]|XAH747-05|Canada|Ontario|658[0n]|BOLD:AAA4426
Sunira bicolorago|[17893]|XAH787-05|Canada|Ontario|658[0n]|BOLD:AAA4426
Sunira bicolorago|[17894]|XAH788-05|Canada|Ontario|658[0n]|BOLD:AAA4426
Sunira bicolorago|[17895]|XAH802-05|Canada|Ontario|658[0n]|BOLD:AAA4426
Sunira bicolorago|[17896]|RDLQF246-06|Canada|Quebec|658[0n]|BOLD:AAA4426
Sunira bicolorago|[17897]|LPOKA678-09|United States|Oklahoma|658[0n]|BOLD:AAA4426
Sunira bicolorago|[17898]|LPOKD606-09|United States|Oklahoma|658[0n]|BOLD:AAA4426
Sunira bicolorago|[17899]|MJMSL089-10|United States|Massachusetts|658[0n]|BOLD:AAA4426
Sunira bicolorago|[17900]|PHSEP393-11|Canada|Ontario|658[0n]|BOLD:AAA4426
Sunira bicolorago|[17901]|PHSEP405-11|Canada|Ontario|658[0n]|BOLD:AAA4426
Sunira bicolorago|[17902]|PHOCT920-11|Canada|Ontario|658[0n]|BOLD:AAA4426
Sunira bicolorago|[17903]|GWOTA075-12|United States|Connecticut|658[0n]|BOLD:AAA4426
Sunira bicolorago|[17904]|GWOTA076-12|United States|Massachusetts|658[0n]|BOLD:AAA4426
Sunira bicolorago|[17905]|HEOCT1002-12|Canada|Ontario|658[0n]|BOLD:AAA4426
Sunira bicolorago|[17906]|ALLEP152-13|Canada|Ontario|658[0n]|BOLD:AAA4426
Sunira bicolorago|[17907]|ALLEP308-13|Canada|Ontario|658[0n]|BOLD:AAA4426
Sunira bicolorago|[17908]|ALLEP309-13|Canada|Ontario|658[0n]|BOLD:AAA4426
Sunira bicolorago|[17909]|ALLEP342-13|Canada|Ontario|658[0n]|BOLD:AAA4426
Sunira bicolorago|[17910]|XAH836-05|Canada|Ontario|658[0n]|BOLD:AAA4426
Sunira bicolorago|[17911]|RDLQB507-05|Canada|Quebec|658[0n]|BOLD:AAA4426
Sunira bicolorago|[17912]|ALLEP402-13|Canada|Ontario|658[0n]|BOLD:AAA4426
Sunira bicolorago|[17913]|ALLEP404-13|Canada|Ontario|658[0n]|BOLD:AAA4426
Sunira bicolorago|[17914]|ALLEP527-14|Canada|Ontario|658[0n]|BOLD:AAA4426
Sunira bicolorago|[17915]|ALLEP528-14|Canada|Ontario|658[0n]|BOLD:AAA4426
Sunira bicolorago|[17916]|PAOCT098-12|Canada|Ontario|603[0n]|BOLD:AAA4426
Sunira bicolorago|[17917]|XAB672-04|Canada|Ontario|658[0n]|BOLD:AAA4426
Sunira bicolorago|[17918]|RDLQ583-07|Canada|Quebec|592[1n]|BOLD:AAA4426
Sunira bicolorago|[17919]|RDNMC306-05|United States|Florida|521[0n]|BOLD:AAA4426
Sunira bicolorago|[17920]|PAOCT105-12|Canada|Ontario|597[0n]|BOLD:AAA4426
Sunira bicolorago|[17921]|XAB669-04|Canada|Ontario|658[0n]|BOLD:AAA4426
Sunira bicolorago|[17922]|PHSEP399-11|Canada|Ontario|658[0n]|BOLD:AAA4426
Sunira bicolorago|[17923]|ALLEP153-13|Canada|Ontario|658[0n]|BOLD:AAA4426
Sunira bicolorago|[17924]|XAD493-04|Canada|Ontario|604[0n]|BOLD:AAA4426
Sunira bicolorago|[17925]|RDLQ579-07|Canada|Quebec|599[0n]|BOLD:AAA4426
Sunira bicolorago|[17926]|PHMNB285-04|Canada|New Brunswick|616[0n]|BOLD:AAA4426
Sunira bicolorago|[17927]|RDLQ580-07|Canada|Quebec|590[0n]|BOLD:AAA4426
Sunira bicolorago|[17928]|XAH634-05|Canada|Ontario|634[0n]|BOLD:AAA4426
Sunira bicolorago|[17929]|RDLQ582-07|Canada|Quebec|592[0n]|BOLD:AAA4426
Sunira bicolorago|[17930]|PAOCT097-12|Canada|Ontario|582[0n]|BOLD:AAA4426
Sunira bicolorago|[17931]|SSROA3809-14|Canada|Ontario|579[0n]|BOLD:AAA4426
Sunira bicolorago|[17932]|SSROA3817-14|Canada|Ontario|579[0n]|BOLD:AAA4426
Sunira bicolorago|[17933]|SMTPI7503-14|Canada|Ontario|582[0n]|BOLD:AAA4426
Sunira bicolorago|[17934]|HEOCT1003-12|Canada|Ontario|658[0n]|BOLD:AAA4426
Sunira bicolorago|[17935]|SMTPJ2698-14|Canada|Ontario|597[0n]|BOLD:AAA4426
Sunira bicolorago|[17936]|RDNM740-05|Canada|Ontario|658[0n]|BOLD:AAA4426
Sunira bicolorago|[17937]|XAH709-05|Canada|Ontario|658[0n]|BOLD:AAA4426
Sunira bicolorago|[17938]|XAH790-05|Canada|Ontario|658[0n]|BOLD:AAA4426
Sunira bicolorago|[17939]|PHNOV485-11|Canada|Ontario|658[0n]|BOLD:AAA4426
Sunira bicolorago|[17940]|SMTPI10017-14|Canada|Ontario|603[0n]|BOLD:AAA4426
Sunira bicolorago|[17941]|SMTPJ5447-14|Canada|Ontario|576[0n]|BOLD:AAA4426
Homoglaea variegata|[17942]|CNCLB531-14|United States|Arizona|658[0n]|BOLD:ACM4590
Homoglaea variegata|[17943]|CNCLB532-14|United States|Arizona|658[0n]|BOLD:ACM4590
Homoglaea variegata|[17944]|CNCLB533-14|United States|Arizona|658[0n]|BOLD:ACM4590
Homoglaea variegata|[17945]|CNCLB534-14|United States|New Mexico|658[0n]|BOLD:ACM4590
Homoglaea variegata|[17946]|CNCLB535-14|United States|Utah|658[0n]|BOLD:ACM4590
Psectraglaea carnosa|[17947]|MJMSL008-10|United States|Massachusetts|658[0n]|BOLD:AAE0651
Psectraglaea carnosa|[17948]|MJMSL007-10|United States|Massachusetts|658[0n]|BOLD:AAE0651
Psectraglaea carnosa|[17949]|RDNMF225-08|Canada|Ontario|658[0n]|BOLD:AAE0651
Psectraglaea carnosa|[17950]|RDNMF224-08|Canada|Ontario|658[0n]|BOLD:AAE0651
Psectraglaea carnosa|[17951]|RDNMF223-08|Canada|Ontario|658[0n]|BOLD:AAE0651
Psectraglaea carnosa|[17952]|RDNMF222-08|Canada|Ontario|658[0n]|BOLD:AAE0651
Psectraglaea carnosa|[17953]|RDNMF221-08|Canada|Ontario|658[0n]|BOLD:AAE0651
Psectraglaea carnosa|[17954]|LNAUT2632-14|United States|Massachusetts|407[0n]|BOLD:AAE0651
Lithomoia germana|[17955]|TTMNB371-06|Canada|New Brunswick|658[0n]|BOLD:AAB3483
Lithomoia germana|[17956]|XAH333-05|Canada|Ontario|658[0n]|BOLD:AAB3483
Lithomoia germana|[17957]|XAH332-05|Canada|Ontario|658[0n]|BOLD:AAB3483
Lithomoia germana|[17958]|RDLQ507-07|Canada|Quebec|601[0n]|BOLD:AAB3483
Lithomoia germana|[17959]|RDLQ508-07|Canada|Quebec|601[0n]|BOLD:AAB3483
Lithomoia germana|[17960]|LBCH2779-10|Canada|British Columbia|658[0n]|BOLD:AAB3483
Lithomoia germana|[17961]|TMNBB227-06|Canada|New Brunswick|658[0n]|BOLD:AAB3483
Lithomoia germana|[17962]|XAH238-05|Canada|Ontario|658[0n]|BOLD:AAB3483
Lithomoia germana|[17963]|LPVIB657-08|Canada|British Columbia|614[0n]|BOLD:AAB3483
Lithomoia germana|[17964]|LBCH2776-10|Canada|British Columbia|658[0n]|BOLD:AAB3483
Lithomoia germana|[17965]|LBCH2838-10|Canada|British Columbia|658[0n]|BOLD:AAB3483
Lithomoia germana|[17966]|LBCH2772-10|Canada|British Columbia|658[0n]|BOLD:AAB3483
Lithomoia germana|[17967]|LBCH2774-10|Canada|British Columbia|658[0n]|BOLD:AAB3483
Lithomoia germana|[17968]|LBCH2860-10|Canada|British Columbia|658[0n]|BOLD:AAB3483
Lithomoia germana|[17969]|LBCH2717-10|Canada|British Columbia|658[0n]|BOLD:AAB3483
Lithomoia germana|[17970]|LBCH2681-10|Canada|British Columbia|658[0n]|BOLD:AAB3483
Lithomoia germana|[17971]|LBCH2506-10|Canada|British Columbia|658[0n]|BOLD:AAB3483
Lithomoia germana|[17972]|TMNBB225-06|Canada|New Brunswick|658[0n]|BOLD:AAB3483
Lithomoia germana|[17973]|XAH362-05|Canada|Ontario|658[0n]|BOLD:AAB3483
Lithomoia germana|[17974]|LPVIB669-08|Canada|British Columbia|633[0n]|BOLD:AAB3483
Lithomoia germana|[17975]|TMNBB226-06|Canada|New Brunswick|658[0n]|BOLD:AAB3483
Lithomoia germana|[17976]|XAH374-05|Canada|Ontario|658[0n]|BOLD:AAB3483
Lithomoia germana|[17977]|XAB413-04|Canada|Ontario|658[0n]|BOLD:AAB3483
Lithomoia germana|[17978]|LALPA710-10|Canada|British Columbia|658[0n]|BOLD:AAB3483
Lithomoia germana|[17979]|LALPA762-10|Canada|British Columbia|658[0n]|BOLD:AAB3483
Lithomoia germana|[17980]|LOWCD177-06|Canada|British Columbia|658[0n]|BOLD:ABZ6598
Lithomoia germana|[17981]|LOWCD181-06|Canada|British Columbia|658[0n]|BOLD:ABZ6598
Lithomoia germana|[17982]|LBCH2570-10|Canada|British Columbia|658[0n]|BOLD:ABZ6598
Lithomoia germana|[17983]|LBCH2609-10|Canada|British Columbia|658[0n]|BOLD:ABZ6598
Lithomoia germana|[17984]|LBCH2773-10|Canada|British Columbia|658[0n]|BOLD:ABZ6598
Lithomoia germana|[17985]|LBCH2778-10|Canada|British Columbia|658[0n]|BOLD:ABZ6598
Lithomoia germana|[17986]|XAH625-05|Canada|Ontario|658[0n]|BOLD:ABZ6598
Lithomoia germana|[17987]|LBCH2775-10|Canada|British Columbia|658[0n]|BOLD:ABZ6598
Lithomoia germana|[17988]|LBCH2777-10|Canada|British Columbia|658[0n]|BOLD:ABZ6598
Lithomoia germana|[17989]|RWWC874-12|United States|Washington|658[0n]|BOLD:ABZ6598
Bellura densa|[17990]|LNCB335-06|United States|North Carolina|658[0n]|BOLD:AAA9615

Lithomoia germana|[17988]|LBCH2777-10|Canada|British Columbia|658[0n]|BOLD:ABZ6598
Lithomoia germana|[17989]|RWWC874-12|United States|Washington|658[0n]|BOLD:ABZ6598
Bellura densa|[17990]|LNCB335-06|United States|North Carolina|658[0n]|BOLD:AAA9615
Bellura brehmei|[17991]|CNCLB2454-14|Canada|Nova Scotia|658[0n]|BOLD:AAA9615
Bellura sp.|[17992]|LNCC1230-11|United States|North Carolina|658[0n]|BOLD:AAA9615
Bellura densa|[17993]|LNCB337-06|United States|North Carolina|658[0n]|BOLD:AAA9615
Bellura sp.|[17994]|LNAUS4133-13|United States|Virginia|658[0n]|BOLD:AAA9615
Bellura sp.|[17995]|LNAUS4134-13|United States|Virginia|658[0n]|BOLD:AAA9615
Bellura sp.|[17996]|LNAUS4136-13|United States|Virginia|658[0n]|BOLD:AAA9615
Bellura|[17997]|LNAUT2651-14|United States|Massachusetts|658[0n]|BOLD:AAA9615
Bellura brehmei|[17998]|LNAUS4123-13|United States|Maryland|658[0n]|BOLD:AAA9615
Bellura brehmei|[17999]|LNAUS4126-13|United States|Maryland|658[0n]|BOLD:AAA9615
Bellura densa|[18000]|LNCB336-06|United States|North Carolina|658[0n]|BOLD:AAA9615
Bellura densa|[18001]|LNCB339-06|United States|North Carolina|658[0n]|BOLD:AAA9615
Bellura densa|[18002]|LNCB338-06|United States|North Carolina|658[0n]|BOLD:AAA9615
Bellura|[18003]|LNAUT822-14|United States|South Carolina|658[0n]|BOLD:AAA9615
Bellura densa|[18004]|RDNMJ045-10|United States|Florida|658[0n]|BOLD:AAA9615
Bellura densa|[18005]|LOFLA376-06|United States|Florida|658[0n]|BOLD:AAA9615
Bellura densa|[18006]|RDNMJ048-10|United States|Florida|658[0n]|BOLD:AAA9615
Bellura sp.|[18007]|CNCLB870-14|United States|Florida|539[0n]|BOLD:AAA9615
Bellura densa|[18008]|RDNMJ050-10|United States|Florida|658[0n]|BOLD:AAA9615
Bellura anoa|[18009]|CNCLB1123-14|United States|Florida|658[0n]|BOLD:AAA9615
Bellura anoa|[18010]|CNCLB1126-14|United States|Florida|658[0n]|BOLD:AAA9615
Bellura densa|[18011]|RDNMJ046-10|United States|Florida|658[0n]|BOLD:AAA9615
Bellura sp.|[18012]|LNAUS4135-13|United States|Virginia|658[0n]|BOLD:AAA9615
Bellura anoa|[18013]|CNCLB1124-14|United States|Florida|658[0n]|BOLD:AAA9615
Bellura|[18014]|LNAUT821-14|United States|South Carolina|658[0n]|BOLD:AAA9615
Bellura sp.|[18015]|CNCLB2456-14|United States|South Carolina|658[0n]|BOLD:AAA9615
Bellura obliqua|[18016]|MILEP089-09|United States|North Carolina|654[0n]|BOLD:AAA9615
Bellura obliqua|[18017]|MILEP090-09|United States|North Carolina|658[0n]|BOLD:AAA9615
Bellura densa|[18018]|LNCC1262-11|United States|North Carolina|658[0n]|BOLD:AAA9615
Bellura brehmei|[18019]|LNAUS4127-13|United States|Maryland|658[0n]|BOLD:AAA9615
Bellura brehmei|[18020]|LNAUS4124-13|United States|Maryland|658[0n]|BOLD:AAA9615
Bellura brehmei|[18021]|LNAUS4125-13|United States|Maryland|658[0n]|BOLD:AAA9615
Bellura densa|[18022]|LOFLC183-06|United States|Florida|658[0n]|BOLD:AAA9615
Bellura anoa|[18023]|CNCLB1125-14|United States|Florida|658[0n]|BOLD:AAA9615
Bellura anoa|[18024]|CNCLB1127-14|United States|Florida|658[0n]|BOLD:AAA9615
Bellura sp.|[18025]|CNCLB875-14|United States|Florida|658[0n]|BOLD:AAA9615
Bellura sp.|[18026]|CNCLB876-14|United States|Florida|658[0n]|BOLD:AAA9615
Bellura sp.|[18027]|CNCLB873-14|United States|Florida|658[0n]|BOLD:AAA9615
Bellura sp.|[18028]|CNCLB874-14|United States|Florida|658[0n]|BOLD:AAA9615
Bellura sp.|[18029]|CNCLB869-14|United States|Florida|658[0n]|BOLD:AAA9615
Bellura sp.|[18030]|CNCLB642-14|United States|Georgia|658[0n]|BOLD:AAA9615
Bellura densa|[18031]|CNCLB639-14|United States|Georgia|658[0n]|BOLD:AAA9615
Bellura sp.|[18032]|LNAUS4129-13|United States|Georgia|658[0n]|BOLD:AAA9615
Bellura sp.|[18033]|LNAUS4128-13|United States|Georgia|658[0n]|BOLD:AAA9615
Bellura densa|[18034]|RDNMJ049-10|United States|Florida|658[0n]|BOLD:AAA9615
Bellura densa|[18035]|RDNMJ044-10|United States|Florida|658[0n]|BOLD:AAA9615
Bellura obliqua|[18036]|LNCB802-09|United States|North Carolina|658[0n]|BOLD:AAA9615
Bellura obliqua|[18037]|MILEP092-09|United States|North Carolina|658[0n]|BOLD:AAA9615
Bellura obliqua|[18038]|MILEP091-09|United States|North Carolina|658[0n]|BOLD:AAA9615
Bellura brehmei sp. 1|[18039]|HKONS516-08|United States|Florida|658[0n]|BOLD:AAA9615
Bellura brehmei sp. 1|[18040]|HKONS515-08|United States|Florida|658[0n]|BOLD:AAA9615
Bellura brehmei sp. 2|[18041]|HKONS533-08|United States|Florida|609[0n]|BOLD:AAA9615
Bellura densa|[18042]|LOFLB715-06|United States|Florida|658[0n]|BOLD:AAA9615
Bellura densa|[18043]|RDNMB929-05|United States|Georgia|532[1n]|BOLD:AAA9615
Noctuidae|[18044]|CNCLB1139-14|United States|Arizona|540[0n]|BOLD:AAA9615
Bellura|[18045]|LNAUT823-14|United States|Florida|658[0n]|BOLD:AAA9615
Bellura sp.|[18046]|CNCLB2455-14|United States|South Carolina|658[0n]|BOLD:AAA9615
Bellura sp.|[18047]|CNCLB868-14|United States|Florida|612[0n]|BOLD:AAA9615
Bellura sp.|[18048]|CNCLB2457-14|United States|South Carolina|658[0n]|BOLD:AAA9615
Bellura sp.|[18049]|RDNMK051-11|United States|Florida|658[0n]|BOLD:ABU6862
Bellura sp.|[18050]|CNCLB872-14|United States|Florida|658[0n]|BOLD:ABU6862
Bellura obliqua|[18051]|LPVIA285-08|Canada|British Columbia|658[0n]|BOLD:AAA9614
Bellura obliqua|[18052]|MILEQ223-11|United States|Alabama|658[0n]|BOLD:AAA9614
Bellura obliqua|[18053]|LALPA188-10|Canada|British Columbia|658[0n]|BOLD:AAA9614
Bellura obliqua|[18054]|LALPA203-10|Canada|British Columbia|658[0n]|BOLD:AAA9614
Bellura obliqua|[18055]|LALPA244-10|Canada|British Columbia|658[0n]|BOLD:AAA9614
Bellura obliqua|[18056]|RWWC265-11|United States|Washington|658[0n]|BOLD:AAA9614
Bellura obliqua|[18057]|RDNMJ146-10|United States|Washington|658[0n]|BOLD:AAA9614
Bellura obliqua|[18058]|BBLPA442-10|Canada|British Columbia|658[0n]|BOLD:AAA9614
Bellura obliqua|[18059]|BBLPA441-10|Canada|British Columbia|658[0n]|BOLD:AAA9614
Bellura obliqua|[18060]|RDNMB927-05|Canada|British Columbia|658[0n]|BOLD:AAA9614
Bellura obliqua|[18061]|RWWA298-09|United States|Washington|658[0n]|BOLD:AAA9614
Bellura obliqua|[18062]|RWWA657-09|United States|Washington|658[0n]|BOLD:AAA9614
Bellura obliqua|[18063]|BBLPA439-10|Canada|British Columbia|641[0n]|BOLD:AAA9614
Bellura obliqua|[18064]|BBLPA440-10|Canada|British Columbia|642[0n]|BOLD:AAA9614
Bellura obliqua|[18065]|BBLPA443-10|Canada|British Columbia|658[0n]|BOLD:AAA9614
Bellura obliqua|[18066]|RWWA600-09|United States|Washington|658[0n]|BOLD:AAA9614
Bellura obliqua|[18067]|RWWB730-10|United States|Washington|658[0n]|BOLD:AAA9614
Bellura obliqua|[18068]|RWWC300-11|United States|Washington|658[0n]|BOLD:AAA9614
Bellura obliqua|[18069]|RWWC453-11|United States|Washington|658[0n]|BOLD:AAA9614
Bellura obliqua|[18070]|LOCBC496-06|United States|California|658[0n]|BOLD:AAA9614
Bellura obliqua|[18071]|LNAUS4130-13|United States|California|658[0n]|BOLD:AAA9614
Bellura obliqua|[18072]|LNAUS4131-13|United States|California|658[0n]|BOLD:AAA9614
Bellura obliqua|[18073]|LNAUS4132-13|United States|California|658[0n]|BOLD:AAA9614
Bellura obliqua|[18074]|RDNMB118-05|Canada|Alberta|658[0n]|BOLD:AAA9614
Bellura obliqua|[18075]|RDNMB931-05|Canada|Alberta|599[0n]|BOLD:AAA9614
Bellura obliqua|[18076]|RDNMJ149-10|Canada|Alberta|658[0n]|BOLD:AAA9614
Bellura obliqua|[18077]|RDNMJ150-10|Canada|Alberta|658[0n]|BOLD:AAA9614
Bellura obliqua|[18078]|CNCLB641-14|United States|Colorado|658[0n]|BOLD:AAA9614
Bellura obliqua|[18079]|CNCLB2543-14|United States|New York|658[0n]|BOLD:AAA9614
Bellura obliqua|[18080]|XAJ420-06|Canada|Ontario|658[0n]|BOLD:AAA9614
Bellura obliqua|[18081]|LPSOB425-08|Canada|Ontario|658[0n]|BOLD:AAA9614
Bellura obliqua|[18082]|LPMN310-08|Canada|Manitoba|658[0n]|BOLD:AAA9614
Bellura obliqua|[18083]|XAI055-05|Canada|Ontario|658[0n]|BOLD:AAA9614
Bellura obliqua|[18084]|XAE366-04|Canada|Ontario|658[0n]|BOLD:AAA9614
Bellura obliqua|[18085]|XAJ671-06|Canada|Ontario|658[0n]|BOLD:AAA9614
Bellura obliqua|[18086]|LPSO848-08|Canada|Ontario|658[0n]|BOLD:AAA9614
Bellura obliqua|[18087]|LPSOB424-08|Canada|Ontario|658[0n]|BOLD:AAA9614
Bellura obliqua|[18088]|BLTIB200-08|Canada|Ontario|658[0n]|BOLD:AAA9614
Bellura obliqua|[18089]|RDNMJ173-10|Canada|Ontario|658[0n]|BOLD:AAA9614
Bellura obliqua|[18090]|CNCLB1756-14|United States|Indiana|658[0n]|BOLD:AAA9614

[illegible]
Bellura obliqua|[18088]|BLTIB200-08|Canada|Ontario|658[0n]|BOLD:AAA9614
Bellura obliqua|[18089]|RDNMJ173-10|Canada|Ontario|658[0n]|BOLD:AAA9614
Bellura obliqua|[18090]|CNCLB1756-14|United States|Indiana|658[0n]|BOLD:AAA9614
Bellura obliqua|[18091]|BBLPA095-10|Canada|Manitoba|658[0n]|BOLD:AAA9614
Bellura obliqua|[18092]|XAB275-04|Canada|Ontario|658[0n]|BOLD:AAA9614
Bellura sp.|[18093]|RDNM975-05|Canada|Ontario|658[0n]|BOLD:AAA9614
Bellura obliqua|[18094]|RDNMB928-05|Canada|Ontario|658[0n]|BOLD:AAA9614
Bellura obliqua|[18095]|XAI056-05|Canada|Ontario|658[0n]|BOLD:AAA9614
Bellura sp.|[18096]|XAK338-06|Canada|Ontario|658[0n]|BOLD:AAA9614
Bellura obliqua|[18097]|RDNMG058-08|Canada|Ontario|658[0n]|BOLD:AAA9614
Bellura obliqua|[18098]|BLTIB198-08|Canada|Ontario|658[0n]|BOLD:AAA9614
Bellura obliqua|[18099]|BBLPA094-10|Canada|Saskatchewan|658[0n]|BOLD:AAA9614
Bellura sp.|[18100]|RDNML025-13|Canada|Ontario|658[0n]|BOLD:AAA9614
Bellura obliqua|[18101]|RDLQB746-05|Canada|Quebec|658[0n]|BOLD:AAA9614
Bellura obliqua|[18102]|LNAUT2652-14|United States|Massachusetts|658[0n]|BOLD:AAA9614
Bellura obliqua|[18103]|XAE464-04|Canada|Ontario|613[1n]|BOLD:AAA9614
Bellura obliqua|[18104]|PHMNB581-04|Canada|New Brunswick|658[0n]|BOLD:AAA9614
Bellura obliqua|[18105]|XAJ539-06|Canada|Ontario|622[0n]|BOLD:AAA9614
Bellura obliqua|[18106]|RDLQG374-06|Canada|Quebec|658[0n]|BOLD:AAA9614
Bellura obliqua|[18107]|XAK155-06|Canada|Ontario|658[0n]|BOLD:AAA9614
Bellura obliqua|[18108]|XAG470-05|Canada|Ontario|658[0n]|BOLD:AAA9614
Bellura sp.|[18109]|RDNM976-05|Canada|Ontario|658[0n]|BOLD:AAA9614
Bellura sp.|[18110]|RDNML026-13|Canada|Ontario|658[0n]|BOLD:AAA9614
Bellura obliqua|[18111]|CNCLA7368-13|Canada|Ontario|658[0n]|BOLD:AAA9614
Bellura obliqua|[18112]|CNCLB2545-14|United States|New York|658[0n]|BOLD:AAA9614
Bellura vulnifica|[18113]|CNCLA7375-13|Canada|Ontario|658[0n]|BOLD:AAD3485
Bellura vulnifica|[18114]|LMDH205-11|United States|Minnesota|658[0n]|BOLD:AAD3485
Bellura sp.|[18115]|RDNMJ043-10|United States|Florida|658[0n]|BOLD:AAD3485
Bellura vulnifica|[18116]|LMDH195-11|United States|Minnesota|658[0n]|BOLD:AAD3485
Bellura vulnifica|[18117]|RDNMJ047-10|United States|Florida|658[0n]|BOLD:AAD3485
Bellura vulnifica|[18118]|RDNMJ042-10|United States|Florida|658[0n]|BOLD:AAD3485
Bellura vulnifica|[18119]|RDNMJ041-10|United States|Florida|658[0n]|BOLD:AAD3485
Bellura sp.|[18120]|LPSO851-08|Canada|Ontario|658[0n]|BOLD:AAD3485
Bellura vulnifica|[18121]|XAK423-06|Canada|Ontario|658[0n]|BOLD:AAD3485
Bellura vulnifica|[18122]|XAJ829-06|Canada|Ontario|658[0n]|BOLD:AAD3485
Bellura sp.|[18123]|LOFLA519-06|United States|Florida|658[0n]|BOLD:AAD3485
Bellura gortynoides|[18124]|RDNMC578-06|Canada|Ontario|658[0n]|BOLD:AAD3485
Bellura vulnifica|[18125]|RDNMB930-05|Canada|Quebec|658[0n]|BOLD:AAD3485
Bellura sp.|[18126]|LOFLA388-06|United States|Florida|658[0n]|BOLD:AAD3485
Bellura vulnifica|[18127]|RDNML371-13|Canada|Ontario|658[0n]|BOLD:AAD3485
Bellura vulnifica|[18128]|RDNML372-13|Canada|Ontario|658[0n]|BOLD:AAD3485
Bellura gortynoides|[18129]|LNAUT817-14|Canada|Nova Scotia|658[0n]|BOLD:AAD3485
Bellura gortynoides|[18130]|LNAUT827-14|United States|Nebraska|658[0n]|BOLD:AAD3485
Bellura|[18131]|CNCLB1754-14|United States|Indiana|658[0n]|BOLD:AAD3485
Bellura gortynoides|[18132]|LNAUT824-14|Canada|Ontario|658[0n]|BOLD:ACD3574
Bellura|[18133]|CNCLB1755-14|United States|Indiana|658[0n]|BOLD:ACD3574
Bellura sp.|[18134]|RDNML027-13|United States|Minnesota|658[0n]|BOLD:ACD3574
Bellura gortynoides|[18135]|CNCLB2712-14|United States|North Carolina|658[0n]|BOLD:ACD3574
Agrochola pulchella|[18136]|RDNMF227-08|Canada|British Columbia|658[0n]|BOLD:AAF7887
Agrochola pulchella|[18137]|RDNMF226-08|United States|Oregon|658[0n]|BOLD:AAF7887
Agrochola pulchella|[18138]|RDNMF228-08|United States|Oregon|658[0n]|BOLD:AAF7887
Agrochola pulchella|[18139]|LBCH2463-10|Canada|British Columbia|658[0n]|BOLD:AAH6129
Agrochola pulchella|[18140]|LBCH2511-10|Canada|British Columbia|658[0n]|BOLD:AAH6129
Agrochola purpurea|[18141]|CGLCA038-10|United States|California|658[0n]|BOLD:AAC1554
Agrochola purpurea|[18142]|RDNMF490-08|United States|Washington|658[0n]|BOLD:AAC1554
Agrochola purpurea|[18143]|NAMUM197-08|United States|California|636[0n]|BOLD:AAC1554
Agrochola purpurea|[18144]|RDNMF489-08|United States|Oregon|658[1n]|BOLD:AAC1554
Agrochola purpurea|[18145]|RDNMF488-08|United States|Washington|648[2n]|BOLD:AAC1554
Agrochola purpurea|[18146]|RDNMF491-08|Canada|British Columbia|652[0n]|BOLD:AAC1554
Agrochola purpurea|[18147]|RDNMF492-08|Canada|British Columbia|658[0n]|BOLD:AAC1554
Agrochola purpurea|[18148]|GMLC017-09|United States|California|658[0n]|BOLD:AAC1554
Agrochola purpurea|[18149]|GMLC031-09|United States|California|658[0n]|BOLD:AAC1554
Agrochola purpurea|[18150]|CGLCA041-10|United States|California|658[0n]|BOLD:AAC1554
Agrochola purpurea|[18151]|GMLC1247-12|United States|California|658[0n]|BOLD:AAC1554
Agrochola purpurea|[18152]|CGLCA039-10|United States|California|658[0n]|BOLD:AAC1554
Agrochola purpurea|[18153]|GMLC085-09|United States|California|658[0n]|BOLD:AAC1554
Agrochola purpurea|[18154]|GMLC052-09|United States|California|658[0n]|BOLD:AAC1554
Agrochola purpurea|[18155]|GMLC044-09|United States|California|658[0n]|BOLD:AAC1554
Agrochola purpurea|[18156]|GMLC043-09|United States|California|658[0n]|BOLD:AAC1554
Agrochola purpurea|[18157]|GMLC006-09|United States|California|658[0n]|BOLD:AAC1554
Agrochola purpurea|[18158]|GMLC005-09|United States|California|658[0n]|BOLD:AAC1554
Agrochola purpurea|[18159]|CGLCA040-10|United States|California|658[0n]|BOLD:AAC1554
Agrochola purpurea|[18160]|GMLC1248-12|United States|California|658[0n]|BOLD:AAC1554
Ipimorpha pleonectusa|[18161]|XAG759-05|Canada|Ontario|658[1n]|BOLD:AAB4652
Ipimorpha pleonectusa|[18162]|XAD089-04|Canada|Ontario|583[0n]|BOLD:AAB4652
Ipimorpha nanaimo|[18163]|RDNMF513-08|Canada|British Columbia|658[1n]|BOLD:AAB4652
Ipimorpha pleonectusa|[18164]|XAG551-05|Canada|Ontario|586[0n]|BOLD:AAB4652
Ipimorpha pleonectusa|[18165]|RDMAB668-06|Canada|Alberta|627[0n]|BOLD:AAB4652
Ipimorpha pleonectusa|[18166]|RDMAB669-06|Canada|Alberta|531[1n]|BOLD:AAB4652
Ipimorpha pleonectusa|[18167]|LPABC990-09|Canada|Alberta|658[0n]|BOLD:AAB4652
Ipimorpha pleonectusa|[18168]|BBLPA435-10|Canada|Alberta|658[0n]|BOLD:AAB4652
Ipimorpha pleonectusa|[18169]|XAG863-05|Canada|Ontario|658[0n]|BOLD:AAB4652
Ipimorpha pleonectusa|[18170]|XAG706-05|Canada|Ontario|658[0n]|BOLD:AAB4652
Ipimorpha pleonectusa|[18171]|XAG387-05|Canada|Ontario|656[0n]|BOLD:AAB4652
Ipimorpha pleonectusa|[18172]|PHMO317-03|Canada|Ontario|639[0n]|BOLD:AAB4652
Ipimorpha pleonectusa|[18173]|XAG974-05|Canada|Ontario|510[1n]|BOLD:AAB4652
Ipimorpha pleonectusa|[18174]|RDNMC021-05|Canada|Alberta|515[2n]|BOLD:AAB4652
Ipimorpha pleonectusa|[18175]|XAD301-04|Canada|Ontario|530[0n]|BOLD:AAB4652
Ipimorpha pleonectusa|[18176]|RDMAB670-06|Canada|Alberta|573[1n]|BOLD:AAB4652
Ipimorpha pleonectusa|[18177]|RDLQF253-06|Canada|Quebec|658[0n]|BOLD:AAB4652
Ipimorpha nanaimo|[18178]|LHLEP191-06|Canada|British Columbia|658[0n]|BOLD:AAB4652
Ipimorpha nanaimo|[18179]|LHLEP192-06|Canada|British Columbia|658[0n]|BOLD:AAB4652
Ipimorpha nanaimo|[18180]|RDNMF514-08|Canada|British Columbia|658[0n]|BOLD:AAB4652
Ipimorpha nanaimo|[18181]|RDNMF515-08|Canada|British Columbia|658[0n]|BOLD:AAB4652
Ipimorpha nanaimo|[18182]|RDNMF516-08|Canada|British Columbia|658[0n]|BOLD:AAB4652
Ipimorpha nanaimo|[18183]|LPVIB066-08|Canada|British Columbia|658[0n]|BOLD:AAB4652
Ipimorpha pleonectusa|[18184]|LPMNB370-09|Canada|Manitoba|658[0n]|BOLD:AAB4652
Ipimorpha pleonectusa|[18185]|LPMNB429-09|Canada|Manitoba|658[0n]|BOLD:AAB4652
Ipimorpha nanaimo|[18186]|LALPA1312-11|Canada|British Columbia|658[0n]|BOLD:AAB4652
Ipimorpha nanaimo|[18187]|NAMUM161-08|United States|California|655[0n]|BOLD:AAB4652
Ipimorpha nanaimo|[18188]|LPVIB064-08|Canada|British Columbia|658[0n]|BOLD:AAB4652
Ipimorpha nanaimo|[18189]|LPVIB516-08|Canada|British Columbia|658[0n]|BOLD:AAB4652
Ipimorpha pleonectusa|[18190]|LPMNB446-09|Canada|Manitoba|658[0n]|BOLD:AAB4652

[illegible]
Ipimorpha nanaimo|[18188]|LPVIB064-08|Canada|British Columbia|658[0n]|BOLD:AAB4652
Ipimorpha nanaimo|[18189]|LPVIB516-08|Canada|British Columbia|658[0n]|BOLD:AAB4652
Ipimorpha pleonectusa|[18190]|LPMNB446-09|Canada|Manitoba|658[0n]|BOLD:AAB4652
Ipimorpha viridipallida|[18191]|RDNMF517-08|United States|Montana|658[0n]|BOLD:AAB4652
Ipimorpha pleonectusa|[18192]|LPVIB065-08|Canada|British Columbia|658[0n]|BOLD:AAB4652
Ipimorpha nanaimo|[18193]|LPVIB517-08|Canada|British Columbia|658[0n]|BOLD:AAB4652
Ipimorpha viridipallida|[18194]|RDNMG599-08|United States|Washington|658[0n]|BOLD:AAB4652
Ipimorpha nanaimo|[18195]|LPVIA840-08|Canada|British Columbia|658[0n]|BOLD:AAB4652
Ipimorpha nanaimo|[18196]|RDNMF512-08|Canada|British Columbia|658[0n]|BOLD:AAB4652
Ipimorpha viridipallida|[18197]|RDNMG598-08|United States|Washington|658[0n]|BOLD:AAB4652
Ipimorpha nanaimo|[18198]|LPVIB518-08|Canada|British Columbia|658[0n]|BOLD:AAB4652
Ipimorpha nanaimo|[18199]|LPVIB519-08|Canada|British Columbia|658[0n]|BOLD:AAB4652
Ipimorpha pleonectusa|[18200]|BBLPC009-09|Canada|New Brunswick|658[0n]|BOLD:AAB4652
Ipimorpha pleonectusa|[18201]|LBCH3789-10|Canada|British Columbia|658[0n]|BOLD:AAB4652
Ipimorpha pleonectusa|[18202]|RDLQB631-05|Canada|Quebec|658[0n]|BOLD:AAB4652
Ipimorpha pleonectusa|[18203]|LOWCD185-06|Canada|British Columbia|658[0n]|BOLD:AAB4652
Ipimorpha pleonectusa|[18204]|LBCH4670-10|Canada|British Columbia|658[0n]|BOLD:AAB4652
Ipimorpha pleonectusa|[18205]|LPABC152-09|Canada|Alberta|614[1n]|BOLD:AAB4652
Ipimorpha pleonectusa|[18206]|XAG705-05|Canada|Ontario|658[0n]|BOLD:AAB4652
Ipimorpha pleonectusa|[18207]|BBLPA438-10|Canada|Saskatchewan|658[0n]|BOLD:AAB4652
Ipimorpha nanaimo|[18208]|BBLPA436-10|Canada|Saskatchewan|658[1n]|BOLD:AAB4652
Ipimorpha pleonectusa|[18209]|BBLPA437-10|Canada|Saskatchewan|658[0n]|BOLD:AAB4652
Ipimorpha pleonectusa|[18210]|LBCH918-10|Canada|British Columbia|658[0n]|BOLD:AAB4652
Ipimorpha pleonectusa|[18211]|RDMAB667-06|Canada|Alberta|555[0n]|BOLD:AAB4652
Ipimorpha pleonectusa|[18212]|RDLQB630-05|Canada|Quebec|523[0n]|BOLD:AAB4652
Ipimorpha pleonectusa|[18213]|CNRMA915-12|Canada|Manitoba|620[0n]|BOLD:AAB4652
Ipimorpha pleonectusa|[18214]|LPMNB431-09|Canada|Manitoba|658[0n]|BOLD:AAB4652
Ipimorpha nanaimo|[18215]|LPVIB661-08|Canada|British Columbia|576[0n]|BOLD:AAB4652
Ipimorpha pleonectusa|[18216]|CNWBE392-13|Canada|Alberta|537[0n]|BOLD:AAB4652
Ipimorpha pleonectusa|[18217]|CNWBE393-13|Canada|Alberta|558[2n]|BOLD:AAB4652
Ipimorpha nanaimo|[18218]|LPVIB276-08|Canada|British Columbia|658[0n]|BOLD:AAB4652
Ipimorpha nanaimo|[18219]|LPVIB063-08|Canada|British Columbia|658[0n]|BOLD:AAB4652
Ipimorpha nanaimo|[18220]|LPVIA305-08|Canada|British Columbia|658[0n]|BOLD:AAB4652
Ipimorpha viridipallida|[18221]|JMMMB589-13|United States|California|606[0n]|BOLD:AAB4652
Lithophane contenta|[18222]|RDNMF209-08|United States|California|658[0n]|BOLD:AAK6635
Lithophane contenta|[18223]|RDNMJ136-10|United States|New Mexico|658[0n]|BOLD:AAV8098
Lithophane abita|[18224]|RDNMD631-06|United States|Louisiana|658[1n]|BOLD:AAJ2386
Lithophane abita|[18225]|RDNMD632-06|United States|Louisiana|658[0n]|BOLD:AAJ2386
Lithophane joannis|[18226]|LNCC612-11|United States|North Carolina|658[0n]|BOLD:AAB1070
Lithophane joannis|[18227]|LNCC614-11|United States|North Carolina|614[0n]|BOLD:AAB1070
Lithophane joannis|[18228]|LNCC433-10|United States|North Carolina|658[0n]|BOLD:AAB1070
Lithophane joannis|[18229]|LNCC615-11|United States|North Carolina|658[0n]|BOLD:AAB1070
Lithophane joannis|[18230]|LNCC613-11|United States|North Carolina|658[0n]|BOLD:AAB1070
Lithophane joannis|[18231]|RDNMJ154-10|United States|Indiana|658[0n]|BOLD:AAB1070
Lithophane joannis|[18232]|LNCC616-11|United States|North Carolina|658[0n]|BOLD:AAB1070
Lithophane patefacta|[18233]|LNC494-06|United States|North Carolina|658[0n]|BOLD:AAB1070
Lithophane patefacta|[18234]|LNCC640-11|United States|North Carolina|614[0n]|BOLD:AAB1070
Lithophane patefacta|[18235]|LNCC630-11|United States|North Carolina|614[0n]|BOLD:AAB1070
Lithophane patefacta|[18236]|LNCC476-11|United States|North Carolina|658[0n]|BOLD:AAB1070
Lithophane patefacta|[18237]|LNCC567-11|United States|North Carolina|658[0n]|BOLD:AAB1070
Lithophane patefacta|[18238]|CNCLB1326-14|United States|Louisiana|658[0n]|BOLD:AAB1070
Lithophane patefacta|[18239]|LNCC568-11|United States|North Carolina|658[0n]|BOLD:AAB1070
Lithophane patefacta|[18240]|LNCC566-11|United States|North Carolina|658[0n]|BOLD:AAB1070
Lithophane patefacta|[18241]|LNCC565-11|United States|North Carolina|658[0n]|BOLD:AAB1070
Lithophane patefacta|[18242]|LNCC564-11|United States|North Carolina|658[0n]|BOLD:AAB1070
Lithophane patefacta|[18243]|LNCC563-11|United States|North Carolina|658[0n]|BOLD:AAB1070
Lithophane patefacta|[18244]|LNCC541-11|United States|North Carolina|658[0n]|BOLD:AAB1070
Lithophane patefacta|[18245]|LNCC539-11|United States|North Carolina|658[0n]|BOLD:AAB1070
Lithophane patefacta|[18246]|PSAT121-10|United States|Florida|658[0n]|BOLD:AAB1070
Lithophane patefacta|[18247]|LNCB976-10|United States|North Carolina|658[0n]|BOLD:AAB1070
Lithophane patefacta|[18248]|LGSMG696-07|United States|Tennessee|658[0n]|BOLD:AAB1070
Lithophane patefacta|[18249]|LSEU133-06|United States|Georgia|658[0n]|BOLD:AAB1070
Lithophane patefacta|[18250]|LNC495-06|United States|North Carolina|656[0n]|BOLD:AAB1070
Lithophane patefacta|[18251]|LNCC478-11|United States|North Carolina|638[0n]|BOLD:AAB1070
Lithophane patefacta|[18252]|PSAT120-10|United States|New Jersey|658[0n]|BOLD:AAB1070
Lithophane patefacta|[18253]|LNCC569-11|United States|North Carolina|658[0n]|BOLD:AAB1070
Lithophane patefacta|[18254]|LNCC570-11|United States|North Carolina|658[0n]|BOLD:AAB1070
Lithophane patefacta|[18255]|LNCC629-11|United States|North Carolina|658[0n]|BOLD:AAB1070
Lithophane patefacta|[18256]|LNCC631-11|United States|North Carolina|658[0n]|BOLD:AAB1070
Lithophane patefacta|[18257]|LNCC632-11|United States|North Carolina|658[0n]|BOLD:AAB1070
Lithophane patefacta|[18258]|LNCC633-11|United States|North Carolina|658[0n]|BOLD:AAB1070
Lithophane patefacta|[18259]|LNCC634-11|United States|North Carolina|658[0n]|BOLD:AAB1070
Lithophane patefacta|[18260]|LNCC635-11|United States|North Carolina|658[0n]|BOLD:AAB1070
Lithophane patefacta|[18261]|LNCC637-11|United States|North Carolina|658[0n]|BOLD:AAB1070
Lithophane patefacta|[18262]|LNCC638-11|United States|North Carolina|658[0n]|BOLD:AAB1070
Lithophane patefacta|[18263]|LNCC639-11|United States|North Carolina|658[0n]|BOLD:AAB1070
Lithophane patefacta|[18264]|RDNMK630-11|United States|North Carolina|658[0n]|BOLD:AAB1070
Lithophane disposita|[18265]|RDNML110-13|United States|New Jersey|658[0n]|BOLD:AAB1070
Lithophane patefacta|[18266]|RDNML113-13|United States|New Jersey|658[0n]|BOLD:AAB1070
Lithophane patefacta|[18267]|RDNML114-13|United States|New Jersey|658[0n]|BOLD:AAB1070
Lithophane patefacta|[18268]|CNCLB1325-14|United States|Louisiana|658[0n]|BOLD:AAB1070
Lithophane patefacta|[18269]|CNCLB1422-14|United States|North Carolina|658[0n]|BOLD:AAB1070
Lithophane semiusta|[18270]|RDLQ157-05|Canada|Quebec|575[0n]|BOLD:AAB1070
Lithophane semiusta|[18271]|RDLQ540-07|Canada|Quebec|658[0n]|BOLD:AAB1070
Lithophane patefacta|[18272]|LNCC542-11|United States|North Carolina|658[0n]|BOLD:AAB1070
Lithophane patefacta|[18273]|LNCC636-11|United States|North Carolina|658[0n]|BOLD:AAB1070
Lithophane patefacta|[18274]|LNCC642-11|United States|North Carolina|658[0n]|BOLD:AAB1070
Lithophane patefacta|[18275]|RDNML112-13|United States|New Jersey|658[5n]|BOLD:AAB1070
Lithophane oriunda|[18276]|XAJ212-06|Canada|Ontario|658[0n]|BOLD:AAB1070
Lithophane oriunda|[18277]|XAF294-05|Canada|Ontario|658[0n]|BOLD:AAB1070
Lithophane oriunda|[18278]|XAE169-04|Canada|Ontario|658[0n]|BOLD:AAB1070
Lithophane oriunda|[18279]|RDLQ510-07|Canada|Quebec|553[0n]|BOLD:AAB1070
Lithophane oriunda|[18280]|RDLQ511-07|Canada|Quebec|577[0n]|BOLD:AAB1070
Lithophane oriunda|[18281]|PHMO047-03|Canada|Ontario|639[0n]|BOLD:AAB1070
Lithophane oriunda|[18282]|XAJ010-06|Canada|Ontario|610[0n]|BOLD:AAB1070
Lithophane oriunda|[18283]|RDLQ512-07|Canada|Quebec|604[0n]|BOLD:AAB1070
Lithophane oriunda|[18284]|LNCC617-11|United States|North Carolina|614[0n]|BOLD:AAB1070
Lithophane disposita|[18285]|LNCC528-11|United States|North Carolina|658[0n]|BOLD:AAB1070
Lithophane disposita|[18286]|LNCB997-10|United States|North Carolina|658[0n]|BOLD:AAB1070
Lithophane disposita|[18287]|LNCB998-10|United States|North Carolina|614[0n]|BOLD:AAB1070
Lithophane sp.|[18288]|RDNML099-13|United States|New Jersey|658[0n]|BOLD:AAB1070
Lithophane disposita|[18289]|RDNML164-13|United States|North Carolina|658[0n]|BOLD:AAB1070
Lithophane bethunei|[18290]|XAJ133-06|Canada|Ontario|658[0n]|BOLD:AAB1070

Lithophane disposita|[18287]|[illegible]|United States|North Carolina|[illegible]|BOLD:AAB1070
Lithophane sp.|[18288]|RDNML099-13|United States|New Jersey|658[0n]|BOLD:AAB1070
Lithophane disposita|[18289]|RDNML164-13|United States|North Carolina|658[0n]|BOLD:AAB1070
Lithophane bethunei|[18290]|XAJ133-06|Canada|Ontario|658[0n]|BOLD:AAB1070
Lithophane patefacta|[18291]|RDNMJ108-10|United States|Indiana|658[0n]|BOLD:AAB1070
Lithophane patefacta|[18292]|XAF207-05|Canada|Ontario|658[0n]|BOLD:AAB1070
Lithophane patefacta|[18293]|RDLQ546-07|Canada|Quebec|658[0n]|BOLD:AAB1070
Lithophane bethunei|[18294]|XAF210-05|Canada|Ontario|658[0n]|BOLD:AAB1070
Lithophane bethunei|[18295]|XAF269-05|Canada|Ontario|658[0n]|BOLD:AAB1070
Lithophane patefacta|[18296]|LNCC628-11|United States|North Carolina|614[0n]|BOLD:AAB1070
Lithophane patefacta|[18297]|XAF197-05|Canada|Ontario|658[0n]|BOLD:AAB1070
Lithophane bethunei|[18298]|RDLQ517-07|Canada|Quebec|658[0n]|BOLD:AAB1070
Lithophane patefacta|[18299]|RDNM849-05|Canada|Ontario|616[0n]|BOLD:AAB1070
Lithophane sp.|[18300]|RDNMK629-11|United States|North Carolina|658[0n]|BOLD:AAB1070
Lithophane patefacta|[18301]|RDNML111-13|United States|New Jersey|658[0n]|BOLD:AAB1070
Lithophane disposita|[18302]|QUNOD342-10|United States|Wisconsin|658[0n]|BOLD:AAB1070
Lithophane patefacta|[18303]|LNCC641-11|United States|North Carolina|658[0n]|BOLD:AAB1070
Lithophane bethunei|[18304]|NAMUM404-09|United States|Maryland|658[0n]|BOLD:AAB1070
Lithophane patefacta|[18305]|LNCB996-10|United States|North Carolina|658[0n]|BOLD:AAB1070
Lithophane patefacta|[18306]|RDLQ547-07|Canada|Quebec|658[0n]|BOLD:AAB1070
Lithophane patefacta|[18307]|RDLQ548-07|Canada|Quebec|658[0n]|BOLD:AAB1070
Lithophane bethunei|[18308]|RDLQ516-07|Canada|Quebec|658[0n]|BOLD:AAB1070
Lithophane disposita|[18309]|XAJ120-06|Canada|Ontario|658[0n]|BOLD:AAB1070
Lithophane patefacta|[18310]|RDNM848-05|Canada|Ontario|658[0n]|BOLD:AAB1070
Lithophane patefacta|[18311]|XAF448-05|Canada|Ontario|658[0n]|BOLD:AAB1070
Lithophane bethunei|[18312]|XAF298-05|Canada|Ontario|658[0n]|BOLD:AAB1070
Lithophane patefacta|[18313]|XAF295-05|Canada|Ontario|658[0n]|BOLD:AAB1070
Lithophane bethunei|[18314]|XAF260-05|Canada|Ontario|658[0n]|BOLD:AAB1070
Lithophane patefacta|[18315]|XAF206-05|Canada|Ontario|658[0n]|BOLD:AAB1070
Lithophane patefacta|[18316]|XAD643-05|Canada|Ontario|658[0n]|BOLD:AAB1070
Lithophane bethunei|[18317]|XAJ015-06|Canada|Ontario|622[0n]|BOLD:AAB1070
Lithophane disposita|[18318]|XAJ016-06|Canada|Ontario|618[0n]|BOLD:AAB1070
Lithophane disposita|[18319]|RDLQ529-07|Canada|Quebec|595[0n]|BOLD:AAB1070
Lithophane patefacta|[18320]|LNCB978-10|United States|North Carolina|658[0n]|BOLD:AAB1070
Lithophane patefacta|[18321]|LNCC540-11|United States|North Carolina|658[0n]|BOLD:AAB1070
Lithophane patefacta|[18322]|RDNML115-13|United States|New Jersey|658[0n]|BOLD:AAB1070
Lithophane pr. patefacta|[18323]|SMTPD1183-13|Canada|Ontario|606[0n]|BOLD:AAB1070
Lithophane lanei|[18324]|RDNM050-05|Canada|Ontario|658[0n]|BOLD:AAB1070
Lithophane lanei|[18325]|RDNM049-05|Canada|Ontario|658[0n]|BOLD:AAB1070
Lithophane lanei|[18326]|RDNM048-05|Canada|Ontario|658[0n]|BOLD:AAB1070
Lithophane lanei|[18327]|RDNM047-05|Canada|Ontario|658[0n]|BOLD:AAB1070
Lithophane lanei|[18328]|XAF262-05|Canada|Ontario|658[0n]|BOLD:AAB1070
Lithophane lanei|[18329]|XAF204-05|Canada|Ontario|658[0n]|BOLD:AAB1070
Lithophane lanei|[18330]|XAF151-05|Canada|Ontario|658[0n]|BOLD:AAB1070
Lithophane lanei|[18331]|RDLQ549-07|Canada|Quebec|596[0n]|BOLD:AAB1070
Lithophane lanei|[18332]|RDLQ550-07|Canada|Quebec|658[0n]|BOLD:AAB1070
Lithophane lanei|[18333]|RDNMJ102-10|United States|Indiana|658[0n]|BOLD:AAB1070
Lithophane innominata|[18334]|RDLQ162-05|Canada|Quebec|625[0n]|BOLD:AAB1070
Lithophane innominata|[18335]|RDLQ530-07|Canada|Quebec|658[0n]|BOLD:AAB1070
Lithophane franclemonti|[18336]|QUNOE399-12|United States|Wisconsin|658[0n]|BOLD:AAB1070
Lithophane sp.|[18337]|LOCT001-05|United States|Connecticut|658[0n]|BOLD:AAB1070
Lithophane bethunei|[18338]|XAF209-05|Canada|Ontario|658[0n]|BOLD:AAB1070
Lithophane bethunei|[18339]|XAF212-05|Canada|Ontario|658[0n]|BOLD:AAB1070
Lithophane hemina|[18340]|LNCC966-11|United States|North Carolina|658[0n]|BOLD:AAB1070
Lithophane hemina|[18341]|RDLQ551-07|Canada|Quebec|658[0n]|BOLD:AAB1070
Lithophane hemina|[18342]|RDLQ539-07|Canada|Quebec|658[0n]|BOLD:AAB1070
Lithophane hemina|[18343]|RDLQ537-07|Canada|Quebec|658[0n]|BOLD:AAB1070
Lithophane hemina|[18344]|RDLQ536-07|Canada|Quebec|658[0n]|BOLD:AAB1070
Lithophane hemina|[18345]|RDLQ535-07|Canada|Quebec|658[0n]|BOLD:AAB1070
Lithophane hemina|[18346]|RDLQ534-07|Canada|Quebec|658[0n]|BOLD:AAB1070
Lithophane hemina|[18347]|RDLQ531-07|Canada|Quebec|658[0n]|BOLD:AAB1070
Lithophane hemina|[18348]|RDLQH031-06|Canada|Quebec|658[0n]|BOLD:AAB1070
Lithophane hemina|[18349]|RDLQH029-06|Canada|Quebec|655[0n]|BOLD:AAB1070
Lithophane hemina|[18350]|RDLQH028-06|Canada|Quebec|658[0n]|BOLD:AAB1070
Lithophane hemina|[18351]|TMNBB242-06|Canada|New Brunswick|658[0n]|BOLD:AAB1070
Lithophane hemina|[18352]|RDNM074-05|Canada|Ontario|658[0n]|BOLD:AAB1070
Lithophane hemina|[18353]|RDNM073-05|Canada|Ontario|658[0n]|BOLD:AAB1070
Lithophane hemina|[18354]|RDNM071-05|Canada|Ontario|658[0n]|BOLD:AAB1070
Lithophane hemina|[18355]|RDNM070-05|Canada|Ontario|658[0n]|BOLD:AAB1070
Lithophane hemina|[18356]|RDNM051-05|Canada|Ontario|658[0n]|BOLD:AAB1070
Lithophane hemina|[18357]|XAF150-05|Canada|Ontario|658[0n]|BOLD:AAB1070
Lithophane hemina|[18358]|RDLQ172-05|Canada|Quebec|658[0n]|BOLD:AAB1070
Lithophane hemina|[18359]|RDLQ170-05|Canada|Quebec|658[0n]|BOLD:AAB1070
Lithophane hemina|[18360]|RDLQ168-05|Canada|Quebec|658[0n]|BOLD:AAB1070
Lithophane hemina|[18361]|RDLQ171-05|Canada|Quebec|561[0n]|BOLD:AAB1070
Lithophane hemina|[18362]|RDLQ532-07|Canada|Quebec|601[0n]|BOLD:AAB1070
Lithophane hemina|[18363]|RDLQ533-07|Canada|Quebec|601[0n]|BOLD:AAB1070
Lithophane hemina|[18364]|RDLQ538-07|Canada|Quebec|595[0n]|BOLD:AAB1070
Lithophane hemina|[18365]|RDLQ552-07|Canada|Quebec|595[0n]|BOLD:AAB1070
Lithophane hemina|[18366]|CNCLB1423-14|United States|North Carolina|658[0n]|BOLD:AAB1070
Lithophane hemina|[18367]|RDNMJ101-10|United States|Indiana|658[0n]|BOLD:AAB1070
Lithophane hemina|[18368]|RDNMJ103-10|United States|Indiana|658[0n]|BOLD:AAB1070
Lithophane hemina|[18369]|LNCC620-11|United States|North Carolina|658[0n]|BOLD:AAB1070
Lithophane hemina|[18370]|CNCLB1426-14|United States|North Carolina|658[0n]|BOLD:AAB1070
Lithophane bethunei|[18371]|RDLQ515-07|Canada|Quebec|658[0n]|BOLD:AAB1070
Lithophane bethunei|[18372]|XAF268-05|Canada|Ontario|658[0n]|BOLD:AAB1070
Lithophane bethunei|[18373]|XAF211-05|Canada|Ontario|658[0n]|BOLD:AAB1070
Lithophane bethunei|[18374]|RDLQ160-05|Canada|Quebec|581[0n]|BOLD:AAB1070
Lithophane bethunei|[18375]|XAF117-05|Canada|Ontario|658[0n]|BOLD:AAB1070
Lithophane bethunei|[18376]|XAF198-05|Canada|Ontario|658[0n]|BOLD:AAB1070
Lithophane bethunei|[18377]|SMTPD2610-13|Canada|Ontario|591[0n]|BOLD:AAB1070
Lithophane bethunei|[18378]|CNCLB2901-14|United States|North Carolina|658[0n]|BOLD:AAB1070
Lithophane innominata|[18379]|XAJ177-06|Canada|Ontario|658[0n]|BOLD:ACE4172
Lithophane innominata|[18380]|LOWCE250-06|Canada|British Columbia|658[0n]|BOLD:ACE4172
Lithophane|[18381]|CNGLF036-13|Canada|British Columbia|590[0n]|BOLD:ACE4172
Lithophane innominata|[18382]|RDNMG172-08|United States|North Carolina|658[0n]|BOLD:ACE4172
Lithophane innominata|[18383]|LSEU127-06|United States|North Carolina|658[0n]|BOLD:ACE4172
Lithophane innominata|[18384]|CNCLB1386-14|United States|North Carolina|624[0n]|BOLD:ACE4172
Lithophane innominata|[18385]|CNCLB1420-14|United States|North Carolina|632[0n]|BOLD:ACE4172
Lithophane petulca|[18386]|LALPA1143-11|Canada|British Columbia|658[0n]|BOLD:ACE4172
Lithophane innominata|[18387]|LNCC1201-11|United States|North Carolina|658[0n]|BOLD:ACE4172
Lithophane petulca|[18388]|RWWC1115-12|United States|Washington|658[0n]|BOLD:ACE4172
Lithophane innominata|[18389]|LNCC1479-13|United States|North Carolina|658[0n]|BOLD:ACE4172
Lithophane innominata|[18390]|LNCC1480-13|United States|North Carolina|658[0n]|BOLD:ACE4172

Lithophane innominata|[18387]|LNCC1201-11|United States|North Carolina|658[0n]|BOLD:ACE4172
Lithophane petulca|[18388]|RWWC1115-12|United States|Washington|658[0n]|BOLD:ACE4172
Lithophane innominata|[18389]|LNCC1479-13|United States|North Carolina|658[0n]|BOLD:ACE4172
Lithophane innominata|[18390]|LNCC1480-13|United States|North Carolina|658[0n]|BOLD:ACE4172
Lithophane innominata|[18391]|LNCC1934-14|United States|North Carolina|658[0n]|BOLD:ACE4172
Lithophane innominata|[18392]|LNCC1935-14|United States|North Carolina|658[0n]|BOLD:ACE4172
Lithophane innominata|[18393]|CNCLB1350-14|United States|North Carolina|658[0n]|BOLD:ACE4172
Lithophane innominata|[18394]|CNCLB1384-14|United States|North Carolina|658[0n]|BOLD:ACE4172
Lithophane innominata|[18395]|CNCLB1385-14|United States|North Carolina|658[0n]|BOLD:ACE4172
Lithophane innominata|[18396]|XAJ014-06|Canada|Ontario|658[0n]|BOLD:ACE4172
Lithophane innominata|[18397]|XAJ056-06|Canada|Ontario|658[0n]|BOLD:ACE4172
Lithophane petulca|[18398]|XAJ093-06|Canada|Ontario|658[0n]|BOLD:ACE4172
Lithophane petulca|[18399]|XAJ259-06|Canada|Ontario|658[0n]|BOLD:ACE4172
Lithophane innominata|[18400]|RDLQH032-06|Canada|Quebec|658[0n]|BOLD:ACE4172
Lithophane innominata|[18401]|RDLQ543-07|Canada|Quebec|658[0n]|BOLD:ACE4172
Lithophane innominata|[18402]|RDLQ544-07|Canada|Quebec|658[0n]|BOLD:ACE4172
Lithophane innominata|[18403]|DUNLP166-08|Canada|British Columbia|658[0n]|BOLD:ACE4172
Lithophane innominata|[18404]|CNCLB1387-14|United States|North Carolina|658[0n]|BOLD:ACE4172
Lithophane innominata|[18405]|CNCLB1418-14|United States|North Carolina|658[0n]|BOLD:ACE4172
Lithophane innominata|[18406]|RDNMG168-08|United States|North Carolina|658[0n]|BOLD:ACE4172
Lithophane innominata|[18407]|RDNMG171-08|United States|North Carolina|658[0n]|BOLD:ACE4172
Lithophane petulca|[18408]|RWWB429-09|United States|Washington|658[0n]|BOLD:ACE4172
Lithophane innominata|[18409]|LALPA033-10|Canada|British Columbia|658[0n]|BOLD:ACE4172
Lithophane innominata|[18410]|LALPA054-10|Canada|British Columbia|658[0n]|BOLD:ACE4172
Lithophane petulca|[18411]|RWWB621-10|United States|Washington|658[0n]|BOLD:ACE4172
Lithophane innominata|[18412]|CNCLB1419-14|United States|North Carolina|658[0n]|BOLD:ACE4172
Lithophane innominata|[18413]|CNCLB1421-14|United States|North Carolina|658[0n]|BOLD:ACE4172
Lithophane petulca|[18414]|TMNBB243-06|Canada|New Brunswick|657[0n]|BOLD:ACE4172
Lithophane petulca|[18415]|XAJ009-06|Canada|Ontario|658[0n]|BOLD:ACE4172
Lithophane innominata|[18416]|TMNBB239-06|Canada|New Brunswick|658[0n]|BOLD:ACE4172
Lithophane innominata|[18417]|TMNBB240-06|Canada|New Brunswick|658[0n]|BOLD:ACE4172
Lithophane innominata|[18418]|LOWCE043-06|Canada|British Columbia|658[0n]|BOLD:ACE4172
Lithophane innominata|[18419]|LOWCE474-06|Canada|British Columbia|658[0n]|BOLD:ACE4172
Lithophane petulca|[18420]|LOWCE025-06|Canada|British Columbia|658[0n]|BOLD:ACE4172
Lithophane petulca|[18421]|LOWCE026-06|Canada|British Columbia|658[0n]|BOLD:ACE4172
Lithophane innominata|[18422]|LSEU128-06|United States|North Carolina|658[0n]|BOLD:ACE4172
Lithophane innominata|[18423]|RDMAB581-06|Canada|Alberta|658[0n]|BOLD:ACE4172
Lithophane innominata|[18424]|LMIS031-05|Canada|Ontario|658[0n]|BOLD:ACE4172
Lithophane innominata|[18425]|LOWCC446-05|Canada|British Columbia|658[0n]|BOLD:ACE4172
Lithophane petulca|[18426]|RDNM045-05|Canada|Ontario|658[0n]|BOLD:ACE4172
Lithophane petulca|[18427]|RDNM072-05|Canada|Ontario|658[0n]|BOLD:ACE4172
Lithophane petulca|[18428]|RDNM044-05|Canada|Ontario|658[0n]|BOLD:ACE4172
Lithophane petulca|[18429]|RDNM043-05|Canada|Ontario|658[0n]|BOLD:ACE4172
Lithophane innominata|[18430]|XAF412-05|Canada|Ontario|658[0n]|BOLD:ACE4172
Lithophane petulca|[18431]|XAF296-05|Canada|Ontario|658[0n]|BOLD:ACE4172
Lithophane innominata|[18432]|XAF208-05|Canada|Ontario|658[0n]|BOLD:ACE4172
Lithophane petulca|[18433]|RDLQ167-05|Canada|Quebec|658[0n]|BOLD:ACE4172
Lithophane innominata|[18434]|RDLQ161-05|Canada|Quebec|658[0n]|BOLD:ACE4172
Lithophane innominata|[18435]|XAJ023-06|Canada|Ontario|658[0n]|BOLD:ACE4172
Lithophane innominata|[18436]|TMNBB238-06|Canada|New Brunswick|656[0n]|BOLD:ACE4172
Lithophane petulca|[18437]|RDLQ159-05|Canada|Quebec|658[1n]|BOLD:ACE4172
Lithophane innominata|[18438]|LOWCC439-05|Canada|British Columbia|594[0n]|BOLD:ACE4172
Lithophane innominata|[18439]|RDNM046-05|Canada|British Columbia|580[0n]|BOLD:ACE4172
Lithophane innominata|[18440]|TMNBB241-06|Canada|New Brunswick|658[0n]|BOLD:ACE4172
Lithophane innominata|[18441]|LOWCC438-05|Canada|British Columbia|564[0n]|BOLD:ACE4172
Lithophane innominata|[18442]|LOWCD170-06|Canada|British Columbia|552[0n]|BOLD:ACE4172
Lithophane innominata|[18443]|RDLQ541-07|Canada|Quebec|598[0n]|BOLD:ACE4172
Lithophane innominata|[18444]|RDLQ542-07|Canada|Quebec|599[0n]|BOLD:ACE4172
Lithophane innominata|[18445]|RDLQ545-07|Canada|Quebec|595[0n]|BOLD:ACE4172
Lithophane innominata|[18446]|LNCC619-11|United States|North Carolina|623[0n]|BOLD:ACE4172
Lithophane innominata|[18447]|SMTPI7504-14|Canada|Ontario|591[0n]|BOLD:ACE4172
Lithophane petulca|[18448]|RWWC131-10|United States|Washington|658[0n]|BOLD:ACE4172
Lithophane innominata|[18449]|LNCC618-11|United States|North Carolina|658[0n]|BOLD:ACE4172
Lithophane innominata|[18450]|CNCLB1424-14|United States|North Carolina|658[0n]|BOLD:ACE4172
Lithophane innominata|[18451]|CNCLB2790-14|United States|North Carolina|658[0n]|BOLD:ACE4172
Lithophane innominata|[18452]|LGSMC456-05|United States|Tennessee|658[0n]|BOLD:ACE4172
Lithophane innominata|[18453]|PMG129-03|Canada|Ontario|617[0n]|BOLD:ACE4172
Lithophane innominata|[18454]|RDNMG169-08|United States|North Carolina|658[0n]|BOLD:ACE4172
Lithophane innominata|[18455]|RDNMG170-08|United States|North Carolina|658[0n]|BOLD:ACE4172
Lithophane innominata|[18456]|LNCC973-11|United States|North Carolina|658[0n]|BOLD:ACE4172
Lithophane innominata|[18457]|CNCLB1383-14|United States|North Carolina|658[0n]|BOLD:ACE4172
Lithophane innominata|[18458]|CNCLB1425-14|United States|North Carolina|658[0n]|BOLD:ACE4172
Lithophane innominata|[18459]|CNCLB2797-14|United States|North Carolina|658[0n]|BOLD:ACE4172
Lithophane signosa|[18460]|RDNML109-13|United States|New Jersey|658[0n]|BOLD:ACF1025
Lithophane signosa|[18461]|RDNMF477-08|United States|New York|609[0n]|BOLD:ACF1025
Lithophane signosa|[18462]|RDNMJ100-10|United States|Indiana|658[0n]|BOLD:ACF1025
Lithophane signosa|[18463]|LNCC477-11|United States|North Carolina|658[0n]|BOLD:ACF1025
Lithophane signosa|[18464]|LNCB977-10|United States|North Carolina|658[0n]|BOLD:ACF1025
Lithophane signosa|[18465]|CNCLB2900-14|United States|North Carolina|658[0n]|BOLD:ACF1025
Lithophane signosa|[18466]|CNCLB2902-14|United States|North Carolina|658[0n]|BOLD:ACF1025
Lithophane fagina|[18467]|XAB692-04|Canada|Ontario|645[0n]|BOLD:AAB3686
Lithophane fagina|[18468]|RDLQF254-06|Canada|Quebec|658[0n]|BOLD:AAB3686
Lithophane fagina|[18469]|RDLQG466-06|Canada|Quebec|658[0n]|BOLD:AAB3686
Lithophane fagina|[18470]|XAH701-05|Canada|Ontario|658[0n]|BOLD:AAB3686
Lithophane fagina|[18471]|XAH746-05|Canada|Ontario|658[0n]|BOLD:AAB3686
Lithophane fagina|[18472]|XAF241-05|Canada|Ontario|658[0n]|BOLD:AAB3686
Lithophane fagina|[18473]|XAF240-05|Canada|Ontario|658[0n]|BOLD:AAB3686
Lithophane fagina|[18474]|XAF201-05|Canada|Ontario|658[0n]|BOLD:AAB3686
Lithophane fagina|[18475]|XAF154-05|Canada|Ontario|658[0n]|BOLD:AAB3686
Lithophane fagina|[18476]|XAF153-05|Canada|Ontario|658[0n]|BOLD:AAB3686
Lithophane fagina|[18477]|XAF027-05|Canada|Ontario|658[0n]|BOLD:AAB3686
Lithophane fagina|[18478]|XAF026-05|Canada|Ontario|658[0n]|BOLD:AAB3686
Lithophane fagina|[18479]|PHMNB778-05|Canada|New Brunswick|658[0n]|BOLD:AAB3686
Lithophane fagina|[18480]|XAE149-04|Canada|Ontario|658[0n]|BOLD:AAB3686
Lithophane fagina|[18481]|XAE039-04|Canada|Ontario|658[0n]|BOLD:AAB3686
Lithophane fagina|[18482]|XAB539-04|Canada|Ontario|658[0n]|BOLD:AAB3686
Lithophane fagina|[18483]|LOWCE466-06|Canada|British Columbia|656[0n]|BOLD:AAB3686
Lithophane fagina|[18484]|PHMO013-03|Canada|Ontario|639[0n]|BOLD:AAB3686
Lithophane fagina|[18485]|PHMO016-03|Canada|Ontario|639[0n]|BOLD:AAB3686
Lithophane fagina|[18486]|PHMO388-03|Canada|Ontario|639[0n]|BOLD:AAB3686
Lithophane fagina|[18487]|LOWCB546-05|Canada|British Columbia|523[4n]|
Lithophane fagina|[18488]|XAJ083-06|Canada|Ontario|574[0n]|BOLD:AAB3686
Lithophane fagina|[18489]|LOWCE023-06|Canada|British Columbia|658[0n]|BOLD:AAB3686
Lithophane fagina|[18490]|XAJ086-06|Canada|Ontario|658[0n]|BOLD:AAB3686

Lithophane fagina|[18487]|LOWCB540-05|Canada|British Columbia|525[4n]|
Lithophane fagina|[18488]|XAJ083-06|Canada|Ontario|574[0n]|BOLD:AAB3686
Lithophane fagina|[18489]|LOWCE023-06|Canada|British Columbia|658[0n]|BOLD:AAB3686
Lithophane fagina|[18490]|XAJ086-06|Canada|Ontario|658[0n]|BOLD:AAB3686
Lithophane fagina|[18491]|LMDH012-11|United States|Minnesota|658[0n]|BOLD:AAB3686
Lithophane fagina|[18492]|LMDH025-11|United States|Minnesota|658[0n]|BOLD:AAB3686
Lithophane fagina|[18493]|CNEIC063-12|Canada|Alberta|633[0n]|BOLD:AAB3686
Lithophane pexata|[18494]|RDLQ518-07|Canada|Quebec|658[0n]|BOLD:AAD9908
Lithophane pexata|[18495]|XAJ089-06|Canada|Ontario|658[0n]|BOLD:AAD9908
Lithophane pexata|[18496]|LOWCC018-05|Canada|British Columbia|658[0n]|BOLD:AAD9908
Lithophane pexata|[18497]|RDLQG462-06|Canada|Quebec|648[1n]|BOLD:AAD9908
Lithophane pexata|[18498]|RDLQ519-07|Canada|Quebec|565[0n]|BOLD:AAD9908
Lithophane dilatocula|[18499]|RDNMB940-05|United States|California|658[0n]|BOLD:AAF7039
Lithophane dilatocula|[18500]|RDNMG947-08|United States|Washington|658[0n]|BOLD:AAF7039
Lithophane dilatocula|[18501]|RDNMG934-08|United States|California|658[0n]|BOLD:AAF7039
Lithophane dilatocula|[18502]|LALPA1105-11|Canada|British Columbia|642[0n]|BOLD:AAF7039
Lithophane amanda|[18503]|XAE168-04|Canada|Ontario|658[0n]|BOLD:AAE0248
Lithophane amanda|[18504]|RDLQ509-07|Canada|Quebec|576[2n]|BOLD:AAE0248
Lithophane amanda|[18505]|RDNMG561-08|Canada|New Brunswick|658[0n]|BOLD:AAE0248
Lithophane amanda|[18506]|RDNMG567-08|Canada|New Brunswick|658[0n]|BOLD:AAE0248
Lithophane amanda|[18507]|RDNMG562-08|Canada|New Brunswick|658[0n]|BOLD:AAE0248
Lithophane amanda|[18508]|CNPAI029-13|Canada|Saskatchewan|658[0n]|BOLD:AAE0248
Lithophane thaxteri|[18509]|RDNMF196-08|Canada|British Columbia|658[0n]|BOLD:AAE0044
Lithophane thaxteri|[18510]|RDNMF195-08|Canada|New Brunswick|658[0n]|BOLD:AAE0044
Lithophane thaxteri|[18511]|RDNMF194-08|Canada|Ontario|658[0n]|BOLD:AAE0044
Lithophane thaxteri|[18512]|RDLQ506-07|Canada|Quebec|609[0n]|BOLD:AAE0044
Lithophane thaxteri|[18513]|DUNLP174-08|Canada|British Columbia|576[0n]|BOLD:AAE0044
Lithophane contra|[18514]|IAWLB580-11|United States|Arizona|632[0n]|BOLD:AAZ0392
Lithophane contra|[18515]|CNCLB134-14|United States|Arizona|658[0n]|BOLD:AAZ0392
Lithophane contra|[18516]|IAWLB615-11|United States|Arizona|658[1n]|BOLD:AAZ0392
Lithophane contra|[18517]|IAWLB579-11|United States|Arizona|658[0n]|BOLD:AAZ0392
Lithophane contra|[18518]|IAWLB616-11|United States|Arizona|603[0n]|BOLD:AAZ0392
Lithophane contra|[18519]|CNCLB135-14|United States|Arizona|658[0n]|BOLD:AAZ0392
Lithophane querquera|[18520]|RDNMC562-06|United States|Georgia|559[0n]|BOLD:ABZ0521
Lithophane querquera|[18521]|RDNM011-05|United States|Georgia|593[0n]|BOLD:ABZ0521
Lithophane querquera|[18522]|RDNMG570-08|United States|New Jersey|658[0n]|BOLD:ABZ0521
Lithophane querquera|[18523]|RDNMG571-08|United States|New Jersey|658[0n]|BOLD:ABZ0521
Lithophane querquera|[18524]|RDNMG572-08|United States|New Jersey|658[0n]|BOLD:ABZ0521
Lithophane scottae|[18525]|RDNM012-05|Canada|Ontario|658[0n]|BOLD:ABZ0521
Lithophane scottae|[18526]|LNCC622-11|United States|North Carolina|622[0n]|BOLD:ABZ0521
Lithophane sp.|[18527]|CNCLA5033-13|United States|Ohio|658[0n]|BOLD:ABZ0521
Lithophane viridipallens|[18528]|LSEU131-06|United States|Georgia|658[0n]|BOLD:ABZ0522
Lithophane viridipallens|[18529]|LSEU132-06|United States|Georgia|658[0n]|BOLD:ABZ0522
Lithophane viridipallens|[18530]|LNCB979-10|United States|North Carolina|658[0n]|BOLD:ABZ0522
Lithophane viridipallens|[18531]|LNCB980-10|United States|North Carolina|658[0n]|BOLD:ABZ0522
Lithophane viridipallens|[18532]|RDNML103-13|United States|New Jersey|658[0n]|BOLD:ABZ0522
Lithophane viridipallens|[18533]|RDNML104-13|United States|New Jersey|658[0n]|BOLD:ABZ0522
Lithophane viridipallens|[18534]|RDNML105-13|United States|New Jersey|658[0n]|BOLD:ABZ0522
Lithophane viridipallens|[18535]|CNCLB2450-14|United States|Texas|658[0n]|BOLD:ABZ0522
Lithophane tepida|[18536]|RDNM120-05|Canada|New Brunswick|658[0n]|BOLD:ABZ0523
Lithophane tepida|[18537]|RDNM121-05|Canada|New Brunswick|658[0n]|BOLD:ABZ0523
Lithophane tepida|[18538]|RDLQF255-06|Canada|Quebec|658[0n]|BOLD:ABZ0523
Lithophane baileyi|[18539]|RDLQ520-07|Canada|Quebec|658[0n]|BOLD:AAD9328
Lithophane baileyi|[18540]|TMNBB244-06|Canada|New Brunswick|658[0n]|BOLD:AAD9328
Lithophane baileyi|[18541]|RDNM116-05|Canada|Ontario|658[0n]|BOLD:AAD9328
Lithophane baileyi|[18542]|RDNM115-05|Canada|Ontario|658[0n]|BOLD:AAD9328
Lithophane baileyi|[18543]|RDNM114-05|Canada|Ontario|658[0n]|BOLD:AAD9328
Lithophane baileyi|[18544]|RDLQ521-07|Canada|Quebec|632[0n]|BOLD:AAD9328
Lithophane baileyi|[18545]|CNPAA513-13|Canada|Saskatchewan|634[0n]|BOLD:AAD9328
Lithophane baileyi|[18546]|ALLEP053-13|Canada|Ontario|658[0n]|BOLD:AAD9328
Lithophane baileyi|[18547]|ALLEP234-13|Canada|Ontario|658[0n]|BOLD:AAD9328
Lithophane baileyi|[18548]|ALLEP287-13|Canada|Ontario|658[0n]|BOLD:AAD9328
Lithophane baileyi|[18549]|RDNML188-13|United States|North Carolina|658[0n]|BOLD:AAD9328
Lithophane baileyi|[18550]|LALPA1353-12|Canada|British Columbia|658[0n]|BOLD:AAD9328
Lithophane baileyi|[18551]|LALPA1305-11|Canada|British Columbia|658[0n]|BOLD:AAD9328
Lithophane baileyi|[18552]|LNCC621-11|United States|North Carolina|658[0n]|BOLD:AAD9328
Lithophane baileyi|[18553]|LALPA768-10|Canada|British Columbia|658[0n]|BOLD:AAD9328
Lithophane baileyi|[18554]|LALPA736-10|Canada|British Columbia|658[0n]|BOLD:AAD9328
Lithophane baileyi|[18555]|DUNLP175-08|Canada|British Columbia|658[0n]|BOLD:AAD9328
Lithophane baileyi|[18556]|RDNM119-05|United States|Oregon|658[0n]|BOLD:AAD9328
Lithophane baileyi|[18557]|RDNM118-05|Canada|British Columbia|658[0n]|BOLD:AAD9328
Lithophane baileyi|[18558]|RDNM117-05|Canada|British Columbia|658[0n]|BOLD:AAD9328
Lithophane baileyi|[18559]|CNCLB2801-14|United States|North Carolina|658[0n]|BOLD:AAD9328
Lithophane baileyi|[18560]|CNCLB2805-14|United States|North Carolina|658[0n]|BOLD:AAD9328
Lithophane baileyi|[18561]|CNCLB2810-14|United States|North Carolina|658[0n]|BOLD:AAD9328
Lithophane tephrina|[18562]|RDNMD630-06|United States|California|658[0n]|BOLD:AAX6386
Lithophane georgii|[18563]|LALPA1028-11|Canada|British Columbia|658[0n]|BOLD:AAC2621
Lithophane georgii|[18564]|RDNMG629-08|United States|Wyoming|658[0n]|BOLD:AAC2621
Lithophane georgii|[18565]|RDNMG630-08|United States|Wyoming|658[0n]|BOLD:AAC2621
Lithophane georgii|[18566]|USLEP635-10|United States|Colorado|658[0n]|BOLD:AAC2621
Lithophane georgii|[18567]|LOWCB544-05|Canada|British Columbia|658[0n]|BOLD:AAC2621
Lithophane georgii|[18568]|LOWCB545-05|Canada|British Columbia|615[1n]|BOLD:AAC2621
Lithophane georgii|[18569]|LOWCE022-06|Canada|British Columbia|658[0n]|BOLD:AAC2621
Lithophane georgii|[18570]|RDNME272-07|United States|Colorado|609[0n]|BOLD:AAC2621
Lithophane georgii|[18571]|LALPA1066-11|Canada|British Columbia|634[0n]|BOLD:AAC2621
Lithophane georgii|[18572]|LOWCE208-06|Canada|British Columbia|658[0n]|BOLD:AAC2621
Lithophane georgii|[18573]|LALPA1029-11|Canada|British Columbia|658[0n]|BOLD:AAC2621
Lithophane georgii|[18574]|LALPA1030-11|Canada|British Columbia|658[0n]|BOLD:AAC2621
Lithophane georgii|[18575]|LALPA1118-11|Canada|British Columbia|658[0n]|BOLD:AAC2621
Lithophane georgii|[18576]|LOPN105-06|United States|Oregon|554[0n]|BOLD:AAC2621
Lithophane georgii|[18577]|LOPN148-06|United States|Oregon|573[1n]|BOLD:AAC2621
Lithophane georgii|[18578]|LOPN106-06|United States|Oregon|592[0n]|BOLD:AAC2621
Lithophane georgii|[18579]|LOPN107-06|United States|Oregon|535[1n]|BOLD:AAC2621
Lithophane georgii|[18580]|LOPN149-06|United States|Oregon|511[5n]|BOLD:AAC2621
Lithophane georgii|[18581]|LOPN150-06|United States|Oregon|572[1n]|BOLD:AAC2621
Lithophane georgii|[18582]|JMMMB539-13|United States|California|600[0n]|BOLD:AAC2621
Lithophane grotei|[18583]|RDNML106-13|United States|New Jersey|658[0n]|BOLD:AAB5821
Lithophane grotei|[18584]|LNCC626-11|United States|North Carolina|614[0n]|BOLD:AAB5821
Lithophane grotei|[18585]|XAF007-05|Canada|Ontario|658[0n]|BOLD:AAB5821
Lithophane grotei|[18586]|PHMO014-03|Canada|Ontario|639[0n]|BOLD:AAB5821
Lithophane grotei|[18587]|XAF001-05|Canada|Ontario|614[0n]|BOLD:AAB5821
Lithophane grotei|[18588]|RDNML107-13|United States|New Jersey|645[0n]|BOLD:AAB5821
Lithophane grotei|[18589]|LNCC625-11|United States|North Carolina|614[0n]|BOLD:AAB5821
Lithophane grotei|[18590]|RDLQH094-06|Canada|Quebec|633[0n]|BOLD:AAB5821

Lithophane grotei|[18587]|XAF001-03|Canada|Ontario|614[0n]|BOLD:AAB5821
Lithophane grotei|[18588]|RDNML107-13|United States|New Jersey|645[0n]|BOLD:AAB5821
Lithophane grotei|[18589]|LNCC625-11|United States|North Carolina|614[0n]|BOLD:AAB5821
Lithophane grotei|[18590]|RDLQH094-06|Canada|Quebec|632[0n]|BOLD:AAB5821
Lithophane grotei|[18591]|RDLQ525-07|Canada|Quebec|658[0n]|BOLD:AAB5821
Lithophane grotei|[18592]|RDLQH034-06|Canada|Quebec|658[0n]|BOLD:AAB5821
Lithophane grotei|[18593]|RDLQH033-06|Canada|Quebec|658[0n]|BOLD:AAB5821
Lithophane grotei|[18594]|TMNBB245-06|Canada|New Brunswick|658[0n]|BOLD:AAB5821
Lithophane grotei|[18595]|XAB546-04|Canada|Ontario|658[0n]|BOLD:AAB5821
Lithophane grotei|[18596]|PHMNB269-04|Canada|New Brunswick|577[0n]|BOLD:AAB5821
Lithophane grotei|[18597]|RDLQ524-07|Canada|Quebec|638[0n]|BOLD:AAB5821
Lithophane grotei|[18598]|LNCC538-11|United States|North Carolina|658[0n]|BOLD:AAB5821
Lithophane grotei|[18599]|LNCC627-11|United States|North Carolina|658[0n]|BOLD:AAB5821
Lithophane grotei|[18600]|XAE013-04|Canada|Ontario|658[0n]|BOLD:AAB5821
Lithophane grotei|[18601]|XAH783-05|Canada|Ontario|658[0n]|BOLD:AAB5821
Lithophane grotei|[18602]|RDLQ526-07|Canada|Quebec|658[0n]|BOLD:AAB5821
Lithophane grotei|[18603]|RDLQ527-07|Canada|Quebec|658[0n]|BOLD:AAB5821
Lithophane grotei|[18604]|RDNMG595-08|Canada|New Brunswick|658[0n]|BOLD:AAB5821
Lithophane grotei|[18605]|LNCB999-10|United States|North Carolina|658[0n]|BOLD:AAB5821
Lithophane grotei|[18606]|LNCC435-10|United States|North Carolina|658[0n]|BOLD:AAB5821
Lithophane grotei|[18607]|LNCC537-11|United States|North Carolina|658[0n]|BOLD:AAB5821
Lithophane grotei|[18608]|LNCC624-11|United States|North Carolina|658[0n]|BOLD:AAB5821
Lithophane grotei|[18609]|LNCC965-11|United States|North Carolina|658[0n]|BOLD:AAB5821
Lithophane grotei|[18610]|RDNML108-13|United States|New Jersey|658[0n]|BOLD:AAB5821
Lithophane laticinerea|[18611]|XAF004-05|Canada|Ontario|658[0n]|BOLD:AAB5821
Lithophane laticinerea|[18612]|XAJ204-06|Canada|Ontario|658[0n]|BOLD:AAB5821
Lithophane laticinerea|[18613]|XAJ020-06|Canada|Ontario|658[0n]|BOLD:AAB5821
Lithophane laticinerea|[18614]|XAH752-05|Canada|Ontario|658[0n]|BOLD:AAB5821
Lithophane laticinerea|[18615]|XAF050-05|Canada|Ontario|658[0n]|BOLD:AAB5821
Lithophane laticinerea|[18616]|XAJ197-06|Canada|Ontario|658[0n]|BOLD:AAB5821
Lithophane laticinerea|[18617]|XAB699-04|Canada|Ontario|658[0n]|BOLD:AAB5821
Lithophane laticinerea|[18618]|XAJ203-06|Canada|Ontario|596[0n]|BOLD:AAB5821
Lithophane laticinerea|[18619]|PHMO006-03|Canada|Ontario|639[0n]|BOLD:AAB5821
Lithophane laticinerea|[18620]|XAJ246-06|Canada|Ontario|658[0n]|BOLD:AAB5821
Lithophane laticinerea|[18621]|RDLQ528-07|Canada|Quebec|658[0n]|BOLD:AAB5821
Lithophane laticinerea|[18622]|RDNMG593-08|Canada|New Brunswick|658[0n]|BOLD:AAB5821
Lithophane laticinerea|[18623]|RDNMG594-08|Canada|New Brunswick|658[0n]|BOLD:AAB5821
Lithophane pertorrida|[18624]|RDNMJ137-10|United States|New Mexico|658[0n]|BOLD:AAB5821
Lithophane pertorrida|[18625]|RDNMF211-08|Canada|British Columbia|658[0n]|BOLD:AAB5821
Lithophane pertorrida|[18626]|RDNMF212-08|Canada|British Columbia|658[0n]|BOLD:AAB5821
Lithophane pertorrida|[18627]|RDNMF213-08|Canada|British Columbia|658[0n]|BOLD:AAB5821
Lithophane pertorrida|[18628]|RDNMF215-08|Canada|British Columbia|658[0n]|BOLD:AAB5821
Lithophane pertorrida|[18629]|LALPA010-10|Canada|British Columbia|658[0n]|BOLD:AAB5821
Lithophane pertorrida|[18630]|LALPA763-10|Canada|British Columbia|658[0n]|BOLD:AAB5821
Lithophane pertorrida|[18631]|JMMMB372-11|United States|California|658[0n]|BOLD:AAB5821
Lithophane pertorrida|[18632]|LALPA1041-11|Canada|British Columbia|658[0n]|BOLD:AAB5821
Lithophane pertorrida|[18633]|LALPA1047-11|Canada|British Columbia|658[0n]|BOLD:AAB5821
Lithophane pertorrida|[18634]|LALPA1057-11|Canada|British Columbia|658[0n]|BOLD:AAB5821
Lithophane pertorrida|[18635]|NAMUM141-08|United States|California|658[0n]|BOLD:AAB5821
Lithophane pertorrida|[18636]|JMMMB416-11|United States|California|658[0n]|BOLD:AAB5821
Lithophane pertorrida|[18637]|LALPA1356-12|Canada|British Columbia|658[0n]|BOLD:AAB5821
Lithophane torrida|[18638]|RDNME269-07|United States|Colorado|658[0n]|BOLD:AAB5821
Lithophane torrida|[18639]|RDNME270-07|United States|Colorado|642[0n]|BOLD:AAB5821
Lithophane unimoda|[18640]|LNCB986-10|United States|North Carolina|658[0n]|BOLD:AAB5821
Lithophane laceyi|[18641]|LNCC530-11|United States|North Carolina|658[0n]|BOLD:AAB5821
Lithophane unimoda|[18642]|LNCB985-10|United States|North Carolina|658[0n]|BOLD:AAB5821
Lithophane unimoda|[18643]|XAH860-05|Canada|Ontario|658[0n]|BOLD:AAB5821
Lithophane unimoda|[18644]|XAJ069-06|Canada|Ontario|658[0n]|BOLD:AAB5821
Lithophane unimoda|[18645]|LNCB989-10|United States|North Carolina|658[0n]|BOLD:AAB5821
Lithophane unimoda|[18646]|LNCB988-10|United States|North Carolina|658[0n]|BOLD:AAB5821
Lithophane laceyi|[18647]|LNCB983-10|United States|North Carolina|658[0n]|BOLD:AAB5821
Lithophane unimoda|[18648]|RDNMG569-08|Canada|New Brunswick|658[0n]|BOLD:AAB5821
Lithophane unimoda|[18649]|XAJ172-06|Canada|Ontario|658[0n]|BOLD:AAB5821
Lithophane unimoda|[18650]|RDMAB698-06|Canada|Alberta|658[0n]|BOLD:AAB5821
Lithophane unimoda|[18651]|XAF411-05|Canada|Ontario|658[0n]|BOLD:AAB5821
Lithophane unimoda|[18652]|XAC098-04|Canada|Ontario|658[0n]|BOLD:AAB5821
Lithophane unimoda|[18653]|XAH822-05|Canada|Ontario|607[0n]|BOLD:AAB5821
Lithophane unimoda|[18654]|RDLQ522-07|Canada|Quebec|593[0n]|BOLD:AAB5821
Lithophane unimoda|[18655]|RDLQ523-07|Canada|Quebec|593[0n]|BOLD:AAB5821
Lithophane unimoda|[18656]|LNCB1000-10|United States|North Carolina|636[0n]|BOLD:AAB5821
Lithophane unimoda|[18657]|LNCB987-10|United States|North Carolina|658[0n]|BOLD:AAB5821
Lithophane laceyi|[18658]|LNCB1001-10|United States|North Carolina|658[0n]|BOLD:AAB5821
Lithophane unimoda|[18659]|LNCC532-11|United States|North Carolina|658[0n]|BOLD:AAB5821
Lithophane laceyi|[18660]|RDNMK628-11|United States|North Carolina|658[0n]|BOLD:AAB5821
Lithophane laceyi|[18661]|RDNML018-13|United States|Louisiana|658[0n]|BOLD:AAB5821
Lithophane unimoda|[18662]|LNCC531-11|United States|North Carolina|658[0n]|BOLD:AAB5821
Lithophane unimoda|[18663]|LNCB984-10|United States|North Carolina|658[0n]|BOLD:AAB5821
Lithophane laceyi|[18664]|LNCB982-10|United States|North Carolina|658[0n]|BOLD:AAB5821
Lithophane unimoda|[18665]|XAC125-04|Canada|Ontario|658[0n]|BOLD:AAB5821
Lithophane laceyi|[18666]|RDNML019-13|United States|Louisiana|658[0n]|BOLD:AAB5821
Lithophane unimoda|[18667]|XAH770-05|Canada|Ontario|658[0n]|BOLD:AAB5821
Lithophane unimoda|[18668]|RDNML096-13|United States|New Jersey|658[0n]|BOLD:AAB5821
Lithophane unimoda|[18669]|RDMAB696-06|Canada|Alberta|616[0n]|BOLD:AAB5821
Lithophane unimoda|[18670]|RDMAB697-06|Canada|Alberta|574[1n]|BOLD:AAB5821
Lithophane unimoda|[18671]|RDMAB699-06|Canada|Alberta|658[0n]|BOLD:AAB5821
Lithophane unimoda|[18672]|RDMAB700-06|Canada|Alberta|658[0n]|BOLD:AAB5821
Lithophane unimoda|[18673]|AHLEP034-10|United States|Pennsylvania|627[0n]|BOLD:AAB5821
Lithophane unimoda|[18674]|XAJ171-06|Canada|Ontario|601[0n]|BOLD:AAB5821
Lithophane unimoda|[18675]|RDLQH084-06|Canada|Quebec|639[0n]|BOLD:AAB5821
Lithophane unimoda|[18676]|PHMO025-03|Canada|Ontario|639[0n]|BOLD:AAB5821
Lithophane unimoda|[18677]|RDMAB701-06|Canada|Alberta|563[1n]|BOLD:AAB5821
Lithophane unimoda|[18678]|RDNML097-13|United States|New Jersey|658[0n]|BOLD:AAB5821
Lithophane unimoda|[18679]|XAJ258-06|Canada|Ontario|658[0n]|BOLD:AAB5821
Lithophane unimoda|[18680]|RDNMG568-08|Canada|New Brunswick|658[0n]|BOLD:AAB5821
Lithophane laceyi|[18681]|LNCC529-11|United States|North Carolina|658[0n]|BOLD:AAB5821
Lithophane unimoda|[18682]|RDNML098-13|United States|New Jersey|658[0n]|BOLD:AAB5821
Lithophane antennata|[18683]|XAF152-05|Canada|Ontario|658[0n]|BOLD:AAB5821
Lithophane antennata|[18684]|XAF052-05|Canada|Ontario|658[0n]|BOLD:AAB5821
Lithophane antennata|[18685]|RDLQ502-07|Canada|Quebec|608[0n]|BOLD:AAB5821
Lithophane antennata|[18686]|LNCC434-10|United States|North Carolina|658[0n]|BOLD:AAB5821
Lithophane antennata|[18687]|XAF258-05|Canada|Ontario|658[0n]|BOLD:AAB5821
Lithophane antennata|[18688]|RDNML101-13|United States|New Jersey|658[0n]|BOLD:AAB5821
Lithophane antennata|[18689]|LNCC623-11|United States|North Carolina|658[0n]|BOLD:AAB5821
[illegible]

Lithophane antennata|[18687]|XAF258-05|Canada|Ontario|658[0n]|BOLD:AAB5821
Lithophane antennata|[18688]|RDNML101-13|United States|New Jersey|658[0n]|BOLD:AAB5821
Lithophane antennata|[18689]|LNCC623-11|United States|North Carolina|658[0n]|BOLD:AAB5821
Lithophane antennata|[18690]|LNCC562-11|United States|North Carolina|658[0n]|BOLD:AAB5821
Lithophane antennata|[18691]|LNCC536-11|United States|North Carolina|658[0n]|BOLD:AAB5821
Lithophane antennata|[18692]|LNCC534-11|United States|North Carolina|658[0n]|BOLD:AAB5821
Lithophane antennata|[18693]|XAJ019-06|Canada|Ontario|658[0n]|BOLD:AAB5821
Lithophane antennata|[18694]|XAF205-05|Canada|Ontario|658[0n]|BOLD:AAB5821
Lithophane antennata|[18695]|RDLQ503-07|Canada|Quebec|563[0n]|BOLD:AAB5821
Lithophane antennata|[18696]|LNCC533-11|United States|North Carolina|647[0n]|BOLD:AAB5821
Lithophane antennata|[18697]|RDNML102-13|United States|New Jersey|658[0n]|BOLD:AAB5821
Lithophane antennata|[18698]|LNCC417-10|United States|North Carolina|658[0n]|BOLD:AAB5821
Lithophane antennata|[18699]|RDNML100-13|United States|New Jersey|658[0n]|BOLD:AAB5821
Lithophane antennata|[18700]|LNCC535-11|United States|North Carolina|658[0n]|BOLD:AAB5821
Lithophane antennata|[18701]|LNCC416-10|United States|North Carolina|658[0n]|BOLD:AAB5821
Lithophane antennata|[18702]|LNCB981-10|United States|North Carolina|658[0n]|BOLD:AAB5821
Lithophane antennata|[18703]|XAH858-05|Canada|Ontario|658[0n]|BOLD:AAB5821
Lithophane antennata|[18704]|SMTPB19916-13|Canada|Ontario|588[0n]|BOLD:AAB5821
Lithophane antennata|[18705]|CNROE002-13|Canada|Ontario|552[0n]|BOLD:AAB5821
Lithophane adipel|[18706]|RDMAB702-06|Canada|Alberta|658[0n]|BOLD:AAC7746
Lithophane lepida|[18707]|RDNMK477-11|United States|New Hampshire|633[1n]|BOLD:AAC7746
Lithophane ponderosa|[18708]|DUNLP169-08|Canada|British Columbia|644[5n]|BOLD:AAC7746
Lithophane ponderosa|[18709]|DUNLP172-08|Canada|British Columbia|650[2n]|BOLD:AAC7746
Lithophane ponderosa|[18710]|RDNMF188-08|United States|Oregon|658[2n]|BOLD:AAC7746
Lithophane ponderosa|[18711]|RDNMF186-08|United States|Oregon|658[1n]|BOLD:AAC7746
Lithophane ponderosa|[18712]|RDNMF187-08|United States|California|647[0n]|BOLD:AAC7746
Lithophane ponderosa|[18713]|RDNMF190-08|United States|California|658[0n]|BOLD:AAC7746
Lithophane ponderosa|[18714]|DUNLP170-08|Canada|British Columbia|658[0n]|BOLD:AAC7746
Lithophane ponderosa|[18715]|DUNLP171-08|Canada|British Columbia|658[0n]|BOLD:AAC7746
Lithophane ponderosa|[18716]|DUNLP173-08|Canada|British Columbia|658[0n]|BOLD:AAC7746
Lithophane ponderosa|[18717]|LBCH5106-10|Canada|British Columbia|658[0n]|BOLD:AAC7746
Lithophane ponderosa|[18718]|JMMMB389-11|United States|California|658[0n]|BOLD:AAC7746
Lithophane ponderosa|[18719]|CNCLB133-14|United States|Oregon|658[0n]|BOLD:AAC7746
Lithophane nasar|[18720]|IAWLB243-11|United States|Arizona|658[0n]|BOLD:AAC7746
Lithophane leeae|[18721]|RDNME848-08|United States|Arizona|658[0n]|BOLD:AAX6385
Lithophane atara|[18722]|RDNMF192-08|Canada|British Columbia|658[0n]|BOLD:ABY9616
Lithophane atara|[18723]|RDNMF191-08|Canada|British Columbia|609[0n]|BOLD:ABY9616
Lithophane atara|[18724]|RDNMF193-08|Canada|British Columbia|609[0n]|BOLD:ABY9616
Lithophane atara|[18725]|DUNLP164-08|Canada|British Columbia|600[3n]|BOLD:ABY9616
Lithophane atara|[18726]|LBCH5156-10|Canada|British Columbia|658[0n]|BOLD:ABY9616
Lithophane sp.|[18727]|PSAT151-10|Mexico||658[0n]|BOLD:ABY9616
Lithophane jefferyi|[18728]|CNCLB132-14|United States|California|658[0n]|BOLD:AAC7746
Lithophane vanduzeei|[18729]|CNCLB137-14|United States|California|658[0n]|BOLD:AAC7746
Lithophane thujae|[18730]|RDNMD633-06|Canada|New Brunswick|658[0n]|BOLD:ABZ4926
Lithophane thujae|[18731]|RDNMF820-08|Canada|New Brunswick|658[0n]|BOLD:ABZ4926
Lithophane thujae|[18732]|RDNMF821-08|Canada|New Brunswick|658[0n]|BOLD:ABZ4926
Lithophane thujae|[18733]|RDNMF822-08|Canada|New Brunswick|658[0n]|BOLD:ABZ4926
Lithophane itata|[18734]|RDNME721-08|United States|Colorado|658[0n]|BOLD:ABZ4930
Lithophane itata|[18735]|RDNMG856-08|Canada|British Columbia|658[0n]|BOLD:ABZ4930
Lithophane itata|[18736]|DUNLP167-08|Canada|British Columbia|658[0n]|BOLD:ABZ4930
Lithophane itata|[18737]|RDNMD629-06|Canada|British Columbia|631[1n]|BOLD:ABZ4930
Lithophane itata|[18738]|RDNMG857-08|Canada|British Columbia|592[0n]|BOLD:ABZ4930
Lithophane gausapata|[18739]|RDNMD628-06|United States|California|658[0n]|BOLD:ACE6388
Lithophane gausapata|[18740]|JMMMB339-11|United States|California|658[0n]|BOLD:ACE6388
Lithophane tarda|[18741]|RDNME862-08|United States|Arizona|658[0n]|BOLD:AAD7657
Lithophane tarda|[18742]|RDNMG186-08|United States|Arizona|658[0n]|BOLD:AAD7657
Lithophane longior|[18743]|RDNMC686-06|United States|California|658[0n]|BOLD:AAD7657
Lithophane longior|[18744]|RDNME587-08|United States|Texas|658[0n]|BOLD:AAD7657
Lithophane longior|[18745]|RDNME588-08|United States|Texas|658[0n]|BOLD:AAD7657
Lithophane longior|[18746]|RDNMH728-09|United States|Texas|658[0n]|BOLD:AAD7657
Lithophane sp.|[18747]|NAMUM387-09|United States|California|658[0n]|BOLD:ABZ4925
Lithophane sp.|[18748]|NAMUM391-09|United States|California|658[0n]|BOLD:ABZ4925
Lithophane subtilis|[18749]|RDNME585-08|United States|Texas|658[0n]|BOLD:ABZ4925
Lithophane boogeri|[18750]|RDNMD627-06|United States|Oregon|658[0n]|BOLD:ABZ4925
Lithophane subtilis|[18751]|RDNMH730-09|United States|California|603[0n]|BOLD:ABZ4925
Lithophane lemmeri|[18752]|RDNMH132-09|United States|New Jersey|658[0n]|BOLD:ACF5438
Lithophane lemmeri|[18753]|RDNMH152-09|United States|New Jersey|658[0n]|BOLD:ACF5438
Lithophane lemmeri|[18754]|RDNMD634-06|United States|Texas|658[0n]|BOLD:ACF5438
Lithophane lemmeri|[18755]|CNCLB2939-14|United States|North Carolina|658[0n]|BOLD:ACF5438
Zotheca tranquilla|[18756]|RDNMF002-08|Canada|British Columbia|658[0n]|BOLD:AAD5204
Zotheca tranquilla|[18757]|LBCW048-08|Canada|British Columbia|658[0n]|BOLD:AAD5204
Zotheca tranquilla|[18758]|LBCW047-08|Canada|British Columbia|658[0n]|BOLD:AAD5204
Zotheca tranquilla|[18759]|LHLEP379-06|Canada|British Columbia|658[0n]|BOLD:AAD5204
Zotheca tranquilla|[18760]|RDNMF093-08|Canada|British Columbia|658[0n]|BOLD:AAD5204
Zotheca tranquilla|[18761]|LBCW049-08|Canada|British Columbia|658[0n]|BOLD:AAD5204
Zotheca tranquilla|[18762]|LALPA419-10|Canada|British Columbia|658[0n]|BOLD:AAD5204
Zotheca tranquilla|[18763]|LPVIA632-08|Canada|British Columbia|658[0n]|BOLD:AAD5204
Zotheca tranquilla|[18764]|LALPA439-10|Canada|British Columbia|658[0n]|BOLD:AAD5204
Zotheca tranquilla|[18765]|LALPA568-10|Canada|British Columbia|658[0n]|BOLD:AAD5204
Zotheca tranquilla|[18766]|LBCH6014-10|Canada|British Columbia|658[0n]|BOLD:AAD5204
Zotheca tranquilla|[18767]|BBLOC1345-11|United States|California|658[0n]|BOLD:AAD5204
Zotheca tranquilla|[18768]|NAMUM154-08|United States|California|658[0n]|BOLD:AAD5204
Zotheca tranquilla|[18769]|BBLOE1401-12|United States|California|658[0n]|BOLD:AAD5204
Eupsilia tristigmata|[18770]|RDLQ563-07|Canada|Quebec|658[0n]|BOLD:ABY3690
Eupsilia tristigmata|[18771]|TMNBB248-06|Canada|New Brunswick|657[0n]|BOLD:ABY3690
Eupsilia tristigmata|[18772]|TMNBB246-06|Canada|New Brunswick|658[0n]|BOLD:ABY3690
Eupsilia tristigmata|[18773]|LOWCE042-06|Canada|British Columbia|658[0n]|BOLD:ABY3690
Eupsilia tristigmata|[18774]|LOWCE041-06|Canada|British Columbia|658[0n]|BOLD:ABY3690
Eupsilia tristigmata|[18775]|LOWCC019-05|Canada|British Columbia|658[0n]|BOLD:ABY3690
Eupsilia tristigmata|[18776]|RDMAB577-06|Canada|Alberta|593[0n]|BOLD:ABY3690
Eupsilia tristigmata|[18777]|PHMO018-03|Canada|Ontario|639[0n]|BOLD:ABY3690
Eupsilia tristigmata|[18778]|RDLQ562-07|Canada|Quebec|596[0n]|BOLD:ABY3690
Eupsilia tristigmata|[18779]|RDLQ565-07|Canada|Quebec|647[0n]|BOLD:ABY3690
Eupsilia tristigmata|[18780]|LALPA004-10|Canada|British Columbia|658[0n]|BOLD:ABY3690
Eupsilia tristigmata|[18781]|LALPA022-10|Canada|British Columbia|658[0n]|BOLD:ABY3690
Eupsilia tristigmata|[18782]|LALPA807-10|Canada|British Columbia|658[0n]|BOLD:ABY3690
Eupsilia vinulenta|[18783]|LNCB959-10|United States|North Carolina|646[0n]|BOLD:AAB4640
Eupsilia vinulenta|[18784]|LNCB973-10|United States|North Carolina|658[0n]|BOLD:AAB4640
Eupsilia vinulenta|[18785]|RDNML124-13|United States|New Jersey|658[0n]|BOLD:AAB4640
Eupsilia morrisoni|[18786]|XAH672-05|Canada|Ontario|658[0n]|BOLD:AAB4640
Eupsilia morrisoni|[18787]|XAJ096-06|Canada|Ontario|658[0n]|BOLD:AAB4640
Eupsilia vinulenta|[18788]|LNCC489-11|United States|North Carolina|658[0n]|BOLD:AAB4640
Eupsilia vinulenta|[18789]|LNCC495-11|United States|North Carolina|658[0n]|BOLD:AAB4640
Eupsilia morrisoni|[18790]|LNCC602-11|United States|North Carolina|658[0n]|BOLD:AAB4640

Eupsilia morrisoni|[18787]|XAJ096-06|Canada|Ontario|658[0n]|BOLD:AAB4640
Eupsilia vinulenta|[18788]|LNCC489-11|United States|North Carolina|658[0n]|BOLD:AAB4640
Eupsilia vinulenta|[18789]|LNCC495-11|United States|North Carolina|658[0n]|BOLD:AAB4640
Eupsilia morrisoni|[18790]|LNCC602-11|United States|North Carolina|658[0n]|BOLD:AAB4640
Eupsilia morrisoni|[18791]|PMG111-03|Canada|Ontario|617[0n]|BOLD:AAB4640
Eupsilia vinulenta|[18792]|BBLCU366-09|United States|Indiana|658[0n]|BOLD:AAB4640
Eupsilia morrisoni|[18793]|LNCC603-11|United States|North Carolina|658[0n]|BOLD:AAB4640
Eupsilia morrisoni|[18794]|UOFTL004-11|Canada|Ontario|658[0n]|
Eupsilia vinulenta|[18795]|LNCC488-11|United States|North Carolina|658[0n]|BOLD:AAB4640
Eupsilia vinulenta|[18796]|LNCC493-11|United States|North Carolina|658[0n]|BOLD:AAB4640
Eupsilia vinulenta|[18797]|LNCC494-11|United States|North Carolina|658[0n]|BOLD:AAB4640
Eupsilia vinulenta|[18798]|LMDH023-11|United States|Minnesota|658[0n]|BOLD:AAB4640
Eupsilia vinulenta|[18799]|LNCC578-11|United States|North Carolina|658[0n]|BOLD:AAB4640
Eupsilia morrisoni|[18800]|LNCC598-11|United States|North Carolina|658[0n]|BOLD:AAB4640
Eupsilia morrisoni|[18801]|PHAPR1313-11|Canada|Ontario|658[0n]|BOLD:AAB4640
Eupsilia morrisoni|[18802]|PHNOV551-11|Canada|Ontario|658[0n]|BOLD:AAB4640
Eupsilia vinulenta|[18803]|RDNML125-13|United States|New Jersey|658[0n]|BOLD:AAB4640
Eupsilia vinulenta|[18804]|RDNML126-13|United States|New Jersey|658[0n]|BOLD:AAB4640
Eupsilia morrisoni|[18805]|XAJ052-06|Canada|Ontario|658[0n]|BOLD:AAB4640
Eupsilia morrisoni|[18806]|XAJ068-06|Canada|Ontario|658[0n]|BOLD:AAB4640
Eupsilia morrisoni|[18807]|XAJ175-06|Canada|Ontario|658[0n]|BOLD:AAB4640
Eupsilia vinulenta|[18808]|LNCB968-10|United States|North Carolina|658[0n]|BOLD:AAB4640
Eupsilia morrisoni|[18809]|XAJ107-06|Canada|Ontario|658[0n]|BOLD:AAB4640
Eupsilia morrisoni|[18810]|XAJ135-06|Canada|Ontario|658[0n]|BOLD:AAB4640
Eupsilia vinulenta|[18811]|RDNML127-13|United States|New Jersey|658[0n]|BOLD:AAB4640
Eupsilia morrisoni|[18812]|XAJ024-06|Canada|Ontario|658[0n]|BOLD:AAB4640
Eupsilia morrisoni|[18813]|TMNBB250-06|Canada|New Brunswick|658[0n]|BOLD:AAB4640
Eupsilia vinulenta|[18814]|TTMNB372-06|Canada|New Brunswick|658[0n]|BOLD:AAB4640
Eupsilia morrisoni|[18815]|LSEU120-06|United States|North Carolina|658[0n]|BOLD:AAB4640
Eupsilia morrisoni|[18816]|LSEU118-06|United States|North Carolina|658[0n]|BOLD:AAB4640
Eupsilia morrisoni|[18817]|XAI028-05|Canada|Ontario|658[0n]|BOLD:AAB4640
Eupsilia morrisoni|[18818]|XAF238-05|Canada|Ontario|658[0n]|BOLD:AAB4640
Eupsilia morrisoni|[18819]|XAF235-05|Canada|Ontario|658[0n]|BOLD:AAB4640
Eupsilia morrisoni|[18820]|LSEU119-06|United States|North Carolina|658[0n]|BOLD:AAB4640
Eupsilia morrisoni|[18821]|XAF202-05|Canada|Ontario|658[0n]|BOLD:AAB4640
Eupsilia morrisoni|[18822]|XAJ112-06|Canada|Ontario|658[0n]|BOLD:AAB4640
Eupsilia morrisoni|[18823]|MEC136-04|Canada|Quebec|658[0n]|BOLD:AAB4640
Eupsilia vinulenta|[18824]|TMNBB247-06|Canada|New Brunswick|658[0n]|BOLD:AAB4640
Eupsilia morrisoni|[18825]|TMNBB249-06|Canada|New Brunswick|632[0n]|BOLD:AAB4640
Eupsilia morrisoni|[18826]|XAJ066-06|Canada|Ontario|643[0n]|BOLD:AAB4640
Eupsilia vinulenta|[18827]|RDLQ561-07|Canada|Quebec|536[0n]|BOLD:AAB4640
Eupsilia morrisoni|[18828]|RDLQ569-07|Canada|Quebec|575[0n]|BOLD:AAB4640
Eupsilia vinulenta|[18829]|LNCC496-11|United States|North Carolina|624[0n]|BOLD:AAB4640
Eupsilia morrisoni|[18830]|LNCC601-11|United States|North Carolina|624[0n]|BOLD:AAB4640
Eupsilia morrisoni|[18831]|XAJ174-06|Canada|Ontario|626[0n]|BOLD:AAB4640
Eupsilia morrisoni|[18832]|RDLQ568-07|Canada|Quebec|586[0n]|BOLD:AAB4640
Eupsilia morrisoni|[18833]|RDNML128-13|United States|New Jersey|658[0n]|BOLD:AAB4640
Eupsilia sidus|[18834]|LNCB970-10|United States|North Carolina|658[0n]|BOLD:ABZ6254
Eupsilia tristigmata|[18835]|LNCB1002-10|United States|North Carolina|658[0n]|BOLD:ABZ6254
Eupsilia tristigmata|[18836]|LNCB1004-10|United States|North Carolina|658[0n]|BOLD:ABZ6254
Eupsilia tristigmata|[18837]|LNCB1005-10|United States|North Carolina|658[0n]|BOLD:ABZ6254
Eupsilia tristigmata|[18838]|XAJ176-06|Canada|Ontario|656[0n]|BOLD:ABZ6254
Eupsilia tristigmata|[18839]|LNCC487-11|United States|North Carolina|658[0n]|BOLD:ABZ6254
Eupsilia tristigmata|[18840]|LNCC492-11|United States|North Carolina|658[0n]|BOLD:ABZ6254
Eupsilia tristigmata|[18841]|LNCC599-11|United States|North Carolina|658[0n]|BOLD:ABZ6254
Eupsilia tristigmata|[18842]|LNCC600-11|United States|North Carolina|658[0n]|BOLD:ABZ6254
Eupsilia tristigmata|[18843]|RDNML129-13|United States|New Jersey|658[0n]|BOLD:ABZ6254
Eupsilia tristigmata|[18844]|RDNML130-13|United States|New Jersey|658[0n]|BOLD:ABZ6254
Eupsilia tristigmata|[18845]|RDNML131-13|United States|New Jersey|658[0n]|BOLD:ABZ6254
Eupsilia sidus|[18846]|RDNML119-13|United States|New Jersey|658[0n]|BOLD:ABZ6254
Eupsilia sidus|[18847]|RDNMF479-08|United States|New York|658[0n]|BOLD:ABZ6254
Eupsilia sidus|[18848]|LNCC498-11|United States|North Carolina|658[0n]|BOLD:ABZ6254
Eupsilia sidus|[18849]|LNCC485-11|United States|North Carolina|658[0n]|BOLD:ABZ6254
Eupsilia sidus|[18850]|RDNML116-13|United States|New Jersey|658[0n]|BOLD:ABZ6254
Eupsilia sidus|[18851]|RDNML117-13|United States|New Jersey|658[0n]|BOLD:ABZ6254
Eupsilia sidus|[18852]|LNCC504-11|United States|North Carolina|658[0n]|BOLD:ABZ6254
Eupsilia sidus|[18853]|RDNML118-13|United States|New Jersey|658[0n]|BOLD:ABZ6254
Eupsilia tristigmata|[18854]|RWWC1123-13|United States|Washington|592[0n]|BOLD:ABZ6254
Eupsilia cirripalea|[18855]|LNCC514-11|United States|North Carolina|658[0n]|BOLD:ABZ6254
Eupsilia cirripalea|[18856]|LNCC481-11|United States|North Carolina|658[0n]|BOLD:ABZ6254
Eupsilia cirripalea|[18857]|LNCB956-10|United States|North Carolina|658[0n]|BOLD:ABZ6254
Eupsilia cirripalea|[18858]|LNCB969-10|United States|North Carolina|658[0n]|BOLD:ABZ6254
Eupsilia cirripalea|[18859]|LNCC480-11|United States|North Carolina|658[0n]|BOLD:ABZ6254
Eupsilia cirripalea|[18860]|LNCC499-11|United States|North Carolina|658[0n]|BOLD:ABZ6254
Eupsilia cirripalea|[18861]|LNCC502-11|United States|North Carolina|658[0n]|BOLD:ABZ6254
Eupsilia cirripalea|[18862]|LNCC518-11|United States|North Carolina|658[0n]|BOLD:ABZ6254
Eupsilia cirripalea|[18863]|LNCC597-11|United States|North Carolina|658[0n]|BOLD:ABZ6254
Eupsilia cirripalea|[18864]|LNCB972-10|United States|North Carolina|658[0n]|BOLD:ABZ6254
Eupsilia cirripalea|[18865]|LNCC510-11|United States|North Carolina|658[0n]|BOLD:ABZ6254
Eupsilia cirripalea|[18866]|RDNMK626-11|United States|North Carolina|658[0n]|BOLD:ABZ6254
Eupsilia cirripalea|[18867]|LNCB954-10|United States|North Carolina|658[0n]|BOLD:ABZ6254
Eupsilia cirripalea|[18868]|LNCB957-10|United States|North Carolina|658[0n]|BOLD:ABZ6254
Eupsilia cirripalea|[18869]|RDNML120-13|United States|New Jersey|658[0n]|BOLD:ABZ6254
Eupsilia cirripalea|[18870]|CNCLB1372-14|United States|Tennessee|658[0n]|BOLD:ABZ6254
Eupsilia cirripalea|[18871]|RDNML121-13|United States|New Jersey|658[0n]|BOLD:ABZ6254
Eupsilia cirripalea|[18872]|LNCC516-11|United States|North Carolina|624[0n]|BOLD:ABZ6254
Eupsilia cirripalea|[18873]|RDNML122-13|United States|New Jersey|658[0n]|BOLD:ABZ6254
Eupsilia cirripalea|[18874]|RDNMK627-11|United States|North Carolina|658[0n]|BOLD:ABZ6254
Eupsilia cirripalea|[18875]|LNCC595-11|United States|North Carolina|658[0n]|BOLD:ABZ6254
Eupsilia cirripalea|[18876]|LNCC519-11|United States|North Carolina|658[0n]|BOLD:ABZ6254
Eupsilia cirripalea|[18877]|LNCC517-11|United States|North Carolina|658[0n]|BOLD:ABZ6254
Eupsilia cirripalea|[18878]|LNCC513-11|United States|North Carolina|658[0n]|BOLD:ABZ6254
Eupsilia cirripalea|[18879]|LNCC511-11|United States|North Carolina|658[0n]|BOLD:ABZ6254
Eupsilia cirripalea|[18880]|LNCC509-11|United States|North Carolina|658[0n]|BOLD:ABZ6254
Eupsilia cirripalea|[18881]|LNCC508-11|United States|North Carolina|658[0n]|BOLD:ABZ6254
Eupsilia cirripalea|[18882]|LNCC506-11|United States|North Carolina|658[0n]|BOLD:ABZ6254
Eupsilia cirripalea|[18883]|LNCC505-11|United States|North Carolina|658[0n]|BOLD:ABZ6254
Eupsilia cirripalea|[18884]|LNCC503-11|United States|North Carolina|658[0n]|BOLD:ABZ6254
Eupsilia cirripalea|[18885]|LNCC501-11|United States|North Carolina|658[0n]|BOLD:ABZ6254
Eupsilia cirripalea|[18886]|LNCC497-11|United States|North Carolina|658[0n]|BOLD:ABZ6254
Eupsilia cirripalea|[18887]|LNCC486-11|United States|North Carolina|658[0n]|BOLD:ABZ6254
Eupsilia cirripalea|[18888]|LNCC484-11|United States|North Carolina|658[0n]|BOLD:ABZ6254
Eupsilia cirripalea|[18889]|LNCC483-11|United States|North Carolina|658[0n]|BOLD:ABZ6254
[illegible]

Eupsilia cirripalea|[18887]|LNCC486-11|United States|North Carolina|658[0n]|BOLD:ABZ6254
Eupsilia cirripalea|[18888]|LNCC484-11|United States|North Carolina|658[0n]|BOLD:ABZ6254
Eupsilia cirripalea|[18889]|LNCC483-11|United States|North Carolina|658[0n]|BOLD:ABZ6254
Eupsilia cirripalea|[18890]|LNCC482-11|United States|North Carolina|658[0n]|BOLD:ABZ6254
Eupsilia cirripalea|[18891]|LNCB1003-10|United States|North Carolina|658[0n]|BOLD:ABZ6254
Eupsilia cirripalea|[18892]|LNCB974-10|United States|North Carolina|658[0n]|BOLD:ABZ6254
Eupsilia cirripalea|[18893]|LNCB971-10|United States|North Carolina|658[0n]|BOLD:ABZ6254
Eupsilia cirripalea|[18894]|LNCC507-11|United States|North Carolina|658[0n]|BOLD:ABZ6254
Eupsilia cirripalea|[18895]|LNCB955-10|United States|North Carolina|658[0n]|BOLD:ABZ6254
Eupsilia cirripalea|[18896]|LNCB958-10|United States|North Carolina|646[0n]|BOLD:ABZ6254
Eupsilia cirripalea|[18897]|LNCC479-11|United States|North Carolina|633[0n]|BOLD:ABZ6254
Eupsilia cirripalea|[18898]|CNCLB1370-14|United States|Tennessee|633[0n]|BOLD:ABZ6254
Eupsilia cirripalea|[18899]|CNCLB1371-14|United States|Tennessee|658[0n]|BOLD:ABZ6254
Eupsilia cirripalea|[18900]|LNCC515-11|United States|North Carolina|658[0n]|BOLD:ABZ6254
Eupsilia cirripalea|[18901]|CNCLB1373-14|United States|Tennessee|658[0n]|BOLD:ABZ6254
Eupsilia cirripalea|[18902]|LNCC500-11|United States|North Carolina|658[0n]|BOLD:ABZ6254
Eupsilia cirripalea|[18903]|LNCC512-11|United States|North Carolina|658[0n]|BOLD:ABZ6254
Eupsilia cirripalea|[18904]|LNCC520-11|United States|North Carolina|658[0n]|BOLD:ABZ6254
Eupsilia cirripalea|[18905]|RDNML123-13|United States|New Jersey|658[0n]|BOLD:ABZ6254
Eupsilia cirripalea|[18906]|CNCLB1374-14|United States|Tennessee|621[0n]|BOLD:ABZ6254
Eupsilia n. sp. nr. sidus|[18907]|LNCC594-11|United States|North Carolina|658[0n]|BOLD:ACE7179
Eupsilia n. sp. nr. sidus|[18908]|LNCC1477-13|United States|North Carolina|658[0n]|BOLD:ACE7179
Eupsilia n. sp. nr. sidus|[18909]|CNCLB1380-14|United States|North Carolina|658[0n]|BOLD:ACE7179
Eupsilia n. sp. nr. sidus|[18910]|RDNMJ312-11|United States|Connecticut|658[0n]|BOLD:ACE7179
Eupsilia n. sp. nr. sidus|[18911]|LNCC592-11|United States|North Carolina|614[0n]|BOLD:ACE7179
Eupsilia n. sp. nr. sidus|[18912]|LNCC596-11|United States|North Carolina|658[0n]|BOLD:ACE7179
Eupsilia n. sp. nr. sidus|[18913]|LNCC593-11|United States|North Carolina|658[0n]|BOLD:ACE7179
Eupsilia n. sp. nr. sidus|[18914]|LNCC1478-13|United States|North Carolina|658[0n]|BOLD:ACE7179
Eupsilia n. sp. nr. sidus|[18915]|CNCLB1381-14|United States|North Carolina|658[0n]|BOLD:ACE7179
Eupsilia n. sp. nr. sidus|[18916]|CNCLB1382-14|United States|North Carolina|658[0n]|BOLD:ACE7179
Eupsilia devia|[18917]|LNCC605-11|United States|North Carolina|658[0n]|BOLD:AAD9847
Eupsilia devia|[18918]|SMTPB17445-13|Canada|Ontario|594[0n]|BOLD:AAD9847
Eupsilia devia|[18919]|PHMO036-03|Canada|Ontario|639[0n]|BOLD:AAD9847
Eupsilia devia|[18920]|RDLQH111-06|Canada|Quebec|652[0n]|BOLD:AAD9847
Eupsilia devia|[18921]|LNCC604-11|United States|North Carolina|614[0n]|BOLD:AAD9847
Eupsilia devia|[18922]|HEAPR3745-12|Canada|Ontario|658[0n]|BOLD:AAD9847
Eupsilia devia|[18923]|RDLQH093-06|Canada|Quebec|658[0n]|BOLD:AAD9847
Eupsilia devia|[18924]|TMNBB251-06|Canada|New Brunswick|658[0n]|BOLD:AAD9847
Eupsilia devia|[18925]|XAF032-05|Canada|Ontario|658[0n]|BOLD:AAD9847
Eupsilia devia|[18926]|SMTPF8855-14|Canada|Ontario|591[0n]|BOLD:AAD9847
Eupsilia devia|[18927]|SMTPF8856-14|Canada|Ontario|561[0n]|BOLD:AAD9847
Eupsilia fringata|[18928]|TML206-14|United States||658[0n]|BOLD:ACO1940
Eupsilia fringata|[18929]|CNCLB1445-14|United States|Washington|658[0n]|BOLD:ACO1940
Phuphena tura|[18930]|CNCLA547-13|United States|Florida|658[0n]|BOLD:ABA7227
Phuphena tura|[18931]|LNAUS4433-13|Cuba|Holguin|658[0n]|BOLD:ABA7227
Phuphena tura|[18932]|LNAUS4434-13|Cuba|Holguin|658[0n]|BOLD:ABA7227
Phuphena tura|[18933]|LNAUS4436-13|Cuba|Holguin|658[0n]|BOLD:ABA7227
Phuphena tura|[18934]|LNAUS4437-13|Cuba|Holguin|658[0n]|BOLD:ABA7227
Speocropia trichroma|[18935]|CNCLB517-14|United States|Florida|658[0n]|BOLD:ACM3994
Speocropia trichroma|[18936]|CNCLB518-14|United States|Florida|658[0n]|BOLD:ACM3994
Speocropia trichroma|[18937]|CNCLB514-14|United States|Florida|658[0n]|BOLD:ACM3994
Speocropia trichroma|[18938]|CNCLB515-14|United States|Florida|658[0n]|BOLD:ACM3994
Speocropia trichroma|[18939]|CNCLB3019-14|United States|Florida|658[0n]|BOLD:ACM3994
Ufeus felsensteini|[18940]|RDNME256-07|United States|Arizona|658[2n]|BOLD:AAF0451
Ufeus felsensteini|[18941]|RDNME257-07|United States|Arizona|658[0n]|BOLD:AAF0451
Ufeus felsensteini|[18942]|RDNME858-08|United States|Arizona|658[0n]|BOLD:AAF0451
Ufeus felsensteini|[18943]|RDNMI117-10|United States|Arizona|658[0n]|BOLD:AAF0451
Ufeus hulstii|[18944]|RDNMG853-08|Canada|British Columbia|592[0n]|BOLD:ACF0561
Ufeus hulstii|[18945]|RDNMG103-08|United States|Washington|658[0n]|BOLD:ACF0561
Ufeus hulstii|[18946]|RDNMG852-08|United States|Washington|642[0n]|BOLD:ACF0561
Ufeus hulstii|[18947]|RDNMG854-08|Canada|British Columbia|658[0n]|BOLD:ACF0561
Ufeus hulstii|[18948]|NAMUM339-08|United States|California|658[0n]|BOLD:ACF0561
Ufeus plicatus|[18949]|RDLQ749-07|Canada|Quebec|635[0n]|BOLD:AAD7193
Ufeus plicatus|[18950]|RDLQ750-07|Canada|Quebec|658[0n]|BOLD:AAD7193
Ufeus faunus|[18951]|NAMUM268-08|United States|California|658[0n]|BOLD:ACE9033
Ufeus faunus|[18952]|LEFIA1412-10|United States|California|670[0n]|BOLD:ACE9033
Ufeus|[18953]|JMMMB345-11|United States|California|658[0n]|BOLD:ACE8179
Ufeus satyricus|[18954]|RDLQ751-07|Canada|Quebec|635[0n]|BOLD:AAB8639
Ufeus satyricus|[18955]|RDNME260-07|Canada|New Brunswick|617[0n]|BOLD:AAB8639
Ufeus satyricus|[18956]|RDNME261-07|Canada|New Brunswick|619[1n]|BOLD:AAB8639
Ufeus satyricus|[18957]|RDNME263-07|United States|Arizona|606[0n]|BOLD:AAB8639
Ufeus satyricus|[18958]|RDNME262-07|United States|Arizona|644[0n]|BOLD:AAB8639
Ufeus satyricus|[18959]|RDNME264-07|United States|Arizona|616[0n]|BOLD:AAB8639
Ufeus satyricus|[18960]|RDNMJ152-10|United States|Arizona|658[0n]|BOLD:AAB8639
Ufeus satyricus|[18961]|LOWCD681-06|Canada|British Columbia|658[0n]|BOLD:AAB8639
Ufeus satyricus|[18962]|LOWCD682-06|Canada|British Columbia|570[0n]|BOLD:AAB8639
Ufeus satyricus|[18963]|LOWCE274-06|Canada|British Columbia|658[0n]|BOLD:AAB8639
Ufeus satyricus|[18964]|CGLCA042-10|United States|California|658[0n]|BOLD:AAB8639
Ufeus satyricus|[18965]|RDNME258-07|Canada|New Brunswick|658[0n]|BOLD:AAB8639
Ufeus satyricus|[18966]|PHMNB268-04|Canada|New Brunswick|658[0n]|BOLD:AAB8639
Ufeus satyricus|[18967]|LOWCD680-06|Canada|British Columbia|565[0n]|BOLD:AAB8639
Ufeus satyricus|[18968]|LOWCD684-06|Canada|British Columbia|589[0n]|BOLD:AAB8639
Ufeus satyricus|[18969]|XAB671-04|Canada|Ontario|658[0n]|BOLD:AAB8639
Ufeus satyricus|[18970]|XAB647-04|Canada|Ontario|658[0n]|BOLD:AAB8639
Ufeus satyricus|[18971]|PHMO363-03|Canada|Ontario|639[0n]|BOLD:AAB8639
Ufeus satyricus|[18972]|LOWCD679-06|Canada|British Columbia|585[1n]|BOLD:AAB8639
Ufeus satyricus|[18973]|LOWCD683-06|Canada|British Columbia|592[0n]|BOLD:AAB8639
Ufeus satyricus|[18974]|RDNME259-07|United States|Nevada|617[1n]|BOLD:AAB8639
Ufeus satyricus|[18975]|RDNMG875-08|United States|Oregon|648[0n]|BOLD:AAB8639
Ufeus satyricus|[18976]|JMMMB341-11|United States|California|658[0n]|BOLD:AAB8639
Ufeus satyricus|[18977]|RDNME689-08|United States|Washington|658[0n]|BOLD:AAB8639
Ufeus satyricus|[18978]|CGLCA054-10|United States|California|658[0n]|BOLD:AAB8639
Ufeus satyricus|[18979]|JMMMB368-11|United States|California|658[0n]|BOLD:AAB8639
Hyppa xylinoides|[18980]|LPSOB714-08|Canada|Ontario|621[0n]|BOLD:ABY9574
Hyppa xylinoides|[18981]|XAG993-05|Canada|Ontario|629[0n]|BOLD:ABY9574
Hyppa xylinoides|[18982]|XAH148-05|Canada|Ontario|658[6n]|BOLD:ABY9574
Hyppa xylinoides|[18983]|XAD367-04|Canada|Ontario|582[0n]|BOLD:ABY9574
Hyppa xylinoides|[18984]|XAF741-05|Canada|Ontario|547[0n]|BOLD:ABY9574
Hyppa xylinoides|[18985]|XAH005-05|Canada|Ontario|637[0n]|BOLD:ABY9574
Hyppa xylinoides|[18986]|TTMNB368-06|Canada|New Brunswick|658[1n]|BOLD:ABY9574
Hyppa xylinoides|[18987]|TTMNB367-06|Canada|New Brunswick|658[1n]|BOLD:ABY9574
Hyppa xylinoides|[18988]|XAJ341-06|Canada|Ontario|658[0n]|BOLD:ABY9574
Hyppa xylinoides|[18989]|XAG347-05|Canada|Ontario|658[0n]|BOLD:ABY9574
[illegible]

Hyppa xylinoides|[18987]|TTMNB367-06|Canada|New Brunswick|658[1n]|BOLD:ABY9574
Hyppa xylinoides|[18988]|XAJ341-06|Canada|Ontario|658[0n]|BOLD:ABY9574
Hyppa xylinoides|[18989]|XAG347-05|Canada|Ontario|658[0n]|BOLD:ABY9574
Hyppa xylinoides|[18990]|XAG876-05|Canada|Ontario|658[0n]|BOLD:ABY9574
Hyppa xylinoides|[18991]|LGSMG997-10|United States|North Carolina|658[0n]|BOLD:ABY9574
Hyppa xylinoides|[18992]|XAJ583-06|Canada|Ontario|658[0n]|BOLD:ABY9574
Hyppa xylinoides|[18993]|XAJ620-06|Canada|Ontario|658[0n]|BOLD:ABY9574
Hyppa xylinoides|[18994]|XAJ672-06|Canada|Ontario|658[0n]|BOLD:ABY9574
Hyppa xylinoides|[18995]|LPSOC086-08|Canada|Ontario|658[0n]|BOLD:ABY9574
Hyppa xylinoides|[18996]|LPSOC323-08|Canada|Ontario|658[0n]|BOLD:ABY9574
Hyppa xylinoides|[18997]|LPSOB326-08|Canada|Ontario|658[0n]|BOLD:ABY9574
Hyppa xylinoides|[18998]|LPSOB454-08|Canada|Ontario|658[0n]|BOLD:ABY9574
Hyppa xylinoides|[18999]|LPSOB976-08|Canada|Ontario|658[0n]|BOLD:ABY9574
Hyppa xylinoides|[19000]|BLTIB163-08|Canada|Ontario|658[0n]|BOLD:ABY9574
Hyppa xylinoides|[19001]|BLTIB223-08|Canada|Ontario|658[0n]|BOLD:ABY9574
Hyppa xylinoides|[19002]|LGSMG996-10|United States|North Carolina|658[0n]|BOLD:ABY9574
Hyppa xylinoides|[19003]|LNCC209-10|United States|North Carolina|658[0n]|BOLD:ABY9574
Hyppa xylinoides|[19004]|XAJ547-06|Canada|Ontario|658[0n]|BOLD:ABY9574
Hyppa xylinoides|[19005]|XAJ548-06|Canada|Ontario|658[0n]|BOLD:ABY9574
Hyppa xylinoides|[19006]|XAJ405-06|Canada|Ontario|658[0n]|BOLD:ABY9574
Hyppa xylinoides|[19007]|XAJ546-06|Canada|Ontario|658[0n]|BOLD:ABY9574
Hyppa xylinoides|[19008]|XAJ265-06|Canada|Ontario|658[0n]|BOLD:ABY9574
Hyppa xylinoides|[19009]|XAJ318-06|Canada|Ontario|658[0n]|BOLD:ABY9574
Hyppa xylinoides|[19010]|TTMNB375-06|Canada|New Brunswick|658[0n]|BOLD:ABY9574
Hyppa xylinoides|[19011]|XAG805-05|Canada|Ontario|658[0n]|BOLD:ABY9574
Hyppa xylinoides|[19012]|MNBB360-05|Canada|New Brunswick|658[0n]|BOLD:ABY9574
Hyppa xylinoides|[19013]|XAF547-05|Canada|Ontario|658[0n]|BOLD:ABY9574
Hyppa xylinoides|[19014]|LOCT315-05|United States|Connecticut|658[0n]|BOLD:ABY9574
Hyppa xylinoides|[19015]|LOCT262-05|United States|Connecticut|658[0n]|BOLD:ABY9574
Hyppa xylinoides|[19016]|PHMNB357-04|Canada|New Brunswick|658[0n]|BOLD:ABY9574
Hyppa xylinoides|[19017]|XAD355-04|Canada|Ontario|658[0n]|BOLD:ABY9574
Hyppa xylinoides|[19018]|XAD235-04|Canada|Ontario|658[0n]|BOLD:ABY9574
Hyppa xylinoides|[19019]|XAB398-04|Canada|Ontario|658[0n]|BOLD:ABY9574
Hyppa xylinoides|[19020]|XAB064-04|Canada|Ontario|658[0n]|BOLD:ABY9574
Hyppa xylinoides|[19021]|XAF522-05|Canada|Ontario|658[0n]|BOLD:ABY9574
Hyppa xylinoides|[19022]|XAJ681-06|Canada|Ontario|656[0n]|BOLD:ABY9574
Hyppa xylinoides|[19023]|TMG116-03|Canada|Ontario|639[0n]|BOLD:ABY9574
Hyppa xylinoides|[19024]|PMG120-03|Canada|Ontario|617[0n]|BOLD:ABY9574
Hyppa xylinoides|[19025]|XAD589-05|Canada|Ontario|642[0n]|BOLD:ABY9574
Hyppa xylinoides|[19026]|RDLQ490-07|Canada|Quebec|612[0n]|BOLD:ABY9574
Hyppa xylinoides|[19027]|JSMAY1452-11|Canada|Ontario|658[0n]|BOLD:ABY9574
Hyppa indistincta|[19028]|LALPA508-10|Canada|British Columbia|658[0n]|BOLD:ABX5989
Hyppa indistincta|[19029]|LALPA737-10|Canada|British Columbia|658[0n]|BOLD:ABX5989
Hyppa indistincta|[19030]|LBCA367-05|Canada|British Columbia|588[1n]|BOLD:ABX5989
Hyppa indistincta|[19031]|LBCH1919-10|Canada|British Columbia|640[0n]|BOLD:ABX5989
Hyppa indistincta|[19032]|LBCC833-05|Canada|British Columbia|658[0n]|BOLD:ABX5989
Hyppa indistincta|[19033]|LBCD095-05|Canada|British Columbia|658[0n]|BOLD:ABX5989
Hyppa indistincta|[19034]|RDMAB548-06|Canada|Alberta|658[0n]|BOLD:ABX5989
Hyppa indistincta|[19035]|LPMN918-08|Canada|Alberta|658[0n]|BOLD:ABX5989
Hyppa indistincta|[19036]|LBCG2393-09|Canada|British Columbia|658[0n]|BOLD:ABX5989
Hyppa indistincta|[19037]|LBCG2394-09|Canada|British Columbia|658[0n]|BOLD:ABX5989
Hyppa indistincta|[19038]|LBCG2772-09|Canada|British Columbia|658[0n]|BOLD:ABX5989
Hyppa indistincta|[19039]|LBCG2836-09|Canada|British Columbia|658[0n]|BOLD:ABX5989
Hyppa indistincta|[19040]|LBCG3053-09|Canada|British Columbia|658[0n]|BOLD:ABX5989
Hyppa indistincta|[19041]|LBCH2256-10|Canada|British Columbia|658[0n]|BOLD:ABX5989
Hyppa indistincta|[19042]|BBLPA445-10|Canada|Alberta|658[0n]|BOLD:ABX5989
Hyppa indistincta|[19043]|BBLPA451-10|Canada|British Columbia|658[0n]|BOLD:ABX5989
Hyppa indistincta|[19044]|LBCA364-05|Canada|British Columbia|658[0n]|BOLD:ABX5989
Hyppa indistincta|[19045]|LBCC429-05|Canada|British Columbia|658[0n]|BOLD:ABX5989
Hyppa indistincta|[19046]|BBLPA453-10|Canada|British Columbia|658[0n]|BOLD:ABX5989
Hyppa indistincta|[19047]|BBLPA454-10|Canada|British Columbia|658[0n]|BOLD:ABX5989
Hyppa brunneicrista|[19048]|RDMAB279-05|Canada|Alberta|509[0n]|BOLD:ABZ2288
Hyppa brunneicrista|[19049]|LCHP379-07|Canada|Manitoba|632[0n]|BOLD:ABZ2288
Hyppa brunneicrista|[19050]|LCHP462-07|Canada|Manitoba|658[0n]|BOLD:ABZ2288
Hyppa brunneicrista|[19051]|LCH245-04|Canada|Manitoba|658[0n]|BOLD:ABZ2288
Hyppa brunneicrista|[19052]|LCHP184-07|Canada|Manitoba|658[0n]|BOLD:ABZ2288
Hyppa brunneicrista|[19053]|LCHP383-07|Canada|Manitoba|658[0n]|BOLD:ABZ2288
Hyppa brunneicrista|[19054]|LCHP481-07|Canada|Manitoba|658[0n]|BOLD:ABZ2288
Hyppa brunneicrista|[19055]|LCHP891-07|Canada|Manitoba|658[0n]|BOLD:ABZ2288
Hyppa brunneicrista|[19056]|LCHP895-07|Canada|Manitoba|658[0n]|BOLD:ABZ2288
Hyppa brunneicrista|[19057]|CHLEP074-09|Canada|Manitoba|658[0n]|BOLD:ABZ2288
Hyppa brunneicrista|[19058]|CHLEP078-09|Canada|Manitoba|658[0n]|BOLD:ABZ2288
Hyppa brunneicrista|[19059]|LCH243-04|Canada|Manitoba|658[0n]|BOLD:ABZ2288
Hyppa brunneicrista|[19060]|LCH244-04|Canada|Manitoba|658[0n]|BOLD:ABZ2288
Hyppa brunneicrista|[19061]|CHLEP083-09|Canada|Manitoba|658[0n]|BOLD:ABZ2288
Hyppa brunneicrista|[19062]|BBLPA447-10|Canada|British Columbia|658[0n]|BOLD:ABZ2288
Hyppa brunneicrista|[19063]|CNWLM2412-13|Canada|Alberta|577[0n]|BOLD:ABZ2288
Hyppa contrasta|[19064]|SSPAG293-13|Canada|Saskatchewan|583[0n]|BOLD:AAA5659
Hyppa contrasta|[19065]|LOWCB934-05|Canada|British Columbia|535[0n]|BOLD:AAA5659
Hyppa contrasta|[19066]|LOWCB933-05|Canada|British Columbia|535[0n]|BOLD:AAA5659
Hyppa contrasta|[19067]|LOWCB922-05|Canada|British Columbia|536[0n]|BOLD:AAA5659
Hyppa contrasta|[19068]|LOWCE839-06|Canada|British Columbia|617[0n]|BOLD:AAA5659
Hyppa contrasta|[19069]|CNWLD1010-12|Canada|Alberta|621[0n]|BOLD:AAA5659
Hyppa contrasta|[19070]|CNJAE970-12|Canada|Alberta|623[0n]|BOLD:AAA5659
Hyppa contrasta|[19071]|LBCA931-05|Canada|British Columbia|658[0n]|BOLD:AAA5659
Hyppa contrasta|[19072]|LBCC673-05|Canada|British Columbia|658[0n]|BOLD:AAA5659
Hyppa contrasta|[19073]|LBCD025-05|Canada|British Columbia|658[0n]|BOLD:AAA5659
Hyppa contrasta|[19074]|LBCD028-05|Canada|British Columbia|658[0n]|BOLD:AAA5659
Hyppa contrasta|[19075]|RDMAB278-05|Canada|Alberta|658[0n]|BOLD:AAA5659
Hyppa contrasta|[19076]|BBLPA448-10|Canada|British Columbia|658[0n]|BOLD:AAA5659
Hyppa contrasta|[19077]|BBLPA449-10|Canada|British Columbia|658[0n]|BOLD:AAA5659
Hyppa contrasta|[19078]|BBLPA450-10|Canada|British Columbia|658[0n]|BOLD:AAA5659
Hyppa contrasta|[19079]|LBCA271-05|Canada|British Columbia|658[0n]|BOLD:AAA5659
Hyppa contrasta|[19080]|LBCA496-05|Canada|British Columbia|658[0n]|BOLD:AAA5659
Hyppa contrasta|[19081]|BBLPA452-10|Canada|British Columbia|658[0n]|BOLD:AAA5659
Hyppa contrasta|[19082]|LBCB938-05|Canada|British Columbia|637[0n]|BOLD:AAA5659
Hyppa contrasta|[19083]|LBCC430-05|Canada|British Columbia|596[0n]|BOLD:AAA5659
Hyppa contrasta|[19084]|LBCB408-05|Canada|British Columbia|658[0n]|BOLD:AAA5659
Hyppa contrasta|[19085]|LBCG2835-09|Canada|British Columbia|658[0n]|BOLD:AAA5659
Hyppa contrasta|[19086]|BBLPB685-10|Canada|British Columbia|658[0n]|BOLD:AAA5659
Hyppa contrasta|[19087]|CNWLE2510-12|Canada|Alberta|632[0n]|BOLD:AAA5659
Hyppa contrasta|[19088]|PHMNB143-04|Canada|New Brunswick|658[0n]|BOLD:AAA5659
Hyppa contrasta|[19089]|LOWCB930-05|Canada|British Columbia|658[0n]|BOLD:AAA5659

Hyppa contrasta|[19087]|CNWLE2510-12|Canada|Alberta|632[0n]|BOLD:AAA5659
Hyppa contrasta|[19088]|PHMNB143-04|Canada|New Brunswick|658[0n]|BOLD:AAA5659
Hyppa contrasta|[19089]|LOWCB930-05|Canada|British Columbia|658[0n]|BOLD:AAA5659
Hyppa contrasta|[19090]|BBLPA446-10|Canada|British Columbia|658[0n]|BOLD:AAA5659
Hyppa contrasta|[19091]|PHMNB718-05|Canada|New Brunswick|658[0n]|BOLD:AAA5659
Hyppa contrasta|[19092]|LOWCB927-05|Canada|British Columbia|658[0n]|BOLD:AAA5659
Hyppa contrasta|[19093]|RDLQB083-05|Canada|Quebec|658[0n]|BOLD:AAA5659
Hyppa contrasta|[19094]|LPMN158-08|Canada|Manitoba|658[0n]|BOLD:AAA5659
Hyppa contrasta|[19095]|BBLPC283-09|Canada|Newfoundland and Labrador|658[0n]|BOLD:AAA5659
Hyppa contrasta|[19096]|BBLPC939-09|Canada|Newfoundland and Labrador|658[0n]|BOLD:AAA5659
Hyppa contrasta|[19097]|BBLPE313-09|Canada|Newfoundland and Labrador|658[0n]|BOLD:AAA5659
Hyppa contrasta|[19098]|LBCH2982-10|Canada|British Columbia|658[0n]|BOLD:AAA5659
Hyppa potamus|[19099]|CNCLA569-13|Canada|Yukon Territory|658[0n]|BOLD:AAA5659
Hyppa contrasta|[19100]|CNWLL021-13|Canada|Alberta|588[0n]|BOLD:AAA5659
Hyppa contrasta|[19101]|LOWCB926-05|Canada|British Columbia|639[0n]|BOLD:AAA5659
Hyppa contrasta|[19102]|PHMNB254-04|Canada|New Brunswick|561[0n]|BOLD:AAA5659
Hyppa contrasta|[19103]|BBLPE150-09|Canada|Nova Scotia|658[0n]|BOLD:AAA5659
Hyppa contrasta|[19104]|BBLPE269-09|Canada|Nova Scotia|658[0n]|BOLD:AAA5659
Hyppa contrasta|[19105]|BBLPE351-09|Canada|Newfoundland and Labrador|658[0n]|BOLD:AAA5659
Hyppa contrasta|[19106]|BBLPE364-09|Canada|Newfoundland and Labrador|658[0n]|BOLD:AAA5659
Hyppa contrasta|[19107]|BBLPE366-09|Canada|Newfoundland and Labrador|658[0n]|BOLD:AAA5659
Hyppa contrasta|[19108]|BBLPE412-09|Canada|Newfoundland and Labrador|658[0n]|BOLD:AAA5659
Hyppa contrasta|[19109]|LBCH2246-10|Canada|British Columbia|658[0n]|BOLD:AAA5659
Hyppa contrasta|[19110]|LALPA655-10|Canada|British Columbia|658[0n]|BOLD:AAA5659
Hyppa contrasta|[19111]|BBLPB686-10|Canada|British Columbia|658[0n]|BOLD:AAA5659
Hyppa contrasta|[19112]|LALPA910-11|Canada|British Columbia|658[0n]|BOLD:AAA5659
Hyppa contrasta|[19113]|LALPA1170-11|Canada|British Columbia|658[0n]|BOLD:AAA5659
Hyppa contrasta|[19114]|LALPA1261-11|Canada|British Columbia|658[0n]|BOLD:AAA5659
Hyppa contrasta|[19115]|RWWC956-12|United States|Washington|658[0n]|BOLD:AAA5659
Hyppa potamus|[19116]|CNCLA565-13|Canada|Yukon Territory|658[0n]|BOLD:AAA5659
Hyppa brunneicrista|[19117]|UAMIC1222-13|United States|Alaska|658[0n]|BOLD:AAA5659
Hyppa contrasta|[19118]|LNCC1736-13|United States|North Carolina|658[0n]|BOLD:AAA5659
Hyppa contrasta|[19119]|BBLEC349-09|Canada|Newfoundland and Labrador|658[0n]|BOLD:AAA5659
Hyppa contrasta|[19120]|BBLPE056-09|Canada|Nova Scotia|658[0n]|BOLD:AAA5659
Hyppa contrasta|[19121]|LSEU138-06|United States|North Carolina|658[0n]|BOLD:AAA5659
Hyppa contrasta|[19122]|LSEU511-06|United States|Georgia|658[0n]|BOLD:AAA5659
Hyppa contrasta|[19123]|LPMN564-08|Canada|Manitoba|655[0n]|BOLD:AAA5659
Hyppa contrasta|[19124]|LPMN818-08|Canada|Manitoba|658[0n]|BOLD:AAA5659
Hyppa contrasta|[19125]|LPAB215-08|Canada|Alberta|658[0n]|BOLD:AAA5659
Hyppa contrasta|[19126]|LPVIB053-08|Canada|British Columbia|658[0n]|BOLD:AAA5659
Hyppa contrasta|[19127]|BBLPA444-10|Canada|Alberta|658[0n]|BOLD:AAA5659
Hyppa contrasta|[19128]|BBLPB684-10|Canada|British Columbia|658[0n]|BOLD:AAA5659
Hyppa contrasta|[19129]|LOWCB929-05|Canada|British Columbia|658[0n]|BOLD:AAA5659
Hyppa contrasta|[19130]|LOWCB931-05|Canada|British Columbia|658[0n]|BOLD:AAA5659
Hyppa contrasta|[19131]|LOWCB932-05|Canada|British Columbia|658[0n]|BOLD:AAA5659
Hyppa contrasta|[19132]|LSEU137-06|United States|North Carolina|658[0n]|BOLD:AAA5659
Hyppa contrasta|[19133]|LOWCB925-05|Canada|British Columbia|658[0n]|BOLD:AAA5659
Hyppa contrasta|[19134]|LOWCB928-05|Canada|British Columbia|658[0n]|BOLD:AAA5659
Hyppa contrasta|[19135]|LOWCB921-05|Canada|British Columbia|658[0n]|BOLD:AAA5659
Hyppa contrasta|[19136]|LOWCB924-05|Canada|British Columbia|658[0n]|BOLD:AAA5659
Hyppa contrasta|[19137]|LOWCB920-05|Canada|British Columbia|658[0n]|BOLD:AAA5659
Hyppa contrasta|[19138]|LBCC013-05|Canada|British Columbia|658[0n]|BOLD:AAA5659
Hyppa contrasta|[19139]|LGSMC698-05|United States|Tennessee|658[0n]|BOLD:AAA5659
Hyppa contrasta|[19140]|LGSMC383-05|United States|Tennessee|658[0n]|BOLD:AAA5659
Hyppa contrasta|[19141]|PHMNB757-05|Canada|New Brunswick|658[0n]|BOLD:AAA5659
Hyppa contrasta|[19142]|PHMNB174-04|Canada|New Brunswick|658[0n]|BOLD:AAA5659
Hyppa contrasta|[19143]|LCH242-04|Canada|Manitoba|658[0n]|BOLD:AAA5659
Hyppa contrasta|[19144]|LGSM674-04|United States|North Carolina|658[0n]|BOLD:AAA5659
Hyppa contrasta|[19145]|LBCB405-05|Canada|British Columbia|658[0n]|BOLD:AAA5659
Hyppa contrasta|[19146]|LGSM673-04|United States|Tennessee|658[0n]|BOLD:AAA5659
Hyppa contrasta|[19147]|RDMAB280-05|Canada|Alberta|658[1n]|BOLD:AAA5659
Hyppa contrasta|[19148]|LOWCB923-05|Canada|British Columbia|649[0n]|BOLD:AAA5659
Hyppa contrasta|[19149]|LCHP830-07|Canada|Manitoba|643[0n]|BOLD:AAA5659
Hyppa contrasta|[19150]|PHMNB034-03|Canada|New Brunswick|639[0n]|BOLD:AAA5659
Hyppa contrasta|[19151]|LOTB095-05|United States|Tennessee|616[0n]|BOLD:AAA5659
Hyppa contrasta|[19152]|BBLPE088-09|Canada|Nova Scotia|634[0n]|BOLD:AAA5659
Hyppa contrasta|[19153]|BBLPE279-09|Canada|Nova Scotia|638[0n]|BOLD:AAA5659
Hyppa contrasta|[19154]|LBCH494-10|Canada|British Columbia|658[0n]|BOLD:AAA5659
Hyppa contrasta|[19155]|LNCC1737-13|United States|North Carolina|658[0n]|BOLD:AAA5659
Acroria pulchra|[19156]|BLPBF292-07|Costa Rica|Guanacaste|590[0n]|BOLD:AAC3645
Acroria pulchra|[19157]|BLPEF6798-14|Costa Rica|Guanacaste|658[0n]|BOLD:AAC3645
Acroria pulchra|[19158]|CNCLB2377-14|Mexico|Chiapas|658[0n]|BOLD:AAC3645
Acroria pulchra|[19159]|CNCLB2423-14|United States|Texas|658[0n]|BOLD:AAC3645
Acroria pulchra|[19160]|BLPCD592-08|Costa Rica|Guanacaste|658[0n]|BOLD:AAC3645
Acroria pulchra|[19161]|BLPDV954-11|Costa Rica|Guanacaste|632[0n]|BOLD:AAC3645
Acroria pulchra|[19162]|MHAUG068-07|Costa Rica|Guanacaste|658[0n]|BOLD:AAC3645
Acroria pulchra|[19163]|BLPBB251-07|Costa Rica|Guanacaste|658[0n]|BOLD:AAC3645
Acroria pulchra|[19164]|BLPAG349-07|Costa Rica|Guanacaste|658[0n]|BOLD:AAC3645
Acroria pulchra|[19165]|BLPAG248-07|Costa Rica|Guanacaste|658[0n]|BOLD:AAC3645
Acroria pulchra|[19166]|BLPAG045-07|Costa Rica|Guanacaste|658[0n]|BOLD:AAC3645
Acroria pulchra|[19167]|BLPAF602-07|Costa Rica|Guanacaste|658[0n]|BOLD:AAC3645
Acroria pulchra|[19168]|BLPAF544-07|Costa Rica|Guanacaste|658[0n]|BOLD:AAC3645
Acroria pulchra|[19169]|BLPAF543-07|Costa Rica|Guanacaste|658[0n]|BOLD:AAC3645
Acroria pulchra|[19170]|MHAUG066-07|Costa Rica|Guanacaste|646[0n]|BOLD:AAC3645
Acroria pulchra|[19171]|LOCRB425-08|Costa Rica|Alajuela|609[0n]|BOLD:AAC3645
Acroria pulchra|[19172]|BLPDU814-11|Costa Rica|Guanacaste|658[0n]|BOLD:AAC3645
Acroria pulchra|[19173]|CNCLB2271-14|Guatemala|San Marcos|658[0n]|BOLD:AAC3645
Acroria pulchra|[19174]|CNCLB2272-14|Guatemala|San Marcos|658[0n]|BOLD:AAC3645
Acroria pulchra|[19175]|CNCLB2376-14|Mexico|Chiapas|658[0n]|BOLD:AAC3645
Acroria pulchra|[19176]|BLPAF601-07|Costa Rica|Guanacaste|658[0n]|BOLD:AAC3645
Acroria pulchra|[19177]|LNAUS4482-13|Dominican Republic|La Vega|540[0n]|BOLD:AAC3645
Acroria pulchra|[19178]|DBFCI057-15|Jamaica||658[0n]|BOLD:AAC3645
Acroria pulchra|[19179]|DBFCI385-15|Jamaica||658[0n]|BOLD:AAC3645
Brachylomia discolor|[19180]|RDNMB947-05|United States|Nevada|658[0n]|BOLD:AAI8358
Brachylomia discolor|[19181]|RDNMB948-05|United States|Wyoming|658[0n]|BOLD:AAI8358
Brachylomia elda|[19182]|RDNMB949-05|United States|Oregon|658[0n]|BOLD:AAJ7979
Brachylomia elda|[19183]|JMMMB393-11|United States|California|658[0n]|BOLD:AAJ7979
Brachylomia obscurifascia|[19184]|RDNM170-05|United States|Oregon|658[0n]|BOLD:AAI8357
Brachylomia obscurifascia|[19185]|RDNM171-05|United States|Oregon|658[0n]|BOLD:AAI8357
Brachylomia curvifascia|[19186]|RDNM172-05|United States|Nevada|658[0n]|BOLD:ABZ1552
Brachylomia curvifascia|[19187]|RDNM173-05|United States|Nevada|658[0n]|BOLD:ABZ1552
Brachylomia curvifascia|[19188]|JMMMB391-11|United States|California|658[0n]|BOLD:ABZ1552
Brachylomia rectifascia|[19189]|RDNM160-05|United States|California|658[0n]|BOLD:AAF6199

Brachylomia curvifascia|[19187]|RDNM173-05|United States|Nevada|658[0n]|BOLD:ABZ1552
Brachylomia curvifascia|[19188]|JMMMB391-11|United States|California|658[0n]|BOLD:ABZ1552
Brachylomia rectifascia|[19189]|RDNM160-05|United States|California|658[0n]|BOLD:AAF6199
Brachylomia rectifascia|[19190]|RDNM161-05|United States|California|658[0n]|BOLD:AAF6199
Brachylomia pallida|[19191]|RDNM166-05|United States|Nevada|658[0n]|BOLD:AAF6199
Brachylomia cascadia|[19192]|RDNM162-05|Canada|British Columbia|580[0n]|BOLD:ABX6835
Brachylomia cascadia|[19193]|RDNM163-05|Canada|British Columbia|658[0n]|BOLD:ABX6835
Brachylomia cascadia|[19194]|RDNM165-05|United States|Washington|658[0n]|BOLD:ABX6835
Brachylomia cascadia|[19195]|RDNMG619-08|Canada|British Columbia|658[0n]|BOLD:ABX6835
Brachylomia cascadia|[19196]|RDNMG620-08|Canada|British Columbia|658[0n]|BOLD:ABX6835
Brachylomia cascadia|[19197]|LALPA923-11|Canada|British Columbia|658[0n]|BOLD:ABX6835
Brachylomia algens|[19198]|RDLQF172-06|Canada|Quebec|658[0n]|BOLD:AAD7480
Brachylomia algens|[19199]|BBLPB766-10|Canada|Saskatchewan|658[0n]|BOLD:AAD7480
Brachylomia algens|[19200]|RDNMG931-08|Canada|Ontario|658[0n]|BOLD:AAD7480
Brachylomia algens|[19201]|CNEIB157-12|Canada|Alberta|635[0n]|BOLD:AAD7480
Brachylomia algens|[19202]|RDNMG933-08|Canada|Ontario|658[0n]|BOLD:ABY6308
Brachylomia algens|[19203]|RDNMG932-08|Canada|Alberta|658[0n]|BOLD:ABY6308
Brachylomia algens|[19204]|CNEIA2066-12|Canada|Alberta|622[0n]|BOLD:ABY6308
Brachylomia algens|[19205]|BBLPB334-10|Canada|Saskatchewan|658[0n]|BOLD:ABY6308
Brachylomia algens|[19206]|RDLQF652-06|Canada|Quebec|637[0n]|BOLD:ABY6308
Brachylomia algens|[19207]|CNEIB156-12|Canada|Alberta|635[0n]|BOLD:ABY6308
Brachylomia algens|[19208]|CNEIF2113-12|Canada|Alberta|661[0n]|BOLD:ABY6308
Brachylomia discinigra|[19209]|RDNMB946-05|United States|Wyoming|658[0n]|BOLD:AAD7477
Brachylomia discinigra|[19210]|RDNMB943-05|United States|Wyoming|658[0n]|BOLD:AAD7477
Brachylomia discinigra|[19211]|RDNMB944-05|Canada|Ontario|658[0n]|BOLD:AAD7477
Brachylomia discinigra|[19212]|RDLQF173-06|Canada|Quebec|658[0n]|BOLD:AAD7477
Brachylomia discinigra|[19213]|RDNMB945-05|Canada|Ontario|658[0n]|BOLD:AAD7477
Brachylomia discinigra|[19214]|CNPAA038-13|Canada|Saskatchewan|632[0n]|BOLD:AAD7477
Brachylomia sierra|[19215]|RDNM168-05|United States|California|658[0n]|BOLD:AAD7482
Brachylomia sierra|[19216]|RDNM167-05|United States|California|658[0n]|BOLD:AAD7482
Brachylomia sierra|[19217]|RDNM169-05|United States|California|552[1n]|BOLD:AAD7482
Brachylomia sierra|[19218]|JMMMB321-11|United States|California|658[0n]|BOLD:AAD7482
Brachylomia sierra|[19219]|JMMMB343-11|United States|California|658[0n]|BOLD:AAD7482
Brachylomia sierra|[19220]|JMMMB353-11|United States|California|658[0n]|BOLD:AAD7482
Brachylomia sierra|[19221]|JMMMB360-11|United States|California|658[0n]|BOLD:AAD7482
Brachylomia sierra|[19222]|JMMMB397-11|United States|California|658[0n]|BOLD:AAD7482
Brachylomia sierra|[19223]|JMMMB599-13|United States|California|576[0n]|BOLD:AAD7482
Brachylomia thula|[19224]|RDNM174-05|United States|Washington|658[0n]|BOLD:AAD7482
Brachylomia thula|[19225]|RDNMG618-08|United States|Washington|658[0n]|BOLD:AAD7482
Brachylomia thula|[19226]|RDMAB1020-09|United States|Oregon|658[0n]|BOLD:AAD7482
Brachylomia thula|[19227]|RWWC1191-13|United States|Washington|593[0n]|BOLD:AAD7482
Achatodes zeae|[19228]|LSEU134-06|United States|Georgia|658[0n]|BOLD:AAD5924
Achatodes zeae|[19229]|RDLQB445-05|Canada|Quebec|658[0n]|BOLD:AAD5924
Achatodes zeae|[19230]|BBLPC554-09|Canada|New Brunswick|658[0n]|BOLD:AAD5924
Achatodes zeae|[19231]|LGSM513-04|United States|Tennessee|658[0n]|BOLD:AAD5924
Achatodes zeae|[19232]|LGSM514-04|United States|North Carolina|658[0n]|BOLD:AAD5924
Achatodes zeae|[19233]|RDLQB576-05|Canada|Quebec|598[0n]|BOLD:AAD5924
Achatodes zeae|[19234]|LGSMG991-10|United States|North Carolina|658[0n]|BOLD:AAD5924
Achatodes zeae|[19235]|LILLA359-11|United States|Illinois|658[0n]|BOLD:AAD5924
Peraniana dissociata|[19236]|CNCLB1116-14|United States|Utah|621[0n]|BOLD:ACM6640
Resapamea stipata|[19237]|TMNBB168-06|Canada|New Brunswick|658[0n]|BOLD:AAB8481
Resapamea stipata|[19238]|TMNBB176-06|Canada|New Brunswick|658[0n]|BOLD:AAB8481
Resapamea stipata|[19239]|TMNBB173-06|Canada|New Brunswick|658[0n]|BOLD:AAB8481
Resapamea stipata|[19240]|TMNBB175-06|Canada|New Brunswick|658[0n]|BOLD:AAB8481
Resapamea stipata|[19241]|TMNBB172-06|Canada|New Brunswick|658[0n]|BOLD:AAB8481
Resapamea stipata|[19242]|TMNBB171-06|Canada|New Brunswick|658[0n]|BOLD:AAB8481
Resapamea stipata|[19243]|TMNBB169-06|Canada|New Brunswick|658[0n]|BOLD:AAB8481
Resapamea stipata|[19244]|TMNBB166-06|Canada|New Brunswick|658[0n]|BOLD:AAB8481
Resapamea stipata|[19245]|TMNBB165-06|Canada|New Brunswick|658[0n]|BOLD:AAB8481
Resapamea stipata|[19246]|TTMNB340-06|Canada|New Brunswick|658[0n]|BOLD:AAB8481
Resapamea stipata|[19247]|TTMNB337-06|Canada|New Brunswick|658[1n]|BOLD:AAB8481
Resapamea stipata|[19248]|TTMNB339-06|Canada|New Brunswick|658[0n]|BOLD:AAB8481
Resapamea stipata|[19249]|TTMNB338-06|Canada|New Brunswick|658[0n]|BOLD:AAB8481
Resapamea stipata|[19250]|TMNBB167-06|Canada|New Brunswick|658[0n]|BOLD:AAB8481
Resapamea stipata|[19251]|TMNBB170-06|Canada|New Brunswick|594[1n]|BOLD:AAB8481
Resapamea stipata|[19252]|TMNBB174-06|Canada|New Brunswick|658[0n]|BOLD:AAB8481
Resapamea stipata|[19253]|RDNMD793-07|United States|South Dakota|658[0n]|BOLD:AAB8481
Hydraecia perobliqua|[19254]|RDNMB932-05|Canada|Manitoba|658[0n]|BOLD:AAF0892
Hydraecia perobliqua|[19255]|RDNMG607-08|Canada|Manitoba|658[0n]|BOLD:AAF0892
Hydraecia perobliqua|[19256]|RDNMG608-08|United States|Washington|658[0n]|BOLD:AAF0892
Hydraecia stramentosa|[19257]|XAD299-04|Canada|Ontario|579[0n]|BOLD:AAI8720
Hydraecia stramentosa|[19258]|RDNMC080-05|Canada|Ontario|658[0n]|BOLD:AAI8720
Hydraecia immanis|[19259]|RDNMB934-05|Canada|Ontario|658[0n]|BOLD:AAF0898
Hydraecia immanis|[19260]|RDNMG609-08|United States|Ohio|658[0n]|BOLD:AAF0898
Hydraecia immanis|[19261]|RDNMG610-08|United States|Ohio|658[0n]|BOLD:AAF0898
Hydraecia medialis|[19262]|RDNM153-05|United States|Wyoming|658[0n]|BOLD:ABY5448
Hydraecia medialis|[19263]|RDNM039-05|Canada|British Columbia|596[2n]|BOLD:ABZ6168
Hydraecia medialis|[19264]|RDNM041-05|United States|Washington|658[0n]|BOLD:ABZ6168
Hydraecia medialis|[19265]|RDNM042-05|United States|Washington|566[1n]|BOLD:ABZ6168
Hydraecia medialis|[19266]|RDNM040-05|Canada|British Columbia|614[1n]|BOLD:ABZ6168
Hydraecia medialis|[19267]|RDNM154-05|United States|Wyoming|658[0n]|BOLD:ABZ6168
Hydraecia medialis|[19268]|RDNM155-05|United States|Wyoming|658[0n]|BOLD:ABZ6168
Hydraecia medialis|[19269]|RDNMB562-05|United States|Colorado|658[0n]|BOLD:ABZ6168
Hydraecia obliqua|[19270]|RDNM038-05|United States|Washington|576[0n]|BOLD:ABY5447
Hydraecia obliqua|[19271]|RDNM037-05|United States|Washington|582[0n]|BOLD:ABY5447
Hydraecia obliqua|[19272]|RDNM273-05|Canada|British Columbia|571[0n]|BOLD:ABY5447
Hydraecia obliqua|[19273]|RDNM274-05|Canada|British Columbia|507[0n]|BOLD:ABY5447
Hydraecia obliqua|[19274]|NAMUM211-08|United States|California|658[0n]|BOLD:ABY5447
Hydraecia obliqua|[19275]|NAMUM286-08|United States|California|658[0n]|BOLD:ABY5447
Hydraecia micacea|[19276]|RDNMD056-06|Finland|Kymenlaakso|591[0n]|BOLD:AAB1631
Hydraecia micacea|[19277]|LEFIA610-10|Finland|South Karelia|658[0n]|BOLD:AAB1631
Hydraecia micacea|[19278]|RDNMB933-05|Canada|Ontario|658[0n]|BOLD:AAB1631
Hydraecia micacea|[19279]|LEFIA611-10|Finland|South Karelia|658[0n]|BOLD:AAB1631
Hydraecia micacea|[19280]|CGUKA996-09|United Kingdom|England|588[0n]|BOLD:AAB1631
Hydraecia micacea|[19281]|XAK609-07|Canada|Ontario|566[0n]|BOLD:AAB1631
Hydraecia micacea|[19282]|CGUKC198-09|United Kingdom|England|641[0n]|BOLD:AAB1631
Hydraecia micacea|[19283]|GWOSI569-10|Germany|Bavaria|581[0n]|BOLD:AAB1631
Hydraecia micacea|[19284]|LEFID980-10|Finland||658[0n]|BOLD:AAB1631
Hydraecia micacea|[19285]|LEFIJ1421-12|Finland||658[0n]|BOLD:AAB1631
Hydraecia micacea|[19286]|CGUKD440-09|United Kingdom|England|634[0n]|BOLD:AAB1631
Hydraecia micacea|[19287]|NORIN080-13|Norway|Akershus|627[0n]|BOLD:AAB1631
Hydraecia micacea|[19288]|RDLQB820-05|Canada|Quebec|618[0n]|BOLD:AAB1631
Hydraecia micacea|[19289]|PMG118-03|Canada|Ontario|617[0n]|BOLD:AAB1631

Hydraecia micacea|[19287]|NORIN080-13|Norway|Akershus|627[0n]|BOLD:AAB1631
Hydraecia micacea|[19288]|RDLQB820-05|Canada|Quebec|618[0n]|BOLD:AAB1631
Hydraecia micacea|[19289]|PMG118-03|Canada|Ontario|617[0n]|BOLD:AAB1631
Hydraecia micacea|[19290]|CGUKB912-09|United Kingdom|Wales|658[0n]|BOLD:AAB1631
Hydraecia micacea|[19291]|LENOA357-11|France|Haute Normandie|658[0n]|BOLD:AAB1631
Hydraecia micacea|[19292]|GBLAC293-13|Germany|Bavaria|658[0n]|BOLD:AAB1631
Hydraecia micacea|[19293]|PHLAH714-12|Austria|Vorarlberg|658[0n]|BOLD:AAB1631
Hydraecia micacea|[19294]|FBLMZ494-12|Germany|Bavaria|658[0n]|BOLD:AAB1631
Hydraecia micacea|[19295]|PHLAH373-12|Austria||658[0n]|BOLD:AAB1631
Hydraecia micacea|[19296]|PHLAH372-12|Austria||658[0n]|BOLD:AAB1631
Hydraecia micacea|[19297]|NLLEA810-12|Netherlands|South Holland|658[0n]|BOLD:AAB1631
Hydraecia micacea|[19298]|GWOSN587-11|Germany|Bavaria|658[0n]|BOLD:AAB1631
Hydraecia micacea|[19299]|ODOPE271-11|Germany|Bavaria|658[0n]|BOLD:AAB1631
Hydraecia micacea|[19300]|GWOSA390-10|Germany|Bavaria|658[0n]|BOLD:AAB1631
Hydraecia micacea|[19301]|LEFIF691-10|Finland||658[0n]|BOLD:AAB1631
Hydraecia micacea|[19302]|LEFIC573-10|Finland|Finland Proper|658[0n]|BOLD:AAB1631
Hydraecia micacea|[19303]|LEFID908-10|Finland||658[0n]|BOLD:AAB1631
Hydraecia micacea|[19304]|LEFIB305-10|Finland|Northern Ostrobothnia|658[0n]|BOLD:AAB1631
Hydraecia micacea|[19305]|CGUKD549-09|United Kingdom|England|658[0n]|BOLD:AAB1631
Hydraecia micacea|[19306]|CGUKB637-09|United Kingdom|England|658[0n]|BOLD:AAB1631
Hydraecia micacea|[19307]|BLTIB935-08|Canada|Ontario|658[0n]|BOLD:AAB1631
Hydraecia micacea|[19308]|XAK510-07|Canada|Ontario|658[0n]|BOLD:AAB1631
Hydraecia micacea|[19309]|RDNMD060-06|Finland|Uusimaa|658[0n]|BOLD:AAB1631
Hydraecia micacea|[19310]|RDNMD059-06|Finland|Uusimaa|658[0n]|BOLD:AAB1631
Hydraecia micacea|[19311]|RDNMD058-06|Finland|Uusimaa|658[0n]|BOLD:AAB1631
Hydraecia micacea|[19312]|RDNMD057-06|Finland|Uusimaa|657[0n]|BOLD:AAB1631
Hydraecia micacea|[19313]|RDNMD055-06|Finland|Aland Islands|658[0n]|BOLD:AAB1631
Hydraecia micacea|[19314]|RDNMD054-06|Finland|Uusimaa|658[0n]|BOLD:AAB1631
Hydraecia micacea|[19315]|XAG382-05|Canada|Ontario|658[0n]|BOLD:AAB1631
Hydraecia micacea|[19316]|NORIN013-13|Norway|Nordland|686[0n]|BOLD:AAB1631
Hydraecia micacea|[19317]|XAK348-06|Canada|Ontario|658[0n]|BOLD:AAB1631
Hydraecia micacea|[19318]|CGUKA997-09|United Kingdom|England|615[0n]|BOLD:AAB1631
Hydraecia micacea|[19319]|CGUKB301-09|United Kingdom|England|646[0n]|BOLD:AAB1631
Hydraecia micacea|[19320]|GBLAC294-13|Germany|Bavaria|658[0n]|BOLD:AAB1631
Hydraecia micacea|[19321]|GBLAF418-14|Germany|Brandenburg|658[0n]|BOLD:AAB1631
Hydraecia micacea|[19322]|GBLAD824-14|Germany|Bavaria|658[0n]|BOLD:AAB1631
Oligia divesta|[19323]|GMLC1084-12|United States|California|658[0n]|BOLD:AAC8262
Oligia divesta|[19324]|GMLC1123-12|United States|California|658[0n]|BOLD:AAC8262
Oligia divesta|[19325]|LOWCB938-05|Canada|British Columbia|658[1n]|BOLD:AAC8262
Oligia divesta|[19326]|LOWCB935-05|Canada|British Columbia|578[1n]|BOLD:AAC8262
Oligia divesta|[19327]|LOWCB937-05|Canada|British Columbia|658[0n]|BOLD:AAC8262
Oligia divesta|[19328]|LOWCB940-05|Canada|British Columbia|658[0n]|BOLD:AAC8262
Oligia divesta|[19329]|BBLSY377-09|United States|Arizona|658[0n]|BOLD:AAC8262
Oligia divesta|[19330]|LPVIB512-08|Canada|British Columbia|658[0n]|BOLD:AAC8262
Oligia divesta|[19331]|LPVIA834-08|Canada|British Columbia|658[0n]|BOLD:AAC8262
Oligia divesta|[19332]|LPVIB257-08|Canada|British Columbia|658[0n]|BOLD:AAC8262
Oligia divesta|[19333]|LBCH6280-10|Canada|British Columbia|658[0n]|BOLD:AAC8262
Oligia divesta|[19334]|LPVIB499-08|Canada|British Columbia|658[0n]|BOLD:AAC8262
Oligia divesta|[19335]|LBCH6290-10|Canada|British Columbia|658[0n]|BOLD:AAC8262
Oligia divesta|[19336]|LBCG2877-09|Canada|British Columbia|658[0n]|BOLD:AAC8262
Oligia divesta|[19337]|LBCH6123-10|Canada|British Columbia|658[0n]|BOLD:AAC8262
Oligia divesta|[19338]|LBCH6124-10|Canada|British Columbia|658[0n]|BOLD:AAC8262
Oligia divesta|[19339]|LBCH6279-10|Canada|British Columbia|658[0n]|BOLD:AAC8262
Oligia divesta|[19340]|LBCH6832-10|Canada|British Columbia|658[0n]|BOLD:AAC8262
Oligia divesta|[19341]|GMLC1168-12|United States|California|658[0n]|BOLD:AAC8262
Oligia divesta|[19342]|LBCH7972-10|Canada|British Columbia|658[0n]|BOLD:AAC8262
Oligia divesta|[19343]|GMLC1218-12|United States|California|658[0n]|BOLD:AAC8262
Oligia divesta|[19344]|GMLC1184-12|United States|California|658[0n]|BOLD:AAC8262
Oligia divesta|[19345]|GMLC1173-12|United States|California|658[0n]|BOLD:AAC8262
Oligia divesta|[19346]|GMLC1158-12|United States|California|658[0n]|BOLD:AAC8262
Oligia divesta|[19347]|LALPA1251-11|Canada|British Columbia|658[0n]|BOLD:AAC8262
Oligia divesta|[19348]|IAWLB261-11|United States|Washington|658[0n]|BOLD:AAC8262
Oligia divesta|[19349]|LALPA694-10|Canada|British Columbia|658[0n]|BOLD:AAC8262
Oligia divesta|[19350]|LALPA650-10|Canada|British Columbia|658[0n]|BOLD:AAC8262
Oligia divesta|[19351]|LPVIB259-08|Canada|British Columbia|658[0n]|BOLD:AAC8262
Oligia divesta|[19352]|RDNMD794-07|United States|Washington|658[0n]|BOLD:AAC8262
Oligia divesta|[19353]|LOWCB936-05|Canada|British Columbia|658[0n]|BOLD:AAC8262
Oligia divesta|[19354]|LOWCB939-05|Canada|British Columbia|658[0n]|BOLD:AAC8262
Oligia divesta|[19355]|LBCH6452-10|Canada|British Columbia|658[0n]|BOLD:AAC8262
Oligia divesta|[19356]|LPVIB638-08|Canada|British Columbia|644[0n]|BOLD:AAC8262
Oligia divesta|[19357]|RDNMB359-05|United States|Washington|608[0n]|BOLD:AAC8262
Oligia divesta|[19358]|LALPA1326-12|Canada|British Columbia|621[0n]|BOLD:AAC8262
Cobalos angelicus|[19359]|LOCBF4012-14|United States|California|600[3n]|BOLD:AAG5837
Cobalos angelicus|[19360]|RDNML209-13|United States|California|658[0n]|BOLD:AAG5837
Cobalos angelicus|[19361]|LOCBF2625-13|United States|California|658[0n]|BOLD:AAG5837
Cobalos angelicus|[19362]|LOCBD008-06|United States|California|658[0n]|BOLD:AAG5837
Cobalos angelicus|[19363]|NAMUM270-08|United States|California|658[0n]|BOLD:AAG5837
Cobalos angelicus|[19364]|LOCBC466-06|United States|California|658[0n]|BOLD:AAG5837
Cobalos angelicus|[19365]|LOCBC048-06|United States|California|658[0n]|BOLD:AAG5837
Cobalos angelicus|[19366]|LOCBC017-06|United States|California|658[0n]|BOLD:AAG5837
Cobalos angelicus|[19367]|LOCBC016-06|United States|California|658[0n]|BOLD:AAG5837
Cobalos angelicus|[19368]|LOCBB156-06|United States|California|658[0n]|BOLD:AAG5837
Cobalos angelicus|[19369]|LOCBB155-06|United States|California|658[0n]|BOLD:AAG5837
Cobalos angelicus|[19370]|LOCBB154-06|United States|California|658[0n]|BOLD:AAG5837
Cobalos angelicus|[19371]|LOCBB153-06|United States|California|658[0n]|BOLD:AAG5837
Cobalos angelicus|[19372]|LOCBB152-06|United States|California|658[0n]|BOLD:AAG5837
Cobalos angelicus|[19373]|CGLCA112-10|United States|California|633[0n]|BOLD:AAG5837
Cobalos angelicus|[19374]|LOCBC015-06|United States|California|614[0n]|BOLD:AAG5837
Cobalos angelicus|[19375]|LOCBF3721-14|United States|California|579[0n]|BOLD:AAG5837
Cobalos angelicus|[19376]|LOCBF3296-14|United States|California|579[0n]|BOLD:AAG5837
Cobalos angelicus|[19377]|LOCBF4013-14|United States|California|588[0n]|BOLD:AAG5837
Cobalos angelicus|[19378]|LOCBF3719-14|United States|California|591[0n]|BOLD:AAG5837
Cobalos angelicus|[19379]|LOCBF3298-14|United States|California|573[0n]|BOLD:AAG5837
Cobalos angelicus|[19380]|LOCBF3696-14|United States|California|588[0n]|BOLD:AAG5837
Cobalos angelicus|[19381]|LOCBF3700-14|United States|California|576[0n]|BOLD:AAG5837
Cobalos angelicus|[19382]|LOCBF3695-14|United States|California|564[0n]|BOLD:AAG5837
Cobalos angelicus|[19383]|LOCBF3295-14|United States|California|564[0n]|BOLD:AAG5837
Cobalos angelicus|[19384]|LOCBF3297-14|United States|California|558[0n]|BOLD:AAG5837
Cobalos angelicus|[19385]|LOCBF3294-14|United States|California|561[0n]|BOLD:AAG5837
Cobalos angelicus|[19386]|LOCBF3702-14|United States|California|555[0n]|BOLD:AAG5837
Cobalos angelicus|[19387]|LOCBF3716-14|United States|California|552[0n]|BOLD:AAG5837
Cobalos angelicus|[19388]|LOCBF3789-14|United States|California|588[0n]|BOLD:AAG5837
Cobalos angelicus|[19389]|LOCBF4010-14|United States|California|600[0n]|BOLD:AAG5837

Cobalos angelicus|[19387]|LOCBF3716-14|United States|California|552[0n]|BOLD:AAG5837
Cobalos angelicus|[19388]|LOCBF3789-14|United States|California|588[0n]|BOLD:AAG5837
Cobalos angelicus|[19389]|LOCBF4010-14|United States|California|600[0n]|BOLD:AAG5837
Cobalos angelicus|[19390]|LOCBF4011-14|United States|California|600[0n]|BOLD:AAG5837
Cobalos angelicus|[19391]|LOCBF4883-15|United States|California|591[0n]|BOLD:AAG5837
Cobalos angelicus|[19392]|LOCBF4931-15|United States|California|591[0n]|BOLD:AAG5837
Photedes defecta|[19393]|XAJ990-06|Canada|Ontario|658[0n]|BOLD:AAC8594
Photedes defecta|[19394]|XAJ880-06|Canada|Ontario|658[0n]|BOLD:AAC8594
Photedes defecta|[19395]|RDLQ471-07|Canada|Quebec|547[2n]|BOLD:AAC8594
Photedes defecta|[19396]|BLTIB946-08|Canada|Ontario|658[0n]|BOLD:AAC8594
Photedes defecta|[19397]|BLTIB998-08|Canada|Ontario|658[0n]|BOLD:AAC8594
Photedes defecta|[19398]|BLTIB794-08|Canada|Ontario|658[0n]|BOLD:AAC8594
Photedes defecta|[19399]|BLTIB963-08|Canada|Ontario|658[0n]|BOLD:AAC8594
Photedes defecta|[19400]|BLTIB1006-08|Canada|Ontario|658[0n]|BOLD:AAC8594
Photedes defecta|[19401]|BLTIB1030-08|Canada|Ontario|658[0n]|BOLD:AAC8594
Photedes defecta|[19402]|LNAUS4461-13|United States|Alaska|658[1n]|BOLD:AAC8594
Photedes enervata|[19403]|LNAUT833-14|Canada|Nova Scotia|658[0n]|BOLD:AAR9607
Photedes enervata|[19404]|LNAUT834-14|Canada|Nova Scotia|658[0n]|BOLD:AAR9607
Photedes enervata|[19405]|LNAUT835-14|Canada|Nova Scotia|658[0n]|BOLD:AAR9607
Photedes enervata|[19406]|LNAUT836-14|Canada|Nova Scotia|658[0n]|BOLD:AAR9607
Photedes sp.|[19407]|CNCLB1604-14|United States|Indiana|658[0n]|BOLD:AAR9607
Photedes enervata|[19408]|RDNMF470-08|Canada|Nova Scotia|658[0n]|BOLD:AAR9607
Photedes enervata|[19409]|CNCLB1764-14|United States|Ohio|658[0n]|BOLD:AAR9607
Parapamea buffaloensis|[19410]|LNC565-06|United States|North Carolina|658[0n]|BOLD:AAF7975
Parapamea buffaloensis|[19411]|RDNMD781-07|United States|Kentucky|658[1n]|BOLD:AAF7975
Parapamea buffaloensis|[19412]|LNC564-06|United States|North Carolina|658[0n]|BOLD:AAF7975
Parapamea buffaloensis|[19413]|RDNMJ031-10|United States|Florida|658[0n]|BOLD:AAF7975
Selicanis cinereola|[19414]|RDNMD788-07|United States|Wyoming|658[0n]|BOLD:AAH5681
Selicanis cinereola|[19415]|CMAZA262-09|United States|Arizona|658[0n]|BOLD:AAH5681
Apameini n. gen.|[19416]|QUNOD122-10|United States|Texas|658[0n]|BOLD:AAH3363
Papaipema appassionata|[19417]|RDLQ296-05|Canada|Quebec|656[0n]|BOLD:AAF1834
Papaipema appassionata|[19418]|RDNMG775-08|Canada|New Brunswick|658[0n]|BOLD:AAF1834
Papaipema appassionata|[19419]|RDNMG776-08|Canada|New Brunswick|658[0n]|BOLD:AAF1834
Papaipema appassionata|[19420]|CNCLB3430-15|United States|Louisiana|658[0n]|BOLD:AAF1834
Papaipema appassionata|[19421]|CNCLB3431-15|United States|Louisiana|658[5n]|BOLD:AAF1834
Apamea vultuosa|[19422]|RWWA144-09|United States|Washington|658[0n]|BOLD:AAB7574
Apamea vultuosa|[19423]|RWWA302-09|United States|Washington|658[0n]|BOLD:AAB7574
Apamea vultuosa|[19424]|RWWA332-09|United States|Washington|658[0n]|BOLD:AAB7574
Apamea vultuosa|[19425]|RWWA334-09|United States|Washington|658[0n]|BOLD:AAB7574
Apamea vultuosa|[19426]|RWWB666-10|United States|Washington|658[0n]|BOLD:AAB7574
Apamea vultuosa|[19427]|BBLPB688-10|Canada|British Columbia|658[0n]|BOLD:AAB7574
Apamea vultuosa|[19428]|RWWC203-11|United States|Washington|658[0n]|BOLD:AAB7574
Apamea vultuosa|[19429]|CNWLL016-13|Canada|Alberta|579[0n]|BOLD:AAB7574
Apamea vultuosa|[19430]|PHMTV412-10|Canada|Ontario|658[0n]|BOLD:AAB7574
Apamea vultuosa|[19431]|RWWC323-11|United States|Washington|658[0n]|BOLD:AAB7574
Apamea vultuosa|[19432]|XAD715-05|Canada|Ontario|658[0n]|BOLD:AAB7574
Apamea vultuosa|[19433]|RDNMC716-06|United States|North Carolina|658[0n]|BOLD:AAB7574
Apamea vultuosa|[19434]|PHJUN4003-11|Canada|Ontario|658[0n]|BOLD:AAB7574
Apamea vultuosa|[19435]|RWWB933-10|United States|Washington|658[0n]|BOLD:AAB7574
Apamea vultuosa|[19436]|RDNMD122-06|United States|Colorado|617[0n]|BOLD:AAB7574
Apamea vultuosa|[19437]|BLTIB275-08|Canada|Ontario|658[0n]|BOLD:AAB7574
Apamea vultuosa|[19438]|RWWB974-10|United States|Washington|658[0n]|BOLD:AAB7574
Apamea vultuosa|[19439]|PHMTV410-10|Canada|Ontario|658[0n]|BOLD:AAB7574
Apamea vultuosa|[19440]|CNWLL019-13|Canada|Alberta|535[3n]|BOLD:AAB7574
Apamea vultuosa|[19441]|CNWBG3068-13|Canada|Alberta|595[2n]|BOLD:AAB7574
Apamea vultuosa|[19442]|RWWA537-09|United States|Washington|658[0n]|BOLD:AAB7574
Apamea vultuosa|[19443]|RDLQB082-05|Canada|Quebec|658[0n]|BOLD:AAB7574
Apamea vultuosa|[19444]|RDNM145-05|Canada|Ontario|658[0n]|BOLD:AAB7574
Apamea vultuosa|[19445]|LOWCD126-06|Canada|British Columbia|600[0n]|BOLD:AAB7574
Apamea vultuosa|[19446]|CNRMD2072-12|Canada|Manitoba|637[0n]|BOLD:AAB7574
Apamea vultuosa|[19447]|CNWLE2400-12|Canada|Alberta|632[0n]|BOLD:AAB7574
Apamea vultuosa|[19448]|SSEIB12896-13|Canada|Alberta|588[0n]|BOLD:AAB7574
Apamea vultuosa|[19449]|SSEIC7313-13|Canada|Alberta|603[0n]|BOLD:AAB7574
Apamea unita|[19450]|RDNMF078-08|United States|Colorado|658[0n]|BOLD:AAJ1441
Apamea unita|[19451]|RDNMG971-08|United States|Colorado|592[0n]|BOLD:AAJ1441
Apamea impulsa|[19452]|LPABC415-09|Canada|Alberta|658[0n]|BOLD:AAD5601
Apamea impulsa|[19453]|RDLQB077-05|Canada|Quebec|658[0n]|BOLD:AAD5601
Apamea impulsa|[19454]|PHMNB190-04|Canada|New Brunswick|616[0n]|BOLD:AAD5601
Apamea impulsa|[19455]|RDLQB078-05|Canada|Quebec|658[0n]|BOLD:AAD5601
Apamea impulsa|[19456]|BBLPC672-09|Canada|Newfoundland and Labrador|658[0n]|BOLD:AAD5601
Apamea impulsa|[19457]|BBLPC673-09|Canada|Newfoundland and Labrador|658[0n]|BOLD:AAD5601
Apamea impulsa|[19458]|LBCH6737-10|Canada|British Columbia|658[0n]|BOLD:AAD5601
Apamea impulsa|[19459]|RWWC1202-13|United States|Washington|661[0n]|BOLD:AAD5601
Apamea remissa|[19460]|LCHQ187-07|Canada|Manitoba|658[0n]|BOLD:AAB1790
Apamea remissa|[19461]|UAMIC1172-13|United States|Alaska|658[0n]|BOLD:AAB1790
Apamea indocilis|[19462]|RWWB920-10|United States|Washington|658[0n]|BOLD:ACE8841
Apamea indocilis|[19463]|RWWB912-10|United States|Washington|658[0n]|BOLD:ACE8841
Apamea indocilis|[19464]|RWWB855-10|United States|Washington|658[0n]|BOLD:ACE8841
Apamea indocilis|[19465]|RWWB745-10|United States|Washington|658[0n]|BOLD:ACE8841
Apamea indocilis|[19466]|RWWA652-09|United States|Washington|658[0n]|BOLD:ACE8841
Apamea indocilis|[19467]|RWWA451-09|United States|Washington|658[0n]|BOLD:ACE8841
Apamea indocilis|[19468]|RDNMC247-05|United States|Washington|658[0n]|BOLD:ACE8841
Apamea indocilis|[19469]|RDMAB1046-09|Canada|British Columbia|619[0n]|BOLD:ACE8841
Apamea indocilis|[19470]|RWWB944-10|United States|Washington|640[0n]|BOLD:ACE8841
Apamea indocilis|[19471]|RWWA392-09|United States|Washington|632[0n]|BOLD:ACE8841
Apamea indocilis|[19472]|RDMAB1047-09|Canada|Alberta|632[0n]|BOLD:ACE8841
Apamea indocilis|[19473]|RWWB964-10|United States|Washington|624[0n]|BOLD:ACE8841
Apamea indocilis|[19474]|LALPA235-10|Canada|British Columbia|658[0n]|BOLD:ACE8841
Apamea indocilis|[19475]|LALPA291-10|Canada|British Columbia|658[0n]|BOLD:ACE8841
Apamea indocilis|[19476]|RWWC406-11|United States|Washington|658[0n]|BOLD:ACE8841
Apamea indocilis|[19477]|RWWC440-11|United States|Washington|658[0n]|BOLD:ACE8841
Apamea indocilis|[19478]|RWWC569-11|United States|Washington|658[0n]|BOLD:ACE8841
Apamea indocilis|[19479]|LCHQ652-08|Canada|Manitoba|658[0n]|BOLD:ACE8841
Apamea indocilis|[19480]|RDNMC245-05|Canada|Ontario|658[0n]|BOLD:ACE8841
Apamea indocilis|[19481]|SSEIB12910-13|Canada|Alberta|564[0n]|BOLD:ACE8841
Apamea indocilis|[19482]|RDNMD265-06|Canada|New Brunswick|658[0n]|BOLD:ACE8841
Apamea indocilis|[19483]|LBCA495-05|Canada|British Columbia|658[0n]|BOLD:ACE8841
Apamea indocilis|[19484]|RDNMD266-06|Canada|British Columbia|654[0n]|BOLD:ACE8841
Apamea indocilis|[19485]|LBCH234-10|Canada|British Columbia|658[0n]|BOLD:ACE8841
Apamea indocilis|[19486]|RDNMC508-06|United States|Colorado|632[0n]|BOLD:ACE8841
Apamea indocilis|[19487]|RDNMD140-06|United States|Colorado|604[0n]|BOLD:ACE8841
Apamea indocilis|[19488]|LNCC491-11|United States|North Carolina|658[0n]|BOLD:ACE8841
Apamea indocilis|[19489]|LNCC490-11|United States|North Carolina|658[0n]|BOLD:ACE8841

Apamea indocilis|[19487]|RDNMD140-06|United States|Colorado|604[0n]|BOLD:ACE8841
Apamea indocilis|[19488]|LNCC491-11|United States|North Carolina|658[0n]|BOLD:ACE8841
Apamea indocilis|[19489]|LNCC490-11|United States|North Carolina|658[0n]|BOLD:ACE8841
Apamea indocilis|[19490]|RDNM947-05|Canada|British Columbia|543[0n]|BOLD:ACE8841
Apamea indocilis|[19491]|RDNMC507-06|Canada|Ontario|532[0n]|BOLD:ACE8841
Apamea indocilis|[19492]|RDNMC246-05|Canada|British Columbia|603[0n]|BOLD:ACE8841
Apamea indocilis|[19493]|TTMNB329-06|Canada|New Brunswick|622[0n]|BOLD:ACE8841
Apamea indocilis|[19494]|CNSLN113-13|Canada|Ontario|552[0n]|BOLD:ACE8841
Apamea indocilis|[19495]|LNCC1738-13|United States|North Carolina|658[0n]|BOLD:ACE8841
Apamea indocilis|[19496]|CNCLB2640-14|United States|North Carolina|658[0n]|BOLD:ACE8841
Apamea sp.|[19497]|NOCNA093-14|United States|Minnesota|658[0n]|BOLD:ACN4957
Apamea unanimis|[19498]|LEFIB638-10|Finland|South Karelia|658[0n]|BOLD:AAA8789
Apamea unanimis|[19499]|GBLAC1135-13|Germany|Bavaria|658[0n]|BOLD:AAA8789
Apamea unanimis|[19500]|XAC289-04|Canada|Ontario|658[0n]|BOLD:AAA8789
Apamea unanimis|[19501]|LPSOB722-08|Canada|Ontario|513[1n]|BOLD:AAA8789
Apamea unanimis|[19502]|LPSOC123-08|Canada|Ontario|658[0n]|BOLD:AAA8789
Apamea unanimis|[19503]|LPSOC366-08|Canada|Ontario|658[0n]|BOLD:AAA8789
Apamea unanimis|[19504]|RDLQF454-06|Canada|Quebec|658[0n]|BOLD:AAA8789
Apamea unanimis|[19505]|LPSOC099-08|Canada|Ontario|658[0n]|BOLD:AAA8789
Apamea unanimis|[19506]|XAJ626-06|Canada|Ontario|658[0n]|BOLD:AAA8789
Apamea unanimis|[19507]|RDLQF058-06|Canada|Quebec|658[0n]|BOLD:AAA8789
Apamea unanimis|[19508]|RDLQF057-06|Canada|Quebec|658[0n]|BOLD:AAA8789
Apamea unanimis|[19509]|RDLQF056-06|Canada|Quebec|658[0n]|BOLD:AAA8789
Apamea unanimis|[19510]|PHMNB531-04|Canada|New Brunswick|658[0n]|BOLD:AAA8789
Apamea unanimis|[19511]|PHMNB364-04|Canada|New Brunswick|658[0n]|BOLD:AAA8789
Apamea unanimis|[19512]|XAE290-04|Canada|Ontario|658[0n]|BOLD:AAA8789
Apamea unanimis|[19513]|XAC186-04|Canada|Ontario|658[0n]|BOLD:AAA8789
Apamea unanimis|[19514]|XAB590-04|Canada|Ontario|658[0n]|BOLD:AAA8789
Apamea unanimis|[19515]|XAB564-04|Canada|Ontario|658[0n]|BOLD:AAA8789
Apamea unanimis|[19516]|XAB507-04|Canada|Ontario|658[0n]|BOLD:AAA8789
Apamea unanimis|[19517]|PHMO166-03|Canada|Ontario|639[0n]|BOLD:AAA8789
Apamea unanimis|[19518]|RDLQB794-05|Canada|Quebec|627[0n]|BOLD:AAA8789
Apamea unanimis|[19519]|LPSOB665-08|Canada|Ontario|644[0n]|BOLD:AAA8789
Apamea unanimis|[19520]|LPSOC392-08|Canada|Ontario|658[0n]|BOLD:AAA8789
Apamea unanimis|[19521]|LPSOB718-08|Canada|Ontario|658[0n]|BOLD:AAA8789
Apamea unanimis|[19522]|LPSOB806-08|Canada|Ontario|658[0n]|BOLD:AAA8789
Apamea unanimis|[19523]|LPSOB820-08|Canada|Ontario|658[0n]|BOLD:AAA8789
Apamea unanimis|[19524]|RDNMG463-08|Canada|New Brunswick|658[0n]|BOLD:AAA8789
Apamea unanimis|[19525]|RDNMG464-08|Canada|New Brunswick|658[0n]|BOLD:AAA8789
Apamea unanimis|[19526]|RDNMG465-08|Canada|Ontario|658[0n]|BOLD:AAA8789
Apamea unanimis|[19527]|BLTIB017-08|Canada|Ontario|658[0n]|BOLD:AAA8789
Apamea unanimis|[19528]|BLTIB188-08|Canada|Ontario|658[0n]|BOLD:AAA8789
Apamea unanimis|[19529]|LPSOD283-09|Canada|Ontario|658[0n]|BOLD:AAA8789
Apamea unanimis|[19530]|BBLPC284-09|Canada|Newfoundland and Labrador|658[0n]|BOLD:AAA8789
Apamea unanimis|[19531]|BBLPC330-09|Canada|Newfoundland and Labrador|658[0n]|BOLD:AAA8789
Apamea unanimis|[19532]|LALPA1171-11|Canada|British Columbia|658[0n]|BOLD:AAA8789
Apamea unanimis|[19533]|LALPA1173-11|Canada|British Columbia|658[0n]|BOLD:AAA8789
Apamea unanimis|[19534]|LENOA538-11|France|Haute Normandie|658[0n]|BOLD:AAA8789
Apamea unanimis|[19535]|LEFIK456-10|Finland||658[0n]|BOLD:AAA8789
Apamea unanimis|[19536]|CGUKB741-09|United Kingdom|England|658[0n]|BOLD:AAA8789
Apamea unanimis|[19537]|CGUKB045-09|United Kingdom|England|658[0n]|BOLD:AAA8789
Apamea unanimis|[19538]|CGUKA069-09|United Kingdom|England|658[0n]|BOLD:AAA8789
Apamea unanimis|[19539]|BLTIB089-08|Canada|Ontario|658[0n]|BOLD:AAA8789
Apamea unanimis|[19540]|LEFIA555-10|Finland|South Karelia|643[0n]|BOLD:AAA8789
Apamea unanimis|[19541]|CGUKC297-09|United Kingdom|England|602[0n]|BOLD:AAA8789
Apamea unanimis|[19542]|PHLAF447-11|Austria|Vorarlberg|658[0n]|BOLD:AAA8789
Apamea unanimis|[19543]|FBLMZ122-12|Germany|Bavaria|658[0n]|BOLD:AAA8789
Apamea unanimis|[19544]|LON1056-12|Norway|Akershus|658[0n]|BOLD:AAA8789
Apamea unanimis|[19545]|GWORZ568-10|Germany|Bavaria|658[0n]|BOLD:AAA8789
Apamea unanimis|[19546]|LON1035-12|Norway|Aust-Agder|658[0n]|BOLD:AAA8789
Apamea unanimis|[19547]|GBLAC820-13|Germany|Bavaria|658[0n]|BOLD:AAA8789
Apamea unanimis|[19548]|GBLAF568-14|Germany|Brandenburg|658[0n]|BOLD:AAA8789
Apamea digitula|[19549]|RDNMB015-05|United States|California|658[0n]|BOLD:AAA4201
Apamea digitula|[19550]|RDNMC701-06|United States|Oregon|562[0n]|BOLD:AAA4201
Apamea digitula|[19551]|RDNMC009-05|United States|Oregon|658[0n]|BOLD:AAA4201
Apamea digitula|[19552]|RDNMC005-05|United States|California|658[0n]|BOLD:AAA4201
Apamea digitula|[19553]|RDNMB019-05|United States|Oregon|658[0n]|BOLD:AAA4201
Apamea digitula|[19554]|RDNMC682-06|United States|California|588[0n]|BOLD:AAA4201
Apamea digitula|[19555]|GMLC664-11|United States|California|658[0n]|BOLD:AAA4201
Apamea digitula|[19556]|GMLC648-11|United States|California|658[0n]|BOLD:AAA4201
Apamea digitula|[19557]|GMLC592-11|United States|California|658[0n]|BOLD:AAA4201
Apamea digitula|[19558]|GMLC584-11|United States|California|658[0n]|BOLD:AAA4201
Apamea digitula|[19559]|GMLC539-11|United States|California|658[0n]|BOLD:AAA4201
Apamea digitula|[19560]|GMLC674-11|United States|California|658[0n]|BOLD:AAA4201
Apamea digitula|[19561]|GMLC1409-12|United States|California|633[0n]|BOLD:AAA4201
Apamea digitula|[19562]|GMLC1424-12|United States|California|601[0n]|BOLD:AAA4201
Apamea sordens|[19563]|RDLQF005-06|Canada|Quebec|658[1n]|BOLD:AAA4201
Apamea sordens|[19564]|RDLQF020-06|Canada|Quebec|658[3n]|BOLD:AAA4201
Apamea sordens|[19565]|RDLQF018-06|Canada|Quebec|658[0n]|BOLD:AAA4201
Apamea sordens|[19566]|RDLQF019-06|Canada|Quebec|658[0n]|BOLD:AAA4201
Apamea sordens|[19567]|RDLQB916-05|Canada|Quebec|658[0n]|BOLD:AAA4201
Apamea sordens|[19568]|RDLQF003-06|Canada|Quebec|658[0n]|BOLD:AAA4201
Apamea sordens|[19569]|PHMNB126-04|Canada|New Brunswick|658[0n]|BOLD:AAA4201
Apamea sordens|[19570]|PHMNB327-04|Canada|New Brunswick|658[0n]|BOLD:AAA4201
Apamea sordens|[19571]|RDLQF023-06|Canada|Quebec|658[0n]|BOLD:AAA4201
Apamea sordens finitima|[19572]|LOCT219-05|United States|Connecticut|658[0n]|BOLD:AAA4201
Apamea sordens finitima|[19573]|LOCT220-05|United States|Connecticut|658[0n]|BOLD:AAA4201
Apamea sordens|[19574]|LPSOC031-08|Canada|Ontario|658[0n]|BOLD:AAA4201
Apamea sordens|[19575]|LPSOC091-08|Canada|Ontario|658[0n]|BOLD:AAA4201
Apamea sordens|[19576]|RDLQF021-06|Canada|Quebec|658[0n]|BOLD:AAA4201
Apamea sordens|[19577]|RDNM966-05|Canada|Ontario|658[0n]|BOLD:AAA4201
Apamea sordens|[19578]|RDLQF017-06|Canada|Quebec|658[0n]|BOLD:AAA4201
Apamea sordens|[19579]|RDLQF012-06|Canada|Quebec|658[0n]|BOLD:AAA4201
Apamea sordens|[19580]|XAI061-05|Canada|Ontario|658[0n]|BOLD:AAA4201
Apamea sordens|[19581]|RDNMB020-05|Canada|New Brunswick|658[0n]|BOLD:AAA4201
Apamea sordens|[19582]|XAF468-05|Canada|Ontario|658[0n]|BOLD:AAA4201
Apamea sordens|[19583]|RDLQF014-06|Canada|Quebec|656[0n]|BOLD:AAA4201
Apamea sordens|[19584]|PHMO099-03|Canada|Ontario|639[0n]|BOLD:AAA4201
Apamea sordens|[19585]|RDLQB806-05|Canada|Quebec|617[0n]|BOLD:AAA4201
Apamea sordens|[19586]|RDLQF004-06|Canada|Quebec|631[0n]|BOLD:AAA4201
Apamea sordens|[19587]|LPSOC316-08|Canada|Ontario|658[0n]|BOLD:AAA4201
Apamea sordens|[19588]|LPSOC362-08|Canada|Ontario|653[0n]|BOLD:AAA4201
Apamea sordens|[19589]|LPSOB699-08|Canada|Ontario|658[0n]|BOLD:AAA4201

Apamea sordens|[19587]|LPSOC316-08|Canada|Ontario|658[0n]|BOLD:AAA4201
Apamea sordens|[19588]|LPSOC362-08|Canada|Ontario|653[0n]|BOLD:AAA4201
Apamea sordens|[19589]|LPSOB699-08|Canada|Ontario|658[0n]|BOLD:AAA4201
Apamea sordens|[19590]|BBLPE346-09|Canada|Newfoundland and Labrador|658[0n]|BOLD:AAA4201
Apamea sordens|[19591]|HPPPC1203-13|Canada|Nova Scotia|580[0n]|BOLD:AAA4201
Apamea sordens|[19592]|RDLQF015-06|Canada|Quebec|632[1n]|BOLD:AAA4201
Apamea sordens|[19593]|XAB506-04|Canada|Ontario|622[0n]|BOLD:AAA4201
Apamea sordens|[19594]|LPSOC327-08|Canada|Ontario|658[0n]|BOLD:AAA4201
Apamea sordens|[19595]|HPPPC1205-13|Canada|Nova Scotia|587[1n]|BOLD:AAA4201
Apamea sordens|[19596]|XAI062-05|Canada|Ontario|508[1n]|BOLD:AAA4201
Apamea sordens|[19597]|LPSOB973-08|Canada|Ontario|658[0n]|BOLD:AAA4201
Apamea sordens|[19598]|RDNMC697-06|United States|Washington|523[1n]|BOLD:AAA4201
Apamea sordens|[19599]|SSWLD1186-13|Canada|Alberta|530[0n]|BOLD:AAA4201
Apamea sordens|[19600]|RDNMB017-05|Canada|British Columbia|524[0n]|BOLD:AAA4201
Apamea sordens|[19601]|RDNM964-05|Canada|British Columbia|557[0n]|BOLD:AAA4201
Apamea sordens|[19602]|RDNMC364-05|United States|Colorado|545[0n]|BOLD:AAA4201
Apamea sordens|[19603]|RWWA187-09|United States|Washington|658[0n]|BOLD:AAA4201
Apamea sordens|[19604]|RWWA502-09|United States|Washington|658[0n]|BOLD:AAA4201
Apamea sordens|[19605]|RWWA466-09|United States|Washington|658[0n]|BOLD:AAA4201
Apamea sordens|[19606]|RWWB704-10|United States|Washington|658[0n]|BOLD:AAA4201
Apamea sordens|[19607]|RWWB729-10|United States|Washington|658[0n]|BOLD:AAA4201
Apamea sordens|[19608]|RWWB750-10|United States|Washington|658[0n]|BOLD:AAA4201
Apamea sordens|[19609]|LALPA217-10|Canada|British Columbia|658[0n]|BOLD:AAA4201
Apamea sordens|[19610]|LALPA301-10|Canada|British Columbia|658[0n]|BOLD:AAA4201
Apamea sordens|[19611]|BBLPB690-10|Canada|Alberta|658[0n]|BOLD:AAA4201
Apamea sordens|[19612]|RWWC937-12|United States|Washington|658[0n]|BOLD:AAA4201
Apamea sordens|[19613]|RDNMD139-06|United States|Colorado|658[0n]|BOLD:AAA4201
Apamea sordens|[19614]|LBCG104-08|Canada|British Columbia|658[0n]|BOLD:AAA4201
Apamea sordens|[19615]|RDLQF016-06|Canada|Quebec|658[0n]|BOLD:AAA4201
Apamea sordens|[19616]|RDNMC700-06|United States|Washington|658[0n]|BOLD:AAA4201
Apamea sordens|[19617]|RDMAB437-05|Canada|Yukon Territory|658[0n]|BOLD:AAA4201
Apamea sordens|[19618]|RDNMC010-05|Canada|Alberta|658[0n]|BOLD:AAA4201
Apamea sordens|[19619]|RDNMC008-05|Canada|Alberta|658[0n]|BOLD:AAA4201
Apamea sordens|[19620]|RDNMC007-05|Canada|Alberta|658[0n]|BOLD:AAA4201
Apamea sordens|[19621]|RDNMC006-05|United States|Oregon|658[0n]|BOLD:AAA4201
Apamea sordens|[19622]|RDNMC004-05|United States|Wyoming|658[0n]|BOLD:AAA4201
Apamea sordens|[19623]|SSWLD6405-13|Canada|Alberta|544[0n]|BOLD:AAA4201
Apamea sordens|[19624]|RDNMC003-05|United States|Wyoming|658[0n]|BOLD:AAA4201
Apamea sordens|[19625]|LBCA503-05|Canada|British Columbia|658[0n]|BOLD:AAA4201
Apamea sordens|[19626]|RDNM965-05|Canada|British Columbia|658[0n]|BOLD:AAA4201
Apamea sordens|[19627]|LPVIB914-08|Canada|British Columbia|642[0n]|BOLD:AAA4201
Apamea sordens|[19628]|SSWLD6406-13|Canada|Alberta|578[0n]|BOLD:AAA4201
Apamea sordens|[19629]|RDNMC699-06|United States|Washington|582[1n]|BOLD:AAA4201
Apamea sordens|[19630]|SSBAD6148-13|Canada|Alberta|629[0n]|BOLD:AAA4201
Apamea sordens|[19631]|RDMAB018-05|Canada|Alberta|576[0n]|BOLD:AAA4201
Apamea sordens|[19632]|RDNMD138-06|United States|Colorado|605[0n]|BOLD:AAA4201
Apamea sordens|[19633]|SSWLD7232-13|Canada|Alberta|569[1n]|BOLD:AAA4201
Apamea sordens|[19634]|CNWLL020-13|Canada|Alberta|558[0n]|BOLD:AAA4201
Apamea sordens|[19635]|HPPPC1204-13|Canada|Nova Scotia|540[0n]|BOLD:AAA4201
Apamea sordens|[19636]|RDLQF011-06|Canada|Quebec|579[3n]|BOLD:AAA4201
Apamea sordens|[19637]|HPPPD1639-13|Canada|Nova Scotia|551[0n]|BOLD:AAA4201
Apamea sordens|[19638]|BBLPB693-10|Canada|Ontario|658[0n]|BOLD:AAA4201
Apamea sordens|[19639]|BLTIB282-08|Canada|Ontario|657[0n]|BOLD:AAA4201
Apamea sordens|[19640]|LPSOC330-08|Canada|Ontario|658[0n]|BOLD:AAA4201
Apamea sordens|[19641]|LPSOC127-08|Canada|Ontario|658[0n]|BOLD:AAA4201
Apamea sordens|[19642]|XAK163-06|Canada|Ontario|658[0n]|BOLD:AAA4201
Apamea sordens|[19643]|XAJ673-06|Canada|Ontario|658[0n]|BOLD:AAA4201
Apamea sordens|[19644]|RDLQF022-06|Canada|Quebec|658[0n]|BOLD:AAA4201
Apamea sordens|[19645]|RDLQF006-06|Canada|Quebec|658[0n]|BOLD:AAA4201
Apamea sordens|[19646]|RDLQF002-06|Canada|Quebec|658[0n]|BOLD:AAA4201
Apamea sordens|[19647]|RDLQF001-06|Canada|Quebec|658[0n]|BOLD:AAA4201
Apamea sordens|[19648]|RDLQB917-05|Canada|Quebec|658[0n]|BOLD:AAA4201
Apamea sordens|[19649]|XAI065-05|Canada|Ontario|658[0n]|BOLD:AAA4201
Apamea sordens|[19650]|RDNMB018-05|Canada|Ontario|658[0n]|BOLD:AAA4201
Apamea sordens|[19651]|RDNMB016-05|Canada|Ontario|658[0n]|BOLD:AAA4201
Apamea sordens|[19652]|XAF802-05|Canada|Ontario|658[0n]|BOLD:AAA4201
Apamea sordens|[19653]|XAC176-04|Canada|Ontario|658[0n]|BOLD:AAA4201
Apamea sordens|[19654]|XAB365-04|Canada|Ontario|658[0n]|BOLD:AAA4201
Apamea sordens|[19655]|XAB350-04|Canada|Ontario|658[0n]|BOLD:AAA4201
Apamea sordens|[19656]|XAI064-05|Canada|Ontario|658[0n]|BOLD:AAA4201
Apamea sordens|[19657]|RBINA5368-13|Canada|Ontario|577[0n]|BOLD:AAA4201
Apamea sordens|[19658]|XAF518-05|Canada|Ontario|617[0n]|BOLD:AAA4201
Apamea sordens|[19659]|PHMO078-03|Canada|Ontario|639[0n]|BOLD:AAA4201
Apamea sordens|[19660]|PHMO122-03|Canada|Ontario|639[0n]|BOLD:AAA4201
Apamea sordens|[19661]|CNCLB2647-14|United States|North Carolina|632[0n]|BOLD:AAA4201
Apamea sordens|[19662]|CNCLB2649-14|United States|North Carolina|658[0n]|BOLD:AAA4201
Apamea maxima|[19663]|LBCH1303-10|Canada|British Columbia|658[0n]|BOLD:AAC7976
Apamea maxima|[19664]|RWWA415-09|United States|Washington|658[0n]|BOLD:AAC7976
Apamea maxima|[19665]|RWWA758-09|United States|Washington|658[0n]|BOLD:AAC7976
Apamea maxima|[19666]|RWWA419-09|United States|Washington|658[0n]|BOLD:AAC7976
Apamea maxima|[19667]|RWWA412-09|United States|Washington|658[0n]|BOLD:AAC7976
Apamea maxima|[19668]|RDNMG460-08|Canada|British Columbia|658[0n]|BOLD:AAC7976
Apamea maxima|[19669]|RDNMG459-08|Canada|British Columbia|658[0n]|BOLD:AAC7976
Apamea maxima|[19670]|RDNMG458-08|Canada|British Columbia|658[0n]|BOLD:AAC7976
Apamea maxima|[19671]|RDNMC253-05|Canada|British Columbia|658[0n]|BOLD:AAC7976
Apamea maxima|[19672]|RDNMC252-05|Canada|British Columbia|597[0n]|BOLD:AAC7976
Apamea robertsoni|[19673]|RDNMC257-05|United States|California|589[0n]|BOLD:AAC7976
Apamea maxima|[19674]|RWWB883-10|United States|Washington|641[0n]|BOLD:AAC7976
Apamea maxima|[19675]|BBLPB249-10|Canada|British Columbia|658[0n]|BOLD:AAC7976
Apamea maxima|[19676]|BBLPB250-10|Canada|British Columbia|658[0n]|BOLD:AAC7976
Apamea maxima|[19677]|RWWC284-11|United States|Washington|658[0n]|BOLD:AAC7976
Apamea maxima|[19678]|RWWC421-11|United States|Washington|658[0n]|BOLD:AAC7976
Franclemontia interrogans|[19679]|LNCC587-11|United States|North Carolina|658[0n]|BOLD:AAY9513
Franclemontia interrogans|[19680]|LNCC1835-13|United States|North Carolina|658[0n]|BOLD:AAY9513
Apamea xylodes|[19681]|RDNMD085-06|United States||658[0n]|BOLD:AAF7818
Apamea xylodes|[19682]|RDNMD086-06|United States||658[0n]|BOLD:AAF7818
Apamea xylodes|[19683]|RDNMD087-06|United States||658[0n]|BOLD:AAF7818
Apamea walshi|[19684]|RDNME899-08|United States|Arizona|658[0n]|BOLD:AAE2903
Apamea walshi|[19685]|RDNMG278-08|United States|Arizona|658[0n]|BOLD:AAE2903
Apamea apamiformis|[19686]|RDNM971-05|Canada|Ontario|658[0n]|BOLD:AAJ1428
Apamea apamiformis|[19687]|RDLQF050-06|Canada|Quebec|658[0n]|BOLD:AAJ1428
Oligia egens|[19688]|RDNM735-05|Canada|Alberta|658[0n]|BOLD:AAE1433
Oligia egens|[19689]|RDNM736-05|Canada|Alberta|658[0n]|BOLD:AAE1433

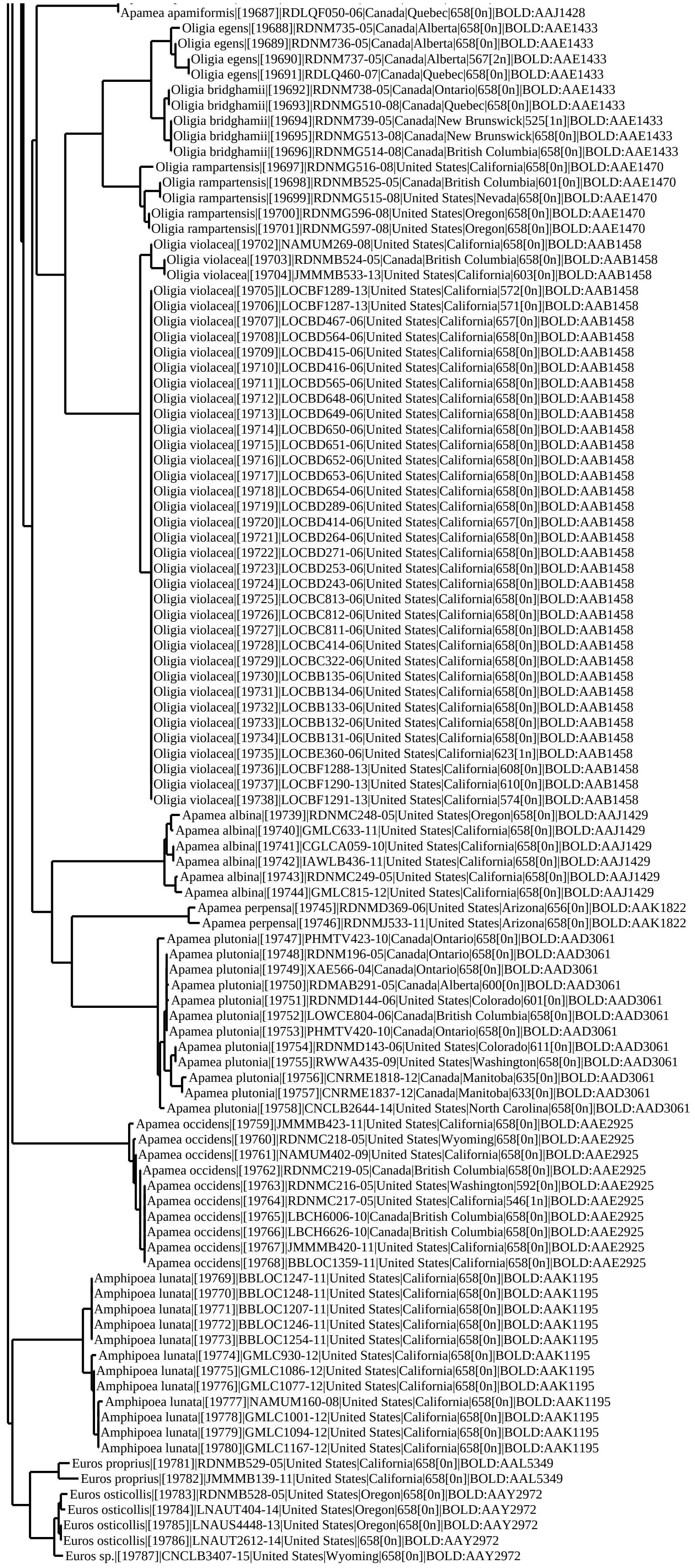
Apamea apamiformis|[19687]|RDLQF050-06|Canada|Quebec|658[0n]|BOLD:AAJ1428
Oligia egens|[19688]|RDNM735-05|Canada|Alberta|658[0n]|BOLD:AAE1433
Oligia egens|[19689]|RDNM736-05|Canada|Alberta|658[0n]|BOLD:AAE1433
Oligia egens|[19690]|RDNM737-05|Canada|Alberta|567[2n]|BOLD:AAE1433
Oligia egens|[19691]|RDLQ460-07|Canada|Quebec|658[0n]|BOLD:AAE1433
Oligia bridghamii|[19692]|RDNM738-05|Canada|Ontario|658[0n]|BOLD:AAE1433
Oligia bridghamii|[19693]|RDNMG510-08|Canada|Quebec|658[0n]|BOLD:AAE1433
Oligia bridghamii|[19694]|RDNM739-05|Canada|New Brunswick|525[1n]|BOLD:AAE1433
Oligia bridghamii|[19695]|RDNMG513-08|Canada|New Brunswick|658[0n]|BOLD:AAE1433
Oligia bridghamii|[19696]|RDNMG514-08|Canada|British Columbia|658[0n]|BOLD:AAE1433
Oligia rampartensis|[19697]|RDNMG516-08|United States|California|658[0n]|BOLD:AAE1470
Oligia rampartensis|[19698]|RDNMB525-05|Canada|British Columbia|601[0n]|BOLD:AAE1470
Oligia rampartensis|[19699]|RDNMG515-08|United States|Nevada|658[0n]|BOLD:AAE1470
Oligia rampartensis|[19700]|RDNMG596-08|United States|Oregon|658[0n]|BOLD:AAE1470
Oligia rampartensis|[19701]|RDNMG597-08|United States|Oregon|658[0n]|BOLD:AAE1470
Oligia violacea|[19702]|NAMUM269-08|United States|California|658[0n]|BOLD:AAB1458
Oligia violacea|[19703]|RDNMB524-05|Canada|British Columbia|658[0n]|BOLD:AAB1458
Oligia violacea|[19704]|JMMMB533-13|United States|California|603[0n]|BOLD:AAB1458
Oligia violacea|[19705]|LOCBF1289-13|United States|California|572[0n]|BOLD:AAB1458
Oligia violacea|[19706]|LOCBF1287-13|United States|California|571[0n]|BOLD:AAB1458
Oligia violacea|[19707]|LOCBD467-06|United States|California|657[0n]|BOLD:AAB1458
Oligia violacea|[19708]|LOCBD564-06|United States|California|658[0n]|BOLD:AAB1458
Oligia violacea|[19709]|LOCBD415-06|United States|California|658[0n]|BOLD:AAB1458
Oligia violacea|[19710]|LOCBD416-06|United States|California|658[0n]|BOLD:AAB1458
Oligia violacea|[19711]|LOCBD565-06|United States|California|658[0n]|BOLD:AAB1458
Oligia violacea|[19712]|LOCBD648-06|United States|California|658[0n]|BOLD:AAB1458
Oligia violacea|[19713]|LOCBD649-06|United States|California|658[0n]|BOLD:AAB1458
Oligia violacea|[19714]|LOCBD650-06|United States|California|658[0n]|BOLD:AAB1458
Oligia violacea|[19715]|LOCBD651-06|United States|California|658[0n]|BOLD:AAB1458
Oligia violacea|[19716]|LOCBD652-06|United States|California|658[0n]|BOLD:AAB1458
Oligia violacea|[19717]|LOCBD653-06|United States|California|658[0n]|BOLD:AAB1458
Oligia violacea|[19718]|LOCBD654-06|United States|California|658[0n]|BOLD:AAB1458
Oligia violacea|[19719]|LOCBD289-06|United States|California|658[0n]|BOLD:AAB1458
Oligia violacea|[19720]|LOCBD414-06|United States|California|658[0n]|BOLD:AAB1458
Oligia violacea|[19721]|LOCBD264-06|United States|California|658[0n]|BOLD:AAB1458
Oligia violacea|[19722]|LOCBD271-06|United States|California|658[0n]|BOLD:AAB1458
Oligia violacea|[19723]|LOCBD253-06|United States|California|658[0n]|BOLD:AAB1458
Oligia violacea|[19724]|LOCBD243-06|United States|California|658[0n]|BOLD:AAB1458
Oligia violacea|[19725]|LOCBC813-06|United States|California|658[0n]|BOLD:AAB1458
Oligia violacea|[19726]|LOCBC812-06|United States|California|658[0n]|BOLD:AAB1458
Oligia violacea|[19727]|LOCBC811-06|United States|California|658[0n]|BOLD:AAB1458
Oligia violacea|[19728]|LOCBC414-06|United States|California|658[0n]|BOLD:AAB1458
Oligia violacea|[19729]|LOCBC322-06|United States|California|658[0n]|BOLD:AAB1458
Oligia violacea|[19730]|LOCBB135-06|United States|California|658[0n]|BOLD:AAB1458
Oligia violacea|[19731]|LOCBB134-06|United States|California|658[0n]|BOLD:AAB1458
Oligia violacea|[19732]|LOCBB133-06|United States|California|658[0n]|BOLD:AAB1458
Oligia violacea|[19733]|LOCBB132-06|United States|California|658[0n]|BOLD:AAB1458
Oligia violacea|[19734]|LOCBB131-06|United States|California|658[0n]|BOLD:AAB1458
Oligia violacea|[19735]|LOCBE360-06|United States|California|623[1n]|BOLD:AAB1458
Oligia violacea|[19736]|LOCBF1288-13|United States|California|608[0n]|BOLD:AAB1458
Oligia violacea|[19737]|LOCBF1290-13|United States|California|610[0n]|BOLD:AAB1458
Oligia violacea|[19738]|LOCBF1291-13|United States|California|574[0n]|BOLD:AAB1458
Apamea albina|[19739]|RDNMC248-05|United States|Oregon|658[0n]|BOLD:AAJ1429
Apamea albina|[19740]|GMLC633-11|United States|California|658[0n]|BOLD:AAJ1429
Apamea albina|[19741]|CGLCA059-10|United States|California|658[0n]|BOLD:AAJ1429
Apamea albina|[19742]|IAWLB436-11|United States|California|658[0n]|BOLD:AAJ1429
Apamea albina|[19743]|RDNMC249-05|United States|California|658[0n]|BOLD:AAJ1429
Apamea albina|[19744]|GMLC815-12|United States|California|658[0n]|BOLD:AAJ1429
Apamea perpensa|[19745]|RDNMD369-06|United States|Arizona|656[0n]|BOLD:AAK1822
Apamea perpensa|[19746]|RDNMJ533-11|United States|Arizona|658[0n]|BOLD:AAK1822
Apamea plutonia|[19747]|PHMTV423-10|Canada|Ontario|658[0n]|BOLD:AAD3061
Apamea plutonia|[19748]|RDNM196-05|Canada|Ontario|658[0n]|BOLD:AAD3061
Apamea plutonia|[19749]|XAE566-04|Canada|Ontario|658[0n]|BOLD:AAD3061
Apamea plutonia|[19750]|RDMAB291-05|Canada|Alberta|600[0n]|BOLD:AAD3061
Apamea plutonia|[19751]|RDNMD144-06|United States|Colorado|601[0n]|BOLD:AAD3061
Apamea plutonia|[19752]|LOWCE804-06|Canada|British Columbia|658[0n]|BOLD:AAD3061
Apamea plutonia|[19753]|PHMTV420-10|Canada|Ontario|658[0n]|BOLD:AAD3061
Apamea plutonia|[19754]|RDNMD143-06|United States|Colorado|611[0n]|BOLD:AAD3061
Apamea plutonia|[19755]|RWWA435-09|United States|Washington|658[0n]|BOLD:AAD3061
Apamea plutonia|[19756]|CNRME1818-12|Canada|Manitoba|635[0n]|BOLD:AAD3061
Apamea plutonia|[19757]|CNRME1837-12|Canada|Manitoba|633[0n]|BOLD:AAD3061
Apamea plutonia|[19758]|CNCLB2644-14|United States|North Carolina|658[0n]|BOLD:AAD3061
Apamea occidens|[19759]|JMMMB423-11|United States|California|658[0n]|BOLD:AAE2925
Apamea occidens|[19760]|RDNMC218-05|United States|Wyoming|658[0n]|BOLD:AAE2925
Apamea occidens|[19761]|NAMUM402-09|United States|California|658[0n]|BOLD:AAE2925
Apamea occidens|[19762]|RDNMC219-05|Canada|British Columbia|658[0n]|BOLD:AAE2925
Apamea occidens|[19763]|RDNMC216-05|United States|Washington|592[0n]|BOLD:AAE2925
Apamea occidens|[19764]|RDNMC217-05|United States|California|546[1n]|BOLD:AAE2925
Apamea occidens|[19765]|LBCH6006-10|Canada|British Columbia|658[0n]|BOLD:AAE2925
Apamea occidens|[19766]|LBCH6626-10|Canada|British Columbia|658[0n]|BOLD:AAE2925
Apamea occidens|[19767]|JMMMB420-11|United States|California|658[0n]|BOLD:AAE2925
Apamea occidens|[19768]|BBLOC1359-11|United States|California|658[0n]|BOLD:AAE2925
Amphipoea lunata|[19769]|BBLOC1247-11|United States|California|658[0n]|BOLD:AAK1195
Amphipoea lunata|[19770]|BBLOC1248-11|United States|California|658[0n]|BOLD:AAK1195
Amphipoea lunata|[19771]|BBLOC1207-11|United States|California|658[0n]|BOLD:AAK1195
Amphipoea lunata|[19772]|BBLOC1246-11|United States|California|658[0n]|BOLD:AAK1195
Amphipoea lunata|[19773]|BBLOC1254-11|United States|California|658[0n]|BOLD:AAK1195
Amphipoea lunata|[19774]|GMLC930-12|United States|California|658[0n]|BOLD:AAK1195
Amphipoea lunata|[19775]|GMLC1086-12|United States|California|658[0n]|BOLD:AAK1195
Amphipoea lunata|[19776]|GMLC1077-12|United States|California|658[0n]|BOLD:AAK1195
Amphipoea lunata|[19777]|NAMUM160-08|United States|California|658[0n]|BOLD:AAK1195
Amphipoea lunata|[19778]|GMLC1001-12|United States|California|658[0n]|BOLD:AAK1195
Amphipoea lunata|[19779]|GMLC1094-12|United States|California|658[0n]|BOLD:AAK1195
Amphipoea lunata|[19780]|GMLC1167-12|United States|California|658[0n]|BOLD:AAK1195
Euros proprius|[19781]|RDNMB529-05|United States|California|658[0n]|BOLD:AAL5349
Euros proprius|[19782]|JMMMB139-11|United States|California|658[0n]|BOLD:AAL5349
Euros osticollis|[19783]|RDNMB528-05|United States|Oregon|658[0n]|BOLD:AAY2972
Euros osticollis|[19784]|LNAUT404-14|United States|Oregon|658[0n]|BOLD:AAY2972
Euros osticollis|[19785]|LNAUS4448-13|United States|Oregon|658[0n]|BOLD:AAY2972
Euros osticollis|[19786]|LNAUT2612-14|United States||658[0n]|BOLD:AAY2972
Euros sp.|[19787]|CNCLB3407-15|United States|Wyoming|658[0n]|BOLD:AAY2972
