# Supplementary material for: Probing planetary biodiversity with DNA barcodes: The Noctuoidea of North America
Source: PLoS One. 2017 Jun 1;12(6):e0178548. doi: 10.1371/journal.pone.0178548 (PMC5453547; doi:10.1371/journal.pone.0178548)
Supplement: S8 Tree — NJ tree based on sequence variation in the barcode region of the cytochrome c oxidase I gene for North American species in the family Noctuidae-3. (PDF) [file pone.0178548.s021.pdf]

BOLD TaxonID Tree

Title	:	Tree Result - Search (10564 records)
Date	:	21-April-2016
Data Type	:	Nucleotide
Distance Model	:	Kimura 2 Parameter
Marker	:	COI-5P

Label	:	Process ID
Label	:	Taxon
Label	:	Country
Label	:	Province/State
Label	:	Sequence Length
Label	:	Barcode Cluster (BIN)

Sequence Count	:	10564
Species count	:	496
Genus count	:	43
Family count	:	1
Unidentified	:	0

BIN Count	:	433

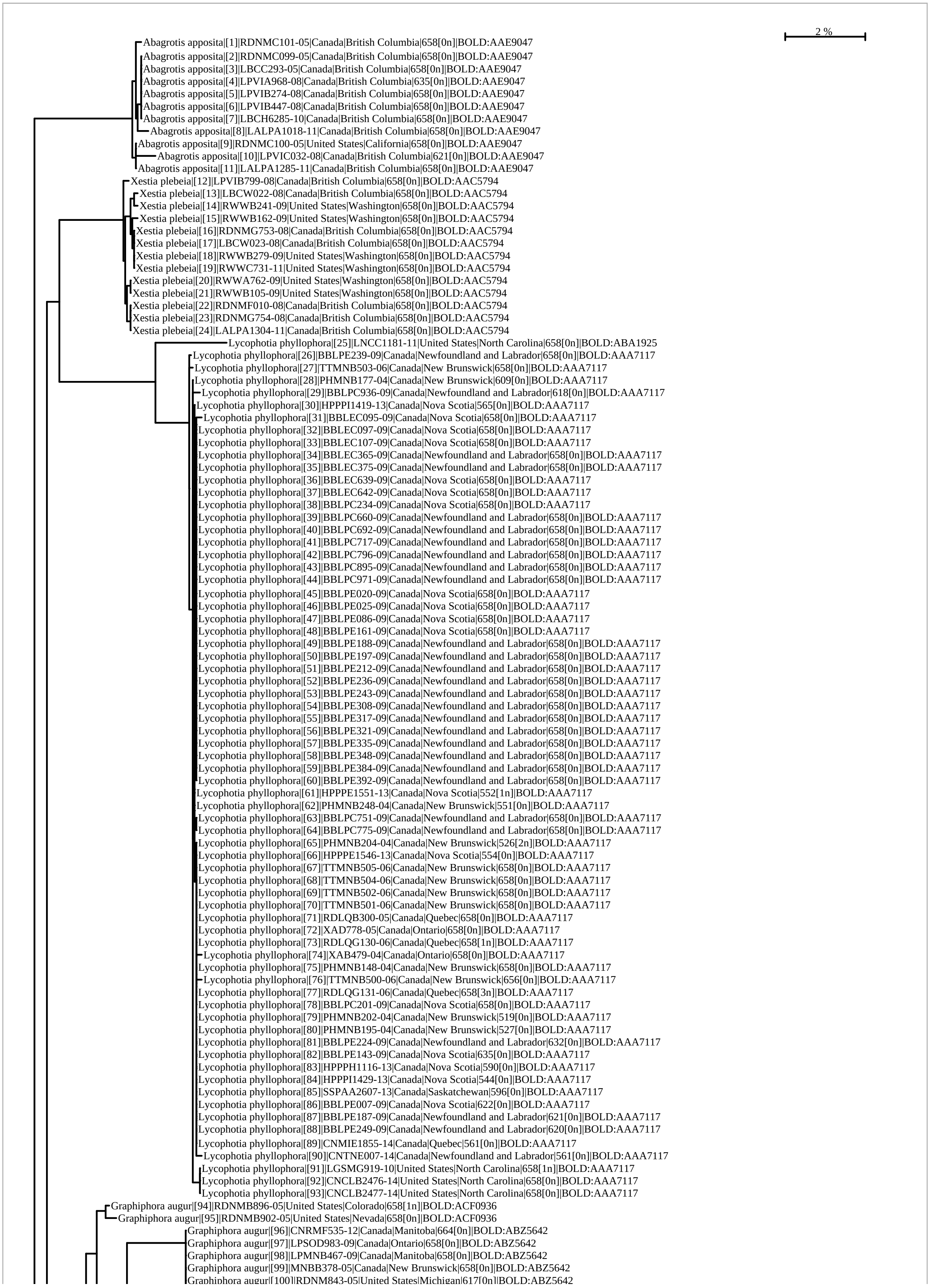
2 %
Abagrotis apposita|[1]|RDNMC101-05|Canada|British Columbia|658[0n]|BOLD:AAE9047
Abagrotis apposita|[2]|RDNMC099-05|Canada|British Columbia|658[0n]|BOLD:AAE9047
Abagrotis apposita|[3]|LBCC293-05|Canada|British Columbia|658[0n]|BOLD:AAE9047
Abagrotis apposita|[4]|LPVIA968-08|Canada|British Columbia|635[0n]|BOLD:AAE9047
Abagrotis apposita|[5]|LPVIB274-08|Canada|British Columbia|658[0n]|BOLD:AAE9047
Abagrotis apposita|[6]|LPVIB447-08|Canada|British Columbia|658[0n]|BOLD:AAE9047
Abagrotis apposita|[7]|LBCH6285-10|Canada|British Columbia|658[0n]|BOLD:AAE9047
Abagrotis apposita|[8]|LALPA1018-11|Canada|British Columbia|658[0n]|BOLD:AAE9047
Abagrotis apposita|[9]|RDNMC100-05|United States|California|658[0n]|BOLD:AAE9047
Abagrotis apposita|[10]|LPVIC032-08|Canada|British Columbia|621[0n]|BOLD:AAE9047
Abagrotis apposita|[11]|LALPA1285-11|Canada|British Columbia|658[0n]|BOLD:AAE9047
Xestia plebeia|[12]|LPVIB799-08|Canada|British Columbia|658[0n]|BOLD:AAC5794
Xestia plebeia|[13]|LBCW022-08|Canada|British Columbia|658[0n]|BOLD:AAC5794
Xestia plebeia|[14]|RWWB241-09|United States|Washington|658[0n]|BOLD:AAC5794
Xestia plebeia|[15]|RWWB162-09|United States|Washington|658[0n]|BOLD:AAC5794
Xestia plebeia|[16]|RDNMG753-08|Canada|British Columbia|658[0n]|BOLD:AAC5794
Xestia plebeia|[17]|LBCW023-08|Canada|British Columbia|658[0n]|BOLD:AAC5794
Xestia plebeia|[18]|RWWB279-09|United States|Washington|658[0n]|BOLD:AAC5794
Xestia plebeia|[19]|RWWC731-11|United States|Washington|658[0n]|BOLD:AAC5794
Xestia plebeia|[20]|RWWA762-09|United States|Washington|658[0n]|BOLD:AAC5794
Xestia plebeia|[21]|RWWB105-09|United States|Washington|658[0n]|BOLD:AAC5794
Xestia plebeia|[22]|RDNMF010-08|Canada|British Columbia|658[0n]|BOLD:AAC5794
Xestia plebeia|[23]|RDNMG754-08|Canada|British Columbia|658[0n]|BOLD:AAC5794
Xestia plebeia|[24]|LALPA1304-11|Canada|British Columbia|658[0n]|BOLD:AAC5794
Lycophotia phyllophora|[25]|LNCC1181-11|United States|North Carolina|658[0n]|BOLD:ABA1925
Lycophotia phyllophora|[26]|BBLPE239-09|Canada|Newfoundland and Labrador|658[0n]|BOLD:AAA7117
Lycophotia phyllophora|[27]|TTMNB503-06|Canada|New Brunswick|658[0n]|BOLD:AAA7117
Lycophotia phyllophora|[28]|PHMNB177-04|Canada|New Brunswick|609[0n]|BOLD:AAA7117
Lycophotia phyllophora|[29]|BBLPC936-09|Canada|Newfoundland and Labrador|618[0n]|BOLD:AAA7117
Lycophotia phyllophora|[30]|HPPPI1419-13|Canada|Nova Scotia|565[0n]|BOLD:AAA7117
Lycophotia phyllophora|[31]|BBLEC095-09|Canada|Nova Scotia|658[0n]|BOLD:AAA7117
Lycophotia phyllophora|[32]|BBLEC097-09|Canada|Nova Scotia|658[0n]|BOLD:AAA7117
Lycophotia phyllophora|[33]|BBLEC107-09|Canada|Nova Scotia|658[0n]|BOLD:AAA7117
Lycophotia phyllophora|[34]|BBLEC365-09|Canada|Newfoundland and Labrador|658[0n]|BOLD:AAA7117
Lycophotia phyllophora|[35]|BBLEC375-09|Canada|Newfoundland and Labrador|658[0n]|BOLD:AAA7117
Lycophotia phyllophora|[36]|BBLEC639-09|Canada|Nova Scotia|658[0n]|BOLD:AAA7117
Lycophotia phyllophora|[37]|BBLEC642-09|Canada|Nova Scotia|658[0n]|BOLD:AAA7117
Lycophotia phyllophora|[38]|BBLPC234-09|Canada|Nova Scotia|658[0n]|BOLD:AAA7117
Lycophotia phyllophora|[39]|BBLPC660-09|Canada|Newfoundland and Labrador|658[0n]|BOLD:AAA7117
Lycophotia phyllophora|[40]|BBLPC692-09|Canada|Newfoundland and Labrador|658[0n]|BOLD:AAA7117
Lycophotia phyllophora|[41]|BBLPC717-09|Canada|Newfoundland and Labrador|658[0n]|BOLD:AAA7117
Lycophotia phyllophora|[42]|BBLPC796-09|Canada|Newfoundland and Labrador|658[0n]|BOLD:AAA7117
Lycophotia phyllophora|[43]|BBLPC895-09|Canada|Newfoundland and Labrador|658[0n]|BOLD:AAA7117
Lycophotia phyllophora|[44]|BBLPC971-09|Canada|Newfoundland and Labrador|658[0n]|BOLD:AAA7117
Lycophotia phyllophora|[45]|BBLPE020-09|Canada|Nova Scotia|658[0n]|BOLD:AAA7117
Lycophotia phyllophora|[46]|BBLPE025-09|Canada|Nova Scotia|658[0n]|BOLD:AAA7117
Lycophotia phyllophora|[47]|BBLPE086-09|Canada|Nova Scotia|658[0n]|BOLD:AAA7117
Lycophotia phyllophora|[48]|BBLPE161-09|Canada|Nova Scotia|658[0n]|BOLD:AAA7117
Lycophotia phyllophora|[49]|BBLPE188-09|Canada|Newfoundland and Labrador|658[0n]|BOLD:AAA7117
Lycophotia phyllophora|[50]|BBLPE197-09|Canada|Newfoundland and Labrador|658[0n]|BOLD:AAA7117
Lycophotia phyllophora|[51]|BBLPE212-09|Canada|Newfoundland and Labrador|658[0n]|BOLD:AAA7117
Lycophotia phyllophora|[52]|BBLPE236-09|Canada|Newfoundland and Labrador|658[0n]|BOLD:AAA7117
Lycophotia phyllophora|[53]|BBLPE243-09|Canada|Newfoundland and Labrador|658[0n]|BOLD:AAA7117
Lycophotia phyllophora|[54]|BBLPE308-09|Canada|Newfoundland and Labrador|658[0n]|BOLD:AAA7117
Lycophotia phyllophora|[55]|BBLPE317-09|Canada|Newfoundland and Labrador|658[0n]|BOLD:AAA7117
Lycophotia phyllophora|[56]|BBLPE321-09|Canada|Newfoundland and Labrador|658[0n]|BOLD:AAA7117
Lycophotia phyllophora|[57]|BBLPE335-09|Canada|Newfoundland and Labrador|658[0n]|BOLD:AAA7117
Lycophotia phyllophora|[58]|BBLPE348-09|Canada|Newfoundland and Labrador|658[0n]|BOLD:AAA7117
Lycophotia phyllophora|[59]|BBLPE384-09|Canada|Newfoundland and Labrador|658[0n]|BOLD:AAA7117
Lycophotia phyllophora|[60]|BBLPE392-09|Canada|Newfoundland and Labrador|658[0n]|BOLD:AAA7117
Lycophotia phyllophora|[61]|HPPPE1551-13|Canada|Nova Scotia|552[1n]|BOLD:AAA7117
Lycophotia phyllophora|[62]|PHMNB248-04|Canada|New Brunswick|551[0n]|BOLD:AAA7117
Lycophotia phyllophora|[63]|BBLPC751-09|Canada|Newfoundland and Labrador|658[0n]|BOLD:AAA7117
Lycophotia phyllophora|[64]|BBLPC775-09|Canada|Newfoundland and Labrador|658[0n]|BOLD:AAA7117
Lycophotia phyllophora|[65]|PHMNB204-04|Canada|New Brunswick|526[2n]|BOLD:AAA7117
Lycophotia phyllophora|[66]|HPPPE1546-13|Canada|Nova Scotia|554[0n]|BOLD:AAA7117
Lycophotia phyllophora|[67]|TTMNB505-06|Canada|New Brunswick|658[0n]|BOLD:AAA7117
Lycophotia phyllophora|[68]|TTMNB504-06|Canada|New Brunswick|658[0n]|BOLD:AAA7117
Lycophotia phyllophora|[69]|TTMNB502-06|Canada|New Brunswick|658[0n]|BOLD:AAA7117
Lycophotia phyllophora|[70]|TTMNB501-06|Canada|New Brunswick|658[0n]|BOLD:AAA7117
Lycophotia phyllophora|[71]|RDLQB300-05|Canada|Quebec|658[0n]|BOLD:AAA7117
Lycophotia phyllophora|[72]|XAD778-05|Canada|Ontario|658[0n]|BOLD:AAA7117
Lycophotia phyllophora|[73]|RDLQG130-06|Canada|Quebec|658[1n]|BOLD:AAA7117
Lycophotia phyllophora|[74]|XAB479-04|Canada|Ontario|658[0n]|BOLD:AAA7117
Lycophotia phyllophora|[75]|PHMNB148-04|Canada|New Brunswick|658[0n]|BOLD:AAA7117
Lycophotia phyllophora|[76]|TTMNB500-06|Canada|New Brunswick|656[0n]|BOLD:AAA7117
Lycophotia phyllophora|[77]|RDLQG131-06|Canada|Quebec|658[3n]|BOLD:AAA7117
Lycophotia phyllophora|[78]|BBLPC201-09|Canada|Nova Scotia|658[0n]|BOLD:AAA7117
Lycophotia phyllophora|[79]|PHMNB202-04|Canada|New Brunswick|519[0n]|BOLD:AAA7117
Lycophotia phyllophora|[80]|PHMNB195-04|Canada|New Brunswick|527[0n]|BOLD:AAA7117
Lycophotia phyllophora|[81]|BBLPE224-09|Canada|Newfoundland and Labrador|632[0n]|BOLD:AAA7117
Lycophotia phyllophora|[82]|BBLPE143-09|Canada|Nova Scotia|635[0n]|BOLD:AAA7117
Lycophotia phyllophora|[83]|HPPPH1116-13|Canada|Nova Scotia|590[0n]|BOLD:AAA7117
Lycophotia phyllophora|[84]|HPPPI1429-13|Canada|Nova Scotia|544[0n]|BOLD:AAA7117
Lycophotia phyllophora|[85]|SSPAA2607-13|Canada|Saskatchewan|596[0n]|BOLD:AAA7117
Lycophotia phyllophora|[86]|BBLPE007-09|Canada|Nova Scotia|622[0n]|BOLD:AAA7117
Lycophotia phyllophora|[87]|BBLPE187-09|Canada|Newfoundland and Labrador|621[0n]|BOLD:AAA7117
Lycophotia phyllophora|[88]|BBLPE249-09|Canada|Newfoundland and Labrador|620[0n]|BOLD:AAA7117
Lycophotia phyllophora|[89]|CNMIE1855-14|Canada|Quebec|561[0n]|BOLD:AAA7117
Lycophotia phyllophora|[90]|CNTNE007-14|Canada|Newfoundland and Labrador|561[0n]|BOLD:AAA7117
Lycophotia phyllophora|[91]|LGSMG919-10|United States|North Carolina|658[1n]|BOLD:AAA7117
Lycophotia phyllophora|[92]|CNCLB2476-14|United States|North Carolina|658[0n]|BOLD:AAA7117
Lycophotia phyllophora|[93]|CNCLB2477-14|United States|North Carolina|658[0n]|BOLD:AAA7117
Graphiphora augur|[94]|RDNMB896-05|United States|Colorado|658[1n]|BOLD:ACF0936
Graphiphora augur|[95]|RDNMB902-05|United States|Nevada|658[0n]|BOLD:ACF0936
Graphiphora augur|[96]|CNRMF535-12|Canada|Manitoba|664[0n]|BOLD:ABZ5642
Graphiphora augur|[97]|LPSOD983-09|Canada|Ontario|658[0n]|BOLD:ABZ5642
Graphiphora augur|[98]|LPMNB467-09|Canada|Manitoba|658[0n]|BOLD:ABZ5642
Graphiphora augur|[99]|MNBB378-05|Canada|New Brunswick|658[0n]|BOLD:ABZ5642
Graphiphora augur|[100]|RDNM843-05|United States|Michigan|617[0n]|BOLD:ABZ5642

[illegible]
Graphiphora augur|[98]|LPMNB467-09|Canada|Manitoba|658[0n]|BOLD:ABZ5642
Graphiphora augur|[99]|MNBB378-05|Canada|New Brunswick|658[0n]|BOLD:ABZ5642
Graphiphora augur|[100]|RDNM843-05|United States|Michigan|617[0n]|BOLD:ABZ5642
Graphiphora augur|[101]|XAG543-05|Canada|Ontario|592[0n]|BOLD:ABZ5642
Graphiphora augur|[102]|RDNMC075-05|Canada|New Brunswick|542[0n]|BOLD:ABZ5642
Graphiphora augur|[103]|SSBAE3531-13|Canada|Alberta|592[0n]|BOLD:ABZ5642
Graphiphora augur|[104]|BLTIB784-08|Canada|Ontario|658[0n]|BOLD:AAA2707
Graphiphora augur|[105]|RDNMB904-05|Canada|Manitoba|658[0n]|BOLD:AAA2707
Graphiphora augur|[106]|XAD573-04|Canada|Ontario|658[0n]|BOLD:AAA2707
Graphiphora augur|[107]|XAG205-05|Canada|Ontario|658[2n]|BOLD:AAA2707
Graphiphora augur|[108]|XAG704-05|Canada|Ontario|658[0n]|BOLD:AAA2707
Graphiphora augur|[109]|XAB002-04|Canada|Ontario|567[0n]|BOLD:AAA2707
Graphiphora augur|[110]|XAG479-05|Canada|Ontario|587[0n]|BOLD:AAA2707
Graphiphora augur|[111]|LMDH189-11|United States|Minnesota|658[0n]|BOLD:AAA2707
Graphiphora augur|[112]|LPABC407-09|Canada|Alberta|658[0n]|BOLD:AAA2707
Graphiphora augur|[113]|RDNMB903-05|Canada|Alberta|658[0n]|BOLD:AAA2707
Graphiphora augur|[114]|MNBB605-05|Canada|New Brunswick|658[0n]|BOLD:AAA2707
Graphiphora augur|[115]|CNEID3344-12|Canada|Alberta|634[0n]|BOLD:AAA2707
Graphiphora augur|[116]|CNWBG3077-13|Canada|Alberta|592[0n]|BOLD:AAA2707
Graphiphora augur|[117]|LPSOD936-09|Canada|Ontario|658[0n]|BOLD:AAA2707
Graphiphora augur|[118]|RDLQ706-07|Canada|Quebec|658[0n]|BOLD:AAA2707
Graphiphora augur|[119]|RDNMB905-05|Canada|Alberta|658[0n]|BOLD:AAA2707
Graphiphora augur|[120]|RDNM844-05|Canada|Ontario|658[0n]|BOLD:AAA2707
Graphiphora augur|[121]|LCHP659-07|Canada|Manitoba|626[0n]|BOLD:AAA2707
Graphiphora augur|[122]|CNWBG3101-13|Canada|Alberta|576[0n]|BOLD:AAA2707
Graphiphora augur|[123]|PHMNB064-03|Canada|New Brunswick|639[0n]|BOLD:AAA2707
Graphiphora augur|[124]|RDNMD051-06|Canada||609[0n]|BOLD:AAA2707
Graphiphora augur|[125]|LCHIP246-07|Canada|Manitoba|650[0n]|BOLD:AAA2707
Graphiphora augur|[126]|JSJUL2389-11|Canada|Ontario|658[0n]|BOLD:AAA2707
Graphiphora augur|[127]|CNWLM2439-13|Canada|Alberta|611[0n]|BOLD:AAA2707
Graphiphora augur|[128]|LOWCC860-05|Canada|British Columbia|658[0n]|BOLD:ACF0936
Graphiphora augur|[129]|LBCH1170-10|Canada|British Columbia|658[0n]|BOLD:ACF0936
Graphiphora augur|[130]|RWWC381-11|United States|Washington|658[0n]|BOLD:ACF0936
Graphiphora augur|[131]|LBCC835-05|Canada|British Columbia|658[0n]|BOLD:ACF0936
Graphiphora augur|[132]|LBCC837-05|Canada|British Columbia|658[0n]|BOLD:ACF0936
Graphiphora augur|[133]|LBCD325-05|Canada|British Columbia|658[0n]|BOLD:ACF0936
Graphiphora augur|[134]|LBCC836-05|Canada|British Columbia|658[0n]|BOLD:ACF0936
Graphiphora augur|[135]|LPABB822-09|Canada|Alberta|658[0n]|BOLD:ACF0936
Graphiphora augur|[136]|LPABC394-09|Canada|Alberta|658[0n]|BOLD:ACF0936
Graphiphora augur|[137]|LPABC430-09|Canada|Alberta|658[0n]|BOLD:ACF0936
Graphiphora augur|[138]|LPABC456-09|Canada|Alberta|658[0n]|BOLD:ACF0936
Graphiphora augur|[139]|LPABC473-09|Canada|Alberta|658[0n]|BOLD:ACF0936
Graphiphora augur|[140]|LBCH4431-10|Canada|British Columbia|658[0n]|BOLD:ACF0936
Graphiphora augur|[141]|LBCH4686-10|Canada|British Columbia|658[0n]|BOLD:ACF0936
Graphiphora augur|[142]|LBCH4685-10|Canada|British Columbia|658[0n]|BOLD:ACF0936
Graphiphora augur|[143]|LBCH4684-10|Canada|British Columbia|658[0n]|BOLD:ACF0936
Graphiphora augur|[144]|LBCH4683-10|Canada|British Columbia|658[0n]|BOLD:ACF0936
Graphiphora augur|[145]|LBCH4682-10|Canada|British Columbia|658[0n]|BOLD:ACF0936
Graphiphora augur|[146]|LBCH4680-10|Canada|British Columbia|658[0n]|BOLD:ACF0936
Graphiphora augur|[147]|LBCH4424-10|Canada|British Columbia|658[0n]|BOLD:ACF0936
Graphiphora augur|[148]|LBCH4423-10|Canada|British Columbia|658[0n]|BOLD:ACF0936
Graphiphora augur|[149]|LBCH4119-10|Canada|British Columbia|658[0n]|BOLD:ACF0936
Graphiphora augur|[150]|LBCH3776-10|Canada|British Columbia|658[0n]|BOLD:ACF0936
Graphiphora augur|[151]|LBCH3371-10|Canada|British Columbia|658[0n]|BOLD:ACF0936
Graphiphora augur|[152]|LBCH3370-10|Canada|British Columbia|658[0n]|BOLD:ACF0936
Graphiphora augur|[153]|LBCH3369-10|Canada|British Columbia|658[0n]|BOLD:ACF0936
Graphiphora augur|[154]|LBCH3368-10|Canada|British Columbia|658[0n]|BOLD:ACF0936
Graphiphora augur|[155]|LBCH3367-10|Canada|British Columbia|658[0n]|BOLD:ACF0936
Graphiphora augur|[156]|LBCH3366-10|Canada|British Columbia|658[0n]|BOLD:ACF0936
Graphiphora augur|[157]|LBCH3365-10|Canada|British Columbia|658[0n]|BOLD:ACF0936
Graphiphora augur|[158]|LBCH3364-10|Canada|British Columbia|658[0n]|BOLD:ACF0936
Graphiphora augur|[159]|LBCH3363-10|Canada|British Columbia|658[0n]|BOLD:ACF0936
Graphiphora augur|[160]|LBCH3362-10|Canada|British Columbia|658[0n]|BOLD:ACF0936
Graphiphora augur|[161]|LBCH3361-10|Canada|British Columbia|658[0n]|BOLD:ACF0936
Graphiphora augur|[162]|LBCH3086-10|Canada|British Columbia|658[0n]|BOLD:ACF0936
Graphiphora augur|[163]|LBCH3072-10|Canada|British Columbia|658[0n]|BOLD:ACF0936
Graphiphora augur|[164]|LBCH2378-10|Canada|British Columbia|658[0n]|BOLD:ACF0936
Graphiphora augur|[165]|LBCH1305-10|Canada|British Columbia|658[0n]|BOLD:ACF0936
Graphiphora augur|[166]|LBCH942-10|Canada|British Columbia|658[0n]|BOLD:ACF0936
Graphiphora augur|[167]|LBCH941-10|Canada|British Columbia|658[0n]|BOLD:ACF0936
Graphiphora augur|[168]|LBCH926-10|Canada|British Columbia|658[0n]|BOLD:ACF0936
Graphiphora augur|[169]|LBCH924-10|Canada|British Columbia|658[0n]|BOLD:ACF0936
Graphiphora augur|[170]|LBCH923-10|Canada|British Columbia|658[0n]|BOLD:ACF0936
Graphiphora augur|[171]|LBCH922-10|Canada|British Columbia|658[0n]|BOLD:ACF0936
Graphiphora augur|[172]|LBCH921-10|Canada|British Columbia|658[0n]|BOLD:ACF0936
Graphiphora augur|[173]|LBCH920-10|Canada|British Columbia|658[0n]|BOLD:ACF0936
Graphiphora augur|[174]|LBCH919-10|Canada|British Columbia|658[0n]|BOLD:ACF0936
Graphiphora augur|[175]|LBCH808-10|Canada|British Columbia|658[0n]|BOLD:ACF0936
Graphiphora augur|[176]|LBCH693-10|Canada|British Columbia|658[0n]|BOLD:ACF0936
Graphiphora augur|[177]|LBCH493-10|Canada|British Columbia|658[0n]|BOLD:ACF0936
Graphiphora augur|[178]|LBCH363-10|Canada|British Columbia|658[0n]|BOLD:ACF0936
Graphiphora augur|[179]|LBCH362-10|Canada|British Columbia|658[0n]|BOLD:ACF0936
Graphiphora augur|[180]|LBCH361-10|Canada|British Columbia|658[0n]|BOLD:ACF0936
Graphiphora augur|[181]|LBCH360-10|Canada|British Columbia|658[0n]|BOLD:ACF0936
Graphiphora augur|[182]|LBCH359-10|Canada|British Columbia|658[0n]|BOLD:ACF0936
Graphiphora augur|[183]|LBCH357-10|Canada|British Columbia|658[0n]|BOLD:ACF0936
Graphiphora augur|[184]|LBCH356-10|Canada|British Columbia|658[0n]|BOLD:ACF0936
Graphiphora augur|[185]|LBCH232-10|Canada|British Columbia|658[0n]|BOLD:ACF0936
Graphiphora augur|[186]|LBCH114-10|Canada|British Columbia|658[0n]|BOLD:ACF0936
Graphiphora augur|[187]|RWWA907-09|United States|Washington|658[0n]|BOLD:ACF0936
Graphiphora augur|[188]|RWWA604-09|United States|Washington|658[0n]|BOLD:ACF0936
Graphiphora augur|[189]|RWWA507-09|United States|Washington|658[0n]|BOLD:ACF0936
Graphiphora augur|[190]|RWWA349-09|United States|Washington|658[0n]|BOLD:ACF0936
Graphiphora augur|[191]|LBCG2312-09|Canada|British Columbia|658[0n]|BOLD:ACF0936
Graphiphora augur|[192]|LBCG2311-09|Canada|British Columbia|658[0n]|BOLD:ACF0936
Graphiphora augur|[193]|LBCG2310-09|Canada|British Columbia|658[0n]|BOLD:ACF0936
Graphiphora augur|[194]|LBCG2305-09|Canada|British Columbia|658[0n]|BOLD:ACF0936
Graphiphora augur|[195]|LBCG1878-09|Canada|British Columbia|658[0n]|BOLD:ACF0936
Graphiphora augur|[196]|LPABC438-09|Canada|Alberta|658[0n]|BOLD:ACF0936
Graphiphora augur|[197]|LPABC425-09|Canada|Alberta|658[0n]|BOLD:ACF0936
Graphiphora augur|[198]|LPABC403-09|Canada|Alberta|658[0n]|BOLD:ACF0936
Graphiphora augur|[199]|LPABC381-09|Canada|Alberta|658[0n]|BOLD:ACF0936
Graphiphora augur|[200]|LPABC351-09|Canada|Alberta|658[0n]|BOLD:ACF0936

[illegible]
Graphiphora augur|[198]|LPABC403-09|Canada|Alberta|658[0n]|BOLD:ACF0936
Graphiphora augur|[199]|LPABC381-09|Canada|Alberta|658[0n]|BOLD:ACF0936
Graphiphora augur|[200]|LPABC351-09|Canada|Alberta|658[0n]|BOLD:ACF0936
Graphiphora augur|[201]|LPABC334-09|Canada|Alberta|658[0n]|BOLD:ACF0936
Graphiphora augur|[202]|LPABC272-09|Canada|Alberta|658[0n]|BOLD:ACF0936
Graphiphora augur|[203]|LBCG766-09|Canada|British Columbia|658[0n]|BOLD:ACF0936
Graphiphora augur|[204]|LPABB603-08|Canada|Alberta|658[0n]|BOLD:ACF0936
Graphiphora augur|[205]|LOWCE784-06|Canada|British Columbia|658[0n]|BOLD:ACF0936
Graphiphora augur|[206]|LOWCD890-06|Canada|British Columbia|658[0n]|BOLD:ACF0936
Graphiphora augur|[207]|LOWCC852-05|Canada|British Columbia|658[0n]|BOLD:ACF0936
Graphiphora augur|[208]|LOWCC187-05|Canada|British Columbia|658[0n]|BOLD:ACF0936
Graphiphora augur|[209]|LOWCC186-05|Canada|British Columbia|658[0n]|BOLD:ACF0936
Graphiphora augur|[210]|LOWCC185-05|Canada|British Columbia|658[0n]|BOLD:ACF0936
Graphiphora augur|[211]|LOWCC184-05|Canada|British Columbia|658[0n]|BOLD:ACF0936
Graphiphora augur|[212]|LOWC840-05|Canada|British Columbia|658[0n]|BOLD:ACF0936
Graphiphora augur|[213]|LOWC839-05|Canada|British Columbia|658[0n]|BOLD:ACF0936
Graphiphora augur|[214]|LOWC838-05|Canada|British Columbia|658[0n]|BOLD:ACF0936
Graphiphora augur|[215]|LOWC837-05|Canada|British Columbia|658[0n]|BOLD:ACF0936
Graphiphora augur|[216]|LOWC836-05|Canada|British Columbia|658[0n]|BOLD:ACF0936
Graphiphora augur|[217]|LOWC835-05|Canada|British Columbia|658[0n]|BOLD:ACF0936
Graphiphora augur|[218]|LOWC834-05|Canada|British Columbia|658[0n]|BOLD:ACF0936
Graphiphora augur|[219]|RDNMB901-05|Canada|British Columbia|658[0n]|BOLD:ACF0936
Graphiphora augur|[220]|RDNMB897-05|United States|Colorado|658[0n]|BOLD:ACF0936
Graphiphora augur|[221]|RDNMB895-05|United States|Colorado|658[0n]|BOLD:ACF0936
Graphiphora augur|[222]|LBCD425-05|Canada|British Columbia|658[0n]|BOLD:ACF0936
Graphiphora augur|[223]|LBCD316-05|Canada|British Columbia|658[0n]|BOLD:ACF0936
Graphiphora augur|[224]|LBCD043-05|Canada|British Columbia|658[0n]|BOLD:ACF0936
Graphiphora augur|[225]|LBCD042-05|Canada|British Columbia|658[0n]|BOLD:ACF0936
Graphiphora augur|[226]|LBCC834-05|Canada|British Columbia|658[0n]|BOLD:ACF0936
Graphiphora augur|[227]|RDNM845-05|Canada|British Columbia|658[0n]|BOLD:ACF0936
Graphiphora augur|[228]|LOWCC188-05|Canada|British Columbia|658[0n]|BOLD:ACF0936
Graphiphora augur|[229]|LOWCD889-06|Canada|British Columbia|658[0n]|BOLD:ACF0936
Graphiphora augur|[230]|LBCH4687-10|Canada|British Columbia|658[0n]|BOLD:ACF0936
Graphiphora augur|[231]|LBCH4688-10|Canada|British Columbia|658[0n]|BOLD:ACF0936
Graphiphora augur|[232]|LBCH4689-10|Canada|British Columbia|658[0n]|BOLD:ACF0936
Graphiphora augur|[233]|RWWB940-10|United States|Washington|658[0n]|BOLD:ACF0936
Graphiphora augur|[234]|LALPA587-10|Canada|British Columbia|658[0n]|BOLD:ACF0936
Graphiphora augur|[235]|RDNMB900-05|Canada|British Columbia|658[2n]|BOLD:ACF0936
Graphiphora augur|[236]|RWWA559-09|United States|Washington|616[0n]|BOLD:ACF0936
Graphiphora augur|[237]|CNWLF2034-12|Canada|Alberta|615[0n]|BOLD:ACF0936
Graphiphora augur|[238]|LOWCD805-06|Canada|British Columbia|595[0n]|BOLD:ACF0936
Graphiphora augur|[239]|LBCD041-05|Canada|British Columbia|658[0n]|BOLD:ACF0936
Graphiphora augur|[240]|LBCH925-10|Canada|British Columbia|658[0n]|BOLD:ACF0936
Graphiphora augur|[241]|LALPA1190-11|Canada|British Columbia|658[0n]|BOLD:ACF0936
Graphiphora augur|[242]|LPABC412-09|Canada|Alberta|658[0n]|BOLD:ACF0936
Graphiphora augur|[243]|ABKWR100-07|United States|Alaska|658[0n]|BOLD:ACF0936
Graphiphora augur|[244]|ABKWR086-07|United States|Alaska|658[0n]|BOLD:ACF0936
Graphiphora augur|[245]|LOWCD892-06|Canada|British Columbia|658[0n]|BOLD:ACF0936
Graphiphora augur|[246]|RDMAB469-05|Canada|Yukon Territory|658[0n]|BOLD:ACF0936
Graphiphora augur|[247]|BBLPB706-10|Canada|British Columbia|658[0n]|BOLD:ACF0936
Graphiphora augur|[248]|LBCH2027-10|Canada|British Columbia|658[0n]|BOLD:ACF0936
Graphiphora augur|[249]|LOWC832-05|Canada|British Columbia|658[0n]|BOLD:ACF0936
Graphiphora augur|[250]|LBCD101-05|Canada|British Columbia|658[0n]|BOLD:ACF0936
Graphiphora augur|[251]|CNWLE2501-12|Canada|Alberta|637[0n]|BOLD:ACF0936
Graphiphora augur|[252]|LPABC264-09|Canada|Alberta|631[0n]|BOLD:ACF0936
Graphiphora augur|[253]|LALPA652-10|Canada|British Columbia|636[0n]|BOLD:ACF0936
Graphiphora augur|[254]|CNWLM031-13|Canada|Alberta|564[0n]|BOLD:ACF0936
Graphiphora augur|[255]|LOWC833-05|Canada|British Columbia|658[0n]|BOLD:ACF0936
Graphiphora augur|[256]|RDNMB898-05|United States|Washington|658[0n]|BOLD:ACF0936
Graphiphora augur|[257]|LBCH4696-10|Canada|British Columbia|643[0n]|BOLD:ACF0936
Graphiphora augur|[258]|CNWLE2408-12|Canada|Alberta|632[0n]|BOLD:ACF0936
Graphiphora augur|[259]|UAMIC516-13|United States|Alaska|635[0n]|BOLD:ACF0936
Graphiphora augur|[260]|LOWCD888-06|Canada|British Columbia|610[0n]|BOLD:ACF0936
Graphiphora augur|[261]|RDNMB899-05|United States|Washington|566[0n]|BOLD:ACF0936
Graphiphora augur|[262]|UAMIC517-13|United States|Alaska|647[0n]|BOLD:ACF0936
Graphiphora augur|[263]|CNWLM040-13|Canada|Alberta|561[0n]|BOLD:ACF0936
Graphiphora augur|[264]|CNWLM2435-13|Canada|Alberta|613[0n]|BOLD:ACF0936
Graphiphora augur|[265]|LPABC270-09|Canada|Alberta|613[0n]|BOLD:ACF0936
Graphiphora augur|[266]|LBCG2306-09|Canada|British Columbia|658[0n]|BOLD:ACF0936
Graphiphora augur|[267]|CNWLE2407-12|Canada|Alberta|601[0n]|BOLD:ACF0936
Graphiphora augur|[268]|LBCH3468-10|Canada|British Columbia|641[0n]|BOLD:ACF0936
Graphiphora augur|[269]|LOWCD138-06|Canada|British Columbia|580[0n]|BOLD:ACF0936
Graphiphora augur|[270]|CNWLE2504-12|Canada|Alberta|634[0n]|BOLD:ACF0936
Graphiphora augur|[271]|RWWA763-09|United States|Washington|631[0n]|BOLD:ACF0936
Graphiphora augur|[272]|CNWLN1038-13|Canada|Alberta|611[0n]|BOLD:ACF0936
Graphiphora augur|[273]|CNWLM2409-13|Canada|Alberta|612[0n]|BOLD:ACF0936
Graphiphora augur|[274]|CNWLM035-13|Canada|Alberta|588[0n]|BOLD:ACF0936
Graphiphora augur|[275]|LOWCC892-05|Canada|British Columbia|586[0n]|BOLD:ACF0936
Graphiphora augur|[276]|LBCH016-10|Canada|British Columbia|643[0n]|BOLD:ACF0936
Graphiphora augur|[277]|LOWCD893-06|Canada|British Columbia|618[0n]|BOLD:ACF0936
Graphiphora augur|[278]|LBCH358-10|Canada|British Columbia|552[0n]|BOLD:ACF0936
Graphiphora augur|[279]|LOWCD804-06|Canada|British Columbia|596[2n]|BOLD:ACF0936
Graphiphora augur|[280]|RDNM846-05|Canada|British Columbia|594[1n]|BOLD:ACF0936
Graphiphora augur|[281]|CNWLM022-13|Canada|Alberta|555[2n]|BOLD:ACF0936
Graphiphora augur|[282]|LOWCD891-06|Canada|British Columbia|606[1n]|BOLD:ACF0936
Graphiphora augur|[283]|CNWLM032-13|Canada|Alberta|546[1n]|BOLD:ACF0936
Graphiphora augur|[284]|CNWLN1043-13|Canada|Alberta|600[0n]|BOLD:ACF0936
Graphiphora augur|[285]|CNWLN1060-13|Canada|Alberta|614[0n]|BOLD:ACF0936
Choephora fungorum|[286]|NAMUM317-08|United States|Georgia|658[0n]|BOLD:AAC7430
Choephora fungorum|[287]|NAMUM318-08|United States|Georgia|658[0n]|BOLD:AAC7430
Choephora fungorum|[288]|RDNMG1032-08|United States|Maryland|658[0n]|BOLD:AAC7430
Choephora fungorum|[289]|LNC479-06|United States|North Carolina|658[0n]|BOLD:AAC7430
Choephora fungorum|[290]|LNC478-06|United States|North Carolina|658[0n]|BOLD:AAC7430
Choephora fungorum|[291]|LPOKA371-08|United States|Oklahoma|658[0n]|BOLD:AAC7430
Choephora fungorum|[292]|LPOKA375-08|United States|Oklahoma|658[0n]|BOLD:AAC7430
Choephora fungorum|[293]|LPOKA610-09|United States|Oklahoma|639[0n]|BOLD:AAC7430
Choephora fungorum|[294]|LPOKA624-09|United States|Oklahoma|658[0n]|BOLD:AAC7430
Choephora fungorum|[295]|LPOKD450-09|United States|Oklahoma|658[0n]|BOLD:AAC7430
Hemipachnobia monochromatea|[296]|TMNBB390-06|Canada|New Brunswick|658[0n]|BOLD:AAE5284
Hemipachnobia monochromatea|[297]|TMNBB391-06|Canada|New Brunswick|658[0n]|BOLD:AAE5284
Hemipachnobia monochromatea|[298]|RDLQF460-06|Canada|Quebec|658[0n]|BOLD:AAE5284
Hemipachnobia monochromatea|[299]|RDLQG138-06|Canada|Quebec|640[2n]|BOLD:AAE5284
[illegible]

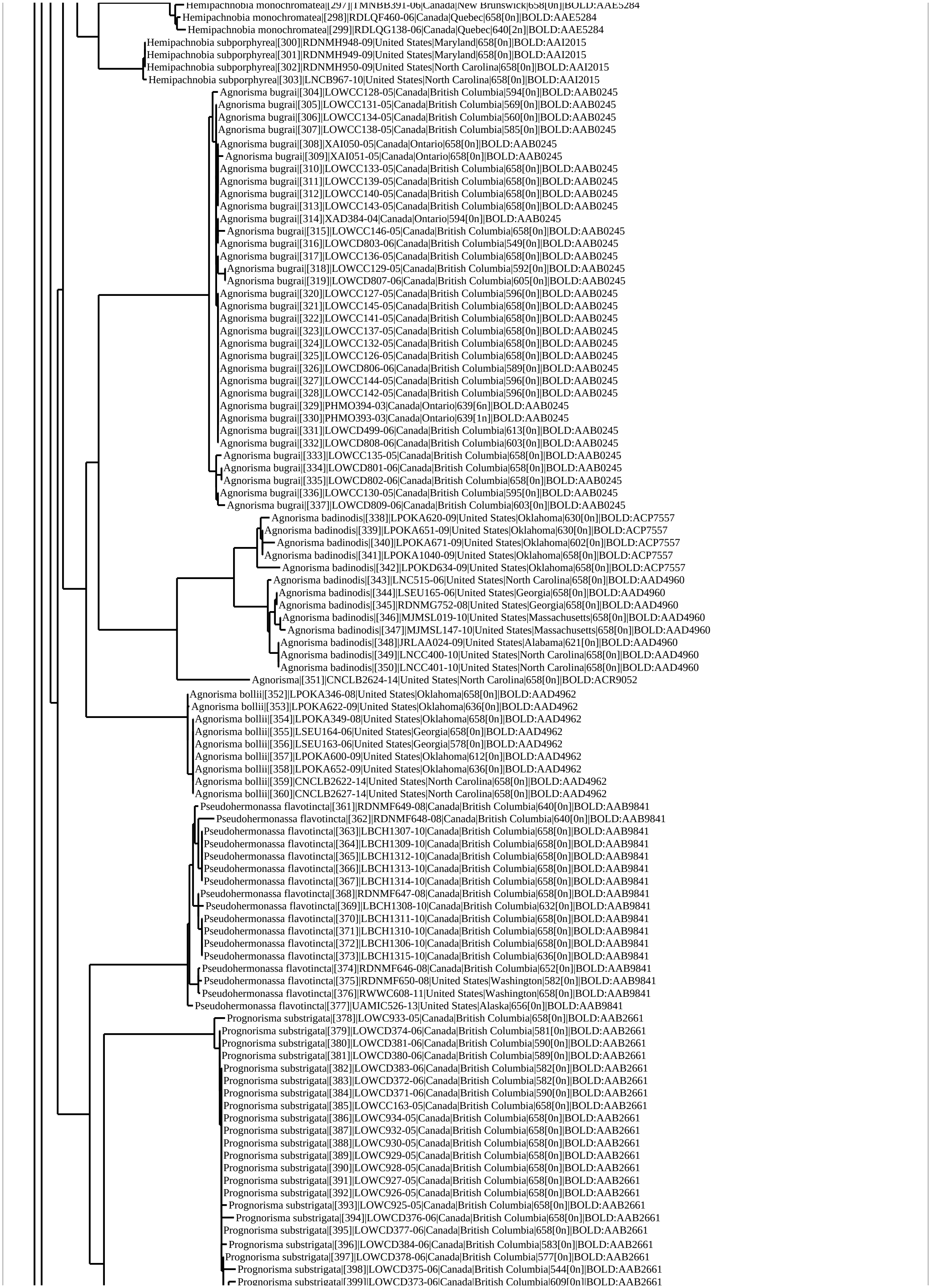

Hemipachnobia monochromatea|[297]|TMNBB391-06|Canada|New Brunswick|658[0n]|BOLD:AAE5284
Hemipachnobia monochromatea|[298]|RDLQF460-06|Canada|Quebec|658[0n]|BOLD:AAE5284
Hemipachnobia monochromatea|[299]|RDLQG138-06|Canada|Quebec|640[2n]|BOLD:AAE5284
Hemipachnobia subporphyrea|[300]|RDNMH948-09|United States|Maryland|658[0n]|BOLD:AAI2015
Hemipachnobia subporphyrea|[301]|RDNMH949-09|United States|Maryland|658[0n]|BOLD:AAI2015
Hemipachnobia subporphyrea|[302]|RDNMH950-09|United States|North Carolina|658[0n]|BOLD:AAI2015
Hemipachnobia subporphyrea|[303]|LNCB967-10|United States|North Carolina|658[0n]|BOLD:AAI2015
Agnorisma bugrai|[304]|LOWCC128-05|Canada|British Columbia|594[0n]|BOLD:AAB0245
Agnorisma bugrai|[305]|LOWCC131-05|Canada|British Columbia|569[0n]|BOLD:AAB0245
Agnorisma bugrai|[306]|LOWCC134-05|Canada|British Columbia|560[0n]|BOLD:AAB0245
Agnorisma bugrai|[307]|LOWCC138-05|Canada|British Columbia|585[0n]|BOLD:AAB0245
Agnorisma bugrai|[308]|XAI050-05|Canada|Ontario|658[0n]|BOLD:AAB0245
Agnorisma bugrai|[309]|XAI051-05|Canada|Ontario|658[0n]|BOLD:AAB0245
Agnorisma bugrai|[310]|LOWCC133-05|Canada|British Columbia|658[0n]|BOLD:AAB0245
Agnorisma bugrai|[311]|LOWCC139-05|Canada|British Columbia|658[0n]|BOLD:AAB0245
Agnorisma bugrai|[312]|LOWCC140-05|Canada|British Columbia|658[0n]|BOLD:AAB0245
Agnorisma bugrai|[313]|LOWCC143-05|Canada|British Columbia|658[0n]|BOLD:AAB0245
Agnorisma bugrai|[314]|XAD384-04|Canada|Ontario|594[0n]|BOLD:AAB0245
Agnorisma bugrai|[315]|LOWCC146-05|Canada|British Columbia|658[0n]|BOLD:AAB0245
Agnorisma bugrai|[316]|LOWCD803-06|Canada|British Columbia|549[0n]|BOLD:AAB0245
Agnorisma bugrai|[317]|LOWCC136-05|Canada|British Columbia|658[0n]|BOLD:AAB0245
Agnorisma bugrai|[318]|LOWCC129-05|Canada|British Columbia|592[0n]|BOLD:AAB0245
Agnorisma bugrai|[319]|LOWCD807-06|Canada|British Columbia|605[0n]|BOLD:AAB0245
Agnorisma bugrai|[320]|LOWCC127-05|Canada|British Columbia|596[0n]|BOLD:AAB0245
Agnorisma bugrai|[321]|LOWCC145-05|Canada|British Columbia|658[0n]|BOLD:AAB0245
Agnorisma bugrai|[322]|LOWCC141-05|Canada|British Columbia|658[0n]|BOLD:AAB0245
Agnorisma bugrai|[323]|LOWCC137-05|Canada|British Columbia|658[0n]|BOLD:AAB0245
Agnorisma bugrai|[324]|LOWCC132-05|Canada|British Columbia|658[0n]|BOLD:AAB0245
Agnorisma bugrai|[325]|LOWCC126-05|Canada|British Columbia|658[0n]|BOLD:AAB0245
Agnorisma bugrai|[326]|LOWCD806-06|Canada|British Columbia|589[0n]|BOLD:AAB0245
Agnorisma bugrai|[327]|LOWCC144-05|Canada|British Columbia|596[0n]|BOLD:AAB0245
Agnorisma bugrai|[328]|LOWCC142-05|Canada|British Columbia|596[0n]|BOLD:AAB0245
Agnorisma bugrai|[329]|PHMO394-03|Canada|Ontario|639[6n]|BOLD:AAB0245
Agnorisma bugrai|[330]|PHMO393-03|Canada|Ontario|639[1n]|BOLD:AAB0245
Agnorisma bugrai|[331]|LOWCD499-06|Canada|British Columbia|613[0n]|BOLD:AAB0245
Agnorisma bugrai|[332]|LOWCD808-06|Canada|British Columbia|603[0n]|BOLD:AAB0245
Agnorisma bugrai|[333]|LOWCC135-05|Canada|British Columbia|658[0n]|BOLD:AAB0245
Agnorisma bugrai|[334]|LOWCD801-06|Canada|British Columbia|658[0n]|BOLD:AAB0245
Agnorisma bugrai|[335]|LOWCD802-06|Canada|British Columbia|658[0n]|BOLD:AAB0245
Agnorisma bugrai|[336]|LOWCC130-05|Canada|British Columbia|595[0n]|BOLD:AAB0245
Agnorisma bugrai|[337]|LOWCD809-06|Canada|British Columbia|603[0n]|BOLD:AAB0245
Agnorisma badinodis|[338]|LPOKA620-09|United States|Oklahoma|630[0n]|BOLD:ACP7557
Agnorisma badinodis|[339]|LPOKA651-09|United States|Oklahoma|630[0n]|BOLD:ACP7557
Agnorisma badinodis|[340]|LPOKA671-09|United States|Oklahoma|602[0n]|BOLD:ACP7557
Agnorisma badinodis|[341]|LPOKA1040-09|United States|Oklahoma|658[0n]|BOLD:ACP7557
Agnorisma badinodis|[342]|LPOKD634-09|United States|Oklahoma|658[0n]|BOLD:ACP7557
Agnorisma badinodis|[343]|LNC515-06|United States|North Carolina|658[0n]|BOLD:AAD4960
Agnorisma badinodis|[344]|LSEU165-06|United States|Georgia|658[0n]|BOLD:AAD4960
Agnorisma badinodis|[345]|RDNMG752-08|United States|Georgia|658[0n]|BOLD:AAD4960
Agnorisma badinodis|[346]|MJMSL019-10|United States|Massachusetts|658[0n]|BOLD:AAD4960
Agnorisma badinodis|[347]|MJMSL147-10|United States|Massachusetts|658[0n]|BOLD:AAD4960
Agnorisma badinodis|[348]|JRLAA024-09|United States|Alabama|621[0n]|BOLD:AAD4960
Agnorisma badinodis|[349]|LNCC400-10|United States|North Carolina|658[0n]|BOLD:AAD4960
Agnorisma badinodis|[350]|LNCC401-10|United States|North Carolina|658[0n]|BOLD:AAD4960
Agnorisma|[351]|CNCLB2624-14|United States|North Carolina|658[0n]|BOLD:ACR9052
Agnorisma bollii|[352]|LPOKA346-08|United States|Oklahoma|658[0n]|BOLD:AAD4962
Agnorisma bollii|[353]|LPOKA622-09|United States|Oklahoma|636[0n]|BOLD:AAD4962
Agnorisma bollii|[354]|LPOKA349-08|United States|Oklahoma|658[0n]|BOLD:AAD4962
Agnorisma bollii|[355]|LSEU164-06|United States|Georgia|658[0n]|BOLD:AAD4962
Agnorisma bollii|[356]|LSEU163-06|United States|Georgia|578[0n]|BOLD:AAD4962
Agnorisma bollii|[357]|LPOKA600-09|United States|Oklahoma|612[0n]|BOLD:AAD4962
Agnorisma bollii|[358]|LPOKA652-09|United States|Oklahoma|636[0n]|BOLD:AAD4962
Agnorisma bollii|[359]|CNCLB2622-14|United States|North Carolina|658[0n]|BOLD:AAD4962
Agnorisma bollii|[360]|CNCLB2627-14|United States|North Carolina|658[0n]|BOLD:AAD4962
Pseudohermonassa flavotincta|[361]|RDNMF649-08|Canada|British Columbia|640[0n]|BOLD:AAB9841
Pseudohermonassa flavotincta|[362]|RDNMF648-08|Canada|British Columbia|640[0n]|BOLD:AAB9841
Pseudohermonassa flavotincta|[363]|LBCH1307-10|Canada|British Columbia|658[0n]|BOLD:AAB9841
Pseudohermonassa flavotincta|[364]|LBCH1309-10|Canada|British Columbia|658[0n]|BOLD:AAB9841
Pseudohermonassa flavotincta|[365]|LBCH1312-10|Canada|British Columbia|658[0n]|BOLD:AAB9841
Pseudohermonassa flavotincta|[366]|LBCH1313-10|Canada|British Columbia|658[0n]|BOLD:AAB9841
Pseudohermonassa flavotincta|[367]|LBCH1314-10|Canada|British Columbia|658[0n]|BOLD:AAB9841
Pseudohermonassa flavotincta|[368]|RDNMF647-08|Canada|British Columbia|658[0n]|BOLD:AAB9841
Pseudohermonassa flavotincta|[369]|LBCH1308-10|Canada|British Columbia|632[0n]|BOLD:AAB9841
Pseudohermonassa flavotincta|[370]|LBCH1311-10|Canada|British Columbia|658[0n]|BOLD:AAB9841
Pseudohermonassa flavotincta|[371]|LBCH1310-10|Canada|British Columbia|658[0n]|BOLD:AAB9841
Pseudohermonassa flavotincta|[372]|LBCH1306-10|Canada|British Columbia|658[0n]|BOLD:AAB9841
Pseudohermonassa flavotincta|[373]|LBCH1315-10|Canada|British Columbia|636[0n]|BOLD:AAB9841
Pseudohermonassa flavotincta|[374]|RDNMF646-08|Canada|British Columbia|652[0n]|BOLD:AAB9841
Pseudohermonassa flavotincta|[375]|RDNMF650-08|United States|Washington|582[0n]|BOLD:AAB9841
Pseudohermonassa flavotincta|[376]|RWWC608-11|United States|Washington|658[0n]|BOLD:AAB9841
Pseudohermonassa flavotincta|[377]|UAMIC526-13|United States|Alaska|656[0n]|BOLD:AAB9841
Prognorisma substrigata|[378]|LOWC933-05|Canada|British Columbia|658[0n]|BOLD:AAB2661
Prognorisma substrigata|[379]|LOWCD374-06|Canada|British Columbia|581[0n]|BOLD:AAB2661
Prognorisma substrigata|[380]|LOWCD381-06|Canada|British Columbia|590[0n]|BOLD:AAB2661
Prognorisma substrigata|[381]|LOWCD380-06|Canada|British Columbia|589[0n]|BOLD:AAB2661
Prognorisma substrigata|[382]|LOWCD383-06|Canada|British Columbia|582[0n]|BOLD:AAB2661
Prognorisma substrigata|[383]|LOWCD372-06|Canada|British Columbia|582[0n]|BOLD:AAB2661
Prognorisma substrigata|[384]|LOWCD371-06|Canada|British Columbia|590[0n]|BOLD:AAB2661
Prognorisma substrigata|[385]|LOWCC163-05|Canada|British Columbia|658[0n]|BOLD:AAB2661
Prognorisma substrigata|[386]|LOWC934-05|Canada|British Columbia|658[0n]|BOLD:AAB2661
Prognorisma substrigata|[387]|LOWC932-05|Canada|British Columbia|658[0n]|BOLD:AAB2661
Prognorisma substrigata|[388]|LOWC930-05|Canada|British Columbia|658[0n]|BOLD:AAB2661
Prognorisma substrigata|[389]|LOWC929-05|Canada|British Columbia|658[0n]|BOLD:AAB2661
Prognorisma substrigata|[390]|LOWC928-05|Canada|British Columbia|658[0n]|BOLD:AAB2661
Prognorisma substrigata|[391]|LOWC927-05|Canada|British Columbia|658[0n]|BOLD:AAB2661
Prognorisma substrigata|[392]|LOWC926-05|Canada|British Columbia|658[0n]|BOLD:AAB2661
Prognorisma substrigata|[393]|LOWC925-05|Canada|British Columbia|658[0n]|BOLD:AAB2661
Prognorisma substrigata|[394]|LOWCD376-06|Canada|British Columbia|658[0n]|BOLD:AAB2661
Prognorisma substrigata|[395]|LOWCD377-06|Canada|British Columbia|658[0n]|BOLD:AAB2661
Prognorisma substrigata|[396]|LOWCD384-06|Canada|British Columbia|583[0n]|BOLD:AAB2661
Prognorisma substrigata|[397]|LOWCD378-06|Canada|British Columbia|577[0n]|BOLD:AAB2661
Prognorisma substrigata|[398]|LOWCD375-06|Canada|British Columbia|544[0n]|BOLD:AAB2661
Prognorisma substrigata|[399]|LOWCD373-06|Canada|British Columbia|609[0n]|BOLD:AAB2661

Prognorisma substrigata|[397]|LOWCD378-06|Canada|British Columbia|577[0n]|BOLD:AAB2661
Prognorisma substrigata|[398]|LOWCD375-06|Canada|British Columbia|544[0n]|BOLD:AAB2661
Prognorisma substrigata|[399]|LOWCD373-06|Canada|British Columbia|609[0n]|BOLD:AAB2661
Prognorisma substrigata|[400]|LOWCD382-06|Canada|British Columbia|610[0n]|BOLD:AAB2661
Prognorisma substrigata|[401]|LOWCD379-06|Canada|British Columbia|546[0n]|BOLD:AAB2661
Prognorisma substrigata|[402]|LOWCD370-06|Canada|British Columbia|610[0n]|BOLD:AAB2661
Prognorisma substrigata|[403]|LOWC931-05|Canada|British Columbia|511[0n]|BOLD:AAB2661
Prognorisma substrigata|[404]|LOWCD385-06|Canada|British Columbia|551[0n]|BOLD:AAB2661
Pseudohermonassa ononensis|[405]|CNCLB977-14|United States|Colorado|658[0n]|BOLD:ACM3894
Pseudohermonassa ononensis|[406]|CNCLB1594-14|Russia||580[0n]|BOLD:ACM3894
Pseudohermonassa ononensis|[407]|CNCLB1593-14|Russia||658[0n]|BOLD:ACM3894
Pseudohermonassa ononensis|[408]|CNCLB1595-14|Russia||585[0n]|BOLD:ACM3894
Pseudohermonassa tenuicula|[409]|TTMNB451-06|Canada|New Brunswick|658[0n]|BOLD:ACE5721
Pseudohermonassa tenuicula|[410]|BBLPC418-09|Canada|New Brunswick|658[0n]|BOLD:ACE5721
Pseudohermonassa tenuicula|[411]|XAD383-04|Canada|Ontario|658[0n]|BOLD:ACE5721
Pseudohermonassa tenuicula|[412]|MNBB482-05|Canada|New Brunswick|658[0n]|BOLD:ACE5721
Pseudohermonassa tenuicula|[413]|MNBB597-05|Canada|New Brunswick|658[0n]|BOLD:ACE5721
Pseudohermonassa tenuicula|[414]|MNBB598-05|Canada|New Brunswick|658[0n]|BOLD:ACE5721
Pseudohermonassa tenuicula|[415]|MNBB667-05|Canada|New Brunswick|658[0n]|BOLD:ACE5721
Pseudohermonassa tenuicula|[416]|RDLQB766-05|Canada|Quebec|658[0n]|BOLD:ACE5721
Pseudohermonassa tenuicula|[417]|RDLQ729-07|Canada|Quebec|658[0n]|BOLD:ACE5721
Pseudohermonassa tenuicula|[418]|RDLQ730-07|Canada|Quebec|658[0n]|BOLD:ACE5721
Pseudohermonassa tenuicula|[419]|BBLPC353-09|Canada|New Brunswick|658[0n]|BOLD:ACE5721
Pseudohermonassa tenuicula|[420]|BBLPC369-09|Canada|New Brunswick|658[0n]|BOLD:ACE5721
Pseudohermonassa tenuicula|[421]|BBLPC390-09|Canada|New Brunswick|658[0n]|BOLD:ACE5721
Pseudohermonassa tenuicula|[422]|BBLPC413-09|Canada|New Brunswick|658[0n]|BOLD:ACE5721
Pseudohermonassa tenuicula|[423]|BBLPC417-09|Canada|New Brunswick|658[0n]|BOLD:ACE5721
Pseudohermonassa tenuicula|[424]|XAD246-04|Canada|Ontario|658[1n]|BOLD:ACE5721
Pseudohermonassa tenuicula|[425]|LOWCD369-06|Canada|British Columbia|586[0n]|BOLD:ACE5721
Pseudohermonassa tenuicula|[426]|LOWCD500-06|Canada|British Columbia|587[0n]|BOLD:ACE5721
Pseudohermonassa tenuicula|[427]|LPMNB232-09|Canada|Manitoba|658[0n]|BOLD:ACE5721
Pseudohermonassa tenuicula|[428]|BBLPB860-10|Canada|Alberta|658[0n]|BOLD:ACE5721
Pseudohermonassa tenuicula|[429]|BBLPB861-10|Canada|Alberta|658[0n]|BOLD:ACE5721
Pseudohermonassa bicarnea|[430]|LSEU173-06|United States|North Carolina|658[0n]|BOLD:AAB1024
Pseudohermonassa bicarnea|[431]|BBLEC429-09|Canada|New Brunswick|658[0n]|BOLD:AAB1024
Pseudohermonassa bicarnea|[432]|BBLPC023-09|Canada|New Brunswick|657[0n]|BOLD:AAB1024
Pseudohermonassa bicarnea|[433]|XAI052-05|Canada|Ontario|658[0n]|BOLD:AAB1024
Pseudohermonassa bicarnea|[434]|XAH220-05|Canada|Ontario|658[0n]|BOLD:AAB1024
Pseudohermonassa bicarnea|[435]|XAG878-05|Canada|Ontario|658[0n]|BOLD:AAB1024
Pseudohermonassa bicarnea|[436]|XAG709-05|Canada|Ontario|658[0n]|BOLD:AAB1024
Pseudohermonassa bicarnea|[437]|XAD243-04|Canada|Ontario|658[0n]|BOLD:AAB1024
Pseudohermonassa bicarnea|[438]|XAD171-04|Canada|Ontario|658[0n]|BOLD:AAB1024
Pseudohermonassa bicarnea|[439]|XAD181-04|Canada|Ontario|593[0n]|BOLD:AAB1024
Pseudohermonassa bicarnea|[440]|XAK421-06|Canada|Ontario|658[0n]|BOLD:AAB1024
Pseudohermonassa bicarnea|[441]|LMDH188-11|United States|Minnesota|658[0n]|BOLD:AAB1024
Pseudohermonassa bicarnea|[442]|PHAUG1796-11|Canada|Ontario|658[0n]|BOLD:AAB1024
Pseudohermonassa bicarnea|[443]|LGSMG910-10|United States|North Carolina|658[0n]|BOLD:AAB1024
Pseudohermonassa bicarnea|[444]|MNBB596-05|Canada|New Brunswick|658[0n]|BOLD:AAB1024
Pseudohermonassa bicarnea|[445]|LNCNW004-06|United States|North Carolina|575[0n]|BOLD:AAB1024
Pseudohermonassa bicarnea|[446]|RDLQF812-06|Canada|Quebec|658[0n]|BOLD:AAB1024
Pseudohermonassa bicarnea|[447]|LGSMG907-10|United States|North Carolina|658[0n]|BOLD:AAB1024
Pseudohermonassa bicarnea|[448]|LGSMG908-10|United States|North Carolina|658[0n]|BOLD:AAB1024
Pseudohermonassa bicarnea|[449]|LGSMG909-10|United States|North Carolina|658[0n]|BOLD:AAB1024
Pseudohermonassa bicarnea|[450]|LSEU172-06|United States|North Carolina|658[0n]|BOLD:AAB1024
Pseudohermonassa bicarnea|[451]|LSEU171-06|United States|North Carolina|658[0n]|BOLD:AAB1024
Pseudohermonassa bicarnea|[452]|LSEU170-06|United States|North Carolina|658[0n]|BOLD:AAB1024
Pseudohermonassa bicarnea|[453]|RDLQB843-05|Canada|Quebec|658[0n]|BOLD:AAB1024
Pseudohermonassa bicarnea|[454]|MNBB595-05|Canada|New Brunswick|658[0n]|BOLD:AAB1024
Pseudohermonassa bicarnea|[455]|MNBB594-05|Canada|New Brunswick|658[0n]|BOLD:AAB1024
Pseudohermonassa bicarnea|[456]|MNBB592-05|Canada|New Brunswick|658[0n]|BOLD:AAB1024
Pseudohermonassa bicarnea|[457]|MNBB591-05|Canada|New Brunswick|658[0n]|BOLD:AAB1024
Pseudohermonassa bicarnea|[458]|MNBB478-05|Canada|New Brunswick|658[0n]|BOLD:AAB1024
Pseudohermonassa bicarnea|[459]|MNBB426-05|Canada|New Brunswick|658[0n]|BOLD:AAB1024
Pseudohermonassa bicarnea|[460]|XAD341-04|Canada|Ontario|658[0n]|BOLD:AAB1024
Pseudohermonassa bicarnea|[461]|XAD176-04|Canada|Ontario|658[0n]|BOLD:AAB1024
Pseudohermonassa bicarnea|[462]|TTMNB452-06|Canada|New Brunswick|658[0n]|BOLD:AAB1024
Pseudohermonassa bicarnea|[463]|TTMNB450-06|Canada|New Brunswick|658[0n]|BOLD:AAB1024
Pseudohermonassa bicarnea|[464]|LNCNW005-06|United States|North Carolina|658[0n]|BOLD:AAB1024
Pseudohermonassa bicarnea|[465]|LNCNW036-06|United States|North Carolina|658[0n]|BOLD:AAB1024
Pseudohermonassa bicarnea|[466]|XAK420-06|Canada|Ontario|658[0n]|BOLD:AAB1024
Pseudohermonassa bicarnea|[467]|LGSMG911-10|United States|North Carolina|658[0n]|BOLD:AAB1024
Pseudohermonassa bicarnea|[468]|CNCLB2967-14|United States|North Carolina|658[0n]|BOLD:AAB1024
Rhyacia clemens|[469]|RDNMH568-09|United States|Oregon|658[0n]|BOLD:AAA4280
Rhyacia clemens|[470]|RDNMG576-08|United States|California|658[0n]|BOLD:AAA4280
Rhyacia clemens|[471]|RDNMG578-08|United States|California|652[6n]|BOLD:AAA4280
Rhyacia clemens|[472]|RDNMH566-09|United States|California|636[0n]|BOLD:AAA4280
Rhyacia quadrangula|[473]|LCHP960-07|Canada|Manitoba|658[0n]|BOLD:AAA4280
Rhyacia quadrangula|[474]|LCHP951-07|Canada|Manitoba|658[0n]|BOLD:AAA4280
Rhyacia quadrangula|[475]|LCHP585-07|Canada|Manitoba|658[0n]|BOLD:AAA4280
Rhyacia clemens|[476]|RDMAB281-05|Canada|Alberta|658[0n]|BOLD:AAA4280
Rhyacia quadrangula|[477]|LCH223-04|Canada|Manitoba|658[0n]|BOLD:AAA4280
Rhyacia quadrangula|[478]|LCH222-04|Canada|Manitoba|658[0n]|BOLD:AAA4280
Rhyacia quadrangula|[479]|LCHIP023-07|Canada|Manitoba|650[0n]|BOLD:AAA4280
Rhyacia quadrangula|[480]|LCHQ480-08|Canada|Manitoba|656[0n]|BOLD:AAA4280
Rhyacia clemens|[481]|RDNMG575-08|United States|Nevada|658[0n]|BOLD:AAA4280
Rhyacia clemens|[482]|RDNMG577-08|United States|California|658[0n]|BOLD:AAA4280
Rhyacia clemens|[483]|RDNMH565-09|United States|Nevada|658[0n]|BOLD:AAA4280
Rhyacia clemens|[484]|RDNMH567-09|United States|Oregon|658[0n]|BOLD:AAA4280
Rhyacia clemens|[485]|JMMMB385-11|United States|California|658[0n]|BOLD:AAA4280
Rhyacia quadrangula|[486]|LCHP851-07|Canada|Manitoba|630[0n]|BOLD:AAA4280
Rhyacia quadrangula|[487]|LCH225-04|Canada|Manitoba|654[0n]|BOLD:AAA4280
Rhyacia quadrangula|[488]|LCH226-04|Canada|Manitoba|632[2n]|BOLD:AAA4280
Rhyacia quadrangula|[489]|LCHP367-07|Canada|Manitoba|640[0n]|BOLD:AAA4280
Rhyacia quadrangula|[490]|LCHQ820-08|Canada|Manitoba|658[0n]|BOLD:AAA4280
Rhyacia quadrangula|[491]|LCHQ819-08|Canada|Manitoba|658[0n]|BOLD:AAA4280
Rhyacia quadrangula|[492]|LCHQ804-08|Canada|Manitoba|658[0n]|BOLD:AAA4280
Rhyacia quadrangula|[493]|LCHQ770-08|Canada|Manitoba|658[0n]|BOLD:AAA4280
Rhyacia quadrangula|[494]|LCHQ751-08|Canada|Manitoba|658[0n]|BOLD:AAA4280
Rhyacia quadrangula|[495]|LCHQ705-08|Canada|Manitoba|657[0n]|BOLD:AAA4280
Rhyacia quadrangula|[496]|LCHQ704-08|Canada|Manitoba|658[0n]|BOLD:AAA4280
Rhyacia quadrangula|[497]|LCHQ670-08|Canada|Manitoba|658[0n]|BOLD:AAA4280
Rhyacia quadrangula|[498]|LCHQ661-08|Canada|Manitoba|658[0n]|BOLD:AAA4280
Rhyacia quadrangula|[499]|LCHQ648-08|Canada|Manitoba|658[0n]|BOLD:AAA4280

Rhyacia quadrangula|[496]|LCHQ704-08|Canada|Manitoba|658[0n]|BOLD:AAA4280
Rhyacia quadrangula|[497]|LCHQ670-08|Canada|Manitoba|658[0n]|BOLD:AAA4280
Rhyacia quadrangula|[498]|LCHQ661-08|Canada|Manitoba|658[0n]|BOLD:AAA4280
Rhyacia quadrangula|[499]|LCHQ648-08|Canada|Manitoba|658[0n]|BOLD:AAA4280
Rhyacia quadrangula|[500]|LCHQ602-08|Canada|Manitoba|658[0n]|BOLD:AAA4280
Rhyacia quadrangula|[501]|LCHQ593-08|Canada|Manitoba|658[0n]|BOLD:AAA4280
Rhyacia quadrangula|[502]|LCHQ572-08|Canada|Manitoba|658[0n]|BOLD:AAA4280
Rhyacia quadrangula|[503]|LCHQ526-08|Canada|Manitoba|658[0n]|BOLD:AAA4280
Rhyacia quadrangula|[504]|LCHQ520-08|Canada|Manitoba|658[0n]|BOLD:AAA4280
Rhyacia quadrangula|[505]|LCHQ503-08|Canada|Manitoba|658[0n]|BOLD:AAA4280
Rhyacia quadrangula|[506]|LCHQ483-08|Canada|Manitoba|657[0n]|BOLD:AAA4280
Rhyacia quadrangula|[507]|LCHQ474-08|Canada|Manitoba|658[0n]|BOLD:AAA4280
Rhyacia quadrangula|[508]|LCHQ432-08|Canada|Manitoba|658[0n]|BOLD:AAA4280
Rhyacia quadrangula|[509]|LCHQ428-08|Canada|Manitoba|658[0n]|BOLD:AAA4280
Rhyacia quadrangula|[510]|LCHQ427-08|Canada|Manitoba|658[0n]|BOLD:AAA4280
Rhyacia quadrangula|[511]|LCHQ395-08|Canada|Manitoba|658[0n]|BOLD:AAA4280
Rhyacia quadrangula|[512]|LCHQ306-08|Canada|Manitoba|658[0n]|BOLD:AAA4280
Rhyacia quadrangula|[513]|LCHQ225-08|Canada|Manitoba|658[0n]|BOLD:AAA4280
Rhyacia quadrangula|[514]|LCHQ186-07|Canada|Manitoba|658[0n]|BOLD:AAA4280
Rhyacia quadrangula|[515]|LCHQ136-07|Canada|Manitoba|658[0n]|BOLD:AAA4280
Rhyacia quadrangula|[516]|LCHQ135-07|Canada|Manitoba|658[0n]|BOLD:AAA4280
Rhyacia quadrangula|[517]|LCHQ122-07|Canada|Manitoba|658[0n]|BOLD:AAA4280
Rhyacia quadrangula|[518]|LCHQ120-07|Canada|Manitoba|658[0n]|BOLD:AAA4280
Rhyacia quadrangula|[519]|LCHQ116-07|Canada|Manitoba|658[0n]|BOLD:AAA4280
Rhyacia quadrangula|[520]|LCHP963-07|Canada|Manitoba|658[0n]|BOLD:AAA4280
Rhyacia quadrangula|[521]|LCHP962-07|Canada|Manitoba|658[0n]|BOLD:AAA4280
Rhyacia quadrangula|[522]|LCHP961-07|Canada|Manitoba|658[0n]|BOLD:AAA4280
Rhyacia quadrangula|[523]|LCHP955-07|Canada|Manitoba|658[0n]|BOLD:AAA4280
Rhyacia quadrangula|[524]|LCHP947-07|Canada|Manitoba|658[0n]|BOLD:AAA4280
Rhyacia quadrangula|[525]|LCHP894-07|Canada|Manitoba|658[0n]|BOLD:AAA4280
Rhyacia quadrangula|[526]|LCHP893-07|Canada|Manitoba|658[0n]|BOLD:AAA4280
Rhyacia quadrangula|[527]|LCHP892-07|Canada|Manitoba|658[0n]|BOLD:AAA4280
Rhyacia quadrangula|[528]|LCHP870-07|Canada|Manitoba|658[0n]|BOLD:AAA4280
Rhyacia quadrangula|[529]|LCHP862-07|Canada|Manitoba|658[0n]|BOLD:AAA4280
Rhyacia quadrangula|[530]|LCHP848-07|Canada|Manitoba|658[0n]|BOLD:AAA4280
Rhyacia quadrangula|[531]|LCHP845-07|Canada|Manitoba|658[0n]|BOLD:AAA4280
Rhyacia quadrangula|[532]|LCHP836-07|Canada|Manitoba|658[0n]|BOLD:AAA4280
Rhyacia quadrangula|[533]|LCHP785-07|Canada|Manitoba|658[0n]|BOLD:AAA4280
Rhyacia quadrangula|[534]|LCHP774-07|Canada|Manitoba|658[0n]|BOLD:AAA4280
Rhyacia quadrangula|[535]|LCHP663-07|Canada|Manitoba|658[0n]|BOLD:AAA4280
Rhyacia quadrangula|[536]|LCHP633-07|Canada|Manitoba|658[0n]|BOLD:AAA4280
Rhyacia quadrangula|[537]|LCHP622-07|Canada|Manitoba|658[0n]|BOLD:AAA4280
Rhyacia quadrangula|[538]|LCHP576-07|Canada|Manitoba|658[0n]|BOLD:AAA4280
Rhyacia quadrangula|[539]|LCHP504-07|Canada|Manitoba|658[0n]|BOLD:AAA4280
Rhyacia quadrangula|[540]|LCHP485-07|Canada|Manitoba|658[0n]|BOLD:AAA4280
Rhyacia quadrangula|[541]|MHLEP124-07|Canada|Manitoba|658[0n]|BOLD:AAA4280
Rhyacia quadrangula|[542]|LCHP354-07|Canada|Manitoba|658[0n]|BOLD:AAA4280
Rhyacia quadrangula|[543]|LCHP310-07|Canada|Manitoba|658[0n]|BOLD:AAA4280
Rhyacia quadrangula|[544]|LCHP278-07|Canada|Manitoba|658[0n]|BOLD:AAA4280
Rhyacia quadrangula|[545]|LCH232-04|Canada|Manitoba|658[0n]|BOLD:AAA4280
Rhyacia quadrangula|[546]|LCH230-04|Canada|Manitoba|658[0n]|BOLD:AAA4280
Rhyacia quadrangula|[547]|LCH228-04|Canada|Manitoba|658[0n]|BOLD:AAA4280
Rhyacia quadrangula|[548]|LCH227-04|Canada|Manitoba|658[0n]|BOLD:AAA4280
Rhyacia quadrangula|[549]|LCH224-04|Canada|Manitoba|658[0n]|BOLD:AAA4280
Rhyacia quadrangula|[550]|LCH221-04|Canada|Manitoba|658[0n]|BOLD:AAA4280
Rhyacia quadrangula|[551]|LCH220-04|Canada|Manitoba|658[0n]|BOLD:AAA4280
Rhyacia quadrangula|[552]|LCH218-04|Canada|Manitoba|658[0n]|BOLD:AAA4280
Rhyacia quadrangula|[553]|LCHQ708-08|Canada|Manitoba|658[0n]|BOLD:AAA4280
Rhyacia quadrangula|[554]|LCHP843-07|Canada|Manitoba|655[0n]|BOLD:AAA4280
Rhyacia quadrangula|[555]|LCH233-04|Canada|Manitoba|654[0n]|BOLD:AAA4280
Rhyacia quadrangula|[556]|LCH231-04|Canada|Manitoba|658[0n]|BOLD:AAA4280
Rhyacia quadrangula|[557]|LCHIP022-07|Canada|Manitoba|650[0n]|BOLD:AAA4280
Rhyacia quadrangula|[558]|LCHP241-07|Canada|Manitoba|634[0n]|BOLD:AAA4280
Rhyacia quadrangula|[559]|LCH219-04|Canada|Manitoba|658[0n]|BOLD:AAA4280
Rhyacia quadrangula|[560]|LCH229-04|Canada|Manitoba|658[0n]|BOLD:AAA4280
Rhyacia quadrangula|[561]|LCHQ304-08|Canada|Manitoba|626[0n]|BOLD:AAA4280
Rhyacia quadrangula|[562]|LCHQ429-08|Canada|Manitoba|656[0n]|BOLD:AAA4280
Rhyacia quadrangula|[563]|LCHQ645-08|Canada|Manitoba|656[0n]|BOLD:AAA4280
Rhyacia quadrangula|[564]|LCHQ720-08|Canada|Manitoba|656[0n]|BOLD:AAA4280
Rhyacia quadrangula|[565]|LCHQ898-08|Canada|Manitoba|656[0n]|BOLD:AAA4280
Rhyacia quadrangula|[566]|GRAFW066-11|Greenland||658[0n]|BOLD:AAA4280
Rhyacia quadrangula|[567]|GRAFW324-11|Greenland||658[0n]|BOLD:AAA4280
Chersotis juncta|[568]|LOWCD502-06|Canada|British Columbia|525[0n]|BOLD:AAB1122
Chersotis juncta|[569]|LPSK463-08|Canada|Saskatchewan|633[0n]|BOLD:AAB1122
Chersotis juncta|[570]|LPABC011-09|Canada|Alberta|658[0n]|BOLD:AAB1122
Chersotis juncta|[571]|LPABC015-09|Canada|Alberta|658[0n]|BOLD:AAB1122
Chersotis juncta|[572]|LOWCD504-06|Canada|British Columbia|658[0n]|BOLD:AAB1122
Chersotis juncta|[573]|LOWCD505-06|Canada|British Columbia|658[0n]|BOLD:AAB1122
Chersotis juncta|[574]|LOWCD696-06|Canada|British Columbia|658[0n]|BOLD:AAB1122
Chersotis juncta|[575]|LOWCD698-06|Canada|British Columbia|658[0n]|BOLD:AAB1122
Chersotis juncta|[576]|LPABB861-09|Canada|Alberta|658[0n]|BOLD:AAB1122
Chersotis juncta|[577]|LPABB863-09|Canada|Alberta|658[0n]|BOLD:AAB1122
Chersotis juncta|[578]|LOWC878-05|Canada|British Columbia|658[0n]|BOLD:AAB1122
Chersotis juncta|[579]|LOWCD503-06|Canada|British Columbia|658[0n]|BOLD:AAB1122
Chersotis juncta|[580]|LOWC876-05|Canada|British Columbia|658[0n]|BOLD:AAB1122
Chersotis juncta|[581]|LOWC877-05|Canada|British Columbia|658[0n]|BOLD:AAB1122
Chersotis juncta|[582]|LPABC175-09|Canada|Alberta|658[0n]|BOLD:AAB1122
Chersotis juncta|[583]|LBCG1351-09|Canada|British Columbia|658[0n]|BOLD:AAB1122
Chersotis juncta|[584]|LOWC875-05|Canada|British Columbia|658[0n]|BOLD:AAB1122
Chersotis juncta|[585]|LOWC874-05|Canada|British Columbia|658[0n]|BOLD:AAB1122
Chersotis juncta|[586]|LOWC873-05|Canada|British Columbia|658[0n]|BOLD:AAB1122
Chersotis juncta|[587]|LOWC872-05|Canada|British Columbia|658[0n]|BOLD:AAB1122
Chersotis juncta|[588]|LOWC871-05|Canada|British Columbia|658[0n]|BOLD:AAB1122
Chersotis juncta|[589]|LOWC870-05|Canada|British Columbia|658[0n]|BOLD:AAB1122
Chersotis juncta|[590]|LOWC869-05|Canada|British Columbia|658[0n]|BOLD:AAB1122
Chersotis juncta|[591]|LOWC868-05|Canada|British Columbia|658[0n]|BOLD:AAB1122
Chersotis juncta|[592]|LOWC867-05|Canada|British Columbia|658[0n]|BOLD:AAB1122
Chersotis juncta|[593]|LOWC866-05|Canada|British Columbia|658[0n]|BOLD:AAB1122
Chersotis juncta|[594]|LOWC865-05|Canada|British Columbia|658[0n]|BOLD:AAB1122
Chersotis juncta|[595]|LOWC864-05|Canada|British Columbia|658[0n]|BOLD:AAB1122
Chersotis juncta|[596]|LOWC863-05|Canada|British Columbia|658[0n]|BOLD:AAB1122
Chersotis juncta|[597]|LOWCD695-06|Canada|British Columbia|595[0n]|BOLD:AAB1122
Chersotis juncta|[598]|LOWCD697-06|Canada|British Columbia|589[0n]|BOLD:AAB1122
[illegible]

Chersotis juncta|[596]|LOWC863-05|Canada|British Columbia|658[0n]|BOLD:AAB1122
Chersotis juncta|[597]|LOWCD695-06|Canada|British Columbia|595[0n]|BOLD:AAB1122
Chersotis juncta|[598]|LOWCD697-06|Canada|British Columbia|589[0n]|BOLD:AAB1122
Chersotis juncta|[599]|UAMIC1174-13|United States|Alaska|658[0n]|BOLD:AAB1122
Protolampra rufipectus|[600]|LOWC881-05|Canada|British Columbia|658[7n]|
Protolampra rufipectus|[601]|LBCD288-05|Canada|British Columbia|618[0n]|BOLD:AAA6777
Protolampra rufipectus|[602]|LPABB369-08|Canada|Alberta|658[0n]|BOLD:AAA6777
Protolampra rufipectus|[603]|LBCH3085-10|Canada|British Columbia|658[0n]|BOLD:AAA6777
Protolampra rufipectus|[604]|LBCH4048-10|Canada|British Columbia|658[0n]|BOLD:AAA6777
Protolampra rufipectus|[605]|LOCBC607-06|United States|California|652[0n]|BOLD:AAA6777
Protolampra rufipectus|[606]|MNBB530-05|Canada|New Brunswick|658[0n]|BOLD:AAA6777
Protolampra rufipectus|[607]|RDLQB633-05|Canada|Quebec|599[0n]|BOLD:AAA6777
Protolampra rufipectus|[608]|RDLQF186-06|Canada|Quebec|658[0n]|BOLD:AAA6777
Protolampra rufipectus|[609]|LOWCD671-06|Canada|British Columbia|658[0n]|BOLD:AAA6777
Protolampra rufipectus|[610]|LOWCD666-06|Canada|British Columbia|658[0n]|BOLD:AAA6777
Protolampra rufipectus|[611]|LOWCD664-06|Canada|British Columbia|657[0n]|BOLD:AAA6777
Protolampra rufipectus|[612]|LOWC888-05|Canada|British Columbia|658[0n]|BOLD:AAA6777
Protolampra rufipectus|[613]|LOWC882-05|Canada|British Columbia|658[0n]|BOLD:AAA6777
Protolampra rufipectus|[614]|LOWCD674-06|Canada|British Columbia|609[0n]|BOLD:AAA6777
Protolampra rufipectus|[615]|LOWCD675-06|Canada|British Columbia|658[0n]|BOLD:AAA6777
Protolampra rufipectus|[616]|LOWCD678-06|Canada|British Columbia|658[0n]|BOLD:AAA6777
Protolampra rufipectus|[617]|LPABC003-09|Canada|Alberta|658[0n]|BOLD:AAA6777
Protolampra rufipectus|[618]|LPABC429-09|Canada|Alberta|658[0n]|BOLD:AAA6777
Protolampra rufipectus|[619]|BBLPB371-10|Canada|British Columbia|658[0n]|BOLD:AAA6777
Protolampra rufipectus|[620]|BBLPB375-10|Canada|Alberta|658[0n]|BOLD:AAA6777
Protolampra rufipectus|[621]|BBLPB373-10|Canada|Alberta|658[0n]|BOLD:AAA6777
Protolampra rufipectus|[622]|LOWC879-05|Canada|British Columbia|658[0n]|BOLD:AAA6777
Protolampra rufipectus|[623]|LOWC883-05|Canada|British Columbia|658[0n]|BOLD:AAA6777
Protolampra rufipectus|[624]|LSEU786-06|United States|Colorado|658[0n]|BOLD:AAA6777
Protolampra rufipectus|[625]|LOWC887-05|Canada|British Columbia|658[0n]|BOLD:AAA6777
Protolampra rufipectus|[626]|LOWCD670-06|Canada|British Columbia|601[0n]|BOLD:AAA6777
Protolampra rufipectus|[627]|LOWCD673-06|Canada|British Columbia|658[0n]|BOLD:AAA6777
Protolampra rufipectus|[628]|LOWCD677-06|Canada|British Columbia|658[0n]|BOLD:AAA6777
Protolampra rufipectus|[629]|LBCH3793-10|Canada|British Columbia|658[0n]|BOLD:AAA6777
Protolampra rufipectus|[630]|LBCH7818-10|Canada|British Columbia|658[0n]|BOLD:AAA6777
Protolampra rufipectus|[631]|BBLPB372-10|Canada|Alberta|658[0n]|BOLD:AAA6777
Protolampra rufipectus|[632]|CNWLG950-12|Canada|Alberta|633[0n]|BOLD:AAA6777
Protolampra rufipectus|[633]|RDLQB637-05|Canada|Quebec|540[1n]|BOLD:ABY5873
Protolampra rufipectus|[634]|LOWCD128-06|Canada|British Columbia|602[1n]|BOLD:ABY5873
Protolampra rufipectus|[635]|LOWCD662-06|Canada|British Columbia|592[0n]|BOLD:ABY5873
Protolampra rufipectus|[636]|LOWCD660-06|Canada|British Columbia|592[0n]|BOLD:ABY5873
Protolampra rufipectus|[637]|LOWCD659-06|Canada|British Columbia|658[0n]|BOLD:ABY5873
Protolampra rufipectus|[638]|LOWCD658-06|Canada|British Columbia|658[0n]|BOLD:ABY5873
Protolampra rufipectus|[639]|LOWC891-05|Canada|British Columbia|658[0n]|BOLD:ABY5873
Protolampra rufipectus|[640]|LOWC890-05|Canada|British Columbia|658[0n]|BOLD:ABY5873
Protolampra rufipectus|[641]|LOWC880-05|Canada|British Columbia|658[0n]|BOLD:ABY5873
Protolampra rufipectus|[642]|RDLQB635-05|Canada|Quebec|600[0n]|BOLD:ABY5873
Protolampra rufipectus|[643]|RDLQB634-05|Canada|Quebec|540[1n]|BOLD:ABY5873
Protolampra rufipectus|[644]|LOWCD661-06|Canada|British Columbia|604[0n]|BOLD:ABY5873
Protolampra rufipectus|[645]|LOWCD667-06|Canada|British Columbia|610[0n]|BOLD:ABY5873
Protolampra rufipectus|[646]|TTMNB490-06|Canada|New Brunswick|658[0n]|BOLD:ABY5873
Protolampra rufipectus|[647]|LBCH927-10|Canada|British Columbia|658[0n]|BOLD:ABY5873
Protolampra rufipectus|[648]|LBCH6287-10|Canada|British Columbia|658[0n]|BOLD:ABY5873
Protolampra rufipectus|[649]|LBCH7552-10|Canada|British Columbia|658[0n]|BOLD:ABY5873
Protolampra rufipectus|[650]|BBLPB374-10|Canada|Alberta|658[0n]|BOLD:ABY5873
Protolampra rufipectus|[651]|BBLPB449-10|Canada|Saskatchewan|658[0n]|BOLD:ABY5873
Protolampra rufipectus|[652]|CNWLM2416-13|Canada|Alberta|613[0n]|BOLD:ABY5873
Protolampra rufipectus|[653]|CNWLN1075-13|Canada|Alberta|613[0n]|BOLD:ABY5873
Protolampra rufipectus|[654]|LBCH6736-10|Canada|British Columbia|658[0n]|BOLD:ACE6699
Protolampra rufipectus|[655]|LBCH1864-10|Canada|British Columbia|658[0n]|BOLD:ACE6699
Protolampra rufipectus|[656]|LPABC796-09|Canada|Alberta|658[0n]|BOLD:ACE6699
Protolampra rufipectus|[657]|LPABB496-08|Canada|Alberta|658[0n]|BOLD:ACE6699
Protolampra rufipectus|[658]|LPMN956-08|Canada|Alberta|658[0n]|BOLD:ACE6699
Protolampra rufipectus|[659]|LOWCD669-06|Canada|British Columbia|658[0n]|BOLD:ACE6699
Protolampra rufipectus|[660]|RDLQB636-05|Canada|Quebec|658[0n]|BOLD:ACE6699
Protolampra rufipectus|[661]|LOWC889-05|Canada|British Columbia|658[0n]|BOLD:ACE6699
Protolampra rufipectus|[662]|LOWC886-05|Canada|British Columbia|658[0n]|BOLD:ACE6699
Protolampra rufipectus|[663]|LOWC885-05|Canada|British Columbia|658[0n]|BOLD:ACE6699
Protolampra rufipectus|[664]|LOWC884-05|Canada|British Columbia|658[0n]|BOLD:ACE6699
Protolampra rufipectus|[665]|LOWCD668-06|Canada|British Columbia|610[0n]|BOLD:ACE6699
Protolampra rufipectus|[666]|CNWLN1065-13|Canada|Alberta|612[0n]|BOLD:ACE6699
Protolampra rufipectus|[667]|BBLPB412-10|Canada|Saskatchewan|658[0n]|BOLD:ACE6699
Protolampra rufipectus|[668]|LBCH7805-10|Canada|British Columbia|658[0n]|BOLD:ACE6699
Protolampra rufipectus|[669]|LOWCD665-06|Canada|British Columbia|599[0n]|BOLD:ACE6699
Protolampra rufipectus|[670]|LOWCD672-06|Canada|British Columbia|590[0n]|BOLD:ACE6699
Protolampra rufipectus|[671]|CNWLN1070-13|Canada|Alberta|586[0n]|BOLD:ACE6699
Protolampra rufipectus|[672]|CNWLN1528-13|Canada|Alberta|591[0n]|BOLD:ACE6699
Aplectoides condita|[673]|RDNM981-05|Canada|Ontario|658[0n]|BOLD:AAA6359
Aplectoides condita|[674]|RDNM982-05|Canada|Ontario|658[0n]|BOLD:AAA6359
Aplectoides condita|[675]|TTMNB562-06|Canada|New Brunswick|658[0n]|BOLD:AAA6359
Aplectoides condita|[676]|TTMNB563-06|Canada|New Brunswick|658[0n]|BOLD:AAA6359
Aplectoides condita|[677]|TTMNB564-06|Canada|New Brunswick|658[0n]|BOLD:AAA6359
Aplectoides condita|[678]|TTMNB565-06|Canada|New Brunswick|658[0n]|BOLD:AAA6359
Aplectoides condita|[679]|TMNBB395-06|Canada|New Brunswick|658[0n]|BOLD:AAA6359
Aplectoides condita|[680]|RDLQF459-06|Canada|Quebec|658[0n]|BOLD:AAA6359
Aplectoides condita|[681]|RDLQF506-06|Canada|Quebec|658[0n]|BOLD:AAA6359
Aplectoides condita|[682]|LPSOD657-09|Canada|Ontario|658[0n]|BOLD:AAA6359
Aplectoides condita|[683]|RDLQB101-05|Canada|Quebec|658[0n]|BOLD:AAA6359
Aplectoides condita|[684]|RDLQF850-06|Canada|Quebec|658[0n]|BOLD:AAA6359
Aplectoides condita|[685]|TMNBB396-06|Canada|New Brunswick|658[0n]|BOLD:AAA6359
Aplectoides condita|[686]|RDLQB102-05|Canada|Quebec|658[0n]|BOLD:AAA6359
Aplectoides condita|[687]|RDLQB100-05|Canada|Quebec|658[0n]|BOLD:AAA6359
Aplectoides condita|[688]|RDLQB099-05|Canada|Quebec|658[0n]|BOLD:AAA6359
Aplectoides condita|[689]|RDLQ736-07|Canada|Quebec|636[0n]|BOLD:AAA6359
Aplectoides condita|[690]|BBLPB765-10|Canada|Saskatchewan|658[0n]|BOLD:AAA6359
Aplectoides condita|[691]|BBLPB771-10|Canada|Saskatchewan|658[0n]|BOLD:AAA6359
Aplectoides condita|[692]|LBCA825-05|Canada|British Columbia|658[0n]|BOLD:AAA6359
Aplectoides condita|[693]|LBCA099-05|Canada|British Columbia|655[0n]|BOLD:AAA6359
Aplectoides condita|[694]|LOWCD599-06|Canada|British Columbia|573[0n]|BOLD:AAA6359
Aplectoides condita|[695]|RDLQG379-06|Canada|Quebec|658[0n]|BOLD:AAA6359
Aplectoides condita|[696]|RDLQG377-06|Canada|Quebec|658[0n]|BOLD:AAA6359
Aplectoides condita|[697]|BBLPB410-10|Canada|Ontario|658[0n]|BOLD:AAA6359
Aplectoides condita|[698]|RDLQG380-06|Canada|Quebec|658[0n]|BOLD:AAA6359

Aplectoides condita|[696]|RDLQG377-06|Canada|Quebec|658[0n]|BOLD:AAA6359
Aplectoides condita|[697]|BBLPB410-10|Canada|Ontario|658[0n]|BOLD:AAA6359
Aplectoides condita|[698]|RDLQG380-06|Canada|Quebec|658[0n]|BOLD:AAA6359
Aplectoides condita|[699]|RDNM983-05|United States|Oregon|658[0n]|BOLD:AAA6359
Aplectoides condita|[700]|LOWCC175-05|Canada|British Columbia|658[0n]|BOLD:AAA6359
Aplectoides condita|[701]|LOWCD597-06|Canada|British Columbia|658[0n]|BOLD:AAA6359
Aplectoides condita|[702]|RDLQG376-06|Canada|Quebec|658[0n]|BOLD:AAA6359
Aplectoides condita|[703]|LALPA196-10|Canada|British Columbia|658[0n]|BOLD:AAA6359
Aplectoides condita|[704]|BBLPC686-09|Canada|Newfoundland and Labrador|658[0n]|BOLD:AAA6359
Aplectoides condita|[705]|BBLEC400-09|Canada|Newfoundland and Labrador|658[0n]|BOLD:AAA6359
Aplectoides condita|[706]|LPSOD895-09|Canada|Ontario|658[0n]|BOLD:AAA6359
Aplectoides condita|[707]|LPSOD851-09|Canada|Ontario|658[0n]|BOLD:AAA6359
Aplectoides condita|[708]|LPSOD850-09|Canada|Ontario|658[0n]|BOLD:AAA6359
Aplectoides condita|[709]|LPSOD740-09|Canada|Ontario|658[0n]|BOLD:AAA6359
Aplectoides condita|[710]|LPSOD457-09|Canada|Ontario|658[0n]|BOLD:AAA6359
Aplectoides condita|[711]|LPSOB317-08|Canada|Ontario|658[0n]|BOLD:AAA6359
Aplectoides condita|[712]|CDIBC003-07|Canada|British Columbia|658[0n]|BOLD:AAA6359
Aplectoides condita|[713]|RDLQG378-06|Canada|Quebec|658[0n]|BOLD:AAA6359
Aplectoides condita|[714]|LOWCD598-06|Canada|British Columbia|658[0n]|BOLD:AAA6359
Aplectoides condita|[715]|LOWCD595-06|Canada|British Columbia|658[0n]|BOLD:AAA6359
Aplectoides condita|[716]|RDNMC337-05|United States|North Carolina|658[0n]|BOLD:AAA6359
Aplectoides condita|[717]|LOWC919-05|Canada|British Columbia|658[0n]|BOLD:AAA6359
Aplectoides condita|[718]|LOWC917-05|Canada|British Columbia|658[0n]|BOLD:AAA6359
Aplectoides condita|[719]|LBCB637-05|Canada|British Columbia|658[0n]|BOLD:AAA6359
Aplectoides condita|[720]|LBCB213-05|Canada|British Columbia|658[0n]|BOLD:AAA6359
Aplectoides condita|[721]|LBCB083-05|Canada|British Columbia|658[0n]|BOLD:AAA6359
Aplectoides condita|[722]|LBCA881-05|Canada|British Columbia|658[0n]|BOLD:AAA6359
Aplectoides condita|[723]|LBCA835-05|Canada|British Columbia|658[0n]|BOLD:AAA6359
Aplectoides condita|[724]|LBCA081-05|Canada|British Columbia|658[0n]|BOLD:AAA6359
Aplectoides condita|[725]|BBLPB463-10|Canada|British Columbia|658[1n]|BOLD:AAA6359
Aplectoides condita|[726]|LBCA082-05|Canada|British Columbia|658[0n]|BOLD:AAA6359
Aplectoides condita|[727]|LBCA826-05|Canada|British Columbia|658[0n]|BOLD:AAA6359
Aplectoides condita|[728]|RDMAB079-05|Canada|Alberta|598[0n]|BOLD:AAA6359
Aplectoides condita|[729]|RDNMC338-05|United States|North Carolina|607[0n]|BOLD:AAA6359
Aplectoides condita|[730]|LOWCD593-06|Canada|British Columbia|585[0n]|BOLD:AAA6359
Aplectoides condita|[731]|RDNM984-05|Canada|British Columbia|560[0n]|BOLD:AAA6359
Aplectoides condita|[732]|LOWC920-05|Canada|British Columbia|559[1n]|BOLD:AAA6359
Aplectoides condita|[733]|LOWCD594-06|Canada|British Columbia|582[0n]|BOLD:AAA6359
Aplectoides condita|[734]|LOWCD596-06|Canada|British Columbia|523[0n]|BOLD:AAA6359
Aplectoides condita|[735]|RDLQG375-06|Canada|Quebec|649[0n]|BOLD:AAA6359
Aplectoides condita|[736]|LPMN759-08|Canada|Manitoba|609[0n]|BOLD:AAA6359
Aplectoides condita|[737]|LNCC1734-13|United States|North Carolina|658[0n]|BOLD:AAA6359
Xestia kolymae|[738]|RDNMF070-08|Canada|Yukon Territory|658[0n]|BOLD:AAF1130
Xestia kolymae|[739]|RDNMG742-08|Canada|Yukon Territory|641[0n]|BOLD:AAF1130
Xestia kolymae|[740]|RDNMG743-08|Canada|Yukon Territory|642[0n]|BOLD:AAF1130
Xestia albuncula|[741]|RDNMD023-06|Russia||658[0n]|BOLD:AAA2274
Xestia albuncula|[742]|RDNMD014-06|Finland|Kymenlaakso|658[0n]|BOLD:AAA2274
Xestia albuncula|[743]|RDNMD022-06|Russia||658[0n]|BOLD:AAA2274
Xestia albuncula|[744]|RDNMD025-06|Russia|Magadan|658[0n]|BOLD:AAA2274
Xestia albuncula|[745]|RDNMF005-08|Canada|Yukon Territory|658[0n]|BOLD:AAA2274
Xestia albuncula|[746]|RDNMG749-08|Canada|Yukon Territory|658[0n]|BOLD:AAA2274
Xestia albuncula|[747]|RDNMG751-08|Canada|Yukon Territory|658[0n]|BOLD:AAA2274
Xestia imperita|[748]|LCHP788-07|Canada|Manitoba|658[0n]|BOLD:ABZ1722
Xestia imperita|[749]|LCHQ894-08|Canada|Manitoba|658[0n]|BOLD:ABZ1722
Xestia imperita|[750]|LCHQ895-08|Canada|Manitoba|632[2n]|BOLD:ABZ1722
Xestia imperita|[751]|LCHP956-07|Canada|Manitoba|658[0n]|BOLD:ABZ1722
Xestia imperita|[752]|LCHQ724-08|Canada|Manitoba|658[0n]|BOLD:ABZ1722
Xestia imperita|[753]|LCHQ546-08|Canada|Manitoba|658[0n]|BOLD:ABZ1722
Xestia imperita|[754]|LPABC174-09|Canada|Alberta|658[1n]|BOLD:ABZ1722
Xestia imperita|[755]|LCHQ911-08|Canada|Manitoba|658[0n]|BOLD:ABZ1722
Xestia imperita|[756]|LCHQ776-08|Canada|Manitoba|658[0n]|BOLD:ABZ1722
Xestia imperita|[757]|LCHQ595-08|Canada|Manitoba|658[0n]|BOLD:ABZ1722
Xestia imperita|[758]|LCHQ586-08|Canada|Manitoba|658[0n]|BOLD:ABZ1722
Xestia imperita|[759]|LCHQ542-08|Canada|Manitoba|658[0n]|BOLD:ABZ1722
Xestia imperita|[760]|LCHQ527-08|Canada|Manitoba|658[0n]|BOLD:ABZ1722
Xestia imperita|[761]|LCHQ489-08|Canada|Manitoba|658[0n]|BOLD:ABZ1722
Xestia imperita|[762]|LCHQ481-08|Canada|Manitoba|658[0n]|BOLD:ABZ1722
Xestia imperita|[763]|LCHQ479-08|Canada|Manitoba|658[0n]|BOLD:ABZ1722
Xestia imperita|[764]|LCHQ196-07|Canada|Manitoba|658[0n]|BOLD:ABZ1722
Xestia imperita|[765]|LCHQ192-07|Canada|Manitoba|658[0n]|BOLD:ABZ1722
Xestia imperita|[766]|LCHQ183-07|Canada|Manitoba|658[0n]|BOLD:ABZ1722
Xestia imperita|[767]|LCHQ143-07|Canada|Manitoba|658[0n]|BOLD:ABZ1722
Xestia imperita|[768]|LCHQ138-07|Canada|Manitoba|658[0n]|BOLD:ABZ1722
Xestia imperita|[769]|LCHQ132-07|Canada|Manitoba|658[0n]|BOLD:ABZ1722
Xestia imperita|[770]|LCHP964-07|Canada|Manitoba|658[0n]|BOLD:ABZ1722
Xestia imperita|[771]|LCHP945-07|Canada|Manitoba|658[0n]|BOLD:ABZ1722
Xestia imperita|[772]|LCHP872-07|Canada|Manitoba|658[0n]|BOLD:ABZ1722
Xestia imperita|[773]|LCHP812-07|Canada|Manitoba|658[0n]|BOLD:ABZ1722
Xestia imperita|[774]|LCHP809-07|Canada|Manitoba|658[0n]|BOLD:ABZ1722
Xestia imperita|[775]|LCHP807-07|Canada|Manitoba|658[0n]|BOLD:ABZ1722
Xestia imperita|[776]|LCHP749-07|Canada|Manitoba|655[0n]|BOLD:ABZ1722
Xestia imperita|[777]|LCHP701-07|Canada|Manitoba|658[0n]|BOLD:ABZ1722
Xestia imperita|[778]|LCHP671-07|Canada|Manitoba|658[0n]|BOLD:ABZ1722
Xestia imperita|[779]|LCHP661-07|Canada|Manitoba|658[0n]|BOLD:ABZ1722
Xestia imperita|[780]|LCHP645-07|Canada|Manitoba|658[0n]|BOLD:ABZ1722
Xestia imperita|[781]|LCHP617-07|Canada|Manitoba|658[0n]|BOLD:ABZ1722
Xestia imperita|[782]|LCHP615-07|Canada|Manitoba|658[0n]|BOLD:ABZ1722
Xestia imperita|[783]|LCHP614-07|Canada|Manitoba|658[0n]|BOLD:ABZ1722
Xestia imperita|[784]|LCHP612-07|Canada|Manitoba|658[0n]|BOLD:ABZ1722
Xestia imperita|[785]|LCHP611-07|Canada|Manitoba|658[0n]|BOLD:ABZ1722
Xestia imperita|[786]|LCHP606-07|Canada|Manitoba|658[0n]|BOLD:ABZ1722
Xestia imperita|[787]|LCHP601-07|Canada|Manitoba|658[0n]|BOLD:ABZ1722
Xestia imperita|[788]|LCHP598-07|Canada|Manitoba|658[0n]|BOLD:ABZ1722
Xestia imperita|[789]|LCHP536-07|Canada|Manitoba|658[0n]|BOLD:ABZ1722
Xestia imperita|[790]|LPABC218-09|Canada|Alberta|658[1n]|BOLD:ABZ1722
Xestia imperita|[791]|LCHP863-07|Canada|Manitoba|658[0n]|BOLD:ABZ1722
Xestia imperita|[792]|LPABC205-09|Canada|Alberta|632[1n]|BOLD:ABZ1722
Xestia imperita|[793]|LCHQ778-08|Canada|Manitoba|656[0n]|BOLD:ABZ1722
Xestia imperita|[794]|LCHQ303-08|Canada|Manitoba|656[0n]|BOLD:ABZ1722
Xestia imperita|[795]|LCHQ185-07|Canada|Manitoba|656[0n]|BOLD:ABZ1722
Xestia imperita|[796]|MHCOL160-07|Canada|Manitoba|653[0n]|BOLD:ABZ1722
Xestia imperita|[797]|LCHQ573-08|Canada|Manitoba|658[0n]|BOLD:ABZ1722
Xestia imperita|[798]|LPABC195-09|Canada|Alberta|597[1n]|BOLD:ABZ1722

Xestia imperita|[illegible]|LCHQ[illegible]-07|Canada|Manitoba|[illegible]|BOLD:ABZ1722
Xestia imperita|[796]|MHCOL160-07|Canada|Manitoba|653[0n]|BOLD:ABZ1722
Xestia imperita|[797]|LCHQ573-08|Canada|Manitoba|658[0n]|BOLD:ABZ1722
Xestia imperita|[798]|LPABC195-09|Canada|Alberta|597[1n]|BOLD:ABZ1722
Xestia imperita|[799]|LPABC220-09|Canada|Alberta|622[0n]|BOLD:ABZ1722
Xestia imperita|[800]|LCHQ383-08|Canada|Manitoba|658[0n]|BOLD:ABZ1722
Xestia imperita|[801]|LCHQ195-07|Canada|Manitoba|658[0n]|BOLD:ABZ1722
Xestia imperita|[802]|LCHQ354-08|Canada|Manitoba|658[0n]|BOLD:ABZ1722
Xestia imperita|[803]|LCHP662-07|Canada|Manitoba|658[0n]|BOLD:ABZ1722
Xestia imperita|[804]|LCHQ555-08|Canada|Manitoba|658[0n]|BOLD:ABZ1722
Xestia imperita|[805]|LCHQ777-08|Canada|Manitoba|658[0n]|BOLD:ABZ1722
Xestia imperita|[806]|LOWCE102-06|Canada|British Columbia|658[0n]|BOLD:ABZ1722
Xestia imperita|[807]|RDLQF165-06|Canada|Quebec|658[0n]|BOLD:ABZ1722
Xestia imperita|[808]|RDLQF163-06|Canada|Quebec|658[0n]|BOLD:ABZ1722
Xestia imperita|[809]|LCHIP223-07|Canada|Manitoba|650[0n]|BOLD:ABZ1722
Xestia imperita|[810]|LCHQ169-07|Canada|Manitoba|658[0n]|BOLD:ABZ1722
Xestia imperita|[811]|LCHP808-07|Canada|Manitoba|658[0n]|BOLD:ABZ1722
Xestia imperita|[812]|LCHQ588-08|Canada|Manitoba|658[0n]|BOLD:ABZ1722
Xestia imperita|[813]|LCHQ194-07|Canada|Manitoba|656[0n]|BOLD:ABZ1722
Xestia imperita|[814]|LCHP658-07|Canada|Manitoba|658[0n]|BOLD:ABZ1722
Xestia imperita|[815]|LCHQ719-08|Canada|Manitoba|658[0n]|BOLD:ABZ1722
Xestia imperita|[816]|LCHQ592-08|Canada|Manitoba|657[0n]|BOLD:ABZ1722
Xestia imperita|[817]|LCHQ583-08|Canada|Manitoba|658[0n]|BOLD:ABZ1722
Xestia imperita|[818]|LCHQ504-08|Canada|Manitoba|658[0n]|BOLD:ABZ1722
Xestia imperita|[819]|LCHQ485-08|Canada|Manitoba|658[0n]|BOLD:ABZ1722
Xestia imperita|[820]|LCHQ476-08|Canada|Manitoba|657[0n]|BOLD:ABZ1722
Xestia imperita|[821]|LCHQ355-08|Canada|Manitoba|658[0n]|BOLD:ABZ1722
Xestia imperita|[822]|LCHQ262-08|Canada|Manitoba|658[0n]|BOLD:ABZ1722
Xestia imperita|[823]|LCHQ226-08|Canada|Manitoba|658[0n]|BOLD:ABZ1722
Xestia imperita|[824]|LCHQ198-07|Canada|Manitoba|658[0n]|BOLD:ABZ1722
Xestia imperita|[825]|LCHQ197-07|Canada|Manitoba|658[0n]|BOLD:ABZ1722
Xestia imperita|[826]|LCHQ193-07|Canada|Manitoba|658[0n]|BOLD:ABZ1722
Xestia imperita|[827]|LCHQ188-07|Canada|Manitoba|658[0n]|BOLD:ABZ1722
Xestia imperita|[828]|LCHQ184-07|Canada|Manitoba|658[0n]|BOLD:ABZ1722
Xestia imperita|[829]|LCHQ145-07|Canada|Manitoba|658[0n]|BOLD:ABZ1722
Xestia imperita|[830]|LCHQ131-07|Canada|Manitoba|658[0n]|BOLD:ABZ1722
Xestia imperita|[831]|LCHQ101-07|Canada|Manitoba|658[0n]|BOLD:ABZ1722
Xestia imperita|[832]|LCHQ098-07|Canada|Manitoba|658[0n]|BOLD:ABZ1722
Xestia imperita|[833]|LCHQ097-07|Canada|Manitoba|658[0n]|BOLD:ABZ1722
Xestia imperita|[834]|LCHQ091-07|Canada|Manitoba|658[0n]|BOLD:ABZ1722
Xestia imperita|[835]|LCHQ090-07|Canada|Manitoba|658[0n]|BOLD:ABZ1722
Xestia imperita|[836]|LCHP982-07|Canada|Manitoba|658[0n]|BOLD:ABZ1722
Xestia imperita|[837]|LCHP953-07|Canada|Manitoba|658[0n]|BOLD:ABZ1722
Xestia imperita|[838]|LCHP950-07|Canada|Manitoba|658[0n]|BOLD:ABZ1722
Xestia imperita|[839]|LCHP944-07|Canada|Manitoba|658[0n]|BOLD:ABZ1722
Xestia imperita|[840]|LCHP819-07|Canada|Manitoba|658[0n]|BOLD:ABZ1722
Xestia imperita|[841]|LCHP813-07|Canada|Manitoba|658[0n]|BOLD:ABZ1722
Xestia imperita|[842]|LCHP810-07|Canada|Manitoba|658[0n]|BOLD:ABZ1722
Xestia imperita|[843]|LCHP806-07|Canada|Manitoba|658[0n]|BOLD:ABZ1722
Xestia imperita|[844]|LCHP797-07|Canada|Manitoba|658[0n]|BOLD:ABZ1722
Xestia imperita|[845]|LCHP670-07|Canada|Manitoba|658[0n]|BOLD:ABZ1722
Xestia imperita|[846]|LCHP668-07|Canada|Manitoba|658[0n]|BOLD:ABZ1722
Xestia imperita|[847]|LCHP667-07|Canada|Manitoba|658[0n]|BOLD:ABZ1722
Xestia imperita|[848]|LCHP660-07|Canada|Manitoba|658[0n]|BOLD:ABZ1722
Xestia imperita|[849]|LCHP620-07|Canada|Manitoba|658[0n]|BOLD:ABZ1722
Xestia imperita|[850]|LCHP618-07|Canada|Manitoba|658[0n]|BOLD:ABZ1722
Xestia imperita|[851]|LCHP603-07|Canada|Manitoba|658[0n]|BOLD:ABZ1722
Xestia imperita|[852]|LCHP602-07|Canada|Manitoba|658[0n]|BOLD:ABZ1722
Xestia imperita|[853]|LCHP600-07|Canada|Manitoba|658[0n]|BOLD:ABZ1722
Xestia imperita|[854]|LCHP599-07|Canada|Manitoba|658[0n]|BOLD:ABZ1722
Xestia imperita|[855]|LCHP594-07|Canada|Manitoba|658[0n]|BOLD:ABZ1722
Xestia imperita|[856]|LCHP535-07|Canada|Manitoba|658[0n]|BOLD:ABZ1722
Xestia imperita|[857]|MHLEP092-07|Canada|Manitoba|658[0n]|BOLD:ABZ1722
Xestia imperita|[858]|MHCOL157-07|Canada|Manitoba|658[0n]|BOLD:ABZ1722
Xestia imperita|[859]|RDLQF171-06|Canada|Quebec|658[0n]|BOLD:ABZ1722
Xestia imperita|[860]|RDLQF170-06|Canada|Quebec|658[0n]|BOLD:ABZ1722
Xestia imperita|[861]|RDLQF169-06|Canada|Quebec|658[0n]|BOLD:ABZ1722
Xestia imperita|[862]|RDLQF168-06|Canada|Quebec|658[0n]|BOLD:ABZ1722
Xestia imperita|[863]|RDLQF167-06|Canada|Quebec|658[0n]|BOLD:ABZ1722
Xestia imperita|[864]|RDLQF166-06|Canada|Quebec|658[0n]|BOLD:ABZ1722
Xestia imperita|[865]|RDLQF164-06|Canada|Quebec|658[0n]|BOLD:ABZ1722
Xestia imperita|[866]|RDLQF162-06|Canada|Quebec|658[0n]|BOLD:ABZ1722
Xestia imperita|[867]|RDLQF160-06|Canada|Quebec|658[0n]|BOLD:ABZ1722
Xestia imperita|[868]|LCH575-04|Canada|Manitoba|658[0n]|BOLD:ABZ1722
Xestia imperita|[869]|RDLQF161-06|Canada|Quebec|658[0n]|BOLD:ABZ1722
Xestia imperita|[870]|LCHP842-07|Canada|Manitoba|651[0n]|BOLD:ABZ1722
Xestia imperita|[871]|LCHQ584-08|Canada|Manitoba|636[0n]|BOLD:ABZ1722
Xestia imperita|[872]|LCHQ100-07|Canada|Manitoba|637[0n]|BOLD:ABZ1722
Xestia imperita|[873]|LCHQ731-08|Canada|Manitoba|636[0n]|BOLD:ABZ1722
Xestia imperita|[874]|LCHQ763-08|Canada|Manitoba|636[0n]|BOLD:ABZ1722
Xestia imperita|[875]|LCHQ775-08|Canada|Manitoba|658[0n]|BOLD:ABZ1722
Xestia imperita|[876]|LCHQ182-07|Canada|Manitoba|658[0n]|BOLD:ABZ1722
Xestia imperita|[877]|LCHQ440-08|Canada|Manitoba|658[0n]|BOLD:ABZ1722
Xestia imperita|[878]|LCHP798-07|Canada|Manitoba|658[0n]|BOLD:ABZ1722
Xestia imperita|[879]|LCHP672-07|Canada|Manitoba|658[0n]|BOLD:ABZ1722
Xestia imperita|[880]|LCHP646-07|Canada|Manitoba|657[0n]|BOLD:ABZ1722
Xestia imperita|[881]|LCHP616-07|Canada|Manitoba|658[0n]|BOLD:ABZ1722
Xestia imperita|[882]|LCHP610-07|Canada|Manitoba|658[0n]|BOLD:ABZ1722
Xestia imperita|[883]|LCHQ486-08|Canada|Manitoba|633[0n]|BOLD:ABZ1722
Xestia imperita|[884]|LCHQ552-08|Canada|Manitoba|658[0n]|BOLD:ABZ1722
Xestia imperita|[885]|LCHQ905-08|Canada|Manitoba|658[0n]|BOLD:ABZ1722
Xestia imperita|[886]|LPABC213-09|Canada|Alberta|658[0n]|BOLD:ABZ1722
Xestia imperita|[887]|LPABB729-08|Canada|Alberta|658[0n]|BOLD:ABZ1722
Xestia imperita|[888]|LCHQ769-08|Canada|Manitoba|658[0n]|BOLD:ABZ1722
Xestia imperita|[889]|LOWCC183-05|Canada|British Columbia|658[0n]|BOLD:ABZ1722
Xestia imperita|[890]|LCHP619-07|Canada|Manitoba|658[0n]|BOLD:ABZ1722
Xestia imperita|[891]|LCHP949-07|Canada|Manitoba|658[0n]|BOLD:ABZ1722
Xestia imperita|[892]|LOWCC174-05|Canada|British Columbia|658[0n]|BOLD:ABZ1722
Xestia imperita|[893]|LPABC256-09|Canada|Alberta|658[1n]|BOLD:ABZ1722
Xestia imperita|[894]|LBCG3318-09|Canada|British Columbia|625[0n]|BOLD:ABZ1722
Xestia fabulosa|[895]|LBCG2490-09|Canada|British Columbia|638[0n]|BOLD:AAA9525
Xestia fabulosa|[896]|LBCH2981-10|Canada|British Columbia|658[0n]|BOLD:AAA9525
Xestia fabulosa|[897]|LCHP538-07|Canada|Manitoba|658[0n]|BOLD:AAA9525
[illegible]

Xestia fabulosa|[895]|LBCG2490-09|Canada|British Columbia|658[0n]|BOLD:AAA9525
Xestia fabulosa|[896]|LBCH2981-10|Canada|British Columbia|658[0n]|BOLD:AAA9525
Xestia fabulosa|[897]|LCHP538-07|Canada|Manitoba|658[0n]|BOLD:AAA9525
Xestia fabulosa|[898]|LBCG2385-09|Canada|British Columbia|658[0n]|BOLD:AAA9525
Xestia fabulosa|[899]|LBCG2387-09|Canada|British Columbia|658[0n]|BOLD:AAA9525
Xestia fabulosa|[900]|LBCG2757-09|Canada|British Columbia|658[0n]|BOLD:AAA9525
Xestia fabulosa|[901]|LBCG3054-09|Canada|British Columbia|658[0n]|BOLD:AAA9525
Xestia fabulosa|[902]|LBCG1904-09|Canada|British Columbia|658[0n]|BOLD:AAA9525
Xestia fabulosa|[903]|LBCG1906-09|Canada|British Columbia|658[0n]|BOLD:AAA9525
Xestia fabulosa|[904]|LBCG2382-09|Canada|British Columbia|658[0n]|BOLD:AAA9525
Xestia fabulosa|[905]|LBCG2383-09|Canada|British Columbia|658[0n]|BOLD:AAA9525
Xestia fabulosa|[906]|LBCG2384-09|Canada|British Columbia|658[0n]|BOLD:AAA9525
Xestia fabulosa|[907]|LBCG2386-09|Canada|British Columbia|658[0n]|BOLD:AAA9525
Xestia fabulosa|[908]|LBCG2388-09|Canada|British Columbia|658[0n]|BOLD:AAA9525
Xestia fabulosa|[909]|LBCG2389-09|Canada|British Columbia|658[0n]|BOLD:AAA9525
Xestia fabulosa|[910]|LBCB804-05|Canada|British Columbia|658[0n]|BOLD:AAA9525
Xestia fabulosa|[911]|LCHP311-07|Canada|Manitoba|658[0n]|BOLD:AAA9525
Xestia fabulosa|[912]|LBCG2390-09|Canada|British Columbia|658[0n]|BOLD:AAA9525
Xestia fabulosa|[913]|LBCG2391-09|Canada|British Columbia|658[0n]|BOLD:AAA9525
Xestia fabulosa|[914]|LBCG2392-09|Canada|British Columbia|658[0n]|BOLD:AAA9525
Xestia fabulosa|[915]|LBCG2756-09|Canada|British Columbia|658[0n]|BOLD:AAA9525
Xestia fabulosa|[916]|LBCG2758-09|Canada|British Columbia|658[0n]|BOLD:AAA9525
Xestia fabulosa|[917]|LBCG2759-09|Canada|British Columbia|658[0n]|BOLD:AAA9525
Xestia fabulosa|[918]|LBCG2760-09|Canada|British Columbia|658[0n]|BOLD:AAA9525
Xestia fabulosa|[919]|LBCG2761-09|Canada|British Columbia|658[0n]|BOLD:AAA9525
Xestia fabulosa|[920]|LCHP588-07|Canada|Manitoba|658[0n]|BOLD:AAA9525
Xestia fabulosa|[921]|LBCG1903-09|Canada|British Columbia|658[0n]|BOLD:AAA9525
Xestia fabulosa|[922]|LBCG2762-09|Canada|British Columbia|658[0n]|BOLD:AAA9525
Xestia fabulosa|[923]|LBCG2763-09|Canada|British Columbia|658[0n]|BOLD:AAA9525
Xestia fabulosa|[924]|LBCG2823-09|Canada|British Columbia|658[0n]|BOLD:AAA9525
Xestia fabulosa|[925]|LBCG2824-09|Canada|British Columbia|658[0n]|BOLD:AAA9525
Xestia fabulosa|[926]|LBCG2825-09|Canada|British Columbia|658[0n]|BOLD:AAA9525
Xestia fabulosa|[927]|LBCG3055-09|Canada|British Columbia|658[0n]|BOLD:AAA9525
Xestia fabulosa|[928]|LBCG3056-09|Canada|British Columbia|658[0n]|BOLD:AAA9525
Xestia fabulosa|[929]|LBCG3057-09|Canada|British Columbia|658[0n]|BOLD:AAA9525
Xestia fabulosa|[930]|LBCH1933-10|Canada|British Columbia|658[0n]|BOLD:AAA9525
Xestia fabulosa|[931]|LBCH2249-10|Canada|British Columbia|658[0n]|BOLD:AAA9525
Xestia fabulosa|[932]|LBCG2539-09|Canada|British Columbia|658[0n]|BOLD:AAA9525
Xestia fabulosa|[933]|LBCH2941-10|Canada|British Columbia|658[0n]|BOLD:AAA9525
Xestia fabulosa|[934]|LBCH2955-10|Canada|British Columbia|658[0n]|BOLD:AAA9525
Xestia fabulosa|[935]|LBCH3027-10|Canada|British Columbia|658[0n]|BOLD:AAA9525
Eueretagrotis attentus|[936]|LSEU169-06|United States|North Carolina|550[2n]|BOLD:AAB3386
Eueretagrotis attentus|[937]|LGSM649-04|United States|Tennessee|658[0n]|BOLD:AAB3386
Eueretagrotis attentus|[938]|BBLEC969-09|Canada|Nova Scotia|658[0n]|BOLD:AAB3386
Eueretagrotis attentus|[939]|BBLEC191-09|Canada|Nova Scotia|577[0n]|BOLD:AAB3386
Eueretagrotis attentus|[940]|TMNBB402-06|Canada|New Brunswick|658[0n]|BOLD:AAB3386
Eueretagrotis attentus|[941]|BBLEC549-09|Canada|Nova Scotia|658[0n]|BOLD:AAB3386
Eueretagrotis attentus|[942]|TTMNB020-06|Canada|New Brunswick|658[0n]|BOLD:AAB3386
Eueretagrotis attentus|[943]|TTMNB019-06|Canada|New Brunswick|658[0n]|BOLD:AAB3386
Eueretagrotis attentus|[944]|TTMNB013-06|Canada|New Brunswick|657[0n]|BOLD:AAB3386
Eueretagrotis attentus|[945]|TTMNB012-06|Canada|New Brunswick|658[0n]|BOLD:AAB3386
Eueretagrotis attentus|[946]|RDLQB298-05|Canada|Quebec|658[0n]|BOLD:AAB3386
Eueretagrotis attentus|[947]|RDLQB295-05|Canada|Quebec|658[0n]|BOLD:AAB3386
Eueretagrotis attentus|[948]|RDLQB297-05|Canada|Quebec|658[0n]|BOLD:AAB3386
Eueretagrotis attentus|[949]|RDLQB296-05|Canada|Quebec|597[0n]|BOLD:AAB3386
Eueretagrotis attentus|[950]|RDLQB299-05|Canada|Quebec|658[0n]|BOLD:AAB3386
Eueretagrotis attentus|[951]|RDLQB294-05|Canada|Quebec|658[0n]|BOLD:AAB3386
Eueretagrotis attentus|[952]|LGSM761-04|United States|North Carolina|658[0n]|BOLD:AAB3386
Eueretagrotis attentus|[953]|TTMNB078-06|Canada|New Brunswick|657[0n]|BOLD:AAB3386
Eueretagrotis attentus|[954]|TMNBB398-06|Canada|New Brunswick|658[0n]|BOLD:AAB3386
Eueretagrotis attentus|[955]|TMNBB399-06|Canada|New Brunswick|658[0n]|BOLD:AAB3386
Eueretagrotis attentus|[956]|TMNBB400-06|Canada|New Brunswick|658[0n]|BOLD:AAB3386
Eueretagrotis attentus|[957]|TMNBB401-06|Canada|New Brunswick|658[0n]|BOLD:AAB3386
Eueretagrotis attentus|[958]|BBLEC116-09|Canada|Nova Scotia|658[0n]|BOLD:AAB3386
Eueretagrotis attentus|[959]|BBLPE617-09|Canada|Nova Scotia|658[0n]|BOLD:AAB3386
Eueretagrotis sigmoides|[960]|LNCC1034-11|United States|North Carolina|658[0n]|BOLD:AAE4603
Eueretagrotis sigmoides|[961]|RDLQ739-07|Canada|Quebec|658[0n]|BOLD:AAE4603
Eueretagrotis sigmoides|[962]|RDLQ738-07|Canada|Quebec|658[0n]|BOLD:AAE4603
Eueretagrotis sigmoides|[963]|RDNMG1034-08|Canada|Ontario|643[0n]|BOLD:AAE4603
Eueretagrotis sigmoides|[964]|RDNMG1035-08|Canada|Ontario|642[0n]|BOLD:AAE4603
Eueretagrotis sigmoides|[965]|LNCC1035-11|United States|North Carolina|658[0n]|BOLD:AAE4603
Eueretagrotis perattentus|[966]|TTMNB498-06|Canada|New Brunswick|658[0n]|BOLD:AAA8152
Eueretagrotis perattentus|[967]|JSJUN2103-11|Canada|Ontario|618[0n]|BOLD:AAA8151
Eueretagrotis perattentus|[968]|RDMAB072-05|Canada|Alberta|611[0n]|BOLD:AAA8151
Eueretagrotis perattentus|[969]|RDMAB071-05|Canada|Alberta|615[0n]|BOLD:AAA8151
Eueretagrotis perattentus|[970]|BBLPB853-10|Canada|British Columbia|658[0n]|BOLD:AAA8151
Eueretagrotis perattentus|[971]|PHMNB424-04|Canada|New Brunswick|658[2n]|BOLD:AAA8151
Eueretagrotis perattentus|[972]|PHMNB001-03|Canada|New Brunswick|639[0n]|BOLD:AAA8151
Eueretagrotis perattentus|[973]|PHMNB368-04|Canada|New Brunswick|658[0n]|BOLD:AAA8151
Eueretagrotis perattentus|[974]|PHMNB467-04|Canada|New Brunswick|658[1n]|BOLD:AAA8151
Eueretagrotis perattentus|[975]|PHMNB684-04|Canada|New Brunswick|658[0n]|BOLD:AAA8151
Eueretagrotis perattentus|[976]|RDLQB307-05|Canada|Quebec|658[0n]|BOLD:AAA8151
Eueretagrotis perattentus|[977]|TTMNB495-06|Canada|New Brunswick|658[0n]|BOLD:AAA8151
Eueretagrotis perattentus|[978]|TTMNB497-06|Canada|New Brunswick|655[0n]|BOLD:AAA8151
Eueretagrotis perattentus|[979]|RDLQG139-06|Canada|Quebec|646[1n]|BOLD:AAA8151
Eueretagrotis perattentus|[980]|LBCB623-05|Canada|British Columbia|658[0n]|BOLD:AAA8151
Eueretagrotis perattentus|[981]|LHLEP393-06|Canada|British Columbia|658[0n]|BOLD:AAA8151
Eueretagrotis perattentus|[982]|LHLEP394-06|Canada|British Columbia|658[0n]|BOLD:AAA8151
Eueretagrotis perattentus|[983]|LHLEP434-06|Canada|British Columbia|658[0n]|BOLD:AAA8151
Eueretagrotis perattentus|[984]|LBCH152-10|Canada|British Columbia|658[0n]|BOLD:AAA8151
Eueretagrotis perattentus|[985]|LBCH153-10|Canada|British Columbia|658[0n]|BOLD:AAA8151
Eueretagrotis perattentus|[986]|LBCH154-10|Canada|British Columbia|658[0n]|BOLD:AAA8151
Eueretagrotis perattentus|[987]|LBCH852-10|Canada|British Columbia|658[0n]|BOLD:AAA8151
Eueretagrotis perattentus|[988]|BLTIB270-08|Canada|Ontario|658[1n]|BOLD:AAA8151
Eueretagrotis perattentus|[989]|XAB093-04|Canada|Ontario|658[0n]|BOLD:AAA8151
Eueretagrotis perattentus|[990]|LMDH174-11|United States|Minnesota|658[0n]|BOLD:AAA8151
Eueretagrotis perattentus|[991]|RDLQB305-05|Canada|Quebec|658[0n]|BOLD:AAA8151
Eueretagrotis perattentus|[992]|BBLPE028-09|Canada|Nova Scotia|658[0n]|BOLD:AAA8151
Eueretagrotis perattentus|[993]|BBLPE006-09|Canada|Nova Scotia|658[0n]|BOLD:AAA8151
Eueretagrotis perattentus|[994]|BBLPC680-09|Canada|Newfoundland and Labrador|658[0n]|BOLD:AAA8151
Eueretagrotis perattentus|[995]|BBLPC168-09|Canada|Nova Scotia|656[0n]|BOLD:AAA8151
Eueretagrotis perattentus|[996]|BLTIB443-08|Canada|Ontario|658[0n]|BOLD:AAA8151
Eueretagrotis perattentus|[997]|TTMNB496-06|Canada|New Brunswick|658[0n]|BOLD:AAA8151

Eueretagrotis perattentus|[995]|BBLPC168-09|Canada|Nova Scotia|656[0n]|BOLD:AAA8151
Eueretagrotis perattentus|[996]|BLTIB443-08|Canada|Ontario|658[0n]|BOLD:AAA8151
Eueretagrotis perattentus|[997]|TTMNB496-06|Canada|New Brunswick|658[0n]|BOLD:AAA8151
Eueretagrotis perattentus|[998]|RDLQB306-05|Canada|Quebec|658[0n]|BOLD:AAA8151
Eueretagrotis perattentus|[999]|RDLQB304-05|Canada|Quebec|658[0n]|BOLD:AAA8151
Eueretagrotis perattentus|[1000]|RDLQB303-05|Canada|Quebec|658[0n]|BOLD:AAA8151
Eueretagrotis perattentus|[1001]|XAD777-05|Canada|Ontario|658[0n]|BOLD:AAA8151
Eueretagrotis perattentus|[1002]|RDLQB301-05|Canada|Quebec|658[0n]|BOLD:AAA8151
Eueretagrotis perattentus|[1003]|RDLQB302-05|Canada|Quebec|658[0n]|BOLD:AAA8151
Eueretagrotis perattentus|[1004]|BBLPE071-09|Canada|Nova Scotia|607[0n]|BOLD:AAA8151
Eueretagrotis perattentus|[1005]|BBLPE136-09|Canada|Nova Scotia|658[0n]|BOLD:AAA8151
Eueretagrotis perattentus|[1006]|BBLPE354-09|Canada|Newfoundland and Labrador|658[0n]|BOLD:AAA8151
Eueretagrotis perattentus|[1007]|BBLPB718-10|Canada|Ontario|658[0n]|BOLD:AAA8151
Eueretagrotis perattentus|[1008]|LNCC1182-11|United States|North Carolina|632[0n]|BOLD:AAA8151
Eueretagrotis perattentus|[1009]|MMNA071-08|United States|North Carolina|657[0n]|BOLD:AAA8151
Eueretagrotis perattentus|[1010]|MMNA072-08|United States|North Carolina|634[0n]|BOLD:AAA8151
Eueretagrotis perattentus|[1011]|MMNA095-08|United States|North Carolina|658[0n]|BOLD:AAA8151
Eueretagrotis perattentus|[1012]|LNCC1031-11|United States|North Carolina|658[0n]|BOLD:AAA8151
Eueretagrotis perattentus|[1013]|MMNA094-08|United States|North Carolina|658[0n]|BOLD:AAA8151
Eueretagrotis perattentus|[1014]|RDNMC341-05|United States|North Carolina|658[0n]|BOLD:AAA8151
Eueretagrotis perattentus|[1015]|RDNMC336-05|United States|North Carolina|611[0n]|BOLD:AAA8151
Eueretagrotis perattentus|[1016]|LNCC1032-11|United States|North Carolina|658[0n]|BOLD:AAA8151
Eueretagrotis perattentus|[1017]|LNCC1033-11|United States|North Carolina|658[0n]|BOLD:AAA8151
Eueretagrotis perattentus|[1018]|CNEIC3022-12|Canada|Alberta|636[0n]|BOLD:AAA8151
Eueretagrotis perattentus|[1019]|CNWLD1012-12|Canada|Alberta|632[0n]|BOLD:AAA8151
Adelphagrotis stellaris|[1020]|LHLEP390-06|Canada|British Columbia|658[0n]|BOLD:AAC2434
Adelphagrotis stellaris|[1021]|RDNMF611-08|United States|Oregon|658[0n]|BOLD:AAC2434
Adelphagrotis stellaris|[1022]|LPVIB256-08|Canada|British Columbia|658[0n]|BOLD:AAC2434
Adelphagrotis stellaris|[1023]|LPVIA611-08|Canada|British Columbia|658[0n]|BOLD:AAC2434
Adelphagrotis stellaris|[1024]|LHLEP357-06|Canada|British Columbia|657[0n]|BOLD:AAC2434
Adelphagrotis stellaris|[1025]|LHLEP356-06|Canada|British Columbia|657[0n]|BOLD:AAC2434
Adelphagrotis stellaris|[1026]|LHLEP435-06|Canada|British Columbia|658[0n]|BOLD:AAC2434
Adelphagrotis stellaris|[1027]|RDNMF612-08|Canada|British Columbia|658[0n]|BOLD:AAC2434
Adelphagrotis stellaris|[1028]|RWWC458-11|United States|Washington|658[0n]|BOLD:AAC2434
Adelphagrotis stellaris|[1029]|LPVIB237-08|Canada|British Columbia|658[0n]|BOLD:AAC2434
Adelphagrotis stellaris|[1030]|IAWLB262-11|United States|Washington|658[0n]|BOLD:AAC2434
Adelphagrotis stellaris|[1031]|RWWC601-11|United States|Washington|658[0n]|BOLD:AAC2434
Adelphagrotis stellaris|[1032]|RWWA813-09|United States|Washington|658[0n]|BOLD:AAC2434
Adelphagrotis stellaris|[1033]|LALPA606-10|Canada|British Columbia|658[0n]|BOLD:AAC2434
Adelphagrotis stellaris|[1034]|RWWA725-09|United States|Washington|658[0n]|BOLD:AAC2434
Adelphagrotis stellaris|[1035]|LPVIB072-08|Canada|British Columbia|658[0n]|BOLD:AAC2434
Adelphagrotis stellaris|[1036]|LPVIB067-08|Canada|British Columbia|658[0n]|BOLD:AAC2434
Adelphagrotis stellaris|[1037]|LPVIA099-08|Canada|British Columbia|658[0n]|BOLD:AAC2434
Adelphagrotis stellaris|[1038]|RDNMF615-08|Canada|British Columbia|658[0n]|BOLD:AAC2434
Adelphagrotis stellaris|[1039]|RDNMF614-08|Canada|British Columbia|658[0n]|BOLD:AAC2434
Adelphagrotis stellaris|[1040]|RDNMF613-08|Canada|British Columbia|658[0n]|BOLD:AAC2434
Adelphagrotis stellaris|[1041]|LBCS265-07|Canada|British Columbia|658[0n]|BOLD:AAC2434
Adelphagrotis stellaris|[1042]|LPVIB085-08|Canada|British Columbia|658[1n]|BOLD:AAC2434
Adelphagrotis stellaris|[1043]|LALPA671-10|Canada|British Columbia|658[1n]|BOLD:AAC2434
Adelphagrotis stellaris|[1044]|LPVIA835-08|Canada|British Columbia|623[0n]|BOLD:AAC2434
Adelphagrotis stellaris|[1045]|RWWC602-11|United States|Washington|658[0n]|BOLD:AAC2434
Adelphagrotis stellaris|[1046]|RWWB179-09|United States|Washington|639[0n]|BOLD:AAC2434
Adelphagrotis stellaris|[1047]|RWWC633-11|United States|Washington|658[0n]|BOLD:AAC2434
Adelphagrotis stellaris|[1048]|GMLC1192-12|United States|California|658[0n]|BOLD:AAC2434
Adelphagrotis indeterminata|[1049]|LOCBB248-06|United States|California|658[0n]|BOLD:AAD2145
Adelphagrotis indeterminata|[1050]|LOCBB249-06|United States|California|658[0n]|BOLD:AAD2145
Adelphagrotis indeterminata|[1051]|LOCBB250-06|United States|California|658[0n]|BOLD:AAD2145
Adelphagrotis indeterminata|[1052]|LOCBB251-06|United States|California|658[0n]|BOLD:AAD2145
Adelphagrotis indeterminata|[1053]|LOCBB252-06|United States|California|658[0n]|BOLD:AAD2145
Adelphagrotis indeterminata|[1054]|NAMUM122-08|United States|California|658[0n]|BOLD:AAD2145
Adelphagrotis indeterminata|[1055]|NAMUM123-08|United States|California|658[0n]|BOLD:AAD2145
Adelphagrotis indeterminata|[1056]|LPVIB558-08|Canada|British Columbia|658[0n]|BOLD:ABZ5978
Adelphagrotis indeterminata|[1057]|RWWB044-09|United States|Washington|658[0n]|BOLD:ABZ5978
Adelphagrotis indeterminata|[1058]|LBCH6275-10|Canada|British Columbia|658[0n]|BOLD:ABZ5978
Adelphagrotis indeterminata|[1059]|LALPA741-10|Canada|British Columbia|658[0n]|BOLD:ABZ5978
Adelphagrotis indeterminata|[1060]|LALPA1209-11|Canada|British Columbia|658[0n]|BOLD:ABZ5978
Adelphagrotis indeterminata|[1061]|LALPA1270-11|Canada|British Columbia|658[0n]|BOLD:ABZ5978
Adelphagrotis indeterminata|[1062]|LALPA1272-11|Canada|British Columbia|658[0n]|BOLD:ABZ5978
Adelphagrotis indeterminata|[1063]|GMLC271-11|United States|California|658[0n]|BOLD:ABZ5978
Adelphagrotis indeterminata|[1064]|GMLC1224-12|United States|California|658[0n]|BOLD:ABZ5978
Setagrotis|[1065]|RDNMG760-08|United States|Colorado|658[0n]|BOLD:AAE1771
Setagrotis radiola|[1066]|RDMAB603-06|Canada|Alberta|658[0n]|BOLD:ABZ0944
Setagrotis radiola|[1067]|RDNMG419-08|United States|Wyoming|658[0n]|BOLD:ABZ0944
Setagrotis radiola|[1068]|RDNMG420-08|United States|Arizona|658[0n]|BOLD:ABZ0944
Setagrotis radiola|[1069]|RDNMG991-08|United States|Colorado|658[0n]|BOLD:ABZ0944
Setagrotis vocalis|[1070]|BBLOC132-11|United States|Texas|658[0n]|BOLD:ACF4384
Setagrotis vocalis|[1071]|NAMUM365-09|United States|California|658[0n]|BOLD:ACF4384
Setagrotis vocalis|[1072]|IAWLB022-10|United States|California|658[0n]|BOLD:ACF4384
Setagrotis vocalis|[1073]|BBLOC143-11|United States|Texas|658[0n]|BOLD:ACF4384
Setagrotis vocalis|[1074]|BBLOC145-11|United States|Texas|658[0n]|BOLD:ACF4384
Setagrotis vocalis|[1075]|RDNMK108-11|United States|Utah|508[0n]|BOLD:AAC3592
Setagrotis pallidicollis|[1076]|LBCG1341-09|Canada|British Columbia|658[4n]|BOLD:AAC3592
Setagrotis pallidicollis|[1077]|RDNMC175-05|United States|California|658[0n]|BOLD:AAC3592
Setagrotis pallidicollis|[1078]|LBCH5725-10|Canada|British Columbia|658[0n]|BOLD:AAC3592
Setagrotis pallidicollis|[1079]|LOWCD676-06|Canada|British Columbia|658[0n]|BOLD:AAC3592
Setagrotis pallidicollis|[1080]|LBCH6306-10|Canada|British Columbia|658[0n]|BOLD:AAC3592
Setagrotis pallidicollis|[1081]|LBCH6119-10|Canada|British Columbia|658[0n]|BOLD:AAC3592
Setagrotis pallidicollis|[1082]|LBCH6019-10|Canada|British Columbia|658[0n]|BOLD:AAC3592
Setagrotis pallidicollis|[1083]|LBCH5800-10|Canada|British Columbia|658[0n]|BOLD:AAC3592
Setagrotis pallidicollis|[1084]|LBCH5716-10|Canada|British Columbia|658[0n]|BOLD:AAC3592
Setagrotis pallidicollis|[1085]|LBCG2881-09|Canada|British Columbia|658[0n]|BOLD:AAC3592
Setagrotis pallidicollis|[1086]|LPABB879-09|Canada|Alberta|658[0n]|BOLD:AAC3592
Setagrotis pallidicollis|[1087]|LPABB639-08|Canada|Alberta|658[0n]|BOLD:AAC3592
Setagrotis pallidicollis|[1088]|LOWC924-05|Canada|British Columbia|658[0n]|BOLD:AAC3592
Setagrotis pallidicollis|[1089]|RDNMC177-05|United States|California|658[0n]|BOLD:AAC3592
Setagrotis pallidicollis|[1090]|RDNMC176-05|United States|Oregon|658[0n]|BOLD:AAC3592
Setagrotis pallidicollis|[1091]|RDNMC094-05|Canada|Alberta|658[0n]|BOLD:AAC3592
Setagrotis pallidicollis|[1092]|LBCB068-05|Canada|British Columbia|658[0n]|BOLD:AAC3592
Setagrotis pallidicollis|[1093]|LBCH6021-10|Canada|British Columbia|648[0n]|BOLD:AAC3592
Setagrotis pallidicollis|[1094]|LOWCD386-06|Canada|British Columbia|579[0n]|BOLD:AAC3592
Setagrotis pallidicollis|[1095]|BBLOC1219-11|United States|California|658[0n]|BOLD:AAC3592
Setagrotis pallidicollis|[1096]|LALPA1257-11|Canada|British Columbia|658[0n]|BOLD:AAC3592
Xestia mustelina|[1097]|LOPN122-06|United States|Oregon|591[0n]|BOLD:AAB7040

Setagrotis pallidicollis|[illegible]
Setagrotis pallidicollis|[1095]|BBLOC1219-11|United States|California|658[0n]|BOLD:AAC3592
Setagrotis pallidicollis|[1096]|LALPA1257-11|Canada|British Columbia|658[0n]|BOLD:AAC3592
Xestia mustelina|[1097]|LOPN122-06|United States|Oregon|591[0n]|BOLD:AAB7040
Xestia mustelina|[1098]|LOPN125-06|United States|Oregon|582[0n]|BOLD:AAB7040
Xestia mustelina|[1099]|LOPN123-06|United States|Oregon|554[1n]|BOLD:AAB7040
Xestia mustelina|[1100]|RWWB169-09|United States|Washington|658[0n]|BOLD:AAB7040
Xestia mustelina|[1101]|LBCD279-05|Canada|British Columbia|658[0n]|BOLD:AAB7040
Xestia mustelina|[1102]|LBCD287-05|Canada|British Columbia|616[0n]|BOLD:AAB7040
Xestia mustelina|[1103]|LBCD289-05|Canada|British Columbia|652[0n]|BOLD:AAB7040
Xestia mustelina|[1104]|DUNLP187-08|Canada|British Columbia|643[0n]|BOLD:AAB7040
Xestia mustelina|[1105]|LOPN121-06|United States|Oregon|602[0n]|BOLD:AAB7040
Xestia mustelina|[1106]|LOPN124-06|United States|Oregon|587[0n]|BOLD:AAB7040
Xestia mustelina|[1107]|LBCG2281-09|Canada|British Columbia|658[0n]|BOLD:AAB7040
Xestia mustelina|[1108]|LBCG2287-09|Canada|British Columbia|658[0n]|BOLD:AAB7040
Xestia mustelina|[1109]|LBCG2288-09|Canada|British Columbia|658[0n]|BOLD:AAB7040
Xestia mustelina|[1110]|LBCG2289-09|Canada|British Columbia|658[0n]|BOLD:AAB7040
Xestia mustelina|[1111]|LBCG2290-09|Canada|British Columbia|658[0n]|BOLD:AAB7040
Xestia mustelina|[1112]|LBCH939-10|Canada|British Columbia|658[0n]|BOLD:AAB7040
Xestia mustelina|[1113]|LBCH943-10|Canada|British Columbia|658[0n]|BOLD:AAB7040
Xestia mustelina|[1114]|LBCH3471-10|Canada|British Columbia|658[0n]|BOLD:AAB7040
Xestia mustelina|[1115]|LBCH3787-10|Canada|British Columbia|658[0n]|BOLD:AAB7040
Xestia mustelina|[1116]|LBCH4116-10|Canada|British Columbia|658[0n]|BOLD:AAB7040
Xestia mustelina|[1117]|LBCH4436-10|Canada|British Columbia|658[0n]|BOLD:AAB7040
Xestia mustelina|[1118]|LBCH4691-10|Canada|British Columbia|658[0n]|BOLD:AAB7040
Xestia mustelina|[1119]|LALPA629-10|Canada|British Columbia|658[0n]|BOLD:AAB7040
Xestia mustelina|[1120]|LALPA668-10|Canada|British Columbia|658[0n]|BOLD:AAB7040
Xestia mustelina|[1121]|LALPA749-10|Canada|British Columbia|658[0n]|BOLD:AAB7040
Xestia mustelina|[1122]|LALPA1306-11|Canada|British Columbia|658[0n]|BOLD:AAB7040
Xestia mustelina|[1123]|LOPN120-06|United States|Oregon|618[4n]|BOLD:ACJ0542
Xestia mustelina|[1124]|JMMMB605-13|United States|California|658[0n]|BOLD:ACJ0542
Abagrotis dodi|[1125]|RDNM672-05|United States|Wyoming|607[0n]|BOLD:AAF3346
Abagrotis dodi|[1126]|RDNMC104-05|United States|Oregon|658[0n]|BOLD:AAF3346
Abagrotis dodi|[1127]|RDNMC105-05|Canada|British Columbia|658[0n]|BOLD:AAF3346
Abagrotis dodi|[1128]|LBCH6545-10|Canada|British Columbia|658[0n]|BOLD:AAF3346
Abagrotis dodi|[1129]|LBCH7554-10|Canada|British Columbia|658[0n]|BOLD:AAF3346
Abagrotis dodi|[1130]|LBCH7557-10|Canada|British Columbia|658[0n]|BOLD:AAF3346
Abagrotis dodi|[1131]|LBCH7887-10|Canada|British Columbia|658[0n]|BOLD:AAF3346
Abagrotis dodi|[1132]|SSBAD6365-13|Canada|Alberta|575[0n]|BOLD:AAF3346
Abagrotis dodi|[1133]|SSBAD6394-13|Canada|Alberta|577[0n]|BOLD:AAF3346
Abagrotis dodi|[1134]|CNWLM026-13|Canada|Alberta|588[0n]|BOLD:AAF3346
Abagrotis mexicana|[1135]|RDNMJ367-11|United States|Arizona|658[0n]|BOLD:AAV9938
Abagrotis mexicana|[1136]|LNAUT919-14|United States|Texas|658[0n]|BOLD:AAV9938
Abagrotis mexicana|[1137]|LNAUT920-14|United States|Texas|658[0n]|BOLD:AAV9938
Abagrotis trigona|[1138]|LBCH6299-10|Canada|British Columbia|658[0n]|BOLD:ABY3181
Abagrotis trigona|[1139]|LBCH6309-10|Canada|British Columbia|658[0n]|BOLD:ABY3181
Abagrotis trigona|[1140]|LBCH6450-10|Canada|British Columbia|658[0n]|BOLD:ABY3181
Abagrotis trigona|[1141]|LBCH7114-10|Canada|British Columbia|658[0n]|BOLD:ABY3181
Abagrotis trigona|[1142]|LBCH7337-10|Canada|British Columbia|658[0n]|BOLD:ABY3181
Abagrotis trigona|[1143]|LBCH7426-10|Canada|British Columbia|658[0n]|BOLD:ABY3181
Abagrotis trigona|[1144]|LBCH7472-10|Canada|British Columbia|658[0n]|BOLD:ABY3181
Abagrotis trigona|[1145]|LBCH7676-10|Canada|British Columbia|658[0n]|BOLD:ABY3181
Abagrotis trigona|[1146]|LBCH7809-10|Canada|British Columbia|658[0n]|BOLD:ABY3181
Abagrotis trigona|[1147]|IAWLB163-10|United States|California|658[0n]|BOLD:ABY3181
Abagrotis trigona|[1148]|LBCH6125-10|Canada|British Columbia|658[0n]|BOLD:ABY3181
Abagrotis trigona|[1149]|LBCH6236-10|Canada|British Columbia|658[0n]|BOLD:ABY3181
Abagrotis trigona|[1150]|LPVIB271-08|Canada|British Columbia|658[0n]|BOLD:ABY3181
Abagrotis trigona|[1151]|LPVIA607-08|Canada|British Columbia|658[0n]|BOLD:ABY3181
Abagrotis trigona|[1152]|RDNMG885-08|United States|Colorado|658[0n]|BOLD:ABY3181
Abagrotis trigona|[1153]|LBCG481-08|Canada|British Columbia|658[0n]|BOLD:ABY3181
Abagrotis trigona|[1154]|LBCG478-08|Canada|British Columbia|658[0n]|BOLD:ABY3181
Abagrotis trigona|[1155]|LOWCE758-06|Canada|British Columbia|658[0n]|BOLD:ABY3181
Abagrotis trigona|[1156]|RDNMC158-05|Canada|Alberta|658[0n]|BOLD:ABY3181
Abagrotis trigona|[1157]|RDNMC157-05|Canada|British Columbia|658[0n]|BOLD:ABY3181
Abagrotis trigona|[1158]|RDNMC155-05|Canada|British Columbia|658[0n]|BOLD:ABY3181
Abagrotis trigona|[1159]|RDNMC154-05|United States|Nevada|658[0n]|BOLD:ABY3181
Abagrotis trigona|[1160]|LBCC340-05|Canada|British Columbia|658[0n]|BOLD:ABY3181
Abagrotis trigona|[1161]|RDNMC156-05|United States|Washington|545[1n]|BOLD:ABY3181
Abagrotis trigona|[1162]|LBCG3248-09|Canada|British Columbia|632[0n]|BOLD:ABY3181
Abagrotis trigona|[1163]|LBCH6272-10|Canada|British Columbia|642[0n]|BOLD:ABY3181
Abagrotis trigona|[1164]|IAWLB165-10|United States|California|658[0n]|BOLD:ABY3181
Abagrotis alternata|[1165]|LPOKD428-09|United States|Oklahoma|658[0n]|BOLD:ABZ1830
Abagrotis alternata|[1166]|LSEU621-06|United States|Georgia|658[0n]|BOLD:ABZ1830
Abagrotis alternata|[1167]|RDLQF269-06|Canada|Quebec|658[0n]|BOLD:ABZ1830
Abagrotis alternata|[1168]|TTMNB570-06|Canada|New Brunswick|658[0n]|BOLD:ABZ1830
Abagrotis alternata|[1169]|TMNBB405-06|Canada|New Brunswick|658[0n]|BOLD:ABZ1830
Abagrotis alternata|[1170]|RDLQ743-07|Canada|Quebec|658[0n]|BOLD:ABZ1830
Abagrotis alternata|[1171]|TMNBB403-06|Canada|New Brunswick|658[0n]|BOLD:ABZ1830
Abagrotis alternata|[1172]|LPOKC611-09|United States|Oklahoma|658[0n]|BOLD:ABZ1830
Abagrotis alternata|[1173]|LPOKD436-09|United States|Oklahoma|658[0n]|BOLD:ABZ1830
Abagrotis alternata|[1174]|TMNBB404-06|Canada|New Brunswick|658[1n]|BOLD:ABZ1830
Abagrotis alternata|[1175]|LNCB310-06|United States|North Carolina|658[0n]|BOLD:ABZ1830
Abagrotis alternata|[1176]|RDLQ744-07|Canada|Quebec|658[0n]|BOLD:ABZ1830
Abagrotis alternata|[1177]|RDLQ004-05|Canada|Quebec|656[0n]|BOLD:ABZ1830
Abagrotis alternata|[1178]|LGSM511-04|United States|Tennessee|561[3n]|BOLD:ABZ1830
Abagrotis alternata|[1179]|LGSM512-04|United States|North Carolina|609[0n]|BOLD:ABZ1830
Abagrotis alternata|[1180]|XAJ815-06|Canada|Ontario|639[0n]|BOLD:ABZ1830
Abagrotis alternata|[1181]|LSEU619-06|United States|Georgia|658[0n]|BOLD:ABZ1830
Abagrotis alternata|[1182]|LSEU620-06|United States|Georgia|658[0n]|BOLD:ABZ1830
Abagrotis alternata|[1183]|LGSMG923-10|United States|North Carolina|658[0n]|BOLD:ABZ1830
Abagrotis alternata|[1184]|LILLA543-11|United States|Illinois|658[0n]|BOLD:ABZ1830
Abagrotis alternata|[1185]|BBLOC1032-11|United States|Texas|658[0n]|BOLD:ABZ1830
Abagrotis alternata|[1186]|LNCC1245-11|United States|North Carolina|658[0n]|BOLD:ABZ1830
Abagrotis scopeops|[1187]|NAMUM284-08|United States|California|658[0n]|BOLD:ACF0138
Abagrotis scopeops|[1188]|RDNMG682-08|United States|California|658[0n]|BOLD:ACF0138
Abagrotis scopeops|[1189]|LALPA1315-11|Canada|British Columbia|658[0n]|BOLD:ACF0138
Abagrotis scopeops|[1190]|LALPA1228-11|Canada|British Columbia|658[0n]|BOLD:ACF0138
Abagrotis scopeops|[1191]|LALPA703-10|Canada|British Columbia|658[0n]|BOLD:ACF0138
Abagrotis scopeops|[1192]|LBCH7814-10|Canada|British Columbia|658[0n]|BOLD:ACF0138
Abagrotis scopeops|[1193]|LBCH7393-10|Canada|British Columbia|658[0n]|BOLD:ACF0138
Abagrotis scopeops|[1194]|LBCH7331-10|Canada|British Columbia|658[0n]|BOLD:ACF0138
Abagrotis scopeops|[1195]|LBCH1776-10|Canada|British Columbia|658[0n]|BOLD:ACF0138
Abagrotis scopeops|[1196]|RDNMG683-08|United States|California|658[0n]|BOLD:ACF0138
[illegible]

Abagrotis scopeops|[1194]|LBCH7331-10|Canada|British Columbia|658[0n]|BOLD:ACF0138
Abagrotis scopeops|[1195]|LBCH1776-10|Canada|British Columbia|658[0n]|BOLD:ACF0138
Abagrotis scopeops|[1196]|RDNMG683-08|United States|California|658[0n]|BOLD:ACF0138
Abagrotis scopeops|[1197]|RDNMG681-08|United States|Utah|658[0n]|BOLD:ACF0138
Abagrotis scopeops|[1198]|RDNMG392-08|Canada|British Columbia|658[0n]|BOLD:ACF0138
Abagrotis scopeops|[1199]|RDNMG391-08|Canada|British Columbia|658[0n]|BOLD:ACF0138
Abagrotis scopeops|[1200]|RDNMG390-08|United States|Utah|658[0n]|BOLD:ACF0138
Abagrotis scopeops|[1201]|RDNMC103-05|United States|Washington|658[0n]|BOLD:ACF0138
Abagrotis scopeops|[1202]|RDNMC102-05|United States|Washington|658[0n]|BOLD:ACF0138
Abagrotis scopeops|[1203]|LALPA1334-12|Canada|British Columbia|632[0n]|BOLD:ACF0138
Abagrotis duanca|[1204]|RDNMC108-05|Canada|British Columbia|658[0n]|BOLD:ACF4293
Abagrotis duanca|[1205]|LBCG1353-09|Canada|British Columbia|658[0n]|BOLD:ACF4293
Abagrotis duanca|[1206]|LBCG193-08|Canada|British Columbia|658[0n]|BOLD:ACF4293
Abagrotis duanca|[1207]|LBCG1349-09|Canada|British Columbia|658[0n]|BOLD:ACF4293
Abagrotis duanca|[1208]|LBCG2872-09|Canada|British Columbia|658[0n]|BOLD:ACF4293
Abagrotis duanca|[1209]|IAWLB062-10|United States|California|658[0n]|BOLD:ACF4293
Abagrotis duanca|[1210]|RDMAB656-06|Canada|Alberta|658[0n]|BOLD:ACF4293
Abagrotis duanca|[1211]|LOWCE759-06|Canada|British Columbia|658[0n]|BOLD:ACF4293
Abagrotis duanca|[1212]|IAWLB063-10|United States|California|658[0n]|BOLD:ACF4293
Abagrotis duanca|[1213]|RDNMC109-05|Canada|British Columbia|658[0n]|BOLD:ACF3564
Abagrotis variata|[1214]|RDNMC334-05|United States|Oregon|603[0n]|BOLD:ABZ7251
Abagrotis variata|[1215]|RDNMF340-08|Canada|British Columbia|658[0n]|BOLD:ABZ7251
Abagrotis variata|[1216]|RDNMF341-08|Canada|British Columbia|658[0n]|BOLD:ABZ7251
Abagrotis variata|[1217]|RDNMC333-05|United States|Oregon|600[0n]|BOLD:ACF5352
Abagrotis variata|[1218]|LPSK436-08|Canada|Saskatchewan|658[0n]|BOLD:ACF5352
Abagrotis variata|[1219]|RDNMG426-08|United States|California|658[0n]|BOLD:ACF5346
Abagrotis variata|[1220]|LALPA724-10|Canada|British Columbia|658[0n]|BOLD:ACF5346
Abagrotis variata|[1221]|LALPA766-10|Canada|British Columbia|658[0n]|BOLD:ACF5346
Abagrotis variata|[1222]|LALPA1275-11|Canada|British Columbia|658[0n]|BOLD:ACF5346
Abagrotis denticulata|[1223]|RDNM655-05|United States|California|658[0n]|BOLD:ABZ1832
Abagrotis denticulata|[1224]|RDNM654-05|United States|California|658[0n]|BOLD:ABZ1832
Abagrotis denticulata|[1225]|GMLC004-09|United States|California|658[0n]|BOLD:ABZ1832
Abagrotis denticulata|[1226]|GMLC1088-12|United States|California|658[0n]|BOLD:ABZ1832
Abagrotis anchocelioides|[1227]|CNSLU011-13|Canada|Ontario|588[0n]|BOLD:ACE6487
Abagrotis anchocelioides|[1228]|CNSLU010-13|Canada|Ontario|588[0n]|BOLD:ACE6487
Abagrotis anchocelioides|[1229]|RDLQ741-07|Canada|Quebec|658[0n]|BOLD:ACE6487
Abagrotis anchocelioides|[1230]|LGSMG924-10|United States|North Carolina|658[0n]|BOLD:ACE6487
Abagrotis anchocelioides|[1231]|LGSM729-04|United States|Tennessee|658[0n]|BOLD:ACE6487
Abagrotis anchocelioides|[1232]|RDLQ005-05|Canada|Quebec|573[0n]|BOLD:ACE6487
Abagrotis anchocelioides|[1233]|RDLQ740-07|Canada|Quebec|658[0n]|BOLD:ACE6487
Abagrotis anchocelioides|[1234]|RDLQ742-07|Canada|Quebec|658[0n]|BOLD:ACE6487
Abagrotis anchocelioides|[1235]|LNCC1544-13|United States|North Carolina|658[0n]|BOLD:ACE6487
Abagrotis forbesi|[1236]|RDNM676-05|United States|California|658[0n]|BOLD:ACF3351
Abagrotis forbesi|[1237]|RDNMC095-05|Canada|British Columbia|658[0n]|BOLD:ACF3351
Abagrotis forbesi|[1238]|LBCH7958-10|Canada|British Columbia|658[0n]|BOLD:ACF3351
Abagrotis forbesi|[1239]|IAWLB161-10|United States|California|658[0n]|BOLD:ACF3351
Abagrotis forbesi|[1240]|RDNMD389-06|United States|California|658[0n]|BOLD:ACF3351
Abagrotis forbesi|[1241]|RDNMD388-06|United States|California|658[0n]|BOLD:ACF3351
Abagrotis forbesi|[1242]|RDNMC098-05|United States|California|658[0n]|BOLD:ACF3351
Abagrotis forbesi|[1243]|RDNM795-05|Canada|British Columbia|533[1n]|BOLD:ACF3351
Abagrotis forbesi|[1244]|RDNMC097-05|United States|California|658[0n]|BOLD:ACF3351
Abagrotis forbesi|[1245]|RDNMC096-05|United States|Washington|658[0n]|BOLD:ACF3351
Abagrotis forbesi|[1246]|RDNMC011-05|United States|Nevada|621[0n]|BOLD:ACF3351
Abagrotis forbesi|[1247]|IAWLB162-10|United States|California|658[0n]|BOLD:ACF3351
Abagrotis forbesi|[1248]|IAWLB164-10|United States|California|658[0n]|BOLD:ACF3351
Abagrotis forbesi|[1249]|BBLOC1263-11|United States|California|658[0n]|BOLD:ACF3351
Abagrotis nefascia|[1250]|RDNM653-05|Canada|British Columbia|636[0n]|BOLD:ABZ7252
Abagrotis nefascia|[1251]|RDNMC138-05|Canada|British Columbia|658[0n]|BOLD:ABZ7252
Abagrotis nefascia|[1252]|LBCH7169-10|Canada|British Columbia|658[0n]|BOLD:ABZ7252
Abagrotis nefascia|[1253]|LBCH7600-10|Canada|British Columbia|658[0n]|BOLD:ABZ7252
Abagrotis vittifrons|[1254]|LBCH6619-10|Canada|British Columbia|658[0n]|BOLD:ACF3351
Abagrotis vittifrons|[1255]|LOWCD686-06|Canada|British Columbia|658[0n]|BOLD:ACF3351
Abagrotis vittifrons|[1256]|LOWCD685-06|Canada|British Columbia|658[0n]|BOLD:ACF3351
Abagrotis vittifrons|[1257]|LOWC916-05|Canada|British Columbia|658[0n]|BOLD:ACF3351
Abagrotis vittifrons|[1258]|LOWC915-05|Canada|British Columbia|658[0n]|BOLD:ACF3351
Abagrotis vittifrons|[1259]|LOWC914-05|Canada|British Columbia|658[0n]|BOLD:ACF3351
Abagrotis vittifrons|[1260]|LOWC913-05|Canada|British Columbia|658[0n]|BOLD:ACF3351
Abagrotis vittifrons|[1261]|RDNMC093-05|Canada|British Columbia|658[0n]|BOLD:ACF3351
Abagrotis vittifrons|[1262]|LOWCD687-06|Canada|British Columbia|609[0n]|BOLD:ACF3351
Abagrotis vittifrons|[1263]|LBCH6835-10|Canada|British Columbia|658[0n]|BOLD:ACF3351
Abagrotis vittifrons|[1264]|LBCH7119-10|Canada|British Columbia|658[0n]|BOLD:ACF3351
Abagrotis vittifrons|[1265]|LBCH7329-10|Canada|British Columbia|658[0n]|BOLD:ACF3351
Abagrotis vittifrons|[1266]|LBCH7504-10|Canada|British Columbia|658[0n]|BOLD:ACF3351
Abagrotis vittifrons|[1267]|LBCH7692-10|Canada|British Columbia|658[0n]|BOLD:ACF3351
Abagrotis vittifrons|[1268]|LBCH7727-10|Canada|British Columbia|658[0n]|BOLD:ACF3351
Abagrotis vittifrons|[1269]|LBCH7881-10|Canada|British Columbia|658[0n]|BOLD:ACF3351
Abagrotis nanalis|[1270]|NAMUM195-08|United States|California|648[0n]|BOLD:ABZ7250
Abagrotis nanalis|[1271]|LBCH7000-10|Canada|British Columbia|658[0n]|BOLD:ABZ7250
Abagrotis nanalis|[1272]|LBCH7780-10|Canada|British Columbia|658[0n]|BOLD:ABZ7250
Abagrotis nanalis|[1273]|LBCH6947-10|Canada|British Columbia|658[0n]|BOLD:ABZ7250
Abagrotis nanalis|[1274]|LBCH6850-10|Canada|British Columbia|658[0n]|BOLD:ABZ7250
Abagrotis nanalis|[1275]|LBCH6834-10|Canada|British Columbia|658[0n]|BOLD:ABZ7250
Abagrotis nanalis|[1276]|LBCH6765-10|Canada|British Columbia|658[0n]|BOLD:ABZ7250
Abagrotis nanalis|[1277]|LBCH6534-10|Canada|British Columbia|658[0n]|BOLD:ABZ7250
Abagrotis nanalis|[1278]|LBCH6000-10|Canada|British Columbia|658[0n]|BOLD:ABZ7250
Abagrotis nanalis|[1279]|LBCG2869-09|Canada|British Columbia|658[0n]|BOLD:ABZ7250
Abagrotis nanalis|[1280]|LBCG1370-09|Canada|British Columbia|658[0n]|BOLD:ABZ7250
Abagrotis nanalis|[1281]|LBCG1369-09|Canada|British Columbia|658[0n]|BOLD:ABZ7250
Abagrotis nanalis|[1282]|RDNMC092-05|Canada|Alberta|658[0n]|BOLD:ABZ7250
Abagrotis nanalis|[1283]|RDNMC091-05|United States|Oregon|545[1n]|BOLD:ABZ7250
Abagrotis nanalis|[1284]|LBCH6919-10|Canada|British Columbia|634[0n]|BOLD:ABZ7250
Abagrotis nanalis|[1285]|LBCH7538-10|Canada|British Columbia|634[0n]|BOLD:ABZ7250
Abagrotis nanalis|[1286]|LBCH7694-10|Canada|British Columbia|643[0n]|BOLD:ABZ7250
Abagrotis nanalis|[1287]|LBCH7882-10|Canada|British Columbia|658[0n]|BOLD:ABZ7250
Abagrotis placida|[1288]|RDNMC728-06|United States|California|658[0n]|BOLD:AAA5965
Abagrotis placida|[1289]|RDNMC150-05|United States|Oregon|658[0n]|BOLD:AAA5965
Abagrotis placida|[1290]|RDNMC114-05|United States|Washington|658[0n]|BOLD:AAA5965
Abagrotis placida|[1291]|RDNM666-05|United States|Oregon|658[0n]|BOLD:AAA5965
Abagrotis placida|[1292]|RDNM665-05|United States|California|658[0n]|BOLD:AAA5965
Abagrotis placida|[1293]|RDNM663-05|United States|California|658[0n]|BOLD:AAA5965
Abagrotis placida|[1294]|RDNMC124-05|United States|Washington|565[0n]|BOLD:AAA5965
Abagrotis placida|[1295]|RDNMC149-05|United States|Oregon|577[0n]|BOLD:AAA5965
Abagrotis placida|[1296]|RDNMC152-05|United States|Oregon|575[0n]|BOLD:AAA5965

Abagrotis placida|[1294]|RDNMC124-05|United States|Washington|565[0n]|BOLD:AAA5965
Abagrotis placida|[1295]|RDNMC149-05|United States|Oregon|577[0n]|BOLD:AAA5965
Abagrotis placida|[1296]|RDNMC152-05|United States|Oregon|575[0n]|BOLD:AAA5965
Abagrotis placida|[1297]|RDNM691-05|United States|Washington|603[0n]|BOLD:AAA5965
Abagrotis placida|[1298]|RDNM693-05|United States|Washington|608[0n]|BOLD:AAA5965
Abagrotis placida|[1299]|RDNMC125-05|United States|Oregon|590[0n]|BOLD:AAA5965
Abagrotis placida|[1300]|RDNMC168-05|United States|Washington|586[0n]|BOLD:AAA5965
Abagrotis placida|[1301]|RDNMC169-05|United States|Washington|576[0n]|BOLD:AAA5965
Abagrotis placida|[1302]|RDNMC734-06|United States|Oregon|577[1n]|BOLD:AAA5965
Abagrotis placida|[1303]|LBCH936-10|Canada|British Columbia|658[0n]|BOLD:AAA5965
Abagrotis placida|[1304]|LBCH4037-10|Canada|British Columbia|658[0n]|BOLD:AAA5965
Abagrotis placida|[1305]|RDNM668-05|United States|Wyoming|658[0n]|BOLD:AAA5965
Abagrotis placida|[1306]|RDNM670-05|United States|Wyoming|658[0n]|BOLD:AAA5965
Abagrotis placida|[1307]|PSAT116-10|United States|Colorado|658[0n]|BOLD:AAA5965
Abagrotis placida|[1308]|RDNMC146-05|Canada|Alberta|577[7n]|
Abagrotis placida|[1309]|RDMAB232-05|Canada|Alberta|658[0n]|BOLD:ACE7993
Abagrotis placida|[1310]|BBLPB556-10|Canada|British Columbia|658[0n]|BOLD:ACE7993
Abagrotis placida|[1311]|LBCH7792-10|Canada|British Columbia|658[0n]|BOLD:ACE7993
Abagrotis placida|[1312]|LBCH7431-10|Canada|British Columbia|658[0n]|BOLD:ACE7993
Abagrotis placida|[1313]|LBCH6659-10|Canada|British Columbia|658[0n]|BOLD:ACE7993
Abagrotis placida|[1314]|LBCH6549-10|Canada|British Columbia|658[0n]|BOLD:ACE7993
Abagrotis placida|[1315]|LBCH6223-10|Canada|British Columbia|658[0n]|BOLD:ACE7993
Abagrotis placida|[1316]|LBCG2584-09|Canada|British Columbia|658[0n]|BOLD:ACE7993
Abagrotis placida|[1317]|LOWCE765-06|Canada|British Columbia|658[0n]|BOLD:ACE7993
Abagrotis placida|[1318]|RDMAB628-06|Canada|Alberta|658[0n]|BOLD:ACE7993
Abagrotis placida|[1319]|RDMAB627-06|Canada|Alberta|658[0n]|BOLD:ACE7993
Abagrotis placida|[1320]|RDMAB626-06|Canada|Alberta|658[0n]|BOLD:ACE7993
Abagrotis placida|[1321]|RDMAB624-06|Canada|Alberta|658[0n]|BOLD:ACE7993
Abagrotis placida|[1322]|RDMAB598-06|Canada|Alberta|658[0n]|BOLD:ACE7993
Abagrotis placida|[1323]|RDMAB234-05|Canada|Alberta|658[0n]|BOLD:ACE7993
Abagrotis placida|[1324]|RDMAB233-05|Canada|Alberta|658[0n]|BOLD:ACE7993
Abagrotis placida|[1325]|RDMAB231-05|Canada|Alberta|658[0n]|BOLD:ACE7993
Abagrotis placida|[1326]|RDNMC170-05|Canada|British Columbia|658[0n]|BOLD:ACE7993
Abagrotis placida|[1327]|RDNMC128-05|Canada|British Columbia|658[0n]|BOLD:ACE7993
Abagrotis placida|[1328]|RDNMC119-05|Canada|British Columbia|658[0n]|BOLD:ACE7993
Abagrotis placida|[1329]|RDNMC111-05|Canada|British Columbia|658[0n]|BOLD:ACE7993
Abagrotis placida|[1330]|LOWCE764-06|Canada|British Columbia|658[0n]|BOLD:ACE7993
Abagrotis placida|[1331]|RDNMC116-05|Canada|Alberta|558[0n]|BOLD:ACE7993
Abagrotis placida|[1332]|RDNMC112-05|Canada|Alberta|570[0n]|BOLD:ACE7993
Abagrotis placida|[1333]|RDNM694-05|Canada|British Columbia|585[0n]|BOLD:ACE7993
Abagrotis placida|[1334]|RDNMC117-05|Canada|Alberta|594[0n]|BOLD:ACE7993
Abagrotis placida|[1335]|RDNMC118-05|Canada|Alberta|574[0n]|BOLD:ACE7993
Abagrotis placida|[1336]|RDMAB625-06|Canada|Alberta|621[0n]|BOLD:ACE7993
Abagrotis placida|[1337]|SSBAD6364-13|Canada|Alberta|581[0n]|BOLD:ACE7993
Abagrotis placida|[1338]|CNWLN1526-13|Canada|Alberta|598[0n]|BOLD:ACE7993
Abagrotis placida|[1339]|CNWLN1072-13|Canada|Alberta|588[0n]|BOLD:AAA5965
Abagrotis placida|[1340]|CNWLN1069-13|Canada|Alberta|613[0n]|BOLD:AAA5965
Abagrotis placida|[1341]|CNWLN1064-13|Canada|Alberta|613[0n]|BOLD:AAA5965
Abagrotis placida|[1342]|RDMAB236-05|Canada|Alberta|658[0n]|BOLD:AAA5965
Abagrotis placida|[1343]|RDMAB235-05|Canada|Alberta|658[0n]|BOLD:AAA5965
Abagrotis placida|[1344]|RDNMC171-05|Canada|Alberta|658[0n]|BOLD:AAA5965
Abagrotis placida|[1345]|RDNMC121-05|Canada|Alberta|658[0n]|BOLD:AAA5965
Abagrotis placida|[1346]|RDNMB287-05|Canada|Manitoba|602[0n]|BOLD:AAA5965
Abagrotis placida|[1347]|RDNMB286-05|Canada|Manitoba|601[0n]|BOLD:AAA5965
Abagrotis placida|[1348]|RDMAB373-05|Canada|Alberta|605[0n]|BOLD:AAA5965
Abagrotis placida|[1349]|RDNMC342-05|Canada|Manitoba|631[0n]|BOLD:AAA5965
Abagrotis placida|[1350]|RDMAB650-06|Canada|Alberta|658[0n]|BOLD:AAA5965
Abagrotis placida|[1351]|RDMAB651-06|Canada|Alberta|658[0n]|BOLD:AAA5965
Abagrotis placida|[1352]|RDMAB654-06|Canada|Alberta|658[0n]|BOLD:AAA5965
Abagrotis placida|[1353]|RDMAB655-06|Canada|Alberta|658[0n]|BOLD:AAA5965
Abagrotis placida|[1354]|LOWCE763-06|Canada|British Columbia|658[0n]|BOLD:AAA5965
Abagrotis placida|[1355]|LOWCE766-06|Canada|British Columbia|658[0n]|BOLD:AAA5965
Abagrotis placida|[1356]|DUNLP190-08|Canada|British Columbia|649[1n]|BOLD:AAA5965
Abagrotis placida|[1357]|CNWLN1076-13|Canada|Alberta|610[0n]|BOLD:AAA5965
Abagrotis placida|[1358]|RDNMC123-05|Canada|Alberta|658[0n]|BOLD:AAA5965
Abagrotis placida|[1359]|RDNMC122-05|Canada|Alberta|658[0n]|BOLD:AAA5965
Abagrotis placida|[1360]|RDNMC120-05|Canada|British Columbia|658[0n]|BOLD:AAA5965
Abagrotis placida|[1361]|RDNM689-05|Canada|British Columbia|658[0n]|BOLD:AAA5965
Abagrotis placida|[1362]|RDNM688-05|Canada|British Columbia|658[0n]|BOLD:AAA5965
Abagrotis placida|[1363]|CNWLF2003-12|Canada|Alberta|637[0n]|BOLD:AAA5965
Abagrotis placida|[1364]|CNWLF2020-12|Canada|Alberta|633[0n]|BOLD:AAA5965
Abagrotis placida|[1365]|RDNM690-05|Canada|British Columbia|599[0n]|BOLD:AAA5965
Abagrotis placida|[1366]|RDNM696-05|Canada|British Columbia|599[0n]|BOLD:AAA5965
Abagrotis placida|[1367]|BBLPB744-10|Canada|British Columbia|658[0n]|BOLD:AAA5965
Abagrotis placida|[1368]|CNWLF2021-12|Canada|Alberta|630[0n]|BOLD:AAA5965
Abagrotis placida|[1369]|CNWLN1068-13|Canada|Alberta|610[0n]|BOLD:AAA5965
Abagrotis placida|[1370]|CNWLN1525-13|Canada|Alberta|598[0n]|BOLD:AAA5965
Abagrotis placida|[1371]|CNWLN1530-13|Canada|Alberta|589[0n]|BOLD:AAA5965
Abagrotis dickeli|[1372]|RDNM695-05|Canada|British Columbia|610[0n]|BOLD:ACF3422
Abagrotis dickeli|[1373]|RDNMC082-05|Canada|British Columbia|658[0n]|BOLD:ACF3422
Abagrotis dickeli|[1374]|RDNMC115-05|Canada|British Columbia|658[0n]|BOLD:ACF3422
Abagrotis dickeli|[1375]|RDNMB272-05|Canada|Manitoba|658[0n]|BOLD:ACE9894
Abagrotis dickeli|[1376]|RDNM667-05|United States|Wyoming|658[0n]|BOLD:ACE9894
Abagrotis dickeli|[1377]|RDNM671-05|United States|Wyoming|611[1n]|BOLD:ACE9894
Abagrotis dickeli|[1378]|RDNMB273-05|Canada|Manitoba|602[0n]|BOLD:ACE9894
Abagrotis dickeli|[1379]|RDNMC127-05|Canada|Saskatchewan|658[0n]|BOLD:ACE9894
Abagrotis turbulenta|[1380]|RDNMC161-05|Canada|British Columbia|658[0n]|BOLD:ACE9894
Abagrotis turbulenta|[1381]|RDNMC162-05|United States|Montana|658[0n]|BOLD:ACE9894
Abagrotis dickeli|[1382]|RDNMC343-05|Canada|Manitoba|658[0n]|BOLD:ACE9894
Abagrotis dickeli|[1383]|RDNMC727-06|Canada|Manitoba|658[0n]|BOLD:ACE9894
Abagrotis turbulenta|[1384]|RDNMG679-08|Canada|British Columbia|658[0n]|BOLD:ACE9894
Abagrotis turbulenta|[1385]|RDNMG680-08|Canada|British Columbia|658[0n]|BOLD:ACE9894
Abagrotis hermina|[1386]|RDNMB942-05|Canada|Alberta|658[0n]|BOLD:ACE7022
Abagrotis hermina|[1387]|RDNMC153-05|Canada|Alberta|658[0n]|BOLD:ACE7022
Abagrotis hermina|[1388]|RDNMC166-05|Canada|Alberta|658[0n]|BOLD:ACE7022
Abagrotis hermina|[1389]|RDMAB599-06|Canada|Alberta|658[0n]|BOLD:ACE7022
Abagrotis hermina|[1390]|LBCD456-05|Canada|British Columbia|658[0n]|BOLD:ACF3428
Abagrotis hermina|[1391]|LBCH7885-10|Canada|British Columbia|644[0n]|BOLD:ACF0135
Abagrotis hermina|[1392]|RDNMC167-05|Canada|Alberta|658[0n]|BOLD:ACF0135
Abagrotis hermina|[1393]|RDMAB237-05|Canada|Alberta|658[0n]|BOLD:ACF0135
Abagrotis hermina|[1394]|RDNMC165-05|Canada|Saskatchewan|655[0n]|BOLD:ACF0135
Abagrotis hermina|[1395]|RDMAB681-06|Canada|Alberta|622[0n]|BOLD:ACF0135
Abagrotis hermina|[1396]|RDNM692-05|Canada|British Columbia|612[0n]|BOLD:ACF0135

[illegible]
Abagrotis hermina|[1394]|RDNMC165-05|Canada|Saskatchewan|655[0n]|BOLD:ACF0135
Abagrotis hermina|[1395]|RDMAB681-06|Canada|Alberta|622[0n]|BOLD:ACF0135
Abagrotis hermina|[1396]|RDNM692-05|Canada|British Columbia|612[0n]|BOLD:ACF0135
Abagrotis hermina|[1397]|RDNMC126-05|Canada|British Columbia|658[0n]|BOLD:ACF0135
Abagrotis hermina|[1398]|BBLPB746-10|Canada|Alberta|658[0n]|BOLD:ACF0135
Abagrotis nefascia|[1399]|BBLEC446-09|Canada|New Brunswick|658[0n]|BOLD:ACF4293
Abagrotis nefascia|[1400]|BBLPC425-09|Canada|New Brunswick|658[0n]|BOLD:ACF4293
Abagrotis cupida|[1401]|RDMAB230-05|Canada|Alberta|600[1n]|BOLD:ACF4293
Abagrotis cupida|[1402]|RDNMB291-05|Canada|Manitoba|566[0n]|BOLD:ACF4293
Abagrotis cupida|[1403]|RDNM723-05|Canada|Ontario|618[0n]|BOLD:ACF4293
Abagrotis cupida|[1404]|RDNM721-05|Canada|Ontario|618[0n]|BOLD:ACF4293
Abagrotis cupida|[1405]|RDMAB243-05|Canada|Alberta|617[0n]|BOLD:ACF4293
Abagrotis cupida|[1406]|RDMAB238-05|Canada|Alberta|658[0n]|BOLD:ACF4293
Abagrotis cupida|[1407]|RDNMC730-06|Canada|Ontario|658[0n]|BOLD:ACF4293
Abagrotis cupida|[1408]|RDNM718-05|Canada|Ontario|609[0n]|BOLD:ACF4293
Abagrotis brunneipennis|[1409]|RDNM683-05|United States|Oregon|612[0n]|BOLD:ACF4293
Abagrotis brunneipennis|[1410]|RDNM682-05|Canada|British Columbia|611[0n]|BOLD:ACF4293
Abagrotis brunneipennis|[1411]|RDNM684-05|United States|Oregon|608[0n]|BOLD:ACF4293
Abagrotis cupida|[1412]|RDNM719-05|Canada|Ontario|607[0n]|BOLD:ACF4293
Abagrotis cupida|[1413]|RDNM720-05|Canada|Ontario|607[0n]|BOLD:ACF4293
Abagrotis cupida|[1414]|RDNMB270-05|Canada|Manitoba|599[0n]|BOLD:ACF4293
Abagrotis brunneipennis|[1415]|RDNM669-05|Canada|British Columbia|658[0n]|BOLD:ACF4293
Abagrotis cupida|[1416]|RDNMB288-05|Canada|Manitoba|601[0n]|BOLD:ACF4293
Abagrotis cupida|[1417]|RDNM722-05|Canada|Ontario|658[0n]|BOLD:ACF4293
Abagrotis cupida|[1418]|RDNMB297-05|Canada|Manitoba|603[0n]|BOLD:ACF4293
Abagrotis cupida|[1419]|RDNMC729-06|Canada|Ontario|658[0n]|BOLD:ACF4293
Abagrotis cupida|[1420]|RDMAB682-06|Canada|Alberta|658[0n]|BOLD:ACF4293
Abagrotis cupida|[1421]|RDNMB271-05|Canada|Manitoba|658[0n]|BOLD:ACF4293
Abagrotis cupida|[1422]|RDNMB269-05|Canada|Manitoba|658[0n]|BOLD:ACF4293
Abagrotis cupida|[1423]|RDNM717-05|Canada|Ontario|658[0n]|BOLD:ACF4293
Abagrotis magnicupida|[1424]|RDNM713-05|United States|Maryland|658[0n]|BOLD:ACF4293
Abagrotis brunneipennis|[1425]|RDNM681-05|Canada|Ontario|658[0n]|BOLD:ACF4293
Abagrotis brunneipennis|[1426]|RDNM680-05|Canada|British Columbia|658[0n]|BOLD:ACF4293
Abagrotis brunneipennis|[1427]|RDNM679-05|Canada|Ontario|658[0n]|BOLD:ACF4293
Abagrotis nefascia|[1428]|RDNM652-05|Canada|British Columbia|658[0n]|BOLD:ACF4293
Abagrotis nefascia|[1429]|RDNM651-05|United States|Oregon|658[0n]|BOLD:ACF4293
Abagrotis cupida|[1430]|RDLQ006-05|Canada|Quebec|658[0n]|BOLD:ACF4293
Abagrotis brunneipennis|[1431]|RDMAB248-05|Canada|Alberta|634[0n]|BOLD:ACF4293
Abagrotis brunneipennis|[1432]|TTMNB494-06|Canada|New Brunswick|658[0n]|BOLD:ACF4293
Abagrotis cupida|[1433]|RDNMB289-05|Canada|Manitoba|658[0n]|BOLD:ACF4293
Abagrotis cupida|[1434]|RDNMB293-05|Canada|Manitoba|658[0n]|BOLD:ACF4293
Abagrotis cupida|[1435]|RDNMB294-05|Canada|Manitoba|658[0n]|BOLD:ACF4293
Abagrotis cupida|[1436]|RDNMB295-05|Canada|Manitoba|658[0n]|BOLD:ACF4293
Abagrotis cupida|[1437]|RDNMB296-05|Canada|Manitoba|658[0n]|BOLD:ACF4293
Abagrotis cupida|[1438]|RDNMB449-05|Canada|Manitoba|658[0n]|BOLD:ACF4293
Abagrotis discoidalis|[1439]|RDNMC106-05|United States|Oregon|658[0n]|BOLD:ACF4293
Abagrotis discoidalis|[1440]|RDNMC107-05|Canada|Alberta|658[0n]|BOLD:ACF4293
Abagrotis nefascia|[1441]|RDNMC135-05|United States|Oregon|658[0n]|BOLD:ACF4293
Abagrotis nefascia|[1442]|RDNMC136-05|United States|Wyoming|658[0n]|BOLD:ACF4293
Abagrotis nefascia|[1443]|RDNMC137-05|Canada|British Columbia|658[0n]|BOLD:ACF4293
Abagrotis nefascia|[1444]|RDNMC139-05|United States|Oregon|658[0n]|BOLD:ACF4293
Abagrotis cupida|[1445]|RDMAB239-05|Canada|Alberta|658[0n]|BOLD:ACF4293
Abagrotis cupida|[1446]|RDMAB241-05|Canada|Alberta|658[0n]|BOLD:ACF4293
Abagrotis brunneipennis|[1447]|RDMAB242-05|Canada|Alberta|658[0n]|BOLD:ACF4293
Abagrotis cupida|[1448]|RDMAB244-05|Canada|Alberta|658[0n]|BOLD:ACF4293
Abagrotis brunneipennis|[1449]|RDMAB245-05|Canada|Alberta|658[0n]|BOLD:ACF4293
Abagrotis brunneipennis|[1450]|RDMAB246-05|Canada|Alberta|658[0n]|BOLD:ACF4293
Abagrotis brunneipennis|[1451]|RDMAB247-05|Canada|Alberta|658[0n]|BOLD:ACF4293
Abagrotis magnicupida|[1452]|LSEU168-06|United States|Georgia|658[0n]|BOLD:ACF4293
Abagrotis cupida|[1453]|RDNMC725-06|Canada|Ontario|658[0n]|BOLD:ACF4293
Abagrotis cupida|[1454]|RDNMC726-06|Canada|Manitoba|658[0n]|BOLD:ACF4293
Abagrotis cupida|[1455]|RDNMC731-06|Canada|Ontario|658[0n]|BOLD:ACF4293
Abagrotis brunneipennis|[1456]|RDMAB653-06|Canada|Alberta|658[0n]|BOLD:ACF4293
Abagrotis brunneipennis|[1457]|TTMNB492-06|Canada|New Brunswick|658[0n]|BOLD:ACF4293
Abagrotis brunneipennis|[1458]|TTMNB493-06|Canada|New Brunswick|658[0n]|BOLD:ACF4293
Abagrotis cupida|[1459]|TMNBB406-06|Canada|New Brunswick|658[0n]|BOLD:ACF4293
Abagrotis cupida|[1460]|TMNBB407-06|Canada|New Brunswick|658[0n]|BOLD:ACF4293
Abagrotis cupida|[1461]|TMNBB408-06|Canada|New Brunswick|658[0n]|BOLD:ACF4293
Abagrotis cupida|[1462]|XAJ855-06|Canada|Ontario|658[0n]|BOLD:ACF4293
Abagrotis discoidalis|[1463]|NAMUM274-08|United States|California|658[0n]|BOLD:ACF4293
Abagrotis nefascia|[1464]|NAMUM278-08|United States|California|658[0n]|BOLD:ACF4293
Abagrotis discoidalis|[1465]|RDNMG395-08|United States|California|658[0n]|BOLD:ACF4293
Abagrotis discoidalis|[1466]|RDNMG396-08|United States|California|658[0n]|BOLD:ACF4293
Abagrotis discoidalis|[1467]|RDNMG397-08|United States|Oregon|658[0n]|BOLD:ACF4293
Abagrotis discoidalis|[1468]|RDNMG398-08|United States|Oregon|658[0n]|BOLD:ACF4293
Abagrotis discoidalis|[1469]|RDNMG574-08|United States|Wyoming|658[0n]|BOLD:ACF4293
Abagrotis brunneipennis|[1470]|LBCH7334-10|Canada|British Columbia|658[0n]|BOLD:ACF4293
Abagrotis discoidalis|[1471]|IAWLB085-10|United States|California|658[0n]|BOLD:ACF4293
Abagrotis discoidalis|[1472]|IAWLB086-10|United States|California|658[0n]|BOLD:ACF4293
Abagrotis discoidalis|[1473]|IAWLB087-10|United States|California|658[0n]|BOLD:ACF4293
Abagrotis discoidalis|[1474]|IAWLB088-10|United States|California|658[0n]|BOLD:ACF4293
Abagrotis discoidalis|[1475]|IAWLB089-10|United States|California|658[0n]|BOLD:ACF4293
Abagrotis magnicupida|[1476]|LNCC083-10|United States|North Carolina|658[0n]|BOLD:ACF4293
Abagrotis magnicupida|[1477]|LNCC084-10|United States|North Carolina|658[0n]|BOLD:ACF4293
Abagrotis brunneipennis|[1478]|LNCC418-10|United States|North Carolina|658[0n]|BOLD:ACF4293
Abagrotis magnicupida|[1479]|LNCC450-10|United States|North Carolina|658[0n]|BOLD:ACF4293
Abagrotis magnicupida|[1480]|LNCC475-10|United States|North Carolina|658[0n]|BOLD:ACF4293
Abagrotis cryptica|[1481]|RDNMJ183-10|United States|Indiana|658[0n]|BOLD:ACF4293
Abagrotis magnicupida|[1482]|LNCC1323-11|United States|North Carolina|658[0n]|BOLD:ACF4293
Abagrotis belfragei|[1483]|CNCLB478-14|United States|Arkansas|658[0n]|BOLD:ACF4293
Abagrotis belfragei|[1484]|CNCLB759-14|United States|Missouri|658[0n]|BOLD:ACF4293
Abagrotis reedi|[1485]|RDNMK474-11|United States|New Mexico|658[1n]|BOLD:ACF3347
Abagrotis reedi|[1486]|RDNM686-05|Canada|British Columbia|579[0n]|BOLD:ACF3347
Abagrotis reedi|[1487]|RDNMC141-05|United States|Oregon|658[0n]|BOLD:ACF3347
Abagrotis reedi|[1488]|RDNM664-05|United States|California|658[0n]|BOLD:ACF3347
Abagrotis reedi|[1489]|LOCBC816-06|United States|California|658[0n]|BOLD:ACF3347
Abagrotis reedi|[1490]|RDNMK470-11|United States|New Mexico|658[0n]|BOLD:ACF3347
Abagrotis reedi|[1491]|RDNMC148-05|Canada|British Columbia|658[0n]|BOLD:ACF3347
Abagrotis reedi|[1492]|RDNMC151-05|Canada|British Columbia|658[0n]|BOLD:ACF3347
Abagrotis reedi|[1493]|LBCH6552-10|Canada|British Columbia|658[0n]|BOLD:ACF3347
Abagrotis reedi|[1494]|LBCH7808-10|Canada|British Columbia|658[0n]|BOLD:ACF3347
Abagrotis reedi|[1495]|LBCH7813-10|Canada|British Columbia|658[0n]|BOLD:ACF3347
[illegible]

Abagrotis reedi|[1493]|LBCH6552-10|Canada|British Columbia|658[0n]|BOLD:ACF3347
Abagrotis reedi|[1494]|LBCH7808-10|Canada|British Columbia|658[0n]|BOLD:ACF3347
Abagrotis reedi|[1495]|LBCH7813-10|Canada|British Columbia|658[0n]|BOLD:ACF3347
Abagrotis reedi|[1496]|RDMAB518-06|Canada|Alberta|658[0n]|BOLD:ACF3347
Abagrotis reedi|[1497]|RDNM687-05|United States|Oregon|658[0n]|BOLD:ACF3347
Abagrotis reedi|[1498]|RDNMC144-05|United States|California|658[0n]|BOLD:ACF3347
Abagrotis reedi|[1499]|LBCH7375-10|Canada|British Columbia|658[0n]|BOLD:ACF3347
Abagrotis reedi|[1500]|RDNMB290-05|Canada|Manitoba|658[0n]|BOLD:ACF3347
Abagrotis reedi|[1501]|RDMAB652-06|Canada|Alberta|658[0n]|BOLD:ACF3347
Abagrotis reedi|[1502]|RDMAB240-05|Canada|Alberta|658[0n]|BOLD:ACF3347
Abagrotis reedi|[1503]|RDNMC143-05|Canada|Saskatchewan|658[0n]|BOLD:ACF3347
Abagrotis reedi|[1504]|RDNMB292-05|Canada|Manitoba|595[0n]|BOLD:ACF3347
Abagrotis reedi|[1505]|CNGRE019-12|Canada|Saskatchewan|636[0n]|BOLD:ACF3347
Abagrotis reedi|[1506]|RDNMC147-05|United States|California|658[0n]|BOLD:ACF3347
Abagrotis reedi|[1507]|LOCBF2631-13|United States|California|658[0n]|BOLD:ACF3347
Abagrotis pulchrata|[1508]|DUNLP147-08|Canada|British Columbia|658[0n]|BOLD:ABZ7253
Abagrotis pulchrata|[1509]|RDNM678-05|United States|Oregon|658[0n]|BOLD:ABZ7253
Abagrotis pulchrata|[1510]|RDNMG393-08|United States|Oregon|658[0n]|BOLD:ABZ7253
Abagrotis pulchrata|[1511]|RDNMG394-08|United States|Oregon|658[0n]|BOLD:ABZ7253
Abagrotis orbis|[1512]|BBLSY670-09|United States|Arizona|658[0n]|BOLD:ACF3347
Abagrotis orbis|[1513]|RDNMG389-08|United States|Colorado|658[0n]|BOLD:ACF3347
Abagrotis orbis|[1514]|AWCLB543-11|United States|Arizona|658[0n]|BOLD:ACF3347
Abagrotis orbis|[1515]|USLEP1302-10|United States|Arizona|658[0n]|BOLD:ACF3347
Abagrotis orbis|[1516]|USLEP290-10|United States|Arizona|658[0n]|BOLD:ACF3347
Abagrotis orbis|[1517]|BBLSX709-09|United States|Arizona|658[0n]|BOLD:ACF3347
Abagrotis orbis|[1518]|BBLSW695-09|United States|Arizona|658[0n]|BOLD:ACF3347
Abagrotis orbis|[1519]|BBLSW225-09|United States|Arizona|658[0n]|BOLD:ACF3347
Abagrotis orbis|[1520]|BBLSW188-09|United States|Arizona|658[0n]|BOLD:ACF3347
Abagrotis orbis|[1521]|RDNMG387-08|Canada|Ontario|658[0n]|BOLD:ACF3347
Abagrotis orbis|[1522]|RDNMG385-08|Canada|Alberta|658[0n]|BOLD:ACF3347
Abagrotis orbis|[1523]|RDNMD390-06|United States|California|658[0n]|BOLD:ACF3347
Abagrotis orbis|[1524]|RDNMC129-05|Canada|Alberta|658[0n]|BOLD:ACF3347
Abagrotis orbis|[1525]|RDNMG388-08|United States|Colorado|658[0n]|BOLD:ACF3347
Abagrotis orbis|[1526]|CMAZA144-09|United States|Arizona|632[0n]|BOLD:ACF3347
Abagrotis orbis|[1527]|IAWLB299-11|United States|Arizona|658[0n]|BOLD:ACF3347
Abagrotis orbis|[1528]|BBLOB1549-11|United States|Arizona|658[0n]|BOLD:ACF3347
Abagrotis baueri|[1529]|LPVIB660-08|Canada|British Columbia|634[0n]|BOLD:ACF3416
Abagrotis baueri|[1530]|RDNM673-05|Canada|British Columbia|658[0n]|BOLD:ACF3416
Abagrotis baueri|[1531]|RDNM674-05|Canada|British Columbia|658[0n]|BOLD:ACF3416
Abagrotis baueri|[1532]|RDNM675-05|United States|Oregon|658[0n]|BOLD:ACF3416
Abagrotis baueri|[1533]|RDNM677-05|United States|Oregon|658[0n]|BOLD:ACF3416
Abagrotis baueri|[1534]|RDNMG386-08|United States|California|658[0n]|BOLD:ACF3416
Abagrotis baueri|[1535]|LALPA729-10|Canada|British Columbia|658[0n]|BOLD:ACF3416
Abagrotis baueri|[1536]|LALPA931-11|Canada|British Columbia|658[0n]|BOLD:ACF3416
Abagrotis baueri|[1537]|LALPA1243-11|Canada|British Columbia|658[0n]|BOLD:ACF3416
Abagrotis rubricundis|[1538]|RDNM662-05|United States|California|616[0n]|BOLD:ACF3643
Abagrotis rubricundis|[1539]|RDNMC173-05|United States|California|658[0n]|BOLD:ACF3643
Abagrotis mirabilis|[1540]|RDNMD305-06|United States|Colorado|658[0n]|BOLD:ACF3643
Abagrotis mirabilis|[1541]|LBCH7325-10|Canada|British Columbia|658[0n]|BOLD:ACF3643
Abagrotis mirabilis|[1542]|LBCH7837-10|Canada|British Columbia|658[0n]|BOLD:ACF3643
Abagrotis mirabilis|[1543]|RDNMD307-06|United States|Colorado|658[0n]|BOLD:ACF3643
Abagrotis mirabilis|[1544]|RDNMD304-06|United States|Colorado|658[0n]|BOLD:ACF3643
Abagrotis mirabilis|[1545]|RDNMD302-06|United States|Colorado|658[0n]|BOLD:ACF3643
Abagrotis mirabilis|[1546]|LOWCD391-06|Canada|British Columbia|658[0n]|BOLD:ACF3643
Abagrotis mirabilis|[1547]|RDNMC164-05|Canada|British Columbia|658[0n]|BOLD:ACF3643
Abagrotis mirabilis|[1548]|RDNMC163-05|Canada|British Columbia|658[0n]|BOLD:ACF3643
Abagrotis mirabilis|[1549]|RDNM660-05|United States|Oregon|658[0n]|BOLD:ACF3643
Abagrotis mirabilis|[1550]|RDNM659-05|Canada|British Columbia|658[0n]|BOLD:ACF3643
Abagrotis mirabilis|[1551]|RDNM658-05|Canada|British Columbia|658[0n]|BOLD:ACF3643
Abagrotis mirabilis|[1552]|RDNM661-05|Canada|British Columbia|616[0n]|BOLD:ACF3643
Abagrotis mirabilis|[1553]|RDNM715-05|United States|Colorado|530[0n]|BOLD:ACF3643
Abagrotis mirabilis|[1554]|LOWCD390-06|Canada|British Columbia|543[0n]|BOLD:ACF3643
Abagrotis mirabilis|[1555]|RDNMD303-06|United States|Colorado|656[0n]|BOLD:ACF3643
Abagrotis mirabilis|[1556]|RDNMD306-06|United States|Colorado|656[0n]|BOLD:ACF3643
Abagrotis mirabilis|[1557]|RDNM714-05|United States|Colorado|518[0n]|
Abagrotis mirabilis|[1558]|RDNM716-05|United States|Colorado|583[4n]|BOLD:ACF3643
Abagrotis mirabilis|[1559]|RDNMD308-06|United States|Colorado|658[0n]|BOLD:ACF3643
Abagrotis mirabilis|[1560]|RDNMD309-06|United States|Colorado|658[0n]|BOLD:ACF3643
Abagrotis mirabilis|[1561]|LBCH6153-10|Canada|British Columbia|658[0n]|BOLD:ACF3643
Abagrotis mirabilis|[1562]|LBCH7239-10|Canada|British Columbia|658[0n]|BOLD:ACF3643
Abagrotis mirabilis|[1563]|BBLPB755-10|Canada|British Columbia|658[0n]|BOLD:ACF3643
Abagrotis glenni|[1564]|RDNM656-05|United States|Oregon|658[0n]|BOLD:ACF3643
Abagrotis glenni|[1565]|RDNMC159-05|United States|Oregon|658[0n]|BOLD:ACF3643
Abagrotis glenni|[1566]|RDNMC160-05|United States|Oregon|658[0n]|BOLD:ACF3643
Abagrotis glenni|[1567]|RDNM657-05|United States|Oregon|530[0n]|BOLD:ACF3643
Abagrotis mirabilis|[1568]|RDNMC140-05|Canada|British Columbia|658[0n]|BOLD:ACF3643
Abagrotis mirabilis|[1569]|DUNLP145-08|Canada|British Columbia|625[1n]|BOLD:ACF3643
Abagrotis mirabilis|[1570]|LBCH6763-10|Canada|British Columbia|658[0n]|BOLD:ACF3643
Abagrotis mirabilis|[1571]|LBCH7101-10|Canada|British Columbia|658[0n]|BOLD:ACF3643
Abagrotis hennei|[1572]|CNCLB975-14|United States|California|658[0n]|BOLD:ACF3643
Abagrotis glenni|[1573]|NAMUM068-08|United States|Arizona|657[0n]|BOLD:ACE4051
Abagrotis petalama|[1574]|RDNM712-05|United States|Oregon|658[0n]|BOLD:ACE4051
Abagrotis alampeta|[1575]|IAWLB298-11|United States|Arizona|658[0n]|BOLD:ACE4051
Abagrotis alampeta|[1576]|IAWLB544-11|United States|Arizona|658[0n]|BOLD:ACE4051
Abagrotis alampeta|[1577]|RDNMJ476-11|United States|Arizona|658[0n]|BOLD:ACE4051
Abagrotis alampeta|[1578]|RDNMJ715-11|United States|Arizona|658[0n]|BOLD:ACE4051
Abagrotis alampeta|[1579]|CMAZA926-12|United States|Arizona|658[0n]|BOLD:ACE4051
Abagrotis hennei|[1580]|CNCLB976-14|United States|California|658[0n]|BOLD:ACE4051
Abagrotis hennei|[1581]|TML193-14|United States||658[0n]|BOLD:ACE4051
Abagrotis bimarginalis|[1582]|NAMUM052-08|United States|Arizona|657[0n]|BOLD:AAJ4476
Abagrotis bimarginalis|[1583]|RDNMJ518-11|United States|Arizona|658[0n]|BOLD:AAJ4476
Abagrotis bimarginalis|[1584]|RDNMJ716-11|United States|Arizona|658[0n]|BOLD:AAJ4476
Abagrotis alcandola|[1585]|LNAUS5289-13|United States|Arizona|658[0n]|BOLD:AAE2248
Abagrotis alcandola|[1586]|CNCLB756-14|||658[0n]|BOLD:AAE2248
Abagrotis alcandola|[1587]|CNCLB757-14|United States|Arizona|658[0n]|BOLD:AAE2248
Abagrotis erratica|[1588]|RDNMC131-05|Canada|British Columbia|635[0n]|BOLD:AAE2248
Abagrotis erratica|[1589]|RDNMC132-05|Canada|British Columbia|581[0n]|BOLD:AAE2248
Abagrotis erratica|[1590]|RDNMC133-05|United States|Nevada|658[0n]|BOLD:AAE2248
Abagrotis erratica|[1591]|RDNMC134-05|United States|Oregon|658[0n]|BOLD:AAE2248
Abagrotis kirkwoodi|[1592]|CNCLB752-14|United States|California|658[0n]|BOLD:AAE2248
Abagrotis erratica|[1593]|RDNMC130-05|United States|Nevada|658[0n]|BOLD:AAE2248
Abagrotis erratica|[1594]|JMMMB182-11|United States|California|658[0n]|BOLD:AAE2248
Abagrotis erratica|[1595]|JMMMB317-11|United States|California|658[0n]|BOLD:AAE2248

Abagrotis erratica|[1593]|RDNMC130-05|United States|Nevada|658[0n]|BOLD:AAE2248
Abagrotis erratica|[1594]|JMMMB182-11|United States|California|658[0n]|BOLD:AAE2248
Abagrotis erratica|[1595]|JMMMB317-11|United States|California|658[0n]|BOLD:AAE2248
Abagrotis kirkwoodi|[1596]|TML194-14|United States||658[1n]|BOLD:AAE2248
Parabagrotis exsertistigma|[1597]|LPABC602-09|Canada|Alberta|632[0n]|BOLD:AAA3352
Parabagrotis exsertistigma|[1598]|LPABC569-09|Canada|Alberta|658[0n]|BOLD:AAA3352
Parabagrotis exsertistigma|[1599]|LPABB465-08|Canada|Alberta|658[0n]|BOLD:AAA3352
Parabagrotis exsertistigma|[1600]|LPABC572-09|Canada|Alberta|658[0n]|BOLD:AAA3352
Parabagrotis exsertistigma|[1601]|LPABC576-09|Canada|Alberta|658[0n]|BOLD:AAA3352
Parabagrotis exsertistigma|[1602]|LPABC604-09|Canada|Alberta|658[0n]|BOLD:AAA3352
Parabagrotis exsertistigma|[1603]|LPAB019-08|Canada|Alberta|658[0n]|BOLD:AAA3352
Parabagrotis exsertistigma|[1604]|LPABC568-09|Canada|Alberta|605[0n]|BOLD:AAA3352
Parabagrotis exsertistigma|[1605]|LPABC570-09|Canada|Alberta|658[0n]|BOLD:AAA3352
Parabagrotis exsertistigma|[1606]|LPABC610-09|Canada|Alberta|658[0n]|BOLD:AAA3352
Parabagrotis exsertistigma|[1607]|LPABC628-09|Canada|Alberta|658[0n]|BOLD:AAA3352
Parabagrotis exsertistigma|[1608]|LPABC632-09|Canada|Alberta|658[0n]|BOLD:AAA3352
Parabagrotis exsertistigma|[1609]|LPABC567-09|Canada|Alberta|658[0n]|BOLD:AAA3352
Parabagrotis exsertistigma|[1610]|LPABC585-09|Canada|Alberta|658[0n]|BOLD:AAA3352
Parabagrotis exsertistigma|[1611]|LPABC605-09|Canada|Alberta|600[0n]|BOLD:AAA3352
Parabagrotis exsertistigma|[1612]|LPABC579-09|Canada|Alberta|658[0n]|BOLD:AAA3352
Parabagrotis exsertistigma|[1613]|LBCH6825-10|Canada|British Columbia|658[0n]|BOLD:AAA3352
Parabagrotis exsertistigma|[1614]|LBCH6823-10|Canada|British Columbia|658[0n]|BOLD:AAA3352
Parabagrotis exsertistigma|[1615]|LBCH6824-10|Canada|British Columbia|658[0n]|BOLD:AAA3352
Parabagrotis exsertistigma|[1616]|LBCH6912-10|Canada|British Columbia|658[0n]|BOLD:AAA3352
Parabagrotis exsertistigma|[1617]|LBCH6935-10|Canada|British Columbia|658[0n]|BOLD:AAA3352
Parabagrotis exsertistigma|[1618]|LPABC565-09|Canada|Alberta|658[0n]|BOLD:AAA3352
Parabagrotis exsertistigma|[1619]|LPABC566-09|Canada|Alberta|658[0n]|BOLD:AAA3352
Parabagrotis exsertistigma|[1620]|LPABC571-09|Canada|Alberta|658[0n]|BOLD:AAA3352
Parabagrotis exsertistigma|[1621]|LPABC574-09|Canada|Alberta|658[0n]|BOLD:AAA3352
Parabagrotis exsertistigma|[1622]|LPABB332-08|Canada|Alberta|658[0n]|BOLD:AAA3352
Parabagrotis exsertistigma|[1623]|LPABB333-08|Canada|Alberta|658[0n]|BOLD:AAA3352
Parabagrotis exsertistigma|[1624]|LPVIC116-08|Canada|British Columbia|658[0n]|BOLD:AAA3352
Parabagrotis exsertistigma|[1625]|LPVIC115-08|Canada|British Columbia|658[0n]|BOLD:AAA3352
Parabagrotis exsertistigma|[1626]|LPABB007-08|Canada|Alberta|658[0n]|BOLD:AAA3352
Parabagrotis exsertistigma|[1627]|LPAB018-08|Canada|Alberta|658[0n]|BOLD:AAA3352
Parabagrotis exsertistigma|[1628]|LPAB017-08|Canada|Alberta|658[0n]|BOLD:AAA3352
Parabagrotis exsertistigma|[1629]|LPAB011-08|Canada|Alberta|658[0n]|BOLD:AAA3352
Parabagrotis exsertistigma|[1630]|LPAB007-08|Canada|Alberta|658[0n]|BOLD:AAA3352
Parabagrotis exsertistigma|[1631]|LBCG418-08|Canada|British Columbia|658[0n]|BOLD:AAA3352
Parabagrotis exsertistigma|[1632]|RDNMD379-06|United States|California|658[0n]|BOLD:AAA3352
Parabagrotis exsertistigma|[1633]|LOWCC182-05|Canada|British Columbia|658[0n]|BOLD:AAA3352
Parabagrotis exsertistigma|[1634]|LPABC609-09|Canada|Alberta|658[0n]|BOLD:AAA3352
Parabagrotis exsertistigma|[1635]|LOWCC181-05|Canada|British Columbia|658[0n]|BOLD:AAA3352
Parabagrotis exsertistigma|[1636]|LPABB358-08|Canada|Alberta|658[0n]|BOLD:AAA3352
Parabagrotis exsertistigma|[1637]|LPABC627-09|Canada|Alberta|658[0n]|BOLD:AAA3352
Parabagrotis exsertistigma|[1638]|LPABC640-09|Canada|Alberta|658[0n]|BOLD:AAA3352
Parabagrotis exsertistigma|[1639]|LPAB006-08|Canada|Alberta|640[0n]|BOLD:AAA3352
Parabagrotis exsertistigma|[1640]|LPABC637-09|Canada|Alberta|632[0n]|BOLD:AAA3352
Parabagrotis exsertistigma|[1641]|LPABC636-09|Canada|Alberta|632[0n]|BOLD:AAA3352
Parabagrotis exsertistigma|[1642]|LPABC626-09|Canada|Alberta|632[0n]|BOLD:AAA3352
Parabagrotis exsertistigma|[1643]|LPABC613-09|Canada|Alberta|632[0n]|BOLD:AAA3352
Parabagrotis exsertistigma|[1644]|LPABC606-09|Canada|Alberta|632[0n]|BOLD:AAA3352
Parabagrotis exsertistigma|[1645]|LPABC601-09|Canada|Alberta|632[0n]|BOLD:AAA3352
Parabagrotis exsertistigma|[1646]|LPABC582-09|Canada|Alberta|632[0n]|BOLD:AAA3352
Parabagrotis exsertistigma|[1647]|LPABC600-09|Canada|Alberta|635[0n]|BOLD:AAA3352
Parabagrotis exsertistigma|[1648]|LPABC623-09|Canada|Alberta|630[0n]|BOLD:AAA3352
Parabagrotis exsertistigma|[1649]|LBCH6914-10|Canada|British Columbia|639[0n]|BOLD:AAA3352
Parabagrotis exsertistigma|[1650]|LBCH6821-10|Canada|British Columbia|639[0n]|BOLD:AAA3352
Parabagrotis exsertistigma|[1651]|LPVIB640-08|Canada|British Columbia|600[0n]|BOLD:AAA3352
Parabagrotis exsertistigma|[1652]|LBCH6915-10|Canada|British Columbia|609[0n]|BOLD:AAA3352
Parabagrotis exsertistigma|[1653]|LBCH7233-10|Canada|British Columbia|637[0n]|BOLD:AAA3352
Parabagrotis exsertistigma|[1654]|LPABC577-09|Canada|Alberta|658[0n]|BOLD:AAA3352
Parabagrotis exsertistigma|[1655]|LPABC578-09|Canada|Alberta|658[0n]|BOLD:AAA3352
Parabagrotis exsertistigma|[1656]|LPABC580-09|Canada|Alberta|658[0n]|BOLD:AAA3352
Parabagrotis exsertistigma|[1657]|LPABC584-09|Canada|Alberta|658[0n]|BOLD:AAA3352
Parabagrotis exsertistigma|[1658]|LPABC587-09|Canada|Alberta|658[0n]|BOLD:AAA3352
Parabagrotis exsertistigma|[1659]|LPABC592-09|Canada|Alberta|658[0n]|BOLD:AAA3352
Parabagrotis exsertistigma|[1660]|LPABB457-08|Canada|Alberta|658[0n]|BOLD:AAA3352
Parabagrotis exsertistigma|[1661]|LPABB458-08|Canada|Alberta|658[0n]|BOLD:AAA3352
Parabagrotis exsertistigma|[1662]|LPABB459-08|Canada|Alberta|658[0n]|BOLD:AAA3352
Parabagrotis exsertistigma|[1663]|LPABB460-08|Canada|Alberta|658[0n]|BOLD:AAA3352
Parabagrotis exsertistigma|[1664]|LPABC593-09|Canada|Alberta|658[0n]|BOLD:AAA3352
Parabagrotis exsertistigma|[1665]|LPABC594-09|Canada|Alberta|658[0n]|BOLD:AAA3352
Parabagrotis exsertistigma|[1666]|LPABC596-09|Canada|Alberta|658[0n]|BOLD:AAA3352
Parabagrotis exsertistigma|[1667]|LPABC597-09|Canada|Alberta|658[0n]|BOLD:AAA3352
Parabagrotis exsertistigma|[1668]|LPABB462-08|Canada|Alberta|658[0n]|BOLD:AAA3352
Parabagrotis exsertistigma|[1669]|LPABB463-08|Canada|Alberta|658[0n]|BOLD:AAA3352
Parabagrotis exsertistigma|[1670]|LPABC599-09|Canada|Alberta|658[0n]|BOLD:AAA3352
Parabagrotis exsertistigma|[1671]|LPABC603-09|Canada|Alberta|658[0n]|BOLD:AAA3352
Parabagrotis exsertistigma|[1672]|LPABC608-09|Canada|Alberta|658[0n]|BOLD:AAA3352
Parabagrotis exsertistigma|[1673]|LPABC611-09|Canada|Alberta|658[0n]|BOLD:AAA3352
Parabagrotis exsertistigma|[1674]|LPABC612-09|Canada|Alberta|658[0n]|BOLD:AAA3352
Parabagrotis exsertistigma|[1675]|LPABC616-09|Canada|Alberta|658[0n]|BOLD:AAA3352
Parabagrotis exsertistigma|[1676]|LPABC617-09|Canada|Alberta|658[0n]|BOLD:AAA3352
Parabagrotis exsertistigma|[1677]|LPABC618-09|Canada|Alberta|658[0n]|BOLD:AAA3352
Parabagrotis exsertistigma|[1678]|LPABC619-09|Canada|Alberta|658[0n]|BOLD:AAA3352
Parabagrotis exsertistigma|[1679]|LPABC622-09|Canada|Alberta|658[0n]|BOLD:AAA3352
Parabagrotis exsertistigma|[1680]|LPABC624-09|Canada|Alberta|658[0n]|BOLD:AAA3352
Parabagrotis exsertistigma|[1681]|LPABC625-09|Canada|Alberta|658[0n]|BOLD:AAA3352
Parabagrotis exsertistigma|[1682]|LPABC629-09|Canada|Alberta|658[0n]|BOLD:AAA3352
Parabagrotis exsertistigma|[1683]|LPABC630-09|Canada|Alberta|658[0n]|BOLD:AAA3352
Parabagrotis exsertistigma|[1684]|LPABC631-09|Canada|Alberta|658[0n]|BOLD:AAA3352
Parabagrotis exsertistigma|[1685]|LPABC633-09|Canada|Alberta|658[0n]|BOLD:AAA3352
Parabagrotis exsertistigma|[1686]|LPABC634-09|Canada|Alberta|658[0n]|BOLD:AAA3352
Parabagrotis exsertistigma|[1687]|LPABC909-09|Canada|Alberta|658[0n]|BOLD:AAA3352
Parabagrotis exsertistigma|[1688]|LPABC926-09|Canada|Alberta|658[0n]|BOLD:AAA3352
Parabagrotis exsertistigma|[1689]|LPABC933-09|Canada|Alberta|658[0n]|BOLD:AAA3352
Parabagrotis exsertistigma|[1690]|LPABC975-09|Canada|Alberta|658[0n]|BOLD:AAA3352
Parabagrotis exsertistigma|[1691]|LBCG1348-09|Canada|British Columbia|658[0n]|BOLD:AAA3352
Parabagrotis exsertistigma|[1692]|LBCH6788-10|Canada|British Columbia|658[0n]|BOLD:AAA3352
Parabagrotis exsertistigma|[1693]|LBCH6818-10|Canada|British Columbia|658[0n]|BOLD:AAA3352
Parabagrotis exsertistigma|[1694]|LBCH6820-10|Canada|British Columbia|658[0n]|BOLD:AAA3352
Parabagrotis exsertistigma|[1695]|LBCH6822-10|Canada|British Columbia|658[0n]|BOLD:AAA3352

[illegible]
Parabagrotis exsertistigma|[1693]|LBCH6818-10|Canada|British Columbia|658[0n]|BOLD:AAA3352
Parabagrotis exsertistigma|[1694]|LBCH6820-10|Canada|British Columbia|658[0n]|BOLD:AAA3352
Parabagrotis exsertistigma|[1695]|LBCH6822-10|Canada|British Columbia|658[0n]|BOLD:AAA3352
Parabagrotis exsertistigma|[1696]|LBCH7029-10|Canada|British Columbia|658[0n]|BOLD:AAA3352
Parabagrotis exsertistigma|[1697]|LBCH7238-10|Canada|British Columbia|658[0n]|BOLD:AAA3352
Parabagrotis exsertistigma|[1698]|LBCH7503-10|Canada|British Columbia|658[0n]|BOLD:AAA3352
Parabagrotis exsertistigma|[1699]|LBCH7942-10|Canada|British Columbia|658[0n]|BOLD:AAA3352
Parabagrotis exsertistigma|[1700]|LBCH7944-10|Canada|British Columbia|658[0n]|BOLD:AAA3352
Parabagrotis exsertistigma|[1701]|LBCH7945-10|Canada|British Columbia|658[0n]|BOLD:AAA3352
Parabagrotis exsertistigma|[1702]|LBCH7948-10|Canada|British Columbia|658[0n]|BOLD:AAA3352
Parabagrotis exsertistigma|[1703]|IAWLB012-10|United States|California|658[0n]|BOLD:AAA3352
Parabagrotis exsertistigma|[1704]|LBCH7943-10|Canada|British Columbia|658[0n]|BOLD:AAA3352
Parabagrotis exsertistigma|[1705]|LPABC614-09|Canada|Alberta|658[0n]|BOLD:AAA3352
Parabagrotis exsertistigma|[1706]|LPABC651-09|Canada|Alberta|658[1n]|
Parabagrotis exsertistigma|[1707]|LBCH7949-10|Canada|British Columbia|658[0n]|BOLD:AAA3352
Parabagrotis exsertistigma|[1708]|LPABC591-09|Canada|Alberta|658[0n]|BOLD:AAA3352
Parabagrotis exsertistigma|[1709]|LPABC595-09|Canada|Alberta|658[0n]|BOLD:AAA3352
Parabagrotis exsertistigma|[1710]|LPABC589-09|Canada|Alberta|658[0n]|BOLD:AAA3352
Parabagrotis exsertistigma|[1711]|LPABC588-09|Canada|Alberta|658[0n]|BOLD:AAA3352
Parabagrotis exsertistigma|[1712]|LPABC586-09|Canada|Alberta|658[0n]|BOLD:AAA3352
Parabagrotis exsertistigma|[1713]|LPABC575-09|Canada|Alberta|658[0n]|BOLD:AAA3352
Parabagrotis exsertistigma|[1714]|LPABB464-08|Canada|Alberta|658[0n]|BOLD:AAA3352
Parabagrotis exsertistigma|[1715]|LPABB461-08|Canada|Alberta|658[0n]|BOLD:AAA3352
Parabagrotis exsertistigma|[1716]|LPABB456-08|Canada|Alberta|658[0n]|BOLD:AAA3352
Parabagrotis exsertistigma|[1717]|LPABC581-09|Canada|Alberta|658[0n]|BOLD:AAA3352
Parabagrotis exsertistigma|[1718]|LPAB077-08|Canada|Alberta|658[0n]|BOLD:AAA3352
Parabagrotis exsertistigma|[1719]|LPAB016-08|Canada|Alberta|658[0n]|BOLD:AAA3352
Parabagrotis exsertistigma|[1720]|RDNMD380-06|United States|California|658[0n]|BOLD:AAA3352
Parabagrotis exsertistigma|[1721]|LPABC583-09|Canada|Alberta|658[0n]|BOLD:AAA3352
Parabagrotis exsertistigma|[1722]|LBCH5107-10|Canada|British Columbia|658[0n]|BOLD:AAA3352
Parabagrotis exsertistigma|[1723]|LPAB009-08|Canada|Alberta|642[0n]|BOLD:AAA3352
Parabagrotis exsertistigma|[1724]|LPABC638-09|Canada|Alberta|632[0n]|BOLD:AAA3352
Parabagrotis exsertistigma|[1725]|RDMAB047-05|Canada|Alberta|635[0n]|BOLD:AAA3352
Parabagrotis exsertistigma|[1726]|LBCH6913-10|Canada|British Columbia|635[0n]|BOLD:AAA3352
Parabagrotis exsertistigma|[1727]|LBCH7313-10|Canada|British Columbia|658[0n]|BOLD:AAA3352
Parabagrotis exsertistigma|[1728]|LPABC598-09|Canada|Alberta|658[0n]|BOLD:AAA3352
Parabagrotis exsertistigma|[1729]|LPABC607-09|Canada|Alberta|658[0n]|BOLD:AAA3352
Parabagrotis exsertistigma|[1730]|LPABC615-09|Canada|Alberta|658[0n]|BOLD:AAA3352
Parabagrotis exsertistigma|[1731]|LPABC620-09|Canada|Alberta|658[0n]|BOLD:AAA3352
Parabagrotis exsertistigma|[1732]|LPABC621-09|Canada|Alberta|658[0n]|BOLD:AAA3352
Parabagrotis exsertistigma|[1733]|LPABC635-09|Canada|Alberta|658[0n]|BOLD:AAA3352
Parabagrotis exsertistigma|[1734]|LPABC639-09|Canada|Alberta|658[0n]|BOLD:AAA3352
Parabagrotis exsertistigma|[1735]|LBCH6144-10|Canada|British Columbia|658[0n]|BOLD:AAA3352
Parabagrotis exsertistigma|[1736]|LBCH6819-10|Canada|British Columbia|658[0n]|BOLD:AAA3352
Parabagrotis exsertistigma|[1737]|LBCH6946-10|Canada|British Columbia|658[0n]|BOLD:AAA3352
Parabagrotis exsertistigma|[1738]|LBCH7133-10|Canada|British Columbia|658[0n]|BOLD:AAA3352
Parabagrotis exsertistigma|[1739]|IAWLB011-10|United States|California|658[0n]|BOLD:AAA3352
Parabagrotis exsertistigma|[1740]|JMMMB364-11|United States|California|658[0n]|BOLD:AAA3352
Parabagrotis exsertistigma|[1741]|BBLOC1365-11|United States|California|658[0n]|BOLD:AAA3352
Parabagrotis sulinaris|[1742]|LHLEP212-06|Canada|British Columbia|658[0n]|BOLD:ABZ1410
Parabagrotis sulinaris|[1743]|LBCH7947-10|Canada|British Columbia|658[0n]|BOLD:ABZ1410
Parabagrotis sulinaris|[1744]|LALPA1253-11|Canada|British Columbia|658[0n]|BOLD:ABZ1410
Parabagrotis sulinaris|[1745]|RWWC104-10|United States|Washington|658[0n]|BOLD:ABZ1410
Parabagrotis sulinaris|[1746]|RWWC088-10|United States|Washington|658[0n]|BOLD:ABZ1410
Parabagrotis sulinaris|[1747]|LALPA798-10|Canada|British Columbia|658[0n]|BOLD:ABZ1410
Parabagrotis sulinaris|[1748]|RWWB881-10|United States|Washington|658[0n]|BOLD:ABZ1410
Parabagrotis sulinaris|[1749]|LBCH7946-10|Canada|British Columbia|658[0n]|BOLD:ABZ1410
Parabagrotis sulinaris|[1750]|LBCH7127-10|Canada|British Columbia|658[0n]|BOLD:ABZ1410
Parabagrotis sulinaris|[1751]|LBCH1102-10|Canada|British Columbia|658[0n]|BOLD:ABZ1410
Parabagrotis sulinaris|[1752]|RWWB339-09|United States|Washington|658[0n]|BOLD:ABZ1410
Parabagrotis sulinaris|[1753]|RWWB335-09|United States|Washington|658[0n]|BOLD:ABZ1410
Parabagrotis sulinaris|[1754]|RWWB325-09|United States|Washington|658[0n]|BOLD:ABZ1410
Parabagrotis sulinaris|[1755]|RWWB270-09|United States|Washington|658[0n]|BOLD:ABZ1410
Parabagrotis sulinaris|[1756]|LBCG2065-09|Canada|British Columbia|658[0n]|BOLD:ABZ1410
Parabagrotis sulinaris|[1757]|LBCG577-09|Canada|British Columbia|658[0n]|BOLD:ABZ1410
Parabagrotis sulinaris|[1758]|LHLEP225-06|Canada|British Columbia|658[0n]|BOLD:ABZ1410
Parabagrotis sulinaris|[1759]|LHLEP215-06|Canada|British Columbia|658[0n]|BOLD:ABZ1410
Parabagrotis sulinaris|[1760]|LHLEP213-06|Canada|British Columbia|658[0n]|BOLD:ABZ1410
Parabagrotis sulinaris|[1761]|RDMAB294-05|Canada|Alberta|658[0n]|BOLD:ABZ1410
Parabagrotis sulinaris|[1762]|LPVIB260-08|Canada|British Columbia|658[0n]|BOLD:ABZ1410
Parabagrotis sulinaris|[1763]|LALPA1303-11|Canada|British Columbia|658[0n]|BOLD:ABZ1410
Parabagrotis sulinaris|[1764]|LPVIB639-08|Canada|British Columbia|637[0n]|BOLD:ABZ1410
Parabagrotis sulinaris|[1765]|LBCH6845-10|Canada|British Columbia|630[0n]|BOLD:ABZ1410
Parabagrotis sulinaris|[1766]|LALPA1322-12|Canada|British Columbia|633[0n]|BOLD:ABZ1410
Parabagrotis formalis|[1767]|RDNMG790-08|United States|Washington|658[0n]|BOLD:ACE7476
Parabagrotis formalis|[1768]|CGLCA037-10|United States|California|658[0n]|BOLD:ACE7476
Parabagrotis formalis|[1769]|RDNMF012-08|United States|California|658[0n]|BOLD:ACE7476
Parabagrotis formalis|[1770]|RDNMG789-08|United States|California|658[0n]|BOLD:ACE7476
Parabagrotis formalis|[1771]|RDNMG897-08|United States|California|658[0n]|BOLD:ACE7476
Parabagrotis formalis|[1772]|CGLCA032-10|United States|California|658[0n]|BOLD:ACE7476
Parabagrotis formalis|[1773]|GMLC579-11|United States|California|658[0n]|BOLD:ACE7476
Parabagrotis formalis|[1774]|GMLC663-11|United States|California|658[0n]|BOLD:ACE7476
Parabagrotis formalis|[1775]|GMLC668-11|United States|California|658[0n]|BOLD:ACE7476
Parabagrotis formalis|[1776]|GMLC724-12|United States|California|658[0n]|BOLD:ACE7476
Parabagrotis formalis|[1777]|RDNMG791-08|United States|Washington|658[0n]|BOLD:ACE7476
Parabagrotis formalis|[1778]|GMLC1171-12|United States|California|658[0n]|BOLD:ACE7476
Parabagrotis formalis|[1779]|GMLC695-11|United States|California|658[0n]|BOLD:ACE7476
Parabagrotis formalis|[1780]|GMLC891-12|United States|California|658[0n]|BOLD:ACE7476
Parabagrotis formalis|[1781]|RDNMG788-08|United States|Washington|658[0n]|BOLD:ACE7476
Parabagrotis formalis|[1782]|RDNMG787-08|United States|California|658[0n]|BOLD:ACE7476
Parabagrotis formalis|[1783]|LPVIB641-08|Canada|British Columbia|630[0n]|BOLD:ACE7476
Parabagrotis formalis|[1784]|GMLC251-11|United States|California|658[0n]|BOLD:ACE7476
Parabagrotis formalis|[1785]|GMLC687-11|United States|California|658[0n]|BOLD:ACE7476
Parabagrotis formalis|[1786]|GMLC1207-12|United States|California|658[0n]|BOLD:ACE7476
Parabagrotis formalis|[1787]|LOCBF327-13|United States|California|590[0n]|BOLD:ACE7476
Parabagrotis cupidissima|[1788]|RDNMC088-05|Canada|British Columbia|658[0n]|BOLD:ABZ2364
Parabagrotis cupidissima|[1789]|RDNMG786-08|United States|Oregon|658[0n]|BOLD:ABZ2364
Parabagrotis cupidissima|[1790]|GMLC952-12|United States|California|658[0n]|BOLD:ABZ2364
Parabagrotis cupidissima|[1791]|GMLC990-12|United States|California|658[0n]|BOLD:ABZ2364
Parabagrotis cupidissima|[1792]|GMLC007-09|United States|California|658[0n]|BOLD:ABZ2364
Parabagrotis cupidissima|[1793]|GMLC728-12|United States|California|634[0n]|BOLD:ABZ2364
Parabagrotis cupidissima|[1794]|GMLC991-12|United States|California|658[0n]|BOLD:ABZ2364
[illegible]

Parabagrotis cupidissima|[1792]|GMLC007-09|United States|California|658[0n]|BOLD:ABZ2364
Parabagrotis cupidissima|[1793]|GMLC728-12|United States|California|634[0n]|BOLD:ABZ2364
Parabagrotis cupidissima|[1794]|GMLC991-12|United States|California|658[0n]|BOLD:ABZ2364
Parabagrotis cupidissima|[1795]|GMLC671-11|United States|California|658[0n]|BOLD:ABZ2364
Parabagrotis cupidissima|[1796]|GMLC708-11|United States|California|658[0n]|BOLD:ABZ2364
Parabagrotis cupidissima|[1797]|GMLC995-12|United States|California|622[0n]|BOLD:ABZ2364
Parabagrotis cupidissima|[1798]|GMLC1104-12|United States|California|658[0n]|BOLD:ABZ2364
Parabagrotis insularis|[1799]|IAWLB456-11|United States|California|658[0n]|BOLD:ABZ2364
Parabagrotis insularis|[1800]|LOCBE090-06|United States|California|658[0n]|BOLD:ABZ2364
Parabagrotis insularis|[1801]|GMLC569-11|United States|California|658[0n]|BOLD:ABZ2364
Parabagrotis insularis|[1802]|LOCBC462-06|United States|California|658[0n]|BOLD:ABZ2364
Parabagrotis insularis|[1803]|LOCBD285-06|United States|California|658[0n]|BOLD:ABZ2364
Parabagrotis insularis|[1804]|RDNMF008-08|United States|California|658[0n]|BOLD:ABZ2364
Parabagrotis insularis|[1805]|IAWLB455-11|United States|California|658[0n]|BOLD:ABZ2364
Parabagrotis insularis|[1806]|GMLC709-11|United States|California|658[0n]|BOLD:ABZ2364
Parabagrotis insularis|[1807]|GMLC814-12|United States|California|658[0n]|BOLD:ABZ2364
Parabagrotis insularis|[1808]|GMLC743-12|United States|California|656[0n]|BOLD:ABZ2364
Parabagrotis insularis|[1809]|JBAZ028-09|United States|California|658[4n]|BOLD:ABZ2364
Parabagrotis insularis|[1810]|GMLC873-12|United States|California|658[0n]|BOLD:ABZ2364
Parabagrotis insularis|[1811]|NAMUM413-09|United States|California|658[0n]|BOLD:ABZ2364
Parabagrotis insularis|[1812]|IAWLB446-11|United States|California|658[0n]|BOLD:ABZ2364
Parabagrotis insularis|[1813]|IAWLB453-11|United States|California|658[0n]|BOLD:ABZ2364
Parabagrotis insularis|[1814]|BBLOE1775-12|United States|California|658[0n]|BOLD:ABZ2364
Parabagrotis insularis|[1815]|GMLC1157-12|United States|California|658[0n]|BOLD:ABZ2364
Parabagrotis insularis|[1816]|GMLC1198-12|United States|California|658[0n]|BOLD:ABZ2364
Parabagrotis insularis|[1817]|BBLOE1774-12|United States|California|658[0n]|BOLD:ABZ2364
Parabagrotis insularis|[1818]|GMLC735-12|United States|California|658[0n]|BOLD:ABZ2364
Parabagrotis insularis|[1819]|BBLOD576-11|United States|California|658[0n]|BOLD:ABZ2364
Parabagrotis insularis|[1820]|IAWLB457-11|United States|California|658[0n]|BOLD:ABZ2364
Parabagrotis insularis|[1821]|IAWLB454-11|United States|California|658[0n]|BOLD:ABZ2364
Parabagrotis insularis|[1822]|GMLC616-11|United States|California|658[0n]|BOLD:ABZ2364
Parabagrotis insularis|[1823]|LOCBE086-06|United States|California|658[1n]|BOLD:ABZ2364
Parabagrotis insularis|[1824]|LOCBC464-06|United States|California|658[0n]|BOLD:ABZ2364
Parabagrotis insularis|[1825]|LOCBB325-06|United States|California|658[0n]|BOLD:ABZ2364
Parabagrotis insularis|[1826]|LOCBB324-06|United States|California|658[0n]|BOLD:ABZ2364
Parabagrotis insularis|[1827]|LOCBB323-06|United States|California|658[0n]|BOLD:ABZ2364
Parabagrotis insularis|[1828]|LOCBB322-06|United States|California|658[0n]|BOLD:ABZ2364
Parabagrotis insularis|[1829]|RWWC074-10|United States|Washington|658[0n]|BOLD:ABZ2364
Parabagrotis insularis|[1830]|LOCB529-06|United States|California|658[0n]|BOLD:ABZ2364
Parabagrotis insularis|[1831]|GMLC717-11|United States|California|632[0n]|BOLD:ABZ2364
Parabagrotis insularis|[1832]|LOCBF470-13|United States|California|619[0n]|BOLD:ABZ2364
Parabagrotis insularis|[1833]|BBLOC936-11|United States|California|658[0n]|BOLD:ABZ2364
Parabagrotis insularis|[1834]|LOCBF949-13|United States|California|558[0n]|BOLD:ABZ2364
Parabagrotis insularis|[1835]|LOCBF950-13|United States|California|571[0n]|BOLD:ABZ2364
Parabagrotis insularis|[1836]|LOCBF1209-13|United States|California|600[0n]|BOLD:ABZ2364
Pronoctua craboi|[1837]|RDMAB342-05|Canada|Alberta|658[0n]|BOLD:AAD8045
Pronoctua craboi|[1838]|RDNMG965-08|United States|Wyoming|643[0n]|BOLD:AAD8045
Pronoctua craboi|[1839]|RDMAB340-05|Canada|Alberta|658[0n]|BOLD:AAD8045
Pronoctua craboi|[1840]|RDNMF432-08|Canada|British Columbia|658[0n]|BOLD:AAD8045
Pronoctua craboi|[1841]|RDNMF433-08|Canada|British Columbia|658[0n]|BOLD:AAD8045
Pronoctua craboi|[1842]|RDNMH290-09|United States|Oregon|658[0n]|BOLD:AAD8045
Pronoctua|[1843]|RDMAB341-05|Canada|Alberta|658[0n]|BOLD:AAD8042
Pronoctua|[1844]|RDMAB629-06|Canada|Alberta|658[0n]|BOLD:AAD8042
Pronoctua|[1845]|RDNMH291-09|United States|Washington|658[0n]|BOLD:AAD8046
Pronoctua|[1846]|CNCLB480-14|United States|California|658[0n]|BOLD:AAD8046
Pronoctua pyrophiloides|[1847]|RDNMH288-09|United States|Colorado|658[0n]|BOLD:AAD8043
Pronoctua pyrophiloides|[1848]|CNCLB481-14|United States|California|658[0n]|BOLD:AAD8043
Pronoctua pyrophiloides|[1849]|RDNMH289-09|United States|Oregon|658[0n]|BOLD:AAD8044
Pronoctua pyrophiloides|[1850]|CNCLB483-14|United States|California|658[0n]|BOLD:AAD8044
Spaelotis clandestina|[1851]|RDLQG362-06|Canada|Quebec|658[0n]|BOLD:ABZ2365
Spaelotis clandestina|[1852]|LPSK119-08|Canada|Saskatchewan|658[0n]|BOLD:ABZ2365
Spaelotis clandestina|[1853]|RDLQF261-06|Canada|Quebec|658[0n]|BOLD:ABZ2365
Spaelotis clandestina|[1854]|LOWCD899-06|Canada|British Columbia|658[0n]|BOLD:ABZ2365
Spaelotis clandestina|[1855]|RDMAB116-05|Canada|Alberta|658[0n]|BOLD:ABZ2365
Spaelotis clandestina|[1856]|LOWC841-05|Canada|British Columbia|658[0n]|BOLD:ABZ2365
Spaelotis clandestina|[1857]|XAD267-04|Canada|Ontario|658[0n]|BOLD:ABZ2365
Spaelotis clandestina|[1858]|PHMNB209-04|Canada|New Brunswick|609[0n]|BOLD:ABZ2365
Spaelotis clandestina|[1859]|PMG162-03|Canada|Ontario|617[0n]|BOLD:ABZ2365
Spaelotis clandestina|[1860]|MNBB529-05|Canada|New Brunswick|658[0n]|BOLD:ABZ2365
Spaelotis clandestina|[1861]|XAB011-04|Canada|Ontario|573[1n]|BOLD:ABZ2365
Spaelotis clandestina|[1862]|MJMSL016-10|United States|Massachusetts|658[0n]|BOLD:ABZ2365
Spaelotis clandestina|[1863]|RDNMC541-06|Canada|Saskatchewan|658[0n]|BOLD:ABZ2365
Spaelotis clandestina|[1864]|PHMO182-03|Canada|Ontario|639[0n]|BOLD:ABZ2365
Spaelotis clandestina|[1865]|RDLQG030-06|Canada|Quebec|658[0n]|BOLD:ABZ2365
Spaelotis clandestina|[1866]|LNCC431-10|United States|North Carolina|658[0n]|BOLD:ABZ2365
Spaelotis clandestina|[1867]|LNCC1176-11|United States|North Carolina|658[0n]|BOLD:ABZ2365
Spaelotis clandestina|[1868]|LOWCD903-06|Canada|British Columbia|658[0n]|BOLD:ABZ2365
Spaelotis clandestina|[1869]|LOWCD898-06|Canada|British Columbia|658[0n]|BOLD:ABZ2365
Spaelotis clandestina|[1870]|LOWCC176-05|Canada|British Columbia|658[0n]|BOLD:ABZ2365
Spaelotis clandestina|[1871]|LOWC843-05|Canada|British Columbia|658[0n]|BOLD:ABZ2365
Spaelotis clandestina|[1872]|LOWCD904-06|Canada|British Columbia|601[0n]|BOLD:ABZ2365
Spaelotis clandestina|[1873]|LOWCD894-06|Canada|British Columbia|553[0n]|BOLD:ABZ2365
Spaelotis clandestina|[1874]|LOWCD911-06|Canada|British Columbia|566[0n]|BOLD:ABZ2365
Spaelotis clandestina|[1875]|UAMIC600-13|United States|Alaska|537[2n]|BOLD:ABZ2365
Spaelotis clandestina|[1876]|BLTIB312-08|Canada|Ontario|658[0n]|BOLD:ABZ2365
Spaelotis clandestina|[1877]|MNBB645-05|Canada|New Brunswick|658[0n]|BOLD:ABZ2365
Spaelotis clandestina|[1878]|LOWC844-05|Canada|British Columbia|658[0n]|BOLD:ABZ2365
Spaelotis clandestina|[1879]|RDLQF542-06|Canada|Quebec|658[0n]|BOLD:ABZ2365
Spaelotis clandestina|[1880]|LBCH7218-10|Canada|British Columbia|658[0n]|BOLD:ABZ2365
Spaelotis clandestina|[1881]|LNCC432-10|United States|North Carolina|658[0n]|BOLD:ABZ2365
Spaelotis clandestina|[1882]|RDNMC540-06|Canada|Saskatchewan|658[0n]|BOLD:ABZ2365
Spaelotis clandestina|[1883]|LOWCC179-05|Canada|British Columbia|658[0n]|BOLD:ABZ2365
Spaelotis clandestina|[1884]|LOWC846-05|Canada|British Columbia|658[0n]|BOLD:ABZ2365
Spaelotis clandestina|[1885]|XAH601-05|Canada|Ontario|658[0n]|BOLD:ABZ2365
Spaelotis clandestina|[1886]|LOWCD902-06|Canada|British Columbia|595[0n]|BOLD:ABZ2365
Spaelotis clandestina|[1887]|LOWCD907-06|Canada|British Columbia|589[0n]|BOLD:ABZ2365
Spaelotis clandestina|[1888]|RDLQG133-06|Canada|Quebec|593[0n]|BOLD:ABZ2365
Spaelotis clandestina|[1889]|BBLPB753-10|Canada|Alberta|658[0n]|BOLD:ABZ2365
Spaelotis velicava|[1890]|BBLSX612-09|United States|Arizona|658[0n]|BOLD:ABZ2365
Spaelotis velicava|[1891]|LNAUT565-14|United States|New Mexico|658[0n]|BOLD:ABZ2365
Spaelotis clandestina|[1892]|LOWCD895-06|Canada|British Columbia|658[0n]|BOLD:ABZ2365
Spaelotis clandestina|[1893]|LOWC845-05|Canada|British Columbia|658[0n]|BOLD:ABZ2365
Spaelotis clandestina|[1894]|LOWCC177-05|Canada|British Columbia|658[0n]|BOLD:ABZ2365

Spaelotis clandestina|[1892]|LOWCD895-06|Canada|British Columbia|658[0n]|BOLD:ABZ2365
Spaelotis clandestina|[1893]|LOWC845-05|Canada|British Columbia|658[0n]|BOLD:ABZ2365
Spaelotis clandestina|[1894]|LOWCC177-05|Canada|British Columbia|658[0n]|BOLD:ABZ2365
Spaelotis clandestina|[1895]|LOWC842-05|Canada|British Columbia|658[0n]|BOLD:ABZ2365
Spaelotis clandestina|[1896]|RDNMC539-06|Canada|Saskatchewan|628[0n]|BOLD:ABZ2365
Spaelotis clandestina|[1897]|LOWCD900-06|Canada|British Columbia|599[0n]|BOLD:ABZ2365
Spaelotis clandestina|[1898]|LBCB071-05|Canada|British Columbia|658[0n]|BOLD:ABZ2365
Spaelotis clandestina|[1899]|XAD271-04|Canada|Ontario|594[0n]|BOLD:ABZ2365
Spaelotis clandestina|[1900]|LOWCD901-06|Canada|British Columbia|593[0n]|BOLD:ABZ2365
Spaelotis clandestina|[1901]|LOWCD908-06|Canada|British Columbia|604[0n]|BOLD:ABZ2365
Spaelotis clandestina|[1902]|LPSK129-08|Canada|Saskatchewan|658[0n]|BOLD:ABZ2365
Spaelotis clandestina|[1903]|LBCH5723-10|Canada|British Columbia|658[0n]|BOLD:ABZ2365
Spaelotis clandestina|[1904]|LBCH7003-10|Canada|British Columbia|658[0n]|BOLD:ABZ2365
Spaelotis clandestina|[1905]|LBCH7955-10|Canada|British Columbia|658[0n]|BOLD:ABZ2365
Spaelotis velicava|[1906]|LNAUT564-14|United States|New Mexico|614[0n]|BOLD:ABZ2365
Spaelotis velicava|[1907]|CNCLB917-14|United States|Nevada|615[0n]|BOLD:ABZ2365
Spaelotis velicava|[1908]|BBLSX600-09|United States|Arizona|658[0n]|BOLD:ABZ2365
Spaelotis velicava|[1909]|BBLSX611-09|United States|Arizona|658[0n]|BOLD:ABZ2365
Spaelotis velicava|[1910]|LNAUT563-14|United States|New Mexico|658[0n]|BOLD:ABZ2365
Spaelotis velicava|[1911]|LNAUT566-14|United States|New Mexico|658[0n]|BOLD:ABZ2365
Spaelotis velicava|[1912]|BBLSX601-09|United States|Arizona|658[0n]|BOLD:ABZ2365
Spaelotis velicava|[1913]|BBLSX605-09|United States|Arizona|658[0n]|BOLD:ABZ2365
Spaelotis velicava|[1914]|BBLSX608-09|United States|Arizona|658[0n]|BOLD:ABZ2365
Spaelotis velicava|[1915]|BBLSX613-09|United States|Arizona|658[0n]|BOLD:ABZ2365
Spaelotis velicava|[1916]|BBLSX614-09|United States|Arizona|658[0n]|BOLD:ABZ2365
Spaelotis velicava|[1917]|CNCLB792-14|United States|Nevada|658[0n]|BOLD:ABZ2365
Spaelotis velicava|[1918]|CNCLB794-14|United States|Nevada|658[0n]|BOLD:ABZ2365
Spaelotis velicava|[1919]|CNCLB918-14|United States|Colorado|658[0n]|BOLD:ABZ2365
Spaelotis velicava|[1920]|LNAUT567-14|United States|New Mexico|658[0n]|BOLD:ABZ2365
Spaelotis velicava|[1921]|LNAUT569-14|United States|Colorado|658[0n]|BOLD:ABZ2365
Spaelotis havilae|[1922]|LNAUT561-14|United States|Nevada|658[0n]|BOLD:ABZ2365
Spaelotis havilae|[1923]|JMMMB299-11|United States|California|658[0n]|BOLD:ABZ2365
Spaelotis bicava|[1924]|RDMAB135-05|Canada|Alberta|658[0n]|BOLD:ABZ2365
Spaelotis bicava|[1925]|RDMAB677-06|Canada|Alberta|658[0n]|BOLD:ABZ2365
Spaelotis bicava|[1926]|RDMAB678-06|Canada|Alberta|658[0n]|BOLD:ABZ2365
Spaelotis bicava|[1927]|LPABB331-08|Canada|Alberta|658[0n]|BOLD:ABZ2365
Spaelotis bicava|[1928]|BBLWU101-09|United States|Colorado|658[0n]|BOLD:ABZ2365
Spaelotis bicava|[1929]|LBCH7192-10|Canada|British Columbia|658[0n]|BOLD:ABZ2365
Spaelotis havilae|[1930]|JMMMB329-11|United States|California|658[0n]|BOLD:ABZ2365
Spaelotis havilae|[1931]|JMMMB395-11|United States|California|658[0n]|BOLD:ABZ2365
Spaelotis havilae|[1932]|BBLOC1392-11|United States|California|658[0n]|BOLD:ABZ2365
Spaelotis havilae|[1933]|CNCLB781-14|United States|Nevada|658[0n]|BOLD:ABZ2365
Spaelotis havilae|[1934]|CNCLB919-14|United States|Oregon|658[0n]|BOLD:ABZ2365
Spaelotis havilae|[1935]|CNCLB920-14|United States|Oregon|658[0n]|BOLD:ABZ2365
Spaelotis havilae|[1936]|LNAUT558-14|United States|Nevada|658[0n]|BOLD:ABZ2365
Spaelotis havilae|[1937]|LNAUT559-14|United States|Nevada|658[0n]|BOLD:ABZ2365
Spaelotis havilae|[1938]|LNAUT560-14|United States|Nevada|658[0n]|BOLD:ABZ2365
Spaelotis havilae|[1939]|LNAUT562-14|United States|Nevada|658[0n]|BOLD:ABZ2365
Spaelotis unicava|[1940]|IAWLB018-10|United States|California|658[0n]|BOLD:ABZ2365
Spaelotis unicava|[1941]|IAWLB133-10|United States|California|658[0n]|BOLD:ABZ2365
Spaelotis unicava|[1942]|BBLSX599-09|United States|Arizona|658[0n]|BOLD:ABZ2365
Spaelotis unicava|[1943]|RDNMD378-06|United States|California|658[0n]|BOLD:ABZ2365
Spaelotis unicava|[1944]|RDNMD377-06|United States|California|658[0n]|BOLD:ABZ2365
Spaelotis unicava|[1945]|NAMUM412-09|United States|California|658[0n]|BOLD:ABZ2365
Spaelotis unicava|[1946]|BBLSX602-09|United States|Arizona|658[0n]|BOLD:ABZ2365
Spaelotis unicava|[1947]|LBCH7181-10|Canada|British Columbia|658[0n]|BOLD:ABZ2365
Spaelotis unicava|[1948]|AWCLB302-10|United States|Arizona|658[0n]|BOLD:ABZ2365
Spaelotis unicava|[1949]|IAWLB433-11|United States|California|658[0n]|BOLD:ABZ2365
Spaelotis quadricava|[1950]|CNCLB791-14|United States|California|658[0n]|BOLD:ABZ2365
Spaelotis unicava|[1951]|CNCLB921-14|United States|Nevada|658[0n]|BOLD:ABZ2365
Spaelotis unicava|[1952]|IAWLB432-11|United States|California|658[0n]|BOLD:ABZ2365
Spaelotis unicava|[1953]|IAWLB065-10|United States|California|658[0n]|BOLD:ABZ2365
Spaelotis unicava|[1954]|IAWLB484-11|United States|Arizona|658[0n]|BOLD:ABZ2365
Spaelotis unicava|[1955]|CNCLB922-14|United States|California|658[0n]|BOLD:ABZ2365
Spaelotis bicava|[1956]|LBCH5474-10|Canada|British Columbia|658[0n]|BOLD:ABZ2365
Spaelotis bicava|[1957]|LOWCC178-05|Canada|British Columbia|564[0n]|BOLD:ABZ2365
Spaelotis bicava|[1958]|LOWCD906-06|Canada|British Columbia|608[0n]|BOLD:ABZ2365
Spaelotis bicava|[1959]|LBCG096-08|Canada|British Columbia|658[0n]|BOLD:ABZ2365
Spaelotis bicava|[1960]|LBCH5714-10|Canada|British Columbia|658[0n]|BOLD:ABZ2365
Spaelotis bicava|[1961]|LALPA817-10|Canada|British Columbia|658[0n]|BOLD:ABZ2365
Spaelotis bicava|[1962]|JMMMB386-11|United States|California|658[0n]|BOLD:ABZ2365
Spaelotis quadricava|[1963]|TML197-14|United States||658[0n]|BOLD:ABZ2365
Tesagrotis corrodera|[1964]|RDNMF337-08|United States|Washington|658[0n]|BOLD:AAE9272
Tesagrotis corrodera|[1965]|RDNMF338-08|Canada|British Columbia|658[0n]|BOLD:AAE9272
Tesagrotis corrodera|[1966]|RDNMF339-08|United States|Oregon|658[0n]|BOLD:AAE9272
Tesagrotis corrodera|[1967]|RDNMF892-08|United States|Oregon|658[0n]|BOLD:AAE9272
Tesagrotis corrodera|[1968]|LBCH7125-10|Canada|British Columbia|658[0n]|BOLD:AAE9272
Tesagrotis corrodera|[1969]|IAWLB001-10|United States|California|658[0n]|BOLD:AAE9272
Tesagrotis corrodera|[1970]|IAWLB002-10|United States|California|658[0n]|BOLD:AAE9272
Tesagrotis corrodera|[1971]|IAWLB003-10|United States|California|658[0n]|BOLD:AAE9272
Tesagrotis corrodera|[1972]|IAWLB004-10|United States|California|658[0n]|BOLD:AAE9272
Tesagrotis corrodera|[1973]|IAWLB005-10|United States|California|658[0n]|BOLD:AAE9272
Tesagrotis piscipellis|[1974]|RDNMF332-08|United States|California|658[0n]|BOLD:AAE9266
Tesagrotis piscipellis|[1975]|LBCD322-05|Canada|British Columbia|658[0n]|BOLD:AAE9266
Tesagrotis piscipellis|[1976]|LBCA599-05|Canada|British Columbia|658[0n]|BOLD:AAE9266
Tesagrotis piscipellis|[1977]|NAMUM166-08|United States|California|621[0n]|BOLD:AAE9266
Tesagrotis piscipellis|[1978]|RDNMF009-08|United States|California|658[0n]|BOLD:AAE9266
Tesagrotis piscipellis|[1979]|RDNMF333-08|United States|California|658[0n]|BOLD:AAE9266
Tesagrotis piscipellis|[1980]|LPABB497-08|Canada|Alberta|658[0n]|BOLD:AAE9266
Tesagrotis piscipellis|[1981]|LBCH5794-10|Canada|British Columbia|658[0n]|BOLD:AAE9266
Tesagrotis piscipellis|[1982]|LBCH7103-10|Canada|British Columbia|658[0n]|BOLD:AAE9266
Tesagrotis atrifrons|[1983]|NAMUM147-08|United States|California|658[0n]|BOLD:ACF3460
Tesagrotis atrifrons|[1984]|IAWLB025-10|United States|California|658[0n]|BOLD:ACF3460
Tesagrotis atrifrons|[1985]|IAWLB026-10|United States|California|658[0n]|BOLD:ACF3460
Tesagrotis atrifrons|[1986]|IAWLB027-10|United States|California|658[0n]|BOLD:ACF3460
Tesagrotis amia|[1987]|CNCLB973-14|United States|Nevada|658[0n]|BOLD:ACM4437
Tesagrotis amia|[1988]|CNCLB974-14|United States|Utah|658[0n]|BOLD:ACM4437
Tesagrotis amia|[1989]|TML198-14|United States||658[0n]|BOLD:ACM4437
Xestia woodi|[1990]|RDNMG693-08|United States|Alaska|642[0n]|BOLD:ABZ1295
Xestia woodi|[1991]|RDNMF065-08|Canada|Yukon Territory|658[0n]|BOLD:ABZ1295
Xestia woodi|[1992]|RDNMG724-08|Canada|Yukon Territory|658[0n]|BOLD:ABZ1295
Xestia lyngei|[1993]|RDNMG694-08|Russia|Magadan|633[0n]|BOLD:AAD0055
Xestia lyngei|[1994]|RDNMG695-08|Canada|Yukon Territory|642[0n]|BOLD:AAD0055

[illegible]
Xestia woodi|[1992]|RDNMG724-08|Canada|Yukon Territory|658[0n]|BOLD:ABZ1295
Xestia lyngei|[1993]|RDNMG694-08|Russia|Magadan|633[0n]|BOLD:AAD0055
Xestia lyngei|[1994]|RDNMG695-08|Canada|Yukon Territory|642[0n]|BOLD:AAD0055
Xestia lyngei|[1995]|RDNMG697-08|Canada|Yukon Territory|658[0n]|BOLD:AAD0055
Xestia lyngei|[1996]|RDNMG696-08|Canada|Yukon Territory|658[0n]|BOLD:AAD0055
Xestia lyngei|[1997]|RDNMF069-08|Canada|Yukon Territory|658[0n]|BOLD:AAD0055
Xestia lyngei|[1998]|RDNMG722-08|Canada|Yukon Territory|642[0n]|BOLD:AAD0055
Xestia lyngei|[1999]|LEFIF308-10|Finland||658[0n]|BOLD:AAD0055
Xestia lyngei|[2000]|LEFIF309-10|Finland||658[0n]|BOLD:AAD0055
Xestia lyngei|[2001]|COLFF454-13|Finland||658[0n]|BOLD:AAD0055
Xestia liquidaria|[2002]|LEFIJ2311-14|Russia|Arkhangelsk|658[0n]|BOLD:AAH9449
Xestia liquidaria|[2003]|RDNMG728-08|Canada|Yukon Territory|658[5n]|BOLD:AAH9449
Xestia liquidaria|[2004]|CNCLB1591-14|Russia||658[0n]|BOLD:AAH9449
Xestia liquidaria|[2005]|CNCLB1592-14|Russia||658[0n]|BOLD:AAH9449
Xestia c-nigrum|[2006]|MNBB425-05|Canada|New Brunswick|658[0n]|BOLD:AAA2144
Xestia c-nigrum|[2007]|LOWC795-05|Canada|British Columbia|658[0n]|BOLD:AAA2144
Xestia c-nigrum|[2008]|XAH087-05|Canada|Ontario|597[13n]|
Xestia c-nigrum|[2009]|LOWC803-05|Canada|British Columbia|658[1n]|BOLD:AAA2144
Xestia c-nigrum|[2010]|LBCB891-05|Canada|British Columbia|615[1n]|BOLD:AAA2144
Xestia c-nigrum|[2011]|BBLPB824-10|Canada|Alberta|658[0n]|BOLD:AAA2144
Xestia c-nigrum|[2012]|RDLQB639-05|Canada|Quebec|573[2n]|BOLD:AAA2144
Xestia c-nigrum|[2013]|MNBB523-05|Canada|New Brunswick|658[0n]|BOLD:AAA2144
Xestia c-nigrum|[2014]|PHMNB247-04|Canada|New Brunswick|537[0n]|BOLD:AAA2144
Xestia c-nigrum|[2015]|PHMNB178-04|Canada|New Brunswick|500[0n]|
Xestia c-nigrum|[2016]|LOWCD783-06|Canada|British Columbia|657[0n]|BOLD:AAA2144
Xestia c-nigrum|[2017]|RWWB747-10|United States|Washington|658[0n]|BOLD:AAA2144
Xestia c-nigrum|[2018]|RWWB809-10|United States|Washington|658[0n]|BOLD:AAA2144
Xestia c-nigrum|[2019]|LGSMG902-10|United States|Tennessee|658[0n]|BOLD:AAA2144
Xestia c-nigrum|[2020]|RWWB445-10|United States|Washington|658[0n]|BOLD:AAA2144
Xestia c-nigrum|[2021]|BBLPC407-09|Canada|New Brunswick|658[0n]|BOLD:AAA2144
Xestia c-nigrum|[2022]|BBLPC430-09|Canada|New Brunswick|658[0n]|BOLD:AAA2144
Xestia c-nigrum|[2023]|XAH530-05|Canada|Ontario|658[0n]|BOLD:AAA2144
Xestia c-nigrum|[2024]|XAH549-05|Canada|Ontario|658[0n]|BOLD:AAA2144
Xestia c-nigrum|[2025]|XAH562-05|Canada|Ontario|658[0n]|BOLD:AAA2144
Xestia c-nigrum|[2026]|XAH605-05|Canada|Ontario|658[0n]|BOLD:AAA2144
Xestia c-nigrum|[2027]|LOWC794-05|Canada|British Columbia|658[0n]|BOLD:AAA2144
Xestia c-nigrum|[2028]|LOWC796-05|Canada|British Columbia|658[0n]|BOLD:AAA2144
Xestia c-nigrum|[2029]|LOWC797-05|Canada|British Columbia|658[0n]|BOLD:AAA2144
Xestia c-nigrum|[2030]|LOWC798-05|Canada|British Columbia|658[0n]|BOLD:AAA2144
Xestia c-nigrum|[2031]|LOWC799-05|Canada|British Columbia|658[0n]|BOLD:AAA2144
Xestia c-nigrum|[2032]|LOWC800-05|Canada|British Columbia|658[0n]|BOLD:AAA2144
Xestia c-nigrum|[2033]|LOWC801-05|Canada|British Columbia|658[0n]|BOLD:AAA2144
Xestia c-nigrum|[2034]|LOWC802-05|Canada|British Columbia|658[0n]|BOLD:AAA2144
Xestia c-nigrum|[2035]|LOWC804-05|Canada|British Columbia|658[0n]|BOLD:AAA2144
Xestia c-nigrum|[2036]|LOWC912-05|Canada|British Columbia|658[0n]|BOLD:AAA2144
Xestia c-nigrum|[2037]|TTMNB441-06|Canada|New Brunswick|658[0n]|BOLD:AAA2144
Xestia c-nigrum|[2038]|TTMNB442-06|Canada|New Brunswick|658[0n]|BOLD:AAA2144
Xestia c-nigrum|[2039]|TTMNB443-06|Canada|New Brunswick|658[0n]|BOLD:AAA2144
Xestia c-nigrum|[2040]|TTMNB444-06|Canada|New Brunswick|658[0n]|BOLD:AAA2144
Xestia c-nigrum|[2041]|TTMNB445-06|Canada|New Brunswick|658[0n]|BOLD:AAA2144
Xestia c-nigrum|[2042]|TTMNB446-06|Canada|New Brunswick|658[0n]|BOLD:AAA2144
Xestia c-nigrum|[2043]|TTMNB447-06|Canada|New Brunswick|658[0n]|BOLD:AAA2144
Xestia c-nigrum|[2044]|TTMNB448-06|Canada|New Brunswick|658[0n]|BOLD:AAA2144
Xestia c-nigrum|[2045]|TTMNB449-06|Canada|New Brunswick|658[0n]|BOLD:AAA2144
Xestia c-nigrum|[2046]|TTMNB566-06|Canada|New Brunswick|658[0n]|BOLD:AAA2144
Xestia c-nigrum|[2047]|LOWCE806-06|Canada|British Columbia|658[0n]|BOLD:AAA2144
Xestia c-nigrum|[2048]|LOWCE810-06|Canada|British Columbia|658[0n]|BOLD:AAA2144
Xestia c-nigrum|[2049]|LOWCE812-06|Canada|British Columbia|658[0n]|BOLD:AAA2144
Xestia c-nigrum|[2050]|LOWCE840-06|Canada|British Columbia|658[0n]|BOLD:AAA2144
Xestia c-nigrum|[2051]|LOWCE858-06|Canada|British Columbia|658[0n]|BOLD:AAA2144
Xestia c-nigrum|[2052]|XAJ549-06|Canada|Ontario|658[0n]|BOLD:AAA2144
Xestia c-nigrum|[2053]|LOCBC163-06|United States|California|658[0n]|BOLD:AAA2144
Xestia c-nigrum|[2054]|XAK337-06|Canada|Ontario|658[0n]|BOLD:AAA2144
Xestia c-nigrum|[2055]|XAK434-06|Canada|Ontario|658[0n]|BOLD:AAA2144
Xestia c-nigrum|[2056]|RDLQF885-06|Canada|Quebec|658[0n]|BOLD:AAA2144
Xestia c-nigrum|[2057]|RDLQ727-07|Canada|Quebec|658[0n]|BOLD:AAA2144
Xestia c-nigrum|[2058]|MMNA085-08|United States|North Carolina|657[0n]|BOLD:AAA2144
Xestia c-nigrum|[2059]|MNBB423-05|Canada|New Brunswick|658[0n]|BOLD:AAA2144
Xestia c-nigrum|[2060]|MNBB424-05|Canada|New Brunswick|658[0n]|BOLD:AAA2144
Xestia c-nigrum|[2061]|MNBB427-05|Canada|New Brunswick|658[0n]|BOLD:AAA2144
Xestia c-nigrum|[2062]|MNBB521-05|Canada|New Brunswick|658[0n]|BOLD:AAA2144
Xestia c-nigrum|[2063]|LPSOC095-08|Canada|Ontario|658[0n]|BOLD:AAA2144
Xestia c-nigrum|[2064]|LPSOC103-08|Canada|Ontario|658[0n]|BOLD:AAA2144
Xestia c-nigrum|[2065]|MNBB386-05|Canada|New Brunswick|658[0n]|BOLD:AAA2144
Xestia c-nigrum|[2066]|MNBB387-05|Canada|New Brunswick|658[0n]|BOLD:AAA2144
Xestia c-nigrum|[2067]|MNBB522-05|Canada|New Brunswick|658[0n]|BOLD:AAA2144
Xestia c-nigrum|[2068]|MNBB524-05|Canada|New Brunswick|658[0n]|BOLD:AAA2144
Xestia c-nigrum|[2069]|LPSOC104-08|Canada|Ontario|658[0n]|BOLD:AAA2144
Xestia c-nigrum|[2070]|LPSOC105-08|Canada|Ontario|658[0n]|BOLD:AAA2144
Xestia c-nigrum|[2071]|LHLEP427-06|Canada|British Columbia|658[0n]|BOLD:AAA2144
Xestia c-nigrum|[2072]|RDLQ726-07|Canada|Quebec|658[0n]|BOLD:AAA2144
Xestia c-nigrum|[2073]|LPSOC114-08|Canada|Ontario|658[0n]|BOLD:AAA2144
Xestia c-nigrum|[2074]|LPSOC115-08|Canada|Ontario|658[0n]|BOLD:AAA2144
Xestia c-nigrum|[2075]|LPSOC116-08|Canada|Ontario|658[0n]|BOLD:AAA2144
Xestia c-nigrum|[2076]|LPSOC291-08|Canada|Ontario|658[0n]|BOLD:AAA2144
Xestia c-nigrum|[2077]|LPSOC292-08|Canada|Ontario|658[0n]|BOLD:AAA2144
Xestia c-nigrum|[2078]|LPSOC293-08|Canada|Ontario|658[0n]|BOLD:AAA2144
Xestia c-nigrum|[2079]|LPSOC108-08|Canada|Ontario|658[0n]|BOLD:AAA2144
Xestia c-nigrum|[2080]|LPSOC112-08|Canada|Ontario|658[0n]|BOLD:AAA2144
Xestia c-nigrum|[2081]|LPSOC294-08|Canada|Ontario|658[0n]|BOLD:AAA2144
Xestia c-nigrum|[2082]|LPSOC295-08|Canada|Ontario|658[0n]|BOLD:AAA2144
Xestia c-nigrum|[2083]|MNBB560-05|Canada|New Brunswick|658[0n]|BOLD:AAA2144
Xestia c-nigrum|[2084]|MNBB590-05|Canada|New Brunswick|658[0n]|BOLD:AAA2144
Xestia c-nigrum|[2085]|MNBB593-05|Canada|New Brunswick|658[0n]|BOLD:AAA2144
Xestia c-nigrum|[2086]|MNBB599-05|Canada|New Brunswick|658[0n]|BOLD:AAA2144
Xestia c-nigrum|[2087]|LPSOC296-08|Canada|Ontario|658[0n]|BOLD:AAA2144
Xestia c-nigrum|[2088]|LPSOC297-08|Canada|Ontario|658[0n]|BOLD:AAA2144
Xestia c-nigrum|[2089]|LPSOC298-08|Canada|Ontario|658[0n]|BOLD:AAA2144
Xestia c-nigrum|[2090]|LPSOC299-08|Canada|Ontario|658[0n]|BOLD:AAA2144
Xestia c-nigrum|[2091]|LPSOC300-08|Canada|Ontario|658[0n]|BOLD:AAA2144
Xestia c-nigrum|[2092]|LPSOC301-08|Canada|Ontario|658[0n]|BOLD:AAA2144
Xestia c-nigrum|[2093]|LPSOC302-08|Canada|Ontario|658[0n]|BOLD:AAA2144
[illegible]

Xestia c-nigrum|[2091]|LPSOC300-08|Canada|Ontario|658[0n]|BOLD:AAA2144
Xestia c-nigrum|[2092]|LPSOC301-08|Canada|Ontario|658[0n]|BOLD:AAA2144
Xestia c-nigrum|[2093]|LPSOC302-08|Canada|Ontario|658[0n]|BOLD:AAA2144
Xestia c-nigrum|[2094]|LPSOC303-08|Canada|Ontario|658[0n]|BOLD:AAA2144
Xestia c-nigrum|[2095]|LPSOC304-08|Canada|Ontario|658[0n]|BOLD:AAA2144
Xestia c-nigrum|[2096]|LPSOC305-08|Canada|Ontario|658[0n]|BOLD:AAA2144
Xestia c-nigrum|[2097]|LPSOC326-08|Canada|Ontario|658[0n]|BOLD:AAA2144
Xestia c-nigrum|[2098]|LPSOC332-08|Canada|Ontario|658[0n]|BOLD:AAA2144
Xestia c-nigrum|[2099]|LPSOC345-08|Canada|Ontario|658[0n]|BOLD:AAA2144
Xestia c-nigrum|[2100]|LPSOC346-08|Canada|Ontario|655[0n]|BOLD:AAA2144
Xestia c-nigrum|[2101]|LPSOC363-08|Canada|Ontario|655[0n]|BOLD:AAA2144
Xestia c-nigrum|[2102]|LPSOB716-08|Canada|Ontario|658[0n]|BOLD:AAA2144
Xestia c-nigrum|[2103]|LPSOB972-08|Canada|Ontario|658[0n]|BOLD:AAA2144
Xestia c-nigrum|[2104]|LPSOB978-08|Canada|Ontario|658[0n]|BOLD:AAA2144
Xestia c-nigrum|[2105]|LPSOB979-08|Canada|Ontario|658[0n]|BOLD:AAA2144
Xestia c-nigrum|[2106]|BLTIB269-08|Canada|Ontario|658[0n]|BOLD:AAA2144
Xestia c-nigrum|[2107]|BLTIB315-08|Canada|Ontario|658[0n]|BOLD:AAA2144
Xestia c-nigrum|[2108]|BLTIB339-08|Canada|Ontario|658[0n]|BOLD:AAA2144
Xestia c-nigrum|[2109]|LPVIC114-08|Canada|British Columbia|658[0n]|BOLD:AAA2144
Xestia c-nigrum|[2110]|LPMNB442-09|Canada|Manitoba|658[0n]|BOLD:AAA2144
Xestia c-nigrum|[2111]|LPMNB443-09|Canada|Manitoba|658[0n]|BOLD:AAA2144
Xestia c-nigrum|[2112]|LPSOD970-09|Canada|Ontario|658[0n]|BOLD:AAA2144
Xestia c-nigrum|[2113]|MNBB666-05|Canada|New Brunswick|658[0n]|BOLD:AAA2144
Xestia c-nigrum|[2114]|MNBB668-05|Canada|New Brunswick|658[0n]|BOLD:AAA2144
Xestia c-nigrum|[2115]|LBCC287-05|Canada|British Columbia|658[0n]|BOLD:AAA2144
Xestia c-nigrum|[2116]|LBCC288-05|Canada|British Columbia|658[0n]|BOLD:AAA2144
Xestia c-nigrum|[2117]|LBCD029-05|Canada|British Columbia|658[0n]|BOLD:AAA2144
Xestia c-nigrum|[2118]|LBCD030-05|Canada|British Columbia|658[0n]|BOLD:AAA2144
Xestia c-nigrum|[2119]|LBCD493-05|Canada|British Columbia|658[0n]|BOLD:AAA2144
Xestia c-nigrum|[2120]|XAH223-05|Canada|Ontario|658[0n]|BOLD:AAA2144
Xestia c-nigrum|[2121]|RWWA055-09|United States|Washington|658[0n]|BOLD:AAA2144
Xestia c-nigrum|[2122]|RWWA374-09|United States|Washington|658[0n]|BOLD:AAA2144
Xestia c-nigrum|[2123]|RWWA501-09|United States|Washington|658[0n]|BOLD:AAA2144
Xestia c-nigrum|[2124]|RWWB090-09|United States|Washington|658[0n]|BOLD:AAA2144
Xestia c-nigrum|[2125]|RWWB333-09|United States|Washington|658[0n]|BOLD:AAA2144
Xestia c-nigrum|[2126]|RWWB343-09|United States|Washington|658[0n]|BOLD:AAA2144
Xestia c-nigrum|[2127]|RWWB352-09|United States|Washington|658[0n]|BOLD:AAA2144
Xestia c-nigrum|[2128]|RWWB353-09|United States|Washington|658[0n]|BOLD:AAA2144
Xestia c-nigrum|[2129]|RWWB378-09|United States|Washington|658[0n]|BOLD:AAA2144
Xestia c-nigrum|[2130]|RWWB405-09|United States|Washington|658[0n]|BOLD:AAA2144
Xestia c-nigrum|[2131]|RWWB411-09|United States|Washington|658[0n]|BOLD:AAA2144
Xestia c-nigrum|[2132]|RWWB420-09|United States|Washington|658[0n]|BOLD:AAA2144
Xestia c-nigrum|[2133]|BBLEC424-09|Canada|New Brunswick|658[0n]|BOLD:AAA2144
Xestia c-nigrum|[2134]|BBLEC430-09|Canada|New Brunswick|658[0n]|BOLD:AAA2144
Xestia c-nigrum|[2135]|BBLEC444-09|Canada|New Brunswick|658[0n]|BOLD:AAA2144
Xestia c-nigrum|[2136]|BBLEC448-09|Canada|New Brunswick|658[0n]|BOLD:AAA2144
Xestia c-nigrum|[2137]|BBLEC684-09|Canada|Nova Scotia|658[0n]|BOLD:AAA2144
Xestia c-nigrum|[2138]|BBLPC050-09|Canada|New Brunswick|658[0n]|BOLD:AAA2144
Xestia c-nigrum|[2139]|BBLPC058-09|Canada|New Brunswick|658[0n]|BOLD:AAA2144
Xestia c-nigrum|[2140]|BBLPC061-09|Canada|New Brunswick|658[0n]|BOLD:AAA2144
Xestia c-nigrum|[2141]|BBLPC063-09|Canada|New Brunswick|658[0n]|BOLD:AAA2144
Xestia c-nigrum|[2142]|BBLPC085-09|Canada|New Brunswick|658[0n]|BOLD:AAA2144
Xestia c-nigrum|[2143]|BBLPC093-09|Canada|New Brunswick|658[0n]|BOLD:AAA2144
Xestia c-nigrum|[2144]|BBLPC347-09|Canada|New Brunswick|658[0n]|BOLD:AAA2144
Xestia c-nigrum|[2145]|BBLPE424-09|Canada|Newfoundland and Labrador|658[0n]|BOLD:AAA2144
Xestia c-nigrum|[2146]|LGSMG898-10|United States|North Carolina|658[0n]|BOLD:AAA2144
Xestia c-nigrum|[2147]|LALPA638-10|Canada|British Columbia|658[0n]|BOLD:AAA2144
Xestia c-nigrum|[2148]|LALPA714-10|Canada|British Columbia|658[0n]|BOLD:AAA2144
Xestia c-nigrum|[2149]|RWWC140-10|United States|Washington|658[0n]|BOLD:AAA2144
Xestia c-nigrum|[2150]|BBLPB825-10|Canada|Alberta|658[0n]|BOLD:AAA2144
Xestia c-nigrum|[2151]|BBLPB826-10|Canada|British Columbia|658[0n]|BOLD:AAA2144
Xestia c-nigrum|[2152]|BBLPB827-10|Canada|British Columbia|658[0n]|BOLD:AAA2144
Xestia c-nigrum|[2153]|BBLPB829-10|Canada|British Columbia|658[0n]|BOLD:AAA2144
Xestia c-nigrum|[2154]|BBLPB830-10|Canada|British Columbia|658[0n]|BOLD:AAA2144
Xestia c-nigrum|[2155]|LALPA1249-11|Canada|British Columbia|658[0n]|BOLD:AAA2144
Xestia c-nigrum|[2156]|GMLC1040-12|United States|California|658[0n]|BOLD:AAA2144
Xestia c-nigrum|[2157]|GMLC1130-12|United States|California|658[0n]|BOLD:AAA2144
Xestia c-nigrum|[2158]|RWWC858-12|United States|Washington|658[0n]|BOLD:AAA2144
Xestia c-nigrum|[2159]|MNBB600-05|Canada|New Brunswick|658[0n]|BOLD:AAA2144
Xestia c-nigrum|[2160]|MNBB608-05|Canada|New Brunswick|658[0n]|BOLD:AAA2144
Xestia c-nigrum|[2161]|MNBB171-05|Canada|New Brunswick|658[0n]|BOLD:AAA2144
Xestia c-nigrum|[2162]|MNBB169-05|Canada|New Brunswick|658[0n]|BOLD:AAA2144
Xestia c-nigrum|[2163]|MNBB119-05|Canada|New Brunswick|658[0n]|BOLD:AAA2144
Xestia c-nigrum|[2164]|XAF549-05|Canada|Ontario|658[0n]|BOLD:AAA2144
Xestia c-nigrum|[2165]|PHMNB745-05|Canada|New Brunswick|658[0n]|BOLD:AAA2144
Xestia c-nigrum|[2166]|XAD500-04|Canada|Ontario|658[0n]|BOLD:AAA2144
Xestia c-nigrum|[2167]|XAD434-04|Canada|Ontario|658[0n]|BOLD:AAA2144
Xestia c-nigrum|[2168]|XAD244-04|Canada|Ontario|658[0n]|BOLD:AAA2144
Xestia c-nigrum|[2169]|XAB363-04|Canada|Ontario|658[0n]|BOLD:AAA2144
Xestia c-nigrum|[2170]|XAB150-04|Canada|Ontario|658[0n]|BOLD:AAA2144
Xestia c-nigrum|[2171]|LGSM045-04|United States|North Carolina|658[0n]|BOLD:AAA2144
Xestia c-nigrum|[2172]|LPABC910-09|Canada|Alberta|658[1n]|BOLD:AAA2144
Xestia c-nigrum|[2173]|BBLPC060-09|Canada|New Brunswick|644[0n]|BOLD:AAA2144
Xestia c-nigrum|[2174]|BBLEC466-09|Canada|New Brunswick|658[0n]|BOLD:AAA2144
Xestia c-nigrum|[2175]|LPSOC109-08|Canada|Ontario|656[0n]|BOLD:AAA2144
Xestia c-nigrum|[2176]|UAMIC529-13|United States|Alaska|654[0n]|BOLD:AAA2144
Xestia c-nigrum|[2177]|LPSOB715-08|Canada|Ontario|609[0n]|BOLD:AAA2144
Xestia c-nigrum|[2178]|BBLEC849-09|Canada|Newfoundland and Labrador|658[0n]|BOLD:AAA2144
Xestia c-nigrum|[2179]|LBCB890-05|Canada|British Columbia|598[0n]|BOLD:AAA2144
Xestia c-nigrum|[2180]|LBCB892-05|Canada|British Columbia|616[0n]|BOLD:AAA2144
Xestia c-nigrum|[2181]|LBCB893-05|Canada|British Columbia|598[0n]|BOLD:AAA2144
Xestia c-nigrum|[2182]|BBLPB828-10|Canada|British Columbia|632[1n]|BOLD:AAA2144
Xestia c-nigrum|[2183]|ABKWR122-07|United States|Alaska|636[0n]|BOLD:AAA2144
Xestia c-nigrum|[2184]|XAF780-05|Canada|Ontario|579[4n]|BOLD:AAA2144
Xestia c-nigrum|[2185]|CNRMH349-12|Canada|Manitoba|629[0n]|BOLD:AAA2144
Xestia c-nigrum|[2186]|UAMIC528-13|United States|Alaska|631[0n]|BOLD:AAA2144
Xestia c-nigrum|[2187]|BBLPC030-09|Canada|New Brunswick|632[0n]|BOLD:AAA2144
Xestia c-nigrum|[2188]|PHMNB003-03|Canada|New Brunswick|639[0n]|BOLD:AAA2144
Xestia c-nigrum|[2189]|PHMO085-03|Canada|Ontario|639[0n]|BOLD:AAA2144
Xestia c-nigrum|[2190]|TTMNB440-06|Canada|New Brunswick|605[0n]|BOLD:AAA2144
Xestia c-nigrum|[2191]|RDLQB707-05|Canada|Quebec|596[0n]|BOLD:AAA2144
Xestia c-nigrum|[2192]|PHMNB222-04|Canada|New Brunswick|538[1n]|BOLD:AAA2144
Xestia c-nigrum|[2193]|MNBB422-05|Canada|New Brunswick|540[0n]|BOLD:AAA2144

Xestia c-nigrum|[2191]|RDLQB707-05|Canada|Quebec|596[0n]|BOLD:AAA2144
Xestia c-nigrum|[2192]|PHMNB222-04|Canada|New Brunswick|538[1n]|BOLD:AAA2144
Xestia c-nigrum|[2193]|MNBB422-05|Canada|New Brunswick|540[0n]|BOLD:AAA2144
Xestia c-nigrum|[2194]|MNBB170-05|Canada|New Brunswick|537[0n]|BOLD:AAA2144
Xestia c-nigrum|[2195]|LOCBF391-13|United States|California|594[0n]|BOLD:AAA2144
Xestia dolosa|[2196]|LNCNW035-06|United States|North Carolina|658[0n]|BOLD:AAA2144
Xestia dolosa|[2197]|LPOKA264-08|United States|Oklahoma|658[0n]|BOLD:AAA2144
Xestia dolosa|[2198]|LPOKA621-09|United States|Oklahoma|658[0n]|BOLD:AAA2144
Xestia dolosa|[2199]|LPOKB150-09|United States|Oklahoma|658[0n]|BOLD:AAA2144
Xestia dolosa|[2200]|LPOKB369-09|United States|Oklahoma|658[0n]|BOLD:AAA2144
Xestia dolosa|[2201]|BBLSX312-09|United States|Oklahoma|658[0n]|BOLD:AAA2144
Xestia dolosa|[2202]|LPOKD440-09|United States|Oklahoma|658[0n]|BOLD:AAA2144
Xestia dolosa|[2203]|LGSMG899-10|United States|Tennessee|658[0n]|BOLD:AAA2144
Xestia dolosa|[2204]|LGSMG900-10|United States|North Carolina|658[0n]|BOLD:AAA2144
Xestia dolosa|[2205]|LSUSA172-06|United States|Kentucky|658[0n]|BOLD:AAA2144
Xestia dolosa|[2206]|LPSOC290-08|Canada|Ontario|658[0n]|BOLD:AAA2144
Xestia dolosa|[2207]|XAH702-05|Canada|Ontario|658[0n]|BOLD:AAA2144
Xestia dolosa|[2208]|LNC952-06|United States|North Carolina|658[0n]|BOLD:AAA2144
Xestia dolosa|[2209]|XAH619-05|Canada|Ontario|658[0n]|BOLD:AAA2144
Xestia dolosa|[2210]|XAH621-05|Canada|Ontario|658[0n]|BOLD:AAA2144
Xestia dolosa|[2211]|LGSM518-04|United States|Tennessee|609[0n]|BOLD:AAA2144
Xestia dolosa|[2212]|LGSMC361-05|United States|Tennessee|658[0n]|BOLD:AAA2144
Xestia dolosa|[2213]|LGSMC363-05|United States|Tennessee|658[0n]|BOLD:AAA2144
Xestia dolosa|[2214]|LGSM517-04|United States|Tennessee|658[0n]|BOLD:AAA2144
Xestia dolosa|[2215]|XAB550-04|Canada|Ontario|658[0n]|BOLD:AAA2144
Xestia dolosa|[2216]|PHMNB278-04|Canada|New Brunswick|658[0n]|BOLD:AAA2144
Xestia dolosa|[2217]|PHMNB598-04|Canada|New Brunswick|658[0n]|BOLD:AAA2144
Xestia dolosa|[2218]|LGSMC360-05|United States|Tennessee|658[0n]|BOLD:AAA2144
Xestia dolosa|[2219]|LGSMC362-05|United States|Tennessee|658[0n]|BOLD:AAA2144
Xestia dolosa|[2220]|LGSMC648-05|United States|Tennessee|658[0n]|BOLD:AAA2144
Xestia dolosa|[2221]|LOTB151-05|United States|Tennessee|658[0n]|BOLD:AAA2144
Xestia dolosa|[2222]|LOTB308-05|United States|Tennessee|658[0n]|BOLD:AAA2144
Xestia dolosa|[2223]|LOCT221-05|United States|Connecticut|658[0n]|BOLD:AAA2144
Xestia dolosa|[2224]|LGSMG901-10|United States|North Carolina|658[0n]|BOLD:AAA2144
Xestia c-nigrum|[2225]|CGUKA789-09|United Kingdom||658[1n]|BOLD:AAA2144
Xestia c-nigrum|[2226]|CGUKD240-09|United Kingdom|England|658[0n]|BOLD:AAA2144
Xestia c-nigrum|[2227]|LEFID939-10|Finland||614[0n]|BOLD:AAA2144
Xestia c-nigrum|[2228]|LEATD170-13|Austria|Tirol|658[0n]|BOLD:AAA2144
Xestia c-nigrum|[2229]|LEATD250-13|Austria|Tirol|658[0n]|BOLD:AAA2144
Xestia c-nigrum|[2230]|GBLAC324-13|Germany|Thuringia|658[0n]|BOLD:AAA2144
Xestia c-nigrum|[2231]|GBLAC335-13|Germany|Saxony|658[0n]|BOLD:AAA2144
Xestia c-nigrum|[2232]|LEATA195-13|Italy|South Tyrol|658[0n]|BOLD:AAA2144
Xestia c-nigrum|[2233]|LEATA289-13|Austria|Tirol|658[0n]|BOLD:AAA2144
Xestia c-nigrum|[2234]|MAMOT3624-13|Pakistan|Khyber Pakhtunkhwa|658[0n]|BOLD:AAA2144
Xestia c-nigrum|[2235]|LEATB748-13|Italy|South Tyrol|658[0n]|BOLD:AAA2144
Xestia c-nigrum|[2236]|MAMOT2313-12|Pakistan||658[0n]|BOLD:AAA2144
Xestia c-nigrum|[2237]|MAMOT2343-12|Pakistan||658[0n]|BOLD:AAA2144
Xestia c-nigrum|[2238]|MAMOT2347-12|Pakistan||658[0n]|BOLD:AAA2144
Xestia c-nigrum|[2239]|MAMOT2623-12|Pakistan||658[0n]|BOLD:AAA2144
Xestia c-nigrum|[2240]|PHLAV017-12|Austria|Vorarlberg|658[0n]|BOLD:AAA2144
Xestia c-nigrum|[2241]|MAMOT2896-12|Pakistan|Khyber Pakhtunkhwa|658[0n]|BOLD:AAA2144
Xestia c-nigrum|[2242]|GWOTL113-13|Germany|Saarland|658[0n]|BOLD:AAA2144
Xestia c-nigrum|[2243]|GWOTL114-13|Germany|Saarland|658[0n]|BOLD:AAA2144
Xestia c-nigrum|[2244]|GBLAB604-13|Germany|Berlin|658[0n]|BOLD:AAA2144
Xestia c-nigrum|[2245]|TRLEP030-13|Turkey|Mersin|658[0n]|BOLD:AAA2144
Xestia c-nigrum|[2246]|MAMOT2303-12|Pakistan||658[0n]|BOLD:AAA2144
Xestia c-nigrum|[2247]|MAMOT2306-12|Pakistan||658[0n]|BOLD:AAA2144
Xestia c-nigrum|[2248]|MAMOT2298-12|Pakistan||658[0n]|BOLD:AAA2144
Xestia c-nigrum|[2249]|MAMOT2299-12|Pakistan||658[0n]|BOLD:AAA2144
Xestia c-nigrum|[2250]|MAMOT2300-12|Pakistan||658[0n]|BOLD:AAA2144
Xestia c-nigrum|[2251]|MAMOT2301-12|Pakistan||658[0n]|BOLD:AAA2144
Xestia c-nigrum|[2252]|MAMOT2293-12|Pakistan||658[0n]|BOLD:AAA2144
Xestia c-nigrum|[2253]|MAMOT2294-12|Pakistan||658[0n]|BOLD:AAA2144
Xestia c-nigrum|[2254]|NLLEA514-12|Netherlands|South Holland|658[0n]|BOLD:AAA2144
Xestia c-nigrum|[2255]|GWOTF337-12|Italy|Basilicata|658[0n]|BOLD:AAA2144
Xestia c-nigrum|[2256]|GWOTG524-12|Germany|Saxony|658[0n]|BOLD:AAA2144
Xestia c-nigrum|[2257]|GWOTG525-12|Georgia||658[0n]|BOLD:AAA2144
Xestia c-nigrum|[2258]|MAMOT2169-12|Pakistan||658[0n]|BOLD:AAA2144
Xestia c-nigrum|[2259]|MAMOT2266-12|Pakistan||658[0n]|BOLD:AAA2144
Xestia c-nigrum|[2260]|MAMOT2269-12|Pakistan||658[0n]|BOLD:AAA2144
Xestia c-nigrum|[2261]|MAMOT2281-12|Pakistan||658[0n]|BOLD:AAA2144
Xestia c-nigrum|[2262]|IBLAO333-12|Spain|Catalonia|658[0n]|BOLD:AAA2144
Xestia c-nigrum|[2263]|NLLEA313-12|Netherlands|South Holland|658[0n]|BOLD:AAA2144
Xestia c-nigrum|[2264]|LENOA181-11|France|Haute Normandie|658[0n]|BOLD:AAA2144
Xestia c-nigrum|[2265]|LENOA182-11|France|Haute Normandie|658[0n]|BOLD:AAA2144
Xestia c-nigrum|[2266]|GWOSZ111-11|Italy|Trentino-Alto Adige|658[0n]|BOLD:AAA2144
Xestia c-nigrum|[2267]|IBLAO323-12|Spain|Castilla-La Mancha|658[0n]|BOLD:AAA2144
Xestia c-nigrum|[2268]|LEFIJ659-10|Finland||658[0n]|BOLD:AAA2144
Xestia c-nigrum|[2269]|MAMOT824-10|Pakistan|Azad Kashmir|658[0n]|BOLD:AAA2144
Xestia c-nigrum|[2270]|MAMOT471-10|Pakistan|Punjab|658[0n]|BOLD:AAA2144
Xestia c-nigrum|[2271]|MAMOT629-10|Pakistan|Punjab|658[0n]|BOLD:AAA2144
Xestia c-nigrum|[2272]|LEFIC673-10|Finland|Finland Proper|658[0n]|BOLD:AAA2144
Xestia c-nigrum|[2273]|GWORZ215-10|Italy|Calabria|658[0n]|BOLD:AAA2144
Xestia c-nigrum|[2274]|GWORL297-09|Germany|Bavaria|658[0n]|BOLD:AAA2144
Xestia c-nigrum|[2275]|LEFID794-10|Finland|Aland Islands|658[0n]|BOLD:AAA2144
Xestia c-nigrum|[2276]|GWOR3951-09|Germany|Bavaria|658[0n]|BOLD:AAA2144
Xestia c-nigrum|[2277]|CGUKB936-09|United Kingdom|Wales|658[0n]|BOLD:AAA2144
Xestia c-nigrum|[2278]|CGUKB727-09|United Kingdom|England|658[0n]|BOLD:AAA2144
Xestia c-nigrum|[2279]|CGUKB249-09|United Kingdom|England|658[0n]|BOLD:AAA2144
Xestia c-nigrum|[2280]|CGUKB043-09|United Kingdom|England|658[0n]|BOLD:AAA2144
Xestia c-nigrum|[2281]|CGUKA811-09|United Kingdom||658[0n]|BOLD:AAA2144
Xestia c-nigrum|[2282]|CGUKA758-09|United Kingdom||658[0n]|BOLD:AAA2144
Xestia c-nigrum|[2283]|CGUKA437-09|United Kingdom|England|658[0n]|BOLD:AAA2144
Xestia c-nigrum|[2284]|CGUKA299-09|United Kingdom|England|658[0n]|BOLD:AAA2144
Xestia c-nigrum|[2285]|CGUKA025-09|United Kingdom|England|658[0n]|BOLD:AAA2144
Xestia c-nigrum|[2286]|MAMOT2335-12|Pakistan||658[0n]|BOLD:AAA2144
Xestia c-nigrum|[2287]|LON272-08|Norway|Akershus|657[0n]|BOLD:AAA2144
Xestia c-nigrum|[2288]|NLLEA513-12|Netherlands|South Holland|658[0n]|BOLD:AAA2144
Xestia c-nigrum|[2289]|FBLMV030-09|Germany|Bavaria|618[0n]|BOLD:AAA2144
Xestia c-nigrum|[2290]|CGUKA564-09|United Kingdom|England|598[0n]|BOLD:AAA2144
Xestia c-nigrum|[2291]|MAMOT2370-12|Pakistan||658[0n]|BOLD:AAA2144
Xestia c-nigrum|[2292]|CGUKA367-09|United Kingdom|England|652[0n]|BOLD:AAA2144
Xestia c-nigrum|[2293]|NOCTU186-13|China|Tibet|630[0n]|BOLD:AAA2144

[illegible]
Xestia c-nigrum|[2291]|MAMOT2370-12|Pakistan||658[0n]|BOLD:AAA2144
Xestia c-nigrum|[2292]|CGUKA367-09|United Kingdom|England|652[0n]|BOLD:AAA2144
Xestia c-nigrum|[2293]|NOCTU186-13|China|Tibet|630[0n]|BOLD:AAA2144
Xestia c-nigrum|[2294]|NOCTU185-13|China|Tibet|630[0n]|BOLD:AAA2144
Xestia c-nigrum|[2295]|NOCTU184-13|China|Tibet|630[0n]|BOLD:AAA2144
Xestia c-nigrum|[2296]|NOCTU183-13|China|Tibet|630[0n]|BOLD:AAA2144
Xestia c-nigrum|[2297]|NOCTU182-13|China|Tibet|630[0n]|BOLD:AAA2144
Xestia c-nigrum|[2298]|NOCTU181-13|China|Tibet|630[0n]|BOLD:AAA2144
Xestia c-nigrum|[2299]|NOCTU180-13|China|Tibet|630[0n]|BOLD:AAA2144
Xestia c-nigrum|[2300]|NOCTU179-13|China|Tibet|630[0n]|BOLD:AAA2144
Xestia c-nigrum|[2301]|NOCTU178-13|China|Tibet|630[0n]|BOLD:AAA2144
Xestia c-nigrum|[2302]|NOCTU177-13|China|Tibet|630[0n]|BOLD:AAA2144
Xestia c-nigrum|[2303]|NOCTU176-13|China|Tibet|630[0n]|BOLD:AAA2144
Xestia c-nigrum|[2304]|NOCTU175-13|China|Tibet|630[0n]|BOLD:AAA2144
Xestia c-nigrum|[2305]|NOCTU174-13|China|Tibet|630[0n]|BOLD:AAA2144
Xestia c-nigrum|[2306]|NOCTU173-13|China|Tibet|630[0n]|BOLD:AAA2144
Xestia c-nigrum|[2307]|NOCTU172-13|China|Tibet|630[0n]|BOLD:AAA2144
Xestia c-nigrum|[2308]|NOCTU171-13|China|Tibet|630[0n]|BOLD:AAA2144
Xestia c-nigrum|[2309]|NOCTU170-13|China|Tibet|630[0n]|BOLD:AAA2144
Xestia c-nigrum|[2310]|NOCTU169-13|China|Tibet|630[0n]|BOLD:AAA2144
Xestia c-nigrum|[2311]|NOCTU168-13|China|Tibet|630[0n]|BOLD:AAA2144
Xestia c-nigrum|[2312]|NOCTU167-13|China|Tibet|630[0n]|BOLD:AAA2144
Xestia c-nigrum|[2313]|NOCTU166-13|China|Tibet|630[0n]|BOLD:AAA2144
Xestia c-nigrum|[2314]|NOCTU165-13|China|Tibet|630[0n]|BOLD:AAA2144
Xestia c-nigrum|[2315]|NOCTU164-13|China|Tibet|630[0n]|BOLD:AAA2144
Xestia c-nigrum|[2316]|NOCTU163-13|China|Tibet|630[0n]|BOLD:AAA2144
Xestia c-nigrum|[2317]|NOCTU159-13|China|Tibet|630[0n]|BOLD:AAA2144
Xestia c-nigrum|[2318]|NOCTU041-13|China|Tibet|630[0n]|BOLD:AAA2144
Xestia c-nigrum|[2319]|NOCTU040-13|China|Tibet|630[0n]|BOLD:AAA2144
Xestia c-nigrum|[2320]|NOCTU039-13|China|Tibet|630[0n]|BOLD:AAA2144
Xestia c-nigrum|[2321]|NOCTU157-13|China|Tibet|630[0n]|BOLD:AAA2144
Xestia c-nigrum|[2322]|NOCTU158-13|China|Tibet|630[1n]|BOLD:AAA2144
Xestia c-nigrum|[2323]|NOCTU038-13|China|Tibet|630[1n]|BOLD:AAA2144
Xestia c-nigrum|[2324]|NOCTU187-13|China|Tibet|630[2n]|BOLD:AAA2144
Xestia c-nigrum|[2325]|NOCTU162-13|China|Tibet|630[0n]|BOLD:AAA2144
Xestia c-nigrum|[2326]|NOCTU161-13|China|Tibet|630[0n]|BOLD:AAA2144
Xestia c-nigrum|[2327]|NOCTU160-13|China|Tibet|630[0n]|BOLD:AAA2144
Xestia c-nigrum|[2328]|NOCTU188-13|China|Tibet|630[3n]|BOLD:AAA2144
Xestia c-nigrum|[2329]|NOCTU189-13|China|Tibet|630[2n]|BOLD:AAA2144
Xestia c-nigrum|[2330]|GBLAF240-14|Germany|Brandenburg|658[0n]|BOLD:AAA2144
Xestia scropulana|[2331]|LCHQ883-08|Canada|Manitoba|658[0n]|BOLD:ABY8431
Xestia inuitica|[2332]|RDNMG708-08|Canada|Nunavut|658[1n]|BOLD:ABY8431
Xestia inuitica|[2333]|RDNMG709-08|Canada|Nunavut|658[2n]|BOLD:ABY8431
Xestia scropulana|[2334]|LCHQ882-08|Canada|Manitoba|658[0n]|BOLD:ABY8431
Xestia scropulana|[2335]|LCHQ885-08|Canada|Manitoba|658[0n]|BOLD:ABY8431
Xestia scropulana|[2336]|LCHQ873-08|Canada|Manitoba|658[0n]|BOLD:ABY8431
Xestia scropulana|[2337]|LCHQ874-08|Canada|Manitoba|658[0n]|BOLD:ABY8431
Xestia scropulana|[2338]|LCHQ887-08|Canada|Manitoba|658[0n]|BOLD:ABY8431
Xestia scropulana|[2339]|LCHQ892-08|Canada|Manitoba|658[0n]|BOLD:ABY8431
Xestia scropulana|[2340]|LCHQ896-08|Canada|Manitoba|658[0n]|BOLD:ABY8431
Xestia scropulana|[2341]|LCHQ913-08|Canada|Manitoba|658[0n]|BOLD:ABY8431
Xestia scropulana|[2342]|LCHQ879-08|Canada|Manitoba|658[0n]|BOLD:ABY8431
Xestia scropulana|[2343]|LCHQ880-08|Canada|Manitoba|658[0n]|BOLD:ABY8431
Xestia scropulana|[2344]|LCHP867-07|Canada|Manitoba|658[0n]|BOLD:ABY8431
Xestia scropulana|[2345]|LCHQ870-08|Canada|Manitoba|658[0n]|BOLD:ABY8431
Xestia scropulana|[2346]|LCHQ876-08|Canada|Manitoba|658[0n]|BOLD:ABY8431
Xestia scropulana|[2347]|LCHQ878-08|Canada|Manitoba|658[0n]|BOLD:ABY8431
Xestia scropulana|[2348]|MHLEP090-07|Canada|Manitoba|658[0n]|BOLD:ABY8431
Xestia scropulana|[2349]|LCHP629-07|Canada|Manitoba|658[0n]|BOLD:ABY8431
Xestia scropulana|[2350]|LCHQ871-08|Canada|Manitoba|658[0n]|BOLD:ABY8431
Xestia scropulana|[2351]|LCHQ872-08|Canada|Manitoba|657[0n]|BOLD:ABY8431
Xestia inuitica|[2352]|RDNMG707-08|Canada|Nunavut|658[0n]|BOLD:ABY8431
Xestia scropulana|[2353]|LCHP214-07|Canada|Manitoba|658[0n]|BOLD:ABY8431
Xestia scropulana|[2354]|LCHP206-07|Canada|Manitoba|658[0n]|BOLD:ABY8431
Xestia scropulana|[2355]|LCHP176-07|Canada|Manitoba|658[0n]|BOLD:ABY8431
Xestia scropulana|[2356]|LCHP174-07|Canada|Manitoba|658[0n]|BOLD:ABY8431
Xestia scropulana|[2357]|LCHP173-07|Canada|Manitoba|658[0n]|BOLD:ABY8431
Xestia scropulana|[2358]|LCHP162-07|Canada|Manitoba|658[0n]|BOLD:ABY8431
Xestia scropulana|[2359]|LCHP161-07|Canada|Manitoba|658[0n]|BOLD:ABY8431
Xestia scropulana|[2360]|LCHP158-07|Canada|Manitoba|658[0n]|BOLD:ABY8431
Xestia scropulana|[2361]|LCHP157-07|Canada|Manitoba|658[0n]|BOLD:ABY8431
Xestia scropulana|[2362]|LCHP007-07|Canada|Manitoba|655[0n]|BOLD:ABY8431
Xestia scropulana|[2363]|LCH256-04|Canada|Manitoba|658[0n]|BOLD:ABY8431
Xestia scropulana|[2364]|LCHQ875-08|Canada|Manitoba|656[0n]|BOLD:ABY8431
Xestia scropulana|[2365]|LCHP160-07|Canada|Manitoba|656[0n]|BOLD:ABY8431
Xestia scropulana|[2366]|LCHP159-07|Canada|Manitoba|656[0n]|BOLD:ABY8431
Xestia scropulana|[2367]|LCHP390-07|Canada|Manitoba|638[0n]|BOLD:ABY8431
Xestia wockei|[2368]|RDNMG732-08|Canada|Yukon Territory|641[0n]|BOLD:ABY8431
Xestia scropulana|[2369]|LCHQ881-08|Canada|Manitoba|658[0n]|BOLD:ABY8431
Xestia scropulana|[2370]|LCHQ884-08|Canada|Manitoba|658[0n]|BOLD:ABY8431
Xestia scropulana|[2371]|LCHQ923-08|Canada|Manitoba|658[0n]|BOLD:ABY8431
Xestia inuitica|[2372]|RDNMF075-08|Canada|Nunavut|551[9n]|
Xestia wockei|[2373]|RDNMG733-08|Canada|Yukon Territory|609[0n]|BOLD:ABY8431
Xestia colorado|[2374]|RDNMG737-08|United States|Colorado|658[0n]|BOLD:ABY8431
Xestia maculata|[2375]|RDMAB1032-09|Canada|Alberta|644[0n]|BOLD:ABY8431
Xestia maculata|[2376]|RDMAB1033-09|Canada|Alberta|644[0n]|BOLD:ABY8431
Xestia maculata|[2377]|RDMAB1034-09|Canada|Alberta|537[0n]|BOLD:ABY8431
Xestia maculata|[2378]|RDMAB1035-09|Canada|Alberta|658[0n]|BOLD:ABY8431
Xestia bolteri|[2379]|CNCLB971-14|United States|Wyoming|658[0n]|BOLD:ACM3882
Xestia bolteri|[2380]|CNCLB972-14|United States|Colorado|658[0n]|BOLD:ACM3882
Xestia bolteri|[2381]|LNAUT546-14|United States|New Mexico|658[0n]|BOLD:ACM3882
Xestia bolteri|[2382]|LNAUT547-14|United States|New Mexico|658[0n]|BOLD:ACM3882
Xestia bolteri|[2383]|LNAUT549-14|United States|New Mexico|658[0n]|BOLD:ACM3882
Xestia bolteri|[2384]|LNAUT550-14|United States|New Mexico|658[0n]|BOLD:ACM3882
Eurois nigra|[2385]|LOWC810-05|Canada|British Columbia|658[0n]|BOLD:AAB0434
Eurois nigra|[2386]|LSEU805-06|United States|Colorado|609[2n]|BOLD:AAB0434
Eurois nigra|[2387]|LSEU806-06|United States|Colorado|658[0n]|BOLD:AAB0434
Eurois nigra|[2388]|IAWLB116-10|United States|California|658[0n]|BOLD:AAB0434
Eurois nigra|[2389]|LPABC495-09|Canada|Alberta|634[0n]|BOLD:AAB0434
Eurois nigra|[2390]|LOWCD785-06|Canada|British Columbia|657[0n]|BOLD:AAB0434
Eurois nigra|[2391]|LOWC814-05|Canada|British Columbia|658[0n]|BOLD:AAB0434
Eurois nigra|[2392]|LPABC029-09|Canada|Alberta|658[1n]|BOLD:AAB0434
[illegible]

Eurois nigra|[2390]|LOWCD785-06|Canada|British Columbia|657[0n]|BOLD:AAB0434
Eurois nigra|[2391]|LOWC814-05|Canada|British Columbia|658[0n]|BOLD:AAB0434
Eurois nigra|[2392]|LPABC029-09|Canada|Alberta|658[1n]|BOLD:AAB0434
Eurois nigra|[2393]|LPABC481-09|Canada|Alberta|658[0n]|BOLD:AAB0434
Eurois nigra|[2394]|LPABC439-09|Canada|Alberta|658[0n]|BOLD:AAB0434
Eurois nigra|[2395]|LPABC432-09|Canada|Alberta|658[0n]|BOLD:AAB0434
Eurois nigra|[2396]|LPABC038-09|Canada|Alberta|658[0n]|BOLD:AAB0434
Eurois nigra|[2397]|LPABB881-09|Canada|Alberta|658[0n]|BOLD:AAB0434
Eurois nigra|[2398]|LPABB718-08|Canada|Alberta|658[0n]|BOLD:AAB0434
Eurois nigra|[2399]|LOWCD784-06|Canada|British Columbia|658[0n]|BOLD:AAB0434
Eurois nigra|[2400]|LPABC496-09|Canada|Alberta|634[0n]|BOLD:AAB0434
Eurois nigra|[2401]|LPABC809-09|Canada|Alberta|658[0n]|BOLD:AAB0434
Eurois nigra|[2402]|LOWCD786-06|Canada|British Columbia|657[0n]|BOLD:AAB0434
Eurois nigra|[2403]|LOWC817-05|Canada|British Columbia|658[0n]|BOLD:AAB0434
Eurois nigra|[2404]|LOWC816-05|Canada|British Columbia|658[0n]|BOLD:AAB0434
Eurois nigra|[2405]|LOWC812-05|Canada|British Columbia|658[0n]|BOLD:AAB0434
Eurois nigra|[2406]|LOWC809-05|Canada|British Columbia|658[0n]|BOLD:AAB0434
Eurois nigra|[2407]|LOWC808-05|Canada|British Columbia|658[0n]|BOLD:AAB0434
Eurois nigra|[2408]|LOWC807-05|Canada|British Columbia|658[0n]|BOLD:AAB0434
Eurois nigra|[2409]|LOWC806-05|Canada|British Columbia|658[0n]|BOLD:AAB0434
Eurois nigra|[2410]|LOWCD787-06|Canada|British Columbia|656[0n]|BOLD:AAB0434
Eurois nigra|[2411]|LOWCD788-06|Canada|British Columbia|657[0n]|BOLD:AAB0434
Eurois nigra|[2412]|LPABB876-09|Canada|Alberta|658[0n]|BOLD:AAB0434
Eurois nigra|[2413]|LPABC499-09|Canada|Alberta|658[0n]|BOLD:AAB0434
Eurois nigra|[2414]|LALPA812-10|Canada|British Columbia|658[0n]|BOLD:AAB0434
Eurois nigra|[2415]|LOWC815-05|Canada|British Columbia|658[0n]|BOLD:AAB0434
Eurois nigra|[2416]|LOWC805-05|Canada|British Columbia|658[0n]|BOLD:AAB0434
Eurois nigra|[2417]|LOWC813-05|Canada|British Columbia|658[0n]|BOLD:AAB0434
Eurois nigra|[2418]|LOWCC832-05|Canada|British Columbia|590[0n]|BOLD:AAB0434
Eurois nigra|[2419]|LOWC811-05|Canada|British Columbia|658[0n]|BOLD:AAB0434
Eurois nigra|[2420]|BBLPA333-10|Canada|Alberta|658[0n]|BOLD:AAB0434
Eurois astricta|[2421]|LBCH1860-10|Canada|British Columbia|658[0n]|BOLD:AAA2221
Eurois astricta|[2422]|LBCB042-05|Canada|British Columbia|658[0n]|BOLD:AAA2221
Eurois astricta|[2423]|LOWCC173-05|Canada|British Columbia|658[0n]|BOLD:AAA2221
Eurois astricta|[2424]|LBCG816-09|Canada|British Columbia|658[0n]|BOLD:AAA2221
Eurois astricta|[2425]|LBCG2883-09|Canada|British Columbia|658[0n]|BOLD:AAA2221
Eurois astricta|[2426]|LBCG2891-09|Canada|British Columbia|658[0n]|BOLD:AAA2221
Eurois astricta|[2427]|LBCH1857-10|Canada|British Columbia|658[0n]|BOLD:AAA2221
Eurois astricta|[2428]|LBCD242-05|Canada|British Columbia|632[0n]|BOLD:AAA2221
Eurois astricta|[2429]|LBCD232-05|Canada|British Columbia|632[0n]|BOLD:AAA2221
Eurois astricta|[2430]|LBCD243-05|Canada|British Columbia|658[0n]|BOLD:AAA2221
Eurois astricta|[2431]|LBCH1675-10|Canada|British Columbia|658[0n]|BOLD:AAA2221
Eurois astricta|[2432]|LBCH2056-10|Canada|British Columbia|658[0n]|BOLD:AAA2221
Eurois astricta|[2433]|LBCG638-09|Canada|British Columbia|658[0n]|BOLD:AAA2221
Eurois astricta|[2434]|LBCD246-05|Canada|British Columbia|616[1n]|BOLD:AAA2221
Eurois astricta|[2435]|LBCD438-05|Canada|British Columbia|658[0n]|BOLD:AAA2221
Eurois astricta|[2436]|LPABC493-09|Canada|Alberta|658[0n]|BOLD:AAA2221
Eurois astricta|[2437]|LBCC325-05|Canada|British Columbia|658[0n]|BOLD:AAA2221
Eurois astricta|[2438]|LBCD241-05|Canada|British Columbia|638[0n]|BOLD:AAA2221
Eurois astricta|[2439]|LBCD271-05|Canada|British Columbia|631[0n]|BOLD:AAA2221
Eurois astricta|[2440]|LBCD419-05|Canada|British Columbia|636[0n]|BOLD:AAA2221
Eurois astricta|[2441]|LBCD244-05|Canada|British Columbia|636[0n]|BOLD:AAA2221
Eurois astricta|[2442]|LBCD420-05|Canada|British Columbia|616[0n]|BOLD:AAA2221
Eurois astricta|[2443]|LBCD237-05|Canada|British Columbia|616[0n]|BOLD:AAA2221
Eurois astricta|[2444]|LBCB037-05|Canada|British Columbia|658[0n]|BOLD:AAA2221
Eurois astricta|[2445]|LBCB045-05|Canada|British Columbia|658[0n]|BOLD:AAA2221
Eurois astricta|[2446]|LPABC400-09|Canada|Alberta|658[0n]|BOLD:AAA2221
Eurois astricta|[2447]|LBCG849-09|Canada|British Columbia|658[0n]|BOLD:AAA2221
Eurois astricta|[2448]|LBCG693-09|Canada|British Columbia|658[0n]|BOLD:AAA2221
Eurois astricta|[2449]|LBCG685-09|Canada|British Columbia|658[0n]|BOLD:AAA2221
Eurois astricta|[2450]|LBCG684-09|Canada|British Columbia|658[0n]|BOLD:AAA2221
Eurois astricta|[2451]|LBCG637-09|Canada|British Columbia|658[0n]|BOLD:AAA2221
Eurois astricta|[2452]|LPABB480-08|Canada|Alberta|658[0n]|BOLD:AAA2221
Eurois astricta|[2453]|LPAB233-08|Canada|Alberta|658[0n]|BOLD:AAA2221
Eurois astricta|[2454]|LPAB210-08|Canada|Alberta|658[0n]|BOLD:AAA2221
Eurois astricta|[2455]|LHLEP389-06|Canada|British Columbia|658[0n]|BOLD:AAA2221
Eurois astricta|[2456]|LBCD253-05|Canada|British Columbia|658[0n]|BOLD:AAA2221
Eurois astricta|[2457]|LBCD250-05|Canada|British Columbia|658[0n]|BOLD:AAA2221
Eurois astricta|[2458]|LBCD249-05|Canada|British Columbia|658[0n]|BOLD:AAA2221
Eurois astricta|[2459]|LBCD247-05|Canada|British Columbia|655[0n]|BOLD:AAA2221
Eurois astricta|[2460]|LBCD240-05|Canada|British Columbia|658[0n]|BOLD:AAA2221
Eurois astricta|[2461]|LBCD239-05|Canada|British Columbia|658[0n]|BOLD:AAA2221
Eurois astricta|[2462]|LBCD238-05|Canada|British Columbia|658[0n]|BOLD:AAA2221
Eurois astricta|[2463]|LBCD223-05|Canada|British Columbia|658[0n]|BOLD:AAA2221
Eurois astricta|[2464]|LBCD222-05|Canada|British Columbia|658[0n]|BOLD:AAA2221
Eurois astricta|[2465]|LBCD044-05|Canada|British Columbia|658[0n]|BOLD:AAA2221
Eurois astricta|[2466]|LBCC827-05|Canada|British Columbia|658[0n]|BOLD:AAA2221
Eurois astricta|[2467]|LBCC826-05|Canada|British Columbia|658[0n]|BOLD:AAA2221
Eurois astricta|[2468]|LBCC750-05|Canada|British Columbia|658[0n]|BOLD:AAA2221
Eurois astricta|[2469]|LBCC671-05|Canada|British Columbia|658[0n]|BOLD:AAA2221
Eurois astricta|[2470]|LBCC324-05|Canada|British Columbia|658[0n]|BOLD:AAA2221
Eurois astricta|[2471]|LBCC323-05|Canada|British Columbia|658[0n]|BOLD:AAA2221
Eurois astricta|[2472]|LBCC024-05|Canada|British Columbia|658[0n]|BOLD:AAA2221
Eurois astricta|[2473]|LBCB615-05|Canada|British Columbia|658[0n]|BOLD:AAA2221
Eurois astricta|[2474]|LBCB046-05|Canada|British Columbia|658[0n]|BOLD:AAA2221
Eurois astricta|[2475]|LBCB044-05|Canada|British Columbia|658[0n]|BOLD:AAA2221
Eurois astricta|[2476]|LBCB043-05|Canada|British Columbia|658[0n]|BOLD:AAA2221
Eurois astricta|[2477]|LBCB040-05|Canada|British Columbia|658[0n]|BOLD:AAA2221
Eurois astricta|[2478]|LBCB039-05|Canada|British Columbia|658[0n]|BOLD:AAA2221
Eurois astricta|[2479]|LBCB038-05|Canada|British Columbia|658[0n]|BOLD:AAA2221
Eurois astricta|[2480]|LBCA756-05|Canada|British Columbia|658[0n]|BOLD:AAA2221
Eurois astricta|[2481]|LBCD329-05|Canada|British Columbia|658[1n]|BOLD:AAA2221
Eurois astricta|[2482]|LBCD216-05|Canada|British Columbia|616[0n]|BOLD:AAA2221
Eurois astricta|[2483]|RDNMD084-06|United States|Idaho|608[0n]|BOLD:AAA2221
Eurois astricta|[2484]|LBCD440-05|Canada|British Columbia|651[0n]|BOLD:AAA2221
Eurois astricta|[2485]|LBCD234-05|Canada|British Columbia|648[1n]|BOLD:AAA2221
Eurois astricta|[2486]|LBCD245-05|Canada|British Columbia|648[0n]|BOLD:AAA2221
Eurois astricta|[2487]|LBCA753-05|Canada|British Columbia|646[0n]|BOLD:AAA2221
Eurois astricta|[2488]|LBCD328-05|Canada|British Columbia|623[0n]|BOLD:AAA2221
Eurois astricta|[2489]|LBCB041-05|Canada|British Columbia|658[0n]|BOLD:AAA2221
Eurois astricta|[2490]|LPABC492-09|Canada|Alberta|605[0n]|BOLD:AAA2221
Eurois astricta|[2491]|LBCG629-09|Canada|British Columbia|658[0n]|BOLD:AAA2221
Eurois astricta|[2492]|LPABC494-09|Canada|Alberta|658[0n]|BOLD:AAA2221

Eurois astricta|[2490]|LPABC492-09|Canada|Alberta|605[0n]|BOLD:AAA2221
Eurois astricta|[2491]|LBCG629-09|Canada|British Columbia|658[0n]|BOLD:AAA2221
Eurois astricta|[2492]|LPABC494-09|Canada|Alberta|658[0n]|BOLD:AAA2221
Eurois astricta|[2493]|LBCG3321-09|Canada|British Columbia|658[0n]|BOLD:AAA2221
Eurois astricta|[2494]|LBCH1090-10|Canada|British Columbia|658[0n]|BOLD:AAA2221
Eurois astricta|[2495]|LBCH1091-10|Canada|British Columbia|658[0n]|BOLD:AAA2221
Eurois astricta|[2496]|LBCH1165-10|Canada|British Columbia|658[0n]|BOLD:AAA2221
Eurois astricta|[2497]|LBCH1236-10|Canada|British Columbia|658[0n]|BOLD:AAA2221
Eurois astricta|[2498]|LBCH1428-10|Canada|British Columbia|658[0n]|BOLD:AAA2221
Eurois astricta|[2499]|LBCH1502-10|Canada|British Columbia|658[0n]|BOLD:AAA2221
Eurois astricta|[2500]|LBCH1565-10|Canada|British Columbia|658[0n]|BOLD:AAA2221
Eurois astricta|[2501]|LBCH1566-10|Canada|British Columbia|658[0n]|BOLD:AAA2221
Eurois astricta|[2502]|LBCH1772-10|Canada|British Columbia|658[0n]|BOLD:AAA2221
Eurois astricta|[2503]|LBCH1854-10|Canada|British Columbia|658[0n]|BOLD:AAA2221
Eurois astricta|[2504]|LBCH1855-10|Canada|British Columbia|658[0n]|BOLD:AAA2221
Eurois astricta|[2505]|LBCH1856-10|Canada|British Columbia|658[0n]|BOLD:AAA2221
Eurois astricta|[2506]|LBCH1858-10|Canada|British Columbia|658[0n]|BOLD:AAA2221
Eurois astricta|[2507]|LBCH1859-10|Canada|British Columbia|658[0n]|BOLD:AAA2221
Eurois astricta|[2508]|LBCH1861-10|Canada|British Columbia|658[0n]|BOLD:AAA2221
Eurois astricta|[2509]|LBCH1996-10|Canada|British Columbia|658[0n]|BOLD:AAA2221
Eurois astricta|[2510]|LBCH2093-10|Canada|British Columbia|658[0n]|BOLD:AAA2221
Eurois astricta|[2511]|LBCH2094-10|Canada|British Columbia|658[0n]|BOLD:AAA2221
Eurois astricta|[2512]|LBCH2101-10|Canada|British Columbia|658[0n]|BOLD:AAA2221
Eurois astricta|[2513]|LBCH2248-10|Canada|British Columbia|658[0n]|BOLD:AAA2221
Eurois astricta|[2514]|LALPA811-10|Canada|British Columbia|658[0n]|BOLD:AAA2221
Eurois astricta|[2515]|BBLPB527-10|Canada|British Columbia|658[0n]|BOLD:AAA2221
Eurois astricta|[2516]|BBLPB528-10|Canada|British Columbia|658[0n]|BOLD:AAA2221
Eurois astricta|[2517]|BBLPB529-10|Canada|British Columbia|658[0n]|BOLD:AAA2221
Eurois astricta|[2518]|BBLPB530-10|Canada|British Columbia|658[0n]|BOLD:AAA2221
Eurois astricta|[2519]|BBLPB531-10|Canada|British Columbia|658[0n]|BOLD:AAA2221
Eurois astricta|[2520]|BBLPB675-10|Canada|British Columbia|658[0n]|BOLD:AAA2221
Eurois astricta|[2521]|GWOSN429-11|Canada|British Columbia|658[0n]|BOLD:AAA2221
Eurois astricta|[2522]|LALPA712-10|Canada|British Columbia|658[0n]|BOLD:AAA2221
Eurois astricta|[2523]|LALPA756-10|Canada|British Columbia|658[0n]|BOLD:AAA2221
Eurois astricta|[2524]|LALPA1280-11|Canada|British Columbia|658[0n]|BOLD:AAA2221
Eurois astricta|[2525]|LALPA1318-12|Canada|British Columbia|632[0n]|BOLD:AAA2221
Eurois astricta|[2526]|LPAB209-08|Canada|Alberta|658[0n]|BOLD:AAA2220
Eurois astricta|[2527]|TTMNB439-06|Canada|New Brunswick|658[0n]|BOLD:AAA2220
Eurois astricta|[2528]|LOWC819-05|Canada|British Columbia|658[0n]|BOLD:AAA2220
Eurois astricta|[2529]|ABKWR096-07|United States|Alaska|658[0n]|BOLD:AAA2220
Eurois astricta|[2530]|LPABB486-08|Canada|Alberta|658[0n]|BOLD:AAA2220
Eurois astricta|[2531]|LCH543-04|Canada|Manitoba|585[1n]|BOLD:AAA2220
Eurois astricta|[2532]|LBCH489-10|Canada|British Columbia|658[0n]|BOLD:AAA2220
Eurois astricta|[2533]|LBCH3764-10|Canada|British Columbia|658[0n]|BOLD:AAA2220
Eurois astricta|[2534]|LBCH4102-10|Canada|British Columbia|658[0n]|BOLD:AAA2220
Eurois astricta|[2535]|LALPA849-11|Canada|British Columbia|658[0n]|BOLD:AAA2220
Eurois astricta|[2536]|LBCH4108-10|Canada|British Columbia|658[0n]|BOLD:AAA2220
Eurois astricta|[2537]|LOWCC865-05|Canada|British Columbia|606[1n]|BOLD:AAA2220
Eurois astricta|[2538]|LPABB874-09|Canada|Alberta|658[0n]|BOLD:AAA2220
Eurois astricta|[2539]|LBCH3480-10|Canada|British Columbia|658[0n]|BOLD:AAA2220
Eurois astricta|[2540]|LBCH3481-10|Canada|British Columbia|658[0n]|BOLD:AAA2220
Eurois astricta|[2541]|LBCH3153-10|Canada|British Columbia|658[0n]|BOLD:AAA2220
Eurois astricta|[2542]|LBCH3327-10|Canada|British Columbia|658[0n]|BOLD:AAA2220
Eurois astricta|[2543]|LBCH3151-10|Canada|British Columbia|658[0n]|BOLD:AAA2220
Eurois astricta|[2544]|LBCH3152-10|Canada|British Columbia|658[0n]|BOLD:AAA2220
Eurois astricta|[2545]|LBCH3148-10|Canada|British Columbia|658[0n]|BOLD:AAA2220
Eurois astricta|[2546]|LBCH3150-10|Canada|British Columbia|658[0n]|BOLD:AAA2220
Eurois astricta|[2547]|LCHQ683-08|Canada|Manitoba|656[0n]|BOLD:AAA2220
Eurois astricta|[2548]|LPABB842-09|Canada|Alberta|658[0n]|BOLD:AAA2220
Eurois astricta|[2549]|LCHQ513-08|Canada|Manitoba|658[0n]|BOLD:AAA2220
Eurois astricta|[2550]|LCHQ559-08|Canada|Manitoba|658[0n]|BOLD:AAA2220
Eurois astricta|[2551]|LCHQ245-08|Canada|Manitoba|658[0n]|BOLD:AAA2220
Eurois astricta|[2552]|LCHQ269-08|Canada|Manitoba|658[0n]|BOLD:AAA2220
Eurois astricta|[2553]|LOWCE851-06|Canada|British Columbia|658[0n]|BOLD:AAA2220
Eurois astricta|[2554]|TMNBB409-06|Canada|New Brunswick|658[0n]|BOLD:AAA2220
Eurois astricta|[2555]|RDLQF348-06|Canada|Quebec|658[0n]|BOLD:AAA2220
Eurois astricta|[2556]|ABKWR058-07|United States|Alaska|658[0n]|BOLD:AAA2220
Eurois astricta|[2557]|ABKWR109-07|United States|Alaska|658[0n]|BOLD:AAA2220
Eurois astricta|[2558]|LCHP752-07|Canada|Manitoba|657[0n]|BOLD:AAA2220
Eurois astricta|[2559]|LCHP754-07|Canada|Manitoba|658[0n]|BOLD:AAA2220
Eurois astricta|[2560]|LCHP926-07|Canada|Manitoba|658[0n]|BOLD:AAA2220
Eurois astricta|[2561]|LCHP928-07|Canada|Manitoba|658[0n]|BOLD:AAA2220
Eurois astricta|[2562]|LCHQ133-07|Canada|Manitoba|658[0n]|BOLD:AAA2220
Eurois astricta|[2563]|LCHQ422-08|Canada|Manitoba|658[0n]|BOLD:AAA2220
Eurois astricta|[2564]|LCHQ471-08|Canada|Manitoba|658[0n]|BOLD:AAA2220
Eurois astricta|[2565]|LCHQ621-08|Canada|Manitoba|658[0n]|BOLD:AAA2220
Eurois astricta|[2566]|LCHQ668-08|Canada|Manitoba|658[0n]|BOLD:AAA2220
Eurois astricta|[2567]|LPMNB503-09|Canada|Manitoba|658[0n]|BOLD:AAA2220
Eurois astricta|[2568]|LPABC025-09|Canada|Alberta|658[0n]|BOLD:AAA2220
Eurois astricta|[2569]|LPABC667-09|Canada|Alberta|658[0n]|BOLD:AAA2220
Eurois astricta|[2570]|LBCG955-09|Canada|British Columbia|658[0n]|BOLD:AAA2220
Eurois astricta|[2571]|LBCG957-09|Canada|British Columbia|658[0n]|BOLD:AAA2220
Eurois astricta|[2572]|BBLPC029-09|Canada|New Brunswick|658[0n]|BOLD:AAA2220
Eurois astricta|[2573]|LBCH928-10|Canada|British Columbia|658[0n]|BOLD:AAA2220
Eurois astricta|[2574]|LBCH929-10|Canada|British Columbia|658[0n]|BOLD:AAA2220
Eurois astricta|[2575]|LBCH930-10|Canada|British Columbia|658[0n]|BOLD:AAA2220
Eurois astricta|[2576]|LBCH931-10|Canada|British Columbia|658[0n]|BOLD:AAA2220
Eurois astricta|[2577]|LBCH3146-10|Canada|British Columbia|658[0n]|BOLD:AAA2220
Eurois astricta|[2578]|LBCH3147-10|Canada|British Columbia|658[0n]|BOLD:AAA2220
Eurois astricta|[2579]|LBCH3763-10|Canada|British Columbia|658[0n]|BOLD:AAA2220
Eurois astricta|[2580]|LBCH3765-10|Canada|British Columbia|658[0n]|BOLD:AAA2220
Eurois astricta|[2581]|LBCH4104-10|Canada|British Columbia|658[0n]|BOLD:AAA2220
Eurois astricta|[2582]|LBCH4105-10|Canada|British Columbia|658[0n]|BOLD:AAA2220
Eurois astricta|[2583]|LBCH3761-10|Canada|British Columbia|658[0n]|BOLD:AAA2220
Eurois astricta|[2584]|LBCH3762-10|Canada|British Columbia|658[0n]|BOLD:AAA2220
Eurois astricta|[2585]|LBCH3759-10|Canada|British Columbia|658[0n]|BOLD:AAA2220
Eurois astricta|[2586]|LBCH3760-10|Canada|British Columbia|658[0n]|BOLD:AAA2220
Eurois astricta|[2587]|LBCH3487-10|Canada|British Columbia|658[0n]|BOLD:AAA2220
Eurois astricta|[2588]|LBCH3758-10|Canada|British Columbia|658[0n]|BOLD:AAA2220
Eurois astricta|[2589]|LBCH4106-10|Canada|British Columbia|658[0n]|BOLD:AAA2220
Eurois astricta|[2590]|LBCH4107-10|Canada|British Columbia|658[0n]|BOLD:AAA2220
Eurois astricta|[2591]|LBCH4407-10|Canada|British Columbia|658[0n]|BOLD:AAA2220
Eurois astricta|[2592]|LBCH4408-10|Canada|British Columbia|658[0n]|BOLD:AAA2220

[illegible]
Eurois astricta|[2590]|LBCH4107-10|Canada|British Columbia|658[0n]|BOLD:AAA2220
Eurois astricta|[2591]|LBCH4407-10|Canada|British Columbia|658[0n]|BOLD:AAA2220
Eurois astricta|[2592]|LBCH4408-10|Canada|British Columbia|658[0n]|BOLD:AAA2220
Eurois astricta|[2593]|LBCH4664-10|Canada|British Columbia|658[0n]|BOLD:AAA2220
Eurois astricta|[2594]|LBCH4667-10|Canada|British Columbia|658[0n]|BOLD:AAA2220
Eurois astricta|[2595]|LBCH6621-10|Canada|British Columbia|658[0n]|BOLD:AAA2220
Eurois astricta|[2596]|LBCH7682-10|Canada|British Columbia|658[0n]|BOLD:AAA2220
Eurois astricta|[2597]|LBCH4409-10|Canada|British Columbia|658[0n]|BOLD:AAA2220
Eurois astricta|[2598]|LBCH4410-10|Canada|British Columbia|658[0n]|BOLD:AAA2220
Eurois astricta|[2599]|LBCH4411-10|Canada|British Columbia|658[0n]|BOLD:AAA2220
Eurois astricta|[2600]|LBCH4412-10|Canada|British Columbia|658[0n]|BOLD:AAA2220
Eurois astricta|[2601]|BBLPB524-10|Canada|Alberta|658[0n]|BOLD:AAA2220
Eurois astricta|[2602]|BBLPB526-10|Canada|Alberta|658[0n]|BOLD:AAA2220
Eurois astricta|[2603]|LBCH4413-10|Canada|British Columbia|658[0n]|BOLD:AAA2220
Eurois astricta|[2604]|LBCH4414-10|Canada|British Columbia|658[0n]|BOLD:AAA2220
Eurois astricta|[2605]|LOWCD259-06|Canada|British Columbia|658[0n]|BOLD:AAA2220
Eurois astricta|[2606]|LOWCD519-06|Canada|British Columbia|658[0n]|BOLD:AAA2220
Eurois astricta|[2607]|LOWCD520-06|Canada|British Columbia|658[0n]|BOLD:AAA2220
Eurois astricta|[2608]|TTMNB010-06|Canada|New Brunswick|658[0n]|BOLD:AAA2220
Eurois astricta|[2609]|TTMNB016-06|Canada|New Brunswick|657[0n]|BOLD:AAA2220
Eurois astricta|[2610]|TTMNB436-06|Canada|New Brunswick|658[0n]|BOLD:AAA2220
Eurois astricta|[2611]|TTMNB437-06|Canada|New Brunswick|658[0n]|BOLD:AAA2220
Eurois astricta|[2612]|TTMNB438-06|Canada|New Brunswick|658[0n]|BOLD:AAA2220
Eurois astricta|[2613]|LOWCC933-05|Canada|British Columbia|658[0n]|BOLD:AAA2220
Eurois astricta|[2614]|RDLQB573-05|Canada|Quebec|658[0n]|BOLD:AAA2220
Eurois astricta|[2615]|LOWC823-05|Canada|British Columbia|658[0n]|BOLD:AAA2220
Eurois astricta|[2616]|LOWC824-05|Canada|British Columbia|658[0n]|BOLD:AAA2220
Eurois astricta|[2617]|BBLPB668-10|Canada|Saskatchewan|658[0n]|BOLD:AAA2220
Eurois astricta|[2618]|LOWC821-05|Canada|British Columbia|658[0n]|BOLD:AAA2220
Eurois astricta|[2619]|LOWC820-05|Canada|British Columbia|658[0n]|BOLD:AAA2220
Eurois astricta|[2620]|LOWC818-05|Canada|British Columbia|658[0n]|BOLD:AAA2220
Eurois astricta|[2621]|MNBB663-05|Canada|New Brunswick|658[0n]|BOLD:AAA2220
Eurois astricta|[2622]|LCH555-04|Canada|Manitoba|658[0n]|BOLD:AAA2220
Eurois astricta|[2623]|LCH554-04|Canada|Manitoba|658[0n]|BOLD:AAA2220
Eurois astricta|[2624]|LCH553-04|Canada|Manitoba|658[0n]|BOLD:AAA2220
Eurois astricta|[2625]|LCH552-04|Canada|Manitoba|658[0n]|BOLD:AAA2220
Eurois astricta|[2626]|LCH546-04|Canada|Manitoba|658[0n]|BOLD:AAA2220
Eurois astricta|[2627]|LPABC199-09|Canada|Alberta|658[0n]|BOLD:AAA2220
Eurois astricta|[2628]|LCHQ470-08|Canada|Manitoba|658[1n]|BOLD:AAA2220
Eurois astricta|[2629]|LBCH3145-10|Canada|British Columbia|658[0n]|BOLD:AAA2220
Eurois astricta|[2630]|LBCH3149-10|Canada|British Columbia|658[0n]|BOLD:AAA2220
Eurois astricta|[2631]|LBCH4103-10|Canada|British Columbia|658[0n]|BOLD:AAA2220
Eurois astricta|[2632]|LBCH932-10|Canada|British Columbia|640[0n]|BOLD:AAA2220
Eurois astricta|[2633]|LBCH3469-10|Canada|British Columbia|641[0n]|BOLD:AAA2220
Eurois astricta|[2634]|UAMIC563-13|United States|Alaska|653[0n]|BOLD:AAA2220
Eurois astricta|[2635]|LCH547-04|Canada|Manitoba|596[0n]|BOLD:AAA2220
Eurois astricta|[2636]|LCHP753-07|Canada|Manitoba|650[0n]|BOLD:AAA2220
Eurois astricta|[2637]|LCH539-04|Canada|Manitoba|650[0n]|BOLD:AAA2220
Eurois astricta|[2638]|LBCH3486-10|Canada|British Columbia|635[0n]|BOLD:AAA2220
Eurois astricta|[2639]|LCH551-04|Canada|Manitoba|615[0n]|BOLD:AAA2220
Eurois astricta|[2640]|LBCH3482-10|Canada|British Columbia|632[0n]|BOLD:AAA2220
Eurois astricta|[2641]|LPABC026-09|Canada|Alberta|621[0n]|BOLD:AAA2220
Eurois astricta|[2642]|LBCH3485-10|Canada|British Columbia|639[0n]|BOLD:AAA2220
Eurois astricta|[2643]|LCH544-04|Canada|Manitoba|627[0n]|BOLD:AAA2220
Eurois astricta|[2644]|LBCH3483-10|Canada|British Columbia|635[0n]|BOLD:AAA2220
Eurois astricta|[2645]|TTMNB015-06|Canada|New Brunswick|635[0n]|BOLD:AAA2220
Eurois astricta|[2646]|LCH548-04|Canada|Manitoba|625[0n]|BOLD:AAA2220
Eurois astricta|[2647]|LOWCD518-06|Canada|British Columbia|609[0n]|BOLD:AAA2220
Eurois astricta|[2648]|LOWC822-05|Canada|British Columbia|585[0n]|BOLD:AAA2220
Eurois astricta|[2649]|LCH540-04|Canada|Manitoba|605[0n]|BOLD:AAA2220
Eurois astricta|[2650]|LCH549-04|Canada|Manitoba|578[0n]|BOLD:AAA2220
Eurois astricta|[2651]|LOWCD517-06|Canada|British Columbia|581[0n]|BOLD:AAA2220
Eurois astricta|[2652]|LOWCD516-06|Canada|British Columbia|580[0n]|BOLD:AAA2220
Eurois astricta|[2653]|LOWCD514-06|Canada|British Columbia|602[0n]|BOLD:AAA2220
Eurois astricta|[2654]|LOWCD140-06|Canada|British Columbia|572[0n]|BOLD:AAA2220
Eurois astricta|[2655]|LCH542-04|Canada|Manitoba|595[0n]|BOLD:AAA2220
Eurois astricta|[2656]|LCH545-04|Canada|Manitoba|586[0n]|BOLD:AAA2220
Eurois astricta|[2657]|LOWCD515-06|Canada|British Columbia|569[0n]|BOLD:AAA2220
Eurois astricta|[2658]|RDLQB572-05|Canada|Quebec|519[1n]|BOLD:AAA2220
Eurois astricta|[2659]|LCH441-04|Canada|Manitoba|561[1n]|BOLD:AAA2220
Eurois astricta|[2660]|LCH541-04|Canada|Manitoba|560[0n]|BOLD:AAA2220
Eurois astricta|[2661]|LCH550-04|Canada|Manitoba|520[4n]|BOLD:AAA2220
Eurois astricta|[2662]|LBCH3484-10|Canada|British Columbia|632[0n]|BOLD:AAA2220
Eurois astricta|[2663]|UAMIC564-13|United States|Alaska|621[0n]|BOLD:AAA2220
Eurois occulta|[2664]|ABKWR126-07|United States|Alaska|658[0n]|BOLD:AAA3312
Eurois occulta|[2665]|LCHQ538-08|Canada|Manitoba|658[0n]|BOLD:AAA3312
Eurois occulta|[2666]|LOWC829-05|Canada|British Columbia|658[0n]|BOLD:AAA3312
Eurois occulta|[2667]|BBLEC459-09|Canada|New Brunswick|658[0n]|BOLD:AAA3312
Eurois occulta|[2668]|LCHQ113-07|Canada|Manitoba|658[0n]|BOLD:AAA3312
Eurois occulta|[2669]|LBCH4392-10|Canada|British Columbia|634[0n]|BOLD:AAA3312
Eurois occulta|[2670]|LBCH7116-10|Canada|British Columbia|658[0n]|BOLD:AAA3312
Eurois occulta|[2671]|LOWCD522-06|Canada|British Columbia|618[0n]|BOLD:AAA3312
Eurois occulta|[2672]|LOWCD525-06|Canada|British Columbia|593[0n]|BOLD:AAA3312
Eurois occulta|[2673]|LPABC189-09|Canada|Alberta|647[1n]|BOLD:AAA3312
Eurois occulta|[2674]|LBCD258-05|Canada|British Columbia|658[0n]|BOLD:AAA3312
Eurois occulta|[2675]|RDLQB575-05|Canada|Quebec|519[0n]|BOLD:AAA3312
Eurois occulta|[2676]|LPABC480-09|Canada|Alberta|612[0n]|BOLD:AAA3312
Eurois occulta|[2677]|LBCH3458-10|Canada|British Columbia|658[0n]|BOLD:AAA3312
Eurois occulta|[2678]|LBCH3459-10|Canada|British Columbia|591[0n]|BOLD:AAA3312
Eurois occulta|[2679]|LBCD439-05|Canada|British Columbia|658[0n]|BOLD:AAA3312
Eurois occulta|[2680]|LOWCD526-06|Canada|British Columbia|658[0n]|BOLD:AAA3312
Eurois occulta|[2681]|LCHP826-07|Canada|Manitoba|658[0n]|BOLD:AAA3312
Eurois occulta|[2682]|LCHP927-07|Canada|Manitoba|658[0n]|BOLD:AAA3312
Eurois occulta|[2683]|LALPA608-10|Canada|British Columbia|658[0n]|BOLD:AAA3312
Eurois occulta|[2684]|LALPA621-10|Canada|British Columbia|658[0n]|BOLD:AAA3312
Eurois occulta|[2685]|LBCH4660-10|Canada|British Columbia|658[0n]|BOLD:AAA3312
Eurois occulta|[2686]|LBCH4663-10|Canada|British Columbia|658[0n]|BOLD:AAA3312
Eurois occulta|[2687]|LBCH4089-10|Canada|British Columbia|658[0n]|BOLD:AAA3312
Eurois occulta|[2688]|LBCH4091-10|Canada|British Columbia|658[0n]|BOLD:AAA3312
Eurois occulta|[2689]|TMNBB368-06|Canada|New Brunswick|658[0n]|BOLD:AAA3312
Eurois occulta|[2690]|LCHP792-07|Canada|Manitoba|658[0n]|BOLD:AAA3312
Eurois occulta|[2691]|LPABC497-09|Canada|Alberta|658[0n]|BOLD:AAA3312
[illegible]

Eurois occulta|[2689]|TMNBB368-06|Canada|New Brunswick|658[0n]|BOLD:AAA3312
Eurois occulta|[2690]|LCHP792-07|Canada|Manitoba|658[0n]|BOLD:AAA3312
Eurois occulta|[2691]|LPABC497-09|Canada|Alberta|658[0n]|BOLD:AAA3312
Eurois occulta|[2692]|LBCG1880-09|Canada|British Columbia|658[0n]|BOLD:AAA3312
Eurois occulta|[2693]|LPABC194-09|Canada|Alberta|658[0n]|BOLD:AAA3312
Eurois occulta|[2694]|LPABC223-09|Canada|Alberta|658[0n]|BOLD:AAA3312
Eurois occulta|[2695]|LBCG848-09|Canada|British Columbia|658[0n]|BOLD:AAA3312
Eurois occulta|[2696]|LBCG850-09|Canada|British Columbia|658[0n]|BOLD:AAA3312
Eurois occulta|[2697]|LPAB206-08|Canada|Alberta|658[0n]|BOLD:AAA3312
Eurois occulta|[2698]|LBCW035-08|Canada|British Columbia|658[0n]|BOLD:AAA3312
Eurois occulta|[2699]|LCHQ659-08|Canada|Manitoba|658[0n]|BOLD:AAA3312
Eurois occulta|[2700]|LCHQ919-08|Canada|Manitoba|658[0n]|BOLD:AAA3312
Eurois occulta|[2701]|TTMNB433-06|Canada|New Brunswick|658[0n]|BOLD:AAA3312
Eurois occulta|[2702]|LOWCE819-06|Canada|British Columbia|658[0n]|BOLD:AAA3312
Eurois occulta|[2703]|LCHQ512-08|Canada|Manitoba|658[0n]|BOLD:AAA3312
Eurois occulta|[2704]|LCHQ539-08|Canada|Manitoba|658[0n]|BOLD:AAA3312
Eurois occulta|[2705]|LBCH1092-10|Canada|British Columbia|658[0n]|BOLD:AAA3312
Eurois occulta|[2706]|LBCH1495-10|Canada|British Columbia|658[0n]|BOLD:AAA3312
Eurois occulta|[2707]|LBCH1827-10|Canada|British Columbia|658[0n]|BOLD:AAA3312
Eurois occulta|[2708]|LBCH1950-10|Canada|British Columbia|658[0n]|BOLD:AAA3312
Eurois occulta|[2709]|BBLEC458-09|Canada|New Brunswick|658[0n]|BOLD:AAA3312
Eurois occulta|[2710]|BBLPC102-09|Canada|New Brunswick|658[0n]|BOLD:AAA3312
Eurois occulta|[2711]|LBCH800-10|Canada|British Columbia|658[0n]|BOLD:AAA3312
Eurois occulta|[2712]|LBCH912-10|Canada|British Columbia|658[0n]|BOLD:AAA3312
Eurois occulta|[2713]|LBCH1995-10|Canada|British Columbia|658[0n]|BOLD:AAA3312
Eurois occulta|[2714]|LBCH2092-10|Canada|British Columbia|658[0n]|BOLD:AAA3312
Eurois occulta|[2715]|LBCH2243-10|Canada|British Columbia|658[0n]|BOLD:AAA3312
Eurois occulta|[2716]|LBCH2636-10|Canada|British Columbia|658[0n]|BOLD:AAA3312
Eurois occulta|[2717]|LBCH2913-10|Canada|British Columbia|658[0n]|BOLD:AAA3312
Eurois occulta|[2718]|LBCH3062-10|Canada|British Columbia|658[0n]|BOLD:AAA3312
Eurois occulta|[2719]|LBCH3326-10|Canada|British Columbia|658[0n]|BOLD:AAA3312
Eurois occulta|[2720]|LBCH3456-10|Canada|British Columbia|658[0n]|BOLD:AAA3312
Eurois occulta|[2721]|LBCH3457-10|Canada|British Columbia|658[0n]|BOLD:AAA3312
Eurois occulta|[2722]|LBCH3461-10|Canada|British Columbia|658[0n]|BOLD:AAA3312
Eurois occulta|[2723]|LBCH3462-10|Canada|British Columbia|658[0n]|BOLD:AAA3312
Eurois occulta|[2724]|LBCH3750-10|Canada|British Columbia|658[0n]|BOLD:AAA3312
Eurois occulta|[2725]|LBCH3751-10|Canada|British Columbia|658[0n]|BOLD:AAA3312
Eurois occulta|[2726]|LBCH3752-10|Canada|British Columbia|658[0n]|BOLD:AAA3312
Eurois occulta|[2727]|LBCH3753-10|Canada|British Columbia|658[0n]|BOLD:AAA3312
Eurois occulta|[2728]|LBCH3754-10|Canada|British Columbia|658[0n]|BOLD:AAA3312
Eurois occulta|[2729]|LBCH3756-10|Canada|British Columbia|658[0n]|BOLD:AAA3312
Eurois occulta|[2730]|LBCH4088-10|Canada|British Columbia|658[0n]|BOLD:AAA3312
Eurois occulta|[2731]|LBCH4389-10|Canada|British Columbia|658[0n]|BOLD:AAA3312
Eurois occulta|[2732]|LBCH4659-10|Canada|British Columbia|658[0n]|BOLD:AAA3312
Eurois occulta|[2733]|LBCH7425-10|Canada|British Columbia|658[0n]|BOLD:AAA3312
Eurois occulta|[2734]|LBCH7758-10|Canada|British Columbia|658[0n]|BOLD:AAA3312
Eurois occulta|[2735]|LALPA622-10|Canada|British Columbia|658[0n]|BOLD:AAA3312
Eurois occulta|[2736]|LALPA623-10|Canada|British Columbia|658[0n]|BOLD:AAA3312
Eurois occulta|[2737]|LALPA631-10|Canada|British Columbia|658[0n]|BOLD:AAA3312
Eurois occulta|[2738]|LALPA715-10|Canada|British Columbia|658[0n]|BOLD:AAA3312
Eurois occulta|[2739]|BBLPA330-10|Canada|British Columbia|658[0n]|BOLD:AAA3312
Eurois occulta|[2740]|BBLPB669-10|Canada|Alberta|658[0n]|BOLD:AAA3312
Eurois occulta|[2741]|BBLPC397-09|Canada|New Brunswick|658[0n]|BOLD:AAA3312
Eurois occulta|[2742]|BBLPC403-09|Canada|New Brunswick|658[0n]|BOLD:AAA3312
Eurois occulta|[2743]|LBCG2828-09|Canada|British Columbia|658[0n]|BOLD:AAA3312
Eurois occulta|[2744]|BBLEC451-09|Canada|New Brunswick|658[0n]|BOLD:AAA3312
Eurois occulta|[2745]|LBCG2826-09|Canada|British Columbia|658[0n]|BOLD:AAA3312
Eurois occulta|[2746]|LBCG2827-09|Canada|British Columbia|658[0n]|BOLD:AAA3312
Eurois occulta|[2747]|BBLPC385-09|Canada|New Brunswick|658[0n]|BOLD:AAA3312
Eurois occulta|[2748]|BBLPC393-09|Canada|New Brunswick|658[0n]|BOLD:AAA3312
Eurois occulta|[2749]|LBCG1935-09|Canada|British Columbia|658[0n]|BOLD:AAA3312
Eurois occulta|[2750]|LBCG2004-09|Canada|British Columbia|658[0n]|BOLD:AAA3312
Eurois occulta|[2751]|TTMNB431-06|Canada|New Brunswick|658[0n]|BOLD:AAA3312
Eurois occulta|[2752]|TTMNB011-06|Canada|New Brunswick|658[0n]|BOLD:AAA3312
Eurois occulta|[2753]|LOWC830-05|Canada|British Columbia|658[0n]|BOLD:AAA3312
Eurois occulta|[2754]|LOWC828-05|Canada|British Columbia|658[0n]|BOLD:AAA3312
Eurois occulta|[2755]|LOWC826-05|Canada|British Columbia|658[0n]|BOLD:AAA3312
Eurois occulta|[2756]|LBCB047-05|Canada|British Columbia|658[0n]|BOLD:AAA3312
Eurois occulta|[2757]|MNBB558-05|Canada|New Brunswick|658[0n]|BOLD:AAA3312
Eurois occulta|[2758]|PHMNB422-04|Canada|New Brunswick|658[0n]|BOLD:AAA3312
Eurois occulta|[2759]|XAE347-04|Canada|Ontario|658[0n]|BOLD:AAA3312
Eurois occulta|[2760]|LOWCD521-06|Canada|British Columbia|658[0n]|BOLD:AAA3312
Eurois occulta|[2761]|LCHQ537-08|Canada|Manitoba|658[0n]|BOLD:AAA3312
Eurois occulta|[2762]|LPMN177-08|Canada|Manitoba|668[0n]|BOLD:AAA3312
Eurois occulta|[2763]|LBCH3463-10|Canada|British Columbia|658[0n]|BOLD:AAA3312
Eurois occulta|[2764]|BBLEC454-09|Canada|New Brunswick|638[0n]|BOLD:AAA3312
Eurois occulta|[2765]|LCHQ112-07|Canada|Manitoba|639[0n]|BOLD:AAA3312
Eurois occulta|[2766]|LPABC210-09|Canada|Alberta|649[0n]|BOLD:AAA3312
Eurois occulta|[2767]|LBCH3460-10|Canada|British Columbia|641[0n]|BOLD:AAA3312
Eurois occulta|[2768]|LBCH4657-10|Canada|British Columbia|641[0n]|BOLD:AAA3312
Eurois occulta|[2769]|LALPA1294-11|Canada|British Columbia|658[0n]|BOLD:AAA3312
Eurois occulta|[2770]|LALPA1336-12|Canada|British Columbia|632[0n]|BOLD:AAA3312
Eurois occulta|[2771]|LBCH7683-10|Canada|British Columbia|658[0n]|BOLD:AAA3312
Eurois occulta|[2772]|LOWC825-05|Canada|British Columbia|658[0n]|BOLD:AAA3312
Eurois occulta|[2773]|LBCG2003-09|Canada|British Columbia|658[0n]|BOLD:AAA3312
Eurois occulta|[2774]|RWWB190-09|United States|Washington|658[0n]|BOLD:AAA3312
Eurois occulta|[2775]|LBCG706-09|Canada|British Columbia|658[0n]|BOLD:AAA3312
Eurois occulta|[2776]|BBLEC484-09|Canada|New Brunswick|658[0n]|BOLD:AAA3312
Eurois occulta|[2777]|LBCH005-10|Canada|British Columbia|658[0n]|BOLD:AAA3312
Eurois occulta|[2778]|LBCH107-10|Canada|British Columbia|658[0n]|BOLD:AAA3312
Eurois occulta|[2779]|LBCH485-10|Canada|British Columbia|658[0n]|BOLD:AAA3312
Eurois occulta|[2780]|LBCH644-10|Canada|British Columbia|658[0n]|BOLD:AAA3312
Eurois occulta|[2781]|LBCH1162-10|Canada|British Columbia|658[0n]|BOLD:AAA3312
Eurois occulta|[2782]|LBCH1163-10|Canada|British Columbia|658[0n]|BOLD:AAA3312
Eurois occulta|[2783]|LBCH2091-10|Canada|British Columbia|658[0n]|BOLD:AAA3312
Eurois occulta|[2784]|LBCH2716-10|Canada|British Columbia|658[0n]|BOLD:AAA3312
Eurois occulta|[2785]|LBCH3757-10|Canada|British Columbia|658[0n]|BOLD:AAA3312
Eurois occulta|[2786]|LBCH4087-10|Canada|British Columbia|658[0n]|BOLD:AAA3312
Eurois occulta|[2787]|LBCH4090-10|Canada|British Columbia|658[0n]|BOLD:AAA3312
Eurois occulta|[2788]|LBCH4092-10|Canada|British Columbia|658[0n]|BOLD:AAA3312
Eurois occulta|[2789]|LBCH4093-10|Canada|British Columbia|658[0n]|BOLD:AAA3312
Eurois occulta|[2790]|LBCH4387-10|Canada|British Columbia|658[0n]|BOLD:AAA3312
Eurois occulta|[2791]|LBCH4391-10|Canada|British Columbia|658[0n]|BOLD:AAA3312

Eurois occulta|[2789]|LBCH4093-10|Canada|British Columbia|658[0n]|BOLD:AAA3312
Eurois occulta|[2790]|LBCH4387-10|Canada|British Columbia|658[0n]|BOLD:AAA3312
Eurois occulta|[2791]|LBCH4391-10|Canada|British Columbia|658[0n]|BOLD:AAA3312
Eurois occulta|[2792]|LBCH4658-10|Canada|British Columbia|658[0n]|BOLD:AAA3312
Eurois occulta|[2793]|LBCH4661-10|Canada|British Columbia|658[0n]|BOLD:AAA3312
Eurois occulta|[2794]|LBCH4662-10|Canada|British Columbia|658[0n]|BOLD:AAA3312
Eurois occulta|[2795]|LBCH7836-10|Canada|British Columbia|658[0n]|BOLD:AAA3312
Eurois occulta|[2796]|LBCG735-09|Canada|British Columbia|658[0n]|BOLD:AAA3312
Eurois occulta|[2797]|BBLEC452-09|Canada|New Brunswick|658[0n]|BOLD:AAA3312
Eurois occulta|[2798]|BBLPA327-10|Canada|Alberta|658[0n]|BOLD:AAA3312
Eurois occulta|[2799]|LBCW034-08|Canada|British Columbia|658[0n]|BOLD:AAA3312
Eurois occulta|[2800]|LCHQ111-07|Canada|Manitoba|658[0n]|BOLD:AAA3312
Eurois occulta|[2801]|LCHQ026-07|Canada|Manitoba|658[0n]|BOLD:AAA3312
Eurois occulta|[2802]|LCHP929-07|Canada|Manitoba|658[0n]|BOLD:AAA3312
Eurois occulta|[2803]|LCHP925-07|Canada|Manitoba|658[0n]|BOLD:AAA3312
Eurois occulta|[2804]|ABKWR134-07|United States|Alaska|658[0n]|BOLD:AAA3312
Eurois occulta|[2805]|TMNBB369-06|Canada|New Brunswick|658[0n]|BOLD:AAA3312
Eurois occulta|[2806]|TTMNB434-06|Canada|New Brunswick|658[0n]|BOLD:AAA3312
Eurois occulta|[2807]|TTMNB432-06|Canada|New Brunswick|658[0n]|BOLD:AAA3312
Eurois occulta|[2808]|LOWCD524-06|Canada|British Columbia|658[0n]|BOLD:AAA3312
Eurois occulta|[2809]|LOWCD523-06|Canada|British Columbia|609[0n]|BOLD:AAA3312
Eurois occulta|[2810]|LCHP621-07|Canada|Manitoba|658[0n]|BOLD:AAA3312
Eurois occulta|[2811]|LOWC831-05|Canada|British Columbia|658[0n]|BOLD:AAA3312
Eurois occulta|[2812]|LBCC825-05|Canada|British Columbia|658[0n]|BOLD:AAA3312
Eurois occulta|[2813]|TTMNB435-06|Canada|New Brunswick|658[0n]|BOLD:AAA3312
Eurois occulta|[2814]|XAE496-04|Canada|Ontario|647[1n]|BOLD:AAA3312
Eurois occulta|[2815]|LCHIP122-07|Canada|Manitoba|650[0n]|BOLD:AAA3312
Eurois occulta|[2816]|LCHIP052-07|Canada|Manitoba|650[0n]|BOLD:AAA3312
Eurois occulta|[2817]|LCHP924-07|Canada|Manitoba|636[0n]|BOLD:AAA3312
Eurois occulta|[2818]|ABKWR114-07|United States|Alaska|636[0n]|BOLD:AAA3312
Eurois occulta|[2819]|BBLEC460-09|Canada|New Brunswick|658[0n]|BOLD:AAA3312
Eurois occulta|[2820]|LBCH7364-10|Canada|British Columbia|633[0n]|BOLD:AAA3312
Eurois occulta|[2821]|ABKWR108-07|United States|Alaska|639[1n]|BOLD:AAA3312
Eurois occulta|[2822]|LOWC827-05|Canada|British Columbia|658[0n]|BOLD:AAA3312
Eurois occulta|[2823]|LBCH4393-10|Canada|British Columbia|658[0n]|BOLD:AAA3312
Eurois occulta|[2824]|LBCH4390-10|Canada|British Columbia|658[0n]|BOLD:AAA3312
Eurois occulta|[2825]|LBCH4388-10|Canada|British Columbia|658[0n]|BOLD:AAA3312
Eurois occulta|[2826]|LBCH4086-10|Canada|British Columbia|658[0n]|BOLD:AAA3312
Eurois occulta|[2827]|LBCH2859-10|Canada|British Columbia|658[0n]|BOLD:AAA3312
Eurois occulta|[2828]|LBCG2005-09|Canada|British Columbia|658[0n]|BOLD:AAA3312
Eurois occulta|[2829]|LBCH3755-10|Canada|British Columbia|658[0n]|BOLD:AAA3312
Eurois occulta|[2830]|BBLPC394-09|Canada|New Brunswick|654[0n]|BOLD:AAA3312
Eurois occulta|[2831]|LBCH3464-10|Canada|British Columbia|636[0n]|BOLD:AAA3312
Eurois occulta|[2832]|LBCH4394-10|Canada|British Columbia|639[0n]|BOLD:AAA3312
Eurois occulta|[2833]|UAMIC562-13|United States|Alaska|649[0n]|BOLD:AAA3312
Eurois occulta|[2834]|PHLAA209-09|Austria|Carinthia|655[0n]|BOLD:AAA3312
Eurois occulta|[2835]|LEFIA583-10|Finland|South Karelia|613[0n]|BOLD:AAA3312
Eurois occulta|[2836]|LEFIA582-10|Finland|South Karelia|612[0n]|BOLD:AAA3312
Eurois occulta|[2837]|GWORZ520-10|Germany|Bavaria|658[0n]|BOLD:AAA3312
Eurois occulta|[2838]|GWOTG558-12|Georgia||658[0n]|BOLD:AAA3312
Eurois occulta|[2839]|GWOTG559-12|Georgia||658[0n]|BOLD:AAA3312
Eurois occulta|[2840]|GRAFW1448-12|Greenland||658[0n]|BOLD:AAA3312
Eurois occulta|[2841]|GRAFW1449-12|Greenland||658[0n]|BOLD:AAA3312
Eurois occulta|[2842]|FBLMV041-09|Germany|Bavaria|658[1n]|BOLD:AAA3312
Eurois occulta|[2843]|PHLAC426-10|Italy|South Tyrol|658[0n]|BOLD:AAA3312
Eurois occulta|[2844]|LEATD161-13|Italy|South Tyrol|658[0n]|BOLD:AAA3312
Eurois occulta|[2845]|LEATD097-13|Austria|Tirol|658[0n]|BOLD:AAA3312
Eurois occulta|[2846]|LON1130-12|Norway|Hedmark|658[0n]|BOLD:AAA3312
Eurois occulta|[2847]|LENOA201-11|France|Provence-Alpes-Cote d'Azur|658[0n]|BOLD:AAA3312
Eurois occulta|[2848]|LEFIB831-10|Finland|Northern Ostrobothnia|658[0n]|BOLD:AAA3312
Eurois occulta|[2849]|PHLAA251-09|Austria|Vorarlberg|658[0n]|BOLD:AAA3312
Eurois occulta|[2850]|GWORO849-09|Germany|Bavaria|658[0n]|BOLD:AAA3312
Eurois occulta|[2851]|LON105-08|Norway|Hedmark|657[0n]|BOLD:AAA3312
Eurois occulta|[2852]|RDNMC543-06|Russia|Buryatiya|658[0n]|BOLD:AAA3312
Eurois occulta|[2853]|GWORL332-09|Germany|Bavaria|626[0n]|BOLD:AAA3312
Eurois occulta|[2854]|LEATB449-13|Austria|Tirol|632[0n]|BOLD:AAA3312
Eurois occulta|[2855]|GBLAC466-13|Germany|Bavaria|615[0n]|BOLD:AAA3312
Eurois occulta|[2856]|FGMLD017-13|Germany|Bavaria|658[0n]|BOLD:AAA3312
Eurois occulta|[2857]|NORIN156-13|Norway|Akershus|657[0n]|BOLD:AAA3312
Paradiarsia littoralis|[2858]|LSEU782-06|United States|Colorado|658[0n]|BOLD:AAA7373
Paradiarsia littoralis|[2859]|LOWCD603-06|Canada|British Columbia|587[0n]|BOLD:AAA7373
Paradiarsia littoralis|[2860]|LOWCD617-06|Canada|British Columbia|658[0n]|BOLD:AAA7373
Paradiarsia littoralis|[2861]|LOWCD607-06|Canada|British Columbia|559[0n]|BOLD:AAA7373
Paradiarsia littoralis|[2862]|LOWCD602-06|Canada|British Columbia|581[0n]|BOLD:AAA7373
Paradiarsia littoralis|[2863]|LOWCD618-06|Canada|British Columbia|608[0n]|BOLD:AAA7373
Paradiarsia littoralis|[2864]|LOWCD611-06|Canada|British Columbia|596[0n]|BOLD:AAA7373
Paradiarsia littoralis|[2865]|LOWCD616-06|Canada|British Columbia|658[0n]|BOLD:AAA7373
Paradiarsia littoralis|[2866]|LOWCD615-06|Canada|British Columbia|658[0n]|BOLD:AAA7373
Paradiarsia littoralis|[2867]|LOWCD613-06|Canada|British Columbia|658[0n]|BOLD:AAA7373
Paradiarsia littoralis|[2868]|LOWCD612-06|Canada|British Columbia|658[0n]|BOLD:AAA7373
Paradiarsia littoralis|[2869]|LOWCD610-06|Canada|British Columbia|658[0n]|BOLD:AAA7373
Paradiarsia littoralis|[2870]|LOWCD609-06|Canada|British Columbia|658[0n]|BOLD:AAA7373
Paradiarsia littoralis|[2871]|LOWCD608-06|Canada|British Columbia|658[0n]|BOLD:AAA7373
Paradiarsia littoralis|[2872]|LOWCD605-06|Canada|British Columbia|657[0n]|BOLD:AAA7373
Paradiarsia littoralis|[2873]|LOWCD604-06|Canada|British Columbia|658[0n]|BOLD:AAA7373
Paradiarsia littoralis|[2874]|LOWCD601-06|Canada|British Columbia|658[0n]|BOLD:AAA7373
Paradiarsia littoralis|[2875]|LOWCD600-06|Canada|British Columbia|658[0n]|BOLD:AAA7373
Paradiarsia littoralis|[2876]|LOWCD606-06|Canada|British Columbia|558[1n]|BOLD:AAA7373
Paradiarsia littoralis|[2877]|LOWCD614-06|Canada|British Columbia|558[0n]|BOLD:AAA7373
Paradiarsia littoralis|[2878]|LOWCD619-06|Canada|British Columbia|600[0n]|BOLD:AAA7373
Paradiarsia littoralis|[2879]|LBCG357-08|Canada|British Columbia|658[0n]|BOLD:AAA7373
Paradiarsia littoralis|[2880]|LBCG364-08|Canada|British Columbia|658[0n]|BOLD:AAA7373
Paradiarsia littoralis|[2881]|LBCG389-08|Canada|British Columbia|658[0n]|BOLD:AAA7373
Paradiarsia littoralis|[2882]|LPABB346-08|Canada|Alberta|658[0n]|BOLD:AAA7373
Paradiarsia littoralis|[2883]|LPABB195-08|Canada|Alberta|658[0n]|BOLD:AAA7373
Paradiarsia littoralis|[2884]|LPABB192-08|Canada|Alberta|658[0n]|BOLD:AAA7373
Paradiarsia littoralis|[2885]|LPMN879-08|Canada|Alberta|658[0n]|BOLD:AAA7373
Paradiarsia littoralis|[2886]|LPABB386-08|Canada|Alberta|658[0n]|BOLD:AAA7373
Paradiarsia littoralis|[2887]|LPABB395-08|Canada|Alberta|658[0n]|BOLD:AAA7373
Paradiarsia littoralis|[2888]|LPABB420-08|Canada|Alberta|658[0n]|BOLD:AAA7373
Paradiarsia littoralis|[2889]|LPABC703-09|Canada|Alberta|658[0n]|BOLD:AAA7373
Paradiarsia littoralis|[2890]|LPSK601-08|Canada|Saskatchewan|658[0n]|BOLD:AAA7373
Paradiarsia littoralis|[2891]|LPSK467-08|Canada|Saskatchewan|658[0n]|BOLD:AAA7373

Paradiarsia littoralis|[2889]|LPABC703-09|Canada|Alberta|658[0n]|BOLD:AAA7373
Paradiarsia littoralis|[2890]|LPSK601-08|Canada|Saskatchewan|658[0n]|BOLD:AAA7373
Paradiarsia littoralis|[2891]|LPSK467-08|Canada|Saskatchewan|658[0n]|BOLD:AAA7373
Paradiarsia littoralis|[2892]|LPSK456-08|Canada|Saskatchewan|658[0n]|BOLD:AAA7373
Paradiarsia littoralis|[2893]|LPMN717-08|Canada|Manitoba|658[0n]|BOLD:AAA7373
Paradiarsia littoralis|[2894]|LPMN711-08|Canada|Manitoba|658[0n]|BOLD:AAA7373
Paradiarsia littoralis|[2895]|LPMN330-08|Canada|Manitoba|658[0n]|BOLD:AAA7373
Paradiarsia littoralis|[2896]|LPSK441-08|Canada|Saskatchewan|658[0n]|BOLD:AAA7373
Paradiarsia littoralis|[2897]|LPSK316-08|Canada|Saskatchewan|658[0n]|BOLD:AAA7373
Paradiarsia littoralis|[2898]|LPSK134-08|Canada|Saskatchewan|658[0n]|BOLD:AAA7373
Paradiarsia littoralis|[2899]|RDMAB076-05|Canada|Alberta|658[0n]|BOLD:AAA7373
Paradiarsia littoralis|[2900]|RDLQB393-05|Canada|Quebec|589[0n]|BOLD:AAA7373
Paradiarsia littoralis|[2901]|LPMN880-08|Canada|Alberta|658[1n]|BOLD:AAA7373
Paradiarsia littoralis|[2902]|BBLPB455-10|Canada|Alberta|658[0n]|BOLD:AAA7373
Paradiarsia littoralis|[2903]|LPAB229-08|Canada|Alberta|658[0n]|BOLD:AAA7373
Paradiarsia littoralis|[2904]|LPABB181-08|Canada|Alberta|658[0n]|BOLD:AAA7373
Paradiarsia littoralis|[2905]|LPABB351-08|Canada|Alberta|658[0n]|BOLD:AAA7373
Paradiarsia littoralis|[2906]|LPABB366-08|Canada|Alberta|658[0n]|BOLD:AAA7373
Paradiarsia littoralis|[2907]|LPABB872-09|Canada|Alberta|657[0n]|BOLD:AAA7373
Paradiarsia littoralis|[2908]|BBLPB370-10|Canada|Alberta|658[0n]|BOLD:AAA7373
Paradiarsia littoralis|[2909]|BBLPB587-10|Canada|Alberta|658[0n]|BOLD:AAA7373
Xestia cinerascens|[2910]|NAMUM130-08|United States|California|655[0n]|BOLD:AAW7524
Xestia vernilis|[2911]|LSEU790-06|United States|Colorado|658[0n]|BOLD:AAB0107
Xestia vernilis|[2912]|RDNMG755-08|United States|Colorado|658[0n]|BOLD:AAB0107
Xestia finatimis|[2913]|LOWCB005-05|Canada|British Columbia|658[3n]|BOLD:AAB0107
Xestia vernilis|[2914]|LPABC184-09|Canada|Alberta|658[0n]|BOLD:AAB0107
Xestia vernilis|[2915]|LPABB864-09|Canada|Alberta|646[0n]|BOLD:AAB0107
Xestia vernilis|[2916]|LPABC216-09|Canada|Alberta|632[1n]|BOLD:AAB0107
Xestia vernilis|[2917]|LPABB720-08|Canada|Alberta|642[0n]|BOLD:AAB0107
Xestia vernilis|[2918]|LPABC219-09|Canada|Alberta|632[1n]|BOLD:AAB0107
Xestia vernilis|[2919]|LPABC192-09|Canada|Alberta|612[0n]|BOLD:AAB0107
Xestia finatimis|[2920]|LOWCD389-06|Canada|British Columbia|570[0n]|BOLD:AAB0107
Xestia finatimis|[2921]|RDNMF425-08|Canada|British Columbia|658[0n]|BOLD:AAB0107
Xestia vernilis|[2922]|LPABB818-09|Canada|Alberta|658[0n]|BOLD:AAB0107
Xestia finatimis|[2923]|LOWCB011-05|Canada|British Columbia|658[0n]|BOLD:AAB0107
Xestia finatimis|[2924]|LOWCB008-05|Canada|British Columbia|658[0n]|BOLD:AAB0107
Xestia finatimis|[2925]|LOWCB007-05|Canada|British Columbia|658[0n]|BOLD:AAB0107
Xestia finatimis|[2926]|LOWCB006-05|Canada|British Columbia|658[0n]|BOLD:AAB0107
Xestia finatimis|[2927]|LOWCB004-05|Canada|British Columbia|658[0n]|BOLD:AAB0107
Xestia finatimis|[2928]|LOWCB003-05|Canada|British Columbia|658[0n]|BOLD:AAB0107
Xestia finatimis|[2929]|LOWCB002-05|Canada|British Columbia|658[0n]|BOLD:AAB0107
Xestia finatimis|[2930]|LOWC939-05|Canada|British Columbia|658[0n]|BOLD:AAB0107
Xestia finatimis|[2931]|LOWC938-05|Canada|British Columbia|658[0n]|BOLD:AAB0107
Xestia finatimis|[2932]|LOWC936-05|Canada|British Columbia|658[0n]|BOLD:AAB0107
Xestia finatimis|[2933]|LOWC935-05|Canada|British Columbia|658[0n]|BOLD:AAB0107
Xestia finatimis|[2934]|LOWC940-05|Canada|British Columbia|658[0n]|BOLD:AAB0107
Xestia finatimis|[2935]|LOWCB010-05|Canada|British Columbia|658[0n]|BOLD:AAB0107
Xestia finatimis|[2936]|LOWCD387-06|Canada|British Columbia|580[0n]|BOLD:AAB0107
Xestia verniloides|[2937]|RDNMG759-08|Canada|British Columbia|642[0n]|BOLD:AAB0107
Xestia verniloides|[2938]|RDNMG758-08|Canada|British Columbia|642[0n]|BOLD:AAB0107
Xestia finatimis|[2939]|LOWCD388-06|Canada|British Columbia|587[0n]|BOLD:AAB0107
Xestia vernilis|[2940]|LPABC196-09|Canada|Alberta|633[0n]|BOLD:AAB0107
Xestia vernilis|[2941]|LPABC214-09|Canada|Alberta|658[0n]|BOLD:AAB0107
Xestia finatimis|[2942]|LALPA672-10|Canada|British Columbia|658[0n]|BOLD:AAB0107
Xestia verniloides|[2943]|RDNMF083-08|Canada|British Columbia|650[0n]|BOLD:AAB0107
Xestia finatimis|[2944]|LALPA1317-11|Canada|British Columbia|658[0n]|BOLD:AAB0107
Xestia infimatis|[2945]|CNCLB1596-14|United States|Washington|658[0n]|BOLD:ABY8915
Xestia infimatis|[2946]|CNCLB1597-14|Canada|British Columbia|658[1n]|BOLD:ABY8915
Xestia finatimis|[2947]|NAMUM126-08|United States|California|657[0n]|BOLD:ABY8915
Xestia vernilis|[2948]|LPABC209-09|Canada|Alberta|658[1n]|BOLD:ABY8915
Xestia finatimis|[2949]|RDNMF424-08|Canada|British Columbia|658[0n]|BOLD:ABY8915
Xestia infimatis|[2950]|LPVIB658-08|Canada|British Columbia|588[0n]|BOLD:ABY8915
Xestia infimatis|[2951]|LPVIB670-08|Canada|British Columbia|632[0n]|BOLD:ABY8915
Xestia infimatis|[2952]|LALPA731-10|Canada|British Columbia|658[1n]|BOLD:ABY8915
Xestia vernilis|[2953]|LPABC217-09|Canada|Alberta|658[0n]|BOLD:ABY8915
Xestia vernilis|[2954]|LPABC008-09|Canada|Alberta|658[0n]|BOLD:ABY8915
Xestia finatimis|[2955]|RDNMF423-08|Canada|British Columbia|658[0n]|BOLD:ABY8915
Xestia vernilis|[2956]|RDNMF053-08|Canada|Alberta|658[0n]|BOLD:ABY8915
Xestia finatimis|[2957]|LOWCB009-05|Canada|British Columbia|658[0n]|BOLD:ABY8915
Xestia finatimis|[2958]|LOWCB001-05|Canada|British Columbia|658[0n]|BOLD:ABY8915
Xestia finatimis|[2959]|LOWC937-05|Canada|British Columbia|658[0n]|BOLD:ABY8915
Xestia vernilis|[2960]|RDNMG756-08|Canada|Alberta|642[0n]|BOLD:ABY8915
Xestia|[2961]|SSBAD6366-13|Canada|Alberta|589[0n]|BOLD:ABY8915
Xestia finatimis|[2962]|CNCLB1598-14|Canada|British Columbia|658[0n]|BOLD:ABY8915
Xestia vernilis|[2963]|CNCLB1599-14|United States|Montana|658[0n]|BOLD:ABY8915
Xestia infimatis|[2964]|RDNMD384-06|United States|California|658[0n]|BOLD:ABY8915
Xestia finatimis|[2965]|LOCBB159-06|United States|California|658[0n]|BOLD:ABY8915
Xestia finatimis|[2966]|LOCBB158-06|United States|California|658[0n]|BOLD:ABY8915
Xestia finatimis|[2967]|CNCLB1600-14|United States|California|658[0n]|BOLD:ABY8915
Xestia lorezi|[2968]|RDNMD001-06|Russia|Magadan|658[0n]|BOLD:AAD2545
Xestia lorezi|[2969]|RDNMG684-08|United States|Alaska|657[0n]|BOLD:AAD2545
Xestia lorezi|[2970]|RDNMF062-08|Canada|Yukon Territory|658[0n]|BOLD:AAD2545
Xestia lorezi|[2971]|RDNMG685-08|Canada|Yukon Territory|642[0n]|BOLD:AAD2545
Pronoctua typica|[2972]|RDNMG677-08|United States|Utah|658[0n]|BOLD:AAD9535
Pronoctua typica|[2973]|RDMAB293-05|Canada|Alberta|658[0n]|BOLD:AAD9535
Pronoctua typica|[2974]|RDNME776-08|Canada|Alberta|658[0n]|BOLD:AAD9535
Pronoctua typica|[2975]|RDNME777-08|Canada|Alberta|658[0n]|BOLD:AAD9535
Pronoctua typica|[2976]|NAMUM315-08|United States|California|658[0n]|BOLD:AAD9535
Pronoctua typica|[2977]|LBCH5719-10|Canada|British Columbia|658[0n]|BOLD:AAD9535
Pronoctua typica|[2978]|LBCH6218-10|Canada|British Columbia|658[0n]|BOLD:AAD9535
Pronoctua typica|[2979]|LBCH7466-10|Canada|British Columbia|658[0n]|BOLD:AAD9535
Xestia quieta|[2980]|RDNMF060-08|Canada|Northwest Territories|658[0n]|BOLD:AAF0710
Xestia quieta|[2981]|LEFIJ365-10|Finland||632[0n]|BOLD:AAF0710
Xestia quieta|[2982]|RDNMG725-08|Canada|Nunavut|658[0n]|BOLD:AAF0710
Xestia quieta|[2983]|RDNMG726-08|Canada|Nunavut|658[0n]|BOLD:AAF0710
Xestia quieta|[2984]|LEFIL447-10|Finland||658[0n]|BOLD:AAF0710
Xestia tecta|[2985]|LCHP188-07|Canada|Manitoba|621[0n]|BOLD:AAC5747
Xestia tecta|[2986]|LCHQ897-08|Canada|Manitoba|658[0n]|BOLD:AAC5747
Xestia tecta|[2987]|LCHQ899-08|Canada|Manitoba|658[0n]|BOLD:AAC5747
Xestia tecta|[2988]|LCHQ900-08|Canada|Manitoba|658[0n]|BOLD:AAC5747
Xestia tecta|[2989]|LCHQ901-08|Canada|Manitoba|658[0n]|BOLD:AAC5747
Xestia tecta|[2990]|LCHQ907-08|Canada|Manitoba|658[0n]|BOLD:AAC5747
[illegible]

Xestia tecta|[2988]|LCHQ900-08|Canada|Manitoba|658[0n]|BOLD:AAC5747
Xestia tecta|[2989]|LCHQ901-08|Canada|Manitoba|658[0n]|BOLD:AAC5747
Xestia tecta|[2990]|LCHQ907-08|Canada|Manitoba|658[0n]|BOLD:AAC5747
Xestia tecta|[2991]|LCHQ908-08|Canada|Manitoba|658[0n]|BOLD:AAC5747
Xestia tecta|[2992]|LCHQ912-08|Canada|Manitoba|658[0n]|BOLD:AAC5747
Xestia tecta|[2993]|LEFIH034-10|Finland||658[0n]|BOLD:AAC5747
Xestia tecta|[2994]|LEFIH035-10|Finland||658[0n]|BOLD:AAC5747
Xestia tecta|[2995]|LEFIH036-10|Finland||658[0n]|BOLD:AAC5747
Xestia perquiritata|[2996]|LOWCC153-05|Canada|British Columbia|569[0n]|BOLD:ACE9300
Xestia perquiritata|[2997]|LOWCC155-05|Canada|British Columbia|633[1n]|BOLD:ACE9300
Xestia perquiritata|[2998]|LBCG1874-09|Canada|British Columbia|658[0n]|BOLD:ACE9300
Xestia perquiritata|[2999]|LBCH3092-10|Canada|British Columbia|658[0n]|BOLD:ACE9300
Xestia perquiritata|[3000]|LBCG1859-09|Canada|British Columbia|658[0n]|BOLD:ACE9300
Xestia perquiritata|[3001]|LBCG1869-09|Canada|British Columbia|658[0n]|BOLD:ACE9300
Xestia perquiritata|[3002]|LBCG1851-09|Canada|British Columbia|658[0n]|BOLD:ACE9300
Xestia perquiritata|[3003]|LBCG1858-09|Canada|British Columbia|658[0n]|BOLD:ACE9300
Xestia perquiritata|[3004]|LOWCC156-05|Canada|British Columbia|658[0n]|BOLD:ACE9300
Xestia perquiritata|[3005]|LOWCC152-05|Canada|British Columbia|658[0n]|BOLD:ACE9300
Xestia perquiritata|[3006]|LOWCC151-05|Canada|British Columbia|658[0n]|BOLD:ACE9300
Xestia perquiritata|[3007]|LOWCC150-05|Canada|British Columbia|658[0n]|BOLD:ACE9300
Xestia perquiritata|[3008]|LOWCC149-05|Canada|British Columbia|658[0n]|BOLD:ACE9300
Xestia perquiritata|[3009]|LOWCC148-05|Canada|British Columbia|658[0n]|BOLD:ACE9300
Xestia perquiritata|[3010]|LOWCC147-05|Canada|British Columbia|658[0n]|BOLD:ACE9300
Xestia perquiritata|[3011]|LOWCC154-05|Canada|British Columbia|540[0n]|BOLD:ACE9300
Xestia perquiritata|[3012]|LOWCE823-06|Canada|British Columbia|624[0n]|BOLD:ACE9300
Xestia perquiritata|[3013]|LBCG732-09|Canada|British Columbia|658[0n]|BOLD:ACE9300
Xestia perquiritata|[3014]|LBCG745-09|Canada|British Columbia|658[0n]|BOLD:ACE9300
Xestia perquiritata|[3015]|LBCG762-09|Canada|British Columbia|658[0n]|BOLD:ACE9300
Xestia perquiritata|[3016]|LBCG763-09|Canada|British Columbia|658[0n]|BOLD:ACE9300
Xestia perquiritata|[3017]|LBCG959-09|Canada|British Columbia|658[0n]|BOLD:ACE9300
Xestia perquiritata|[3018]|LBCG998-09|Canada|British Columbia|658[0n]|BOLD:ACE9300
Xestia perquiritata|[3019]|LBCG1849-09|Canada|British Columbia|658[0n]|BOLD:ACE9300
Xestia perquiritata|[3020]|LBCH3796-10|Canada|British Columbia|658[0n]|BOLD:ACE9300
Xestia perquiritata|[3021]|LBCG2838-09|Canada|British Columbia|658[0n]|BOLD:ACE9312
Xestia perquiritata|[3022]|LBCG2765-09|Canada|British Columbia|658[0n]|BOLD:ACE9312
Xestia perquiritata|[3023]|LBCG2368-09|Canada|British Columbia|658[0n]|BOLD:ACE9312
Xestia perquiritata|[3024]|LBCB756-05|Canada|British Columbia|658[0n]|BOLD:ACE9312
Xestia perquiritata|[3025]|LBCB359-05|Canada|British Columbia|658[0n]|BOLD:ACE9312
Xestia perquiritata|[3026]|LBCD187-05|Canada|British Columbia|658[0n]|BOLD:ACE9312
Xestia perquiritata|[3027]|LBCB360-05|Canada|British Columbia|658[0n]|BOLD:ACE9312
Xestia perquiritata|[3028]|LBCG1900-09|Canada|British Columbia|658[0n]|BOLD:ACE9312
Xestia perquiritata|[3029]|LBCG1901-09|Canada|British Columbia|658[0n]|BOLD:ACE9312
Xestia perquiritata|[3030]|LBCG2039-09|Canada|British Columbia|658[0n]|BOLD:ACE9312
Xestia perquiritata|[3031]|LBCG2369-09|Canada|British Columbia|658[0n]|BOLD:ACE9312
Xestia perquiritata|[3032]|LBCG2370-09|Canada|British Columbia|658[0n]|BOLD:ACE9312
Xestia perquiritata|[3033]|LBCG2371-09|Canada|British Columbia|658[0n]|BOLD:ACE9312
Xestia perquiritata|[3034]|LBCG2373-09|Canada|British Columbia|658[0n]|BOLD:ACE9312
Xestia perquiritata|[3035]|LBCG2375-09|Canada|British Columbia|658[0n]|BOLD:ACE9312
Xestia perquiritata|[3036]|LBCG2376-09|Canada|British Columbia|658[0n]|BOLD:ACE9312
Xestia perquiritata|[3037]|LBCG2377-09|Canada|British Columbia|658[0n]|BOLD:ACE9312
Xestia perquiritata|[3038]|LBCG2378-09|Canada|British Columbia|658[0n]|BOLD:ACE9312
Xestia perquiritata|[3039]|LBCG2379-09|Canada|British Columbia|658[0n]|BOLD:ACE9312
Xestia perquiritata|[3040]|LBCG2380-09|Canada|British Columbia|658[0n]|BOLD:ACE9312
Xestia perquiritata|[3041]|LBCG2381-09|Canada|British Columbia|658[0n]|BOLD:ACE9312
Xestia perquiritata|[3042]|LBCG2764-09|Canada|British Columbia|658[0n]|BOLD:ACE9312
Xestia perquiritata|[3043]|LBCG2766-09|Canada|British Columbia|658[0n]|BOLD:ACE9312
Xestia perquiritata|[3044]|LBCG2767-09|Canada|British Columbia|658[0n]|BOLD:ACE9312
Xestia perquiritata|[3045]|LBCG2839-09|Canada|British Columbia|658[0n]|BOLD:ACE9312
Xestia perquiritata|[3046]|LBCG3036-09|Canada|British Columbia|658[0n]|BOLD:ACE9312
Xestia perquiritata|[3047]|LBCG3048-09|Canada|British Columbia|658[0n]|BOLD:ACE9312
Xestia perquiritata|[3048]|LBCH1108-10|Canada|British Columbia|658[0n]|BOLD:ACE9312
Xestia perquiritata|[3049]|LBCH1429-10|Canada|British Columbia|658[0n]|BOLD:ACE9312
Xestia perquiritata|[3050]|LBCH1438-10|Canada|British Columbia|658[0n]|BOLD:ACE9312
Xestia perquiritata|[3051]|LBCH1444-10|Canada|British Columbia|658[0n]|BOLD:ACE9312
Xestia perquiritata|[3052]|LBCH1541-10|Canada|British Columbia|658[0n]|BOLD:ACE9312
Xestia perquiritata|[3053]|LBCH1543-10|Canada|British Columbia|658[0n]|BOLD:ACE9312
Xestia perquiritata|[3054]|LBCH1460-10|Canada|British Columbia|658[0n]|BOLD:ACE9312
Xestia perquiritata|[3055]|LBCH1519-10|Canada|British Columbia|658[0n]|BOLD:ACE9312
Xestia perquiritata|[3056]|LBCH1568-10|Canada|British Columbia|658[0n]|BOLD:ACE9312
Xestia perquiritata|[3057]|LBCH1580-10|Canada|British Columbia|658[0n]|BOLD:ACE9312
Xestia perquiritata|[3058]|LBCH2099-10|Canada|British Columbia|658[0n]|BOLD:ACE9312
Xestia perquiritata|[3059]|LBCH2266-10|Canada|British Columbia|658[0n]|BOLD:ACE9312
Xestia perquiritata|[3060]|LBCH2267-10|Canada|British Columbia|658[0n]|BOLD:ACE9312
Xestia perquiritata|[3061]|LBCH2268-10|Canada|British Columbia|658[0n]|BOLD:ACE9312
Xestia perquiritata|[3062]|LBCH2269-10|Canada|British Columbia|658[0n]|BOLD:ACE9312
Xestia perquiritata|[3063]|LBCH2270-10|Canada|British Columbia|658[0n]|BOLD:ACE9312
Xestia perquiritata|[3064]|LBCH2271-10|Canada|British Columbia|658[0n]|BOLD:ACE9312
Xestia perquiritata|[3065]|LBCH2272-10|Canada|British Columbia|658[0n]|BOLD:ACE9312
Xestia perquiritata|[3066]|LBCG1902-09|Canada|British Columbia|658[0n]|BOLD:ACE9312
Xestia perquiritata|[3067]|LBCG2014-09|Canada|British Columbia|658[0n]|BOLD:ACE9312
Xestia perquiritata|[3068]|LBCG1892-09|Canada|British Columbia|658[0n]|BOLD:ACE9312
Xestia perquiritata|[3069]|LBCG1886-09|Canada|British Columbia|658[0n]|BOLD:ACE9312
Xestia perquiritata|[3070]|LBCG595-09|Canada|British Columbia|658[0n]|BOLD:ACE9312
Xestia perquiritata|[3071]|LBCD149-05|Canada|British Columbia|658[0n]|BOLD:ACE9312
Xestia perquiritata|[3072]|LBCC674-05|Canada|British Columbia|658[0n]|BOLD:ACE9312
Xestia perquiritata|[3073]|LBCB803-05|Canada|British Columbia|658[0n]|BOLD:ACE9312
Xestia perquiritata|[3074]|LBCB757-05|Canada|British Columbia|658[0n]|BOLD:ACE9312
Xestia perquiritata|[3075]|LBCB485-05|Canada|British Columbia|658[0n]|BOLD:ACE9312
Xestia perquiritata|[3076]|LBCB358-05|Canada|British Columbia|658[0n]|BOLD:ACE9312
Xestia perquiritata|[3077]|LBCA305-05|Canada|British Columbia|658[0n]|BOLD:ACE9312
Xestia perquiritata|[3078]|LBCB582-05|Canada|British Columbia|652[0n]|BOLD:ACE9312
Xestia perquiritata|[3079]|LBCA737-05|Canada|British Columbia|655[0n]|BOLD:ACE9312
Xestia perquiritata|[3080]|LBCA736-05|Canada|British Columbia|658[0n]|BOLD:ACE9312
Xestia perquiritata|[3081]|LBCG2374-09|Canada|British Columbia|658[0n]|BOLD:ACE9312
Xestia perquiritata|[3082]|LBCH1439-10|Canada|British Columbia|658[0n]|BOLD:ACE9312
Xestia perquiritata|[3083]|LBCH1542-10|Canada|British Columbia|640[0n]|BOLD:ACE9312
Xestia perquiritata|[3084]|LBCH2128-10|Canada|British Columbia|636[0n]|BOLD:ACE9312
Xestia perquiritata|[3085]|BBLPB660-10|Canada|British Columbia|658[0n]|BOLD:ACE9312
Xestia perquiritata|[3086]|LCH241-04|Canada|Manitoba|658[0n]|BOLD:AAA4307
Xestia perquiritata|[3087]|LCHP534-07|Canada|Manitoba|658[0n]|BOLD:AAA4307
Xestia perquiritata|[3088]|LCHP906-07|Canada|Manitoba|658[0n]|BOLD:AAA4307
Xestia perquiritata|[3089]|RDLQB640-05|Canada|Quebec|584[0n]|BOLD:AAA4307
Xestia perquiritata|[3090]|RDLQB641-05|Canada|Quebec|566[1n]|BOLD:AAA4307

Xestia perquiritata|[3088]|LCHP906-07|Canada|Manitoba|658[0n]|BOLD:AAA4307
Xestia perquiritata|[3089]|RDLQB640-05|Canada|Quebec|584[0n]|BOLD:AAA4307
Xestia perquiritata|[3090]|RDLQB641-05|Canada|Quebec|566[1n]|BOLD:AAA4307
Xestia perquiritata|[3091]|ABKWR103-07|United States|Alaska|658[0n]|BOLD:AAA4307
Xestia perquiritata|[3092]|LCHP587-07|Canada|Manitoba|658[0n]|BOLD:AAA4307
Xestia perquiritata|[3093]|ABKWR056-07|United States|Alaska|658[0n]|BOLD:AAA4307
Xestia perquiritata|[3094]|BBLPB521-10|Canada|Ontario|658[0n]|BOLD:AAA4307
Xestia perquiritata|[3095]|BBLPB522-10|Canada|Ontario|658[0n]|BOLD:AAA4307
Xestia perquiritata|[3096]|UAMIC524-13|United States|Alaska|604[0n]|BOLD:AAA4307
Xestia perquiritata|[3097]|UAMIC525-13|United States|Alaska|658[0n]|BOLD:AAA4307
Xestia okakensis|[3098]|RDNMG689-08|Canada|Alberta|642[0n]|BOLD:AAB9912
Xestia okakensis|[3099]|RDNMG690-08|Canada|Alberta|658[0n]|BOLD:AAB9912
Xestia okakensis|[3100]|CHLEP209-09|Canada|Manitoba|658[0n]|BOLD:AAB9912
Xestia okakensis|[3101]|CHLEP155-09|Canada|Manitoba|658[0n]|BOLD:AAB9912
Xestia okakensis|[3102]|LCHP411-07|Canada|Manitoba|658[0n]|BOLD:AAB9912
Xestia okakensis|[3103]|LCHP210-07|Canada|Manitoba|658[0n]|BOLD:AAB9912
Xestia okakensis|[3104]|LCHP008-07|Canada|Manitoba|656[0n]|BOLD:AAB9912
Xestia okakensis|[3105]|CHLEP211-09|Canada|Manitoba|642[0n]|BOLD:AAB9912
Xestia okakensis|[3106]|LCHP259-07|Canada|Manitoba|644[0n]|BOLD:AAB9912
Xestia okakensis|[3107]|CHLEP210-09|Canada|Manitoba|636[0n]|BOLD:AAB9912
Xestia okakensis|[3108]|CHLEP203-09|Canada|Manitoba|637[0n]|BOLD:AAB9912
Xestia okakensis|[3109]|LCHP006-07|Canada|Manitoba|635[0n]|BOLD:AAB9912
Xestia okakensis|[3110]|LCHP165-07|Canada|Manitoba|634[0n]|BOLD:AAB9912
Xestia okakensis|[3111]|CHLEP087-09|Canada|Manitoba|635[0n]|BOLD:AAB9912
Xestia okakensis|[3112]|CHLEP216-09|Canada|Manitoba|634[0n]|BOLD:AAB9912
Parabarrovia keelei|[3113]|RDNMG704-08|Canada|Yukon Territory|641[0n]|BOLD:ACE9951
Parabarrovia keelei|[3114]|RDNMF073-08|Canada|Yukon Territory|658[0n]|BOLD:ABZ2143
Parabarrovia keelei|[3115]|RDNMG705-08|Canada|Yukon Territory|658[0n]|BOLD:ABZ2143
Parabarrovia omilaki|[3116]|RDNMF081-08|United States|Alaska|658[0n]|BOLD:ABZ2144
Parabarrovia omilaki|[3117]|RDNMG706-08|United States|Alaska|658[0n]|BOLD:ABZ2144
Parabarrovia ogilviensis|[3118]|RDNMG701-08|Canada|Yukon Territory|642[0n]|BOLD:AAD5961
Parabarrovia ogilviensis|[3119]|RDNMG703-08|Canada|Yukon Territory|641[2n]|BOLD:AAD5961
Parabarrovia ogilviensis|[3120]|RDNMF074-08|Canada|Yukon Territory|658[0n]|BOLD:AAD5961
Parabarrovia ogilviensis|[3121]|RDNMG699-08|Canada|Yukon Territory|658[0n]|BOLD:AAD5961
Parabarrovia ogilviensis|[3122]|RDNMG700-08|Canada|Yukon Territory|658[0n]|BOLD:AAD5961
Parabarrovia ogilviensis|[3123]|RDNMG702-08|Canada|Yukon Territory|658[0n]|BOLD:AAD5961
Parabarrovia ogilviensis|[3124]|RDNMG780-08|United States|Oregon|658[0n]|BOLD:AAD5961
Xestia homogena|[3125]|LCHQ190-07|Canada|Manitoba|658[0n]|BOLD:AAA3385
Xestia homogena|[3126]|LCHQ099-07|Canada|Manitoba|658[0n]|BOLD:AAA3385
Xestia homogena|[3127]|RDNMD037-06|Canada|Manitoba|658[0n]|BOLD:AAA3385
Xestia homogena|[3128]|LCH319-04|Canada|Manitoba|658[0n]|BOLD:AAA3385
Xestia homogena|[3129]|LCH246-04|Canada|Manitoba|658[0n]|BOLD:AAA3385
Xestia homogena|[3130]|LCH447-04|Canada|Manitoba|658[0n]|BOLD:AAA3385
Xestia homogena|[3131]|LCH593-04|Canada|Manitoba|595[1n]|BOLD:AAA3385
Xestia homogena|[3132]|RDNMD038-06|Canada|Quebec|614[0n]|BOLD:AAA3385
Xestia homogena|[3133]|RDNMD040-06|United States|New Hampshire|616[0n]|BOLD:AAA3385
Xestia homogena|[3134]|LCHP805-07|Canada|Manitoba|658[0n]|BOLD:AAA3385
Xestia homogena|[3135]|LCHQ488-08|Canada|Manitoba|632[0n]|BOLD:AAA3385
Xestia homogena|[3136]|LSEU809-06|United States|Colorado|658[0n]|BOLD:AAA3385
Xestia homogena|[3137]|LBCG872-09|Canada|British Columbia|658[0n]|BOLD:AAA3385
Xestia homogena|[3138]|LBCH1448-10|Canada|British Columbia|658[0n]|BOLD:AAA3385
Xestia homogena|[3139]|LBCG967-09|Canada|British Columbia|658[0n]|BOLD:AAA3385
Xestia homogena|[3140]|LBCG1016-09|Canada|British Columbia|658[0n]|BOLD:AAA3385
Xestia homogena|[3141]|LBCG1018-09|Canada|British Columbia|658[0n]|BOLD:AAA3385
Xestia homogena|[3142]|LBCG1853-09|Canada|British Columbia|658[0n]|BOLD:AAA3385
Xestia homogena|[3143]|LBCG1862-09|Canada|British Columbia|658[0n]|BOLD:AAA3385
Xestia homogena|[3144]|LBCG1875-09|Canada|British Columbia|658[0n]|BOLD:AAA3385
Xestia homogena|[3145]|LBCG768-09|Canada|British Columbia|658[0n]|BOLD:AAA3385
Xestia homogena|[3146]|LBCG962-09|Canada|British Columbia|658[0n]|BOLD:AAA3385
Xestia homogena|[3147]|LBCG963-09|Canada|British Columbia|658[0n]|BOLD:AAA3385
Xestia homogena|[3148]|LBCG964-09|Canada|British Columbia|658[0n]|BOLD:AAA3385
Xestia homogena|[3149]|LBCG1856-09|Canada|British Columbia|658[0n]|BOLD:AAA3385
Xestia homogena|[3150]|LBCG1861-09|Canada|British Columbia|658[0n]|BOLD:AAA3385
Xestia homogena|[3151]|LBCG734-09|Canada|British Columbia|658[0n]|BOLD:AAA3385
Xestia homogena|[3152]|LBCG767-09|Canada|British Columbia|658[0n]|BOLD:AAA3385
Xestia homogena|[3153]|LBCH3470-10|Canada|British Columbia|658[0n]|BOLD:AAA3385
Xestia homogena|[3154]|LBCG2055-09|Canada|British Columbia|658[0n]|BOLD:AAA3385
Xestia homogena|[3155]|LBCH1094-10|Canada|British Columbia|658[0n]|BOLD:AAA3385
Xestia homogena|[3156]|LBCG1014-09|Canada|British Columbia|658[0n]|BOLD:AAA3385
Xestia homogena|[3157]|LBCH2109-10|Canada|British Columbia|658[0n]|BOLD:AAA3385
Xestia homogena|[3158]|LBCH2108-10|Canada|British Columbia|658[0n]|BOLD:AAA3385
Xestia homogena|[3159]|LBCH2107-10|Canada|British Columbia|658[0n]|BOLD:AAA3385
Xestia homogena|[3160]|LBCH2106-10|Canada|British Columbia|658[0n]|BOLD:AAA3385
Xestia homogena|[3161]|LBCH2104-10|Canada|British Columbia|658[0n]|BOLD:AAA3385
Xestia homogena|[3162]|LBCH2103-10|Canada|British Columbia|658[0n]|BOLD:AAA3385
Xestia homogena|[3163]|LBCH1998-10|Canada|British Columbia|658[0n]|BOLD:AAA3385
Xestia homogena|[3164]|LBCH1951-10|Canada|British Columbia|658[0n]|BOLD:AAA3385
Xestia homogena|[3165]|LBCH1680-10|Canada|British Columbia|658[0n]|BOLD:AAA3385
Xestia homogena|[3166]|LBCH1679-10|Canada|British Columbia|658[0n]|BOLD:AAA3385
Xestia homogena|[3167]|LBCH1584-10|Canada|British Columbia|658[0n]|BOLD:AAA3385
Xestia homogena|[3168]|LBCH1537-10|Canada|British Columbia|658[0n]|BOLD:AAA3385
Xestia homogena|[3169]|LBCH1536-10|Canada|British Columbia|658[0n]|BOLD:AAA3385
Xestia homogena|[3170]|LBCH1535-10|Canada|British Columbia|658[0n]|BOLD:AAA3385
Xestia homogena|[3171]|LBCH1533-10|Canada|British Columbia|658[0n]|BOLD:AAA3385
Xestia homogena|[3172]|LBCH1532-10|Canada|British Columbia|658[0n]|BOLD:AAA3385
Xestia homogena|[3173]|LBCH1531-10|Canada|British Columbia|658[0n]|BOLD:AAA3385
Xestia homogena|[3174]|LBCH1530-10|Canada|British Columbia|658[0n]|BOLD:AAA3385
Xestia homogena|[3175]|LBCH1521-10|Canada|British Columbia|658[0n]|BOLD:AAA3385
Xestia homogena|[3176]|LBCH1455-10|Canada|British Columbia|658[0n]|BOLD:AAA3385
Xestia homogena|[3177]|LBCH1453-10|Canada|British Columbia|658[0n]|BOLD:AAA3385
Xestia homogena|[3178]|LBCH1452-10|Canada|British Columbia|658[0n]|BOLD:AAA3385
Xestia homogena|[3179]|LBCH1451-10|Canada|British Columbia|658[0n]|BOLD:AAA3385
Xestia homogena|[3180]|LBCH1450-10|Canada|British Columbia|658[0n]|BOLD:AAA3385
Xestia homogena|[3181]|LBCH1443-10|Canada|British Columbia|658[0n]|BOLD:AAA3385
Xestia homogena|[3182]|LBCH1441-10|Canada|British Columbia|658[0n]|BOLD:AAA3385
Xestia homogena|[3183]|LBCH1440-10|Canada|British Columbia|658[0n]|BOLD:AAA3385
Xestia homogena|[3184]|LBCH1435-10|Canada|British Columbia|658[0n]|BOLD:AAA3385
Xestia homogena|[3185]|LBCH1235-10|Canada|British Columbia|658[0n]|BOLD:AAA3385
Xestia homogena|[3186]|LBCH1100-10|Canada|British Columbia|658[0n]|BOLD:AAA3385
Xestia homogena|[3187]|LBCH1098-10|Canada|British Columbia|658[0n]|BOLD:AAA3385
Xestia homogena|[3188]|LBCH1097-10|Canada|British Columbia|658[0n]|BOLD:AAA3385
Xestia homogena|[3189]|LBCH1096-10|Canada|British Columbia|658[0n]|BOLD:AAA3385
Xestia homogena|[3190]|LBCG2057-09|Canada|British Columbia|658[0n]|BOLD:AAA3385

Xestia homogena|[3188]|LBCH1097-10|Canada|British Columbia|658[0n]|BOLD:AAA3385
Xestia homogena|[3189]|LBCH1096-10|Canada|British Columbia|658[0n]|BOLD:AAA3385
Xestia homogena|[3190]|LBCG2057-09|Canada|British Columbia|658[0n]|BOLD:AAA3385
Xestia homogena|[3191]|LBCG2056-09|Canada|British Columbia|658[0n]|BOLD:AAA3385
Xestia homogena|[3192]|LBCG2054-09|Canada|British Columbia|658[0n]|BOLD:AAA3385
Xestia homogena|[3193]|LBCG2052-09|Canada|British Columbia|658[0n]|BOLD:AAA3385
Xestia homogena|[3194]|LBCG2051-09|Canada|British Columbia|658[0n]|BOLD:AAA3385
Xestia homogena|[3195]|LBCG2050-09|Canada|British Columbia|658[0n]|BOLD:AAA3385
Xestia homogena|[3196]|LBCG2048-09|Canada|British Columbia|658[0n]|BOLD:AAA3385
Xestia homogena|[3197]|LBCG2047-09|Canada|British Columbia|658[0n]|BOLD:AAA3385
Xestia homogena|[3198]|LBCG2046-09|Canada|British Columbia|658[0n]|BOLD:AAA3385
Xestia homogena|[3199]|LBCG2044-09|Canada|British Columbia|658[0n]|BOLD:AAA3385
Xestia homogena|[3200]|LBCG2042-09|Canada|British Columbia|658[0n]|BOLD:AAA3385
Xestia homogena|[3201]|LBCG2018-09|Canada|British Columbia|658[0n]|BOLD:AAA3385
Xestia homogena|[3202]|LBCG1871-09|Canada|British Columbia|658[0n]|BOLD:AAA3385
Xestia homogena|[3203]|LBCG1860-09|Canada|British Columbia|658[0n]|BOLD:AAA3385
Xestia homogena|[3204]|LBCG1855-09|Canada|British Columbia|658[0n]|BOLD:AAA3385
Xestia homogena|[3205]|LBCG1854-09|Canada|British Columbia|658[0n]|BOLD:AAA3385
Xestia homogena|[3206]|LBCG1850-09|Canada|British Columbia|658[0n]|BOLD:AAA3385
Xestia homogena|[3207]|LBCG1017-09|Canada|British Columbia|658[0n]|BOLD:AAA3385
Xestia homogena|[3208]|LBCG995-09|Canada|British Columbia|658[0n]|BOLD:AAA3385
Xestia homogena|[3209]|LBCG994-09|Canada|British Columbia|658[0n]|BOLD:AAA3385
Xestia homogena|[3210]|LBCG972-09|Canada|British Columbia|658[0n]|BOLD:AAA3385
Xestia homogena|[3211]|LBCG971-09|Canada|British Columbia|658[0n]|BOLD:AAA3385
Xestia homogena|[3212]|LBCG958-09|Canada|British Columbia|658[0n]|BOLD:AAA3385
Xestia homogena|[3213]|LBCG956-09|Canada|British Columbia|658[0n]|BOLD:AAA3385
Xestia homogena|[3214]|LBCG949-09|Canada|British Columbia|658[0n]|BOLD:AAA3385
Xestia homogena|[3215]|LPABC428-09|Canada|Alberta|658[0n]|BOLD:AAA3385
Xestia homogena|[3216]|LBCG860-09|Canada|British Columbia|658[0n]|BOLD:AAA3385
Xestia homogena|[3217]|LBCG858-09|Canada|British Columbia|658[0n]|BOLD:AAA3385
Xestia homogena|[3218]|LBCG833-09|Canada|British Columbia|658[0n]|BOLD:AAA3385
Xestia homogena|[3219]|LBCG737-09|Canada|British Columbia|658[0n]|BOLD:AAA3385
Xestia homogena|[3220]|LBCG650-09|Canada|British Columbia|658[0n]|BOLD:AAA3385
Xestia homogena|[3221]|LBCG594-09|Canada|British Columbia|658[0n]|BOLD:AAA3385
Xestia homogena|[3222]|LBCG593-09|Canada|British Columbia|658[0n]|BOLD:AAA3385
Xestia homogena|[3223]|LBCG591-09|Canada|British Columbia|658[0n]|BOLD:AAA3385
Xestia homogena|[3224]|LBCG579-09|Canada|British Columbia|658[0n]|BOLD:AAA3385
Xestia homogena|[3225]|LBCG570-09|Canada|British Columbia|658[0n]|BOLD:AAA3385
Xestia homogena|[3226]|LBCD186-05|Canada|British Columbia|658[0n]|BOLD:AAA3385
Xestia homogena|[3227]|LBCD185-05|Canada|British Columbia|658[0n]|BOLD:AAA3385
Xestia homogena|[3228]|LBCD184-05|Canada|British Columbia|658[0n]|BOLD:AAA3385
Xestia homogena|[3229]|LBCD183-05|Canada|British Columbia|658[0n]|BOLD:AAA3385
Xestia homogena|[3230]|LBCD144-05|Canada|British Columbia|658[0n]|BOLD:AAA3385
Xestia homogena|[3231]|LBCD143-05|Canada|British Columbia|658[0n]|BOLD:AAA3385
Xestia homogena|[3232]|LBCD102-05|Canada|British Columbia|658[0n]|BOLD:AAA3385
Xestia homogena|[3233]|LBCC874-05|Canada|British Columbia|658[0n]|BOLD:AAA3385
Xestia homogena|[3234]|LBCC670-05|Canada|British Columbia|658[0n]|BOLD:AAA3385
Xestia homogena|[3235]|LBCC563-05|Canada|British Columbia|658[0n]|BOLD:AAA3385
Xestia homogena|[3236]|LBCG590-09|Canada|British Columbia|658[0n]|BOLD:AAA3385
Xestia homogena|[3237]|LBCC875-05|Canada|British Columbia|658[5n]|BOLD:AAA3385
Xestia homogena|[3238]|LBCH1101-10|Canada|British Columbia|658[0n]|BOLD:AAA3385
Xestia homogena|[3239]|LBCH1099-10|Canada|British Columbia|658[0n]|BOLD:AAA3385
Xestia homogena|[3240]|LBCH1095-10|Canada|British Columbia|658[0n]|BOLD:AAA3385
Xestia homogena|[3241]|LBCH1449-10|Canada|British Columbia|621[0n]|BOLD:AAA3385
Xestia homogena|[3242]|LBCH1454-10|Canada|British Columbia|658[0n]|BOLD:AAA3385
Xestia homogena|[3243]|LBCH1534-10|Canada|British Columbia|658[0n]|BOLD:AAA3385
Xestia homogena|[3244]|LBCH2105-10|Canada|British Columbia|658[0n]|BOLD:AAA3385
Xestia homogena|[3245]|LBCH2110-10|Canada|British Columbia|658[0n]|BOLD:AAA3385
Xestia homogena|[3246]|BBLPB287-10|Canada|British Columbia|658[0n]|BOLD:AAA3385
Xestia homogena|[3247]|BBLPB288-10|Canada|British Columbia|658[0n]|BOLD:AAA3385
Xestia homogena|[3248]|BBLPB289-10|Canada|British Columbia|658[0n]|BOLD:AAA3385
Xestia homogena|[3249]|BBLPB290-10|Canada|British Columbia|658[0n]|BOLD:AAA3385
Xestia lupa|[3250]|RDNMF063-08|Canada|Yukon Territory|658[0n]|BOLD:ACE6805
Xestia lupa|[3251]|RDNMG746-08|Canada|Alberta|642[0n]|BOLD:ACE6805
Xestia lupa|[3252]|RDNMG747-08|Canada|Yukon Territory|642[0n]|BOLD:ACE6805
Xestia lupa|[3253]|RDNMG748-08|Canada|Yukon Territory|658[0n]|BOLD:ACE6805
Xestia aequaeva|[3254]|RDNMD048-06|||658[0n]|BOLD:AAD9296
Xestia alaskae|[3255]|RDNMD046-06|Russia|Magadan|658[0n]|BOLD:AAD9296
Xestia aequaeva|[3256]|RDNMD049-06|Russia||658[0n]|BOLD:AAD9296
Xestia alaskae|[3257]|RDNMF061-08|Canada|Yukon Territory|658[0n]|BOLD:AAD9296
Xestia thula|[3258]|RDNMF067-08|Canada|Nunavut|641[0n]|BOLD:AAD9296
Xestia aequaeva|[3259]|RDNMG686-08|Canada|Yukon Territory|652[0n]|BOLD:AAD9297
Xestia aequaeva|[3260]|RDNMG688-08|United States|Alaska|658[0n]|BOLD:AAD9297
Xestia alaskae|[3261]|RDNMD047-06|Russia||658[0n]|BOLD:AAF4067
Xestia alaskae|[3262]|UAMIC482-13|United States|Alaska|658[0n]|BOLD:AAF4067
Xestia aequaeva|[3263]|RDNMF068-08|Canada|Yukon Territory|658[0n]|BOLD:AAD9297
Xestia aequaeva|[3264]|RDNMG687-08|Canada|Yukon Territory|640[0n]|BOLD:AAD9297
Xestia aequaeva|[3265]|CNCLB1501-14|Canada|Yukon Territory|596[0n]|BOLD:AAD9297
Xestia aequaeva|[3266]|CNCLB1502-14|Canada|Yukon Territory|619[0n]|BOLD:AAD9297
Xestia aequaeva|[3267]|CNCLB1503-14|Canada|Yukon Territory|571[0n]|BOLD:AAD9297
Xestia atrata|[3268]|LCHP505-07|Canada|Manitoba|658[0n]|BOLD:AAE1738
Xestia atrata|[3269]|LCHP275-07|Canada|Manitoba|658[0n]|BOLD:AAE1738
Xestia atrata|[3270]|LCHP258-07|Canada|Manitoba|656[0n]|BOLD:AAE1738
Xestia atrata|[3271]|LCHP628-07|Canada|Manitoba|658[0n]|BOLD:AAE1738
Xestia atrata|[3272]|LCHQ046-07|Canada|Manitoba|658[0n]|BOLD:AAE1738
Xestia atrata|[3273]|CNCLB1584-14|Russia||658[0n]|BOLD:ACK6551
Xestia atrata|[3274]|LCHP256-07|Canada|Manitoba|658[0n]|BOLD:AAC1499
Xestia atrata|[3275]|LCHP276-07|Canada|Manitoba|657[0n]|BOLD:AAC1499
Xestia atrata|[3276]|LCHP636-07|Canada|Manitoba|658[0n]|BOLD:AAC1499
Xestia atrata|[3277]|LCHP519-07|Canada|Manitoba|658[0n]|BOLD:AAC1499
Xestia atrata|[3278]|LCHP408-07|Canada|Manitoba|658[0n]|BOLD:AAC1499
Xestia atrata|[3279]|LCHP257-07|Canada|Manitoba|658[0n]|BOLD:AAC1499
Xestia atrata|[3280]|LCHP388-07|Canada|Manitoba|658[0n]|BOLD:AAC1499
Xestia atrata|[3281]|LCHP631-07|Canada|Manitoba|658[0n]|BOLD:AAC1499
Xestia atrata|[3282]|LCHP386-07|Canada|Manitoba|649[0n]|BOLD:AAC1499
Xestia atrata|[3283]|LCHQ096-07|Canada|Manitoba|658[0n]|BOLD:AAC1499
Xestia atrata|[3284]|LCHQ625-08|Canada|Manitoba|658[0n]|BOLD:AAC1499
Xestia atrata|[3285]|RDNMF607-08|Canada|Manitoba|658[0n]|BOLD:AAC1499
Xestia atrata|[3286]|CHLEP309-09|Canada|Manitoba|658[0n]|BOLD:AAC1499
Xestia atrata|[3287]|CNCLB1582-14|Canada|Quebec|658[0n]|BOLD:AAC1499
Xestia atrata|[3288]|LEFIF312-10|Finland||658[0n]|BOLD:AAC1499
Xestia atrata|[3289]|LEFIF313-10|Finland||631[0n]|BOLD:AAC1499
Xestia atrata|[3290]|RDNMG745-08|Canada|Yukon Territory|658[0n]|BOLD:AAC1499

Xestia atrata|[3287]|CNCLB1582-14|Canada|Quebec|658[0n]|BOLD:AAC1499
Xestia atrata|[3288]|LEFIF312-10|Finland||658[0n]|BOLD:AAC1499
Xestia atrata|[3289]|LEFIF313-10|Finland||631[0n]|BOLD:AAC1499
Xestia atrata|[3290]|RDNMG745-08|Canada|Yukon Territory|658[0n]|BOLD:AAC1499
Xestia atrata|[3291]|LEFID251-10|Finland||658[0n]|BOLD:AAC1499
Xestia atrata|[3292]|CNCLB1583-14|Canada|Ontario|658[0n]|BOLD:AAC1499
Xestia atrata|[3293]|CNCLB1585-14|Russia||658[0n]|BOLD:AAC1499
Xestia ursae|[3294]|LCHQ910-08|Canada|Manitoba|658[0n]|BOLD:AAB0146
Xestia ursae|[3295]|LCHQ877-08|Canada|Manitoba|658[0n]|BOLD:AAB0146
Xestia ursae|[3296]|LCHQ189-07|Canada|Manitoba|658[0n]|BOLD:AAB0146
Xestia ursae|[3297]|LCHQ128-07|Canada|Manitoba|658[0n]|BOLD:AAB0146
Xestia ursae|[3298]|LCHP954-07|Canada|Manitoba|658[0n]|BOLD:AAB0146
Xestia ursae|[3299]|LCHP905-07|Canada|Manitoba|658[0n]|BOLD:AAB0146
Xestia ursae|[3300]|LCHP856-07|Canada|Manitoba|658[0n]|BOLD:AAB0146
Xestia ursae|[3301]|LCHP750-07|Canada|Manitoba|655[0n]|BOLD:AAB0146
Xestia ursae|[3302]|LCHP713-07|Canada|Manitoba|658[0n]|BOLD:AAB0146
Xestia ursae|[3303]|LCHP703-07|Canada|Manitoba|658[0n]|BOLD:AAB0146
Xestia ursae|[3304]|LCHP649-07|Canada|Manitoba|658[0n]|BOLD:AAB0146
Xestia ursae|[3305]|LCHP634-07|Canada|Manitoba|658[0n]|BOLD:AAB0146
Xestia ursae|[3306]|LCHP630-07|Canada|Manitoba|658[0n]|BOLD:AAB0146
Xestia ursae|[3307]|LCHP607-07|Canada|Manitoba|658[0n]|BOLD:AAB0146
Xestia ursae|[3308]|LCHP595-07|Canada|Manitoba|658[0n]|BOLD:AAB0146
Xestia ursae|[3309]|LCHP586-07|Canada|Manitoba|658[0n]|BOLD:AAB0146
Xestia ursae|[3310]|LCHP582-07|Canada|Manitoba|658[0n]|BOLD:AAB0146
Xestia ursae|[3311]|LCHP537-07|Canada|Manitoba|658[0n]|BOLD:AAB0146
Xestia ursae|[3312]|LCHP514-07|Canada|Manitoba|658[0n]|BOLD:AAB0146
Xestia ursae|[3313]|LCHP463-07|Canada|Manitoba|658[0n]|BOLD:AAB0146
Xestia ursae|[3314]|LCHP461-07|Canada|Manitoba|658[0n]|BOLD:AAB0146
Xestia ursae|[3315]|LCHP421-07|Canada|Manitoba|658[0n]|BOLD:AAB0146
Xestia ursae|[3316]|LCHP413-07|Canada|Manitoba|658[0n]|BOLD:AAB0146
Xestia ursae|[3317]|LCHP389-07|Canada|Manitoba|658[0n]|BOLD:AAB0146
Xestia ursae|[3318]|LCHP387-07|Canada|Manitoba|658[0n]|BOLD:AAB0146
Xestia ursae|[3319]|LCHP274-07|Canada|Manitoba|658[0n]|BOLD:AAB0146
Xestia ursae|[3320]|LCHP273-07|Canada|Manitoba|658[0n]|BOLD:AAB0146
Xestia ursae|[3321]|LCHP222-07|Canada|Manitoba|658[0n]|BOLD:AAB0146
Xestia ursae|[3322]|LCHP166-07|Canada|Manitoba|658[0n]|BOLD:AAB0146
Xestia ursae|[3323]|LCHP164-07|Canada|Manitoba|658[0n]|BOLD:AAB0146
Xestia ursae|[3324]|LCHP409-07|Canada|Manitoba|638[0n]|BOLD:AAB0146
Xestia ursae|[3325]|LCHP464-07|Canada|Manitoba|658[0n]|BOLD:AAB0146
Xestia ursae|[3326]|LCHQ903-08|Canada|Manitoba|658[0n]|BOLD:AAB0146
Xestia ursae|[3327]|LCHQ904-08|Canada|Manitoba|658[0n]|BOLD:AAB0146
Xestia ursae|[3328]|CNCLB1586-14|Russia||658[0n]|BOLD:AAB0146
Xestia ursae|[3329]|CNCLB1587-14|Russia||658[0n]|BOLD:AAB0146
Xestia intermedia|[3330]|RDNME519-08|Canada|Yukon Territory|658[0n]|BOLD:AAE4575
Xestia intermedia|[3331]|RDNMF048-08|Canada|British Columbia|658[0n]|BOLD:AAE4575
Xestia intermedia|[3332]|RDNMG691-08|Canada|Yukon Territory|642[0n]|BOLD:AAE4575
Xestia intermedia|[3333]|RDNMG692-08|Canada|Yukon Territory|642[0n]|BOLD:AAE4575
Xestia fergusoni|[3334]|CNCLB485-14|Canada|Yukon Territory|550[0n]|BOLD:ACQ8186
Xestia fergusoni|[3335]|CNCLB2546-14|United States|Alaska|658[1n]|BOLD:ACQ8186
Xestia bryanti|[3336]|RDNMF046-08|Canada|British Columbia|658[0n]|BOLD:AAE4633
Xestia bryanti|[3337]|RDNMG531-08|Canada|British Columbia|658[0n]|BOLD:AAE4633
Xestia bryanti|[3338]|RDNMG532-08|Canada|British Columbia|658[0n]|BOLD:AAE4633
Xestia bryanti|[3339]|RDNMG533-08|Canada|British Columbia|658[0n]|BOLD:AAE4633
Xestia laxa|[3340]|RDNMF071-08|Canada|Yukon Territory|658[0n]|BOLD:AAH9453
Xestia laxa|[3341]|RDNMG727-08|Canada|Yukon Territory|642[0n]|BOLD:AAH9453
Xestia mixta|[3342]|LCHQ030-07|Canada|Manitoba|642[0n]|BOLD:ABY7781
Xestia mixta|[3343]|LCHP234-07|Canada|Manitoba|658[0n]|BOLD:ABY7781
Xestia mixta|[3344]|LCHP669-07|Canada|Manitoba|658[0n]|BOLD:ABY7781
Xestia mixta|[3345]|LCHP699-07|Canada|Manitoba|658[0n]|BOLD:ABY7781
Xestia mixta|[3346]|LCHP647-07|Canada|Manitoba|658[0n]|BOLD:ABY7781
Xestia mixta|[3347]|LCHP665-07|Canada|Manitoba|658[0n]|BOLD:ABY7781
Xestia mixta|[3348]|LCHP624-07|Canada|Manitoba|658[0n]|BOLD:ABY7781
Xestia mixta|[3349]|LCHP604-07|Canada|Manitoba|658[0n]|BOLD:ABY7781
Xestia mixta|[3350]|LCHP596-07|Canada|Manitoba|658[0n]|BOLD:ABY7781
Xestia mixta|[3351]|LCHP517-07|Canada|Manitoba|658[0n]|BOLD:ABY7781
Xestia mixta|[3352]|LCHP516-07|Canada|Manitoba|658[0n]|BOLD:ABY7781
Xestia mixta|[3353]|LCHP513-07|Canada|Manitoba|658[0n]|BOLD:ABY7781
Xestia mixta|[3354]|LCHP494-07|Canada|Manitoba|658[0n]|BOLD:ABY7781
Xestia mixta|[3355]|LCHP484-07|Canada|Manitoba|658[0n]|BOLD:ABY7781
Xestia mixta|[3356]|LCHP480-07|Canada|Manitoba|658[0n]|BOLD:ABY7781
Xestia mixta|[3357]|LCHP309-07|Canada|Manitoba|658[0n]|BOLD:ABY7781
Xestia mixta|[3358]|LCHP748-07|Canada|Manitoba|655[0n]|BOLD:ABY7781
Xestia mixta|[3359]|LCHP757-07|Canada|Manitoba|658[0n]|BOLD:ABY7781
Xestia mixta|[3360]|LCHP767-07|Canada|Manitoba|658[0n]|BOLD:ABY7781
Xestia mixta|[3361]|LCHQ025-07|Canada|Manitoba|658[0n]|BOLD:ABY7781
Xestia mixta|[3362]|LCHQ027-07|Canada|Manitoba|658[0n]|BOLD:ABY7781
Xestia mixta|[3363]|LCHQ031-07|Canada|Manitoba|658[0n]|BOLD:ABY7781
Xestia mixta|[3364]|LCHQ034-07|Canada|Manitoba|658[0n]|BOLD:ABY7781
Xestia mixta|[3365]|LCHQ092-07|Canada|Manitoba|658[0n]|BOLD:ABY7781
Xestia mixta|[3366]|LCHQ095-07|Canada|Manitoba|658[0n]|BOLD:ABY7781
Xestia speciosa|[3367]|LCHP773-07|Canada|Manitoba|658[0n]|BOLD:ACF2639
Xestia speciosa|[3368]|LCHP865-07|Canada|Manitoba|658[0n]|BOLD:ACF2639
Xestia speciosa|[3369]|LCHQ475-08|Canada|Manitoba|657[0n]|BOLD:ACF2639
Xestia speciosa|[3370]|LCHQ700-08|Canada|Manitoba|658[0n]|BOLD:ACF2639
Xestia speciosa|[3371]|LCHQ701-08|Canada|Manitoba|658[0n]|BOLD:ACF2639
Xestia speciosa|[3372]|RDNMD007-06|Russia||658[0n]|BOLD:ACE4665
Xestia speciosa|[3373]|RDNMD006-06|Russia||658[0n]|BOLD:ACE4665
Xestia speciosa|[3374]|RDNMD008-06|Russia||658[0n]|BOLD:ACE4665
Xestia speciosa|[3375]|LBCG873-09|Canada|British Columbia|658[0n]|BOLD:AAA2273
Xestia speciosa|[3376]|LBCG2012-09|Canada|British Columbia|636[0n]|BOLD:AAA2273
Xestia speciosa|[3377]|LBCH1431-10|Canada|British Columbia|658[0n]|BOLD:AAA2273
Xestia speciosa|[3378]|LBCG2045-09|Canada|British Columbia|658[0n]|BOLD:AAA2273
Xestia speciosa|[3379]|LBCG2037-09|Canada|British Columbia|658[0n]|BOLD:AAA2273
Xestia speciosa|[3380]|LBCG2031-09|Canada|British Columbia|658[0n]|BOLD:AAA2273
Xestia speciosa|[3381]|LBCG2028-09|Canada|British Columbia|658[0n]|BOLD:AAA2273
Xestia speciosa|[3382]|LBCG2016-09|Canada|British Columbia|658[0n]|BOLD:AAA2273
Xestia speciosa|[3383]|LBCG2015-09|Canada|British Columbia|658[0n]|BOLD:AAA2273
Xestia speciosa|[3384]|LBCG580-09|Canada|British Columbia|658[0n]|BOLD:AAA2273
Xestia speciosa|[3385]|LBCH1436-10|Canada|British Columbia|638[0n]|BOLD:AAA2273
Xestia speciosa|[3386]|LBCG999-09|Canada|British Columbia|658[0n]|BOLD:AAA2273
Xestia speciosa|[3387]|LBCG1857-09|Canada|British Columbia|658[0n]|BOLD:AAA2273
Xestia speciosa|[3388]|LBCH4425-10|Canada|British Columbia|658[0n]|BOLD:AAA2273
Xestia speciosa|[3389]|LBCG1015-09|Canada|British Columbia|658[0n]|BOLD:AAA2273

Xestia speciosa|[3387]|LBCG1857-09|Canada|British Columbia|658[0n]|BOLD:AAA2273
Xestia speciosa|[3388]|LBCH4425-10|Canada|British Columbia|658[0n]|BOLD:AAA2273
Xestia speciosa|[3389]|LBCG1015-09|Canada|British Columbia|658[0n]|BOLD:AAA2273
Xestia speciosa|[3390]|LBCG2009-09|Canada|British Columbia|658[0n]|BOLD:AAA2273
Xestia speciosa|[3391]|LBCG2010-09|Canada|British Columbia|658[0n]|BOLD:AAA2273
Xestia speciosa|[3392]|LBCG2011-09|Canada|British Columbia|658[0n]|BOLD:AAA2273
Xestia speciosa|[3393]|LBCG2013-09|Canada|British Columbia|658[0n]|BOLD:AAA2273
Xestia speciosa|[3394]|LBCG2017-09|Canada|British Columbia|658[0n]|BOLD:AAA2273
Xestia speciosa|[3395]|LBCG2019-09|Canada|British Columbia|658[0n]|BOLD:AAA2273
Xestia speciosa|[3396]|LBCG2020-09|Canada|British Columbia|658[0n]|BOLD:AAA2273
Xestia speciosa|[3397]|LBCG2021-09|Canada|British Columbia|658[0n]|BOLD:AAA2273
Xestia speciosa|[3398]|LBCG2022-09|Canada|British Columbia|658[0n]|BOLD:AAA2273
Xestia speciosa|[3399]|LBCG2023-09|Canada|British Columbia|658[0n]|BOLD:AAA2273
Xestia speciosa|[3400]|LBCG2024-09|Canada|British Columbia|658[0n]|BOLD:AAA2273
Xestia speciosa|[3401]|LBCG2025-09|Canada|British Columbia|658[0n]|BOLD:AAA2273
Xestia speciosa|[3402]|LBCG2026-09|Canada|British Columbia|658[0n]|BOLD:AAA2273
Xestia speciosa|[3403]|LBCG2027-09|Canada|British Columbia|658[0n]|BOLD:AAA2273
Xestia speciosa|[3404]|LBCG2029-09|Canada|British Columbia|658[0n]|BOLD:AAA2273
Xestia speciosa|[3405]|LBCG2030-09|Canada|British Columbia|658[0n]|BOLD:AAA2273
Xestia speciosa|[3406]|LBCG2032-09|Canada|British Columbia|658[0n]|BOLD:AAA2273
Xestia speciosa|[3407]|LBCG2033-09|Canada|British Columbia|658[0n]|BOLD:AAA2273
Xestia speciosa|[3408]|LBCG2034-09|Canada|British Columbia|658[0n]|BOLD:AAA2273
Xestia speciosa|[3409]|LBCG2035-09|Canada|British Columbia|658[0n]|BOLD:AAA2273
Xestia speciosa|[3410]|LBCG2036-09|Canada|British Columbia|658[0n]|BOLD:AAA2273
Xestia speciosa|[3411]|LBCG2038-09|Canada|British Columbia|658[0n]|BOLD:AAA2273
Xestia speciosa|[3412]|LBCG2041-09|Canada|British Columbia|658[0n]|BOLD:AAA2273
Xestia speciosa|[3413]|LBCG2043-09|Canada|British Columbia|658[0n]|BOLD:AAA2273
Xestia speciosa|[3414]|LBCG2049-09|Canada|British Columbia|658[0n]|BOLD:AAA2273
Xestia speciosa|[3415]|LBCG2053-09|Canada|British Columbia|658[0n]|BOLD:AAA2273
Xestia speciosa|[3416]|LBCH109-10|Canada|British Columbia|658[0n]|BOLD:AAA2273
Xestia speciosa|[3417]|LBCH218-10|Canada|British Columbia|658[0n]|BOLD:AAA2273
Xestia speciosa|[3418]|LBCH3795-10|Canada|British Columbia|658[0n]|BOLD:AAA2273
Xestia speciosa|[3419]|LBCH4109-10|Canada|British Columbia|658[0n]|BOLD:AAA2273
Xestia speciosa|[3420]|LBCH4110-10|Canada|British Columbia|658[0n]|BOLD:AAA2273
Xestia speciosa|[3421]|LBCH4426-10|Canada|British Columbia|658[0n]|BOLD:AAA2273
Xestia speciosa|[3422]|BBLPB651-10|Canada|British Columbia|658[0n]|BOLD:AAA2273
Xestia speciosa|[3423]|BBLPB652-10|Canada|British Columbia|658[0n]|BOLD:AAA2273
Xestia speciosa|[3424]|BBLPB653-10|Canada|British Columbia|658[0n]|BOLD:AAA2273
Xestia speciosa|[3425]|BBLPB654-10|Canada|British Columbia|658[0n]|BOLD:AAA2273
Xestia speciosa|[3426]|BBLPB657-10|Canada|British Columbia|658[0n]|BOLD:AAA2273
Xestia speciosa|[3427]|BBLPB658-10|Canada|British Columbia|658[0n]|BOLD:AAA2273
Xestia speciosa|[3428]|LBCG2007-09|Canada|British Columbia|658[0n]|BOLD:AAA2273
Xestia speciosa|[3429]|LBCG2008-09|Canada|British Columbia|658[0n]|BOLD:AAA2273
Xestia speciosa|[3430]|LBCG1877-09|Canada|British Columbia|658[0n]|BOLD:AAA2273
Xestia speciosa|[3431]|LBCG2006-09|Canada|British Columbia|658[0n]|BOLD:AAA2273
Xestia speciosa|[3432]|LBCG1873-09|Canada|British Columbia|658[0n]|BOLD:AAA2273
Xestia speciosa|[3433]|LBCG1876-09|Canada|British Columbia|658[0n]|BOLD:AAA2273
Xestia speciosa|[3434]|LBCG1868-09|Canada|British Columbia|658[0n]|BOLD:AAA2273
Xestia speciosa|[3435]|LBCG1872-09|Canada|British Columbia|658[0n]|BOLD:AAA2273
Xestia speciosa|[3436]|LBCG1863-09|Canada|British Columbia|658[0n]|BOLD:AAA2273
Xestia speciosa|[3437]|LBCG1867-09|Canada|British Columbia|658[0n]|BOLD:AAA2273
Xestia speciosa|[3438]|LBCG1002-09|Canada|British Columbia|658[0n]|BOLD:AAA2273
Xestia speciosa|[3439]|LBCG1019-09|Canada|British Columbia|658[0n]|BOLD:AAA2273
Xestia speciosa|[3440]|LBCG997-09|Canada|British Columbia|658[0n]|BOLD:AAA2273
Xestia speciosa|[3441]|LBCG1001-09|Canada|British Columbia|658[0n]|BOLD:AAA2273
Xestia speciosa|[3442]|LBCG969-09|Canada|British Columbia|658[0n]|BOLD:AAA2273
Xestia speciosa|[3443]|LBCG996-09|Canada|British Columbia|658[0n]|BOLD:AAA2273
Xestia speciosa|[3444]|LBCG966-09|Canada|British Columbia|658[0n]|BOLD:AAA2273
Xestia speciosa|[3445]|LBCG968-09|Canada|British Columbia|658[0n]|BOLD:AAA2273
Xestia speciosa|[3446]|LBCG960-09|Canada|British Columbia|658[0n]|BOLD:AAA2273
Xestia speciosa|[3447]|LBCG965-09|Canada|British Columbia|658[0n]|BOLD:AAA2273
Xestia speciosa|[3448]|LBCG953-09|Canada|British Columbia|658[0n]|BOLD:AAA2273
Xestia speciosa|[3449]|LBCG954-09|Canada|British Columbia|658[0n]|BOLD:AAA2273
Xestia speciosa|[3450]|LPABC279-09|Canada|Alberta|658[0n]|BOLD:AAA2273
Xestia speciosa|[3451]|LPABC392-09|Canada|Alberta|658[0n]|BOLD:AAA2273
Xestia speciosa|[3452]|LBCG855-09|Canada|British Columbia|658[0n]|BOLD:AAA2273
Xestia speciosa|[3453]|LBCG870-09|Canada|British Columbia|658[0n]|BOLD:AAA2273
Xestia speciosa|[3454]|LBCG853-09|Canada|British Columbia|658[0n]|BOLD:AAA2273
Xestia speciosa|[3455]|LBCG854-09|Canada|British Columbia|658[0n]|BOLD:AAA2273
Xestia speciosa|[3456]|LBCG851-09|Canada|British Columbia|658[0n]|BOLD:AAA2273
Xestia speciosa|[3457]|LBCG852-09|Canada|British Columbia|658[0n]|BOLD:AAA2273
Xestia speciosa|[3458]|LBCG829-09|Canada|British Columbia|658[0n]|BOLD:AAA2273
Xestia speciosa|[3459]|LBCG839-09|Canada|British Columbia|658[0n]|BOLD:AAA2273
Xestia speciosa|[3460]|LBCG822-09|Canada|British Columbia|658[0n]|BOLD:AAA2273
Xestia speciosa|[3461]|LBCG823-09|Canada|British Columbia|658[0n]|BOLD:AAA2273
Xestia speciosa|[3462]|LBCG736-09|Canada|British Columbia|658[0n]|BOLD:AAA2273
Xestia speciosa|[3463]|LBCG761-09|Canada|British Columbia|658[0n]|BOLD:AAA2273
Xestia speciosa|[3464]|LBCG819-09|Canada|British Columbia|658[0n]|BOLD:AAA2273
Xestia speciosa|[3465]|LBCG820-09|Canada|British Columbia|658[0n]|BOLD:AAA2273
Xestia speciosa|[3466]|LBCG826-09|Canada|British Columbia|658[0n]|BOLD:AAA2273
Xestia speciosa|[3467]|LBCG827-09|Canada|British Columbia|658[0n]|BOLD:AAA2273
Xestia speciosa|[3468]|LBCG2314-09|Canada|British Columbia|658[0n]|BOLD:AAA2273
Xestia speciosa|[3469]|LBCG2372-09|Canada|British Columbia|658[0n]|BOLD:AAA2273
Xestia speciosa|[3470]|LBCH496-10|Canada|British Columbia|658[0n]|BOLD:AAA2273
Xestia speciosa|[3471]|LBCH497-10|Canada|British Columbia|658[0n]|BOLD:AAA2273
Xestia speciosa|[3472]|LBCH498-10|Canada|British Columbia|658[0n]|BOLD:AAA2273
Xestia speciosa|[3473]|LBCH499-10|Canada|British Columbia|658[0n]|BOLD:AAA2273
Xestia speciosa|[3474]|LBCH500-10|Canada|British Columbia|658[0n]|BOLD:AAA2273
Xestia speciosa|[3475]|LBCH501-10|Canada|British Columbia|658[0n]|BOLD:AAA2273
Xestia speciosa|[3476]|LBCH502-10|Canada|British Columbia|658[0n]|BOLD:AAA2273
Xestia speciosa|[3477]|LBCH503-10|Canada|British Columbia|658[0n]|BOLD:AAA2273
Xestia speciosa|[3478]|LBCH648-10|Canada|British Columbia|658[0n]|BOLD:AAA2273
Xestia speciosa|[3479]|LBCH815-10|Canada|British Columbia|658[0n]|BOLD:AAA2273
Xestia speciosa|[3480]|LBCH915-10|Canada|British Columbia|658[0n]|BOLD:AAA2273
Xestia speciosa|[3481]|LBCH935-10|Canada|British Columbia|658[0n]|BOLD:AAA2273
Xestia speciosa|[3482]|LBCH1107-10|Canada|British Columbia|658[0n]|BOLD:AAA2273
Xestia speciosa|[3483]|LBCH1166-10|Canada|British Columbia|658[0n]|BOLD:AAA2273
Xestia speciosa|[3484]|LBCG592-09|Canada|British Columbia|658[0n]|BOLD:AAA2273
Xestia speciosa|[3485]|LBCG641-09|Canada|British Columbia|658[0n]|BOLD:AAA2273
Xestia speciosa|[3486]|LBCG651-09|Canada|British Columbia|658[0n]|BOLD:AAA2273
Xestia speciosa|[3487]|LBCG692-09|Canada|British Columbia|658[0n]|BOLD:AAA2273
Xestia speciosa|[3488]|LBCG708-09|Canada|British Columbia|658[0n]|BOLD:AAA2273
Xestia speciosa|[3489]|LBCG709-09|Canada|British Columbia|658[0n]|BOLD:AAA2273

Xestia speciosa|[3487]|LBCG692-09|Canada|British Columbia|658[0n]|BOLD:AAA2273
Xestia speciosa|[3488]|LBCG708-09|Canada|British Columbia|658[0n]|BOLD:AAA2273
Xestia speciosa|[3489]|LBCG709-09|Canada|British Columbia|658[0n]|BOLD:AAA2273
Xestia speciosa|[3490]|LBCG710-09|Canada|British Columbia|658[0n]|BOLD:AAA2273
Xestia speciosa|[3491]|LBCG733-09|Canada|British Columbia|658[0n]|BOLD:AAA2273
Xestia speciosa|[3492]|LBCH1434-10|Canada|British Columbia|658[0n]|BOLD:AAA2273
Xestia speciosa|[3493]|LBCH1442-10|Canada|British Columbia|658[0n]|BOLD:AAA2273
Xestia speciosa|[3494]|LBCH1523-10|Canada|British Columbia|658[0n]|BOLD:AAA2273
Xestia speciosa|[3495]|LBCH1524-10|Canada|British Columbia|658[0n]|BOLD:AAA2273
Xestia speciosa|[3496]|LBCH1525-10|Canada|British Columbia|658[0n]|BOLD:AAA2273
Xestia speciosa|[3497]|LBCH1526-10|Canada|British Columbia|658[0n]|BOLD:AAA2273
Xestia speciosa|[3498]|LBCH1527-10|Canada|British Columbia|658[0n]|BOLD:AAA2273
Xestia speciosa|[3499]|LBCH1528-10|Canada|British Columbia|658[0n]|BOLD:AAA2273
Xestia speciosa|[3500]|LBCH1529-10|Canada|British Columbia|658[0n]|BOLD:AAA2273
Xestia speciosa|[3501]|LBCH1544-10|Canada|British Columbia|658[0n]|BOLD:AAA2273
Xestia speciosa|[3502]|LBCH1545-10|Canada|British Columbia|658[0n]|BOLD:AAA2273
Xestia speciosa|[3503]|LBCH1676-10|Canada|British Columbia|658[0n]|BOLD:AAA2273
Xestia speciosa|[3504]|LBCH1677-10|Canada|British Columbia|658[0n]|BOLD:AAA2273
Xestia speciosa|[3505]|LBCH1932-10|Canada|British Columbia|658[0n]|BOLD:AAA2273
Xestia speciosa|[3506]|LBCH1971-10|Canada|British Columbia|658[0n]|BOLD:AAA2273
Xestia speciosa|[3507]|LBCH2130-10|Canada|British Columbia|658[0n]|BOLD:AAA2273
Xestia speciosa|[3508]|LBCH3087-10|Canada|British Columbia|658[0n]|BOLD:AAA2273
Xestia speciosa|[3509]|LBCH3089-10|Canada|British Columbia|658[0n]|BOLD:AAA2273
Xestia speciosa|[3510]|LBCH3466-10|Canada|British Columbia|658[0n]|BOLD:AAA2273
Xestia speciosa|[3511]|LBCH3788-10|Canada|British Columbia|658[0n]|BOLD:AAA2273
Xestia speciosa|[3512]|LBCH4427-10|Canada|British Columbia|658[0n]|BOLD:AAA2273
Xestia speciosa|[3513]|LBCH4681-10|Canada|British Columbia|658[0n]|BOLD:AAA2273
Xestia speciosa|[3514]|BBLPB655-10|Canada|British Columbia|658[0n]|BOLD:AAA2273
Xestia speciosa|[3515]|BBLPB656-10|Canada|British Columbia|658[0n]|BOLD:AAA2273
Xestia speciosa|[3516]|LBCH1430-10|Canada|British Columbia|658[0n]|BOLD:AAA2273
Xestia speciosa|[3517]|LBCH1433-10|Canada|British Columbia|658[0n]|BOLD:AAA2273
Xestia speciosa|[3518]|LBCH1238-10|Canada|British Columbia|658[0n]|BOLD:AAA2273
Xestia speciosa|[3519]|LBCH1427-10|Canada|British Columbia|658[0n]|BOLD:AAA2273
Xestia speciosa|[3520]|LBCG587-09|Canada|British Columbia|658[0n]|BOLD:AAA2273
Xestia speciosa|[3521]|LBCG586-09|Canada|British Columbia|658[0n]|BOLD:AAA2273
Xestia speciosa|[3522]|LBCG584-09|Canada|British Columbia|658[0n]|BOLD:AAA2273
Xestia speciosa|[3523]|LBCG581-09|Canada|British Columbia|658[0n]|BOLD:AAA2273
Xestia speciosa|[3524]|LBCG578-09|Canada|British Columbia|658[0n]|BOLD:AAA2273
Xestia speciosa|[3525]|LBCG575-09|Canada|British Columbia|658[0n]|BOLD:AAA2273
Xestia speciosa|[3526]|LBCG574-09|Canada|British Columbia|658[0n]|BOLD:AAA2273
Xestia speciosa|[3527]|LBCG572-09|Canada|British Columbia|658[0n]|BOLD:AAA2273
Xestia speciosa|[3528]|LPABC197-09|Canada|Alberta|658[0n]|BOLD:AAA2273
Xestia speciosa|[3529]|LBCD452-05|Canada|British Columbia|658[0n]|BOLD:AAA2273
Xestia speciosa|[3530]|LBCH018-10|Canada|British Columbia|658[0n]|BOLD:AAA2273
Xestia speciosa|[3531]|LBCG1870-09|Canada|British Columbia|658[0n]|BOLD:AAA2273
Xestia speciosa|[3532]|LBCG2040-09|Canada|British Columbia|658[0n]|BOLD:AAA2273
Xestia speciosa|[3533]|LBCH1437-10|Canada|British Columbia|643[0n]|BOLD:AAA2273
Xestia speciosa|[3534]|LBCH347-10|Canada|British Columbia|625[0n]|BOLD:AAA2273
Xestia speciosa|[3535]|LBCH1522-10|Canada|British Columbia|636[0n]|BOLD:AAA2273
Xestia speciosa|[3536]|BBLPB659-10|Canada|British Columbia|658[0n]|BOLD:AAA2273
Xestia xanthographa|[3537]|CGUKB253-09|United Kingdom|England|658[0n]|BOLD:AAA6806
Xestia xanthographa|[3538]|LENOA194-11|France|Haute Normandie|658[1n]|BOLD:AAA6806
Xestia xanthographa|[3539]|CGUKC106-09|United Kingdom|England|658[0n]|BOLD:AAA6806
Xestia xanthographa|[3540]|CGUKA281-09|United Kingdom|England|658[0n]|BOLD:AAA6806
Xestia xanthographa|[3541]|GWOTA090-12|Spain|Comunidad Valenciana|577[0n]|BOLD:AAA6806
Xestia xanthographa|[3542]|LENOA193-11|France|Haute Normandie|658[0n]|BOLD:AAA6806
Xestia xanthographa|[3543]|RWWC063-10|United States|Washington|658[0n]|BOLD:AAA6806
Xestia xanthographa|[3544]|RWWC023-10|United States|Washington|658[0n]|BOLD:AAA6806
Xestia xanthographa|[3545]|RWWC022-10|United States|Washington|658[0n]|BOLD:AAA6806
Xestia xanthographa|[3546]|RWWC013-10|United States|Washington|658[0n]|BOLD:AAA6806
Xestia xanthographa|[3547]|RWWC001-10|United States|Washington|658[0n]|BOLD:AAA6806
Xestia xanthographa|[3548]|LEFIK427-10|Finland||658[0n]|BOLD:AAA6806
Xestia xanthographa|[3549]|LALPA704-10|Canada|British Columbia|658[0n]|BOLD:AAA6806
Xestia xanthographa|[3550]|LALPA682-10|Canada|British Columbia|658[0n]|BOLD:AAA6806
Xestia xanthographa|[3551]|LALPA640-10|Canada|British Columbia|658[0n]|BOLD:AAA6806
Xestia xanthographa|[3552]|LALPA607-10|Canada|British Columbia|658[0n]|BOLD:AAA6806
Xestia xanthographa|[3553]|LEFIF677-10|Finland||658[0n]|BOLD:AAA6806
Xestia xanthographa|[3554]|LEFIC504-10|Finland|Aland Islands|658[0n]|BOLD:AAA6806
Xestia xanthographa|[3555]|PHLAA693-09|Italy||658[0n]|BOLD:AAA6806
Xestia xanthographa|[3556]|GWORO984-09|Germany|Bavaria|658[0n]|BOLD:AAA6806
Xestia xanthographa|[3557]|RWWB307-09|United States|Washington|658[0n]|BOLD:AAA6806
Xestia xanthographa|[3558]|CGUKA1010-09|United Kingdom|England|657[0n]|BOLD:AAA6806
Xestia xanthographa|[3559]|RWWB277-09|United States|Washington|658[0n]|BOLD:AAA6806
Xestia xanthographa|[3560]|RWWB276-09|United States|Washington|658[0n]|BOLD:AAA6806
Xestia xanthographa|[3561]|RWWB273-09|United States|Washington|658[0n]|BOLD:AAA6806
Xestia xanthographa|[3562]|RWWB231-09|United States|Washington|658[0n]|BOLD:AAA6806
Xestia xanthographa|[3563]|RWWB230-09|United States|Washington|658[0n]|BOLD:AAA6806
Xestia xanthographa|[3564]|RWWB214-09|United States|Washington|658[0n]|BOLD:AAA6806
Xestia xanthographa|[3565]|RWWB208-09|United States|Washington|658[0n]|BOLD:AAA6806
Xestia xanthographa|[3566]|RWWB188-09|United States|Washington|658[0n]|BOLD:AAA6806
Xestia xanthographa|[3567]|RWWB165-09|United States|Washington|658[0n]|BOLD:AAA6806
Xestia xanthographa|[3568]|CGUKD448-09|United Kingdom|England|658[0n]|BOLD:AAA6806
Xestia xanthographa|[3569]|CGUKB921-09|United Kingdom|Wales|658[0n]|BOLD:AAA6806
Xestia xanthographa|[3570]|CGUKB618-09|United Kingdom|England|658[0n]|BOLD:AAA6806
Xestia xanthographa|[3571]|CGUKB368-09|United Kingdom|England|658[0n]|BOLD:AAA6806
Xestia xanthographa|[3572]|CGUKA718-09|United Kingdom||658[0n]|BOLD:AAA6806
Xestia xanthographa|[3573]|LPVIB824-08|Canada|British Columbia|658[0n]|BOLD:AAA6806
Xestia xanthographa|[3574]|LPVIB805-08|Canada|British Columbia|658[0n]|BOLD:AAA6806
Xestia xanthographa|[3575]|LPVIB746-08|Canada|British Columbia|658[0n]|BOLD:AAA6806
Xestia xanthographa|[3576]|LPVIB744-08|Canada|British Columbia|658[0n]|BOLD:AAA6806
Xestia xanthographa|[3577]|LPVIB702-08|Canada|British Columbia|658[0n]|BOLD:AAA6806
Xestia xanthographa|[3578]|LPVIB572-08|Canada|British Columbia|658[0n]|BOLD:AAA6806
Xestia xanthographa|[3579]|LPVIB557-08|Canada|British Columbia|658[0n]|BOLD:AAA6806
Xestia xanthographa|[3580]|LPVIB556-08|Canada|British Columbia|658[0n]|BOLD:AAA6806
Xestia xanthographa|[3581]|LPVIB504-08|Canada|British Columbia|658[0n]|BOLD:AAA6806
Xestia xanthographa|[3582]|LPVIB004-08|Canada|British Columbia|658[0n]|BOLD:AAA6806
Xestia xanthographa|[3583]|LBCW043-08|Canada|British Columbia|658[0n]|BOLD:AAA6806
Xestia xanthographa|[3584]|LBCW042-08|Canada|British Columbia|658[0n]|BOLD:AAA6806
Xestia xanthographa|[3585]|RDNMF011-08|Canada|British Columbia|658[0n]|BOLD:AAA6806
Xestia xanthographa|[3586]|LHLEP441-06|Canada|British Columbia|658[0n]|BOLD:AAA6806
Xestia xanthographa|[3587]|LHLEP392-06|Canada|British Columbia|658[0n]|BOLD:AAA6806
Xestia xanthographa|[3588]|LHLEP391-06|Canada|British Columbia|658[0n]|BOLD:AAA6806
Xestia xanthographa|[3589]|LHLEP[illegible]-06|Canada|British Columbia|658[0n]|BOLD:AAA6806

Xestia xanthographa|[3586]|LHLEP441-06|Canada|British Columbia|658[0n]|BOLD:AAA6806
Xestia xanthographa|[3587]|LHLEP392-06|Canada|British Columbia|658[0n]|BOLD:AAA6806
Xestia xanthographa|[3588]|LHLEP391-06|Canada|British Columbia|658[0n]|BOLD:AAA6806
Xestia xanthographa|[3589]|LHLEP207-06|Canada|British Columbia|658[0n]|BOLD:AAA6806
Xestia xanthographa|[3590]|LHLEP206-06|Canada|British Columbia|658[0n]|BOLD:AAA6806
Xestia xanthographa|[3591]|LHLEP205-06|Canada|British Columbia|658[0n]|BOLD:AAA6806
Xestia xanthographa|[3592]|LHLEP204-06|Canada|British Columbia|658[0n]|BOLD:AAA6806
Xestia xanthographa|[3593]|LHLEP203-06|Canada|British Columbia|658[0n]|BOLD:AAA6806
Xestia xanthographa|[3594]|LHLEP202-06|Canada|British Columbia|658[0n]|BOLD:AAA6806
Xestia xanthographa|[3595]|LHLEP201-06|Canada|British Columbia|658[0n]|BOLD:AAA6806
Xestia xanthographa|[3596]|LHLEP200-06|Canada|British Columbia|658[0n]|BOLD:AAA6806
Xestia xanthographa|[3597]|LHLEP199-06|Canada|British Columbia|658[0n]|BOLD:AAA6806
Xestia xanthographa|[3598]|LHLEP198-06|Canada|British Columbia|658[0n]|BOLD:AAA6806
Xestia xanthographa|[3599]|LHLEP197-06|Canada|British Columbia|658[0n]|BOLD:AAA6806
Xestia xanthographa|[3600]|LHLEP196-06|Canada|British Columbia|658[0n]|BOLD:AAA6806
Xestia xanthographa|[3601]|LHLEP195-06|Canada|British Columbia|658[0n]|BOLD:AAA6806
Xestia xanthographa|[3602]|LHLEP194-06|Canada|British Columbia|658[0n]|BOLD:AAA6806
Xestia xanthographa|[3603]|LHLEP193-06|Canada|British Columbia|658[0n]|BOLD:AAA6806
Xestia xanthographa|[3604]|LPVIB650-08|Canada|British Columbia|572[0n]|BOLD:AAA6806
Xestia xanthographa|[3605]|LPVIB668-08|Canada|British Columbia|633[0n]|BOLD:AAA6806
Xestia xanthographa|[3606]|LPVIB659-08|Canada|British Columbia|632[0n]|BOLD:AAA6806
Xestia xanthographa|[3607]|CGUKB019-09|United Kingdom|England|630[0n]|BOLD:AAA6806
Xestia xanthographa|[3608]|LPVIB653-08|Canada|British Columbia|636[0n]|BOLD:AAA6806
Xestia xanthographa|[3609]|LPVIB647-08|Canada|British Columbia|636[0n]|BOLD:AAA6806
Xestia xanthographa|[3610]|LPVIB597-08|Canada|British Columbia|636[0n]|BOLD:AAA6806
Xestia xanthographa|[3611]|CGUKA303-09|United Kingdom|England|636[0n]|BOLD:AAA6806
Xestia xanthographa|[3612]|LPVIB651-08|Canada|British Columbia|610[0n]|BOLD:AAA6806
Xestia xanthographa|[3613]|LPVIB655-08|Canada|British Columbia|573[0n]|BOLD:AAA6806
Xestia xanthographa|[3614]|CGUKA832-09|United Kingdom||635[0n]|BOLD:AAA6806
Xestia xanthographa|[3615]|LPVIB648-08|Canada|British Columbia|634[0n]|BOLD:AAA6806
Xestia xanthographa|[3616]|LPVIB656-08|Canada|British Columbia|623[0n]|BOLD:AAA6806
Xestia xanthographa|[3617]|LPVIB652-08|Canada|British Columbia|623[0n]|BOLD:AAA6806
Xestia xanthographa|[3618]|LPVIB649-08|Canada|British Columbia|623[0n]|BOLD:AAA6806
Xestia xanthographa|[3619]|CGUKB018-09|United Kingdom|England|603[0n]|BOLD:AAA6806
Xestia xanthographa|[3620]|RWWB232-09|United States|Washington|624[0n]|BOLD:AAA6806
Xestia xanthographa|[3621]|RWWB296-09|United States|Washington|620[1n]|BOLD:AAA6806
Xestia xanthographa|[3622]|LENOA195-11|France|Haute Normandie|658[0n]|BOLD:AAA6806
Xestia xanthographa|[3623]|RWWC695-11|United States|Washington|658[0n]|BOLD:AAA6806
Xestia xanthographa|[3624]|RWWC752-11|United States|Washington|658[0n]|BOLD:AAA6806
Xestia xanthographa|[3625]|RWWC791-11|United States|Washington|658[0n]|BOLD:AAA6806
Xestia xanthographa|[3626]|LALPA1245-11|Canada|British Columbia|658[0n]|BOLD:AAA6806
Xestia xanthographa|[3627]|NLLEA1042-12|Netherlands|South Holland|658[0n]|BOLD:AAA6806
Xestia xanthographa|[3628]|GWOTF339-12|Italy|Basilicata|658[0n]|BOLD:AAA6806
Xestia xanthographa|[3629]|LEATC567-13|Austria|Vorarlberg|658[0n]|BOLD:AAA6806
Xestia xanthographa|[3630]|GBLAC316-13|Germany|Thuringia|658[0n]|BOLD:AAA6806
Xestia xanthographa|[3631]|GBLAB605-13|Germany|Brandenburg|658[0n]|BOLD:AAA6806
Xestia xanthographa|[3632]|GWOTL256-13|Germany|Saarland|658[0n]|BOLD:AAA6806
Xestia xanthographa|[3633]|GWOTL255-13|Germany|Saarland|658[0n]|BOLD:AAA6806
Xestia xanthographa|[3634]|GWOTL117-13|Germany|Saarland|658[0n]|BOLD:AAA6806
Xestia xanthographa|[3635]|GWOTL116-13|Germany|Saarland|658[0n]|BOLD:AAA6806
Xestia xanthographa|[3636]|IBLAO675-12|Spain|Castilla-La Mancha|658[0n]|BOLD:AAA6806
Xestia xanthographa|[3637]|NLLEA1198-12|Netherlands|Overijssel|658[0n]|BOLD:AAA6806
Xestia xanthographa|[3638]|LON891-11|Norway||657[0n]|BOLD:AAA6806
Xestia xanthographa|[3639]|IBLAO080-11|Spain|Murcia|658[0n]|BOLD:AAA6806
Xestia xanthographa|[3640]|GWORK384-09|Germany|Bavaria|658[0n]|BOLD:AAA6806
Xestia xanthographa|[3641]|LON276-08|Norway|Akershus|657[0n]|BOLD:AAA6806
Xestia xanthographa|[3642]|CGUKA282-09|United Kingdom|England|632[0n]|BOLD:AAA6806
Xestia xanthographa|[3643]|NLLEA1104-12|Netherlands|South Holland|615[0n]|BOLD:AAA6806
Xestia xanthographa|[3644]|RWWC1279-13|United States|Washington|594[0n]|BOLD:AAA6806
Xestia xanthographa|[3645]|GBLAC353-13|Germany|Saxony|658[0n]|BOLD:AAA6806
Xestia xanthographa|[3646]|GBLAF570-14|Germany|Brandenburg|658[0n]|BOLD:AAA6806
Xestia xanthographa|[3647]|GBLAA1013-14|Germany|North Rhine-Westphalia|658[0n]|BOLD:AAA6806
Xestia xanthographa|[3648]|ABOLA180-14|Austria||658[0n]|BOLD:AAA6806
Xestia conchis|[3649]|NAMUM057-08|United States|Arizona|657[1n]|BOLD:AAF0960
Xestia conchis|[3650]|RDNMD859-07|United States|New Mexico|655[0n]|BOLD:AAF0960
Xestia conchis|[3651]|RDNMD858-07|United States|Arizona|655[0n]|BOLD:AAF0960
Xestia conchis|[3652]|RDNMJ723-11|United States|Arizona|658[0n]|BOLD:AAF0960
Xestia conchis|[3653]|IAWLB551-11|United States|Arizona|658[0n]|BOLD:AAF0960
Xestia conchis|[3654]|IAWLB552-11|United States|Arizona|658[0n]|BOLD:AAF0960
Xestia conchis|[3655]|IAWLB553-11|United States|Arizona|658[0n]|BOLD:AAF0960
Xestia conchis|[3656]|IAWLB554-11|United States|Arizona|658[0n]|BOLD:AAF0960
Xestia oblata|[3657]|LCH522-04|Canada|Manitoba|658[0n]|BOLD:AAA5964
Xestia oblata|[3658]|LCH521-04|Canada|Manitoba|658[1n]|BOLD:AAA5964
Xestia oblata|[3659]|RDLQB094-05|Canada|Quebec|658[0n]|BOLD:AAA5964
Xestia oblata|[3660]|RDLQB093-05|Canada|Quebec|589[0n]|BOLD:AAA5964
Xestia oblata|[3661]|RDLQB095-05|Canada|Quebec|658[0n]|BOLD:AAA5964
Xestia oblata|[3662]|RDLQB096-05|Canada|Quebec|658[0n]|BOLD:AAA5964
Xestia oblata|[3663]|RDLQB097-05|Canada|Quebec|658[0n]|BOLD:AAA5964
Xestia oblata|[3664]|RDLQB098-05|Canada|Quebec|658[0n]|BOLD:AAA5964
Xestia oblata|[3665]|BBLEC096-09|Canada|Nova Scotia|658[0n]|BOLD:AAA5964
Xestia oblata|[3666]|BBLPC584-09|Canada|Nova Scotia|658[0n]|BOLD:AAA5964
Xestia oblata|[3667]|BBLPC225-09|Canada|Nova Scotia|658[0n]|BOLD:AAA5964
Xestia oblata|[3668]|BBLPC211-09|Canada|Nova Scotia|658[0n]|BOLD:AAA5964
Xestia oblata|[3669]|BBLPC224-09|Canada|Nova Scotia|638[0n]|BOLD:AAA5964
Xestia oblata|[3670]|BBLPC606-09|Canada|Nova Scotia|639[0n]|BOLD:AAA5964
Xestia oblata|[3671]|BBLPC896-09|Canada|Newfoundland and Labrador|658[0n]|BOLD:AAA5964
Xestia oblata|[3672]|BBLPC935-09|Canada|Newfoundland and Labrador|658[0n]|BOLD:AAA5964
Xestia oblata|[3673]|BBLPE018-09|Canada|Nova Scotia|658[0n]|BOLD:AAA5964
Xestia oblata|[3674]|BBLPE350-09|Canada|Newfoundland and Labrador|658[0n]|BOLD:AAA5964
Xestia oblata|[3675]|BBLPE383-09|Canada|Newfoundland and Labrador|658[0n]|BOLD:AAA5964
Xestia oblata|[3676]|LOWCC885-05|Canada|British Columbia|588[0n]|BOLD:AAA5964
Xestia oblata|[3677]|LOWC132-05|Canada|British Columbia|590[0n]|BOLD:AAA5964
Xestia oblata|[3678]|BBLPB611-10|Canada|Alberta|658[0n]|BOLD:AAA5964
Xestia oblata|[3679]|BBLPB610-10|Canada|British Columbia|658[0n]|BOLD:AAA5964
Xestia oblata|[3680]|BBLPB608-10|Canada|British Columbia|658[0n]|BOLD:AAA5964
Xestia oblata|[3681]|BBLPB607-10|Canada|British Columbia|658[0n]|BOLD:AAA5964
Xestia oblata|[3682]|BBLPB503-10|Canada|British Columbia|658[0n]|BOLD:AAA5964
Xestia oblata|[3683]|LBCH348-10|Canada|British Columbia|658[0n]|BOLD:AAA5964
Xestia oblata|[3684]|LPABB350-08|Canada|Alberta|658[0n]|BOLD:AAA5964
Xestia oblata|[3685]|LPABB072-08|Canada|Alberta|658[0n]|BOLD:AAA5964
Xestia oblata|[3686]|LPAB010-08|Canada|Alberta|658[0n]|BOLD:AAA5964
Xestia oblata|[3687]|LPMN581-08|Canada|Manitoba|658[0n]|BOLD:AAA5964
Xestia oblata|[3688]|LOWCE843-06|Canada|British Columbia|658[0n]|BOLD:AAA5964

Xestia oblata|[3686]|LPAB010-08|Canada|Alberta|658[0n]|BOLD:AAA5964
Xestia oblata|[3687]|LPMN581-08|Canada|Manitoba|658[0n]|BOLD:AAA5964
Xestia oblata|[3688]|LOWCE843-06|Canada|British Columbia|658[0n]|BOLD:AAA5964
Xestia oblata|[3689]|LOWCD793-06|Canada|British Columbia|657[0n]|BOLD:AAA5964
Xestia oblata|[3690]|LOWC131-05|Canada|British Columbia|658[0n]|BOLD:AAA5964
Xestia oblata|[3691]|LOWC130-05|Canada|British Columbia|658[0n]|BOLD:AAA5964
Xestia oblata|[3692]|LOWC129-05|Canada|British Columbia|658[0n]|BOLD:AAA5964
Xestia oblata|[3693]|LBCC640-05|Canada|British Columbia|658[0n]|BOLD:AAA5964
Xestia oblata|[3694]|LBCD317-05|Canada|British Columbia|658[0n]|BOLD:AAA5964
Xestia oblata|[3695]|BBLPB609-10|Canada|British Columbia|658[0n]|BOLD:AAA5964
Xestia oblata|[3696]|LCH284-04|Canada|Manitoba|658[0n]|BOLD:AAA5964
Xestia oblata|[3697]|CNWLM037-13|Canada|Alberta|531[3n]|BOLD:AAA5964
Xestia oblata|[3698]|LPABC076-09|Canada|Alberta|658[1n]|BOLD:AAA5964
Xestia oblata|[3699]|LOWCD794-06|Canada|British Columbia|570[0n]|BOLD:AAA5964
Xestia oblata|[3700]|LOWC124-05|Canada|British Columbia|591[0n]|BOLD:AAA5964
Xestia oblata|[3701]|LOWCD790-06|Canada|British Columbia|658[0n]|BOLD:AAA5964
Xestia oblata|[3702]|LOWCD791-06|Canada|British Columbia|658[0n]|BOLD:AAA5964
Xestia oblata|[3703]|LOWC123-05|Canada|British Columbia|658[0n]|BOLD:AAA5964
Xestia oblata|[3704]|LOWC122-05|Canada|British Columbia|658[0n]|BOLD:AAA5964
Xestia oblata|[3705]|LOWC121-05|Canada|British Columbia|658[0n]|BOLD:AAA5964
Xestia oblata|[3706]|LOWC120-05|Canada|British Columbia|658[0n]|BOLD:AAA5964
Xestia oblata|[3707]|LBCD313-05|Canada|British Columbia|658[0n]|BOLD:AAA5964
Xestia oblata|[3708]|LBCC341-05|Canada|British Columbia|658[0n]|BOLD:AAA5964
Xestia oblata|[3709]|LBCB550-05|Canada|British Columbia|658[0n]|BOLD:AAA5964
Xestia oblata|[3710]|LOWC125-05|Canada|British Columbia|582[0n]|BOLD:AAA5964
Xestia oblata|[3711]|LOWC126-05|Canada|British Columbia|578[0n]|BOLD:AAA5964
Xestia oblata|[3712]|LOWC127-05|Canada|British Columbia|658[0n]|BOLD:AAA5964
Xestia oblata|[3713]|LOWC128-05|Canada|British Columbia|658[0n]|BOLD:AAA5964
Xestia oblata|[3714]|LOWCD789-06|Canada|British Columbia|657[0n]|BOLD:AAA5964
Xestia oblata|[3715]|LOWCD795-06|Canada|British Columbia|657[0n]|BOLD:AAA5964
Xestia oblata|[3716]|LOWCD796-06|Canada|British Columbia|657[0n]|BOLD:AAA5964
Xestia oblata|[3717]|LOWCD797-06|Canada|British Columbia|657[0n]|BOLD:AAA5964
Xestia oblata|[3718]|LPMN903-08|Canada|Alberta|658[0n]|BOLD:AAA5964
Xestia oblata|[3719]|LPAB088-08|Canada|Alberta|658[0n]|BOLD:AAA5964
Xestia oblata|[3720]|LPABB367-08|Canada|Alberta|658[0n]|BOLD:AAA5964
Xestia oblata|[3721]|LPABB474-08|Canada|Alberta|658[0n]|BOLD:AAA5964
Xestia oblata|[3722]|LPABC803-09|Canada|Alberta|658[0n]|BOLD:AAA5964
Xestia oblata|[3723]|LPABC929-09|Canada|Alberta|658[0n]|BOLD:AAA5964
Xestia oblata|[3724]|LBCH1567-10|Canada|British Columbia|658[0n]|BOLD:AAA5964
Xestia oblata|[3725]|LBCH1867-10|Canada|British Columbia|658[0n]|BOLD:AAA5964
Xestia oblata|[3726]|LBCH2029-10|Canada|British Columbia|658[0n]|BOLD:AAA5964
Xestia oblata|[3727]|LBCH6220-10|Canada|British Columbia|658[0n]|BOLD:AAA5964
Xestia oblata|[3728]|LALPA507-10|Canada|British Columbia|658[0n]|BOLD:AAA5964
Xestia oblata|[3729]|LALPA612-10|Canada|British Columbia|658[0n]|BOLD:AAA5964
Xestia oblata|[3730]|BBLPB606-10|Canada|British Columbia|658[0n]|BOLD:AAA5964
Xestia oblata|[3731]|LALPA896-11|Canada|British Columbia|658[0n]|BOLD:AAA5964
Xestia oblata|[3732]|LALPA1234-11|Canada|British Columbia|658[0n]|BOLD:AAA5964
Xestia oblata|[3733]|LOWCD792-06|Canada|British Columbia|658[0n]|BOLD:AAA5964
Xestia oblata|[3734]|CNWLN1059-13|Canada|Alberta|614[0n]|BOLD:AAA5964
Xestia normanianus|[3735]|XAH135-05|Canada|Ontario|658[1n]|BOLD:ABY7124
Xestia normanianus|[3736]|BBLEC935-09|Canada|Nova Scotia|658[0n]|BOLD:ABY7124
Xestia normanianus|[3737]|BBLEC186-09|Canada|Nova Scotia|638[0n]|BOLD:ABY7124
Xestia normanianus|[3738]|BBLEC943-09|Canada|Nova Scotia|621[4n]|BOLD:ABY7124
Xestia normanianus|[3739]|LSEU161-06|United States|North Carolina|658[0n]|BOLD:AAA8650
Xestia normanianus|[3740]|XAG898-05|Canada|Ontario|658[0n]|BOLD:AAA8650
Xestia normanianus|[3741]|LNCNW001-06|United States|North Carolina|658[0n]|BOLD:AAA8650
Xestia normanianus|[3742]|LPSOD972-09|Canada|Ontario|658[0n]|BOLD:AAA8650
Xestia normanianus|[3743]|BBLEC947-09|Canada|Nova Scotia|614[0n]|BOLD:AAA8650
Xestia normanianus|[3744]|TTMNB464-06|Canada|New Brunswick|658[0n]|BOLD:AAA8650
Xestia normanianus|[3745]|BBLEC942-09|Canada|Nova Scotia|607[0n]|BOLD:AAA8650
Xestia normanianus|[3746]|XAD174-04|Canada|Ontario|658[0n]|BOLD:AAA8650
Xestia normanianus|[3747]|XAD359-04|Canada|Ontario|572[1n]|BOLD:AAA8650
Xestia normanianus|[3748]|HESEP1384-12|Canada|Ontario|604[0n]|BOLD:AAA8650
Xestia normanianus|[3749]|XAH050-05|Canada|Ontario|658[1n]|BOLD:AAA8650
Xestia normanianus|[3750]|XAG446-05|Canada|Ontario|658[1n]|BOLD:AAA8650
Xestia normanianus|[3751]|BBLEC040-09|Canada|New Brunswick|658[0n]|BOLD:AAA8650
Xestia normanianus|[3752]|LNCNW003-06|United States|North Carolina|658[0n]|BOLD:AAA8650
Xestia normanianus|[3753]|LPMNB440-09|Canada|Manitoba|658[0n]|BOLD:AAA8650
Xestia normanianus|[3754]|BBLEC014-09|Canada|New Brunswick|658[0n]|BOLD:AAA8650
Xestia normanianus|[3755]|BBLEC036-09|Canada|New Brunswick|658[0n]|BOLD:AAA8650
Xestia normanianus|[3756]|BBLEC044-09|Canada|New Brunswick|658[0n]|BOLD:AAA8650
Xestia normanianus|[3757]|BBLEC047-09|Canada|New Brunswick|658[0n]|BOLD:AAA8650
Xestia normanianus|[3758]|BBLEC495-09|Canada|New Brunswick|658[0n]|BOLD:AAA8650
Xestia normanianus|[3759]|BBLEC898-09|Canada|Nova Scotia|658[0n]|BOLD:AAA8650
Xestia normanianus|[3760]|BBLEC920-09|Canada|Nova Scotia|658[0n]|BOLD:AAA8650
Xestia normanianus|[3761]|BBLPC086-09|Canada|New Brunswick|658[0n]|BOLD:AAA8650
Xestia normanianus|[3762]|PHAUG1795-11|Canada|Ontario|658[0n]|BOLD:AAA8650
Xestia normanianus|[3763]|NAMUM349-08|United States|Maryland|658[0n]|BOLD:AAA8650
Xestia normanianus|[3764]|LNCNW002-06|United States|North Carolina|658[0n]|BOLD:AAA8650
Xestia normanianus|[3765]|TMNBB411-06|Canada|New Brunswick|658[0n]|BOLD:AAA8650
Xestia normanianus|[3766]|TTMNB463-06|Canada|New Brunswick|658[0n]|BOLD:AAA8650
Xestia normanianus|[3767]|TTMNB462-06|Canada|New Brunswick|658[0n]|BOLD:AAA8650
Xestia normanianus|[3768]|LSEU160-06|United States|North Carolina|658[0n]|BOLD:AAA8650
Xestia normanianus|[3769]|RDLQB644-05|Canada|Quebec|658[0n]|BOLD:AAA8650
Xestia normanianus|[3770]|XAH136-05|Canada|Ontario|658[0n]|BOLD:AAA8650
Xestia normanianus|[3771]|RDLQB643-05|Canada|Quebec|577[0n]|BOLD:AAA8650
Xestia normanianus|[3772]|XAG804-05|Canada|Ontario|658[0n]|BOLD:AAA8650
Xestia normanianus|[3773]|XAG802-05|Canada|Ontario|658[0n]|BOLD:AAA8650
Xestia normanianus|[3774]|XAD376-04|Canada|Ontario|658[0n]|BOLD:AAA8650
Xestia normanianus|[3775]|XAD375-04|Canada|Ontario|658[0n]|BOLD:AAA8650
Xestia normanianus|[3776]|BBLPC108-09|Canada|New Brunswick|633[0n]|BOLD:AAA8650
Xestia normanianus|[3777]|BBLEC515-09|Canada|New Brunswick|633[0n]|BOLD:AAA8650
Xestia normanianus|[3778]|PHAUG1788-11|Canada|Ontario|611[0n]|BOLD:AAA8650
Xestia normanianus|[3779]|PAAUG2539-12|Canada|Ontario|528[0n]|
Xestia normanianus|[3780]|SSFDB2379-14|Canada|New Brunswick|591[0n]|BOLD:AAA8650
Xestia smithii|[3781]|XAH126-05|Canada|Ontario|658[0n]|BOLD:AAA2590
Xestia smithii|[3782]|BBLPC374-09|Canada|New Brunswick|658[0n]|BOLD:AAA2590
Xestia smithii|[3783]|JSAUG1680-11|Canada|Ontario|658[0n]|BOLD:AAA2590
Xestia smithii|[3784]|TMNBB371-06|Canada|New Brunswick|658[0n]|BOLD:AAA2590
Xestia smithii|[3785]|RDLQG063-06|Canada|Quebec|658[0n]|BOLD:AAA2590
Xestia smithii|[3786]|MJMSL148-10|United States|Massachusetts|658[0n]|BOLD:AAA2590
Xestia smithii|[3787]|XAG994-05|Canada|Ontario|637[0n]|BOLD:AAA2590
Xestia smithii|[3788]|LPMNB329-09|Canada|Manitoba|658[0n]|BOLD:AAA2590

Xestia smithii|[3786]|MJMSL148-10|United States|Massachusetts|658[0n]|BOLD:AAA2590
Xestia smithii|[3787]|XAG994-05|Canada|Ontario|637[0n]|BOLD:AAA2590
Xestia smithii|[3788]|LPMNB329-09|Canada|Manitoba|658[0n]|BOLD:AAA2590
Xestia smithii|[3789]|LPMNB482-09|Canada|Manitoba|658[0n]|BOLD:AAA2590
Xestia smithii|[3790]|BBLEC007-09|Canada|New Brunswick|658[0n]|BOLD:AAA2590
Xestia smithii|[3791]|PHAUG1783-11|Canada|Ontario|658[0n]|BOLD:AAA2590
Xestia smithii|[3792]|PHAUG1785-11|Canada|Ontario|658[0n]|BOLD:AAA2590
Xestia smithii|[3793]|PHAUG1792-11|Canada|Ontario|658[0n]|BOLD:AAA2590
Xestia smithii|[3794]|PHAUG1794-11|Canada|Ontario|658[0n]|BOLD:AAA2590
Xestia smithii|[3795]|PHAUG1791-11|Canada|Ontario|658[0n]|BOLD:AAA2590
Xestia smithii|[3796]|LBCH951-10|Canada|British Columbia|658[0n]|BOLD:AAA2590
Xestia smithii|[3797]|LPABC014-09|Canada|Alberta|634[1n]|BOLD:AAA2590
Xestia smithii|[3798]|LOWC785-05|Canada|British Columbia|658[0n]|BOLD:AAA2590
Xestia smithii|[3799]|LOWC788-05|Canada|British Columbia|658[0n]|BOLD:AAA2590
Xestia smithii|[3800]|LOWC789-05|Canada|British Columbia|658[0n]|BOLD:AAA2590
Xestia smithii|[3801]|LOWCD819-06|Canada|British Columbia|657[0n]|BOLD:AAA2590
Xestia smithii|[3802]|LOWCD821-06|Canada|British Columbia|657[0n]|BOLD:AAA2590
Xestia smithii|[3803]|LOWCD834-06|Canada|British Columbia|658[0n]|BOLD:AAA2590
Xestia smithii|[3804]|LPABB516-08|Canada|Alberta|658[0n]|BOLD:AAA2590
Xestia smithii|[3805]|LBCH1103-10|Canada|British Columbia|658[0n]|BOLD:AAA2590
Xestia smithii|[3806]|LHLEP569-06|Canada|British Columbia|658[0n]|BOLD:AAA2590
Xestia smithii|[3807]|LBCH3345-10|Canada|British Columbia|658[0n]|BOLD:AAA2590
Xestia smithii|[3808]|RWWB053-09|United States|Washington|658[0n]|BOLD:AAA2590
Xestia smithii|[3809]|RWWB109-09|United States|Washington|658[0n]|BOLD:AAA2590
Xestia smithii|[3810]|RWWC028-10|United States|Washington|658[0n]|BOLD:AAA2590
Xestia smithii|[3811]|RWWC010-10|United States|Washington|658[0n]|BOLD:AAA2590
Xestia smithii|[3812]|LALPA738-10|Canada|British Columbia|658[0n]|BOLD:AAA2590
Xestia smithii|[3813]|LALPA725-10|Canada|British Columbia|658[0n]|BOLD:AAA2590
Xestia smithii|[3814]|LALPA686-10|Canada|British Columbia|658[0n]|BOLD:AAA2590
Xestia smithii|[3815]|LALPA656-10|Canada|British Columbia|658[0n]|BOLD:AAA2590
Xestia smithii|[3816]|LBCH4649-10|Canada|British Columbia|658[0n]|BOLD:AAA2590
Xestia smithii|[3817]|LBCH4647-10|Canada|British Columbia|658[0n]|BOLD:AAA2590
Xestia smithii|[3818]|LBCH4644-10|Canada|British Columbia|658[0n]|BOLD:AAA2590
Xestia smithii|[3819]|LBCH4643-10|Canada|British Columbia|658[0n]|BOLD:AAA2590
Xestia smithii|[3820]|LBCH4352-10|Canada|British Columbia|658[0n]|BOLD:AAA2590
Xestia smithii|[3821]|LBCH4350-10|Canada|British Columbia|658[0n]|BOLD:AAA2590
Xestia smithii|[3822]|LBCH4346-10|Canada|British Columbia|658[0n]|BOLD:AAA2590
Xestia smithii|[3823]|LBCH4100-10|Canada|British Columbia|658[0n]|BOLD:AAA2590
Xestia smithii|[3824]|LBCH4099-10|Canada|British Columbia|658[0n]|BOLD:AAA2590
Xestia smithii|[3825]|LBCH4026-10|Canada|British Columbia|658[0n]|BOLD:AAA2590
Xestia smithii|[3826]|LBCH4024-10|Canada|British Columbia|658[0n]|BOLD:AAA2590
Xestia smithii|[3827]|LBCH3794-10|Canada|British Columbia|658[0n]|BOLD:AAA2590
Xestia smithii|[3828]|LBCH3792-10|Canada|British Columbia|658[0n]|BOLD:AAA2590
Xestia smithii|[3829]|LBCH3532-10|Canada|British Columbia|658[0n]|BOLD:AAA2590
Xestia smithii|[3830]|LBCH3531-10|Canada|British Columbia|658[0n]|BOLD:AAA2590
Xestia smithii|[3831]|LBCH3529-10|Canada|British Columbia|658[0n]|BOLD:AAA2590
Xestia smithii|[3832]|LBCH3525-10|Canada|British Columbia|658[0n]|BOLD:AAA2590
Xestia smithii|[3833]|LBCH3352-10|Canada|British Columbia|658[0n]|BOLD:AAA2590
Xestia smithii|[3834]|LBCH3347-10|Canada|British Columbia|658[0n]|BOLD:AAA2590
Xestia smithii|[3835]|LBCH3079-10|Canada|British Columbia|658[0n]|BOLD:AAA2590
Xestia smithii|[3836]|LBCH3077-10|Canada|British Columbia|658[0n]|BOLD:AAA2590
Xestia smithii|[3837]|LBCH3076-10|Canada|British Columbia|658[0n]|BOLD:AAA2590
Xestia smithii|[3838]|LBCH3075-10|Canada|British Columbia|658[0n]|BOLD:AAA2590
Xestia smithii|[3839]|LBCH3074-10|Canada|British Columbia|658[0n]|BOLD:AAA2590
Xestia smithii|[3840]|LBCH3073-10|Canada|British Columbia|658[0n]|BOLD:AAA2590
Xestia smithii|[3841]|LBCH2705-10|Canada|British Columbia|658[0n]|BOLD:AAA2590
Xestia smithii|[3842]|LBCH963-10|Canada|British Columbia|658[0n]|BOLD:AAA2590
Xestia smithii|[3843]|LBCH957-10|Canada|British Columbia|658[0n]|BOLD:AAA2590
Xestia smithii|[3844]|LBCH956-10|Canada|British Columbia|658[0n]|BOLD:AAA2590
Xestia smithii|[3845]|RWWB195-09|United States|Washington|658[0n]|BOLD:AAA2590
Xestia smithii|[3846]|RWWB172-09|United States|Washington|658[0n]|BOLD:AAA2590
Xestia smithii|[3847]|RWWB099-09|United States|Washington|658[0n]|BOLD:AAA2590
Xestia smithii|[3848]|RWWB082-09|United States|Washington|658[0n]|BOLD:AAA2590
Xestia smithii|[3849]|RWWB063-09|United States|Washington|658[0n]|BOLD:AAA2590
Xestia smithii|[3850]|RWWB060-09|United States|Washington|658[0n]|BOLD:AAA2590
Xestia smithii|[3851]|RWWB037-09|United States|Washington|658[0n]|BOLD:AAA2590
Xestia smithii|[3852]|RWWA988-09|United States|Washington|658[0n]|BOLD:AAA2590
Xestia smithii|[3853]|RWWA981-09|United States|Washington|658[0n]|BOLD:AAA2590
Xestia smithii|[3854]|RWWA960-09|United States|Washington|658[0n]|BOLD:AAA2590
Xestia smithii|[3855]|RWWA959-09|United States|Washington|658[0n]|BOLD:AAA2590
Xestia smithii|[3856]|RWWA882-09|United States|Washington|658[0n]|BOLD:AAA2590
Xestia smithii|[3857]|RWWA844-09|United States|Washington|658[0n]|BOLD:AAA2590
Xestia smithii|[3858]|LBCG2313-09|Canada|British Columbia|658[0n]|BOLD:AAA2590
Xestia smithii|[3859]|LBCG2303-09|Canada|British Columbia|658[0n]|BOLD:AAA2590
Xestia smithii|[3860]|LBCG2285-09|Canada|British Columbia|658[0n]|BOLD:AAA2590
Xestia smithii|[3861]|LPVIB571-08|Canada|British Columbia|658[0n]|BOLD:AAA2590
Xestia smithii|[3862]|LPVIB569-08|Canada|British Columbia|658[0n]|BOLD:AAA2590
Xestia smithii|[3863]|LPVIB506-08|Canada|British Columbia|658[0n]|BOLD:AAA2590
Xestia smithii|[3864]|LPVIB505-08|Canada|British Columbia|658[0n]|BOLD:AAA2590
Xestia smithii|[3865]|LPVIB261-08|Canada|British Columbia|658[0n]|BOLD:AAA2590
Xestia smithii|[3866]|ABKWR065-07|United States|Alaska|658[0n]|BOLD:AAA2590
Xestia smithii|[3867]|LHLEP440-06|Canada|British Columbia|658[0n]|BOLD:AAA2590
Xestia smithii|[3868]|LHLEP415-06|Canada|British Columbia|658[0n]|BOLD:AAA2590
Xestia smithii|[3869]|LOWCD823-06|Canada|British Columbia|658[0n]|BOLD:AAA2590
Xestia smithii|[3870]|LBCD426-05|Canada|British Columbia|658[0n]|BOLD:AAA2590
Xestia smithii|[3871]|LOWCD830-06|Canada|British Columbia|657[0n]|BOLD:AAA2590
Xestia smithii|[3872]|LBCG2299-09|Canada|British Columbia|621[0n]|BOLD:AAA2590
Xestia smithii|[3873]|LBCH4645-10|Canada|British Columbia|643[0n]|BOLD:AAA2590
Xestia smithii|[3874]|LALPA544-10|Canada|British Columbia|637[0n]|BOLD:AAA2590
Xestia smithii|[3875]|RWWC638-11|United States|Washington|658[0n]|BOLD:AAA2590
Xestia smithii|[3876]|RWWC691-11|United States|Washington|658[0n]|BOLD:AAA2590
Xestia smithii|[3877]|RWWC722-11|United States|Washington|658[0n]|BOLD:AAA2590
Xestia smithii|[3878]|LALPA1237-11|Canada|British Columbia|658[0n]|BOLD:AAA2590
Xestia smithii|[3879]|LALPA1248-11|Canada|British Columbia|658[0n]|BOLD:AAA2590
Xestia smithii|[3880]|LALPA1258-11|Canada|British Columbia|658[0n]|BOLD:AAA2590
Xestia smithii|[3881]|UAMIC527-13|United States|Alaska|658[0n]|BOLD:AAA2590
Xestia smithii|[3882]|UAMIC611-13|United States|Alaska|658[0n]|BOLD:AAA2590
Xestia smithii|[3883]|LBCG2293-09|Canada|British Columbia|658[0n]|BOLD:AAA2590
Xestia smithii|[3884]|LBCG2307-09|Canada|British Columbia|658[0n]|BOLD:AAA2590
Xestia smithii|[3885]|LBCH1674-10|Canada|British Columbia|658[0n]|BOLD:AAA2590
Xestia smithii|[3886]|LBCH3349-10|Canada|British Columbia|658[0n]|BOLD:AAA2590
Xestia smithii|[3887]|LBCH4097-10|Canada|British Columbia|658[0n]|BOLD:AAA2590
Xestia smithii|[3888]|LBCH4101-10|Canada|British Columbia|658[0n]|BOLD:AAA2590

Xestia smithii|[3885]|LBCH1674-10|Canada|British Columbia|658[0n]|BOLD:AAA2590
Xestia smithii|[3886]|LBCH3349-10|Canada|British Columbia|658[0n]|BOLD:AAA2590
Xestia smithii|[3887]|LBCH4097-10|Canada|British Columbia|658[0n]|BOLD:AAA2590
Xestia smithii|[3888]|LBCH4101-10|Canada|British Columbia|658[0n]|BOLD:AAA2590
Xestia smithii|[3889]|LBCH4345-10|Canada|British Columbia|658[0n]|BOLD:AAA2590
Xestia smithii|[3890]|BBLPB580-10|Canada|Alberta|658[0n]|BOLD:AAA2590
Xestia smithii|[3891]|BBLPB586-10|Canada|British Columbia|658[0n]|BOLD:AAA2590
Xestia smithii|[3892]|LBCG2300-09|Canada|British Columbia|658[0n]|BOLD:AAA2590
Xestia smithii|[3893]|XAG973-05|Canada|Ontario|638[0n]|BOLD:AAA2590
Xestia smithii|[3894]|LGSMG903-10|United States|North Carolina|658[0n]|BOLD:AAA2590
Xestia smithii|[3895]|XAH124-05|Canada|Ontario|658[0n]|BOLD:AAA2590
Xestia smithii|[3896]|LGSMG906-10|United States|North Carolina|658[0n]|BOLD:AAA2590
Xestia smithii|[3897]|LGSMG905-10|United States|North Carolina|658[0n]|BOLD:AAA2590
Xestia smithii|[3898]|LGSMG904-10|United States|North Carolina|658[0n]|BOLD:AAA2590
Xestia smithii|[3899]|LNCNW007-06|United States|North Carolina|658[0n]|BOLD:AAA2590
Xestia smithii|[3900]|LSEU158-06|United States|North Carolina|658[0n]|BOLD:AAA2590
Xestia smithii|[3901]|PHAUG1781-11|Canada|Ontario|658[1n]|BOLD:AAA2590
Xestia smithii|[3902]|LOWC783-05|Canada|British Columbia|658[0n]|BOLD:AAA2590
Xestia smithii|[3903]|LOWCD822-06|Canada|British Columbia|658[0n]|BOLD:AAA2590
Xestia smithii|[3904]|BBLPB417-10|Canada|Alberta|658[0n]|BOLD:AAA2590
Xestia smithii|[3905]|PHAUG1782-11|Canada|Ontario|658[1n]|BOLD:AAA2590
Xestia smithii|[3906]|LBCG2274-09|Canada|British Columbia|658[0n]|BOLD:AAA2590
Xestia smithii|[3907]|LBCH4646-10|Canada|British Columbia|636[0n]|BOLD:AAA2590
Xestia smithii|[3908]|BBLPB561-10|Canada|Saskatchewan|658[0n]|BOLD:AAA2590
Xestia smithii|[3909]|BBLPB560-10|Canada|Saskatchewan|658[0n]|BOLD:AAA2590
Xestia smithii|[3910]|BBLPB558-10|Canada|Alberta|658[0n]|BOLD:AAA2590
Xestia smithii|[3911]|BBLPB462-10|Canada|Alberta|658[0n]|BOLD:AAA2590
Xestia smithii|[3912]|BBLPB444-10|Canada|Saskatchewan|658[0n]|BOLD:AAA2590
Xestia smithii|[3913]|BBLPB415-10|Canada|Alberta|658[0n]|BOLD:AAA2590
Xestia smithii|[3914]|BBLPB414-10|Canada|Alberta|658[0n]|BOLD:AAA2590
Xestia smithii|[3915]|BBLPB413-10|Canada|Saskatchewan|658[0n]|BOLD:AAA2590
Xestia smithii|[3916]|BBLPB395-10|Canada|Saskatchewan|658[0n]|BOLD:AAA2590
Xestia smithii|[3917]|BBLPB394-10|Canada|Saskatchewan|658[0n]|BOLD:AAA2590
Xestia smithii|[3918]|BBLPB384-10|Canada|Saskatchewan|658[0n]|BOLD:AAA2590
Xestia smithii|[3919]|BBLPA829-10|Canada|Saskatchewan|658[0n]|BOLD:AAA2590
Xestia smithii|[3920]|LBCH4648-10|Canada|British Columbia|658[0n]|BOLD:AAA2590
Xestia smithii|[3921]|LBCH4642-10|Canada|British Columbia|658[0n]|BOLD:AAA2590
Xestia smithii|[3922]|LBCH4641-10|Canada|British Columbia|658[0n]|BOLD:AAA2590
Xestia smithii|[3923]|LBCH4351-10|Canada|British Columbia|658[0n]|BOLD:AAA2590
Xestia smithii|[3924]|LBCH4349-10|Canada|British Columbia|658[0n]|BOLD:AAA2590
Xestia smithii|[3925]|LBCH4347-10|Canada|British Columbia|658[0n]|BOLD:AAA2590
Xestia smithii|[3926]|LBCH4098-10|Canada|British Columbia|658[0n]|BOLD:AAA2590
Xestia smithii|[3927]|LBCH4096-10|Canada|British Columbia|658[0n]|BOLD:AAA2590
Xestia smithii|[3928]|LBCH4095-10|Canada|British Columbia|658[0n]|BOLD:AAA2590
Xestia smithii|[3929]|LBCH4094-10|Canada|British Columbia|658[0n]|BOLD:AAA2590
Xestia smithii|[3930]|LBCH4039-10|Canada|British Columbia|658[0n]|BOLD:AAA2590
Xestia smithii|[3931]|LBCH3791-10|Canada|British Columbia|658[0n]|BOLD:AAA2590
Xestia smithii|[3932]|LBCH3790-10|Canada|British Columbia|658[0n]|BOLD:AAA2590
Xestia smithii|[3933]|LBCH3530-10|Canada|British Columbia|658[0n]|BOLD:AAA2590
Xestia smithii|[3934]|LBCH3528-10|Canada|British Columbia|658[0n]|BOLD:AAA2590
Xestia smithii|[3935]|LBCH3527-10|Canada|British Columbia|658[0n]|BOLD:AAA2590
Xestia smithii|[3936]|LBCH3526-10|Canada|British Columbia|658[0n]|BOLD:AAA2590
Xestia smithii|[3937]|LBCH3351-10|Canada|British Columbia|658[0n]|BOLD:AAA2590
Xestia smithii|[3938]|LBCH3350-10|Canada|British Columbia|658[0n]|BOLD:AAA2590
Xestia smithii|[3939]|LBCH3348-10|Canada|British Columbia|658[0n]|BOLD:AAA2590
Xestia smithii|[3940]|LBCH3346-10|Canada|British Columbia|658[0n]|BOLD:AAA2590
Xestia smithii|[3941]|LBCH3343-10|Canada|British Columbia|658[0n]|BOLD:AAA2590
Xestia smithii|[3942]|LBCH3078-10|Canada|British Columbia|658[0n]|BOLD:AAA2590
Xestia smithii|[3943]|LBCH2863-10|Canada|British Columbia|658[0n]|BOLD:AAA2590
Xestia smithii|[3944]|LBCH2840-10|Canada|British Columbia|658[0n]|BOLD:AAA2590
Xestia smithii|[3945]|LBCH2822-10|Canada|British Columbia|658[0n]|BOLD:AAA2590
Xestia smithii|[3946]|LBCH2791-10|Canada|British Columbia|658[0n]|BOLD:AAA2590
Xestia smithii|[3947]|LBCH2718-10|Canada|British Columbia|658[0n]|BOLD:AAA2590
Xestia smithii|[3948]|LBCH1774-10|Canada|British Columbia|658[0n]|BOLD:AAA2590
Xestia smithii|[3949]|LBCH1498-10|Canada|British Columbia|658[0n]|BOLD:AAA2590
Xestia smithii|[3950]|LBCH955-10|Canada|British Columbia|658[0n]|BOLD:AAA2590
Xestia smithii|[3951]|LBCH954-10|Canada|British Columbia|658[0n]|BOLD:AAA2590
Xestia smithii|[3952]|LBCH953-10|Canada|British Columbia|658[0n]|BOLD:AAA2590
Xestia smithii|[3953]|LBCH952-10|Canada|British Columbia|658[0n]|BOLD:AAA2590
Xestia smithii|[3954]|LBCH123-10|Canada|British Columbia|658[0n]|BOLD:AAA2590
Xestia smithii|[3955]|LBCG2309-09|Canada|British Columbia|658[0n]|BOLD:AAA2590
Xestia smithii|[3956]|LBCG2308-09|Canada|British Columbia|658[0n]|BOLD:AAA2590
Xestia smithii|[3957]|LBCG2304-09|Canada|British Columbia|658[0n]|BOLD:AAA2590
Xestia smithii|[3958]|LBCG2302-09|Canada|British Columbia|658[0n]|BOLD:AAA2590
Xestia smithii|[3959]|LBCG2298-09|Canada|British Columbia|658[0n]|BOLD:AAA2590
Xestia smithii|[3960]|LBCG2295-09|Canada|British Columbia|658[0n]|BOLD:AAA2590
Xestia smithii|[3961]|LBCG2294-09|Canada|British Columbia|658[0n]|BOLD:AAA2590
Xestia smithii|[3962]|LBCG2292-09|Canada|British Columbia|658[0n]|BOLD:AAA2590
Xestia smithii|[3963]|LBCG2291-09|Canada|British Columbia|658[0n]|BOLD:AAA2590
Xestia smithii|[3964]|LBCG2286-09|Canada|British Columbia|658[0n]|BOLD:AAA2590
Xestia smithii|[3965]|LPSOD937-09|Canada|Ontario|658[0n]|BOLD:AAA2590
Xestia smithii|[3966]|LPABC398-09|Canada|Alberta|658[0n]|BOLD:AAA2590
Xestia smithii|[3967]|LPABC021-09|Canada|Alberta|658[0n]|BOLD:AAA2590
Xestia smithii|[3968]|LPMNB506-09|Canada|Manitoba|658[0n]|BOLD:AAA2590
Xestia smithii|[3969]|LBCG631-09|Canada|British Columbia|658[0n]|BOLD:AAA2590
Xestia smithii|[3970]|TTMNB467-06|Canada|New Brunswick|658[0n]|BOLD:AAA2590
Xestia smithii|[3971]|TTMNB466-06|Canada|New Brunswick|658[0n]|BOLD:AAA2590
Xestia smithii|[3972]|TTMNB465-06|Canada|New Brunswick|658[0n]|BOLD:AAA2590
Xestia smithii|[3973]|LOWCD836-06|Canada|British Columbia|658[0n]|BOLD:AAA2590
Xestia smithii|[3974]|LOWCD835-06|Canada|British Columbia|658[0n]|BOLD:AAA2590
Xestia smithii|[3975]|LOWCD832-06|Canada|British Columbia|658[0n]|BOLD:AAA2590
Xestia smithii|[3976]|LOWCD831-06|Canada|British Columbia|658[0n]|BOLD:AAA2590
Xestia smithii|[3977]|LOWCD829-06|Canada|British Columbia|658[0n]|BOLD:AAA2590
Xestia smithii|[3978]|LOWCD828-06|Canada|British Columbia|657[0n]|BOLD:AAA2590
Xestia smithii|[3979]|LOWCD827-06|Canada|British Columbia|658[0n]|BOLD:AAA2590
Xestia smithii|[3980]|LOWCD826-06|Canada|British Columbia|657[0n]|BOLD:AAA2590
Xestia smithii|[3981]|LOWCD825-06|Canada|British Columbia|658[0n]|BOLD:AAA2590
Xestia smithii|[3982]|LOWCD824-06|Canada|British Columbia|658[0n]|BOLD:AAA2590
Xestia smithii|[3983]|LOWCD817-06|Canada|British Columbia|657[0n]|BOLD:AAA2590
Xestia smithii|[3984]|LOWCD816-06|Canada|British Columbia|657[0n]|BOLD:AAA2590
Xestia smithii|[3985]|LOWCD813-06|Canada|British Columbia|657[0n]|BOLD:AAA2590
Xestia smithii|[3986]|LOWCD811-06|Canada|British Columbia|657[0n]|BOLD:AAA2590
Xestia smithii|[3987]|LOWCD810-06|Canada|British Columbia|657[0n]|BOLD:AAA2590
[illegible]

Xestia smithii|[3985]|LOWCD813-06|Canada|British Columbia|657[0n]|BOLD:AAA2590
Xestia smithii|[3986]|LOWCD811-06|Canada|British Columbia|657[0n]|BOLD:AAA2590
Xestia smithii|[3987]|LOWCD810-06|Canada|British Columbia|657[0n]|BOLD:AAA2590
Xestia smithii|[3988]|RDLQB652-05|Canada|Quebec|658[0n]|BOLD:AAA2590
Xestia smithii|[3989]|LOWCC161-05|Canada|British Columbia|658[0n]|BOLD:AAA2590
Xestia smithii|[3990]|LOWCC160-05|Canada|British Columbia|658[0n]|BOLD:AAA2590
Xestia smithii|[3991]|LOWC793-05|Canada|British Columbia|658[0n]|BOLD:AAA2590
Xestia smithii|[3992]|LOWC792-05|Canada|British Columbia|658[0n]|BOLD:AAA2590
Xestia smithii|[3993]|LOWC791-05|Canada|British Columbia|658[0n]|BOLD:AAA2590
Xestia smithii|[3994]|LOWC786-05|Canada|British Columbia|658[0n]|BOLD:AAA2590
Xestia smithii|[3995]|LOWC784-05|Canada|British Columbia|658[0n]|BOLD:AAA2590
Xestia smithii|[3996]|XAH195-05|Canada|Ontario|658[0n]|BOLD:AAA2590
Xestia smithii|[3997]|LBCD312-05|Canada|British Columbia|658[0n]|BOLD:AAA2590
Xestia smithii|[3998]|LBCD106-05|Canada|British Columbia|658[0n]|BOLD:AAA2590
Xestia smithii|[3999]|LOWCD820-06|Canada|British Columbia|656[0n]|BOLD:AAA2590
Xestia smithii|[4000]|LOWCD818-06|Canada|British Columbia|656[0n]|BOLD:AAA2590
Xestia smithii|[4001]|LOWCD815-06|Canada|British Columbia|656[0n]|BOLD:AAA2590
Xestia smithii|[4002]|LOWCD814-06|Canada|British Columbia|656[0n]|BOLD:AAA2590
Xestia smithii|[4003]|LOWCD833-06|Canada|British Columbia|564[0n]|BOLD:AAA2590
Xestia smithii|[4004]|LBCH7793-10|Canada|British Columbia|658[0n]|BOLD:AAA2590
Xestia smithii|[4005]|BBLPB563-10|Canada|Saskatchewan|658[0n]|BOLD:AAA2590
Xestia smithii|[4006]|BBLPB573-10|Canada|Saskatchewan|658[0n]|BOLD:AAA2590
Xestia smithii|[4007]|BBLPB579-10|Canada|Alberta|658[0n]|BOLD:AAA2590
Xestia smithii|[4008]|BBLPB581-10|Canada|Alberta|658[0n]|BOLD:AAA2590
Xestia smithii|[4009]|BBLPB583-10|Canada|Saskatchewan|658[0n]|BOLD:AAA2590
Xestia smithii|[4010]|LALPA1259-11|Canada|British Columbia|658[0n]|BOLD:AAA2590
Xestia smithii|[4011]|JSAUG1681-11|Canada|Ontario|658[0n]|BOLD:AAA2590
Xestia smithii|[4012]|LPABC013-09|Canada|Alberta|658[1n]|BOLD:AAA2590
Xestia smithii|[4013]|CNWBG3062-13|Canada|Alberta|607[0n]|BOLD:AAA2590
Xestia smithii|[4014]|RDLQB628-05|Canada|Quebec|580[0n]|BOLD:AAA2590
Xestia smithii|[4015]|LBCH958-10|Canada|British Columbia|643[0n]|BOLD:AAA2590
Xestia smithii|[4016]|SSEID2462-13|Canada|Alberta|603[0n]|BOLD:AAA2590
Xestia smithii|[4017]|CNEIF2106-12|Canada|Alberta|603[0n]|BOLD:AAA2590
Xestia smithii|[4018]|LOWCD812-06|Canada|British Columbia|657[0n]|BOLD:AAA2590
Xestia smithii|[4019]|CNFNT129-14|Canada|Quebec|585[0n]|BOLD:AAA2590
Xestia smithii|[4020]|LOWC790-05|Canada|British Columbia|658[0n]|BOLD:AAA2590
Xestia smithii|[4021]|CNGBO073-14|Canada|Ontario|584[0n]|BOLD:AAA2590
Xestia smithii|[4022]|NGNAY2797-14|Canada|British Columbia|579[0n]|BOLD:AAA2590
Xestia smithii|[4023]|CNGBO072-14|Canada|Ontario|583[0n]|BOLD:AAA2590
Xestia smithii|[4024]|CNTNH1258-14|Canada|Newfoundland and Labrador|564[3n]|BOLD:AAA2590
Xestia smithii|[4025]|CNTNG186-14|Canada|Newfoundland and Labrador|576[0n]|BOLD:AAA2590
Xestia smithii|[4026]|LPMNB504-09|Canada|Manitoba|658[0n]|BOLD:AAA2590
Xestia smithii|[4027]|XAH464-05|Canada|Ontario|658[0n]|BOLD:AAA2590
Xestia smithii|[4028]|XAH130-05|Canada|Ontario|658[0n]|BOLD:AAA2590
Xestia smithii|[4029]|LPSOD960-09|Canada|Ontario|658[0n]|BOLD:AAA2590
Xestia smithii|[4030]|PHAUG1790-11|Canada|Ontario|658[0n]|BOLD:AAA2590
Xestia smithii|[4031]|CNWBG3170-13|Canada|Alberta|576[0n]|BOLD:AAA2590
Xestia smithii|[4032]|BBLEC507-09|Canada|New Brunswick|634[0n]|BOLD:AAA2590
Xestia smithii|[4033]|BBLPB416-10|Canada|Alberta|620[0n]|BOLD:AAA2590
Xestia smithii|[4034]|CNFNH153-14|Canada|Quebec|573[0n]|BOLD:AAA2590
Xestia smithii|[4035]|CNWBC132-13|Canada|Alberta|582[0n]|BOLD:AAA2590
Xestia smithii|[4036]|CNEIF2105-12|Canada|Alberta|576[0n]|BOLD:AAA2590
Xestia smithii|[4037]|LSEU159-06|United States|North Carolina|658[0n]|BOLD:AAA2590
Xestia smithii|[4038]|XAH008-05|Canada|Ontario|616[0n]|BOLD:AAA2590
Xestia smithii|[4039]|CNFNH132-14|Canada|Quebec|560[0n]|BOLD:AAA2590
Xestia smithii|[4040]|CNTNH1259-14|Canada|Newfoundland and Labrador|567[0n]|BOLD:AAA2590
Xestia smithii|[4041]|CNTNK351-14|Canada|Newfoundland and Labrador|567[0n]|BOLD:AAA2590
Xestia smithii|[4042]|RDLQ722-07|Canada|Quebec|658[0n]|BOLD:AAA2590
Xestia smithii|[4043]|LNCNW006-06|United States|North Carolina|658[0n]|BOLD:AAA2590
Xestia smithii|[4044]|TTMNB468-06|Canada|New Brunswick|658[0n]|BOLD:AAA2590
Xestia smithii|[4045]|LOWC787-05|Canada|British Columbia|658[0n]|BOLD:AAA2590
Xestia smithii|[4046]|XAH276-05|Canada|Ontario|658[0n]|BOLD:AAA2590
Xestia smithii|[4047]|XAH273-05|Canada|Ontario|658[0n]|BOLD:AAA2590
Xestia smithii|[4048]|XAH194-05|Canada|Ontario|658[0n]|BOLD:AAA2590
Xestia smithii|[4049]|XAH125-05|Canada|Ontario|658[0n]|BOLD:AAA2590
Xestia smithii|[4050]|XAG734-05|Canada|Ontario|658[0n]|BOLD:AAA2590
Xestia smithii|[4051]|LSEU157-06|United States|North Carolina|657[0n]|BOLD:AAA2590
Xestia smithii|[4052]|RDLQ723-07|Canada|Quebec|652[0n]|BOLD:AAA2590
Xestia smithii|[4053]|LPSOD967-09|Canada|Ontario|658[0n]|BOLD:AAA2590
Xestia smithii|[4054]|BBLEC439-09|Canada|New Brunswick|658[0n]|BOLD:AAA2590
Xestia smithii|[4055]|MJMSL210-10|United States|Massachusetts|658[0n]|BOLD:AAA2590
Xestia smithii|[4056]|BBLPB584-10|Canada|Alberta|658[0n]|BOLD:AAA2590
Xestia smithii|[4057]|LALPA1235-11|Canada|British Columbia|658[0n]|BOLD:AAA2590
Xestia smithii|[4058]|JSAUG1679-11|Canada|Ontario|658[0n]|BOLD:AAA2590
Xestia smithii|[4059]|JSAUG1682-11|Canada|Ontario|658[0n]|BOLD:AAA2590
Xestia smithii|[4060]|PHAUG1789-11|Canada|Ontario|658[0n]|BOLD:AAA2590
Xestia smithii|[4061]|CNCLB2949-14|United States|North Carolina|658[0n]|BOLD:AAA2590
Coenophila opacifrons|[4062]|RDLQB650-05|Canada|Quebec|658[0n]|BOLD:AAD2661
Coenophila opacifrons|[4063]|RDLQB816-05|Canada|Quebec|622[0n]|BOLD:AAD2661
Coenophila opacifrons|[4064]|RDLQF311-06|Canada|Quebec|658[0n]|BOLD:AAD2661
Coenophila opacifrons|[4065]|RDLQB821-05|Canada|Quebec|658[0n]|BOLD:AAD2661
Coenophila opacifrons|[4066]|RDLQB814-05|Canada|Quebec|621[0n]|BOLD:AAD2661
Coenophila opacifrons|[4067]|RDLQB815-05|Canada|Quebec|658[0n]|BOLD:AAD2661
Coenophila opacifrons|[4068]|RDLQF879-06|Canada|Quebec|658[0n]|BOLD:AAD2661
Coenophila opacifrons|[4069]|LBCH4036-10|Canada|British Columbia|658[0n]|BOLD:AAD2661
Coenophila opacifrons|[4070]|LBCH4475-10|Canada|British Columbia|658[0n]|BOLD:AAD2661
Protolampra brunneicollis|[4071]|XAD350-04|Canada|Ontario|604[1n]|BOLD:AAC5112
Protolampra brunneicollis|[4072]|LBCH7392-10|Canada|British Columbia|658[0n]|BOLD:AAC5112
Protolampra brunneicollis|[4073]|MJMSL031-10|United States|Massachusetts|658[0n]|BOLD:AAC5112
Protolampra brunneicollis|[4074]|LGSM677-04|United States|North Carolina|537[5n]|BOLD:AAC5112
Protolampra brunneicollis|[4075]|XAH275-05|Canada|Ontario|658[0n]|BOLD:AAC5112
Protolampra brunneicollis|[4076]|XAD248-04|Canada|Ontario|649[0n]|BOLD:AAC5112
Protolampra brunneicollis|[4077]|LPOKC618-09|United States|Oklahoma|658[0n]|BOLD:AAC5112
Protolampra brunneicollis|[4078]|MJMSL030-10|United States|Massachusetts|658[1n]|BOLD:AAC5112
Protolampra brunneicollis|[4079]|XAD286-04|Canada|Ontario|572[0n]|BOLD:AAC5112
Protolampra brunneicollis|[4080]|XAH040-05|Canada|Ontario|636[0n]|BOLD:AAC5112
Protolampra brunneicollis|[4081]|XAH197-05|Canada|Ontario|658[0n]|BOLD:AAC5112
Protolampra brunneicollis|[4082]|RDLQB819-05|Canada|Quebec|658[0n]|BOLD:AAC5112
Protolampra brunneicollis|[4083]|LSUSA109-06|United States|Kentucky|658[0n]|BOLD:AAC5112
Protolampra brunneicollis|[4084]|XAJ814-06|Canada|Ontario|658[0n]|BOLD:AAC5112
Protolampra brunneicollis|[4085]|LNCC115-10|United States|North Carolina|658[0n]|BOLD:AAC5112
Protolampra brunneicollis|[4086]|MJMSL056-10|United States|Massachusetts|658[0n]|BOLD:AAC5112
Xestia badicollis|[4087]|RDLQB675-05|Canada|Quebec|501[0n]|

Protolampra brunneicollis|[4085]|LNCC115-10|United States|North Carolina|658[0n]|BOLD:AAC5112
Protolampra brunneicollis|[4086]|MJMSL056-10|United States|Massachusetts|658[0n]|BOLD:AAC5112
Xestia badicollis|[4087]|RDLQB675-05|Canada|Quebec|501[0n]|
Xestia badicollis|[4088]|RDNMB098-05|United States|Tennessee|658[8n]|
Xestia badicollis|[4089]|TTMNB472-06|Canada|New Brunswick|658[4n]|BOLD:AAA5962
Xestia badicollis|[4090]|RDLQF181-06|Canada|Quebec|658[0n]|BOLD:AAA5962
Xestia badicollis|[4091]|RDLQF182-06|Canada|Quebec|658[0n]|BOLD:AAA5962
Xestia badicollis|[4092]|RDLQF184-06|Canada|Quebec|658[0n]|BOLD:AAA5962
Xestia badicollis|[4093]|LPSOD963-09|Canada|Ontario|658[0n]|BOLD:AAA5962
Xestia badicollis|[4094]|LPSOD971-09|Canada|Ontario|658[0n]|BOLD:AAA5962
Xestia badicollis|[4095]|LPSOD985-09|Canada|Ontario|658[0n]|BOLD:AAA5962
Xestia badicollis|[4096]|RDLQF178-06|Canada|Quebec|658[0n]|BOLD:AAA5962
Xestia badicollis|[4097]|RDLQF180-06|Canada|Quebec|658[0n]|BOLD:AAA5962
Xestia badicollis|[4098]|LPSOD993-09|Canada|Ontario|658[0n]|BOLD:AAA5962
Xestia badicollis|[4099]|RDLQF288-06|Canada|Quebec|658[2n]|BOLD:AAA5962
Xestia badicollis|[4100]|RDNMB097-05|United States|Tennessee|658[0n]|BOLD:AAA5962
Xestia badicollis|[4101]|BBLEC517-09|Canada|New Brunswick|613[0n]|BOLD:AAA5962
Xestia dilucida|[4102]|LNC449-05|United States|North Carolina|658[0n]|BOLD:AAA5962
Xestia dilucida|[4103]|LNC450-05|United States|North Carolina|658[0n]|BOLD:AAA5962
Xestia dilucida|[4104]|LNC451-05|United States|North Carolina|658[0n]|BOLD:AAA5962
Xestia dilucida|[4105]|LNC477-06|United States|North Carolina|658[0n]|BOLD:AAA5962
Xestia dilucida|[4106]|RDNML165-13|United States|North Carolina|658[0n]|BOLD:AAA5962
Xestia dilucida|[4107]|RDNML166-13|United States|North Carolina|658[0n]|BOLD:AAA5962
Xestia dilucida|[4108]|LNCC1535-13|United States|North Carolina|658[0n]|BOLD:AAA5962
Xestia dilucida|[4109]|LNCC1537-13|United States|North Carolina|658[0n]|BOLD:AAA5962
Xestia praevia|[4110]|RDNMC383-05|Canada|Ontario|658[1n]|BOLD:AAA5962
Xestia badicollis|[4111]|RDNMB105-05|United States|Tennessee|658[1n]|BOLD:AAA5962
Xestia badicollis|[4112]|RDLQF183-06|Canada|Quebec|658[1n]|BOLD:AAA5962
Xestia badicollis|[4113]|RDNMB093-05|United States|Tennessee|658[0n]|BOLD:AAA5962
Xestia badicollis|[4114]|RDNMC385-05|Canada|Ontario|658[0n]|BOLD:AAA5962
Xestia badicollis|[4115]|RDNMC386-05|Canada|Ontario|658[0n]|BOLD:AAA5962
Xestia badicollis|[4116]|TTMNB469-06|Canada|New Brunswick|658[0n]|BOLD:AAA5962
Xestia badicollis|[4117]|TTMNB470-06|Canada|New Brunswick|658[0n]|BOLD:AAA5962
Xestia badicollis|[4118]|TTMNB473-06|Canada|New Brunswick|658[0n]|BOLD:AAA5962
Xestia badicollis|[4119]|TTMNB474-06|Canada|New Brunswick|658[0n]|BOLD:AAA5962
Xestia badicollis|[4120]|TMNBB373-06|Canada|New Brunswick|658[0n]|BOLD:AAA5962
Xestia badicollis|[4121]|RDLQF179-06|Canada|Quebec|658[0n]|BOLD:AAA5962
Xestia badicollis|[4122]|RDLQF185-06|Canada|Quebec|658[0n]|BOLD:AAA5962
Xestia badicollis|[4123]|RDLQF287-06|Canada|Quebec|658[0n]|BOLD:AAA5962
Xestia badicollis|[4124]|BBLEC432-09|Canada|New Brunswick|658[0n]|BOLD:AAA5962
Xestia badicollis|[4125]|BBLEC504-09|Canada|New Brunswick|658[0n]|BOLD:AAA5962
Xestia badicollis|[4126]|BBLEC514-09|Canada|New Brunswick|658[0n]|BOLD:AAA5962
Xestia badicollis|[4127]|BBLPC018-09|Canada|New Brunswick|658[0n]|BOLD:AAA5962
Xestia badicollis|[4128]|BBLPC019-09|Canada|New Brunswick|658[0n]|BOLD:AAA5962
Xestia badicollis|[4129]|BBLPC547-09|Canada|New Brunswick|658[0n]|BOLD:AAA5962
Xestia badicollis|[4130]|LGSMG912-10|United States|North Carolina|658[0n]|BOLD:AAA5962
Xestia elimata|[4131]|LSEU162-06|United States|Georgia|658[0n]|BOLD:AAA5962
Xestia elimata|[4132]|RDNMB106-05|United States|Tennessee|658[0n]|BOLD:AAA5962
Xestia badicollis|[4133]|BBLPC378-09|Canada|New Brunswick|632[0n]|BOLD:AAA5962
Xestia elimata|[4134]|RDNMI047-10|United States|Florida|658[1n]|BOLD:AAA5962
Xestia praevia|[4135]|RDLQB525-05|Canada|Quebec|658[1n]|BOLD:AAA5962
Xestia badicollis|[4136]|RDNMB094-05|United States|Tennessee|658[0n]|BOLD:AAA5962
Xestia praevia|[4137]|BBLPC152-09|Canada|Nova Scotia|658[0n]|BOLD:AAA5962
Xestia praevia|[4138]|BBLEC977-09|Canada|Nova Scotia|658[0n]|BOLD:AAA5962
Xestia praevia|[4139]|BBLEC917-09|Canada|Nova Scotia|658[0n]|BOLD:AAA5962
Xestia praevia|[4140]|BBLEC687-09|Canada|Nova Scotia|658[0n]|BOLD:AAA5962
Xestia praevia|[4141]|RDLQF798-06|Canada|Quebec|658[0n]|BOLD:AAA5962
Xestia praevia|[4142]|RDLQF377-06|Canada|Quebec|658[0n]|BOLD:AAA5962
Xestia praevia|[4143]|RDLQF376-06|Canada|Quebec|658[0n]|BOLD:AAA5962
Xestia praevia|[4144]|LSEU156-06|United States|North Carolina|658[0n]|BOLD:AAA5962
Xestia praevia|[4145]|RDNMC384-05|Canada|Ontario|658[0n]|BOLD:AAA5962
Xestia praevia|[4146]|RDLQB739-05|Canada|Quebec|658[0n]|BOLD:AAA5962
Xestia elimata|[4147]|RDNMB102-05|United States|Tennessee|658[0n]|BOLD:AAA5962
Xestia praevia|[4148]|BBLEC697-09|Canada|Nova Scotia|658[0n]|BOLD:AAA5962
Xestia praevia|[4149]|BBLPC461-09|Canada|New Brunswick|658[0n]|BOLD:AAA5962
Xestia badicollis|[4150]|BBLEC527-09|Canada|New Brunswick|594[0n]|BOLD:AAA5962
Xestia elimata|[4151]|RDNMB103-05|United States|Tennessee|575[1n]|BOLD:AAA5962
Xestia badicollis|[4152]|TMNBB372-06|Canada|New Brunswick|658[0n]|BOLD:AAA5962
Xestia badicollis|[4153]|BBLPC036-09|Canada|New Brunswick|621[0n]|BOLD:AAA5962
Xestia elimata|[4154]|LNCC1322-11|United States|North Carolina|636[0n]|BOLD:AAA5962
Xestia praevia|[4155]|LNCC1507-13|United States|North Carolina|658[0n]|BOLD:AAA5962
Xestia elimata|[4156]|LNCC405-10|United States|North Carolina|658[0n]|BOLD:AAA5962
Xestia elimata|[4157]|LNC516-06|United States|North Carolina|601[0n]|BOLD:AAA5962
Xestia elimata|[4158]|MJMSL055-10|United States|Massachusetts|658[0n]|BOLD:AAA5962
Xestia elimata|[4159]|LNCC1538-13|United States|North Carolina|658[0n]|BOLD:AAA5962
Xestia badicollis|[4160]|RDNMB090-05|United States|Tennessee|658[6n]|BOLD:AAA5962
Xestia badicollis|[4161]|RDNMB092-05|United States|Tennessee|658[8n]|
Xestia badicollis|[4162]|RDNMB091-05|United States|Tennessee|639[12n]|
Xestia badicollis|[4163]|TMNBB370-06|Canada|New Brunswick|658[8n]|
Xestia badicollis|[4164]|RDNMB107-05|United States|Tennessee|658[1n]|BOLD:AAA5962
Xestia badicollis|[4165]|TTMNB471-06|Canada|New Brunswick|658[3n]|BOLD:AAA5962
Xestia badicollis|[4166]|RDNMB096-05|United States|Tennessee|658[1n]|BOLD:AAA5962
Xestia badicollis|[4167]|RDNMB095-05|United States|Tennessee|658[0n]|BOLD:AAA5962
Xestia badicollis|[4168]|RDNMB101-05|United States|Tennessee|658[0n]|BOLD:AAA5962
Xestia badicollis|[4169]|LGSMG913-10|United States|Tennessee|658[0n]|BOLD:AAA5962
Xestia dilucida|[4170]|RDNML149-13|United States|North Carolina|612[0n]|BOLD:AAA5962
Xestia dilucida|[4171]|LNC357-05|United States|North Carolina|503[0n]|BOLD:AAA5962
Xestia dilucida|[4172]|TMNBB387-06|Canada|New Brunswick|656[2n]|BOLD:AAA5962
Xestia dilucida|[4173]|TTMNB484-06|Canada|New Brunswick|658[0n]|BOLD:AAA5962
Xestia dilucida|[4174]|TMNBB374-06|Canada|New Brunswick|658[0n]|BOLD:AAA5962
Xestia dilucida|[4175]|TMNBB375-06|Canada|New Brunswick|658[0n]|BOLD:AAA5962
Xestia dilucida|[4176]|TMNBB377-06|Canada|New Brunswick|658[0n]|BOLD:AAA5962
Xestia dilucida|[4177]|TMNBB378-06|Canada|New Brunswick|658[0n]|BOLD:AAA5962
Xestia dilucida|[4178]|TMNBB379-06|Canada|New Brunswick|658[0n]|BOLD:AAA5962
Xestia dilucida|[4179]|TMNBB381-06|Canada|New Brunswick|658[0n]|BOLD:AAA5962
Xestia dilucida|[4180]|TMNBB382-06|Canada|New Brunswick|658[0n]|BOLD:AAA5962
Xestia dilucida|[4181]|TMNBB384-06|Canada|New Brunswick|658[0n]|BOLD:AAA5962
Xestia dilucida|[4182]|TMNBB385-06|Canada|New Brunswick|658[0n]|BOLD:AAA5962
Xestia dilucida|[4183]|TMNBB386-06|Canada|New Brunswick|658[0n]|BOLD:AAA5962
Xestia dilucida|[4184]|TMNBB389-06|Canada|New Brunswick|658[0n]|BOLD:AAA5962
Xestia dilucida|[4185]|RDLQF285-06|Canada|Quebec|658[0n]|BOLD:AAA5962
Xestia dilucida|[4186]|RDLQF286-06|Canada|Quebec|658[0n]|BOLD:AAA5962
Xestia dilucida|[4187]|RDLQF289-06|Canada|Quebec|658[0n]|BOLD:AAA5962

Xestia dilucida|[4184]|[illegible]|Canada|New Brunswick|658[0n]|BOLD:AAA5962
Xestia dilucida|[4185]|RDLQF285-06|Canada|Quebec|658[0n]|BOLD:AAA5962
Xestia dilucida|[4186]|RDLQF286-06|Canada|Quebec|658[0n]|BOLD:AAA5962
Xestia dilucida|[4187]|RDLQF289-06|Canada|Quebec|658[0n]|BOLD:AAA5962
Xestia dilucida|[4188]|RDLQF290-06|Canada|Quebec|658[0n]|BOLD:AAA5962
Xestia dilucida|[4189]|RDLQF291-06|Canada|Quebec|658[0n]|BOLD:AAA5962
Xestia dilucida|[4190]|RDLQF292-06|Canada|Quebec|658[0n]|BOLD:AAA5962
Xestia dilucida|[4191]|RDLQF293-06|Canada|Quebec|658[0n]|BOLD:AAA5962
Xestia dilucida|[4192]|RDLQF295-06|Canada|Quebec|658[0n]|BOLD:AAA5962
Xestia dilucida|[4193]|RDLQF298-06|Canada|Quebec|658[0n]|BOLD:AAA5962
Xestia dilucida|[4194]|RDLQF299-06|Canada|Quebec|658[0n]|BOLD:AAA5962
Xestia dilucida|[4195]|RDLQF300-06|Canada|Quebec|658[0n]|BOLD:AAA5962
Xestia dilucida|[4196]|RDLQF301-06|Canada|Quebec|658[0n]|BOLD:AAA5962
Xestia dilucida|[4197]|RDLQF304-06|Canada|Quebec|658[0n]|BOLD:AAA5962
Xestia dilucida|[4198]|RDLQF305-06|Canada|Quebec|658[0n]|BOLD:AAA5962
Xestia dilucida|[4199]|RDLQF310-06|Canada|Quebec|658[0n]|BOLD:AAA5962
Xestia dilucida|[4200]|RDLQF873-06|Canada|Quebec|658[0n]|BOLD:AAA5962
Xestia dilucida|[4201]|RDLQF874-06|Canada|Quebec|658[0n]|BOLD:AAA5962
Xestia dilucida|[4202]|RDLQF875-06|Canada|Quebec|658[0n]|BOLD:AAA5962
Xestia dilucida|[4203]|RDLQF876-06|Canada|Quebec|658[0n]|BOLD:AAA5962
Xestia dilucida|[4204]|RDLQF877-06|Canada|Quebec|658[0n]|BOLD:AAA5962
Xestia dilucida|[4205]|MJMSL032-10|United States|Massachusetts|658[0n]|BOLD:AAA5962
Xestia dilucida|[4206]|MJMSL033-10|United States|Massachusetts|658[0n]|BOLD:AAA5962
Xestia dilucida|[4207]|MJMSL034-10|United States|Massachusetts|658[0n]|BOLD:AAA5962
Xestia dilucida|[4208]|MJMSL052-10|United States|Massachusetts|658[0n]|BOLD:AAA5962
Xestia dilucida|[4209]|MJMSL053-10|United States|Massachusetts|658[0n]|BOLD:AAA5962
Xestia dilucida|[4210]|MJMSL146-10|United States|Massachusetts|658[0n]|BOLD:AAA5962
Xestia dilucida|[4211]|TTMNB482-06|Canada|New Brunswick|658[0n]|BOLD:AAA5962
Xestia dilucida|[4212]|TTMNB483-06|Canada|New Brunswick|658[0n]|BOLD:AAA5962
Xestia dilucida|[4213]|LNCC403-10|United States|North Carolina|658[0n]|BOLD:AAA5962
Xestia dilucida|[4214]|TTMNB481-06|Canada|New Brunswick|658[0n]|BOLD:AAA5962
Xestia dilucida|[4215]|TTMNB480-06|Canada|New Brunswick|658[0n]|BOLD:AAA5962
Xestia dilucida|[4216]|TTMNB479-06|Canada|New Brunswick|658[0n]|BOLD:AAA5962
Xestia dilucida|[4217]|TTMNB478-06|Canada|New Brunswick|658[0n]|BOLD:AAA5962
Xestia dilucida|[4218]|TTMNB477-06|Canada|New Brunswick|658[0n]|BOLD:AAA5962
Xestia dilucida|[4219]|TTMNB476-06|Canada|New Brunswick|658[0n]|BOLD:AAA5962
Xestia dilucida|[4220]|TTMNB475-06|Canada|New Brunswick|658[0n]|BOLD:AAA5962
Xestia dilucida|[4221]|RDLQB775-05|Canada|Quebec|658[0n]|BOLD:AAA5962
Xestia dilucida|[4222]|MJMSL054-10|United States|Massachusetts|658[0n]|BOLD:AAA5962
Xestia dilucida|[4223]|RDLQB764-05|Canada|Quebec|658[0n]|BOLD:AAA5962
Xestia dilucida|[4224]|RDNMI048-10|United States|Florida|658[0n]|BOLD:AAA5962
Xestia dilucida|[4225]|LNCC402-10|United States|North Carolina|658[1n]|BOLD:AAA5962
Xestia badicollis|[4226]|GWOTA064-12|United States|Massachusetts|658[0n]|BOLD:AAA5962
Xestia dilucida|[4227]|TMNBB383-06|Canada|New Brunswick|658[0n]|BOLD:AAA5962
Xestia dilucida|[4228]|RDLQF306-06|Canada|Quebec|658[1n]|BOLD:AAA5962
Xestia dilucida|[4229]|RDLQF302-06|Canada|Quebec|658[1n]|BOLD:AAA5962
Xestia dilucida|[4230]|TMNBB380-06|Canada|New Brunswick|658[0n]|BOLD:AAA5962
Xestia dilucida|[4231]|RDLQF303-06|Canada|Quebec|658[2n]|BOLD:AAA5962
Xestia dilucida|[4232]|RDLQF297-06|Canada|Quebec|643[0n]|BOLD:AAA5962
Xestia dilucida|[4233]|RDLQF296-06|Canada|Quebec|638[2n]|BOLD:AAA5962
Xestia dilucida|[4234]|TMNBB376-06|Canada|New Brunswick|658[0n]|BOLD:AAA5962
Xestia dilucida|[4235]|TMNBB388-06|Canada|New Brunswick|658[0n]|BOLD:AAA5962
Xestia dilucida|[4236]|RDLQF307-06|Canada|Quebec|632[0n]|BOLD:AAA5962
Xestia dilucida|[4237]|RDLQF294-06|Canada|Quebec|630[0n]|BOLD:AAA5962
Xestia dilucida|[4238]|RDLQF308-06|Canada|Quebec|653[2n]|BOLD:AAA5962
Xestia dilucida|[4239]|RDLQF309-06|Canada|Quebec|632[0n]|BOLD:AAA5962
Xestia dilucida|[4240]|GWOTA071-12|United States|Massachusetts|658[0n]|BOLD:AAA5962
Xestia dilucida|[4241]|LNCC1536-13|United States|North Carolina|658[0n]|BOLD:AAA5962
Xestia dilucida|[4242]|LNC355-05|United States|North Carolina|527[1n]|BOLD:AAA5962
Xestia elimata|[4243]|RDNMB104-05|United States|Tennessee|658[0n]|BOLD:AAA5962
Xestia dilucida|[4244]|QUNOD666-11|United States|Michigan|658[0n]|BOLD:AAA5962
Xestia dilucida|[4245]|QUNOD667-11|United States|Michigan|658[0n]|BOLD:AAA5962
Xestia dilucida|[4246]|RDNMB110-05|United States|Tennessee|658[0n]|BOLD:AAA5962
Xestia dilucida|[4247]|RDLQB647-05|Canada|Quebec|549[0n]|BOLD:AAA5962
Xestia dilucida|[4248]|QUNOD668-11|United States|Michigan|658[0n]|BOLD:AAA5962
Xestia dilucida|[4249]|CNCLB2966-14|United States|North Carolina|658[0n]|BOLD:AAA5962
Cerastis salicarum|[4250]|LOWCE028-06|Canada|British Columbia|658[1n]|BOLD:AAC4727
Cerastis salicarum|[4251]|LOWC923-05|Canada|British Columbia|658[0n]|BOLD:AAC4727
Cerastis salicarum|[4252]|LOWCE344-06|Canada|British Columbia|658[0n]|BOLD:AAC4727
Cerastis salicarum|[4253]|LOWCE500-06|Canada|British Columbia|616[0n]|BOLD:AAC4727
Cerastis salicarum|[4254]|LOWCE463-06|Canada|British Columbia|622[0n]|BOLD:AAC4727
Cerastis salicarum|[4255]|LOWCE508-06|Canada|British Columbia|618[0n]|BOLD:AAC4727
Cerastis salicarum|[4256]|RDLQH098-06|Canada|Quebec|626[0n]|BOLD:AAC4727
Cerastis salicarum|[4257]|LOWCE246-06|Canada|British Columbia|658[0n]|BOLD:AAC4727
Cerastis salicarum|[4258]|LOWCE027-06|Canada|British Columbia|658[0n]|BOLD:AAC4727
Cerastis salicarum|[4259]|LOWC922-05|Canada|British Columbia|658[0n]|BOLD:AAC4727
Cerastis salicarum|[4260]|RDLQH096-06|Canada|Quebec|606[0n]|BOLD:AAC4727
Cerastis salicarum|[4261]|RDLQH097-06|Canada|Quebec|639[0n]|BOLD:AAC4727
Cerastis salicarum|[4262]|LMDH018-11|United States|Minnesota|658[0n]|BOLD:AAC4727
Cerastis fishii|[4263]|LSEU166-06|United States|North Carolina|658[0n]|BOLD:AAE1522
Cerastis fishii|[4264]|LNC647-06|United States|North Carolina|658[0n]|BOLD:AAE1522
Cerastis fishii|[4265]|LNC646-06|United States|North Carolina|658[0n]|BOLD:AAE1522
Cerastis fishii|[4266]|TMNBB393-06|Canada|New Brunswick|658[0n]|BOLD:AAE1522
Cerastis fishii|[4267]|RDLQ732-07|Canada|Quebec|632[0n]|BOLD:AAE1522
Cerastis tenebrifera|[4268]|TMNBB392-06|Canada|New Brunswick|658[1n]|BOLD:AAC1487
Cerastis tenebrifera|[4269]|LGSMG707-07|United States|Tennessee|658[0n]|BOLD:AAC1487
Cerastis tenebrifera|[4270]|LGSMG708-07|United States|Tennessee|658[0n]|BOLD:AAC1487
Cerastis tenebrifera|[4271]|LGSMG706-07|United States|Tennessee|658[0n]|BOLD:AAC1487
Cerastis tenebrifera|[4272]|LPOKA944-09|United States|Oklahoma|658[0n]|BOLD:AAC1487
Cerastis tenebrifera|[4273]|LSEU167-06|United States|Georgia|658[0n]|BOLD:AAC1487
Cerastis tenebrifera|[4274]|LGSMG705-07|United States|Tennessee|658[0n]|BOLD:AAC1487
Cerastis tenebrifera|[4275]|LNCC611-11|United States|North Carolina|658[0n]|BOLD:AAC1487
Cerastis tenebrifera|[4276]|LNC721-06|United States|North Carolina|658[0n]|BOLD:AAC1487
Cerastis tenebrifera|[4277]|TTMNB485-06|Canada|New Brunswick|658[0n]|BOLD:AAC1487
Cerastis tenebrifera|[4278]|XAJ210-06|Canada|Ontario|658[0n]|BOLD:AAC1487
Cerastis tenebrifera|[4279]|TMNBB394-06|Canada|New Brunswick|658[0n]|BOLD:AAC1487
Cerastis tenebrifera|[4280]|LNC720-06|United States|North Carolina|658[0n]|BOLD:AAC1487
Cerastis tenebrifera|[4281]|RDLQ733-07|Canada|Quebec|646[0n]|BOLD:AAC1487
Cerastis tenebrifera|[4282]|RDLQ734-07|Canada|Quebec|658[0n]|BOLD:AAC1487
Cerastis tenebrifera|[4283]|LMDH043-11|United States|Minnesota|658[0n]|BOLD:AAC1487
Cerastis tenebrifera|[4284]|LPOKE283-11|United States|Oklahoma|658[0n]|BOLD:AAC1487
Cerastis cornuta|[4285]|GMLC154-09|United States|California|658[0n]|BOLD:ACF5044
Cerastis cornuta|[4286]|GMLC1312-12|United States|California|615[0n]|BOLD:ACF5044
[illegible]

Cerastis tenebrifera|[4284]|LPOKE283-11|United States|Oklahoma|658[0n]|BOLD:AAC1487
Cerastis cornuta|[4285]|GMLC154-09|United States|California|658[0n]|BOLD:ACF5044
Cerastis cornuta|[4286]|GMLC1312-12|United States|California|615[0n]|BOLD:ACF5044
Cerastis cornuta|[4287]|GMLC1336-12|United States|California|618[0n]|BOLD:ACF5044
Cerastis cornuta|[4288]|GMLC217-11|United States|California|658[0n]|BOLD:ACF5044
Cerastis cornuta|[4289]|GMLC1270-12|United States|California|658[0n]|BOLD:ACF5044
Cerastis cornuta|[4290]|GMLC1306-12|United States|California|664[0n]|BOLD:ACF5044
Cerastis cornuta|[4291]|GMLC1311-12|United States|California|664[0n]|BOLD:ACF5044
Cerastis cornuta|[4292]|GMLC1381-12|United States|California|607[0n]|BOLD:ACF5044
Cerastis robertsoni|[4293]|CNCLB981-14|United States|California|618[0n]|BOLD:ACF5044
Cerastis robertsoni|[4294]|CNCLB980-14|United States|California|599[0n]|BOLD:ACF5044
Cerastis robertsoni|[4295]|CNCLB982-14|United States|California|658[0n]|BOLD:ACF5044
Cerastis enigmatica|[4296]|LALPA037-10|Canada|British Columbia|637[1n]|BOLD:AAE1521
Cerastis enigmatica|[4297]|RDNMF354-08|Canada|British Columbia|658[0n]|BOLD:AAE1521
Cerastis enigmatica|[4298]|RDNMG1026-08|Canada|British Columbia|658[0n]|BOLD:AAE1521
Cerastis enigmatica|[4299]|RDNMF353-08|Canada|British Columbia|658[0n]|BOLD:AAE1521
Cerastis enigmatica|[4300]|RDNMG1027-08|Canada|British Columbia|658[0n]|BOLD:AAE1521
Cerastis enigmatica|[4301]|RWWB487-10|United States|Washington|629[0n]|BOLD:AAE1521
Cerastis enigmatica|[4302]|RWWB482-10|United States|Washington|658[0n]|BOLD:AAE1521
Cerastis enigmatica|[4303]|RDNMG1028-08|Canada|British Columbia|658[0n]|BOLD:AAE1521
Cerastis enigmatica|[4304]|RWWB483-10|United States|Washington|658[0n]|BOLD:AAE1521
Cerastis enigmatica|[4305]|RWWB488-10|United States|Washington|658[0n]|BOLD:AAE1521
Cerastis enigmatica|[4306]|RWWB569-10|United States|Washington|658[0n]|BOLD:AAE1521
Cerastis enigmatica|[4307]|RWWB585-10|United States|Washington|658[0n]|BOLD:AAE1521
Cerastis enigmatica|[4308]|LALPA053-10|Canada|British Columbia|658[0n]|BOLD:AAE1521
Cerastis enigmatica|[4309]|LALPA075-10|Canada|British Columbia|658[0n]|BOLD:AAE1521
Cerastis enigmatica|[4310]|LALPA1056-11|Canada|British Columbia|658[0n]|BOLD:AAE1521
Cerastis gloriosa|[4311]|CNCLB983-14|United States|Washington|658[0n]|BOLD:ACM4461
Cerastis gloriosa|[4312]|CNCLB984-14|United States|Washington|658[0n]|BOLD:ACM4461
Anaplectoides prasina|[4313]|PHMNB740-05|Canada|New Brunswick|658[0n]|BOLD:AAA2948
Anaplectoides prasina|[4314]|LBCS367-07|Canada|British Columbia|658[0n]|BOLD:AAA2948
Anaplectoides prasina|[4315]|LPABC023-09|Canada|Alberta|658[0n]|BOLD:AAA2948
Anaplectoides prasina|[4316]|BBLPB661-10|Canada|Saskatchewan|658[0n]|BOLD:AAA2948
Anaplectoides prasina|[4317]|RDNMC002-05|Canada|British Columbia|658[0n]|BOLD:AAA2948
Anaplectoides prasina|[4318]|LBCH4678-10|Canada|British Columbia|658[0n]|BOLD:AAA2948
Anaplectoides prasina|[4319]|LOWC853-05|Canada|British Columbia|658[0n]|BOLD:AAA2948
Anaplectoides prasina|[4320]|LOWCD585-06|Canada|British Columbia|586[0n]|BOLD:AAA2948
Anaplectoides prasina|[4321]|LBCH3143-10|Canada|British Columbia|658[0n]|BOLD:AAA2948
Anaplectoides prasina|[4322]|BBLPB662-10|Canada|Alberta|658[0n]|BOLD:AAA2948
Anaplectoides prasina|[4323]|MNBB323-05|Canada|New Brunswick|658[0n]|BOLD:AAA2948
Anaplectoides prasina|[4324]|MNBB349-05|Canada|New Brunswick|658[0n]|BOLD:AAA2948
Anaplectoides prasina|[4325]|BBLEC790-09|Canada|Newfoundland and Labrador|658[0n]|BOLD:AAA2948
Anaplectoides prasina|[4326]|BBLEC422-09|Canada|New Brunswick|658[0n]|BOLD:AAA2948
Anaplectoides prasina|[4327]|BBLEC272-09|Canada|Nova Scotia|658[0n]|BOLD:AAA2948
Anaplectoides prasina|[4328]|LPMNB419-09|Canada|Manitoba|658[0n]|BOLD:AAA2948
Anaplectoides prasina|[4329]|RDLQG373-06|Canada|Quebec|658[0n]|BOLD:AAA2948
Anaplectoides prasina|[4330]|RDLQG031-06|Canada|Quebec|658[0n]|BOLD:AAA2948
Anaplectoides prasina|[4331]|TMNBB410-06|Canada|New Brunswick|658[0n]|BOLD:AAA2948
Anaplectoides prasina|[4332]|TMNBB397-06|Canada|New Brunswick|658[0n]|BOLD:AAA2948
Anaplectoides prasina|[4333]|TTMNB487-06|Canada|New Brunswick|658[0n]|BOLD:AAA2948
Anaplectoides prasina|[4334]|TTMNB486-06|Canada|New Brunswick|658[0n]|BOLD:AAA2948
Anaplectoides prasina|[4335]|XAI053-05|Canada|Ontario|658[0n]|BOLD:AAA2948
Anaplectoides prasina|[4336]|RDNMC001-05|Canada|Ontario|658[0n]|BOLD:AAA2948
Anaplectoides prasina|[4337]|MNBB651-05|Canada|New Brunswick|658[0n]|BOLD:AAA2948
Anaplectoides prasina|[4338]|MNBB612-05|Canada|New Brunswick|658[0n]|BOLD:AAA2948
Anaplectoides prasina|[4339]|MNBB514-05|Canada|New Brunswick|658[0n]|BOLD:AAA2948
Anaplectoides prasina|[4340]|MNBB481-05|Canada|New Brunswick|658[0n]|BOLD:AAA2948
Anaplectoides prasina|[4341]|MNBB438-05|Canada|New Brunswick|658[0n]|BOLD:AAA2948
Anaplectoides prasina|[4342]|MNBB437-05|Canada|New Brunswick|658[0n]|BOLD:AAA2948
Anaplectoides prasina|[4343]|MNBB359-05|Canada|New Brunswick|658[0n]|BOLD:AAA2948
Anaplectoides prasina|[4344]|MNBB351-05|Canada|New Brunswick|658[0n]|BOLD:AAA2948
Anaplectoides prasina|[4345]|MNBB350-05|Canada|New Brunswick|658[0n]|BOLD:AAA2948
Anaplectoides prasina|[4346]|MNBB242-05|Canada|New Brunswick|658[0n]|BOLD:AAA2948
Anaplectoides prasina|[4347]|MNBB175-05|Canada|New Brunswick|658[0n]|BOLD:AAA2948
Anaplectoides prasina|[4348]|MNBB174-05|Canada|New Brunswick|658[0n]|BOLD:AAA2948
Anaplectoides prasina|[4349]|MNBB173-05|Canada|New Brunswick|658[0n]|BOLD:AAA2948
Anaplectoides prasina|[4350]|MNBB118-05|Canada|New Brunswick|658[0n]|BOLD:AAA2948
Anaplectoides prasina|[4351]|MNBB105-05|Canada|New Brunswick|658[0n]|BOLD:AAA2948
Anaplectoides prasina|[4352]|MNBB104-05|Canada|New Brunswick|658[0n]|BOLD:AAA2948
Anaplectoides prasina|[4353]|MNBB103-05|Canada|New Brunswick|658[0n]|BOLD:AAA2948
Anaplectoides prasina|[4354]|MNBB102-05|Canada|New Brunswick|658[0n]|BOLD:AAA2948
Anaplectoides prasina|[4355]|MNBB101-05|Canada|New Brunswick|658[0n]|BOLD:AAA2948
Anaplectoides prasina|[4356]|MNBB100-05|Canada|New Brunswick|658[0n]|BOLD:AAA2948
Anaplectoides prasina|[4357]|MNBB099-05|Canada|New Brunswick|658[0n]|BOLD:AAA2948
Anaplectoides prasina|[4358]|MNBB098-05|Canada|New Brunswick|658[0n]|BOLD:AAA2948
Anaplectoides prasina|[4359]|MNBB096-05|Canada|New Brunswick|658[0n]|BOLD:AAA2948
Anaplectoides prasina|[4360]|PHMNB102-04|Canada|New Brunswick|658[0n]|BOLD:AAA2948
Anaplectoides prasina|[4361]|XAC611-04|Canada|Ontario|658[0n]|BOLD:AAA2948
Anaplectoides prasina|[4362]|XAC482-04|Canada|Ontario|658[0n]|BOLD:AAA2948
Anaplectoides prasina|[4363]|LGSM654-04|United States|Tennessee|658[0n]|BOLD:AAA2948
Anaplectoides prasina|[4364]|MNBB176-05|Canada|New Brunswick|658[0n]|BOLD:AAA2948
Anaplectoides prasina|[4365]|MNBB097-05|Canada|New Brunswick|658[0n]|BOLD:AAA2948
Anaplectoides prasina|[4366]|PMG090-03|Canada|Ontario|617[0n]|BOLD:AAA2948
Anaplectoides prasina|[4367]|PHMNB065-03|Canada|New Brunswick|639[0n]|BOLD:AAA2948
Anaplectoides prasina|[4368]|LOWCD583-06|Canada|British Columbia|586[0n]|BOLD:AAA2948
Anaplectoides prasina|[4369]|XAC568-04|Canada|Ontario|596[0n]|BOLD:AAA2948
Anaplectoides prasina|[4370]|LGSM653-04|United States|Tennessee|546[4n]|BOLD:AAA2948
Anaplectoides prasina|[4371]|MNBB287-05|Canada|New Brunswick|591[0n]|BOLD:AAA2948
Anaplectoides prasina|[4372]|MNBB244-05|Canada|New Brunswick|575[0n]|BOLD:AAA2948
Anaplectoides prasina|[4373]|LOWCD589-06|Canada|British Columbia|600[0n]|BOLD:AAA2948
Anaplectoides prasina|[4374]|MNBB095-05|Canada|New Brunswick|554[0n]|BOLD:AAA2948
Anaplectoides prasina|[4375]|MNBB215-05|Canada|New Brunswick|540[0n]|BOLD:AAA2948
Anaplectoides prasina|[4376]|LOWCD590-06|Canada|British Columbia|575[0n]|BOLD:AAA2948
Anaplectoides prasina|[4377]|LPVIB632-08|Canada|British Columbia|634[0n]|BOLD:AAA2948
Anaplectoides prasina|[4378]|BBLPC026-09|Canada|New Brunswick|656[0n]|BOLD:AAA2948
Anaplectoides prasina|[4379]|BBLPC099-09|Canada|New Brunswick|658[0n]|BOLD:AAA2948
Anaplectoides prasina|[4380]|BBLPC113-09|Canada|New Brunswick|658[0n]|BOLD:AAA2948
Anaplectoides prasina|[4381]|BBLPC598-09|Canada|Nova Scotia|658[0n]|BOLD:AAA2948
Anaplectoides prasina|[4382]|BBLPC620-09|Canada|Nova Scotia|658[0n]|BOLD:AAA2948
Anaplectoides prasina|[4383]|LGSMG845-10|United States|North Carolina|658[0n]|BOLD:AAA2948
Anaplectoides prasina|[4384]|LGSMG915-10|United States|North Carolina|658[0n]|BOLD:AAA2948
Anaplectoides prasina|[4385]|LGSMG916-10|United States|North Carolina|658[0n]|BOLD:AAA2948
Anaplectoides prasina|[4386]|LGSMG917-10|United States|North Carolina|658[0n]|BOLD:AAA2948

Anaplectoides prasina|[4384]|LGSMG915-10|United States|North Carolina|658[0n]|BOLD:AAA2948
Anaplectoides prasina|[4385]|LGSMG916-10|United States|North Carolina|658[0n]|BOLD:AAA2948
Anaplectoides prasina|[4386]|LGSMG917-10|United States|North Carolina|658[0n]|BOLD:AAA2948
Anaplectoides prasina|[4387]|LBCB888-05|Canada|British Columbia|640[1n]|BOLD:AAA2948
Anaplectoides prasina|[4388]|LBCC751-05|Canada|British Columbia|658[1n]|BOLD:AAA2948
Anaplectoides prasina|[4389]|LHLEP402-06|Canada|British Columbia|658[0n]|BOLD:AAA2948
Anaplectoides prasina|[4390]|LHLEP403-06|Canada|British Columbia|658[0n]|BOLD:AAA2948
Anaplectoides prasina|[4391]|LOWCD591-06|Canada|British Columbia|658[0n]|BOLD:AAA2948
Anaplectoides prasina|[4392]|LOWCD592-06|Canada|British Columbia|658[0n]|BOLD:AAA2948
Anaplectoides prasina|[4393]|LBCH3333-10|Canada|British Columbia|658[0n]|BOLD:AAA2948
Anaplectoides prasina|[4394]|LBCH3465-10|Canada|British Columbia|658[0n]|BOLD:AAA2948
Anaplectoides prasina|[4395]|LBCH3766-10|Canada|British Columbia|658[0n]|BOLD:AAA2948
Anaplectoides prasina|[4396]|LBCH4111-10|Canada|British Columbia|658[0n]|BOLD:AAA2948
Anaplectoides prasina|[4397]|LOWCD587-06|Canada|British Columbia|658[0n]|BOLD:AAA2948
Anaplectoides prasina|[4398]|LOWCD588-06|Canada|British Columbia|658[0n]|BOLD:AAA2948
Anaplectoides prasina|[4399]|LOWCD584-06|Canada|British Columbia|658[0n]|BOLD:AAA2948
Anaplectoides prasina|[4400]|LOWCD586-06|Canada|British Columbia|658[0n]|BOLD:AAA2948
Anaplectoides prasina|[4401]|LBCW054-08|Canada|British Columbia|658[0n]|BOLD:AAA2948
Anaplectoides prasina|[4402]|LPGVA599-08|Canada|British Columbia|658[0n]|BOLD:AAA2948
Anaplectoides prasina|[4403]|LBCG818-09|Canada|British Columbia|658[0n]|BOLD:AAA2948
Anaplectoides prasina|[4404]|LPABC339-09|Canada|Alberta|658[0n]|BOLD:AAA2948
Anaplectoides prasina|[4405]|LBCG2315-09|Canada|British Columbia|658[0n]|BOLD:AAA2948
Anaplectoides prasina|[4406]|LBCH013-10|Canada|British Columbia|658[0n]|BOLD:AAA2948
Anaplectoides prasina|[4407]|LBCH110-10|Canada|British Columbia|658[0n]|BOLD:AAA2948
Anaplectoides prasina|[4408]|LBCH224-10|Canada|British Columbia|658[0n]|BOLD:AAA2948
Anaplectoides prasina|[4409]|LBCH345-10|Canada|British Columbia|658[0n]|BOLD:AAA2948
Anaplectoides prasina|[4410]|LBCH490-10|Canada|British Columbia|658[0n]|BOLD:AAA2948
Anaplectoides prasina|[4411]|LBCH803-10|Canada|British Columbia|658[0n]|BOLD:AAA2948
Anaplectoides prasina|[4412]|LBCH933-10|Canada|British Columbia|658[0n]|BOLD:AAA2948
Anaplectoides prasina|[4413]|LBCH934-10|Canada|British Columbia|658[0n]|BOLD:AAA2948
Anaplectoides prasina|[4414]|LBCH1426-10|Canada|British Columbia|658[0n]|BOLD:AAA2948
Anaplectoides prasina|[4415]|LBCH1563-10|Canada|British Columbia|658[0n]|BOLD:AAA2948
Anaplectoides prasina|[4416]|LBCH2242-10|Canada|British Columbia|658[0n]|BOLD:AAA2948
Anaplectoides prasina|[4417]|LBCH3144-10|Canada|British Columbia|658[0n]|BOLD:AAA2948
Anaplectoides prasina|[4418]|LBCH3328-10|Canada|British Columbia|658[0n]|BOLD:AAA2948
Anaplectoides prasina|[4419]|LBCH4415-10|Canada|British Columbia|658[0n]|BOLD:AAA2948
Anaplectoides prasina|[4420]|LBCH4416-10|Canada|British Columbia|658[0n]|BOLD:AAA2948
Anaplectoides prasina|[4421]|LOWC851-05|Canada|British Columbia|658[0n]|BOLD:AAA2948
Anaplectoides prasina|[4422]|LOWC852-05|Canada|British Columbia|658[0n]|BOLD:AAA2948
Anaplectoides prasina|[4423]|LBCH4417-10|Canada|British Columbia|658[0n]|BOLD:AAA2948
Anaplectoides prasina|[4424]|LBCH4418-10|Canada|British Columbia|658[0n]|BOLD:AAA2948
Anaplectoides prasina|[4425]|LOWC854-05|Canada|British Columbia|658[0n]|BOLD:AAA2948
Anaplectoides prasina|[4426]|LOWC855-05|Canada|British Columbia|658[0n]|BOLD:AAA2948
Anaplectoides prasina|[4427]|LHLEP404-06|Canada|British Columbia|658[0n]|BOLD:AAA2948
Anaplectoides prasina|[4428]|LBCS654-07|Canada|British Columbia|658[0n]|BOLD:AAA2948
Anaplectoides prasina|[4429]|LBCH4419-10|Canada|British Columbia|658[0n]|BOLD:AAA2948
Anaplectoides prasina|[4430]|LBCH4420-10|Canada|British Columbia|658[0n]|BOLD:AAA2948
Anaplectoides prasina|[4431]|LBCH4421-10|Canada|British Columbia|658[0n]|BOLD:AAA2948
Anaplectoides prasina|[4432]|LBCH4422-10|Canada|British Columbia|658[0n]|BOLD:AAA2948
Anaplectoides prasina|[4433]|LBCH4671-10|Canada|British Columbia|658[0n]|BOLD:AAA2948
Anaplectoides prasina|[4434]|LBCH4672-10|Canada|British Columbia|658[0n]|BOLD:AAA2948
Anaplectoides prasina|[4435]|LBCH4673-10|Canada|British Columbia|658[0n]|BOLD:AAA2948
Anaplectoides prasina|[4436]|LBCH4674-10|Canada|British Columbia|658[0n]|BOLD:AAA2948
Anaplectoides prasina|[4437]|LBCH4675-10|Canada|British Columbia|658[0n]|BOLD:AAA2948
Anaplectoides prasina|[4438]|LBCH4676-10|Canada|British Columbia|658[0n]|BOLD:AAA2948
Anaplectoides prasina|[4439]|LBCH4677-10|Canada|British Columbia|658[0n]|BOLD:AAA2948
Anaplectoides prasina|[4440]|LBCH6524-10|Canada|British Columbia|658[0n]|BOLD:AAA2948
Anaplectoides prasina|[4441]|LALPA720-10|Canada|British Columbia|658[0n]|BOLD:AAA2948
Anaplectoides prasina|[4442]|LALPA764-10|Canada|British Columbia|658[0n]|BOLD:AAA2948
Anaplectoides prasina|[4443]|LALPA801-10|Canada|British Columbia|658[0n]|BOLD:AAA2948
Anaplectoides prasina|[4444]|LALPA803-10|Canada|British Columbia|658[0n]|BOLD:AAA2948
Anaplectoides prasina|[4445]|LBCD094-05|Canada|British Columbia|658[0n]|BOLD:AAA2948
Anaplectoides prasina|[4446]|LBCD093-05|Canada|British Columbia|658[0n]|BOLD:AAA2948
Anaplectoides prasina|[4447]|LBCD092-05|Canada|British Columbia|658[0n]|BOLD:AAA2948
Anaplectoides prasina|[4448]|LBCC829-05|Canada|British Columbia|658[0n]|BOLD:AAA2948
Anaplectoides prasina|[4449]|LBCC752-05|Canada|British Columbia|658[0n]|BOLD:AAA2948
Anaplectoides prasina|[4450]|LBCC326-05|Canada|British Columbia|658[0n]|BOLD:AAA2948
Anaplectoides prasina|[4451]|LBCC014-05|Canada|British Columbia|658[0n]|BOLD:AAA2948
Anaplectoides prasina|[4452]|LBCB614-05|Canada|British Columbia|658[0n]|BOLD:AAA2948
Anaplectoides prasina|[4453]|LBCB613-05|Canada|British Columbia|658[0n]|BOLD:AAA2948
Anaplectoides prasina|[4454]|LBCD330-05|Canada|British Columbia|650[0n]|BOLD:AAA2948
Anaplectoides prasina|[4455]|LOWC847-05|Canada|British Columbia|658[0n]|BOLD:AAA2948
Anaplectoides prasina|[4456]|LOWC848-05|Canada|British Columbia|658[0n]|BOLD:AAA2948
Anaplectoides prasina|[4457]|LOWC849-05|Canada|British Columbia|658[0n]|BOLD:AAA2948
Anaplectoides prasina|[4458]|LOWC850-05|Canada|British Columbia|658[0n]|BOLD:AAA2948
Anaplectoides prasina|[4459]|LALPA804-10|Canada|British Columbia|658[0n]|BOLD:AAA2948
Anaplectoides prasina|[4460]|LBCG007-08|Canada|British Columbia|658[0n]|BOLD:AAA2948
Anaplectoides prasina|[4461]|LPABC278-09|Canada|Alberta|545[0n]|BOLD:AAA2948
Anaplectoides prasina|[4462]|CNWBG3125-13|Canada|Alberta|575[0n]|BOLD:AAA2948
Anaplectoides brunneomedia|[4463]|LGSM476-04|United States|North Carolina|658[0n]|BOLD:AAA3756
Anaplectoides brunneomedia|[4464]|RDNMC330-05|United States|North Carolina|600[0n]|BOLD:AAA3756
Anaplectoides brunneomedia|[4465]|RDNMC331-05|United States|North Carolina|609[0n]|BOLD:AAA3756
Anaplectoides pressus|[4466]|LPABC489-09|Canada|Alberta|658[0n]|BOLD:AAA3756
Anaplectoides pressus|[4467]|LBCH025-10|Canada|British Columbia|658[0n]|BOLD:AAA3756
Anaplectoides pressus|[4468]|LPABC452-09|Canada|Alberta|658[0n]|BOLD:AAA3756
Anaplectoides pressus|[4469]|LPABC459-09|Canada|Alberta|658[0n]|BOLD:AAA3756
Anaplectoides pressus|[4470]|BBLPE363-09|Canada|Newfoundland and Labrador|658[0n]|BOLD:AAA3756
Anaplectoides pressus|[4471]|LPABC271-09|Canada|Alberta|641[0n]|BOLD:AAA3756
Anaplectoides pressus|[4472]|LOWC894-05|Canada|British Columbia|658[0n]|BOLD:AAA3756
Anaplectoides pressus|[4473]|LBCH237-10|Canada|British Columbia|658[0n]|BOLD:AAA3756
Anaplectoides pressus|[4474]|CNRME1821-12|Canada|Manitoba|636[0n]|BOLD:AAA3756
Anaplectoides pressus|[4475]|LBCB076-05|Canada|British Columbia|658[0n]|BOLD:AAA3756
Anaplectoides pressus|[4476]|LALPA1256-11|Canada|British Columbia|658[0n]|BOLD:AAA3756
Anaplectoides pressus|[4477]|LALPA874-11|Canada|British Columbia|658[0n]|BOLD:AAA3756
Anaplectoides pressus|[4478]|BBLPB869-10|Canada|British Columbia|658[0n]|BOLD:AAA3756
Anaplectoides pressus|[4479]|BBLPB856-10|Canada|British Columbia|658[0n]|BOLD:AAA3756
Anaplectoides pressus|[4480]|BBLPB704-10|Canada|British Columbia|658[0n]|BOLD:AAA3756
Anaplectoides pressus|[4481]|BBLPB408-10|Canada|Saskatchewan|658[0n]|BOLD:AAA3756
Anaplectoides pressus|[4482]|LBCH4693-10|Canada|British Columbia|658[0n]|BOLD:AAA3756
Anaplectoides pressus|[4483]|LBCH4692-10|Canada|British Columbia|658[0n]|BOLD:AAA3756
Anaplectoides pressus|[4484]|LBCH4435-10|Canada|British Columbia|658[0n]|BOLD:AAA3756
Anaplectoides pressus|[4485]|LBCH4432-10|Canada|British Columbia|658[0n]|BOLD:AAA3756
Anaplectoides pressus|[4486]|LBCH4115-10|Canada|British Columbia|658[0n]|BOLD:AAA3756

Anaplectoides pressus|[4483]|LBCH4092-10|Canada|British Columbia|658[0n]|BOLD:AAA3756
Anaplectoides pressus|[4484]|LBCH4435-10|Canada|British Columbia|658[0n]|BOLD:AAA3756
Anaplectoides pressus|[4485]|LBCH4432-10|Canada|British Columbia|658[0n]|BOLD:AAA3756
Anaplectoides pressus|[4486]|LBCH4115-10|Canada|British Columbia|658[0n]|BOLD:AAA3756
Anaplectoides pressus|[4487]|LBCH4114-10|Canada|British Columbia|658[0n]|BOLD:AAA3756
Anaplectoides pressus|[4488]|LBCH3467-10|Canada|British Columbia|658[0n]|BOLD:AAA3756
Anaplectoides pressus|[4489]|LBCH3093-10|Canada|British Columbia|658[0n]|BOLD:AAA3756
Anaplectoides pressus|[4490]|LBCH3091-10|Canada|British Columbia|658[0n]|BOLD:AAA3756
Anaplectoides pressus|[4491]|LBCH3090-10|Canada|British Columbia|658[0n]|BOLD:AAA3756
Anaplectoides pressus|[4492]|LBCH2396-10|Canada|British Columbia|658[0n]|BOLD:AAA3756
Anaplectoides pressus|[4493]|LBCH967-10|Canada|British Columbia|658[0n]|BOLD:AAA3756
Anaplectoides pressus|[4494]|LBCH813-10|Canada|British Columbia|658[0n]|BOLD:AAA3756
Anaplectoides pressus|[4495]|LBCH812-10|Canada|British Columbia|658[0n]|BOLD:AAA3756
Anaplectoides pressus|[4496]|LBCH811-10|Canada|British Columbia|658[0n]|BOLD:AAA3756
Anaplectoides pressus|[4497]|LBCH697-10|Canada|British Columbia|658[0n]|BOLD:AAA3756
Anaplectoides pressus|[4498]|LBCH495-10|Canada|British Columbia|658[0n]|BOLD:AAA3756
Anaplectoides pressus|[4499]|LBCH352-10|Canada|British Columbia|658[0n]|BOLD:AAA3756
Anaplectoides pressus|[4500]|LBCH236-10|Canada|British Columbia|658[0n]|BOLD:AAA3756
Anaplectoides pressus|[4501]|LBCH235-10|Canada|British Columbia|658[0n]|BOLD:AAA3756
Anaplectoides pressus|[4502]|LBCH055-10|Canada|British Columbia|658[0n]|BOLD:AAA3756
Anaplectoides pressus|[4503]|LBCH028-10|Canada|British Columbia|658[0n]|BOLD:AAA3756
Anaplectoides pressus|[4504]|LBCH027-10|Canada|British Columbia|658[0n]|BOLD:AAA3756
Anaplectoides pressus|[4505]|LBCH026-10|Canada|British Columbia|658[0n]|BOLD:AAA3756
Anaplectoides pressus|[4506]|LBCH024-10|Canada|British Columbia|658[0n]|BOLD:AAA3756
Anaplectoides pressus|[4507]|LBCH023-10|Canada|British Columbia|658[0n]|BOLD:AAA3756
Anaplectoides pressus|[4508]|LBCH022-10|Canada|British Columbia|658[0n]|BOLD:AAA3756
Anaplectoides pressus|[4509]|LBCH021-10|Canada|British Columbia|658[0n]|BOLD:AAA3756
Anaplectoides pressus|[4510]|BBLPC897-09|Canada|Newfoundland and Labrador|658[0n]|BOLD:AAA3756
Anaplectoides pressus|[4511]|BBLPC656-09|Canada|Newfoundland and Labrador|658[0n]|BOLD:AAA3756
Anaplectoides pressus|[4512]|BBLPC645-09|Canada|Newfoundland and Labrador|658[0n]|BOLD:AAA3756
Anaplectoides pressus|[4513]|BBLPC244-09|Canada|Nova Scotia|658[0n]|BOLD:AAA3756
Anaplectoides pressus|[4514]|BBLPC241-09|Canada|Nova Scotia|658[0n]|BOLD:AAA3756
Anaplectoides pressus|[4515]|BBLPC155-09|Canada|Nova Scotia|658[0n]|BOLD:AAA3756
Anaplectoides pressus|[4516]|BBLEC855-09|Canada|Newfoundland and Labrador|658[0n]|BOLD:AAA3756
Anaplectoides pressus|[4517]|LBCG2771-09|Canada|British Columbia|658[0n]|BOLD:AAA3756
Anaplectoides pressus|[4518]|LPABC478-09|Canada|Alberta|658[0n]|BOLD:AAA3756
Anaplectoides pressus|[4519]|LPABC470-09|Canada|Alberta|658[0n]|BOLD:AAA3756
Anaplectoides pressus|[4520]|LPABC465-09|Canada|Alberta|658[0n]|BOLD:AAA3756
Anaplectoides pressus|[4521]|LPABC462-09|Canada|Alberta|658[0n]|BOLD:AAA3756
Anaplectoides pressus|[4522]|LPABC461-09|Canada|Alberta|658[0n]|BOLD:AAA3756
Anaplectoides pressus|[4523]|LPABC455-09|Canada|Alberta|658[0n]|BOLD:AAA3756
Anaplectoides pressus|[4524]|LPABC451-09|Canada|Alberta|658[0n]|BOLD:AAA3756
Anaplectoides pressus|[4525]|LPABC450-09|Canada|Alberta|658[0n]|BOLD:AAA3756
Anaplectoides pressus|[4526]|LPABC441-09|Canada|Alberta|658[0n]|BOLD:AAA3756
Anaplectoides pressus|[4527]|LPABC424-09|Canada|Alberta|658[0n]|BOLD:AAA3756
Anaplectoides pressus|[4528]|LPABC421-09|Canada|Alberta|658[0n]|BOLD:AAA3756
Anaplectoides pressus|[4529]|LPABC417-09|Canada|Alberta|658[0n]|BOLD:AAA3756
Anaplectoides pressus|[4530]|LPABC393-09|Canada|Alberta|658[0n]|BOLD:AAA3756
Anaplectoides pressus|[4531]|LPABC391-09|Canada|Alberta|658[0n]|BOLD:AAA3756
Anaplectoides pressus|[4532]|LPABC377-09|Canada|Alberta|658[0n]|BOLD:AAA3756
Anaplectoides pressus|[4533]|LPABC372-09|Canada|Alberta|658[0n]|BOLD:AAA3756
Anaplectoides pressus|[4534]|LPABC371-09|Canada|Alberta|658[0n]|BOLD:AAA3756
Anaplectoides pressus|[4535]|LPABC350-09|Canada|Alberta|658[0n]|BOLD:AAA3756
Anaplectoides pressus|[4536]|LPABC332-09|Canada|Alberta|658[0n]|BOLD:AAA3756
Anaplectoides pressus|[4537]|LBCG845-09|Canada|British Columbia|658[0n]|BOLD:AAA3756
Anaplectoides pressus|[4538]|LBCG571-09|Canada|British Columbia|658[0n]|BOLD:AAA3756
Anaplectoides pressus|[4539]|LPAB244-08|Canada|Alberta|658[0n]|BOLD:AAA3756
Anaplectoides pressus|[4540]|LOWCE844-06|Canada|British Columbia|658[0n]|BOLD:AAA3756
Anaplectoides pressus|[4541]|LOWCE841-06|Canada|British Columbia|658[0n]|BOLD:AAA3756
Anaplectoides pressus|[4542]|LOWCE822-06|Canada|British Columbia|658[0n]|BOLD:AAA3756
Anaplectoides pressus|[4543]|LOWCE786-06|Canada|British Columbia|658[0n]|BOLD:AAA3756
Anaplectoides pressus|[4544]|LOWCE785-06|Canada|British Columbia|658[0n]|BOLD:AAA3756
Anaplectoides pressus|[4545]|TTMNB489-06|Canada|New Brunswick|658[0n]|BOLD:AAA3756
Anaplectoides pressus|[4546]|TTMNB488-06|Canada|New Brunswick|658[0n]|BOLD:AAA3756
Anaplectoides pressus|[4547]|LOWCD582-06|Canada|British Columbia|658[0n]|BOLD:AAA3756
Anaplectoides pressus|[4548]|LOWCD581-06|Canada|British Columbia|658[0n]|BOLD:AAA3756
Anaplectoides pressus|[4549]|LOWCD580-06|Canada|British Columbia|658[0n]|BOLD:AAA3756
Anaplectoides pressus|[4550]|LOWCD579-06|Canada|British Columbia|658[0n]|BOLD:AAA3756
Anaplectoides pressus|[4551]|LOWCD577-06|Canada|British Columbia|658[0n]|BOLD:AAA3756
Anaplectoides pressus|[4552]|LOWCD576-06|Canada|British Columbia|658[0n]|BOLD:AAA3756
Anaplectoides pressus|[4553]|LOWCD575-06|Canada|British Columbia|658[0n]|BOLD:AAA3756
Anaplectoides pressus|[4554]|RDLQB293-05|Canada|Quebec|658[0n]|BOLD:AAA3756
Anaplectoides pressus|[4555]|RDLQB292-05|Canada|Quebec|658[0n]|BOLD:AAA3756
Anaplectoides pressus|[4556]|RDLQB290-05|Canada|Quebec|658[0n]|BOLD:AAA3756
Anaplectoides pressus|[4557]|LOWC918-05|Canada|British Columbia|658[0n]|BOLD:AAA3756
Anaplectoides pressus|[4558]|LOWC893-05|Canada|British Columbia|658[0n]|BOLD:AAA3756
Anaplectoides pressus|[4559]|LOWC892-05|Canada|British Columbia|658[0n]|BOLD:AAA3756
Anaplectoides pressus|[4560]|LOWC862-05|Canada|British Columbia|658[0n]|BOLD:AAA3756
Anaplectoides pressus|[4561]|LOWC860-05|Canada|British Columbia|658[0n]|BOLD:AAA3756
Anaplectoides pressus|[4562]|LOWC859-05|Canada|British Columbia|658[0n]|BOLD:AAA3756
Anaplectoides pressus|[4563]|LOWC856-05|Canada|British Columbia|658[0n]|BOLD:AAA3756
Anaplectoides pressus|[4564]|LBCC644-05|Canada|British Columbia|658[0n]|BOLD:AAA3756
Anaplectoides pressus|[4565]|LBCC059-05|Canada|British Columbia|658[0n]|BOLD:AAA3756
Anaplectoides pressus|[4566]|LBCB755-05|Canada|British Columbia|658[0n]|BOLD:AAA3756
Anaplectoides pressus|[4567]|RDNM979-05|Canada|British Columbia|658[0n]|BOLD:AAA3756
Anaplectoides pressus|[4568]|RDNM978-05|United States|Oregon|658[0n]|BOLD:AAA3756
Anaplectoides pressus|[4569]|RDNM977-05|Canada|Ontario|658[0n]|BOLD:AAA3756
Anaplectoides pressus|[4570]|RDLQB291-05|Canada|Quebec|658[0n]|BOLD:AAA3756
Anaplectoides pressus|[4571]|LPABC193-09|Canada|Alberta|619[0n]|BOLD:AAA3756
Anaplectoides pressus|[4572]|LOWC857-05|Canada|British Columbia|608[0n]|BOLD:AAA3756
Anaplectoides pressus|[4573]|LOWC861-05|Canada|British Columbia|658[0n]|BOLD:AAA3756
Anaplectoides pressus|[4574]|LOWCD578-06|Canada|British Columbia|546[0n]|BOLD:AAA3756
Anaplectoides pressus|[4575]|LOWC858-05|Canada|British Columbia|578[0n]|BOLD:AAA3756
Anaplectoides pressus|[4576]|LPABC488-09|Canada|Alberta|635[0n]|BOLD:AAA3756
Anaplectoides pressus|[4577]|LOWC921-05|Canada|British Columbia|601[0n]|BOLD:AAA3756
Anaplectoides pressus|[4578]|LPABC490-09|Canada|Alberta|612[0n]|BOLD:AAA3756
Anaplectoides pressus|[4579]|LPABC482-09|Canada|Alberta|623[0n]|BOLD:AAA3756
Anaplectoides pressus|[4580]|BBLPB703-10|Canada|British Columbia|621[0n]|BOLD:AAA3756
Anaplectoides pressus|[4581]|LGSMG914-10|United States|North Carolina|658[0n]|BOLD:AAA3756
Anaplectoides pressus|[4582]|RDNMD343-06|United States|North Carolina|658[0n]|BOLD:AAA3756
Anaplectoides pressus|[4583]|RDNMD342-06|United States|North Carolina|658[0n]|BOLD:AAA3756
Anaplectoides pressus|[4584]|RDNM980-05|United States|Oregon|658[0n]|BOLD:AAA3756
Anaplectoides pressus|[4585]|LGSM477-04|United States|North Carolina|658[0n]|BOLD:AAA3756
[illegible]

Anaplectoides pressus|[4583]|RDNMD342-06|United States|North Carolina|658[0n]|BOLD:AAA3756
Anaplectoides pressus|[4584]|RDNM980-05|United States|Oregon|658[0n]|BOLD:AAA3756
Anaplectoides pressus|[4585]|LGSM477-04|United States|North Carolina|658[0n]|BOLD:AAA3756
Anaplectoides pressus|[4586]|CNWBG3128-13|Canada|Alberta|603[0n]|BOLD:AAA3756
Noctua pronuba|[4587]|LEFIA581-10|Finland|South Karelia|658[0n]|BOLD:AAA2632
Noctua pronuba|[4588]|NLLEA786-12|Netherlands|South Holland|658[0n]|BOLD:AAA2632
Noctua pronuba|[4589]|LOCT314-05|United States|Connecticut|658[0n]|BOLD:AAA2632
Noctua pronuba|[4590]|GWOSZ109-11|Italy|Trentino-Alto Adige|658[0n]|BOLD:AAA2632
Noctua pronuba|[4591]|LBCS639-07|Canada|British Columbia|647[0n]|BOLD:AAA2632
Noctua pronuba|[4592]|BCMI035-11|Israel||658[0n]|BOLD:AAA2632
Noctua pronuba|[4593]|LENOA114-11|France|Haute Normandie|658[0n]|BOLD:AAA2632
Noctua pronuba|[4594]|AWCLB329-10|United States|California|658[0n]|BOLD:AAA2632
Noctua pronuba|[4595]|LEFIC780-10|Finland|Finland Proper|658[0n]|BOLD:AAA2632
Noctua pronuba|[4596]|CGUKA568-09|United Kingdom|England|658[0n]|BOLD:AAA2632
Noctua pronuba|[4597]|LBCS246-07|Canada|British Columbia|658[0n]|BOLD:AAA2632
Noctua pronuba|[4598]|LBCS181-07|Canada|British Columbia|658[0n]|BOLD:AAA2632
Noctua pronuba|[4599]|LOCBE116-06|United States|California|658[0n]|BOLD:AAA2632
Noctua pronuba|[4600]|CNWLD1009-12|Canada|Alberta|633[0n]|BOLD:AAA2632
Noctua pronuba|[4601]|CGUKB722-09|United Kingdom|England|658[0n]|BOLD:AAA2632
Noctua pronuba|[4602]|LEATC646-13|Italy|South Tyrol|658[0n]|BOLD:AAA2632
Noctua pronuba|[4603]|IBLAO286-12|Spain|Murcia|658[0n]|BOLD:AAA2632
Noctua pronuba|[4604]|BCMI423-11|Israel||658[0n]|BOLD:AAA2632
Noctua pronuba|[4605]|BBLEC418-09|Canada|New Brunswick|658[0n]|BOLD:AAA2632
Noctua pronuba|[4606]|GWORO843-09|Germany|Bavaria|658[0n]|BOLD:AAA2632
Noctua pronuba|[4607]|GWORL292-09|Germany|Bavaria|658[0n]|BOLD:AAA2632
Noctua pronuba|[4608]|LBCS366-07|Canada|British Columbia|658[0n]|BOLD:AAA2632
Noctua pronuba|[4609]|PHMNB084-03|Canada|New Brunswick|639[0n]|BOLD:AAA2632
Noctua pronuba|[4610]|JBAZ031-09|United States|California|658[0n]|BOLD:AAA2632
Noctua pronuba|[4611]|GBLAC330-13|Germany|Thuringia|658[0n]|BOLD:AAA2632
Noctua pronuba|[4612]|LALPA121-10|Canada|British Columbia|658[0n]|BOLD:AAA2632
Noctua pronuba|[4613]|LCHP814-07|Canada|Manitoba|658[0n]|BOLD:AAA2632
Noctua pronuba|[4614]|LNCNW012-06|United States|North Carolina|608[0n]|BOLD:AAA2632
Noctua pronuba|[4615]|HPPPD1636-13|Canada|Nova Scotia|548[0n]|BOLD:AAA2632
Noctua pronuba|[4616]|CGUKB073-09|United Kingdom|England|658[0n]|BOLD:AAA2632
Noctua pronuba|[4617]|GWOR4045-09|Germany|Bavaria|658[0n]|BOLD:AAA2632
Noctua pronuba|[4618]|GWOSP677-11|Tunisia||658[0n]|BOLD:AAA2632
Noctua pronuba|[4619]|LEATB703-13|Italy|South Tyrol|658[0n]|BOLD:AAA2632
Noctua pronuba|[4620]|TRLEP056-13|Turkey|Mersin|658[0n]|BOLD:AAA2632
Noctua pronuba|[4621]|LBCS104-07|Canada|British Columbia|658[0n]|BOLD:AAA2632
Noctua pronuba|[4622]|LPSO732-08|Canada|Ontario|658[0n]|BOLD:AAA2632
Noctua pronuba|[4623]|BBLEC455-09|Canada|New Brunswick|658[0n]|BOLD:AAA2632
Noctua pronuba|[4624]|CGUKA349-09|United Kingdom|England|658[0n]|BOLD:AAA2632
Noctua pronuba|[4625]|CGUKA712-09|United Kingdom||658[0n]|BOLD:AAA2632
Noctua pronuba|[4626]|GWOTL103-13|Germany|Saarland|658[0n]|BOLD:AAA2632
Noctua pronuba|[4627]|PPGB342-12|Canada|Ontario|669[0n]|
Noctua pronuba|[4628]|LEUAE1113-12|United Arab Emirates|Fujairah|665[0n]|BOLD:AAA2632
Noctua pronuba|[4629]|GBLAC403-13|Germany|Bavaria|658[0n]|BOLD:AAA2632
Noctua pronuba|[4630]|LEATD235-13|Austria|Tirol|658[0n]|BOLD:AAA2632
Noctua pronuba|[4631]|LEATC134-13|Austria|Tirol|658[0n]|BOLD:AAA2632
Noctua pronuba|[4632]|TRLEP058-13|Turkey|Mersin|658[0n]|BOLD:AAA2632
Noctua pronuba|[4633]|GBLAB596-13|Germany|Brandenburg|658[0n]|BOLD:AAA2632
Noctua pronuba|[4634]|UAMIC649-13|United States|Alaska|658[0n]|BOLD:AAA2632
Noctua pronuba|[4635]|UAMIC648-13|United States|Alaska|658[0n]|BOLD:AAA2632
Noctua pronuba|[4636]|PHLAV335-12|Austria|Vorarlberg|658[0n]|BOLD:AAA2632
Noctua pronuba|[4637]|FBLMZ509-12|Germany|Bavaria|658[0n]|BOLD:AAA2632
Noctua pronuba|[4638]|RWWC850-12|United States|Washington|658[0n]|BOLD:AAA2632
Noctua pronuba|[4639]|IBLAO669-12|Spain|Castilla-La Mancha|658[0n]|BOLD:AAA2632
Noctua pronuba|[4640]|GMLC1208-12|United States|California|658[0n]|BOLD:AAA2632
Noctua pronuba|[4641]|NLLEA510-12|Netherlands|South Holland|658[0n]|BOLD:AAA2632
Noctua pronuba|[4642]|NLLEA207-12|Netherlands|South Holland|658[0n]|BOLD:AAA2632
Noctua pronuba|[4643]|PHJUN3992-11|Canada|Ontario|658[0n]|BOLD:AAA2632
Noctua pronuba|[4644]|BCMI426-11|Israel||658[0n]|BOLD:AAA2632
Noctua pronuba|[4645]|RWWC266-11|United States|Washington|658[0n]|BOLD:AAA2632
Noctua pronuba|[4646]|MPSC047-11|Portugal|Minho|658[0n]|BOLD:AAA2632
Noctua pronuba|[4647]|GMLC619-11|United States|California|658[0n]|BOLD:AAA2632
Noctua pronuba|[4648]|LENOA112-11|France|Haute Normandie|658[0n]|BOLD:AAA2632
Noctua pronuba|[4649]|LENOA111-11|France|Haute Normandie|658[0n]|BOLD:AAA2632
Noctua pronuba|[4650]|RWWC156-10|United States|Washington|658[0n]|BOLD:AAA2632
Noctua pronuba|[4651]|AWCLB380-10|United States|California|658[0n]|BOLD:AAA2632
Noctua pronuba|[4652]|AWCLB378-10|United States|California|658[0n]|BOLD:AAA2632
Noctua pronuba|[4653]|AWCLB330-10|United States|California|658[0n]|BOLD:AAA2632
Noctua pronuba|[4654]|LALPA122-10|Canada|British Columbia|658[0n]|BOLD:AAA2632
Noctua pronuba|[4655]|GWORZ204-10|Italy|Calabria|658[0n]|BOLD:AAA2632
Noctua pronuba|[4656]|LBCH7424-10|Canada|British Columbia|658[0n]|BOLD:AAA2632
Noctua pronuba|[4657]|LBCH7326-10|Canada|British Columbia|658[0n]|BOLD:AAA2632
Noctua pronuba|[4658]|LGSMG922-10|United States|North Carolina|658[0n]|BOLD:AAA2632
Noctua pronuba|[4659]|LGSMG921-10|United States|North Carolina|658[0n]|BOLD:AAA2632
Noctua pronuba|[4660]|LGSMG920-10|United States|North Carolina|658[0n]|BOLD:AAA2632
Noctua pronuba|[4661]|BBLPC438-09|Canada|New Brunswick|658[0n]|BOLD:AAA2632
Noctua pronuba|[4662]|BBLPC402-09|Canada|New Brunswick|658[0n]|BOLD:AAA2632
Noctua pronuba|[4663]|BBLPC396-09|Canada|New Brunswick|658[0n]|BOLD:AAA2632
Noctua pronuba|[4664]|BBLPC392-09|Canada|New Brunswick|658[0n]|BOLD:AAA2632
Noctua pronuba|[4665]|BBLEC456-09|Canada|New Brunswick|658[0n]|BOLD:AAA2632
Noctua pronuba|[4666]|BBLEC441-09|Canada|New Brunswick|658[0n]|BOLD:AAA2632
Noctua pronuba|[4667]|BBLEC423-09|Canada|New Brunswick|658[0n]|BOLD:AAA2632
Noctua pronuba|[4668]|BBLEC420-09|Canada|New Brunswick|658[0n]|BOLD:AAA2632
Noctua pronuba|[4669]|BBLEC419-09|Canada|New Brunswick|658[0n]|BOLD:AAA2632
Noctua pronuba|[4670]|BBLEC416-09|Canada|New Brunswick|658[0n]|BOLD:AAA2632
Noctua pronuba|[4671]|BBLWU077-09|United States|Colorado|658[0n]|BOLD:AAA2632
Noctua pronuba|[4672]|RWWB218-09|United States|Washington|658[0n]|BOLD:AAA2632
Noctua pronuba|[4673]|RWWA897-09|United States|Washington|658[0n]|BOLD:AAA2632
Noctua pronuba|[4674]|RWWA194-09|United States|Washington|656[0n]|BOLD:AAA2632
Noctua pronuba|[4675]|LBCG3749-09|Canada|British Columbia|658[0n]|BOLD:AAA2632
Noctua pronuba|[4676]|LBCG3748-09|Canada|British Columbia|658[0n]|BOLD:AAA2632
Noctua pronuba|[4677]|LBCG3747-09|Canada|British Columbia|658[0n]|BOLD:AAA2632
Noctua pronuba|[4678]|LBCG3745-09|Canada|British Columbia|658[0n]|BOLD:AAA2632
Noctua pronuba|[4679]|UDLEP153-09|United States|Pennsylvania|658[0n]|BOLD:AAA2632
Noctua pronuba|[4680]|LPMNB466-09|Canada|Manitoba|658[0n]|BOLD:AAA2632
Noctua pronuba|[4681]|GMLC109-09|United States|California|658[0n]|BOLD:AAA2632
Noctua pronuba|[4682]|GMLC072-09|United States|California|658[0n]|BOLD:AAA2632
Noctua pronuba|[4683]|CGUKB950-09|United Kingdom|Wales|658[0n]|BOLD:AAA2632
Noctua pronuba|[4684]|CGUKA794-09|United Kingdom||658[0n]|BOLD:AAA2632
Noctua pronuba|[4685]|CGUKA435-09|United Kingdom|England|658[0n]|BOLD:AAA2632

Noctua pronuba|[4683]|CGUKB950-09|United Kingdom|Wales|658[0n]|BOLD:AAA2632
Noctua pronuba|[4684]|CGUKA794-09|United Kingdom||658[0n]|BOLD:AAA2632
Noctua pronuba|[4685]|CGUKA435-09|United Kingdom|England|658[0n]|BOLD:AAA2632
Noctua pronuba|[4686]|CGUKA251-09|United Kingdom|England|658[0n]|BOLD:AAA2632
Noctua pronuba|[4687]|TTNFS176-09|Serbia||658[0n]|BOLD:AAA2632
Noctua pronuba|[4688]|LBCW045-08|Canada|British Columbia|658[0n]|BOLD:AAA2632
Noctua pronuba|[4689]|LON580-08|Norway|Ostfold|657[0n]|BOLD:AAA2632
Noctua pronuba|[4690]|BLTIB1000-08|Canada|Ontario|658[0n]|BOLD:AAA2632
Noctua pronuba|[4691]|BLTIB783-08|Canada|Ontario|658[0n]|BOLD:AAA2632
Noctua pronuba|[4692]|BLTIB704-08|Canada|Ontario|658[0n]|BOLD:AAA2632
Noctua pronuba|[4693]|LPSOB946-08|Canada|Ontario|658[0n]|BOLD:AAA2632
Noctua pronuba|[4694]|LPSOB798-08|Canada|Ontario|658[0n]|BOLD:AAA2632
Noctua pronuba|[4695]|LPSOB079-08|Canada|Ontario|658[0n]|BOLD:AAA2632
Noctua pronuba|[4696]|LCHQ658-08|Canada|Manitoba|658[0n]|BOLD:AAA2632
Noctua pronuba|[4697]|LBCS638-07|Canada|British Columbia|658[0n]|BOLD:AAA2632
Noctua pronuba|[4698]|LBCS637-07|Canada|British Columbia|658[0n]|BOLD:AAA2632
Noctua pronuba|[4699]|LBCS439-07|Canada|British Columbia|658[0n]|BOLD:AAA2632
Noctua pronuba|[4700]|LBCS438-07|Canada|British Columbia|658[0n]|BOLD:AAA2632
Noctua pronuba|[4701]|LBCS365-07|Canada|British Columbia|658[0n]|BOLD:AAA2632
Noctua pronuba|[4702]|LBCS362-07|Canada|British Columbia|658[0n]|BOLD:AAA2632
Noctua pronuba|[4703]|LBCS247-07|Canada|British Columbia|658[0n]|BOLD:AAA2632
Noctua pronuba|[4704]|LBCS245-07|Canada|British Columbia|658[0n]|BOLD:AAA2632
Noctua pronuba|[4705]|LBCS244-07|Canada|British Columbia|658[0n]|BOLD:AAA2632
Noctua pronuba|[4706]|LBCS243-07|Canada|British Columbia|658[0n]|BOLD:AAA2632
Noctua pronuba|[4707]|LBCS183-07|Canada|British Columbia|658[0n]|BOLD:AAA2632
Noctua pronuba|[4708]|LBCS107-07|Canada|British Columbia|658[0n]|BOLD:AAA2632
Noctua pronuba|[4709]|LBCS106-07|Canada|British Columbia|658[0n]|BOLD:AAA2632
Noctua pronuba|[4710]|LBCS105-07|Canada|British Columbia|658[0n]|BOLD:AAA2632
Noctua pronuba|[4711]|LBCS027-07|Canada|British Columbia|658[0n]|BOLD:AAA2632
Noctua pronuba|[4712]|LBCS025-07|Canada|British Columbia|658[0n]|BOLD:AAA2632
Noctua pronuba|[4713]|LBCS001-07|Canada|British Columbia|658[0n]|BOLD:AAA2632
Noctua pronuba|[4714]|LCHP771-07|Canada|Manitoba|658[0n]|BOLD:AAA2632
Noctua pronuba|[4715]|LHLEP061-06|Canada|British Columbia|658[0n]|BOLD:AAA2632
Noctua pronuba|[4716]|LHLEP024-06|Canada|British Columbia|658[0n]|BOLD:AAA2632
Noctua pronuba|[4717]|RDLQF474-06|Canada|Quebec|658[0n]|BOLD:AAA2632
Noctua pronuba|[4718]|XAK448-06|Canada|Ontario|658[0n]|BOLD:AAA2632
Noctua pronuba|[4719]|XAJ684-06|Canada|Ontario|658[0n]|BOLD:AAA2632
Noctua pronuba|[4720]|LOCBB214-06|United States|California|658[0n]|BOLD:AAA2632
Noctua pronuba|[4721]|XAH450-05|Canada|Ontario|658[0n]|BOLD:AAA2632
Noctua pronuba|[4722]|XAH336-05|Canada|Ontario|658[0n]|BOLD:AAA2632
Noctua pronuba|[4723]|XAG911-05|Canada|Ontario|658[0n]|BOLD:AAA2632
Noctua pronuba|[4724]|MNBB576-05|Canada|New Brunswick|658[0n]|BOLD:AAA2632
Noctua pronuba|[4725]|MNBB575-05|Canada|New Brunswick|658[0n]|BOLD:AAA2632
Noctua pronuba|[4726]|MNBB475-05|Canada|New Brunswick|658[0n]|BOLD:AAA2632
Noctua pronuba|[4727]|MNBB381-05|Canada|New Brunswick|658[0n]|BOLD:AAA2632
Noctua pronuba|[4728]|MNBB380-05|Canada|New Brunswick|658[0n]|BOLD:AAA2632
Noctua pronuba|[4729]|MNBB131-05|Canada|New Brunswick|658[0n]|BOLD:AAA2632
Noctua pronuba|[4730]|XAF573-05|Canada|Ontario|658[0n]|BOLD:AAA2632
Noctua pronuba|[4731]|XAD098-04|Canada|Ontario|658[0n]|BOLD:AAA2632
Noctua pronuba|[4732]|CGUKA040-09|United Kingdom|England|658[0n]|BOLD:AAA2632
Noctua pronuba|[4733]|CNPPB1403-12|Canada|Ontario|601[0n]|BOLD:AAA2632
Noctua pronuba|[4734]|CNBPF461-13|Canada|Ontario|632[0n]|BOLD:AAA2632
Noctua pronuba|[4735]|BBLPC035-09|Canada|New Brunswick|632[0n]|BOLD:AAA2632
Noctua pronuba|[4736]|LPSO933-08|Canada|Ontario|658[0n]|BOLD:AAA2632
Noctua pronuba|[4737]|LPSO731-08|Canada|Ontario|658[0n]|BOLD:AAA2632
Noctua pronuba|[4738]|LBCS108-07|Canada|British Columbia|658[0n]|BOLD:AAA2632
Noctua pronuba|[4739]|LBCS026-07|Canada|British Columbia|658[0n]|BOLD:AAA2632
Noctua pronuba|[4740]|XAJ812-06|Canada|Ontario|658[0n]|BOLD:AAA2632
Noctua pronuba|[4741]|LNCNW056-06|United States|North Carolina|658[0n]|BOLD:AAA2632
Noctua pronuba|[4742]|TTMNB430-06|Canada|New Brunswick|658[0n]|BOLD:AAA2632
Noctua pronuba|[4743]|MNBB132-05|Canada|New Brunswick|658[0n]|BOLD:AAA2632
Noctua pronuba|[4744]|LOCT085-05|United States|Connecticut|658[0n]|BOLD:AAA2632
Noctua pronuba|[4745]|PHMNB182-04|Canada|New Brunswick|533[0n]|BOLD:AAA2632
Noctua pronuba|[4746]|LOCT045-05|United States|Connecticut|647[0n]|BOLD:AAA2632
Noctua pronuba|[4747]|LHLEP023-06|Canada|British Columbia|654[0n]|BOLD:AAA2632
Noctua pronuba|[4748]|DUNLP176-08|Canada|British Columbia|654[0n]|BOLD:AAA2632
Noctua pronuba|[4749]|RWWA163-09|United States|Washington|658[0n]|BOLD:AAA2632
Noctua pronuba|[4750]|RWWA807-09|United States|Washington|658[0n]|BOLD:AAA2632
Noctua pronuba|[4751]|BBLCU086-09|United States|Kansas|658[0n]|BOLD:AAA2632
Noctua pronuba|[4752]|BBLEC132-09|Canada|New Brunswick|658[0n]|BOLD:AAA2632
Noctua pronuba|[4753]|BBLEC564-09|Canada|Nova Scotia|658[0n]|BOLD:AAA2632
Noctua pronuba|[4754]|BBLPC100-09|Canada|New Brunswick|658[0n]|BOLD:AAA2632
Noctua pronuba|[4755]|LBCH7117-10|Canada|British Columbia|658[0n]|BOLD:AAA2632
Noctua pronuba|[4756]|LPSO631-08|Canada|Ontario|658[0n]|BOLD:AAA2632
Noctua pronuba|[4757]|PHLPM055-11|Portugal|Madeira|658[0n]|BOLD:AAA2632
Noctua pronuba|[4758]|GMLC523-11|United States|California|658[0n]|BOLD:AAA2632
Noctua pronuba|[4759]|LEFIA580-10|Finland|South Karelia|658[0n]|BOLD:AAA2632
Noctua pronuba|[4760]|LALPA706-10|Canada|British Columbia|658[0n]|BOLD:AAA2632
Noctua pronuba|[4761]|GMLC561-11|United States|California|658[0n]|BOLD:AAA2632
Noctua pronuba|[4762]|BBLPC395-09|Canada|New Brunswick|658[0n]|BOLD:AAA2632
Noctua pronuba|[4763]|BBLEC485-09|Canada|New Brunswick|658[0n]|BOLD:AAA2632
Noctua pronuba|[4764]|BBLEC453-09|Canada|New Brunswick|658[0n]|BOLD:AAA2632
Noctua pronuba|[4765]|RWWA328-09|United States|Washington|658[0n]|BOLD:AAA2632
Noctua pronuba|[4766]|GMLC046-09|United States|California|658[0n]|BOLD:AAA2632
Noctua pronuba|[4767]|LBCW044-08|Canada|British Columbia|658[0n]|BOLD:AAA2632
Noctua pronuba|[4768]|BLTIB311-08|Canada|Ontario|658[0n]|BOLD:AAA2632
Noctua pronuba|[4769]|LCHQ320-08|Canada|Manitoba|658[0n]|BOLD:AAA2632
Noctua pronuba|[4770]|MNBB643-05|Canada|New Brunswick|658[0n]|BOLD:AAA2632
Noctua pronuba|[4771]|LBCS363-07|Canada|British Columbia|658[0n]|BOLD:AAA2632
Noctua pronuba|[4772]|LBCS182-07|Canada|British Columbia|658[0n]|BOLD:AAA2632
Noctua pronuba|[4773]|LENOA113-11|France|Haute Normandie|658[0n]|BOLD:AAA2632
Noctua pronuba|[4774]|XAJ948-06|Canada|Ontario|658[0n]|BOLD:AAA2632
Noctua pronuba|[4775]|LPSOB705-08|Canada|Ontario|609[0n]|BOLD:AAA2632
Noctua pronuba|[4776]|PHMNB229-04|Canada|New Brunswick|609[0n]|BOLD:AAA2632
Noctua pronuba|[4777]|PHMO004-03|Canada|Ontario|639[0n]|BOLD:AAA2632
Noctua pronuba|[4778]|RWWB216-09|United States|Washington|631[0n]|BOLD:AAA2632
Noctua pronuba|[4779]|PMG138-03|Canada|Ontario|617[0n]|BOLD:AAA2632
Noctua pronuba|[4780]|LBCS364-07|Canada|British Columbia|658[0n]|BOLD:AAA2632
Noctua pronuba|[4781]|GMLC754-12|United States|California|622[0n]|BOLD:AAA2632
Noctua pronuba|[4782]|CGUKB241-09|United Kingdom|England|638[0n]|BOLD:AAA2632
Noctua pronuba|[4783]|GWOTG523-12|Germany|Saxony|658[0n]|BOLD:AAA2632
Noctua pronuba|[4784]|GMLC609-11|United States|California|651[0n]|BOLD:AAA2632
Noctua pronuba|[4785]|BBLEC682-09|Canada|Nova Scotia|642[0n]|BOLD:AAA2632

[illegible]
Noctua pronuba|[4783]|GWOTG523-12|Germany|Saxony|658[0n]|BOLD:AAA2632
Noctua pronuba|[4784]|GMLC609-11|United States|California|651[0n]|BOLD:AAA2632
Noctua pronuba|[4785]|BBLEC682-09|Canada|Nova Scotia|642[0n]|BOLD:AAA2632
Noctua pronuba|[4786]|BBLEC049-09|Canada|New Brunswick|647[0n]|BOLD:AAA2632
Noctua pronuba|[4787]|XAH097-05|Canada|Ontario|638[0n]|BOLD:AAA2632
Noctua pronuba|[4788]|BBLPC101-09|Canada|New Brunswick|634[0n]|BOLD:AAA2632
Noctua pronuba|[4789]|XAD173-04|Canada|Ontario|616[0n]|BOLD:AAA2632
Noctua pronuba|[4790]|PHMO100-03|Canada|Ontario|639[0n]|BOLD:AAA2632
Noctua pronuba|[4791]|XAC570-04|Canada|Ontario|602[0n]|BOLD:AAA2632
Noctua pronuba|[4792]|AWCLB379-10|United States|California|621[0n]|BOLD:AAA2632
Noctua pronuba|[4793]|CNWLM2436-13|Canada|Alberta|612[0n]|BOLD:AAA2632
Noctua pronuba|[4794]|NORIN064-13|Norway|Akershus|640[0n]|BOLD:AAA2632
Noctua pronuba|[4795]|GBLAD247-14|Germany|Saxony|658[0n]|BOLD:AAA2632
Noctua pronuba|[4796]|GBLAF274-14|Germany|Brandenburg|658[0n]|BOLD:AAA2632
Cryptocala acadiensis|[4797]|LPSK240-08|Canada|Saskatchewan|658[0n]|BOLD:AAA4464
Cryptocala acadiensis|[4798]|LPABC275-09|Canada|Alberta|613[0n]|BOLD:AAA4464
Cryptocala acadiensis|[4799]|BBLPB507-10|Canada|Alberta|658[0n]|BOLD:AAA4464
Cryptocala acadiensis|[4800]|PHMNB046-03|Canada|New Brunswick|639[0n]|BOLD:AAA4464
Cryptocala acadiensis|[4801]|BBLEC099-09|Canada|Nova Scotia|631[0n]|BOLD:AAA4464
Cryptocala acadiensis|[4802]|LPSK239-08|Canada|Saskatchewan|658[0n]|BOLD:AAA4464
Cryptocala acadiensis|[4803]|BBLEC019-09|Canada|New Brunswick|650[0n]|BOLD:AAA4464
Cryptocala acadiensis|[4804]|BBLPC681-09|Canada|Newfoundland and Labrador|658[0n]|BOLD:AAA4464
Cryptocala acadiensis|[4805]|BBLPE337-09|Canada|Newfoundland and Labrador|658[0n]|BOLD:AAA4464
Cryptocala acadiensis|[4806]|BBLPE356-09|Canada|Newfoundland and Labrador|658[0n]|BOLD:AAA4464
Cryptocala acadiensis|[4807]|BBLPE394-09|Canada|Newfoundland and Labrador|658[0n]|BOLD:AAA4464
Cryptocala acadiensis|[4808]|LPMNB534-09|Canada|Manitoba|658[0n]|BOLD:AAA4464
Cryptocala acadiensis|[4809]|BBLPB504-10|Canada|Alberta|658[0n]|BOLD:AAA4464
Cryptocala acadiensis|[4810]|BBLPC114-09|Canada|New Brunswick|658[0n]|BOLD:AAA4464
Cryptocala acadiensis|[4811]|LPABB216-08|Canada|Alberta|658[0n]|BOLD:AAA4464
Cryptocala acadiensis|[4812]|BBLPB510-10|Canada|British Columbia|658[0n]|BOLD:AAA4464
Cryptocala acadiensis|[4813]|MNBB450-05|Canada|New Brunswick|658[0n]|BOLD:AAA4464
Cryptocala acadiensis|[4814]|BBLPB508-10|Canada|British Columbia|658[0n]|BOLD:AAA4464
Cryptocala acadiensis|[4815]|LPABC140-09|Canada|Alberta|658[0n]|BOLD:AAA4464
Cryptocala acadiensis|[4816]|BBLPB401-10|Canada|Alberta|658[0n]|BOLD:AAA4464
Cryptocala acadiensis|[4817]|LOWC906-05|Canada|British Columbia|658[0n]|BOLD:AAA4464
Cryptocala acadiensis|[4818]|LPABC517-09|Canada|Alberta|658[0n]|BOLD:AAA4464
Cryptocala acadiensis|[4819]|LPSK237-08|Canada|Saskatchewan|658[0n]|BOLD:AAA4464
Cryptocala acadiensis|[4820]|LPABC526-09|Canada|Alberta|629[0n]|BOLD:AAA4464
Cryptocala acadiensis|[4821]|BBLPE331-09|Canada|Newfoundland and Labrador|658[0n]|BOLD:AAA4464
Cryptocala acadiensis|[4822]|BBLPB402-10|Canada|Alberta|658[0n]|BOLD:AAA4464
Cryptocala acadiensis|[4823]|BBLPB397-10|Canada|British Columbia|658[0n]|BOLD:AAA4464
Cryptocala acadiensis|[4824]|LOWC897-05|Canada|British Columbia|658[0n]|BOLD:AAA4464
Cryptocala acadiensis|[4825]|BBLPC481-09|Canada|New Brunswick|658[0n]|BOLD:AAA4464
Cryptocala acadiensis|[4826]|LOWC901-05|Canada|British Columbia|658[0n]|BOLD:AAA4464
Cryptocala acadiensis|[4827]|LPABC317-09|Canada|Alberta|658[0n]|BOLD:AAA4464
Cryptocala acadiensis|[4828]|LPABC524-09|Canada|Alberta|658[0n]|BOLD:AAA4464
Cryptocala acadiensis|[4829]|BBLPB513-10|Canada|British Columbia|658[0n]|BOLD:AAA4464
Cryptocala acadiensis|[4830]|BBLPB512-10|Canada|British Columbia|658[0n]|BOLD:AAA4464
Cryptocala acadiensis|[4831]|BBLPB506-10|Canada|Alberta|658[0n]|BOLD:AAA4464
Cryptocala acadiensis|[4832]|BBLPB404-10|Canada|Alberta|658[0n]|BOLD:AAA4464
Cryptocala acadiensis|[4833]|BBLPB403-10|Canada|Alberta|658[0n]|BOLD:AAA4464
Cryptocala acadiensis|[4834]|BBLPB399-10|Canada|Alberta|658[0n]|BOLD:AAA4464
Cryptocala acadiensis|[4835]|BBLPB396-10|Canada|British Columbia|658[0n]|BOLD:AAA4464
Cryptocala acadiensis|[4836]|LBCH4023-10|Canada|British Columbia|658[0n]|BOLD:AAA4464
Cryptocala acadiensis|[4837]|LBCH3786-10|Canada|British Columbia|658[0n]|BOLD:AAA4464
Cryptocala acadiensis|[4838]|LBCH3492-10|Canada|British Columbia|658[0n]|BOLD:AAA4464
Cryptocala acadiensis|[4839]|LBCH3490-10|Canada|British Columbia|658[0n]|BOLD:AAA4464
Cryptocala acadiensis|[4840]|LBCH3488-10|Canada|British Columbia|658[0n]|BOLD:AAA4464
Cryptocala acadiensis|[4841]|LBCH3335-10|Canada|British Columbia|658[0n]|BOLD:AAA4464
Cryptocala acadiensis|[4842]|LBCH3084-10|Canada|British Columbia|658[0n]|BOLD:AAA4464
Cryptocala acadiensis|[4843]|LBCH937-10|Canada|British Columbia|658[0n]|BOLD:AAA4464
Cryptocala acadiensis|[4844]|BBLPE359-09|Canada|Newfoundland and Labrador|658[0n]|BOLD:AAA4464
Cryptocala acadiensis|[4845]|BBLPC214-09|Canada|Nova Scotia|658[0n]|BOLD:AAA4464
Cryptocala acadiensis|[4846]|BBLPC104-09|Canada|New Brunswick|658[0n]|BOLD:AAA4464
Cryptocala acadiensis|[4847]|LBCG2272-09|Canada|British Columbia|658[0n]|BOLD:AAA4464
Cryptocala acadiensis|[4848]|LBCG2255-09|Canada|British Columbia|658[0n]|BOLD:AAA4464
Cryptocala acadiensis|[4849]|LBCG2254-09|Canada|British Columbia|658[0n]|BOLD:AAA4464
Cryptocala acadiensis|[4850]|LBCG2253-09|Canada|British Columbia|658[0n]|BOLD:AAA4464
Cryptocala acadiensis|[4851]|LBCG2252-09|Canada|British Columbia|658[0n]|BOLD:AAA4464
Cryptocala acadiensis|[4852]|LPABC472-09|Canada|Alberta|658[0n]|BOLD:AAA4464
Cryptocala acadiensis|[4853]|LPABC447-09|Canada|Alberta|658[0n]|BOLD:AAA4464
Cryptocala acadiensis|[4854]|LPABC446-09|Canada|Alberta|658[0n]|BOLD:AAA4464
Cryptocala acadiensis|[4855]|LPABC409-09|Canada|Alberta|658[0n]|BOLD:AAA4464
Cryptocala acadiensis|[4856]|LPABC328-09|Canada|Alberta|658[0n]|BOLD:AAA4464
Cryptocala acadiensis|[4857]|LPABC316-09|Canada|Alberta|658[0n]|BOLD:AAA4464
Cryptocala acadiensis|[4858]|LPABC009-09|Canada|Alberta|658[0n]|BOLD:AAA4464
Cryptocala acadiensis|[4859]|LPABC006-09|Canada|Alberta|658[0n]|BOLD:AAA4464
Cryptocala acadiensis|[4860]|LPMNB277-09|Canada|Manitoba|658[0n]|BOLD:AAA4464
Cryptocala acadiensis|[4861]|LPMNB259-09|Canada|Manitoba|658[0n]|BOLD:AAA4464
Cryptocala acadiensis|[4862]|LPMNB258-09|Canada|Manitoba|658[0n]|BOLD:AAA4464
Cryptocala acadiensis|[4863]|LPMNB257-09|Canada|Manitoba|658[0n]|BOLD:AAA4464
Cryptocala acadiensis|[4864]|LPMNB244-09|Canada|Manitoba|658[0n]|BOLD:AAA4464
Cryptocala acadiensis|[4865]|LPABB491-08|Canada|Alberta|658[0n]|BOLD:AAA4464
Cryptocala acadiensis|[4866]|LPABB253-08|Canada|Alberta|658[0n]|BOLD:AAA4464
Cryptocala acadiensis|[4867]|LPABB144-08|Canada|Alberta|658[0n]|BOLD:AAA4464
Cryptocala acadiensis|[4868]|LOWCE078-06|Canada|British Columbia|658[0n]|BOLD:AAA4464
Cryptocala acadiensis|[4869]|LOWCD694-06|Canada|British Columbia|658[0n]|BOLD:AAA4464
Cryptocala acadiensis|[4870]|LOWCD691-06|Canada|British Columbia|658[0n]|BOLD:AAA4464
Cryptocala acadiensis|[4871]|LOWCD689-06|Canada|British Columbia|658[0n]|BOLD:AAA4464
Cryptocala acadiensis|[4872]|LOWCD688-06|Canada|British Columbia|658[0n]|BOLD:AAA4464
Cryptocala acadiensis|[4873]|LOWC911-05|Canada|British Columbia|658[0n]|BOLD:AAA4464
Cryptocala acadiensis|[4874]|LOWC910-05|Canada|British Columbia|658[0n]|BOLD:AAA4464
Cryptocala acadiensis|[4875]|LOWC909-05|Canada|British Columbia|658[0n]|BOLD:AAA4464
Cryptocala acadiensis|[4876]|LOWC908-05|Canada|British Columbia|658[0n]|BOLD:AAA4464
Cryptocala acadiensis|[4877]|LOWC907-05|Canada|British Columbia|658[0n]|BOLD:AAA4464
Cryptocala acadiensis|[4878]|LOWC905-05|Canada|British Columbia|658[0n]|BOLD:AAA4464
Cryptocala acadiensis|[4879]|LOWC904-05|Canada|British Columbia|658[0n]|BOLD:AAA4464
Cryptocala acadiensis|[4880]|LOWC903-05|Canada|British Columbia|658[0n]|BOLD:AAA4464
Cryptocala acadiensis|[4881]|LOWC902-05|Canada|British Columbia|658[0n]|BOLD:AAA4464
Cryptocala acadiensis|[4882]|MNBB133-05|Canada|New Brunswick|658[0n]|BOLD:AAA4464
Cryptocala acadiensis|[4883]|LOWC900-05|Canada|British Columbia|658[0n]|BOLD:AAA4464
Cryptocala acadiensis|[4884]|LOWC898-05|Canada|British Columbia|658[0n]|BOLD:AAA4464
[illegible]

Cryptocala acadiensis|[4882]|MNBB133-05|Canada|New Brunswick|658[0n]|BOLD:AAA4464
Cryptocala acadiensis|[4883]|LOWC900-05|Canada|British Columbia|658[0n]|BOLD:AAA4464
Cryptocala acadiensis|[4884]|LOWC898-05|Canada|British Columbia|658[0n]|BOLD:AAA4464
Cryptocala acadiensis|[4885]|BBLPC042-09|Canada|New Brunswick|621[0n]|BOLD:AAA4464
Cryptocala acadiensis|[4886]|BBLPC062-09|Canada|New Brunswick|658[0n]|BOLD:AAA4464
Cryptocala acadiensis|[4887]|LOWCD693-06|Canada|British Columbia|658[0n]|BOLD:AAA4464
Cryptocala acadiensis|[4888]|LPABC512-09|Canada|Alberta|611[0n]|BOLD:AAA4464
Cryptocala acadiensis|[4889]|LBCH3493-10|Canada|British Columbia|658[0n]|BOLD:AAA4464
Cryptocala acadiensis|[4890]|LOWC896-05|Canada|British Columbia|658[0n]|BOLD:AAA4464
Cryptocala acadiensis|[4891]|LOWC895-05|Canada|British Columbia|658[0n]|BOLD:AAA4464
Cryptocala acadiensis|[4892]|LPMNB499-09|Canada|Manitoba|658[0n]|BOLD:AAA4464
Cryptocala acadiensis|[4893]|LPABC324-09|Canada|Alberta|608[0n]|BOLD:AAA4464
Cryptocala acadiensis|[4894]|LOWCD690-06|Canada|British Columbia|584[0n]|BOLD:AAA4464
Cryptocala acadiensis|[4895]|LOWCD692-06|Canada|British Columbia|584[0n]|BOLD:AAA4464
Cryptocala acadiensis|[4896]|RDLQF695-06|Canada|Quebec|609[0n]|BOLD:AAA4464
Cryptocala acadiensis|[4897]|LOWC899-05|Canada|British Columbia|555[1n]|BOLD:AAA4464
Cryptocala acadiensis|[4898]|LPABC247-09|Canada|Alberta|621[0n]|BOLD:AAA4464
Cryptocala acadiensis|[4899]|LPABC257-09|Canada|Alberta|624[0n]|BOLD:AAA4464
Cryptocala acadiensis|[4900]|LPABC471-09|Canada|Alberta|634[0n]|BOLD:AAA4464
Cryptocala acadiensis|[4901]|LPABC511-09|Canada|Alberta|634[0n]|BOLD:AAA4464
Cryptocala acadiensis|[4902]|LBCH3783-10|Canada|British Columbia|658[0n]|BOLD:AAA4464
Cryptocala acadiensis|[4903]|BBLPB398-10|Canada|Alberta|658[0n]|BOLD:AAA4464
Cryptocala acadiensis|[4904]|BBLPB509-10|Canada|British Columbia|658[0n]|BOLD:AAA4464
Cryptocala acadiensis|[4905]|GWOSN431-11|Canada|British Columbia|658[0n]|BOLD:AAA4464
Noctua comes|[4906]|GWORZ207-10|Italy|Basilicata|658[0n]|BOLD:AAA2633
Noctua comes|[4907]|GWOR4307-09|Italy|Sardinia|658[0n]|BOLD:AAA2633
Noctua comes|[4908]|GMGMD010-14|Germany|Rhineland-Palatinate|579[0n]|BOLD:AAA2633
Noctua comes|[4909]|GWOR4311-09|Greece|Crete|658[0n]|BOLD:AAA2633
Noctua comes|[4910]|GWOR4312-09|Turkey|Antalya|658[0n]|BOLD:AAA2633
Noctua comes|[4911]|GWOR4306-09|Greece||658[0n]|BOLD:AAA2633
Noctua comes|[4912]|TRLEP055-13|Turkey|Mersin|658[0n]|BOLD:AAA2633
Noctua comes|[4913]|TRLEP059-13|Turkey|Mersin|658[0n]|BOLD:AAA2633
Noctua comes|[4914]|LEATD063-13|Italy|South Tyrol|658[0n]|BOLD:AAA2633
Noctua comes|[4915]|GBLAB654-13|United Kingdom|Scotland|658[0n]|BOLD:AAA2633
Noctua comes|[4916]|LENOA121-11|France|Haute Normandie|658[0n]|BOLD:AAA2633
Noctua comes|[4917]|GWOR4310-09|Croatia||658[0n]|BOLD:AAA2633
Noctua comes|[4918]|SMTPI988-14|Canada|Ontario|611[0n]|BOLD:AAA2633
Noctua comes|[4919]|LEFIF304-10|Finland|Aland Islands|658[0n]|BOLD:AAA2633
Noctua comes|[4920]|LEFIF643-10|Finland||658[0n]|BOLD:AAA2633
Noctua comes|[4921]|LEFIF644-10|Finland||658[0n]|BOLD:AAA2633
Noctua comes|[4922]|CGUKB955-09|United Kingdom|Wales|658[0n]|BOLD:AAA2633
Noctua comes|[4923]|GMGMK188-14|Germany|Rhineland-Palatinate|588[0n]|BOLD:AAA2633
Noctua comes|[4924]|CGUKB242-09|United Kingdom|England|643[0n]|BOLD:AAA2633
Noctua comes|[4925]|GMGMD004-14|Germany|Rhineland-Palatinate|588[0n]|BOLD:AAA2633
Noctua comes|[4926]|CGUKC376-09|United Kingdom|England|609[0n]|BOLD:AAA2633
Noctua comes|[4927]|GMGMD014-14|Germany|Rhineland-Palatinate|588[0n]|BOLD:AAA2633
Noctua comes|[4928]|GMGMD050-14|Germany|Rhineland-Palatinate|588[0n]|BOLD:AAA2633
Noctua comes|[4929]|GMGMD015-14|Germany|Rhineland-Palatinate|588[0n]|BOLD:AAA2633
Noctua comes|[4930]|GBLAB653-13|United Kingdom|Scotland|658[0n]|BOLD:AAA2633
Noctua comes|[4931]|NLLEA373-12|Netherlands|South Holland|658[0n]|BOLD:AAA2633
Noctua comes|[4932]|GWORO980-09|Germany|Bavaria|658[0n]|BOLD:AAA2633
Noctua comes|[4933]|GBLAA178-14|Germany|Baden-Wuerttemberg|658[0n]|BOLD:AAA2633
Noctua comes|[4934]|GMGMM096-14|Germany|Rhineland-Palatinate|579[0n]|BOLD:AAA2633
Noctua comes|[4935]|ABOLA063-14|Austria||658[0n]|BOLD:AAA2633
Noctua comes|[4936]|GBLAC341-13|Germany|Saxony|658[0n]|BOLD:AAA2633
Noctua comes|[4937]|GBLAC315-13|Germany|Thuringia|658[0n]|BOLD:AAA2633
Noctua comes|[4938]|GBLAB655-13|United Kingdom|Scotland|658[0n]|BOLD:AAA2633
Noctua comes|[4939]|PHLAI266-13|Austria|Vorarlberg|658[0n]|BOLD:AAA2633
Noctua comes|[4940]|GWOTL105-13|Germany|Saarland|658[0n]|BOLD:AAA2633
Noctua comes|[4941]|GWOTL104-13|Germany|Saarland|658[0n]|BOLD:AAA2633
Noctua comes|[4942]|PHLAH368-12|Austria|Tirol|658[0n]|BOLD:AAA2633
Noctua comes|[4943]|PHLAH367-12|Austria|Tirol|658[0n]|BOLD:AAA2633
Noctua comes|[4944]|NLLEA1103-12|Netherlands|South Holland|658[0n]|BOLD:AAA2633
Noctua comes|[4945]|NLLEA1101-12|Netherlands|South Holland|658[0n]|BOLD:AAA2633
Noctua comes|[4946]|NLLEA1045-12|Netherlands|South Holland|658[0n]|BOLD:AAA2633
Noctua comes|[4947]|NLLEA384-12|Netherlands|South Holland|658[0n]|BOLD:AAA2633
Noctua comes|[4948]|PHLSA555-11|Spain|Comunidad Valenciana|658[0n]|BOLD:AAA2633
Noctua comes|[4949]|LALPA1194-11|Canada|British Columbia|658[0n]|BOLD:AAA2633
Noctua comes|[4950]|PHLAE465-11|France|Provence-Alpes-Cote d'Azur|658[0n]|BOLD:AAA2633
Noctua comes|[4951]|LENOA120-11|France|Haute Normandie|658[0n]|BOLD:AAA2633
Noctua comes|[4952]|IBLAO161-11|Spain|Castilla-La Mancha|658[0n]|BOLD:AAA2633
Noctua comes|[4953]|IBLAO040-11|Spain|Catalonia|658[0n]|BOLD:AAA2633
Noctua comes|[4954]|IBLAO039-11|Spain|Murcia|658[0n]|BOLD:AAA2633
Noctua comes|[4955]|PHLAC574-10|Italy|South Tyrol|658[0n]|BOLD:AAA2633
Noctua comes|[4956]|LALPA799-10|Canada|British Columbia|658[0n]|BOLD:AAA2633
Noctua comes|[4957]|LALPA789-10|Canada|British Columbia|658[0n]|BOLD:AAA2633
Noctua comes|[4958]|LALPA767-10|Canada|British Columbia|658[0n]|BOLD:AAA2633
Noctua comes|[4959]|LALPA761-10|Canada|British Columbia|658[0n]|BOLD:AAA2633
Noctua comes|[4960]|FBLMV009-09|Germany|Bavaria|658[0n]|BOLD:AAA2633
Noctua comes|[4961]|FBLMV008-09|Germany|Bavaria|658[0n]|BOLD:AAA2633
Noctua comes|[4962]|RWWB349-09|United States|Washington|658[0n]|BOLD:AAA2633
Noctua comes|[4963]|RWWB337-09|United States|Washington|658[0n]|BOLD:AAA2633
Noctua comes|[4964]|RWWB222-09|United States|Washington|658[0n]|BOLD:AAA2633
Noctua comes|[4965]|RWWB186-09|United States|Washington|658[0n]|BOLD:AAA2633
Noctua comes|[4966]|GWORL294-09|Germany|Bavaria|658[0n]|BOLD:AAA2633
Noctua comes|[4967]|CGUKD380-09|United Kingdom|England|658[0n]|BOLD:AAA2633
Noctua comes|[4968]|GWOR4308-09|Italy||658[0n]|BOLD:AAA2633
Noctua comes|[4969]|GWOR4046-09|Germany|Bavaria|658[0n]|BOLD:AAA2633
Noctua comes|[4970]|CGUKB341-09|United Kingdom|England|658[0n]|BOLD:AAA2633
Noctua comes|[4971]|CGUKA810-09|United Kingdom||658[0n]|BOLD:AAA2633
Noctua comes|[4972]|CGUKA797-09|United Kingdom||658[0n]|BOLD:AAA2633
Noctua comes|[4973]|CGUKA777-09|United Kingdom||658[0n]|BOLD:AAA2633
Noctua comes|[4974]|CGUKA436-09|United Kingdom|England|658[0n]|BOLD:AAA2633
Noctua comes|[4975]|CGUKA389-09|United Kingdom|England|658[0n]|BOLD:AAA2633
Noctua comes|[4976]|CGUKA339-09|United Kingdom|England|658[0n]|BOLD:AAA2633
Noctua comes|[4977]|CGUKA274-09|United Kingdom|England|658[0n]|BOLD:AAA2633
Noctua comes|[4978]|LBCW046-08|Canada|British Columbia|658[0n]|BOLD:AAA2633
Noctua comes|[4979]|DUNLP177-08|Canada|British Columbia|658[0n]|BOLD:AAA2633
Noctua comes|[4980]|LHLEP129-06|Canada|British Columbia|658[0n]|BOLD:AAA2633
Noctua comes|[4981]|LHLEP127-06|Canada|British Columbia|658[0n]|BOLD:AAA2633
Noctua comes|[4982]|LHLEP126-06|Canada|British Columbia|658[0n]|BOLD:AAA2633
Noctua comes|[4983]|LHLEP124-06|Canada|British Columbia|658[0n]|BOLD:AAA2633
Noctua comes|[4984]|LMH033-06|Canada|British Columbia|658[0n]|BOLD:AAA2633

Noctua comes|[4982]|LHLEP126-06|Canada|British Columbia|658[0n]|BOLD:AAA2633
Noctua comes|[4983]|LHLEP124-06|Canada|British Columbia|658[0n]|BOLD:AAA2633
Noctua comes|[4984]|LMH033-06|Canada|British Columbia|658[0n]|BOLD:AAA2633
Noctua comes|[4985]|GBLAB597-13|Germany|Brandenburg|658[0n]|BOLD:AAA2633
Noctua comes|[4986]|LHLEP125-06|Canada|British Columbia|658[0n]|BOLD:AAA2633
Noctua comes|[4987]|LHLEP128-06|Canada|British Columbia|658[0n]|BOLD:AAA2633
Noctua comes|[4988]|LPVIB825-08|Canada|British Columbia|658[0n]|BOLD:AAA2633
Noctua comes|[4989]|LALPA1302-11|Canada|British Columbia|658[0n]|BOLD:AAA2633
Noctua comes|[4990]|LON613-08|Norway|Ostfold|656[0n]|BOLD:AAA2633
Noctua comes|[4991]|FBLMV414-09|Germany|Bavaria|647[0n]|BOLD:AAA2633
Noctua comes|[4992]|GBLAA271-14|Germany|Schleswig-Holstein|658[0n]|BOLD:AAA2633
Noctua comes|[4993]|GBLAF574-14|Germany|Brandenburg|621[0n]|BOLD:AAA2633
Noctua comes|[4994]|GWOTL233-13|Germany|Saarland|629[0n]|BOLD:AAA2633
Noctua comes|[4995]|GWOR4309-09|Germany|Thuringia|620[0n]|BOLD:AAA2633
Noctua comes|[4996]|CGUKC064-09|United Kingdom|England|617[0n]|BOLD:AAA2633
Noctua comes|[4997]|SMTPI1798-14|Canada|British Columbia|531[0n]|BOLD:AAA2633
Noctua comes|[4998]|GWORB3838-14|Germany|Bavaria|658[0n]|BOLD:AAA2633
Noctua comes|[4999]|ABOLA525-14|Austria||658[0n]|BOLD:AAA2633
Noctua comes|[5000]|GMGMM052-14|Germany|Rhineland-Palatinate|579[0n]|BOLD:AAA2633
Noctua comes|[5001]|SMTPI1799-14|Canada|British Columbia|576[0n]|BOLD:AAA2633
Noctua comes|[5002]|LON318-08|Norway|Vest-Agder|657[1n]|BOLD:AAA2633
Noctua comes|[5003]|RWWC1409-14|United States|Washington|579[0n]|BOLD:AAA2633
Diarsia rosaria freemani|[5004]|LCH296-04|Canada|Manitoba|658[1n]|BOLD:ACE5645
Diarsia rosaria freemani|[5005]|LPMN794-08|Canada|Manitoba|658[0n]|BOLD:ACE5645
Diarsia rosaria freemani|[5006]|LPABC404-09|Canada|Alberta|658[0n]|BOLD:ACE5645
Diarsia rosaria freemani|[5007]|LPABC449-09|Canada|Alberta|658[0n]|BOLD:ACE5645
Diarsia rosaria freemani|[5008]|LCH559-04|Canada|Manitoba|589[0n]|BOLD:ACE5645
Diarsia rosaria freemani|[5009]|LCH272-04|Canada|Manitoba|652[0n]|BOLD:ACE5645
Diarsia rosaria freemani|[5010]|LPMN336-08|Canada|Manitoba|658[0n]|BOLD:ACE5645
Diarsia rosaria freemani|[5011]|LCH280-04|Canada|Manitoba|658[0n]|BOLD:ACE5645
Diarsia rosaria freemani|[5012]|LCH275-04|Canada|Manitoba|658[0n]|BOLD:ACE5645
Diarsia rosaria freemani|[5013]|LCH267-04|Canada|Manitoba|658[0n]|BOLD:ACE5645
Diarsia rosaria freemani|[5014]|LOWCC166-05|Canada|British Columbia|544[0n]|BOLD:ACE5645
Diarsia rosaria freemani|[5015]|LOWCD883-06|Canada|British Columbia|658[0n]|BOLD:ACE5645
Diarsia rosaria freemani|[5016]|LPABB866-09|Canada|Alberta|658[0n]|BOLD:ACE5645
Diarsia rosaria freemani|[5017]|LPABC442-09|Canada|Alberta|658[0n]|BOLD:ACE5645
Diarsia rosaria freemani|[5018]|BBLPE316-09|Canada|Newfoundland and Labrador|658[0n]|BOLD:ACE5645
Diarsia rosaria rosaria|[5019]|LSEU813-06|United States|Colorado|658[0n]|BOLD:AAA5639
Diarsia rosaria rosaria|[5020]|LSEU814-06|United States|Colorado|658[0n]|BOLD:AAA5639
Diarsia rosaria rosaria|[5021]|ABKWR143-07|United States|Alaska|649[1n]|BOLD:AAA5639
Diarsia rosaria rosaria|[5022]|ABKWR087-07|United States|Alaska|658[0n]|BOLD:AAA5639
Diarsia rosaria rosaria|[5023]|ABKWR061-07|United States|Alaska|658[0n]|BOLD:AAA5639
Diarsia rosaria rosaria|[5024]|ABKWR052-07|United States|Alaska|658[0n]|BOLD:AAA5639
Diarsia rosaria rosaria|[5025]|LBCB492-05|Canada|British Columbia|658[0n]|BOLD:AAA5639
Diarsia rosaria rosaria|[5026]|ABKWR104-07|United States|Alaska|658[2n]|BOLD:AAA5639
Diarsia rosaria rosaria|[5027]|ABKWR142-07|United States|Alaska|658[0n]|BOLD:AAA5639
Diarsia rosaria rosaria|[5028]|LBCG830-09|Canada|British Columbia|658[0n]|BOLD:AAA5639
Diarsia rosaria rosaria|[5029]|BBLPB617-10|Canada|British Columbia|614[0n]|BOLD:AAA5639
Diarsia rosaria rosaria|[5030]|LPABC181-09|Canada|Alberta|640[3n]|BOLD:AAA5639
Diarsia rosaria rosaria|[5031]|LPABB826-09|Canada|Alberta|658[0n]|BOLD:AAA5639
Diarsia rosaria rosaria|[5032]|LBCD054-05|Canada|British Columbia|658[0n]|BOLD:AAA5639
Diarsia rosaria rosaria|[5033]|LPABB635-08|Canada|Alberta|658[0n]|BOLD:AAA5639
Diarsia rosaria rosaria|[5034]|LPABC075-09|Canada|Alberta|658[0n]|BOLD:AAA5639
Diarsia rosaria rosaria|[5035]|LPABC319-09|Canada|Alberta|658[0n]|BOLD:AAA5639
Diarsia rosaria rosaria|[5036]|LBCG2469-09|Canada|British Columbia|658[0n]|BOLD:AAA5639
Diarsia rosaria rosaria|[5037]|LBCG2472-09|Canada|British Columbia|658[0n]|BOLD:AAA5639
Diarsia rosaria rosaria|[5038]|LBCG3029-09|Canada|British Columbia|658[0n]|BOLD:AAA5639
Diarsia rosaria rosaria|[5039]|LBCH1683-10|Canada|British Columbia|658[0n]|BOLD:AAA5639
Diarsia rosaria rosaria|[5040]|BBLPB554-10|Canada|British Columbia|658[0n]|BOLD:AAA5639
Diarsia rosaria rosaria|[5041]|BBLPB555-10|Canada|British Columbia|658[0n]|BOLD:AAA5639
Diarsia rosaria rosaria|[5042]|LBCH1775-10|Canada|British Columbia|658[0n]|BOLD:AAA5639
Diarsia rosaria rosaria|[5043]|LBCH1865-10|Canada|British Columbia|658[0n]|BOLD:AAA5639
Diarsia rosaria rosaria|[5044]|LBCH2202-10|Canada|British Columbia|658[0n]|BOLD:AAA5639
Diarsia rosaria rosaria|[5045]|LBCH2250-10|Canada|British Columbia|658[0n]|BOLD:AAA5639
Diarsia rosaria rosaria|[5046]|BBLPB574-10|Canada|British Columbia|658[0n]|BOLD:AAA5639
Diarsia rosaria rosaria|[5047]|BBLPB575-10|Canada|British Columbia|658[0n]|BOLD:AAA5639
Diarsia rosaria rosaria|[5048]|BBLPB576-10|Canada|British Columbia|658[0n]|BOLD:AAA5639
Diarsia rosaria rosaria|[5049]|BBLPB577-10|Canada|British Columbia|658[0n]|BOLD:AAA5639
Diarsia rosaria rosaria|[5050]|BBLPB578-10|Canada|British Columbia|658[0n]|BOLD:AAA5639
Diarsia rosaria rosaria|[5051]|BBLPB592-10|Canada|British Columbia|658[0n]|BOLD:AAA5639
Diarsia rosaria rosaria|[5052]|BBLPB594-10|Canada|British Columbia|658[0n]|BOLD:AAA5639
Diarsia rosaria rosaria|[5053]|BBLPB595-10|Canada|British Columbia|658[0n]|BOLD:AAA5639
Diarsia rosaria rosaria|[5054]|BBLPB596-10|Canada|British Columbia|658[0n]|BOLD:AAA5639
Diarsia rosaria rosaria|[5055]|BBLPB601-10|Canada|British Columbia|658[0n]|BOLD:AAA5639
Diarsia rosaria rosaria|[5056]|BBLPB602-10|Canada|British Columbia|658[0n]|BOLD:AAA5639
Diarsia rosaria rosaria|[5057]|BBLPB616-10|Canada|British Columbia|658[0n]|BOLD:AAA5639
Diarsia rosaria rosaria|[5058]|BBLPB618-10|Canada|British Columbia|658[0n]|BOLD:AAA5639
Diarsia rosaria rosaria|[5059]|BBLPB619-10|Canada|British Columbia|658[0n]|BOLD:AAA5639
Diarsia rosaria rosaria|[5060]|LBCB493-05|Canada|British Columbia|658[0n]|BOLD:AAA5639
Diarsia rosaria rosaria|[5061]|LBCC669-05|Canada|British Columbia|658[0n]|BOLD:AAA5639
Diarsia rosaria rosaria|[5062]|BBLPB849-10|Canada|British Columbia|658[0n]|BOLD:AAA5639
Diarsia rosaria rosaria|[5063]|LALPA1159-11|Canada|British Columbia|658[0n]|BOLD:AAA5639
Diarsia rosaria rosaria|[5064]|LOWCC868-05|Canada|British Columbia|514[1n]|
Diarsia rosaria rosaria|[5065]|LBCH1317-10|Canada|British Columbia|632[0n]|BOLD:AAA5639
Diarsia rosaria rosaria|[5066]|LOWCD886-06|Canada|British Columbia|543[0n]|BOLD:AAA5639
Diarsia rosaria rosaria|[5067]|LBCW080-08|Canada|British Columbia|658[0n]|BOLD:AAA5639
Diarsia rosaria rosaria|[5068]|RWWB686-10|United States|Washington|658[1n]|BOLD:AAA5639
Diarsia rosaria rosaria|[5069]|RWWB183-09|United States|Washington|658[0n]|BOLD:AAA5639
Diarsia rosaria rosaria|[5070]|RWWB139-09|United States|Washington|658[0n]|BOLD:AAA5639
Diarsia rosaria rosaria|[5071]|RWWB054-09|United States|Washington|658[0n]|BOLD:AAA5639
Diarsia rosaria rosaria|[5072]|RWWA964-09|United States|Washington|658[0n]|BOLD:AAA5639
Diarsia rosaria rosaria|[5073]|RWWA826-09|United States|Washington|658[0n]|BOLD:AAA5639
Diarsia rosaria rosaria|[5074]|RWWA255-09|United States|Washington|658[0n]|BOLD:AAA5639
Diarsia rosaria rosaria|[5075]|RWWA107-09|United States|Washington|658[0n]|BOLD:AAA5639
Diarsia rosaria rosaria|[5076]|RWWA018-09|United States|Washington|658[0n]|BOLD:AAA5639
Diarsia rosaria rosaria|[5077]|RWWA009-09|United States|Washington|658[0n]|BOLD:AAA5639
Diarsia rosaria rosaria|[5078]|RWWA008-09|United States|Washington|658[0n]|BOLD:AAA5639
Diarsia rosaria rosaria|[5079]|RWWA007-09|United States|Washington|658[0n]|BOLD:AAA5639
Diarsia rosaria rosaria|[5080]|RWWA004-09|United States|Washington|658[0n]|BOLD:AAA5639
Diarsia rosaria rosaria|[5081]|RWWA003-09|United States|Washington|658[0n]|BOLD:AAA5639
Diarsia rosaria rosaria|[5082]|RWWB249-09|United States|Washington|634[0n]|BOLD:AAA5639
Diarsia rosaria rosaria|[5083]|RWWB288-09|United States|Washington|658[0n]|BOLD:AAA5639
Diarsia rosaria rosaria|[5084]|RWWB377-09|United States|Washington|658[0n]|BOLD:AAA5639

[illegible]
Diarsia rosaria rosaria|[5082]|RWWB249-09|United States|Washington|634[0n]|BOLD:AAA5639
Diarsia rosaria rosaria|[5083]|RWWB288-09|United States|Washington|658[0n]|BOLD:AAA5639
Diarsia rosaria rosaria|[5084]|RWWB377-09|United States|Washington|658[0n]|BOLD:AAA5639
Diarsia rosaria rosaria|[5085]|RWWB611-10|United States|Washington|658[0n]|BOLD:AAA5639
Diarsia rosaria rosaria|[5086]|RWWB613-10|United States|Washington|658[0n]|BOLD:AAA5639
Diarsia rosaria rosaria|[5087]|RWWB633-10|United States|Washington|658[0n]|BOLD:AAA5639
Diarsia rosaria rosaria|[5088]|RWWC060-10|United States|Washington|658[0n]|BOLD:AAA5639
Diarsia rosaria rosaria|[5089]|RWWC067-10|United States|Washington|658[0n]|BOLD:AAA5639
Diarsia rosaria rosaria|[5090]|RWWC072-10|United States|Washington|658[0n]|BOLD:AAA5639
Diarsia rosaria rosaria|[5091]|RWWC197-11|United States|Washington|658[0n]|BOLD:AAA5639
Diarsia rosaria rosaria|[5092]|RWWC232-11|United States|Washington|658[0n]|BOLD:AAA5639
Diarsia rosaria rosaria|[5093]|LOWCD884-06|Canada|British Columbia|598[0n]|BOLD:AAA5639
Diarsia rosaria rosaria|[5094]|LOWCC167-05|Canada|British Columbia|658[0n]|BOLD:AAA5639
Diarsia rosaria rosaria|[5095]|LOWCC168-05|Canada|British Columbia|658[0n]|BOLD:AAA5639
Diarsia rosaria rosaria|[5096]|LOWCC171-05|Canada|British Columbia|658[0n]|BOLD:AAA5639
Diarsia rosaria rosaria|[5097]|LOWCD885-06|Canada|British Columbia|658[0n]|BOLD:AAA5639
Diarsia rosaria rosaria|[5098]|LHLEP426-06|Canada|British Columbia|658[0n]|BOLD:AAA5639
Diarsia rosaria rosaria|[5099]|LALPA1158-11|Canada|British Columbia|658[0n]|BOLD:AAA5639
Diarsia rosaria rosaria|[5100]|LBCG309-08|Canada|British Columbia|658[1n]|BOLD:AAA5639
Diarsia rosaria rosaria|[5101]|LBCH1104-10|Canada|British Columbia|658[0n]|BOLD:AAA5639
Diarsia rosaria rosaria|[5102]|BBLPB615-10|Canada|British Columbia|658[0n]|BOLD:AAA5639
Diarsia rosaria rosaria|[5103]|LBCH2894-10|Canada|British Columbia|658[0n]|BOLD:AAA5639
Diarsia rosaria rosaria|[5104]|LBCH2903-10|Canada|British Columbia|658[0n]|BOLD:AAA5639
Diarsia rosaria rosaria|[5105]|LBCH2985-10|Canada|British Columbia|658[0n]|BOLD:AAA5639
Diarsia rosaria rosaria|[5106]|LOWCE761-06|Canada|British Columbia|604[0n]|BOLD:AAA5639
Diarsia rosaria rosaria|[5107]|LOWCD882-06|Canada|British Columbia|604[0n]|BOLD:AAA5639
Diarsia rosaria rosaria|[5108]|LOWCD887-06|Canada|British Columbia|596[0n]|BOLD:AAA5639
Diarsia rosaria rosaria|[5109]|LOWCE753-06|Canada|British Columbia|600[0n]|BOLD:AAA5639
Diarsia rosaria rosaria|[5110]|LBCH2896-10|Canada|British Columbia|658[0n]|BOLD:AAA5639
Diarsia rosaria rosaria|[5111]|LBCH2897-10|Canada|British Columbia|658[0n]|BOLD:AAA5639
Diarsia rosaria rosaria|[5112]|LBCH2201-10|Canada|British Columbia|658[0n]|BOLD:AAA5639
Diarsia rosaria rosaria|[5113]|LBCH2895-10|Canada|British Columbia|658[0n]|BOLD:AAA5639
Diarsia rosaria rosaria|[5114]|LBCH2898-10|Canada|British Columbia|658[0n]|BOLD:AAA5639
Diarsia rosaria rosaria|[5115]|LBCH2900-10|Canada|British Columbia|658[0n]|BOLD:AAA5639
Diarsia rosaria rosaria|[5116]|LBCH543-10|Canada|British Columbia|658[0n]|BOLD:AAA5639
Diarsia rosaria rosaria|[5117]|LBCH2087-10|Canada|British Columbia|658[0n]|BOLD:AAA5639
Diarsia rosaria rosaria|[5118]|LBCH410-10|Canada|British Columbia|658[0n]|BOLD:AAA5639
Diarsia rosaria rosaria|[5119]|LBCH528-10|Canada|British Columbia|658[0n]|BOLD:AAA5639
Diarsia rosaria rosaria|[5120]|LBCH409-10|Canada|British Columbia|658[0n]|BOLD:AAA5639
Diarsia rosaria rosaria|[5121]|LBCH149-10|Canada|British Columbia|658[0n]|BOLD:AAA5639
Diarsia rosaria rosaria|[5122]|LBCG315-08|Canada|British Columbia|658[0n]|BOLD:AAA5639
Diarsia rosaria rosaria|[5123]|LBCG308-08|Canada|British Columbia|658[1n]|BOLD:AAA5639
Diarsia rosaria rosaria|[5124]|LBCG180-08|Canada|British Columbia|658[0n]|BOLD:AAA5639
Diarsia rosaria rosaria|[5125]|LBCG150-08|Canada|British Columbia|658[0n]|BOLD:AAA5639
Diarsia rosaria rosaria|[5126]|LOWCE762-06|Canada|British Columbia|658[0n]|BOLD:AAA5639
Diarsia rosaria rosaria|[5127]|LOWCE760-06|Canada|British Columbia|658[0n]|BOLD:AAA5639
Diarsia rosaria rosaria|[5128]|LOWCE751-06|Canada|British Columbia|658[0n]|BOLD:AAA5639
Diarsia rosaria rosaria|[5129]|LOWCE750-06|Canada|British Columbia|658[0n]|BOLD:AAA5639
Diarsia rosaria rosaria|[5130]|LBCD055-05|Canada|British Columbia|658[0n]|BOLD:AAA5639
Diarsia rosaria rosaria|[5131]|LOWCC169-05|Canada|British Columbia|510[0n]|BOLD:AAA5639
Diarsia rosaria rosaria|[5132]|LOWCC170-05|Canada|British Columbia|658[0n]|BOLD:AAA5639
Diarsia rosaria rosaria|[5133]|LBCH2902-10|Canada|British Columbia|658[0n]|BOLD:AAA5639
Diarsia rosaria rosaria|[5134]|LBCH2901-10|Canada|British Columbia|658[0n]|BOLD:AAA5639
Diarsia rosaria rosaria|[5135]|LBCH2904-10|Canada|British Columbia|658[0n]|BOLD:AAA5639
Diarsia rosaria rosaria|[5136]|LBCH3006-10|Canada|British Columbia|658[0n]|BOLD:AAA5639
Diarsia rosaria rosaria|[5137]|LBCH3007-10|Canada|British Columbia|658[0n]|BOLD:AAA5639
Diarsia rosaria rosaria|[5138]|LBCH3008-10|Canada|British Columbia|658[0n]|BOLD:AAA5639
Diarsia rosaria rosaria|[5139]|LBCH6005-10|Canada|British Columbia|658[0n]|BOLD:AAA5639
Diarsia rosaria rosaria|[5140]|BBLPB593-10|Canada|British Columbia|658[0n]|BOLD:AAA5639
Diarsia rosaria rosaria|[5141]|LALPA1227-11|Canada|British Columbia|658[0n]|BOLD:AAA5639
Diarsia rosaria rosaria|[5142]|LALPA1323-12|Canada|British Columbia|601[0n]|BOLD:AAA5639
Diarsia rubifera|[5143]|RDLQF799-06|Canada|Quebec|609[0n]|BOLD:ABX5264
Diarsia rubifera|[5144]|BBLEC008-09|Canada|New Brunswick|621[0n]|BOLD:ABX5264
Diarsia rubifera|[5145]|RDLQ719-07|Canada|Quebec|658[0n]|BOLD:ABX5264
Diarsia rubifera|[5146]|RDLQ720-07|Canada|Quebec|632[0n]|BOLD:ABX5264
Diarsia rubifera|[5147]|BBLEC004-09|Canada|New Brunswick|621[0n]|BOLD:ABX5264
Diarsia rubifera|[5148]|RDLQB651-05|Canada|Quebec|587[1n]|BOLD:ABX5264
Diarsia rubifera|[5149]|RDLQ716-07|Canada|Quebec|627[0n]|BOLD:ABX5264
Diarsia rubifera|[5150]|LGSMG874-10|United States|North Carolina|658[0n]|BOLD:ABX5264
Diarsia rubifera|[5151]|RDLQB832-05|Canada|Quebec|620[0n]|BOLD:ABX5264
Diarsia rubifera|[5152]|LGSM040-04|United States|North Carolina|658[0n]|BOLD:ABX5264
Diarsia rubifera|[5153]|LGSM043-04|United States|North Carolina|658[1n]|BOLD:ABX5264
Diarsia rubifera|[5154]|LGSMG934-10|United States|North Carolina|658[0n]|BOLD:ABX5264
Diarsia rubifera|[5155]|LNCC251-10|United States|North Carolina|658[0n]|BOLD:ABX5264
Diarsia rubifera|[5156]|BBLPB552-10|Canada|Alberta|658[0n]|BOLD:ABX5264
Diarsia rubifera|[5157]|BBLEC017-09|Canada|New Brunswick|626[0n]|BOLD:ABX5264
Diarsia rubifera|[5158]|BBLEC516-09|Canada|New Brunswick|658[0n]|BOLD:ABX5264
Diarsia rubifera|[5159]|BBLPB553-10|Canada|Alberta|658[0n]|BOLD:ABX5264
Diarsia rubifera|[5160]|LPSOD938-09|Canada|Ontario|658[0n]|BOLD:ABX5264
Diarsia rubifera|[5161]|LPSOD941-09|Canada|Ontario|658[0n]|BOLD:ABX5264
Diarsia rubifera|[5162]|LPMNB508-09|Canada|Manitoba|658[0n]|BOLD:ABX5264
Diarsia rubifera|[5163]|LPMNB470-09|Canada|Manitoba|658[0n]|BOLD:ABX5264
Diarsia rubifera|[5164]|LPMNB468-09|Canada|Manitoba|658[0n]|BOLD:ABX5264
Diarsia rubifera|[5165]|LPGVA608-08|Canada|British Columbia|658[0n]|BOLD:ABX5264
Diarsia rubifera|[5166]|RDLQ715-07|Canada|Quebec|658[0n]|BOLD:ABX5264
Diarsia rubifera|[5167]|LHLEP424-06|Canada|British Columbia|658[0n]|BOLD:ABX5264
Diarsia rubifera|[5168]|RDLQF372-06|Canada|Quebec|658[0n]|BOLD:ABX5264
Diarsia rubifera|[5169]|RDLQF371-06|Canada|Quebec|658[0n]|BOLD:ABX5264
Diarsia rubifera|[5170]|RDLQF175-06|Canada|Quebec|658[0n]|BOLD:ABX5264
Diarsia rubifera|[5171]|LPSOD961-09|Canada|Ontario|658[0n]|BOLD:ABX5264
Diarsia rubifera|[5172]|TTMNB491-06|Canada|New Brunswick|658[0n]|BOLD:ABX5264
Diarsia rubifera|[5173]|BBLPC037-09|Canada|New Brunswick|634[0n]|BOLD:ABX5264
Diarsia rubifera|[5174]|BBLPB566-10|Canada|Alberta|658[0n]|BOLD:ABX5264
Diarsia rubifera|[5175]|CNEIE1814-12|Canada|Alberta|606[0n]|BOLD:ABX5264
Diarsia rubifera|[5176]|CNEIF2115-12|Canada|Alberta|603[0n]|BOLD:ABX5264
Diarsia rubifera|[5177]|CNEIF2116-12|Canada|Alberta|600[0n]|BOLD:ABX5264
Diarsia rubifera|[5178]|LBCG2259-09|Canada|British Columbia|658[0n]|BOLD:ABX5264
Diarsia rubifera|[5179]|CNEIF2108-12|Canada|Alberta|594[0n]|BOLD:ABX5264
Diarsia rubifera|[5180]|LBCH3355-10|Canada|British Columbia|658[0n]|BOLD:ABX5264
Diarsia rubifera|[5181]|BBLPB359-10|Canada|Alberta|658[0n]|BOLD:ABX5264
Diarsia rubifera|[5182]|LBCG2264-09|Canada|British Columbia|658[0n]|BOLD:ABX5264
Diarsia rubifera|[5183]|BBLPB450-10|Canada|Alberta|658[0n]|BOLD:ABX5264
[illegible]

Diarsia rubifera|[5181]|BBLPB359-10|Canada|Alberta|658[0n]|BOLD:ABX5264
Diarsia rubifera|[5182]|LBCG2264-09|Canada|British Columbia|658[0n]|BOLD:ABX5264
Diarsia rubifera|[5183]|BBLPB450-10|Canada|Alberta|658[0n]|BOLD:ABX5264
Diarsia rubifera|[5184]|BBLPB568-10|Canada|Alberta|658[0n]|BOLD:ABX5264
Diarsia rubifera|[5185]|BBLPB354-10|Canada|Alberta|645[0n]|BOLD:ABX5264
Diarsia rubifera|[5186]|LBCH4019-10|Canada|British Columbia|658[0n]|BOLD:ABX5264
Diarsia rubifera|[5187]|BBLPB353-10|Canada|Alberta|658[0n]|BOLD:ABX5264
Diarsia rubifera|[5188]|BBLPB567-10|Canada|Alberta|658[0n]|BOLD:ABX5264
Diarsia rubifera|[5189]|LBCG2258-09|Canada|British Columbia|658[0n]|BOLD:ABX5264
Diarsia rubifera|[5190]|LBCH3357-10|Canada|British Columbia|658[0n]|BOLD:ABX5264
Diarsia rubifera|[5191]|BBLPB550-10|Canada|Alberta|658[0n]|BOLD:ABX5264
Diarsia rubifera|[5192]|BBLPB551-10|Canada|Alberta|658[0n]|BOLD:ABX5264
Diarsia rubifera|[5193]|LBCH962-10|Canada|British Columbia|658[0n]|BOLD:ABX5264
Diarsia rubifera|[5194]|LBCH3106-10|Canada|British Columbia|658[0n]|BOLD:ABX5264
Diarsia rubifera|[5195]|LBCH960-10|Canada|British Columbia|658[0n]|BOLD:ABX5264
Diarsia rubifera|[5196]|LBCH961-10|Canada|British Columbia|658[0n]|BOLD:ABX5264
Diarsia rubifera|[5197]|LBCG2277-09|Canada|British Columbia|658[0n]|BOLD:ABX5264
Diarsia rubifera|[5198]|LBCH946-10|Canada|British Columbia|658[0n]|BOLD:ABX5264
Diarsia rubifera|[5199]|LBCG2268-09|Canada|British Columbia|658[0n]|BOLD:ABX5264
Diarsia rubifera|[5200]|LBCG2270-09|Canada|British Columbia|658[0n]|BOLD:ABX5264
Diarsia rubifera|[5201]|LBCH4750-10|Canada|British Columbia|658[0n]|BOLD:ABX5264
Diarsia rubifera|[5202]|LALPA728-10|Canada|British Columbia|658[0n]|BOLD:ABX5264
Diarsia rubifera|[5203]|LBCG2266-09|Canada|British Columbia|658[0n]|BOLD:ABX5264
Diarsia rubifera|[5204]|LBCG2267-09|Canada|British Columbia|658[0n]|BOLD:ABX5264
Diarsia rubifera|[5205]|LBCH3518-10|Canada|British Columbia|658[0n]|BOLD:ABX5264
Diarsia rubifera|[5206]|LBCH3799-10|Canada|British Columbia|658[0n]|BOLD:ABX5264
Diarsia rubifera|[5207]|LBCG2265-09|Canada|British Columbia|658[0n]|BOLD:ABX5264
Diarsia rubifera|[5208]|LBCG2262-09|Canada|British Columbia|658[0n]|BOLD:ABX5264
Diarsia rubifera|[5209]|LBCG2261-09|Canada|British Columbia|658[0n]|BOLD:ABX5264
Diarsia rubifera|[5210]|LBCG2260-09|Canada|British Columbia|658[0n]|BOLD:ABX5264
Diarsia rubifera|[5211]|LBCG2257-09|Canada|British Columbia|658[0n]|BOLD:ABX5264
Diarsia rubifera|[5212]|LBCG2251-09|Canada|British Columbia|658[0n]|BOLD:ABX5264
Diarsia rubifera|[5213]|LBCG2250-09|Canada|British Columbia|658[0n]|BOLD:ABX5264
Diarsia rubifera|[5214]|LHLEP425-06|Canada|British Columbia|658[0n]|BOLD:ABX5264
Diarsia rubifera|[5215]|LOWCE789-06|Canada|British Columbia|658[0n]|BOLD:ABX5264
Diarsia rubifera|[5216]|LOWCE788-06|Canada|British Columbia|658[0n]|BOLD:ABX5264
Diarsia rubifera|[5217]|LOWCE787-06|Canada|British Columbia|658[0n]|BOLD:ABX5264
Diarsia rubifera|[5218]|LBCH3491-10|Canada|British Columbia|636[0n]|BOLD:ABX5264
Diarsia rubifera|[5219]|LBCH4722-10|Canada|British Columbia|644[0n]|BOLD:ABX5264
Diarsia rubifera|[5220]|BBLPB582-10|Canada|Alberta|658[1n]|BOLD:ABX5264
Diarsia rubifera|[5221]|LBCH3360-10|Canada|British Columbia|658[0n]|BOLD:ABX5264
Diarsia rubifera|[5222]|LBCH3505-10|Canada|British Columbia|658[0n]|BOLD:ABX5264
Diarsia rubifera|[5223]|LBCH4015-10|Canada|British Columbia|658[0n]|BOLD:ABX5264
Diarsia rubifera|[5224]|LBCH4734-10|Canada|British Columbia|658[0n]|BOLD:ABX5264
Diarsia rubifera|[5225]|LBCH4748-10|Canada|British Columbia|658[0n]|BOLD:ABX5264
Diarsia rubifera|[5226]|LBCH4749-10|Canada|British Columbia|658[0n]|BOLD:ABX5264
Diarsia rubifera|[5227]|LBCH3142-10|Canada|British Columbia|658[0n]|BOLD:ABX5264
Diarsia rubifera|[5228]|LBCH3342-10|Canada|British Columbia|658[0n]|BOLD:ABX5264
Diarsia rubifera|[5229]|BBLPB355-10|Canada|Alberta|658[0n]|BOLD:ABX5264
Diarsia rubifera|[5230]|BBLPB356-10|Canada|Alberta|658[0n]|BOLD:ABX5264
Diarsia rubifera|[5231]|BBLPB461-10|Canada|Alberta|658[0n]|BOLD:ABX5264
Diarsia rubifera|[5232]|BBLPB549-10|Canada|Alberta|658[0n]|BOLD:ABX5264
Diarsia rubifera|[5233]|LBCH3354-10|Canada|British Columbia|658[0n]|BOLD:ABX5264
Diarsia rubifera|[5234]|LBCH3356-10|Canada|British Columbia|658[0n]|BOLD:ABX5264
Diarsia rubifera|[5235]|LBCH3358-10|Canada|British Columbia|658[0n]|BOLD:ABX5264
Diarsia rubifera|[5236]|LBCH3359-10|Canada|British Columbia|658[0n]|BOLD:ABX5264
Diarsia rubifera|[5237]|BBLPB571-10|Canada|Alberta|658[0n]|BOLD:ABX5264
Diarsia rubifera|[5238]|BBLPB745-10|Canada|Alberta|658[0n]|BOLD:ABX5264
Diarsia rubifera|[5239]|LBCH4717-10|Canada|British Columbia|658[0n]|BOLD:ABX5264
Diarsia rubifera|[5240]|SSEIA7705-13|Canada|Alberta|608[0n]|BOLD:ABX5264
Diarsia calgary|[5241]|LBCH138-10|Canada|British Columbia|658[0n]|BOLD:ACE3480
Diarsia calgary|[5242]|LBCH150-10|Canada|British Columbia|658[0n]|BOLD:ACE3480
Diarsia calgary|[5243]|LBCH253-10|Canada|British Columbia|658[0n]|BOLD:ACE3480
Diarsia calgary|[5244]|LBCH521-10|Canada|British Columbia|658[0n]|BOLD:ACE3480
Diarsia calgary|[5245]|LBCH041-10|Canada|British Columbia|658[0n]|BOLD:ACE3480
Diarsia calgary|[5246]|LBCH049-10|Canada|British Columbia|658[0n]|BOLD:ACE3480
Diarsia calgary|[5247]|LBCH671-10|Canada|British Columbia|658[0n]|BOLD:ACE3480
Diarsia calgary|[5248]|LBCH2899-10|Canada|British Columbia|658[0n]|BOLD:ACE3480
Diarsia calgary|[5249]|CNWLE2399-12|Canada|Alberta|614[0n]|BOLD:ABX6134
Diarsia calgary|[5250]|BBLPB443-10|Canada|Alberta|658[0n]|BOLD:ABX6134
Diarsia calgary|[5251]|RDMAB255-05|Canada|Alberta|658[0n]|BOLD:ABX6134
Diarsia calgary|[5252]|RDMAB264-05|Canada|Alberta|658[0n]|BOLD:ABX6134
Diarsia calgary|[5253]|LOWCD294-06|Canada|British Columbia|658[0n]|BOLD:ABX6134
Diarsia calgary|[5254]|LOWCE752-06|Canada|British Columbia|658[0n]|BOLD:ABX6134
Diarsia calgary|[5255]|BBLPB452-10|Canada|British Columbia|658[0n]|BOLD:ABX6134
Diarsia calgary|[5256]|CNWLE2516-12|Canada|Alberta|636[0n]|BOLD:ABX6134
Diarsia calgary|[5257]|SSWLB054-13|Canada|Alberta|577[0n]|BOLD:ABX6134
Diarsia calgary|[5258]|SSWLB055-13|Canada|Alberta|592[0n]|BOLD:ABX6134
Diarsia esurialis|[5259]|LBCH523-10|Canada|British Columbia|658[0n]|BOLD:ACF1489
Diarsia esurialis|[5260]|LBCH849-10|Canada|British Columbia|658[0n]|BOLD:ACF1489
Diarsia esurialis|[5261]|LBCH846-10|Canada|British Columbia|658[0n]|BOLD:ACF1489
Diarsia esurialis|[5262]|LBCH829-10|Canada|British Columbia|658[0n]|BOLD:ACF1489
Diarsia esurialis|[5263]|LBCH681-10|Canada|British Columbia|658[0n]|BOLD:ACF1489
Diarsia esurialis|[5264]|LBCH668-10|Canada|British Columbia|658[0n]|BOLD:ACF1489
Diarsia esurialis|[5265]|LBCH657-10|Canada|British Columbia|658[0n]|BOLD:ACF1489
Diarsia esurialis|[5266]|LBCH517-10|Canada|British Columbia|658[0n]|BOLD:ACF1489
Diarsia esurialis|[5267]|LBCH407-10|Canada|British Columbia|658[0n]|BOLD:ACF1489
Diarsia esurialis|[5268]|LBCH399-10|Canada|British Columbia|658[0n]|BOLD:ACF1489
Diarsia esurialis|[5269]|LBCH264-10|Canada|British Columbia|658[0n]|BOLD:ACF1489
Diarsia esurialis|[5270]|LBCH261-10|Canada|British Columbia|658[0n]|BOLD:ACF1489
Diarsia esurialis|[5271]|LBCH131-10|Canada|British Columbia|658[0n]|BOLD:ACF1489
Diarsia esurialis|[5272]|LBCH043-10|Canada|British Columbia|658[0n]|BOLD:ACF1489
Diarsia esurialis|[5273]|LBCS136-07|Canada|British Columbia|658[0n]|BOLD:ACF1489
Diarsia esurialis|[5274]|LBCS135-07|Canada|British Columbia|658[0n]|BOLD:ACF1489
Diarsia esurialis|[5275]|LBCH1937-10|Canada|British Columbia|636[0n]|BOLD:ACF1489
Diarsia esurialis|[5276]|LBCH2387-10|Canada|British Columbia|658[0n]|BOLD:ACF1489
Diarsia esurialis|[5277]|LBCH2392-10|Canada|British Columbia|658[0n]|BOLD:ACF1489
Diarsia esurialis|[5278]|LBCH2906-10|Canada|British Columbia|658[0n]|BOLD:ACF1489
Diarsia esurialis|[5279]|LBCH2961-10|Canada|British Columbia|658[0n]|BOLD:ACF1489
Diarsia esurialis|[5280]|LBCH3041-10|Canada|British Columbia|658[0n]|BOLD:ACF1489
Diarsia esurialis|[5281]|LBCH3047-10|Canada|British Columbia|658[0n]|BOLD:ACF1489
Diarsia esurialis|[5282]|RWWB884-10|United States|Washington|658[0n]|BOLD:ACF1489
Diarsia esurialis|[5283]|RWWB976-10|United States|Washington|658[0n]|BOLD:ACF1489

Diarsia esurialis|[5281]|LBCH3047-10|Canada|British Columbia|658[0n]|BOLD:ACF1489
Diarsia esurialis|[5282]|RWWB884-10|United States|Washington|658[0n]|BOLD:ACF1489
Diarsia esurialis|[5283]|RWWB976-10|United States|Washington|658[0n]|BOLD:ACF1489
Diarsia esurialis|[5284]|LBCH265-10|Canada|British Columbia|658[0n]|BOLD:ABX6710
Diarsia esurialis|[5285]|LBCH968-10|Canada|British Columbia|658[0n]|BOLD:ABX6710
Diarsia esurialis|[5286]|LBCH2393-10|Canada|British Columbia|658[0n]|BOLD:ABX6710
Diarsia esurialis|[5287]|ABKWR141-07|United States|Alaska|641[0n]|BOLD:ABX6710
Diarsia esurialis|[5288]|ABKWR140-07|United States|Alaska|637[0n]|BOLD:ABX6710
Diarsia esurialis|[5289]|LBCH156-10|Canada|British Columbia|658[0n]|BOLD:ABX6710
Diarsia esurialis|[5290]|LBCH044-10|Canada|British Columbia|658[0n]|BOLD:ABX6710
Diarsia esurialis|[5291]|LBCH047-10|Canada|British Columbia|658[0n]|BOLD:ABX6710
Diarsia esurialis|[5292]|LBCH048-10|Canada|British Columbia|658[0n]|BOLD:ABX6710
Diarsia esurialis|[5293]|LBCH051-10|Canada|British Columbia|658[0n]|BOLD:ABX6710
Diarsia esurialis|[5294]|LBCH130-10|Canada|British Columbia|658[0n]|BOLD:ABX6710
Diarsia esurialis|[5295]|LBCH132-10|Canada|British Columbia|658[0n]|BOLD:ABX6710
Diarsia esurialis|[5296]|LBCH133-10|Canada|British Columbia|658[0n]|BOLD:ABX6710
Diarsia esurialis|[5297]|LBCH139-10|Canada|British Columbia|658[0n]|BOLD:ABX6710
Diarsia esurialis|[5298]|LBCH140-10|Canada|British Columbia|658[0n]|BOLD:ABX6710
Diarsia esurialis|[5299]|LBCH142-10|Canada|British Columbia|658[0n]|BOLD:ABX6710
Diarsia esurialis|[5300]|LBCH145-10|Canada|British Columbia|658[0n]|BOLD:ABX6710
Diarsia esurialis|[5301]|LBCH157-10|Canada|British Columbia|658[0n]|BOLD:ABX6710
Diarsia esurialis|[5302]|LBCH247-10|Canada|British Columbia|658[0n]|BOLD:ABX6710
Diarsia esurialis|[5303]|LBCH250-10|Canada|British Columbia|658[0n]|BOLD:ABX6710
Diarsia esurialis|[5304]|LBCH255-10|Canada|British Columbia|658[0n]|BOLD:ABX6710
Diarsia esurialis|[5305]|LBCH257-10|Canada|British Columbia|658[0n]|BOLD:ABX6710
Diarsia esurialis|[5306]|LBCH259-10|Canada|British Columbia|658[0n]|BOLD:ABX6710
Diarsia esurialis|[5307]|LBCH260-10|Canada|British Columbia|658[0n]|BOLD:ABX6710
Diarsia esurialis|[5308]|LBCH263-10|Canada|British Columbia|658[0n]|BOLD:ABX6710
Diarsia esurialis|[5309]|LBCH267-10|Canada|British Columbia|658[0n]|BOLD:ABX6710
Diarsia esurialis|[5310]|LBCH391-10|Canada|British Columbia|658[0n]|BOLD:ABX6710
Diarsia esurialis|[5311]|LBCH405-10|Canada|British Columbia|658[0n]|BOLD:ABX6710
Diarsia esurialis|[5312]|LBCH406-10|Canada|British Columbia|658[0n]|BOLD:ABX6710
Diarsia esurialis|[5313]|LBCH412-10|Canada|British Columbia|658[0n]|BOLD:ABX6710
Diarsia esurialis|[5314]|LBCH519-10|Canada|British Columbia|658[0n]|BOLD:ABX6710
Diarsia esurialis|[5315]|LBCH524-10|Canada|British Columbia|658[0n]|BOLD:ABX6710
Diarsia esurialis|[5316]|LBCH527-10|Canada|British Columbia|658[0n]|BOLD:ABX6710
Diarsia esurialis|[5317]|LBCH534-10|Canada|British Columbia|658[0n]|BOLD:ABX6710
Diarsia esurialis|[5318]|LBCH541-10|Canada|British Columbia|658[0n]|BOLD:ABX6710
Diarsia esurialis|[5319]|LBCH658-10|Canada|British Columbia|658[0n]|BOLD:ABX6710
Diarsia esurialis|[5320]|LBCH665-10|Canada|British Columbia|658[0n]|BOLD:ABX6710
Diarsia esurialis|[5321]|LBCH673-10|Canada|British Columbia|658[0n]|BOLD:ABX6710
Diarsia esurialis|[5322]|LBCH834-10|Canada|British Columbia|658[0n]|BOLD:ABX6710
Diarsia esurialis|[5323]|LBCH837-10|Canada|British Columbia|658[0n]|BOLD:ABX6710
Diarsia esurialis|[5324]|LBCH845-10|Canada|British Columbia|658[0n]|BOLD:ABX6710
Diarsia esurialis|[5325]|LBCH851-10|Canada|British Columbia|658[0n]|BOLD:ABX6710
Diarsia esurialis|[5326]|LBCH2389-10|Canada|British Columbia|658[0n]|BOLD:ABX6710
Diarsia esurialis|[5327]|LBCH2391-10|Canada|British Columbia|658[0n]|BOLD:ABX6710
Diarsia esurialis|[5328]|LBCH2394-10|Canada|British Columbia|658[0n]|BOLD:ABX6710
Diarsia esurialis|[5329]|LBCH2395-10|Canada|British Columbia|658[0n]|BOLD:ABX6710
Diarsia esurialis|[5330]|LBCH2893-10|Canada|British Columbia|658[0n]|BOLD:ABX6710
Diarsia esurialis|[5331]|LBCH2905-10|Canada|British Columbia|658[0n]|BOLD:ABX6710
Diarsia esurialis|[5332]|LBCH2914-10|Canada|British Columbia|658[0n]|BOLD:ABX6710
Diarsia esurialis|[5333]|LBCH2916-10|Canada|British Columbia|658[0n]|BOLD:ABX6710
Diarsia esurialis|[5334]|LBCH2918-10|Canada|British Columbia|658[0n]|BOLD:ABX6710
Diarsia esurialis|[5335]|LBCH2936-10|Canada|British Columbia|658[0n]|BOLD:ABX6710
Diarsia esurialis|[5336]|LBCH2938-10|Canada|British Columbia|658[0n]|BOLD:ABX6710
Diarsia esurialis|[5337]|LBCH2963-10|Canada|British Columbia|658[0n]|BOLD:ABX6710
Diarsia esurialis|[5338]|LBCH2964-10|Canada|British Columbia|658[0n]|BOLD:ABX6710
Diarsia esurialis|[5339]|LBCH3005-10|Canada|British Columbia|658[0n]|BOLD:ABX6710
Diarsia esurialis|[5340]|LBCH3043-10|Canada|British Columbia|658[0n]|BOLD:ABX6710
Diarsia esurialis|[5341]|LBCH3046-10|Canada|British Columbia|658[0n]|BOLD:ABX6710
Diarsia esurialis|[5342]|LBCH3050-10|Canada|British Columbia|658[0n]|BOLD:ABX6710
Diarsia esurialis|[5343]|LBCH147-10|Canada|British Columbia|658[0n]|BOLD:ABX6710
Diarsia esurialis|[5344]|LBCH160-10|Canada|British Columbia|658[0n]|BOLD:ABX6710
Diarsia esurialis|[5345]|LBCH659-10|Canada|British Columbia|658[0n]|BOLD:ABX6710
Diarsia esurialis|[5346]|LBCH848-10|Canada|British Columbia|658[0n]|BOLD:ABX6710
Diarsia esurialis|[5347]|LBCH3042-10|Canada|British Columbia|658[0n]|BOLD:ABX6710
Diarsia esurialis|[5348]|LBCH2987-10|Canada|British Columbia|658[0n]|BOLD:ABX6710
Diarsia esurialis|[5349]|LBCH2984-10|Canada|British Columbia|658[0n]|BOLD:ABX6710
Diarsia esurialis|[5350]|LBCH2962-10|Canada|British Columbia|658[0n]|BOLD:ABX6710
Diarsia esurialis|[5351]|LBCH2937-10|Canada|British Columbia|658[0n]|BOLD:ABX6710
Diarsia esurialis|[5352]|LBCH2935-10|Canada|British Columbia|658[0n]|BOLD:ABX6710
Diarsia esurialis|[5353]|LBCH2917-10|Canada|British Columbia|658[0n]|BOLD:ABX6710
Diarsia esurialis|[5354]|LBCH2915-10|Canada|British Columbia|658[0n]|BOLD:ABX6710
Diarsia esurialis|[5355]|LBCH2390-10|Canada|British Columbia|658[0n]|BOLD:ABX6710
Diarsia esurialis|[5356]|LBCH2388-10|Canada|British Columbia|658[0n]|BOLD:ABX6710
Diarsia esurialis|[5357]|LBCH850-10|Canada|British Columbia|658[0n]|BOLD:ABX6710
Diarsia esurialis|[5358]|LBCH847-10|Canada|British Columbia|658[0n]|BOLD:ABX6710
Diarsia esurialis|[5359]|LBCH836-10|Canada|British Columbia|658[0n]|BOLD:ABX6710
Diarsia esurialis|[5360]|LBCH691-10|Canada|British Columbia|658[0n]|BOLD:ABX6710
Diarsia esurialis|[5361]|LBCH663-10|Canada|British Columbia|658[0n]|BOLD:ABX6710
Diarsia esurialis|[5362]|LBCH661-10|Canada|British Columbia|658[0n]|BOLD:ABX6710
Diarsia esurialis|[5363]|LBCH660-10|Canada|British Columbia|658[0n]|BOLD:ABX6710
Diarsia esurialis|[5364]|LBCH656-10|Canada|British Columbia|658[0n]|BOLD:ABX6710
Diarsia esurialis|[5365]|LBCH655-10|Canada|British Columbia|658[0n]|BOLD:ABX6710
Diarsia esurialis|[5366]|LBCH545-10|Canada|British Columbia|658[0n]|BOLD:ABX6710
Diarsia esurialis|[5367]|LBCH533-10|Canada|British Columbia|658[0n]|BOLD:ABX6710
Diarsia esurialis|[5368]|LBCH532-10|Canada|British Columbia|658[0n]|BOLD:ABX6710
Diarsia esurialis|[5369]|LBCH531-10|Canada|British Columbia|658[0n]|BOLD:ABX6710
Diarsia esurialis|[5370]|LBCH522-10|Canada|British Columbia|658[0n]|BOLD:ABX6710
Diarsia esurialis|[5371]|LBCH520-10|Canada|British Columbia|658[0n]|BOLD:ABX6710
Diarsia esurialis|[5372]|LBCH411-10|Canada|British Columbia|658[0n]|BOLD:ABX6710
Diarsia esurialis|[5373]|LBCH403-10|Canada|British Columbia|658[0n]|BOLD:ABX6710
Diarsia esurialis|[5374]|LBCH402-10|Canada|British Columbia|658[0n]|BOLD:ABX6710
Diarsia esurialis|[5375]|LBCH400-10|Canada|British Columbia|658[0n]|BOLD:ABX6710
Diarsia esurialis|[5376]|LBCH396-10|Canada|British Columbia|658[0n]|BOLD:ABX6710
Diarsia esurialis|[5377]|LBCH274-10|Canada|British Columbia|658[0n]|BOLD:ABX6710
Diarsia esurialis|[5378]|LBCH273-10|Canada|British Columbia|658[0n]|BOLD:ABX6710
Diarsia esurialis|[5379]|LBCH272-10|Canada|British Columbia|658[0n]|BOLD:ABX6710
Diarsia esurialis|[5380]|LBCH271-10|Canada|British Columbia|658[0n]|BOLD:ABX6710
Diarsia esurialis|[5381]|LBCH270-10|Canada|British Columbia|658[0n]|BOLD:ABX6710
Diarsia esurialis|[5382]|LBCH269-10|Canada|British Columbia|658[0n]|BOLD:ABX6710
Diarsia esurialis|[5383]|LBCH268-10|Canada|British Columbia|658[0n]|BOLD:ABX6710

Diarsia esurialis|[5381]|LBCH270-10|Canada|British Columbia|658[0n]|BOLD:ABX6710
Diarsia esurialis|[5382]|LBCH269-10|Canada|British Columbia|658[0n]|BOLD:ABX6710
Diarsia esurialis|[5383]|LBCH268-10|Canada|British Columbia|658[0n]|BOLD:ABX6710
Diarsia esurialis|[5384]|LBCH266-10|Canada|British Columbia|658[0n]|BOLD:ABX6710
Diarsia esurialis|[5385]|LBCH262-10|Canada|British Columbia|658[0n]|BOLD:ABX6710
Diarsia esurialis|[5386]|LBCH258-10|Canada|British Columbia|658[0n]|BOLD:ABX6710
Diarsia esurialis|[5387]|LBCH256-10|Canada|British Columbia|658[0n]|BOLD:ABX6710
Diarsia esurialis|[5388]|LBCH254-10|Canada|British Columbia|658[0n]|BOLD:ABX6710
Diarsia esurialis|[5389]|LBCH252-10|Canada|British Columbia|658[0n]|BOLD:ABX6710
Diarsia esurialis|[5390]|LBCH161-10|Canada|British Columbia|658[0n]|BOLD:ABX6710
Diarsia esurialis|[5391]|LBCH159-10|Canada|British Columbia|658[0n]|BOLD:ABX6710
Diarsia esurialis|[5392]|LBCH158-10|Canada|British Columbia|658[0n]|BOLD:ABX6710
Diarsia esurialis|[5393]|LBCH155-10|Canada|British Columbia|658[0n]|BOLD:ABX6710
Diarsia esurialis|[5394]|LBCH151-10|Canada|British Columbia|658[0n]|BOLD:ABX6710
Diarsia esurialis|[5395]|LBCH148-10|Canada|British Columbia|658[0n]|BOLD:ABX6710
Diarsia esurialis|[5396]|LBCH146-10|Canada|British Columbia|658[0n]|BOLD:ABX6710
Diarsia esurialis|[5397]|LBCH144-10|Canada|British Columbia|658[0n]|BOLD:ABX6710
Diarsia esurialis|[5398]|LBCH143-10|Canada|British Columbia|658[0n]|BOLD:ABX6710
Diarsia esurialis|[5399]|LBCH136-10|Canada|British Columbia|658[0n]|BOLD:ABX6710
Diarsia esurialis|[5400]|LBCH135-10|Canada|British Columbia|658[0n]|BOLD:ABX6710
Diarsia esurialis|[5401]|LBCH134-10|Canada|British Columbia|658[0n]|BOLD:ABX6710
Diarsia esurialis|[5402]|LBCH054-10|Canada|British Columbia|658[0n]|BOLD:ABX6710
Diarsia esurialis|[5403]|LBCH050-10|Canada|British Columbia|658[0n]|BOLD:ABX6710
Diarsia esurialis|[5404]|LBCH046-10|Canada|British Columbia|658[0n]|BOLD:ABX6710
Diarsia esurialis|[5405]|LBCH045-10|Canada|British Columbia|658[0n]|BOLD:ABX6710
Diarsia esurialis|[5406]|LBCH042-10|Canada|British Columbia|658[0n]|BOLD:ABX6710
Diarsia esurialis|[5407]|LBCH040-10|Canada|British Columbia|658[0n]|BOLD:ABX6710
Diarsia esurialis|[5408]|LBCH039-10|Canada|British Columbia|658[0n]|BOLD:ABX6710
Diarsia esurialis|[5409]|LBCH038-10|Canada|British Columbia|658[0n]|BOLD:ABX6710
Diarsia esurialis|[5410]|LBCH037-10|Canada|British Columbia|658[0n]|BOLD:ABX6710
Diarsia esurialis|[5411]|LBCH036-10|Canada|British Columbia|658[0n]|BOLD:ABX6710
Diarsia esurialis|[5412]|LBCH035-10|Canada|British Columbia|658[0n]|BOLD:ABX6710
Diarsia esurialis|[5413]|LBCH034-10|Canada|British Columbia|658[0n]|BOLD:ABX6710
Diarsia esurialis|[5414]|LBCG442-08|Canada|British Columbia|658[0n]|BOLD:ABX6710
Diarsia esurialis|[5415]|LBCH141-10|Canada|British Columbia|658[0n]|BOLD:ABX6710
Diarsia esurialis|[5416]|LBCH518-10|Canada|British Columbia|658[0n]|BOLD:ABX6710
Diarsia esurialis|[5417]|ABKWR079-07|United States|Alaska|658[0n]|BOLD:ABX6710
Diarsia esurialis|[5418]|LBCH550-10|Canada|British Columbia|658[0n]|BOLD:ABX6710
Diarsia esurialis|[5419]|LBCH033-10|Canada|British Columbia|658[0n]|BOLD:ABX6710
Diarsia esurialis|[5420]|LBCH3044-10|Canada|British Columbia|658[0n]|BOLD:ABX6710
Diarsia esurialis|[5421]|LBCH3045-10|Canada|British Columbia|658[0n]|BOLD:ABX6710
Diarsia esurialis|[5422]|LBCH3048-10|Canada|British Columbia|658[0n]|BOLD:ABX6710
Diarsia esurialis|[5423]|LBCH3049-10|Canada|British Columbia|658[0n]|BOLD:ABX6710
Diarsia esurialis|[5424]|LBCH3784-10|Canada|British Columbia|658[0n]|BOLD:ABX6710
Diarsia esurialis|[5425]|RWWA525-09|United States|Washington|627[0n]|BOLD:ABX6710
Diarsia esurialis|[5426]|RWWA569-09|United States|Washington|658[0n]|BOLD:ABX6710
Diarsia esurialis|[5427]|RWWA571-09|United States|Washington|658[0n]|BOLD:ABX6710
Diarsia esurialis|[5428]|RWWA583-09|United States|Washington|658[0n]|BOLD:ABX6710
Diarsia esurialis|[5429]|RWWA670-09|United States|Washington|658[0n]|BOLD:ABX6710
Diarsia esurialis|[5430]|RWWA686-09|United States|Washington|658[0n]|BOLD:ABX6710
Diarsia esurialis|[5431]|RWWA854-09|United States|Washington|658[0n]|BOLD:ABX6710
Diarsia esurialis|[5432]|LALPA311-10|Canada|British Columbia|658[0n]|BOLD:ABX6710
Diarsia esurialis|[5433]|LALPA963-11|Canada|British Columbia|658[0n]|BOLD:ABX6710
Diarsia esurialis|[5434]|LALPA962-11|Canada|British Columbia|658[0n]|BOLD:ABX6710
Diarsia esurialis|[5435]|UAMIC553-13|United States|Alaska|624[0n]|BOLD:ABX6710
Diarsia esurialis|[5436]|UAMIC554-13|United States|Alaska|632[1n]|BOLD:ABX6710
Diarsia esurialis|[5437]|UAMIC559-13|United States|Alaska|593[0n]|BOLD:ABX6710
Diarsia esurialis|[5438]|RWWA552-09|United States|Washington|658[1n]|BOLD:ABX6710
Diarsia esurialis|[5439]|RWWA471-09|United States|Washington|658[0n]|BOLD:ABX6710
Diarsia esurialis|[5440]|RWWA830-09|United States|Washington|658[0n]|BOLD:ABX6710
Diarsia esurialis|[5441]|RWWC1193-13|United States|Washington|592[0n]|BOLD:ABX6710
Diarsia jucunda|[5442]|ABKWR083-07|United States|Alaska|658[0n]|BOLD:AAB0038
Diarsia jucunda|[5443]|LNCC1029-11|United States|North Carolina|658[0n]|BOLD:AAB0038
Diarsia jucunda|[5444]|BBLEC700-09|Canada|Nova Scotia|658[0n]|BOLD:AAB0038
Diarsia jucunda|[5445]|BBLEC016-09|Canada|New Brunswick|658[0n]|BOLD:AAB0038
Diarsia jucunda|[5446]|RDNMC340-05|United States|North Carolina|658[0n]|BOLD:AAB0038
Diarsia jucunda|[5447]|LNCC1180-11|United States|North Carolina|658[0n]|BOLD:AAB0038
Diarsia jucunda|[5448]|BBLPC587-09|Canada|Nova Scotia|658[0n]|BOLD:AAB0038
Diarsia jucunda|[5449]|ABKWR105-07|United States|Alaska|658[1n]|BOLD:AAB0038
Diarsia dislocata|[5450]|UAMIC551-13|United States|Alaska|642[0n]|BOLD:AAB0038
Diarsia dislocata|[5451]|UAMIC552-13|United States|Alaska|634[0n]|BOLD:AAB0038
Diarsia jucunda|[5452]|CNKOG1301-14|Canada|New Brunswick|564[0n]|BOLD:AAA1521
Diarsia dislocata|[5453]|RDLQG136-06|Canada|Quebec|658[0n]|BOLD:AAA1521
Diarsia dislocata|[5454]|LBCH383-10|Canada|British Columbia|658[0n]|BOLD:AAA1521
Diarsia dislocata|[5455]|LBCH549-10|Canada|British Columbia|658[0n]|BOLD:AAA1521
Diarsia dislocata|[5456]|LBCH966-10|Canada|British Columbia|658[0n]|BOLD:AAA1521
Diarsia dislocata|[5457]|LBCH3511-10|Canada|British Columbia|658[0n]|BOLD:AAA1521
Diarsia dislocata|[5458]|LBCH4745-10|Canada|British Columbia|658[0n]|BOLD:AAA1521
Diarsia dislocata|[5459]|CNKOG1303-14|Canada|New Brunswick|564[0n]|BOLD:AAA1521
Diarsia dislocata|[5460]|LPABB869-09|Canada|Alberta|631[0n]|BOLD:AAA1521
Diarsia dislocata|[5461]|LCH300-04|Canada|Manitoba|658[0n]|BOLD:AAA1521
Diarsia dislocata|[5462]|LPABC437-09|Canada|Alberta|658[0n]|BOLD:AAA1521
Diarsia dislocata|[5463]|LCHQ028-07|Canada|Manitoba|658[0n]|BOLD:AAA1521
Diarsia dislocata|[5464]|LCHP482-07|Canada|Manitoba|658[0n]|BOLD:AAA1521
Diarsia dislocata|[5465]|LCHP652-07|Canada|Manitoba|658[0n]|BOLD:AAA1521
Diarsia dislocata|[5466]|LBCH4030-10|Canada|British Columbia|658[0n]|BOLD:AAA1521
Diarsia dislocata|[5467]|BBLPE233-09|Canada|Newfoundland and Labrador|614[0n]|BOLD:AAA1521
Diarsia dislocata|[5468]|CNGMG037-14|Canada|Newfoundland and Labrador|590[0n]|BOLD:AAA1521
Diarsia dislocata|[5469]|LBCH239-10|Canada|British Columbia|658[0n]|BOLD:AAA1521
Diarsia dislocata|[5470]|BBLPC938-09|Canada|Newfoundland and Labrador|632[0n]|BOLD:AAA1521
Diarsia dislocata|[5471]|LCH314-04|Canada|Manitoba|632[0n]|BOLD:AAA1521
Diarsia dislocata|[5472]|LBCH839-10|Canada|British Columbia|636[0n]|BOLD:AAA1521
Diarsia dislocata|[5473]|LCH288-04|Canada|Manitoba|658[0n]|BOLD:AAA1521
Diarsia dislocata|[5474]|LCH292-04|Canada|Manitoba|658[0n]|BOLD:AAA1521
Diarsia dislocata|[5475]|LCHP279-07|Canada|Manitoba|658[0n]|BOLD:AAA1521
Diarsia dislocata|[5476]|LCH271-04|Canada|Manitoba|615[0n]|BOLD:AAA1521
Diarsia dislocata|[5477]|LCHP518-07|Canada|Manitoba|658[0n]|BOLD:AAA1521
Diarsia dislocata|[5478]|LBCH3508-10|Canada|British Columbia|658[0n]|BOLD:AAA1521
Diarsia dislocata|[5479]|LBCH4044-10|Canada|British Columbia|658[0n]|BOLD:AAA1521
Diarsia dislocata|[5480]|LBCH4025-10|Canada|British Columbia|658[0n]|BOLD:AAA1521
Diarsia dislocata|[5481]|LBCH4017-10|Canada|British Columbia|658[0n]|BOLD:AAA1521
Diarsia dislocata|[5482]|LBCH3824-10|Canada|British Columbia|658[0n]|BOLD:AAA1521

Diarsia dislocata|[5480]|LBCH4025-10|Canada|British Columbia|658[0n]|BOLD:AAA1521
Diarsia dislocata|[5481]|LBCH4017-10|Canada|British Columbia|658[0n]|BOLD:AAA1521
Diarsia dislocata|[5482]|LBCH3824-10|Canada|British Columbia|658[0n]|BOLD:AAA1521
Diarsia dislocata|[5483]|LBCH3810-10|Canada|British Columbia|658[0n]|BOLD:AAA1521
Diarsia dislocata|[5484]|LBCH3802-10|Canada|British Columbia|658[0n]|BOLD:AAA1521
Diarsia dislocata|[5485]|LBCH3800-10|Canada|British Columbia|658[0n]|BOLD:AAA1521
Diarsia dislocata|[5486]|LBCH3524-10|Canada|British Columbia|658[0n]|BOLD:AAA1521
Diarsia dislocata|[5487]|LBCH3353-10|Canada|British Columbia|658[0n]|BOLD:AAA1521
Diarsia dislocata|[5488]|LBCH3141-10|Canada|British Columbia|658[0n]|BOLD:AAA1521
Diarsia dislocata|[5489]|LBCH3130-10|Canada|British Columbia|658[0n]|BOLD:AAA1521
Diarsia dislocata|[5490]|LBCH3126-10|Canada|British Columbia|658[0n]|BOLD:AAA1521
Diarsia dislocata|[5491]|LBCH3118-10|Canada|British Columbia|658[0n]|BOLD:AAA1521
Diarsia dislocata|[5492]|LBCH825-10|Canada|British Columbia|658[0n]|BOLD:AAA1521
Diarsia dislocata|[5493]|LBCH677-10|Canada|British Columbia|658[0n]|BOLD:AAA1521
Diarsia dislocata|[5494]|LBCH529-10|Canada|British Columbia|658[0n]|BOLD:AAA1521
Diarsia dislocata|[5495]|LBCH513-10|Canada|British Columbia|658[0n]|BOLD:AAA1521
Diarsia dislocata|[5496]|LBCH398-10|Canada|British Columbia|658[0n]|BOLD:AAA1521
Diarsia dislocata|[5497]|LBCH373-10|Canada|British Columbia|658[0n]|BOLD:AAA1521
Diarsia dislocata|[5498]|LBCH369-10|Canada|British Columbia|658[0n]|BOLD:AAA1521
Diarsia dislocata|[5499]|LBCH238-10|Canada|British Columbia|658[0n]|BOLD:AAA1521
Diarsia dislocata|[5500]|LBCG2263-09|Canada|British Columbia|658[0n]|BOLD:AAA1521
Diarsia dislocata|[5501]|LBCG2247-09|Canada|British Columbia|658[0n]|BOLD:AAA1521
Diarsia dislocata|[5502]|LCHP900-07|Canada|Manitoba|658[0n]|BOLD:AAA1521
Diarsia dislocata|[5503]|LCHP866-07|Canada|Manitoba|658[0n]|BOLD:AAA1521
Diarsia dislocata|[5504]|LCHP650-07|Canada|Manitoba|658[0n]|BOLD:AAA1521
Diarsia dislocata|[5505]|LCHP638-07|Canada|Manitoba|658[0n]|BOLD:AAA1521
Diarsia dislocata|[5506]|LCHP583-07|Canada|Manitoba|658[0n]|BOLD:AAA1521
Diarsia dislocata|[5507]|LCHP392-07|Canada|Manitoba|658[0n]|BOLD:AAA1521
Diarsia dislocata|[5508]|LCHP255-07|Canada|Manitoba|658[0n]|BOLD:AAA1521
Diarsia dislocata|[5509]|LCH587-04|Canada|Manitoba|658[0n]|BOLD:AAA1521
Diarsia dislocata|[5510]|LCH313-04|Canada|Manitoba|658[0n]|BOLD:AAA1521
Diarsia dislocata|[5511]|LCH312-04|Canada|Manitoba|658[0n]|BOLD:AAA1521
Diarsia dislocata|[5512]|LCH305-04|Canada|Manitoba|658[0n]|BOLD:AAA1521
Diarsia dislocata|[5513]|LCH303-04|Canada|Manitoba|658[0n]|BOLD:AAA1521
Diarsia dislocata|[5514]|LCH286-04|Canada|Manitoba|658[0n]|BOLD:AAA1521
Diarsia dislocata|[5515]|LCH282-04|Canada|Manitoba|658[0n]|BOLD:AAA1521
Diarsia dislocata|[5516]|LCH306-04|Canada|Manitoba|658[0n]|BOLD:AAA1521
Diarsia dislocata|[5517]|LCHP825-07|Canada|Manitoba|652[0n]|BOLD:AAA1521
Diarsia dislocata|[5518]|LCHQ487-08|Canada|Manitoba|634[0n]|BOLD:AAA1521
Diarsia dislocata|[5519]|LBCH3497-10|Canada|British Columbia|635[0n]|BOLD:AAA1521
Diarsia dislocata|[5520]|LBCH3510-10|Canada|British Columbia|644[0n]|BOLD:AAA1521
Diarsia dislocata|[5521]|LBCH4442-10|Canada|British Columbia|643[0n]|BOLD:AAA1521
Diarsia dislocata|[5522]|LBCH4443-10|Canada|British Columbia|658[0n]|BOLD:AAA1521
Diarsia dislocata|[5523]|LBCH4448-10|Canada|British Columbia|658[0n]|BOLD:AAA1521
Diarsia dislocata|[5524]|LBCH4450-10|Canada|British Columbia|658[0n]|BOLD:AAA1521
Diarsia dislocata|[5525]|LBCH4453-10|Canada|British Columbia|658[0n]|BOLD:AAA1521
Diarsia dislocata|[5526]|LBCH4463-10|Canada|British Columbia|658[0n]|BOLD:AAA1521
Diarsia dislocata|[5527]|LBCH4464-10|Canada|British Columbia|658[0n]|BOLD:AAA1521
Diarsia dislocata|[5528]|LBCH4702-10|Canada|British Columbia|658[0n]|BOLD:AAA1521
Diarsia dislocata|[5529]|LBCH4708-10|Canada|British Columbia|658[0n]|BOLD:AAA1521
Diarsia dislocata|[5530]|LBCH4718-10|Canada|British Columbia|658[0n]|BOLD:AAA1521
Diarsia dislocata|[5531]|LBCH4738-10|Canada|British Columbia|658[0n]|BOLD:AAA1521
Diarsia dislocata|[5532]|LBCH4740-10|Canada|British Columbia|658[0n]|BOLD:AAA1521
Diarsia dislocata|[5533]|LBCH4747-10|Canada|British Columbia|658[0n]|BOLD:AAA1521
Diarsia dislocata|[5534]|LPABC263-09|Canada|Alberta|566[1n]|BOLD:AAA1521
Diarsia dislocata|[5535]|LCH277-04|Canada|Manitoba|617[0n]|BOLD:AAA1521
Diarsia dislocata|[5536]|LBCH3506-10|Canada|British Columbia|642[0n]|BOLD:AAA1521
Diarsia dislocata|[5537]|LBCH4737-10|Canada|British Columbia|658[0n]|BOLD:AAA1521
Diarsia dislocata|[5538]|LBCH4736-10|Canada|British Columbia|658[0n]|BOLD:AAA1521
Diarsia dislocata|[5539]|LBCH4735-10|Canada|British Columbia|658[0n]|BOLD:AAA1521
Diarsia dislocata|[5540]|LBCH4733-10|Canada|British Columbia|658[0n]|BOLD:AAA1521
Diarsia dislocata|[5541]|LBCH4731-10|Canada|British Columbia|658[0n]|BOLD:AAA1521
Diarsia dislocata|[5542]|LBCH4730-10|Canada|British Columbia|658[0n]|BOLD:AAA1521
Diarsia dislocata|[5543]|LBCH4729-10|Canada|British Columbia|658[0n]|BOLD:AAA1521
Diarsia dislocata|[5544]|LBCH4724-10|Canada|British Columbia|658[0n]|BOLD:AAA1521
Diarsia dislocata|[5545]|LBCH4723-10|Canada|British Columbia|658[0n]|BOLD:AAA1521
Diarsia dislocata|[5546]|LBCH4720-10|Canada|British Columbia|658[0n]|BOLD:AAA1521
Diarsia dislocata|[5547]|LBCH4719-10|Canada|British Columbia|658[0n]|BOLD:AAA1521
Diarsia dislocata|[5548]|LBCH4716-10|Canada|British Columbia|658[0n]|BOLD:AAA1521
Diarsia dislocata|[5549]|LBCH4715-10|Canada|British Columbia|658[0n]|BOLD:AAA1521
Diarsia dislocata|[5550]|LBCH4714-10|Canada|British Columbia|658[0n]|BOLD:AAA1521
Diarsia dislocata|[5551]|LBCH4711-10|Canada|British Columbia|658[0n]|BOLD:AAA1521
Diarsia dislocata|[5552]|LBCH4710-10|Canada|British Columbia|658[0n]|BOLD:AAA1521
Diarsia dislocata|[5553]|LBCH4709-10|Canada|British Columbia|658[0n]|BOLD:AAA1521
Diarsia dislocata|[5554]|LBCH4707-10|Canada|British Columbia|658[0n]|BOLD:AAA1521
Diarsia dislocata|[5555]|LBCH4705-10|Canada|British Columbia|658[0n]|BOLD:AAA1521
Diarsia dislocata|[5556]|LBCH4704-10|Canada|British Columbia|658[0n]|BOLD:AAA1521
Diarsia dislocata|[5557]|LBCH4703-10|Canada|British Columbia|658[0n]|BOLD:AAA1521
Diarsia dislocata|[5558]|LBCH4700-10|Canada|British Columbia|658[0n]|BOLD:AAA1521
Diarsia dislocata|[5559]|LBCH4699-10|Canada|British Columbia|658[0n]|BOLD:AAA1521
Diarsia dislocata|[5560]|LBCH4697-10|Canada|British Columbia|658[0n]|BOLD:AAA1521
Diarsia dislocata|[5561]|LBCH4690-10|Canada|British Columbia|658[0n]|BOLD:AAA1521
Diarsia dislocata|[5562]|LBCH4477-10|Canada|British Columbia|658[0n]|BOLD:AAA1521
Diarsia dislocata|[5563]|LBCH4476-10|Canada|British Columbia|658[0n]|BOLD:AAA1521
Diarsia dislocata|[5564]|LBCH4474-10|Canada|British Columbia|658[0n]|BOLD:AAA1521
Diarsia dislocata|[5565]|LBCH4472-10|Canada|British Columbia|658[0n]|BOLD:AAA1521
Diarsia dislocata|[5566]|LBCH4471-10|Canada|British Columbia|658[0n]|BOLD:AAA1521
Diarsia dislocata|[5567]|LBCH4470-10|Canada|British Columbia|658[0n]|BOLD:AAA1521
Diarsia dislocata|[5568]|LBCH4469-10|Canada|British Columbia|658[0n]|BOLD:AAA1521
Diarsia dislocata|[5569]|LBCH4468-10|Canada|British Columbia|658[0n]|BOLD:AAA1521
Diarsia dislocata|[5570]|LBCH4467-10|Canada|British Columbia|658[0n]|BOLD:AAA1521
Diarsia dislocata|[5571]|LBCH4462-10|Canada|British Columbia|658[0n]|BOLD:AAA1521
Diarsia dislocata|[5572]|LBCH4461-10|Canada|British Columbia|658[0n]|BOLD:AAA1521
Diarsia dislocata|[5573]|LBCH4459-10|Canada|British Columbia|658[0n]|BOLD:AAA1521
Diarsia dislocata|[5574]|LBCH4458-10|Canada|British Columbia|658[0n]|BOLD:AAA1521
Diarsia dislocata|[5575]|LBCH4457-10|Canada|British Columbia|658[0n]|BOLD:AAA1521
Diarsia dislocata|[5576]|LBCH4456-10|Canada|British Columbia|658[0n]|BOLD:AAA1521
Diarsia dislocata|[5577]|LBCH4455-10|Canada|British Columbia|658[0n]|BOLD:AAA1521
Diarsia dislocata|[5578]|LBCH4454-10|Canada|British Columbia|658[0n]|BOLD:AAA1521
Diarsia dislocata|[5579]|LBCH4451-10|Canada|British Columbia|658[0n]|BOLD:AAA1521
Diarsia dislocata|[5580]|LBCH4447-10|Canada|British Columbia|658[0n]|BOLD:AAA1521
Diarsia dislocata|[5581]|LBCH4446-10|Canada|British Columbia|658[0n]|BOLD:AAA1521
Diarsia dislocata|[5582]|LBCH4445-10|Canada|British Columbia|658[0n]|BOLD:AAA1521

Diarsia dislocata|[5580]|LBCH4447-10|Canada|British Columbia|658[0n]|BOLD:AAA1521
Diarsia dislocata|[5581]|LBCH4446-10|Canada|British Columbia|658[0n]|BOLD:AAA1521
Diarsia dislocata|[5582]|LBCH4445-10|Canada|British Columbia|658[0n]|BOLD:AAA1521
Diarsia dislocata|[5583]|LBCH4444-10|Canada|British Columbia|658[0n]|BOLD:AAA1521
Diarsia dislocata|[5584]|LBCH4045-10|Canada|British Columbia|658[0n]|BOLD:AAA1521
Diarsia dislocata|[5585]|LBCH4043-10|Canada|British Columbia|658[0n]|BOLD:AAA1521
Diarsia dislocata|[5586]|LBCH4041-10|Canada|British Columbia|658[0n]|BOLD:AAA1521
Diarsia dislocata|[5587]|LBCH4040-10|Canada|British Columbia|658[0n]|BOLD:AAA1521
Diarsia dislocata|[5588]|LBCH4038-10|Canada|British Columbia|658[0n]|BOLD:AAA1521
Diarsia dislocata|[5589]|LBCH4035-10|Canada|British Columbia|658[0n]|BOLD:AAA1521
Diarsia dislocata|[5590]|LBCH4034-10|Canada|British Columbia|658[0n]|BOLD:AAA1521
Diarsia dislocata|[5591]|LBCH4033-10|Canada|British Columbia|658[0n]|BOLD:AAA1521
Diarsia dislocata|[5592]|LBCH4032-10|Canada|British Columbia|658[0n]|BOLD:AAA1521
Diarsia dislocata|[5593]|LBCH4031-10|Canada|British Columbia|658[0n]|BOLD:AAA1521
Diarsia dislocata|[5594]|LBCH4029-10|Canada|British Columbia|658[0n]|BOLD:AAA1521
Diarsia dislocata|[5595]|LBCH4027-10|Canada|British Columbia|658[0n]|BOLD:AAA1521
Diarsia dislocata|[5596]|LBCH4022-10|Canada|British Columbia|658[0n]|BOLD:AAA1521
Diarsia dislocata|[5597]|LBCH4021-10|Canada|British Columbia|658[0n]|BOLD:AAA1521
Diarsia dislocata|[5598]|LBCH4020-10|Canada|British Columbia|658[0n]|BOLD:AAA1521
Diarsia dislocata|[5599]|LBCH4018-10|Canada|British Columbia|658[0n]|BOLD:AAA1521
Diarsia dislocata|[5600]|LBCH3823-10|Canada|British Columbia|658[0n]|BOLD:AAA1521
Diarsia dislocata|[5601]|LBCH3822-10|Canada|British Columbia|658[0n]|BOLD:AAA1521
Diarsia dislocata|[5602]|LBCH3821-10|Canada|British Columbia|658[0n]|BOLD:AAA1521
Diarsia dislocata|[5603]|LBCH3820-10|Canada|British Columbia|658[0n]|BOLD:AAA1521
Diarsia dislocata|[5604]|LBCH3819-10|Canada|British Columbia|658[0n]|BOLD:AAA1521
Diarsia dislocata|[5605]|LBCH3818-10|Canada|British Columbia|658[0n]|BOLD:AAA1521
Diarsia dislocata|[5606]|LBCH3817-10|Canada|British Columbia|658[0n]|BOLD:AAA1521
Diarsia dislocata|[5607]|LBCH3816-10|Canada|British Columbia|658[0n]|BOLD:AAA1521
Diarsia dislocata|[5608]|LBCH3815-10|Canada|British Columbia|658[0n]|BOLD:AAA1521
Diarsia dislocata|[5609]|LBCH3814-10|Canada|British Columbia|658[0n]|BOLD:AAA1521
Diarsia dislocata|[5610]|LBCH3813-10|Canada|British Columbia|658[0n]|BOLD:AAA1521
Diarsia dislocata|[5611]|LBCH3812-10|Canada|British Columbia|658[0n]|BOLD:AAA1521
Diarsia dislocata|[5612]|LBCH3809-10|Canada|British Columbia|658[0n]|BOLD:AAA1521
Diarsia dislocata|[5613]|LBCH3808-10|Canada|British Columbia|658[0n]|BOLD:AAA1521
Diarsia dislocata|[5614]|LBCH3807-10|Canada|British Columbia|658[0n]|BOLD:AAA1521
Diarsia dislocata|[5615]|LBCH3806-10|Canada|British Columbia|658[0n]|BOLD:AAA1521
Diarsia dislocata|[5616]|LBCH3805-10|Canada|British Columbia|658[0n]|BOLD:AAA1521
Diarsia dislocata|[5617]|LBCH3804-10|Canada|British Columbia|658[0n]|BOLD:AAA1521
Diarsia dislocata|[5618]|LBCH3803-10|Canada|British Columbia|658[0n]|BOLD:AAA1521
Diarsia dislocata|[5619]|LBCH3801-10|Canada|British Columbia|658[0n]|BOLD:AAA1521
Diarsia dislocata|[5620]|LBCH3798-10|Canada|British Columbia|658[0n]|BOLD:AAA1521
Diarsia dislocata|[5621]|LBCH3523-10|Canada|British Columbia|658[0n]|BOLD:AAA1521
Diarsia dislocata|[5622]|LBCH3522-10|Canada|British Columbia|658[0n]|BOLD:AAA1521
Diarsia dislocata|[5623]|LBCH3521-10|Canada|British Columbia|658[0n]|BOLD:AAA1521
Diarsia dislocata|[5624]|LBCH3520-10|Canada|British Columbia|658[0n]|BOLD:AAA1521
Diarsia dislocata|[5625]|LBCH3517-10|Canada|British Columbia|658[0n]|BOLD:AAA1521
Diarsia dislocata|[5626]|LBCH3516-10|Canada|British Columbia|658[0n]|BOLD:AAA1521
Diarsia dislocata|[5627]|LBCH3515-10|Canada|British Columbia|658[0n]|BOLD:AAA1521
Diarsia dislocata|[5628]|LBCH3513-10|Canada|British Columbia|658[0n]|BOLD:AAA1521
Diarsia dislocata|[5629]|LBCH3512-10|Canada|British Columbia|658[0n]|BOLD:AAA1521
Diarsia dislocata|[5630]|LBCH3509-10|Canada|British Columbia|658[0n]|BOLD:AAA1521
Diarsia dislocata|[5631]|LBCH3507-10|Canada|British Columbia|658[0n]|BOLD:AAA1521
Diarsia dislocata|[5632]|LBCH3502-10|Canada|British Columbia|658[0n]|BOLD:AAA1521
Diarsia dislocata|[5633]|LBCH3501-10|Canada|British Columbia|658[0n]|BOLD:AAA1521
Diarsia dislocata|[5634]|LBCH3500-10|Canada|British Columbia|658[0n]|BOLD:AAA1521
Diarsia dislocata|[5635]|LBCH3499-10|Canada|British Columbia|658[0n]|BOLD:AAA1521
Diarsia dislocata|[5636]|LBCH3498-10|Canada|British Columbia|658[0n]|BOLD:AAA1521
Diarsia dislocata|[5637]|LBCH3496-10|Canada|British Columbia|658[0n]|BOLD:AAA1521
Diarsia dislocata|[5638]|LBCH3495-10|Canada|British Columbia|658[0n]|BOLD:AAA1521
Diarsia dislocata|[5639]|LBCH3494-10|Canada|British Columbia|658[0n]|BOLD:AAA1521
Diarsia dislocata|[5640]|LBCH3341-10|Canada|British Columbia|658[0n]|BOLD:AAA1521
Diarsia dislocata|[5641]|LBCH3340-10|Canada|British Columbia|658[0n]|BOLD:AAA1521
Diarsia dislocata|[5642]|LBCH3339-10|Canada|British Columbia|658[0n]|BOLD:AAA1521
Diarsia dislocata|[5643]|LBCH3338-10|Canada|British Columbia|658[0n]|BOLD:AAA1521
Diarsia dislocata|[5644]|LBCH3337-10|Canada|British Columbia|658[0n]|BOLD:AAA1521
Diarsia dislocata|[5645]|LBCH3139-10|Canada|British Columbia|658[0n]|BOLD:AAA1521
Diarsia dislocata|[5646]|LBCH3138-10|Canada|British Columbia|658[0n]|BOLD:AAA1521
Diarsia dislocata|[5647]|LBCH3137-10|Canada|British Columbia|658[0n]|BOLD:AAA1521
Diarsia dislocata|[5648]|LBCH3135-10|Canada|British Columbia|658[0n]|BOLD:AAA1521
Diarsia dislocata|[5649]|LBCH3134-10|Canada|British Columbia|658[0n]|BOLD:AAA1521
Diarsia dislocata|[5650]|LBCH3133-10|Canada|British Columbia|658[0n]|BOLD:AAA1521
Diarsia dislocata|[5651]|LBCH3132-10|Canada|British Columbia|658[0n]|BOLD:AAA1521
Diarsia dislocata|[5652]|LBCH3131-10|Canada|British Columbia|658[0n]|BOLD:AAA1521
Diarsia dislocata|[5653]|LBCH3129-10|Canada|British Columbia|658[0n]|BOLD:AAA1521
Diarsia dislocata|[5654]|LBCH3128-10|Canada|British Columbia|658[0n]|BOLD:AAA1521
Diarsia dislocata|[5655]|LBCH3127-10|Canada|British Columbia|658[0n]|BOLD:AAA1521
Diarsia dislocata|[5656]|LBCH3125-10|Canada|British Columbia|658[0n]|BOLD:AAA1521
Diarsia dislocata|[5657]|LBCH3124-10|Canada|British Columbia|658[0n]|BOLD:AAA1521
Diarsia dislocata|[5658]|LBCH3122-10|Canada|British Columbia|658[0n]|BOLD:AAA1521
Diarsia dislocata|[5659]|LBCH3121-10|Canada|British Columbia|658[0n]|BOLD:AAA1521
Diarsia dislocata|[5660]|LBCH3120-10|Canada|British Columbia|658[0n]|BOLD:AAA1521
Diarsia dislocata|[5661]|LBCH3119-10|Canada|British Columbia|658[0n]|BOLD:AAA1521
Diarsia dislocata|[5662]|LBCH3117-10|Canada|British Columbia|658[0n]|BOLD:AAA1521
Diarsia dislocata|[5663]|LBCH3116-10|Canada|British Columbia|658[0n]|BOLD:AAA1521
Diarsia dislocata|[5664]|LBCH3114-10|Canada|British Columbia|658[0n]|BOLD:AAA1521
Diarsia dislocata|[5665]|LBCH3111-10|Canada|British Columbia|658[0n]|BOLD:AAA1521
Diarsia dislocata|[5666]|LBCH3109-10|Canada|British Columbia|658[0n]|BOLD:AAA1521
Diarsia dislocata|[5667]|LBCH3108-10|Canada|British Columbia|658[0n]|BOLD:AAA1521
Diarsia dislocata|[5668]|LBCH3107-10|Canada|British Columbia|658[0n]|BOLD:AAA1521
Diarsia dislocata|[5669]|LBCH3105-10|Canada|British Columbia|658[0n]|BOLD:AAA1521
Diarsia dislocata|[5670]|LBCH3104-10|Canada|British Columbia|658[0n]|BOLD:AAA1521
Diarsia dislocata|[5671]|LBCH3103-10|Canada|British Columbia|658[0n]|BOLD:AAA1521
Diarsia dislocata|[5672]|LBCH3102-10|Canada|British Columbia|658[0n]|BOLD:AAA1521
Diarsia dislocata|[5673]|LBCH3101-10|Canada|British Columbia|658[0n]|BOLD:AAA1521
Diarsia dislocata|[5674]|LBCH3100-10|Canada|British Columbia|658[0n]|BOLD:AAA1521
Diarsia dislocata|[5675]|LBCH3098-10|Canada|British Columbia|658[0n]|BOLD:AAA1521
Diarsia dislocata|[5676]|LBCH3097-10|Canada|British Columbia|658[0n]|BOLD:AAA1521
Diarsia dislocata|[5677]|LBCH3096-10|Canada|British Columbia|658[0n]|BOLD:AAA1521
Diarsia dislocata|[5678]|LBCH3095-10|Canada|British Columbia|658[0n]|BOLD:AAA1521
Diarsia dislocata|[5679]|LBCH3094-10|Canada|British Columbia|658[0n]|BOLD:AAA1521
Diarsia dislocata|[5680]|LBCH3080-10|Canada|British Columbia|658[0n]|BOLD:AAA1521
Diarsia dislocata|[5681]|LBCH2385-10|Canada|British Columbia|658[0n]|BOLD:AAA1521
Diarsia dislocata|[5682]|LBCH2383-10|Canada|British Columbia|658[0n]|BOLD:AAA1521

[illegible]
Diarsia dislocata|[5680]|LBCH3080-10|Canada|British Columbia|658[0n]|BOLD:AAA1521
Diarsia dislocata|[5681]|LBCH2385-10|Canada|British Columbia|658[0n]|BOLD:AAA1521
Diarsia dislocata|[5682]|LBCH2383-10|Canada|British Columbia|658[0n]|BOLD:AAA1521
Diarsia dislocata|[5683]|LBCH2382-10|Canada|British Columbia|658[0n]|BOLD:AAA1521
Diarsia dislocata|[5684]|LBCH2381-10|Canada|British Columbia|658[0n]|BOLD:AAA1521
Diarsia dislocata|[5685]|LBCH2380-10|Canada|British Columbia|658[0n]|BOLD:AAA1521
Diarsia dislocata|[5686]|LBCH2379-10|Canada|British Columbia|658[0n]|BOLD:AAA1521
Diarsia dislocata|[5687]|LBCH965-10|Canada|British Columbia|658[0n]|BOLD:AAA1521
Diarsia dislocata|[5688]|LBCH964-10|Canada|British Columbia|658[0n]|BOLD:AAA1521
Diarsia dislocata|[5689]|LBCH959-10|Canada|British Columbia|658[0n]|BOLD:AAA1521
Diarsia dislocata|[5690]|LBCH950-10|Canada|British Columbia|658[0n]|BOLD:AAA1521
Diarsia dislocata|[5691]|LBCH949-10|Canada|British Columbia|658[0n]|BOLD:AAA1521
Diarsia dislocata|[5692]|LBCH948-10|Canada|British Columbia|658[0n]|BOLD:AAA1521
Diarsia dislocata|[5693]|LBCH947-10|Canada|British Columbia|658[0n]|BOLD:AAA1521
Diarsia dislocata|[5694]|LBCH940-10|Canada|British Columbia|658[0n]|BOLD:AAA1521
Diarsia dislocata|[5695]|LBCH844-10|Canada|British Columbia|658[0n]|BOLD:AAA1521
Diarsia dislocata|[5696]|LBCH843-10|Canada|British Columbia|658[0n]|BOLD:AAA1521
Diarsia dislocata|[5697]|LBCH842-10|Canada|British Columbia|658[0n]|BOLD:AAA1521
Diarsia dislocata|[5698]|LBCH841-10|Canada|British Columbia|658[0n]|BOLD:AAA1521
Diarsia dislocata|[5699]|LBCH840-10|Canada|British Columbia|658[0n]|BOLD:AAA1521
Diarsia dislocata|[5700]|LBCH838-10|Canada|British Columbia|658[0n]|BOLD:AAA1521
Diarsia dislocata|[5701]|LBCH833-10|Canada|British Columbia|658[0n]|BOLD:AAA1521
Diarsia dislocata|[5702]|LBCH832-10|Canada|British Columbia|658[0n]|BOLD:AAA1521
Diarsia dislocata|[5703]|LBCH831-10|Canada|British Columbia|658[0n]|BOLD:AAA1521
Diarsia dislocata|[5704]|LBCH830-10|Canada|British Columbia|658[0n]|BOLD:AAA1521
Diarsia dislocata|[5705]|LBCH828-10|Canada|British Columbia|658[0n]|BOLD:AAA1521
Diarsia dislocata|[5706]|LBCH826-10|Canada|British Columbia|658[0n]|BOLD:AAA1521
Diarsia dislocata|[5707]|LBCH690-10|Canada|British Columbia|658[0n]|BOLD:AAA1521
Diarsia dislocata|[5708]|LBCH689-10|Canada|British Columbia|658[0n]|BOLD:AAA1521
Diarsia dislocata|[5709]|LBCH686-10|Canada|British Columbia|658[0n]|BOLD:AAA1521
Diarsia dislocata|[5710]|LBCH685-10|Canada|British Columbia|658[0n]|BOLD:AAA1521
Diarsia dislocata|[5711]|LBCH684-10|Canada|British Columbia|658[0n]|BOLD:AAA1521
Diarsia dislocata|[5712]|LBCH683-10|Canada|British Columbia|658[0n]|BOLD:AAA1521
Diarsia dislocata|[5713]|LBCH682-10|Canada|British Columbia|658[0n]|BOLD:AAA1521
Diarsia dislocata|[5714]|LBCH680-10|Canada|British Columbia|658[0n]|BOLD:AAA1521
Diarsia dislocata|[5715]|LBCH679-10|Canada|British Columbia|658[0n]|BOLD:AAA1521
Diarsia dislocata|[5716]|LBCH676-10|Canada|British Columbia|658[0n]|BOLD:AAA1521
Diarsia dislocata|[5717]|LBCH675-10|Canada|British Columbia|658[0n]|BOLD:AAA1521
Diarsia dislocata|[5718]|LBCH674-10|Canada|British Columbia|658[0n]|BOLD:AAA1521
Diarsia dislocata|[5719]|LBCH672-10|Canada|British Columbia|658[0n]|BOLD:AAA1521
Diarsia dislocata|[5720]|LBCH669-10|Canada|British Columbia|658[0n]|BOLD:AAA1521
Diarsia dislocata|[5721]|LBCH667-10|Canada|British Columbia|658[0n]|BOLD:AAA1521
Diarsia dislocata|[5722]|LBCH666-10|Canada|British Columbia|658[0n]|BOLD:AAA1521
Diarsia dislocata|[5723]|LBCH664-10|Canada|British Columbia|658[0n]|BOLD:AAA1521
Diarsia dislocata|[5724]|LBCH662-10|Canada|British Columbia|658[0n]|BOLD:AAA1521
Diarsia dislocata|[5725]|LBCH548-10|Canada|British Columbia|658[0n]|BOLD:AAA1521
Diarsia dislocata|[5726]|LBCH547-10|Canada|British Columbia|658[0n]|BOLD:AAA1521
Diarsia dislocata|[5727]|LBCH546-10|Canada|British Columbia|658[0n]|BOLD:AAA1521
Diarsia dislocata|[5728]|LBCH544-10|Canada|British Columbia|658[0n]|BOLD:AAA1521
Diarsia dislocata|[5729]|LBCH542-10|Canada|British Columbia|658[0n]|BOLD:AAA1521
Diarsia dislocata|[5730]|LBCH539-10|Canada|British Columbia|658[0n]|BOLD:AAA1521
Diarsia dislocata|[5731]|LBCH538-10|Canada|British Columbia|658[0n]|BOLD:AAA1521
Diarsia dislocata|[5732]|LBCH536-10|Canada|British Columbia|658[0n]|BOLD:AAA1521
Diarsia dislocata|[5733]|LBCH535-10|Canada|British Columbia|658[0n]|BOLD:AAA1521
Diarsia dislocata|[5734]|LBCH530-10|Canada|British Columbia|658[0n]|BOLD:AAA1521
Diarsia dislocata|[5735]|LBCH526-10|Canada|British Columbia|658[0n]|BOLD:AAA1521
Diarsia dislocata|[5736]|LBCH525-10|Canada|British Columbia|658[0n]|BOLD:AAA1521
Diarsia dislocata|[5737]|LBCH516-10|Canada|British Columbia|658[0n]|BOLD:AAA1521
Diarsia dislocata|[5738]|LBCH515-10|Canada|British Columbia|658[0n]|BOLD:AAA1521
Diarsia dislocata|[5739]|LBCH511-10|Canada|British Columbia|658[0n]|BOLD:AAA1521
Diarsia dislocata|[5740]|LBCH510-10|Canada|British Columbia|658[0n]|BOLD:AAA1521
Diarsia dislocata|[5741]|LBCH508-10|Canada|British Columbia|658[0n]|BOLD:AAA1521
Diarsia dislocata|[5742]|LBCH408-10|Canada|British Columbia|658[0n]|BOLD:AAA1521
Diarsia dislocata|[5743]|LBCH404-10|Canada|British Columbia|658[0n]|BOLD:AAA1521
Diarsia dislocata|[5744]|LBCH401-10|Canada|British Columbia|658[0n]|BOLD:AAA1521
Diarsia dislocata|[5745]|LBCH397-10|Canada|British Columbia|658[0n]|BOLD:AAA1521
Diarsia dislocata|[5746]|LBCH395-10|Canada|British Columbia|658[0n]|BOLD:AAA1521
Diarsia dislocata|[5747]|LBCH393-10|Canada|British Columbia|658[0n]|BOLD:AAA1521
Diarsia dislocata|[5748]|LBCH392-10|Canada|British Columbia|658[0n]|BOLD:AAA1521
Diarsia dislocata|[5749]|LBCH390-10|Canada|British Columbia|658[0n]|BOLD:AAA1521
Diarsia dislocata|[5750]|LBCH388-10|Canada|British Columbia|658[0n]|BOLD:AAA1521
Diarsia dislocata|[5751]|LBCH387-10|Canada|British Columbia|658[0n]|BOLD:AAA1521
Diarsia dislocata|[5752]|LBCH386-10|Canada|British Columbia|658[0n]|BOLD:AAA1521
Diarsia dislocata|[5753]|LBCH385-10|Canada|British Columbia|658[0n]|BOLD:AAA1521
Diarsia dislocata|[5754]|LBCH382-10|Canada|British Columbia|658[0n]|BOLD:AAA1521
Diarsia dislocata|[5755]|LBCH381-10|Canada|British Columbia|658[0n]|BOLD:AAA1521
Diarsia dislocata|[5756]|LBCH380-10|Canada|British Columbia|658[0n]|BOLD:AAA1521
Diarsia dislocata|[5757]|LBCH379-10|Canada|British Columbia|658[0n]|BOLD:AAA1521
Diarsia dislocata|[5758]|LBCH378-10|Canada|British Columbia|658[0n]|BOLD:AAA1521
Diarsia dislocata|[5759]|LBCH377-10|Canada|British Columbia|658[0n]|BOLD:AAA1521
Diarsia dislocata|[5760]|LBCH375-10|Canada|British Columbia|658[0n]|BOLD:AAA1521
Diarsia dislocata|[5761]|LBCH374-10|Canada|British Columbia|658[0n]|BOLD:AAA1521
Diarsia dislocata|[5762]|LBCH372-10|Canada|British Columbia|658[0n]|BOLD:AAA1521
Diarsia dislocata|[5763]|LBCH371-10|Canada|British Columbia|658[0n]|BOLD:AAA1521
Diarsia dislocata|[5764]|LBCH370-10|Canada|British Columbia|658[0n]|BOLD:AAA1521
Diarsia dislocata|[5765]|LBCH368-10|Canada|British Columbia|658[0n]|BOLD:AAA1521
Diarsia dislocata|[5766]|LBCH367-10|Canada|British Columbia|658[0n]|BOLD:AAA1521
Diarsia dislocata|[5767]|LBCH366-10|Canada|British Columbia|658[0n]|BOLD:AAA1521
Diarsia dislocata|[5768]|LBCH365-10|Canada|British Columbia|658[0n]|BOLD:AAA1521
Diarsia dislocata|[5769]|LBCH364-10|Canada|British Columbia|658[0n]|BOLD:AAA1521
Diarsia dislocata|[5770]|LBCH251-10|Canada|British Columbia|658[0n]|BOLD:AAA1521
Diarsia dislocata|[5771]|LBCH249-10|Canada|British Columbia|658[0n]|BOLD:AAA1521
Diarsia dislocata|[5772]|LBCH248-10|Canada|British Columbia|658[0n]|BOLD:AAA1521
Diarsia dislocata|[5773]|LBCH245-10|Canada|British Columbia|658[0n]|BOLD:AAA1521
Diarsia dislocata|[5774]|LBCH244-10|Canada|British Columbia|658[0n]|BOLD:AAA1521
Diarsia dislocata|[5775]|LBCH243-10|Canada|British Columbia|658[0n]|BOLD:AAA1521
Diarsia dislocata|[5776]|LBCH242-10|Canada|British Columbia|658[0n]|BOLD:AAA1521
Diarsia dislocata|[5777]|LBCH241-10|Canada|British Columbia|658[0n]|BOLD:AAA1521
Diarsia dislocata|[5778]|LBCH240-10|Canada|British Columbia|658[0n]|BOLD:AAA1521
Diarsia dislocata|[5779]|LBCH128-10|Canada|British Columbia|658[0n]|BOLD:AAA1521
Diarsia dislocata|[5780]|LBCH126-10|Canada|British Columbia|658[0n]|BOLD:AAA1521
Diarsia dislocata|[5781]|LBCH125-10|Canada|British Columbia|658[0n]|BOLD:AAA1521
[illegible]

Diarsia dislocata|[5779]|LBCH128-10|Canada|British Columbia|658[0n]|BOLD:AAA1521
Diarsia dislocata|[5780]|LBCH126-10|Canada|British Columbia|658[0n]|BOLD:AAA1521
Diarsia dislocata|[5781]|LBCH125-10|Canada|British Columbia|658[0n]|BOLD:AAA1521
Diarsia dislocata|[5782]|LBCH124-10|Canada|British Columbia|658[0n]|BOLD:AAA1521
Diarsia dislocata|[5783]|LBCH053-10|Canada|British Columbia|658[0n]|BOLD:AAA1521
Diarsia dislocata|[5784]|LBCH031-10|Canada|British Columbia|658[0n]|BOLD:AAA1521
Diarsia dislocata|[5785]|LBCH029-10|Canada|British Columbia|658[0n]|BOLD:AAA1521
Diarsia dislocata|[5786]|LBCG2296-09|Canada|British Columbia|658[0n]|BOLD:AAA1521
Diarsia dislocata|[5787]|LBCG2284-09|Canada|British Columbia|658[0n]|BOLD:AAA1521
Diarsia dislocata|[5788]|LBCG2282-09|Canada|British Columbia|658[0n]|BOLD:AAA1521
Diarsia dislocata|[5789]|LBCG2280-09|Canada|British Columbia|658[0n]|BOLD:AAA1521
Diarsia dislocata|[5790]|LBCG2279-09|Canada|British Columbia|658[0n]|BOLD:AAA1521
Diarsia dislocata|[5791]|LBCG2278-09|Canada|British Columbia|658[0n]|BOLD:AAA1521
Diarsia dislocata|[5792]|LBCG2276-09|Canada|British Columbia|658[0n]|BOLD:AAA1521
Diarsia dislocata|[5793]|LBCG2275-09|Canada|British Columbia|658[0n]|BOLD:AAA1521
Diarsia dislocata|[5794]|LBCG2273-09|Canada|British Columbia|658[0n]|BOLD:AAA1521
Diarsia dislocata|[5795]|LBCG2269-09|Canada|British Columbia|658[0n]|BOLD:AAA1521
Diarsia dislocata|[5796]|LBCG2248-09|Canada|British Columbia|658[0n]|BOLD:AAA1521
Diarsia dislocata|[5797]|LPABC474-09|Canada|Alberta|658[0n]|BOLD:AAA1521
Diarsia dislocata|[5798]|LPABC440-09|Canada|Alberta|658[0n]|BOLD:AAA1521
Diarsia dislocata|[5799]|LPABC422-09|Canada|Alberta|658[0n]|BOLD:AAA1521
Diarsia dislocata|[5800]|LPABC418-09|Canada|Alberta|658[0n]|BOLD:AAA1521
Diarsia dislocata|[5801]|LPABC406-09|Canada|Alberta|658[0n]|BOLD:AAA1521
Diarsia dislocata|[5802]|LPABC405-09|Canada|Alberta|658[0n]|BOLD:AAA1521
Diarsia dislocata|[5803]|LPABC402-09|Canada|Alberta|658[0n]|BOLD:AAA1521
Diarsia dislocata|[5804]|LPABC401-09|Canada|Alberta|658[0n]|BOLD:AAA1521
Diarsia dislocata|[5805]|LPABC395-09|Canada|Alberta|658[0n]|BOLD:AAA1521
Diarsia dislocata|[5806]|LPABC390-09|Canada|Alberta|658[0n]|BOLD:AAA1521
Diarsia dislocata|[5807]|LPABC382-09|Canada|Alberta|658[0n]|BOLD:AAA1521
Diarsia dislocata|[5808]|LPABC376-09|Canada|Alberta|658[0n]|BOLD:AAA1521
Diarsia dislocata|[5809]|LPABC375-09|Canada|Alberta|658[0n]|BOLD:AAA1521
Diarsia dislocata|[5810]|LPABC368-09|Canada|Alberta|658[0n]|BOLD:AAA1521
Diarsia dislocata|[5811]|LPABC364-09|Canada|Alberta|658[0n]|BOLD:AAA1521
Diarsia dislocata|[5812]|LPABC349-09|Canada|Alberta|658[0n]|BOLD:AAA1521
Diarsia dislocata|[5813]|LPABC338-09|Canada|Alberta|658[0n]|BOLD:AAA1521
Diarsia dislocata|[5814]|LPABC019-09|Canada|Alberta|658[0n]|BOLD:AAA1521
Diarsia dislocata|[5815]|LPABC017-09|Canada|Alberta|658[0n]|BOLD:AAA1521
Diarsia dislocata|[5816]|LPABC002-09|Canada|Alberta|658[0n]|BOLD:AAA1521
Diarsia dislocata|[5817]|LPABB899-09|Canada|Alberta|658[0n]|BOLD:AAA1521
Diarsia dislocata|[5818]|LPABB152-08|Canada|Alberta|658[0n]|BOLD:AAA1521
Diarsia dislocata|[5819]|LPAB227-08|Canada|Alberta|658[0n]|BOLD:AAA1521
Diarsia dislocata|[5820]|LCHQ144-07|Canada|Manitoba|658[0n]|BOLD:AAA1521
Diarsia dislocata|[5821]|LCHP899-07|Canada|Manitoba|658[0n]|BOLD:AAA1521
Diarsia dislocata|[5822]|LCHP868-07|Canada|Manitoba|658[0n]|BOLD:AAA1521
Diarsia dislocata|[5823]|LCHP786-07|Canada|Manitoba|658[0n]|BOLD:AAA1521
Diarsia dislocata|[5824]|LCHP704-07|Canada|Manitoba|658[0n]|BOLD:AAA1521
Diarsia dislocata|[5825]|LCHP702-07|Canada|Manitoba|658[0n]|BOLD:AAA1521
Diarsia dislocata|[5826]|LCHP525-07|Canada|Manitoba|658[0n]|BOLD:AAA1521
Diarsia dislocata|[5827]|LCHP523-07|Canada|Manitoba|658[0n]|BOLD:AAA1521
Diarsia dislocata|[5828]|LCHP522-07|Canada|Manitoba|658[0n]|BOLD:AAA1521
Diarsia dislocata|[5829]|LCHP521-07|Canada|Manitoba|658[0n]|BOLD:AAA1521
Diarsia dislocata|[5830]|LCHP479-07|Canada|Manitoba|658[0n]|BOLD:AAA1521
Diarsia dislocata|[5831]|LCHP288-07|Canada|Manitoba|658[0n]|BOLD:AAA1521
Diarsia dislocata|[5832]|LCHP163-07|Canada|Manitoba|658[0n]|BOLD:AAA1521
Diarsia dislocata|[5833]|LCH578-04|Canada|Manitoba|658[0n]|BOLD:AAA1521
Diarsia dislocata|[5834]|LCH558-04|Canada|Manitoba|658[0n]|BOLD:AAA1521
Diarsia dislocata|[5835]|LCH316-04|Canada|Manitoba|658[0n]|BOLD:AAA1521
Diarsia dislocata|[5836]|LCH315-04|Canada|Manitoba|658[0n]|BOLD:AAA1521
Diarsia dislocata|[5837]|LCH311-04|Canada|Manitoba|658[0n]|BOLD:AAA1521
Diarsia dislocata|[5838]|LCH310-04|Canada|Manitoba|658[0n]|BOLD:AAA1521
Diarsia dislocata|[5839]|LCH308-04|Canada|Manitoba|658[0n]|BOLD:AAA1521
Diarsia dislocata|[5840]|LCH307-04|Canada|Manitoba|658[0n]|BOLD:AAA1521
Diarsia dislocata|[5841]|LCH304-04|Canada|Manitoba|658[0n]|BOLD:AAA1521
Diarsia dislocata|[5842]|LCH302-04|Canada|Manitoba|658[0n]|BOLD:AAA1521
Diarsia dislocata|[5843]|LCH298-04|Canada|Manitoba|658[0n]|BOLD:AAA1521
Diarsia dislocata|[5844]|LCH297-04|Canada|Manitoba|658[0n]|BOLD:AAA1521
Diarsia dislocata|[5845]|LCH294-04|Canada|Manitoba|658[0n]|BOLD:AAA1521
Diarsia dislocata|[5846]|LCH291-04|Canada|Manitoba|658[0n]|BOLD:AAA1521
Diarsia dislocata|[5847]|LCH290-04|Canada|Manitoba|658[0n]|BOLD:AAA1521
Diarsia dislocata|[5848]|LCH285-04|Canada|Manitoba|658[0n]|BOLD:AAA1521
Diarsia dislocata|[5849]|LCH278-04|Canada|Manitoba|658[0n]|BOLD:AAA1521
Diarsia dislocata|[5850]|LCH276-04|Canada|Manitoba|658[0n]|BOLD:AAA1521
Diarsia dislocata|[5851]|LCH273-04|Canada|Manitoba|658[0n]|BOLD:AAA1521
Diarsia dislocata|[5852]|LCH269-04|Canada|Manitoba|658[0n]|BOLD:AAA1521
Diarsia dislocata|[5853]|LCH268-04|Canada|Manitoba|658[0n]|BOLD:AAA1521
Diarsia dislocata|[5854]|LCH266-04|Canada|Manitoba|658[0n]|BOLD:AAA1521
Diarsia dislocata|[5855]|LCH265-04|Canada|Manitoba|658[0n]|BOLD:AAA1521
Diarsia dislocata|[5856]|LCHQ142-07|Canada|Manitoba|658[1n]|BOLD:AAA1521
Diarsia dislocata|[5857]|LPABC018-09|Canada|Alberta|658[0n]|BOLD:AAA1521
Diarsia dislocata|[5858]|LBCH129-10|Canada|British Columbia|658[0n]|BOLD:AAA1521
Diarsia dislocata|[5859]|LBCH376-10|Canada|British Columbia|658[0n]|BOLD:AAA1521
Diarsia dislocata|[5860]|LBCH394-10|Canada|British Columbia|658[0n]|BOLD:AAA1521
Diarsia dislocata|[5861]|LBCH3110-10|Canada|British Columbia|658[0n]|BOLD:AAA1521
Diarsia dislocata|[5862]|LBCH3123-10|Canada|British Columbia|658[0n]|BOLD:AAA1521
Diarsia dislocata|[5863]|LBCH3504-10|Canada|British Columbia|658[0n]|BOLD:AAA1521
Diarsia dislocata|[5864]|LCH283-04|Canada|Manitoba|654[0n]|BOLD:AAA1521
Diarsia dislocata|[5865]|LCH579-04|Canada|Manitoba|594[0n]|BOLD:AAA1521
Diarsia dislocata|[5866]|LBCH4452-10|Canada|British Columbia|647[0n]|BOLD:AAA1521
Diarsia dislocata|[5867]|LCHP515-07|Canada|Manitoba|634[0n]|BOLD:AAA1521
Diarsia dislocata|[5868]|TTCFW642-08|Canada|Alberta|631[0n]|
Diarsia dislocata|[5869]|LBCH3797-10|Canada|British Columbia|644[0n]|BOLD:AAA1521
Diarsia dislocata|[5870]|LBCH4465-10|Canada|British Columbia|650[0n]|BOLD:AAA1521
Diarsia dislocata|[5871]|LBCH4728-10|Canada|British Columbia|649[0n]|BOLD:AAA1521
Diarsia dislocata|[5872]|LBCH3519-10|Canada|British Columbia|658[0n]|BOLD:AAA1521
Diarsia dislocata|[5873]|LBCH3140-10|Canada|British Columbia|658[0n]|BOLD:AAA1521
Diarsia dislocata|[5874]|LBCH3113-10|Canada|British Columbia|658[0n]|BOLD:AAA1521
Diarsia dislocata|[5875]|LBCH3112-10|Canada|British Columbia|658[0n]|BOLD:AAA1521
Diarsia dislocata|[5876]|LBCH835-10|Canada|British Columbia|658[0n]|BOLD:AAA1521
Diarsia dislocata|[5877]|LBCH827-10|Canada|British Columbia|658[0n]|BOLD:AAA1521
Diarsia dislocata|[5878]|LBCH824-10|Canada|British Columbia|658[0n]|BOLD:AAA1521
Diarsia dislocata|[5879]|LBCH688-10|Canada|British Columbia|658[0n]|BOLD:AAA1521
Diarsia dislocata|[5880]|LBCH687-10|Canada|British Columbia|658[0n]|BOLD:AAA1521
Diarsia dislocata|[5881]|LBCH678-10|Canada|British Columbia|658[0n]|BOLD:AAA1521

Diarsia dislocata|[5879]|LBCH688-10|Canada|British Columbia|658[0n]|BOLD:AAA1521
Diarsia dislocata|[5880]|LBCH687-10|Canada|British Columbia|658[0n]|BOLD:AAA1521
Diarsia dislocata|[5881]|LBCH678-10|Canada|British Columbia|658[0n]|BOLD:AAA1521
Diarsia dislocata|[5882]|LBCH551-10|Canada|British Columbia|658[0n]|BOLD:AAA1521
Diarsia dislocata|[5883]|LBCH540-10|Canada|British Columbia|658[0n]|BOLD:AAA1521
Diarsia dislocata|[5884]|LBCH512-10|Canada|British Columbia|658[0n]|BOLD:AAA1521
Diarsia dislocata|[5885]|LBCH509-10|Canada|British Columbia|658[0n]|BOLD:AAA1521
Diarsia dislocata|[5886]|LBCH384-10|Canada|British Columbia|658[0n]|BOLD:AAA1521
Diarsia dislocata|[5887]|LBCH246-10|Canada|British Columbia|658[0n]|BOLD:AAA1521
Diarsia dislocata|[5888]|LBCH127-10|Canada|British Columbia|658[0n]|BOLD:AAA1521
Diarsia dislocata|[5889]|LBCG2271-09|Canada|British Columbia|658[0n]|BOLD:AAA1521
Diarsia dislocata|[5890]|LBCH030-10|Canada|British Columbia|658[0n]|BOLD:AAA1521
Diarsia dislocata|[5891]|LBCH032-10|Canada|British Columbia|658[0n]|BOLD:AAA1521
Diarsia dislocata|[5892]|LBCH052-10|Canada|British Columbia|658[0n]|BOLD:AAA1521
Diarsia dislocata|[5893]|LBCH692-10|Canada|British Columbia|658[0n]|BOLD:AAA1521
Diarsia dislocata|[5894]|LBCH3099-10|Canada|British Columbia|658[0n]|BOLD:AAA1521
Diarsia dislocata|[5895]|LBCH3115-10|Canada|British Columbia|658[0n]|BOLD:AAA1521
Diarsia dislocata|[5896]|LBCH3514-10|Canada|British Columbia|658[0n]|BOLD:AAA1521
Diarsia dislocata|[5897]|LBCH3336-10|Canada|British Columbia|658[0n]|BOLD:AAA1521
Diarsia dislocata|[5898]|LBCH4441-10|Canada|British Columbia|644[0n]|BOLD:AAA1521
Diarsia dislocata|[5899]|LBCH4712-10|Canada|British Columbia|658[0n]|BOLD:AAA1521
Diarsia dislocata|[5900]|LBCH4721-10|Canada|British Columbia|658[0n]|BOLD:AAA1521
Diarsia dislocata|[5901]|LBCH4725-10|Canada|British Columbia|658[0n]|BOLD:AAA1521
Diarsia dislocata|[5902]|LBCH4727-10|Canada|British Columbia|658[0n]|BOLD:AAA1521
Diarsia dislocata|[5903]|LBCH4732-10|Canada|British Columbia|658[0n]|BOLD:AAA1521
Diarsia dislocata|[5904]|LBCG2283-09|Canada|British Columbia|658[0n]|BOLD:AAA1521
Diarsia dislocata|[5905]|LBCG2297-09|Canada|British Columbia|658[0n]|BOLD:AAA1521
Diarsia dislocata|[5906]|LBCH389-10|Canada|British Columbia|658[0n]|BOLD:AAA1521
Diarsia dislocata|[5907]|LBCH514-10|Canada|British Columbia|658[0n]|BOLD:AAA1521
Diarsia dislocata|[5908]|LBCH537-10|Canada|British Columbia|658[0n]|BOLD:AAA1521
Diarsia dislocata|[5909]|LBCH670-10|Canada|British Columbia|658[0n]|BOLD:AAA1521
Diarsia dislocata|[5910]|LBCH2384-10|Canada|British Columbia|658[0n]|BOLD:AAA1521
Diarsia dislocata|[5911]|LBCH2386-10|Canada|British Columbia|658[0n]|BOLD:AAA1521
Diarsia dislocata|[5912]|LBCH3136-10|Canada|British Columbia|658[0n]|BOLD:AAA1521
Diarsia dislocata|[5913]|LBCH3503-10|Canada|British Columbia|658[0n]|BOLD:AAA1521
Diarsia dislocata|[5914]|LBCH4028-10|Canada|British Columbia|658[0n]|BOLD:AAA1521
Diarsia dislocata|[5915]|LBCH4042-10|Canada|British Columbia|658[0n]|BOLD:AAA1521
Diarsia dislocata|[5916]|LBCH4449-10|Canada|British Columbia|658[0n]|BOLD:AAA1521
Diarsia dislocata|[5917]|LBCH4460-10|Canada|British Columbia|658[0n]|BOLD:AAA1521
Diarsia dislocata|[5918]|LBCH4698-10|Canada|British Columbia|658[0n]|BOLD:AAA1521
Diarsia dislocata|[5919]|LBCH4701-10|Canada|British Columbia|658[0n]|BOLD:AAA1521
Diarsia dislocata|[5920]|LBCH4706-10|Canada|British Columbia|658[0n]|BOLD:AAA1521
Diarsia dislocata|[5921]|LBCH4726-10|Canada|British Columbia|658[0n]|BOLD:AAA1521
Diarsia dislocata|[5922]|LBCH4741-10|Canada|British Columbia|658[0n]|BOLD:AAA1521
Diarsia dislocata|[5923]|LBCH4742-10|Canada|British Columbia|658[0n]|BOLD:AAA1521
Diarsia dislocata|[5924]|LBCH4743-10|Canada|British Columbia|658[0n]|BOLD:AAA1521
Diarsia dislocata|[5925]|LBCH4744-10|Canada|British Columbia|658[0n]|BOLD:AAA1521
Diarsia dislocata|[5926]|LBCH4746-10|Canada|British Columbia|658[0n]|BOLD:AAA1521
Diarsia dislocata|[5927]|LBCH6748-10|Canada|British Columbia|658[0n]|BOLD:AAA1521
Diarsia dislocata|[5928]|BBLPB343-10|Canada|Alberta|658[0n]|BOLD:AAA1521
Diarsia dislocata|[5929]|BBLPB620-10|Canada|British Columbia|658[0n]|BOLD:AAA1521
Diarsia dislocata|[5930]|BBLPE250-09|Canada|Newfoundland and Labrador|658[0n]|BOLD:AAA1521
Diarsia dislocata|[5931]|BBLPE221-09|Canada|Newfoundland and Labrador|658[0n]|BOLD:AAA1521
Diarsia dislocata|[5932]|BBLPE114-09|Canada|Nova Scotia|658[0n]|BOLD:AAA1521
Diarsia dislocata|[5933]|BBLPE061-09|Canada|Nova Scotia|658[0n]|BOLD:AAA1521
Diarsia dislocata|[5934]|BBLPC926-09|Canada|Newfoundland and Labrador|658[0n]|BOLD:AAA1521
Diarsia dislocata|[5935]|BBLPC840-09|Canada|Newfoundland and Labrador|658[0n]|BOLD:AAA1521
Diarsia dislocata|[5936]|BBLPC300-09|Canada|Newfoundland and Labrador|658[0n]|BOLD:AAA1521
Diarsia dislocata|[5937]|LPABC074-09|Canada|Alberta|658[0n]|BOLD:AAA1521
Diarsia dislocata|[5938]|LPABB894-09|Canada|Alberta|658[0n]|BOLD:AAA1521
Diarsia dislocata|[5939]|LCHP874-07|Canada|Manitoba|658[0n]|BOLD:AAA1521
Diarsia dislocata|[5940]|LCHP818-07|Canada|Manitoba|658[0n]|BOLD:AAA1521
Diarsia dislocata|[5941]|LCHP651-07|Canada|Manitoba|658[0n]|BOLD:AAA1521
Diarsia dislocata|[5942]|LCHP648-07|Canada|Manitoba|658[0n]|BOLD:AAA1521
Diarsia dislocata|[5943]|LCHP487-07|Canada|Manitoba|658[0n]|BOLD:AAA1521
Diarsia dislocata|[5944]|LCHP486-07|Canada|Manitoba|658[0n]|BOLD:AAA1521
Diarsia dislocata|[5945]|LCHP207-07|Canada|Manitoba|658[0n]|BOLD:AAA1521
Diarsia dislocata|[5946]|LCH577-04|Canada|Manitoba|658[0n]|BOLD:AAA1521
Diarsia dislocata|[5947]|LCH309-04|Canada|Manitoba|658[0n]|BOLD:AAA1521
Diarsia dislocata|[5948]|LCH301-04|Canada|Manitoba|658[0n]|BOLD:AAA1521
Diarsia dislocata|[5949]|LCH299-04|Canada|Manitoba|658[0n]|BOLD:AAA1521
Diarsia dislocata|[5950]|LCH295-04|Canada|Manitoba|658[0n]|BOLD:AAA1521
Diarsia dislocata|[5951]|LCH293-04|Canada|Manitoba|658[0n]|BOLD:AAA1521
Diarsia dislocata|[5952]|LCH289-04|Canada|Manitoba|658[0n]|BOLD:AAA1521
Diarsia dislocata|[5953]|LCH287-04|Canada|Manitoba|658[0n]|BOLD:AAA1521
Diarsia dislocata|[5954]|LCH281-04|Canada|Manitoba|658[0n]|BOLD:AAA1521
Diarsia dislocata|[5955]|LCH279-04|Canada|Manitoba|658[0n]|BOLD:AAA1521
Diarsia dislocata|[5956]|LCH270-04|Canada|Manitoba|658[0n]|BOLD:AAA1521
Diarsia dislocata|[5957]|LCH576-04|Canada|Manitoba|658[1n]|BOLD:AAA1521
Diarsia dislocata|[5958]|LCH274-04|Canada|Manitoba|645[0n]|BOLD:AAA1521
Diarsia dislocata|[5959]|BBLPE230-09|Canada|Newfoundland and Labrador|634[0n]|BOLD:AAA1521
Diarsia dislocata|[5960]|BBLPE244-09|Canada|Newfoundland and Labrador|622[0n]|BOLD:AAA1521
Diarsia dislocata|[5961]|LBCH4713-10|Canada|British Columbia|643[0n]|BOLD:AAA1521
Diarsia dislocata|[5962]|CNKOP133-14|Canada|New Brunswick|567[0n]|BOLD:AAA1521
Diarsia dislocata|[5963]|CNTNG187-14|Canada|Newfoundland and Labrador|591[0n]|BOLD:AAA1521
Diarsia jucunda|[5964]|RDLQ721-07|Canada|Quebec|562[0n]|BOLD:AAA1521
Diarsia jucunda|[5965]|LGSM628-04|United States|Tennessee|609[0n]|BOLD:AAA1521
Diarsia jucunda|[5966]|BBLEC276-09|Canada|Nova Scotia|629[0n]|BOLD:AAA1521
Diarsia jucunda|[5967]|RDLQ711-07|Canada|Quebec|658[0n]|BOLD:AAA1521
Diarsia jucunda|[5968]|RDLQ712-07|Canada|Quebec|646[0n]|BOLD:AAA1521
Diarsia jucunda|[5969]|LNCC1846-13|United States|North Carolina|658[0n]|BOLD:AAA1521
Diarsia jucunda|[5970]|BBLPE024-09|Canada|Nova Scotia|621[0n]|BOLD:AAA1521
Diarsia jucunda|[5971]|BBLPE182-09|Canada|Nova Scotia|636[0n]|BOLD:AAA1521
Diarsia jucunda|[5972]|LNCC1030-11|United States|North Carolina|658[0n]|BOLD:AAA1521
Diarsia jucunda|[5973]|BBLPE123-09|Canada|Nova Scotia|658[0n]|BOLD:AAA1521
Diarsia jucunda|[5974]|BBLPC448-09|Canada|New Brunswick|658[0n]|BOLD:AAA1521
Diarsia jucunda|[5975]|BBLPC094-09|Canada|New Brunswick|658[0n]|BOLD:AAA1521
Diarsia jucunda|[5976]|BBLEC280-09|Canada|Nova Scotia|658[0n]|BOLD:AAA1521
Diarsia jucunda|[5977]|TTMNB499-06|Canada|New Brunswick|658[0n]|BOLD:AAA1521
Diarsia jucunda|[5978]|RDNMC339-05|United States|North Carolina|658[0n]|BOLD:AAA1521
Diarsia jucunda|[5979]|RDLQB908-05|Canada|Quebec|658[0n]|BOLD:AAA1521
Diarsia jucunda|[5980]|LGSM627-04|United States|Tennessee|658[0n]|BOLD:AAA1521
Diarsia jucunda|[5981]|BBLEC322-09|Canada|Nova Scotia|658[0n]|BOLD:AAA1521

[illegible]
Diarsia jucunda|[5979]|RDLQB908-05|Canada|Quebec|658[0n]|BOLD:AAA1521
Diarsia jucunda|[5980]|LGSM627-04|United States|Tennessee|658[0n]|BOLD:AAA1521
Diarsia jucunda|[5981]|BBLEC322-09|Canada|Nova Scotia|658[0n]|BOLD:AAA1521
Diarsia jucunda|[5982]|BBLEC303-09|Canada|Nova Scotia|645[0n]|BOLD:AAA1521
Diarsia jucunda|[5983]|BBLEC338-09|Canada|Nova Scotia|622[0n]|BOLD:AAA1521
Diarsia jucunda|[5984]|LNCC1711-13|United States|North Carolina|632[1n]|BOLD:AAA1521
Diarsia jucunda|[5985]|LNCC1712-13|United States|North Carolina|658[0n]|BOLD:AAA1521
Diarsia jucunda|[5986]|LNCC1713-13|United States|North Carolina|658[0n]|BOLD:AAA1521
Diarsia jucunda|[5987]|LNCC1714-13|United States|North Carolina|658[0n]|BOLD:AAA1521
Diarsia jucunda|[5988]|LNCC1715-13|United States|North Carolina|658[0n]|BOLD:AAA1521
Diarsia jucunda|[5989]|LNCC1716-13|United States|North Carolina|658[0n]|BOLD:AAA1521
Diarsia jucunda|[5990]|LNCC1717-13|United States|North Carolina|658[0n]|BOLD:AAA1521
Diarsia jucunda|[5991]|LNCC1724-13|United States|North Carolina|658[0n]|BOLD:AAA1521
Diarsia jucunda|[5992]|LNCC1725-13|United States|North Carolina|658[0n]|BOLD:AAA1521
Diarsia jucunda|[5993]|LNCC1726-13|United States|North Carolina|658[0n]|BOLD:AAA1521
Diarsia jucunda|[5994]|LNCC1727-13|United States|North Carolina|658[0n]|BOLD:AAA1521
Diarsia jucunda|[5995]|RDLQB254-05|Canada|Quebec|658[0n]|BOLD:AAA1521
Diarsia jucunda|[5996]|BBLEC106-09|Canada|Nova Scotia|658[0n]|BOLD:AAA1521
Diarsia jucunda|[5997]|BBLEC261-09|Canada|Nova Scotia|658[0n]|BOLD:AAA1521
Diarsia jucunda|[5998]|BBLEC270-09|Canada|Nova Scotia|658[0n]|BOLD:AAA1521
Diarsia jucunda|[5999]|BBLEC331-09|Canada|Nova Scotia|658[0n]|BOLD:AAA1521
Diarsia jucunda|[6000]|BBLPC589-09|Canada|Nova Scotia|658[0n]|BOLD:AAA1521
Diarsia jucunda|[6001]|BBLPE031-09|Canada|Nova Scotia|658[0n]|BOLD:AAA1521
Diarsia jucunda|[6002]|BBLPE039-09|Canada|Nova Scotia|658[0n]|BOLD:AAA1521
Diarsia jucunda|[6003]|BBLPE085-09|Canada|Nova Scotia|658[0n]|BOLD:AAA1521
Diarsia jucunda|[6004]|CNGMF1918-14|Canada|Newfoundland and Labrador|593[0n]|BOLD:AAA1521
Diarsia jucunda|[6005]|CNGMF1920-14|Canada|Newfoundland and Labrador|577[0n]|BOLD:AAA1521
Diarsia jucunda|[6006]|RDLQ710-07|Canada|Quebec|617[0n]|BOLD:AAA1521
Diarsia jucunda|[6007]|MNBB611-05|Canada|New Brunswick|658[0n]|BOLD:AAA1521
Diarsia jucunda|[6008]|BBLEC330-09|Canada|Nova Scotia|658[0n]|BOLD:AAA1521
Diarsia jucunda|[6009]|BBLPE026-09|Canada|Nova Scotia|658[0n]|BOLD:AAA1521
Diarsia jucunda|[6010]|CNTNG188-14|Canada|Newfoundland and Labrador|588[0n]|BOLD:AAA1521
Euxoa castanea|[6011]|LOWCD400-06|Canada|British Columbia|594[0n]|BOLD:ACF2028
Euxoa castanea|[6012]|LOWCD394-06|Canada|British Columbia|595[0n]|BOLD:ACF2028
Euxoa castanea|[6013]|CNWBG3120-13|Canada|Alberta|611[0n]|BOLD:ACF2028
Euxoa castanea|[6014]|RDNMD321-06|United States|Colorado|603[0n]|BOLD:ACF2028
Euxoa castanea|[6015]|LOWCD405-06|Canada|British Columbia|605[0n]|BOLD:ACF2028
Euxoa castanea|[6016]|LOWCD404-06|Canada|British Columbia|606[0n]|BOLD:ACF2028
Euxoa castanea|[6017]|LPABC341-09|Canada|Alberta|636[0n]|BOLD:ACF2028
Euxoa castanea|[6018]|RDNMB325-05|Canada|British Columbia|615[0n]|BOLD:ACF2028
Euxoa castanea|[6019]|BBLPB837-10|Canada|British Columbia|626[0n]|BOLD:ACF2028
Euxoa castanea|[6020]|LOWCD397-06|Canada|British Columbia|603[0n]|BOLD:ACF2028
Euxoa castanea|[6021]|LOWCD399-06|Canada|British Columbia|626[0n]|BOLD:ACF2028
Euxoa castanea|[6022]|LOWCD401-06|Canada|British Columbia|619[0n]|BOLD:ACF2028
Euxoa castanea|[6023]|LBCG3249-09|Canada|British Columbia|638[0n]|BOLD:ACF2028
Euxoa castanea|[6024]|BBLPB839-10|Canada|British Columbia|658[1n]|BOLD:ACF2028
Euxoa castanea|[6025]|LOWCD406-06|Canada|British Columbia|658[0n]|BOLD:ACF2028
Euxoa castanea|[6026]|LOWCD407-06|Canada|British Columbia|658[0n]|BOLD:ACF2028
Euxoa castanea|[6027]|LOWCD409-06|Canada|British Columbia|658[0n]|BOLD:ACF2028
Euxoa castanea|[6028]|LPABC344-09|Canada|Alberta|658[0n]|BOLD:ACF2028
Euxoa castanea|[6029]|LBCH6365-10|Canada|British Columbia|658[0n]|BOLD:ACF2028
Euxoa castanea|[6030]|LBCH7784-10|Canada|British Columbia|658[0n]|BOLD:ACF2028
Euxoa castanea|[6031]|BBLPB736-10|Canada|Alberta|658[0n]|BOLD:ACF2028
Euxoa castanea|[6032]|BBLPB838-10|Canada|British Columbia|658[0n]|BOLD:ACF2028
Euxoa castanea|[6033]|BBLPB840-10|Canada|Alberta|658[0n]|BOLD:ACF2028
Euxoa castanea|[6034]|BBLPB841-10|Canada|Alberta|658[0n]|BOLD:ACF2028
Euxoa castanea|[6035]|BBLPB842-10|Canada|Alberta|658[0n]|BOLD:ACF2028
Euxoa castanea|[6036]|BBLPB843-10|Canada|Alberta|658[0n]|BOLD:ACF2028
Euxoa castanea|[6037]|RDNM352-05|Canada|British Columbia|603[0n]|BOLD:ACF2028
Euxoa castanea|[6038]|RDNMB323-05|Canada|British Columbia|597[1n]|BOLD:ACF2028
Euxoa castanea|[6039]|LOWCD403-06|Canada|British Columbia|600[0n]|BOLD:ACF2028
Euxoa castanea|[6040]|LOWCD408-06|Canada|British Columbia|599[0n]|BOLD:ACF2028
Euxoa castanea|[6041]|LPABC159-09|Canada|Alberta|653[2n]|BOLD:ACF2028
Euxoa castanea|[6042]|LOWCD395-06|Canada|British Columbia|599[0n]|BOLD:ACF2028
Euxoa castanea|[6043]|RDNMB330-05|Canada|British Columbia|595[1n]|BOLD:ACF2028
Euxoa castanea|[6044]|LOWCD410-06|Canada|British Columbia|609[0n]|BOLD:ACF2028
Euxoa castanea|[6045]|LOWCD411-06|Canada|British Columbia|595[0n]|BOLD:ACF2028
Euxoa castanea|[6046]|LOWCD398-06|Canada|British Columbia|595[0n]|BOLD:ACF2028
Euxoa castanea|[6047]|LOWCD396-06|Canada|British Columbia|607[0n]|BOLD:ACF2028
Euxoa castanea|[6048]|LOWCD392-06|Canada|British Columbia|609[0n]|BOLD:ACF2028
Euxoa castanea|[6049]|RDNMB328-05|Canada|British Columbia|599[0n]|BOLD:ACF2028
Euxoa idahoensis group|[6050]|RDNMD318-06|United States|Colorado|658[0n]|BOLD:ACF2028
Euxoa castanea|[6051]|RDNM351-05|Canada|British Columbia|567[0n]|BOLD:ACF2028
Euxoa castanea|[6052]|RDNMB329-05|Canada|British Columbia|557[0n]|BOLD:ACF2028
Euxoa castanea|[6053]|RDNMB322-05|Canada|British Columbia|549[0n]|BOLD:ACF2028
Euxoa castanea|[6054]|RDNMB324-05|Canada|Alberta|519[3n]|BOLD:ACF2028
Euxoa castanea|[6055]|LOWCD393-06|Canada|British Columbia|613[0n]|BOLD:ACF2028
Euxoa castanea|[6056]|RDNMD326-06|United States|Colorado|658[0n]|BOLD:ACF2028
Euxoa castanea|[6057]|RDNMD317-06|United States|Colorado|658[0n]|BOLD:ACF2028
Euxoa castanea|[6058]|LOWCD402-06|Canada|British Columbia|612[0n]|BOLD:ACF2028
Euxoa idahoensis group|[6059]|RDNMC317-05|United States|Oregon|658[0n]|BOLD:ACF2028
Euxoa castanea|[6060]|LBCH6020-10|Canada|British Columbia|658[0n]|BOLD:ACF2028
Euxoa castanea|[6061]|LBCH6494-10|Canada|British Columbia|658[0n]|BOLD:ACF2028
Euxoa castanea|[6062]|LBCG3312-09|Canada|British Columbia|658[0n]|BOLD:ACF2028
Euxoa castanea|[6063]|LBCH6749-10|Canada|British Columbia|658[0n]|BOLD:ACF2028
Euxoa castanea|[6064]|RDNMD876-07|United States|Wyoming|655[0n]|BOLD:ACF2028
Euxoa castanea|[6065]|RDNMD875-07|United States|Wyoming|655[0n]|BOLD:ACF2028
Euxoa castanea|[6066]|RDNMB670-05|Canada|British Columbia|658[0n]|BOLD:ACF2028
Euxoa castanea|[6067]|RDNMB667-05|United States|Wyoming|658[0n]|BOLD:ACF2028
Euxoa castanea|[6068]|RDNMB664-05|United States|Wyoming|658[0n]|BOLD:ACF2028
Euxoa castanea|[6069]|RDNMB318-05|United States|Wyoming|658[0n]|BOLD:ACF2028
Euxoa furtivus|[6070]|BBLPB832-10|Canada|Alberta|658[0n]|BOLD:ACF2028
Euxoa furtivus|[6071]|LBCH7693-10|Canada|British Columbia|658[0n]|BOLD:ACF2028
Euxoa furtivus|[6072]|LBCH6495-10|Canada|British Columbia|658[0n]|BOLD:ACF2028
Euxoa furtivus|[6073]|LBCH6492-10|Canada|British Columbia|658[0n]|BOLD:ACF2028
Euxoa furtivus|[6074]|LBCH6371-10|Canada|British Columbia|658[0n]|BOLD:ACF2028
Euxoa furtivus|[6075]|LBCG2876-09|Canada|British Columbia|658[0n]|BOLD:ACF2028
Euxoa furtivus|[6076]|LPSK570-08|Canada|Saskatchewan|658[0n]|BOLD:ACF2028
Euxoa furtivus|[6077]|LPSK505-08|Canada|Saskatchewan|658[0n]|BOLD:ACF2028
Euxoa furtivus|[6078]|LOWCD335-06|Canada|British Columbia|657[0n]|BOLD:ACF2028
Euxoa furtivus|[6079]|RDMAB495-06|Canada|Alberta|658[0n]|BOLD:ACF2028
Euxoa furtivus|[6080]|RDNMC332-05|Canada|Alberta|658[0n]|BOLD:ACF2028
[illegible]

Euxoa furtivus|[6078]|LOWCD335-06|Canada|British Columbia|657[0n]|BOLD:ACF2028
Euxoa furtivus|[6079]|RDMAB495-06|Canada|Alberta|658[0n]|BOLD:ACF2028
Euxoa furtivus|[6080]|RDNMC332-05|Canada|Alberta|658[0n]|BOLD:ACF2028
Euxoa furtivus|[6081]|RDNMB676-05|Canada|British Columbia|658[0n]|BOLD:ACF2028
Euxoa furtivus|[6082]|RDNMB668-05|Canada|Alberta|658[0n]|BOLD:ACF2028
Euxoa furtivus|[6083]|RDNMB665-05|Canada|Alberta|658[0n]|BOLD:ACF2028
Euxoa furtivus|[6084]|RDNM350-05|Canada|British Columbia|658[0n]|BOLD:ACF2028
Euxoa furtivus|[6085]|LOWCD334-06|Canada|British Columbia|606[0n]|BOLD:ACF2028
Euxoa furtivus|[6086]|LOWCD337-06|Canada|British Columbia|597[0n]|BOLD:ACF2028
Euxoa furtivus|[6087]|LOWCD331-06|Canada|British Columbia|610[0n]|BOLD:ACF2028
Euxoa furtivus|[6088]|LOWCD340-06|Canada|British Columbia|601[0n]|BOLD:ACF2028
Euxoa furtivus|[6089]|LOWCD332-06|Canada|British Columbia|549[0n]|BOLD:ACF2028
Euxoa furtivus|[6090]|RDNMB669-05|Canada|Alberta|543[0n]|BOLD:ACF2028
Euxoa furtivus|[6091]|RDNMB680-05|Canada|British Columbia|528[0n]|BOLD:ACF2028
Euxoa furtivus|[6092]|BBLPB833-10|Canada|Alberta|658[0n]|BOLD:ACF2028
Euxoa castanea|[6093]|RDNMD879-07|United States|Wyoming|655[0n]|BOLD:ACF2028
Euxoa castanea|[6094]|RDNMJ824-11|United States|Wyoming|658[0n]|BOLD:ACF2028
Euxoa castanea|[6095]|RDNMB326-05|Canada|British Columbia|579[0n]|BOLD:ACF2028
Euxoa castanea|[6096]|RDNMD325-06|United States|Colorado|658[0n]|BOLD:ACF2028
Euxoa furtivus|[6097]|RDNMC318-05|United States|Nevada|600[0n]|BOLD:ACF2028
Euxoa furtivus|[6098]|RDNMB311-05|Canada|Alberta|599[0n]|BOLD:ACF2028
Euxoa furtivus|[6099]|RDNMB314-05|United States|Wyoming|613[0n]|BOLD:ACF2028
Euxoa furtivus|[6100]|RDNMB313-05|United States|Wyoming|658[0n]|BOLD:ACF2028
Euxoa furtivus|[6101]|RDNMB312-05|Canada|Alberta|658[0n]|BOLD:ACF2028
Euxoa furtivus|[6102]|RDNMB310-05|Canada|Alberta|658[0n]|BOLD:ACF2028
Euxoa furtivus|[6103]|RDNM336-05|United States|Wyoming|658[0n]|BOLD:ACF2028
Euxoa furtivus|[6104]|RDNMB315-05|Canada|Alberta|615[0n]|BOLD:ACF2028
Euxoa furtivus|[6105]|RDNMB675-05|Canada|Alberta|658[0n]|BOLD:ACF2028
Euxoa furtivus|[6106]|RDNMD319-06|United States|Colorado|658[0n]|BOLD:ACF2028
Euxoa furtivus|[6107]|RDNMD320-06|United States|Colorado|658[0n]|BOLD:ACF2028
Euxoa furtivus|[6108]|RDNMD877-07|United States|Wyoming|655[0n]|BOLD:ACF2028
Euxoa furtivus|[6109]|RDNMD878-07|United States|Wyoming|655[0n]|BOLD:ACF2028
Euxoa furtivus|[6110]|RDNMJ825-11|United States|Wyoming|658[0n]|BOLD:ACF2028
Euxoa cinnabarina|[6111]|CNCLB2028-14|United States|California|307[0n]|
Euxoa cinnabarina|[6112]|CNCLB2029-14|United States|California|658[0n]|BOLD:ACF2028
Euxoa cinnabarina|[6113]|CNCLB2030-14|United States|California|658[0n]|BOLD:ACF2028
Euxoa cinnabarina|[6114]|CNCLB2031-14|United States|California|658[0n]|BOLD:ACF2028
Euxoa castanea|[6115]|RDNM337-05|Canada|Saskatchewan|567[0n]|BOLD:ACF2028
Euxoa castanea|[6116]|RDNM338-05|Canada|Saskatchewan|535[0n]|BOLD:ACF2028
Euxoa castanea|[6117]|RDNM339-05|Canada|Saskatchewan|511[0n]|BOLD:ACF2028
Euxoa idahoensis|[6118]|RDNM344-05|United States|Nevada|658[0n]|BOLD:ACF2028
Euxoa idahoensis|[6119]|RDNM343-05|United States|Nevada|658[0n]|BOLD:ACF2028
Euxoa idahoensis|[6120]|RDNM345-05|United States|Washington|567[0n]|BOLD:ACF2028
Euxoa idahoensis|[6121]|RDNMB673-05|United States|Washington|568[0n]|BOLD:ACF2028
Euxoa idahoensis|[6122]|RDNMB316-05|United States|Washington|597[0n]|BOLD:ACF2028
Euxoa idahoensis|[6123]|RDNMB327-05|United States|Washington|592[0n]|BOLD:ACF2028
Euxoa idahoensis|[6124]|RDNM353-05|United States|Washington|606[0n]|BOLD:ACF2028
Euxoa idahoensis|[6125]|RDNMB674-05|United States|Washington|605[0n]|BOLD:ACF2028
Euxoa idahoensis|[6126]|RDNMB677-05|United States|Nevada|658[0n]|BOLD:ACF2028
Euxoa idahoensis|[6127]|RDNMB678-05|United States|Nevada|658[0n]|BOLD:ACF2028
Euxoa idahoensis|[6128]|LBCH7952-10|Canada|British Columbia|658[0n]|BOLD:ACF2028
Euxoa costata|[6129]|RDNM354-05|United States|Washington|658[0n]|BOLD:ACF2028
Euxoa costata|[6130]|RDNM355-05|United States|Oregon|658[0n]|BOLD:ACF2028
Euxoa costata|[6131]|RDNM356-05|United States|California|658[0n]|BOLD:ACF2028
Euxoa costata|[6132]|RDNMF838-08|Canada|British Columbia|658[0n]|BOLD:ACF2028
Euxoa nr. idahoensis sp. 2 black|[6133]|RDNM333-05|United States|Wyoming|658[0n]|BOLD:ACF2028
Euxoa nr. idahoensis sp. 2 black|[6134]|RDNM334-05|United States|Wyoming|658[0n]|BOLD:ACF2028
Euxoa nr. idahoensis sp. 2 black|[6135]|RDNM335-05|United States|Wyoming|658[0n]|BOLD:ACF2028
Euxoa nr. idahoensis sp. 2 black|[6136]|RDNMD322-06|United States|Colorado|658[0n]|BOLD:ACF2028
Euxoa idahoensis|[6137]|RDNM330-05|Canada|Alberta|658[0n]|BOLD:ACF2028
Euxoa idahoensis|[6138]|RDNM332-05|United States|Wyoming|658[0n]|BOLD:ACF2028
Euxoa idahoensis|[6139]|RDNM328-05|Canada|Alberta|658[0n]|BOLD:ACF2028
Euxoa idahoensis|[6140]|RDNM340-05|Canada|Alberta|578[0n]|BOLD:ACF2028
Euxoa idahoensis|[6141]|RDNM341-05|Canada|Alberta|658[0n]|BOLD:ACF2028
Euxoa idahoensis|[6142]|RDNMD323-06|United States|Colorado|658[0n]|BOLD:ACF2028
Euxoa idahoensis|[6143]|RDNMD324-06|United States|Colorado|658[0n]|BOLD:ACF2028
Euxoa idahoensis|[6144]|RDNMC179-05|United States|Oregon|658[0n]|BOLD:ACF2028
Euxoa idahoensis|[6145]|RDNMC316-05|United States|Oregon|658[0n]|BOLD:ACF2028
Euxoa idahoensis|[6146]|RDNMB671-05|United States|Oregon|658[0n]|BOLD:ACF2028
Euxoa idahoensis|[6147]|RDNMC321-05|United States|Oregon|604[0n]|BOLD:ACF2028
Euxoa idahoensis|[6148]|RDNMC319-05|United States|Oregon|518[0n]|BOLD:ACF2028
Euxoa idahoensis|[6149]|RDNMC320-05|United States|Oregon|567[0n]|BOLD:ACF2028
Euxoa idahoensis|[6150]|RDNMC322-05|United States|Oregon|593[0n]|BOLD:ACF2028
Euxoa idahoensis|[6151]|BBLPB835-10|Canada|British Columbia|658[0n]|BOLD:ACF2028
Euxoa idahoensis|[6152]|RDNM331-05|Canada|Alberta|658[3n]|BOLD:ACF2028
Euxoa idahoensis|[6153]|LBCG194-08|Canada|British Columbia|658[0n]|BOLD:ACF2028
Euxoa idahoensis|[6154]|LBCH6004-10|Canada|British Columbia|658[0n]|BOLD:ACF2028
Euxoa idahoensis|[6155]|LBCH6230-10|Canada|British Columbia|658[0n]|BOLD:ACF2028
Euxoa idahoensis|[6156]|LBCG1069-09|Canada|British Columbia|658[0n]|BOLD:ACF2028
Euxoa idahoensis|[6157]|LBCG2583-09|Canada|British Columbia|658[0n]|BOLD:ACF2028
Euxoa idahoensis|[6158]|LBCG251-08|Canada|British Columbia|658[0n]|BOLD:ACF2028
Euxoa idahoensis|[6159]|LPAB226-08|Canada|Alberta|658[0n]|BOLD:ACF2028
Euxoa idahoensis|[6160]|RDNMB679-05|Canada|British Columbia|658[0n]|BOLD:ACF2028
Euxoa idahoensis|[6161]|RDNMB662-05|Canada|British Columbia|658[0n]|BOLD:ACF2028
Euxoa idahoensis|[6162]|RDNMB659-05|Canada|British Columbia|658[0n]|BOLD:ACF2028
Euxoa idahoensis|[6163]|RDNMB319-05|Canada|British Columbia|658[0n]|BOLD:ACF2028
Euxoa idahoensis|[6164]|LOWCD336-06|Canada|British Columbia|656[0n]|BOLD:ACF2028
Euxoa idahoensis|[6165]|RDNMB317-05|Canada|British Columbia|597[1n]|BOLD:ACF2028
Euxoa idahoensis|[6166]|LOWCD343-06|Canada|British Columbia|658[0n]|BOLD:ACF2028
Euxoa idahoensis|[6167]|LOWCD341-06|Canada|British Columbia|598[0n]|BOLD:ACF2028
Euxoa idahoensis|[6168]|LPABB823-09|Canada|Alberta|658[0n]|BOLD:ACF2028
Euxoa idahoensis|[6169]|RDNM329-05|Canada|Alberta|506[0n]|
Euxoa idahoensis|[6170]|RDNM346-05|Canada|British Columbia|658[0n]|BOLD:ACF2028
Euxoa idahoensis|[6171]|RDNMB660-05|Canada|British Columbia|616[0n]|BOLD:ACF2028
Euxoa idahoensis|[6172]|LOWCD344-06|Canada|British Columbia|638[0n]|BOLD:ACF2028
Euxoa idahoensis|[6173]|RDNMB661-05|Canada|British Columbia|606[0n]|BOLD:ACF2028
Euxoa idahoensis|[6174]|LPABB875-09|Canada|Alberta|658[0n]|BOLD:ACF2028
Euxoa idahoensis|[6175]|LBCG3250-09|Canada|British Columbia|638[0n]|BOLD:ACF2028
Euxoa idahoensis|[6176]|LBCG3268-09|Canada|British Columbia|631[0n]|BOLD:ACF2028
Euxoa idahoensis|[6177]|LOWCD339-06|Canada|British Columbia|602[0n]|BOLD:ACF2028
Euxoa idahoensis|[6178]|LBCH6505-10|Canada|British Columbia|658[0n]|BOLD:ACF2028
Euxoa idahoensis|[6179]|LBCH6544-10|Canada|British Columbia|658[0n]|BOLD:ACF2028
Euxoa idahoensis|[6180]|LBCH6743-10|Canada|British Columbia|658[0n]|BOLD:ACF2028

Euxoa idahoensis|[6178]|LBCH6505-10|Canada|British Columbia|658[0n]|BOLD:ACF2028
Euxoa idahoensis|[6179]|LBCH6544-10|Canada|British Columbia|658[0n]|BOLD:ACF2028
Euxoa idahoensis|[6180]|LBCH6743-10|Canada|British Columbia|658[0n]|BOLD:ACF2028
Euxoa idahoensis|[6181]|BBLPB834-10|Canada|Alberta|658[0n]|BOLD:ACF2028
Euxoa idahoensis|[6182]|BBLPB836-10|Canada|British Columbia|658[0n]|BOLD:ACF2028
Euxoa furtivus|[6183]|RDNMC313-05|United States|Nevada|571[0n]|BOLD:ACF2028
Euxoa furtivus|[6184]|RDNM347-05|United States|Washington|562[0n]|BOLD:ACF2028
Euxoa furtivus|[6185]|RDNM348-05|United States|Washington|552[0n]|BOLD:ACF2028
Euxoa furtivus|[6186]|RDNMB672-05|United States|Washington|518[0n]|BOLD:ACF2028
Euxoa furtivus|[6187]|RDNM349-05|United States|Washington|658[0n]|BOLD:ACF2028
Euxoa furtivus|[6188]|RDNMB663-05|Canada|British Columbia|658[0n]|BOLD:ACF2028
Euxoa furtivus|[6189]|RDNMC178-05|United States|California|658[0n]|BOLD:ACF2028
Euxoa furtivus|[6190]|RDNMC314-05|United States|Oregon|658[0n]|BOLD:ACF2028
Euxoa furtivus|[6191]|RDNMC315-05|United States|Oregon|658[0n]|BOLD:ACF2028
Euxoa furtivus|[6192]|LBCG189-08|Canada|British Columbia|658[0n]|BOLD:ACF2028
Euxoa foeminalis|[6193]|CNCLB2033-14|United States|Arizona|658[0n]|BOLD:ACF2028
Euxoa foeminalis|[6194]|CNCLB2035-14|United States|Arizona|658[0n]|BOLD:ACF2028
Euxoa foeminalis|[6195]|CNCLB2034-14|United States|Arizona|658[0n]|BOLD:ACF2028
Euxoa foeminalis|[6196]|CNCLB1723-14|United States|Arizona|658[0n]|BOLD:ACF2028
Euxoa foeminalis|[6197]|CNCLB2032-14|United States|Arizona|658[0n]|BOLD:ACF2028
Euxoa foeminalis|[6198]|CNCLB2036-14|United States|Arizona|658[0n]|BOLD:ACF2028
Euxoa scholastica|[6199]|RDNMF700-08|United States|Montana|658[0n]|BOLD:ACF3210
Euxoa scholastica|[6200]|XAJ818-06|Canada|Ontario|658[0n]|BOLD:ACF3210
Euxoa scholastica|[6201]|RDNMF701-08|Canada|Ontario|658[0n]|BOLD:ACF3210
Euxoa pallidimacula|[6202]|RDNM985-05|United States|Oregon|658[0n]|BOLD:ABZ9160
Euxoa pallidimacula|[6203]|RDNM986-05|United States|Oregon|658[0n]|BOLD:ABZ9160
Euxoa pallidimacula|[6204]|RDNM987-05|United States|Oregon|658[0n]|BOLD:ABZ9160
Euxoa pallidimacula|[6205]|RDNMB332-05|United States|Washington|538[1n]|BOLD:ABZ9160
Euxoa pallidimacula|[6206]|RDNM988-05|United States|Washington|538[0n]|BOLD:ABZ9160
Euxoa setonia|[6207]|RDNMB841-05|Canada|Alberta|658[0n]|BOLD:ACE5989
Euxoa setonia|[6208]|RDNMB843-05|Canada|Alberta|658[0n]|BOLD:ACE5989
Euxoa setonia|[6209]|RDNMB844-05|Canada|Alberta|658[0n]|BOLD:ACE5989
Euxoa setonia|[6210]|RDMAB104-05|Canada|Alberta|658[0n]|BOLD:ACE5989
Euxoa pallidimacula|[6211]|RDNMB333-05|United States|Washington|564[3n]|BOLD:ABZ9160
Euxoa pallidimacula|[6212]|LBCH5512-10|Canada|British Columbia|658[0n]|BOLD:ABZ9160
Euxoa setonia|[6213]|LBCH5699-10|Canada|British Columbia|658[0n]|BOLD:ABZ9160
Euxoa setonia|[6214]|LPABB423-08|Canada|Alberta|658[0n]|BOLD:ABZ9160
Euxoa setonia|[6215]|LPABB345-08|Canada|Alberta|658[0n]|BOLD:ABZ9160
Euxoa setonia|[6216]|RDNMF860-08|United States|Oregon|658[0n]|BOLD:ABZ9160
Euxoa setonia|[6217]|RDNMF859-08|United States|Oregon|658[0n]|BOLD:ABZ9160
Euxoa setonia|[6218]|RDNMF858-08|United States|Oregon|658[0n]|BOLD:ABZ9160
Euxoa setonia|[6219]|LOWCE310-06|Canada|British Columbia|658[0n]|BOLD:ABZ9160
Euxoa setonia|[6220]|LBCH5700-10|Canada|British Columbia|647[0n]|BOLD:ABZ9160
Euxoa setonia|[6221]|BBLPB695-10|Canada|British Columbia|658[0n]|BOLD:ABZ9160
Euxoa mojave|[6222]|CNCLB2044-14|United States|California|525[0n]|
Euxoa mojave|[6223]|CNCLB2045-14|United States|California|658[0n]|BOLD:ACR1562
Euxoa mojave|[6224]|CNCLB2047-14|United States|California|658[0n]|BOLD:ACR1562
Euxoa mojave|[6225]|CNCLB2048-14|United States|California|658[0n]|BOLD:ACR1562
Euxoa clausa|[6226]|RDNMF699-08|United States|Colorado|658[0n]|BOLD:ACF2028
Euxoa auripennis|[6227]|LOWCD450-06|Canada|British Columbia|653[0n]|BOLD:ACF3242
Euxoa auripennis|[6228]|LOWCD427-06|Canada|British Columbia|530[0n]|BOLD:ACF3242
Euxoa auripennis|[6229]|LOWCD435-06|Canada|British Columbia|568[0n]|BOLD:ACF3242
Euxoa auripennis|[6230]|LOWCD438-06|Canada|British Columbia|532[0n]|BOLD:ACF3242
Euxoa auripennis|[6231]|LOWCD432-06|Canada|British Columbia|617[0n]|BOLD:ACF3242
Euxoa auripennis|[6232]|LOWCD442-06|Canada|British Columbia|613[0n]|BOLD:ACF3242
Euxoa auripennis|[6233]|LOWCD446-06|Canada|British Columbia|611[0n]|BOLD:ACF3242
Euxoa auripennis|[6234]|LOWCD441-06|Canada|British Columbia|612[0n]|BOLD:ACF3242
Euxoa auripennis|[6235]|LOWCD430-06|Canada|British Columbia|611[0n]|BOLD:ACF3242
Euxoa auripennis|[6236]|LOWCD436-06|Canada|British Columbia|610[0n]|BOLD:ACF3242
Euxoa auripennis|[6237]|LOWCD440-06|Canada|British Columbia|608[0n]|BOLD:ACF3242
Euxoa auripennis|[6238]|LOWCD439-06|Canada|British Columbia|608[0n]|BOLD:ACF3242
Euxoa auripennis|[6239]|LBCH7048-10|Canada|British Columbia|632[0n]|BOLD:ACF3242
Euxoa auripennis|[6240]|LOWCD431-06|Canada|British Columbia|602[0n]|BOLD:ACF3242
Euxoa auripennis|[6241]|LOWCD437-06|Canada|British Columbia|599[0n]|BOLD:ACF3242
Euxoa auripennis|[6242]|LOWCD429-06|Canada|British Columbia|600[0n]|BOLD:ACF3242
Euxoa auripennis|[6243]|LOWCD443-06|Canada|British Columbia|658[1n]|BOLD:ACF3242
Euxoa auripennis|[6244]|LOWCD444-06|Canada|British Columbia|595[0n]|BOLD:ACF3242
Euxoa auripennis|[6245]|LBCH7687-10|Canada|British Columbia|658[0n]|BOLD:ACF3242
Euxoa auripennis|[6246]|LBCH7549-10|Canada|British Columbia|658[0n]|BOLD:ACF3242
Euxoa auripennis|[6247]|LBCH6785-10|Canada|British Columbia|658[0n]|BOLD:ACF3242
Euxoa auripennis|[6248]|LBCH6500-10|Canada|British Columbia|658[0n]|BOLD:ACF3242
Euxoa auripennis|[6249]|LBCH6491-10|Canada|British Columbia|658[0n]|BOLD:ACF3242
Euxoa auripennis|[6250]|LBCH6487-10|Canada|British Columbia|658[0n]|BOLD:ACF3242
Euxoa auripennis|[6251]|LBCH6229-10|Canada|British Columbia|658[0n]|BOLD:ACF3242
Euxoa auripennis|[6252]|LOWCD447-06|Canada|British Columbia|658[0n]|BOLD:ACF3242
Euxoa auripennis|[6253]|LOWCD798-06|Canada|British Columbia|592[0n]|BOLD:ACF3242
Euxoa auripennis|[6254]|LOWCD445-06|Canada|British Columbia|598[0n]|BOLD:ACF3242
Euxoa auripennis|[6255]|LOWCD448-06|Canada|British Columbia|586[0n]|BOLD:ACF3242
Euxoa auripennis|[6256]|LOWCD449-06|Canada|British Columbia|531[0n]|BOLD:ACF3242
Euxoa auripennis|[6257]|LOWCD434-06|Canada|British Columbia|531[0n]|BOLD:ACF3242
Euxoa auripennis|[6258]|LOWCD433-06|Canada|British Columbia|611[0n]|BOLD:ACF3242
Euxoa auripennis|[6259]|LBCH7783-10|Canada|British Columbia|658[0n]|BOLD:ACF3242
Euxoa auripennis|[6260]|LBCH7787-10|Canada|British Columbia|658[0n]|BOLD:ACF3242
Euxoa auripennis|[6261]|LBCH7965-10|Canada|British Columbia|658[0n]|BOLD:ACF3242
Euxoa auripennis|[6262]|LBCH7967-10|Canada|British Columbia|658[0n]|BOLD:ACF3242
Euxoa auripennis|[6263]|LBCH7969-10|Canada|British Columbia|658[0n]|BOLD:ACF3242
Euxoa redimicula|[6264]|RDNMF867-08|Canada|Ontario|658[0n]|BOLD:ACF3242
Euxoa redimicula|[6265]|RDNMF289-08|Canada|Ontario|658[0n]|BOLD:ACF3242
Euxoa redimicula|[6266]|RDNMF290-08|Canada|Ontario|658[0n]|BOLD:ACF3242
Euxoa redimicula|[6267]|RDNMF291-08|Canada|Ontario|658[0n]|BOLD:ACF3242
Euxoa redimicula|[6268]|RDNMF868-08|Canada|Ontario|658[0n]|BOLD:ACF3242
Euxoa redimicula|[6269]|RDNMF869-08|Canada|Ontario|658[0n]|BOLD:ACF3242
Euxoa redimicula|[6270]|RDNMF870-08|Canada|Ontario|658[0n]|BOLD:ACF3242
Euxoa redimicula|[6271]|LNCC346-10|United States|North Carolina|658[0n]|BOLD:ACF3242
Euxoa declarata|[6272]|LOWCE737-06|Canada|British Columbia|613[0n]|BOLD:ACF3242
Euxoa declarata|[6273]|LBCH6789-10|Canada|British Columbia|658[0n]|BOLD:ACF3242
Euxoa declarata|[6274]|LOWCE731-06|Canada|British Columbia|617[0n]|BOLD:ACF3242
Euxoa declarata|[6275]|LOWCE733-06|Canada|British Columbia|612[0n]|BOLD:ACF3242
Euxoa declarata|[6276]|LOWCE738-06|Canada|British Columbia|656[0n]|BOLD:ACF3242
Euxoa declarata|[6277]|LOWCE730-06|Canada|British Columbia|608[0n]|BOLD:ACF3242
Euxoa declarata|[6278]|LOWCE735-06|Canada|British Columbia|658[0n]|BOLD:ACF3242
Euxoa declarata|[6279]|LBCH1624-10|Canada|British Columbia|658[0n]|BOLD:ACF3242
Euxoa declarata|[6280]|LBCH6842-10|Canada|British Columbia|658[0n]|BOLD:ACF3242

[illegible]
Euxoa declarata|[6278]|LOWCE735-06|Canada|British Columbia|658[0n]|BOLD:ACF3242
Euxoa declarata|[6279]|LBCH1624-10|Canada|British Columbia|658[0n]|BOLD:ACF3242
Euxoa declarata|[6280]|LBCH6842-10|Canada|British Columbia|658[0n]|BOLD:ACF3242
Euxoa declarata|[6281]|LBCH7544-10|Canada|British Columbia|658[0n]|BOLD:ACF3242
Euxoa declarata|[6282]|LBCH7962-10|Canada|British Columbia|658[0n]|BOLD:ACF3242
Euxoa declarata|[6283]|CMAZA1015-12|United States|Arizona|658[0n]|BOLD:ACF3242
Euxoa rockburnei|[6284]|LPVIC049-08|Canada|British Columbia|617[0n]|BOLD:ACF3242
Euxoa rockburnei|[6285]|RDNMF691-08|United States|California|612[0n]|BOLD:ACF3242
Euxoa rockburnei|[6286]|RDNMF688-08|United States|Washington|609[0n]|BOLD:ACF3242
Euxoa rockburnei|[6287]|RDNMF689-08|United States|Washington|658[0n]|BOLD:ACF3242
Euxoa rockburnei|[6288]|RDNMF690-08|United States|Washington|658[0n]|BOLD:ACF3242
Euxoa rockburnei|[6289]|RDNMF692-08|United States|California|658[0n]|BOLD:ACF3242
Euxoa rockburnei|[6290]|LBCH7132-10|Canada|British Columbia|658[0n]|BOLD:ACF3242
Euxoa rockburnei|[6291]|JMMMB376-11|United States|California|658[0n]|BOLD:ACF3242
Euxoa rockburnei|[6292]|JMMMB382-11|United States|California|658[0n]|BOLD:ACF3242
Euxoa campestris|[6293]|LOWCE732-06|Canada|British Columbia|614[0n]|BOLD:ACF3242
Euxoa campestris|[6294]|RDLQ697-07|Canada|Quebec|658[0n]|BOLD:ACF3242
Euxoa campestris|[6295]|RDLQ696-07|Canada|Quebec|658[0n]|BOLD:ACF3242
Euxoa campestris|[6296]|RDLQB442-05|Canada|Quebec|658[0n]|BOLD:ACF3242
Euxoa campestris|[6297]|RDLQF283-06|Canada|Quebec|658[0n]|BOLD:ACF3242
Euxoa campestris|[6298]|RDLQ695-07|Canada|Quebec|658[3n]|BOLD:ACF3242
Euxoa campestris|[6299]|RDLQF817-06|Canada|Quebec|658[0n]|BOLD:ACF3242
Euxoa campestris|[6300]|BBLEC509-09|Canada|New Brunswick|658[0n]|BOLD:ACF3242
Euxoa campestris|[6301]|BBLPC122-09|Canada|New Brunswick|649[0n]|BOLD:ACF3242
Euxoa campestris|[6302]|LOWCE740-06|Canada|British Columbia|618[0n]|BOLD:ACF3242
Euxoa campestris|[6303]|LOWCE736-06|Canada|British Columbia|619[0n]|BOLD:ACF3242
Euxoa campestris|[6304]|LOWCE741-06|Canada|British Columbia|617[0n]|BOLD:ACF3242
Euxoa campestris|[6305]|LOWCE734-06|Canada|British Columbia|614[0n]|BOLD:ACF3242
Euxoa campestris|[6306]|LBCD457-05|Canada|British Columbia|657[0n]|BOLD:ACF3242
Euxoa campestris|[6307]|LOWCE729-06|Canada|British Columbia|658[0n]|BOLD:ACF3242
Euxoa campestris|[6308]|LOWCE739-06|Canada|British Columbia|658[0n]|BOLD:ACF3242
Euxoa campestris|[6309]|RDLQF142-06|Canada|Quebec|658[0n]|BOLD:ACF3242
Euxoa campestris|[6310]|RDLQF143-06|Canada|Quebec|658[0n]|BOLD:ACF3242
Euxoa campestris|[6311]|RDLQF144-06|Canada|Quebec|658[0n]|BOLD:ACF3242
Euxoa campestris|[6312]|LBCH7961-10|Canada|British Columbia|658[0n]|BOLD:ACF3242
Euxoa campestris|[6313]|BBLPB729-10|Canada|Alberta|658[0n]|BOLD:ACF3242
Euxoa campestris|[6314]|BBLPB730-10|Canada|Alberta|658[0n]|BOLD:ACF3242
Euxoa campestris|[6315]|BBLPB859-10|Canada|Alberta|658[0n]|BOLD:ACF3242
Euxoa campestris|[6316]|BBLPB862-10|Canada|Alberta|658[0n]|BOLD:ACF3242
Euxoa campestris|[6317]|BBLPB864-10|Canada|Alberta|658[0n]|BOLD:ACF3242
Euxoa campestris|[6318]|BBLPB865-10|Canada|Alberta|658[0n]|BOLD:ACF3242
Euxoa campestris|[6319]|RDNMD892-07|United States|Utah|613[0n]|BOLD:ACF3242
Euxoa campestris|[6320]|UAMIC518-13|United States|Alaska|643[0n]|BOLD:ACF3242
Euxoa campestris|[6321]|ABKWR112-07|United States|Alaska|650[0n]|BOLD:ACF3242
Euxoa campestris|[6322]|ABKWR123-07|United States|Alaska|632[0n]|BOLD:ACF3242
Euxoa campestris|[6323]|UAMIC519-13|United States|Alaska|632[0n]|BOLD:ACF3242
Euxoa rockburnei|[6324]|RDNMJ717-11|United States|Arizona|658[0n]|BOLD:ACF3242
Euxoa rockburnei|[6325]|CNCLA545-13|United States|Arizona|658[0n]|BOLD:ACF3242
Euxoa oblongistigma|[6326]|RDNMF891-08|Canada|Alberta|658[0n]|BOLD:ACE8316
Euxoa oblongistigma|[6327]|RDMAB554-06|Canada|Alberta|658[0n]|BOLD:ACE8316
Euxoa oblongistigma|[6328]|RDMAB996-09|Canada|Alberta|651[0n]|BOLD:ACE8316
Euxoa oblongistigma|[6329]|RDMAB997-09|Canada|Alberta|651[0n]|BOLD:ACE8316
Euxoa oblongistigma|[6330]|RDMAB998-09|Canada|Alberta|602[0n]|BOLD:ACE8316
Euxoa oblongistigma|[6331]|LBCH6787-10|Canada|British Columbia|658[0n]|BOLD:ACE8316
Euxoa oblongistigma|[6332]|LBCH6846-10|Canada|British Columbia|658[0n]|BOLD:ACE8316
Euxoa oblongistigma|[6333]|LBCH7723-10|Canada|British Columbia|658[0n]|BOLD:ACE8316
Euxoa oblongistigma|[6334]|LBCH7873-10|Canada|British Columbia|658[0n]|BOLD:ACE8316
Euxoa oblongistigma|[6335]|LBCH7968-10|Canada|British Columbia|658[0n]|BOLD:ACE8316
Euxoa agema|[6336]|RDNMF661-08|United States|Colorado|578[1n]|BOLD:ACE8316
Euxoa agema|[6337]|RDNMF662-08|United States|Colorado|609[0n]|BOLD:ACE8316
Euxoa agema|[6338]|RDNMF660-08|United States|Colorado|544[0n]|BOLD:ACE8316
Euxoa agema|[6339]|RDNMF658-08|United States|Colorado|658[0n]|BOLD:ACE8316
Euxoa agema|[6340]|RDNMF659-08|United States|California|640[0n]|BOLD:ACE8316
Euxoa agema|[6341]|IAWLB071-10|United States|California|658[0n]|BOLD:ACE8316
Euxoa agema|[6342]|IAWLB072-10|United States|California|658[0n]|BOLD:ACE8316
Euxoa olivalis|[6343]|BBLOC1284-11|United States|California|658[0n]|BOLD:ACE9577
Euxoa olivalis|[6344]|LBCH6277-10|Canada|British Columbia|658[0n]|BOLD:ACE9577
Euxoa olivalis|[6345]|BBLOC1000-11|United States|California|658[0n]|BOLD:ACE9577
Euxoa olivalis|[6346]|LPSK482-08|Canada|Saskatchewan|658[0n]|BOLD:ACE9577
Euxoa olivalis|[6347]|LPSK572-08|Canada|Saskatchewan|658[0n]|BOLD:ACE9577
Euxoa olivalis|[6348]|RDNMG977-08|United States|Nevada|658[0n]|BOLD:ACE9577
Euxoa olivalis|[6349]|LBCG3254-09|Canada|British Columbia|639[0n]|BOLD:ACE9577
Euxoa olivalis|[6350]|LOWCE771-06|Canada|British Columbia|658[0n]|BOLD:ACE9577
Euxoa olivalis|[6351]|RDNMF898-08|Canada|British Columbia|658[0n]|BOLD:ACE9577
Euxoa olivalis|[6352]|LBCG2112-09|Canada|British Columbia|658[0n]|BOLD:ACE9577
Euxoa olivalis|[6353]|LBCG3313-09|Canada|British Columbia|658[0n]|BOLD:ACE9577
Euxoa olivalis|[6354]|LBCH6057-10|Canada|British Columbia|658[0n]|BOLD:ACE9577
Euxoa olivalis|[6355]|LBCH6364-10|Canada|British Columbia|658[0n]|BOLD:ACE9577
Euxoa olivalis|[6356]|LBCH6751-10|Canada|British Columbia|658[0n]|BOLD:ACE9577
Euxoa olivalis|[6357]|BBLOC127-11|United States|Texas|658[0n]|BOLD:ACE9577
Euxoa olivalis|[6358]|BBLOC1285-11|United States|California|658[0n]|BOLD:ACE9577
Euxoa olivalis|[6359]|RDNMG978-08|United States|Nevada|658[0n]|BOLD:ACE9577
Euxoa olivalis|[6360]|LBCH5554-10|Canada|British Columbia|658[0n]|BOLD:ACE9577
Euxoa olivalis|[6361]|LBCH6455-10|Canada|British Columbia|658[0n]|BOLD:ACE9577
Euxoa olivalis|[6362]|BBLOC1385-11|United States|California|658[0n]|BOLD:ACE9577
Euxoa olivalis|[6363]|BBLOC1270-11|United States|California|658[0n]|BOLD:ACE9577
Euxoa olivalis|[6364]|BBLOC1250-11|United States|California|658[0n]|BOLD:ACE9577
Euxoa olivalis|[6365]|BBLOC1001-11|United States|California|658[0n]|BOLD:ACE9577
Euxoa olivalis|[6366]|BBLOC999-11|United States|California|658[0n]|BOLD:ACE9577
Euxoa olivalis|[6367]|BBLOC147-11|United States|Texas|658[0n]|BOLD:ACE9577
Euxoa olivalis|[6368]|NAMUM273-08|United States|California|658[0n]|BOLD:ACE9577
Euxoa olivalis|[6369]|BBLOC1387-11|United States|California|632[0n]|BOLD:ACE9577
Euxoa olivalis|[6370]|BBLOC1728-11|United States|California|658[0n]|BOLD:ACE9577
Euxoa sp.|[6371]|LOCBC801-06|United States|California|658[0n]|BOLD:ACF3235
Euxoa sp.|[6372]|LOCBC800-06|United States|California|658[0n]|BOLD:ACF3235
Euxoa sp.|[6373]|LOCBC817-06|United States|California|653[0n]|BOLD:ACF3235
Euxoa sp.|[6374]|LOCBC612-06|United States|California|657[0n]|BOLD:ACF3235
Euxoa sp.|[6375]|LOCBC613-06|United States|California|658[0n]|BOLD:ACF3235
Euxoa sp.|[6376]|LOCBC815-06|United States|California|658[0n]|BOLD:ACF3235
Euxoa sp.|[6377]|LOCBD268-06|United States|California|658[0n]|BOLD:ACF3235
Euxoa sp.|[6378]|LOCBC409-06|United States|California|658[0n]|BOLD:ACF3235
Euxoa sp.|[6379]|LOCBC618-06|United States|California|606[0n]|BOLD:ACF3235
[illegible]

Euxoa sp.|[6377]|LOCBD268-06|United States|California|658[0n]|BOLD:ACF3235
Euxoa sp.|[6378]|LOCBC409-06|United States|California|658[0n]|BOLD:ACF3235
Euxoa sp.|[6379]|LOCBC618-06|United States|California|606[0n]|BOLD:ACF3235
Euxoa sp.|[6380]|JBAZ050-09|United States|California|658[0n]|BOLD:ACF3235
Euxoa antica|[6381]|LNAUS2839-13|United States|Utah|658[0n]|BOLD:ACI3790
Euxoa antica|[6382]|LNAUS2840-13|United States|Utah|658[0n]|BOLD:ACI3790
Euxoa antica|[6383]|LNAUS2841-13|United States|Colorado|658[0n]|BOLD:ACI3790
Euxoa antica|[6384]|LNAUS2842-13|United States|Colorado|658[0n]|BOLD:ACI3790
Euxoa antica|[6385]|LNAUS2843-13|United States|Colorado|658[0n]|BOLD:ACI3790
Euxoa terrenus|[6386]|LBCG3031-09|Canada|British Columbia|658[0n]|BOLD:ACF3235
Euxoa terrenus|[6387]|LBCH5822-10|Canada|British Columbia|658[0n]|BOLD:ACF3235
Euxoa terrenus|[6388]|LBCH5869-10|Canada|British Columbia|658[0n]|BOLD:ACF3235
Euxoa terrenus|[6389]|LBCH5943-10|Canada|British Columbia|658[0n]|BOLD:ACF3235
Euxoa terrenus|[6390]|LBCD280-05|Canada|British Columbia|614[0n]|BOLD:ACF3235
Euxoa terrenus|[6391]|LOWCD494-06|Canada|British Columbia|583[0n]|BOLD:ACF3235
Euxoa terrenus|[6392]|LBCH5598-10|Canada|British Columbia|658[0n]|BOLD:ACF3235
Euxoa terrenus|[6393]|LOWCD498-06|Canada|British Columbia|580[0n]|BOLD:ACF3235
Euxoa terrenus|[6394]|LBCH6002-10|Canada|British Columbia|658[0n]|BOLD:ACF3235
Euxoa terrenus|[6395]|LBCH5866-10|Canada|British Columbia|658[0n]|BOLD:ACF3235
Euxoa terrenus|[6396]|LBCH5865-10|Canada|British Columbia|658[0n]|BOLD:ACF3235
Euxoa terrenus|[6397]|LBCH5860-10|Canada|British Columbia|658[0n]|BOLD:ACF3235
Euxoa terrenus|[6398]|LBCH5859-10|Canada|British Columbia|658[0n]|BOLD:ACF3235
Euxoa terrenus|[6399]|LBCH5858-10|Canada|British Columbia|658[0n]|BOLD:ACF3235
Euxoa terrenus|[6400]|LBCH5855-10|Canada|British Columbia|658[0n]|BOLD:ACF3235
Euxoa terrenus|[6401]|LBCH5730-10|Canada|British Columbia|658[0n]|BOLD:ACF3235
Euxoa terrenus|[6402]|LBCH5724-10|Canada|British Columbia|658[0n]|BOLD:ACF3235
Euxoa terrenus|[6403]|LBCH5721-10|Canada|British Columbia|658[0n]|BOLD:ACF3235
Euxoa terrenus|[6404]|LBCH5713-10|Canada|British Columbia|658[0n]|BOLD:ACF3235
Euxoa terrenus|[6405]|LBCH5672-10|Canada|British Columbia|658[0n]|BOLD:ACF3235
Euxoa terrenus|[6406]|LBCH5599-10|Canada|British Columbia|658[0n]|BOLD:ACF3235
Euxoa terrenus|[6407]|LBCH5595-10|Canada|British Columbia|658[0n]|BOLD:ACF3235
Euxoa terrenus|[6408]|LBCH5593-10|Canada|British Columbia|658[0n]|BOLD:ACF3235
Euxoa terrenus|[6409]|LBCH5591-10|Canada|British Columbia|658[0n]|BOLD:ACF3235
Euxoa terrenus|[6410]|LBCH5425-10|Canada|British Columbia|658[0n]|BOLD:ACF3235
Euxoa terrenus|[6411]|LOWCE318-06|Canada|British Columbia|658[0n]|BOLD:ACF3235
Euxoa terrenus|[6412]|LOWCE317-06|Canada|British Columbia|658[0n]|BOLD:ACF3235
Euxoa terrenus|[6413]|LOWCE316-06|Canada|British Columbia|658[0n]|BOLD:ACF3235
Euxoa terrenus|[6414]|LOWCE315-06|Canada|British Columbia|658[0n]|BOLD:ACF3235
Euxoa terrenus|[6415]|LOWCE314-06|Canada|British Columbia|658[0n]|BOLD:ACF3235
Euxoa terrenus|[6416]|LOWCE313-06|Canada|British Columbia|658[0n]|BOLD:ACF3235
Euxoa terrenus|[6417]|LOWCE312-06|Canada|British Columbia|658[0n]|BOLD:ACF3235
Euxoa terrenus|[6418]|LOWCE311-06|Canada|British Columbia|658[0n]|BOLD:ACF3235
Euxoa terrenus|[6419]|LOWCE297-06|Canada|British Columbia|658[0n]|BOLD:ACF3235
Euxoa terrenus|[6420]|LOWCD496-06|Canada|British Columbia|658[0n]|BOLD:ACF3235
Euxoa terrenus|[6421]|LOWCD495-06|Canada|British Columbia|658[0n]|BOLD:ACF3235
Euxoa terrenus|[6422]|LOWCD497-06|Canada|British Columbia|588[0n]|BOLD:ACF3235
Euxoa terrenus|[6423]|LOWCE300-06|Canada|British Columbia|612[0n]|BOLD:ACF3235
Euxoa terrenus|[6424]|LOWCE309-06|Canada|British Columbia|615[0n]|BOLD:ACF3235
Euxoa terrenus|[6425]|RDNMG639-08|United States|Washington|651[0n]|BOLD:ACF3235
Euxoa terrenus|[6426]|LBCH6003-10|Canada|British Columbia|658[0n]|BOLD:ACF3235
Euxoa terrenus|[6427]|LBCH6222-10|Canada|British Columbia|658[0n]|BOLD:ACF3235
Euxoa terrenus|[6428]|LBCH6443-10|Canada|British Columbia|658[0n]|BOLD:ACF3235
Euxoa terrenus|[6429]|IAWLB438-11|United States|California|658[0n]|BOLD:ACF3235
Euxoa terrenus|[6430]|IAWLB439-11|United States|California|658[0n]|BOLD:ACF3235
Euxoa terrenus|[6431]|LALPA984-11|Canada|British Columbia|658[0n]|BOLD:ACF3235
Euxoa franclemonti|[6432]|CNCLB989-14|United States|Colorado|658[0n]|BOLD:ACF3235
Euxoa franclemonti|[6433]|IAWLB242-11|United States|Arizona|658[0n]|BOLD:ACF3235
Euxoa franclemonti|[6434]|IAWLB241-11|United States|Arizona|658[0n]|BOLD:ACF3235
Euxoa franclemonti|[6435]|CNCLB1507-14|United States|Arizona|658[0n]|BOLD:ACF3235
Euxoa absona|[6436]|CNCLB1508-14|United States|Idaho|588[0n]|BOLD:ACF3235
Euxoa unica|[6437]|RDNMF698-08|Canada|Saskatchewan|658[0n]|BOLD:ACE7161
Euxoa intrita|[6438]|LBCH2088-10|Canada|British Columbia|658[0n]|BOLD:ACE7161
Euxoa intrita|[6439]|RDNMG377-08|Canada|Saskatchewan|658[0n]|BOLD:ACE7161
Euxoa intrita|[6440]|LOWCD316-06|Canada|British Columbia|658[0n]|BOLD:ACE7161
Euxoa intrita|[6441]|LOWCD317-06|Canada|British Columbia|592[0n]|BOLD:ACE7161
Euxoa intrita|[6442]|LOWCD311-06|Canada|British Columbia|580[1n]|BOLD:ACE7161
Euxoa intrita|[6443]|LOWCD312-06|Canada|British Columbia|594[0n]|BOLD:ACE7161
Euxoa intrita|[6444]|LOWCD314-06|Canada|British Columbia|599[0n]|BOLD:ACE7161
Euxoa intrita|[6445]|LOWCD318-06|Canada|British Columbia|608[0n]|BOLD:ACE7161
Euxoa intrita|[6446]|LOWCD319-06|Canada|British Columbia|609[0n]|BOLD:ACE7161
Euxoa intrita|[6447]|LOWCD313-06|Canada|British Columbia|586[2n]|BOLD:ACE7161
Euxoa intrita|[6448]|LOWCD315-06|Canada|British Columbia|580[0n]|BOLD:ACE7161
Euxoa intrita|[6449]|RDNMF028-08|Canada|Alberta|658[0n]|BOLD:ACE7161
Euxoa intrita|[6450]|LOWCD320-06|Canada|British Columbia|607[0n]|BOLD:ACE7161
Euxoa intrita|[6451]|RDNMF895-08|Canada|British Columbia|658[0n]|BOLD:ACE7161
Euxoa intrita|[6452]|BBLPB565-10|Canada|Alberta|658[0n]|BOLD:ACE7161
Euxoa permixta|[6453]|RDNMJ122-10|United States|Washington|658[0n]|BOLD:ABZ9342
Euxoa permixta|[6454]|LNAUS2881-13|United States|Colorado|658[0n]|BOLD:ABZ9342
Euxoa bicollaris|[6455]|IAWLB449-11|United States|California|658[0n]|BOLD:ACE8317
Euxoa bicollaris|[6456]|RDNM995-05|United States|Oregon|658[0n]|BOLD:ACE8317
Euxoa bicollaris|[6457]|RDNM994-05|United States|California|658[0n]|BOLD:ACE8317
Euxoa bicollaris|[6458]|RDNM791-05|United States|Oregon|658[0n]|BOLD:ACE8317
Euxoa bicollaris|[6459]|RDNM790-05|United States|Oregon|658[0n]|BOLD:ACE8317
Euxoa bicollaris|[6460]|RDNM792-05|United States|California|616[0n]|BOLD:ACE8317
Euxoa bicollaris|[6461]|LOCBD542-06|United States|California|658[0n]|BOLD:ACE8317
Euxoa bicollaris|[6462]|LBCH5811-10|Canada|British Columbia|658[0n]|BOLD:ACE8317
Euxoa bicollaris|[6463]|LBCH5942-10|Canada|British Columbia|658[0n]|BOLD:ACE8317
Euxoa bicollaris|[6464]|IAWLB447-11|United States|California|658[0n]|BOLD:ACE8317
Euxoa bicollaris|[6465]|IAWLB448-11|United States|California|658[0n]|BOLD:ACE8317
Euxoa bicollaris|[6466]|IAWLB450-11|United States|California|658[0n]|BOLD:ACE8317
Euxoa satis|[6467]|LBCH6286-10|Canada|British Columbia|658[0n]|BOLD:ACF3240
Euxoa satis|[6468]|LBCH6288-10|Canada|British Columbia|658[0n]|BOLD:ACF3240
Euxoa satis|[6469]|LBCH6045-10|Canada|British Columbia|658[0n]|BOLD:ACF3240
Euxoa satis|[6470]|LBCH6155-10|Canada|British Columbia|658[0n]|BOLD:ACF3240
Euxoa satis|[6471]|LBCH5876-10|Canada|British Columbia|658[0n]|BOLD:ACF3240
Euxoa satis|[6472]|LBCH5944-10|Canada|British Columbia|658[0n]|BOLD:ACF3240
Euxoa satis|[6473]|LBCG1091-09|Canada|British Columbia|658[0n]|BOLD:ACF3240
Euxoa satis|[6474]|LBCG1090-09|Canada|British Columbia|658[0n]|BOLD:ACF3240
Euxoa satis|[6475]|LBCG1065-09|Canada|British Columbia|658[0n]|BOLD:ACF3240
Euxoa satis|[6476]|RDMAB1016-09|Canada|British Columbia|658[0n]|BOLD:ACF3240
Euxoa satis|[6477]|LBCG876-09|Canada|British Columbia|658[0n]|BOLD:ACF3240
Euxoa satis|[6478]|RDNMF811-08|United States|Washington|658[0n]|BOLD:ACF3240
Euxoa satis|[6479]|LOWCD487-06|Canada|British Columbia|658[0n]|BOLD:ACF3240

Euxoa satis|[6477]|LBCG876-09|Canada|British Columbia|658[0n]|BOLD:ACF3240
Euxoa satis|[6478]|RDNMF811-08|United States|Washington|658[0n]|BOLD:ACF3240
Euxoa satis|[6479]|LOWCD487-06|Canada|British Columbia|658[0n]|BOLD:ACF3240
Euxoa satis|[6480]|RDNMC067-05|United States|Oregon|658[0n]|BOLD:ACF3240
Euxoa satis|[6481]|RDNMB644-05|United States|Oregon|658[0n]|BOLD:ACF3240
Euxoa satis|[6482]|RDNMB641-05|Canada|British Columbia|658[0n]|BOLD:ACF3240
Euxoa satis|[6483]|RDNM782-05|United States|Oregon|658[0n]|BOLD:ACF3240
Euxoa satis|[6484]|RDNM781-05|United States|Oregon|658[0n]|BOLD:ACF3240
Euxoa satis|[6485]|RDMAB1017-09|Canada|British Columbia|638[0n]|BOLD:ACF3240
Euxoa satis|[6486]|RDNM804-05|Canada|British Columbia|605[1n]|BOLD:ACF3240
Euxoa satis|[6487]|RDNMB640-05|Canada|British Columbia|509[3n]|BOLD:ACF3240
Euxoa satis|[6488]|LOWCD488-06|Canada|British Columbia|541[0n]|BOLD:ACF3240
Euxoa satis|[6489]|LOWCD490-06|Canada|British Columbia|596[0n]|BOLD:ACF3240
Euxoa satis|[6490]|LBCH6289-10|Canada|British Columbia|634[0n]|BOLD:ACF3240
Euxoa satis|[6491]|LBCH6379-10|Canada|British Columbia|658[0n]|BOLD:ACF3240
Euxoa satis|[6492]|LBCH6752-10|Canada|British Columbia|658[0n]|BOLD:ACF3240
Euxoa satis|[6493]|LBCH7553-10|Canada|British Columbia|658[0n]|BOLD:ACF3240
Euxoa satis|[6494]|LNAUS2880-13|United States|Colorado|658[0n]|BOLD:ACF3240
Euxoa excogita|[6495]|RDMAB1007-09|United States|Washington|588[0n]|BOLD:ACF0818
Euxoa excogita|[6496]|LBCH5867-10|Canada|British Columbia|658[0n]|BOLD:ACF0818
Euxoa brunneigera|[6497]|RDNM794-05|United States|Nevada|658[0n]|BOLD:ACF3238
Euxoa brunneigera|[6498]|LOCBC397-06|United States|California|658[0n]|BOLD:ACF3238
Euxoa brunneigera|[6499]|RDNMD387-06|United States|California|658[0n]|BOLD:ACF3238
Euxoa brunneigera|[6500]|RDNM793-05|United States|California|658[0n]|BOLD:ACF3238
Euxoa brunneigera|[6501]|RDNM803-05|United States|Washington|658[0n]|BOLD:ACF3238
Euxoa brunneigera|[6502]|RDNM805-05|United States|Oregon|658[0n]|BOLD:ACF3238
Euxoa brunneigera|[6503]|CGLCA180-10|United States|California|658[0n]|BOLD:ACF3238
Euxoa brunneigera|[6504]|LBCH6753-10|Canada|British Columbia|658[0n]|BOLD:ACF3238
Euxoa brunneigera|[6505]|RDNMJ119-10|United States|California|658[0n]|BOLD:ACF3238
Euxoa brunneigera|[6506]|RDNMJ120-10|United States|California|658[0n]|BOLD:ACF3238
Euxoa brunneigera|[6507]|IAWLB451-11|United States|California|658[0n]|BOLD:ACF3238
Euxoa brunneigera|[6508]|IAWLB452-11|United States|California|658[0n]|BOLD:ACF3238
Euxoa satis|[6509]|RDNMB637-05|United States|Oregon|658[7n]|
Euxoa satis|[6510]|RDNMB634-05|United States|Oregon|557[0n]|BOLD:ACF3238
Euxoa satis|[6511]|RDNMB633-05|United States|Oregon|658[0n]|BOLD:ACF3238
Euxoa satis|[6512]|GMLC865-12|United States|California|658[0n]|BOLD:ACF3238
Euxoa selenis|[6513]|LOCBF1214-13|United States|California|554[0n]|BOLD:ACF3238
Euxoa selenis|[6514]|LOCBF1215-13|United States|California|588[0n]|BOLD:ACF3238
Euxoa satis|[6515]|LOCBC180-06|United States|California|658[2n]|BOLD:ACF3238
Euxoa satis|[6516]|LOCBC181-06|United States|California|615[0n]|BOLD:ACF3238
Euxoa satis|[6517]|BBLSW333-09|United States|Arizona|658[0n]|BOLD:ACF3238
Euxoa selenis|[6518]|LNAUS2882-13|United States|California|658[0n]|BOLD:ACF3238
Euxoa selenis|[6519]|LNAUS2883-13|United States|California|658[0n]|BOLD:ACF3238
Euxoa selenis|[6520]|LNAUS2884-13|United States|California|658[0n]|BOLD:ACF3238
Euxoa selenis|[6521]|LNAUS2885-13|United States|California|658[0n]|BOLD:ACF3238
Euxoa selenis|[6522]|LNAUS2886-13|United States|California|658[0n]|BOLD:ACF3238
Euxoa satis|[6523]|LBCH6022-10|Canada|British Columbia|658[0n]|BOLD:ACF3238
Euxoa satis|[6524]|LBCC012-05|Canada|British Columbia|658[0n]|BOLD:ACF3238
Euxoa satis|[6525]|RDNM789-05|United States|Oregon|552[0n]|BOLD:ACF3238
Euxoa satis|[6526]|RDNMC327-05|United States|Oregon|583[0n]|BOLD:ACF3238
Euxoa satis|[6527]|LPSK584-08|Canada|Saskatchewan|658[0n]|BOLD:ACF3238
Euxoa satis|[6528]|LPMN930-08|Canada|Alberta|658[0n]|BOLD:ACF3238
Euxoa satis|[6529]|LBCG1070-09|Canada|British Columbia|658[0n]|BOLD:ACF3238
Euxoa satis|[6530]|LBCG1078-09|Canada|British Columbia|658[0n]|BOLD:ACF3238
Euxoa satis|[6531]|LBCG1100-09|Canada|British Columbia|658[0n]|BOLD:ACF3238
Euxoa satis|[6532]|JMMMB429-11|United States|California|658[0n]|BOLD:ACF3238
Euxoa satis|[6533]|RDNMB638-05|Canada|British Columbia|658[0n]|BOLD:ACF3238
Euxoa satis|[6534]|RDNM783-05|United States|Oregon|658[0n]|BOLD:ACF3239
Euxoa satis|[6535]|RDNMB636-05|United States|Oregon|592[0n]|BOLD:ACF3239
Euxoa satis|[6536]|RDNMB642-05|United States|Oregon|553[0n]|BOLD:ACF3239
Euxoa satis|[6537]|RDNMB643-05|United States|Oregon|584[1n]|BOLD:ACF3239
Euxoa satis|[6538]|BBLPB698-10|Canada|British Columbia|636[0n]|BOLD:ACF3239
Euxoa inyoca|[6539]|CNCLB1638-14|United States|California|658[0n]|BOLD:ACF0812
Euxoa inyoca|[6540]|CNCLB1639-14|United States|California|658[0n]|BOLD:ACF0812
Euxoa brunneigera|[6541]|LBCH7815-10|Canada|British Columbia|658[0n]|BOLD:ACF0812
Euxoa brunneigera|[6542]|LBCH6550-10|Canada|British Columbia|658[0n]|BOLD:ACF0812
Euxoa brunneigera|[6543]|LBCH6529-10|Canada|British Columbia|658[0n]|BOLD:ACF0812
Euxoa brunneigera|[6544]|LBCH6027-10|Canada|British Columbia|658[0n]|BOLD:ACF0812
Euxoa brunneigera|[6545]|LOWCD485-06|Canada|British Columbia|658[0n]|BOLD:ACF0812
Euxoa brunneigera|[6546]|LOWCD486-06|Canada|British Columbia|536[0n]|BOLD:ACF0812
Euxoa brunneigera|[6547]|LOWCD493-06|Canada|British Columbia|577[0n]|BOLD:ACF0812
Euxoa piniae|[6548]|CNCLB1658-14|United States|California|658[0n]|BOLD:ACF0812
Euxoa infausta|[6549]|RDMAB318-05|Canada|Alberta|658[0n]|BOLD:ACF3238
Euxoa infausta|[6550]|LBCG476-08|Canada|British Columbia|658[0n]|BOLD:ACF3238
Euxoa infausta|[6551]|RDNM868-05|Canada|British Columbia|658[0n]|BOLD:ACF3238
Euxoa infausta|[6552]|RDNM869-05|Canada|Saskatchewan|658[0n]|BOLD:ACF3238
Euxoa infausta|[6553]|LBCH5864-10|Canada|British Columbia|658[0n]|BOLD:ACF3238
Euxoa excogita|[6554]|RDMAB1008-09|United States|Oregon|658[0n]|BOLD:ACF3238
Euxoa excogita|[6555]|LBCG505-08|Canada|British Columbia|658[0n]|BOLD:ACF3238
Euxoa excogita|[6556]|RDNM802-05|United States|Oregon|658[0n]|BOLD:ACF3238
Euxoa excogita|[6557]|RDNM801-05|United States|Oregon|658[0n]|BOLD:ACF3238
Euxoa excogita|[6558]|RDMAB1006-09|Canada|British Columbia|651[0n]|BOLD:ACF3238
Euxoa excogita|[6559]|RDMAB1009-09|Canada|British Columbia|635[0n]|BOLD:ACF3238
Euxoa excogita|[6560]|LBCG2879-09|Canada|British Columbia|658[0n]|BOLD:ACF3238
Euxoa excogita|[6561]|LBCH5793-10|Canada|British Columbia|658[0n]|BOLD:ACF3238
Euxoa excogita|[6562]|LBCH6372-10|Canada|British Columbia|658[0n]|BOLD:ACF3238
Euxoa excogita|[6563]|LBCH6548-10|Canada|British Columbia|658[0n]|BOLD:ACF3238
Euxoa excogita|[6564]|LBCH6551-10|Canada|British Columbia|658[0n]|BOLD:ACF3238
Euxoa|[6565]|CNWLF2001-12|Canada|Alberta|636[0n]|BOLD:ACF3238
Euxoa infausta|[6566]|RDNM870-05|United States|Oregon|605[0n]|BOLD:ACF3238
Euxoa satis|[6567]|LOWCD492-06|Canada|British Columbia|596[0n]|BOLD:ACF3238
Euxoa satis|[6568]|RDNMB639-05|United States|Oregon|556[0n]|BOLD:ACF3238
Euxoa satis|[6569]|LBCD455-05|Canada|British Columbia|658[0n]|BOLD:ACF3238
Euxoa infausta|[6570]|JMMMB361-11|United States|California|658[1n]|BOLD:ACF3238
Euxoa satis|[6571]|LBCD463-05|Canada|British Columbia|658[0n]|BOLD:ACF3238
Euxoa satis|[6572]|LOWCD484-06|Canada|British Columbia|548[0n]|BOLD:ACF3238
Euxoa satis|[6573]|LOWCD489-06|Canada|British Columbia|658[0n]|BOLD:ACF3238
Euxoa satis|[6574]|LOWCD491-06|Canada|British Columbia|658[0n]|BOLD:ACF3238
Euxoa satis|[6575]|LBCH6553-10|Canada|British Columbia|658[0n]|BOLD:ACF3238
Euxoa infausta|[6576]|JMMMB327-11|United States|California|658[0n]|BOLD:ACF3238
Euxoa infausta|[6577]|JMMMB332-11|United States|California|658[0n]|BOLD:ACF3238
Euxoa infausta|[6578]|JMMMB405-11|United States|California|658[0n]|BOLD:ACF3238
Euxoa infausta|[6579]|JMMMB411-11|United States|California|658[0n]|BOLD:ACF3238

[illegible]
Euxoa infausta|[6577]|JMMMB332-11|United States|California|658[0n]|BOLD:ACF3238
Euxoa infausta|[6578]|JMMMB405-11|United States|California|658[0n]|BOLD:ACF3238
Euxoa infausta|[6579]|JMMMB411-11|United States|California|658[0n]|BOLD:ACF3238
Euxoa infausta|[6580]|JMMMB413-11|United States|California|658[0n]|BOLD:ACF3238
Euxoa|[6581]|CNWLM046-13|Canada|Alberta|588[0n]|BOLD:ACF3238
Euxoa faulkneri|[6582]|RDNM787-05|United States|California|658[0n]|BOLD:ACF3238
Euxoa faulkneri|[6583]|RDNM788-05|United States|California|658[0n]|BOLD:ACF3238
Euxoa piniae|[6584]|CNCLB1659-14|United States|California|658[0n]|BOLD:ACF3238
Euxoa scotogrammoides|[6585]|RDNM824-05|United States|California|550[1n]|BOLD:ACE8542
Euxoa scotogrammoides|[6586]|RDNM991-05|United States|Oregon|658[0n]|BOLD:ACE8542
Euxoa scotogrammoides|[6587]|RDNM990-05|United States|Oregon|658[0n]|BOLD:ACE8542
Euxoa scotogrammoides|[6588]|RDNM826-05|United States|Oregon|658[0n]|BOLD:ACE8542
Euxoa scotogrammoides|[6589]|RDNM825-05|United States|Oregon|658[0n]|BOLD:ACE8542
Euxoa scotogrammoides|[6590]|RDNM992-05|United States|Washington|601[0n]|BOLD:ACE8542
Euxoa tocoyae|[6591]|NAMUM298-08|United States|California|658[0n]|BOLD:ACE8542
Euxoa tocoyae|[6592]|RDNMJ117-10|United States|California|658[0n]|BOLD:ACE8542
Euxoa tocoyae|[6593]|RDNMJ118-10|United States|California|658[0n]|BOLD:ACE8542
Euxoa tocoyae|[6594]|IAWLB225-11|United States|California|658[0n]|BOLD:ACE8542
Euxoa tocoyae|[6595]|IAWLB226-11|United States|California|658[0n]|BOLD:ACE8542
Euxoa serricornis|[6596]|BBLSY591-09|United States|Arizona|589[0n]|BOLD:ACE9578
Euxoa serricornis|[6597]|BBLSY641-09|United States|Arizona|589[0n]|BOLD:ACE9578
Euxoa serricornis|[6598]|USLEP090-10|United States|Arizona|658[0n]|BOLD:ACE9578
Euxoa serricornis|[6599]|USLEP702-10|United States|Arizona|658[0n]|BOLD:ACE9578
Euxoa serricornis|[6600]|BBLSY654-09|United States|Arizona|518[0n]|BOLD:ACE9578
Euxoa serricornis|[6601]|BBLSW269-09|United States|Arizona|658[0n]|BOLD:ACE9578
Euxoa serricornis|[6602]|BBLSY747-09|United States|Arizona|658[0n]|BOLD:ACE9578
Euxoa serricornis|[6603]|BBLSY748-09|United States|Arizona|658[0n]|BOLD:ACE9578
Euxoa serricornis|[6604]|BBLSY749-09|United States|Arizona|658[0n]|BOLD:ACE9578
Euxoa serricornis|[6605]|CGLCA160-10|United States|California|630[0n]|BOLD:ACE9578
Euxoa serricornis|[6606]|BBLSY728-09|United States|Arizona|632[0n]|BOLD:ACE9578
Euxoa serricornis|[6607]|NAMUM388-09|United States|California|658[0n]|BOLD:ACE9578
Euxoa serricornis|[6608]|CGLCA029-10|United States|California|658[0n]|BOLD:ACE9578
Euxoa serricornis|[6609]|BBLSY653-09|United States|Arizona|658[0n]|BOLD:ACE9578
Euxoa serricornis|[6610]|BBLSY723-09|United States|Arizona|658[0n]|BOLD:ACE9578
Euxoa serricornis|[6611]|IAWLB214-11|United States|Arizona|658[0n]|BOLD:ACE9578
Euxoa serricornis|[6612]|USLEP141-10|United States|Arizona|637[0n]|BOLD:ACE9578
Euxoa serricornis|[6613]|BBLSW250-09|United States|Arizona|658[0n]|BOLD:ACE9578
Euxoa serricornis|[6614]|NAMUM389-09|United States|California|658[0n]|BOLD:ACE9578
Euxoa serricornis|[6615]|BBLSW191-09|United States|Arizona|643[0n]|BOLD:ACE9578
Euxoa serricornis|[6616]|BBLSY662-09|United States|Arizona|658[0n]|BOLD:ACE9578
Euxoa serricornis|[6617]|BBLSY729-09|United States|Arizona|658[0n]|BOLD:ACE9578
Euxoa serricornis|[6618]|USLEP140-10|United States|Arizona|658[0n]|BOLD:ACE9578
Euxoa serricornis|[6619]|USLEP142-10|United States|Arizona|658[0n]|BOLD:ACE9578
Euxoa serricornis|[6620]|USLEP143-10|United States|Arizona|658[0n]|BOLD:ACE9578
Euxoa serricornis|[6621]|IAWLB215-11|United States|Arizona|658[0n]|BOLD:ACE9578
Euxoa serricornis|[6622]|IAWLB498-11|United States|Arizona|658[0n]|BOLD:ACE9578
Euxoa basalis|[6623]|RDNMF031-08|Canada|Alberta|658[0n]|BOLD:ABZ9144
Euxoa basalis|[6624]|RDNMG984-08|United States|Wyoming|658[0n]|BOLD:ABZ9144
Euxoa basalis|[6625]|LPSK594-08|Canada|Saskatchewan|658[0n]|BOLD:ABZ9144
Euxoa basalis|[6626]|RDNMF294-08|Canada|Saskatchewan|658[0n]|BOLD:ABZ9144
Euxoa basalis|[6627]|RDNMF295-08|Canada|Alberta|658[0n]|BOLD:ABZ9144
Euxoa basalis|[6628]|RDNMF844-08|United States|Nevada|658[0n]|BOLD:ABZ9144
Euxoa basalis|[6629]|RDNMF845-08|United States|Nevada|658[0n]|BOLD:ABZ9144
Euxoa hollemani|[6630]|RDNMG998-08|United States|Oregon|658[0n]|BOLD:ABZ9892
Euxoa hollemani|[6631]|RDNMF329-08|Canada|British Columbia|658[0n]|BOLD:ABZ9892
Euxoa hollemani|[6632]|RDNMF328-08|Canada|British Columbia|658[0n]|BOLD:ABZ9892
Euxoa hollemani|[6633]|JMMMB333-11|United States|California|658[0n]|BOLD:ABZ9892
Euxoa hollemani|[6634]|JMMMB359-11|United States|California|658[0n]|BOLD:ABZ9892
Euxoa hollemani|[6635]|JMMMB390-11|United States|California|658[0n]|BOLD:ABZ9892
Euxoa choris|[6636]|LBCH6377-10|Canada|British Columbia|513[0n]|
Euxoa choris|[6637]|RDNMF434-08|United States|Nevada|658[0n]|BOLD:ABZ9440
Euxoa choris|[6638]|LBCH6370-10|Canada|British Columbia|658[0n]|BOLD:ABZ9440
Euxoa choris|[6639]|LBCH6735-10|Canada|British Columbia|642[0n]|BOLD:ABZ9440
Euxoa choris|[6640]|JMMMB375-11|United States|California|658[0n]|BOLD:ABZ9440
Euxoa choris|[6641]|BBLOC206-11|United States|Texas|658[0n]|BOLD:ABZ9440
Euxoa choris|[6642]|LBCH6733-10|Canada|British Columbia|658[0n]|BOLD:ABZ9440
Euxoa choris|[6643]|LBCH6734-10|Canada|British Columbia|658[0n]|BOLD:ABZ9440
Euxoa choris|[6644]|LBCH7756-10|Canada|British Columbia|658[0n]|BOLD:ABZ9440
Euxoa choris|[6645]|IAWLB132-10|United States|California|658[0n]|BOLD:ABZ9440
Euxoa choris|[6646]|LBCH6730-10|Canada|British Columbia|658[0n]|BOLD:ABZ9440
Euxoa choris|[6647]|LBCH6732-10|Canada|British Columbia|658[0n]|BOLD:ABZ9440
Euxoa choris|[6648]|LBCH6728-10|Canada|British Columbia|658[0n]|BOLD:ABZ9440
Euxoa choris|[6649]|LBCH6729-10|Canada|British Columbia|658[0n]|BOLD:ABZ9440
Euxoa choris|[6650]|LBCH6661-10|Canada|British Columbia|658[0n]|BOLD:ABZ9440
Euxoa choris|[6651]|LBCH6543-10|Canada|British Columbia|658[0n]|BOLD:ABZ9440
Euxoa choris|[6652]|LBCH6530-10|Canada|British Columbia|658[0n]|BOLD:ABZ9440
Euxoa choris|[6653]|LBCH6444-10|Canada|British Columbia|658[0n]|BOLD:ABZ9440
Euxoa choris|[6654]|LBCH6378-10|Canada|British Columbia|658[0n]|BOLD:ABZ9440
Euxoa choris|[6655]|LBCH6041-10|Canada|British Columbia|658[0n]|BOLD:ABZ9440
Euxoa choris|[6656]|LBCH5857-10|Canada|British Columbia|658[0n]|BOLD:ABZ9440
Euxoa choris|[6657]|LBCG3283-09|Canada|British Columbia|658[0n]|BOLD:ABZ9440
Euxoa choris|[6658]|LBCG2882-09|Canada|British Columbia|658[0n]|BOLD:ABZ9440
Euxoa choris|[6659]|RDNMG828-08|United States|Washington|658[0n]|BOLD:ABZ9440
Euxoa choris|[6660]|RDNMG657-08|Canada|Alberta|658[0n]|BOLD:ABZ9440
Euxoa choris|[6661]|RDNMF435-08|United States|Oregon|658[0n]|BOLD:ABZ9440
Euxoa choris|[6662]|RDNMG658-08|United States|Washington|622[17n]|
Euxoa choris|[6663]|LBCG2109-09|Canada|British Columbia|658[0n]|BOLD:ABZ9440
Euxoa choris|[6664]|LBCH6731-10|Canada|British Columbia|658[0n]|BOLD:ABZ9440
Euxoa choris|[6665]|BBLOC208-11|United States|Texas|658[0n]|BOLD:ABZ9440
Euxoa plagigera|[6666]|BBLPB811-10|Canada|Alberta|658[0n]|BOLD:ACF2029
Euxoa plagigera|[6667]|LBCH7366-10|Canada|British Columbia|658[0n]|BOLD:ACF2029
Euxoa plagigera|[6668]|LOWCE661-06|Canada|British Columbia|602[0n]|BOLD:ACF2029
Euxoa plagigera|[6669]|LBCG3252-09|Canada|British Columbia|632[0n]|BOLD:ACF2029
Euxoa plagigera|[6670]|BBLPB807-10|Canada|Alberta|658[0n]|BOLD:ACF2029
Euxoa plagigera|[6671]|LOWCE671-06|Canada|British Columbia|627[0n]|BOLD:ACF2029
Euxoa plagigera|[6672]|LBCH7788-10|Canada|British Columbia|658[0n]|BOLD:ACF2029
Euxoa plagigera|[6673]|LBCH7688-10|Canada|British Columbia|658[0n]|BOLD:ACF2029
Euxoa plagigera|[6674]|LBCH7685-10|Canada|British Columbia|658[0n]|BOLD:ACF2029
Euxoa plagigera|[6675]|LBCH7312-10|Canada|British Columbia|658[0n]|BOLD:ACF2029
Euxoa plagigera|[6676]|LBCH7237-10|Canada|British Columbia|658[0n]|BOLD:ACF2029
Euxoa plagigera|[6677]|LBCH6747-10|Canada|British Columbia|658[0n]|BOLD:ACF2029
Euxoa plagigera|[6678]|LBCH6658-10|Canada|British Columbia|658[0n]|BOLD:ACF2029
[illegible]

Euxoa plagigera|[6676]|LBCH7257-10|Canada|British Columbia|658[0n]|BOLD:ACF2029
Euxoa plagigera|[6677]|LBCH6747-10|Canada|British Columbia|658[0n]|BOLD:ACF2029
Euxoa plagigera|[6678]|LBCH6658-10|Canada|British Columbia|658[0n]|BOLD:ACF2029
Euxoa plagigera|[6679]|LBCH6657-10|Canada|British Columbia|658[0n]|BOLD:ACF2029
Euxoa plagigera|[6680]|LBCH6656-10|Canada|British Columbia|658[0n]|BOLD:ACF2029
Euxoa plagigera|[6681]|LBCH6655-10|Canada|British Columbia|658[0n]|BOLD:ACF2029
Euxoa plagigera|[6682]|LBCH6654-10|Canada|British Columbia|658[0n]|BOLD:ACF2029
Euxoa plagigera|[6683]|LBCH6653-10|Canada|British Columbia|658[0n]|BOLD:ACF2029
Euxoa plagigera|[6684]|LBCH6652-10|Canada|British Columbia|658[0n]|BOLD:ACF2029
Euxoa plagigera|[6685]|LBCH6503-10|Canada|British Columbia|658[0n]|BOLD:ACF2029
Euxoa plagigera|[6686]|LBCH6502-10|Canada|British Columbia|658[0n]|BOLD:ACF2029
Euxoa plagigera|[6687]|LBCH6498-10|Canada|British Columbia|658[0n]|BOLD:ACF2029
Euxoa plagigera|[6688]|LBCH6496-10|Canada|British Columbia|658[0n]|BOLD:ACF2029
Euxoa plagigera|[6689]|LBCH6493-10|Canada|British Columbia|658[0n]|BOLD:ACF2029
Euxoa plagigera|[6690]|LBCH6454-10|Canada|British Columbia|658[0n]|BOLD:ACF2029
Euxoa plagigera|[6691]|LBCH6449-10|Canada|British Columbia|658[0n]|BOLD:ACF2029
Euxoa plagigera|[6692]|LBCH6375-10|Canada|British Columbia|658[0n]|BOLD:ACF2029
Euxoa plagigera|[6693]|LBCH6373-10|Canada|British Columbia|658[0n]|BOLD:ACF2029
Euxoa plagigera|[6694]|LBCH6363-10|Canada|British Columbia|658[0n]|BOLD:ACF2029
Euxoa plagigera|[6695]|LBCH6361-10|Canada|British Columbia|658[0n]|BOLD:ACF2029
Euxoa plagigera|[6696]|LBCH6354-10|Canada|British Columbia|658[0n]|BOLD:ACF2029
Euxoa plagigera|[6697]|LBCH6278-10|Canada|British Columbia|658[0n]|BOLD:ACF2029
Euxoa plagigera|[6698]|LBCH6232-10|Canada|British Columbia|658[0n]|BOLD:ACF2029
Euxoa plagigera|[6699]|LBCH6231-10|Canada|British Columbia|658[0n]|BOLD:ACF2029
Euxoa plagigera|[6700]|LBCH6096-10|Canada|British Columbia|658[0n]|BOLD:ACF2029
Euxoa plagigera|[6701]|LBCG2875-09|Canada|British Columbia|658[0n]|BOLD:ACF2029
Euxoa plagigera|[6702]|LBCG479-08|Canada|British Columbia|658[0n]|BOLD:ACF2029
Euxoa plagigera|[6703]|LOWCE673-06|Canada|British Columbia|658[0n]|BOLD:ACF2029
Euxoa plagigera|[6704]|LOWCE669-06|Canada|British Columbia|658[0n]|BOLD:ACF2029
Euxoa plagigera|[6705]|LOWCE667-06|Canada|British Columbia|658[0n]|BOLD:ACF2029
Euxoa plagigera|[6706]|LOWCE666-06|Canada|British Columbia|658[0n]|BOLD:ACF2029
Euxoa plagigera|[6707]|LOWCE662-06|Canada|British Columbia|658[0n]|BOLD:ACF2029
Euxoa plagigera|[6708]|LOWCE660-06|Canada|British Columbia|658[0n]|BOLD:ACF2029
Euxoa plagigera|[6709]|LOWCE659-06|Canada|British Columbia|658[0n]|BOLD:ACF2029
Euxoa plagigera|[6710]|LOWCE658-06|Canada|British Columbia|658[0n]|BOLD:ACF2029
Euxoa plagigera|[6711]|LOWCE656-06|Canada|British Columbia|658[0n]|BOLD:ACF2029
Euxoa plagigera|[6712]|LOWCE654-06|Canada|British Columbia|658[0n]|BOLD:ACF2029
Euxoa plagigera|[6713]|LOWCE653-06|Canada|British Columbia|658[0n]|BOLD:ACF2029
Euxoa plagigera|[6714]|LOWCE652-06|Canada|British Columbia|658[0n]|BOLD:ACF2029
Euxoa plagigera|[6715]|LOWCE670-06|Canada|British Columbia|609[0n]|BOLD:ACF2029
Euxoa plagigera|[6716]|LOWCE655-06|Canada|British Columbia|654[0n]|BOLD:ACF2029
Euxoa plagigera|[6717]|LBCH6651-10|Canada|British Columbia|639[0n]|BOLD:ACF2029
Euxoa plagigera|[6718]|LOWCE663-06|Canada|British Columbia|610[0n]|BOLD:ACF2029
Euxoa plagigera|[6719]|LOWCE672-06|Canada|British Columbia|612[0n]|BOLD:ACF2029
Euxoa plagigera|[6720]|LOWCE668-06|Canada|British Columbia|607[0n]|BOLD:ACF2029
Euxoa plagigera|[6721]|LOWCE657-06|Canada|British Columbia|617[0n]|BOLD:ACF2029
Euxoa plagigera|[6722]|LBCH7803-10|Canada|British Columbia|648[0n]|BOLD:ACF2029
Euxoa plagigera|[6723]|LBCH7876-10|Canada|British Columbia|658[0n]|BOLD:ACF2029
Euxoa plagigera|[6724]|LBCH7877-10|Canada|British Columbia|658[0n]|BOLD:ACF2029
Euxoa plagigera|[6725]|LBCH7966-10|Canada|British Columbia|658[0n]|BOLD:ACF2029
Euxoa plagigera|[6726]|LBCH7970-10|Canada|British Columbia|658[0n]|BOLD:ACF2029
Euxoa plagigera|[6727]|BBLPB808-10|Canada|Alberta|658[0n]|BOLD:ACF2029
Euxoa plagigera|[6728]|BBLPB809-10|Canada|Alberta|658[0n]|BOLD:ACF2029
Euxoa plagigera|[6729]|BBLPB810-10|Canada|Alberta|658[0n]|BOLD:ACF2029
Euxoa plagigera|[6730]|BBLPB866-10|Canada|British Columbia|658[0n]|BOLD:ACF2029
Euxoa plagigera|[6731]|LOWCE664-06|Canada|British Columbia|658[0n]|BOLD:ACF2029
Euxoa plagigera|[6732]|JMMMB607-13|United States|California|597[0n]|BOLD:ACF2029
Euxoa rufula|[6733]|LSEU808-06|United States|Colorado|658[0n]|BOLD:ACE8541
Euxoa rufula|[6734]|RDNMF038-08|Canada|Alberta|658[0n]|BOLD:ACE8541
Euxoa rufula|[6735]|RDNMG636-08|Canada|British Columbia|658[0n]|BOLD:ACE8541
Euxoa rufula|[6736]|RDNMG637-08|Canada|British Columbia|658[0n]|BOLD:ACE8541
Euxoa rufula|[6737]|RDNMG638-08|Canada|British Columbia|658[0n]|BOLD:ACE8541
Euxoa lafontainei|[6738]|RDNME736-08|United States|New Mexico|658[0n]|BOLD:AAA6217
Euxoa lafontainei|[6739]|RDNME737-08|United States|New Mexico|658[0n]|BOLD:AAA6217
Euxoa simulata|[6740]|RDNMG642-08|United States|California|658[0n]|BOLD:AAD7748
Euxoa simulata|[6741]|RDNMG640-08|United States|California|658[0n]|BOLD:AAD7748
Euxoa simulata|[6742]|RDNMG641-08|Canada|British Columbia|658[0n]|BOLD:AAD7748
Euxoa simulata|[6743]|RDNMF326-08|United States|Nevada|658[0n]|BOLD:AAD7748
Euxoa simulata|[6744]|RDNMF327-08|United States|Washington|658[0n]|BOLD:AAD7748
Euxoa simulata|[6745]|RDNMG643-08|United States|Nevada|658[0n]|BOLD:AAD7748
Euxoa ustulata|[6746]|LNAUS2907-13|United States|Oregon|658[0n]|BOLD:ACI3810
Euxoa cursoria|[6747]|RDNMB013-05|Canada|Alberta|581[0n]|BOLD:ACE9573
Euxoa cursoria|[6748]|RDMAB1003-09|Canada|Alberta|658[0n]|BOLD:ACE9573
Euxoa cursoria|[6749]|RDNMG656-08|Canada|Alberta|658[0n]|BOLD:ACE9573
Euxoa cursoria|[6750]|RDMAB1004-09|Canada|Alberta|644[0n]|BOLD:ACE9573
Euxoa cursoria|[6751]|RDMAB1002-09|Canada|Alberta|654[0n]|BOLD:ACE9573
Euxoa cursoria|[6752]|RDMAB1005-09|Canada|Alberta|633[2n]|BOLD:ACE9573
Euxoa ochrogaster|[6753]|UAMIC570-13|United States|Alaska|658[0n]|BOLD:ACE9574
Euxoa ochrogaster|[6754]|BBLPB799-10|Canada|Alberta|658[0n]|BOLD:ACE9574
Euxoa ochrogaster|[6755]|BBLPB798-10|Canada|Alberta|658[0n]|BOLD:ACE9574
Euxoa ochrogaster|[6756]|BBLPB797-10|Canada|Alberta|658[0n]|BOLD:ACE9574
Euxoa ochrogaster|[6757]|BBLPB796-10|Canada|Alberta|658[0n]|BOLD:ACE9574
Euxoa ochrogaster|[6758]|BBLPB795-10|Canada|Alberta|658[0n]|BOLD:ACE9574
Euxoa ochrogaster|[6759]|BBLPB794-10|Canada|Alberta|658[0n]|BOLD:ACE9574
Euxoa ochrogaster|[6760]|BBLPB614-10|Canada|Alberta|658[0n]|BOLD:ACE9574
Euxoa ochrogaster|[6761]|BBLPB613-10|Canada|British Columbia|658[0n]|BOLD:ACE9574
Euxoa ochrogaster|[6762]|BBLPB564-10|Canada|Alberta|658[0n]|BOLD:ACE9574
Euxoa ochrogaster|[6763]|BBLPB559-10|Canada|Alberta|658[0n]|BOLD:ACE9574
Euxoa ochrogaster|[6764]|LALPA691-10|Canada|British Columbia|658[0n]|BOLD:ACE9574
Euxoa ochrogaster|[6765]|LALPA688-10|Canada|British Columbia|658[0n]|BOLD:ACE9574
Euxoa ochrogaster|[6766]|LALPA677-10|Canada|British Columbia|658[0n]|BOLD:ACE9574
Euxoa ochrogaster|[6767]|RDLQ700-07|Canada|Quebec|658[0n]|BOLD:ACE9574
Euxoa ochrogaster|[6768]|RDLQ699-07|Canada|Quebec|658[0n]|BOLD:ACE9574
Euxoa ochrogaster|[6769]|LOWCD324-06|Canada|British Columbia|657[0n]|BOLD:ACE9574
Euxoa ochrogaster|[6770]|LOWCD322-06|Canada|British Columbia|614[0n]|BOLD:ACE9574
Euxoa ochrogaster|[6771]|LOWCD321-06|Canada|British Columbia|554[0n]|BOLD:ACE9574
Euxoa ochrogaster|[6772]|LOWCD323-06|Canada|British Columbia|598[0n]|BOLD:ACE9574
Euxoa ochrogaster|[6773]|CNWLN1074-13|Canada|Alberta|605[0n]|BOLD:ACE9574
Euxoa ochrogaster|[6774]|CNWLN1524-13|Canada|Alberta|600[0n]|BOLD:ACE9574
Euxoa scandens|[6775]|XAE453-04|Canada|Ontario|602[0n]|BOLD:ABZ9001
Euxoa scandens|[6776]|RDLQF369-06|Canada|Quebec|658[0n]|BOLD:ABZ9001
Euxoa scandens|[6777]|RDNMF876-08|Canada|Ontario|658[0n]|BOLD:ABZ9001
Euxoa scandens|[6778]|XAE443-04|Canada|Ontario|658[0n]|BOLD:ABZ9001

Euxoa scandens|[6776]|RDLQF369-06|Canada|Quebec|658[0n]|BOLD:ABZ9001
Euxoa scandens|[6777]|RDNMF876-08|Canada|Ontario|658[0n]|BOLD:ABZ9001
Euxoa scandens|[6778]|XAE443-04|Canada|Ontario|658[0n]|BOLD:ABZ9001
Euxoa scandens|[6779]|RDMAB095-05|Canada|Alberta|658[0n]|BOLD:ABZ9001
Euxoa scandens|[6780]|RDNMF897-08|Canada|Saskatchewan|658[0n]|BOLD:ABZ9001
Euxoa quebecensis|[6781]|LBCD466-05|Canada|British Columbia|616[0n]|BOLD:ACF0815
Euxoa quebecensis|[6782]|LBCD016-05|Canada|British Columbia|658[0n]|BOLD:ACF0815
Euxoa quebecensis|[6783]|RDLQB087-05|Canada|Quebec|658[0n]|BOLD:ACF0815
Euxoa quebecensis|[6784]|RDMAB434-05|Canada|Yukon Territory|658[0n]|BOLD:ACF0815
Euxoa quebecensis|[6785]|LPABB378-08|Canada|Alberta|658[0n]|BOLD:ACF0815
Euxoa quebecensis|[6786]|LPABB597-08|Canada|Alberta|658[0n]|BOLD:ACF0815
Euxoa quebecensis|[6787]|LPABC713-09|Canada|Alberta|658[0n]|BOLD:ACF0815
Euxoa quebecensis|[6788]|BBLPB376-10|Canada|Alberta|658[0n]|BOLD:ACF0815
Euxoa quebecensis|[6789]|BBLPB377-10|Canada|British Columbia|658[0n]|BOLD:ACF0815
Euxoa quebecensis|[6790]|BBLPB378-10|Canada|British Columbia|658[0n]|BOLD:ACF0815
Euxoa quebecensis|[6791]|BBLPB379-10|Canada|British Columbia|658[0n]|BOLD:ACF0815
Euxoa quebecensis|[6792]|BBLPB381-10|Canada|Alberta|658[0n]|BOLD:ACF0815
Euxoa quebecensis|[6793]|BBLPB382-10|Canada|Alberta|658[0n]|BOLD:ACF0815
Euxoa quebecensis|[6794]|RDLQB085-05|Canada|Quebec|658[0n]|BOLD:ACF0815
Euxoa quebecensis|[6795]|RDLQB086-05|Canada|Quebec|658[0n]|BOLD:ACF0815
Euxoa quebecensis|[6796]|RDLQB088-05|Canada|Quebec|658[0n]|BOLD:ACF0815
Euxoa quebecensis|[6797]|RDLQG363-06|Canada|Quebec|658[0n]|BOLD:ACF0815
Euxoa quebecensis|[6798]|RDLQG364-06|Canada|Quebec|658[0n]|BOLD:ACF0815
Euxoa quebecensis|[6799]|BBLPB411-10|Canada|Ontario|658[0n]|BOLD:ACF0815
Euxoa edictalis|[6800]|RDNMF679-08|United States|Oregon|658[0n]|BOLD:ABZ9140
Euxoa edictalis|[6801]|RDNMF677-08|United States|Wyoming|658[0n]|BOLD:ABZ9140
Euxoa edictalis|[6802]|RDNMF678-08|United States|Wyoming|658[0n]|BOLD:ABZ9140
Euxoa edictalis|[6803]|RDNMF680-08|United States|California|658[0n]|BOLD:ABZ9140
Euxoa edictalis|[6804]|RDNMF681-08|Canada|British Columbia|641[0n]|BOLD:ABZ9140
Euxoa edictalis|[6805]|RDNMF682-08|Canada|British Columbia|622[0n]|BOLD:ABZ9140
Euxoa edictalis|[6806]|IAWLB412-11|United States|California|658[0n]|BOLD:ABZ9140
Euxoa edictalis|[6807]|IAWLB413-11|United States|California|658[0n]|BOLD:ABZ9140
Euxoa edictalis|[6808]|IAWLB414-11|United States|California|658[0n]|BOLD:ABZ9140
Euxoa inconcinna|[6809]|IAWLB527-11|United States|Arizona|658[0n]|BOLD:ACE7158
Euxoa inconcinna|[6810]|RDNME1033-08|United States|Arizona|658[0n]|BOLD:ACE7158
Euxoa inconcinna|[6811]|BBLSZ065-09|United States|Arizona|658[0n]|BOLD:ACE7158
Euxoa inconcinna|[6812]|IAWLB542-11|United States|Arizona|658[0n]|BOLD:ACE7158
Euxoa inconcinna|[6813]|BBLOE1488-12|United States|New Mexico|658[0n]|BOLD:ACE7158
Euxoa inconcinna|[6814]|BBLSY827-09|United States|Arizona|633[0n]|BOLD:ACE7158
Euxoa inconcinna|[6815]|NAMUM063-08|United States|Arizona|657[0n]|BOLD:ACE7158
Euxoa inconcinna|[6816]|BBLSY671-09|United States|Arizona|658[0n]|BOLD:ACE7158
Euxoa inconcinna|[6817]|RDNMJ720-11|United States|Arizona|658[0n]|BOLD:ACE7158
Euxoa inconcinna|[6818]|IAWLB191-11|United States|Arizona|658[0n]|BOLD:ACE7158
Euxoa inconcinna|[6819]|IAWLB526-11|United States|Arizona|658[0n]|BOLD:ACE7158
Euxoa inconcinna|[6820]|IAWLB543-11|United States|Arizona|658[0n]|BOLD:ACE7158
Euxoa inconcinna|[6821]|LNAUS2906-13|United States|New Mexico|658[0n]|BOLD:ACE7158
Euxoa auxiliaris|[6822]|LPSK110-08|Canada|Saskatchewan|658[0n]|BOLD:ABZ9343
Euxoa auxiliaris|[6823]|CNWLM2432-13|Canada|Alberta|613[0n]|BOLD:ABZ9343
Euxoa auxiliaris|[6824]|NAMUM288-08|United States|Colorado|658[0n]|BOLD:ABZ9343
Euxoa auxiliaris|[6825]|LPSK146-08|Canada|Saskatchewan|658[0n]|BOLD:ABZ9343
Euxoa auxiliaris|[6826]|BBLWU158-09|United States|Colorado|658[0n]|BOLD:ABZ9343
Euxoa auxiliaris|[6827]|BBLWU056-09|United States|Colorado|658[0n]|BOLD:ABZ9343
Euxoa auxiliaris|[6828]|BBLWU159-09|United States|Colorado|658[0n]|BOLD:ABZ9343
Euxoa auxiliaris|[6829]|BBLCU088-09|United States|Kansas|658[0n]|BOLD:ABZ9343
Euxoa auxiliaris|[6830]|USLEP1144-10|United States|Colorado|658[0n]|BOLD:ABZ9343
Euxoa auxiliaris|[6831]|CNGRB518-12|Canada|Saskatchewan|627[0n]|BOLD:ABZ9343
Euxoa auxiliaris|[6832]|BBLWU097-09|United States|Colorado|658[0n]|BOLD:ABZ9343
Euxoa auxiliaris|[6833]|USLEP1145-10|United States|Colorado|658[0n]|BOLD:ABZ9343
Euxoa auxiliaris|[6834]|BBLSW410-09|United States|New Mexico|658[0n]|BOLD:ABZ9343
Euxoa auxiliaris|[6835]|AWCLB104-10|United States|Arizona|658[0n]|BOLD:ABZ9343
Euxoa auxiliaris|[6836]|BBLWU061-09|United States|Colorado|658[0n]|BOLD:ABZ9343
Euxoa auxiliaris|[6837]|BBLSX882-09|United States|Oklahoma|658[0n]|BOLD:ABZ9343
Euxoa auxiliaris|[6838]|BBLSX615-09|United States|Arizona|658[0n]|BOLD:ABZ9343
Euxoa auxiliaris|[6839]|BBLSX604-09|United States|Arizona|658[0n]|BOLD:ABZ9343
Euxoa auxiliaris|[6840]|BBLSW409-09|United States|New Mexico|658[0n]|BOLD:ABZ9343
Euxoa auxiliaris|[6841]|BBLSW408-09|United States|New Mexico|658[0n]|BOLD:ABZ9343
Euxoa auxiliaris|[6842]|LPOKB129-09|United States|Oklahoma|658[0n]|BOLD:ABZ9343
Euxoa auxiliaris|[6843]|BBLWU099-09|United States|Colorado|658[0n]|BOLD:ABZ9343
Euxoa auxiliaris|[6844]|USLEP1142-10|United States|Colorado|658[0n]|BOLD:ABZ9343
Euxoa auxiliaris|[6845]|IAWLB577-11|United States|Arizona|658[0n]|BOLD:ABZ9343
Euxoa auxiliaris|[6846]|CNGRB515-12|Canada|Saskatchewan|640[0n]|BOLD:ABZ9343
Euxoa auxiliaris|[6847]|CNGRB516-12|Canada|Saskatchewan|638[0n]|BOLD:ABZ9343
Euxoa auxiliaris|[6848]|CNWLA688-12|Canada|Alberta|632[0n]|BOLD:ABZ9343
Euxoa auxiliaris|[6849]|BBLCU087-09|United States|Kansas|658[0n]|BOLD:ABZ9343
Euxoa auxiliaris|[6850]|LPOKD623-09|United States|Oklahoma|658[0n]|BOLD:ABZ9343
Euxoa auxiliaris|[6851]|AWCLB099-10|United States|Arizona|658[0n]|BOLD:ABZ9343
Euxoa auxiliaris|[6852]|AWCLB544-11|United States|Arizona|658[0n]|BOLD:ABZ9343
Euxoa auxiliaris|[6853]|LPOKD805-10|United States|Oklahoma|658[0n]|BOLD:ABZ9343
Euxoa auxiliaris|[6854]|CMAZA361-10|United States|Arizona|658[0n]|BOLD:ABZ9343
Euxoa auxiliaris|[6855]|BBLWU143-09|United States|Colorado|658[0n]|BOLD:ABZ9343
Euxoa auxiliaris|[6856]|BBLWU190-09|United States|Colorado|658[0n]|BOLD:ABZ9343
Euxoa auxiliaris|[6857]|IAWLB064-10|United States|California|658[0n]|BOLD:ABZ9343
Euxoa auxiliaris|[6858]|USLEP1141-10|United States|Arizona|658[0n]|BOLD:ABZ9343
Euxoa auxiliaris|[6859]|CGLCA036-10|United States|California|658[0n]|BOLD:ABZ9343
Euxoa auxiliaris|[6860]|USLEP771-10|United States|Colorado|658[0n]|BOLD:ABZ9343
Euxoa auxiliaris|[6861]|BBLWU091-09|United States|Colorado|658[0n]|BOLD:ABZ9343
Euxoa auxiliaris|[6862]|BBLWU141-09|United States|Colorado|658[0n]|BOLD:ABZ9343
Euxoa auxiliaris|[6863]|BBLWU072-09|United States|Colorado|658[0n]|BOLD:ABZ9343
Euxoa auxiliaris|[6864]|BBLWU078-09|United States|Colorado|658[0n]|BOLD:ABZ9343
Euxoa auxiliaris|[6865]|BBLWU068-09|United States|Colorado|658[0n]|BOLD:ABZ9343
Euxoa auxiliaris|[6866]|BBLWU070-09|United States|Colorado|658[0n]|BOLD:ABZ9343
Euxoa auxiliaris|[6867]|LPABC177-09|Canada|Alberta|658[0n]|BOLD:ABZ9343
Euxoa auxiliaris|[6868]|RDNMG983-08|United States|Colorado|658[0n]|BOLD:ABZ9343
Euxoa auxiliaris|[6869]|LPSK012-08|Canada|Saskatchewan|658[0n]|BOLD:ABZ9343
Euxoa auxiliaris|[6870]|NAMUM289-08|United States|Colorado|658[0n]|BOLD:ABZ9343
Euxoa auxiliaris|[6871]|LPSK112-08|Canada|Saskatchewan|658[0n]|BOLD:ABZ9343
Euxoa auxiliaris|[6872]|LSEU788-06|United States|Colorado|658[0n]|BOLD:ABZ9343
Euxoa auxiliaris|[6873]|LOCBC499-06|United States|California|658[0n]|BOLD:ABZ9343
Euxoa auxiliaris|[6874]|BBLWU093-09|United States|Colorado|658[0n]|BOLD:ABZ9343
Euxoa auxiliaris|[6875]|BBLWU075-09|United States|Colorado|658[0n]|BOLD:ABZ9343
Euxoa auxiliaris|[6876]|USLEP1143-10|United States|Colorado|658[2n]|BOLD:ABZ9343
Euxoa auxiliaris|[6877]|CGLCA118-10|United States|California|658[0n]|BOLD:ABZ9343
Euxoa auxiliaris|[6878]|AWCLB542-11|United States|Arizona|658[0n]|BOLD:ABZ9343

Euxoa auxiliaris|[6876]|USLEP1143-10|United States|Colorado|658[2n]|BOLD:ABZ9343
Euxoa auxiliaris|[6877]|CGLCA118-10|United States|California|658[0n]|BOLD:ABZ9343
Euxoa auxiliaris|[6878]|AWCLB542-11|United States|Arizona|658[0n]|BOLD:ABZ9343
Euxoa auxiliaris|[6879]|BBLSX610-09|United States|Arizona|658[0n]|BOLD:ABZ9343
Euxoa auxiliaris|[6880]|LPSK494-08|Canada|Saskatchewan|658[0n]|BOLD:ABZ9343
Euxoa auxiliaris|[6881]|RDNMG982-08|United States|Colorado|658[0n]|BOLD:ABZ9343
Euxoa auxiliaris|[6882]|NAMUM290-08|United States|Colorado|658[0n]|BOLD:ABZ9343
Euxoa auxiliaris|[6883]|BBLSX603-09|United States|Arizona|632[0n]|BOLD:ABZ9343
Euxoa auxiliaris|[6884]|CNGRB519-12|Canada|Saskatchewan|630[0n]|BOLD:ABZ9343
Euxoa auxiliaris|[6885]|CNWLF2016-12|Canada|Alberta|629[0n]|BOLD:ABZ9343
Euxoa auxiliaris|[6886]|SSWLA3864-13|Canada|Alberta|604[0n]|BOLD:ABZ9343
Euxoa auxiliaris|[6887]|LPABB004-08|Canada|Alberta|658[0n]|BOLD:ABZ9343
Euxoa auxiliaris|[6888]|BBLWU189-09|United States|Colorado|658[0n]|BOLD:ABZ9343
Euxoa auxiliaris|[6889]|BBLSY561-09|United States|New Mexico|658[0n]|BOLD:ABZ9343
Euxoa auxiliaris|[6890]|BBLWU098-09|United States|Colorado|658[0n]|BOLD:ABZ9343
Euxoa auxiliaris|[6891]|BBLWU066-09|United States|Colorado|637[0n]|BOLD:ABZ9343
Euxoa auxiliaris|[6892]|BBLWU074-09|United States|Colorado|658[0n]|BOLD:ABZ9343
Euxoa auxiliaris|[6893]|BBLWU001-09|United States|Colorado|620[0n]|BOLD:ABZ9343
Euxoa auxiliaris|[6894]|BBLWU067-09|United States|Colorado|658[0n]|BOLD:ABZ9343
Euxoa auxiliaris|[6895]|BBLWU073-09|United States|Colorado|658[0n]|BOLD:ABZ9343
Euxoa auxiliaris|[6896]|BBLWU071-09|United States|Colorado|658[0n]|BOLD:ABZ9343
Euxoa auxiliaris|[6897]|BBLWU069-09|United States|Colorado|658[0n]|BOLD:ABZ9343
Euxoa auxiliaris|[6898]|BBLWU059-09|United States|Colorado|658[0n]|BOLD:ABZ9343
Euxoa auxiliaris|[6899]|BBLSY669-09|United States|Arizona|658[0n]|BOLD:ABZ9343
Euxoa auxiliaris|[6900]|BBLSX744-09|United States|Arizona|658[0n]|BOLD:ABZ9343
Euxoa auxiliaris|[6901]|BBLWU076-09|United States|Colorado|633[0n]|BOLD:ABZ9343
Euxoa auxiliaris|[6902]|BBLWU096-09|United States|Colorado|658[0n]|BOLD:ABZ9343
Euxoa auxiliaris|[6903]|BBLCU089-09|United States|Kansas|658[0n]|BOLD:ABZ9343
Euxoa auxiliaris|[6904]|CMAZA342-10|United States|Arizona|658[0n]|BOLD:ABZ9343
Euxoa auxiliaris|[6905]|AWCLB470-10|United States|Arizona|658[0n]|BOLD:ABZ9343
Euxoa auxiliaris|[6906]|USLEP291-10|United States|Arizona|658[0n]|BOLD:ABZ9343
Euxoa auxiliaris|[6907]|RDNMK634-11|United States|New Mexico|658[0n]|BOLD:ABZ9343
Euxoa auxiliaris|[6908]|CNWLK013-13|Canada|Alberta|588[0n]|BOLD:ABZ9343
Euxoa auxiliaris|[6909]|LSEU787-06|United States|Colorado|658[0n]|BOLD:ABZ9343
Euxoa auxiliaris|[6910]|CNWLN1078-13|Canada|Alberta|582[0n]|BOLD:ABZ9343
Euxoa terrealis|[6911]|LNAUS2903-13|United States|New Mexico|658[0n]|BOLD:ACI3834
Euxoa terrealis|[6912]|LNAUS2904-13|United States|New Mexico|658[0n]|BOLD:ACI3834
Euxoa terrealis|[6913]|LNAUS2905-13|United States|New Mexico|658[0n]|BOLD:ACI3834
Euxoa adumbrata|[6914]|LEFIL023-10|Russia|Bashkortostan|658[6n]|BOLD:ACF0816
Euxoa adumbrata|[6915]|LEFIL026-10|Russia|Chelyabinsk|658[0n]|BOLD:ACF0816
Euxoa adumbrata|[6916]|LEFIL027-10|Finland||658[0n]|BOLD:ACF0816
Euxoa adumbrata|[6917]|LEFIL028-10|Finland||658[0n]|BOLD:ACF0816
Euxoa adumbrata|[6918]|GWOSI803-10|Sweden||658[0n]|BOLD:ACF0816
Euxoa adumbrata|[6919]|LEFIJ1016-11|Finland||658[0n]|BOLD:ACF0816
Euxoa adumbrata|[6920]|LEFIJ1017-11|Finland||658[0n]|BOLD:ACF0816
Euxoa adumbrata|[6921]|LEFIJ1184-11|Finland||658[0n]|BOLD:ACF0816
Euxoa adumbrata|[6922]|LBCH7004-10|Canada|British Columbia|658[0n]|BOLD:ACF0816
Euxoa adumbrata|[6923]|LMDH159-11|United States|Minnesota|658[0n]|BOLD:ACF0816
Euxoa adumbrata|[6924]|LEFIJ173-10|Greenland||658[0n]|BOLD:ACF0816
Euxoa adumbrata|[6925]|BBLPB750-10|Canada|Alberta|658[0n]|BOLD:ACF0816
Euxoa adumbrata|[6926]|LBCH7598-10|Canada|British Columbia|658[0n]|BOLD:ACF0816
Euxoa adumbrata|[6927]|LBCH6829-10|Canada|British Columbia|658[0n]|BOLD:ACF0816
Euxoa adumbrata|[6928]|LPABB357-08|Canada|Alberta|658[0n]|BOLD:ACF0816
Euxoa adumbrata|[6929]|LCHQ589-08|Canada|Manitoba|655[0n]|BOLD:ACF0816
Euxoa adumbrata|[6930]|LCHQ141-07|Canada|Manitoba|658[0n]|BOLD:ACF0816
Euxoa adumbrata|[6931]|LCHQ094-07|Canada|Manitoba|658[0n]|BOLD:ACF0816
Euxoa adumbrata|[6932]|LCHP946-07|Canada|Manitoba|658[0n]|BOLD:ACF0816
Euxoa adumbrata|[6933]|LCHP827-07|Canada|Manitoba|658[0n]|BOLD:ACF0816
Euxoa adumbrata|[6934]|LCHP751-07|Canada|Manitoba|655[0n]|BOLD:ACF0816
Euxoa adumbrata|[6935]|LCH262-04|Canada|Manitoba|658[0n]|BOLD:ACF0816
Euxoa adumbrata|[6936]|GMGLQ195-13|Greenland||594[0n]|BOLD:ACF0816
Euxoa adumbrata|[6937]|GMGLQ197-13|Greenland||577[0n]|BOLD:ACF0816
Euxoa adumbrata|[6938]|GMGLQ198-13|Greenland||607[0n]|BOLD:ACF0816
Euxoa flavidens|[6939]|RDNMD873-07|United States|Utah|655[0n]|BOLD:ACE9833
Euxoa flavidens|[6940]|RDNMD891-07|United States|Arizona|655[0n]|BOLD:ACE9833
Euxoa flavidens|[6941]|RDNMJ718-11|United States|Arizona|658[0n]|BOLD:ACE9833
Euxoa flavidens|[6942]|RDNMJ719-11|United States|Arizona|658[0n]|BOLD:ACE9833
Euxoa tristicula|[6943]|RDNM357-05|United States|Oregon|516[0n]|BOLD:ABZ9161
Euxoa tristicula|[6944]|RDNMC086-05|United States|Nevada|658[0n]|BOLD:ABZ9161
Euxoa tristicula|[6945]|RDNM359-05|Canada|Alberta|658[0n]|BOLD:ABZ9161
Euxoa tristicula|[6946]|RDNM358-05|Canada|Alberta|658[0n]|BOLD:ABZ9161
Euxoa tristicula|[6947]|RDMAB043-05|Canada|Alberta|647[0n]|BOLD:ABZ9161
Euxoa tristicula|[6948]|RDMAB044-05|Canada|Alberta|634[0n]|BOLD:ABZ9161
Euxoa tristicula|[6949]|LPSK122-08|Canada|Saskatchewan|636[0n]|BOLD:ABZ9161
Euxoa tristicula|[6950]|LPSK130-08|Canada|Saskatchewan|658[0n]|BOLD:ABZ9161
Euxoa tristicula|[6951]|LPSK138-08|Canada|Saskatchewan|658[0n]|BOLD:ABZ9161
Euxoa tristicula|[6952]|LPSK516-08|Canada|Saskatchewan|658[0n]|BOLD:ABZ9161
Euxoa tristicula|[6953]|LALPA871-11|Canada|British Columbia|658[0n]|BOLD:ABZ9161
Euxoa occidentalis|[6954]|LBCH7957-10|Canada|British Columbia|658[0n]|BOLD:ACF3236
Euxoa comosa|[6955]|RDNM305-05|United States|Oregon|658[0n]|BOLD:ACF3236
Euxoa occidentalis|[6956]|LBCH6369-10|Canada|British Columbia|658[0n]|BOLD:ACF3236
Euxoa comosa|[6957]|RDNMB848-05|United States|Oregon|658[0n]|BOLD:ACF3236
Euxoa comosa|[6958]|RDNMB781-05|United States|Nevada|658[0n]|BOLD:ACF3236
Euxoa occidentalis|[6959]|LBCH6446-10|Canada|British Columbia|658[0n]|BOLD:ACF3236
Euxoa comosa|[6960]|RDNM304-05|United States|Oregon|658[0n]|BOLD:ACF3236
Euxoa occidentalis|[6961]|LBCG477-08|Canada|British Columbia|658[0n]|BOLD:ACF3236
Euxoa occidentalis|[6962]|LOWCE775-06|Canada|British Columbia|658[0n]|BOLD:ACF3236
Euxoa comosa|[6963]|RDNMB847-05|United States|Oregon|658[0n]|BOLD:ACF3236
Euxoa occidentalis|[6964]|RDMAB1018-09|United States|Oregon|651[0n]|BOLD:ACF3236
Euxoa comosa|[6965]|RDNMB798-05|United States|Washington|573[1n]|BOLD:ACF3236
Euxoa occidentalis|[6966]|RDMAB1019-09|United States|Oregon|635[0n]|BOLD:ACF3236
Euxoa occidentalis|[6967]|LBCG2887-09|Canada|British Columbia|658[0n]|BOLD:ACF3236
Euxoa occidentalis|[6968]|LBCG2889-09|Canada|British Columbia|658[0n]|BOLD:ACF3236
Euxoa occidentalis|[6969]|LBCH6374-10|Canada|British Columbia|658[0n]|BOLD:ACF3236
Euxoa occidentalis|[6970]|LBCH6460-10|Canada|British Columbia|658[0n]|BOLD:ACF3236
Euxoa occidentalis|[6971]|LBCH6539-10|Canada|British Columbia|658[0n]|BOLD:ACF3236
Euxoa occidentalis|[6972]|LBCH6828-10|Canada|British Columbia|658[0n]|BOLD:ACF3236
Euxoa occidentalis|[6973]|LBCH7539-10|Canada|British Columbia|658[0n]|BOLD:ACF3236
Euxoa occidentalis|[6974]|LBCH7545-10|Canada|British Columbia|658[0n]|BOLD:ACF3236
Euxoa occidentalis|[6975]|LBCH7789-10|Canada|British Columbia|658[0n]|BOLD:ACF3236
Euxoa occidentalis|[6976]|JMMMB424-11|United States|California|658[0n]|BOLD:ACF3236
Euxoa comosa|[6977]|RDNM366-05|United States|Wyoming|658[0n]|BOLD:ACF2026
[illegible]

Euxoa occidentalis|[6975]|LBCH7789-10|Canada|British Columbia|658[0n]|BOLD:ACF3236
Euxoa occidentalis|[6976]|JMMMB424-11|United States|California|658[0n]|BOLD:ACF3236
Euxoa comosa|[6977]|RDNM366-05|United States|Wyoming|658[0n]|BOLD:ACF2026
Euxoa comosa|[6978]|RDNM367-05|United States|Wyoming|658[0n]|BOLD:ACF2026
Euxoa comosa|[6979]|RDNMB785-05|United States|Oregon|658[0n]|BOLD:ACF2026
Euxoa comosa|[6980]|RDNMB825-05|Canada|Alberta|658[0n]|BOLD:ACF2026
Euxoa comosa|[6981]|RDNMB823-05|Canada|Alberta|658[0n]|BOLD:ACF2026
Euxoa comosa|[6982]|RDNM302-05|Canada|Alberta|658[0n]|BOLD:ACF2026
Euxoa comosa|[6983]|RDNMB840-05|Canada|Alberta|581[1n]|BOLD:ACF2026
Euxoa comosa|[6984]|RDMAB559-06|Canada|Alberta|658[0n]|BOLD:ACF2026
Euxoa comosa|[6985]|RDNME277-07|United States|Colorado|609[0n]|BOLD:ACF2026
Euxoa comosa|[6986]|RDNME279-07|United States|Colorado|609[0n]|BOLD:ACF2026
Euxoa comosa|[6987]|RDNMC071-05|United States|Colorado|658[0n]|BOLD:ACF2026
Euxoa comosa|[6988]|RDNMC070-05|United States|Colorado|658[0n]|BOLD:ACF2026
Euxoa comosa|[6989]|RDNMB838-05|Canada|Alberta|658[0n]|BOLD:ACF2026
Euxoa comosa|[6990]|RDNMB837-05|Canada|Alberta|658[0n]|BOLD:ACF2026
Euxoa comosa|[6991]|RDNMB830-05|Canada|Alberta|658[0n]|BOLD:ACF2026
Euxoa comosa|[6992]|RDNMB828-05|Canada|Alberta|658[0n]|BOLD:ACF2026
Euxoa comosa|[6993]|RDNMB827-05|Canada|Alberta|658[0n]|BOLD:ACF2026
Euxoa comosa|[6994]|RDNMB826-05|Canada|Alberta|658[0n]|BOLD:ACF2026
Euxoa comosa|[6995]|RDNMB802-05|Canada|Alberta|658[0n]|BOLD:ACF2026
Euxoa comosa|[6996]|RDNME278-07|United States|Colorado|643[0n]|BOLD:ACF2026
Euxoa comosa|[6997]|RDNME280-07|United States|Colorado|605[0n]|BOLD:ACF2026
Euxoa comosa|[6998]|RDNM303-05|Canada|Alberta|658[0n]|BOLD:ACF2027
Euxoa comosa|[6999]|RDNMB824-05|Canada|Alberta|658[0n]|BOLD:ACF2027
Euxoa comosa|[7000]|JMMMB538-13|United States|California|543[0n]|
Euxoa comosa|[7001]|RDNMB822-05|United States|Washington|542[0n]|BOLD:ACE5080
Euxoa comosa|[7002]|RDNM319-05|United States|Washington|658[0n]|BOLD:ACE5080
Euxoa comosa|[7003]|RDNMC068-05|United States|Washington|600[0n]|BOLD:ACE5080
Euxoa comosa|[7004]|RDNMC072-05|United States|Colorado|658[0n]|BOLD:ACE5080
Euxoa comosa|[7005]|RDNMB784-05|Canada|British Columbia|616[0n]|BOLD:ACE5080
Euxoa comosa|[7006]|RDNM325-05|Canada|Ontario|658[0n]|BOLD:ACE5080
Euxoa comosa|[7007]|RDNM315-05|United States|Nevada|658[0n]|BOLD:ACE5080
Euxoa comosa|[7008]|RDNMB786-05|United States|California|579[1n]|BOLD:ACE5080
Euxoa comosa|[7009]|RDNMB835-05|United States|Nevada|520[0n]|BOLD:ACE5080
Euxoa comosa|[7010]|RDNMB834-05|United States|Nevada|658[0n]|BOLD:ACE5080
Euxoa comosa|[7011]|RDNMB800-05|Canada|British Columbia|658[0n]|BOLD:ACE5080
Euxoa comosa|[7012]|RDNMC060-05|Canada|British Columbia|546[1n]|BOLD:ACE5080
Euxoa comosa|[7013]|RDNM310-05|United States|Nevada|658[0n]|BOLD:ACE5080
Euxoa comosa|[7014]|RDNM306-05|United States|Oregon|658[0n]|BOLD:ACE5080
Euxoa comosa|[7015]|RDNMB791-05|Canada|British Columbia|563[1n]|BOLD:ACE5080
Euxoa comosa|[7016]|RDNM317-05|United States|Nevada|658[0n]|BOLD:ACE5080
Euxoa comosa|[7017]|RDNMB794-05|United States|Washington|513[0n]|BOLD:ACE5080
Euxoa comosa|[7018]|RDNMB832-05|United States|Nevada|658[0n]|BOLD:ACE5080
Euxoa comosa|[7019]|RDNMB833-05|United States|Nevada|658[0n]|BOLD:ACE5080
Euxoa comosa|[7020]|RDNMB783-05|Canada|British Columbia|658[0n]|BOLD:ACE5080
Euxoa comosa|[7021]|RDNMB797-05|Canada|British Columbia|658[0n]|BOLD:ACE5080
Euxoa comosa|[7022]|RDNM308-05|United States|Nevada|658[0n]|BOLD:ACE5080
Euxoa comosa|[7023]|RDNMB792-05|Canada|British Columbia|658[0n]|BOLD:ACE5080
Euxoa comosa|[7024]|RDNM316-05|United States|Nevada|658[0n]|BOLD:ACE5080
Euxoa comosa|[7025]|RDNM307-05|United States|Nevada|658[0n]|BOLD:ACE5080
Euxoa comosa|[7026]|RDNM314-05|United States|Nevada|658[0n]|BOLD:ACE5080
Euxoa comosa|[7027]|RDNMC046-05|United States|Nevada|657[0n]|BOLD:ACE5080
Euxoa comosa|[7028]|RDNMC053-05|Canada|British Columbia|658[0n]|BOLD:ACE5080
Euxoa comosa|[7029]|RDNMC058-05|United States|Washington|658[0n]|BOLD:ACE5080
Euxoa comosa|[7030]|IAWLB016-10|United States|California|658[0n]|BOLD:ACE5080
Euxoa comosa|[7031]|LPABB867-09|Canada|Alberta|636[0n]|BOLD:ACE5080
Euxoa comosa|[7032]|RDNMC049-05|Canada|British Columbia|658[0n]|BOLD:ACE5080
Euxoa comosa|[7033]|RDMAB676-06|Canada|Alberta|657[0n]|BOLD:ACE5080
Euxoa comosa|[7034]|RDNMB818-05|Canada|Alberta|658[0n]|BOLD:ACE5080
Euxoa comosa|[7035]|RDNMB817-05|Canada|Alberta|658[0n]|BOLD:ACE5080
Euxoa comosa|[7036]|RDNMB816-05|Canada|British Columbia|658[0n]|BOLD:ACE5080
Euxoa comosa|[7037]|RDNMB814-05|Canada|Alberta|658[0n]|BOLD:ACE5080
Euxoa comosa|[7038]|RDNMB813-05|Canada|Alberta|658[0n]|BOLD:ACE5080
Euxoa comosa|[7039]|RDNM321-05|Canada|British Columbia|658[0n]|BOLD:ACE5080
Euxoa comosa|[7040]|RDNMB788-05|United States|Nevada|658[0n]|BOLD:ACE5080
Euxoa comosa|[7041]|RDNMC050-05|United States|Nevada|658[0n]|BOLD:ACE5080
Euxoa comosa|[7042]|LPABB611-08|Canada|Alberta|658[0n]|BOLD:ACE5080
Euxoa comosa|[7043]|RDNMC041-05|Canada|British Columbia|551[1n]|BOLD:ACE5080
Euxoa comosa|[7044]|RDNMC043-05|Canada|British Columbia|594[0n]|BOLD:ACE5080
Euxoa comosa|[7045]|RDNMB795-05|Canada|British Columbia|658[0n]|BOLD:ACE5080
Euxoa comosa|[7046]|RDNMC044-05|Canada|British Columbia|520[0n]|BOLD:ACE5080
Euxoa comosa|[7047]|RDNMB815-05|Canada|Alberta|614[0n]|BOLD:ACE5080
Euxoa comosa|[7048]|RDNM327-05|Canada|New Brunswick|567[0n]|BOLD:ACE5080
Euxoa comosa|[7049]|RDNMC212-05|Canada|New Brunswick|599[2n]|BOLD:ACE5080
Euxoa comosa|[7050]|RDNMC076-05|Canada|New Brunswick|658[0n]|BOLD:ACE5080
Euxoa comosa|[7051]|RDLQF177-06|Canada|Quebec|658[0n]|BOLD:ACE5080
Euxoa comosa|[7052]|BBLPC199-09|Canada|Nova Scotia|658[0n]|BOLD:ACE5080
Euxoa comosa|[7053]|RDNMC056-05|United States|Washington|587[0n]|BOLD:ACE5080
Euxoa comosa|[7054]|LALPA632-10|Canada|British Columbia|658[0n]|BOLD:ACE5080
Euxoa comosa|[7055]|RDNMC085-05|Canada|Alberta|658[0n]|BOLD:ACE5080
Euxoa comosa|[7056]|RDNMC084-05|Canada|Alberta|658[0n]|BOLD:ACE5080
Euxoa comosa|[7057]|LBCH6537-10|Canada|British Columbia|658[0n]|BOLD:ACE5080
Euxoa comosa|[7058]|LBCH6538-10|Canada|British Columbia|658[0n]|BOLD:ACE5080
Euxoa comosa|[7059]|BBLPB756-10|Canada|Alberta|658[0n]|BOLD:ACE5080
Euxoa comosa|[7060]|RDNMB780-05|Canada|British Columbia|589[0n]|BOLD:ACE5080
Euxoa comosa|[7061]|RDNMB789-05|United States|Washington|499[0n]|
Euxoa comosa|[7062]|RDNMB793-05|Canada|British Columbia|546[1n]|BOLD:ACE5080
Euxoa comosa|[7063]|RDNMC059-05|Canada|British Columbia|507[0n]|BOLD:ACE5080
Euxoa comosa|[7064]|RDNMC052-05|Canada|British Columbia|658[1n]|BOLD:ACE5080
Euxoa comosa|[7065]|RDNMB790-05|Canada|British Columbia|658[0n]|BOLD:ACE5080
Euxoa comosa|[7066]|RDNMC057-05|United States|Oregon|568[0n]|BOLD:ACE5080
Euxoa comosa|[7067]|RDNMG660-08|Canada|British Columbia|649[0n]|BOLD:ACE5080
Euxoa comosa|[7068]|RDNM313-05|United States|California|658[0n]|BOLD:ACE5080
Euxoa comosa|[7069]|RDNM312-05|United States|California|658[0n]|BOLD:ACE5080
Euxoa comosa|[7070]|RDNM309-05|United States|Washington|550[0n]|BOLD:ACE5080
Euxoa comosa|[7071]|RDNM320-05|United States|Washington|579[0n]|BOLD:ACE5080
Euxoa comosa|[7072]|RDNMB779-05|United States|Washington|594[0n]|BOLD:ACE5080
Euxoa comosa|[7073]|RDNMC045-05|United States|Washington|657[0n]|BOLD:ACE5080
Euxoa comosa|[7074]|RDNMC047-05|United States|Washington|658[0n]|BOLD:ACE5080
Euxoa comosa|[7075]|RDNMB799-05|United States|Washington|586[0n]|BOLD:ACE5080
Euxoa comosa|[7076]|RDNMB796-05|United States|Oregon|534[0n]|BOLD:ACE5080
Euxoa comosa|[7077]|RDNMC048-05|United States|Washington|658[0n]|BOLD:ACE5080

Euxoa comosa|[7075]|RDNMB799-05|United States|Washington|586[0n]|BOLD:ACE5080
Euxoa comosa|[7076]|RDNMB796-05|United States|Oregon|534[0n]|BOLD:ACE5080
Euxoa comosa|[7077]|RDNMC048-05|United States|Washington|658[0n]|BOLD:ACE5080
Euxoa comosa|[7078]|LALPA997-11|Canada|British Columbia|658[0n]|BOLD:ACE5080
Euxoa comosa|[7079]|RDNMC055-05|United States|Washington|658[3n]|BOLD:ACE5080
Euxoa comosa|[7080]|RDNM301-05|Canada|Alberta|658[0n]|BOLD:ACE5080
Euxoa comosa|[7081]|RDNMB801-05|Canada|Alberta|658[0n]|BOLD:ACE5080
Euxoa comosa|[7082]|RDNMB810-05|Canada|Alberta|658[0n]|BOLD:ACE5080
Euxoa comosa|[7083]|RDNMB850-05|Canada|Alberta|658[0n]|BOLD:ACE5080
Euxoa comosa|[7084]|RDNMB861-05|Canada|Alberta|658[0n]|BOLD:ACE5080
Euxoa comosa|[7085]|RDNMC051-05|United States|California|658[0n]|BOLD:ACE5080
Euxoa comosa|[7086]|RDNMB811-05|United States|Nevada|658[0n]|BOLD:ACE5080
Euxoa comosa|[7087]|RDNMB807-05|Canada|Alberta|658[0n]|BOLD:ACE5080
Euxoa comosa|[7088]|RDNM318-05|United States|Nevada|658[0n]|BOLD:ACE5080
Euxoa comosa|[7089]|RDNMC061-05|Canada|British Columbia|585[2n]|BOLD:ACE5080
Euxoa comosa|[7090]|LBCH7956-10|Canada|British Columbia|658[0n]|BOLD:ACE5080
Euxoa comosa|[7091]|BBLPB342-10|Canada|Alberta|658[0n]|BOLD:ACE5080
Euxoa comosa|[7092]|CNWLO770-13|Canada|Alberta|602[0n]|BOLD:ACE5080
Euxoa lucida|[7093]|RDNMB864-05|United States|Wyoming|658[0n]|BOLD:ACE5080
Euxoa lucida|[7094]|RDNMB865-05|United States|Wyoming|658[0n]|BOLD:ACE5080
Euxoa lucida|[7095]|RDNMB866-05|United States|Wyoming|658[0n]|BOLD:ACE5080
Euxoa comosa|[7096]|RDNM311-05|United States|Nevada|658[0n]|BOLD:ACE5080
Euxoa comosa|[7097]|RDNMB812-05|United States|Nevada|658[0n]|BOLD:ACE5080
Euxoa comosa|[7098]|RDNMC042-05|United States|Nevada|658[0n]|BOLD:ACE5080
Euxoa comosa|[7099]|RDNMB782-05|United States|California|658[0n]|BOLD:ACE5080
Euxoa comosa|[7100]|RDNMB836-05|United States|Nevada|658[0n]|BOLD:ACE5080
Euxoa comosa|[7101]|RDNMC069-05|United States|Colorado|658[0n]|BOLD:ACE5080
Euxoa comosa|[7102]|RDNMB839-05|Canada|Alberta|658[0n]|BOLD:ACE5080
Euxoa comosa|[7103]|RDNM324-05|Canada|Alberta|658[0n]|BOLD:ACE5080
Euxoa comosa|[7104]|RDMAB666-06|Canada|Alberta|573[1n]|BOLD:ACE5080
Euxoa comosa|[7105]|RDNM322-05|Canada|Alberta|658[0n]|BOLD:ACE5080
Euxoa comosa|[7106]|RDMAB317-05|Canada|Alberta|594[1n]|BOLD:ACE5080
Euxoa comosa|[7107]|BAWG216-12|Canada|Alberta|658[0n]|BOLD:ACE5080
Euxoa comosa|[7108]|RDNM323-05|Canada|Alberta|658[0n]|BOLD:ACE5080
Euxoa comosa|[7109]|RDNMB803-05|Canada|Alberta|658[0n]|BOLD:ACE5080
Euxoa comosa|[7110]|RDNMB804-05|Canada|Alberta|658[0n]|BOLD:ACE5080
Euxoa comosa|[7111]|RDNMB805-05|Canada|Alberta|658[0n]|BOLD:ACE5080
Euxoa comosa|[7112]|RDNMB806-05|Canada|Alberta|658[0n]|BOLD:ACE5080
Euxoa comosa|[7113]|RDNMB808-05|Canada|Alberta|658[0n]|BOLD:ACE5080
Euxoa comosa|[7114]|RDNMB809-05|Canada|Alberta|658[0n]|BOLD:ACE5080
Euxoa comosa|[7115]|RDNMB829-05|Canada|Alberta|658[0n]|BOLD:ACE5080
Euxoa comosa|[7116]|RDNMB842-05|Canada|Alberta|658[0n]|BOLD:ACE5080
Euxoa comosa|[7117]|RDNMB845-05|Canada|Alberta|658[0n]|BOLD:ACE5080
Euxoa comosa|[7118]|RDMAB315-05|Canada|Alberta|658[0n]|BOLD:ACE5080
Euxoa comosa|[7119]|RDMAB316-05|Canada|Alberta|658[0n]|BOLD:ACE5080
Euxoa comosa|[7120]|BAWG230-12|Canada|Alberta|658[0n]|BOLD:ACE5080
Euxoa lineifrons|[7121]|RDNM368-05|United States|Wyoming|658[0n]|BOLD:ACE5080
Euxoa lineifrons|[7122]|RDNMB856-05|United States|Wyoming|658[0n]|BOLD:ACE5080
Euxoa fumalis|[7123]|RDNMB821-05|Canada|Ontario|658[0n]|BOLD:ACE5080
Euxoa fumalis|[7124]|RDNM326-05|Canada|New Brunswick|520[3n]|BOLD:ACE5080
Euxoa fumalis|[7125]|RDNM152-05|Canada|Ontario|658[0n]|BOLD:ACE5080
Euxoa fumalis|[7126]|RDNMB819-05|Canada|Ontario|658[0n]|BOLD:ACE5080
Euxoa fumalis|[7127]|RDNMB820-05|Canada|Ontario|658[0n]|BOLD:ACE5080
Euxoa fumalis|[7128]|TMNBB367-06|Canada|New Brunswick|658[0n]|BOLD:ACE5080
Euxoa comosa|[7129]|RDNMB846-05|United States|Oregon|658[0n]|BOLD:ACE5080
Euxoa comosa|[7130]|RDNMC073-05|United States|Colorado|658[0n]|BOLD:ACE5080
Euxoa comosa|[7131]|RDNMC074-05|United States|Colorado|658[0n]|BOLD:ACE5080
Euxoa comosa|[7132]|RDNMB787-05|United States|Nevada|658[0n]|BOLD:ACE5080
Euxoa comosa|[7133]|RDNMC054-05|United States|Nevada|658[0n]|BOLD:ACE5080
Euxoa guadalupensis|[7134]|CNCLB2038-14|United States|Texas|658[0n]|BOLD:ACE5080
Euxoa emma|[7135]|RDNM777-05|United States|Washington|658[0n]|BOLD:ACE8685
Euxoa annulipes|[7136]|RDNM780-05|United States|Oregon|658[0n]|BOLD:ACE8685
Euxoa annulipes|[7137]|RDNM779-05|United States|Oregon|658[0n]|BOLD:ACE8685
Euxoa annulipes|[7138]|RDNMC325-05|United States|Oregon|553[0n]|BOLD:ACE8685
Euxoa oncocnemoides|[7139]|TML191-14|United States||658[0n]|BOLD:ACE8685
Euxoa nomas|[7140]|RDMAB976-09|Canada|Alberta|658[0n]|BOLD:ACF3241
Euxoa nomas|[7141]|RDNMF035-08|Canada|Alberta|658[0n]|BOLD:ACF3241
Euxoa nomas|[7142]|RDMAB977-09|Canada|Alberta|632[0n]|BOLD:ACF3241
Euxoa nomas|[7143]|RDMAB978-09|Canada|Alberta|590[0n]|BOLD:ACF3241
Euxoa wilsoni|[7144]|NAMUM234-08|United States|California|658[0n]|BOLD:ACF3241
Euxoa wilsoni|[7145]|RWWC686-11|United States|Washington|658[0n]|BOLD:ACF3241
Euxoa wilsoni|[7146]|RWWC692-11|United States|Washington|658[0n]|BOLD:ACF3241
Euxoa wilsoni|[7147]|RWWB058-09|United States|Washington|658[0n]|BOLD:ACF3241
Euxoa wilsoni|[7148]|RWWB166-09|United States|Washington|658[0n]|BOLD:ACF3241
Euxoa wilsoni|[7149]|RWWB250-09|United States|Washington|658[0n]|BOLD:ACF3241
Euxoa wilsoni|[7150]|RWWB261-09|United States|Washington|658[0n]|BOLD:ACF3241
Euxoa wilsoni|[7151]|RWWA987-09|United States|Washington|658[0n]|BOLD:ACF3241
Euxoa wilsoni|[7152]|RWWB048-09|United States|Washington|658[0n]|BOLD:ACF3241
Euxoa wilsoni|[7153]|RWWB210-09|United States|Washington|658[0n]|BOLD:ACF3241
Euxoa wilsoni|[7154]|RWWB227-09|United States|Washington|658[0n]|BOLD:ACF3241
Euxoa wilsoni|[7155]|RWWA951-09|United States|Washington|658[0n]|BOLD:ACF3241
Euxoa wilsoni|[7156]|RWWA971-09|United States|Washington|658[0n]|BOLD:ACF3241
Euxoa wilsoni|[7157]|RWWA941-09|United States|Washington|658[0n]|BOLD:ACF3241
Euxoa wilsoni|[7158]|RWWA891-09|United States|Washington|658[0n]|BOLD:ACF3241
Euxoa wilsoni|[7159]|RWWA877-09|United States|Washington|658[0n]|BOLD:ACF3241
Euxoa wilsoni|[7160]|RWWA837-09|United States|Washington|658[0n]|BOLD:ACF3241
Euxoa wilsoni|[7161]|RWWA824-09|United States|Washington|658[0n]|BOLD:ACF3241
Euxoa wilsoni|[7162]|RWWA801-09|United States|Washington|658[0n]|BOLD:ACF3241
Euxoa wilsoni|[7163]|RWWA745-09|United States|Washington|658[0n]|BOLD:ACF3241
Euxoa wilsoni|[7164]|RDNMG648-08|Canada|British Columbia|658[0n]|BOLD:ACF3241
Euxoa wilsoni|[7165]|RDNMG647-08|United States|Washington|658[0n]|BOLD:ACF3241
Euxoa wilsoni|[7166]|RDNMG646-08|United States|Washington|658[0n]|BOLD:ACF3241
Euxoa wilsoni|[7167]|RDNMF317-08|Canada|British Columbia|658[0n]|BOLD:ACF3241
Euxoa wilsoni|[7168]|RDNMF316-08|Canada|British Columbia|658[0n]|BOLD:ACF3241
Euxoa wilsoni|[7169]|RWWB255-09|United States|Washington|637[0n]|BOLD:ACF3241
Euxoa wilsoni|[7170]|RWWC746-11|United States|Washington|658[0n]|BOLD:ACF3241
Euxoa macrodentata|[7171]|RDNMF702-08|Canada|Yukon Territory|658[0n]|BOLD:ACF3241
Euxoa perpolita|[7172]|RDLQB497-05|Canada|Quebec|658[0n]|BOLD:ACF3241
Euxoa perpolita|[7173]|RDLQB498-05|Canada|Quebec|658[0n]|BOLD:ACF3241
Euxoa perpolita|[7174]|RDLQF176-06|Canada|Quebec|658[0n]|BOLD:ACF3241
Euxoa perpolita|[7175]|RDNMG1016-08|Canada|Ontario|658[0n]|BOLD:ACF3241
Euxoa perpolita|[7176]|RDNMG1018-08|Canada|Ontario|658[0n]|BOLD:ACF3241
Euxoa perpolita|[7177]|LBCH7680-10|Canada|British Columbia|658[0n]|BOLD:ACF3241

Euxoa perpolita|[7175]|RDNMG1016-08|Canada|Ontario|658[0n]|BOLD:ACF3241
Euxoa perpolita|[7176]|RDNMG1018-08|Canada|Ontario|658[0n]|BOLD:ACF3241
Euxoa perpolita|[7177]|LBCH7680-10|Canada|British Columbia|658[0n]|BOLD:ACF3241
Euxoa perpolita|[7178]|LBCH7681-10|Canada|British Columbia|658[0n]|BOLD:ACF3241
Euxoa ridingsiana|[7179]|BBLPB726-10|Canada|Alberta|658[0n]|BOLD:ACF3241
Euxoa ridingsiana|[7180]|BBLPB731-10|Canada|Alberta|658[0n]|BOLD:ACF3241
Euxoa ridingsiana|[7181]|BBLPB733-10|Canada|Alberta|658[0n]|BOLD:ACF3241
Euxoa ridingsiana|[7182]|LBCH7785-10|Canada|British Columbia|658[0n]|BOLD:ACF3241
Euxoa ridingsiana|[7183]|LBCH6501-10|Canada|British Columbia|658[0n]|BOLD:ACF3241
Euxoa ridingsiana|[7184]|LBCG1103-09|Canada|British Columbia|658[0n]|BOLD:ACF3241
Euxoa ridingsiana|[7185]|RDNMF775-08|Canada|Alberta|658[0n]|BOLD:ACF3241
Euxoa ridingsiana|[7186]|RDNMF774-08|Canada|Alberta|658[0n]|BOLD:ACF3241
Euxoa ridingsiana|[7187]|RDNMF773-08|Canada|Alberta|658[0n]|BOLD:ACF3241
Euxoa ridingsiana|[7188]|RDNMF772-08|Canada|Alberta|658[0n]|BOLD:ACF3241
Euxoa ridingsiana|[7189]|RDNMF771-08|Canada|Saskatchewan|658[0n]|BOLD:ACF3241
Euxoa ridingsiana|[7190]|RDNMF769-08|Canada|Alberta|658[0n]|BOLD:ACF3241
Euxoa ridingsiana|[7191]|RDNMF768-08|Canada|Alberta|658[0n]|BOLD:ACF3241
Euxoa ridingsiana|[7192]|RDNMG560-08|Canada|British Columbia|658[0n]|BOLD:ACF3241
Euxoa ridingsiana|[7193]|RDNMG501-08|Canada|Alberta|658[0n]|BOLD:ACF3241
Euxoa ridingsiana|[7194]|RDNMG500-08|Canada|Alberta|658[0n]|BOLD:ACF3241
Euxoa ridingsiana|[7195]|RDNMG499-08|Canada|British Columbia|658[0n]|BOLD:ACF3241
Euxoa ridingsiana|[7196]|LOWCE722-06|Canada|British Columbia|658[0n]|BOLD:ACF3241
Euxoa ridingsiana|[7197]|LBCH7964-10|Canada|British Columbia|658[0n]|BOLD:ACF3241
Euxoa ridingsiana|[7198]|BBLPB722-10|Canada|Alberta|658[0n]|BOLD:ACF3241
Euxoa ridingsiana|[7199]|BBLPB723-10|Canada|Alberta|658[0n]|BOLD:ACF3241
Euxoa ridingsiana|[7200]|BBLPB724-10|Canada|Alberta|658[0n]|BOLD:ACF3241
Euxoa ridingsiana|[7201]|BBLPB725-10|Canada|Alberta|658[0n]|BOLD:ACF3241
Euxoa ridingsiana|[7202]|BBLPB732-10|Canada|Alberta|658[0n]|BOLD:ACF3241
Euxoa ridingsiana|[7203]|BBLPB734-10|Canada|Alberta|658[0n]|BOLD:ACF3241
Euxoa ridingsiana|[7204]|BBLPB735-10|Canada|Alberta|658[0n]|BOLD:ACF3241
Euxoa ridingsiana|[7205]|BBLPB737-10|Canada|Alberta|658[0n]|BOLD:ACF3241
Euxoa ridingsiana|[7206]|BBLPB738-10|Canada|Alberta|658[0n]|BOLD:ACF3241
Euxoa taura|[7207]|RDNMF040-08|Canada|Alberta|658[0n]|BOLD:ACF3241
Euxoa taura|[7208]|RDNMF027-08|Canada|Alberta|658[0n]|BOLD:ACF3241
Euxoa perolivalis|[7209]|LPABB424-08|Canada|Alberta|658[0n]|BOLD:ACF3241
Euxoa perolivalis|[7210]|LPABB405-08|Canada|Alberta|658[0n]|BOLD:ACF3241
Euxoa perolivalis|[7211]|RDNMF030-08|Canada|Alberta|658[0n]|BOLD:ACF3241
Euxoa perolivalis|[7212]|RDMAB973-09|United States|Wyoming|636[0n]|BOLD:ACF3241
Euxoa perolivalis|[7213]|RDMAB974-09|Canada|Alberta|635[0n]|BOLD:ACF3241
Euxoa perolivalis|[7214]|RDMAB975-09|Canada|Alberta|635[0n]|BOLD:ACF3241
Euxoa taura|[7215]|RDMAB1022-09|Canada|Alberta|658[0n]|BOLD:ACF3241
Euxoa manitobana|[7216]|RDMAB488-06|Canada|Alberta|636[0n]|BOLD:ACF3241
Euxoa|[7217]|CNGRD1029-12|Canada|Saskatchewan|632[0n]|BOLD:ACF3241
Euxoa taura|[7218]|RDMAB1021-09|Canada|Alberta|620[0n]|BOLD:ACF3241
Euxoa taura|[7219]|RDMAB1023-09|Canada|Alberta|635[0n]|BOLD:ACF3241
Euxoa|[7220]|CNWBG3165-13|Canada|Alberta|588[0n]|BOLD:ACF3241
Euxoa ridingsiana|[7221]|LOWCE719-06|Canada|British Columbia|602[0n]|BOLD:ACF3241
Euxoa ridingsiana|[7222]|LBCH6783-10|Canada|British Columbia|645[0n]|BOLD:ACF3241
Euxoa montana|[7223]|CNCLB778-14|United States|Colorado|510[0n]|BOLD:ACF3241
Euxoa montana|[7224]|CNCLB992-14|United States|Colorado|658[0n]|BOLD:ACF3241
Euxoa maimes|[7225]|LOWCE715-06|Canada|British Columbia|658[0n]|BOLD:ACF3241
Euxoa maimes|[7226]|LOWCE718-06|Canada|British Columbia|604[0n]|BOLD:ACF3241
Euxoa maimes|[7227]|RDMAB596-06|Canada|Alberta|658[0n]|BOLD:ACF3241
Euxoa maimes|[7228]|RDMAB597-06|Canada|Alberta|658[0n]|BOLD:ACF3241
Euxoa maimes|[7229]|LOWCE717-06|Canada|British Columbia|658[0n]|BOLD:ACF3241
Euxoa maimes|[7230]|LBCH6784-10|Canada|British Columbia|658[0n]|BOLD:ACF3241
Euxoa aberrans|[7231]|RDNMG453-08|Canada|British Columbia|658[0n]|BOLD:ACF3241
Euxoa aberrans|[7232]|RDNMG454-08|Canada|British Columbia|658[0n]|BOLD:ACF3241
Euxoa aberrans|[7233]|RDNMG455-08|Canada|British Columbia|658[0n]|BOLD:ACF3241
Euxoa aberrans|[7234]|LBCH7002-10|Canada|British Columbia|658[0n]|BOLD:ACF3241
Euxoa aberrans|[7235]|LBCH7054-10|Canada|British Columbia|658[0n]|BOLD:ACF3241
Euxoa flavicollis|[7236]|RDNMG495-08|Canada|British Columbia|639[0n]|BOLD:ACF3241
Euxoa flavicollis|[7237]|RDNMG494-08|Canada|British Columbia|658[0n]|BOLD:ACF3241
Euxoa flavicollis|[7238]|RDNMG493-08|United States|Washington|658[0n]|BOLD:ACF3241
Euxoa flavicollis|[7239]|LPSK393-08|Canada|Saskatchewan|658[0n]|BOLD:ACF3241
Euxoa flavicollis|[7240]|LPSK145-08|Canada|Saskatchewan|658[0n]|BOLD:ACF3241
Euxoa flavicollis|[7241]|LPSK137-08|Canada|Saskatchewan|658[0n]|BOLD:ACF3241
Euxoa flavicollis|[7242]|RDNMF037-08|Canada|Alberta|658[0n]|BOLD:ACF3241
Euxoa flavicollis|[7243]|LOWCE714-06|Canada|British Columbia|658[0n]|BOLD:ACF3241
Euxoa ridingsiana|[7244]|RDNMG502-08|United States|Washington|652[0n]|BOLD:ACE9576
Euxoa flavicollis|[7245]|RDNMF770-08|Canada|Saskatchewan|658[0n]|BOLD:ACF3241
Euxoa flavicollis|[7246]|LBCH6091-10|Canada|British Columbia|658[0n]|BOLD:ACF3241
Euxoa flavicollis|[7247]|LBCH7963-10|Canada|British Columbia|658[0n]|BOLD:ACF3241
Euxoa riversii|[7248]|CNCLB2051-14|United States|California|307[0n]|
Euxoa riversii|[7249]|CNCLB2049-14|United States|California|658[0n]|BOLD:ACF3241
Euxoa riversii|[7250]|LNAUS2891-13|United States|California|540[1n]|BOLD:ACF3241
Euxoa riversii|[7251]|CNCLB2050-14|United States|California|658[1n]|BOLD:ACF3241
Euxoa riversii|[7252]|CNCLB2052-14|United States|California|658[0n]|BOLD:ACF3241
Euxoa melura|[7253]|CNCLB763-14|United States|Nevada|658[0n]|BOLD:ACM3876
Euxoa melura|[7254]|CNCLB764-14|United States|Washington|658[0n]|BOLD:ACM3876
Euxoa lewisi|[7255]|LSEU807-06|United States|Colorado|658[0n]|BOLD:ACE5991
Euxoa lewisi|[7256]|RDNMF309-08|United States|Wyoming|658[0n]|BOLD:ACE5991
Euxoa lewisi|[7257]|RDNMF032-08|Canada|Alberta|658[0n]|BOLD:ACE5991
Euxoa lewisi|[7258]|RDNMH1031-09|Canada|British Columbia|658[0n]|BOLD:ACE5991
Euxoa lewisi|[7259]|LBCH2200-10|Canada|British Columbia|658[0n]|BOLD:ACE5991
Euxoa lewisi|[7260]|BBLPB871-10|Canada|British Columbia|658[0n]|BOLD:ACE5991
Euxoa lewisi juliae|[7261]|CNCLB1775-14|United States|California|658[0n]|BOLD:ACE5991
Euxoa atomaris|[7262]|RDNM275-05|Canada|British Columbia|609[0n]|BOLD:ABX5971
Euxoa atomaris|[7263]|RDNM279-05|United States|Washington|544[0n]|BOLD:ABX5971
Euxoa atomaris|[7264]|RDNM278-05|United States|Washington|563[0n]|BOLD:ABX5971
Euxoa atomaris|[7265]|RDNM280-05|United States|Washington|562[0n]|BOLD:ABX5971
Euxoa atomaris|[7266]|LBCH7330-10|Canada|British Columbia|658[0n]|BOLD:ABX5971
Euxoa atomaris|[7267]|LBCH7333-10|Canada|British Columbia|658[0n]|BOLD:ABX5971
Euxoa atomaris|[7268]|RDNM277-05|United States|Oregon|658[0n]|BOLD:ABX5971
Euxoa atomaris|[7269]|LBCH7234-10|Canada|British Columbia|658[0n]|BOLD:ABX5971
Euxoa atomaris|[7270]|RDNM276-05|United States|Oregon|658[0n]|BOLD:ABX5971
Euxoa atomaris|[7271]|LOCBB330-06|United States|California|658[0n]|BOLD:ABX5971
Euxoa atomaris|[7272]|LOCBB327-06|United States|California|658[0n]|BOLD:ABX5971
Euxoa atomaris|[7273]|LOCBB326-06|United States|California|658[0n]|BOLD:ABX5971
Euxoa atomaris|[7274]|LOCBB328-06|United States|California|658[0n]|BOLD:ABX5971
Euxoa atomaris|[7275]|LOCBB329-06|United States|California|658[0n]|BOLD:ABX5971
Euxoa atomaris|[7276]|RDNMD385-06|United States|California|658[0n]|BOLD:ABX5971
[illegible]

Euxoa atomaris|[7274]|LOCBB328-06|United States|California|658[0n]|BOLD:ABX5971
Euxoa atomaris|[7275]|LOCBB329-06|United States|California|658[0n]|BOLD:ABX5971
Euxoa atomaris|[7276]|RDNMD385-06|United States|California|658[0n]|BOLD:ABX5971
Euxoa atomaris|[7277]|RDNMD386-06|United States|California|658[0n]|BOLD:ABX5971
Euxoa atomaris|[7278]|LBCH7807-10|Canada|British Columbia|658[0n]|BOLD:ABX5971
Euxoa extranea|[7279]|RDNMF318-08|United States|Washington|658[0n]|BOLD:ABZ9891
Euxoa extranea|[7280]|RDNMF319-08|United States|Oregon|658[0n]|BOLD:ABZ9891
Euxoa extranea|[7281]|RDNMF320-08|United States|Oregon|658[0n]|BOLD:ABZ9891
Euxoa extranea|[7282]|RDNMF839-08|United States|California|658[0n]|BOLD:ABZ9891
Euxoa extranea|[7283]|RDNMF848-08|United States|Oregon|658[0n]|BOLD:ABZ9891
Euxoa extranea|[7284]|IAWLB013-10|United States|California|658[0n]|BOLD:ABZ9891
Euxoa extranea|[7285]|IAWLB014-10|United States|California|658[0n]|BOLD:ABZ9891
Euxoa extranea|[7286]|IAWLB015-10|United States|California|658[0n]|BOLD:ABZ9891
Euxoa sp.|[7287]|RDNMB857-05|United States|Wyoming|658[0n]|BOLD:ABZ8981
Euxoa sp.|[7288]|RDNMB858-05|United States|Wyoming|658[0n]|BOLD:ABZ8981
Euxoa divergens|[7289]|RDNM776-05|Canada|British Columbia|583[0n]|BOLD:ACE5082
Euxoa divergens|[7290]|LPABB125-08|Canada|Alberta|638[0n]|BOLD:ACE5082
Euxoa divergens|[7291]|RDNMD310-06|United States|Colorado|658[0n]|BOLD:ACE5082
Euxoa divergens|[7292]|LOWCE294-06|Canada|British Columbia|658[0n]|BOLD:ACE5082
Euxoa divergens|[7293]|RDMAB267-05|Canada|Alberta|658[0n]|BOLD:ACE5082
Euxoa divergens|[7294]|RDMAB256-05|Canada|Alberta|658[0n]|BOLD:ACE5082
Euxoa divergens|[7295]|RDNMD313-06|United States|Colorado|643[0n]|BOLD:ACE5082
Euxoa divergens|[7296]|RDNMD314-06|United States|Colorado|658[0n]|BOLD:ACE5082
Euxoa divergens|[7297]|LPABB124-08|Canada|Alberta|658[0n]|BOLD:ACE5082
Euxoa divergens|[7298]|LPABB343-08|Canada|Alberta|658[0n]|BOLD:ACE5082
Euxoa divergens|[7299]|LPABB352-08|Canada|Alberta|658[0n]|BOLD:ACE5082
Euxoa divergens|[7300]|LPABB380-08|Canada|Alberta|658[0n]|BOLD:ACE5082
Euxoa divergens|[7301]|LPABB421-08|Canada|Alberta|658[0n]|BOLD:ACE5082
Euxoa divergens|[7302]|LPABB616-08|Canada|Alberta|658[0n]|BOLD:ACE5082
Euxoa divergens|[7303]|LPABB856-09|Canada|Alberta|658[0n]|BOLD:ACE5082
Euxoa divergens|[7304]|LPABC573-09|Canada|Alberta|658[0n]|BOLD:ACE5082
Euxoa divergens|[7305]|LPABC590-09|Canada|Alberta|658[0n]|BOLD:ACE5082
Euxoa divergens|[7306]|LBCG2585-09|Canada|British Columbia|658[0n]|BOLD:ACE5082
Euxoa divergens|[7307]|LSEU816-06|United States|Colorado|658[0n]|BOLD:ACE5082
Euxoa divergens|[7308]|RDMAB036-05|Canada|Alberta|611[0n]|BOLD:ACE5082
Euxoa divergens|[7309]|RDNMB624-05|Canada|New Brunswick|658[1n]|BOLD:ACE5082
Euxoa divergens|[7310]|LBCG1350-09|Canada|British Columbia|658[0n]|BOLD:ACE5082
Euxoa divergens|[7311]|RDMAB532-06|Canada|Alberta|658[0n]|BOLD:ACE5082
Euxoa divergens|[7312]|RDMAB113-05|Canada|Alberta|658[0n]|BOLD:ACE5082
Euxoa divergens|[7313]|RDMAB109-05|Canada|Alberta|658[0n]|BOLD:ACE5082
Euxoa divergens|[7314]|RDMAB108-05|Canada|Alberta|658[0n]|BOLD:ACE5082
Euxoa divergens|[7315]|RDNMB623-05|Canada|New Brunswick|658[0n]|BOLD:ACE5082
Euxoa divergens|[7316]|RDNM784-05|Canada|Quebec|658[0n]|BOLD:ACE5082
Euxoa divergens|[7317]|LOWCD421-06|Canada|British Columbia|621[0n]|BOLD:ACE5082
Euxoa divergens|[7318]|PHMNB201-04|Canada|New Brunswick|574[0n]|BOLD:ACE5082
Euxoa divergens|[7319]|LOWCD422-06|Canada|British Columbia|604[0n]|BOLD:ACE5082
Euxoa divergens|[7320]|LOWCD414-06|Canada|British Columbia|604[0n]|BOLD:ACE5082
Euxoa divergens|[7321]|RDMAB132-05|Canada|Alberta|631[0n]|BOLD:ACE5082
Euxoa divergens|[7322]|LOWCD420-06|Canada|British Columbia|601[0n]|BOLD:ACE5082
Euxoa divergens|[7323]|LOWCD412-06|Canada|British Columbia|594[0n]|BOLD:ACE5082
Euxoa divergens|[7324]|LOWCD413-06|Canada|British Columbia|589[0n]|BOLD:ACE5082
Euxoa divergens|[7325]|LOWCD423-06|Canada|British Columbia|593[0n]|BOLD:ACE5082
Euxoa divergens|[7326]|LOWCD426-06|Canada|British Columbia|656[0n]|BOLD:ACE5082
Euxoa divergens|[7327]|LOWCE325-06|Canada|British Columbia|658[0n]|BOLD:ACE5082
Euxoa divergens|[7328]|LOWCE326-06|Canada|British Columbia|658[0n]|BOLD:ACE5082
Euxoa divergens|[7329]|RDLQF147-06|Canada|Quebec|658[0n]|BOLD:ACE5082
Euxoa divergens|[7330]|LPSK139-08|Canada|Saskatchewan|658[0n]|BOLD:ACE5082
Euxoa divergens|[7331]|LPSK234-08|Canada|Saskatchewan|658[0n]|BOLD:ACE5082
Euxoa divergens|[7332]|LPMN738-08|Canada|Manitoba|658[0n]|BOLD:ACE5082
Euxoa divergens|[7333]|LBCG1342-09|Canada|British Columbia|658[0n]|BOLD:ACE5082
Euxoa divergens|[7334]|LBCG1347-09|Canada|British Columbia|658[0n]|BOLD:ACE5082
Euxoa divergens|[7335]|LBCG2870-09|Canada|British Columbia|658[0n]|BOLD:ACE5082
Euxoa divergens|[7336]|LBCH5253-10|Canada|British Columbia|658[0n]|BOLD:ACE5082
Euxoa divergens|[7337]|LBCH5480-10|Canada|British Columbia|658[0n]|BOLD:ACE5082
Euxoa divergens|[7338]|LBCH6011-10|Canada|British Columbia|658[0n]|BOLD:ACE5082
Euxoa divergens|[7339]|BBLPB721-10|Canada|Ontario|658[0n]|BOLD:ACE5082
Euxoa divergens|[7340]|RDNMB620-05|Canada|Saskatchewan|509[0n]|BOLD:ACE5082
Euxoa divergens|[7341]|RDNM773-05|Canada|Saskatchewan|507[0n]|BOLD:ACE5082
Euxoa divergens|[7342]|RDLQF148-06|Canada|Quebec|658[0n]|BOLD:ACE5082
Euxoa divergens|[7343]|SSJAE1894-13|Canada|Alberta|614[1n]|BOLD:ACE5082
Euxoa divergens|[7344]|LOWCD419-06|Canada|British Columbia|593[0n]|BOLD:ACE5082
Euxoa divergens|[7345]|LOWCD418-06|Canada|British Columbia|576[0n]|BOLD:ACE5082
Euxoa divergens|[7346]|LOWCD416-06|Canada|British Columbia|579[0n]|BOLD:ACE5082
Euxoa divergens|[7347]|RDMAB037-05|Canada|Alberta|628[0n]|BOLD:ACE5082
Euxoa divergens|[7348]|LOWCD338-06|Canada|British Columbia|563[2n]|BOLD:ACE5082
Euxoa divergens|[7349]|LOWCD342-06|Canada|British Columbia|558[1n]|BOLD:ACE5082
Euxoa divergens|[7350]|RDLQF849-06|Canada|Quebec|658[0n]|BOLD:ACE5082
Euxoa divergens|[7351]|LPSK013-08|Canada|Saskatchewan|658[0n]|BOLD:ACE5082
Euxoa divergens|[7352]|LPSK125-08|Canada|Saskatchewan|658[0n]|BOLD:ACE5082
Euxoa divergens|[7353]|LPSK140-08|Canada|Saskatchewan|658[0n]|BOLD:ACE5082
Euxoa divergens|[7354]|LPSK144-08|Canada|Saskatchewan|658[0n]|BOLD:ACE5082
Euxoa divergens|[7355]|LPSK232-08|Canada|Saskatchewan|658[0n]|BOLD:ACE5082
Euxoa divergens|[7356]|LPSK235-08|Canada|Saskatchewan|658[0n]|BOLD:ACE5082
Euxoa divergens|[7357]|LPSK250-08|Canada|Saskatchewan|658[0n]|BOLD:ACE5082
Euxoa divergens|[7358]|LPMN174-08|Canada|Manitoba|658[0n]|BOLD:ACE5082
Euxoa divergens|[7359]|LPMN345-08|Canada|Manitoba|658[0n]|BOLD:ACE5082
Euxoa divergens|[7360]|LPMN577-08|Canada|Manitoba|658[0n]|BOLD:ACE5082
Euxoa divergens|[7361]|LPSK565-08|Canada|Saskatchewan|658[0n]|BOLD:ACE5082
Euxoa divergens|[7362]|LPSK574-08|Canada|Saskatchewan|658[0n]|BOLD:ACE5082
Euxoa divergens|[7363]|LPABB398-08|Canada|Alberta|658[0n]|BOLD:ACE5082
Euxoa divergens|[7364]|LPABB613-08|Canada|Alberta|658[0n]|BOLD:ACE5082
Euxoa divergens|[7365]|LPABB620-08|Canada|Alberta|658[0n]|BOLD:ACE5082
Euxoa divergens|[7366]|LPSOD335-09|Canada|Ontario|658[0n]|BOLD:ACE5082
Euxoa divergens|[7367]|LBCG1343-09|Canada|British Columbia|658[0n]|BOLD:ACE5082
Euxoa sinelinea|[7368]|RDNMH397-09|Canada|Alberta|658[0n]|BOLD:ACE5082
Euxoa sinelinea|[7369]|RDNMH398-09|Canada|Alberta|658[0n]|BOLD:ACE5082
Euxoa divergens|[7370]|LBCH5945-10|Canada|British Columbia|658[0n]|BOLD:ACE5082
Euxoa divergens|[7371]|LBCH6009-10|Canada|British Columbia|658[0n]|BOLD:ACE5082
Euxoa divergens|[7372]|RDNMD311-06|United States|Colorado|658[0n]|BOLD:ACE5082
Euxoa divergens|[7373]|RDNMD316-06|United States|Colorado|658[0n]|BOLD:ACE5082
Euxoa divergens|[7374]|RDNMI116-10|Canada|Yukon Territory|658[0n]|BOLD:ACE5082
Euxoa divergens|[7375]|BBLPB727-10|Canada|Alberta|658[0n]|BOLD:ACE5082
Euxoa divergens|[7376]|LOWCE327-06|Canada|British Columbia|658[0n]|BOLD:ACE5082

Euxoa divergens|[7374]|RDNMI116-10|Canada|Yukon Territory|658[0n]|BOLD:ACE5082
Euxoa divergens|[7375]|BBLPB727-10|Canada|Alberta|658[0n]|BOLD:ACE5082
Euxoa divergens|[7376]|LOWCE327-06|Canada|British Columbia|658[0n]|BOLD:ACE5082
Euxoa divergens|[7377]|LOWCE328-06|Canada|British Columbia|658[0n]|BOLD:ACE5082
Euxoa divergens|[7378]|LOWCE329-06|Canada|British Columbia|658[0n]|BOLD:ACE5082
Euxoa divergens|[7379]|LOWCE330-06|Canada|British Columbia|658[0n]|BOLD:ACE5082
Euxoa divergens|[7380]|BBLPB858-10|Canada|British Columbia|658[0n]|BOLD:ACE5082
Euxoa divergens|[7381]|CNCLB987-14|United States|California|658[0n]|BOLD:ACE5082
Euxoa divergens|[7382]|LOWCE320-06|Canada|British Columbia|658[0n]|BOLD:ACE5082
Euxoa divergens|[7383]|LOWCE321-06|Canada|British Columbia|658[0n]|BOLD:ACE5082
Euxoa divergens|[7384]|LOWCD425-06|Canada|British Columbia|657[0n]|BOLD:ACE5082
Euxoa divergens|[7385]|LOWCE319-06|Canada|British Columbia|658[0n]|BOLD:ACE5082
Euxoa sinelinea|[7386]|RDLQB289-05|Canada|Quebec|658[0n]|BOLD:ACE5082
Euxoa divergens|[7387]|RDMAB435-05|Canada|Yukon Territory|658[0n]|BOLD:ACE5082
Euxoa sinelinea|[7388]|RDLQB288-05|Canada|Quebec|658[0n]|BOLD:ACE5082
Euxoa sinelinea|[7389]|RDLQB287-05|Canada|Quebec|658[0n]|BOLD:ACE5082
Euxoa sinelinea|[7390]|RDLQB283-05|Canada|Quebec|658[0n]|BOLD:ACE5082
Euxoa divergens|[7391]|RDLQB282-05|Canada|Quebec|658[0n]|BOLD:ACE5082
Euxoa divergens|[7392]|RDNMB621-05|Canada|Saskatchewan|658[0n]|BOLD:ACE5082
Euxoa divergens|[7393]|RDNMB619-05|Canada|British Columbia|658[0n]|BOLD:ACE5082
Euxoa divergens|[7394]|RDNMB617-05|Canada|Alberta|658[0n]|BOLD:ACE5082
Euxoa divergens|[7395]|RDNMB616-05|Canada|Alberta|658[0n]|BOLD:ACE5082
Euxoa sinelinea|[7396]|RDLQB285-05|Canada|Quebec|658[1n]|BOLD:ACE5082
Euxoa divergens|[7397]|RDNMD315-06|United States|Colorado|601[0n]|BOLD:ACE5082
Euxoa divergens|[7398]|LOWCD415-06|Canada|British Columbia|599[0n]|BOLD:ACE5082
Euxoa divergens|[7399]|LOWCD333-06|Canada|British Columbia|592[0n]|BOLD:ACE5082
Euxoa sinelinea|[7400]|RDLQB286-05|Canada|Quebec|603[0n]|BOLD:ACE5082
Euxoa sinelinea|[7401]|RDLQB284-05|Canada|Quebec|598[0n]|BOLD:ACE5082
Euxoa divergens|[7402]|LOWCD417-06|Canada|British Columbia|551[0n]|BOLD:ACE5082
Euxoa divergens|[7403]|LOWCD424-06|Canada|British Columbia|592[0n]|BOLD:ACE5082
Euxoa divergens|[7404]|RDLQ687-07|Canada|Quebec|597[0n]|BOLD:ACE5082
Euxoa divergens|[7405]|BBLPE466-09|Canada|Newfoundland and Labrador|654[0n]|BOLD:ACE5082
Euxoa divergens|[7406]|CNCLB988-14|United States|California|658[0n]|BOLD:ACE5082
Euxoa divergens|[7407]|RDNMB622-05|United States|Washington|658[0n]|BOLD:ACF2023
Euxoa divergens|[7408]|SSBAD6410-13|Canada|Alberta|613[0n]|BOLD:ACF2023
Euxoa divergens|[7409]|RDNMB613-05|United States|Washington|658[0n]|BOLD:ACF2023
Euxoa divergens|[7410]|RDNMB614-05|United States|Washington|555[2n]|BOLD:ACF2023
Euxoa divergens|[7411]|RDNMB615-05|United States|Oregon|517[0n]|BOLD:ACF2023
Euxoa divergens|[7412]|LBCH1953-10|Canada|British Columbia|658[0n]|BOLD:ACF2023
Euxoa divergens|[7413]|LBCH2251-10|Canada|British Columbia|658[0n]|BOLD:ACF2023
Euxoa divergens|[7414]|RDNMB618-05|Canada|British Columbia|523[0n]|BOLD:ACF2023
Euxoa divergens|[7415]|RDNM774-05|Canada|British Columbia|522[0n]|BOLD:ACF2023
Euxoa divergens|[7416]|RDNM775-05|United States|Oregon|658[0n]|BOLD:ACF2023
Euxoa divergens|[7417]|RDNMD312-06|United States|Colorado|658[0n]|BOLD:ACF2023
Euxoa divergens|[7418]|LPABB831-09|Canada|Alberta|658[0n]|BOLD:ACF2023
Euxoa divergens|[7419]|LBCH1445-10|Canada|British Columbia|658[0n]|BOLD:ACF2023
Euxoa divergens|[7420]|LBCH1863-10|Canada|British Columbia|658[0n]|BOLD:ACF2023
Euxoa divergens|[7421]|RWWC1189-13|United States|Washington|579[0n]|BOLD:ACF2023
Euxoa leuschneri|[7422]|CNCLB985-14|United States|California|658[0n]|BOLD:ACE5082
Euxoa leuschneri|[7423]|CNCLB986-14|United States|California|658[0n]|BOLD:ACE5082
Euxoa leuschneri|[7424]|TML190-14|United States||658[0n]|BOLD:ACE5082
Euxoa sp.|[7425]|BBLSY541-09|United States|New Mexico|658[0n]|BOLD:AAH5396
Euxoa vernalis|[7426]|RDNME1032-08|United States|Arizona|658[0n]|BOLD:AAY4203
Euxoa hyperborea|[7427]|RDNMF321-08|United States|Alaska|658[0n]|BOLD:ABZ9739
Euxoa hyperborea|[7428]|RDNMF322-08|United States|Alaska|640[0n]|BOLD:ABZ9739
Euxoa muldersi|[7429]|RDNMH1034-09|Canada|Nunavut|658[0n]|BOLD:ABZ9739
Euxoa muldersi|[7430]|RDNMH1035-09|Canada|Nunavut|658[1n]|BOLD:ABZ9739
Euxoa trifasciata|[7431]|CNCLB761-14|United States|California|658[0n]|BOLD:ACM4614
Euxoa trifasciata|[7432]|CNCLB779-14|United States|Oregon|658[0n]|BOLD:ACM4614
Euxoa chimoensis|[7433]|RDNMC207-05|Canada|Quebec|512[0n]|BOLD:ACF3481
Euxoa chimoensis|[7434]|LCH261-04|Canada|Manitoba|658[0n]|BOLD:ACF3481
Euxoa chimoensis|[7435]|MECB945-05|Canada|Quebec|524[0n]|BOLD:ACF3481
Euxoa chimoensis|[7436]|LCHP524-07|Canada|Manitoba|658[0n]|BOLD:ACF3481
Euxoa chimoensis|[7437]|LCHP416-07|Canada|Manitoba|658[0n]|BOLD:ACF3481
Euxoa chimoensis|[7438]|LCHP852-07|Canada|Manitoba|643[0n]|BOLD:ACF3481
Euxoa chimoensis|[7439]|RDNMF862-08|Canada|Manitoba|658[0n]|BOLD:ACF3481
Euxoa chimoensis|[7440]|RDNMF863-08|Canada|Manitoba|658[0n]|BOLD:ACF3481
Euxoa chimoensis|[7441]|RDNMF864-08|Canada|Manitoba|658[0n]|BOLD:ACF3481
Euxoa dissona|[7442]|RDLQ415-05|Canada|Quebec|618[0n]|BOLD:ACE5992
Euxoa dissona|[7443]|RDNMG1036-08|Canada|Manitoba|658[0n]|BOLD:ACE5992
Euxoa dissona|[7444]|MECB946-05|Canada|Manitoba|578[0n]|BOLD:ACE5992
Euxoa dissona|[7445]|RDNMG1037-08|Canada|Manitoba|641[0n]|BOLD:ACE5992
Euxoa dissona|[7446]|RDNMG1038-08|Canada|Manitoba|658[0n]|BOLD:ACE5992
Euxoa dissona|[7447]|CHLEP208-09|Canada|Manitoba|658[0n]|BOLD:ACE5992
Euxoa churchillensis|[7448]|RDNMG976-08|Canada|Nunavut|658[0n]|BOLD:ACE5992
Euxoa churchillensis|[7449]|RDNMF325-08|Canada|Nunavut|658[0n]|BOLD:ACE5992
Euxoa churchillensis|[7450]|RDNMF324-08|Canada|Nunavut|658[0n]|BOLD:ACE5992
Euxoa churchillensis|[7451]|RDNMF323-08|Canada|Nunavut|658[0n]|BOLD:ACE5992
Euxoa churchillensis|[7452]|RDNMG974-08|Canada|Northwest Territories|642[0n]|BOLD:ACE5992
Euxoa churchillensis|[7453]|RDNMG975-08|Canada|Northwest Territories|592[0n]|BOLD:ACE5992
Euxoa churchillensis alpina|[7454]|CNCLB1721-14|United States|Colorado|649[1n]|BOLD:ACE5992
Euxoa westermanni|[7455]|LCH250-04|Canada|Manitoba|658[0n]|BOLD:ABZ8446
Euxoa westermanni|[7456]|LCHP581-07|Canada|Manitoba|658[0n]|BOLD:ABZ8446
Euxoa westermanni|[7457]|LCHQ478-08|Canada|Manitoba|658[0n]|BOLD:ABZ8446
Euxoa westermanni|[7458]|LCHP873-07|Canada|Manitoba|658[0n]|BOLD:ABZ8446
Euxoa westermanni|[7459]|LCHP871-07|Canada|Manitoba|658[0n]|BOLD:ABZ8446
Euxoa westermanni|[7460]|LCHP840-07|Canada|Manitoba|658[0n]|BOLD:ABZ8446
Euxoa westermanni|[7461]|LCHP608-07|Canada|Manitoba|658[0n]|BOLD:ABZ8446
Euxoa westermanni|[7462]|LCHP417-07|Canada|Manitoba|658[0n]|BOLD:ABZ8446
Euxoa westermanni|[7463]|LCH255-04|Canada|Manitoba|658[0n]|BOLD:ABZ8446
Euxoa westermanni|[7464]|LCH254-04|Canada|Manitoba|658[0n]|BOLD:ABZ8446
Euxoa westermanni|[7465]|LCH253-04|Canada|Manitoba|658[0n]|BOLD:ABZ8446
Euxoa westermanni|[7466]|LCH252-04|Canada|Manitoba|658[0n]|BOLD:ABZ8446
Euxoa westermanni|[7467]|LCH251-04|Canada|Manitoba|658[0n]|BOLD:ABZ8446
Euxoa westermanni|[7468]|LCHP948-07|Canada|Manitoba|639[0n]|BOLD:ABZ8446
Euxoa westermanni|[7469]|LCHQ702-08|Canada|Manitoba|658[0n]|BOLD:ABZ8446
Euxoa austrina|[7470]|CNCLB2023-14|United States|California|307[0n]|
Euxoa austrina|[7471]|CNCLB1744-14|United States|California|573[1n]|BOLD:AAF6228
Euxoa altens|[7472]|RDNMJ123-10|United States|Washington|658[0n]|BOLD:AAF6228
Euxoa altens|[7473]|IAWLB095-10|United States|California|658[0n]|BOLD:AAF6228
Euxoa altens|[7474]|IAWLB094-10|United States|California|658[0n]|BOLD:AAF6228
Euxoa altens|[7475]|IAWLB093-10|United States|California|658[0n]|BOLD:AAF6228
Euxoa altens|[7476]|IAWLB092-10|United States|California|658[0n]|BOLD:AAF6228

Euxoa altens|[7474]|IAWLB094-10|United States|California|658[0n]|BOLD:AAF6228
Euxoa altens|[7475]|IAWLB093-10|United States|California|658[0n]|BOLD:AAF6228
Euxoa altens|[7476]|IAWLB092-10|United States|California|658[0n]|BOLD:AAF6228
Euxoa altens|[7477]|IAWLB023-10|United States|California|658[0n]|BOLD:AAF6228
Euxoa altens|[7478]|LBCH2199-10|Canada|British Columbia|658[0n]|BOLD:AAF6228
Euxoa altens|[7479]|BBLSX606-09|United States|Arizona|658[0n]|BOLD:AAF6228
Euxoa altens|[7480]|RDNMG659-08|United States|Washington|658[0n]|BOLD:AAF6228
Euxoa altens|[7481]|RDNMF018-08|United States|California|652[0n]|BOLD:AAF6228
Euxoa austrina|[7482]|TML186-14|United States||570[0n]|BOLD:AAF6228
Euxoa austrina|[7483]|CNCLB1742-14|United States|California|658[0n]|BOLD:AAF6228
Euxoa austrina|[7484]|CNCLB2025-14|United States|California|658[0n]|BOLD:AAF6228
Euxoa austrina|[7485]|CNCLB2026-14|United States|California|658[0n]|BOLD:AAF6228
Euxoa austrina|[7486]|CNCLB2027-14|United States|California|307[0n]|
Euxoa bivittata|[7487]|CNCLB1716-14|United States|California|658[1n]|BOLD:ACU0854
Euxoa bivittata|[7488]|CNCLB3149-15|United States|California|658[0n]|BOLD:ACU0854
Euxoa bivittata|[7489]|CNCLB3150-15|United States|California|658[0n]|BOLD:ACU0854
Euxoa nr. aurulenta|[7490]|RDNM287-05|United States|Nevada|658[0n]|BOLD:AAC8211
Euxoa nr. aurulenta|[7491]|RDNM288-05|United States|Nevada|658[0n]|BOLD:AAC8211
Euxoa nr. aurulenta|[7492]|RDNM289-05|United States|Nevada|658[0n]|BOLD:AAC8211
Euxoa aurulenta|[7493]|RDNM284-05|Canada|Saskatchewan|658[0n]|BOLD:AAC8211
Euxoa aurulenta|[7494]|RDNM281-05|United States|Washington|658[0n]|BOLD:AAC8211
Euxoa aurulenta|[7495]|RDNM282-05|United States|Washington|658[0n]|BOLD:AAC8211
Euxoa aurulenta|[7496]|RDNM283-05|United States|Washington|658[0n]|BOLD:AAC8211
Euxoa aurulenta|[7497]|RDNM285-05|Canada|Saskatchewan|658[0n]|BOLD:AAC8211
Euxoa aurulenta|[7498]|RDNM286-05|Canada|Saskatchewan|658[0n]|BOLD:AAC8211
Euxoa aurulenta|[7499]|RDNME595-08|United States|Utah|658[0n]|BOLD:AAC8211
Euxoa aurulenta|[7500]|RDNME596-08|United States|Utah|658[0n]|BOLD:AAC8211
Euxoa aurulenta|[7501]|RDNME738-08|United States|Utah|658[0n]|BOLD:AAC8211
Euxoa olivia|[7502]|AWCLB627-11|United States|Arizona|594[1n]|BOLD:AAD3112
Euxoa olivia|[7503]|IAWLB483-11|United States|Arizona|658[0n]|BOLD:AAD3112
Euxoa olivia|[7504]|LOCBB341-06|United States|California|658[0n]|BOLD:AAD3112
Euxoa olivia|[7505]|LOCBB216-06|United States|California|658[0n]|BOLD:AAD3112
Euxoa olivia|[7506]|LOCBB217-06|United States|California|631[0n]|BOLD:AAD3112
Euxoa olivia|[7507]|NAMUM239-08|United States|California|656[0n]|BOLD:AAD3112
Euxoa olivia|[7508]|RDNMD880-07|United States|Utah|655[0n]|BOLD:AAD3112
Euxoa olivia|[7509]|AWCLB474-10|United States|Arizona|658[0n]|BOLD:AAD3112
Euxoa olivia|[7510]|IAWLB495-11|United States|Arizona|658[0n]|BOLD:AAD3112
Euxoa olivia|[7511]|AWCLB103-10|United States|Arizona|658[0n]|BOLD:AAD3112
Euxoa olivia|[7512]|LBCH7228-10|Canada|British Columbia|658[0n]|BOLD:AAD3112
Euxoa olivia|[7513]|LOCBB215-06|United States|California|658[0n]|BOLD:AAD3112
Euxoa olivia|[7514]|LOWCE665-06|Canada|British Columbia|658[0n]|BOLD:AAD3112
Euxoa olivia|[7515]|LBCH7314-10|Canada|British Columbia|658[0n]|BOLD:AAD3112
Euxoa olivia|[7516]|LALPA816-10|Canada|British Columbia|658[0n]|BOLD:AAD3112
Euxoa olivia|[7517]|RDNMK365-11|United States|New Mexico|658[0n]|BOLD:AAD3112
Euxoa vetusta|[7518]|RDNMF300-08|Canada|British Columbia|658[0n]|BOLD:ABZ9012
Euxoa vetusta|[7519]|LBCW056-08|Canada|British Columbia|658[0n]|BOLD:ABZ9012
Euxoa vetusta|[7520]|RWWB264-09|United States|Washington|658[0n]|BOLD:ABZ9012
Euxoa vetusta|[7521]|RWWB282-09|United States|Washington|658[0n]|BOLD:ABZ9012
Euxoa vetusta|[7522]|RDNMF301-08|Canada|British Columbia|658[0n]|BOLD:ABZ9012
Euxoa vetusta|[7523]|RWWB221-09|United States|Washington|658[0n]|BOLD:ABZ9012
Euxoa vetusta|[7524]|LALPA1247-11|Canada|British Columbia|658[0n]|BOLD:ABZ9012
Euxoa vetusta|[7525]|RDNMF299-08|United States|Oregon|658[0n]|BOLD:ABZ9012
Euxoa vetusta|[7526]|RWWB237-09|United States|Washington|658[0n]|BOLD:ABZ9012
Euxoa vetusta|[7527]|LALPA1244-11|Canada|British Columbia|658[0n]|BOLD:ABZ9012
Euxoa vetusta|[7528]|LALPA1269-11|Canada|British Columbia|658[0n]|BOLD:ABZ9012
Euxoa vetusta|[7529]|LALPA1293-11|Canada|British Columbia|658[0n]|BOLD:ABZ9012
Euxoa vetusta|[7530]|RWWC1070-12|United States|Washington|658[0n]|BOLD:ABZ9012
Euxoa sp.|[7531]|RDNM807-05|Canada|Ontario|658[0n]|BOLD:ACE5988
Euxoa sp.|[7532]|RDNME732-08|United States|New Mexico|658[0n]|BOLD:ACE5988
Euxoa sp.|[7533]|RDNME733-08|United States|New Mexico|658[0n]|BOLD:ACE5988
Euxoa vallus|[7534]|RDNMB011-05|Canada|British Columbia|658[0n]|BOLD:ACE5988
Euxoa vallus|[7535]|LSEU811-06|United States|Colorado|658[0n]|BOLD:ACE5988
Euxoa vallus|[7536]|LSEU810-06|United States|Colorado|658[0n]|BOLD:ACE5988
Euxoa vallus|[7537]|LSEU812-06|United States|Colorado|658[0n]|BOLD:ACE5988
Euxoa macleani|[7538]|RDNMF856-08|Canada|British Columbia|658[0n]|BOLD:ACE5988
Euxoa macleani|[7539]|RDNM996-05|Canada|British Columbia|658[0n]|BOLD:ACE5988
Euxoa macleani|[7540]|RDNM997-05|Canada|British Columbia|658[0n]|BOLD:ACE5988
Euxoa macleani|[7541]|RDNM999-05|Canada|British Columbia|656[0n]|BOLD:ACE5988
Euxoa macleani|[7542]|RDNMF852-08|Canada|British Columbia|658[0n]|BOLD:ACE5988
Euxoa macleani|[7543]|RDNMF853-08|Canada|British Columbia|658[0n]|BOLD:ACE5988
Euxoa macleani|[7544]|RDNMH1032-09|Canada|British Columbia|658[0n]|BOLD:ACE5988
Euxoa apopsis|[7545]|RDNMH1033-09|Canada|British Columbia|658[0n]|BOLD:ACE5988
Euxoa pestula|[7546]|RDNMF039-08|Canada|Alberta|658[0n]|BOLD:ACE5988
Euxoa pestula|[7547]|RDMAB979-09|Canada|Alberta|632[0n]|BOLD:ACE5988
Euxoa pestula|[7548]|RDNMF656-08|United States|Washington|658[0n]|BOLD:ACE5988
Euxoa pestula|[7549]|RDNMF657-08|United States|Washington|658[0n]|BOLD:ACE5988
Euxoa pestula|[7550]|RDMAB980-09|Canada|Alberta|635[0n]|BOLD:ACE5988
Euxoa pestula|[7551]|RDMAB981-09|Canada|Alberta|635[0n]|BOLD:ACE5988
Euxoa simona|[7552]|RDNMG670-08|United States|Oregon|658[0n]|BOLD:ACE5988
Euxoa simona|[7553]|RDNMF653-08|United States|California|658[0n]|BOLD:ACE5988
Euxoa simona|[7554]|RDNMF654-08|United States|Oregon|658[0n]|BOLD:ACE5988
Euxoa simona|[7555]|RDNMG1041-08|United States|Oregon|643[0n]|BOLD:ACE5988
Euxoa simona|[7556]|IAWLB124-10|United States|California|658[0n]|BOLD:ACE5988
Euxoa pleuritica|[7557]|RDMAB489-06|Canada|Alberta|658[0n]|BOLD:ACE5988
Euxoa pleuritica|[7558]|RDNMF880-08|Canada|Alberta|658[0n]|BOLD:ACE5988
Euxoa pleuritica|[7559]|RDNMF881-08|Canada|Alberta|658[0n]|BOLD:ACE5988
Euxoa pleuritica|[7560]|RDNMF882-08|Canada|Alberta|658[0n]|BOLD:ACE5988
Euxoa sp.|[7561]|RDNMK625-11|United States|Colorado|658[0n]|BOLD:ACE5129
Euxoa catenula|[7562]|RDNMD881-07|United States|Utah|655[0n]|BOLD:ABZ8589
Euxoa catenula|[7563]|LBCH6979-10|Canada|British Columbia|658[0n]|BOLD:ABZ8589
Euxoa catenula|[7564]|LBCH6980-10|Canada|British Columbia|658[0n]|BOLD:ABZ8589
Euxoa catenula|[7565]|LBCH6977-10|Canada|British Columbia|658[0n]|BOLD:ABZ8589
Euxoa catenula|[7566]|LBCH6974-10|Canada|British Columbia|658[0n]|BOLD:ABZ8589
Euxoa catenula|[7567]|LBCH6898-10|Canada|British Columbia|658[0n]|BOLD:ABZ8589
Euxoa catenula|[7568]|LBCH6981-10|Canada|British Columbia|639[0n]|BOLD:ABZ8589
Euxoa catenula|[7569]|LBCH6761-10|Canada|British Columbia|658[0n]|BOLD:ABZ8589
Euxoa catenula|[7570]|LBCH6760-10|Canada|British Columbia|658[0n]|BOLD:ABZ8589
Euxoa catenula|[7571]|LPVIB804-08|Canada|British Columbia|658[0n]|BOLD:ABZ8589
Euxoa catenula|[7572]|RDNM297-05|United States|Washington|658[0n]|BOLD:ABZ8589
Euxoa catenula|[7573]|RDNM296-05|United States|Washington|658[0n]|BOLD:ABZ8589
Euxoa catenula|[7574]|RDNM295-05|United States|Washington|658[0n]|BOLD:ABZ8589
Euxoa catenula|[7575]|RDNM293-05|United States|Oregon|658[0n]|BOLD:ABZ8589
Euxoa catenula|[7576]|RDNM291-05|United States|Oregon|658[0n]|BOLD:ABZ8589

Euxoa catenula|[7573]|RDNM296-05|United States|Washington|658[0n]|BOLD:ABZ8589
Euxoa catenula|[7574]|RDNM295-05|United States|Washington|658[0n]|BOLD:ABZ8589
Euxoa catenula|[7575]|RDNM293-05|United States|Oregon|658[0n]|BOLD:ABZ8589
Euxoa catenula|[7576]|RDNM291-05|United States|Oregon|658[0n]|BOLD:ABZ8589
Euxoa catenula|[7577]|LBCH6978-10|Canada|British Columbia|658[0n]|BOLD:ABZ8589
Euxoa catenula|[7578]|LBCH7351-10|Canada|British Columbia|640[0n]|BOLD:ABZ8589
Euxoa catenula|[7579]|LBCH6897-10|Canada|British Columbia|658[0n]|BOLD:ABZ8589
Euxoa catenula|[7580]|LBCH7502-10|Canada|British Columbia|658[0n]|BOLD:ABZ8589
Euxoa catenula|[7581]|RDNM292-05|United States|Washington|548[0n]|BOLD:ABZ8589
Euxoa catenula|[7582]|LBCH6982-10|Canada|British Columbia|658[0n]|BOLD:ABZ8589
Euxoa catenula|[7583]|RDNMD293-06|United States|Colorado|656[0n]|BOLD:ABZ8589
Euxoa catenula|[7584]|LBCH7252-10|Canada|British Columbia|658[0n]|BOLD:ABZ8589
Euxoa catenula|[7585]|RDMAB555-06|Canada|Alberta|658[0n]|BOLD:ABZ8589
Euxoa catenula|[7586]|RDNM290-05|Canada|Alberta|658[0n]|BOLD:ABZ8589
Euxoa catenula|[7587]|RDNM294-05|Canada|British Columbia|580[0n]|BOLD:ABZ8589
Euxoa catenula|[7588]|LPVIB798-08|Canada|British Columbia|658[0n]|BOLD:ABZ8589
Euxoa catenula|[7589]|LBCH6826-10|Canada|British Columbia|658[0n]|BOLD:ABZ8589
Euxoa catenula|[7590]|LBCH6975-10|Canada|British Columbia|658[0n]|BOLD:ABZ8589
Euxoa catenula|[7591]|LBCH6976-10|Canada|British Columbia|658[0n]|BOLD:ABZ8589
Euxoa catenula|[7592]|LBCH7618-10|Canada|British Columbia|658[0n]|BOLD:ABZ8589
Euxoa servitus|[7593]|LSEU789-06|United States|Colorado|658[0n]|BOLD:ABZ9439
Euxoa servitus|[7594]|LBCH7543-10|Canada|British Columbia|658[0n]|BOLD:ABZ9439
Euxoa servitus|[7595]|LBCG191-08|Canada|British Columbia|658[0n]|BOLD:ABZ9439
Euxoa servitus|[7596]|LBCG197-08|Canada|British Columbia|658[0n]|BOLD:ABZ9439
Euxoa servitus|[7597]|LBCG3272-09|Canada|British Columbia|642[0n]|BOLD:ABZ9439
Euxoa servitus|[7598]|LBCG3270-09|Canada|British Columbia|642[0n]|BOLD:ABZ9439
Euxoa servitus|[7599]|LBCG3267-09|Canada|British Columbia|641[0n]|BOLD:ABZ9439
Euxoa servitus|[7600]|LBCG3263-09|Canada|British Columbia|641[0n]|BOLD:ABZ9439
Euxoa servitus|[7601]|LBCG3258-09|Canada|British Columbia|641[0n]|BOLD:ABZ9439
Euxoa servitus|[7602]|LBCG3265-09|Canada|British Columbia|638[0n]|BOLD:ABZ9439
Euxoa servitus|[7603]|LBCG3256-09|Canada|British Columbia|638[0n]|BOLD:ABZ9439
Euxoa servitus|[7604]|LBCH6485-10|Canada|British Columbia|658[0n]|BOLD:ABZ9439
Euxoa servitus|[7605]|LBCH6484-10|Canada|British Columbia|658[0n]|BOLD:ABZ9439
Euxoa servitus|[7606]|LBCH6362-10|Canada|British Columbia|658[0n]|BOLD:ABZ9439
Euxoa servitus|[7607]|LBCH6360-10|Canada|British Columbia|658[0n]|BOLD:ABZ9439
Euxoa servitus|[7608]|LBCH6359-10|Canada|British Columbia|658[0n]|BOLD:ABZ9439
Euxoa servitus|[7609]|LBCH6358-10|Canada|British Columbia|658[0n]|BOLD:ABZ9439
Euxoa servitus|[7610]|LBCH6356-10|Canada|British Columbia|658[0n]|BOLD:ABZ9439
Euxoa servitus|[7611]|LBCH6355-10|Canada|British Columbia|658[0n]|BOLD:ABZ9439
Euxoa servitus|[7612]|LBCH5958-10|Canada|British Columbia|658[0n]|BOLD:ABZ9439
Euxoa servitus|[7613]|LBCH5957-10|Canada|British Columbia|658[0n]|BOLD:ABZ9439
Euxoa servitus|[7614]|LBCH5956-10|Canada|British Columbia|658[0n]|BOLD:ABZ9439
Euxoa servitus|[7615]|LBCG3317-09|Canada|British Columbia|658[0n]|BOLD:ABZ9439
Euxoa servitus|[7616]|LBCG3314-09|Canada|British Columbia|658[0n]|BOLD:ABZ9439
Euxoa servitus|[7617]|LBCG3269-09|Canada|British Columbia|658[0n]|BOLD:ABZ9439
Euxoa servitus|[7618]|LBCG3266-09|Canada|British Columbia|658[0n]|BOLD:ABZ9439
Euxoa servitus|[7619]|LBCG3262-09|Canada|British Columbia|658[0n]|BOLD:ABZ9439
Euxoa servitus|[7620]|LBCG3260-09|Canada|British Columbia|658[0n]|BOLD:ABZ9439
Euxoa servitus|[7621]|LBCG3259-09|Canada|British Columbia|658[0n]|BOLD:ABZ9439
Euxoa servitus|[7622]|LBCG3255-09|Canada|British Columbia|658[0n]|BOLD:ABZ9439
Euxoa servitus|[7623]|LBCG3253-09|Canada|British Columbia|658[0n]|BOLD:ABZ9439
Euxoa servitus|[7624]|LBCG2894-09|Canada|British Columbia|658[0n]|BOLD:ABZ9439
Euxoa servitus|[7625]|LBCG2893-09|Canada|British Columbia|658[0n]|BOLD:ABZ9439
Euxoa servitus|[7626]|LBCG2892-09|Canada|British Columbia|658[0n]|BOLD:ABZ9439
Euxoa servitus|[7627]|LBCG2110-09|Canada|British Columbia|658[0n]|BOLD:ABZ9439
Euxoa servitus|[7628]|LBCG198-08|Canada|British Columbia|658[0n]|BOLD:ABZ9439
Euxoa servitus|[7629]|LBCG196-08|Canada|British Columbia|658[0n]|BOLD:ABZ9439
Euxoa servitus|[7630]|LBCG195-08|Canada|British Columbia|658[0n]|BOLD:ABZ9439
Euxoa servitus|[7631]|LBCG192-08|Canada|British Columbia|658[0n]|BOLD:ABZ9439
Euxoa servitus|[7632]|LOWCE645-06|Canada|British Columbia|658[0n]|BOLD:ABZ9439
Euxoa servitus|[7633]|LOWCE644-06|Canada|British Columbia|658[0n]|BOLD:ABZ9439
Euxoa servitus|[7634]|LOWCE638-06|Canada|British Columbia|658[0n]|BOLD:ABZ9439
Euxoa servitus|[7635]|LOWCE324-06|Canada|British Columbia|658[0n]|BOLD:ABZ9439
Euxoa servitus|[7636]|LOWCD859-06|Canada|British Columbia|658[0n]|BOLD:ABZ9439
Euxoa servitus|[7637]|LOWCD853-06|Canada|British Columbia|658[0n]|BOLD:ABZ9439
Euxoa servitus|[7638]|LOWCD852-06|Canada|British Columbia|658[0n]|BOLD:ABZ9439
Euxoa servitus|[7639]|LOWCD800-06|Canada|British Columbia|658[0n]|BOLD:ABZ9439
Euxoa servitus|[7640]|LBCG2111-09|Canada|British Columbia|658[0n]|BOLD:ABZ9439
Euxoa servitus|[7641]|LBCG3261-09|Canada|British Columbia|636[0n]|BOLD:ABZ9439
Euxoa servitus|[7642]|LBCG3264-09|Canada|British Columbia|632[0n]|BOLD:ABZ9439
Euxoa servitus|[7643]|LBCG3271-09|Canada|British Columbia|616[0n]|BOLD:ABZ9439
Euxoa servitus|[7644]|LOWCD858-06|Canada|British Columbia|610[0n]|BOLD:ABZ9439
Euxoa servitus|[7645]|LOWCD854-06|Canada|British Columbia|608[1n]|BOLD:ABZ9439
Euxoa servitus|[7646]|LOWCE631-06|Canada|British Columbia|607[0n]|BOLD:ABZ9439
Euxoa servitus|[7647]|LOWCD850-06|Canada|British Columbia|607[0n]|BOLD:ABZ9439
Euxoa servitus|[7648]|LOWCD851-06|Canada|British Columbia|579[0n]|BOLD:ABZ9439
Euxoa servitus|[7649]|LOWCD856-06|Canada|British Columbia|585[0n]|BOLD:ABZ9439
Euxoa servitus|[7650]|LBCH6486-10|Canada|British Columbia|658[0n]|BOLD:ABZ9439
Euxoa servitus|[7651]|LBCH6488-10|Canada|British Columbia|658[0n]|BOLD:ABZ9439
Euxoa servitus|[7652]|LBCH6489-10|Canada|British Columbia|658[0n]|BOLD:ABZ9439
Euxoa servitus|[7653]|LBCH6490-10|Canada|British Columbia|658[0n]|BOLD:ABZ9439
Euxoa servitus|[7654]|LBCH6499-10|Canada|British Columbia|658[0n]|BOLD:ABZ9439
Euxoa servitus|[7655]|BBLPB814-10|Canada|Alberta|658[0n]|BOLD:ABZ9439
Euxoa servitus|[7656]|BBLPB815-10|Canada|Alberta|658[0n]|BOLD:ABZ9439
Euxoa servitus|[7657]|LPABC810-09|Canada|Alberta|658[0n]|BOLD:ACF3237
Euxoa servitus|[7658]|LPABC754-09|Canada|Alberta|658[0n]|BOLD:ACF3237
Euxoa servitus|[7659]|LPABC794-09|Canada|Alberta|658[0n]|BOLD:ACF3237
Euxoa servitus|[7660]|LPABC836-09|Canada|Alberta|658[0n]|BOLD:ACF3237
Euxoa servitus|[7661]|LPABC838-09|Canada|Alberta|658[0n]|BOLD:ACF3237
Euxoa servitus|[7662]|LPABC949-09|Canada|Alberta|658[0n]|BOLD:ACF3237
Euxoa servitus|[7663]|LOWCD799-06|Canada|British Columbia|658[0n]|BOLD:ABZ9439
Euxoa servitus|[7664]|LOWCD855-06|Canada|British Columbia|607[0n]|BOLD:ABZ9439
Euxoa servitus|[7665]|LOWCD857-06|Canada|British Columbia|612[0n]|BOLD:ABZ9439
Euxoa servitus|[7666]|LPAB087-08|Canada|Alberta|658[0n]|BOLD:ABZ9439
Euxoa servitus|[7667]|LPABB844-09|Canada|Alberta|658[0n]|BOLD:ABZ9439
Euxoa servitus|[7668]|LPABC084-09|Canada|Alberta|658[0n]|BOLD:ABZ9439
Euxoa servitus|[7669]|LPABC814-09|Canada|Alberta|658[0n]|BOLD:ABZ9439
Euxoa servitus|[7670]|LPABC837-09|Canada|Alberta|658[0n]|BOLD:ABZ9439
Euxoa servitus|[7671]|LPABC839-09|Canada|Alberta|658[0n]|BOLD:ABZ9439
Euxoa servitus|[7672]|BBLPB812-10|Canada|Alberta|658[0n]|BOLD:ABZ9439
Euxoa servitus|[7673]|BBLPB813-10|Canada|Alberta|658[0n]|BOLD:ABZ9439
Euxoa servitus|[7674]|BBLPB816-10|Canada|Alberta|658[0n]|BOLD:ABZ9439
Euxoa silens|[7675]|RDNMF307-08|United States|California|658[0n]|BOLD:AAC7725

Euxoa servitus|[7673]|BBLPB813-10|Canada|Alberta|658[0n]|BOLD:ABZ9439
Euxoa servitus|[7674]|BBLPB816-10|Canada|Alberta|658[0n]|BOLD:ABZ9439
Euxoa silens|[7675]|RDNMF307-08|United States|California|658[0n]|BOLD:AAC7725
Euxoa silens|[7676]|LOWCD329-06|Canada|British Columbia|557[0n]|BOLD:AAC7725
Euxoa silens|[7677]|LOWCD327-06|Canada|British Columbia|601[0n]|BOLD:AAC7725
Euxoa silens|[7678]|LOWCD330-06|Canada|British Columbia|612[0n]|BOLD:AAC7725
Euxoa silens|[7679]|LBCH5824-10|Canada|British Columbia|658[0n]|BOLD:AAC7725
Euxoa silens|[7680]|LBCH6058-10|Canada|British Columbia|658[0n]|BOLD:AAC7725
Euxoa silens|[7681]|LBCH5718-10|Canada|British Columbia|658[0n]|BOLD:AAC7725
Euxoa silens|[7682]|LBCH5729-10|Canada|British Columbia|658[0n]|BOLD:AAC7725
Euxoa silens|[7683]|BBLSX616-09|United States|Arizona|658[0n]|BOLD:AAC7725
Euxoa silens|[7684]|BBLSX609-09|United States|Arizona|658[0n]|BOLD:AAC7725
Euxoa silens|[7685]|BBLSX607-09|United States|Arizona|658[0n]|BOLD:AAC7725
Euxoa silens|[7686]|NAMUM272-08|United States|California|658[0n]|BOLD:AAC7725
Euxoa silens|[7687]|RDNMF308-08|Canada|British Columbia|658[0n]|BOLD:AAC7725
Euxoa silens|[7688]|RDNMF091-08|United States|Utah|658[0n]|BOLD:AAC7725
Euxoa silens|[7689]|RDNMF017-08|United States|Colorado|658[0n]|BOLD:AAC7725
Euxoa silens|[7690]|LOWCD328-06|Canada|British Columbia|658[0n]|BOLD:AAC7725
Euxoa silens|[7691]|LOWCD326-06|Canada|British Columbia|658[0n]|BOLD:AAC7725
Euxoa silens|[7692]|IAWLB146-10|United States|California|658[0n]|BOLD:AAC7725
Euxoa silens|[7693]|BBLOC148-11|United States|Texas|658[0n]|BOLD:AAC7725
Euxoa pimensis|[7694]|BBLSY731-09|United States|Arizona|658[0n]|BOLD:ACF0582
Euxoa pimensis|[7695]|BBLSX719-09|United States|Arizona|658[0n]|BOLD:ACF0582
Euxoa pimensis|[7696]|BBLSY045-09|United States|Arizona|658[0n]|BOLD:ACF0582
Euxoa pimensis|[7697]|RDNME1031-08|United States|Arizona|658[0n]|BOLD:ACF0582
Euxoa pimensis|[7698]|LOCBC385-06|United States|California|658[0n]|BOLD:ACF0582
Euxoa pimensis|[7699]|BBLSY308-09|United States|Arizona|634[0n]|BOLD:ACF0582
Euxoa pimensis|[7700]|BBLSY313-09|United States|Arizona|646[0n]|BOLD:ACF0582
Euxoa pimensis|[7701]|BBLSY312-09|United States|Arizona|597[0n]|BOLD:ACF0582
Euxoa pimensis|[7702]|BBLSY721-09|United States|Arizona|658[0n]|BOLD:ACF0582
Euxoa pimensis|[7703]|BBLSY727-09|United States|Arizona|658[0n]|BOLD:ACF0582
Euxoa pimensis|[7704]|IAWLB244-11|United States|Arizona|658[0n]|BOLD:ACF0582
Euxoa pimensis|[7705]|IAWLB245-11|United States|Arizona|658[0n]|BOLD:ACF0582
Euxoa pimensis|[7706]|CMAZA1054-12|United States|Arizona|658[0n]|BOLD:ACF0582
Euxoa immixta|[7707]|LNAUS2857-13|United States|Maryland|623[0n]|BOLD:ACI3709
Euxoa immixta|[7708]|LNAUS2858-13|United States|Texas|476[0n]|
Euxoa tessellata|[7709]|RDNMB626-05|United States|Washington|500[0n]|
Euxoa tessellata|[7710]|RDNM808-05|United States|Washington|565[1n]|BOLD:ACF2025
Euxoa tessellata|[7711]|BBLOC1389-11|United States|California|615[0n]|BOLD:ACF2025
Euxoa tessellata|[7712]|RDNM809-05|United States|Nevada|658[0n]|BOLD:ACF2025
Euxoa tessellata|[7713]|LBCG1099-09|Canada|British Columbia|658[0n]|BOLD:ACF2025
Euxoa tessellata|[7714]|LBCG1101-09|Canada|British Columbia|658[0n]|BOLD:ACF2025
Euxoa tessellata|[7715]|LBCG2888-09|Canada|British Columbia|658[0n]|BOLD:ACF2025
Euxoa tessellata|[7716]|LBCH5810-10|Canada|British Columbia|658[0n]|BOLD:ACF2025
Euxoa tessellata|[7717]|LBCH5856-10|Canada|British Columbia|658[0n]|BOLD:ACF2025
Euxoa tessellata|[7718]|LBCH5914-10|Canada|British Columbia|658[0n]|BOLD:ACF2025
Euxoa tessellata|[7719]|LBCH7686-10|Canada|British Columbia|658[0n]|BOLD:ACF2025
Euxoa tessellata|[7720]|IAWLB061-10|United States|California|658[0n]|BOLD:ACF2025
Euxoa tessellata|[7721]|JMMMB404-11|United States|California|658[0n]|BOLD:ACF2025
Euxoa tessellata|[7722]|BBLOC1391-11|United States|California|658[0n]|BOLD:ACF2025
Euxoa tessellata|[7723]|JMMMB529-13|United States|California|606[0n]|BOLD:ACF2025
Euxoa tessellata|[7724]|LOWCE777-06|Canada|British Columbia|575[0n]|BOLD:ACF2025
Euxoa tessellata|[7725]|LOWCE774-06|Canada|British Columbia|658[0n]|BOLD:ACF2025
Euxoa tessellata|[7726]|LOWCE779-06|Canada|British Columbia|658[0n]|BOLD:ACF2025
Euxoa tessellata|[7727]|LOWCE780-06|Canada|British Columbia|658[0n]|BOLD:ACF2025
Euxoa tessellata|[7728]|LOWCE783-06|Canada|British Columbia|658[0n]|BOLD:ACF2025
Euxoa tessellata|[7729]|RDNMD327-06|United States|Colorado|658[0n]|BOLD:ACF2025
Euxoa tessellata|[7730]|RDNMD328-06|United States|Colorado|658[0n]|BOLD:ACF2025
Euxoa tessellata|[7731]|LBCH6756-10|Canada|British Columbia|658[0n]|BOLD:ACF2025
Euxoa tessellata|[7732]|LBCH6541-10|Canada|British Columbia|658[0n]|BOLD:ACF2025
Euxoa tessellata|[7733]|RDNMB625-05|Canada|British Columbia|551[0n]|BOLD:ACF2025
Euxoa tessellata|[7734]|LOWCE781-06|Canada|British Columbia|658[0n]|BOLD:ACF2025
Euxoa tessellata|[7735]|LOWCE323-06|Canada|British Columbia|658[0n]|BOLD:ACF2025
Euxoa tessellata|[7736]|LOWCE782-06|Canada|British Columbia|658[0n]|BOLD:ACF2025
Euxoa tessellata|[7737]|LBCH6156-10|Canada|British Columbia|658[0n]|BOLD:ACF2025
Euxoa tessellata|[7738]|LBCH6376-10|Canada|British Columbia|658[0n]|BOLD:ACF2025
Euxoa tessellata|[7739]|LBCH7804-10|Canada|British Columbia|658[0n]|BOLD:ACF2025
Euxoa tessellata|[7740]|JMMMB608-13|United States|California|606[0n]|BOLD:ACF2025
Euxoa tessellata|[7741]|LOCBF901-13|United States|California|594[0n]|BOLD:ACF2025
Euxoa tessellata|[7742]|BBLPB344-10|Canada|British Columbia|577[0n]|BOLD:ACF2025
Euxoa tessellata|[7743]|JMMMB597-13|United States|California|576[0n]|BOLD:ACF2025
Euxoa tessellata|[7744]|CNWBC134-13|Canada|Alberta|574[0n]|BOLD:ACF2025
Euxoa tessellata|[7745]|LOCBF899-13|United States|California|558[3n]|BOLD:ACF2025
Euxoa tessellata|[7746]|BBLSW369-09|United States|Arizona|658[0n]|BOLD:ACF2025
Euxoa tessellata|[7747]|LBCG2880-09|Canada|British Columbia|658[0n]|BOLD:ACF2025
Euxoa tessellata|[7748]|LBCG503-08|Canada|British Columbia|658[0n]|BOLD:ACF2025
Euxoa tessellata|[7749]|LBCG482-08|Canada|British Columbia|658[0n]|BOLD:ACF2025
Euxoa tessellata|[7750]|LOCBD260-06|United States|California|658[0n]|BOLD:ACF2025
Euxoa tessellata|[7751]|LOWCE778-06|Canada|British Columbia|658[0n]|BOLD:ACF2025
Euxoa tessellata|[7752]|LOWCE776-06|Canada|British Columbia|658[0n]|BOLD:ACF2025
Euxoa tessellata|[7753]|LBCA608-05|Canada|British Columbia|633[0n]|BOLD:ACF2025
Euxoa tessellata|[7754]|CGLCA113-10|United States|California|632[0n]|BOLD:ACF2025
Euxoa tessellata|[7755]|LBCH5597-10|Canada|British Columbia|658[0n]|BOLD:ACF2025
Euxoa tessellata|[7756]|LBCH5863-10|Canada|British Columbia|658[0n]|BOLD:ACF2025
Euxoa tessellata|[7757]|LBCH6023-10|Canada|British Columbia|658[0n]|BOLD:ACF2025
Euxoa tessellata|[7758]|LBCH6744-10|Canada|British Columbia|658[0n]|BOLD:ACF2025
Euxoa tessellata|[7759]|BBLPB349-10|Canada|Alberta|658[0n]|BOLD:ACF2025
Euxoa tessellata|[7760]|BBLPB863-10|Canada|British Columbia|658[0n]|BOLD:ACF2025
Euxoa tessellata|[7761]|IAWLB440-11|United States|California|658[0n]|BOLD:ACF2025
Euxoa tessellata|[7762]|GMLC773-12|United States|California|658[0n]|BOLD:ACF2025
Euxoa tessellata|[7763]|GMLC919-12|United States|California|658[0n]|BOLD:ACF2025
Euxoa tessellata|[7764]|LOCBF900-13|United States|California|558[1n]|BOLD:ACF2025
Euxoa tessellata|[7765]|LOCBF1212-13|United States|California|583[0n]|BOLD:ACF2025
Euxoa tessellata|[7766]|CNWLM2411-13|Canada|Alberta|605[0n]|BOLD:ACF2025
Euxoa tessellata|[7767]|NGNAX947-14|Canada|British Columbia|591[0n]|BOLD:ACF2025
Euxoa tessellata|[7768]|CNGRL488-13|Canada|Saskatchewan|598[0n]|BOLD:ACF2025
Euxoa tessellata|[7769]|RDNMB630-05|Canada|New Brunswick|601[0n]|BOLD:ACF2025
Euxoa tessellata|[7770]|RDLQB421-05|Canada|Quebec|590[0n]|BOLD:ACF2025
Euxoa tessellata|[7771]|XAJ819-06|Canada|Ontario|658[0n]|BOLD:ACF2025
Euxoa tessellata|[7772]|XAJ838-06|Canada|Ontario|658[0n]|BOLD:ACF2025
Euxoa tessellata|[7773]|XAJ847-06|Canada|Ontario|658[0n]|BOLD:ACF2025
Euxoa tessellata|[7774]|RDLQF833-06|Canada|Quebec|658[0n]|BOLD:ACF2025
Euxoa tessellata|[7775]|CNGRJ073-13|Canada|Saskatchewan|583[0n]|BOLD:ACF2025

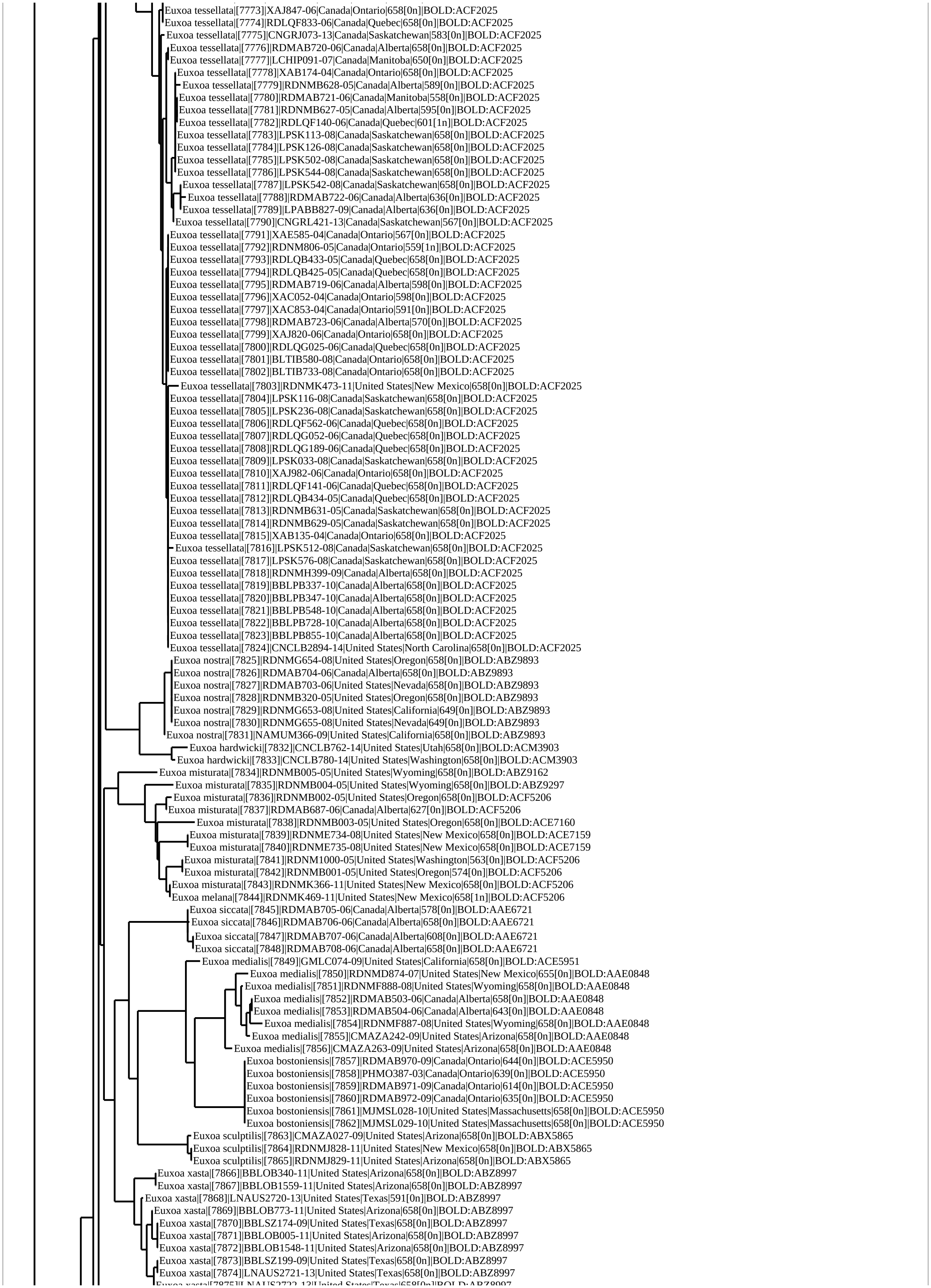
Euxoa tessellata|[7773]|XAJ847-06|Canada|Ontario|658[0n]|BOLD:ACF2025
Euxoa tessellata|[7774]|RDLQF833-06|Canada|Quebec|658[0n]|BOLD:ACF2025
Euxoa tessellata|[7775]|CNGRJ073-13|Canada|Saskatchewan|583[0n]|BOLD:ACF2025
Euxoa tessellata|[7776]|RDMAB720-06|Canada|Alberta|658[0n]|BOLD:ACF2025
Euxoa tessellata|[7777]|LCHIP091-07|Canada|Manitoba|650[0n]|BOLD:ACF2025
Euxoa tessellata|[7778]|XAB174-04|Canada|Ontario|658[0n]|BOLD:ACF2025
Euxoa tessellata|[7779]|RDNMB628-05|Canada|Alberta|589[0n]|BOLD:ACF2025
Euxoa tessellata|[7780]|RDMAB721-06|Canada|Manitoba|558[0n]|BOLD:ACF2025
Euxoa tessellata|[7781]|RDNMB627-05|Canada|Alberta|595[0n]|BOLD:ACF2025
Euxoa tessellata|[7782]|RDLQF140-06|Canada|Quebec|601[1n]|BOLD:ACF2025
Euxoa tessellata|[7783]|LPSK113-08|Canada|Saskatchewan|658[0n]|BOLD:ACF2025
Euxoa tessellata|[7784]|LPSK126-08|Canada|Saskatchewan|658[0n]|BOLD:ACF2025
Euxoa tessellata|[7785]|LPSK502-08|Canada|Saskatchewan|658[0n]|BOLD:ACF2025
Euxoa tessellata|[7786]|LPSK544-08|Canada|Saskatchewan|658[0n]|BOLD:ACF2025
Euxoa tessellata|[7787]|LPSK542-08|Canada|Saskatchewan|658[0n]|BOLD:ACF2025
Euxoa tessellata|[7788]|RDMAB722-06|Canada|Alberta|636[0n]|BOLD:ACF2025
Euxoa tessellata|[7789]|LPABB827-09|Canada|Alberta|636[0n]|BOLD:ACF2025
Euxoa tessellata|[7790]|CNGRL421-13|Canada|Saskatchewan|567[0n]|BOLD:ACF2025
Euxoa tessellata|[7791]|XAE585-04|Canada|Ontario|567[0n]|BOLD:ACF2025
Euxoa tessellata|[7792]|RDNM806-05|Canada|Ontario|559[1n]|BOLD:ACF2025
Euxoa tessellata|[7793]|RDLQB433-05|Canada|Quebec|658[0n]|BOLD:ACF2025
Euxoa tessellata|[7794]|RDLQB425-05|Canada|Quebec|658[0n]|BOLD:ACF2025
Euxoa tessellata|[7795]|RDMAB719-06|Canada|Alberta|598[0n]|BOLD:ACF2025
Euxoa tessellata|[7796]|XAC052-04|Canada|Ontario|598[0n]|BOLD:ACF2025
Euxoa tessellata|[7797]|XAC853-04|Canada|Ontario|591[0n]|BOLD:ACF2025
Euxoa tessellata|[7798]|RDMAB723-06|Canada|Alberta|570[0n]|BOLD:ACF2025
Euxoa tessellata|[7799]|XAJ820-06|Canada|Ontario|658[0n]|BOLD:ACF2025
Euxoa tessellata|[7800]|RDLQG025-06|Canada|Quebec|658[0n]|BOLD:ACF2025
Euxoa tessellata|[7801]|BLTIB580-08|Canada|Ontario|658[0n]|BOLD:ACF2025
Euxoa tessellata|[7802]|BLTIB733-08|Canada|Ontario|658[0n]|BOLD:ACF2025
Euxoa tessellata|[7803]|RDNMK473-11|United States|New Mexico|658[0n]|BOLD:ACF2025
Euxoa tessellata|[7804]|LPSK116-08|Canada|Saskatchewan|658[0n]|BOLD:ACF2025
Euxoa tessellata|[7805]|LPSK236-08|Canada|Saskatchewan|658[0n]|BOLD:ACF2025
Euxoa tessellata|[7806]|RDLQF562-06|Canada|Quebec|658[0n]|BOLD:ACF2025
Euxoa tessellata|[7807]|RDLQG052-06|Canada|Quebec|658[0n]|BOLD:ACF2025
Euxoa tessellata|[7808]|RDLQG189-06|Canada|Quebec|658[0n]|BOLD:ACF2025
Euxoa tessellata|[7809]|LPSK033-08|Canada|Saskatchewan|658[0n]|BOLD:ACF2025
Euxoa tessellata|[7810]|XAJ982-06|Canada|Ontario|658[0n]|BOLD:ACF2025
Euxoa tessellata|[7811]|RDLQF141-06|Canada|Quebec|658[0n]|BOLD:ACF2025
Euxoa tessellata|[7812]|RDLQB434-05|Canada|Quebec|658[0n]|BOLD:ACF2025
Euxoa tessellata|[7813]|RDNMB631-05|Canada|Saskatchewan|658[0n]|BOLD:ACF2025
Euxoa tessellata|[7814]|RDNMB629-05|Canada|Saskatchewan|658[0n]|BOLD:ACF2025
Euxoa tessellata|[7815]|XAB135-04|Canada|Ontario|658[0n]|BOLD:ACF2025
Euxoa tessellata|[7816]|LPSK512-08|Canada|Saskatchewan|658[0n]|BOLD:ACF2025
Euxoa tessellata|[7817]|LPSK576-08|Canada|Saskatchewan|658[0n]|BOLD:ACF2025
Euxoa tessellata|[7818]|RDNMH399-09|Canada|Alberta|658[0n]|BOLD:ACF2025
Euxoa tessellata|[7819]|BBLPB337-10|Canada|Alberta|658[0n]|BOLD:ACF2025
Euxoa tessellata|[7820]|BBLPB347-10|Canada|Alberta|658[0n]|BOLD:ACF2025
Euxoa tessellata|[7821]|BBLPB548-10|Canada|Alberta|658[0n]|BOLD:ACF2025
Euxoa tessellata|[7822]|BBLPB728-10|Canada|Alberta|658[0n]|BOLD:ACF2025
Euxoa tessellata|[7823]|BBLPB855-10|Canada|Alberta|658[0n]|BOLD:ACF2025
Euxoa tessellata|[7824]|CNCLB2894-14|United States|North Carolina|658[0n]|BOLD:ACF2025
Euxoa nostra|[7825]|RDNMG654-08|United States|Oregon|658[0n]|BOLD:ABZ9893
Euxoa nostra|[7826]|RDMAB704-06|Canada|Alberta|658[0n]|BOLD:ABZ9893
Euxoa nostra|[7827]|RDMAB703-06|United States|Nevada|658[0n]|BOLD:ABZ9893
Euxoa nostra|[7828]|RDNMB320-05|United States|Oregon|658[0n]|BOLD:ABZ9893
Euxoa nostra|[7829]|RDNMG653-08|United States|California|649[0n]|BOLD:ABZ9893
Euxoa nostra|[7830]|RDNMG655-08|United States|Nevada|649[0n]|BOLD:ABZ9893
Euxoa nostra|[7831]|NAMUM366-09|United States|California|658[0n]|BOLD:ABZ9893
Euxoa hardwicki|[7832]|CNCLB762-14|United States|Utah|658[0n]|BOLD:ACM3903
Euxoa hardwicki|[7833]|CNCLB780-14|United States|Washington|658[0n]|BOLD:ACM3903
Euxoa misturata|[7834]|RDNMB005-05|United States|Wyoming|658[0n]|BOLD:ABZ9162
Euxoa misturata|[7835]|RDNMB004-05|United States|Wyoming|658[0n]|BOLD:ABZ9297
Euxoa misturata|[7836]|RDNMB002-05|United States|Oregon|658[0n]|BOLD:ACF5206
Euxoa misturata|[7837]|RDMAB687-06|Canada|Alberta|627[0n]|BOLD:ACF5206
Euxoa misturata|[7838]|RDNMB003-05|United States|Oregon|658[0n]|BOLD:ACE7160
Euxoa misturata|[7839]|RDNME734-08|United States|New Mexico|658[0n]|BOLD:ACE7159
Euxoa misturata|[7840]|RDNME735-08|United States|New Mexico|658[0n]|BOLD:ACE7159
Euxoa misturata|[7841]|RDNM1000-05|United States|Washington|563[0n]|BOLD:ACF5206
Euxoa misturata|[7842]|RDNMB001-05|United States|Oregon|574[0n]|BOLD:ACF5206
Euxoa misturata|[7843]|RDNMK366-11|United States|New Mexico|658[0n]|BOLD:ACF5206
Euxoa melana|[7844]|RDNMK469-11|United States|New Mexico|658[1n]|BOLD:ACF5206
Euxoa siccata|[7845]|RDMAB705-06|Canada|Alberta|578[0n]|BOLD:AAE6721
Euxoa siccata|[7846]|RDMAB706-06|Canada|Alberta|658[0n]|BOLD:AAE6721
Euxoa siccata|[7847]|RDMAB707-06|Canada|Alberta|608[0n]|BOLD:AAE6721
Euxoa siccata|[7848]|RDMAB708-06|Canada|Alberta|658[0n]|BOLD:AAE6721
Euxoa medialis|[7849]|GMLC074-09|United States|California|658[0n]|BOLD:ACE5951
Euxoa medialis|[7850]|RDNMD874-07|United States|New Mexico|655[0n]|BOLD:AAE0848
Euxoa medialis|[7851]|RDNMF888-08|United States|Wyoming|658[0n]|BOLD:AAE0848
Euxoa medialis|[7852]|RDMAB503-06|Canada|Alberta|658[0n]|BOLD:AAE0848
Euxoa medialis|[7853]|RDMAB504-06|Canada|Alberta|643[0n]|BOLD:AAE0848
Euxoa medialis|[7854]|RDNMF887-08|United States|Wyoming|658[0n]|BOLD:AAE0848
Euxoa medialis|[7855]|CMAZA242-09|United States|Arizona|658[0n]|BOLD:AAE0848
Euxoa medialis|[7856]|CMAZA263-09|United States|Arizona|658[0n]|BOLD:AAE0848
Euxoa bostoniensis|[7857]|RDMAB970-09|Canada|Ontario|644[0n]|BOLD:ACE5950
Euxoa bostoniensis|[7858]|PHMO387-03|Canada|Ontario|639[0n]|BOLD:ACE5950
Euxoa bostoniensis|[7859]|RDMAB971-09|Canada|Ontario|614[0n]|BOLD:ACE5950
Euxoa bostoniensis|[7860]|RDMAB972-09|Canada|Ontario|635[0n]|BOLD:ACE5950
Euxoa bostoniensis|[7861]|MJMSL028-10|United States|Massachusetts|658[0n]|BOLD:ACE5950
Euxoa bostoniensis|[7862]|MJMSL029-10|United States|Massachusetts|658[0n]|BOLD:ACE5950
Euxoa sculptilis|[7863]|CMAZA027-09|United States|Arizona|658[0n]|BOLD:ABX5865
Euxoa sculptilis|[7864]|RDNMJ828-11|United States|New Mexico|658[0n]|BOLD:ABX5865
Euxoa sculptilis|[7865]|RDNMJ829-11|United States|Arizona|658[0n]|BOLD:ABX5865
Euxoa xasta|[7866]|BBLOB340-11|United States|Arizona|658[0n]|BOLD:ABZ8997
Euxoa xasta|[7867]|BBLOB1559-11|United States|Arizona|658[0n]|BOLD:ABZ8997
Euxoa xasta|[7868]|LNAUS2720-13|United States|Texas|591[0n]|BOLD:ABZ8997
Euxoa xasta|[7869]|BBLOB773-11|United States|Arizona|658[0n]|BOLD:ABZ8997
Euxoa xasta|[7870]|BBLSZ174-09|United States|Texas|658[0n]|BOLD:ABZ8997
Euxoa xasta|[7871]|BBLOB005-11|United States|Arizona|658[0n]|BOLD:ABZ8997
Euxoa xasta|[7872]|BBLOB1548-11|United States|Arizona|658[0n]|BOLD:ABZ8997
Euxoa xasta|[7873]|BBLSZ199-09|United States|Texas|658[0n]|BOLD:ABZ8997
Euxoa xasta|[7874]|LNAUS2721-13|United States|Texas|658[0n]|BOLD:ABZ8997
Euxoa xasta|[7875]|LNAUS2722-13|United States|Texas|658[0n]|BOLD:ABZ8997

Euxoa xasta|[7872]|BBLOB1540-11|United States|Arizona|658[0n]|BOLD:ABZ8997
Euxoa xasta|[7873]|BBLSZ199-09|United States|Texas|658[0n]|BOLD:ABZ8997
Euxoa xasta|[7874]|LNAUS2721-13|United States|Texas|658[0n]|BOLD:ABZ8997
Euxoa xasta|[7875]|LNAUS2722-13|United States|Texas|658[0n]|BOLD:ABZ8997
Euxoa niveilinea|[7876]|RDNMF088-08|United States|Colorado|658[0n]|BOLD:AAE6689
Euxoa niveilinea|[7877]|RDNMF016-08|United States|Colorado|658[0n]|BOLD:AAE6689
Euxoa niveilinea|[7878]|RDNMG417-08|United States|Colorado|658[0n]|BOLD:AAE6689
Euxoa niveilinea|[7879]|RDNMG418-08|United States|Colorado|658[0n]|BOLD:AAE6689
Euxoa niveilinea|[7880]|RDNMJ121-10|United States|New Mexico|658[0n]|BOLD:ABX5403
Euxoa citricolor|[7881]|RDMAB553-06|Canada|Alberta|658[0n]|BOLD:ACE4732
Euxoa citricolor|[7882]|RDMAB590-06|Canada|Alberta|658[0n]|BOLD:ACE4732
Euxoa citricolor|[7883]|RDNMB611-05|United States|Wyoming|658[0n]|BOLD:ACE4732
Euxoa citricolor|[7884]|RDNMB612-05|United States|Oregon|542[1n]|BOLD:ACE4732
Euxoa citricolor|[7885]|RDNMG665-08|United States|Oregon|658[0n]|BOLD:ACE4732
Euxoa citricolor|[7886]|RDNMG666-08|United States|Oregon|658[0n]|BOLD:ACE4732
Euxoa citricolor|[7887]|RDNMG667-08|United States|Nevada|658[0n]|BOLD:ACE4732
Euxoa mimallonis|[7888]|RDNMB648-05|Canada|Saskatchewan|658[0n]|BOLD:AAC3453
Euxoa biformata|[7889]|RDNMF834-08|United States|Washington|658[0n]|BOLD:AAC3453
Euxoa shasta|[7890]|RDNMB656-05|United States|California|583[1n]|BOLD:AAC3453
Euxoa shasta|[7891]|RDNMB658-05|United States|California|658[0n]|BOLD:AAC3453
Euxoa shasta|[7892]|RDNMB657-05|United States|Nevada|658[0n]|BOLD:AAC3453
Euxoa mimallonis|[7893]|RDNMB651-05|United States|California|658[0n]|BOLD:AAC3453
Euxoa mimallonis|[7894]|RDNM797-05|United States|Colorado|658[0n]|BOLD:AAC3453
Euxoa mimallonis|[7895]|RDNM796-05|United States|Colorado|658[0n]|BOLD:AAC3453
Euxoa intermontana|[7896]|RDNMB654-05|United States|Nevada|614[0n]|BOLD:AAC3453
Euxoa mimallonis|[7897]|RDMAB295-05|Canada|Alberta|658[0n]|BOLD:AAC3453
Euxoa biformata|[7898]|RDNMF851-08|United States|Washington|658[0n]|BOLD:AAC3453
Euxoa shasta|[7899]|IAWLB155-10|United States|California|658[0n]|BOLD:AAC3453
Euxoa mimallonis|[7900]|RDNM798-05|United States|Nevada|658[0n]|BOLD:AAC3453
Euxoa mimallonis|[7901]|RDNM799-05|United States|Nevada|658[0n]|BOLD:AAC3453
Euxoa mimallonis|[7902]|RDNMB649-05|Canada|Alberta|658[0n]|BOLD:AAC3453
Euxoa mimallonis|[7903]|RDNMB650-05|United States|California|658[0n]|BOLD:AAC3453
Euxoa intermontana|[7904]|RDNMG985-08|United States|Colorado|658[0n]|BOLD:AAC3453
Euxoa mimallonis|[7905]|LBCH6273-10|Canada|British Columbia|658[0n]|BOLD:AAC3453
Euxoa mimallonis|[7906]|LBCH6660-10|Canada|British Columbia|658[0n]|BOLD:AAC3453
Euxoa biformata|[7907]|RDNMF850-08|United States|Washington|658[0n]|BOLD:AAC3453
Euxoa mimallonis|[7908]|RDLQB604-05|Canada|Quebec|658[0n]|BOLD:AAC3453
Euxoa biformata|[7909]|RDNMB653-05|United States|Washington|587[0n]|BOLD:AAC3453
Euxoa biformata|[7910]|IAWLB154-10|United States|California|658[0n]|BOLD:AAC3453
Euxoa mimallonis|[7911]|RDNMB647-05|Canada|British Columbia|658[0n]|BOLD:AAC3453
Euxoa mimallonis|[7912]|RDNMB646-05|United States|Wyoming|658[0n]|BOLD:AAC3453
Euxoa mimallonis|[7913]|RDNMB645-05|United States|Wyoming|658[0n]|BOLD:AAC3453
Euxoa biformata|[7914]|RDNMB652-05|United States|Washington|658[43n]|BOLD:AAC3453
Euxoa biformata|[7915]|RDNMB655-05|United States|Washington|650[0n]|BOLD:AAC3453
Euxoa intermontana|[7916]|RDNMG427-08|United States|Colorado|658[0n]|BOLD:AAC3453
Euxoa biformata|[7917]|RDNMF854-08|United States|Washington|658[0n]|BOLD:AAC3453
Euxoa mimallonis|[7918]|LBCH6527-10|Canada|British Columbia|658[0n]|BOLD:AAC3453
Euxoa mimallonis|[7919]|LBCH6528-10|Canada|British Columbia|658[0n]|BOLD:AAC3453
Euxoa mimallonis|[7920]|LBCH6759-10|Canada|British Columbia|658[0n]|BOLD:AAC3453
Euxoa mimallonis|[7921]|LBCH6827-10|Canada|British Columbia|658[0n]|BOLD:AAC3453
Euxoa mimallonis|[7922]|LBCH7540-10|Canada|British Columbia|658[0n]|BOLD:AAC3453
Euxoa intermontana|[7923]|LNAUS2897-13|United States|Colorado|658[0n]|BOLD:AAC3453
Euxoa sp.|[7924]|IAWLB501-11|United States|Arizona|658[0n]|BOLD:ABZ9139
Euxoa sp.|[7925]|IAWLB500-11|United States|Arizona|658[0n]|BOLD:ABZ9139
Euxoa sp.|[7926]|IAWLB502-11|United States|Arizona|658[0n]|BOLD:ABZ9139
Euxoa septentrionalis|[7927]|RDNMF024-08|United States|Colorado|652[0n]|BOLD:ABZ8716
Euxoa septentrionalis|[7928]|LBCH7130-10|Canada|British Columbia|658[0n]|BOLD:AAC8054
Euxoa septentrionalis|[7929]|RDNMF297-08|United States|Oregon|658[0n]|BOLD:AAC8054
Euxoa septentrionalis|[7930]|JMMMB425-11|United States|California|658[0n]|BOLD:AAC8054
Euxoa septentrionalis|[7931]|NAMUM131-08|United States|California|658[0n]|BOLD:AAC8054
Euxoa septentrionalis|[7932]|RDNMF298-08|United States|California|658[0n]|BOLD:AAC8054
Euxoa septentrionalis|[7933]|GMLC051-09|United States|California|658[0n]|BOLD:AAC8054
Euxoa septentrionalis|[7934]|CGLCA114-10|United States|California|658[0n]|BOLD:AAC8054
Euxoa septentrionalis|[7935]|LOCBB338-06|United States|California|658[0n]|BOLD:AAC8054
Euxoa septentrionalis|[7936]|LOCBB339-06|United States|California|658[0n]|BOLD:AAC8054
Euxoa septentrionalis|[7937]|LOCBB340-06|United States|California|658[0n]|BOLD:AAC8054
Euxoa septentrionalis|[7938]|LOCBB342-06|United States|California|658[0n]|BOLD:AAC8054
Euxoa septentrionalis|[7939]|JMMMB388-11|United States|California|658[0n]|BOLD:AAC8054
Euxoa septentrionalis|[7940]|GMLC1166-12|United States|California|658[0n]|BOLD:AAC8054
Euxoa quadridentata|[7941]|RDMAB552-06|Canada|Alberta|658[0n]|BOLD:ABZ9158
Euxoa quadridentata|[7942]|LOWCD107-06|Canada|British Columbia|570[1n]|BOLD:ABZ9158
Euxoa quadridentata|[7943]|LOWCD102-06|Canada|British Columbia|615[0n]|BOLD:ABZ9158
Euxoa quadridentata|[7944]|LOWCD103-06|Canada|British Columbia|615[0n]|BOLD:ABZ9158
Euxoa quadridentata|[7945]|LOWCE633-06|Canada|British Columbia|658[0n]|BOLD:ABZ9158
Euxoa quadridentata|[7946]|LOWCD105-06|Canada|British Columbia|614[5n]|BOLD:ABZ9158
Euxoa quadridentata|[7947]|LOWCE643-06|Canada|British Columbia|658[0n]|BOLD:ABZ9158
Euxoa quadridentata|[7948]|LOWCD096-06|Canada|British Columbia|603[0n]|BOLD:ABZ9158
Euxoa quadridentata|[7949]|LOWCE634-06|Canada|British Columbia|658[0n]|BOLD:ABZ9158
Euxoa quadridentata|[7950]|LOWCE635-06|Canada|British Columbia|658[0n]|BOLD:ABZ9158
Euxoa quadridentata|[7951]|LOWCE629-06|Canada|British Columbia|658[0n]|BOLD:ABZ9158
Euxoa quadridentata|[7952]|LOWCE630-06|Canada|British Columbia|658[0n]|BOLD:ABZ9158
Euxoa quadridentata|[7953]|LOWCD109-06|Canada|British Columbia|658[0n]|BOLD:ABZ9158
Euxoa quadridentata|[7954]|LOWCD110-06|Canada|British Columbia|658[0n]|BOLD:ABZ9158
Euxoa quadridentata|[7955]|LOWCD108-06|Canada|British Columbia|657[0n]|BOLD:ABZ9158
Euxoa quadridentata|[7956]|LOWCD106-06|Canada|British Columbia|657[0n]|BOLD:ABZ9158
Euxoa quadridentata|[7957]|LOWCD104-06|Canada|British Columbia|658[0n]|BOLD:ABZ9158
Euxoa quadridentata|[7958]|LOWCD101-06|Canada|British Columbia|658[0n]|BOLD:ABZ9158
Euxoa quadridentata|[7959]|LOWCD099-06|Canada|British Columbia|658[0n]|BOLD:ABZ9158
Euxoa quadridentata|[7960]|LOWCD098-06|Canada|British Columbia|658[0n]|BOLD:ABZ9158
Euxoa quadridentata|[7961]|LOWCD097-06|Canada|British Columbia|658[0n]|BOLD:ABZ9158
Euxoa quadridentata|[7962]|LOWCD094-06|Canada|British Columbia|658[0n]|BOLD:ABZ9158
Euxoa quadridentata|[7963]|LOWCD093-06|Canada|British Columbia|658[0n]|BOLD:ABZ9158
Euxoa quadridentata|[7964]|LOWCD090-06|Canada|British Columbia|657[0n]|BOLD:ABZ9158
Euxoa quadridentata|[7965]|LOWCD091-06|Canada|British Columbia|622[0n]|BOLD:ABZ9158
Euxoa quadridentata|[7966]|LOWCD092-06|Canada|British Columbia|612[0n]|BOLD:ABZ9158
Euxoa quadridentata|[7967]|LOWCD095-06|Canada|British Columbia|609[0n]|BOLD:ABZ9158
Euxoa quadridentata|[7968]|LOWCD100-06|Canada|British Columbia|612[0n]|BOLD:ABZ9158
Euxoa quadridentata|[7969]|LOWCD111-06|Canada|British Columbia|614[0n]|BOLD:ABZ9158
Euxoa quadridentata|[7970]|LOWCE632-06|Canada|British Columbia|613[0n]|BOLD:ABZ9158
Euxoa quadridentata|[7971]|LOWCE637-06|Canada|British Columbia|656[0n]|BOLD:ABZ9158
Euxoa quadridentata|[7972]|LOWCE636-06|Canada|British Columbia|658[0n]|BOLD:ABZ9158
Euxoa quadridentata|[7973]|LOWCE639-06|Canada|British Columbia|658[0n]|BOLD:ABZ9158
Euxoa quadridentata|[7974]|LOWCE640-06|Canada|British Columbia|658[0n]|BOLD:ABZ9158
[illegible]

Euxoa quadridentata|[7972]|LOWCE636-06|Canada|British Columbia|658[0n]|BOLD:ABZ9158
Euxoa quadridentata|[7973]|LOWCE639-06|Canada|British Columbia|658[0n]|BOLD:ABZ9158
Euxoa quadridentata|[7974]|LOWCE640-06|Canada|British Columbia|658[0n]|BOLD:ABZ9158
Euxoa quadridentata|[7975]|LOWCE641-06|Canada|British Columbia|658[0n]|BOLD:ABZ9158
Euxoa quadridentata|[7976]|LOWCE642-06|Canada|British Columbia|658[0n]|BOLD:ABZ9158
Euxoa quadridentata|[7977]|LOWCE646-06|Canada|British Columbia|658[0n]|BOLD:ABZ9158
Euxoa quadridentata|[7978]|LOWCE647-06|Canada|British Columbia|658[0n]|BOLD:ABZ9158
Euxoa quadridentata|[7979]|LOWCE648-06|Canada|British Columbia|658[0n]|BOLD:ABZ9158
Euxoa quadridentata|[7980]|LOWCE649-06|Canada|British Columbia|658[0n]|BOLD:ABZ9158
Euxoa quadridentata|[7981]|LBCH6843-10|Canada|British Columbia|658[0n]|BOLD:ABZ9158
Euxoa perexcellens|[7982]|RDMAB296-05|Canada|Alberta|539[3n]|BOLD:ABZ9415
Euxoa bifasciata|[7983]|RDNM785-05|United States|Oregon|548[1n]|BOLD:AAE6701
Euxoa bifasciata|[7984]|RDNM786-05|United States|California|658[0n]|BOLD:AAE6701
Euxoa bifasciata|[7985]|RDNM898-05|United States|Washington|658[0n]|BOLD:AAE6701
Euxoa bifasciata|[7986]|RDNMB849-05|United States|Oregon|658[0n]|BOLD:AAE6701
Euxoa perexcellens|[7987]|RDNMG1040-08|United States|Washington|592[0n]|BOLD:ABZ9159
Euxoa perexcellens|[7988]|RDNMG1039-08|United States|Washington|590[0n]|BOLD:ABZ9159
Euxoa perexcellens|[7989]|NAMUM218-08|United States|California|609[0n]|BOLD:ABZ9159
Euxoa perexcellens|[7990]|RWWC714-11|United States|Washington|658[0n]|BOLD:ABZ9159
Euxoa perexcellens|[7991]|NAMUM235-08|United States|California|585[0n]|BOLD:ABZ9159
Euxoa perexcellens|[7992]|JMMMB316-11|United States|California|658[0n]|BOLD:ABZ9159
Euxoa perexcellens|[7993]|RWWB170-09|United States|Washington|658[0n]|BOLD:ABZ9159
Euxoa perexcellens|[7994]|RWWC736-11|United States|Washington|658[0n]|BOLD:ABZ9159
Euxoa perexcellens|[7995]|RWWC803-11|United States|Washington|658[0n]|BOLD:ABZ9159
Euxoa perexcellens|[7996]|RWWC849-12|United States|Washington|658[0n]|BOLD:ABZ9159
Euxoa velleripennis|[7997]|XAH222-05|Canada|Ontario|658[0n]|BOLD:ABZ9143
Euxoa velleripennis|[7998]|XAH435-05|Canada|Ontario|658[0n]|BOLD:ABZ9143
Euxoa velleripennis|[7999]|XAH346-05|Canada|Ontario|658[0n]|BOLD:ABZ9143
Euxoa velleripennis|[8000]|XAB454-04|Canada|Ontario|658[0n]|BOLD:ABZ9143
Euxoa velleripennis|[8001]|XAD439-04|Canada|Ontario|658[0n]|BOLD:ABZ9143
Euxoa velleripennis|[8002]|PHMO386-03|Canada|Ontario|639[0n]|BOLD:ABZ9143
Euxoa velleripennis|[8003]|XAH131-05|Canada|Ontario|658[0n]|BOLD:ABZ9143
Euxoa velleripennis|[8004]|XAH243-05|Canada|Ontario|658[0n]|BOLD:ABZ9143
Euxoa velleripennis|[8005]|XAH272-05|Canada|Ontario|658[0n]|BOLD:ABZ9143
Euxoa velleripennis|[8006]|XAH242-05|Canada|Ontario|658[0n]|BOLD:ABZ9143
Euxoa velleripennis|[8007]|XAH620-05|Canada|Ontario|627[0n]|BOLD:ABZ9143
Euxoa lillooet|[8008]|RDNMF674-08|Canada|British Columbia|636[0n]|BOLD:AAD3167
Euxoa lillooet|[8009]|RDNMF671-08|United States|Oregon|658[0n]|BOLD:AAD3167
Euxoa lillooet|[8010]|RDNMF672-08|United States|Oregon|658[0n]|BOLD:AAD3167
Euxoa lillooet|[8011]|RDNMF673-08|United States|Oregon|658[0n]|BOLD:AAD3167
Euxoa lillooet|[8012]|RDNMF675-08|Canada|British Columbia|658[0n]|BOLD:AAD3167
Euxoa lillooet|[8013]|RDNMF676-08|Canada|British Columbia|658[0n]|BOLD:AAD3167
Euxoa lillooet|[8014]|RDNMF896-08|Canada|British Columbia|658[0n]|BOLD:AAD3167
Euxoa latro|[8015]|JMMMB596-13|United States|California|658[0n]|BOLD:ACJ0355
Euxoa atristrigata|[8016]|RDNMF668-08|United States|Colorado|588[0n]|BOLD:ACE5990
Euxoa atristrigata|[8017]|RDNMF670-08|United States|Montana|658[0n]|BOLD:ACE5990
Euxoa atristrigata|[8018]|RDNMF666-08|United States|Arizona|658[0n]|BOLD:ACE5990
Euxoa atristrigata|[8019]|RDNMF667-08|United States|New Mexico|640[1n]|BOLD:ACE5990
Euxoa atristrigata|[8020]|LBCH6847-10|Canada|British Columbia|634[0n]|BOLD:ACE5990
Euxoa atristrigata|[8021]|LBCH6943-10|Canada|British Columbia|658[0n]|BOLD:ACE5990
Euxoa atristrigata|[8022]|LBCH7746-10|Canada|British Columbia|658[0n]|BOLD:ACE5990
Euxoa pallipennis|[8023]|RDNMG672-08|United States|Oregon|658[0n]|BOLD:ABX6450
Euxoa pallipennis|[8024]|RDNMG671-08|United States|Oregon|658[0n]|BOLD:ABX6450
Euxoa pallipennis|[8025]|RDNMF023-08|United States|Colorado|658[0n]|BOLD:ABX6450
Euxoa pallipennis|[8026]|RDNMG673-08|United States|Oregon|641[0n]|BOLD:ABX6450
Euxoa pallipennis|[8027]|RDNMK361-11|United States|New Mexico|658[0n]|BOLD:ABX6450
Euxoa baja|[8028]|RDNML211-13|United States|California|658[0n]|BOLD:ABX6450
Euxoa munis|[8029]|RDNMF029-08|Canada|Alberta|658[0n]|BOLD:ABX5442
Euxoa munis|[8030]|NAMUM281-08|United States|California|658[0n]|BOLD:ABX5442
Euxoa munis|[8031]|RDNMG496-08|Canada|Alberta|658[0n]|BOLD:ABX5442
Euxoa munis|[8032]|RDNMG497-08|Canada|Alberta|658[0n]|BOLD:ABX5442
Euxoa aequalis|[8033]|RDNM364-05|Canada|Alberta|658[0n]|BOLD:ABZ9912
Euxoa aequalis|[8034]|RDNM365-05|Canada|Alberta|523[0n]|BOLD:ABZ9912
Euxoa aequalis|[8035]|RDNM363-05|Canada|Alberta|658[0n]|BOLD:ABZ9912
Euxoa aequalis|[8036]|RDNM361-05|United States|Washington|570[0n]|BOLD:ABZ9912
Euxoa aequalis|[8037]|RDNM360-05|United States|Washington|590[1n]|BOLD:ABZ9912
Euxoa aequalis|[8038]|RDNM362-05|United States|Washington|587[1n]|BOLD:ABZ9912
Euxoa aequalis|[8039]|RDNMC083-05|Canada|Alberta|658[0n]|BOLD:ABZ9912
Euxoa aequalis|[8040]|LTOL1329-12|United States|California|658[0n]|BOLD:ABZ9912
Euxoa laetificans|[8041]|RDMAB993-09|United States|Oregon|636[0n]|BOLD:ABZ8980
Euxoa laetificans|[8042]|RDNMB666-05|United States|Wyoming|658[0n]|BOLD:ABZ8980
Euxoa laetificans|[8043]|RDMAB994-09|Canada|Alberta|636[0n]|BOLD:ABZ8980
Euxoa laetificans|[8044]|RDMAB595-06|Canada|Alberta|551[0n]|BOLD:ABZ8980
Euxoa laetificans|[8045]|RDMAB995-09|Canada|Alberta|645[0n]|BOLD:ABZ8980
Euxoa henrietta|[8046]|GMLC1232-12|United States|California|658[0n]|BOLD:ABZ8999
Euxoa henrietta|[8047]|GMLC1243-12|United States|California|658[0n]|BOLD:ABZ8999
Euxoa henrietta|[8048]|JMMMB532-13|United States|California|658[0n]|BOLD:ABZ8999
Euxoa recula|[8049]|NAMUM242-08|United States|California|657[0n]|BOLD:ABZ9142
Euxoa cicatricosa|[8050]|RDNMF302-08|United States|Oregon|658[0n]|BOLD:ABZ9142
Euxoa cicatricosa|[8051]|RDMAB556-06|Canada|Alberta|658[0n]|BOLD:ABZ9142
Euxoa cicatricosa|[8052]|RDNMF303-08|United States|Washington|640[0n]|BOLD:ABZ9142
Euxoa cicatricosa|[8053]|RDNMG795-08|Canada|British Columbia|593[2n]|BOLD:ABZ9142
Euxoa recula|[8054]|RDNMG676-08|United States|Oregon|658[0n]|BOLD:ABZ9142
Euxoa recula|[8055]|RDNMG798-08|United States|Nevada|658[0n]|BOLD:ABZ9142
Euxoa cicatricosa|[8056]|RDNMG1001-08|United States|Washington|658[0n]|BOLD:ABZ9142
Euxoa cicatricosa|[8057]|RDNMG898-08|United States|Colorado|658[0n]|BOLD:ABZ9142
Euxoa cicatricosa|[8058]|RDNMF304-08|United States|Oregon|658[0n]|BOLD:ABZ9142
Euxoa cicatricosa|[8059]|RDNMF015-08|United States|Colorado|658[0n]|BOLD:ABZ9142
Euxoa cicatricosa|[8060]|RDNMG796-08|Canada|British Columbia|641[0n]|BOLD:ABZ9142
Euxoa detersa|[8061]|BBLEC490-09|Canada|New Brunswick|658[0n]|BOLD:ABZ9142
Euxoa detersa|[8062]|BBLEC498-09|Canada|New Brunswick|658[0n]|BOLD:ABZ9142
Euxoa detersa|[8063]|BBLEC502-09|Canada|New Brunswick|658[0n]|BOLD:ABZ9142
Euxoa detersa|[8064]|BBLEC503-09|Canada|New Brunswick|658[0n]|BOLD:ABZ9142
Euxoa detersa|[8065]|BBLEC512-09|Canada|New Brunswick|658[0n]|BOLD:ABZ9142
Euxoa detersa|[8066]|RDNMG650-08|Canada|Ontario|658[0n]|BOLD:ABZ9142
Euxoa detersa|[8067]|LNC475-06|United States|North Carolina|658[0n]|BOLD:ABZ9142
Euxoa detersa|[8068]|LNC476-06|United States|North Carolina|658[0n]|BOLD:ABZ9142
Euxoa detersa|[8069]|RDNMG649-08|United States|Indiana|658[0n]|BOLD:ABZ9142
Euxoa detersa|[8070]|RDNMG651-08|Canada|Ontario|658[0n]|BOLD:ABZ9142
Euxoa detersa|[8071]|RDNMG652-08|Canada|Ontario|649[4n]|BOLD:ABZ9142
Euxoa detersa|[8072]|BBLEC537-09|Canada|New Brunswick|658[0n]|BOLD:ABZ9142
Euxoa nevada|[8073]|RDNMF335-08|United States|Washington|658[0n]|BOLD:AAD3157
Euxoa nevada|[8074]|RDNMF021-08|United States|Wyoming|658[0n]|BOLD:AAD3157

Euxoa detersa|[8072]|BBLEC537-09|Canada|New Brunswick|658[0n]|BOLD:ABZ9142
Euxoa nevada|[8073]|RDNMF335-08|United States|Washington|658[0n]|BOLD:AAD3157
Euxoa nevada|[8074]|RDNMF021-08|United States|Wyoming|658[0n]|BOLD:AAD3157
Euxoa nevada|[8075]|RDNMF090-08|United States|Wyoming|658[0n]|BOLD:AAD3157
Euxoa nevada|[8076]|RDNMF334-08|United States|Oregon|658[0n]|BOLD:AAD3157
Euxoa nevada|[8077]|RDNMG981-08|United States|Wyoming|658[0n]|BOLD:AAD3157
Euxoa nevada|[8078]|RDNMF849-08|United States|Washington|658[0n]|BOLD:AAD3157
Euxoa nevada|[8079]|RDNMF871-08|United States|Washington|658[0n]|BOLD:AAD3157
Euxoa nevada|[8080]|LBCH7053-10|Canada|British Columbia|658[0n]|BOLD:AAD3157
Euxoa nevada|[8081]|LBCH7056-10|Canada|British Columbia|658[0n]|BOLD:AAD3157
Euxoa moerens|[8082]|RDNMF022-08|United States|Colorado|658[0n]|BOLD:ABZ9000
Euxoa dargo|[8083]|RDNMF436-08|United States|Washington|658[1n]|BOLD:AAE0511
Euxoa dargo|[8084]|RDNMF020-08|United States|Colorado|658[0n]|BOLD:AAE0511
Euxoa dargo|[8085]|RDNMF437-08|United States|Washington|658[0n]|BOLD:AAE0511
Euxoa dargo|[8086]|RDNMG999-08|Canada|Alberta|658[0n]|BOLD:AAE0511
Euxoa dargo|[8087]|RDNMG1000-08|United States|Washington|658[0n]|BOLD:AAE0511
Euxoa satiens|[8088]|RDNMF348-08|United States|California|658[0n]|BOLD:AAF6167
Euxoa satiens|[8089]|RDNMF346-08|United States|Wyoming|658[0n]|BOLD:AAF6167
Euxoa satiens|[8090]|RDNMF347-08|United States|Wyoming|658[0n]|BOLD:AAF6167
Euxoa satiens|[8091]|LBCH6920-10|Canada|British Columbia|635[0n]|BOLD:AAF6167
Euxoa satiens|[8092]|LBCH6812-10|Canada|British Columbia|658[0n]|BOLD:AAF6167
Euxoa satiens|[8093]|LBCH6811-10|Canada|British Columbia|658[0n]|BOLD:AAF6167
Euxoa satiens|[8094]|LBCH6810-10|Canada|British Columbia|658[0n]|BOLD:AAF6167
Euxoa satiens|[8095]|LBCH6447-10|Canada|British Columbia|658[0n]|BOLD:AAF6167
Euxoa satiens|[8096]|LBCH6813-10|Canada|British Columbia|636[0n]|BOLD:AAF6167
Euxoa satiens|[8097]|LBCH6814-10|Canada|British Columbia|658[0n]|BOLD:AAF6167
Euxoa satiens|[8098]|LBCH6815-10|Canada|British Columbia|658[0n]|BOLD:AAF6167
Euxoa satiens|[8099]|LBCH6816-10|Canada|British Columbia|658[0n]|BOLD:AAF6167
Euxoa satiens|[8100]|LBCH6817-10|Canada|British Columbia|658[0n]|BOLD:AAF6167
Euxoa satiens|[8101]|LBCH6889-10|Canada|British Columbia|658[0n]|BOLD:AAF6167
Euxoa satiens|[8102]|LBCH6890-10|Canada|British Columbia|658[0n]|BOLD:AAF6167
Euxoa satiens|[8103]|LBCH6891-10|Canada|British Columbia|658[0n]|BOLD:AAF6167
Euxoa satiens|[8104]|LBCH6892-10|Canada|British Columbia|658[0n]|BOLD:AAF6167
Euxoa satiens|[8105]|LBCH6893-10|Canada|British Columbia|658[0n]|BOLD:AAF6167
Euxoa satiens|[8106]|LBCH6894-10|Canada|British Columbia|658[0n]|BOLD:AAF6167
Euxoa satiens|[8107]|LBCH6895-10|Canada|British Columbia|658[0n]|BOLD:AAF6167
Euxoa satiens|[8108]|LBCH6896-10|Canada|British Columbia|658[0n]|BOLD:AAF6167
Euxoa satiens|[8109]|LBCH7005-10|Canada|British Columbia|658[0n]|BOLD:AAF6167
Euxoa satiens|[8110]|LBCH7006-10|Canada|British Columbia|658[0n]|BOLD:AAF6167
Euxoa satiens|[8111]|LBCH7007-10|Canada|British Columbia|658[0n]|BOLD:AAF6167
Euxoa satiens|[8112]|LBCH7008-10|Canada|British Columbia|658[0n]|BOLD:AAF6167
Euxoa satiens|[8113]|LBCH7009-10|Canada|British Columbia|658[0n]|BOLD:AAF6167
Euxoa satiens|[8114]|LBCH7010-10|Canada|British Columbia|658[0n]|BOLD:AAF6167
Euxoa satiens|[8115]|LBCH7011-10|Canada|British Columbia|658[0n]|BOLD:AAF6167
Euxoa satiens|[8116]|LBCH7012-10|Canada|British Columbia|658[0n]|BOLD:AAF6167
Euxoa subandera|[8117]|RDNMF665-08|Canada|British Columbia|618[0n]|BOLD:AAE6682
Euxoa subandera|[8118]|RDNMF663-08|United States|California|658[0n]|BOLD:AAE6682
Euxoa subandera|[8119]|RDNMF664-08|United States|Nevada|658[0n]|BOLD:AAE6682
Euxoa subandera|[8120]|NAMUM279-08|United States|California|658[0n]|BOLD:AAE6682
Euxoa moerens|[8121]|RDNMF815-08|United States|Oregon|658[0n]|BOLD:ABZ9003
Euxoa moerens|[8122]|RDNMF817-08|United States|Oregon|658[0n]|BOLD:ABZ9003
Euxoa murdocki|[8123]|NAMUM240-08|United States|California|658[0n]|BOLD:ABZ9002
Euxoa murdocki|[8124]|RDNMF875-08|United States|Oregon|658[0n]|BOLD:ABZ9002
Euxoa murdocki|[8125]|RDNMF306-08|United States|Oregon|658[0n]|BOLD:ABZ9002
Euxoa murdocki|[8126]|RDNMG986-08|United States|Utah|658[0n]|BOLD:ABZ9002
Euxoa murdocki|[8127]|RDNMF873-08|Canada|British Columbia|658[0n]|BOLD:ABZ9002
Euxoa murdocki|[8128]|RDNMF874-08|United States|Washington|658[0n]|BOLD:ABZ9002
Euxoa murdocki|[8129]|RDNMF036-08|United States|Oregon|658[0n]|BOLD:ABZ9002
Euxoa murdocki|[8130]|RDNMF305-08|United States|Oregon|658[0n]|BOLD:ABZ9002
Euxoa murdocki|[8131]|JMMMB319-11|United States|California|658[0n]|BOLD:ABZ9002
Euxoa pluralis|[8132]|RDNMF311-08|United States|Oregon|658[0n]|BOLD:ABZ9141
Euxoa pluralis|[8133]|RDNMF013-08|United States|Colorado|658[0n]|BOLD:ABZ9141
Euxoa pluralis|[8134]|RDNMF310-08|United States|Washington|658[0n]|BOLD:ABZ9141
Euxoa pluralis|[8135]|NAMUM271-08|United States|California|658[0n]|BOLD:ABZ9141
Euxoa pluralis|[8136]|LBCH5698-10|Canada|British Columbia|658[0n]|BOLD:ABZ9141
Euxoa pluralis|[8137]|IAWLB082-10|United States|California|658[0n]|BOLD:ABZ9141
Euxoa pluralis|[8138]|IAWLB083-10|United States|California|658[0n]|BOLD:ABZ9141
Euxoa pluralis|[8139]|IAWLB084-10|United States|California|658[0n]|BOLD:ABZ9141
Euxoa infracta|[8140]|RDNMF019-08|United States|Colorado|658[0n]|BOLD:ACE8540
Euxoa infracta|[8141]|RDNMG979-08|United States|Colorado|658[0n]|BOLD:ABZ9442
Euxoa infracta|[8142]|RDNMG800-08|Canada|British Columbia|590[1n]|BOLD:ABZ9442
Euxoa infracta|[8143]|LBCC010-05|Canada|British Columbia|658[0n]|BOLD:ABZ9442
Euxoa infracta|[8144]|RDNMF350-08|Canada|British Columbia|658[0n]|BOLD:ABZ9442
Euxoa infracta|[8145]|RDNMG799-08|Canada|British Columbia|658[0n]|BOLD:ABZ9442
Euxoa infracta|[8146]|LBCG1095-09|Canada|British Columbia|658[0n]|BOLD:ABZ9442
Euxoa infracta|[8147]|LBCH6750-10|Canada|British Columbia|658[0n]|BOLD:ABZ9442
Euxoa infracta|[8148]|LBCH7434-10|Canada|British Columbia|658[0n]|BOLD:ABZ9442
Euxoa infracta|[8149]|LBCH7546-10|Canada|British Columbia|658[0n]|BOLD:ABZ9442
Euxoa infracta|[8150]|LBCH7547-10|Canada|British Columbia|658[0n]|BOLD:ABZ9442
Euxoa infracta|[8151]|LBCH7678-10|Canada|British Columbia|658[0n]|BOLD:ABZ9442
Euxoa infracta|[8152]|LBCD458-05|Canada|British Columbia|658[0n]|BOLD:ABZ9442
Euxoa infracta|[8153]|LBCH7820-10|Canada|British Columbia|658[0n]|BOLD:ABZ9442
Euxoa infracta|[8154]|LBCH7960-10|Canada|British Columbia|658[2n]|BOLD:ABZ9442
Euxoa infracta|[8155]|LBCH7436-10|Canada|British Columbia|658[0n]|BOLD:ABZ9442
Euxoa dodi|[8156]|RDNMG980-08|United States|Wyoming|658[0n]|BOLD:ACF0932
Euxoa dodi|[8157]|RDNMF818-08|Canada|Alberta|658[0n]|BOLD:ACF0932
Euxoa dodi|[8158]|RDNMF819-08|Canada|Alberta|658[0n]|BOLD:ACF0932
Euxoa dodi|[8159]|RDNMF889-08|United States|Wyoming|658[0n]|BOLD:ACF0932
Euxoa dodi|[8160]|RDNMF890-08|Canada|Alberta|658[0n]|BOLD:ACF0932
Euxoa dodi|[8161]|CNGRG006-12|Canada|Saskatchewan|630[0n]|BOLD:ACF0932
Euxoa difformis|[8162]|LOWCE742-06|Canada|British Columbia|579[1n]|BOLD:ABZ9157
Euxoa difformis group|[8163]|RDNME1030-08|United States|Arizona|658[0n]|BOLD:ACF0986
Euxoa difformis|[8164]|LPVIB806-08|Canada|British Columbia|658[0n]|BOLD:ACE8314
Euxoa difformis|[8165]|LPVIB823-08|Canada|British Columbia|658[0n]|BOLD:ACE8314
Euxoa difformis|[8166]|RDNMB831-05|United States|Oregon|658[0n]|BOLD:ACE8314
Euxoa difformis|[8167]|LBCH7332-10|Canada|British Columbia|658[0n]|BOLD:ACE8314
Euxoa difformis|[8168]|LBCH7055-10|Canada|British Columbia|658[0n]|BOLD:ACE8314
Euxoa difformis|[8169]|RDNMC323-05|United States|Oregon|538[0n]|BOLD:ACE8314
Euxoa difformis|[8170]|RDNMF812-08|United States|Oregon|658[0n]|BOLD:ACE8314
Euxoa difformis|[8171]|RDNMF814-08|United States|Oregon|658[0n]|BOLD:ACE8314
Euxoa difformis|[8172]|LBCH7261-10|Canada|British Columbia|658[0n]|BOLD:ACE8314
Euxoa difformis|[8173]|LBCH7264-10|Canada|British Columbia|658[0n]|BOLD:ACE8314
Euxoa difformis|[8174]|LPVIB624-08|Canada|British Columbia|658[0n]|BOLD:ACE8314

Euxoa difformis|[8171]|RDNMF814-08|United States|Oregon|658[0n]|BOLD:ACE8314
Euxoa difformis|[8172]|LBCH7261-10|Canada|British Columbia|658[0n]|BOLD:ACE8314
Euxoa difformis|[8173]|LBCH7264-10|Canada|British Columbia|658[0n]|BOLD:ACE8314
Euxoa difformis|[8174]|LPVIB624-08|Canada|British Columbia|658[0n]|BOLD:ACE8314
Euxoa difformis|[8175]|LPVIB626-08|Canada|British Columbia|627[0n]|BOLD:ACE8314
Euxoa difformis|[8176]|LPVIB745-08|Canada|British Columbia|658[0n]|BOLD:ACE8314
Euxoa difformis|[8177]|LPVIB807-08|Canada|British Columbia|658[0n]|BOLD:ACE8314
Euxoa difformis|[8178]|LPVIB808-08|Canada|British Columbia|658[0n]|BOLD:ACE8314
Euxoa difformis|[8179]|LPVIB809-08|Canada|British Columbia|658[0n]|BOLD:ACE8314
Euxoa difformis|[8180]|LPVIB822-08|Canada|British Columbia|658[0n]|BOLD:ACE8314
Euxoa difformis|[8181]|LPVIB837-08|Canada|British Columbia|658[0n]|BOLD:ACE8314
Euxoa difformis|[8182]|LBCH7262-10|Canada|British Columbia|658[0n]|BOLD:ACE8314
Euxoa difformis|[8183]|LBCH7251-10|Canada|British Columbia|658[0n]|BOLD:ACE8314
Euxoa difformis|[8184]|LBCH7220-10|Canada|British Columbia|658[0n]|BOLD:ACE8314
Euxoa difformis|[8185]|LBCH6790-10|Canada|British Columbia|658[0n]|BOLD:ACE8314
Euxoa difformis|[8186]|LPVIB811-08|Canada|British Columbia|658[0n]|BOLD:ACE8314
Euxoa difformis|[8187]|LPVIB810-08|Canada|British Columbia|658[0n]|BOLD:ACE8314
Euxoa difformis|[8188]|LPVIB625-08|Canada|British Columbia|658[0n]|BOLD:ACE8314
Euxoa difformis|[8189]|RDNMF813-08|United States|Oregon|658[0n]|BOLD:ACE8314
Euxoa difformis|[8190]|LPVIB628-08|Canada|British Columbia|629[0n]|BOLD:ACE8314
Euxoa difformis|[8191]|LPVIB630-08|Canada|British Columbia|634[0n]|BOLD:ACE8314
Euxoa difformis|[8192]|LPVIB631-08|Canada|British Columbia|634[0n]|BOLD:ACE8314
Euxoa difformis|[8193]|LBCH7263-10|Canada|British Columbia|658[0n]|BOLD:ACE8314
Euxoa difformis|[8194]|LBCH7265-10|Canada|British Columbia|658[0n]|BOLD:ACE8314
Euxoa difformis|[8195]|LBCH7959-10|Canada|British Columbia|658[0n]|BOLD:ACE8314
Euxoa difformis|[8196]|LALPA1296-11|Canada|British Columbia|658[0n]|BOLD:ACE8314
Euxoa difformis|[8197]|LALPA740-10|Canada|British Columbia|658[0n]|BOLD:ACE8314
Euxoa difformis|[8198]|LALPA1324-12|Canada|British Columbia|630[0n]|BOLD:ACE8314
Euxoa difformis|[8199]|LALPA1338-12|Canada|British Columbia|632[0n]|BOLD:ACE8314
Euxoa cinereopallidus|[8200]|RDMAB587-06|Canada|Alberta|658[0n]|BOLD:ABZ9422
Euxoa cinereopallidus|[8201]|RDMAB586-06|Canada|Alberta|658[0n]|BOLD:ABZ9422
Euxoa cinereopallidus|[8202]|RDNMG994-08|United States|Washington|658[0n]|BOLD:ABZ9422
Euxoa cinereopallidus|[8203]|RDNMG995-08|United States|Washington|658[0n]|BOLD:ABZ9422
Euxoa cinereopallidus|[8204]|RDNMG996-08|United States|Oregon|658[0n]|BOLD:ABZ9422
Euxoa teleboa|[8205]|RDMAB562-06|Canada|Alberta|616[1n]|BOLD:ABZ8386
Euxoa teleboa|[8206]|RDMAB563-06|Canada|Alberta|658[0n]|BOLD:ABZ8386
Euxoa teleboa|[8207]|RDMAB561-06|Canada|Alberta|658[0n]|BOLD:ABZ8386
Euxoa teleboa|[8208]|RDMAB560-06|Canada|Alberta|658[0n]|BOLD:ABZ8386
Euxoa teleboa|[8209]|RDNMF883-08|Canada|Alberta|658[0n]|BOLD:ABZ8386
Euxoa teleboa|[8210]|RDNMF884-08|Canada|Alberta|658[0n]|BOLD:ABZ8386
Euxoa teleboa|[8211]|RDNMF885-08|United States|Wyoming|658[0n]|BOLD:ABZ8386
Euxoa cona|[8212]|RDNMG668-08|United States|Oregon|658[0n]|BOLD:ACF5840
Euxoa cona|[8213]|RDNMG669-08|United States|Oregon|658[0n]|BOLD:ACF5840
Euxoa tronellus|[8214]|RDNM298-05|United States|Nevada|658[0n]|BOLD:ABX5591
Euxoa tronellus|[8215]|RDNM300-05|United States|Nevada|658[0n]|BOLD:ABX5591
Euxoa tronellus|[8216]|RDNMB851-05|Canada|Alberta|658[0n]|BOLD:ABX5591
Euxoa tronellus|[8217]|RDNMB852-05|Canada|Alberta|658[0n]|BOLD:ABX5591
Euxoa tronellus|[8218]|RDNMB853-05|Canada|Alberta|658[0n]|BOLD:ABX5591
Euxoa tronellus|[8219]|RDNMB854-05|Canada|Alberta|658[0n]|BOLD:ABX5591
Euxoa tronellus|[8220]|RDNMB860-05|Canada|Alberta|658[0n]|BOLD:ABX5591
Euxoa tronellus|[8221]|RDNMB862-05|Canada|Alberta|658[0n]|BOLD:ABX5591
Euxoa tronellus|[8222]|RDNMB855-05|Canada|Alberta|658[0n]|BOLD:ABX5591
Euxoa tronellus|[8223]|RDNMB859-05|Canada|Alberta|658[0n]|BOLD:ABX5591
Euxoa tronellus|[8224]|RDNMB863-05|Canada|Alberta|658[0n]|BOLD:ABX5591
Euxoa tronellus|[8225]|RDNMK362-11|United States|New Mexico|658[0n]|BOLD:ABX5591
Euxoa tronellus|[8226]|RDNME712-08|United States|New Mexico|658[0n]|BOLD:ABX5591
Euxoa tronellus|[8227]|RDNM299-05|United States|Oregon|658[0n]|BOLD:ABX5591
Euxoa tronellus|[8228]|RDNME731-08|United States|New Mexico|658[0n]|BOLD:ABX5591
Euxoa tronellus|[8229]|RDNMK360-11|United States|New Mexico|658[0n]|BOLD:ABX5591
Euxoa tronellus|[8230]|RDNMK471-11|United States|New Mexico|658[0n]|BOLD:ABX5591
Euxoa albipennis|[8231]|LOWCE745-06|Canada|British Columbia|595[0n]|BOLD:AAE6702
Euxoa albipennis|[8232]|LOWCE743-06|Canada|British Columbia|595[0n]|BOLD:AAE6702
Euxoa albipennis|[8233]|LBCH7235-10|Canada|British Columbia|658[0n]|BOLD:AAE6702
Euxoa albipennis|[8234]|LBCH7548-10|Canada|British Columbia|658[0n]|BOLD:AAE6702
Euxoa albipennis|[8235]|NGNAW111-14|Canada|British Columbia|549[0n]|BOLD:AAE6702
Euxoa albipennis|[8236]|RDLQB770-05|Canada|Quebec|658[0n]|BOLD:AAE6702
Euxoa albipennis|[8237]|RDLQF240-06|Canada|Quebec|630[0n]|BOLD:AAE6702
Euxoa albipennis|[8238]|LBCH7677-10|Canada|British Columbia|658[0n]|BOLD:AAE6702
Euxoa albipennis|[8239]|LBCH7819-10|Canada|British Columbia|658[0n]|BOLD:AAE6702
Euxoa albipennis|[8240]|LBCH6448-10|Canada|British Columbia|658[0n]|BOLD:AAE6702
Euxoa albipennis|[8241]|LBCH7050-10|Canada|British Columbia|658[0n]|BOLD:AAE6702
Euxoa albipennis|[8242]|LBCH7236-10|Canada|British Columbia|658[0n]|BOLD:AAE6702
Euxoa albipennis|[8243]|LBCH7689-10|Canada|British Columbia|658[0n]|BOLD:AAE6702
Euxoa albipennis|[8244]|NGAAE028-14|Canada|British Columbia|534[0n]|BOLD:AAE6702
Euxoa albipennis|[8245]|CNCLB1657-14|United States|California|658[0n]|BOLD:AAE6702
Euxoa inscripta|[8246]|CNCLB1662-14|United States|Wyoming|658[0n]|BOLD:ACP3521
Euxoa inscripta|[8247]|CNCLB1663-14|United States|Wyoming|658[0n]|BOLD:ACP3521
Euxoa punctigera|[8248]|JMMMB377-11|United States|California|658[0n]|BOLD:ACE9834
Euxoa punctigera|[8249]|RDNMG559-08|United States|Oregon|658[0n]|BOLD:ABZ9016
Euxoa punctigera|[8250]|LBCH6274-10|Canada|British Columbia|658[0n]|BOLD:ABZ9016
Euxoa punctigera|[8251]|RDNMB635-05|United States|Oregon|658[0n]|BOLD:ABZ9016
Euxoa spumata|[8252]|RDNMF694-08|Canada|Saskatchewan|621[0n]|BOLD:ABZ9016
Euxoa spumata|[8253]|RDNMF693-08|United States|Montana|609[0n]|BOLD:ABZ9016
Euxoa spumata|[8254]|RDNMF695-08|Canada|Saskatchewan|609[0n]|BOLD:ABZ9016
Euxoa spumata|[8255]|RDNMF696-08|United States|Montana|658[0n]|BOLD:ABZ9016
Euxoa stigmatalis|[8256]|LNAUS2899-13|United States|Arizona|658[0n]|BOLD:ABZ9016
Euxoa punctigera|[8257]|LBCH6367-10|Canada|British Columbia|658[0n]|BOLD:ABZ9016
Euxoa punctigera|[8258]|LBCH6442-10|Canada|British Columbia|658[0n]|BOLD:ABZ9016
Euxoa punctigera|[8259]|LBCH6742-10|Canada|British Columbia|658[0n]|BOLD:ABZ9016
Euxoa punctigera|[8260]|LBCH6746-10|Canada|British Columbia|658[0n]|BOLD:ABZ9016
Euxoa punctigera|[8261]|LBCH7129-10|Canada|British Columbia|658[0n]|BOLD:ABZ9016
Euxoa punctigera|[8262]|LBCH7131-10|Canada|British Columbia|658[0n]|BOLD:ABZ9016
Euxoa punctigera|[8263]|LBCH7541-10|Canada|British Columbia|658[0n]|BOLD:ABZ9016
Euxoa punctigera|[8264]|LBCH7550-10|Canada|British Columbia|658[0n]|BOLD:ABZ9016
Euxoa punctigera|[8265]|LBCH7791-10|Canada|British Columbia|658[0n]|BOLD:ABZ9016
Euxoa punctigera|[8266]|NAMUM236-08|United States|California|658[0n]|BOLD:ABZ9016
Euxoa punctigera|[8267]|RDNMG558-08|United States|California|658[0n]|BOLD:ABZ9016
Euxoa punctigera|[8268]|LBCH6745-10|Canada|British Columbia|658[0n]|BOLD:ABZ9016
Euxoa punctigera|[8269]|RDNMG557-08|Canada|British Columbia|658[0n]|BOLD:ABZ9016
Euxoa punctigera|[8270]|LBCH7950-10|Canada|British Columbia|658[0n]|BOLD:ABZ9016
Euxoa punctigera|[8271]|IAWLB017-10|United States|California|658[0n]|BOLD:ABZ9016
Euxoa punctigera|[8272]|IAWLB090-10|United States|California|658[0n]|BOLD:ABZ9016
Euxoa punctigera|[8273]|IAWLB091-10|United States|California|658[0n]|BOLD:ABZ9016
[illegible]

Euxoa punctigera|[8271]|IAWLB017-10|United States|California|658[0n]|BOLD:ABZ9016
Euxoa punctigera|[8272]|IAWLB090-10|United States|California|658[0n]|BOLD:ABZ9016
Euxoa punctigera|[8273]|IAWLB091-10|United States|California|658[0n]|BOLD:ABZ9016
Euxoa punctigera|[8274]|BBLOC1267-11|United States|California|658[0n]|BOLD:ABZ9016
Euxoa cana|[8275]|CNCLB990-14|United States|Nevada|658[1n]|BOLD:ABZ9016
Euxoa stigmatalis|[8276]|RDNMJ521-11|United States|Arizona|658[0n]|BOLD:ABZ9016
Euxoa stigmatalis|[8277]|LNAUS2898-13|United States|Arizona|658[0n]|BOLD:ABZ9016
Euxoa stigmatalis|[8278]|LNAUS2900-13|United States|Arizona|658[0n]|BOLD:ABZ9016
Euxoa stigmatalis|[8279]|LNAUS2901-13|United States|Arizona|658[0n]|BOLD:ABZ9016
Euxoa spumata|[8280]|RDNMF697-08|United States|North Dakota|640[1n]|BOLD:ABZ9016
Euxoa punctigera|[8281]|LBCH7806-10|Canada|British Columbia|658[0n]|BOLD:ABZ9016
Euxoa stigmatalis|[8282]|LNAUS2845-13|United States|Arizona|658[0n]|BOLD:ABZ9016
Euxoa cana|[8283]|CNCLB991-14|United States|Nevada|658[0n]|BOLD:ABZ9016
Euxoa stygialis|[8284]|NAMUM065-08|United States|Arizona|656[0n]|BOLD:ABX6499
Euxoa conjuncta|[8285]|CNCLB1712-14|United States|New Mexico|658[0n]|BOLD:ACP4141
Euxoa mitis|[8286]|RDMAB589-06|Canada|Alberta|658[0n]|BOLD:AAE0993
Euxoa mitis|[8287]|RDMAB689-06|Canada|Alberta|561[1n]|BOLD:AAE0993
Euxoa mitis|[8288]|RDNMF808-08|Canada|Alberta|658[0n]|BOLD:AAE0993
Euxoa mitis|[8289]|RDNMF809-08|United States|Wyoming|658[0n]|BOLD:AAE0993
Euxoa mitis|[8290]|RDNMF810-08|United States|Oregon|658[0n]|BOLD:AAE0993
Euxoa brevipennis|[8291]|RDNMG456-08|United States|Oregon|658[1n]|BOLD:AAE6690
Euxoa brevipennis|[8292]|NAMUM241-08|United States|California|658[0n]|BOLD:AAE6690
Euxoa brevipennis|[8293]|RDMAB588-06|Canada|Alberta|658[0n]|BOLD:AAE6690
Euxoa brevipennis|[8294]|RDNMG457-08|Canada|Alberta|658[0n]|BOLD:AAE6690
Euxoa brevipennis|[8295]|JMMMB606-13|United States|California|658[0n]|BOLD:AAE6690
Euxoa oberfoelli|[8296]|RDNMF683-08|United States|Kansas|658[0n]|BOLD:AAF6163
Euxoa oberfoelli|[8297]|RDNMF684-08|Canada|Saskatchewan|658[0n]|BOLD:AAF6163
Euxoa oberfoelli|[8298]|RDNMF687-08|United States|Montana|658[0n]|BOLD:AAF6163
Euxoa obeliscoides|[8299]|LBCH7575-10|Canada|British Columbia|658[0n]|BOLD:ABZ9441
Euxoa obeliscoides|[8300]|RDNMB867-05|Canada|Alberta|658[0n]|BOLD:ABZ9441
Euxoa obeliscoides|[8301]|RDMAB579-06|Canada|Alberta|658[0n]|BOLD:ABZ9441
Euxoa obeliscoides|[8302]|LBCH7580-10|Canada|British Columbia|658[0n]|BOLD:ABZ9441
Euxoa obeliscoides|[8303]|RDNMB008-05|United States|Washington|658[0n]|BOLD:ABZ9441
Euxoa obeliscoides|[8304]|LOWCE724-06|Canada|British Columbia|658[0n]|BOLD:ABZ9441
Euxoa obeliscoides|[8305]|LOWCE723-06|Canada|British Columbia|658[0n]|BOLD:ABZ9441
Euxoa obeliscoides|[8306]|RDNMB007-05|Canada|Alberta|594[0n]|BOLD:ABZ9441
Euxoa obeliscoides|[8307]|RDNMB006-05|Canada|Alberta|658[0n]|BOLD:ABZ9441
Euxoa obeliscoides|[8308]|LOWCE725-06|Canada|British Columbia|616[0n]|BOLD:ABZ9441
Euxoa obeliscoides|[8309]|LBCH6738-10|Canada|British Columbia|658[0n]|BOLD:ABZ9441
Euxoa obeliscoides|[8310]|LBCH6909-10|Canada|British Columbia|658[0n]|BOLD:ABZ9441
Euxoa obeliscoides|[8311]|LBCH7100-10|Canada|British Columbia|658[0n]|BOLD:ABZ9441
Euxoa obeliscoides|[8312]|LBCH7128-10|Canada|British Columbia|658[0n]|BOLD:ABZ9441
Euxoa obeliscoides|[8313]|LBCH7240-10|Canada|British Columbia|658[0n]|BOLD:ABZ9441
Euxoa obeliscoides|[8314]|LBCH7505-10|Canada|British Columbia|658[0n]|BOLD:ABZ9441
Euxoa obeliscoides|[8315]|LBCH7534-10|Canada|British Columbia|658[0n]|BOLD:ABZ9441
Euxoa obeliscoides|[8316]|LBCH7574-10|Canada|British Columbia|658[0n]|BOLD:ABZ9441
Euxoa obeliscoides|[8317]|LBCH7576-10|Canada|British Columbia|658[0n]|BOLD:ABZ9441
Euxoa obeliscoides|[8318]|LBCH7577-10|Canada|British Columbia|658[0n]|BOLD:ABZ9441
Euxoa obeliscoides|[8319]|LBCH7578-10|Canada|British Columbia|658[0n]|BOLD:ABZ9441
Euxoa obeliscoides|[8320]|LBCH7579-10|Canada|British Columbia|658[0n]|BOLD:ABZ9441
Euxoa obeliscoides|[8321]|LBCH7581-10|Canada|British Columbia|658[0n]|BOLD:ABZ9441
Euxoa obeliscoides|[8322]|LBCH7782-10|Canada|British Columbia|658[0n]|BOLD:ABZ9441
Euxoa obeliscoides|[8323]|RDLQ703-07|Canada|Quebec|658[0n]|BOLD:ABZ9441
Euxoa obeliscoides|[8324]|JMMMB383-11|United States|California|658[0n]|BOLD:ABZ9441
Euxoa coconino|[8325]|CNCLB1720-14|United States|Arizona|658[0n]|BOLD:ACU0853
Euxoa luctuosa|[8326]|RDNMG970-08|United States|Colorado|658[0n]|BOLD:ABZ9740
Euxoa luctuosa|[8327]|RDNMK372-11|United States|New Mexico|658[0n]|BOLD:ABZ9740
Euxoa cincta|[8328]|CNCLB770-14|United States|Arizona|618[0n]|BOLD:ACM4380
Euxoa cincta|[8329]|CNCLB771-14|United States|New Mexico|658[0n]|BOLD:ACM4380
Euxoa cincta|[8330]|CNCLB772-14|United States|Texas|658[0n]|BOLD:ACM4380
Euxoa cincta|[8331]|CNCLB2037-14|United States|Texas|658[0n]|BOLD:ACM4380
Euxoa messoria|[8332]|LALPA1333-12|Canada|British Columbia|612[0n]|BOLD:AAA2634
Euxoa messoria|[8333]|RDNMK363-11|United States|New Mexico|658[0n]|BOLD:AAA2634
Euxoa messoria|[8334]|RDNMC732-06|United States|Texas|540[0n]|BOLD:AAA2634
Euxoa messoria|[8335]|RDNMK364-11|United States|New Mexico|658[0n]|BOLD:AAA2634
Euxoa messoria|[8336]|RDNMK472-11|United States|New Mexico|658[0n]|BOLD:AAA2634
Euxoa messoria|[8337]|LBCH7811-10|Canada|British Columbia|658[0n]|BOLD:AAA2634
Euxoa messoria|[8338]|RDNML016-13|Canada|Nova Scotia|658[0n]|BOLD:AAA2634
Euxoa messoria|[8339]|RDNML017-13|Canada|Nova Scotia|658[0n]|BOLD:AAA2634
Euxoa messoria|[8340]|LOWCE772-06|Canada|British Columbia|658[0n]|BOLD:AAA2634
Euxoa messoria|[8341]|RWWC142-10|United States|Washington|658[0n]|BOLD:AAA2634
Euxoa messoria|[8342]|LBCH1972-10|Canada|British Columbia|658[0n]|BOLD:AAA2634
Euxoa messoria|[8343]|RWWB318-09|United States|Washington|658[0n]|BOLD:AAA2634
Euxoa messoria|[8344]|LOWCE773-06|Canada|British Columbia|658[0n]|BOLD:AAA2634
Euxoa messoria|[8345]|LHLEP443-06|Canada|British Columbia|658[0n]|BOLD:AAA2634
Euxoa messoria|[8346]|LOWCE322-06|Canada|British Columbia|658[0n]|BOLD:AAA2634
Euxoa messoria|[8347]|XAH007-05|Canada|Ontario|658[0n]|BOLD:AAA2634
Euxoa messoria|[8348]|PHMO339-03|Canada|Ontario|639[0n]|BOLD:AAA2634
Euxoa messoria|[8349]|PHMO336-03|Canada|Ontario|639[0n]|BOLD:AAA2634
Euxoa messoria|[8350]|LBCH6755-10|Canada|British Columbia|615[1n]|BOLD:AAA2634
Euxoa messoria|[8351]|LBCH7374-10|Canada|British Columbia|658[0n]|BOLD:AAA2634
Euxoa messoria|[8352]|LBCH7555-10|Canada|British Columbia|658[0n]|BOLD:AAA2634
Euxoa messoria|[8353]|LBCH7816-10|Canada|British Columbia|658[0n]|BOLD:AAA2634
Euxoa messoria|[8354]|LBCH7817-10|Canada|British Columbia|658[0n]|BOLD:AAA2634
Euxoa messoria|[8355]|LBCH7971-10|Canada|British Columbia|658[0n]|BOLD:AAA2634
Euxoa messoria|[8356]|JMMMB378-11|United States|California|658[0n]|BOLD:AAA2634
Euxoa messoria|[8357]|JMMMB392-11|United States|California|658[0n]|BOLD:AAA2634
Euxoa messoria|[8358]|JMMMB409-11|United States|California|658[0n]|BOLD:AAA2634
Euxoa messoria|[8359]|JMMMB427-11|United States|California|658[0n]|BOLD:AAA2634
Euxoa messoria|[8360]|ABCBF179-12|Canada|British Columbia|658[0n]|
Euxoa messoria|[8361]|JMMMB415-11|United States|California|658[0n]|BOLD:AAA2634
Euxoa messoria|[8362]|LBCH7433-10|Canada|British Columbia|658[0n]|BOLD:AAA2634
Euxoa messoria|[8363]|RDLQ698-07|Canada|Quebec|658[0n]|BOLD:AAA2634
Euxoa messoria|[8364]|LOWCD663-06|Canada|British Columbia|658[0n]|BOLD:AAA2634
Euxoa messoria|[8365]|RDLQB269-05|Canada|Quebec|658[0n]|BOLD:AAA2634
Euxoa messoria|[8366]|MBNOC004-13|Canada|Manitoba|623[0n]|BOLD:AAA2634
Euxoa messoria|[8367]|NGNAP071-14|Canada|British Columbia|576[0n]|BOLD:AAA2634
Euxoa camalpa|[8368]|RDNMG261-08|United States|Texas|658[0n]|BOLD:AAL4844
Euxoa camalpa|[8369]|BBLSY931-09|United States|Texas|658[0n]|BOLD:AAH5421
Euxoa camalpa|[8370]|RDNMJ124-10|United States|Texas|658[0n]|BOLD:AAV7167
Euxoa maderensis|[8371]|CNCLB2042-14|United States|Arizona|658[0n]|BOLD:ACR1650
Euxoa maderensis|[8372]|CNCLB2040-14|United States|Arizona|658[0n]|BOLD:ACR1650
Euxoa maderensis|[8373]|CNCLB2039-14|United States|Arizona|658[0n]|BOLD:ACR1650

Euxoa maderensis|[8371]|CNCLB2042-14|United States|Arizona|658[0n]|BOLD:ACR1650
Euxoa maderensis|[8372]|CNCLB2040-14|United States|Arizona|658[0n]|BOLD:ACR1650
Euxoa maderensis|[8373]|CNCLB2039-14|United States|Arizona|658[0n]|BOLD:ACR1650
Euxoa maderensis|[8374]|CNCLB2041-14|United States|Arizona|307[0n]|
Euxoa maderensis|[8375]|CNCLB2043-14|United States|Arizona|307[0n]|
Euxoa violaris|[8376]|GWOTA065-12|United States|Massachusetts|658[0n]|BOLD:ABV4968
Euxoa bochus|[8377]|LOWCB672-05|Canada|British Columbia|658[0n]|BOLD:AAC8204
Euxoa bochus|[8378]|LOWCB677-05|Canada|British Columbia|658[0n]|BOLD:AAC8204
Euxoa bochus|[8379]|LOWCB676-05|Canada|British Columbia|658[0n]|BOLD:AAC8204
Euxoa bochus|[8380]|LOWCB675-05|Canada|British Columbia|658[0n]|BOLD:AAC8204
Euxoa bochus|[8381]|LOWCB673-05|Canada|British Columbia|658[0n]|BOLD:AAC8204
Euxoa bochus|[8382]|LOWCB671-05|Canada|British Columbia|657[0n]|BOLD:AAC8204
Euxoa bochus|[8383]|LOWCB669-05|Canada|British Columbia|658[0n]|BOLD:AAC8204
Euxoa bochus|[8384]|LOWCB670-05|Canada|British Columbia|504[0n]|BOLD:AAC8204
Euxoa bochus|[8385]|LOWCB674-05|Canada|British Columbia|650[0n]|BOLD:AAC8204
Euxoa bochus|[8386]|LOWCB678-05|Canada|British Columbia|577[0n]|BOLD:AAC8204
Euxoa bochus|[8387]|LBCH6531-10|Canada|British Columbia|658[0n]|BOLD:AAC8204
Euxoa bochus|[8388]|LBCH7226-10|Canada|British Columbia|658[0n]|BOLD:AAC8204
Euxoa bochus|[8389]|LBCH6628-10|Canada|British Columbia|658[0n]|BOLD:AAC8204
Euxoa bochus|[8390]|LBCH7760-10|Canada|British Columbia|658[0n]|BOLD:AAC8204
Hemieuxoa rudens|[8391]|GMLC953-12|United States|California|622[0n]|BOLD:AAC1265
Hemieuxoa rudens|[8392]|LOCBB286-06|United States|California|615[0n]|BOLD:AAC1265
Hemieuxoa rudens|[8393]|CMAZA245-09|United States|Arizona|658[0n]|BOLD:AAC1265
Hemieuxoa rudens|[8394]|CMAZA280-09|United States|Arizona|658[0n]|BOLD:AAC1265
Hemieuxoa rudens|[8395]|USLEP144-10|United States|Colorado|658[0n]|BOLD:AAC1265
Hemieuxoa rudens|[8396]|USLEP431-10|United States|Texas|658[0n]|BOLD:AAC1265
Hemieuxoa rudens|[8397]|CMAZA1071-12|United States|Arizona|658[0n]|BOLD:AAC1265
Hemieuxoa rudens|[8398]|GMLC1113-12|United States|California|658[0n]|BOLD:AAC1265
Hemieuxoa rudens|[8399]|CMAZA1040-12|United States|Arizona|658[0n]|BOLD:AAC1265
Hemieuxoa rudens|[8400]|CMAZA1044-12|United States|Arizona|658[0n]|BOLD:AAC1265
Hemieuxoa rudens|[8401]|GMLC1160-12|United States|California|658[0n]|BOLD:AAC1265
Hemieuxoa rudens|[8402]|GMLC1253-12|United States|California|658[0n]|BOLD:AAC1265
Hemieuxoa rudens|[8403]|CMAZA577-10|United States|Arizona|658[0n]|BOLD:AAC1265
Hemieuxoa rudens|[8404]|RDNMK367-11|United States|New Mexico|658[0n]|BOLD:AAC1265
Hemieuxoa rudens|[8405]|CMAZA167-09|United States|Arizona|658[0n]|BOLD:AAC1265
Hemieuxoa rudens|[8406]|BBLSX651-09|United States|Oklahoma|658[0n]|BOLD:AAC1265
Hemieuxoa rudens|[8407]|LPOKB1021-09|United States|Oklahoma|658[0n]|BOLD:AAC1265
Hemieuxoa rudens|[8408]|LPOKA980-09|United States|Oklahoma|658[0n]|BOLD:AAC1265
Hemieuxoa rudens|[8409]|RDNMD889-07|United States|Arizona|655[0n]|BOLD:AAC1265
Hemieuxoa rudens|[8410]|RDNMD888-07|United States|New Mexico|655[0n]|BOLD:AAC1265
Hemieuxoa rudens|[8411]|RDNMD657-06|United States|New Mexico|658[0n]|BOLD:AAC1265
Hemieuxoa rudens|[8412]|LOCBB287-06|United States|California|658[0n]|BOLD:AAC1265
Hemieuxoa rudens|[8413]|LOCBB285-06|United States|California|658[0n]|BOLD:AAC1265
Hemieuxoa rudens|[8414]|LOCBB284-06|United States|California|658[0n]|BOLD:AAC1265
Hemieuxoa rudens|[8415]|LOCBB183-06|United States|California|658[0n]|BOLD:AAC1265
Hemieuxoa rudens|[8416]|LOCBB160-06|United States|California|658[0n]|BOLD:AAC1265
Hemieuxoa rudens|[8417]|LOCBB184-06|United States|California|656[0n]|BOLD:AAC1265
Hemieuxoa rudens|[8418]|LOCBB347-06|United States|California|622[0n]|BOLD:AAC1265
Hemieuxoa rudens|[8419]|LOCBE091-06|United States|California|611[0n]|BOLD:AAC1265
Hemieuxoa rudens|[8420]|GMLC1172-12|United States|California|658[0n]|BOLD:AAC1265
Hemieuxoa rudens|[8421]|GMLC1254-12|United States|California|658[0n]|BOLD:AAC1265
Hemieuxoa rudens|[8422]|GMLC1299-12|United States|California|636[0n]|BOLD:AAC1265
Hemieuxoa rudens|[8423]|GMLC1304-12|United States|California|614[0n]|BOLD:AAC1265
Protogygia album|[8424]|JBAZ072-09|United States|California|658[0n]|BOLD:AAH4575
Protogygia album|[8425]|LNAUS4790-13|United States|California|658[0n]|BOLD:AAH4575
Protogygia album|[8426]|LNAUS4791-13|United States|California|658[0n]|BOLD:AAH4575
Protogygia album|[8427]|NAMUM184-08|United States|California|646[0n]|BOLD:AAH4575
Protogygia album|[8428]|LNAUS4789-13|United States|California|658[0n]|BOLD:AAH4575
Protogygia album|[8429]|LNAUS4792-13|United States|California|658[0n]|BOLD:AAH4575
Protogygia pallida|[8430]|RDNME1003-08|United States|New Mexico|658[0n]|BOLD:AAX8406
Protogygia pallida|[8431]|LNAUS4788-13|United States|New Mexico|658[0n]|BOLD:AAX8406
Protogygia pallida|[8432]|RDNMK467-11|United States|New Mexico|658[0n]|BOLD:AAX8406
Protogygia pallida|[8433]|LNAUS4793-13|United States|New Mexico|658[0n]|BOLD:AAX8406
Dichagyris timbor|[8434]|CNCLB700-14|Mexico|Oaxaca|658[0n]|BOLD:ACM4314
Dichagyris timbor|[8435]|CNCLB701-14|Mexico||658[0n]|BOLD:ACM4314
Dichagyris timbor|[8436]|CNCLB702-14|Mexico||658[0n]|BOLD:ACM4314
Dichagyris timbor|[8437]|CNCLB703-14|Mexico||658[0n]|BOLD:ACM4314
Dichagyris pyrsogramma|[8438]|CNCLB704-14|Mexico||658[0n]|BOLD:ACM4703
Dichagyris pyrsogramma|[8439]|CNCLB705-14|Mexico||658[0n]|BOLD:ACM4703
Dichagyris pyrsogramma|[8440]|CNCLB706-14|Mexico||658[0n]|BOLD:ACM4703
Dichagyris pyrsogramma|[8441]|CNCLB707-14|Mexico||658[0n]|BOLD:ACM4703
Dichagyris pyrsogramma|[8442]|CNCLB708-14|Mexico||645[3n]|BOLD:ACM4703
Protogygia pectinata|[8443]|RDNMG428-08|United States|Utah|658[0n]|BOLD:AAH7648
Protogygia pectinata|[8444]|RDNMG429-08|United States|Utah|658[0n]|BOLD:AAH7648
Protogygia whitesandsensis|[8445]|RDNMH091-09|United States|New Mexico|658[0n]|BOLD:AAH7648
Protogygia milleri|[8446]|JMMMB324-11|United States|California|658[0n]|BOLD:AAN3362
Protogygia milleri|[8447]|RDNMJ338-11|United States|California|658[0n]|BOLD:AAN3362
Protogygia milleri|[8448]|RDNMJ337-11|United States|California|658[0n]|BOLD:AAN3362
Protogygia milleri|[8449]|IAWLB030-10|United States|California|658[0n]|BOLD:AAN3362
Protogygia milleri|[8450]|IAWLB029-10|United States|California|658[0n]|BOLD:AAN3362
Protogygia milleri|[8451]|IAWLB028-10|United States|California|658[0n]|BOLD:AAN3362
Protogygia milleri|[8452]|JMMMB407-11|United States|California|658[0n]|BOLD:AAN3362
Protogygia biclavis|[8453]|LNAUS4797-13|United States|California|658[0n]|BOLD:ACJ6478
Protogygia biclavis|[8454]|LNAUS4794-13|United States|California|658[0n]|BOLD:ACJ6478
Protogygia biclavis|[8455]|LNAUS4795-13|United States|California|658[0n]|BOLD:ACJ6478
Protogygia biclavis|[8456]|LNAUS4796-13|United States|California|658[0n]|BOLD:ACJ6478
Protogygia biclavis|[8457]|LNAUS4798-13|United States|California|658[0n]|BOLD:ACJ6478
Protogygia polingi|[8458]|LNAUS4800-13|United States|California|658[0n]|BOLD:ACI8439
Protogygia polingi|[8459]|LNAUS4799-13|United States|California|658[0n]|BOLD:ACI8439
Protogygia polingi|[8460]|LNAUS4801-13|United States|California|658[0n]|BOLD:ACI8439
Protogygia polingi|[8461]|LOCBF352-13|United States|California|658[0n]|BOLD:ACI8439
Protogygia polingi|[8462]|LOCBF1039-13|United States|California|658[0n]|BOLD:ACI8439
Protogygia polingi|[8463]|LNAUS4802-13|United States|California|658[0n]|BOLD:ACI8439
Protogygia comstocki|[8464]|RDNMC220-05|United States|California|617[0n]|BOLD:AAE8183
Protogygia comstocki|[8465]|RDNMC221-05|United States|Oregon|658[0n]|BOLD:AAE8183
Protogygia comstocki|[8466]|RDNMC222-05|United States|Oregon|658[0n]|BOLD:AAE8183
Protogygia comstocki|[8467]|RDNMC223-05|United States|California|658[0n]|BOLD:AAE8183
Protogygia alberta|[8468]|RDNMB592-05|Canada|Alberta|658[0n]|BOLD:ACE7812
Protogygia arena|[8469]|RDNMB593-05|United States|Oregon|658[0n]|BOLD:ACE9082
Protogygia arena|[8470]|RDNMB594-05|United States|Oregon|603[0n]|BOLD:ACE9082
Protogygia elevata|[8471]|RDNMB595-05|United States|Utah|610[0n]|BOLD:ACE9083
Protogygia elevata|[8472]|RDNMB596-05|United States|Utah|614[0n]|BOLD:ACE9083
Protogygia enalaga|[8473]|RDNMC224-05|United States|Oregon|564[0n]|BOLD:AAE8189

[illegible]
Protogygia elevata|[8471]|RDNMB595-05|United States|Utah|610[0n]|BOLD:ACE9083
Protogygia elevata|[8472]|RDNMB596-05|United States|Utah|614[0n]|BOLD:ACE9083
Protogygia enalaga|[8473]|RDNMC224-05|United States|Oregon|564[0n]|BOLD:AAE8189
Protogygia enalaga|[8474]|RDNMC225-05|United States|California|605[2n]|BOLD:AAE8189
Protogygia enalaga|[8475]|RDMAB137-05|Canada|Alberta|658[0n]|BOLD:AAE8189
Protogygia enalaga|[8476]|RDNMC226-05|Canada|Alberta|658[0n]|BOLD:AAE8189
Protogygia postera|[8477]|RDNMC228-05|Canada|Alberta|582[0n]|BOLD:AAF6326
Protogygia postera|[8478]|RDNMC229-05|United States|California|566[2n]|BOLD:AAF6326
Protogygia postera|[8479]|RDNMC227-05|United States|California|525[1n]|BOLD:AAF6326
Protogygia postera|[8480]|BBLOC209-11|United States|Texas|658[0n]|BOLD:AAF6326
Protogygia postera|[8481]|BBLOC954-11|United States|Texas|658[0n]|BOLD:AAF6326
Protogygia postera|[8482]|BBLOC995-11|United States|California|658[0n]|BOLD:AAF6326
Protogygia postera|[8483]|BBLOC1277-11|United States|California|658[0n]|BOLD:AAF6326
Protogygia postera|[8484]|BBLOC1278-11|United States|California|658[0n]|BOLD:AAF6326
Protogygia postera|[8485]|BBLOC1281-11|United States|California|658[0n]|BOLD:AAF6326
Protogygia postera|[8486]|BBLOC1366-11|United States|California|658[0n]|BOLD:AAF6326
Protogygia postera|[8487]|BBLOC1376-11|United States|California|658[0n]|BOLD:AAF6326
Protogygia lagena|[8488]|RDNMC233-05|United States|California|658[0n]|BOLD:AAC5097
Protogygia lagena|[8489]|RDNMC236-05|United States|California|588[1n]|BOLD:AAC5097
Protogygia lagena|[8490]|RDNMC237-05|United States|California|658[0n]|BOLD:AAC5097
Protogygia querula|[8491]|RDNMF045-08|Canada|Alberta|658[0n]|BOLD:AAC5097
Protogygia querula|[8492]|RDNMG739-08|Canada|Alberta|658[0n]|BOLD:AAC5097
Protogygia querula|[8493]|RDNMG738-08|United States|Utah|658[0n]|BOLD:AAC5097
Protogygia querula|[8494]|RDNMG740-08|United States|Utah|658[0n]|BOLD:AAC5097
Protogygia querula|[8495]|RDNMG741-08|United States|Utah|658[0n]|BOLD:AAC5097
Protogygia lagena|[8496]|RDNMC230-05|United States|California|616[0n]|BOLD:AAC5097
Protogygia lagena|[8497]|RDNMC231-05|United States|California|618[0n]|BOLD:AAC5097
Protogygia lagena|[8498]|RDNMC232-05|United States|California|549[1n]|BOLD:AAC5097
Protogygia lagena|[8499]|RDNMC234-05|United States|California|657[0n]|BOLD:AAC5097
Protogygia lagena|[8500]|RDNMB775-05|United States|California|658[0n]|BOLD:AAC5097
Protogygia lagena|[8501]|IAWLB126-10|United States|California|658[0n]|BOLD:AAC5097
Protogygia lagena|[8502]|IAWLB127-10|United States|California|658[0n]|BOLD:AAC5097
Protogygia lagena|[8503]|IAWLB128-10|United States|California|658[0n]|BOLD:AAC5097
Protogygia lagena|[8504]|IAWLB129-10|United States|California|658[0n]|BOLD:AAC5097
Protogygia lagena|[8505]|RDNM756-05|United States|California|532[0n]|BOLD:ACF0017
Protogygia lagena|[8506]|RDNMB774-05|United States|California|658[0n]|BOLD:ACF0017
Protogygia lagena|[8507]|RDNMB777-05|United States|California|658[0n]|BOLD:ACF0017
Protogygia lagena|[8508]|RDNMC239-05|United States|California|658[0n]|BOLD:ACF0017
Protogygia lagena|[8509]|RDNMC240-05|United States|California|658[0n]|BOLD:ACF0017
Protogygia lagena|[8510]|RDNMC241-05|United States|California|574[2n]|BOLD:ACF0017
Protogygia lagena|[8511]|RDNMB776-05|United States|California|556[1n]|BOLD:ACE7807
Protogygia lagena|[8512]|RDNM757-05|United States|California|658[0n]|BOLD:ACE7807
Protogygia lagena|[8513]|RDNMC235-05|United States|California|555[0n]|BOLD:ACE7807
Protogygia rufescens|[8514]|RDNML373-13|United States|Utah|658[0n]|BOLD:ACE7807
Protogygia rufescens|[8515]|RDNML374-13|United States|Arizona|658[0n]|BOLD:ACE7807
Eucoptocnemis dollii|[8516]|CNCLB959-14|United States|New Mexico|658[0n]|BOLD:ACM4196
Eucoptocnemis dollii|[8517]|CNCLB960-14|United States|New Mexico|658[0n]|BOLD:ACM4196
Eucoptocnemis canescens|[8518]|CNCLB1509-14|United States|Nevada|658[0n]|BOLD:ACP0195
Eucoptocnemis canescens|[8519]|CNCLB1510-14|United States|Nevada|563[0n]|BOLD:ACP0195
Actebia fennica|[8520]|BBLPB831-10|Canada|Alberta|658[0n]|BOLD:AAD5720
Actebia fennica|[8521]|LBCH4405-10|Canada|British Columbia|658[0n]|BOLD:AAD5720
Actebia fennica|[8522]|LCHP943-07|Canada|Manitoba|658[0n]|BOLD:AAD5720
Actebia fennica|[8523]|ABKWR124-07|United States|Alaska|658[0n]|BOLD:AAD5720
Actebia fennica|[8524]|RDLQG059-06|Canada|Quebec|658[0n]|BOLD:AAD5720
Actebia fennica|[8525]|RDLQG022-06|Canada|Quebec|658[0n]|BOLD:AAD5720
Actebia fennica|[8526]|RDLQF247-06|Canada|Quebec|641[0n]|BOLD:AAD5720
Actebia fennica|[8527]|LPVIB629-08|Canada|British Columbia|636[0n]|BOLD:AAD5720
Actebia fennica|[8528]|LEFIJ368-10|Finland||594[0n]|BOLD:AAD5720
Actebia fennica|[8529]|LEFIJ886-10|Finland||658[0n]|BOLD:AAD5720
Actebia fennica|[8530]|LALPA868-11|Canada|British Columbia|658[0n]|BOLD:AAD5720
Actebia fennica|[8531]|ALLEP289-13|Canada|Ontario|658[0n]|BOLD:AAD5720
Eucoptocnemis elingua|[8532]|CNCLB1511-14|United States|California|658[0n]|BOLD:ACP0196
Eucoptocnemis elingua|[8533]|CNCLB1512-14|United States|California|566[2n]|BOLD:ACP0196
Ochropleura implecta|[8534]|LGSM427-04|United States|North Carolina|658[0n]|BOLD:AAA3073
Ochropleura implecta|[8535]|LGSMG892-10|United States|North Carolina|658[0n]|BOLD:AAA3073
Ochropleura implecta|[8536]|LGSMG893-10|United States|North Carolina|658[0n]|BOLD:AAA3073
Ochropleura implecta|[8537]|LOCT087-05|United States|Connecticut|658[0n]|BOLD:AAA3073
Ochropleura implecta|[8538]|XAG735-05|Canada|Ontario|658[3n]|BOLD:AAA3073
Ochropleura implecta|[8539]|LPSO498-08|Canada|Ontario|658[0n]|BOLD:AAA3073
Ochropleura implecta|[8540]|LPSOC334-08|Canada|Ontario|658[0n]|BOLD:AAA3073
Ochropleura implecta|[8541]|XAE184-04|Canada|Ontario|658[0n]|BOLD:AAA3073
Ochropleura implecta|[8542]|LOCT088-05|United States|Connecticut|658[0n]|BOLD:AAA3073
Ochropleura implecta|[8543]|LPSOB989-08|Canada|Ontario|658[0n]|BOLD:AAA3073
Ochropleura implecta|[8544]|BLTIB135-08|Canada|Ontario|658[0n]|BOLD:AAA3073
Ochropleura implecta|[8545]|BLTIB251-08|Canada|Ontario|658[0n]|BOLD:AAA3073
Ochropleura implecta|[8546]|LPSOB080-08|Canada|Ontario|658[0n]|BOLD:AAA3073
Ochropleura implecta|[8547]|LPSOB821-08|Canada|Ontario|658[0n]|BOLD:AAA3073
Ochropleura implecta|[8548]|XAK300-06|Canada|Ontario|656[0n]|BOLD:AAA3073
Ochropleura implecta|[8549]|XAH267-05|Canada|Ontario|617[0n]|BOLD:AAA3073
Ochropleura implecta|[8550]|XAD776-05|Canada|Ontario|658[0n]|BOLD:AAA3073
Ochropleura implecta|[8551]|LPSOC117-08|Canada|Ontario|658[0n]|BOLD:AAA3073
Ochropleura implecta|[8552]|LPSOC119-08|Canada|Ontario|658[0n]|BOLD:AAA3073
Ochropleura implecta|[8553]|LPSOC387-08|Canada|Ontario|658[0n]|BOLD:AAA3073
Ochropleura implecta|[8554]|BLTIB098-08|Canada|Ontario|658[0n]|BOLD:AAA3073
Ochropleura implecta|[8555]|BLTIB213-08|Canada|Ontario|658[0n]|BOLD:AAA3073
Ochropleura implecta|[8556]|BLTIB280-08|Canada|Ontario|658[0n]|BOLD:AAA3073
Ochropleura implecta|[8557]|BLGSM037-09|Canada|Ontario|658[0n]|BOLD:AAA3073
Ochropleura implecta|[8558]|BLGSM044-09|Canada|Ontario|658[0n]|BOLD:AAA3073
Ochropleura implecta|[8559]|BLGSM063-09|Canada|Ontario|658[0n]|BOLD:AAA3073
Ochropleura implecta|[8560]|LGSMG891-10|United States|North Carolina|658[0n]|BOLD:AAA3073
Ochropleura implecta|[8561]|BLTIB110-08|Canada|Ontario|658[1n]|BOLD:AAA3073
Ochropleura implecta|[8562]|LPSOB822-08|Canada|Ontario|658[0n]|BOLD:AAA3073
Ochropleura implecta|[8563]|LPSO436-08|Canada|Ontario|658[0n]|BOLD:AAA3073
Ochropleura implecta|[8564]|XAK318-06|Canada|Ontario|658[0n]|BOLD:AAA3073
Ochropleura implecta|[8565]|LGSM426-04|United States|North Carolina|658[0n]|BOLD:AAA3073
Ochropleura implecta|[8566]|XAB071-04|Canada|Ontario|562[0n]|BOLD:AAA3073
Ochropleura implecta|[8567]|PMG139-03|Canada|Ontario|617[0n]|BOLD:AAA3073
Ochropleura implecta|[8568]|LSEU618-06|United States|Georgia|621[0n]|BOLD:AAA3073
Ochropleura implecta|[8569]|RDLQG224-06|Canada|Quebec|632[0n]|BOLD:AAA3073
Ochropleura implecta|[8570]|LPSO841-08|Canada|Ontario|658[0n]|BOLD:AAA3073
Ochropleura implecta|[8571]|LPSOC110-08|Canada|Ontario|658[0n]|BOLD:AAA3073
Ochropleura implecta|[8572]|LPSOB986-08|Canada|Ontario|658[0n]|BOLD:AAA3073
[illegible]

Ochropleura implecta|[8570]|LPSO841-08|Canada|Ontario|658[0n]|BOLD:AAA3073
Ochropleura implecta|[8571]|LPSOC110-08|Canada|Ontario|658[0n]|BOLD:AAA3073
Ochropleura implecta|[8572]|LPSOB986-08|Canada|Ontario|658[0n]|BOLD:AAA3073
Ochropleura implecta|[8573]|BLTIB222-08|Canada|Ontario|658[0n]|BOLD:AAA3073
Ochropleura implecta|[8574]|BLTIB247-08|Canada|Ontario|658[0n]|BOLD:AAA3073
Ochropleura implecta|[8575]|BLTIB506-08|Canada|Ontario|658[0n]|BOLD:AAA3073
Ochropleura implecta|[8576]|BLTIB1023-08|Canada|Ontario|658[0n]|BOLD:AAA3073
Ochropleura implecta|[8577]|BLGSM043-09|Canada|Ontario|658[0n]|BOLD:AAA3073
Ochropleura implecta|[8578]|BLGSM050-09|Canada|Ontario|658[0n]|BOLD:AAA3073
Ochropleura implecta|[8579]|LNCC217-10|United States|North Carolina|658[0n]|BOLD:AAA3073
Ochropleura implecta|[8580]|MNBB128-05|Canada|New Brunswick|606[2n]|BOLD:AAA3073
Ochropleura implecta|[8581]|RWWA348-09|United States|Washington|658[0n]|BOLD:AAA3073
Ochropleura implecta|[8582]|LHLEP069-06|Canada|British Columbia|658[0n]|BOLD:AAA3073
Ochropleura implecta|[8583]|PHMNB607-04|Canada|New Brunswick|658[0n]|BOLD:AAA3073
Ochropleura implecta|[8584]|BBLPE274-09|Canada|Nova Scotia|658[0n]|BOLD:AAA3073
Ochropleura implecta|[8585]|BBLPE407-09|Canada|Newfoundland and Labrador|633[0n]|BOLD:AAA3073
Ochropleura implecta|[8586]|LPGVA605-08|Canada|British Columbia|632[0n]|BOLD:AAA3073
Ochropleura implecta|[8587]|LPGVA606-08|Canada|British Columbia|627[0n]|BOLD:AAA3073
Ochropleura implecta|[8588]|LPMN741-08|Canada|Manitoba|609[0n]|BOLD:AAA3073
Ochropleura implecta|[8589]|BBLEC387-09|Canada|Newfoundland and Labrador|658[0n]|BOLD:AAA3073
Ochropleura implecta|[8590]|BBLPC726-09|Canada|Newfoundland and Labrador|658[0n]|BOLD:AAA3073
Ochropleura implecta|[8591]|BBLPC695-09|Canada|Newfoundland and Labrador|658[0n]|BOLD:AAA3073
Ochropleura implecta|[8592]|BBLPC694-09|Canada|Newfoundland and Labrador|658[0n]|BOLD:AAA3073
Ochropleura implecta|[8593]|BBLPC662-09|Canada|Newfoundland and Labrador|658[0n]|BOLD:AAA3073
Ochropleura implecta|[8594]|BBLPC600-09|Canada|Nova Scotia|658[0n]|BOLD:AAA3073
Ochropleura implecta|[8595]|BBLPC599-09|Canada|Nova Scotia|658[0n]|BOLD:AAA3073
Ochropleura implecta|[8596]|BBLPC593-09|Canada|Nova Scotia|658[0n]|BOLD:AAA3073
Ochropleura implecta|[8597]|BBLPC257-09|Canada|Nova Scotia|658[0n]|BOLD:AAA3073
Ochropleura implecta|[8598]|BBLPC254-09|Canada|Nova Scotia|658[0n]|BOLD:AAA3073
Ochropleura implecta|[8599]|BBLEC666-09|Canada|Nova Scotia|658[0n]|BOLD:AAA3073
Ochropleura implecta|[8600]|BBLEC624-09|Canada|Nova Scotia|658[0n]|BOLD:AAA3073
Ochropleura implecta|[8601]|BBLEC603-09|Canada|Nova Scotia|658[0n]|BOLD:AAA3073
Ochropleura implecta|[8602]|RWWA258-09|United States|Washington|658[0n]|BOLD:AAA3073
Ochropleura implecta|[8603]|RWWA253-09|United States|Washington|658[0n]|BOLD:AAA3073
Ochropleura implecta|[8604]|LPABC815-09|Canada|Alberta|658[0n]|BOLD:AAA3073
Ochropleura implecta|[8605]|LPVIA114-08|Canada|British Columbia|658[0n]|BOLD:AAA3073
Ochropleura implecta|[8606]|LPGVA607-08|Canada|British Columbia|658[0n]|BOLD:AAA3073
Ochropleura implecta|[8607]|LPGVA603-08|Canada|British Columbia|658[0n]|BOLD:AAA3073
Ochropleura implecta|[8608]|LPABB056-08|Canada|Alberta|658[0n]|BOLD:AAA3073
Ochropleura implecta|[8609]|LPMN805-08|Canada|Manitoba|658[0n]|BOLD:AAA3073
Ochropleura implecta|[8610]|LPMN727-08|Canada|Manitoba|658[0n]|BOLD:AAA3073
Ochropleura implecta|[8611]|LPSOB996-08|Canada|Ontario|658[0n]|BOLD:AAA3073
Ochropleura implecta|[8612]|LHLEP235-06|Canada|British Columbia|658[0n]|BOLD:AAA3073
Ochropleura implecta|[8613]|LHLEP234-06|Canada|British Columbia|658[0n]|BOLD:AAA3073
Ochropleura implecta|[8614]|LHLEP233-06|Canada|British Columbia|658[0n]|BOLD:AAA3073
Ochropleura implecta|[8615]|LHLEP232-06|Canada|British Columbia|658[0n]|BOLD:AAA3073
Ochropleura implecta|[8616]|LHLEP231-06|Canada|British Columbia|658[0n]|BOLD:AAA3073
Ochropleura implecta|[8617]|LHLEP230-06|Canada|British Columbia|658[0n]|BOLD:AAA3073
Ochropleura implecta|[8618]|LHLEP229-06|Canada|British Columbia|658[0n]|BOLD:AAA3073
Ochropleura implecta|[8619]|LHLEP228-06|Canada|British Columbia|658[0n]|BOLD:AAA3073
Ochropleura implecta|[8620]|LHLEP227-06|Canada|British Columbia|658[0n]|BOLD:AAA3073
Ochropleura implecta|[8621]|LHLEP226-06|Canada|British Columbia|658[0n]|BOLD:AAA3073
Ochropleura implecta|[8622]|LHLEP224-06|Canada|British Columbia|658[0n]|BOLD:AAA3073
Ochropleura implecta|[8623]|LHLEP223-06|Canada|British Columbia|658[0n]|BOLD:AAA3073
Ochropleura implecta|[8624]|LHLEP222-06|Canada|British Columbia|658[0n]|BOLD:AAA3073
Ochropleura implecta|[8625]|LHLEP221-06|Canada|British Columbia|658[0n]|BOLD:AAA3073
Ochropleura implecta|[8626]|LHLEP220-06|Canada|British Columbia|658[0n]|BOLD:AAA3073
Ochropleura implecta|[8627]|LHLEP219-06|Canada|British Columbia|658[0n]|BOLD:AAA3073
Ochropleura implecta|[8628]|LHLEP218-06|Canada|British Columbia|658[0n]|BOLD:AAA3073
Ochropleura implecta|[8629]|LHLEP217-06|Canada|British Columbia|658[0n]|BOLD:AAA3073
Ochropleura implecta|[8630]|LHLEP071-06|Canada|British Columbia|658[0n]|BOLD:AAA3073
Ochropleura implecta|[8631]|LHLEP070-06|Canada|British Columbia|658[0n]|BOLD:AAA3073
Ochropleura implecta|[8632]|RDLQF546-06|Canada|Quebec|658[0n]|BOLD:AAA3073
Ochropleura implecta|[8633]|RDLQF456-06|Canada|Quebec|658[0n]|BOLD:AAA3073
Ochropleura implecta|[8634]|RDLQF270-06|Canada|Quebec|658[0n]|BOLD:AAA3073
Ochropleura implecta|[8635]|RDLQF225-06|Canada|Quebec|658[0n]|BOLD:AAA3073
Ochropleura implecta|[8636]|TTMNB044-06|Canada|New Brunswick|658[0n]|BOLD:AAA3073
Ochropleura implecta|[8637]|RDLQB537-05|Canada|Quebec|658[0n]|BOLD:AAA3073
Ochropleura implecta|[8638]|RDLQB536-05|Canada|Quebec|658[0n]|BOLD:AAA3073
Ochropleura implecta|[8639]|LBCC294-05|Canada|British Columbia|658[0n]|BOLD:AAA3073
Ochropleura implecta|[8640]|MNBB451-05|Canada|New Brunswick|658[0n]|BOLD:AAA3073
Ochropleura implecta|[8641]|LBCA933-05|Canada|British Columbia|658[0n]|BOLD:AAA3073
Ochropleura implecta|[8642]|LBCA812-05|Canada|British Columbia|658[0n]|BOLD:AAA3073
Ochropleura implecta|[8643]|LBCA507-05|Canada|British Columbia|658[0n]|BOLD:AAA3073
Ochropleura implecta|[8644]|XAD775-05|Canada|Ontario|658[0n]|BOLD:AAA3073
Ochropleura implecta|[8645]|PHMNB608-04|Canada|New Brunswick|658[0n]|BOLD:AAA3073
Ochropleura implecta|[8646]|PHMNB383-04|Canada|New Brunswick|658[0n]|BOLD:AAA3073
Ochropleura implecta|[8647]|PHMNB378-04|Canada|New Brunswick|658[0n]|BOLD:AAA3073
Ochropleura implecta|[8648]|LCH264-04|Canada|Manitoba|658[0n]|BOLD:AAA3073
Ochropleura implecta|[8649]|BBLEC864-09|Canada|Newfoundland and Labrador|654[0n]|BOLD:AAA3073
Ochropleura implecta|[8650]|RWWA505-09|United States|Washington|654[0n]|BOLD:AAA3073
Ochropleura implecta|[8651]|RDLQF224-06|Canada|Quebec|655[0n]|BOLD:AAA3073
Ochropleura implecta|[8652]|TTMNB090-06|Canada|New Brunswick|656[0n]|BOLD:AAA3073
Ochropleura implecta|[8653]|RDLQG214-06|Canada|Quebec|654[0n]|BOLD:AAA3073
Ochropleura implecta|[8654]|LHLEP072-06|Canada|British Columbia|652[0n]|BOLD:AAA3073
Ochropleura implecta|[8655]|LBCA375-05|Canada|British Columbia|649[0n]|BOLD:AAA3073
Ochropleura implecta|[8656]|LPGVA635-08|Canada|British Columbia|649[0n]|BOLD:AAA3073
Ochropleura implecta|[8657]|RDLQB703-05|Canada|Quebec|579[2n]|BOLD:AAA3073
Ochropleura implecta|[8658]|LPGVA604-08|Canada|British Columbia|626[0n]|BOLD:AAA3073
Ochropleura implecta|[8659]|PHMNB674-04|Canada|New Brunswick|624[0n]|BOLD:AAA3073
Ochropleura implecta|[8660]|BBLPC145-09|Canada|Nova Scotia|658[0n]|BOLD:AAA3073
Ochropleura implecta|[8661]|PHMNB025-03|Canada|New Brunswick|639[0n]|BOLD:AAA3073
Ochropleura implecta|[8662]|XAF781-05|Canada|Ontario|503[3n]|
Ochropleura implecta|[8663]|RDLQB731-05|Canada|Quebec|593[2n]|BOLD:AAA3073
Ochropleura implecta|[8664]|XAJ840-06|Canada|Ontario|619[0n]|BOLD:AAA3073
Ochropleura implecta|[8665]|LPGVA634-08|Canada|British Columbia|633[0n]|BOLD:AAA3073
Ochropleura implecta|[8666]|BBLEC842-09|Canada|Newfoundland and Labrador|634[0n]|BOLD:AAA3073
Ochropleura implecta|[8667]|BBLPC664-09|Canada|Newfoundland and Labrador|634[0n]|BOLD:AAA3073
Ochropleura implecta|[8668]|PHMNB753-05|Canada|New Brunswick|650[0n]|BOLD:AAA3073
Ochropleura implecta|[8669]|BBLPC771-09|Canada|Newfoundland and Labrador|620[0n]|BOLD:AAA3073
Ochropleura implecta|[8670]|BBLPE081-09|Canada|Nova Scotia|658[0n]|BOLD:AAA3073
Ochropleura implecta|[8671]|BBLPE116-09|Canada|Nova Scotia|658[0n]|BOLD:AAA3073
Ochropleura implecta|[8672]|BBLPE277-09|Canada|Nova Scotia|658[0n]|BOLD:AAA3073

Ochropleura implecta|[8670]|BBLPE081-09|Canada|Nova Scotia|658[0n]|BOLD:AAA3073
Ochropleura implecta|[8671]|BBLPE116-09|Canada|Nova Scotia|658[0n]|BOLD:AAA3073
Ochropleura implecta|[8672]|BBLPE277-09|Canada|Nova Scotia|658[0n]|BOLD:AAA3073
Ochropleura implecta|[8673]|BBLPE353-09|Canada|Newfoundland and Labrador|658[0n]|BOLD:AAA3073
Ochropleura implecta|[8674]|BBLPE390-09|Canada|Newfoundland and Labrador|658[0n]|BOLD:AAA3073
Ochropleura implecta|[8675]|LBCH1501-10|Canada|British Columbia|658[0n]|BOLD:AAA3073
Ochropleura implecta|[8676]|LBCH2986-10|Canada|British Columbia|658[0n]|BOLD:AAA3073
Ochropleura implecta|[8677]|LBCH3004-10|Canada|British Columbia|658[0n]|BOLD:AAA3073
Ochropleura implecta|[8678]|RWWB751-10|United States|Washington|658[0n]|BOLD:AAA3073
Ochropleura implecta|[8679]|RWWB848-10|United States|Washington|658[0n]|BOLD:AAA3073
Ochropleura implecta|[8680]|LALPA185-10|Canada|British Columbia|658[0n]|BOLD:AAA3073
Ochropleura implecta|[8681]|LALPA251-10|Canada|British Columbia|658[0n]|BOLD:AAA3073
Ochropleura implecta|[8682]|LALPA405-10|Canada|British Columbia|658[0n]|BOLD:AAA3073
Ochropleura implecta|[8683]|BBLPB457-10|Canada|British Columbia|658[0n]|BOLD:AAA3073
Ochropleura implecta|[8684]|BBLPB846-10|Canada|British Columbia|658[0n]|BOLD:AAA3073
Ochropleura implecta|[8685]|BBLPB847-10|Canada|British Columbia|658[0n]|BOLD:AAA3073
Ochropleura implecta|[8686]|BBLPB848-10|Canada|British Columbia|658[0n]|BOLD:AAA3073
Ochropleura implecta|[8687]|BBLPB850-10|Canada|Ontario|658[0n]|BOLD:AAA3073
Ochropleura implecta|[8688]|RWWC627-11|United States|Washington|658[0n]|BOLD:AAA3073
Anicla tenuescens|[8689]|RDNMF594-08|United States|Nebraska|522[3n]|
Anicla tenuescens|[8690]|RDNMF593-08|United States|Nebraska|658[0n]|BOLD:AAE5009
Anicla tenuescens|[8691]|RDNMF592-08|United States|Colorado|658[0n]|BOLD:AAE5009
Anicla tenuescens|[8692]|RDNMF595-08|United States|Wyoming|658[0n]|BOLD:AAE5009
Anicla lubricans|[8693]|HKONB388-09|United States|Texas|658[0n]|BOLD:AAC0401
Anicla lubricans|[8694]|HKONB390-09|United States|Texas|658[0n]|BOLD:AAC0401
Anicla lubricans|[8695]|HKONB391-09|United States|Louisiana|658[0n]|BOLD:AAC0401
Anicla lubricans|[8696]|LNCB791-09|United States|North Carolina|658[0n]|BOLD:AAC0401
Anicla lubricans|[8697]|LSEU177-06|United States|Georgia|658[0n]|BOLD:AAC0401
Anicla lubricans|[8698]|HKONS504-08|United States|Florida|658[0n]|BOLD:AAC0401
Anicla lubricans|[8699]|LNCB792-09|United States|North Carolina|658[0n]|BOLD:AAC0401
Anicla sullivani|[8700]|HKONS521-08|United States|Florida|658[0n]|BOLD:ABZ0306
Anicla sullivani|[8701]|HKONS522-08|United States|Florida|658[0n]|BOLD:ABZ0306
Anicla sullivani|[8702]|HKONS523-08|United States|Florida|658[0n]|BOLD:ABZ0306
Anicla sullivani|[8703]|HKONS524-08|United States|Florida|658[0n]|BOLD:ABZ0306
Anicla sullivani|[8704]|HKONS525-08|United States|Florida|658[0n]|BOLD:ABZ0306
Anicla sullivani|[8705]|HKONS526-08|United States|Florida|658[0n]|BOLD:ABZ0306
Anicla sullivani|[8706]|LNC832-06|United States|North Carolina|656[0n]|BOLD:ABZ0306
Anicla sullivani|[8707]|MILEP052-09|United States|North Carolina|658[0n]|BOLD:ABZ0306
Anicla lubricans|[8708]|HKONS510-08|United States|Florida|658[0n]|BOLD:ACE4818
Anicla lubricans|[8709]|HKONS509-08|United States|Florida|658[0n]|BOLD:ACE4818
Anicla lubricans|[8710]|HKONS511-08|United States|Florida|658[3n]|BOLD:ACE4818
Anicla lubricans|[8711]|HKONB389-09|United States|Texas|658[0n]|BOLD:ACE4818
Anicla lubricans|[8712]|LNCB682-09|United States|North Carolina|600[0n]|BOLD:ACE4818
Anicla simplicius|[8713]|LPOKA149-08|United States|Oklahoma|658[0n]|BOLD:ACE5562
Anicla simplicius|[8714]|RDNMK075-11|United States|Florida|658[0n]|BOLD:ACE5562
Anicla lubricans|[8715]|HKONS505-08|United States|Florida|658[0n]|BOLD:ACF1404
Anicla lubricans|[8716]|HKONS508-08|United States|Florida|658[1n]|BOLD:ACF1404
Anicla lubricans|[8717]|LNC831-06|United States|North Carolina|658[0n]|BOLD:ACF1404
Anicla lubricans|[8718]|LNC834-06|United States|North Carolina|658[0n]|BOLD:ACF1404
Anicla lubricans|[8719]|HKONS501-08|United States|Florida|658[0n]|BOLD:ACF1404
Anicla lubricans|[8720]|HKONS502-08|United States|Florida|658[0n]|BOLD:ACF1404
Anicla lubricans|[8721]|HKONS503-08|United States|Florida|658[0n]|BOLD:ACF1404
Anicla lubricans|[8722]|HKONS506-08|United States|Florida|658[0n]|BOLD:ACF1404
Anicla lubricans|[8723]|HKONS507-08|United States|Florida|658[0n]|BOLD:ACF1404
Anicla lubricans|[8724]|HKONS512-08|United States|Florida|658[0n]|BOLD:ACF1404
Anicla lubricans|[8725]|LNCB697-09|United States|North Carolina|658[0n]|BOLD:ACF1404
Anicla lubricans|[8726]|LNCB813-09|United States|North Carolina|658[0n]|BOLD:ACF1404
Anicla lubricans|[8727]|LNCC015-10|United States|North Carolina|658[0n]|BOLD:ACF1404
Anicla illapsa|[8728]|LNCB629-09|United States|North Carolina|658[0n]|BOLD:ACF5764
Anicla illapsa|[8729]|IAWLB389-11|United States|Virginia|658[0n]|BOLD:ACF5764
Anicla illapsa|[8730]|LOFLC113-06|United States|Florida|655[0n]|BOLD:ACF5764
Anicla illapsa|[8731]|RDNML007-13|United States|Florida|658[0n]|BOLD:ACF5764
Anicla illapsa|[8732]|LNCC407-10|United States|North Carolina|658[0n]|BOLD:ACF5764
Anicla illapsa|[8733]|LNCC1646-13|United States|North Carolina|658[0n]|BOLD:ACF5764
Anicla illapsa|[8734]|LNCC406-10|United States|North Carolina|658[0n]|BOLD:ACF5764
Anicla illapsa|[8735]|NCCH102-11|Canada|Ontario|658[0n]|BOLD:ACF5764
Anicla illapsa|[8736]|LOTB109-05|United States|Tennessee|658[0n]|BOLD:ACF5764
Anicla illapsa|[8737]|LGSMC693-05|United States|Tennessee|658[0n]|BOLD:ACF5764
Anicla illapsa|[8738]|LNCC1645-13|United States|North Carolina|658[0n]|BOLD:ACF5764
Anicla illapsa|[8739]|LNCC1264-11|United States|North Carolina|658[0n]|BOLD:ACF5764
Anicla illapsa|[8740]|LNCC1243-11|United States|North Carolina|658[0n]|BOLD:ACF5764
Anicla illapsa|[8741]|LNCC299-10|United States|North Carolina|658[0n]|BOLD:ACF5764
Anicla illapsa|[8742]|LNCC298-10|United States|North Carolina|658[0n]|BOLD:ACF5764
Anicla illapsa|[8743]|LNCC297-10|United States|North Carolina|658[0n]|BOLD:ACF5764
Anicla illapsa|[8744]|LPOKC738-09|United States|Oklahoma|658[0n]|BOLD:ACF5764
Anicla illapsa|[8745]|XAJ617-06|Canada|Ontario|658[0n]|BOLD:ACF5764
Anicla illapsa|[8746]|LOTB140-05|United States|Tennessee|658[0n]|BOLD:ACF5764
Anicla illapsa|[8747]|MEC404-04|Canada|Quebec|658[0n]|BOLD:ACF5764
Anicla illapsa|[8748]|XAC627-04|Canada|Ontario|658[0n]|BOLD:ACF5764
Anicla illapsa|[8749]|XAC188-04|Canada|Ontario|658[0n]|BOLD:ACF5764
Anicla illapsa|[8750]|XAC178-04|Canada|Ontario|658[0n]|BOLD:ACF5764
Anicla illapsa|[8751]|LGSM724-04|United States|Tennessee|658[0n]|BOLD:ACF5764
Anicla illapsa|[8752]|LGSM723-04|United States|Tennessee|658[0n]|BOLD:ACF5764
Anicla illapsa|[8753]|LOTB317-05|United States|Tennessee|651[0n]|BOLD:ACF5764
Anicla illapsa|[8754]|BLTIB379-08|Canada|Ontario|638[0n]|BOLD:ACF5764
Anicla illapsa|[8755]|LNCC1644-13|United States|North Carolina|658[0n]|BOLD:ACF5764
Anicla illapsa|[8756]|LNCC1647-13|United States|North Carolina|658[0n]|BOLD:ACF5764
Anicla illapsa|[8757]|LNCC1648-13|United States|North Carolina|658[0n]|BOLD:ACF5764
Anicla exuberans|[8758]|LOWCE767-06|Canada|British Columbia|658[1n]|BOLD:AAC5892
Anicla exuberans|[8759]|LOWCD538-06|Canada|British Columbia|658[1n]|BOLD:AAC5892
Anicla exuberans|[8760]|LOWCE768-06|Canada|British Columbia|658[0n]|BOLD:AAC5892
Anicla exuberans|[8761]|LOWCE298-06|Canada|British Columbia|658[0n]|BOLD:AAC5892
Anicla exuberans|[8762]|LOWCE296-06|Canada|British Columbia|658[0n]|BOLD:AAC5892
Anicla exuberans|[8763]|LOWCE295-06|Canada|British Columbia|658[0n]|BOLD:AAC5892
Anicla exuberans|[8764]|LOWCE769-06|Canada|British Columbia|623[0n]|BOLD:AAC5892
Anicla exuberans|[8765]|LBCG412-08|Canada|British Columbia|658[0n]|BOLD:AAC5892
Anicla exuberans|[8766]|LBCG413-08|Canada|British Columbia|658[0n]|BOLD:AAC5892
Anicla exuberans|[8767]|LBCG414-08|Canada|British Columbia|658[0n]|BOLD:AAC5892
Anicla exuberans|[8768]|LBCH5511-10|Canada|British Columbia|658[0n]|BOLD:AAC5892
Anicla exuberans|[8769]|LBCH5513-10|Canada|British Columbia|658[0n]|BOLD:AAC5892
Anicla exuberans|[8770]|LBCH5570-10|Canada|British Columbia|658[0n]|BOLD:AAC5892
Anicla exuberans|[8771]|LBCH5571-10|Canada|British Columbia|658[0n]|BOLD:AAC5892
Anicla exuberans|[8772]|LBCH6040-10|Canada|British Columbia|658[0n]|BOLD:AAC5892

[illegible]
Anicla exuberans|[8770]|LBCH5570-10|Canada|British Columbia|658[0n]|BOLD:AAC5892
Anicla exuberans|[8771]|LBCH5571-10|Canada|British Columbia|658[0n]|BOLD:AAC5892
Anicla exuberans|[8772]|LBCH6040-10|Canada|British Columbia|658[0n]|BOLD:AAC5892
Anicla exuberans|[8773]|LBCH6042-10|Canada|British Columbia|658[0n]|BOLD:AAC5892
Anicla exuberans|[8774]|LBCH6043-10|Canada|British Columbia|658[0n]|BOLD:AAC5892
Anicla exuberans|[8775]|LBCH6095-10|Canada|British Columbia|658[0n]|BOLD:AAC5892
Anicla exuberans|[8776]|RDNME633-08|United States|Arizona|658[0n]|BOLD:AAC5892
Anicla exuberans|[8777]|BBLSW414-09|United States|New Mexico|658[0n]|BOLD:AAC5892
Anicla exuberans|[8778]|RDNMK370-11|United States|New Mexico|658[0n]|BOLD:AAC5892
Anicla digna|[8779]|BBLSY115-09|United States|Texas|658[0n]|BOLD:AAH5334
Anicla digna|[8780]|BBLSW954-09|United States|Texas|658[0n]|BOLD:AAH5334
Anicla digna|[8781]|USLEP1162-10|United States|Texas|658[0n]|BOLD:AAH5334
Anicla digna|[8782]|BBLSY117-09|United States|Texas|658[0n]|BOLD:AAH5334
Anicla digna|[8783]|BBLSY123-09|United States|Texas|658[0n]|BOLD:AAH5334
Anicla digna|[8784]|BBLSY124-09|United States|Texas|658[0n]|BOLD:AAH5334
Anicla digna|[8785]|USLEP1163-10|United States|Texas|658[0n]|BOLD:AAH5334
Anicla digna|[8786]|RDNMK369-11|United States|New Mexico|658[0n]|BOLD:AAH5334
Anicla digna|[8787]|BBLSY118-09|United States|Texas|636[0n]|BOLD:AAH5334
Anicla digna|[8788]|BBLSY119-09|United States|Texas|658[0n]|BOLD:AAH5334
Anicla digna|[8789]|BBLSY121-09|United States|Texas|658[0n]|BOLD:AAH5334
Anicla digna|[8790]|BBLSY122-09|United States|Texas|658[0n]|BOLD:AAH5334
Anicla digna|[8791]|BBLSY120-09|United States|Texas|658[0n]|BOLD:AAH5334
Anicla digna|[8792]|BBLSY129-09|United States|Texas|658[0n]|BOLD:AAH5334
Anicla digna|[8793]|BBLSY222-09|United States|Texas|658[0n]|BOLD:AAH5334
Anicla digna|[8794]|USLEP809-10|United States|Texas|658[0n]|BOLD:AAH5334
Anicla digna|[8795]|BBLSY116-09|United States|Texas|652[0n]|BOLD:AAH5334
Anicla digna|[8796]|BBLSY223-09|United States|Texas|658[0n]|BOLD:AAH5334
Anicla digna|[8797]|BBLSY224-09|United States|Texas|658[0n]|BOLD:AAH5334
Anicla digna|[8798]|USLEP810-10|United States|Texas|658[0n]|BOLD:AAH5334
Anicla digna|[8799]|RDNMK368-11|United States|New Mexico|658[0n]|BOLD:AAH5334
Anicla digna|[8800]|RDNMK371-11|United States|New Mexico|658[0n]|BOLD:AAH5334
Anicla espoetia|[8801]|RDNMJ714-11|United States|Arizona|658[0n]|BOLD:ABZ7532
Anicla forbesi|[8802]|RDNMG941-08|Canada|Ontario|658[0n]|BOLD:ACF5752
Anicla forbesi|[8803]|RDNMG942-08|Canada|Ontario|658[0n]|BOLD:ACF5752
Anicla forbesi|[8804]|RDLQB679-05|Canada|Quebec|658[0n]|BOLD:ACF5752
Anicla forbesi|[8805]|RDLQ705-07|Canada|Quebec|658[0n]|BOLD:ACF5752
Anicla forbesi|[8806]|RDNMG943-08|Canada|Ontario|658[0n]|BOLD:ACF5752
Anicla tepperi|[8807]|LPSK131-08|Canada|Saskatchewan|658[0n]|BOLD:AAC5355
Anicla tepperi|[8808]|LPSK545-08|Canada|Saskatchewan|658[0n]|BOLD:AAC5355
Anicla tepperi|[8809]|LPSK398-08|Canada|Saskatchewan|658[0n]|
Anicla tepperi|[8810]|RDNMF095-08|Canada|Alberta|658[0n]|BOLD:AAC5355
Anicla tepperi|[8811]|CNGRD1032-12|Canada|Saskatchewan|636[0n]|BOLD:AAC5355
Anicla tepperi|[8812]|LPSK504-08|Canada|Saskatchewan|658[0n]|BOLD:AAC5355
Anicla tepperi|[8813]|RDNMC289-05|United States|Colorado|587[0n]|BOLD:AAC5355
Anicla tepperi|[8814]|LPSK141-08|Canada|Saskatchewan|658[0n]|BOLD:AAC5355
Anicla tepperi|[8815]|LPSK248-08|Canada|Saskatchewan|658[0n]|BOLD:AAC5355
Anicla tepperi|[8816]|RDNMG443-08|United States|Wyoming|658[0n]|BOLD:AAC5355
Anicla tepperi|[8817]|RDNMG444-08|United States|Wyoming|658[0n]|BOLD:AAC5355
Anicla tepperi|[8818]|RDNMG445-08|United States|Wyoming|658[0n]|BOLD:AAC5355
Anicla tepperi|[8819]|CNGRJ081-13|Canada|Saskatchewan|549[0n]|BOLD:AAC5355
Anicla beata|[8820]|LNAUS4384-13|United States|New Mexico|658[0n]|BOLD:AAH5395
Anicla beata|[8821]|LNAUS4382-13|United States|New Mexico|658[0n]|BOLD:AAH5395
Anicla beata|[8822]|LNAUS4381-13|United States|New Mexico|658[0n]|BOLD:AAH5395
Anicla beata|[8823]|BBLWU156-09|United States|Colorado|658[0n]|BOLD:AAH5395
Anicla beata|[8824]|BBLWU102-09|United States|Colorado|658[0n]|BOLD:AAH5395
Anicla beata|[8825]|BBLWU100-09|United States|Colorado|658[0n]|BOLD:AAH5395
Anicla beata|[8826]|BBLWU191-09|United States|Colorado|658[0n]|BOLD:AAH5395
Anicla beata|[8827]|BBLSY540-09|United States|New Mexico|658[0n]|BOLD:AAH5395
Anicla beata|[8828]|LNAUS4383-13|United States|New Mexico|647[0n]|BOLD:AAH5395
Anicla beata|[8829]|LNAUS4385-13|United States|New Mexico|658[0n]|BOLD:AAH5395
Anicla beata|[8830]|CNCLB184-14|United States|Wyoming|658[0n]|BOLD:AAH5395
Anicla biformata|[8831]|RDNME950-08|United States|Texas|658[0n]|BOLD:AAF1319
Anicla biformata|[8832]|CMAZA028-09|United States|Arizona|658[0n]|BOLD:AAF1319
Anicla biformata|[8833]|RDNMD886-07|United States|Arizona|655[0n]|BOLD:AAF1319
Anicla biformata|[8834]|RDNMD887-07|United States|Arizona|655[0n]|BOLD:AAF1319
Anicla biformata|[8835]|RDNMJ125-10|United States|New Mexico|658[0n]|BOLD:AAF1319
Anicla mus|[8836]|USLEP295-10|United States|Colorado|658[0n]|BOLD:AAN6807
Anicla mus|[8837]|CNCLB183-14|United States|New Mexico|658[0n]|BOLD:AAN6807
Anicla mus|[8838]|CNCLB187-14|United States|Colorado|658[0n]|BOLD:AAN6807
Anicla infecta|[8839]|LOFLC186-06|United States|Florida|658[0n]|BOLD:ACE9897
Anicla infecta|[8840]|BBLOB1307-11|United States|Florida|658[0n]|BOLD:ACE9897
Anicla infecta|[8841]|BBLOC1037-11|United States|Texas|658[0n]|BOLD:ACE9897
Anicla infecta|[8842]|USLEP1192-10|United States|Florida|658[0n]|BOLD:ACE9897
Anicla infecta|[8843]|LILLA797-11|United States|Illinois|658[0n]|BOLD:ACE9897
Anicla infecta|[8844]|LPOKA961-09|United States|Oklahoma|658[0n]|BOLD:ACE9897
Anicla infecta|[8845]|LPOKA668-09|United States|Oklahoma|658[0n]|BOLD:ACE9897
Anicla infecta|[8846]|LOFLB487-06|United States|Florida|658[0n]|BOLD:ACE9897
Anicla infecta|[8847]|LOFLB126-06|United States|Florida|658[0n]|BOLD:ACE9897
Anicla infecta|[8848]|LNC485-06|United States|North Carolina|658[0n]|BOLD:ACE9897
Anicla infecta|[8849]|LPOKA616-09|United States|Oklahoma|639[0n]|BOLD:ACE9897
Anicla infecta|[8850]|LNC486-06|United States|North Carolina|650[0n]|BOLD:ACE9897
Anicla infecta|[8851]|LPOKA518-09|United States|Oklahoma|632[0n]|BOLD:ACE9897
Anicla infecta|[8852]|BBLSW846-09|United States|Texas|632[0n]|BOLD:ACE9897
Anicla infecta|[8853]|LGSMG894-10|United States|Tennessee|658[0n]|BOLD:ACE9897
Anicla infecta|[8854]|LGSMG895-10|United States|North Carolina|658[0n]|BOLD:ACE9897
Anicla infecta|[8855]|USLEP292-10|United States|Florida|658[0n]|BOLD:ACE9897
Anicla infecta|[8856]|USLEP300-10|United States|Florida|658[0n]|BOLD:ACE9897
Anicla infecta|[8857]|USLEP632-10|United States|Florida|658[0n]|BOLD:ACE9897
Anicla infecta|[8858]|USLEP642-10|United States|Florida|658[0n]|BOLD:ACE9897
Anicla infecta|[8859]|USLEP1148-10|United States|Florida|658[0n]|BOLD:ACE9897
Anicla infecta|[8860]|BBLOE1435-12|United States|Arkansas|658[0n]|BOLD:ACE9897
Anicla cemolia|[8861]|CNCLB185-14|United States|Florida|658[0n]|BOLD:ACM4137
Anicla cemolia|[8862]|CNCLB186-14|United States|Florida|658[0n]|BOLD:ACM4137
Anicla cemolia|[8863]|LNAUT940-14|United States|Florida|658[0n]|BOLD:ACM4137
Anicla cemolia|[8864]|LNAUT941-14|United States|Florida|658[0n]|BOLD:ACM4137
Dichagyris socorro|[8865]|CMAZA800-10|United States|Arizona|658[0n]|BOLD:AAP3530
Dichagyris socorro|[8866]|RDNMJ821-11|United States|Arizona|658[0n]|BOLD:AAP3530
Dichagyris polycala|[8867]|RDNMK861-13|United States|Arizona|658[0n]|BOLD:AAZ0699
Dichagyris salina|[8868]|RDNMJ665-11|United States|Arizona|658[0n]|BOLD:AAX9336
Dichagyris salina|[8869]|RDNMK932-13|Mexico|Sonora|658[0n]|BOLD:AAX9336
Dichagyris arabella|[8870]|CNCLB2017-14|Guatemala||622[0n]|BOLD:ACR1291
Dichagyris arabella|[8871]|CNCLB2019-14|Mexico||307[0n]|
[illegible]

Dichagyris salina|[8869]|RDNMK932-13|Mexico|Sonora|658[0n]|BOLD:AAX9336
Dichagyris arabella|[8870]|CNCLB2017-14|Guatemala||622[0n]|BOLD:ACR1291
Dichagyris arabella|[8871]|CNCLB2019-14|Mexico||307[0n]|
Dichagyris arabella|[8872]|CNCLB962-14|United States|Texas|550[0n]|BOLD:ACQ1225
Dichagyris arabella|[8873]|CNCLB2021-14|United States|Texas|307[0n]|
Dichagyris arabella|[8874]|CNCLB2020-14|United States|Texas|475[1n]|
Dichagyris arabella|[8875]|CNCLB2022-14|United States|New Mexico|475[0n]|
Agrotis apicalis|[8876]|RDNML328-13|United States|Florida|658[1n]|BOLD:ACD9077
Agrotis apicalis|[8877]|RDNML329-13|United States|Florida|658[0n]|BOLD:ACD9077
Agrotis apicalis|[8878]|LNAUS2760-13|Jamaica||658[0n]|BOLD:ACD9077
Agrotis ipsilon|[8879]|LGSM492-04|United States|North Carolina|609[0n]|BOLD:AAA3364
Agrotis ipsilon|[8880]|SMTPD1181-13|Canada|Ontario|591[0n]|BOLD:AAA3364
Agrotis ipsilon|[8881]|XAB709-04|Canada|Ontario|658[0n]|BOLD:AAA3364
Agrotis ipsilon|[8882]|XAD508-04|Canada|Ontario|577[0n]|BOLD:AAA3364
Agrotis ipsilon|[8883]|PHMNB276-04|Canada|New Brunswick|569[0n]|BOLD:AAA3364
Agrotis ipsilon|[8884]|XAB677-04|Canada|Ontario|571[0n]|BOLD:AAA3364
Agrotis ipsilon|[8885]|SMTPB17386-13|Canada|Ontario|592[0n]|BOLD:AAA3364
Agrotis ipsilon|[8886]|LGSMC653-05|United States|Tennessee|614[0n]|BOLD:AAA3364
Agrotis ipsilon|[8887]|LPOKB329-09|United States|Oklahoma|627[0n]|BOLD:AAA3364
Agrotis ipsilon|[8888]|MHCOL167-07|Canada|Manitoba|625[0n]|BOLD:AAA3364
Agrotis ipsilon|[8889]|MHCOL156-07|Canada|Manitoba|658[0n]|BOLD:AAA3364
Agrotis ipsilon|[8890]|MHCOL284-07|Canada|Manitoba|658[0n]|BOLD:AAA3364
Agrotis ipsilon|[8891]|MHCOL360-07|Canada|Manitoba|658[0n]|BOLD:AAA3364
Agrotis ipsilon|[8892]|MHCOL366-07|Canada|Manitoba|658[0n]|BOLD:AAA3364
Agrotis ipsilon|[8893]|MHLEP015-07|Canada|Manitoba|658[0n]|BOLD:AAA3364
Agrotis ipsilon|[8894]|MHLEP078-07|Canada|Manitoba|658[0n]|BOLD:AAA3364
Agrotis ipsilon|[8895]|MHLEP085-07|Canada|Manitoba|658[0n]|BOLD:AAA3364
Agrotis ipsilon|[8896]|MHLEP093-07|Canada|Manitoba|658[0n]|BOLD:AAA3364
Agrotis ipsilon|[8897]|LBCS028-07|Canada|British Columbia|658[0n]|BOLD:AAA3364
Agrotis ipsilon|[8898]|LBCS184-07|Canada|British Columbia|658[0n]|BOLD:AAA3364
Agrotis ipsilon|[8899]|LPSO022-08|Canada|Ontario|658[0n]|BOLD:AAA3364
Agrotis ipsilon|[8900]|BLTIB545-08|Canada|Ontario|658[0n]|BOLD:AAA3364
Agrotis ipsilon|[8901]|BLTIB830-08|Canada|Ontario|658[0n]|BOLD:AAA3364
Agrotis ipsilon|[8902]|LPOKA521-09|United States|Oklahoma|658[0n]|BOLD:AAA3364
Agrotis ipsilon|[8903]|LOCT104-05|United States|Connecticut|658[0n]|BOLD:AAA3364
Agrotis ipsilon|[8904]|MNBB179-05|Canada|New Brunswick|658[0n]|BOLD:AAA3364
Agrotis ipsilon|[8905]|LPOKA525-09|United States|Oklahoma|658[0n]|BOLD:AAA3364
Agrotis ipsilon|[8906]|LPSOD1045-09|Canada|Ontario|658[0n]|BOLD:AAA3364
Agrotis ipsilon|[8907]|LPOKA670-09|United States|Oklahoma|658[0n]|BOLD:AAA3364
Agrotis ipsilon|[8908]|LPOKA676-09|United States|Oklahoma|658[0n]|BOLD:AAA3364
Agrotis ipsilon|[8909]|LPOKA934-09|United States|Oklahoma|658[0n]|BOLD:AAA3364
Agrotis ipsilon|[8910]|LPOKB323-09|United States|Oklahoma|658[0n]|BOLD:AAA3364
Agrotis ipsilon|[8911]|MNBB374-05|Canada|New Brunswick|658[0n]|BOLD:AAA3364
Agrotis ipsilon|[8912]|MNBB428-05|Canada|New Brunswick|658[0n]|BOLD:AAA3364
Agrotis ipsilon|[8913]|LPOKB1002-09|United States|Oklahoma|658[0n]|BOLD:AAA3364
Agrotis ipsilon|[8914]|BBLSY416-09|United States|Arizona|658[0n]|BOLD:AAA3364
Agrotis ipsilon|[8915]|BBLEC543-09|Canada|Nova Scotia|658[0n]|BOLD:AAA3364
Agrotis ipsilon|[8916]|BBLPC033-09|Canada|New Brunswick|658[0n]|BOLD:AAA3364
Agrotis ipsilon|[8917]|BBLPC508-09|Canada|New Brunswick|658[0n]|BOLD:AAA3364
Agrotis ipsilon|[8918]|CMAZA226-09|United States|Arizona|658[0n]|BOLD:AAA3364
Agrotis ipsilon|[8919]|MNBB477-05|Canada|New Brunswick|658[0n]|BOLD:AAA3364
Agrotis ipsilon|[8920]|MNBB550-05|Canada|New Brunswick|658[0n]|BOLD:AAA3364
Agrotis ipsilon|[8921]|MNBB474-05|Canada|New Brunswick|658[0n]|BOLD:AAA3364
Agrotis ipsilon|[8922]|MNBB476-05|Canada|New Brunswick|658[0n]|BOLD:AAA3364
Agrotis ipsilon|[8923]|LGSMG881-10|United States|North Carolina|658[0n]|BOLD:AAA3364
Agrotis ipsilon|[8924]|LALPA175-10|Canada|British Columbia|658[0n]|BOLD:AAA3364
Agrotis ipsilon|[8925]|JGLL060-10|Canada|Manitoba|658[0n]|BOLD:AAA3364
Agrotis ipsilon|[8926]|JGLL061-10|Canada|Manitoba|658[0n]|BOLD:AAA3364
Agrotis ipsilon|[8927]|JGLL062-10|Canada|Manitoba|658[0n]|BOLD:AAA3364
Agrotis ipsilon|[8928]|LILLA632-11|United States|Illinois|658[0n]|BOLD:AAA3364
Agrotis ipsilon|[8929]|LILLA833-11|United States|Illinois|658[0n]|BOLD:AAA3364
Agrotis ipsilon|[8930]|GMLC597-11|United States|California|658[0n]|BOLD:AAA3364
Agrotis ipsilon|[8931]|RWWC227-11|United States|Washington|658[0n]|BOLD:AAA3364
Agrotis ipsilon|[8932]|BBLOD900-11|United States|California|658[0n]|BOLD:AAA3364
Agrotis ipsilon|[8933]|BBLOD955-11|United States|Texas|658[0n]|BOLD:AAA3364
Agrotis ipsilon|[8934]|BBLOD967-11|United States|Texas|658[0n]|BOLD:AAA3364
Agrotis ipsilon|[8935]|BBLOD968-11|United States|Texas|658[0n]|BOLD:AAA3364
Agrotis ipsilon|[8936]|BBLOD1661-11|United States|Texas|658[0n]|BOLD:AAA3364
Agrotis ipsilon|[8937]|XAH615-05|Canada|Ontario|658[0n]|BOLD:AAA3364
Agrotis ipsilon|[8938]|XAH638-05|Canada|Ontario|658[0n]|BOLD:AAA3364
Agrotis ipsilon|[8939]|CMAZA900-12|United States|Arizona|658[0n]|BOLD:AAA3364
Agrotis ipsilon|[8940]|CMAZA909-12|United States|Arizona|658[0n]|BOLD:AAA3364
Agrotis ipsilon|[8941]|TTMNB421-06|Canada|New Brunswick|658[0n]|BOLD:AAA3364
Agrotis ipsilon|[8942]|LOCB744-06|United States|California|658[0n]|BOLD:AAA3364
Agrotis ipsilon|[8943]|LOCBB236-06|United States|California|658[0n]|BOLD:AAA3364
Agrotis ipsilon|[8944]|LOCBB237-06|United States|California|658[0n]|BOLD:AAA3364
Agrotis ipsilon|[8945]|XAH667-05|Canada|Ontario|658[0n]|BOLD:AAA3364
Agrotis ipsilon|[8946]|LNC572-06|United States|North Carolina|658[0n]|BOLD:AAA3364
Agrotis ipsilon|[8947]|LNC573-06|United States|North Carolina|658[0n]|BOLD:AAA3364
Agrotis ipsilon|[8948]|TTMNB420-06|Canada|New Brunswick|658[0n]|BOLD:AAA3364
Agrotis ipsilon|[8949]|LBCS250-07|Canada|British Columbia|658[0n]|BOLD:AAA3364
Agrotis ipsilon|[8950]|LTOL929-08|United States|Maryland|658[0n]|BOLD:AAA3364
Agrotis ipsilon|[8951]|MHCOL107-07|Canada|Manitoba|658[0n]|BOLD:AAA3364
Agrotis ipsilon|[8952]|MHCOL114-07|Canada|Manitoba|658[0n]|BOLD:AAA3364
Agrotis ipsilon|[8953]|XAJ813-06|Canada|Ontario|658[0n]|BOLD:AAA3364
Agrotis ipsilon|[8954]|LOCBE106-06|United States|California|658[0n]|BOLD:AAA3364
Agrotis ipsilon|[8955]|LNCNW114-06|United States|North Carolina|658[0n]|BOLD:AAA3364
Agrotis ipsilon|[8956]|RDNMD382-06|United States|California|658[0n]|BOLD:AAA3364
Agrotis ipsilon|[8957]|LOTB312-05|United States|Tennessee|658[0n]|BOLD:AAA3364
Agrotis ipsilon|[8958]|LOCT058-05|United States|Connecticut|658[0n]|BOLD:AAA3364
Agrotis ipsilon|[8959]|LOTB235-05|United States|Tennessee|658[0n]|BOLD:AAA3364
Agrotis ipsilon|[8960]|LOTB310-05|United States|Tennessee|658[0n]|BOLD:AAA3364
Agrotis ipsilon|[8961]|LOCBB238-06|United States|California|658[0n]|BOLD:AAA3364
Agrotis ipsilon|[8962]|LOCBC158-06|United States|California|658[0n]|BOLD:AAA3364
Agrotis ipsilon|[8963]|LOCB822-06|United States|California|658[0n]|BOLD:AAA3364
Agrotis ipsilon|[8964]|LOCBB235-06|United States|California|658[0n]|BOLD:AAA3364
Agrotis ipsilon|[8965]|XAB775-04|Canada|Ontario|658[0n]|BOLD:AAA3364
Agrotis ipsilon|[8966]|XAB774-04|Canada|Ontario|658[0n]|BOLD:AAA3364
Agrotis ipsilon|[8967]|XAB750-04|Canada|Ontario|658[0n]|BOLD:AAA3364
Agrotis ipsilon|[8968]|PHMNB286-04|Canada|New Brunswick|658[0n]|BOLD:AAA3364
Agrotis ipsilon|[8969]|XAD374-04|Canada|Ontario|658[0n]|BOLD:AAA3364
Agrotis ipsilon|[8970]|XAC035-04|Canada|Ontario|658[0n]|BOLD:AAA3364
Agrotis ipsilon|[8971]|XAB690-04|Canada|Ontario|658[0n]|BOLD:AAA3364

Agrotis ipsilon|[8969]|XAD374-04|Canada|Ontario|658[0n]|BOLD:AAA3364
Agrotis ipsilon|[8970]|XAC035-04|Canada|Ontario|658[0n]|BOLD:AAA3364
Agrotis ipsilon|[8971]|XAB690-04|Canada|Ontario|658[0n]|BOLD:AAA3364
Agrotis ipsilon|[8972]|XAB667-04|Canada|Ontario|658[0n]|BOLD:AAA3364
Agrotis ipsilon|[8973]|LPOKA629-09|United States|Oklahoma|643[0n]|BOLD:AAA3364
Agrotis ipsilon|[8974]|LPOKA042-08|United States|Oklahoma|656[0n]|BOLD:AAA3364
Agrotis ipsilon|[8975]|MHCOL368-07|Canada|Manitoba|650[0n]|BOLD:AAA3364
Agrotis ipsilon|[8976]|PMG086-03|Canada|Ontario|617[0n]|BOLD:AAA3364
Agrotis ipsilon|[8977]|MHCOL007-07|Canada|Manitoba|646[0n]|BOLD:AAA3364
Agrotis ipsilon|[8978]|RDLQF818-06|Canada|Quebec|551[0n]|BOLD:AAA3364
Agrotis ipsilon|[8979]|MHCOL113-07|Canada|Manitoba|628[0n]|BOLD:AAA3364
Agrotis ipsilon|[8980]|PHMO170-03|Canada|Ontario|639[0n]|BOLD:AAA3364
Agrotis ipsilon|[8981]|XAB710-04|Canada|Ontario|589[0n]|BOLD:AAA3364
Agrotis ipsilon|[8982]|LOCB742-06|United States|California|620[0n]|BOLD:AAA3364
Agrotis ipsilon|[8983]|LOCBC169-06|United States|California|623[0n]|BOLD:AAA3364
Agrotis ipsilon|[8984]|LPOKA191-08|United States|Oklahoma|623[0n]|BOLD:AAA3364
Agrotis ipsilon|[8985]|LPOKC617-09|United States|Oklahoma|630[0n]|BOLD:AAA3364
Agrotis ipsilon|[8986]|PHLCH800-11|Canada|Manitoba|632[0n]|BOLD:AAA3364
Agrotis ipsilon|[8987]|GMLC1298-12|United States|California|631[0n]|BOLD:AAA3364
Agrotis ipsilon|[8988]|GMLC1300-12|United States|California|633[0n]|BOLD:AAA3364
Agrotis ipsilon|[8989]|GMLC968-12|United States|California|658[0n]|BOLD:AAA3364
Agrotis ipsilon|[8990]|RWWC926-12|United States|Washington|658[0n]|BOLD:AAA3364
Agrotis ipsilon|[8991]|SMTPD2879-13|Canada|Ontario|603[0n]|BOLD:AAA3364
Agrotis ipsilon|[8992]|SMTPD5182-13|Canada|Ontario|603[0n]|BOLD:AAA3364
Agrotis gladiaria|[8993]|LPOKA603-09|United States|Oklahoma|603[0n]|BOLD:ACF0067
Agrotis gladiaria|[8994]|LPOKD620-09|United States|Oklahoma|633[0n]|BOLD:ACF0067
Agrotis gladiaria|[8995]|LPOKA341-08|United States|Oklahoma|658[0n]|BOLD:ACF0067
Agrotis gladiaria|[8996]|LPOKA647-09|United States|Oklahoma|658[0n]|BOLD:ACF0067
Agrotis gladiaria|[8997]|LPOKA542-09|United States|Oklahoma|639[0n]|BOLD:ACF0067
Agrotis gladiaria|[8998]|LPOKA635-09|United States|Oklahoma|621[0n]|BOLD:ACF0067
Agrotis gladiaria|[8999]|XAD435-04|Canada|Ontario|575[0n]|BOLD:ACF0067
Agrotis gladiaria|[9000]|XAD385-04|Canada|Ontario|595[0n]|BOLD:ACF0067
Agrotis gladiaria|[9001]|XAD377-04|Canada|Ontario|595[0n]|BOLD:ACF0067
Agrotis gladiaria|[9002]|XAD380-04|Canada|Ontario|548[0n]|BOLD:ACF0067
Agrotis gladiaria|[9003]|XAD369-04|Canada|Ontario|593[0n]|BOLD:ACF0067
Agrotis gladiaria|[9004]|XAD378-04|Canada|Ontario|658[0n]|BOLD:ACF0067
Agrotis gladiaria|[9005]|PHMO331-03|Canada|Ontario|639[0n]|BOLD:ACF0067
Agrotis gladiaria|[9006]|PHMO340-03|Canada|Ontario|639[0n]|BOLD:ACF0067
Agrotis gladiaria|[9007]|XAD387-04|Canada|Ontario|658[0n]|BOLD:ACF0067
Agrotis gladiaria|[9008]|XAH354-05|Canada|Ontario|658[0n]|BOLD:ACF0067
Agrotis gladiaria|[9009]|XAH361-05|Canada|Ontario|658[0n]|BOLD:ACF0067
Agrotis gladiaria|[9010]|XAH391-05|Canada|Ontario|658[0n]|BOLD:ACF0067
Agrotis gladiaria|[9011]|XAH412-05|Canada|Ontario|658[0n]|BOLD:ACF0067
Agrotis gladiaria|[9012]|XAH459-05|Canada|Ontario|658[0n]|BOLD:ACF0067
Agrotis gladiaria|[9013]|XAH460-05|Canada|Ontario|658[0n]|BOLD:ACF0067
Agrotis gladiaria|[9014]|XAD381-04|Canada|Ontario|658[0n]|BOLD:ACF0067
Agrotis gladiaria|[9015]|MJMSL020-10|United States|Massachusetts|658[0n]|BOLD:ACF0067
Agrotis gladiaria|[9016]|MJMSL025-10|United States|Massachusetts|658[0n]|BOLD:ACF0067
Agrotis gladiaria|[9017]|MJMSL026-10|United States|Massachusetts|658[0n]|BOLD:ACF0067
Agrotis daedalus|[9018]|RDNMG421-08|United States|South Dakota|658[0n]|BOLD:AAF1591
Agrotis daedalus|[9019]|RDNMF041-08|Canada|Alberta|658[0n]|BOLD:AAF1591
Agrotis daedalus|[9020]|RDNMG422-08|United States|Colorado|658[0n]|BOLD:AAF1591
Agrotis rileyana|[9021]|RDMAB306-05|Canada|Alberta|658[0n]|BOLD:ACF3230
Agrotis rileyana|[9022]|RDMAB594-06|Canada|Alberta|658[0n]|BOLD:ACF3230
Agrotis rileyana|[9023]|RDNMG879-08|Canada|Alberta|658[0n]|BOLD:ACF3230
Agrotis rileyana|[9024]|RDNMG880-08|Canada|Alberta|658[0n]|BOLD:ACF3230
Agrotis ruta|[9025]|LCHQ325-08|Canada|Manitoba|658[0n]|BOLD:AAA1629
Agrotis ruta|[9026]|LCHQ562-08|Canada|Manitoba|658[0n]|BOLD:AAA1629
Agrotis ruta|[9027]|LCHQ622-08|Canada|Manitoba|658[0n]|BOLD:AAA1629
Agrotis ruta|[9028]|LCHQ631-08|Canada|Manitoba|658[0n]|BOLD:AAA1629
Agrotis ruta|[9029]|LCHP762-07|Canada|Manitoba|658[2n]|BOLD:AAA1629
Agrotis ruta|[9030]|LCHQ423-08|Canada|Manitoba|616[0n]|BOLD:AAA1629
Agrotis ruta|[9031]|LCHQ221-08|Canada|Manitoba|658[0n]|BOLD:AAA1629
Agrotis ruta|[9032]|LCHIP095-07|Canada|Manitoba|650[0n]|BOLD:AAA1629
Agrotis ruta|[9033]|LCHQ341-08|Canada|Manitoba|656[0n]|BOLD:AAA1629
Agrotis ruta|[9034]|LCHQ344-08|Canada|Manitoba|658[0n]|BOLD:AAA1629
Agrotis ruta|[9035]|LCHQ348-08|Canada|Manitoba|658[0n]|BOLD:AAA1629
Agrotis ruta|[9036]|LCHQ426-08|Canada|Manitoba|658[0n]|BOLD:AAA1629
Agrotis ruta|[9037]|LCHQ577-08|Canada|Manitoba|658[0n]|BOLD:AAA1629
Agrotis ruta|[9038]|LCHQ582-08|Canada|Manitoba|658[0n]|BOLD:AAA1629
Agrotis ruta|[9039]|LCHQ636-08|Canada|Manitoba|658[0n]|BOLD:AAA1629
Agrotis ruta|[9040]|LCHQ644-08|Canada|Manitoba|658[0n]|BOLD:AAA1629
Agrotis ruta|[9041]|LCHQ647-08|Canada|Manitoba|658[0n]|BOLD:AAA1629
Agrotis ruta|[9042]|LCHQ671-08|Canada|Manitoba|658[0n]|BOLD:AAA1629
Agrotis ruta|[9043]|LCHQ684-08|Canada|Manitoba|657[0n]|BOLD:AAA1629
Agrotis ruta|[9044]|LCHQ694-08|Canada|Manitoba|658[0n]|BOLD:AAA1629
Agrotis ruta|[9045]|LCHQ416-08|Canada|Manitoba|658[1n]|BOLD:AAA1629
Agrotis ruta|[9046]|LCHQ540-08|Canada|Manitoba|656[1n]|BOLD:AAA1629
Agrotis ruta|[9047]|LCHQ813-08|Canada|Manitoba|658[0n]|BOLD:AAA1629
Agrotis ruta|[9048]|LCHQ654-08|Canada|Manitoba|658[0n]|BOLD:AAA1629
Agrotis ruta|[9049]|LCHQ514-08|Canada|Manitoba|632[0n]|BOLD:AAA1629
Agrotis ruta|[9050]|LCHIP024-07|Canada|Manitoba|650[0n]|BOLD:AAA1629
Agrotis ruta|[9051]|LCHQ332-08|Canada|Manitoba|656[0n]|BOLD:AAA1629
Agrotis ruta|[9052]|LCHQ339-08|Canada|Manitoba|658[0n]|BOLD:AAA1629
Agrotis ruta|[9053]|LCHQ425-08|Canada|Manitoba|658[0n]|BOLD:AAA1629
Agrotis ruta|[9054]|LCHQ519-08|Canada|Manitoba|658[0n]|BOLD:AAA1629
Agrotis ruta|[9055]|LCHQ239-08|Canada|Manitoba|658[0n]|BOLD:AAA1629
Agrotis ruta|[9056]|LCHQ240-08|Canada|Manitoba|658[0n]|BOLD:AAA1629
Agrotis ruta|[9057]|LCHQ237-08|Canada|Manitoba|658[0n]|BOLD:AAA1629
Agrotis ruta|[9058]|LCHQ238-08|Canada|Manitoba|658[0n]|BOLD:AAA1629
Agrotis ruta|[9059]|LCHQ213-08|Canada|Manitoba|658[0n]|BOLD:AAA1629
Agrotis ruta|[9060]|LCHQ212-08|Canada|Manitoba|658[0n]|BOLD:AAA1629
Agrotis ruta|[9061]|LCHQ211-08|Canada|Manitoba|658[0n]|BOLD:AAA1629
Agrotis ruta|[9062]|LCHQ210-08|Canada|Manitoba|658[0n]|BOLD:AAA1629
Agrotis ruta|[9063]|LCHQ209-08|Canada|Manitoba|657[0n]|BOLD:AAA1629
Agrotis ruta|[9064]|LCHQ208-08|Canada|Manitoba|658[0n]|BOLD:AAA1629
Agrotis ruta|[9065]|LCHQ803-08|Canada|Manitoba|645[0n]|BOLD:AAA1629
Agrotis ruta|[9066]|LCHP747-07|Canada|Manitoba|655[0n]|BOLD:AAA1629
Agrotis ruta|[9067]|MHLEP094-07|Canada|Manitoba|658[0n]|BOLD:AAA1629
Agrotis ruta|[9068]|MHLEP089-07|Canada|Manitoba|658[0n]|BOLD:AAA1629
Agrotis ruta|[9069]|LCHQ328-08|Canada|Manitoba|658[0n]|BOLD:AAA1629
Agrotis ruta|[9070]|LCHQ321-08|Canada|Manitoba|658[0n]|BOLD:AAA1629
Agrotis ruta|[9071]|LCHQ808-08|Canada|Manitoba|656[0n]|BOLD:AAA1629

[illegible]
Agrotis ruta|[9069]|LCHQ328-08|Canada|Manitoba|658[0n]|BOLD:AAA1629
Agrotis ruta|[9070]|LCHQ321-08|Canada|Manitoba|658[0n]|BOLD:AAA1629
Agrotis ruta|[9071]|LCHQ808-08|Canada|Manitoba|656[0n]|BOLD:AAA1629
Agrotis ruta|[9072]|LCHQ685-08|Canada|Manitoba|656[0n]|BOLD:AAA1629
Agrotis ruta|[9073]|LCHQ665-08|Canada|Manitoba|656[0n]|BOLD:AAA1629
Agrotis ruta|[9074]|LCHQ656-08|Canada|Manitoba|655[0n]|BOLD:AAA1629
Agrotis ruta|[9075]|LCHQ650-08|Canada|Manitoba|655[0n]|BOLD:AAA1629
Agrotis ruta|[9076]|LCHQ626-08|Canada|Manitoba|656[0n]|BOLD:AAA1629
Agrotis ruta|[9077]|LCHQ585-08|Canada|Manitoba|655[0n]|BOLD:AAA1629
Agrotis ruta|[9078]|LCHQ473-08|Canada|Manitoba|655[0n]|BOLD:AAA1629
Agrotis ruta|[9079]|LCHQ420-08|Canada|Manitoba|621[0n]|BOLD:AAA1629
Agrotis ruta|[9080]|LCHQ414-08|Canada|Manitoba|635[0n]|BOLD:AAA1629
Agrotis ruta|[9081]|LCHQ263-08|Canada|Manitoba|658[0n]|BOLD:AAA1629
Agrotis ruta|[9082]|LCHQ598-08|Canada|Manitoba|658[0n]|BOLD:AAA1629
Agrotis ruta|[9083]|LCHIP096-07|Canada|Manitoba|639[0n]|BOLD:AAA1629
Agrotis ruta|[9084]|LCHQ418-08|Canada|Manitoba|632[0n]|BOLD:AAA1629
Agrotis ruta|[9085]|LCHQ667-08|Canada|Manitoba|632[0n]|BOLD:AAA1629
Agrotis ruta|[9086]|LCHIP128-07|Canada|Manitoba|650[0n]|BOLD:AAA1629
Agrotis ruta|[9087]|LCHIP112-07|Canada|Manitoba|650[0n]|BOLD:AAA1629
Agrotis ruta|[9088]|LCHIP097-07|Canada|Manitoba|650[0n]|BOLD:AAA1629
Agrotis ruta|[9089]|LCHQ415-08|Canada|Manitoba|649[0n]|BOLD:AAA1629
Agrotis ruta|[9090]|LCHQ643-08|Canada|Manitoba|643[0n]|BOLD:AAA1629
Agrotis ruta|[9091]|LCHQ805-08|Canada|Manitoba|646[0n]|BOLD:AAA1629
Agrotis ruta|[9092]|LCHQ814-08|Canada|Manitoba|648[0n]|BOLD:AAA1629
Agrotis ruta|[9093]|LCHQ674-08|Canada|Manitoba|658[0n]|BOLD:AAA1629
Agrotis ruta|[9094]|LCHQ675-08|Canada|Manitoba|658[0n]|BOLD:AAA1629
Agrotis ruta|[9095]|LCHQ676-08|Canada|Manitoba|657[0n]|BOLD:AAA1629
Agrotis ruta|[9096]|LCHQ677-08|Canada|Manitoba|658[0n]|BOLD:AAA1629
Agrotis ruta|[9097]|LCHQ679-08|Canada|Manitoba|658[0n]|BOLD:AAA1629
Agrotis ruta|[9098]|LCHQ680-08|Canada|Manitoba|658[0n]|BOLD:AAA1629
Agrotis ruta|[9099]|LCHQ672-08|Canada|Manitoba|658[0n]|BOLD:AAA1629
Agrotis ruta|[9100]|LCHQ673-08|Canada|Manitoba|657[0n]|BOLD:AAA1629
Agrotis ruta|[9101]|LCHQ642-08|Canada|Manitoba|658[0n]|BOLD:AAA1629
Agrotis ruta|[9102]|LCHQ649-08|Canada|Manitoba|658[0n]|BOLD:AAA1629
Agrotis ruta|[9103]|LCHQ223-08|Canada|Manitoba|658[0n]|BOLD:AAA1629
Agrotis ruta|[9104]|LCHQ224-08|Canada|Manitoba|658[0n]|BOLD:AAA1629
Agrotis ruta|[9105]|LCHQ258-08|Canada|Manitoba|658[0n]|BOLD:AAA1629
Agrotis ruta|[9106]|LCHQ259-08|Canada|Manitoba|658[0n]|BOLD:AAA1629
Agrotis ruta|[9107]|LCHQ334-08|Canada|Manitoba|658[0n]|BOLD:AAA1629
Agrotis ruta|[9108]|LCHQ335-08|Canada|Manitoba|658[0n]|BOLD:AAA1629
Agrotis ruta|[9109]|LCHQ629-08|Canada|Manitoba|658[0n]|BOLD:AAA1629
Agrotis ruta|[9110]|LCHQ630-08|Canada|Manitoba|658[0n]|BOLD:AAA1629
Agrotis ruta|[9111]|LCHQ617-08|Canada|Manitoba|658[0n]|BOLD:AAA1629
Agrotis ruta|[9112]|LCHQ618-08|Canada|Manitoba|658[0n]|BOLD:AAA1629
Agrotis ruta|[9113]|LCHQ619-08|Canada|Manitoba|658[0n]|BOLD:AAA1629
Agrotis ruta|[9114]|LCHQ620-08|Canada|Manitoba|657[0n]|BOLD:AAA1629
Agrotis ruta|[9115]|LCHQ623-08|Canada|Manitoba|658[0n]|BOLD:AAA1629
Agrotis ruta|[9116]|LCHQ624-08|Canada|Manitoba|658[0n]|BOLD:AAA1629
Agrotis ruta|[9117]|LCHQ214-08|Canada|Manitoba|658[0n]|BOLD:AAA1629
Agrotis ruta|[9118]|LCHQ215-08|Canada|Manitoba|658[0n]|BOLD:AAA1629
Agrotis ruta|[9119]|LCHQ218-08|Canada|Manitoba|658[0n]|BOLD:AAA1629
Agrotis ruta|[9120]|LCHQ219-08|Canada|Manitoba|658[0n]|BOLD:AAA1629
Agrotis ruta|[9121]|LCHQ250-08|Canada|Manitoba|658[0n]|BOLD:AAA1629
Agrotis ruta|[9122]|LCHQ251-08|Canada|Manitoba|658[0n]|BOLD:AAA1629
Agrotis ruta|[9123]|LCHQ329-08|Canada|Manitoba|658[0n]|BOLD:AAA1629
Agrotis ruta|[9124]|LCHQ330-08|Canada|Manitoba|658[0n]|BOLD:AAA1629
Agrotis ruta|[9125]|LCHQ331-08|Canada|Manitoba|658[0n]|BOLD:AAA1629
Agrotis ruta|[9126]|LCHQ333-08|Canada|Manitoba|658[0n]|BOLD:AAA1629
Agrotis ruta|[9127]|LCHQ627-08|Canada|Manitoba|658[0n]|BOLD:AAA1629
Agrotis ruta|[9128]|LCHQ628-08|Canada|Manitoba|658[0n]|BOLD:AAA1629
Agrotis ruta|[9129]|LCHQ216-08|Canada|Manitoba|658[0n]|BOLD:AAA1629
Agrotis ruta|[9130]|LCHQ217-08|Canada|Manitoba|658[0n]|BOLD:AAA1629
Agrotis ruta|[9131]|LCHQ326-08|Canada|Manitoba|658[0n]|BOLD:AAA1629
Agrotis ruta|[9132]|LCHQ327-08|Canada|Manitoba|658[0n]|BOLD:AAA1629
Agrotis ruta|[9133]|LCHQ613-08|Canada|Manitoba|658[0n]|BOLD:AAA1629
Agrotis ruta|[9134]|LCHQ614-08|Canada|Manitoba|658[0n]|BOLD:AAA1629
Agrotis ruta|[9135]|LCHQ615-08|Canada|Manitoba|658[0n]|BOLD:AAA1629
Agrotis ruta|[9136]|LCHQ616-08|Canada|Manitoba|658[0n]|BOLD:AAA1629
Agrotis ruta|[9137]|LCHQ307-08|Canada|Manitoba|657[0n]|BOLD:AAA1629
Agrotis ruta|[9138]|LCHQ322-08|Canada|Manitoba|658[0n]|BOLD:AAA1629
Agrotis ruta|[9139]|LCHQ323-08|Canada|Manitoba|658[0n]|BOLD:AAA1629
Agrotis ruta|[9140]|LCHQ324-08|Canada|Manitoba|658[0n]|BOLD:AAA1629
Agrotis ruta|[9141]|LCHQ600-08|Canada|Manitoba|658[0n]|BOLD:AAA1629
Agrotis ruta|[9142]|LCHQ601-08|Canada|Manitoba|658[0n]|BOLD:AAA1629
Agrotis ruta|[9143]|LCHQ521-08|Canada|Manitoba|658[0n]|BOLD:AAA1629
Agrotis ruta|[9144]|LCHQ522-08|Canada|Manitoba|658[0n]|BOLD:AAA1629
Agrotis ruta|[9145]|LCHQ587-08|Canada|Manitoba|658[0n]|BOLD:AAA1629
Agrotis ruta|[9146]|LCHQ599-08|Canada|Manitoba|658[0n]|BOLD:AAA1629
Agrotis ruta|[9147]|LCHQ563-08|Canada|Manitoba|658[0n]|BOLD:AAA1629
Agrotis ruta|[9148]|LCHQ569-08|Canada|Manitoba|658[0n]|BOLD:AAA1629
Agrotis ruta|[9149]|LCHQ570-08|Canada|Manitoba|657[0n]|BOLD:AAA1629
Agrotis ruta|[9150]|LCHQ571-08|Canada|Manitoba|658[0n]|BOLD:AAA1629
Agrotis ruta|[9151]|LCHQ264-08|Canada|Manitoba|658[0n]|BOLD:AAA1629
Agrotis ruta|[9152]|LCHQ265-08|Canada|Manitoba|658[0n]|BOLD:AAA1629
Agrotis ruta|[9153]|LCHQ266-08|Canada|Manitoba|658[0n]|BOLD:AAA1629
Agrotis ruta|[9154]|LCHQ267-08|Canada|Manitoba|658[0n]|BOLD:AAA1629
Agrotis ruta|[9155]|LCHQ574-08|Canada|Manitoba|658[0n]|BOLD:AAA1629
Agrotis ruta|[9156]|LCHQ575-08|Canada|Manitoba|658[0n]|BOLD:AAA1629
Agrotis ruta|[9157]|LCHQ220-08|Canada|Manitoba|658[0n]|BOLD:AAA1629
Agrotis ruta|[9158]|LCHQ222-08|Canada|Manitoba|658[0n]|BOLD:AAA1629
Agrotis ruta|[9159]|LCHQ252-08|Canada|Manitoba|658[0n]|BOLD:AAA1629
Agrotis ruta|[9160]|LCHQ253-08|Canada|Manitoba|658[0n]|BOLD:AAA1629
Agrotis ruta|[9161]|LCHQ254-08|Canada|Manitoba|658[0n]|BOLD:AAA1629
Agrotis ruta|[9162]|LCHQ255-08|Canada|Manitoba|658[0n]|BOLD:AAA1629
Agrotis ruta|[9163]|LCHQ256-08|Canada|Manitoba|658[0n]|BOLD:AAA1629
Agrotis ruta|[9164]|LCHQ257-08|Canada|Manitoba|658[0n]|BOLD:AAA1629
Agrotis ruta|[9165]|LCHQ268-08|Canada|Manitoba|658[0n]|BOLD:AAA1629
Agrotis ruta|[9166]|LCHQ270-08|Canada|Manitoba|658[0n]|BOLD:AAA1629
Agrotis ruta|[9167]|LCHQ576-08|Canada|Manitoba|657[0n]|BOLD:AAA1629
Agrotis ruta|[9168]|LCHQ578-08|Canada|Manitoba|658[0n]|BOLD:AAA1629
Agrotis ruta|[9169]|LCHQ579-08|Canada|Manitoba|658[0n]|BOLD:AAA1629
Agrotis ruta|[9170]|LCHQ581-08|Canada|Manitoba|658[0n]|BOLD:AAA1629
[illegible]

Agrotis ruta|[9168]|LCHQ578-08|Canada|Manitoba|658[0n]|BOLD:AAA1629
Agrotis ruta|[9169]|LCHQ579-08|Canada|Manitoba|658[0n]|BOLD:AAA1629
Agrotis ruta|[9170]|LCHQ581-08|Canada|Manitoba|658[0n]|BOLD:AAA1629
Agrotis ruta|[9171]|LCHQ560-08|Canada|Manitoba|658[0n]|BOLD:AAA1629
Agrotis ruta|[9172]|LCHQ561-08|Canada|Manitoba|657[0n]|BOLD:AAA1629
Agrotis ruta|[9173]|LCHQ553-08|Canada|Manitoba|658[0n]|BOLD:AAA1629
Agrotis ruta|[9174]|LCHQ558-08|Canada|Manitoba|658[0n]|BOLD:AAA1629
Agrotis ruta|[9175]|LCHQ550-08|Canada|Manitoba|658[0n]|BOLD:AAA1629
Agrotis ruta|[9176]|LCHQ551-08|Canada|Manitoba|658[0n]|BOLD:AAA1629
Agrotis ruta|[9177]|LCHQ500-08|Canada|Manitoba|658[0n]|BOLD:AAA1629
Agrotis ruta|[9178]|LCHQ501-08|Canada|Manitoba|658[0n]|BOLD:AAA1629
Agrotis ruta|[9179]|LCHQ502-08|Canada|Manitoba|658[0n]|BOLD:AAA1629
Agrotis ruta|[9180]|LCHQ515-08|Canada|Manitoba|658[0n]|BOLD:AAA1629
Agrotis ruta|[9181]|LCHQ541-08|Canada|Manitoba|658[0n]|BOLD:AAA1629
Agrotis ruta|[9182]|LCHQ543-08|Canada|Manitoba|658[0n]|BOLD:AAA1629
Agrotis ruta|[9183]|LCHQ544-08|Canada|Manitoba|658[0n]|BOLD:AAA1629
Agrotis ruta|[9184]|LCHQ545-08|Canada|Manitoba|658[0n]|BOLD:AAA1629
Agrotis ruta|[9185]|LCHQ548-08|Canada|Manitoba|658[0n]|BOLD:AAA1629
Agrotis ruta|[9186]|LCHQ549-08|Canada|Manitoba|658[0n]|BOLD:AAA1629
Agrotis ruta|[9187]|LCHQ241-08|Canada|Manitoba|658[0n]|BOLD:AAA1629
Agrotis ruta|[9188]|LCHQ242-08|Canada|Manitoba|658[0n]|BOLD:AAA1629
Agrotis ruta|[9189]|LCHQ243-08|Canada|Manitoba|658[0n]|BOLD:AAA1629
Agrotis ruta|[9190]|LCHQ244-08|Canada|Manitoba|658[0n]|BOLD:AAA1629
Agrotis ruta|[9191]|LCHQ260-08|Canada|Manitoba|658[0n]|BOLD:AAA1629
Agrotis ruta|[9192]|LCHQ261-08|Canada|Manitoba|658[0n]|BOLD:AAA1629
Agrotis ruta|[9193]|LCHQ431-08|Canada|Manitoba|658[0n]|BOLD:AAA1629
Agrotis ruta|[9194]|LCHQ466-08|Canada|Manitoba|658[0n]|BOLD:AAA1629
Agrotis ruta|[9195]|LCHQ467-08|Canada|Manitoba|658[0n]|BOLD:AAA1629
Agrotis ruta|[9196]|LCHQ468-08|Canada|Manitoba|657[0n]|BOLD:AAA1629
Agrotis ruta|[9197]|LCHQ469-08|Canada|Manitoba|658[0n]|BOLD:AAA1629
Agrotis ruta|[9198]|LCHQ472-08|Canada|Manitoba|658[0n]|BOLD:AAA1629
Agrotis ruta|[9199]|LCHQ246-08|Canada|Manitoba|658[0n]|BOLD:AAA1629
Agrotis ruta|[9200]|LCHQ247-08|Canada|Manitoba|658[0n]|BOLD:AAA1629
Agrotis ruta|[9201]|LCHQ248-08|Canada|Manitoba|658[0n]|BOLD:AAA1629
Agrotis ruta|[9202]|LCHQ249-08|Canada|Manitoba|658[0n]|BOLD:AAA1629
Agrotis ruta|[9203]|LCHQ516-08|Canada|Manitoba|658[0n]|BOLD:AAA1629
Agrotis ruta|[9204]|LCHQ518-08|Canada|Manitoba|658[0n]|BOLD:AAA1629
Agrotis ruta|[9205]|LCHQ421-08|Canada|Manitoba|657[0n]|BOLD:AAA1629
Agrotis ruta|[9206]|LCHQ424-08|Canada|Manitoba|658[0n]|BOLD:AAA1629
Agrotis ruta|[9207]|LCHQ394-08|Canada|Manitoba|658[0n]|BOLD:AAA1629
Agrotis ruta|[9208]|LCHQ419-08|Canada|Manitoba|658[0n]|BOLD:AAA1629
Agrotis ruta|[9209]|LCHQ392-08|Canada|Manitoba|658[0n]|BOLD:AAA1629
Agrotis ruta|[9210]|LCHQ393-08|Canada|Manitoba|658[0n]|BOLD:AAA1629
Agrotis ruta|[9211]|LCHQ382-08|Canada|Manitoba|658[0n]|BOLD:AAA1629
Agrotis ruta|[9212]|LCHQ391-08|Canada|Manitoba|658[0n]|BOLD:AAA1629
Agrotis ruta|[9213]|LCHQ352-08|Canada|Manitoba|658[0n]|BOLD:AAA1629
Agrotis ruta|[9214]|LCHQ353-08|Canada|Manitoba|658[0n]|BOLD:AAA1629
Agrotis ruta|[9215]|LCHQ350-08|Canada|Manitoba|658[0n]|BOLD:AAA1629
Agrotis ruta|[9216]|LCHQ351-08|Canada|Manitoba|658[0n]|BOLD:AAA1629
Agrotis ruta|[9217]|LCHQ347-08|Canada|Manitoba|658[0n]|BOLD:AAA1629
Agrotis ruta|[9218]|LCHQ349-08|Canada|Manitoba|658[0n]|BOLD:AAA1629
Agrotis ruta|[9219]|LCHQ345-08|Canada|Manitoba|658[0n]|BOLD:AAA1629
Agrotis ruta|[9220]|LCHQ346-08|Canada|Manitoba|658[0n]|BOLD:AAA1629
Agrotis ruta|[9221]|LCHQ342-08|Canada|Manitoba|658[0n]|BOLD:AAA1629
Agrotis ruta|[9222]|LCHQ343-08|Canada|Manitoba|658[0n]|BOLD:AAA1629
Agrotis ruta|[9223]|LCHQ338-08|Canada|Manitoba|658[0n]|BOLD:AAA1629
Agrotis ruta|[9224]|LCHQ340-08|Canada|Manitoba|658[0n]|BOLD:AAA1629
Agrotis ruta|[9225]|LCHQ336-08|Canada|Manitoba|658[0n]|BOLD:AAA1629
Agrotis ruta|[9226]|LCHQ337-08|Canada|Manitoba|658[0n]|BOLD:AAA1629
Agrotis ruta|[9227]|LCHQ632-08|Canada|Manitoba|658[0n]|BOLD:AAA1629
Agrotis ruta|[9228]|LCHQ633-08|Canada|Manitoba|658[0n]|BOLD:AAA1629
Agrotis ruta|[9229]|LCHQ635-08|Canada|Manitoba|658[0n]|BOLD:AAA1629
Agrotis ruta|[9230]|LCHQ637-08|Canada|Manitoba|658[0n]|BOLD:AAA1629
Agrotis ruta|[9231]|LCHQ638-08|Canada|Manitoba|657[0n]|BOLD:AAA1629
Agrotis ruta|[9232]|LCHQ639-08|Canada|Manitoba|658[0n]|BOLD:AAA1629
Agrotis ruta|[9233]|LCHQ640-08|Canada|Manitoba|658[0n]|BOLD:AAA1629
Agrotis ruta|[9234]|LCHQ641-08|Canada|Manitoba|658[0n]|BOLD:AAA1629
Agrotis ruta|[9235]|LCHQ651-08|Canada|Manitoba|658[0n]|BOLD:AAA1629
Agrotis ruta|[9236]|LCHQ655-08|Canada|Manitoba|658[0n]|BOLD:AAA1629
Agrotis ruta|[9237]|LCHQ657-08|Canada|Manitoba|658[0n]|BOLD:AAA1629
Agrotis ruta|[9238]|LCHQ662-08|Canada|Manitoba|658[0n]|BOLD:AAA1629
Agrotis ruta|[9239]|LCHQ663-08|Canada|Manitoba|658[0n]|BOLD:AAA1629
Agrotis ruta|[9240]|LCHQ664-08|Canada|Manitoba|658[0n]|BOLD:AAA1629
Agrotis ruta|[9241]|LCHQ666-08|Canada|Manitoba|658[0n]|BOLD:AAA1629
Agrotis ruta|[9242]|LCHQ669-08|Canada|Manitoba|657[0n]|BOLD:AAA1629
Agrotis ruta|[9243]|LCHQ681-08|Canada|Manitoba|657[0n]|BOLD:AAA1629
Agrotis ruta|[9244]|LCHQ682-08|Canada|Manitoba|658[0n]|BOLD:AAA1629
Agrotis ruta|[9245]|LCHQ686-08|Canada|Manitoba|658[0n]|BOLD:AAA1629
Agrotis ruta|[9246]|LCHQ687-08|Canada|Manitoba|658[0n]|BOLD:AAA1629
Agrotis ruta|[9247]|LCHQ688-08|Canada|Manitoba|658[0n]|BOLD:AAA1629
Agrotis ruta|[9248]|LCHQ689-08|Canada|Manitoba|658[0n]|BOLD:AAA1629
Agrotis ruta|[9249]|LCHQ690-08|Canada|Manitoba|658[0n]|BOLD:AAA1629
Agrotis ruta|[9250]|LCHQ691-08|Canada|Manitoba|658[0n]|BOLD:AAA1629
Agrotis ruta|[9251]|LCHQ692-08|Canada|Manitoba|658[0n]|BOLD:AAA1629
Agrotis ruta|[9252]|LCHQ693-08|Canada|Manitoba|658[0n]|BOLD:AAA1629
Agrotis ruta|[9253]|LCHQ695-08|Canada|Manitoba|658[0n]|BOLD:AAA1629
Agrotis ruta|[9254]|LCHQ696-08|Canada|Manitoba|657[0n]|BOLD:AAA1629
Agrotis ruta|[9255]|LCHQ697-08|Canada|Manitoba|658[0n]|BOLD:AAA1629
Agrotis ruta|[9256]|LCHQ698-08|Canada|Manitoba|658[0n]|BOLD:AAA1629
Agrotis ruta|[9257]|LCHQ699-08|Canada|Manitoba|658[0n]|BOLD:AAA1629
Agrotis ruta|[9258]|LCHQ807-08|Canada|Manitoba|658[0n]|BOLD:AAA1629
Agrotis ruta|[9259]|LCHQ809-08|Canada|Manitoba|658[0n]|BOLD:AAA1629
Agrotis ruta|[9260]|LCHQ810-08|Canada|Manitoba|658[0n]|BOLD:AAA1629
Agrotis ruta|[9261]|LCHQ811-08|Canada|Manitoba|658[0n]|BOLD:AAA1629
Agrotis ruta|[9262]|LCHQ812-08|Canada|Manitoba|658[0n]|BOLD:AAA1629
Agrotis ruta|[9263]|LCHQ815-08|Canada|Manitoba|658[0n]|BOLD:AAA1629
Agrotis ruta|[9264]|LCHQ816-08|Canada|Manitoba|658[0n]|BOLD:AAA1629
Agrotis ruta|[9265]|LCHQ817-08|Canada|Manitoba|632[0n]|BOLD:AAA1629
Agrotis robustior|[9266]|RDNMF202-08|Canada|Alberta|658[0n]|BOLD:ACF3665
Agrotis robustior|[9267]|RDNMF203-08|Canada|Alberta|658[0n]|BOLD:ACF3665
Agrotis robustior|[9268]|RDNMF330-08|Canada|Alberta|658[0n]|BOLD:ACF3665
Agrotis robustior|[9269]|RDNMF331-08|Canada|Alberta|658[0n]|BOLD:ACF3665
Agrotis orthogonia|[9270]|RDNMG401-08|Canada|Alberta|658[0n]|BOLD:ABZ7032

Agrotis robustior|[9268]|RDNMF330-08|Canada|Alberta|658[0n]|BOLD:ACF3665
Agrotis robustior|[9269]|RDNMF331-08|Canada|Alberta|658[0n]|BOLD:ACF3665
Agrotis orthogonia|[9270]|RDNMG401-08|Canada|Alberta|658[0n]|BOLD:ABZ7032
Agrotis orthogonia|[9271]|RDNMG402-08|Canada|Alberta|658[0n]|BOLD:ABZ7032
Agrotis orthogonia|[9272]|RDNMF014-08|United States|Colorado|658[0n]|BOLD:ABZ7032
Agrotis orthogonia|[9273]|RDNMF351-08|United States|Colorado|658[0n]|BOLD:ABZ7032
Agrotis orthogonia|[9274]|RDNMG403-08|United States|Colorado|658[0n]|BOLD:ABZ7032
Agrotis orthogonia|[9275]|RDNMG761-08|United States|Colorado|658[0n]|BOLD:ABZ7032
Agrotis orthogonia|[9276]|RDNMJ362-11|United States|New Mexico|658[0n]|BOLD:ABZ7032
Agrotis vetusta|[9277]|LNC491-06|United States|North Carolina|658[0n]|BOLD:ACF0067
Agrotis vetusta|[9278]|LNC490-06|United States|North Carolina|658[0n]|BOLD:ACF0067
Agrotis vetusta|[9279]|LOCT007-05|United States|Connecticut|658[0n]|BOLD:ACF0067
Agrotis vetusta|[9280]|RDLQB602-05|Canada|Quebec|658[0n]|BOLD:ACF0067
Agrotis vetusta|[9281]|RDLQF276-06|Canada|Quebec|658[0n]|BOLD:ACF0067
Agrotis vetusta|[9282]|BBLEC489-09|Canada|New Brunswick|658[0n]|BOLD:ACF0067
Agrotis vetusta|[9283]|BBLEC530-09|Canada|New Brunswick|658[0n]|BOLD:ACF0067
Agrotis vetusta|[9284]|CMAZA231-09|United States|Arizona|658[0n]|BOLD:ACF0067
Agrotis vetusta|[9285]|BBLPB317-10|Canada|Alberta|622[0n]|BOLD:ACF0067
Agrotis vetusta|[9286]|AWCLB471-10|United States|Arizona|658[0n]|BOLD:ACF0067
Agrotis vetusta|[9287]|BBLPB318-10|Canada|Saskatchewan|658[0n]|BOLD:ACF0067
Agrotis vetusta|[9288]|RDLQB603-05|Canada|Quebec|658[0n]|BOLD:ACF0067
Agrotis vetusta|[9289]|LOCBB228-06|United States|California|658[0n]|BOLD:ACF0067
Agrotis vetusta|[9290]|AWCLB479-11|United States|Arizona|658[0n]|BOLD:ACF0067
Agrotis vetusta|[9291]|LOWC103-05|Canada|British Columbia|658[0n]|BOLD:ACF0067
Agrotis vetusta|[9292]|AWCLB096-10|United States|Arizona|658[0n]|BOLD:ACF0067
Agrotis vetusta|[9293]|AWCLB480-11|United States|Arizona|658[0n]|BOLD:ACF0067
Agrotis vetusta|[9294]|CMAZA279-09|United States|Arizona|658[0n]|BOLD:ACF0067
Agrotis vetusta|[9295]|AWCLB472-10|United States|Arizona|658[0n]|BOLD:ACF0067
Agrotis vetusta|[9296]|AWCLB478-11|United States|Arizona|658[0n]|BOLD:ACF0067
Agrotis vetusta|[9297]|AWCLB483-11|United States|Arizona|658[0n]|BOLD:ACF0067
Agrotis venerabilis|[9298]|GMLC001-09|United States|California|658[0n]|BOLD:ABZ1938
Agrotis venerabilis|[9299]|RDNMC310-05|United States|Colorado|607[0n]|BOLD:ABZ1938
Agrotis venerabilis|[9300]|RDNMD884-07|United States|Arizona|655[0n]|BOLD:ABZ1938
Agrotis venerabilis|[9301]|CMAZA288-09|United States|Arizona|658[0n]|BOLD:ABZ1938
Agrotis venerabilis|[9302]|LBCH7017-10|Canada|British Columbia|658[0n]|BOLD:ABZ1938
Agrotis venerabilis|[9303]|LPOKA360-08|United States|Oklahoma|658[0n]|BOLD:ABZ1938
Agrotis venerabilis|[9304]|LBCH6809-10|Canada|British Columbia|658[0n]|BOLD:ABZ1938
Agrotis venerabilis|[9305]|LBCH6841-10|Canada|British Columbia|658[0n]|BOLD:ABZ1938
Agrotis venerabilis|[9306]|LBCH6848-10|Canada|British Columbia|658[0n]|BOLD:ABZ1938
Agrotis venerabilis|[9307]|LBCH6901-10|Canada|British Columbia|658[0n]|BOLD:ABZ1938
Agrotis venerabilis|[9308]|LBCH6937-10|Canada|British Columbia|658[0n]|BOLD:ABZ1938
Agrotis venerabilis|[9309]|LBCH6938-10|Canada|British Columbia|658[0n]|BOLD:ABZ1938
Agrotis venerabilis|[9310]|LBCH6942-10|Canada|British Columbia|658[0n]|BOLD:ABZ1938
Agrotis venerabilis|[9311]|LBCH7018-10|Canada|British Columbia|658[0n]|BOLD:ABZ1938
Agrotis venerabilis|[9312]|LBCH7134-10|Canada|British Columbia|658[0n]|BOLD:ABZ1938
Agrotis venerabilis|[9313]|LBCH7135-10|Canada|British Columbia|658[0n]|BOLD:ABZ1938
Agrotis venerabilis|[9314]|LBCH7139-10|Canada|British Columbia|658[0n]|BOLD:ABZ1938
Agrotis venerabilis|[9315]|LBCH7141-10|Canada|British Columbia|658[0n]|BOLD:ABZ1938
Agrotis venerabilis|[9316]|LBCH7242-10|Canada|British Columbia|658[0n]|BOLD:ABZ1938
Agrotis venerabilis|[9317]|LBCH7243-10|Canada|British Columbia|658[0n]|BOLD:ABZ1938
Agrotis venerabilis|[9318]|LBCH7245-10|Canada|British Columbia|658[0n]|BOLD:ABZ1938
Agrotis venerabilis|[9319]|LBCH7246-10|Canada|British Columbia|658[0n]|BOLD:ABZ1938
Agrotis venerabilis|[9320]|LBCH7247-10|Canada|British Columbia|658[0n]|BOLD:ABZ1938
Agrotis venerabilis|[9321]|LBCH7248-10|Canada|British Columbia|658[0n]|BOLD:ABZ1938
Agrotis venerabilis|[9322]|LBCH7249-10|Canada|British Columbia|658[0n]|BOLD:ABZ1938
Agrotis venerabilis|[9323]|LBCH7304-10|Canada|British Columbia|658[0n]|BOLD:ABZ1938
Agrotis venerabilis|[9324]|LBCH7305-10|Canada|British Columbia|658[0n]|BOLD:ABZ1938
Agrotis venerabilis|[9325]|LBCH7306-10|Canada|British Columbia|658[0n]|BOLD:ABZ1938
Agrotis venerabilis|[9326]|LBCH7307-10|Canada|British Columbia|658[0n]|BOLD:ABZ1938
Agrotis venerabilis|[9327]|LBCH7309-10|Canada|British Columbia|658[0n]|BOLD:ABZ1938
Agrotis venerabilis|[9328]|LBCH7310-10|Canada|British Columbia|658[0n]|BOLD:ABZ1938
Agrotis venerabilis|[9329]|LBCH7311-10|Canada|British Columbia|658[0n]|BOLD:ABZ1938
Agrotis venerabilis|[9330]|LBCH7137-10|Canada|British Columbia|658[0n]|BOLD:ABZ1938
Agrotis venerabilis|[9331]|LBCH7138-10|Canada|British Columbia|658[0n]|BOLD:ABZ1938
Agrotis venerabilis|[9332]|LBCH7019-10|Canada|British Columbia|658[0n]|BOLD:ABZ1938
Agrotis venerabilis|[9333]|LBCH7066-10|Canada|British Columbia|658[0n]|BOLD:ABZ1938
Agrotis venerabilis|[9334]|LBCH6807-10|Canada|British Columbia|658[0n]|BOLD:ABZ1938
Agrotis venerabilis|[9335]|LBCH6808-10|Canada|British Columbia|658[0n]|BOLD:ABZ1938
Agrotis venerabilis|[9336]|LBCH6939-10|Canada|British Columbia|658[0n]|BOLD:ABZ1938
Agrotis venerabilis|[9337]|LBCH6940-10|Canada|British Columbia|658[0n]|BOLD:ABZ1938
Agrotis venerabilis|[9338]|LBCH7690-10|Canada|British Columbia|658[0n]|BOLD:ABZ1938
Agrotis venerabilis|[9339]|LBCH6804-10|Canada|British Columbia|658[0n]|BOLD:ABZ1938
Agrotis venerabilis|[9340]|LBCH6781-10|Canada|British Columbia|658[0n]|BOLD:ABZ1938
Agrotis venerabilis|[9341]|LBCH6780-10|Canada|British Columbia|658[0n]|BOLD:ABZ1938
Agrotis venerabilis|[9342]|LBCH6779-10|Canada|British Columbia|658[0n]|BOLD:ABZ1938
Agrotis venerabilis|[9343]|LBCH6778-10|Canada|British Columbia|658[0n]|BOLD:ABZ1938
Agrotis venerabilis|[9344]|LBCH6777-10|Canada|British Columbia|658[0n]|BOLD:ABZ1938
Agrotis venerabilis|[9345]|LBCH6776-10|Canada|British Columbia|658[0n]|BOLD:ABZ1938
Agrotis venerabilis|[9346]|LBCH6775-10|Canada|British Columbia|658[0n]|BOLD:ABZ1938
Agrotis venerabilis|[9347]|LOWC111-05|Canada|British Columbia|658[0n]|BOLD:ABZ1938
Agrotis venerabilis|[9348]|LBCH7140-10|Canada|British Columbia|658[0n]|BOLD:ABZ1938
Agrotis venerabilis|[9349]|LOWC104-05|Canada|British Columbia|658[0n]|BOLD:ABZ1938
Agrotis venerabilis|[9350]|LOWCD881-06|Canada|British Columbia|601[0n]|BOLD:ABZ1938
Agrotis venerabilis|[9351]|LBCH7244-10|Canada|British Columbia|634[0n]|BOLD:ABZ1938
Agrotis venerabilis|[9352]|LOWC116-05|Canada|British Columbia|590[2n]|BOLD:ABZ1938
Agrotis venerabilis|[9353]|LBCH6802-10|Canada|British Columbia|641[0n]|BOLD:ABZ1938
Agrotis venerabilis|[9354]|LBCH6803-10|Canada|British Columbia|641[0n]|BOLD:ABZ1938
Agrotis venerabilis|[9355]|LBCH6806-10|Canada|British Columbia|641[0n]|BOLD:ABZ1938
Agrotis venerabilis|[9356]|LBCH7308-10|Canada|British Columbia|630[0n]|BOLD:ABZ1938
Agrotis venerabilis|[9357]|LOWC115-05|Canada|British Columbia|599[2n]|BOLD:ABZ1938
Agrotis venerabilis|[9358]|LOWCD878-06|Canada|British Columbia|574[1n]|BOLD:ABZ1938
Agrotis venerabilis|[9359]|LOWCD877-06|Canada|British Columbia|658[1n]|BOLD:ABZ1938
Agrotis venerabilis|[9360]|LOWC113-05|Canada|British Columbia|658[1n]|BOLD:ABZ1938
Agrotis venerabilis|[9361]|LOWC110-05|Canada|British Columbia|590[3n]|BOLD:ABZ1938
Agrotis venerabilis|[9362]|LOWC112-05|Canada|British Columbia|609[0n]|BOLD:ABZ1938
Agrotis venerabilis|[9363]|LOWC105-05|Canada|British Columbia|658[0n]|BOLD:ABZ1938
Agrotis venerabilis|[9364]|LOWC107-05|Canada|British Columbia|658[0n]|BOLD:ABZ1938
Agrotis venerabilis|[9365]|LOWCD876-06|Canada|British Columbia|658[0n]|BOLD:ABZ1938
Agrotis venerabilis|[9366]|LOWCD880-06|Canada|British Columbia|658[0n]|BOLD:ABZ1938
Agrotis venerabilis|[9367]|LBCH7136-10|Canada|British Columbia|658[0n]|BOLD:ABZ1938
Agrotis venerabilis|[9368]|BBLPB845-10|Canada|Saskatchewan|658[0n]|BOLD:ABZ1938
Agrotis venerabilis|[9369]|LMDH010-11|United States|Minnesota|658[0n]|BOLD:ABZ1938
Agrotis venerabilis|[9370]|LOCBB319-06|United States|California|658[0n]|BOLD:ABZ1938

Agrotis venerabilis|[9368]|BBLPB845-10|Canada|Saskatchewan|658[0n]|BOLD:ABZ1938
Agrotis venerabilis|[9369]|LMDH010-11|United States|Minnesota|658[0n]|BOLD:ABZ1938
Agrotis venerabilis|[9370]|LOCBB319-06|United States|California|658[0n]|BOLD:ABZ1938
Agrotis venerabilis|[9371]|LOCBB320-06|United States|California|658[0n]|BOLD:ABZ1938
Agrotis venerabilis|[9372]|LOCBB317-06|United States|California|658[0n]|BOLD:ABZ1938
Agrotis venerabilis|[9373]|LOCBB318-06|United States|California|658[0n]|BOLD:ABZ1938
Agrotis venerabilis|[9374]|LOCBB321-06|United States|California|658[0n]|BOLD:ABZ1938
Agrotis venerabilis|[9375]|LPOKA350-08|United States|Oklahoma|658[0n]|BOLD:ABZ1938
Agrotis venerabilis|[9376]|LPOKA659-09|United States|Oklahoma|658[0n]|BOLD:ABZ1938
Agrotis venerabilis|[9377]|LPOKA601-09|United States|Oklahoma|616[0n]|BOLD:ABZ1938
Agrotis venerabilis|[9378]|LOWC108-05|Canada|British Columbia|598[2n]|BOLD:ABZ1938
Agrotis venerabilis|[9379]|LOWC117-05|Canada|British Columbia|590[3n]|BOLD:ABZ1938
Agrotis venerabilis|[9380]|LOWC106-05|Canada|British Columbia|658[0n]|BOLD:ABZ1938
Agrotis venerabilis|[9381]|LOWC109-05|Canada|British Columbia|590[4n]|BOLD:ABZ1938
Agrotis venerabilis|[9382]|LOWC118-05|Canada|British Columbia|604[1n]|BOLD:ABZ1938
Agrotis venerabilis|[9383]|RDLQF267-06|Canada|Quebec|658[2n]|BOLD:ABZ1938
Agrotis venerabilis|[9384]|LBCH7014-10|Canada|British Columbia|658[0n]|BOLD:ABZ1938
Agrotis venerabilis|[9385]|LBCH7015-10|Canada|British Columbia|658[0n]|BOLD:ABZ1938
Agrotis venerabilis|[9386]|LBCH6941-10|Canada|British Columbia|658[0n]|BOLD:ABZ1938
Agrotis venerabilis|[9387]|LBCH7013-10|Canada|British Columbia|658[0n]|BOLD:ABZ1938
Agrotis venerabilis|[9388]|LBCH6906-10|Canada|British Columbia|658[0n]|BOLD:ABZ1938
Agrotis venerabilis|[9389]|LBCH6931-10|Canada|British Columbia|658[0n]|BOLD:ABZ1938
Agrotis venerabilis|[9390]|LBCH7016-10|Canada|British Columbia|658[0n]|BOLD:ABZ1938
Agrotis venerabilis|[9391]|LBCH6905-10|Canada|British Columbia|658[0n]|BOLD:ABZ1938
Agrotis venerabilis|[9392]|LBCH6904-10|Canada|British Columbia|658[0n]|BOLD:ABZ1938
Agrotis venerabilis|[9393]|LBCH6903-10|Canada|British Columbia|658[0n]|BOLD:ABZ1938
Agrotis venerabilis|[9394]|LBCH6902-10|Canada|British Columbia|658[0n]|BOLD:ABZ1938
Agrotis venerabilis|[9395]|LBCH6899-10|Canada|British Columbia|658[0n]|BOLD:ABZ1938
Agrotis venerabilis|[9396]|LBCH6805-10|Canada|British Columbia|658[0n]|BOLD:ABZ1938
Agrotis venerabilis|[9397]|LBCH6774-10|Canada|British Columbia|658[0n]|BOLD:ABZ1938
Agrotis venerabilis|[9398]|TMNBB364-06|Canada|New Brunswick|658[0n]|BOLD:ABZ1938
Agrotis venerabilis|[9399]|TTMNB416-06|Canada|New Brunswick|658[0n]|BOLD:ABZ1938
Agrotis venerabilis|[9400]|LOWCD879-06|Canada|British Columbia|658[0n]|BOLD:ABZ1938
Agrotis venerabilis|[9401]|LOWC119-05|Canada|British Columbia|658[0n]|BOLD:ABZ1938
Agrotis venerabilis|[9402]|LBCH6936-10|Canada|British Columbia|636[0n]|BOLD:ABZ1938
Agrotis venerabilis|[9403]|LBCH6900-10|Canada|British Columbia|639[0n]|BOLD:ABZ1938
Agrotis venerabilis|[9404]|LBCH7020-10|Canada|British Columbia|646[0n]|BOLD:ABZ1938
Agrotis venerabilis|[9405]|LPOKA359-08|United States|Oklahoma|658[0n]|BOLD:ABZ1938
Agrotis venerabilis|[9406]|RDNMC309-05|United States|Florida|587[0n]|BOLD:ABZ1938
Agrotis venerabilis|[9407]|TTMNB417-06|Canada|New Brunswick|658[0n]|BOLD:ABZ1938
Agrotis venerabilis|[9408]|RDLQF279-06|Canada|Quebec|658[0n]|BOLD:ABZ1938
Agrotis venerabilis|[9409]|LPOKA354-08|United States|Oklahoma|658[0n]|BOLD:ABZ1938
Agrotis venerabilis|[9410]|LPOKA643-09|United States|Oklahoma|658[0n]|BOLD:ABZ1938
Agrotis venerabilis|[9411]|LPOKA646-09|United States|Oklahoma|658[0n]|BOLD:ABZ1938
Agrotis venerabilis|[9412]|LPOKD618-09|United States|Oklahoma|658[0n]|BOLD:ABZ1938
Agrotis venerabilis|[9413]|MJMSL022-10|United States|Massachusetts|602[0n]|BOLD:ABZ1938
Agrotis venerabilis|[9414]|TTMNB415-06|Canada|New Brunswick|658[0n]|BOLD:ABZ1938
Agrotis venerabilis|[9415]|TTMNB418-06|Canada|New Brunswick|658[0n]|BOLD:ABZ1938
Agrotis venerabilis|[9416]|TTMNB419-06|Canada|New Brunswick|658[0n]|BOLD:ABZ1938
Agrotis venerabilis|[9417]|MJMSL021-10|United States|Massachusetts|658[0n]|BOLD:ABZ1938
Agrotis venerabilis|[9418]|MJMSL023-10|United States|Massachusetts|658[0n]|BOLD:ABZ1938
Agrotis venerabilis|[9419]|MJMSL024-10|United States|Massachusetts|658[0n]|BOLD:ABZ1938
Agrotis venerabilis|[9420]|MJMSL050-10|United States|Massachusetts|658[0n]|BOLD:ABZ1938
Agrotis venerabilis|[9421]|MJMSL051-10|United States|Massachusetts|658[0n]|BOLD:ABZ1938
Agrotis venerabilis|[9422]|XAB412-04|Canada|Ontario|580[2n]|BOLD:ABZ1938
Agrotis venerabilis|[9423]|XAH313-05|Canada|Ontario|658[1n]|BOLD:ABZ1938
Agrotis venerabilis|[9424]|XAH566-05|Canada|Ontario|658[0n]|BOLD:ABZ1938
Agrotis venerabilis|[9425]|XAH531-05|Canada|Ontario|658[0n]|BOLD:ABZ1938
Agrotis venerabilis|[9426]|XAH529-05|Canada|Ontario|658[0n]|BOLD:ABZ1938
Agrotis venerabilis|[9427]|XAH448-05|Canada|Ontario|658[0n]|BOLD:ABZ1938
Agrotis venerabilis|[9428]|XAH411-05|Canada|Ontario|658[0n]|BOLD:ABZ1938
Agrotis venerabilis|[9429]|XAH390-05|Canada|Ontario|658[0n]|BOLD:ABZ1938
Agrotis venerabilis|[9430]|XAH387-05|Canada|Ontario|658[0n]|BOLD:ABZ1938
Agrotis venerabilis|[9431]|XAH316-05|Canada|Ontario|658[0n]|BOLD:ABZ1938
Agrotis venerabilis|[9432]|XAD386-04|Canada|Ontario|658[0n]|BOLD:ABZ1938
Agrotis venerabilis|[9433]|XAB441-04|Canada|Ontario|658[0n]|BOLD:ABZ1938
Agrotis venerabilis|[9434]|XAB428-04|Canada|Ontario|658[0n]|BOLD:ABZ1938
Agrotis venerabilis|[9435]|XAH436-05|Canada|Ontario|658[0n]|BOLD:ABZ1938
Agrotis venerabilis|[9436]|XAH363-05|Canada|Ontario|658[0n]|BOLD:ABZ1938
Agrotis venerabilis|[9437]|PHMO341-03|Canada|Ontario|639[0n]|BOLD:ABZ1938
Agrotis venerabilis|[9438]|PHMO347-03|Canada|Ontario|639[0n]|BOLD:ABZ1938
Agrotis venerabilis|[9439]|SMTPD3725-13|Canada|Ontario|603[0n]|BOLD:ABZ1938
Agrotis longicornis|[9440]|CNCLB180-14|United States|Nevada|658[0n]|BOLD:ACM4685
Agrotis longicornis|[9441]|CNCLB181-14|United States|Oregon|658[0n]|BOLD:ACM4685
Agrotis longicornis|[9442]|CNCLB182-14|United States|Oregon|658[0n]|BOLD:ACM4685
Agrotis buchholzi|[9443]|LNCB306-06|United States|North Carolina|658[0n]|BOLD:ACE4378
Agrotis buchholzi|[9444]|LNCB307-06|United States|North Carolina|658[0n]|BOLD:ACE4378
Agrotis buchholzi|[9445]|LNCB308-06|United States|North Carolina|658[0n]|BOLD:ACE4378
Agrotis buchholzi|[9446]|LNCB309-06|United States|North Carolina|658[0n]|BOLD:ACE4378
Agrotis buchholzi|[9447]|MILEP469-10|United States|New Jersey|658[0n]|BOLD:ACE4378
Agrotis buchholzi|[9448]|MILEP470-10|United States|New Jersey|658[0n]|BOLD:ACE4378
Agrotis buchholzi|[9449]|MILEP471-10|United States|New Jersey|658[0n]|BOLD:ACE4378
Agrotis buchholzi|[9450]|MILEP472-10|United States|New Jersey|658[0n]|BOLD:ACE4378
Agrotis antica|[9451]|LBCG378-08|Canada|British Columbia|658[0n]|BOLD:ACE9952
Agrotis antica|[9452]|RDNMD882-07|United States|Wyoming|655[0n]|BOLD:ACE9952
Agrotis antica|[9453]|RDNMD883-07|United States|Wyoming|655[0n]|BOLD:ACE9952
Agrotis antica|[9454]|IAWLB067-10|United States|California|658[0n]|BOLD:ACE9952
Agrotis antica|[9455]|IAWLB068-10|United States|California|658[0n]|BOLD:ACE9952
Agrotis antica|[9456]|RDNMK038-11|United States|Colorado|658[0n]|BOLD:ACE9952
Agrotis antica|[9457]|RDNMK039-11|United States|Colorado|658[0n]|BOLD:ACE9952
Agrotis antica|[9458]|RDNMK041-11|United States|Colorado|658[0n]|BOLD:ACE9952
Agrotis vancouverensis|[9459]|RWWA093-09|United States|Washington|614[0n]|BOLD:ACE9952
Agrotis vancouverensis|[9460]|RWWB756-10|United States|Washington|658[0n]|BOLD:ACE9952
Agrotis vancouverensis|[9461]|RWWA651-09|United States|Washington|658[0n]|BOLD:ACE9952
Agrotis vancouverensis|[9462]|RWWA601-09|United States|Washington|658[0n]|BOLD:ACE9952
Agrotis vancouverensis|[9463]|GMLC504-11|United States|California|658[0n]|BOLD:ACE9952
Agrotis vancouverensis|[9464]|RWWA425-09|United States|Washington|658[0n]|BOLD:ACE9952
Agrotis vancouverensis|[9465]|RWWB119-09|United States|Washington|658[0n]|BOLD:ACE9952
Agrotis vancouverensis|[9466]|RWWC256-11|United States|Washington|658[0n]|BOLD:ACE9952
Agrotis vancouverensis|[9467]|RWWB844-10|United States|Washington|642[0n]|BOLD:ACE9952
Agrotis vancouverensis|[9468]|RWWA202-09|United States|Washington|658[0n]|BOLD:ACE9952
Agrotis vancouverensis|[9469]|RWWA183-09|United States|Washington|658[0n]|BOLD:ACE9952

Agrotis vancouverensis|[9467]|RWWB844-10|United States|Washington|642[0n]|BOLD:ACE9952
Agrotis vancouverensis|[9468]|RWWA202-09|United States|Washington|658[0n]|BOLD:ACE9952
Agrotis vancouverensis|[9469]|RWWA183-09|United States|Washington|658[0n]|BOLD:ACE9952
Agrotis vancouverensis|[9470]|RWWB948-10|United States|Washington|658[0n]|BOLD:ACE9952
Agrotis vancouverensis|[9471]|RWWA550-09|United States|Washington|658[0n]|BOLD:ACE9952
Agrotis vancouverensis|[9472]|RWWC261-11|United States|Washington|653[0n]|BOLD:ACE9952
Agrotis vancouverensis|[9473]|RWWC325-11|United States|Washington|658[0n]|BOLD:ACE9952
Agrotis vancouverensis|[9474]|RWWA070-09|United States|Washington|615[0n]|BOLD:ACE9952
Agrotis vancouverensis|[9475]|RWWA805-09|United States|Washington|658[0n]|BOLD:ACE9952
Agrotis vancouverensis|[9476]|RWWB764-10|United States|Washington|658[0n]|BOLD:ACE9952
Agrotis vancouverensis|[9477]|RWWC539-11|United States|Washington|658[0n]|BOLD:ACE9952
Agrotis vancouverensis group|[9478]|LOCBC284-06|United States|California|540[1n]|BOLD:ACE9952
Agrotis vancouverensis group|[9479]|LOCBC269-06|United States|California|658[0n]|BOLD:ACE9952
Agrotis vancouverensis group|[9480]|LOCBC392-06|United States|California|658[0n]|BOLD:ACE9952
Agrotis vancouverensis group|[9481]|LOCBC275-06|United States|California|658[0n]|BOLD:ACE9952
Agrotis vancouverensis group|[9482]|LOCBD300-06|United States|California|658[0n]|BOLD:ACE9952
Agrotis vancouverensis group|[9483]|LOCBC273-06|United States|California|658[0n]|BOLD:ACE9952
Agrotis vancouverensis group|[9484]|LOCBC276-06|United States|California|612[0n]|BOLD:ACE9952
Agrotis vancouverensis group|[9485]|LOCBD269-06|United States|California|658[0n]|BOLD:ACE9952
Agrotis vancouverensis group|[9486]|LOCBD232-06|United States|California|658[0n]|BOLD:ACE9952
Agrotis vancouverensis group|[9487]|GMLC563-11|United States|California|658[0n]|BOLD:ACE9952
Agrotis vancouverensis group|[9488]|GMLC525-11|United States|California|658[0n]|BOLD:ACE9952
Agrotis vancouverensis group|[9489]|LOCBD656-06|United States|California|658[0n]|BOLD:ACE9952
Agrotis vancouverensis group|[9490]|LOCBD566-06|United States|California|658[0n]|BOLD:ACE9952
Agrotis vancouverensis group|[9491]|LOCBD562-06|United States|California|658[0n]|BOLD:ACE9952
Agrotis vancouverensis group|[9492]|LOCBD559-06|United States|California|658[0n]|BOLD:ACE9952
Agrotis vancouverensis group|[9493]|LOCBD419-06|United States|California|658[0n]|BOLD:ACE9952
Agrotis vancouverensis group|[9494]|LOCBD411-06|United States|California|658[0n]|BOLD:ACE9952
Agrotis vancouverensis group|[9495]|LOCBD410-06|United States|California|658[0n]|BOLD:ACE9952
Agrotis vancouverensis group|[9496]|LOCBD273-06|United States|California|658[0n]|BOLD:ACE9952
Agrotis vancouverensis group|[9497]|LOCBD174-06|United States|California|658[0n]|BOLD:ACE9952
Agrotis vancouverensis group|[9498]|LOCBD065-06|United States|California|658[0n]|BOLD:ACE9952
Agrotis vancouverensis group|[9499]|LOCBD064-06|United States|California|658[0n]|BOLD:ACE9952
Agrotis vancouverensis group|[9500]|LOCBC814-06|United States|California|658[0n]|BOLD:ACE9952
Agrotis vancouverensis group|[9501]|LOCBC803-06|United States|California|658[0n]|BOLD:ACE9952
Agrotis vancouverensis group|[9502]|LOCBC412-06|United States|California|658[0n]|BOLD:ACE9952
Agrotis vancouverensis group|[9503]|LOCBC411-06|United States|California|658[0n]|BOLD:ACE9952
Agrotis vancouverensis group|[9504]|LOCBC410-06|United States|California|658[0n]|BOLD:ACE9952
Agrotis vancouverensis group|[9505]|LOCBC286-06|United States|California|658[0n]|BOLD:ACE9952
Agrotis vancouverensis group|[9506]|LOCBC285-06|United States|California|658[0n]|BOLD:ACE9952
Agrotis vancouverensis group|[9507]|LOCBC281-06|United States|California|658[0n]|BOLD:ACE9952
Agrotis vancouverensis group|[9508]|LOCBC280-06|United States|California|657[0n]|BOLD:ACE9952
Agrotis vancouverensis group|[9509]|LOCBC279-06|United States|California|656[0n]|BOLD:ACE9952
Agrotis vancouverensis group|[9510]|LOCBC274-06|United States|California|658[0n]|BOLD:ACE9952
Agrotis vancouverensis group|[9511]|LOCBC272-06|United States|California|658[0n]|BOLD:ACE9952
Agrotis vancouverensis group|[9512]|LOCBC271-06|United States|California|658[0n]|BOLD:ACE9952
Agrotis vancouverensis group|[9513]|LOCBC270-06|United States|California|658[0n]|BOLD:ACE9952
Agrotis vancouverensis group|[9514]|RDNM817-05|United States|California|658[0n]|BOLD:ACE9952
Agrotis vancouverensis group|[9515]|LOCBC277-06|United States|California|657[0n]|BOLD:ACE9952
Agrotis vancouverensis group|[9516]|LOCBC278-06|United States|California|645[0n]|BOLD:ACE9952
Agrotis vancouverensis group|[9517]|LOCBC283-06|United States|California|636[0n]|BOLD:ACE9952
Agrotis vancouverensis group|[9518]|LOCBC268-06|United States|California|642[0n]|BOLD:ACE9952
Agrotis vancouverensis group|[9519]|LOCBC282-06|United States|California|623[0n]|BOLD:ACE9952
Agrotis vancouverensis group|[9520]|GMLC507-11|United States|California|658[0n]|BOLD:ACE9952
Agrotis vancouverensis group|[9521]|GMLC540-11|United States|California|646[0n]|BOLD:ACE9952
Agrotis vancouverensis group|[9522]|GMLC508-11|United States|California|658[0n]|BOLD:ACE9952
Agrotis vancouverensis group|[9523]|GMLC512-11|United States|California|658[0n]|BOLD:ACE9952
Agrotis vancouverensis group|[9524]|GMLC538-11|United States|California|658[0n]|BOLD:ACE9952
Agrotis vancouverensis group|[9525]|GMLC555-11|United States|California|658[0n]|BOLD:ACE9952
Agrotis vancouverensis group|[9526]|GMLC556-11|United States|California|658[0n]|BOLD:ACE9952
Agrotis vancouverensis group|[9527]|GMLC688-11|United States|California|658[0n]|BOLD:ACE9952
Agrotis vancouverensis group|[9528]|GMLC412-11|United States|California|658[0n]|BOLD:ACE9952
Agrotis vancouverensis group|[9529]|GMLC439-11|United States|California|658[0n]|BOLD:ACE9952
Agrotis vancouverensis group|[9530]|GMLC690-11|United States|California|658[0n]|BOLD:ACE9952
Agrotis obliqua|[9531]|LBCD053-05|Canada|British Columbia|658[0n]|BOLD:ACE9952
Agrotis obliqua|[9532]|LSEU815-06|United States|Colorado|658[0n]|BOLD:ACE9952
Agrotis obliqua|[9533]|RDNME438-08|Canada|Alberta|658[0n]|BOLD:ACE9952
Agrotis obliqua|[9534]|RDNME439-08|Canada|Alberta|658[0n]|BOLD:ACE9952
Agrotis obliqua|[9535]|LPABB373-08|Canada|Alberta|658[0n]|BOLD:ACE9952
Agrotis obliqua|[9536]|LBCG3030-09|Canada|British Columbia|658[0n]|BOLD:ACE9952
Agrotis obliqua|[9537]|BBLPB612-10|Canada|British Columbia|658[0n]|BOLD:ACE9952
Agrotis obliqua|[9538]|BBLPB719-10|Canada|Alberta|658[0n]|BOLD:ACE9952
Agrotis obliqua|[9539]|BBLPB844-10|Canada|Alberta|658[0n]|BOLD:ACE9952
Agrotis obliqua|[9540]|RDNMK040-11|United States|Colorado|658[0n]|BOLD:ACE9952
Agrotis obliqua|[9541]|RDNMK623-11|United States|Colorado|658[0n]|BOLD:ACE9952
Agrotis obliqua|[9542]|RDNMK624-11|United States|Colorado|658[0n]|BOLD:ACE9952
Agrotis obliqua|[9543]|RDNMK632-11|United States|Colorado|658[0n]|BOLD:ACE9952
Agrotis sp.|[9544]|IAWLB066-10|United States|California|658[0n]|BOLD:ABZ0371
Agrotis sp.|[9545]|JMMMB396-11|United States|California|658[0n]|BOLD:ABZ0371
Agrotis sp.|[9546]|LBCH5207-10|Canada|British Columbia|658[0n]|BOLD:ABZ0371
Agrotis sp.|[9547]|LBCH5278-10|Canada|British Columbia|658[0n]|BOLD:ABZ0371
Agrotis sp.|[9548]|JMMMB410-11|United States|California|658[0n]|BOLD:ABZ0371
Agrotis arenarius|[9549]|RDNMG399-08|Canada|Nova Scotia|640[0n]|BOLD:ACF2766
Agrotis arenarius|[9550]|RDNMF077-08|Canada|Nova Scotia|618[1n]|BOLD:ACF2766
Agrotis arenarius|[9551]|RDNMG400-08|Canada|Nova Scotia|640[1n]|BOLD:ACF2766
Agrotis stigmosa|[9552]|RDNMG409-08|United States|Colorado|658[0n]|BOLD:ABZ7028
Agrotis stigmosa|[9553]|RDMAB999-09|Canada|Alberta|639[0n]|BOLD:ABZ7028
Agrotis obliqua|[9554]|LCH258-04|Canada|Manitoba|658[0n]|BOLD:ABZ7027
Agrotis obliqua|[9555]|RDMAB352-05|Canada|Alberta|604[0n]|BOLD:ABZ7027
Agrotis obliqua|[9556]|RDMAB349-05|Canada|Alberta|603[0n]|BOLD:ABZ7027
Agrotis obliqua|[9557]|RDMAB355-05|Canada|Alberta|538[1n]|BOLD:ABZ7027
Agrotis obliqua|[9558]|RDNMF200-08|Canada|Alberta|658[0n]|BOLD:ABZ7027
Agrotis obliqua|[9559]|RDMAB1000-09|Canada|Alberta|658[0n]|BOLD:ABZ7027
Agrotis obliqua|[9560]|RDMAB1001-09|Canada|Alberta|658[0n]|BOLD:ABZ7027
Agrotis obliqua|[9561]|RDLQG395-06|Canada|Quebec|658[0n]|BOLD:ABZ7027
Agrotis obliqua|[9562]|RDLQG394-06|Canada|Quebec|658[0n]|BOLD:ABZ7027
Agrotis obliqua|[9563]|RDLQG393-06|Canada|Quebec|658[0n]|BOLD:ABZ7027
Agrotis obliqua|[9564]|RDLQ685-07|Canada|Quebec|591[0n]|BOLD:ABZ7027
Agrotis obliqua|[9565]|LPSOD392-09|Canada|Ontario|658[0n]|BOLD:ABZ7027
Agrotis vancouverensis|[9566]|LOWCD919-06|Canada|British Columbia|575[0n]|BOLD:ABZ7030
Agrotis vancouverensis|[9567]|LHLEP381-06|Canada|British Columbia|582[0n]|BOLD:ABZ7030
Agrotis vancouverensis|[9568]|RDMAB348-05|Canada|Alberta|578[0n]|BOLD:ABZ7030
Agrotis vancouverensis|[9569]|RDNM724-05|United States|California|503[2n]|

Agrotis vancouverensis|[9567]|LHLEP381-06|Canada|British Columbia|582[0n]|BOLD:ABZ7030
Agrotis vancouverensis|[9568]|RDMAB348-05|Canada|Alberta|578[0n]|BOLD:ABZ7030
Agrotis vancouverensis|[9569]|RDNM724-05|United States|California|503[2n]|
Agrotis vancouverensis|[9570]|RDNM725-05|United States|Oregon|563[0n]|BOLD:ABZ7030
Agrotis volubilis|[9571]|XAE217-04|Canada|Ontario|617[0n]|BOLD:ABZ7030
Agrotis volubilis|[9572]|PMG087-03|Canada|Ontario|617[0n]|BOLD:ABZ7030
Agrotis vancouverensis|[9573]|LMH006-06|Canada|British Columbia|658[1n]|BOLD:ABZ7030
Agrotis vancouverensis|[9574]|LHLEP399-06|Canada|British Columbia|658[0n]|BOLD:ABZ7030
Agrotis vancouverensis|[9575]|LHLEP400-06|Canada|British Columbia|658[0n]|BOLD:ABZ7030
Agrotis vancouverensis|[9576]|LHLEP397-06|Canada|British Columbia|658[0n]|BOLD:ABZ7030
Agrotis vancouverensis|[9577]|LHLEP398-06|Canada|British Columbia|658[0n]|BOLD:ABZ7030
Agrotis vancouverensis|[9578]|LPVIB838-08|Canada|British Columbia|658[0n]|BOLD:ABZ7030
Agrotis vancouverensis|[9579]|LPVIB961-08|Canada|British Columbia|658[0n]|BOLD:ABZ7030
Agrotis vancouverensis|[9580]|LPVIA289-08|Canada|British Columbia|658[0n]|BOLD:ABZ7030
Agrotis vancouverensis|[9581]|LPVIA290-08|Canada|British Columbia|658[0n]|BOLD:ABZ7030
Agrotis vancouverensis|[9582]|LHLEP395-06|Canada|British Columbia|658[0n]|BOLD:ABZ7030
Agrotis vancouverensis|[9583]|LHLEP396-06|Canada|British Columbia|658[0n]|BOLD:ABZ7030
Agrotis vancouverensis|[9584]|LPVIA095-08|Canada|British Columbia|658[0n]|BOLD:ABZ7030
Agrotis vancouverensis|[9585]|LPVIA096-08|Canada|British Columbia|658[0n]|BOLD:ABZ7030
Agrotis vancouverensis|[9586]|LHLEP380-06|Canada|British Columbia|658[0n]|BOLD:ABZ7030
Agrotis vancouverensis|[9587]|LHLEP382-06|Canada|British Columbia|658[0n]|BOLD:ABZ7030
Agrotis vancouverensis|[9588]|RDMAB357-05|Canada|Alberta|658[0n]|BOLD:ABZ7030
Agrotis vancouverensis|[9589]|LOWCD918-06|Canada|British Columbia|658[0n]|BOLD:ABZ7030
Agrotis vancouverensis|[9590]|RDMAB122-05|Canada|Alberta|658[0n]|BOLD:ABZ7030
Agrotis vancouverensis|[9591]|RDMAB350-05|Canada|Alberta|658[0n]|BOLD:ABZ7030
Agrotis vancouverensis|[9592]|RDMAB105-05|Canada|Alberta|658[0n]|BOLD:ABZ7030
Agrotis vancouverensis|[9593]|RDMAB102-05|Canada|Alberta|658[0n]|BOLD:ABZ7030
Agrotis vancouverensis|[9594]|RDNM727-05|Canada|Alberta|658[0n]|BOLD:ABZ7030
Agrotis vancouverensis|[9595]|RDNM726-05|Canada|Alberta|658[0n]|BOLD:ABZ7030
Agrotis volubilis|[9596]|XAD640-05|Canada|Ontario|658[0n]|BOLD:ABZ7030
Agrotis volubilis|[9597]|XAE097-04|Canada|Ontario|658[0n]|BOLD:ABZ7030
Agrotis volubilis|[9598]|XAB254-04|Canada|Ontario|658[0n]|BOLD:ABZ7030
Agrotis volubilis|[9599]|XAB231-04|Canada|Ontario|658[0n]|BOLD:ABZ7030
Agrotis volubilis|[9600]|XAB219-04|Canada|Ontario|658[0n]|BOLD:ABZ7030
Agrotis volubilis|[9601]|LPSOB693-08|Canada|Ontario|652[0n]|BOLD:ABZ7030
Agrotis vancouverensis|[9602]|LMH016-06|Canada|British Columbia|649[1n]|BOLD:ABZ7030
Agrotis volubilis|[9603]|TMG146-03|Canada|Ontario|639[0n]|BOLD:ABZ7030
Agrotis vancouverensis|[9604]|LPVIA266-08|Canada|British Columbia|643[0n]|BOLD:ABZ7030
Agrotis vancouverensis|[9605]|LPVIB908-08|Canada|British Columbia|647[0n]|BOLD:ABZ7030
Agrotis vancouverensis|[9606]|LPVIB928-08|Canada|British Columbia|637[0n]|BOLD:ABZ7030
Agrotis vancouverensis|[9607]|RDMAB356-05|Canada|Alberta|609[0n]|BOLD:ABZ7030
Agrotis vancouverensis|[9608]|RDMAB354-05|Canada|Alberta|608[0n]|BOLD:ABZ7030
Agrotis vancouverensis|[9609]|LOWCC199-05|Canada|British Columbia|602[0n]|BOLD:ABZ7030
Agrotis vancouverensis|[9610]|LPVIC096-08|Canada|British Columbia|658[0n]|BOLD:ABZ7030
Agrotis vancouverensis|[9611]|LPVIA097-08|Canada|British Columbia|658[0n]|BOLD:ABZ7030
Agrotis vancouverensis|[9612]|LPVIA278-08|Canada|British Columbia|657[0n]|BOLD:ABZ7030
Agrotis volubilis|[9613]|BLTIB121-08|Canada|Ontario|658[0n]|BOLD:ABZ7030
Agrotis vancouverensis|[9614]|LPABB005-08|Canada|Alberta|658[0n]|BOLD:ABZ7030
Agrotis volubilis|[9615]|BLTIB041-08|Canada|Ontario|658[0n]|BOLD:ABZ7030
Agrotis volubilis|[9616]|BLTIB111-08|Canada|Ontario|658[0n]|BOLD:ABZ7030
Agrotis volubilis|[9617]|LPSOB339-08|Canada|Ontario|658[0n]|BOLD:ABZ7030
Agrotis vancouverensis|[9618]|LPAB014-08|Canada|Alberta|658[0n]|BOLD:ABZ7030
Agrotis volubilis|[9619]|LPSOB218-08|Canada|Ontario|658[0n]|BOLD:ABZ7030
Agrotis volubilis|[9620]|LPSOB231-08|Canada|Ontario|658[0n]|BOLD:ABZ7030
Agrotis volubilis|[9621]|LPSOB214-08|Canada|Ontario|658[0n]|BOLD:ABZ7030
Agrotis volubilis|[9622]|LPSOB216-08|Canada|Ontario|658[0n]|BOLD:ABZ7030
Agrotis vancouverensis|[9623]|LBCG037-08|Canada|British Columbia|658[0n]|BOLD:ABZ7030
Agrotis volubilis|[9624]|LPSOC094-08|Canada|Ontario|658[0n]|BOLD:ABZ7030
Agrotis volubilis|[9625]|KPOEC039-08|Canada|Ontario|658[0n]|BOLD:ABZ7030
Agrotis volubilis|[9626]|LPSO134-08|Canada|Ontario|658[0n]|BOLD:ABZ7030
Agrotis vancouverensis|[9627]|RDNME567-08|United States|California|658[0n]|BOLD:ABZ7030
Agrotis volubilis|[9628]|RDNMF201-08|Canada|Ontario|658[0n]|BOLD:ABZ7030
Agrotis vancouverensis|[9629]|LHLEP410-06|Canada|British Columbia|658[0n]|BOLD:ABZ7030
Agrotis vancouverensis|[9630]|RDNME561-08|United States|California|658[0n]|BOLD:ABZ7030
Agrotis vancouverensis|[9631]|LHLEP401-06|Canada|British Columbia|658[0n]|BOLD:ABZ7030
Agrotis vancouverensis|[9632]|LHLEP406-06|Canada|British Columbia|658[0n]|BOLD:ABZ7030
Agrotis vancouverensis|[9633]|LPVIC024-08|Canada|British Columbia|658[0n]|BOLD:ABZ7030
Agrotis vancouverensis|[9634]|LPABB403-08|Canada|Alberta|658[0n]|BOLD:ABZ7030
Agrotis vancouverensis|[9635]|LPABB454-08|Canada|Alberta|658[0n]|BOLD:ABZ7030
Agrotis vancouverensis|[9636]|LPABB819-09|Canada|Alberta|658[0n]|BOLD:ABZ7030
Agrotis volubilis|[9637]|LPSOD333-09|Canada|Ontario|658[0n]|BOLD:ABZ7030
Agrotis vancouverensis|[9638]|IAWLB069-10|United States|California|658[0n]|BOLD:ABZ7030
Agrotis vancouverensis|[9639]|IAWLB070-10|United States|California|658[0n]|BOLD:ABZ7030
Agrotis vancouverensis|[9640]|IAWLB077-10|United States|California|658[0n]|BOLD:ABZ7030
Agrotis vancouverensis|[9641]|LALPA161-10|Canada|British Columbia|658[0n]|BOLD:ABZ7030
Agrotis vancouverensis|[9642]|LALPA214-10|Canada|British Columbia|658[0n]|BOLD:ABZ7030
Agrotis vancouverensis|[9643]|LALPA406-10|Canada|British Columbia|658[0n]|BOLD:ABZ7030
Agrotis vancouverensis|[9644]|LALPA425-10|Canada|British Columbia|658[0n]|BOLD:ABZ7030
Agrotis vancouverensis|[9645]|BBLPB691-10|Canada|Alberta|658[0n]|BOLD:ABZ7030
Agrotis vancouverensis|[9646]|BBLPB716-10|Canada|Alberta|658[0n]|BOLD:ABZ7030
Agrotis vancouverensis|[9647]|BBLPB717-10|Canada|Alberta|658[0n]|BOLD:ABZ7030
Agrotis vancouverensis|[9648]|BBLPB720-10|Canada|Alberta|658[0n]|BOLD:ABZ7030
Agrotis vancouverensis|[9649]|BBLPB851-10|Canada|British Columbia|658[0n]|BOLD:ABZ7030
Agrotis vancouverensis|[9650]|JMMMB365-11|United States|California|658[0n]|BOLD:ABZ7030
Agrotis vancouverensis|[9651]|LALPA1128-11|Canada|British Columbia|658[0n]|BOLD:ABZ7030
Agrotis vancouverensis|[9652]|LALPA1165-11|Canada|British Columbia|658[0n]|BOLD:ABZ7030
Agrotis gravis|[9653]|RDNMG405-08|Canada|British Columbia|658[0n]|BOLD:AAE4279
Agrotis gravis|[9654]|RDNMF198-08|United States|Oregon|658[0n]|BOLD:AAE4279
Agrotis gravis|[9655]|RDNMG406-08|United States|Oregon|658[0n]|BOLD:AAE4279
Agrotis gravis|[9656]|RDNMG404-08|United States|Washington|658[0n]|BOLD:AAE4279
Agrotis gravis|[9657]|RDNMF199-08|United States|Washington|658[0n]|BOLD:AAE4279
Agrotis gravis|[9658]|RWWC1124-13|United States|Washington|565[0n]|BOLD:AAE4279
Agrotis striata|[9659]|CNCLB2011-14|United States|Arizona|658[0n]|BOLD:ACR1187
Agrotis striata|[9660]|CNCLB2012-14|United States|New Mexico|658[0n]|BOLD:ACR1187
Agrotis striata|[9661]|CNCLB2010-14|United States|Arizona|658[0n]|BOLD:ACR1187
Agrotis striata|[9662]|CNCLB2013-14|Mexico||658[0n]|BOLD:ACR1187
Actebia balanitis|[9663]|LBCH6844-10|Canada|British Columbia|658[0n]|BOLD:AAB2919
Actebia balanitis|[9664]|LPABB417-08|Canada|Alberta|658[0n]|BOLD:AAB2919
Actebia balanitis|[9665]|LPABB415-08|Canada|Alberta|658[0n]|BOLD:AAB2919
Actebia balanitis|[9666]|LPABB406-08|Canada|Alberta|658[0n]|BOLD:AAB2919
Actebia balanitis|[9667]|LPABB385-08|Canada|Alberta|658[0n]|BOLD:AAB2919
Actebia balanitis|[9668]|LPABB337-08|Canada|Alberta|658[0n]|BOLD:AAB2919
Actebia balanitis|[9669]|LPABB334-08|Canada|Alberta|658[0n]|BOLD:AAB2919

[illegible]
Actebia balanitis|[9667]|LPABB385-08|Canada|Alberta|658[0n]|BOLD:AAB2919
Actebia balanitis|[9668]|LPABB337-08|Canada|Alberta|658[0n]|BOLD:AAB2919
Actebia balanitis|[9669]|LPABB334-08|Canada|Alberta|658[0n]|BOLD:AAB2919
Actebia balanitis|[9670]|LPSK127-08|Canada|Saskatchewan|658[0n]|BOLD:AAB2919
Actebia balanitis|[9671]|LOWCE308-06|Canada|British Columbia|658[0n]|BOLD:AAB2919
Actebia balanitis|[9672]|LOWCE307-06|Canada|British Columbia|658[0n]|BOLD:AAB2919
Actebia balanitis|[9673]|LOWCE306-06|Canada|British Columbia|658[0n]|BOLD:AAB2919
Actebia balanitis|[9674]|LOWCE304-06|Canada|British Columbia|658[0n]|BOLD:AAB2919
Actebia balanitis|[9675]|LOWCE303-06|Canada|British Columbia|658[0n]|BOLD:AAB2919
Actebia balanitis|[9676]|LOWCE302-06|Canada|British Columbia|658[0n]|BOLD:AAB2919
Actebia balanitis|[9677]|LOWCE301-06|Canada|British Columbia|658[0n]|BOLD:AAB2919
Actebia balanitis|[9678]|LOWCD910-06|Canada|British Columbia|658[0n]|BOLD:AAB2919
Actebia balanitis|[9679]|LOWCD897-06|Canada|British Columbia|658[0n]|BOLD:AAB2919
Actebia balanitis|[9680]|LOWCD512-06|Canada|British Columbia|658[0n]|BOLD:AAB2919
Actebia balanitis|[9681]|LOWCD511-06|Canada|British Columbia|658[0n]|BOLD:AAB2919
Actebia balanitis|[9682]|LOWCD508-06|Canada|British Columbia|658[0n]|BOLD:AAB2919
Actebia balanitis|[9683]|LOWCD507-06|Canada|British Columbia|656[0n]|BOLD:AAB2919
Actebia balanitis|[9684]|LOWCD513-06|Canada|British Columbia|594[0n]|BOLD:AAB2919
Actebia balanitis|[9685]|LOWCD506-06|Canada|British Columbia|578[0n]|BOLD:AAB2919
Actebia balanitis|[9686]|LOWCD905-06|Canada|British Columbia|611[0n]|BOLD:AAB2919
Actebia balanitis|[9687]|LPABC221-09|Canada|Alberta|632[0n]|BOLD:AAB2919
Actebia balanitis|[9688]|LOWCD510-06|Canada|British Columbia|607[0n]|BOLD:AAB2919
Actebia balanitis|[9689]|LOWCD509-06|Canada|British Columbia|597[0n]|BOLD:AAB2919
Actebia balanitis|[9690]|LBCH6921-10|Canada|British Columbia|601[0n]|BOLD:AAB2919
Dichagyris grandipennis|[9691]|RDNMJ842-11|United States|Arizona|658[0n]|BOLD:AAV6611
Dichagyris grandipennis|[9692]|RDNMJ843-11|United States|Arizona|658[0n]|BOLD:AAV6611
Eucoptocnemis dapsilis|[9693]|LSEU175-06|United States|Georgia|658[0n]|BOLD:AAJ0914
Eucoptocnemis dapsilis|[9694]|LSEU176-06|United States|Georgia|658[0n]|BOLD:AAJ0914
Eucoptocnemis fimbriaris|[9695]|LNC474-06|United States|North Carolina|658[1n]|BOLD:AAE4847
Eucoptocnemis fimbriaris|[9696]|LNC631-06|United States|North Carolina|658[0n]|BOLD:AAE4847
Eucoptocnemis fimbriaris|[9697]|GWOTA063-12|United States|Massachusetts|658[0n]|BOLD:AAE4847
Eucoptocnemis fimbriaris|[9698]|LNC630-06|United States|North Carolina|658[0n]|BOLD:AAE4847
Eucoptocnemis fimbriaris|[9699]|LNC473-06|United States|North Carolina|658[0n]|BOLD:AAE4847
Eucoptocnemis fimbriaris|[9700]|LNCC1540-13|United States|North Carolina|658[0n]|BOLD:AAE4847
Feltia tricosa|[9701]|RDLQB760-05|Canada|Quebec|620[1n]|BOLD:ACE9865
Feltia tricosa|[9702]|RDLQ689-07|Canada|Quebec|632[0n]|BOLD:ACE9867
Feltia subgothica|[9703]|RDNMG836-08|United States|Colorado|658[0n]|BOLD:ACE9865
Feltia subgothica|[9704]|XAD579-04|Canada|Ontario|528[0n]|BOLD:ACE9865
Feltia subgothica|[9705]|RDLQ692-07|Canada|Quebec|658[0n]|BOLD:ACE9865
Feltia subgothica|[9706]|XAK422-06|Canada|Ontario|658[0n]|BOLD:ACE9865
Feltia subgothica|[9707]|XAK347-06|Canada|Ontario|658[0n]|BOLD:ACE9865
Feltia subgothica|[9708]|XAG851-05|Canada|Ontario|658[0n]|BOLD:ACE9865
Feltia subgothica|[9709]|XAG651-05|Canada|Ontario|658[0n]|BOLD:ACE9865
Feltia subgothica|[9710]|XAG096-05|Canada|Ontario|658[0n]|BOLD:ACE9865
Feltia subgothica|[9711]|XAG017-05|Canada|Ontario|658[0n]|BOLD:ACE9865
Feltia subgothica|[9712]|PHMO260-03|Canada|Ontario|639[0n]|BOLD:ACE9865
Feltia subgothica|[9713]|PHMO382-03|Canada|Ontario|639[1n]|BOLD:ACE9865
Feltia subgothica|[9714]|XAG241-05|Canada|Ontario|636[0n]|BOLD:ACE9865
Feltia subgothica|[9715]|RDLQ688-07|Canada|Quebec|607[0n]|BOLD:ACE9865
Feltia subgothica|[9716]|LPOKA281-08|United States|Oklahoma|658[0n]|BOLD:ACE9865
Feltia tricosa|[9717]|LGSMG883-10|United States|North Carolina|658[0n]|BOLD:ACE9865
Feltia tricosa|[9718]|LGSMG886-10|United States|North Carolina|658[0n]|BOLD:ACE9865
Feltia tricosa|[9719]|RDLQB438-05|Canada|Quebec|593[0n]|BOLD:ACE9865
Feltia tricosa|[9720]|LNCNW040-06|United States|North Carolina|658[0n]|BOLD:ACE9865
Feltia tricosa|[9721]|LNCC235-10|United States|North Carolina|658[0n]|BOLD:ACE9865
Feltia tricosa|[9722]|LNCC1257-11|United States|North Carolina|658[0n]|BOLD:ACE9865
Feltia tricosa|[9723]|BLTIB797-08|Canada|Ontario|658[0n]|BOLD:ACE9865
Feltia tricosa|[9724]|BLTIB750-08|Canada|Ontario|658[0n]|BOLD:ACE9865
Feltia tricosa|[9725]|PHMO279-03|Canada|Ontario|639[0n]|BOLD:ACE9865
Feltia tricosa|[9726]|RDLQB756-05|Canada|Quebec|617[0n]|BOLD:ACE9865
Feltia tricosa|[9727]|BLTIB798-08|Canada|Ontario|658[0n]|BOLD:ACE9865
Feltia tricosa|[9728]|XAK482-07|Canada|Ontario|603[0n]|BOLD:ACE9865
Feltia tricosa|[9729]|BLTIB749-08|Canada|Ontario|658[0n]|BOLD:ACE9865
Feltia tricosa|[9730]|BLTIB1013-08|Canada|Ontario|658[1n]|BOLD:ACE9865
Feltia tricosa|[9731]|LNCB508-07|United States|North Carolina|658[0n]|BOLD:ACE9865
Feltia tricosa|[9732]|XAC724-04|Canada|Ontario|658[0n]|BOLD:ACE9865
Feltia tricosa|[9733]|PHMO233-03|Canada|Ontario|639[0n]|BOLD:ACE9865
Feltia tricosa|[9734]|RDLQ691-07|Canada|Quebec|642[0n]|BOLD:ACE9865
Feltia tricosa|[9735]|LPOKA409-09|United States|Oklahoma|658[0n]|BOLD:ACE9865
Feltia tricosa|[9736]|XAG109-05|Canada|Ontario|637[0n]|BOLD:ACE9865
Feltia tricosa|[9737]|PMG113-03|Canada|Ontario|617[0n]|BOLD:ACE9865
Feltia tricosa|[9738]|RDLQB725-05|Canada|Quebec|515[3n]|BOLD:ACE9865
Feltia tricosa|[9739]|RDLQB757-05|Canada|Quebec|658[0n]|BOLD:ACE9865
Feltia tricosa|[9740]|BLTIB1129-08|Canada|Ontario|656[0n]|BOLD:ACE9865
Feltia tricosa|[9741]|XAG095-05|Canada|Ontario|658[0n]|BOLD:ACE9865
Feltia tricosa|[9742]|XAG309-05|Canada|Ontario|658[0n]|BOLD:ACE9865
Feltia tricosa|[9743]|XAJ817-06|Canada|Ontario|658[0n]|BOLD:ACE9865
Feltia tricosa|[9744]|BLTIB775-08|Canada|Ontario|658[0n]|BOLD:ACE9865
Feltia tricosa|[9745]|JSAUG1676-11|Canada|Ontario|658[0n]|BOLD:ACE9865
Feltia herilis|[9746]|LPOKA555-09|United States|Oklahoma|637[0n]|BOLD:ACE9865
Feltia herilis|[9747]|XAG880-05|Canada|Ontario|658[0n]|BOLD:ACE9865
Feltia herilis|[9748]|LPMNB566-09|Canada|Manitoba|658[0n]|BOLD:ACE9865
Feltia herilis|[9749]|LPOKA468-09|United States|Oklahoma|658[0n]|BOLD:ACE9865
Feltia herilis|[9750]|LGSMG884-10|United States|North Carolina|658[0n]|BOLD:ACE9865
Feltia herilis|[9751]|XAG858-05|Canada|Ontario|617[0n]|BOLD:ACE9865
Feltia herilis|[9752]|LPMN991-09|Canada|Manitoba|658[0n]|BOLD:ACE9865
Feltia herilis|[9753]|LPMNB438-09|Canada|Manitoba|636[0n]|BOLD:ACE9865
Feltia herilis|[9754]|LNCB507-07|United States|North Carolina|658[0n]|BOLD:ACE9865
Feltia herilis|[9755]|PHMO306-03|Canada|Ontario|639[0n]|BOLD:ACE9865
Feltia herilis|[9756]|BLTIB581-08|Canada|Ontario|621[0n]|BOLD:ACE9865
Feltia herilis|[9757]|LPOKA571-09|United States|Oklahoma|633[0n]|BOLD:ACE9865
Feltia herilis|[9758]|LGSMG887-10|United States|North Carolina|634[0n]|BOLD:ACE9865
Feltia herilis|[9759]|RDLQ690-07|Canada|Quebec|656[0n]|BOLD:ACE9865
Feltia herilis|[9760]|LPMN963-08|Canada|Alberta|658[0n]|BOLD:ACE9865
Feltia herilis|[9761]|LPMNB435-09|Canada|Manitoba|658[0n]|BOLD:ACE9865
Feltia herilis|[9762]|LPMNB505-09|Canada|Manitoba|658[0n]|BOLD:ACE9865
Feltia herilis|[9763]|LPMNB433-09|Canada|Manitoba|658[0n]|BOLD:ACE9865
Feltia herilis|[9764]|LPMNB434-09|Canada|Manitoba|658[0n]|BOLD:ACE9865
Feltia herilis|[9765]|LPMN999-09|Canada|Manitoba|658[0n]|BOLD:ACE9865
Feltia herilis|[9766]|LPOKA544-09|United States|Oklahoma|658[0n]|BOLD:ACE9865
Feltia herilis|[9767]|LPOKA288-08|United States|Oklahoma|658[0n]|BOLD:ACE9865
Feltia herilis|[9768]|LPMN989-09|Canada|Manitoba|658[0n]|BOLD:ACE9865
[illegible]

Feltia herilis|[9766]|LPOKA544-09|United States|Oklahoma|658[0n]|BOLD:ACE9865
Feltia herilis|[9767]|LPOKA288-08|United States|Oklahoma|658[0n]|BOLD:ACE9865
Feltia herilis|[9768]|LPMN989-09|Canada|Manitoba|658[0n]|BOLD:ACE9865
Feltia herilis|[9769]|BLTIB751-08|Canada|Ontario|658[0n]|BOLD:ACE9865
Feltia herilis|[9770]|BLTIB903-08|Canada|Ontario|658[0n]|BOLD:ACE9865
Feltia herilis|[9771]|LPABC928-09|Canada|Alberta|658[0n]|BOLD:ACE9865
Feltia herilis|[9772]|LPMNB565-09|Canada|Manitoba|658[0n]|BOLD:ACE9865
Feltia herilis|[9773]|RWWA940-09|United States|Washington|658[0n]|BOLD:ACE9865
Feltia herilis|[9774]|BBLPC352-09|Canada|New Brunswick|658[0n]|BOLD:ACE9865
Feltia herilis|[9775]|LPOKD605-09|United States|Oklahoma|658[0n]|BOLD:ACE9865
Feltia herilis|[9776]|LGSMG885-10|United States|North Carolina|658[0n]|BOLD:ACE9865
Feltia herilis|[9777]|LGSMG888-10|United States|North Carolina|658[0n]|BOLD:ACE9865
Feltia herilis|[9778]|BBLPB820-10|Canada|Alberta|658[0n]|BOLD:ACE9865
Feltia herilis|[9779]|BBLPB823-10|Canada|Alberta|658[0n]|BOLD:ACE9865
Feltia herilis|[9780]|LNCC1299-11|United States|North Carolina|658[0n]|BOLD:ACE9865
Feltia herilis|[9781]|LNCC1300-11|United States|North Carolina|658[0n]|BOLD:ACE9865
Feltia herilis|[9782]|RWWC589-11|United States|Washington|658[0n]|BOLD:ACE9865
Feltia herilis|[9783]|LPMNB236-09|Canada|Manitoba|658[0n]|BOLD:ACE9865
Feltia herilis|[9784]|LPMNB241-09|Canada|Manitoba|658[0n]|BOLD:ACE9865
Feltia herilis|[9785]|TMNBB366-06|Canada|New Brunswick|658[0n]|BOLD:ACE9865
Feltia herilis|[9786]|XAK418-06|Canada|Ontario|658[0n]|BOLD:ACE9865
Feltia herilis|[9787]|TMNBB365-06|Canada|New Brunswick|658[0n]|BOLD:ACE9865
Feltia herilis|[9788]|TTMNB428-06|Canada|New Brunswick|658[0n]|BOLD:ACE9865
Feltia herilis|[9789]|TTMNB427-06|Canada|New Brunswick|658[0n]|BOLD:ACE9865
Feltia herilis|[9790]|RDLQB541-05|Canada|Quebec|658[0n]|BOLD:ACE9865
Feltia herilis|[9791]|RDLQB509-05|Canada|Quebec|658[0n]|BOLD:ACE9865
Feltia herilis|[9792]|XAH368-05|Canada|Ontario|658[0n]|BOLD:ACE9865
Feltia herilis|[9793]|XAH244-05|Canada|Ontario|658[0n]|BOLD:ACE9865
Feltia herilis|[9794]|XAH123-05|Canada|Ontario|658[0n]|BOLD:ACE9865
Feltia herilis|[9795]|XAD274-04|Canada|Ontario|658[0n]|BOLD:ACE9865
Feltia herilis|[9796]|XAD172-04|Canada|Ontario|658[0n]|BOLD:ACE9865
Feltia herilis|[9797]|XAC794-04|Canada|Ontario|658[0n]|BOLD:ACE9865
Feltia herilis|[9798]|LPOKA307-08|United States|Oklahoma|658[0n]|BOLD:ACE9865
Feltia herilis|[9799]|RWWB209-09|United States|Washington|658[0n]|BOLD:ACE9865
Feltia herilis|[9800]|BBLPB822-10|Canada|British Columbia|658[0n]|BOLD:ACE9865
Feltia herilis|[9801]|LALPA1242-11|Canada|British Columbia|658[0n]|BOLD:ACE9865
Feltia herilis|[9802]|CNRMF522-12|Canada|Manitoba|632[0n]|BOLD:ACE9865
Feltia jaculifera|[9803]|LPOKA576-09|United States|Oklahoma|658[0n]|BOLD:ACW5313
Feltia jaculifera|[9804]|LNCNW045-06|United States|North Carolina|658[0n]|BOLD:ACW5313
Feltia jaculifera|[9805]|LNCB509-07|United States|North Carolina|658[0n]|BOLD:ACW5313
Feltia jaculifera|[9806]|CNPPJ1453-12|Canada|Ontario|644[0n]|BOLD:ACW5313
Feltia jaculifera|[9807]|RDNM698-05|Canada|Ontario|617[0n]|BOLD:ACW5313
Feltia jaculifera|[9808]|LPMNB356-09|Canada|Manitoba|658[0n]|BOLD:ACW5313
Feltia jaculifera|[9809]|CNPPJ1455-12|Canada|Ontario|644[0n]|BOLD:ACW5313
Feltia jaculifera|[9810]|RDNM699-05|Canada|Alberta|513[2n]|BOLD:AAA3351
Feltia jaculifera|[9811]|RDNM703-05|Canada|Alberta|519[0n]|BOLD:AAA3351
Feltia jaculifera|[9812]|XAD357-04|Canada|Ontario|513[0n]|BOLD:AAA3351
Feltia jaculifera|[9813]|RDLQB727-05|Canada|Quebec|525[0n]|BOLD:AAA3351
Feltia jaculifera|[9814]|RDNM701-05|Canada|British Columbia|514[0n]|BOLD:AAA3351
Feltia jaculifera|[9815]|RDNM705-05|Canada|British Columbia|506[0n]|BOLD:AAA3351
Feltia jaculifera|[9816]|LOWCD574-06|Canada|British Columbia|524[0n]|BOLD:AAA3351
Feltia jaculifera|[9817]|RDNMD551-06|United States|Colorado|658[0n]|BOLD:AAA3351
Feltia jaculifera|[9818]|RDNM704-05|United States|Oregon|658[0n]|BOLD:AAA3351
Feltia jaculifera|[9819]|LBCH6120-10|Canada|British Columbia|658[0n]|BOLD:AAA3351
Feltia jaculifera|[9820]|LBCH7198-10|Canada|British Columbia|658[0n]|BOLD:AAA3351
Feltia jaculifera|[9821]|LBCH7200-10|Canada|British Columbia|658[0n]|BOLD:AAA3351
Feltia jaculifera|[9822]|LBCH7589-10|Canada|British Columbia|658[0n]|BOLD:AAA3351
Feltia jaculifera|[9823]|LOWCB704-05|Canada|British Columbia|658[0n]|BOLD:AAA3351
Feltia jaculifera|[9824]|LBCH7033-10|Canada|British Columbia|658[0n]|BOLD:AAA3351
Feltia jaculifera|[9825]|LBCH6910-10|Canada|British Columbia|632[0n]|BOLD:AAA3351
Feltia jaculifera|[9826]|LBCH7441-10|Canada|British Columbia|658[0n]|BOLD:AAA3351
Feltia jaculifera|[9827]|LBCH7323-10|Canada|British Columbia|658[0n]|BOLD:AAA3351
Feltia jaculifera|[9828]|LBCH7322-10|Canada|British Columbia|658[0n]|BOLD:AAA3351
Feltia jaculifera|[9829]|LBCH7319-10|Canada|British Columbia|658[0n]|BOLD:AAA3351
Feltia jaculifera|[9830]|LBCH7459-10|Canada|British Columbia|640[0n]|BOLD:AAA3351
Feltia jaculifera|[9831]|LBCH7462-10|Canada|British Columbia|636[0n]|BOLD:AAA3351
Feltia jaculifera|[9832]|LBCH7725-10|Canada|British Columbia|658[0n]|BOLD:AAA3351
Feltia jaculifera|[9833]|LOWCB703-05|Canada|British Columbia|593[0n]|BOLD:AAA3351
Feltia jaculifera|[9834]|LOWCD561-06|Canada|British Columbia|658[0n]|BOLD:AAA3351
Feltia jaculifera|[9835]|LBCH7456-10|Canada|British Columbia|658[0n]|BOLD:AAA3351
Feltia jaculifera|[9836]|XAH140-05|Canada|Ontario|658[0n]|BOLD:AAA3351
Feltia jaculifera|[9837]|LOWCD567-06|Canada|British Columbia|598[2n]|BOLD:AAA3351
Feltia jaculifera|[9838]|LOWCB705-05|Canada|British Columbia|658[0n]|BOLD:AAA3351
Feltia jaculifera|[9839]|LBCH7874-10|Canada|British Columbia|658[0n]|BOLD:AAA3351
Feltia jaculifera|[9840]|LBCH7872-10|Canada|British Columbia|658[0n]|BOLD:AAA3351
Feltia jaculifera|[9841]|LBCH7871-10|Canada|British Columbia|658[0n]|BOLD:AAA3351
Feltia jaculifera|[9842]|LBCH7786-10|Canada|British Columbia|658[0n]|BOLD:AAA3351
Feltia jaculifera|[9843]|LBCH7726-10|Canada|British Columbia|658[0n]|BOLD:AAA3351
Feltia jaculifera|[9844]|LBCH7722-10|Canada|British Columbia|658[0n]|BOLD:AAA3351
Feltia jaculifera|[9845]|LBCH7721-10|Canada|British Columbia|658[0n]|BOLD:AAA3351
Feltia jaculifera|[9846]|LBCH7720-10|Canada|British Columbia|658[0n]|BOLD:AAA3351
Feltia jaculifera|[9847]|LBCH7719-10|Canada|British Columbia|658[0n]|BOLD:AAA3351
Feltia jaculifera|[9848]|LBCH7588-10|Canada|British Columbia|658[0n]|BOLD:AAA3351
Feltia jaculifera|[9849]|LBCH7586-10|Canada|British Columbia|658[0n]|BOLD:AAA3351
Feltia jaculifera|[9850]|LBCH7585-10|Canada|British Columbia|658[0n]|BOLD:AAA3351
Feltia jaculifera|[9851]|LBCH7584-10|Canada|British Columbia|658[0n]|BOLD:AAA3351
Feltia jaculifera|[9852]|LBCH7582-10|Canada|British Columbia|658[0n]|BOLD:AAA3351
Feltia jaculifera|[9853]|LBCH7537-10|Canada|British Columbia|658[0n]|BOLD:AAA3351
Feltia jaculifera|[9854]|LBCH7536-10|Canada|British Columbia|658[0n]|BOLD:AAA3351
Feltia jaculifera|[9855]|LBCH7535-10|Canada|British Columbia|658[0n]|BOLD:AAA3351
Feltia jaculifera|[9856]|LBCH7533-10|Canada|British Columbia|658[0n]|BOLD:AAA3351
Feltia jaculifera|[9857]|LBCH7532-10|Canada|British Columbia|658[0n]|BOLD:AAA3351
Feltia jaculifera|[9858]|LBCH7531-10|Canada|British Columbia|658[0n]|BOLD:AAA3351
Feltia jaculifera|[9859]|LBCH7530-10|Canada|British Columbia|658[0n]|BOLD:AAA3351
Feltia jaculifera|[9860]|LBCH7463-10|Canada|British Columbia|658[0n]|BOLD:AAA3351
Feltia jaculifera|[9861]|LBCH7461-10|Canada|British Columbia|658[0n]|BOLD:AAA3351
Feltia jaculifera|[9862]|LBCH7460-10|Canada|British Columbia|658[0n]|BOLD:AAA3351
Feltia jaculifera|[9863]|LBCH7458-10|Canada|British Columbia|658[0n]|BOLD:AAA3351
Feltia jaculifera|[9864]|LBCH7457-10|Canada|British Columbia|658[0n]|BOLD:AAA3351
Feltia jaculifera|[9865]|LBCH7444-10|Canada|British Columbia|658[0n]|BOLD:AAA3351
Feltia jaculifera|[9866]|LBCH7443-10|Canada|British Columbia|658[0n]|BOLD:AAA3351
Feltia jaculifera|[9867]|LBCH7442-10|Canada|British Columbia|658[0n]|BOLD:AAA3351
Feltia jaculifera|[9868]|LBCH7440-10|Canada|British Columbia|658[0n]|BOLD:AAA3351

Feltia jaculifera|[9866]|LBCH7443-10|Canada|British Columbia|658[0n]|BOLD:AAA3351
Feltia jaculifera|[9867]|LBCH7442-10|Canada|British Columbia|658[0n]|BOLD:AAA3351
Feltia jaculifera|[9868]|LBCH7440-10|Canada|British Columbia|658[0n]|BOLD:AAA3351
Feltia jaculifera|[9869]|LBCH7439-10|Canada|British Columbia|658[0n]|BOLD:AAA3351
Feltia jaculifera|[9870]|LBCH7438-10|Canada|British Columbia|658[0n]|BOLD:AAA3351
Feltia jaculifera|[9871]|LBCH7437-10|Canada|British Columbia|658[0n]|BOLD:AAA3351
Feltia jaculifera|[9872]|LBCH7367-10|Canada|British Columbia|658[0n]|BOLD:AAA3351
Feltia jaculifera|[9873]|LBCH7321-10|Canada|British Columbia|658[0n]|BOLD:AAA3351
Feltia jaculifera|[9874]|LBCH7320-10|Canada|British Columbia|658[0n]|BOLD:AAA3351
Feltia jaculifera|[9875]|LBCH7317-10|Canada|British Columbia|658[0n]|BOLD:AAA3351
Feltia jaculifera|[9876]|LBCH7199-10|Canada|British Columbia|658[0n]|BOLD:AAA3351
Feltia jaculifera|[9877]|LBCH7197-10|Canada|British Columbia|658[0n]|BOLD:AAA3351
Feltia jaculifera|[9878]|LBCH7196-10|Canada|British Columbia|658[0n]|BOLD:AAA3351
Feltia jaculifera|[9879]|LBCH7195-10|Canada|British Columbia|658[0n]|BOLD:AAA3351
Feltia jaculifera|[9880]|LBCH7194-10|Canada|British Columbia|658[0n]|BOLD:AAA3351
Feltia jaculifera|[9881]|LBCH7193-10|Canada|British Columbia|658[0n]|BOLD:AAA3351
Feltia jaculifera|[9882]|LBCH7126-10|Canada|British Columbia|658[0n]|BOLD:AAA3351
Feltia jaculifera|[9883]|LBCH7102-10|Canada|British Columbia|658[0n]|BOLD:AAA3351
Feltia jaculifera|[9884]|LBCH7099-10|Canada|British Columbia|658[0n]|BOLD:AAA3351
Feltia jaculifera|[9885]|LBCH7038-10|Canada|British Columbia|658[0n]|BOLD:AAA3351
Feltia jaculifera|[9886]|LBCH7036-10|Canada|British Columbia|658[0n]|BOLD:AAA3351
Feltia jaculifera|[9887]|LBCH7035-10|Canada|British Columbia|658[0n]|BOLD:AAA3351
Feltia jaculifera|[9888]|LBCH7034-10|Canada|British Columbia|658[0n]|BOLD:AAA3351
Feltia jaculifera|[9889]|LBCH7032-10|Canada|British Columbia|658[0n]|BOLD:AAA3351
Feltia jaculifera|[9890]|LBCH7031-10|Canada|British Columbia|658[0n]|BOLD:AAA3351
Feltia jaculifera|[9891]|LBCH6908-10|Canada|British Columbia|658[0n]|BOLD:AAA3351
Feltia jaculifera|[9892]|LBCH6786-10|Canada|British Columbia|658[0n]|BOLD:AAA3351
Feltia jaculifera|[9893]|LBCH6504-10|Canada|British Columbia|658[0n]|BOLD:AAA3351
Feltia jaculifera|[9894]|LBCH6445-10|Canada|British Columbia|658[0n]|BOLD:AAA3351
Feltia jaculifera|[9895]|LBCH6121-10|Canada|British Columbia|658[0n]|BOLD:AAA3351
Feltia jaculifera|[9896]|LBCG3316-09|Canada|British Columbia|658[0n]|BOLD:AAA3351
Feltia jaculifera|[9897]|LBCG3315-09|Canada|British Columbia|658[0n]|BOLD:AAA3351
Feltia jaculifera|[9898]|LBCG2895-09|Canada|British Columbia|658[0n]|BOLD:AAA3351
Feltia jaculifera|[9899]|LBCG1104-09|Canada|British Columbia|658[0n]|BOLD:AAA3351
Feltia jaculifera|[9900]|LPABB575-08|Canada|Alberta|658[0n]|BOLD:AAA3351
Feltia jaculifera|[9901]|LBCG190-08|Canada|British Columbia|658[0n]|BOLD:AAA3351
Feltia jaculifera|[9902]|LOWCD572-06|Canada|British Columbia|658[0n]|BOLD:AAA3351
Feltia jaculifera|[9903]|LOWCD571-06|Canada|British Columbia|658[0n]|BOLD:AAA3351
Feltia jaculifera|[9904]|LOWCD570-06|Canada|British Columbia|658[0n]|BOLD:AAA3351
Feltia jaculifera|[9905]|LOWCD569-06|Canada|British Columbia|657[0n]|BOLD:AAA3351
Feltia jaculifera|[9906]|LOWCD566-06|Canada|British Columbia|658[0n]|BOLD:AAA3351
Feltia jaculifera|[9907]|LOWCD563-06|Canada|British Columbia|658[0n]|BOLD:AAA3351
Feltia jaculifera|[9908]|LOWCD562-06|Canada|British Columbia|658[0n]|BOLD:AAA3351
Feltia jaculifera|[9909]|LOWCD560-06|Canada|British Columbia|657[0n]|BOLD:AAA3351
Feltia jaculifera|[9910]|LOWCD559-06|Canada|British Columbia|658[0n]|BOLD:AAA3351
Feltia jaculifera|[9911]|LOWCD558-06|Canada|British Columbia|658[0n]|BOLD:AAA3351
Feltia jaculifera|[9912]|LOWCD557-06|Canada|British Columbia|658[0n]|BOLD:AAA3351
Feltia jaculifera|[9913]|LOWCD556-06|Canada|British Columbia|658[0n]|BOLD:AAA3351
Feltia jaculifera|[9914]|LOWCD113-06|Canada|British Columbia|658[0n]|BOLD:AAA3351
Feltia jaculifera|[9915]|LOWCC165-05|Canada|British Columbia|658[0n]|BOLD:AAA3351
Feltia jaculifera|[9916]|LOWCC164-05|Canada|British Columbia|658[0n]|BOLD:AAA3351
Feltia jaculifera|[9917]|LOWCB706-05|Canada|British Columbia|658[0n]|BOLD:AAA3351
Feltia jaculifera|[9918]|LOWCB702-05|Canada|British Columbia|658[0n]|BOLD:AAA3351
Feltia jaculifera|[9919]|LOWCB701-05|Canada|British Columbia|658[0n]|BOLD:AAA3351
Feltia jaculifera|[9920]|LOWCB699-05|Canada|British Columbia|658[0n]|BOLD:AAA3351
Feltia jaculifera|[9921]|LOWCB698-05|Canada|British Columbia|658[0n]|BOLD:AAA3351
Feltia jaculifera|[9922]|XAH532-05|Canada|Ontario|658[0n]|BOLD:AAA3351
Feltia jaculifera|[9923]|XAH128-05|Canada|Ontario|658[0n]|BOLD:AAA3351
Feltia jaculifera|[9924]|XAH039-05|Canada|Ontario|658[0n]|BOLD:AAA3351
Feltia jaculifera|[9925]|RDNM697-05|United States|Oregon|658[0n]|BOLD:AAA3351
Feltia jaculifera|[9926]|LOWCD565-06|Canada|British Columbia|658[0n]|BOLD:AAA3351
Feltia jaculifera|[9927]|XAD284-04|Canada|Ontario|658[0n]|BOLD:AAA3351
Feltia jaculifera|[9928]|XAH129-05|Canada|Ontario|658[1n]|BOLD:AAA3351
Feltia jaculifera|[9929]|LBCH7316-10|Canada|British Columbia|636[0n]|BOLD:AAA3351
Feltia jaculifera|[9930]|LOWCD501-06|Canada|British Columbia|599[0n]|BOLD:AAA3351
Feltia jaculifera|[9931]|LBCH6911-10|Canada|British Columbia|633[0n]|BOLD:AAA3351
Feltia jaculifera|[9932]|RDNM706-05|Canada|British Columbia|609[0n]|BOLD:AAA3351
Feltia jaculifera|[9933]|XAD177-04|Canada|Ontario|592[0n]|BOLD:AAA3351
Feltia jaculifera|[9934]|LOWCD573-06|Canada|British Columbia|612[0n]|BOLD:AAA3351
Feltia jaculifera|[9935]|LOWCD568-06|Canada|British Columbia|605[0n]|BOLD:AAA3351
Feltia jaculifera|[9936]|LBCH7037-10|Canada|British Columbia|639[0n]|BOLD:AAA3351
Feltia jaculifera|[9937]|LBCH7583-10|Canada|British Columbia|643[0n]|BOLD:AAA3351
Feltia jaculifera|[9938]|LBCH7587-10|Canada|British Columbia|658[0n]|BOLD:AAA3351
Feltia jaculifera|[9939]|LOWCB700-05|Canada|British Columbia|658[0n]|BOLD:AAA3351
Feltia jaculifera|[9940]|LBCH7875-10|Canada|British Columbia|658[0n]|BOLD:AAA3351
Feltia jaculifera|[9941]|LBCH7878-10|Canada|British Columbia|658[0n]|BOLD:AAA3351
Feltia jaculifera|[9942]|MJMSL027-10|United States|Massachusetts|658[0n]|BOLD:AAA3351
Feltia jaculifera|[9943]|LOWCD564-06|Canada|British Columbia|658[0n]|BOLD:AAA3351
Feltia jaculifera|[9944]|XAH551-05|Canada|Ontario|658[2n]|BOLD:AAA3351
Feltia jaculifera|[9945]|XAD175-04|Canada|Ontario|658[0n]|BOLD:AAA3351
Feltia jaculifera|[9946]|XAH149-05|Canada|Ontario|658[0n]|BOLD:AAA3351
Feltia jaculifera|[9947]|XAH461-05|Canada|Ontario|658[0n]|BOLD:AAA3351
Feltia jaculifera|[9948]|TTMNB423-06|Canada|New Brunswick|658[0n]|BOLD:AAA3351
Feltia jaculifera|[9949]|TTMNB424-06|Canada|New Brunswick|658[0n]|BOLD:AAA3351
Feltia jaculifera|[9950]|LPABC918-09|Canada|Alberta|658[0n]|BOLD:AAA3351
Feltia jaculifera|[9951]|BBLEC131-09|Canada|New Brunswick|658[0n]|BOLD:AAA3351
Feltia jaculifera|[9952]|BBLEC467-09|Canada|New Brunswick|658[0n]|BOLD:AAA3351
Feltia jaculifera|[9953]|XAD170-04|Canada|Ontario|578[0n]|BOLD:AAA3351
Feltia jaculifera|[9954]|XAD290-04|Canada|Ontario|518[0n]|BOLD:AAA3351
Feltia jaculifera|[9955]|XAH376-05|Canada|Ontario|658[0n]|BOLD:AAA3351
Feltia jaculifera|[9956]|LBCH7724-10|Canada|British Columbia|658[0n]|BOLD:AAA3351
Feltia jaculifera|[9957]|XAG881-05|Canada|Ontario|564[0n]|BOLD:AAA3351
Feltia jaculifera|[9958]|XAH315-05|Canada|Ontario|607[1n]|BOLD:AAA3351
Feltia jaculifera|[9959]|XAB678-04|Canada|Ontario|658[0n]|BOLD:AAA3351
Feltia jaculifera|[9960]|XAH587-05|Canada|Ontario|658[0n]|BOLD:AAA3351
Feltia jaculifera|[9961]|BBLPB819-10|Canada|Alberta|658[0n]|BOLD:AAA3351
Feltia jaculifera|[9962]|LPMN955-08|Canada|Alberta|658[0n]|BOLD:AAA3351
Feltia jaculifera|[9963]|RDLQF207-06|Canada|Quebec|658[0n]|BOLD:AAA3351
Feltia jaculifera|[9964]|TTMNB426-06|Canada|New Brunswick|658[0n]|BOLD:AAA3351
Feltia jaculifera|[9965]|TTMNB425-06|Canada|New Brunswick|658[0n]|BOLD:AAA3351
Feltia jaculifera|[9966]|TTMNB422-06|Canada|New Brunswick|658[0n]|BOLD:AAA3351
Feltia jaculifera|[9967]|RDLQB759-05|Canada|Quebec|658[0n]|BOLD:AAA3351
Feltia jaculifera|[9968]|XAH431-05|Canada|Ontario|658[0n]|BOLD:AAA3351

[illegible]
Feltia jaculifera|[9966]|TTMNB422-06|Canada|New Brunswick|658[0n]|BOLD:AAA3351
Feltia jaculifera|[9967]|RDLQB759-05|Canada|Quebec|658[0n]|BOLD:AAA3351
Feltia jaculifera|[9968]|XAH431-05|Canada|Ontario|658[0n]|BOLD:AAA3351
Feltia jaculifera|[9969]|XAH416-05|Canada|Ontario|658[0n]|BOLD:AAA3351
Feltia jaculifera|[9970]|XAH360-05|Canada|Ontario|658[0n]|BOLD:AAA3351
Feltia jaculifera|[9971]|XAH299-05|Canada|Ontario|658[0n]|BOLD:AAA3351
Feltia jaculifera|[9972]|XAH282-05|Canada|Ontario|658[0n]|BOLD:AAA3351
Feltia jaculifera|[9973]|XAH139-05|Canada|Ontario|658[0n]|BOLD:AAA3351
Feltia jaculifera|[9974]|XAH138-05|Canada|Ontario|658[0n]|BOLD:AAA3351
Feltia jaculifera|[9975]|XAD470-04|Canada|Ontario|658[0n]|BOLD:AAA3351
Feltia jaculifera|[9976]|XAB430-04|Canada|Ontario|658[0n]|BOLD:AAA3351
Feltia jaculifera|[9977]|XAH463-05|Canada|Ontario|658[0n]|BOLD:AAA3351
Feltia jaculifera|[9978]|XAH089-05|Canada|Ontario|658[2n]|BOLD:AAA3351
Feltia jaculifera|[9979]|XAB429-04|Canada|Ontario|616[0n]|BOLD:AAA3351
Feltia jaculifera|[9980]|XAH417-05|Canada|Ontario|658[0n]|BOLD:AAA3351
Feltia jaculifera|[9981]|XAH462-05|Canada|Ontario|603[0n]|BOLD:AAA3351
Feltia jaculifera|[9982]|LPABB115-08|Canada|Alberta|650[0n]|BOLD:AAA3351
Feltia jaculifera|[9983]|LPABB274-08|Canada|Alberta|658[0n]|BOLD:AAA3351
Feltia jaculifera|[9984]|LPABB624-08|Canada|Alberta|658[0n]|BOLD:AAA3351
Feltia jaculifera|[9985]|LPABB625-08|Canada|Alberta|658[0n]|BOLD:AAA3351
Feltia jaculifera|[9986]|LPMNB439-09|Canada|Manitoba|658[0n]|BOLD:AAA3351
Feltia jaculifera|[9987]|LPABC931-09|Canada|Alberta|658[0n]|BOLD:AAA3351
Feltia jaculifera|[9988]|BBLPB821-10|Canada|Alberta|658[0n]|BOLD:AAA3351
Feltia jaculifera|[9989]|BAWG181-12|Canada|Alberta|658[0n]|BOLD:AAA3351
Feltia evanidalis|[9990]|NAMUM129-08|United States|California|612[0n]|BOLD:AAA3351
Feltia jaculifera|[9991]|LPMNB437-09|Canada|Manitoba|658[0n]|BOLD:AAA3351
Feltia jaculifera|[9992]|RDNM700-05|Canada|British Columbia|571[2n]|BOLD:AAA3351
Feltia jaculifera|[9993]|LBCH7318-10|Canada|British Columbia|658[0n]|BOLD:AAA3351
Feltia jaculifera|[9994]|RDNMD552-06|United States|Colorado|658[0n]|BOLD:AAA3351
Feltia jaculifera|[9995]|RDNMD553-06|United States|Colorado|658[0n]|BOLD:AAA3351
Feltia jaculifera|[9996]|LPABC840-09|Canada|Alberta|658[0n]|BOLD:AAA3351
Feltia jaculifera|[9997]|BAWG171-12|Canada|Alberta|658[0n]|BOLD:AAA3351
Feltia jaculifera|[9998]|BAWG234-12|Canada|Alberta|658[0n]|BOLD:AAA3351
Feltia inyoca|[9999]|CNCLB749-14|United States|California|658[0n]|BOLD:AAA3351
Feltia manifesta|[10000]|LSEU522-06|United States|Georgia|658[0n]|BOLD:AAE6661
Feltia manifesta|[10001]|LSEU520-06|United States|Georgia|658[0n]|BOLD:AAE6661
Feltia manifesta|[10002]|LSEU521-06|United States|Georgia|658[0n]|BOLD:AAE6661
Feltia manifesta|[10003]|LSEU523-06|United States|Georgia|658[0n]|BOLD:AAE6661
Feltia mollis|[10004]|LOWCB692-05|Canada|British Columbia|658[0n]|BOLD:ABY6006
Feltia mollis|[10005]|LOWCB689-05|Canada|British Columbia|564[0n]|BOLD:ABY6006
Feltia mollis|[10006]|LOWCB688-05|Canada|British Columbia|649[0n]|BOLD:ABY6006
Feltia mollis|[10007]|LOWCB686-05|Canada|British Columbia|562[1n]|BOLD:ABY6006
Feltia mollis|[10008]|LOWCD913-06|Canada|British Columbia|658[0n]|BOLD:ABY6006
Feltia mollis|[10009]|LOWCB697-05|Canada|British Columbia|658[0n]|BOLD:ABY6006
Feltia mollis|[10010]|LOWCB696-05|Canada|British Columbia|658[0n]|BOLD:ABY6006
Feltia mollis|[10011]|LOWCB695-05|Canada|British Columbia|658[0n]|BOLD:ABY6006
Feltia mollis|[10012]|LOWCB694-05|Canada|British Columbia|658[0n]|BOLD:ABY6006
Feltia mollis|[10013]|LOWCB693-05|Canada|British Columbia|658[0n]|BOLD:ABY6006
Feltia mollis|[10014]|LOWCB691-05|Canada|British Columbia|658[0n]|BOLD:ABY6006
Feltia mollis|[10015]|LOWCB690-05|Canada|British Columbia|658[0n]|BOLD:ABY6006
Feltia mollis|[10016]|LOWCB687-05|Canada|British Columbia|658[0n]|BOLD:ABY6006
Feltia mollis|[10017]|LOWCD912-06|Canada|British Columbia|609[0n]|BOLD:ABY6006
Feltia mollis|[10018]|LOWCD914-06|Canada|British Columbia|585[0n]|BOLD:ABY6006
Feltia mollis|[10019]|LOWCD915-06|Canada|British Columbia|589[0n]|BOLD:ABY6006
Feltia mollis|[10020]|LOWCD916-06|Canada|British Columbia|658[0n]|BOLD:ABY6006
Feltia mollis|[10021]|LBCH5954-10|Canada|British Columbia|658[0n]|BOLD:ABY6006
Feltia mollis|[10022]|LPABC022-09|Canada|Alberta|658[0n]|BOLD:ABY6006
Feltia mollis|[10023]|LCH628-05|Canada|Manitoba|658[0n]|BOLD:ABY6006
Feltia mollis|[10024]|LBCH5955-10|Canada|British Columbia|658[0n]|BOLD:ABY6006
Feltia mollis|[10025]|BBLPB867-10|Canada|Alberta|658[0n]|BOLD:ABY6006
Feltia nigrita|[10026]|RDNMG713-08|Canada|Yukon Territory|642[0n]|BOLD:ABY6005
Feltia nigrita|[10027]|RDNMG711-08|Canada|Yukon Territory|658[0n]|BOLD:ABY6005
Feltia nigrita|[10028]|RDNMG712-08|Canada|Yukon Territory|658[0n]|BOLD:ABY6005
Feltia nigrita|[10029]|RDNMG710-08|Canada|Saskatchewan|658[0n]|BOLD:ABY6005
Feltia nigrita|[10030]|RDNMG714-08|Canada|Alberta|658[0n]|BOLD:ABY6005
Feltia nigrita|[10031]|RDNMF788-08|Canada|Alberta|658[0n]|BOLD:ABY6005
Feltia nigrita|[10032]|RDNMF789-08|Canada|Alberta|658[0n]|BOLD:ABY6005
Feltia nigrita|[10033]|RDNMF790-08|Canada|Alberta|658[0n]|BOLD:ABY6005
Feltia nigrita|[10034]|RDNMF791-08|Canada|Alberta|658[0n]|BOLD:ABY6005
Feltia nigrita|[10035]|RDNMF792-08|Canada|Alberta|658[0n]|BOLD:ABY6005
Feltia nigrita|[10036]|RDNMF793-08|Canada|Alberta|658[0n]|BOLD:ABY6005
Feltia nigrita|[10037]|RDNMF794-08|Canada|British Columbia|658[0n]|BOLD:ABY6005
Feltia nigrita|[10038]|BBLPB754-10|Canada|Alberta|658[0n]|BOLD:ABY6005
Feltia boreana|[10039]|RDNMF066-08|Canada|Yukon Territory|658[0n]|BOLD:AAD9414
Feltia boreana|[10040]|RDNMG718-08|Canada|Northwest Territories|645[0n]|BOLD:AAD9414
Feltia boreana|[10041]|RDNMG719-08|Canada|Northwest Territories|642[1n]|BOLD:AAD9414
Feltia boreana|[10042]|RDNMG720-08|Canada|Northwest Territories|633[2n]|BOLD:AAD9414
Feltia boreana|[10043]|RDNMG721-08|Canada|Yukon Territory|642[0n]|BOLD:AAD9414
Feltia boreana|[10044]|RDNMG734-08|Canada|Yukon Territory|642[0n]|BOLD:AAD9414
Feltia beringiana|[10045]|RDNMF059-08|Canada|Yukon Territory|658[0n]|BOLD:ABY9079
Feltia beringiana|[10046]|RDNMG736-08|Canada|Yukon Territory|642[0n]|BOLD:ABY9079
Feltia woodiana|[10047]|RDNMF058-08|Canada|Yukon Territory|658[0n]|BOLD:ACE9137
Feltia woodiana|[10048]|RDNMG715-08|Canada|Yukon Territory|641[0n]|BOLD:ACE9137
Feltia woodiana|[10049]|RDNMG716-08|Canada|Yukon Territory|642[0n]|BOLD:ACE9137
Feltia woodiana|[10050]|RDNMG717-08|Canada|Yukon Territory|658[0n]|BOLD:ACE9137
Feltia troubridgei|[10051]|RDNMI191-12|Canada|Yukon Territory|614[0n]|BOLD:ACE9137
Feltia floridensis|[10052]|RDNMC305-05|United States|Florida|417[0n]|
Feltia floridensis|[10053]|RDNMC724-06|United States|Florida|658[0n]|BOLD:AAE6660
Feltia geniculata|[10054]|RDLQF282-06|Canada|Quebec|658[0n]|BOLD:AAE6660
Feltia geniculata|[10055]|RDLQB772-05|Canada|Quebec|658[0n]|BOLD:AAE6660
Feltia geniculata|[10056]|LSEU174-06|United States|Georgia|571[1n]|BOLD:AAE6660
Feltia geniculata|[10057]|TTMNB429-06|Canada|New Brunswick|658[0n]|BOLD:AAE6660
Feltia geniculata|[10058]|RDNMJ099-10|United States|Arkansas|658[0n]|BOLD:AAE6660
Feltia austrina|[10059]|CNCLB742-14|United States|Texas|658[0n]|BOLD:ACM4373
Feltia austrina|[10060]|CNCLB745-14|United States|Texas|604[0n]|BOLD:ACM4373
Feltia austrina|[10061]|LNAUT921-14|United States|Texas|658[0n]|BOLD:ACM4373
Feltia austrina|[10062]|CNCLB743-14|United States|Texas|658[0n]|BOLD:ACM4373
Feltia austrina|[10063]|CNCLB744-14|United States|Texas|658[0n]|BOLD:ACM4373
Feltia austrina|[10064]|CNCLB746-14|United States|Texas|658[0n]|BOLD:ACM4373
Feltia austrina|[10065]|LNAUT923-14|United States|Texas|658[0n]|BOLD:ACM4373
Dichagyris triphaenoides|[10066]|RDNMJ844-11|United States|Arizona|646[0n]|BOLD:AAV6613
Dichagyris triphaenoides|[10067]|RDNMJ845-11|United States|Arizona|645[0n]|BOLD:AAV6613
[illegible]

Feltia austrina|[10065]|LNAUT923-14|United States|Texas|658[0n]|BOLD:ACM4373
Dichagyris triphaenoides|[10066]|RDNMJ844-11|United States|Arizona|646[0n]|BOLD:AAV6613
Dichagyris triphaenoides|[10067]|RDNMJ845-11|United States|Arizona|645[0n]|BOLD:AAV6613
Dichagyris kyune|[10068]|NOCNA032-14|United States|Arizona|658[0n]|BOLD:ACM4105
Dichagyris kyune|[10069]|CNCLB961-14|United States|Arizona|658[0n]|BOLD:ACM4105
Dichagyris larga|[10070]|RDNME629-08|United States|Arizona|658[0n]|BOLD:AAI9531
Dichagyris larga|[10071]|RDNMJ516-11|United States|Arizona|658[0n]|BOLD:AAI9531
Dichagyris cyminopristes|[10072]|LNAUT928-14|United States|Texas|616[0n]|BOLD:AAI9531
Dichagyris larga|[10073]|NAMUM084-08|United States|Arizona|658[0n]|BOLD:AAI9531
Dichagyris larga|[10074]|RDNMH851-09|United States|Arizona|658[0n]|BOLD:AAI9531
Dichagyris larga|[10075]|RDNMJ728-11|United States|Arizona|658[0n]|BOLD:AAI9531
Dichagyris cyminopristes|[10076]|LNAUT929-14|Mexico|Nuevo Leon|658[0n]|BOLD:AAI9531
Dichagyris cyminopristes|[10077]|LNAUT930-14|Mexico|Nuevo Leon|658[0n]|BOLD:AAI9531
Dichagyris cyminopristes|[10078]|LNAUT931-14|Mexico|Nuevo Leon|658[0n]|BOLD:AAI9531
Agrotis malefida|[10079]|BBLSX062-09|United States|Texas|658[0n]|BOLD:AAE5299
Agrotis malefida|[10080]|LNC483-06|United States|North Carolina|658[0n]|BOLD:AAE5299
Agrotis malefida|[10081]|USLEP1146-10|United States|Texas|658[0n]|BOLD:AAE5299
Agrotis malefida|[10082]|LPOKB1000-09|United States|Oklahoma|658[0n]|BOLD:AAE5299
Agrotis malefida|[10083]|BBLSW918-09|United States|Texas|658[0n]|BOLD:AAE5299
Agrotis malefida|[10084]|BBLOB1320-11|United States|Florida|658[0n]|BOLD:AAE5299
Agrotis malefida|[10085]|BBLOC1695-11|United States|Texas|658[0n]|BOLD:AAE5299
Feltia repleta|[10086]|BLPCD352-08|Costa Rica|Guanacaste|658[0n]|BOLD:AAH3365
Feltia repleta|[10087]|GWOSB014-10|Ecuador|Pichincha|658[0n]|BOLD:AAH3365
Feltia repleta|[10088]|GWOSU309-11|Ecuador|Pichincha|658[0n]|BOLD:AAH3365
Feltia repleta|[10089]|GWOSU308-11|Ecuador|Pichincha|658[0n]|BOLD:AAH3365
Feltia repleta|[10090]|BLPCD521-08|Costa Rica|Guanacaste|658[0n]|BOLD:AAH3365
Feltia repleta|[10091]|BLPDQ303-10|Costa Rica|Alajuela|658[0n]|BOLD:AAH3365
Feltia repleta|[10092]|HKONB144-08|United States|Texas|658[0n]|BOLD:AAH3365
Feltia repleta|[10093]|HKONB145-08|United States|Texas|658[0n]|BOLD:AAH3365
Feltia repleta|[10094]|GWORT494-10|Ecuador|Pichincha|658[0n]|BOLD:AAH3365
Feltia repleta|[10095]|BLPEF4986-13|Costa Rica|Guanacaste|658[0n]|BOLD:AAH3365
Dichagyris longivesica|[10096]|CNCLB1747-14|Mexico|Durango|658[0n]|BOLD:ACP7339
Dichagyris neaphronus|[10097]|CNCLB1748-14|Mexico|Durango|658[0n]|BOLD:ACP7340
Eucoptocnemis rufula|[10098]|RDNMJ822-11|United States|Arizona|649[0n]|BOLD:AAV7030
Eucoptocnemis rufula|[10099]|RDNMJ823-11|United States|Arizona|658[0n]|BOLD:AAV7030
Feltia subterranea|[10100]|BBLSW342-09|United States|Arizona|658[0n]|BOLD:AAB6793
Feltia subterranea|[10101]|CMAZA1022-12|United States|Arizona|658[0n]|BOLD:AAB6793
Feltia subterranea|[10102]|CMAZA271-09|United States|Arizona|658[0n]|BOLD:AAB6793
Feltia subterranea|[10103]|RDNMD885-07|United States|Arizona|655[0n]|BOLD:AAB6793
Feltia subterranea|[10104]|LOCBF424-13|United States|California|632[0n]|BOLD:AAB6793
Feltia subterranea|[10105]|LOCBF425-13|United States|California|611[0n]|BOLD:AAB6793
Feltia subterranea|[10106]|BBLSW334-09|United States|Arizona|647[0n]|BOLD:AAB6793
Feltia subterranea|[10107]|BBLOB450-11|United States|Florida|658[0n]|BOLD:AAB6793
Feltia subterranea|[10108]|LGSMG882-10|United States|North Carolina|658[0n]|BOLD:AAB6793
Feltia subterranea|[10109]|LPOKA886-09|United States|Oklahoma|658[0n]|BOLD:AAB6793
Feltia subterranea|[10110]|BBLOC651-11|United States|Arkansas|622[0n]|BOLD:AAB6793
Feltia subterranea|[10111]|BBLOC652-11|United States|Arkansas|622[0n]|BOLD:AAB6793
Feltia subterranea|[10112]|LGSM681-04|United States|Tennessee|527[1n]|BOLD:AAB6793
Feltia subterranea|[10113]|LNC482-06|United States|North Carolina|586[0n]|BOLD:AAB6793
Feltia subterranea|[10114]|BBLOC654-11|United States|Arkansas|621[0n]|BOLD:AAB6793
Feltia subterranea|[10115]|BBLOC876-11|United States|Arkansas|658[0n]|BOLD:AAB6793
Feltia subterranea|[10116]|BBLOC1049-11|United States|Arkansas|658[0n]|BOLD:AAB6793
Feltia subterranea|[10117]|LPOKB141-09|United States|Oklahoma|658[0n]|BOLD:AAB6793
Feltia subterranea|[10118]|LYPAP595-09|Mexico|Quintana Roo|658[0n]|BOLD:AAB6793
Feltia subterranea|[10119]|BBLSW841-09|United States|Texas|658[0n]|BOLD:AAB6793
Feltia subterranea|[10120]|BBLSW847-09|United States|Texas|658[0n]|BOLD:AAB6793
Feltia subterranea|[10121]|LGSMG890-10|United States|North Carolina|658[0n]|BOLD:AAB6793
Feltia subterranea|[10122]|BBLOB501-11|United States|Florida|658[0n]|BOLD:AAB6793
Feltia subterranea|[10123]|BBLOB1674-11|United States|Florida|658[0n]|BOLD:AAB6793
Feltia subterranea|[10124]|BBLOB1689-11|United States|Florida|658[0n]|BOLD:AAB6793
Feltia subterranea|[10125]|BBLOC881-11|United States|Arkansas|658[0n]|BOLD:AAB6793
Feltia subterranea|[10126]|BBLOC1034-11|United States|Texas|658[0n]|BOLD:AAB6793
Feltia subterranea|[10127]|BBLOC1036-11|United States|Texas|658[0n]|BOLD:AAB6793
Feltia subterranea|[10128]|BBLOC1041-11|United States|Arkansas|658[0n]|BOLD:AAB6793
Feltia subterranea|[10129]|LOFLA735-06|United States|Florida|658[0n]|BOLD:AAB6793
Feltia subterranea|[10130]|LOFLB115-06|United States|Florida|658[0n]|BOLD:AAB6793
Feltia subterranea|[10131]|LOFLB269-06|United States|Florida|658[0n]|BOLD:AAB6793
Feltia subterranea|[10132]|LOFLB479-06|United States|Florida|658[0n]|BOLD:AAB6793
Feltia subterranea|[10133]|LOFLB512-06|United States|Florida|658[0n]|BOLD:AAB6793
Feltia subterranea|[10134]|BLPCJ503-08|Costa Rica|Guanacaste|658[0n]|BOLD:AAB6793
Feltia subterranea|[10135]|BBLSX698-09|United States|Texas|658[0n]|BOLD:AAB6793
Feltia subterranea|[10136]|LGSMG889-10|United States|North Carolina|658[0n]|BOLD:AAB6793
Feltia subterranea|[10137]|BBLOC1042-11|United States|Arkansas|658[0n]|BOLD:AAB6793
Feltia subterranea|[10138]|BBLOC1043-11|United States|Arkansas|658[0n]|BOLD:AAB6793
Feltia subterranea|[10139]|BBLOC1045-11|United States|Arkansas|658[0n]|BOLD:AAB6793
Feltia subterranea|[10140]|BBLOC1060-11|United States|Arkansas|658[0n]|BOLD:AAB6793
Feltia subterranea|[10141]|LNC481-06|United States|North Carolina|658[0n]|BOLD:AAB6793
Feltia subterranea|[10142]|LOFLA687-06|United States|Florida|658[0n]|BOLD:AAB6793
Feltia subterranea|[10143]|BBLOE1361-12|United States|Florida|658[0n]|BOLD:AAB6793
Feltia subterranea|[10144]|BBLSW335-09|United States|Arizona|658[0n]|BOLD:AAB6793
Feltia subterranea|[10145]|GMLC1178-12|United States|California|658[0n]|BOLD:AAB6793
Feltia subterranea|[10146]|GMLC1279-12|United States|California|658[0n]|BOLD:AAB6793
Feltia subterranea|[10147]|GBGL6796-09|||624[0n]|BOLD:AAB6793
Feltia subterranea|[10148]|MOTAR022-12|Argentina|Entre Rios|634[0n]|BOLD:AAB6793
Feltia subterranea|[10149]|BBLOD953-11|United States|Texas|658[0n]|BOLD:AAB6793
Feltia subterranea|[10150]|LOCBF2616-13|United States|California|658[0n]|BOLD:AAB6793
Feltia subterranea|[10151]|LOCBF2605-13|United States|California|658[0n]|BOLD:AAB6793
Feltia subterranea|[10152]|GMLC1112-12|United States|California|658[0n]|BOLD:AAB6793
Feltia subterranea|[10153]|GMLC951-12|United States|California|658[0n]|BOLD:AAB6793
Feltia subterranea|[10154]|CMAZA1064-12|United States|Arizona|658[0n]|BOLD:AAB6793
Feltia subterranea|[10155]|BBLOD956-11|United States|Texas|658[0n]|BOLD:AAB6793
Feltia subterranea|[10156]|BBLOD950-11|United States|California|658[0n]|BOLD:AAB6793
Feltia subterranea|[10157]|BBLOD587-11|United States|California|658[0n]|BOLD:AAB6793
Feltia subterranea|[10158]|AWCLB416-10|United States|Arizona|658[0n]|BOLD:AAB6793
Feltia subterranea|[10159]|AWCLB273-10|United States|Arizona|658[0n]|BOLD:AAB6793
Feltia subterranea|[10160]|CMAZA002-09|United States|Arizona|658[0n]|BOLD:AAB6793
Feltia subterranea|[10161]|BBLSX684-09|United States|Arizona|658[0n]|BOLD:AAB6793
Feltia subterranea|[10162]|BBLSW777-09|United States|Arizona|658[0n]|BOLD:AAB6793
Feltia subterranea|[10163]|BBLSW322-09|United States|Arizona|658[0n]|BOLD:AAB6793
Feltia subterranea|[10164]|LOCBC451-06|United States|California|658[0n]|BOLD:AAB6793
Feltia subterranea|[10165]|RDNMD383-06|United States|California|658[0n]|BOLD:AAB6793
Feltia subterranea|[10166]|LOCBF3468-14|United States|California|579[0n]|BOLD:AAB6793
Feltia subterranea|[10167]|GMLC1072-12|United States|California|658[0n]|BOLD:AAB6793

Feltia subterranea|[10165]|RDNMD383-06|United States|California|658[0n]|BOLD:AAB6793
Feltia subterranea|[10166]|LOCBF3468-14|United States|California|579[0n]|BOLD:AAB6793
Feltia subterranea|[10167]|GMLC1072-12|United States|California|658[0n]|BOLD:AAB6793
Feltia subterranea|[10168]|LOCBE089-06|United States|California|658[0n]|BOLD:AAB6793
Feltia subterranea|[10169]|GMLC1377-12|United States|California|636[0n]|BOLD:AAB6793
Feltia subterranea|[10170]|LOCBC661-06|United States|California|630[0n]|BOLD:AAB6793
Feltia subterranea|[10171]|LOCBF426-13|United States|California|626[0n]|BOLD:AAB6793
Feltia subterranea|[10172]|LOCBF427-13|United States|California|621[0n]|BOLD:AAB6793
Feltia subterranea|[10173]|LOCBF049-13|United States|California|581[0n]|BOLD:AAB6793
Feltia subterranea|[10174]|LOCBF428-13|United States|California|602[0n]|BOLD:AAB6793
Feltia subterranea|[10175]|LOCBF3531-14|United States|California|579[0n]|BOLD:AAB6793
Dichagyris dubitata|[10176]|CNCLB963-14|Mexico|Baja California|658[0n]|BOLD:ACM4229
Copablepharon spiritum|[10177]|RDNMB113-05|United States|Washington|658[0n]|BOLD:ACF1458
Copablepharon spiritum|[10178]|RDNM238-05|United States|Nevada|658[0n]|BOLD:ACF1458
Copablepharon spiritum|[10179]|RDNM235-05|United States|Washington|658[0n]|BOLD:ACF1458
Copablepharon spiritum|[10180]|RDNMB576-05|United States|California|567[1n]|BOLD:ACF1458
Copablepharon spiritum|[10181]|RDNMB577-05|United States|California|658[0n]|BOLD:ACF1458
Copablepharon spiritum|[10182]|RDNM237-05|United States|Nevada|658[0n]|BOLD:ABX6338
Copablepharon spiritum|[10183]|RDNMB114-05|United States|Nevada|658[0n]|BOLD:ABX6338
Copablepharon spiritum|[10184]|RDNM233-05|United States|Washington|658[0n]|BOLD:ABX6338
Copablepharon spiritum|[10185]|RDNM234-05|United States|Washington|658[0n]|BOLD:ABX6338
Copablepharon spiritum|[10186]|RDNMB575-05|United States|Colorado|504[0n]|BOLD:ABX6338
Copablepharon spiritum|[10187]|RDNMB578-05|United States|Colorado|658[0n]|BOLD:ABX6338
Copablepharon albisericea|[10188]|RDNME710-08|United States|Colorado|658[0n]|BOLD:ACF2120
Copablepharon spiritum|[10189]|RDNM236-05|United States|Colorado|566[0n]|BOLD:ACF2120
Copablepharon spiritum group|[10190]|RDNMG423-08|United States|Colorado|658[0n]|BOLD:ACF2120
Copablepharon albisericea|[10191]|RDNMG891-08|United States|Colorado|658[0n]|BOLD:ACF2120
Copablepharon albisericea|[10192]|RDNMG892-08|United States|Colorado|658[0n]|BOLD:ACF2120
Copablepharon sp.|[10193]|LNAUS4775-13|United States|Colorado|658[0n]|BOLD:ACF2120
Copablepharon spiritum group|[10194]|RDNMB014-05|United States|Colorado|658[0n]|BOLD:ACF2120
Copablepharon spiritum group|[10195]|RDNMB581-05|United States|Utah|658[0n]|BOLD:ACF2120
Copablepharon sp.|[10196]|LNAUS4774-13|United States|Colorado|658[0n]|BOLD:ACF2120
Copablepharon sp.|[10197]|LNAUS4776-13|United States|Colorado|658[0n]|BOLD:ACF2120
Copablepharon sp.|[10198]|LNAUS4773-13|United States|Colorado|658[0n]|BOLD:ACF2120
Copablepharon sp.|[10199]|LNAUS4777-13|United States|Colorado|658[0n]|BOLD:ACF2120
Copablepharon robertsoni|[10200]|CGLCA034-10|United States|California|658[0n]|BOLD:ACP6834
Copablepharon robertsoni|[10201]|CGLCA035-10|United States|California|658[0n]|BOLD:ACP6834
Copablepharon robertsoni|[10202]|CGLCA147-10|United States|California|658[0n]|BOLD:ACP6834
Copablepharon canariana|[10203]|LNAUS4758-13|United States|Nevada|658[0n]|BOLD:ABW3097
Copablepharon canariana|[10204]|LNAUS4756-13|United States|Nevada|546[0n]|BOLD:ABW3097
Copablepharon canariana|[10205]|BBLOC1372-11|United States|California|658[0n]|BOLD:ABW3097
Copablepharon canariana|[10206]|LNAUS4757-13|United States|Nevada|658[0n]|BOLD:ABW3097
Copablepharon canariana|[10207]|LNAUS4759-13|United States|Nevada|658[0n]|BOLD:ABW3097
Copablepharon canariana|[10208]|LNAUS4760-13|United States|Nevada|658[0n]|BOLD:ABW3097
Copablepharon fuscum|[10209]|RDNM711-05|United States|Washington|526[0n]|
Copablepharon fuscum|[10210]|RDNMB571-05|United States|Washington|658[0n]|BOLD:ABY9004
Copablepharon atrinotum|[10211]|RDNM710-05|United States|Nevada|658[0n]|BOLD:ABY9004
Copablepharon atrinotum|[10212]|RDNM709-05|United States|California|658[0n]|BOLD:ABY9004
Copablepharon absidum|[10213]|RDNM232-05|United States|Washington|658[0n]|BOLD:ABY9004
Copablepharon absidum|[10214]|RDNM229-05|United States|Washington|544[0n]|BOLD:ABY9004
Copablepharon absidum|[10215]|RDNM231-05|United States|Washington|580[0n]|BOLD:ABY9004
Copablepharon fuscum|[10216]|RDNMB572-05|United States|Washington|609[0n]|BOLD:ABY9004
Copablepharon grandis|[10217]|RDNME985-08|United States|New Mexico|658[7n]|
Copablepharon grandis|[10218]|RDNMF007-08|United States|Colorado|658[0n]|BOLD:ABY9003
Copablepharon grandis|[10219]|RDNMF094-08|United States|Colorado|658[0n]|BOLD:ABY9003
Copablepharon grandis|[10220]|RDNMG424-08|United States|Colorado|658[0n]|BOLD:ABY9003
Copablepharon grandis|[10221]|RDNMG425-08|United States|Colorado|658[0n]|BOLD:ABY9003
Copablepharon sanctaemonicae|[10222]|CNCLB965-14|United States|California|399[0n]|
Copablepharon sanctaemonicae|[10223]|CNCLB966-14|United States|California|658[1n]|BOLD:ABY9003
Copablepharon viridisparsa hopfingeri|[10224]|RDNM218-05|United States|Washington|658[0n]|BOLD:AA...
Copablepharon viridisparsa hopfingeri|[10225]|RDNM219-05|United States|Washington|658[0n]|BOLD:AA...
Copablepharon viridisparsa hopfingeri|[10226]|RDNM220-05|United States|Washington|658[0n]|BOLD:AA...
Copablepharon viridisparsa hopfingeri|[10227]|RDNMG895-08|United States|Washington|658[0n]|BOLD:A...
Copablepharon viridisparsa gilvum|[10228]|RDNM213-05|United States|California|658[0n]|BOLD:AAC4953
Copablepharon viridisparsa gilvum|[10229]|IAWLB006-10|United States|California|658[0n]|BOLD:AAC4953
Copablepharon viridisparsa gilvum|[10230]|IAWLB007-10|United States|California|658[0n]|BOLD:AAC4953
Copablepharon viridisparsa gilvum|[10231]|IAWLB008-10|United States|California|658[0n]|BOLD:AAC4953
Copablepharon viridisparsa gilvum|[10232]|IAWLB009-10|United States|California|658[0n]|BOLD:AAC4953
Copablepharon viridisparsa gilvum|[10233]|IAWLB010-10|United States|California|658[0n]|BOLD:AAC4953
Copablepharon viridisparsa|[10234]|RDNMB565-05|United States|Oregon|658[0n]|BOLD:AAC4953
Copablepharon viridisparsa gilvum|[10235]|RDNM217-05|United States|Nevada|658[0n]|BOLD:AAC4953
Copablepharon viridisparsa gilvum|[10236]|RDNM216-05|United States|Nevada|658[0n]|BOLD:AAC4953
Copablepharon viridisparsa gilvum|[10237]|RDNM215-05|United States|Nevada|658[0n]|BOLD:AAC4953
Copablepharon viridisparsa gilvum|[10238]|RDNM214-05|United States|California|658[0n]|BOLD:AAC4953
Copablepharon viridisparsa ravum|[10239]|RDNM208-05|United States|Washington|658[0n]|BOLD:AAC4953
Copablepharon viridisparsa ravum|[10240]|RDNM206-05|United States|California|565[0n]|BOLD:AAC4953
Copablepharon viridisparsa ravum|[10241]|RDNM205-05|United States|California|658[0n]|BOLD:AAC4953
Copablepharon viridisparsa gilvum|[10242]|RDNM212-05|United States|California|658[0n]|BOLD:AAC4953
Copablepharon viridisparsa ravum|[10243]|RDNM207-05|United States|California|543[0n]|BOLD:AAC4953
Copablepharon viridisparsa ravum|[10244]|RDNM211-05|United States|Washington|554[1n]|BOLD:AAC4953
Copablepharon viridisparsa|[10245]|RDNMB566-05|United States|Oregon|517[0n]|BOLD:AAC4953
Copablepharon viridisparsa ravum|[10246]|BBLOC1383-11|United States|California|658[0n]|BOLD:AAC4953
Copablepharon viridisparsa|[10247]|RDNM221-05|Canada|Alberta|658[0n]|BOLD:AAC4953
Copablepharon viridisparsa|[10248]|RDNMJ624-11|Canada|Saskatchewan|658[1n]|BOLD:AAC4953
Copablepharon viridisparsa|[10249]|RDNMB568-05|United States|Colorado|614[0n]|BOLD:AAC4953
Copablepharon viridisparsa|[10250]|RDNMB564-05|United States|Wyoming|658[0n]|BOLD:AAC4953
Copablepharon viridisparsa|[10251]|RDNM226-05|United States|Wyoming|658[0n]|BOLD:AAC4953
Copablepharon viridisparsa|[10252]|RDNM225-05|United States|Wyoming|658[0n]|BOLD:AAC4953
Copablepharon viridisparsa|[10253]|RDNM224-05|United States|Wyoming|658[0n]|BOLD:AAC4953
Copablepharon viridisparsa|[10254]|RDNM223-05|Canada|Alberta|658[0n]|BOLD:AAC4953
Copablepharon viridisparsa|[10255]|RDNM222-05|Canada|Alberta|658[0n]|BOLD:AAC4953
Copablepharon viridisparsa|[10256]|RDNMB563-05|United States|Colorado|554[1n]|BOLD:AAC4953
Copablepharon viridisparsa|[10257]|RDNMB567-05|United States|Wyoming|615[0n]|BOLD:AAC4953
Copablepharon viridisparsa|[10258]|RDNMB569-05|United States|Colorado|658[0n]|BOLD:AAC4953
Copablepharon viridisparsa gilvum|[10259]|RDNMG894-08|United States|Wyoming|658[0n]|BOLD:AAC4953
Copablepharon flavum|[10260]|CNCLB964-14|United States|Colorado|658[0n]|BOLD:AAC4953
Copablepharon opleri|[10261]|CNCLB967-14|United States|Utah|658[0n]|BOLD:ACP6829
Copablepharon opleri|[10262]|CNCLB968-14|United States|Utah|658[0n]|BOLD:ACP6829
Copablepharon serrata|[10263]|LNAUS4771-13|United States|California|599[0n]|BOLD:ACP6831
Copablepharon pictum|[10264]|CNCLB969-14|United States|Colorado|658[0n]|BOLD:ACP6830
Copablepharon pictum|[10265]|CNCLB970-14|United States|Colorado|658[0n]|BOLD:ACP6830
Copablepharon nevada|[10266]|RDNM251-05|United States|Nevada|658[0n]|BOLD:ABX6554
Copablepharon mustelini|[10267]|IAWL995-09|United States|California|658[0n]|BOLD:ACP6832

[illegible]
Copablepharon pictum|[10265]|CNCLB970-14|United States|Colorado|658[0n]|BOLD:ACP6830
Copablepharon nevada|[10266]|RDNM251-05|United States|Nevada|658[0n]|BOLD:ABX6554
Copablepharon mustelini|[10267]|IAWL995-09|United States|California|658[0n]|BOLD:ACP6832
Copablepharon mustelini|[10268]|IAWL996-09|United States|California|658[0n]|BOLD:ACP6832
Copablepharon mustelini|[10269]|IAWL997-09|United States|California|658[0n]|BOLD:ACP6832
Copablepharon mustelini|[10270]|CNCLB1450-14|United States|California|658[0n]|BOLD:ACP6832
Copablepharon mustelini|[10271]|CNCLB1760-14|United States|California|658[0n]|BOLD:ACP6832
Copablepharon longipenne serraticornis|[10272]|RDNMB573-05|United States|New Mexico|658[0n]|BOLD_...
Copablepharon longipenne serraticornis|[10273]|RDNMB574-05|United States|New Mexico|658[0n]|BOLD_...
Copablepharon serratigrande|[10274]|RDNME690-08|United States|New Mexico|658[0n]|BOLD:ACP6833
Copablepharon longipenne|[10275]|RDNMB582-05|United States|Utah|658[0n]|BOLD:ACP6833
Copablepharon longipenne|[10276]|RDNMB580-05|United States|Utah|658[0n]|BOLD:ACP6833
Copablepharon longipenne|[10277]|RDNMG108-08|United States|Wyoming|647[0n]|BOLD:ACP6833
Copablepharon longipenne|[10278]|RDNMG109-08|United States|Wyoming|658[0n]|BOLD:ACP6833
Copablepharon longipenne|[10279]|RDNMG889-08|United States|Wyoming|658[0n]|BOLD:ACP6833
Copablepharon longipenne|[10280]|RDNMG888-08|United States|Colorado|658[0n]|BOLD:ACP6833
Copablepharon sp.|[10281]|RDNMK475-11|United States|New Mexico|658[0n]|BOLD:ACP6833
Copablepharon longipenne|[10282]|RDNM249-05|Canada|Saskatchewan|658[0n]|BOLD:ACP6833
Copablepharon longipenne|[10283]|RDNM248-05|Canada|Saskatchewan|658[0n]|BOLD:ACP6833
Copablepharon longipenne|[10284]|RDNM250-05|Canada|Saskatchewan|658[0n]|BOLD:ACP6833
Copablepharon michiganensis|[10285]|RDNM252-05|United States|Michigan|658[0n]|BOLD:ACP6833
Copablepharon michiganensis|[10286]|RDNM253-05|United States|Michigan|593[0n]|BOLD:ACP6833
Copablepharon alaskensis|[10287]|LNAUS4754-13|United States|Alaska|658[0n]|BOLD:ACP6833
Copablepharon alaskensis|[10288]|LNAUS4755-13|United States|Alaska|658[0n]|BOLD:ACP6833
Copablepharon mutans|[10289]|RDNM244-05|United States|Washington|658[0n]|BOLD:ACP6833
Copablepharon columbia|[10290]|RDNM245-05|United States|Washington|658[0n]|BOLD:ACP6833
Copablepharon columbia|[10291]|RDNM246-05|United States|Washington|658[0n]|BOLD:ACP6833
Copablepharon mutans|[10292]|RDNMB111-05|United States|Washington|658[0n]|BOLD:ACP6833
Copablepharon columbia|[10293]|RDNMB112-05|United States|Washington|658[0n]|BOLD:ACP6833
Copablepharon mutans|[10294]|RDNM242-05|United States|Washington|658[0n]|BOLD:ACP6833
Copablepharon mutans|[10295]|RDNM243-05|United States|Washington|658[0n]|BOLD:ACP6833
Copablepharon mutans|[10296]|LNAUS4761-13|United States|Washington|658[0n]|BOLD:ACP6833
Copablepharon mutans|[10297]|LNAUS4762-13|United States|Washington|658[0n]|BOLD:ACP6833
Copablepharon gillaspyi|[10298]|CNCLB1777-14|United States|Texas|658[0n]|BOLD:ACP6833
Copablepharon gillaspyi|[10299]|CNCLB2016-14|United States|Texas|658[0n]|BOLD:ACP6833
Dichagyris madida|[10300]|RDNMJ820-11|United States|Arizona|658[0n]|BOLD:AAX9338
Dichagyris madida|[10301]|CNCLB711-14|United States|Arizona|658[0n]|BOLD:AAX9338
Dichagyris lobato|[10302]|CNCLB709-14|United States|Arizona|658[0n]|BOLD:ACM3942
Dichagyris lobato|[10303]|CNCLB710-14|United States|Arizona|658[0n]|BOLD:ACM3942
Dichagyris lobato|[10304]|CNCLB712-14|United States|Arizona|658[0n]|BOLD:ACM3942
Richia parentalis|[10305]|RDNMD868-07|United States|Arizona|613[0n]|BOLD:AAI0926
Richia parentalis|[10306]|RDNMD869-07|United States|New Mexico|653[0n]|BOLD:AAI0926
Richia chortalis|[10307]|RDNMK916-13|United States|Arizona|658[0n]|BOLD:AAH5618
Richia chortalis|[10308]|CMAZA056-09|United States|Arizona|658[0n]|BOLD:AAH5618
Richia chortalis|[10309]|CMAZA099-09|United States|Arizona|658[0n]|BOLD:AAH5618
Richia chortalis|[10310]|RDNME622-08|United States|Colorado|658[0n]|BOLD:AAH5618
Richia chortalis|[10311]|CMAZA032-09|United States|Arizona|658[0n]|BOLD:AAH5618
Richia chortalis|[10312]|CMAZA052-09|United States|Arizona|658[0n]|BOLD:AAH5618
Richia chortalis|[10313]|CMAZA118-09|United States|Arizona|658[0n]|BOLD:AAH5618
Richia chortalis|[10314]|CMAZA232-09|United States|Arizona|658[0n]|BOLD:AAH5618
Richia chortalis|[10315]|CMAZA233-09|United States|Arizona|658[0n]|BOLD:AAH5618
Richia chortalis|[10316]|CNCLB2104-14|United States|Arizona|658[0n]|BOLD:AAH5618
Dichagyris capota|[10317]|RDNMJ339-11|United States|Arizona|658[0n]|BOLD:AAI9532
Dichagyris capota|[10318]|RDNMH846-09|United States|Arizona|658[0n]|BOLD:AAI9532
Dichagyris capota|[10319]|NAMUM097-08|United States|Arizona|658[0n]|BOLD:AAI9532
Dichagyris capota|[10320]|RDNMJ340-11|United States|Arizona|658[0n]|BOLD:AAI9532
Dichagyris capota|[10321]|IAWLB294-11|United States|Arizona|658[0n]|BOLD:AAI9532
Striacosta albicosta|[10322]|RDNMD890-07|United States|New Mexico|655[0n]|BOLD:AAF3338
Striacosta albicosta|[10323]|CMAZA562-10|United States|Arizona|658[0n]|BOLD:AAF3338
Striacosta albicosta|[10324]|NAMUM066-08|United States|Arizona|657[0n]|BOLD:AAF3338
Striacosta albicosta|[10325]|RDNME626-08|United States|Arizona|658[0n]|BOLD:AAF3338
Striacosta albicosta|[10326]|CMAZA862-12|United States|Arizona|658[0n]|BOLD:AAF3338
Dichagyris mizteca|[10327]|RDNMJ405-11|United States|Arizona|658[0n]|BOLD:AAX9346
Dichagyris variabilis|[10328]|CGLCA065-10|United States|California|658[0n]|BOLD:AAD0257
Dichagyris variabilis|[10329]|JMMMB330-11|United States|California|658[0n]|BOLD:AAD0257
Dichagyris variabilis|[10330]|LPVIB266-08|Canada|British Columbia|658[0n]|BOLD:AAD0257
Dichagyris variabilis|[10331]|LPVIB267-08|Canada|British Columbia|658[0n]|BOLD:AAD0257
Dichagyris variabilis|[10332]|LPVIB268-08|Canada|British Columbia|658[0n]|BOLD:AAD0257
Dichagyris variabilis|[10333]|LPVIB445-08|Canada|British Columbia|658[0n]|BOLD:AAD0257
Dichagyris variabilis|[10334]|LPVIB446-08|Canada|British Columbia|658[0n]|BOLD:AAD0257
Dichagyris variabilis|[10335]|LPVIB573-08|Canada|British Columbia|658[0n]|BOLD:AAD0257
Dichagyris variabilis|[10336]|LBCH7150-10|Canada|British Columbia|658[0n]|BOLD:AAD0257
Dichagyris variabilis|[10337]|LBCH7744-10|Canada|British Columbia|658[0n]|BOLD:AAD0257
Dichagyris variabilis|[10338]|LBCH7929-10|Canada|British Columbia|658[0n]|BOLD:AAD0257
Dichagyris variabilis|[10339]|LBCH7933-10|Canada|British Columbia|658[0n]|BOLD:AAD0257
Dichagyris variabilis|[10340]|LBCH7221-10|Canada|British Columbia|658[0n]|BOLD:AAD0257
Dichagyris variabilis|[10341]|LOWCB684-05|Canada|British Columbia|658[0n]|BOLD:AAD0257
Dichagyris variabilis|[10342]|LOWCB685-05|Canada|British Columbia|658[0n]|BOLD:AAD0257
Dichagyris variabilis|[10343]|LOWCB683-05|Canada|British Columbia|658[0n]|BOLD:AAD0257
Dichagyris variabilis|[10344]|LOWCB682-05|Canada|British Columbia|658[0n]|BOLD:AAD0257
Dichagyris variabilis|[10345]|LOWCB681-05|Canada|British Columbia|658[0n]|BOLD:AAD0257
Dichagyris variabilis|[10346]|LOWCB680-05|Canada|British Columbia|658[0n]|BOLD:AAD0257
Dichagyris variabilis|[10347]|LOWCB679-05|Canada|British Columbia|658[0n]|BOLD:AAD0257
Dichagyris variabilis|[10348]|LOWCD917-06|Canada|British Columbia|608[0n]|BOLD:AAD0257
Dichagyris variabilis|[10349]|LBCH7092-10|Canada|British Columbia|644[0n]|BOLD:AAD0257
Dichagyris variabilis|[10350]|LBCH7470-10|Canada|British Columbia|658[0n]|BOLD:AAD0257
Dichagyris variabilis|[10351]|LBCH7500-10|Canada|British Columbia|658[0n]|BOLD:AAD0257
Dichagyris variabilis|[10352]|LBCH7926-10|Canada|British Columbia|658[0n]|BOLD:AAD0257
Dichagyris variabilis|[10353]|LBCH7927-10|Canada|British Columbia|658[0n]|BOLD:AAD0257
Dichagyris variabilis|[10354]|LBCH7928-10|Canada|British Columbia|658[0n]|BOLD:AAD0257
Dichagyris variabilis|[10355]|LBCH7930-10|Canada|British Columbia|658[0n]|BOLD:AAD0257
Dichagyris variabilis|[10356]|LBCH7931-10|Canada|British Columbia|658[0n]|BOLD:AAD0257
Dichagyris variabilis|[10357]|LBCH7932-10|Canada|British Columbia|658[0n]|BOLD:AAD0257
Dichagyris variabilis|[10358]|LALPA1252-11|Canada|British Columbia|658[0n]|BOLD:AAD0257
Dichagyris reliqua|[10359]|RDNMI193-12|Canada|Manitoba|627[0n]|BOLD:ACD0448
Dichagyris reliqua|[10360]|RDNMI194-12|United States|Michigan|601[0n]|BOLD:ACD0448
Dichagyris longidens|[10361]|CNCLB713-14|United States|Arizona|658[1n]|BOLD:ACM3916
Dichagyris longidens|[10362]|CNCLB714-14|United States|Arizona|658[0n]|BOLD:ACM3916
Dichagyris longidens|[10363]|CNCLB715-14|United States|Arizona|658[0n]|BOLD:ACM3916
Dichagyris longidens|[10364]|CNCLB717-14|United States|Arizona|658[0n]|BOLD:ACM3916
Dichagyris ruckesi|[10365]|RDNMG884-08|United States|Colorado|658[0n]|BOLD:AAX9337
Dichagyris broui|[10366]|CNCLB139-14|United States|Louisiana|658[0n]|BOLD:ACM4298
[illegible]

Dichagyris longidens|[10364]|CNCLB717-14|United States|Arizona|658[0n]|BOLD:ACM3916
Dichagyris ruckesi|[10365]|RDNMG884-08|United States|Colorado|658[0n]|BOLD:AAX9337
Dichagyris broui|[10366]|CNCLB139-14|United States|Louisiana|658[0n]|BOLD:ACM4298
Dichagyris broui|[10367]|CNCLB138-14|United States|Louisiana|658[0n]|BOLD:ACM4298
Dichagyris broui|[10368]|CNCLB1262-14|United States|Louisiana|658[0n]|BOLD:ACM4298
Dichagyris broui|[10369]|CNCLB1263-14|United States|Louisiana|658[0n]|BOLD:ACM4298
Dichagyris grotei|[10370]|RDNMD862-07|United States|New Mexico|655[0n]|BOLD:AAE0889
Dichagyris grotei|[10371]|RDNMD863-07|United States|New Mexico|655[0n]|BOLD:AAE0889
Dichagyris grotei|[10372]|NAMUM058-08|United States|Arizona|656[1n]|BOLD:AAE0889
Dichagyris cataclivis|[10373]|RDNMD865-07|United States|Arizona|653[0n]|BOLD:ACE8858
Dichagyris cataclivis|[10374]|NAMUM079-08|United States|Arizona|657[0n]|BOLD:ACE8858
Dichagyris cataclivis|[10375]|CMAZA791-10|United States|Arizona|658[0n]|BOLD:ACE8858
Dichagyris cataclivis|[10376]|RDNMD864-07|United States|Arizona|655[0n]|BOLD:AAF5699
Dichagyris cataclivis|[10377]|RDNMJ114-10|United States|Arizona|658[0n]|BOLD:AAF5699
Dichagyris cataclivis|[10378]|RDNMJ115-10|United States|Arizona|658[0n]|BOLD:AAF5699
Dichagyris acclivis|[10379]|RDNMF600-08|United States|Kansas|658[0n]|BOLD:AAE0890
Dichagyris acclivis|[10380]|RDNMG213-08|United States|Maryland|658[0n]|BOLD:AAE0890
Dichagyris acclivis|[10381]|RDNMF599-08|United States|Texas|658[0n]|BOLD:AAE0890
Dichagyris acclivis|[10382]|NAMUM325-08|United States|Maryland|658[0n]|BOLD:AAE0890
Dichagyris acclivis|[10383]|NAMUM326-08|United States|Maryland|658[0n]|BOLD:AAE0890
Dichagyris acclivis|[10384]|RDNMF596-08|United States|Pennsylvania|658[0n]|BOLD:AAE0890
Dichagyris acclivis|[10385]|RDNMG212-08|United States|Maryland|658[0n]|BOLD:AAE0890
Dichagyris acclivis|[10386]|LILLA796-11|United States|Illinois|658[0n]|BOLD:AAE0890
Dichagyris proclivis|[10387]|RDNMD866-07|United States|Arizona|655[0n]|BOLD:AAI9535
Dichagyris proclivis|[10388]|RDNMD867-07|United States|Arizona|655[0n]|BOLD:AAI9535
Dichagyris proclivis|[10389]|RDNMJ734-11|United States|Arizona|658[0n]|BOLD:AAI9535
Dichagyris proclivis|[10390]|CMAZA863-12|United States|Arizona|658[0n]|BOLD:AAI9535
Dichagyris proclivis|[10391]|CMAZA1008-12|United States|Arizona|658[0n]|BOLD:AAI9535
Dichagyris proclivis|[10392]|CMAZA561-10|United States|Arizona|658[0n]|BOLD:AAI9535
Dichagyris neoclivis|[10393]|LNAUS2761-13|United States|Texas|658[0n]|BOLD:AAI9535
Richia herculeana|[10394]|LNAUS4804-13|Mexico|Hidalgo|658[0n]|BOLD:ABW2960
Richia herculeana|[10395]|LNAUS4805-13|Mexico|Hidalgo|658[0n]|BOLD:ABW2960
Richia herculeana|[10396]|LNAUS4806-13|Mexico|Hidalgo|658[0n]|BOLD:ABW2960
Richia praefixa|[10397]|LNAUS5285-13|United States|New Mexico|658[0n]|BOLD:ACK3813
Richia praefixa|[10398]|LNAUS5286-13|United States|New Mexico|658[0n]|BOLD:ACK3813
Richia praefixa|[10399]|LNAUS5284-13|United States|New Mexico|658[0n]|BOLD:ACK3813
Richia praefixa|[10400]|LNAUS5287-13|United States|New Mexico|658[0n]|BOLD:ACK3813
Richia praefixa|[10401]|LNAUS5288-13|United States|New Mexico|658[0n]|BOLD:ACK3813
Richia praefixa|[10402]|CNCLB978-14|United States|Wyoming|658[0n]|BOLD:ACK3813
Richia praefixa|[10403]|CNCLB979-14|United States|Wyoming|658[0n]|BOLD:ACK3813
Richia palliviridis|[10404]|CNCLB1745-14|Mexico|Oaxaca|658[0n]|BOLD:ACP7111
Richia bicollaris|[10405]|CNCLB1746-14|Mexico|Nuevo Leon|658[0n]|BOLD:ACP7110
Richia serano|[10406]|RDNMD860-07|United States|Arizona|655[0n]|BOLD:AAF8023
Richia serano|[10407]|RDNMD861-07|United States|New Mexico|655[0n]|BOLD:AAF8023
Richia serano|[10408]|NAMUM053-08|United States|Arizona|657[0n]|BOLD:AAF8023
Richia furva|[10409]|CNCLB1749-14|Mexico|Oaxaca|658[0n]|BOLD:ACP6775
Richia nigratopis|[10410]|CNCLB1750-14|Mexico|Michoacan|658[0n]|BOLD:ACP6850
Peridroma saucia|[10411]|LPOKB998-09|United States|Oklahoma|658[0n]|BOLD:AAA6377
Peridroma saucia|[10412]|LCHP394-07|Canada|Manitoba|658[0n]|BOLD:AAA6377
Peridroma saucia|[10413]|LBCG856-09|Canada|British Columbia|658[0n]|BOLD:AAA6377
Peridroma saucia|[10414]|SELCA664-14|United States|California|547[0n]|BOLD:AAA6377
Peridroma saucia|[10415]|LOCT057-05|United States|Connecticut|618[0n]|BOLD:AAA6377
Peridroma saucia|[10416]|CNPPF1352-12|Canada|Ontario|618[0n]|BOLD:AAA6377
Peridroma saucia|[10417]|LPOKE601-12|United States|Oklahoma|612[0n]|BOLD:AAA6377
Peridroma saucia|[10418]|XAB693-04|Canada|Ontario|611[0n]|BOLD:AAA6377
Peridroma saucia|[10419]|LGSMG896-10|United States|North Carolina|658[0n]|BOLD:AAA6377
Peridroma saucia|[10420]|CNPPE1030-12|Canada|Ontario|632[0n]|BOLD:AAA6377
Peridroma saucia|[10421]|JMMMB541-13|United States|California|576[0n]|BOLD:AAA6377
Peridroma saucia|[10422]|CNPPA3924-12|Canada|Ontario|606[0n]|BOLD:AAA6377
Peridroma saucia|[10423]|GMRMC002-14|United States|California|532[0n]|BOLD:AAA6377
Peridroma saucia|[10424]|CNWLI320-12|Canada|Alberta|632[0n]|BOLD:AAA6377
Peridroma saucia|[10425]|GMRMF148-14|United States|California|546[0n]|BOLD:AAA6377
Peridroma saucia|[10426]|CNPPB2161-12|Canada|Ontario|600[0n]|BOLD:AAA6377
Peridroma saucia|[10427]|SMTPD3161-13|Canada|Ontario|576[0n]|BOLD:AAA6377
Peridroma saucia|[10428]|SMTPD1565-13|Canada|Ontario|576[0n]|BOLD:AAA6377
Peridroma saucia|[10429]|SMTPD1564-13|Canada|Ontario|576[0n]|BOLD:AAA6377
Peridroma saucia|[10430]|GMRMD001-14|United States|California|593[0n]|BOLD:AAA6377
Peridroma saucia|[10431]|LNCC668-11|United States|North Carolina|658[0n]|BOLD:AAA6377
Peridroma saucia|[10432]|GMRMD002-14|United States|California|592[0n]|BOLD:AAA6377
Peridroma saucia|[10433]|SMTPI2726-14|Canada|Ontario|591[0n]|BOLD:AAA6377
Peridroma saucia|[10434]|LPOKA1015-09|United States|Oklahoma|658[0n]|BOLD:AAA6377
Peridroma saucia|[10435]|FBLMX154-11|Germany|Bavaria|658[0n]|BOLD:AAA6377
Peridroma saucia|[10436]|SDRAP008-14|United States|California|588[0n]|BOLD:AAA6377
Peridroma saucia|[10437]|CNWLH462-12|Canada|Alberta|632[0n]|BOLD:AAA6377
Peridroma saucia|[10438]|MAMOT2879-12|Pakistan|Khyber Pakhtunkhwa|633[0n]|BOLD:AAA6377
Peridroma saucia|[10439]|CNPPE1021-12|Canada|Ontario|632[0n]|BOLD:AAA6377
Peridroma saucia|[10440]|RWWB911-10|United States|Washington|636[0n]|BOLD:AAA6377
Peridroma saucia|[10441]|RDLQ707-07|Canada|Quebec|658[3n]|BOLD:AAA6377
Peridroma saucia|[10442]|LPOKE446-12|United States|Oklahoma|630[0n]|BOLD:AAA6377
Peridroma saucia|[10443]|RWWA022-09|United States|Washington|630[0n]|BOLD:AAA6377
Peridroma saucia|[10444]|RWWC157-10|United States|Washington|658[0n]|BOLD:AAA6377
Peridroma saucia|[10445]|BBLPB692-10|Canada|Ontario|658[0n]|BOLD:AAA6377
Peridroma saucia|[10446]|MAMOT296-10|Pakistan|Punjab|658[0n]|BOLD:AAA6377
Peridroma saucia|[10447]|RWWC132-10|United States|Washington|658[0n]|BOLD:AAA6377
Peridroma saucia|[10448]|LPOKA930-09|United States|Oklahoma|658[0n]|BOLD:AAA6377
Peridroma saucia|[10449]|LPOKA946-09|United States|Oklahoma|658[0n]|BOLD:AAA6377
Peridroma saucia|[10450]|LOCBE107-06|United States|California|658[0n]|BOLD:AAA6377
Peridroma saucia|[10451]|LOCBE108-06|United States|California|658[0n]|BOLD:AAA6377
Peridroma saucia|[10452]|LOCBE109-06|United States|California|658[0n]|BOLD:AAA6377
Peridroma saucia|[10453]|DQGM003-08|Taiwan|Taichung City|658[0n]|BOLD:AAA6377
Peridroma saucia|[10454]|DQGM093-08|Taiwan|Taichung City|658[0n]|BOLD:AAA6377
Peridroma saucia|[10455]|LBCW055-08|Canada|British Columbia|658[0n]|BOLD:AAA6377
Peridroma saucia|[10456]|LPOKA978-09|United States|Oklahoma|658[0n]|BOLD:AAA6377
Peridroma saucia|[10457]|LPOKA1006-09|United States|Oklahoma|658[0n]|BOLD:AAA6377
Peridroma saucia|[10458]|LPOKB116-09|United States|Oklahoma|658[0n]|BOLD:AAA6377
Peridroma saucia|[10459]|LPOKB127-09|United States|Oklahoma|658[0n]|BOLD:AAA6377
Peridroma saucia|[10460]|LPOKB137-09|United States|Oklahoma|658[0n]|BOLD:AAA6377
Peridroma saucia|[10461]|LPOKB157-09|United States|Oklahoma|658[0n]|BOLD:AAA6377
Peridroma saucia|[10462]|LPOKB181-09|United States|Oklahoma|658[0n]|BOLD:AAA6377
Peridroma saucia|[10463]|LPOKB185-09|United States|Oklahoma|658[0n]|BOLD:AAA6377
Peridroma saucia|[10464]|LPOKB968-09|United States|Oklahoma|658[0n]|BOLD:AAA6377
Peridroma saucia|[10465]|LPOKB986-09|United States|Oklahoma|658[0n]|BOLD:AAA6377
Peridroma saucia|[10466]|LPOKB987-09|United States|Oklahoma|658[0n]|BOLD:AAA6377

Peridroma saucia|[10464]|LPOKB968-09|United States|Oklahoma|658[0n]|BOLD:AAA6377
Peridroma saucia|[10465]|LPOKB986-09|United States|Oklahoma|658[0n]|BOLD:AAA6377
Peridroma saucia|[10466]|LPOKB987-09|United States|Oklahoma|658[0n]|BOLD:AAA6377
Peridroma saucia|[10467]|LPOKB999-09|United States|Oklahoma|658[0n]|BOLD:AAA6377
Peridroma saucia|[10468]|RDNMD381-06|United States|California|658[0n]|BOLD:AAA6377
Peridroma saucia|[10469]|LOCBC460-06|United States|California|658[0n]|BOLD:AAA6377
Peridroma saucia|[10470]|RWWA158-09|United States|Washington|656[0n]|BOLD:AAA6377
Peridroma saucia|[10471]|LNCB630-09|United States|North Carolina|658[0n]|BOLD:AAA6377
Peridroma saucia|[10472]|LNCB631-09|United States|North Carolina|658[0n]|BOLD:AAA6377
Peridroma saucia|[10473]|RWWA592-09|United States|Washington|658[0n]|BOLD:AAA6377
Peridroma saucia|[10474]|BBLSW299-09|United States|Oklahoma|658[0n]|BOLD:AAA6377
Peridroma saucia|[10475]|BBLSW336-09|United States|Arizona|658[0n]|BOLD:AAA6377
Peridroma saucia|[10476]|BBLSW337-09|United States|Arizona|658[0n]|BOLD:AAA6377
Peridroma saucia|[10477]|BBLSW338-09|United States|Arizona|658[0n]|BOLD:AAA6377
Peridroma saucia|[10478]|BBLSW803-09|United States|Oklahoma|658[0n]|BOLD:AAA6377
Peridroma saucia|[10479]|BBLSX105-09|United States|Oklahoma|658[0n]|BOLD:AAA6377
Peridroma saucia|[10480]|BBLSX886-09|United States|Oklahoma|658[0n]|BOLD:AAA6377
Peridroma saucia|[10481]|LPOKC626-09|United States|Oklahoma|658[0n]|BOLD:AAA6377
Peridroma saucia|[10482]|CGUKA1005-09|United Kingdom|England|657[0n]|BOLD:AAA6377
Peridroma saucia|[10483]|LGSMG897-10|United States|North Carolina|658[0n]|BOLD:AAA6377
Peridroma saucia|[10484]|BBLSW339-09|United States|Arizona|658[0n]|BOLD:AAA6377
Peridroma saucia|[10485]|BBLSW526-09|United States|Oklahoma|658[0n]|BOLD:AAA6377
Peridroma saucia|[10486]|RWWB439-10|United States|Washington|658[0n]|BOLD:AAA6377
Peridroma saucia|[10487]|RWWB455-10|United States|Washington|658[0n]|BOLD:AAA6377
Peridroma saucia|[10488]|LEFID224-10|Finland||658[0n]|BOLD:AAA6377
Peridroma saucia|[10489]|GWORZ213-10|Italy|Basilicata|658[0n]|BOLD:AAA6377
Peridroma saucia|[10490]|RWWB574-10|United States|Washington|658[0n]|BOLD:AAA6377
Peridroma saucia|[10491]|USLEP320-10|United States|Texas|658[0n]|BOLD:AAA6377
Peridroma saucia|[10492]|USLEP650-10|United States|Texas|658[0n]|BOLD:AAA6377
Peridroma saucia|[10493]|USLEP1147-10|United States|Texas|658[0n]|BOLD:AAA6377
Peridroma saucia|[10494]|LPOKD681-10|United States|Oklahoma|658[0n]|BOLD:AAA6377
Peridroma saucia|[10495]|RWWB639-10|United States|Washington|658[0n]|BOLD:AAA6377
Peridroma saucia|[10496]|GWOSB420-10|Taiwan|Nantou County|658[0n]|BOLD:AAA6377
Peridroma saucia|[10497]|JGLL050-10|Canada|Manitoba|658[0n]|BOLD:AAA6377
Peridroma saucia|[10498]|LOCRF205-10|Costa Rica|Cartago|658[0n]|BOLD:AAA6377
Peridroma saucia|[10499]|LEFIL166-10|Spain|Andalusia|658[0n]|BOLD:AAA6377
Peridroma saucia|[10500]|LEFIL536-10|Bulgaria||658[0n]|BOLD:AAA6377
Peridroma saucia|[10501]|JMMMB349-11|United States|California|658[0n]|BOLD:AAA6377
Peridroma saucia|[10502]|JMMMB421-11|United States|California|658[0n]|BOLD:AAA6377
Peridroma saucia|[10503]|AWCLB592-11|United States|Arizona|658[0n]|BOLD:AAA6377
Peridroma saucia|[10504]|IBLAO073-11|Spain|Murcia|658[0n]|BOLD:AAA6377
Peridroma saucia|[10505]|LENOA159-11|France|Haute Normandie|658[0n]|BOLD:AAA6377
Peridroma saucia|[10506]|LENOA161-11|France|Haute Normandie|658[0n]|BOLD:AAA6377
Peridroma saucia|[10507]|LENOA162-11|Spain|Andalusia|658[0n]|BOLD:AAA6377
Peridroma saucia|[10508]|PHLPM060-11|Portugal|Madeira|658[0n]|BOLD:AAA6377
Peridroma saucia|[10509]|LILLA533-11|United States|Illinois|658[0n]|BOLD:AAA6377
Peridroma saucia|[10510]|MAMOT1207-11|Pakistan||658[0n]|BOLD:AAA6377
Peridroma saucia|[10511]|LALPA1124-11|Canada|British Columbia|658[0n]|BOLD:AAA6377
Peridroma saucia|[10512]|PHLAF398-11|Macedonia||658[0n]|BOLD:AAA6377
Peridroma saucia|[10513]|CMAZA878-12|United States|Arizona|658[0n]|BOLD:AAA6377
Peridroma saucia|[10514]|GMLC1281-12|United States|California|658[0n]|BOLD:AAA6377
Peridroma saucia|[10515]|IBLAO658-12|Spain|Murcia|658[0n]|BOLD:AAA6377
Peridroma saucia|[10516]|RWWC868-12|United States|Washington|658[0n]|BOLD:AAA6377
Peridroma saucia|[10517]|MAMOT2229-12|Pakistan||658[0n]|BOLD:AAA6377
Peridroma saucia|[10518]|MAMOT2233-12|Pakistan||658[0n]|BOLD:AAA6377
Peridroma saucia|[10519]|MAMOT2248-12|Pakistan||658[0n]|BOLD:AAA6377
Peridroma saucia|[10520]|MAMOT2249-12|Pakistan||658[0n]|BOLD:AAA6377
Peridroma saucia|[10521]|MAMOT2250-12|Pakistan||658[0n]|BOLD:AAA6377
Peridroma saucia|[10522]|MAMOT2254-12|Pakistan||658[0n]|BOLD:AAA6377
Peridroma saucia|[10523]|MAMOT2255-12|Pakistan||658[0n]|BOLD:AAA6377
Peridroma saucia|[10524]|MAMOT2286-12|Pakistan||658[0n]|BOLD:AAA6377
Peridroma saucia|[10525]|MAMOT2290-12|Pakistan||658[0n]|BOLD:AAA6377
Peridroma saucia|[10526]|MAMOT2426-12|Pakistan||658[0n]|BOLD:AAA6377
Peridroma saucia|[10527]|MAMOT2859-12|Pakistan|Punjab|658[0n]|BOLD:AAA6377
Peridroma saucia|[10528]|BAWG096-12|Canada|Alberta|658[0n]|BOLD:AAA6377
Peridroma saucia|[10529]|TRLEP022-13|Turkey|Mersin|658[0n]|BOLD:AAA6377
Peridroma saucia|[10530]|LOCBC060-06|United States|California|658[0n]|BOLD:AAA6377
Peridroma saucia|[10531]|LNCB207-06|United States|North Carolina|658[0n]|BOLD:AAA6377
Peridroma saucia|[10532]|LOCBB863-06|United States|California|658[0n]|BOLD:AAA6377
Peridroma saucia|[10533]|LSUSA169-06|United States|Kentucky|658[0n]|BOLD:AAA6377
Peridroma saucia|[10534]|LOCBB263-06|United States|California|658[0n]|BOLD:AAA6377
Peridroma saucia|[10535]|LOCBB262-06|United States|California|658[0n]|BOLD:AAA6377
Peridroma saucia|[10536]|LOCBB261-06|United States|California|658[0n]|BOLD:AAA6377
Peridroma saucia|[10537]|LOCBB259-06|United States|California|658[0n]|BOLD:AAA6377
Peridroma saucia|[10538]|LOCBB258-06|United States|California|658[0n]|BOLD:AAA6377
Peridroma saucia|[10539]|LOCB821-06|United States|California|658[0n]|BOLD:AAA6377
Peridroma saucia|[10540]|XAH834-05|Canada|Ontario|658[0n]|BOLD:AAA6377
Peridroma saucia|[10541]|LGSMC881-05|United States|North Carolina|658[0n]|BOLD:AAA6377
Peridroma saucia|[10542]|RWWB588-10|United States|Washington|658[0n]|BOLD:AAA6377
Peridroma saucia|[10543]|XAB689-04|Canada|Ontario|658[0n]|BOLD:AAA6377
Peridroma saucia|[10544]|RWWB370-09|United States|Washington|658[0n]|BOLD:AAA6377
Peridroma saucia|[10545]|LEFIL167-10|Spain|Andalusia|658[0n]|BOLD:AAA6377
Peridroma saucia|[10546]|LOCBB260-06|United States|California|656[0n]|BOLD:AAA6377
Peridroma saucia|[10547]|LOCB743-06|United States|California|658[0n]|BOLD:AAA6377
Peridroma saucia|[10548]|LOCBF063-13|United States|California|600[0n]|BOLD:AAA6377
Peridroma saucia|[10549]|LPOKE271-11|United States|Oklahoma|596[0n]|BOLD:AAA6377
Peridroma saucia|[10550]|MHCOL120-07|Canada|Manitoba|603[0n]|BOLD:AAA6377
Peridroma saucia|[10551]|GMRMD003-14|United States|California|583[0n]|BOLD:AAA6377
Peridroma saucia|[10552]|CNPPH808-12|Canada|Ontario|637[0n]|BOLD:AAA6377
Peridroma saucia|[10553]|SDRVF1664-14|United States|California|594[0n]|BOLD:AAA6377
Peridroma saucia|[10554]|SMTPI2725-14|Canada|Ontario|576[0n]|BOLD:AAA6377
Peridroma saucia|[10555]|LOCBB864-06|United States|California|644[0n]|BOLD:AAA6377
Peridroma saucia|[10556]|LGSMG697-07|United States|Tennessee|634[0n]|BOLD:AAA6377
Peridroma saucia|[10557]|XAC150-04|Canada|Ontario|626[0n]|BOLD:AAA6377
Peridroma saucia|[10558]|LOCRA425-07|Costa Rica|Alajuela|584[0n]|BOLD:AAA6377
Peridroma saucia|[10559]|LOCRA426-07|Costa Rica|Alajuela|593[0n]|BOLD:AAA6377
Peridroma saucia|[10560]|LPOKA1027-09|United States|Oklahoma|612[0n]|BOLD:AAA6377
Peridroma saucia|[10561]|CNPKA023-14|Canada|Ontario|578[0n]|BOLD:AAA6377
Peridroma saucia|[10562]|GMRMA238-14|United States|California|581[0n]|BOLD:AAA6377
Peridroma saucia|[10563]|SMTPI2740-14|Canada|Ontario|575[0n]|BOLD:AAA6377
Peridroma saucia|[10564]|LEATH616-14|Italy|South Tyrol|658[0n]|BOLD:AAA6377
